# Finding Potential Therapeutic Targets against *Shigella flexneri* through Proteome Exploration

**DOI:** 10.3389/fmicb.2016.01817

**Published:** 2016-11-22

**Authors:** Mohammad Uzzal Hossain, Md. Arif Khan, Abu Hashem, Md. Monirul Islam, Mohammad Neaz Morshed, Chaman Ara Keya, Md. Salimullah

**Affiliations:** ^1^Department of Biotechnology and Genetic Engineering, Life Science Faculty, Mawlana Bhashani Science and Technology UniversityTangail, Bangladesh; ^2^Department of Science and Humanities, Military Institute of Science and Technology, Mirpur CantonmentDhaka, Bangladesh; ^3^Microbial Biotechnology Division, National Institute of BiotechnologySavar, Bangladesh; ^4^Department of Biochemistry and Microbiology, North South UniversityBashundhara, Dhaka, Bangladesh; ^5^Molecular Biotechnology Division, National Institute of BiotechnologySavar, Bangladesh

**Keywords:** *S. flexneri*, drug target, therapeutics, metabolic proteins, proteome

## Abstract

**Background:** *Shigella flexneri* is a gram negative bacteria that causes the infectious disease “shigellosis.” *S. flexneri* is responsible for developing diarrhea, fever, and stomach cramps in human. Antibiotics are mostly given to patients infected with shigella. Resistance to antibiotics can hinder its treatment significantly. Upon identification of essential therapeutic targets, vaccine and drug could be effective therapy for the treatment of shigellosis.

**Methods:** The study was designed for the identification and qualitative characterization for potential drug targets from *S. flexneri* by using the subtractive proteome analysis. A set of computational tools were used to identify essential proteins those are required for the survival of *S. flexneri*. Total proteome (13,503 proteins) of *S. flexneri* was retrieved from NCBI and further analyzed by subtractive channel analysis. After identification of the metabolic proteins we have also performed its qualitative characterization to pave the way for the identification of promising drug targets.

**Results:** Subtractive analysis revealed that a list of 53 targets of *S. flexneri* were human non-homologous essential metabolic proteins that might be used for potential drug targets. We have also found that 11 drug targets are involved in unique pathway. Most of these proteins are cytoplasmic, can be used as broad spectrum drug targets, can interact with other proteins and show the druggable properties. The functionality and drug binding site analysis suggest a promising effective way to design the new drugs against *S. flexneri*.

**Conclusion:** Among the 53 therapeutic targets identified through this study, 13 were found highly potential as drug targets based on their physicochemical properties whilst only one was found as vaccine target against *S. flexneri*. The outcome might also be used as module as well as circuit design in systems biology.

## Introduction

In developing countries, *S. flexnari* is the foremost reason of bacillary dysentery among the four species of Shigella. Annually, 1.1 million deaths occur out of 164.7 million cases of Shigellosis worldwide and children <5 years of age are the worst victim of it (Bardhan et al., 2010). Depending on the combinations of antigenic determinants existing on the O antigen of the cell envelope lipopolysaccharide (LPS)(2–14), *S. flexneri* is divided into 19 serotypes viz. serotypes 1a, 1b, 1c, 1d, 2a, 2b, 3a, 3b, 4a, 4av, 4b, 5a, 5b, X, Xv, Y, Yv, F6, and 7b (Simmons and Romanowska, 1987; Kotloff et al., 1999; Stagg et al., 2009; Ye et al., 2010; Foster et al., 2011; Sun et al., 2011, 2012; Luo et al., 2012; Perepelov et al., 2012).

Shigella, as Gram-negative facultative human pathogens, cause intestinal infections with sign and symptoms such as fever, abdominal cramps and watery, or bloody diarrhea. Recent evidence suggests that the third prominent reason of global infant mortality is diarrhea (Black et al., 2010). Children below the age of five are mostly affected by Shigella (Kotloff et al., 1999; Peng et al., 2002). Majority of endemic dysentery are caused by *S. flexneri* in regions of the world having less facility of sanitation. Also, improper use of antibiotic rendered resistance to shigella. Therefore, antimicrobials development could be the better alternatives for the prevention of antibiotic-resistant Shigella as it is non-toxic, cheap, easy to apply and produce life-long immunity (Kärnell et al., 1995; Coster et al., 1999; Mukhopadhaya et al., 2003, 2006; Katz et al., 2004; Ranallo et al., 2005; Paterson, 2006). Recently, it has been reported that Shigella serotypes including *S. flexneri* 2a, 3a, *S. dysenteriae* 1, and *S. sonnei* are targeted for vaccine development. In developing countries, the first three are more widespread while the last serotypes found in regions where sanitation standard is high (Jennison and Verma, 2004; Mukhopadhaya et al., 2006).

Antibiotic is mostly given for the treatment with shigella infection. But the high failure rate is increasing day by day due to acquired resistance to commonly used antibiotics (Nessar et al., 2012). To combat these infections new drugs discovery are necessary due to emerging multi-drug resistance and absence of optimal treatment. Therefore, to identify novel drug target(s) are one of the key ways of drug discovery. The present study is aimed to identify potential drug targets in *S. flexneri* by subtractive proteome analysis. The traditional way of drug discovery needs more times, expensive experiments and also laborious efforts whereas the computational way could be effective alternative way which could accelerate of drug discovery process within a very short time. The identification of drug targets has been growing more by coupling of “omics” data viz., genomics, proteomics, and metabolomics and the utilization of computational approaches. The sequencing of genome and revealing of proteome of disease causing organisms are advancing the search of drug targets in the field of drug research based on essential genes of specific pathogen, interacting factors of host-pathogen, proteins persistence, resistance genes/resistance-associated proteins, metabolic pathways, prediction of gene expression levels (Galperin and Koonin, 1999; Yeh et al., 2004; Briken, 2008; Raman et al., 2008; Barh et al., 2011; Vetrivel et al., 2011). To identify novel drug targets these approaches have already been utilized in several life threatening pathogens, including *Mycobacterium tuberculosis* (Anishetty et al., 2005; Asif et al., 2009), *M. leprae* (Shanmugam and Natarajan, 2010), *M. ulcerans* (Butt et al., 2012), *Helicobacter pylori* (Sarkar et al., 2012), *Streptococcus pneumonia* (Singh et al., 2007), *Yersinia pestis* (Sharma and Pan, 2012), and *Pseudomonas aeruginosa* (Sakharkar et al., 2004). The foremost criteria for the identification of promising therapeutic candidates are essentiality and selectivity/specificity. To avoid the undesired interactions of host-pathogen which could occur to death of the pathogen by mediating inhibition of important proteins, the targets must be specific as drugs interact to host proteins. The present study integrates the various computational methods for the identification and characterization of the drug targets of *S. flexneri* which enables us to identify 53 potential therapeutic targets based on essentiality and specificity. The qualitative characterization of 53 therapeutic candidates predicts the uniqueness in metabolic pathway, capability to act as a broad spectrum target, the cellular location, cellular function, functional association with metabolic proteins, and druggability properties.

## Materials and methods

An *in silico* systematic method consists of three stages is applied to classify and illustrate possible drug targets against *S. flexneri*. At the stage I, the protein dataset was collected from the NCBI–FTP site for the analysis. Clarification of protein datasets through subtractive channel of analysis was done in stage II and possible drug targets found from stage I and II were qualitatively characterized in stage III.

### Stage I: mining of protein datasets

The whole protein sequences of *S. flexneri* 2a strain 2457T were retrieved from NCBI (http://www.ncbi.nlm.nih.gov/) protein database as FASTA format.

### Stage II: subtractive channel of analysis

Protein datasets were further selected and subjected to be qualified by passing through a sequence of subtractive proteome analysis. In this process, highly discerning and effective drug targets could be identified.

#### Identification of paralog proteins

Paralog proteins were identified by exposing *S. flexneri* proteins to CD-hit suite (Huang et al., 2010). The CDHIT server can be utilized for the “sequence identity” among the proteins (Fasta file). In this server users can exploit 10–90% sequence identity in the “sequence identity cut-off” box depending on their requirement (Huang et al., 2010). It has been widely accepted to set 60% sequence identity as cut-off to maintain a rigid criteria to remove duplicate proteins (Dutta et al., 2006; Barh and Kumar, 2009; Rahman et al., 2014; Mondal et al., 2015; Hasan et al., 2016). Therefore, we have used CDHIT server (http://weizhong-lab.ucsd.edu/cdhit_suite/cgi-bin/index.cgi?cmd=cd-hit) in which 0.6 (60%) was manually set in “sequence identity cut-off” box for the stringent selection of duplicate proteins. The duplicates were omitted and for further selection those were designated from the remaining set of non-paralog proteins that have in excess of 100 amino acids.

#### Identification of orthologs in gut flora

The non-duplicate proteins that were described in the preceding step, analyzed to explore their similarity with the proteome of human gut microbiota (Fujimura et al., 2010). BLASTing (Altschul et al., 1990) of those proteins against the gut flora proteome available from literature with 0.0001 as *e*-value threshold helped to further escape from the orthologs.

#### Identification of orthologs in human proteome

BLASTp (Altschul et al., 1990) was done for the qualified proteins resulted from stage I against non-redundant database of *H. sapiens* with an estimated threshold value of 0.0001. Those proteins are selected for the next step that shows no hits for the above mentioned *e*-value.

#### Essentiality analysis

To identify essential proteins by BLASTp searching against Database of Essential Genes (DEG; Zhang and Lin, 2009), non-homologous proteins were screened. Protein alignments associated with expect value of <0.0001(Barh et al., 2011; Sharma and Pan, 2012) were considered as more significant hits.

#### Metabolic pathway analysis

Metabolic pathway analysis was done by KAAS server at KEGG to classify the possible targets of the human non-homologous essential proteins of *S. flexneri* which has been assimilated from DEG (Moriya et al., 2007). Functional annotation of genes is obtained through KAAS by *comparing* BLAST beside manually created KEGG GENES database. The result comprises of KO (KEGG Orthology) task that identify the metabolic proteins.

### Stage III: qualitative characterization of the short-listed targets

#### Detection of proteins involved in unique pathways

KEGG (Kyoto Encyclopedia of Genes and Genomes) Genome Database (Kanehisa et al., 2014) was applied to find the distinctive metabolic pathways of *S. flexneri* in contrast to *H. sapiens*. In order to compare the metabolic pathway three letter codes that are particular for host and pathogen was placed in the Genome Comparison and Combination box. Eventually KEGG Genome generated pathway maps were recognized as distinctive pathways. Human non-homologous metabolic proteins that were selected formerly were subjected to screening to disclose their association in those distinctive pathways.

#### Cellular localization analysis

In this study, PSORTb 3.0.2(Yu et al., 2010), CELLO 2.0(Yu et al., 2006), Signal IP 4.1(Petersen et al., 2011), and Phobius (Käll et al., 2007) were used to identify the location of the short-listed proteins in various modules like SVM, S-TMHMM, and SCL-BLAST with proteins of known localization from bacteria (Gram-positive and negative) and archaea as training set. These server were used for the accurate localization of identified targets.

#### Broad spectrum analysis

BLASTp (Altschul et al., 1990) search was done beside a wide-range of pathogenic bacteria to explore proteins with an estimated threshold value of 0.005 to find broad spectrum targets. In the broad spectrum analysis, overall 240 disease-causing bacteria from different genus including with other serotypes of *S. flexneri* were used. Cluster of Orthologous Groups of proteins (COG) search was used to identify homologs of the short listed targets in other pathogenic bacteria by means of COGnitor from NCBI that matches the query sequence with the COG database.

#### Interactome analysis

STRING 9.0 (Szklarczyk et al., 2011) was used to build a protein-protein interaction network for each of the authorized targets. All interactors with low along with medium confidence score (<0.700) were removed from the network to escape false positives and false negatives.

#### Functionality analysis

INTERPROSCAN, a tool that incorporates various protein signature recognition methods and databases, was used to predict the role of the hypothetical proteins from the list of possible targets (Mulder and Apweiler, 2007).

#### Binding site analysis

Local meta-threading-server (LOMETS; Wu and Zhang, 2007) was used for the selection of best template from 9 locally-installed threading programs (FFAS-3D, HHsearch, MUSTER, pGenTHREADER, PPAS, PRC, PROSPECT2, SP3, and SPARKS-X). Then, Modeller 9.17 (Webb and Sali, 2014) was used to build the 3D model of metabolic hypothetical proteins. A set of tools Procheck (Laskowski et al., 1993), Verify3D (Eisenberg et al., 1997), and proSA (Wiederstein and Sippl, 2007) were utilized for the model quality assessment. Thereafter, COFACTOR server (Roy et al., 2012) was also employed to reveal out the binding site of 6 metabolic hypothetical proteins.

#### Druggability analysis

In the current study, a similarity search was done in contrast to Drug Bank 3.0 target collection for all of the target (Knox et al., 2011).

#### Docking analysis

We have performed Autodock vina (Trott and Olson, 2010) for the analysis of binding affinity to the druggable targets. We retrieved all the interacting drugs (.pdb files) from 6 druggable targets which were found by virtual screening in Drugbank database. Then we generated the .pdbqt files of these 6 druggable targets for docking experiments. Blind docking was performed for the identification of most effective binding site of these drugs. A grid box parameter for covering the whole protein was set for all docking runs.

Furtheremore, we have performed ProtParam (http://web.expasy.org/protparam/) and VaxiJen (http://www.ddg-pharmfac.net/vaxijen/VaxiJen/VaxiJen.html) server for the identification of most suitable drug/vaccine target.

## Results and discussion

### Data acquisition

A number of potential therapeutic targets in *S. flexneri* is detected and categorized through an *in silico* method. The strategy applied a hierarchy of subtractive analysis where functionally essential proteins of *S. flexneri* derived from whole proteome of 13,503 proteins dataset (Drug Target, Data Sheet S1). The number of subtractive channel was used to select the potential candidates that could serve either drug or vaccine for therapeutics treatment. Overall, the strategy composed of stages of data mining, subtractive channel of analysis and qualitative characterization.

### Subtractive channel of analysis

#### Subtraction of duplicate and mini proteins

CD-HIT suite identified the duplicate or paralog proteins from the proteome. This tool sorted out 4559 proteins as non-duplicate at sequence identity upto 60% (Drug Target, Data Sheet S2). Proteins that showed above 60% matching were considered as duplicates or paralogs in this analysis. We have chosen 60% similarity as a cut-off to maintain a very stringent selection criteria for the identification of the most effective therapeutic targets. Also, we considered to keep one protein from two identical sequences (>60% similarity) as they might be similar for protein domain, motifs, binding site etc. The proteins found from non-duplicate analysis have various length distribution. Proteins were excluded from the analysis having length of less than 100 amino acids known as mini proteins (Wang et al., 2008; Kumar et al., 2010; Barh et al., 2011). Prokaryotic genomes contain a high level of mini proteins which have key role in numerous biological phenomenon as well as regulatory purposes (Kumar et al., 2010). These mini proteins were deleted from the non-duplicate proteins as they are less likely to represent the essential therapeutics candidate (Drug Target, Data Sheet S3). In addition to this, the larger amino acid sequence has the probability to be involved in essential metabolic pathways (Haag et al., 2012).

#### Subtraction of orthologs in gut flora

**Figure 1:**
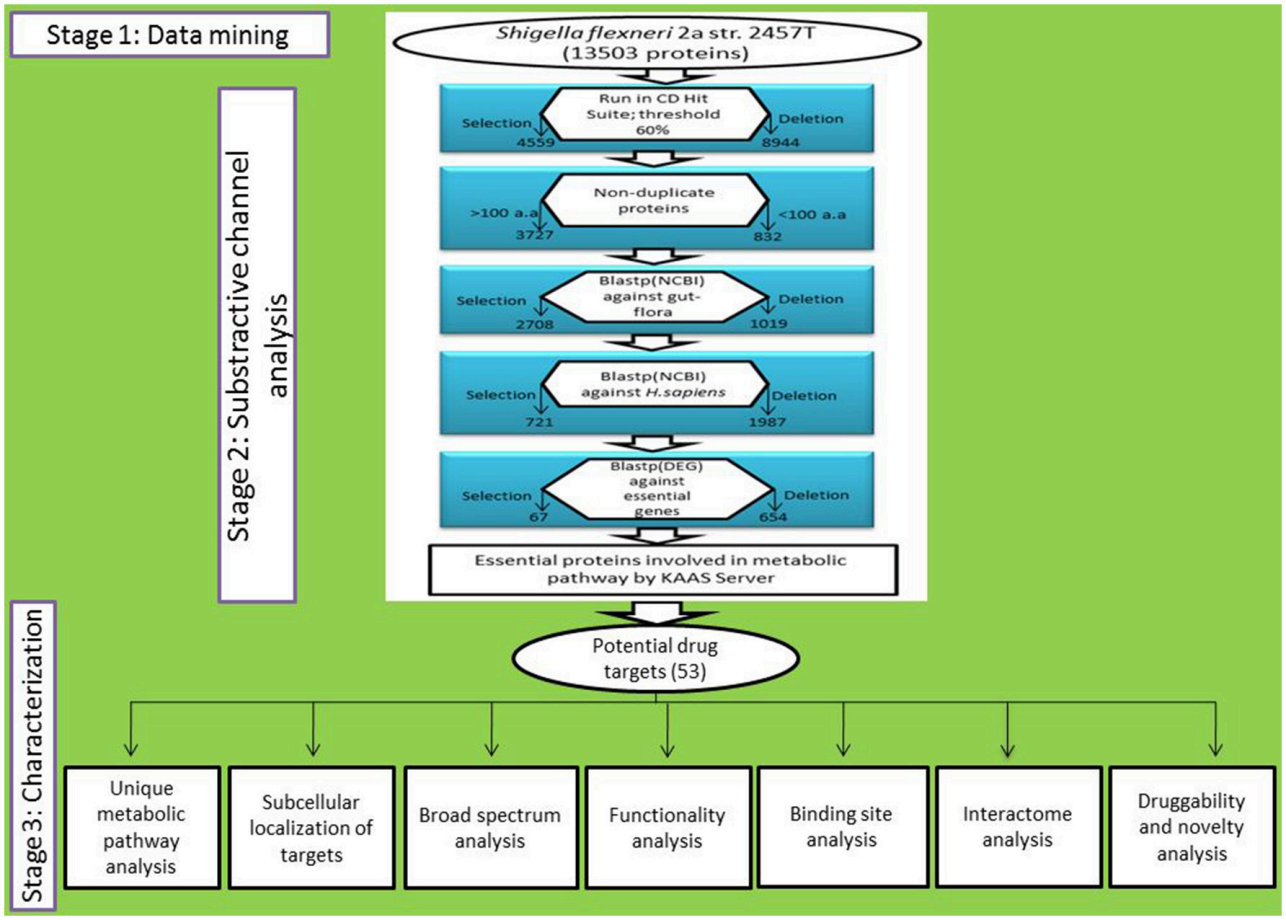
**Schematic representation of whole work**.

A critical step in this study is to identify proteins that are non-homologous to gut flora proteins for circumventing extreme lethal effects in host. It is reported that around 10^14^ (Kärnell et al., 1995) microorganisms exist in the gastrointestinal tract of a normal healthy human (Fujimura et al., 2010). As gut microbiota maintains a symbiotic relationship, it helps in metabolism by fermenting indigestible food particles along with defense from colonization of pathogenic bacteria in gut (Rabizadeh and Sears, 2008). If gut flora proteins are spoiled accidentally it may decline the microbiota which may cause harm to the host. Therefore, Human gut flora proteins were subjected to analyze for the assurance of non-similarity with our selected proteins. BLASTp was employed to recognize the gut homologs with our selected proteins and gut flora proteins. Eventually, we have identified 2708 gut flora similar proteins (Drug Target, Data Sheet S4 and Figure 1).

#### Subtraction of orthologs in human proteome

Identification of pathogen specific protein is the main goal of this analysis. The importance of this step is to reduce unwanted cross reactivity of the drug and thus to inhibit its binding to the active sites of the homologous proteins in host (Sarkar et al., 2012). In *in silico* drug target identification method, the first step is considered as the filtration of homologous proteins to human proteome (Anishetty et al., 2005; Sarkar et al., 2012). This non-similarity analysis was carried out for the gut flora non-similarity proteins datasets. BLASTp was used for similarity search in contrast to whole proteome of *H. sapiens* (host) with *e*-value threshold of 0.0001 (Altschul et al., 1990). Those Proteins were considered as close homologs that exhibit hits with proteins of human proteome. From the analysis of 2708 input proteins 1987 homologous proteins were omitted and 721 proteins were nominated that are non-homologous to human (Drug Target, Data Sheet S5 and Figure 1).

#### Identification of essential genes in *S. flexneri*

DEG server was used for the screening the essential genes of S. *flexneri* from human proteome non-homologous protein list with an estimated value of 0.0001. DEG 6.1 is a storehouse of genes necessary for the survival of an organism. It comprises 10,618 essential genes from prokaryotic and eukaryotic organisms. These types of proteins were considered as essential and it was clearly demonstrated that similar proteins which are crucial in one organism are likely to be essential in another. A potential drug target possesses a crucial feature for the existence of the pathogen and must be an indispensable protein (Sarkar et al., 2012). The 67 proteins (Drug Target, Data Sheet S6 and Figure 1) out of 721 non-homologous input proteins were nominated for the consecutive analysis and considered as vital for the existence of the pathogen as they have homologs with not more than the given threshold value (Supplementary Table, S1). Proteins showing no hit against DEG were omitted from the analysis and regarded as non-essential.

#### Metabolic pathway analysis

The output of this server assists to identify the potential drug targets by revealing the KEGG pathways as well as KO (KEGG orthology) assignments. About 53 proteins are involved in metabolic pathways obtained from the essential proteins (Drug Target, Data Sheet S7 and Figure 1). These 53 proteins (Supplementary Table, S2) have the key role in metabolism for the bacterial survival.

### Qualitative characterization of metabolic pathway proteins

#### Unique pathway analysis

Besides the identification of metabolic pathway proteins we have also analyzed the unique metabolic pathway proteins which answers the disputable question whether the metabolic pathway proteins are also present or not in host. Here, we have found 11 unique metabolic pathway proteins that are only present in the bacterial metabolic pathway (Table 1). These unique proteins were found in the pathways: Purine metabolism, Pyrimidine metabolism, Fructose and mannose metabolism, Amino sugar and nucleotide sugar metabolism, Lipopolysaccharide biosynthesis, Pyruvate metabolism, Propanoate metabolism Butanoate metabolism, Lysine biosynthesis, Terpenoid backbone biosynthesis, Phosphotransferase system (PTS), Peptidoglycan biosynthesis, Flagellar assembly, Arginine and proline metabolism and bacterial pathogenic cycle.

**Table 1:** **Proteins involved in unique pathways**.

**SN**	**Accession no**.	**Name of protein**	**KO number (gene name)**	**Pathways**
1	NP_839575.1	DNA-directed RNA polymerase subunit alpha	K03040	Purine metabolism, Pyrimidine metabolism
2	NP_838872.1	Mannose-specific IIA component	K02794(manX)	Fructose and mannose metabolism, Amino sugar, and nucleotide sugar metabolism
3	NP_838706.1	Arabinose-5-phosphate isomerase	K06041	Lipopolysaccharide biosynthesis
4	NP_838628.1	Formate C-acetyltransferase	K00656 (pflD)	Pyruvate metabolism, Propanoate metabolism Butanoate metabolism
5	NP_835770.1	4-hydroxy-tetrahydrodipicolinate reductase	K00215(dapB)	Lysine biosynthesis
6	NP_835768.1	4-hydroxy-3-methylbut-2-enyl diphosphate reductase	K03527 (ispH, lytB)	Terpenoid backbone biosynthesis
7	AAP19547.1	PTS system, ascorbate-specific IIC component	K03475	Phosphotransferase system (PTS)
8	EFS15406.1	penicillin-binding protein 1C	K05367 (pbpC)	Peptidoglycan biosynthesis
9	EFS14577.1	Flagellar FliJ protein	K02413 (fliJ)	Flagellar assembly
10	EFS13661.1	Arginine N-succinyltransferase	K00673 (astA)	Arginine and proline metabolism
11	EFS12253.1	RNA polymerase nonessential primary-like sigma factor	K03087	Bacterial pathogenic cycle

#### Subcellular localization

Proteins can be found in five possible subcellular locations, specifically, cytoplasm, periplasm, plasma membrane, outer membrane, and extracellular. The importance of the localization study is to depict the protein as drug or vaccine target. Surface membrane proteins and cytoplasmic proteins can be used as vaccine and drug targets respectively (Barh et al., 2011). Protein databases like UniProt contain information about subcellular location of some proteins. PSORTb 3.0.2 (Yu et al., 2010), CELLO 2.0 (Yu et al., 2006), SignalIP (Petersen et al., 2011), and Phobius (Käll et al., 2007) tools were utilized for the prediction of subcellular localization.

Therefore, we have analyzed the essential human non-homologous metabolic pathway proteins (53) for the prediction of their subcellular localization. From these analysis we have found 29 targets as cytoplasmic, 14 targets as inner membrane, 6 targets as periplasmic, 3 targets as outer membrane and only 1 target as extracellular (Table 2).

**Table 2:** **Subcellular localization of drug targets**.

**Cytoplasmic**	**Inner membrane**	**Periplasmic**	**Outer membrane**	**Extracellular**
NP_839574.2 NP_839575.1 NP_839064.1 NP_838943.1 NP_838894.1 NP_838872.1 NP_838722.1 NP_838706.1 NP_838628.1 NP_837679.1 NP_837604.1 NP_837597.1 NP_837443.1 NP_836675.1 NP_836672.1 NP_836278.1 NP_835876.1 NP_835770.1 NP_835873.1 NP_835768.1 AAP19293.1 AAP18497.1 AAP16677.1 EFS15144.1 EFS15122.1 EFS13661.1 EFS12253.1 EFS11712.1 EFS10930.1	NP_836827.2 (TM;6) NP_839768.1 (SP), (TM;1) NP_839521.1 (TM;12) NP_839165.1 NP_836948.1 (TM;5) NP_836937.1 (TM;6) NP_836465.1 (TM;5) AAP19547.1 (TM;12) EFS15406.1 EFS14933.1TM;2) EFS13325.1 EFS11623.1 (TM;10) EFS10693.1 (TM;7) NP_837444.1	NP_839600.1 (SP) NP_837676.1 NP_836681.1 (SP), (TM;1) EFS14577.1 EFS13874.1 (TM;1) EFS12950.1 (SP)	NP_837438.1 EFS15865.1 EFS15439.1	EFS11306.1

*Here, SP, Signal Peptide; TM, Transmembrane Helices*.

#### Broadspectrum analysis

The progress of drug resistance can be reduced greatly by such types of broadspectrum and pathogen specific targets analysis (Raman et al., 2008). In this study, list of pathogenic bacteria described in literature was well-thought out (Griffith et al., 2007; Raman et al., 2008; Shenai et al., 2009). It is theorized from the similarity analysis in contrast to each of the pathogen that close homologs existing in more number of pathogens are more expected to be a “promising broad spectrum target” (Raman et al., 2008). Therefore, we have also analyzed broadspectrum targets by comparing the sequences analysis between the 240 pathogens shigella species as well as other clinically important bacterial pathogens with the selected 53 essential metabolic proteins (Drug Target, Data Sheet S7). This was done by the BLASTp analysis. About 25 targets were found to have close identity with less than 10 bacterial pathogens, 10 targets in less than 20 bacteria, 8 targets in less than 30, 7 less than 40 bacteria, and 3 in less than 50 bacterial pathogens (Table 3). From these results, it is concluded that these 3 (NP_836465.1, NP_836278.1 and NP_835876.1) or 7 (NP_837443.1, NP_837438.1, NP_836948.1, NP_836937.1, NP_836681.1, NP_836675.1 and NP_836672.1) targets are the exclusive proteins of Shigella.

**Table 3:** **Broad spectrum analysis of essential proteins**.

**Hit pathogenic bacterial protein (240 Pathogenic bacteria)**				
=>10	=>20	=>30	=>40	=>50
EFS11712.1 EFS11623.1 EFS11306.1 EFS10930.1 EFS10693.1 NP_836827.2 NP_839574.2 NP_839768.1 NP_839600.1 NP_839575.1 EFS13874.1 EFS13661.1 EFS13325.1 EFS12950.1 NP_839521.1 NP_839165.1 NP_839064.1 NP_838943.1 NP_838894.1 EFS12253.1 NP_838872.1 NP_838722.1 NP_838706.1 NP_838628.1 NP_837679.1	NP_837679.1 NP_837676.1 NP_837604.1 NP_837597.1 NP_837444.1 EFS15406.1 EFS15144.1 EFS15122.1 EFS14933.1 EFS14577.1	NP_835770.1 NP_835768.1 AAP19547.1 AAP19293.1 AAP18497.1 AAP16677.1 EFS15865.1 EFS15439.1	NP_837443.1 NP_837438.1 NP_836948.1 NP_836937.1 NP_836681.1 NP_836675.1 NP_836672.1	NP_836465.1 NP_836278.1 NP_835876.1

#### Interactome analysis

**Figure 2:**
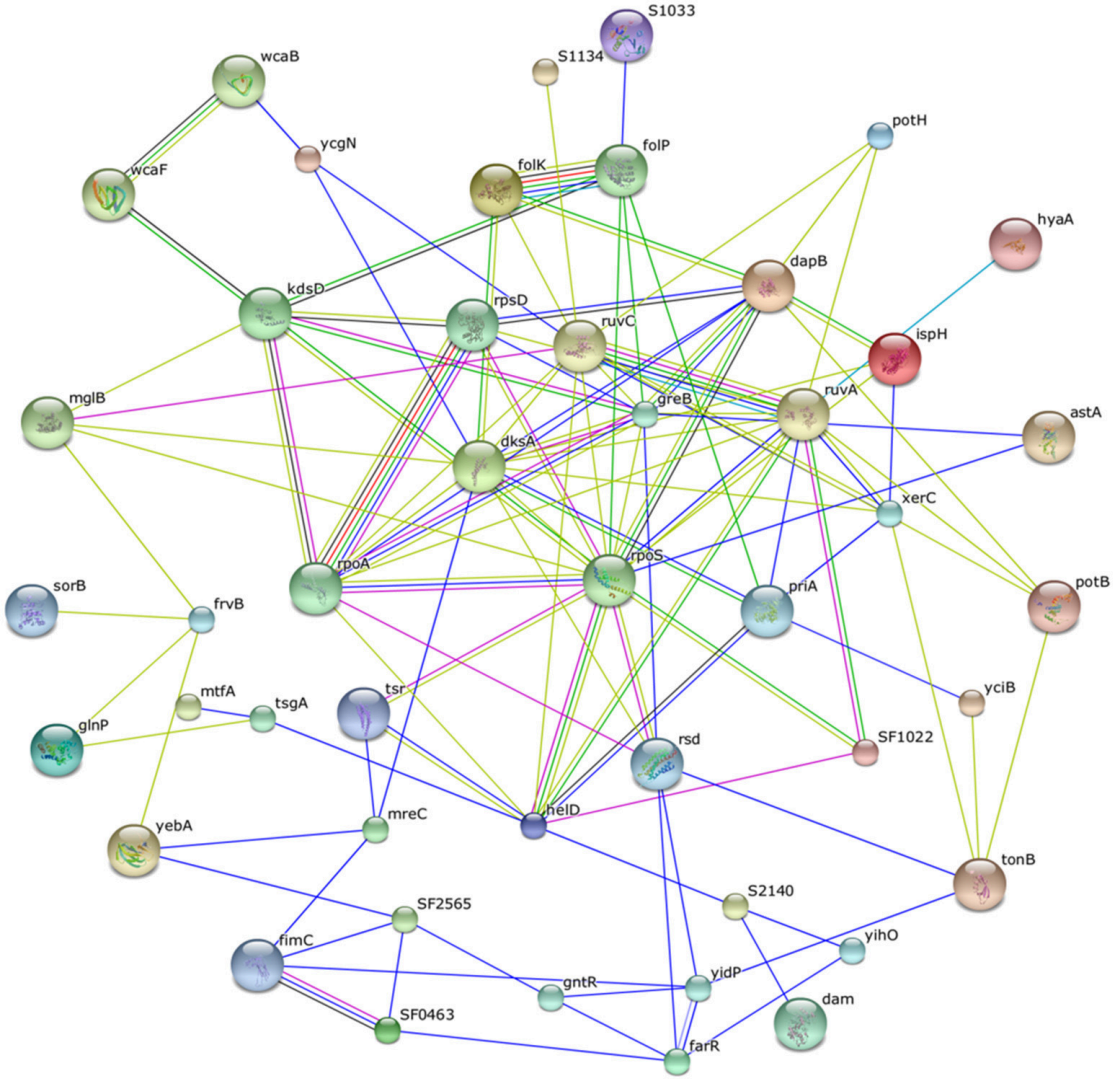
**Interactome analysis of the final metabolic proteins**.

After data mining, finally identified protein data analyzed and depicted a PPI network which is shown in (Figure 2). The networking was established by STRING analysis. In STRING, one protein is interacted with a number of proteins and showed the strength of interaction as score. The interacting score depends on Neighborhood in the genome, Gene fusions, Co-occurrence across genomes, Co-Expression, Association in curated databases and text mining (Jensen et al., 2009). Protein network contains high confidence interactors with score more than or equal to 0.700. Based on the variation in the critical network parameter values the potentiality of the targets was determined. In the bacterial metabolic system, the significance of the query protein was figured out from the number of interacting proteins (nodes) and interactions (edges) interrupted on its deletion (Kushwaha and Shakya, 2010). In low confidence score (0.150), all of the proteins interacted with each other. In highest confidence score (0.900), 30S ribosomal protein (rpsD), DNA-directed RNA polymerase subunit alpha (rpoA), RNA polymerase sigma factor RpoS (RpoS), RNA polymerase-binding transcription factor (dksA), anti-RNA polymerase sigma 70 factor (rsd), Holliday junction resolvase (ruvC), Holliday junction DNA helicase (RuvA), 7,8-dihydropteroate synthase (folP), and 2-amino-4-hydroxy-6-hydroxymethyldihydropteridine pyrophosphokinase (folK) were shown to interact with each other (Figure 2). The highest confidence score of interaction found in between folP and folk which was 0.999. The homologous genes of those proteins are neighbors of other species and in addition, putative homologs are reported to interact in other species.

#### Functionality analysis

From the subtractive analysis we have found 6 hypothetical proteins (Drug Target, Data Sheet S8) which are uncharacterized proteins from the list of 53 potential targets. Attempts were made to characterize their function. To predict their function we have used INTERPROSCAN for the characterization of their functional domain, Molecular function, and Biological process (Table 4). But Molecular function and Biological process also was none predicted in some of the cases.

**Table 4:** **Functionality analysis of Hypothetical proteins**.

**Name of hypothetical protein**	**Gene ontology**	**Functional domain**	**Molecular function**	**Biological process**
NP_839521.1	GO:0055085	Major facilitator superfamily (4–385)	None predicted	Transmembrane transport
NP_837604.1	GO:0008237	Metallopeptidase, catalytic domain (44–257)	Metallopeptidase activity	None predicted
NP_837438.1	None predicted	Duplicated Hybrid motif (169–408)	None predicted	None predicted
NP_836675.1	GO:0048037	NAD(P)-binding domain, coA-binding protein	Cofactor binding	None predicted
AAP19547.1	GO:0009401	Panther (21–480)	None predicted	phosphoenolpyruvate-dependent sugar phosphotransferase system
AAP16677.1	None predicted	Putative zinc- or iron-chelating domain containing protein	None predicted	None predicted

#### Binding site analysis

We have selected the best template from the Local meta-threading-server (LOMETS; Wu and Zhang, 2007) where more than one threading program showed same template with higher score (Align Length, Coverage, Z score and Confidence score; Supplementary Figure S1). After selecting the template we have prepared the required files for sequence of interest and template (.ali, .pir, .bin, and python file). We have run python programming based Modeller software (Modeller 9.17) for generating the number of models (Webb and Sali, 2014). Then we have followed the both DOPE (Discrete Optimized Protein Energy) and GA341 method for selecting the best model from the number of generated models. As GA341 is not as good as DOPE at differentiating between high and low quality model, we have calculated the DOPE score as it is designed for selecting the best structure from a collection of models built by MODELLER. We have selected the best model which shown the lowest DOPE score as it is reported that lower the DOPE score the better is the model. Thereafter, we have checked the quality assessment of every model built by Modeller. We have utilized Ramachandran Plot (Laskowski et al., 1993), Verify3D (Eisenberg et al., 1997), and ProSA (Wiederstein and Sippl, 2007) for the model assessment. All the three tools showed satisfactory results which are summarized in Supplementary Table, S3, Figures, S2, S3. Through these results we have confirmed the predicted model of each hypothetical protein as high-quality. All the predicted model showed above 90% residues in favorable region (Supplementary Table, S3). Verify3D and ProSA also confirmed that the built model are good (Supplementary Figures, S2, S3). We have also analyzed the binding site and binding site residues of these hypothetical proteins (Table 5 and Supplementary Figures, S4–S9).

**Table 5:** **Predicted Binding site for hypothetical proteins and their interacting residues**.

**Hypothetical Protein**	**Cscore^LB^**	**PDB Hit**	**TM-score**	**RMSD^a^**	**IDEN^a^**	**Cov**.	**BS-score**	**Lig. Name**	**Predicted binding site residues**
NP_839521.1	0.01	3mk7D	0.335	7.03	0.035	0.628	0.84	CA	64, 66, 71, 115
	0.01	2z1qA	0.386	5.90	0.086	0.575	0.62	FAD	34, 37, 38, 45, 94
	0.01	1n38A	0.355	6.67	0.037	0.575	0.73	CH1	277, 278, 309, 312, 313, 314, 375
	0.01	2fonB	0.386	5.96	0.084	0.567	0.62	FAD	16, 77, 78, 118, 120, 123
NP_837604.1	0.74	3khiA	0.801	0.18	0.807	0.802	1.58	ZN	124, 161, 165, 224
	0.01	3kllA	0.428	6.35	0.054	0.741	0.88	MAL	217, 226, 227, 228, 229
NP_837438.1	0.24	2gu1A	0.721	0.56	0.417	0.725	1.48	ZN	314, 318, 395
	0.01	1fiqA	0.143	5.82	0.032	0.207	0.87	FES	345, 346, 363, 384, 385, 386, 387, 392
	0.01	2ckjC	0.329	6.76	0.048	0.523	0.83	FES	345, 346, 363, 384, 386, 387, 389, 392
	0.01	2ckjA	0.330	6.76	0.048	0.523	0.88	FES	330, 331, 332, 334, 350, 351, 358, 359
	0.01	3eubS	0.160	5.78	0.060	0.232	0.91	FES	331, 332, 333, 352, 353, 356, 357
NP_836675.1	0.45	3q9uA	0.692	2.46	0.409	0.826	1.38	COA	37, 38, 40, 42, 45, 46, 64, 65, 66, 67, 68, 96, 97, 100, 104, 119, 120, 143, 145, 146
	0.03	1pgqA	0.603	3.22	0.115	0.774	0.93	2AM	63, 64, 65, 94, 95, 104, 108
AAP19547.1	0.01	3eubS	0.163	4.76	0.055	0.211	0.84	FES	352, 353, 377, 378, 379, 382, 384
	0.01	2ckjC	0.330	7.93	0.031	0.579	0.86	FES	384, 386, 387, 390, 401, 402, 403, 404
	0.01	2ckjB	0.314	7.20	0.039	0.504	0.83	FES	225, 387, 389, 392, 394, 399
	0.01	2ckjA	0.324	7.81	0.028	0.558	0.86	FES	41, 42, 43, 47, 65, 66, 67, 68
AAP16677.1	0.57	3o7bA	0.646	3.48	0.145	0.903	0.90	SAH	64, 65, 66, 83, 84, 86, 98, 111, 112, 113, 114, 115, 116, 118, 121

Here, Cscore^LB^ (Confidence score of predicted binding site) predicts a reliable ligand-binding site ranging in between [0 and 1]. BS-score measures between template binding site and predicted binding site. BS-score >1 reflects a significant binding site between the predicted and template. TM-score measures the global structural similarity between query and template protein. RMSD^a^ ensures the structurally aligned by TM-align. IDEN^a^ measures the percentage of sequence identity in the structurally aligned region. Cov. embodies the coverage of global structural alignment.

#### Druggability analysis

High binding affinity to the drug like molecule is a major feature of a “druggable target.” Drug Bank contains 6816 experimental and FDA-approved drugs, 4326 drug targets and 169 drug enzymes/carriers. The module estimates the degree of similarity by using the similarity search option BLASTp program (Mondal et al., 2015). Presence of targets acts as assign for their druggable property. In contrast, its absence designates the novelty of the target and therefore, categorized as “novel target” (Knox et al., 2011). From the selected 53 targets we have found 6 targets (Drug Target, Data Sheet S9) which showed the similarity with approved, investigational, and experimental drugs when the BLASTp search was done against DrugBank database with cut-off parameters (Table 6). We have observed that target EFS15406.1 showed the highest similarity with 15 drugs which could act as inhibitors (both approved and investigational). The docking energy also revealed that the interacting drugs have better binding affinity to their respective targets (Table 6). Therefore, out of 53 therapeutic targets, 47 could be considered as novel drug targets as the remaining 6 targets showed druggable properties.

**Table 6:** **Druggability properties of metabolic human non-homologous essential proteins**.

**Protein ID**	**DrugBank ID**	**Drug name**	**Drug group**	**Pharmacological action**	**Docking energy (Kcal/mol)**	**Chemical formula**
NP_835873.1	DB00233	Aminosalicylic Acid	Approved	Unknown	−7.8	C_7_H_7_NO_3_
NP_835770.1	DB04267	Dipicolinic Acid	Experimental	Unknown	−8.1	C_7_H_5_NO_4_
AAP18497.1	DB00259	Sulfanilamide	Approved	Inhibitor	−7.1	C_6_H_8_N_2_O_2_S
	DB00264	Sulfisoxazole	Approved, Vet_approved	Inhibitor	−7.5	C_11_H_13_N_3_O_3_S
	DB00576	Sulfamethizole	Approved, Vet_approved	Inhibitor	−8.7	C_9_H_10_N_4_O_2_S_2_
	DB00634	Sulfacetamide	Approved	Inhibitor	−6.9	C_8_H_10_N_2_O_3_S
	DB01015	Sulfamethoxazole	Approved	Inhibitor	−8.0	C_10_H_11_N_3_O_3_S
	DB01298	Sulfacytine	Approved	Inhibitor	−7.8	C_12_H_14_N_4_O_3_S
	DB01581	Sulfamerazine	Approved, Vet_approved	Inhibitor	−8.8	C_11_H_12_N_4_O_2_S
	DB01582	Sulfamethazine	Approved, Vet_approved	Inhibitor	−6.5	C_12_H_14_N_4_O_2_S
	DB06729	Sulfaphenazole	Approved	Inhibitor	−7.0	C_15_H_14_N_4_O_2_S
EFS15406.1	DB00274	Cefmetazole	Approved	Inhibitor	−8.3	C_15_H_17_N_7_O_5_S_3_
	DB00303	Ertapenem	Approved, Investigational	Inhibitor	−8.4	C_22_H_25_N_3_O_7_S
	DB00430	Cefpiramide	Approved	Inhibitor	−6.9	C_25_H_24_N_8_O_7_S_2_
	DB00438	Ceftazidime	Approved	Inhibitor	−8.0	C_22_H_22_N_6_O_7_S_2_
	DB01327	Cefazolin	Approved	Inhibitor	−7.7	C_14_H_14_N_8_O_4_S_3_
	DB01328	Cefonicid	Approved	Inhibitor	−6.8	C_18_H_18_N_6_O_8_S_3_
	DB01329	Cefoperazone	Approved	Inhibitor	−7.8	C_25_H_27_N_9_O_8_S
	DB01331	Cefoxitin	Approved	Inhibitor	−7.9	C_16_H_17_N_3_O_7_S_2_
	DB01332	Ceftizoxime	Approved	Inhibitor	−6.7	C_13_H_13_N_5_O_5_S_2_
	DB01333	Cefradine	Approved	Inhibitor	−7.2	C_16_H_19_N_3_O_4_S
	DB01414	Cefacetrile	Approved	Inhibitor	−7.0	C_13_H_13_N_3_O_6_S
	DB01415	Ceftibuten	Approved	Inhibitor	−6.1	C_15_H_14_N_4_O_6_S
	DB01598	Imipenem	Approved	Inhibitor	−6.6	C_12_H_17_N_3_O_4_S
	DB04570	Latamoxef	Approved	Inhibitor	−7.9	C_20_H_20_N_6_O_9_S
	DB06211	Doripenem	Approved, Investigational	Antagonist, Inhibitor	−6.8	C_15_H_24_N_4_O_6_S
EFS13874.1	DB02767	3-Hydroxy-Myristic Acid	Experimental	Unknown	−7.2	C_14_H_28_O_3_
	DB04147	Lauryl Dimethylamine-N-Oxide	Experimental	Unknown	−6.9	C_14_H_31_NO
	DB08231	MYRISTIC ACID	Experimental	Unknown	−7.5	C_14_H_28_O
EFS11712.1	DB01862	Isopropyl beta-D-thiogalactopyranoside	Experimental	Unknown	−6.0	C_9_H_18_O_5_S
	DB08297	ORTHONITROPHENYL-BETA-D-FUCOPYRANOSIDE	Experimental	Unknown	−7.2	C_12_H_15_NO_7_

#### Implications for drug and vaccine development

**Figure 3:**
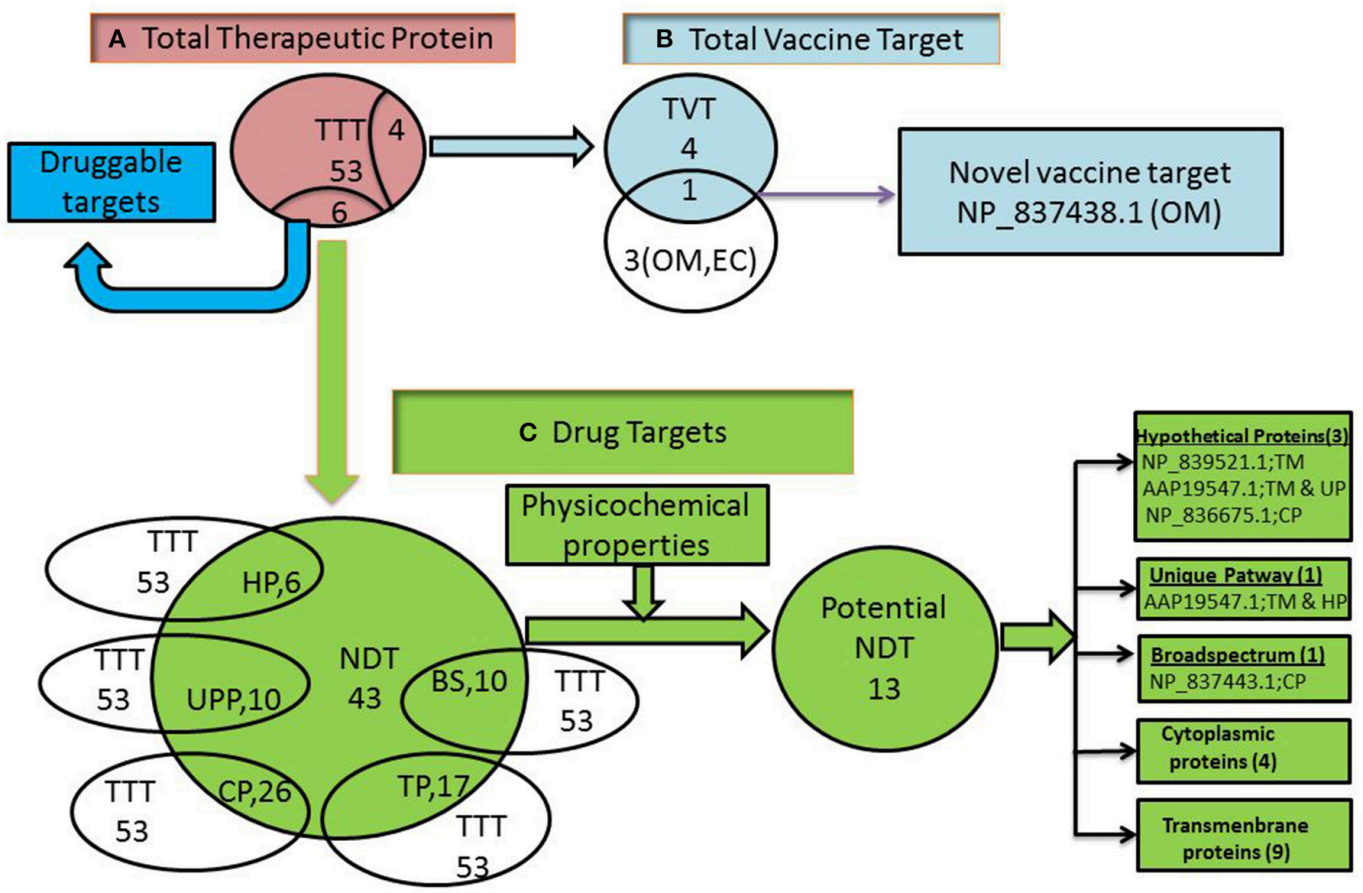
**Prioritization of total therapeutic proteins (53)**. TTT, Total therapeutic targets; TVT, Total vaccine target; NDT, Novel drug targets; HP, Hypothetical proteins; UPP, Unique pathway proteins; OM, Outer membrane proteins; EC, Extracellular proteins; CP, Cytoplasmic proteins; TP, Transmembrane proteins; BS, Broadspectrum. Here, cytoplasmic proteins, 4 (NP_837679.1, NP_837676.1, NP_837443.1, NP_836675.1,) and Transmembrane proteins, 9 (NP_836827.2, NP_839521.1, NP_837444.1, NP_836948.1, AAP19547.1, EFS14933.1, EFS13325.1, EFS11623.1, EFS10693.1).

The 53 targets identified through the current study for vaccine and drug design against *S. flexneri* will make inroad for the development of effective drug(s) and vaccine(s) (Figure 3). Among the 53 targets 43 were found as novel drug targets (Figure 3 and Table 6). The development of such therapeutics might be targeted based on their qualitative characteristics. *S. flexneri* specific unique metabolic pathway might be a target for drug development (Table 1). Drug and vaccine development might also consider cellular location of the targets: Bacterial surface appears to be important for the immunogenicity and surface proteins are more accessible for vaccination. Therefore, four (4) surface proteins NP_837438.1, EFS15865.1, EFS15439.1, and EFS11306.1 might be targeted for the vaccine design.

However, after identification of this vaccine targets we have analyzed their probable antigen and allergenecity assessment. And we have found NP_837438.1 could be an ideal target for the epitope based peptide vaccine as it showed probable antigenecity and non-allergenecity (Supplementary Table, S4).

Membrane proteins are the gateways to the cell: many nutrients, ions, waste products, and even DNA and proteins enter and leave cells via proteins which are tightly controlled, maintaining the integrity of the cell. Drugs often target membrane proteins; therefore, understanding their molecular structure helps us design better drugs to cure diseases. We have found 14 targets identified through this analysis can be suitable for drug design (Table 2).

Besides, Membrane localized proteins are sometimes difficult to purify and assay (Duffield et al., 2010) and therefore, cytoplasmic proteins are more favorable as drug targets. We have found 35 targets which might be suitable for drug design (Table 2).

A promising drug target should have better physicochemical properties such as increased hydrophobicities, *in vivo* half-lives, propensity for being membrane bound and the stability of protein (Bull and Doig, 2015). Our analysis also revealed 14 targets (NP_836827.2, NP_839521.1, NP_837679.1, NP_837676.1, NP_837444.1, NP_837443.1, NP_836948.1, NP_836675.1, NP_835770.1, AAP19547.1, EFS14933.1, EFS13325.1, EFS11623.1, EFS10693.1) among the 49 suitable drug targets which showed protein stability, increased half -life propensity, greater hydrophobicity etc. (Supplementary Table, S5). We observed 13 targets as novel candidate where only NP_835770.1 target showed druggable (Figure 3).

Also, the functions of each identified targets including hypothetical targets may be exploited for the identification or design drug/vaccine for effective therapy against *S. flexneri*. In the perspectives of broadspectrum analysis we have identified some targets which might be used as “promising broadspectrum targets” as they cover the targets more than 40–50 pathogenic bacteria including *S. flexneri*. From the 13 novel drug targets we investigated NP_837443.1 as the most suitable drug candidate which covers the target similarity of 40 pathogenic bacteria including Shigella (Figure 3). The identification of the suitable target(s) is the key device for drug discovery. Our identified promising target (NP_837443.1) plays an important role in Holliday junction which exchanges the segments of genetic information during recombination as well as Helicase activity for the survival of *S. flexneri*. For the drug discovery against this target we need to identify the conserved domain responsible for Holliday junction. The effective binding site and its active residues of this domain should also be identified for the drug design. Finally, drug-target binding affinity of this binding site, ADMET (Absorption, Distribution, Metabolism, and Excretion), QSAR (Quantitative Structural Activity Relationship), and toxicity measurement will lead the way to inhibit the function of this target protein. Further, animal model experiment must be needed to confirm the drug efficacy against this target.

As the selection of effective therapeutics targets are the crucial things for the vaccine and drug design (Hasan et al., 2015; Khan et al., 2015; Oany et al., 2015; Hossain et al., 2016a,b; Hossain and Oany, 2016d) as well as for the building of networking (protein-protein interaction; Hossain et al., 2016c), our subtractive strategy will have a great impact in therapeutics discovery against *S. flexneri*.

## Conclusion

Subtractive channel analysis in proteome of *S. flexneri* 2a str. 2445T suggest 53 drug targets and further with their qualitative characterization stage explicates unique metabolic pathways, localization of targets, broadspectrum, functionality, interactome, and druggable properties. This study might facilitate the development of drug and vaccines against *S. flexneri* as well as come to be handy in clinical interest with identification of drug candidates against other pathogens.

## Author contributions

MS: Conceived, designed, and guided the study, analyzed the data, helped in drafting and performed critical revision. CK: Guided the study, acquisition and analyzed the data, helped in drafting the manuscript. MM and AH: Guided the study, analyzed the data, and helped in drafting the manuscript. MK and MI: Helped in Bioinformatics analysis and drafted the manuscript. MH: Helped to design the study, performed bioinformatics analysis, drafted, and developed the manuscript and performed critical revision. All authors have approved the manuscript.

### Conflict of interest statement

The authors declare that the research was conducted in the absence of any commercial or financial relationships that could be construed as a potential conflict of interest.
