# Supplementary material for: Finding Potential Therapeutic Targets against Shigella flexneri through Proteome Exploration
Source: Front Microbiol. 2016 Nov 22;7:1817. doi: 10.3389/fmicb.2016.01817 (PMC5118456; doi:10.3389/fmicb.2016.01817)
Supplement: Supplementary file 1 [file Table1.PDF]

Supplementary Table, S1: List of Essential genes.

Query Protein	No. of homologs in DEG	DEG AC Number
gi|161486506|ref|NP_836827.2| spermidine/putrescine ABC transporter [Shigellaflexneri 2a str. 2457T]	4	DEG10180369; DEG10030729; DEG10180188; DEG10180247;
gi|161486375|ref|NP_839574.2| 30S ribosomal protein S4 [Shigellaflexneri 2a str. 2457T]	1	DEG10030513;
gi|30065597|ref|NP_839768.1| methyl-accepting chemotaxis protein I, serine sensor receptor [Shigellaflexneri 2a str. 2457T]	3	DEG10030094; DEG10030096; DEG10180242;
gi|30065585|ref|NP_839756.1| carbon starvation protein [Shigellaflexneri 2a str. 2457T]	1	DEG10110041;
gi|30065429|ref|NP_839600.1| periplasmic chaperone [Shigellaflexneri 2a str. 2457T]	2	DEG10180340; DEG10180123;
gi|30065404|ref|NP_839575.1| DNA-directed RNA polymerase subunit alpha [Shigellaflexneri 2a str. 2457T]	2	DEG10110177; DEG10030512;
gi|30065350|ref|NP_839521.1| hypothetical protein S4380 [Shigellaflexneri 2a str. 2457T]	1	DEG10110081;
gi|30064994|ref|NP_839165.1| transcriptional regulator [Shigellaflexneri 2a str. 2457T]	2	DEG10030707; DEG10180541;
gi|30064893|ref|NP_839064.1| site-specific tyrosine recombinaseXerC [Shigellaflexneri 2a str. 2457T]	1	DEG10180554;

gi|30064772|ref|NP_838943.1| primosome assembly protein PriA [Shigellaflexneri 2a str. 2457T]	1	DEG10180563;
gi|30064723|ref|NP_838894.1| anti-RNA polymerase sigma 70 factor [Shigellaflexneri 2a str. 2457T]	1	DEG10180573;
gi|30064701|ref|NP_838872.1| sorbose-permease PTS system IIB component [Shigellaflexneri 2a str. 2457T]	1	DEG10180464;
gi|30064551|ref|NP_838722.1| IS600 orf [Shigellaflexneri 2a str. 2457T]	5	DEG10030032; DEG10030343; DEG10030685; DEG10030738; DEG10030632;
gi|30064535|ref|NP_838706.1| D-arabinose 5-phosphate isomerase [Shigellaflexneri 2a str. 2457T]	1	DEG10310189;
gi|30064457|ref|NP_838628.1| formateacetyltransferase 3 [Shigellaflexneri 2a str. 2457T]	1	DEG10180399;
gi|30063781|ref|NP_837952.1| hypothetical protein S2579 [Shigellaflexneri 2a str. 2457T]	1	DEG10180364;
gi|30063512|ref|NP_837683.1| transporter [Shigellaflexneri 2a str. 2457T]	3	DEG10110045; DEG10180408; DEG10110108;
gi|30063508|ref|NP_837679.1| colanic acid biosynthesis acetyltransferaseWcaB	1	DEG10180533;

[Shigellaflexneri 2a str. 2457T]		
gi|30063505|ref|NP_837676.1| colanic acid biosynthesis acetyltransferaseWcaF [Shigellaflexneri 2a str. 2457T]	1	DEG10110123;
gi|30063433|ref|NP_837604.1| hypothetical protein S2148 [Shigellaflexneri 2a str. 2457T]	1	DEG10180331;
gi|30063426|ref|NP_837597.1| crossover junction endodeoxyribonuclease [Shigellaflexneri 2a str. 2457T]	1	DEG10180103;
gi|30063273|ref|NP_837444.1| Holliday junction resolvase [Shigellaflexneri 2a str. 2457T]	2	DEG10310228; DEG10180312;
gi|30063272|ref|NP_837443.1| Holliday junction DNA helicase RuvA [Shigellaflexneri 2a str. 2457T]	1	DEG10030355;
gi|30063267|ref|NP_837438.1| hypothetical protein S1932 [Shigellaflexneri 2a str. 2457T]	1	DEG10310161;
gi|30063205|ref|NP_837376.1| amino acid/amine transport protein [Shigellaflexneri 2a str. 2457T]	1	DEG10110081;
gi|30062777|ref|NP_836948.1| intracellular septation protein A [Shigellaflexneri 2a str. 2457T]	1	DEG10180217;
gi|30062766|ref|NP_836937.1| oligopeptide transport permease [Shigellaflexneri 2a str. 2457T]	3	DEG10180348; DEG10180213; DEG10180255;
gi|30062510|ref|NP_836681.1| hydrogenase-1 small subunit [Shigellaflexneri 2a str. 2457T]	1	DEG10180159;
gi|30062504|ref|NP_836675.1| hypothetical protein	1	DEG10180158;

S1033 [Shigellaflexneri 2a str. 2457T]		
gi|30062501|ref|NP_836672.1| DNA helicase IV [Shigellaflexneri 2a str. 2457T]	2	DEG10110204; DEG10180549;
gi|30062294|ref|NP_836465.1| glutamine ABC transporter permease [Shigellaflexneri 2a str. 2457T]	4	DEG10180115; DEG10110116; DEG10180133; DEG10110134;
gi|30062107|ref|NP_836278.1| DNA-binding transcriptional repressor MngR [Shigellaflexneri 2a str. 2457T]	2	DEG10030707; DEG10180541;
gi|30061705|ref|NP_835876.1| DnaK transcriptional regulator DksA [Shigellaflexneri 2a str. 2457T]	1	DEG10030123;
gi|30061702|ref|NP_835873.1| 2-amino-4-hydroxy-6-hydroxymethyldihydropteridine pyrophosphokinase [Shigellaflexneri 2a str. 2457T]	1	DEG10180033;
gi|30061599|ref|NP_835770.1| dihydrodipicolinatereductase [Shigellaflexneri 2a str. 2457T]	2	DEG10180009; DEG10030471;
gi|30061597|ref|NP_835768.1| 4-hydroxy-3-methylbut-2-enyl diphosphatereductase [Shigellaflexneri 2a str. 2457T]	3	DEG10310114; DEG10030149; DEG10180008;
gi|30043828|gb|AAP19547.1| hypothetical protein S4618 [Shigellaflexneri 2a str. 2457T]	1	DEG10110054;
gi|30043573|gb|AAP19293.1| transcription elongation factor and transcript cleavage factor [Shigellaflexneri 2a str. 2457T]	1	DEG10310029;
gi|30042898|gb|AAP18621.1| hypothetical protein	1	DEG10180579;

S3575 [Shigellaflexneri 2a str. 2457T]		
gi|30042774|gb|AAP18497.1| 7,8-dihydropteroate synthase [Shigellaflexneri 2a str. 2457T]	1	DEG10030131;
gi|30040947|gb|AAP16677.1| hypothetical protein S1258 [Shigellaflexneri 2a str. 2457T]	1	DEG10030225;
gi|313651501|gb|EFS15897.1| uvrD/REP helicase family protein [Shigellaflexneri 2a str. 2457T]	2	DEG10110204; DEG10180549;
gi|313651469|gb|EFS15865.1| DNA adenine methylase family protein [Shigellaflexneri 2a str. 2457T]	1	DEG10030542;
gi|313651164|gb|EFS15563.1| outer membrane usher papC domain protein [Shigellaflexneri 2a str. 2457T]	1	DEG10110026;
gi|313651162|gb|EFS15561.1| chaperone protein pmfD [Shigellaflexneri 2a str. 2457T]	2	DEG10180340; DEG10180123;
gi|313651039|gb|EFS15439.1| ABC transporter periplasmic-binding protein yphF [Shigellaflexneri 2a str. 2457T]	2	DEG10180343; DEG10110127;
gi|313651006|gb|EFS15406.1| penicillin-binding 1C domain protein [Shigellaflexneri 2a str. 2457T]	1	DEG10310062;
gi|313650743|gb|EFS15144.1| chaperone torD domain protein [Shigellaflexneri 2a str. 2457T]	1	DEG10180165;
gi|313650721|gb|EFS15122.1| phoH-like family protein [Shigellaflexneri 2a str. 2457T]	1	DEG10030200;
gi|313650575|gb|EFS14978.1| drug resistance MFS transporter [Shigellaflexneri 2a str. 2457T]	1	DEG10110091;

gi|313650527|gb|EFS14933.1| binding--dependent transport systems inner membrane component domain protein [Shigellaflexneri 2a str. 2457T]	1	DEG10180247;
gi|313650353|gb|EFS14762.1| HTH-type transcriptional regulator dicA [Shigellaflexneri 2a str. 2457T]	1	DEG10180263;
gi|313650164|gb|EFS14577.1| flagellar export protein FliJ [Shigellaflexneri 2a str. 2457T]	1	DEG10180327;
gi|313649443|gb|EFS13874.1| protein tonB [Shigellaflexneri 2a str. 2457T]	1	DEG10180215;
gi|313649227|gb|EFS13661.1| arginine N-succinyltransferase [Shigellaflexneri 2a str. 2457T]	1	DEG10180298;
gi|313648972|gb|EFS13409.1| major Facilitator Superfamily protein [Shigellaflexneri 2a str. 2457T]	1	DEG10110081;
gi|313648888|gb|EFS13325.1| glucuronide transporter [Shigellaflexneri 2a str. 2457T]	1	DEG10180060;
gi|313648508|gb|EFS12950.1| rod shape-determining protein MreC [Shigellaflexneri 2a str. 2457T]	1	DEG10180485;
gi|313647807|gb|EFS12253.1| RNA polymerase sigma factor rpoS [Shigellaflexneri 2a str. 2457T]	5	DEG10030098; DEG10180457; DEG10110169; DEG10030019; DEG10180521;
gi|313647791|gb|EFS12238.1| drug resistance MFS transporter [Shigellaflexneri 2a str. 2457T]	2	DEG10110091; DEG10110128;
gi|313647260|gb|EFS11712.1| HTH-type	1	DEG10110163;

transcriptional regulator gntR [Shigellaflexneri 2a str. 2457T]		
gi|313647169|gb|EFS11623.1| fructose-like PTS system EIIBC component [Shigellaflexneri 2a str. 2457T]	1	DEG10180365;
gi|313646928|gb|EFS11385.1| bacterial extracellular solute-binding s, family 5 Middle family protein [Shigellaflexneri 2a str. 2457T]	1	DEG10030704;
gi|313646849|gb|EFS11306.1| outer membrane usher sfmD domain protein [Shigellaflexneri 2a str. 2457T]	1	DEG10110026;
gi|313646468|gb|EFS10930.1| IS222, transposase ORFA [Shigellaflexneri 2a str. 2457T]	4	DEG10030031; DEG10030299; DEG10030344; DEG10030633;
gi|313646413|gb|EFS10875.1| hypothetical protein SF2457T_5328 [Shigellaflexneri 2a str. 2457T]	1	DEG10180593;
gi|313646231|gb|EFS10693.1| amino acid carrier family protein [Shigellaflexneri 2a str. 2457T]	1	DEG10110061;
