# Supplementary material for: Finding Potential Therapeutic Targets against Shigella flexneri through Proteome Exploration
Source: Front Microbiol. 2016 Nov 22;7:1817. doi: 10.3389/fmicb.2016.01817 (PMC5118456; doi:10.3389/fmicb.2016.01817)
Supplement: Supplementary file 2 [file Table2.PDF]

Supplementary Table, S2: List of Metabolic pathway proteins.

Target ID	KO Number	Function
NP_836827.2	K11071	potB; spermidine/putrescine transport system permease protein
NP_839574.2	K02986	RP-S4, rpsD; small subunit ribosomal protein S4
NP_839768.1	K05874	tsr; methyl-accepting chemotaxis protein I, serine sensor receptor
NP_839756.1	N/A	N/A
NP_839600.1	K07346	fimC; fimbrial chaperone protein
NP_839575.1	K03040	rpoA; DNA-directed RNA polymerase subunit alpha [EC:2.7.7.6]
NP_839521.1	K06141	tsgA; MFS transporter, TsgA protein
NP_839165.1	K03482	yidP; GntR family transcriptional regulator, glv operon transcriptional regulator
NP_839064.1	K03733	xerC; integrase/recombinase XerC
NP_838943.1	K04066	priA; primosomal protein N' (replication factor Y) (superfamily II helicase) [EC:3.6.4.-]
NP_838894.1	K07740	rsd; regulator of sigma D
NP_838872.1	K02794	PTS-Man-EIIB, manX; PTS system, mannose-specific IIB component [EC:2.7.1.191]
NP_838722.1	K07497	putative transposase
NP_838706.1	K06041	kdsD, kpsF; arabinose-5-phosphate isomerase [EC:5.3.1.13]
NP_838628.1	K00656	pflD; formate C-acetyltransferase [EC:2.3.1.54]
NP_837952.1	N/A	N/A
NP_837683.1	N/A	N/A
NP_837679.1	K03819	wcaB; putative colanic acid biosynthesis acetyltransferase WcaB [EC:2.3.1.-]
NP_837676.1	K03818	wcaF; putative colanic acid biosynthesis acetyltransferase

		WcaF [EC:2.3.1.-]
NP_837604.1	K09933	mtfA; MtfA peptidase
NP_837597.1	K01160	rusA; crossover junction endodeoxyribonuclease RusA [EC:3.1.22.4]
NP_837444.1	K01159	ruvC; crossover junction endodeoxyribonuclease RuvC [EC:3.1.22.4]
NP_837443.1	K03550	ruvA; holliday junction DNA helicase RuvA [EC:3.6.4.12]
NP_837438.1	K19304	mepM; murein DD-endopeptidase [EC:3.4.24.-]
NP_837376.1	N/A	N/A
NP_836948.1	K06190	ispZ; intracellular septation protein
NP_836937.1	K15582	oppC; oligopeptide transport system permease protein
NP_836681.1	K06282	hyaA, hybO; hydrogenase small subunit [EC:1.12.99.6]
NP_836675.1	K06929	uncharacterized protein
NP_836672.1	K03658	helD; DNA helicase IV [EC:3.6.4.12]
NP_836465.1	K10037	glnP; glutamine transport system permease protein
NP_836278.1	K11922	mngR, farR; GntR family transcriptional regulator, mannosyl-D-glycerate transport/metabolism system repressor
NP_835876.1	K06204	dksA; DnaK suppressor protein
NP_835873.1	K00950	folK; 2-amino-4-hydroxy-6-hydroxymethyldihydropteridine diphosphokinase [EC:2.7.6.3]
NP_835770.1	K00215	dapB; 4-hydroxy-tetrahydrodipicolinate reductase [EC:1.17.1.8]
NP_835768.1	K03527	ispH, lytB; 4-hydroxy-3-methylbut-2-en-1-yl diphosphate reductase [EC:1.17.7.4]
AAP19547.1	K03475	PTS-Ula-EIIC, ulaA, sgaT; PTS system, ascorbate-specific IIC component
AAP19293.1	K04760	greB; transcription elongation factor GreB
AAP18621.1	N/A	N/A
AAP18497.1	K00796	folP; dihydropteroate synthase [EC:2.5.1.15]

AAP16677.1	K09160	uncharacterized protein
EFS15897.1	N/A	N/A
EFS15865.1	K06223	dam; DNA adenine methylase [EC:2.1.1.72]
EFS15563.1	N/A	N/A
EFS15561.1	N/A	N/A
EFS15439.1	K02058	ABC.SS.S; simple sugar transport system substrate-binding protein
EFS15406.1	K05367	pbpC; penicillin-binding protein 1C [EC:2.4.1.-]
EFS15144.1	K03533	torD; TorA specific chaperone
EFS15122.1	K06217	phoH, phoL; phosphate starvation-inducible protein PhoH and related proteins
EFS14978.1	N/A	N/A
EFS14933.1	K02054	ABC.SP.P1; putative spermidine/putrescine transport system permease protein
EFS14762.1	N/A	N/A
EFS14577.1	K02413	fliJ; flagellar FliJ protein
EFS13874.1	K03832	tonB; periplasmic protein TonB
EFS13661.1	K00673	astA; arginine N-succinyltransferase [EC:2.3.1.109]
EFS13409.1	N/A	N/A
EFS13325.1	K16139	uidB, gusB; glucuronide carrier protein
EFS12950.1	K03570	mreC; rod shape-determining protein MreC
EFS12253.1	K03087	SIG2, rpoS; RNA polymerase nonessential primary-like sigma factor
EFS12238.1	N/A	N/A
EFS11712.1	K06145	gntR; LacI family transcriptional regulator, gluconate utilization system Gnt-I transcriptional repressor
EFS11623.1	K11203	PTS-Fru2-EIIC; PTS system, fructose-specific IIC-like component
EFS11385.1	N/A	N/A
EFS11306.1	K07354	sfmD; outer membrane usher protein

EFS10930.1	K07483	transposase
EFS10875.1	N/A	N/A
EFS10693.1	K03310	TC.AGCS; alanine or glycine:cation symporter, AGCS family
