# Supplementary material for: Finding Potential Therapeutic Targets against Shigella flexneri through Proteome Exploration
Source: Front Microbiol. 2016 Nov 22;7:1817. doi: 10.3389/fmicb.2016.01817 (PMC5118456; doi:10.3389/fmicb.2016.01817)
Supplement: Supplementary file 3 [file Table3.PDF]

Supplementary Table, S3_1: Model (NP_839521.1) quality assessment of Ramachandran plot.

**Ramachandran plot statistics**	**Residues**	**%**
Residues in the most favored regions [A,B,L]	331	92.%
Residues in the additional allowed regions [a,b,l,p]	18	5.2%
Residues in the generously allowed regions [a,b,l,p]	5	1.4%
Residues in the disallowed regions [xx]	3	0.9%
Number of non-glycine and non-proline residues	347	100%
Number of end residues (excl. Gly and Pro)	2	
Number of glycine residues	33	
Number of proline residues	11	
Total number of residues	393	

Supplementary Table, S3_2: Model (NP_837604.1) quality assessment of Ramachandran plot.

**Ramachandran plot statistics**	**Residues**	**%**
Residues in the most favored regions [A,B,L]	233	92.8%
Residues in the additional allowed regions [a,b,l,p]	16	6.4%
Residues in the generously allowed regions [a,b,l,p]	0	0.0%
Residues in the disallowed regions [xx]	2	0.8%
Number of non-glycine and non-proline residues	251	100%
Number of end residues (excl. Gly and Pro)	2	
Number of glycine residues	11	
Number of proline residues	14	
Total number of residues	278	

Supplementary Table, S3_3: Model (NP_837438.1) quality assessment of Ramachandran plot.

**Ramachandran plot statistics**	**Residues**	**%**
Residues in the most favored regions [A,B,L]	347	90.6%
Residues in the additional allowed regions [a,b,l,p]	33	8.6%
Residues in the generously allowed regions [a,b,l,p]	2	0.5%
Residues in the disallowed regions [xx]	1	0.3%
Number of non-glycine and non-proline residues	383	100%
Number of end residues (excl. Gly and Pro)	2	
Number of glycine residues	37	
Number of proline residues	18	
Total number of residues	440	

Supplementary Table, S3_4: Model (NP_836675.1) quality assessment of Ramachandran plot.

**Ramachandran plot statistics**	**Residues**	**%**
Residues in the most favored regions [A,B,L]	120	90.2%
Residues in the additional allowed regions [a,b,l,p]	13	9.8%
Residues in the generously allowed regions [a,b,l,p]	0	0.0%
Residues in the disallowed regions [xx]	0	0.0%
Number of non-glycine and non-proline residues	133	100%
Number of end residues (excl. Gly and Pro)	2	
Number of glycine residues	13	

Number of proline residues	7	
Total number of residues	155	

Supplementary Table, S3_5: Model (AAP19547.1) quality assessment of Ramachandran plot.

**Ramachandran plot statistics**	**Residues**	**%**
Residues in the most favored regions [A,B,L]	413	97.2%
Residues in the additional allowed regions [a,b,l,p]	11	2.6%
Residues in the generously allowed regions [a,b,l,p]	0	0.0%
Residues in the disallowed regions [xx]	1	0.2%
Number of non-glycine and non-proline residues	425	100%
Number of end residues (excl. Gly and Pro)	2	
Number of glycine residues	44	
Number of proline residues	13	
Total number of residues	484	

Supplementary Table, S3_6: Model (AAP16677.1) quality assessment of Ramachandran plot.

**Ramachandran plot statistics**	**Residues**	**%**
Residues in the most favored regions [A,B,L]	127	91.4%
Residues in the additional allowed regions [a,b,l,p]	10	7.2%
Residues in the generously allowed regions [a,b,l,p]	1	0.7%
Residues in the disallowed regions [xx]	1	0.7%
Number of non-glycine and non-proline residues	139	100%
Number of end residues (excl. Gly and Pro)	2	

Number of glycine residues	5	
Number of proline residues	7	
Total number of residues	153	
