# Supplementary material for: Finding Potential Therapeutic Targets against Shigella flexneri through Proteome Exploration
Source: Front Microbiol. 2016 Nov 22;7:1817. doi: 10.3389/fmicb.2016.01817 (PMC5118456; doi:10.3389/fmicb.2016.01817)
Supplement: Supplementary file 4 [file Table4.PDF]

Supplementary Table, S4: Identification of epitope based peptide Vaccine Target.

**Target ID**	**Vaxijen Score**	**Allergenecity**
NP_837438.1	0.5711 (Probable Antigen)	Non-Allergen
EFS15865.1	0.4115 (Non-Antigen)	Non-Allergen
EFS15439.1	0.5969 (Probable Antigen)	Probable Allergen
EFS11306.1	0.4684 (Non-Antigen)	Non-Allergen
