# Supplementary material for: Finding Potential Therapeutic Targets against Shigella flexneri through Proteome Exploration
Source: Front Microbiol. 2016 Nov 22;7:1817. doi: 10.3389/fmicb.2016.01817 (PMC5118456; doi:10.3389/fmicb.2016.01817)
Supplement: Supplementary file 5 [file Table5.PDF]

Supplementary Table, S5: Analysis of Physiochemical properties for the identification of suitable drug targets.

Protein name	Molecular weight	Theoretical PI	Extinction coefficient ($M^{-1}CM^{-1}$)	Estimated half-life	Instability index	Aliphatic index	Grand average of hydropathicity (GRAVY)
NP_836827.2	32208.29	9.58	46535	30 hours (mammalian reticulocytes, in vitro).<br><br>>20 hours (yeast, in vivo).<br><br>>10 hours (Escherichia coli, in vivo).	26.57 (stable)	141.23	0.920
NP_839574.2	23469.09	10.05	17420	30 hours (mammalian reticulocytes, in vitro).<br><br>>20 hours (yeast, in vivo).<br><br>>10 hours (Escherichia coli, in vivo).	27.09 (stable)	86.17	-0.664

NP_839768.1	59878.52	4.91	42400	30 hours (mammalian reticulocytes, in vitro).<br><br>>20 hours (yeast, in vivo).<br><br>>10 hours (Escherichia coli, in vivo).	34.80 (stable)	94.46	-0.124
NP_839600.1	26689.00	9.47	22920	30 hours (mammalian reticulocytes, in vitro).<br><br>>20 hours (yeast, in vivo).<br><br>>10 hours (Escherichia coli, in vivo).	36.30 (stable)	91.91	-0.165
NP_839575.1	36511.72	4.97	13200	30 hours (mammalian reticulocytes, in vitro).<br><br>>20 hours (yeast, in vivo).<br><br>>10 hours (Escherichia coli, in vivo).	41.59 (unstable)	106.93	-0.228

NP_839521.1	43261.54	9.21	67630	30 hours (mammalian reticulocytes, in vitro).<br><br>>20 hours (yeast, in vivo).<br><br>>10 hours (Escherichia coli, in vivo).	35.66 (stable)	112.65	0.874
NP_839165.1	25583.91	9.43	18910	30 hours (mammalian reticulocytes, in vitro).<br><br>>20 hours (yeast, in vivo).<br><br>>10 hours (Escherichia coli, in vivo).	47.79 (unstable)	107.05	-0.019
NP_839064.1	33809.68	9.40	41940	30 hours (mammalian reticulocytes, in vitro).<br><br>>20 hours (yeast, in vivo).<br><br>>10 hours (Escherichia coli, in vivo).	38.42 (stable)	96.28	-0.379

NP_838943.1	81618.88	9.05	105475	30 hours (mammalian reticulocytes, in vitro).<br><br>>20 hours (yeast, in vivo).<br><br>>10 hours (Escherichia coli, in vivo).	47.92 (unstable)	97.98	-0.183
NP_838894.1	18242.85	5.65	22920	30 hours (mammalian reticulocytes, in vitro).<br><br>>20 hours (yeast, in vivo).<br><br>>10 hours (Escherichia coli, in vivo).	24.28 (stable)	108.04	-0.277
NP_838872.1	18404.16	6.28	16960	30 hours (mammalian reticulocytes, in vitro).<br><br>>20 hours (yeast, in vivo).<br><br>>10 hours (Escherichia coli, in vivo).	44.91 (unstable)	109.94	-0.129

NP_838722.1	41121.31	9.97	49195	30 hours (mammalian reticulocytes, in vitro).<br><br>>20 hours (yeast, in vivo).<br><br>>10 hours (Escherichia coli, in vivo).	46.77 (unstable)	83.85	-0.549
NP_838706.1	35226.11	6.22	7365	30 hours (mammalian reticulocytes, in vitro).<br><br>>20 hours (yeast, in vivo).<br><br>>10 hours (Escherichia coli, in vivo).	43.12 (unstable)	101.37	0.222

NP_838628.1	85908.52	5.48	92390	30 hours (mammalian reticulocytes, in vitro).<br><br>>20 hours (yeast, in vivo).<br><br>>10 hours (Escherichia coli, in vivo).	33.29 (stable)	85.33	-0.290
NP_837679.1	17615.54	8.91	22710	30 hours (mammalian reticulocytes, in vitro).<br><br>>20 hours (yeast, in vivo).<br><br>>10 hours (Escherichia coli, in vivo).	26.52 (stable)	112.47	0.348
NP_837676.1	19962.09	9.40	46075	30 hours (mammalian reticulocytes, in vitro).<br><br>>20 hours (yeast, in vivo).<br><br>>10 hours (Escherichia	25.51 (stable)	99.01	0.199

				coli, in vivo).			
NP_837604.1	31700.74	4.63	55835	30 hours (mammalian reticulocytes, in vitro).<br><br>>20 hours (yeast, in vivo).<br><br>>10 hours (Escherichia coli, in vivo).	52.35 (unstable)	91.62	-0.144
NP_837597.1	13841.10	9.83	11460	30 hours (mammalian reticulocytes, in vitro).<br><br>>20 hours (yeast, in vivo).<br><br>>10 hours (Escherichia coli, in vivo).	56.12 (unstable)	107.19	-0.322

NP_837444.1	18794.83	9.59	7575	30 hours (mammalian reticulocytes, in vitro).<br><br>>20 hours (yeast, in vivo).<br><br>>10 hours (Escherichia coli, in vivo).	34.84 (stable)	102.08	0.055
NP_837443.1	22115.71	5.72	5960	30 hours (mammalian reticulocytes, in vitro).<br><br>>20 hours (yeast, in vivo).<br><br>>10 hours (Escherichia coli, in vivo).	36.01 (stable)	102.86	0.044
NP_837438.1	49057.75	9.53	52370	30 hours (mammalian reticulocytes, in vitro).<br><br>>20 hours (yeast, in vivo).<br><br>>10 hours (Escherichia coli, in vivo).	38.28 (stable)	82.48	-0.458
NP_836948.1	20820.21	9.65	50420	30 hours (mammalian reticulocytes, in vitro).<br><br>>20 hours (yeast, in vivo).	32.47 (stable)	129.61	0.894

				>10 hours (Escherichia coli, in vivo).			
NP_836937.1	33031.96	5.43	34950	30 hours (mammalian reticulocytes, in vitro).<br><br>>20 hours (yeast, in vivo).<br><br>>10 hours (Escherichia coli, in vivo).	40.65 (unstable)	118.08	0.672
NP_836681.1	40681.36	6.74	57255	30 hours (mammalian reticulocytes, in vitro).<br><br>>20 hours (yeast, in vivo).<br><br>>10 hours (Escherichia coli, in vivo).	43.56 (unstable)	73.76	-0.205

NP_836675.1	16654.38	5.33	18450	30 hours (mammalian reticulocytes, in vitro).<br><br>>20 hours (yeast, in vivo).<br><br>>10 hours (Escherichia coli, in vivo).	26.77 (stable)	107.61	0.179
NP_836672.1	77904.14	8.56	121140	30 hours (mammalian reticulocytes, in vitro).<br><br>>20 hours (yeast, in vivo).<br><br>>10 hours (Escherichia coli, in vivo).	42.97 (unstable)	89.24	-0.409
NP_836465.1	24364.31	10.17	44460	30 hours (mammalian reticulocytes, in vitro).<br><br>>20 hours (yeast, in vivo).<br><br>>10 hours (Escherichia coli, in vivo).	40.21 (unstable)	138.08	0.877

NP_836278.1	36693.94	6.73	33015	30 hours (mammalian reticulocytes, in vitro).<br><br>>20 hours (yeast, in vivo).<br><br>>10 hours (Escherichia coli, in vivo).	55.12 (unstable)	86.56	-0.511
NP_835876.1	17527.75	5.06	10220	30 hours (mammalian reticulocytes, in vitro).<br><br>>20 hours (yeast, in vivo).<br><br>>10 hours (Escherichia coli, in vivo).	44.67 (unstable)	74.37	-0.866
NP_835873.1	17953.56	5.21	22460	30 hours (mammalian reticulocytes, in vitro).<br><br>>20 hours (yeast, in vivo).<br><br>>10 hours (Escherichia coli, in vivo).	50.51 (unstable)	95.16	-0.255

NP_835770.1	28770.64	5.45	8605	30 hours (mammalian reticulocytes, in vitro).<br><br>>20 hours (yeast, in vivo).<br><br>>10 hours (Escherichia coli, in vivo).	21.80 (stable)	92.34	0.021
NP_835768.1	34755.51	5.17	20190	30 hours (mammalian reticulocytes, in vitro).<br><br>>20 hours (yeast, in vivo).<br><br>>10 hours (Escherichia coli, in vivo).	30.10 (stable).	97.75	-0.169
AAP19547.1	52875.11	8.06	81860	30 hours (mammalian reticulocytes, in vitro).<br><br>>20 hours (yeast, in vivo).<br><br>>10 hours (Escherichia coli, in vivo).	34.15 (stable).	116.69	0.866

AAP19293.1	19966.80	8.82	36900	30 hours (mammalian reticulocytes, in vitro).<br><br>>20 hours (yeast, in vivo).<br><br>>10 hours (Escherichia coli, in vivo).	33.60 (stable).	82.53	-0.709
AAP18497.1	32417.24	5.84	11585	30 hours (mammalian reticulocytes, in vitro).<br><br>>20 hours (yeast, in vivo).<br><br>>10 hours (Escherichia coli, in vivo).	33.42 (stable).	97.88	-0.011
AAP16677.1	17910.37	4.90	37970	30 hours (mammalian reticulocytes, in vitro).<br><br>>20 hours (yeast, in vivo).<br><br>>10 hours (Escherichia coli, in vivo).	52.54 (unstable).	72.09	-0.508

EFS15865.1	29559.57	8.69	30495	30 hours (mammalian reticulocytes, in vitro).<br><br>>20 hours (yeast, in vivo).<br><br>>10 hours (Escherichia coli, in vivo).	46.23 (unstable).	78.21	-0.402
EFS15439.1	20173.46	9.34	16055	30 hours (mammalian reticulocytes, in vitro).<br><br>>20 hours (yeast, in vivo).<br><br>>10 hours (Escherichia coli, in vivo).	27.40 (stable).	101.73	-0.002
EFS15406.1	34464.86	9.97	69440	30 hours (mammalian reticulocytes, in vitro).<br><br>>20 hours (yeast, in vivo).<br><br>>10 hours (Escherichia coli, in vivo).	51.21 (unstable).	94.69	-0.272

EFS15144.1	16143.46	5.14	14565	30 hours (mammalian reticulocytes, in vitro).<br><br>>20 hours (yeast, in vivo).<br><br>>10 hours (Escherichia coli, in vivo).	53.95 (unstable).	97.06	-0.085
EFS15122.1	29298.67	7.61	14815	30 hours (mammalian reticulocytes, in vitro).<br><br>>20 hours (yeast, in vivo).<br><br>>10 hours (Escherichia coli, in vivo).	51.98 (unstable).	89.01	-0.220
EFS14933.1	12217.63	9.39	5960	30 hours (mammalian reticulocytes, in vitro).<br><br>>20 hours (yeast, in vivo).<br><br>>10 hours (Escherichia coli, in vivo).	39.33 (stable)	125.65	0.870

EFS14577.1	13732.50	9.34	26470	30 hours (mammalian reticulocytes, in vitro).<br><br>>20 hours (yeast, in vivo).<br><br>>10 hours (Escherichia coli, in vivo).	43.02 (unstable)	66.93	-1.117
EFS13874.1	26593.89	9.62	15470	30 hours (mammalian reticulocytes, in vitro).<br><br>>20 hours (yeast, in vivo).<br><br>>10 hours (Escherichia coli, in vivo).	58.91 (unstable)	79.88	-0.439
EFS13661.1	38427.81	6.02	37275	30 hours (mammalian reticulocytes, in vitro).<br><br>>20 hours (yeast, in vivo).<br><br>>10 hours (Escherichia coli, in vivo).	32.57 (stable)	84.24	-0.204

EFS13325.1	30094.81	9.33	38765	30 hours (mammalian reticulocytes, in vitro).<br><br>>20 hours (yeast, in vivo).<br><br>>10 hours (Escherichia coli, in vivo).	22.93 (stable).	121.14	0.843
EFS12950.1	39572.46	8.51	17545	30 hours (mammalian reticulocytes, in vitro).<br><br>>20 hours (yeast, in vivo).<br><br>>10 hours (Escherichia coli, in vivo).	46.63 (unstable).	98.86	-0.145
EFS12253.1	29587.84	5.96	36440	30 hours (mammalian reticulocytes, in vitro).<br><br>>20 hours (yeast, in vivo).<br><br>>10 hours (Escherichia coli, in vivo).	51.19 (unstable).	94.80	-0.520

EFS11712.1	36421.98	6.20	19035	30 hours (mammalian reticulocytes, in vitro).<br><br>>20 hours (yeast, in vivo).<br><br>>10 hours (Escherichia coli, in vivo).	43.14 (unstable).	96.07	-0.103
EFS11623.1	51307.76	8.76	48485	30 hours (mammalian reticulocytes, in vitro).<br><br>>20 hours (yeast, in vivo).<br><br>>10 hours (Escherichia coli, in vivo).	39.36 (stable).	114.72	0.704
EFS11306.1	19064.66	4.89	20190	30 hours (mammalian reticulocytes, in vitro).<br><br>>20 hours (yeast, in vivo).<br><br>>10 hours (Escherichia coli, in vivo).	31.14 (stable).	87.97	-0.035

EFS10930.1	15233.47	9.67	12490	30 hours (mammalian reticulocytes, in vitro).<br><br>>20 hours (yeast, in vivo).<br><br>>10 hours (Escherichia coli, in vivo).	51.21 (unstable).	70.76	-0.844
EFS10693.1	36984.64	9.58	65680	30 hours (mammalian reticulocytes, in vitro).<br><br>>20 hours (yeast, in vivo).<br><br>>10 hours (Escherichia coli, in vivo).	27.71 (stable).	103.55	0.756
