# Supplementary material for: Finding Potential Therapeutic Targets against Shigella flexneri through Proteome Exploration
Source: Front Microbiol. 2016 Nov 22;7:1817. doi: 10.3389/fmicb.2016.01817 (PMC5118456; doi:10.3389/fmicb.2016.01817)
Supplement: Supplementary file 9 [file DataSheet4.PDF]

>gi|229089130|ref|NP_839492.3| peptidoglycan synthetase [Shigella flexneri 2a str. 2457T]

MKFVKYFLILAVCCILLGAGSIYGLYRYIEPQLPDVVTLKDVRLQIPMQIYSADGELIAQYGEKRRIPVT

LDQIPPEMVKAFIATEDSRFYEHHGVDPVGIFRAASVALFSGHASQGASTITQQLARNFFLSPERTMMRK

IKEVFLAIRIEQLLTKDEILELYLNKIYLGYRAYGVGAAAQVYFGKTVDQLTLNEMAVIAGLPKAPSTFN

PLYSMDRAVARRNVVLSRMLDEGYITQQQFDQTRTEAINANYHAPEIAFSAPYLSEMVRQEMYNRYGESA

YEDGYRIYTTITRKVQQAAQQAVRNNVLDYDMRHGYRGPANVLWKVGESAWDNNKITDTLKALPTYGPLL

PAAVTSANPQGATAMLADGSTVALSMEGVRWARPYRSDTQQGPTPRKVTDVLQTGQQIWVRQVGDAWWLA

QVPEVNSALVSINPQNGAVMALVGGFDFNQSKFNRATQALRQVGSNIKPFLYTAAMDKGLTLASMLNDVP

ISRWDAGAGSDWQPKNSPPQYAGPIRLRQGLGQSKNVVMVRAMRAMGVDYAAEYLQRFGFPAQNIVHTES

LALGSASFTPMQVARGYAVMANGGFLVDPWFISKIENDQGGVIFEAKPKVACPECDIPVIYGDTQKSNVL

ENNDVEDVAISREQQNVSVPMPQLEQANQALVAKTGAQEYAPHVINTPLAFLIKSALNTNIFGEPGWQGT

GWRAGRDLQRRDIGGKTGTTNSSKDAWFSGYGPGVVTSVWIGFDDHRRNLGHTTASGAIKDQISGYEGGA

KSAQPAWDAYMKAVLEGVPEQPLTPPPGIVTVNIDRSTGQLANGGNSREEYFIEGTQPTQQAVHEVGTTI

IDNGEAQELF

>gi|161486509|ref|NP_836789.2| ribonuclease E [Shigella flexneri 2a str. 2457T]

MKRMLINATQQEELRVALVDGQRLYDLDIESPGHEQKKANIYKGKITRIEPSLEAAFVDYGAERHGFLPL

KEIAREYFPANYSAHGRPNIKDVLREGQEVIVQIDKEERGNKGAALTTFISLAGSYLVLMPNNPRAGGIS

RRIEGDDRTELKEALASLELPEGMGLIVRTAGVGKSAEALQWDLSFRLKHWEAIKKAAESRPAPFLIHQE

SNVIVRAFRDYLRQDIGEILIDNPKVLELARQHIAALGRPDFSSKIKLYTGEIPLFSHYQIESQIESAFQ

REVRLPSGGSIVIDSTEALTAIDINSARATRGGDIEETAFNTNLEAADEIARQLRLRDLGGLIVIDFIDM

TPVRHQRAVENRLREAVRQDRARIQISHISRFGLLEMSRQRLSPSLGESSHHVCPRCSGTGTVRDNESLS

LSILRLIEEEALKENTQEVHAIVPVPIASYLLNEKRSAVNAIETRQDGVRCVIVPNDQMETPHYHVLRVR

KGEETPTLSYMLPKLHEEAMALPSEEEFAERKRPEQPALATFAMPDVPPAPTPAEPAATVVAPAPKAATA

TPAAPAQPGLLSRFFGALKALFSGGEEAKPTEQPTPKAEAKPERQQDRRKPRQSNRRDRNERRDTRSERT

EGSDNREENRRNRRQAQQQTAETRESRQQAEVTEKARTTDEQQAPRRERSRRRNDDKRQAQQEAKALNVE

EQSVQETEQEERVRPVQPRRKQRQLNQKVRYEQSVAEEAVVAPVVEETAAAEPIVQEAPAPRTELVKVPL

PVVAQTAPEQQEENNADNRDNGGMPRRSRRSPRHLRVSGQRRRRYRDERYPTQSPMPLTVACASPELASG

KVWIRYPIVRPQDVQVEEQREQEEVQVQPMVTEVPVAAAVEPVVSAPVVEEMAEVVEAPVPVAEPQPEVV

ETTHPEVIAAAVTEQPQVITESDVAVAQEVAEHAEPMVEPQEETADADIEEVAETAEVVVAEPEVVAQPA

APVVAEVAAEVETVAAVEPEITVEHNHATAPMTRAPAPEYVPEAPRHSDWQRPTFAFEGKGAAGGHTATH

HASAAPARPQPVE

>gi|161486507|ref|NP_836795.2| glycerol-3-phosphate acyltransferase PlsX [Shigella flexneri 2a str. 2457T]

MTRLTLALDVMGGDFGPSVTVPAALQALNSNSQLTLLLVGNPDAITPLLAKADFEQRSRLQIIPAQSVIA

SDARPSQAIRASRGSSMRMALELVKEGRAQACVSAGNTGALMGLAKLLLKPLEGIERPALVTVLPHQQKG

KTVVLDLGANVDCDSTMLVQFAIMGSVLAEEVVEIPNPRVALLNIGEEEVKGLDSIRDASAVLKTIPSIN

YIGYLEANELLTGKTDVLVCDGFTGNVTLKTMEGVVRMFLSLLKSQGEGKKRSWWLLLLKRWLQKSLTRR

FSHLNPDQYNGACLLGLRGTVIKSHGAANQRAFAVAIEQAVQAVQRQVPQRIAARLESVYPAGFELLDGG

KSGTLR

>gi|161486506|ref|NP_836827.2| spermidine/putrescine ABC transporter [Shigella flexneri 2a str. 2457T]

MKNTSKFQNVVIVTIVGWLVLFVFLPNLMIIGTSFLTRDDASFVKMVFTLDNYTRLLDPLYFEVLLHSLN

MALIATLACLVLGYPFAWFLAKLPHKVRPLLLFLLIVPFWTNSLIRIYGLKIFLSTKGYLNEFLLWLGVI

DTPIRIMFTPSAVIIGLVYILLPFMVMPLYSSIEKLDKPLLEAARDLGASKLQTFIRIIIPLTMPGIIAG

CLLVMLPAMGLFYVSDLMGGAKNLLIGNVIKVQFLNIRDWPFGAATSITLTIVMGLMLLVYWRASRLLNK

KVELE

>gi|161486464|ref|NP_837855.2| UDP-4-amino-4-deoxy-L-arabinose--oxoglutarate aminotransferase [Shigella flexneri 2a str. 2457T]

MSEFLPFSRPAMGVEELAAVKEVLESGWITTGPKNQALEQAFCQLTGNQHAIAVSSATAGMHITLMALEI

GKGDEVITPSLTWVSTLNMISLLGATPVMVDVDRDTLMVTPEAIESAITPRTKAIIPVHYAGAPADIDAI

RAIGERYGIAVIEDAAHAVGTYYKGRHIGAKGTAIFSFHAIKNITCAEGGLIVTDNENLARQLRMLKFHG

LGVDAYDRQTWGRAPQAEVLTPGYKYNLTDINAAIALTQLVKLEHLNTRRREIAQQYQQALAALPFQPLS

LPAWPHVHAWHLFIIRVDEQRCGISRDALMEALKERGIGTGLHFRAAHTQKYYRERFPTLSLPNTEWNSE

RICSLPLFPDMTTADADRVITALQQLAGQ

>gi|161486462|ref|NP_837867.2| menaquinone-specific isochorismate synthase [Shigella flexneri 2a str. 2457T]

MQSLTTALENLLRHLSQEIPATPGIRVIDIPFPLKDAFDALSWLASQQTYPQFYWQQRNGDEEAAVLGAI

TRFTSLDQAQRFLRQHPEHADLRIWGLNAFDPSQGNLLLPRLEWRRCGGKATLRLTLFSESSLQHDAIQA

KEFIATLVSIKPLPGLHLTTTREQHWPDKTGWTQLIELATKTIAEGELDKVVLARATDLHFASPVNAAAM

MAASRRLNLNCYHFYMAFDGENAFLGSSPERLWRRRDKALRTEALAGTVANHPDDKQAQQLGEWLMADDK

NQRENMLVVEDICQRLQADTQTLDVLPPQVLRLRKVQHLRRCIWTSLNKADDVICLHQLQPTAAVAGLPR

DLARQFIARHEPFTREWYAGSAGYLSLQQSEFCVSLRSAKISGNVVRLYAGAGIVRGSDPEQEWQEIDNK

AAGLRTLLQME

>gi|161486447|ref|NP_838238.2| D-arabinose 5-phosphate isomerase [Shigella flexneri 2a str. 2457T]

MSEALLNTGRQTLMLELQEASRLPERLGDDFVRAANIILHCEGKVVVSGIGKSGHIGKKIAATLASTGTP

AFFVHPAEALHGDLGMIESRDVMLFISYSGGAKELDLIIPRLEDKSIALLAMTGKPTSPLGLAAKAVLDI

SVEREACPMHLAPTSSTVNTLMMGDALAMAVMQARGFNEEDFARSHPAGALGARLLNKVHHLMRRDDAIP

QVALTASVMDAMLELSRTGLGLVAVCDAQQQVQGVFTDGDLRRWLVGGGALTTPVNEAMTTGGTTLQAQS

RAIDAKEVLMKRKITAAPVVDENGKLTGAINLQDFYQAGII

>gi|161486433|ref|NP_838629.2| propionate/acetate kinase [Shigella flexneri 2a str. 2457T]

MRKEMNEFPVVLVINCGSSSIKFSVLDASDCEVLMSGIADGINSENAFLSVNGGEPAPLAHHSYEGALKA

IAFELEKRNLNDSVALIGHRIAHGGSIFTESAIITDEVIDNIRRVSPLAPLHNYANLSGIESAQQLFPGV

TQVAVFDTSFHQTMAPEAYLYGLPWKYYEELGVRRYGFHGTSHRYVSQRAHSLLNLAEDDSGLVVAHLGN

GASICAVRNGQSVDTSMGMTPLEGLMMGTRSGDVDFGAMSWVASQTNQSLGDLERVVNKESGLLGISGLS

SDLRVLEKAWHEGHERAQLAIKTFVHRIARHIAGHAASLRRLDGIIFTGGIGENSSLIRRLVMEHLAVLG

VEIDTEMNNRSNSFGERIVSSENAHVICVVIPTNEEKMIALDAIHLGKVNAPAEFA

>gi|161486412|ref|NP_838913.2| glutamate racemase [Shigella flexneri 2a str. 2457T]

MATKLQDGNTPCLAATPSEPRPTVLVFDSGVGGLSVYDEIRHLLPDLHYIYAFDNVAFPYGEKSEAFIVE

RVVAIVTAVQERYPLALAVVACNTASTVSLPALREKFDFPVVGVVPAIKPAARLTANGIVGLLATRGTVK

RSYTHELIARFANECQIEMLGSAEMVELAEAKLHGEDVSLDALKRILRPWLRMKEPPDTVVLGCTHFPLL

QEELLQVLPEGTRLVDSGAAIARRTAWLLEHEAPDAKSADANIAFCMAMTPEAEQLLPVLQRYGFETLEK

LAVLG

>gi|161486375|ref|NP_839574.2| 30S ribosomal protein S4 [Shigella flexneri 2a str. 2457T]

MARYLGPKLKLSRREGTDLFLKSGVRAIDTKCKIEQAPGQHGARKPRLSDYGVQLREKQKVRRIYGVLER

QFRNYYKEAARLKGNTGENLLALLEGRLDNVVYRMGFGATRAEARQLVSHKAIMVNGRVVNIASYQVSPN

DVVSIREKAKKQSRVKAALELAEQREKPTWLEVDAGKMEGTFKRKPERSDLSADINEHLIVELYSK

>gi|30065637|ref|NP_839808.1| two-component response regulator [Shigella flexneri 2a str. 2457T]

MQTPHILIVEDELVTRNTLKSIFEAEGYDVFEATDGAEMHQILSEYDINLVIMDINLPGKNGLLLARELR

EQANVALMFLTGRDNEVDKILGLEIGADDYITKPFNPRELTIRARNLLSRTMNLGTVSEERRSVESYKFN

GWELDINSRSLIGPDGEQYKLPRSEFRAMLHFCENPGKIQSRAELLKKMTGRELKPHDRTVDVTIRRIRK

HFESTPDTPEIIATIHGEGYRFCGDLED

>gi|30065635|ref|NP_839806.1| sensory histidine kinase CreC [Shigella flexneri 2a str. 2457T]

MRIGMRLLLGYFLLVAVAAWFVLAIFVKEVKPGVRRATEGTLIDTATLLAELARPDLLSGDPTHGQLAQA

FNQLQHRPFRANIGGINKVRNEYHVYMTDAHGKVLFDSANKAVGQDYSRWNDVWLTLRGQYGARSTLQNP

ADPESSVMYVAAPIMGGSRLIGVLSVGKPNAAMAPVIKRSERRILWASAILLGIALVIGAGMVWWINRSI

ARLTRYADSVTDNKPVPLPDLGSSELRKLAQALESMRVKLEGKNYIEQYVYALTHELKSPLAAIRGAAEI
LREGPPPEVVARFTDNILTQNARMQALVETLLRQARLENRQEVVLTAVDVAALFRRVSEARTVQLAEKNI
TLHVMPTEVNVAAEPALLDQALGNLLDNAIDFTPESGCITLSAEVDQEHVTLKVLDTGSGIPDYALSRIF
ERFYSLPRANGQKSSGLGLAFVSEVARLFNGEVTLRNVQEGGVLASLRLHRHFT

>gi|30065634|ref|NP_839805.1| DNA-binding response regulator CreB [Shigella flexneri 2a str. 2457T]
MQRETVWLVEDEQGIADTLVYMLQQEGFAVEVFERGLPVLDKARQQVPDVMILDVGLPDISGFELCRQLL
ALHPALPVLFLTARSEEVDRLLGLEIGADDYVAKPFSPREVCARVRTLLRRVKKFSTPSPVIRIGHFELN
EPAAQISWFDTPLTLTRYEFLLLKTLLKSPGRVWSRQQLMDSVWEDAQDTYDRTVDIHIKTLRAKLRAIN
PDLSPINTHRGMGYSLRGL

>gi|30065632|ref|NP_839803.1| right origin-binding protein [Shigella flexneri 2a str. 2457T]
MDQAGIIRDLLIWLEGHLDQPLSLDNVAAKAGYSKWHLQRMFKDVTGHAIGAYIRARRLSKSAVALRLTA
RPILDIALQYRFDSQQTFTRAFKKQFAQTPALYRRSPEWSAFGIRPPLRLGEFTMPEHKFVTLEDTPLIG
VTQSYSCSLEQISDFRHEMRYQFWHDFLGNAPTIPPVLYGLNETRPSQDKDDEQEVFYTTALAQDQADGY
VLTGHPVMLQGGEYVMFTYEGLGTGVQEFILTVYGTCMPMLNLTRRKGQDIERYYPAEDAKAGDRPINLR
CELLIPIRR

>gi|30065622|ref|NP_839793.1| purine nucleoside phosphorylase [Shigella flexneri 2a str. 2457T]

MATPHINAEMGDFADVVLMPGDPLRAKYIAETFLEDAREVNNVRGMLGFTGTYKGRKISVMGHGMGIPSC

SIYTKELITDFGVKKIIRVGSCGAVLPHVKLRDVVIGMGACTDSKVNRIRFKDHDFAAIADFDMVRNAVD

AAKALGVDARVGNLFSADLFYSPDGEMFDVMEKYGILGVEMEAAGIYGVAAEFGAKALTICTVSDHIRTH

EQTTAAERQTTFNDMIKIALESVLLGDKE

>gi|30065603|ref|NP_839774.1| primosomal protein DnaI [Shigella flexneri 2a str. 2457T]

MSSRVLTPDVVGIDALVHDHQTVLAKAEGGVVAVFANNAPAFYAVTPARLAELLALEEKLARPGSDVALD

DQLYQEPQAAPVAVPMGKFAMYPDWQPDADFIRLAALWGVALREPVTTEELASFIAYWQAEGKVFHHVQW

QQKLARSLQIGRASNGGLPKRDVNTVSEPDSQIPPGFRG

>gi|30065602|ref|NP_839773.1| DNA replication protein DnaC [Shigella flexneri 2a str. 2457T]

MKNVGDLMQRLQKMMPAHIKPAFKTGEELLAWQKEQGAIRSAALERENRAMKMQRTFNRSGIRPLHQNCS

FENYRVECEGQMNALSKARQYVEEFDGNIASFIFSGKPGTGKNHLAAAICNELLLRGKSVLIITVADIMS

AMKDTFRNSGTSEEQLLNDLSNVDLLVIDEIGVQTESKYEKVIINQIVDRRSSSKRPTGMLTNSNMEEMT

KLLGERVMDRMRLGNSLWVIFNWDSYRSRVTGKEY

>gi|30065597|ref|NP_839768.1| methyl-accepting chemotaxis protein I, serine sensor receptor [Shigella flexneri 2a str. 2457T]

MLKRIKIVTSLLLVLAVFGLLQLTSGGLFFNALKNDKENFTVLQTIRQQQPTLNGSWVALLQTRNTLNRA

GIRYMMDQNNIGSGSTVAELMQSASISLKQAEKNWADYEALPRDPRQSTAAAAEIKRNYDIYHNALAELI
QLLGAGKINEFFDQPTQGYQDGFEKQYVAYMEQNDRLYDIAVSDNNASYSQAMWILVGVMIVVLAVIFAV
WFGIKASLVAPMNRLIDSIRHIAGGDLVKPIEVDGSNEMGQLAESLRHMQGELMRTVGDVRNGANAIYSG
ASEIATGNNDLSSRTEQQAASLEETAASMEQLTATVKQNAENARQASHLALSASETAQRGGKVVDNVVQT
MRDISTSSQKIADIISVIDGITFQTNILALNAAVEAARAGEQGRGFAVVAGEVRNLAQRSAQAVREIKSL
IEDSVGKVDVGSTLVESAGETMAEIVSAVTRVTDIMGEIASASDEQSRGIDQVGLAVAEMDRVTQQNAAL
VEESAAAAAALEEQASRLTEAVAVFRIQQQQQQRETSAVVKTVTPATPRKMAVADSGENWETF

>gi|30065585|ref|NP_839756.1| carbon starvation protein [Shigella flexneri 2a str. 2457T]
MPGFTMDTKKLFKHIPWVILGIIGAFCLAVVALRRGEHVSALWIVVASVSVYLVAYRYYSLYIAQKVMKL
DPTRATPAVINNDGLNYVPTNRYVLFGHHFAAIAGAGPLVGPVLAAQMGYLPGTLWLLAGVVLAGAVQDF
MVLFISSRRNGASLGEMIKEEMGPVPGTIALFGCFLIMIIILAVLALIVVKALAESPWGVFTVCSTVPIA
LFMGIYMRFIRPGRVGEVSVIGIVLLVASIYFGGVIAHDPYWGPALTFKDTTITFALIGYAFVSALLPVW
LILAPRDYLATFLKIGVIVGLALGIVVLNPELKMPAMTQYIDGTGPLWKGALFPFLFITIACGAVSGFHA
LISSGTTPKLLANETDARFIGYGAMLMESFVAIMALVAASIIEPGLYFAMNTPPAGLGITMPNLHEMGGE
NAPIIMAQLKDVTAHAAATVSSWGFVISPEQILQTAKDIGEPSVLNRAGGAPTLAVGIAHVFHKVLPMAD
MGFWYHFGILFEALFILTALDAGTRSGRFMLQDLLGNFIPFLKKTDSLVAGIIGTAGCVGLWGYLLYQGV
VDPLGGVKSLWPLFGISNQMLAAVALVLGTVVLIKMKRTQYIWVTVVPAVWLLICTTWALGLKLFSTNPQ
MEGFFYMASQYKEKIANGTDLTAQQIANMNHIVVNNYTNAGLSILFLIVVYSIIFYGFKTWLAVRNSDKR
TDKETPYVPIPEGGVKISSHH

>gi|30065572|ref|NP_839743.1| primosomal replication protein N [Shigella flexneri 2a str. 2457T]

MTNRLVLSGTVCRTPLRKVSPSGIPHCQFVLEHRSVQEEAGFHRQAWCQMPVIVSGHENQAITHSITVGS

RITVQGFISCHKAKNGLSKMVLHAEQIELIDSGD

>gi|30065571|ref|NP_839742.1| 30S ribosomal protein S6 [Shigella flexneri 2a str. 2457T]

MRHYEIVFMVHPDQSEQVPGMIERYTAAITGAEGKIHRLEDWGRRQLAYPINKLHKAHYVLMNVEAPQEV

IDELETTFRFNDAVIRSMVMRTKHAVTEASPMVKAKDERRERRDDFANETADDAEAGDSEE

>gi|30065566|ref|NP_839737.1| L-ascorbate-specific enzyme IIA component of PTS [Shigella flexneri 2a str. 2457T]

MKLHDSLAENKSIRLQAEAETWQDAVKIGVDLLVAADVVEPRYYQAILDAVEQHGPYFVLAPGLAMPHGR

PEEGVKKTGFALVTLKKPLEFNHEDNDPVDILITMAAVDANTHQEVGIMQIVNLFEDEENFDRLRACRTE

QEVLDLIDRTNAAA

>gi|30065544|ref|NP_839715.1| RNA-binding protein Hfq [Shigella flexneri 2a str. 2457T]

MAKGQSLQDPFLNALRRERVPVSIYLVNGIKLQGQIESFDQFVILLKNTVSQMVYKHAISTVVPSRPVSH

HSNNAGGGTSSNYHHGSSAQNTSAQQDSEETE

>gi|30065541|ref|NP_839712.1| N-acetylmuramoyl-l-alanine amidase II [Shigella flexneri 2a str. 2457T]

MMYRIRNWLVATLLLLCTPVGAATLSDIQVSNGNQQARITLSFIGDPDYAFSHQSKRTVALDIKQTGMIQ

GLPLLFSGNNLVKAIRSGTPKDAQTLRLVVDLTENGKTEAVKRQNGSNYTVVFTINADVPPPPPPPPVVA

KRVETPAVVAPRVSEPARNPFKTESNRTTGVISSNTVTRPAARATANTGDKIIIAIDAGHGGQDPGAIGP

GGTREKNVTIAIARKLRTLLNDDPMFKGVLTRDGDYFISVMGRSDVAHKQNANFLVSIHADAAPNRSATG

ASVWVLSNRRANSEMASWLEQHEKLSELLGGAGDVLANSQSDPYLSQAVLDLQFGHSQRVGYDVATSMIS

QLQRIGEIHKRRPEHASLGVLRSPDIPSVLVETGFISNNSEERLLASDDYQQQLAEAIYKGLRNYFLAHP

MQSAPQGATAQTASTVTTPDRTLPN

>gi|30065540|ref|NP_839711.1| ATPase [Shigella flexneri 2a str. 2457T]

MMNRVIPLPDEQATLDLGERVAKACDGATVIYLYGDLGAGKTTFSRGFLQALGHQGNVKSPTYTLVEPYT

LDNLMVYHFDLYRLADPEELEFMGIRDYFANDAICLVEWPQQGTGVLPDPDVEIHIDYQAQGREARVSAV

SSAGELLLARLAG

>gi|30065524|ref|NP_839695.1| elongation factor P [Shigella flexneri 2a str. 2457T]

MATYYSNDFRAGLKIMLDGEPYAVEASEFVKPGKGQAFARVKLRRLLTGTRVEKTFKSTDSAEGADVVDM

NLTYLYNDGEFWHFMNNETFEQLSADAKAIGDNAKWLLDQAECIVTLWNGQPISVTPPNFVELEIVDTDP

GLKGDTAGTGGKPATLSTGAVVKVPLFVQIGEVIKVDTRSGEYVSRVK

>gi|30065498|ref|NP_839669.1| iron-sulfur cluster repair di-iron protein [Shigella flexneri 2a str. 2457T]

MAYRDQPLGELALSIPRASALFRKYDMDYCCGGKQTLARAAARKELDVEVIEAELAKLAEQPIEKDWRSA

PLAEIIDHIIVRYHDRHREQLPELILQATKVERVHADKPSVPKGLTKYLTMLHEELSSHMMKEEQILFPM

IKQGMGSQAMGPISVMESEHDEAGELLEVIKHTTNNVTPPPEACTTWKAMYNGINELIDDLMDHISLENN

VLFPRALAGE

>gi|30065490|ref|NP_839661.1| transporter [Shigella flexneri 2a str. 2457T]

MLNSILVILCLIAVSAFFSMSEISLAASRKIKLKLLADEGNINAQRVLNMQENPGMFFTVVQIGLNAVAI

LGGIVGDAAFSPAFHSLFSRYMSAELSEQLSFILSFSLVTGMFILFADLTPKRIGMIAPEAVALRIINPM

RFCLYVCTPLVWFFNGLANMIFRIFKLPMVRKDDITSDDIYAVVEAGALAGVLRKQEHELIENVFELESR

TVPSSMTPRENVIWFDLHEDEQSLKNKVAEHPHSKFLVCNEDIDHIIGYVDSKDLLNRVLANQSLALNSG

VQIRNTLIVPDTLTLSEALESFKTAGEDFAVIMNEYALVVGIITLNDVMTTLMGDLVGQGLEEQIVARDE

NSWLIDGGTPIDDVMRVLDIDEFPQSGNYETIGGFMMFMLRKIPKRTDSVKFAGYKFEVVDIDNYRIDQL

LVTRIDSKATALSPKLPDAKDKEESVA

>gi|30065486|ref|NP_839657.1| hypothetical protein S4529 [Shigella flexneri 2a str. 2457T]

MRIFVYGSLRHKQGNSHWMTNAQLLGDFSIDNYQLYSLGHYPGAVPGNGTVHGEVYRIDNATLAELDALR

TRGGEYARQLIQTPYGSAWMYVYQRPVDGLKLIESGDWLDRDK

>gi|30065484|ref|NP_839655.1| inorganic pyrophosphatase [Shigella flexneri 2a str. 2457T]

MSLLNVPAGKDLPEDIYVVIEIPANADPIKYEIDKESGALFVDRFMSTAMFYPCNYGYINHTLSLDGDPV

DVLVPTPYPLQPGSVIRCRPVAVLKMTDEAGEDAKLVAVPHSKLSKEYDHIKDVNDLPELLKAQIAHFFE

HYKDLEKGKWVKVEGWENAEAAKAEIVASFERAKNK

>gi|30065482|ref|NP_839653.1| transport system permease [Shigella flexneri 2a str. 2457T]

MMPQSLPDTTPPKRRFHWPTGMPQLAALLLVLLVDSLVAPHFWQVVLQDGRLFGSPIDILNRAAPVALLA

IGITLVIATGGIDLSVGAVMAIAGATTAAMTVAGFSLPIVLLSALGTGILAGLWNGILVAILKIQPFVAT

LILMVAGRGVAQLITAGQIVTFNSPDLSWFGSGSLLFLPTPVIIAVLTLLLFWLLTRKTALGMFIEVVGI

NIRAAKNAGVNTRIIVMLTYVLSGLCAAIAGIIVAADIRGADANNAGLWLELDAILAVVIGGGSLMGGRF

NLLLSVVGALIIQGMNTGILLSGFPPEMNQVVKAVVVLCVLIVQSQRFISLIKGVRSHDKT

>gi|30065479|ref|NP_839650.1| ligase [Shigella flexneri 2a str. 2457T]

MRIHILGICGTFMGGLAMLARQLGHEVTGSDANVYPPMSTLLEKQGIELIQGYDASQLEPQPDLVIIGNA

MTRGNPCVEAVLEKNIPYMSGPQWLHDFVLRDRWVLAVAGTHGKTTTAGMATWILEQCGYKPGFVIGGVP

GNFEVSARLGESDFFVIEADEYDCAFFDKRSKFVHYCPRTLILNNLEFDHADIFDDLKAIQKQFHHLVRI

VPGQGRIIWPENDINLKQTMAMGCWSEQELVGEQGHWQAKKLTTDASEWEVLLDGEKVGEVKWSLVGEHN

MHNGLMAIAAARHVGVAPADAANALGSFINARRRLELRGEANGVTVYDDFAHHPTAILATLAALRGKVGG

TARIIAVLEPRSNTMKMGICKDDLAPSLGRADEVFLLQPAHIPWQVAEVAEACVQPAHWSGDVDTLADMV

VKTAQPGDHILVMSNGGFGGIHQKLLDGLAKKAEAAQ

>gi|30065472|ref|NP_839643.1| trehalose repressor [Shigella flexneri 2a str. 2457T]

MQNRLTIKDIARLSGVGKSTVSRVLNNESGVSQRTRERVEAVMNQHGFSPSRSARAMRGQSDKVVAIIVT

RLDSLSENLAVQTMLPAFYEQGYDPIMMENQFSPQLVAEHLGVLKRRNIDGVVLFGFTGITEEMLAHWQS

SLVLLARDAKGFASVCYDDEGAIKILMQRLYDQGHRNISYLGVPHSDVTTGKRRHEAYLAFCKAHKLHPV

AALPGLAMKQGYENVAKVITPETTALLCATDTLALGASKYLQEQRIDTLQLASVGNTPLMKFLHPEIVTV

DPGYAEAGRQAACQLIAQVTGRSEPQQIIIPATLS

>gi|30065469|ref|NP_839640.1| aspartate carbamoyltransferase regulatory subunit [Shigella flexneri 2a str. 2457T]

MTHDNKLQVEAIKRGTVIDHIPAQIGFKLLSLFKLTETDQRITIGLNLPSGEMGRKDLIKIENTFLSEEQ

VDQLALYAPQATVNRIDNYEVVGKSRPSLPERIDNVLVCPNSNCISHAEPVSSSFAVRKRANDIALKCKY

CEKEFSHNVVLAN

>gi|30065454|ref|NP_839625.1| DNA polymerase III subunit chi [Shigella flexneri 2a str. 2457T]

MKNATFYLLDNDTTVDGLSAVEQLVCEIAAERWRSGKRVLIACEDEKQAYRLDEALWARPAESFVPHNLA

GEGPRGSAPVEIAWPQKRSSSPRDILISLRTSFADFATAFTEVVDFVPYEDSLKQLARERYKAYRVAGFN

LNTATWK

>gi|30065452|ref|NP_839623.1| hypothetical protein S4489 [Shigella flexneri 2a str. 2457T]

MIIIRYLVRETLKSQLAILFILLLIFFCQKLVRILGAAVDGDIPANLVLSLLGLGVPEMAQLILPLSLFL

GLLMTLGKLYTESEITVMHACGLSKAVLVKAAMILAVFTAIVAAVNVMWAGPWSSRHQDEVLAEAKANPG

MAALAQGQFQQATNGSSVLFIESVDGSDFKDVFLAQIRPKGNARPSVVVADSGHLTQLRDGSQVVTLNQG

TRFEGTALLRDFRITDFQDYQAIIGHQAVALDPNDTDQMDMRTLWNTDTDRARAELNWRITLVFTVFMMA

LMVVPLSVVNPRQGRVLSMLPAMLLYLLFFLIQTSLKSNGGKGKLDPTLWMWTVNLIYLALAIVLNLWDT

VPVRRLRASFSRKGAV

>gi|30065451|ref|NP_839622.1| hypothetical protein S4488 [Shigella flexneri 2a str. 2457T]

MQPFGVLDRYIGKTIFTTIMMTLFMLVSLSGIIKFVDQLKKAGQGSYDALGAGMYTLLSVPKDVQIFFPM

AALLGALLGLGMLAQRSELVVMQASGFTRMQVALSVMKTAIPLVLLTMAIGEWVAPQGEQMARNYRAQAM

YGGSLLSTQQGLWAKDGNNFVYIERVKGDEVLGGISIYAFNENRRLQSVRYAATAKFDPEHKVWRLSQVD

ESDLTNPKQITGSQTVSGTWKTDLTPDKLGVVALDPDALSISGLHNYVKYLKSSGQDAGRYQLNMWSKIF

QPLSVAVMMLMALSFIFGPLRSVPMGVRVVTGISFGFVFYVLDQIFGPLTLVYGIPPIIGALLPSASFFL

ISLWLLMRKS

>gi|30065432|ref|NP_839603.1| tyrosine recombinase [Shigella flexneri 2a str. 2457T]

MSKRRYLTGKEVQAMMQAVCYGATGARDYCLILLAYRHGMRISELLDLHYQDLDLNEGRINIRRLKNGFS

TVHPLRFDEREAVERWTQERANWKGADRTDAIFISRRGSRLSRQQAYRIIRDAGIEAGTVTQTHPHMLRH

ACGYELAERGADTRLIQDYLGHRNIRHTVRYTASNAARFAGLWERNNLINEKLKREEV

>gi|30065429|ref|NP_839600.1| periplasmic chaperone [Shigella flexneri 2a str. 2457T]

MSNKNVNVRKSQEITFCLLAGILMFMAMMVAGRAEAGVALGATRVIYPAGQKQVQLAVTNNDENSTYLIQ

SWVENADGVKDGRFIVTPPLFAMKGKKENTLRILDATNNQLPQDRESLFWMNVKAIPSMDKSKLTENMLQ

LAIISRIKLYYRPAKLALPPDQAAEKLRFRRSANSLTLINPTPYYLTVTELNAGTRVLENALVPPMGEST

VKLPSDAGSNITYRTINDYGALTPKMTGVME

>gi|30065423|ref|NP_839594.1| fructuronate transporter [Shigella flexneri 2a str. 2457T]

MHVLNILWVVFGIGLMLILNLKFKINSMVALLVAALSVGMLAGMDLMSLLHTMKAGFGNTLGELAIIVVF

GAVIGKLMVDSGAAHQIAHTLLARLGLRYVQLSVIIIGLIFGLAMFYEVAFIMLAPLVIVIAAEAKIPFL

KLAIPAVAAATTAHSLFPPQPGPVALVNAYGADMGMVYIYGVLVTIPSVICAGLILPKFLGNLERPTPSF

LKADQPVDMNNLPSFGVSILVPLIPAIIMISTTIANIWLVKDTPAWEVVNFIGSSPIAMFIAMVVAFVLF

GTARGHDMQWVMNAFESAVKSIAMVILIIGAGGVLKQTIIDTGIGDTIGMLMSHGNISPYIMAWLITVLI

RLATGQGVVSAMTAAGIISAAILDPATGQLVGVNPALLVLATAAGSNTLTHINDASFWLFKGYFDLSVKD

TLKTWGLLELVNSVVGLIIVLIISMVA

>gi|30065420|ref|NP_839591.1| DNA-binding transcriptional repressor UxuR [Shigella flexneri 2a str. 2457T]

MKSVTSAQRPYQEVGAMIHDLIIKTPYNPGERLPPEREIAEMLDVTRTVVREALIMLEIKGLVEVRRGAG

IYVLDSSGSHNTDSPDANVCNDAGPFELLQARQLLESNIAEFAALQATREDIVKMRQALQLEERELASSA

PGSSESGDMQFHLAIAEATHNSMLVELFRQSWQWRENNPMWIQLHSHLDDSLYRKEWLGDHKQILAALIK

KDARAAKLAMWQHLENVKQRLLEFSNVDDIYFDGYLFDSWPLDKVDA

>gi|30065404|ref|NP_839575.1| DNA-directed RNA polymerase subunit alpha [Shigella flexneri 2a str. 2457T]

MQGSVTEFLKPRLVDIEQVSSTHAKVTLEPLERGFGHTLGNALRRILLSSMPGCAVTEVEIDGVLHEYST

KEGVQEDILEILLNLKGLAVRVQGKDEVILTLNKSGIGPVTAADITHDGDVEIVKPQHVICHLTDENASI

SMRIKVQRGRGYVPASTRIHSEEDERPIGRLLVDACYSPVERIAYNVEAARVEQRTDLDKLVIEMETNGT

IDPEEAIRRAATILAEQLEAFVDLRDVRQPEVKEEKPEFDPILLRPVDDLELTVRSANCLKAEAIHYIGD

LVQRTEVELLKTPNLGKKSLTEIKDVLASRGLSLGMRLENWPPASIADE

>gi|30065399|ref|NP_839570.1| preprotein translocase subunit SecY [Shigella flexneri 2a str. 2457T]

MAKQPGLDFQSAKGGLGELKRRLLFVIGALIVFRIGSFIPIPGIDAAVLAKLLEQQRGTIIEMFNMFSGG

ALSRASIFALGIMPYISASIIIQLLTVVHPTLAEIKKEGESGRRKISQYTRYGTLVLAIFQSIGIATGLP

NMPGMQGLVINPGFAFYFTAVVSLVTGTMFLMWLGEQITERGIGNGISIIIFAGIVAGLPPAIAHTIEQA

RQGDLHFLVLLLVAVLVFAVTFFVVFVERGQRRIVVNYAKRQQGRRVYAAQSTHLPLKVNMAGVIPAIFA

SSIILFPATIASWFGGGTGWNWLTTISLYLQPGQPLYVLLYASAIIFFCFFYTALVFNPRETADNLKKSG

AFVPGIRPGEQTAKYIDKVMTRLTLVGALYITFICLIPEFMRDAMKVPFYFGGTSLLIVVVVIMDFMAQV

QTLMMSSQYESALKKANLKGYGR

>gi|30065394|ref|NP_839565.1| 50S ribosomal protein L6 [Shigella flexneri 2a str. 2457T]

MSRVAKAPVVVPAGVDVKINGQVITIKGKNGELTRTLNDAVEVKHADNTLTFGPRDGYADGWAQAGTARA

LLNSMVIGVTEGFTKKLQLVGVGYRAAVKGNVINLSLGFSHPVDHQLPAGITAECPTQTEIVLKGADKQV

IGQVAADLRAYRRPEPYKGKGVRYADEVVRTKEAKKK

>gi|30065390|ref|NP_839561.1| 50S ribosomal protein L24 [Shigella flexneri 2a str. 2457T]

MAAKIRRDDEVIVLTGKDKGKRGKVKNVLSSGKVIIEGINLVKKHQKPVPALNQPGGIVEKEAAIQVSNV

AIFNAATGKADRVGFRFEDGKKVRFFKSNSETIK

>gi|30065370|ref|NP_839541.1| sulfur relay protein TusC [Shigella flexneri 2a str. 2457T]

MKRIAFVFSTAPHGTAAGREGLDALLATSALTDDLAVFFIADGVFQLLSGQKPDAVLARDYIATFKLLSL

YDIEQCWVCAASLRERGLDPQTPFVVEATPLEADALRRELANYDVILRF

>gi|30065369|ref|NP_839540.1| sulfur transfer complex subunit TusD [Shigella flexneri 2a str. 2457T]

MRFAIVVTGPAYGTQQASSAFQFAQALIAEGHKLSSVFFYREGVYNANQLTSPASDEFDLVRGWQQLNAQ

HGVALNICVAAALRRGIVDETEAGRLGLASSNLQPGFTLSGLGALAEASLTCDRVVQF

>gi|30065365|ref|NP_839536.1| FKBP-type peptidyl-prolyl cis-trans isomerase [Shigella flexneri 2a str. 2457T]

MKVAKDLVVSLAYQVRTEDGVLVDESPVSAPLDYLHGHGSLISGLETALEGHEVGDKFDVAVGANDAYGQ

YDENLVQRVPKDVFMGVDELQVGMRFLAETDQGPVPVEITAVEDDHVVVDGNHMLAGQNLKFNVEVVAIR

EATEEELAHGHVHGAHDHHHDHDHDGCCGGHGHDHGHEHGGEGCCGGKGNGGCGCH

>gi|30065358|ref|NP_839529.1| hypothetical protein S4388 [Shigella flexneri 2a str. 2457T]

MQARVKWVEGLTFLGESASGHQILMDGNSGDKAPSPMEMVLMAAGGCSAIDVVSILQKGRQDVVDCEVKL

TSERREEAPRLFTHINLHFIVTGRDLKDAAVARAVDLSAEKYCSVALMLEKAVNITHSYEVVAA

>gi|30065353|ref|NP_839524.1| cell filamentation protein Fic [Shigella flexneri 2a str. 2457T]

MSDKFGEGRDLYLYPGLDIMRNRLNIRQQQRLEQAAYEMTALRAATIELGPLVRGLPHLRTIHRQLYQDI

FDWAGQLREVDIYQGDTPFCHFAYIEKEGNALMQDLEEEGYLVGLEKAKFVERLAHYYCEINVLHPFRVG

SGLAQRIFFEQLAIHAGYQLSWQGIEKEAWNQANQSGAMGDLTALQMIFSKVVSEAGESE

>gi|30065350|ref|NP_839521.1| hypothetical protein S4380 [Shigella flexneri 2a str. 2457T]

MTNSNRIKLTWISFLSYALTGALVIVTGMVMGNIADYFNLPVSSMSNTFTFLNAGILISIFLNAWLMEIV

PLKTQLRFGFLLMVLAVAGLMFSHSLALFSTAMFILGVVSGITMSIGTFLITQMYEGRQRGSRLLFTDSF

FSMAGMIFPMIAAFLLARSIEWYWVYACIGLVYVAIFILTFGCEFPALGKRAPKTDAPVEKEKWGIGVLF

LSVAALCYILGQLGFISWVPEYAKGLGMSLNDAGTLVSNFWMSYMVGMWAFSFILRFFDLQRILTVLAGL

AAILMYVFNTGTPVHMAWSILALGFFSSAIYTTIITLGSQQTKVPSPKLVNFVLTCGTIGTMLTFVVTGT

IVEHSGPQAALLTANGLYAVVFVMCFLLGFVSRHRQHNTLTSH

>gi|30065342|ref|NP_839513.1| fructoselysine 6-kinase [Shigella flexneri 2a str. 2457T]

MKTLATIGDNCVDIYPQLNKAFSGGNAVNVAVYCTRYGIQPGCITWVGDDDYGTKLKQDLARMGVDISHV

HTKHGVTAQTQVELHDNDRVFGDYTEGVMADFALSEEDYAWLAQYDIVHAAIWGHAEDAFPQLHAAGKLT

AFDFSDKWDSPLWQTLVPHLDFAFASAPQEDEALRLKMKAIVARGAGTVIVTLGENGSIAWDGAQFWRQA

PEPVTVIDTMGAGDSFIAGFLCGWSAGMTLPQAMAQGTACAAKTIQYHGAW

>gi|30065341|ref|NP_839512.1| DNA-binding transcriptional regulator FrlR [Shigella flexneri 2a str. 2457T]

MLLLAGKRPYNPSNSFFISGSIMSATDRYSHQLLYATVRQRLLDDIAQGVYQAGQQIPTENELCTQYNVS

RITIRKAISDLVADGVLIRWQGKGTFVQSQKVENALLTVSGFTDFGVSQGKSTKEKVIEQERVSAAPFCE

KLNIPGNSEVFHLCRVMYLDKEPLFIDSSWIPLSRYPDFDEIYVEGSSTYQLFQERFDTRVVSDKKTIDI

FAATRPQAKWLKCELGEPLFRISKIAFDQNDKPVHVSELFCRANRITLTIDNKRH

>gi|30065329|ref|NP_839500.1| DNA adenine methylase [Shigella flexneri 2a str. 2457T]

MKKNRAFLKWAGGKYPLLDDIKRHLPKGECLVEPFVGAGSVFLNTDFSRYILADINSDLISLYNIVKMRT

DEYVQAARELFVPETNCAEVYYQFREEFNKSQDPFRRAVLFLYLNRYGYNGLCRYNLRGEFNVPFGRYKK

PYFPEAELYHFAEKAQNAFFYCESYADSMERADDASVVYCDPPYAPLSATANFTAYHTNSFTLEQQAHLA

EIAEGLVERHIPVLISNHDTMLTREWYQRAKLHVVKVRRSISSNGGTRKKVDELLALYKPGVVSPAKK

>gi|30065327|ref|NP_839498.1| 3-dehydroquinate synthase [Shigella flexneri 2a str. 2457T]

MERIVVTLGERSYPITIASGLFNEPASFLPLKSGEQVMLVTSETLAPLYLDKVRGVLEQAGVNVDSVILP

DGEQYKSLAVLDTVFTALLQKPHGRDTTLVALGGGVVGDLTGFAAASYQRGVRFIQVPTTLLSQVDSSVG

GKTAVNHPLGKNMIGAFYQPASVVVDLDCLKTLPPRELASGLAEVIKYGIILDGAFFNWLEENLDALLRL

DGPAMAYCIRRCCELKAEVVAADERETGLRALLNLGHTFGHAIEAEMGYGNWLHGEAVAAGMVMAARTSE

RLGQFSSAETQRIITLLTRAGLPVNGPREMSAQAYLPHMLRDKKVLAGEMRLILPLAIGKSEVRSGVSHE

LVLNAIADCQSA

>gi|30065313|ref|NP_839484.1| osmolarity sensor protein [Shigella flexneri 2a str. 2457T]

MRRLRFSPRSSFARTLLLIVTLLFASLVTTYLVVLNFAILPSLQQFNKVLAYEVRMLMTDKLQLEDGTQL

VVPPAFRREIYRELGISLYSNEAAEEAGLRWAQHYEFLSHQMAQQLGGPTEVRVEVNKSSPVVWLKTWLS

PNIWVRVPLTEIHQGDFSPLFRYTLAIMLLAIGGAWLFIRIQNRPLVDLEHAALQVGKGIIPPPLREYGA

SEVRSVTRAFNHMAAGVKQLADDRTLLMAGVSHDLRTPLTRIRLATEMMSEQDGYLAESINKDIEECNAI

IEQFIDYLRTGQEMPMEMADLNAVLGEVIAAESGYEREIETALYPGSIEVKMHPLSIKRAVANMVVNAAR

YGNGWIKVSSGTEPNRAWFQVEDDGPGIAPEQRKHLFQPFVRGDSARTISGTGLGLAIVQRIVDNHNGML

ELGTSERGGLSIRAWLPVPVTRAQGTTKEG

>gi|30065312|ref|NP_839483.1| osmolarity response regulator [Shigella flexneri 2a str. 2457T]

MQENYKILVVDDDMRLRALLERYLTEQGFQVRSVANAEQMDRLLTRESFHLMVLDLMLPGEDGLSICRRL

RSQSNPMPIIMVTAKGEEVDRIVGLEIGADDYIPKPFNPRELLARIRAVLRRQANELPGAPSQEEAVIAF

GKFKLNLGTREMFREDEPMPLTSGEFAVLKALVSHPREPLSRDKLMNLARGREYSAMERSIDVQISRLRR

MVEEDPAHPRYIQTVWGLGYVFVPDGSKA

>gi|30065306|ref|NP_839477.1| hypothetical protein S4333 [Shigella flexneri 2a str. 2457T]

MSKKQSSTPHDALFKLFLRQPETARDFLAFHLPAPIHALCDMKTLKLESRSFIDDDLRESYSDVLWSVKT

EQGPGYIYCLIEHQSTSNKLIAFRMMRYAIAAMQNHLDAGYKTLPMVVPLLFYHGIESPYPYSLCWLDCF

ADPKLARQLYASAFPLIDVTVMPDDEIMQHRRMALLELIQKHIRQRDLMGLVEQMACLLSSGYANDRQIK

GLFNYILQTGDAVRFNDFIDGVAERSPKHKESLMTIAERLRQEGEQSKALHIAKIMLESGVPLADIMRFT

GLSEEELAAASRLAP

>gi|30065300|ref|NP_839471.1| high-affinity transport permease for gluconate [Shigella flexneri 2a str. 2457T]

MPLVIVAIGVILLLLLMIRFKMNGFIALVLVALAVGLMQGMPLDKVIGSIKAGVGGTLGSLALIMGFGAM

LGKMLADCGGAQRIATTLIAKFGKKHIQWAVVLTGFTVGFALFYEVGFVLMLPLVFTIAASANIPLLYVG

VPMAAALSVTHGFLPPHPGPTAIATIFNADMGKTLLYGTILAIPTVILAGPVYARVLKGIDKPIPEGLYS

AKTFSEEEMPSFGVSVWTSLVPVVLMAMRAIAEMILPKGHAFLPVAEFLGDPVMATLIAVLIAMFTFGLN

RGRSMDQINDTLVSSIKIIAMMLLIIGGGGAFKQVLVDSGVDKYIASMMHETNISPLLMAWSIAAVLRIA

LGSATVAAITAGGIAAPLIATTGVSPELMVIAVGSGSVIFSHVNDPGFWLFKEYFNLTIGETIKSWSMLE

TIISVCGLVGCLLLNMVI

>gi|30065295|ref|NP_839466.1| 2-component regulator [Shigella flexneri 2a str. 2457T]

MRKTVAFGFVGTVLDYAGRGSQRWSKWRPSLCIYQQESLVIDRLELLHDARSRSLFETLKRDIASVSPET

EVVGVEIELHNPWDFEEVYACLHDFARGYEFQPEKEDYLIHITTGTHVAQICWFLLAEARYLPARLIQSS

TPRKKEQPRGPGEVTIIDLDLSRYNAIASRFAEERQQTLDFLKSGIATRNPHFNRMIEQIEKVAIKSRAP

ILLNGPTGAGKSFLARRIFELKQARHQFSGAFVEVNCATLRGDTAMSTLFGHVKGAFTGARESREGLLRS

ANGGMLFLDEIGELGADEQAMLLKAIEEKTFYPFGSDRQVSSDFQLIAGTVRDLRQLVAEGKFREDLYAR

INLWTFTLPGLRQRQEDIEPNLDYEVERHATLTGDSVRFNTEARRAWLAFATSPQATWRGNFRELSASVT

RMATFATSGRITLDVVEDEINRLRYNWQESRPSALTALLGAEAENIDLFDRMQLEHVIAICRQAKSLSAA

GRQLFDVSRQGKASVNDADRLRKYLARFGLTWEAVQDQHSSS

>gi|30065283|ref|NP_839454.1| aspartate-semialdehyde dehydrogenase [Shigella flexneri 2a str. 2457T]

MKNVGFIGWRGMVGSVLMQRMVEERDFDAIRPVFFSTSQLGQAAPSFGGTTGTLQDAFDLEALKALDIIV

TCQGGDYTNEIYPKLRESGWQGYWIDAASSLRMKDDAIIILDPVNQDVITDGLNNGIRTFVGGNCTVSLM

LMSLGGLFANDLVDWVSVATYQAASGGGARHMRELLTQMGHLYGHVADELATPSSAILDIERKVTTLTRS

GELPVDNFGVPLAGSLIPWIDKQLDNGQSREEWKGQAETNKILNTSSVIPVDGLCVRVGALRCHSQAFTI

KLKKDVSIPTVEELLAAHNPWAKVVPNDREITMRELTPAAVTGTLTTPVGRLRKLNMGPEFLSAFTVGDQ

LLWGAAEPLRRMLRQLA

>gi|30065281|ref|NP_839452.1| low affinity gluconate transporter [Shigella flexneri 2a str. 2457T]

MKARMHAFLALMVVSMGAGLFSGMPLDKIAATMEKGMGGTLGFLAVVVALGAMFGKILYETGAVDQIAVK

MLKSFGHSRAHYAIGLAGLVCALPLFFEVAIVLLISVAFSMARHTGTNLVKLVIPLFAGVAAAAAFLVPG

PAPMLLASQMNADFGWMILIGLCAAIPGMIIAGPLWGNFISRYVELHIPDDISEPHLGEGKMPSFGFSLS

LILLPLVLVGLKTIAARFVPEGSTAYEWFEFIGHPFTAILVACLVAIYGLAMRQGMPKDKVMEICGHALQ

PAGIILLVIGAGGVFKQVLVDSGVGPALGEALTGMGLPIAITCFVLAAAVRIIQGAATVACLTAVGLVMP

VIEQLNYSGAQMAALSICIAGGSIVVSHVNDAGFWLFGKFTGATEAETLKTWTMMETILGTVGAIVGMIA

FQLLS

>gi|30065262|ref|NP_839433.1| RNA polymerase factor sigma-32 [Shigella flexneri 2a str. 2457T]

MTDKMQSLALAPVGNLDSYIRAANAWPMLSADEERALAEKLHYHGDLEAAKTLILSHLRFVVHIARNYAG

YGLPQADLIQEGNIGLMKAVRRFNPEVGVRLVSFAVHWIKAEIHEYVLRNWRIVKVATTKAQRKLFFNLR

KTKQRLGWFNQDEVEMVARELGVTSKDVREMESRMAAQDMTFDLSSDDDSDSQPMAPVLYLQDKSSNFAD

GIEDDNWEEQAANRLTDAMHGLDERSQDIIRARWLDEDNKSTLQELADRYGVSAERVRQLEKNAMKKLRA

AIEA

>gi|30065258|ref|NP_839429.1| 16S rRNA m(2)G966-methyltransferase [Shigella flexneri 2a str. 2457T]

MKKPNHSGSGQIRIIGGQWRGRKLPVPDSPGLRPTTDRVRETLFNWLAPVIVDAQCLDCFAGSGALGLEA

LSRYAAGATLIEMDRVVSQQLIKNLATLKAGNARVVNSNAMSFLAQKGTPHNIVFVDPPFRRGLLEETIN

LLEDNGWLADEALIYVESEVENGLPTVPANWSLHREKVAGQVAYRLYQHEAQGESDAD

>gi|30065256|ref|NP_839427.1| receptor [Shigella flexneri 2a str. 2457T]

MSKPPLFFIVIIGLIVVAASFRFMQQRREKADNDMAPLQQKLVVVSNKREKPINDRRSRQQEVTPAGTSM

RYEASFKPQSGGMEQTFRLDAQQYHALTVGDKGTLSYKGTRFVSFVGEQ

>gi|30065247|ref|NP_839418.1| periplasmic binding protein for nickel [Shigella flexneri 2a str. 2457T]

MLSTLRRTLFALLACASFIVHAAAPDEITTAWPVNVGPLNPHLYTPNQMFAQSMVYEPLVKYQADGSVIP

WLAKSWTHSEDGKTWTFTLRDDVKFSNGEPFDAEAAAENFRAVLDNRQRHAWLELANQIVDVKALNKTEL

QITLKSAYYPFLQELALPRPFRFIAPSQFKNHETMNGIKAPIGTGPWILQESKLNQYDVFVRNENYWGEK

PAIKKITFNVIPDPTTRAVAFETGDIDLLYGNEGLLPLDTFARFSQNPAYHTQLSQPIETVMLALNTAKA

PTNELAVREALNYAVNKKSLIDNALYGTQQVADTLFAPSVPYANLGLKPRQYDPQKAKELLEKAGWTLPA

GKDIREKNGQPLRIELSFIGTDALSKSMAEIIQADMRQIGADVSLIGEEESSIYARQRDGRFGMIFHRTW

GAPYDPHAFLSSMRVPSHADFQAQQGLADKPLIDKEIGEVLATHDETQRQALYRDILTRLHDEAVYLPIS

YISMMVVSKPELGNIPYAPIATEIPFEQIKPVKP

>gi|30065245|ref|NP_839416.1| nickel transporter permease NikC [Shigella flexneri 2a str. 2457T]

MNFFLSSRWSVRLALIIIALLALIALTSQWWQPYDPQAIDLPSCLLSPDAQHWLGTDHLGRDIFSRLMAA

TRVSLGSVMACLLLVLTLGLVIGGSAGLIGGHVDQATMRVADMFMTFPTSILSFFMVGVLGTGLTNVIIA

IALSHWAWYARMVRSLVISLRQREFVLASRLSGAGHVRVFVDHLAGAVIPSLLVLATLDIGHMMLHVAGM

SFLGLGVTAPTAEWGVMINDARQYIWTQPLQMFWPGLALFISVMAFNLVGDALRDHLDPHLVTEHAH

>gi|30065230|ref|NP_839401.1| fimbrial protein remnant [Shigella flexneri 2a str. 2457T]

MFYRLSLILIMALLAGQLSAQEWSFDSSQLEGNVSADTVAMFNQGEQLPGNYRVEIYLNGEKVDVGEFPF

HRPESPEEKELVPCLTVDDLIHYGIKIDKSSSDTDNKKNQCFKWNSIEGLKVNYDFDSQRVQITVPQLYL

QDKKSSLAPVSLWNEGVAAFRMVYQTNIDISKQNDNQSTTRNSRYGRFTPGFNLGAWRFRSSVTWSKELG

QSERWQRGYMWFERGINAIKSRLTLGESYTSSEVFDSIPFRGGMLATDDAMTPPEDSYYTPVVHGIAQSE

AQVIIKQNGQIIFTRSVPPGPFALDNLPTLAVGGELDVTVRESNGEEQYFSVPFQTPAIALHEGYFKYSV

MGGNIKKKV

>gi|30065221|ref|NP_839392.1| universal stress protein [Shigella flexneri 2a str. 2457T]

MAYKHILIAVDLSPESKVLVEKAVSMARPYNAKVSLIHVDVNYSDLYTGLIDVNLGDMQKRISEETHHAL

TELSTNAGYPITETLSGSGDLGQVLVDAIKKYDMDLVVCGHHQDFWSKLMSSARQLINTVHVDMLIVPLR

DEEE

>gi|30065214|ref|NP_839385.1| arsenate reductase [Shigella flexneri 2a str. 2457T]

MSNITIYHNPACGTSRNTLEMIRNSGTEPTIIHYLETPPTRDELVKLIADMGISVRALLRKNVEPYEELG

LAEDKFTDDRLIDFMLQHPILINRPIVVTPLGTRLCRPSEVVLEILPDAQKGAFSKEDGEKVVDEAGKRL

K

>gi|30065208|ref|NP_839379.1| outer membrane protein induced after carbon starvation [Shigella flexneri 2a str. 2457T]

MVLIFVDKDSNMNMTKGALILSLSFLLAACSSIPQNIKGNNQPDIQKSFVAVHNQPGLYVGQQARFGGKV

INVINGKTDTLLEIAVLPLDSYAKPDIEANYQGRLLARQSGFLDPVNYRNHFVTILGTIQGEQPGFINKV

PYNFLEVNMQGIQVWHLREVVNTTYNLWDYGYGAFWPEPGWGAPYYTNAVSQVTPELVK

>gi|30065178|ref|NP_839349.1| dipeptide transporter [Shigella flexneri 2a str. 2457T]

MSQVTENKVISAPVPMTPLQEFWHYFKRNKGAVVGLVYVAIVLFIAIFANWIAPYNPADQFRDALLAPPA

WQEGGSMAHLLGTDDVGRDVLSRLMYGARLSLLVGCLVVVLSLIMGVILGLIAGYFGGLVDNIIMRVVDI

MLALPSLLLALVLVAIFGPSIGNAALALTFVALPHYVRLTRAAVLVEVNRDYVTASRVAGAGAMRQMFIN

IFPNCLAPLIVQASLGFSNAILDMAALGFLGMGAQPPTPEWGTMLSDVLQFAQSAWWVVTFPGLAILLTV

LAFNLMGDGLRDALDPKLKQ

>gi|30065176|ref|NP_839347.1| dipeptide transport protein [Shigella flexneri 2a str. 2457T]

MRISLKKSGMLKLGLSLVAMTVAASVQAKTLVYCSEGSPEGFNPQLFTSGTTYDASSVPLYNRLVEFKIG

TTEVIPGLAEKWEVSEDGKTYTFHLRKGVKWHDNKEFKPTREMNADDVVFSFDRQKNAQNPYHKVSGGSY

EYFEGMGLPELISEVKKVDDNTVQFVLTRPEAPFLADLAMDFASILSKEYADAMMKAGTPEKLDLNPIGT

GPFQLQQYQKDSRIRYKAFDGYWGTKPQIDTLVFSITPDASVRYAKLQKNECQVMPYPNPADIARMKQDK

SINLMEMPGLNVGYLSYNVQKKPLDDVKVRQALTYAVNKDAIIKAVYQGAGVSAKNLIPPTMWGYNDDVQ

DYTYDPEKAKALLKEAGLEKGFSIDLWAMPVQRPYNPNARRMAEMIQADWAKVGVQAKIVTYEWGEYLKR

AKDGEHQTVMMGWTGDNGDPDNFFATLFSCAASEQGSNYSKWCYKPFEDLIQPARATDDHNKRVELYKQA

QVVMHDQAPALIIAHSTVFEPVRKEVKGYVVDPLGKHHFENVSIE

>gi|30065171|ref|NP_839342.1| hypothetical protein S4185 [Shigella flexneri 2a str. 2457T]

MIREAQRSELPAILELWLESTTWGHPFIKANYWRDCIPLVRDAYLANAQNWVWEEDGKLLGFVSIMEGRF

LAAMFVAPKAVRRGIGKALMQYVQQRYPHLMLEVYQKNQPAIDFYRAQGFHIVDCAWQDETQLPTWIMSW

PVVQTL

>gi|30065164|ref|NP_839335.1| IS150 transposase [Shigella flexneri 2a str. 2457T]

MKVLNELRQFYPLDELLRAAEIPRSTFYYHLKALSKPDKYADVKKRIGEIYHENRGRYGYRRVTLSLHRE

GKQINHKAVQRLMGTLSLKAAIKVKRYRSYRGEVGQTAPNVLQRDFKATRPNEKWVTDVTEFAVNGRKLY

LSPVIDLFNNEVISYSLSERPVMNMVENMLDQAFKKLNPHEHPVLHSDQGWQYRMRRYQNILKEHGIKQS

MSRKGNCLDNAVVECFFGTLKSECFYLDEFSNISELKDAVTEYIEYYNSRRISLKLKGLTPIEYRNQTYM

PRV

>gi|30065159|ref|NP_839330.1| DNA-binding transcriptional regulator GadX [Shigella flexneri 2a str. 2457T]

MQSLHGNCLIAYARHKYILTMVNGEYRYFNGGDLVFADASQIRVDKCVENFVLVSRDTLSLFLPMLKEEA

LNLHAHKKISSLLVHHCSRDIPVFQEVAQLSQNKNLRYAEMLRKRALIFALLSVFLEDEHFIPLLLNVLQ

PNMRTRVCTVINNNIAHEWTLARIASELLMSPSLLKKKLREEETSYSQLLTECRMQRALQLIVIHGFSIK

RVAVSCGYHSVSYFIYVFRNYYGMTPTEYQERSAQGLPNRDSAASIVAQGNFYGTNRSAEGIRL

>gi|30065158|ref|NP_839329.1| ARAC-type regulatory protein [Shigella flexneri 2a str. 2457T]

MTHVCSVILIRRSFDIYHEQQKISLHNESILLLEKNLADDFAFCSPDTRRLDIDELTVCHYLQNIRQLPR

NLGLHSKDRLLINQSPPMPLVTAIFDSFNESGVNSPILSNMLYLSCLSMFSHKKELIPLLFNSISTVSGK

VERLISFDIAKRWYLRDIAERMYTSESLIKKKLQDENTCFSKILLASRMSMARRLLELRQIPLHTIAEKC

GYSSTSYFINTFRQYYGVTPHQFAQHSPGTFS

>gi|30065154|ref|NP_839325.1| glycyl-tRNA synthetase subunit beta [Shigella flexneri 2a str. 2457T]

MSEKTFLVEIGTEELPPKALRSLAESFAANFTAELDNAGLAHGTVQWFAAPRRLALKVANLAEAQPDREI

EKRGPAIAQAFDAEGKPSKAAEGWARGCGITVDQAERLTTDKGEWLLYRAHVKGESTEALLPNMVATSLA

KLPIPKLMRWGASDVHFVRPVHTVTLLLGDKVIPATILGIQSDRVIRGHRFMGEPEFTIDNADQYPEILR

ERGKVIADYEERKAKIKADAEEAARKIGGNADLSESLLEEVASLVEWPVVLTAKFEEKFLAVPAEALVYT

MKGDQKYFPVYANDGKLLPNFIFVANIESKDPQQIISGNEKVVRPRLADAEFFFNTDRKKRLEDNLPRLQ

TVLFQQQLGTLRDKTDRIQALAGWIAEQIGADVNHATRAGLLSKCDLMTNMVFEFTDTQGVMGMHYARHD

GEAEDVAVALNEQYQPRFAGDDLPSNPVACALAIADKMDTLAGIFGIGQHPKGDKDPFALRRAALGVLRI

IVEKNLNLDLQTLTEEAVRLYGDKLTNANVVDDVIDFMLGRFRAWYQDEGYTVDTIQAVLARRPTRPADF

DARMKAVSHFRTLEAAAALAAANKRVSNILAKSDEVLSDRVNASTLKEPEEIKLAMQVVVLRDKLEPYFA

EGRYQDALVELAELREPVDAFFDKVMVMVDDKELRLNRLTMLEKLRELFLRVADISLLQ

>gi|30065153|ref|NP_839324.1| glycyl-tRNA synthetase subunit alpha [Shigella flexneri 2a str. 2457T]

MQKFDTRTFQGLILTLQDYWARQGCTIVQPLDMEVGAGTSHPMTCLRALGPEPMAAAYVQPSRRPTDGRY

GENPNRLQHYYQFQVVIKPSPDNIQELYLGSLKELGMDPTIHDIRFVEDNWENPTLGAWGLGWEVWLNGM

EVTQFTYFQQVGGLECKPVTGEITYGLERLAMYIQGVDSVYDLVWSDGPLGKTTYGDVFHQNEVEQSTYN

FEYADVDFLFTCFEQYEKEAQQLLALENPLPLPAYERILKAAHSFNLLDARKAISVTERQRYILRIRTLT

KAVAEAYYASREALGFPMCNKDK

>gi|30065148|ref|NP_839319.1| D-xylose transporter subunit XylF [Shigella flexneri 2a str. 2457T]

MKIKNILLTLCTSLLLTNVAAHAKEVKIGMAIDDLRLERWQKDRDIFVKKAESLGAKVFVQSANGNEETQ

MSQIENMINRGVDVLVIIPYNGQVLSNVVKKAKQEGIKVLAYDRMINDADIDFYISFDNEKVGELQAKAL

VDIVPQGNYFLMGGSPVDNNAKLFRAGQMKVLKPYVDSGKIKVVGDQWVDGWLPENALKIMENALTANNN

KIDAVVASNDATAGGAIQALSAQGLSGKVAISGQDADLAGIKRIAAGTQTMTVYKPITLLANTAAEIAVE

LGNGQEPKADTSLNNGLKDVPSRLLTPIDVNKNNIKDTVIKDGFHKESEL

>gi|30065117|ref|NP_839288.1| serine acetyltransferase [Shigella flexneri 2a str. 2457T]

MSCEELEIVWNNIKAEARTLADCEPMLASFYHATLLKHENLGSALSYMLANKLSSPIMPAIAIREVVEEA

YAADPEMIASAACDIQAVRTRDPAVDKYSTPLLYLKGFHALQAYRIGHWLWNQGRRALAIFLQNQVSVTF

QVDIHPAAKIGRGIMLDHATGIVVGETAVIENDVSILQSVTLGGTGKSGGDRHPKIREGVMIGAGAKILG

NIEVGRGAKIGAGSVVLQPVPPHTTAAGVPARIVGKPDSDKPSMDMDQHFNGINHTFEYGDGI

>gi|30065115|ref|NP_839286.1| preprotein translocase subunit SecB [Shigella flexneri 2a str. 2457T]

MSEQNNTEMTFQIQRIYTKDISFEAPNAPHVFQKDWQPEVKLDLDTASSQLADDVYEVVLRVTVTASLGE

ETAFLCEVQQGGIFSIAGIEGTQMAHCLGAYCPNILFPYARECITSMVSRGTFPQLNLAPVNFDALFMNY

LQQQAGEGTEEHQDA

>gi|30065113|ref|NP_839284.1| hypothetical protein S4118 [Shigella flexneri 2a str. 2457T]

MQEIMQFVGRHPILSIAWIALLVAVLVTTFKSLTSKVKVITRGEATRLINKEDAVVVDLRQRDDFRKGHI

AGSINLLPSEIKANNVGELEKHKDKSVIVVDGSGMQCQEPANALTKAGFAQVFVLKEGVAGWAGENLPLV

RGK

>gi|30065112|ref|NP_839283.1| phosphoglyceromutase [Shigella flexneri 2a str. 2457T]

MSVSKKPMVLVILDGYGYREEQQDNAIFSAKTPVMDALWANRPHTLIDASGLEVGLPDRQMGNSEVGHVN

LGAGRIVYQDLTRLDVEIKDRAFFANPVLAGAVHKAKNAGKAVHIMGLLSAGGVHSHEDHIMAMVELAAE

RGAEKIYLHAFLDGRDTPPRSAESSLKKFEEKFAALGKGRVASIIGRYYAMDRDNRWDRVEKAYDLLTLA

QGEFQADTAVAGLQAAYARDENDEFVKATVIRAEGQPDAAMEDGDALIFMNFRADRAREITRAFVNADFD

GFARKKVVNVDFVMLTEYAADIKTAVAYPPASLVNTFGEWMAKNDKTQLRISETEKYAHVTFFFNGGVEE

SFKGEDRILINSPKVATYDLQPEMSSAELTEKLVAAIKSGKYDTIICNYPNGDMVGHTGVMEAAVKAVEA

LDHCVEEVAKAVESVGGQLLITADHGNAEQMRDPATGQAHTAHTNLPVPLIYVGDKNVKAVAGGKLSDIA

PTMLSLMGMEIPQEMTGKPLFIVE

>gi|30065102|ref|NP_839273.1| ADP-heptose--LPS heptosyltransferase [Shigella flexneri 2a str. 2457T]

MGDVLHTLPALTDAQQAIPGIKFDWVVEEGFAQIPSWHAAVERVIPVAIRRWRKAWFSAPIKAERKAFRE

ALQAENYDAVIDAQGLVKSAALVTRLAHGVKHGLDWQTAREPLASLFYNCKHHIAKQQHAVERTRELFAK

SLGYSKPQTQGDYAIAQHFLTNLPTDAGEYAVFLHATTRDDKHWPEEHWRELIGLLADSGIRIKLPWGAP

HEEERAKRLAEGFAYVEVLPKMSLEGVARVLAGAKFVVSVDTGLSHLTAALDRPNITVYGPTDPGLIGGY

GKNQVECRSTSMSLADLPAQTVFQNLNLEIITNKLTSEIR

>gi|30065101|ref|NP_839272.1| lipid A-core, surface polymer ligase [Shigella flexneri 2a str. 2457T]

MTSTLFFSLEKKNWIAYWNRALVFLFITTYFLGGITRYKHLIVILMTITTIVYLCKRPKHYLSLFKTFLF

GSVAILTIAALLSLLQSPDAGASMKEVFKAIIENTLLCTIAIPVILRDEKREDVEKIVFFSFISALGLRC

FSELITYYKDYQQGIMPFADYRHRSISDSMVFLFPALLNLWLIKSAKYRISFVVLSVIFIFLILGTLSRG

AWLSVLVIGLIWILMFKQWKLLLVGVMVSIIALSVIFTHKEMTAKLTYKLQQTNSSYRYANGTQGSALDL

ILENPVIGYGYGNVAYKDVYNKRVIDYPEWTFRQSIGPHNFALFIWFGTGLLGLVSLMMLYCAILKECIK

NGVKNKYRSPYNAYYIILLSFIGYFVIRGNVEQIEPNLLGVYAGLLLAMKNK

>gi|30065091|ref|NP_839262.1| 3-deoxy-D-manno-octulosonic-acid transferase [Shigella flexneri 2a str. 2457T]

MLELLYTALLYLIQPLIWIRLWVRGRKAPAYRKRWGERYGFYRHPLKPGGIMLHSVSVGETLAAIPLVRA

LRHRYPDLPITVTTMTPTGSERVQSAFGKDVQHVYLPYDLPDALNRFLNKVDPKLVLIMETELWPNLIAA

LHKRKIPLVIANARLSARSAAGYAKLGKFVRRLLRRITLIAAQNEEDGARFVALGAKNNQVTVTGSLKFD

ISVTPQLAAKAVTLRRQWAPHRPVWIATSTHEGEESVVIAAHQALLQQFPNLLLILVPRHPERFPDAINL

VRQAGLSYITRSSGEVPSTSTQVVVGDTMGELMLLYGIADLAFVGGSLVERGGHNPLEAAAHAIPVLMGP

HTFNFKDICARLEQASGLITVTDATTLAKEVSSLLTDAVYRSFYGRHAVEVLYQNQGALQRLLQLLEPYL

PPKTH

>gi|30065090|ref|NP_839261.1| phosphopantetheine adenylyltransferase [Shigella flexneri 2a str. 2457T]

MQKRAIYPGTFDPITNGHIDIVTRATQMFDHVILAIAASPSKKPMFTLEERVALAQQATAHLGNVEVVGF

SDLMANFARNQHATVLIRGLRAVADFEYEMQLAHMNRHLMPELESVFLMPSKEWSFISSSLVKEVARHQG

DVTHFLPENVHQALMAKLA

>gi|30065077|ref|NP_839248.1| NAD-dependent DNA ligase LigB [Shigella flexneri 2a str. 2457T]

MKVWMAILISILCWQSSVWAVCPAWSPARAQEEISRLQQQIKQWDDDYWKEGKSEVEDGVYDQLSARLTQ

WQRCFVSEPRDVMMPPLNGAVMHPVAHTGVRKMADKNALSLWMRERSDLWVQPKVDGVAVTLVYRDGKLN

KAISRGNGLKGEDWTQKVSLISAVLQTVSGPLANSTLQGEIFLQREGHIQQQMGGINARAKVAGLMMRQG

NSDTLNSLAVFVWAWPDGPQLMTDRLKELATAGFTLTQRYTRAVKNADEVARVRNEWWKAKLPFVTDGVV

VRGAKEPESRHWLPGQAEWLVAWKYQPVAQVAEVKAIQFAVGKSGKISVVASLAPVMLDDKKVQRVNIGS

VRRWQEWDIAPGDQILVSLAGQGIPRIDDVVWRGAERTKPTPPENRFNPLTCYFASDVCQEQFISRLVWL

GSKQVLGLDGIGEAGWRALHQTHRFEHIFSWLLLTPEQLQNTPGIAKSKSAQLWHQFNLARNQPFTRWVM

AMGIPLTRAALNASDERSWSQLLFSTEQFWQQLPGTGSGRARQVIEWKENAQIKKLGSWLAAQQITGFEP

>gi|30065045|ref|NP_839216.1| long polar fimbriae [Shigella flexneri 2a str. 2457T]

MMTTRIVVGLTAGTCLIFSQNLMAEVSVFNPALLEINHQSGVDIRQFNRANLMPPGVYSVDIFINGKMFE

RQDVTFVQDNPDADLHACFIAIKKTLSSFGIKVDALKSFNDVDETVCLDPAPRIEGSSWQFDSDKLQLNI

SIHQIYMDAMAYDYISPTRWDEGINALTINYDFSGSHTLRSDYGSQETDTSYLNLRNGLNIGPWRLRNYS

TLNTSDGRAEYNSISTWIQRDIAALRSQIMIGDTWTASDIFDSTQIRGARLYTDNDMLPASQNGFAPVVR

GIAKSNATVIIRQNGYVIYQSAVPQGAFEITDLNTASTGGDLDVTIKEEDGSEQRFTQPYASLAILKREG

LTDVDVSVGELRDEDGFTPDVLQAQILHGFSHGITLYGGMQAAENYGSAALGVGKDLGALGAISFDVTHA

RANFSHDDTETGQSYRFLYSKLFDDTDTSLRLVGYRYSTEGYYTLNEWASRRNSPEDFWETGNRRSRVEG

TLTQSLGRDYGNLYLTLSRQQYWHTDDVERLMQFGYSSSWKRLSWNVSWSYSNTARQGTGNNHASDNTSE

QIYMLSLSVPLSGWWGNSYATYSVSQNDNSGSSHQLGLSGTALERNNLSWNLMQSYNSHDDEVGGNMSLT

YDGSYGTVNGSYNYSQNSQRLNYGIRGGILAHSEGVTLSQELGETIALVKAPGAAGLEIDNMRGAATDWR

GYTVKTQLNPYDENRVAISDNYFSKSNIELDNTVVTMVPTRGAVVKAEFVTHVGYRVLFRVLNANGKPVP

FGAIAAIQDASLADSGIVGDRGELYLSGLPEKGQVTLSWGENASTKCIFNYSFSTPESESGLIEQGVTCH

>gi|30065043|ref|NP_839214.1| phosphate ABC transporter substrate-binding protein [Shigella flexneri 2a str. 2457T]

MKVMRTTVATVVAATLSMSAFSVFAEASLTGAGATFPAPVYAKWADTYQKETGNKVNYQGIGSSGGVKQI

IANTVDFGASDAPLSDEKLAQEGLFQFPTVIGGVVLAVNIPGLKSGELVLDGKTLGDIYLGKIKKWDDEA

IAKLNPGLKLPSQNIAVVRRADGSGTSFVFTSYLAKVNEEWKNNVGTGSTVKWPIGLGGKGNDGIAAFVQ

RLPGAIGYVEYAYAKQNNLAYTKLISADGKPVSPTEENFANAAKGADWSKTFAQDLTNQKGEDAWPITST

TFILIHKDQKKPEQGTEVLKFFDWAYKTGAKQANDLDYASLPDSVVEQVRAAWKTNIKDSSGKPLY

>gi|30065032|ref|NP_839203.1| xylanase [Shigella flexneri 2a str. 2457T]

MNIKIAALTLAIASGISAQWAIAADMPASPAPTIPVKQYVTQVNADNSVTFRYFAPGAKNVSVVVGVPVP

DNIHPMTKDEAGVWSWRTPILKGNLYEYFFNVDGVRSIDTGTAMTKPQRQVNSSMILVPGSYLDTRSVAH

GDLIAITYHSNALQSERQMYVWTPPGYTGMGEPLPVLYFYHGFGDTGRSAIDQGRIPQIMDNLLAEGKIK

PMLVVIPDTETDAKGIIPEDFVPQERRKVFYPLNAKAADRELMNDIIPLISKRFSVRKDADGRALAGLSQ

GGYQALVSGMNHLESFGWLATFSGVTTTTVPDEGVAARLNDPAAINQQLRNFTVVVGDKDVVTGKDIAGL

KTELEQKKIKFDYQEYPGLNHEMDVWRPAYAAFVQKFKIRH

>gi|30065010|ref|NP_839181.1| chromosomal replication initiation protein [Shigella flexneri 2a str. 2457T]

MSLSLWQQCLARLQDELPATEFSMWIRPLQAELSDNTLALYAPNRFVLDWVRDKYLNNINGLLTSFCGAD

APQLRFEVGTKPVTQTPQAAVTSNVAAPAQVAQTQPQRAAPSTRSGWDNVPAPAEPTYRSNVNVKHTFDN

FVEGKSNQLARAAACQVADNPGGAYNPLFLYGGTGLGKTHLLHAVGNGIMARKPNAKVVYMHSERFVQDM

VKALQNNAIEEFKRYYRSVDALLIDDIQFFANKERSQEEFFHTFNALLEGNQQIILTSDRYPKEINGVED

RLKSRFGWGLTVAIEPPELETRVAILMKKADENDIRLPGEVAFFIAKRLRSNVRELEGALNRVIANANFT

GRAITIDFVREALRDLLALQEKLVTIDNIQKTVAEYYKIKVADLLSKRRSRSVARPRQMAMALAKELTNH

SLPEIGDAFGGRDHTTVLHACRKIEQLREESHDIKEDFSNLIRTLSS

>gi|30065008|ref|NP_839179.1| recombination protein F [Shigella flexneri 2a str. 2457T]

MSLTRLLIRDFRNIETADLALSPGFNFLVGANGSGKTSVLEAIYTLGHGRAFRSLQIGRVIRHEQEAFVL

HGRLQGEERETAIGLTKDKQGDSKVRIDGTDGHKVAELAHLMPMQLITPEGFTLLNGGPKYRRAFLDWGC

FHNEPGFFTAWSNLKRLLKQRNAALRQVTRYEQLRPWDKELIPLAEQISTWRAEYSAGIAADMDDTCKQF

LPEFSLTFSFQRGWEKETEYAEVLERNFERDRQLTYTAHGPHKADLRIRADGAPVEDTLSRGQLKLLMCA

LRLAQGEFLTRESGRRCLYLIDDFASELDDERRGLLASRLKATQSQVFVSAISAEHVIDMSDENSKMFTV

EKGKITD

>gi|30065003|ref|NP_839174.1| regulator protein for dgo operon [Shigella flexneri 2a str. 2457T]

MTLNKTDRIVITLGKQIVHGKYVPGSPLPAEAELCEEFATSRNIIREVFRSLMAKRLIEMKRYRGAFVAP

RNQWNYLDTDVLQWVLENDYDPRLISAMSEVRNLVEPAIARWAAERATSSDLAQIESALNEMIANNQDRE

AFNEADIRYHEAVLQSVHNPVLQQLSIAISSLQRAVFERTWMGDEANMPQTLQEHKALFDAIRHQDGDAA

EQAALTMIASSTRRLKEIT

>gi|30064994|ref|NP_839165.1| transcriptional regulator [Shigella flexneri 2a str. 2457T]

MIYKSIAERLRIRLNSADFTLNSLLPGEKKLAEEFAVSRMTIRKAIDLLVAWGLVVRRHGSGTYLVRKDV

LHQTASLTGLVEVLKRQGKTVTSQVLIFEIMPAPPAIASQLRIQINEQIYFSRRVRFVEGKPLMLEDSYM

PVKLFRNLSLQHLEGSKFEYIEQECGILIGGNYESLMPVLADRLLARQMKVAEHTPLLRITSLSYSESGE

FLNYSVMFRNASEY

>gi|30064992|ref|NP_839163.1| ARAC-type regulatory protein [Shigella flexneri 2a str. 2457T]

MNGKLQSSDVKNETPYNIPLLINENVISSGISLISLWHTYADEHYRVIWPRDKKKPLIANSWVAVYTVQG

CGKILLKNGEQITLHSNCIIFLKPMDIHSYHCEGLVWEQYWMEFTPTSMMDIPVGQQSVIYNGEIYNQEL

TEVAELITSPEAIKNNLAVAFLTKIIYQWICLMSADGKKDPQRRQIEKLIATLHASLQQRWSVADMAATI

PCSEAWLRRLFLRYTGKTPKEYYLDARLDLALSLLKQQGNSVGEVADTLNFFDSFHFSKAFKHKFGYAPS

AVLKNTDQHPTDASPHN

>gi|30064990|ref|NP_839161.1| hypothetical protein S3984 [Shigella flexneri 2a str. 2457T]

MGIIAQNKISSLGMLFGAIALMMGIIHFSFGPFSAPPPTLESIVADKTAEIKRGLLAGIKGEKITTVEKK

EDMDIDKILDQSGIALAIAALLCAFIGGMRKENRWGIRGALVFGIGIVCSILLIFLIFSFLTGGSLV

>gi|30064986|ref|NP_839157.1| multidrug resistance protein D [Shigella flexneri 2a str. 2457T]

MLVLLVAVGQMAQTIYIPAIADMARDLNVREGAVQSVMGAYLLTYGVSQLFYGPISDRVGRRPVILVGMS

IFMLATLVAVTTSSLTVLIAASAMQGMGTGVGGVMARTLPRDLYERTQLRHANSLLNMGILVSPLLAPLI

GGLLDTMWNWRACYLFLLVLCAGVTFSMARWMPETRPVDAPRTRLLTSYKTLFGNSGFNCYLLMLIGGLA

GIAAFEACSGVLMGAVLGLSSMTVSILFILPIPAAFFGAWFAGRPNKRFSTLMWQSVICCLLAGLLMWIP

DWFGVMNVWTLLVPAALFFFGAGMLFPLATSGAMEPFPFLAGTAGALVGGLQNIGSGVLASLSAMLPQTG

QGSLGLLMTLMGLLIVLCWLPLATRMSHQGQPV

>gi|30064977|ref|NP_839148.1| hypothetical protein S3971 [Shigella flexneri 2a str. 2457T]

MIIITEPLLSFVLQKQGIKSPPMDKKMNNDNTDYVSNESGTLSRLFKLPQHGTTVRTELIAGMTTFLTMV

YIVFVNPQILGAAQMDPKVVFVTTCLIAGIGSIAMGIFANLPVALAPAMGLNAFFAFVVVGAMGISWQTG

MGAIFWGAIGLFLLTLFRIRYWMISNIPLSLRIGITSGIGLFIALMGLKNTGVIVANKDTLVMIGDLSSH

GVLLGILGFFIITVLSSRHFHAAVLVSIVVTSCCGLFFGDVHFSGVYSIPPDISGVIGEVDLSGALTLEL

AGIIFSFMLINLFDSSGTLIGVTDKAGLIDSNGKFPNMNKALYVDSVSSVAGAFIGTSSVTAYIESTSGV

AVGGRTGLTAVVVGVMFLLVMFFSPLVAMVPPYATAGALIFVGVLMTSSLARVNWDDFTESVPAFITTVM

MPFTFSITEGIALGFMSYCIMKVCTGRWRDLNLCVVVVAALFALKIILVD

>gi|30064965|ref|NP_839136.1| bifunctional N-acetylglucosamine-1-phosphate uridyltransferase/glucosamine-1-phosphate acetyltransferase [Shigella flexneri 2a str. 2457T]

MLNNAMSVVILAAGKGTRMYSDLPKVLHTLAGKAMVQHVIDAANELGAAHVHLVYGHGGDLLKQALKDDN

LNWVLQAEQLGTGHAMQQAAPFFADDEDILMLYGDVPLISVETLQRLRDAKPQGGIGLLTVKLDDPTGYG

RITRENGKVTGIVEHKDATDEQRQIQEINTGILIANGADMKRWLAKLTNNNAQGEYYITDIIALAYQEGR

EIVAVHPQRLSEVEGVNNRLQLSRLERVYQSEQAEKLLLAGVMLRDPARFDLRGTLTHGRDVEIDTNVII

EGNVTLGHRVKIGTGCVIKNSVIGDDCEISPYTVVEDANLAAACTIGPFARLRPGAELLEGAHVGNFVEM

KKARLGKGTKAGHLTYLGDAEIGDNVNIGAGTITCNYDGANKFKTIIGDDVFVGSDTQLVAPVTVGKGAT

IAAGTTVTRNVGENALAISRVPQTQKEGWRRPVKKK

>gi|30064964|ref|NP_839135.1| F0F1 ATP synthase subunit epsilon [Shigella flexneri 2a str. 2457T]

MTYHLDVVSAEQQMFSGLVEKIQVTGSEGELGIYPGHAPLLTAIKPGMIRIVKQHGHEEFIYLSGGILEV

QPGNVTVLADTAIRGQDLDEARAMEAKRKAEEHISSSHGDVDYAQASAELAKAIAQLRVIELTKKAM

>gi|30064959|ref|NP_839130.1| F0F1 ATP synthase subunit B [Shigella flexneri 2a str. 2457T]

MNLNATILGQAIAFVLFVLFCMKYVWPPLMAAIEKRQKEIADGLASAERAHKDLDLAKASATDQLKKAKA

EAQVIIEQANKRRSQILDEAKAEAEQERTKIVAQAQAEIEAERKRAREELRKQVAILAVAGAEKIIERSV

DEAANSDIVDKLVAEL

>gi|30064957|ref|NP_839128.1| F0F1 ATP synthase subunit A [Shigella flexneri 2a str. 2457T]

MASENMTPQDYIGHHLNNLQLDLRTFSLVDPQNPPATFWTINIDSMFFSVVLGLLFLVLFRSVAKKATSG

VPGKFQTAIELVIGFVNGSVKDMYHGKSKLIAPLALTIFVWVFLMNLMDLLPIDLLPYIAEHVLGLPALR

VVPSADVNVTLSMALGVFILILFYNIKMKGIGGFTKELTLQPFNHWAFIPVNLILEGVSLLSKPVSLGLR

LFGNMYAGELIFILIAGLLPWWSQWILNVPWAIFHILIITLQAFIFMVLTIVYLSMASEEH

>gi|30064941|ref|NP_839112.1| transcriptional repressor RbsR [Shigella flexneri 2a str. 2457T]

MKDVARLAGVSTSTVSHVINKDRFVSEAITAKVEAAIKELNYAPSALARSLKLNQTHTIGMLITASTNPF

YSELVRGVERSCFERGYSLVLCNTEGDEQRMNRNLETLMQKRVDGLLLLCTETHQPSREIMQRYPTVPTV

MMDWAPFDGDSDLIQDNSLLGGDLATQYLIDKGHTRIACITGPLDKTPARLRLEGYRAAMKRAGLNIPDG

YEVTGDFEFNGGFDAMRQLLSHPLRPQAVFTGNDAMAVGVYQALYQAELQVPQDIAVIGYDDIELASFMT

PPLTTIHQPKDELGELAIDVLIHRITQPTLQQQRLQLTPILMERGSA

>gi|30064937|ref|NP_839108.1| hypothetical protein S3920 [Shigella flexneri 2a str. 2457T]

MAESFTTTNRYFDNKHYPRGFSRHGDFTIKEAQLLERHGYAFNELDLGKREPVTEEEKLFVAVCRGEREP

VTEAERVWSKYMTRIKRPKRFHTLSGGKPQVEGAEDYTDSDD

>gi|30064927|ref|NP_839098.1| ATP-dependent DNA helicase Rep [Shigella flexneri 2a str. 2457T]

MRLNPGQQQAVEFVTGPCLVLAGAGSGKTRVITNKIAHLIRGCGYQARHIAAVTFTNKAAREMKERVGQT

LGRKEAHGLMISTFHTLGLDIIKREYAALGMKANFSLFDDTDQLALLKELTEGLIEDDKVLLQQLISTIS

NWKNDLKTPSQAAASAIGERDRIFAHCYGLYDAHLKACNVLDFDDLILLPTLLLQRNEEVRERWQNKIRY

LLVDEYQDTNTSQYELVKLLVGSRARFTVVGDDDQSIYSWRGARPQNLVLLSQDFPALKVIKLEQNYRSS

GRILKAANILIANNPHVFEKRLFSELGYGTELKVLSANNEEHEAERVTGELIAHHFVNKTQYKDYAILYR

GNYQSRVFEKFLMQNRIPYKISGGTSFFSRPEIKDLLAYLRVLTNPDDDSAFLRIVNTPKREIGPATLKK

LGEWAMTRNKSMFTASFDMGLSQTLSGRGYEALTRFTHWLAEIQRLAEREPIAAVRDLIHGMDYESWLYE

TSPSTKAAEMRMKNVNQLFSWMTEMLEGSELDEPMTLTQVVTRFTLRDMMERGESEEELDQVQLMTLHAS

KGLEFPYVYMVGMEEGFLPHQSSIDEDNIDEERRLAYVGITRAQKELTFTLCKERRQYGELVRPEPSRFL

LELPQDDLIWEQERKVVSAEERMQKGQSHLANLKAMMAAKRGK

>gi|30064926|ref|NP_839097.1| guanosine pentaphosphate phosphohydrolase [Shigella flexneri 2a str. 2457T]

MGSTSSLYAAIDLGSNSFHMLVVREVAGSIQTLTRIKRKVRLAAGLNSENDLSNEAMERGWQCLRLFAER

LQDIPPSQIRVVATATLRLAVNAGDFIAKAQEILGCPVQVISGEEEARLIYQGVAHTTGGADQRLVVDIG

GASTELVTGTGAQTTSLFSLSMGCVTWLERYFADRNLGQENFDAAEKAAREVLRPVADELRYHGWKVCVG

ASGTVQALQEIMMAQGMDERITLEKLQQLKQRAIHCGRLEELEIDGLTLERALVFPSGLAILIAIFTELN

IQCMTLAGGALREGLVYGMLHLAVEQDIRSRTLRNIQRRFMIDIDQAQRVAKVAANFFDQVENEWHLEAI

SRDLLISACQLHEIGLSVDFKQAPQHAAYLVRNLDLPGFTPAQKKLLATLLLNQTNPVDLSSLHQQNAVP

PRVAEQLCRLLRLAIIFASRRRDDLVPEMTLQANHELLTLTLPQGWLTQHPLGKEIIAQENQWQSYVHWP

LEVH

>gi|30064922|ref|NP_839093.1| transcription termination factor Rho [Shigella flexneri 2a str. 2457T]

MNLTELKNTPVSELITLGENMGLENLARMRKQDIIFAILKQHAKSGEDIFGDGVLEILQDGFGFLRSADS

SYLAGPDDIYVSPSQIRRFNLRTGDTISGKIRPPKEGERYFALLKVNEVNFDKPENARNKILFENLTPLH

ANSRLRMERGNGSTEDLTARVLDLASPIGRGQRGLIVAPPKAGKTMLLQNIAQSIAYNHPDCVLMVLLID

ERPEEVTEMQRLVKGEVVASTFDEPASRHVQVAEMVIEKAKRLVEHKKDVIILLDSITRLARAYNTVVPA

SGKVLTGGVDANALHRPKRFFGAARNVEEGGSLTIIATALIDTGSKMDEVIYEEFKGTGNMELHLSRKIA

EKRVFPAIDYNRSGTRKEELLTTQEELQKMWILRKIIHPMGEIDAMEFLINKLAMTKTNDDFFEMMKRS

>gi|30064914|ref|NP_839085.1| TDP-4-oxo-6-deoxy-D-glucose transaminase [Shigella flexneri 2a str. 2457T]

MIPFNAPPVVGTELDYMQSAMGSGKLCGDGGFTRRCQQWLEQRFGSAKVLLTPSCTASLEMAALLLDIQP

GDEVIMPSYTFVSTANAFVLRGAKIVFVDVRPDTMNIDETLIEAAITDKTRVIVPVHYAGVACEMDTIMA

LAKKHNLFVVEDAAQGVMSTYKGRALGTIGHIGCFSFHETKNYTAGGEGGATLINDKALIERAEIIREKG

TNRSQFFRGQVDKYTWRDIGSSYLMSDLQAAYLWAQLEAADRINQQRLALWQNYYDTLAPLAKAGRIELP

SIPDGCVQNAHMFYIKLRDIDDRSALINFLKEAEIMAVFHYIPLHGCPAGEHFGEFHGEDRYTTKESERL

LRLPLFYNLSPVNQRTVIATLLNYFS

>gi|30064911|ref|NP_839082.1| common antigen polymerase [Shigella flexneri 2a str. 2457T]

MSLLQFSGLFVVWLLCTLFIATLTWFEFRRVRFNFNVFFSLLFLLTFFFGFPLTSVLVFRFDVGVAPPEI

LLQVLLSAGCFYAVYYVTYKTRLRKRVADVPRRPLFTMNRVETNLTWVILMGIALVSVGIFFMHNGFLLF

RLNSYSQIFSSEVSGVALKRFFYFFIPAMLVVYFLRQDSKAWLFFLVSTVAFGLLTYMIVGGTRANIIIA

FAIFLFIGIIRGWISLWMLAAAGVLGIVGMFWLALKRYGMNVSGDEAFYTFLYLTRDTFSPWENLALLLQ

NYDNIDFQGLAPIVRDFYVFIPSWLWPGRPSMVLNSANYFTWEVLNNHSGLAISPTLIGSLVVMGGALFI

PLGAIVVGLIIKWFDWLYELGNRETNRYKAAILHSFCFGAIFNMIVLAREGLDSFVSRVVFFIVVFGACL

MIAKLLYWLFESAGLIHKRTKSSLRTQVEG

>gi|30064903|ref|NP_839074.1| uroporphyrinogen-III synthase [Shigella flexneri 2a str. 2457T]

MSILVTRPSPAGEELVSRLRTLGQVAWHFPLIEFSPGRQLPQLADQLAALGESDLLFALSQHAVAFAQSQ

LHQQDRKWPQLPTYFAIGRTTALALHTVSGQKILYPQDREISEVLLQLPELQNIAGKRALILRGNGGREL

IGDTLTVRGAEVTFCECYQRCAIHYDGAEEAMRWQSREVTMVVVTSGEMLQQLWSLIPQWYREHWLLHCR

LLVVSERLAKLARELGWQDIKVADNADNDALLRALQ

>gi|30064893|ref|NP_839064.1| site-specific tyrosine recombinase XerC [Shigella flexneri 2a str. 2457T]

MTDLHTDVERYLRYLSVERQLSPITLLNYQRQLEAIINFASENGLQSWQQCDAAMVRNFAVRSRRKGLGA

ASLALRLSALRSFFDWLVSQNELKANPAKGVSAPKAPRHLPKNIDVDDMNRLLDIDINDPLAVRDRAMLE

VMYGAGLRLSELVGLDIKHLDLESGEVWVMGKGSKERRLPIGRNAVAWIEHWLDLRDLFGSEDDALFLSK

LGKRISARNVQKRFAEWGIKQGLNNHVHPHKLRHSFATHMLESSGDLRGVQELLGHANLSTTQIYTHLDF

QHLASVYDAAHPRAKRGK

>gi|30064891|ref|NP_839062.1| DNA-dependent helicase II [Shigella flexneri 2a str. 2457T]

MDVSYLLDSLNDKQREAVAAPRSNLLVLAGAGSGKTRVLVHRIAWLMSVENCSPYSIMAVTFTNKAAAEM

RHRIGQLMGTSQGGMWVGTFHGLAHRLLRAHHMDANLPQDFQILDSEDQLRLLKRLIKAMNLDEKQWPPR

QAMWYINSQKDEGLRPHHIQSYGNPVEQTWQKVYQAYQEACDRAGLVDFAELLLRAHELWLNKPHILQHY

RERFTNILVDEFQDTNNIQYAWIRLLAGDTGKVMIVGDDDQSIYGWRGAQVENIQRFLNDFPGAEIIRLE

QNYRSTSNILSAANALIENNNGRLGKKLWTDGADGEPISLYCAFNELDEARFVVNRIKTWQDNGGALAEC

AILYRSNAQSRVLEEALLQASMPYRIYGGMRFFERQEIKDALSYLRLIANRNDDAAFERVVNTPTRGIGD

RTLDVVRQTSRDRQLTLWQACRELLQEKALAGRAASALQRFMELIDALAQETADMPLHVQTDRVIKDSGL

RTMYEQEKGEKGQTRIENLEELVTATRQFSYNEEDEDLMPLQAFLSHAALEAGEGQADTWQDAVQLMTMH

SAKGLEFPQVFIVGMEEGMFPSQMSLDEGGRLEEERRLAYVGVTRAMQKLTLTYAETRRLYGKEVYHRPS

RFIGELPEECVEEVRLRATVSRPVSHQRMGTPMVENDSGYKLGQRVRHAKFGEGTIVNMEGSGEHSRLQV

AFQGQGIKWLVAAYARLESV

>gi|30064887|ref|NP_839058.1| magnesium/nickel/cobalt transporter CorA [Shigella flexneri 2a str. 2457T]

MLSAFQLENNRLTRLEVEESQPLVNAVWIDLVEPDDDERLRVQSELGQSLATRPELEDIEASARFFEDDD

GLHIHSFFFFEDAEDHAGNSTVAFTIRDGRLFTLRERELPAFRLYRMRARSQSMVDGNAYELLLDLFETK

IEQLADEIENIYSDLEQLSRVIMEGHQGDEYDEALSTLAELEDIGWKVRLCLMDTQRALNFLVRKARLPG

GQLEQAREILRDIESLLPHNESLFQKVNFLMQAAMGFINIEQNRIIKIFSVVSVVFLPPTLVASSYGMNF

EFMPELKWSFGYPGAIIFMILAGLAPYLYFKRKNWL

>gi|30064869|ref|NP_839040.1| hypothetical protein S3842 [Shigella flexneri 2a str. 2457T]

MPFKPLVTAGIESLLNTFLYRSPALKTARSRLLGKVLRVEVKGFSTSLILVFSERQVDVLGEWAGDADCT

VIAYASVLPKLRDRQQLTALIRSGELEVQGDIQVVQNFVALADLAEFDPAELLAPYTGDIAAEGISKAMR

GGAKFLHHGIKRQQRYVAEAITEEWRMAPGPLEVAWFAEETAAVERAVDALTKRLEKLEAK

>gi|30064862|ref|NP_839033.1| 3-octaprenyl-4-hydroxybenzoate carboxy-lyase [Shigella flexneri 2a str. 2457T]

MDAMKYNDLRDFLTLLEQQGELKRITLPVDPHLEITEIADRTLRAGGPALLFENPKGYSMPVLCNLFGTP

KRVAMGMGQEDVSALREVGKLLAFLKEPEPPKGFRDLFDKLPQFKQVLNMPTKRLRGAPCQQKIVSGDDV

DLNRIPIMTCWPEDAAPLITWGLTVTRGPHKERQNLGIYRQQLIGKNKLIMRWLSHRGGALDYQEWCAAH

PGERFPVSVALGADPATILGAVTPVPDTLSEYAFAGLLRGTKTEVVKCISNDLEVPASAEIVLEGYIDPG

EMAPEGPYGDHTGYYNEVDNFPVFTVTHITQREDAIYHSTYTGRPPDEPAVLGVALNEVFVPILQKQFPE

IVDFYLPPEGCSYRLAVVTIKKQYAGHAKRVMMGVWSFLRQFMYTKFVIVCDDDVNARDWNDVIWAITTR

MDPARDTVLVENTPIDYLDFASPVSGLGSKMGLDATNKWPGETQREWGRPIKKDPDVVAHIDAIWDELAI

FNNGKSA

>gi|30064855|ref|NP_839026.1| protoporphyrinogen oxidase [Shigella flexneri 2a str. 2457T]

MKTLILFSTRDGQTREIASYLASELKELGIQTDVANVHRIEEPQWENYDRVVIGASIRYGHYHSAFQEFV

KKHATRLNSMPSAFYSVNLVARKPEKRTPQTNSYARKFLMNSQWRPDRCAVIAGALRYPRYRWYDRFMLK

LIMKMSGGETDTRKEVVYTDWEQVANFAREIAHLTDKPTLK

>gi|30064850|ref|NP_839021.1| periplasmic protein disulfide isomerase I [Shigella flexneri 2a str. 2457T]

MKKIWLALAGLVLAFSASAAQYEDGKQYTTLEKPVAGAPQVLEFFSFFCPHCYQFEEVLHISDNVKKKLP

EGVKMTKYHVNFMGGDLGKDLTQAWAVAMALGVEDKVTVPLFEGVQKTQTIRSASDIRDVFINAGIKGEE

YDAAWNSFVVKSLVAQQEKAAADVQLRGVPAMFVNGKYQLNPQGMDTSNMDVFVQQYADTVKYLSEEK

>gi|30064849|ref|NP_839020.1| hypothetical protein S3815 [Shigella flexneri 2a str. 2457T]

MDIQSFAVLSGNIYMIRKSATGVIVALAVIWGGGTWYTGTQIQPGIEKFIKDFNDAKKKGEHAYDMTLSY

KNFDKGFFNSRFQMQMTFDNGAPDLNIKPGQKVVFDVDVEHGPLPITMLMHGNVIPALAAAKVNLVNNEL

TQPLFIAAKNKSPVEATLRFAFGGSFSTTLDVAPAEYGKFSFGEGQFTFNGDGSSLSNLDIEGKVEDIVL

QLSPMNKVTAKSFTIDSLARLEEKKFPVGESESKFNQINIINHGEDVAQIDAFVAKTRLDRVKDKDYINV

NLTYELDKLTKGNQQLGSGEWSLIAESIDPSAVRQFIIQYNIAMQKQLAAHPELANDEVALQEVNAALFK

EYLPLLQKSEPTIKQPVRWKNALGELNANLDISIADPAKSSSSTNKDIKSLNFDVKLPLNVVTETAKQLN

LSEGMDAEKAQKQADKQISGMMTLGQMFQLITIDNNTASLQLRYTPGKVVFNGQEMSEEEFMSRAGRFVH

>gi|30064843|ref|NP_839014.1| nitrogen regulation protein NR(I) [Shigella flexneri 2a str. 2457T]

MQRGIVWVVDDDSSIRWVLERALAGAGLTCTTFENGAEVLEALASKTPDVLLSDIRMPGMDGLALLKQIK

QRHPMLPVIIMTAHSDLDAAVSAYQQGAFDYLPKPFDIDEAVALVERAISHYQEQQQPRNIQLNGPTTDI

IGEAQAMQDVFRIIGRLSRSSISVLINGESGTGKELVAHALHRHSPRTKAPFIALNMAAIPKDLIESELF

GHEKGAFTGANTIRQGRFEQADGGTLFLDEIGDMPLDVQTRLLRVLADGQFYRVGGYAPVKVDVRIIAAT

HQNLEQRVQEGKFREDLFHRLNVIRVHLPPLRERREDIPRLARHFLQVAARELGVEAKLLHPETEAALTR

LAWPGNVRQLENTCRWLTVMAAGQEVLIQDLPGELFESTVAESTSQMQPDSWATLLAQWADRALRSGHQN

LLSEAQPELERTLLTTALRHTQGHKQEAARLLGWGRNTLTRKLKELGME

>gi|30064842|ref|NP_839013.1| nitrogen regulation protein NR(II) [Shigella flexneri 2a str. 2457T]

MATGTQPDAGQILNSLINSILLIDDNLAIHYANPAAQQLLAQSSRKLFGTPLPELLSYFSLNIELMQESL

EAGQGFTDNEVTLVIDGRSHILSVTAQRMPDGMILLEMAPMDNQRRLSQEQLQHAQQVAARDLVRGLAHE

IKNPLGGLRGAAQLLSKALPDPSLLEYTKVIIEQADRLRNLVDRLLGPQLPGTRVTESIHKVAERVVTLV

SMELPDNVRLIRDYDPSLPELAHDPDQIEQVLLNIVRNALQALGPEGGEIILRTRTAFQLTLHGERYRLA

ARIDVEDNGPGIPPHLQDTLFYPMVSGREGGTGLGLSIARNLIDQHSGKIEFTSWPGHTEFSVYLPIRK

>gi|30064839|ref|NP_839010.1| transcriptional regulator [Shigella flexneri 2a str. 2457T]

MAENQSTVENAKEKLDRWLKDGITTPGGKLPSERELGELLGIKRMTLRQALLNLEAESKIFRKDRKGWFV

TQPRFNYSPELSASFQRAAIEQGREPSWGFTEKNRTSDIPETLAPLIAVTPSTELYRITGWGALEGHKVF

YHETYINPEVAPGFIEQLENHSFSAVWEKCYQKETVVKKLIFKPVRMPGDISKYLGGSAGMPAILIEKHR

ADQQGNIVQIDIEYWRFEAVDLIINL

>gi|30064814|ref|NP_838985.1| formate dehydrogenase-O, iron-sulfur subunit [Shigella flexneri 2a str. 2457T]

MAYQSQDIIRRSATNGLTPAPQARDFQEEVAKLIDVTTCIGCKACQVACSEWNDIRDTVGNNIGVYDNPN

DLSAKSWTVMRFSEVEQNDKLEWLIRKDGCMHCSDPGCLKACPAEGAIIQYANGIVDFQSEQCIGCGYCI

AGCPFDIPRLNPEDNRVYKCTLCVDRVVVGQEPACVKTCPTGAIHFGTKESMKTLASERVAELKTRGYDN

AGLYDPAGVGGTHVMYVLHHADKPNLYHGLPENPEISETVKFWKGIWKPLAAVGFAATFAASIFHYVGVG

PNRADEEENNLHEEKDEERK

>gi|30064804|ref|NP_838975.1| rhamnulose-1-phosphate aldolase, partial [Shigella flexneri 2a str. 2457T]

MPLLANTPFIVTGSGKFFRNVQLDPAANLGVVKVDSDGAGYHILWGLTNEAVPTSELPAHFLSHCERIKA

TNGKDRVIMHCHATNLIALTYVLENDTAVFTRQLWEGSTECLVVFPDGVGILPWMVPGTDEIGQATAQEM

QKHSLVLWPFHGVFGSGPTLDEAFGLIDTAEKSAQVLVKVYSMGGMKQTISREELIALGKRFGVTPLASA

LAL

>gi|30064794|ref|NP_838965.1| two-component sensor protein [Shigella flexneri 2a str. 2457T]

MIGSLTARIFAIFWLTLVLVLMLVLMLPKLDSRQMTELLDSEQRQGLMIEQHVEAELANDPPNDLMWWRR

LFRAIDKWAPPGQRLLLVTTEGRVIGAERSEMQIIRNFIGQADNADHPQKKKYGRVELVGPFSVRDGEDN

YQLYLIRPASSSQSDFINLLFDRPLLLLIVTMLVSTPLLLWLAWSLAKPARKLKNAADEVAQGNLRQHPE

LEAGPQEFLAAGASFNQMVTALERMMTSQQRLLSDISHELRTPLTRLQLGTALLRRRSGESKELERIETE

AQRLDSMINDLLVMSRNQQKNALVSETIKANQLWSEVLDNAAFEAEQMGKSLTVNFPPGPWPLYGNPNAL

ESALENIVRNALRYSHTKIEVGFAVDKDGITITVDDDGPGVSPEDREQIFRPFYRTDEARDRESGGTGLG

LAIVETAIQQHRGWVKAEDSPLGGLRLVIWLPLYKRS

>gi|30064793|ref|NP_838964.1| DNA-binding transcriptional regulator CpxR [Shigella flexneri 2a str. 2457T]

MNKILLVDDDRELTSLLKELLEMEGFNVIVAHDGEQALDLLDDSIDLLLLDVMMPKKNGIDTLKALRQTH

QTPVIMLTARGSELDRVLGLELGADDYLPKPFNDRELVARIRAILRRSHWSEQQQNNDNGSPTLEVDALV

LNPGRQEASFDGQTLELTGTEFTLLYLLAQHLGQVVSREHLSQEVLGKRLTPFDRAIDMHISNLRRKLPD

RKDGHPWFKTLRGRGYLMVSAS

>gi|30064784|ref|NP_838955.1| universal stress protein UspD [Shigella flexneri 2a str. 2457T]

MAYKHIGVAISGNEEDALLVNKALELARHNDAHLTLIHIDDGLSELYPGIYFPATEDILQLLKNKSDNKL

YKLTKNIQWPKTKLRIERGEMPETLLEIMQKEQCDLLVCGHHHSFINRLMPAYRGMINKMSADLLIVPFI

DK

>gi|30064775|ref|NP_838946.1| ATP-dependent protease peptidase subunit [Shigella flexneri 2a str. 2457T]

MTTIVSVRRNGHVVIAGDGQATLGNTVMKGNVKKVRRLYNDKVIAGFAGGTADAFTLFELFERKLEMHQG

HLVKAAVELAKDWRTDRMLRKLEALLAVADETASLIITGNGDVVQPENDLIAIGSGGPYAQAAARALLEN

TELSAREIAEKALDIAGDICIYTNHFHTIEELSYKA

>gi|30064773|ref|NP_838944.1| DNA-binding transcriptional regulator CytR [Shigella flexneri 2a str. 2457T]

MKAKKQETAATMKDVALKAKVSTATVSRALMNPDKVSQATRNRVEKAAREVGYLPQPMGRNVKRNESRTI

LVIVPDICDPFFSEIIRGIEVTAANHGYLVLIGDCAHQNQQEKTFIDLIITKQIDGMLLLGSRLPFDASI

EEQRNLPPMVMANEFAPELELPTVHIDNLTAAFDAVNYLYEQGHKRIGCIAGPEEMPLCHYRLQGYVQAL

RRCGIMVDPQYIARGDFTFEAGSKAMQQLLDLPQPPTAVFCHSDVMALGALSQAKRQGLKVPEDLSIIGF

DNIDLTQFCDPPLTTIAQPRYEIGREAMLLLLDQMQGQHVGSGSRLMDCELIIRGSTRALP

>gi|30064772|ref|NP_838943.1| primosome assembly protein PriA [Shigella flexneri 2a str. 2457T]

MPVAHVALPVPLPRTFDYLLPEGMTVKAGCRVRVPFGKQQERIGIVVSVSDASELPLTELKAVVEVLDGE

PVFTHSVWRLLLWAADYYHHPIGDVLFHALPILLRQGRPAANAPMWYWFATEQGQAVDLNSLKRSPKQQQ

ALAALRQGKIWRDQVATLEFNDAALQALRKKGLCDLASETPEFSDWRTNYAVSGERLRLNTEQATAVGAI

HSAADTFSAWLLAGVTGSGKTEVYLSVLENVLAQGKQALVMVPEIGLTPQTIARFRERFNAPVEVLHSGL

NDSERLSAWLKAKNGEAAIVIGTRSALFTPFKNLGVIVIDEEHDSSYKQQEGWRYHARDLAVYRAHSEQI

PIILGSATPALETLCNVQQKKYRLLRLTRRAGNARPAIQHVLDLKGQKVQAGLAPALITRMRQHLQANNQ

VILFLNRRGFAPALLCHDCGWIAECPRCDHYYTLHQAQQHLRCHHCDSQRPVPRQCPSCGSTHLVPVGLG

TEQLEQTLAPLFPDVPISRIDRDTTSRKGALEQQLAEVHRGGARILIGTQMLAKGHHFPDVTLVALLDVD

GALFSADFRSAERFAQLYTQVAGRAGRAGKQGEVVLQTHHPEHPLLQTLLYKGYDAFAEQALAERRMMQL

PPWTSHVIVRAEDHNNQHAPLFLQQLRNLILSSPLADDKLWVLGPVPALAPKRGGRWRWQILLQHPSRVR

LQHIISGTLALINTIPDSRKVKWVLDVDPIEG

>gi|30064767|ref|NP_838938.1| bifunctional aspartate kinase II/homoserine dehydrogenase II [Shigella flexneri 2a str. 2457T]

MSVIAQAGAKGRQLHKFGGSSLADVKCYLRVAGIMAEFSQPDDMMVVSAAGSTTNQLINWLKLSQTDRLS

AHQVQQTLRRYQCDLISGLLPAEEADSLISAFVSDLERLAALLDSGINDAVYAEVVGHGEVWSARLMSAV

LNQQGLPAAWLDAREFLRAERAAQPQVDEGLSYPLLQQLLVQHPSKRLVVTGFISRNNAGETVLLGRNGS

DYSATQIGALAGVSRVTIWSDVAGVYSADPRKVKDACLLPLLRLDEASELARLAAPVLHARTLQPVSGSE

IDLQLRCSYTPDQGSTRIERVLASGTGARIVTSHDDVCLIEFQVPASQDFKLAHKEIDQILKRAQVRPLA

VGVHNDRQLLQFCYTSEVADSALKILDEAGLPGELRLRQGLALVAMVGAGVTRNPLHCHRFWQQLKGQPV

EFTWQSDDGISLVAVLRTGPTESLIQGLHQSVFRAEKRIGLVLFGKGNIGSRWLELFAREQSTLSARTGF

EFVLAGVVDSRRSLLSYDGLDASRALAFFNDEAVEQDEESLFLWMRAHPYDDLVVLDVTASQQLADQYLD

FASHGFHVISANKLAGASDSNKYRQIHDAFEKTGRHWLYNATVGAGLPINHTVRDLIDSGDTILSISGIF

SGTLSWLFLQFDGSVPFTELVDQAWQQGLTEPDPRDDLSGKDVMRKLVILAREAGYNIEPDQVRVESLVP

AHCEGGSIDHFFENGDELNEQMVQRLEAAREMGLVLRYVARFDANGKARVGVEAVREDYPLASLLPCDNV

FAIESRWYRDNPLVIRGPGAGRDVTAGAIQSDINRLAQLL

>gi|30064765|ref|NP_838936.1| catalase; hydroperoxidase HPI(I) [Shigella flexneri 2a str. 2457T]

MSTSDDIHNTTATGKCPFHQGGHDQSAGAGTTTRDWWPNQLRVDLLNQHSNRSNPLGEDFDYRKEFSKLD

YYGLKKDLKALLTESQPWWPADWGSYAGLFIRMAWHGAGTYRSIDGRGGAGRGQQRFAPLNSWPDNVSLD

KARRLLWPIKQKYGQKISWADLFILAGNVALENSGFRTFGFGAGREDVWEPDLDVNWGDEKAWLTHRHPE

ALAKAPLGATEMGLIYVNPEGPDHSGEPLSAAAAIRATFGNMGMNDEETVALIAGGHTLGKTHGAGPTSN

VGPDPEAAPIEEQGLGWASTYGSGVGADAITSGLEVVWTQTPTQWSNYFFENLFKYEWVQTRSPAGAIQF

EAVDAPEIIPDPFDPSKKRKPTMLVTDLTLRFDPEFEKISRRFLNDPQAFNEAFARAWFKLTHRDMGPKS

RYIGPEVPKEDLIWQDPLPQPIYNPTEQDIIDLKFAIADSGLSVSELVSVAWASASTFRGGDKRGGANGA

RLALMPQRDWDVNAAAVRALPVLEKIQKESGKASLADIIVLAGVVGVEKAASAAGLSIHVPFAPGRVDAR

QDQTDIEMFELLEPIADGFRNYRARLDVSTTESLLIDKAQQLTLTAPEMTALVGGMRVLGANFDGSKNGV

FTDRVGVLSNDFFVNLLDMRYEWKATDESKELFEGRDRETGEVKYTASRADLVFGSNSVLRAVAEVYASS

DAHEKFVKDFVAAWVKVMNLDRFDLL

>gi|30064763|ref|NP_838934.1| fructose-like phosphotransferase EIIB subunit 2 [Shigella flexneri 2a str. 2457T]

MTKIIAVTACPSGVAHTYMAAEALESAAKAKGWEVKVETQGSIGLENELTAEDVASADMVILTKDIGIKF

EERFAGKTIVRVNISDAVKRADAIMSKIEAHLAQTA

>gi|30064760|ref|NP_838931.1| fructose-like phosphotransferase EIIB subunit 3 [Shigella flexneri 2a str. 2457T]

MAYLVAVTACVSGVAHTYMAAERLEKLCQLEKWGVSIETQGALGTENRLADEDIRRADVALLITDIELAG

AERFEHCRYVQCSIYAFLREPQRVMSAVRKVLSAPQQTHLILE

>gi|30064755|ref|NP_838926.1| N-acetyl-gamma-glutamyl-phosphate reductase [Shigella flexneri 2a str. 2457T]

MLNTLIVGASGYAGAELVTYVNRHPHMNITALTVSAQSNDAGKLISDLHPQLKGIVELPLQPMSDISEFS

PGVDVVFLATAHEVSHDLAPQFLEAGCVVFDLSGAFRVNDATFYEKYYGFTHQYPELLEQAAYGLAEWCG

NKLKEANLIAVPGCYPTAAQLALKPLIDADLLDLNQWPVINATSGVSGAGRKAAISNSFCEVSLQPYGVF

THRHQPEIATHLGADVIFTPHLGNFPRGILETITCRLKSGVTQAQVAQALQQAYAHKPLVRLYDKGVPAL

KNVVGLPFCDIGFAVQGEHLIIVATEDNLLKGAAAQAVQCANIRFGYAETQSLI

>gi|30064741|ref|NP_838912.1| UDP-N-acetylenolpyruvoylglucosamine reductase [Shigella flexneri 2a str. 2457T]

MNHSLKPWNTFGIDHNAQHIVCAEDEQQLLNAWQHATAEGQPVLILGEGSNVLFLEDYRGTVIINRIKGI

EIHDEPDAWYLHVGAGENWHRLVKYTLQEGMPGLENLALIPGCVGSSPIQNIGAYGVELQRVCAYVDCVE

LATGKQVRLTAKECRFGYRDSIFKHEYQDRFAIVAVGLRLPKEWQPVLTYGDLTRLDPTTVTPQQVFNAV

CHMRTTKLPDPKVNGNAGSFFKNPVVSAETAKALLAQFPTAPNYPQAGGSVKLAAGWLIDQCQLKGMQMG

GAAVHRQQALVLINEDNAKSEDVVQLAHHVRQKVGEKFNVWLEPEVRFIGASGEVSAVETIS

>gi|30064736|ref|NP_838907.1| preprotein translocase subunit SecE [Shigella flexneri 2a str. 2457T]

MSANTEAQGSGRGLEAMKWVVVVALLLAAIVGNYLYRDIMLPLRALAVVILIAAAGGVALLTTKGKATIA

FAREARTEVRKVIWPTRQETLHTTLIVAAVTAVMSLILWGLDGILVRLVSFITGLRF

>gi|30064735|ref|NP_838906.1| transcription antitermination protein NusG [Shigella flexneri 2a str. 2457T]

MSEAPKKRWYVVQAFSGFEGRVATSLREHIKLHNMEDLFGEVMVPTEEVVEIRGGQRRKSERKFFPGYVL

VQMVMNDASWHLVRSVPRVMGFIGGTSDRPAPISDKEVDAIMNRLQQVGDKPRPKTLFEPGEMVRVNDGP

FADFNGVVEEVDYEKSRLKVSVSIFGRATPVELDFSQVEKA

>gi|30064733|ref|NP_838904.1| 50S ribosomal protein L1 [Shigella flexneri 2a str. 2457T]

MAKLTKRMRVIREKVDATKQYDINEAIALLKELATAKFVESVDVAVNLGIDARKSDQNVRGATVLPHGTG

RSVRVAVFTQGANAEAAKAAGAELVGMEDLADQIKKGEMNFDVVIASPDAMRVVGQLGQVLGPRGLMPNP

KVGTVTPNVAEAVKNAKAGQVRYRNDKNGIIHTTIGKVDFDADKLKENLEALLVALKKAKPTQAKGVYIK

KVSISTTMGAGVAVDQAGLSASVN

>gi|30064732|ref|NP_838903.1| 50S ribosomal protein L10 [Shigella flexneri 2a str. 2457T]

MALNLQDKQAIVAEVSEVAKGALSAVVADSRGVTVDKMTELRKAGREAGVYMRVVRNTLLRRAVEGTPFE

CLKDAFVGPTLIAYSMEHPGAAARLFKEFAKANAKFEVKAAAFEGELIPASQIDRLATLPTYEEAIARLM

ATMKEASAGKLVRTLAAVRDAKEAA

>gi|30064723|ref|NP_838894.1| anti-RNA polymerase sigma 70 factor [Shigella flexneri 2a str. 2457T]

MLNQLDNLTERVRGSNKLVDRWLHVRKHLLVAYYNLVGIKPGKESYMRLNEKALDDFCQSLVDYLSAGHF

SIYERILHKLEGNGQLARAAKIWPQLEANTQQIMDYYDSSLETAIDHDNYLEFQQVLSDIGEALEARFVL

EDKLILLVLDAARVKHPA

>gi|30064715|ref|NP_838886.1| sensor protein ZraS [Shigella flexneri 2a str. 2457T]

MRFMQRSKDSLAKWLSAILPVVIVGLVGLFAVTVIRDYGRETAAARQTLLEKGSVLIRALESGSRVGMGM

RMHHAQQQALLEEMAGQPGVRWFAVTDEQGTIVMHSNSGMVGKQLYSPQEMQQLHPGDEEAWRRIDSADG

EPVLEIYRQFQPMFAAGMYRMRHMQQYAATPQAIFIAFDASNIVSAEDREQRNTLIILFALATVLLASVL

SFFWYRRYLRSRQLLQDEMKRKEKLVALGHLAAGVAHEIRNPLSSIKGLAKYFAERAPAGGEAHQLAQVM

AKEADRLNRVVSELLELVKPTHLALQAVDLNTLINHSLQLVSQDANSREIQLRFTANDTLPEIQADPDRL

TQVLLNLYLNAIQAIGQHGVISVTASESGAGVKISVTDSGKGIAADQLEAIFTPYFTTKAEGTGLGLAVV

HNIVEQHGGTIQVASQEGKGSTFTLWLPVNITRKDPQG

>gi|30064701|ref|NP_838872.1| sorbose-permease PTS system IIB component [Shigella flexneri 2a str. 2457T]

MNITLARIDDRLIHGQVTTVWSKVANAQRIIICNDEVYNDEVRRTLLRQAAPPGMKVNVVNIEKAVAVYH

NPQYQDETVFYLFTRPQDALAMVRQGVKIGTLNIGGMAWRPGKKQLTKAVSLDDDDINAFHELNNLGVIL

DLRVVASDPSINIIDKINEQLIAN

>gi|30064697|ref|NP_838868.1| 23S rRNA pseudouridine synthase F [Shigella flexneri 2a str. 2457T]

MLPDSSVRLNKYISESGICSRREADRYIEQGNVFLNGKRATIGDQVKPGDIVKVNGQLIEPREAEDLVLI

ALNKPVGIVSTTEDGERDNIVDFVNHSKRVFPIGRLDKDSQGLIFLTNHGDLVNKILRAGNDHEKEYLVT

VDKPITDEFIRGMGAGVPILGTVTKKCKVKKEAPFVFRITLVQGLNRQIRRMCEHFGYEVKKLERTRIMN

VSLSGIPLGEWRDLTDDELIDLFKLIENSSSEAKPKAKAKPKTAGIKRPVVKMEKTAEKGGRPASNGKRF

TSPGRKKKGR

>gi|30064694|ref|NP_838865.1| sensory histidine kinase DcuS [Shigella flexneri 2a str. 2457T]

MRHSLPYHILRKRPMKLSTTVILMVSAVLFSVLLVVHLIYFSQISDMTRDGLANKALAVARTLADSPEIR

QGLQKKPQESGIQAIAEAVRKRNDLLFIVVTDMQSLRYSHPEAQRIGQPFKGDDILNALNGEENVAINRG

FLAQALRVFTPIYDENHKQIGVVAIGLELSRVTQQINDSRWSIIWSVLFGMLVGLIGTCILVKVLKKILF

GLEPYEISTLFEQRQAMLQSIKEGVVAVDDRGEVTLINDAAQELLNYRKSQDDEKLSTLSHSWSQVVDVS

EVLRDGTPRRDEEITIKDRLLLINTVPVRSNGVIIGAISTFRDKTEVRKLMQRLDGLVNYADALRERSHE

FMNKLHVILGLLHLKSYKQLEDYILKTANNYQEEIGSLLGKIKSPVIAGFLISKINRATDLGHTLILNSE

SQLPDSGSEDQVATLITTLGNLIENALEALGPEPGGEISVTLHYRHGWLHCEVNDDGPGIAPDKIDHIFD

KGVSTKGSERGVGLALVKQQVENLGGSIAVESEPGIFTQFFVQIPWDGERSNR

>gi|30064690|ref|NP_838861.1| hypothetical protein S3628 [Shigella flexneri 2a str. 2457T]

MTRTLKPLILNTGALALTLILIYTGISAHDKLTWLLEVTPVIIVVPLLLATARRYPLTLLLYTLIFFHAI

ILMVGGQYTYAKVPVGFEVQEWLGLSRNPYDKLGHFFQGLVPALVAREILVRGMYVRGRKMVAFLVCCVA

LAISAMYELIEWWAALAMGQGADDFLGTQGDQWDTQSDMFCALLGALTTVILLARFHCRQLRRYGLITG

>gi|30064685|ref|NP_838856.1| ARAC-type regulatory protein [Shigella flexneri 2a str. 2457T]

MRICSDQPCIVLLTEKDVWIRVNGKEPISLKANHMALLNCENNIIDVSSLNNTLVAHISHDIIKDYLRFL

NKDLSQIPVWQRSATPILTLPCLTPDVFRVAAQHSMMPAETESEKERTRALLFTVLSRFLDSKKFLSLMM

YMLRNCVSDSVYQIIESDIHKDWNLSMVASCLCLSPSLLKKKLKSENTSYSQIITTCRMRYAVNELMMDG

KNISQVSQSCGYNSTSYFISVFKDFYGMTPLHYVSQHRERTVA

>gi|30064682|ref|NP_838853.1| DNA-binding transcriptional regulator BasR [Shigella flexneri 2a str. 2457T]

MKILIVEDDTLLLQGLILAAQTEGYACDGVTTARMAEQSLEDGHYSLVVLDLGLPDEDGLHFLARIRQKK

YTLPVLILTARDTLTDKIAGLDVGADDYLVKPFALEELHARIRALLRRHNNQGESELIVGNLTLNMGRRQ

VWMGGEELILTPKEYALLSRLMLKAGSPVHREILYNDIYNWDNEPSTNTLEVHIHNLRDKVGKARIRTVR

GFGYMLVANEEN

>gi|30064681|ref|NP_838852.1| sensor protein BasS/PmrB [Shigella flexneri 2a str. 2457T]

MNLMRFLRRPISLRQRLILTIGAILLVFELISVFWLWHESTEQIQLFEQALRDNRNNDRHIMREIREAVA

SLIVPGVFMVSLTLFICYQAVRRITRPLAELQKELEARTADNLTPIAIHSATLEIEAVVSALNDLVSRLT

STLDNERLFTADVAHELRTPLAGVRLHLELLAKTHHIDVAPLVARLDQMMESVSQLLQLARAGQSFSSGN

YQHVKLLEDVILPSYDELSTMLDQRQQTLLLPESAADITVQGDATLLRMLLRNLVENAHRYSPQGSNIMI

KLQEDGGAVMAVEDEGPGIDESKCGELSKAFVRMDSRYGGIGLGLSIVSRITQLHHGQFFLQNRQETSGT

RAWVRLKKDQNVANQI

>gi|30064661|ref|NP_838832.1| formate-dependent nitrite reductase [Shigella flexneri 2a str. 2457T]

MTWSRRQFLTGVGVLAAVSGTAGRVVAKTLNINGVRYGMVHDESLCIGCTACMDACREVNKVPEGVSRLT

IIRSEPQGEFPDVKYRFFRKSCQHCDHAPCVDVCPTGASFRDAASGIVDVNPDLCVGCQYCIAACPYRVR

FIHPVTKTADKCDFCRKTNLQAGKLPACVEACPTKALTFGNLDDPNSEISQLLRQKPTYRYKLALGTKPK

LYRVPFKYGEVSQ

>gi|30064654|ref|NP_838825.1| hypothetical protein S3591 [Shigella flexneri 2a str. 2457T]

MSTPSARTGGSLDAWFKISQRGSTVRQEVVAGLTTFLAMVYSVIVVPGMLGKAGFPPAAVFVATCLVAGL

GSIVMGLWANLPLAIGCAISLTAFTAFSLVLGQHISVPVALGAVFLMGVLFTVISATGIRSWILRNLPHG

VAHGTGIGIGLFLLLIAANGVGLVIKNPLDGLPVALGDFATFPVIMSLAGLAVIIGLEKLKVPGGILLTI

IGISIVGLIFDPNVHFSGVFAMPSLSDENGNSLIGSLDIMGALNPVVLPSVLALVMTAVFDATGTIRAVA

GQANLLDKDGQIIDGGKALTTDSMSSVFSGLVGAAPAAVYIESAAGTAAGGKTGLTAITVGVLFLLILFL

SPLSYLVPGYATAPALMYVGLLMLSNVAKIDFADFVDAMAGLVTAVFIVLTCNIVTGIMIGFATLVIGRL

VSGEWRKLNIGTVVIAVALVTFYAGGWAI

>gi|30064652|ref|NP_838823.1| DNA-binding transcriptional regulator SoxS [Shigella flexneri 2a str. 2457T]

MSHQKIIQDLIAWIDVHIDQPLNIDVVAKKSGYSKWYLQRMFRTVTHQTLGDYIRQRRLLLAAVELRTTE

RPIFDIAMDLGYVSQQTFSRVFRRQFDRTPSDYRHRL

>gi|30064642|ref|NP_838813.1| alanine racemase [Shigella flexneri 2a str. 2457T]

MQAATVVINRRALRHNLQRLRELAPASKMVAVVKANAYGHGLLETARTLPDADAFGVARLEEALRLRAGG

ITKPVLLLEGFFDARDLPTISAQHFHTAVHNEEQLAALEEASLDEPVTVWMKLDTGMHRLGVRPEQAEAF

YHRLTQCKNVRQPVNIVSHFARADEPKCGATEKQLAIFNTFCEGKPGQRSIAASGGILLWPQSHFDWVRP

GIILYGVSPLEDRSTGADFGCQPVMSLTSSLIAVREHKAGEPVGYGGTWVSERDTRLGVVAMGYGDGYPR

AAPSGTPVLVNGREVPIVGRVAMDMICVDLGPQAQDKAGDPVILWGEGLPVERIAEMTKVSAYELITRLT

SRVAMKYVD

>gi|30064641|ref|NP_838812.1| replicative DNA helicase [Shigella flexneri 2a str. 2457T]

MAGNKPFNKQQAEPRERDPQVAGLKVPPHSIEAEQSVLGGLMLDNERWDDVAERVVADDFYTRPHRHIFT

EMARLQESGSPIDLITLAESLERQGQLDSVGGFAYLAELSKNTPSAANISAYADIVRERAVVREMISVAN

EIAEAGFDPQGRTSEDLLDLAESRVFKIAESRANKDEGPKNIADVLDATVARIEQLFQQPHDGVTGVNTG

YDDLNKKTAGLQPSDLIIVAARPSMGKTTFAMNLVENAAMLQDKPVLIFSLEMPSEQIMMRSLASLSRVD

QTKIRTGQLDDEDWARISGTMGILLEKRNIYIDDSSGLTPTEVRSRARRIAREHGGIGLIMIDYLQLMRV

PALSDNRTLEIAEISRSLKALAKELNVPVVALSQLNRSLEQRADKRPVNSDLRESGSIEQDADLIMFIYR

DEVYHENSDLKGIAEIIIGKQRNGPIGTVRLTFNGQWSRFDNYAGPQYDDE

>gi|30064637|ref|NP_838808.1| hypothetical protein S3573 [Shigella flexneri 2a str. 2457T]

MWVNKYTDDCTDEDLNDRDFIASVVDRAIFHFAINSICNPGDNKDAMPIEQCTFDVETKNGLPSTVQLFY

EESKDNEPLANIHFQAIGSGFLTFVNACQEHDDNSLKLFASLLISLSYSSAYADLSETVYINENNESYLK

AQFEKLYQRDMKKYLGEMKRLADGGEMNFDGYLDKMSHLVNEGTLDPDILSKMRDAAPQLISFAKSFDPT

SKEEIKILTDTSKLIYDLFGVKSEK

>gi|30064621|ref|NP_838792.1| phosphate-starvation-inducible protein PsiE [Shigella flexneri 2a str. 2457T]

MTSLSRPRVEFISTILQTVLNLGLLCLGLILVVFLGKETVHLADVLFAPEQTSKYELVEGLVVYFLYFEF

IALIVKYFQSGFHFPLRYFVYIGITAIVRLIIVDHKSPLDVLIYSAAILLLVITLWLCNSKRLKRE

>gi|30064615|ref|NP_838786.1| aspartate kinase III [Shigella flexneri 2a str. 2457T]

MSEIVVSKFGGTSVADFDAMNRSADIVLSDANVRLVVLSASAGITNLLVALAEGLEPGERFEKLDAIRNI

QFAILERLRYPNVIREEIERLLENITVLAEAAALATSPALTDELVSHGELMSTLLFVEILRERDVQAQWF

DVRKVMRTNDRFGRAEPDVAALAELAALQLLPRLNEGLVITQGFIGSENKGRTTTLGRGGSDYTAALLAE

ALHASRVDIWTDVPGIYTTDPRVVSAAKRIDEIAFAEAAEMATFGAKVLHPATLLPAVRSDIPVFVGSSK

DPRAGGTLVCNKTENPPLFRALALRRNQTLLTLHSLNMLHSRGFLAEVFGILARHNISVDLITTSEVSVA

LTLDTTGSTSTGDTLLTQSLLMELSALCRVEVEEGLALVALIGNDLSKACGVGKEVFGVLEPFNIRMICY

GASSHNLCFLVPGEDAEQVVQKLHSNLFE

>gi|30064601|ref|NP_838772.1| transferase [Shigella flexneri 2a str. 2457T]

MKCFTLSSQRRRIIGIPIFCTSIFLIFFSVCCFFTLFAAIAHKTVLFCILKDLHKANNAPKVISKSFIAE

VSMSDVLRPYRDLFPQIGQRVMIDDSSVVIGDVRLADDVGIWPLVVIRGDVHYVQIGARTNIQDGSMLHV

THKSSYNPDGNPLTIGEDVTVGHKVMLHGCTIGNRVLVGMGSILLDGAIVEDDVMIGAGSLVPQNKRLES

GYLYLGSPVKQIRPLSDEEKAGLRYSANNYVKWKDEYLDQGNQTQP

>gi|30064599|ref|NP_838770.1| transport system permease [Shigella flexneri 2a str. 2457T]

MTKVLLSQPSRPASHNSSRAMVWVRKNLFSSWSNSLLTIGCIWLMWELIPPLLNWAFLQANWVGSTRADC

TKAGACWVFIHERFGQFMYGLYPHDQRWRINLALLIGLVSIAPMFWKILPHRGRYIAAWAVIYPLIVWWL

MYGGFFGLERVETRQWGGLTLTLIIASVGIAGALPWGILLALGRRSHMPIVRILSVIFIEFWRGVPLITV

LFMSSVMLPLFMAEGTSIDKLIRALVGVILFQSAYVAEVVRGGLQALPKGQYEAAESLALGYWKTQGLVI

LPQALKLVIPGLVNTIIALFKDTSLVIIIGLFDLFSSVQQATVDPAWLGMSTEGYVFAALIYWIFCFSMS

RYSQHLEKRFNTGRTPH

>gi|30064580|ref|NP_838751.1| rod shape-determining protein MreD [Shigella flexneri 2a str. 2457T]

MASYRSQGRWVIWLSFLIALLLQIMPWPDNLIVFRPNWVLLILLYWILALPHRVNVGTGFVMGAILDLIS
GSMLGVRVLAMSIIAYLVALKYQLFRNLALWQQALVVMLLSLVVDIIVFWAEFLVINVSFRPEVFWSSVV
NGVLWPWIFLLMRKVRQQFAVQ

>gi|30064578|ref|NP_838749.1| ribonuclease G [Shigella flexneri 2a str. 2457T]
MTAELLVNVTPSETRVAYIDGGILQEIHIEREARRGIVGNIYKGRVSRVLPGMQAAFVDIGLDKAAFLHA
SDIMPHTECVAGEEQKQFTVRDISELVRQGQDLMVQVVKDPLGTKGARLTTDITLPSRYLVFMPGASHVG
VSQRIESESERERLKKVVAEYCDEQGGFIIRTAAEGVGEAELASDAAYLKRVWTKVMERKKRPQTRYQLY
GELALAQRVLRDFADAELDRIRVDSRLTYEALLEFTSEYIPEMTSKLEHYTGRQPIFDLFDVENEIQRAL
ERKVELKSGGYLIIDQTEAMTTVDINTGAFVGHRNLDDTIFNTNIEATQAIARQLRLRNLGGIIIIDFID
MNNEDHRRRVLHSLEQALSKDRVKTSVNGFSALGLVEMTRKRTRESIEHVLCNECPTCHGRGTVKTVETV
CYEIMREIVRVHHAYDSDRFLVYASPAVAEALKGEESHSLAEVEIFVGKQVKVQIEPLYNQEQFDVVMM

>gi|30064569|ref|NP_838740.1| arginine repressor [Shigella flexneri 2a str. 2457T]
MRSSAKQEELVKAFKALLKEEKFSSQGEIVAALQEQGFDNINQSKVSRMLTKFGAVRTRNAKMEMVYCLP
AELGVPTTSSPLKNLVLDIDYNDAVVVIHTSPGAAQLIARLLDSLGKAEGILGTIAGDDTIFTTPANGFT
VKDLYEAILELFDQEL

>gi|30064566|ref|NP_838737.1| cytochrome d ubiquinol oxidase subunit III [Shigella flexneri 2a str. 2457T]
MTWEYALIGLVVGIIIGAVAMRFGNRKLRQQQALQYELEKNKAELDEYREELVSHFARSAELLDTMAHDY

RQLYQHMAKSSSSLLPELSAEANPFRNRLAESEASNDQAPVQVPRDYSEGASGLLRTGAKRD

>gi|30064561|ref|NP_838732.1| ClpXP protease specificity-enhancing factor [Shigella flexneri 2a str. 2457T]

MDLSQLTPRRPYLLRAFYEWLLDNQLTPHLVVDVTLPGVQVPMEYARDGQIVLNIAPRAVGNLELANDEV

RFNARFGGIPRQVSVPLAAVLAIYARENGAGTMFEPEAAYDEDTSIMNDEEASADNETVMSVIDGDKPDH

DDDTHPDDEPPQPPRGGRPALRVVK

>gi|30064557|ref|NP_838728.1| transcriptional regulator NanR [Shigella flexneri 2a str. 2457T]

MNAFDSQTEDSSPAIGRNLRSRPLARKKLSEMVEEELEQMIRRREFGEGEQLPSERELMAFFNVGRPSVR

EALAALKRKGLVQINNGERARVSRPSADTIIGELSGMAKDFLSHPGGIAHFEQLRLFFESSLVRYAAEHA

TDEQIDLLAKALEINSQSLDNNAAFIRSDVDFHRVLAEIPGNPIFMAIHVALLDWLIAARPTVTDQALHE

HNNVSYQQHIAIVDAIRRHDPDEADRALQSHLNSVSATWHAFGQTTNKKK

>gi|30064554|ref|NP_838725.1| N-acetylmannosamine-6-phosphate 2-epimerase [Shigella flexneri 2a str. 2457T]

MSLLAQLDQKIAANGGLIVSCQPVPDSPLDKPEIVAAMALAAEQAGAVAIRIEGVANLQATRAVVSVPII

GIVKRDLEDSPVRITAYIEDVDALAQAGADIIAIDGTDRPRPVPVETLLARIHHHGLLAMTDCSTPEDGL

ACQKLGAEIIGTTLSGYTTPETPEEPDLALVKTLSEAGCRVIAEGRYNTPAQAADAMRHGAWAVTVGSAI

TRLEHICQWYNTAMKKAVL

>gi|30064551|ref|NP_838722.1| IS600 orf [Shigella flexneri 2a str. 2457T]

MAHIRTRETYGTRRLQTELAENGIIVGRDRLARLRKELRLRCKQKRKFRATTNPNHNLPVAPNLLNQTFA

PTAPNQVWVADLTYVATQEGWLYLAGIKDVYTCEIVGYAMGERMTKELTGKALFMALRSQRPPAGLIHHS

DRGSQYCAYDYRVIQEQSGLKTSMSRKGNCYDNAPMESFWGTLKNESLSHYRFNNRDEAISVIREYIEIF

YNRQRRHSRLGNISPAAFRENIIRWLLKKRTNGSVRYCQYTSKVAMIYIEQLELIHKSGDVLYPVKITRK

SSGKTAFHLVPFGLNKTHDLLEVEDASEAIRLVIDERHSIRCSTLTATITNKKGKRIKRTGIYSIKGVNI

KEYNVR

>gi|30064542|ref|NP_838713.1| PTS IIA-like nitrogen-regulatory protein PtsN [Shigella flexneri 2a str. 2457T]

MTNNDTTLQLSSVLNRECTRSRVHCQSKKRALEIISELAAKQLSLPPQVVFEAILTREKMGSTGIGNGIA

IPHGKLEEDTLRAVGVFVQLETPIAFDAIDNQPVDLLFALLVPADQTKTHLHTLSLVAKRLADKTICRRL

RAAQSDEELYQIITDTEGTPDEA

>gi|30064540|ref|NP_838711.1| RNA polymerase factor sigma-54 [Shigella flexneri 2a str. 2457T]

MKQGLQLRLSQQLAMTPQLQQAIRLLQLSTLELQQELQQALESNPLLEQIDTHEEIDTRETQDSETLDTA

DALEQKEMPEELPLDASWDTIYTAGTPSGTSGDYIDDELPVYQGETTQTLQDYLMWQVELTPFSDTDRAI

ATSIVDAVDETGYLTVPLEDILESMGDEEIDIDEVEAVLKRIQRFDPVGVAAKDLRDCLLIQLSQFDKTT

PWLEEARLIISDHLDLLANHDFRTLMRVTRLKEDVLKEAVNLIQSLDPRPGQSIQTGEPEYVIPDVLVRK

HNGHWTVELNSDSIPRLQINQHYASMCNNARNDGDSQFIRSNLQDAKWLIKSLESRNDTLLRVSRCIVEQ

QQAFFEQGEEYMKPMVLADIAQAVEMHESTISRVTTQKYLHSPRGIFELKYFFSSHVNTEGGGEASSTAI

RALVKKLIAAENPAKPLSDSKLTSLLSEQGIMVARRTVAKYRESLSIPPSNQRKQLV

>gi|30064535|ref|NP_838706.1| D-arabinose 5-phosphate isomerase [Shigella flexneri 2a str. 2457T]

MSHVELQPGFDFQQAGKEVLAIERECLAELDQYINQNFTLACEKMFWCKGKVVVMGMGKSGHIGRKMAAT

FASTGTPSFFVHPSEAAHGDLGMVTPQDVVIAISNSGESSEITALIPVLKRLHVPLICITGRPESSMARA

ADVHLCVKVAKEACPLGLAPTSSTTATLVMGDALAVALLKARGFTAEDFALSHPGGALGRKLLLRVNDIM

HTGDEIPHVKKTASLRDALLEVTRKNLGMTVICDDNMMIEGIFTDGDLRRVFDMGVDVRQLSIADVMTPG

GIRVRPGILAVEALNLMQSRHITSVMVADGDHLLGVLHMHDLLRAGVV

>gi|30064527|ref|NP_838698.1| UDP-N-acetylglucosamine 1-carboxyvinyltransferase [Shigella flexneri 2a str. 2457T]

MDKFRVQGPTKLQGEVTISGAKNAALPILFAALLAEEPVEIQNVPKLKDVDTSMKLLSQLGAKVERNGSV

HIDARDVNVFCAPYDLVKTMRASIWALGPLVARFGQGQVSLPGGCTIGARPVDLHISGLEQLGATIKLEE

GYVKASVDGRLKGAHIVMDKVSVGATVTIMCAATLAEGTTIIENAAREPEIVDTANFLITLGAKISGQGT

DRIVIEGVERLGGGVYRVLPDRIETGTFLVAAAISRGKIICRNAQPDTLDAVLAKLRDAGADIEVGEDWI

SLDMHGKRPKAVNVRTAPHPAFPTDMQAQFTLLNLVAEGTGFITETVFENRFMHVPELSRMGAHAEIESN

TVICHGVEKLSGAQVMATDLRASASLVLAGCIAEGTTVVDRIYHIDRGYERIEDKLRALGANIERVKGE

>gi|30064524|ref|NP_838695.1| 50S ribosomal protein L21 [Shigella flexneri 2a str. 2457T]

MYAVFQSGGKQHRVSEGQTVRLEKLDIATGETVEFAEVLMIANGEEVKIGVPFVDGGVIKAEVVAHGRGE

KVKIVKFRRRKHYRKQQGHRQWFTDVKITGISA

>gi|30064519|ref|NP_838690.1| transcription elongation factor GreA [Shigella flexneri 2a str. 2457T]

MQAIPMTLRGAEKLREELDFLKSVRRPEIIAAIAEAREHGDLKENAEYHAAREQQGFCEGRIKDIEAKLS

NAQVIDVTKMPNNGRVIFGATVTVLNLDSDEEQTYRIVGDDEADFKQNLISVNSPIARGLIGKEEDDVVV

IKTPGGEVEFEVIKVEYL

>gi|30064513|ref|NP_838684.1| preprotein translocase subunit SecG [Shigella flexneri 2a str. 2457T]

MYEALLVVFLIVAIGLVGLIMLQQGKGADMGASFGAGASATLFGSSGSGNFMTRMTALLATLFFIISLVL

GNINSNKTNRGSEWENLSAPAKTEQTQPAAPAKPTSDIPN

>gi|30064509|ref|NP_838680.1| transcription elongation factor NusA [Shigella flexneri 2a str. 2457T]

MNKEILAVVEAVSNEKALPREKIFEALESALATATKKKYEQEIDVRVQIDRKSGDFDTFRRWLVVDEVTQ

PTKEITLEAARYEDESLNLGDYVEDQIESVTFDRITTQTAKQVIVQKVREAERAMVVDQFREHEGEIITG

VVKKVNRDNISLDLGNNAEAVILREDMLPRENFRPGDRVRGVLYSVRPEARGAQLFVTRSKPEMLIELFR

IEVPEIGEEVIEIKAAARDPGSRAKIAVKTNDKRIDPVGACVGMRGARVQAVSTELGGERIDIVLWDDNP

AQFVINAMAPADVASIVVDEDKHTMDIAVEAGNLAQAIGRNGQNVRLASQLSGWELNVMTVDDLQAKHQA

EAHAAIDTFTKYLDIDEDFATVLVEEGFSTLEELAYVPMKELLEIEGLDEPTVEALRERAKNALATIAQA

QEESLGDNKPADDLLNLEGVDRDLAFKLAARGVCTLEDLAEQGIDDLADIEGLTDEKAGALIMAARNICW

FGDEA

>gi|30064507|ref|NP_838678.1| ribosome-binding factor A [Shigella flexneri 2a str. 2457T]

MAKEFGRPQRVAQEMQKEIALILQREIKDPRLGMMTTVSGVEMSRDLAYAKVYVTFLNDKDEDAVKAGIK

ALQEASGFIRSLLGKAMRLRIVPELTFFYDNSLVEGMRMSNLVTSVVKHDEERRVNPDDSKED

>gi|30064496|ref|NP_838667.1| hypothetical protein S3414 [Shigella flexneri 2a str. 2457T]

MLIRVEIPIDAPGIDALLRRSFESDAEAKLVHDLREDGFLTLGLVATDDEGQVIGYVAFSPVDVQGEDLQ

WVGMAPLAVDEKYRGQGLARQLVYEGLDSLNEFGYAAVVTLGDPALYSRFGFELAAHHDLRCRWPGTESA

FLVHRLADDALNGVTGLVEYHEHFNRF

>gi|30064494|ref|NP_838665.1| hypothetical protein S3412 [Shigella flexneri 2a str. 2457T]

METLIAISRWLAKQHVVTWCVQQEGELWCANAFYLFDAQKVAFYILTEEKTRHAQMSGPQAAVAGTVNGQ

PKTVALIRGVQFKGEIRRLEGEESDLARKAYNRRFPVARMLSAPVWEIRLDEIKFTDNTLGFGKKMIWLR

DSGTEQA

>gi|30064489|ref|NP_838660.1| DnaA initiator-associating protein DiaA [Shigella flexneri 2a str. 2457T]

MQERIKACFTESIQTQIAAAEALPDAISRAAMTLVQSLLNGNKILCCGNGTSAANAQHFAASMINRFETE

RPSLPAIALNTDNVVLTAIANDRLHDEVYAKQVRALGHAGDVLLAISTRGNSRDIVKAVEAAVTRDMTIV

ALTGYDGGELAGLLGPQDVEIRIPSHRSARIQEMHMLTVNCLCDLIDNTLFPHQDD

>gi|30064482|ref|NP_838653.1| chaperone [Shigella flexneri 2a str. 2457T]

MSKRTFAVILTLLCSFCIGQALAGGIVLQRTRVIYDASRKEAALPVANKGAETPYLLQSWVDNIDGKSRA

PFIITPPLFRLEASDDSSLRIIKTADNLPENKESLFYINVRAIPAKKKSDDVNANELTLVFKTRVKMFYR

PAHLKGRVNDAWKSLEFKRSDHSLNIYNPTEYYVVFAGLAVDKTDLTSKIEYIAPGEHKQLPLPASGGKN

VKWAAINDYGGSSGTETRPLQ

>gi|30064473|ref|NP_838644.1| transport enzyme subunit [Shigella flexneri 2a str. 2457T]

MLSIILTGHGGFASGMEKAMKQILGDQSQFIAIDFPETSSAALLTSQLEEAIAQLDCEDGIVFLTDLLGG

TPFRVASTLAMQKPGCEVITGTNLQLLLEMVLEREGLSGEEFRVQALECGHRGLTSLVDELGRCHEECPV

EEGI

>gi|30064470|ref|NP_838641.1| N-acetylgalactosamine-specific PTS system transporter subunit IIB [Shigella flexneri 2a str. 2457T]

MKANKQNKEEHAMPNIVLSRIDERLIHGQVGVQWVGFAGANLVLVANDEVAEDPVQQNLMEMVLAEGIAV

RFWTLQKVIDNIHRAADRQKILLVCKTPADFLTLVKGGVPVNRINVGNMHYANGKQQIAKTVSVDAGDIA

AFNDLKAAGVECFVQGVPTEPAVDLFKLL

>gi|30064462|ref|NP_838633.1| DNA-binding transcriptional activator TdcR [Shigella flexneri 2a str. 2457T]

MTGITIFYGDNIIRYVVNTKKGLRPYFKQLPDNYQAKFELNLMSKFSNFIINKPFSAINTAARHIFSRYL

LENKHLFYQYFKISNTGIDHLEQLINVNFFSSDRTSFCECNRFP

>gi|30064457|ref|NP_838628.1| formate acetyltransferase 3 [Shigella flexneri 2a str. 2457T]

MKVDIDTSDKLYADAWLGFKGTDWKSEINVRDFIQHNYTPYEGDESFLAEATPATTELWEKVMEGIRIEN

ATHAPVDFDTNIATTITAHDAGYINQPLEKIVGLQTDAPLKRALHPFGGINMIKSSFHAYGREMDSEFEY

LFTDLRKTHNQGVFDVYSPDMLRCRKSGVLTGLPDGYGRGRIIGDYRRVALYGISYLVRERELQFADLQS

RLEKGEDLEATIRLREELAEHRHALLQIQEMAAKYGFDISRPAQNAQEAVQWLYFAYLAAVKSQNGGAMS

LGRTASFLDIYIERDFKAGVLNEQQAQELIDHFIMKIRMVRFLRTPEFDSLFSGDPIWATEVIGGMGLDG

RTLVTKNSFRYLHTLHTMGPAPEPNLTILWSEELPIAFKKYAAQVSIVTSSLQYENDDLMRTDFNSDDYA

IACCVSPMVIGKQMQFFGARANLAKTLLYAINGGVDEKLKIQVGPKTAPLMDDVLDYDKVMDSLDHFMDW

LAVQYISALNIIHYMHDKYSYEASLMALHDRDVYRTMACGIAGLSVATDSLSAIKYARVKPIRDENGLAV

DFEIDGEYPQYGNNDERVDSIACDLVERFMKKIKALPTYRNAVPTQSILTITSNVVYGQKTGNTPDGRRA

GTPFAPGANPMHGRDRKGAVASLTSVAKLPFTYAKDGISYTFSIVPAALGKEDPVRKTNLVGLLDGYFHH

EADVEGGQHLNVNVMNREMLLDAIEHPEKYPNLTIRVSGYAVRFNALTREQQQDVISRTFTQAL

>gi|30064443|ref|NP_838614.1| hypothetical protein S3347 [Shigella flexneri 2a str. 2457T]

MSLRQLAWSGTVLLLVGTLLLAWSAVRQQESTLAIRAVHQGTTMPDGFSIWHHLDAHGIPFKSITPKNDT

LLITFDSSDQSAAAKAVLDRTLPHGYIIAQQDNNSQAMQWLTRLRDNSHRFG

>gi|30064442|ref|NP_838613.1| hypothetical protein S3346 [Shigella flexneri 2a str. 2457T]

MELLTQLLQALWAQDFETLANPSMIGMLYFVLFVILFLENGLLPAAFLPGDSLLVLVGVLIAKGAMGYPQ

TILLLTVAASLGCWVSYIQGRWLGNTRTVQNWLSHLPAHYHQRAHHLFHKHGLSALLIGRFIAFVRTLLP

TIAGLSGLNNARFQFFNWMSGLLWVLILTTLGYMLGKTPVFLKYEDQLMSCLMLLPVVLLVFGLAGSLVV

LWKKKYGNRG

>gi|30064429|ref|NP_838600.1| hypothetical protein S3333 [Shigella flexneri 2a str. 2457T]

MLRAFARLLLRICFSRRTLKIACLLLLVAGATIFIADRVMVNASKQLTWGDVNAVPARNVGLLLGARPGN

RYFTRRIDTAAALYHAGKVKWLLVSGDNGRKNYDEASGMQQALIAKGVPAKVIFCDYAGFSTLDSVVRAK

KVFGENHITIISQEFHNQRTIWLAKQYGIDAIGFNAPDLNMKHGFYTQLREKLARVSAVIDAKILHRQPK

YLGPSVMIGPFSEHGCPAKE

>gi|30064419|ref|NP_838590.1| DNA-binding transcriptional repressor EbgR [Shigella flexneri 2a str. 2457T]

MATLKDIAIEAGVSLATVSRVLNDDPTLNVKEETKHRILEIAEKLEYKTSSARKLQTGAVNQHHILAIYS

YQQELEINDPYYLAIRHGIETQCEKLAIELTNCYEHNGLPDIKNVTGILIVGKPTPALRAAASALTDNIC

FIDFHEPGSGYDAVDIDLARISKEIIDFYINQGVNRIGFIGGEDEPGKADIREVAFAEYGRLKQVVREED

IWRGGFSSSSGYELAKQMLAREDYPKALFVASDSIAIGVLRAIHERGLNIPQDISLISVNDIPTARFTFP

PLSTVRIHSEMMGSQGVNLVYEKARDGRALPLLVFVPSKLKLRGTTR

>gi|30064414|ref|NP_838585.1| DNA primase [Shigella flexneri 2a str. 2457T]

MAGRIPRVFINDLLARTDIVDLIDARVKLKKQGKNFHACCPFHNEKTPSFTVNGEKQFYHCFGCGAHGNA

IDFLMNYDKLEFVETVEELAAMHNLEVPFEAGSGPSQIERHQRQTLYQLMDGLNTFYQQSLQQPVATSAR

QYLEKRGLSHEVIARFAIGFAPPGWDNVLKRFGGNPENRQSLIDAGMLVTNDQGRSYDRFRERVMFPIRD

KRGRVIGFGGRVLGNDTPKYLNSPETDIFHKGRQLYGLYEAQQDNAEPNRLLVVEGYMDVVALAQYGINY

AVASLGTSTTADHIQLLFRATNNVICCYDGDRAGRDAAWRALETALPYMTDGRQLRFMFLPDGEDPDTLV

RKEGKEAFEARMEQAMPLSAFLFNSLMPQVDLSTPDGRARLSTLALPLISQVPGETLRIYLRQELGNKLG

ILDDSQLERLMPKAAESGVSRPVPQLKRTTMRILIGLLVQNPELATLVPPLENLDENKLPGLGLFRELVN

TCLSQPGLTTGQLLEHYRGTNNAATLEKLSMWDDIADKNIAEQTFTDSLNHMFDSLLELRQEELIARERT

HGLSNEERLELWTLNQELAKK

>gi|30064406|ref|NP_838577.1| bifunctional dihydroneopterin aldolase/dihydroneopterin triphosphate 2'-epimerase [Shigella flexneri 2a str. 2457T]

MDIVFIEQLSVITTIGVYDWEQTIEQKLVFDIEMAWDNRKAAKSDDVADCLSYADIAETVVSHVEGARFA

LVERVAEEVAELLLARFNSPWVRIKLSKPGAVARAANVGVIIERGNNLKENN

>gi|30064391|ref|NP_838562.1| 3,4-dihydroxy-2-butanone 4-phosphate synthase [Shigella flexneri 2a str. 2457T]

MNQTLLSSFGTPFERVENALAALREGRGVMVLDDEDRENEGDMIFPAETMTVEQMALTIRHGSGIVCLCI

TEDRRKQLDLPMMVENNTSAYGTGFTVTIEAAEGVTTGVSAADRITTVRAAIADGAKPSDLNRPGHVFPL

RAQAGGVLTRGGHTEATIDLMTLAGFKPAGVLCELTNDDGTMARAPECIEFANKHNMALVTIEDLVAYRQ

AHERKAS

>gi|30064383|ref|NP_838554.1| hypothetical protein S3278 [Shigella flexneri 2a str. 2457T]

MKRYTPDFPEMMRLCEMNFSQLRRLLPRNDAPGETVSYQVANAQYRLTIVESTRYTTLVTIEQTAPAISY

WSLPSMTVRLYHDAMVAEVCSSQQIFRFKARYDYPNKKLHQRDEKHQINQFLADWLRYCLAHGAMAIPVY

>gi|30064377|ref|NP_838548.1| hypothetical protein S3272 [Shigella flexneri 2a str. 2457T]

MLKQKIKTIFEALLYIMLTYWLIDSFFAFNKYDWMLESGGNICSIPSVSGEDRILQAMIAAFFLLTPLII

LILRKLFMREMFEFWLYVFSLVICLVCGWWLFWGRFIFCY

>gi|30064376|ref|NP_838547.1| sensor protein QseC [Shigella flexneri 2a str. 2457T]

MKFTQRLSLRVRLTLIFLILASVTWLLSSFVAWKQTTDNVDELFDTQLMLFAKRLSTLDLNEINAADRMA

QTPNKLKHGHVDDDALTFAIFTHDGRMVLNDGDNGEDIPYSYQREGFADGQLVGEDDPWRFVWMTSPDGK

YRIVVGQEWEYREDMALAIVAGQLIPWLVALPIMLIIMMVLLGRELAPLNKLALALRMRDPDSEKPLNAT

GVPSEVRPLVESLNQLFARTHAMMVRERRFTSDAAHELRSPLTALKVQTEVAQLSDDDPQARKKALLQLH

SGIDRATRLVDQLLTLSRLDSLDNLQDVAEIPLEDLLQSSVMDIYHTAQQAKIDVRLTLNAHGIKRTGQP

LLLSLLVRNLLDNGPGVTPEALARIGERFYRPPGQTATGSGLGLSIVQRIAKLHDMNVEFGNAEQGGFEA

KVSW

>gi|30064375|ref|NP_838546.1| DNA-binding transcriptional regulator QseB [Shigella flexneri 2a str. 2457T]

MRILLIEDDMLIGDGIKTGLSKMGFSVDWFTQGRQGKEALYSAPYDAVILDLTLPGMDGRDILREWREKG

QREPVLNLTARDALAERVEGLRLGADDYLCKPFALIEVAARLEALMRRTNGQASNELRHGNVMLDPGKRI

ATLAGEPLTLKPKEFALLELLMRNAGRVLPRKLIEEKLYTWDEEVTSNAVEVHVHHLRRKLGSDFIRTVH

GIGYTLGEK

>gi|30064360|ref|NP_838531.1| biopolymer transport protein ExbB [Shigella flexneri 2a str. 2457T]

MGNNLMQTDLSVWGMYQYADIVVKCVMIGLILASVVTWAIFFSKSVEFFNQKRRLKREQQLLAEARSLNQ

ANDIAADFGSKSLSLHLLNEAQNELELSEGSDDNEGIKERTSFRLERRVAAVGRQMGRGNGYLATIGAIS

PFVGLFGTVWGIMNSFIGIAQTQTTNLAVVAPGIAEALLATAIGLVAAIPAVVIYNVFARQIGGFKAMLG

DVAAQVLLLQSRDLDLEASAAAHPVRVAQKLRAG

>gi|30064359|ref|NP_838530.1| biopolymer transport protein ExbD [Shigella flexneri 2a str. 2457T]

MAMHLNENLDDNGEMHDINVTPFIDVMLVLLIIFMVAAPLATVDVKVNLPASTSTPQPRPEKPVYLSVKA

DNSMFIGNDPVTDETMITALNALTEGKKDTTIFFRADKTVDYETLMKVMDTLHQAGYLKIGLVGEETAKA

K

>gi|30064356|ref|NP_838527.1| hypothetical protein S3250 [Shigella flexneri 2a str. 2457T]

MERFLENAMYASRWLLAPVYFGLSLALVALALKFFQEIIHVLPNIFSMAESDLILVLLSLVDMTLVGGLL

VMVMFSGYENFVSQLDISENKEKLKWLGKMDATSLKNKVAASIVAISSIHLLRVFMDAKNVPDNKLMWYV

IIHLTFVLSAFVMGYLDRLTRHNH

>gi|30064352|ref|NP_838523.1| hydrogenase 2 small subunit [Shigella flexneri 2a str. 2457T]

MTGDNTLIHSHGINRRDFMKLCAALAATMGLSSKAAAEMAESVTNPQRPPVIWIGAQECTGCTESLLRAT

HPTVENLVLETISLEYHEVLSAAFGHQVEENKHNALEKYKGQYVLVVDGSIPLKDNGIYCMVAGEPIVDH

IRKAAEGSAAIIAIGSCSAWGGVAAAGVNPTGAVSLQEVLPGKTVINIPGCPPNPHNFLATVAHIITYGK

PPKLDDKNRPTFAYGRLIHEHCERRPHFDAGRFAKEFGDEGHREGWCLYHLGCKGPETYGNCSTLQFCDV

GGVWPVAIGHPCYGCNEKGIGFHKGIHQLANVENQTPRSQKPDVNAKEGGNVSAGAIGLLGGVVGLVAGV

SVMAVRELGRQQKKDNADSRGE

>gi|30064351|ref|NP_838522.1| hydrogenase 2 protein HybA [Shigella flexneri 2a str. 2457T]

MNRRNFIKAASCGALLTGALPSVSHAAAENRPPIPGSLGMLYDSTLCVGCQACVTKCQDINFPERNPQGE

QTWSNNDKLSPYTNNIIQVWTSGTGVNKDQEENGYAYIKKQCMHCVDPNCVSVCPVSALKKDPKTGIVHY

DKDVCTGCRYCMVACPYNVPKYDYNNPFGALHKCELCNQKGVERLDKGGLPGCVEVCPAGAVIFGTREEL

MAEAKKRLALKPGSEYHYPRQTLKSGDTYLHTVPKYYPHLYGEKEGGGTQVLVLTGVPYENLDLPKLDDL

STGARSENIQHTLYKGMMLPLAVLAGLTVLVRRNTKNDHHDGGDDHES

>gi|30064350|ref|NP_838521.1| hydrogenase 2 b cytochrome subunit [Shigella flexneri 2a str. 2457T]

MSHDPQPLGGKIISKPVMIFGPLIVICMLLIVKRLVFGLGSVSDLNGGFPWGVWIAFDLLIGTGFACGGW

ALAWAVYVFNRGQYHPLVRPALLASLFGYSLGGLSITIDVGRYWNLPYFYIPGHFNVNSVLFETAVCMTI

YIGVMALEFAPALFERLGWKVSLQRLNKVMFFIIALGALLPTMHQSSMGSLMISAGYKVHPLWQSYEMLP

LFSLLTAFIMGFSIVIFEGSLVQAGLRGNGPDEKSLFVKLTNTISVLLAIFIGLRFGELIYRDKLSLAFA

GDFYSVMFWIEVLLMLFPLVVLRVAKLRNDSRMLFLSALSALLGCATWRLTYSLVAFNPGGGYAYFPTWE

ELLISIGFVAIEICAYIVLIRLLPILPPLKQNDHNRHEASKA

>gi|30064348|ref|NP_838519.1| hydrogenase 2 maturation endopeptidase [Shigella flexneri 2a str. 2457T]

MRILVLGVGNILLTDEAIGVRIVEALEQRYILPDYVEILDGGTAGMELLGDMANRDHLIIADAIVSKKNT

PGTMMILRDEEVPALFTNKISPHQLGLADVLSALRFTGEFPKKLTLVGVIPESLEPHIGLTPTVEAMIEP

ALEQVLAALRESGVEAIPREAIHD

>gi|30064335|ref|NP_838506.1| hypothetical protein S3228 [Shigella flexneri 2a str. 2457T]

MKLVEHYIMRGTRRLVLIIVGFLIFIFASYSAQRYLTEAANGTLALDVVLDIVFYKVLIALEMLLPVGLY

VSVGVTLGQMYTDSEITAISAAGGSPGRLYKAVLYLAIPLSIFVTLLSMYGRPWAYAQIYQLEQQSQSEL

DVRQLRAKKFNTNDNGRMILSQTVDQDNNRLTDALIYTSTANRTRIFRAGSVDVVDPSPEKPTVMLHNGT

AYLLDHQGRDDNEQIYRNLQLHLNPLDQSPNVKRKAKSVTELARSAFPADHAELQWRQSRGLTALLMALL

AISLSRVKPRQGRFSTLLPLTLLFIAIFYGGDVCRTLVANGAIPLIPGLWLVPGLMLMGLLILVARDFSL

LQKFSR

>gi|30064334|ref|NP_838505.1| hypothetical protein S3227 [Shigella flexneri 2a str. 2457T]

MNVFSRYLIRHLFLGFAAAAGLLLPLFTTFNLINELDGVSPGGYRWTQAVLVVLMTLPRTLVELSPFIAL

LGGIVGLGQLSKNSELTAIRSTGFSIFRIALVALVAGILWTVSLGAIDEWVASPLQQQALQIKSTATALG

EDDDITGNMLWARRGNEFVTVKSLNEQGQPVGVEIFHYRDDLSLESYIYARSATIEDDKTWVLHGVNHKK

WLNGKETLETLDNLAWQSAFTSMNLEELSMPGNTFSVRQLNHYIHYLQETGQPSSEYHLALWEKLGQPIL

TLAMILLAVPFTFSAPRSPGMGSRLAVGVIVGLLTWISYQIMVNLGLLFALSAPVTALGLPVAFVLVALS

LVYWYDRQH

>gi|30064328|ref|NP_838499.1| DNA-binding transcriptional regulator GlcC [Shigella flexneri 2a str. 2457T]

MKDERRPICEVVAESIERLIIDGVLKVGQPLPSERRLCEKLGFSRSALREGLTVLRGRGIIETAQGRDSH

VARLNREQDTSPLIHLFSTQPRTLYDLLDVRALLEGESARLAATLGTQADFVVITRCYEKMLAASENHKE

ISLIEHAQLDHAFHLAICQASHNQVLVFTLQSLTDLMFNSVFASVNDLYHRPQQKKQIDRQHARIYNAVL

QRLPHVAQRAARDHVRTVKKNLHDIELEGHHLIRSAVPLEMNKVGM

>gi|30064320|ref|NP_838491.1| IS3 orfB [Shigella flexneri 2a str. 2457T]

MKYVFIEKHQAEFSIKAMCRVLRVARSGWYTWCQRRTRISTRQQFRQHCDSVVLAAFTRSKQRYGAPRLT

DELRAQGYPFNVKTVAASLRRQGLRAKASRKFSPVSYRAHGLPVSENLLEQDFYASGPNQKWPGDITYLR

TDEGWLYLAVVIDLWSRAVIGWSMSPRMTAQLPCDALQMALWRRKRPRNVIVHTDRGGQYCSADYQAQLK

RHNLRGSMSAKGCCYDNACVESFFHSLKVECIHGEHFISREIMRATVFNYIECDYNRWRRHSWCGGLSPE

QFENQNLA

>gi|30064316|ref|NP_838487.1| hypothetical protein S3204 [Shigella flexneri 2a str. 2457T]

MNTLPDTHVREASGCPSPITIWQTLLTRLLDQHYGLTLNDTPFADERVIEQHIEAGISLCDAVNFLVEKY

ALVRTDQPGFSAGAPSQLINSIDILRARRATGLMTRDNYRTVNNITRGKHPEAKQ

>gi|30064281|ref|NP_838452.1| tRNA (guanine-N(7)-)-methyltransferase [Shigella flexneri 2a str. 2457T]

MKNDVISPEFDENGRPLRRIRSFVRRQGRLTKGQEHALENYWPVMGVEFSEDMLDFPALFGREAPVTLEI

GFGMGASLVAMAKDRPEQDFLGIEVHSPGVGACLASAHEEGLSNLRVMCHDAVEVLHKMIPDNSLRMVQL

FFPDPWHKARHNKRRIVQVPFAELVKSKLQLGGVFHMATDWEPYAEHMLEVMSSIDGYKNLSESNDYVPR

PASRPVTKFEQRGHRLGHGVWDLMFERVK

>gi|30064268|ref|NP_838439.1| resistance protein [Shigella flexneri 2a str. 2457T]

MNTLTFLLSTVIELYTMVLLLRIWMQCAHCDFYTPFSQFVVKVAQPIIGPLRRVIPAMGPIDSASLLVAY

ILSFIKAIVLFEVVTFLPIIWIAGLLILLKTIGLLIFWVLLVMAIMSWVSQGRSPIEYVLIQLADPLLRP

IRRLLPAMGGIDFSPMILVLLLYAINMGVAEVLQATGNMLLPGLWMAL

>gi|30064265|ref|NP_838436.1| Holliday junction resolvase-like protein [Shigella flexneri 2a str. 2457T]

MSGTLLAFDFGTKSIGVAVGQRITGTARPLPAIKAQDGTPDWNLIERLLKEWQPDEIIVGLPLNMDGTEQ

PLTARARKFANRIHGRFGVEVKLHDERLSTVEARSGLFEQGGYRALNKGKVDSASAVIILESYFEQGY

>gi|30064263|ref|NP_838434.1| glutathione synthetase [Shigella flexneri 2a str. 2457T]

MIKLGIVMDPIANINIKKDSSFAMLLEAQRRGYELHYMEMGDLYLINGEARAHTRTLNVKQNYEEWFSFV

GEQDLPLADLDVILMRKDPPFDTEFIYATYILERAEEKGTLIVNKPQSLRDCNEKLFTAWFSDLTPETLV

TRNXAQLKAFWEKHSDIILKPLDGMGGASIFRVKEGDPNLGVIAETLTEHGTCYCMAQNYLPAIKDGDKR

VLVVDGEPVPYCLARIPQGGETRGNLAAGGRGEPRPLTESDWKIARQIGPTLKEKGLIFVGLDIIGDRLT

EINVTSPTCIREIEAEFPVSITGMLMDAIEARLQQQ

>gi|30064249|ref|NP_838420.1| hypothetical protein S3128 [Shigella flexneri 2a str. 2457T]

MLACCAICSPVRCAFPYTETERRFTCKRRIFRIILTLCGRENMKIRALLVAMSVATVLTGCQNMDSNGLL

SSGAEAFQAYSLSDAQVKTLSDQACQEMDSKATIAPANSEYAKRLTTIANALGNNINGQPVNYKVYMAKD

VNAFAMANGCIRVYSGLMDMMTDNEVEAVIGHEMGHVALGHVKKGMQVALGTNAVRVAAASAGGIVGSLS

QSQLGDLGEKLVNSQFSQRQEAEADDYSYDLLHQRGISPAGLATSFEKLAKLEEGRQSSMFDDHPASAER

AQHIRDRISADGIK

>gi|30064233|ref|NP_838404.1| fructose-bisphosphate aldolase [Shigella flexneri 2a str. 2457T]

MSKIFDFVKPGVITGDDVQKVFQVAKENNFALPAVNCVGTDSINAVLETAAKVKAPVIVQFSNGGASFIA

GKGVKSDVPQGAAILGAISGAHHVHQMAEHYGVPVILHTDHCAKKLLPWIDGLLDAGEKHFAATGKPLFS

SHMIDLSEESLQENIEICSKYLERMSKIGMTLEIELGCTGGEEDGVDNSHMDASALYTQPEDVDYAYTEL

SKISPRFTIAASFGNVHGVYKPGNVVLTPTILRDSQEYVSKKHNLPHNSLNFVFHGGSGSTAQEIKDSVS

YGVVKMNIDTDTQWATWEGVLNYYKANEAYLQGQLGNPKGEDQPNKKYYDPRVWLRAGQTSMIARLEKAF

QELNAIDVL

>gi|30064231|ref|NP_838402.1| arginine exporter protein [Shigella flexneri 2a str. 2457T]

MFSYYFQGLALGAAMILPLGPQNAFVMNQGIRRQYHIMIALLCAISDLVLICAGIFGGSALLMQSPWLLA

LVTWGGVVFLLWYGFGAFKTAMSSNIELASAEVLKQGRWKIIATMLAVTWLNPHVYLDTFVVLGSLGGQL

DVEPKRWFALGTISASFLWFFGLAILAAWLAPRLRTAKSQRIINLVVGCVMWFIALQLARDGIAHAQALF

S

>gi|30064220|ref|NP_838391.1| hypothetical protein S3094 [Shigella flexneri 2a str. 2457T]

MSIQNEMPGYNEMNQYLNQQGTGLTPAEMHGLISGMICGGNDDSSWLPLLHDLTNEGMAFGHELAQALRK

MHSATSDALQDDGFLFQLYLPDGDDVSVFDRADALAGWVNHFLLGLGVTQPKLDKVTGETGEAIDDLRNI

AQLGYDEDEDQEELEMSLEEIIEYVRVAALLCHDTFTHPQPTAPEVQKPTLH

>gi|30064207|ref|NP_838378.1| flavodoxin FldB [Shigella flexneri 2a str. 2457T]

MNMGLFYGSSTCYTEMAAEKIRDIIGPELVTLHNLKDDSPKLMEQYDVLILGIPTWDFGEIQEDWEAVWD

QLDDLNLEGKIIALYGLGDQLGYGEWFLDALGMLHDKLSTKGVKFVGYWPTEGYEFTSPKPVIADGQLFV

GLALDETNQYDLSDERIQSWCEQILNEMAEHYA

>gi|30064206|ref|NP_838377.1| site-specific tyrosine recombinase XerD [Shigella flexneri 2a str. 2457T]

MKQELARIEQFLDALWLEKNLAENTLNAYRRDLSMMVEWLHHRGLTLATAQSDDLQALLAERLEGGYKAT

SSARLLSAVRRLFQYLYREKFREDDPSAHLASPKLPQRLPKDLSEAQVERLLQAPLIDQPLELRDKAMLE

VLYATGLRVSELVGLTMSDISLRQGVVRVIGKGNKERLVPLGEEAVYWLETYLEHGRPWLLNGVSIDVLF

PSQRAQQMTRQTFWHRIKHYAVLAGIDSEKLSPHVLRHAFATHLLNHGADLRVVQMLLGHSDLSTTQIYT

HVATERLRQLHQQHHPRA

>gi|30064178|ref|NP_838349.1| DNA-binding transcriptional regulator GalR [Shigella flexneri 2a str. 2457T]

MATIKDVARLAGVSVATVSRVINNSPKASEASRLAVHSAMESLSYHPNANARALAQQTTETVGLIVGDVS

DPFFGAMVKAVEQVAYHTGNFLLIGNGYHNEQKERQAIEQLIRHRCAALVVHAKMIPDADLASLMKQMPG

MVLINRILPGFENRCIALDDRYGAWLATRHLIQQGHTRIGYLCSNHSISDAEDRLQGYYDALAESGIPAN

DRLVTFGEPDESGGEQAMTELLGRGRNFTAVACYNDSMAAGAMGVLNDNGIDVPGEISLIGFDDVLVSRY

VRPRLTTVRYPIVTMATQAAELALALADNRPLPEITNVFSPTLVRRHSVSTPSLEASHHATSD

>gi|30064173|ref|NP_838344.1| transporter [Shigella flexneri 2a str. 2457T]

MLFAWITDPNAWLALGTLTLLEIVLGIDNIIFLSLVVAKLPTAQRAHARRLGLAGAMVMRLALLASISWV

TRLTNPLFTIFSQEISARDLILLLGGLFLIWKASKEIHESIEGEEEGLKTRVSSFLGAIVQIMLLDIIFS

LDSVITAVGLSDHLFIMMAAVVIAVGVMMFAARSIGDFVERHPSVKMLALSFLILVGFTLILESFDIHVP

KGYIYFAMFFSIAVESLNLIRNKKNPL

>gi|30064171|ref|NP_838342.1| dinucleoside polyphosphate hydrolase [Shigella flexneri 2a str. 2457T]

MIDDDGYRPNVGIVICNRQGQVMWARRFGQHSWQFPQGGINPGESAEQAMYRELFEEVGLSRKDVRILAS

TRNWLRYKLPKRLVRWDTKPVCIGQKQKWFLLQLVSGDAEINMQTSSTPEFDGWRWVSYWYPVRQVVSFK

RDVYRRVMKEFASVVMSLQENTPKPQNASAYRRKRG

>gi|30064169|ref|NP_838340.1| prolipoprotein diacylglyceryl transferase [Shigella flexneri 2a str. 2457T]

MTSSYLHFPEFDPVIFSIGPVALHWYGLMYLVGFIFAMWLATRRANRPGSGWTKNEVENLLYAGFLGVFL

GGRIGYVLFYNFPQFMADPLYLFRVWDGGMSFHGGLIGVIVVMIIFARRTKRSFFQVSDFIAPLIPFGLG

AGRLGNFINGELWGRVDPNFPFAMLFPGSRTEDILLLQTNPQWQSIFDTYGVLPRHPSQLYELLLEGVVL

FIILNLYIRKPRPMGAVSGLFLIGYGAFRIIVEFFRQPDAQFTGAWVQYISMGQILSIPMIVAGVIMMVW

AYRRSPQQHVS

>gi|30064164|ref|NP_838335.1| exonuclease V subunit gamma [Shigella flexneri 2a str. 2457T]

MLRVYHSNRLDVLEALMEFIVERERLDDPFEPEMILVQSTGMAQWLQMTLSQKFGIAANIDFPLPASFIW

DMFVRVLPEIPKESAFNKQSMSWKLMTLLPQLLEREDFTLLRHYLTDDSDKRKLFQLSSKAADLFDQYLV

YRPDWLAQWETGHLVEGLGEAQAWQAPLWKALVEYTHELGQPRWHRANLYQRFIETLESATTCPPGLPSR

VFICGISALPPVYLQALQALGKHIEIHLLFTNPCRYYWGDIKDPAYLAKLLTRQRRHSFEDRELPLFRDS

ENAGQLFNSDGEQDVGNSLLASWGKLGRDYIYLLSDLESSQELDAFVDVTPDNLLHNIQSDILELENRAV

AGVNIEEFSRSDNKRPLDPLDSSITFHVCHSPQREVEVLHDRLLAMLEEAPTLTPRDIIVMVADIDSYSP

FIQAVFGSAPADRYLPYAISDRRARQSHPVLEAFISLLSLPDSRFVSEDVLALLDVPVLAARFDITEEGL

RYLRQWVNESGIRWGIDDDNVRELELPATGQHTWRFGLTRMLLGYAMESAQGEWQSVLPYDESSGLIAEL

VGHLASLLMQLNIWRRGLAQERPLEEWLPVCRDMLNAFFLPDAETEAAMTLIEQQWQAIIAEGLGAQYGD

AVPLSLLRDELAQRLDQERISQRFLAGPVNICTLMPMRSIPFKVVCLLGMNDGVYPRQLAPLGFDLMSQK
PKRGDRSRRDDDRYLFLEALISAQQKLYISYIGRSIQDNSERFPSVLVQELIDYIGQSHYLPGDEALNCD
ESEARVKAHLTCLHTRMPFDPQNYQPGERQSYAREWLPAASQAGKAHSEFVQPLPFTLPETVPLETLQRF
WAHPVRAFFQMRLQVNFHTEDSEIPDTEPFILEGLSRYQINQQLLNALVEQDDAERLFRRFRAAGDLPYG
AFGEIFWETQCQEMQQLADRVIACRQPGQSMEIDLACNGVQITGWLPQVQPDGLLRWRPSLLSVAQGMQL
WLEHLVYCASGGNGESRLFLRKDGEWRFPPLAAEQALHYLSQLIEGYREGMSAPLLVLPESGGAWLKTCY
DAQNDAMLDDDSTLQKARTKFLQAYEGNMMVRGEGDDIWYQRLWRQLTPETMETIVEQSQRFLLPLFRFN
QS

>gi|30064159|ref|NP_838330.1| amidase [Shigella flexneri 2a str. 2457T]
MTDYASFAKVSGQISRLLVTQLRFLLLGRGMSGSNTAISRRRLLQGAGAMWLLSVSQVSLAAVSQVVAVR
VWPASSYTRVTVESNRQLKYKQFALSNPERVVVDIEDVNLNSVLKGMAAQIRADDPFIKSARVGQFDPQT
VRMVFELKQNVKPQLFALAPVAGFKERLVMDLYPANAQDMQDPLLALLEDYNKGDLEKQVPPAQSGPQPG
KAGRDRPIVIMLDPGHGGEDSGAVGKYKTREKDVVLQIARRLRSLIEEEGNMKVYMTRNEDIFIPLQVRV
AKAQKQRADLFVSIHADAFTSRQPSGSSVFALSTKGATSTAAKYLAQTQNASDLIGGVSKSGDRYVDHTM
FDMVQSLTIADSLKFGKAVLNKLGKINKLHKNQVEQAGFAVLKAPDIPSILVETAFISNVEEERKLKTAT
FQQEVAESILAGIKAYFADGATLARRG

>gi|30064156|ref|NP_838327.1| hypothetical protein S3020 [Shigella flexneri 2a str. 2457T]

MTNPQFAGHPFGTTVTAETLRNTFAPLSQWEDKYRQLIMLGKQLPALPDELKAQAKEIAGCENRVWLGYT

VAENGKMHFFGNSEGRIVRGLLAVLLTAVEGKTAAELQAQSPLALFDELGLRAQLSASRSQGLNALSEAI

IAATKQV

>gi|30064152|ref|NP_838323.1| hypothetical protein S3016 [Shigella flexneri 2a str. 2457T]

MTSRFMLIFAAISGFIFVALGAFGAHVLSKTMGAVEMGWIQTGLEYQAFHTLAILGLAVAMQRRISIWFY

WSSVFLALGTVLFSGSLYCLALSHLRLWAFVTPVGGVSFLAGWALMLVGAIRLKRKGVSHE

>gi|30064144|ref|NP_838315.1| exonuclease IX [Shigella flexneri 2a str. 2457T]

MRSLFLFSQPAIACSGIECYPYRLIFKGVIVAVHLLIVDALNLIRRIHAVQGSPCVETCQHALDQLIMHS

QPTHAVAVFDDENRSSGWRHQRLPDYKADRPPMPEELHDEMPALRAAFEQRGVPCWSASGNEADDLAATL

AVKVTQAGHQATIVSTDKGYCQLLSPTLRIRDYFQKRWLDAPFIDKEFGVQPQQLPDYWGLAGISSSKVP

GVAGIGPKSATQLLVEFQSLEGIYENLDAVAEKWRKKLETHKEMAFLCRDIARLQTDLHIDGNLQQLRLV

R

>gi|30064143|ref|NP_838314.1| L-serine dehydratase (deaminase), L-SD2 [Shigella flexneri 2a str. 2457T]

MISVFDIFKIGIGPSSSHTVGPMKAGKQFTDDLIARNLLKDVTRVVVDVYGSLSLTGKGHHTDIAIIMGL

AGNLPDTVDIDSIPGFIQDVNTHGRLMLANGQHEVEFPVDQCMNFHADNLSLHENGMRITALAGDKVVYS

QTYYSIGGGFIVDEEHFGQQDSAPVEVPYPYSSAADLQKHCQETGLSLSGLMMKNELALHSKEELEQHLA

NVWEVMRGGIERGISTEGVLPGKLRVPRRAAALRRMLVSQDKTTTDPMAVVDWINMFALAVNEENAAGGR

VVTAPTNGACGIIPAVLAYYDKFIREVNANSLARYLLVASAIGSLYKMNASISGAEVGCQGEVGVACSMA

AAGLAELLGASPAQVCIAAEIAMEHNLGLTCDPVAGQVQVPCIERNAIAAVKAVNAARMALRRTSEPRVC

LDKVIETMYETGKDMNAKYRETSRGGLAMKIVACD

>gi|30064131|ref|NP_838302.1| hybrid sensory histidine kinase BarA [Shigella flexneri 2a str. 2457T]

MTNYSLRARMMILILAPTVLIGLLLSIFFVVHRYNDLQRQLEDAGASIIEPLAVSTEYGMSLQNRESIGQ

LISVLHRRHSDIVRAISVYDENNRLFVTSNFHLDPSSMQLGSNVPFPRQLTVTRDGDIMILRTPIISESY

SPDESPSSDAKNSQNMLGYIALELDLKSVRLQQYKEIFISCVMMLFCIGIALIFGWRLMRDVTGPIRNMV

NTVDRIRRGQLDSRVEGFMLGELDMLKNGINSMAMSLAAYHEEMQHNIDQATSDLRETLEQMEIQNVELD

LAKKRAQEAARIKSEFLANMSHELRTPLNGVIGFTRLTLKTELTPTQRDHLNTIERSANNLLAIINDVLD

FSKLEAGKLILESIPFPLRSTLDEVVTLLAHSSHDKGLELTLNIKSDVPDNVIGDPLRLQQIITNLVGNA

IKFTENGNIDILVEKRALSNTKVQIEVQIRDTGIGIPERDQSRLFQAFRQADASISRRHGGTGLGLVITQ

KLVNEMGGDISFHSQPNRGSTFWFHINLDLNPNIIIEGPSIQCLAGKRLAYVEPNSAAAQCTLDILSETP

LEVVYSPTFSALPPAHYDMMLLGIAVTFREPLTMQHERLAKAVSMTDFLMLALPCHAQVNAEKLKQDGIG

ACLLKPLTPTRLLPALTEFCHHKQNTLLPVTDESKLAMTVMAVDDNPANLKLIGALLEDMVQHVELCDSG

HQAVERAKQMPFDLILMDIQMPDMDGIRACELIHQLPHQRQTPVIAVTAHAMAGQKEKLLGAGMSDYLAK

PIEEERLHNLLLRYKPGSGISSRVVTPEVNEIVVNPNATLDWQLALRQAAGKTDLARDMLQMLLDFLPEV

RNKVEEQLAGENPEGLVDLIHKLHGSCGYSGVPRMKNLCQLIEQQLRSGTKEEDLEPELLELLDEMDNVA

REASKILG

>gi|30064129|ref|NP_838300.1| GDP/GTP pyrophosphokinase [Shigella flexneri 2a str. 2457T]

MVAVRSAHINKAGEFDPEKWIASLGITSQKSCECLAETWAYCLQQTQGHPDASLLLWRGVEMVEILSTLS

MDIDTLRAALLFPLADANVVSEDVLRESVGKSVVNLIHGVRDMAAIRQLKATHTDSVSSEQVDNVRRMLL

AMVDDFRCVVIKLAERIAHLREVKDAPEDERVLAAKECTNIYAPLANRLGIGQLKWELEDYCFRYLHPTE

YKRIAKLLHERRLDREHYIEEFVGHLRAEMKAEGVKAEVYGRPKHIYSIWRKMQKKNLAFDELFDVRAVR

IVAERLQDCYAALGIVHTHYRHLPDEFDDYVANPKPNGYQSIHTVVLGPGGKTVEIQIRTKQMHEDAELG

VAAHWKYKEGAAAGGARSGHEDRIAWLRKLIAWQEEMADSGEMLDEVRSQVFDDRVYVFTPKGDVVDLPA

GSTPLDFAYHIHSDVGHRCIGAKIGGRIVPFTYQLQMGDQIEIITQKQPNPSRDWLNPNLGYVTTSRGRS

KIHAWFRKQDRDKNILAGRQILDDELEHLGISLKEAEKHLLPRYNFNDVDELLAAIGGGDIRLNQMVNFL

QSQFNKPSAEEQDAAALKQLQQKSYTPQNRSKDNGRVVVEGVGNLMHHIARCCQPIPGDEIVGFITQGRG

ISVHRADCEQLAELRSHAPERIVDAVWGESYSAGYSLVVRVVANDRSGLLRDITTILANEKVNVLGVASR

SDTKQQLATIDMTIEIYNLQVLGRVLGKLNQVPDVIDARRLHGS

>gi|30064105|ref|NP_838276.1| cell division protein FtsB [Shigella flexneri 2a str. 2457T]

MGKLTLLLLAILVWLQYSLWFGKNGIHDYTRVNDDVAALQATNAKLKARNDQLFAEIDDLNGGQEALEER

ARNELSMTRPGETFYRLVPDASKRAQSAGQNNR

>gi|30064103|ref|NP_838274.1| 2-C-methyl-D-erythritol 2,4-cyclodiphosphate synthase [Shigella flexneri 2a str. 2457T]

MRIGHGFDVHAFGGEGPIIIGGVRIPYEKGLLAHSDGDVALHALTDALLGAAALGDIGKLFPDTDPAFKG

ADSRELLREAWRRIQAKGYTLGNVDVTIIAQAPKMLPHIPQMRVFIAEDLGCHMDDVNVKATTTEKLGFT

GRGEGIACEAVALLIKATK

>gi|30064097|ref|NP_838268.1| phenylacrylic acid decarboxylase-like protein [Shigella flexneri 2a str. 2457T]

MKLIVGMTGATGAPLGVALLQALREMPNVETHLVMSKWAKTTIELETPYSARDVAALADFSHNPADQAAI

ISSGSFRTDGMIVIPCSMKTLAGIRAGYADGLVGRAADVVLKEGRKLVLVPREMPLSTIHLENMLALSRM

GVAMVPPMPAFYNHPETVDDIVHHVVARVLDQFGLEHPHARRWQGLPQARNFSQENE

>gi|30064096|ref|NP_838267.1| 4-hydroxybenzoate decarboxylase, partial [Shigella flexneri 2a str. 2457T]

MAFDDLRSFLQALDDHGQLLKISEEVNAEPDLAAAANATGRIGDGAPALWFDNIRGFTDARVAMNTIGSW

QNHAISLGLPPNTPVKKQIDEFIRRWDNFPIAPERRANPAWAQNTVDGEEINLFDILPLFRLNDGDGGFY

LDKACVVSRDPLDPDNFGKQNVGIYRMEVKGKRKLGLQPVPMHDIALHLHKAEERGEDLPIAITLGNDPI

ITLMGATPLKYDQSEYEMAGALRESPYPIATAPLTGFDVPWGSEVILEGVIESRKREIEGPFGEFTGHYS

GGRNMTVVRIDKVSYRSKPIFESLYLGMPWTEIDYL

>gi|30064086|ref|NP_838257.1| hypothetical protein S2941 [Shigella flexneri 2a str. 2457T]

MSGKRISREKLTIKKMIDLYQAKCPQASAEPEHYEALFVYAQKRLDKCVFGEEKPACKQCPVHCYQPAKR

EEMKQIMRWAGPRMLWRHPILTVRHLIDDKRPVPELPEKYRPKKPRE

>gi|30064085|ref|NP_838256.1| formate hydrogen-lyase transcriptional activator for fdhF, hyc and hyp operons [Shigella flexneri 2a str. 2457T]

MSYTPMSDLGQQGLFDITRTLLQQPDLASLCEALSQLVKRSALADNAAIVLWQAQTQRASYYASREKDTP

IKYEDETVLAHGPVRSILSRPDTLHCSYEEFCETWPQLAAGGLYPKFGHYCLMPLAAGGHIFGGCEFIRY

DERPWSEKEFNRLQTFTQIVSVVTEQIQSRVVNNVDYELLCRERDNFRILVAITNAVLSRLDMDELVSEV

AKEIHYYFDIDDISIVLRSHRKNKLNIYSTHYLDKQHPAHEQSEVDEAGTLTERVFKSKEMLLINLYERD

DLAPYERMLFDTWGNQIQTLCLLPLMSGDTMLGVLKLAQCEEKVFTTTNLNLLRQIAERVAIAVDNALAY

QEIHRLKERLVDENLALTEQLNNVDSEFGEIIGRSEAMYSVLKQVEMVAQSDSTVLILGETGSGKELIAR

AIHNLSGRNNRRMVKMNCAAMPAGLLESDLFGHERGAFTGANAQRIGRFELADKSSLFLDEVGDMPLELQ

PKLLRVLQEQEFERLGSNKIIQTDVRLIAATNRDLKKMVADREFRSDLYYRLNVFPIHLPPLRERPEDIP

LLAKAFTFKIARRLGRNIDSIPAETLRTLSNMEWPGNVRELENVIERAVLLTRGNVLQLSLPDIALPEPE

TPPAATVVAQEGEDEYQLIVRVLKETNSVVAGPKGAAQRLGLKRTTLLSRMKRLGIDKSALI

>gi|30064078|ref|NP_838249.1| small subunit of hydrogenase-3, iron-sulfur protein (part of formate hydrogenlyase (FHL) complex) [Shigella flexneri 2a str. 2457T]

MNRFVIADSTLCIGCHTCEAACSETHRQHGLQSMPRLRVMLNEKESAPQLCHHCEDAPCAVVCPVNAITR

VDGAVQLNESLCVSCKLCGIACPFGAIEFSGSRPLDIPANANTPKAPPAPPAPARVSTLLDWVPGIRAIA

VKCDLCSFDEQGPACVRMCPTKALHLVDNTDIARVSKRKRELTFNTDFGDLTLFQQAQSGEAK

>gi|30064073|ref|NP_838244.1| ascBF operon repressor [Shigella flexneri 2a str. 2457T]

MTTMLEVAKRAGVSKATVSRVLSGNGYVSQETKDRVFQAVEESSYRPNLLARNLSAKSTQTLGLVVTNTL

YHGIYFSELLFHAARMAEEKGRQLLLADGKHSAEEERQAIQYLLDLRCDAIMIYPRFLSVDEIDDIIDAH

SQPIMVLNRRLRNNSSHSVWCDHKQTSFNAVAELINAGHQEIAFLTGSMDSPTSIERLAGYKDALSQHGI

ALNEKLIANGKWTPASGAEGVETLLERGAKFSALVASNDDMAIGAIKALHERGVAVPEQVSVIGFDDIAI

APYIVPALSSVKIPVTEMIQEIIGRLIFMLDGGDFSPPKTFSGKLIRRDSLIALSR

>gi|30064072|ref|NP_838243.1| electron transport protein HydN [Shigella flexneri 2a str. 2457T]

MNRFIIADASKCIGCRTCEVACVVSHQENQDCASLTPETFLPRIHVIKGVNISTATVCRQCEDAPCANVC

PNGAISRDKGFVPVMQERCIGCKTCVVACPYGAMEVVVRPVIRNSGAGLNVRADKAEANKCDLCNHREDG

PACMAACPTHALICVDRNKLEQLSAEKRRRTALMF

>gi|30064051|ref|NP_838222.1| multidrug resistant protein emrB [Shigella flexneri 2a str. 2457T]

MQQQKPLEGAQLVIMTIALSLATFMQVLNSTIANVAIPTIAGNLGSSLSQGTWVITSFGVANAISIPLTG

WLAKRVGEVKLFLWSTIAFAIASWACGVSSSLNMLIFFRVIQGIVAGPLIPLSQSLLLNNYPPAKRSIAL

ALWSMTVIVAPICGPILGGYISDNYHWGWIFFINVPIGVAVVLMTLQTLRGRETRTERRRIDAVGLALLV

IGIGSLQIMLDRGKELDCFSSQEIIILTVVAVVAICFLIVWELTDDNPIVDLSLFKSRNFTIGCLCISLA

YMLYFGAIVLLPQLLQEVYGYTATWAGLASAPVGIIPVILSPIIGRFAHKLDMRRLVTFSFIMYAVCFYW

RAYTFEPGMDFCASAWPQFIQGFAVVCFFMPLTTITLSGLPPERLAAASSLSNFTRTLAGSIGTSITTTM

WTNRESMHHAQLTESVNPFNPNAQAMYSQLEGLGMTQQQASGWIAQQITNQGLIISANEIFWMSAGIFLV

LLGLVWFAKPPFGAGGGGGGAH

>gi|30064043|ref|NP_838214.1| ribonucleotide-diphosphate reductase subunit beta [Shigella flexneri 2a str. 2457T]

MKLSRISAINWNKISDDKDLEVWNRLTSNFWLPEKVPLSNDIPAWQTLTVVEQQLTMRVFTGLTLLDTLQ

NVIGAPSLMPDALTPHEEAVLSNISFMEAVHARSYSSIFSTLCQTKDVDAAYAWSEENAPLQRKAQIIQQ

HYRGDDPLKKKIASVFLESFLFYSGFWLPMYFSSRGKLTNTADLIRLIIRDEAVHGYYIGYKYQKNMEKI

SLGQREELKSFAFDLLLELYDNELQYTDELYAETPWADDVKAFLCYNTNKALMNLGYEPLFPAEMAEVNP

AILAALSPNADENHDFFSGSGSSYVMGKAVETEDEDWNF

>gi|30064041|ref|NP_838212.1| ribonucleotide reductase stimulatory protein [Shigella flexneri 2a str. 2457T]

MSQLVYFSSSSENTQRFIERLGLPAVRIPLNERERIQVDEPYILIVPSYGGGGTAGAVPRQVIRFLNDEH

NRALLRGVIASGNRNFGEAYGRAGDVIARKCGVPWLYRFELMGTQSDIENVRKGVTEFWQRQPQNA

>gi|30064037|ref|NP_838208.1| DNA binding protein, nucleoid-associated [Shigella flexneri 2a str. 2457T]

MSVMLQSLNNIRTLRAMAREFSIDVLEEMLEKFRVVTKERREEEEQQRELAERQEKISTWLELMKADGI

NPEELLGNSSAAAPRAGKKRQPRPAKYKFTDVNGETKTWTGQGRTPKPIAQALAEGKSLDDFLI

>gi|30064036|ref|NP_838207.1| hypothetical protein S2882 [Shigella flexneri 2a str. 2457T]

MALTTISPHDAQELIARGAKLIDIRDADEYLREHIPEADLAPLSVLEQSGLPPKLRREQIIFHCQAGKRT

SNNADKLAAIAAPAEIFLLEDGIDGWKRAGLPVAVNKSQPLPLMRQVQIAAGGLILNGVVLGYTVNSGFF

LLSGFVGAGLLFAGISGFCGMARLLDKMPWNQRA

>gi|30064031|ref|NP_838202.1| LysM domain/BON superfamily protein [Shigella flexneri 2a str. 2457T]

MGLFNFVKDAGEKLWDAVTGQHDKDDQAKKVQEHLSKTGIPDADKVNIQIADGKATVTGDGLSQEAKEKI

LVAVGNISGIASVDDQVKTATPATASQFYTVKSGDTLSAISKQVYGNANLYNKIFEANKPMLKSPDKIYP

GQVLRIPEE

>gi|30064007|ref|NP_838178.1| 16S rRNA-processing protein RimM [Shigella flexneri 2a str. 2457T]

MSKQLTAQAPVDPIVLGKMGSSYGIRGWLRVFSSTEDAESIFDYQPWFIQKAGQWQQVQLESWKHHNQDM

IIKLKGVDDRDAANLLTNCEIVVDSSQLPQLEEGDYYWKDLMGCQVVTTEGYDLGKVVDMMETGSNDVLV

IKANLKDAFGIKERLVPFLDGQVIKKVDLTTRSIEVDWDPGF

>gi|30064006|ref|NP_838177.1| tRNA (guanine-N(1)-)-methyltransferase [Shigella flexneri 2a str. 2457T]

MWIGIISLFPEMFRAITDYGVTGRAVKNGLLSIQSWSPRDFTHDRHRTVDDRPYGGGPGMLMMVQPLRDA

IHAAKAAAGEGAKVIYLSPQGRKLDQAGVSELATNQKLILVCGRYEGIDERVIQTEIDEEWSIGDYVLSG

GELPAMTLIDSVSRFIPGVLGHEASATEDSFAEGLLDCPHYTRPEVLEGMEVPPVLLSGNHAEIRRWRLK

QSLGRTWLRRPELLENLALTEEQARLLAEFKTEHAQQQHKHDGMA

>gi|30064005|ref|NP_838176.1| 50S ribosomal protein L19 [Shigella flexneri 2a str. 2457T]

MSNIIKQLEQEQMKQDVPSFRPGDTVEVKVWVVEGSKKRLQAFEGVVIAIRNRGLHSAFTVRKISNGEGV

ERVFQTHSPVVDSISVKRRGAVRKAKLYYLRERTGKAARIKERLN

>gi|30064001|ref|NP_838172.1| phospho-2-dehydro-3-deoxyheptonate aldolase [Shigella flexneri 2a str. 2457T]

MHKDALNNVHITDEQVLMTPEQLKAAFPLSLQQEAQIADSRKTISDIIAGRDPRLLVVCGPCSIHDPETA

LEYARRFKALAAEVSDSLYLVMRVYFEKPRTTVGWKGLINDPHMDGSFDVEAGLQIARKLLLELVNMGLP

LATEALDPNSPQYLGDLFSWSAIGARTTESQTHREMASGLSMPVGFKNGTDGSLATAINAMRAAAQPHCF

VGINQAGQVALLQTQGNPDGHVILRGGKAPNYSPADVAQCEKEMEQAGLRPSLMVDCSHGNSNKDYRRQP

AVAESVVAQIKDGNRSIIGLMIESNIHEGNQSSEQPRSEMKYGVSVTDACISWEMTDALLREIHQDLNGQ

LTARVA

>gi|30063999|ref|NP_838170.1| bifunctional chorismate mutase/prephenate dehydratase [Shigella flexneri 2a str. 2457T]

MTSENPLLALREKISALDEKLLALLAERRELAVEVGKAKLLSHRPVRDIDRERDLLERLITLGKAHHLDA

HYITRLFQLIIEDSVLTQQALLQQHLNKINPHSARIAFLGPKGSYSHLAARQYAARHFEQFIESGCAKFA

DIFNQVETGQADYAVVPIENTSSGAINDVYDLLQHTSLSIVGEMTLTIDHCLLVSGTTDLSTINTVYSHP

QPFQQCSKFLNRYPHWKIEYTESTSAAMEKVAQAKSPHVAALGSEAGGTLYGLQVLERIEANQRQNFTRF

VVLARKAINVSDQVPAKTTLLMATGQQAGALVEALLVLRNHNLIMTRLESRPIHGNPWEEMFYLDIQANL

ESAEMQKALKELGEITRSMKVLGCYPSENVVPVDPT

>gi|30063990|ref|NP_838161.1| outer membrane protein assembly complex subunit YfiO [Shigella flexneri 2a str. 2457T]

MTRMKYLVAAATLSLFLAGCSGSKEEVPDNPPNEIYATAQQKLQDGNWRQAITQLEALDNRYPFGPYSQQ

VQLDLIYAYYKNADLPLAQAAIDRFIRLNPTHPNIDYVMYMRGLTNMALDDSALQGFFGVDRSDRDPQHA

RAAFSDFSKLVRGYPNSQYTTDATKRLVFLKDRLAKYEYSVAEYYTERGAWVAVVNRIEGMLRDYPDTQA

TRDALPLMENAYRQMQMNAQAEKVAKIIAANSSNT

>gi|30063980|ref|NP_838151.1| autonomous glycyl radical cofactor GrcA [Shigella flexneri 2a str. 2457T]

MITGIQITKAANADLLNSFWLLDSEKGEARCIVAKAGYAEDEVVAVSKLGDIEYREVPVEVKPEVRVEGG

QHLNVNVLRRETLEDAVKHPEKYPQLTIRVSGYAVRFNSLTPEQQRDVIARTFTESL

>gi|30063975|ref|NP_838146.1| RNA polymerase sigma factor RpoE [Shigella flexneri 2a str. 2457T]

MSEQLTDQVLVERVQKGDQKAFNLLVVRYQHKVASLVSRYVPSGDVPDVVQEAFIKAYRALDSFRGDSAF

YTWLYRIAVNTAKNYLVAQGRRPPSSDVDAIEAENFESGGALKEISNPENLMLSEELRQIVFRTIESLPE

DLRMAITLRELDGLSYEEIAAIMDCPVGTVRSRIFRAREAIDNKVQPLIRR

>gi|30063974|ref|NP_838145.1| anti-RNA polymerase sigma factor SigE [Shigella flexneri 2a str. 2457T]

MQKEQLSALMDGETLDSELLNELAHNPEMQKTWESYHLIRDSMRGDTPEVLHFDISSRVMAAIEEEPVRQ

SATLIPEAQPAPHQWQKMPFWQKVRPWAAQLTQMGVAACVSLAVIVGVQHYNGQSETSQQPETPVFNTLP

MMGKASPVSLGVPSEATANNGQQQQVQEQRRRINAMLQDYELQRRLHSEQLQFEQAQTQQAAVQVPGIQT

LGTQSQ

>gi|30063970|ref|NP_838141.1| signal peptidase I [Shigella flexneri 2a str. 2457T]

MANMFALILVIATLVTGILWCVDKFFFAPKRRERQAAAQAAAGDSLDKATLKKVAPKPGWLETGASVFPV

LAIVLIVRSFIYEPFQIPSGSMMPTLLIGDFILVEKFAYGIKDPIYQKTLIETGHPKRGDIVVFKYPEDP

KLDYIKRAVGLPGDKVTYDPVSKELTIQPGCSSGQACENALPVTYSNVEPSDFVQTFSRRNGGEATSGFF

EVPKNETKENGIRLSERKETLGDVTHRILTVPIAQDQVGMYYQQPGQQLATWIVPPGQYFMMGDNRDNSA

DSRYWGFVPEANLVGRATAIWMSFDKQEGEWPTGVRLSRIGGIH

>gi|30063966|ref|NP_838137.1| pyridoxine 5'-phosphate synthase [Shigella flexneri 2a str. 2457T]

MAELLLGVNIDHIATLRNARGTAYPDPVQAAFIAEQAGADGITVHLREDRRHITDRDVRILRQTLDTRMN

LEMAVTEEMLAIAVETKPHFCCLVPEKRQEVTTEGGLDVAGQREKIRDACKRLADAGIQVSLFIDADEEQ

IKAAAEVGAPFIEIHTGCYADAKTDAEQAQELVRIAKAATFAASLGLKVNAGHGLTYHNVKAIAAIPEMH

ELNIGHAIIGRAVMTGLKDAVAEMKRLMLEARG

>gi|30063965|ref|NP_838136.1| 4'-phosphopantetheinyl transferase [Shigella flexneri 2a str. 2457T]

MAILGLGTDIVEIARIEAVIARSGERLARRVLSDNEWAIWKTHHQPVRFLAKRFAVKEAAAKAFGTGIRN

GLAFNQFEVFNDELGKPRLRLWGEALKLAEKLGVVNMHVTLADERHYACATVIIES

>gi|30063943|ref|NP_838114.1| 2-component sensor protein [Shigella flexneri 2a str. 2457T]

MLYLNFPSVSIRLFASDESNTLKRWPVFPRSLRQLVMLAFLLILLPLLVLAWQAWQSLNALSDQAALVNR

TTLIDARRSEAMTNAALEMERSYRQYCVLDDPTLAKVYQSQRKRYSEMLDAHAGVLPDDKLYQALRQDLN

NLAQLQCNNSGPDAAAAARLEAFASANTEMVQATRTVVFSRGQQLQREIAERGQYFGWQSLVLFLVSLVM

VLLFTRMIIGPVKNIERMINRLGEGRSLGNSVSFCGPSELRSVGQRILWLSERLSWLESQRHQFLRHLSH

ELKTPLASMREGTELLADQVVGPLTPEQKEVVSILDSSSCNLQKLIEQLLDYNRKQADSAVELENVELAP

LVETVVSAHSLPARAKMMHTDVDLKATACLAEPMLLMSVLDNLYSNAVHYGAESGNICLRSSLHGARVYI

DVINTGTPIPQEERAMIFEPFFQGSHQRKGAVKGSGLGLSIARDCIRRMQGELYLVDESGQDVCFRIELP

SSKNTK

>gi|30063941|ref|NP_838112.1| 2-component transcriptional regulator [Shigella flexneri 2a str. 2457T]

MSHKPAHLLLVDDDPGLLKLLGLRLTSEGYSVVTAESGAEGLRVLNREKVDLVISDLRMDEMDGMQLFAE

IQKVQPGMPVIILTAHGSIPDAVAATQQGVFSFLTKPVDKDALYQAIDDALEQSAPATDERWREAIVTRS

PLMLRLLEQARLVAQSDVSVLINGQSGTGKEIFAQAIHNASPRNSKPFIAINCGALPEQLLESELFGHAR

GAFTGAVSNREGLFQAAEGGTLFLDEIGDMPAPLQVKLLRVLQERKVRPLGSNRDIDINVRIISATHRDL

SKAMARGEFREDLYYRLNVVSLKIPALAERTEDIPLLANHLLRQAAERHKPFVRAFSTDAMKRLMTASWP

GNVRQLVNVIEQCVALTSSPVISDALVEQALEGENTALPTFVEARNHFELNYLRKLLQITKGNVTHAARM

AGRNRTEFYKLLSRHELDANDFKE

>gi|30063940|ref|NP_838111.1| nitrogen regulatory protein P-II 1 [Shigella flexneri 2a str. 2457T]

MKKIDAIIKPFKLDDVREALAEVGITGMTVTEVKGFGRQKGHTELYRGAEYMVDFLPKVKIEIVVPDDIV

DTCVDTIIRTAQTGKIGDGKIFVFDVARVIRIRTGEEDDAAI

>gi|30063929|ref|NP_838100.1| 3-phenylpropionate dioxygenase subunit beta [Shigella flexneri 2a str. 2457T]

MSAQVSLELHHRISQFLFHEASLLDDWKFRDWLAQLDEEIRYTMRTTVNAQTRDRRKGVQPPTTWIFNDT

KDQLERRIARLETGMAWAEEPPSRTRHLISNCQVSETDIPNVFAVRVNYLLYRAQKERDETFYVGTRFDK

VRRLEDDNWRLLERDIVLDQAVITSHNLSVLF

>gi|30063907|ref|NP_838078.1| 4-hydroxy-3-methylbut-2-en-1-yl diphosphate synthase [Shigella flexneri 2a str. 2457T]

MHNQAPIQRRKSTRIYVGNVPIGDGAPIAVQSMTNTRTTDVEATVNQIKALERVGADIVRVSVPTMDAAE

AFKLIKQRVNVPLVADIHFDYRIALKVAEYGVDCLRINPGNIGNEERIRMVVDCARDKNIPIRIGVNAGS

LEKDLQEKYGEPTPQALLESAMRHVDHLDRLNFDQFKVSVKASDVFLAVESYRLLAKQIDQPLHLGITEA

GGARSGAVKSAIGLGLLLSEGIGDTLRVSLAADPVEEIKVGFDILKSLRIRSRGINFIACPTCSRQEFDV

IGTVNALEQRLEDIITPMDVSIIGCVVNGPGEALVSTLGVTGGNKKSGLYEDGVRKDRLDNNDMIDQLEA

RIRAKASQLDEARRIDVQQVGKIIT

>gi|30063883|ref|NP_838054.1| phosphoglucosamine mutase [Shigella flexneri 2a str. 2457T]

MVRKYFGTDGIRGKANEGAMTAETALRVGMAAGRVFRRGDHRHRVVIGKDTRLSGYMLEPALTAGFTSMG

MDVFLFGPLPTTYRKNKRPFHPSPTALDRS

>gi|30063882|ref|NP_838053.1| dihydropteroate synthase [Shigella flexneri 2a str. 2457T]

MNKSLIIFGIVNITSDSFSDGGRYLAPDAAIAQARKLMAEGADVIDLGPASSNPDAAPVSSDTEIARIAP

VLDALKADGIPVSLDSYQPATQAYALSRGVAYLNDIRGFPDAAFYPQLAKSSAKLVVMHSVQDGQADRRE

APAGDIMDHIAAFFDARIAALTGAGIKRNRLVLDPGMGFFLGAAPETSLSVLARFDELRLRFDLPVLLSV

SRKSFLRALTGRGPGDVGAATLAAELAAAAGGADFIRTHEPRPLRDGLAVLAALKETARIR

>gi|30063876|ref|NP_838047.1| exopolyphosphatase [Shigella flexneri 2a str. 2457T]

MPIHDKSPRPQEFAAVDLGSNSFHMVIARVVDGAMQIIGRLKQRVHLADGLGPDNMLSEEAMTRGLNCLS

LFAERLQGFSPASVCIVGTHTLRQALNATDFLKRAEKVIPYPIEIISGNEEARLIFMGVEHTQPEKGRKL

VIDIGGGSTELVIGENFEPILVESRRMGCVSFAQLYFPGGVINKENFQRARMAAAQKLETLTWQFRIQGW

NVAMGASGTIKAAHEVLMEMGEKDGIITPERLEKLVKEILRHRNFASLSLPGLSEERKTVFVPGLAILCG

VFDALAIRELRLSDGALREGVLYEMEGRFRHQDVRSRTASSLANQYHIDSEQARRVLDTTMQMYEQWREQ

QPKLAHPQLEALLRWAAMLHEVGLNINHSGLHRHSAYILQNSDLPGFNQEQQLMMATLVRYHRKAIKLDD

LPRFTLFKKKQFLPLIQLLRLGVLLNNQRQATTTPPTLTLITDDSHWTLRFPHDWFSQNALVLLDLEKEQ

EYWEGVAGWRLKIEEESTPEIAA

>gi|30063870|ref|NP_838041.1| DNA replication initiation factor [Shigella flexneri 2a str. 2457T]

MVNFSRFCEILVEVSLNTPAQLSLPLYLPDDETFASFWPGDNSSLLAALQNVLRQEHSGYIYLWAREGAG

RSHLLHAACAELSQRGDAVGYVPLDKRTWFVPEVLDGMEHLSLVCIDNIECIAGDELWEMAIFDLYNRIL

ESGKTRLLITGDRPPRQLNLGLPDLASRLDWGQIYKLQPLSDEDKLQALQLRARLRGFELPEDVGRFLLK

RLDREMRTLFMTLDQLDRASITAQRKLTIPFVKEILKL

>gi|30063869|ref|NP_838040.1| oxidoreductase [Shigella flexneri 2a str. 2457T]

MTKQVKIYHNPRCSKSRETLNLLKENGVEPEVVLYLETPADAATLRDLLKMLGMNSARELMRQKEDLYKE

LNLADSSLSEEALIQAMVDNPKLMERPIMVANGKARIGRPPEQVLEIVG

>gi|30063859|ref|NP_838030.1| hydrogenase 4 Fe-S subunit [Shigella flexneri 2a str. 2457T]

MNRFVVAEPLWCTGCNTCLAACSDVHKTQGLQQHPRLALAKTSTITAPVVCHHCEEAPCLQVCPVNAISQ

RDDAIQLNESLCIGCKLCAVVCPFGAISASGSRPVNAHAQYVFQAEGSLKDGEENVPPQHALLRWEPGVQ

TVAVKCDLCDFLPEGPACVRACPNQALRLITDDSLQRQMKEKQRLAASWFANGGESPLSLTQEQH

>gi|30063850|ref|NP_838021.1| hypothetical protein S2664 [Shigella flexneri 2a str. 2457T]

MVTLYGIKNCDTIKKARRWLEANNIDYRFHDYRVDGLDSELLNGFINELGWEALLNTRGTTWRKLDETTR

NKITDAASAAALMTEMPAIIKRPLLCAPGKPMLLGFSDSSYQQFFHEV

>gi|30063839|ref|NP_838010.1| iso-IS1 ORF2 [Shigella flexneri 2a str. 2457T]

MAFICELDEQWSYVGSKARQHWLGYAYNTKTGGVLAYTFGPRTDQTCRELLALLTPFNIGMLTSDDWGSY

GREVPKNKHLTGKIFTQRIERNNLTLRTRIKRLGRKTICFSRSVEIHEKVIGAFIEKHMFY

>gi|30063830|ref|NP_838001.1| hypothetical protein S2641 [Shigella flexneri 2a str. 2457T]

MPALDLIRPSVTAMRVIASVNAEFARELKLPPHIRSLGLISADSDDVTYIAADEATKQAMVEVVYGRSLY

AGAAHGPSPTAGEVLIMLGGPNPAEVRAGLDAMVAHIENGAAFQWANDAENTAFLAHVVSRTGSYLSSTA

GITLGDPMAYLVAPPLEATYGIDAALKSADVQLVTYVPPPSETNYSAAFLTGSQAACKAACNAFTDAVLE

IARNPIQRA

>gi|30063827|ref|NP_837998.1| N-acetylmuramoyl-l-alanine amidase I [Shigella flexneri 2a str. 2457T]

MSTFKPLKTLTSRRQVLKAGLAALTLSGMSQAIAKDEPLKTSNGHSKPKAKKSGGKRVVVLDPGHGGIDT

GAIGRNGSKEKHVVLAIAKNVRSILRNHGIDARLTRSGDTFIPLYDRVEIAHKHGADLFMSIHADGFTNP

KAAGASVFALSNRGASSAMAKYLSERENRADEVAGKKATDKDHLLQQVLFDLVQTDTIKNSLTLGSHILK

KIKPVHKLHSRNTEQAAFVVLKSPSVPSVLVETSFITNPEEERLLGTAAFRQKIATAIAEGVISYFHWFD

NQKAHSKKR

>gi|30063817|ref|NP_837988.1| sulfate/thiosulfate transporter subunit [Shigella flexneri 2a str. 2457T]

MFAVSSRRVLPGFTLSLGTSLLFVCLILLLPLSALVMQLSEMSWAQYWEVITNPQVVAAYKVTLLSAFVA

SIFNGVFGLLMAWILTRYRFPGRTLLDALMDLPFALPTAVAGLTLASLFSVNGFYGEWLAKFDIKVTYTW

LGIAVAMAFTSIPFVVRTVQPVLEELGPEYEEAAETLGATRWQSFCKVVLPELSPALVAGVALSFTRSLG

EFGAVIFIAGNIAWKTEVTSLMIFVRLQEFDYPAASAIASVILAASLLLLFSINTLQSRFGRRVVGH

>gi|30063791|ref|NP_837962.1| PTS system enzyme IIB component [Shigella flexneri 2a str. 2457T]

MSKKLIALCACPMGLAHTFMAAQVLEEAAVEAGYEVKIETQGADGIQNRLTAQDIAEATIIIHSVAVTPE

DNERFESRDVYEITLQDAIKNAAGIIKEIEEMIASEQQ

>gi|30063781|ref|NP_837952.1| hypothetical protein S2579 [Shigella flexneri 2a str. 2457T]

MKRLIMATMVTAILASSTVWAADNAPVAAQQQTQQTQKTAAAERISEQGLYAMRDVQVARLALFHGDPEK

AKELTNEASALLSDDSTEWAKFAKPGKKTNLNDDQYIVINASVGISESYVATPEKEAAIKIANEKMAKGD

KKGAMEELRLAGVGVMENQYLMPLKQTRNALADAQKLLDKKQYYEANLALKGAEDGIIVDSEALFVN

>gi|30063765|ref|NP_837936.1| long-chain fatty acid outer membrane transporter [Shigella flexneri 2a str. 2457T]

MSQKTLFTKSALAVAVALISTQAWSAGFQLNEFSSSGLGRAYSGEGAIADDAGNVSRNPALITMFDRPTF

SAGAVYIDPDVNISGTSPSGRSLKADNIAPTAWVPNMHFVAPINDQFGWGASITSNYGLATEFNDTYAGG

SVGGTTDLETMNLNLSGAYRLNNAWSFGLGFNAIYARAKIERFAGDLGQLVAGQIMQSPAGQTPQGQALA

ATANGIDSNTKIAHLNGNQWGFGWNAGILYELDKNNRYALTYRSEVKIDFKGNYSSDLNRAFNNYGLPIP

TATGGATQSGYLTLNLPEMWEVSGYNRVDPQWAIHYSLAYTSWSQFQQLKATSTSGDTLFQKHEGFKDAY

RIALGTTYYYDDNWTFRTGIAFDDSPVPAQNRSISIPDQDRFWLSAGTTYAFNKDASVDVGVSYMHGQSV

KINEGPYQFESEGKAWLFGTNFNYAF

>gi|30063743|ref|NP_837914.1| hypothetical protein S2528 [Shigella flexneri 2a str. 2457T]

MDLIYFLIDFILHIDVHLAELVAEYGVWVYAILFLILFCETGLVVMPFLPGDSLLFVAGALASLETNDLN

VHMMVVLMLIAAIVGDAVNYTIGRLFGEKLFSNPNSKIFRRSYLDKTHQFYEKHGGKTIILARFVPIVRT

FAPFVAGMGHMSYRHFAAYNVIGALLWVLLFTYAGYFFGTIPMVQDNLKLLIVGIIVVSILPGVIEIIRH

KRAAARAAK

>gi|30063739|ref|NP_837910.1| colicin V production protein [Shigella flexneri 2a str. 2457T]

MVWIDYAIIAVIAFSSLVSLIHGFVREALSLVTWGCAFFVASHYYTYLSVWFTGFEDELVRNGIAIAVLF

IATLIVGAIVNFVIGQLVEKTGLSGTDRVLGVCFGALRGVLIVAAILFFLDSFTGVSKSEDWSKSQLIPQ

FSFIIRWFFDYLQSSSSFLPRA

>gi|30063737|ref|NP_837908.1| 3-octaprenyl-4-hydroxybenzoate carboxy-lyase [Shigella flexneri 2a str. 2457T]

MKRLIVGISGASGAIYGVRLLQVLRDVTDIETHLVMSQAARQTLSLETDFSLREVQALADVTLDARDISA

SISSGSFQTLGMVILPCSIKTLSGIVHSYTDGLLTRAADVVLKERRPLVLCVRETPLHLGHLRLMTQAAE

IGAVIMPPVPAFYHRPQSLDDVINQTVNRVLDQFAITLPEDLFARWQGA

>gi|30063734|ref|NP_837905.1| histidine transport system permease [Shigella flexneri 2a str. 2457T]

MLYGFSGVILQGALVTLELAISSVVLAVIIGLIGAGGKLSQNRLSGLIFEGYTTLIRGVPDLVLMLLIFY

GLQIALNMVTEAMGVGQIDIDPMVAGIITLGFIYGAYFTETFRGAFMAVPKGHIEAATAFGFTRGQVFRR

IMFPAMMRYALPGIGNNWQVILKSTALVSLLGLEDVVKATQLAGKSTWEPFYFAIVCGVIYLVFTTVSNG

VLLFLERRYSVGVKRADL

>gi|30063733|ref|NP_837904.1| histidine transport, membrane protein M [Shigella flexneri 2a str. 2457T]

MIEILHEYWKPLLWTDGYRFTGVAITLWLLILSVVIGGVLALFLAIGRVSSNKYIQFPIWLFTYIFRGTP

LYVQLLVFYSGMYTLEIVKGTEFLNAFFRSGLNCTVLALTLNTCAYTTEIFAGAIRSVPHGEIEAARAYG

FSTFKMYRCIILPSALRIALPAYSNEVILMLHSTALAFTATVPDLLKIARDINAATYQPFTAFGIAAVLY

LIISYVLISLFRRAEKRWLQHVKPSSTH

>gi|30063722|ref|NP_837893.1| acetate kinase [Shigella flexneri 2a str. 2457T]

MSSKLVLVLNCGSSSLKFAIIDAVNGEEYLSGLAECFHLPEARIKWKMDGNKQEAALGAGAAHSEALNFI

VNTILAQKPELSAQLTAIGHRIVHGGEKYTSSVVIDESVIQGIKDAASFAPLHNPAHLIGIEEALKSFPQ

LKDKNVAVFDTAFHQTMPEESYLYALPYNLYKEHGIRRYGAHGTSHFYVTQEAAKMLNKPVEELNIITCH

LGNGGSVSAIRNGKCVDTSMGLTPLEGLVMGTRSGDIDPAIIFHLHDTLGMSVDAINKLLTKESGLLGLT

EVTSDCRYVEDNYATKEDAKRAMDVYCHRLAKYIGAYTALMDGRLDAVAFTGGIGENAAMVRELSLGKLG

VLGFEVDHERNLAARFGKSGFINKEGTRPAVVIPTNEELVIAQDASRLTA

>gi|30063698|ref|NP_837869.1| hypothetical protein S2480 [Shigella flexneri 2a str. 2457T]

MIEWQDLHHSELSVSQLYALLQLRCAVFVVEQNCPYQDIDGDDLTGDNRHILGWKNDELVAYARILKSDD

DLEPVVIGRVIVSEALRGEKVGQQLMSKTLETCTHHWPDKPVYLGAQAHLQNFYQSFGFIPVTEVYEEDG

IPHIGMAREVIQA

>gi|30063678|ref|NP_837849.1| hypothetical protein S2459 [Shigella flexneri 2a str. 2457T]

MTESTTSSPHDAVFKTFIFTPETARDFLEIHLPEPLRKLCNLQTLRLEPTSFIEKSLRAYYSDVLWSVET

SDGDGYIYCVIEHQSSAEKNMAFRPMRYATAAMQSHLDKGYDRVPLVVPLLFYHGETSPYPYSLNWLDEF

DDPQLARQLYTEAFPLVDITIVPDDEIMQHRRIALLELIQKHIRDHDLIGMVDRITTLLVRGFTNDSQLQ

TLFNYLLQCGDTSRFTRFIQEIAERSPLQKERLMTIAERLRQEGHQIGWQEGKLVGLQQGKLEGLQEGMH

EQAIKIALRMLEQGIDRDQVLAATQLSEADLAANNH

>gi|30063657|ref|NP_837828.1| hybrid sensory kinase in two-component regulatory system with RcsB and YojN [Shigella flexneri 2a str. 2457T]

MKYLASFRTTLKASRYMFRALALVLWLLIAFSSVFYIVNALHQRESEIRQEFNLSSDQAQRFIQRTSDVM

KELKYIAENRLSAENGVLSPRGRETQADVPAFEPLFADSDCSAMSNTWRGSLESLAWFMRYWRDNFSAAY

DLNRVFLIGSDNLCMANFGLRDMPVERDTALKALHERINKYRNAPQDDSGSNLYWISEGPRPCVGYFYAL

TPVYLANRLQALLGVEQTIRMENFFLPGTLPMGVTILDENGHTLISLTGPESKIKGDPRWMQERSWFGYT

EGFRELVLKKNLPPSSLSIVYSVPVDKVLERIRMLILNAILLNVLAGAALFTLARMYERRIFIPAESDAL

RLEEHEQFNRKIVASAPVGICILRTADGVNILSNELAHTYLNMLTHEDRQRLTQIICGQQVNFVDVLTSN

NTNLQISFVHSRYRNENVAICVLVDVSSRVKMEESLQEMAQAAEQASQSKSMFLATVSHELRTPLYGIIG

NLDLLQTKELPKGVDRLVTAMNNSSSLLLKIISDILDFSKIESEQLKIEPREFSPREVMNHITANYLPLV

VRKQLGLYCFIEPDVPVALNGDPMRLQQVISNLLSNAIKFTDTGCIVLHVRADGDYLSIRVRDTGVGIPA

KEVVRLFDPFFQVGTGVQRNFQGTGLGLAICEKLISMMDGDISVDSEPGMGSQFTVRIPLYGAQYPQKKG

VEGLSGKRCWLAVRNASLCQFLETSLQRSGIVVTTYEGQEPTPEDVLITDEVVSKKWQGRAVVTFCRRHI

GIPLEKAPGEWVHSVAAPHELPALLARIYLIEMESDDPANALPSTDKAVSDNDDMMILVVDDHPINRRLL

ADQLGSLGYQCKTANDGVDALNVLSKNHIDIVLSDVNMPNMDGYRLTQRIRQLGLTLPVIGVTANALAEE

KQRCLESGMDSCLSKPVTLDVIKQTLTVYAERVRKSRDS

>gi|30063654|ref|NP_837825.1| outer membrane porin protein C [Shigella flexneri 2a str. 2457T]

MKVKVLSLLVPALLVAGAANAAEVYNKDGNKLDLYGKVDGLHYFSDDKSVDGDQTYMRLGFKGETQVTDQ

LTGYGQWEYQIQGNSAENENNSWTRVAFAGLKFQDVGSFDYGRNYGVVYDVTSWTDVLPEFGGDTYGSDN

FMQQRGNGFATYRSTDFFGLVDGLNFAVQYQGKNGSPEGEGMTNNGREALRQNGDGVGGSITYDYEGFGI

GAAVSSSKRTDDQNFGLNRYDERYIGNGDRAETYTGGLKYDANNIYLAAQYTQTYNATRVGNLGWANKAQ

NFEAVAQYQFDFGLRPSLAYLQSKGKNLGVINGRNYDDEDILKYVDVGATYYFNKNMSTYVDYKINLLDD

NQFTRDAGINTDNIVALGLVYQF

>gi|30063644|ref|NP_837815.1| quinol dehydrogenase membrane component [Shigella flexneri 2a str. 2457T]

MANRKRDAGREALEKKGWWRSHRWLVLRRLCQFFVLGMFLSGPWFGVWILHGNYSSSLLFDTVPLTDPLM

TLQSLASGHLPATVALTGAVIITVLYALAGKRLFCSWVCPLNPITDLANWLRRRFDLNQSATIPRHIRYV

LLVVILVGSALTGTLIWEWINPVSLMGRSLVMGFGSGALLILALFLFDLLVVEHGWCGHICPVGALYGVL

GSKGVITVAASDRQKCNRCMDCFHVCPEPHVLRAPVLDEQSPVQVTSRDCMTCGRCVDVCSEDVFTITTR

WSSGAKS

>gi|30063637|ref|NP_837808.1| cytochrome c-type biogenesis protein CcmE [Shigella flexneri 2a str. 2457T]

MNIRRKNRLWIACAVLAGLALTIGLVLYALRSNIDLFYTPGEILYGKRETQQMPEVGQRLRVGGMVMPGS

VQRDPNSLKVTFTIYDAEGSVDVSYEGILPDLFREGQGVVVQGELEKGNHILAKEVLAKHDENYTPPEVE

KAMEANHRRPASVYKDPAS

>gi|30063626|ref|NP_837797.1| 16S rRNA pseudouridylate synthase A [Shigella flexneri 2a str. 2457T]

MRLDKFIAQQLGVSRAIAGREIRGNRVTVDGEIVRNAAFKLLPEHDVAYDGNPLAQQHGPRYFMLNKPQG

YVCSTDDPDHPTVLYFLDEPVAWKLHAAGRLDIDTTGLVLMTDDGQWSHRITSPRHHCEKTYLVTLESPV

ADDTAEQFAKGVQLHNEKDLTKPAVLEVITPTQVRLTISEGRYHQVKRMFAAVGNHVVELHRERIGGITL

DADLAPGEYRPLTEEEIASVV

>gi|30063625|ref|NP_837796.1| bicyclomycin/multidrug efflux system [Shigella flexneri 2a str. 2457T]

MTTRQHSSFAIVFILGLLAMLMPLSIDMYLPALPVISAQFGVSAGSTQMTLSTYILGFALGQLIYGPMAD

SFGRKPVVLGGTLVFAAAAVACALAQTIDQLIVMRFFHGLAAAAASVVINALMRDIYPKEEFSRMMSFVM

LVTTIALLMAPIVGGWVLVWLSWHYIFWILALAAILASAMIFFLIKETLPPERRQPFHIRTTIGNFAALF

RHKRVLSYMLASGFSFAGMFSFLSAGPFVYIEINHVAPENFGYYFALNIVFLFVMTIFNSRFVRRIGALN

MFRSGLWIQFIMAAWMVISAPLGLGFWSLVVGVAAFVGCVSMVSSNAMAVILDEFPHMAGTASSLAGTFR

FGIGAIVGALLSLATFNSAWPMIWSIAFCATSSILFCLYASRPKKR

>gi|30063622|ref|NP_837793.1| transport system permease [Shigella flexneri 2a str. 2457T]

MSRLSPVNQARWARFRHNRRGYWSLWIFLVLFGLSLCSELIANDKPLLVRYDGSWYFPLLKNYSESDFGG

PLASQADYQDPWLKQRLENNGWVLWAPIRFGATSINFATDKPFPSPPSRQNWLGTDANGGDVLARILYGT

RISVLFGLMLTLCSSVMGVLAGALQGYYGGKVDLWGQRFIEVWSGMPTLFLIILLSSVVQPNFWWLLAIT

VLFGWMSLVGVVRAEFLRTRNFDYIRAAQALGVSDRSIILRHMLPNAMVATLTFLPFILCSSITTLTSLD

FLGFGLPLGSPSLGELLLQGKNNLQAPWLGITAFLSVAILLSLLIFIGEAVRDAFDPNKAV

>gi|30063618|ref|NP_837789.1| outer membrane lipoprotein [Shigella flexneri 2a str. 2457T]

MVKSQPILRYILRGIPAIAVAVLLSACSANNTAKNMHPETRAVGSETSSLQASQDEFENLVRNVDVKSRI

MDQYADWKGVRYRLGGSTKKGIDCSGFVQRTFREQFGLELPRSTYEQQEMGKSVSRSNLRTGDLVLFRAG

STGRHVGIYIGNNQFVHASTSSGVIISSMNEPYWKKRYNEARRVLSRS

>gi|30063604|ref|NP_837775.1| DNA-binding transcriptional activator YeiL [Shigella flexneri 2a str. 2457T]

MSESAFKDCFSTDVSADTRLFHFLARDYIVQEGQQPSWLFYLTRGRARLYATLANGRVSLIDFFAAPCFI

GEIELIDKDHEPRAVQAIEECWCLALPMKHYRPLLLNDTLFLRKLCVTLSHKNYRNIVSLTQNQSFPLVN

RLAAFILLSQEGDLYHEKHTQAAEYLGVSYRHLLYVLAQFIHDGLLIKRKDI

>gi|30063594|ref|NP_837765.1| DNA-binding transcriptional regulator GalS [Shigella flexneri 2a str. 2457T]

MITIRDVARQAGVSVATVSRVLNNSTLVSADTREAVMKAVSELDYRPNANAQALATQVSDTIGVVVMDVS

DAFFGALVKAVDLVAQQHQKYVLIGNSYHEAEKERHAIEVLIRQRCNALIVHSKALSDDELAQFMDNIPG

MVLINRVVPGYAHRCVCLDNLSGARMATRMLLNNGHQRIGYLSSSHGIEDDAMRKAGWMSALKEQDIIPP

ESWIGTGTPDMPGGEAAMVELLGRNLQLTAVFAYNDNMAAGALTALKDNGIAIPLHLSIIGFDDIPIARY

TDPQLTTVRYPIASMAKLATELALQGAAGNIDPRASHCFMPTLVRRHSVATRQNAAAITNSTNQAM

>gi|30063593|ref|NP_837764.1| galactose-binding transport protein; receptor for galactose taxis [Shigella flexneri 2a str. 2457T]

MNKKVLTLSAVMASMLFGAAAHAADTRIGVTIYKYDDNFMSVVRKAIEQDAKAAPDVQLLMNDSQNDQSK

QNDQIDVLLAKGVKALAINLVDPAAAGTVIEKARGQNVPVVFFNKEPSRKALDSYDKAYYVGTDSKESGI

IQGDLIAKHWAANQGWDLNKDGQIQFVLLKGEPGHPDAEARTTYVIKELNDKGIKTEQLQLDTAMWDTAQ

AKDKMDAWLSGPNANKIEVVIANNDAMAMGAVEALKAHNKSSIPVFGVDALPEALALVKSGALAGTVLND

ANNQAKATFDLAKNLADGKGAADGTNWKIDNKVVRVPYVGVDKDNLAEFSKK

>gi|30063588|ref|NP_837759.1| hypothetical protein S2358 [Shigella flexneri 2a str. 2457T]

MLKRVFLSLLVLIGLLLLTVLGLDRWMSWKTAPYIYDELQDLPYRQVGVVLGTAKYYRTGVINQYYRYRI

QGAINAYNSGKVNYLLLSGDNALQSYNEPMTMRKDLIAAGVDPSDIVLDYAGFRTLDSIVRTRKVFDTND

FIIITQRFHCERALFIALHMGIQAQCYAVPSPKDMLSVRIREFAARFGTLADLYIFKREPRFLGPLVPIP

AMHQVPEDAQGYPAVTPEQLLELQKKQGK

>gi|30063546|ref|NP_837717.1| fimbrial-like protein [Shigella flexneri 2a str. 2457T]

MKRSIIAAAVFSSFFMSAGVFAADVDTGTLTIKGNIAESPCKFEAGGDSVSINMPTVPTTVFEGKAKYST

YDDAVGVTSSMLKISCPKEVAGVKLSLITNDKITGNDKAIASSNDTVGDNSDVLDVSAPFNIESYKTAEG

QYAIPFKAKYLKLTDNSVQSGDVLSSLVMRVAQD

>gi|30063545|ref|NP_837716.1| outer membrane protein [Shigella flexneri 2a str. 2457T]

MLRMTPLASAIVALLLGIEAYAAEETFDTHFMIGGMKDQQVANIRLDDNQPLPGQYDIDIYVNKQWRGKY

EIIVKDNPQETCLSREVIKRLGINSDNFASGKQCLTFEQLVQGGSYSWDIGVFRLDFSVPQAWVEELESG

YVPPENWERGINAFYTSYYVSQYYSDYKASGNNKSTYVRFNSGLNLLEWQLHSDASFSKTNNNPGVWKSN

TLYLERGFAQFLGTLRVGDMYTSSDIFDSVRFSGVRLFRDMQMLPNSKQNFTPRVQGIAQSNALVTIEQN

GFVVYQKEVPPGPFAITDLQLAGGGADLDVSVKEADGSVTTYLVPYAAVPNMLQPGVSKYDFAAGRSHIE

GASKQSDFVQAGYQYGFNNLLTLYGGTMVANNYYAFTLGTGWNTRIGAISVDATKSHSKQDNGDVFDGQS

YQIAYNKFVSQTSTRFGLAAWRYSSRDYRTFNDHVWANNKDNYRRDENDIYDIADYYQNDFGRKNSFSAN

MSQSLPEGWGSVSLSTLWRDYWGRSGSSKDYQLSYSNNWRRISYTLAASQAYGENHHEEKRFNIFISIPC

DWGDDVTTPRRQIYMSNSTTFDDQGFASNNTGLSGTVGSRDQFNYGVNLSHQHQGNETTAGANLTWNAPV

ATVNGSYSQSSTYRQTGASVSGGIVAWSGGVNLANRLSETFAVMNAPGIKDAYVNGQKYRTTNRNGVVVY

DGMTPYRENHLMLDVSQSDSEAELRGNRKIAAPYRGAVVLVNFDTDQRKPWFIKALRADGQPLTFGYEVN

DIHGHNIGVVGQGSQLFIRTNEIPPSVNVAIDKQQGLSCTITFGKEIDESRNYICQ

>gi|30063542|ref|NP_837713.1| phosphomethylpyrimidine kinase [Shigella flexneri 2a str. 2457T]

MKRINALTIAGTDPSGGAGIQADLKTFSALGAYGCSVITALVAQNTRGVQSVYRIEPDFVAAQLDSVFSD

VRIDTTKIGMLAETDIVEAVAERLQRYQIQNVVLDTVMLAKSGDPLLSPSAVATLRSRLLPQVSLITPNL

PEAAALLDAPHARTEQEMLEQGRSLLAMGCGAVLMKGGHLDDEQSPDWLFTREGEQRFTAPRIMTKNTHG

TGCTLSAALAALRPRHTNWADTVQEAKIWLSSALAQADTLEVGHGIGPVHHFHAWW

>gi|30063540|ref|NP_837711.1| transcriptional regulator [Shigella flexneri 2a str. 2457T]

MEQAHTQLIAQLNERILAADNTPLYIKFAETVKNAVRSGVLEHGNILPGERDLSQLTGVSRITVRKAMQA

LEEEGVVTRSRGYGTQINNIFEYSLKEARGFSQQVVLRGKKPDTLWVNKRVVKCPEEVAQQLAVEAGSDV

FLLKRIRYVDEEAVSIEESWVPAHLIHDVDAIGISLYDYFRSQHIYPQRTRSRVSARMPDAEFQSHIQLD

SKIPVLVIKQVALDQQQRPIEYSISHCRSDLYVFVCEE

>gi|30063537|ref|NP_837708.1| tagatose-bisphosphate aldolase [Shigella flexneri 2a str. 2457T]

MYVVSTKQMLNNAQRGGYAVPAFNIHNLETMQVVVETAANLHAPVIIAGTPGTFTHAGTENLLALVSAMA

KQYHHPLAIHLDHHTKFDDIAQKVRSGVRSVMIDASHLPFAQNISRVKEVVDFCHRFDVSVEAELGQLGG

QEDDVQVNEVDALYTNPAQAREFAEATGIDSLAVAIGTAHGMYASAPVLDFSRLENIRQWVNLPLVLHGA

SGLSTKDIQQTIKLGICKINVATELKNAFLQSLKNYLTEHPEATDPRDYLQSAKSAMRDVVSKVIADCGC

EGRA

>gi|30063534|ref|NP_837705.1| galactitol-specific PTS system component IIB [Shigella flexneri 2a str. 2457T]

MKRKIIVACGGAVATSTMAAEEIKELCQSHNIPVELIQCRVNEIETYMDGVHLICTTARVDRSFGDIPLV

HGMPFVSGVGIEALQNKILTILQG

>gi|30063525|ref|NP_837696.1| hypothetical protein S2270 [Shigella flexneri 2a str. 2457T]

MLFSISNFNQGVIMAGWFELSKSSDNQFRFVLKAGNGETILTSELYTSKASAEKGIASVRSNSPQEERYE

KKTASNGKFYFNLKAANHQIIGSSQMYATAQSRETGIASVKANGTSQTVKDNT

>gi|30063524|ref|NP_837695.1| DNA-binding transcriptional regulator BaeR [Shigella flexneri 2a str. 2457T]

MTELPIDENTPRILIVEDEPKLGQLLIDYLRAASYAPTLISHGDQVLAYVRQTPPDLILLDLMLPGTDGL

TLCREIRRFSDVPIVMVTAKIEEIDRLLGLEIGADDYICKPYSPREVVARVKTILRRCKPQRELQQQDAE

SPLIIDEGRFQASWRGKMLDLTPAEFRLLKTLSHEPGKVFSREQLLNHLYDDYRVVTDRTIDSHIKNLRR

KLESLDAEQSFIRAVYGVGYRWEADACRIV

>gi|30063523|ref|NP_837694.1| signal transduction histidine-protein kinase BaeS [Shigella flexneri 2a str. 2457T]

MKFWRPGITGKLFLAIFATCIVLLISMHWAVRISFERGFIDYIKHGNEQRLQLLSDALGEQYAQHGNWRF

LRNNDRFVFQILRSFEHDNSEDKPGPGMPPHGWRTQFWVVDQNNKVLVGPRAPIPPDGTRRPILVNGAEV

GAVIASPVERLTRNTDINFDKQQRQTSWLIVALATLLAALATFLLARGLLAPVKRLVDGTHKLAAGDFTT

RVTPTSEDELGKLAQDFNQLASTLEKNQQMRRDFMADISHELRTPLAVLRGELEAIQDGVRKFTPETVAS

LQAEVGTLTKLVDDLHQLSMSDEGALAYQKAPVDLIPLLEVAGGAFRERFASRGLKLQFSLPDSITVFGD

RDRLMQLFNNLLENSLRYTDSGGSLKISAEQHDKTVRLTFADSAPGVSDDQLQKLFERFYRTEGSRNRAS

GGSGLGLAICLNIVEAHNGRIIAAHSPFGGVSITVELPLERDLQREV

>gi|30063522|ref|NP_837693.1| multidrug efflux system protein MdtE [Shigella flexneri 2a str. 2457T]

MTDLPDSTRWQLWIVAFGFFMQSLDTTIVNTALPSMAQSLGESPLHMHMVIVSYVLTVAVMLPASGWLAD

KVGVRNIFFTAIVLFTLGSLFCALSGTLNELLLARALQGVGGAMMVPVGRLTVMKIVPREQYMAAMTFVT

LPGQVGPLLGPALGGLLVEYASWHWIFLINIPVGIIGAIATLMLMPNYTMQTRRFDLSGFLLLAVGMAVL

TLALDGSKGTGLSPLAIAGLVAVGVVALVLYLLHARNNNRALFSLKLFRTRTFSLGLAGSFAGRIGSGML

PFMTPVFLQIGLGFSPFHAGLMMIPMVLGSMGMKRIVVQVVNRFGYRRVLVATTLGLSLVTLLFMTTALL

GWYYVLPFVLFLQGMVNSTRFSSMNTLTLKDLPDNLASSGNSLLSMIMQLSMSIGVTIAGLLLGLFGSQH

ISVDSGTTQTVFMYTWLSMAFIIALPAFIFARVPNDTHQNVAISRRKRSAQ

>gi|30063521|ref|NP_837692.1| multidrug efflux system subunit MdtC [Shigella flexneri 2a str. 2457T]

MKFFALFIYRPVATILLSVAITLCGILGFRMLPVAPLPQVDFPVIMVSASLPGASPETMASSVATPLERS

LGRIAGVSEMTSSSSLGSTRIILQFDFDRDINGAARDVQAAINAAQSLLPSGMPSRPTYRKANPSDAPIM

ILTLTSDTYSQGELYDFASTQLAPTISQIDGVGDVDVGGSSLPAVRVGLTPQALFNQGVSLDDVRTAISN

ANVRKPQGALEDGTHRWQIQTNDELKTAAEYQPLIIHYNNGGAVRLGDVATVTDSVQDVRNAGMTNAKPA

ILLMIRKLPEANIIQTVDSIRAKLPELQETIPAAIDLQIAQDRSPTIRASLEEVEQTLIISVALVILVVF

LFLRSGRATIIPAVAVPVSLIGTFAAMYLCGFSLNNLSLMALTIATGFVVDDAIVVLENIARHLEAGMKP

LQAALQGTREVGFTVLSMSLSLVAVFLPLLLMGGLPGRLLREFAVTLSVAIGISLLVSLTLTPMMCGWML

KASKPREQKRLRGFGRMLVALQQGYGKSLKWVLNHTRLVGVVLLGTIALNISIPKTFFPEQDTGVLMGGI

QADQSISFQAMRGKLQDFMKIIRDDPAVDNVTGFTGGSRVNSGMMFITLKPRDERSETAQQIIDRLRVKL

AKEPGANLFLMAVQDIRVGGRQSNASYQYTLLSDDLAALREWEPKIRKKLATLPELADVNSDQQDNGAEM

NLVYDRDTMARLGIDVQAANSLLNNAFGQRQISTIYQPMNQYKVVMEVDPRYTQDISALEKMFVINNEGK

AIPLSYFAKWQPANAPLSVNHQGLSAASTISFNLPTGKSLSDASAAIDRAMTQLGVPSTVRGSFAGTAQV

FQETMNSQVILIIAAIATVYIVLGILYESYVHPLTILSTLPSAGVGALLALELFNAPFSLIALIGIMLLI

GIVKKNAIMMVDFALEAQRHGNLTPQEAIFQACLLRFRPIMMTTLAALFGALPLVLSGGDGSELRQPLEI

TIVGGLVMSQLLTLYTTPVVYLFFDRLRLRFSRKPKQTVTE

>gi|30063519|ref|NP_837690.1| hypothetical protein S2262 [Shigella flexneri 2a str. 2457T]

MSEQITFATSDFASNPEPRCPCILLLDVSGSMSGRPINELNTGLVTFRDELLADSLALKRVELGIVTFGP

VHVEQPFTSAANFFPPILFAQGDTPMGAAITKALDMVEERKREYRANGISYYRPWIFLITDGAPIDEWQA

AANKVFRGEEDKRFAFFSIGVQGADMKTLAQISVRQPLPLQGLQFRELFSWLSSSLRSVSRSTPGTEVVL

EAPKGWTSV

>gi|30063512|ref|NP_837683.1| transporter [Shigella flexneri 2a str. 2457T]

MEWIADPSIWAGLITLIVIELVLGIDNLVFIAILAEKLPPKQRDRARVTGLLLAMLMRLLLLASISWLVT

LTQPLFSFRSFTFSARDLIMLFGGFFLLFKATMELNERLEGKDSNNPTQRKGAKFWGVVTQIVVLDAIFS

LDSVITAVGMVDHLLVMMAAVVIAISLMLMASKPLTQFVNSHPTIVILCLSFLLMIGFSLVAEGFGFVIP

KGYLYAAIGFSVMIEALNQLAIFNRRRFLSANQTLRQRTTEAVMRLLSGQKEDAELDAETASMLVDHGNQ

QIFNPQERRMIERVLNLNQRTVSSIMTSRHDIEHIDLNAPEEEIRQLLERNQHTRLVVTDGDDAEDLLGV

VHVIDLLQQSLRGEPLNLRVLIRQPLVFPETLPLLPALEQFRNARTHFAFVVDEFGSVEGIVTLSDVTET

IAGNLPNEVEEIDARHDIQKNADGSWTANGHMPLEDLVQYVPLPLDEKREYHTIAGLLMEYLQRIPKPGE

EVQVGDYLLKTLQVESHRVQKVQIIPLRKDGEMEYEV

>gi|30063508|ref|NP_837679.1| colanic acid biosynthesis acetyltransferase WcaB [Shigella flexneri 2a str. 2457T]

MLEDLRANSWSLRPCCMVLAYRVAHFCSVWRKKNVLNNLWAAPLLVLYRIITECFFGYEIQAAATIGRRF

TIHHGYAVVINKNVVAGDDFTIRHGVTIGNRGADNMACPHIGNGVELGANVIILGDITLGNNVTVGAGSV

VLDSVPDNALVVGEKARVKVIK

>gi|30063505|ref|NP_837676.1| colanic acid biosynthesis acetyltransferase WcaF [Shigella flexneri 2a str. 2457T]

MQDLSGFSVPKGFRGGNAIKVQLWWAVQATIFAWSPQVLYRWRAFLLRLFGAKIGKNVVIRPSVKITYPW

KLTLGDYAWVGDDVNLYTLGEITIGAHSVISQKSYLCTGSHDHASQHFTINATPIVIGEKCWLATDVFVA

PGVTIGDGTVVGARSSVFKSLPANVVCRGNPAVVIRERVETE

>gi|30063476|ref|NP_837647.1| bifunctional phosphoribosyl-AMP cyclohydrolase/phosphoribosyl-ATP [Shigella flexneri 2a str. 2457T]

MLTEQQRRELDWEKTDGLMPVIVQHAVSGEVLMLGYMNPEALDKTIESGKVTFFSRTKQRLWIKGETSGN

FLNVVSIAPDCDNDTLLVLANPIGPTCHKGTSSCFGNTAHQWLFLYQLEQLLAERKYADPETSYTAKLYA

SGTKRIAQKVGEEGVETALAATVHDRFELTNEASDLMYHLLVLLQDQDLDLTTVIENLHKRHQ

>gi|30063472|ref|NP_837643.1| imidazole glycerol-phosphate dehydratase/histidinol phosphatase [Shigella flexneri 2a str. 2457T]

MSQKYLFIDRDGTLISEPPSDFQVDRFDKLAFEPGVIPELLKLQKAGYKLVMITNQDGLGTQSFPQADFD

GPHNLMMQIFTSQGVQFDEVLICPHLPADECDCRKPKVKLVEGYLAEQAMDRANSYVIGDRATDIQLAEN

MGINGLRYDRETLNWPMIGEQLTKRDRYAHVVRNTKETQIDVQVWLDREGGSKINTGVGFFDHMLDQIAT

HGGFRMEINVKGDLYIDDHHTVEDTGLALGEALKIALGDKRGICRFGFVLPMDECLARCALDISGRPHLE

YKAEFTYQRVGDLSTEMIEHFFRSLSYTMGVTLHLKTKGKNDHHRVESLFKAFGRTLRQAIRVEGDTLPS

SKGVL

>gi|30063462|ref|NP_837633.1| transport system permease [Shigella flexneri 2a str. 2457T]

MFSMILSGLICGALLGFVMQRGRFCLTGGFRDMYIAKNNRMFYALLIAISAQSVGVFALIQAGLLTYEAG

AFPWLGTVIGGYLFGLGIVLAGGCATGTWYRAGEGLIGSWIALFTYMVMSAVMRSPHASGLNQTLQHYTT

EHNSIAETFNLSVWPLVTVLLVITLWVVMKELKKPKLKVATLPPRRTGIAHILFEKRWHPFVTAVLIGLI

ALLAWPLSEATGRMFGLGVTSPTANILQFLVAGDVKYINWGVFLVLGIFVEAFIAAKASREFRVRAADAQ

TTLRSGLGGVLMGFGASIAGGCSIGNGLVMTAMMTWQGWIGLVFMILGVWTASWLVYVRPQRKARLATAA

AN

>gi|30063460|ref|NP_837631.1| exonuclease I [Shigella flexneri 2a str. 2457T]

MTDTDKQPTFLFHDYETFGTHPALDRPAQFAAIRTDDEFNVIGEPEVFYCKPADDYLPRPGAVLITGITP

QEARAKGENEAAFAARIHSLFTVPKTCILGYNNVRFDDEVTRNVFYRNFYDPYAWSWQHDNSRWDLLDVM

RACYALRPEGINWPENDDGLPSFRLEHLTKANGIEHSNAHDAMADVYATIAMAKLVKTRQPRLFDYLFTH

RNKHKLMALIDVPQMKPLAHVSGMFGAWRGNTSWVAPLAWHPENRNAVIMADLAGDISPLLELDIDTLRE

RLYTAKADLGDNAAVPVKLVHINKCPVLAQANTLRPEDADRLGINRQHCLDNLKILRENPQVREKVVAIF

AEAEPFTPSDNVDAQLYNGFFSDADRAAMKIVLETEPRNLPALDITFVDKRIEKLLFNYRARNFPGTLDY

AEQQRWLEHRHQVFTPEFLQGYADELQMLVQQYADDKEKVALLKALWQYAEEIV

>gi|30063433|ref|NP_837604.1| hypothetical protein S2148 [Shigella flexneri 2a str. 2457T]

MQFCSSDEFASKTMIKWPWKVQESAHQTALPWQEALSIPLLTCLTEQEQSKLVALAERFLQQKRLVPLQG

FELNSLRSCRIALLFCLPVLELGLEWLDGFHEVLIYPAPFVVDDEWEDDIGLVHNQRIVQSGQSWQQGPI

VLNWLDIQDSFDASGFNLIIHEVAHKLDTRNGDRASGVPFISLREVAGWEHDLHAAMNNIQEEIELVGEN

AASIDAYAASDPAECFAVLSEYFFSAPELFAPRFPSLWQRFCQFYQQDPLQRLHHANDTDSFSATNVH

>gi|30063426|ref|NP_837597.1| crossover junction endodeoxyribonuclease [Shigella flexneri 2a str. 2457T]

MTERIEFVLPYPPTVNTYWRRRGSTYFVSKAGERYRRDVALIVRQQRLKLNLSGRLAIKIIAEPPDKRRR

DLDNILKAPLDALTHAGLLIDDEQFDEINIVRGQLVPGGRLGIKITELGCA

>gi|30063407|ref|NP_837578.1| transcriptional regulatory protein YedW [Shigella flexneri 2a str. 2457T]

MKILLIEDNQRTQEWVTQGLSEAGYVIDAVSDGRDGLYLALKDDYALIILDIMLPGMDGWQILQTLRTAK

QTPVICLTARDSVDDRVRGLDSGANDYLVKPFSFSELLARVRAQLRQHHALNSTLEISGLKMDSVSQSVS

RDNISITLTRKEFQLLWLLASRAGEIIPRTVIASEIWGINFDSDTNTVDVAIRRLRAKVDDPFPEKLIAT

IRGMGYSFVAVKK

>gi|30063406|ref|NP_837577.1| 2-component sensor protein [Shigella flexneri 2a str. 2457T]

MKRLSITVRLTLLFIFLQSVAGAGIVWTLYNGLASELKWRDDTTLINRTAQIKQLLIDGVNPDTLPVYFN

RMMDVSQDILIIHSDGINKIVNRTNVSDDMLNNIPASETISAAGIYRSIINDTEIDALRINIDEVSPSLT

VTVAKLASARHNMLEQYKINSIIICIVAIILCSVLSPLLIRTGVREIKKLSGVTEALNYNDSRVPVEVNA

LPRELKPLGQALNKMHHALVKDFERLSQFADDLAHELRTPINALLGQNQVTLSQIRSIAEYQKTIAGNIE

ELENISRLTENILFLARADKNNVLVKLDSLSLNKEVENLLDYLEYLSDEKEICFKVKCNQQIFADKILLQ

RMLSNLIVNAIRYSPEKSRIHITSFLDANGSLNIDIASPGTKINEPEKLFRRFWRGDNSRHSVGQGLGLS

LVKAIAELHGGSATYHYLSKHNVFRITLPQRN

>gi|30063399|ref|NP_837570.1| outer membrane pore protein [Shigella flexneri 2a str. 2457T]

MTLRCLLNPWRFSERILTDRENGEKGDNSQDTSYARVGVKGETQINPEMTGYGQFELDLEASNRHNPDQT

RLAYAGLSYKDFGSFDYSRNVGVAYDAEAFTDMFVEWGGDSWAGTDLFMTNRTNGVATYRNTDFFGMVEG

LNFALQYQGKNEGTGNYKANGDGHGLSATYTIDGFSFAGAYANSDRTDWQSGDGKGERAEVWALSTKYDA

NNVYAAVMYGESHNMNSDDGDVVNKTQNFEAVLQYQFDFGLRPSIGYSYSKALDVAGWICPYISRHLLSL

NPLQARCRRYSRGER

>gi|30063397|ref|NP_837568.1| ISEhe3 orfB [Shigella flexneri 2a str. 2457T]

MLDVHPSGFYAWLQQPHSQRHQADLRLTGQIKQFWLESGCVYGYRKIHLDLRDSGQQCGVNRVWRLMKRV

GIKAQVGYRSPRARKGEASIVSPNRLQRQFNPDAPDERWVTDITYIRTHEGWLYLAVVVDLFSRKIIGWS

MQSRMTKDIVLNALLMAVWWRNPEKQVLVHSDQGSQYTSHEWQSFLKSHGLEGSMSRRGNCHDNAVAESF

FQLLKRERIKKKIYGTREEARSDIFDYIEMFYNSKRRHGSSEQMSPTEYENQYYQRLGSV

>gi|30063385|ref|NP_837556.1| flagellar motor switch protein FliN [Shigella flexneri 2a str. 2457T]

MSDMNNPADDNNGAMDDLWAEALSEQKSTSEKSAADAVFQQFGGGDVSGTLQDIDLIMDIPVKLTVELGR

TRMTIKELLRLTQGSVVALDGLAGEPLDILINGYLIAQGEVVVVADKYGVRITDIITPSERMRRLSR

>gi|30063383|ref|NP_837554.1| flagellar basal body-associated protein FliL [Shigella flexneri 2a str. 2457T]

MTDYAISKKSKRSLWIPILVFITLAACASAGYSYWHSHQVAADDKAQQRVVPSPVFYALDTFTVNLGDAD

RVLYIGITLRLKDEATRSRLSEYLPEVRSRLLLLFSRQDAAVLATEEGKKNLIAEIKTTLSTPLVAGQPK

QDVTDVLYTAFILR

>gi|30063377|ref|NP_837548.1| multidrug efflux protein [Shigella flexneri 2a str. 2457T]

MNPYIYLGGAILAEVIGTTLMKFSEGFTRLWPSVGTIICYCASFWLLAQTLAYIPTGIAYAIWSGVGIVL

ISLLSWGFFGQRLDLPAIIGMMLICAGVLVINLLSRSAPH

>gi|30063371|ref|NP_837542.1| inner membrane protein [Shigella flexneri 2a str. 2457T]

MSWQQFKHAWLIKFWAPIPAVIAAGILSTYYFGITGTFWAVTGEFTRWGGQLLQLFGVHAEEWGYFKIIH

LEGSPLTRIDGMMILGMFGGCFAAALWANNVKLRMPRSRIRIMQAIIGGIIAGFGARLAMGCNLAAFFTG

IPQFSLHAWFFAIATAIGSWFGARFTLLPIFRIPVKMQKVSAASPLTQKPDQARRRFRLGMLVFFGMLGW

ALLTAMNQPKLGLAMLFGVGFGLLIERAQICFTSAFRDMWITGRTHMAKAIIIGMAVSAIGIFSYVQLGV

EPKIMWAGPNAVIGGLLFGFGIVLAGGCETGWMYRAVEGQVHYWWVGLGNVIGSTILAYYWDDFAPALAT

DWDKINLLKTFGPMGGLLVTYLLLFAALMLIIGWEKRFFRREAPQTAKEIA

>gi|30063365|ref|NP_837536.1| flagellin [Shigella flexneri 2a str. 2457T]

MAQVINTNSLSLITQNNINKNQSALSSSIERLSSGLRINSAKDDAAGQAIANRFTSNIKGLTQAARNAND

GISVAQTTEGALSEINNNLQRIRELTVQASTGTNSDSDLDSIQDEIKSRLDEIDRVSGQTQFNGVNVLAK

DGSMKIQVGANDGQTITIDLKKIDSDTLGLNGFNVNGGGAVANTAASKADLVAANATVVGNKYTVSAGYD

AAKASDLLAGVSDGDTVQATINNGFGTAASATNYKYDSASKSYSFDTTTASAADVQKYLTPGVGDTAKGT

ITIDGSAQDVQISSDGKITASNGDKLYIDTTGRLTKNGSGASLTEASLSTLAANNTKATTIDIGGTSISF

TGNSTTPDTITYSVTGAKVDQAAFDKAVSTSGNNVDFTTAGYSVNGTTGAVTKGVDSVYVDNNEALTTSD

TVDFYLQDDGSVTNGSGKAVYKDADGKLTTDAETKAATTADPLKALDEAISSIDKFRSSLGAVQNRLDSA

VTNLNNTTTNLSEAQSRIQDADYATEVSNMSKAQIIQQAGNSVLAKANQVPQQVLSLLQG

>gi|30063362|ref|NP_837533.1| transport system permease (former yecC) [Shigella flexneri 2a str. 2457T]

MQESIQLVIDSLPFLLKGAGYTLQLSIGGMFFGLLLGFILALMRLSPIWPVRWLARFYISIFRGTPLIAQ

LFMIYYGLPQFGIELDPIPSAMIGLSLNTAAYAAETLRAAISSIDKGQWEAAASIGMTPWQTMRRAILPQ

AARVALPPLSNSFISLVKDTSLAATIQVPELFRQAQLITSRTLEVFTMYLAASLIYWIMATVLSTLQNHF

ENQLNRQEREPK

>gi|30063342|ref|NP_837513.1| flagellar motor protein MotA [Shigella flexneri 2a str. 2457T]

MLILLGYLVVLGTVFGGYLMTGGSLGALYQPAELVIIAGAGIGSFIVGNNGKAIKGTLKALPLLFRRSKY

TKAMYMDLLALLYRLMAKSRQMGMFSLERDIENPRESEIFASYPRILADSVMLDFIVDYLRLIISGHMNT

FEIEALMDEEIETHESEAEVPANSLALVGDSLPAFGIVAAVMGGVHALGSADRPAAELGALIAHAMVGTF

LGILLAYGFISPLASVLRQKSAETSKMMQCVKVTLLSNLNGYAPPIAVEFGRKTLYSSERPSFIELEEHV

RAVKNPQQQTTTEEA

>gi|30063341|ref|NP_837512.1| flagellar motor protein MotB [Shigella flexneri 2a str. 2457T]

MKNQAHPIIVVKRRKAKSHGAAHGSWKIAYADFMTAMMAFFLVMWLISISSPKELIQIAEYFRTPLATAV

TGGDRISNSESPIPGGGDDYTQSQGEVNKQPNIEELKKRMEQSRLRKLRGDLDQLIESDPKLRALRLHLK

IDLVQEGLRIQIIDSQNRPMFRTGSADVEPYMRDILRAIAPVLNGIPNRISLSGHTDDFPYASGEKGYSN

WELSADRANASRRELMVGGLDSGKVLRVVGMAATMRLSDRGPDDAVNRRISLLVLNKQAEQAILHENAES

QNEPVSALEKPEVAPQVSVPTMPSAEPR

>gi|30063340|ref|NP_837511.1| chemotaxis protein CheA [Shigella flexneri 2a str. 2457T]

MSIDISDFYQTFFDEADELLADMEQHLLVLQPEAPDAEQLNAIFRAAHSIKGGAGTFGFSVLQETTHLME

NLLDEARRGEMQLNTDIINLFLETKDIMQEQLDAYKQSQEPDAASYDYICQALRQLALEAKGETPSAVTR

LSVVAKSEPQDEQSRSQLPRRIILSRLKAGEVDLLEEELGHLTTLNDVVKGADSLSAILPGDIAEDDITA

VLCFVIEADQITFETVEVSPKIATPPVLKLAAEQAPTGRVEREKTTRSSESTSIRVAVEKVDQLINLVGE

LVITQSMLAQRSSELDPVNHGDLITSMGQLQRNARDLQESVMSIRMMPMEYVFSRYPRLVRDLAGKLGKQ

VELTLVGSSTELDKSLIERIIDPLTHLVRNSLDHGIELPEKRLAAGKNSVGNLILSAEHQGGNICIEVTD

DGAGLNRERILAKAASQGLTVSENMSDDEVAMLIFAPGFSTAEQVTDVSGRGVGMDVVKRNIQEMGGHVE

IQSKQGTGTTIRILLPLTLAILDGMSVRVADEVFILPLNAVMESLQPREADLHPLAGGERVLEVRGEYLP

IVELWKVFNVAGAKTEATQGIVVILQSGGRRYALLVDQLIGQHQVVVKNLESNYRKVPGISAATILGDGS

VALIVDVSALQAINREQRMANTAA

>gi|30063339|ref|NP_837510.1| purine-binding chemotaxis protein [Shigella flexneri 2a str. 2457T]

MTGMTNVTKLASEPSGQEFLVFTLGDEEYGIDILKVQEIRGYDQVTRIANTPAFIKGVTNLRGVIVPIVD

LRIKFSQVDVDYNDNTVVIVLNLGQRVVGIVVDGVSDVLSLTAEQIRPAPEFAVTLSTEYLTGLGALGDR

MLILVNIEKLLNSEEMALLDSAASEVA

>gi|30063338|ref|NP_837509.1| methyl-accepting chemotaxis protein II, aspartate sensor receptor [Shigella flexneri 2a str. 2457T]

MINRIRVVTLLVMVLGVFALLQLISGSLFFSSLHHSQKSFVVSNQLREQQGELTSTWDLMLQTRINLSRS

AVRMMMDSSNQQSNAKVELLDSARKTLAQAATHYKKFKSMAPLPEMVATSRNFDEKYKNYYTALTELIDY

LDYGNTGAYFAQPTQGMQNAMGEAFAQYALSSEKLYRDIVTDNADDYRFAQWQLAVIALVVVLILLVAWY

GIRRMLLTPLAKIIAHICEIAGGNLANTLTIDGRSEMGDLAQSVSHMQRSLTDTVTHVREGSDAIYAGTR

EIAAGNTDLSSRTEQQASALEETAASMEQLTATVKQNADNARQASQLAQSASDTAQHGGKVVDGVVKTMH

EIADSSKKIADIISVIDGIAFQTNILALNAAVEAARAGEQGRGFAVVAGEVRNLASRSALAAKEIKALIE

DSVSRIDTGSVLVESAGETMNNIVNAVTRVTDIMGEIASASDEQSRGIDQVALAVSEMDRVTQQNASLVQ

ESAAAAAALEEQASRLTQAVSAFRLAASPLTNKPQTPSRPASEQPPAQPRLRITEQDPNWETF

>gi|30063337|ref|NP_837508.1| methyl-accepting protein IV [Shigella flexneri 2a str. 2457T]

MFNRIRISTTLFLILILCGILQIGSNGMSFWAFRDDLQRLNHGEQSNQQRAALAQTRAVMLQASTALNKA

GTLTALSYPADDIKTLMTTARASLTQSTTLFKSFMAMTAGNEHVRALQKETEKSFARWHNDLEHQATWLE

SNQLSDFLTAPVQGSQNAFDVNFEAWQLEINHVLEAASAQSQRNYQISALVFISMIIVAAIYISSALWWT

RKMIVQPLAIIGSHFDSIAAGNLARPIAVYGRNEITAIFASLKTMQQALRGTVSDVRKGSHEMHIGIAEI

VAGNNDLSSRTEQQAASLAQTAASMEQLTATVGQNADNARQASELAKNAATTAQAGGVQVSTMTHTMQEI

ATSSQKIGDIISVIDGIAFQTNILALNAAVEAARAGEQGRGFAVVAGEVRNLASRSAQAAKEIKGLIEES

VNRVQQGSKLVNNAAATMTDIVSSVTRVNDIMGEIASASEEQRRGIEQVAQAVSQMDQVTQQNSSLVEEA

AVATEQLANQADHLSSRVAVFTLEEHEVARHESAQLQIAPVVS

>gi|30063336|ref|NP_837507.1| chemotaxis methyltransferase CheR [Shigella flexneri 2a str. 2457T]

MTSSLPCGQTSLLLQMTERLALSDAHFRRISQLIYQRAGIVLADHKRDMVYNRLVRRLRSLGLTDFGHYL

NLLESNQHSGEWQAFINSLTTNLTAFFREAHHFPLLADHARRRSGEDRVWSAAASTGEEPYSIAMTLADT

LGTVPGRWKVFASDIDTEVLEKARSGIYRHEELKNLTPQQLQRYFMRGTGPHEGLVRVRQELANYVDFAP

LNLLAKQYTVPGPFDAIFCRNVMIYFDQTTQQEILRRFVPLLKPDGLLFAGHSENFSHLERRFTLRGQTV

YALSKD

>gi|30063334|ref|NP_837505.1| chemotaxis regulatory protein CheY [Shigella flexneri 2a str. 2457T]

MADKELKFLVVDDFSTMRRIVRNLLKELGFNNVEEAEDGVDALNKLQAGGYGFVISDWNMPNMDGLELLK

TIRADGAMSALPVLMVTAEAKKENIIAAAQAGASGYVVKPFTAATLEEKLNKIFEKLGM

>gi|30063319|ref|NP_837490.1| enzyme [Shigella flexneri 2a str. 2457T]

MIDFGNFYSLIAKNHLSHWLETLPAQIANWQREQQHGLFKQWSNTVEFLPEIKPYRLDLLHSVTAESEEP

LSTGQIKRIETLMRNLMPWRKGPFSLYGVNIDTEWRSDWKWDRVLPHLSDLTGRTILDVGCGSGYHMWRM

IGAGAHLAVGIDPTQLFLCQFEAVRKLLGNDQRAHLLPLGIEQLPALKAFDTVFSMGVLYHRRSPLEHLW

QLKDQLVNEGELVLETLVIDGDENTVLVPGDRYAQMRNVYFIPSALALKNWLKKCGFVDIRIVDVCVTTT

EEQRRTEWMVTESLSDFLDPHDPSKTVEGYPAPKRAVLIARKP

>gi|30063294|ref|NP_837465.1| Iron transport protein [Shigella flexneri 2a str. 2457T]

MLLGCLALTCSIAFQASATEKFKVITTFTIIADMAKNVAGDAAEVSSITKPGAEIHEYQPTPGDIKRAQG

AQLILANGMNLELWFQRFYQHLNGVPEVIVSSGVTPVGITEGPYEGKPNPHAWMSPDNALIYVDNIRDAL

IKYDPANAQTYQRNADTYKAKITQTLAPLRKQITELPENQRWMVTSEGAFSYLARDLGLKELYLWPINAD

QQGTPQQVRKVVDIVKKNHIPAVFSESTISDKPARQVARETGAHYGGVLYVDSLSTENGPVPTYIDLLKV

TTSTLVQGIKAGKREK

>gi|30063273|ref|NP_837444.1| Holliday junction resolvase [Shigella flexneri 2a str. 2457T]

MAIILGIDPGSRVTGYGVIRQVGRQLSYLGSGCIRTKVDDLPSRLKLIYAGVTEIITQFQPDYFAIEQVF

MAKNADSALKLGQARGVAIVAAVNQELPVFEYAARQVKQTVVGMGSAEKSQVQHMVRTLLKLPANPQADA

ADALAIAITHCHVSQNAMQMSESRLNLTRGRLR

>gi|30063272|ref|NP_837443.1| Holliday junction DNA helicase RuvA [Shigella flexneri 2a str. 2457T]

MIGRLRGIIIEKQPPLVLIEVGGVGYEVHMPMTCFYELPEAGQEAIVFTHFVVREDAQLLYGFNNKQERT

LFKELIKTNGVGPKLALAILSGMSAQQFVNAVEREEVGALVKLPGIGKKTAERLIVEMKDRFKGLHGDLF

TPAADLVLTSPASPATDDAEQEAVAALVALGYKPQEASRMVSKIARPDTSSETLIREALRAAL

>gi|30063267|ref|NP_837438.1| hypothetical protein S1932 [Shigella flexneri 2a str. 2457T]

MQQIARSVALAFNNLPRPHRVMLGSLTVLTLAVAVWRPYVYHRDATPIVKTIELEQNEIRSLLPEASEPI

DQAAQEDEAIPQDELDDKIAGEAGVHEYVVSTGDTLSSILNQYGIDMGDITQLAAADKELRNLKIGQQLS

WTLTADGELQRLTWEVSRRETRTYDRTAANGFKMTSEMQQGEWVNNLLKGTVGGSFVASARNAGLTSAEV

SAVIKAMQWQMDFRKLKKGDEFAVLMSREMLDGKREQSQLLGVRLRSEGKDYYAIRAEDGKFYDRNGTGL

AKGFLRFPTAKQFRISSNFNPRRTNPVTGRVAPHRGVDFAMPQGTPVLSVGDGEVVVAKRSGAAGYYVAI

RHGRSYTTRYMHLRKILVKPGQKVKRGDRIALSGNTGRSTGPHLHYEVWINQQAVNPLTAKLPRTEGLTG

SDRREFLAQAKEIVPQLRFD

>gi|30063258|ref|NP_837429.1| hypothetical protein S1922 [Shigella flexneri 2a str. 2457T]

MANWLNQLQSLLGQSSSSTSSSADQGLGKLLVSGALGGLAGLLVANKSARKLLTKYGTNALLVGGGAVAG

TVLWNKYKDKIRAAHQDEPQFGAQSTPLDERTERLILALVFAAKSDGHIDANERAAIDQQLREAGVEEQG

RVLIEQAIEQPLDPQRLATGVRNEEEALEIYFLSCAAIDIDHFMERSYLNALGDALKIPQDVREGIERDL

EQQKRTLAE

>gi|30063252|ref|NP_837423.1| hypothetical protein S1915 [Shigella flexneri 2a str. 2457T]

MMKKSILAFLLLTSSAAALAAPQVITVSRFEVGKDKWAFNREEVMLTCRPGNALYVINPSTLVQYPLNDI

AQKEVASGKTNAQPISVIQIDDPNNPGEKMSLAPFIERAEKLC

>gi|30063246|ref|NP_837417.1| transport system permease [Shigella flexneri 2a str. 2457T]

MHSERAPFFLKLAAWGGVVFLHFPILIIAAYAFNTEDAAFSFPPQGLTLRWFSVAAQRSDILDAVTLSLK

VAALATLIALVLGTLAAAALWRRDFFGKNAISLLLLLPIALPGIVTGLALLTAFKTINLEPGFFTIVVGH

ATFCVVVVFNNVIARFRRTSWSLVEASMDLGANGWQTFRYVVLPNLSSALLAGGMLAFALSFDEIIVTTF

TAGHERTLPLWLLNQLGRPRDVPVTNVVALLVMLVTTLPNLGAWWLTREGDNGQ

>gi|30063242|ref|NP_837413.1| hypothetical protein S1902 [Shigella flexneri 2a str. 2457T]

MNQSLTLAFLIAAGIGLVVQNTLMARITQTSSTILIAMLLNSLVGIVLFVSILWFKQGMAGFGELVSSVR

WWTLIPGLLGSFFVFASISGYQNVGAATTIAVLVASQLIGGLVLDIFRSHGVPLRALFGPICGAILLVVG

AWLVARRSF

>gi|30063241|ref|NP_837412.1| resistance protein [Shigella flexneri 2a str. 2457T]

MSIRFARKADCAAIAEIYNHAVLYTAAIWNDQTVDADNRIAWFEARTIAGYPVLVSEEDGVVTGYASFGD

WRSFDGFRHTVEHSVYVHPDHQGKGLGRKLLSRLIDEARDCGKHVMVAGIESQNQASLHLHQSLGFVVTA

QMPQVGTKFGHWLDLTFMQLQLDERTEPDAIG

>gi|30063226|ref|NP_837397.1| hypothetical protein S1881 [Shigella flexneri 2a str. 2457T]

MDYCCVGRNNNDSVPREVTLLRVKTLMKMANHPRPGDIIQESLDELNVSLREFARAMEIAPSTASRLLTG

KAALTPEMAIKLSVVIGSSPQMWLNLQNAWSLAEAEKTVVVSRLRRLVTQ

>gi|30063219|ref|NP_837390.1| ATP-dependent peptide transporter membrane subunit [Shigella flexneri 2a str. 2457T]

MMLSEETSAVRPQKQTRFNGAKLVWMLKGSPLTVTGAVIIVLMRLMMIFSPWLATHDPNAIDLTARLLPP

SAAHWFGTDEVGRDLFSRVLVGSQQSILAGLVVVAIAGMIGSLLGCLSGVLGGRADAIIMRIMDIMLSIP

SLVLTMALAAALGPSLFNAMLAIAIVRIPFYVRLARGQALVVRQYTYVQAAKTFGASRWHLINWHILRNS

LPPLIVQASLDIGSAILMAATLGFIGLGAQQPSAEWGAMVANGRNYVLDQWWYCAFPGAAILLTSVGFNL

FGDGIRDLLDPKAGGKQS

>gi|30063205|ref|NP_837376.1| amino acid/amine transport protein [Shigella flexneri 2a str. 2457T]

MTSLAEKFSTDNAGIAYLISGIGLGRLISILFFGVISDKFGRRAVILMAVIMYLLFFFGIPACPNLTLAY

GLAVCVGIANSALDTGGYPALMECFPKASGSAVILVKAMVSFGQMFYPMLVSYMLLNNIWYGYGLIIPGI

LFVLITLMLLKSKFPSQLVDASVANELPQMNSKPLVWLEGVSSVLFGVAAFSTFYVIVVWMPKYAMAFAG

MSEAEALKTISYYSMGSLVCVFIFAALLKKMVRPIWANVFNSALATITAAIIYLYPSPLVCNAGAFVIGF

SAAGGILQLGVSVMSEFFPKSKAKVTSIYMMMGGLANFVIPLITGYLSNIGLQYIIVLDFTFALLALITA

IIVFSRYYRVFIIPENDVRFGERKFSTRLNTIKHRG

>gi|30063200|ref|NP_837371.1| hypothetical protein S1848 [Shigella flexneri 2a str. 2457T]

MIWKRKITLEALNAMGEGNMVGFLDIRFEHIGDDTLEATMPVDSRTKQPFGLLHGGASVVLAESIGSVAG

YLCTEGEQKVVGLEINANHVRSAREGRVRGICKPLHLGSRHQVWQIEIFDEKGRLCCSSRLTTAIL

>gi|30063193|ref|NP_837364.1| cysteine desufuration protein SufE [Shigella flexneri 2a str. 2457T]

MALLPDKEKLLRNFLRCANWEEKYLYIIELGQRLPELRAEDGSPQNSIQGCQSQVWIVMRQNAQGIIELQ

GDSDAAIVKGLIAVVFILYDQMTPQDIVNFDVRPWFEKMALTQHLTPSRSQGLEAMIRAIRAKAAALS

>gi|30063186|ref|NP_837357.1| hypothetical protein S1834 [Shigella flexneri 2a str. 2457T]

MNPVDRPLLDIGLTRLEFLRISGKGLAGLTIAPALLSLLGCKQEDIDSGTVGLINTPKGVLVTQRARCTG

CHRCEISCTNFNDGSVGTFFSRIKIHRNYFFGDNGVGSGGGLYGDLNYTADTCRQCKEPQCMNVCPIGAI

TWQQKEGCITVDHKRCIGCSACTTACPWMMATVNTESKKSSKCVLCGECANACPTGALKIIEWKDITV

>gi|30063184|ref|NP_837355.1| oxidoreductase, Fe-S subunit [Shigella flexneri 2a str. 2457T]

MSWIGWTVAATALGDNQMSFTRRKFVLGMGTVIFFTGSASSLLANTRQEKEVRYAMIHDESRCNGCNICA

RACRKTNHVPAQGSRLSIAHIPVTDNDNETQYHFFRQSCQHCEDAPCIDVCPTGASWRDEQGIVRVEKSQ

CIGCSYCIGACPYLVRYLNPVTKVADKCDFCAESRLAKGFPPICVSACPEHALIFGREDSPEIQAWLQDN

KYYQYQLPGTGKPHLYRRFGQHLIKKENV

>gi|30063181|ref|NP_837352.1| hypothetical protein S1827 [Shigella flexneri 2a str. 2457T]

MATLLQLHFAFNGPFGDAMAEQLKPLAESINQEPGFLWKVWTESEKNHEAGGIYLFTDEKSALAYLEKHT

ARLKNLGVEEVVAKVFDVNEPLSQINQAKLA

>gi|30063178|ref|NP_837349.1| riboflavin synthase subunit alpha [Shigella flexneri 2a str. 2457T]

MFTGIVQGTAKLVSIDEKPNFRTHVVELPDHMLDGLETGASVAHNGCCLTVTEINGNHVSFDLMKETLRI

TNLGDLKVGDWVNVERAAKFSDEIGGHLMSGHIMTTAEVAKILTSENNRQIWFKVQDSQLMKYILYKGFI

GIDGISLTVGEVTPTRFCVHLIPETLERTTLGKKKLGARVNIEIDPQTQAVVDTVERVLAARENAMNQPG

TEA

>gi|30063176|ref|NP_837347.1| inner membrane transport protein YdhC [Shigella flexneri 2a str. 2457T]

MQPGKRFLVWLAGLSVLGFLATDMYLPAFAAIQADLQTPASAVSASLSLFLAGFAAAQLLWGPLSDRYGR

KPVLLIGLTIFALGSLGMLWVENAATLLVLRFVQAVGVCAAAVIWQALVTDYYPSQKVNRIFATIMPLVG

LSPALAPLLGSWLLVHFSWQAIFATLFAITVVLILPIFWLKPTTKARNNSQDGLTFTDLLRSKTYRGNVL

IYAACSASFFAWLTGSPFILSEMGYSPAVIGLSYVPQTIAFLIGGYGCRAALQKWQGKQLLPWLLVLFAV

SVIATWAAGFISHVSLVEILIPFCVMAIANGAIYPIVVAQALRPFPHATGRAAALQNTLQLGLCFLASLV

VSWLISISTPLLTTTSVMLSTVVLVALGYMMQRCEEVDCPNHGNAEVAHSESH

>gi|30063171|ref|NP_837342.1| lipoprotein [Shigella flexneri 2a str. 2457T]

MARINRISITLCALLFTTLPLTPMAHASKQARESSATTHITKKADKKKSTATTKKTQKASQKTAKKAASK

SMTKSKTASSVKKSSITASKNAKTRSKHAVNKTASASFTEKCTKRKGYKSHCVKVKNAASGTLADAHKAK

VQKATKVAMNKLMQQIGKPYRWGGSSPRTGFDCSGLVYYAYKDLVKIRIPRTANEMYHLRDAGPIERSEL

KNGDLVFFRTQGRGTADHVGVYVGNGKFIQSPRTGQEIQITSLSEDYWQRHYVGARRVMTPKTLR

>gi|30063168|ref|NP_837339.1| ribonuclease T [Shigella flexneri 2a str. 2457T]

MSDNAQLTGLCDRFRGFYPVVIDVETAGFNAKTDALLEIAAITLKMDEQGWLMPDTTLHFHVEPFVGANL

QPEALAFNGIDPNDPDRGAVSEYEALHEIFKVVRKGIKASGCNRAIMVAHNANFDHSFMMAAAERASLKR

NPFHPFATFDTAALAGLALGQTVLSKACQTAGMDFDSTQAHSALYDTERTAVLFCEIVNRWKRLGGWPLP

AAEEV

>gi|30063164|ref|NP_837335.1| hypothetical protein S1808 [Shigella flexneri 2a str. 2457T]

MFASIMFGVFVHALECSRPVGLVQAKLLLWTLTASIMVIILSEEGEVAEQLEFFPVQSPCRGICQSDERG

FCRGCFRSRDERFNWNKMSDGEKQEVLRLCRQRLMRKLRANKPASSDEPEQPSLF

>gi|30063146|ref|NP_837317.1| electron transport complex protein RnfG [Shigella flexneri 2a str. 2457T]

MLKTIRKHGITLALFAAGSTGLTAAINQMTKTTIAEQASLQQKTLFDQVLPAERYNNALAQSCYLVTAPE

LGKGEHRVYIAKQDDKPVAAVLEATAPDGYSGAIQLLVGADFNGTVLGTRVTEHHETPGLGDKIELRLSD

WLTHFAGKKISGADDAHWAVKKDGGDFDQFTGATITPRAVVNAVKRAGLYAQTLPAQLSQLPACGE

>gi|30063145|ref|NP_837316.1| electron transport complex protein RnfD [Shigella flexneri 2a str. 2457T]

MVFRIASSPYTHNQRQTSRIMLLVLLAAVPGIAAQLRFFGWGTLVQILLASVSALLAEALVLKLRKQSVA

ATLKDNSALLTGLLLAVSIPPLAPWWMVVLGTVFAVIIAKQLYGGLGQNPFNPAMIGYVVLLISFPVQMT

SWLPPHEIAVNIPGFIDAIQVIFSGHTASGGDMNTLRLGIDGISQATPLDTFKTSVRAGHSVEQIMQYPI

YSGILAGAGWQWVNLAWLAGGVWLLWQKAIRWHIPLSFLVTLALCATLGWLFSPETLAAPQIHLLSGATM

LGAFFILTDPVTASTTNRGRLMFGALAGLLVWLIRSFGGYPDGVAFAVLLANITVPLIDYYTRPRVYGHR

KG

>gi|30063142|ref|NP_837313.1| Na(+)-translocating NADH-quinone reductase subunit E [Shigella flexneri 2a str. 2457T]

MTDYLLLFVGTVLVNNFVLVKFLGLCPFMGVSKKLETAMGMGLATTFVMTLTSICAWLIDTWILIPLNLI

YLRTLAFILVIAVVVQFTEMVVRKTSPVLYRLLGIFLPLITTNCAVLGVALLNINLGHNFLQSALYGFSA

AVGFSLVMVLFAAIRERLAVADVPAPFRGNAIALITAGLMSLAFMGFSGLVKL

>gi|30063135|ref|NP_837306.1| DNA-binding transcriptional repressor MalI [Shigella flexneri 2a str. 2457T]

MATAKKITIHDVALAAGVSVSTVSLVLNGKGRISTATGERVNAAIEELGFVRNRQASALRGGQSGVIGLI

VRDLSAPFYAELTAGLTEALEAQGRMVFLLHGGKDGEQLAQRFSLLLNQGVDGVVIAGAAGSSDDLRRMA

EEKAIPVIFASRASYLDDVDTVRPDNMQAAQLLTEHLIRNGHQRIAWLGGQSSSLTRAERVGGYCATLLK

FGLPFHSDWVLECTSSQKQAAEAITALLRHNPTISAVVCYNETIAMGAWFGLLKAGRQSGESGVDRYFEQ

QVSLAAFTDATPTTLDDIPVTWASTPARELGITLADRMMQKITHEETHSRNLIIPARLIAAK

>gi|30063132|ref|NP_837303.1| repressor for uid operon [Shigella flexneri 2a str. 2457T]

MMDNMQTEAQPTRTRILNAAREIFSENGFHSASMKAICKSCAISPGTLYHHFISKEALIQAIILQDQERA

LARFREPIEGIHFVDYMVESIVSLTHEAFGQRALVVEIMAEGMRNPQVAAMLKNKHMTITEFVAQRMRDA

QQKGEISPDINTAMTSRLLLDLTYGVLADIEAEDLAREASFAQGLRAMIGGILTAS

>gi|30063125|ref|NP_837296.1| DNA replication terminus site-binding protein [Shigella flexneri 2a str. 2457T]

MARYDLVDRLNTTFRQMEQELATFAAHLEQHKLLVARVFSLPEVKKEDEHNPLNRIEVKQHLGNDAQSLA

LRHFRHLFIQQQSENRSSKAAVRLPGVLCYQVDNLSQAALVSHIQHINKLKTTFEHIVTVESELPTAARF

EWVHRHLPGLITLNAYRTLTVLHDPATLRFGWANKHIIKNLHRDEVLAQLEKSLKSPRSVAPWTREEWQR

KLEREYQDIAALPQNAKLKIKRPVKVQPIARVWYKGDQKQVQHACPTPLIALINRDNGAGVPDVGELLNY

DADNVQHRYKPQAQPLRLIIPRLHLYVAD

>gi|30063124|ref|NP_837295.1| sensor protein RstB [Shigella flexneri 2a str. 2457T]

MKKLFIQFYLLLFVCFLVMSLLVGLVYKFTAERAGKQSLDDLMNSSLYLMRSELREIPPHDWGKTLKEMD

LNLSFDLRVEPLSKYHLDDISMHRLRGGEIVALDDQYTFLQRIPRSHYVLAVGPVPYLYYLHQMRLLDIA

LIAFIAISLAFPVFIWMRPHWQDMLKLEAAAQRFGDGHLNERIHFDEGSSFERLGIAFNQMADNINALIA

SKKQLIDGIAHELRTPLVRLRYRLEMSDNLSAAESQALNRDISQLEALIEELLTYARLDRPQNELHLSEP

DLPLWLSTHLADIQAVTPDKTVRIKTLMQGHYAALDMRLMERVLDNLLNNALRYCHSTVETSLLLSGNRA

TLIVEDDGPGIAPENREHIFEPFVRLDPSRDRSTGGCGLGLAIVHSIALTMGGTVNCDISELGGARFSFS

WPLWHNIPQFTSA

>gi|30063122|ref|NP_837293.1| DNA-binding transcriptional regulator RstA [Shigella flexneri 2a str. 2457T]

MNTIVFVEDDAEVGSLIAAYLAKHDMQVTVEPRGDQAEETILRENPDLVLLDIMLPGKDGMTICRDLRAK

WSGPIVLLTSLDSDMNHILALEMGACDYILKTTPPAVLLARLRLHLRQNEQATLTKGLQETSLTPYKALH

FGTLTIDPINRVVTLANTEISLSTADFELLWELATHAGQIMDRDALLKNLRGVSYDGLDRSVDVAISRLR

KKLLDNAAEPYRIKTVRNKGYLFAPHAWDSDNKQSDD

>gi|30063113|ref|NP_837284.1| multidrug efflux system protein MdtI [Shigella flexneri 2a str. 2457T]

MAQFEWVHAAWLALAIVLEIVANVFLKFSDGFRRKIFGLLSLAAVLAAFSALSQAVKGIDLSVVYALWGG

FGIAATLAAGWILFGQRLNRKGWIGLVLLLAGMIMVKLA

>gi|30063104|ref|NP_837275.1| oxidoreductase, Fe-S subunit [Shigella flexneri 2a str. 2457T]

MTTQYGFFIDSSRCTGCKTCELACKDFKDLGPEVSFRRIYEYAGGDWQEDNGIWHQNVFAYYLSISCNHC

DDPACTKVCPSGAMHKREDGFVVVDEDVCIGCRYCHMACPYGAPQYNAEKGHMTKCDGCYSRVAEGKQPI

CVESCPLRALEFGPIEELRQKHGTLAAVAPLPRAHFTKPNIVIKPNANSRPTGDTTGYLANPEEV

>gi|30063095|ref|NP_837266.1| hypothetical protein S1723 [Shigella flexneri 2a str. 2457T]

MKLSTCCAALLLALASPAVLAAPGSCERIQSDISQRIINNGVPESSFTLSIVPNDQVDQPDSQVVGHCAN

DTHKILYNRTTSGNVSAPAQSTQDGAPAEPQ

>gi|30063092|ref|NP_837263.1| hypothetical protein S1719 [Shigella flexneri 2a str. 2457T]

MIKTTLLFFATALCEIIGCFLPWLWLKRNASIWLLLPAGISLALFVWLLTLHPAASGRVYAAYGGVYVCT

ALMWLRVVDGVKLSLYDWTGALIALCGMLIIVAGWGRT

>gi|30063074|ref|NP_837245.1| hypothetical protein S1697 [Shigella flexneri 2a str. 2457T]

MQSLDPLFARLSRSKFRSRFRLGMKERQYCLEKGAPVIEQHAADFVAKRLAPALPANDGKQTPMRGHPVF

IAQHATATCCRGCLAKWHNIPQGVSLSEEQQRYIVAVIYHWLVV

>gi|30063068|ref|NP_837239.1| DNA-binding transcriptional activator MarA [Shigella flexneri 2a str. 2457T]

MSRRNTDAITIHSILDWIEDNLESPLSLEKVSERSGYSKWHLQRMFKKETGHSLGQYIRSRKMTEIAQKL

KESNEPILYLAERYGFESQQTLTRTFKNYFDVPPHKYRMTNMQGESRFLHPLNHYNS

>gi|30063065|ref|NP_837236.1| transporter [Shigella flexneri 2a str. 2457T]

MATLPFMTIYLSRQYSLSVDLIGYAMTIALTIGVVFSLGFGILADKFDKKRYMLLAITAFASGFIAIPLV

NNVTLVVLFFALINCAYSVFATVLKAWFADNLSSTSKTKIFSINYTMLNIGWTIGPPLGTLLVMQSINLP

FWLAAICSAFPMLFIQIWVKRSEKIIATETGSVWSPKVLLQDKALLWFTCSGFLASFVSGAFASCISQYV

MVIADGDFAEKVVAVVLPVNAAMVVTLQYSVGRRLNPANIRALMTAGTLCFVIGLVGFIFSGNNLLLWGM

SAAVFTVGEIIYAPGEYMLIDHIAPPGMKASYFSAQSLGWLGAAINPLVSGVVLTSLPPSSLFVILALVI

IAAWVLMLKGIRARPWGQPALC

>gi|30063039|ref|NP_837210.1| transcriptional regulator YdeO [Shigella flexneri 2a str. 2457T]

MSLVCSVIFIHHAFNANILDKDYAFSDGEILMVDNAVRTHFEPYERHFKEIGFNENTIKKYLQCTNIQTV

TVPVPAKFLRASNVPTGLLNEMIAYLNSEERNHHNFSELLLFSCLSIFAACKGFITLLTNGVLSVSGKVR

NIVNMKLAHPWKLKDICDCLYISESLLKKKLKQEQTTFSQILLDARMQHAKNLIRVEGSVNKIAEQCGYA

STSYFIYAFRKHFGNSPKRVSKEYRCQRHTGMNTGNTMSALAI

>gi|30063029|ref|NP_837200.1| phospho-2-dehydro-3-deoxyheptonate aldolase [Shigella flexneri 2a str. 2457T]

MNRTDELRTARIESLVTPAELALRYPVTPGVATHVTDSRRRIEKILNGEDKRLLVIIGPCSIHDLTAAME

YATRLQSLRNQYQSRLEIVMRTYFEKPRTVVGWKGLISDPDLNGSYRVNHGLELARKLLLQVNELGVPTA

TEFLDMVTGQFIADLISWGAIGARTTESQIHREMASALSCPVGFKNGTDGNTRIAVDAIRAARASHMFLS

PDKNGQMTIYQTSGNPYGHIIMRGGKKPNYHADDIAAACDTLHEFDLPEHLVVDFSHGNCQKQHRRQLEV

CEDICQQIRNGSTEIAGIMAESFLREGTQKIVGGQPLTYGQSITDPCLGWEDTERLVEKLAFAVDTRF

>gi|30063025|ref|NP_837196.1| lipoprotein [Shigella flexneri 2a str. 2457T]

MRFCLILITALFLAGCSHHKAPPPNARLSDSITVIAGLNDQLQSWHGTPYRYGGMTRRGVDCSGFVVVTM

RDRFDLHLPRETKQQASIGTQIDKDELLPGDLVFFKTGSGQNGLHVGIYDTNNQFIHASTSKGVMRSSLD

NVYWQKNFWQARRI

K

>gi|30063015|ref|NP_837186.1| translation initiation factor IF-3 [Shigella flexneri 2a str. 2457T]

MSLREALEKAEEAGVDLVEISPNAEPPVCRIMDYGKFLYEKSKSSKEQKKKQKVIQVKEIKFRPSTDEGD

YQVKLRSLIRFLEEGDKAKITLRFRGREMAHQQIGMEVLNRVKDDLQELAVVESFPTKIEGRQMIMVLAP

KKKQ

>gi|30063011|ref|NP_837182.1| hypothetical protein S1625 [Shigella flexneri 2a str. 2457T]

MLAGGMFASLNAAADDSVFTVMDDPASAKKPFEGNLNAGYLAQSGNTKSSSLTADTTMTWYGQTTAWSLW

GNASNTSSNDERSSEKYAAGGRSRFNLTDYDYLFGQASWLTDRYNGYRERDVLTAGYGRQFLNGPVHSFR

FEFGPGVRYDKYTDNASETQPLGYASGAYAWQLTDNAKFTQGVSVFGAEDTTLNSESALNVAINEHFGLK

VAYNVTWNSEPPESAPEHTDRRTTLSLGYSM

>gi|30062996|ref|NP_837167.1| N,N'-diacetylchitobiose-specific PTS system transporter subunit IIA [Shigella flexneri 2a str. 2457T]

MMDLDNIPDTQTEAEELEEVVMGLIINSGQARSLAYAALKQAKQGDFAAAKAMMDQSRMALNEAHLVQTK

LIEGDAGEGKMKVSLVLVHAQDHLMTSMLARELITELIELHEKLKA

>gi|30062995|ref|NP_837166.1| N,N'-diacetylchitobiose-specific PTS system transporter subunit IIB [Shigella flexneri 2a str. 2457T]

MEKKHIYLFCSAGMSISLLVSKMRAQAEKYEVPVIIEAFPETLAGEKGQNADVVLLGPQIAYMLPEIQRL

LPNKPVEVIDSLLYGKVDGLGVLKAAVAAIKKAAAN

>gi|30062994|ref|NP_837165.1| DNA-binding transcriptional activator OsmE [Shigella flexneri 2a str. 2457T]

MNKNMAGILSAAAVLTMLAGCTAYDRTKDQFVQPVVKDVKKGMSRAQVAQIAGKPSSEVSMIHARGTCQT

YILGQRDGKAETYFVALDDTGHVINSGYQTCAEYDTDPQAAK

>gi|30062993|ref|NP_837164.1| NAD synthetase [Shigella flexneri 2a str. 2457T]

MTLQQQIIKALGAKPQINAEEEIRRSVDFLKSYLQTYPFIKSLVLGISGGQDSTLAGKLCQMAINELRQE

TRNESLQFIAVRLPYGVQADEQDCQDAIAFIQPDRVLTVNIKGAVLASEQALREAGIELSDFVRGNEKAR

ERMKAQYSIAGMTSGVVVGTDHAAEAITGFFTKYGDGGTDINPLYRLNKRQGKQLLTALGCPEHLYKKAP

TADLEDDRPSLPDEVALGVTYDNIDDYLEGKNLPEQVARTIENWYLKTEHKRRPPINVFDDFWKK

>gi|30062992|ref|NP_837163.1| nucleotide excision repair endonuclease [Shigella flexneri 2a str. 2457T]

MVRRLTSPRLEFEAAAIYEYPEHLHSFLNDLPTRPGVYLFHGESDTMPLYIGKSVNIRSRVLSHLRTPDE

AAMLRQSRRISWICTAGEIGALLLEARLIKEQQPLFNKRLRRNRQLCALQLNEKRVDVVYAKEVDFSRAP

NLFGLFANRRAALQALQSIADEQKLCYGLLGLEPLSRGRACFRSALKRCAGACCGKESHEEHALRLRQSL

ERLRVVCWPWQGAVALKEQHPEMTQYHIIQNWLWLGAVNSLEEATTLIRTPAGFDHDGYKILCKPLLSGN

YEITELDPANDQRAS

>gi|30062991|ref|NP_837162.1| hypothetical protein S1601 [Shigella flexneri 2a str. 2457T]

MEYFDMRKMSVNLWRNAAGETREICTFPPAKRDFYWRASIASIAANGEFSLFPGMERIVTLLEGGEMLLE

SADRFNHTLKPLQPFAFTADQVVKAKLTAGQMSMDFNIMTRLDVCKAKVRIAERTFTTFGSRGGVVFVIN

GAWQLGDKLLTTDQGVCWFDGRHTLRLLQPQGKLLFSEINWLAGHSPDQVQ

>gi|30062982|ref|NP_837153.1| hypothetical protein S1590 [Shigella flexneri 2a str. 2457T]

MGLPPLSKIPFILRPQAWLHRRHYGEVLSPIRWWGRIPFIFYLVSMFVGWLERKRSPLDPVVRSLVSARI

AQMCLCEFCVDITSMKVAERTGSTDKLLAVADWRQSPLFSDEERLALEYAEAASVTPPTVDDALRTRLAA

HFDAQALTELTALIGLQNLSARFNSAMDIPAQGLCRIPEKRS

>gi|30062979|ref|NP_837150.1| cytochrome oxidase [Shigella flexneri 2a str. 2457T]

MLDRHLHPRIKPLLHQCVRVLDKPGITPDGLTLVGFAIGVLALPFLALGWYLAALVVILLNRLLDGLDGA

LARRRGLTDAGGFLDISLDFLFYALVPFGFILAAPEQNALAGGWLLFAFIGTGSSFLAFAALAAKHQIDN

PGYAHKSFYYLGGLTEGTETILLFVLGCLFPAWFAWFAWIFGALCWMTTFTRVWSGYLTLKSLQRQ

>gi|30062978|ref|NP_837149.1| pyrimidine (deoxy)nucleoside triphosphate pyrophosphohydrolase [Shigella flexneri 2a str. 2457T]

MKMIEVVAAIIERDGKILLAQRPAQSDQAGLWEFAGGKVELDESQQQALVRELNEELDIEATVGEYVASH

QREVSGRIIHLHAWHVPDFHGTLQAHEHQALVWCSPEEALQYPLAPADIPLLEAFMALRAARAAD

>gi|30062972|ref|NP_837143.1| hypothetical protein S1573 [Shigella flexneri 2a str. 2457T]

MDALELLINRRSASRLAEPAPTGEQLQNILRAGMRAPDHKSMQPWHFFVIEGEGCERFSAVLEQGAIAAG

SDDKAIDKARNAPFRAPLIITVVAKCKENHKVPRWEQEMSAGCAVMAMQMAAVAQGFGGIWRSGALTESP

VVREAFGCREQDKIVGFLYLGTPQLKASTSINVPDPTPFVTYF

>gi|30062964|ref|NP_837135.1| aldolase [Shigella flexneri 2a str. 2457T]

MLADIRYWENDATNKYYAIAHFNVWNAEMLMGVKDAAEEAKSPVIISFSTGFVGNTSFEDFSHMMVSMAQ

KATVPVITHWDHGRSIEIIHNAWTHGMNSLMRDASAFDFEENIRLTKEAVDFFHPLGIPVEAELGHVGNE

TVYEEALAGYHYTDPDQAAEFVERTGCDSLAVAIGNQHGVYTSEPQLNFEVVKRVRDAVSVPLVLHGASG

ISDADIKTAISLGIAKINIHTELCQAAMVAVKENQDQPFLHLEREVRKAVKERALEKIELFGSDGKAE

>gi|30062950|ref|NP_837121.1| amino acid/amine transport protein [Shigella flexneri 2a str. 2457T]

MTCSTSLSGKNRIVLIAGILMIATTLRVTFTGAAPLLDTIRSAYSLTTAQTGLLTTLPLLAFALISPLAA

PVARRFGMERSLFAALLLICAGIAIRSLPSPYLLFGGTAVIGGGIALGNVLLPGLIKRDFPHSVARLTGA

YSLTMGAAAALGSAMVVPLALNGFGWQGALFMLMCFPLLALFLWLPQWRSQQHANLSTSRALHTRGIWRS

PLAWQVTLFLGINSLVYYVIIGWLPAILISHGYSEAQAGSLHGLLQLATAAPGLLIALFLHHVKDQRGIA

AFVALMCAVGAVGLCFMPAHAITWTLLFGFGSGATMILGLTFIGLRQVLRIRRRHSRGWHNPSGICWQPV

GRR

>gi|30062949|ref|NP_837120.1| hypothetical protein S1547 [Shigella flexneri 2a str. 2457T]

MKGRTNTMNIQCKRVYDPAEQSDGYRVLVDRLWPRGIKKTDLALDEWDKEITPSTELRKAFHGEVVDFAT

FREQYRAELAQHEQEGKRLADIAKKQPLTLLYAAKNTTQNHALVLADWLRSL

>gi|30062940|ref|NP_837111.1| outer membrane protein [Shigella flexneri 2a str. 2457T]

MAVQKNVIKGILAGTFALMLSGCVTVPDAIKGSSTTPQQDLVRVMSAPQLYVGQEARFGGKVVAVQNQQG

KTRLEIATVPLDSGARPTLGEPSRGRIYADVNGFLDPVDFRGQLVTVVGPITGAVDGKIGNTPYKFMVMQ

VTGYKRWHLTQQVIMPPQPIDPWFYGGRGWPYGYGGWGWYNPGPARVQTVVTE

>gi|30062939|ref|NP_837110.1| hypothetical protein S1536 [Shigella flexneri 2a str. 2457T]

MRILAIDTATEACSVALWNDGTVNAHFELCPREHTQRILPMVQDILTTSGTSLTDINALAYGRGPGSFTG

VRIGIGIAQGLALGAELPMIGVSTLMTMAQGAWRKNGATRVLSAIDARMGEVYWAEYQRDENGIWHGEET

EAVLKPELVHERMQQLSGEWVTVGTGWQAWPDLGKESGLVLRDGEVLLPAAEDMLPIACQMFAEGKTVAV

EHAEPVYLRNNVAWKKLPGKE

>gi|30062937|ref|NP_837108.1| hypothetical protein S1534 [Shigella flexneri 2a str. 2457T]

MAVFAARKWKYNEDKMMTIVRIDAEARWSDVVIHNNTLYYTGVPENLDADAFEQTANTLAQIDAVLEKQG

SNKSSILDATIFLADKNDFAAMNKAWDAWVVAGHAPVRCTVQAGLMNPKYKVEIKIVAAV

>gi|30062934|ref|NP_837105.1| para-aminobenzoate synthase component I [Shigella flexneri 2a str. 2457T]

MKTLSPTVITLPWRQDAAEFYFSRLSHLPWAMLLHSGYADHPYSRFDIVVADPICTLTTLGKETVVSESE

KRTTTTDDPLQVLQQVLDRADIRPTHNEDLPFQGGALGLFGYDLGRRFESLPEIAEQDIVLPDMAVGIYD

WALIVDHQRHTVSLLSHNDVNARRAWLESQQFSPQEDFTLTSDWQSNMTHEQYGEKFRQVQEYLHSGDCY

QVNLAQRFHATYSGDEWQAFLQLNQANRAPFSAFLRLEQGAILSLSPERFILCDNSEIQTRPIKGTLPRL

PDPQEDSKQAEKLANSAKDRAENLMIVDLMRNDIGRVAVAGSVKVPELFVVEPFPAVHHLVSTITARLPE

QLHASDLLRAAFPGGSITGAPKVRAMEIIDELEPQRRNAWCGSIGYLSFCGNMDTSITIRTLTAINGQIY

CSAGGGIVADSQEEAEYQETFDKVNKILRQLEK

>gi|30062930|ref|NP_837101.1| transporter [Shigella flexneri 2a str. 2457T]

MEFLMDPSIWAGLLTLVVLEIVLGIDNLVFIAILADKLPPKQRDKARLLGLSLALIMRLGLLSLISWMVT

LTKPLFTVMDFSFSGRDLIMLFGGIFLLFKATTELHERLENRDHDSGHGKGYASFWVVVTQIVILDAVFS

LDAVITAVGMVNHLPVMMAAVVIAMAVMLLASKPLTRFVNQHPTVVVLCLSFLLMIGLSLVAEGFGFHIP

KGYLYAAIGFSIIIEVFNQIARRNFIRHQSTLPLRARTADAILRLMGGKRQANVQHDADNPMPIPEGAFA

EEERYMINGVLTLASRSLRGIMTPRGEISWVDANLGVDEIREQLLSSPHSLFPVCRGELDEIIGIVRAKE

LLVALEEGVDVAAIASASPAIIVPETLDPINLLGVLRRARGSFVIVTNEFGVVQGLVTPLDVLEAIAGEF

PDADETPEIITDGNGWLVKGGTDLHALQQALDVEHLADDDDIATVAGLVISANGHIPRVGDVIDVGPLHI

TIFEANDYRVDLVRIVKEQPAHDEDE

>gi|30062929|ref|NP_837100.1| PTS enzyme IIAB [Shigella flexneri 2a str. 2457T]

MTIAIVIGTHGWAAEQLLKTAEMLLGEQENVGWIDFVPGENAETLIEKYNAQLAKLDTTKGVLFLVDTWG

GSPFNAASRIVVDKEHYEVIAGVNIPMLVETLMARDDDPSFDELVALAVETGREGVKALKAKPVEKAAPA

PAAAAPKAAPTPAKPMGPNDYMVIGLARIDDRLIHGQVATRWTKETNVSRIIVVSDEVAADTVRKTLLTQ

VAPPGVTAHVVDVAKMIRVYNNPKYAGERVMLLFTNPTDVERLVEGGVKITSVNVGGMAFRQGKTQVNNA

VSVDEKDIEAFKKLNARGIELEVRKVSTDPKLKMMDLISKIDK

>gi|30062916|ref|NP_837087.1| transporter [Shigella flexneri 2a str. 2457T]

MVAIHLLPVSYNSATSTVNISARIIPLLIIHQRYKIPMPKVQADGLPLPQRYGAILTIVIGISMAVLDGA

IANVALPTIATDLHATPASSIWVVNAYQIAIVISLLSFSFLGDMFGYRRIYKCGLVVFLLSSLFCALSDS

LQMLTLARVIQGFGGAALMSVNTALIRLIYPQRFLGRGMGINSFIVAVSSAAGPTIAAAILSIASWKWLF

LINVPLGIIALLLAMRFLPPNGSRASKPRFDLPSAVMNALTFGLLITALSGFAQGQSLTLIAAELVVMVV

VGIFFIRRQLSLPVPLLPVDLLRIPLFSLSICTSVCSFCAQMLAMVSLPFYLQTVLGRSEVETGLLLTPW

PLATMVMAPLAGYLIERVHAGLLGALGLFIMATGLFSLVLLPASPADINIIWPMILCGAGFGLFQSPNNH

TIITSAPRERSGGASGMLGTARLLGQSSGAALVALMLNQFGDNGTHVSLMAAAILAVIAACVSGLRITQP

RARA

>gi|30062898|ref|NP_837069.1| ribosomal-protein-L7/L12-serine acetyltransferase [Shigella flexneri 2a str. 2457T]

MTETIKVSESLELHAVAESHVTPLYQLICKNKTWLQQSLNWPQFVQSEEDTRKTVQGNVMLHQRGYAKMF

MIFKEDELIGVISFNRIEPLNKTAEIGYWLDESHQGQGIISQALQTLIHHYAQSGELRRFVIKCRVDNPQ

SNQVALRNGFILEGCLKQAEFLNDAYDDVNLYARIIDS

>gi|30062892|ref|NP_837063.1| methyl-accepting chemotaxis protein III, ribose sensor receptor [Shigella flexneri 2a str. 2457T]

MNTTPSQRLGFLHHIRLVPLFACILGGILVLFALSSALAGYFLWQADRDQRDVTAEIEIRTGLANSSDFL

RSARINMIQAGAASRIAEMEAMKRNIAQAESEIKQSQQGYRAYQNRPVKTPADEAFDTELNQRFQAYITG

MQPMLKYAKNGMFEAIINHESEQIRPLDNAYTDILNKAVKIRSTRANQLAELAHQRTRLGGMFMIGAFVL

ALVMTLITFMVLRRIVIRPLQHAAQRIEKIASGDLTMNDEPAGRNEIGRLSRHLQQMQHSLGMTVGTVRQ

GAEEIYRGTSEISAGNADLSSRTEEQAAAIEQTAASMEQLTATVKQNADNAHHASKLAQEASIKASDGGQ

TVSGVVKTMGAISTSSKKISEITAVINSIAFQTNILALNAAVEAARAGEQGRGFAVVASEVRTLASRSAQ

AAKEIEGLISESVRLIDLGSDEVATAGKTMSTIVDAVASVTHIMQEIAAASDEQSRGITQVSQAISEMDK

VTQQNASLVEEASAAAVSLEEQAARLTEAVNVFRLNKHSVSAEPRGAGEPVSFATV

>gi|30062884|ref|NP_837055.1| azoreductase [Shigella flexneri 2a str. 2457T]

MSKVLVLKSSILAGYSQSNQLSDYFVEQWSEKHSADEITVRDLAANPIPVLDGELVGALRPSDAPLTPRQ

QEALALSDELIAELKAHDVIVIAAPMYNFNISTQLKNYFDLVARAGVTFRYTENGPEGLVTGKKAIVITS

RGGIHKDGPTDLVTPYLSTFLGFIGITDVKFVFAEGIAYGPEMAAKAQSDAKAAIDSIVAA

>gi|30062880|ref|NP_837051.1| phosphatidate cytidylyltransferase [Shigella flexneri 2a str. 2457T]

MISGPRWMTLTFFTLISFLALKEYCTLISVHFSRWLYWVIPLNYLLIGFNCFELFLLFIPLTGFLILATW

RVFVGDPSGFQHTVSAIFWGWIMTVFALSHAAWLLMLPTINIQGGALLVLFLLALTESNDIAQYLWGKSC

GRRKVVPKVSPGKTLEGLLGGVITTMIASLIIGPLLTPLNTLQALLAGLLIGISGFCGDVVMSAIKRDIG

VKDSGKLLLGHGGLLDRIDSLIFTAPVFFYFIRYCCY

>gi|30062850|ref|NP_837021.1| transport periplasmic protein [Shigella flexneri 2a str. 2457T]

MSVRGKLMKHSVSVTCCALLVSSISLSYAAEVPSGTVLAEKQELVRHIKDEPASLDPAKAVGLPEIQVIR

DLFEGLVNQNEKGEIVPGVATQWKSNDNRIWTFTLRDNAKWADGTPVTAQDFVYSWQRLVDPKTLSPFAW

FAALAGINNAQAIIDGKATPDQLGVTAVDAHTLKIQLDKPLPWFVNLTANFAFFPVQKANVESGKEWTKP

GNLIGNGAYVLKERVVNEKLVVVPNTHYWDNAKTVLQKVTFLPINQESAATKRYLAGDIDITESFPKNMY

QKLLKDIPGQVYTPPQLGTYYYAFNTQKGPTADQRVRLALSMTIDRRLMTEKVLGTGEKPAWHFIPDVTA

GFTPEPSPFEQMSQEELNAQAKTLLSAAGYGPQKPLKLTLLYNTSENHQKIAIAVASMWKKNLGVDVKLQ

NQEWKTYIDSRNTGNFDVIRASWVGDYNEPSTFLTLLTSMHSGNISRFNNPAYDKVLAQASTENTVKARN

ADYNAAEKILMEQAPIAPIYQYTNGRLIKPWLKGYPINNPEDVAYSRTMYIVKH

>gi|30062844|ref|NP_837015.1| murein peptide amidase A [Shigella flexneri 2a str. 2457T]

MTVTRSRAERGAFPPGTEHYGRSLLGAPLIWFPAPAASRESGLILAGTHGDENSSVVTLSCALRTLTPSL

RRHHVVLCVNPDGCQLGLRANANGVDLNRNFPAANWKEGETVYRWNSAAEERDVVLLTGDKPGSEPETQA

LCQLIHRIQPAWVVSFHDPLACIEDPRHNELGEWLAQSFELPLVTSVGYETPGSFGSWCADLNLHCITAE

FPPISSDEASEKYLFAMANLLRWHPKDAIRPS

>gi|30062842|ref|NP_837013.1| thiol peroxidase [Shigella flexneri 2a str. 2457T]

MSQTVHFQGNPVTVANSIPQAGSKAQTFTLVAKDLSDVTLGQFAGKRKVLNIFPSIDTGVCAASVRKFNQ

LATEIDNTVVLCISADLPFAQSRFCGAEGLNNVITLSTFRNAEFLQAYGVAIADGPLKGLAARAVVVIDE

NDNVIFSQLVDEITTEPDYEAALAVLKA

>gi|30062841|ref|NP_837012.1| DNA-binding transcriptional regulator TyrR [Shigella flexneri 2a str. 2457T]

MRLEVFCEDRLGLTRELLDLLVLRGIDLRGIEIDPIGRIYLNFAELEFESFSSLMAEIRRIAGVTDVRTV

PWMPSEREHLALSALLEALPEPVLSVDMKSKVDMANPASCQLFGQKLDRLRNHTAAQLINGFNFLRWLES

EPQDSHNEHVVINGQNFLMEITPVYLQDENDQHVLTGAVVMLRSTIRMGRQLQNVAAQDVSAFSQIVAVS

PKMKHVVEQAQKLAMLSAPLLITGDTGTGKDLFAYACHQASPRAGKPYLALNCASIPEDAVESELFGHAP

EGKKGFFEQANGGSVLLDEIGEMSPRMQAKLLRFLNDGTFRRVGEDHEVHVDVRVICATQKNLVELVQKG

VFREDLYYRLNVLTLNLPPLRDCPQDIMPLTELFVARFADEQGVPRPKLAADLNTVLTRYAWPGNVRQLK

NAIYRALTQLDGYELRPQDILLPDYDAATVAVGEDAMEGSLDEITSRFERSVLTQLYRNYPSTRKLAKRL

GVSHTAIANKLREYGLSQKKNEE

>gi|30062840|ref|NP_837011.1| hypothetical protein S1411 [Shigella flexneri 2a str. 2457T]

MTEPLKPRIDFDGPLEVEQNPKFRAQQTFDENQAQNFAPATLDEAQEEEGQVEAVMDAALRPKRSLWRKM

VMGGLALFGASVVGQGIQWTMNAWQTQDWVALGGCAAGALIIGAGVGSVVTEWRRLWRLRQRAHERDEAR

DLLHSHGTGKGRAFCEKLAQQAGIDQSHPALQRWYASIHETQNDREVVSLYAHLVQPVLDAQARREISRS

AAESTLMIAVSPLALVDMAFIAWRNLRLINRIATLYGIELGYYSRLRLFKLVLLNIAFAGASELVREVGM

DWMSQDLAARLSTRAAQGIGAGLLTVRLGIKAMELCRPLPWIDDDKPRLGDFRRQLIGQVKETLQKGKTP

>gi|30062838|ref|NP_837009.1| LACI-type transcriptional regulator [Shigella flexneri 2a str. 2457T]

MSPTIYDIARVAGVSKSTVSRVLNKQTNISPEAREKVLRAIEELQYQPNKLARALTSSGFDAIMVISTRS

TKTTAGNPFFSEVLHAITAKAEEEGFDVILQTSHNPAEDLQKCESKIKQKMIKGIIMLSSPADESFFAQL

DKYDIPVVVIGKVEGQYAHVYSVDTDNFGDSIALTDALIESGHQNIACLHAPLDVHVSVDRVNGYKQSLS

AHNIAVRDKWIVDGGYTHETALQAARQLLSQSPLPEAVFATDSLKLMSIYRAAAEKNIAIPQQLAVVGYS

NETLSFILTPAPGGIDVPTQELGQQSCELLFRLISGKPSPQNITVATHMTLK

>gi|30062832|ref|NP_837003.1| transport system permease [Shigella flexneri 2a str. 2457T]

MATNKRTLSRIGFYCGLALFLIITLFPFFVMLMTSFKSAKEAISLHPTLLPQQWTLEHYVDIFNPMIFPF

VDYFRNSMVVSVVSSVVAVFLGILGAYALSRLRFKGRMTINASFYTVYMFSGILLVVPLFKIITALGIYD

TEMALIITMVTQTLPTAVFMLKSYFDTIPDEIEEAAMMDGLNRLQIIFRITVPLAMSGLISVFVYCFMVA

WNDYLFASIFLSSASNFTLPVGLNALFSTPDYIWGRMMAASLVTALPVVIMYALLERFIKSGLTAGGVKG

>gi|30062828|ref|NP_836999.1| thiosulfate:cyanide sulfurtransferase [Shigella flexneri 2a str. 2457T]

MAIFIHRRTLTCLKKGLLALALVFSLPVFAAEHWIDVRVPEQYQQEHVQGAINIPLKEVKERIATAVPDK

NDTVKVYCNAGRQSGQAKELLSEMGYTHVENAGGLKDIAMPKVKG

>gi|30062826|ref|NP_836997.1| DNA-binding transcriptional activator PspC [Shigella flexneri 2a str. 2457T]

MAGINLNKKLWRIPQQGMVRGVCAGIANYFDVPVKLVRILVVLSIFFGLALFTLVAYIILSFALDPMPDN

MAFGEQLPSSSELLDEVDRELAASETRLREMERYVTSDTFTLRSRFRQL

>gi|30062824|ref|NP_836995.1| phage shock protein PspA [Shigella flexneri 2a str. 2457T]

MGIFSRFADIVNANINALLEKAEDPQKLVRLMIQEMEDTLVEVRSTSARALAEKKQLTRRIEQASAREVE
WQEKAELALLKEREDLARAALIEKQKLTDLIKSLEHEVTLVDDTLARMKKEIGELENKLSETRARQQALM
LRHQAANSSRDVRRQLDSGKLDEAMARFESFERRIDQMEAEAESHSFGKQKSLDDQFAELKADDAISEQL
AQLKAKMKQDNQ

>gi|30062815|ref|NP_836986.1| peptide transport periplasmic protein [Shigella flexneri 2a str. 2457T]

MRQVLSSLLVIAGLVSGQAIAAPESPPHADIRDSGFVYCVSGQVNTFNPSKASSGLIVDTLAAQFYDRLL
DVDPYTYRLMPELAESWEVLDNGATYRFHLRRDVPFQKTDWFTPTRKMNADDVVFTFQRIFDRNNPWHNV
NGSNFPYFDSLQFADNVKSVRKLDNHTVEFRLAQPDASFLWHLATHYASVMSAEYARKLEKEDRQEQLDR
QPVGTGPYQLSEYRAGQFIRLQRHDDFWRGKPLMPQVVVDLGSGGTGRLSKLLTGECDVLAWPAASQLSI
LRDDPRLRLTLRPGMNVAYLAFNTAKPPLNNPAVRHALALAINNQRLMQSIYYGTAETAAFILPRASWAY
DNEAKITEYNPAKSREQLKALGLENLTLKLWVPTRSQAWNPSPLKTAELIQADMAQVGVKVVIVPVEGRF
QEARLMDMSHDLTLSGWATDSNDPDSFFRPLLSCAAIHSQTNLAHWCNPKFDSVLRKALSSQQLAARIEA
YDEAQSILAQELPILPLASSLRLQAYRYDIKGLVLSPFGNASFAGVYREKQDEVKKP

>gi|30062813|ref|NP_836984.1| peptide transport permease [Shigella flexneri 2a str. 2457T]

MPYDSVYSEKRPPGTLRTAWRKFYSDASAMVGLYGCAGLAVLCIFGGWFAPYGIDQQFLGYQLLPPSWSR

YGEVSFFLGTDDLGRDVLSRLLSGAAPTVGGAFVVTLAATICGLVLGTFAGATHGLRSAVLNHILDTLLA

IPSLLLAIIVVAFAGPSLSHAMFAVWLALLPRMVRSIYSMVHDELEKEYVIAARLDGASTLNILWFAVMP

NITAGLVTEITRALSMAILDIAALGFLDLGAQLPSPEWGAMLGDALELIYVAPWTVMLPGAAIMISVLLV

NLLGDGVRRAIIAGVE

>gi|30062804|ref|NP_836975.1| translation initiation factor Sui1 [Shigella flexneri 2a str. 2457T]

MMSDSNSRLVYSTETGRIDEPKAAPVRPKGDGAVRIQRQTSGRKGKGVCLITGIDLDDAELTKLAAELKK

KCGCGGAVKDGVIEIQGDKRDLLKSLLEAKGMKVKLAGG

>gi|30062803|ref|NP_836974.1| orotidine 5'-phosphate decarboxylase [Shigella flexneri 2a str. 2457T]

MTLTASSSSRAVTNSPVVVALDYHNCDDALSFVDKIDPRDCRLKVGKEMFTLFGPQFVRELQQRGFDIFL

DLKFHDIPNTAAHAVAAAADLGVWMVNVHASGGARMMTAAREALVPFGKDAPLLIAVTVLTSMEASDLAD

LGVTLSPADYAERLAALTQKCDLDGVVCSAQEAVRFKQVFGQEFKLVTPGIRPQGSEAGDQRRIMTPEQA

LAAGVDYMVIGRPVTQSVDPAQTLKAINASLQRSA

>gi|30062802|ref|NP_836973.1| tetratricopeptide repeat protein [Shigella flexneri 2a str. 2457T]

MLELLFLLLPVAAAYGWYMGRRSAQQNKQDEANRLSRDYVAGVNFLLSNQQDKAVDLFLDMLKEDTGTVE

AHLTLGNLFRSRGEVDRAIRIHQTLMESASLTYEQRLLAIQQLGRDYMAAGLYDRAEDMFNQLTDETDFR

IGALQQLLQIYQATSEWQKAIDVAERLVKLGKDKQRVEIAHFYCELALQHMASDDLDRAMTLLKKGAAAD

KNSARVSIMMGRVFMAKGEYAKAVESLQRVISQDRELVSETLEMLQTCYQQLGKTAEWAEFLQRAVEENT

GADAELMLADIIEARDGSEAAQVYITRQLQRHPTMRVFHKLMDYHLNEAEEGRAKESLMVLRDMVGEKVR

SKPRYSCQKCGFTAYTLYWHCPSCRAWSTIKPIRGLDGL

>gi|30062801|ref|NP_836972.1| hypothetical protein S1366 [Shigella flexneri 2a str. 2457T]

MKYLLIFLLVLAIFVISVTLGAQNDQQVTFNYLLAQGEYRISTLLAVLFAAGFAIGWLICGLFWLRVRVS

LARAERKIKRLENQLSPATDVAVVPHSSAAKE

>gi|30062799|ref|NP_836970.1| GTP cyclohydrolase II [Shigella flexneri 2a str. 2457T]

MQLKRVAEAKLPTPWGDFLMVGFEELATGHDHVALVYGDISGHTPVLARVHSECLTGDALFSLRCDCGFQ

LEAALTQIAEEGRGILLYHRQEGRNIGLLNKIRAYALQDQGYDTVEANHQLGFAADERDFTLCADMFKLL

GVNEVRLLTNNPKKVEILTEAGINIVERVPLIVGRNPNNEHYLDTKAEKMGHLLNK

>gi|30062792|ref|NP_836963.1| cob(I)yrinic acid a,c-diamide adenosyltransferase [Shigella flexneri 2a str. 2457T]

MSDERYQQRQQRVKEKVDARVAQAQDERGIIIVFTGNGKGKTTAAFGTATRAVGHGKKVGVVQFIKGTWP

NGERNLLEPHGVEFQVMATGFTWDTQNRESDTAACREVWQHAKRMLADSSLDMVLLDELTYMVAYDYLPL

EEVVQALNERPHQQTVIITGRGCHRDILELADTVSELRPIRHAFDAGVKAQIGIDY

>gi|30062791|ref|NP_836962.1| 23S rRNA pseudouridylate synthase B [Shigella flexneri 2a str. 2457T]

MSEKLQKVLARAGHGSRREIESIIEAGRVSVDGKIAKLGDRVEVTPGLKIRIDGHLISVRESAEQICRVL

AYYKPEGELCTRNDPEGRPTVFDRLPKLRGARWIAVGRLDVNTCGLLLFTTDGELANRLMHPSREVEREY

AVRVFGQVDDAKLRDLSRGVQLEDGPAAFKTIKFSGGEGINQWYNVTLTEGRNREVRRLWEAVGVQVSRL

IRVRYGDIPLPKGLPRGGWTELDLAQTNYLRELVELPPETSSKVAVEKDRRRMKANQIRRAVKRHSQVSG

SRRSGGRNNG

>gi|30062787|ref|NP_836958.1| anthranilate synthase component I [Shigella flexneri 2a str. 2457T]

MQTQKPTLELLTCEGAYRDNPTALFHQLCGNRPATLLLESADIDSKDDLKSLLLIDSALRITALGDTVTI

QALSGNGEALLALLDNALPAGVESEQSPNCRVLRFPPVSPLLDEDARLCSLSVFDAFRLLQNLLNVPKEE

REAMFFGGLFSYDLVAGFEDLPQLSAENNCPDFCFYLAETLMVIDHQKKSTRIQASLFAPNEEEKQRLTA

RLNELRQQLTEAAPPLPVVSVPHMRCECNQSDEEFGGVVRLLQKAIRAGEIFQVVPSRRFSLPCPSPLAA

YYVLKKSNPSPYMFFMQDNDFTLFGASPESSLKYDATSRQIEIYPIAGTRPRGRRADGSLDRDLDSRIEL

EMRTDHKELSEHLMLVDLARNDLARICTPGSRYIADLTKVDRYSYVMHLVSRVVGELRHDLDALHAYRAC

MNMGTLSGAPKVRAMQLIAEAEGRRRGSYGGAVGYFTAHGDLDTCIVIRSALVENGIATVQAGAGVVLDS

VPQSEADETRNKARAVLRAIATAHHAQETF

>gi|30062777|ref|NP_836948.1| intracellular septation protein A [Shigella flexneri 2a str. 2457T]

MKQFLDFLPLVVFFAFYKIYDIYAATAALIVATAIVLIYSWVRFRKVEKMALITFVLVVVFGGLTLFFHN

DEFIKWKVTVIYALFAGALLVSQWVMKKPLIQRMLSKELTLPQPVWSKLNLAWAVFFILCGLANIYIAFW

LPQNIWVNFKVFGLTALTLIFTLLSGIYIYRHMPQEDKS

>gi|30062766|ref|NP_836937.1| oligopeptide transport permease [Shigella flexneri 2a str. 2457T]

MMLSKKNSETLENFSEKLEVEGRSLWQDARRRFMHNRAAVASLIVLVLIALFVILAPMLSQFAYDDTDWA

MMSSAPDMESGHYFGTDSSGRDLLVRVAIGGRISLMVGVAAALVAVVVGTLYGSLSGYLGGKVDSVMMRL

LEILNSFPFMFFVILLVTFFGQNILLIFVAIGMVSWLDMARIVRGQTLSLKRKEFIEAAQVGGVSTPGIV

IRHIVPNVLGVVVVYASLLVPSMILFESFLSFLGLGTQEPLSSWGALLSDGANSMEVSPWLLLFPAGFLV

VTLFCFNFIGDGLRDALDPKDR

>gi|30062764|ref|NP_836935.1| periplasmic oligopeptide binding protein [Shigella flexneri 2a str. 2457T]

MTNITKRSLVAAGVLAALMAGNVALAADVPAGVTLAEKQTLVRNNGSEVQSLDPHKIEGVPESNISRDLF

EGLLVSDLDGHPAPGVAESWDNKDAKVWTFHLRKDAKWSDGTPVTAQDFVYSWQRSVDPNTASPYASYLQ

YGHIAGIDEILEGKKPITDLGVKAIDDHTLEVTLSEPVPYFYKLLVHPSTSPVPKAAIEKFGEKWTQPGN

IVTNGAYTLKDWVVNERIVLERSPTYWNNAKTVINQVTYLPIASEVTDVNRYRSGEIDMTYNNMPIELFQ

KLKKEIPDEVHVDPYLCTYYYEINNQKPPFNDVRVRTALKLGMDRDIIVNKVKAQGDMPAYGYTPPYTDG

AKLTQPEWFGWSQEKRNEEAKKLLAEAGYTADKPLTINLRYNTSDLHKKLAIAASSLWKKNIGVNVKLVN

QEWKTFLDTRHQGTFDVARAGWCADYNEPTSFLNTMLSNSSMNTAHYKSPAFDSIMAETLKVTDEAQRTA

LYTKAEQQLDKDSAIVPVYYYVNARLVKPWVGGYTGKDPLDNTYTRNMYIVKH

>gi|30062758|ref|NP_836929.1| thymidine kinase [Shigella flexneri 2a str. 2457T]

MAQLYFYYSAMNAGKSTALLQSSYNYQERGMRTVVYTAEIDDRFGAGKVSSRIGLSSPAKLFNQNSSLFD

EIRAEHEQQAIHCVLVDECQFLTRQQVYELSEVVDQLDIPVLCYGLRTDFRGELFIGSQYLLAWSDKLVE

LKTICFCGRKASMVLRLDQAGRPYNEGEQVVIGGNERYVSVCRKHYKEALQVGSLTAIQERHRHD

>gi|30062757|ref|NP_836928.1| global DNA-binding transcriptional dual regulator H-NS [Shigella flexneri 2a str. 2457T]

MSEALKILNNIRTLRAQARECTLETLEEMLEKLEVVVNERREEESAAAAEVEERTRKLQQYREMLIADGI

DPNELLNSLAAVKSGTKAKRAQRPAKYSYVDENGETKTWTGQGRTPAVIKKAMDEQGKSLDDFLIKQ

>gi|30062756|ref|NP_836927.1| UTP--glucose-1-phosphate uridylyltransferase subunit GalU [Shigella flexneri 2a str. 2457T]

MAAINTKVKKAVIPVAGLGTRMLPATKAIPKEMLPLVDKPLIQYVVNECIAAGITEIVLVTHSSKNSIEN

HFDTSFELEAMLEKRVKRQLLDEVQSICPPHVTIMQVRQGLAKGLGHAVLCAHPVVGDEPVAVILPDVIL

DEYESDLSQDNLAEMIRRFDETGHSQIMVEPVADVTAYGVVDCKGVELAPGESVPMVGVVEKPKADVAPS

NLAIVGRYVLSADIWPLLAKTPPGAGDEIQLTDAIDMLIEKETVEAYHMKGKSHDCGNKLGYMQAFVEYG

IRHNTLGTEFKAWLEEEMGIKK

>gi|30062755|ref|NP_836926.1| response regulator of RpoS [Shigella flexneri 2a str. 2457T]

MTQPLVGKQILIVEDEQVFRSLLDSWFSSLGATTVLAADGVDALELLGGFTPDLMICDIAMPRMNGLKLL
EHIRNRGDQTPVLVISATENMADIAKALRLGVEDVLLKPVKDLNRLREMVFACLYPSMFNSRVEEEERLF
RDWDAMVDNPAAAAKLLQELQPPVQQVISHCRVNYRQLVAADKPGLVLDIAALSENDLAFYCLDVTRAGH
NGVLAALLLRALFNGLLQEQLAHQNQRLPELGALLKQVNHLLRQANLPGQFPLLVGYYHRELKNLILVSA
GLNATLNTGEHQVQISNGVPLGTLGNAYLNQLSQRCDAWQCQIWGTGGRLRLMLSAE

>gi|30062749|ref|NP_836920.1| nitrate reductase 1, beta subunit [Shigella flexneri 2a str. 2457T]
MKIRSQVGMVLNLDKCIGCHTCSVTCKNVWTSREGVEYAWFNNVETKPGQGFPTDWENQEKYKGGWIRKI
NGKLQPRMGNRAMLLGKIFANPHLPGIDDYYEPFDFDYQNLHTAPEGSKSQPIARPRSLITGERMAKIEK
GPNWEDDLGGEFDKLAKDKNFDNIQKAMYSQFENTFMMYLPRLCEHCLNPACVATCPSGAIYKREEDGIV
LIDQDKCRGWRMCITGCPYKKIYFNWKSGKSEKCIFCYPRIEAGQPTVCSETCVGRIRYLGVLLYDADAI
ERAASTENEKDLYQRQLDVFLDPNDPKVIEQAIKDGIPLSVIEAAQQSPVYKMAMEWKLALPLHPEYRTL
PMVWYVPPLSPIQSAADAGELGSNGILPDVESLRIPVQYLANLLTAGDTKPVLRALKRMLAMRHYKRAET
VDGKVDTRALEEVGLTEAQAQEMYRYLAIANYEDRFVVPSSHRELAREAFPEKNGCGFTFGDGCHGSDTK
FNLFNSRRIDAIDVTSKTEPHP

>gi|30062745|ref|NP_836916.1| transcriptional regulator NarL [Shigella flexneri 2a str. 2457T]
MSNQEPATILLIDDHPMLRTGVKQLISMAPDITVVGEASNGEQGIELAESLDPDLILLDLNMPGMNGLET
LDKLREKSLSGRIVVFSVSNHEEDVVTALKRGADGYLLKDMEPEDLLKALHQAAAGEMVLSEALTPVLAA

SLRANRATTERDVNQLTPRERDILKLIAQGLPNKMIARRLDITESTVKVHVKHMLKKMKLKSRVEAAVWV

HQERIF

>gi|30062739|ref|NP_836910.1| 2-dehydro-3-deoxyphosphooctonate aldolase [Shigella flexneri 2a str. 2457T]

MKQKVVSIGDINVANDLPFVLFGGMNVLESRDLAMRICEHYVTVTQKLGIPYVFKASFDKANRSSIHSYR

GPGLEEGMKIFQELKQTFGVKIITDVHEPSQAQPVADVVDVIQLPAFLARQTDLVEAMAKTGAVINVKKP

QFVSPGQMGNIVDKFKEGGNEKVILCDRGANFGYDNLVVDMLGFSIMKKVSGNSPVIFDVTHALQCRDPF

GAASGGRRAQVAELARAGMAVGLAGLFIEAHPDPEHAKCDGPSALPLAKLEPFLKQMKAIDDLVKGFEEL

DTSK

>gi|30062737|ref|NP_836908.1| transcriptional regulator [Shigella flexneri 2a str. 2457T]

MTSFSTLLSVHLISIALSVGLLTLRFWLRYQKHPQAFARWTRIVPPVVDTVLLLSGIALMAKAHILPFSG

QAQWLTEKLFGVIIYIVLGFIALDYRRMHSQQARIIAFPLALVVLYIIIKLATTKVPLLG

>gi|30062733|ref|NP_836904.1| outer membrane lipoprotein LolB [Shigella flexneri 2a str. 2457T]

MPLPDFRLIRLLPLAALVLTACSVTTPKGPGKSPDSPQWRQHQQDVRNLNQYQTRGAFAYISDQQKVYAR

FFWQQTGQDRYRLLLTNPLGSTELELNAQPGNVQLVDNKGQRYTADDAEEMIGKLTGMPIPLNSLRQWIL

GLPGDATDYKLDDQYRLSEITYSQNGKNWKVVYGGYDTKTQPAMPANMELTDGGQRIKLKMDNWIVK

>gi|30062732|ref|NP_836903.1| 4-diphosphocytidyl-2-C-methyl-D-erythritol kinase [Shigella flexneri 2a str. 2457T]

MRTQWPSPAKLNLFLYITGQRADGYHTLQTLFQFLDYGDTISIELRDDGDIRLLTPVEGVEHEDNLIVRA

ARLLMKTAADSGRLPTGSGANISIDKRLPMGGGLGGGSSNAATVLVALNHLWQCGLSMDELAEMGLTLGA

DVPVFVRGHAAFAEGVGEILTPVDPPEKWYLVAHPGVSIPTPVIFKDPELPRNTPKRSIETLLKCEFSND

CEVIARKRFREVDAVLSWLLEYAPSRLTGTGACVFAEFDTESEARQVLEQAPEWLNGFVAKGVNLSPLHR

AML

>gi|30062725|ref|NP_836896.1| DNA-binding transcriptional regulator DhaR [Shigella flexneri 2a str. 2457T]

MRDMSGAFNNDGRGISPLIATSWERCNKLMKRETWNVPHQAQGVTFASIYRRKKAMLTLGQAALEDAWEY

MAPRECALFILDETACILSRNGDPQTLQQLSALGFNDGTYCAEGIIGTCALSLAAISGQAVKTMADQHFK

QALWNWAFCATPLFDSKGRLTGTIALACPVEQTTAADLPLTLAIAREVGNLLLTDSLLAETNRHLNQLNA

LLESMDDGVISWDEQGNLQFINAQAARVLRLDATASQGRAITELLTLPAVLQQAIKLAHPLKHVEATFES

QHQFIDAVITLKPIIETQGTSFILLLHPVEQMRQLMTSQLGKVSHTFAHMPQDDPQTRRLIHFGRQAARS

SFPVLLCGEEGVGKALLSQAIHNESERAAGPYIAVNCELYGDAALAEEFIGGDRTDNENGRLSRLELAHG

GTLFLEKIEYLAVELQSALLQVIKQGVITRLDARRLIPIDVKVIATTTADLAMLVEQNRFSRQLYYALHA

FEITIPPLRMRRGSIPALVNNKLRSLEKRFSTRLKIDDDALARLVSCAWPGNDFELYSVIENLALSSDNG

RIRVSDLPEHLFTEQATDDVSATRLSTSLSFAEVEKEAIINAAQVTGGRIQEMSALLGIGRTTLWRKMKQ

HGIDAGQFKRRV

>gi|30062707|ref|NP_836878.1| alanine racemase [Shigella flexneri 2a str. 2457T]

MTRPIQASLDLQALKQNLSIVRQAAPHARVWSVVKANAYGHGIERIWSALGATDGFALLNLEEAITLRER

GWKGPILMLEGFFHAQDLEIYDQHRLTTCVHSNWQLKALQNARLKAPLDIYLKVNSGMNRLGFQSDRVLT

VWQQLRAMANVGEMTLMSHFAEAEHPDGISGAMARIEQAAEGLECRRSLSNSAATLWHPEAHFDWVRPGI

ILYGASPSGQWRDIANTGLRPVMTLSSEIIGVQTLKAGERVGYGGRYTARDEQRIGIVAAGYADGYPRHA

PTGAPVLVDGVRTMTVGTVSMDMLAVDLTPCPQAGIGTPVELWGKEIKIDDVAAAAGTVGYELMCALALR

VPVVTV

>gi|30062706|ref|NP_836877.1| D-amino acid dehydrogenase small subunit [Shigella flexneri 2a str. 2457T]

MRVVILGSGVVGVASAWYLNQAGHEVTVIDREPGAALETSAANAGQISPGYAAPWAAPGVPLKAIKWMFQ

RHAPLAVRLDGTQFQLKWMWQMLRNCDTSHYMENKGRMVRLAEYSRDCLKALRAETNIQYEGRQGGTLQL

FRTEQQYENATRDIAVLEDAGVPYQLLESSRLAEVDPALAEVAHKLTGGLQLPNDETGDCQLFTQNLARM

AEQAGVKFRFNTPVDQLLCDGEQIYGVKFGDEVIKADAYVMAFGSYSTAMLKGIVDIPVYPLKGYSLTIP

IAQEDGAPVSTILDETYKIAITRFDNRIRVGGMAEIVGFNTELLQPRRETLEMVVRDLYPRGGHVEQATF

WTGLRPMTPDGTPVVGSTRFKNLWLNTGHGTLGWTMACGSGQLLSDLLSGRTPAIPYEDLSVARYSRGFT

PSRPGHLHGAHS

>gi|30062683|ref|NP_836854.1| isocitrate dehydrogenase [Shigella flexneri 2a str. 2457T]

MESKVVVPAQGKKITLQNGKLNVPENPIIPYIEGDGIGVDVTPAMLKVVDAAVEKAYKGERKISWMEIYT

GEKSTQVYGQDVWLPAETLDLIREYRVAIKGPLTTPVGGGIRSLNVALRQELDLYICLRPVRYYQGTPSP

VKHPELTDMVIFRENSEDIYAGIEWKADSADAEKVIKFLREEMGVKKIRFPEHCGIGIKPCSEEGTKRLV

RAAIEYAIANDRDSVTLVHKGNIMKFTEGAFKDWGYQLAREEFGGELIDGGPWLKVKNPNTGKEIVIKDV

IADAFLQQILLRPAEYDVIACMNLNGDYISDALAAQVGGIGIAPGANIGDECALFEATHGTAPKYAGQDK

VNPGSIILSAEMMLRHMGWTEAADLIVKGMEGAINAKTVTYDFERLMEGAKLLKCSEFGDAIIKNM

>gi|30062677|ref|NP_836848.1| DNA-binding transcriptional regulator PhoP [Shigella flexneri 2a str. 2457T]

MRVLVVEDNALLRHHLKVQIQDAGHQVDDAEDAKEADYYLNEHLPDIAIVDLGLPDEDGLSLIRRWRSND

VSLPILVLTARESWQDKVEVLSAGADDYVTKPFHIEEVMARMQALMRRNSGLASQVISLPPFQVDLSRRE

LSINDEVIKLTAFEYTIMETLIRNNGKVVSKDSLMLQLYPDAELRESHTIDVLMGRLRKKIQAQYPQEVI

TTVRGQGYLFELR

>gi|30062676|ref|NP_836847.1| sensor protein PhoQ [Shigella flexneri 2a str. 2457T]

MKKLLRLFFPLSLRVRFLLATAAVVLVLSLAYGMVALIGYSVSFDKTTFRLLRGESNLFYTLAQWENNKL

HVELPENIDKQSPTMTLIYDENGQLLWAQRDVPWLMKMIQPDWLKSNGFHEIEADVNDTSLLLSGDHSIQ

QQLQEVREDDDDAEMTHSVAVNVYPATSRMPKLTIVVVDTIPVELKSSYMVWSWFIYVLSANLLLVIPLL

WVAAWWSLRPIEALAKEVRELEEHNRELLNPATTRELTSLVRNLNRLLKSERERYDKYRTTLTDLTHSLK

TPLAVLQSTLRSLRSEKMSVSDAEPVMLEQISRISQQIGYYLHRASMRGGTLLSRELHPVAPLLDNLTSA

LNKVYQRKGVNISLDISPEISFVGEQNDFVEVMGNVLDNACKYCLEFVEISARQTDEHLYIVVEDDGPGI

PLSKREVIFDRGQRVDTLRPGQGVGLAVAREITEQYEGKIVAGESMLGGARMEVIFGRQHSAPKDE

>gi|30062655|ref|NP_836826.1| spermidine/putrescine ABC transporter [Shigella flexneri 2a str. 2457T]

MIGRLLRGGFMTAIYAYLYIPIIILIVNSFNSSRFGINWQGFTTKWYSLLMNNDSLLQAAQHSLTMAVFS

ATFATLIGSLTAVALYRYRFRGKPFVSGMLFVVMMSPDIVMAISLLVLFMLLGIQLGFWSLLFSHITFCL

PFVVVTVYSRLKGFDVRMLEAAKDLGASEFTILRKIILPLAMPAVAAGWVLSFTLSMDDVVVSSFVTGPS

YEILPLKIYSMVKVGVSPEVNALATILLVLSLVMVIASQLIARDKTKGNTGDVK

>gi|30062654|ref|NP_836825.1| spermidine/putrescine ABC transporter substrate-binding protein [Shigella flexneri 2a str. 2457T]

MKKWSRHLLAAGALALGMSAAHADDNNTLYFYNWTEYVPPGLLEQFTKETGIKVIYSTYESNETMYAKLK

TYKDGAYDLVVPSTYYVDKMRKEGMIQKIDKSKLTNFSNLDPDMLNKPFDPNNDYSIPYIWGATAIGVNG

DAVDPKSVTSWADLWKPEYKGSLLLTDDAREVFQMALRKLGYSGNTTDPKEIEAAYNELKKLMPNVAAFN

SDNPANPYMEGEVNLGMIWNGSAFVARQAGTPIDVVWPKEGGIFWMDSLAIPANAKNKEGALKLINFLLR

PDVAKQVAETIGYPTPNLAARKLLSPEVANDKTLYPDAETIKNGEWQNDVGAASSIYEEYYQKLKAGR

>gi|30062651|ref|NP_836822.1| outer membrane-specific lipoprotein transporter subunit LolE [Shigella flexneri 2a str. 2457T]

MAMPLSLLIGLRFSRGRRRGGMVSLISVISTIGIALGVAVLIVGLSAMNGFERELNNRILAVVPHGEIEA

VNQPWTNWQEALDNVQKVPGIAAAAPYINFTGLVESGANLRAIQVKGVNPQQEQRLSALPSFVQGDAWRN

FKAGEQQIIIGKGVADALKVKQGDWVSIMIPNSNPEHKLMQPKRVRLHIAGILQLSGQLDHSFAMIPLAD

AQQYLDMGSSVSGIALKMTDVFNANKLVRDAGEVTNSYVYIKSWIGTYGYMYRDIQMIRAIMYLAMVLVI

GVACFNIVSTLVMAVKDKSGDIAVLRTLGAKDGLIRAIFVWYGLLAGLFGSLCGVVIGVVVSLQLTPIIE

WIEKLIGHQFLSSDIYFIDFLPSELHWLDVFYVLVTALLLSLLASWYPARRASNIDPARVLSGQ

>gi|30062649|ref|NP_836820.1| outer membrane-specific lipoprotein transporter subunit LolC [Shigella flexneri 2a str. 2457T]

MYQPVALFIGLRYMRGRAVDRFGRFVSWLSTIGITLGVMALVTVLSVMNGFERELQNNILGLMPQAILSS

EHGSLNPQQLPETAVKLDGVNRVAPITTGDVVLQSARSVAVGVMLGIDPAQKDPLTPYLVNVKQTDLEPG

KYNVILGEQLASQLGVNRGDQIRVMVPSASQFTPMGRIPSQRLFNVIGTFAANSEVDGYEMLVNIEDASR

LMRYPAANITGWRLWLDEPLRVDSLSQQKLPEGSKWQDWRDRKGELFQAVRMEKNMMGLLLSLIVAVAAF

NIITSLGLMVMEKQGEVAILQTQGLTPRQIMMVFMVQGASAGIIGAILGAALGALLASQLNNLMPIIGVL

LDGAALPVAIEPLQVIVIALVAMAIALLSTLYPSWRAAATQPAEALRYE

>gi|30062644|ref|NP_836815.1| hypothetical protein S1195 [Shigella flexneri 2a str. 2457T]

MGSGLVNGGDYFYNNLSFTVTRYNGIMATDSTQCVKKSRGRPKVFDRDAALDKAMKLFWQHGYEATSLAD

LVEATGAKAPTLYAEFTNKEGLFRAVLDRYIDRFAAKHEAQLFCEEKSVESALADYFAAIANCFTSKDTP

AGCFMINNCTTLSPDSGDIANTLKSRHAMQERTLQQFLCQRQARGEIPTHCDVTHLAEFLNCIIQGMSIS

AREGASLEKLMQIAGTTLRLWPELVK

>gi|30062641|ref|NP_836812.1| hypothetical protein S1192 [Shigella flexneri 2a str. 2457T]

MIIYLHGFDSNSPGNHEKVLQLQFIDPDVRLISYSTRHPKHDMQHLLKEVDKMLQLNVDERPLICGVGLG

GYWAERIGFLCDIRQVIFNPNLFPYENMEGKIDRPEEYADIATKCVTNFREKNRDRCLVILSRNDEALNS

QRTSEELHHYYEIVWDEEQTHKFKNISPHLQRIKAFKTLG

>gi|30062633|ref|NP_836804.1| DNA polymerase III subunit delta' [Shigella flexneri 2a str. 2457T]

MRWYPWLRPDFEKLVASYQAGRGHHALLIQALPGMGDDALIYALSRYLLCQQPQGHKSCGHCRGCQLMQA

GTHPDYYTLAPEKGKNTLGIDAVREVTEKLNEHARLGGAKVVWVTDAALLTDAAANALLKTLEEPPAETW

FFLATREPERLLATLRSRCRLHYLAPPPEQYAVTWLSREVTMSQDALLAALRLSAGSPGAALALFQGDNW

QARETLCQALAYSVPSGDWYSLLAALNHEQAPARLHWLATLLMDALKRHHGAAQVTNVDVPGLVAELANH

LSPSRLQAILGDVCHIREQLMSVTGINRELLITDLLLRIEHYLQPGVVLPVPHL

>gi|30062625|ref|NP_836796.1| 3-oxoacyl-ACP synthase [Shigella flexneri 2a str. 2457T]

MYTKIIGTGSYLPEQVRTNADLEKMVDTSDEWIVTRTGIRERHIAAPNETVSTMGFEAATRAIEMAGIEK

DQIGLIVVATTSATHAFPSAACQIQSMLDIKGCPAFDVAAACAGFTYALSVADQYVKSGAVKYALVVGSD

VLARTCDPTDRGTIIIFGDGAGAAVLAASEEPGIISTHLHADGSYGELLTLPNADRVNPENSIHLTMAGN

EVFKVAVTELAHIVDETLAANNLDRSQLDWLVPHQANLRIISATAKKLGMSMDNVVVTLDRHGNTSAASV

PCALDEAVRDGRIKPGQLVLLEAFGGGFTWGSALVRF

>gi|30062619|ref|NP_836790.1| hypothetical protein S1169 [Shigella flexneri 2a str. 2457T]

MSSRVANLTVIVSSKRRRVSVARFSCGKTAQLSKKQTGYYSPEIFPSTGKDCNPQPANCLKDQYVLRHCC

VDDRSGKMGYSVKFLVLTRMDTETASLFHCKPCYSKMTFTIYHPLTHSFFTSCW

>gi|30062607|ref|NP_836778.1| virulence factor [Shigella flexneri 2a str. 2457T]

MNLLKSLAAVSSMTMFSRVLGFARDAIVARIFGAGMATDAFFVAFKLPNLLRRIFAEGAFSQAFVPILAE

YKSKQGEDATRVFVSYVSGLLTLALAVVTVAGMLAAPWVIMVTAPGFADTADKFALTSQLLKITFPYILL

ISLASLVGAILNTWNRFSIPAFAPTLLNISMIGFALFAAPYFNPPVLALAWAVTVGGILQLVYQLPHLKK

IGMLVLPRINFHDAGAMRVVKQMGPAILGVSVSQISLIINTIFASFLASGSVSWMYYADRLMEFPSGVLG

VALGTILLPSLSKSFASGNHDEYNRLMDWGLRLCFLLALPSAVALGILSGPLTVSLFQYGKFTAFDALMT

QRALIAYSVGLIGLIVVKVLAPGFYSRQDIKTPVKIAIVTLILTQLMNLAFIGPLKHAGLSLSIGLAACL

NASLLYWQLRKQKIFTPQPGWMAFLLRLVVAVLVMSGVLLGMLHIMPEWSLGTMPWRLLRLMAVVLAGIA

AYFAALAVLGFKVKEFARRTV

>gi|30062594|ref|NP_836765.1| hypothetical protein S1140 [Shigella flexneri 2a str. 2457T]

MKKSLLGLTFASLMFSAGSAVAADYKIDKEGQHAFVNFRIQHLGYSWLYGTFKDFDGTFTFDEKNPAADK

VNVTINTTSVDTNHAERDKHLRSADFLNTTKYPQATFTSTSVKKDGDELDITGDLTLNGVTKPVTLEAKL

IGQGDDPWGGKRAGFEAEGKIKLKDFNIKTDLGPASQEVDLIISVEGVQQK

>gi|30062563|ref|NP_836734.1| curli assembly protein CsgE [Shigella flexneri 2a str. 2457T]

MKRYLRWIVAAEFLFAAGNLHAVEVEVPGLLTDHTVSSIGHDFYRAFSDKWESDYTGNLTINERPSARWG

SWITITVNQDVIFQTFLFPLKRDSEKTVVFALIQTEEALNRRQINQALLSTDDLAHDEF

>gi|30062561|ref|NP_836732.1| hypothetical protein S1103 [Shigella flexneri 2a str. 2457T]

MAAFSAIMRGMNILLSIAITTGILSGIWGWVAVSLGLLSWAGFLGCTAYFACPQGGLKGLAISAATLLSG

VVWAMVIIYGSALAPHLEILGYVITGIVAFLMCIQAKQLLLSFVPGTFIGACATFAGQGDWKLVLPSLAL

GLVFGYAMKNSGLWLAARSAKTAHREQEIKNKA

>gi|30062559|ref|NP_836730.1| hydrolase [Shigella flexneri 2a str. 2457T]

MYPVDLHMHTVASTHAYSTLSDYIAQAKQKGIKLFAITDHGPDMEDAPHHWHFINMRIWPRVVDGVGILR

GIEANIKNVDGEIDCSGKMFDSLDLIIAGFHEPVFAPHDKATNTQAMISTIASGNVHIISHPGNPKYEID

VKAVAEAAAKHQVALEINNSSFLHSRKGSEDNCRAVAAAVRDAGGWVALGSDSHTAFTMGEFEECLKILD

AVDFPLERILNVSPRRLLNFLESRGMAPIAEFADL

>gi|30062548|ref|NP_836719.1| synthetase [Shigella flexneri 2a str. 2457T]

MPRPSLCADSGGGMMTTLTARPEAITFDPQQSAQIVVDMQNAYATPGGYLDLAGFDVSTTRPVIANIQTA

VTAARAAGMLIIWFQNGWDEQYVEAGGPGSPNFHKSNALKTMRKQPQLQGKLLAKGSWDYQLVDELVPQP

GDIVLPKPRYSGFFNTPLDSILRSRGIRHLVFTGIATNVCVESTLRDGFFLEHFGVVLEDATHQAGPEFA

QKAALFNIETFFGWVSDVETFCDALSPTSFARIA

>gi|30062536|ref|NP_836707.1| chaperone-modulator protein CbpM [Shigella flexneri 2a str. 2457T]

MANVTVTFTITEFCLHTGISEEELNEIVGLGVVEPREIQETTWVFDDHAAIVVQRAVRLRHELALDWPGI

AVALTLMDDIAHLKQENRLLRQRLSRFVAHP

>gi|30062532|ref|NP_836703.1| DNA-binding transcriptional regulator TorR [Shigella flexneri 2a str. 2457T]

MPHHIVIVEDKPVTQARLQSYFTQEGYTVSVTASGAGLREIMQNQPVDLILLDINLPDENGLMLTRALRE

RSTVGIILVTGRSDRIDRIVGLEMGADDYVTKPLELRELVVRVKNLLWRIDLARQAQPHTQDYCYRFAGY

CLNVSRHTLERDGEPIKLTRAEYEMLVAFVTNPGEILSRERLLRMLSARRVENPDLRTVDVLIRRLRHKL

SADLLVTQHGEGYFLAADVC

>gi|30062530|ref|NP_836701.1| hypothetical protein S1062 [Shigella flexneri 2a str. 2457T]

MAEKKRTRWQRRPGTTGGKLPWNDWRNATTWRKATQFLLLAMNIYIAITFWYWVRYYETAGSTTFVARPG

GIEGWLPIAGLMNLKYSLATGQLPSVHAAAMLLLVAFIVISLLLKKAFCSWLCPVGTLSELIGDLGNKLF

GRQCVLPRWLDIPLRGVKYLLLSLFLYIALLMPAQAIHYFMLSPYSVVMDVKMLDFFRHMGTATLISVTV

LLIASLFIRHAWCRYLCPYGALMGVVSLLSPFKIRRNAESCIDCGKCAKKCPSRIPVDKLIQVRTVECTG

CMTCVESCPVASTLTFSLQKPAANKKAFALSGWLMTLLVLGIMFAVIGYAMYAGVWQSPVPEELYRRLIP

QAPMIGH

>gi|30062517|ref|NP_836688.1| third cytochrome oxidase, subunit II [Shigella flexneri 2a str. 2457T]

MFDYETLRFIWWLLIGVILVVFMISDGFDMGIGCLLPLVARNDDERRIVINSVGAHWEGNQVWLILAGGA

LFAAWPRVYAAAFSGFYVAMILVLCSLFFRPLAFDYRGKIADARWRKMWDAGLVIGSLVPPVVFGIAFGN

LLLGVPFAFTPQLRVEYLGSFWQLLTPFPLLCGLLSLGMVILQGGVWLQLKTVGVIHLRSQLATKRAALL

VMLCFLLAGYWLWVGIDGFVLLAQDANGPSHPLMKLVAVLPGAWMNNFVESPVLWIFPLLGFFCPLLTVM

AIYRGRPGWGFLMASLMQFGVIFTAGITLFPFVMPSSVSPISSLTLWDSTSSQLTLSIMLVIVLIFLPIV

LLYTLWSYYKMWGRMTTETLRRNKNELY

>gi|30062513|ref|NP_836684.1| hydrogenase 1 maturation protease [Shigella flexneri 2a str. 2457T]

MSEQHVVVMGLGNLLWADEGFGVRVAERLYAHYHWPEYVEIVDGGTQGLNLLGYVESASHLLILDAIDYG

LEPGTLRTYAGERIPAYLSAKKMSLHQNSFSEVLALADIRGHLPAHIALVGLQPAMLDDYGGSLSELARE

QLPAAEQSALAQLAAWGSVPQPANESRCLNYDCLSMENYEGVRLRQYRMTQEEQG

>gi|30062512|ref|NP_836683.1| hydrogenase 1 b-type cytochrome subunit [Shigella flexneri 2a str. 2457T]

MQQKSDNVVSHYVFEAPVRIWHWLTVLCMAVLMVTGYFIGKPLPSVSGEATYLFYMGYIRLIHFSAGMIF

TVVLLMRIYWAFVGNRYSRELFIVPVWRKSWWQGVWYEIRWYLFLAKRPSADIGHNPIAQAAMFGYFLMS

VFMIITGFALYSEHSQYAIFAPFRYVVEFFYWTGGNSMDIHSWHRLGMWLIGAFVIGHVYMALREDIMSD

DTVISTMVNGYRSHKFGKISNKERS

>gi|30062510|ref|NP_836681.1| hydrogenase-1 small subunit [Shigella flexneri 2a str. 2457T]

MNNEETFYQAMRRQGVTRRSFLKYCSLAATSLGLGAGMAPKIAWALENKPRIPVVWIHGLECTCCTESFI

RSAHPLAKDVILSLISLDYDDTLMAAAGTQAEEVFEDIITQYNGKYILAVEGNPPLGEQGMFCISSGRPF

IEKLKRAAAGASAIIAWGTCASWGCVQAARPNPTQATPIDKVITDKPIIKVPGCPPIPDVMSAIITYMVT

FDRLPDVDRMGRPLMFYGQRIHDKCYRRAHFDAGEFVQSWDDDAARKGYCLYKMGCKGPTTYNACSSTRW

NDGVSFPIQSGHGCLGCAENGFWDRGSFYSRVVDIPQMGTHSTADTVGLTALGVVAAAVGVHAVASAVDQ

RRRHNQQPTETEHQPGNEDKQA

>gi|30062504|ref|NP_836675.1| hypothetical protein S1033 [Shigella flexneri 2a str. 2457T]

MVGMSALSYTLLNSLEEIMKETDIAGILTSTHTIALVGASDKPDRPSYRVMKYLLDQGYHVIPVSPKVAG

KTLLGQQGYGTLADVPEKVDMVDVFRNSEAAWGVAQEAIAIGAKTLWMQLGVINEQAAVLARDAGLNVVM

DRCPAIEIPRLGLAK

>gi|30062501|ref|NP_836672.1| DNA helicase IV [Shigella flexneri 2a str. 2457T]

MELKATTLGKRLAQHPYDRAVILNAGIKVSGDRHEYLIPFNQLLAIHCKRGLVWGELEFVLPDEKVVRLH

GTEWGETQRFYHHLDAHWRRWSGEMSEIASGVLRQQLDLIATRTGENKWLTREQTSGVQQQIRQALSALP

LPVNRLEEFDNCREAWRKCQAWLKDIESARLQHNQAYTEAMLTEYADFFRQVESSPLNPAQARAVVNGEH

SLLVLAGAGSGKTSVLVARAGWLLARGEASPEQILLLAFGRKAAEEMDERIRERLHTEDITARTFHALAL

HIIQQGSKKVPIVSKLENDTAARHELFIAEWRKQCSEKKAQAKGWRQWLTEEMQWSVPEGNFWDDEKLQR

RLASRLDRWVSLMRMHGGAQAEMIASAPEEIRDLFSKRIKLMAPLLKAWKGALKAENAVDFSGLIHQAIV

ILEKGRFISPWKHILVDEFQDISPQRAALLAALRKQNSQTTLFAVGDDWQAIYRFSGAQMSLTTAFHENF

GEGDRCDLDTTYRFNSRIGEVANRFIQQNPGQLKKPLNSLTNGDKKAVTLLDESQLDALLDKLSGYAKPE

ERILILARYHHMRPASLEKAATRWPKLQIDFMTIHASKGQQADYVIIVGLQEGSGGFPAAARESIMEEAL

LPPVEDFPDAEERRLMYVALTRARHRVWALFNKENPSPFVEILKNLDVPVARKP

>gi|30062496|ref|NP_836667.1| hypothetical protein S1025 [Shigella flexneri 2a str. 2457T]

MKSLSYKRIYKSQEYLATLGTIEYRSLFGSYSLTVDDTVFAMVSDGELYLRACEQSAQYCVKHPPVWLTY

KKCGRSVTLNYYRVDESLWRNQLKLVRLSKYSLDAALKEKSTRNTRERLKDLPNMSFHLEAILGEVGRLA

P

>gi|30062493|ref|NP_836664.1| hypothetical protein S1022 [Shigella flexneri 2a str. 2457T]

MKYQQLENLESGWKWKYLVKKHREGELITRYIEASAAQEAVDVLLSLENEPVLVNGWIDKHMNPELVNRM

KQTIRARRKRHFNAEHQHTRKKSIDLEFIVWQRLAGLAQRRGKTLSETIVQLIEDAENKEKYANKMSSLK

QDLQALLGKE

>gi|30062491|ref|NP_836662.1| 3-hydroxydecanoyl-ACP dehydratase [Shigella flexneri 2a str. 2457T]

MVDKRESYTKEDLLASGHGELFGAKGPQLPAPNMLMIDRVVKMTETGGNFDKGYVEAELDINPDLWFFGC

HFIGDPVMPGCLGLDAMWQLVGFYLGWLGGEGKGRALGVGEVKFTGQVLPTAKKVTYRIHFKRIVNRRLI

MGLADGEVLVDCRLIYTASDLKVGLFQDTSAF

>gi|30062483|ref|NP_836654.1| hypothetical protein S1012 [Shigella flexneri 2a str. 2457T]

MLLRFYRVGERQMRIKPDDNWRWYYDEEHDRMMLDLANGMLFRSRFARKMLTPDAFSPAGFCVDDAALYF

SFEEKCRDFNLSKDQKAELVLNALVAIRYLKPQMPKSWHFVSHGEMWVPMPGDAACVWLSDTHEQVNLLV

VESGENAALCLLAQPCVVIAGRAMQLGDAIKIMNDRLKPQVNVDSFSLEQAV

>gi|30062477|ref|NP_836648.1| outer membrane protein [Shigella flexneri 2a str. 2457T]

MYRTHRQHSLLSSGGVPSFIGGLVVFVSAAFNAQAETWFDPAFFKDDPSMVADLSRFEKGQKITPGVYRV

DIVLNQTIVDTRNVNFVEITPEKGIAACLTTESLDAMGVNTDAFPAFKQLDKQVCVPLAEIIPDASVTFN

VNKLRLEISVPQIAIKSNARGYVPPERWDEGINALLLGYSFSGANSIHSSADSDSGDSYFLNLNSGVNLG

PWRLRNNSTWSRSSGQTAEWKNLSSYLQRAVIPLKGELTVGDDYTAGDFFDSVSFRGVQLASDDNMLPDS

LKGFAPVVRGIAKSNAQITIKQNGYTIYQTYVSPGAFEISDLYSTSSSGDLLVEIKEADGSVNSYSVPFS

SVPLLQRQGRIKYAVTLAKYRTNSNEQQESKFAQTTLQWGGPWGTTWYGGGQYAEYYRAAMFGLGFNLGD

FGAISFDATQAKSTLADQSEHKGQSYRFLYAKTLNQLGTNFQLMGYRYSTSGFYTLSDTMYKHMDGYEFN

DGDDEDTPMWSRYYNLFYTKRGKLQVNISQQLGEYGSFYLSGSQQTYWHTDQQDRLLQFGYNTQIKDLSL

GVSWNYSKSRGQPDADQVFALNFSLPLNLLLPRSNDSYTRKKNYAWMTSNTSIDNEGHITQNLGLTETLL

DDGNLSYSVQQGYNSEGKTANGSASMDYKGAFADARVGYNYSDNGSQQQLNYALSGSLVAHSQGITLGQS

LGETNVLIAAPGAENTRVANSTGLKTDWRGYTVVPYATSYRENRIALDAASLKRNVDLENAVVNVVPTKG

ALVLAEFNAHAGARVLMKTSKQGIPLRFGAIATLDGIQTNSGIIDDDGSLYMSGLPAQGAITVRWGEAPD

QICHISYQLTEQQINSAITRMDAICR

>gi|30062474|ref|NP_836645.1| IS1 orfB, A [Shigella flexneri 2a str. 2457T]

MASISIRCPSCSATEGVVRNGKSTAGHQRYLCSHCRKTWQLQFTYTASQPGTHQKIIDMAMNGVGCRASA

RIMGVGLYTVLRHLKNSAESVTSRIQPGSDVIVCAEMDEHWGYVGAKSRQRWLFYAYDRIRRTVVAHVFG

ERTLATLERLLSLLSAFEVVVWMTDGCPLYESRLKGKLHVISKRYTQRIERHNLNLRQHLARLVRKSLSF

SKSVELHDKAIGHYLNIKHYQ

>gi|30062464|ref|NP_836635.1| outer membrane protein F [Shigella flexneri 2a str. 2457T]

MMKRNILAVIVPALLVAGTANAAEIYNKDGNKVDLYGKTVGLHYFSKGNGENSYGGNGDMTYARLGFKGE

TQINSDLTGYGQWEYNFQGNNSEGADAQTGNKTRLAFAGLKYADVGSFDYGRNYGVVYDALGYTDMLPEF

GGDTAYSDDFFVGRVGGVATYRNSNFFGLVDGLNFAVQYLGKNERDTARRSNGDGVGGSISYEYEGFGIV

GAYGAADRTNLQEAQPLGNGKKAEQWATGLKYDANNIYLAANYGETRNATPITNKFTNTSGFANKTQDVL

LVAQYQFDFGLRPSIAYTKSKAKDVEGIGDVDLVNYFEVGATYYFNKNMSTYVDYIINQIDSDNKLGVGS

DDTVAVGIVYQF

>gi|30062461|ref|NP_836632.1| hypothetical protein S0987 [Shigella flexneri 2a str. 2457T]

MDKFDANRRKLLALGGVALGAAILPTPAFATLSTPRPRILTLNNLHTGESIKAEFFDGRGYIQEELAKLN

HFFRDYRANKIKSIDPGLFDQLYRLQGLLGTRKPVQLISGYRSIDTNNELRARSRGVAKKSYHTKGQAMD

FHIEGIALSNIRKAALSMRAGGVGYYPRSNFVHIDTGPARHW

>gi|30062458|ref|NP_836629.1| cell division protein MukB [Shigella flexneri 2a str. 2457T]

MIERGKFRSLTLINWNGFFARTFDLDELVTTLSGGNGAGKSTTMAAFVTALIPDLTLLHFRNTTEAGATS

GSRDKGLHGKLKAGVCYSMLDTINSRHQRVVVGVRLQQVAGRDRKVDIKPFAIQGLPMSVQPTQLVTETL

NERQARVLPLNELKDKLEAMEGVQFKQFNSITDYHSLMFDLGIIARRLRSASDRSKFYRLIEASLYGGIS

SAITRSLRDYLLPENSGVRKAFQDMEAALRENRMTLEAIRVTQSDRDLFKHLISEATNYVAADYMRHANE

RRVHLDKALEFRRELHTSRQQLAAEQYKHVDMARELAEHNGAEGDLEADYQAASDHLNLVQTALRQQEKI

ERYEVDLDELQIRLEEQNEVVAEAIERQEENEARAEAAELEVDELKSQLADYQQALDVQQTRAIQYNQAI

AALNRAKELCHLPDLTADSAAEWLETFQAKELEATEKMLSLEQKMSMAQTAHSQFEQAYQLVVAINGPLA

RNEAWDVARELLREGVDQRHLAEQVQPLRMRLSELEQRLREQQEAERLLADFCKRQGKNFDIDELEALHQ

ELEARIASLSDSVSNAREERMALRQEQEQLQSRIQSLMQRAPVWLAAQNSLNQLSEQCGEEFSSSQDVTE

YLQQLLEREREAIVERDEVGARKNAVDEEIERLSQPGGSEDQRLNALAERFGGVLLSEIYDDVSLEDAPY

FSALYGPSRHAIVVPDLSQVTEHLEGLTDCPEDLYLIEGDPQSFDDSVFSVDELEKAVVVKIADRQWRYS

RFPEVPLFGRAARESRIESLHAEREVLSERFATLSFDVQKTQRLHQAFSRFIGSHLAVAFESDPEAEIRQ

LNSRRVELERALSNHENDNQQQRIQFEQAKEGVTALNRILPRLNLLADDSLADRVDEIRERLDEAQEAAR

FVQQFGNQLAKLEPIVSVLQSDPEQFEQLKEDYAYSQQMQRDARQQAFALTEVVQRRAHFSYSDSAEMLS

GNSDLNEKLRERLEQAEAERTRAREALRGHAAQLSQYNQVLASLKSSYDTKKELLNDLQRELQDIGVRAD

SGAEERARIRRDELHAQLSNNRSRRNQLEKALTFCEAEMDNLTRKLRKLERDYFEMREQVVTAKAGWCAV

MRMVKDNGVERRLHRRELAYLSADDLRSMSDKALGALRLAVADNEHLRDVLRMSEDPKRPERKIQFFVAV

YQHLRERIRQDIIRTDDPVEAIEQMEIELSRLTEELTSREQKLAISSRSVANIIRKTIQREQNRIRMLNQ

GLQNVSFGQVNSVRLNVNVRETHAMLLDVLSEQHEQHQDLFNSNRLTFSEALAKLYQRLNPQIDMGQRTP

QTIGEELLDYRNYLEMEVEVNRGSDGWLRAESGALSTGEAIGTGMSILVMVVQSWEDESRRLRGKDISPC

RLLFLDEAARLDARSIATLFELCERLQMQLIIAAPENISPEKGTTYKLVRKVFQNTEHVHVVGLRGFAPQ

LPETLLGRDEAPSQAS

>gi|30062457|ref|NP_836628.1| condesin subunit E [Shigella flexneri 2a str. 2457T]

MPVKLAQALANPLFPALDSALRSGRHIGLDELDNHAFLMDFQEYLEEFYARYNVELIRAPEGFFYLRPRS

TTLIPRSVLSELDMMVGKILCYLYLSPERLANEGIFTQQELYDELLTLADEAKLLKLVNNRSTGSDVDRQ

KLQEKVRSSLNRLRRLGMVWFMGHDSSKFRITESVFRFGADVRAGDDPREAQRRLIRDGEAMPIENHLQL

NDETEESQPDSGEEE

>gi|30062456|ref|NP_836627.1| condesin subunit F [Shigella flexneri 2a str. 2457T]

MSEFSQTVPELVAWARKNDFSISLPVDRLSFLLAVATLNGERLDGEMSEGELVDAFRHVSDAFEQTSETI

GVRANNAINDMVRQRLLNRFTSEQAEGNAIYRLTPLGIGITDYYIRQREFSTLRLSMQLSIVAGELKRAA

DAAEEGGDEFHWHRNVYAPLKYSVAEIFDSIDLTQRLMDEQQQQVKDDIAQLLNKDWRAAISSCELLLSE

TSGTLRELQDTLEAAGDKLQANLLRIQDATMTHDDLHFVDRLVFDLQSKLDRIISWGQQSIDLWIGYDRH

VHKFIRTAIDMDKNRVFAQRLRQSVQTYFDEPWALTYANADRLLDMRDEEMALRDEEVTGELPEDLEYEE

FNEIREQLAAIIEEQLAVYKTRQVPLDLGLVVREYLSQYPRARHFDVARIVIDQAVRLGVAQADFTGLPA

KWQPINDYGAKVQAHVIDKY

>gi|30062449|ref|NP_836620.1| tetraacyldisaccharide 4'-kinase [Shigella flexneri 2a str. 2457T]

MIEKIWSGESPLWRLLLPLSWLYGLVSGAIRLCYKLKLKRAWRAPVPVVVVGNLTAGGNGKTPVVVWLVE

QLQQRGIRVGVVSRGYGGKAESYPLLLSADTTTAQAGDEPVLIYQRTDAPVAVSPVRSDAVKAILAQHPD

VQIIVTDDGLQHYRLARDVEIVVIDGVRRFGNGWWLPAGPMRERAGRLKSVDAVIVNGGVPRSGEIPMHL

LPGQAVNLRTGTRCDVAQLEHVVAMAGIGHPPRFFATLKMCGVQPEKCVPLADHQSLNHADVSALVSAGQ

TLVMTEKDAVKCRAFAEENWWYLPVDAQLSGDEPAKLLTQLTSLASGN

>gi|30062448|ref|NP_836619.1| lipid transporter ATP-binding/permease [Shigella flexneri 2a str. 2457T]

MHNDKDLSTWQTFRRLWPTIAPFKAGLIVAGVALILNAASDTFMLSLLKPLLDDGFGKTDRSVLVWMPLV

VIGLMILRGITSYVSSYCISWVSGKVVMTMRRRLFGHMMGMPVSFFDKQSTGTLLSRITYDSEQVASSSS

GALITVVREGASIIGLFIMMFYYSWQLSIILIVLAPIVSIAIRVVSKRFRNISKNMQNTMGQVTTSAEQM

LKGHKEVLIFGGQEVETKRFDKVSNRMRLQGMKMVSASSISDPIIQLIASLALAFVLYAASFPSVMDNLT

AGTITVVFSSMIALMRPLKSLTNVNAQFQRGMAACQTLFTILDSEQEKDEGKRVIERATGDVEFRNVTFT

YPGRDVPALRNINLKIPAGKTVALVGRSGSGKSTIASLITRFYDIDEGEILMDGHDLREYTLASLRNQVA

LVSQNVHLFNDTVANNIAYARTEQYSREQIEEAARMAYAMDFINKMDNGLDTVIGENGVLLSGGQRQRIA

IARALLRDSPILILDEATSALDTESERAIQAALDELQKNRTSLVIAHRLSTIEKADEIVVVEDGVIVERG

THNDLLEHRGVYAQLHKMQFGQ

>gi|30062445|ref|NP_836616.1| cytidylate kinase [Shigella flexneri 2a str. 2457T]

MTAIAPVITIDGPSGAGKGTLCKAMAEALQWHLLDSGAIYRVLALAALHHHVDVASEDALVPLASHLDVR

FVSTNGNLEVILEGEDVSGEIRTQEVANAASQVAAFPRVREALLRRQRAFRELPGLIADGRDMGTVVFPD

APVKIFLDASSEERAHRRMLQLQEKGFSVNFERLLAEIKERDDRDRNRAVAPLVPAADALVLDSTTLSIE

QLIEKALQYARQKLALA

>gi|30062442|ref|NP_836613.1| 3-phosphoshikimate 1-carboxyvinyltransferase [Shigella flexneri 2a str. 2457T]

MESLTLQPIARVDGTINLPGSKSVSNRALLLAALAHGKTVLTNLLDSDDVRHMLNALTALGLSYTLSADR

TRCEIIGNGGPLHAEGALELFLGNAGTAMRPLAAALCLDSNDIVLTGEPRMKERPIGHLVDALRLGGAKI

TYLEQENYPPLRLQGGFTGGNVDVDGSVSSQFLTALLMTAPLAPEDTVIRIKGDLVSKPYIDITLNLMKT

FGVEIENQHYQQFVVKGGQSYQSPGTYLVEGDASSASYFLAAAAIKGGTVKVTGIGRNSMQGDIRFADVL

EKMGATICWGDDYISCTRGELNAIDMDMNHIPDAAMTIATAALFAKGTTTLRNIYNWRVKETDRLFAMAT

ELRKVGAEVEEGHDYIRITPPEKLNFAEIATYNDHRMAMCFSLVALSDTPVTILDPKCTAKTFPDYFEQL

ARISQAA

>gi|30062428|ref|NP_836599.1| IS629 orfB [Shigella flexneri 2a str. 2457T]

MPLLDKLREQYRVGPLCSELHIAPSTYYHCQQQRHHPDKRSARAQRDDWLKKEIQRVYDENHKVYGVRKV

WRQLLREGIRVARCTVARLMEVMGLAGVLRGKKVRTTISRKAVAAGDRVNRQFVAERPDQLWVADFTYVS

TWQGFVYVAFIIDVFAGYIVGWRVSSSMETTFVLDALEQALWARRPSGTVHHSDKGSQYVSLAYTQRLKE

AGLLASTGSTGDSYDNAMAESINGLYKAEVIHRKSWKNRAEVELATLTWVDWYNNRRLLERLGHIPPAEA

EKAYYASIGNDDLAA

>gi|30062400|ref|NP_836571.1| bacteriophage protein [Shigella flexneri 2a str. 2457T]

MKIRHEHIESVLLALAAEKGQAWVANAITEEYLRQGGGELSLVPGKDWNNQQNIYHRWLKGETKAQREKI

QKLIPAVLAILPRELRHRLCIFDTLERRALLAAQEALSTAIDAHDDAVQAVYRKAHFSGGGSPGDSVVVH

>gi|30062399|ref|NP_836570.1| bacteriophage protein [Shigella flexneri 2a str. 2457T]

MASNWIKLEVITPDKPEIFRLAEILNIDPDAALGKVIRFWAWADQQMIDGNADCNARGVTKSAIDRITFM

SGFADALIQVGWLVENDGGLSLPNFERHNGKSSKKRAVTNERVTKIRELKRKGNAASVTQTDQKALPVEE

EEEDLNTDLPLNPPRQKRASKKFEPEAIELPDWLPETLWHEWVRFRQALRKPIRTEQGDTGTGKIPSAGF

YT

>gi|30062384|ref|NP_836555.1| hypothetical protein S0897 [Shigella flexneri 2a str. 2457T]

MTKPYVRLDKNDAAVLLVDHQAGLLSLVRDIEPDKFKNNVLALGDLAKYFNLPTILTTSFETGPNGPLVL

ELKAQFPDAPYIARPGNINAWDNEDFVKAVKATGKKQLIIAGVVTEVCVAFPALSAIEEGFDVFVVTDAS

GTFNEITRHSAWDRMSQAGAQLMTWFGVACELHRDWRNDIEGLATLFSNHIPDYRNLMTSYDTLTKQK

>gi|30062378|ref|NP_836549.1| outer-membrane lipoprotein carrier protein [Shigella flexneri 2a str. 2457T]

MMKKIAITCALLSSLVASSVWADAASDLKSRLDKVSSFHASFTQKVTDGSGAAVQEGQGDLWVKRPNLFN

WHMTQPDESILVSDGKTLWFYNPFVEQATATWLKDATGNTPFMLIARNQSSDWQQYNIKQNGDDFVLTPK

ASNGNLKQFTINVGRDGTIHQFSAVEQDDQRSSYQLKSQQNGAVDAAKFTFTPPQGVTVDDQRK

>gi|30062377|ref|NP_836548.1| DNA translocase FtsK [Shigella flexneri 2a str. 2457T]

MSQEYTEDKEVTLTKLSSGRRLLEALLILIVLFAVWLMAALLSFNPSDPSWSQTAWHEPIHNLGGMPGAW

LADTLFFIFGVMAYTIPVIIVGGCWFAWRHQSSDEYIDYFAVSLRIIGVLALILTSCGLAAINADDIWYF

ASGGVIGSLLSTTLQPLLHSSGGTIALLCVWAAGLTLFTGWSWVTIAEKLGGWILNILTFASNRTRRDDT

WVDEDEYEDDEEYEDENHGKQHESRRARILRGALARRKRLAEKFINPMGRQTDAALFSGKRMDDDEEITY

TARGVAADPDDVLFSGNRATQPEYDEYDPLLNSAPITEPVAVAAAATTATQSWAAPVEPVTQTPPVASVD

VPPSQPTVAWQPVPGPQTGEPVIAPAPEGYPQQSQYAQPAVQYNEPLQQPVQPQQPYYAPAAEQPAQQPY

YAPAPEQPVAGNAWQAEEQQSTFAPLSTYQTEQTYQQPAAQEPLYQQPQPVEQQPVVEPEPVVEETKPAR

PPLYYFEEVEEKRAREREQLAAWYQPIPEPVKEPEPIKSSLKAPSVAAVPPVEAAAAVSPLASGVKKATL

ATGAAATVAAPVFSLANSGGPRPQVKEGIGPQLPRPKRIRVPTRRELASYGIKLPSQRAAEEKAREAQRN

QYDSGDQYNDDEIDAMQQDELARQFAQTQQQRYGEQYQHDVPVNAEDADAAAEAELARQFAQTQQQRYSG

EQPAGANPFSLDDFEFSPMKALLDDGPHEPLFTPIVEPVQQPQQPVAPQQQYQQPQQPVAPQQQYQQPQQ

QVAPQPQYQQPQQPVAPQQQYQQPQQPVAPQPQYQQPQQPVAPQPQYQQPQQPVAPQPQDTLLHPLLMRN

GDSRPLHKPTTPLPSLDLLTPPPSEVEPVDTFALEQMARLVEARLADFRIKADVVNYSPGPVITRFELNL

APGVKAARISNLSRDLARSLSTVAVRVVEVIPGKPYVGLELPNKKRQTVYLREVLDNAKFRDNPSPLTVV

LGKDIAGEPVVADLAKMPHLLVAGTTGSGKSVGVNAMILSMLYKAQPEDVRFIMIDPKMLELSVYEGIPH

LLTEVVTDMKDAANALRWCVNEMERRYKLMSALGVRNLAGYNEKIAEADRMMRPIPDPYWKPGDSMDAQH

PVLKKEPYIVVLVDEFADLMMTVGKKVEELIARLAQKARAAGIHLVLATQRPSVDVITGLIKANIPTRIA

FTVSSKIDSRTILDQAGAESLLGMGDMLYSGPNSTLPVRVHGAFVRDQEVHAVVQDWKARGRPQYVDGIT

SDSESEGGAGGFDGAEELDPLFDQAVQFVTEKRKASISGVQRQFRIGYNRAARIIEQMEAQGIVSEQGHN

GNREVLAPPPFD

>gi|30062369|ref|NP_836540.1| ATP-dependent Clp protease adaptor protein ClpS [Shigella flexneri 2a str. 2457T]

MGKTNDWLDFDQLAEEKVRDALKPPSMYKVILVNDDYTPMEFVIDVLQKFFSYDVERATQLMLAVHYQGK

AICGVFTAEVAETKVAMVNKYARENEHPLLCTLEKA

>gi|30062351|ref|NP_836522.1| regulator [Shigella flexneri 2a str. 2457T]

MRRVFWLVAAALLLAGCAGEKGIVEKEGYQLDTRHQAQAAYPRIKVLVIHYTADDFDSSLATLTDKQVSS

HYLIPAVPPRYNGKPRIWQLVPEQELAWHAGISAWRGATRLNDTSIGIELENRGWQKSSGVKYFAPFEQA

QIQALIPLAKDIIARYHIKPENVVAHADIAPQRKDDPGPLFPWQQLAQQGIGAWPDAQRVNFYLAGRAPH

TPVDTASLLELLARYGYDVKPDMTPREQRRVIMAFQMHFRPTLYNGEADAETQAIAEALLEKYGQD

>gi|30062347|ref|NP_836518.1| lipoprotein [Shigella flexneri 2a str. 2457T]

MRYRSLSLLIPCALLLSACTTVTPAYKDNGPRTGSCVQGGPDSVAQQFYDYRIQHRSNDITALRPYLSDK

LATQLSDASRDNSHRELLSSDPFSSRTTLPDSAHVASASTIPNRDARNIPLRVDLKQGDQGWQDEVLMIQ

EGQCWVIDDVRYLGGSVHATAGTLRQSIENR

>gi|30062344|ref|NP_836515.1| arginine transporter permease subunit ArtQ [Shigella flexneri 2a str. 2457T]

MNEFFPLASAAGMTVGLAVCALIVGLALAMFFAVWESAKWRPVAWAGSALVTILRGLPEILVVLFIYFGS

SQLLLTLSDGFTINLGFVQIPVQMDIENFDVSPFLCGVIALSLLYAAYASQTLRGALKAVPVGQWESGQA

LGLSKSAIFFRLVMPQMWRHALPGLGNQWLVLLKDTALVSLISVNDLMLQTKSIATRTQEPFTWYIVAAA

IYLVITLLSQYILKRIDLRATRFERRPS

>gi|30062343|ref|NP_836514.1| arginine transporter permease subunit ArtM [Shigella flexneri 2a str. 2457T]

MFEYLPELMKGLHTSLTLTVASLIVALILALIFTIILTLKTPVLVWLVRGYITLFTGTPLLVQIFLIYYG

PGQFPTLQEYPALWHLLSEPWLCALIALSLNSAAYTTQLFYGAIRAIPEGQWQSCSALGMSKKDTLAILL

PYAFKRSLSSYSNEVVLVFKSTSLAYTITLMEVMGYSQLLYGRTYDVMVFGAAGIIYLVVNGLLTLMMRL

IERKALAFERRN

>gi|30062340|ref|NP_836511.1| hypothetical protein S0854 [Shigella flexneri 2a str. 2457T]

MEDETLGFFKKTSSSHARLNVPALVQVAALAIIMIRGLDVLMIFNTLGVRGIGEFIHRSVQTWSLTLVFL

SSLVLVFIEIWCAFSLVKGRRWARWLYLLTQITAASYLWAASLGYGYPELFSIPGESKREIFHSLMLQKL

PDMLILMLLFVPSTSRRFFQLQ

>gi|30062339|ref|NP_836510.1| putrescine transporter subunit: membrane component of ABC superfamily [Shigella flexneri 2a str. 2457T]

MNNLPVVRSPWRIVILLLGFTFLYAPMLMLVIYSFNSSKLVTVWGGWSTRWYGELLRDDAMMSAVGSSLT

IAACAATAAAILGTIAAVVLVRFGRFRGSNGFAFMITAPLVMPDVITGLSLLLLFVALAHAIGWPADRGM

LTIWLAHVTFCTAYVAVVISSRLRELDRSIEEAAMDLGATPLKVFFVITLPMIMPAIISGWLLAFTLSLD

DLVIASFVSGPGATTLPMLVFSSVRMGVNPEINALATLILGAVGIVGFIAWYLMARAEKQRIRDIQRARR

G

>gi|30062338|ref|NP_836509.1| putrescine transporter subunit: membrane component of ABC superfamily [Shigella flexneri 2a str. 2457T]

MNTLEPAAQSKPPGGFKLWLSQLQMKHGRKLVIALPYIWLILLFLLPFLIVFKISLAEMARAIPPYTELM

EWADGQLSITLNLGNFLQLTDDPLYFDAYLQSLQVAAISTFCCLLIGYPLAWAVAHSKPSTRNILLLLVI

LPSWTSFLIRVYAWMGILKNNGVLNNFLLWLGVIDQPLTILHTNLAVYIGIVYAYVPFMVLPIYTALIRI

DYSLVEAALDLGARPLKTFFTVIVPLTKGGIIAGSMLVFIPAVGEFVIPELLGGPDSIMIGRVLWQEFFN

NRDWPVASAVAIIMLLLLIVPIMWFHKHQQKSVGEHG

>gi|30062328|ref|NP_836499.1| DEOR-type transcriptional regulator [Shigella flexneri 2a str. 2457T]

MRRANDPQRREKIIQATLEAVKLYGIHAVTHRKIATLAGVPLGSMTYYFSGIDELLLEAFSSFTEIMSRQ

YQAFFSDVSDAQGACQAITDMIYSSQVATPDNMELMYQLYALASRKPLLKTVMQNWMQRSQQTLEQWFEP

GTARALDAFIEGMTLHFVTDRKPLSREEILRMVERVAG

>gi|30062324|ref|NP_836495.1| chloramphenicol resistance pump Cmr [Shigella flexneri 2a str. 2457T]

MQNKLASGARLGRQALLFPLCLVLYEFSTYIGNDMIQPGMLAVVEQYQAGIDWVPTSMTAYLAGGMFLQW

LLGPLSDRIGRRPVMLAGVVWFIVTCLAILLAQNIEQFTLLRFLQGISFCFIGAVGYAAIQESFEEAVCI

KITALMANVALIAPLLGPLVGAAWIHVLPWEGMFVLFAALAAISFFGLQRAMPETAMRIGEKLSLKELGR

DYKLVLKNGRFVAGALGFVSLPLLAWIAQSPIIIITGEQLSSYEYGLLQVPIFGALIAGNLLLARLTSRR

TVRSLIIMGGWPIMIGLLVAAAATVISSHAYLWMTAGLSIYAFGIGLANAGLVRLTLFASDMSKGTVSAA

MGMLQMLIFTVGIEISKHAWLNGGNGLFNLFNLVNGILWLSLMVIFLKDKQMGNSHEG

>gi|30062315|ref|NP_836486.1| transport system permease [Shigella flexneri 2a str. 2457T]

MRLFNWRRQAVLNAMPLVKPDQVRTPWHEFWRRFRRQHMAMTAALFVILLIVVAIFARWIAPYDAENYFD

YDNLNNGPSLQHWFGVDSLGRDIFSRVLVGAQISLAAGVFAVFIGVAIGTLLGLLAGYYEGWWDRLIMRI

CDVLFAFPGILLAIAVVAVLGSGIANVIIAVAIFSIPAFARLVRGNTLVLKQQTFIESARSIGASDMTIL

LRHILPGTVSSIVVFFTMRIGTSIISAASLSFLGLGAQPPTPEWGAMLNEARADMVIAPHVAVFPALAIF

LTVLAFNLLGDGLRDALDPKIKG

>gi|30062313|ref|NP_836484.1| transporter [Shigella flexneri 2a str. 2457T]

MARAVHRSGLVALGIATALMASCAFAAKDVVVAVGSNFTTLDPYDANDTLSQAVAKSFYQGLFGLDKEMK

LKNVLAESYTVSDDGITYTVKLREGIKFQDGTDFNAVAVKANLDRASDPANHLKRYNLYKNIAKTEAIDP

TTVKITLKQPFSAFINILAHPATAMISPTALEKYGKEIGFHPVGTGPYELDTWNQTDFVKVKKFAGYWQP

GLPKLDSITWRPVADNNTRAAMLQTGEAQFAFPIPYEQATLLEKNKNIELMASPSIMQRYISMNVTQKPF

DNPKVREALNYAINRPALVKVAFAGYATPATGVVPPSIAYAQSYKPWPYDPVKARELLKEAGYPNGFSTT

LWSSHNHSTAQKVLQFTQQQLAQVGIKAQVTAMDAGQRAAEVEGKGQKESGVRMFYTGWSASTGEADWAL

SPLFASQNWPPTLFNTAFYSNKQVDDFLAQALKTNDPAEKTRLYKAAQDIIWQESPWIPLVVEKLVSAHS

KNLTGFWIMPDTGFSFEDADLQ

>gi|30062306|ref|NP_836477.1| formate acetyltransferase [Shigella flexneri 2a str. 2457T]

MTTLKLDTLSDRIKAHKNALVHIVKPPVCTERAQHYTEMYQQHLDKPIPVRRALALAHHLANRTIWIKHD

ELIIGNQASEVRAAPIFPEYTVSWIEKEIDDLADRPGAGFAVSEENKRVLHEVCPWWRGQTVQDRCYGMF

TDEQKGLLATGIIKAEGNMTSGDAHLAVNFPLLLEKGLDGLREKVAERRSRINLTVLEDLHGEQFLKAID

IMLVAVSEHIERFAALARAMAATETRESRRDELLAMAENCDLIAHQPPQTFWQALQLCYFIQLILQIESN

GHSVSFGRMDQYLYPYYRRDVELNQTLDREHAIELLHSCWLKLLEVNKIRSGSHSKASAGSPLYQNVTIG

GQNLVDGQPMDAVNPLSYAILESCGRLRSTQPNLSVRYHAGMSNDFLDACVHVIRCGFGMPAFNNDEIVI

PEFIKLGIEPQDAYDYAAIGCIETAVGGKWGYRCTGMSFINFARVMLAALEGGRDATSGKVFLPQEKALS

AGNFNNFDEVMDAWDTQIRYYTRKSIEIEYVVDTMLEENVHDILCSALVDDCIERAKSIKQGGAKYDWVS

GLQVGIANLGNSLAAVKKLVFEQGAIGQQQLAAALADDFDGLTHEQLRQRLINGAPKYGNDDDTVDTLLA

RAYQTYIDELKQYHNPRYGRGPVGGNYYAGTSSISANVPFGAQTMATPDGRKAHTPLAEGASPASGTDHL

GPTAVIGSVGKLPTAAILGGVLLNQKLNPATLENESDKQKLMILLRTFFEVHKGWHIQYNIVSRETLLEA

KKHPDQYRDLVVRVAGYSAFFTALSPDAQDDIIARTEHML

>gi|30062300|ref|NP_836471.1| manganese transport regulator MntR [Shigella flexneri 2a str. 2457T]

MSRRAGTPIAKKVTQLVNVEEHVEGFRQVREAHRRELIDDYVELISDLIREVGEARQVDMAARLGVSQPT

VAKMLKRLATMGLIEMIPWRGVFLTAEGEKLAQESRERHQIVENFLLVLGVSPEIARRDAEGMEHHVSEE

TLDAFRLFTQKHGAK

>gi|30062294|ref|NP_836465.1| glutamine ABC transporter permease [Shigella flexneri 2a str. 2457T]

MQFDWSAIWPAIPLLIEGAKMTLWISVLGLAGGLVIGLLAGFARTFGGWIANHVALVFIEVIRGTPIVVQ

VMFIYFALPMAFNDLRIDPFTAAVVTIMINSGAYIAEITRGAVLSIHKGFREAGLALGLSRWETIRYVIL

PLALRRMLPPLGNQWIISIKDTSLFIVIGVAELTRQGQEIIAGNFRALEIWSAVAVFYLIITLVLSFILR

RLERRMKIL

>gi|30062283|ref|NP_836454.1| hypothetical protein S0789 [Shigella flexneri 2a str. 2457T]

MPVRAQRIQHVMQDTIINFYSTSDDYGDFSNFAARPIKVDGNTWPTSEHYFQAQKFLDEKYREEIRRVSS

PMVAARMGRNRSKPLRKNWESVKEQVMRKALRAKFEQHAELRVLLLATAPAKLVEHTENDAYWGDGGNGK

GKNRLGYLLMELREQLAIEK

>gi|30062276|ref|NP_836447.1| hypothetical protein S0782 [Shigella flexneri 2a str. 2457T]

MKWQQRVRVATGLSCWQIMLHLLVVALLVVGWMSKTLVHVGVGLCALYCVTVVMMLVFQRHPEQRWREVA

DVLEELTTTWYFGAALIVLWLLSRVLENNFLLAIAGLAILAGPAVVSLLAKDKKLHHLTSKHRVRR

>gi|30062275|ref|NP_836446.1| hypothetical protein S0781 [Shigella flexneri 2a str. 2457T]

MPDQTQQFSFKVLTINIHKGFTAFNRRFILPELRDAVRTVSADIVCLQEVMGAHEVHPLHVENWPDTSHY

EFLADTMWSDFAYGRNAVYPEGHHGNAVLSRYPIEHYENRDVSVDGAEKRGVLYCRIVPPMTGKAIHVMC

VHLGLREAHRQAQLAMLAEWVNELPDGEPVLVAGDFNDWRQKANHPLKVQAGLDEIFTRAHGRPARTFPV

QFPLLRLDRIYVKNASASAPTALPLRTWRHLSDHAPLSAEIHL

>gi|30062273|ref|NP_836444.1| hypothetical protein S0779 [Shigella flexneri 2a str. 2457T]

MSKSHPRWRLAKKILTWLFFIAVIVLLVVYAKKVDWEEVWKVIRDYNRVALLSAVGLVVVSYLIYGCYDL

LARFYCGHKLAKRQVMLVSFICYAFNLTLSTWVGGIGMRYRLYSRLGLPGSTITRISSLSITTNWLGYIL

LAGIIFTAGVVELPDYWYVDQTTLRILGIGLLMIIAVYLWFCAFAKHRHMTIKGQKLVLPSWKFALAQML

ISSVNWMVMGAIIWLLLGQSVNYFFVLGVLLVSSIAGVIVHIPAGIGVLEAVFIALLAGEHTSKGSIIAA

LLAYRVLYYFIPLLLALICYLLLESQAKKLRAKNEAAM

>gi|30062260|ref|NP_836431.1| biotin synthase [Shigella flexneri 2a str. 2457T]

MAHRPRWTLSQVTELFEKPLLDLLFEAQQVHRQHFEPRQVQVSTLLSIKTGACPEDCKYCPQSSRYKTGL

EAERLMEVEQVLESARKAKAAGSTRFCMGAAWKNPHERDMPYLEQMVQGVKAMGLEACMTLGTLSESQAQ

RLANAGLDYYNHNLDTSPEFYGNIITTRTYQERLDTLEKVRDAGIKVCSGGIVGLGETVKDRAGLLLQLA

NLPTPPESVPINMLVKVKGTPLADNDDVDAFDFIRTIAVARIMMPTSYVRLSAGREQMNEQTQAMCFMAG

ANSIFYGCKLLTTPNPEEDKDLQLFRKLGLNPQQTAVLAGDNEQQQRLGQALMTPDTDEYYNAAAL

>gi|30062224|ref|NP_836395.1| helicase [Shigella flexneri 2a str. 2457T]

MTTPVWRNDDLEGAVIGAFFLRGADPEVMDILATLPADVFSVRAYQDIYTGICRQARVSGVIDPVLLCNE

MPELAPVITDTGRKTWVKSSLEHYVAALRRNAALRDAEKTLNEALQKLRDAHTCEAAEDALKDAQNMMVT

LSTGKGVIQPVHIDDVLPEVVERVECRNQGLEKSRTLMTGIDELDAKTGGMEPGDLVFIAARPSMGKTEL

ALDIIDKVTEQGHGVLLFTMEMANIQIGERMVSAAGGMPVSRLKSVAHFEDEDWTRFSQGVGRMTGRNIW

MVDQANLAIDEICATTKHHLIKYPETALVVVDYLGLIKTRTTGRHDLAVGEISKGLKGLAKSGGFPLIAL

SQLSRGVESRPNKRPMNSDLKNSGEIEADADIILMLYRDEVYNPDTQARGIAEINITKQRNGSLGTIYRR

FYNGHFLPVDQESARVLSTPMKPGNPRRYSNKRTDSSKMERFF

>gi|30062223|ref|NP_836394.1| replication protein DnaC [Shigella flexneri 2a str. 2457T]

MMTFNLREQQKRLQARMDELRAEIAFAQKGEKPWPYRSCLMREGRGYCEKHGEYHTHILVWSDRNGEDRE

EISCCPDCLIAEANDLTMELSSIKAEELTDNAGIALRFRDCEFDNYLEVNPGAARNLAACRRYAENWPDM

LENGTSLVMTGSCGTGKNHLAVAMAKHIIRNYLASVEITDVMRLTRAVKNCWRNDSEKTADEVIERYASM

DLLIIDEVGVQFGSAAEMAILQEIINARYESILPTILISNLSPEELWAFISPRIADRITDGGRNWLSFNW

PSYRSRIRGVAA

>gi|30062186|ref|NP_836357.1| camphor resistance protein CrcB [Shigella flexneri 2a str. 2457T]

MLQLLLAVFIGGGTGSVARWLLSMRFNPLHQAIPLGTLAANLIGAFIIGMGFAWFSRMTNIDPVWKVLIT

TGFCGGLTTFSTFSAEVVFLLQEGRFGWALLNVFVNLLGSFAMTALAFWLFSASTVH

>gi|30062177|ref|NP_836348.1| cell wall shape-determining protein [Shigella flexneri 2a str. 2457T]

MTDNPNKKTFWDKVHLDPTMLLILLALLVYSALVIWSASGQDIGMMERKIGQIAMGLVIMVVMAQIPPRV

YEGWAPYLYIICIILLVAVDAFGAISKGAQRWLDLGIVRFQPSEIAKIAVPLMVARFINRDVCPPSLKNT

GIALVLIFMPTLLVAAQPDLGTSILVALSGLFVLFLSGLSWRLIGVAVVLVAAFIPILWFFLMHDYQRQR

VMMLLDPESDPLGAGYHIIQSKIAIGSGGLRGKGWLHGTQSQLEFLPERHTDFIFAVLAEELGLVGILIL

LALYILLIMRGLWIAARAQTTFGRVMAGGLMLILFVYVFVNIGMVSGILPVVGVPLPLVSYGGSALIVLM

AGFGIVMSIHTHRKMLSKSV

>gi|30062176|ref|NP_836347.1| penicillin-binding protein 2 [Shigella flexneri 2a str. 2457T]

MKLQNSFRDYTAESALFVRRALVAFLGILLLTGVLIANLYNLQIVRFTDYQTRSNENRIKLVPIAPSRGI

IYDRNGIPLALNRTIYQIEMMPEKVDNVQQTLDALRSVVDLTDDDIAAFRKERARSHRFTSIPVKTNLTE

VQVARFAVNQYRFPGVEVKGYKRRYYPYGSALTHVIGYVSKINDKDVERLNNDGKLANYAATHDIGKLGI

ERYYEDVLHGQTGYEEVEVNNRGRVIRQLKEVPPQAGHDIYLTLDLKLQQYIETLLAGSRAAVVVTEPRT

GGVLALVSTPSYDPNLFVDGISSKDYSALLNDPNTPLVNRATQGVYPPASTVKPYVAVSALSAGVITRNT

TLFDPGWWQLPGSEKRYRDWKKWGHGRLNVTRSLEESADTFFYQVAYDMGIDRLSEWMGKFGYGHYTGID

LAEERSGNMPTREWKQKRFKKPWYQGDTIPVGIGQGYWTATPIQMSKALMILINDGIVKVPHLLMSTAED

GKQVPWVQPHEPPVGDIHSGYWELAKDGMYGVANRPNGTANKYFASAPYKIAAKSGTAQVFGLKANETYN

AHKIAERLRDHKLMTAFAPYNNPQVAVAMILENGGAGPAVGTLMRQILDHIMLGDNNTDLPAENPAVAAA

EDH

>gi|30062175|ref|NP_836346.1| rRNA large subunit methyltransferase [Shigella flexneri 2a str. 2457T]

MKLQLVAVGTKMPDWVQTGFTEYLRRFPKDMPFELIEIPAGKRGKNADIKRILDKEGELMLAAAGKNRIV

TLDIPGKPWDTPQLAAELERWKLDGRDVSLLIGGPEGLSPACKAAAEQSWSLSALTLPHPLVRVLVAESL

YRAWSITTNHPYHRE

>gi|30062171|ref|NP_836342.1| DNA polymerase III subunit delta [Shigella flexneri 2a str. 2457T]

MIRLYPEQLRAQLNEGLRAAYLLLGNDPLLLQESQDAVRQVAAAQGFEEHHTFSIDPNTDWNAIFSLCQA

MSLFASRQTLLLLLPENGPNAAINEQLLTLTGLLHDDLLLIVRGNKLSKAQENAAWFTALANRSVQVTCQ

TPEQAQLPRWVAVRAKQLNLELDDAANQVLCYCYEGNLLALAQALERLSLLWPDGKLTLPRVEQAVNDAA

HFTPFHWVDALLMGKSKRALHILQQLRLEGSEPVILLRTLQRELLLLVNLKRQSAHTPLRALFDKHRVWQ

NRRGMMGEALNRLSQPQLRQAVQLLTRTELTLKQDYGQSVWAELEGLSLLLCHKPLADVFIDG

>gi|30062170|ref|NP_836341.1| LPS-assembly lipoprotein RlpB [Shigella flexneri 2a str. 2457T]

MRYLATLLLSLAVLITAGCGWHLRDTTQVPSTMKVMILDSGDPNGPLSRAVRNQLRLNGVELLDKETTRK

DVPSLRLGKVSIAKDTASVFRNGQTAEYQMIMTVNATVLIPGRDIYPISAKVFRSFFDNPQMALAKDNEQ

DMIVKEMYDRAAEQLIRKLPSIRAADIRSDEEQTSTTTDTPATPARVSTMLGN

>gi|30062159|ref|NP_836330.1| glutamate/aspartate transport system permease [Shigella flexneri 2a str. 2457T]

MYEFDWSSIVPSLPYLLDGLVITLKITVTAVVIGILWGTMLAVMRLSSFAPVSWFAKAYVNVFRSIPLVM

VLLWFYLIVPGFLQNVLGLSPKNDIRLISAMVAFSMFEAAYYSEIIRAGIQSISRGQSSAALALGMTHWQ

SMKLIILPQAFRAMVPLLLTQGIVLFQDTSLVYVLSLADFFRTASTIGERDGTQVEMILFAGFVYFVISL

SASLLVSYLKRRTA

>gi|30062158|ref|NP_836329.1| glutamate/aspartate transport system permease [Shigella flexneri 2a str. 2457T]

MSIDWNWGIFLQQAPFGNTTYLGWVWSGFQVTIALSICAWIIAFLVGSFFGILRTVPNRFLSGLGTLYVE

LFRNVPLIVQFFTWYLVIPELLPEKIGMWFKAELDPNIQFFLSSMLCLGLFTAARVCEQVRAAIQSLPRG

QKNAALAMGLTLPQTYRYVLLPNAYRVIVPPMTSEMMNLVKNSAIASTIGLVDMAAQAGKLLDYSAHAWE

SFTAITLAYVLINAFIMLVMTLVERKVRLPGNMGGK

>gi|30062154|ref|NP_836325.1| metalloprotease [Shigella flexneri 2a str. 2457T]

MSQVILDLQLACEDNSGLPEESQFQTWLNAVIPQFQEESEVTIRVVDTAESHSLNLTYRGKDKPTNVLSF

PFEVPPGMEMSLLGDLVICRQVVEKEAQEQGKPLEAHWAHMVVHGSLHLLGYDHIEDDEAEEMEALETEI

MLALGYEDPYIAEKE

>gi|30062153|ref|NP_836324.1| ATP-binding protein in pho regulon [Shigella flexneri 2a str. 2457T]

MFSFRRHTDKRNGLNIDTREITLEPADNARLLSLCGPFDDNIKQLERRLGIEINRRDNHFKLTGRPICVT

AAADILRSLYVDTAPMRGQIQDIEPEQIHLAIKEARVLEQSAESVPEYGKAVNIKTKRGVIKPRTPNQAQ

YIANILDHDITFGVGPAGTGKTYLAVAAAVDALERQEIRRILLTRPAVEAGEKLGFLPGDLSQKVDPYLR

PLYDALFEMLGFEKVEKLIERNVIEVAPLAYMRGRTLNDAFIILDESQNTTIEQMKMFLTRIGFNSKAVI

TGDVTQIDLPRNTKSGLRHAIEVLADVEEISFNFFHSEDVVRHPVVARIVNAYEAWEEAEQKRKAALAAE

RKREEQEQK

>gi|30062143|ref|NP_836314.1| hypothetical protein S0622 [Shigella flexneri 2a str. 2457T]

MKKLILIAMMASGLVACAQSTAPQEDSRLKEAYSACINTAQGSPEKIEACQSVLNVLKKEKQHQQFADQE

SVRVLDYQQCLRATQTGHDQAVKADCDKVWQEIRSNNK

>gi|30062142|ref|NP_836313.1| ferric uptake regulator [Shigella flexneri 2a str. 2457T]

MTDNNTALKKAGLKVTLPRLKILEVLQEPDNHHVSAEDLYKRLIDMGEEIGLATVYRVLNQFDDAGIVTR

HNFEGGKSVFELTQQHHHDHLICLDCGKVIEFSDDSIEARQREIAAKHGIRLTNHSLYLYGHCAEGDCRE

DEHAHEGK

>gi|30062141|ref|NP_836312.1| flavodoxin FldA [Shigella flexneri 2a str. 2457T]

MAITGIFFGSDTGNTENIAKMIQKQLGKDVADVHDIAKSSKEDLEAYDILLLGIPTWYYGEAQCDWDDFF

PTLEEIDFNGKLVALFGCGDQEDYAEYFCDALGTIRDIIEPRGATIVGHWPTAGYHFEASKGLADDDHFV

GLAIDEDRQPELTAERVEKWVKQISEELHLDEILNA

>gi|30062138|ref|NP_836309.1| replication initiation regulator SeqA [Shigella flexneri 2a str. 2457T]

MKTIEVDDELYSYIASHTKHIGESASDILRRMLKFSAASQPAAPVTKEVRVASPAIVEAKPVKTIKDKVR

AMRELLLSDEYAEQKRAVNRFMLLLSTLYSLDAQAFAEATESLHGRTRVYFAADEQTLLKNGNQTKPKHV

PGTPYWVITNTNTGRKCSMIEHIMQSMQFPAELIEKVCGTI

>gi|30062130|ref|NP_836301.1| Rhs-family protein [Shigella flexneri 2a str. 2457T]

MEEAFWAARQGDALLHTSFMADVLGAVLEVAANVVIDALIVGTTSLLATSVGITMGCTAVVLTGFVAGVA

MVYTGVTEKVSEACSALANMLFPPQIEGYILTGSGDTWINSKPAARAAATAASRQDIEAQEAQAKAEQEE

AQRQEDARTFRDVAGEYLEMAGAIALAVNPVTGPVLMSRALHDQLSTEEGRSEMVDGVTHFVSELWQPTV

ASAAPGSTPTPDDKIDCHKHPSSLTQFLAQKKAAFLDAPLGTALNLLNPGGMLETAFQGVNAVIGSISNL

FKGDDEPPAAEYIAEGARDVRINSQPATRSGARCTCEARVVNEPGNGAFVSPDVRIGGPLLVVRDIRSGR

SQITLVATVALMFLRPGKMLSKIACFAASVGMSMLTQKATSALPHPVNAATGAKYLADDDDFDFSLPGHF

PLDWQRVYSSRDERTEGMFGQGWSVMYEVSLVCTPGSADENCMTFVSGMGRRLDMEAVLPGGGFYSPGEG

LAVRRGEQGHWLISSDDGQCFLFEADPHHPQRQRLKMLGDRNSNCLNLYYDDRGRIVEISGEQQRPCIRL

YYELAAHPRRVTQIYQHFPETAPLLLRRYSYDEAGHLNGVYDSTGHLLREFAYDENHCMTLHRQPGGEGY

YYQWGWYEGPDDAAWRVTGHHTDSGEQYRLAWDLAQRRLCVTDGLGRTRYHQWDAQNQVTAYQDEAGQVT

TFRWSDEERLLLGMTDPQGGKWRYVYDLQGHITETHDPLGRVEQAQWHPVWHQPETEVDAAGNLLGRRAG

ERATAENSVVPFNRLMSYRGVHYRYDEFGRAVEKEGRSGTQSYRYDAEHRMVEVTTARGTYRYVYDALGR

RTEKQHISPDGKPYNRTKFLWDGMRLAQESRPEGTGSLYIYRDQGSYEPLARVDKAGKEGPNRILYFHTD

VNGAPEEMTDSDGKIVWETGYQVWGNTIQEKDHGGVEQNLRYQGQYLDRETGLHYNLHRYYDPDVGRFMV

TDPIGLRGGLNLYSYAPNPLKYADPLGLTPCAVSNQKANRLLDSSETKVTVRSRSDAEQLFMDRYLGHNY

KNMTGESGPSTKNLMEYLTENKTKAGSYHWDDIKDPSVTKPSYRVSGHGPGNPDGDLPHLQVHQHGGSVR

HIFFPWET

>gi|30062120|ref|NP_836291.1| chaperone [Shigella flexneri 2a str. 2457T]

MTFMKGLPLLLLVASLCSHAALQPDRTRIVFNANDKATSLRVDNRSDKLPYLAYSWLENEKGEKSDDLLV

ALPPIQRLEPKATTQVRIVKQASTTKLPGDRETLFFYNMREIPPAPEKNSDHAVLQVAIQSRIKVFWRPA

ALRKKAGEKVELQLQVSQQGNQLTLKNPTAYYLTIAYLGRNEKGVLPGFKTVMVAPFSTVNTNTGNYSGS

QFYLGYMDDYGALRMTTLNCSGQCHLQAVEAKK

>gi|30062119|ref|NP_836290.1| outer membrane protein [Shigella flexneri 2a str. 2457T]

MDTVNIYRLSFVSCLVVAMPCALAVEFNLNVLDKSMRDRIDISLLKEKGVIAPGEYFVSVAVNNNQISNE

QKINWHKNDDKTIPCINDLLVDKFGLKPEVRQSLPLINQCVDFSSRPEMLFNFDQANQQLNISIPQAWLA

WHSENWTPPSTWKEGVAGVLMDYNLFASSYRPQDGSSSTNLNAYGTAGINTGAWRLRSDYQLNHTDSDDN

HEQSGEISRTYLFRPLPQLGSKLTLGETDFSPNIFDGFSYTGAALASDDRMLPWELRGYAPQISGIAQTN

ATVTISQSGRVIYQKKVPPGPFIIDDLNQSVQGTLDVKVTEEDGRVNNFQVSAASTPFLTRQGQVRYKLA

AGQPRPSMSHQTENETFFSNEVSWGMLSNTSLYSGLLLSGDDYHSAAMGIGQNMLWLGALSFDVTWASSH

FDTQQDERGLSYRFNYSKQVDATNSTISLAAYRFSDRHFHSYANYLDHKYNDSDAQDEKQTISLSVGQPI

TPLNLNLYANLLHQTWWNADASTTANITAGFNVDIGDWRDISISTSFNTTHYEDKDRDNQIYLSISLPFG

NGGRVGYDMQNSSHSTTHRMSWNDTLDERNSWGMSAGLQSDRPDNGAQVSGNYQHLSSAGEWDISGTYAA

NDYSSVSSSWSGSFTATQYGAAFHRRSSTNEPRLMVSTDGVADIPVQGNLDYTNHFGIAVVPLISSYQPS

TVAVNMNDLPDGVTVAENVIKETWIEGAIGYKSLASRSGKDVNVIIRNASGQFPPLGADIRQDDSGISVG

MVGEEGHAWLSGVAENQKFTVVWGDSQHCSLHLPEHMEDTANRLILPCH

>gi|30062107|ref|NP_836278.1| DNA-binding transcriptional repressor MngR [Shigella flexneri 2a str. 2457T]

MGHKPLYRQIADRIREQIARGELKPGDALPTESALQTEFGVSRVTVRQALRQLVEQQILESIQGSGTYVK

EERVNYDIFQLTSFDEKLSDRHVDTHSEVLIFEVIPADDFLQQQLQITVQDRVWHVKRVRYRKQKPMALE

ETWMPLALFPDLTWQVMENSKYHFIEEVKKMVIDRSEQEIIPLMPTEEMSRLLNISQTKPILEKVSRGYL

VDGRVFEYSRNAFNTDDYKFTLIAQPKIIAISTKRPSYDGQPCGNRQPFAIPVEGSGEGYFLLQIFISEQ

FSVLHRYPPGCERFSRLRLPEQRISHPQSLSHRQ

>gi|30062106|ref|NP_836277.1| 2-O-a-mannosyl-D-glycerate specific PTS transporten components IIABC [Shigella flexneri 2a str. 2457T]

MVLFCRAHWRDYKNDQVRIMMNLTTLTHRDALCLNARFTSREEAIHALTQRLAALGKISSTEQFLKEVYR

RESLGPTALSEGLAVPHGKTAAVKEAAFAVATLSEPLQWEGVDGPEAVDLVVLLAIPLNEAGTTHMQLLT

ALTTRLADDEIRARIQSATTPDELLSALDDKGGTQPSASFSNAPTIVCVTACPAGIAHTYMAAEYLEKAG

RKLGVNVYVEKQGANGIEGRLTADQLNSATACIFAAEVAIKESERFNGIPALSVPVAEPIRHAEALIQQS

LTLERGDETRTLQQDTQPVKSVKTELKQALLSGISFAVPLIVAGGTVLAVAVLLSQIFGLQDLFNEENSW

LWMYRKLGGGLLGILMVPVLAAYTAYSLADKPALAPGFAAGLAANMIGSGFLGAVVGGLIAGYLIRWMKN

HLRLSSKFNGFLTFYLYPVLGTLGAGSLMLFVVGEPVAWINNSLTAWLNGLSGSNALLLGAILGFMCSFD

LGGPVNKAAYAFCLGAMANGVYGPYAIFASVKMVSAFTVTASTMLAPRLFKEFEIETGKSTWLLGLAGIT

EGAIPMAIEDPLRVIGSFVLGSMVTGAIVGAMNIGLSTPGAGIFSLFLLHDNGAGGVMAAIGWFGAALVG

AAISTAILLIWRRHAVKHGNYLTDGVMP

>gi|30062104|ref|NP_836275.1| cytochrome d terminal oxidase, polypeptide subunit I [Shigella flexneri 2a str. 2457T]

MMLDIVELSRLQFALTAMYHFLFVPLTLGMAFLLAIMETVYVLSGKQIYKDMTKFWGKLFGINFALGVAT

GLTMEFQFGTNWSYYSHYVGDIFGAPLAIEGLMAFFLESTFVGLFFFGWDRLGKVQHMCVTWLVALGSNL

SALWILVANGWMQNPIASDFNFETMRMEMVSFSELVLNPVAQVKFVHTVASGYVTGAMFILGISAWYMLK

GRDFAFAKRSFAIAASFGMAAVLSVIVLGDESGYEMGDVQKTKLAAIEAEWETQPAPAAFTLFGIPDQEE

ETNKFAIQIPYALGIIATRSVDTPVIGLKELMVQHEERIRNGMKAYSLLEQLRSGSTDQTVRDQFNSMKK

DLGYGLLLKRYTPNVADATEAQIQQATKDSIPRVAPLYFAFRIMVACGFLLLAIIALSFWSVIRNRIGEK

KWLLRAALYGIPLPWIAVEAGWFVAEYGRQPWAIGEVLPTAVANSSLTAGDLIFSMVLICGLYTLFLVAE

LFLMFKFARLGPSSLKTGRYHFEQSSTTTQPAR

>gi|30062103|ref|NP_836274.1| cytochrome d terminal oxidase polypeptide subunit II [Shigella flexneri 2a str. 2457T]

MIDYEVLRFIWWLLVGVLLIGFAVTDGFDMGVGMLTRFLGRNDTERRIMINSIAPHWDGNQVWLITAGGA

LFAAWPMVYAAAFSGFYVAMILVLASLFFRPVGFDYRSKIEETRWRNMWDWGIFIGSFVPPLVIGVAFGN

LLQGVPFNVDEYLRLYYTGNFFQLLNPFGLLAGVVSVGMIITQGATYLQMRTVGELHLRTRATAQVAALV

TLVCFALAGVWVMYGIDGYVVKSTMDHYAASNPLNKEVVREAGAWLVNFNNTPILWAIPALGVVLLLLTI

LTARMDKAAWAFVFSSLTLACIILTAGIAMFPFVMPSSTMMNASLTMWDATSSQLTLNVMTWVAVVLVPI

ILLYTAWCYWKMFGRITKEDIERNTHSLY

>gi|30062101|ref|NP_836272.1| acyl-CoA thioester hydrolase YbgC [Shigella flexneri 2a str. 2457T]

MNTTLFRWPVRVYYEDTDAGGVVYHASYVAFYERARTEMLRHHHFSQQALMAERVAFVVRKMTVEYYAPA

RLDDMLEIQTEITSMRGTSLVFTQRIVNAENTLLNEAEVLVVCVDPLKMKPRALPKSIVAEFKQ

>gi|30062100|ref|NP_836271.1| colicin uptake protein TolQ [Shigella flexneri 2a str. 2457T]

MTDMNILDLFLKASLLVKLIMLILIGFSIASWAIIIQRTRILNAAAREAEAFEDKFWSGIELSRLYQESQ

GKRDNLTGSEQIFYSGFKEFVRLHRANSHAPEAVVEGASRAMRISMNRELENLETHIPFLGTVGSISPYI

GLFGTVWGIMHAFIALGAVKQATLQMVAPGIAEALIATAIGLFAAIPAVMAYNRLNQRVNKLELNYDNFM

EEFTAILHRQAFTVSESNKG

>gi|30062097|ref|NP_836268.1| translocation protein TolB [Shigella flexneri 2a str. 2457T]

MKQALRVAFGFLILWASVLHAEVRIVIDSGVDSGRPIGVVPFQWAGPGAAPEDIGGIVAADLRNSGKFNP

LDRARLPQQPGSAQEVQPAAWSALGIDAVVVGQVTPNPDGSYNVAYQLVDTGGAPGTVLAQNSYKVNKQW

LRYAGHTASDEVFEKLTGIKGAFRTRIAYVVQTNGGQFPYELRVSDYDGYNQFVVHRSPQPLMSPAWSPD

GSKLAYVTFESGRSALVIQTLANGAVRQVASFPRHNGAPAFSPDGSKLAFALSKTGSLNLYVMDLASGQI

RQVTDGRSNNTEPTWFPDSQNLAFTSDQAGRPQVYKVNINGGAPQRITWEGSQNQDADVSSDGKFMVMVS

SNGGQQHIAKQDLATGGVQVLSSTFLDETPSLAPNGTMVIYSSSQGMGSVLNLVSTDGRFKARLPATDGQ

VKFPAWSPYL

>gi|30062096|ref|NP_836267.1| peptidoglycan-associated outer membrane lipoprotein [Shigella flexneri 2a str. 2457T]

MQLNKVLKGLMIALPVMAIAACSSNKNASNDGSEGMLGAGTGMDANGGNGNMSSEEQARLQMQQLQQNNI

VYFDLDKYDIRSDFAQMLDAHANFLRSNPSYKVTVEGHADERGTPEYNISLGERRANAVKMYLQGKGVSA

DQISIVSYGKEKPAVLGHDEAAYSKNRRAVLVY

>gi|30062090|ref|NP_836261.1| phospho-2-dehydro-3-deoxyheptonate aldolase [Shigella flexneri 2a str. 2457T]

MNYQNDDLRIKEIKELLPPVALLEKFPATENAANTVAHARKAIHKILKGNDDRLLVVIGPCSIHDPVAAK

EYATRLLALREELKDELEIVMRVYFEKPRTTVGWKGLINDPHMDNSFQINDGLRIARKLLLDINDSGLPA

AGEFLDMITPQYLADLMSWGAIGARTTESQVHRELASGLSCPVGFKNGTDGTIKVAIDAINAAGAPHCFL

SVTKWGHSAIVNTSGNGDCHIILRGGKEPNYSAKHVAEVKEGLNKAGLPAQVMIDFSHANSSKQFKKQMD

VCADVCQQIAGGEKAIIGVMVESHLVEGNQSLDSGEPLAYGKSITDACIGWEDTDALLRQLVNAVKARRG

>gi|30062080|ref|NP_836251.1| molybdate ABC transporter permease [Shigella flexneri 2a str. 2457T]

MILTDPEWQAVLLSLKVSSLAVLFSLPFGIFFAWLLVRCTFPGKALLDSVLHLPLVLPPVVVGYLLLVSM

GRRGFIGERLYDWFGITFAFSWRGAVLAAAVMSFPLMVRAIRLALEGVDVKLEQAARTLGAGRWRVFFTI

TLPLTLPGIIVGTVLAFARSLGEFGATITFVSNIPSETRTIPSAMYTLIQTPGGESGAARLCIISIALAM

ISLLISEWLARISRERAGR

>gi|30062078|ref|NP_836249.1| phosphotransferase [Shigella flexneri 2a str. 2457T]

MTTRVIALDLDGTLLTPKKTLLPSSIEALARAREAGYQLIIVTGRHHVAIHPFYQALALDTPAICCNGTY

LYDYHAKTVLEADPMPVNKALQLIEMLNEHHIHGLMYVDDAMVYEHPTGHVIRTSNWAQTLPPEQRPTFT

QVASLAETTQQVNAVWKFALTHDDLPQLQHFGKHVEHELGLECEWSWHDQVDIAHGGNSKGKRLTKWVEA

QGWSMENVVAFGDNFNDISMLEAAGTGVAMGNADDAVKARANIVIGDNTTDSIAQFIYSHLI

>gi|30062068|ref|NP_836239.1| nucleoside diphosphate kinase regulator [Shigella flexneri 2a str. 2457T]

MSRPTIIINDLDAERIDILLEQPAYAGLPIADALNAELDRAQMCSPEEMPHDVVTMNSRVKFRNLSDGEV

RVRTLVYPAKMTDSNTQLSVMAPVGAALLGLRVGDSIHWELPGGVATHLEVLELEYQPEAAGDYLL

>gi|30062054|ref|NP_836225.1| hypothetical protein S0517 [Shigella flexneri 2a str. 2457T]

MIWKRHLTLDELNATSDNTMVAHLGIVYTLLGDDVLEAEMPVDTRTHQPFGLLHGGASAALAETLGSMAG

FMMTRDGQCVVGTELNATHHRPVSEGKVRGVCQPLHLGRQNQSWEIVVFDEQGRRCCTCRLGTAVLG

>gi|30062050|ref|NP_836221.1| iron-enterobactin transporter periplasmic binding protein [Shigella flexneri 2a str. 2457T]

MRLAPLYRNALLLTGLLLSGIAVVQAADWPRQITDSRGTHTLESQPQRIVSTSVTLTGSLLAIDAPVIAS

GATTPNNRVADDQGFLRQWSKVAKERKLQRLYIGEPSAEAVAAQMPDLILISATGGDSALALYDQLSTIA

PTLIINYDDKSWQSLLTQLGEITGHEKQAAERIAQFDKQLAAAKEQIKLPPQPVTAIVYTAAAHSANLWT

PESAQGQMLEQLGFTLAKLPAGLNASQSQGKRHDIIQLGGENLAAGLNGESLFLFAGDQKDADAIYANPL

LAHLPAVQNKQVYALGTETFRLDYYSAMQVLERLKALF

>gi|30062029|ref|NP_836200.1| inner membrane component for iron transport [Shigella flexneri 2a str. 2457T]

MIEWIIRRSVANRFLVLMGALFLSIWGTWTIINTPVDALPDLSDVQVIIKTSYPGQAPQIVENQVTYPLT

TTMLSVPGAKTVRGFSQFGDSYVYVIFEDGTDPYWARSRVLEYLNQVQGKLPAGVSAELGPDATGVGWIY

EYALVDRSGKHDLADLRSLQDWFLKYELKTIPDVAEVASVGGVVKEYQVVIDPQRLAQYGISLAEVKSAL

DASNQEAGGSSIELAEAEYMVRASGYLQTLDDFNHIVLKASENGVPVYLRDVAKVQIGPKMRRGIAELNG

EGEVAGGVVILRSGKNAREVIAAVKDKLETLKSSLPEGVEIVTTYDRSQLIDRAIDNLSGKLLEEFIVVA

VVCAPFLWHVRSALVAIISLPLGLCIAFIVMPFQGLNANIMSLGGIAIAVGAMVDAAIVMIENAHKRLEE

WQHQHPDATLDNKTRWQVITDASVEVGPALFISLLIITLSFIPIFTLEGQEGRLFGPLAFTKTYAMAGAA

LLAIVVIPILMGYWIRGKIPPESSNPLNRFLIRVYHPLLLKVLHWPKTTLLVAALSVLTVLWPLNKVGGE

FLPQINEGDLLYMPSTLPGISAAEAASMLQKTDKLIMSVPEVARVFGKTGKAETATDSAPLEMVETTIQL

KPQDQWRPGMTMDKIIEELDNTVRLPGLANLWVPPIRNRIDMLSTGIKSPIGIKVSGTVLADIDAMAEQI

EEVARTVPGVASALAERLEGGRYINVEINREKAARYGMTVADVQLFVTSAVGGAMVGETVEGIARYPINL

RYPQSWRDSPQALRQLPILTPMKQQITLADVADVKVSTGPSMLKTENARPTSWIYIDARDRDMVSVVHDL

QKAIAEKVQLKPGTSVAFSGQFELLERANHKLKLMVPMTLMIIFVLLYLAFRRVGEALLIISSVPFALVG

GIWLLWWMGFHLSVATGTGFIALAGVAAEFGVVMLMYLRHAIEAEPSLNNPQTFSEQKLDEALYHGAVLR

VRPKAMTVAVIIAGLLPILWGTGAGSEVMSRIAAPMIGGMITAPLLSLFIIPAAYKLMWLHRHRVRN

>gi|30062021|ref|NP_836192.1| DNA-binding transcriptional activator CusR [Shigella flexneri 2a str. 2457T]

MKLLIVEDEKKTGEYLTKGLTEAGFVVDLADNGLNGYHLAMTGDYDLIILDIMLPDVNGWDIVRMLRSAN

KGMPILLLTALGTIEHRVKGLELGADDYLVKPFAFAELLARVRTLLRRGAAVIIESQFQVADLMVDLVSR

KVTRSGTRITLTSKEFTLLEFFLRHQGEVLPRSLIASQVWDMNFDSDTNAIDVAVKLLRGKIDNDFEPKL

IQTVRGVGYMLEVPDGQ

>gi|30062020|ref|NP_836191.1| sensor kinase CusS [Shigella flexneri 2a str. 2457T]

MVSKPFQRPFSLATRLTFFISLATIAAFFAFAWIIIHSVKVHFAEQDINDLKEISATLERVLNHPDETQA

RRLMTLEDIVSGYSNVLISLADSHGKTVYHSPGAPDIREFARDAIPDKDARGGEVYLLSGPTIMMPGHGH

GHMEHSNWRMINLPVGPLVDGKPIYTLYIALSIDFHLHYINDLMNKLIMTASVISILIVFIVLLAVHKGH

APIRSVSRQIQNITSKDLDVRLDPQTVPIELEQLVLSFNHMIERIEDVFTRQSNFSADIAHEIRTPITNL

ITQTEIALSQSRSQKELEDVLYSNLEELTRMAKMVSDMLFLAQADNNQLIPEKKMLNLADEVGKVFDFFE

ALAEDRGVELRFVGDKRQVAGDPLMLRRALSNLLSNALRYTPTRETIVVRCQTVDHQVQVSVENPGTPIA

PEHLPRLFDRFYRVDPSRQRKGEGSGIGLAIVKSIVVAHKGTVAVTSDARGTRFVITLPA

>gi|30062018|ref|NP_836189.1| envelope protein [Shigella flexneri 2a str. 2457T]

MQLSSSEPCVVILTEKEVEVSVNNHATFTLPKNYLAAFACNNNVIELSTLNHVLITHINRNIINDYLLFL

NKKLTCVKPWSRLATPVIACHSRTPEVFRLATNHSKQQSSKPCEAELTRALLFTVLSNFLEQSRFIALLM

YILRSSVRDSVCRIIQSDIQHYWNLRIVASSLCLSPSLLKKKLKNENTSYSQIVTECRMRYAVQMLLMDN

KNITQVAQLCGYSSTSYFISVFKAFYSLTPLNYLAKQRQKVMW

>gi|30062015|ref|NP_836186.1| chaperone [Shigella flexneri 2a str. 2457T]

MMTKIKLLMLMIFYLIISASAHAAGGIALGATRIIYPADAKQTAVWIKNSHTNELFLVNSWIENSSGVKE

KSFIITPPLFVSEPKSENTLRIIYTGPPLAADRESLFWMNVKTIPSVDKNALNGRNVLQLAILSRMKLFL

RPIQLQELPAEAPDTLKFSRSGNYINVHNPSPFYVTLVNLQVGSQKLGNAMAAPRVSSQIPLPSGVQGKL

KFQTVNDYGSVTPVREVNLN

>gi|30062008|ref|NP_836179.1| UDP-2,3-diacylglucosamine hydrolase [Shigella flexneri 2a str. 2457T]

MATLFIADLHLCVEEPAITAGFLRFLAGEARKADALYILGDLFEAWIGDDDPNPLHRQMAAAIKAVSDSG

VPCYFIHGNRDFLLGKRFARESGMTLLPEEKVLELYGRRVLIMHGDTLCTDDAGYQAFRAKVHKPWLQML

FLALPLFVRKRIAARMRANSKEANSSKSLAIMDVNQNAVVSAMEKHQVQWLIHGHTHRPAVHELIANQQP

AFRVVLGAWHTEGSMVKVTADDVELIHFPF

>gi|30062005|ref|NP_836176.1| carbamate kinase [Shigella flexneri 2a str. 2457T]

MKTLVVALGGNALLQRGEALTAENQYRNIASAVPALARLARSYRLAIVHGNGPQVGLLALQNLAWKEVEP

YPLDVLVAESQGMIGYMLAQSLSAQPQMPPVTTVLTRIEVSPDDPAFLQPEKFIGPVYQPEEQEALEAAY

GWQMKRDGKYLRRVVASPQPRKILDSEAIELLLKEGHVVICSGGGGVPVTEDGAGSEAVIDKDLAAALLA

EQINADGLVILTDADAVYENWGTPQQRAIRHATPDELAPFAKADGSMGPKVTAVSGYVRSRSKPAWIGAL

SRIEETLAGEAGTCISL

>gi|30061997|ref|NP_836168.1| ureidoglycolate hydrolase [Shigella flexneri 2a str. 2457T]

MKLQVLPLSQEAFSAYGDVIETQQRDFFHINNGLVERYHDLALVEILEQDRTLISINRAQPANLPLTIHE

LERHPLGTQAFIPMKGEVFVVVVALGDDKPDLSTLRAFITNGEQGVNYHRNVWHHPLFAWQRVTDFLTID

RGGSDNCDVESIPEQELCFA

>gi|30061987|ref|NP_836158.1| DNA-binding transcriptional regulator CueR [Shigella flexneri 2a str. 2457T]

MNISDVAKITGLTSKAIRFYEEKGLVTPPMRSENGYRTYTQQHLNELTLLRQARQVGFNLEESGELVNLF

NDPQRHSADVKRRTLEKVAEIERHIEELQSMRDQLLALANACPGDDSADCPIIENLSGCCHHRAG

>gi|30061983|ref|NP_836154.1| hypothetical protein S0435 [Shigella flexneri 2a str. 2457T]

MIQYVLASLFTGKQQLKTMKQATRKPTTPGDILLYEYLEPLDLKINELAELLHVHRNRVSALINNNRKLT

TEMAFRLAKVFDTTVDFRLNLQAAVDLWEVENNMRTQEELGRIETVAEYLARREERAKKVA

>gi|30061982|ref|NP_836153.1| ligase [Shigella flexneri 2a str. 2457T]

MDLLYRVKTLWAALRGNHYTWPAIDITLPGNRHFHLIGSIHMGSHDMAPLPTRLLKKLKNADALIVEADV

STSDTPFANLPACEALEERISEEQLQNLQHISQEMGISPSLFSTQPLWQIAMVLQATQAQKLGLRAEYGI

DYQLLQAAKQQHKPVIELEGAENQIAMLLQLPDKGLALLDDTLTHWHTNARLLQQMMSWWLNAPPQNNDI

TLPNTFSQSLYDVLMHQRNLAWRDKLRAMPPGRYVVAVGALHLYGEGNLPQMLR

>gi|30061971|ref|NP_836142.1| hypothetical protein S0423 [Shigella flexneri 2a str. 2457T]

MFGKGGLGNLMKQAQQMQEKMQKMQEEIAQLEVTGESGAGLVKVTINGAHNCRRVEIDPSLLEDDKEMLE

DLVAAAFNDAARRIEETQKEKMASVSSGMQLPPGFKMPF

>gi|30061968|ref|NP_836139.1| hypothetical protein S0420 [Shigella flexneri 2a str. 2457T]

MQRIILIIIGWLAVVLGTLGVVLPVLPTTPFILLAAWCFARSSPRFHAWLLYRSWFGSYLRFWQKHHAMP

RGVKPRAILLILLTFAISLWFVQMPWVRIMLLVILACLLFYMWRIPVIDEKQEKH

>gi|30061967|ref|NP_836138.1| primosomal replication protein N'' [Shigella flexneri 2a str. 2457T]

MKTALLLEKLEGQLATLRQRCAPVSQFATLSARFDRHLFQTRATTLQSCLDEAGDNLAALRHAVEQQQLP

QVAWLAEHLAAQLEAIAREASAWSLREWDSAPPKISRWQRKRIQHQDFERRLREMVAERRARLARVTDLV

EQQTLHREVKAYEARLARCRHALEKIENRLARLTR

>gi|30061962|ref|NP_836133.1| acriflavine resistance protein [Shigella flexneri 2a str. 2457T]

MPNFFIDRPIFAWVIAIIIMLAGGLAILKLPVAQYPTIAPPAVTISASYPGADAKTVQDTVTQVIEQNMN

GIDNLMYMSSNSDSTGTVQITLTFESGTDADIAQVQVQNKLQLAMPLLPQEVQQQGVSVEKSSSSFLMVV

GVINTDGTMTQEDISDYVAANMKDAISRTSGVGDVQLFGSQYAMRIWMNPNELNKFQLTPVDVITAIKAQ

NAQVAAGQLGGTPPVKGQQLNASIIAQTRLTSTEEFGKILLKVNQDGSRVLLRDVAKIELGGENYDIIAE

FNGQPASGLGIKLATGANALDTAAAIRAELAKMEPFFPSGLKIVYPYDTTPFVKISIHEVVKTLVEAIIL

VFLVMYLFLQNFRATLIPTIAVPVVLLGTFAVLAAFGFSINTLTMFGMVLAIGLLVDDAIVVVENVERVM

AEEGLPPKEATRKSMGQIQGALVGIAMVLSAVFVPMAFFGGSTGAIYRQFSITIVSAMALSVLVALILTP

ALCATMLKPIAKGDHGEGKKGFFGWLNRMFEKSTHHYTDSVGGILRSTGRYLVLYLIIVVGMAYLFVRLP

SSFLPDEDQGVFMTMVQLPAGATQERTQKVLNEVTHYYLTKEKNNVESVFAVNGFGFAGRGQNTGIAFVS

LKDWADRPGEENKVEAITMRATRAFSQIKDAMVFAFNLPAIVELGTATGFDFELIDQAGLGHEKLTQARN

QLLAEAAKHPDMLTSVRPNGLEDTPQFKIDIDQEKAQALGVSINDINTTLGAAWGGSYVNDFIDRGRVKK

VYVMSEAKYRMLPDDIGDWYVRAADGQMVPFSAFSSSRWEYGSPRLERYNGLPSMEILGQAAPGKSTGEA

MELMEQLASKLPTGVGYDWTGMSYQERLSGNQAPSLYAISLIVVFLCLAALYESWSTPFSVMLVVPLGVI

GALLAATFRGLTNDVYFQVGLLTTIGLSAKNAILIVEFAKDLMDKEGKGLIEATLDAVRMRLRPILMTSL

AFILGVMPLVISTGAGSGAQNAVGTGVMGGMVTATVLAIFFVPVFFVVVRRRFSRKNEDIEHSHTVDHH

>gi|30061957|ref|NP_836128.1| hypothetical protein S0408 [Shigella flexneri 2a str. 2457T]

MKYVDGFVVAVPADKKDAYREMAAKAAPLFKEFGALRIVECWASDVPDGKVTDFRMAVKAEENEEVVFSW

IEYPSKEVRDAANQKMMSDPRMKEFGESMPFDGKRMIYGGFESIIDE

>gi|30061947|ref|NP_836118.1| hypothetical protein S0397 [Shigella flexneri 2a str. 2457T]

MEKEMARLVAFDMDGTLLMPDHHLGEKTLSTLARLRERDITLTFATGRHALEMQHILGALSLDAYLITGN

GTRVHSLEGELLHRDDLPADVAELVLYQQWDTRASMHIFNDDGWFTGKESPALLQVFVYSGFRYQIIDVK

KMPLGSVTKICFCGDHDDLTRLQIQLYEALGERAHLCFSATDCLEVLPVGCNKGAALTVRTQHLGLSLRD

CMAFGDAMNDREMLGSVGSGFIMGNAMPQLRAELPHLPVIGHCRNQAVSHYLTHWLDYPHLPYSPE

>gi|30061943|ref|NP_836114.1| peptidyl-prolyl cis-trans isomerase (rotamase D) [Shigella flexneri 2a str. 2457T]

MMDSLRTAANSLVLKIIFGIIIVSFILTGVSGYLIGGGNNYAAKVNDQEISRGQFENAFNSERNRMQQQL

GDQYSELAANEGYMKTLRQQVLNRLIDEALLDQYARELKLGISDEQVKQAIFATPAFQVDGKFDNSRYNG

ILNQMGMTADQYAQALRNQLTTQQLINGVAGTDFMLKGETDELAALVAQQRVVREATIDVNALAAKQPVT

EQEIASYYEQNKNNFMTPEQFRVSYIKLVAATMQQPVSDADIQSYYDQHQDQFTQPQRTRYSIIQTKTED

EAKAVLDELNKGGDFAALAKEKSADIISARNGGDMGWLEDATIPDELKNAGLKEKGQLSGVIKSSVGFLI

VRLDDIQPAKVKSLDEVRDDIAAKVKHEKALDAYYALQQKVSDAASNDTESLAGAEQAAGVKATQTGWFS

KDNLPEELNFKPVADAIFNGGLVGENGASGINSDIITVDGDRAFVLRVSEHKPEAVKPLADVQEQVKALV

QHNKAEQQAKVDAEKLLVDLKAGKGAEAMQAAGLKFGEPKTLSRSGRDPISQAAFALPLPAKDKPSYGMA

TDMQGNVVLLALDEVKQGSMPEDQKKAMVQGITQNNAQIVFEALMSNLRKEAKIKIGDALEQQ

>gi|30061928|ref|NP_836099.1| cytochrome o ubiquinol oxidase subunit IV [Shigella flexneri 2a str. 2457T]

MSHSTDHSGASHGSVKTYMTGFILSIILTVIPFWMVMTGAASPAVILGTILAMAVVQVLVHLVCFLHMNT

KSDEGWNMTAFVFTVLIIAILVVGSIWIMWNLNYNMMMH

>gi|30061913|ref|NP_836084.1| phosphatidylglycerophosphatase A [Shigella flexneri 2a str. 2457T]

MTILSRHKDVAKSRLKMSNPWHLLAVGFGSGLSPIVPGSMGSLAAIPFWYLMTFLPWQLYSLVVMLGICI

GVYLCHQTAKDMGVHDHGSIVWDEFIGMWITLMALPTNDWQWVAAGFVIFRILDMWKPWPIRWFDRNVHG

GMGIMIDDIVAGVISAGILYFIGHHWPLGILS

>gi|30061912|ref|NP_836083.1| thiamine monophosphate kinase [Shigella flexneri 2a str. 2457T]

MACGEFSLIARYFDRVRSSRLDVELGIGDDCALLNIPEKQTLAISTDTLVAGNHFLPDIDPADLAYKALA

VNLSDLAAMGADPAWLTLALTLPDVDEAWLESFSDSLFDLLNYYDMQLIGGDTTRGPLSMTLGIHGFVPM

GRALTRSGAKPGDWIYVTGTPGDSAAGLAILQNRLQVADAKDADYLIKRHLRPSPRILQGQALRDLANSA

IDLSDGLISDLGHIVKASDCGARIDLALLPFSDALSRHVEPEQALRWALSGGEDYELCFTVPELNRGALD

VALGHLGVPFTCIGQMTADIEGLCFIRDGEPVTFDWKGYDHFVTP

>gi|30061911|ref|NP_836082.1| transcription antitermination protein NusB [Shigella flexneri 2a str. 2457T]

MKPAARRRARECAVQALYSWQLSQNDIADVEYQFLAEQDVKDVDVLYFRELLAGVATNTAYLDGLMKPYL

SRLLEELGQVEKAVLRIALYELSKRSDVPYKVAINEAIELAKSFGAEDSHKFVNGVLDKAAPVIRPNKK

>gi|30061910|ref|NP_836081.1| 6,7-dimethyl-8-ribityllumazine synthase [Shigella flexneri 2a str. 2457T]

MNIIEANVATPDARVAITIARFNNFINDSLLEGAIDALKRIGQVKDENITVVWVPGAYELPLAAGALAKT

GKYDAVIALGTVIRGGTAHFEYVAGGASNGLAHVAQDSEIPVAFGVLTTESIEQAIERAGTKAGNKGAEA

ALTALEMINVLKAIKA

>gi|30061909|ref|NP_836080.1| bifunctional diaminohydroxyphosphoribosylaminopyrimidine deaminase/5-amino-6-(5-phosphoribosylamino)uracil reductase [Shigella flexneri 2a str. 2457T]

MQDEYYMARALKLAQRGRFTTHPNPNVGCVIVKDGEIVGEGYHQRAGEPHAEVHALRMAGEKAKGATAYV

TLEPCSHHGRTPPCCDALIAAGVARVVAAMQDPNPQVAGRGLYRLQQAGIDVSHGLMMSEAEQLNKGFLK

RMRTGFPYIQLKLGASLDGRTAMASGESQWITSPQARRDVQRQRAQSHAILTSSATVLADDPALTVRWSE

LDEQTQALYPQQNLRQPVRIVIDSQNRVTPEHRIVQQPGETWFARTQEDSREWPETVRTLLIPEHKGHLD

LVVLMMQLGKQQINSIWVEAGPTLAGVLLQAGLVDELIVYIAPKLLGSDARGLCSLPGLEKLADAPQFKF

KEIRHVGPDVCLHLVGA

>gi|30061904|ref|NP_836075.1| preprotein translocase subunit SecF [Shigella flexneri 2a str. 2457T]

MAQEYTVEQLNHGRKVYDFMRWDYWAFGISGLLLIAAIVIMGVRGFNWGLDFTGGTVIEITLEKPAEIDV

MRDALQKAGFEEPMLQNFGSSHDIMVRMPPAEGETGGQVLGSQVLKVINESTNQNAAVKRIEFVGPSVGA

DLAQTGAMALMAALLSILVYVGFRFEWRLAAGVVIALTHDVIITLGILSLFHIEIDLTIVASLMSVIGYS

LNDSIVVSDRIRENFRKIRRGTPYEIFNVSLTQTLHRTLITSGTTLMVILMLYLFGGPVLEGFSLTMLIG

VSIGTASSIYVASALALKLGMKREHMLQQKVEKEGADQPSILP

>gi|30061903|ref|NP_836074.1| preprotein translocase subunit SecD [Shigella flexneri 2a str. 2457T]

MLNRYPLWKYVMLIVVIVIGLLYALPNLFGEDPAVQITGARGVAASEQTLIQVQKTLQEEKITAKSVALE

EGAILARFDSTDTQLRAREALMGVMGDKYVVALNLAPATPRWLAAIHAEPMKLGLDLRGGVHFLMEVDMD

TALGKLQEQNIDSLRSDLREKGIPYTTVRKENNYGLSITFRDAKARDEAIAYLSKRHPDLVISSQGSNQL

RAVMSDARLSEAREYAVQQNINILRNRVNQLGVAEPVVQRQGADRIVVELPGIQDTARAKEILGATATLE

FRLVNTNVDQAAAASGRVPGDSEVKQTREGQPVVLYKRVILTGDHITDSTSSQDEYNQPQVNISLDSAGG

NIMSNFTKDNIGKPMATLFVEYKDSGKKDANGRAVLVKQEEVINIANIQSRLGNSFRITGINNPNEARQL

SLLLRAGALIAPIQIVEERTIGPTLGMQNIEQGLEACLAGLLVSILFMIIFYKKFGLIATSALIANLILI

VGIMSLLPGATLSMPGIAGIVLTLAVAVDANVLINERIKEELSNGRTVQQAIDEGYRGAFSSIFDANITT

LIKVIILYAVGTGAIKGFAITTGIGVATSMFTAIVGTRAIVNLLYGGKRVKKLSI

>gi|30061902|ref|NP_836073.1| preprotein translocase subunit YajC [Shigella flexneri 2a str. 2457T]

MSFFISDAVAATGAPAQGSPMSLILMLVVFGLIFYFMILRPQQKRTKEHKKLMDSIAKGDEVLTNGGLVG
RVTKVAENGYIAIALNDTTEVVIKRDFVAAVLPKGTMKAL

>gi|30061896|ref|NP_836067.1| phosphate regulon sensor protein [Shigella flexneri 2a str. 2457T]
MLERLSWKRLVLELLLCCLPAFILGAFFGYLPWFLLASVTGLLIWHFWNLLRLSWWLWVDRSMTPPPGRG
SWEPLLYGLHQMQLRNKKRRRELGNLIKRFRSGAESLPDAVVLTTEEGGIFWCNGLAQQILGLRWPEDNG
QNILNLLRYPEFTQYLKTRDFSRPLNLVLNTGRHLEIRVMPYTHKQLLMVARDVTQMHQLEGARRNFFAN
VSHELRTPLTVLQGYLEMMDEQPLEGAVREKALHTMREQTQRMEGLVKQLLTLSKIEAAPTQLLNEKVDV
PMMLRVVEREAQTLSQKKQTFTFEIDNGLKVSGNEDQLRSAISNLVYNAVNHTPEGTHITVRWQRVPHGA
EFSVEDNGPGIAPEHIPRLTERFYRVDKARSRQTGGSGLGLAIVKHAVNHHESRLNIESTVGKGTRFSFV
IPERLIAKNSD

>gi|30061895|ref|NP_836066.1| transcriptional regulator PhoB [Shigella flexneri 2a str. 2457T]
MARRILVVEDEAPIREMVCFVLEQNGFQPVEAEDYDSAVNQLNEPWPDLILLDWMLPGGSGIQFIKHLKR
ESMTRDIPVVMLTARGEEEDRVRGLETGADDYITKPFSPKELVARIKAVMRRISPMAVEEVIKMQGLSLN
PTSHRVMAGEEPLEMGPTEFKLLHFFMTHPERVYSREQLLNHVWGTNVYVEDRTVDVHIRRLRKALEPGG
HDRMVQTVRGTGYRFSTRF

>gi|30061847|ref|NP_836018.1| hypothetical protein S0298 [Shigella flexneri 2a str. 2457T]

MTTIKLIVNSVSKSERESIIAALHGQSIFNGGGLSPLNKISPSHPPKPATVAVPEETEKKARDVNEKTAL

LKKKSATELGELATSINTIARDAHMEANLEMEIVPQGLRVLIKDDQNRNMFERGSAQIMPFFKTLLVELA

PVFDSLYNKIIITGHTDAMAYKNNIYNNWNLSGDRALSARRVLEEAGMPEDKVMQVSAMADQMLLDAKNP

QSAGNRRIEIMVLTKSASDTLYQYFGQHGDKVVQPLVQKLDKQQVLSQRMR

>gi|30061841|ref|NP_836012.1| phosphoheptose isomerase [Shigella flexneri 2a str. 2457T]

MYQDLIRNELNEAAETLANFLKDDANIHAIQRAAVLLADSFKAGGKVLSCGNGGSHCDAMHFAEELTGRY

RENRPGYPAIAISDVSHISCVGNDFGFNDIFSRYVEAVGREGDVLLGISTSGNSANVIKAIAAAREKGMK

VITLTGKDGGKMAGTADIEIRVPHFGYADRIQEIHIKVIHILIQLIEKEMVK

>gi|30061837|ref|NP_836008.1| Rhs-family protein [Shigella flexneri 2a str. 2457T]

MTARDYIWNADGEVGGINDKLRGCLVFSYDRSGWLTSRTGQMYDHDHYYYDKAGNLLTDEYQGAVMDNRL

PGYGRDRYRYNEWGELTERRDQQLEWNAQGQLTRVISSNSETRYQYDALGRRISKATSNLHTDRGERSRT

TFVWEGFRLLQETTWQGKRTYLYDAEQPYTPVAAITGRGESQKIWYYHTDLTGTVHEVTAPDGTLVWAGY

QAGFGENRGDISNSGAYFEQPQRLPGQYFDEETGLHYNLFRYYAPECGRFVSQDPIGLNGGLNLYAYAPN

PLGWIDPLGLNSLGIDSNNIFRGDGNYKGGGIGQPKSGTISAQDIIDHL

>gi|30061808|ref|NP_835979.1| hypothetical protein S0256 [Shigella flexneri 2a str. 2457T]

MADFTLSKSLFSGKYRNASSTPGNIAYALFVLFCFWAGAQLLNLLVHAPGVYERLMQVQETGRPRVEIGL

GVGTIFGLIPFLAGCLIFAVVALWLHWRHRRQ

>gi|30061806|ref|NP_835977.1| D-alanyl-alanine synthetase A [Shigella flexneri 2a str. 2457T]

MEKLRVGIVFGGKSAEHEVSLQSAKNIVDAIDKSRFDVVLLGIDKQGQWHVSDASNYLLNADDPAHIALR

PSATSLAQVPGKHEHQLIDAQNGQPLPTVDVIFPIVHGTLGEDGSLQGMLRVANLPFVGSDVLASAACMD

KDVTKRLLRDAGLNIAPFITLTRANRHNISFAEVESKLGLPLFVKPANQGSSVGVSKVTSEEQYAIAVDL

AFEFDHKVIVEQGIKGREIECAVLGNDNPQASTCGEIVLTSDFYAYDTKYIDEDGAKVVVPAAIAPEIND

KIRAIAVQAYQTLGCAGMARVDVFLTPENEVVINEINTLPGFTNISMYPKLWQASGLGYTDLITRLIELA

LERHAADNALKTTM

>gi|30061786|ref|NP_835957.1| IS911 orfA [Shigella flexneri 2a str. 2457T]

MTRWVKQLRDERQGKTPKASPITPEQIEIRELRKKLQRIEMENEILKRLRALDVRLPEQFSIIGKLRAHY

PVVTLCHVFGVHRSSYRYWKNRPEKPDGRRAVLRSQVLELHGISHGSAGARSIATMATRRGYQMGRWLAG

RLMKELGLVSCQQPTHRYKRGGHEHVAIPNYLERQFAVTEPNQVWCGDVTYIWTGKRWAYLAVVLDLFAR

KPVGWAMSFSPDSRLTMKALEMAWETRGKPGGVMFHSDQGSHYTSRQFRQLLWRYQIRQSMSRRGNCWDN

SPMERFFRSLKNEWMPVVGYVSFSEAAHAITDYIVGYYSALRPHEYNGGLPPNESENRYWKNSNSVASFC

>gi|30061782|ref|NP_835953.1| hypothetical protein S0230 [Shigella flexneri 2a str. 2457T]

MTQKYELIVKGIRNFENKVTVTLALRDKKRFDGEIFDLDISLDRVEGAALEFYEAAARRSIRQVFLDVAA

GLCEGDELLPETRPCSEARYTIKINSSDNSITGC

>gi|30061771|ref|NP_835942.1| outer membrane usher protein [Shigella flexneri 2a str. 2457T]

MDPRLLEYYNRELSYLRETGAEFAARHPKVAARLGMQGTDIADPYVERMVEAFSFLTARTQLKIDAEFPR

FTQRLLEVVSPNYVTPTPSMSVAQLHPDTEEGDLAKGFTVPRDTAFFSAIPEGESTACQFRSSQDVTLWP

LAIEEARLTAAPPDMPALHRYLPANIHVAGALRITLRTFGELTFSQLAGLDRLPFYLCGEERTASHLLEL

LHTSAIAPLAGIPGHFDGALDVNLQQPVMYEGLEPDQGLLPLAWNVFHGHNLLHEYFACPERFYFFTPTG

LSAGLQKIDGGVAEIVILLNRLPPDWLIHQTNAAQFSLFCTPVINLFPRVTARIDVTHSTTEQHLVVDRT

HPLDYEVFSVQEVEGLETDTTRKMAFRPLYHTRNNDEGNHGRYFSLRREPRRLSENARRYGTRTPYTGSE

VFLSLVDQYEAPYPENLRHITITAMVTNRDLPCLIARNGRDDLTVDAAIPVAGVGLIRPPRSPQPPMAER

EMAWRLIRQLSFNYLPLADLDHRTGGQALRDLLNLFIPAHDSPQSRQVRSLIGCKTTPVTRRLPGSGLLV

YGRGVSCELTVDEEGFSGISPYLFGLVLEHYIARHVSINTFSQMTLHSMQRGKIMTWPVRAGQRGSV

>gi|30061767|ref|NP_835938.1| DNA polymerase III subunit epsilon [Shigella flexneri 2a str. 2457T]

MSTAITRQIVLDTETTGMNQIGAHYEGHKIIEIGAVEVVNRRLTGNNFHVYLKPDRLVDPEAFGVHGIAD

EFLLDKPTFAEVADEFMDYIRGAELVIHNAAFDIGFMDYEFSLLKRDIPKTNTFCKVTDSLAVARKMFPG

KRNSLDALCARYEIDNSKRTLHGALLDAQILAEVYLAMTGGQTSMAFAMEGETQQQQGEATIQRIVRQAS

KLRVVFATDEELAAHEARLDLVQKKGGSCLWRA

>gi|30061761|ref|NP_835932.1| hypothetical protein S0203 [Shigella flexneri 2a str. 2457T]

MRKNTYAMRYVAGQPAERILPPGSFASIGQALPPGEPLSTEERIRILVWNIYKQQRAEWLSVLKNYGKDA

HLVLLQEAQTTPELVQFATANYLAADQVPAFVLPQHPSGVMTLSAAHPVYCCPLREREPILRLAKSALVT

VYPLPDTRLLMVVNIHAVNFSLGVDVYSKQLLPIGDQIAHHSGPVIMAGDFNAWSRRRMNALYRFAREMS

LRQVRFTDDQRRRAFGRPLDFVFYRGLNVSEASVLVTRASDHNPLLVEFSPGKPDK

>gi|30061757|ref|NP_835928.1| D,D-heptose 1,7-bisphosphate phosphatase [Shigella flexneri 2a str. 2457T]

MAKSVPAIFLDRDGTINVDHGYVHEIDNFEFIDGVIDAMRELKKMGFALVVVTNQSGIARGKFTEAQFET

LTEWMDWSLADRDVDLDGIYYCPHHPQGSVEEFRQVCDCRKPHPGMLLSARDYLHIDMAASYMVGDKLED

MQAAVAANVGTKVLVRTGKPITPEAENAADWVLNSLADLPQAIKKQQKPAQ

>gi|30061745|ref|NP_835916.1| tRNA(Ile)-lysidine synthetase [Shigella flexneri 2a str. 2457T]

MTLTLNRQLLTSRQILVAFSGGLDSTVLLHQLVQWRTENPGVTLRAIHVHHGLSANADAWVTHCENVCQQ

WQVPLVVERVQLAQEGLGIEAQARQARYQAFARTLLPGEVLVTAQHLDDQCETFLLALKRGSGPAGLSAM

AEVSEFAGTRLIRPLLARTRGELEQWALAHGLRWIEDESNQDDSYDRNFLRLRVVPLLQQRWPHFAEATA

RSATLCAEQESLLDELLADDLAHCQTSQGTLQIAPMLAMSDARRAAIIRRWLAGQNAPMPSRDALVRIWQ

EVALAREDASPCLRLGAFEIRRYQSQLWWIKSVTGQSETIVLWQTWLQPLELPAGLGTVQLTAGGDIRPP

RADEAVSVRFKAPGLLHIVGRNGGRKLKKIWQELGVPPWLRDTTPLLFYGETLIAAAGVFVTQEGVAEGE

NGVSFVWQKTLS

>gi|30061741|ref|NP_835912.1| DNA polymerase III subunit alpha [Shigella flexneri 2a str. 2457T]

MSEPRFVHLRVHSDYSMIDGLAKTAPLVKKAAALGMPALAITDFTNLCGLVKFYGAGHGAGIKPIVGADF

NVQCDLLGDELTHLTVLAANNTGYQNLALLISKAYQRGYGAAGPIIDRDWLIELNEGLILLSGGRMGDVG

RSLLRGNSALVDECVAFYEEHFPDRYFLELIRTGRPDEESYLHAAVELAEARGLPVVATNDVRFIDSSDF

DAHEIRVAIHDGFTLDDPKRPRNYSPQQYMRSEEEMCELFADIPEALANTVEIAKRCNVTVRLGEYFLPQ

FPTGDMSTEDYLVKRAKEGLEERLAFLFPDEEERLKRRPEYDERLETELQVINQMGFPGYFLIVMEFIQW

SKDNGVPVGPGRGSGAGSLVAYALKITDLDPLEFDLLFERFLNPERVSMPDFDVDFCMEKRDQVIEHVAD

MYGRDAVSQIITFGTMAAKAVIRDVGRVLGHPYGFVDRISKLIPPDPGMTLAKAFEAEPQLPEIYEADEE

VKALIDMARKLEGVTRNAGKHAGGVVIAPTKITDFAPLYCDEEGKHPVTQFDKSDVEYAGLVKFDFLGLR

TLTIINWALEMINKRRAKNGEPPLDIAAIPLDDKKSFDMLQRSETTAVFQLESRGMKDLIKRLQPDCFED

MIALVALFRPGPLQSGMVDNFIDRKHGREEISYPDVQWQHESLKSVLEPTYGIILYQEQVMQIAQVLSGY

TLGGADMLRRAMGKKKPEEMAKQRSVFAEGAEKNGINAELAMKIFDLVEKFAGYGFNKSHSAAYALVSYQ

TLWLKAHYPAEFMAAVMTADMDNTEKVVGLVDECWRMGLKILPPDINSGLYHFHVNDDGEIVYGIGAIKG

VGEGPIEAIIEARNKGGYFRELFDLCARTDTKKLNRRVLEKLIMSGAFDRLGPHRAALMNSLGDALKAAD

QHAKAEAIGQADMFGVLAEEPEQIEQSYASCQPWPEQVVLDGERETLGLYLTGHPINQYLKEIERYVGGV

RLKDMHPTERGKVITAAGLVVAARVMVTKRGNRIGICTLDDRSGRLEVMLFTDALDKYQQLLEKDRILIV

SGQVSFDDFSGGLKMTAREVMDIDEAREKYARGLAISLTDRQIDDQLLNRLRQSLEPHRSGTIPVHLYYQ

RADARARLRFGATWRVSPSDRLLNDLRGLIGSEQVELEFD

>gi|30061739|ref|NP_835910.1| lipid-A-disaccharide synthase [Shigella flexneri 2a str. 2457T]

MTDQRPLTIALVAGETSGDILGAGLIRALKERVPNARFVGVAGPRMQAEGCEAWYEMEELAVMGIVEVLG

RLRRLLHIRADLTKRFGELKPDVFVGIDAPDFNITLEGNLKKQGIKTIHYVSPSVWAWRQKRVFKIGRAT

DLVLAFLPFEKAFYDKYNVPCRFIGHTMADAMPLDPDKNGARDVLGIPYDAHCLALLPGSRGAEVEMLSA

DFLKTAQLLRQTYPDLEIVVPLVNAKRREQFERIKAEVAPDLSVHLLDGMGREAMVASDAALLASGTAAL

ECMLAKCPMVVGYRMKPFTFWLAKRLVKTDYVSLPNLLAGRELVKELLQEECEPQKLAAALLPLLANGKT

SHAMHDTFRELHQQIRCNADEQAAQAVLELAQ

>gi|30061738|ref|NP_835909.1| UDP-N-acetylglucosamine acyltransferase [Shigella flexneri 2a str. 2457T]

MIDKSAFVHPTAIVEEGASIGANAHIGPFCIVGPHVEIGEGTVLKSHVVVNGHTKIGRDNEIYQFASIGE

VNQDLKYAGEPTRVEIGDRNRIRESVTIHRGTVQGGGLTKVGSDNLLMINAHIAHDCTVGNRCILANNAT

LAGHVSVDDFAIIGGMTAVHQFCIIGAHVMVGGCSGVAQDVPPYVIAQGNHATPFGVNIEGLKRRGFSRE

AITAIRNAYKLIYRSGKTLDEVKPEIAELAETYPEVKAFTDFFARSTRGLIR

>gi|30061737|ref|NP_835908.1| (3R)-hydroxymyristoyl-ACP dehydratase [Shigella flexneri 2a str. 2457T]

MTTNTHTLQIEEILELLPHRFPFLLVDRVLDFEEGRFLRAVKNVSVNEPFFQGHFPGKPIFPGVLILEAM

AQATGILAFKSVGKLEPGELYYFAGIDEARFKRPVVPGDQMIMEVTFEKTRRGLTRFKGVALVDGKVVCE

ATMMCARSREA

>gi|30061736|ref|NP_835907.1| UDP-3-O-[3-hydroxymyristoyl] glucosamine N-acyltransferase [Shigella flexneri 2a str. 2457T]

MPSIRLADLAQQLDAELHGDGDIVITGVASMQSAQTGHITFMVNPKYREHLGLCQASAVVMTQDDLPFAK

SAALVVKNPYLTYARMAQILDTTPQPAQNIAPSAVIDATAKLGNNVSIGANAVIESGVELGDNVIIGAGC

FVGKNSKIGAGSRLWANVTIYHEIQIGQNCLIQSGTVVGADGFGYANDRGNWVKIPQIGRVIIGDRVEIG

ACTTIDRGALDDTVIGNGVIIDNQCQIAHNVVIGDNTAVAGGVIMAGSLKIGRYCMIGGASVINGHMEIC

DKVTVTGMGMVMRPITEPGVYSSGIPLQPNKVWRKTAALVMNIDDMSKRLKSLERKVNQQD

>gi|30061735|ref|NP_835906.1| periplasmic chaperone [Shigella flexneri 2a str. 2457T]

MKKWLLAAGLGLALATSAQAADKIAIVNMGSLFQQVAQKTGVSNTLENEFKGRASELQRMETDLQAKMKK

LQSMKAGSDRTKLEKDVMAQRQTFAQKAQAFEQDRARRSNEERGKLVTRIQTAVKSVANSQDIDLVVDAN

AVAYNSSDVKDITADVLKQVK

>gi|30061733|ref|NP_835904.1| zinc metallopeptidase RseP [Shigella flexneri 2a str. 2457T]

MLSFLWDLASFIVALGVLITVHEFGHFWVARRCGVRVERFSIGFGKALWRRTDKLGTEYVIALIPLGGYV

KMLDERAEPVVPELRHHAFNNKSVGQRAAIIAAGPVANFIFAIFAYWLVFIIGVPGVRPVVGEIAANSIA

AEAQIAPGTELKAVDGIETPDWDAVRLQLVDKIGDESTTITVAPFDSDQRRDVKLDLRHWAFEPDKEDPV

SSLGIRPRGPQIEPVLENVQPNSAASKAGLQAGDRIVKVDGQPLTQWVTFVMLVRDNPGKSLALEIERQG

SPLSLTLIPESKPGNGKAIGFVGIEPKVIPLPDEYKVVRQYGPFNAIVEATDKTWQLMKLTVSMLGKLIT

GDVKLNNLSGPISIAKGAGMTAELGVVYYLPFLALISVNLGIINLFPLPVLDGGHLLFLAIEKIKGGPVS

ERVQDFCYRIGSILLVLLMGLALFNDFSRL

>gi|30061730|ref|NP_835901.1| 1-deoxy-D-xylulose 5-phosphate reductoisomerase [Shigella flexneri 2a str. 2457T]

MKQLTILGSTGSIGCSTLDVVRHNPEHFRVVALVAGKNVTRMVEQCLEFSPRYAVMDDEASAKLLKTMLQ

QQGSRTEVLSGQQAACDMAALEEVDQVMAAIVGAAGLLPTLAAIRAGKTILLANKESLVTCGRLFMDAVK

QSKAQLLPVDSEHNAIFQSLPQPIQHNLGYADLEQNGVVSILLTGSGGPFRETPLRDLATMTPDQACRHP

NWSMGRKISVDSATMMNKGLEYIEARWLFNASASQMEVLIHPQSVIHSMVRYQDGSVLAQLGEPDMRTPI

AHTMAWPNRVNSGVKPLDFCKLSALTFAAPDYERYPCLKLAMEAFEQGQAATTALNAANEITVAAFLAQQ

IRFTDIAALNLSVLEKMDMREPQCVDDVLSVDANASEVARKEVMRLAS

>gi|30061728|ref|NP_835899.1| uridylate kinase [Shigella flexneri 2a str. 2457T]

MATNAKPVYKRILLKLSGEALQGTEGFGIDASILDRMAQEIKELVELGIQVGVVIGGGNLFRGAGLAKAG

MNRVVGDHMGMLATVMNGLAMRDALHRAYVNARLMSAIPLNGVCDSYSWAEAISLLRNNRVVILSAGTGN

PFFTTDSAACLRGIEIEADVVLKATKVDGVFTADPAKDPTATMYEQLTYSEVLEKELKVMDLAAFTLARD

HKLPIRVFNMNKPGALRRVVMGEKEGTLITE

>gi|30061724|ref|NP_835895.1| PII uridylyl-transferase [Shigella flexneri 2a str. 2457T]

MNTLPEQYANTALSTLPGQPQNPCAWPRDELTVCGIKAHIDTFQRWLGDAFDNGISAEQLIEARTEFIDQ

LLQRLWIEAGFSQIADLALVAVGGYGRGELHPLSDIDLLILSRKKLPDDQAQKVGELLTLLWDVKLEVGH

SVRTLEECMLEGLSDLTVATNLIESRLLIGDVALFLELQKHIFSEGFWPSDKFYAAKVEEQNQRHQRYHG

TSYNLEPDIKSSPGGLRDIHTLQWVARRHFGATSLDEMVGFGFLTSAERAELNECLHILWRIRFALHLVV

SRYDNRLLFDRQLSVAQRLNYSGEGNEPVERMMKDYFRVTRRVSELNQMLLQLFDEAILALPADEKPRPL

DDEFQLRGTLIDLRDETLFMRQPEAILRMFYTMVRNSAITGIYSTTLRQLRHARRHLQQPLCNIPQARKL

FLSILRHPGAVRRGLLPMHRHSVLGAYMPQWSHIVGQMQFDLFHAYTVDEHTIRVMLKLESFASEETRQR

HPLCVDVWPRLPSTELIFIAALFHDIAKGRGGDHSILGAQDVVHFAELHGLNSRETQLVAWLVRQHLLMS

VTAQRRDIQDPEVIKQFAEEVQTENRLRYLVCLTVADICATNETLWNSWKQSLLRELYFATEKQLRRGMQ

NTPDMRERVRHHQLQALALLRMDNIDEEALHQIWSRCRANYFVRHSPNQLAWHARHLLQHDLSKPLVLLS

PQATRGGTEIFIWSPDRPYLFAAVCAELDRRNLSVHDAQIFTTRDGMAMDTFIVLEPDGSPLSADRHEVI

RFGLEQVLTQSSWQPPQPRRQPAKLRHFTVETEVTFLPTHTDRKSFLELIALDQPGLLARVGKIFADLGI

SLHGARITTIGERVEDLFIIATADRRALNNELQQEVHQRLTEALNPNDKG

>gi|30061723|ref|NP_835894.1| 2,3,4,5-tetrahydropyridine-2,6-carboxylate N-succinyltransferase [Shigella flexneri 2a str. 2457T]

MQQLQNIIETAFERRAEITPANADTVTREAVNQVIALLDSGALRVAEKIDGQWVTHQWLKKAVLLSFRIN

DNQVIEGAESRYFDKVPMKFADYDEARFQKEGFRVVPPAAVRQGAFIARNTVLMPSYVNIGAYVDEGTMV

DTWATVGSCAQIGKNVHLSGGVGIGGVLEPLQANPTIIEDNCFIGARSEVVEGVIVEEGSVISMGVYIGQ

STRIYDRETGEIHYGRVPAGSVVVSGNLPSKDGKYSLYCAVIVKKVDAKTRGKVGINELLRTID

>gi|30061719|ref|NP_835890.1| 5'-methylthioadenosine/S-adenosylhomocysteine nucleosidase [Shigella flexneri 2a str. 2457T]

MKIGIIGAMEEEVTLLRDKIENRQTISLGGCEIYTGQLNGTEVALLKSGIGKVAAALGATLLLEHCKPDV

IINTGSAGGLAPTLKVGDIVVSDEARYHDADVTAFGYEYGQLPGCPAGFKADDKLIAAAEACIAELNLNA

VRGLIVSGDAFINGSVGLAKIRHNFPQAIAVEMEATAIAHVCHNFNVPFVVVRAISDVADQQSHLSFDEF

LAVAAKQSSLMVESLVQKLAHG

>gi|30061709|ref|NP_835880.1| penicillin-binding protein 1b [Shigella flexneri 2a str. 2457T]

MAGNDREPIGRKGKPTRPVKQKVSRRRYEDDDDYDDYDDYEDEEPMPRKGKGKGKGRKPRGKRGWLWLLL

KLAIVFAVLIAIYGVYLDQKIRSRIDGKVWQLPAAVYGRMVNLEPDMTISKNEMVKLLEATQYRQVSKMT

RPGEFTVQANSIEMIRRPFDFPDSKEGQVRARLTFDGDHLATIVNMENNRQFGFFRLDPRLITMISSPNG

EQRLFVPRSGFPDLLVDTLLATEDRHFYEHDGISLYSIGRAVLANLTAGRTVQGASTLTQQLVKNLFLSS

ERSYWRKANEAYMALIMDARYSKDRILELYMNEVYLGQSGDNEIRGFPLASLYYFGRPVEELSLDQQALL

VGMVKGASIYNPWRNPKLALERRNLVLRLLQQQQIIDQELYDMLSARPLGVQPRGGVISPQPAFMQLVRQ

ELQAKLGDKVKDLSGVKIFTTFDSVAQDAAEKAAVEGIPALKKQRKLSDLETAIVVVDRFSGEVRAMVGG

SEPQFAGYNRAMQARRSIGSLAKPATYLTALSQPKIYRLNTWIADAPIALRQPNGQVWSPQNDDRRYSES

GRVMLVDALTRSMNVPTVNLGMALGLPAVTETWIKLGVPKDQLHPVPAMLLGALNLTPIEVAQAFQTIAS

GGNRAPLSALRSVIAEDGKVLYQSFPQAERAVPAQAAYLTLWTMQQVVQRGTGRQLGAKYPNLHLAGKTG

TTNNNVDTWFAGIDGSTVTITWVGRDNNQPTKLYGASGAMSIYQRYLANQTPTPLNLVPPEDIADMGVDY

DGNFVCSGGMRVLPVWTSDPQSLCQQSEMQQQPSGNPFDQSSQPQQQPQQQPAQQEQKDSDGVAGWIKDM

FGSN

>gi|30061705|ref|NP_835876.1| DnaK transcriptional regulator DksA [Shigella flexneri 2a str. 2457T]

MQEGQNRKTSSLSILAIAGVEPYQEKPGEEYMNEAQLAHFRRILEAWRNQLRDEVDRTVTHMQDEAANFP

DPVDRAAQEEEFSLELRNRDRERKLIKKIEKTLKKVEDEDFGYCESCGVEIGIRRLEARPTADLCIDCKT

LAEIREKQMAG

>gi|30061702|ref|NP_835873.1| 2-amino-4-hydroxy-6-hydroxymethyldihydropteridine pyrophosphokinase [Shigella flexneri 2a str. 2457T]

MTVAYIAIGSNLASPLEQVNAALKALGDIPESRILAVSSFYRTPPLGPQDQPDYLNAAVALETSPAPEEL

LNHTQRIELQQGRVRKAERWGPRTLDLDIMLFGNEVINTERLTVPHYDMKNRGFMLWPLFEIAPELAFPD

GETLREVLHTRAFDKLSKW

>gi|30061699|ref|NP_835870.1| 3-methyl-2-oxobutanoate hydroxymethyltransferase [Shigella flexneri 2a str. 2457T]

MKPTTIASLQKYKQDKKRFATITAYDYSFAKLFADEGLNVMLVGDSLGMTVQGHDSTLPVTVADIAYHTA

AVRRGAPNCLLLADLPFMAYATPEQAFENAATVMRAGANMVKIEGGEWLVETVKMLTERAVPVCGHLGLT

PQSVNIFGGYKVQGRGNEASDRLLSDALALEAAGAQLLVLECVPVELAKRITEALAIPVIGIGAGNVTDG

QILVMHDAFGITGGHIPKFAKNFLAETGDIRAAVRQYMAEVESGVYPGEEHSFH

>gi|30061698|ref|NP_835869.1| pantoate--beta-alanine ligase [Shigella flexneri 2a str. 2457T]

MLIIETLPLLRQQIRRLRMEGKRVALVPTMGNLHNGHMKLVDEAKARADVVVVSIFVNPMQFDRPEDLAR

YPRTLQEDCEKLNKRKVDLVFAPSVKEIYPNGTETHTYVDVPGLSTMLEGASRPGHFRGVSTIVSKLFNL

VQPDIACFGEKDFQQLALIRKMVADMGFDIEIVGVPIMRAKDGLALSSRNGYLTAEQRKIAPGLYKVLSS

IADKLQAGERDLDEIITIAGQELNEKGFRADDIQIRDADTLLEVSETSKRAVILVAAWLGDARLIDNKMV

ELA

>gi|30061697|ref|NP_835868.1| hypothetical protein S0131 [Shigella flexneri 2a str. 2457T]

MDAPSTTPHDAVFKQFLMHAETARDFLEIHLPVELRELCDLNTLHLESGSFIEECLKGHSTDVLYSMQMQ

GNPGYLHVVIEHQSKPDKKMAFRMMRYSIAAMHRHLEAGHDKLPLVVPILFYQGEATPYPLSMCWFDMFY

SPELARRVYNSPFPLVDITITPDDEIMQHRRIAILELLQKHIRQRDLMLLLEQLVTLIDEGYTSGSQLVA

MQNYMLQRGHTEQADLFYGVLRDRETGGKSMMTLAQWFEEKGIEKGIQQGRQEERQEFALRLLSKGMSRE

DVAEMANLPLAEIDKVINLI

>gi|30061694|ref|NP_835865.1| PTS enzyme II B component [Shigella flexneri 2a str. 2457T]

MLGWVITCHDDRAQEILDALEKKHGALLQCRAVNFWRGLSSNMLSRMMCDALHEADSGEGVIFLTDIAGA

PPYRVASLLSHKHSRCEVISGVTLPLIEQMMACRETMTSSEFRECIVELGGPEVSSLWHQQQKNPPFVLK

HNLYEY

>gi|30061691|ref|NP_835862.1| carbonic anhydrase [Shigella flexneri 2a str. 2457T]

MKDIDTLISNNALWSKMLVEEDPGFFEKLAQAQKPRFLWIGCSDSRVPAERLTGLEPGELFVHRNVANLV

IHTDLNCLSVVQYAVDVLEVEHIIICGHYGCGGVQAAVENPELGLINNWLLHIRDIWFKHSSLLGEMPQE

RRLDTLCELNVMEQVYNLGHSTIMQSAWKRGQKVTIHGWAYGIHDGLLRDLDVTATNRETLEQRYRHGIS

NLKLKHANHK

>gi|30061689|ref|NP_835860.1| glucose dehydrogenase [Shigella flexneri 2a str. 2457T]

MAINNTASRRLLVTLTALFAALCGLYLLIGGGWLVAIGGSWYYPIAGLVMLGVAWMLWRSKRAALWLYAA

LLLGTMIWGVWEVGFDFWALTPRSDILVFFGIWLILPFVWRRLVIPASGAVAALVVALLISGGILTWAGF

NDPQEINGTLSADATPAEAISPVADQDWPAYGRNQEGQRFSPLKQIHADNVHKLKEAWVFRTGDVKQPND

PGEITNEVTPIKVGDTLYLCTAHQRLFALDAASGKEKWHYDPELKTNESFQHVTCRGVSYHEAKAETASP

EVMADCPRRIILPVNDGRLIAINAENGKLCETFANKGVLNLQSNMPDTKPGLYEPTSPPIITDKTIVMAG

SVTDNFSTRETSGVIRGFDVNTGELLWAFDPGAKDPNAIPSDEHTFTFNSPNSWAPAAYDAKLDLVYLPM

GVTTPDIWGGNRTPEQERYASSILALNATTGKLAWSYQTVHHDLWDMDLPAQPTLADITVNGQKVPIIYA

PAKTGNIFVLDRRNGELVVPAPEKPVPQGAAKGDYVTPTQPFSELSFRPTKDLSGADMWGATMFDQLVCR

VMFHQMRYEGIFTPPSEQGTLVFPGNLGMFEWGGISVDPNREVAIANPMALPFVSKLLPRGPGNPMEQPK

DAKGTGTESGIQPQYGVPYGVTLNPFLSPFGLPCKQPAWGYISALDLKTNEVVWKKRIGTPQDSMPFPMP

VPVPFNMGMPMLGGPISTAGNVLFIAATADNYLRAYNMSNGEKLWQGRLPAGGQATPMTYEVNGKQYVVI

SAGGHGSFGTKMGDYIVAYALPDDVK

>gi|30061687|ref|NP_835858.1| hypothetical protein S0121 [Shigella flexneri 2a str. 2457T]

MSIVLPLTGRSSRRHNLIDNNGRRLARSVLTFIFFKPLVEAMKTFFRTVLFGSLIAVCANSYALSESEAE

DMADLTAVFVFLKNDCGYQNLSNGQIRRALVFFAQQNQWDLSNYDTFDMKALGEDSYRDLSGIGIPVAKK

CKALARDSLSLLAYVK

>gi|30061683|ref|NP_835854.1| bifunctional aconitate hydratase 2/2-methylisocitrate dehydratase [Shigella flexneri 2a str. 2457T]

MLEEYRKHVAERAAEGIAPKPLDANQMAALVELLKNPPAGEEEFLLDLLTNRVPPGVDEAAYVKAGFLAA

IAKGEAKSPLLTPEKAIELLGTMQGGYNIHPLIDALDDAKLAPIAAKALSHTLLMFDNFYDVEEKAKAGN

EYAKQVMQSWADAEWFLNRPALAEKLTVTVFKVTGETNTDDLSPAPDAWSRPDIPLHALAMLKNAREGIE

PDQPGVVGPIKQIEALQQKGFPLAYVGDVVGTGSSRKSATNSVLWFMGDDIPHVPNKRGGGLCLGGKIAP

IFFNTMEDAGALPIEVNVSNLNMGDVIDVYPYKGEVRNHETGELLATFELKTDVLIDEVRAGGRIPLIIG

RGLTTKAREALGLPHSDVFRQAKDVAESDRGFSLAQKMVGRACGVKGIRPGAYCEPKMTSVGSQDTTGPM

TRDELKDLACLGFSADLVMQSFCHTAAYPKPVDVNTHHTLPDFIMNRGGVSLRPGDGVIHSWLNRMLLPD

TVGTGGDSHTRFPIGISFPAGSGLVAFAAATGVMPLDMPESVLVRFKGKMQPGITLRDLVHAIPLYAIKQ

GLLTVEKKGKKNIFSGRILEIEGLPDLKVEQAFELTDASAERSAAGCTIKLNKEPIIEYLSSNIVLLKWM

IAEGYGDRRTLERRIQGMEKWLANPELLEADADAEYAAVIDIDLAEIKEPILCAPNDPDDARPLSAVQGE

KIDEVFIGSCMTNIGHFRAAGKLLDAHKGQLPTRLWVAPPTRMDAAQLTEEGYYSVFGKSGARIEIPGCS

LCMGNQARVADGATVVSTSTRNFPNRLGTGANVFLASAELAAVAALIGKLPTPEEYQTYVAQVDKTAVDT

YRYLNFNQLSQYTEKADGVIFQTAV

>gi|30061679|ref|NP_835850.1| transcriptional regulator PdhR [Shigella flexneri 2a str. 2457T]

MAYSKIRQPKLSDVIEQQLEFLILEGTLRPGEKLPPERELAKQFDVSRPSLREAIQRLEAKGLLLRRQGG

GTFVQSSLWQSFSDPLVELLSDHPESQYDLLETRHALEGIAAYYAALRSTDEDKERIRELHHAIELAQQS

GDLDAESNAVLQYQIAVTEAAHNVVLLHLLRCMEPMLAQNVRQNFELLYSRREMLPLVSSHRTRIFEAIM

VGKPEEAREASHRHLAFIEEILLDRSREESRRERSLRRLEQRKN

>gi|30061676|ref|NP_835847.1| N-acetyl-anhydromuranmyl-L-alanine amidase [Shigella flexneri 2a str. 2457T]

MLLEQGWLVGARRVPSPHYDCRLDDETPTLLVVHNISLPPGEFGGPWIDALFTGTIDPQAHPFFAEIAHL

RVSAHCLIRRDGEIVQYVPFDKRAWHAGVSQYQGRERCNDFSIGIELEGTDTLAYTDAQYQQLAAVTRAL

IDRYPDIANNMTGHCDIAPDRKTDPGPAFDWARFRALVSKETT

>gi|30061666|ref|NP_835837.1| nucleoside triphosphate pyrophosphohydrolase [Shigella flexneri 2a str. 2457T]

MKKLQIAVGIIRNENNEIFITRRAADAHMANKLEFPGGKIEMGETPEQAVVRELQEEVGITPQHFSLFEK

LEYEFPDRHITLWFWLVESWEGVPWGKEGQPGEWMSLVGLNADDFPPANEPVIAKLKRL

>gi|30061665|ref|NP_835836.1| preprotein translocase subunit SecA [Shigella flexneri 2a str. 2457T]

MLIKLLTKVFGSRNDRTLRRMRKVVNIINAMEPEMEKLSDEELKGKTAEFRARLEKGEVLENLIPEAFAV

VREASKRVFGMRHFDVQLLGGMVLNERCIAEMRTGEGKTLTATLPAYLNALTGKGVHVVTVNDYLAQRDA

ENNRPLFEFLGLTVGINLPGMPAPAKREAYAADITYGTNNEYGFDYLRDNMAFSPEERVQRKLHYALVDE

VDSILIDEARTPLIISGPAEDSSEMYKRVNKIIPHLIRQEKEDSETFQGEGHFSVDEKSRQVNLTERGLV

LIEELLVKEGIMDEGESLYSPANIMLMHHVTAALRAHALFTRDVDYIVKDGEVIIVDEHTGRTMQGRRWS

DGLHQAVEAKEGVQIQNENQTLASITFQNYFRLYEKLAGMTGTADTEAFEFSSIYKLDTVVVPTNRPMIR

KDLPDLVYMTEAEKIQAIIEDIKERTAKGQPVLVGTISIEKSELVSNELTKAGIKHNVLNAKFHANEAAI

VAQAGYPAAVTIATNMAGRGTDIVLGGSWQAEVAALENPTAEQIEKIKADWQVRHDAVLEAGGLHIIGTE

RHESRRIDNQLRGRSGRQGDAGSSRFYLSMEDALMRIFASDRVSGMMRKLGMKPGEAIEHPWVTKAIANA

QRKVESRNFDIRKQLLEYDDVANDQRRAIYSQRNELLDVSDVSETINSIREDVFKATIDAYIPPQSLEEM

WDIPGLQERLKNDFDLDLPITEWLDKEPELHEETLRERILAQSIEVYQRKEEVVGAEMMRHFEKGVMLQT

LDSLWKEHLAAMDYLRQGIHLRGYAQKDPKQEYKRESFSMFAAMLESLKYEVISTLSKVQVRMPEEVEEL

EQQRRMEAERLAQMQQLSHQDDDSAAAAALAAQTGERKVGRNDPCPCGSGKKYKQCHGRLQ

>gi|30061663|ref|NP_835834.1| UDP-3-O-[3-hydroxymyristoyl] N-acetylglucosamine deacetylase [Shigella flexneri 2a str. 2457T]

MIKQRTLKRIVQATGVGLHTGKKVTLTLRPAPANTGVIYRRTDLNPPVDFPADAKSVRDTMLCTCLVNEH

DVRISTVEHLNAALAGLGIDNIVIEVNAPEIPIMDGSAAPFVYLLLDAGIDELNCAKKFVRIKETVRVED

GDKWAEFKPYNGFSLDFTIDFNHPAIDSSNQRYAMNFSADAFMRQISRARTFGFMRDIEYLQSRGLCLGG

SFDCAIVVDDYRVLNEDGLRFEDEFVRHKMLDAIGDLFMCGHNIIGAFTAYKSGHALNNKLLQAVLAKQE

AWEYVTFQDDAELPLAFKAPSAVLA

>gi|30061662|ref|NP_835833.1| cell division protein FtsZ [Shigella flexneri 2a str. 2457T]

MFEPMELTNDAVIKVIGVGGGGGNAVEHMVRERIEGVEFFAVNTDAQALRKTAVGQTIQIGSGITKGLGA
GANPEVGRNAADEDRDALRAALEGADMVFIAAGMGGGTGTGAAPVVAEVAKDLGILTVAVVTKPFNFEGK
KRMAFAEQGITELSKHVDSLITIPNDKLLKVLGRGISLLDAFGAANDVLKGAVQGIAELITRPGLMNVDF
ADVRTVMSEMGYAMMGSGVASGEDRAEEAAEMAISSPLLEDIDLSGARGVLVNITAGFDLRLDEFETVGN
TIRAFASDNATVVIGTSLDPDMNDELRVTVVATGIGMDKRPEITLVTNKQVQQPVMDRYQQHGMAPLTQE
QKPVAKVVNDNAPQTAKEPDYLDIPAFLRKQAD

>gi|30061661|ref|NP_835832.1| cell division protein FtsA [Shigella flexneri 2a str. 2457T]
MIKATDRKLVVGLEIGTAKVAALVGEVLPDGMVNIIGVGSCPSRGMDKGGVNDLESVVKCVQRAIDQAEL
MADCQISSVYLALSGKHISCQNEIGMVPISEEEVTQEDVENVVHTAKSVRVRDEHRVLHVIPQEYAIDYQ
EGIKNPVGLSGVRMQAKVHLITCHNDMAKNIVKAVERCGLKVDQLIFAGLASSYSVLTEDERELGVCVVD
IGGGTMDIAVYTGGALRHTKVIPYAGNVVTSDIAYAFGTPPSDAEAIKVRHGCALGSIVGKDESVEVPSV
GGRPPRSLQRQTLAEVIEPRYTELLNLVNEEILQLQEKLRQQGVKHHLAAGIVLTGGAAQIEGLAACAQR
VFHTQVRIGAPLNITGLTDYAQEPYYSTAVGLLHYGKESHLNGEAEVEKRVTASVGSWIKRLNSWLRKEF

>gi|30061660|ref|NP_835831.1| cell division protein FtsQ [Shigella flexneri 2a str. 2457T]
MSQAALNTRNSEEEVSSRRNNGTRLAGILFLLTVLTTVLVSGWVVLSWMEDAQRLPLSKLVLTGERHYTR
NDDIRQSILALGEPGTFMTQDVNIIQTQIEQRLPWIKQVSVRKQWPDELKIHLVEYVPIARWNDQHMVDA
EGNTFSVPPDRTSKQVLPMLYGPEGSANEVLQGYREMGQMLAKDRFTLKEAAMTARRSWQLTLNNDIKLN
LGRGDTMKRLVRFVELYPVLQQQAQTDGKRISYVDLRYDSGAAVGWAPLPPEESTQQQNQAQAEQQ

>gi|30061659|ref|NP_835830.1| D-alanine--D-alanine ligase [Shigella flexneri 2a str. 2457T]
MTDKIAVLLGGTSAEREVSLNSGAAVLAGLREGGIDAYPVDPKEVDVTQLKSMGFQKVFIALHGPGGEDG
TLQGMLELMGLPYTGSGVMASALSIDKLRSKLLWQGAGLPVAPWVALTRAEFEKGLSDKQLAEISALGLP
VIVKPSREGSSVGMSKVVAENALQDALRLAFQHDEEVLIEKWLSGPEFTVAILGEEILPSIRIQPSGTFY

DYEAKYLSDETQYFCPAGLEASQEANLQALVLKAWTTLGCKGWGRIDVMLDSDGQFYLLEANTSPGMTSH

SLVPMAARQAGMSFSQLVVRILELAD

>gi|30061658|ref|NP_835829.1| UDP-N-acetylmuramate--L-alanine ligase [Shigella flexneri 2a str. 2457T]

MNTQQLAKLRSIVPEMRRVRHIHFVGIGGAGMGGIAEVLANEGYQISGSDLAPNPVTQQLLNLGATIYFN

HRPENVRDASVVVVSSAISADNPEIVAAHEARIPVIRRAEMLAELMRFRHGIAIAGTHGKTTTTAMVSSI

YAEAGLDPTFVNGGLVKAAGVHARLGHGRYLIAEADESDASFLHLQPMVAIVTNIEADHMDTYQGDFENL

KQTFINFLHNLPFYGRAVMCVDDPVIRELLPRVGRQTTTYGFSEDADVRVEDYQQIGPQGHFTLLRQDKE

PMCVTLNAPGRHNALNAAAAVAVATEEGIDDEAILRALESFQGTGRRFDFLGEFPLEPVNGKSGTAMLVD

DYGHHPTEVDATIKAARAGWPDKNLVMLFQPHRFTRTRDLYDDFANVLTQVDTLLMLEVYPAGEAPIPGA

DSRSLCRTIRGRGKIDPILVPDPAQVAEMLAPVLTGNDLILVQGAGNIGKIARSLAEIKLKPQTPEEEQH

D

>gi|30061657|ref|NP_835828.1| undecaprenyldiphospho-muramoylpentapeptide beta-N-acetylglucosaminyltransferase [Shigella flexneri 2a str. 2457T]

MSGQGKRLMVMAGGTGGHVFPGLAVAHYLMAQGWQVRWLGTADRMEADLVPKHGIEIDFIRISGLRGKGI

KALIAAPLRIFNAWRQARAIMKAYKPDVVLGMGGYVSGPGGLAAWSLGIPVVLHEQNGIAGLTNKWLAKI

ATKVMQAFPGAFPNAEVVGNPVRTDVLALPLPQQRLAGREGPVRVLVVGGSQGARILNQTMPQVAAKLGD

SVTIWHQSGKGSQQSVEQAYAEAGQPQHKVTEFIDDMAAAYAWADVVVCRSGALTVSEIAAAGLPALFVP

FQHKDRQQYWNALPLEKAGAAKIIEQPQLSVDAVANTLAGWSRETLLTMAERARAASIPDATERVANEVS

RAARA

>gi|30061656|ref|NP_835827.1| cell division protein FtsW [Shigella flexneri 2a str. 2457T]

MRLSLPRLKMPRLPGFSILVWISTALKGWVMGSREKDTDSLIMYDRTLLWLTFGLAAIGFIMVTSASMPI

GQRLTNDPFFFAKRDGVYLILAFILAIITLRLPMEFWQRYSATMLLGSIILLMIVLVVGSAVKGASRWID

LGLLRIQPAELTKLSLFCYIANYLVRKGDEVRNNLRGFLKPMGVILVLAVLLLAQPDLGTVVVLFVTTLA

MLFLAGAKLWQFIAIIGMGISAVVLLILAEPYRIRRVTAFWNPWEDPFGSGYQLTQSLMAFGRGELWGQG

LGNSVQKLEYLPEAHTDFIFAIIGEELGYVGVVLALLMVFFVAFRAMSIGRKALEIDHRFSGFLACSIGI

WFSFQALVNVGAAAGMLPTKGLTLPLISYGGSSLLIMSTAIMMLLRIDYETRLEKAQAFVRGSR

>gi|30061655|ref|NP_835826.1| UDP-N-acetylmuramoyl-L-alanyl-D-glutamate synthetase [Shigella flexneri 2a str. 2457T]

MADYQGKNVVIIGLGLTGLSCVDFFLARGVTPRVMDTRMTPPGLDKLPEAVERHTGSLNDEWLMAADLIV

ASPGIALAHPSLSAAADAGIEIVGDIELFCREAQAPIVAITGSNGKSTVTTLVGEMAKAAGVNVGVGGNI

GLPALMLLDDECELYVLELSSFQLETTSSLQAVAATILNVTEDHMDRYPFGLQQYRAAKLRIYENAKVCV

VNADDALTMPIRGADERCVSFGVNMGDCHLNHQQGETWLRVKGEKVLNVKEMKLSGQHNYTNALVALALA

DAAGLPRASSLKALTTFTGLPHRFEVVLEHNGVRWINDSKATNVGSTEAALNGLHVDGTLHLLLGGDGKS

ADFSPLVRYLNGDNVRLYCFGRDGAQLAALRPEVAEQTETMEQAMRLLAPRVQPGDMVLLSPACASLDQF

KNFEQRGNEFARLAKELG

>gi|30061654|ref|NP_835825.1| phospho-N-acetylmuramoyl-pentapeptide-transferase [Shigella flexneri 2a str. 2457T]

MLVWLAEHLVKYYSGFNVFSYLTFRAIVSLLTALFISLWMGPRMIAHLQKLSFGQVVRNDGPESHFSKRG

TPTMGGIMILTAIVISVLLWAYPSNPYVWCVLVVLVGYGVIGFVDDYRKVVRKDTKGLIARWKYFWMSVI

ALGVAFALYLAGKDTPATQLVVPFFKDVMPQLGLFYILLAYFVIVGTGNAVNLTDGLDGLAIMPTVFVAG

GFALVAWATGNMNFASYLHIPYLRHAGELVIVCTAIVGAGLGFLWFNTYPAQVFMGDVGSLALGGALGII

AVLLRQEFLLVIMGGVFVVETLSVILQVGSFKLRGQRIFRMAPIHHHYELKGWPEPRVIVRFWIISLMLV

LIGLATLKVR

>gi|30061653|ref|NP_835824.1| UDP-N-acetylmuramoyl-tripeptide--D-alanyl-D-alanine ligase [Shigella flexneri 2a str. 2457T]

MISVTLSQLTDILNGELQGADITLDAVTTDTRKLTPGCLFVALKGERFDAHDFADQAKAGGAGALLVSRP

LDIDLPQLIVKDTRLAFGELAAWVRQQVPALVVALTGSSGKTSVKEMTAAILSQCGNTLYTAGNLNNDIG

VPMTLLRLTPEYDYAVIELGANHQGEIAWTVSLTRPEAALVNNLAAAHLEGFGSLAGVAKAKGEIFSGLP

ENGIAIMNADNNDWLNWQSVIGSRKVWRFSPNAANSDFTATNIHVTSHGMEFTLQTPTGSVDVLLPLPGR

HNIANALAAAALSMSVGATLDAIKAGLANLKAVPGRLFPIQLAENQLLLDDSYNANVGSMTAAVQVLAEM

PGYRVLVVGDMAELGAESEACHVQVGEAAKAAGIDRVLSMGKQSHAISTASGVGEHFADKTALITRLKSL

IAEQQVITILVKGSRSAAMEEVVRALQENGTC

>gi|30061652|ref|NP_835823.1| UDP-N-acetylmuramoylalanyl-D-glutamate--2,6-diaminopimelate ligase [Shigella flexneri 2a str. 2457T]

MADRNLRDLLAPWVPDAPSRALREMTLDSRVAAAGDLFVAVVGHQADGRRYIPQAIAQGVAAIIAEAKDE

ATDGEIREMHGVPVIYLSQLNERLSALAGRFYHEPSDNLRLVGVTGTNGKTTTTQLLAQWSQLLGETSAV

MGTVGNGLLGKVIPTENTTGSAVDVQHELAGLVDQGATFCAMEVSSHGLVQHRVAALKFPASVFTNLSRD

HLDYHGDMEHYEAAKWLLYSEHHCGQAIINADDEVGRRWLAKLPDAVAVSMEDHINPNCHGRWLKATEVN

YHDSGATIRFSSSWGDGEIESHLMGAFNVSNLLLALATLLALGYPLADLLKTAARLQPVCGRMEVFTAPG

KPTVVVDYAHTPDALEKALQAARLHCAGKLWCVFGCGGDRDKGKRPLMGAIAEEFADVAVVTDDNPRTEE

PRAIINDILAGMLDAGHAKVMEGRAEAVTCAVMQAKENDVVLVAGKGHEDYQIVGNQRLDYSDRVTVARL

LGGIA

>gi|30061651|ref|NP_835822.1| penicillin-binding protein 3; peptidoglycan synthetase [Shigella flexneri 2a str. 2457T]

MKAAAKTQKPKRQEEHANFISWRFALLCGCILLALAFLLGRVAWLQVISPDMLVKEGDMRSLRVQQVSTS

RGMITDRSGRPLAVSVPVKAIWADPKEVHDAGGISVGDRWKALANALNIPLDQLSARINANPKGRFIYLA

RQVNSDMADYIKKLKLPGIHLREESRRYYPSGEVTAHLIGFTNVDSQGIEGVEKSFDKWLTGQPGERIVR

KDRYGRVIEDISSTDSQAAHNLALSIDERLQALVYRELNNAVAFNKAESGSAVLVDVNTGEVLAMANSPS

YNPNNLSGTPKEAMRNRTITDVFEPGSTVKPMVVMTALQRGVVRENSVLNTIPYRINGHEIKDVARYSEL

TLTGVLQKSSNVGVSKLALTMPSSALVDTYSRFGLGKATNLGLVGERSGLYPQKQRWSDIERATFSFGYG

LMVTPLQLARVYATIGSYGIYRPLSITKVDPPVPGERVFPESIVRTVVHMMESVALPGGGGVKAAIKGYR

IAIKTGTAKKVGPDGRYINKYIAYTAGVAPASQPRFALVVVINDPQAGKYYGGAVSAPVFGAIMGGVLRT

MNIEPDALTTGDKNEFVINQGEGTGGRS

>gi|30061650|ref|NP_835821.1| cell division protein FtsL [Shigella flexneri 2a str. 2457T]

MISRVTEALSKVKGSMGSHERHALPGVIGDDLLRFGKLPLCLFICIILTAVTVVTTAHHTRLLTAQREQL

VLERDALDIEWRNLILEENALGDHSRVERIATEKLQMQHVDPSQENIVVQK

>gi|30061645|ref|NP_835816.1| DNA-binding transcriptional regulator FruR [Shigella flexneri 2a str. 2457T]

MKLDEIARLAGVSRTTASYVINGKAKQYRVSDKTVEKVMAVVREHNYHPNAVAAGLRAGRTRSIGLVIPD

LENTSYTRIANYLERQARQRGYQLLIACSEDQPDNEMRCIEHLLQRQVDAIIVSTSLPPEHPFYQRWAND

PFPIVALDRALDREHFTSVVGADQDDAEMLAEELRKFPAETVLYLGALPELSVSFLREQGFRTAWKDDPR

EVHFLYANSYEREAAAQLFEKWLETHPMPQALFTTSFALLQGVMDVTLRRDGKLPSDLAIATFGDNELLD

FLQCPVLAVAQRHRDVAERVLEIVLASLDEPRKPKPGLTRIKRNLYRRGVLSRS

>gi|30061634|ref|NP_835805.1| transcriptional regulator SgrR [Shigella flexneri 2a str. 2457T]

MPSARLQQQFIRLWQCCEGKSQDTTLNELAALLSCSRRHMRTLLNTMQDRGWLTWEAEVGRGKRSRLTFL

YTGLALQQQRAEDLLEQDRIDQLVQLVGDKATVRQMLVSHLGRSFRQGRHILRVLYYRPLRNLLPGSALR

RSETHIARQIFSSLTRINEENGELEADIAHHWQQISPLHWRFFLRPGVHFHHGRELEMDDVIASLKRINT

LPLYSHITDIVSPTPWTLDIHLTQPDRWLPLLLGQVPAMILPCEWETLSNFASHPIGTGPYAVIRNSTNQ

LKIQAFDDFFGYRALIDEVNVWVLPEIADEPAGGLMLKGPQGEKKEIESRLEEGCYYLLFDSRTHRGANQ

QVRDWVSYVLSPTNLVYFAEEQYQQLWFPAYGLLPRWHHARTIKSEKPAGLESLTLTFYQDHSEHRVIAG

IMQQILASHQVTLEIKEISYDQWHEGEIESDIWLNSANFTLPLDFSLFAHLCEVPLLQHCIPIDWQADAA

RWRNGEMNLANWCQQLVASKAMVPLIHHWLIIQGQRSMRGLRMNTLGWFDFKSAWFAPPDP

>gi|30061632|ref|NP_835803.1| thiamine transporter membrane protein [Shigella flexneri 2a str. 2457T]

MATRRQPLIPGWLIPGVSAATLVVAVALAAFLALWWNAPQGNWVAVWQDSYLWHVVRFSFWQAFLSALLS

VVPAIFLARALYRRRFPGRLALLRLCAMTLILPVLVAVFGILSVYGRQGWLASLCQSLGLEWTFSPYGLQ

GILLAHVFFNLPMASRLLLQALENIPGEQRQLAAQLGMRGWHFFRFVEWPWLRRQIPPVAALIFMLCFAS

FATVLSLGGGPQATTIELAIYQALSYDYDPARAAMLALLQMVCCLGLVLLSQRLSKAIAPGTTLLQGWRD

PDDRLHSRICDTVLIVLALLLLLPPLLAVIVDGVNRQLPEVLAQPVLWQALWTSLRIALAAGVLCVVLTM

MLLWSSRELRARQKMLAGQALEMSGMLILAMPGIVLATGFFLLLNNTIGLPQSADGIVIFTNALMAIPYA

LKVLENPMRDITARYSMLCQSLGIEGWSRLKVVELRALKRPLAQALAFACVLSIGDFGVVALFGNDDFRT

LPFYLYQQIGSYRSQDGAVTALILLLLCFLLFTVIEKLPGRNVKTD

>gi|30061630|ref|NP_835801.1| hypothetical protein S0062 [Shigella flexneri 2a str. 2457T]

MQALLEHFITQSTVYSLMAVVLVAFLESLALVGLILPGTVLMAGLGALIGSGELSFWHAWLAGIVGCLLG

DWISFWLGWRFKKPLHRWSFLKKNKALLDKTEHALHQHSMFTILVGRFVGPTRPLVPMVAGMLDLPVAKF

ITPNIIGCLLWPPFYFLPGILAGAAIDIPAGMQSGEFKWLLLATAVFLWVGGWLCWRLWRSGKATDRLSH

YLSRGRLLWLTPLISAIGVVALVVLIRHPLMPVYIDILRKVVGV

>gi|30061621|ref|NP_835792.1| organic solvent tolerance protein [Shigella flexneri 2a str. 2457T]

MKKRIPTLLATMIATALYSQQGLAADLASQCMLGVPSYDRPLVQGDTNDLPVTINADHAKGDYPDDAVFT

GSVDIMQGNSRLQADEVQLHQKEAPGQPEPVRTVDALGNVHYDDNQVILKGPKGWANLNTKDTNVWEGDY

QMVGRQGRGKADLMKQRGENRYTILDNGSFTSCLPGSDTWSVVGSEIIHDREEQVAEIWNARFKVGPVPI

FYSPYLQLPVGDKRRSGFLIPNAKYTTTNYFEFYLPYYWNIAPNMDATITPHYMHRRGNIMWENEFRYLS

QAGAGLMELDYLPSDKVYEDEHPNDDSSRRWLFYWNHSGVMDQVWRFNVDYTKVSDPSYFNDFDNKYGSS

TDGYATQKFSVGYAVQNFNATVSTKQFQVFSEQNTSSYSAEPQLDVNYYQNDVGPFDTRIYGQAVHFVNT

RDDMPEATRVHLEPTINLPLSNNWGSINTEAKFLATHYQQTNLDWYNSRNTTKLDESVNRVMPQFKVDGK

MVFERDMEMLAPGYTQTLEPRAQYLYVPYRDQSDIYNYDSSLLQSDYSGLFRDRTYGGLDRIASANQVTT

GVTSRIYDDAAVERFNISVGQIYYFTESRTGDDNITWENDDKTGSLVWAGDTYWRISERWGLRGGIQYDT

RLDNVATSNSSIEYRRDEDRLVQLNYHYASPEYIQATLPKYYSTAEQYKNGISQVGAVASRPIADRWSIV

GAYYYDTNANKQADSMLGVQYSSCCYAIRVGYERKLNGWDNDKQHAVYDNAIGFNIELRGLSSNYGLGTQ

EMLRSNILPYQNTL

>gi|30061619|ref|NP_835790.1| 4-hydroxythreonine-4-phosphate dehydrogenase [Shigella flexneri 2a str. 2457T]

MVKTQRVVITPGEPAGIGPDLVVQLAQREWPVELVVCADATLLTDRAAMLGLPLTLRPYSPNSPAQPQTT

GTLTLLPVALRESVTAGQLAIENGHYVVETLARACDGCLNGEFAALITGPVHKGVINDASIPFTGHTEFF

EERSQAKKVVMMLATEELRVALATTHLPLRDIADAITPALLHEVIAILHHDLRTKFGIAEPRILVCGLNP

HAGEGGHMGTEEIDTIIPVLDELRAQGMKLNGPLPADTLFQPKYLDNADAVLAMYHDQGLPVLKYQGFGR

GVNITLGLPFIRTSVDHGTALELAGRGEADVGSFITALNLAIKMIVNTQ

>gi|30061603|ref|NP_835774.1| carnitine operon protein CaiE [Shigella flexneri 2a str. 2457T]

MERTLTTVSYYAFEGLIPVVHPTAFVHPSAVLIGDVIVGAGVYIGPLASLRGDYGRLIVQAGANIQDGCI

MHGYCDTDTIVGENGHIGHGAILHGCVIGRDALVGMNSVIMDGAVIGEESIVAAMSFVKAGFSGEKRQLL

MGTPARAVRSVSDDELHWKRLNTKEYQDLVGRCHASLHETQPLRQMEENRPRLQGTTDVTPKR

>gi|30061599|ref|NP_835770.1| dihydrodipicolinate reductase [Shigella flexneri 2a str. 2457T]

MHDANIRVAIAGAGGRMGRQLIQAALALEGVQLGAALEREGSSLLGSDAGELAGAGKTGVTVQSSLDAIK

DDFDVFIDFTRPEGTLNHLAFCRQHGKGMVIGTTGFDEAGKQAIRDAAADIAIVFAANFSVGVNVMLKLL

EKAAKVMGDYTDIEIIEAHHRHKVDAPSGTALAMGEAIAHALDKDLKDCAVYSREGHTGERVPGTIGFAT

VRAGDIVGEHTAMFADIGERLEITHKASSRMTFANGAVRSALWLSGKESGLFDMRDVLDLNNL

>gi|30061597|ref|NP_835768.1| 4-hydroxy-3-methylbut-2-enyl diphosphate reductase [Shigella flexneri 2a str. 2457T]

MQILLANPRGFCAGVDRAISIVENALAIYGAPIYVRHEVVHNRYVVDSLRERGAIFIEQISEVPDGAILI

FSAHGVSQAVRNEAKSRDLTVFDATCPLVTKVHMEVARASRRGEESILIGHAGHPEVEGTMGQYSNPEGG

MYLVESPDDVWKLTVKNEEKLSFMTQTTLSVDDTSDVIDALRKRFPKIVGPRKDDICYATTNRQEAVRAL

AEQAEVVLVVGSKNSSNSNRLAELAQRMGKHAFLIDDAKDIQEEWVKEVKCVGVTAGASAPDILVQNVVA

RLQQLGGGEAIPLEGREENIVFEVPKELRVDIREVD

>gi|30061595|ref|NP_835766.1| lipoprotein signal peptidase [Shigella flexneri 2a str. 2457T]

MSQSICSTGLRWLWLVVVVLIIDLGSKYLILQNFALGDTVPLFPSLNLHYARNYGAAFSFLADSGGWQRW

FFAGIAIGISVLLAVMMYRSKATQKLNNIAYALIIGGALGNLFDRLWHGFVVDMIDFYVGDWHFATFNLA

DTAICVGAALIVLEGFLPSKAKKQ

>gi|30061593|ref|NP_835764.1| bifunctional riboflavin kinase/FMN adenylyltransferase [Shigella flexneri 2a str. 2457T]

MKLIRGIHNLSQAPQEGCVLTIGNFDGVHRGHRALLQGLQEEGRKRNLPVMVMLFEPQPLELFATDKAPA

RLTRLREKLRYLAECGVDYVLCVRFDRRFAALTAQNFISDLLVKHLRVKFLAVGDDFRFGAGREGDFLLL

QKAGMEYGFDITSTQTFCEGGVRISSTAVRQALADDNLALAESLLGHPFAISGRVVHGDELGRTIGFPTA

NVPLRRQVSPVKGVYAVEVLGLGEKPLPGVANIGTRPTVAGIRQQLEVHLLDVAMDLYGRHIQVVLRKKI

RNEQRFASLDELKAQIARDELTAREFFGLTKPA

>gi|30061587|ref|NP_835758.1| pH-dependent sodium/proton antiporter [Shigella flexneri 2a str. 2457T]

MKHLHRFFSSDASGGIILIIAAILAMIMANSGATSGWYHDFLETPVQLRVGSLEINKNMLLWINDALMAV

FFLLVGLEVKRELMQGSLASLRQAAFPVIAAIGGMIVPALLYLAFNYADPITREGWAIPAATDIAFALGV

LALLGSRVPLVLKIFLMALAIIDDLGAIIIIALFYTNDLSMASLGVAAVAIAVLAVLNLCGVRRTGVYIL

VGVVLWTAVLKSGVHATLAGVIVGFFIPLKEKHGRSPAKRLEHVLHPWVAYLILPLFAFANAGVSLQGVT

LDGLTSILPLGIIAGLLIGKPLGISLFCWLALRLKLAHLPEGTTYQQIMVVGILCGIGFTMSIFIASLAF

GSVDPELINWAKLGILVGSISSAVIGYSWLRVRLRPSV

>gi|30061573|ref|NP_835744.1| bifunctional aspartokinase I/homeserine dehydrogenase I [Shigella flexneri 2a str. 2457T]

MRVLKFGGTSVANAERFLRVADILESNARQGQVATVLSAPAKITNHLVAMIEKTISGQDALPNISDAERI

FAELLTGLAAAQPGFPLAQLKTFVDQEFAQIKHVLHGISLLGQCPDSINAALICRGEKMSIAIMAGVLEA

RGHNVTVIDPVEKLLAVGHYLESTVDIAESTRRIAASRIPADHMVLMAGFTAGNEKGELVVLGRNGSDYS

AAVLAACLRADCCEIWTDVDGVYTCDPRQVPDARLLKSMSYQEAMELSYFGAKVLHPRTITPIAQFQIPC

LIKNTGNPQAPGTLIGASRDEDELPVKGISNLNNMAMFSVSGPGMKGMVGMAARVFAAMSRARISVVLIT

QSSSEYSISFCVPQSDCVRAERAMQEEFYLELKEGLLEPLAVTERLAIISVVGDGMRTLRGISAKFFAAL

ARANINIVAIAQGSSERSISVVVNNDDATTGVRVTHQMLFNTDQVIEVFVIGVGGVGGALLEQLKRQQSW

LKNKHIDLRVCGVANSKALLTSVHGLNLENWQEELAQAKEPFNLGRLIRLVKEYHLLNPVIVDCTSSQAV

ADQYADFLREGFHVVTPNKKANTSSMDYYHQLRYAAEKSRRKFLYDTNVGAGLPVIENLQNLLNAGDELV

KFSGILSGSLSYIFGKLDEGMSFSEATTLAREMGYTEPDPRDDLSGMDVARKLLILARETGRELELADIE

IEPVLPAEFNAEGDVAAFMANLSQLDDLFAARVAKARDEGKVLRYVGNIDEDGVCRVKIAEVDGNDPLFK

VKNGENALAFYSHYYQPLPLVLRGYGAGNDVTAAGVFADLLRTLSWKLGV

>gi|30043893|gb|AAP19612.1| soluble lytic murein transglycosylase [Shigella flexneri 2a str. 2457T]

MYLHLEDALVEKAKQVTWRLLAAGVCLLTVSSVARADSLDEQRSRYAQIKQAWDNRQMDVVEQMMPGLKD

YPLYPYLEYRQITDDLMNQPAVTVTNFVRANPTLPPARTLQSRFVNELARREDWRGLLAFSPEKPGTTEA

QCNYYYAKWNTGQSEEAWQGAKELWLTGKSQPNACDKLFSVWRASGKQDPLAYLERIRLAMKAGNTGLVT

VLAGQMPADYQTIASAIISLANNPNTVLTFARTTGATDFTRQMAAVAFASVARQDAENARLMIPSLAQAQ

QLNEDQIQELRDIVAWRLMGNDVTDEQAKWRDDAIMRSQSTSLIERRVRMALGTGDRRGLNTWLARLPME

AKEKDEWRYWQADLLLERGREAEAKEILHQLIQQRGFYPMVAAQRIGEEYELKIDKAPQNVDSTLTQGPE

MARVRELMYWNLDNTARSEWANLVKSKSKTEQAQLARYAFNNQWWDLSVQATIAGKLWDHLEERFPLAYN

DLFKRYTSGKEIPQSYAMAIARQESAWNPKVKSPVGASGLMQIMPGTATHTVKMFSIPGYSSPGQLLDPE

TNINIGTSYLQYVYQQFGNNRIFSSAAYNAGPGRVRTWLGNSAGRIDAVAFVESIPFSETRGYVKNVLAY

DAYYRYFMGDKPTLMSATEWGRRY

>gi|30043828|gb|AAP19547.1| hypothetical protein S4618 [Shigella flexneri 2a str. 2457T]

MHNIPGVRNTRLPLLQEIVMEILYNIFTVFFNQVMTNAPLLLGIVTCLGYILLRKSVSVIIKGTIKTIIG

FMLLQAGSGILTSTFKPVVAKMSEVYGINGAISDTYASMMATIDRMGDAYSWVGYAVLLALALNICYVLL

RRITGIRTIMLTGHIMFQQAGLIAVTLFIFGYSMWTTIICTAILVSLYWGITSNMMYKPTQEVTDGCGFS

IGHQQQFASLIAYKVAPFLGKKEESVEDLKLPGWLNIFHDNIVSTAIVMTIFFGAILLSFGIDTVQAMAG

KVHWTVYILQTGFSFAVAIFIITQGVRMFVAELSEAFNGISQRLIPGAVLAIDCAAIYSFAPNAVVWGFM

WGTIGQLIAVGILVACGSSILIIPGFIPMFFSNATIGVFANHFGGWRAALKICLVMGMIEIFGCVWVVKL

TGMSAWMGMADWSILAPPMMQGFFSIGIAFMAVIIVIALAYMFFAGRALRAEEDAEKQLAEQSA

>gi|30043573|gb|AAP19293.1| transcription elongation factor and transcript cleavage factor [Shigella flexneri 2a str. 2457T]

MRIIKQTKGINEMKTPLVTREGYEKLKQELNYLWREERPEVTKKVTWAASLGDRSENADYQYNKKRLREI

DRRVRYLTKCLENLKIVDYSPQQEGKVFFGAWVEIENDDGVTHRFRIVGYDEIFGRKDYISIDSPMARAL

LKKEVGDLAVVNTPAGEANWYVNAIEYVKP

>gi|30043155|gb|AAP18877.1| diaminopimelate epimerase [Shigella flexneri 2a str. 2457T]

MMQFSKMHGLGNDFMVVDAVTQNVFFSPELIRRLADRHLGVGFDQLLVVEPPYDPELDFHYRIFNADGSE

VAQCGNGARCFARFVRLKGLTNKRDIRVSTANGRMVLTVTDDDLVRVNMGEPNFEPSAVPFRANKAEKTY

IMRAAEQTILCGVVSMGNPHCVIQVDDVDTAAVETLGPVLESHERFPERANIGFMQVVKREHIRLRVYER

GAGETQACGSGACAAVAVGIQQGLLDEEVRVELPGGRLDIAWKGPGHPLYMTGPAVHVYDGFIHL

>gi|30042987|gb|AAP18709.1| thiamin biosynthesis protein, thiazole moiety [Shigella flexneri 2a str. 2457T]

MSSGRNISCRMATRSCFFRLLQGVEMLRIADKTFDSHLFTGTGKFASSQLMVEAIRASGSQLVTLAMKRV

DLRQHNDAILEPLIAAGVALLPNTSGAKTAEEAIFAAHLAREALGTNWLKLEIHPDARWLLPDPIETLKA

AETLVQQGFVVLPYCGADPVLCKRLEEVGCAAVMPLGAPIGSNQGLETRAMLEIIIQQATVPVVVDAGIG

VPSHATQALEMGADAVLVNTAIAVADDPVNMAKAFRLAVEVGLLARQSGPGSRSYFAHATSPLTGFLEAS

A

>gi|30042976|gb|AAP18698.1| hypothetical protein S3661 [Shigella flexneri 2a str. 2457T]

MSKNDSLPAAGESFLLVYHARLPVISAFHRWHGRCNTRSKTTTGGLTMKRNTKIALVMMALSAMAMGSTS

AFAHGGHGMWQQNAAPLTSEQQTAWQKIHNDFYAQSSALQQQLVTKRYEYNALLAANPPDSSKINAVAKE

MENLRQSLDELRVKRDIAMAEAGIPRGAGMGMGYGGCGGGGHMGMGHW

>gi|30042898|gb|AAP18621.1| hypothetical protein S3575 [Shigella flexneri 2a str. 2457T]

MKMPIKRVLTLTCWNTRSNWWRISVNFSPKSSQIHHALRTVAGRFAVKSIDYFWHDSCNASKRFHIWESI

MLELLFVIGFFVMLMVTGVSLLGIIAALVVATAIMFLGGMLALMIKLLPWLLLAIAVVWVIKAIKAPKMP

KYQRYDRWRY

>gi|30042774|gb|AAP18497.1| 7,8-dihydropteroate synthase [Shigella flexneri 2a str. 2457T]

MLRGFFLSIHTRDNIMKLFAQGTSLDLSHPHVMGILNVTPDSFSDGGTHNSLIDAVKHANLMINAGATII

DVGGESTRPGAAEVSVEEELQRVIPVVEAIAQRFEVWISVDTSKPEVIRESAKVGAHIINDIRSLSEPGA

LEAAAETGLPVCLMHMQGNPKTMQEAPKYDDVFAEVNRYFIEQIARCEQAGIAKEKLLLDPGFGFGKNLS

HNYSLLARLAEFHHFNLPLLVGMSRKSMIGQLLNVGPSERLSGSLACAVIAAMQGAHIIRVHDVKETVEA

MRVVEATLSAKENKRYE

>gi|30042769|gb|AAP18492.1| hypothetical protein S3428 [Shigella flexneri 2a str. 2457T]

MGLSTLEQKLTEMITAPVEALGFELVGIEFIRGRTSTLRIYIDSEDGINVDDCADVSHQVSAVLDVEDPI

TVAYNLEVSSPGLDRPLFTAEHYARFVGEEVTLVLRMAVQNRRKWQGVIKAVDGEMITVTVEGKDEVFAL

SNIQKANLVPHF

>gi|30042537|gb|AAP18261.1| hypothetical protein S3159 [Shigella flexneri 2a str. 2457T]

MGLNVREGEIMAKNRSRRLRKKMHIDEFQELGFSVAWRFPEGTSEEQIDKIVDDFINEVIEPNKLAFDGS

GYLAWEGLICMQEIGKCTEEHQAIVRKWLEECKLDEVRTSELFDVWWD

>gi|30042521|gb|AAP18245.1| hypothetical protein S3143 [Shigella flexneri 2a str. 2457T]

MTAPFFPRILGAVAFLNQETEPLTMNLQHHFLIAMPALQDPIFRRSVVYICEHNTNGAMGIIVNKPLENL

KIEGILEKLKITPEPRDESIRLDKPVMLGGPLAEDRGFILHTPPSNFASSIRISDNTVMTTSRDVLETLG

TDKQPSDVLVALGYASWEKGQLEQEILDNAWLTAPADLNILFKTPIADRWREAAKLIGVDILTMPGVAGH

A

>gi|30042324|gb|AAP18049.1| putative 2-component transcriptional regulator [Shigella flexneri 2a str. 2457T]

MSSQNDYQSNGRYDNIYSQIDSEAKMSFSVDVLANIAIELQRGIGHQDRFQRLITTLRQVLECDASALLR

YDSRQFIPLAIDGLAKDVLGRRFALEGHPRLEAIARAGDVVRFPADSELPDPYDGLIPGQESLKVHACVG

LPLFVGQNLIGALTLDGMQPDQFDVFSDEELRLIAALAAGALSNALLIEQLESQNMLPGDATPFEAVKQT

QMIGLSPGMTQLKKEIEIVAASDLNVLISGETGTGKELVAKAIHEASPRAVNPLVYLNCAALPESVAESE

LFGHVKGAFTGAISNRSGKFEMADNGTLFLDEIGELSLALQAKLLRVLQYGDIQRVGDDRSLRVDVRVLA

ATNRDLREEVLAGRFRADLFHRLSVFPLSVPPLRERGDDVILLAGYFCEQCRLRQGLSRVVLSAGARNLL

QHYSFPGNVRELEHAIHRAVVLARATRNGDEVILEAQHFAFPEVTLPPPEAAAVPVVKQNLREATEAFQR

ETIRQALAQNHHNWAACARMLETDVANLHRLAKRLGLKD

>gi|30041867|gb|AAP17594.1| putative elongation factor [Shigella flexneri 2a str. 2457T]

MQGVLAGLPSGIPGIGLEIEGAVQHAPQPGRHSIRCSLKVNHRIDERAQYHLFCTSKSANTTCPKGPSRV

LCRDIFDIFDYRNFSMPRANEIKKGMVLNYNGKLLLAKDIDIQSPTARGAATLYKMRFSDVRTGLKVEER

FKGDDIVDTVTLTRRYVDFSYVDGNEYVFMDKEDYTPYTFTKDQIEEELLFMPEGGMPDMQVLTWDGQLL

ALELPQTVDLEIVETAPGIKGASASARNKPATLSTGLVIQVPEYLSPGEKIRIHIEERRYMGRAD

>gi|30041520|gb|AAP17248.1| putative adhesin [Shigella flexneri 2a str. 2457T]

MIGRIMLHKKTLLFAALSAALWGGATQAADAAVVASLKPVGFIASAIADGVTETEVLLPDGASEHDYSLR

PSDVKRLQNADLVVWVGPEMEAFMQKPVSKLPEAKQVTIAQLEDVKPLLMKSIHGDDDDHDHAEKSDEDH

HHGDFNMHLWLSPEIARATAVAIHGKLVELMPQSRAKLDANLKDFEAQLASTETQVGNELAPLKGKGYFV

FHDAYGYFEKQFGLTPLGHFTVNPEIQPGAQRLHEIRTQLVEQKATCVFAEPQFRPAVVESVARGTSVRM

GTLDPLGTNIKLGKTSYSEFLSQLANQYASCLKGD

>gi|30041136|gb|AAP16866.1| cytochrome b(561) [Shigella flexneri 2a str. 2457T]

MLTENYVKAGNVMENKYSRLQISIHWLVFLLVIAAYCAMEFRGFFPRSDRPLINMIHVSCGISILVLMVV

RLLLRLKYPTPPIIPKPKPMMTGLAHLGHLVIYLLFIALPVIGLVMMYNRGNPWFAFGLTMPYASEANFE

RVDSLKSWHETLANLGYFVIGLHAAAALAHHYFWKDNTLLRMMPRKRS

>gi|30041071|gb|AAP16801.1| psp operon transcriptional activator [Shigella flexneri 2a str. 2457T]

MANFIMAEYKDNLLGEANSFLEVLEQVSHLAPLDKPVLIIGERGTGKELIASRLHYLSSRWQGPFISLNC

AALNENLLDSELFGHEAGAFTGAQKRHPGRFERADGGTLFLDELATAPMMVQEKLLRVIEYGELERVGGS

QPLQVNVRLVCATNADLPAMVNEGTFRADLLDRLAFDVVQLPPLRERESDIMLMAEHFAIQMCREIKLPL

FPGFTERARETLLNYRWPGNIRELKNVVERSVYRHGTSDYPLDDIIIDPFKRRPPEEAIAVSENTSLPTL

PLDLREFQMQQEKELLQLSLQQGKYNQKRAAELLGLTYHQFRALLKKHQI

>gi|30040947|gb|AAP16677.1| hypothetical protein S1258 [Shigella flexneri 2a str. 2457T]

MAEHLMSDVPFWQSKTLDEMSDAEWESLCDGCGQCCLHKLMDEDTDEIYFTNVACRQLNIKTCQCRNYER

RFEFEPDCIKLTRENLPTFEWLPMTCAYRLLAEGKDLPAWHPLLTGSKAAMHGERISVRHIAVKESEVID

WQDHILNKPDWAQ

>gi|30040754|gb|AAP16485.1| putative sulfite reductase [Shigella flexneri 2a str. 2457T]

MRGFVFCYTAASLIKVVIIMLIFEGKEIETDTEGYLKESSQWSEPLAVVIAENEGISLSPEHWEVVRFVR

DFYLEFNTSPAIRMLVKAMANKFGEEKGNSRYLYRLFPKGPAKQATKIAGLPKPVKCI

>gi|30040748|gb|AAP16479.1| methylglyoxal synthase [Shigella flexneri 2a str. 2457T]

MYIMELTTRTLPARKHIALVAHDHCKQMLMSWVERHQPLLEQHVLYATGTTGNLISRATGMNVNAMLSGP

MGGDQQVGALISEGKIDVLIFFWDPLNAVPHDPDVKALLRLATVWNIPVATNVATADFIIQSPHFNDAVD

ILIPDYQRYLADRLK

>gi|30039906|gb|AAP15640.1| hypothetical protein S0096 [Shigella flexneri 2a str. 2457T]

MLWTSGFNDKICALNTFEFDRDGNNVSGILTRWRQFGKRYFWPHLLLGMVAASLGLPALSNAAEPNAPAK

ATTRNHEPSAKVNFGQLALLEANTRRPNSNYSVDYWHQHAIRTVIRHLSFAMAPQTLPVAEESLPLQAQH

LALLDTLSALLTQEGTPSEKGYRIDYAHFTPQAKFSTPVWISQAQGIRAGPQRLT

>gi|60594515|pdb|1Y2I|E Chain E, Crystal Structure Of Mcsg Target Apc27401 From Shigella Flexneri

MHHHHHHSSGVDLGTENLYFQSNAXQFSTTPTLEGLTIVEYCGVVTGEAILGANIFRDFFAGIRDIVGGR

SGAYEKELRKAREIAFEELGSQARALGADAVVGIDIDYETVGQNGSXLXVSVSGTAVKTRRNI

>gi|313651501|gb|EFS15897.1| uvrD/REP helicase family protein [Shigella flexneri 2a str. 2457T]

MGKPTDEQRVIIENANANNMVIAAPGSGKSFTMIEAVISILRQFPYAKVGMVTFTRAATNSLAEKLKRRL

SKKDQDRVLVNTFHGFIRMQLDMVNWKGKMLISSAQRSVIHRALKESGAPFRYPDAEFAIDAIGREMDTD

IISVRHTRQQIHLFNTYQAICQKDHVADLNALSRFVVGQMYSGKMQPLNLTHLVVDEVQDTDSIQYAWIS

LHTRAGVNTSIVGDDDQAIYSFRASGGVKIFQQFEKQFRPNIFYLNTCFRCEPEILKVAGALIEKNVYRY

AKDLRSAKGGGGKVHFRSYVDMDEQIQGILNLINQDPIGWAILSRGNAHLDQLESLIEQPVLRYGGKSFW

DEKETSDVLHLMAFFRHSNDVRLMKRVLALFGENEEVLDQTALSMKGRKVTFGELNIPNESSLETRTLHS

NFTRFTQETREKVEIEKRFANLIKWMELSSIKMRTQKGSPSLSRIALDTCKQWAEKTGWQNMINRAAAMC

LGPKKKDEEYTPDKVVLSTLHGSKGLEWKNVIIMSCNADQIPSKRSVGQEAIEEERRLLFVGFTRAEQQL

HVMWYGDPSFFLSECAEDKLKEAAKSRTESPLTE

>gi|313651489|gb|EFS15885.1| parB family protein [Shigella flexneri 2a str. 2457T]

MSDEQHIGNDKSRYINAPKRTEVIHRSGLQGLKGQPRLKKLFTLHNGRKLEAEHIIVPAEKVELETTVHP

LNPRNQEALSVNAVRDILKDIEARGVDTEGVAVKRNGVYLLIEGSRRRFCCIQSAKELPLWVLPDDVNAD

DINSIISATQTSRRFSYREVGLKYLREMEEHGFVTNEELANYHGISHVSVSKRVQAAKINSNLIALFPDY

EAIPNSYYNRLFRLQKYIEKNLFSLEEVVENTREEIRDLDISDIAEAQKTVMEKITTVVEKLDIKPPSKG

WDTRELATFTNKDKYARISKSSSGRKIRFEFNRMNRELIDEIEKFIKSKLSEMNN

>gi|313651488|gb|EFS15884.1| parA [Shigella flexneri 2a str. 2457T]

MNLIDKIALVGQRMKSEQISLKESLLVSSRVSVSDDSVDGVDRLIYNHCLNKKNLSDFFGKSRVTFNKIL

ADLEEKELVGAPIYQNKNHLYTRWDVQKIMDALNYPRYSDYYCSRTIVTQNHKGGTGKSTTSGVLAVAAA

LDLHLNARILLIEWDPQGSIGSGMIQSVAEDDVFLTAIDAILGVYEEDSDYRKYLDLGYSEEQIIEGMPF

STHLPNLDVITAFPTDARFKDKYWQCSREERTELLLRFKEVILPVLKSKYDLIIIDTPPEDSPITWAADE

AADGILVAVSPREYDYASTTDFMLTISERFKQSPSKGENLSWFKVLAVNVDDKSPYEKIVLDKLVRTVQE

LFMSANIKNSEAFKAAASRGRTVLDIKKSEELCSPKQLDVAEESVMAVYQQFINEIKSFSVKQGGNV

>gi|313651469|gb|EFS15865.1| DNA adenine methylase family protein [Shigella flexneri 2a str. 2457T]

MPFLSKHFPHDKSRRWVEPFIGGGAVFLNMFATEALLADSNPDLINLYRNIQRNKPAFIREVQLLAERHF

EEEDYYVLRNTFNSTSFDDAPLQRAAIFYAMNRLGYNGLCRYNLKRKFSVPWGKRYQFSLDIQKVDYLSF

RLSSVELKTADFGQTLEFAGGGDQIYCDPPYDKISKTSFVSYDGIPFDKSAHVKLADMLVDANRKGASVA

ISNSMTPFTLELYEERGFDIHTHNAYRSVGSQSKSRKKEIEILAVLR

>gi|313651164|gb|EFS15563.1| outer membrane usher papC domain protein [Shigella flexneri 2a str. 2457T]

MLPPNLRGYAPDISGVAHTTAKVTVSQMGRVIYETQVPAGPFRIQDLGDSVSGTLHIRIEEQNGQVQEYD

ISTASMPYLTRPGQVRYKIMMGRPQEWGHHVEGEFFSGAEASWGIANGWSLYGGALGDENYQSAALGVGR

DLSTFGAVAFDVTHSHTKLDKDTAYGKGSLDGNSFRVSYSKDFDQLNSRVTFAGYRFSEENFMTMSEYLD

ASDSGMVRTGNDKEMYTATYNQNFRDAGVSVYLNYTRHTYWDREEQTNYNIMLSHYFNMGSIRNVSISMT

GYRYEYDNQADKGMYISLSMPWGDNSTVSYNGTMAVGRTAVRSVISAVSMTRLTIS

>gi|313651163|gb|EFS15562.1| outer membrane usher papC domain protein [Shigella flexneri 2a str. 2457T]

MNVGTSDKHTSVDGYYSHDGSLAQVDLSANYHEGQYTSAGLSLQGGATLTAHGGALHRTQNMGGTRLLID

ADGVADVPVEGNGAAVYTNMFGKAVVSDVNNYYRNQAYIDLNKLPENAEATQSVVQATLTEGAIGYRKFA

VISGQKAMAVLRLQDGSHPPFGAEVKNDNEQTVGLVDDDGNVYLAGVKPGEHMSVFWSGVAHCDINLPDP

LPADLFNGLLLPCQHKGNVAPVVPDDIKPVIQEQTQQVTPTNPPVSVSANQ

>gi|313651162|gb|EFS15561.1| chaperone protein pmfD [Shigella flexneri 2a str. 2457T]

MSDLLCSAKLGATTLALLLSAASLSAQASVTPDRTRLIFNESDKSISVTLRNNDPKLPYLAQSWIEDEKG

NKISSPLTVLPPVQRIDSMMNGQVKVQGMPDINKLPADRESLFYFNVREIPPKSNKANTLQIALQTRIKL

FWRPKALENVSMKNP

>gi|313651039|gb|EFS15439.1| ABC transporter periplasmic-binding protein yphF [Shigella flexneri 2a str. 2457T]

MPKKMRTTRNLLLMATLLGSALFARAADKEMTIGAIYLDTQGYYAGVRQGVQDAAKDSSVQVQLIETNAQ

GDISKESTFVDTLVARNVDAIILSAVSENGSSRTVRRASEAGIPVICYNTCINQKGVDKYVSAYLVGDPL

EFGKNWVTLPPIILLPIKLTSRKLPSSIAKPLKFVCSDVKDLKKY

>gi|313651006|gb|EFS15406.1| penicillin-binding 1C domain protein [Shigella flexneri 2a str. 2457T]

MLDNLIEARYLEALINYEDRWFWKHPGVNPFSVARAAWQDLTSGRVISGGSTLTMQVARLLDPHPKTFGG

KILQLWRALQLEWHLSKREILILYLNRAPFGGTLQGIGAASWAYLGKSPANLSYSEAAMLAVLPQVPSRL

RPDRWPERAEAARNKVLERMAAQGVWSREQVKESREEPIWLAPRQMPQLAPLFSRMMLGKSKSDKIVTTL

DAGLQRRLEELAQNWKGRLPPRSSLAMIVVNHTDMRVRGWVGSVDLNDDSRFGHVDMVNAIRSPGSVLKP

FVYGLALDEGLIHPASLLQDVPRHR

>gi|313650949|gb|EFS15349.1| hydrogenase-4 transcriptional activator [Shigella flexneri 2a str. 2457T]

MAMSDEAMFAPPQGITIEAVNGMLAERLAQKHGKASLLRAFIPLPPPFSPVQLIELHVLKSNFYYRYHDD

GSDVTATTEYQGEMVDYSRYAVLLGSSGMAELRFIRTHGSRFTPQDCTLFNWLARIITPVLQSWLNDEAQ

QVALRLLEKDRDHHRVLVDITNAVLSHLDLDDLIADVAREIHHFFGLASVSMVLGDHRKNEKFSLWCSDL

SASHCACLPHNMPGDSVLLTQTLQTRQPTLTHRADDLFLWQRDPLLLLLASNGCESALLIPLTFGNHTPG

ALLLAHTSSTLFSEENCQLLQHIADRIAIAVGNADACRRMTDLQESLQQENHQLSEQLLSNLGVGDIIYQ

SQAMEDLLQQVDIVAKSDSTVLICGETGTGKEVIARAIHQLSPRHDKPLVKINCAAIPASLLESELFGHD

KGAFTGAINTHRGRFEIADGGTLFLDEIGDLPLELQPKLLRVLQEREIERLGGSRTIPVNVRVIAATNRD

LWQMVEDRQFRSDLFYRLNVFPLELPPLRDRPEDIPLLAKHFTQKMARHMNRSIDAIPTEALRQLMSWDW

PGNVRELENVIERAVLLTRGNSLNLHLNVRQSRLLPTLNEDSALRSSMA

>gi|313650935|gb|EFS15335.1| glycine cleavage system transcriptional repressor [Shigella flexneri 2a str. 2457T]

MTLSSQHYLVITALGADRPGIVNTITRHVSSCGCNIEDSRLAMLGEEFTFIMLLSGSWNAITLIESTLPL

KGAELDLLIVMKRTTARPRPPMPASVWVQVDVADSPHLIERFTALFDAHHMNIAELVSRTQPAENERAAQ

LHIQITAHSPASADAANIEQAFKALCTELNAQGSINVVNYSQHDEQDGVK

>gi|313650908|gb|EFS15308.1| glutamate racemase [Shigella flexneri 2a str. 2457T]

MFSAESPIGVIDSGVGGLTSVKEIINLLPGEDIIYCGDNMNAPYGNRSADDIISLTKKMLTFLQSRNVKL

VAVACNTISSTLESEEYSGYAKSFPFPILSIIEPAVEDVIRQQYKNVGIIATEFTIKTGCHKELIKKLNS

TINVFGEPSKNLAMLIEEGNLNAPAILNDIKKHVNHLISLHPVNEIILGCTHYPIVQNFFEAVAPDIKFI

NPAHDQAISIKNHLGQLNLLNSSNIGTLQINTSGSMEIYKTVLGELSITKPHTFSIRQF

>gi|313650880|gb|EFS15280.1| sulfate ABC transporter, permease protein CysW [Shigella flexneri 2a str. 2457T]

MAEVTQLKRYDARPINWGKWFLIGIGMLVSAFILLVPMIYIFVQAFSKGLMPVLQNLADPDMLHAIWLTV

MIALIAVPVNLVFGILLAWLVTRFNFPGRQLLLTLLDIPFAVSPVVAGLVYLLFYGSNGPLGGWLDEHNL

QIMFSWPGMVLVTIFVTCPFVVRELVPVMLSQGSQEDEAAILLGASGWQMFRRVTLPNIRWALLYGVVLT

NARAIGEFGAVSVVSGSIRGETLSLPLQIELLEQDYNTVGSFTAAALLTLMAIITLFLKSMLQWRLENQE

KRAQQEEHHEH

>gi|313650867|gb|EFS15267.1| cell division protein ZipA [Shigella flexneri 2a str. 2457T]

MMQDLRLILIIVGAIAIIALLVHGFWTSRKERSSMFRDRPLKRMKSKRDDDSYDEDVEDDEGVGEVRVHR

VNHAPANAQEHEAARPSPQHQYLPPYASAQPRQPVQQPPEAQVPPQHAPRPAQPVQQPAYQPQPEQPLQQ

PVSPQVAPAPQPVHSAPQPAQQAFQPAEPVAAPQPEPVAEPAPVMDKPKRKEAVIIMNVAAHHGSELNGE

LLLNSIQQAGFIFGDMNIYHRHLSPDGSGPALFSLANMVKPGTFDPEMKDFTTPGVTIFMQVPSYGDELQ

NFKLMLQSAQHIADEVGGVVLDDQRRMMTPQKLREYQDIIREVKDANA

>gi|313650753|gb|EFS15154.1| TMAO reductase sytem sensor TorS [Shigella flexneri 2a str. 2457T]

MLQALREQGFDTTAIEQQEQEISRSLRQQGELVGRRLQLRQQQRQLSQQIAAADEIARLEQGQANNATTS

AGATQAGIYDLIEQDQRQAAESALDRLIDIDLEYVNQMNELRLSALRVQQMVMNLGLEQIQKNAPTLEKQ

LNNAVKILQRRQIRIEDPGVRAQVATTLTTVSQYSDLLALFQQDSEISNHLQTLAQNNIAQFAQFSSEVS

QLVDTIELHNQHGLAHLEKASARGQYSLLLLGMVSLCALIMILWRVVYRSFTRPLAEQTQALQRLLDGDI

DSPFPETAGVRELDTIGRLMDAFRSSVHALNRHREQLAAQVKARTAELQELVIEHRQARAEAEKASQAKS

AFLTAMSHEIRTPLYGILGTAQLLADNPALNAQRDDLQAITDSGESLLTILNDILDYSAIEAGGKNVSVS

DEPFEPRPLLESTLQLMSGRVKGRPIRLATAIADDVPSALMGDPRRIRQVITNLLSNALRFTDEGHIILR

SRTDGEQWLVEVEDSGCGIDPAKLAEIFQPFVQVSGKRGGTGLGLTISSRLAQAMGGELSATSTPEVGSC

FCLRLPLRVATAPVPKTANQAVRLDGLRLLLIEDNPLTQRITVEMLNTSGAQVVAIGNAAQALETLQNSE

PFAAALVDFDLPDIDGITLARQLAQQYPSLVLIGFSAHVIDETLRQRTSSLFRGIIPKPVPREVLGQLLA

HYLQLQANNDLPLDVSQLNEDAQLMGTEKIHEWLALFKQHALPLLDEIDIARASQDSEKIKRAAHQLKSS

CSSLGMRSASQLCAQLEQQPLSAPLPHEEITRSVAALEA

>gi|313650743|gb|EFS15144.1| chaperone torD domain protein [Shigella flexneri 2a str. 2457T]

MAALPATLTVRDDARLELAADFCGLFLMTDKQAALPYASAYKQDEQEIKRLLVEAGMETSGNFNEPADHL

AIYLELLSYLHFSLGEGTVPARRIDSLRQKTLTALRQWLPEFAARCRQYDSFGFYAALSQLLLVLVECDH

QNR

>gi|313650721|gb|EFS15122.1| phoH-like family protein [Shigella flexneri 2a str. 2457T]

MGRQKAVIKARREAKRVLRRDSRSHKQREEESVTSLVQMSGVEAIGMARDSRDTSPILARNEAQLHYLQA

IESKQLIFATGEAGCGKTWISAAKAAEALIHKDVDRIIVTRPVLQADEDLGFLPGDIAEKFAPYFRPVYD

LLVRRLGASFMQYCLRPEIGKVETAPFAYMRGRTFENAVVILDEAQNVTAAQMKMFLTRLGENVTVIVNG

DITQCDLPRGVCSGLSDALERFEEDEMVGIVRFGKEDCVRSALCQRTLHAYS

>gi|313650715|gb|EFS15116.1| putative dehydrogenase [Shigella flexneri 2a str. 2457T]

MDIIFYHPTFDTQWWIEALRKAIPQARVRAWKSGDNDSADYVLVWHPPVEMLAGRDLKAVFALGAGVDSI

LSKLQAHPEMLNPSVPLFRLEDTGMGEQMQEYAVSQVLHWFRRFDDYRIQQNSSHWQPLPEYHREDFTIG

ILGAGVLGCKVAQSLQT

>gi|313650596|gb|EFS14999.1| heat shock hslJ domain protein [Shigella flexneri 2a str. 2457T]

MKKVAAFVALSLLMAGCVSNDKIAVTPEQLQHHRFVLESVNGKPVTSDKNPPEISFGEKMMISGSMCNRF

SGEGKLSNGELTAKGLAMTRMMCANPQLNELDNTVMTPTY

>gi|313650577|gb|EFS14980.1| smr domain protein [Shigella flexneri 2a str. 2457T]

MLDKLRSGKYPQQASLNLLRQPVEECRKMVFSFIQQALADGLRNVLIIHGKGREDKSHANIVRSYVARWL

TEFDDVQAYCTALPHHGGSGACYVALRKTAQAKQENWERHAKRSR

>gi|313650575|gb|EFS14978.1| drug resistance MFS transporter [Shigella flexneri 2a str. 2457T]

MSMRKHIAFASMCMGLFIAQLDIQIVSSSLNEIGGGLSAGKDEMAWLQTSYLIAEIIVIPLSGWLSRVFS

TRWLFTLSAGIFTLMSIACGLAWNIQIMILFRALQGAAGASMIPLVFTMAFIYYQGKELGLAAAVVSALA

SLSPTLGPTLGGWLTDNLDWRWLFYINILPGIYLVLSIPFLVNFDKPDLSLLKVADYPSIILLAMTLGCL

EYTLEEGARWGWLDDNTILLTSVLALVSFILFAARTLTISNPIMDLHAFKDKNFTLGCFFSFSGGVGIFS

TVYLIPVFLGQIRGLNAEEIGFAVCTTGIFQLFSVPFYFWLSKKINLRWLLMAGLGGFVFSMYLFTPITH

EWGWQELLFPQAIRGISQQFAMAPIVTLTLGGIPKERLKLASGVFNLTRNLGGAIGIALCGSILNNRTNF

HFSRMGEKMVSVPHTVNDFISRSALFFNRSRSDQTSEILASTKLLSQLMLREAQTMAFSVPFC

>gi|313650527|gb|EFS14933.1| binding--dependent transport systems inner membrane component domain protein [Shigella flexneri 2a str. 2457T]

MILPVQAALERLPPSLLQASADLGARPRQTFRYVVLPLAIPGIAAGSIFTFSLTLGDFIVPQLVGPPGYF

IGNMVYSQQGAIGNMPMAAAFTLVPIILIALYLAFVKRLGAFDAL

>gi|313650353|gb|EFS14762.1| HTH-type transcriptional regulator dicA [Shigella flexneri 2a str. 2457T]

MKNETFGARLLHRRKKLKLSQAALGKLVKVAHVTISQWERDETQPAGKRLFALSQALQCSPTWLLFGDED

KQPGEPIPDNQPAILTEDQKELLQLFDALPESEQKALLSEMRARVENFNKLFEELLKARKRSANK

>gi|313650164|gb|EFS14577.1| flagellar export protein FliJ [Shigella flexneri 2a str. 2457T]

MAEEQLKMLIDYQNEYRNNLNSDMSAGMTSNRWINYQQFIQTLEKAITQHRQQLNQWTQKVDIALNSWRE

KKQRLQAWQTLQERQSTAALLAENRLDQKKMDEFAQRAAMRKPE

>gi|313650116|gb|EFS14529.1| L-arabinose-binding periplasmic domain protein [Shigella flexneri 2a str. 2457T]

MHKFTKALAAIGLAAVMSQSAMAENLKLGFLVKQPEEPWFQTEWKFADKAGKDLGFEVIKIAVPDGEKTL

NAIDSLAASGAKGFVICTPDPKLGSAIVAKARGYDMKVIAVDDQFVNAKGKPMDTVPLVMMAATKIGERQ

G

>gi|313650115|gb|EFS14528.1| L-arabinose-binding periplasmic protein [Shigella flexneri 2a str. 2457T]

MQKRGWDVKESAVMAITANELDTARRRTTGSMDALKAAGFPEKQIYQVPTKSNDIPGAFDAANSMLVQHP

EVKHWLIVGMNDSTVLGGVRATEGQGFKAADIIGIGINGVDAVSELSKAQATGFYGSLLPSPDVHGYKSS

EMLYNWVAKDVETPKFTEVTDVVLITRDNFKEELEKKGLGGK

>gi|313649993|gb|EFS14411.1| outer membrane usher fimD domain protein [Shigella flexneri 2a str. 2457T]

MGDGYTQGDIFDGINFRGAQLASDDNMLPDSQRGFAPVIHGIARGTAQVTIKQNGYGIYNSTVPPGPFTI

NDIYAAGNSGDLQVTIKEADGSTQIFTVPYSSVPLLQREGHTRYSITAGEYRSGNAQQEKPRFFQSTLLH

GLPAGWTIYGGTQLADRYRAFNFGIGKNMEALGALSVDMTQANSTLPDDSQHDGQSVRFLYNKSLNESGT

NIQLVGYRYSTSGYFNFADTTYSRMNGYNIETQDGVIQVKPKFTDYYNLAYNKRGKLQLTVTQQLGRTST

LYLSGSHQTYWGTSNVDEQFQAGLNTAFEDINWTLSYSLTKNAWQKGRDQMLALNVNIPFSHWLRSDSKS

QWRHASASYSMSHDLNGRMTNLAGVYGTLLEDNNLSYSVQTGYAGGGDGNSGSTGYATLNYRGGYGNANI

GYSHSDDIKQLYYGVSGGVLAHANGVTLGQPLNDTVVLVKAPGAKDAKVENQTGVRTDWRGYAVLPYATE

YRENRVALDTNTLADNVDLDNAVANVVPTRGAIVRAEFKARVGIKLLMTLTHNNKPLPFGAMVTSESSQS

SGIVADNGQVYLSGMPLAGKVQVKWGEEENAHCVANYQLPPESQQQLLTQLSAECR

>gi|313649991|gb|EFS14409.1| outer membrane usher fimD domain protein [Shigella flexneri 2a str. 2457T]

MAGFFVRLVVACAFAAQAPLSSADLYFNLRFLADDPQAVADLSRFENGQELPPGTYRVDIYLNNGYMATR

DVTFNTGDSEQGIVPCLTRAQLASMGLNTASVAGMNLLADDACVPLTTMVQDATAHLDVGQQRLNLTIPQ

AFMSNRARGYIPPELWDPGINAGLLNYNFSGNSVQNRVMLPTY

>gi|313649905|gb|EFS14325.1| putative colanic acid polymerase domain protein [Shigella flexneri 2a str. 2457T]

MSTSIRICSYLLLPLIYLLVNVKIAQLGESFPITIVTFLPVLLLLFLERISIKKLMIALGIGAGLTAFNY

LFGQSLDAGKYVTSTMLFVYIVIIGMVWSIRFKTISPHNHRKILRFFYLVVGLVVALAAVEMAQIILTG

GSSIMESISKYLIYSNSYVLNFIKFGGKRTTALYFEPAFFALALISIWLSIKQFGIKTPKTDALILAGII

LSGSFSGVMTFILFYLLEWAFQYLNKEAIKKKLPLALISLAVFRVGVVIAFPYISTRLGDLGTG

>gi|313649879|gb|EFS14299.1| multidrug resistance protein mdtB [Shigella flexneri 2a str. 2457T]

MRPVATTLLMVAILLAGIIGYRALPVSALPEVDYPTIQVVTLYPGASPDVMTSAVTAPLERQFGQMSGLK

QMSSQSSGGASVITLQFQLTLPLNVAEQEVQAAINAATNLLPSDLPNPPVYSKVNPADPPIMTLAVTSTA

MPMTQVEDMVETRVAQKISQISGVGLVTLSGGQRPAVRVKLNAQAIAALGLTSETVRTAITGANVNSAKG

SLDGPSRAVTLSANDQMQSAEEYRQLIIAYQNGAPIRLGDVATVEQGAENSWLGAWANKEQAIVMNVQRQ

PGANIISTADSIRQMLPQLTESLPKSVKVTVLSDRTTNIRASVDDTQFELMMAIALVVMIIYLFLRNIPA

TIIPGVAVPLSLIGTFAVMVFLDFSINNLTLMALTIATGFVVDDAIVVIENISRYIEKGEKPLAAALKGA

GEIGFTIISLTFSLIAVLIPLLFMGDIVGRLFREFAITLAVAILISAVVSLTLTPMMCARMLSQESLRKQ

NRFSRASEKMFDRIIAAYGRGLAKVLNHPWLTLSVALSTLLLSVLLWVFIPKGFFPVQDNGIIQGTLQAP

QSSSFANMAQRQRQVADVILQDPAVQSLTSFVGVDGTNPSLNSARLQINLKPLDERDDRVQKVIARLQTA

VDKVPGVDLFLQPTQDLTIDTQVSRTQYQFTLQATSLDALSTWVPQLMEKLQQLPQLSDVSSDWQDKGLV

AYVNVDRDSASRLGISMADVDNALYNAFGQRLISTIYTQANQYRVVLEHNTENTPGLAALDTIRLTSSDG

GVVPLSSIAKIEQRFAPLSINHLDQFPVTTISFNVPDNYSLGDAVQAIMDTEKTLNLPVDITTQF

>gi|313649845|gb|EFS14265.1| gram-negative pili assembly chaperone [Shigella flexneri 2a str. 2457T]

MVLPAHAGIVIYGTRIIYPAENKEVMVQLMNQGNRSSLLQAWIDDGDTSLPPEKIQVPFMLTPPVAKIGA

NSGQQVKIKIMPNKLPTNKESIFYLNVLYIPPNSPEQEGKNALKFAMQNRIKLFYRPAGIAPVNKATFKK

LLVNRSGNGLVIKNDSANWVTISDVKANNVKVNYETIMIAPLESQSVNIKSNNANNWYLTIINDHGNYIS

DKI

>gi|313649670|gb|EFS14094.1| KDP operon transcriptional regulatory protein kdpE [Shigella flexneri 2a str. 2457T]

MTNVLIVEDEQAIRRFLRTALEGDGMRVFEAETLQRGLLEAATRKPDLIILDLGLPDGDGIEFIRDLRQW

SPVPVIVLSARSEESDKIAALDAGADDYLSKPFGIGELQARLRVALRRHSATPPPPRPIRW

>gi|313649669|gb|EFS14093.1| sensor kdpD domain protein [Shigella flexneri 2a str. 2457T]

MYVETPALHRLPEKKRRAILSALRLAQELGAETATLSDPAEEKAVVRYAREHNLGKIILGRPASRRWWRR

ETFADRLARIAPDLDQVLVALDEPPARTINNAPDSRSFKDKWRVQIQGCVVAAALCAVITLIAMQWLMAF

DAANLVMLYLLGVVVALFYGRWPSVVATVINVVSFDLFFIAPRGTLAVSDVQYLLTFAVMLTVGLVIGNL

TAGVRYQARVARYREQRTRHLYEMSKALAVGRSPQDIAATSEQFIASTFHARSQVLLPDDNGKLQPLTHP

QGITPWDDAIAQWSFDKGLPAGAGTDTLPGVPYQILPLKSGEKTYGLVVVEPGNLRQLMIPEQQRLLETF

TLLVANALERLTLTASEEQARMASEREQIRNALLAALSHDLRTPLTVLFGQAEILTLDLASEGSPHARQA

SEIRQHVLNTTRLVNNLLDMARIQSGGFNLKKEWLTLEEVVGSALQMPEPGLSSPINLSLPEPLTLIHVD

GPLFERVLINLLENAVKYAGAQAEIGIDAHVEGENLQLDVWDNGPGLPPGQEQTIFDKFARGNKESAVPG

VGLGLAICRAIVDVHGGTITAFNRPEGGACFRVTLPQQTAPELEDFHEDM

>gi|313649443|gb|EFS13874.1| protein tonB [Shigella flexneri 2a str. 2457T]

MIMTSITLDLPRRFPWPTLLSVCIHGAVVAGLLYTSVHQVIELPAPAQPISVTMVAPADLEPPQAVQPPP

EPVVEPEPEPEPIPEPPKEAPVVIEKPKPKPKPKPVKKVQEQPKRDVKPVESRPASPFENTAPARPTS

STATAATSKPVTSVASGPRALSRNQPQYPARAQALRIEGQVKVKFDVTPDGRVDNVQILSAKPANMFERE

VKNAMRRWRYEPGKPGSGIVVNILFKINGTTEIQ

>gi|313649333|gb|EFS13765.1| DNA replication protein dnaC [Shigella flexneri 2a str. 2457T]

MKNIATGGVLERIRRLTPPHVTAPFRTVAEWREWQLAEGQKRCEEINRLNRQLRVEKILNRSGIQPLHRK

CSFANYRAQNDGQRHALSQAKSIADELMTGCTNFVFSGKPGTGKNHLAAAIGNRLMAKGRSVIIVTVSDV

MSVLHESYDNGKSGEKFLQELCGVDLLVLDEIGMQRDTKNEQVVLNQIVDRRTASLRGVGMLTNINHAAM

NTLLGERVMDRMVMNGGRWVNFNWESWRPNVSHSRVVK

>gi|313649332|gb|EFS13764.1| uncharacterized ydaU domain protein [Shigella flexneri 2a str. 2457T]

MAGVLEGRLSGGTAAEFCNSAVVALQAAGLDVCREYPVPERGDGCGGRIDIVVTDRNGVRCGIELDRNSP

RQKSLLKIGAVETGICVLRRSDIARHTEQGILVIGGAVRQKKFDPLSVDLPDWLSETLWHEWVQFRQALR

KPIRTELGANGAIRELEKFRQQGFTPEQVIRHSIANEYQGLFAPKGVRPETLLRQVNTVSLPDSAIPPGF

RG

>gi|313649230|gb|EFS13664.1| N-succinylarginine dihydrolase domain protein [Shigella flexneri 2a str. 2457T]

MAIEVPATQVSVSDAVSTYLFNSQLLSRDDGSMMLVLPQECREHAGVWGYLNELLAADNPISELKVFDLR

ESMANGGGPACLRLRVVLTEEERRAVNPAVMMNDTLFNALNDWVDRYYRDRLTAADLADPQLLREGREAL

DVLSQLLNLGSVYPFQREGGGNG

>gi|313649229|gb|EFS13663.1| N-succinylarginine dihydrolase [Shigella flexneri 2a str. 2457T]

MNAWEVNFDGLVGLTHHYAGLSFGNEASTRHRFQVSNPRLAAKQGLLKMKALADAGFPQAVIPPHERPFI

PVLRQLGFSGSDEQVLEKVARQAPHWLSSVSSASPMWVANAATIAPSADTLDGKVHLTVANLNNKFHRSL

EAPVTESLLKAIFKDEEKFSVHSALPQVALLGDEGAANHNRLGGHYGEPGMLLFVYGREEGNDTRPSRYP

ARQTREASEAVARLNQVNPQQVIFAQQNPDVIDQSVFHNDVIAVSNRQVLFCHQQAFARQSQLLARG

>gi|313649227|gb|EFS13661.1| arginine N-succinyltransferase [Shigella flexneri 2a str. 2457T]

MMVIRPVERSDVSALMQLASKTGGGLTSLPANEATLSARIERAIKTWQGELPKSEQGYVFVLEDSETGTV

AGICAIEVAVGLNDPWYNYRVGTLVHASKELNVYNALPTLFLSNDHTGSSELCTLFLDPDWRKEGNGYLL

SKSRFMFMAAFRDKFNDKVVAEMRGVIDEHGYSPFWQSLGKRFFSMDFSRADFLCGTGQKAFIAELMPKH

PIYTHFLSQEAQDVIGQVHPQTAPARAVLEKEGFRYRNYIDIFDGGPTLECDIDRVRAIRKSRLVEVAEG

QPAQGDFPACLVANENYHHFRVVLARTDPATERLILTAAQLDALKCHAGDRVRLVRLCAEEKTA

>gi|313649122|gb|EFS13556.1| serine/threonine-phosphatase 1 domain protein [Shigella flexneri 2a str. 2457T]

MKQPAPVYQRIAGHQWRHIWLSGDIHGCLEQLRRKLWHCRFDPWRDLLISVGDVIDRGPQSLRCLQLLEQ

HWVRAVRGNHEQMAMDALAFQQMSLWLMNGGDWFIALADNQQKQAKTALEKCQHLPFILEVHSRTGKHVI

AHADYPDDV

>gi|313648972|gb|EFS13409.1| major Facilitator Superfamily protein [Shigella flexneri 2a str. 2457T]

MKNPYYPTALGLYFNYLVHGMGVILMSLNMASLETLWQTNAAGVSIVISSLGIGRLSVLLFAGLLSDRFG

RRPFIMLGMCCYMAFFFDILQTNNIIIAYVFGFLAGMANSFLDAGTYPSLMEAFPRSPGTANILIKAFVS

SGQFLLPLIISLLVWAELWFGWSFMIAAGIMFINALFLYRCTFPPHPGRRLPVIKKTTSSTEHRCSIIDL

ASYTLYGYISMATFYLVSQWLAQYGQFVAGMSYTMSIKLLSIYTVGSLLCVFITAPLIRNTVRPTTLLML

YTFISFIALFTVCLHPTFYVVIIFAFVIGFTSAGGVVQIGLTLMAERFPYAKGKATGIYYSTGSIATFTI

PLITAHLSQRSIADIMWFDTAIGFLLALFIGLRSRKKTRHHSLKENVAPGG

>gi|313648888|gb|EFS13325.1| glucuronide transporter [Shigella flexneri 2a str. 2457T]

MGLGLCYSLVNIPYGSLATAMTQQPQSRARLGAARGIAASLTFVCLAFLIGPSIKNSSPEEMVSVYHFWT

IVLVIAGMVLYFICFKSTRENVVRIVAQPSLKISLQTLKRNRPLFMLCIGALCVLISTFAVSASSLFYVR

YVLNDTGLFTVLVLVQNLVGTVASAPLVPGMVARIGKKNTFLIGALLGTCGYLLFFWVSVWSLPVALVAL

AIASIGQGVTMTVMWALEADTVEYGEYLTGVRIEGLTYSLFSFTRKCGQAIGGSIPAFILGLSGYIANQV

>gi|313648885|gb|EFS13322.1| hypothetical protein SF2457T_2545 [Shigella flexneri 2a str. 2457T]

MNKSLVAVGVIVALGVVWTGGAWYTGKKIETHLEDMVAQANAQLKLTAPESNLEVSYQNYHRGVFSSQLQ

LLVKPIAGKENPWIKSGQSVIFNESVDHGPFPLAQLKKLNLIPSMASIQTTLVNNEVSKPLFDMAKGETP

FEINSRIGYSGDSSSDISLNPLNYEQKDEKVAFSGGEFQLNADRDGKAISLSGEAQSGRIDAVNEYNQKV

QLTFNNLKTDGSSTLGSFGERVGNQKLSLEKMTISVEGKELALLEGMEISGKSDLVNDGKTINSQLDYSL

NSLKVQNQDLGSGKLTLKVGQIDGEAWHQFSQQYNAQTQALLAQPEIANNPELYQEKVTEAFFSALPLML

KGDPVITIAPLSWKNSQGESALNLSLFLKDPATTKEAPQTLAQEVDRSVKSLDAKLTIPVDMATELMTQV

AKLEGYQEDQAKKLAKQQVEGASAMGQMFRLTTLQDNTITTSLQYANGQITLNGQKMSLEDFVGMFAMPA

LNVPAVPAIPQQ

>gi|313648856|gb|EFS13295.1| phthalate permease family domain protein [Shigella flexneri 2a str. 2457T]

MDVDVSTSVAGNKPQRIRRIQTVTLVLLFMAGIVNFLDRSSLSVAGEAIRGELGLSATEFGVLLSAFSLS

YGFSQLPSGILLDRFGPRIVLGAGLIFWSLMQALTGMVNSFSHFILMRIGLGGNAANLLI

>gi|313648747|gb|EFS13187.1| uncharacterized protein yjbS [Shigella flexneri 2a str. 2457T]

MNISYVNSNKTTSLPVELDALNNKDISYAKDFFLYIETQLKIAKDFCRPGEEVSSSIASKVFHAFIDLVN

KIRGKKDFMYICTLCCFAEEVKGDYSHYRTFLFDIGNQYKVKLTQSGKKSSL

>gi|313648622|gb|EFS13063.1| general L-amino acid transport system permease aapQ domain protein [Shigella flexneri 2a str. 2457T]

MAIVLSVGLFRFNKTYQIKTGQLRRSWPIAAVLIIGLPLLAQWLFGAALHWDVPALQGFNFRGGMVLIPE

LAALTLALSVYTSAFIAEIIRAGIQAVPYGQHEAARSLGLPNPVTLRQVIIPQALRVIIPPLTSQYLNIV

KNSSLAAVIGYPDMVSLFAGTVLNQTGQAIETIAMTMSVYLIISLTISLLMNIYNRRIAIVER

>gi|313648616|gb|EFS13058.1| uncharacterized outer membrane usher yraJ domain protein [Shigella flexneri 2a str. 2457T]

MTSQKDRPTQHEMRLDGSLLDDGRLSYSLEQSLDDDNNHNSSLNASYRSPYGTFSAGYSYGNDSSQYNYG

VTGGVVIHPHGVTLSQYLGNAFALIDANGASGVRIQNYPGIATDPFGYAVVPYLTTYQENRLSVDTTQLP

DNVDLEQTTQFVVPNRGAMVAARFNANIGYRVLVTVSDRNGKPLPFGALASNDDTGQQSIVDEGGILYLS

GISSKSQSWTVRWGNQADQQCQFAFSTPDSEPTTSVLQGTAQCH

>gi|313648508|gb|EFS12950.1| rod shape-determining protein MreC [Shigella flexneri 2a str. 2457T]

MKPIFSRGPSLQIRLILAVLVALGIIIADSRLGTFSQIRTYMDTAVSPFYFVSNAPRELLDGISQTLASR

DQLELENRALRQELLLKNSELLMLGQYKQENARLRELLGSPLRQDEQKMVTQVISTVNDPYSDQVVIDKG

SVNGVYEGQPVISDKGVVGQVVAVAKLTSRVLLICDATHALPIQVLRNDIRVIAAGNGCTDDLQLEHLPA

NTDIRVGDVLVTSGLGGRFPEGYPVAVVSSVKLDTQRAYTVIQARPTAGLQRLRYLLLLWGADRNGANPM

TPEEVHRVANERLMQMMPQVLPSPDAMGPKLPEPATGIAQPTPQQPATGNAATAPVAPTQPAANRSPQRA

TPPQSGAQPPARAPGGQ

>gi|313648438|gb|EFS12881.1| aerotaxis receptor domain protein [Shigella flexneri 2a str. 2457T]

MEAARAGEQGKGFAVVAGEVRHLASRSANAANDIRKLIDASADKVQSGSQQVHAAGRTMEDIVAQVKNVT

QLIAQISHSTLEQADGLSSLTRAVDELNLITQKNAELVEESAQVSAMVKHRASRLEDAVTVLH

>gi|313648437|gb|EFS12880.1| aerotaxis receptor [Shigella flexneri 2a str. 2457T]

MSSHPYVTQQNTPLADDTTLMSTTDLQSYITHANDTFVQVSGFTLQELQGQPHNMVRHPDMPKAAFADMW

FTLKKGEPWSGIVKNRRKNGDHYWVRANAVPMVREGKISGYMSIRTRATDEEIAAVEPLYKALNSGRTSK

RIHKGLVVRKGWLGKLPSLPLRWRTRGVMTLMFILLAAMLWFVAAPVVTYFLCVLVVLLASACFEWQIVR

PIENVARQALKVATGERNSVEHLNRSDELGLTLRAVGQLGLMCRWLINDVSSQVSSVRNGSETLAKGTDE

LNEHTQQTVDNVQQTVATMNQMAASVKQNSATASAADKLSITASNAAVQGGEAMTTVIKTMDDIADST

>gi|313648213|gb|EFS12658.1| serine/threonine-protein phosphatase 2 [Shigella flexneri 2a str. 2457T]

MQQEAIVLLLKFHHLPHIIEITNDIIKYVIAHADYPGDEYLFGKEIAESELLWPVYRVQKSLNGELQQIN

GADYFIFGHMMFDNIQTFANQIYIDTGSPKSGRLSFYKIR

>gi|313647807|gb|EFS12253.1| RNA polymerase sigma factor rpoS [Shigella flexneri 2a str. 2457T]

MSQNTLKVHDLNEDAEFDENGVEVFDEKALVEEEPSDNDLAEEELLSQGATQRVLDATQLYLGEIGYSPL

LTAEEEVYFARRALRGDVASRRRMIESNLRLVVKIARRYGNRGLALLDLIEEGNLGLIRAVEKFDPERGF

RFSTYATWWIRQTIERAIMNQTRTIRLPIHIVKELNVYLRTARELSHKLDHEPSAEEIAEQLDKPVDDVS

RMLRLNERITSVDTPWVVIPKKRCWTSWPMKKRTVRKIPRKMTI

>gi|313647791|gb|EFS12238.1| drug resistance MFS transporter [Shigella flexneri 2a str. 2457T]

MSDKKKRSMSGLPWIAAMAFFMQALDATILNTALPAIAHSLNRSPLAMQSAIISYTLTVAMLIPVSGWLA

DRFGTRRIFTLAVSLFTLGSLACALSNSLPQLVVFRVIQGIGDAMMMPVARPALLRAYPRNELLPVLNFV

AMPGLVGPILGPVLGGVLVTWATWHWIFLINIPIGIAGLLYARKHMPNFTTARRRFDITGFLLFGLSLVL

FSSGIELFGEKIVASWIALTVIVTSIGLLLLYILHARRTPNPLISLDLFKTRTFSIGIVGNIATRLGTGC

VPFLMPLMLQVGFGYQAFIAGCMMAPTALGSIIAKSMVTQVLRRLGYRHTLVGITVIIGLMIAQFSLQSP

AMAIWMLILPLFILGMAMSTQFTAMNTITLADLTDDNASSGNSVLAVTQQLSISLGVAVSAAVLRIYEGM

EGTTTVEQFHYTFITMGIITVASAAMFMLLKTTDGNNLIKRQRKSMPNRVPSESE

>gi|313647790|gb|EFS12237.1| bacterial regulatory s, gntR family protein [Shigella flexneri 2a str. 2457T]

MPLSAQQLAAQKNLSYVLAEKLAQRILKGEYEPGTILPGEIELGEQFGVSRTAVREAVKTLTAKGMVLPR

PRIGTRVMPQSNWNFLDQELLTWWITEENFHQVIDHFLVMRICLEPQACLLAATVGTAEQKAHLNTLMAE

MAALKENFRRERWIEVDMAWHEHIYEMSANPFLTSFASLFHSVYHTYFTSITSDTVIKLDLHQAIVDAII

QSDGDAAFKACQALLRSPDK

>gi|313647468|gb|EFS11918.1| sugar (Glycoside-Pentoside-Hexuronide) transporter family protein [Shigella flexneri 2a str. 2457T]

MGGVITNDPTQRISLQSWRFVLATAGGMLSTVLMMPLVNLIGGDNKPLGFQGGIVVLSVVAFMMLAFCFF

TTKERVEAPPTTTSMREDLRDIWQNDQWRIVGLLTIFNILAVCVRGGAMMYYVTWILGTPEVFVAFLTTY

CVGNLIGSALAKPLTDWKCKVTIFWWTNALLAVISLAMFFVPMQASITMFVFIFVIGVLHQLVTPIQWVM

MFDTVDYGEWCNGKRLTGISFAGTLFVLKLGLAFGGALIGWMLAYGGYDAAEKAQNSATISIIIALFTIV

PAICYLLSAIIAKRYYSLTTHNLKTVMEQLAQGKRRCQQQFTSQEVQN

>gi|313647260|gb|EFS11712.1| HTH-type transcriptional regulator gntR [Shigella flexneri 2a str. 2457T]

MKKKRPVLQDVADRVGVTKMTVSRFLRNPEQVSVALRGKIAAALDELGYIPNRAPDILSNATSRAIGVLL

PSLTNQVFAEVLRGIESVTDAHGYQTMLAHYGYKPEMEQERLESMLSWNIDGLILTERTHTPRTLKMIEV

AGIPVVELMDSQSPCLDIAVGFDNFEAARQMTTAIIARGHRHIAYLGARLDERTIIKQKGYEQAMLDAGL

VPYSVMVEQSSSYSSGIELIRQARREYPQLDGVFCTNDDLAVGAAFECQRLGLKVPDDMAIAGFHGHDIG

QVMEPRLASVLTPRERMGSIGAERLLARIRGESVTPKMLDLGFTLSPGGSI

>gi|313647208|gb|EFS11660.1| fimbrial Usher family protein [Shigella flexneri 2a str. 2457T]

MSITTNRYASGYATLTEAVSAQDERNRKRDKNSHDGSTISLSQPLGNIGNLNFNTTRYNSSRGTGNTRST

SLSYSTVWRGITFSINWAKNDLLTSHKWKVDRKLSVGISVPLSLGDENQIYASSQMSRSGEQGNNYQVSL

SGQNSGGVWWDVATNITNAHQSQPKSTMNIVQVGKNGSYGQFSSHYSSSENMKQLGANLSGGILITRDGL

TFGQNVDGTLALIEAPGATGVNVNGWPGLSTDFRGYAILPVQPYRRDDVILDEKTIGKNYDLPQTSQLVV

PTAGAVVPATLAVKSGDKGLVTLKQKEGKPIPFGAVISYSKDTENMAGIVGEDGIAYVSGLSAEGEFNVK

WGYSKDQSCIAKYQLPAKKSASGLYQIAATCL

>gi|313647169|gb|EFS11623.1| fructose-like PTS system EIIBC component [Shigella flexneri 2a str. 2457T]

MAMESSLRIVAITNCPAGIAHTYMVAEALEQKARSLGHTIKVETQGSSGVENRLSSEEIAAADYVILATG

RGLSGDDRARFAGKKVYEIAISLALKNIDQIFSELPTNSQLFAADSGVKLGKQEVQSGSVMSHLMAGVSA

ALPFVIGGGILVALANMLVQFGLPYTDMSKGAPSFTWVVESIGYLGFTFMIPIMGAYIASSIADKPAFAP

AFLVCYLANDKALLGTQSGAGFLGAVVLGLAIGYFVFWFRKVRLGKALQPLLGSMLIPFVTLLVFGVLTY

YVIGPVMSDLMGGLLHFLNTIPPSMKFAAAFLVGAMLAFDMGGPINKTAWFFCFSLLEKHIYDWYAIVGV

VALMPPVAAGLATFIAPKLFTRQEKEAASSAIVVGATVATEPAIPYALAAPLPMITANTLAGGITGVLVI

AFGIKRLAPGLGIFDPLIGLMSPVGSFYLVLAIGLALNISFIIVLKGLWLRRKAKAAQQELVHEH

>gi|313647131|gb|EFS11586.1| homoserine/Threonine efflux family protein [Shigella flexneri 2a str. 2457T]

MTLEWWFAYLLTSIILSLSPGSGAINTMTTSLNHGYRGAVASIAGLQTGLAIHIVLVGVGLGTLFSRSVI

AFEVLKWAGAAYLIWLGIQQWRAAGAIDLKSLASTQSRRHLFQRAVFVNLTNPKSIVFLAALFPQFIMPQ

QPQLMQYIVLGVTTIVVDIIVMIGYATLAQRIALWIKGPKQMKALNKIFGSLFMLVGALLASARHA

>gi|313647046|gb|EFS11502.1| putative isochorismate synthase [Shigella flexneri 2a str. 2457T]

MERQAIPEQTTFEQMGARAAALTATPQVDKVVLSRLIDITTDAAIDSGVLLERLIAQNPVSYNFHVPLAD

GGVLLGASPELLLRKDGERFSSIPLAGSARRQPDEVLDREAGNRLLASEKDRHEHELGTQAMKEVLRERS

SELHVPSSPQLITTPTLWHLATPFEGKANSQENALTLACLLHPTPALSGFPHQAATQVIAELEPFDRELF

GGIVGWCDSEGNGEWVVTIRCAKLRENQVRLFAGAGIVPASSPLGEWRETGVKLSTMLNVFGLH

>gi|313646928|gb|EFS11385.1| bacterial extracellular solute-binding s, family 5 Middle family protein [Shigella flexneri 2a str. 2457T]

MRQAQEAGWLEWQAQSGRGKRGQLRFLVTPESLRNAMMEQALETGKQQDVLELAQLAPGELRTLLQPFMG

GQWQNDTPTLRIPYYRPLEPLQPGFLPGRAEQHLAGQIFSGLTRFDNNTQRPIGDLAHHWETSTDRLRWD

FYLRSTLHWHNGDAVKASHLHQRLLMLLQLPALDQLFISVKRIEVTHPQCLTFFLHRPDYWLAHRLASYC

SHLAHPQFPLIGTGPFRLTQFTAELVRLESHDYYHLRHPLLKAVEYWITPPLFEKDLGTSCRHPVQITIG

KPEELQRVSQVSSGISLGFCYLTLRKSPRLSLWQARKVISIIHQSGLLQTLEVGENLITASHALLPGWTI

PHWQVPDEVKLPKTLTLVYHLPIELHTMAERLQATLAAEGCELTIIFHNAKNWDDTTLLAHADLMMGDRL

IGEAPEYTLEQWLRCDPLWPHVFDAPAYAHLQSTLDAVQVMPDEENRFNALKAVFSQLMADATLTPLFNY

HYRISAPPGVNGVRLTPRGWFEFTEAWLPAPSQ

>gi|313646861|gb|EFS11318.1| uncharacterized ylbF domain protein [Shigella flexneri 2a str. 2457T]

MAQGIRLGRFTVKQPQRYCLLRITPPSHPQPLAAAWMQRAEETGLFGPLAMAASDPLPAELRQFRHCFQA

ALNGVKTDWRHWLGKGPGLTPSHDDTLSGMLLAAWYYGALDARAGRPFFACSDNLQLVTTAVSVSYLRYA

AQGYFASPLLHFVHALSCPKRTAVAIDSLLALGHTSGADTLLGFWLGQQLLQGKP

>gi|313646849|gb|EFS11306.1| outer membrane usher sfmD domain protein [Shigella flexneri 2a str. 2457T]

MKIPTTTDIPQRYTWCLAGICYSSLAILPSFLSYAESYFNPAFLLENGTSVADLSRFERGNHQPAGVYRV

DLWRNDEFIGSQDIVFESTTENTGDKSGGLMPCFNQVLLERIGLNSSAFPELAQQQNNKCINLLKAVPDA

TINFDFAAMRLNITIPQIALLSSAHGVMTPTY

>gi|313646468|gb|EFS10930.1| IS222, transposase ORFA [Shigella flexneri 2a str. 2457T]

MDRAVRMVKWHTEFGHLNRGDMLTSEQHRCSNEKRNFSAEFKRESAQLVVDQKYTVADAAKAMDVGLSTM

TRWVKQLRDERQGKTPKASPITPEQIEIRELRKKLQRIEMENEILKKATALLMSDSLNSSR

>gi|313646413|gb|EFS10875.1| hypothetical protein SF2457T_5328 [Shigella flexneri 2a str. 2457T]

MLPRIRHNNFIGAVELFVKSSHTKTHSNDFFNNIQHAFKKKDWVSNYDSLLTLRESFRCATQIDKNSYQV

LSSKNETVNAMDNFLISFSLKDNGAEYTITLRGSGFEYEEIPITINEYNSFMDFKNREFPLEQNRRLYAC

DILQKKQSDIPKRIKGYIRQAFGDVSFGYALLEDVVSKLKRGKFELQIPGGGIKECDGWYIYEKIIDDNF

AIVIESLGFALKIYGGDERFRNGSSVVLEDEDYSLIYNFLVNAGCQQVELAEQVDAIVSANLAADSNITK

EKICEKYKSTIEAFKKEQLALPVLVRCKNSET

>gi|313646231|gb|EFS10693.1| amino acid carrier family protein [Shigella flexneri 2a str. 2457T]

MPVFFSFINSVLWGSVMIYLLFGAGCWFTFRTGFVQFRYIRQFGKSLKNSIHPQPGGLTSFQSLCTSLAA

RVGSGNLAGVALAITAGGPGAVFWMWVAAFIGMATSFAECSLAQLYKERDVNGQFRGGPAWYMARGLGMR

WMGVLFAVFLLIAYGIIFSGVQANAVARALSFSFDFPPLVTGIILAVFALLAITRGLHGVARLMQGFVPL

MAIIWVLTSLVICVINIGQLPHVIWSIFESAFGWQEAAGGAAGYTLSQAITNGFQRSMFSNEVGMGSTPN

AAAAAASWPPHPAAQGIVQMIGIFIDTLVICTASAMLILLAGNGTTYMPLEGIQLIQKAMRVLMGS
