# Supplementary material for: Finding Potential Therapeutic Targets against Shigella flexneri through Proteome Exploration
Source: Front Microbiol. 2016 Nov 22;7:1817. doi: 10.3389/fmicb.2016.01817 (PMC5118456; doi:10.3389/fmicb.2016.01817)
Supplement: Supplementary file 10 [file DataSheet5.PDF]

>gi|229089135|ref|NP_836175.2| allantoate amidohydrolase [Shigella flexneri 2a str. 2457T]

MVVHCRIIHRITHRIACGEITHSRQAIEETLPWLSSFGADPTGGMTRLLYSPEWLETQQQFKKRMAASGL

ETRFDEVGNLYGRLSGTEYPQEVVLSGSHIDTVVNGGNLDGQFGALAAWLAIDWLKTQYGAPLRTVEVVA

MAEEEGSRFPYVFWGSKNIFGLANPDDVRNICDAKGNSFADAMKACGFTLPNAPLTPRQAIKAFVELHIE

QGCVLESNGQSIGVVNAIVGQRRYTVTLNGESNHAGTTPMGYRRDTVYAFSRICHQSIEKAKKMGDPLVL

TFGKVEPRPNTVNVVPGKTTFTIDCRHTDAAVLRDFTQQLENDMRAICDEMDIGIDIDLWMDEEPVPMNK

ELVATLTELCEREKLNYRVMHSGAGHDAQIFAPRVPTCMIFIPSINGISHNPAERTNITDLAEGVKTLAL

MLYQLAWQE

>gi|229089132|ref|NP_839340.2| outer membrane lipoprotein [Shigella flexneri 2a str. 2457T]

MFILLAAVVSGALAVSGCTTNPYTGEREAGKSAIGAGLGSLVGAGIGALSSSKKDRGKGALIGAAAGAAL

GGGVGYYMDVQEAKLRDKMRGTGVSVTRSGDNIILNMPNNVTFDSSSAPLKPAGANTLTGVAMVLKEYPK

TAVNVIGYTDSTGGHDLNMRLSQQRADSVASALITQGVDASRIRTQGLGPANPIASNSTAEGKAQNRRVE

ITLSPL

>gi|229089131|ref|NP_839360.2| phosphodiesterase [Shigella flexneri 2a str. 2457T]

MAMVAAVVLVFVFIFCTVLLFHLVQQNRYNTATQLESIARSVREPLSSAILKGDIPEAEAILASIKPAGV

VSRADVVLPNQFQALRKSFIPERPVPVMVTRLFELPVQISLGVYSLERPANPQPIAYLVLQADSFRMYKF

VMSTLSTLVTIYLLLSLILTVAISWCINRLILHPLRNIARELNAIPAQELVGHQLALPRLHQDDEIGMLV

RSYNLNQQLLQRHYEEQNENAMRFPVSDLPNKALLMEMLEQVVARKQTTALMIITCETLRDTAGVLKEAQ

REILLLTLVEKLKSVLSPRMILAQISGYDFAVIANGVQEPWHAITLGQQVLTIMSERLPIERIQLRPHCS

IGVAMFYGDLTAEQLYSRAISAAFTARHKGKNQIQFFDPQQMEAAQKRLTEESDILNALENHQFAIWLQP

QVEMTSGKLVSAEVLLRIQQPDGSWDLPDGLIDRIECCGLMVTVGHWVLEESCRLLAAWQERGIMLPLSV

NLSALQLMHPNMVADMLELLTRYRIQPGTLILEVTESRRIDDPHAAVAILRPLRNAGVRVALDDFGMGYA

GLRQLQHMKSLPIDVLKIDKMFVEGLPEDSSMIAAIIMLAQSLNLQMIAEGVETEAQRDWLAKAGVGIAQ

GFLFARPLPIEIFEESYLEEK

>gi|229089130|ref|NP_839492.3| peptidoglycan synthetase [Shigella flexneri 2a str. 2457T]

MKFVKYFLILAVCCILLGAGSIYGLYRYIEPQLPDVVTLKDVRLQIPMQIYSADGELIAQYGEKRRIPVT

LDQIPPEMVKAFIATEDSRFYEHHGVDPVGIFRAASVALFSGHASQGASTITQQLARNFFLSPERTMMRK

IKEVFLAIRIEQLLTKDEILELYLNKIYLGYRAYGVGAAAQVYFGKTVDQLTLNEMAVIAGLPKAPSTFN

PLYSMDRAVARRNVVLSRMLDEGYITQQQFDQTRTEAINANYHAPEIAFSAPYLSEMVRQEMYNRYGESA

YEDGYRIYTTITRKVQQAAQQAVRNNVLDYDMRHGYRGPANVLWKVGESAWDNNKITDTLKALPTYGPLL

PAAVTSANPQGATAMLADGSTVALSMEGVRWARPYRSDTQQGPTPRKVTDVLQTGQQIWVRQVGDAWWLA

QVPEVNSALVSINPQNGAVMALVGGFDFNQSKFNRATQALRQVGSNIKPFLYTAAMDKGLTLASMLNDVP

ISRWDAGAGSDWQPKNSPPQYAGPIRLRQGLGQSKNVVMVRAMRAMGVDYAAEYLQRFGFPAQNIVHTES

LALGSASFTPMQVARGYAVMANGGFLVDPWFISKIENDQGGVIFEAKPKVACPECDIPVIYGDTQKSNVL

ENNDVEDVAISREQQNVSVPMPQLEQANQALVAKTGAQEYAPHVINTPLAFLIKSALNTNIFGEPGWQGT

GWRAGRDLQRRDIGGKTGTTNSSKDAWFSGYGPGVVTSVWIGFDDHRRNLGHTTASGAIKDQISGYEGGA

KSAQPAWDAYMKAVLEGVPEQPLTPPPGIVTVNIDRSTGQLANGGNSREEYFIEGTQPTQQAVHEVGTTI

IDNGEAQELF

>gi|229089129|ref|NP_839775.2| hypothetical protein S4664 [Shigella flexneri 2a str. 2457T]

MGVIEFLLALAQDMILAAIPAVGFAMVFNVPVRALRWCALLGSIGHGSRMILMTSGLNIEWSTFMASMLV

GTIGIQWSRWYLAHPKVFTVAAVIPMFPGISAYTAMISSVKISQLGYSEPLMITLLTNFLTASSIVGALS

IGLSIPGLWLYRKRPRV

>gi|161486509|ref|NP_836789.2| ribonuclease E [Shigella flexneri 2a str. 2457T]

MKRMLINATQQEELRVALVDGQRLYDLDIESPGHEQKKANIYKGKITRIEPSLEAAFVDYGAERHGFLPL

KEIAREYFPANYSAHGRPNIKDVLREGQEVIVQIDKEERGNKGAALTTFISLAGSYLVLMPNNPRAGGIS

RRIEGDDRTELKEALASLELPEGMGLIVRTAGVGKSAEALQWDLSFRLKHWEAIKKAAESRPAPFLIHQE

SNVIVRAFRDYLRQDIGEILIDNPKVLELARQHIAALGRPDFSSKIKLYTGEIPLFSHYQIESQIESAFQ

REVRLPSGGSIVIDSTEALTAIDINSARATRGGDIEETAFNTNLEAADEIARQLRLRDLGGLIVIDFIDM

TPVRHQRAVENRLREAVRQDRARIQISHISRFGLLEMSRQRLSPSLGESSHHVCPRCSGTGTVRDNESLS

LSILRLIEEEALKENTQEVHAIVPVPIASYLLNEKRSAVNAIETRQDGVRCVIVPNDQMETPHYHVLRVR

KGEETPTLSYMLPKLHEEAMALPSEEEFAERKRPEQPALATFAMPDVPPAPTPAEPAATVVAPAPKAATA

TPAAPAQPGLLSRFFGALKALFSGGEEAKPTEQPTPKAEAKPERQQDRRKPRQSNRRDRNERRDTRSERT

EGSDNREENRRNRRQAQQQTAETRESRQQAEVTEKARTTDEQQAPRRERSRRRNDDKRQAQQEAKALNVE

EQSVQETEQEERVRPVQPRRKQRQLNQKVRYEQSVAEEAVVAPVVEETAAAEPIVQEAPAPRTELVKVPL

PVVAQTAPEQQEENNADNRDNGGMPRRSRRSPRHLRVSGQRRRRYRDERYPTQSPMPLTVACASPELASG

KVWIRYPIVRPQDVQVEEQREQEEVQVQPMVTEVPVAAAVEPVVSAPVVEEMAEVVEAPVPVAEPQPEVV

ETTHPEVIAAAVTEQPQVITESDVAVAQEVAEHAEPMVEPQEETADADIEEVAETAEVVVAEPEVVAQPA

APVVAEVAAEVETVAAVEPEITVEHNHATAPMTRAPAPEYVPEAPRHSDWQRPTFAFEGKGAAGGHTATH

HASAAPARPQPVE

>gi|161486507|ref|NP_836795.2| glycerol-3-phosphate acyltransferase PlsX [Shigella flexneri 2a str. 2457T]

MTRLTLALDVMGGDFGPSVTVPAALQALNSNSQLTLLLVGNPDAITPLLAKADFEQRSRLQIIPAQSVIA

SDARPSQAIRASRGSSMRMALELVKEGRAQACVSAGNTGALMGLAKLLLKPLEGIERPALVTVLPHQQKG

KTVVLDLGANVDCDSTMLVQFAIMGSVLAEEVVEIPNPRVALLNIGEEEVKGLDSIRDASAVLKTIPSIN

YIGYLEANELLTGKTDVLVCDGFTGNVTLKTMEGVVRMFLSLLKSQGEGKKRSWWLLLLKRWLQKSLTRR

FSHLNPDQYNGACLLGLRGTVIKSHGAANQRAFAVAIEQAVQAVQRQVPQRIAARLESVYPAGFELLDGG

KSGTLR

>gi|161486506|ref|NP_836827.2| spermidine/putrescine ABC transporter [Shigella flexneri 2a str. 2457T]

MKNTSKFQNVVIVTIVGWLVLFVFLPNLMIIGTSFLTRDDASFVKMVFTLDNYTRLLDPLYFEVLLHSLN

MALIATLACLVLGYPFAWFLAKLPHKVRPLLLFLLIVPFWTNSLIRIYGLKIFLSTKGYLNEFLLWLGVI

DTPIRIMFTPSAVIIGLVYILLPFMVMPLYSSIEKLDKPLLEAARDLGASKLQTFIRIIIPLTMPGIIAG

CLLVMLPAMGLFYVSDLMGGAKNLLIGNVIKVQFLNIRDWPFGAATSITLTIVMGLMLLVYWRASRLLNK

KVELE

>gi|161486501|ref|NP_836924.2| hypothetical protein S1319 [Shigella flexneri 2a str. 2457T]

MSQLCPCGSAVEYSLCCHPYVSGEKVAPDPEHLMRSRYCAFVMQDADYLIKTWHPSCGAAALRAELMTGF

AHTEWLGLTVFEHCWQDADNIGFVSFVARFTEGGKTGAIIERSRFLKENGQWYYIDGTRPQFGRNDPCPC

GSGKKFKKCCGQ

>gi|161486492|ref|NP_837237.2| O-acetylserine/cysteine export protein [Shigella flexneri 2a str. 2457T]

MSRKDGVLALLVVVVWGLNFVVIKVGLHNMPPLMLAGLRFMLVAFPAIFFVARPKVPLNLLLGYGLTISF

AQFAFLFCAINFGMPAGLASLVLQAQAFFTIVLGAFTFGERLHGKQLAGIALAIFGVLVLIEDSLNGQHV

AMLGFMLTLAAAFSWACGNIFNKKIMSHSTRPAVMSLVIWSALIPIIPFFVVSLILDGSASMIHSLVTID

MTTILSLMYLAFVATIVGYGIWGTLLGRYETWRVAPLSLLVPVVGLASAALLLDERLTGLQFLGAVLIMT

GLYINVFGLRWRKAVKVGS

>gi|161486491|ref|NP_837240.2| DNA-binding transcriptional repressor MarR [Shigella flexneri 2a str. 2457T]

MKSTSDLFNEIIPLGRLIHMVNQKKDRLLNEYLSPLDITAAQFKVLCSIRCAACITPVELKKVLSVDLGA

LTRMLDRLVCKGWVERLPNPNDKRGVLVKLTTSGAAICEQCHQLVGQDLHQELTKNLTADEVATLEHLLK

KVLP

>gi|161486483|ref|NP_837301.2| outer membrane porin protein [Shigella flexneri 2a str. 2457T]

MRKIVAMAVICLTAASGLTSAYAAQLADDEAGLRIRLKNELRRADKPSAGAGRDIYAWVQGGLLDFNSGY

YSNIVGVEGGAYYVYKLGARADMSTRWYLDGDKSFGFALGAVKIKPSENSLLKLGRFGTDYSYGSLPYRI

PLMVGSSQRTLPTVSEGALGYWALTPNIDLWGMWRSRVFLWTDSTTGIRDEGVYNSQTGKYDKHRARSFL

AASWYDDTSRYSLGASVQKDVSNQIQSILEKSIPLDPNYTLKGELLGFYAQLEGLSRNTSQPNETALVSG

QLTWNAPWGSVFGSGGYLRHAMNGAVVDTDIGYPFSLSLDRNREGMQSWQLGANYRVTPQFTLTFAPIVT

RGYESSKRDVRIEGAGILGGMNYRVSEGPLQGMNFFLAADKGREKRDGSTLGDRLNYWDVKMSIQYDFML

K

>gi|161486474|ref|NP_837528.2| excinuclease ABC subunit C [Shigella flexneri 2a str. 2457T]

MSDQFDAKAFLKTVTSQPGVYRMYDAGGTVIYVGKAKDLKKRLSSYFRSNLASRKTEALVAQIQQIDVTV

THTETEALLLEHNYIKLYQPRYNVLLRDDKSYPFIFLSGDTHPRLAMHRGAKHAKGEYFGPFPNGYAVRE

TLALLQKIFPIRQCENSVYRNRSRPCLQYQIGRCLGPCVEGLVSEEEYAQQVEYVRLFLSGKDDQVLTQL

ISRMETASQNLEFEEAACIRDQIQAVRRVTEKQFVSNTGDDLDVIGVAFDAGMACVHVLFIRQGKVLGSR

SYFPKVPGGTELSEVVETFVGQFYLQGSQMRTLPGEILLDFNLSDKTLLADSLSELAGRKINVQTKPRGD

RARYLKLARTNAATALTSKLSQQSTVHQRLTALASVLKLPEVKRMECFDISHTMGEQTVASCVVFDANGP

LRAEYRRYNITGITPGDDYAAMNQVLRRRYGKAIDDSKIPDVILIDGGKGQLAQAKNVFAELDVSWDKNH

PLLLGVAKGADRKAGLETLFFEPEGEGFSLPPDSPALHVIQHIRDESHDHAIGGHRKKRAKVKNTSSLET

IEGVGPKRRQMLLKYMGGLQGLRNASVEEIAKVPGISQGLAEKIFWSLKH

>gi|161486472|ref|NP_837557.2| flagellar biosynthesis protein FliO [Shigella flexneri 2a str. 2457T]

MNNHATVQSSAPVSAEPLLQVSGALIAIIALILAAAWLVKRLGFAPKRTGVNGLKISASASLGARERVVV

VDVEDARLVLGVTAGQINLLHKLPPSAPTEEIPQTDFQSVMKNLLKRGGRS

>gi|161486468|ref|NP_837752.2| D-alanyl-D-alanine endopeptidase [Shigella flexneri 2a str. 2457T]

MLIMPKFRVSLFSLALMLAVPFAPQAVAKTAAATTASQPEIASGSAMIVDLNTNKVIYSNHPDLVRPIAS

ISKLMTAMVVLDARLPLDEKLKVDISQTPEMKGVYSRVRLNSEISRKDMLLLALMSSENRAAASLAHHYP

GGYKAFIKAMNAKAKSLGMNNTRFVEPTGLSVHNVSTARDLTKLLIASKQYPLIGQLSTTREDMATFSNP

TYTLPFRNTNHLVYRDNWNIQLTKTGFTNAAGHCLVMRTVINNKPVALVVMDAFGKYTHFADASRLRTWI

ETGKVMPVPAAALSYKKQKAAQMAAAGQTAQND

>gi|161486464|ref|NP_837855.2| UDP-4-amino-4-deoxy-L-arabinose--oxoglutarate aminotransferase [Shigella flexneri 2a str. 2457T]

MSEFLPFSRPAMGVEELAAVKEVLESGWITTGPKNQALEQAFCQLTGNQHAIAVSSATAGMHITLMALEI

GKGDEVITPSLTWVSTLNMISLLGATPVMVDVDRDTLMVTPEAIESAITPRTKAIIPVHYAGAPADIDAI

RAIGERYGIAVIEDAAHAVGTYYKGRHIGAKGTAIFSFHAIKNITCAEGGLIVTDNENLARQLRMLKFHG

LGVDAYDRQTWGRAPQAEVLTPGYKYNLTDINAAIALTQLVKLEHLNTRRREIAQQYQQALAALPFQPLS

LPAWPHVHAWHLFIIRVDEQRCGISRDALMEALKERGIGTGLHFRAAHTQKYYRERFPTLSLPNTEWNSE

RICSLPLFPDMTTADADRVITALQQLAGQ

>gi|161486462|ref|NP_837867.2| menaquinone-specific isochorismate synthase [Shigella flexneri 2a str. 2457T]

MQSLTTALENLLRHLSQEIPATPGIRVIDIPFPLKDAFDALSWLASQQTYPQFYWQQRNGDEEAAVLGAI

TRFTSLDQAQRFLRQHPEHADLRIWGLNAFDPSQGNLLLPRLEWRRCGGKATLRLTLFSESSLQHDAIQA

KEFIATLVSIKPLPGLHLTTTREQHWPDKTGWTQLIELATKTIAEGELDKVVLARATDLHFASPVNAAAM

MAASRRLNLNCYHFYMAFDGENAFLGSSPERLWRRRDKALRTEALAGTVANHPDDKQAQQLGEWLMADDK

NQRENMLVVEDICQRLQADTQTLDVLPPQVLRLRKVQHLRRCIWTSLNKADDVICLHQLQPTAAVAGLPR

DLARQFIARHEPFTREWYAGSAGYLSLQQSEFCVSLRSAKISGNVVRLYAGAGIVRGSDPEQEWQEIDNK

AAGLRTLLQME

>gi|161486458|ref|NP_837911.2| hypothetical protein S2525 [Shigella flexneri 2a str. 2457T]

MASKFQNRLVGTIVLVALGVIVLPGLLDGQKKHYQDEFAAIPLVPKAGDRDEPDMMPAATQALPTQPPEG

AAEEVRAGDAAAPSLDPATIAANNTEFEPEPAPVAPPKPKPVEPPKPKVEVPPAPKPEPKPVVEEKAAPT

GKAYVVQLGALKNADKVNEIVGKLRGAGYRVYTSPSTPVQGKITRILVGPDASKDKLKGSLGELKQLSGL

SGVVMGYTPN

>gi|161486454|ref|NP_838118.2| hypothetical protein S2779 [Shigella flexneri 2a str. 2457T]

MATHERRVVFFDLDGTLHQQDMFGSFLRYLLRRQPLNALLVLPLLPIIAIALLIKGRAARWPMSLLLWGC

TFGHSEARLQTLQADFVRWFRDNVTAFPLVQERLTTYLLSSDADIWLITGSPQPLVEAVYFDTPWLPRVN

LIASQIQRGYGGWVLTMRCLGHEKVAQLERKIGTPLRLYSGYSDSNQDNPLLYFCQHRWRVTPRGELQQL

E

>gi|161486447|ref|NP_838238.2| D-arabinose 5-phosphate isomerase [Shigella flexneri 2a str. 2457T]

MSEALLNTGRQTLMLELQEASRLPERLGDDFVRAANIILHCEGKVVVSGIGKSGHIGKKIAATLASTGTP

AFFVHPAEALHGDLGMIESRDVMLFISYSGGAKELDLIIPRLEDKSIALLAMTGKPTSPLGLAAKAVLDI

SVEREACPMHLAPTSSTVNTLMMGDALAMAVMQARGFNEEDFARSHPAGALGARLLNKVHHLMRRDDAIP

QVALTASVMDAMLELSRTGLGLVAVCDAQQQVQGVFTDGDLRRWLVGGGALTTPVNEAMTTGGTTLQAQS

RAIDAKEVLMKRKITAAPVVDENGKLTGAINLQDFYQAGII

>gi|161486445|ref|NP_838337.2| hypothetical protein S3032 [Shigella flexneri 2a str. 2457T]

MNREKGVSSLALVLMLLVLGSLLLQGMSQQDRSFASRVSMESQSLRRQAIVQSALAWGKMHSWQTQPAVQ

CSQYAGTDAQVCLRLLADNEALLIAGYEGVSLWRTGEVIDGKIVFSPRGWSDFCPLKEGALCQLP

>gi|161486433|ref|NP_838629.2| propionate/acetate kinase [Shigella flexneri 2a str. 2457T]

MRKEMNEFPVVLVINCGSSSIKFSVLDASDCEVLMSGIADGINSENAFLSVNGGEPAPLAHHSYEGALKA

IAFELEKRNLNDSVALIGHRIAHGGSIFTESAIITDEVIDNIRRVSPLAPLHNYANLSGIESAQQLFPGV

TQVAVFDTSFHQTMAPEAYLYGLPWKYYEELGVRRYGFHGTSHRYVSQRAHSLLNLAEDDSGLVVAHLGN

GASICAVRNGQSVDTSMGMTPLEGLMMGTRSGDVDFGAMSWVASQTNQSLGDLERVVNKESGLLGISGLS

SDLRVLEKAWHEGHERAQLAIKTFVHRIARHIAGHAASLRRLDGIIFTGGIGENSSLIRRLVMEHLAVLG

VEIDTEMNNRSNSFGERIVSSENAHVICVVIPTNEEKMIALDAIHLGKVNAPAEFA

>gi|161486418|ref|NP_838840.2| multidrug efflux system protein MdtO [Shigella flexneri 2a str. 2457T]

MSALNSLPLPVVRLLAFFHEELSERRPGRVPQIVQLWVGCLLVILISMTFEIPFVALSLAVLFYGIQSNA

FYTKFVAILFVVATVLEIGSLFLIYKWSYGEPLIRLIIAGPILMGCMFLMRTHRLGLVFFAVAIYGQTFP

AMLDYPEVVVRLTLWCIVVGLYPTLLMTLIGVLWFPNRAITQMHQALNDRLDDAISHLTDSLAPLPETRI

EREALALQKLNVFCLADDANWRTQSAWWQSCVATVTYIYSTLNRYDPTSFADSQAIIEFRQKLASEINKL

QHAVAEGQCWQSDWRLSESEAVAARECNLENICQTLLQLGQMNPNTPPTPAAKPPSMVADAFTNPDYIRY

AVKTLLACLICYTFYSGVDWEGIHTCMLTCVIVANPNVGSSYQKMVLRFGGAFCGAILALLFTLLVMPWL

DNIVELLFVLAPIFLLGAWIATSSERSSYIGTQMVVTFALATLENVFGPVYDLVEIRDRALGIIIGTVVS

AMIYTFVWPESEARTLPQKLAGALGMLSKVMRIPRQQEVTALRTYLQIRIGLHAAFNACEEMCQRVALER

QLDSEERALLIERSQTVIRQGRDILHAWDATWNSAQALDNALQPDRAGQFADALEKYAAGLATALSRSPQ

ITLEETPTSQAILPTLLKQEQHVCQLFARLPDWTAPALTPATEQAQGATQ

>gi|161486412|ref|NP_838913.2| glutamate racemase [Shigella flexneri 2a str. 2457T]

MATKLQDGNTPCLAATPSEPRPTVLVFDSGVGGLSVYDEIRHLLPDLHYIYAFDNVAFPYGEKSEAFIVE

RVVAIVTAVQERYPLALAVVACNTASTVSLPALREKFDFPVVGVVPAIKPAARLTANGIVGLLATRGTVK

RSYTHELIARFANECQIEMLGSAEMVELAEAKLHGEDVSLDALKRILRPWLRMKEPPDTVVLGCTHFPLL

QEELLQVLPEGTRLVDSGAAIARRTAWLLEHEAPDAKSADANIAFCMAMTPEAEQLLPVLQRYGFETLEK

LAVLG

>gi|161486410|ref|NP_838963.2| periplasmic repressor CpxP [Shigella flexneri 2a str. 2457T]

MRIVTAAVMASTLAVSSLSHAAEVGSGDNWHPGEELTQRSTQSHMFDGISLTEHQRQQMRDLMQQARHEQ

PPVNVSELETMHRLVTAENFDENAVRAQAEKMANEQIARQVEMAKVRNQMYRLLTPEQQAVLNEKHQQRM

EQLRDVTQWQKSSSLKLLSSSNSRSQ

>gi|161486402|ref|NP_839121.2| hypothetical protein S3943 [Shigella flexneri 2a str. 2457T]

MLTLDTLNVMLAVSEEGLIEEMIIALLASPQLAVFFEKFPRLKAAITDDVPRWREALRSRLKDARVPPEL

TEEVMCYQQSQLLSTPQFIVQLPQILDLLHRLNSPWAEQARQLVDANSTITSALHTLFLQRWRLSLIVQA

TTLNQQLLEEEREQLLSEVQERMTLSGQLEPILADNNTAAGRLWDMSAGQLKRGDYQFIVKYGEFLNEQP

ELKRLAEQLGRSREAKSIPRNDAQMETFRTMVREPATVPEQVDGLQQSDDILRLLPPELATLGITELEYE

FYRRLVEKQLLTYRLHGESWREKVFERPVVHKDYDEQPRGPFIVCVDTSGSMGGFNEQCAKAFCLALMRI

ALAENRRCYIMLFSTEIVRYELSGPQGIEQAIRFLSQQFRGGTDLASCFRAIMERLQSREWFDADAVVIS

DFIAQRLPDDVTSKVKELQRVHQHRFHAVAMSAHGKPGIMRIFDHIWRFDTGMRSRLLRRWRR

>gi|161486390|ref|NP_839257.2| DNA repair protein RadC [Shigella flexneri 2a str. 2457T]

MKVKNNAQLLMPREKMLKFGISALTDVELLALFLRTGTRGKDVLTLAKEMLENFGSLYGLLTSEYEQFSG
VHGIGVAKFAQLKGIAELARRYYNVRMREESPLLSPEMTREFLQSQLTGEEREIFMVIFLDSQHRVITHS
RLFSGTLNHVEVHPREIIREAIKINASALILAHNHPSGCAEPSKADKLITERIIKSCQFMDLRVLDHIVI
GRGEYVSFAERGWI

>gi|161486389|ref|NP_839282.2| hypothetical protein S4116 [Shigella flexneri 2a str. 2457T]

MRGKAINTMTRAVKPRRFAIRPIIYASVLSAGVLLCAFSAHADERDQLKSIQADIAAKERAVRQKQQQRA
SLLAQLKKQEEAISEATRKLRETQNTLNQLNKQIDEMNASIAKLEQQKAAQERSLAAQLDAAFRQGEHTG
IQLILSGEESQRGQRLQAYFGYLNQARQETIAQLKQTREEVTMQHAELEEKQSEQQTLLYEQRAQQAKLT
QALNERKKTLAGLESSIQQGQQQLSELRANESRLRNSIARAEAAAKARAEREAREAQAVRERQKEATRKG
TTYKPTESEKSLMSRTGGLGAPRGQAFWPVRGPTLHRYGEQLQGELRWKGMVIGASEGTEVKAIADGRVI
LADWLQGYGLVVVVEHGKGDMSLYGYNQSALVSVGSQVRAGQPIALVGSSGGQGRPSLYFEIRRQGQAVN
PQPWLGR

>gi|161486375|ref|NP_839574.2| 30S ribosomal protein S4 [Shigella flexneri 2a str. 2457T]

MARYLGPKLKLSRREGTDLFLKSGVRAIDTKCKIEQAPGQHGARKPRLSDYGVQLREKQKVRRIYGVLER
QFRNYYKEAARLKGNTGENLLALLEGRLDNVVYRMGFGATRAEARQLVSHKAIMVNGRVVNIASYQVSPN
DVVSIREKAKKQSRVKAALELAEQREKPTWLEVDAGKMEGTFKRKPERSDLSADINEHLIVELYSK

>gi|161486371|ref|NP_839686.2| FxsA [Shigella flexneri 2a str. 2457T]

MRWLPFIAIFLYVYIEISIFIQVAHVLGVLLTLVLVIFTSVIGMSLVRNQGFKNFVLMQQKMAAGENPAA

EMIKSVSLIIAGLLLLLPGFFTDFLGLLLLLPPVQKHLTVKLMPHLRFSRMPGGGFSAGTGGGNTFDGEY

QRKDDDRDRLDHKDDRQD

>gi|161486370|ref|NP_839707.2| ribosome-associated GTPase [Shigella flexneri 2a str. 2457T]

MSKNKLSKGQQRRVNANHQRRLKTSKEKPDYDDNLFGEPDEGIVISRFGMHADVESADGDVHRCNIRRTI

RSLVTGDRVVWRPGKPAAEGVNVKGIVEAVHERTSVLTRPDFYDGVKPIAANIDQIVIVSAILPELSLNI

IDRYLVACETLQIEPIIVLNKIDLLDDEGMAFVNEQMDIYRNIGYRVLMVSSHTQDGLKPLEEALTGRIS

IFAGQSGVGKSSLLNALLGLQKEILTNDVSDNSGLGQHTTTAARLYHFPHGGDVIDSPGVREFGLWHLEP

EQITQGFVEFHDYLGLCKYRDCKHDTDPGCAIREAVEEGKIAETRFENYHRILESMAQVKTRKNFSDTDD

>gi|161486365|ref|NP_839771.2| phosphoglycerol transferase I [Shigella flexneri 2a str. 2457T]

MSELLSFALFLASVLIYAWKAGRNTWWFAATLTVLGLFVVLNITLFASDYFTGDGINDAVLYTLTNSLTG

AGVSKYILPGIGIVLGLTAVFGALGWILRRRRHHPHHFGYSLLALLLALGSVDASPAFRQITELVKSQSR

DGDPDFAAYYKEPSKTIPDPKLNLVYIYGESLERTYFDNEAFPDLTPELGALKNEGLDFSHTQQLPGTDY

TIAGMVASQCGIPLFAPFEGNASASVSSFFPQNICLGDILKNSGYQNYFVQGANLRFAGKDVFLKSHGFD

HLYGSEELKSVVADPHYRNDWGFYDDTVLDEAWKKFEELSRSGQRFSLFTLTVDTHHPDGFISRTCNRKK

YDFDGKPNQSFSAVSCSQENIATFINKIKASPWFKDTVIVVSSDHLAMNNTAWKYLNKQDRNNLFFVIRG

DKPQQKTLAVKRNTMDNGATVLDILGGDNYLGLGRSSLSGQSMSEIFLNIKEKTLAWKPDIIRLWKFPKE

MKEFTIDQQKNMIAFSGSHFRLPLLLRVSDKRVEPLPESEYSAPLRFQLADFAPRDNFVWVDSCYKMAQL

WAPELALSTDWCVSQGQLGGQQIVQHVDKTTWKSKTAFKDTVIDMARYKGNVDTLKIVDNDIRYKADSFI

FNVAGAPEEVKQFSGISRPESWGRWSNAQLGDEVKIEYKHPLPKKFDLVITAKAYGNNASRPIPVRVGNE

EQTLVLGNEVTTTTLHFDNPTDADTLVIVPPEPVSTNEGNILGHSPRKLGIGMVEIKVVEREG

>gi|161486364|ref|NP_839778.2| DNA-binding transcriptional activator BglJ [Shigella flexneri 2a str. 2457T]

MSSIGIESLFRKFAGNPYKLHTYTSQESFQDAMSRISFAAVIFSFSAMRSERREGLSCLTELAIKFPRTR

RLVIVDDDIEARLIGSLSPSPLDGVLSKASTLEIFHQELFLSLNGVRQATDRLNNQWYINQSRTLSPTER
EILRFMSRGYSMTQIAEQLKRNIKTIRAHKFNVMSKLGVSSDAGLLEAADILLCMRHCEASNVLHPY

>gi|30065639|ref|NP_839810.1| RNA methyltransferase [Shigella flexneri 2a str. 2457T]
MRITIILVAPARAENIGAAARAMKTMGFSELRIVDSQAHLEPATRWVAHGSGDIIDNIKVFPTLAESLHD
VDFTVATTARSRAKYHYYATPVELVPLLEEKSSWMSHAALVFGREDSGLTNEELALADVLTGVPMVADYP
SLNLGQAVKVYCYQLATLIQQPAKSDTTADQHQLQALRERVMALLTTLAVADDIKLVDWLQQRLGLLEQR
DTAMLHRLLHDIEKNITK

>gi|30065637|ref|NP_839808.1| two-component response regulator [Shigella flexneri 2a str. 2457T]
MQTPHILIVEDELVTRNTLKSIFEAEGYDVFEATDGAEMHQILSEYDINLVIMDINLPGKNGLLLARELR
EQANVALMFLTGRDNEVDKILGLEIGADDYITKPFNPRELTIRARNLLSRTMNLGTVSEERRSVESYKFN
GWELDINSRSLIGPDGEQYKLPRSEFRAMLHFCENPGKIQSRAELLKKMTGRELKPHDRTVDVTIRRIRK
HFESTPDTPEIIATIHGEGYRFCGDLED

>gi|30065636|ref|NP_839807.1| hypothetical protein S4703 [Shigella flexneri 2a str. 2457T]
MLKSPLFWKMTTLFGAVLLLLIPIMLIRQVIVERADYRSDVEDAIRQSTSGPQKLVGPLIAIPVTELYTV
QEEDKTVERKRSFIHFWLPESLMVDGNQNVEERKIGIYTGQVWHSDLTLKADFDVSRLSELNAPNIILGK
PFIVISVGDARGIGVVKAPEVNGTALTIEPGTGLEQGGQGVHIPLPEGDWRKQNLQLNMALNLSGTGDLS
VVPAGRNSEMTLTSNWPHPSFLGDFLPAKREASESGFQAQWQSSWFANNLGERFASGNDTGWENFPAFSV
AVTTPADQYQLTDRATKYAILLIALTFMAFFVFETLTAQRLHPMQYLLVGLSLVMFYLLLLALSEHIGFT
VAWIIASLIGALMNGIYLQAVLKGWRNSMLFTLALLLLDGVMWGLLNSADSALLLGTSVLVVALAGMMFV
TRNIDWYAFSLPKMKASKEVTTDDELRIWK

>gi|30065635|ref|NP_839806.1| sensory histidine kinase CreC [Shigella flexneri 2a str. 2457T]

MRIGMRLLLGYFLLVAVAAWFVLAIFVKEVKPGVRRATEGTLIDTATLLAELARPDLLSGDPTHGQLAQA
FNQLQHRPFRANIGGINKVRNEYHVYMTDAHGKVLFDSANKAVGQDYSRWNDVWLTLRGQYGARSTLQNP
ADPESSVMYVAAPIMGGSRLIGVLSVGKPNAAMAPVIKRSERRILWASAILLGIALVIGAGMVWWINRSI
ARLTRYADSVTDNKPVPLPDLGSSELRKLAQALESMRVKLEGKNYIEQYVYALTHELKSPLAAIRGAAEI
LREGPPPEVVARFTDNILTQNARMQALVETLLRQARLENRQEVVLTAVDVAALFRRVSEARTVQLAEKNI
TLHVMPTEVNVAAEPALLDQALGNLLDNAIDFTPESGCITLSAEVDQEHVTLKVLDTGSGIPDYALSRIF
ERFYSLPRANGQKSSGLGLAFVSEVARLFNGEVTLRNVQEGGVLASLRLHRHFT

>gi|30065634|ref|NP_839805.1| DNA-binding response regulator CreB [Shigella flexneri 2a str. 2457T]

MQRETVWLVEDEQGIADTLVYMLQQEGFAVEVFERGLPVLDKARQQVPDVMILDVGLPDISGFELCRQLL
ALHPALPVLFLTARSEEVDRLLGLEIGADDYVAKPFSPREVCARVRTLLRRVKKFSTPSPVIRIGHFELN
EPAAQISWFDTPLTLTRYEFLLLKTLLKSPGRVWSRQQLMDSVWEDAQDTYDRTVDIHIKTLRAKLRAIN
PDLSPINTHRGMGYSLRGL

>gi|30065633|ref|NP_839804.1| hypothetical protein S4700 [Shigella flexneri 2a str. 2457T]

MKYKHLILSLSLIMLGPLAHAEEIGSVDTVFKMIGPDHKIVVEAFDDPDVKNVTCYVSRAKTGGIKGGLG
LAEDTSDAAISCQQVGPIELSDRIKNGKAQGEVVFKKRTSLVFKSLQVVRFYDAKRNALAYLAYSDKVVE
GSPKNAISAVPVMPWRQ

>gi|30065632|ref|NP_839803.1| right origin-binding protein [Shigella flexneri 2a str. 2457T]

MDQAGIIRDLLIWLEGHLDQPLSLDNVAAKAGYSKWHLQRMFKDVTGHAIGAYIRARRLSKSAVALRLTA
RPILDIALQYRFDSQQTFTRAFKKQFAQTPALYRRSPEWSAFGIRPPLRLGEFTMPEHKFVTLEDTPLIG
VTQSYSCSLEQISDFRHEMRYQFWHDFLGNAPTIPPVLYGLNETRPSQDKDDEQEVFYTTALAQDQADGY
VLTGHPVMLQGGEYVMFTYEGLGTGVQEFILTVYGTCMPMLNLTRRKGQDIERYYPAEDAKAGDRPINLR

CELLIPIRR

>gi|30065630|ref|NP_839801.1| Trp operon repressor [Shigella flexneri 2a str. 2457T]

MAQQSPYSAAMAEQRHQEWLRFVDLLKNAYQNDLHLPLLNLMLTPDEREALGTRVRIVEELLSGEMSQRE

LKNELGAGIATITRGSNSLKAAPVELRQWLEEVLLKSD

>gi|30065626|ref|NP_839797.1| DNA repair protein RadA [Shigella flexneri 2a str. 2457T]

MAKAPKRAFVCNECGADYPRWQGQCSACHAWNTITEVRLAASPTVARNERLSGYAGSAGVAKVQKLSDIS

LEELPRFSTGFKEFDRVLGGGVVPGSAILIGGNPGAGKSTLLLQTLCKLAQQMKTLYVTGEESLQQVAMR

AHRLGLPTDNLNMLSETSIEQICLIAEEEQPKLMVIDSIQVMHMADVQSSPGSVAQVRETAAYLTRFAKT

RGVAIVMVGHVTKDGSLAGPKVLEHCIDCSVLLDGDADSRFRTLRSHKNRFGAVNELGVFAMTEQGLREV

SNPSAIFLSRGDEVTSGSSVMVVWEGTRPLLVEIQALVDHSIMANPRRVAVGLEQNRLAILLAVLHRHGG

LQMADQDVFVNVVGGVKVTETSADLALLLAMVSSLRDRPLPQDLVVFGEVGLAGEIRPVPSGQERISEAA

KHGFRRAIVPAANVPKKAPEGMQIFGVKKLSDALSVFDDL

>gi|30065624|ref|NP_839795.1| hypothetical protein S4690 [Shigella flexneri 2a str. 2457T]

MARTKLKFRLHRAVIVLFCLALLVALMQGASWFSQNHQRQRNPQLEELARTLARQVTLNVAPLMRTDSPD

EKRIQAILDQLTDESRILDAGVYDEQGDLIARSGESVEVRDRLALDGKKAGGYFNQQIVEPIAGKNGPLG

YLRLTLDTHTLATEAQQVDNTTNILRLMLLLSLAIGVVLTRTLLQGKRTRWQQSPFLLTASKPVPEEEES

EKKE

>gi|30065622|ref|NP_839793.1| purine nucleoside phosphorylase [Shigella flexneri 2a str. 2457T]

MATPHINAEMGDFADVVLMPGDPLRAKYIAETFLEDAREVNNVRGMLGFTGTYKGRKISVMGHGMGIPSC

SIYTKELITDFGVKKIIRVGSCGAVLPHVKLRDVVIGMGACTDSKVNRIRFKDHDFAAIADFDMVRNAVD

AAKALGVDARVGNLFSADLFYSPDGEMFDVMEKYGILGVEMEAAGIYGVAAEFGAKALTICTVSDHIRTH

EQTTAAERQTTFNDMIKIALESVLLGDKE

>gi|30065621|ref|NP_839792.1| phosphopentomutase [Shigella flexneri 2a str. 2457T]

MKRAFIMVLDSFGIGATEDAERFGDVGADTLGHIAEACAKGEADNGRKGPLNLPNLTRLGLAKAHEGSTG

FIPAGMDGNAEVIGAYAWAHEMSSGKDTPSGHWEIAGVPVLFEWGYFSDHENSFPQELLDKLVERANLPG

YLGNCHSSGTVILDQLGEEHMKTGKPIFYTSADSVFQIACHEETFGLDKLYELCEIAREELTNGGYNIGR

VIARPFIGDKAGNFQRTGNRHDLAVEPPAPTVLQKLVDEKHGQVVSVGKIADIYANCGITKKVKATGLDA

LFDATIKEMKEAGDNTIVFTNFVDFDSSWGHRRDVAGYAAGLELFDRRLPELMSLLRDDDILILTADHGC

DPTWTGTDHTREHIPVLVYGPKVKPGSLGHRETFADIGQTLAKYFGTSDMEYGKAMF

>gi|30065618|ref|NP_839789.1| hypothetical protein S4683 [Shigella flexneri 2a str. 2457T]

MPTSHENALQQRCQQIVTSPVLSPEQKRHFLALEAENNLPYPQLPAEARRALDEGVICDMFEGHAPYKPR

YVLPDYARFLANGSEWLELEGAKDLDDALSLLTILYHHVPSVTSMPVYLGQLDALLQPYVRILTQNEIDV

RIKRFWRYLDRTLPDAFMHANIGPSDSPITRAILRADAQVSPNLTFIYDPEITPDDLLLEVAKNICECSK

PHIANGPVHDKIFTKGGYGIVSCYNSLPLAGGGSTLVRLNLKAIAERSESLDDFFTRTLPHYCQQQIAII

DARCEFLYQQSHFFENSFLVKEGLINPERFVPMFGMYGLAEAVNLLCEKEGIAARYGKEAAANEVGYRIS

AQLAEFVANTPVKYGWQKRAMLHAQSGISSDIGTTPGARLPYGDEPDPITHLQTVAPHHAYYYSGISDIL

TLDETIKRNPQALVQLCLGAFKAGMREFTANVSGNDLVRVTGYMVRLSDLEKYRAEGSRTNTTWLGEEAA

RNTRILERQPRVISHEQQMRFSQ

>gi|30065617|ref|NP_839788.1| activating enzyme [Shigella flexneri 2a str. 2457T]

MNSRCALVSKIIPFSCVDGPGSRLALFLQGCNLRCKNCHNPWTMGRCNDCGECVPQCPHQALQIVDGKVV

WNAVVCEQCDTCLKMCPQHATPMAQSMSVDEVLSHVRKAVLFIEGITVSGGEATTQLPFVVALFTAIKND

PQLRHLTCLVDSNGMLSETGWEKLLPVCDGAMLDLKAWGSECHQQLTGRDNQQIKRSIYLLAERGKLAEL

RLLMIPGQVDYLQHIEELAAFIKGLGDVPVRLNAFHAHGVYGEAQSWASATPEDVEPLADALKVRGVSRL

IFPALYL

>gi|30065615|ref|NP_839786.1| hypothetical protein S4680 [Shigella flexneri 2a str. 2457T]

MGQRIPVTLGNIAPLSLRPFQPGRIALVCEGGGQRGIFTAGVLDEFMRAQFNPFDLYLGTSAGAQNLSAF

ICNQPGYARKVIMRYTTKREFFDPLRFVRGGNLIDLDWLVEATASQMPLQMDTAARLFDSGKSFYMCACR

QDDYAPNYFLPTKQNWLDVIRASSAIPGFYRSGVSLEGINYLDGGISDAIPVKEAARQGAKTLVVIRTVP

SQMYYTPQWFKRMERWLGDSSLQPLVNLVQHHETSYRDIQQFIEKPPGKLRISEIYPPKPLHSIALGSRI

PALREDYKLGRLCGRYFLATVGKLLTEKAPLTRHLVPVVTPESIVIPPAPVANDTLVAEVSDAPQANDPT

FNNEDLA

>gi|30065614|ref|NP_839785.1| periplasmic protein [Shigella flexneri 2a str. 2457T]

MTMTRLKISKTLLAVMLTSAVATGSAYAENNAQTTNESAGQKVDSSMNKVGNFMDDSAITAKVKAALVDH

DNIKSTDISVKTDQKVVTLSGFVESQAQAEEAVKVAKGVEGVTSVSDKLHVRDAKEGSVKGYAGDTATTS

EIKAKLLADDIVPSRKVKVETTDGVVQLSGTVDSQAQSDRAESIAKAVDGVKSVKNDLKTK

>gi|30065610|ref|NP_839781.1| DNA polymerase III subunit psi [Shigella flexneri 2a str. 2457T]

MTSRRDWQLQQLGITQWSLRRPGALQGEIAIASPAHVRLVMVANDLPALTDPLVSDVLRALTVSPDQVLQ

LTPEKIAMLPQGSRCNSWRLGTDEPLSLEGAQVASPALTELRANPTARAALWQQICTYEHDFFPRND

>gi|30065609|ref|NP_839780.1| 16S ribosomal RNA m2G1207 methyltransferase [Shigella flexneri 2a str. 2457T]

MSAFTPASEVLLRHSDDFEQSRILFAGDLQDDLPARLDTAASRAHTQQFHHWQVLSCQMGDNARFSLVAT

ANDVADCDTLIYYWPKNKPEAQFQLMNLLSLLPVGTDIFVVGENRSGVRSAEQMLADYAPLNKVDSARRC

GLYFGRLEKQPVFDADKFWGEYSVDGLTVKTLPGVFSRDGLDVGSQLLLSTLTPHTKGKVLDVGCGAGVL

SVAFARHSPKIRLTLCDVSAPAVEASRATLAANGVEGEVFASNVFSEVKGCFDMIISNPPFHDGMQTSLD

AAQTLIRGAVRHLNSGGELRIVANAFLPYPDVLDETFGFHEVIAQTGRFKVYRAIMTRQAKKG

>gi|30065608|ref|NP_839779.1| ferric iron reductase involved in ferric hydroximate transport [Shigella flexneri 2a str. 2457T]

MAYRSAPLYEDVIWRTHLQPQDAGLAQAVRAMIAKHREHLLEFIRLDEPAPLNAMTLAQWSSPNALSSLL

AVYSDHIYRNQPTMIRENKPLISLWAQWYIGLMVPPLMLALLTQEKALDVSPEHFHAEFHETGRVACFWV

DVCEDKNATPHSPQQRMETLISQALVPVVQALEATGEINGKLIWSNTGYLINWYLTEMKQLLGEATVESL

RHALFFEKTLTNGEDNPLWRTVVLRDGLLVRRTCCQRYRLPDVQQCGYCTLK

>gi|30065603|ref|NP_839774.1| primosomal protein DnaI [Shigella flexneri 2a str. 2457T]

MSSRVLTPDVVGIDALVHDHQTVLAKAEGGVVAVFANNAPAFYAVTPARLAELLALEEKLARPGSDVALD

DQLYQEPQAAPVAVPMGKFAMYPDWQPDADFIRLAALWGVALREPVTTEELASFIAYWQAEGKVFHHVQW

QQKLARSLQIGRASNGGLPKRDVNTVSEPDSQIPPGFRG

>gi|30065602|ref|NP_839773.1| DNA replication protein DnaC [Shigella flexneri 2a str. 2457T]

MKNVGDLMQRLQKMMPAHIKPAFKTGEELLAWQKEQGAIRSAALERENRAMKMQRTFNRSGIRPLHQNCS

FENYRVECEGQMNALSKARQYVEEFDGNIASFIFSGKPGTGKNHLAAAICNELLLRGKSVLIITVADIMS

AMKDTFRNSGTSEEQLLNDLSNVDLLVIDEIGVQTESKYEKVIINQIVDRRSSSKRPTGMLTNSNMEEMT

KLLGERVMDRMRLGNSLWVIFNWDSYRSRVTGKEY

>gi|30065601|ref|NP_839772.1| hypothetical protein S4661 [Shigella flexneri 2a str. 2457T]

MMMKTIKHLLCCAIAASALISTGVHAASWKDALSSAASELGNQNSTTQEGGWSLASLTNLLSSGNQALSA

DNMNNAAGILQYCAKQKLASVTDAENIKNQVLEKLGLNSEEQKEDTNYLDGIQGLLKTKDGQQLNLDNIG

TTPLAEKVKTKACDLVLKQGLNFIS

>gi|30065597|ref|NP_839768.1| methyl-accepting chemotaxis protein I, serine sensor receptor [Shigella flexneri 2a str. 2457T]

MLKRIKIVTSLLLVLAVFGLLQLTSGGLFFNALKNDKENFTVLQTIRQQQPTLNGSWVALLQTRNTLNRA

GIRYMMDQNNIGSGSTVAELMQSASISLKQAEKNWADYEALPRDPRQSTAAAAEIKRNYDIYHNALAELI

QLLGAGKINEFFDQPTQGYQDGFEKQYVAYMEQNDRLYDIAVSDNNASYSQAMWILVGVMIVVLAVIFAV

WFGIKASLVAPMNRLIDSIRHIAGGDLVKPIEVDGSNEMGQLAESLRHMQGELMRTVGDVRNGANAIYSG

ASEIATGNNDLSSRTEQQAASLEETAASMEQLTATVKQNAENARQASHLALSASETAQRGGKVVDNVVQT

MRDISTSSQKIADIISVIDGITFQTNILALNAAVEAARAGEQGRGFAVVAGEVRNLAQRSAQAVREIKSL

IEDSVGKVDVGSTLVESAGETMAEIVSAVTRVTDIMGEIASASDEQSRGIDQVGLAVAEMDRVTQQNAAL

VEESAAAAAALEEQASRLTEAVAVFRIQQQQQQQRETSAVVKTVTPATPRKMAVADSGENWETF

>gi|30065596|ref|NP_839767.1| regulator of the 4HPA-hydroxylase operon [Shigella flexneri 2a str. 2457T]

MHDSLTIALLQAREAAMSYFRPIVKRHNLTEQQWRIVRILAESPSMDFHDLAYRACILRPSLTGILTRME

RDGLVLRLKPINDQRKLYISLTKEGQALYNRAQTQIEEAYRQIEAQFTAEKMQQLTHLLEEFIALGNSRQ

EDIPGDNE

>gi|30065593|ref|NP_839764.1| homoprotocatechuate dyoxygenase [Shigella flexneri 2a str. 2457T]

MGKLALAAKITHVPSMYLSELPGKNHGCRQGAIDGHKEISKRCREMGVDTIIVFDTHWLVNSAYHINCAD

HFEGVYTSNELPHFIRDMTYNYEGNPELGQLIAEEALKLGVRAKAHNIPSLKLEYGTLVPMRYMNEDKHF

KVVSISAFCTVHDFADSRKLGEAILKAIEQYDGTVAVLASGSLSHRFIDDQRAEEGMNSYTREFDRQMDE

RVVKLWREGQFKEFCNMLPEYADYCYGEGNMHDTVMLLGMLGWDKYDGKVEFITELFPSSGTGQVNAVFP

LPA

>gi|30065592|ref|NP_839763.1| 5-carboxymethyl-2-hydroxymuconate delta-isomerase [Shigella flexneri 2a str. 2457T]

MPHFIVECTENIREEARLPELFASVNTALAATGIFPLGGIRSRAHWIDTWQMADGKHDYAFVHMTLKIGS

GRSLESRQEVGEMLFDLIKTHFASLMESRYLALSFEIAELHPTLNFKQNNVHALFK

>gi|30065591|ref|NP_839762.1| 2-oxo-hepta-3-ene-1,7-dioic acid hydratase [Shigella flexneri 2a str. 2457T]

MFDKHTHTLIAQRLDQAEKQREQIRAISLDYPEITIEDAYAVQREWVRLKIAESRTLKGHKIGLTSKAMQ

ASSQISEPDYGALLDDMFFHDGSDIPTDRFIVPRIEVELAFVLAKPLRGPNCTLFDVYNATDYVIPALEL

IDARCHNIDPETQRPRKVFDTISDNAANAGVILGGRPIKPDELDLRWISALMYRNGVIEETGVAAGVLNH

PANGVAWLANKLAPYDVQLETGQIILGGSFTLPVPARKGDTFHVDYGNMGSISCRFV

>gi|30065590|ref|NP_839761.1| 2,4-dihydroxyhept-2-ene-1,7-dioic acid aldolase [Shigella flexneri 2a str. 2457T]

MENSFKAALKAGRPQIGLWLGLSSSYSAELLAGPGFDWLLIDGEHAPNNVQTVLTQLQAIAPYPSQPVVR

PSWNDPVQIKQLLDVGTQTLLVPMVQNADEARKAVRATRYPPAGIRGVGSALARASRWNRIPDYLQKAND

QMCVLVQIETREAMKNLPQILDVEGVDGVFIGPADLSADMGYAGNPQHPEVQAAIEQAIVQIREAGKAPG

ILIANEQLAKRYLELGALFVAVGVDTTLLARAAEALAARFGNSSSISMAKQNNNSVY

>gi|30065588|ref|NP_839759.1| 4-hydroxyphenylacetate 3-monooxygenase operon regulatory protein [Shigella flexneri 2a str. 2457T]

MSSEAIFEHGYENIDLVREYDARYAGEDVHYETFARLANFFGRDMRPHWHDRYFQLHYLVTGRITLQLDE

HFYSVRAPLFVLTPPSVPHTFFTNPDSDGHVLTVRQELIWPLVEKLWPGSGDAAMLQGICLSLEGMEHTL

AALNHYWPIISLEFQQNKQGREILLQSLAQSIFTILLREAPPNDISTCSVRGEMCLFQKFNRLIDENYRQ

HLMVPEYATMLGMSESRLTELCRRFANQSPKRLIFERVSREARRLLLYSEQSINQIALDLGYKDPAYFAR

FFNRMMGCSPTQFRGR

>gi|30065586|ref|NP_839757.1| component C of the 4HPA-hydroxylase [Shigella flexneri 2a str. 2457T]

MQLDEQRLRFHDAMASLSAAVNIITTEGDAGQCGITATAVCSVTDTPPSLMVCINANSAMNPVFQGNGKL

CVNVLNHEQELMARHFAGMTGMAMEERFSLSCWQKGPLAQPVLKGSLASLEGEIRDVQAIGTHLVYLVEI

KNIILSAEGHGLIYFKRRFHPVMLEMEAAI

>gi|30065585|ref|NP_839756.1| carbon starvation protein [Shigella flexneri 2a str. 2457T]

MPGFTMDTKKLFKHIPWVILGIIGAFCLAVVALRRGEHVSALWIVVASVSVYLVAYRYYSLYIAQKVMKL

DPTRATPAVINNDGLNYVPTNRYVLFGHHFAAIAGAGPLVGPVLAAQMGYLPGTLWLLAGVVLAGAVQDF

MVLFISSRRNGASLGEMIKEEMGPVPGTIALFGCFLIMIIILAVLALIVVKALAESPWGVFTVCSTVPIA

LFMGIYMRFIRPGRVGEVSVIGIVLLVASIYFGGVIAHDPYWGPALTFKDTTITFALIGYAFVSALLPVW

LILAPRDYLATFLKIGVIVGLALGIVVLNPELKMPAMTQYIDGTGPLWKGALFPFLFITIACGAVSGFHA

LISSGTTPKLLANETDARFIGYGAMLMESFVAIMALVAASIIEPGLYFAMNTPPAGLGITMPNLHEMGGE

NAPIIMAQLKDVTAHAAATVSSWGFVISPEQILQTAKDIGEPSVLNRAGGAPTLAVGIAHVFHKVLPMAD

MGFWYHFGILFEALFILTALDAGTRSGRFMLQDLLGNFIPFLKKTDSLVAGIIGTAGCVGLWGYLLYQGV

VDPLGGVKSLWPLFGISNQMLAAVALVLGTVVLIKMKRTQYIWVTVVPAVWLLICTTWALGLKLFSTNPQ

MEGFFYMASQYKEKIANGTDLTAQQIANMNHIVVNNYTNAGLSILFLIVVYSIIFYGFKTWLAVRNSDKR

TDKETPYVPIPEGGVKISSHH

>gi|30065580|ref|NP_839751.1| hypothetical protein S4635 [Shigella flexneri 2a str. 2457T]

MSVEKLIVDHMETWTSALQTRSTAGRGSSGKIDLYGIKKLRELILELAVRGKLVPQDPNDEPASDLLKRI

AAEKAELVKQGKIKKQKPLPEISEEEKPFELPEGWEWVHLPDIYCSISESSRKIKSSEILPEGKYPVIEQ

SQEFISGYCNNECLLIKLNNPVIVFGDHTRNIKFIDFDFVVGADGVKILSPILICERFFFWQLRSFKLDV

RGYARHFKVLNSCLFALPPIAEQERIVEKVSSLMSLCDQLEQQSLTSLDAHQQLVETLLGTLTDSQNTAE

LAENWARISEHFDTLFTTEASVDALKQTILQLAVMGKLVPQDPNDEPASELLKRIAQEKAQLVKEGKIQK

PLPPISDEEKPFELPEGWEWCRIGNIVNIKSELVSPKDYLNLYQVAPDIIEKGTGRVISKRTVKESGVKG

PNSRFYKGQIVYSKIRPSLSKVFLAEYNGLCSADMYPLDCYINPNYLLKYILSIPFLMQVKKAENRIKMP

KLNSDSFYNIIVAIPPYNEQQAIFDKINSIEAVCNGLISYIGIYHKTQLHLADALTDAAIN

>gi|30065579|ref|NP_839750.1| hypothetical protein S4633 [Shigella flexneri 2a str. 2457T]

MLFDHLRDEVMRLDAGITQEVLKLYIAFKAETNFVDVVPQKSRLRLSLNMQFHELVDPKGIAKDVTNVGR
WGNGDVEIGFSDLAQLPYIMGLIRQAFEKQMESALV

>gi|30065572|ref|NP_839743.1| primosomal replication protein N [Shigella flexneri 2a str. 2457T]
MTNRLVLSGTVCRTPLRKVSPSGIPHCQFVLEHRSVQEEAGFHRQAWCQMPVIVSGHENQAITHSITVGS
RITVQGFISCHKAKNGLSKMVLHAEQIELIDSGD

>gi|30065571|ref|NP_839742.1| 30S ribosomal protein S6 [Shigella flexneri 2a str. 2457T]
MRHYEIVFMVHPDQSEQVPGMIERYTAAITGAEGKIHRLEDWGRRQLAYPINKLHKAHYVLMNVEAPQEV
IDELETTFRFNDAVIRSMVMRTKHAVTEASPMVKAKDERRERRDDFANETADDAEAGDSEE

>gi|30065568|ref|NP_839739.1| L-xylulose 5-phosphate 3-epimerase [Shigella flexneri 2a str. 2457T]
MLSKQIPLGIYEKALPAGECWLERLRLAKTLGFDFVEMSVDETNERLSRLDWSREQRLALVNAIVETGVR
VPSMCLSAHRRFPLGSEDDAVRAQGLEIMRKAIQFAQDVGIRVIQLAGYDVYYQEANNETRRRFRDGLKE
SVEMASRAQVTLAMEIMDYPLMNSISKALGYAHYLNNPWFQLYPDIGNLSAWDNDVQMELQAGIGHIVAV
HVKDTKPGVFKNVPFGEGVVDFERCFETLKQSGYCGPYLIEMWSETAEDPAAEVVKACDWVKARMAKAGM
VEAA

>gi|30065567|ref|NP_839738.1| 3-keto-L-gulonate-6-phosphate decarboxylase [Shigella flexneri 2a str. 2457T]

MSLPMLQVALDNQTMDSAYETTRLIAEEVDIIEVGTILCVGEGVRAVRDLKALYPHKIVLADAKIADAGK

ILSRMCFEANADWVTVICCADINTAKGALDVAKEFNGDVQIELTGYWTWEQAQQWRDAGIQQVVYHRSRD

AQAAGVAWGEADITAIKRLSDMGFKVTVTGGLALEDLPLFKGIPIHVFIAGRSIRDAASPVEAARQFKRS

IAELWG

>gi|30065566|ref|NP_839737.1| L-ascorbate-specific enzyme IIA component of PTS [Shigella flexneri 2a str. 2457T]

MKLHDSLAENKSIRLQAEAETWQDAVKIGVDLLVAADVVEPRYYQAILDAVEQHGPYFVLAPGLAMPHGR

PEEGVKKTGFALVTLKKPLEFNHEDNDPVDILITMAAVDANTHQEVGIMQIVNLFEDEENFDRLRACRTE

QEVLDLIDRTNAAA

>gi|30065565|ref|NP_839736.1| L-ascorbate-specific enzyme IIB component of PTS [Shigella flexneri 2a str. 2457T]

MTVRILAVCGNGQGSSMIMKMKVDQFLTQSNIDHTVNSCAVGEYKSELSGADIIIASTHIAGEITVTGNK

YVVGVRNMLSPADFGPKLLEVIKAHFPQDVK

>gi|30065563|ref|NP_839734.1| L-ascorbate 6-phosphate lactonase [Shigella flexneri 2a str. 2457T]

MAMSKVKSITRESWILSTFPEWGSWLNEEIEQEQVAPGTFAMWWLGCTGIWLKSEGGTNVCVDFWCGTGK

QSHGNPLMKQGHQMQRMAGVKKLQPNLRTTPFVLDPFAIRQIDAVLATHDHNDHIDVNVAAAVMQNCADD

VPFIGPKTCVDLWIGWGVPKERCIVVKPGDVVKVKDIEIHALDAFDRTALITLPADQKAAGVLPDGMDDR

AVNYLFKTPGGSLYHSGDSHYSNYYAKHGNEHQIDVALGSYGENPRGITDKMTSADMLRMGEALNAKVVI

PFHHDIWSNFQADPQEIRVLWEIKKDRLKYGFKPFIWQVGGKFTWPLDKDNFEYHYPRGFDDCFTIEPDL

PFKSFL

>gi|30065562|ref|NP_839733.1| transcriptional repressor UlaR [Shigella flexneri 2a str. 2457T]

MTEAQRHQILLEMLAQLGFVTVEKVVERLGISPATARRDINKLGESGKLKKVRNGAEAITQQRPRWTPMN

LHQAQNHDEKVRIAKAASQLVNPGESVVINCGSTAFLLGREMCGKPVQIITNYLPLANYLIDQEHDSVII

MGGQYNKSQSITLSPQGSENSLYAGHWMFTSGKGLTAEGLYKTDMLTAMAEQKMLSVVGKLVVLVDSSKI

GERAGMLFSRADQIDMLITGKNANPEILQQLEAQGVSILRV

>gi|30065561|ref|NP_839732.1| esterase [Shigella flexneri 2a str. 2457T]

MIEIESRELADIPVLHAYPVGQKDTPLPCVIFYHGFTSSSLVYSYFAVALAQAGLRVIMPDAPDHGSRFS

GDEARRLNQFWQILLQSMQEFTTLRAAIAEENWLLDDRLAVGGASMGAMTALGITARHPTVKCTASMMGS

GYFTSLARSLFPPLIPETAAQQNEFNKIVAPLAEWEATNHLEQLGDRPLLLWHGLDDDVVPADESLRLQQ

ALSETGRDKLLTCSWQPGVRHRITPEALDAAVTFFRQHL

>gi|30065559|ref|NP_839730.1| hypothetical protein S4613 [Shigella flexneri 2a str. 2457T]

MGKRKMELTMKQLLASPSLQLVTYPASATAQSAEFASADCVTGLNEIGQISVSNISGDPQDVERIVALKA

DEQGASWYRIITMYEDQQPDNWRVQAILYA

>gi|30065556|ref|NP_839727.1| synthetase/amidase [Shigella flexneri 2a str. 2457T]

MLRHNVPVRRDLDQIAANNGFDFHIIDNEIYWDESRAYRFTLRQIEEQIEKPTAELHQMCLEVVDRAVKD

EEILTQLAIPPLYWDVIAESWRARDPSLYGRMDFAWCGNAPVKLLEYNADTPTSLYESAYFQWLWLEDAR

RSGIIPRDADQYNAIQERLISRFSELYSRELFYFCCCQDTDEDRSTVLYLQDCAQQAGQESRFIYIEDLG

LGVGGVLTDLDDNVIQRAFKLYPLEWMMRDDNGPLLRKRREQWVEPLWKSILSNKGLMPLLWRFFPGHPN

LLASWFEGEKSQIAAGESYVRKPIYSREGGNVTIFDGQNNVVDHADGDYADEPMIYQAFQPLPRFGDSYT

LIGSWIVDDEACGMGIREDNTLITKDTSRFVPHYIAG

>gi|30065555|ref|NP_839726.1| hypothetical protein S4607 [Shigella flexneri 2a str. 2457T]

MARKRKSRNNSKIGHGAISRIGRPNNPFEPRRNRYAQKYLTLALMGGAAFFVLKGCSDSSDVDNDGDGTF

YATVQDCIDDGNNADICARGWNNAKAAFYADVPKNMTQQNCQSKYENCYYDNVEQSWIPVVSGFLLSRVI

RKDRDEPFVYNSGGSSFASRPVWRNTSGDYSWRFGSGKKESYSSGGFTTRKASTVSRGGYGRSSSARGHW

GG

>gi|30065550|ref|NP_839721.1| transcriptional repressor NsrR [Shigella flexneri 2a str. 2457T]

MQLTSFTDYGLRALIYMASLPEGRMTSISEVTDVYGVSRNHMVKIINQLSRAGYVTAVRGKNGGIRLGKP

ASAIRIGDVVRELEPLSLVNCSSEFCHITPACRLKQALSKAVQSFLTELDNYTLADLVEENQPLYKLLLV

E

>gi|30065544|ref|NP_839715.1| RNA-binding protein Hfq [Shigella flexneri 2a str. 2457T]

MAKGQSLQDPFLNALRRERVPVSIYLVNGIKLQGQIESFDQFVILLKNTVSQMVYKHAISTVVPSRPVSH

HSNNAGGGTSSNYHHGSSAQNTSAQQDSEETE

>gi|30065541|ref|NP_839712.1| N-acetylmuramoyl-l-alanine amidase II [Shigella flexneri 2a str. 2457T]

MMYRIRNWLVATLLLLCTPVGAATLSDIQVSNGNQQARITLSFIGDPDYAFSHQSKRTVALDIKQTGMIQ

GLPLLFSGNNLVKAIRSGTPKDAQTLRLVVDLTENGKTEAVKRQNGSNYTVVFTINADVPPPPPPPPVVA

KRVETPAVVAPRVSEPARNPFKTESNRTTGVISSNTVTRPAARATANTGDKIIIAIDAGHGGQDPGAIGP

GGTREKNVTIAIARKLRTLLNDDPMFKGVLTRDGDYFISVMGRSDVAHKQNANFLVSIHADAAPNRSATG

ASVWVLSNRRANSEMASWLEQHEKLSELLGGAGDVLANSQSDPYLSQAVLDLQFGHSQRVGYDVATSMIS

QLQRIGEIHKRRPEHASLGVLRSPDIPSVLVETGFISNNSEERLLASDDYQQQLAEAIYKGLRNYFLAHP

MQSAPQGATAQTASTVTTPDRTLPN

>gi|30065540|ref|NP_839711.1| ATPase [Shigella flexneri 2a str. 2457T]

MMNRVIPLPDEQATLDLGERVAKACDGATVIYLYGDLGAGKTTFSRGFLQALGHQGNVKSPTYTLVEPYT

LDNLMVYHFDLYRLADPEELEFMGIRDYFANDAICLVEWPQQGTGVLPDPDVEIHIDYQAQGREARVSAV

SSAGELLLARLAG

>gi|30065538|ref|NP_839709.1| hypothetical protein S4590 [Shigella flexneri 2a str. 2457T]

MSEPLDLNQLAQKIKQWGLELGFQQVGITDTDLSESEPKLQAWLDKQYHGEMDWMARHGMLRARPHELLP

GTLRVISVRMNYLPANAAFASTLKNPKLGYVSRYALGRDYHKLLRNRLKKLGEMIQQHCVSLNFRPFVDS

APILERPLAEKAGLGWTGKHSLILNREAGSFFFLGELLVDIPLPVDQPVEEGCGKCVACMTICPTGAIVE

PYTVDARRCISYLTIELEGALPEELRPLMGNRIYGCDDCQLICPWNRYSQLTTEDDFSPRKPLHAPELIE

LFAWSEEKFLKVTEGSAIRRIGHLRWLRNIAVALGNAPWDETILTALESRKGEHPLLDEHIAWAIAQQIE

RRNACIVEVQLPKKQRLVRVIEKGLPRDA

>gi|30065534|ref|NP_839705.1| hypothetical protein S4581 [Shigella flexneri 2a str. 2457T]

MRLIITFLMAWCLSWGAYAATAPDSKQITQELEQAKAAKPAQPEVVEALQSALNALEERKGSLERIKQYQ

QVIDNYPKLSATLRAQLNNIRDEPRSVSPGMSTDALNQEILQVSSQLLDKSRQAQQEQERAREIADSLNQ

LPQQQTDARRQLNEIERRLGTLTGNTPLNQAQNFALQSDSARLKALVDELELAQLSANNRQELARLRSEL

AEKESQQLDAYLQALRNQLNSQRQLEAERALESTELLAENSADLPKDIVAQFKINRELSAALNQQAQRME

LVASQQRQAASQTLQVRQALNTLREQSQWLGSSNLLGEALRAQVARLPEMPKPQQLDTEMAQLRVQRLRY

EDLLNKQPLLRQIHQADGQPLTAEQNRILEAQLRTQRELLNSLLQGGDTLLLELTKLKVSNRQLEDALKE

VNEATHRYLFWASDVRPMTIAWPLEIAQDLRRLISLDTFSQLGKASVMMLTSKETILPLFGALILVGCSI

YSRRYFTRFLERSAAKVGKVTQDHFWLTLRTLFWSILVASPLPVLWMTLGYGLREAWPYPLAVAIGDGVT

ATVPLLWVVMICATFARPNGLFIAHFGWPRERVSRGMRYYLMSIGLIMALMMFDNLDDREFSGSLGRLCF

ILICGALAVVTLSLKKAGIPLYLNKEGSGDNITNHMLWNMMIGAPLVAILASAVGYLATAQALLARLETS

VAIWFLLLVVYHVIRRWMLIQRRRLAFDRAKHRRAEMLAQRARGEEEAHHHSSPEGAIEVDESEVDLDAI

SAQSLRLVRSILMLIALLSVIVLWSEIHSAFGFLENISLWDVTSTVQGVESLEPITLGAVLIAILVFIIT

TQLVRNLPALLELAILQHLDLTPGTGYAITTITKYLLMLIGGLVGFSMIGIEWSKLQWLVAALGVGLGFG

LQEIFANFISGLIILFEKPIRIGDTVTIRDLTGSVTKINTRATTISDWDRKEIIVPNKAFITEQFINWSL

SDSVTRVVLTIPAPADANSEEVTEILLTAARRCSLVIDNPAPEVFLVDLQQGIQIFELRIYAAEMGHRMP

LRHEIHQLILAGFHAHGIDMPFPPFQMRLESLNGKQTGRTLTSAGKGRQAGSL

>gi|30065529|ref|NP_839700.1| fumarate reductase subunit C [Shigella flexneri 2a str. 2457T]

MTTKRKPYVRPMTSTWWKKLPFYRFYMLREGTAVPAVWFSIELIFALFALKNGPEAWAGFVDFLQNPVIV

IINLITLTAALLHTKTWFELAPKAANIIVKDEKMGPEPIIKSLWAVTVVATIVILFVALYW

>gi|30065528|ref|NP_839699.1| fumarate reductase subunit D [Shigella flexneri 2a str. 2457T]

MINPNPKRSDEPVFWGLFGAGGMWSAIIAPVMILLVGILLPLGLFPGDALSYERVLAFAQSFIGRVFLFL

MIVLPLWCGLHRMHHAMHDLKIHVPAGKWVFYGLAAILTVVTLIGVVTI

>gi|30065525|ref|NP_839696.1| suppresser of groEL [Shigella flexneri 2a str. 2457T]

MPFVFSAIVIKVIVEIPLPPGKISVQPSALQDDLQTPLFTGDGPKFPEPDMSWIILVIAGLLEVVWAVGL

KYTHGFSRLTPSVITVTAMIVSMALLAWAMKSLPVGTAYAVWTGIGAVGAAITGIVLLGESANPMRLASL

ALIVLGIIGLKLSTH

>gi|30065524|ref|NP_839695.1| elongation factor P [Shigella flexneri 2a str. 2457T]

MATYYSNDFRAGLKIMLDGEPYAVEASEFVKPGKGQAFARVKLRRLLTGTRVEKTFKSTDSAEGADVVDM

NLTYLYNDGEFWHFMNNETFEQLSADAKAIGDNAKWLLDQAECIVTLWNGQPISVTPPNFVELEIVDTDP

GLKGDTAGTGGKPATLSTGAVVKVPLFVQIGEVIKVDTRSGEYVSRVK

>gi|30065523|ref|NP_839694.1| hypothetical protein S4569 [Shigella flexneri 2a str. 2457T]

MAHIVTLNTPSREDWLTQLADVVTDPDELLRLLNIDADEKLLAGRSAKKLFALRVPRSFIDRMEKGNPDD

PLLRQVLTSQDEFVVASGFSTDPLEEQHSVVPGLLHKYHNRALLLVKGGCAVNCRYCFRRHFPYAENQGN

KRNWQTALEYVAAHPELDEMIFSGGDPLMAKEHELDWLLTQLEAIPHIKRLRIHSRLPIVIPARITEALV

ERFARSTLQILLVNHINHANEVDETFRQAMAKLRRVGVTLLNQSVLLRGVNDNAQTLANLSNALFDAGVM

PYYLHVLDKVQGAAHFMVSDDEARQIMRELLTLVSGYLVPKLAREIGGEPSKTPLDLQLRQQ

>gi|30065520|ref|NP_839691.1| hypothetical protein S4566 [Shigella flexneri 2a str. 2457T]

MAISIKGVNTGVIRKSNNFIALALKIKEPRNKESLFFMSVMELRDLLIALESRLHQKHKLDAAARLQYEQ

ARDKVIKKMAENIPEILVDELKNADINRRVNTLELTDNQGENLTFVLTLHDGSKCELVVNELQIEMLARA

IIHAINNAEMRELALRITSLLDFLPLYDVDCQDNGNLEYDTYSQPEWKHNLFDHYLAVLYRFKDESGKEQ

FSGAVVKTREATPGKEIEAITRRMLDFSPRLKKLAGVPCQVYVRTVAANNAQPLTQDQCLRALHHLRVQS

TSKTAPQAK

>gi|30065519|ref|NP_839690.1| hypothetical protein S4565 [Shigella flexneri 2a str. 2457T]

MASSSLIMGNNMHVKYLAGIVGAALLMAGCSSSNELSAAGQSVRIVDEQPGAECQLIGTATGKQSNWLSG

QHGEEGGSMRGAANDLRNQAAAMGGNVIYGISSPSQGMLSSFVPTDSQIIGQVYKCPN

>gi|30065513|ref|NP_839684.1| anaerobic C4-dicarboxylate transporter [Shigella flexneri 2a str. 2457T]

MLVVELIIVLLAIFLGARLGGIGIGFAGGLGVLVLAAIGVKPGNIPFDVISIIMAVIAAISAMQVAGGLD

YLVHQTEKLLRRNPKYITILAPIVTYFLTIFAGTGNISLATLPVIAEVAKEQGVKPCRPLSTAVVSAQIA

ITASPISAAVVYMSSVMEGHGISYLHLLSVVIPSTLLAVLVMSFLVTMLFNSKLSDDPIYRKRLEEGLVE

LRGEKQIEIKSGAKTSVWLFLLGVVGVVIYAIINSPSMGLVEKPLMNTTNAILIIMLSVATLTTVICKVD

TDNILNSSTFKAGMSACICILGVAWLGDTFVSNNIDWIKDTAGEVIQGHPWLLAVIFFFASALLYSQAAT

AKALMPMALALNVSPLTAVASFAAVSGLFILPTYPTLVAAVQMDDTGTTRIGKFVFNHPFFIPGTLGVAL

AVCFGFVLGSFML

>gi|30065511|ref|NP_839682.1| thiol:disulfide interchange protein precursor [Shigella flexneri 2a str. 2457T]

MAQRIFTLILLLCSTSVFAGLFDAPGRSQFVPADQAFAFDFQQNQHDLNLTWQIKDGYYLYRKQIRITPE

HAKIADEQLPQGVWHEDEFYGKSEIYRDRLTLPVTINQASAGATLTVTYQGCADAGFCYPPETKTVPLSE

VVANNAASQPVSVSQQEQHTAQLPFSALWALLIGIGIAFTPCVLPMYPLISGIVLGGKQRLSTARALLLT

FIYVQGMALTYTALGLVVAAAGLQFQAALQHPYVLIGLAIVFTLLAMSMFGLFTLQLPSSLQTRLTLMSN

RQQGGSPSGVFVMGAIAGLICSPCTTAPLSAILLYIAQSGNMWPGGGTLYLYALGMGLPLMLITVFGNRL

LPKSGPWMEQVKTAFGFVILALPVFLLERVIGDVWGLRLWSALGVAFFGWAFITSLQAKRGWMRVVQIIL

LAAALVSVRPLQDWAFGATHTAQTQTHLNFTQIKTVDELNQALVEAKGKPVMLDLYADWCVACKEFEKYT

FSDPQVQKALADTVLLQANVTANDAQDVALLKHLNVLGLPTILFFDGQGQEHPQARVTGFMDAETFSAHL

RDRQP

>gi|30065510|ref|NP_839681.1| transcriptional regulator [Shigella flexneri 2a str. 2457T]

MQREDVLGEALKLLELQGIANTTLEMVAERVDYPLDELRRFWPDKEAILYDALRYLSQQIDVWRRQLMLD

ETQTAEQKLLARYQALSECVKNNRYPGCLFIAACTFYPDPGHPIHQLADQQKSAAYDFTHELLTTLEVDD

PAMVAKQMELVLEGCLSRMLVNRSQADVDTAHRLAEDILRFARCRQGGALT

>gi|30065503|ref|NP_839674.1| hypothetical protein S4548 [Shigella flexneri 2a str. 2457T]

MRDLLQRPDLFSINTATLGYKTPLPAIIDACAARGIGAIAPWRRELQGEDLQQITRQLAASNMSVSGLCR

STYYTAPTLAERKLAIDDNRRALDDAAVLNAACYMQVVGGLPTGTKDLYEAREQVKQGIRQLLPHSKDVG

VPIALEPLHPMTAADRSCLCTLRQALDWCDELDPDGEFGLGVAVDVYHVWWDPDLASQILRAGKRILAFH

VSDWLMPTTDLVNDRGMPGDGVINIPSIRRLVENAGFNGAIELEIFSPYWWQKDINSTGNDSNLLIVFYV

QIMPDDLVMQLHRF

>gi|30065500|ref|NP_839671.1| hypothetical protein S4545 [Shigella flexneri 2a str. 2457T]

MIEEQDVEQEETMPGRFELKPTLEKVWHAPDNFRFMDPLPPMHRRGIIIAAIVLVVGFLLPSDDTPNASV

VTREAQLDIQSQSQPPTEEQPQTTQTQPFQPDSGIDNQWRSYRVEPGKTMAQLFRDHGLPATDVYAMAQV

EGAGKPLSNLQNGQMVKIRQNASGVVTGLTIDTGNNQQVLFTRQPDGSFIRAR

>gi|30065498|ref|NP_839669.1| iron-sulfur cluster repair di-iron protein [Shigella flexneri 2a str. 2457T]

MAYRDQPLGELALSIPRASALFRKYDMDYCCGGKQTLARAAARKELDVEVIEAELAKLAEQPIEKDWRSA

PLAEIIDHIIVRYHDRHREQLPELILQATKVERVHADKPSVPKGLTKYLTMLHEELSSHMMKEEQILFPM

IKQGMGSQAMGPISVMESEHDEAGELLEVIKHTTNNVTPPPEACTTWKAMYNGINELIDDLMDHISLENN

VLFPRALAGE

>gi|30065497|ref|NP_839668.1| transmembrane subunit [Shigella flexneri 2a str. 2457T]

MISGVLYALLAGLMWGLIFVGPLIVPEYPAMLQSMGRYLALGLIALPIAWLGRVRLRQLARRDWLTALML

TMMGNLIYYFCLASAIQRTGAPVSTMIIGTLPVVIPVFANLLYSQRDGKLAWGKLAPALICIGIGLASVN

IAELNHGLPDFDWARYTSGIVLALVSVVCWAWYALRNARWLRENPDKHPMMWATAQALVTLPVSLIGYLV

ACYWLNIQTPDFSLPFGPRPLVFISLMVAIAVLCSWVGALCWNVASQRLPTVILGPLIVFETLAGLLYTF

LLRQQMPPLMTLSGIALLVIGVVIAVRAKPEKPLTESVSES

>gi|30065496|ref|NP_839667.1| oxidoreductase [Shigella flexneri 2a str. 2457T]

MIAITGATGQLGHYIIESLMKTVPASQIVAIVRNPAKAQALAAQGITVRQADYSDEAALTSALQGVEKLL

LISSSEVGQRAPQHRNVINAAKAAGVKFIAYTSLLHADTSPLGLADEHIETEKMLADSGIVYTLLRNGWY

SENYLASAPAALEHGVFIGAAGDGKIASATRADYATAAARVISEAGHEGKVYELAGDSAWTLTQLAAELT

KQSGKQVTYQNLSEADFAAALKSVGLPDGLADMLADSDVGASKGGLFDNSKTLSKLIGCPTTTLAESVSH

LFNVNN

>gi|30065495|ref|NP_839666.1| hypothetical protein S4540 [Shigella flexneri 2a str. 2457T]

MGSLHRFVLCLNTLTPKLTFSKYVQKGKYEMSQVSLSQQLKEGNLFAEQCPSREVLKHVTSRWGVLILVA

LREGTHRFSDLRRKIGGVSEKMLAQSLQALEQDGFLNRIAYPVVPPHVEYSLTPLGEQVSEKVAALADWI

ELNLPEVLAVRDERAA

>gi|30065492|ref|NP_839663.1| hypothetical protein S4535 [Shigella flexneri 2a str. 2457T]

MTLRKILALTCLLLPMMASTHQFEIGQRVPPIGITDRGELVLDKDQFSYKTWNSAQLVGKVRVLQHIAGR

TSAKEKNATLIEAIKSAKLPHDRYQTTTIVNTDDAIPGSGMFVRSSLESNKKLYPWSQFIVDSNGVARGA

WQLDEESSAVVVLDKDGRVQWAKDGALTPEEVQQVMDLLQKLLK

>gi|30065490|ref|NP_839661.1| transporter [Shigella flexneri 2a str. 2457T]

MLNSILVILCLIAVSAFFSMSEISLAASRKIKLKLLADEGNINAQRVLNMQENPGMFFTVVQIGLNAVAI

LGGIVGDAAFSPAFHSLFSRYMSAELSEQLSFILSFSLVTGMFILFADLTPKRIGMIAPEAVALRIINPM

RFCLYVCTPLVWFFNGLANMIFRIFKLPMVRKDDITSDDIYAVVEAGALAGVLRKQEHELIENVFELESR

TVPSSMTPRENVIWFDLHEDEQSLKNKVAEHPHSKFLVCNEDIDHIIGYVDSKDLLNRVLANQSLALNSG

VQIRNTLIVPDTLTLSEALESFKTAGEDFAVIMNEYALVVGIITLNDVMTTLMGDLVGQGLEEQIVARDE

NSWLIDGGTPIDDVMRVLDIDEFPQSGNYETIGGFMMFMLRKIPKRTDSVKFAGYKFEVVDIDNYRIDQL

LVTRIDSKATALSPKLPDAKDKEESVA

>gi|30065488|ref|NP_839659.1| hypothetical protein S4531 [Shigella flexneri 2a str. 2457T]

MRYIRQLCCVSLLCLSGSAVAANVRLQVEGLSGQLEKNVRAQLSTIESDEVTPDRRFRARVDDAIREGLK

ALGYYQPTIEFDLRPPPKKGRQVLIAKVTPGVPVLIGGTDVVLRGGARTDKDYLKLLDTRPAIGTVLNQG

DYENFKKSLTSIALRKGYFDSEFTKAQLGIALGLHKAFWDIDYNSGERYRFGHVTFEGSQIRDEYLQNLV

PFKEGDEYESKDLAELNRRLSATGWFNSVVVAPQFDKARETKVLPLTGVVSPRTENTIETGVGYSTDVGP

RVKATWKKPWMNSYGHSLTTSTSISAPEQTLDFSYKMPLLKNPLEQYYLVQGGFKRTDLNDTESDSTTLV
ASRYWDLSSGWQRAINLRWSLDHFTQGEITNTTMLFYPGVMISRTRSRGGLMPTWGDSQRYSIDYSNTAW
GSDVDFSVFQAQHVWIRTLYDRHRFVTRGTLGWIETGDFDKVPPDLRFFVGGDRSIRGYKYKSIAPKYAN
GDLKGASKLITGSLEYQYNVTGKWWGAVFVDSGEAVSDIRRSDFKTGTGVGVRWESPVGPIKLDFAVPVA
DKDEHGLQFYIGLGPEL

>gi|30065487|ref|NP_839658.1| hypothetical protein S4530 [Shigella flexneri 2a str. 2457T]
MSLWKKISLGVVIVILLLLGSVAFLVGTTSGLHLVFKAADRWVPGLDIGKVTGGWRDLTLSDVRYEQLGV
AVKAGNLHLGVGLECLWNSSVCINDLALKDIQVNIDSKKMPPSEQVEEEEDSGPLDLSTPYPITLTRVAL
DNVNIKIDDTTVSVMDFTSGLNWQEKTLTLKPTSLKGLLIALPKVVEVAQEEVVEPKIENPQPEEKPLGE
TLKDLFSRPVLPEMTDVHLPLNLNIEEFKGEQLRVTGDTDITVRTMLLKVSSIDGNTKLDALDIDSNQGI
VNASGTAQLSDNWPVDITLNSTLNVEPLKGEKVKLKVGGALREQLEIGVNLSGPVDMDLRAQTRLAEAGL
PLNVEVNSKQLYWPFTGEKQYQADDLKLKLTGKMTDYTLSMRTAVKGQEIPPATITLDAKGNEQQVNLDK
LTVAALEGKTELKALLDWQQAISWRGELTLNGINTAKEFPEWPSKLNGLIKTRGSLYGGTWQMEVPELKL
TGNVKQNKVNVDGTLKGNSYMQWMIPGLHLELGPNSAEVKGELGVKDLNLDATINAPGLDNALPGLGGTA
KGLVKVRGTVEAPQLLADITARGLRWQELSVAQVRVEGDIKSTDQIAGKLDVRVEQISQPDVNINLVTLN
AKGSEKQHELQLRIQGEPVSGQLNLAGSFDRKEERWKGTLSNTRFQTPVGPWSLTRDIALDYRNKEQKIS
IGPHCWLNPNAELCVPQTIDAGAEGRAVVNLNRFDLAMLKPFMPETTQASGIFTGKADVAWDTTKEGLPQ
GSITLSGRNVQVTQTVNDAALPVAFQTLNLTAELRNNRAELGWTIRLTNNGQFDGQVQVTDPQGRRNLGG
NVNIRNFNLAMINPIFTRGEKAAGMVSANLRLGGDVQSPQLFGQLQVTGVDIDGNFMPFDMQPSQLAVNF
NGMRSTLAGTVRTQQGEIYLNGDADWSQIENWRARVTAKGSKVRITVPPMVRMDVSPDVVFEATPNLFTL
DGRVDVPWARIVVHDLPESAVGVSSDVVMLNDNLQPEEPKTASIPINSNLIVHVGNNVRIDAFGLKARLT
GDLNVVQDKQGLGLNGQINIPEGRFHAYGQDLIVRKGELLFSGPPDQPYLNIEAIRNPDATEDDVIAGVR
VTGLADEPKAEIFSDPAMSQQAALSYLLRGQGLESDQSDSAAMTSMLIGLGVAQSGQIVGKIGETFGVSN
LALDTLGVGDSSQVVVSGYVLPGLQVKYGVGIFDSIATLTLRYRLMPKLYLEAVSGVDQALDLLYQFEF

>gi|30065486|ref|NP_839657.1| hypothetical protein S4529 [Shigella flexneri 2a str. 2457T]

MRIFVYGSLRHKQGNSHWMTNAQLLGDFSIDNYQLYSLGHYPGAVPGNGTVHGEVYRIDNATLAELDALR

TRGGEYARQLIQTPYGSAWMYVYQRPVDGLKLIESGDWLDRDK

>gi|30065484|ref|NP_839655.1| inorganic pyrophosphatase [Shigella flexneri 2a str. 2457T]

MSLLNVPAGKDLPEDIYVVIEIPANADPIKYEIDKESGALFVDRFMSTAMFYPCNYGYINHTLSLDGDPV

DVLVPTPYPLQPGSVIRCRPVAVLKMTDEAGEDAKLVAVPHSKLSKEYDHIKDVNDLPELLKAQIAHFFE

HYKDLEKGKWVKVEGWENAEAAKAEIVASFERAKNK

>gi|30065482|ref|NP_839653.1| transport system permease [Shigella flexneri 2a str. 2457T]

MMPQSLPDTTPPKRRFHWPTGMPQLAALLLVLLVDSLVAPHFWQVVLQDGRLFGSPIDILNRAAPVALLA

IGITLVIATGGIDLSVGAVMAIAGATTAAMTVAGFSLPIVLLSALGTGILAGLWNGILVAILKIQPFVAT

LILMVAGRGVAQLITAGQIVTFNSPDLSWFGSGSLLFLPTPVIIAVLTLLLFWLLTRKTALGMFIEVVGI

NIRAAKNAGVNTRIIVMLTYVLSGLCAAIAGIIVAADIRGADANNAGLWLELDAILAVVIGGGSLMGGRF

NLLLSVVGALIIQGMNTGILLSGFPPEMNQVVKAVVVLCVLIVQSQRFISLIKGVRSHDKT

>gi|30065479|ref|NP_839650.1| ligase [Shigella flexneri 2a str. 2457T]

MRIHILGICGTFMGGLAMLARQLGHEVTGSDANVYPPMSTLLEKQGIELIQGYDASQLEPQPDLVIIGNA

MTRGNPCVEAVLEKNIPYMSGPQWLHDFVLRDRWVLAVAGTHGKTTTAGMATWILEQCGYKPGFVIGGVP

GNFEVSARLGESDFFVIEADEYDCAFFDKRSKFVHYCPRTLILNNLEFDHADIFDDLKAIQKQFHHLVRI

VPGQGRIIWPENDINLKQTMAMGCWSEQELVGEQGHWQAKKLTTDASEWEVLLDGEKVGEVKWSLVGEHN

MHNGLMAIAAARHVGVAPADAANALGSFINARRRLELRGEANGVTVYDDFAHHPTAILATLAALRGKVGG

TARIIAVLEPRSNTMKMGICKDDLAPSLGRADEVFLLQPAHIPWQVAEVAEACVQPAHWSGDVDTLADMV

VKTAQPGDHILVMSNGGFGGIHQKLLDGLAKKAEAAQ

>gi|30065478|ref|NP_839649.1| hypothetical protein S4518 [Shigella flexneri 2a str. 2457T]

MTKQPEDWLDDVPGDDIEDEDDEIIWVSKSEIKRDAEELKRLGAEIVDLGKNALDKIPLDADLRPAIELA

QRIKMEGRRRQLQLIGKMLRQRDVEPIRQALDKLKNRHNQQVVLFHKLENLRDRLIDQGDDAIAEVLNLW

PDADRQQLRTLIRNAKKEKEGNKPPKSARQIFQYLRELAENEG

>gi|30065477|ref|NP_839648.1| peptidase PmbA [Shigella flexneri 2a str. 2457T]

MALAMKVISQVEAQRKILEEAVSTALELASGKSDGAEVAVSKTTGISVSTRYGEVENVEFNSDGALGITV

YHQNRKGSASSTDLSPQAIARTVQAALDIARYTSPDPCAGVADKELLAFDAPNLDLFHPAEVSPDEAIEL

AARAEQAALQADKRITNTEGGSFNSHYGVKVFGNSHGMLQGYCSTRHSLFSCVIAEENGDMERDYAYTIG

RAMSDLQTPEWVGADCARRTLSRLSPRKLSTMKAPVIFANEVATGLFGHLVGAIAGGSVYRKSTFLLDSL

GKQILPDWLTIEEHPHLLKGLASTPFDSEGVRTERRDIIKDGILTQWLLTSYSARKLGLKSTGHAGGIHN

WRIAGQGLSFEQMLKEMGTGLVVTELMGQGVSAITGDYSRGAAGFWVENGEIQYPVSEITIAGNLKDMWR

NIVTVGNDIETRSNIQCGSVLLPEMKIAGQ

>gi|30065476|ref|NP_839647.1| anaerobic ribonucleotide reductase-activating protein [Shigella flexneri 2a str. 2457T]

MNYHQYYPVDIVNGPGTRCTLFVSGCVHECPGCYNKSTWRVNSGQPFTKAMEDKIIKDLNDTRIKRQGIS

LSGGDPLHPQNVPDILKLVKRIRAECPGKDIWVWTGYKLDELNAAQMQVVDLINVLVDGKFVQDLKDPSL

IWRGSSNQVVHHLR

>gi|30065475|ref|NP_839646.1| anaerobic ribonucleoside triphosphate reductase [Shigella flexneri 2a str. 2457T]

MTPHVMKRDGCKVPFKSERIKEAILRAAKAAEVDDADYCATVAAVVNEQMQGRNQVDINEIQTAVENQLM

SGPYKQLARAYIEYRHDRDIEREKRGRLNQEIRGLVEQTNASLLNENANKDSKVIPTQRDLLAGIVAKHY

ARQHLLPRDVVQAHERGDIHYHDLDYSPFFPMFNCMLIDLKGMLTQGFKMGNAEIEPPKSISTATAVTAQ

IIAQVASHIYGGTTINRIDEVLAPFVTASYNKHRKTAEEWNIPDAEGYANSRTIKECYDAFQSLEYEVNT

LHTANGQTPFVTFGFGLGTSWESRLIQESILRNRIAGLGKNRKTAVFPKLVFAIRDGLNHKKGDPNYDIK

QLALECASKRMYPDILNYDQVVNVTGSFKTPMGCRSFLGVWENENGEQIHDGRNNLGVISLNLPRIALEA

KGDEATFWKLLDERLVLARKALMTRIARLEGVKARVAPILYMEGACGVRLNADDDVSEIFKNGRASISLG

YIGIHETINALFSGEHVYDNEKLRAKGIAIVERLRQAVDQWKEETGYGFSLYSTPSENLCDRFCRLDTAE

FGVVPGVTDKGYYTNSFHLDVEKKVNPYDKIDFEAPYPPLANGGFICYGEYPNIQHNLKALEDVWDYSYQ

HVPYYGTNTPIDECYECGFTGEFECTSKGFTCPKCGNHDASRVSVTRRVCGYLGSPDARPFNAGKQEEVK

RRVKHLGNGQIG

>gi|30065473|ref|NP_839644.1| trehalose(maltose)-specific PTS system components IIBC [Shigella flexneri 2a str. 2457T]

MSKINQTDIDRLIELVGGRGNIATVSHCITRLRFVLNQPANARPKEIEQLPMVKGCFTNAGQFQVVIGTN

VGDYYQALIASTGQAQVDKEQVKKAARQNMKWHEQLISHFAEIFFPLLPALISGGLILGFRNVIGDLPMS

NGQTLAQMYPSLQTIYDFLWLIGEAIFFYLPVGICWSAVKKMGGTPILGIVLGVTLVSPQLMNAYLLGQQ

LPEVWNFGMFSIAKVGYQAQVIPALLAGLALGVIETRLKRIVPDYLYLVVVPVCSLILAVFLAHALIGPF

GRMIGDGVAFAVRHLMTGSFAPIGAALFGFLYAPLVITGVHQTTLAIDLQMIQSMGGTPVWPLIALSNIA

QGSAVIGIIISSRKHNEREISVPAAISAWLGVTEPAMYGINLKYRFPMLCAMIGSGLAGLLCGLNGVMAN

GIGVGGLPGILSIQPSYWQVFALAMAIAIIIPIVLTSFIYQRKYRLGTLDIV

>gi|30065472|ref|NP_839643.1| trehalose repressor [Shigella flexneri 2a str. 2457T]

MQNRLTIKDIARLSGVGKSTVSRVLNNESGVSQRTRERVEAVMNQHGFSPSRSARAMRGQSDKVVAIIVT

RLDSLSENLAVQTMLPAFYEQGYDPIMMENQFSPQLVAEHLGVLKRRNIDGVVLFGFTGITEEMLAHWQS

SLVLLARDAKGFASVCYDDEGAIKILMQRLYDQGHRNISYLGVPHSDVTTGKRRHEAYLAFCKAHKLHPV

AALPGLAMKQGYENVAKVITPETTALLCATDTLALGASKYLQEQRIDTLQLASVGNTPLMKFLHPEIVTV

DPGYAEAGRQAACQLIAQVTGRSEPQQIIIPATLS

>gi|30065469|ref|NP_839640.1| aspartate carbamoyltransferase regulatory subunit [Shigella flexneri 2a str. 2457T]

MTHDNKLQVEAIKRGTVIDHIPAQIGFKLLSLFKLTETDQRITIGLNLPSGEMGRKDLIKIENTFLSEEQ

VDQLALYAPQATVNRIDNYEVVGKSRPSLPERIDNVLVCPNSNCISHAEPVSSSFAVRKRANDIALKCKY

CEKEFSHNVVLAN

>gi|30065466|ref|NP_839637.1| hypothetical protein S4506 [Shigella flexneri 2a str. 2457T]

MSNTLNHTSSRQIVRHYTHSQKRCKHLMQYFVSANGLFELKVKIYAFLFSMILEGKCRSVSIIADISCFF

LFHFHAIRNAFYSIHPTYRAEGENERLTLLLIAQGYALSL

>gi|30065465|ref|NP_839636.1| hypothetical protein S4504 [Shigella flexneri 2a str. 2457T]

MVERTAVFPAGRHSLYAEHRYSAAIRSGDLLFVSGQVGSREDGTPEPDFQQQVRLAFDNLHATLAAAGCT

FDDIIDVTSFHTDPEKQSEDIMTVKNEIFSAPPYPNWTAVGVTWLAGFDFEIKVIARIPEL

>gi|30065461|ref|NP_839632.1| hypothetical protein S4500 [Shigella flexneri 2a str. 2457T]

MIIGNIHNLQPWLPQELRQAIEHIKAHVTAETPKGKHDIEGNRLFYLISEDMTEPYEARRAEYHARYLDI

QIVLKGQEGMTFSTQPAGAPDTDWLADKDIAFLPEGVDEKTVILNEGDFVVFYPGEVHKPLCAVGAPAQV

RKAVVKMLMA

>gi|30065457|ref|NP_839628.1| hypothetical protein S4495 [Shigella flexneri 2a str. 2457T]

MANPEQLEEQREETRLIIEELLEDGSDPDALYTIEHHLSADDLETLEKAAVEAFKLGYEVTDPEELEVED

GDIVICCDILSECALNADLIDAQVEQLMTLAEKFDVEYDGWGTYFEDPNGEDGDDEDFVDEDDDGVRH

>gi|30065456|ref|NP_839627.1| hypothetical protein S4493 [Shigella flexneri 2a str. 2457T]

MAQVINEMDVPSHSFVFHGTGERYFLICVVNVLLTIITLGIYLPWALMKCKRYLYANMEVNGQRFSYGIT

GGNVFVSCLVFVFFYFAILMTVSADMPLVGCVLTLLLLVLLIFMAAKGLRYQALMTSLNGVRFSFNCSLK

GFWWVTFFLPILMAIGMGTVFFISTKMLHANSSSSVIISVVLMAIVGIVSIGIFNGTLYSLVMSFLWSNT

SFGIHRFKVKLDTAYCIKYAILAFLALLPFLAVAGYIIFDQILNEYDSSGYANDDIENLQQFMEMQRKMI

IAQLIYYFGIAVSTSYLTVSLRNHFMSNLSLNDGRIRFRSTLTYHGMLYRMCALVVISGITGGLAYPLLK

IWMIDWQAKNTYLLGDLDDLPLINKEEQPDKGFLASISRGVMPSLPFL

>gi|30065454|ref|NP_839625.1| DNA polymerase III subunit chi [Shigella flexneri 2a str. 2457T]

MKNATFYLLDNDTTVDGLSAVEQLVCEIAAERWRSGKRVLIACEDEKQAYRLDEALWARPAESFVPHNLA

GEGPRGSAPVEIAWPQKRSSSPRDILISLRTSFADFATAFTEVVDFVPYEDSLKQLARERYKAYRVAGFN

LNTATWK

>gi|30065452|ref|NP_839623.1| hypothetical protein S4489 [Shigella flexneri 2a str. 2457T]

MIIIRYLVRETLKSQLAILFILLLIFFCQKLVRILGAAVDGDIPANLVLSLLGLGVPEMAQLILPLSLFL

GLLMTLGKLYTESEITVMHACGLSKAVLVKAAMILAVFTAIVAAVNVMWAGPWSSRHQDEVLAEAKANPG

MAALAQGQFQQATNGSSVLFIESVDGSDFKDVFLAQIRPKGNARPSVVVADSGHLTQLRDGSQVVTLNQG

TRFEGTALLRDFRITDFQDYQAIIGHQAVALDPNDTDQMDMRTLWNTDTDRARAELNWRITLVFTVFMMA

LMVVPLSVVNPRQGRVLSMLPAMLLYLLFFLIQTSLKSNGGKGKLDPTLWMWTVNLIYLALAIVLNLWDT

VPVRRLRASFSRKGAV

>gi|30065451|ref|NP_839622.1| hypothetical protein S4488 [Shigella flexneri 2a str. 2457T]

MQPFGVLDRYIGKTIFTTIMMTLFMLVSLSGIIKFVDQLKKAGQGSYDALGAGMYTLLSVPKDVQIFFPM

AALLGALLGLGMLAQRSELVVMQASGFTRMQVALSVMKTAIPLVLLTMAIGEWVAPQGEQMARNYRAQAM

YGGSLLSTQQGLWAKDGNNFVYIERVKGDEVLGGISIYAFNENRRLQSVRYAATAKFDPEHKVWRLSQVD

ESDLTNPKQITGSQTVSGTWKTDLTPDKLGVVALDPDALSISGLHNYVKYLKSSGQDAGRYQLNMWSKIF

QPLSVAVMMLMALSFIFGPLRSVPMGVRVVTGISFGFVFYVLDQIFGPLTLVYGIPPIIGALLPSASFFL

ISLWLLMRKS

>gi|30065450|ref|NP_839621.1| hypothetical protein S4487 [Shigella flexneri 2a str. 2457T]

MSEPLLIARTPDTELFLLPGMANRHGLITGATGTGKTVTLQKLAESLSEIGVPVFMADVKGDLTGIAQAG

TASEKLLTRLKNIGVNDWQPHANPVVVWDIFGEKGHTVRATVSDLGPLLLARLLNLNDVQSGVLNIIFRI

ADDQGLLLLDFKDLRAITQYIGDNAKSFQNQYGNISSASVGAIQRGLLSLEQQGAAHFFGEPMLDIKDWM

RTDANGKGVINILSAEKLYQMPKLYAASLLWMLSELYEQLPEAGDLEKPKLVFFFDEAHLLFNDAPQVLL

DKIEQVIRLIRSKGVGVWFVSQNPSDIPDNVFGQLGNRVQHALRAFTPKDQKAVKTAAQTMRANPAFDTE

KAIQELGTGEALISFLDAKGSPSVVERAMVIAPCSRMGPVTEDERNGLINHSPVYGKYEDEVDRESAYEM

LQKGFQASTEQQNNPPVKGKEVAVDDGILGGLKDILFGTTGPRGGKKDGVVQTMAKSAARQVTNQIVRGD

VGEFAGGEKKISQT

>gi|30065439|ref|NP_839610.1| DeoR family transcriptional regulator [Shigella flexneri 2a str. 2457T]

MLEVSVMTIRRDLHQEDEPLPLTLLGGYIVMVNKPAPSMPVIHDVPKNHRDDLPIAILAAGMVNENDLIF

FDNGQEIPLVISMIPDAITFTGICYSHRVFVALNEKPNVTAILCGGTYRARSDAFYDASNSSPLDSLNPR

KIFISASGVHNHFGVSWFNPEDLATKRKVMNRGLRKILLARHALFDEVASASLAPISAFDVLISDRPLPA

DYVTHCQNGSVKIITPDSEDE

>gi|30065433|ref|NP_839604.1| recombinase; regulator for fimA [Shigella flexneri 2a str. 2457T]

MRNKADNKKRNFLTHSEIESLLKAANTGPHATRNYCLILLCFIHGFRASEICRLRISDIDLKAKCIYIHR

LKKGFSTTHPLLNKEVQALKNWLSIRTSYPHAESEWVFLSRKGNPLSRQQFYHIISTSGGNAGLSLEIHP

HMLRYSCGFALANMGIDTRLI

>gi|30065432|ref|NP_839603.1| tyrosine recombinase [Shigella flexneri 2a str. 2457T]

MSKRRYLTGKEVQAMMQAVCYGATGARDYCLILLAYRHGMRISELLDLHYQDLDLNEGRINIRRLKNGFS

TVHPLRFDEREAVERWTQERANWKGADRTDAIFISRRGSRLSRQQAYRIIRDAGIEAGTVTQTHPHMLRH

ACGYELAERGADTRLIQDYLGHRNIRHTVRYTASNAARFAGLWERNNLINEKLKREEV

>gi|30065431|ref|NP_839602.1| major type 1 subunit fimbrin (pilin) [Shigella flexneri 2a str. 2457T]

MKIKTLAIVVLSALSLSSAAALADTTTVNGGTIHFKGEVVNAACAVDAGSVDQTVQLGQVRTASLKQAGA

TSSAVGFNIQLNDCDTTVATKAAVAFLGTAIDATRTDVLALQSSAAGSATNVGVQILDRTGNALTLDGAT

FSAQTTLNNGTNTIPFQARYYAIGEATPGAANADATFKVQYQ

>gi|30065430|ref|NP_839601.1| fimbrial protein [Shigella flexneri 2a str. 2457T]

MRMQPSRFSINNLPRFKDVITGRDAHPCAIKITMKRKRLFLLASLLPMFALAGNKWNTTLPGGNMQFQGV

IIAETCRIEAGDKQMTVNMGQISSNRFHAAGEDSAPVPFVIHLRECSTVVSERVGVAFHGVADGKNPDVL

SVGEGPGIASNIGVALFDDEGNLVPINRPPANWKRLYSGSTSLHFIAKYRATGRRVTGGIANAQAWFSLT

YQ

>gi|30065429|ref|NP_839600.1| periplasmic chaperone [Shigella flexneri 2a str. 2457T]

MSNKNVNVRKSQEITFCLLAGILMFMAMMVAGRAEAGVALGATRVIYPAGQKQVQLAVTNNDENSTYLIQ

SWVENADGVKDGRFIVTPPLFAMKGKKENTLRILDATNNQLPQDRESLFWMNVKAIPSMDKSKLTENMLQ

LAIISRIKLYYRPAKLALPPDQAAEKLRFRRSANSLTLINPTPYYLTVTELNAGTRVLENALVPPMGEST

VKLPSDAGSNITYRTINDYGALTPKMTGVME

>gi|30065426|ref|NP_839597.1| minor fimbrial subunit, precursor polypeptide [Shigella flexneri 2a str. 2457T]

MRNKPFYLLCAFLWLAVSRVLAADSTITIRGYVRDNGCSVAAESTNFTVDLMENAAKQFNNIGATTPVVP

FRILLSPCGNAVSAVKVGFTGVADSHNANLLALENTVSAAAGLGIQLLNEQQNQIPLNAPSSAISWTTLT

PGKPNTLNFYARLMATQVPVTAGHINATATFTLEYQ

>gi|30065425|ref|NP_839596.1| minor fimbrial subunit, precursor polypeptide [Shigella flexneri 2a str. 2457T]

MKWCKRGYVLAAMLALASATIQAADVTITVNGKVVAKPCTVSTTNATVDLGDLYSFSLMSAGAASAWHDV

ALELTNCPVGTSRVTASFSGAADSTGYYKNQGTAQNIQLELQDDSGNTLNTGATKTVQVDDSSQSAHFPL

QVRALTVNGGATQGTIQAVISITYTYS

>gi|30065424|ref|NP_839595.1| minor fimbrial subunit, D-mannose specific adhesin [Shigella flexneri 2a str. 2457T]

MKRAITLFAVLLMGWSVNAWSFACKTANGTAIPIGGGSANVYVNLAPVVNVGQNLVVDLSTQIFCHNDYP

ETITDYVTLQRGSAYGGVLSNFSGTVKYSGSSYPFPTTSETPRVVYNSRTDKPWPVALYLTPVSSAGGVA

IKAGSLIAVLILRQTNNYNSDDFQFVWNIYANNDVVVPTGGCDVSARDVTVTLPDYPGSVPIPLTVYCAK

SQNLGYYLSGTTADAGNSIFTNTASFSPAQGVGVQLTRNGTIIPANNTVSLGAVGTSAVSLGLTANYART

GGQVTAGNVQSIIGVTFVYQ

>gi|30065423|ref|NP_839594.1| fructuronate transporter [Shigella flexneri 2a str. 2457T]

MHVLNILWVVFGIGLMLILNLKFKINSMVALLVAALSVGMLAGMDLMSLLHTMKAGFGNTLGELAIIVVF

GAVIGKLMVDSGAAHQIAHTLLARLGLRYVQLSVIIIGLIFGLAMFYEVAFIMLAPLVIVIAAEAKIPFL

KLAIPAVAAATTAHSLFPPQPGPVALVNAYGADMGMVYIYGVLVTIPSVICAGLILPKFLGNLERPTPSF

LKADQPVDMNNLPSFGVSILVPLIPAIIMISTTIANIWLVKDTPAWEVVNFIGSSPIAMFIAMVVAFVLF

GTARGHDMQWVMNAFESAVKSIAMVILIIGAGGVLKQTIIDTGIGDTIGMLMSHGNISPYIMAWLITVLI

RLATGQGVVSAMTAAGIISAAILDPATGQLVGVNPALLVLATAAGSNTLTHINDASFWLFKGYFDLSVKD

TLKTWGLLELVNSVVGLIIVLIISMVA

>gi|30065422|ref|NP_839593.1| mannonate dehydratase [Shigella flexneri 2a str. 2457T]

MEQTWRWYGPNDPVSLADVRQAGATGVVTALHHIPNGEVWSVEEILKRKAIVEDAGLVWSVVESVPIHED

IKTHTGNYEQWIANYQQTLRNLAQCGIRTVCYNFMPVLDWTRTDLEYVLPDGSKALRFDQIEFAAFEMHI

LKRPGAEADYTEEEIAQAAERFATMSDEDKARLTRNIIAGLPGAEEGYTLDQFRKHLELYKDIDKAKLRE

NFAVFLKAIIPVAEEVGVRMAVHPDDPPRPILGLPRIVSTIEDMQWMVDTVNSMANGFTMCTGSYGVRAD

NDLVDMIKQFGPRIYFTHLRSTMREDNPKTFHEAAHLNGDVDMYKVVKAIVEEEHRRKAEGKEDLIPMRP

DHGHQMLDDLKKKTNPGYSAIGRLKGLAEVRGVELAIQRAFFSR

>gi|30065421|ref|NP_839592.1| D-mannonate oxidoreductase [Shigella flexneri 2a str. 2457T]

MTTIVDSNLPVARPSWDHSRLESRIVHLGCGAFHRAHQALYTHHLLESTDSDWGICEVNLMPGNDRMLIE

NLKKQQLLYTVAEKGAESTELKIIGSMKEALHPEIDGCEGILNAMARPQTAIVSLTVTEKGYCADAASGQ

LDLNNPLIKHDLENPTAPKSAIGYIVEALRLRREKGLKAFTVMSCDNVRENGHVAKVAVLGLAQARDPQL

AAWIEENVTFPCTMVDRIVPAATPETLQEIADQLGVYDPCAIACEPFRQWVIEDNFVNGRPDWDKVGTQF

VADVVPFEMMKLRMLNGSHSFLAYLGYLGGYETIADTMTNPDYRKAAFALMMQEQAPTLSMPEGTDLNAY

ATLLIERFSNPSLRHRTWQIAMDGSQKLPQRLLDPVRLHLQNGGSWRHLALGVAGWMGYTQGVDEQGNAI

DVVDPMLAEFQKINAQYQGADRVKALLGLSGIFADDLPQNADFVGAVTSAYQQLCERGARECVAAL

>gi|30065420|ref|NP_839591.1| DNA-binding transcriptional repressor UxuR [Shigella flexneri 2a str. 2457T]

MKSVTSAQRPYQEVGAMIHDLIIKTPYNPGERLPPEREIAEMLDVTRTVVREALIMLEIKGLVEVRRGAG

IYVLDSSGSHNTDSPDANVCNDAGPFELLQARQLLESNIAEFAALQATREDIVKMRQALQLEERELASSA

PGSSESGDMQFHLAIAEATHNSMLVELFRQSWQWRENNPMWIQLHSHLDDSLYRKEWLGDHKQILAALIK

KDARAAKLAMWQHLENVKQRLLEFSNVDDIYFDGYLFDSWPLDKVDA

>gi|30065417|ref|NP_839588.1| hypothetical protein S4448 [Shigella flexneri 2a str. 2457T]

MRKMMRQSLQAVLPEISGNKTSLLRKSVCSDLLTLFNSPHSALPSLLVSGMPEWQVHNPSDKHLQSWYCR
QLRSALLFHEPRIAALQVNLKEAYCHTLAISLEIMLYHDDEPLTFDLVWDNGGWRSATLENVS

>gi|30065416|ref|NP_839587.1| DNA-binding transcriptional regulator [Shigella flexneri 2a str. 2457T]
MDDCGAVLHNIETKWLYDFLTLEKCRNFSQAAVSRNVSQPAFSRRIRALEQAIGVELFNRQVTPLQLSEQ
GKIFHSQIRHLLQQLESNLAELRGGSDYAQRKIKIAAAHSLSLGLLPSIISQMPPLFTWAIEAIDVDEAV
DKLREGQSDCIFSFHDEDLLEAPFDHIRLFESQLFPVCASDEHGEALFNLAQPHFPLLNYSRNSYMVRLI
NRTLTRHSELSFSTFFVSSMSELLKQVALDGCGIAWLPEYAIQQEIRSGQLVVLNRDEQVIPIQAYAYRM
NTRMNPVAERFWRELRELEIVLS

>gi|30065415|ref|NP_839586.1| isoaspartyl dipeptidase [Shigella flexneri 2a str. 2457T]
MIDYTAAGFTLLQGAHLYAPEDRGICDVLVANGKIIAVASNIPSDIVPNCTVVDLSGQILCPGFIDQHVH
LIGGGGEAGPTTRTPEVALSRLTEAGVTSVVGLLGTDSISRHPESLLAKTRALNEEGISAWMLTGAYHVP
SRTITGSVEKDVAIIDRVIGVKCAISDHRSAAPDVYHLANMAAESRVGGLLGGKPGVTVFHMGDSKKALK
PIYDLLENCDVPISKLLPTHVNRNVPLFEQALEFARKGGTIDITSSIDEPVAPAEGIARAVQAGIPLARV
TLSSDGNGSQPFFDDEGNLTHIGVAGFETLLETVQVLVKDYDFSISDALRPLTSSVAGFLNMSGKGEILP
GNDADLLVMTPELRIEQVYARGKLMVKDGKACVKGTFETA

>gi|30065414|ref|NP_839585.1| hypothetical protein S4445 [Shigella flexneri 2a str. 2457T]
MTTQVRKNVMDMFIDGARRGFTIATTNLLPNVVMAFVIIQALKITGLLDWVGHICEPVMALWGLPGEAAT
VLLAALMSMGGAVGVAASLATADALTGHDVTVLLPAMYLMGNPVQNVGRCLGTAEVNAKYYPHIITVCVI
NALLSIWVMQLIV

>gi|30065413|ref|NP_839584.1| hypothetical protein S4444 [Shigella flexneri 2a str. 2457T]
MGIVMTQQGDAVAGELATEKVGIKGYLAFFLTIIFFPGVFSGTDSWWRVFDFSVLNGSFGQLPGANGATT

SFRGVGGAGAKDGFLFALELAPSVILSLGIISITDGLGGLRAAQQLMTPVLKPLLGIPGICSLALIANLQ

NTDAAAGMTKELAQEGEITERDKVIFAAYQTSGSAIITNYFSSGVAVFAFLGTSVIVPLAVILVFKFVGA

NILRVWLNFEERRNPTQGAQA

>gi|30065407|ref|NP_839578.1| ISSfl3 orfB [Shigella flexneri 2a str. 2457T]

MITLPTGTKIWIIAGITDMRCGFNGLASKVQNTLKDDPFSGHIFVFRGRSGKMVKILWADRDGLCLFTKR

LAGDPGRESAPDASSVIHATGGDRVATSQTDRTAWHPDITRDKTRE

>gi|30065406|ref|NP_839577.1| ISSfl3 orfA [Shigella flexneri 2a str. 2457T]

MNSQTTKDIPCFRSYLPDALRLRFEDKLTIRAIAQRLGLSHSTIHTLFQRFLASGIAWPLPDSVSFAQLD

AILYANRKKELTEPQIREGSWRKERRTSYSREFKVRLAKQALQPGAVVARIAREHDINDNLLFKWKSQYE

DGLLSDDDIQECMPVPVALTDTPEPTRPVTNPFWRNKHDERPEGAPGNVPRCELHLKSGVVKLFDPLTPE

LLRALIREMKGGIR

>gi|30065404|ref|NP_839575.1| DNA-directed RNA polymerase subunit alpha [Shigella flexneri 2a str. 2457T]

MQGSVTEFLKPRLVDIEQVSSTHAKVTLEPLERGFGHTLGNALRRILLSSMPGCAVTEVEIDGVLHEYST

KEGVQEDILEILLNLKGLAVRVQGKDEVILTLNKSGIGPVTAADITHDGDVEIVKPQHVICHLTDENASI

SMRIKVQRGRGYVPASTRIHSEEDERPIGRLLVDACYSPVERIAYNVEAARVEQRTDLDKLVIEMETNGT

IDPEEAIRRAATILAEQLEAFVDLRDVRQPEVKEEKPEFDPILLRPVDDLELTVRSANCLKAEAIHYIGD

LVQRTEVELLKTPNLGKKSLTEIKDVLASRGLSLGMRLENWPPASIADE

>gi|30065399|ref|NP_839570.1| preprotein translocase subunit SecY [Shigella flexneri 2a str. 2457T]

MAKQPGLDFQSAKGGLGELKRRLLFVIGALIVFRIGSFIPIPGIDAAVLAKLLEQQRGTIIEMFNMFSGG

ALSRASIFALGIMPYISASIIIQLLTVVHPTLAEIKKEGESGRRKISQYTRYGTLVLAIFQSIGIATGLP

NMPGMQGLVINPGFAFYFTAVVSLVTGTMFLMWLGEQITERGIGNGISIIIFAGIVAGLPPAIAHTIEQA

RQGDLHFLVLLLVAVLVFAVTFFVVFVERGQRRIVVNYAKRQQGRRVYAAQSTHLPLKVNMAGVIPAIFA

SSIILFPATIASWFGGGTGWNWLTTISLYLQPGQPLYVLLYASAIIFFCFFYTALVFNPRETADNLKKSG

AFVPGIRPGEQTAKYIDKVMTRLTLVGALYITFICLIPEFMRDAMKVPFYFGGTSLLIVVVVIMDFMAQV

QTLMMSSQYESALKKANLKGYGR

>gi|30065394|ref|NP_839565.1| 50S ribosomal protein L6 [Shigella flexneri 2a str. 2457T]

MSRVAKAPVVVPAGVDVKINGQVITIKGKNGELTRTLNDAVEVKHADNTLTFGPRDGYADGWAQAGTARA

LLNSMVIGVTEGFTKKLQLVGVGYRAAVKGNVINLSLGFSHPVDHQLPAGITAECPTQTEIVLKGADKQV

IGQVAADLRAYRRPEPYKGKGVRYADEVVRTKEAKKK

>gi|30065390|ref|NP_839561.1| 50S ribosomal protein L24 [Shigella flexneri 2a str. 2457T]

MAAKIRRDDEVIVLTGKDKGKRGKVKNVLSSGKVIIEGINLVKKHQKPVPALNQPGGIVEKEAAIQVSNV

AIFNAATGKADRVGFRFEDGKKVRFFKSNSETIK

>gi|30065377|ref|NP_839548.1| bacterioferritin [Shigella flexneri 2a str. 2457T]

MKGDVKIINYLNKLLGNELVAINQYFLHARMFKNWGLMRLNDVEYHESIDEMKHADKYIERILFLEGIPN

LQDLGKLGIGEDVEEMLQSDLRLELEGAKDLREAIAYADSVHDYVSRDMMIEILADEEGHIDWLETELDL

IGKIGLQNYLQSQIKVKD

>gi|30065370|ref|NP_839541.1| sulfur relay protein TusC [Shigella flexneri 2a str. 2457T]

MKRIAFVFSTAPHGTAAGREGLDALLATSALTDDLAVFFIADGVFQLLSGQKPDAVLARDYIATFKLLSL

YDIEQCWVCAASLRERGLDPQTPFVVEATPLEADALRRELANYDVILRF

>gi|30065369|ref|NP_839540.1| sulfur transfer complex subunit TusD [Shigella flexneri 2a str. 2457T]

MRFAIVVTGPAYGTQQASSAFQFAQALIAEGHKLSSVFFYREGVYNANQLTSPASDEFDLVRGWQQLNAQ

HGVALNICVAAALRRGIVDETEAGRLGLASSNLQPGFTLSGLGALAEASLTCDRVVQF

>gi|30065368|ref|NP_839539.1| hypothetical protein S4398 [Shigella flexneri 2a str. 2457T]

MSRSLLTNETSELDLLDQRPFDQTDFDILKSYEAVVDGLAMLIGSHCEIVLHSLQDLKCSAIRIANGEHT

GRKIGSPITDLALRMLHDMTGADSSVSKCYFTRAKSGVLMKSLTIAIRNREQRVIGLLCINMNLDVPFSQ

IMSTFVPPETPDVGSSVNFASSVEDLVTQTLEFTIEEVNADRNVSNNAKNRQIVLNLYEKGIFDIKDAIN

QVADRLNISKHTVYLYIRQFKSGDFQGQDK

>gi|30065365|ref|NP_839536.1| FKBP-type peptidyl-prolyl cis-trans isomerase [Shigella flexneri 2a str. 2457T]

MKVAKDLVVSLAYQVRTEDGVLVDESPVSAPLDYLHGHGSLISGLETALEGHEVGDKFDVAVGANDAYGQ

YDENLVQRVPKDVFMGVDELQVGMRFLAETDQGPVPVEITAVEDDHVVVDGNHMLAGQNLKFNVEVVAIR

EATEEELAHGHVHGAHDHHHDHDHDGCCGGHGHDHGHEHGGEGCCGGKGNGGCGCH

>gi|30065359|ref|NP_839530.1| phosphoribulokinase [Shigella flexneri 2a str. 2457T]

MSAKHPVIAVTGSSGAGTTTTSLAFRKIFAQLNLHAAEVEGDSFHRYTRPEMDMAIRKARDAGRHISYFG

PEANDFGLLEQTFIEYGQSGKGKSRKYLHTYDEAVPWNQVPGTFTPWQPLPEPTDVLFYEGLHGGVVTPQ

HNVAQHVDLLVGVVPIVNLEWIQKLIRDTSERGHSREAVMDSVVRSMEDYINYITPQFSRTHLNFQRVPT

VDTSNPFAAKGIPSLDESFVVIHFRNLEGIDFPWLLAMLQGSFISHINTLVVPGGKMGLAMELIMLPLVQ

RLMEGKKIE

>gi|30065358|ref|NP_839529.1| hypothetical protein S4388 [Shigella flexneri 2a str. 2457T]

MQARVKWVEGLTFLGESASGHQILMDGNSGDKAPSPMEMVLMAAGGCSAIDVVSILQKGRQDVVDCEVKL

TSERREEAPRLFTHINLHFIVTGRDLKDAAVARAVDLSAEKYCSVALMLEKAVNITHSYEVVAA

>gi|30065356|ref|NP_839527.1| hypothetical protein S4386 [Shigella flexneri 2a str. 2457T]

MWRRLIYHPDINYALRQTLVLCLPVAVGLMLGELRFGLLFSLVPACCNIAGLDTPHKRFFKHLIIGASLF

ATCSLLTQLLLVKDVPLPFLLTGLTLVLGVTAELGPLHAKLLPASLLAAIFTLSLAGYMPVWEPLLIYAL

GTLWYGLFNWFWFWIWREQPLRESLSLLYRELADYCEAKYSLLTQHTDPEKALPPLLVRQQKAVDLITQC

YQQMHMLSAQNNTDYKRMLRIFQEALDLQEHISVSLHQPEEVQKLVERSHAEEVIRWNAQTVAARLRVLA

DDILYHRLPTRFTMEKQIGALEKIARQHPDNPVGQFCYWHFSRIARVLRTQKPLYARDLLADKQRRMPLL

PALKSYLSLKSPALRNAGRLSVMLSVASLMGTVLHLPKSYWILMTVLLVTQNGYGATRLRIVNRSVGTVV

GLIIAGVALHFKIPESYTLTLMLITTLASYLILRKNYGWATVGFTITAVYTLQLLWLNGEQYILPRLIDT

IIGCLIAFGGTVWLWPQWQSGLLRKNAHDALEAYQEAIRLILSEDPQPTPLAWQRMRVNQAHNTLYNSLN

QAMQEPAFNSHYLADMKLWVTHSQFIVEHINAMTTLAREHRALPPELAQEYLQSCEIAIQRCQQRLEYDE

PGSSGDANIMDAPEMQPHEGAAGTLEQHLQRVIGHLNTMHTISSMAWRQRPHHGIWLSRKLRDSKA

>gi|30065353|ref|NP_839524.1| cell filamentation protein Fic [Shigella flexneri 2a str. 2457T]

MSDKFGEGRDLYLYPGLDIMRNRLNIRQQQRLEQAAYEMTALRAATIELGPLVRGLPHLRTIHRQLYQDI

FDWAGQLREVDIYQGDTPFCHFAYIEKEGNALMQDLEEEGYLVGLEKAKFVERLAHYYCEINVLHPFRVG

SGLAQRIFFEQLAIHAGYQLSWQGIEKEAWNQANQSGAMGDLTALQMIFSKVVSEAGESE

>gi|30065350|ref|NP_839521.1| hypothetical protein S4380 [Shigella flexneri 2a str. 2457T]

MTNSNRIKLTWISFLSYALTGALVIVTGMVMGNIADYFNLPVSSMSNTFTFLNAGILISIFLNAWLMEIV

PLKTQLRFGFLLMVLAVAGLMFSHSLALFSTAMFILGVVSGITMSIGTFLITQMYEGRQRGSRLLFTDSF

FSMAGMIFPMIAAFLLARSIEWYWVYACIGLVYVAIFILTFGCEFPALGKRAPKTDAPVEKEKWGIGVLF

LSVAALCYILGQLGFISWVPEYAKGLGMSLNDAGTLVSNFWMSYMVGMWAFSFILRFFDLQRILTVLAGL

AAILMYVFNTGTPVHMAWSILALGFFSSAIYTTIITLGSQQTKVPSPKLVNFVLTCGTIGTMLTFVVTGT

IVEHSGPQAALLTANGLYAVVFVMCFLLGFVSRHRQHNTLTSH

>gi|30065348|ref|NP_839519.1| nitrite reductase small subunit [Shigella flexneri 2a str. 2457T]

MSQWKDICKIDDILPETGVCALLGDKQVAIFRPYHSDQVFAISNIDPFFESSVLSRGLIAEHQGELWVAS

PLKKQRFRLSDGLCMEDEQFSVKHYEARVKDGVVQLRG

>gi|30065346|ref|NP_839517.1| siroheme synthase [Shigella flexneri 2a str. 2457T]

MDHLPIFCQLRDRDCLIVGGGDVAERKARLLLDAGARLTVNALAFIPQFTAWADAGMLTLVEGPFDESLL

DTCWLAIAATDDDTLNQRVSEAAEARRIFCNVVDAPKAASFIMPSIIDRSPLMVAVSSGGTSPVLARLLR

EKLESLLPLHLGQVAKYAGQLRGRVKQQFATMSERRRFWEKLFVNDRLAQSLANNDQKAITETTEQLINE

PLDHRGEVVLVGAGPGDAGLLTLKGLQQIQQADVVVYDRLVSDDIMNLVRRDADRVFVGKRAGYHCVPQE

EINQILLREAQKGKRVVRLKGGDPFIFGRGGEELETLCNAGIPFSVVPGITAASGCSAYSGIPLTHRDYA

QSVRLITGHLKTGGELDWENLAAEKQTLVFYMGLNQAATIQQKLIEHGMPGEMPVAIVENGTAVTQRVID

GTLTQLGELAQQMNSPSLIIIGRVVGLRDKLNWFSNH

>gi|30065343|ref|NP_839514.1| fructoselysine 3-epimerase [Shigella flexneri 2a str. 2457T]

MKTGMFTCGHQRLPIEHAFRDASELGYDGIEIWGGRPHAFAPDLKAGGIKQIKALAQTYQMPIIGYTPET

NGYPYNMMLGDEHMRRESLDMIKLAIDMAKEMNAGYTLISAAHAGYLTPPNVIWGRLAENLSELCEYAEN

IGMDLILEPLTPYESNVVCNANDVLHALALVPSPRLFSMVDICAPYVQAEPVMSYFDKLGDKLRHLHIVD

SDGASDTHYIPGEGKMPLRELMRDIIDRGYEGYSTVELVTMYMNEPRLYARQALERFRALLPEDER

>gi|30065342|ref|NP_839513.1| fructoselysine 6-kinase [Shigella flexneri 2a str. 2457T]

MKTLATIGDNCVDIYPQLNKAFSGGNAVNVAVYCTRYGIQPGCITWVGDDDYGTKLKQDLARMGVDISHV

HTKHGVTAQTQVELHDNDRVFGDYTEGVMADFALSEEDYAWLAQYDIVHAAIWGHAEDAFPQLHAAGKLT

AFDFSDKWDSPLWQTLVPHLDFAFASAPQEDEALRLKMKAIVARGAGTVIVTLGENGSIAWDGAQFWRQA

PEPVTVIDTMGAGDSFIAGFLCGWSAGMTLPQAMAQGTACAAKTIQYHGAW

>gi|30065341|ref|NP_839512.1| DNA-binding transcriptional regulator FrlR [Shigella flexneri 2a str. 2457T]

MLLLAGKRPYNPSNSFFISGSIMSATDRYSHQLLYATVRQRLLDDIAQGVYQAGQQIPTENELCTQYNVS

RITIRKAISDLVADGVLIRWQGKGTFVQSQKVENALLTVSGFTDFGVSQGKSTKEKVIEQERVSAAPFCE

KLNIPGNSEVFHLCRVMYLDKEPLFIDSSWIPLSRYPDFDEIYVEGSSTYQLFQERFDTRVVSDKKTIDI

FAATRPQAKWLKCELGEPLFRISKIAFDQNDKPVHVSELFCRANRITLTIDNKRH

>gi|30065340|ref|NP_839511.1| hypothetical protein S4368 [Shigella flexneri 2a str. 2457T]

MKTFPLQSLTLAEAQQKQFALVDTICRHFPGSEFLAGGDLGLTPSLNQPRITQRVEQVLADAFHAQAAAL

VQGAGTGAIRAGLAALLKPGQRLLVHDAPVYPTTRVIIEQMGLTLITADFNDLSALKQVVDEQQPDAALV

QHTRQQPQDSYVLADVLATLRAAGVPALTDDNYAVMKVARIGCECGANVSTFSCFKLFGPEGVGAVVGDA

DVISRIRATLYSGGSQIQGSQALEVLRGLVFAPLIHAVKAGVSERLLALLNGGAVAEVKSAVIANAQSKV

LIVEFHQLIAARVLEEAQKRGALPYPVGAESKYEIPPLFYRLSGTFRQANPQLEHCAIRINPNRSGEETV

LRILRESIASI

>gi|30065339|ref|NP_839510.1| transport system permease [Shigella flexneri 2a str. 2457T]

MDLYIQIIVVACLTGMTSLLAHRSAAVFHDGIHPILPQLIEGYMNRREAGSIAFGLSIGFVASVGISFTL

KTGLLNAWLLFLPTDILGVLAINSLMAFGLGAIWGVLILTCLLPVNQLLTALPVDVLGSLGELSSPVVSA

FALFPLVAIFYQFGWKQSLVAAVVVLMTRVVVVRYFPHLNPESIEIFIGMVMLLGIAITHDLRHRDENDI

DASGLSVFEERTSRIIKNLPYIAIVGALIAAVASMKIFAGSEVSIFTLEKAYSAGVTPEQSQTLINQAAL

AEFMRGLGFVPLIATTALATGVYAVAGFTFVYAVGYLSPNPMVAAVLGAVVISAEVLLLRSIGKWLGRYP

SVRNASDNIRNAMNMLMEVALLVGSIFAAIKMAGYTGFSIAVAIYFLNESLGRPVQKMAAPVVAVMITGI

LLNVLYWLGLFVPA

>gi|30065338|ref|NP_839509.1| hypothetical protein S4366 [Shigella flexneri 2a str. 2457T]

MKIPLNFSNKDRLMKKIGVAGLQREQIKKTIEATAPGCFEVFIHNDMEAAMKVKSGQLDYYIGACNTGAG

AALSIAIAVIGYNKSCTIAKPGIKAKDEHIAKMIAEGKVAFGLSVEHVEHAIPMLINHLK

>gi|30065336|ref|NP_839507.1| mutase [Shigella flexneri 2a str. 2457T]

MARFVVLVIDSFGVGAMKDVTLVRPQDAGANTCGHILSQLPHLQLPTLEKLGLINTLGYAPGDMQPSDSA

TWGVAELQHEGGDTFMGHQEILGTRPLPPLRMPFRDVIDRVEQALVSAGWQVERRGDDLQFLSVNQAVAI

GDNLEADLGQVYNITANLSVISFDDAIKIGRIVRDQVQVGRVITFGGLLTNSQRILDAAESKEGRFIGIN

APRSGAYDNGFQVVHMGYGVDEKVQVPQKLYEAGVPTVLVGKVADIVSNPYGVSWQNLVDSQRIMDITLD

EFNTHPTAFICINIQETDLAGHAEDVARYAERLQVVDRNLARLVEAMQPDDCLVVMADHGNDPTIGHSHH

TREVVPVLVYQQGLVATQLGVRTTLSDVGATVCEFFRAPPPQNGRSFLSSLRFAGDTL

>gi|30065335|ref|NP_839506.1| hypothetical protein S4363 [Shigella flexneri 2a str. 2457T]

MFVEALKRQNPALISAALSLWQQGKIAPDSWVIDVDQILENGKRLIETARLYGIELYLMTKQFGRNPWLA

EKLLALGYSGIVAVDYKEARVMRRAGLPVAHQGHLVQIPCHQVADAVEQGTDIITVFTLDKAREVSAAAV

KAGRVQPVLLKVYSDDDFLYPGQESGFVQHSLHEVIAEIKKLPGLHLAGLTHFPCLLWDEAAGKVLPTPN

LHTLVQARDQLAKSGIAIEQLNVPSATSCTSLPLLAEYGATHAEPGHALTGTIPANQQGDQPERIAMLWL

SEISHHFRGDSYCYGGGYYRRGHAQHALVFTPENQKITETNLKTVDDSSIDYTLPLAGEYPVSSAVVLCF

RTQIFVTRSDVVLVSGIQRGEPEIVGRYDSLGNPLEA

>gi|30065334|ref|NP_839505.1| hypothetical protein S4362 [Shigella flexneri 2a str. 2457T]

MGNRNPVIKRKASDMETRLNLLCEAGVIDKDVCKGMMQVVNVLEKECHLPVRSEQGTMAMTHMASALMRS

RRGEEIEPLDNELLAELAQSSHWQAVVQLHQELLKEFALEVNPCEEGYFLANLYGLWMAANEEV

>gi|30065331|ref|NP_839502.1| phosphoglycolate phosphatase [Shigella flexneri 2a str. 2457T]

MNKFEDIRGVAFDLDGTLVDSAPGLAAAVDMALYALELPVAGEERVITWIGNGADVLMERALTWARQERA

TLRKTMGKPPVDDDIPAEEQVRILRKLFDRYYGEVAEEGTFLFPHVADTLGALQAKGLPLGLVTNKPTPF

VAPLLEALDIAKYFSVVIGGDDVQNKKPHPDPLLLVAERMGIAPQQMLFVGDSRNDIQAAKAAGCPSVGL

TYGYNYGEAIDLSQPDVIYQSINDLLPALGLPHSENQESKND

>gi|30065329|ref|NP_839500.1| DNA adenine methylase [Shigella flexneri 2a str. 2457T]

MKKNRAFLKWAGGKYPLLDDIKRHLPKGECLVEPFVGAGSVFLNTDFSRYILADINSDLISLYNIVKMRT

DEYVQAARELFVPETNCAEVYYQFREEFNKSQDPFRRAVLFLYLNRYGYNGLCRYNLRGEFNVPFGRYKK

PYFPEAELYHFAEKAQNAFFYCESYADSMERADDASVVYCDPPYAPLSATANFTAYHTNSFTLEQQAHLA

EIAEGLVERHIPVLISNHDTMLTREWYQRAKLHVVKVRRSISSNGGTRKKVDELLALYKPGVVSPAKK

>gi|30065328|ref|NP_839499.1| hypothetical protein S4356 [Shigella flexneri 2a str. 2457T]

MDEFKPEDELKPDPSDRRTGRSRQSSERSERTERGEPQINFDDIELDDTDDRRPTRAQKERNEEPEIEEE

IDESEDETVDEERVERRPRKRKKAASKPASRQYMMMGVGILVLLLLIIGIGSALKAPSTTSSDQTASGEK

SIDLAGNATDQANGVQPAPGTTSAENTQQDVSLPPISSTPTQGQTPVATDGQQRVEVQGDLNNALTQPQN

QQQLNNVAVNSTLPTEPATVAPVRNGNASRDTAKTQTAERPSTTRPVRQQAVIEPKKPQATVKTEPKPVA

QTPKRTEPEPAAPVASTKAPAATSTPAPKETATTAPVQTASPAQTTATPAAGGKTAGNVGSLKSAPSSHY

TLQLSSSSNYDNLNGWAKKENLKNYVVYETTRNGQPWYVLVSGVYASKEEAKKAVSTLPADVQAKNPWAK

PLRQVQADLK

>gi|30065327|ref|NP_839498.1| 3-dehydroquinate synthase [Shigella flexneri 2a str. 2457T]

MERIVVTLGERSYPITIASGLFNEPASFLPLKSGEQVMLVTSETLAPLYLDKVRGVLEQAGVNVDSVILP

DGEQYKSLAVLDTVFTALLQKPHGRDTTLVALGGGVVGDLTGFAAASYQRGVRFIQVPTTLLSQVDSSVG

GKTAVNHPLGKNMIGAFYQPASVVVDLDCLKTLPPRELASGLAEVIKYGIILDGAFFNWLEENLDALLRL

DGPAMAYCIRRCCELKAEVVAADERETGLRALLNLGHTFGHAIEAEMGYGNWLHGEAVAAGMVMAARTSE

RLGQFSSAETQRIITLLTRAGLPVNGPREMSAQAYLPHMLRDKKVLAGEMRLILPLAIGKSEVRSGVSHE

LVLNAIADCQSA

>gi|30065325|ref|NP_839496.1| outer membrane porin HofQ [Shigella flexneri 2a str. 2457T]

MKQWIAALLLMLIPGVQAAKPQKVTLMVDDVPVAQVLQALAEQEKLNLVVSPDVSGTVSLHLTDVPWKQA

LQTVVKSAGLITRQEGNILSVHSIAWQNDNIARQEAEQARAQANLPLENRSITLQYADAGELAKAGEKLL

SAKGSMTVDKRTNRLLLRDNKTALSALEQWVAQMDLPVGQVELSAHIVTINEKSLRELGVKWTLADAQHA

GGVGQVTTLGSDLSVATATTHVGFNIGRINGRLLDLELSALEQKQQLDIIASPRLLASHLQPASIKQESE

IPYQVSSGESGATSVEFKEAVLGMEVTPTVLQKGRIRLKLHISQNVPGQVLQQADGEVLAIDKQEIETQV

EVKSGETLALGGIFTRKNKSGQDSVPLLGDIPWFGQLFRHDGKEDERRELVVFITPRLVSSE

>gi|30065324|ref|NP_839495.1| hypothetical protein S4351 [Shigella flexneri 2a str. 2457T]

MNMFFDWWFATSPRLRQFCWAFWLLMLVTLIFLSSTHHEERDALIRLRASHHQQWAALYRLVDTTPFSEE

KTLPFSPLDFQLSGAQLVSWHPSAQGGELALKTLWEAVPSAFTRLAERNVSVSRFSLSVEGDDLLFTLQL

EMPHEG

>gi|30065323|ref|NP_839494.1| hypothetical protein S4350 [Shigella flexneri 2a str. 2457T]

MNPPINFLPWRQQRRTAFLRFWLLMFVAPLLLAVGITLILRLTGSAEARIDAVLLQAEQQLARSLQITKP

RLLEQQQLREQRSQRQRQRQFTRDWQSALEALAALLPEHAWLTTISWQQGTLEIKGLTTSITALNALETS

LRQDASFHLNQRGATQQDAQGRWQFEYQLTRKVSDEHVL

>gi|30065322|ref|NP_839493.1| hypothetical protein S4349 [Shigella flexneri 2a str. 2457T]

MTGDKEIITMTFKIWQIGLHLQQQEAAAVAIVRGAKECFLQRWWRLPLAHDIIKDGRIVDAQQLAKTLLP

WSRELPQRHHIMLAFPASRTLQRSFPRPSMSLGEREQTAWLSGTMARELDMDPDSLRFDYSEDSLSPAYN

VTAAQSKELATLLTLAERLRVHVSAITPDASALQRFLPFLPSHQQCLAWRDNEQWLWATRYSWGRKLAVG

MTSAKELAAALSVDPDSVAICGEGGFDPWEAVSVRQPPLPPPGGDFAIALGLALGKAY

>gi|30065319|ref|NP_839490.1| dehydrogenase [Shigella flexneri 2a str. 2457T]

MSTIVIFLAALLACSLLAGWLIKVRSRRRQLPWTNAFADAQTRKLTPEERSAVENYLESLTQVLQVPGPT

EASAAPISLALNAESNNVMMLTHAITRYGISTDDPNKWRYYLDSVEVHLPPFWEQYINDENTVELIHTDS

LPLVISLNGHTLQEYMQETRGYALQPVPSTQASIRGEESEQIELLNIRKETHEEYALSRPRGLREALLIV

ASFLMFFFCLITPDVFVPWLAGGALLLLGAGLWGLFAPPAKSSLREIHCLRGTPRRWGLFGENDQEQINN

ISLGIIDLVYPAHWQPYIAQDLGQQTDIDIYLDRHVVRQGRYLSLHDEVKNFPLQHWLRSTIIAAGSLLV

LFMLLFWIPLDMPLKFTLSWMKGAQTIEATSVKQLADAGVRVGDTLRISGTGMCNIRTSGTWSAKTNSPF

LPFDCSQIIWNDARSLPLPESELVNKATALTEAVNRQLHPKPEDESRVSASLRSAIQKSGMVLLDDFGDI

VLKTADLCSAKDDCVRLKNALVNLGNSKDWDALVKRANAGKLDGVNVLLRPVSAESLDNLVATSTAPFIT

HETARAAQSLNSPAPGGFLIVSDEGSDFVDQPWPSASLYDYPPQEQWNAFQKLAQMLMHTPFNAEGIVTK

IFTDANGTQHIGLHPIPDRSGLWRYLSTTLLLLTMLGSAIYNGVQAWRRYQRHRTRMMEIQAYYESCLNP

QLITPSESLIE

>gi|30065318|ref|NP_839489.1| phosphatase [Shigella flexneri 2a str. 2457T]

MLPCRANCFTLEISLMHINIAWQDVDTVLLDMDGTLLDLAFDNYFWQKLVPETWGAKNGVTPQEAMEYMR

QQYHDVQHTLNWYCLDYWSEQLGLDICAMTTEMGPRAVLREDTIPFLEALKASGKQRILLTNAHPHNLAV

KLEHTGLDAHLDLLLSTHTFGYPKEDQRLWHAVAEATGLKAERTLFIDDSEAILDAAAQFGIRYCLGVTN

PDSGIAEKQYQRHPSLNDYRRLIPSLM

>gi|30065317|ref|NP_839488.1| ribosome-associated heat shock protein Hsp15 [Shigella flexneri 2a str. 2457T]

MKEKPAVEVRLDKWLWAARFYKTRALAREMIEGGKVHYNGQRSKPSKIVELNATLTLRQGNDERTVIVKA

ITEQRRPASEAALLYEETAESVEKREKMAMARKLNALTMPHPDRRPDKKERRDLLRFKHGDSE

>gi|30065316|ref|NP_839487.1| Hsp33-like chaperonin [Shigella flexneri 2a str. 2457T]

MPQHDQLHRYLFENFAVRGELVTVSETLQQILENHDYPQPVKNVLAELLVATSLLTATLKFDGDITVQLQ

GDGPMNLAVINGNNNQQMRGVARVQGEIPENADLKTLVGNGYVVITITPSEGERYQGVVGLEGDTLAACL

EDYFMRSEQLPTRLFIRTGDVDGKPAAGGMLLQVMPAQNTQQDDFDHLATLTETIKTEELLTLPANEVLW

RLYHEEEVTVYDPQDVEFKCTCSRERCADALKTLPDEEVDSILAEDGEIDMHCDYCGNHYLFNAMDIAEI

RNNASPADP

>gi|30065315|ref|NP_839486.1| transporter [Shigella flexneri 2a str. 2457T]

MDNVELSPATRWGMIATGLLQGMIATGLLQGLVCYLLIAWLAGKNHSWIVYGVPATVAFSSVLLFSVISF

KQKRLWGWLALVFIATTGMSGWLKWQTDGMNPWRAEKAIWDFGCYLLLMAMLLLPWIQQSLRIRNGSSRY

SYFYQSVWHNVLILLVIFLSNGLTWLVLLLWGELFKLVGITFFNTLFFATDWFMYLTFGLVTALAVILAR

TQSRLIDSIQKLFTLIATGLLPLVSLLTLMFIITLPFTGLSAISRHISAAGLLLTLAFLQLILMAIVRDP

QKASLPWTGPLRCLIKTALLVAPLYVFVATWGLWLRVAQYGWTVDRLQGALAELVLLVWSLGYFVSIVWR

NGQNPLVLQGKVNLAVSLLVLVILVLLNSPVLDSMRISVNSHMARYQSGKNTPDQVTIYMLEQSGRYGRA

ALESLKSDAGFMKDPKRARDLLMALDGEQHLQEQVSEKVFAENVLIAPGSVKPDATFWSALIQDRYNVMT

CIEKDACVLVEQDLNSDGQAERILFAFNDDRVIVYGFDSDRKEWDALDMSLLPNEITKEKLLTAAKDGKL

GTRPKAWRDLTVDGETLEINLSK

>gi|30065314|ref|NP_839485.1| phosphoenolpyruvate carboxykinase [Shigella flexneri 2a str. 2457T]

MRVNNGLPPQELEAYGISDVHDIVYNPSYDLLYQEELDPSLTGYERGVLTNLGAVAVDTGIFTGRSPKDK

YIVRDDTTRDTFWWADKGKGKNDNKPLSPETWQHLKGLVTRQLSGKRLFVVDAFCGANPDTRLSVRFITE

VAWQAHFVKNMFIRPSDEELAGFKPDFIVMNGAKCTNPQWKEQGLNSENFVAFNLTERMQLIGGTWYGGE

MKKGMFSMMNYLLPLKGIASMHCSANVGEKGDVAVFFGLSGTGKTTLSTDPKRRLIGDDEHGWDDDGVFN

FEGGCYAKTIKLSKEAEPEIYNAIRRDALLENVTVREDGTIDFDDGSKTENTRVSYPIYHIDNIVKPVSK

AGHATKVIFLTADAFGVLPPVSRLTADQTQYHFLSGFTAKLAGTERGITEPTPTFSACFGAAFLSLHPTQ

YAEVLVKRMQAAGAQAYLVNTGWNGTGKRISIKDTRAIIDAILNGSLDNAETFTLPMFNLAIPTELPGVD

TKILDPRNTYASPEQWQEKAETLAKLFIDNFDKYTDTPAGAALVAAGPKL

>gi|30065313|ref|NP_839484.1| osmolarity sensor protein [Shigella flexneri 2a str. 2457T]

MRRLRFSPRSSFARTLLLIVTLLFASLVTTYLVVLNFAILPSLQQFNKVLAYEVRMLMTDKLQLEDGTQL

VVPPAFRREIYRELGISLYSNEAAEEAGLRWAQHYEFLSHQMAQQLGGPTEVRVEVNKSSPVVWLKTWLS

PNIWVRVPLTEIHQGDFSPLFRYTLAIMLLAIGGAWLFIRIQNRPLVDLEHAALQVGKGIIPPPLREYGA

SEVRSVTRAFNHMAAGVKQLADDRTLLMAGVSHDLRTPLTRIRLATEMMSEQDGYLAESINKDIEECNAI

IEQFIDYLRTGQEMPMEMADLNAVLGEVIAAESGYEREIETALYPGSIEVKMHPLSIKRAVANMVVNAAR

YGNGWIKVSSGTEPNRAWFQVEDDGPGIAPEQRKHLFQPFVRGDSARTISGTGLGLAIVQRIVDNHNGML

ELGTSERGGLSIRAWLPVPVTRAQGTTKEG

>gi|30065312|ref|NP_839483.1| osmolarity response regulator [Shigella flexneri 2a str. 2457T]

MQENYKILVVDDDMRLRALLERYLTEQGFQVRSVANAEQMDRLLTRESFHLMVLDLMLPGEDGLSICRRL

RSQSNPMPIIMVTAKGEEVDRIVGLEIGADDYIPKPFNPRELLARIRAVLRRQANELPGAPSQEEAVIAF

GKFKLNLGTREMFREDEPMPLTSGEFAVLKALVSHPREPLSRDKLMNLARGREYSAMERSIDVQISRLRR

MVEEDPAHPRYIQTVWGLGYVFVPDGSKA

>gi|30065306|ref|NP_839477.1| hypothetical protein S4333 [Shigella flexneri 2a str. 2457T]

MSKKQSSTPHDALFKLFLRQPETARDFLAFHLPAPIHALCDMKTLKLESRSFIDDDLRESYSDVLWSVKT

EQGPGYIYCLIEHQSTSNKLIAFRMMRYAIAAMQNHLDAGYKTLPMVVPLLFYHGIESPYPYSLCWLDCF

ADPKLARQLYASAFPLIDVTVMPDDEIMQHRRMALLELIQKHIRQRDLMGLVEQMACLLSSGYANDRQIK

GLFNYILQTGDAVRFNDFIDGVAERSPKHKESLMTIAERLRQEGEQSKALHIAKIMLESGVPLADIMRFT

GLSEEELAAASRLAP

>gi|30065300|ref|NP_839471.1| high-affinity transport permease for gluconate [Shigella flexneri 2a str. 2457T]

MPLVIVAIGVILLLLLMIRFKMNGFIALVLVALAVGLMQGMPLDKVIGSIKAGVGGTLGSLALIMGFGAM

LGKMLADCGGAQRIATTLIAKFGKKHIQWAVVLTGFTVGFALFYEVGFVLMLPLVFTIAASANIPLLYVG

VPMAAALSVTHGFLPPHPGPTAIATIFNADMGKTLLYGTILAIPTVILAGPVYARVLKGIDKPIPEGLYS

AKTFSEEEMPSFGVSVWTSLVPVVLMAMRAIAEMILPKGHAFLPVAEFLGDPVMATLIAVLIAMFTFGLN

RGRSMDQINDTLVSSIKIIAMMLLIIGGGGAFKQVLVDSGVDKYIASMMHETNISPLLMAWSIAAVLRIA

LGSATVAAITAGGIAAPLIATTGVSPELMVIAVGSGSVIFSHVNDPGFWLFKEYFNLTIGETIKSWSMLE

TIISVCGLVGCLLLNMVI

>gi|30065299|ref|NP_839470.1| 4-alpha-glucanotransferase [Shigella flexneri 2a str. 2457T]

MESKRLDNAALAAGISPNYINAHGKPQSISAETKRRLLDAMHQRTATKVAVTPVPNVMVYTSGKKMPMVV

EGSGEYSWQLTTEEGTQYKGHVTGGKAFNLPTKLPEGYHTLTLTQDDQRAHCRVIVAPKRCYEPQALLNK

QKLWGACVQLYTLRSEKNWGIGDFGDLKAMLVDVAKRGGSFIGLNPIHALYPANPESASPYSPSSRRWLN

VIYIDVNAVEDFHLSEEAQAWWQLPTTQQTLQQARDADWVDYSTVTALKMTALRMAWKGFAQRDDEQMAA

FRQFVAEQGDSLFWQAAFDALHAQQVKEDEMRWGWPAWPEMYKNVDSPEVRQFCEEHRDDVDFYLWLQWL

AYSQFAACWEISQGYEMPIGLYRDLAVGVAEGGAETWCDRELYCLKASVGAPPDILGPLGQNWGLPPMDP

HIITARAYEPFIELLRANMQNCGALRIDHVMSMLRLWWIPYGETADQGAYVHYPVDDLLSILALESKRHR

CMVIGEDLGTVPVEIVGKLRSSGVYSYKVLYFENDHEKTFRAPKAYPEQSMAVAATHDLPTLRGYWESGD

LTLGKTLGLYPDEVVLRGLYQDRELAKQGLLDALHKYGCLPKRAGHKASLMSMTPTLNRGLQRYIADSNS

ALLGLQPEDWLDMAEPVNIPGTSYQYKNWRRKLSATLESMFADDGVNKLLKDLDRRRRAAAKKK

>gi|30065295|ref|NP_839466.1| 2-component regulator [Shigella flexneri 2a str. 2457T]

MRKTVAFGFVGTVLDYAGRGSQRWSKWRPSLCIYQQESLVIDRLELLHDARSRSLFETLKRDIASVSPET

EVVGVEIELHNPWDFEEVYACLHDFARGYEFQPEKEDYLIHITTGTHVAQICWFLLAEARYLPARLIQSS

TPRKKEQPRGPGEVTIIDLDLSRYNAIASRFAEERQQTLDFLKSGIATRNPHFNRMIEQIEKVAIKSRAP

ILLNGPTGAGKSFLARRIFELKQARHQFSGAFVEVNCATLRGDTAMSTLFGHVKGAFTGARESREGLLRS

ANGGMLFLDEIGELGADEQAMLLKAIEEKTFYPFGSDRQVSSDFQLIAGTVRDLRQLVAEGKFREDLYAR

INLWTFTLPGLRQRQEDIEPNLDYEVERHATLTGDSVRFNTEARRAWLAFATSPQATWRGNFRELSASVT

RMATFATSGRITLDVVEDEINRLRYNWQESRPSALTALLGAEAENIDLFDRMQLEHVIAICRQAKSLSAA

GRQLFDVSRQGKASVNDADRLRKYLARFGLTWEAVQDQHSSS

>gi|30065294|ref|NP_839465.1| DNA-binding transcriptional repressor GlpR [Shigella flexneri 2a str. 2457T]

MKQTQRHNGIIELVKQQGYVSTEELVEHFSVSPQTIRRDLNELAEQNLILRHHGGAALPSSSVNTPWHDR

KATQTEEKERIARKVAEQIPNGSTLFIDIGTTPEAVAHALLNHSNLRIVTNNLNVANTLMVKEDFRIILA

GGELRSRDGGIIGEATLDFISQFRLDFGILGISGIDSDGSLLEFDYHEVRTKRAIIENSRHVMLVVDHSK

FGRNAMVNMGSISMVDAVYTDAPPPVSVMQVLTDHHIQLELC

>gi|30065292|ref|NP_839463.1| thiosulfate sulfurtransferase [Shigella flexneri 2a str. 2457T]

MDQFECINVADAHQKLQEKEAVLVDIRDPQSFAMGHAVQAFHLTNDTLGAFMRDNDFDTPVMVMCYHGNS

SKGAAQYLLQQGYDVVYSIDGGFEAWQRQFPAEVAYGA

>gi|30065287|ref|NP_839458.1| glycogen synthase [Shigella flexneri 2a str. 2457T]

MQVLHVCSEMFPLLKTGGLADVIGALPAAQIADGVDARVLLPAFPDIRRGVTDAQVVSRRDTFAGHITLL

FGHYNGVGIYLIDAPHLYDRPGSPYHDTNLFAYTDNVLRFALLGWVGAEMASGLDPFWRPDVVHVHDWHA

GLAPAYLAARGRPAKSVFTVHNLAYQGMFYAHHMNDIQLPWSFFNIHGLEFNGQISFLKAGLYYADHITA

VSPTYAREITEPQFAYGMEGLLQQRHREGRLSGVLNGVDEKIWSPETDLLLASRYTRDTLEDKAENKSQL

QIAMGLKVDDKVPLFAVVSRLTSQKGLDLVLEALPGLLEQGGQLALLGAGDPVLQEGFLAAAAEYPGQVG

VQIGYHEAFSHRIMGGADVILVPSRFEPCGLTQLYGLKYGTLPLVRRTGGLADTVSDCSLENLADGVASG

FVFEDSNAWSLLRAIRRAFVLWSRPSLWRFVQRQAMAMDFSWQVAAKSYRELYYRLK

>gi|30065283|ref|NP_839454.1| aspartate-semialdehyde dehydrogenase [Shigella flexneri 2a str. 2457T]

MKNVGFIGWRGMVGSVLMQRMVEERDFDAIRPVFFSTSQLGQAAPSFGGTTGTLQDAFDLEALKALDIIV

TCQGGDYTNEIYPKLRESGWQGYWIDAASSLRMKDDAIIILDPVNQDVITDGLNNGIRTFVGGNCTVSLM

LMSLGGLFANDLVDWVSVATYQAASGGGARHMRELLTQMGHLYGHVADELATPSSAILDIERKVTTLTRS

GELPVDNFGVPLAGSLIPWIDKQLDNGQSREEWKGQAETNKILNTSSVIPVDGLCVRVGALRCHSQAFTI

KLKKDVSIPTVEELLAAHNPWAKVVPNDREITMRELTPAAVTGTLTTPVGRLRKLNMGPEFLSAFTVGDQ

LLWGAAEPLRRMLRQLA

>gi|30065282|ref|NP_839453.1| dITP- and XTP- hydrolase [Shigella flexneri 2a str. 2457T]

MNEIISAAVLLILIMDPLGNLPIFMSVLKHTEPKRRRAIMVRELLIALLVMLVFLFAGEKILAFLSLRAE

TVSISGGIILFLIAIKMIFPSASGNSSGLPAGEEPFIVPLAIPLVAGPTILATLMLLSHQYPNQMGHLVI

ALLLAWGGTFVILLQSSLFLRLLGEKGVNALERLMGLILVMMATQMFLDGIRMWMKG

>gi|30065281|ref|NP_839452.1| low affinity gluconate transporter [Shigella flexneri 2a str. 2457T]

MKARMHAFLALMVVSMGAGLFSGMPLDKIAATMEKGMGGTLGFLAVVVALGAMFGKILYETGAVDQIAVK

MLKSFGHSRAHYAIGLAGLVCALPLFFEVAIVLLISVAFSMARHTGTNLVKLVIPLFAGVAAAAAFLVPG

PAPMLLASQMNADFGWMILIGLCAAIPGMIIAGPLWGNFISRYVELHIPDDISEPHLGEGKMPSFGFSLS

LILLPLVLVGLKTIAARFVPEGSTAYEWFEFIGHPFTAILVACLVAIYGLAMRQGMPKDKVMEICGHALQ

PAGIILLVIGAGGVFKQVLVDSGVGPALGEALTGMGLPIAITCFVLAAAVRIIQGAATVACLTAVGLVMP

VIEQLNYSGAQMAALSICIAGGSIVVSHVNDAGFWLFGKFTGATEAETLKTWTMMETILGTVGAIVGMIA

FQLLS

>gi|30065274|ref|NP_839445.1| hypothetical protein S4297 [Shigella flexneri 2a str. 2457T]

MKRLLILTALLPFVGFAQPINTLNNPNQPGYQIPSQQRMQTQMQTQQIQQKGMLNQQLKTQTQLQQQHLE

NQINNNSQRVLQSQPGERNPARQQMLPNTNGGMLNSNRNPDSSLNQQHMLPERRNGDMLNQPSTPQPDIP

LKTIGP

>gi|30065272|ref|NP_839443.1| glycerol-3-phosphate transporter membrane protein [Shigella flexneri 2a str. 2457T]

MIENRPWLTIFSHTMLILGIAVILFPLYVAFVAATLDKQAVYAAPMTLIPGTHLLENIHNIWVNGVGTNS

APFWRMLLNSFVMAFSITLGKITVSMLSAFAIVWFRFPLRNLFFWMIFITLMLPVEVRIFPTVEVIANLK

MLDSYAGLTLPLMASATATFLFRQFFMTLPDELVEAARIDGASPMRFFCDIVFPLSKTNLAALFVITFIY

GWNQYLWPLLIITDVDLGTTVAGIKGMIATGEGTTEWNSVMAAMLLTLIPPVVIVLVMQRAFVRGLVDSE

K

>gi|30065271|ref|NP_839442.1| glycerol-3-phosphate transporter permease [Shigella flexneri 2a str. 2457T]

MSSSRPVFRSRWLPYLLVAPQLIITVIFFIWPAGEALWYSLQSVDPFGFSSQFVGLDNFVTLFHDSYYLD

SFWTTIKFSTFVTVSGLLVSLFFAALVEYIVRGSRFYQTLMLLPYAVAPAVAAVLWIFLFNPGRGLITHF

LAEFGYDWNHAQNSGQAMFLVVFASVWKQISYNFLFFYAALQSIPRSLIEAAAIDGAGPIRRFFKIALPL

IAPVSFFLLVVNLVYAFFDTFPVIDAATSGGPVQATTTLIYKIYREGFTGLDLASSAAQSMVLMFLVIVL

TVVQFRYVESKVRYQ

>gi|30065270|ref|NP_839441.1| glycerol-3-phosphate transporter periplasmic binding protein [Shigella flexneri 2a str. 2457T]

MKPLHYTASALALGLALMGNAQAVTTIPFWHSMEGELGKEVDSLAQRFNAENPDYKIVPTYKGNYEQNLS

AGIAVFRTGNAPAILQVYEVGTATMMASKAIKPVYDVFKEAGIQFDESQFVPTVSGYYSDSKTGHLLSQP

FNSSTSVLYYNKDAFKKAGLDPEQPPKTWQDLADYSAKLKASGIKCGYASGWQGWIQLENFSAWNGLPFA

SKNNGFDGTDAVLEFNKPEQVKHIAMLEEMNKKGDFSYVGRKDESTEKFYNGDCAMTTASSGSLANIREY

AKFNYGVGMMPYDADAKDAPQNAIIGGASLWVMQGKDKETYTGVAKFLDFLAKPENAAEWHQKTGYLPIT

KAAYDLTREQGFYEKNPGADTATRQMLNKPPLPFTKGLRLGNMPQIRVIVDEELESVWTGKKTPQQALDT

AVERGNQLLRRFEKSTKS

>gi|30065267|ref|NP_839438.1| leucine/isoleucine/valine transporter permease subunit [Shigella flexneri 2a str. 2457T]

MKPMHIAMALLSAAMFFVLAGVFMGVQLELDGTKLVVDTASDIRWQWMFIGTAVVFFFQLLRPAFQKGLK

SVSGPKFILPAIDGSTVKQKLFLVALLVLVVAWPFMVSRGTVDIATLTMIYIILGLGLNVVVGLSGLLVL

GYGGFYAIGAYTFALLNHYYGLGFWTCLPIAGLMAAAAGFLLGFPVLRLRGDYLAIVTLGFGEIVRILLL

NNTEITGGPNGISQIPKPTLFGLEFSRTAREGGWDTFSNFFGLKYDPSDRVIFLYLVALLLVVLSLFVIN

RLLRMPLGRAWEALREDEIACRSLGLSPRRIKLTAFTISAAFAGFAGTLFAARQGFVSPESFTFAESAFV

LAIVVLGGMGSQFAVILAAVLLVVSRELMRDFNEYSMLMLGGLMVLMMIWRPQGLLPMTRPQLKLKNGAA

KGEQA

>gi|30065266|ref|NP_839437.1| branched-chain amino acid ABC transporter permease [Shigella flexneri 2a str. 2457T]

MSEQFLYFLQQMFNGVTLGSTYALIAIGYTMVYGIIGMINFAHGEVYMIGSYVSFMIIAALMMMGIDTGW

LLVAAGFVGAIVIASAYGWSIERVAYRPVRNSKRLIALISAIGMSIFLQNYVSLTEGSRDVALPSLFNGQ

WVVGHSENFSASITTMQAVIWIVTFLAMLALTIFIRYSRMGRACRACAEDLKMASLLGINTDRVIALTFV

IGAAMAAVAGVLLGQFYCVINPYIGFMAGMKAFTAAVLGGIGSIPGAMIGGLILGIAEALSSAYLSTEYK

DVVSFALLILVLLVMPTGILGRPEVEKV

>gi|30065264|ref|NP_839435.1| hypothetical protein S4286 [Shigella flexneri 2a str. 2457T]

MKLTIIRLENFSDQDRIDLQKIWPEYSPSSLQVDDNHRIYAARFNERLLAAVRVTLSGTEGALDSLRVRE

VTRRRGVGQYLLEEVLRNNPGVSCWWMADAGVEDRGVMTAFMQALGFTAQQGGWEKR

>gi|30065262|ref|NP_839433.1| RNA polymerase factor sigma-32 [Shigella flexneri 2a str. 2457T]

MTDKMQSLALAPVGNLDSYIRAANAWPMLSADEERALAEKLHYHGDLEAAKTLILSHLRFVVHIARNYAG

YGLPQADLIQEGNIGLMKAVRRFNPEVGVRLVSFAVHWIKAEIHEYVLRNWRIVKVATTKAQRKLFFNLR

KTKQRLGWFNQDEVEMVARELGVTSKDVREMESRMAAQDMTFDLSSDDDSDSQPMAPVLYLQDKSSNFAD

GIEDDNWEEQAANRLTDAMHGLDERSQDIIRARWLDEDNKSTLQELADRYGVSAERVRQLEKNAMKKLRA

AIEA

>gi|30065261|ref|NP_839432.1| cell division protein FtsX [Shigella flexneri 2a str. 2457T]

MNKRDAINHIRQFGGRLDRFRKSVGGSGDGGRNAPKRAKSSPKPVNRKTNVFNEQVRYAFHGALQDLKSK

PFATFLTVMVIAISLTLPSVCYMVYKNVNQAATQYYPSPQITVYLQKTLDDDAAAGVVAQLQAEQGVEKV

NYLSREDALGEFRNWSGFGGALDMLEENPLPAVAVVIPKLDFQGTESLNTLRDRITQINGIDEVRMDDSW

FARLAALTGLVGRVSAMIGVLMVAAVFLVIGNSVRLSIFARRDSINVQKLIGATDGFILRPFLYGGALLG

FSGALLSLILSEILVLRLSSAVAEVAQVFGTKFDINGLSFDECLLLLLVCSMIGWVAAWLATVQHLRHFT

PE

>gi|30065258|ref|NP_839429.1| 16S rRNA m(2)G966-methyltransferase [Shigella flexneri 2a str. 2457T]

MKKPNHSGSGQIRIIGGQWRGRKLPVPDSPGLRPTTDRVRETLFNWLAPVIVDAQCLDCFAGSGALGLEA

LSRYAAGATLIEMDRVVSQQLIKNLATLKAGNARVVNSNAMSFLAQKGTPHNIVFVDPPFRRGLLEETIN

LLEDNGWLADEALIYVESEVENGLPTVPANWSLHREKVAGQVAYRLYQHEAQGESDAD

>gi|30065256|ref|NP_839427.1| receptor [Shigella flexneri 2a str. 2457T]

MSKPPLFFIVIIGLIVVAASFRFMQQRREKADNDMAPLQQKLVVVSNKREKPINDRRSRQQEVTPAGTSM

RYEASFKPQSGGMEQTFRLDAQQYHALTVGDKGTLSYKGTRFVSFVGEQ

>gi|30065255|ref|NP_839426.1| enzyme [Shigella flexneri 2a str. 2457T]

MLWSFIAVCLSAWLSVDASYRGPTWQRWVFKPLTLLLLLLLAWQAPMFDAISYLVLAGLCASLLGDALTL

LPRQRLMYAIGAFFLSHLLYTIYFASQMTLSFFWPLPLVLLVLGALLLAIIWTRLEEYRWPICTFIGMTL

VMVWLAGELWFFRPTAPALSAFVGASLLFISNFVWLGSHYRRRFRADNAIAAACYFAGHFLIVRSLYL

>gi|30065252|ref|NP_839423.1| hypothetical protein S4274 [Shigella flexneri 2a str. 2457T]

MNVFSQTQRYKALFWLSLFHLLVITSSNYLVQLPVSIFGFHTTWGAFSFPFIFLATDLTVRIFGAPLARR

IIFAVMIPALLISYVISSLFYMGSWQGFGALAHFNLFVARIATASFMAYALGQILDVHVFNRLRQSRRWW

LAPTASTLFGNVSDTLAFFFIAFWRSPDAFMAEHWMEIALVDYCFKVLISIVFFLPMYGVLLNMLLKRLA

DKSEINALQAS

>gi|30065249|ref|NP_839420.1| hypothetical protein S4271 [Shigella flexneri 2a str. 2457T]

METPQPDKTGMHILLKLASLVVILAGIHAAADIIVQLLLALFFAIVLNPLVTWFIRRGVQRPVAITIVVV

VMLIALTALVGVLAASFNEFISMLPKFNKELTRKLFKLQEMLPFLNLHMSPERMLQRMDSEKVVTFTTAL

MTGLSGAMASVLLLVMTVVFMLFEVRHVPYKMRFALNNPQIHIAGLHRALKGVSHYLALKTLLSLWTGVI

VWLGLALMGVQFALMWAVLAFLLNYVPNIGAVISAVPPMIQVLLFNGVYECILVGALFLVVHMVIGNILE

PRMMGHRLGMSTMVVFLSLLIWGWLLGPVGMLLSVPLTSVCKIWMETTKGGSKLAILLGPGRPKSRLPG

>gi|30065248|ref|NP_839419.1| holo-(acyl carrier protein) synthase 2 [Shigella flexneri 2a str. 2457T]

MYRIVLGKVSTLSAAPLPPGLREQAPQGPRRERWLAGRTLLSHTLSPLPEIIYGEQGKPAFAPETPLWFN

LSHSGDDIALLLSDEGEVGCDIEVIRPRVNWRWLANAVFSLGEHAEMDAVHPDQQLEMFWRIWTRKEAIV

KQRGGSAWQIVSVDSTYHSSLSVSHCQLENLSLAICTPTPFTLTADSVQWIDSVN

>gi|30065247|ref|NP_839418.1| periplasmic binding protein for nickel [Shigella flexneri 2a str. 2457T]

MLSTLRRTLFALLACASFIVHAAAPDEITTAWPVNVGPLNPHLYTPNQMFAQSMVYEPLVKYQADGSVIP

WLAKSWTHSEDGKTWTFTLRDDVKFSNGEPFDAEAAAENFRAVLDNRQRHAWLELANQIVDVKALNKTEL

QITLKSAYYPFLQELALPRPFRFIAPSQFKNHETMNGIKAPIGTGPWILQESKLNQYDVFVRNENYWGEK

PAIKKITFNVIPDPTTRAVAFETGDIDLLYGNEGLLPLDTFARFSQNPAYHTQLSQPIETVMLALNTAKA

PTNELAVREALNYAVNKKSLIDNALYGTQQVADTLFAPSVPYANLGLKPRQYDPQKAKELLEKAGWTLPA

GKDIREKNGQPLRIELSFIGTDALSKSMAEIIQADMRQIGADVSLIGEEESSIYARQRDGRFGMIFHRTW

GAPYDPHAFLSSMRVPSHADFQAQQGLADKPLIDKEIGEVLATHDETQRQALYRDILTRLHDEAVYLPIS

YISMMVVSKPELGNIPYAPIATEIPFEQIKPVKP

>gi|30065246|ref|NP_839417.1| nickel transporter permease NikB [Shigella flexneri 2a str. 2457T]

MLRYVLRRFLLLIPMVLAASVIIFLMLRLGTGDPALDYLRLSNLPPTPEMLASTRTMLGLDQPLYVQYGT

WLWKALHLDFGISFASQRPVLDDMLNFLPATLELAGAALVLILLTSVPLGIWAARHRDRLPDFAVRFIAF

LGVSMPNFWLAFLLVMAFSVYLQWLPAMGYGSWQHIILPAVSIAFMSLAINARLLRASMLDVAGQRHVTW

ARLRGLNDKQTERRHILRNASLPMITAVGMHIGELIGGTMIIENIFAWPGVGRYAVSAIFNRDYPVIQCF

TLMMVVVFVVCNLIVDLLNAALDPRIRRHEGAHA

>gi|30065245|ref|NP_839416.1| nickel transporter permease NikC [Shigella flexneri 2a str. 2457T]

MNFFLSSRWSVRLALIIIALLALIALTSQWWQPYDPQAIDLPSCLLSPDAQHWLGTDHLGRDIFSRLMAA

TRVSLGSVMACLLLVLTLGLVIGGSAGLIGGHVDQATMRVADMFMTFPTSILSFFMVGVLGTGLTNVIIA

IALSHWAWYARMVRSLVISLRQREFVLASRLSGAGHVRVFVDHLAGAVIPSLLVLATLDIGHMMLHVAGM

SFLGLGVTAPTAEWGVMINDARQYIWTQPLQMFWPGLALFISVMAFNLVGDALRDHLDPHLVTEHAH

>gi|30065242|ref|NP_839413.1| nickel responsive regulator [Shigella flexneri 2a str. 2457T]

MQRVTITLDDDLLETLDSLSQRRGYNNRSEAIRDILRSALAQEATQQHGTQGFAVLSYVYEHEKRDLASR

IVSTQHHHHDLSVATLHVHINHDDCLEIAVLKGDMGDVQHFADDVIAQRGVRHGHLQCLPKED

>gi|30065241|ref|NP_839412.1| hypothetical protein S4263 [Shigella flexneri 2a str. 2457T]

MIKLKTPNSMEIAEQPAVITYVPELNAFRGKFLGLSGYCDFVSDSIQGLQKEGELSLREYLEDCKAAGIE

PYARTEKIKTFTLRYPESLSERLNNAAAQQQVSVNTYIIETLNERLNHL

>gi|30065240|ref|NP_839411.1| transporter [Shigella flexneri 2a str. 2457T]

MRHLRNIFNLGIKELRSLLGDKAMLTLIVFSFTVSVYSSATVTPGSLNLAPIAIADMDQSQLSNRIVNSF

YRPWFLPPEMITADEMDAGLDAGRYTFAINIPPNFQRDVLAGRQPDIQVNVDATRMSQAFTGNGYIQNII

NGEVNSFVARYRDNSEPLVSLETRMRFNPNLDPAWFGGVMAIINNITMLAIVLTGSALIREREHGTVEHL

LVMPITPFEIMMAKIWSMGLVVLVVSGLSLVLMVKGVLGVPIEGSIPLFMLGVALSLFATTSIGIFMGTI

ARSMPQLGLLVILVLLPLQMLSGGSTPRESMPQMVQDIMLTMPTTHFVSLAQAILYRGAGFEIVWPQFLT

LMAIGGAFFTIALLRFRKTIGTMA

>gi|30065238|ref|NP_839409.1| hypothetical protein S4260 [Shigella flexneri 2a str. 2457T]

MDKSKRHLAWWVVGALAVAAVVAWWLLRPAGVPEGFAVSNGRIEATEVDIASKIAGRIDTILVKEGQFVR

EGEVLAKMDTRVLQEQRLEAIAQIKEAQSAVAAAQALLEQRQSETRAAQSLVNQRQAELDSVAKRHTRSR

SLAQRGAISAQQLDDDRAAAESARAALESAKAQVSASKAAIEAARTNIIQAQTRVEAAQATERRIAADID

DSELKAPRDGRVQYRVAEPGEVLAAGGRVLNMVDLSDVYMTFFLPTEQAGTLKLGGEARLILDAAPDLRI

PATISFVASVAQFTPKTVETSDERLKLMFRVKARIPPELLQQHLEYVKTGLPGVAWVRVNEELPWPDDLV

VRLPQ

>gi|30065237|ref|NP_839408.1| hypothetical protein S4259 [Shigella flexneri 2a str. 2457T]

MKIGTVAGTNGSTTTIATNDMVQEHVTNFTKELFGYIANGIGDDISSIARTMLGEVVEKIDDWQIERFQQ

SIQDDKISFTIQTNHSEKYSMLSGMRAHILRRNNCYQFIVTINSKNYGCPLDNTDINWCSIVYLLNNMTV

NDNANDVAVTESYKPIWNWEISQYNVFDIKFETIIKPQFADRTYFSNCSPVDPTSTRPTYFGDTDGSVGA

VLYALFATGHLGIMAEGENFLSQLLNIEDEVLNVLLRENFNEQLDTNVNTIISILNRRDNVLESLQPYLV

INKDAVTPCTFLGDQTGDRFSNICGDQFIIDLLKRIMSINENVHVLAGNHETNCNGNYMQNFTRMKPLDE

DTYDGIKDYPVCFYDPKYKIMANHHGITFDDQRKRYIIGPITVSIDEMTNALDPVELAEIINKKHHAIIN

GKKFKTSRAISCRSFNRYFSVSTDYRPKLEALLACSQMLGINQVVAHNGNGGRERIGETGTVLGLNARDS

KHAGRMFSMHNCQINPGAGPEITTPWKSYQHEKNRNGLMPLIRRRTMLQL

>gi|30065236|ref|NP_839407.1| hypothetical protein S4838 [Shigella flexneri 2a str. 2457T]
MTKLMQFVQRCYYMTNKKMYFILILVFTLLQVCFFALWKARDGSTTSLECTSTLTRNAKTDHSLYYSANL
SVILKKDGSGSFTIVGLTDEDTPRKFSHSYFFTYKIDSNGRISGNAKAKVSGLENQIKDENFRLNFLDAS
LTGKGNARLSKFNNVYIFSIPGLIINTCAPI

>gi|30065235|ref|NP_839406.1| hypothetical protein S4837 [Shigella flexneri 2a str. 2457T]
MERRCFFIINDAIIFDPNKCSLSNVNTQDEINFPATAGCILAVLLESNGGAVDRNSIIDSVLERFSFDLS
NNTLNQYISLLRKSFKNIGVEENVILTIPKVGFYISENITISKQSYDDSHLQEDVVEKVERKRSIPLFRI
LISLVVVIVIIEMFAVAFKDTYSITDYPLVKSGKIDTCDLYLSENLNEIGLAKSQIEAAEISKKHLPCVP
GANFIYDVNSMHYLLGEGQRYLSRCIKSSNDETYAICSEVLLYD

>gi|30065234|ref|NP_839405.1| hypothetical protein S4836 [Shigella flexneri 2a str. 2457T]
MLSKITQELLKMNNVLLMRPDDSEFMIPDDWSGLNAKGITYEQDNRVTESFLPFKSFYCSPVCTTVELIS
QNNCFAMGIRYLLEKKRDLRLNHFKAFSHKCETKPDVLIMTGCTVADTYSIFSRIVEENIMKTMTPIVII
TGHHSEDLCSLASKLGGVKIISPTEKIAKIENAINDAFNSKPKKIRFLTDILSPQQIETMKMVAQGRSVN
EISAAMNISIKTVYAHKVRAINKLGIKKKVYEALFYKALIQFN

>gi|30065233|ref|NP_839404.1| hypothetical protein S4255 [Shigella flexneri 2a str. 2457T]
MKKTLLAIMLAGTAFASQAGTLVSQGTEASANLTLTKPIVVNNTIQPVKGVYSGTLTAWTPLATGIVGAS
DGQSHDYAVTFPDDIYAESSTSADAVISGDNNPDHKLKVSLTTLEQDPPSAASEEIGGKRYMMLKNTGTG
GAYRVVSHMKEQVVEPDSYTIRTQAYIYAE

>gi|30065230|ref|NP_839401.1| fimbrial protein remnant [Shigella flexneri 2a str. 2457T]

MFYRLSLILIMALLAGQLSAQEWSFDSSQLEGNVSADTVAMFNQGEQLPGNYRVEIYLNGEKVDVGEFPF

HRPESPEEKELVPCLTVDDLIHYGIKIDKSSSDTDNKKNQCFKWNSIEGLKVNYDFDSQRVQITVPQLYL

QDKKSSLAPVSLWNEGVAAFRMVYQTNIDISKQNDNQSTTRNSRYGRFTPGFNLGAWRFRSSVTWSKELG

QSERWQRGYMWFERGINAIKSRLTLGESYTSSEVFDSIPFRGGMLATDDAMTPPEDSYYTPVVHGIAQSE

AQVIIKQNGQIIFTRSVPPGPFALDNLPTLAVGGELDVTVRESNGEEQYFSVPFQTPAIALHEGYFKYSV

MGGNIKKKV

>gi|30065224|ref|NP_839395.1| hypothetical protein S4245 [Shigella flexneri 2a str. 2457T]

MERFDAIIIGAGAAGMFCSALAGQVGRRVLLIDNGKILGRKILMSGGGRCNFTNLYVEPGAYLSQNPHFC

KSALARFTQWDFIDLVNKHGIAWHEKTLGQLFCDDSAQQIVDMLVDECEKGNVTFRLRSEVLSVAKDETG

FTLDLNGMTVGCEKLVIATGGLSMPGLGASPFGYKIAEQFGLNVLPTRAGLVPFTLHKPLLEELQVLAGV

AVPSVITAENGTVFRENLLFTHRGLSGPAVLQISSYWQPGEFVSINLLPDVDLETFLNEQRNAHPNQSLK

NTLAVHLPKRLVERLQQLGQIPDVSLKQLNVCDQQALISTLTDWRVQPNGTEGYRTAEVTLGGVDTNELS

SRTMEARKVPGLYFIGEVMDVTGWLGGYNFQWAWSSAWACAQDLASIVK

>gi|30065222|ref|NP_839393.1| universal stress protein UspB [Shigella flexneri 2a str. 2457T]

MISTVALFWALCVVCIVNMARYFSSLRALLVVLRNCDPLLYQYVDGGGFFTSHGQPNKQVRLVWYIYAQR

YRDHHDDEFIRRCERVRRQFILTSALCGLVVVSLIALMIWH

>gi|30065221|ref|NP_839392.1| universal stress protein [Shigella flexneri 2a str. 2457T]

MAYKHILIAVDLSPESKVLVEKAVSMARPYNAKVSLIHVDVNYSDLYTGLIDVNLGDMQKRISEETHHAL

TELSTNAGYPITETLSGSGDLGQVLVDAIKKYDMDLVVCGHHQDFWSKLMSSARQLINTVHVDMLIVPLR

DEEE

>gi|30065220|ref|NP_839391.1| methyltransferase [Shigella flexneri 2a str. 2457T]

MKICLIDETGAGDGALSVLAARWGLEHDEDNLMALVLTPEHLELRKRDEPKLGGIFVDFVGGAMAHRRKF

GGGRGEAVAKAVGIKGDYLPDVVDATAGLGRDAFVLASVGCRVRMLERNPVVAALLDDGLARGYADAEIG

GWLQERLQLIHASSLTALTDITPRPQVVYLDPMFPHKQKSALVKKEMRVFQSLVGPDLDADGLLEPARLL

ATKRVVVKRPDYAPPLANVATPNAVVTKGHRFDIYAGTPV

>gi|30065218|ref|NP_839389.1| hypothetical protein S4238 [Shigella flexneri 2a str. 2457T]

MLSYRHSFHAGNHADVLKHTVQSLIIESLKEKDKPFLYLDTHAGAGRYQLGSEHAERTGEYLEGIARIWQ

QDDLPAELEAYINVVKHFNRSGQLRYYPGSPLIARQLLREQDSLQLTELHPSDYPLLRSGFQKDSRARVE

KADGFQQLKAKLPPVSRRGLILIDPPYEMKTDYQAVVSGIAEGYKRFATGTYALWYPVVLRQQIKRMIHD

LEATGIRKILQIELAVLPDSDRRGMTASGMIVINPPWKLEQQMNNVLSWLHSKLVPAGTGHATVSWIVPE

>gi|30065214|ref|NP_839385.1| arsenate reductase [Shigella flexneri 2a str. 2457T]

MSNITIYHNPACGTSRNTLEMIRNSGTEPTIIHYLETPPTRDELVKLIADMGISVRALLRKNVEPYEELG

LAEDKFTDDRLIDFMLQHPILINRPIVVTPLGTRLCRPSEVVLEILPDAQKGAFSKEDGEKVVDEAGKRL

K

>gi|30065213|ref|NP_839384.1| arsenical pump membrane protein [Shigella flexneri 2a str. 2457T]

MLLAGAIFVLTIVLVIWQPKGLGIGWSATLGAVLALVTGVVHPGDIPVVWNIVWNATAAFIAVIIISLLL

DESGFFEWAALHVSRWGNGRGRLLFTWIVLLGAAVAALFANDGAALILTPIVIAMLLALGFSKGTTLAFV

MAAGFIADTASLPLIVSNLVNIVSADFFGLGFREYASVMVPVDIAAIVATLVMLHLYFRKDIPQNYDMAL

LKSPAEAIKDPATFKTGWVVLLLLLVGFFVLEPLGIPVSAIAAVGALILFVVAKRGHAINTGKVLRGAPW

QIVIFSLGMYLVVYGLRNAGLTEYLSGVLNVLADNGLWAATLGTGFLTAFLSSIMNNMPTVLVGALSIDG

STASGVIKEAMVYANVIGCDLGPKITPIGSLATLLWLHVLSQKNMTISWGYYFRTGIIMTLPVLFVTLAA

LALRLSFTL

>gi|30065212|ref|NP_839383.1| DNA-binding transcriptional repressor ArsR [Shigella flexneri 2a str. 2457T]

MLPIQLFKILADETRLGIVLLLSELGELCVCDLCTALDQSQPKISRHLALLRESGLLLDRKQGKWVHYRL

SPHIPAWAAKIIDEAWRCEQEKIQAIVRNLARQNCSGDSKNICS

>gi|30065208|ref|NP_839379.1| outer membrane protein induced after carbon starvation [Shigella flexneri 2a str. 2457T]

MVLIFVDKDSNMNMTKGALILSLSFLLAACSSIPQNIKGNNQPDIQKSFVAVHNQPGLYVGQQARFGGKV

INVINGKTDTLLEIAVLPLDSYAKPDIEANYQGRLLARQSGFLDPVNYRNHFVTILGTIQGEQPGFINKV

PYNFLEVNMQGIQVWHLREVVNTTYNLWDYGYGAFWPEPGWGAPYYTNAVSQVTPELVK

>gi|30065207|ref|NP_839378.1| hypothetical protein S4226 [Shigella flexneri 2a str. 2457T]

MFLIITRDTMFFTAMKNILSKGNVVHIQNEEEIDVMLHQNAFVIIDTLMNNVFHSNFLTQIERLKPVHVI

VFSPFNIKRCLGKVPVTFVPRTITIIDFVALINGSYCSVPEANVSLSRKQHQVLSCIANQMTTEDILEKL

KISLKTFYCHKHNIMMILNLKRINELVRHQHIDYLV

>gi|30065206|ref|NP_839377.1| acid-resistance protein [Shigella flexneri 2a str. 2457T]

MGYKMNISSLRKAFIFMGAVAALSLVNAQSALAANESAKDMTCQEFIDLNPKAMTPVAWWMLHEETVYKG

GDTVTLNETDLTQIPKVIEYCKKNPQKNLYTFKNQASNDLPN

>gi|30065205|ref|NP_839376.1| acid-resistance protein [Shigella flexneri 2a str. 2457T]

MKKVLGVILGGLLLLPVVSNAADAQKAADNKKPVNSWTCEDFLAVDESFQPTAVGFAEALNNKDKPEDAV

LDVQGIATVTPAIVQACTQDKQANFKDKVKGEWDKIKKDM

>gi|30065204|ref|NP_839375.1| acid-resistance membrane protein [Shigella flexneri 2a str. 2457T]

MLYIDKATILKFDLEMLKKHRRAIQFIAVLLFIVGLLCISFPFVSGDILSTVVGALLICSGIALIVGLFS

NRSHNFWPVLSGFLVAVAYLLIGYFFIRAPELGIFAIAAFIAGLFCVAGVIRLMSWYRQRSMKGSWLQLV

IGVLDIVIAWIFLGATPMVSVTLVSTLVGIELIFSAASLFSFASLFVKQQ

>gi|30065203|ref|NP_839374.1| hypothetical protein S4221 [Shigella flexneri 2a str. 2457T]

MIFLMTKDSFLLQGFWQLKDNHEMIKINSLSEIKKVGNKPFKVIIDTYHNHILDEEAIKFLEKLDAERII

VLAPYHISKLKAKAPIYFVSRKESIKNLLEITYGKHLPHKNSQLCFSHNQFKIMQLILKNKNESNITSTL

NISQQTLKIQKFNIMYKLKLRRMSDIVTLGITSYF

>gi|30065198|ref|NP_839369.1| regulator [Shigella flexneri 2a str. 2457T]

MQIVMFDRQSIFIHGIKISLQQRIPGVSIQGVSQADELWQKLESYPEALVMLDGDQDGEFCYWLLQKTVV

QFPEVKVLITATDCNKRWLQEVIHFNVLAIVPRDSTVETFALAVNSAAMGMMFLPGDWRTTPEKDIKDLK

SLSARQREILTMLAAGESNKEIGRALNISTGTVKAHLESLYRRLEVKNRTQAAMMLNISS

>gi|30065197|ref|NP_839368.1| transcriptional regulator LYSR-type [Shigella flexneri 2a str. 2457T]

MLEKTINNAICALLFRCEQQSVKEMDKIHAMQLFIKVAELESFSRAADFFALPKGSVSRQIQALEHQLGT

QLLQRTTRRVKLTPEGMTYYQRAKDVLSNLSELDGLFQQDATSISGKLRIDIPSGIAKNLLLPRLSEFLY

QHPGIELELSSHNRPVDILHDGFDCVIRTGALPEDGVIARPLGKLTMVNCASPHYLTRFGYPQSPDDLTS

HAIVRYTPHLGVHPLGFEVASVNGVQWFKSGGMLTVNSSENYLAAGLAGLGIIQIPRIAVREALRAGRLI

EVLPGYRAEPLSLSLVYPQRRELSRRVNLFMQWLAGVMKEHLD

>gi|30065196|ref|NP_839367.1| hypothetical protein S4213 [Shigella flexneri 2a str. 2457T]

MTQENEIKRPTQDLEHEPIKQLDNSEKGGKVSQALETVTTTAEKVQRQPVIAHLIRATERFNDRLGNQFG

AAITYFSFLSMIPILMVSFAAGGFVLASHPMLLQDIFDKILQNISDPTLAATLKNTINTAVQQRTTVGLV

GLAVALYSGINWMGNLREAIRAQSRDVWERSPQDQEKFWVKYLRDFISLIGLLIALIVTLSITSVAGSAQ

QMIISALHLNSIEWLKPTWRLIGLAISIFANYLLFFWIFWRLPRHRPRKKALIRGTFLAAIGFEVIKIVM

TYTLPSLMKSPSGAAFGSVLGLMAFFYFFARLTLFCAAWIATAEYKDDPRMPGKTQP

>gi|30065195|ref|NP_839366.1| transporter [Shigella flexneri 2a str. 2457T]

MQATATTLDHEQEYTPINSRNKVLVASLIGTAIEFFDFYIYATAAVIVFPHIFFPQGDPTAATLQSLATF

AIAFVARPIGSAVFGHFGDRVGRKATLVASLLTMGISTVVIGLLPGYATIGIFAPLLLALARFGQGLGLG

GEWGGAALLATENAPPRKRALYGSFPQLGAPIGFFFANGTFLLLSWLLTDEQFMSWGWRVPFIFSAVLVI

IGLYVRVSLHESPVFEKVAKAKKQVKIPLGTLLTKHVRVTVLGTFIMLATYTLFYIMTVYSMTFSTAAAP

VGLGLPRNEVLWMLMMAVIGFGVMVPVAGLLADAFGRRKSMVIITTLIILFALFAFNPLLGSGNPILVFA

FLLLGLSLMGLTFGPMGALLPELFPTEVRYTGASFSYNVSSILGASVAPYIAAWLQANYGLGAVGLYLAA

MAGLTLIALLLTHETRHQSL

>gi|30065194|ref|NP_839365.1| hypothetical protein S4211 [Shigella flexneri 2a str. 2457T]

MSKAGKITAAISGAFLLLIVVAIILIATFDWNRLKPTINQKVSAELNRPFAIRGNLGVVWERQKQETGWR

SWVPWPHVHAEDIILGNPPDIPEVTMVHLPRVEATLAPLALLTKTVWLPWIKLEKPDARLIRLSEKNNNW

TFNLANDDNKDANAKPSAWSFRLDNILFDQGRIAIDDKVSKADLEIFVDPLGKPLPFSEVTGSKGKADKE

KVGDYIFGLKAQGRYNGEPLTGTGKIGGMLALRGEGTPFPVQADFRSGNTRVAFDGVVNDPMKMGGVDLR

LKFSGDSLGDLYELTGVLLPDTPPFETDGRLVAKIDTEKSSVFDYRGFNGRIGDSDIHGSLVYTTGKPRP

KLEGDVESRQLRLADLGPLIGVDSGKGAEKSKRSEQKKGEKSVQPAGKVLPYDRFETDKWDVMDADVRFK
GRRIEHGSSLPISDLSTHIILKNADLRLQPLKFGMAGGSIAANIHLEGDKKPMQGRADIQARRLKLKELM
PDVELMQKTLGEMNGDAELRGSGNSVAALLGNSNGNLKLLMNDGLVSRNLMEIVGLNVGNYIVGAIFGDD
EVRVNCAAANLNIANGVARPQIFAFDTENALINVTGTASFASEQLDLTIDPESKGIRIITLRSPLYVRGT
FKNPQAGVKAGPLIARGAVAAALATLVTPAAALLALISPSEGEANQCRTILSQMKK

>gi|30065193|ref|NP_839364.1| EAL domain-containing protein [Shigella flexneri 2a str. 2457T]
MIRQVIQRISNPEASIESLQERRFWLQCERAYTWQPIYQTCGRLMAVELLTVVTHPLNPSQRLPPDRYFT
EITVSHRMEVVKEQIDLLAQKADFFVEHGLLASVNIDGPTLIALRQQPKILRQIERLPWLRFELVEHIRL
PKDSTFASMCEFGPLWLDDFGTGMANFSALSEVRYDYIKIARELFVMLRQSPEGRTLFSQLLHLMNRYCR
GVIVEGVETPEEWRDVQNSPAFAAQGWFLSRPAPMETLNTAVLAL

>gi|30065192|ref|NP_839363.1| ketodeoxygluconokinase [Shigella flexneri 2a str. 2457T]
MFKSDAARTRQRLSARKGRTLPPGRGKFPHSTTESFRNTFWLKKIMEHCFNMVDQQTTTAQTNANFLQIR
FTTMSKKIAVIGECMIELSEKGADVKRGFGGDTLNTSVYIARQVDPAALTVHYVTALGTDSFSQQMLDAW
HGENVDTSLTQRMENRLPGLYYIETDSTGERTFYYWRNEAAAKFWLASEQSAAICEELANFDYLYLSGIS
LAILSPTSREKLLSLLRECRAKGGKVIFDNNYRPRLWASKEETQQVYQQMLECTDIAFLTLDDEDALWGQ
QPVEDVIARTHNAGVKEVVVKRGADSCLVSIAGEALVDVPAVKLPKEKVIDTTAAGDSFSAGYLAVRLTG
GSAENAAKRGHLTASTVIQYRGAIIPREAMPA

>gi|30065191|ref|NP_839362.1| hypothetical protein S4208 [Shigella flexneri 2a str. 2457T]
MQGIKIRLLAGGLLMMATAGYVQADALQPDPAWQQGTLSNGLQWQVLTTPQRPSDRVEIRLLVNTGSLAE
STQQSGYSHAIPRIALTQSGGLDAAQARSLWQQGIDPKRPMPPVIVSYDTTLFNLSLPNNRNDLLKEALS
YLANATGKLTITPETINHALQSQDMVATWPADTKEGWWRYRLKGSTLLGHDPADPLKQPVEAEKIKDFYQ
KWYTPDAMTLLVVGNVDARSVVDQINKTFGELKGKRETPAPVPTLSPLRAEAVSIMTDAVRQDRLSIMWD

TPWQPIRESAALLRYWRADLAREALFWHVQQALSASDSKDIGLGFDCRVLYLRAQCAINIESPNDKLNSN

LNLVARELAKVRDKGLPEEEFNALVAQKKLELQKLFAAYARADTDILMGQRMRSLQNQVVDIAPEQYQKL

RQDFLNSLTVEMLNQDLRQQLSNDMALILLQPKGEPEFNMKALQAAWDQIMAPSTAAATTSVATDDVHPE

VTDIPPAQ

>gi|30065188|ref|NP_839359.1| endo-1,4-D-glucanase [Shigella flexneri 2a str. 2457T]

MNVLRSGIVTMLLLAAFSVQAACTWPAWEQFKKDYISQEGRVIDPSDARKITTSEGQSYGMFFALAANDR

AAFDNILDWTQNNLAQGSLKERLPAWLWGKKENSKWEVLDSNSASDGDVWMAWSLLEAGRLWKEQRYTDI

GSALLKRIAREEVVTVPGLGSMLLPGKVGFAEDNSWRFNPSYMPPTLAQYFTRFGAPWTTLRETNQHLLL

ETAPKGFSPDWVRYEKDKGWQLKAEKTLISSYDAIRVYMWVGMMPDSDPQKARMLNRFKPMATFTEKNGY

PPEKVDVATGKAQGKGPVGFSAAMLPFLQNRDAQAVQRQRVADNFPGSDTYYNYVLTLFGQGWDQHRFRF

STKGELLPDWGQECANSH

>gi|30065186|ref|NP_839357.1| cell division protein [Shigella flexneri 2a str. 2457T]

MAVLGLQGVRGGVGTTTITAALAWSLQMLGENVLVVDACPDNLLRLSFNVDFTHRQGWARAMLDGQDWRD

AGLRYTSQLDLLPFGQLSIEEQENPQHWQTRLSDICSGLQQLKASGRYQWILIDLPRDASQITHQLLSLC

EHSLAIVNVDANCHIRLHQQALPDGAHILINDFRIGSQVQDDIYQLWLQSQRRLLPMLIHRDEAMAECLA

AKQPVGEYRSDALAAEEILTLANWCLLNYSGLKTPVGSAS

>gi|30065184|ref|NP_839355.1| protease [Shigella flexneri 2a str. 2457T]

MRDIVDPVFSIGISSLWDELRHMPAGGVWWFNVDRHEDAISLANQTIASQAETAHVAVISMDSDPAKIFQ

LDDSQGPEKIKLFSMLNHEKGLYYLARDLQCSIDPHNYLFILVCANNAWQNIPAERLRSWLDKMNKWSRL

NHCSLLVINPGNNNDKQFSLLLEEYRSLFGLASLRFQGDQHLLDIAFWCNEKGVSARQQLSVQQQNGIWT

LVQSEEAEIQPRSDEKRILSNVAVLEGAPPLSEHWQLFNNNEVLFNEARTAQAATVVFSLQQNAQIEPLA

RSIHTLRRQRGSAMKILVRENTASLRATDERLLLACGANMVIPWNAPLSRCLTMIESVQGQKFSRYVPED

ITTLLSMTQPLKLRGFQKWDVFCNAVNNMMNNPLLPAHGKGVLVALRPVPGIRVEQALTLCRPNRTGDIM

TIGGNRLVLFLSFCRINDLDTALNHIFPLPTGDIFSNRMVWFEDDQISAELVQMRLLAPEQWGMPLPLTQ

SSKPVINAEHDGRHWRRIPEPMRLLDDAVERSS

>gi|30065182|ref|NP_839353.1| hypothetical protein S4197 [Shigella flexneri 2a str. 2457T]

MTQFTQNTAMPSSLWQYWRGLSGWNFYFLVKFGLLWAGYLNFHPLLNLVFAAFLLMPIPRYSLHRLRHWI

ALPIGFALFWHDIWLPGPESIMSQGSQVAGFSTDYLIDLVTRFINWQMIGAIFVLLVAWLFLSQWIRITV

FVVAILLWLNVLTLAGPSFSLWPAGQPTTTVTTTGGNAAATVAATGGAPVVGDMPAQTAPPTTANLNAWL

NNFYNAEAKRKSTFPSSLPADAQPFELLVINICSLSWSDIEAAGLMSHPLWSHFDIDFKNFNSATSYSGP

AAIRLLRASCGQTSHTNLYQPANNDCYLFDNLSKLGFTQHLMMGHNGQFGGFLKEVRENGGMQTELMDQT

NLPVILLGFDGSPVYDDTAVLNRWLDVTEKDKNSRSATFYNTLPLHDGNHYPGVSKTADYKARAQKFFDE

LDAFFTELEKSGRKVMVVVVPEHGGALKGDRMQVSGLRDIPSPSITDVPVGVKFFGMKAPHQGAPIVIDQ

PSSFLAISDLVVRVLDGKIFTEDNVDWKKLTSGLPQTAPVSENSNAVVIQYQDKPYVRLNGGDWVPYPQ

>gi|30065181|ref|NP_839352.1| transporter protein [Shigella flexneri 2a str. 2457T]

MQHNTLSKHNQKLPFTRYDFGWVLLCIGMAIGAGTVLMPVQIGLKGIWVFITAAIIAYPVTWVVQDIYLK

TLSESDSCNDYTDIISHYLGKNWGIFLGVIYFLMIIHGIFIYSLSVVFDSASYLKTFGLTDADLSQSLLY

KVAIFAVLIAIASGGERLLFKISGPMVVVKVGIIVVFGFAMIPHWNFANITAFPQASVFFRDVLLTIPFC

FFSAVFIQVLNPMNIAYRKREADKVLATRLALRTHRISYITLIAVILFFAFSFTFSISHEEAVSAFEQNI

SALALAAQVIPGHIIHITSTVLNIFAVLTAFFGIYLGFHEAIKGIILNLLSRIIDTKKINSRMLTLAICA

FIVITLTIWVSFRVSVLVFFQLGSPLYGIVSCLIPFFLIYKVAQLEKLRGFKAWLILLYGILLCLSPLLK

LIE

>gi|30065178|ref|NP_839349.1| dipeptide transporter [Shigella flexneri 2a str. 2457T]

MSQVTENKVISAPVPMTPLQEFWHYFKRNKGAVVGLVYVAIVLFIAIFANWIAPYNPADQFRDALLAPPA

WQEGGSMAHLLGTDDVGRDVLSRLMYGARLSLLVGCLVVVLSLIMGVILGLIAGYFGGLVDNIIMRVVDI

MLALPSLLLALVLVAIFGPSIGNAALALTFVALPHYVRLTRAAVLVEVNRDYVTASRVAGAGAMRQMFIN

IFPNCLAPLIVQASLGFSNAILDMAALGFLGMGAQPPTPEWGTMLSDVLQFAQSAWWVVTFPGLAILLTV

LAFNLMGDGLRDALDPKLKQ

>gi|30065177|ref|NP_839348.1| dipeptide transporter permease DppB [Shigella flexneri 2a str. 2457T]

MLQFILRRLGLVIPTFIGITLLTFAFVHMIPGDPVMIMAGERGISPERHVQLLAELGLDKPMWQQYLHYI

WGVMHGDLGISMKSRIPVWEEFVPRFQATLELGVCAMIFATAVGIPVGVLAAVKRGSIFDHTAVGLALTG

YSMPIFWWGMMLIMLVSVHWNLTPVSGRVSDMVFLDDSNPLTGFMLIDTAIWGEDGNFIDAVAHMILPAI

VLGTIPLAVIVRMTRSSMLEVLGEDYIRTARAKGLTRMRVIIVHALRNAMLPVVTVIGLQVGTLLAGAIL

TETIFSWPGLGRWLIDALQRRDYPVVQGGVLLVATMIILVNLLVDLLYGVVNPRIRHKK

>gi|30065176|ref|NP_839347.1| dipeptide transport protein [Shigella flexneri 2a str. 2457T]

MRISLKKSGMLKLGLSLVAMTVAASVQAKTLVYCSEGSPEGFNPQLFTSGTTYDASSVPLYNRLVEFKIG

TTEVIPGLAEKWEVSEDGKTYTFHLRKGVKWHDNKEFKPTREMNADDVVFSFDRQKNAQNPYHKVSGGSY

EYFEGMGLPELISEVKKVDDNTVQFVLTRPEAPFLADLAMDFASILSKEYADAMMKAGTPEKLDLNPIGT

GPFQLQQYQKDSRIRYKAFDGYWGTKPQIDTLVFSITPDASVRYAKLQKNECQVMPYPNPADIARMKQDK

SINLMEMPGLNVGYLSYNVQKKPLDDVKVRQALTYAVNKDAIIKAVYQGAGVSAKNLIPPTMWGYNDDVQ

DYTYDPEKAKALLKEAGLEKGFSIDLWAMPVQRPYNPNARRMAEMIQADWAKVGVQAKIVTYEWGEYLKR

AKDGEHQTVMMGWTGDNGDPDNFFATLFSCAASEQGSNYSKWCYKPFEDLIQPARATDDHNKRVELYKQA

QVVMHDQAPALIIAHSTVFEPVRKEVKGYVVDPLGKHHFENVSIE

>gi|30065173|ref|NP_839344.1| lipase [Shigella flexneri 2a str. 2457T]

MIIKKSGGRWQLSLLASVVISAFFLNTAYAWQQEYIVDTQPGHSTERYTWDSDHQPDYNDILSQRIQSSQ

RALGLEVNLAEETPVDVTSSMSMGWNFPLYEQVTTGPVAALHYDGTTTSMYNEFGDSTTTLTDPLWHASV

SSLGWRVDSRLGDLRPWAQISYNQQFGENIWKAQSGLSRMTATNQNGNWLDVTVGADMLLNQNIAAYAAL

TQAENTTNNSDYLYTMGVSARF

>gi|30065172|ref|NP_839343.1| 3-methyladenine DNA glycosylase [Shigella flexneri 2a str. 2457T]

MERCGWVSQGPLYIAYHDNEWGVPETDSKKLFEMICFEGQQAGLSWITVLKKRENYRAYFHQFDPVKVAA

MQEEDVERLVQDAGIIRHRGKIQAIIGNARAYLQMEQNGEPFPDFVWSFVNHQPQVTQATTLSEIPTSTS

ASDALSKALKKRGFKFVGTTICYSFMQACGLVNDHVVGCCCYLGNKP

>gi|30065171|ref|NP_839342.1| hypothetical protein S4185 [Shigella flexneri 2a str. 2457T]

MIREAQRSELPAILELWLESTTWGHPFIKANYWRDCIPLVRDAYLANAQNWVWEEDGKLLGFVSIMEGRF

LAAMFVAPKAVRRGIGKALMQYVQQRYPHLMLEVYQKNQPAIDFYRAQGFHIVDCAWQDETQLPTWIMSW

PVVQTL

>gi|30065167|ref|NP_839338.1| hypothetical protein S4181 [Shigella flexneri 2a str. 2457T]

MILPGRLRRKGILQACPGLSLSRQTRVCRCALFLGERSKKMATGKSCSRWFAPLAALLMVVSLSGCFDKE

GDQRKAFIDFLQNTVMRSGERLPTLTADQKKQFGPFVSDYAILYGYSQQVNQAMDSGLRPVVDSVNAIRV

PQDYVTQSGPLREMNGSLGVLAQQLQNAKLQADAAHSALKQSDDLKPVFDQAFTKVVTTPADALQPLIPA

AQTFTQQLVMVGDYIAQQGTQVSFVANGIQFPTSQQASEYNKLIAPLPAQHQAFNQAWTTAVTATQ

>gi|30065164|ref|NP_839335.1| IS150 transposase [Shigella flexneri 2a str. 2457T]

MKVLNELRQFYPLDELLRAAEIPRSTFYYHLKALSKPDKYADVKKRIGEIYHENRGRYGYRRVTLSLHRE

GKQINHKAVQRLMGTLSLKAAIKVKRYRSYRGEVGQTAPNVLQRDFKATRPNEKWVTDVTEFAVNGRKLY

LSPVIDLFNNEVISYSLSERPVMNMVENMLDQAFKKLNPHEHPVLHSDQGWQYRMRRYQNILKEHGIKQS

MSRKGNCLDNAVVECFFGTLKSECFYLDEFSNISELKDAVTEYIEYYNSRRISLKLKGLTPIEYRNQTYM

PRV

>gi|30065161|ref|NP_839332.1| cytochrome C peroxidase [Shigella flexneri 2a str. 2457T]

MKMVSRITAIGLAGVAICYLGLSGYVWYHDNKRSKQADVQASAVSENNKVLGFLREKGCDYCHTPSAELP

AYYYIPGAKQLMDYDIKLGYKSFNLEAVRAALLADKPVSQSDLNKIEWVMQYETMPPTRYTALHWAGKVS

DEERAEILAWIAKQRAEYYASNDTAPEHRNEPVQPIPQKLPTDAQKVALGFALYHDPRLSADSTISCAHC

HTLNAGGVDGRKTSIGVGGAVGPINAPTVFNSVFNVEQFWDGRAATLQDQAGGPPLNPIEMASKSWDEII

AKLEKDPQLKAQFLEVYPQGFSGENITDAIAEFEKTLITPDSPFDKWLRGDENALTAQQKKGYQLFKDNK

CATCHGGIILGGRSFEPLGLKKDFNFGEITAADIGRMNVTKEERDKLRQKVPGLRNVALTAPYFHRGDVP

TLDGAVKLMLRYQVGKELPQEDVDDIVAFLHSLNGVYTPYMQDKQ

>gi|30065159|ref|NP_839330.1| DNA-binding transcriptional regulator GadX [Shigella flexneri 2a str. 2457T]

MQSLHGNCLIAYARHKYILTMVNGEYRYFNGGDLVFADASQIRVDKCVENFVLVSRDTLSLFLPMLKEEA

LNLHAHKKISSLLVHHCSRDIPVFQEVAQLSQNKNLRYAEMLRKRALIFALLSVFLEDEHFIPLLLNVLQ

PNMRTRVCTVINNNIAHEWTLARIASELLMSPSLLKKKLREEETSYSQLLTECRMQRALQLIVIHGFSIK

RVAVSCGYHSVSYFIYVFRNYYGMTPTEYQERSAQGLPNRDSAASIVAQGNFYGTNRSAEGIRL

>gi|30065158|ref|NP_839329.1| ARAC-type regulatory protein [Shigella flexneri 2a str. 2457T]

MTHVCSVILIRRSFDIYHEQQKISLHNESILLLEKNLADDFAFCSPDTRRLDIDELTVCHYLQNIRQLPR

NLGLHSKDRLLINQSPPMPLVTAIFDSFNESGVNSPILSNMLYLSCLSMFSHKKELIPLLFNSISTVSGK

VERLISFDIAKRWYLRDIAERMYTSESLIKKKLQDENTCFSKILLASRMSMARRLLELRQIPLHTIAEKC

GYSSTSYFINTFRQYYGVTPHQFAQHSPGTFS

>gi|30065154|ref|NP_839325.1| glycyl-tRNA synthetase subunit beta [Shigella flexneri 2a str. 2457T]

MSEKTFLVEIGTEELPPKALRSLAESFAANFTAELDNAGLAHGTVQWFAAPRRLALKVANLAEAQPDREI

EKRGPAIAQAFDAEGKPSKAAEGWARGCGITVDQAERLTTDKGEWLLYRAHVKGESTEALLPNMVATSLA

KLPIPKLMRWGASDVHFVRPVHTVTLLLGDKVIPATILGIQSDRVIRGHRFMGEPEFTIDNADQYPEILR

ERGKVIADYEERKAKIKADAEEAARKIGGNADLSESLLEEVASLVEWPVVLTAKFEEKFLAVPAEALVYT

MKGDQKYFPVYANDGKLLPNFIFVANIESKDPQQIISGNEKVVRPRLADAEFFFNTDRKKRLEDNLPRLQ

TVLFQQQLGTLRDKTDRIQALAGWIAEQIGADVNHATRAGLLSKCDLMTNMVFEFTDTQGVMGMHYARHD

GEAEDVAVALNEQYQPRFAGDDLPSNPVACALAIADKMDTLAGIFGIGQHPKGDKDPFALRRAALGVLRI

IVEKNLNLDLQTLTEEAVRLYGDKLTNANVVDDVIDFMLGRFRAWYQDEGYTVDTIQAVLARRPTRPADF

DARMKAVSHFRTLEAAAALAAANKRVSNILAKSDEVLSDRVNASTLKEPEEIKLAMQVVVLRDKLEPYFA

EGRYQDALVELAELREPVDAFFDKVMVMVDDKELRLNRLTMLEKLRELFLRVADISLLQ

>gi|30065153|ref|NP_839324.1| glycyl-tRNA synthetase subunit alpha [Shigella flexneri 2a str. 2457T]

MQKFDTRTFQGLILTLQDYWARQGCTIVQPLDMEVGAGTSHPMTCLRALGPEPMAAAYVQPSRRPTDGRY

GENPNRLQHYYQFQVVIKPSPDNIQELYLGSLKELGMDPTIHDIRFVEDNWENPTLGAWGLGWEVWLNGM

EVTQFTYFQQVGGLECKPVTGEITYGLERLAMYIQGVDSVYDLVWSDGPLGKTTYGDVFHQNEVEQSTYN

FEYADVDFLFTCFEQYEKEAQQLLALENPLPLPAYERILKAAHSFNLLDARKAISVTERQRYILRIRTLT

KAVAEAYYASREALGFPMCNKDK

>gi|30065152|ref|NP_839323.1| hypothetical protein S4164 [Shigella flexneri 2a str. 2457T]

MQPKIYWIDNLRGIACLMVVMIHTTTWYVTNAHSVSPVTWDIANVLNSASRVSVPLFFMISGYLFFGERS

AQPRHFLRIGLCLIFYSAIALLYIALFTSINMELALKNLLQKPVFYHLWFFFAIAVIYLVSPLIQVKNVG

GKMLLVLMVVIGIIANPNTVPQKIDGFEWLPINLYINGDTFYYILYGMLGRAIGMMDTQHKALSWVSAAL

FATGVFIISRGTLYELQWRGNFADTWYLYCGPMVFICAIALLTLVKNTLDTRTIRGLGLISRHSLGIYGF

HALIIHALRTRGIELKNWPILDIIWIFCATLAASLLLSMLVQRIDRNRLVS

>gi|30065151|ref|NP_839322.1| hypothetical protein S4163 [Shigella flexneri 2a str. 2457T]

MDNKISTYSPAFSIVSWIALVGGIVTYLLGLWNAEMQLNEKGYYFAVLVLGLFSAASYQKTVRDKYEGIP

TTSIYYMTCLTVFIISVALLMVGLWNATLLLSEKGFYGLAFFLSLFGAVAVQKNIRDAGINPPKETQVTQ

EEYSE

>gi|30065150|ref|NP_839321.1| hypothetical protein S4162 [Shigella flexneri 2a str. 2457T]

MKTSKTVAKLLFVVGALVYLVGLWISCPLLSGKGYFLGVLMTATFGNYAYLRAEKLGQLDNFFTHICQLV

ALITIGLLFIGVLNAPINAYEMVIYPIAFFVCLFGQMRLFRSV

>gi|30065148|ref|NP_839319.1| D-xylose transporter subunit XylF [Shigella flexneri 2a str. 2457T]

MKIKNILLTLCTSLLLTNVAAHAKEVKIGMAIDDLRLERWQKDRDIFVKKAESLGAKVFVQSANGNEETQ

MSQIENMINRGVDVLVIIPYNGQVLSNVVKKAKQEGIKVLAYDRMINDADIDFYISFDNEKVGELQAKAL

VDIVPQGNYFLMGGSPVDNNAKLFRAGQMKVLKPYVDSGKIKVVGDQWVDGWLPENALKIMENALTANNN

KIDAVVASNDATAGGAIQALSAQGLSGKVAISGQDADLAGIKRIAAGTQTMTVYKPITLLANTAAEIAVE

LGNGQEPKADTSLNNGLKDVPSRLLTPIDVNKNNIKDTVIKDGFHKESEL

>gi|30065146|ref|NP_839317.1| xylose transporter membrane component [Shigella flexneri 2a str. 2457T]

MSKSNPSEVKLAVPTSGGFSGLKSLNLQVFVMIAAIIAIMLFFTWTTDGAYLSARNVSNLLRQTAITGIL

AVGMVFVIISAEIDLSVGSMMGLLGGVAAICDVWLGWPLPLTIIVTLVLGLLLGAWNGWWVAYRKVPSFI

VTLAGMLAFRGILIGITNGTTVSPTSAAMSQIGQSYLPASTGFIIGALGLMAFVGWQWRGRMRRQALGLQ

SPASTAVVGRQALTAIIVLGAIWLLNDYRGVPTPVLLLTLLLLGGMFMATRTAFGRRIYAIGGNLEAARL

SGINVERTKLAVFAINGLMVAIAGLILSSRLGAGSPSAGNIAELDAIAACVIGGTSLAGGVGSVAGAVMG

AFIMASLDNGMSMMDVPTFWQYIVKGAILLLAVWMDSATKRRS

>gi|30065145|ref|NP_839316.1| hypothetical protein S4155 [Shigella flexneri 2a str. 2457T]

MILTPIRRYGAMILMLLTLVFSSEVLAKTHTTTASQKSHLTKASNKQVSSKQEYSRNSAKSSSLPDLRKY

PSGTPRKKAFLRTVMPYITSQNAAITAERNWLISKQYQGQWAPAERARLKDIAKRYKVKWSGNTRKIPWN

TLLERVDIIPTSMVATMAAAESGWGTSKLARNNNNLFGMKCMKGRCTNAPGKVKGYSQFSSVKESVSAYV

TNLNTHPAYSSFRKSRAQLRKADQEVTATAMIHKLKGYSTKGKSYNNYLFAMYQDNQRLIAAHM

>gi|30065141|ref|NP_839312.1| regulator [Shigella flexneri 2a str. 2457T]

MGKEVMGKKENEMAQEKERPAGSQSLFRGLMLIEILSNYPNGCPLAHLSELAGLNKSTVHRLLQGLQSCG

YVTTAPAAGSYRLTTKFIAVGQKALSSLNIIHIAAPHLEALNIATGETINFSSREDDHAILIYKLEPTTG

MLRTRAYIGQHMPLYCSAMGKIYMAFGHPDYVKSYWENHQHEIQPLTRNTITELPAMFDELAHIRESGAA

MDREENELGVSCIAVPVFDIHGRVPYAVSISLSTSRLKQVGEKNLLKPLRETAQAISNELGFTVRDDLGA

IT

>gi|30065140|ref|NP_839311.1| 2,3-diketo-L-gulonate reductase [Shigella flexneri 2a str. 2457T]

MKVTFEQLKAAFNRVLISRGVDSETADACAEMFARTTESGVYSHGVNRFPRFIQQLENGDIIPDAQPKRI

TSLGAIEQWDAQRSIGNLTAKKMMDRAIELAADHGIGLVALRNANHWMRGGSYGWQAAEKGYIGICWTNS

IAVMPPWGAKECRIGTNPLIVAIPSTPITMVDMSMSMFSYGMLEVNRLAGRQLPVDGGFDDEGNLTKEPG

VIEKNRRILPMGYWKGSGMSIVLDMIATLLSDGASVAEVTQDNSDEYGISQIFIAIEVDKLIDGPTRDAK

LQRIMDYVTTAERADENQAIRLPGHEFTTLLAENRRNGITVDDSVWAKIQAL

>gi|30065137|ref|NP_839308.1| hypothetical protein S4145 [Shigella flexneri 2a str. 2457T]

MAVLIFLGCLLGGIAIGLPIAWALLLCGAALMFWLDMFDVQIMAQTLVNGADSFSLLAIPFFVLAGEIMN

AGGLLKRIVDLPMKLVGHKPGGLGYVGVLAAMIMASLSGSAVADTAAVAALLVPMMRSANYPVNRAAGLI

ASGGIIAPIIPPSIPFIIFGVSSGLSISKLFMAGIAPGIMMGATLMLTWWRQASRLNLPRQQKATMQEIW

HSFVSGIWALFLPVIIIGGFRSGLFTPTEAGAVAAFYALFVATVIYREMTFATLWHVLIGAAKTTSVVMF

LVASAQVSAWLITIAELPMMVSDLLQPLVDSPRLLFIVIMVAILIVGMVMDLTPTVLILTPVLMPLVKEA

GIDPIYFGVMFIINCSIGLITPPIGNVLNVISGVAKLKFDDAVRGVFPYVLVLYSLLVVFVFIPDLIILP

LKWMVMLPTY

>gi|30065131|ref|NP_839302.1| selenocysteine synthase [Shigella flexneri 2a str. 2457T]

MTTETRSLYSQLPAIDRLLRDSSFLSLRDTYGHTRVVELLRQMLDEAREVIRGSQTLPAWCENWAQEVDA

RLTKEAQSALRPVINLTGTVLHTNLGRALQAEAAVEAVAQAMRSPVTLEYDLDDAGRGHRDRALAQLLCR

ITGAEDACIVNNNAAAVLLMLAATASGKEVVVSRGELVEIGGAFRIPDVMRQAGCTLHEVGTTNRTHAND

YRQAVNENTALLMKVHTSNYSIQGFTKAIDEAELVALGKELDVPVVTDLGSGSLVDLSQYGLPKEPMPQE

LIAAGVSLVSFSGDKLLGGPQAGIIVGKKEMIARLQSHPLKRALRADKMTLAALEATLRLYLHPEALSEK

LPTLRLLTRSAEVIQIQAQRLQAPLVAHYGAEFAVQVMPCLSQIGSGSLPVDRLPSAALTFTPHDGRGSH

LESLAARWRELPVPVIGRIYDGRLWLDLRCLEDEQRFLEMLLK

>gi|30065129|ref|NP_839300.1| hypothetical protein S4137 [Shigella flexneri 2a str. 2457T]

MDLLIVLTYVALATAALGGVFLVSGLILLMNYNHPYTFTAQKAVIAIPITPQVTGIVTEVTDKNNQLIQK

GEVLFKLDPVRYQARVDRLQADLMTATHNIKTLRAQLTEAQANTTQVSAERDRLFKNYQRYLKGSQAAVN

PFSERDIDDARQNFLAQDALVKGSVAEQAQIQSQLDSMVNGEQSQIVSLRAQLTEAKYNLEQTVIRAPSN

GYVTQVLIRPGTYAAALPLRPVMVFIPEQKRQIVAQFRQNSLLRLKPGDDAEVVFNALPGQVFHGKLTSI

LPVVPGGSYQAQGVLQSLTVVPGTDGVLGTIELDPNDDIDALPDGIYAQVAVYSDHFSHVSVMRKVLLRM

TSWMHYLYLDH

>gi|30065128|ref|NP_839299.1| hypothetical protein S4136 [Shigella flexneri 2a str. 2457T]

MFLNYFALGVLIFVFLVIFYGIIAIHDIPYLIAKKRNHPHADAIHTAGWVSLFTLHVIWPFLWIWATLYQ

PERGWGMQSHVASQEKATEPEIAALSDRISRLEHQLAAEKKTDYSTFPEI

>gi|30065127|ref|NP_839298.1| PTS system, mannitol-specific enzyme IIABC components [Shigella flexneri 2a str. 2457T]

MSSDIKIKVQSFGRFLSNMVMPNIGAFIAWGIITALFIPTGWLPNETLAKLVGPMITYLLPLLIGYTGGK

LVGGKRGGVVGAITTMGVIVGADMPMFLGSMIAGPLGGWCIKHFDRWVDGKIKSGFEMLVNNFSAGIIGM

ILAILAFLGIGPIVEALSKMLAAGVNFMVVHDMLPLASIFVEPAKILFLNNAINHGIFSPLGIQQSHELG

KSIFFLIEANPGPGMGVLLAYMFFGRGSAKQSAGGAAIIHFLGGIHEIYFPYVLMNPRLILAVILGGMTG

VFTLTILGGGLVSPASPGSILAVLAMTPKGAYFANIAGVCAAMAVSFVVSAILLKTSKVKEEDDIEAATR

RMQDMKAESKGTSPLSAGDVTNDLSHVRKIIVACDAGMGSSAMGAGVLRKKIQDAGLSQISVTNSAINNL

PPDVDLVITHRDLTERAMRQVPQAQHISLTNFLDSGLYTSLTERLVAAQRHTENEVKVKDSLKDSFDDSS

ANLFKLGAENIFLGRKAATKEEAIRFAGEQLVKGGYVEPEYVQAMLDREKLTPTYLGESIAVPHGTVEAK

DRVLKTGVVFCQYPEGVRFGEEEDDIARLVIGIAARNNEHIQVITSLTNALDDESVIERLAHTTSVDEVL

ELLAGRK

>gi|30065126|ref|NP_839297.1| mannitol-1-phosphate 5-dehydrogenase [Shigella flexneri 2a str. 2457T]

MKALHFGAGNIGRGFIGKLLADAGIQLTFADVNQVVLDALNARHSYQVHVVGETEQVDTVSGVNAVSSIG

DDVVDLIAQVDLVTTAVGPVVLERIAPAIAKGLVKRKEQGNESPLNIIACENMVRGTTQLKGHVMNALPE

DAKAWVEEHVGFVDSAVDRIVPPSASATNDPLEVTVETFSEWIVDKTQFKGALPNIPGMELTDNLMAFVE

RKLFTLNTGHAITAYLGKLAGHQTIRDAILDEKIRAVVKGAMEESGAVLIKRYGFDADKHAAYIQKILGR

FENPYLKDDVERVGRQPLRKLSAGDRLIKPLLGTLEYSLPHKNLIQGIAGAMHFRSEDDPQAQELAALIA

DKGPQAALAQISGLDANSEVVSEAVTAYKAMQ

>gi|30065125|ref|NP_839296.1| mannitol repressor protein [Shigella flexneri 2a str. 2457T]

MVDQAQDTLRPNNRLSDMQATMEQTQAVENRVLERLNAGKTVRSFLITAVELLTEAVNLLVLQVFRKDDY

AVKYAVEPLLDGDGPLGDLSVRLKLIYGLGVINRQEYEDAELLMALREELNHDGNEYAFTDDEILGPFGE

LHCVAALPPPPQFEPADSSLYAMQIQRYQQAVRSTMVLSLTELISKISLKKAFQK

>gi|30065124|ref|NP_839295.1| hypothetical protein S4132 [Shigella flexneri 2a str. 2457T]

MKEVEKNEIKRLSDRLDAIRHQQADLSLVEAADKYAELEKEKATLEAEIARLREVHSQKLSKEAQKLMKM

PFQRAITKKEQADMGKLKKSVRGLVVVHPMTALGREMGLEEMTGFSKTTF

>gi|30065121|ref|NP_839292.1| L-lactate permease [Shigella flexneri 2a str. 2457T]

MNLWQQNYDPAGNIWLSSLIASLPILFFFFALIKLKLKGYVAASWTVAIALAVALLFYKMPVANALASVV

YGFFYGLWPIAWIIIAAVFVYKISVKTGQFDIIRSSILSITPDQRLQMLIVGFCFGAFLEGAAGFGAPVA

ITAALLVGLGFKPLYAAGLCLIVNTAPVAFGAMGIPILVAGQVTGIDSFEIGQMVGRQLPFMTIIVLFWI

MAIMDGWRGIKETWPAVVVAGGSFAIAQYLSSNFIGPELPDIISSLVSLLCLTLFLKRWQPVRVFRFGDL

GASQVDMTLAHTGYTAGQVLRAWTPFLFLTATVTLWSIPPFKALFASGGALYEWVINIPVPYLNKLVARM

PPVVSEATAYAAVFKFDWFSATGTAILFAALLSIVWLKMKPSDAISTFGSTLKELALPIYSIGMVLAFAF

ISNYSGLSSTLALALAHTGHAFTFFSPFLGWLGVFLTGSDTSSNALFAALQATAAQQIGVSDLLLVAANT

TGGVTGKMISPQSIAIACAAVGLVGKESDLFRFTVKHSLIFTCMVGVITTLQAYVLTWMIP

>gi|30065120|ref|NP_839291.1| DNA-binding transcriptional repressor LldR [Shigella flexneri 2a str. 2457T]

MIVLPRRLSDEVADRVRALIDEKNLEAGMKLPAERQLAMQLGVSRNSLREALAKLVSEGVLLSRRGGGTF

IRWRHDTWSEQNIVQPLKTLMADDPDYSFDILEARYAIEASTAWHAAMRATPGDKEKIQLCFEATLSEDP

DIASQADVRFHLAIAEASHNIVLLQTMRGFFDVLQSSVKHSRQRMYLVPPVFSQLTEQHQAVIDAIFAGD

ADGARKAMMAHLSFVHTTMKRFDEDQARHARITRLPGEHNEHSREKNA

>gi|30065118|ref|NP_839289.1| tRNA/rRNA methyltransferase YibK [Shigella flexneri 2a str. 2457T]

MLNIVLYEPEIPPNTGNIIRLCANTGFRLHIIEPMGFAWDDKRLRRAGLDYHEFTAVTRHHDYRAFLEAE

NPQRLFALTTKGTPAHSAVSYQDGDYLMFGPETRGLPASILDALPAEQKIRIPMVPDSRSMNLSNAVSVV

VYEAWRQLGYPGALLRD

>gi|30065117|ref|NP_839288.1| serine acetyltransferase [Shigella flexneri 2a str. 2457T]

MSCEELEIVWNNIKAEARTLADCEPMLASFYHATLLKHENLGSALSYMLANKLSSPIMPAIAIREVVEEA

YAADPEMIASAACDIQAVRTRDPAVDKYSTPLLYLKGFHALQAYRIGHWLWNQGRRALAIFLQNQVSVTF

QVDIHPAAKIGRGIMLDHATGIVVGETAVIENDVSILQSVTLGGTGKSGGDRHPKIREGVMIGAGAKILG

NIEVGRGAKIGAGSVVLQPVPPHTTAAGVPARIVGKPDSDKPSMDMDQHFNGINHTFEYGDGI

>gi|30065115|ref|NP_839286.1| preprotein translocase subunit SecB [Shigella flexneri 2a str. 2457T]

MSEQNNTEMTFQIQRIYTKDISFEAPNAPHVFQKDWQPEVKLDLDTASSQLADDVYEVVLRVTVTASLGE

ETAFLCEVQQGGIFSIAGIEGTQMAHCLGAYCPNILFPYARECITSMVSRGTFPQLNLAPVNFDALFMNY

LQQQAGEGTEEHQDA

>gi|30065113|ref|NP_839284.1| hypothetical protein S4118 [Shigella flexneri 2a str. 2457T]

MQEIMQFVGRHPILSIAWIALLVAVLVTTFKSLTSKVKVITRGEATRLINKEDAVVVDLRQRDDFRKGHI

AGSINLLPSEIKANNVGELEKHKDKSVIVVDGSGMQCQEPANALTKAGFAQVFVLKEGVAGWAGENLPLV

RGK

>gi|30065112|ref|NP_839283.1| phosphoglyceromutase [Shigella flexneri 2a str. 2457T]

MSVSKKPMVLVILDGYGYREEQQDNAIFSAKTPVMDALWANRPHTLIDASGLEVGLPDRQMGNSEVGHVN

LGAGRIVYQDLTRLDVEIKDRAFFANPVLAGAVHKAKNAGKAVHIMGLLSAGGVHSHEDHIMAMVELAAE

RGAEKIYLHAFLDGRDTPPRSAESSLKKFEEKFAALGKGRVASIIGRYYAMDRDNRWDRVEKAYDLLTLA

QGEFQADTAVAGLQAAYARDENDEFVKATVIRAEGQPDAAMEDGDALIFMNFRADRAREITRAFVNADFD

GFARKKVVNVDFVMLTEYAADIKTAVAYPPASLVNTFGEWMAKNDKTQLRISETEKYAHVTFFFNGGVEE

SFKGEDRILINSPKVATYDLQPEMSSAELTEKLVAAIKSGKYDTIICNYPNGDMVGHTGVMEAAVKAVEA

LDHCVEEVAKAVESVGGQLLITADHGNAEQMRDPATGQAHTAHTNLPVPLIYVGDKNVKAVAGGKLSDIA

PTMLSLMGMEIPQEMTGKPLFIVE

>gi|30065110|ref|NP_839281.1| hypothetical protein S4115 [Shigella flexneri 2a str. 2457T]

MLAMPSAISVAVLPDSPHAREMATKAHNSGHEVLIHLPMAPLSKQPLEKNTLRPEMSSDEIERIIRSAVN

NVPYAVGINNHMGSKMTSNLFGMQKVMQALERYNLYFLDSVTIGNTQAMRAAQGTGVKVIKRKVFLDDSQ

NEADIRVQFNRAIDLARRNGSTIAIGHPHPSTVRVLQQMVYNLPPDITLVKASSLLNEPQVDTSTPPKNA

VPDAPRNPFRGVKLCKPKKPIEPVYANRFFEVLSESISQSTLIVYFQHQWQGWGKQPEAAKFNASAN

>gi|30065103|ref|NP_839274.1| ADP-heptose--LPS heptosyltransferase [Shigella flexneri 2a str. 2457T]

MKILVIGPSWVGDMMMSQSLYRTLQARYPQAIIDVMAPAWCRPLLSRMPEVNEAIPMPLGHGALEIGERR

KLGHSLREKRYDRAYVLPNSFKSALVPFFAGIPHRTGWRGEMRYGLLNDVRVLDKEAWPLMVERYVALAY

DKGIMRTAQDLPQPLLWPQLQVSEGEKSYTCNQFSLSSERPMIGFCPGAEFGPAKRWPHYHYAELAKQLI

DEGYQVVLFGSAKDHEAGNEILAALNTEQQAWCRNLAGETQLDQAVILIAACKAIVTNDSGLMHVAAALN

RPLVALYGPSSPDFTPPLSHKARVIRLITGYHKVRKGDAAEGYHQSLIDITPQRVLEELNALLLQEEA

>gi|30065102|ref|NP_839273.1| ADP-heptose--LPS heptosyltransferase [Shigella flexneri 2a str. 2457T]

MGDVLHTLPALTDAQQAIPGIKFDWVVEEGFAQIPSWHAAVERVIPVAIRRWRKAWFSAPIKAERKAFRE

ALQAENYDAVIDAQGLVKSAALVTRLAHGVKHGLDWQTAREPLASLFYNCKHHIAKQQHAVERTRELFAK

SLGYSKPQTQGDYAIAQHFLTNLPTDAGEYAVFLHATTRDDKHWPEEHWRELIGLLADSGIRIKLPWGAP

HEEERAKRLAEGFAYVEVLPKMSLEGVARVLAGAKFVVSVDTGLSHLTAALDRPNITVYGPTDPGLIGGY

GKNQVECRSTSMSLADLPAQTVFQNLNLEIITNKLTSEIR

>gi|30065101|ref|NP_839272.1| lipid A-core, surface polymer ligase [Shigella flexneri 2a str. 2457T]

MTSTLFFSLEKKNWIAYWNRALVFLFITTYFLGGITRYKHLIVILMTITTIVYLCKRPKHYLSLFKTFLF

GSVAILTIAALLSLLQSPDAGASMKEVFKAIIENTLLCTIAIPVILRDEKREDVEKIVFFSFISALGLRC

FSELITYYKDYQQGIMPFADYRHRSISDSMVFLFPALLNLWLIKSAKYRISFVVLSVIFIFLILGTLSRG

AWLSVLVIGLIWILMFKQWKLLLVGVMVSIIALSVIFTHKEMTAKLTYKLQQTNSSYRYANGTQGSALDL

ILENPVIGYGYGNVAYKDVYNKRVIDYPEWTFRQSIGPHNFALFIWFGTGLLGLVSLMMLYCAILKECIK

NGVKNKYRSPYNAYYIILLSFIGYFVIRGNVEQIEPNLLGVYAGLLLAMKNK

>gi|30065100|ref|NP_839271.1| lipopolysaccharide 1,2-N- acetylglucosaminetransferase [Shigella flexneri 2a str. 2457T]

MVDKIIFTVTPIFSIPPRGAAAVETWMYQVAQRTNFPNRIVCIKNPGYSNYTFVNDNCSIHRVGFSRIYK

RLFQKWTRLDPLPYSQRILNIAHDFPITKESVIVVHNSMKLYTQIRKLAPQARVVIHMHNAFEPKLLEQN

VKMIVPSLYLKKYYQSYLANADIEIVPNGIDLETYQSNFQPITRSELNISPEEKIIFYAGRIVPDKGILL

LMQSFEKLAAAHKNLKLVVIGDYTEMSKSDKGAYQRNVREIAKRLKDRCIMLGSIPPEKMYCYYPLADLV

VIPSQFQEPFCMVAIEAMGAGKPVLVSTRGGMTEFVKENTTGFHLKEPMTADSISSDILKTLANPELTAV

AKQGQDFVFDHYSWDGVTQRFEEVIHNWFE

>gi|30065099|ref|NP_839270.1| UDP-glucose:(galactosyl) LPS alpha1,2-glucosyltransferase [Shigella flexneri 2a str. 2457T]

MDFKHLTQFKDIIELDKRPVKLDERETFNVSWGIDENYQVGAAISIASILEKNKQNKFTFHIIADYLDKE

YIELLSQLATKYQTVIKLYHIDSEPLKALPQSNIWPVSIYYRLLSFDYFSARLDSLLYLDADIVCKGSLN

ELIALEFKDEYGAVVIDVDAMQSKSAERLCNEDFNGSYFNSGVMYINLREWLKQRLTEKFFDLLSDESII

KKLKYPDQDILNLMFLHHAKILPRKYNCIYTIKSEFEEKNSEYYTRFINDDTVFIHYTGITKPWHDWANY

ASADYFRNIYNISPWRNIPYKKAVKKHEHKEKYKHLLYQKKFLDGVFTAIKYNVMKG

>gi|30065098|ref|NP_839269.1| lipopolysaccharide core biosynthesis protein [Shigella flexneri 2a str. 2457T]

MIYNKTINGLKVFIKDNDPFYEQVLNDFLTCRVKTLKVFRSIDDTKVILIDTARGPLVLKVYAPKHKMTE

RFLKSCIKKDYYENLIYQTDRVRGEGIQSINDYFLLAERKTLNFAHYYIMLIEYIEGVGLNEYLEISEDL

KDQLSESIKELHQHGMVSGDPHKGNFIVSEKGLRLIDLSGKKTTAVLKAKDRIDLERHYNIKNELKDFGY

TYLIFKKKIKKVIRDVKVKLGLKSK

>gi|30065097|ref|NP_839268.1| UDP-D-galactose:(glucosyl)lipopolysaccharide- alpha-1,3-D-galactosyltransferase [Shigella flexneri 2a str. 2457T]

MSQLNDSDIILFEYNFHYQNIRSKNTLDIAFGIDRNFLFGCGVAIASILLNNREISCEFHVFTDYISDKD

KLYFSDLAKQYNSRINIYVINCDKLKSLPSTKNWTYATYFRFIIADYFYHKHEKILYLDADIACKGSIKE

LLDYQFSTNEIAAVVAERDIEWWQNRASVLTTPQLASGYFNAGFLLINIDEWNLNNISSKAIEMLRDPDW

VSKITHLDQDVLNVLLNGKVKFISEKYNTRYSINYELKDKVDNPVNDDTVFIHYVGPTKPWHEWADYPVS

RSFLIAKAASPWSKEDLLKPVNSNQYRYCAKHKFKQKHYMAGIFNYLKYYKEKCF

>gi|30065096|ref|NP_839267.1| lipopolysaccharide core biosynthesis protein [Shigella flexneri 2a str. 2457T]

MVWMVELKEPFATLWRGKDPFEEVKTLQGEVFRELETRRTLRFEMAGKSYFLKWHRGTTLKEIIKNLLSL

RMPVLGADREWNAIHRLRDVGVDTMYGVAFGEKGMNPLTRTSFIITEDLTPTISLEDYCADWATNPPDVR

VKRMLIKRVATMVRDMHAAGINHRDCYICHFLLHLPFSGKEEELKISVIDLHRAQLRTRVPGRWRDKDLI

GLYFSSMNIGLTQRDIWRFMKVYFAAPLKDILKQEQGLLSQAEAKATKIRERTIRKSL

>gi|30065095|ref|NP_839266.1| LPS alpha1,3-glucosyltransferase [Shigella flexneri 2a str. 2457T]

MIVAFCLYKYFPFGGLQRDFMRIAQTVAARGHHVRVYTQSWEGECPDVFEVIKVPVKSHTNHGRNAEYFA

WVQKHLREHPVDKVVGFNKMPGLDVYYAADVCYAEKVAQEKGFFYRLTSRYRHYAAFERATFEQGKPTQL

LMLTDKQIADFQKHYQTEAERFHILPPGIYPDRKYSQQPANSREIFRKKNGITEQQYLLLQVGSDFTRKG

ADRSIEALASLPDSLRHNTLLYVVGQDKPRKFEALAEKRGVRSNVHFFSGRNDVSELMAAADLLLHPAYQ

EAAGIVLLEAITAGLPVLTTAVCGYAHYIVDANCGEAIAEPFRQETLNEILRKALTQSSLRQAWAENARH

YADTQDLYSLPEKAADIITGGLDG

>gi|30065094|ref|NP_839265.1| lipopolysaccharide core biosynthesis protein [Shigella flexneri 2a str. 2457T]

MDKPFRRILLIKMRFHGDMLLTTPVISSLKKNYPDAKIDVLLYQDTIPILSENPEINALYGIKNKKAKAS

EKIANFFHLIKVLRANKYDLIVNLTDQWMVAILVRLLNARVKISQDYHHRQSAFWRKSFTHLVPLQGGNV

VESNLSVLTPLGLDSLVKQTTMSYPPASWKRMRRELDHAGVGQNYVVIQPTARQIFKCWDNAKFSAVIDA

LHARGYEVVLTSGPDKDDLACVNEIAQGCQTPPVTALAGKVTFPELGALIDHAQLFIGVDSAPAHIAAAV

NTPLISLFGATDHIFWRPWSNNMIQFWAGDYREMPTRDQRDRNEMYLSVIPAADVIAAVDKLLPSSTTGT

SL

>gi|30065091|ref|NP_839262.1| 3-deoxy-D-manno-octulosonic-acid transferase [Shigella flexneri 2a str. 2457T]

MLELLYTALLYLIQPLIWIRLWVRGRKAPAYRKRWGERYGFYRHPLKPGGIMLHSVSVGETLAAIPLVRA

LRHRYPDLPITVTTMTPTGSERVQSAFGKDVQHVYLPYDLPDALNRFLNKVDPKLVLIMETELWPNLIAA

LHKRKIPLVIANARLSARSAAGYAKLGKFVRRLLRRITLIAAQNEEDGARFVALGAKNNQVTVTGSLKFD

ISVTPQLAAKAVTLRRQWAPHRPVWIATSTHEGEESVVIAAHQALLQQFPNLLLILVPRHPERFPDAINL

VRQAGLSYITRSSGEVPSTSTQVVVGDTMGELMLLYGIADLAFVGGSLVERGGHNPLEAAAHAIPVLMGP

HTFNFKDICARLEQASGLITVTDATTLAKEVSSLLTDAVYRSFYGRHAVEVLYQNQGALQRLLQLLEPYL

PPKTH

>gi|30065090|ref|NP_839261.1| phosphopantetheine adenylyltransferase [Shigella flexneri 2a str. 2457T]

MQKRAIYPGTFDPITNGHIDIVTRATQMFDHVILAIAASPSKKPMFTLEERVALAQQATAHLGNVEVVGF

SDLMANFARNQHATVLIRGLRAVADFEYEMQLAHMNRHLMPELESVFLMPSKEWSFISSSLVKEVARHQG

DVTHFLPENVHQALMAKLA

>gi|30065080|ref|NP_839251.1| hypothetical protein S4085 [Shigella flexneri 2a str. 2457T]

MIRSMTAYARREIKGEWGSATWEMRSVNQRYLETYFRLPEQFRSLEPVVRERIRSRLTRGKVECTLRYEP

DVSAQGELILNEKLAKQLVTAANWVKMKSDEGEINPVDILRWPGVMAAQEQDLDAIAAEILAALDGTLDD

FIVARETEGQALKALIEQRLEGVTAEVVKVRAHMPEILQWQRERLVAKLEDAQVQLENNRLEQELVLLAQ

RIDVAEELDRLEAHVKETYNILKKKEAVGRRLDFMMQEFNRESNTLASKSINAEVTNSAIELKVLIEQMR

EQIQNIE

>gi|30065079|ref|NP_839250.1| DNA-damage-inducible protein D [Shigella flexneri 2a str. 2457T]

MNEHHQPFEEIKLINANGAEQWSARQLGKLLGYSEYRHFIPVLTRAKEACENSGHTIDDHFEEILDMVKI

GSNAKRALKDIVLSRYACYLVVQNGDPAKPVIAAGQTYFTIQTRRQELADDEAFKQLREDEKRLFLRNEL

KEHNKQLVEAAQQAGVATATDFAIFQNHGYQGLYGGLDQKAIHQLKGLKKSQKILDHMGSTELAANLFRA

TQTEEKLKRDSVNSKQQANTTHFDVGSKVRQTIQELGGTIPEELPTPQVSIKQLENSVKITEKK

>gi|30065078|ref|NP_839249.1| hypothetical protein S4083 [Shigella flexneri 2a str. 2457T]

MTMRTNRYPARQIFRERTMLLHILYLVGITAEAMTGALAAGRRRMDTFGVIIIATATAIGGGSVRDILLG

HYPLGWVKHPEYVIIVATAAVLTTIVAPVMPYLRKVFLVLDALGLVVFSIIGAQVALDMGHGPIIAVVAA

VTTGVFGGVLRDMFCKRIPLVFQKELYAGVSFASAVLYIALQHYVSNHDVVIISTLVFGFFARLLALRLK

LGLPVFYYSHEGH

>gi|30065077|ref|NP_839248.1| NAD-dependent DNA ligase LigB [Shigella flexneri 2a str. 2457T]

MKVWMAILISILCWQSSVWAVCPAWSPARAQEEISRLQQQIKQWDDDYWKEGKSEVEDGVYDQLSARLTQ

WQRCFVSEPRDVMMPPLNGAVMHPVAHTGVRKMADKNALSLWMRERSDLWVQPKVDGVAVTLVYRDGKLN

KAISRGNGLKGEDWTQKVSLISAVLQTVSGPLANSTLQGEIFLQREGHIQQQMGGINARAKVAGLMMRQG

NSDTLNSLAVFVWAWPDGPQLMTDRLKELATAGFTLTQRYTRAVKNADEVARVRNEWWKAKLPFVTDGVV

VRGAKEPESRHWLPGQAEWLVAWKYQPVAQVAEVKAIQFAVGKSGKISVVASLAPVMLDDKKVQRVNIGS

VRRWQEWDIAPGDQILVSLAGQGIPRIDDVVWRGAERTKPTPPENRFNPLTCYFASDVCQEQFISRLVWL

GSKQVLGLDGIGEAGWRALHQTHRFEHIFSWLLLTPEQLQNTPGIAKSKSAQLWHQFNLARNQPFTRWVM

AMGIPLTRAALNASDERSWSQLLFSTEQFWQQLPGTGSGRARQVIEWKENAQIKKLGSWLAAQQITGFEP

>gi|30065071|ref|NP_839242.1| glutamate transport protein [Shigella flexneri 2a str. 2457T]

MFHLDTLATLVAATLTLLLGRKLVHSVSFLKKYTIPEPVAGGLLVALALLVLKKSMGWEVNFDMSLRDPL

MLAFFATIGLNANIASLRAGGRVVGIFLIVVVGLLVMQNAIGIGMASLLGLDPLMGLLAGSITLSGGHGT

GAAWSKLFIERYGFTNATEVAMACATFGLVLGGLIGGPVARYLVKHSTTPNGIPDDQEVPTAFEKPDVGR

MITSLVLIETIALIAICLTVGKIVAQLLAGTAFELPTFVCVLFVGVILSNGLSIMGFYRVFERAVSVLGN

VSLSLFLAMALMGLKLWELASLALPMLAILVVQTIFMALYAIFVTWRMMGKNYDAAVLAAGHCGFGLGAT

PTAIANMQAITERFGPSHMAFLVVPMVGAFFIDIVNALVIKLYLMLPIFAG

>gi|30065069|ref|NP_839240.1| hypothetical protein S4074 [Shigella flexneri 2a str. 2457T]

MKFIGKLLLYILIALLVVIAGLYFLLQTRWGAEHISAWVSENSDYHLAFGAMDHRFSAPSHIVLENVTFG

RDGQPAPLVAKSVDIALSSRQLTEPRHVDTILLENGTLNLTDQTAPLPFKADRLQLRDMAFNSPNSEWKL

SAQRVNGGVVPWSPEAGKVLGTKAQIQFSAGSLSLNDVPATNVLIEGSIDNDRVTLTNLGADIARGTLTG

NAQRNADGSWQVENLRMADIRLQSEKSLTDFFAPLRSVPSLQIGRLEVIDARLQGPDWAVTDLDLSLRNM

TFSKDDWQTQEGKLSMNASEFIYGSLHLFDPIINAEFSPQGVALRQFTSRWEGGMVRTSGNWLRDGKTLI

LDDAAIAGLEYTLPKNWQQLWMETTPGWLNSLQLKRFSASRNLIIDIDPDFPWQLTALDGYGANLTLVTD

HKWGVWSGSANLNAAAATFNRVDVRRPSLALTANSSTVNISELSAFTEKGILEATASVSQTPQRQTHISL

NGRGVPVNILQQWGWPELPLTGDGNIQLTASGDIQANAPLKPTVSGQLHAVNAAKQQVTQTMNAGVVSSG

EVTSTEPVR

>gi|30065067|ref|NP_839238.1| integrase [Shigella flexneri 2a str. 2457T]

MNSSKAGGCHGINDIKVKTAKPKDKPYKLADGGGMYLLINTNGSKYWRMKYRFAGKEKMLSIGVYPDVTL

ADAREKRSEARKLLAAGGDPGEAKKEEKIAQQMSLKNTFEAIAREWHQLKADRWSLRYRDEIIDTFEKDI

FPYIGKRPIAEIKPMKLLEALRKMEKRGALEKMRKVRQRCGEVFRYAIVTGRADYNPAPDLASALATPKK

VHFPFLTANELPHFLNDLAGYTGSIITKTATQIIMLTGVRTQELRFARWEDIDFETKLWEIPAEVMKMKR

PHIVPLSEQVIMLFKQLEPISKHHPLVFIGRNDPRKPISKESINQVIELLGYKGRLTGHGFRHTMSTILH

EQGFNSAWIEMQLAHVDKNSIRGTYNHALYLDGRREMMQWYADYIDSLSSRES

>gi|30065066|ref|NP_839237.1| hypothetical protein S4834 [Shigella flexneri 2a str. 2457T]

MNDRLCFEVHDNKGYFVFPDTWFGPLLGEFEEVLDAYDADEISETSYINKLRRLAQREPDFIDIHAHLAY

AFLEQNAPRKALNAALKGLAAGNRIIPESFCGEIIWMHPENRPYLRALYAAILANVHLQRHQDAVMLTDK

ILAYNPEDNQGARWLLGSELLRTGDHERAFSVLKEHADEFSPYWYELGLLHFLNGEHVKAATAFRHGFAT

NTYIAEMLCGNLHPFPLAVWHDFSGSLDTAEDYYATYSPLWGQYPEALLFVNWLYNHSSVLHERSEIIKC

AEMLIQEDDFEICESILRQQEHLWKRIDKTLSEEIVQKCRNMNGEYIWPWILPFSAAGMKHSSIQYQ

>gi|30065062|ref|NP_839233.1| hypothetical protein S4831 [Shigella flexneri 2a str. 2457T]

MMLFSIIIMLLSPKITHANEKVICQNSASFVTKEFICGTILVSLILSSLFCYISISPSIFMHTYGLSTFS

YSIIFSFSVLFFIIGNQLSKIEKLSNLWFLLPNSQFYRITNKWFKNRICRIYYRFYSNNKRWYNQFPVSN

RKRQRCWSRNYFRSWFTNNFITVNSRNRHCRK

>gi|30065060|ref|NP_839231.1| IS629 orfA [Shigella flexneri 2a str. 2457T]

MTKNTRFSPEVRQRAVRMVLESQGEYDSQWATICSIAPKIGCTPETLRVRVRQHERDTGGGDGGLTTAER

QRLKELERENRELRRSNDILRQASAYFAKAEFDRLWKK

>gi|30065057|ref|NP_839228.1| colV-immunity protein [Shigella flexneri 2a str. 2457T]

MKRWFKYPVTFDLHYTVSHRLIFAPARFLFLLVVTILNAYDHSILAPVWLCFNSAFRADKQLLAGILLVL

MSVPLFTSADFHIPSNRAYSLSLDYSIVRLKFFLNNNRSTAGRSSPFFITHCTDNHNLISRN

>gi|30065054|ref|NP_839225.1| hypothetical protein S4829 [Shigella flexneri 2a str. 2457T]

MPCFTAMRAEIALMSGSAFAVTHHAFSSGAGRTSDGNGSHASTLKSPSYTKSVSWQHYPWSETKAAYRCF

SNDKVSANKIMTPHKENITERAQQFKRVLVLQDTTELNYSGQKEKQGVGPKKHKDERNLFLHPQLVISES

GVCLGVYDDYQWFRDELKTSKNTRQEICNDLLHKKHVSEKETWRWVEGYNKATELARQCPDTHVLSISDR

EGDFYDLFERAAQTPGIKADWLVRMKFNNRATLNINGKRDHRLLHERIMEITPQQLVEFTIPDGRGQQAR

TLPDMLSSDAGPLSYPLIYSSKVMIISLFDSTVMPDKYRHNGK

>gi|30065053|ref|NP_839224.1| membrane transport protein [Shigella flexneri 2a str. 2457T]

MSPGIEDTPQKPLSCWPLAFSAGLLGIGQNGLLVVLPVLVIQTNLSLSVWAALLMLGSMLFLPSSPWWGK

QISRTGSKPVVLWALGGYGISFTLLGLGSVLMATSAITTAVGLGILIIARIAYGLTVSAMVPACQVWALQ

RAGEGNRMAALATISSGLSCGRLFGPLCAAAMLAIHPLAPLGLLMAAPVLALLMLLRLPGTPPQPTPECK

SVSLKRDCLPYLLCAILLAAAVSMMQLGLSPALTRQFATDTTAISQQVAWLLGLSAVAALIAQFGVLRPQ

RLTPVALLLSAGVLMSGGLAIMLSEQLWLFYPGCAVLSFGAALATPAYQLLLNDKLADGAGAGWLATSHT

LGYGLCALLVPLVSKTGVAIALIMAALFATILFTIVSVFIWHYRTIK

>gi|30065052|ref|NP_839223.1| siderophore biosynthesis protein [Shigella flexneri 2a str. 2457T]

MRIIIDIIVIILLCGSCLTMTLPSEKPATDVAAQCFLNALIRETTDWKLTEYPPDELLIPLDEQKSLHFR

VAYFSPTQHHRFAFPARLVTASGSYPVDFTTLSRLIIDKLRHQLFLPVPLCETFHQRVLESHVHTQQAID

ARHDWAALREKALNFGEAEQALLTGHAFHPAPKSHEPFNRREAERYLPDMAPHFPLRWFSVDKTQIAGES

LHLNLQQRLTRFAAENAPQLLNELSDNQWLFPLHPWQGEYLLQQGWCQALVAKGLIKDLGEAGTSWLPTT

SSRSLYCATSRDMIKFSLSVRLTNSIRTLSVKEVKRGMRLARLAQTDGWQMLQVRFPTFRVMQEDGWAGL

LDLNGNIMQESLFALRENLLVDQPKSQTNVLVSLTQAAPDGGDSLLVSAVKRLSDRLGITVQQAAHAWVD

AYCQQVLKPLFTAEADYGLVLLAHQQNILVQMLGDLPVGFIYRDCQGSAFMPHATDWLDSIGEAQAENIF

THEQLLRYFPYYLLVNSTFAVTAALGAAGLDSESNLMARVRASLAEVRDQVTHKTCLNYVLESPYWNVKG

NFFCYLNDHNENTIVDPSVIYFDFANPLQAQEV

>gi|30065051|ref|NP_839222.1| siderophore biosynthesis protein [Shigella flexneri 2a str. 2457T]

MSEANIIHSRYGLRCEKLDKPLNLGWGLDNSAVLHCPGELPTGWLCDALDQIFIAAPQLSAVALPWAEWR

EEPQALTLFGQVKSDIIHRTAFWQLPLWLSSPANRASGEMVFDAEREIYFPQRPPRPQGEVYRRYDPRIR

RMLSFRIADPVSDAERFTRWMNDPRVEYFWEQSGSLEVQTAYLERQLTGKHAFPLIGCFDDRPFSYFEIY

WAAEDRIGRHYSWQPFDRGLHLLVGEQQWRGAHYVQSWLRGLTHYLLLDEPRTQRTVLEPRTDNQRLFRH

LEPAGYRTIKEFDFPHKRSRMVMADRHHFFTEVGL

>gi|30065050|ref|NP_839221.1| siderophore biosynthesis protein [Shigella flexneri 2a str. 2457T]

MNHKDWDFVNRRLVAKMLSEMEYEQVFHAESQGDDHYCINLPGAQWRFIAERGIWGWLWIDAQTLRCTDE

PVLAQTLLMQLKPVLSMSDATVAEHMQDLYATLLGDLQLLKARRGLSASDLINLDADRLQCLLSGHPKFV

FNKGRRGWGKEALERYAPEYTNTFRLHWLAVKREHMIWRCDNDLDIQQLLTAAMDPQEFTRFSQVWQENG

LDHNWLPLPVHPWQWQQKIATDFIADFAEGRMVSLGEFGDQWLAQQSLRTLTNASRRGGLDIKLPLTIYN

TSCYRGIPGRYIAAGPLASRWLQQVFATDATLVQSGAVILGEPAAGYVSHEGYAALARAPYRYQEMLGVI

WRENPCRWLKPDESPVLMATLMECDENNQPLAGAYIDRSGLDAETWLTQLFRVVVVPLYHLLCRYGVALI

AHGQNITLAMKEGVPQRVLLKDFQGDMRLVKEAFPEMDSLPQEVRDVTSRLSADYLIHDLQTGHFVTVLR

FISPLMVRLGVPERRFYQLLAAVLSDYMNKHPQMAERFALFSLFRPQIIRVVLNPVKLTWPDLDGGSRML

PNYLENLQNPLWLVTQEYES

>gi|30065049|ref|NP_839220.1| lysine:N6-hydroxylase [Shigella flexneri 2a str. 2457T]

MKKSVDFIGVGTGPFNLSIAALSHQIEELNCLFFDEHPHFSWHPGMLVPDCHMQTVFLKDLVSAVAPTNP

YSFVNYLVKHKKFYRFLTSRLRTVSREEFSDYLRWAAEDMNNLYFSHTVENIDFDKKSRLFLVQTSRGEY

FAHNICLGTGKQPYLPPCVKHVTQSCFHASEMNLRRPDLSGKRITVVGGGQSGADLFLNALRGEWGEAAE

INWVSRRNNFNALDEAAFADEYFTPEYISGFSGLKEDIRHQLLDEQKMTSDGITADSLLTIYRELYHRFE

VLRKPRNIRLLPSRSVTTLESSGPGWRLLMEHHLDQGRESLESDVVIFATGYRSALPQILPSLMPLITMH

DKNTFKVRDDFTLEWSGPKENNIFAVNASMQTHGIAEPQLSLMAWRSARILNRVLGRDLFDLSMPPALIQ

WRSGSRKKPQPEAASLTHYTANIQE

>gi|30065048|ref|NP_839219.1| ferric siderophore receptor [Shigella flexneri 2a str. 2457T]

MRKKYMPRALGPLLLVVLSPAVAQQNDDNEIIVSASRSNRTVAEMAQTTWVIENAELEQQIQGGKELKDA

LAQLIPGLDVSSQSRTNYGMNMRGRPLVVLIDGVRLNSSRSDSRQLDSVDPFNIDHIEVISGATALYGGG

STGGLINIVTKKGQPETMMEFEAGTKSGFNSSKDHDERIAGAVSGGNDHISGRLSVAYQKFGGWFDGNGD

ATLLDNTQTGLQHSNRLDIMGTGTLNIDESRQLQLITQYYKSQGDDNYGLNLGKGFSAISGSSTPYVSKG

LNSDRIPGTERHLISLQYSDSDFLGQELVGQVYYRDESLRYYPFPTVNANKQATAFSSSQQDTDQYGMKL

TLNSQLMDGWQITWGLDAEHERFTSNQMFFDLAQASASGGLNNHKIYTTGRYPSYDITNLAAFLQSSYDI

NDIFTVSGGVRYQYTENRVDDFIDYTQQQKIATGKAISADAIPGGSVDYDNFLFNAGLLMHITERQQAWF

NFSQGVALPDPGKYYGRGIYGAAVNGHLPLTKSVNVSDSKLEGVKVDSYELGWRFIGDNLRTQIAAYYSL

SNKSVERNKDLTISVKDDRRRIYGVEGAVDYLIPDTDWSTGVNFNVLKTESKVNGQWQKYDVKESSPSKA

TAYINWAPEPWSLRVQSTTSFDVSDAEGNDINGYTTVDFISSWQLPVGTLSFSVENLFDRDYTTVWGQRA

PLYYSPGYGPASLYDYKGRGRTFGLNYSVLF

>gi|30065045|ref|NP_839216.1| long polar fimbriae [Shigella flexneri 2a str. 2457T]

MMTTRIVVGLTAGTCLIFSQNLMAEVSVFNPALLEINHQSGVDIRQFNRANLMPPGVYSVDIFINGKMFE

RQDVTFVQDNPDADLHACFIAIKKTLSSFGIKVDALKSFNDVDETVCLDPAPRIEGSSWQFDSDKLQLNI

SIHQIYMDAMAYDYISPTRWDEGINALTINYDFSGSHTLRSDYGSQETDTSYLNLRNGLNIGPWRLRNYS

TLNTSDGRAEYNSISTWIQRDIAALRSQIMIGDTWTASDIFDSTQIRGARLYTDNDMLPASQNGFAPVVR

GIAKSNATVIIRQNGYVIYQSAVPQGAFEITDLNTASTGGDLDVTIKEEDGSEQRFTQPYASLAILKREG

LTDVDVSVGELRDEDGFTPDVLQAQILHGFSHGITLYGGMQAAENYGSAALGVGKDLGALGAISFDVTHA

RANFSHDDTETGQSYRFLYSKLFDDTDTSLRLVGYRYSTEGYYTLNEWASRRNSPEDFWETGNRRSRVEG

TLTQSLGRDYGNLYLTLSRQQYWHTDDVERLMQFGYSSSWKRLSWNVSWSYSNTARQGTGNNHASDNTSE

QIYMLSLSVPLSGWWGNSYATYSVSQNDNSGSSHQLGLSGTALERNNLSWNLMQSYNSHDDEVGGNMSLT

YDGSYGTVNGSYNYSQNSQRLNYGIRGGILAHSEGVTLSQELGETIALVKAPGAAGLEIDNMRGAATDWR

GYTVKTQLNPYDENRVAISDNYFSKSNIELDNTVVTMVPTRGAVVKAEFVTHVGYRVLFRVLNANGKPVP

FGAIAAIQDASLADSGIVGDRGELYLSGLPEKGQVTLSWGENASTKCIFNYSFSTPESESGLIEQGVTCH

>gi|30065043|ref|NP_839214.1| phosphate ABC transporter substrate-binding protein [Shigella flexneri 2a str. 2457T]

MKVMRTTVATVVAATLSMSAFSVFAEASLTGAGATFPAPVYAKWADTYQKETGNKVNYQGIGSSGGVKQI

IANTVDFGASDAPLSDEKLAQEGLFQFPTVIGGVVLAVNIPGLKSGELVLDGKTLGDIYLGKIKKWDDEA

IAKLNPGLKLPSQNIAVVRRADGSGTSFVFTSYLAKVNEEWKNNVGTGSTVKWPIGLGGKGNDGIAAFVQ

RLPGAIGYVEYAYAKQNNLAYTKLISADGKPVSPTEENFANAAKGADWSKTFAQDLTNQKGEDAWPITST

TFILIHKDQKKPEQGTEVLKFFDWAYKTGAKQANDLDYASLPDSVVEQVRAAWKTNIKDSSGKPLY

>gi|30065041|ref|NP_839212.1| phosphate transporter permease subunit PtsA [Shigella flexneri 2a str. 2457T]

MAMVEMQTTAALAESRRKMQARRRLKNRIALTLSMATMAFGLFWLIWILMSTITRGIDGMSLALFTEMTP

PPNTEGGGLANALAGSGLLILWATVFGTPLGIMAGIYLAEYGRKSWLAEVIRFINDILLSAPSIVVGLFV

YTIVVAQMEHFSGWAGVIALALLQVPIVIRTTENMLKLVPDSLREAAYALGTPKWKMISAITLKASVSGI

MTGILLAIARIAGETAPLLFTALSNQFWSTDMMQPIANLPVTIFKFAMSPFAEWQQLAWAGVLIITLCVL

LLNILARVVFAKNKHG

>gi|30065039|ref|NP_839210.1| transcriptional regulator PhoU [Shigella flexneri 2a str. 2457T]

MDSLNLNKHISGQFNAELESIRTQVMTMGGMVEQQLSDAITAMHNQDSDLAKRVIEGDKNVNMMEVAIDE

ACVRIIAKRQPTASDLRLVMVISKTIAELERIGDVADKICRTALEKFSQQHQPLLVSLESLGRHTIQMLH

DVLDAFARMDIDEAVRIYREDKKVDQEYEGIVRQLMTYMMEDSRTIPSVLTALFCARSIERIGDRCQNIC

EFIFYYVKGQDFRHVGGDELDKLLAGKDSDK

>gi|30065038|ref|NP_839209.1| transcriptional antiterminator BglG [Shigella flexneri 2a str. 2457T]

MNMQITKILNNNVVVVIDDQQREKVVMGRGIGFQKRAGERINSSGIEKEYALSSHELNGRLSELLSHIPL

EVMATCDRLISLAQERLGKLQDSIYISLTDHCQFAIKRFQQNVLLWDIQRLYPKEFQLGEEALTIIDKRL

GVQLPKDEVGFIAMHLVSAQMSGNMEDVAGVTQLMREMLQLIKFQFSLNYQEESLSYQRLVTHLKFLSWR

ILEHASINDSDESLQQAVKQNYPQAWQCAERIAIFIGLQYQRKISPAEIMFLAINIERVRKEH

>gi|30065037|ref|NP_839208.1| beta-glucoside-specific PTS system components IIABC [Shigella flexneri 2a str. 2457T]

MTELARKIVAGVGGADNIVSLMHCATRLRFKLKDESKAQAEVLKKTPGIIMVVESGGQFQVVIGNHVADV

FLAVNSVAGLGEKAQQAPENDEKGNLLNRFVYVISGIFTPLIGLMAATGILKGMLALALTFQWTTEQSGT

YLILFSASDALFGFFPIILGYTAGKRFGGNPFTAMVIGGALVHPLILTAFENGQKADAPGLDFLGIPVTL

LNYSSSVIPIIFSAWLCSILERRLNAWLPSAIKNFFTPLLCLMVITPVTFLLVGQLSTWISEMIAAGYLW

LYQAVPAFAGAVMGGFWQIFVMFGLHRGLVPLCINNFTVLGYDTMIPLLMPAIMAQVGAALGVFLCERDA

QKKVVAGSAALTGLFGITEPAVYGVNLPRKYPFVIACISGALGATIIGYAQTKVYSFGLPSIFTFMQTIP

STGIDFTVWASVIGGVIAIGCAFVGTLMLHFITSKRQPAQGAPQEKTPEVITPPEQGGICSPMTGEIVPL

IHVADTTFASGLLGKGIAILPSVGEVRSPVAGRIASLFATLHAIGIESDDGVEILIHVGIDTVKLDGKFF

SAHVNVGDKVNTGDRLISFDIPAIREAGFDLTTPVLISNSDDFTDVLPHGTAQISAGEPLLSIIR

>gi|30065033|ref|NP_839204.1| receptor protein [Shigella flexneri 2a str. 2457T]

MAPLAFSAQSLAESLTVEQRLELLEKALRETQSELKKYKDEEKKKYTPATVNRSVSTNDQGYAANPFPTS

SAAKPDAVLVKNEEKNASETGSIYSSMTLKDFSKFVKDEIGFSYNGYYRSGWGTASHGSPKSWAIGSLGR

FGNEYSGWFDLQLKQRVYNENGKRVDAVVMIDGNVGQQYSTGWFGDNAGGENFMQFSDMYVTTKGFLPFA

PEADFWVGKHGAPKIEIQMLDWKTQRTDAAAGVGLENWKVGPGKIDIALVREDVDDYDRSLQNKQQINTH

TIDLRYKDIPLWDKATLMVSGRYVTANESASEKDNQDNNGYYDWKDTWMFGTSLTQKFDKGGFNEFSFLV

ANNSIARNFGRYAGASPFTTFNGRYYGDHTGGTAVRLTSQGEAYIGDHFIVANAIVYSFGNNIYSYETGA

HSDFESIRAVVRPAYIWDQYNQTGVELGYFTQQNKDANSNKFNESGYKTTLFHTFKVNTSMLTSRLEIRF

YATYIKALENELDGFTFEDNKDAQFAVGAQAEIWW

>gi|30065032|ref|NP_839203.1| xylanase [Shigella flexneri 2a str. 2457T]

MNIKIAALTLAIASGISAQWAIAADMPASPAPTIPVKQYVTQVNADNSVTFRYFAPGAKNVSVVVGVPVP

DNIHPMTKDEAGVWSWRTPILKGNLYEYFFNVDGVRSIDTGTAMTKPQRQVNSSMILVPGSYLDTRSVAH

GDLIAITYHSNALQSERQMYVWTPPGYTGMGEPLPVLYFYHGFGDTGRSAIDQGRIPQIMDNLLAEGKIK

PMLVVIPDTETDAKGIIPEDFVPQERRKVFYPLNAKAADRELMNDIIPLISKRFSVRKDADGRALAGLSQ

GGYQALVSGMNHLESFGWLATFSGVTTTTVPDEGVAARLNDPAAINQQLRNFTVVVGDKDVVTGKDIAGL

KTELEQKKIKFDYQEYPGLNHEMDVWRPAYAAFVQKFKIRH

>gi|30065030|ref|NP_839201.1| ISSfl3 orfC [Shigella flexneri 2a str. 2457T]

MNNELPDDIELLKAMLRKQQSRLRQYACQVAGYEQEIERLKAQLDRLRRMLFGQSSEKKRHKLENQIRQA

EKRLSELENRLNTARNLLEDASSVTDSPDTSPPSENPIASKPESPGRKSSRKPLPAELPRETHRLLPAET

SCPACGGVLKEMGETISEQLDIINTAFKVIETIRPKLACSRCDVIVQAPLPPKPIERGYASAGLLARILV

SKYMEHIPLYRQSEIYARQGVELSRNTMVRWVSEMADKLRPLYIALNDYVLEAGKVHADDTPVKVLAPGN

GKTKTGRLWVYVRDDRNAGSSLPAAVWFAYSADRKGEHPQLHLAKYQGVLQADAYAGYNVLYETGRVKEA

GCLAHARRKIHDEDVRRPTEMTQEALRRIAELYDIEAEIRGSPAEERLAVRKARSVQLMQSLYDWIQLQR

KTLSKHAKMAKAFDYILNHWNALNEFCRDGRVEIDNNIGENALRSVAVGRKNYLFFGSDKGGESAAIIYS

LLVTCKQNEVEPEDWLREVIEKLNDWPSNQVHELLPWNFSSVK

>gi|30065027|ref|NP_839198.1| inner membrane protein [Shigella flexneri 2a str. 2457T]

MSVSRRVIHHGLYFAVLGPLIGVLFLVLYIFFAKEPLVLLVIIQVLPLFLLLSITTGAIPALLTGVMVAC

LPEKIGSQKNYRCLAGGIGGVVITEIYCAVIVHIKGMASSELFENILSGDSLVVRIIPALLAGVVMSRII

TRLPGLDISCPETDSLS

>gi|30065026|ref|NP_839197.1| 6-phosphogluconate phosphatase [Shigella flexneri 2a str. 2457T]

MSRIEAVFFDCDGTLVDSEVICSRAYVTMFQEFGITLDPEEVFKRFKGVKLYEIIDIVSLEHGVTLAKTE

AEHVYRAEVARLFDSAIEGAGALLSAITAPMCVVSNGPNNKMQHSMGKLNMLHYFPDKLFSGYDIQRWKP

DPALMFHAAKAMNVNVENCILVDDSVAGAQSGIDAGMEVFYFCADPHNKPIVHPKVTTFTHLSQLPELWK
ARGWDITA

>gi|30065024|ref|NP_839195.1| hypothetical protein S4025 [Shigella flexneri 2a str. 2457T]
MSEKLQVVTLLGSLRKGSFNGMVARTLPKIAPASMEVNALPSIADIPLYDADVQQEEGFPATVEALAEQI
RQADGVVIVTPEYNYSVPGGLKNAIDWLSRLPDQPLAGKPVLIQTSSMGVIGGARCQYHLRQILVFLDAM
VMNKPEFMGGVIQNKVDPQTGEVIDQGTLDHLTGQLTAFGEFIQRVKI

>gi|30065023|ref|NP_839194.1| hypothetical protein S4024 [Shigella flexneri 2a str. 2457T]
MATHFARGILTEGHLISVRLPSQCHQEARNIPPHRQSRFLASRGLLAELMFMLYGIGELPEIVTLPKGKP
VFSDKNLPSFSISYAGNMVGVALTTEGECGLDMELQRATRGFHSPHAPDNHTFSSNESLWISKQNDPNEA
RAQLITLRRSVLKLTGDVLNDDPRDLQLLPIAGRLKCAHVNHVEALCDAEDVLVWSVAVTPAIEKLSVWE
LDGKHSWKSLPDIHSRANNPTSRMMRFAQLSTVKSFSPN

>gi|30065019|ref|NP_839190.1| tryptophan permease TnaB [Shigella flexneri 2a str. 2457T]
MTDQAEKKHSAFWGVMVIAGTVIGGGMFALPVDLAGAWFFWGAFILIIAWFSMLHSGLLLLEANLNYPVG
SSFNTITKDLIGNTWNIISGITVAFVLYILTYAYISANGAIISETISMNLGYHANPRIVGICTAIFVASV
LWISSLAASRITSLFLGLKIISFVIVFGSFFFQVDYSILRDATSTTAGTSYFPYIFMALPVCLASFGFHG
NIPSLIICYGKRKDKLIKSVVFGSLLALVIYLFWLYCTMGNIPRESFKAIISSGGNIDSLVKSFLGTKQH
GIIEFCLLVFSNLAVASSFFGVTLGLFDYLADLFKIDNSHGGRFKTVLLTFLPPALLYLIFPNGFIYGIG
GAGLCATIWAVIIPAVLAIKARKKFPNQMFTVWGGNLIPAIVILFGITVILCWFGNVFNVLPKFG

>gi|30065018|ref|NP_839189.1| tryptophanase [Shigella flexneri 2a str. 2457T]

MENFKHLPEPFRIRVIEPVKRTTRAYREEAIIKSGMNPFLLDSEDVFIDLLTDSGTGAVTQSMQAAMMRG

DEAYSGSRSYYALAESVKNIFGYQYTIPTHQGRGAEQIYIPVLIKKREQEKGLDRSKMVAFSNYFFDTTQ

GHSQINGCTVRNVYIKEAFDTGVRYDFKGNFDLEGLERGIEEVGPNNVPYIVATITSNSAGGQPVSLANL

KAMYSIAKKYDIPVVMDSARFAENAYFIKQREAEYKDWTIEQITRETYKYADMLAMSAKKDAMVPMGGLL

CMKDDSFFDVYTECRTLCVVQEGFPTYGGLEGGAMERLAVGLYDGMNLDWLAYRIAQVQYLVDGLEEIGV

VCQQAGGHAAFVDAGKLLPHIPADQFPAQALACELYKVAGIRAVEIGSFLLGRDPKTGKQLPCPAELLRL

TIPRATYTQTHMDFIIEAFKHVKENAANIKGLTFTYEPKVLRHFTAKLKEV

>gi|30065012|ref|NP_839183.1| ribonuclease P [Shigella flexneri 2a str. 2457T]

MVKLAFPRELRLLTPSQFTFVFQQPQRAGTPQITILGRLNSLGHPRIGLTVAKKNVRRAHERNRIKRLTR

ESFRLRQHELPAMDFVVVAKKGVADLDNRALSEALEKLWRRHCRLARGS

>gi|30065010|ref|NP_839181.1| chromosomal replication initiation protein [Shigella flexneri 2a str. 2457T]

MSLSLWQQCLARLQDELPATEFSMWIRPLQAELSDNTLALYAPNRFVLDWVRDKYLNNINGLLTSFCGAD

APQLRFEVGTKPVTQTPQAAVTSNVAAPAQVAQTQPQRAAPSTRSGWDNVPAPAEPTYRSNVNVKHTFDN

FVEGKSNQLARAAACQVADNPGGAYNPLFLYGGTGLGKTHLLHAVGNGIMARKPNAKVVYMHSERFVQDM

VKALQNNAIEEFKRYYRSVDALLIDDIQFFANKERSQEEFFHTFNALLEGNQQIILTSDRYPKEINGVED

RLKSRFGWGLTVAIEPPELETRVAILMKKADENDIRLPGEVAFFIAKRLRSNVRELEGALNRVIANANFT

GRAITIDFVREALRDLLALQEKLVTIDNIQKTVAEYYKIKVADLLSKRRSRSVARPRQMAMALAKELTNH
SLPEIGDAFGGRDHTTVLHACRKIEQLREESHDIKEDFSNLIRTLSS

>gi|30065009|ref|NP_839180.1| DNA polymerase III subunit beta [Shigella flexneri 2a str. 2457T]
MKFTVEREHLLKPLQQVSGPLGGRPTLPILGNLLLQVADGTLSLTGTDLEMEMVARVALVQPHEPGATTV
PARKFFDICRGLPEGAEIAVQLEGERMLVRSGRSRFSLSTLPAADFPNLDDWQSEVEFTLPQATMKRLIE
ATQFSMAHQDVRYYLNGMLFETEGEELRTVATDGHRLAVCSMPIGQSLPSHSVIVPRKGVIELMRMLDGG
DNLLRVQIGSNNIRAHVGDFIFTSKLVDGRFPDYRRVLPKNPDKHLEAGCDLLKQAFARAAILSNEKFRG
VRLYVSENQLKITANNPEQEEAEEILDVTYSGAEMEIGFNVSYVLDVLNALKCENVRMMLTDSVSSVQIE
DAASQSAAYVVMPMRL

>gi|30065008|ref|NP_839179.1| recombination protein F [Shigella flexneri 2a str. 2457T]
MSLTRLLIRDFRNIETADLALSPGFNFLVGANGSGKTSVLEAIYTLGHGRAFRSLQIGRVIRHEQEAFVL
HGRLQGEERETAIGLTKDKQGDSKVRIDGTDGHKVAELAHLMPMQLITPEGFTLLNGGPKYRRAFLDWGC
FHNEPGFFTAWSNLKRLLKQRNAALRQVTRYEQLRPWDKELIPLAEQISTWRAEYSAGIAADMDDTCKQF
LPEFSLTFSFQRGWEKETEYAEVLERNFERDRQLTYTAHGPHKADLRIRADGAPVEDTLSRGQLKLLMCA
LRLAQGEFLTRESGRRCLYLIDDFASELDDERRGLLASRLKATQSQVFVSAISAEHVIDMSDENSKMFTV
EKGKITD

>gi|30065005|ref|NP_839176.1| sugar phosphatase [Shigella flexneri 2a str. 2457T]
MAIKLIAIDMDGTLLLPDHTISPAVKNAIAAARARGVNVVLTTGRPYAGVHNYLKELHMEQPGDYCITYN
GALVQKAADGSTVAQTALSYDDYRFLEKLSREVGSHFHALDRTTLYTANRDISYYTVHESFVATIPLVFC
EAEKMDPNTQFLKVMMIDEPAILDQAIARIPQEVKEKYTVLKSAPYFLEILDKRVNKGTGVKSLADVLGI
KPEEIMAIGDQENDIAMIEYAGVGVAMDNAIPSVKEVANFVTKSNLEDGVAFAIEKYVLN

>gi|30065003|ref|NP_839174.1| regulator protein for dgo operon [Shigella flexneri 2a str. 2457T]

MTLNKTDRIVITLGKQIVHGKYVPGSPLPAEAELCEEFATSRNIIREVFRSLMAKRLIEMKRYRGAFVAP

RNQWNYLDTDVLQWVLENDYDPRLISAMSEVRNLVEPAIARWAAERATSSDLAQIESALNEMIANNQDRE

AFNEADIRYHEAVLQSVHNPVLQQLSIAISSLQRAVFERTWMGDEANMPQTLQEHKALFDAIRHQDGDAA

EQAALTMIASSTRRLKEIT

>gi|30065002|ref|NP_839173.1| 2-oxo-3-deoxygalactonate kinase [Shigella flexneri 2a str. 2457T]

MTARYIAIDWGSTNLRAWLYQGDHCLESRQSEAGVTRLNGKSPAAVLAEVTTDWREENTPVVMAGMVGSN

VGWKVAPYLSVPARFSSIGEQLTSVGDNIWIIPGLCVSHDDNHNVMRGEETQLIGARALAPSSLYVMPGT

HCKWVQADSQQINDFRTVMTGELHHLLLNHSLIGAGLPPQENSADAFAAGLERGLNAPAILPQLFEVRAS

HVLGTLPREQVSEFLSGLLIGAEVASMRDYVTHQHAITLVAGTSLTARYQQAFQAMGCDVAAVAGDTAFQ

AGIRSIAHAVAN

>gi|30065000|ref|NP_839171.1| oxidoreductase [Shigella flexneri 2a str. 2457T]

MEHFDVAIIGLGPAGSALARKLAGKMQVIALDKKHQCGTEGFSKPCGGLLAPDAQRSFIRDGLTLPVDVI

ANPQIFSVKTVDVAASLTRNYQRSYININRHAFDLWMKSLIPASVEVYHDSLCRKIWREDDKWHVIFRAD

GWEQHITARYLVGADGANSMVRRHLYPDHQIRKYVAIQQWFAEKHPVPFYSCIFDNAITDCYSWSISKDG

YFIFGGAYPMKDGQTRFTTLKEKMSAFQFQFGKAVKSEKCTVLFPSRWQDFVCGKDNAFLIGEAAGFISA

SSLEGISYALDSAEILRAVFLKQPEKSNAAYWRATHKLRLKLFGKIVKSRCLTAPALRKWIMRSGVAHIP

QLKDYPTRFTSPTSRM

>gi|30064999|ref|NP_839170.1| hypothetical protein S3996 [Shigella flexneri 2a str. 2457T]

MMAGTVLYQDRAMKQITFAPRNHLLTNTNTNTNTNTNTWTPDSQWLVFDVRPSGASFTGETIERVNIHTG

EVEVIYRASQGAHVGVVTVHPKSEKYVFIHGPENPDETWHYDFHHRRGVIAEGGKVSNLDAMDITAPYTP

GALRGGSHVHVFSPNGERGSFTYNDHVMHQLDPALDLRNVGVAAPFGPVNVQKQHPREYSGSHWCVLVSK

TTPTPQPGSDEINRAYEEGWVGNHALAFIGDTLSPKGEKVPELFIVELPQDEASWKSAGDAPLSGTETTL

PAPPRGVVQRRLTFTHHRAYPGLVNVPRHWVRCNPQGTQIAFLMRDDNGIVQLWLISPQGGEPRKLTHNK

TDIQSAFNWHPSGEWLGFVLDNRIVCAHAQSGEVEYLTENHANPPSADAVVFSPDGQWLAWMEGGQLWIT

ETDR

>gi|30064996|ref|NP_839167.1| heat shock chaperone IbpB [Shigella flexneri 2a str. 2457T]

MRNFDLSPLMRQWIGFDKLANALQNAGESQSFPPYNIEKSDDNHYRITLALAGFRQEDLEIQLEGTRLSV

KGTPEQPKEEKKWLHQGLMNQPFSLSFTLAENMEVSGATFVNGLLHIDLIRNEPEPIAAQRIAISERPAL

NS

>gi|30064995|ref|NP_839166.1| hypothetical protein S3992 [Shigella flexneri 2a str. 2457T]

MSDIALTVSILALVAVVGLFIGNVKFRGIGLGIGGVLFGGIIVGHFVSQAGMTLSSDMLHVIQEFGLILF

VYTIGIQVGPGFFASLRVSGLRLNLFAVLIVIIGGLVTAILHKLFDIPLPVVLGIFSGAVTNTPALGAGQ

QILRDLGTPMEMVDQMGMSYAMAYPFGICGILFTMWMLRVIFRVNVETEAQQHESSRTNGGALIKTINIR

VENPNLHDLAIKDVPILNGDKIICSRLKREETLKVPSPDTIIQLGDLLHLVGQPADLHNAQLVIGQEVDT

SLSTKGTDLRVERVVVTNENVLGKRIRDLHFKERYDVVISRLNRAGVELVASGDISLQFGDILNLVGRPS

AIDAVANVLGNAQQKLQQVQMLPVFIGIGLGVLLGSIPVFVPGFPAALKLGLAGGPLIMALILGRIGSIG

KLYWFMPPSANLALRELGIVLFLSVVGLKSGGDFVNTLVNGEGLSWIGYGALITAVPLITVGILARMLAK

MNYLTMCGMLAGSMTDPPALAFANNLHPTSGAAALSYATVYPLVMFLRIITPQLLAVLFWSIG

>gi|30064994|ref|NP_839165.1| transcriptional regulator [Shigella flexneri 2a str. 2457T]

MIYKSIAERLRIRLNSADFTLNSLLPGEKKLAEEFAVSRMTIRKAIDLLVAWGLVVRRHGSGTYLVRKDV

LHQTASLTGLVEVLKRQGKTVTSQVLIFEIMPAPPAIASQLRIQINEQIYFSRRVRFVEGKPLMLEDSYM

PVKLFRNLSLQHLEGSKFEYIEQECGILIGGNYESLMPVLADRLLARQMKVAEHTPLLRITSLSYSESGE

FLNYSVMFRNASEY

>gi|30064992|ref|NP_839163.1| ARAC-type regulatory protein [Shigella flexneri 2a str. 2457T]

MNGKLQSSDVKNETPYNIPLLINENVISSGISLISLWHTYADEHYRVIWPRDKKKPLIANSWVAVYTVQG

CGKILLKNGEQITLHSNCIIFLKPMDIHSYHCEGLVWEQYWMEFTPTSMMDIPVGQQSVIYNGEIYNQEL

TEVAELITSPEAIKNNLAVAFLTKIIYQWICLMSADGKKDPQRRQIEKLIATLHASLQQRWSVADMAATI

PCSEAWLRRLFLRYTGKTPKEYYLDARLDLALSLLKQQGNSVGEVADTLNFFDSFHFSKAFKHKFGYAPS

AVLKNTDQHPTDASPHN

>gi|30064990|ref|NP_839161.1| hypothetical protein S3984 [Shigella flexneri 2a str. 2457T]

MGIIAQNKISSLGMLFGAIALMMGIIHFSFGPFSAPPPTLESIVADKTAEIKRGLLAGIKGEKITTVEKK

EDMDIDKILDQSGIALAIAALLCAFIGGMRKENRWGIRGALVFGIGIVCSILLIFLIFSFLTGGSLV

>gi|30064989|ref|NP_839160.1| hypothetical protein S3983 [Shigella flexneri 2a str. 2457T]

MKISRLGEAPDYRFSLANERTFLAWIRTALGFLAAGVGLDQLAPDFATPVIRELLALLLCLFSGGLAMYG

YLRWLRNEKAMRLKEDLPYTNSLLIISLILMVVAVIVMGLVLYAG

>gi|30064988|ref|NP_839159.1| hypothetical protein S3982 [Shigella flexneri 2a str. 2457T]

MPDSRKARRIADPGLQPERTSLAWFRTMLGYGALMALAIKHNWHQAGMLFWISIGILAIVALILWHYTRN

RNLMDVTNSDFSQFHVVRDKFLISLAVLSLAILFAVTHIHQLIVFIERVA

>gi|30064987|ref|NP_839158.1| transcriptional regulator [Shigella flexneri 2a str. 2457T]

MTGSQVIDAEEDRHKLVVEYKDALQPADFYHNFKQRGIRSVQLIPYLEFDDRGDLTAASVTAELWGKFLI

ALFECWVRADISRISIELFDATLQKWCGSENPQPRRDCQACDWHRLCPHARQETPDSVLCAGYQAFYSYS

APHMRVMRDLIKQHRSPMELMTMLR

>gi|30064986|ref|NP_839157.1| multidrug resistance protein D [Shigella flexneri 2a str. 2457T]

MLVLLVAVGQMAQTIYIPAIADMARDLNVREGAVQSVMGAYLLTYGVSQLFYGPISDRVGRRPVILVGMS

IFMLATLVAVTTSSLTVLIAASAMQGMGTGVGGVMARTLPRDLYERTQLRHANSLLNMGILVSPLLAPLI

GGLLDTMWNWRACYLFLLVLCAGVTFSMARWMPETRPVDAPRTRLLTSYKTLFGNSGFNCYLLMLIGGLA

GIAAFEACSGVLMGAVLGLSSMTVSILFILPIPAAFFGAWFAGRPNKRFSTLMWQSVICCLLAGLLMWIP

DWFGVMNVWTLLVPAALFFFGAGMLFPLATSGAMEPFPFLAGTAGALVGGLQNIGSGVLASLSAMLPQTG

QGSLGLLMTLMGLLIVLCWLPLATRMSHQGQPV

>gi|30064982|ref|NP_839153.1| DNA-binding transcriptional activator UhpA [Shigella flexneri 2a str. 2457T]

MITVALIDDHLIVRSGFAQLLGLEPDLQVVAEFGSGREALAGLPGRGVQVCICDISMPDISGLELLSQLP

KGMATIMLSVHDSPALVEQALNAGARGFLSKRCSPDELIAAVHTVATGGCYLTPDIAIKLASGRQDPLTK

RERQVAEKLAQGMAVKEIAAELGLSPKTVHVHRANLMEKLGVSNDVELARRMFDGW

>gi|30064981|ref|NP_839152.1| sensory histidine kinase UhpB [Shigella flexneri 2a str. 2457T]

MKTLFSRLITVIACFFIFSAAWFCLWSISLHLVERPDMAVLLFPFGLRLGLMLQCPRGYWPVLLGAEWLL

IYWLTQAVGLTHFPLLMIGSLLTLLPVALISRYRHQRDWRTLLLQGAALTAAALLQSLPWLWHGKESWNA

LLLTLTGGLTLAPICLVFWHYLANNTWLPLGPSLVSQPINWRGRHLVWYLLLFVISLWLQLGLPDELSRF

TPFCLALPIIALAWHYGWQGALIATLMNAIALIASQTWRDHLVDLLLSLLVQSLTGLLLGAGIQRLRELN

QSLQKELARNQHLAERLLETEESVRRDVARELHDDIGQTITAIRTQAGIVQRLAADNASVKQSGGQLIEQL

SLGVYDAVRRLLGRLRPRQLDDLTLEQAIRSLMREMELEGRGIVSHLEWRIDESALSENQRVTLFRVCQE

GLNNIVKHADASAVTLQGWQQDERLMLVIEDDGSGLPPGSGQQGFGLTGMRERVTALGGTLHISCLHGTR

VSVSLPQRYV

>gi|30064978|ref|NP_839149.1| cryptic adenine deaminase [Shigella flexneri 2a str. 2457T]

MNNSINHKFHHISRAEYQELLAVSRGDAVADYIIDNVSILDLINAGEISGPIVIKGRYIAGVGAEYADTP

ALQRIDARGATAVPGFIDAHLHIESSMMTPVTFETATLPRGLTTVICDPHEIVNVMGEAGFAWFARCAEQ

ARQNQYLQVSSCVPALEGCDVNGASFTLEQMLAWRDHPQVTGLAEMMDYPGVINGQNALLDKLDAFRYLT

LDGHCPGLGGKELNAYIAAGIENCHESYQLEEGRRKLQLGMSLMIREGSAASNLNALAPLINEFNSPQCM

LCTDDRNPWEIAHEGHIDALIRRLIEQHNVPLHVAYRVASWSTARHFGLNHLGLLAPGKQADIVLLSDAR

KVTVQQVLVKGEPIDAQTLQAEESARLALSAPPYGNTIARQPVSASDFALQFTPGKRYRVIDVIHNELIT

HSRSSVYSENGFDRDDVCFIAVLERYGQRLAPACGLLGGFGLNEGALAATVSHDSHNIVVIGRSAEEMAL

AVNQVIQDGGGLCVVRNGQVQSHLPLPIAGLMSTDTAQSLAEQIDALKAAARECGPLPDEPFIQMAFLSL

PVIPALKLTSQGLFDGEKFAFTTLEVTE

>gi|30064977|ref|NP_839148.1| hypothetical protein S3971 [Shigella flexneri 2a str. 2457T]

MIIITEPLLSFVLQKQGIKSPPMDKKMNNDNTDYVSNESGTLSRLFKLPQHGTTVRTELIAGMTTFLTMV

YIVFVNPQILGAAQMDPKVVFVTTCLIAGIGSIAMGIFANLPVALAPAMGLNAFFAFVVVGAMGISWQTG

MGAIFWGAIGLFLLTLFRIRYWMISNIPLSLRIGITSGIGLFIALMGLKNTGVIVANKDTLVMIGDLSSH

GVLLGILGFFIITVLSSRHFHAAVLVSIVVTSCCGLFFGDVHFSGVYSIPPDISGVIGEVDLSGALTLEL

AGIIFSFMLINLFDSSGTLIGVTDKAGLIDSNGKFPNMNKALYVDSVSSVAGAFIGTSSVTAYIESTSGV

AVGGRTGLTAVVVGVMFLLVMFFSPLVAMVPPYATAGALIFVGVLMTSSLARVNWDDFTESVPAFITTVM

MPFTFSITEGIALGFMSYCIMKVCTGRWRDLNLCVVVVAALFALKIILVD

>gi|30064976|ref|NP_839147.1| hypothetical protein S3970 [Shigella flexneri 2a str. 2457T]

MSSDYAGELMIWIMLATLAVVFVVGFRVLTSGARKAIRRLSDRLNIDVVPVESMVDQMGKSAGDEFLRYL

HRPDESHLQNAAQVLLIWQIVIVDDSEQNLLQWHRILQKARLSAPITDAQVRLALGFLRETEPEMQDINA

FQMRYNAFFQPAEGVHWLH

>gi|30064968|ref|NP_839139.1| major fimbrial subunit [Shigella flexneri 2a str. 2457T]

MKRNIIGGAFTLASLMLAGHALAEDGVVNFVGEIVDTTCEVTSDTADQIVPLGKVSKNAFSGVGSLASPQ

KFSIKLENCPATYTQAAVRFDGTEAPGGDGDLKVGTPLTAGNPGDFTGTGQAIAATGVGIRIFNQSDNSQ

VKLYNDSAYTAIDAEGKAEMKFIARYVATNATVTAGTANADSQFTVEYKK

>gi|30064965|ref|NP_839136.1| bifunctional N-acetylglucosamine-1-phosphate uridyltransferase/glucosamine-1-phosphate acetyltransferase [Shigella flexneri 2a str. 2457T]

MLNNAMSVVILAAGKGTRMYSDLPKVLHTLAGKAMVQHVIDAANELGAAHVHLVYGHGGDLLKQALKDDN

LNWVLQAEQLGTGHAMQQAAPFFADDEDILMLYGDVPLISVETLQRLRDAKPQGGIGLLTVKLDDPTGYG

RITRENGKVTGIVEHKDATDEQRQIQEINTGILIANGADMKRWLAKLTNNNAQGEYYITDIIALAYQEGR

EIVAVHPQRLSEVEGVNNRLQLSRLERVYQSEQAEKLLLAGVMLRDPARFDLRGTLTHGRDVEIDTNVII

EGNVTLGHRVKIGTGCVIKNSVIGDDCEISPYTVVEDANLAAACTIGPFARLRPGAELLEGAHVGNFVEM

KKARLGKGTKAGHLTYLGDAEIGDNVNIGAGTITCNYDGANKFKTIIGDDVFVGSDTQLVAPVTVGKGAT

IAAGTTVTRNVGENALAISRVPQTQKEGWRRPVKKK

>gi|30064964|ref|NP_839135.1| F0F1 ATP synthase subunit epsilon [Shigella flexneri 2a str. 2457T]

MTYHLDVVSAEQQMFSGLVEKIQVTGSEGELGIYPGHAPLLTAIKPGMIRIVKQHGHEEFIYLSGGILEV

QPGNVTVLADTAIRGQDLDEARAMEAKRKAEEHISSSHGDVDYAQASAELAKAIAQLRVIELTKKAM

>gi|30064959|ref|NP_839130.1| F0F1 ATP synthase subunit B [Shigella flexneri 2a str. 2457T]

MNLNATILGQAIAFVLFVLFCMKYVWPPLMAAIEKRQKEIADGLASAERAHKDLDLAKASATDQLKKAKA

EAQVIIEQANKRRSQILDEAKAEAEQERTKIVAQAQAEIEAERKRAREELRKQVAILAVAGAEKIIERSV

DEAANSDIVDKLVAEL

>gi|30064957|ref|NP_839128.1| F0F1 ATP synthase subunit A [Shigella flexneri 2a str. 2457T]

MASENMTPQDYIGHHLNNLQLDLRTFSLVDPQNPPATFWTINIDSMFFSVVLGLLFLVLFRSVAKKATSG

VPGKFQTAIELVIGFVNGSVKDMYHGKSKLIAPLALTIFVWVFLMNLMDLLPIDLLPYIAEHVLGLPALR

VVPSADVNVTLSMALGVFILILFYNIKMKGIGGFTKELTLQPFNHWAFIPVNLILEGVSLLSKPVSLGLR

LFGNMYAGELIFILIAGLLPWWSQWILNVPWAIFHILIITLQAFIFMVLTIVYLSMASEEH

>gi|30064955|ref|NP_839126.1| 16S rRNA methyltransferase GidB [Shigella flexneri 2a str. 2457T]

MLNKLSLLLKDAGISLTDHQKNQLIAYVNMLHKWNKAYNLTSVRDPNEMLVRHILDSIVVAPYLQGERFI

DVGTGPGLPGIPLSIVRPEAHFTLLDSLGKRVRFLRQVQHELKLENIEPVQSRVEEFPSEPPFDGVISRA

FASLNDMVSWCHHLPGEQGRFYALKGQMPEDEIALLPEEYQVESVVKLQVPALDGERHLVVIKANKI

>gi|30064952|ref|NP_839123.1| DNA-binding transcriptional regulator AsnC [Shigella flexneri 2a str. 2457T]

MENYLIDNLDRGILEALMGNARTAYAELAKQFGVSPGTIHVRVEKMKQAGIITGARIDVSPKQLGYDVGC

FIGIILKSAKDYPSALAKLESLDEVTEAYYTTGHYSIFIKVMCRSIDALQHVLINKIQTIDEIQSTETLI

VLQNPIMRTIKP

>gi|30064951|ref|NP_839122.1| asparagine synthetase AsnA [Shigella flexneri 2a str. 2457T]

MKTAYIAKQRQISFVKSHFSRQLEERLGLIEVQAPILSRVGDGTQDNLSGCEKAVQVKVKALPDAQFEVV

HSLAKWKRQTLGQHDFSAGEGLYTHMKALRPDEDRLSPLHSVYVDQWDWERVMGDGERQLSTLKSTVEAI

WAGIKATEAAVNEEFGLAPFLPDQIHFVHSQELLSRYPDLDAKGRERAIAKDLGAVFLVGIGGKLSDGHR

HDVRAPDYDDWSTPSELGHAGLNGDILVWNPVLEDAFELSSMGIRVDADTLKHQLALTGDEDRLQLEWHQ

ALLRGEMPQTIGGGIGQSRLTMLLLQLPHIGQVQCGVWPAAVRESVPSLL

>gi|30064947|ref|NP_839118.1| potassium transport protein Kup [Shigella flexneri 2a str. 2457T]

MSTDNKQSLPAITLAAIGVVYGDIGTSPLYTLRECLSGQFGFGVERDAVFGFLSLIFWLLIFVVSIKYLT

FVMRADNAGEGGILTLMSLAGRNTSARTTSMLVIMGLIGGSFFYGEVVITPAISVMSAIEGLEIVAPQLD

TWIVPLSIIVLTLLFMIQKHGTAMVGKLFAPIMLTWFLILAGLGLRSIIANPEVLHALNPMWAVHFFLEY

KTVSFIALGAVVLSITGGEVLYADMGHFGKFSIRLAWFTVVLPSLTLNYFGQGALLLKNPEAIKNPFFLL

APDWALIPLLIIAALATVIASQAVISGVFSLTRQAVRLGYLSPMRIIHTSEMESGQIYIPFVNWMLYVAV

VIVIVSFEHSSNLAAAYGIAVTGTMVLTSILSTTVARQNWHWNKYFVALILIAFLCVDIPLFTANLDKLL

SGGWLPLSLGTVMFIVMTTWKSERFRLLRRMHEHGNSLEAMIASLEKSPPVRVPGTAVYMSRAINVIPFA

LMHNLKHNKVLHERVILLTLRTEDAPYVHNVRRVQIEQLSPTFWRVVASYGWRETPNVEEVFHRCGLEGL

SCRMMETSFFMSHESLILGKRPWYLRLRGKLYLLLQRNALRAPDQFEIPPNRVIELGTQVEI

>gi|30064943|ref|NP_839114.1| ribose ABC transporter permease [Shigella flexneri 2a str. 2457T]

MITQTVSGRRYFTKAWLMEQKSLIALLVLIAIVSTLSPNFFTINNLFNILQQTSVNAIMAVGMTLVILTS

GIDLSVGSLLALTGAVAASIVGIEVNALVAVAAALTLGAAIGAVTGVIVAKGRVQAFIATLVMMLLLRGV

TMVYTNGSPVNTGFTENADLFGWFGIGRPLGVPTPVWIMGIVFLAAWYMLHHTRLGRYIYALGGNEAATR

LSGINVNKIKIIVYSLCGLLASLAGIIEVARLSSAQPTAGTGYELDAIAAVVLGGTSLAGGKGRIVGTLI

GALILGFLNNGLNLLGVSSYYQMIVKAVVILLAVLVDNKKQ

>gi|30064941|ref|NP_839112.1| transcriptional repressor RbsR [Shigella flexneri 2a str. 2457T]

MKDVARLAGVSTSTVSHVINKDRFVSEAITAKVEAAIKELNYAPSALARSLKLNQTHTIGMLITASTNPF

YSELVRGVERSCFERGYSLVLCNTEGDEQRMNRNLETLMQKRVDGLLLLCTETHQPSREIMQRYPTVPTV

MMDWAPFDGDSDLIQDNSLLGGDLATQYLIDKGHTRIACITGPLDKTPARLRLEGYRAAMKRAGLNIPDG

YEVTGDFEFNGGFDAMRQLLSHPLRPQAVFTGNDAMAVGVYQALYQAELQVPQDIAVIGYDDIELASFMT

PPLTTIHQPKDELGELAIDVLIHRITQPTLQQQRLQLTPILMERGSA

>gi|30064938|ref|NP_839109.1| transcriptional regulator HdfR [Shigella flexneri 2a str. 2457T]

MDTELLKTFLEVSRTRHFGRAAESLYLTQSAVSFRIRQLENQLGVNLFTRHRNNIRLTAAGEKLLPYAET

LMSTWQAARKEVAHTSRHNEFSIGASASLWECMLNQWLGRLYQNQDAHTGLQFEARIAQRQSLVKQLHER
QLDLLITTEAPKMDEFSSQLLGYFTLALYTSAPSKLKGDLNYLRLEWGPDFQQHEAGLIGADEVPILTTS
SAELAQQQIAMLNGCTWLPVSWARKKGGLHTVVDSTTLSRPLYAIWLQNSDKNALIRDLLKINVLDEVY

>gi|30064937|ref|NP_839108.1| hypothetical protein S3920 [Shigella flexneri 2a str. 2457T]
MAESFTTTNRYFDNKHYPRGFSRHGDFTIKEAQLLERHGYAFNELDLGKREPVTEEEKLFVAVCRGEREP
VTEAERVWSKYMTRIKRPKRFHTLSGGKPQVEGAEDYTDSDD

>gi|30064932|ref|NP_839103.1| dihydroxy-acid dehydratase [Shigella flexneri 2a str. 2457T]
MPKYRSATTTHGRNMAGARALWRATGMTDADFGKPIIAVVNSFTQFVPGHVHLRDLGKLVAEQIEAAGGV
AKEFNTIAVDDGIAMGHGGMLYSLPSRELIADSVEYMVNAHCADAMVCISNCDKITPGMLMASLRLNIPV
IFVSGGPMEAGKTKLSDQIIKLDLVDAMIQGADPKVSDSQSDQVERSACPTCGSCSGMFTANSMNCLTEA
LGLSQPGNGSLLATHADRKQLFLNAGKRIVELTKRYYEQNDESALPRNIASKAAFENAMTLDIAMGGSTN
TVLHLLAAAQEAEIDFTMSDIDKLSRKVPQLCKVAPSTQKYHMEDVHRAGGVIGILGELDRAGLLNRDVK
NVLGLTLPQTLEQYDVMLTQDDAVKNMFRAGPAGIRTTQAFSQDCRWDSLDDDRANGCIRSLEHAYSKDG
GLAVLYGNFAENGCIVKTAGVDDSILKFTGPAKVYESQDDAVEAILGGKVVAGDVVVIRYEGPKGGPGMQ
EMLYPTSFLKSMGLGKACALITDGRFSGGTSGLSIGHVSPEAASGGSIGLIEDGDLIAIDIPNRGIQLQV
SDAELAARREAQEARGDKAWTPKNRERQVSFALCAYASLATSADKGAVRDKSKLGG

>gi|30064930|ref|NP_839101.1| DNA-binding transcriptional regulator IlvY [Shigella flexneri 2a str. 2457T]

MDLRDLKTFLHLAESRHFGRSARAMHVSPSTLSRQIQRLEEDLGQPLFVRDNRTVTLTEAGEELRVFAQQ

TLLQYQQLRHTIDQQGPLLYGELHIFCSVTAAYSHLPPILDRFRAEHPSVEIKLTTGDAADAMEKVVTGE

ADLAIAGKPETLPGAVAFSMLENLAVVLIAPALPCPVRNQVSVEKPDWSTVPFIMADQGPVRRRIELWFR

RNKISNPMIYATVGGHEAMVSMVALGCGVALLPEVVLENSPEPVRNRVMILERSDEKTPFELGVCAQKKR

LHEPLIEAFWKILPNH

>gi|30064929|ref|NP_839100.1| ketol-acid reductoisomerase [Shigella flexneri 2a str. 2457T]

MANYFNTLNLRQQLAQLGKCRFMGRDEFADGASYLQGKKVVIVGCGAQGLNQGLNMRDSGLDISYALRKE

AIAEKRASWRKATENGFKVGTYEELIPQADLVVNLTPDKQHSDVVRTVQPLMKDGAALGYSHGFNIVEVG

EQIRKDITVVMVAPKCPGTEVREEYKRGFGVPTLIAVHPENDPKGEGMAIAKAWAAATGGHRAGVLESSF

VAEVKSDLMGEQTILCGMLQAGSLLCFDKLVEEGTDPAYAEKLIQFGWETITEALKQGGITLMMDRLSNP

AKLRAYALSEQLKEIMAPLFQKHMDDIISGEFSSGMMADWANDDKKLLTWREETGKTAFETAPQYEGKIG

EQEYFDKGVLMIAMVKAGVELAFETMVDSGIIEESAYYESLHELPLIANTIVRKRLYEMNVVISDTAEYG

NYLFSYACVPLLKPFMAELQPGDLGKAIPEGAVDNAQLRDVNEAIRSHAIEQVGKKLRGYMTDMKRIAVA

G

>gi|30064927|ref|NP_839098.1| ATP-dependent DNA helicase Rep [Shigella flexneri 2a str. 2457T]

MRLNPGQQQAVEFVTGPCLVLAGAGSGKTRVITNKIAHLIRGCGYQARHIAAVTFTNKAAREMKERVGQT

LGRKEAHGLMISTFHTLGLDIIKREYAALGMKANFSLFDDTDQLALLKELTEGLIEDDKVLLQQLISTIS

NWKNDLKTPSQAAASAIGERDRIFAHCYGLYDAHLKACNVLDFDDLILLPTLLLQRNEEVRERWQNKIRY

LLVDEYQDTNTSQYELVKLLVGSRARFTVVGDDDQSIYSWRGARPQNLVLLSQDFPALKVIKLEQNYRSS

GRILKAANILIANNPHVFEKRLFSELGYGTELKVLSANNEEHEAERVTGELIAHHFVNKTQYKDYAILYR

GNYQSRVFEKFLMQNRIPYKISGGTSFFSRPEIKDLLAYLRVLTNPDDDSAFLRIVNTPKREIGPATLKK

LGEWAMTRNKSMFTASFDMGLSQTLSGRGYEALTRFTHWLAEIQRLAEREPIAAVRDLIHGMDYESWLYE

TSPSTKAAEMRMKNVNQLFSWMTEMLEGSELDEPMTLTQVVTRFTLRDMMERGESEEELDQVQLMTLHAS

KGLEFPYVYMVGMEEGFLPHQSSIDEDNIDEERRLAYVGITRAQKELTFTLCKERRQYGELVRPEPSRFL

LELPQDDLIWEQERKVVSAEERMQKGQSHLANLKAMMAAKRGK

>gi|30064926|ref|NP_839097.1| guanosine pentaphosphate phosphohydrolase [Shigella flexneri 2a str. 2457T]

MGSTSSLYAAIDLGSNSFHMLVVREVAGSIQTLTRIKRKVRLAAGLNSENDLSNEAMERGWQCLRLFAER

LQDIPPSQIRVVATATLRLAVNAGDFIAKAQEILGCPVQVISGEEEARLIYQGVAHTTGGADQRLVVDIG

GASTELVTGTGAQTTSLFSLSMGCVTWLERYFADRNLGQENFDAAEKAAREVLRPVADELRYHGWKVCVG

ASGTVQALQEIMMAQGMDERITLEKLQQLKQRAIHCGRLEELEIDGLTLERALVFPSGLAILIAIFTELN

IQCMTLAGGALREGLVYGMLHLAVEQDIRSRTLRNIQRRFMIDIDQAQRVAKVAANFFDQVENEWHLEAI

SRDLLISACQLHEIGLSVDFKQAPQHAAYLVRNLDLPGFTPAQKKLLATLLLNQTNPVDLSSLHQQNAVP

PRVAEQLCRLLRLAIIFASRRRDDLVPEMTLQANHELLTLTLPQGWLTQHPLGKEIIAQENQWQSYVHWP

LEVH

>gi|30064922|ref|NP_839093.1| transcription termination factor Rho [Shigella flexneri 2a str. 2457T]

MNLTELKNTPVSELITLGENMGLENLARMRKQDIIFAILKQHAKSGEDIFGDGVLEILQDGFGFLRSADS

SYLAGPDDIYVSPSQIRRFNLRTGDTISGKIRPPKEGERYFALLKVNEVNFDKPENARNKILFENLTPLH

ANSRLRMERGNGSTEDLTARVLDLASPIGRGQRGLIVAPPKAGKTMLLQNIAQSIAYNHPDCVLMVLLID

ERPEEVTEMQRLVKGEVVASTFDEPASRHVQVAEMVIEKAKRLVEHKKDVIILLDSITRLARAYNTVVPA

SGKVLTGGVDANALHRPKRFFGAARNVEEGGSLTIIATALIDTGSKMDEVIYEEFKGTGNMELHLSRKIA

EKRVFPAIDYNRSGTRKEELLTTQEELQKMWILRKIIHPMGEIDAMEFLINKLAMTKTNDDFFEMMKRS

>gi|30064921|ref|NP_839092.1| UDP-GlcNAc:undecaprenylphosphate GlcNAc-1-phosphate transferase [Shigella flexneri 2a str. 2457T]

MNLLTVSTDLISIFLFTTLFLFFARKVAKKVGLVDKPNFRKRHQGLIPLVGGISVYAGICFTFGIVDYYI

PHASLYLACAGVLVFIGALDDRFDISVKIRATIQAAVGIVMMVFGKLYLSSLGYIFGSWEMVLGPFGYFL

TLFAVWAAINAFNMVDGIDGLLGGLSCVSFAAIGMILWFDGQTSLAIWCFAMIAAILPYIMLNLGILGRR

YKVFMGDAGSTLIGFTVIWILLETTQGKTHPISPVTALWIIAIPLMDMVAIMYRRLRKGMSPFSPDRQHI

HHLIMRAGFTSRQAFVLITLAAALLASIGVLAEYSHFVPEWVMLVLFLLAFFLYGYCIKRAWKVARFIKR

VKRRLRRNRGGSPNLTK

>gi|30064920|ref|NP_839091.1| lipopolysaccharide biosynthesis protein WzzE [Shigella flexneri 2a str. 2457T]

MTQPMPGKPAEDAENELDIRGLFRTLWAGKLWIIGMGLAFALIALAYTFFARQEWSSTAITDRPTVNMLG

GYYSQQQFLRNLDVRSNMASADQPSVMDEAYKEFVMQLASWDTRREFWLQTDYYKQRMVGNSKADAALLD

EMINNIQFIPGDFTRAVNDSVKLIAETAPDANNLLRQYVAFASQRAASHLNDELKGAWAARTIQMKAQVK

RQEEVAKAIYDRRMNSIEQALKIAEQHNISRSATDVPAEELPDSEMFLLGRPMLQARLENLQAVGPAFDL

DYDQNRAMLNTLNVGPTLDPRFQTYRYLRTPEEPVKRDSPRRAFLMIMWGIVGGLIGAGVALTRRCSK

>gi|30064915|ref|NP_839086.1| TDP-fucosamine acetyltransferase [Shigella flexneri 2a str. 2457T]

MQAKIAASNTGELDALQQLGFSLVEGEVDLALPVNNVSDSGAVVAQETDIPALRQLASAAFAQSRFRAPW

YAPDASGRFYAQWIENAVRGTFDHQCLILRAASGDIRGYVSLRELNATDARIGLLAGRGAGAELMQTALN

WAYARGKTTLRVATQMGNTAALKRYIQSGANVESTAYWLYR

>gi|30064914|ref|NP_839085.1| TDP-4-oxo-6-deoxy-D-glucose transaminase [Shigella flexneri 2a str. 2457T]

MIPFNAPPVVGTELDYMQSAMGSGKLCGDGGFTRRCQQWLEQRFGSAKVLLTPSCTASLEMAALLLDIQP

GDEVIMPSYTFVSTANAFVLRGAKIVFVDVRPDTMNIDETLIEAAITDKTRVIVPVHYAGVACEMDTIMA

LAKKHNLFVVEDAAQGVMSTYKGRALGTIGHIGCFSFHETKNYTAGGEGGATLINDKALIERAEIIREKG

TNRSQFFRGQVDKYTWRDIGSSYLMSDLQAAYLWAQLEAADRINQQRLALWQNYYDTLAPLAKAGRIELP

SIPDGCVQNAHMFYIKLRDIDDRSALINFLKEAEIMAVFHYIPLHGCPAGEHFGEFHGEDRYTTKESERL

LRLPLFYNLSPVNQRTVIATLLNYFS

>gi|30064913|ref|NP_839084.1| cytochrome [Shigella flexneri 2a str. 2457T]

MSLAKASLWTAASTLVKIGAGLLVGKLLAVSFGPAGLGLAANFRQLITVLGVLAGAGIFNGVTKYVAQYH

DNPQQLRRVVGTSSAMVLGFSTLMALVFVLAAAPISQGLFGNTDYQGLVRLVALVQMGIAWGNLLLALMK

GFRDAAGNALSLIVGSLIGVLAYYVSYRLGGYEGALLGLALIPALVVIPAAIMLIKRGVIPLSYLKPSWD

NGLAGQLSKFTLMALITSVTLPVAYIMMRKLLAAQYSWDEVGIWQGVSSISDAYLQFITASFSVYLLPTL

SRLTEKRDITREVVKSLKFVLPAVAAASFTVWLLRDFAIWLLLSNKFTAMRDLFAWQLVGDVLKVGAYVF

GYLVIAKASLRFYILAEVSQFTLLMVFAHWLIPAHGALGAAQAYMATYIVYFSLCCGVFLLWRRRA

>gi|30064912|ref|NP_839083.1| 4-alpha-L-fucosyltransferase [Shigella flexneri 2a str. 2457T]

MTVLIHVLGSDIPHHNRTVLRFFNDALAATSEHAREFMVVGKDDGLSDSCPALSVQFFPGKKSLAEAVIA

KAKANRQQRFFFHGQFNPTLWLALLSGGIKPSQFFWHIWGADLYELSSGLRYKLFYPLRRLAQKRVGCVF

ATRGDLSFFAKTHPKVRGELLYFPTRMDPSLNTMANDRQREGKMTILVGNSGDRSNEHVAALRAVHQQFG

DTVKVVVPMGYPPNNEAYIEEVRQAGLELFSEENLQILSEKLEFDAYLALLRQCDLGYFIFARQQGIGTL

CLLIQAGIPCVLNRENPFWQDMTEQHLPVLFTTDDLNEDIVREAQRQLASADKNTIAFFSPNYLQGWQRA

LAIAAGEVA

>gi|30064911|ref|NP_839082.1| common antigen polymerase [Shigella flexneri 2a str. 2457T]

MSLLQFSGLFVVWLLCTLFIATLTWFEFRRVRFNFNVFFSLLFLLTFFFGFPLTSVLVFRFDVGVAPPEI

LLQVLLSAGCFYAVYYVTYKTRLRKRVADVPRRPLFTMNRVETNLTWVILMGIALVSVGIFFMHNGFLLF

RLNSYSQIFSSEVSGVALKRFFYFFIPAMLVVYFLRQDSKAWLFFLVSTVAFGLLTYMIVGGTRANIIIA

FAIFLFIGIIRGWISLWMLAAAGVLGIVGMFWLALKRYGMNVSGDEAFYTFLYLTRDTFSPWENLALLLQ

NYDNIDFQGLAPIVRDFYVFIPSWLWPGRPSMVLNSANYFTWEVLNNHSGLAISPTLIGSLVVMGGALFI

PLGAIVVGLIIKWFDWLYELGNRETNRYKAAILHSFCFGAIFNMIVLAREGLDSFVSRVVFFIVVFGACL

MIAKLLYWLFESAGLIHKRTKSSLRTQVEG

>gi|30064910|ref|NP_839081.1| UDP-N-acetyl-D-mannosaminuronic acid transferase [Shigella flexneri 2a str. 2457T]

MNNNTTAPTYTLRGLQLIGWRDMQHALDYLFADGQLKQGTLVAINAEKMLTIEDNAEVRELINAAEFKYA

DGISIVRSVRKKYPQAQVSRVAGADLWEELMARAGKEGTPVFLVGGKPEVLAQTEAKLRNQWNVNIVGSQ

DGYFKPEQRQALFERIHASGAQIVTVAMGSPKQEIFMRDCRLVHPDALYMGVGGTYDVFTGHVKRAPKIW

QTLGLEWLYRLLSQPSRIKRQLRLLRYLRWHYTGNL

>gi|30064908|ref|NP_839079.1| arylsulfatase regulator [Shigella flexneri 2a str. 2457T]

MLQQVPTRAFHVIAKPSGSDCNLNCDYCFYLEKQSLYREKPVTHMDDDTLEAYIRHYIAASELQNEVAFT

WQGGEPTLLGLEFYRRAVALQAKYGAGRKISNSFQTNGVLLDDEWCAFLAENNFLVGLSLDGPAEIHNQY

RVTKGGRPTHKLVMRALTLLQKHHVDYNVLVCVNRTSAQQPLQVYDFLCDAGVEFIQFIPVVERLADETA

VRDGLKLHAPGDIQGELTEWSVRPDEFGEFLVAIFDHWIKRDVGKIFVMNIEWAFANFVGAPGAVCHHQP

TCGRSVIVEHNGDVYACDHYVYPQYRLGNMHQQTIAEMIDSPQQQVFGEDKFKQLPAQCRSCNVLKACWG

GCPKHRFMLDASGKPGLNYLCAGYQRYFRHLPPYLKAMADLLAHGRPASDIMQAHLMVVNK

>gi|30064905|ref|NP_839076.1| protoheme IX biogenesis protein [Shigella flexneri 2a str. 2457T]

MLKVLLLFVLLIAGIVVGPMIAGHQGYVLIQTDNYNIETSVTGLAIILILAMVVLFAIEWLLRRIFRTGA

HTRGWFVGRKRRRARKQTEQALLKLAEGDYQQVEKLMAKNADHAEQPVVNYLLAAEAAQQRGDEARANQH

LERAAELAGNDTIPVEITRVRLQLARNENHAARHGVDKLLEVTPRHPEVLRLAEQAYIRTGAWSSLLDII

PSMAKAHVGDEEHRAMLEQQAWIGLMDQARADNGSEGLRNWWKNQSRKTRHQVALQVAMAEHLIECDDHD

TAQQIIIDGLKRQYDDRLLLPIPRLKTNNPEQLEKVLRQQIKNVGDRPLLWSTLGQSLMKHGEWQEASLA

FRAALKQRPDAYDYAWLADALDRLHKPEEAAAMRRDGLMLTLQNNPPQ

>gi|30064904|ref|NP_839075.1| uroporphyrinogen III C-methyltransferase [Shigella flexneri 2a str. 2457T]

MTEQEKTSAVVEETREAVDTTSQPVATEKKSKNNTALILSAVAIAIALAAGVGLYGWGKQQAVNQTATSD

ALANQLTALQKAQESQKAELEGIIKQQAAQLKQANRQQETLAKQLDEVQQKIATISGSDAKTWLLAQADF

LVKLAGRKLWSDQDVTTSAALLKSADASLADMNDPSLITVRRAITDDIASLSAVSQVDYDGIILKLNQLS

NQVDNLRLADNDSDGSPMDSDGEELSSSISEWRINLQKSWQNFMDNFITIRRRDDTAVPLLAPNQDIYLR

ENIRSRLLVAAQAVPRHQEETYRQALENVSTWVRAYYDTDDATTKAFLDEVDQLSQQNISMDLPETLQSQ

AMLEKLMQTRVRNLLAQPAAGTTEAKPAPAPAPAPAPQGDTPAAAPQGE

>gi|30064903|ref|NP_839074.1| uroporphyrinogen-III synthase [Shigella flexneri 2a str. 2457T]

MSILVTRPSPAGEELVSRLRTLGQVAWHFPLIEFSPGRQLPQLADQLAALGESDLLFALSQHAVAFAQSQ

LHQQDRKWPQLPTYFAIGRTTALALHTVSGQKILYPQDREISEVLLQLPELQNIAGKRALILRGNGGREL

IGDTLTVRGAEVTFCECYQRCAIHYDGAEEAMRWQSREVTMVVVTSGEMLQQLWSLIPQWYREHWLLHCR

LLVVSERLAKLARELGWQDIKVADNADNDALLRALQ

>gi|30064901|ref|NP_839072.1| adenylate cyclase [Shigella flexneri 2a str. 2457T]

MYLYIETLKQRLDAINQLRVDRALAAMGPAFQQVYSLLPTLLHYHHPRMPGYLDGNVPKGICLYTPDETQ

RHYLNELELYRGMSVQDPPKGELPITGVYTMGSTSSVGQSCSSDLDIWVCHQSWLDSEERQLLQRKCSLL

ESWAASLGVEVSFFLIDENRFRHNESGSLGGEDCGSTQHILLLDEFYRTAVRLAGKRILWNMVPCDEEEH

YDDYVMTLYAQGVLTPNEWLDLGGLSSLSAEEYFGASLWQLYKSIDSPYKAVLKTLLLEAYSWEYPNPRL

LAKDIKQRLHDGEIVSFGLDPYCMMLERVTEYLTAIEDFTRLDLVRRCFYLKVCEKLSRERACVGWRRAV

LSQLVSEWGWDEARLAMLDNRANWKIDQVREAHNELLDAMMQSYRNLIRFARRNNLSVSASPQDIGVLTR

KLYAAFEALPGKVTLVNPQISPDLSEPNLTFIYVPPGRANRSGWYLYNRAPNIESIISHQPLEYNRYLNK

LVAWAWFNGLLTSRTRLYIKGNGIVDLPKLQEMVADVSHHFPLRLPAPTPKALYSPCEIRHLAIIVNLEY

DPTAAFRNQVVHFDFRKLDVFSFGENQNCLVGSVDLLYRNSWNEVRTLHFNGEQSMIEALKTILGKMHQD

AAPPDSVEVFCYSQHLRGLIRTRVQQLVSECIELRLSSTRQETGRFKALRVSGQTWGLFFERLNVSVQKL

ENAIEFYGAISHNKLHGLSVQVETNHVKLPAVVDGFASEGIIQFFFEETQDENGFNIYILDESNRVEVYH

HCEGSKEELVRDVSRFYSSSHDRFTYGSSFINFNLPQFYQIVKVDGREQVIPFRTKSIGNLPPANQDHDT

PLLQQYFS

>gi|30064894|ref|NP_839065.1| hypothetical protein S3868 [Shigella flexneri 2a str. 2457T]

MKQPGEELQETLTELDDRAVVDYLIKNPEFFIRNARAVEAIRVPHPVRGTVSLVEWHMARARNHIHVLEE

NMALLMEQAIANEGLFYRLLYLQRSLTAASSLDDMLMRFHRWARDLGLAGASLRLFPDRWRLGAPSNHTH

LALSRQSFEPLRIQRLGQEQHYLGPLNGPELLVVLPEAKAVGSVAMSMLGSDADLGVVLFTSRDASHYQQ

GQGTQLLHEIALMLPELLERWIERV

>gi|30064893|ref|NP_839064.1| site-specific tyrosine recombinase XerC [Shigella flexneri 2a str. 2457T]

MTDLHTDVERYLRYLSVERQLSPITLLNYQRQLEAIINFASENGLQSWQQCDAAMVRNFAVRSRRKGLGA

ASLALRLSALRSFFDWLVSQNELKANPAKGVSAPKAPRHLPKNIDVDDMNRLLDIDINDPLAVRDRAMLE

VMYGAGLRLSELVGLDIKHLDLESGEVWVMGKGSKERRLPIGRNAVAWIEHWLDLRDLFGSEDDALFLSK

LGKRISARNVQKRFAEWGIKQGLNNHVHPHKLRHSFATHMLESSGDLRGVQELLGHANLSTTQIYTHLDF

QHLASVYDAAHPRAKRGK

>gi|30064891|ref|NP_839062.1| DNA-dependent helicase II [Shigella flexneri 2a str. 2457T]

MDVSYLLDSLNDKQREAVAAPRSNLLVLAGAGSGKTRVLVHRIAWLMSVENCSPYSIMAVTFTNKAAAEM

RHRIGQLMGTSQGGMWVGTFHGLAHRLLRAHHMDANLPQDFQILDSEDQLRLLKRLIKAMNLDEKQWPPR

QAMWYINSQKDEGLRPHHIQSYGNPVEQTWQKVYQAYQEACDRAGLVDFAELLLRAHELWLNKPHILQHY

RERFTNILVDEFQDTNNIQYAWIRLLAGDTGKVMIVGDDDQSIYGWRGAQVENIQRFLNDFPGAEIIRLE

QNYRSTSNILSAANALIENNNGRLGKKLWTDGADGEPISLYCAFNELDEARFVVNRIKTWQDNGGALAEC

AILYRSNAQSRVLEEALLQASMPYRIYGGMRFFERQEIKDALSYLRLIANRNDDAAFERVVNTPTRGIGD

RTLDVVRQTSRDRQLTLWQACRELLQEKALAGRAASALQRFMELIDALAQETADMPLHVQTDRVIKDSGL

RTMYEQEKGEKGQTRIENLEELVTATRQFSYNEEDEDLMPLQAFLSHAALEAGEGQADTWQDAVQLMTMH

SAKGLEFPQVFIVGMEEGMFPSQMSLDEGGRLEEERRLAYVGVTRAMQKLTLTYAETRRLYGKEVYHRPS

RFIGELPEECVEEVRLRATVSRPVSHQRMGTPMVENDSGYKLGQRVRHAKFGEGTIVNMEGSGEHSRLQV

AFQGQGIKWLVAAYARLESV

>gi|30064888|ref|NP_839059.1| hypothetical protein S3862 [Shigella flexneri 2a str. 2457T]

MAHRLLIGKGMITLNLKRIFLTLTLLPLFAVAADDCALSDPTLTVQAYTVNPQTERVKMYWQKANGEAWG

TLHALLADINSQGQVQMAMNGGIYDESYAPLGLYIENGQQKV

>gi|30064887|ref|NP_839058.1| magnesium/nickel/cobalt transporter CorA [Shigella flexneri 2a str. 2457T]

MLSAFQLENNRLTRLEVEESQPLVNAVWIDLVEPDDDERLRVQSELGQSLATRPELEDIEASARFFEDDD

GLHIHSFFFFEDAEDHAGNSTVAFTIRDGRLFTLRERELPAFRLYRMRARSQSMVDGNAYELLLDLFETK

IEQLADEIENIYSDLEQLSRVIMEGHQGDEYDEALSTLAELEDIGWKVRLCLMDTQRALNFLVRKARLPG

GQLEQAREILRDIESLLPHNESLFQKVNFLMQAAMGFINIEQNRIIKIFSVVSVVFLPPTLVASSYGMNF

EFMPELKWSFGYPGAIIFMILAGLAPYLYFKRKNWL

>gi|30064886|ref|NP_839057.1| hypothetical protein S3860 [Shigella flexneri 2a str. 2457T]

MSKEYMNDDSLSEKWKYRFNFYDQHGFPGFWGATPEYKAAFKALKVRQRLTIQMNFIAFFCSWIYLFVLG

LWKKAIIVLLLGILSLFVGALIGVNILGIAVAAYVAVNTNKWFYEKEVKGLNTWSL

>gi|30064885|ref|NP_839056.1| hypothetical protein S3859 [Shigella flexneri 2a str. 2457T]

MLTGDSHKDVKFMLRMFIPTSNGKISRHRYIFLFILINFIFAFLIIFFNDGEAGFLVIVSTIALHYLVIN

MNCQRLRDSGFIYIKIYVFGTLAVYIISIITMIAEDFACSGNGSMIFLICYFSTFSMLMLAPTDSSKQ

>gi|30064884|ref|NP_839055.1| hypothetical protein S3858 [Shigella flexneri 2a str. 2457T]

MDAKQTRQGVLLALAAYFIWGIAPAYFKLIYYVPADEILTHRVIWSFFFMVVLMSICRQWSYLKTLIQTP

QKIFMLAVSAVLIGGNWLLFIWAVNNHHMLEASLGYFINPLVNIVLGMIFLGERFRRMQWLAVILAICGV

LVQLWTFGSLPIIALGLAFSFAFYGLVRKKIAVEAQTGMLIETMWLLPVAAIYLFAIADSSTSHMGQNPM

SLNLLLIAAGIVTTVPLLCFTAAATRLRLSTLGFFQYIGPTLMFLLAVTFYGEKPGADKMVTFTFIWVAL

AIFVMDAIYTQRRTSK

>gi|30064882|ref|NP_839053.1| phospholipase A [Shigella flexneri 2a str. 2457T]

MRTLQGWLLPVFMLPMAVYAQEATVKEVHDAPAVRGSIIANMLQEHDNPFTLYPYDTNYLIYTQTSDLNK

EAIASYDWAENARKDEVKFQLSLAFPLWRGILGPNSVLGASYTQKSWWQLSNSEESSPFRETNYEPQLFL

GFATDYRFAGWTLRDVEMGYNHDSNGRSDPTSRSWNRLYTRLMAENGNWLVEVKPWYVVGNTDDNPDITK

YMGYYQLKIGYHLGDAVLSAKGQYNWNTGYGGAELGLSYPITKHVRLYTQVYSGYGESLIDYNFNQTRVG

VGVMLNDLF

>gi|30064877|ref|NP_839048.1| sugar phosphatase [Shigella flexneri 2a str. 2457T]

MYQVVASDLDGTLLSPDHTLSPYAKETLKLLTARGINFVFATGRHHVDVGQIRDNLEIKSYMITSNGARV

HDLDGNLIFAHNLDRDIASDLFGVVNDNPDIITNVYRDDEWFMNRHRPEEMRFFKEAVFKYALYEPGLLE

PEGISKVFFTCDSHEQLLPLEQAINARWGDRVNVSFSTLTCLEVMAGGVSKGHALEAVAKKLGYSLKDCI

AFGDGMNDAEMLSMAGKGCIMGSAHQRLKDLHPELEVIGTNADDAVPHYLRKLYLS

>gi|30064876|ref|NP_839047.1| hypothetical protein S3850 [Shigella flexneri 2a str. 2457T]

MALLIITTILWAFSFSFYGEYLAGHVDSYFAVLVRVGLAALVFLPFLRTRSNSLKTVGLYMLVGAMQLGV

MYMLSFRAYLYLTVSELLLFTVLTPLYITLIYDIMSKRRLRWGYAFSALLAVIGAGIIRYDQVTDHFWTG

LLLVQLSNITFAIGMVGYKRLMETRPMPQYNAFAWFYLGAFLVAVIAWFLLGNAQKMPQTTLQWGILVFL

GVVASGIGYFMWNYGATQVDAGTLGIMNNMHVPAGLLVNLAIWHQQPHWPTFITGALVILASLWVHRKWV

APRSSQTADDRRRDCALSE

>gi|30064875|ref|NP_839046.1| regulator for metE and metH [Shigella flexneri 2a str. 2457T]

MIEVKHLKTLQALRNCGSLAAAAATLHQTQSALSHQFSDLEQRLGFRLFVRKSQPLRFTPQGEILLQLAN

QVLPQISQALQACNEPQQTRLRIAIECHSCIQWLTPALGNFHKNWPQVEMDFKSGVTFDPQPALQQGELD

LVMTSDILPRSGLHYSPMFDYEVRLVLAPDHPLAAKTRITPEDLASETLLIYPVQRSRLDVWRHFLQPAG

VSPSLKSVDNTLLLIQMVAARMGIAALPHWVVESFERQGLVVTKTLGEGLWSRLYAAVRDGEQRQPVTEA

FIRSARNHACDHLPFVKSAERPTYDAPTVRPGSPARL

>gi|30064874|ref|NP_839045.1| 5-methyltetrahydropteroyltriglutamate/homocysteine S-methyltransferase [Shigella flexneri 2a str. 2457T]

MTILNHTLGFPRVGLRRELKKAQESYWAGNSTREELLTVGRELRARHWDQQKQAGIDLLPVGDFAWYDHV

LTTSLLLGNVPPRHQNKDGSVDIDTLFRIGRGRAPTGEPAAAAEMTKWFNTNYHYMVPEFVKGQQFKLTW

TQLLEEVDEALALSHNVKPVLLGPVTYLWLGKVKGEQFDRLSLLNDILPVYQQVLAELAKRGIEWVQIDE

PALVLELPQAWLDAYKPAYDALQGQVKLLLTTYFEGVTPNLDTITALPVQGLHVDLVHGKDNVVELHKRL

PSDWLLSAGLINGRNVWRADLTEKYAQIKDIVGKRDLWVASSCSLLHSPIDLSVETRLDAEVKSWFAFAL

QKCHELALLRDALNSGDTAALAEWSAPIQARRHSTRVHNPAVEKRLAAITAQDSQRANVYEVRAEAQRAR

FKLPAWPTTTIGSFPQTTEIRTLRLDFKKGNLDANNYRTGIAEHIKQAIVEQERLGLDVLVHGEAERNDM

VEYFGEHLDGFVFTQNGWVQSYGSRCVKPPIVIGDISRPAPITVEWAKYAQSLTDKPVKGMLTGPVTILC

WSFPREDVSRETIAKQIALALRDEVADLEAAGIGIIQIDEPALREGLPLRRSDWDAYLQWGVEAFRINAA

VAKDDTQIHTHMCYCEFNDIMDSIAALDADVITIETSRSDMELLESFEEFDYPNEIGPGVYDIHSPNVPS

VEWIEALLKKAAKRIPAERLWVNPDCGLKTRGWPETRAALANMVQAAQNLRRG

>gi|30064873|ref|NP_839044.1| hypothetical protein S3847 [Shigella flexneri 2a str. 2457T]

MNIAAVAGSPTGDMIEIGQHLRVRHFCYQTAKQRGDIGKIIRIAFAEVKFRCDSQIPLQRQTATNVADMF

MHAKNFLHYNDHWQWAIALLWSGMVSRHVIPL

>gi|30064871|ref|NP_839042.1| DNA recombination protein RmuC [Shigella flexneri 2a str. 2457T]

MVYAVIALVGVAIGWLFASYQHAQQKAEQLAEREEMVAELSAAKQQITQSEHWRAECELLNNEVRSLQSI

NTSLEADLREVTTRMEAAQQHADDKIRQMINSEQRLSEQFENLANRIFEHSNRRVDEQNRQSLNSLLSPL

REQLDGFRRQVQDSFGKEAQERHTLTHEIRNLQQLNAQMAQEAINLTRALKGDNKTQGNWGEVVLTRVLE

ASGLREGYEYETQVSIENDARSRMQPDVIVRLPQGKDVVIDAKMTLVAYERYFNAEDDYTRESALQEHIA

SVRNHIRLLGRKDYQQLPGLRTLDYVLMFIPVEPAFLLALDRQPELITEALKNNIMLVSPTTLLVALRTI

ANLWRYEHQSRNAQQIADRASKLYDKMRLFIDDMSAIGQSLDKAQDNYRQAMKKLSSGRGNVLAQAEAFR

GLGVEIKREINPDLAEQAVSQDEEYRLRSVPEQPNDEAYQRDDEYNQQSR

>gi|30064869|ref|NP_839040.1| hypothetical protein S3842 [Shigella flexneri 2a str. 2457T]

MPFKPLVTAGIESLLNTFLYRSPALKTARSRLLGKVLRVEVKGFSTSLILVFSERQVDVLGEWAGDADCT

VIAYASVLPKLRDRQQLTALIRSGELEVQGDIQVVQNFVALADLAEFDPAELLAPYTGDIAAEGISKAMR

GGAKFLHHGIKRQQRYVAEAITEEWRMAPGPLEVAWFAEETAAVERAVDALTKRLEKLEAK

>gi|30064867|ref|NP_839038.1| twin arginine translocase A [Shigella flexneri 2a str. 2457T]

MRLCLIIIYHRGTCMGGISIWQLLIIAVIVVLLFGTKKLGSIGSDLGASIKGFKKAMSDDEPKQDKTSQD

ADFTAKTIADKQADTNQEQAKTEDAKRHDKEQV

>gi|30064866|ref|NP_839037.1| sec-independent translocase [Shigella flexneri 2a str. 2457T]

MFDIGFSELLLVFIIGLVVLGPQRLPVAVKTVAGWIRALRSLATTVQNELTQELKLQEFQDSLKKVEKAS

LTNLTPELKASMDELRQAAESMKRSYVANDPEKASDEAHTIHNPVVKDNEAAHEGVTPAAAQTQASSPEQ

KPETTPEPVVKPAADAEPKTAAPSPSSSDKP

>gi|30064865|ref|NP_839036.1| twin-arginine protein translocation system subunit TatC [Shigella flexneri 2a str. 2457T]

MSVEDTQPLITHLIELRKRLLNCIIAVIVIFLCLVYFANDIYHLVSAPLIKQLPQGSTMIATDVASPFFT

PIKLTFMVSLILSAPVILYQVWAFIAPALYKHERRLVVPLLVSSSLLFYIGMAFAYFVVFPLAFGFLANT

APEGVQVSTDIASYLSFVMALFMAFGVSFEVPVAIVLLCWMGITSSEDLRKKRPYVLVGAFVVGMLLTPP

DVFSQTLLAIPMYCLFEIGVFFSRFYVGKGRNREEENDAEAESEKTEE

>gi|30064863|ref|NP_839034.1| transcriptional activator RfaH [Shigella flexneri 2a str. 2457T]

MQSWYLLYCKRGQLQRAQEHLERQAVNCLAPMITLEKIVRGKRTAVSEPLFPNYLFVEFDPEVIHTTTIS

ATRGVSHFVRFGASPAIVPSAVIHQLSVYKPKDIVDPAPPYPGDKVIITEGAFEGFQAIFTEPDGEARSM

LLLNLINKEIKHSVKNTEFRKL

>gi|30064862|ref|NP_839033.1| 3-octaprenyl-4-hydroxybenzoate carboxy-lyase [Shigella flexneri 2a str. 2457T]

MDAMKYNDLRDFLTLLEQQGELKRITLPVDPHLEITEIADRTLRAGGPALLFENPKGYSMPVLCNLFGTP

KRVAMGMGQEDVSALREVGKLLAFLKEPEPPKGFRDLFDKLPQFKQVLNMPTKRLRGAPCQQKIVSGDDV

DLNRIPIMTCWPEDAAPLITWGLTVTRGPHKERQNLGIYRQQLIGKNKLIMRWLSHRGGALDYQEWCAAH

PGERFPVSVALGADPATILGAVTPVPDTLSEYAFAGLLRGTKTEVVKCISNDLEVPASAEIVLEGYIDPG

EMAPEGPYGDHTGYYNEVDNFPVFTVTHITQREDAIYHSTYTGRPPDEPAVLGVALNEVFVPILQKQFPE

IVDFYLPPEGCSYRLAVVTIKKQYAGHAKRVMMGVWSFLRQFMYTKFVIVCDDDVNARDWNDVIWAITTR

MDPARDTVLVENTPIDYLDFASPVSGLGSKMGLDATNKWPGETQREWGRPIKKDPDVVAHIDAIWDELAI

FNNGKSA

>gi|30064861|ref|NP_839032.1| FMN reductase [Shigella flexneri 2a str. 2457T]

MTTLSCKVTSVAAITDTVYRVRIVPDAAFSFRAGQYLMVVMDERDKRPFSMASTPDEKGFIELHIGASEI

NLYAKAVMDRILKDHQIVVDLPHGEAWLRDDEERPMILIAGGTGFSYARSILLTALARNPNRDITIYWGG

REEQHLYDLCELEALSLKHLGLQVVPVVEQPEAGWRGRTGTVLTAVLQDHGTLAEHDIYIAGRFEMAKIA

RDLFCSERNAREDRLFGDAFAFI

>gi|30064857|ref|NP_839028.1| hypothetical protein S3828 [Shigella flexneri 2a str. 2457T]

MESWLIPAAPVTVVEEIKKSRFITMLAHTDGVEAAKAFVESVRAEHPDARHHCVAWVAGAPDDSQQLGFS

DDGEPAGTAGKPMLAQLMGSGVGEITAVVVRYYGGILLGTGGLVKAYGGGVNQALRQLTTQRKTPLTEYT

LQCEYSQLTGIEALLGQCDGKIINSDYQAFVLLRVALPAAKVAEFSAKLADFSRGSLQLLAIEE

>gi|30064856|ref|NP_839027.1| potassium transporter [Shigella flexneri 2a str. 2457T]

MHFRAITRIVGLLVILFSGTMIIPGLVALIYRDGAGRAFTQTFFVALAIGSMLWWPNRKEKGELKSREGF

LIVVLFWTVLGSVGALPFIFSESPNLTITDAFFESFSGLTTTGATTLVGLDSLPHAILFYRQMLQWFGGM

GIIVLAVAILPILGVGGMQLYRAEMPGPLKDNKMRPRIAETAKTLWLIYVLLTVACALALWFAGMDAFDA

IGHSFATIAIGGFSTHDASIGYFDSPTINTIIAIFLLISGCNYGLHFSLLSGRSLKVYWRDPEFRMFIGV

QFTLVVICTLVLWFHNVYSSALMTINQAFFQVVSMATTAGFTTDSIARWPLFLPVLLLCSAFIGGCAGST

GGGLKVIRILLLFKQGNRELKRLVHPNAVYSIKLGNRALPERILEAVWGFFSAYALVFIVSMLAIIATGV

DDFSAFASVVATLNNLGPGLGVVADNFTSMNPVAKWILIANMLFGRLEVFTLLVLFTPTFWRE

>gi|30064855|ref|NP_839026.1| protoporphyrinogen oxidase [Shigella flexneri 2a str. 2457T]

MKTLILFSTRDGQTREIASYLASELKELGIQTDVANVHRIEEPQWENYDRVVIGASIRYGHYHSAFQEFV

KKHATRLNSMPSAFYSVNLVARKPEKRTPQTNSYARKFLMNSQWRPDRCAVIAGALRYPRYRWYDRFMLK

LIMKMSGGETDTRKEVVYTDWEQVANFAREIAHLTDKPTLK

>gi|30064854|ref|NP_839025.1| molybdopterin-guanine dinucleotide biosynthesis protein B [Shigella flexneri 2a str. 2457T]

MAGKTMIPLLAFAAWSGTGKTTLLKKLIPALCARGIRPGLIKHTHHDMDVDKPGKDSYELRKAGAVQTIV

ASQQRWALMTETPDEEELDLHFLASRMDTSKLDLILVEGFKHEEIAKIVLFRDGAGHRPEELVIDRHVIA

VASDVPLNLDVALLDINDVEGLADFVVEWMQKQNG

>gi|30064853|ref|NP_839024.1| molybdopterin-guanine dinucleotide biosynthesis protein A [Shigella flexneri 2a str. 2457T]

MNLMTTITGVVLAGGKARRMGGVDKGLLDLNGKPLWQHVADALKTQLSHVVVNANRHQEIYQAGGLKVIE

DSLADYPGPLAGMLSVMQQEAGEWFLFCPCDTPYIPHDLAARLNHQRKDAPVVWVHDGERDHPTIALVNR

AIEPLLLEYLQAGERRVMAFMRLAGGHAVDFSDHKDAFVNVNTPEELARWQEKR

>gi|30064851|ref|NP_839022.1| serine/threonine protein kinase [Shigella flexneri 2a str. 2457T]

MNNSAFTFQTLHPDTIMDALFKQGIRVDSGLTPLNSYENRVYQFQDEERRRFVVKFYRPERWTADQILEE

HQFALQLVNDEVPVAAPVAFNGQTLLNHQGFYFAVFPSVGGRQFEADNIDQMEAVGRYLGRMHQTGRKQL

FIHRPTIGLNEYLIEPRKLFEDATLIPSGLKAAFLKATDELIAAVTAHWREDFTVLRLHGDCHAGNILWR

DGPMFVDLDDARNGPAIQDLWMLLNGDKAQQRMQLETIIEAYEEFSEFDTAEIGLIEPLRAMRLVYYLAW

LMRHWADPAFPKNFPWLTGEDYWLRQTATFIEQAKVLQEPPLQLTPMY

>gi|30064850|ref|NP_839021.1| periplasmic protein disulfide isomerase I [Shigella flexneri 2a str. 2457T]

MKKIWLALAGLVLAFSASAAQYEDGKQYTTLEKPVAGAPQVLEFFSFFCPHCYQFEEVLHISDNVKKKLP

EGVKMTKYHVNFMGGDLGKDLTQAWAVAMALGVEDKVTVPLFEGVQKTQTIRSASDIRDVFINAGIKGEE

YDAAWNSFVVKSLVAQQEKAAADVQLRGVPAMFVNGKYQLNPQGMDTSNMDVFVQQYADTVKYLSEEK

>gi|30064849|ref|NP_839020.1| hypothetical protein S3815 [Shigella flexneri 2a str. 2457T]

MDIQSFAVLSGNIYMIRKSATGVIVALAVIWGGGTWYTGTQIQPGIEKFIKDFNDAKKKGEHAYDMTLSY
KNFDKGFFNSRFQMQMTFDNGAPDLNIKPGQKVVFDVDVEHGPLPITMLMHGNVIPALAAAKVNLVNNEL
TQPLFIAAKNKSPVEATLRFAFGGSFSTTLDVAPAEYGKFSFGEGQFTFNGDGSSLSNLDIEGKVEDIVL
QLSPMNKVTAKSFTIDSLARLEEKKFPVGESESKFNQINIINHGEDVAQIDAFVAKTRLDRVKDKDYINV
NLTYELDKLTKGNQQLGSGEWSLIAESIDPSAVRQFIIQYNIAMQKQLAAHPELANDEVALQEVNAALFK
EYLPLLQKSEPTIKQPVRWKNALGELNANLDISIADPAKSSSSTNKDIKSLNFDVKLPLNVVTETAKQLN
LSEGMDAEKAQKQADKQISGMMTLGQMFQLITIDNNTASLQLRYTPGKVVFNGQEMSEEEFMSRAGRFVH

>gi|30064845|ref|NP_839016.1| hypothetical protein S3810 [Shigella flexneri 2a str. 2457T]
MKPSSSNSRSKGHAKARRKTREELDQEARDRKRQKKRRGHAPGSRAAGGNTTSGSKGQNAPKDPRIGSKT
PIPLGVTEKVTKQHKPKSEKPMLSPQAELELLETDERLDALLERLEAGETLSAEEQSWVDAKLDRIDELM
QKLGLSYDDDEEEEEDEKQEDMMRLLRGN

>gi|30064843|ref|NP_839014.1| nitrogen regulation protein NR(I) [Shigella flexneri 2a str. 2457T]
MQRGIVWVVDDDSSIRWVLERALAGAGLTCTTFENGAEVLEALASKTPDVLLSDIRMPGMDGLALLKQIK
QRHPMLPVIIMTAHSDLDAAVSAYQQGAFDYLPKPFDIDEAVALVERAISHYQEQQQPRNIQLNGPTTDI
IGEAQAMQDVFRIIGRLSRSSISVLINGESGTGKELVAHALHRHSPRTKAPFIALNMAAIPKDLIESELF
GHEKGAFTGANTIRQGRFEQADGGTLFLDEIGDMPLDVQTRLLRVLADGQFYRVGGYAPVKVDVRIIAAT
HQNLEQRVQEGKFREDLFHRLNVIRVHLPPLRERREDIPRLARHFLQVAARELGVEAKLLHPETEAALTR
LAWPGNVRQLENTCRWLTVMAAGQEVLIQDLPGELFESTVAESTSQMQPDSWATLLAQWADRALRSGHQN
LLSEAQPELERTLLTTALRHTQGHKQEAARLLGWGRNTLTRKLKELGME

>gi|30064842|ref|NP_839013.1| nitrogen regulation protein NR(II) [Shigella flexneri 2a str. 2457T]

MATGTQPDAGQILNSLINSILLIDDNLAIHYANPAAQQLLAQSSRKLFGTPLPELLSYFSLNIELMQESL

EAGQGFTDNEVTLVIDGRSHILSVTAQRMPDGMILLEMAPMDNQRRLSQEQLQHAQQVAARDLVRGLAHE

IKNPLGGLRGAAQLLSKALPDPSLLEYTKVIIEQADRLRNLVDRLLGPQLPGTRVTESIHKVAERVVTLV

SMELPDNVRLIRDYDPSLPELAHDPDQIEQVLLNIVRNALQALGPEGGEIILRTRTAFQLTLHGERYRLA

ARIDVEDNGPGIPPHLQDTLFYPMVSGREGGTGLGLSIARNLIDQHSGKIEFTSWPGHTEFSVYLPIRK

>gi|30064839|ref|NP_839010.1| transcriptional regulator [Shigella flexneri 2a str. 2457T]

MAENQSTVENAKEKLDRWLKDGITTPGGKLPSERELGELLGIKRMTLRQALLNLEAESKIFRKDRKGWFV

TQPRFNYSPELSASFQRAAIEQGREPSWGFTEKNRTSDIPETLAPLIAVTPSTELYRITGWGALEGHKVF

YHETYINPEVAPGFIEQLENHSFSAVWEKCYQKETVVKKLIFKPVRMPGDISKYLGGSAGMPAILIEKHR

ADQQGNIVQIDIEYWRFEAVDLIINL

>gi|30064838|ref|NP_839009.1| hypothetical protein S3803 [Shigella flexneri 2a str. 2457T]

MVTINNARKILQRVDTLPLYLHAYAFHLNMRLERVLPADLLDIASENNLRGVKIHVLDGERFSLGNMDDK

ELSAFGDKARRLNLDIHIETSASDKASIDEAVAIALKTGATSVRFYPRYEGNLRDVLSIIANDIAYVRET

YQDSGLTFTIEQHEDLKSHELVSLVKESEMESLSLLFDFANMINANEHPIDALKTMAPHITQVHIKDALI

VKEQGGLGHKACISGQGDMPFKALLTHLICLGDDEPQVTAYGLEEEVDYYASAFRFEDEDDNPWIPYRQM

SETPLPENHLLDARLRKEKEDAINQINHVRNVLQQIKQEANHLLNH

>gi|30064837|ref|NP_839008.1| resistance protein, partial [Shigella flexneri 2a str. 2457T]

MLTKKKWALFSLLTLCGGTIYKLPSLKDAFYIPMQEYFHLTNGQIGNAMSVNSFVTTVGFFLSIYFADKL

PRRYTMSFSLIATGLLGVYLTTMPGYWGILFVWALFGVTCDMMNWPVLLKSVSRLGNSEQQGRLFGFFET

GRGIVDTVVAFSALAVFTWFGSGLLGFKAGIWFYSLIVIAVGIIIFFVLNDKEEAPSVEVKKEDGASQNT

SMTSVLRDKTIWLIAFNVFFVYAVYCGLTFFIPFLKNIYLLPVALVGAYGIINQYCLKMIGGPIGGMISD

KILKSPSKYLCYTFIISTAALVLLIMLPHESMPVYLGMACTLGFGAIVFTQRAVFFAPIGEAKIGNAANL

LI

>gi|30064832|ref|NP_839003.1| aldose-1-epimerase [Shigella flexneri 2a str. 2457T]

MQITNMHCSGQTVSLAAGDYHATIVTVGAGLAELTFQGCHLVIPHKPEEMPLAHLGKVLIPWPNRIANGC

YRYQGQEYQLPINEHSSKAAIHGLLAWRDWQISELTTTSVTLTAFLPPSYGYPFMLASQVVYSLNAHTGL

SVEIASQNIGTVAAPYGVGIHPYLTCNLTSVDEYLFQLPANQVYAVDEHVNPTTLHHVDELDLNFTQAKK

IAATKIDHTFKTANDLWEMTITHPQQALSVSLCSDQPWVQVYSGEKLQRQGLAVEPMSCPPNAFNSGIDL

LLLEPGKPHRLFFNIYGQRK

>gi|30064831|ref|NP_839002.1| hypothetical protein S3794 [Shigella flexneri 2a str. 2457T]

MKWFNTLSHNRWLEQETDRIFDFGKNSVVPTGFGWLGNKGQIKEEMGTHLWITARMLHVYSVAAAMGRPG

AYALVDHGIKAMNGALRDKKYGGWYACVNDEGVVDASKQGYQHFFALLGAASAVTTGHPEARKLLDYTIE

IIEKYFWSEEEQMCLESWDEAFSKTEEYRGGNANMHAVEAFLIVYDVTHDKKWLDRAIRVASVIIHDVAR

NNHYRVNEHFDTQWNPLPDYNKDNPAHRFRAFGGTPGHWIEWGRLMLHIHAALEARCEQPPAWLLEDAKG

LFNATVRDAWAPDGADGIVYTVDWEGKPVVRERVRWPIVEAMGTAYALYTVTGDRQYETWYQTWWEYCIK

YLMDYENGSWWQELDADNKVITKVWDGKQDIYHLLHCLVIPRIPLAPGMAPAVAAGLLDINAK

>gi|30064830|ref|NP_839001.1| aldolase [Shigella flexneri 2a str. 2457T]

MNKYTINDITRASGGFAMLAVDQREAMRMMFAAAGAPAPVADSVLTDFKVNAAKALSPYASAILVDQQFC

YRQVVEQNAIAKSCAMIVAADEFIPGNGIPVDSVVIDRKINPLQIKQDGGKALKLLVLWRSDEDAQQRLD

MVKEFNELCHSHGLVSIIEPVVRPPRRGDKFDREQAIIDAAKELGDSGADLYKVEMPLYGKGPQQELLCA

SQRLNDHINMPWVILSSSVDEKLFPRAVRVAMTAGASGFLAGRAVWASVVGLPDNELMLRDVCAPKLQQL

GDIVDEMMAKRR

>gi|30064827|ref|NP_838998.1| DEOR-type transcriptional regulator [Shigella flexneri 2a str. 2457T]

MSLTELTGNPRHDQLLMLIAERGYMNIDELANLLDVSTQTVRRDIRKLSEQGLITRHHGGAGRASSVVNT

AFEQREVSQTEKKKAIAEAVADYIPDGSTIFITIGTTVEHVARALLNHNHLRIITNSLRVAHILYHNPRF

EVMVPGGTLRSHNSGIIGPSAASFVADFRADYLVTSVGAIESDGALMEFDVNEANVVKTMMAHARNILLV

ADHTKYHASAAVEIGNVAQVTALFTDELPPAALKSRLQDSQIEIILPQEDA

>gi|30064825|ref|NP_838996.1| ribonuclease BN [Shigella flexneri 2a str. 2457T]

MLKTIQDKARHRTRPLWAWLKLLWQRIDEDNMTTLAGNLAYVSLLSLVPLVAVVFALFAAFPMFSDVSIQ

LRHFIFANFLPATGDVIQRYIEQFVANSNKMTAVGACGLIVTALLLMYSIDSALNTIWRSKRARPKIYSF

AVYWMILTLGPLLAGASLAISSYLLSLRWASDLNTVIDNVLRIFPLLLSWISFWLLYSIVPTIRVPNRDA

IVGAFVAALLFEAGKKGFALYITMFPSYQLIYGVLAVIPILFVWVYWTWCIVLLGAEITVTLGEYRKLKQ

AAEQEEDDEP

>gi|30064823|ref|NP_838994.1| acetyltransferase [Shigella flexneri 2a str. 2457T]

MSQLPGLSRETRESIAMYHLQVPQTEEELERYYHFRWEMLRKPLHQPKGSERDAWDAMAHHQMVVDEQGN

LVAVGRLYINADNEASIRFMVVHPDVQDKGLGTLMAMTLESVARQEGVKRVTCSAREDAVEFFAKLGFVN

QGEITTPTTTPIRHFLMIKPVATLDDILHRGDWCAQLQQAWYEHIPLSEKMGVRIQQYTGQKFITTMPET

GNQNPHHTLFAGSLFSLATLTGWGLIWLMLRERHLGGTIILADAHIRYSKPISGKPHAVADLGALSGDLD

RLARGRKARVQMQVEIFGDETPGAVFEGTYIVLPAKPFGPYEEGGNEEE

>gi|30064819|ref|NP_838990.1| hypothetical protein S4827 [Shigella flexneri 2a str. 2457T]

MCERMKGGISMFNHTTKTADNIEHNLQLSFLVGSRPSITNKYSAKDVGPDILALPVNVYHADFKIKINNA

VEKASSIVLCAVQKNLHAARKVLSGIIGNVPKNNKPKTIIFDLRFLSAIKQKVFLGSEKPLFVKHTAMMR

EACKELPQSVEYVPLACEAHSHRSVALAKTVAAPIMATGVGLALMYATSSGPYYAQSLSGSPDIEMIKNR

MAELFNQSDISVRNKYSPAFDKWNELVDKLNHERNTVPFACLTTILATAINETLEGDTNVAVVMGCKSAK

DRTISIVLGNSMLQTLLEKRLADSGEIENLFDQQGYFSCDSLTPEELMMLKDLFDIRVLHVSNKFNVGLQ

GNINTDVLQDSFFKNVDFIHEYNSYTVGMGV

>gi|30064816|ref|NP_838987.1| formate dehydrogenase accessory protein FdhE [Shigella flexneri 2a str. 2457T]

MSIRIIPQDELGSSEKRTADMIPPLLFPRLKNLYNRRAERLRELAENNPLGDYLRFAALIAHAQKVVLYD

HPLEMDLTARIKEASAQGKPPLDIHVLPRDKHWQKLLMALIAELKPEMSGPALAVIENLEKASTQELEDM

ASALFASDFSSVSSDKAPFIWAALSLYWAQMANLIPGKARAEYGEQRQYCPVCGSMPVSSMVQIGTTQGL

RYLHCNLCETEWHVVRVKCSNCEQSGKLHYWSLDDEQAAIKAESCDDCGTYLKILYQEKEPKVEAVADDL

ASLVLDARMEQEGYARSSINPFLFPGEGE

>gi|30064815|ref|NP_838986.1| formate dehydrogenase-O subunit gamma [Shigella flexneri 2a str. 2457T]

MKRRDTIVRYTAPERINHWITAFCFILAAVSGLGFLFPSFNWLMQIMGTPQLARILHPFVGVVMFASFII

MFFRYWHQNLINRDDIFWAKNIRKIVVNEEVGDTGRYNFGQKCVFWAAIIFLVLLLVSGVIIWRPYFAPA

FSIPVIRFALMLHSFAAVALIVVIMVHIYAALWVKGTITAMVEGWVTSAWAKKHHPRWYREVRKTTEKKA

E

>gi|30064814|ref|NP_838985.1| formate dehydrogenase-O, iron-sulfur subunit [Shigella flexneri 2a str. 2457T]

MAYQSQDIIRRSATNGLTPAPQARDFQEEVAKLIDVTTCIGCKACQVACSEWNDIRDTVGNNIGVYDNPN

DLSAKSWTVMRFSEVEQNDKLEWLIRKDGCMHCSDPGCLKACPAEGAIIQYANGIVDFQSEQCIGCGYCI

AGCPFDIPRLNPEDNRVYKCTLCVDRVVVGQEPACVKTCPTGAIHFGTKESMKTLASERVAELKTRGYDN

AGLYDPAGVGGTHVMYVLHHADKPNLYHGLPENPEISETVKFWKGIWKPLAAVGFAATFAASIFHYVGVG

PNRADEEENNLHEEKDEERK

>gi|30064813|ref|NP_838984.1| formate dehydrogenase-O, major subunit [Shigella flexneri 2a str. 2457T]

MQVSRRQFFKICAGGMAGTTAAALGFAPSVALAETRQYKLLRTRETRNTCTYCSVGCGLLMYSLGDGAKN

AKASIFHIEGDPDHPVNRGALCPKGAGLVDFIHSESRLKFPEYRAPGSDKWQQISWEEAFDRIAKLMKED

RDANYIAQNAEGVTVNRWLSTGMLCASASSNETGYLTQKFSRALGMLAVDNQARVUHGPTVASLAPTFGR

GAMTNHWVDIKNANLVVVMGGNAAEAHPVGFRWAMEAKIHNGAKLIVIDPRFTRTAAVADYYAPIRSGTD

IAFLSGVLLYLLNNEKFNHEYTEAYTNASLIVREDYGFEDGLFTGYDAEKRKYDKSSWTYELDENGFAKR

DTTLQHPRCVWNLLKQHVSRYTPDVVENICGTPKDAFLKVCEYIAETSAHDKTASFLYALGWTQHSVGAQ

NIRTMAMIQLLLGNMGMAGGGVNALRGHSNIQGLTDLGLLSQSLPGYMTLPSEKQTDLQTYLTANTPKPL

LEGQVNYWGNYPKFFVSMMKAFFGDKATAENSWGFDWLPKWDKGYDVLQYFEMMKEGKVNGYICQGFNPV

ASFPNKNKVIGCLSKLKFLVTIDPLNTETSNFWQNHGELNEVDSSKIQTEVFRLPSTCFAEENGSIVNSG

RWLQWHWKGADAPGIALTDGEILSGIFLRLRKMYAEQGGANPDQVLNMTWNYAIPHEPKSEEVAMESNGK

ALADITDPATGAVIVKKGQQLSSFAQLRDDGTTSCGCWIFAGSWTPEGNQMARRDNADPSGLGNTLGWAW

AWPLNRRILYNRASADPQGNPWDPKRQLLKWDGTKWTGWDIPDYSAAPPGSGVGPFIMQQEGMGRLFALD

KMAEGPFPEHYEPFETPLGTNPLHPNVISNPAARIFKDDAEALGKADKFPYVGTTYRLTEHFHYWTKHAL

LNAILQPEQFVEIGESLANKLGIAQGDTVKVSSNRGYIKAKAVVTKRIRTLKANGKDIDTIGIPIHWGYE

GVAKKGFIANTLTPFVGDANTQTPEFKSFLVNVEKV

>gi|30064812|ref|NP_838983.1| formate dehydrogenase accessory protein [Shigella flexneri 2a str. 2457T]

MKKTQRKEIENVTNITGVRQIELWRRDDLQHPRLDEVAEEVPVALVYNGISHVVMMASPKDLEYFALGFS

LSEGIIESPRDIFGMDVVPSCNGLEVQIELSSRRFMGLKERRRALAGRTGCGVCGVEQLNDIGKPVQPLP

FTQTFDLNKLDDALRHLNDFQPVGRLTGCTHAAAWMLPSGELVGGHEDVGRHVALDKLLGRRSQEGESWQ

QGAVLVSSRASYEMVQKSAMCGVEILFAVSAATTLAVEVAERCNLTLVGFCKPGRATVYTHPQRLSN

>gi|30064809|ref|NP_838980.1| frv operon regulatory protein, partial [Shigella flexneri 2a str. 2457T]

MLNERQLKIVDLLEQQPRTPGELAQQTGVSGRTILRDIDYLNFTLNGKARIFASGSAGYQLEIFERRSFF

QLLQKHDNDDRLLALLLLNTFTPRAQLASALNLPETWVAERLPQLKQRYERTCCLASRPGLGHFIDETEE

KLVILLANLLRKDPFLIPLAGITRDNLQHLSTACDNQHRWPLIQGDYLSSLILAIYALRNQLTDEWPQYP

VMLPTY

>gi|30064808|ref|NP_838979.1| fructose-specific phosphotransferase system protein FrvX [Shigella flexneri 2a str. 2457T]

MNIELLQQLCEASAVSGDEQEVRDILINTLEPCVNEITFDGLGSFVARKGNKGPKVAVVGHMDEVGFMVT

HIDESGFLRFTTIGGWWNQSMLNHRVTIRTHKGVKIPGVIGFVAPHALTEKQRQQPLSFDEMFIDIGANS

RDEVEKRGVAMGDFISPEANFACWGEDKVVGKALDNRIGCAMMAELLQTVNNPEITLYGVGSVEEEVGLR

GAQTSAEHIKPDVVIVLDTAVAGDVPGIDNIKYPLKLGQGPGLMLFDKRYFPNQKLVAALKNCAAHNDLP

LQFSTMKTGATDGGRYNVMGGGRPVVALCLPTRYLHANSGMISKADYDALLTLIRGFLTTLTAEKVNAFS

QFRQVD

>gi|30064806|ref|NP_838977.1| fructose-like phosphotransferase system subunit EIIA [Shigella flexneri 2a str. 2457T]

MAALTASCIDLNIQGNGAYSVLKQLATIALQNGFITDSHQFLQTLLLREKMHSTGFGSGVAVPHGKSACV

KQPFVLFARKAQAIDWKASDGEDVNCWICLGVPQSGEEDQVKIIGTLCRKIIHQEFIHQLQQGDTDQVLA

LLNQTLSS

>gi|30064805|ref|NP_838976.1| hypothetical protein S3770 [Shigella flexneri 2a str. 2457T]

MIRKAFVMQVNPDAHEEYQRRHNPIWPELEAVLKSHGVHNYAIYLDKARNLLFAMVEIESEERWNAVAST

DVCQRWWKYMTDVMPANPDNSPVSSELQEVFYLP

>gi|30064804|ref|NP_838975.1| rhamnulose-1-phosphate aldolase, partial [Shigella flexneri 2a str. 2457T]

MPLLANTPFIVTGSGKFFRNVQLDPAANLGVVKVDSDGAGYHILWGLTNEAVPTSELPAHFLSHCERIKA

TNGKDRVIMHCHATNLIALTYVLENDTAVFTRQLWEGSTECLVVFPDGVGILPWMVPGTDEIGQATAQEM

QKHSLVLWPFHGVFGSGPTLDEAFGLIDTAEKSAQVLVKVYSMGGMKQTISREELIALGKRFGVTPLASA

LAL

>gi|30064803|ref|NP_838974.1| L-rhamnose isomerase [Shigella flexneri 2a str. 2457T]

MTTQLEQAWELAKQRFAAVGIDVEEALRQLDRLPVSMHCWQGDDVSGFENPEGSLTGGIQATGNYPGKAR

NASELRADLEQAMRLIPGPKRLNLHAIYLESDTPVSRDQIKPEHFKNWVEWAKANQLGLDFNPSCFSHPL

SADGFTLSHPDDSIRQFWIDHCKASRRVSAYFGEQLGTPSVMNIWIPDGMKDITVDRLAPRQRLLAALDE

VISEKLNPAHHIDAVESKLFGIGAESYTVGSNEFYMGYATSRQTALCLDAGHFHPTEVISDKISAAMLYV

PQLLLHVSRPVRWDSDHVVLLDDETQAIASEIVRHDLFDRVHIGLDFFDASINRIAAWVIGTRNMKKALL

RALLEPTAELRKLEAAGDYTARLALLEEQKSLPRQAVWEMYCQHHDTPAGSEWLESVRAYEKAILSQRG

>gi|30064802|ref|NP_838973.1| rhamnulokinase [Shigella flexneri 2a str. 2457T]

MTFRNCVAVDLGASSGRVMLARYERECRSLTLREIHRFNNGLHSQNGYVTWDVDSLESAIRLGLNKVCEE
GIRIDSIGIDTWGVDFVLLDQQGQRVGLPVAYRDSRTNGLMPQAQQQLGKRDIYQRSGIQFLPFNTLYQL
RALTEQQPELIPHIAHALLMPDYFSYRLTGKMNWEYTNATTTQLVNINSDDWDESLLAWSGANKAWFGRP
THPGNVIGHWICPQGNEIPVVAVASHDTASAVIASPLNGSRAAYLSSGTWSLMGFESQTPFTNDTALAAN
ITNEGGAEGRYRVLKNIMGLWLLQRVLQERQINDLPALIAATQALPACRFIINPNDDRFINPDEMCSEIQ
AACRETAQPIPGSDAELARCIFDSLALLYADVLHELAQLRGEDFSQLHIVGGGCQNTLLNQLCADACGIR
VIAGPVEASTLGNIGIELMTLDELNNVDDFRQVVSTTANLTTFTPNPDSEIAHYVAQIHSTRQTKELCA

>gi|30064801|ref|NP_838972.1| transcriptional activator RhaS [Shigella flexneri 2a str. 2457T]
MTVLHSVDFFPSCNASVAIEPRLPQADFPEHHHDFHEIVIVEHGTGIHVFNGQPYTITGGTVCFVRDHDR
HLYEHTDNLCLTNVLYRSPDRFQFLAGLNQLLPQELDGQYPSHWRVNHSVLQQVRQLVAQMEQQEGENDL
PSTASREILFMQLLLLLRKSSLQENLENSASRLNLLLAWLEDHFADEVNWDAVADQFSLSLRTLHRQLKQ
QTGLTPQRYLNRLRLMKARHLLRHSEASVTDIAYRCGFSDSNHFSTLFRREFNWSPRDIRQGRDGFLQ

>gi|30064800|ref|NP_838971.1| transcriptional activator RhaR [Shigella flexneri 2a str. 2457T]
MAFCNNANLLNVFVRHIANNQLRSLAEVATVAHQLKLLKDDFFASDQQAVAVADRYPQDVFAEHTHDFCE
LVIVWRGNGLHVLNDRPYRITRGDLFYIHADDKHSYASVNDLVLQNIIYCPERLKLNLDWQGAIPGFSAS
AGQPHWRLGSVGMAQARQVIGQLEHESSQHVPFANEMAELLFGQLVMLLNRHRYTSDSLPPTSSETLLDK
LITRLAASLKSPFALDKFCDEASCSERVLRQQFRQQTGMTINQYLRQVRVCHAQYLLQHSRLLISDISTE
CGFEDSNYFSVVFTRETGMTPSQWRHLNSQKD

>gi|30064798|ref|NP_838969.1| 2-keto-3-deoxygluconate permease [Shigella flexneri 2a str. 2457T]
MQIKRSIEKIPGGMMLVPLFLGALCHTFSPGAGKYFGSFTNGMITGTVPILAVWFFCMGASIKLSATGTV
LRKSGTLVVTKIAVAWVVAAIASRIIPEHGVEVGFFAGLSTLALVAAMDMTNGGLYASIMQQYGTKEEAG
AFVLMSLESGPLMTMIILGTAGIASFEPHVFVGAVLPFLVGFALGNLDPELREFFSKAVQTLIPFFAFAL

GNTIDLTVIAQTGLLGILLGVAVIIVTGIPLIIADKLIGGGDGTAGIAASSSAGAAVATPVLIAEMVPAF

KPMAPAATSLVATAVIVTSILVPILTSIWSRKVKARAAKIEILGTVK

>gi|30064795|ref|NP_838966.1| hypothetical protein S3759 [Shigella flexneri 2a str. 2457T]

MRYPVDVYTGKIQAYPEGKPSAIAKIQVDGELMLTELGLEGDEQAEKKVHGGPDRALCHYPREHYLYWVR

EFPEQAELFVAPAFGENLSTDGLTESNVHMGDIFRWGEALIQVSQPRSPCYKLNYHFDISDIAQLMQNTG

KVGWLYSVIAPGKVSADAPLELVSRVSDVTVQEAAAIAWHMPFDDDQYHRLLSAAGLSKSWTRTMQKRRL

SGKIEDFSRRLWGK

>gi|30064794|ref|NP_838965.1| two-component sensor protein [Shigella flexneri 2a str. 2457T]

MIGSLTARIFAIFWLTLVLVLMLVLMLPKLDSRQMTELLDSEQRQGLMIEQHVEAELANDPPNDLMWWRR

LFRAIDKWAPPGQRLLLVTTEGRVIGAERSEMQIIRNFIGQADNADHPQKKKYGRVELVGPFSVRDGEDN

YQLYLIRPASSSQSDFINLLFDRPLLLLIVTMLVSTPLLLWLAWSLAKPARKLKNAADEVAQGNLRQHPE

LEAGPQEFLAAGASFNQMVTALERMMTSQQRLLSDISHELRTPLTRLQLGTALLRRRSGESKELERIETE

AQRLDSMINDLLVMSRNQQKNALVSETIKANQLWSEVLDNAAFEAEQMGKSLTVNFPPGPWPLYGNPNAL

ESALENIVRNALRYSHTKIEVGFAVDKDGITITVDDDGPGVSPEDREQIFRPFYRTDEARDRESGGTGLG

LAIVETAIQQHRGWVKAEDSPLGGLRLVIWLPLYKRS

>gi|30064793|ref|NP_838964.1| DNA-binding transcriptional regulator CpxR [Shigella flexneri 2a str. 2457T]

MNKILLVDDDRELTSLLKELLEMEGFNVIVAHDGEQALDLLDDSIDLLLLDVMMPKKNGIDTLKALRQTH

QTPVIMLTARGSELDRVLGLELGADDYLPKPFNDRELVARIRAILRRSHWSEQQQNNDNGSPTLEVDALV

LNPGRQEASFDGQTLELTGTEFTLLYLLAQHLGQVVSREHLSQEVLGKRLTPFDRAIDMHISNLRRKLPD

RKDGHPWFKTLRGRGYLMVSAS

>gi|30064789|ref|NP_838960.1| sulfate transporter subunit [Shigella flexneri 2a str. 2457T]

MNKWGVGLTFLLAATSVMAKDIQLLNVSYDPTRELYEQYNKAFSAHWKQQTGDNVVIRQSHGGSGKQATS

VINGIEADVVTLALAYDVDAIAERGRIDKEWIKRLPDNSAPYTSTIVFLVRKGNPKQIHDWNDLIKPGVS

VITPNPKSSGGARWNYLAAWGYALHHNNNDQAKAQDFVRALYKNVEVLDSGARGSTNTFVERGIGDVLIA

WENEALLAANELGKDKFEIVTPSESILAEPTVSVVDKVVEKKGTKEVAEAYLKYLYSPEGQEIAAKNYYR

PRDAEVAKKYENAFPKLKLFTIDEEFGGWTKAQKEHFANGGTFDQISKR

>gi|30064788|ref|NP_838959.1| CDP-diacylglycerol pyrophosphatase [Shigella flexneri 2a str. 2457T]

MKKAGLLFLVMIVIAVVAAGIGYWKLTGEESDTLRKIVLEECLPNQQQNQNPSPCAEVKPNAGYVVLKDL

NGPLQYLLMPTYRINGTESPLLTDPSTPNFFWLAWQARDFMSKKYGQPVPDRAVSLAINSRTGHTQNHFH

IHISCIRPDVREQLDKNLANISSRWLPLPGGLRGHEYLARRVTESELVQRSPFMMLAEEVPEAREHMGSY

GLAMVRQSDNSFVLLATQRNLLTLNRASAEEIQDHECEILR

>gi|30064786|ref|NP_838957.1| hypothetical protein S3749 [Shigella flexneri 2a str. 2457T]

MKPGCTLFFLLCSALTVTTTAHAQTPDTATTAPYLLAGAPTFDLSISQFREDFNSQNPSLPLNEFRAIDS

SPDKANLTRAASKINENLYASTALERGTLKIKSIQMTWLPIQGPEQKAAKAKAQEYMAAVIRTLTPLMTK

TQSQKKLQSLLTAGKNKRYYTETEGALRYVVADNGEKGLTFAVEPIKLALSESLEGLNK

>gi|30064785|ref|NP_838956.1| hypothetical protein S3747 [Shigella flexneri 2a str. 2457T]

MKDVVDKCSTKGCAIDIGTVIDNDNCTSKFSRFFATREEAESFMTKLKELAAAASSADEGASVAYKIKDL

EGQVELDAAFTFSCQAEMIIFELSLRSLA

>gi|30064784|ref|NP_838955.1| universal stress protein UspD [Shigella flexneri 2a str. 2457T]

MAYKHIGVAISGNEEDALLVNKALELARHNDAHLTLIHIDDGLSELYPGIYFPATEDILQLLKNKSDNKL

YKLTKNIQWPKTKLRIERGEMPETLLEIMQKEQCDLLVCGHHHSFINRLMPAYRGMINKMSADLLIVPFI

DK

>gi|30064782|ref|NP_838953.1| fructose 1,6-bisphosphatase II [Shigella flexneri 2a str. 2457T]

MRRELAIEFSRVTESAALAGYKWLGRGDKNTADGAAVNAMRIMLNQVNIDGTIVIGEGEIDEAPMLYIGE

KVGTGRGDAVDIAVDPIEGTRMTAMGQANALAVLAVGDKGCFLNAPDMYMEKLIVGPGAKGTIDLNLPLA

DNLRNVAAALGKPLSELTVTILAKPRHDAVIAEMQQLGVRVFAIPDGDVAASILTCMPDSEVDVLYGIGG

APEGVVSAAVIRALDGDMNGRLLARHDVKGDNEENRRIGEQELARCKAMGIEAGKVLRLGDMARSDNVIF

SATGITKGDLLEGISRKGNIATTETLLIRGKSRTIRRIQSIHYLDRKDPEMQVHIL

>gi|30064778|ref|NP_838949.1| ribonuclease activity regulator protein RraA [Shigella flexneri 2a str. 2457T]

MKYDTSELCDIYQEDVNVVEPLFSNFGGRASFGGQIITVKCFEDNGLLYDLLEQNGRGRVLVVDGGGSVR

RALVDAELARLAVQNEWEGLVIYGAVRQVDDLEELDIGIQAMAAIPVGAAGEGIGESDVRVNFGGVTFFS

GDHLYADNTGIILSEDPLDIE

>gi|30064775|ref|NP_838946.1| ATP-dependent protease peptidase subunit [Shigella flexneri 2a str. 2457T]

MTTIVSVRRNGHVVIAGDGQATLGNTVMKGNVKKVRRLYNDKVIAGFAGGTADAFTLFELFERKLEMHQG

HLVKAAVELAKDWRTDRMLRKLEALLAVADETASLIITGNGDVVQPENDLIAIGSGGPYAQAAARALLEN

TELSAREIAEKALDIAGDICIYTNHFHTIEELSYKA

>gi|30064774|ref|NP_838945.1| essential cell division protein FtsN [Shigella flexneri 2a str. 2457T]

MAQRDYVRRSQPAPSRRKKSTSRKKQRNLPAVSPAMVAIAAAVLVTFIGGLYFITHHKKEESETLQSQKV

TGNGLPPKPEERWRYIKELESRQPGVRAPTEPSAGGEVKTPEQLTPEQRQLLEQMQADMRQQPTQLVEVP

WNEQTPEQRQQTLQRQRQAQQLAEQQRLAQQSRTTEQSWQQQTRTSQAAPVQAQPRQSKPASTQQPYQDL

LQTPAHTTAQSKPQQAAPVARAADAPKPTAEKKDERRWMVQCGSFRGAEQAETVRAQLAFEGFDSKITTN

NGWNRVVIGPVKGKENADSTLNRLKMAGHTNCIRLAAGG

>gi|30064773|ref|NP_838944.1| DNA-binding transcriptional regulator CytR [Shigella flexneri 2a str. 2457T]

MKAKKQETAATMKDVALKAKVSTATVSRALMNPDKVSQATRNRVEKAAREVGYLPQPMGRNVKRNESRTI

LVIVPDICDPFFSEIIRGIEVTAANHGYLVLIGDCAHQNQQEKTFIDLIITKQIDGMLLLGSRLPFDASI

EEQRNLPPMVMANEFAPELELPTVHIDNLTAAFDAVNYLYEQGHKRIGCIAGPEEMPLCHYRLQGYVQAL

RRCGIMVDPQYIARGDFTFEAGSKAMQQLLDLPQPPTAVFCHSDVMALGALSQAKRQGLKVPEDLSIIGF

DNIDLTQFCDPPLTTIAQPRYEIGREAMLLLLDQMQGQHVGSGSRLMDCELIIRGSTRALP

>gi|30064772|ref|NP_838943.1| primosome assembly protein PriA [Shigella flexneri 2a str. 2457T]

MPVAHVALPVPLPRTFDYLLPEGMTVKAGCRVRVPFGKQQERIGIVVSVSDASELPLTELKAVVEVLDGE

PVFTHSVWRLLLWAADYYHHPIGDVLFHALPILLRQGRPAANAPMWYWFATEQGQAVDLNSLKRSPKQQQ

ALAALRQGKIWRDQVATLEFNDAALQALRKKGLCDLASETPEFSDWRTNYAVSGERLRLNTEQATAVGAI

HSAADTFSAWLLAGVTGSGKTEVYLSVLENVLAQGKQALVMVPEIGLTPQTIARFRERFNAPVEVLHSGL

NDSERLSAWLKAKNGEAAIVIGTRSALFTPFKNLGVIVIDEEHDSSYKQQEGWRYHARDLAVYRAHSEQI

PIILGSATPALETLCNVQQKKYRLLRLTRRAGNARPAIQHVLDLKGQKVQAGLAPALITRMRQHLQANNQ

VILFLNRRGFAPALLCHDCGWIAECPRCDHYYTLHQAQQHLRCHHCDSQRPVPRQCPSCGSTHLVPVGLG

TEQLEQTLAPLFPDVPISRIDRDTTSRKGALEQQLAEVHRGGARILIGTQMLAKGHHFPDVTLVALLDVD

GALFSADFRSAERFAQLYTQVAGRAGRAGKQGEVVLQTHHPEHPLLQTLLYKGYDAFAEQALAERRMMQL

PPWTSHVIVRAEDHNNQHAPLFLQQLRNLILSSPLADDKLWVLGPVPALAPKRGGRWRWQILLQHPSRVR

LQHIISGTLALINTIPDSRKVKWVLDVDPIEG

>gi|30064770|ref|NP_838941.1| peptidoglycan peptidase [Shigella flexneri 2a str. 2457T]

MKNRLLILSLLVSVPAFAWQPQTGDIIFQISRSSQSKAIQLATHSDYSHTGMLVMRNKKPYVFEAVGPVK

YTPLKQWIAHGEKGNYVVRRVEGGLSVEQQQKLTQTAKRYLGKPYDFSFSWSDDRQYCSEVVWKVYQNAL

GMRVGEQQKLKEFDLSNPLVQAKLKERYGKNIPLEETVVSPQAVFDAPQLTTVAKEWPLFSW

>gi|30064769|ref|NP_838940.1| transcriptional repressor protein MetJ [Shigella flexneri 2a str. 2457T]

MAEWSGEYISPYAEHGKKSEQVKKITVSIPLKVLKILTDERTRRQVNNLRHATNSELLCEAFLHAFTGQP

LPDDADLRKERSDEIPEAAKEIMREMGINPETWEY

>gi|30064767|ref|NP_838938.1| bifunctional aspartate kinase II/homoserine dehydrogenase II [Shigella flexneri 2a str. 2457T]

MSVIAQAGAKGRQLHKFGGSSLADVKCYLRVAGIMAEFSQPDDMMVVSAAGSTTNQLINWLKLSQTDRLS

AHQVQQTLRRYQCDLISGLLPAEEADSLISAFVSDLERLAALLDSGINDAVYAEVVGHGEVWSARLMSAV

LNQQGLPAAWLDAREFLRAERAAQPQVDEGLSYPLLQQLLVQHPSKRLVVTGFISRNNAGETVLLGRNGS

DYSATQIGALAGVSRVTIWSDVAGVYSADPRKVKDACLLPLLRLDEASELARLAAPVLHARTLQPVSGSE

IDLQLRCSYTPDQGSTRIERVLASGTGARIVTSHDDVCLIEFQVPASQDFKLAHKEIDQILKRAQVRPLA

VGVHNDRQLLQFCYTSEVADSALKILDEAGLPGELRLRQGLALVAMVGAGVTRNPLHCHRFWQQLKGQPV

EFTWQSDDGISLVAVLRTGPTESLIQGLHQSVFRAEKRIGLVLFGKGNIGSRWLELFAREQSTLSARTGF

EFVLAGVVDSRRSLLSYDGLDASRALAFFNDEAVEQDEESLFLWMRAHPYDDLVVLDVTASQQLADQYLD

FASHGFHVISANKLAGASDSNKYRQIHDAFEKTGRHWLYNATVGAGLPINHTVRDLIDSGDTILSISGIF

SGTLSWLFLQFDGSVPFTELVDQAWQQGLTEPDPRDDLSGKDVMRKLVILAREAGYNIEPDQVRVESLVP

AHCEGGSIDHFFENGDELNEQMVQRLEAAREMGLVLRYVARFDANGKARVGVEAVREDYPLASLLPCDNV

FAIESRWYRDNPLVIRGPGAGRDVTAGAIQSDINRLAQLL

>gi|30064765|ref|NP_838936.1| catalase; hydroperoxidase HPI(I) [Shigella flexneri 2a str. 2457T]

MSTSDDIHNTTATGKCPFHQGGHDQSAGAGTTTRDWWPNQLRVDLLNQHSNRSNPLGEDFDYRKEFSKLD

YYGLKKDLKALLTESQPWWPADWGSYAGLFIRMAWHGAGTYRSIDGRGGAGRGQQRFAPLNSWPDNVSLD

KARRLLWPIKQKYGQKISWADLFILAGNVALENSGFRTFGFGAGREDVWEPDLDVNWGDEKAWLTHRHPE

ALAKAPLGATEMGLIYVNPEGPDHSGEPLSAAAAIRATFGNMGMNDEETVALIAGGHTLGKTHGAGPTSN

VGPDPEAAPIEEQGLGWASTYGSGVGADAITSGLEVVWTQTPTQWSNYFFENLFKYEWVQTRSPAGAIQF

EAVDAPEIIPDPFDPSKKRKPTMLVTDLTLRFDPEFEKISRRFLNDPQAFNEAFARAWFKLTHRDMGPKS

RYIGPEVPKEDLIWQDPLPQPIYNPTEQDIIDLKFAIADSGLSVSELVSVAWASASTFRGGDKRGGANGA

RLALMPQRDWDVNAAAVRALPVLEKIQKESGKASLADIIVLAGVVGVEKAASAAGLSIHVPFAPGRVDAR

QDQTDIEMFELLEPIADGFRNYRARLDVSTTESLLIDKAQQLTLTAPEMTALVGGMRVLGANFDGSKNGV

FTDRVGVLSNDFFVNLLDMRYEWKATDESKELFEGRDRETGEVKYTASRADLVFGSNSVLRAVAEVYASS

DAHEKFVKDFVAAWVKVMNLDRFDLL

>gi|30064764|ref|NP_838935.1| fructose-like permease EIIC subunit 2 [Shigella flexneri 2a str. 2457T]

MNELVQILKNTRQHLMTGVSHMIPFVVSGGILLAVSVMLYGKGAVPDAVADPNLKKLFDIGVAGLTLMVP

FLAAYIGYSIAERSALAPCAIGAWVGNSFGAGFFGALIAGIIGGIVVHYLKKIPVHKVLRSVMPIFIIPI

IGTLITAGIMMWGLGEPVGALTNSLTQWLQGMQQGSIVMLAVIMGLMLAFDMGGPVNKVAYAFMLICVAQ

GVYTVVAIAAVGICIPPLGMGLATLIGRKNFSAEERETGKAALVMGCVGVTEGAIPFAAADPLRVIPSIM

VGSVCGAVTAALVGAQCYAGWGGLIVLPVVEGKLGYIAAVAVGAVVTAVCVNVLKSLARKNGSSTDEKED

DLDLDFEIN

>gi|30064763|ref|NP_838934.1| fructose-like phosphotransferase EIIB subunit 2 [Shigella flexneri 2a str. 2457T]

MTKIIAVTACPSGVAHTYMAAEALESAAKAKGWEVKVETQGSIGLENELTAEDVASADMVILTKDIGIKF

EERFAGKTIVRVNISDAVKRADAIMSKIEAHLAQTA

>gi|30064762|ref|NP_838933.1| formate acetyltransferase 2 [Shigella flexneri 2a str. 2457T]

MTNRISRLKTALFANTREISLERALLYTASHRQTEGEPVILRRAKATAYILEHVEISIRDEELIAGNRTV

KPRAGIMSPEMDPYWLLKELDQFPTRPQDRFAISEEDKRIYREELFPYWEKRSMKDFINGQMTDEVKAAT

NTQIFSINQTDKGQGHIIIDYPRLLNHGLGELVAQMQQHCQQQPENHFYQAALLLLEASQKHILRYAELA

ETMAANCTDAQRREELLTIAEISRHNAQHKPQTFWQACQLFWYMNIILQYESNASSLSLGRFDQYMLPFY

QASLTQGEDPAFLKELLESLWVKCNDIVLLRSTSSARYFAGFPTGYTALLGGLTENGRSAVNVLSFLCLD

AYQSVQLPQPNLGVRTNALIDTPFLMKTAETIRLGTGIPQIFNDEVVVPAFLNRGVSLEDARDYSVVGCV

ELSIPGRTYGLHDIAMFNLLKVMEICLHENEGNAALTYEGLLEQIRAKISHYITLMVEGSNICDIGHRDW

APVPLLSSFISDCLEKGRDITDGGARYNFSGVQGIGIANLSDSLHALKGMVFEQQRLSFDELLSVLKANF

ATPEGEKVRARLINRFEKYGNDIDEVDNISAELLRHYCKEVEKYQNPRGGYFTPGSYTVSAHVPLGSVVG

ATPDGRFAGEQLADGGLSPMLGQDAQGPTAVLKSVSKLDNTLLSNGTLLNVKFTPATLEGEAGLRKLADF

LRVFTQLKLQHIQFNVVNADTLREAQQRPQDYAGLVVRVAGYSAFFVELSKEIQDDIIRRTAHQL

>gi|30064761|ref|NP_838932.1| pyruvate formate lyase II activase [Shigella flexneri 2a str. 2457T]

MTSSAGQRISCNVVETRRDDVARIFNIQRYSLNDGEGIRTVVFFKGCPHLCPWCANPESISGKIQTVRRE

AKCLHCAKCLRDADECPSGAFERIGRDISLDALEREVMKDDIFFRTSGGGVTLSGGEVLMQAEFATRFLQ

RLRLWGVSCAIETAGDAPASKLLPLAKLCDEVLFDLKIMDATQARDVVKMNLPRVLENLRLLVSEGVNVI

PRLPLIPGFTLSRENMQQALDVLIPLKIRQIHLLPFHQYGEPKYRLLGKTWSMKEVPAPSSADVTTMREM

AERAGFQVTVGG

>gi|30064760|ref|NP_838931.1| fructose-like phosphotransferase EIIB subunit 3 [Shigella flexneri 2a str. 2457T]

MAYLVAVTACVSGVAHTYMAAERLEKLCQLEKWGVSIETQGALGTENRLADEDIRRADVALLITDIELAG

AERFEHCRYVQCSIYAFLREPQRVMSAVRKVLSAPQQTHLILE

>gi|30064759|ref|NP_838930.1| ARAC-type regulatory protein [Shigella flexneri 2a str. 2457T]

MYHDVSYLLSRLINGPLSLRQIYFASSNGPVPDLAYQVDFPRLEIVLEGEFVDTGAGATLVPGDVLYVAA

GGWNFPQWKTPATTFSVLFGKQQLGFSVVQWDGKQYQNLAKQHVARRGPRIGSFLLQTLNEMQMQPQEQQ

TARLIVASLLSHCRDLLGSQIQTASRSQALFEAIRDYIDERYASALTRESVAQAFYISPNYLSHLFQKTG

AIGFNEYLNHTRLEHAKTLLKGYDLKVKEVAHACGFVDSNYFCRLFRKNTEHSPSEYRRQYHSQLTEKPT

TPE

>gi|30064758|ref|NP_838929.1| hypothetical protein S3714 [Shigella flexneri 2a str. 2457T]

MHSTEVQAKPLSSWKALGWALLYFWFFSTLLQAIIYISGYSGTNGIRDSLLFSSLWLIPVFLFPKRIKII

AAVIGVVLWTASLAALCYYVIYGQEFSQSVLFVMFETNTNEASEYLSQYFSLKIVLIALAYTAVAVLLWT

RLRPVYIPKPWRYVVSFALLYGLILHPIAMNTFIKNKPFEKTLDNLASRMEPAAPWQFLTGYYQYRQQLN

SLTKLLNENNALPPLANFKDESGNEPRTLVLVIGESTQRGRMSLYGYPRETTPELDALHKTDPNLTVFNN

VVTSRPYTIEILQQALTFANEKNPDLYLTQPSLMNMMKQAGYKTFWITNQQTMTARNTMLTVFSRQTDKQ

YYMNQQRTQSAREYDTNVLKPFQDVLNDPAPKKLIIVHLLGTHIKYKYRYPENQGKFDGNTDHVPPGLSA

EELESYNDYDNANLYNDHVVASLIKDFKAADPNGFLVYFSDHGEEVYDTPPHKTQGRNEDNPTRHMYTIP

FLLWTSEKWQATHPRDFSQDVDRKYSLAELIHTWSDLAGLSYDGYDPTRSVVNPQFKETTRWIGNPYKKN

ALIDYDTLPYGDQVGNQ

>gi|30064757|ref|NP_838928.1| phosphoenolpyruvate carboxylase [Shigella flexneri 2a str. 2457T]

MNEQYSALRSNVSMLGKVLGETIKDALGEHILERVETIRKLSKSSRAGNDANRQELLTTLQNLSNDELLP

VARAFSQFLNLANTAEQYHSISPKGEAASNPEVIARTLRKLKNQPELSEDTIKKAVESLSLELVLTAHPT

EITRRTLIHKMVEVNACLKQLDNKDIADYERNQLMRRLRQLIAQSWHTDEIRKLRPSPVDEAKWGFAVVE

NSLWQGVPNYLRELNEQLEENLGYKLPVEFVPVRFTSWMGGDRDGNPNVTADITRHVLLLSRWKATDLFL

KDIQVLVSELSMVEATPELLALVGEEGAAEPYRYLMKNLRSRLMATQAWLEARLKGEELPKPEGLLTQNE

ELWEPLYACYQSLQACGMGIIANGDLLDTLRRVKCFGVPLVRIDIRQESTRHTEALGELTRYLGIGDYES

WSEADKQAFLIRELNSKRPLLPRNWQPSAETREVLDTCQVIAEAPQGSIAAYVISMAKTPSDVLAVHLLL

KEAGIGFAMPVAPLFETLDDLNNANDVMTQLLNIDWYRGLIQGKQMVMIGYSDSAKDAGVMAASWAQYQA

QDALIKTCEKAGIELTLFHGRGGSIGRGGAPAHAALLSQPPGSLKGGLRVTEQGEMIRFKYGLPEITVSS

LSLYTGAILEANLLPPPEPKESWRRIMDELSVISCDLYRGYVRENKDFVPYFRSATPEQELGKLPLGSRP

AKRRPTGGVESLRAIPWIFAWTQNRLMLPAWLGAGTALQKVVEDGKQSELEAMCRDWPFFSTRLGMLEMV

FAKADLWLAEYYDQRLVDKALWPLGKELRNLQEEDIKVVLAIANDSHLMADLPWIAESIQLRNIYTDPLN

VLQAELLHRSRQAEKEGQEPDPRVEQALMVTIAGIAAGMRNTG

>gi|30064755|ref|NP_838926.1| N-acetyl-gamma-glutamyl-phosphate reductase [Shigella flexneri 2a str. 2457T]

MLNTLIVGASGYAGAELVTYVNRHPHMNITALTVSAQSNDAGKLISDLHPQLKGIVELPLQPMSDISEFS

PGVDVVFLATAHEVSHDLAPQFLEAGCVVFDLSGAFRVNDATFYEKYYGFTHQYPELLEQAAYGLAEWCG

NKLKEANLIAVPGCYPTAAQLALKPLIDADLLDLNQWPVINATSGVSGAGRKAAISNSFCEVSLQPYGVF

THRHQPEIATHLGADVIFTPHLGNFPRGILETITCRLKSGVTQAQVAQALQQAYAHKPLVRLYDKGVPAL

KNVVGLPFCDIGFAVQGEHLIIVATEDNLLKGAAAQAVQCANIRFGYAETQSLI

>gi|30064754|ref|NP_838925.1| acetylglutamate kinase [Shigella flexneri 2a str. 2457T]

MNPLIIKLGGVLLDSEEALERLFSALVNYRESHQRPLVIVHGGGCVVDELMKGLNLPVKKKNGLRVTPAD

QIDIITGALAGTANKTLLAWAKKHQIAAVGLFLGDGDSVKVTQLDEELGHVGLAQPGSPKLINSLLENGY

LPVVSSIGVTDEGQLMNVNADQAATALAATLGADLILLSDVSGILDGKGQRIAEMTAAKAEQLIEQGIIT

DGMIVKVNAALDAARTLGRPVDIASWRYAEQLPALFNGMPMGTRILA

>gi|30064748|ref|NP_838919.1| DNA-binding transcriptional regulator OxyR [Shigella flexneri 2a str. 2457T]

MNIRDLEYLVALAEHRHFRRAADSCHVSQPTLSGQIRKLEDELGVMLLERTSRKVLFTQAGMLLVDQART

VLREVKVLKEMASQQGETMSGPLHIGLIPTVGPYLLPHIIPMLHQTFPKLEMYLHEAQTHQLLAQLDSGK

LDCVILALVKESEAFIEVPLFDEPMLLAIYEDHPWANRECVLMADLAGEKLLMLEDGHCLRDQAMGFCFE

AGADEDTHFRATSLETLRNMVAAGSGITLLPALAVPPERKRDGVVYLPCIKPEPCRTIGLVYRPGSPLRS

RYEQLAEAIRARMDGHFDKVLKQAV

>gi|30064746|ref|NP_838917.1| DNA-binding transcriptional repressor FabR [Shigella flexneri 2a str. 2457T]

MFILWYSASSTFGKDSDIVMGVRAQQKEKTRRSLVEAAFSQLSAERSFASLSLREVAREAGIAPTSFYRH

FRDVDELGLTMVDESGLMLRQLMRQARQRIAKGGSVIRTSVSTFMEFIGNNPNAFRLLLRERSGTSAAFR

AAVAREIQHFIAELADYLELENHMPRAFTEAQAEAMVTIVFSAGAEALDVGVEQRRQLEERLVLQLRMIS

KGAYYWYRREQEKTAIIPGNVKDE

>gi|30064745|ref|NP_838916.1| hypothetical protein S3698 [Shigella flexneri 2a str. 2457T]

MKQANQDRGTLLLALVAGLSINGTFAALFSSIVPFSVFPIISLVLTVYCLHQRYLNRTMSVGLPGLAAAC

FILGVLLYSTVVRAEYPDIGSNFFPAVLSVIMVFWIGAKMRNRKQEVAE

>gi|30064743|ref|NP_838914.1| vitamin B12/cobalamin outer membrane transporter [Shigella flexneri 2a str. 2457T]

MIKKASLLTACSVTAFSAWAQDTSPDTLVVTAIRFEQPRSTVLAPTTVVTRQDIDRWQSTSVNDVLRRLP

GVDITQNGGSGQLSSIFIRGTNASHVLVLIDGVRLNLAGVSGSADLSQFPIALVQRIEYIRGPRSAVYGS

DAIGGVVNIITTRDEPGTEISAGWGSNSYQNYDVSTQQQLGDKTRVTLLGDYAHTHGYDVVAYGNTGTQA

QTDNDGFLSKTLYGALEHNFTDAWSGFVRGYGYDNRTNYDAYYSPGSPLLDTRKLYSQSWDAGLRYNGEL

IKSQLITSYSHSKDYNYDPHYGRYDSSATLDEMKQYTVQWANNVIVGHGSIGAGVDWQKQTTTPGTGYVE

DGYDQRNTGIYLTGLQQVGDFTFEGAARSDDNSQFGRHGTWQTSAGWEFIEGYRFIASYGTSYKALNLGQ

LYGFYGNPNLDPEKSKQWEGAFEGLTAGVNWRISGYRNDVSDLIDYDDHTLKYYNEGKARIKGVEATANF

DTGPLTHTVSYDYVDARNAITDTPLLRRAKQQVKYQLDWQLYDFDWGITYQYLGTRYDKDYSSYPYQTVK

MGGVSLWDLAVAYPVTSHLTVRGKIANLFDKDYETVYGYQTAGREYTLSGSYTF

>gi|30064741|ref|NP_838912.1| UDP-N-acetylenolpyruvoylglucosamine reductase [Shigella flexneri 2a str. 2457T]

MNHSLKPWNTFGIDHNAQHIVCAEDEQQLLNAWQHATAEGQPVLILGEGSNVLFLEDYRGTVIINRIKGI

EIHDEPDAWYLHVGAGENWHRLVKYTLQEGMPGLENLALIPGCVGSSPIQNIGAYGVELQRVCAYVDCVE

LATGKQVRLTAKECRFGYRDSIFKHEYQDRFAIVAVGLRLPKEWQPVLTYGDLTRLDPTTVTPQQVFNAV

CHMRTTKLPDPKVNGNAGSFFKNPVVSAETAKALLAQFPTAPNYPQAGGSVKLAAGWLIDQCQLKGMQMG

GAAVHRQQALVLINEDNAKSEDVVQLAHHVRQKVGEKFNVWLEPEVRFIGASGEVSAVETIS

>gi|30064736|ref|NP_838907.1| preprotein translocase subunit SecE [Shigella flexneri 2a str. 2457T]

MSANTEAQGSGRGLEAMKWVVVVVALLLAAIVGNYLYRDIMLPLRALAVVILIAAAGGVALLTTKGKATIA

FAREARTEVRKVIWPTRQETLHTTLIVAAVTAVMSLILWGLDGILVRLVSFITGLRF

>gi|30064735|ref|NP_838906.1| transcription antitermination protein NusG [Shigella flexneri 2a str. 2457T]

MSEAPKKRWYVVQAFSGFEGRVATSLREHIKLHNMEDLFGEVMVPTEEVVEIRGGQRRKSERKFFPGYVL

VQMVMNDASWHLVRSVPRVMGFIGGTSDRPAPISDKEVDAIMNRLQQVGDKPRPKTLFEPGEMVRVNDGP

FADFNGVVEEVDYEKSRLKVSVSIFGRATPVELDFSQVEKA

>gi|30064733|ref|NP_838904.1| 50S ribosomal protein L1 [Shigella flexneri 2a str. 2457T]

MAKLTKRMRVIREKVDATKQYDINEAIALLKELATAKFVESVDVAVNLGIDARKSDQNVRGATVLPHGTG

RSVRVAVFTQGANAEAAKAAGAELVGMEDLADQIKKGEMNFDVVIASPDAMRVVGQLGQVLGPRGLMPNP

KVGTVTPNVAEAVKNAKAGQVRYRNDKNGIIHTTIGKVDFDADKLKENLEALLVALKKAKPTQAKGVYIK

KVSISTTMGAGVAVDQAGLSASVN

>gi|30064732|ref|NP_838903.1| 50S ribosomal protein L10 [Shigella flexneri 2a str. 2457T]

MALNLQDKQAIVAEVSEVAKGALSAVVADSRGVTVDKMTELRKAGREAGVYMRVVRNTLLRRAVEGTPFE

CLKDAFVGPTLIAYSMEHPGAAARLFKEFAKANAKFEVKAAAFEGELIPASQIDRLATLPTYEEAIARLM

ATMKEASAGKLVRTLAAVRDAKEAA

>gi|30064728|ref|NP_838899.1| thiamine biosynthesis protein ThiH [Shigella flexneri 2a str. 2457T]

MKTFSNRWRQLDWDDIRLRINGKTAADVERALNASQLTRDDMMALLSPAASGYLEQLAQQAQRLTRQRFG

NTVSFYVPLYLSNLCANDCTYCGFSMSNRIKRKTLDEADIARESAAIREMGFEHLLLVTGEHQAKVGMDY

FRRHLPALREQFSSLQMEVQPLAETEYAELKQLGLDGVMVYQETYHEATYTRHHLKGKKQDFFWRLETPD

RLGRAGIDKIGLGALIGLSDNWRVDCYMVAEHLLWLQQHYWQSRYSVSFPRLRPCTGGIEPASIMDERQL

VQTICAFRLLAPEIELSLSTRESPWFRDRVIPLAINNVSAFSKTQPGGYADNHPELEQFSPHDDRRPEAV

AAVLTAQGLQPVWKDWDSYLGRASQRL

>gi|30064725|ref|NP_838896.1| thiamine-phosphate pyrophosphorylase [Shigella flexneri 2a str. 2457T]

MYQPDFPPVPFRLGLYPVVDSVQWIERLLDAGVRTLQLRIKDRRDEEVEADVVAAIALGRRYNARLFIND

YWRLAIKHQAYGVHLGQEDLQATDLNAIRAAGLRLGVSTHDDMEIDVALAARPSYIALGHVFPTQTKQMP

SAPQGLEQLARHVERLADYPTVAIGGISLARAPAVIATGVGSIAVVSAITQAADWRLATAQLLEIAGVGD

E

>gi|30064724|ref|NP_838895.1| thiamine biosynthesis protein ThiC [Shigella flexneri 2a str. 2457T]

MSATKLTRREQRARAQHFIDTLEGTAFPNSKRIYITGTHPGVRVPMREIQLSPTLIGGSKEQPQYEENEA

IPVYDTSGPYGDPQIAINVQQGLAKLRQPWIDARADTEELTVRSSDYTRTRLADDGLDELRFSGLLTPKR

AKTGRRVTQLHYARQGIITPEMEFIAIRENMGRERIRSEVLRHQHPGMSFGAHLPENITAEFVRDEVAAG

RAIIPANINHPESEPMIIGRNFLVKVNANIGNSAVTSSIEEEVEKLVWSTRWGADTVMDLSTGRYIHETR

EWILRNSPVPIGTVPIYQALEKVNGIAEDLTWEAFRDTLLEQAEQGVDYFTIHAGVLLRYVPMTAKRLTG

IVSRGGSIMAKWCLSHHQENFLYQHFREICEICAAYDVSLSLGDGLRPGSIQDANDEAQFAELHTLGELT

KIAWEYDVQVMIEGPGHVPMQMIRRNMTEELEHCHEAPFYTLGPLTTDIAPGYDHFTSGIGAAMIGWFGC

AMLCYVTPKEHLGLPNKEDVKQGLITYKIAAHAADLAKGHPGAQIRDNAMSKARFEFRWEDQFNLALDPF

TARAYHDETLPQESGKVAHFCSMCGPKFCSMKISQEVRDYAAAQTIEVGMADMSENFRARGGEIYLHKEE

A

>gi|30064723|ref|NP_838894.1| anti-RNA polymerase sigma 70 factor [Shigella flexneri 2a str. 2457T]

MLNQLDNLTERVRGSNKLVDRWLHVRKHLLVAYYNLVGIKPGKESYMRLNEKALDDFCQSLVDYLSAGHF

SIYERILHKLEGNGQLARAAKIWPQLEANTQQIMDYYDSSLETAIDHDNYLEFQQVLSDIGEALEARFVL

EDKLILLVLDAARVKHPA

>gi|30064719|ref|NP_838890.1| hypothetical protein S3664 [Shigella flexneri 2a str. 2457T]

MLQNPIHLRLERLESWQHVTFMACLCERMYPNYAMFCQQTGFGDGQIYRRILDLIWETLTVKDAKVNFDS

QLEKFEEAIPSADDFDLYGVYPAIDACVALSELVHSRLSGETLEHAVEVSKTSITTVAMLEMTQAGREMG

DEELKENPAVEQEWDIQWEIFRLLAECEERDIELIKGLRADLRESGESNIGIIFQQ

>gi|30064717|ref|NP_838888.1| hypothetical protein S3662 [Shigella flexneri 2a str. 2457T]

MNSFNEGVVSPLLSFWRRSLMLAGALFLTACSHNSSLPPFTASGFAEDQGAVRIWRKDSGDNVHLLAVFS

PWRSGDTTTREYRWQGDNLTLININVYSKPPVNIRARFDDRGDLSFMQRESDGEKQQLSNDQIDLYRYRA

DQIRQISDALRQGRVVLRQGRWHAMEQTVTTCEGQTIKPDLDSQAIAHIERRQSRSSVDVSVAWLEAPEG

SQLLLVANSDFCRWQPNEKTF

>gi|30064715|ref|NP_838886.1| sensor protein ZraS [Shigella flexneri 2a str. 2457T]

MRFMQRSKDSLAKWLSAILPVVIVGLVGLFAVTVIRDYGRETAAARQTLLEKGSVLIRALESGSRVGMGM

RMHHAQQQALLEEMAGQPGVRWFAVTDEQGTIVMHSNSGMVGKQLYSPQEMQQLHPGDEEAWRRIDSADG

EPVLEIYRQFQPMFAAGMYRMRHMQQYAATPQAIFIAFDASNIVSAEDREQRNTLIILFALATVLLASVL

SFFWYRRYLRSRQLLQDEMKRKEKLVALGHLAAGVAHEIRNPLSSIKGLAKYFAERAPAGGEAHQLAQVM

AKEADRLNRVVSELLELVKPTHLALQAVDLNTLINHSLQLVSQDANSREIQLRFTANDTLPEIQADPDRL

TQVLLNLYLNAIQAIGQHGVISVTASESGAGVKISVTDSGKGIAADQLEAIFTPYFTTKAEGTGLGLAVV

HNIVEQHGGTIQVASQEGKGSTFTLWLPVNITRKDPQG

>gi|30064714|ref|NP_838885.1| transcriptional regulatory protein ZraR [Shigella flexneri 2a str. 2457T]

MTHDNIDILVVDDDISHCTILQALLRGWGYNVALANSGRQALEQVREQVFDLVLCDVRMAEMDGIATLKE

IKALNPAIPVLIMTAYSSVETAVEALKTGAQDYLIKPLDFDNLQATLEKALAHTHSIDAETPAVTASQFG

MVGKSPAMQHLLSEIALVAPSEATVLIHGDSGTGKELVARAIHASSARSEKPLVTLNCAALNESLLESEL

FGHEKGAFTGADKRREGRFVEADGGTLFLDEIGDISPMMQVRLLRAIQEREVQRVGSNQTISVDVRLIAA

THRDLAAEVNAGRFRQDLYYRLNVVAIEVPSLRQRREDIPLLAGHFLQRFAERNRKAVKGFTPQAMDLLI

HYDWPGNIRELENAVERAVVLLTGEYISERELPLAIASTPIPLGQSQDIQPLVEVEKEVILAALEKTGGN

KTEAARQLGITRKTLLAKLSR

>gi|30064711|ref|NP_838882.1| hypothetical protein S3652 [Shigella flexneri 2a str. 2457T]

MVISIRRSRHEEGEELVAIWCRSVDATHDFLSAEYRAELEELVRSFLPEAPLWVAVNEREQPVGFMLLSG

QHMDALFIDPDVCGCGVGRMLVEHALSMAPELTTNVNEQNEQAVGFYKKVGFKVTGRSEVDDLGKPYPLL

NLAYVGE

>gi|30064710|ref|NP_838881.1| homoserine O-succinyltransferase [Shigella flexneri 2a str. 2457T]

MPIRVPDELPAVNFLREENVFVMTTSRASGQEIRPLKVLILNLMPKKIETENQFLRLLSNSPLQVDIQLL

RIDSRESRNTPAEHLNNFYCNFEDIQEQNFDGLIVTGAPLGLVEFNDVAYWPQIKQVLEWSKDHVTSTLF
VCWAVQAALNILYGIPKQTRTDKLSGVYEHHILHPHALLTRGFDDSFLAPHSRYADFPAALIRDYTDLEI
LAETEEGDAYLFASKDKRIAFVTGHPEYDAQTLAQEYLRDVEAGLDPDVPYNYFPHNDPQNKPRASWRSH
GNLLFTNWLNYYVYQITPYDLRHMNPTLD

>gi|30064709|ref|NP_838880.1| isocitrate lyase [Shigella flexneri 2a str. 2457T]
MKTRTQQIEELQKEWTQPRWEGITRPYSAEDVVKLRGSVNPECTLAQLGAAKMWRLLHGESKKGYINNLG
ALTGGQALQQAKAGIEAVYLSGWQVAADANLAASMYPDQSLYPANSVPAVVERINNTFRRADQIQWSVGI
EPGDPRYVDYFLPIVADAEAGFGGVLNAFELMKAMIEAGAAAVHFEDQLASVKKCGHMGGKVLVPTQEAI
QKLVAARLAADVTGVPTLLVARTDADAADLITSDCDPYDSEFITGERTSEGFFRTHAGIEQAISRGLAYA
PYADLVWCETSTPDLELARRFAQAIHAKYPGKLLAYNCSPSFNWQKNLDDKTIASFQQQLSDMGYKFQFI
TLAGIHSMWFNMFDLANAYAQGEGMKHYVEKVQQPEFAAAKDGYTFVSHQQEVGTGYFDKVTTIIQGGTS
SVTALTGSTEESQF

>gi|30064706|ref|NP_838877.1| alpha helix protein [Shigella flexneri 2a str. 2457T]
MLTLLHLLSAVALLVWGTHIVRTGVMRVFGARLRTVLSRSVEKKPLAFCAGIGVTALVQSSNATTMLVTS
FVAQDLVALAPALVIVLGADVGTALMARILTFDLSWLSPLLIFIGVIFFLGRKQSRAGQLGRVGIGLGLI
LLALELIVQAVTPITQANGVQVIFASLTGDILLDALIGAMFAIISYSSLAAVLLTATLTAAGIISFPVAL
CLVIGANLGSGLLAMLNNSAANAAARRVALGSLLFKLVGSLIILPFVHLLAETMGKLSLPKAELVIYFHV
FYNLVRCLVMLPFVDPMARFCKTIIRDEPELDTQLRPKHLDVSALDTPTLALANAARETLRIGDAMEQMM
EGLNKVMHGEPRQEKELRKLADDINVLYTAIKLYLARMPKEELAEEESRRWAEIIEMSLNLEQASDIVER
MGSEIADKSLAARRAFSLDGLKELDALYEQLLSNLKLAMSVFFSGDVTSARRLRRSKHRFRILNRRYSHA
HVDRLHQQNVQSIETSSLHLGLLGDMQRLNSLFCSVAYSVLEQPDEDEGRDEY

>gi|30064705|ref|NP_838876.1| peptidase E [Shigella flexneri 2a str. 2457T]

MELLLLSNSTLPGKAWLEHALPLIAEQLQGRRSAVFIPFAGVTQTWDDYTEKTAAVLAPLGVSVTGIHSV

VDPVAAIENAEIVIVGGGNTFQLLKQCRERGLLAPITDVVKRGALYIGWSAGANLACPTIRTTNDMPIVD

PQGFDALNLFPLQINPHFTNALPEGHKGETREQRIRELLVVAPELTIIGLPEGNWITVSKGHATLGGPNT

TYVFKAGEEAVPLEAGHRF

>gi|30064704|ref|NP_838875.1| L-sorbose-1-P-reductase [Shigella flexneri 2a str. 2457T]

MKTTALRLYGKRDLRLETFDLPEMQEDEILATVVTDSLCLSSWKEANLGENHKKVPDDVTTNPIIIGHEF

CGDILAVGKKWQHKFQPGQRYVIQANLQLPDRPDCPGYSFPWVDGEATHVVIPNEVMEQYCLLAYDGETY

FEGSLVEPLSCVIGAFNANYHLQEGSYNHTMGIRPQGHTLILGGTGPMGLLAIDYALHGPVNPSLLVITD

TDNDKLSYARKHYPSEPQTLIHYLNATDAAFDTLMALSGGHGFDDIFVFVPNEGLVTLASSLLATDGCLN

FFAGPQDKHFSAPINFYDVHYAFTHYVGTSGGNTDDMRAAVKLIEEKKVQAAKVVTHILGLNAAGETTLE

LPAVGGGKKLVYTGKYLPLTSLTQIQDQALAAILARHQGIWSGEVEQYLLAHAEAISHD

>gi|30064701|ref|NP_838872.1| sorbose-permease PTS system IIB component [Shigella flexneri 2a str. 2457T]

MNITLARIDDRLIHGQVTTVWSKVANAQRIIICNDEVYNDEVRRTLLRQAAPPGMKVNVVNIEKAVAVYH

NPQYQDETVFYLFTRPQDALAMVRQGVKIGTLNIGGMAWRPGKKQLTKAVSLDDDDINAFHELNNLGVIL

DLRVVASDPSINIIDKINEQLIAN

>gi|30064700|ref|NP_838871.1| sorbose-permease PTS system IIA component [Shigella flexneri 2a str. 2457T]

MVNAIFCAHGKLACAMLESVQMVYGNANVEAVAFVPGENAGDIAVKLEKLVSIHNQDEWLIAVDLQCGSP

WNAAAMLAMRNPRLRVISGLSLPLALELVDNQDSMNVDELCEHLTQIAKQTCVVWKQLATTEEDF

>gi|30064698|ref|NP_838869.1| sor-operon regulator [Shigella flexneri 2a str. 2457T]

MENSDDIRLIVKIAQLYYEQDMTQAQIARELGIYRTTISRLLKRGRDQGMVTIAINYDYNENLWLEQQLK

QKFGLKDVVVVSGNDEDEETQLAMMGLHGAQLLDRLLEPGDIVGFSWGRAVSALVENLPQAGQSRQLICV

PIIGGPSGKLESRYHVNTLTYSAAAKLKGESHLADFPALLDNPLIRNGIMQSQHFKTISAYWDNLDIALV

GIGSPAIRDGANWHAFYGGEESDDLNARQVAGDICSRFFDIHGAMVETNMSEKTLSIEMNKLKQARYSIG

IAMSEEKYSGIIGALRGKYINCLVTNSSTAELLLK

>gi|30064697|ref|NP_838868.1| 23S rRNA pseudouridine synthase F [Shigella flexneri 2a str. 2457T]

MLPDSSVRLNKYISESGICSRREADRYIEQGNVFLNGKRATIGDQVKPGDIVKVNGQLIEPREAEDLVLI

ALNKPVGIVSTTEDGERDNIVDFVNHSKRVFPIGRLDKDSQGLIFLTNHGDLVNKILRAGNDHEKEYLVT

VDKPITDEFIRGMGAGVPILGTVTKKCKVKKEAPFVFRITLVQGLNRQIRRMCEHFGYEVKKLERTRIMN

VSLSGIPLGEWRDLTDDELIDLFKLIENSSSEAKPKAKAKPKTAGIKRPVVKMEKTAEKGGRPASNGKRF

TSPGRKKKGR

>gi|30064694|ref|NP_838865.1| sensory histidine kinase DcuS [Shigella flexneri 2a str. 2457T]

MRHSLPYHILRKRPMKLSTTVILMVSAVLFSVLLVVHLIYFSQISDMTRDGLANKALAVARTLADSPEIR

QGLQKKPQESGIQAIAEAVRKRNDLLFIVVTDMQSLRYSHPEAQRIGQPFKGDDILNALNGEENVAINRG

FLAQALRVFTPIYDENHKQIGVVAIGLELSRVTQQINDSRWSIIWSVLFGMLVGLIGTCILVKVLKKILF

GLEPYEISTLFEQRQAMLQSIKEGVVAVDDRGEVTLINDAAQELLNYRKSQDDEKLSTLSHSWSQVVDVS

EVLRDGTPRRDEEITIKDRLLLINTVPVRSNGVIIGAISTFRDKTEVRKLMQRLDGLVNYADALRERSHE

FMNKLHVILGLLHLKSYKQLEDYILKTANNYQEEIGSLLGKIKSPVIAGFLISKINRATDLGHTLILNSE

SQLPDSGSEDQVATLITTLGNLIENALEALGPEPGGEISVTLHYRHGWLHCEVNDDGPGIAPDKIDHIFD

KGVSTKGSERGVGLALVKQQVENLGGSIAVESEPGIFTQFFVQIPWDGERSNR

>gi|30064693|ref|NP_838864.1| DNA-binding transcriptional activator DcuR [Shigella flexneri 2a str. 2457T]

MINVLIIDDDAMVAELNRRYVAQIPGFQCCGTASTLEKAKEIIFNSDAPIDLILLDIYMQKENGLDLLPV

LHNARCKSDVIVISSAADAATIKDSLHYGVVDYLIKPFQASRFEEALTGWRQKKMALEKHQYYDQAELDQ

LIHGSSSNEQDPRRLPKGLTPQTLRTLCQWIDAHQDYEFSTDELANEVNISRVSCRKYLIWLVNCHILFT

SIHYGVTGRPVYRYRIQAEHYSLLKQYCQ

>gi|30064692|ref|NP_838863.1| anaerobic C4-dicarboxylate transporter [Shigella flexneri 2a str. 2457T]

MLFTIQLIIILICLFYGARKGGIALGLLGGIGLVILVFVFHLQPGKPPVDVMLVIIAVVAASATLQASGG

LDVMLQIAEKLLRRNPKYVSIVAPFVTCTLTILCGTGHVVYTILPIIYDVAIKNNIRPERPMAASSIGAQ

MGIIASPVSVAVVSLVAMLGNVTFDGRHLEFLDLLAITIPSTLIGILAIGIFSWFRGKDLDKDEEFQKFI

SVPENREYVYGDTATLLDKKLPKSNWLAMWIFLGAIAVVALLGADSDLRPSFGGKPLSMVLVIQMFMLLT

GALIIILTKTNPASISKNEVFRSGMIAIVAVYGIAWMAETMFGAHMSEIQGVLGEMVKEYPWAYAIVLLL

VSKFVNSQAAALAAIVPVALAIGVDPAYIVASAPACYGYYILPTYPSDLAAIQFDRSGTTHIGRFVINHS

FILPGLIGVSVSCVFGWIFAAMYGFL

>gi|30064691|ref|NP_838862.1| fumarase B, fumarate hydratase class I [Shigella flexneri 2a str. 2457T]

MSNKPFIYQAPFPMGKDNTEYYLLTSDYVSVADFDGETILKVEPEALTLLAQQAFHDASFMLRPAHQKQV

AAILHDPEASENDKYVALQFLRNSEIAAKGVLPTCQDTGTAIIVGKKGQRVWTGGGDEEALSKGVYNTYI

EDNLRYSQNAALDMYKEVNTGTNLPAQIDLYAVDGDEYKFLCVAKGGGSANKTYLYQETKALLTPGKLKN

FLVEKMRTLGTAACPPYHIAFVIGGTSAENNLKTVKLASAHYYDELPTEGNEHGQAFRDVQLEQELLEEA

QKLGLGAQFGGKYFAHDIRVIRLPRHGASCPVGMGVSCSADRNIKAKINREGIWIEKLEHNLGQYIPQEL

RQAGEGEAVKVDLNRPMKEILAQLSQYPVSTRLSLTGTIIVGRDIAHAKLKELIDAGKELPQYIKDHPIY

YAGPAKTPAGYPSGSLGPTTAGRMDSYVDLLQSHGGSMIMLAKGNRSQQVTDACHKHGGFYLGSIGGPAA

VLAQQSIKHLECVAYPELGMEAIWKIEVEDFPAFILVDDKGNDFFQQIVNKQCANCTK

>gi|30064690|ref|NP_838861.1| hypothetical protein S3628 [Shigella flexneri 2a str. 2457T]

MTRTLKPLILNTGALALTLILIYTGISAHDKLTWLLEVTPVIIVVPLLLATARRYPLTLLLYTLIFFHAI

ILMVGGQYTYAKVPVGFEVQEWLGLSRNPYDKLGHFFQGLVPALVAREILVRGMYVRGRKMVAFLVCCVA

LAISAMYELIEWWAALAMGQGADDFLGTQGDQWDTQSDMFCALLGALTTVILLARFHCRQLRRYGLITG

>gi|30064688|ref|NP_838859.1| alpha-galactosidase [Shigella flexneri 2a str. 2457T]

MMSAPKITFIGAGSTIFVKNILGDVFHREALKTAHIALMDIDPTRLEESHIVVRKLMDSAGASGKITCHT

QQKEALEDADFVVVAFQIGGYEPCTVTDFEVCKRHGLEQTIADTLGPGGIMRALRTIPHLWQICENMTEV

CPDATMLNYVNPMAMNTWAMYARYPYIKQVGLCHSVQGTAEELARDLNIDPATLRYRCAGINHMAFYLEL

ERKTADGSYVNLYPELLAAYEAGQAPKPNIHGNTRCQNIVRYEMFKKLGYFVTESSEHFAEYTPWFIKLG

REDLIERYKVPLDEYPKRCVEQLANWHKELEEYKNASRIDIKPSREYASTIMNAIWTGEPSVIYGNVRND

GLIDNLPQGCCVEVACLVDANGIQPTKVGTLPSHLAALMQTNINVQTLLTEAILTENRDRVYHAAMMDPH

TAAVLGIDEIYALVDDLIAAHGDWLPSWLHR

>gi|30064687|ref|NP_838858.1| DNA-binding transcriptional regulator MelR [Shigella flexneri 2a str. 2457T]

MNTDTFMCSSDEKQTRSPLSLYSEYQRMEIEFRAPHIMPTSHWHGQVEVNVPFDGDVEYLINNEKVNINQ

GHITLFWACTPHQLTDTGTCQSMAIFNLPMHLFLSWPLDKDLINHVTHGMVIKSLATQQLSPFEVRRWQQ

ELNSPNEQIRQLAIDEIGLMLKRFSLSSWEPILVNKTSRTHKNSVSRHAQFYVSQMLGFIAENYDQALTI

NDVAEHVKLNANYAMGIFQRVMQLTMKQYITAMRINHVRALLSDTDKSILDIALTAGFRSSSRFYSTFGK

YVGMSPQQYRKLSQQRRQTFPG

>gi|30064685|ref|NP_838856.1| ARAC-type regulatory protein [Shigella flexneri 2a str. 2457T]

MRICSDQPCIVLLTEKDVWIRVNGKEPISLKANHMALLNCENNIIDVSSLNNTLVAHISHDIIKDYLRFL

NKDLSQIPVWQRSATPILTLPCLTPDVFRVAAQHSMMPAETESEKERTRALLFTVLSRFLDSKKFLSLMM

YMLRNCVSDSVYQIIESDIHKDWNLSMVASCLCLSPSLLKKKLKSENTSYSQIITTCRMRYAVNELMMDG

KNISQVSQSCGYNSTSYFISVFKDFYGMTPLHYVSQHRERTVA

>gi|30064683|ref|NP_838854.1| cell division protein [Shigella flexneri 2a str. 2457T]

MRTLFDGNTVMLKRLLKRPSLNLLAWLLLAAFYISICLNIAFFKQVLQALPLDSLHNVLVFLSMPVVAFS

VINIVLTLSSFLWLNRPLACLFILIGAAAQYFIMTYGIVIDRSMIANIIDTTPAESYALMTPQMLLTLGF

SGVLAELIACWLKIKPATSRLRSVLFRGANILVSVLLILLVAALFYKDYASLFRNNKELVKSLSPSNSIV

ASWSWYSHQRLANLPLVRIGEDAHRNPLMQNEKRKNLTILIVGETSRAENFSLNGYPRETNPRLAKDNVV

YFPNTASCGTATAVSVPCMFSDMPREHYKEELAQHQEGVLDIIQRAGINVLWNDNDGGCKGACDRVPHQN

VTALNLPDQCINGECYDEVLFHGLEEYINNLQGDGVIVLHTIGSHGPTYYNRYPPQFRKFTPTCDTNEIQ

TCTKEQLVNTYDNTLVYVDYIVDKAINLLKEHQDKFTTSLVYLSDHGESLGENGIYLHGLPYAIAPDSQK

QVPMLLWLSEDYQKRYQVDQNCLQKQAQTQHYSQDNLFSTLLGLTGVETKYYQAADDILQTCRRVSK

>gi|30064682|ref|NP_838853.1| DNA-binding transcriptional regulator BasR [Shigella flexneri 2a str. 2457T]

MKILIVEDDTLLLQGLILAAQTEGYACDGVTTARMAEQSLEDGHYSLVVLDLGLPDEDGLHFLARIRQKK

YTLPVLILTARDTLTDKIAGLDVGADDYLVKPFALEELHARIRALLRRHNNQGESELIVGNLTLNMGRRQ

VWMGGEELILTPKEYALLSRLMLKAGSPVHREILYNDIYNWDNEPSTNTLEVHIHNLRDKVGKARIRTVR

GFGYMLVANEEN

>gi|30064681|ref|NP_838852.1| sensor protein BasS/PmrB [Shigella flexneri 2a str. 2457T]

MNLMRFLRRPISLRQRLILTIGAILLVFELISVFWLWHESTEQIQLFEQALRDNRNNDRHIMREIREAVA
SLIVPGVFMVSLTLFICYQAVRRITRPLAELQKELEARTADNLTPIAIHSATLEIEAVVSALNDLVSRLT
STLDNERLFTADVAHELRTPLAGVRLHLELLAKTHHIDVAPLVARLDQMMESVSQLLQLARAGQSFSSGN
YQHVKLLEDVILPSYDELSTMLDQRQQTLLLPESAADITVQGDATLLRMLLRNLVENAHRYSPQGSNIMI
KLQEDGGAVMAVEDEGPGIDESKCGELSKAFVRMDSRYGGIGLGLSIVSRITQLHHGQFFLQNRQETSGT
RAWVRLKKDQNVANQI

>gi|30064680|ref|NP_838851.1| proline/glycine betaine transporter [Shigella flexneri 2a str. 2457T]
MLKRKKVKPITLRDVTIIDDGKLRKAITAASLGNAMEWFDFGVYGFVAYALGKVFFPGADPSVQMVAALA
TFSVPFLIRPLGGLFFGMLGDKYGRQKILAITIVIMSISTFCIGLIPSYDTIGIWAPILLLICKMAQGFS
VGGEYTGASIFVAEYSPDRKRGFMGSWLDFGSIAGFVLGAGVVVLISTIVGEANFLDWGWRIPFFIALPL
GIIGLYLRHALEETPAFQQHVDKLEQGDREGLQDGPKVSFKEIATKYWRSLLTCIGLVIATNVTYYMLLT
YMPSYLSHNLHYSEDHGVLIIIAIMIGMLFVQPVMGLLSDRFGRRPFVLLGSVALFVLAIPAFILINSNV
IGLIFAGLLMLAVILNCFMGVMASTLPAMFPTHIRYSALAAAFNISVLVAGLTPTLAAWLVESSQNLMMP
AYYLMVVAVIGLITGVTMKETANRPLKGATPAASDIQEAKEILVEHYDNIEQKIDNIDHEIADLQAKRTR
LVQQHPRIDE

>gi|30064678|ref|NP_838849.1| hypothetical protein S3616 [Shigella flexneri 2a str. 2457T]
MYTQTLYELSQEAERLLQLSRQQLQLLEKMPLSVPGDDAPQLALPWSQPNIAERHAMLNNELCKISRLEM
VLAIVGTMKAGKSTTINAIVGTEVLPNRNRPMTALPTLIRHTPGQKEPVLHFSHVAPIDCLIQQLQQRLR
DCDIKHLTDVLEIDKDMRALMQRIENGVAFEKYYLGAQPIFHCLKSLNDLVRLAKALDVDFPFSAYAAIE
HIPVIEVEFVHLAGLESYPGQLTLLDTPGPNEAGQPHLQKMLNQQLARASAVLAVLDYTQLKSISDEEVR
EAILAVGQSVPLYVLVNKFDQQDRNSDDADQVRALISGTLMKGCITPQQIFPVSSMWGYLANRARYELAN
NGKLPAPEQQRWVEDFAHAALGRRWRHADLADLEHIRHAADQLWEDSLFAQPIQALLHAAYANASLYALR
SAAHKLLNYAQQAREYLDFRAHGLNVACEQLRQNIHQVEESLQLLQLNQAQVSGEIKHEIELALTSANHF

LRQQQDAVNAQLAALFQDDSEPLSEIRTRCETLLQTAQNTISRDFTLRFAEMESTLCRVLTDVIRPIEQQ

VKMELSESGFRPGFHFPVFHGVVPHFNTRQLFSEVISRQEATDEQSTRLGVVRETFSRWLNQPDWGRGNE

KSPTETVDYSVLQRALSAEVDLYCQQMAKVLAEQVDESVTAGMNTFFAEFASCLTELQTRLRESLALRQQ

NESVVRLMQQQLQQTVMTHSWIYTDAQLLRDDIQTLFTAERY

>gi|30064677|ref|NP_838848.1| hypothetical protein S3615 [Shigella flexneri 2a str. 2457T]

MSLPHCPKCNSEYTYEDNGMYICPECAYEWNDAEPAQESDELIVKDANGNLLADGDSVTIIKDLKVKGSS

SMLKIGTKVKNIRLVEGDHNIDCKIDGFGPMKLKSEFVKKN

>gi|30064676|ref|NP_838847.1| hypothetical protein S3614 [Shigella flexneri 2a str. 2457T]

MPLSPYLSFAGNCSDAIAYYQRTLGAELLYKISFGEMPKSAQDSAENCPSGMQFPDTAIAHANVRIAGSD

IMMSDAIPSGKASYSGFTLVLDSQQVEEGKRWFDNLAANGKIEMAWQETFWAHGFGKVTDKFGVPWMINV

VKQQPTQ

>gi|30064674|ref|NP_838845.1| periplasmic binding protein component of phosphonate transporter [Shigella flexneri 2a str. 2457T]

MNAKIIASLAFTSMFSLSTLLTPAHAEEQEKALNFGIISTESQQNLKPQWTPFLQDMEKKLGVKVNAFFA

PDYAGIIQGMRFNKVDIAWYGNLSAMEAVDRANGQVFAQTVAADGSPGYWSVLIVNKDSPINNLNDLLAK

RKDLTFGNGDPNSTSGFLVPGYYVFAKNNISASDFKRTVNAGHETNALAVANKQVDVATNNTENLDKLKT

SAPEKLKELKVIWKSPLILGDPIVWRKNLSETTKDKIYDFFMNYGKTPEEKAVLERLGWAPFRASSDLQL

VPIRQLALFKEIQSVKGNKGLNEQDKLAKTTEIQAQLDDLDRLNNALSAMSSVSKAVQ

>gi|30064670|ref|NP_838841.1| multidrug resistance protein MdtN [Shigella flexneri 2a str. 2457T]

MESTPKKAPRSKFPALLVVALALVALVFVIWRVDSAPSTNDAYASADTIDVVPEVSGRIVELAVTDNQAV

KQGDLLFRIDPRPYEANLAKAEASLAALDKQIMLTQRSVDAQQFGADSVNATVEKARAAAKQATDTLRRT

EPLLKEGFVSAEDVDRARTAQRAAEADLNAVLLQAQSAASAVSGVDALVAQRAAVEADIALTKLHLEMTT

VRAPFDGRIISLKTSVGQFASAMRPIFTLIDTRHWYVIANFRETDLKNIRSGTPATIRLMSDSGKTFEGK

VDSIGYGVLPDDGGLVLGGLPKVSRSINWVRVAQRFPVKIMVDKPDPEMFRIGASAVANLEPQ

>gi|30064668|ref|NP_838839.1| outer membrane efflux protein MdtP [Shigella flexneri 2a str. 2457T]

MINRQLSRLLLCSILGSTTLISGCALVRKDSAPHQQLKPEQIKLADDIHLASSGWPQAQWWKQLNDPQLD

ALIQRTLSGSHTLAEAKLREEKAQSQADLLDAGSQLQVAALGMLNRQRVSANGFLSPYAMDAPALGMDGP

YYTEATVGLFAGLDLDLWGVHRSAVAAAIGAHNAALAETAAVELSLTTGVAQLYYSMQASYQMLDLLEQT

RDVIDYAVKAHQSKVAHGLEAQVPFHGARAQILAVDKQIAAVKGQITETRESLRALIGAGASDMPEIKPV

ALPRVQTGIPATLSYELLARRPDLQAMRWYVQASLDQVDSARALFYPSFDIKAFFGLDAIHLDTLFKKTS

RQFNFIPGLKLPLFDGGRLNANLEGTRAASNMMIERYNQSVLNAVRDVAVNGTRLQTLNDEREMQAEHVE

ATRFTQRAAEAAYQRGLTSRLQATEARLPVLAEEMSLLMLDSRRVIQSIQLMKSLGGGYQAAPVVEKK

>gi|30064665|ref|NP_838836.1| formate-dependent nitrite reductase complex subunit NrfG [Shigella flexneri 2a str. 2457T]

MKQPQIPVKMLTTLTILMVFLCVGSYLLSPKWQAVRAEYQRQRDPLHQFASQQTPEAQLQALQDKIRANP

QNSEQWALLGEYYLWQNDYSNSLLAYRQALQLRGENAELYAALATVLYYQASQHMTAQTRAMIDKALALD

SNEITALMLLASDAFMQANYAQAIELWQKVMDLNSPRINRTQLVESINMAKLLQRRSD

>gi|30064664|ref|NP_838835.1| formate-dependent nitrite reductase complex subunit NrfF [Shigella flexneri 2a str. 2457T]

MNKGLLTLLLLFTCFARAQVVDTWQFANPQQQQQALNIASQLRCPQCQNQNLLESNAPVAVSMRHQVYSM

VSEGKNEVEIIGWMTERYGDFVRYNPPLTGQTLVLWALPVVLLLLMALILWRVRAKR

>gi|30064663|ref|NP_838834.1| heme lyase subunit NrfE [Shigella flexneri 2a str. 2457T]

MLTPLTAFAGVRLRWPAMMRLTCIGILAQFALLLLAFGVLTYCFLISDFSVIYVAQHSYSLLSWELKLAA

VWGGHEGSLLLWVLLLSAWSALFAWHYRQQSDPLFPLTLAVLSLMLAALLLFVVLWSDPFVRIFPPAIEG

RDLNPMLQHPGLIFHPPLLYLGYGGLMVAASVALASLLRGEFDAASARICWRWALPGWSALTAGIILGSW

WAYCELGWGGWWFWDPVENASLLPWLSATALLHSLSLTRQRGIFRHWSLLLAIVTLMLSLLGTLIVRSGI

LVSVHAFALDNVRAVPLFSLFALISLASLALYGWRARDGGPAVRFSGLSREMLILATLLLFCAVLLIVLV

GTLYPMIYGLLGWGRLSVGAPYFNRATLPFGLLMLVVIVLATFVSGKRVQLPALVAHAGVLLFAAGIVVS

SVSRQEISLNLQPGQQVTLAGYTFRFERLDLQARGNYTSEKAIVALFDHQQRIGELTPERRFYEARRQQM

MEPSIRWNGIHDWYAVMGEKTRADRYAFRLYVQSGVRWIWGGGLLMIAGALLSGWRGKKRDE

>gi|30064662|ref|NP_838833.1| formate-dependent nitrate reductase, transmembrane protein [Shigella flexneri 2a str. 2457T]

MTQTSAFHFESLVWDWPIAIYLFLIGISAGLVTLAVLLRRFYPQAGGADSTLLRTTLIVGPGAVILGLLI

LVFHLTRPWTFWKLMFHYSFTSVMSMGVMLFQLYMVVLVLWLAKIFEHDLLALQQRWLPKLGIVQKVLSL

LTPVHRGLETLMLVLAVLLGAYTGFLLSALKSYPFLNNPSLPVLLLFSGISSGAAVALIAMAIRQRSNPH

STEAHFVHRMEIPVVWGEIFLLVAFFVGLALGDDGKVRALVAALGGGFWTWWFWLGVAGLGLIVPMLLKP

WVNRSSGIPAVLAACGASLVGVLMLRFFILYAGQLTVA

>gi|30064661|ref|NP_838832.1| formate-dependent nitrite reductase [Shigella flexneri 2a str. 2457T]

MTWSRRQFLTGVGVLAAVSGTAGRVVAKTLNINGVRYGMVHDESLCIGCTACMDACREVNKVPEGVSRLT

IIRSEPQGEFPDVKYRFFRKSCQHCDHAPCVDVCPTGASFRDAASGIVDVNPDLCVGCQYCIAACPYRVR

FIHPVTKTADKCDFCRKTNLQAGKLPACVEACPTKALTFGNLDDPNSEISQLLRQKPTYRYKLALGTKPK

LYRVPFKYGEVSQ

>gi|30064660|ref|NP_838831.1| cytochrome c nitrite reductase pentaheme subunit [Shigella flexneri 2a str. 2457T]

MSVLRSLLTAGVLASGLLWSLNGITATPAVQASGDRYEVTQQRNPDAACLDCHKPDTEGMHGKHASVINP

NNKLPVTCTNCHGQASPQHREGVKDVMRFNEPMYKVGEQNSVCMSCHLPEQLQKAFWPHDVHVTKVACAS

CHSLHPQQDTMQTLSDKGRIKICVDCHSDQRTNPNFNPASVPLLKEQP

>gi|30064659|ref|NP_838830.1| cytochrome c552 [Shigella flexneri 2a str. 2457T]

MTRIKINARRIFSLLIPFFFFTSVHAEQTAAPAKPVTVEAKNETFAPQHHDQYLSWKATSEQSERVDALA

EDPRLVILWAGYPFSRDYNKPRGHAFAVTDVRETLRTGAPKNAEDGPLPMACWSCKSPDVARLIQKDGED

GYFHGKWARGGPEIVNNLGCADCHNTASPEFAKGKPELTLSRPYAARAMEAIGKPFEKAGRFDQQSMVCG

QCHVEYYFDGKNKAVKFPWDDGMKVENMEQYYDKIAFSDWTNSLSKTPMLKAQHPEYETWTAGIHGKNNV

TCIDCHMPKVQNAEGKLYTDHKIGNPFDNFAQTCANCHTQDKAALQKVVAERKQSINDLKIKVEDQLVHA

HFEAKAALDAGATEAEMKPIQDDIRHAQWRWDLAIASHGIHMHAPEEGLRMLGTAMDKAADARTKLARLL

ATKGITHEIQIPDISTKEKAQQAIGLNMEQIKAEKQDFIKTVIPQWEEQARKNGLLSQ

>gi|30064658|ref|NP_838829.1| hypothetical protein S3595 [Shigella flexneri 2a str. 2457T]

MNGTIYQRIEDNAHFRELVEKRQRFATILSIIMLAVYIGFILLIAFAPGWLGTPLNPNTSVTRGIPIGVG

VIVISFVLTGIYIWRANGEFDRLNNEVLHEVQAS

>gi|30064654|ref|NP_838825.1| hypothetical protein S3591 [Shigella flexneri 2a str. 2457T]

MSTPSARTGGSLDAWFKISQRGSTVRQEVVAGLTTFLAMVYSVIVVPGMLGKAGFPPAAVFVATCLVAGL

GSIVMGLWANLPLAIGCAISLTAFTAFSLVLGQHISVPVALGAVFLMGVLFTVISATGIRSWILRNLPHG

VAHGTGIGIGLFLLLIAANGVGLVIKNPLDGLPVALGDFATFPVIMSLAGLAVIIGLEKLKVPGGILLTI

IGISIVGLIFDPNVHFSGVFAMPSLSDENGNSLIGSLDIMGALNPVVLPSVLALVMTAVFDATGTIRAVA

GQANLLDKDGQIIDGGKALTTDSMSSVFSGLVGAAPAAVYIESAAGTAAGGKTGLTAITVGVLFLLILFL

SPLSYLVPGYATAPALMYVGLLMLSNVAKIDFADFVDAMAGLVTAVFIVLTCNIVTGIMIGFATLVIGRL

VSGEWRKLNIGTVVIAVALVTFYAGGWAI

>gi|30064653|ref|NP_838824.1| redox-sensing activator of soxS [Shigella flexneri 2a str. 2457T]

MEKKLPRIKALLTPGEVAKRSGVAVSALHFYESKGLITSIRNSGNQRRYKRDVLRYVAIIKIAQRIGIPL

ATIGEAFGVLPEGHTLSAKEWKQLSSQWREELDRRIHTLVALRDELDGCIGCGCLSRSDCPLRNPGDRLG

EEGTGARLLEDEQN

>gi|30064652|ref|NP_838823.1| DNA-binding transcriptional regulator SoxS [Shigella flexneri 2a str. 2457T]

MSHQKIIQDLIAWIDVHIDQPLNIDVVAKKSGYSKWYLQRMFRTVTHQTLGDYIRQRRLLLAAVELRTTE

RPIFDIAMDLGYVSQQTFSRVFRRQFDRTPSDYRHRL

>gi|30064651|ref|NP_838822.1| hypothetical protein S3587 [Shigella flexneri 2a str. 2457T]

MYHASREQRRAVQGIGSFQRNCLMATLTTGVVLLRWQLLSAVMMFLASTLNIRFRRSDYVGLAVISSGLG

VVSACWFAMGLLGITMADITAIWHNIESVMIEEMNQTPPQWPMILT

>gi|30064646|ref|NP_838817.1| hypothetical protein S3582 [Shigella flexneri 2a str. 2457T]

MTISELLQYCMAKPSAEQSVHNDWKATQIKVEDVLFAMVKEVENRPAVSLKTSPELAELLRQQHSDVRPS

RHLNKAHWSTVYLDGSLPDSQIYYLVDASYQQAVNLLPEEKRKLLVQL

>gi|30064645|ref|NP_838816.1| hypothetical protein S3581 [Shigella flexneri 2a str. 2457T]

MWYQKTLTLSAKSRGFHLVTDEILNQLADMPRVNIGLLHLLLQHTSASLTLNENCDPTVRHDMECFFLRT
VPDNGNYEHDYEGADDMPSHIKSSMLGTSLVLPVHKGRIQTGTWQGIWLGEHRIHGGSRRIIATLQGE

>gi|30064644|ref|NP_838815.1| acid phosphatase/phosphotransferase [Shigella flexneri 2a str. 2457T]
MRKITQAISAVCLLFALNSSAVALASSPSPLNPGTNVARLAEQAPIHWVSVAQIENSLAGRPPMAVGFDI
DDTVLFSSPGFWRGKKTFSPESEDYLKNPVFWEKMNNGWDEFSIPKEVARQLIDMHVRRGDAIFFVTGRS
PTKTETVSKTLADNFHIPATNMNPVIFAGDKPGQNTKSQWLQDKNIRIFYGDSDNDITAARDVGARGIRI
LRASNSTYKPLPQAGAFGEEVIVNSEY

>gi|30064642|ref|NP_838813.1| alanine racemase [Shigella flexneri 2a str. 2457T]
MQAATVVINRRALRHNLQRLRELAPASKMVAVVKANAYGHGLLETARTLPDADAFGVARLEEALRLRAGG
ITKPVLLLEGFFDARDLPTISAQHFHTAVHNEEQLAALEEASLDEPVTVWMKLDTGMHRLGVRPEQAEAF
YHRLTQCKNVRQPVNIVSHFARADEPKCGATEKQLAIFNTFCEGKPGQRSIAASGGILLWPQSHFDWVRP
GIILYGVSPLEDRSTGADFGCQPVMSLTSSLIAVREHKAGEPVGYGGTWVSERDTRLGVVAMGYGDGYPR
AAPSGTPVLVNGREVPIVGRVAMDMICVDLGPQAQDKAGDPVILWGEGLPVERIAEMTKVSAYELITRLT
SRVAMKYVD

>gi|30064641|ref|NP_838812.1| replicative DNA helicase [Shigella flexneri 2a str. 2457T]
MAGNKPFNKQQAEPRERDPQVAGLKVPPHSIEAEQSVLGGLMLDNERWDDVAERVVADDFYTRPHRHIFT
EMARLQESGSPIDLITLAESLERQGQLDSVGGFAYLAELSKNTPSAANISAYADIVRERAVVREMISVAN
EIAEAGFDPQGRTSEDLLDLAESRVFKIAESRANKDEGPKNIADVLDATVARIEQLFQQPHDGVTGVNTG
YDDLNKKTAGLQPSDLIIVAARPSMGKTTFAMNLVENAAMLQDKPVLIFSLEMPSEQIMMRSLASLSRVD
QTKIRTGQLDDEDWARISGTMGILLEKRNIYIDDSSGLTPTEVRSRARRIAREHGGIGLIMIDYLQLMRV
PALSDNRTLEIAEISRSLKALAKELNVPVVALSQLNRSLEQRADKRPVNSDLRESGSIEQDADLIMFIYR

DEVYHENSDLKGIAEIIIGKQRNGPIGTVRLTFNGQWSRFDNYAGPQYDDE

>gi|30064637|ref|NP_838808.1| hypothetical protein S3573 [Shigella flexneri 2a str. 2457T]

MWVNKYTDDCTDEDLNDRDFIASVVDRAIFHFAINSICNPGDNKDAMPIEQCTFDVETKNGLPSTVQLFY

EESKDNEPLANIHFQAIGSGFLTFVNACQEHDDNSLKLFASLLISLSYSSAYADLSETVYINENNESYLK

AQFEKLYQRDMKKYLGEMKRLADGGEMNFDGYLDKMSHLVNEGTLDPDILSKMRDAAPQLISFAKSFDPT

SKEEIKILTDTSKLIYDLFGVKSEK

>gi|30064633|ref|NP_838804.1| LexA repressor [Shigella flexneri 2a str. 2457T]

MKALTARQQEVFDLIRDHISQTGMPPTRAEIAQRLGFRSPNAAEEHLKALARKGVIEIVSGASRGIRLLQ

EEEEGLPLVGRVAAGEPLLAQQHIEGHYQVDPSLFKPNADFLLRVSGMSMKDIGIMDGDLLAVHKTQDVR

NGQVVVARIDDEVTVKRLKKQGNKVELLPENSEFKPIVVDLRQQSFTIEGLAVGVIRNGDWL

>gi|30064632|ref|NP_838803.1| diacylglycerol kinase [Shigella flexneri 2a str. 2457T]

MANNTTGFTRIIKAAGYSWKGLRAAWINEAAFRQEGVAVLLAVVIACWLDVDAITRVLLISSVMLVMIVE

ILNSAIEAVVDRIGSEYHELSGRAKDMGSAAVLIAIIVAVITWCILLWSHFG

>gi|30064629|ref|NP_838800.1| chorismate pyruvate lyase [Shigella flexneri 2a str. 2457T]

MSHPALTQLRALRYFKEIPALDPQLLDWLLLEDSMTKRFEQQGKTVSVTMIREGFVEQNEIPEELPLLPK

ESRYWLREILLCADGEPWLAGRTVVPVSTLSGPELALQKLGKTPLGRYLFTSSTLTRDFIEIGRDAGLWG

RRSRLRLSGKPLLLTELFLPASPLY

>gi|30064628|ref|NP_838799.1| maltose regulon periplasmic protein [Shigella flexneri 2a str. 2457T]

MKMNKSLIALCLSAGLLASAPGISLADVNYVPQNTSDAPAIPSAALQQLTWTPVDQSKTQTTQLATGGQQ

LNVPGISGPVAAYSVPANIGELTLTLTSEVNKQTSVFAPNVLILDQNMTPSAFFPSSYFTYQELGVMSAD

RLEGVMRLTPALGQQKLYVLVFTTEKDLQQTTQLLDPAKAYAKGVGNSIPDIPDPVARHTSDGLLKLKVK

TNSSSSVLVGPLFGSSAPAPVTVGNTAAPAVAAPAPVPVKKSEPMLNDTESYFNTAIKNAVAKGDVDKAL

KLLDEAERLGSTSARSTFISSVKGKG

>gi|30064627|ref|NP_838798.1| maltoporin [Shigella flexneri 2a str. 2457T]

MITLRKLPLAVAVAAGVMSAQAMAVDFHGYARSGIGWTGSGGEQQCFQTTGAQSKYRLGNECETYAELKL

GQEVWKEGDKSFYFDTNVAYSVAQQNDWEATDPAFREANVQGKNLIEWLPGSTIWAGKRFYQRHDVHMID

FYYWDISGPGAGLENIDVGFGKLSLAATRSSEAGGSSSFASNNIYDYTNETANDVFDVRLAQMEINPGGT

LELGVDYGRANLRDNYRLVDGASKDGWLFTAEHTQSVLKGFNKFVVQYATDSMTSQGKGLSQGSGVAFDN

EKFAYNINNNGHMLRILDHGAISMGDNWDMMYVGMYQDINWDNDNGTKWWTVGIRPMYKWTPIMSTVMEI

GYDNVESQRTGDKNNQYKITLAQQWQAGDSIWSRPAIRVFATYAKWDEKWGYDYNGDSKVNPNYGKAVPA

DFNGGSFGRGDSDEWTFGAQMEIWW

>gi|30064625|ref|NP_838796.1| maltose ABC transporter periplasmic protein [Shigella flexneri 2a str. 2457T]

MKIKTGARILALSALTTMMFSASALAKIEEGKLVIWINGDKGYNGLAEVGKKFEKDTGIKVTVEHPDKLE

EKFPQVAATGDGPDIIFWAHDRFGGYAQSGLLAEITPDKAFQDKLYPFTWDAVRYNGKLIAYPIAVEALS

LIYNKDLLPNPPKTWEEIPALDKELKAKGKSALMFNLQEPYFTWPLIAADGGYAFKYENGKYDIKDVGVD

NAGAKAGLTFLVDLIKNKHMNADTDYSIAEAAFNKGETAMTINGPWAWSNIDTSKVNYGVTVLPTFKGQP

SKPFVGVLSTGINAASPNKELAKEFLENYLLTDEGLEAVNKDKPLGAVALKSYEEELAKDPRIAATMENA

QKGEIMPNIPQMSAFWYAVRTAVINAASGRQTVDEALKDAQTRITK

>gi|30064624|ref|NP_838795.1| maltose transporter membrane protein [Shigella flexneri 2a str. 2457T]

MDVIKKKHWWQSDALKWSVLGLLGLLVGYLVVLMYAQGEYLFAITTLILSSAGLYIFANRKAYAWRYVYP

GMAGMGLFVLFPLVCTIAIAFTNYSSTNQLTFERAQEVLLDRSWQAGKTYNFGLYPAGDEWQLALSDGET

GKNYLSDAFKFGGEQKLQLKETTAQPEGERANLRVITQNRQALSDITAILPDGNKVMMSSLRQFSGTQPL

YTLDGDGTLTNNQSGVKYRPNNQIGFYQSITADGNWGDEKLSPGYTVTTGWKNFTRVFTDEGIQKPFLAI

FVWTVVFSLITVFLTVAVGMVLACLVQWEALRGKAVYRVLLILPYAVPSFISILIFKGLFNQSFGEINMM

LSALFGVKPAWFSDPTTARTMLIIVNTWLGYPYMMILCMGLLKAIPDDLYEASAMDGAGPFQNFFKITLP

LLIKPLTPLMIASFAFNFNNFVLIQLLTNGGPDRLGTTTPAGYTDLLVNYTYRIAFEGGGGQDFGLAAAI

ATLIFLLVGALAIVNLKATRMKFD

>gi|30064623|ref|NP_838794.1| maltose transporter permease [Shigella flexneri 2a str. 2457T]

MAMVQPKSQKARLFITHLLLLLFIAAIMFPLLMVVAISLRQGNFATGSLIPEQISWDHWKLALGFSVEQA

DGRITPPPFPVLLWLWNSVKVAGISAIGIVALSTTCAYAFARMRFPGKATLLKGMLIFQMFPAVLSLVAL

YALFDRLGEYIPFIGLNTHGGVIFAYLGGIALHVWTIKGYFETIDSSLEEAAALDGATPWQAFRLVLLPL

SVPILAVVFILSFIAAITEVPVASLLLRDVNSYTLAVGMQQYLNPQNYLWGDFAAAAVMSALPITIVFLL

AQRWLVNGLTAGGVKG

>gi|30064621|ref|NP_838792.1| phosphate-starvation-inducible protein PsiE [Shigella flexneri 2a str. 2457T]

MTSLSRPRVEFISTILQTVLNLGLLCLGLILVVFLGKETVHLADVLFAPEQTSKYELVEGLVVYFLYFEF

IALIVKYFQSGFHFPLRYFVYIGITAIVRLIIVDHKSPLDVLIYSAAILLLVITLWLCNSKRLKRE

>gi|30064620|ref|NP_838791.1| hypothetical protein S3555 [Shigella flexneri 2a str. 2457T]

MKKRHLLSLLALGISTACYGETYPAPIGPSQSDFGGVGLLQTPTARMAREGELSLNYRDNDQYRYYSASV

QLFPWLETTLRYTDVRTRQYSSVEAFSGDQTYKDKAFDLKLRLWEESYWLPQVAVGARDIGGTGLFDAEY

LVASKAWGPFDFTLGLGWGYLGTSGNVKNPLCSASDKYCYRDNSYKQAGSIDGSQMFHGPASLFGGVEYQ

TPWQPLRLKLEYEGNNYQQDFAGKLEQKSKFNVGAIYRVTDWADVNLSYERGNTFMFGVTLRTNFNDLRP

SYIDNARPQYQPQPQDAILQHSVVANQLTLLKYNAGLADPQIQAKGDTLYVTGEQVKYRDSREGIIRANR

IVMNDLPDGIKTIRITENRLNMPQVTTETDVASLKNHLAGEPLGHETTLAHKRVEPVVPKSTEQGWYIDK

SRFDFHIDPVLNQSVGGPENFYMYQLGVMGTADLWLTDHLLTTGSLFANLANNYDKFNYTNPPQDSHLPR

VRTHVREYVQNDVYVNNLQANYFQHLGNGFYGQVYGGYLETMFGGAGAEVLYRPLDSNWAFGLDANYVKQ

RDWRSAKDMMKFTDYSVKTGHLTAYWTPSFAQDVLVKASVGQYLAGDKGGTLEIAKRFDSGVVVGGYATI

TNVSKEEYGEGDFTKGVYVSVPLDLFSSGPTRSRAAIGWTPLTRDGGQQLGRKFQLYDMTSDRSVNFR

>gi|30064619|ref|NP_838790.1| hypothetical protein S3554 [Shigella flexneri 2a str. 2457T]

MIKQTIVALLLSVGASSVFAAGTVKVFSNGSSEAKTLTGAEHLIDLVGQPRLANSWWPGAVISEELATAA

ALRQQQALLTRLAEQGADSSTDDAAAINALRQQIQALKVTGRQKINLDPDIVRVAERGNPPLQGNYTLWV

GPPPSTVTLFGLISHPGNQPFTPGRDVASYLSDQSLLSGADRSYAWVVVYPDGRTQKAPVAYWNKRHVEPM

PGSIIYVGLADSVWSETPDALNADILQTLTQRIPQ

>gi|30064618|ref|NP_838789.1| hypothetical protein S3553 [Shigella flexneri 2a str. 2457T]

MVILPLWRRVVKRPALILICLLLQACSATTKELGNSLWDSLFGTPGVQLTDDDIQNMPYASQYMQLNGGP

QLFVVLAFAEDGQQKWVTQDQATLVTQHGRLVKTLLGGDNLIEVNNLAADPLIKPAQIVDGATWTRTMGW

TEYQQVRYATARSVFKWDGTDTVKVGSDETLVRVLDEEVSTDQARWHNRYWIDSEGQIRQSEQYLGADYF

PVKTTLIKAAKQ

>gi|30064615|ref|NP_838786.1| aspartate kinase III [Shigella flexneri 2a str. 2457T]

MSEIVVSKFGGTSVADFDAMNRSADIVLSDANVRLVVLSASAGITNLLVALAEGLEPGERFEKLDAIRNI

QFAILERLRYPNVIREEIERLLENITVLAEAAALATSPALTDELVSHGELMSTLLFVEILRERDVQAQWF

DVRKVMRTNDRFGRAEPDVAALAELAALQLLPRLNEGLVITQGFIGSENKGRTTTLGRGGSDYTAALLAE

ALHASRVDIWTDVPGIYTTDPRVVSAAKRIDEIAFAEAAEMATFGAKVLHPATLLPAVRSDIPVFVGSSK

DPRAGGTLVCNKTENPPLFRALALRRNQTLLTLHSLNMLHSRGFLAEVFGILARHNISVDLITTSEVSVA

LTLDTTGSTSTGDTLLTQSLLMELSALCRVEVEEGLALVALIGNDLSKACGVGKEVFGVLEPFNIRMICY

GASSHNLCFLVPGEDAEQVVQKLHSNLFE

>gi|30064611|ref|NP_838782.1| potassium transporter peripheral membrane component [Shigella flexneri 2a str. 2457T]

MKIIILGAGQVGGTLAENLVGENNDITVVDTNGERLRTLQDKFDLRVVQGHGSHPRVLREAGADDADMLV

AVTSSDETNMVACQVAYSLFNTPNRIARIRSPDYVRDADKLFHSDAVPIDHLIAPEQLVIDNIYRLIEYP

GALQVVNFAEGKVSLAVVKAYYGGPLIGNALSTMREHMPHIDTRVAAIFRHDRPIRPQGSTIVEAGDEVF

FIAASQHIRAVMSELQRLEKPYKRIMLVGGGNIGAGLARRLEKDYSVKLIERNQQRAAELAEKLQNTIVF

FGDASDQELLAEEHIDQVDLFIAVTNDDEANIMSAMLAKRMGAKKVMVLIQRRAYVDLVQGSVIDIAISP

QQATISALLSHVRKADIVGVSSLRRGVAEAIEAVAHGDESTSRVVGRVIDEIKLPPGTIIGAVVRGNDVM

IANDNLRIEQGDHVIMFLTDKKFITDVERLFQPSPFFL

>gi|30064607|ref|NP_838778.1| DNA protecting protein DprA [Shigella flexneri 2a str. 2457T]

MVDIEIWLRLMSISSLYGDDMVRIAHWLAKQSHIDAVVLQQTGLTLRQAQRFLSFPRKSIESSLCWLEQP

NHHLIPADSEFYPPQLQATTDYPGALFVEGELHALHSFQLAVVGSRAHSWYGERWGRLFCETLATCGVTI

TSGLARGIDGVVHKAALQVNGVSIAVLGNGLNTIHPRRHARLAASLFEQGGALVSEFPLDVPPLAYNFPR

RNRIISGLSKGVLVVEAALRSGSLVTARCALEQGREVFALPGPIGNPGSEGPHWLIKQGAILVTEPEEIL

ENLQFGLHWLPDAPENSFYSPDQQDVALPFPELLANVGDEVTPVDVVAERAGQPVPEVVTQLLELELAGW

IAAVPGGYVRLRRACHVRRTNVFV

>gi|30064605|ref|NP_838776.1| DNA topoisomerase [Shigella flexneri 2a str. 2457T]

MRNNESCPKCGAELVIRSGKHGPFLGCSQYPACDYVRPLKSSADGHIVKVLEGQVCPVCGANLVLRQGRF

GMFIGCSNYPECEHTELIDKPDETAITCPQCRTGHLVQRRSRYGKTFHSCDRYPECQFAINFKPIAGECP

ECHYPLLIEKKTAQGVKHFCASKQCGKPVSAE

>gi|30064603|ref|NP_838774.1| shikimate 5-dehydrogenase [Shigella flexneri 2a str. 2457T]

METYAVFGNPIAHSKSPFIHQQFAQQLNIEHPYGRVLAPINDFINTLNAFFRAGGKGANVTVPFKEEAFA

RADELTERAALAGAVNTLKRLEDGRLLGDNTDGVGLLSDLERLSFIRPGLRILLIGAGGASRGVLLPLLS

LDCAVTITNRTVSRAEELAKLFAHTGSIQALGMDELEGHEFDLIINATSSGISGDIPAIPSSLIHPGIYC

YDMFYQKGKTPFLAWCEQRGSKRNADGLGMLVAQAAHAFLLWHGVLPDVEPVIKQLQEELSA

>gi|30064601|ref|NP_838772.1| transferase [Shigella flexneri 2a str. 2457T]

MKCFTLSSQRRRIIGIPIFCTSIFLIFFSVCCFFTLFAAIAHKTVLFCILKDLHKANNAPKVISKSFIAE

VSMSDVLRPYRDLFPQIGQRVMIDDSSVVIGDVRLADDVGIWPLVVIRGDVHYVQIGARTNIQDGSMLHV

THKSSYNPDGNPLTIGEDVTVGHKVMLHGCTIGNRVLVGMGSILLDGAIVEDDVMIGAGSLVPQNKRLES

GYLYLGSPVKQIRPLSDEEKAGLRYSANNYVKWKDEYLDQGNQTQP

>gi|30064599|ref|NP_838770.1| transport system permease [Shigella flexneri 2a str. 2457T]

MTKVLLSQPSRPASHNSSRAMVWVRKNLFSSWSNSLLTIGCIWLMWELIPPLLNWAFLQANWVGSTRADC

TKAGACWVFIHERFGQFMYGLYPHDQRWRINLALLIGLVSIAPMFWKILPHRGRYIAAWAVIYPLIVWWL

MYGGFFGLERVETRQWGGLTLTLIIASVGIAGALPWGILLALGRRSHMPIVRILSVIFIEFWRGVPLITV

LFMSSVMLPLFMAEGTSIDKLIRALVGVILFQSAYVAEVVRGGLQALPKGQYEAAESLALGYWKTQGLVI

LPQALKLVIPGLVNTIIALFKDTSLVIIIGLFDLFSSVQQATVDPAWLGMSTEGYVFAALIYWIFCFSMS

RYSQHLEKRFNTGRTPH

>gi|30064596|ref|NP_838767.1| periplasmic binding transport protein [Shigella flexneri 2a str. 2457T]

MQCGISDGLPRFSYADADGKFSGIDVDVCRGVAAAVFGDDTKVKYTPLTAKERFTALQSGEVDLLSRNTT

WTSSRDAGMGMAFTGVTYYDGIGFLTHDKAGLKSAKELDGATVCIQAGTDTELNVADYFKANNMKYTPVT

FDRSDESAKALESGRCDTLASDQSQLYALRIKLSNPAEWIVLPEVISKEPLGPVVRRGDDEWFSIVRWTL

FAMLNAEEMGINSQNVDEKAANPATPDMAHLLGKEGDYGKDLKLDNKWAYNIIKQVSNYSEIFERNVGSE

SPLKIKRGQNNLWNNGGIQYAPPVR

>gi|30064593|ref|NP_838764.1| methyltransferase [Shigella flexneri 2a str. 2457T]

MRTGCEPTRFGNEAKTIIHGDAFAELKKLPTESVDLLFADPPYNIGKNFDGLIEAWKEDLFIDWLFEVIA

ECHRVLKKQGSMYIMNSTENMPFIDLQCRKLFTIKSRIVWSYDSSGVQAKKHYGSMYEPILMMVKDAKNY

TFNGDAILVEAKTGSQRALIDYRKNPPQPYNHQKVPGNVWDFPRVRYLMDEYENHPTQKPKALLKRIILA

SSNPGDIVLDPFAGSFTTGAVAIASGRKFIGIEINSEYIKMGLRRLDVASHYSAEELAKVKKRKTGNLSK

RSRLSEVDPDLIAK

>gi|30064583|ref|NP_838754.1| regulatory protein CsrD [Shigella flexneri 2a str. 2457T]

MRLTTKFSAFVTLLTGLTIFVTLLGCSLSFYNAIQYKFSHRVQAVATAIDTHLVSNDFSALRPQITELMV

SADIVRVDLLHGDKQVYTLARNGSYRPVGTSDLFRELSVPLIKHPGMSLRLVYQDPMGNYFHSLMTTAPL

TGAIGFIILMLFLAVRWLQRQLAGQELLETRATRILNGERGSNVLGTIYEWPPRTSSALDTLLREIQNAR

EQHSRLDTLIRSYAAQDVKTGLNNRLFFDNQLATLLEDQEKVGTHGIVMMIRLPDFNMLSDTWGHSQVEE

QFFTLTNLLSTFMMRYPGALLARYHRSDFAALLPHRTLKEAESIASQLIKAVDTLPNNKMLDRDDMIHIG

ICAWRSGQDTEQVMEHAESATRNAGLQGGNSWAIYDDSLPEKGRGNVRWRTLIEQMLSRGGPRLYQKPAV

TREGQVHHRELMCRIFDGNEEVSSAEYMPMVLQFGLSEEYDRLQISRLIPLLRYWPEENLAIQVTVESLI

RPRFQRWLRDTLMQCEKSQRKRIIIELAEADVGQHISRLQPVIRLVNALGVRVAVNQAGLTLVSTSWIKE

LNVELLKLHPGLVRNIEKRTENQLLVQSLVEACSGTSTHVYATGVRSRSEWQTLIQRGVTGGQGDFFASS

QPLDTNVKKYSQRYSV

>gi|30064580|ref|NP_838751.1| rod shape-determining protein MreD [Shigella flexneri 2a str. 2457T]

MASYRSQGRWVIWLSFLIALLLQIMPWPDNLIVFRPNWVLLILLYWILALPHRVNVGTGFVMGAILDLIS

GSMLGVRVLAMSIIAYLVALKYQLFRNLALWQQALVVMLLSLVVDIIVFWAEFLVINVSFRPEVFWSSVV

NGVLWPWIFLLMRKVRQQFAVQ

>gi|30064578|ref|NP_838749.1| ribonuclease G [Shigella flexneri 2a str. 2457T]

MTAELLVNVTPSETRVAYIDGGILQEIHIEREARRGIVGNIYKGRVSRVLPGMQAAFVDIGLDKAAFLHA

SDIMPHTECVAGEEQKQFTVRDISELVRQGQDLMVQVVKDPLGTKGARLTTDITLPSRYLVFMPGASHVG

VSQRIESESERERLKKVVAEYCDEQGGFIIRTAAEGVGEAELASDAAYLKRVWTKVMERKKRPQTRYQLY

GELALAQRVLRDFADAELDRIRVDSRLTYEALLEFTSEYIPEMTSKLEHYTGRQPIFDLFDVENEIQRAL

ERKVELKSGGYLIIDQTEAMTTVDINTGAFVGHRNLDDTIFNTNIEATQAIARQLRLRNLGGIIIIDFID

MNNEDHRRRVLHSLEQALSKDRVKTSVNGFSALGLVEMTRKRTRESIEHVLCNECPTCHGRGTVKTVETV

CYEIMREIVRVHHAYDSDRFLVYASPAVAEALKGEESHSLAEVEIFVGKQVKVQIEPLYNQEQFDVVMM

>gi|30064576|ref|NP_838747.1| protease TldD [Shigella flexneri 2a str. 2457T]

MSLNLVSEQLLAANGLKHQDLFAILGQLAERRLDYGDLYFQSSYHESWVLEDRIIKDGSYNIDQGVGVRA

ISGEKTGFAYADQISLLALEQSAQAARTIVRDSGDGKVQTLGAVEHSPLYTSVDPLQSMSREEKLDILRR

VDKVAREADKRVQEVTASLSGVYELILVAATDGTLAADVRPLVRLSVSVLVEEDGKRERGASGGGGRFGY

EFFLADLDGEVRADAWAKEAVRMALVNLSAVAAPAGTMPVVLGAGWPGVLLHEAVGHGLEGDFNRRGTSV

FSGQVGELVASELCTVVDDGTMVDRRGSVAIDDEGTPGQYNVLIENGILKGYMQDKLNARLMGMTPTGNG

RRESYAHLPMPRMTNTYMLPGKSTPQEIIESVEYGIYAPNFGGGQVDITSGKFVFSTSEAYLIENGKVTK

PVKGATLIGSGIETMQQISMVGNDLKLDNGVGVCGKEGQSLPVGVGQPTLKVDNLTVGGTA

>gi|30064573|ref|NP_838744.1| p-hydroxybenzoic acid efflux subunit AaeA [Shigella flexneri 2a str. 2457T]

MKTLIRKFSRTAITVVLVILAFIAIFNAWVYYTESPWTRDARFSADVVAIAPDVSGLITQVNVHDNQLVK

KGQILFTIDQPRYQKALEEAQADVAYYQVLAQEKRQEAGRRNRLGVQAMSREEIDQANNVLQTVLHQLAK

AQATRDLAKLDLERTVIRAPADGWVTNLNVYTGEFITRGSTAVALVKQNSFYVLAYMEETKLEGVRPGYR

AEITPLGSNKVLKGTVDSVAAGVTNASSTRDDKGMATIDSDLEWVRLAQRVPVRIRLDNQQENIWPAGTT

ATVVVTGKQDRDESQDSFFRKMAHRLREFG

>gi|30064572|ref|NP_838743.1| p-hydroxybenzoic acid efflux subunit AaeB [Shigella flexneri 2a str. 2457T]

MGIFSIANQHIRFAVKLATAIVLALFVGFHFQLETPRWAVLTAAIVAAGPAFAAGGEPYSGAIRYRGFLR

IIGTFIGCIAGLVIIIAMIRAPLLMILVCCIWAGFCTWISSLVRIENSYAWGLAGYTALIIVITIQPEPL

LTPQFAVERCSEIVIGIVCAIMADLLFSPRSIKQEVDRELESLLVAQYQLMQLCIKHGDGEVVDKAWGDL

VRRTTALQGMRSNLNMESSRWARANRRLKAINTLSLTLITQSCETYLIQNTRPELITDTFREFFDTPVET

AQDVHKQLKRLRRVIAWTGERETPVTIYSWVAAATRYQLLKRGVISNTKINATEEEILQGEPEVKVESAE

RHHAMVNFWRTTLSCILGTLFWLWTGWTSGSGAMVMIAVVTSLAMRLPNPRMVAIDFIYGTLAALPLGLL

YFLVIIPNTQQSMLLLCISLAVLGFFLGIEVQKRRLGSMGALASTINIIVLDNPMTFHFSQFLDSALGQI

VGCVLAFTVILLVRDKSRDRTGRVLLNQFVSAAVSAMTTNVARRKENHLPALYQQLFLLMNKFPGDLPKF

RLALTMIIAHQRLRDAPIPVNEDLSAFHRQMRRTADHVISARSDDKRRRYFGQLLEELEIYQEKLRIWQA

PPQVTEPVHRLAGMLHKYQHALTDS

>gi|30064570|ref|NP_838741.1| hypothetical protein S3493 [Shigella flexneri 2a str. 2457T]

MGHETKAQLKDYVEVKIMKIKTTVAALSVLSVLSFGAFAADSIDAAQAQNREAIGTVSVSGVASSPMDIR

EMLNKKAEEKGATAYQITEARSGDTWHATAELYK

>gi|30064569|ref|NP_838740.1| arginine repressor [Shigella flexneri 2a str. 2457T]

MRSSAKQEELVKAFKALLKEEKFSSQGEIVAALQEQGFDNINQSKVSRMLTKFGAVRTRNAKMEMVYCLP

AELGVPTTSSPLKNLVLDIDYNDAVVVIHTSPGAAQLIARLLDSLGKAEGILGTIAGDDTIFTTPANGFT

VKDLYEAILELFDQEL

>gi|30064566|ref|NP_838737.1| cytochrome d ubiquinol oxidase subunit III [Shigella flexneri 2a str. 2457T]

MTWEYALIGLVVGIIIGAVAMRFGNRKLRQQQALQYELEKNKAELDEYREELVSHFARSAELLDTMAHDY

RQLYQHMAKSSSSLLPELSAEANPFRNRLAESEASNDQAPVQVPRDYSEGASGLLRTGAKRD

>gi|30064561|ref|NP_838732.1| ClpXP protease specificity-enhancing factor [Shigella flexneri 2a str. 2457T]

MDLSQLTPRRPYLLRAFYEWLLDNQLTPHLVVDVTLPGVQVPMEYARDGQIVLNIAPRAVGNLELANDEV

RFNARFGGIPRQVSVPLAAVLAIYARENGAGTMFEPEAAYDEDTSIMNDEEASADNETVMSVIDGDKPDH

DDDTHPDDEPPQPPRGGRPALRVVK

>gi|30064560|ref|NP_838731.1| cryptic C4-dicarboxylate transporter DcuD [Shigella flexneri 2a str. 2457T]

MFGIIISVIVLITMGYLILKNYKPQVVLAAAGVFLMMCGVWLGFGGVLDPAKSSGYLIVDIYNEILRMLS

NRIAGLGLSIMAVGGYARYMERIGASCAMVSLLSRPLKLIRSPYIILSATYVIGQIMAQFITSASGLGML

LMVTLFPTLVSLGVSRLSAVAVIATTMSIEWGILETNSIFAAQVAGMKIATYFFHYQLPVASCVIISVAI

SHFFVQRAFDKKDKNINHEQAEQKTLDNVPPLYYAILPVMPLILMLGSLFLAHVGLMKSELHLVVVMLLS

LTVTMFVEFFRKHNLRETMDDVQAFFDGMGTQFVNVVTLVVAGEIFAKGLTTIGTVDAVIRGAEHSGLGG

IGVMIIMALVIAICAIVMGSGNAPFMSFASLIPNIAAGLHVPAVVMIMPMHFATTLARAVSPITAVVVVT

SGIAGVSPFAVVKRTAIPMAVGFVVNMIATITLFY

>gi|30064557|ref|NP_838728.1| transcriptional regulator NanR [Shigella flexneri 2a str. 2457T]

MNAFDSQTEDSSPAIGRNLRSRPLARKKLSEMVEEELEQMIRRREFGEGEQLPSERELMAFFNVGRPSVR

EALAALKRKGLVQINNGERARVSRPSADTIIGELSGMAKDFLSHPGGIAHFEQLRLFFESSLVRYAAEHA

TDEQIDLLAKALEINSQSLDNNAAFIRSDVDFHRVLAEIPGNPIFMAIHVALLDWLIAARPTVTDQALHE

HNNVSYQQHIAIVDAIRRHDPDEADRALQSHLNSVSATWHAFGQTTNKKK

>gi|30064554|ref|NP_838725.1| N-acetylmannosamine-6-phosphate 2-epimerase [Shigella flexneri 2a str. 2457T]

MSLLAQLDQKIAANGGLIVSCQPVPDSPLDKPEIVAAMALAAEQAGAVAIRIEGVANLQATRAVVSVPII

GIVKRDLEDSPVRITAYIEDVDALAQAGADIIAIDGTDRPRPVPVETLLARIHHHGLLAMTDCSTPEDGL

ACQKLGAEIIGTTLSGYTTPETPEEPDLALVKTLSEAGCRVIAEGRYNTPAQAADAMRHGAWAVTVGSAI

TRLEHICQWYNTAMKKAVL

>gi|30064552|ref|NP_838723.1| hypothetical protein S3474 [Shigella flexneri 2a str. 2457T]

MMMGEVQSLPSAGLHPALQDALTLALAARPQEKAPGRYELQGDNIFMNVMTFNTQSPVEKKAELHEQYID

IQLLLNGEERILFGMAGTARQCEEFHHEDDYQLCSAIENEQTIILKPGMFAVFMPGEPHKPGCVVGEPGE

IKKVVVKVKADLMA

>gi|30064551|ref|NP_838722.1| IS600 orf [Shigella flexneri 2a str. 2457T]

MAHIRTRETYGTRRLQTELAENGIIVGRDRLARLRKELRLRCKQKRKFRATTNPNHNLPVAPNLLNQTFA

PTAPNQVWVADLTYVATQEGWLYLAGIKDVYTCEIVGYAMGERMTKELTGKALFMALRSQRPPAGLIHHS

DRGSQYCAYDYRVIQEQSGLKTSMSRKGNCYDNAPMESFWGTLKNESLSHYRFNNRDEAISVIREYIEIF

YNRQRRHSRLGNISPAAFRENIIRWLLKKRTNGSVRYCQYTSKVAMIYIEQLELIHKSGDVLYPVKITRK

SSGKTAFHLVPFGLNKTHDLLEVEDASEAIRLVIDERHSIRCSTLTATITNKKGKRIKRTGIYSIKGVNI

KEYNVR

>gi|30064545|ref|NP_838716.1| hypothetical protein S3465 [Shigella flexneri 2a str. 2457T]

MIRLSEQSPLGTGRHRKCYAHPEDAQRCIKIVYHRGDGGDKEIRRELKYYAHLGRRLKDWSGIPRYHGTV

ETDCGTGYVYDVIADFDGKPSITLTEFAEQCRYEEDIAQLRQLLKQLKRYLQDNRIVTMSLKPQNILCHR

ISESEVIPVVCDNIGESTLIPLATWSKWCCLRKQERLWKRFIAQPALAIALQKDLQPRESKTLALTSREA

>gi|30064543|ref|NP_838714.1| hypothetical protein S3463 [Shigella flexneri 2a str. 2457T]

MVLMIVSGRSGSGKSVALRALEDMGFYCVDNLPVVLLPDLARTLADREISAAVSIDVRNMPESPEIFEQA

MSNLPDAFSPQLLFLDADRNTLIRRYSDTRRLHPLSSKNLSLESAIDKESDLLEPLRSRADLIVDTSEMS

VHELAEMLRTRLLGKRERELTMVFESFGFKHGIPIDADYVFDVRFLPNPHWDPKLRPMTGLDKPVAAFLD

RHTEVHNFIYQTRSYLELWLPMLETNNRSYLTVAIGCTGGKHRSVYIAEQLADYFRSRGKNVQSRHRTLE

KRKP

>gi|30064542|ref|NP_838713.1| PTS IIA-like nitrogen-regulatory protein PtsN [Shigella flexneri 2a str. 2457T]

MTNNDTTLQLSSVLNRECTRSRVHCQSKKRALEIISELAAKQLSLPPQVVFEAILTREKMGSTGIGNGIA

IPHGKLEEDTLRAVGVFVQLETPIAFDAIDNQPVDLLFALLVPADQTKTHLHTLSLVAKRLADKTICRRL

RAAQSDEELYQIITDTEGTPDEA

>gi|30064540|ref|NP_838711.1| RNA polymerase factor sigma-54 [Shigella flexneri 2a str. 2457T]

MKQGLQLRLSQQLAMTPQLQQAIRLLQLSTLELQQELQQALESNPLLEQIDTHEEIDTRETQDSETLDTA

DALEQKEMPEELPLDASWDTIYTAGTPSGTSGDYIDDELPVYQGETTQTLQDYLMWQVELTPFSDTDRAI

ATSIVDAVDETGYLTVPLEDILESMGDEEIDIDEVEAVLKRIQRFDPVGVAAKDLRDCLLIQLSQFDKTT

PWLEEARLIISDHLDLLANHDFRTLMRVTRLKEDVLKEAVNLIQSLDPRPGQSIQTGEPEYVIPDVLVRK

HNGHWTVELNSDSIPRLQINQHYASMCNNARNDGDSQFIRSNLQDAKWLIKSLESRNDTLLRVSRCIVEQ

QQAFFEQGEEYMKPMVLADIAQAVEMHESTISRVTTQKYLHSPRGIFELKYFFSSHVNTEGGGEASSTAI

RALVKKLIAAENPAKPLSDSKLTSLLSEQGIMVARRTVAKYRESLSIPPSNQRKQLV

>gi|30064538|ref|NP_838709.1| lipopolysaccharide transport periplasmic protein LptA [Shigella flexneri 2a str. 2457T]

MKFKTNKLSLNLVLASSLLAASIPAFAVTGDTDQPIHIESDQQSLDMQGNVVTFTGNVIVTQGTIKINAD

KVVVTRPGGEQGKEVIDGYGKPATFYQMQDNGKPVEGHASQMHYELAKDFVVLTGNAYLQQVDSNIKGDK

ITYLVKEQKMQAFSDKGKRVTTVLVPSQLQDKNNKGQTPAQKKGN

>gi|30064537|ref|NP_838708.1| hypothetical protein S3457 [Shigella flexneri 2a str. 2457T]

MSKARRWVIIVLSLAVLVMIGINMAEKDDTAQVVVNNNDPTYKSEHTDTLVYNPEGALSYRLIAQHVEYY

SDQAVSWFTQPVLTTFDKDKIPTWSVKADKAKLTNDRMLYLYGHVEVNALVPDSQLRRITTDNAQINLVT

QDVTSEDLVTLYGTTFNSSGLKMRGNLRSKNAELIEKVRTSYEIQNKQTQP

>gi|30064535|ref|NP_838706.1| D-arabinose 5-phosphate isomerase [Shigella flexneri 2a str. 2457T]

MSHVELQPGFDFQQAGKEVLAIERECLAELDQYINQNFTLACEKMFWCKGKVVVMGMGKSGHIGRKMAAT

FASTGTPSFFVHPSEAAHGDLGMVTPQDVVIAISNSGESSEITALIPVLKRLHVPLICITGRPESSMARA

ADVHLCVKVAKEACPLGLAPTSSTTATLVMGDALAVALLKARGFTAEDFALSHPGGALGRKLLLRVNDIM

HTGDEIPHVKKTASLRDALLEVTRKNLGMTVICDDNMMIEGIFTDGDLRRVFDMGVDVRQLSIADVMTPG

GIRVRPGILAVEALNLMQSRHITSVMVADGDHLLGVLHMHDLLRAGVV

>gi|30064532|ref|NP_838703.1| integral membrane protein [Shigella flexneri 2a str. 2457T]

MLLNALASLGHKGIKTLRTFGRAGLMLFNALVGKPEFRKHAPLLVRQLYNVGVLSMLIIVVSGVFIGMVL

GLQGYLVLTTYSAETSLGMLVALSLLRELGPVVAALLFAGRAGSALTAEIGLMRATEQLSSMEMMAVDPL

RRVISPRFWAGVISLPLLTVIFVAVGIWGGSLVGVSWKGIDSGFFWSAMQNAVDWRMDLVNCLIKSVVFA

ITVTWISLFNGYDAIPTSAGISRATTRTVVHSSLAVLGLDFVLTALMFGN

>gi|30064531|ref|NP_838702.1| cytoplasmic membrane protein [Shigella flexneri 2a str. 2457T]

MQTKKNEIWVGIFLLAALLAALFVCLKAANVTSIRTESTYTLYATFDNIGGLKARSPVSIGGVVVGRVAD

ITLDPKTYLPRVTLEIEQRYNHIPDTSSLSIRTSGLLGEQYLALNVGFEDPELGTAILKDGDTIQDTKSA

MVLEDLIGQFLYGSKGDDNKNSGDAPAAAPGNNETTEPVGTTK

>gi|30064530|ref|NP_838701.1| hypothetical protein S3450 [Shigella flexneri 2a str. 2457T]

MFKRLMMVALLVIAPLSAATAADQTNPYKLMDEAAQKTFDRLKNEQPQIRANPDYLRTIVDQELLPYVQV

KYAGALVLGQYYKSATPAQREAYFAAFREYLKQAYGQALAMYHGQTYQIAPEQPLGDKTIVPIRVTIIDP

NGRPPVRLDFQWRKNSQTGNWQAYDMIAEGVSMITTKQNEWGTLLRTKGIDGLTAQLKSISQQKITLEEK

K

>gi|30064529|ref|NP_838700.1| hypothetical protein S3449 [Shigella flexneri 2a str. 2457T]

MGNAAAYQRYRRPDCATEIDFSTENHSGREKIMSESLSWMQTGDTLALSGELDQDVLLPLWEMREEAVKG

ITCIDLSRVSRVDTGGLALLLHLIDLAKKQGNNVTLQGVNDKVYTLAKLYNLPADVLPR

>gi|30064527|ref|NP_838698.1| UDP-N-acetylglucosamine 1-carboxyvinyltransferase [Shigella flexneri 2a str. 2457T]

MDKFRVQGPTKLQGEVTISGAKNAALPILFAALLAEEPVEIQNVPKLKDVDTSMKLLSQLGAKVERNGSV

HIDARDVNVFCAPYDLVKTMRASIWALGPLVARFGQGQVSLPGGCTIGARPVDLHISGLEQLGATIKLEE

GYVKASVDGRLKGAHIVMDKVSVGATVTIMCAATLAEGTTIIENAAREPEIVDTANFLITLGAKISGQGT

DRIVIEGVERLGGGVYRVLPDRIETGTFLVAAAISRGKIICRNAQPDTLDAVLAKLRDAGADIEVGEDWI

SLDMHGKRPKAVNVRTAPHPAFPTDMQAQFTLLNLVAEGTGFITETVFENRFMHVPELSRMGAHAEIESN

TVICHGVEKLSGAQVMATDLRASASLVLAGCIAEGTTVVDRIYHIDRGYERIEDKLRALGANIERVKGE

>gi|30064524|ref|NP_838695.1| 50S ribosomal protein L21 [Shigella flexneri 2a str. 2457T]

MYAVFQSGGKQHRVSEGQTVRLEKLDIATGETVEFAEVLMIANGEEVKIGVPFVDGGVIKAEVVAHGRGE

KVKIVKFRRRKHYRKQQGHRQWFTDVKITGISA

>gi|30064522|ref|NP_838693.1| hypothetical protein S3442 [Shigella flexneri 2a str. 2457T]

MKQQAGIGILLALTTAICWGALPIAMKQVLEVMEPPTIVFYRFLMASIGLGAILAVKKRLPPLRVFRKPR

WLILLAVATAGLFGNFILFSSSLQYLSPTASQVIGQLSPVGMMVASVFILKEKMRSTQVVGALMLLSGLV

MFFNTSLVEIFTKLTDYTWGVIFGVGAATVWVSYGVAQKVLLRRLASPQILFLLYTLCTIALFPLAKPGV

IAQLSHWQLACLIFCGLNTLVGYGALAEAMARWQAAQVSAIITLTPLFTLFFSDLLSLAWPDFFARPMLN

LLGYLGAFVVVAGAMYSAIGHRIWGGLRKHTTVVSQPRAGE

>gi|30064520|ref|NP_838691.1| D-alanyl-D-alanine carboxypeptidase [Shigella flexneri 2a str. 2457T]

MRFSRFIIGLTSCIAFSVQAANVDEYVTQLPAGANLALMVQKVGASAPAIDYHSQQMALPASTQKVITAL

AALIQLGPDFRFTTTLETKGNVENGVLKGDLVARFGADPTLKRQDIRNMVATLKKSGVNQIDGNVLIDTS

IFASHDKAPGWPWNDMTQCFSAPPAAAIVDRNCFSVSLYSAPKPGDMAFIRVASYYPVTMFSQVRTLPRG

SAEAQYCELDVVPSDLNRFTLTGCLPQRSEPLPLAFAVQDGASYAGAILKDELKQAGITWSGTLLRQTQV

NEPGTVVASKQSAPLHDLLKIMLKKSDNMIADTVFRMIGHARFNVPGTWRAGSDAVRQILRQQAGVDIGN

TIIADGSGLSRHNLIAPATMMQVLQYIAQHDNELNFISMLPLAGYDGSLQYRAGLHQAGVDGKVSAKTGS

LQGVYNLAGFITTASGQRMAFVQYLSGYAVKPADQRNRRIPLVRFESRLYKDIYQNN

>gi|30064519|ref|NP_838690.1| transcription elongation factor GreA [Shigella flexneri 2a str. 2457T]

MQAIPMTLRGAEKLREELDFLKSVRRPEIIAAIAEAREHGDLKENAEYHAAREQQGFCEGRIKDIEAKLS

NAQVIDVTKMPNNGRVIFGATVTVLNLDSDEEQTYRIVGDDEADFKQNLISVNSPIARGLIGKEEDDVVV

IKTPGGEVEFEVIKVEYL

>gi|30064513|ref|NP_838684.1| preprotein translocase subunit SecG [Shigella flexneri 2a str. 2457T]

MYEALLVVFLIVAIGLVGLIMLQQGKGADMGASFGAGASATLFGSSGSGNFMTRMTALLATLFFIISLVL

GNINSNKTNRGSEWENLSAPAKTEQTQPAAPAKPTSDIPN

>gi|30064509|ref|NP_838680.1| transcription elongation factor NusA [Shigella flexneri 2a str. 2457T]

MNKEILAVVEAVSNEKALPREKIFEALESALATATKKKYEQEIDVRVQIDRKSGDFDTFRRWLVVDEVTQ

PTKEITLEAARYEDESLNLGDYVEDQIESVTFDRITTQTAKQVIVQKVREAERAMVVDQFREHEGEIITG

VVKKVNRDNISLDLGNNAEAVILREDMLPRENFRPGDRVRGVLYSVRPEARGAQLFVTRSKPEMLIELFR

IEVPEIGEEVIEIKAAARDPGSRAKIAVKTNDKRIDPVGACVGMRGARVQAVSTELGGERIDIVLWDDNP

AQFVINAMAPADVASIVVDEDKHTMDIAVEAGNLAQAIGRNGQNVRLASQLSGWELNVMTVDDLQAKHQA

EAHAAIDTFTKYLDIDEDFATVLVEEGFSTLEELAYVPMKELLEIEGLDEPTVEALRERAKNALATIAQA

QEESLGDNKPADDLLNLEGVDRDLAFKLAARGVCTLEDLAEQGIDDLADIEGLTDEKAGALIMAARNICW

FGDEA

>gi|30064507|ref|NP_838678.1| ribosome-binding factor A [Shigella flexneri 2a str. 2457T]

MAKEFGRPQRVAQEMQKEIALILQREIKDPRLGMMTTVSGVEMSRDLAYAKVYVTFLNDKDEDAVKAGIK

ALQEASGFIRSLLGKAMRLRIVPELTFFYDNSLVEGMRMSNLVTSVVKHDEERRVNPDDSKED

>gi|30064503|ref|NP_838674.1| lipoprotein NlpI [Shigella flexneri 2a str. 2457T]

MKPFLRWCFVATALTLAGCSNTSWRKSEVLAVPLQPTLQQEVILARMEQILASRALTDDERAQLLYERGV

LYDSLGLRALARNDFSQALAIRPDMPEVFNYLGIYLTQAGNFDAAYEAFDSVLELDPTYNYAHLNRGIAL

YYGGRDKLAQDDLLAFYQDDPNDPFRSLWLYLAEQKLDEKQAKEVLKQHFEKSDKEQWGWNIVEFYLGNI

SEQTLMERLKADATDNTSLAEHLSETNFYLGKYYLSLGDLDSATALFKLAVANNVHNFVEHRYALLELSL

LGQDQDDLAESDQQ

>gi|30064501|ref|NP_838672.1| tryptophan permease [Shigella flexneri 2a str. 2457T]

MATLTTTQTSPSLLGGVVIIGGTIIGAGMFSLPVVMSGAWFFWSMAALIFTWFCMLHSGLMILEANLNYR

IGSSFDTITKDLLGKGWNVVNGISIAFVLYILTYAYISASGSILHHTFAEMSLNVPARAAGFGFALLVAF

VVWLSTKAVSRMTAIVLGAKVITFFLTFGSLLGHVQPATLFNVAESNASYAPYLLMTLPFCLASFGYHGN

VPSLMKYYGKDPKTIVKCLVYGTLMALALYTIWLLATMGNIPRPEFIGIAEKGGNIDVLVQALSGVLNSR

SLDLLLVVFSNFAVASSFLGVTLGLFDYLADLFGFDDSAVGRLKTALLTFAPPVVGGLLFPNGFLYAIGY

AGLAATIWAAIVPALLARASRKRFGSPKFRVWGGKPMIALILVFGVGNALVHILSSFNLLPVYQ

>gi|30064500|ref|NP_838671.1| hypothetical protein S3418 [Shigella flexneri 2a str. 2457T]

MTDKTIAFSLLDLAPIPEGSSAREAFSHSLDLARLAEKRGYHRYWLAEHHNMTGIASAATSVLIGYLAAN

TTTLHLGSGGVMLPNHLPLVIAEQFGTLNTLYPGRIDLGLGRAPGSDQRTMMALRRHMSGDIDNFPRDVA

ELVDWFDARDPNPHVRPVPGYGEKIPVWLLGSSLYSAQLAAQLGLPFAFASHFAPDMLFQALHLYRSNFK

PSARLEKPYAMVCINIIAADSNRDAEFLFTSMQQAFVKLRRGETGQLPPPIQNMDQFWSPSEQYDVQQAL

SMSLVGDKAKVRHGLQSILRETDADEIMVNGQIFDHQARLHSFELAMDVKEELLG

>gi|30064499|ref|NP_838670.1| hypothetical protein S3417 [Shigella flexneri 2a str. 2457T]

MKYSLGPVLWYWPKETLEDFYQQAAASSADVIYLGEAVCSKRRATKVGDCLEMAKSLAGSGKQIVLSTLA

LVQASSELGELKRYVENGEFLIEASDLGVVNMCAERKLPFVAGHALNCYNAVTLKILLKQGMMRWCMPVE

LSRDWLVNLLNQCDELGIRNQFEVEVLSYGHLPLAYSARCFTARSEDRPKDECETCCIKYPNGRNVLSQE

NQQVFVLNGIQTMSGYVYNLGNELASMQGLVDVVRLSPQGTDTFAMLDAFRANENGAVPLPLTANSDCNG

YWRRLAGLELQA

>gi|30064498|ref|NP_838669.1| collagenase [Shigella flexneri 2a str. 2457T]

MELLCPAGNLPALKAAIENGADAVYIGLKDDTNARHFAGLNFTEKKLQEAVSFVHQHRRKLHIAINTFAH

PDGYARWQRAVDMAAQLGADALILADLAMLEYAAERYPHIERHVSVQASATNEEAINFYHRHFDVARVVL

PRVLSIHQVKQLARVTPVPLEVFAFGSLCIMSEGRCYLSSYLTGESPNTVGACSPARFVRWQQTPQGLES

RLNEVLIDRYQDGENAGYPTLCKGRYLVDGERYHALEEPTSLNTLELLPELMAANIASVKIEGRQRSPAY

VSQAAKVWRQAIDRCKADPQNFVPQSAWMETLGSMSEGTQTTLGAYHRKWQ

>gi|30064497|ref|NP_838668.1| hypothetical protein S3415 [Shigella flexneri 2a str. 2457T]

MLDKLRSRIVHLGPSLLSVPVKLTPFALKRQVLEQVLSWQFRQALDDGELEFLEGRWLSIHVRDIDLQWF

TSVVNGKLVVSQNTQADVSFSADASDLLMIAARKQDPDTLFFQRRLVIEGDTELGVYVKNLMDAIELEQM

PKALRMMLLQLADFVEAGMKTAPETKQTSVGEPC

>gi|30064496|ref|NP_838667.1| hypothetical protein S3414 [Shigella flexneri 2a str. 2457T]

MLIRVEIPIDAPGIDALLRRSFESDAEAKLVHDLREDGFLTLGLVATDDEGQVIGYVAFSPVDVQGEDLQ

WVGMAPLAVDEKYRGQGLARQLVYEGLDSLNEFGYAAVVTLGDPALYSRFGFELAAHHDLRCRWPGTESA

FLVHRLADDALNGVTGLVEYHEHFNRF

>gi|30064494|ref|NP_838665.1| hypothetical protein S3412 [Shigella flexneri 2a str. 2457T]

METLIAISRWLAKQHVVTWCVQQEGELWCANAFYLFDAQKVAFYILTEEKTRHAQMSGPQAAVAGTVNGQ

PKTVALIRGVQFKGEIRRLEGEESDLARKAYNRRFPVARMLSAPVWEIRLDEIKFTDNTLGFGKKMIWLR

DSGTEQA

>gi|30064491|ref|NP_838662.1| hypothetical protein S3409 [Shigella flexneri 2a str. 2457T]

MTGQSSSQAATPIQWWKPALFFLVVIAGLWYVKWQPYYGKAFTAAETHSIGKSILAQADANPWQAALDYA

MIYFLAVWKAAVLGVILGSLIQVLIPRDWLLRTLGQSRFRGTLLGTLFSLPGMMCTCCAAPVAAGMRRQQ

VSMGGALAFWMGNPVLNPATLVFMGFVLGWGFAAIRLVAGLVMVLLIATLVQKWVRETPQTQAPVEIDIP

EAQSGFFSRWGRALWTLFWSTIPVYILAVLVLGAARVWLFPHADGAVDNSLMWVVAMAVAGCLFVIPTAA

EIPIVQTMMLAGMGTAPALALLMTLPAVSLPSLIMLRKAFPAKALWLTGAMVAVSGVIVGGLALLF

>gi|30064490|ref|NP_838661.1| hypothetical protein S3408 [Shigella flexneri 2a str. 2457T]

MKALSPIAVLISALLLQGCVAAAVVGTAAVGTKAATDPRSVGTQVDDGTLEVRVNSALSKDEQIKKETRI

NVTAYQGKVLLVGQSPNAELSARAKQIAMGVDGANEVYNEIRQGQPIGLGEASNDTWITTKVRSQLLTSD

LVKSSNVKVTTENGEVFLMGLVTEREAKAAADIASRVSGVKRVTTAFTFIK

>gi|30064489|ref|NP_838660.1| DnaA initiator-associating protein DiaA [Shigella flexneri 2a str. 2457T]

MQERIKACFTESIQTQIAAAEALPDAISRAAMTLVQSLLNGNKILCCGNGTSAANAQHFAASMINRFETE

RPSLPAIALNTDNVVLTAIANDRLHDEVYAKQVRALGHAGDVLLAISTRGNSRDIVKAVEAAVTRDMTIV

ALTGYDGGELAGLLGPQDVEIRIPSHRSARIQEMHMLTVNCLCDLIDNTLFPHQDD

>gi|30064488|ref|NP_838659.1| hypothetical protein S3406 [Shigella flexneri 2a str. 2457T]

MATVPTRSGSPRQLTTKQTGDAWEAQARRWLEGKGLRFIAANVNERGGEIDLIMREGLTTVFVEVRYRRS

ALYGGAAASVTRSKQHKLLQTARLWLARHNGSFDTVDCRFDVVAFTGNEVEWIKDAFNDHS

>gi|30064487|ref|NP_838658.1| glycosylase [Shigella flexneri 2a str. 2457T]

MVPSTFSRLKAARCLPVVLAALIFAGCGTHTPDQSTAYMQGTAQADSAFYLQQMQQSSDDTRINWQLLAI

RALVKEGKTGQAVELFNQLPQELNDSQRREKTLLAAEIKLAQKDFAGAQNLLAKITPADLEQNQQARYWQ

AKIDASQGRPSIDLLRALIAQEPLLGAKEKQQNIDATWQALSSMTQEQANTLVINADENILQGWLDLQRV

WFDNRNDPDMMKAGIADWQKRYPNNPGAKMLPTQLVNVKAFKPASTNKIALLLPLNGQAAVFGRTIQQGF

EAAKNIGTQPVAAQVAAAPAADVAEQPQPQTADSVASPAQASVSDLTGDQPAAQPVPVSAPATSTAAVSA

PANPSAELKIYDTSSQPLSQILSQVQQDGASIVVGPLLKNNVEELLKSNTPLNVLALNQPENIENRVNIC

YFALSPEDEARDAARHIRDQGKQAPLVLIPRSALGDRVANAFAQEWQKLGGGTVLQQKFGSTSELRAGVN

GGSGIALTGSPITPRATTDSGMTTNNPTLQTTPTDDQFTNNGGRVDAVYIVATPGEIAFIKPMIAMRNGS

QSGATLYASSRSAQGTAGPDFRLEMEGLQYSEIPMLAGGNLPLMQQALSAVNNDYSLARMYAMGVDAWSL

ANHFSQMRQVQGFEINGNTGSLTANPDCVINRKLSWLQYQQGQVVPAS

>gi|30064486|ref|NP_838657.1| hypothetical protein S3404 [Shigella flexneri 2a str. 2457T]

MKQHQSADNSQGQLYIVPTPIGNLADITQRTLEVLQAVDLIAAEDTRHTGLLLQHFGINARLFALHDHNE

QQKAETLLAKLQEGQNIALVSDAGTPLINDPGYHLVRTCREAGIRVVPLPGPCAAITALSAAGLPSDRFC

YEGFLPAKSKGRRDALKAIEAEPRTLIFYESTHRLLDSLEDIVAVLGESRYVVLARELTKTWETIHGAPV

GELLAWVKEDENRRKGEMVLIVEGHKAQEEDLPADALRTLALLQAELPLKKAAALAAEIHGVKKNALYKY

ALEQQG

>gi|30064485|ref|NP_838656.1| fimbrial protein [Shigella flexneri 2a str. 2457T]

MKRAPLITGLLLISTSCAYASSGGCGADSTSGATNYSSVVDDVTVNQTDNVTGREFTSATLSSTNWQYAC

SCSAGKAVKLVYMVSPVLTTTGHQTGYYKLNDSLDIKTTLQANDIPGLTTDQVVSVNTRFTQIKSSTVYS

AATQTGVCQGDTSRYGPVNIGANTTFTLYVTKPFLGSMTIPKTDIAVIKGAWVDGMGSPSTGDFHDLVKL

SIQGNLTAPQSCKINQGDVIKVNFGFINGQKFTTRNAMPDGFTPVDFDITYDCGDTSKIKNSLQMRIDGT

TGVVDQYNLVARRRSSDNVPDVGIRIENLGGGVANIPFQNGILPVDPSGHGTVNMRAWPVNLVGGELETG

KFQGTATITVMVR

>gi|30064482|ref|NP_838653.1| chaperone [Shigella flexneri 2a str. 2457T]

MSKRTFAVILTLLCSFCIGQALAGGIVLQRTRVIYDASRKEAALPVANKGAETPYLLQSWVDNIDGKSRA

PFIITPPLFRLEASDDSSLRIIKTADNLPENKESLFYINVRAIPAKKKSDDVNANELTLVFKTRVKMFYR

PAHLKGRVNDAWKSLEFKRSDHSLNIYNPTEYYVVFAGLAVDKTDLTSKIEYIAPGEHKQLPLPASGGKN

VKWAAINDYGGSSGTETRPLQ

>gi|30064478|ref|NP_838649.1| N-acetylgalactosamine-specific PTS system transporter subunit IID [Shigella flexneri 2a str. 2457T]

MGSEISKKDITRLGFRSSLLQASFNYERMQAGGFTWAMLPILKKIYKDDKPGLSAAMKDNLEFINTHPNL

VGFLMGLLISMEEKGENRDTIKGLKVALFGPIAGIGDAIFWFTLLPIMAGICSSFASQGNLLGPILFFAV

YLLIFFLRVGWTHVGYSVGVKAIDKVRENSQMIARSATILGITVIGGLIASYVHINVVTSFAIDSTHSVA

LQQDFFDKVFPNILPMAYTLLMYYFLRVKKAHPVLLIGVTFVLSIVCSAFGIL

>gi|30064477|ref|NP_838648.1| N-acetylgalactosamine-specific PTS system transporter subunit IIC [Shigella flexneri 2a str. 2457T]

MHEITLLQGLSLAALVFVLGIDFWLEALFLFRPIIVCTLTGAILGDIQTGLITGGLTELAFAGLTPAGGV

QPPNPIMAGLMTTVIAWSTGVDAKTAIGLGLPFSLLMQYVILFFYSAFSLFMTKADKCAKEADTAAFSRL

NWTTMLIVASAYAVIAFLCTYLAQGAMQALVKAMPAWLTHGFEVAGGILPAVGFGLLLRVMFKAQYIPYL

IAGFLFVCYIRVSNLLPVAVLGAGFAVYEFFNAKSRQQAQPQPVASKNEEEDYSNGI

>gi|30064473|ref|NP_838644.1| transport enzyme subunit [Shigella flexneri 2a str. 2457T]

MLSIILTGHGGFASGMEKAMKQILGDQSQFIAIDFPETSSAALLTSQLEEAIAQLDCEDGIVFLTDLLGG

TPFRVASTLAMQKPGCEVITGTNLQLLLEMVLEREGLSGEEFRVQALECGHRGLTSLVDELGRCHEECPV

EEGI

>gi|30064472|ref|NP_838643.1| transport enzyme subunit [Shigella flexneri 2a str. 2457T]

MASNQTTLPNVSENEETLLTGVNENVYEDRSIAAELTKKDINRVAWRSMLLQASFNYERMQASGWLYGLL

PALKKIHTNKRDLARAMKGHMGFFNTHPFLVTFVIGIILAMERSKQDVNSIQSTKIAVGAPLGGIGDAMF

WLTLLPICGGIGASLALQGSILGAVVFIVLFNVVHLGLRFGLAHYAYRMGVAAIPLIKANTKKVGHAASI

VGMTVIGALVATYVRLSTTLEITAGDAVVKLQADVIDKLMPAFLPLVYTLTMFWLVRRG

>gi|30064471|ref|NP_838642.1| N-acetylgalactosameine-specific IIC component 2 [Shigella flexneri 2a str. 2457T]

MEISLLQAFALGIIAFIAGLDMFNGLTHMHRPVVLGPLVGLVLGDLHTGILTGDTLELVWMGLAPLAGAQ

PPNVIIGTIVGTAFAITTGVKPDVAVGVAVPFAVAVQMGITFLFSVMSGVMSRCDRMAENADTRGIERVN

YLALLALGTFYFLCAFLPIYFGAEHAKTIIDVLPQRLIDGLGVAGGIMPAIGFAVLLKIMMKNVYIPYFI

LGFVAATWLKLPVLAIAAAALAMALIDLLRKSPEPTQPAAQKEEFEDGI

>gi|30064470|ref|NP_838641.1| N-acetylgalactosamine-specific PTS system transporter subunit IIB [Shigella flexneri 2a str. 2457T]

MKANKQNKEEHAMPNIVLSRIDERLIHGQVGVQWVGFAGANLVLVANDEVAEDPVQQNLMEMVLAEGIAV

RFWTLQKVIDNIHRAADRQKILLVCKTPADFLTLVKGGVPVNRINVGNMHYANGKQQIAKTVSVDAGDIA

AFNDLKAAGVECFVQGVPTEPAVDLFKLL

>gi|30064469|ref|NP_838640.1| tagatose 6-phosphate kinase 2 [Shigella flexneri 2a str. 2457T]

MKHLTEMVRQHKAGKTNAIYAVCSAHPLVLEAAIRYASANQTPLLIEATSNQVDQFGGYTGMTPADFRGF

VCQLADSLNFPQDALILGGDHLGPNRWQNLPAAQAMANADDLIKSYVAAGFKKIHLDCSMSCQDDPIPLT

DDIVAERAARLAKVAEETCREHFGEADLEYVIGTEVPVPGGAHETLSELAVTTPDAARATLEAHRHAFEK

QGLNAIWPRIIALVVQPGVEFDHTNVIDYQPAKASALSQMVENYETLIFEAHSTDYQTPQSLRQLVIDHF

AILKVGPALTFALREALFSLAAIEEELVPAKACSGLRQVLENVMLDRPEYWQSHYHGDGNARRLARGYSY

SDRVRYYWPDSQIDDAFAHLVRNLADSPIPLPLISQYLPLQYVKVRSGELQPTPRELIINHIQDILAQYH

TACEGQ

>gi|30064468|ref|NP_838639.1| DNA-binding transcriptional regulator AgaR [Shigella flexneri 2a str. 2457T]

MSNTDASGEKRVTGTSERREQIIQRLRQQGSVQVNDLSALYGVSTVTIRNDLAFLEKQGIAVRAYGGALI

CDSTTPSVEPSVEDKSALNTAMKRSVAKAAVELIQPGHRVILDSGTTTFEIARLMRKYTDVIAMTNGMNV

ANALLEAEGVELLMTGGHLRRQSQSFYGDQAEQSLQNYHFDMLFLGVDAIDLERGVSTHNEDEARLSRRM

CEVAERIIVVTDSSKFNRSSLHKIIDTQRIDMIIVDEGIPADSLEGLRKAGVEVILVGDHASSL

>gi|30064467|ref|NP_838638.1| hydrolase [Shigella flexneri 2a str. 2457T]

MANIEIRQETPTAFYIKVHDTDNVAIIVNDNGLKAGTRFPDGLELIEHIPQGHKVALLDIPANGEIIRYG

EVIGYAVRAIPRGSWIDESMVVLPEAPPLHTLPLATKVPEPLPPLEGYTFEGYRNADGSVGTKNLLGITT

SVHCVAGVVDYVVKIIERDLLPKYPNVDGVVGLNHLYGCGVAINAPAAVVPIRTIHNISLNPNFGGEVMV

IGLGCEKLQPERLLTGTDDVQAIPVESASIVSLQDEKHVGFQSMVEDILQVAERHLQKLNQRQRETCPAS

ELVVGMHCGGSDAFSGVTANPAVGYASDLLVRCGATVMFSEVTEVRDAIHLLTPRAVNEEVGKRLLEEME

WYDNYLNMGKTDRSANPSPGNKKGGLANVVEKALGSIAKSGKSAIVEVLSPGQRPTKRGLIYAATPASDF

VCGTQQVASGITVQVFTTGRGTPYGLMAVPVIKMAPRTELANRWFDLMDINAGTIATGEETIEEEGWKLF

HFILDVASGKKKTFSDQWGLHNQLAVFNPAPVT

>gi|30064462|ref|NP_838633.1| DNA-binding transcriptional activator TdcR [Shigella flexneri 2a str. 2457T]

MTGITIFYGDNIIRYVVNTKKGLRPYFKQLPDNYQAKFELNLMSKFSNFIINKPFSAINTAARHIFSRYL

LENKHLFYQYFKISNTGIDHLEQLINVNFFSSDRTSFCECNRFP

>gi|30064461|ref|NP_838632.1| DNA-binding transcriptional activator TdcA [Shigella flexneri 2a str. 2457T]

MSTILLPKTQHLVVFQEVIRSGSIGSAAKELGLTQPAVSKIINDIEDYFGVELVVRKNTGVTLTPAGQLL

LSRSESITREMKNMVNEISGMSSEAVVEVSFGFPSLIGFTFMSGMINKFKEVFPKAQVSMYEAQLSSFLP

AIRDGRLDFAIGTLSAEMKLQDLHVEPLFESEFVLVASKSRTCTGTTTLESLKNEQWVLPQTNMGYYSEL

LTTLQRNGISIENIVKTDSVVTIYNLVLNADFLTVIPCDMTSPFGSNQFITIPVEETLPVAQYAAVWSKN

YRIKKAASVLVELAKEYSSYNGCRRRQLIEVG

>gi|30064459|ref|NP_838630.1| threonine/serine transporter TdcC [Shigella flexneri 2a str. 2457T]

MSTSDSIVSSQTKQSSWRKSDTTWTLGLFGTAIGAGVLFFPIRAGFGGLIPILLMLVLAYPIAFYCHRAL

ARLCLSGSNPSGNITETVEEHFGKTGGVVITFLYFFAICPLLWIYGVTITNTFMTFWENQLGFAPLNRGF

VALFLLLLMAFVIWFGKDLMVKVMSYLVWPFIASLVLISLSLIPYWNSAVIDQVDLGSLSLTGHDGILIT

VWLGISIMVFSFNFSPIVSSFVVSKREEYEKDFGRDFTERKCSQIISRASMLMVAVVMFFAFSCLFTLSP

ANMAEAKAQNIPVLSYLANHFASMTGTKTTFAITLEYAASIIALVAIFKSFFGHYLGTLEGLNGLILKFG

YKGDKTKVSLGKLNTLSMIFIMGSTWVVAYANPNILDLIEAMGAPIIASLLCLLPMYAIRKAPSLAKYRG

RLDNVFVTVIGLLTILNIVYKLF

>gi|30064457|ref|NP_838628.1| formate acetyltransferase 3 [Shigella flexneri 2a str. 2457T]

MKVDIDTSDKLYADAWLGFKGTDWKSEINVRDFIQHNYTPYEGDESFLAEATPATTELWEKVMEGIRIEN

ATHAPVDFDTNIATTITAHDAGYINQPLEKIVGLQTDAPLKRALHPFGGINMIKSSFHAYGREMDSEFEY

LFTDLRKTHNQGVFDVYSPDMLRCRKSGVLTGLPDGYGRGRIIGDYRRVALYGISYLVRERELQFADLQS

RLEKGEDLEATIRLREELAEHRHALLQIQEMAAKYGFDISRPAQNAQEAVQWLYFAYLAAVKSQNGGAMS

LGRTASFLDIYIERDFKAGVLNEQQAQELIDHFIMKIRMVRFLRTPEFDSLFSGDPIWATEVIGGMGLDG

RTLVTKNSFRYLHTLHTMGPAPEPNLTILWSEELPIAFKKYAAQVSIVTSSLQYENDDLMRTDFNSDDYA

IACCVSPMVIGKQMQFFGARANLAKTLLYAINGGVDEKLKIQVGPKTAPLMDDVLDYDKVMDSLDHFMDW

LAVQYISALNIIHYMHDKYSYEASLMALHDRDVYRTMACGIAGLSVATDSLSAIKYARVKPIRDENGLAV

DFEIDGEYPQYGNNDERVDSIACDLVERFMKKIKALPTYRNAVPTQSILTITSNVVYGQKTGNTPDGRRA

GTPFAPGANPMHGRDRKGAVASLTSVAKLPFTYAKDGISYTFSIVPAALGKEDPVRKTNLVGLLDGYFHH

EADVEGGQHLNVNVMNREMLLDAIEHPEKYPNLTIRVSGYAVRFNALTREQQQDVISRTFTQAL

>gi|30064454|ref|NP_838625.1| transport system permease [Shigella flexneri 2a str. 2457T]

MEIASNKGVIADASTPAGRAGMSESEWREAIKFDSTDTGWVIMSIGMAIGAGIVFLPVQVGLMGLWVFLL

SSVIGYPAMYLFQRLFINTLAESPECKDYPSVISGYLGKNWGILLGALYFVMLVIWMFVYSTAITNDSAS

YLHTFGVTEGLLSDSPFYGLVLICILVAISSRGEKLLFKISTDMVLTKLLVVAALGVSMVGMWHLYNVGS

LPPLGLLVKNAIITLPFTLTSILFIQTLSPMVISYRSREKSIEVARHKALRAMNIAFGILFVTVFFYAVS

FTLAMGHDEAVKAYEQNISALAIAAQFISGDGAAWVKVVSVILNIFAVMTAFFGVYLGFREATQGIVMNI

LRRKMPAEKINENLVQRGIMIFAILLAWSAIVLNAPVLSFTSICSPIFGMVGCLIPAWLVYKVPALHKYK

GMSLYLIIVTGLLLCVSLFLAFS

>gi|30064450|ref|NP_838621.1| hypothetical protein S3357 [Shigella flexneri 2a str. 2457T]

MITTRTARQCGQADYGWLQARYTFSFGHYFDPKLLGYASLRVLNQEVLAPGAAFQPRTYPKVDILNVILD

GEAEYRDSEGNHVQASAGEALLLSTQPGVSYSEHNLSKDKPLTRMQLWLDTCPQRENPLIQKLALNMDKQ

QLIASPEGAMGSLQLRQQVWLHHIVLDKGESANFQLHGPRAYLQSIHGKFHALTHHEEKAALTCGDGAFI

RDEANITLVADSPLRALLIDLPV

>gi|30064449|ref|NP_838620.1| LYSR-type transcriptional regulator [Shigella flexneri 2a str. 2457T]

MAKERALTLEALRVMDAIDRRGSFAAAADELGRVPSALSYTMQKLEEELDVVLFDRSGHRTKFTNVGRML

LERGRVLLEAADKLTTDAEALARGWETHLTIVTEALVPTPAFFPLIDKLAAKANTQLAIITEVLAGAWER

LEQGRADIVIAPDMHFRSSSEINSRKLYTLMNVYVAAPDHPIHQEPEPLSEVTRVKYRGIAVADTARERP

VLTVQLLDKQPRLTVSTIEDKRQALLAGLGVATMPYPMVEKDIAEGRLRVVSPESTSEIDIIMAWRRDSM

GEAKSWCLREIPKLFAGK

>gi|30064448|ref|NP_838619.1| cytochrome [Shigella flexneri 2a str. 2457T]

MDWYLKVLKNYVGFRGRARRKEYWMFILVNIIFTFVLGLLDKMLGWQRAGGEGILTIIYGILVFLPWWAV

QFRRLHDTDRSAWWALLFLIPFIGWLIIIVFNCQAGTPGENRFGPDPKLEQE

>gi|30064447|ref|NP_838618.1| transferase [Shigella flexneri 2a str. 2457T]

MGQLIDGVWHDTWYDTKSTGGKFQRSASAFRNWLTADGAPGPTGTGGFIAEKDRYHLYVSLACPWAHRTL

IMRKLKGLEPFISVSVVNPLMLENGWTFDDSFPGATGDTLYQHEFLYQLYLHADPHYSGRVTVPVLWDKK

NHTIVSNESAEIIRMFNTAFDALGAKAGDYYPPALQTKIDELNGWIYDTVNNGVYKAGFATSQQAYDEAV

AKVFESLARLEQILGQHRYLTGNQLTEADIRLWTTLVRFDPVYVTHFKCDKHRISDYLNLYGFLRDIYQM

PGIAETVNFDHIRNHYFRSHKTINPTGIISIGPWQDLDEPHGRDVRFG

>gi|30064445|ref|NP_838616.1| hypothetical protein S3350 [Shigella flexneri 2a str. 2457T]

MADTHHAQGPGKSVLGIGQRIVSIMVEMVETRLRLAVVELEEEKANLFQLLLMLGLTMLFAAFGLMSLMV

LIIWAVDPQYRLNAMIATTVVLLLLALIGGIWTLRKSRKSTLLRHTRHELANDRQLLEEESREQ

>gi|30064443|ref|NP_838614.1| hypothetical protein S3347 [Shigella flexneri 2a str. 2457T]

MSLRQLAWSGTVLLLVGTLLLAWSAVRQQESTLAIRAVHQGTTMPDGFSIWHHLDAHGIPFKSITPKNDT

LLITFDSSDQSAAAKAVLDRTLPHGYIIAQQDNNSQAMQWLTRLRDNSHRFG

>gi|30064442|ref|NP_838613.1| hypothetical protein S3346 [Shigella flexneri 2a str. 2457T]

MELLTQLLQALWAQDFETLANPSMIGMLYFVLFVILFLENGLLPAAFLPGDSLLVLVGVLIAKGAMGYPQ

TILLLTVAASLGCWVSYIQGRWLGNTRTVQNWLSHLPAHYHQRAHHLFHKHGLSALLIGRFIAFVRTLLP

TIAGLSGLNNARFQFFNWMSGLLWVLILTTLGYMLGKTPVFLKYEDQLMSCLMLLPVVLLVFGLAGSLVV

LWKKKYGNRG

>gi|30064441|ref|NP_838612.1| DNA-binding transcriptional repressor ExuR [Shigella flexneri 2a str. 2457T]

MEITEPRRLYQQLAADLKERIEQGVYLVGDKLPAERFIADEKNVSRTVVREAIIMLEVEGYVEVRKGSGI

HVVSNQPRHQQAADNNMEFANYGPFELLQARQLIESNIAEFAATQVTKQDIMKLMAIQEQARGEQCFRDS

EWDLQFHIQVALATQNSALAAIVEKMWTQRSHNPYWKKLHEHIDSRTVDNWCDDHDQILKALIRKDPHAA

KLAMWQHLENTKIMLFNETSDDFEFNADRYLFAENPVVHLDTATSGSK

>gi|30064435|ref|NP_838606.1| glucuronate isomerase [Shigella flexneri 2a str. 2457T]

MTPFMTEDFLLDTEFARRLYHDYAKDQPIFDYHCHLPPQQIAEDYRFKNLYDIWLKGDHYKWRAMRTNGV

AERLCTGDASDREKFDAWAATVPHTIGNPLYHWTHLELRRPFGITGKLLSPSTADEIWNECNELLAQDNF

SARGIMQQMNVKMVGTTDDPIDSLEHHAEIAKDGSFTIKVLPSWRPDKAFNIEQATFNDYMAKLGEVSDT

DIRRFADLQTALTKRLDHFAAHGCKVSDHALDVVMFAEANEAELDSILARRLAGETLSEHEVAQFKTAVL

VFLGAEYARRGWVQQYHIGALRNNNLRQFKLLGPDVGFDSINDRPMAEELSKLLSKQNEENLLPKTILYC

LNPRDNEVLGTMIGNFQGEGMPGKMQFGSGWWFNDQKDGMERQMTQLAQLGLLSRFVGMLTDSRSFLSYT

RHEYFRRILCQMIGRWVEAGEAPADINLLGEMVKNICFNNARDYFAIELN

>gi|30064434|ref|NP_838605.1| altronate hydrolase [Shigella flexneri 2a str. 2457T]

MQYIKIHALDNVAVALADLAEGTEVSVDNQTVTLRQDVARGHKFALTDIAKGANVIKYGLPIGYALADIA

AGEHVHAHNTRTNLSDLDQYRYQLDFQDLPAQAADPEVQIYRRANGDVGVRNELWILPTVGCVNGIARQI

QNRFLKETNNAEGTDGVFLFSHTYGCSQLGDDHINTRTMLQNMVRHPNAGAVLVIGLGCENNQVAAFRET

LGDIDPERVHFMICQQQDDEIEAGIEHLHQLYNVMRNDKREPGKLSELKFGLECGGSDGLSGITANPMLG

RFSDYVIANGGTTVLTEVPEMFGAEQLLMDHCRDEATFEKLVTMVNDFKQYFIAHDQPIYENPSPGNKAG

GITTLEDKSLGCTQKAGSSVVVDVLRYGERLKTPGLNLLSAPGNDAVATSALAGAGCHMVLFSTGRGTPY

GGFVPTVKIATNSELAAKKKHWIDFDAGQLIHGKAMPQLLEEFIDTIVEFANGKQTCNERNDFRELAIFK

SGVTL

>gi|30064433|ref|NP_838604.1| hypothetical protein S3337 [Shigella flexneri 2a str. 2457T]

MTAYWLAQGVGVIAFLIGITTFFNRDERRFKKQLSVYSAVIGVHFFLLGTYPAGASAILNAIRTLITLRT

RSLWVMAIFIVLTGGIGLAKFHHPVELLPVIGTIVSTWALFRCKGLTMRCVMWFSTCCWVIHNFWAGSIG

GTMIEGSFLLMNGLNIIRFWRMQKRGIDPFKVEKTPPAIDERG

>gi|30064431|ref|NP_838602.1| transporter [Shigella flexneri 2a str. 2457T]

MNTVGTPLLWGGFAVVVAIMLAIDLLLQGRRGAHAMTMKQAAAWSLVWVTLSLLFNAAFWWYLVQTEGRA

VADPQALAFLTGYLIEKSLAVDNVFVWLMLFSYFSVPAALQRRVLVYGVLGAIVLRTIMIFTGSWLISQF

DWILYIFGAFLLFTGVKMALAHEDESGIGDKPLVRWLRGHLRMTDTIDNEHFFVRKNGLLYATPLMLVLI

LVELSDVIFAVDSIPAIFAVTTDPFIVLTSNLFAILGLRAMYFLLAGVAERFSMLKYGLAVILVFIGIKM

LIVDFYHIPIAVSLGVVFGILVMTFIINAWVNYRHDKQRGE

>gi|30064429|ref|NP_838600.1| hypothetical protein S3333 [Shigella flexneri 2a str. 2457T]

MLRAFARLLLRICFSRRTLKIACLLLLVAGATIFIADRVMVNASKQLTWGDVNAVPARNVGLLLGARPGN

RYFTRRIDTAAALYHAGKVKWLLVSGDNGRKNYDEASGMQQALIAKGVPAKVIFCDYAGFSTLDSVVRAK

KVFGENHITIISQEFHNQRTIWLAKQYGIDAIGFNAPDLNMKHGFYTQLREKLARVSAVIDAKILHRQPK

YLGPSVMIGPFSEHGCPAKE

>gi|30064428|ref|NP_838599.1| hypothetical protein S3332 [Shigella flexneri 2a str. 2457T]

MSIPFFSFSWAVMSNLTYLQGYPEQLLSQVRTLINEQRLGDVLAKRYPGTHDYATDKALWQYTQDLKNQF

LRNAPPINKVMYDNKIHVLKNALGLHTAVSRVQGSKLKAKAEIRVATVFRNAPEPFLRMIVVHELAHLKE

KEHNKAFYQLCCHMEPQYHQLEFDTRLWLTQLSLGQDKI

>gi|30064427|ref|NP_838598.1| enzyme [Shigella flexneri 2a str. 2457T]

MSHLDNGFRSLTLQRFPATDDVNPLQAWEAADEYLLQQLDDTEILGPVLILNDAFGALSCALAEHKPYSI

GDSYISELATRENLRLNGIDESSVKFLDSTADYPQQPGVVLIKVPKTLALLEQQLRALRKVVTSDTRIIA

GAKARDIHTSTLELFEKVLGPTTTTLAWKKARLINCTFNEPPLADAPQTVSWKLEGTDWTIHNHANVFSR

TGLDIGARFFMQHLPENLEGEIVDLGCGNGVIGLTLLDKNPQAKVVFVDESPMAVASSRLNVETNMPEAL

DRSEFMINNALSGVEPFRFNAVLCNPPFHQQHALTDNVAWEMFHYARRCLKINGELYIVANRHLDYFHKL

KKIFGNCTTIATNNKFVVLKAVKLGRRR

>gi|30064426|ref|NP_838597.1| hypothetical protein S3330 [Shigella flexneri 2a str. 2457T]

MHLITQKALKDAAEKYPQHKTELVALGNTIAKGYFKKPESLKAVFPSLDNFKYLDKHYVFNVGGNELRVV

AMVFFESQKCYIREVMTHKEYDFFTAVHRTKGKK

>gi|30064425|ref|NP_838596.1| hypothetical protein S3329 [Shigella flexneri 2a str. 2457T]

MIAIADILQAGEKLTAVAPFLAGIQNEEQYTQALELVDHLLLNDPENPLLDLVCAKITAWEESAPEFAEF

NAMAQAMPGGIAVIRTLMDQYGLTLSDLPEIGSKSMVSRVLSGKRKLTLEHAKKLATRFGISPALFID

>gi|30064424|ref|NP_838595.1| NADPH dehydrogenase [Shigella flexneri 2a str. 2457T]

MSYPSLFAPLDLGFTTLKNRVLMGSMHTGLEEYPDGAERLAAFYAERARHGVALIVSGGIAPDLTGVGME

GGAMLNDASQIPHHRTITEAVHQEGGKIALQILHTGRYSYQPHLVAPSALQAPINRFVPHELTHEEILQL

IDDFARCAQLAREAGYDGVEVMGSEGYLINEFLTLRTNQRSDQWGGDYRNRMRFAVEVVRTVRERVGNDF

IIIYRLSMLDLVEGGGTFAETVELAQAIEAAGATIINTGIGWHEARIPTIATPVPRGAFSWVTRKLKGHV

SLPLVTTNRINDPQVADDILSRGDADIVSMARPFLADAELLSKAQSGRADEINTCIGCNQGCLDQIFVGK

VTSCLVNPRACHETKMPILPAVQKKNLAVVGAGPAGLAFAINAAARGHQVTLFDAHSEIGGQFNIAKQIP

GKEEFYETLRYYRRMIEVTGVTLKLNHTVTADQLQAFDETILASGIVPRVPPIDGIDHPKVLSYLDVLRD

KAPVGNKVAIIGCGGIGFDTAMYLSQPGESTSQNIARFCNEWGIDSSLQQAGGLSPQGMQIPRSPRQIVM

LQRKASKPGQGLGKTTGWIHRTTLLSRGVKMIPGVSYQKIDDDGLHVVINGETQVLAVDNVVICAGQEPN

RALAQPLIDSGKTVHLIGGCDVAMELDARRAIAQGTRLALEI

>gi|30064423|ref|NP_838594.1| glycosyl hydrolase [Shigella flexneri 2a str. 2457T]

MKIKTILTPVTCALLISFSAHAANADNYKNVINRTGAPQYMKDYDYDDHQRFNPFFDLGAWHGHLLPDGP

NTMGGFPGVALLTEEYINFMASNFDRLTVWQDGKKVNFTLEAYSIPGALVQKLTAKDVQVEMTLRFATPR

TSLLETKIISNKPLDLVWDGELLEKLEAKEGKPLSDKTIAGEYPDYQRKISATRDGLKVTFGKVRATWDL

LTSGESEYQVHKSLPVQTEINGNRFTSKAHINGSTTLYTTYSHLLTAQEVSKEQMQIRDILARPAFYLTA

SQQRWEEYLKKGLTNPDATPEQTRVAVKAIETLNGNWRSPGGAVNYNTVTPSVTGRWFSGNQTWPWDTWK

QAFAMAHFNPDIAKENIRAVFSWQIQPGDSVRPQDVGFVPDLIAWNLSPERGGDGGNWNERNTKPSLAAW

SVMEVYNVTQDKAWLAEMYPKLVAYHDWWLRNRDHNGNGVPEYGATRDKAHNTESGEMLFTVKKGDKEET

QSGLNNYARVVEKGQYDSLEIPAQVAASWESGRDDAAVFGFIDKEQLDKYVANGGKRSDWTVKFAENRSQ

DGTLLGYSLLQESVDQASYMYSDNHYLAEMATILGKPEEAKRYRQLAQQLADYINTCMFDPTTQFYYDVR

IEDKPLANGCAGKPIVERGKGPEGWSPLFNGAATQANADPVVKVMLDPKEFNTFVPLGTAALTNPAFGAD

IYWRGRVWVDQFWFGLKGMERYGYRDDALKLADTFFRHAKGLTADGPIQENYNPLTGAQQGAPNFSWSAA

HLYMLYNDFFRKQ

>gi|30064422|ref|NP_838593.1| oxidoreductase [Shigella flexneri 2a str. 2457T]

MSDTKRNTIGKFGLLSLTFAAVYSFNNVINNNIELGLASAPMFFLATIFYFIPFCLIIAEFVSLNKNSEA

GVYAWVKSSLGGRWAFITAYTYWFVNLFFFTSLLPRVIAYASYAFLGYEYIMTPVATTVISMVLFAFSTW

VSTNGAKMLGPITSVTSTLMLLLTLSYILLAGTALVGGVQPADPITVDAMIPNFNWAFLGVTTWIFMAAG

GAESVAVYVNDVKGGSKSFVKVIILAGIFIGVLYSVSSVLINVFVSSKELKFTGGSVQVFHGMAAYFGLP

EALMNRFVGLVSFTAMFGSLLMWTATPVKIFFSEIPEGIFGKKTVELNENGVPARAAWIQFLIVIPLMII

PMLGSNTVQDLMNTIINMTAAASMLPPLFIMLAYLNLRAKLDHLPRDFRMGSRRTGIIVVSMLIAIFAVG

FVASTFPTGANILTIIFYNVGGIVIFLGFAWWKYSKYIKGLTAEERHIEATPASNVD

>gi|30064421|ref|NP_838592.1| cryptic beta-D-galactosidase subunit beta [Shigella flexneri 2a str. 2457T]

MRIIDNLEQFRQIYASGKKWQRCVEAIENIDNIQPGVAHSIGDSLTYRVENDSATDALFTGHRRYFEVHY

YLQGQQKIEYAPKETLQVVEYYRDETDREYLKGCGETVEVHEGQIVICDIHEAYRFICNNAVKKVVLKVT

IEDGYFHNK

>gi|30064419|ref|NP_838590.1| DNA-binding transcriptional repressor EbgR [Shigella flexneri 2a str. 2457T]

MATLKDIAIEAGVSLATVSRVLNDDPTLNVKEETKHRILEIAEKLEYKTSSARKLQTGAVNQHHILAIYS

YQQELEINDPYYLAIRHGIETQCEKLAIELTNCYEHNGLPDIKNVTGILIVGKPTPALRAAASALTDNIC

FIDFHEPGSGYDAVDIDLARISKEIIDFYINQGVNRIGFIGGEDEPGKADIREVAFAEYGRLKQVVREED

IWRGGFSSSSGYELAKQMLAREDYPKALFVASDSIAIGVLRAIHERGLNIPQDISLISVNDIPTARFTFP

PLSTVRIHSEMMGSQGVNLVYEKARDGRALPLLVFVPSKLKLRGTTR

>gi|30064417|ref|NP_838588.1| hypothetical protein S3318 [Shigella flexneri 2a str. 2457T]

MSHHHEGCCKHEGQPRHEGCCKGEKSEHEHCGHGHQHEHGQCCGGRHGRGGGRRQRFFGHGELRLVILDI

LSRDDSHGYELIKAIENLTQGNYTPSPGVIYPTLDFLQEQSLITIREEEGGKKQIALTEQGAQWLEENRE

QVEMIEERIKARCVGAALRQNPQMKRALDNFKAVLDLRVNQSDISDAQIKKIIAVIDRAAFDITQLD

>gi|30064414|ref|NP_838585.1| DNA primase [Shigella flexneri 2a str. 2457T]

MAGRIPRVFINDLLARTDIVDLIDARVKLKKQGKNFHACCPFHNEKTPSFTVNGEKQFYHCFGCGAHGNA

IDFLMNYDKLEFVETVEELAAMHNLEVPFEAGSGPSQIERHQRQTLYQLMDGLNTFYQQSLQQPVATSAR

QYLEKRGLSHEVIARFAIGFAPPGWDNVLKRFGGNPENRQSLIDAGMLVTNDQGRSYDRFRERVMFPIRD

KRGRVIGFGGRVLGNDTPKYLNSPETDIFHKGRQLYGLYEAQQDNAEPNRLLVVEGYMDVVALAQYGINY

AVASLGTSTTADHIQLLFRATNNVICCYDGDRAGRDAAWRALETALPYMTDGRQLRFMFLPDGEDPDTLV

RKEGKEAFEARMEQAMPLSAFLFNSLMPQVDLSTPDGRARLSTLALPLISQVPGETLRIYLRQELGNKLG

ILDDSQLERLMPKAAESGVSRPVPQLKRTTMRILIGLLVQNPELATLVPPLENLDENKLPGLGLFRELVN

TCLSQPGLTTGQLLEHYRGTNNAATLEKLSMWDDIADKNIAEQTFTDSLNHMFDSLLELRQEELIARERT

HGLSNEERLELWTLNQELAKK

>gi|30064410|ref|NP_838581.1| L(+)-tartrate dehydratase subunit beta [Shigella flexneri 2a str. 2457T]

MKKILTTPIKAEDLQDIRVGDVIYLTGTLVTCRDVCHRRLIELKRPIPYDLNGKAIFHAGPIVRKNGDKW

EMVSVGPTTSMRMESFEREFIEQTGVKLVVGKGGMGPLTEEGCQKFKALHVIFPAGCAVLAATQVEEIEE

VHWTELGMPESLWVCRVKEFGPLIVSIDTHGNNLIAENKKLFAERRDPIVEEICEHVHYIK

>gi|30064409|ref|NP_838580.1| tartrate dehydratase subunit alpha [Shigella flexneri 2a str. 2457T]

MSESNKQQAVNKLTEIVANFTAMISTRMPDDVVDKLKQLKDAETSSMGKIIYHTMFDNMQKAIDLNRPAC

QDTGEIMFFVKVGSRFPLLGELQSILKQAVEEATVKAPLRHNAVEIFDEVNTGKNTGSGVPWVTWDIIPD

NDDAEIEVYMAGGGCTLPGRSKVLMPSEGYEGVVKFVFENISTLAVNACPPVLVGVGIATSVETAAVLSR

KAILRPIGSRHPNPKAAELELRLEEGLNRLGIGPQGLTGNSSVMGASSRHAAGSCRSHL

>gi|30064408|ref|NP_838579.1| transcriptional activator TtdR [Shigella flexneri 2a str. 2457T]

MLNSWPLAKDLQVLVEIVHSGSFSAAAATLGQTPAFVTKRIQILENTLATTLLNRSARGVALTESGQRCY

EHALEILTQYQRLVDDVTQIKTRPEGMIRIGCSFGFGRSHIAPAITELMRNYPELQVHFELFDRQIDLVQ

DNIDLDIRINDEIPDYYIAHLLTKNKRILCAAPEYLQKYPQPQSLQELSRHDCLVTKERDMTHGIWELGN

GQEKKSVKVSGHLSSNSGEIVLQWALEGKGIMLRSEWDVLPFLESGKLVQVLPEYAQSANIWAVYRELLY

RSMKFVSARNFWRHGASNGWASPMKAIRSCRSTRNHSGSFSRFLLNFVQIFVS

>gi|30064407|ref|NP_838578.1| glycerol-3-phosphate acyltransferase PlsY [Shigella flexneri 2a str. 2457T]

MSAIAPGMILIAYLCGSISSAILVCRLCGLPDPRTSGSGNPGATNVLRIGGKGAAVAVLIFDVLKGMLPV

WGAYELGVSPFWLGLIAIAACLGHIWPVFFGFKGGKGVATAFGAIAPISWDLTGVMAGTWLLTVLLSGYS

SLGAIVSALIAPFYVWWFKPQFTFPVSMLSCLILLRHHDNIQRLWRRQETKIWTKFKRKREKDPE

>gi|30064406|ref|NP_838577.1| bifunctional dihydroneopterin aldolase/dihydroneopterin triphosphate 2'-epimerase [Shigella flexneri 2a str. 2457T]

MDIVFIEQLSVITTIGVYDWEQTIEQKLVFDIEMAWDNRKAAKSDDVADCLSYADIAETVVSHVEGARFA

LVERVAEEVAELLLARFNSPWVRIKLSKPGAVARAANVGVIIERGNNLKENN

>gi|30064405|ref|NP_838576.1| undecaprenyl pyrophosphate phosphatase [Shigella flexneri 2a str. 2457T]

MSDMHSLLIAAILGVVEGLTEFLPVSSTGHMIIVGHLLGFEGDTAKTFEVVIQLGSILAVVVMFWRRLFG

LIGIHFGRPLQHEGESKGRLTLIHILLGMIPAVVLGLLFHDTIKSLFNPINVMYALVVGGLLLIAAECLK

PKEPRAPGLDDMTYRQAFMIGCFQCLALWPGFSRSGATISGGMLMGVSRYAASEFSFLLAVPMMMGATAL

DLYKSWGFLTTGDIPMFAVGFITAFVVALIAIKTFLQLIKRISFIPFAIYRFIVAAAVYVVFF

>gi|30064403|ref|NP_838574.1| signal transduction protein [Shigella flexneri 2a str. 2457T]

MPKLRLIGLTLLALSATAVSHAEETRYVSDELNTWVRSGPGDHYRLVGTVNAGEEVTLLQTDANTNYAQV

KDSSGRTAWIPLKQLSTEPSLRSRVPDLENQVKTLTDKLTNIDNTWNQRTAEMQQKVAQSDSVINGLKEE

NQKLKNELIVAQKKVDAASVQLDDKQRTIIMQWFMYGGGVLGLGLLLGLVLPHLIPSRKRKDRWMN

>gi|30064402|ref|NP_838573.1| hypothetical protein S3300 [Shigella flexneri 2a str. 2457T]

MAQEIELKFIVNHSAVEALRDHLNTLGGEHHDPVQLLNIYYETPDNWLRRHDMGLRIRGENGRYEMTMKV

AGRVTGGLHQRPEYNVALSEPTLDLAQLPTEIWPNGELPADLASRVQSLFSTDFYREKWLVEIDGSRIEI

ALDQGEVKAGEFAEPICELELELLSGDTRAVLKLANQLVSQTGLRQGSLSKAARGYHLAQGNPAREIKPT

TILHVAAKADVEQGLEAALELVLAQWQYHEELWVRGNDAAKEQVLAAISLVRHTLMLFGGIVPRKASTHL

RDLLTQCEATIASAVSAVTAVYSTETAMAKLALTEWLVSKAWQPFLDAKAQGKISDSFKRFADIHLSRHA

AELKSVFCQPLGDRYRDQLPRLMRDIDSILLLAGYYDPVVAQAWLENWQGLRHAIATGQRIEIEHFRNEA

NNQEPFWLHSGKR

>gi|30064401|ref|NP_838572.1| bifunctional glutamine-synthetase adenylyltransferase/deadenyltransferase [Shigella flexneri 2a str. 2457T]

MKPLSSPLQQYWQTVVERLPELLAEESLSAQAKSVLTFSDFVQDSVIAHPEWLTELESQPPQADEWQHYS

VWLQEALSNVSDEAGLMRELRLFRRRIMVRIAWAQTLALVTEESILQQLSYLAETLIVAARDWLYDACCR

EWGTPCNAQGEAQPLLILGMGKLGGGELNFSSDIDLIFAWPEHGCTQGGRRELDNAQFFTRMGQRLIKVL

DQPTQDGFVYRVDMRLRPFGESGPLVLSFAALEDYYQEQGRDWERYAMVKARIMGDSEGVYANELRAMLR

PFVFRRYIDFSVIQSLRNMKGMIAREVRRRGLTDNIKLGAGGIREIEFIVQVFQLIRGGREPLLQSRSLL

PTLSAIAALHLLSENDAEQLRVAYLFLRRLENLLQSINDEQTQTLPSDELNRARLAWAMDFADWPQLTGA

LTAHMTNVRRVFNELIGDDESETQEESLSEQWRELWQDALQEDDTTPVLAHLSEDDRKQVLTMIADFRKE

LDKRTIGPRGRQVLDHLMPHLLSDVCAREDAAVTLSRITALLVGIVTRTTYLELLSEFPAALKHLISLCA

ASPMIASQLARYPLLLDELLDPNTLYQPTATDAYRDELRQYLLRVPEDDEEQQLEALRQFKQAQLLRIAA

ADIAGTLPVMKVSDHLTWLAEAMIDAVVQQAWGQMVARYGKPNHLNEREGRGFAVVGYGKLGGWELGYSS

DLDLIFLHDCPMDAMTDGEREIDGRQFYLRLAQRIMHLFSTRTSSGILYEVDARLRPSGAAGMLVTSAEA

FADYQKNEAWTWEHQALVRARVVYGDPQLTAHFDAVRREIMTLPREGKTLQTEVREMREKMRAHLGNKHR

NRFDIKADEGGITDIEFITQYLVLRYAHEKPKLTRWSDNVRILELLAQNDIMEEQEAMALTRAYTTLRDE

LHHLALQELPGHVSEDCFTAERDLVRASWQKWLVEE

>gi|30064392|ref|NP_838563.1| hypothetical protein S3287 [Shigella flexneri 2a str. 2457T]

MTYPYRTTMVLNTYQYRETTMIDPKKIEQIARQVHESMPKGIREFGEDVEKKIRQTLQAQLTRLDLVSRE

EFDVQTQVLLRTREKLALLEQRISELENRSTEIKKQPDPETLPPTL

>gi|30064391|ref|NP_838562.1| 3,4-dihydroxy-2-butanone 4-phosphate synthase [Shigella flexneri 2a str. 2457T]

MNQTLLSSFGTPFERVENALAALREGRGVMVLDDEDRENEGDMIFPAETMTVEQMALTIRHGSGIVCLCI

TEDRRKQLDLPMMVENNTSAYGTGFTVTIEAAEGVTTGVSAADRITTVRAAIADGAKPSDLNRPGHVFPL

RAQAGGVLTRGGHTEATIDLMTLAGFKPAGVLCELTNDDGTMARAPECIEFANKHNMALVTIEDLVAYRQ

AHERKAS

>gi|30064389|ref|NP_838560.1| hypothetical protein S3284 [Shigella flexneri 2a str. 2457T]

MTPLVKDIIMSSTRMPALFLGHGSPMNVLEDNLYTRSWQKLGMTLPRPQAIVVVSAHWFTRGTGVTAMET

PPTIHDFGGFPQALYDTHYPAPGSPALAQRLVELLAPIPVTLDKEAWGFDHGSWGVLIKMYPDADIPMVQ

LSIDSSKPAAWHFEMGRKLAALRDEGIMLVASGNVVHNLRTVKWHGDSSPYPWATSFNEYVKANLTWQGP

VEQHPLVNYLDHEGGALSNPTPEHYLPLLYVLGVWDGQEPITIPVDGIEMGSLSMLSVQIG

>gi|30064388|ref|NP_838559.1| synthetase/amidase [Shigella flexneri 2a str. 2457T]

MERVSITERPDWREKAHEYGFNFHTMYGEPYWCEDAYYKLTLAQVEKLEEVTAELHQMCLKVVEKVIASD

ELMTKFRIPKHTWSFVRQSWLTHQPSLYSRLDLAWDGTGEPKLLENNADTPTSLYEAAFFQWIWLEDQLN

AGNLPEGSDQFNSLQEKLIDRFVELREQYGFQLLHLTCCRDTVEDRGTIQYLQDCATEAEIATEFLYIDE

IGLGEKGQFTDLQDQVISNLFKLYPWEFMLREMFSTKLEDAGVRWLEPAWKSIISNKALLPLLWEMFPNH

PNLLPAYFAEDDHPQMEKYVVKPIFSREGANVSIIENGKTIEAAEGPYGEEGMIVQQFHPLPKFGDSYML

IGSWLVNDQPAGIGIREDRALITQDMSRFYPHIFVE

>gi|30064384|ref|NP_838555.1| ADP-ribose pyrophosphatase NudF [Shigella flexneri 2a str. 2457T]

MLKPDNLPVTFGKNDVEIIARETLYRGFFSLDLYRFRHRLFNGQMSHEVRREIFERGHAAVLLPFDPVRD

EVVLIEQIRIAAYDTSETPWLLEMVAGMIEEGESVEDVARREAIEEAGLIVKRTKPVLSFLASPGGTSER

SSIMVGEVDATTASGIHGLADENEDIRVHVVSREQAYQWVEEGKIDNAASVIALQWLQLHHQALKNEWA

>gi|30064383|ref|NP_838554.1| hypothetical protein S3278 [Shigella flexneri 2a str. 2457T]

MKRYTPDFPEMMRLCEMNFSQLRRLLPRNDAPGETVSYQVANAQYRLTIVESTRYTTLVTIEQTAPAISY

WSLPSMTVRLYHDAMVAEVCSSQQIFRFKARYDYPNKKLHQRDEKHQINQFLADWLRYCLAHGAMAIPVY

>gi|30064382|ref|NP_838553.1| cyclic 3',5'-adenosine monophosphate phosphodiesterase [Shigella flexneri 2a str. 2457T]

MESLLTLPLAGEARGRILQITDTHLFAQKHEALLGVNTWESYQAVLEAIRPHQHEFDLIVATGDLAQDQS

SAAYQHFAEGIASFRAPCVWLPGNHDFQPAMYSALQDAGISPAKRVFIGEQWQILLLDSQVFGVPHGELS

EFQLEWLERKLADAPERHTLLLLHHHPLPAGCSWLDQHSLRNAGELDTVLAKFPHVKYLLCGHIHQELDL

DWNGRRLLATPSTCVQFKPHCSNFTLDTIAPGWRTLELHADGTLTTEVHRLADTRFQPDTASEGY

>gi|30064381|ref|NP_838552.1| esterase YqiA [Shigella flexneri 2a str. 2457T]

MSTLLYLHGFNSSPRSAKASLLKNWLAEHHPDVEMIIPQLPPYPSDAAELLESIVLEHGGDSLGIVGSSL

GGYYATWLSQCFMLPAVVVNPAVRPFELLTDYLGQNENPYTGQQYVLESRHIYDLKVMQIDPLEAPDLIW

LLQQTGDEVLDYRQAVAYYASCRQTVIEGGNHAFTGFEDYFNPIVDFLGLHHL

>gi|30064379|ref|NP_838550.1| hypothetical protein S3274 [Shigella flexneri 2a str. 2457T]

MLTVIAEIRTRPGQHHRQAVLDQFAKIVPTVLKEEGCHGYAPMVDCAAGVSFQSMAPDSIVMIEQWESIA

HLEAHLQTPHMKAYSEAVKGDVLEMNIRILQPGI

>gi|30064378|ref|NP_838549.1| modulator of drug activity B [Shigella flexneri 2a str. 2457T]

MSNILIINGAKKFAHSNGQLNDTLTEVADGTLRDLGHDVRIVRADSDYNVKAEVQNFLWADVVIWQMPGW

WMGAPWTVKKYIDDVFTEGHGTLYASDGRTRKDPSKKYGSGSLVQDKKYMLSLTWNAPMEAFTEKDQFFH

GVGVDGVYLPFHKANQFLGMEPLPTFIANDVIKMPDVPRYTEEYRKHLVEIFG

>gi|30064377|ref|NP_838548.1| hypothetical protein S3272 [Shigella flexneri 2a str. 2457T]

MLKQKIKTIFEALLYIMLTYWLIDSFFAFNKYDWMLESGGNICSIPSVSGEDRILQAMIAAFFLLTPLII

LILRKLFMREMFEFWLYVFSLVICLVCGWWLFWGRFIFCY

>gi|30064376|ref|NP_838547.1| sensor protein QseC [Shigella flexneri 2a str. 2457T]

MKFTQRLSLRVRLTLIFLILASVTWLLSSFVAWKQTTDNVDELFDTQLMLFAKRLSTLDLNEINAADRMA

QTPNKLKHGHVDDDALTFAIFTHDGRMVLNDGDNGEDIPYSYQREGFADGQLVGEDDPWRFVWMTSPDGK

YRIVVGQEWEYREDMALAIVAGQLIPWLVALPIMLIIMMVLLGRELAPLNKLALALRMRDPDSEKPLNAT

GVPSEVRPLVESLNQLFARTHAMMVRERRFTSDAAHELRSPLTALKVQTEVAQLSDDDPQARKKALLQLH

SGIDRATRLVDQLLTLSRLDSLDNLQDVAEIPLEDLLQSSVMDIYHTAQQAKIDVRLTLNAHGIKRTGQP

LLLSLLVRNLLDNGPGVTPEALARIGERFYRPPGQTATGSGLGLSIVQRIAKLHDMNVEFGNAEQGGFEA

KVSW

>gi|30064375|ref|NP_838546.1| DNA-binding transcriptional regulator QseB [Shigella flexneri 2a str. 2457T]

MRILLIEDDMLIGDGIKTGLSKMGFSVDWFTQGRQGKEALYSAPYDAVILDLTLPGMDGRDILREWREKG

QREPVLNLTARDALAERVEGLRLGADDYLCKPFALIEVAARLEALMRRTNGQASNELRHGNVMLDPGKRI

ATLAGEPLTLKPKEFALLELLMRNAGRVLPRKLIEEKLYTWDEEVTSNAVEVHVHHLRRKLGSDFIRTVH

GIGYTLGEK

>gi|30064374|ref|NP_838545.1| hypothetical protein S3269 [Shigella flexneri 2a str. 2457T]

MKKFAAVIAVMALCSAPVMAAEQGGFSGPSVTQSQAGGFQGPNGSVTTVESAKSLRDDTWVTLRGNIVER

ISDDLYVFKDASGTINVDIDHKRWNGVTVTPKDTVEIQGEVDKDWNSVEIDVKQIRKVNP

>gi|30064373|ref|NP_838544.1| hypothetical protein S3268 [Shigella flexneri 2a str. 2457T]

MTNLTLDVNIIDFPSIPVAMLPHRCSPELLNYSVAKFIMWRKETGLSPVNQSQTFGVAWDAPATTAPEAF

RFDICGSVSEPIPDNRYGVSNGELTGGRYAVARHVGELDDISHTIWGIIRHWLPASGEKMRKAPILFHYT
NLAEGVTEQRLETDVYVPLA

>gi|30064370|ref|NP_838541.1| repressor protein for FtsI [Shigella flexneri 2a str. 2457T]
MSLSRRQFIQASGIALCAGAVPLKASAAGQQQPLPVPPLLESRRGQPLFMTVQRAHWSFTPGTRASVWGI
NGRYLGPTIRVWKGDDVKLIYSNRLTENVSMTVAGLQVPGPLMGGPARMMSPNADWAPVLPIRQNAATLW
YHANTPNRTAQQVYNGLAGMWLVEDEVSKSLPIPNHYGVDDFPVIIQDKRLDNFGTPEYNEPGSGGFVGD
TLLVNGVQSPYVEVSRGWVRLRLLNASNSRRYQLQMSDGRPLHVISGDQGFLPAPVSVKQLSLAPGERRE
ILVDMSNGDEVSITCGEAASIVDRIRGFFEPSSILVSTLVLTLRPTGLLPLVTDSLPMRLLPTEIMAGSP
IRSRDISLGDDPGINGQLWDVNRIDVTAQQGTWERWTVRADEPQAFHIEGVMFQIRNVNGAMPFPEDRGW
KDTVWVDGQVELLVYFGQPSWAHFPFYFNSQTLEMADRGSIGQLLVNPVP

>gi|30064367|ref|NP_838538.1| hypothetical protein S3261 [Shigella flexneri 2a str. 2457T]
MKIILLFLAALASFTVHAQPPSQTVEQTVRQIYQNYKSDASTPYFGETGERAITSARIQQALTLNDNLTL
PGNIGWLDYDPVCDCQDFGDLVLESVAITQTDVDHADAVVRFRIFKDDKEKTTQTLKMVAENGRWVIDDI
VSNHGSVLQAVNSENEKTLAALASLQKEQPESFVAELFEHIADYSWPWTWVVSDSYRQAVNAFYKTTFKT
ANNPDEDMQIERQFIYDNPICFGEESLFSRVDEIRVLEKTADSARIHVRFTLTNGNNEEQELVLQRREGK
WEIADFIRPNSGSLLKQIEAKTAARLKQ

>gi|30064364|ref|NP_838535.1| ARAC-type regulatory protein [Shigella flexneri 2a str. 2457T]

MRVGVCRLKLFITCSLCWANMRAENDLPDVFHCDRQRPGSQCSFLPMSCLFLQSAGEMLQNCAQSNCRII

PKKLRDMKREEICRLLADKVNKLKNKENSLSELLPDVRLLYGETPFARTPVMYEPGIIILFSGHKIGYIN

ERVFRYDANEYLLLTVPLPFECETYATSEVPLAGLRLNVDILQLQELLMDIGEDEHFQPSMAASGINSAT

LSEEILCAAERLLDVMERPLDARILGKQIIREILYYVLTGPCGGALLALVSRQTHFSLISRVLKRIENKY

TENLSVEQLAAEANMSVSAFHHNFKSVTSTSPLQYLKNYRLHKARMMIIHDGMKASAAAMRVGYESASQF

SREFKRYFGVTPGEDAARMRAMQGN

>gi|30064360|ref|NP_838531.1| biopolymer transport protein ExbB [Shigella flexneri 2a str. 2457T]

MGNNLMQTDLSVWGMYQYADIVVKCVMIGLILASVVTWAIFFSKSVEFFNQKRRLKREQQLLAEARSLNQ

ANDIAADFGSKSLSLHLLNEAQNELELSEGSDDNEGIKERTSFRLERRVAAVGRQMGRGNGYLATIGAIS

PFVGLFGTVWGIMNSFIGIAQTQTTNLAVVAPGIAEALLATAIGLVAAIPAVVIYNVFARQIGGFKAMLG

DVAAQVLLLQSRDLDLEASAAAHPVRVAQKLRAG

>gi|30064359|ref|NP_838530.1| biopolymer transport protein ExbD [Shigella flexneri 2a str. 2457T]

MAMHLNENLDDNGEMHDINVTPFIDVMLVLLIIFMVAAPLATVDVKVNLPASTSTPQPRPEKPVYLSVKA

DNSMFIGNDPVTDETMITALNALTEGKKDTTIFFRADKTVDYETLMKVMDTLHQAGYLKIGLVGEETAKA

K

>gi|30064356|ref|NP_838527.1| hypothetical protein S3250 [Shigella flexneri 2a str. 2457T]

MERFLENAMYASRWLLAPVYFGLSLALVALALKFFQEIIHVLPNIFSMAESDLILVLLSLVDMTLVGGLL

VMVMFSGYENFVSQLDISENKEKLKWLGKMDATSLKNKVAASIVAISSIHLLRVFMDAKNVPDNKLMWYV

IIHLTFVLSAFVMGYLDRLTRHNH

>gi|30064352|ref|NP_838523.1| hydrogenase 2 small subunit [Shigella flexneri 2a str. 2457T]

MTGDNTLIHSHGINRRDFMKLCAALAATMGLSSKAAAEMAESVTNPQRPPVIWIGAQECTGCTESLLRAT

HPTVENLVLETISLEYHEVLSAAFGHQVEENKHNALEKYKGQYVLVVDGSIPLKDNGIYCMVAGEPIVDH

IRKAAEGSAAIIAIGSCSAWGGVAAAGVNPTGAVSLQEVLPGKTVINIPGCPPNPHNFLATVAHIITYGK

PPKLDDKNRPTFAYGRLIHEHCERRPHFDAGRFAKEFGDEGHREGWCLYHLGCKGPETYGNCSTLQFCDV

GGVWPVAIGHPCYGCNEKGIGFHKGIHQLANVENQTPRSQKPDVNAKEGGNVSAGAIGLLGGVVGLVAGV

SVMAVRELGRQQKKDNADSRGE

>gi|30064351|ref|NP_838522.1| hydrogenase 2 protein HybA [Shigella flexneri 2a str. 2457T]

MNRRNFIKAASCGALLTGALPSVSHAAAENRPPIPGSLGMLYDSTLCVGCQACVTKCQDINFPERNPQGE

QTWSNNDKLSPYTNNIIQVWTSGTGVNKDQEENGYAYIKKQCMHCVDPNCVSVCPVSALKKDPKTGIVHY

DKDVCTGCRYCMVACPYNVPKYDYNNPFGALHKCELCNQKGVERLDKGGLPGCVEVCPAGAVIFGTREEL

MAEAKKRLALKPGSEYHYPRQTLKSGDTYLHTVPKYYPHLYGEKEGGGTQVLVLTGVPYENLDLPKLDDL

STGARSENIQHTLYKGMMLPLAVLAGLTVLVRRNTKNDHHDGGDDHES

>gi|30064350|ref|NP_838521.1| hydrogenase 2 b cytochrome subunit [Shigella flexneri 2a str. 2457T]

MSHDPQPLGGKIISKPVMIFGPLIVICMLLIVKRLVFGLGSVSDLNGGFPWGVWIAFDLLIGTGFACGGW

ALAWAVYVFNRGQYHPLVRPALLASLFGYSLGGLSITIDVGRYWNLPYFYIPGHFNVNSVLFETAVCMTI

YIGVMALEFAPALFERLGWKVSLQRLNKVMFFIIALGALLPTMHQSSMGSLMISAGYKVHPLWQSYEMLP

LFSLLTAFIMGFSIVIFEGSLVQAGLRGNGPDEKSLFVKLTNTISVLLAIFIGLRFGELIYRDKLSLAFA

GDFYSVMFWIEVLLMLFPLVVLRVAKLRNDSRMLFLSALSALLGCATWRLTYSLVAFNPGGGYAYFPTWE

ELLISIGFVAIEICAYIVLIRLLPILPPLKQNDHNRHEASKA

>gi|30064349|ref|NP_838520.1| hydrogenase 2 large subunit [Shigella flexneri 2a str. 2457T]

MSQRITIDPVTRIEGHLRIDCEIENGVVSKAWASGTMWRGMEEIVKNRDPRDAWMIVQRICGVCTTTHAL

SSVRAAESALNIDVPVNAQYIRNIILAAHTTHDHIVHFYQLSALDWVDITSALQADPTKASEMLKGVSTW

HLNSPEEFTKVQNKIKDLVASGQLGIFANGYWGHPAMKLPPEVNLIAVAHYLQALECQRDANRVVALLGG

KTPHIQNLAVGGVANPINLDGLGVLNLERLMYIKSFIDKLSDFVEQVYKVDTAVIAAFYPEWLTRGKGAV

NYLSVPEFPTDSKNGSFLFPGGYIENADLSSYRPITSHSDEYLIKGIQESAKHSWYKDEAPQAPWEGTTI

PAYDGWSDDGKYSWVKSPTFYGKTVEVGPLANMLVKLAAGRESTQNKLNEIVAIYQKLTGNTLEVAQLHS

TLGRIIGRTVHCCELQDILQNQYSALITNIGKGDHTTFVKPNIPATGEFKGVGFLEAPRGMLSHWMVIKD

GIISNYQAVVPSTWNSGPRNFNDDVGPYEQSLVGTPVADPNKPLEVVRTIHSFDPCMACAVHVVDADGNE

VVSVKVL

>gi|30064348|ref|NP_838519.1| hydrogenase 2 maturation endopeptidase [Shigella flexneri 2a str. 2457T]

MRILVLGVGNILLTDEAIGVRIVEALEQRYILPDYVEILDGGTAGMELLGDMANRDHLIIADAIVSKKNT

PGTMMILRDEEVPALFTNKISPHQLGLADVLSALRFTGEFPKKLTLVGVIPESLEPHIGLTPTVEAMIEP

ALEQVLAALRESGVEAIPREAIHD

>gi|30064347|ref|NP_838518.1| hydrogenase 2-specific chaperone [Shigella flexneri 2a str. 2457T]

MTEEIAGFQTSPKAQVQAAFEEIARRSMHDLSFLHPSMPVYVSDFTLFEGQWTGCVITPWMLSAVIFPGP

DQLWPLRKVSEKIGLQLPYGTMTFTVGELDGVSQYLSCSLMSPLSHSMSIEEGQRLTDDCARMILSLPVT

NPDVPHAGRRALLFGRRRGENA

>gi|30064346|ref|NP_838517.1| hydrogenase nickel incorporation protein HybF [Shigella flexneri 2a str. 2457T]

MHELSLCQSAVEIIQRQAEQHDVKRVTAVWLEIGALSCVEESAVRFSFEIVCHGTVAQGCDLHIVYKPAQ

AWCWDCSQVVEIHQHDAQCPLCHGERLRVDTGDSLIVKSIEVE

>gi|30064343|ref|NP_838514.1| bifunctional glutathionylspermidine amidase/glutathionylspermidine synthetase [Shigella flexneri 2a str. 2457T]

MSKGTTSQDAPFGTLLGYAPGGVAIYSSDYSSLDPQEYEDDAVFRSYIDDEYMGHKWQCVEFARRFLFLN

YGVVFTDVGMAWEIFSLRFLREVVNDNILPLQAFPNGSPRAPVAGALLIWDKGGEFKDTGHVAIITQLHG

NKVRIAEQNVIHSPLPQGQQWTRELEMVVENGCYTLKDTFDDTTILGWMIQTEDTEYSLPQPEIAGELLK

ISGARLENKGQFDGKWLDEKDPLQNAYVQANGQVINQDPYHYYTITESAEQELIKATNELHLMYLHATDK

VLKDDNLLALFDIPKILWPRLRLSWQRRRHHMITGRMDFCMDERGLKVYEYNADSASCHTEAGLILERWA

EQGYKGNGFNPAEGLINELAGAWKHSRARPFVHIMQDKDIEENYHAQFMEQALHQAGFETRILRGLDELG

WDAAGQLIDGEGRLVNCVWKTWAWETAFDQIREVSDREFAAVPIRTGHPQNEVRLIDVLLRPEVLVFEPL

WTVIPGNKAILPILWSLFPHHRYLLDTDFTVNDELVKTGYAVKPIAGRCGSNIDLVSHHEEVLDKTSGKF

AEQKNIYQQLWCLPKVDGKYIQVCTFTVGGNYGGTCLRGDESLVIKKESDIEPLIVVKK

>gi|30064341|ref|NP_838512.1| hypothetical protein S3234 [Shigella flexneri 2a str. 2457T]

MQSITPPLIAVIGSDASGKSTVCEHLITVVEKYGAAERVHLGKQAGNVGRAVTKLPLMGKSLHKTIERNQ

VKTAKKLPGPVPALVITAFVARRLLRFRHMLACRRRGLIVLTDRYPQDQIPGAYDGTVFPPNVEGGRFVS
WLASQERKAFHWMASHKPDLVIKLNVDLEVACARKPDHKRESLARKIAITPQLTFGGAQLVDIDANQPLE
QVLVDAEKAITDFMTARGYH

>gi|30064340|ref|NP_838511.1| hypothetical protein S3233 [Shigella flexneri 2a str. 2457T]
MNQRNMSIINSTPVRVIAIVGCDGSGKSTLTASLVNELAARMPTEHIYLGQSSGRIGEWISQLPVIGAPC
GCYLRSKAAHVHEKPLTPPGNITALVIYLLSCWRAYKFRKMLCKSQQGYLLITDRYPQVEVPGFRFDGPQ
LAKTTGGNGWIKMLRQRELKLYQWMASYLPVLLIRLGIDEQTAFARKPDHQLAALQEKIAVTPQLTFNGA
KILELDGRQPADEIMQASLRAIHAALS

>gi|30064339|ref|NP_838510.1| hypothetical protein S3232 [Shigella flexneri 2a str. 2457T]
MDALQTQTVNSTTAPQPNYIPGLIAVVGCDGTGKSTLTTDLVKSLQQHWQTERRYLGLLSGEDGDKIKRL
PLVGVWLERRLAAKSSKTQSMKTKSPALWAAVIMYCFSLRRMANLRKVQRLAQSGVLVVSDRFPQAEISG
FYYDGPGIGVERATGKISMFLAQRERSLYQKMAQYRPELIIRLGIDIDTAISRKPDHEYAELQDKIGVMS
TIGYNGTKILEIDSRAPYSEVLEQVQKAVSLVAIVSDRRSLT

>gi|30064338|ref|NP_838509.1| hypothetical protein S3231 [Shigella flexneri 2a str. 2457T]
MAGFNIKHWFADGAFRTIIRNSAWLGSSNVESALLGLLALSCAGKGMTPAMFGVLVIVQSYAKSISDFIK
FQTWQLVVQYGTPALTNNNPQQFRNVVSFSFSLDIVSGAVAIVGGIALLPFLSHSLGLDDQSFWLAALYC
TLIPSMASSTPTGILRAVDRFDLIAVQQATKPFLRAAGSVAAWYFDFGFVGFVIAWYVSNLVGGTMYWWF
AARELRRRNIHNAFKLNLFESARHIKGAWSFVWSTNIAHSIWSARNSCSTVLVGIVLGPAAAGLFKIAMT
FFDAAGTPAGLLGKSFYPEVMRLDPRTTRPWLLGVKSGLLAGGIGILVALAVLIVGKPLISLVFGVKYLE
AYDLIQVMLGAIVISMLGFPQESLLLMAGKQRAFLVAQTIASIGYIVLLFMFCHLFGVLGAAFAYFGGQC
LDVALSLIPTLKAFFQRHSLLYNAAGEKS

>gi|30064337|ref|NP_838508.1| hypothetical protein S3230 [Shigella flexneri 2a str. 2457T]

MMRKYFPLEASERLFVAIEEDDVVDAQVSLPPTIALSCTTEIIHDNYALCLQFWLNGVDRQELLRLVRKQ

AKGDELTADERKQFKYMRARYKHLRFAQRLYLKKHQAGFLFGKTTVFLGRFQDGFRNGKKNIVSYYGNLL

RIYLSSPVWSLVNYSLRHSQLESVSSFIAYRQKQMHTLKEIIAKPRLTGREFHDVRKIISQQVSYYDTLR

SLDPENKEALQISRFLAAINGLMGDKHDDMVADDMENRQSYYAPLALDSDICQRLELLISRFPL

>gi|30064336|ref|NP_838507.1| hypothetical protein S3229 [Shigella flexneri 2a str. 2457T]

MSSRLIIALIIMLLAPGVQAHNFVTGKTVTPVYIQEGGELLLNSDDEIHYQKWNSTQLAGKVRIIQYIAG

RKSAKKKNSLLIKAVEAANFPQDRFQPTTIVNTDDAIFGTGYFVVGKIEKNKRRYPWAQFVIDGNGQGRV

AWRLPEQSSTILVLNKAGQIQWAKDGSLTPEEVDHVIALAQKLINE

>gi|30064335|ref|NP_838506.1| hypothetical protein S3228 [Shigella flexneri 2a str. 2457T]

MKLVEHYIMRGTRRLVLIIVGFLIFIFASYSAQRYLTEAANGTLALDVVLDIVFYKVLIALEMLLPVGLY

VSVGVTLGQMYTDSEITAISAAGGSPGRLYKAVLYLAIPLSIFVTLLSMYGRPWAYAQIYQLEQQSQSEL

DVRQLRAKKFNTNDNGRMILSQTVDQDNNRLTDALIYTSTANRTRIFRAGSVDVVDPSPEKPTVMLHNGT

AYLLDHQGRDDNEQIYRNLQLHLNPLDQSPNVKRKAKSVTELARSAFPADHAELQWRQSRGLTALLMALL

AISLSRVKPRQGRFSTLLPLTLLFIAIFYGGDVCRTLVANGAIPLIPGLWLVPGLMLMGLLILVARDFSL

LQKFSR

>gi|30064334|ref|NP_838505.1| hypothetical protein S3227 [Shigella flexneri 2a str. 2457T]

MNVFSRYLIRHLFLGFAAAAGLLLPLFTTFNLINELDGVSPGGYRWTQAVLVVLMTLPRTLVELSPFIAL

LGGIVGLGQLSKNSELTAIRSTGFSIFRIALVALVAGILWTVSLGAIDEWVASPLQQQALQIKSTATALG

EDDDITGNMLWARRGNEFVTVKSLNEQGQPVGVEIFHYRDDLSLESYIYARSATIEDDKTWVLHGVNHKK

WLNGKETLETLDNLAWQSAFTSMNLEELSMPGNTFSVRQLNHYIHYLQETGQPSSEYHLALWEKLGQPIL

TLAMILLAVPFTFSAPRSPGMGSRLAVGVIVGLLTWISYQIMVNLGLLFALSAPVTALGLPVAFVLVALS

LVYWYDRQH

>gi|30064328|ref|NP_838499.1| DNA-binding transcriptional regulator GlcC [Shigella flexneri 2a str. 2457T]

MKDERRPICEVVAESIERLIIDGVLKVGQPLPSERRLCEKLGFSRSALREGLTVLRGRGIIETAQGRDSH

VARLNREQDTSPLIHLFSTQPRTLYDLLDVRALLEGESARLAATLGTQADFVVITRCYEKMLAASENHKE

ISLIEHAQLDHAFHLAICQASHNQVLVFTLQSLTDLMFNSVFASVNDLYHRPQQKKQIDRQHARIYNAVL

QRLPHVAQRAARDHVRTVKKNLHDIELEGHHLIRSAVPLEMNKVGM

>gi|30064327|ref|NP_838498.1| hypothetical protein S3217 [Shigella flexneri 2a str. 2457T]

MKTKVILSQQMASAIIAAGQEEAQKNNWSVSIAVADDGGHLLALSRMDDCAPIAAYISQEKARTAALGRR

ETKGYEEMVNNGRTAFVTAPLLTSLEGGVPVVVDGQIIGAVGVSGLTGAQDAQVAKVAAAVLAK

>gi|30064326|ref|NP_838497.1| malate synthase G [Shigella flexneri 2a str. 2457T]

MSQTITQGRLRIDANFKRFVDGEVLPGVELDAAAFWHNVDEIVHDLAPENRQLLAERDRIQAALDEWHRS

NPGPVKDKAAYKSFLRELGYLVPQPERVTVETTGIDSEITSQAGPQLVVPAMNARYALNAANARWGSLYD

ALYGSDIIPQEGAMVSGYDPQRGEQVIAWVRRFLDESLPLENGSYQDVVAFKVVDKQLRIQLKNGKETTL

RTPAQFVGYRGDAAAPTCILLKNNGLHIELQIDANGRIGKDDPAHINDVIVEAAISTILDCEDSVAAVDA

EDKILLYRNLLGLMQGTLQEKMEKNGRQIVRKLNDDRQYTAADGSEISLHGRSLLFIRNVGHLMTIPVIW

DSEGNEIPEGILDGVMTGAIALYDLKVQKNSRTGSVYIVKPKMHGPQEVAFANKLFTRIETMLGMAPNTL

KMGIMDEERRTSLNLRSCIAQARNRVAFINTGFLDRTGDEMHSVMEAGPMLRKNQMKSTPWIKAYERNNV

LSGLFCGLRGKAQIGKGMWAMPDLMADMYSQKGDQLRAGANTAWVPSPTAATLHALHYHHTNVQSVQANI

AQTEFSAEFEPLLDDLLTIPVAENANWSAQEIQQELDNNVQGILGYVVRWVEQGIGCSKVPDIHNVALME

DRATLRISSQHIANWLRHGILTKEQVQASLENMAKVVDQQNAGDPAYRPMAGNFANSCAFKAASDLIFLG

VKQPNGYTEPLLHAWRLREKESH

>gi|30064325|ref|NP_838496.1| permease [Shigella flexneri 2a str. 2457T]

MVTWTQMYMPMGGLGLSALVALIPIIFFFVALAVLRLKGHVAGAITLILSILIAIFAFKMPIDMAFAAAG

YGFIYGLWPIAWIIVAAVFLYKLTVASGQFDIIRSSVISITDDQRLQVLLIGFSFGALLEGAAGFGATVA

FGALGVPILVAGQVTGIDPFHIGAMAGRQLPFLSVLVPFWLVAMMDGWKGVKETWPAALVAGGSFAVTQF

FTSNYIGPELPDITSALVSIVSLALFLKVWRPKNTETAISMGQSAGAMVVNKPSSGGPVPSEYSLGQIIR

AWSPFLILTVLVTIWTMKPFKALFAPGGAFYSLVINFQIPHLHQQVLKAAPIVAQPTPMDAVFKFDPLSA

GGTAIFIAAIISIFILGVGIKKGIGVFAEMLISLKWPILSIGMVLAFAFVTNYSGMSTTLALVLAGTGVM

FPFFSPFLGWLGVFLTGSDTSSNALFGSLQSTTAQQINVSDTLLVAANTSGGVTGKMISPQSIAVACAAT

GMVGRESELFRYTVKHSLIFASVITLLQAYVFTGMLVS

>gi|30064322|ref|NP_838493.1| hypothetical protein S3212 [Shigella flexneri 2a str. 2457T]

MSDDRFYFSLGQGVKFYPASRRITTSTGNVINLSENGYRFLLLLLQGESDKQHIINEVWREQKGSVSDSS

YYGQIYMLRKALDVAGLSSSLIKTIPRKGVRYLGKVEKHVCENERDSDEDSSSYTNDSEAREVNTDANIP

ENDNSQEFSTEELLPIKHTDKNIEWYRTKQWSVFISVLTVLAVCWLTTLVFLVLLFLNQDK

>gi|30064320|ref|NP_838491.1| IS3 orfB [Shigella flexneri 2a str. 2457T]

MKYVFIEKHQAEFSIKAMCRVLRVARSGWYTWCQRRTRISTRQQFRQHCDSVVLAAFTRSKQRYGAPRLT

DELRAQGYPFNVKTVAASLRRQGLRAKASRKFSPVSYRAHGLPVSENLLEQDFYASGPNQKWPGDITYLR

TDEGWLYLAVVIDLWSRAVIGWSMSPRMTAQLPCDALQMALWRRKRPRNVIVHTDRGGQYCSADYQAQLK

RHNLRGSMSAKGCCYDNACVESFFHSLKVECIHGEHFISREIMRATVFNYIECDYNRWRRHSWCGGLSPE

QFENQNLA

>gi|30064318|ref|NP_838489.1| hypothetical protein S3207 [Shigella flexneri 2a str. 2457T]

MSEITASRPEVVNGHTDVICSTSIRHILAVRKSTLLQIDTLIRQLAEVSAMTESIRGKTALDWAMKQDFR

CGCWLMEKPETAMKAITRNLDRELWRDLMQRSGMLSLMDAQTRDTWYRSLEYDNFPEISEANILSTFEQL

HQNKDEVFERGVINVFKGLSWDYKTNSPCKFGSKIIVNNLVRWDRWGFHLITGQQTDRLVDLERMLHLFS

GKPIPDNRENITIRLDGHIQSVQGKERYEDEMFIIKYFKKGSAHITFKRLELIDRINDIIARYFPSVLSA

>gi|30064317|ref|NP_838488.1| hypothetical protein S3205 [Shigella flexneri 2a str. 2457T]

MKLALTLEADSVNVQALNMGRIVVDVDGVNLSELINKVSENGYSLRVVDKSDQHATSTPPPLTTLTCIRC

STAHITETDNAWLYSLSHQTNDDGETEWIHFTGSGYLLRTDAWSYPVLRLKRLGLSRTFRRLVVTLTRRY

GVSLIHLDAGAECLPGFPTFDW

>gi|30064316|ref|NP_838487.1| hypothetical protein S3204 [Shigella flexneri 2a str. 2457T]

MNTLPDTHVREASGCPSPITIWQTLLTRLLDQHYGLTLNDTPFADERVIEQHIEAGISLCDAVNFLVEKY

ALVRTDQPGFSAGAPSQLINSIDILRARRATGLMTRDNYRTVNNITRGKHPEAKQ

>gi|30064315|ref|NP_838486.1| structural protein [Shigella flexneri 2a str. 2457T]

MSDTLPGTTPPDDNHDRPWWGLPCTVTPCFGARLVQEGNRLHYLADRAGIRGRFSDVDAYHLDQAFPLLM

KQLELMLTGGELNPRHQHTVTLYAKGLTCEADTLGSCGYVYLAVYPTPAAPATTV

>gi|30064313|ref|NP_838484.1| RADC family DNA repair protein [Shigella flexneri 2a str. 2457T]

MVAGTMQQLSFLPGEMTTRERSLILRALKTLDRHLHEPGVAFTSTHAAREWLILNMAGLEREEFRVLYLN

NQNQLIAGETLFTGTINRTEVHPREVIKRALYHNAAAVVLAHNHPSGEVPPSKADRLITERLVQALALVD

IRVPDHLIVGGSQVFSFAEHGLL

>gi|30064312|ref|NP_838483.1| hypothetical protein S3200 [Shigella flexneri 2a str. 2457T]

MKTLSQNTTSSACAPETDLQQLVATLVPDEQRISFWPQHFGLIPQWVTLEPRVFGWMDRLCEDYCGGIWN

LYTLNNGGAFMAPEPDDDDDETWVLFNAMNGNRAEMSPEAAGIAACLMTYSHHACRTECYAMTVHYYRLR

DYALQHPECSAIMRIID

>gi|30064311|ref|NP_838482.1| hypothetical protein S3199 [Shigella flexneri 2a str. 2457T]

MQLVSRFGYANQIRRDRPLTHEELMHHVPGIFGEEKHTSRSQNYTYIPTITVLESLQREGFQPFFACQTR

VRDPGRRGYTKHMLRLRRAGEINGEHVPEIILLNSHDGTSSYQMLPGYFRFVCQNGCVCGQSLGEVRVPH

RGNVVEKVIEGAYEVVGVFDRIEEKRDAMQSLVLPPPARQALAQAALTYRYGDEHQPVTTADILTPRRRE

DYGKDLWSTYQTIQENMLKGGISGRSAKGKRIHTRAIHNIDTDIKLNRALWVMAETLLESLR

>gi|30064310|ref|NP_838481.1| hypothetical protein S3197 [Shigella flexneri 2a str. 2457T]

MIHLFKTCMITAFILGLTWSAPLRAQDQRYISIRNTDTIWLPGNICAYQFRLDNGGNGEGFGPLTITLQL

KDKYGQTLVTRKMETEAFGDSNATRTTDAFLETECVENVATTEIIKATEESNGHRVSLPLSVFDPQDYHP

LLITVSGKNVN

>gi|30064306|ref|NP_838477.1| hypothetical protein S3193 [Shigella flexneri 2a str. 2457T]

MNPSDAIEAIEKPLSSLPYSLSRHILEHLRKLTSHEPVIGIMGKSGAGKSSLCNALFQGEVTPVSDVHAG

TREVQRFRLSGHGHSMVITDLPGVGESRDRDAEYEALYRDILPELDLVLWLIKADDRALSVDEYFWRHIL

QCGHQQVLFVVTQADKTEPCHEWDMAGIQPSPAQEQNIREKTEAVFRLFRPVHPVVAVSARTGWELDTLV

SALMTALPDHAASPLMTRLQDELRTESVRSQAREQFTGAVDRIFDTAESVCVASVVRTALRAVRDTVVSV

ARAVWNWIFF

>gi|30064305|ref|NP_838476.1| hypothetical protein S4826 [Shigella flexneri 2a str. 2457T]

MNNEQMLIKCRDPRKCRFYAEYLNHKYPNEFPHFAPDNPQNCIRNVNLFLKEKYCNVSPPPFGTFGTTEE

EALNNEINELEIIAGQAILPEESFLWLKTDELATFFTWTTIYLSKEDHTKFPKINHNSSGIKNGIEVRKI

RTVTDETLNTYKKSNLPDNPSSHNERIKVIISYFDSCHSLINKNAKSIYLEELRQAWLKKKSACQKFKWL

LPDDDEMCQWFWTRLKQKQDEKHPVHNPVTRWFIPSSTKEYYLASLVAFALWKEAPDTIELFRTRINHAW

HQKKQKDKYRSDGKKAINIHIRSNVKEMLDELRTVYGLRTGEFFEMLITEKYLEHKRRK

>gi|30064303|ref|NP_838474.1| hypothetical protein S3191 [Shigella flexneri 2a str. 2457T]

MQQLENRCDLLLIQHQKWMTSVTRLIVAHGMGSPHLHGYHRLTLAHFFLPEKGSVISVAPQGLYQVVNPG

TPPFIPAIQEGLMTSIQTHEIMLLTHFNLGGVLLSELHRLGENRLANRLNSLLRRFDDRDLYHTLIWLCW

YDLMCAHSMQPWTEELKHKSHAELENWAVARKREKRELELMIDEYLLYAC

>gi|30064299|ref|NP_838470.1| hypothetical protein S3187 [Shigella flexneri 2a str. 2457T]

MQTYNTNPAYEMAPQLLEHFNQHLDSLFGVYSKLLPFRMDFAYRKNTLSYRCACRYAMCAEMLQLINEVG

EKLVGYAWVMEYTERKGLHIHFVGYLNGQSHRSSYLVSRLMGDIWRRVTEGNGYYHWCRFNKNYPVNINH

VIHYSDHKAVNDLRYAISYLAKREQKECGIILKCSGLPEKSNRGRPRLDSPLPGICALV

>gi|30064298|ref|NP_838469.1| hypothetical protein S3186 [Shigella flexneri 2a str. 2457T]

MYAKSFLALDGNGRLTGARTAQTAPYDSYTCHLCGSALRYHPQYDTERPWFEHADEGLTEHGHECPYVRP

ERREIRLIKRLQQFVPDTLPVVRKASWHCRQCHHDYYGEQYCTHCQTGRFSEEGGAE

>gi|30064297|ref|NP_838468.1| hypothetical protein S3185 [Shigella flexneri 2a str. 2457T]

MNGIKTGLGITPGEHIISADSALSRNIRQCFCLSCHGRLILQTDAQGAWFEHDLHALSEQQKADCVVLNP

EKSHPYIEDMAMFLSPLPVVLEWHCVMCEQFFHGKKFCEACGTGIYCRAVCTKSVYSYQPDLFEDCGGAS

T

>gi|30064293|ref|NP_838464.1| serine protease precurser [Shigella flexneri 2a str. 2457T]

MQDDFDAPVDFVSGLGPLNWTYDKTSGTGTLSQGSKNWTMHGQKDNDLNAGKNLVFSGQNGAIILKDSVT

QGAGYLEFKDSYTVSAESGKTWTGAGIITDKGTNVTWKVNGVAGDNLHKLGEGTLTINGTGVNPGGLKTG

DGIVVLNQQADTAGNIQAFSSVNLASGRPTVVLGDARQVNPDNISWGYRGGKLDLNGNAVTFTRLQAADY

GAVITNNAQQKSQLLLDLKAQDTNVSEPTIGNISPFGGTGTPGNLYSMILNSQTRFYILKSASYGNTLWG

NSLNDPAQWEFVGMNKNKAVQTVKDRILAGRAKQPVIFHGQLTGNMDVAIPQVPGGRKVIFDGSVNLPEG

TLSQDSGTLIFQGHPVIHASISGSAPVSLNQKDWENRQFTMKTLSLKDADFHLSRNASLNSDIKSDNSHI

TLGSDRAFVDKNDGTGNYVIPEEGTSVPDTVNDRSQYEGNITLNHNSALDIGSRFTGGIDAYDSAVSITS

PDVLLTAPGAFAGSSLTVHDGGHLTALNGLFSDGHIQAGKNGKITLSGTPVKDTANQYAPAVYLTDGYDL

TGDNAALEITRGAHASGDIHASAASTVTIGSDTPAELASAETAASAFAGSLLEGYNAAFNGAITGGRADV

SMHNALWTLGGDSAIHSLTVRNSRISSEGDRTFRTLTVNKLDATGSDFVLRTDLKNADKINVTEKATGSD

NSLNVSFMNNPAQGQALNIPLVTAPAGTSAEMFKAGTRVTGFSRVTPTLHVDTSGGNTKWILDGFKAEAD

KAAAAKADSFMNAGYKNFMTEVNNLNKRMGDLRDTNGDAGAWARIMSGAGSADGGYSDNYTHVQVGFDKK

HELDGVDLFTGVTMTYTDSSADSHAFSGKTKSVGGGLYASALFESGAYIDLIGKYIHHDNDYTGNFASLG

TKHYNTHSWYAGAETGYRYHLTEDTFIEPQAELVYGAVSGKTFRWKDGDMDLSMKNRDFSPLVGRTGVEL

GKTFSGKDWSVTARAGTSWQFDLLNNGETVLRDASGEKRIKGEKDSRMLFNVGMNAQIKDNMRFGLEFEK

SAFGKYNVDNAVNANFRYMF

>gi|30064291|ref|NP_838462.1| serine protease [Shigella flexneri 2a str. 2457T]

MNKIYSLKYSHITGGLVAVSELTRKVSVGTSRKKVILGIILSSIYGSYGETAFAAMLDINNIWTRDYLDL

AQNRGEFRPGATNVQLMMKDGKIFHFPELPVPDFSAVSNKGATTSIGGAYSVTATHNGTQHHAITTQSWD

QTAYKASNRVSSGDFSVHRLNKFVVETTGVTESADFSLSPEDAMKRYGVNYNGKEQIIGFRAGAGTTSTI

LNGKQYLFGQNYNPDLLSASLFNLDWKNKSYIYTNRTPFKNSPIFGDSGSGSYLYDKEQQKWVFHGVTST

VGFISSTNIAWTNYSLFNNILVNNLKKNFTNTMQLDGKKQELSSIIKDKDLSVSGGGVLTLKQDTDLGIG

GLIFDKNQTYKVYGKDKSYKGAGIDIDNNTTVEWNVKGVAGDNLHKIGSGTLDVKIAQGNNLKIGNGTVI

LSAEKAFNKIYMAGGKGTVKINAKDALSESGNGEIYFTRNGGTLDLNGYDQSFQKIAATDAGTTVTNSNV

KQSTLSLTNTDAYMYHGNVSGNISINHIINTTQQHNNNANLIFDGSVDIKNDISVRNAQLTLQGHATEHA

IFKEGNNNCPIPFLCQKDYSAAIKDQESTVNKRYNTEYKSNNQIASFSQPDWESRKFNFRKLNLENATLS

IGRDANVKGHIEAKNSQIVLGNKTAYIDMFSGRNITGEGFGFRQQLRSGDSAGESSFNGSLSAQNSKITV

GDKSTVTMTGALSLINTDLIINKGATVTAQGKMYVDKAIELAGTLTLTGTPTENNKYSPAIYMSDGYNMT

EDGATLKAQNYAWVNGNIKSDKKASILFGVDQYKEDNLDKTTHTPLATGLLGGFDTSYTGGIDAPAASAS

MYNTLWRVNGQSALQSLKTRDSLLLFSNIENSGFHTVTVNTLDATNTAVIMRADLSQSVNQSDKLIVKNQ

LTGSNNSLSVDIQKVGNNNSGLNVDLITAPKGSNKEIFKASTQAIGFSNISPVISTKEDQEHTTWTLTGY

KVAENTASSGAAKSYMSGNYKAFLTEVNNLNKRMGDLRDTNGEAGAWARIMSGAGSASGGYSDNYTHVQI

GVDKKHELDGLDLFTGLTMTYTDSHASSNAFSGKTKSVGAGLYASAIFDSGAYIDLISKYVHHDNEYSAT

FAGLGTKDYSSHSLYVGAEAGYRYHVTEDSWIEPQAELVYGAVSGKRFDWQDRGMSVTMKDKDFNPLIGR

TGVDVGKSFSGKDWKVTARAGLGYQFDLFANGETVLRDASGEKRIKGEKDGRILMNVGLNAEIRDNLRFG

LEFEKSAFGKYNVDNAINANFRYSF

>gi|30064290|ref|NP_838461.1| hypothetical protein S4823 [Shigella flexneri 2a str. 2457T]

MVKSHGTVSVDGKVSDADLTYLEEVANSTGQEVDKSRLTSQACARTALITDVGIALATELETAGQKWSLG

FPPKFQRVDLFNYNVLVRNYDSSAFKGDRYHNTKNGINADIGASTDLDDNWTLGLVAQNLISRSIETKEV

NGITETFRIRPQVTAGVSWHNAMFTTAFDVDLTPASGFTSDSNRQFAAIGTEFNAWKWAQLRAGYRQNLA

GNDGSAFTAGVGISPFDVVHLDVAGLIGTDNTYGAVAQFQFTF

>gi|30064289|ref|NP_838460.1| superfamily I DNA helicase [Shigella flexneri 2a str. 2457T]

MDENALGFASYWRNSLADAESGKGSFKRKDAQNFTHWHGIAAGRLDEAIVSKFFEGEKDDVETVDVILRP

KVYFRLLQHGKDRSAGAPDIVTPIVTPALLSREGFLYPTPATSIPRDLLEPLPKGAFSIGEIGQYDKYKT

THTTFSINFDDSVDKTAETDEEREARYAALQQEWRQYLYDSERLLKSVAGDWIEKPEQYELAEHGYIVKT

AQSGGASSHILSLYDHLLVCNKDVPLFNRFASREVHAAESLLAPGAKFSDRLGHSGDKFPLAKAQRDALS

HFLDARHGDILAVNGPPGTGKTTLVLSIIATQWARAALEKSEPPVIIATSTNNQAVTNIIEAFGKDFSQG

SGAMAGRWLPELKSFGAYFPSSSRKAEAAKKYQTEDFFNQVESKEYVEDALLFYLEKAKAAFPGKECSSP

EKVIELLHGQLAAKSEQLIRLNATWQTLSQIRAARELIANDIEQYLDNLNKLLSGQEQKVTLLKSAKTEW

KKYRAGESLIYSLFSWLPAVRNKRQYQIQLFLEDKLGALIAGNQWSDPETIERNIDGLLNSAEREQTTYR

QQIDSAHEIVLKEQQAVQEWQRLAFDLGYEGDEELSFSQADELADTQIRFPAFLLTTHYWEGRWLMDMAS

IDDLQDEKKKKGAKGVTARWQRRMKLTPCVVMTCYMLPGNMQISEHKGQRKFEKSYLYDFADLLIVDEAG

QVLPEVAAASFALAKKALVIGDTEQIPPIWSIAPAIDVGNMLAEKILSGSTQEEITEKYTAIADLGKSAA

SGSVMKIAQFASRYQYDPELARGMYLYEHRRCYDNIIGYCNTLCYHGKLLPKRGREESNLMPAMGYLHID

GKGELASSGSRYNLLEAETIAVWLAENQQNIEAHYGKSLHEVVGIVTPFSAQVSTIKQVLGKQDISTGTN

EKSLTVGTVHSLQGAERAIVIFSPVYSKHEDGGFIDSDNSMLNVAVSRAKDSFLVFGDMDLFEVQPASSP

RGLLAKYLFESEKNALSFDYKERKDLKTAGTKIYTLHGVEQHDNFLNQTFENTSKHITIISPWLTWQRLE

QTGFLDSMIAACSRGINVTIVTDRSYNTEHNDFEKRKEKQQNFKAALEKLNALGIATKLVNRVHSKIVIG

DDGLLCVGSFNWFSATREARYERYDTSMVYCGDNLKGEIEAIYNSLERRQV

>gi|30064288|ref|NP_838459.1| P4-type integrase [Shigella flexneri 2a str. 2457T]

MALTDAKIRAAKPTDKAYKLTDGAGMFLLVHPNGSRYWRLRYRILGKEKTLALGVYPEVSLSEARTKRDE

ARKLISEGIDPCEQKRVKKVVPDLQLSFEHIARRWHASNKQWAQSHSDKVLKSLETHVFPFIGNRDITTL

NTPDLLIPVRAAEAKQIYEIASRLQQRISAVMRYAVQSGIIRYNPALDMAGALTTVKRQHRPALELSRLP

ELLSRIDGYKGQPVTRLAVMLNLLVFIRSSELRYARWSEIDIDNSMWTIPAEREPLPGVKFSHRGSKMRT

PHLVPLSKQVVAILAELQTWAGENGLIFTGAHDPRKPISENTVNKALRVMGYDTTQEVCGHGFRAMACSA

LIESGLWSRDAVERQMSHQERNGVRAAYIHKAEHLEERRLMLQWWADFLDANREKGISPFEYAKINNPLK

>gi|30064287|ref|NP_838458.1| transporter [Shigella flexneri 2a str. 2457T]

MVIGPFINASAVLLGGVLGALLSQRLPERIRVSMTSIFGLASLGIGILLVVKCANLPAMVLATLLGALIG

EICLLEKGVNTAVAKAQNLFRHSRKKPAHESFIQNYVAIIVLFCASGTGIFGAMNEGMTGDPSILIAKSF

LDFFTAMIFACSLGIAVSVISIPLLIIQLTLAWAAALILPLITPSMMADFSAVGGLLLLATGLRICGIKM

FPVVNMLPALLLAMPLSAAWTAWFA

>gi|30064286|ref|NP_838457.1| ornithine decarboxylase [Shigella flexneri 2a str. 2457T]

MGQGFPPCPVFLLPRNGFALMKSMNIAASSELVSRLSSHRRVVALGDTDFTDVAAVVITAADSRSGILAL

LKRTGFHLPVFLYSEHAVELPAGVTAVINGNEQQWLELESAACQYEENLLPPFYDTLTQYVEMGNSTFAC

PGHQHGAFFKKHPAGRHFYDFFGENVFRADMCNADVKLGDLLIHEGSAKDAQKFAAKVFHADKTYFVLNG

TSAANKVVTNALLTRGDLVLFDRNNHKSNHHGALIQAGTTPVYLEASRNPFGFIGGIDAHCFNEEYLRQQ

IRDVAPEKADLPRPFRLTIIQLGTYDGTVYNARQVIDTIGHLCDYILFDSAWVGYEQFISMMADSSPLLL

ELNENDPGIFVTQLVHKQQAGFSQTSQIHKKDNHIRGQARFCPHKRLNNAFMLHASTSPFYPLFAALDVN

AKIHEGESGRRLWAECVELGIESRKAILARCKLFRPFIPPVVDGKLWQDYPTSVLASDRRFFSFEPGAKW

HGFEGYAADQYFVDPCKLLLTTPGINAETGEYSDFGVPATILAHYLRENGIVPEKCDLNSILFLLTPAES

HEKLAQLVAMLAQFEQHIEDDSSLAEVLPSVYNKYPVRYRDYTLRQLCQEMHDLYVSFDVKDLQKAMFRQ

QSFPSVVMNPQDAHSAYIRGEVELVRIRDAEGRIAAEGALPYPPGVLCVVPGEVWGGAVQRYFLALEEGV

NLLPGFSPELQGVYSETDADGMKRLYGYVLK

>gi|30064285|ref|NP_838456.1| permease for nucleosides [Shigella flexneri 2a str. 2457T]

MCFFKHSSASRCEEINMNLKLQLKILSFLQFCLWGSWLTTLGSYMFVTLKFDGASIGAVYSSLGIAAVFM

PALLGIVADKWLSAKWVYAICHTIGAITLFMAAQVTTPEAMFLVILINSFAYMPTLGLINTISYYRLQNA

GMDIVTDFPPIRIWGTIGFIMAMWVVSLSGFELSHMQLYIGAALSAILVLFTLTLPHIPVAKQQANQSWT

TLLGLDAFALFKNKRMAIFFIFSMLLGAELQITNMFGNTFLHSFDKDPMFASSFIVQHASIIMSISQISE

TLFILTIPFFLSRYGIKNVMMISIVAWILRFALFAYGDPTPFGTVLLVLSMIVYGCAFDFFNISGSVFVE

KEVSPAIRASAQGMFLMMTNGFGCILGGIVSGKVVEMYTQNGITDWQTVWLIFAGYSVVLAFAFMAMFKY

KHVRVPTGTQTVSH

>gi|30064284|ref|NP_838455.1| murein transglycosylase C [Shigella flexneri 2a str. 2457T]

MMKKYLALALIAPLLISCSTTKKGDTYNEAWVKDTNGFDILMGQFAHNIENIWGFKEVVIAGPKDYVKYT

DQYQTRSHINFDDGTITIETIAGTEPAAHLRRAIIKTLLMGDDPSSVDLYSDVDDITISKEPFLYGQVVD

NTGQPIRWEGRASNFADYLLKNRLKSRSNGLRIIYSVTINMVPNHLDKRAHKYLGMVRQASRKYGVDESL

ILAIMQTESSFNPYAVSRSDALGLMQVVQNTAGKDVFRSQGKSGTPSRSFLFDPASNIDTGTAYLAMLNN

VYLGGIDNPTSRRYAVITAYNGGAGSVLRVFSNDKIQAANIINTMTPGDVYQTLTTRHPSAESRRYLYKV

NTAQKSYRRR

>gi|30064281|ref|NP_838452.1| tRNA (guanine-N(7)-)-methyltransferase [Shigella flexneri 2a str. 2457T]

MKNDVISPEFDENGRPLRRIRSFVRRQGRLTKGQEHALENYWPVMGVEFSEDMLDFPALFGREAPVTLEI

GFGMGASLVAMAKDRPEQDFLGIEVHSPGVGACLASAHEEGLSNLRVMCHDAVEVLHKMIPDNSLRMVQL

FFPDPWHKARHNKRRIVQVPFAELVKSKLQLGGVFHMATDWEPYAEHMLEVMSSIDGYKNLSESNDYVPR

PASRPVTKFEQRGHRLGHGVWDLMFERVK

>gi|30064277|ref|NP_838448.1| alpha helix chain [Shigella flexneri 2a str. 2457T]

MKKQWIVGTALLMLMTGNAWADGEPPTENILKDQFKKQYHGILKLDAISLKNLDAKGNQATWSAEGDVSS

SDDLYTWVGQLADYELLEQTWTKDKPVKFSAMLTSKGTPASGWSVNFYSFQAAASDRGRVVDDIKTNNKY

LIVNSEDFNYRFSQLESALNTQKNSIPALEKEVKALDKQMVAAQKAADAYWGKDANGKQMTREEAFKKIH

QQRDEFNKQNDSEAFAVKYDKEVYQPAIAACHKQSEECYEVPIQQKRDFDINEQRRQTFLQSQKLSRKLQ

DDWVTLEKGQYPLTMKVSEINSKKVAILMKIDDINQANER

>gi|30064276|ref|NP_838447.1| hypothetical protein S3155 [Shigella flexneri 2a str. 2457T]

MDSYIAITLFGCFFVLVFIGVPISFSIGIATVASMLLMFPWDIAAITVSQRLANGLDNFALLAIPFFIFA

GTLMNSGGIAIRLINLAQVMVGRVPGSLGHVNVLANMMFGSISGSAVAAAAAVGGTLNPIQTKEGYDPAF

STAVNVSSCITGLLIPPSNVLIVFSLTAGGVSVASLFMAGYLPGILMGLAVMIVCGIIAKRRGYPLSERA

TFAQACKAFLDALPSLLLVFIVMGGILGGIFTATEASAIAVVYTFILSVLIYREVKWRHLPKLILESVVT

TSIVLLLIGFSVGMSWAMTNADIPYMISDALMGISDNPLIILLLINIVLLIVGIFMDMTPAVLIFTPIFL

PIAQELGMDPVHFGIMMVANLCIGLLTPPVGSALFVGCSISGVKIQHLIKPLLLFYAALLVALMMITYIP

QISLFIPQLLGLM

>gi|30064274|ref|NP_838445.1| hypothetical protein S3153 [Shigella flexneri 2a str. 2457T]

MIFNRLKLAVDRVIATFSVAVMLALVVCVVWQVFSRYVLNQPSTLTDELARFLMIWVGLLGAAYTVGAQR

HLSIDLFALALNKRKQLLLSIVINVLILGFAGSVIVTGGLKLIDKTLATSQVSAAMQIPMGYVYIILPLS

GVVMMFYALCFINQSIQQLKQPAQEAS

>gi|30064273|ref|NP_838444.1| hypothetical protein S3152 [Shigella flexneri 2a str. 2457T]

MDSTKDKGFFALSAYVAGTRSFYAKKPITKPEDLKGLKIRVQPSPTTIKMIELMGGSPTPISFGEVYTAM

QQGVVDGAENNVPSWMQTRHIEIAKVFSEDEHASIPDFLVISTKTWNKLTPEQQQILETAAKKSEAYQQK

LWEKIDADTRAQAKAMGGEIVKVDKAPFRKAVQPLFDDFKKDPKQAALLEKFDNAAQ

>gi|30064272|ref|NP_838443.1| hypothetical protein S3151 [Shigella flexneri 2a str. 2457T]

MKMRSFTRSLVCASLLALVSTGVNAAEKVALKLAHNLERSHVVHQSFEELAKEVKQLSKGNMVIRIYPSS

QMGNARETMELLQNGALDMTKGSASDLESFDNIYAIYNLPFLLSCFW

>gi|30064268|ref|NP_838439.1| resistance protein [Shigella flexneri 2a str. 2457T]

MNTLTFLLSTVIELYTMVLLLRIWMQCAHCDFYTPFSQFVVKVAQPIIGPLRRVIPAMGPIDSASLLVAY

ILSFIKAIVLFEVVTFLPIIWIAGLLILLKTIGLLIFWVLLVMAIMSWVSQGRSPIEYVLIQLADPLLRP

IRRLLPAMGGIDFSPMILVLLLYAINMGVAEVLQATGNMLLPGLWMAL

>gi|30064266|ref|NP_838437.1| transporter [Shigella flexneri 2a str. 2457T]

MPGYAQYRSFSEDLSMNMEEIVALSVKHNVSDLHLCSAWPARWRIRGRMEAAPFDAPDVEELLREWLDDD

QRAILLENGQLDFAVSLAENQRLRGSAFAQRQGISLALRLLPSHCPQLEQLGAPPVLPELLKSENGLILV

TGATGSGKSTTLAAMVGYLNQHADAHILTLEDPVEYLYASQRCLIQQREIGLHCMTFASGLRAALREDPD

VILLGELRDSETIRLALTAAETGHLVLATLHTRGAAQAVERLVDSFPAQEKDPVRNQLAGSLRAVLSQKL

EVDKQEGRVALFELLINTPAVGNLIREGKTHQLPHVIQTGQKVGMLTFQQSYQQRVGEGRL

>gi|30064265|ref|NP_838436.1| Holliday junction resolvase-like protein [Shigella flexneri 2a str. 2457T]

MSGTLLAFDFGTKSIGVAVGQRITGTARPLPAIKAQDGTPDWNLIERLLKEWQPDEIIVGLPLNMDGTEQ

PLTARARKFANRIHGRFGVEVKLHDERLSTVEARSGLFEQGGYRALNKGKVDSASAVIILESYFEQGY

>gi|30064263|ref|NP_838434.1| glutathione synthetase [Shigella flexneri 2a str. 2457T]

MIKLGIVMDPIANINIKKDSSFAMLLEAQRRGYELHYMEMGDLYLINGEARAHTRTLNVKQNYEEWFSFV

GEQDLPLADLDVILMRKDPPFDTEFIYATYILERAEEKGTLIVNKPQSLRDCNEKLFTAWFSDLTPETLV

TRNXAQLKAFWEKHSDIILKPLDGMGGASIFRVKEGDPNLGVIAETLTEHGTCYCMAQNYLPAIKDGDKR

VLVVDGEPVPYCLARIPQGGETRGNLAAGGRGEPRPLTESDWKIARQIGPTLKEKGLIFVGLDIIGDRLT

EINVTSPTCIREIEAEFPVSITGMLMDAIEARLQQQ

>gi|30064262|ref|NP_838433.1| 16S ribosomal RNA methyltransferase RsmE [Shigella flexneri 2a str. 2457T]

MRIPRIYHPEPLTSHSHIALCEDAANHIGRVLRMGPGQALQLFDGSNQVFDAEITSASKKSVEVKVLEGQ

IDDRESPLHIHLGQVMSRGEKMEFTIQKSIELGVSLITPLFSERCGVKLDSERLNKKLQQWQKIAIAACE

QCGRNRVPEIRPAMDLEAWCAEQDEGLKLNLHPRASNSINTLPLPVERVRLLIGPEGGLSADEIAMTARY

QFTDILLGPRVLRTETTALTAITALQVRFGDLG

>gi|30064261|ref|NP_838432.1| DNA-specific endonuclease I [Shigella flexneri 2a str. 2457T]

MYRYLSIAAVVLSAAFSGPTLAEGINSFSQAKAAAVKVHADAPGTFYCGCKINWQGKKGVVDLQSCGYQV

RKNENRASRVEWEHIVPAWQFGHQRQCWQDGGRKNCAKDPVYRKMESDMHNLQPSVGEVNGDRGNFMYSQ

WNGGEGQYGQCAMKVDFKEKAAEPPARARGAIARTYFYMRDQYNLTLSRQQTQLFNAWNKMYPVTDWECE

RDERIAKVQGNHNPYVQRACQARKS

>gi|30064260|ref|NP_838431.1| hypothetical protein S3139 [Shigella flexneri 2a str. 2457T]

MKTSRLPIAIQQAVMRRLREKLAQANLKLGRNYPEPKLSYTQRGTSAGTAWLESYEIRLNPVLLLENSEA

FIEEVVPHELAHLLVWKHFGRVAPHGKEWKWMMESVLGVPARRTHQFELQSVRRNTFPYRCKCQEHQLTV

RRHNRVVRGEAIYRCVHCGEQLVAK

>gi|30064254|ref|NP_838425.1| arginine decarboxylase [Shigella flexneri 2a str. 2457T]

MSDDMSMGLPSSAGEHGVLRSMQEVAMSSQEASKMLRTYNIAWWGNNYYDVNELGHISVCPDPDVPEARV

DLAQLVKTREAQGQRLPALFCFPQILQHRLRSINAAFKRARESYGYNGDYFLVYPIKVNQHRRVIESLIH

SGEPLGLEAGSKAELMAVLAHAGMTRSVIVCNGYKDREYIRLALIGEKMGHKVYLVIEKMSEIAIVLDEA

ERLNVVPRLGVRARLASQGSGKWQSSGGEKSKFGLAATQVLQLVETLREAGRLDSLQLLHFHLGSQMANI

RDIATGVRESARFYVELHKLGVNIQCFDVGGGLGVDYEGTRSQSDCSVNYGLNEYANNIIWAIGDACEEN

GLPHPTVITESGRAVTEHHTVLVSNIIGVERNEYTVPTAPVEDAPRALQSMWETWQEMHEPGTRRSLREW

LHDSQMDLHDIHIGYSSGTFSLQERAWAEQLYLSMCHEVQKQLDPQNRAHRPIIDELQERMADKMYVNFS

LFQSMPDAWGIDQLFPVLPLEGLDQVPERRAVLLDITCDSDGAIDHYIDGDGIATTMPMPEYDPENPPML

GFFMVGAYQEILGNMHNLFGDTEAVDVFVFPDGSVEVELSDEGDTVADMLQYVQLDPKTLLTQFRDQVKK

TDLDAELQQQFLEEFEAGLYGYTYLEDE

>gi|30064251|ref|NP_838422.1| lipoprotein, partial [Shigella flexneri 2a str. 2457T]

MNYQLYVRNYSRNTNYEIVATADGLDVLNGKQGSLNNNGYIVNAGDSLVIKGFRKDKHTEAAFQFANVAD

SYAANSAQGDVRNTGVIGFAAFELQGPAQNALPPCSGQAFPADNNGYAPPPCRK

>gi|30064249|ref|NP_838420.1| hypothetical protein S3128 [Shigella flexneri 2a str. 2457T]

MLACCAICSPVRCAFPYTETERRFTCKRRIFRIILTLCGRENMKIRALLVAMSVATVLTGCQNMDSNGLL

SSGAEAFQAYSLSDAQVKTLSDQACQEMDSKATIAPANSEYAKRLTTIANALGNNINGQPVNYKVYMAKD

VNAFAMANGCIRVYSGLMDMMTDNEVEAVIGHEMGHVALGHVKKGMQVALGTNAVRVAAASAGGIVGSLS

QSQLGDLGEKLVNSQFSQRQEAEADDYSYDLLHQRGISPAGLATSFEKLAKLEEGRQSSMFDDHPASAER

AQHIRDRISADGIK

>gi|30064247|ref|NP_838418.1| serine protease [Shigella flexneri 2a str. 2457T]

MKAPVSLTSFRPQKSLAIAIGVLAVVVLPFLSYYTVNEGERGILLRYGKIVKVAEPGLGFKIPFMESVEK

ISTRNQAVVYQGLQAYSRDQQPAQMTVSVSFHIKPSEAGAVYTTYNTIEALKDRLIVRQLPTQLENIFGQ

YTAISAVQDRTKLVQDLQNAMRKAVVGPVVIDGVQIENIDFSDAYEKSIENRMKAEVAIATRKQNLETEK

IQAQIAVTQAQAEADSKLAAAKAEAETIRVRGAAEAETIRLKSAAEAEAIRLRGEALRDNPGLVALTTAE

RWDGKLPDTMIPGSTVPFISTK

>gi|30064244|ref|NP_838415.1| oxidoreductase, partial [Shigella flexneri 2a str. 2457T]

MGLPSGYSAGYSYEYFGGNATYMIIPEIAINLGCVLPYHGSYFAAASLAEPMCCIIGAYHANYHTTQYVY

EHRMGVKPGGNIALLACAGPMGIGAIDYAINGGIQPSRVVVVDIDDKRLAQVQKLLPVDLAASKGIELVY

VNTKGMSDPIQMLRALTGDVGFDDIFVYAAVPAVVEMADELLAEDGCLNFFAGLTDKNFKVPFNFYNVHY

NSTHVVGTSGGSTDDMKEAIALSATGQLQPSFMVTHIGGLDAVPETVLNLPDIPGGKKLIYNGVTMPLTA

IADFAEKGKTDPLFKELARLVEETHGIWNEQAEKYLLAQFGVDIGEAAQ

>gi|30064243|ref|NP_838414.1| fructose-1,6-bisphosphatase II-like protein [Shigella flexneri 2a str. 2457T]

MMSLAWPLFRVTEQAALAAWPQTGCGDKNKIDGLAVTAMRQALNDVAFRGRVVIGEGEIDHAPMLWIGEE

VGKGDGPEVDIAVDPIEGTRMVAMGQSNALAVMAFAPRDSLLHAPDMYMKKLVVNRLAAGAIDLSLPLAD

NLRNVARALGKPLDKLRMVTLDKPRLSAAIEEATQLGVKVFALPDGDVAASVLTCWQDNPYDVMYTIGGA

PEGVISACAVKALGGDMQAELIDFCQAKGDYTENRQIAEQERKRCKAMGVDVNRVYSLDELVRGNDILFS

ATGVTGGELVNGIQQTANGVRTQTLLIGGADQTCNIIDSLH

>gi|30064239|ref|NP_838410.1| lipoprotein, partial [Shigella flexneri 2a str. 2457T]

MQYVPVQCFIRALFASRLPPGVAYLFAGPLLVVEQLKRQLAIIHEAQRARGVPLDEGWYQRLRAMPALII

PLTHNALNDLAVRGAALDMRAFRIHNRRTTLWAPADSTLQRVARYTMILLMLTEFGAWIWLR

>gi|30064238|ref|NP_838409.1| lipoprotein, partial [Shigella flexneri 2a str. 2457T]

MHPFTSLTLWALAACTTLILPAQTILPIYSAATFFCLIALKATRRRAKYVVWLMFSLGAGLWLVHGGWLP

EWLSGTPRSPERWSHAITLWLLILAIVSTSQL

>gi|30064237|ref|NP_838408.1| inner membrane protein [Shigella flexneri 2a str. 2457T]

MARSHFSSQALVLIVISIAINMIGGQLASMVKLPIFLDSIGTLISAVLLGPVIGMLTGLLTNLLWGLLTD

PIAAAFAPVAMVIGLVSGWLARAGWFRTLPKVVVSGVIITLAVTVVAVPLRTALFGGVTGSGADLFVAWM

HSMGQNLVESVAITVIGANLVDKILTAVIVWLLLRQLPIRTTRHFPAMAAVR

>gi|30064233|ref|NP_838404.1| fructose-bisphosphate aldolase [Shigella flexneri 2a str. 2457T]

MSKIFDFVKPGVITGDDVQKVFQVAKENNFALPAVNCVGTDSINAVLETAAKVKAPVIVQFSNGGASFIA

GKGVKSDVPQGAAILGAISGAHHVHQMAEHYGVPVILHTDHCAKKLLPWIDGLLDAGEKHFAATGKPLFS

SHMIDLSEESLQENIEICSKYLERMSKIGMTLEIELGCTGGEEDGVDNSHMDASALYTQPEDVDYAYTEL

SKISPRFTIAASFGNVHGVYKPGNVVLTPTILRDSQEYVSKKHNLPHNSLNFVFHGGSGSTAQEIKDSVS

YGVVKMNIDTDTQWATWEGVLNYYKANEAYLQGQLGNPKGEDQPNKKYYDPRVWLRAGQTSMIARLEKAF

QELNAIDVL

>gi|30064232|ref|NP_838403.1| mechanosensitive channel MscS [Shigella flexneri 2a str. 2457T]

MEDLNVVDSINGAGSWLVANQALLLSYAVNIVAALAIIIVGLIIARMISNAVNRLMISRKIDATVADFLS

ALVRYGIIAFTLIAALGRVGVQTASVIAVLGAAGLAVGLALQGSLSNLAAGVLLVMFRPFRAGEYVDLGG

VAGTVLSVQIFSTTMRTADGKIIVIPNGKIIAGNIINFSREPVRRNEFIIGVAYDSDIDQVKQILTNIIQ

SEDRILKDREMTVRLNELGASSINFVVRVWSNSGDLQNVYWDVLERIKREFDAAGISFPYPQMDVNFKRV

KEDKAA

>gi|30064231|ref|NP_838402.1| arginine exporter protein [Shigella flexneri 2a str. 2457T]

MFSYYFQGLALGAAMILPLGPQNAFVMNQGIRRQYHIMIALLCAISDLVLICAGIFGGSALLMQSPWLLA

LVTWGGVVFLLWYGFGAFKTAMSSNIELASAEVLKQGRWKIIATMLAVTWLNPHVYLDTFVVLGSLGGQL

DVEPKRWFALGTISASFLWFFGLAILAAWLAPRLRTAKSQRIINLVVGCVMWFIALQLARDGIAHAQALF

S

>gi|30064230|ref|NP_838401.1| hypothetical protein S3107 [Shigella flexneri 2a str. 2457T]

MKFKVIALAALMGISGMAAQANELPDGPHIVTSGTASVDAVPDIATLAIEVNVAAKDAATAKKQADERVA

QYISFLELNQIAKKDISSANLRTQPDYDYQDGKSILKGYRAVRTVEVTLRQLDKLNSLLDGALKAGLNEI

RSVSLGVAQPDAYKDKARKAAIDNAIHQAQELANGFHRKLGPVYSVRYHVSNYQPSPMVRMMKADAAPVS

AQETYEQAAIQFDDQVDVVFQLEPVDQQPAKTPAAQ

>gi|30064229|ref|NP_838400.1| LYSR-type transcriptional regulator [Shigella flexneri 2a str. 2457T]

MDIFISKKMRNFILLAQTNNIARAAEKIHMTASPFGKSIAALEEQIGYTLFTRKDNNISLNKAGQELYQK

LFPVYQRLSAIDNEIHNSGRRSREIVIGIDNTYPTIIFDQLISLGDKYEGVTAQPVEFSENGVIDNLFDR

QLDFIISPQHVSARVQELENLTISELPPLRLGFLVSRRYEERQEQELLQELPWLQMRFQNRANFEAMIDA

NMRPCGINPTIIYRPYSFMAKISAVERGHFLTVIPHFAWRLVNPATLKYFDAPHRPMYMQEYLYSIRNHR

YTATIFSILLKIVTGQTINPASSRLQLNYGVSRRRG

>gi|30064225|ref|NP_838396.1| chromosome replication initiation inhibitor protein [Shigella flexneri 2a str. 2457T]

MKRPDYRTLQALDAVIRERGFERAAQKLCITQSAVSQRIKQLENMFGQPLLVRTVPPRPTEQGQKLLALL

RQVELLEEEWLGDEQTGSTPLLLSLAVNADSLATWLLPALAPVLADSPIRLNLQVEDETRTQERLRRGEV

VGAVSIQHQALPSCLVDKLGALDYLFVSSKPFAEKYFPNGVTRSALLKAPVVAFDHLDDMHQAFLQQNFD

LPPGSVPCHIVNSSEAFVQLARQGTTCCMIPHLQIEKELASGELIDLTPGLFQRRMLYWHRFAPESRMMR

KVTDALLDYGHKVLRQD

>gi|30064221|ref|NP_838392.1| Z-ring-associated protein [Shigella flexneri 2a str. 2457T]

MSAQPVDIQIFGRSLRVNCPPDQRDALNQAADDLNQRLQDLKERTRVTNTEQLVFIAALNISYELAQEKA

KTRDYAASMEQRIRMLQQTIEQALLEQGRITEKTNQNFE

>gi|30064220|ref|NP_838391.1| hypothetical protein S3094 [Shigella flexneri 2a str. 2457T]

MSIQNEMPGYNEMNQYLNQQGTGLTPAEMHGLISGMICGGNDDSSWLPLLHDLTNEGMAFGHELAQALRK

MHSATSDALQDDGFLFQLYLPDGDDVSVFDRADALAGWVNHFLLGLGVTQPKLDKVTGETGEAIDDLRNI

AQLGYDEDEDQEELEMSLEEIIEYVRVAALLCHDTFTHPQPTAPEVQKPTLH

>gi|30064208|ref|NP_838379.1| hypothetical protein S3081 [Shigella flexneri 2a str. 2457T]

MVLWQSDLRVSWRAQWLSLLIHGLVAAVILLMPWPLSYTPLWMVLLSLVVFDCVRSQRCINARQGEIRLL

MDGRLRWQGQEWSIVKAPWMIKSGMMLRLRSDGGKRQHLWLAADSMDEAEWRDLRRILLQQETQR

>gi|30064207|ref|NP_838378.1| flavodoxin FldB [Shigella flexneri 2a str. 2457T]

MNMGLFYGSSTCYTEMAAEKIRDIIGPELVTLHNLKDDSPKLMEQYDVLILGIPTWDFGEIQEDWEAVWD

QLDDLNLEGKIIALYGLGDQLGYGEWFLDALGMLHDKLSTKGVKFVGYWPTEGYEFTSPKPVIADGQLFV

GLALDETNQYDLSDERIQSWCEQILNEMAEHYA

>gi|30064206|ref|NP_838377.1| site-specific tyrosine recombinase XerD [Shigella flexneri 2a str. 2457T]

MKQELARIEQFLDALWLEKNLAENTLNAYRRDLSMMVEWLHHRGLTLATAQSDDLQALLAERLEGGYKAT

SSARLLSAVRRLFQYLYREKFREDDPSAHLASPKLPQRLPKDLSEAQVERLLQAPLIDQPLELRDKAMLE

VLYATGLRVSELVGLTMSDISLRQGVVRVIGKGNKERLVPLGEEAVYWLETYLEHGRPWLLNGVSIDVLF

PSQRAQQMTRQTFWHRIKHYAVLAGIDSEKLSPHVLRHAFATHLLNHGADLRVVQMLLGHSDLSTTQIYT

HVATERLRQLHQQHHPRA

>gi|30064205|ref|NP_838376.1| thiol:disulfide interchange protein DsbC [Shigella flexneri 2a str. 2457T]

MKKGFMLFTLLAAFSGFAQADDAAIQQTLAKMGIKSSDIQPAPVAGMKTVLTNSGVLYITDDGKHIIQGP

MYDVSGTAPVNVTNKMLLKQLNALEKEMIVYKAPQEKHVITVFTDITCGYCHKLHEQMADYNALGITVRY

LAFPRQGLDSDAEKEMKAIWCAKDKNKAFDDVMAGKSVAPASCDVDIADHYALGVQLGVSGTPAVVLSNG

TLVPGYQPPKDMKEFLDEHQKMTSGK

>gi|30064204|ref|NP_838375.1| ssDNA exonuclease RecJ [Shigella flexneri 2a str. 2457T]

MKQQIQLRRREVDETADLPAELPPLLRRLYASRGVRSAQELERSVKGMLPWQQLSGVEKAVEILYNAFRE

GTRIIVVGDFDADGATSTALSVLAMRSLGCSNIDYLVPNRFEDGYGLSPEVVDQAHARGAQLIVTVDNGI

SSHAGVEHARSLGIPVIVTDHHLPGDTLPAAEAIINPNLRDCNFPSKSLAGVGVAFYLMLALRTFLRDQG

WFDERGIAIPNLAELLDLVALGTVADVVPLDANNRILTWQGMSRIRAGKCRPGIKALLEVANRDAQKLAA

SDLGFALGPRLNAAGRLDDMSVGVALLLCDNIGEARVLANELDALNQTRKEIEQGMQVEALTLCEKLERS

RDTLPGGLAMYHPEWHQGVVGILASRIKERFHRPVIAFAPAGDGTLKGSGRSIQGLHMRDALERLDTLYP

GMMLKFGGHAMAAGLSLEEDKFELFQQRFGELVTEWLAPSLLQGEVVSDGPLSPAEMTMEVAQLLRDAGP

WGQMFPEPLFDGHFRLLQQRLVGERHLKVMVEPVGGGPLLDGIAFNVDTALWPDNGVREVQLAYKLDINE

FRGNRSLQIIIDNIWPI

>gi|30064194|ref|NP_838365.1| lipoprotein [Shigella flexneri 2a str. 2457T]

MFFKRGKILSAGRLNKKSLGIVMFLSVGLLLAGCSGSKSSDTGTYSGSVYTVKRGDTLYRISRTTGTSVK

ELARLNGISPPYTIEVGQKLKLGGAKSSSSTRKSTAKSTTKTASVTPSSAVPKSSWPPVGQRCWLWPTTG

KVIMPYSTADGGNKGIDISAPRGTPIYAAGAGKVVYVGNQLRGYGNLIMIKHSEDYITAYAHNDTMLVNN

GQSVKAGQKIATMGSTDAASVRLHFQIRYRATAIDPLRYLPPQGSKPKC

>gi|30064193|ref|NP_838364.1| integrase [Shigella flexneri 2a str. 2457T]

MLKSRTYLYQRNGVFYIRLRMKTTSRLTASLPSHNRYKLASVSLRTKDRRTAMAHSRHIKSALKAIHADN

PNASYEELREHLKTIVEWELSVSRDDLNDPESYQLYVDQYDDIKSNLREAVATERLTVDQHRYINDVIGV

LKACQDRLNGDSSGLLSYLEPETGSLRPSVSLSVLAEPEVPEPKALTLASLIEQYEQENAQNWKPATLSE

NRASHSTLIEIFDYLDIQDVGKATRADMLRVREVLQQLPKNRKQRFKSMPLSDLLNRESKTDCLDVVTIN

NKYLIKMAAVFKWAVRNDLIAKNLTEGLELKVPQRKASDARDAFSPEQVGQLLVAAKAYSQKTSGKPYHY

YVTALAAITGARLNEVAQLQVKDVRTTEAGTVFIHINEDDSSLPGKSIKNAHSDRCVPLVDGAYGFVLAD

FMSLVEDRRKTEGDNAMVFNGLKLMKNGYGEQVSKWFNRTLLPKVLADRSGLAFHSFRHTVATQLKQHGV

ELAYAQAIMGHSSGSITYDRYAKEVEVETLKEKLAESLSVKKIDGK

>gi|30064192|ref|NP_838363.1| hypothetical protein S3063 [Shigella flexneri 2a str. 2457T]

MASEDWQREHHCRTGIIFTAFAIEAMFIFYRKQVDPGYDKTQKECRKTMHKNTLKLCGINNYMGTKPYQI

IKECLEVRDAIAHGDSYTSSFNFSADHLDNQDDIAKVSWR

>gi|30064191|ref|NP_838362.1| hypothetical protein S3062 [Shigella flexneri 2a str. 2457T]

MSQQEISIINLDQLVSMTSVEIAELTGKEHKHVLRDIRNMVEELNGAKTEHCSTLSSELNGSKFGLVGEE

VYKDAKGESRTMYRLDRKHTFILVAGYSVHLRAKCYDHIQTLERRVLQLEDQKKRAAIQSANRRGVTWGD

YCKTYGLPAQKLMTALLQHRGLFRKNPISNEWSVNPKYSDCFRIIKPSDQKFSAGGYNFRFNAKGLEVFG

KPEMVDKMRGILIAFTGTDQQKQEHLLKLAQSGKVEGI

>gi|30064190|ref|NP_838361.1| hypothetical protein S3060 [Shigella flexneri 2a str. 2457T]

MMFNNNNWKLSVTDINLYENTVSLDGQSYPLSLAIKTLIPGYLSGLPSTSREAMELLEALAEAGVTIGNF

FSNDLMTAYQRRQMNKRAEAERIAKELASQKERTREMFMTEDEWQKELQRREQVKAERRTYGENLRSATH

SAGRSRAAIVADLESGGNWMDSL

>gi|30064189|ref|NP_838360.1| hypothetical protein S3059 [Shigella flexneri 2a str. 2457T]

MNMTKNAFQFGIEPVRITDTDNIQVNEGLPTNADPQVYALQLAKTVKAMLNGVLKDAQDNIPFPVEVLPT

RNSLPTPIIAHTLADRSVLVPVRGGKRPEVVTAPSGTEITVEPIEQAILVSHQTKLWDQKSTTGFTQGTL

QQDALNICDNVIRTINSKMVDVLESSKLLKTVELPALTGSLTAKADAIMDALYENTESSFGSEVSDYGII

AHESHLKALSRLAAKQGFGGEDAIVDMLGTDVAYYNGEDRGVFMMAKRFTALSFGCFRHDGESITVVLSR

DGDSQSHDLEILGKVFVVAEAATTIKMGTGSATAVLPVVKRLSFTKEAN

>gi|30064186|ref|NP_838357.1| transporter protein [Shigella flexneri 2a str. 2457T]

MVLFITALVAWPLTYWPHKALCQFILSSKTSAGEGITGAVTHYYGKKIGNLITTLYFIAFFVVVLIYAVA

ITNSLTEQLAKHMVIDLRIRMLVSLGVVLILNLIFLMGRHATIRVMGFLVFPLIAYFLFLSIYLVGSWQP

DLLTTQVEFNQNTLHQIWISIPVMVFAFSHTPIISTFAIDRREKYGEHAMDKCKKIMKVAYLIICISVLF

FVFSCLLSIPSSYIEAAKEEGVTILSALSMLPNAPAWLSISGIIVAVVAMSKSFLGTYFGVIEGATEVVK

TTLQQVGVKKSRAFNHALSIMLVSLITFIVCCINPNAISMIYAISGPLIAMILFIMPTLSTYLIPALKPW

RSIGNLITLIVGILCVSVMFFS

>gi|30064184|ref|NP_838355.1| 5-keto-4-deoxyuronate isomerase [Shigella flexneri 2a str. 2457T]

MDVRQSIHGAHAKTLDTQGLRNEFLVEKVFVADEYTMVYSHIDRIIVGGIMPITKTVSVGGEVGKQLGVS

YFLERRELGVINIGGAGTITVDGQCYEIGHRDALYVGKGAKEVVFASIDTATPAKFYYNCAPAHTTYPTK

KVTPDEVSPVTLGDNLTSNRRTINKYFVPDVLETCQLSMGLTELAPGNLWNTMPCHTHERRMEVYFYFNM

DDDACVFHMMGQPQETRHIVMHNEQAVISPSWSIHSGVGTKAYTFIWGMVGENQVFNDMDHVGVKDLR

>gi|30064181|ref|NP_838352.1| racemase [Shigella flexneri 2a str. 2457T]

MKTIGLLGGMSWESTIPYYRLINEGIKQRLGGLHSAQMLLHSVDFHEIEECQRRGEWDKTGDILAEAALG

LQRAGAEGIVLCTNTMHKVADAIESRCTLPFLHIADATGRAITGAGMTRVALLGTRYTMEQDFYRGRLTE

QFSINCLIPEADERAKINQIIFEELCLGQFTEASRAYYAQVIARLAEQGAQGVIFGCTEIGLLVPEERSV

LPVFDTAAIHAEDAVAFMLS

>gi|30064180|ref|NP_838351.1| DNA-binding transcriptional regulator LysR [Shigella flexneri 2a str. 2457T]

MAAVNLRHIEIFHAVMTAGSLTEAAHLLHTSQPTVSRELARFEKVIGLKLFERVRGRLHPTVQGLRLFEE

VQRSWYGLDRIVSAAESLREFRQGELSIACLPVFSQSFLPQLLQPFLARYPDVSLNIVPQESPLLEEWLS

AQRHDLGLTETLHTPTGTERTELLSLDEVCVLPPGHPLAVKKVLTPDDFQGENYISLSRTDSYRQLLDQL

FTEHQVKRRMIVETHSAASVCAMVRAGVGISVVNPLTALDYAASGLVVRRFSIAVPFTVSLIRPLHRPSS

ALVQAFSGHLQAGLPKLVTSLDAILSSATTA

>gi|30064178|ref|NP_838349.1| DNA-binding transcriptional regulator GalR [Shigella flexneri 2a str. 2457T]

MATIKDVARLAGVSVATVSRVINNSPKASEASRLAVHSAMESLSYHPNANARALAQQTTETVGLIVGDVS

DPFFGAMVKAVEQVAYHTGNFLLIGNGYHNEQKERQAIEQLIRHRCAALVVHAKMIPDADLASLMKQMPG

MVLINRILPGFENRCIALDDRYGAWLATRHLIQQGHTRIGYLCSNHSISDAEDRLQGYYDALAESGIPAN

DRLVTFGEPDESGGEQAMTELLGRGRNFTAVACYNDSMAAGAMGVLNDNGIDVPGEISLIGFDDVLVSRY

VRPRLTTVRYPIVTMATQAAELALALADNRPLPEITNVFSPTLVRRHSVSTPSLEASHHATSD

>gi|30064176|ref|NP_838347.1| lysophospholipid transporter LplT [Shigella flexneri 2a str. 2457T]

MSESVHTNTSLWSKGMKAVIVAQFLSAFGDNALLFATLALLKAQFYPEWSQPILQMVFVGAYILLAPFVG

QVADSFAKGRVMMFANGLKLLGAASICFGINPFLGYTLVGVGAAACSPAKYGILGELTTGSKLVKANGLM

EASAIAAILLGSVAGGVLADWHVLVALAACALAYGGAVVANIYIPKLAARPGQSWNLINMTRSFLNACTS

LWCNGETRFSLVGTSLFWGAGVTLRFLLVLWVPVALGITDNATPTYLNAMVAIGIVVGAGAAAKLVTLET

VSRCMPAGILIGVVVPIFSLQHELLPAYALLMLIGVLGGFFVVPLNALLQERGKKSVGAGNAIAVQNLGE

NSAMLLMLGIYSLAVMVGIPVVPIGIGFGALFALAITALWIWQRRH

>gi|30064173|ref|NP_838344.1| transporter [Shigella flexneri 2a str. 2457T]

MLFAWITDPNAWLALGTLTLLEIVLGIDNIIFLSLVVAKLPTAQRAHARRLGLAGAMVMRLALLASISWV

TRLTNPLFTIFSQEISARDLILLLGGLFLIWKASKEIHESIEGEEEGLKTRVSSFLGAIVQIMLLDIIFS

LDSVITAVGLSDHLFIMMAAVVIAVGVMMFAARSIGDFVERHPSVKMLALSFLILVGFTLILESFDIHVP

KGYIYFAMFFSIAVESLNLIRNKKNPL

>gi|30064172|ref|NP_838343.1| DNA mismatch repair protein [Shigella flexneri 2a str. 2457T]

MSQPRPLLSPPETEEQLLAQAQQLSGYTLGELAALDGLVTPENLKRDKGWIGVLLEIWLGASAGSKPEQD

FAALGVELKTIPVDSLGRPLETTFVCVARLTGNSGVTWETSHVRHKLKRVLWIPVEGERSIPLAQRRVGS

PLLWSPNEEEDRQLREDWEELMDMIVLGQVERITARHGEYLQIRPKAANAKALTEAIGARGERILTLPRG

FYLKKNFTSALLARHFLIQ

>gi|30064171|ref|NP_838342.1| dinucleoside polyphosphate hydrolase [Shigella flexneri 2a str. 2457T]

MIDDDGYRPNVGIVICNRQGQVMWARRFGQHSWQFPQGGINPGESAEQAMYRELFEEVGLSRKDVRILAS

TRNWLRYKLPKRLVRWDTKPVCIGQKQKWFLLQLVSGDAEINMQTSSTPEFDGWRWVSYWYPVRQVVSFK

RDVYRRVMKEFASVVMSLQENTPKPQNASAYRRKRG

>gi|30064170|ref|NP_838341.1| fused phosphoenolpyruvate-protein phosphotransferase PtsP/GAF domain [Shigella flexneri 2a str. 2457T]

MLTRLREIVEKVASAPRLNEALNILVTDICLAMDTEVCSVYLADHDRRCYYLMATRGLKKPRGRTVTLAF

DEGIVGLVGRLAEPINLADAQKHPSFKYIPSVKEERFRAFLGVPIIQRRQLLGVLVVQQRELRQYDESEE

SFLVTLATQMAAILSQSQLTALFGQYRQTRIRALPAAPGVAIAEGWQDATLPLMEQVYQASTLDPALERE

RLTGALEEAANEFRRYSKRFAAGAQKETAAIFDLYSHLLSDTRLRRELFAEVDKGSVAEWAVKTVIEKFA

EQFAALSDNYLKERAGDLRALGQRLLFHLDDANQGPNAWPERFILVADELSATTLAELPQDRLVGVVVRD

GAANSHAAIMVRALGIPTVMGADIQPSVLHRRTLIVDGYRGELLVDPEPVLLQEYQRLISEEIELSRLAE

DDVNLPAQLKSGERIKVMLNAGLSPEHEEKLGSCIDGIGLYRTEIPFMLQSGFPSEEEQVAQYQGMLQMF

NDKPVTLRTLDVGADKQLPYMPISEENPCLGWRGIRITLDQPEIFLIQVRAMLRANAATGNLNILLPMVT

SLDEVDEARRLIERAGREVEEMIGYEIPKPRIGIMLEVPSMVFMLPHLAKRVDFISVGTNDLTQYILAVD

RNNTRVANIYDSLHPAMLRALAMIAREAEIHGIDLRLCGEMAGDPMCVAILIGLGYRHLSMNGRSVARVK

YLLRRIDFAEAENLAQRSLEAQLATEVRHQVAAFMERRGMGGLIRGGL

>gi|30064169|ref|NP_838340.1| prolipoprotein diacylglyceryl transferase [Shigella flexneri 2a str. 2457T]

MTSSYLHFPEFDPVIFSIGPVALHWYGLMYLVGFIFAMWLATRRANRPGSGWTKNEVENLLYAGFLGVFL

GGRIGYVLFYNFPQFMADPLYLFRVWDGGMSFHGGLIGVIVVMIIFARRTKRSFFQVSDFIAPLIPFGLG

AGRLGNFINGELWGRVDPNFPFAMLFPGSRTEDILLLQTNPQWQSIFDTYGVLPRHPSQLYELLLEGVVL

FIILNLYIRKPRPMGAVSGLFLIGYGAFRIIVEFFRQPDAQFTGAWVQYISMGQILSIPMIVAGVIMMVW

AYRRSPQQHVS

>gi|30064167|ref|NP_838338.1| hypothetical protein S3033 [Shigella flexneri 2a str. 2457T]

MPVKEQGFSLLEVLIAMAISSVLLLGAARFLPALQRESLTSTRKLALEDEIWLRVFTVAKHLQRAGYCHG

SCTGEGLEIVGQGDCIIVQWDANSNGIWDREPVKESDQIGFRLKEHVLETLRGATSCEGKGWDKVTNPDA

IIIDTFQVVRQDVSGFSPVLTVNMRAASKSEPQTVVDASYSVTGFNL

>gi|30064165|ref|NP_838336.1| hypothetical protein S3031 [Shigella flexneri 2a str. 2457T]

MSASLKNQQGFSLPEVMVAMVLMVLMVLMVLMVMIVTALSGIQRTSMNSLASRNQYQQLWRHGWQQTQLR

AISPPANWQVNRMQTSQAGCVSISVTLVSPGGREGEMTRLHCPNCQ

>gi|30064164|ref|NP_838335.1| exonuclease V subunit gamma [Shigella flexneri 2a str. 2457T]

MLRVYHSNRLDVLEALMEFIVERERLDDPFEPEMILVQSTGMAQWLQMTLSQKFGIAANIDFPLPASFIW

DMFVRVLPEIPKESAFNKQSMSWKLMTLLPQLLEREDFTLLRHYLTDDSDKRKLFQLSSKAADLFDQYLV

YRPDWLAQWETGHLVEGLGEAQAWQAPLWKALVEYTHELGQPRWHRANLYQRFIETLESATTCPPGLPSR

VFICGISALPPVYLQALQALGKHIEIHLLFTNPCRYYWGDIKDPAYLAKLLTRQRRHSFEDRELPLFRDS

ENAGQLFNSDGEQDVGNSLLASWGKLGRDYIYLLSDLESSQELDAFVDVTPDNLLHNIQSDILELENRAV

AGVNIEEFSRSDNKRPLDPLDSSITFHVCHSPQREVEVLHDRLLAMLEEAPTLTPRDIIVMVADIDSYSP

FIQAVFGSAPADRYLPYAISDRRARQSHPVLEAFISLLSLPDSRFVSEDVLALLDVPVLAARFDITEEGL

RYLRQWVNESGIRWGIDDDNVRELELPATGQHTWRFGLTRMLLGYAMESAQGEWQSVLPYDESSGLIAEL

VGHLASLLMQLNIWRRGLAQERPLEEWLPVCRDMLNAFFLPDAETEAAMTLIEQQWQAIIAEGLGAQYGD

AVPLSLLRDELAQRLDQERISQRFLAGPVNICTLMPMRSIPFKVVCLLGMNDGVYPRQLAPLGFDLMSQK

PKRGDRSRRDDDRYLFLEALISAQQKLYISYIGRSIQDNSERFPSVLVQELIDYIGQSHYLPGDEALNCD

ESEARVKAHLTCLHTRMPFDPQNYQPGERQSYAREWLPAASQAGKAHSEFVQPLPFTLPETVPLETLQRF

WAHPVRAFFQMRLQVNFHTEDSEIPDTEPFILEGLSRYQINQQLLNALVEQDDAERLFRRFRAAGDLPYG

AFGEIFWETQCQEMQQLADRVIACRQPGQSMEIDLACNGVQITGWLPQVQPDGLLRWRPSLLSVAQGMQL

WLEHLVYCASGGNGESRLFLRKDGEWRFPPLAAEQALHYLSQLIEGYREGMSAPLLVLPESGGAWLKTCY

DAQNDAMLDDDSTLQKARTKFLQAYEGNMMVRGEGDDIWYQRLWRQLTPETMETIVEQSQRFLLPLFRFN

QS

>gi|30064162|ref|NP_838333.1| exonuclease V subunit beta [Shigella flexneri 2a str. 2457T]

MSDVAETLDPLRLPLQGERLIEASAGTGKTFTIAALYLRLLLGLGGSAAFPRPLIVEELLVVTFTEAATA

ELRGRIRSNIHELRIACLRETTDNPLYERLLEEIDDKAQAAQWLLLAERQMDEAAVFTIHGFCQRMLNLN

AFESGMLFEQQLIEDESLLRYQACADFWRRHCYPLPREIAQVVFETWKGPQALLRDINRYLQGEPPVIKA

PPPDDETLASRHAQIVARIDTVKQQWRDAVGELDALIESSGIDRRKFNRSNQAKWIEKISAWAEEETNSY

QLPESLEKFSQRFLEDRTKAGGETPRHPLFEAIDQLLAEPLSIRDLLITRALAEIRETVAREKRRRGELG

FDDMLSRLDSALRSESGEVLAAAIRTRFPVAMIDEFQDTDPQQYRIFRRIWHHQPETALLLIGDPKQAIY

AFRGADIFTYMKARSEVHAHYTLDTNWRSAPGMVNSVNKLFSQTDDTFMFREIPFIPVKSAGKNQALRFV

FKGETQPAMKMWLMEGESCGVGDYQSTMAQVCAAQIRDWLQAGQRGEALLMNGDDARPVRASDISVLVRS

RQEAAQVRDALTLLEIPSVYLSNRDSVFETLEAQEMLWLLQAVMTPERENTLRSALATSMMGLNALDIET

LNNDEHAWDAVVEEFDGYRQIWRKRGVMPMLRALMSARNIAENLLATAGGERRLTDILHISELLQEAGTQ

LESEHALVRWLSQHILEPDSNASSQQMRLESDKHLVQIVTIHKSKGLEYPLVWLPFITNFRVQDQAFYHD

RHSFEAVLDLNAAPESVDLAEVERLAEDLRLLYVALTRSVWHCSLGVAPLVRRRGDKKGDTDVHQSALGR

LLQKGEPQDAAGLRTCIEALCDDDIAWQTAQTGDNQPWQVNDALTAELNARTLQRLPGDNWRVTSYSGLQ

QRGHGIAQDLMPRLDVDAAGVVSVVEEPTLTPHQFPRGASPGTFLHSLFEDLDFTQPVDPNWVQEKLELG

GFESQWEPVLTEWITAVLQAPLNETGVSLSQLSDRDKQVEMEFYLPISEPLIASQLDALIRQFDPLSAGC

PPLEFMQVRGMLKGFIDLVFRHEGRYYLLDYKSNWLGEDSSAYTQQAMAAAMQAHRYDLQYQLYTLALHR

YLRHRIADYDYDLHFGGVIYLFLRGVDKEHPQQGIYTTRPNAGLIALMDEMFAGMTLEEA

>gi|30064161|ref|NP_838332.1| exonuclease V subunit alpha [Shigella flexneri 2a str. 2457T]

MKLQKQLLEAVEHKQLRPLDVQFALTVAGDEHPAVTLAAALLSHDAGEGHVCLPLSRLENNEASNPLLAT

CVSEIGELQNWEECLLASQAVSRGDEPTPMILCGDRLYLNRMWCNERTVARFFNEVNHTIEVDEALLAQT

LDKLFPVSDEINWQKVAAAVALTRRISVISGGPGTGKTTTVAKLLAALIQMADGERCRIRLAAPTGKAAA

RLTESLGKALRQLPLTDEQKKRIPEDASTLHRLLGAQPGSQRLRHHAGNPLHLDVLVVDEASMIDLPMMS

RLIDALPDHARVIFLGDRDQLASVEAGAVLGDICAYANAGFTAERARQLSRLTGTHVPAGTGTEAASLRD

SLCLLQKSYRFGSDSGIGQLAAAINRGDKTAVKTVFQQDFTDIEKRLLQSGEDYIAMLEEALAGYGRYLD

LLQARAEPDLIIQAFNEYQLLCALREGPFGVAGLNERIEQFMQQKRKIHRHPHSRWYEGRPVMIARNDSA

LGLFNGDIGIALDRGQGTRVWFAMPDGNIKSVQPSRLPEHETTWAMTVHKSQGSEFDHAALILPSQRTPV

VTRELVYTAVTRARRRLSLYADERILSAAIATRTERRSGLAALFSSRE

>gi|30064160|ref|NP_838331.1| N-acetylglutamate synthase [Shigella flexneri 2a str. 2457T]

MVKERKTELVEGFRHSVPYINTHRGKTFVIMLGGEAIEHENFSSIVNDIGLLHSLGIRLVVVYGARPQID

ANLAAHHHEPLYHKNIRVTDAKTLELVKQAAGTLQLDITARLSMSLNNTPLQGAHINVVSGNFIIAQPLG

VDDGVDYCHSGRIRRIDEDALHRQLESGAIVLMGPVAVSVTGESFNLTSEEIATQLAIKLKAEKMIGFCS

SQGVTNDDGDIVSELFPNEAQARVEAQEEKGDYNSGTVRFLRGAVKACRSGVRRCHLISYQEDGALLQEL

FSRDGIGTQIVMESAEQIRRATINDIGGILELIRPLEQQGILVRRSREQLEMEIDKFTIIQRDNTTIACA

ALYPFPEEKIGEMACVAVHPDYRSSSRGEVLLERIAAQAKQSGLSKLFVLTTRSIHWFQERGFTPVDIDL

LPESKKQLYNYQRKSKVLMADLG

>gi|30064159|ref|NP_838330.1| amidase [Shigella flexneri 2a str. 2457T]

MTDYASFAKVSGQISRLLVTQLRFLLLGRGMSGSNTAISRRRLLQGAGAMWLLSVSQVSLAAVSQVVAVR

VWPASSYTRVTVESNRQLKYKQFALSNPERVVVDIEDVNLNSVLKGMAAQIRADDPFIKSARVGQFDPQT

VRMVFELKQNVKPQLFALAPVAGFKERLVMDLYPANAQDMQDPLLALLEDYNKGDLEKQVPPAQSGPQPG

KAGRDRPIVIMLDPGHGGEDSGAVGKYKTREKDVVLQIARRLRSLIEEEGNMKVYMTRNEDIFIPLQVRV

AKAQKQRADLFVSIHADAFTSRQPSGSSVFALSTKGATSTAAKYLAQTQNASDLIGGVSKSGDRYVDHTM

FDMVQSLTIADSLKFGKAVLNKLGKINKLHKNQVEQAGFAVLKAPDIPSILVETAFISNVEEERKLKTAT

FQQEVAESILAGIKAYFADGATLARRG

>gi|30064158|ref|NP_838329.1| murein transglycosylase A [Shigella flexneri 2a str. 2457T]

MKGRWVKYLLMGTVVAMLAACSSKPTDRGQQYKDGKFTQPFSLVNQPDAVGAPINAGDFAEQINHIRNSS

PRLYGNQSNVYNAVQEWLRAGGDTRNMRQFGIDAWQMEGADNYGNVQFTGYYTPVIQARHTRQGEFQYPI

YRMPPKRGRLPSRAEIYAGALGDKYILAYSNSLMDNFIMDVQGSGYIDFGDGSPLNFFSYAGKNGHAYRS

IGKVLIDRGEVKKEDMSMQAIRHWGETHSEAEVRELLEQNPSFVFFKPQSFAPVKGASAVPLVGRASVAS

DRSIIPPGTTLLAEVPLLDNNGKFNGQYELRLMVALDVGGAIKGQHFDIYQGIGPEAGHRAGWYNHYGRV

WVLKTAPGAGNVFSG

>gi|30064156|ref|NP_838327.1| hypothetical protein S3020 [Shigella flexneri 2a str. 2457T]

MTNPQFAGHPFGTTVTAETLRNTFAPLSQWEDKYRQLIMLGKQLPALPDELKAQAKEIAGCENRVWLGYT

VAENGKMHFFGNSEGRIVRGLLAVLLTAVEGKTAAELQAQSPLALFDELGLRAQLSASRSQGLNALSEAI

IAATKQV

>gi|30064153|ref|NP_838324.1| DNA-binding transcriptional activator GcvA [Shigella flexneri 2a str. 2457T]

MSKRLPPLNALRVFDAAARHLSFTRAAEELFVTQAAVSHQIKSLEDFLGLKLFRRRNRSLLLTEEGQSYF

LDIKEIFSQLTEATRKLQARSAKGALTVSLLPSFAIHWLVPRLSSFNSAYPGIDVRIQAVDRQEDKLADD

VDVAIFYGRGNWPGLRVEKLYAEYLLPVCSPLLLTGEKPLKTPEDLAKHTLLHDASRRDWQTYTRQLGFN

HINVQQGPIFSHSAMVLQAAIHGQGVALANNVMAQSEIEAGRLVCPFNDVLVSKNAFYLVCHDSQAELGK

IAAFRQWILAKAAAEQEKFRFRYEQ

>gi|30064152|ref|NP_838323.1| hypothetical protein S3016 [Shigella flexneri 2a str. 2457T]

MTSRFMLIFAAISGFIFVALGAFGAHVLSKTMGAVEMGWIQTGLEYQAFHTLAILGLAVAMQRRISIWFY

WSSVFLALGTVLFSGSLYCLALSHLRLWAFVTPVGGVSFLAGWALMLVGAIRLKRKGVSHE

>gi|30064151|ref|NP_838322.1| RNA 2'-O-ribose methyltransferase [Shigella flexneri 2a str. 2457T]

MNKVVLLCRPGFEKECAAEITDKAGQREIFGFARVKENAGYVIYECYQPDDGDKLIRELPFSSLIFARQW

FVVGELLQHLPPEDRITPIVGMLQGVVEKGGELRVEVADTNESKELLKFCRKFTVPLRAALRDAGVLANY

ETPKRPVVHVFFIAPGCCYTGYSYSNNNSPFYMGIPRLKFPAEAPSRSTLKLEEAFHVFIPADEWDERLA

NGMWAVDLGACPGGWTYQLVKRNMWVYSVDNGPMAQSLMDTGQVTWLREDGFKFRPTRSNISWMVCDMVE

KPAKVAALMAQWLVNGWCRETIFNLKLPMKKRYEEVSHNLAYIQAQLDEHGINAQIQARQLYHDREEVTV

HVRRIWAAVGGRRDER

>gi|30064150|ref|NP_838321.1| DNA-binding transcriptional activator FucR [Shigella flexneri 2a str. 2457T]

MKAARQQAIVDLLLNHTSLTTEALSEQLKVSKETIRRDLNELQTQGKILRNHGRAKYIHRQNQDSGDPFH

IRLKSHYAHKADIAREALAWIEEGMVIALDASSTCWYLARQLPDINIQVFTNSHPICHELGKRERIQLIS

SGGTLERKYGCYVNPSLISQLKSLEIDLFIFSCEGIDSSGALWDSNAINADYKSMLLKRAAQSLLLIDKS

KFNRSGEARIGHLDEVTHIISDERQVATSLVTA

>gi|30064148|ref|NP_838319.1| L-fucose isomerase [Shigella flexneri 2a str. 2457T]

MKKISLPKIGIRPVIDGRRMGVRESLEEQTMNMAKATAALLTEKLRHACGAAVECVISDTCIAGMAEAAA

CEEKFSSQNVGLTITVTPCWCYGSETIDMDPTRPKAIWGFNGTERPGAVYLAAALAAHSQKGIPAFSIYG

HDVQDADDTSIPADVEEKLLRFARAGLAVASMKGKSYLSLGGVSMGIAGSIVDHNFFESWLGMKVQAVDM

TELRRRIDQKIYDEAELEMALAWADKNFRYGEDENNKQYQRNAEQSRAVLRESLLMAMCIRDMMQGNSKL

ADIGRVEESLGYNAIAAGFQGQRHWTDQYPNGDTAEAILNSSFDWNGVREPFVVATENDSLNGVAMLMGH

QLTGTAQVFADVRTYWSPEAIERVTGHKLDGLAEHGIIHLINSGSAALDGSCKQRDSEGNPTMKPHWEIS

QQEADACLAATEWCPAIHEYFRGGGYSSRFLTEGGVPFTMTRVNIIKGLGPVLQIAEGWSVELPKDVHDI

LNKRTNSTWPTTWFAPRLTGKGPFTDVYSVMANWGANHGVLTIGHVGADFITLASMLRIPVCMHNVEETK

VYRPSAWAAHGMDIEGQDYRACQNYGPLYKR

>gi|30064147|ref|NP_838318.1| L-fucose transporter [Shigella flexneri 2a str. 2457T]

MGNTSIQTQSYRAVDKDAGQSRSYIIPFALLCSLFFLWAVANNLNDILLPQFQQAFTLTNFQAGLIQSAF

YFGYFIIPIPAGILMKKLSYKAGIITGLFLYALGAALFWPAAEIMNYTLFLVGLFIIAAGLGCLETAANP

FVTVLGPESSSHFRLNLAQTFNSFGAIIAVVFGQSLILSNVPHQSQDVLDKMSPEQLSAYKHSLVLSVQT

PYMIIVAIVLLVALLIMLTKFPALQSDNHSDAKQGSFSASLSRLARIRHWRWAVLAQFCYVGAQTACWSY

LIRYAVEEILGMTAGFAANYLTGTMVCFFIGRFTGTWLISRFAPHKVLAAYALIAMALCLISAFAGGHVG

LIALTLCSAFMSIQYPTIFSLGIKNLGQDTKYGSSFIVMTIIGGGIVTPVMGFVSDAAGNIPTAELIPAL

CFAVIFIFARFRSQTATN

>gi|30064144|ref|NP_838315.1| exonuclease IX [Shigella flexneri 2a str. 2457T]

MRSLFLFSQPAIACSGIECYPYRLIFKGVIVAVHLLIVDALNLIRRIHAVQGSPCVETCQHALDQLIMHS

QPTHAVAVFDDENRSSGWRHQRLPDYKADRPPMPEELHDEMPALRAAFEQRGVPCWSASGNEADDLAATL

AVKVTQAGHQATIVSTDKGYCQLLSPTLRIRDYFQKRWLDAPFIDKEFGVQPQQLPDYWGLAGISSSKVP

GVAGIGPKSATQLLVEFQSLEGIYENLDAVAEKWRKKLETHKEMAFLCRDIARLQTDLHIDGNLQQLRLV

R

>gi|30064143|ref|NP_838314.1| L-serine dehydratase (deaminase), L-SD2 [Shigella flexneri 2a str. 2457T]

MISVFDIFKIGIGPSSSHTVGPMKAGKQFTDDLIARNLLKDVTRVVVDVYGSLSLTGKGHHTDIAIIMGL

AGNLPDTVDIDSIPGFIQDVNTHGRLMLANGQHEVEFPVDQCMNFHADNLSLHENGMRITALAGDKVVYS

QTYYSIGGGFIVDEEHFGQQDSAPVEVPYPYSSAADLQKHCQETGLSLSGLMMKNELALHSKEELEQHLA

NVWEVMRGGIERGISTEGVLPGKLRVPRRAAALRRMLVSQDKTTTDPMAVVDWINMFALAVNEENAAGGR
VVTAPTNGACGIIPAVLAYYDKFIREVNANSLARYLLVASAIGSLYKMNASISGAEVGCQGEVGVACSMA
AAGLAELLGASPAQVCIAAEIAMEHNLGLTCDPVAGQVQVPCIERNAIAAVKAVNAARMALRRTSEPRVC
LDKVIETMYETGKDMNAKYRETSRGGLAMKIVACD

>gi|30064142|ref|NP_838313.1| serine transporter [Shigella flexneri 2a str. 2457T]
METTQTSTIASKDSRSAWRKTDTMWMLGLYGTAIGAGVLFLPINAGVGGMIPLIIMAILAFPMTFFAHRG
LTRFVLSGKNPGEDITEVVEEHFGIGAGKLITLLYFFAIYPILLVYSVAITNTVESFMSHQLGMTPPPRA
ILSLILIVGMMTIVRFGEQMIVKAMSILVFPFVGVLMLLALYLIPQWNGAALETLSLDTASATGNGLWMT
LWLAIPVMVFSFNHSPIISSFAVAKREEYGDMAEQKCSKILAFAHIMMVLTVMFFVFSCVLSLTPADLAA
AKEQNISILSYLANHFNAPVIAWMAPIIAIIAITKSFLGHYLGAREGFNGMVIKSLRGKGKSIEINKLNR
ITALFMLVTTWIVATLNPSILGMIETLGGPIIAMILFLMPMYAIQKVPAMRKYSGHISNVFVVVMGLIAI
SAIFYSLFS

>gi|30064141|ref|NP_838312.1| IS4 orf [Shigella flexneri 2a str. 2457T]
MHIGQALDLVSRYDSLRNPLTSLGDYLDPELISRCLAESGTVTLRKRRLPLEMMVWCIVGMALERKEPLH
QIVNRLDIMLPGNRPFVAPSAVIQARQRLGSEAVRRVFTKTAQLWHNATPHPHWCGLTLLAIDGVFWRTP
DTPENDAAFPRQTHAGNPALYPQVKMVCQMELTSHLLTAAAFGTMKNSENELAEQLIEQTGDNTLTLMDK
GYYSLGLLNGWSLAGEHRHWMIPLRKGAQYEELRKLGKGDHLVKLKTSPQARKKWPGLGNEVTARLLTVT
RKGKVCHLLTSMTDAMRFPGEEMADLYSNRWEIELGYREIKQTMQLSRLTLRSKKPELVEQELWGVLLAY
NLVRYQMIKMAEHLKGYWPNQLSFSESCGMVMRMLMTLQGASPGRIPELMRDLASMGQLVKLPTRRGRAF
PRVVKERPWKYPTAPKKRPVRLLN

>gi|30064140|ref|NP_838311.1| hypothetical protein S3003 [Shigella flexneri 2a str. 2457T]
MITHISPLGSMDMLSQLEVDMLKRTASSDLYQLFRNCSLAVLNSGSLTDNSKELLSRFENFDINVLRRER

GVKLELINPPEEAFVDGRIIRALQANLFAVLRDILFVYGQIHNTVRFPNLNLDNSVHITNLVFSILRNAR

ALHVGEAPNMVVCWGGHSINENEYLYARRVGNQLGLRELNICTGCGPGAMEAPMKGAAVGHAQQRYKDSR

FIGMTEPSIIAAEPPNPLVNELIIMPDIEKRLEAFVRIAHGIIIFPGGVGTAEELLYLLGILMNPANKDQ

VLPLILTGPKESADYFRVLDEFVVHTLGENARRHYRIIIDDAAEVARQMKKSMPLVKENRRDTGDAYSFN

WSMRIAPDLQMPFEPSHENMANLKLYPDQPVEVLAADLRRAFSGIVAGNVKEVGIRAIEEFGPYKINGDK

EIMRRMDDLLQGFVAQHRMKLPGSAYIPCYEICT

>gi|30064139|ref|NP_838310.1| 7-cyano-7-deazaguanine reductase [Shigella flexneri 2a str. 2457T]

MSSYANHQALAGLTLGKSTDYRDTYDASLLQGVPRSLNRDPLGLKADNLPFHGTDIWTLYELSWLNAKGL

PQVAVGHVELDYTSVNLIESKSFKLYLNSFNQTRFNNWDEVRQTLERDLSTCAQGKISVALYRLDELEGQ

PIGHFNGTCIDDQDITIDNYEFTTDYLENATSGEKVVEETLVSHLLKSNCLITHQPDWGSIQIQYRGRQI

DREKLLRYLVSFRHHNEFHEQCVERIFNDLLRFCQPEKLSVYARYTRRGGLDINPWRSNSDFVPSTTRLV

RQ

>gi|30064138|ref|NP_838309.1| SecY interacting protein Syd [Shigella flexneri 2a str. 2457T]

MDDLTAQALKDFTARYCDAWHEEHKSWPLSEELYGVPSPCIISTTEDAVYWQPQPFTGEQNVNAVERAFD

IVIQPTIHTFYTTQFAGDMHAQFGDIKLTLLQTWSEDDFRRVQENLIGHLVTQKRLKLPPTLFIATLEEE

LEVISVCNLSGEVCKETLGTRKRTNLASNLAEFLNQLKPLL

>gi|30064137|ref|NP_838308.1| hypothetical protein S2999 [Shigella flexneri 2a str. 2457T]

MTTHDRVRLQLQALEALLREHQHWRNDEPQPHQFNSTQPFFMDTMEPLEWLQWGLIPRMHDLLNNNQPLP

GAFAVAPYYEMALATDHPQRALILAELEKLDALFADDAS

>gi|30064133|ref|NP_838304.1| glucarate dehydratase [Shigella flexneri 2a str. 2457T]

MATQSSPVITDMKVIPVAGHDSMLLNIGGAHNAYFTRNIVVLTDNAGHTGIGEAPGGEVIYQTLVDAIPM

VLGQEVARLNKVVQQVHKGNQAADFDTFGKGAWTFELRVNAVAALEAALLDLLGKALNVPVCELLGPGKQ

RETITVLGYLFYIGDRTKTDLPYLENTPGNHEWYQLRHQKAMNSEAVVRLAEASQDRYGFKDFKLKGGVL

PGEQEIDTVRALKKSFPDARITVDPNGAWLLDEAISLCKGLNDVLTYAEDPCGAEQGFSGREVMAEFRRA

TGLPVATNMIATNWREMGHAVMLNAVDIPLADPHFWTLSGAVRVAQLCDDWGLTWGCHSNNHFDISLAIF

THVGAAAPGNPTAIDTHWIWQEGDCRLTKNPLEIKNGKIAVPDAPGLGVELDWEQVQKAHEAYKRLPCGA

RNDAGPMQYLIPGWTFDRKRPVFGRH

>gi|30064131|ref|NP_838302.1| hybrid sensory histidine kinase BarA [Shigella flexneri 2a str. 2457T]

MTNYSLRARMMILILAPTVLIGLLLSIFFVVHRYNDLQRQLEDAGASIIEPLAVSTEYGMSLQNRESIGQ

LISVLHRRHSDIVRAISVYDENNRLFVTSNFHLDPSSMQLGSNVPFPRQLTVTRDGDIMILRTPIISESY

SPDESPSSDAKNSQNMLGYIALELDLKSVRLQQYKEIFISCVMMLFCIGIALIFGWRLMRDVTGPIRNMV

NTVDRIRRGQLDSRVEGFMLGELDMLKNGINSMAMSLAAYHEEMQHNIDQATSDLRETLEQMEIQNVELD

LAKKRAQEAARIKSEFLANMSHELRTPLNGVIGFTRLTLKTELTPTQRDHLNTIERSANNLLAIINDVLD

FSKLEAGKLILESIPFPLRSTLDEVVTLLAHSSHDKGLELTLNIKSDVPDNVIGDPLRLQQIITNLVGNA

IKFTENGNIDILVEKRALSNTKVQIEVQIRDTGIGIPERDQSRLFQAFRQADASISRRHGGTGLGLVITQ

KLVNEMGGDISFHSQPNRGSTFWFHINLDLNPNIIIEGPSIQCLAGKRLAYVEPNSAAAQCTLDILSETP

LEVVYSPTFSALPPAHYDMMLLGIAVTFREPLTMQHERLAKAVSMTDFLMLALPCHAQVNAEKLKQDGIG

ACLLKPLTPTRLLPALTEFCHHKQNTLLPVTDESKLAMTVMAVDDNPANLKLIGALLEDMVQHVELCDSG

HQAVERAKQMPFDLILMDIQMPDMDGIRACELIHQLPHQRQTPVIAVTAHAMAGQKEKLLGAGMSDYLAK

PIEEERLHNLLLRYKPGSGISSRVVTPEVNEIVVNPNATLDWQLALRQAAGKTDLARDMLQMLLDFLPEV

RNKVEEQLAGENPEGLVDLIHKLHGSCGYSGVPRMKNLCQLIEQQLRSGTKEEDLEPELLELLDEMDNVA

REASKILG

>gi|30064129|ref|NP_838300.1| GDP/GTP pyrophosphokinase [Shigella flexneri 2a str. 2457T]

MVAVRSAHINKAGEFDPEKWIASLGITSQKSCECLAETWAYCLQQTQGHPDASLLLWRGVEMVEILSTLS

MDIDTLRAALLFPLADANVVSEDVLRESVGKSVVNLIHGVRDMAAIRQLKATHTDSVSSEQVDNVRRMLL

AMVDDFRCVVIKLAERIAHLREVKDAPEDERVLAAKECTNIYAPLANRLGIGQLKWELEDYCFRYLHPTE

YKRIAKLLHERRLDREHYIEEFVGHLRAEMKAEGVKAEVYGRPKHIYSIWRKMQKKNLAFDELFDVRAVR

IVAERLQDCYAALGIVHTHYRHLPDEFDDYVANPKPNGYQSIHTVVLGPGGKTVEIQIRTKQMHEDAELG

VAAHWKYKEGAAAGGARSGHEDRIAWLRKLIAWQEEMADSGEMLDEVRSQVFDDRVYVFTPKGDVVDLPA

GSTPLDFAYHIHSDVGHRCIGAKIGGRIVPFTYQLQMGDQIEIITQKQPNPSRDWLNPNLGYVTTSRGRS

KIHAWFRKQDRDKNILAGRQILDDELEHLGISLKEAEKHLLPRYNFNDVDELLAAIGGGDIRLNQMVNFL

QSQFNKPSAEEQDAAALKQLQQKSYTPQNRSKDNGRVVVEGVGNLMHHIARCCQPIPGDEIVGFITQGRG

ISVHRADCEQLAELRSHAPERIVDAVWGESYSAGYSLVVRVVANDRSGLLRDITTILANEKVNVLGVASR

SDTKQQLATIDMTIEIYNLQVLGRVLGKLNQVPDVIDARRLHGS

>gi|30064128|ref|NP_838299.1| nucleoside triphosphate pyrophosphohydrolase [Shigella flexneri 2a str. 2457T]

MNQIDRLLTIMQRLRDPENGCPWDKEQTFATIAPYTLEETYEVLDAIAREDFDDLRGELGDLLFQVVFYA

QMAQEEGRFDFNDICAAISDKLERRHPHVFADSSAENSSEVLARWEQIKTEERAQKAQHSALDDIPRSLP

ALMRAQKIQKRCANVGFDWTTLGPVVDKVYEEIDEVMYEARQAVVDQAKLEEEMGDLLFATVNLARHLGT

KAEIALQKVNEKFERRFREVERIVAARGLEMTGVDLETMEEVWQQVKRQEIDL

>gi|30064123|ref|NP_838294.1| hypothetical protein S2984 [Shigella flexneri 2a str. 2457T]

MQYPINEMFQTLQGEGYFTGVPAIFIRLQGCPVGCAWCDTKHTWEKLEDREVSLFSILAKTKESDKWGAA

SSEDLLAVIGRQGYTARHVVITGGEPCIHDLLPLTDLLEKNGFSCQIETSGTHEVRCTPNTWVTVSPKLN

MRGGYEVLSQALERANEIKHPVGRVRDIEALDELLATLTDDKPRVIALQPISQKDDATRLCIETCIARNW

RLSMQTHKYLNIA

>gi|30064112|ref|NP_838283.1| sulfite reductase subunit beta [Shigella flexneri 2a str. 2457T]

MSEKHPGPLVVEGKLTDAERMKLESNYLRGTIAEDLNDGLTGGFKGDNFLLIRFHGMYQQDDRDIRAERA

EQKLEPRHAMLLRCRLPGGVITTKQWQAIDKFAGENTIYGSIRLTNRQTFQFHGILKKNVKPVHQMLHSV

GLDALATANDMNRNVLCTSNPYESQLHAEAYEWAKKISEHLLPRTRAYAEIWLDQEKVATTDEEPILGQT

YLPRKFKTTVVIPPQNDIDLHANDMNFVAIAENGKLVGFNLLVGGGLSIEHGNKKTYARTASEFGYLPLE

HTLAVAEAVVTTQRDWGNRTDRKNAKTKYTLERVGVETFKAEVERRAGIKFEPIRPYEFTGRGDRIGWVK

GIDDNWHLTLFIENGRILDYPGRPLKTGLLEIAKIHKGDFRITANQNLIIAGVPESEKAKIEKIAKESGL

MNAVTPQRENSMACVSFPTCPLAMAEAERFLPSFIDNIDNLMVKHGVSDEHIVMRVTGCPNGCGRAMLAE

VGLVGKAPGRYNLHLGGNRIGTRIPRMYKENITEPEILASLDELIGRWAKEREAGEGFGDFTVRAGIIRP

VLDPARDLWD

>gi|30064111|ref|NP_838282.1| phosphoadenosine phosphosulfate reductase [Shigella flexneri 2a str. 2457T]

MSKLDLNALNELPKVDRILALAETNAELEKLDAEGRVAWALDNLPGEYVLSSSFGIQAAVSLHLVNQIHP

DIPVILTDTGYLFPETYRFIDELTDKLKLNLKVYRATESAAWQEARYGKLWEQGVEGIEKYNDINKVEPM

NRALKELNAQTWFAGLRREQSGSRANLPVLAIQRGVFKVLPIIDWDNRTIYQYLQKHGLKYHPLWDEGYL

SVGDTHTTRKWEPGMAEEETRFFGLKRECGLHEG

>gi|30064110|ref|NP_838281.1| alkaline phosphatase isozyme conversion aminopeptidase [Shigella flexneri 2a str. 2457T]

MFSALRHRTAALALGVCFILPVHASSPKPGDFANTQARHIATFFPGRMTGTPAEMLSADYIRQQFQQMGY

RSDIRTFNSRYIYTARDNRKNWHNVTGSTVIAAHEGKAPQQIIIMAHLDTYAPLSDADADANLGGLTLQG

IDDNAAGLGVMLELAERLKNTPTEYGIRFVATSGEEEGKLGAENLLKRMSDTEKKNTLLVINLDNLIVGD

KLYFNSGVKTPEAVRKLTRDRALAIARSHGIAATTNPGLNKNYPKGTGCCNDAEIFDKAGIAVLSVEATN

WNLGNKDGYQQRAKTPAFPAGNSWHDVRLDNQQHIDKALPGRIERRCRDVMRIMLPLVKELAKAS

>gi|30064109|ref|NP_838280.1| sulfate adenylyltransferase subunit 2 [Shigella flexneri 2a str. 2457T]

MAQKRLTHLRQLEAESIHIIREVAAEFSNPVMLYSIGKDSSVMLHLARKAFYPGTLPFPLLHVDTGWKFR

EMYEFRDRTAKAYGCELLVHKNPEGVAMGINPFVHGSAKHTDIMKTEGLKQALNKYGFDAAFGGARRDEE

KSRAKERIYSFRDRFHRWDPKNQRPELWHNYNGQINKGESIRVFPLSNWTEQDIWQYIWLENIDIVPLYL

AAERPVLERDGMLMMIDDNRIDLQPGEVIKKRMVRFRTLGCWPLTGAVESNAQTLPEIIEEMLVSTTSER

QGRVIDRDQAGSMELKKRQGYF

>gi|30064106|ref|NP_838277.1| hypothetical protein S2965 [Shigella flexneri 2a str. 2457T]

MRNSHNITLTNNDSLTEDEETTWSLPGAVVGFISWLFALAMPMLIYGSNTLFFFLYTWPFFLALMPVAVV

VGIALHSLMDGKLRYSIVFTLVTVGIMFGALFMWLLG

>gi|30064105|ref|NP_838276.1| cell division protein FtsB [Shigella flexneri 2a str. 2457T]

MGKLTLLLLAILVWLQYSLWFGKNGIHDYTRVNDDVAALQATNAKLKARNDQLFAEIDDLNGGQEALEER

ARNELSMTRPGETFYRLVPDASKRAQSAGQNNR

>gi|30064103|ref|NP_838274.1| 2-C-methyl-D-erythritol 2,4-cyclodiphosphate synthase [Shigella flexneri 2a str. 2457T]

MRIGHGFDVHAFGGEGPIIIGGVRIPYEKGLLAHSDGDVALHALTDALLGAAALGDIGKLFPDTDPAFKG
ADSRELLREAWRRIQAKGYTLGNVDVTIIAQAPKMLPHIPQMRVFIAEDLGCHMDDVNVKATTTEKLGFT
GRGEGIACEAVALLIKATK

>gi|30064101|ref|NP_838272.1| stationary phase survival protein SurE [Shigella flexneri 2a str. 2457T]
MRILLSNYDGVHAPGIQTLAKALREFADVQVVAPDRNRSGASNSLTLESSLRTFTFENGDIAVQMGTPTD
CVYLGVNALMRPRPDIVVSGINAGPNLGDDVIYSGTVAAAMEGRHLGFPALAVSLDGHKHYDTAAAVTCS
ILRALCKEPLRTGRILNINVQDLPLDQIKGIRVTRCGTRHPADQVIPQQDPRGNTLYWIGPPGGKCDAGP
GTDFAAVDEGYVSITPLHVDLTAHSAQDVVSDWLNSVGVGTQW

>gi|30064099|ref|NP_838270.1| lipoprotein NlpD [Shigella flexneri 2a str. 2457T]
MSAGSPKFTVRRIAALSLVSLWLAGCSDTSNPPAPVSSVNGNAPANTNSGMLITPPPKMGTTSTAQQPQI
QPVQQPQIQATQQPQIQPVQPVAQQPVQMENGRIVYNRQYGNIPKGSYSGSTYTVKKGDTLFYIAWITGN
DFRDLAQRNNIQAPYALNVGQTLQVGNASGTPITGGNAITQADAAEQGVVIKPAQNSTVAVASQPTITYS
ESSGEQSANKMLPNNKPTATTVTAPVTVPTASTTEPTVSSTSTSTPISTWRWPTEGKVIETFGASEGGNK
GIDIAGSKGQAIIATADGRVVYAGNALRGYGNLIIIKHNDDYLSAYAHNDTMLVREQQEVKAGQKIATMG
STGTSSTRLHFEIRYKGKSVNPLRYLPQR

>gi|30064098|ref|NP_838269.1| transcriptional regulator [Shigella flexneri 2a str. 2457T]
MALRNKAFHQLRQLFQQHTARWQHELPDLTKPQYAVMRAIADKPGIEQVALIEAAVSTKATLAEMLARME
NRGLVRREHDVADKRRRFVWLTAEGEKLLAAAIPIGDSVDEEFLGRLSGEEQELFMQLVRKMMST

>gi|30064097|ref|NP_838268.1| phenylacrylic acid decarboxylase-like protein [Shigella flexneri 2a str. 2457T]
MKLIVGMTGATGAPLGVALLQALREMPNVETHLVMSKWAKTTIELETPYSARDVAALADFSHNPADQAAI

ISSGSFRTDGMIVIPCSMKTLAGIRAGYADGLVGRAADVVLKEGRKLVLVPREMPLSTIHLENMLALSRM

GVAMVPPMPAFYNHPETVDDIVHHVVARVLDQFGLEHPHARRWQGLPQARNFSQENE

>gi|30064096|ref|NP_838267.1| 4-hydroxybenzoate decarboxylase, partial [Shigella flexneri 2a str. 2457T]

MAFDDLRSFLQALDDHGQLLKISEEVNAEPDLAAAANATGRIGDGAPALWFDNIRGFTDARVAMNTIGSW

QNHAISLGLPPNTPVKKQIDEFIRRWDNFPIAPERRANPAWAQNTVDGEEINLFDILPLFRLNDGDGGFY

LDKACVVSRDPLDPDNFGKQNVGIYRMEVKGKRKLGLQPVPMHDIALHLHKAEERGEDLPIAITLGNDPI

ITLMGATPLKYDQSEYEMAGALRESPYPIATAPLTGFDVPWGSEVILEGVIESRKREIEGPFGEFTGHYS

GGRNMTVVRIDKVSYRSKPIFESLYLGMPWTEIDYL

>gi|30064095|ref|NP_838266.1| regulatory protein [Shigella flexneri 2a str. 2457T]

MRKAPQGSFPADGKEPIIERIFRLVERYPSRNAAARAWGINEGTLKNYYNRRDYAPIPRPYQLKKIAESE

NVTLEWLMTGEGDVELKDSKEPKKENADNVDAQILMFLSFLKPQEKQQVADVLGRKGAEQLLILLDEHIQ

ELHALVGVRRAIALSLGNLPDEKVREIYALSEAKGDHLNLPQKAASA

>gi|30064093|ref|NP_838264.1| phage transposase [Shigella flexneri 2a str. 2457T]

MQSYVTVNDLLGVPGMPATTKGIRQALQRFSRDLGDVSRRREGTKAIEYHIDCLPEITRKALRERYVEQL

VATENNVSEVKAVTRKTRNPDAVQAIEAYRGSPQLMEERLNALTENQRWVSEARAALVVEVLKLESAGNP

GRLKAINFLVEKARKGELPERLQQAAVNANAKRGANRTISRDPLYQWVLKYNQSQNAERLLLLAPGKRD

EIKPEEISWLPEFLAQYRQVNGRPMSEAYEDFVAEWQRRHADEPYMLEVMPSYDVVRYAMKKLPEVVKQK

GRVTGSEYRQLEGFTRRDWTAMPVNYVWIGDGHGMKLKCAHPIHGRPFSPEVTFVIDGGTRFVVGWSLDL

AENVFAVAGAIQHGIRNHGKPFLYYSDNGSGETADMLDKEVVGILPRLGIKHPTGIAGNPQGRGIIERLN

RTLPMRIARRYRTYFGKGADRESLRVLNRDLRSAFNALQQDKPLNDRQKAAMRELPSWAELIEAIREGVE

WYNNRPHSELPMKPNGRHYSPTEFRKKRQAEEDTEIEWLSDLELRDMFRPMVERPVRRCEIQWLNNIYYA

PELRDEHGRKVLISYDIHDAERITVRRKDGSFICEAIWNGNKRAAFAVSAEYHKQQQRIKGMRKRAEEKI

RDAEDEGIQILEHKQAEPWLSNVYRPVGNVVAVQQPEYEEEHDEEFERDFRLGMQKLFAMQEEDDPLA

>gi|30064090|ref|NP_838261.1| DEOR-type transcriptional regulator [Shigella flexneri 2a str. 2457T]

MSAGQRKEQKLIPVERRQIILEMVAEKGIVSIAELTDRINVSHMTIRRDLQKLEQQGAVVLVSGGVQSPG

RVAHEPSHQVKTALAMTQKAAIGKLAASLVLPGSCIYLDAGTTTLAIAQHLIHMESLTVVTNDFVIANYL

LDNSNCTIIHTGGAVCRENRSCVGEAAATMLCSLMIDQAFISASSWSVRGISTPAEDKVTVKRAIASASR

QRVLVCDATKYGQVATWLALPLSEFDQIITDDGLPESASRALAKQDLSLLVAKNE

>gi|30064086|ref|NP_838257.1| hypothetical protein S2941 [Shigella flexneri 2a str. 2457T]

MSGKRISREKLTIKKMIDLYQAKCPQASAEPEHYEALFVYAQKRLDKCVFGEEKPACKQCPVHCYQPAKR

EEMKQIMRWAGPRMLWRHPILTVRHLIDDKRPVPELPEKYRPKKPRE

>gi|30064085|ref|NP_838256.1| formate hydrogen-lyase transcriptional activator for fdhF, hyc and hyp operons [Shigella flexneri 2a str. 2457T]

MSYTPMSDLGQQGLFDITRTLLQQPDLASLCEALSQLVKRSALADNAAIVLWQAQTQRASYYASREKDTP

IKYEDETVLAHGPVRSILSRPDTLHCSYEEFCETWPQLAAGGLYPKFGHYCLMPLAAGGHIFGGCEFIRY

DERPWSEKEFNRLQTFTQIVSVVTEQIQSRVVNNVDYELLCRERDNFRILVAITNAVLSRLDMDELVSEV

AKEIHYYFDIDDISIVLRSHRKNKLNIYSTHYLDKQHPAHEQSEVDEAGTLTERVFKSKEMLLINLYERD

DLAPYERMLFDTWGNQIQTLCLLPLMSGDTMLGVLKLAQCEEKVFTTTNLNLLRQIAERVAIAVDNALAY

QEIHRLKERLVDENLALTEQLNNVDSEFGEIIGRSEAMYSVLKQVEMVAQSDSTVLILGETGSGKELIAR

AIHNLSGRNNRRMVKMNCAAMPAGLLESDLFGHERGAFTGANAQRIGRFELADKSSLFLDEVGDMPLELQ

PKLLRVLQEQEFERLGSNKIIQTDVRLIAATNRDLKKMVADREFRSDLYYRLNVFPIHLPPLRERPEDIP

LLAKAFTFKIARRLGRNIDSIPAETLRTLSNMEWPGNVRELENVIERAVLLTRGNVLQLSLPDIALPEPE

TPPAATVVAQEGEDEYQLIVRVLKETNSVVAGPKGAAQRLGLKRTTLLSRMKRLGIDKSALI

>gi|30064083|ref|NP_838254.1| hydrogenase isoenzyme HypD [Shigella flexneri 2a str. 2457T]

MRFVDEYRAPEQVMQLIEHLRERASHLSYTAERPLRIMEVCGGHTHAIFKFGLDQLLPENVEFIHGPGCP

VCVLPMGRIDTCVEIASHPEVIFCTFGDAMRVPGKQGSLLQAKARGADVRIVYSPMDALKLAQENPTRKV

VFFGLGFETTMPTTAITLQQAKARDVQNFYFFCQHITLIPTLRSLLEQPDNGIDAFLAPGHVSMVIGTDA

YNFIASDFHRPLVVAGFEPLDLLQGVVMLVEQKIAAHSKVENQYRRVVPDAGNLLAQQAIADVFCVNGDS

EWRGLGVIESSGVHLTPDYQRFDAEAHFRPAPQQVCDDPRARCGEVLTGKCKPHQCPLFGNTCNPQTAFG

ALMVSSEGACAAWYQYRQQESEA

>gi|30064081|ref|NP_838252.1| hydrogenase nickel incorporation protein HypB [Shigella flexneri 2a str. 2457T]

MCTTCGCGEGNLYIEGDEHNPHSAFRSAPFAPAARPQMKITGIKAPEFTPSQTEEGDLHYGHGEAGTHTP

GMSQRRMLEVEIDVLDKNNRLAERNRARFAARKQLVLNLVSSPGSGKTTLLTETLMRLKDSVPCAVIEGD

QQTVNDAARIRATGTPAIQVNTGKGCHLDAQMIADAAPRLPLDDNGILFIENVGNLVCPASFDLGEKHKV

AVLSVTEGEDKPLKYPHMFAAASLMLLNKVDLLPYLNFDVEKCIACAREVNPEIEIILISATSGEGMDQW

LNWLETQRCA

>gi|30064080|ref|NP_838251.1| hydrogenase nickel incorporation protein [Shigella flexneri 2a str. 2457T]

MGGDMHEITLCQRALELIEQQAAKHGAKRVTGVWLKIGAFSCVETSSLAFCFDLVCRGSVAEGCKLHLEE

QEAECWCETCQQYVTLLTQRVRRCPQCHGDMLQIVADDGLQIRRIEIDQE

>gi|30064079|ref|NP_838250.1| formate hydrogenlyase regulatory protein HycA [Shigella flexneri 2a str. 2457T]

MTIWEISEKADYIAQRHRRLQDQWHIYCNSLVQGITLSKARLHHAMSCAPDKELCFVLFEHFRIYVTLAD

GFNSHTIEYYVETKDGEDKQRIAQAQLSIDGMIDGKVNIRDREQVLEHYLEKIAGVYDSLYTAIENNVPV

NLSQLVKGQSPAA

>gi|30064078|ref|NP_838249.1| small subunit of hydrogenase-3, iron-sulfur protein (part of formate hydrogenlyase (FHL) complex) [Shigella flexneri 2a str. 2457T]

MNRFVIADSTLCIGCHTCEAACSETHRQHGLQSMPRLRVMLNEKESAPQLCHHCEDAPCAVVCPVNAITR

VDGAVQLNESLCVSCKLCGIACPFGAIEFSGSRPLDIPANANTPKAPPAPPAPARVSTLLDWVPGIRAIA

VKCDLCSFDEQGPACVRMCPTKALHLVDNTDIARVSKRKRELTFNTDFGDLTLFQQAQSGEAK

>gi|30064073|ref|NP_838244.1| ascBF operon repressor [Shigella flexneri 2a str. 2457T]

MTTMLEVAKRAGVSKATVSRVLSGNGYVSQETKDRVFQAVEESSYRPNLLARNLSAKSTQTLGLVVTNTL

YHGIYFSELLFHAARMAEEKGRQLLLADGKHSAEEERQAIQYLLDLRCDAIMIYPRFLSVDEIDDIIDAH

SQPIMVLNRRLRNNSSHSVWCDHKQTSFNAVAELINAGHQEIAFLTGSMDSPTSIERLAGYKDALSQHGI

ALNEKLIANGKWTPASGAEGVETLLERGAKFSALVASNDDMAIGAIKALHERGVAVPEQVSVIGFDDIAI

APYIVPALSSVKIPVTEMIQEIIGRLIFMLDGGDFSPPKTFSGKLIRRDSLIALSR

>gi|30064072|ref|NP_838243.1| electron transport protein HydN [Shigella flexneri 2a str. 2457T]

MNRFIIADASKCIGCRTCEVACVVSHQENQDCASLTPETFLPRIHVIKGVNISTATVCRQCEDAPCANVC

PNGAISRDKGFVPVMQERCIGCKTCVVACPYGAMEVVVRPVIRNSGAGLNVRADKAEANKCDLCNHREDG

PACMAACPTHALICVDRNKLEQLSAEKRRRTALMF

>gi|30064071|ref|NP_838242.1| transcriptional regulatory protein [Shigella flexneri 2a str. 2457T]

MAKNTSCGVQLRIRGKVQGVGFRPFVWQLAQQLNLHGDVCNDGDGVEVRLLEDPETFLVQLHQHCPPLAR

IDSVEREPFIWSQLPTEFTIRQSTGGTMNTQIVPDAATCPACLAEMNTPGERRYRYPFINCTHCGPRFTI

IRAMPYDRPFTVMAAFPLCPACDKEYRDPLDRRFHAQPVACPECGPHLEWVSHGEHAEQEAALQAAIAQL

KMGNIVAIKGIGGFHLACDARNSNAVATLRERKHRPAKPLAVMLPVAESLPDAARQLLTTPAAPIVLVDK

KYVPELCDDIAPGLNEVGVMLPANPLQHLLLQELQCPLVMTSGNLSGKPPAISNEQALADLQGIADGFLL

HNRDIVQRMDDSVVRESGEMLRRSRGYVPDALVLPPGFKNVPPVLCLGADLKNTFCLVRGEQAVLSQHLG

DLSDDGIQMQWREALRLMQNIYDFTPQYIVHDVHPGYVSSQWASEMNLPTQTVLHHHAHAAACLAEHQWP

LDGGDVIALTLDGIGMGENGALWGGECLRVNYRECEHLGGLPAVALPGGDLAAKQPWRNLLAQCLRFVPE

WQNYPETASVQQQNWSVLARAIERGINAPLASSCGRLFDAVAAALGCAPATLSYEGEAACALEALAASCD

GVTHPVTMPRVDNQLDLATFWQQWLNWQALVNQRAWAFHDALAQGFAALMREQATMRGITTLVFSGGVIH

NRLLRARLAHYLADFTLLFPQSLPAGDGGLSLGQGVIAAARWLAGEVQNG

>gi|30064069|ref|NP_838240.1| anaerobic nitric oxide reductase flavorubredoxin [Shigella flexneri 2a str. 2457T]

MSIVVKNNIHWVGQRDWEVRDFHGTEYKTLRGSSYNSYLIREEKNVLIDTVDHKFSREFVQNLRNEIDLA

DIDYIVINHAEEDHAGALTELMAQIPDTPIYCTANAIDSINGHHHHPEWNFNVVKTGDTLDIGNGKQLIF

VETPMLHWPDSMMTYLTGDAVLFSNDAFGQHYCDEHLFNDEVDQTELFEQCQRYYANILTPFSRLVTPKI

TEILGFNLPVDMIATSHGVVWRDNPTQIVELYLKWAADYQEDRITIFYDTMSNNTRMMADAIAQGIAETD

PRVAVKIFNVARSDKNEILTNVFRSKGVLVGTSTMNNVMMPKIAGLVEEMTGLRFRNKRASAFGSHGWSG

GAMDRLSTRLQDAGFEMSLSLKAKWRPDQDALELCREHGREIARQWALAPLPQSTVNTVVKEETSATTTA

DLGPRMQCSVCQWIYDPAKGEPMQDVAPGTPWSEVPDNFLCPECSLGKDVFDELASEAK

>gi|30064066|ref|NP_838237.1| DNA-bindng transcriptional repressor SrlR [Shigella flexneri 2a str. 2457T]

MKPRQRQAAILEYLQKQGKCSVEELAQYFDTTGTTIRKDLVILEHAGTVIRTYGGVVLNKEESDPPIDHK

TLINTHKKELIAEAAVSFIHDGDSIILDAGSTVLQIVPLLSRFNNITVMTNSLHIVNALSELDNEQTILM

PGGTFRKKSASFHGQLAENAFEHFTFDKLFMGTDGIDLNAGVTTFNEVYTVSKAMCNAAREVILMADSSK

FGRKSPNVVCSLESVDKLITDAGIDPAFRQALEEKGIDVIITGESNE

>gi|30064065|ref|NP_838236.1| DNA-binding transcriptional activator GutM [Shigella flexneri 2a str. 2457T]

MVSALITVAVIAWCAQLALGGWQISRFNRAFDTLCQQGRVGVDRSSGRFKPRVVVAIALDDQQCIVDTLF

MKGLTVFARPQKIPAITGMHAGDLQPDVIFPHDPLSQNALSLALKLKRG

>gi|30064063|ref|NP_838234.1| glucitol/sorbitol-specific PTS system component IIA [Shigella flexneri 2a str. 2457T]

MTVIYQTTITRIGASATDALSDQMLITFREGAPADLEEYCFIHCHGELKGALHPGLQFSLGQHRYPVTAV

GSVAEDNLRELGHVTLRFDGLNEAEFPGTVHVAGPVPDDIAPGSVLKFESVKE

>gi|30064062|ref|NP_838233.1| PTS system, glucitol/sorbitol-specific IIB component and second of two IIC components [Shigella flexneri 2a str. 2457T]

MTRIRIEKGTGGWGGPLELEATPGKKIVYITAGTRPAIVDKLAQLTGWQAIDGFKEGEPAEAEIGVAVID

CGGTLRCGIYPKRRIPTINIHSTGKSGPLAQYIVEDIYVSGVKEENITVVGDATPQPSSVGRDYDTSKKI

TEQSDGLLAKVGMGMGSAVAVLFQSGRDTIDTVLKTILPFMAFVSALIGIIMASGLGDWIAHGLAPLASH

PLGLVMLALICSFPLLSPFLGPGAVIAQVIGVLIGVQIGLGNIPPHLALPALFAINAQAACDFIPVGLSL

AEARQDTVRVGVPSVLVSRFLTGAPTVLIAWFVSGFIYQ

>gi|30064061|ref|NP_838232.1| murein hydrolase B [Shigella flexneri 2a str. 2457T]

MFKRRYVTLLPLFVLLAACSSKPKPTETETTTGTPSGGFLLEPQHNVMQMGGDFANNPNAQQFIDKMVNK

HGFDRQQLQEILSQAKRLDSVLRLMDNQAPTTSVKPPSGPNGAWLRYRKKFITPDNVQNGVVFWNQYEDA

LNRAWQVYGVPPEIIVGIIGVETRWGRVMGKTRILDALATLSFNYPRRAEYFSGELETFLLMARDEQDDP

LNLKGSFAGAMGYGQFMPSSYKQYAVDFSGDGHINLWDPVDAIGSVANYFKAHGWVKGDQVAIMANGQAP

GLPNGFKTKYSISQLAAAGLTPQQPLGNHQQASLLRLDVGTGYQYWYGLPNFYTITRYNHSTHYAMAVWQ

LGQAVALARVQ

>gi|30064060|ref|NP_838231.1| competence damage-inducible protein A [Shigella flexneri 2a str. 2457T]

MTDSELMQLSEQVGQALKARGATVTTAESCTGGWVAKVITDIAGSSAWFERGFVTYSNEAKAQMIGVREE

TLAQHGAVSEPVVVEMAIGALKAARADYAVSISGIAGPDGGSEEKPVGTVWFAFATARGEGITRRECFSG

DRDAVRRQATAYALQTLWQQFLQNT

>gi|30064058|ref|NP_838229.1| recombination regulator RecX [Shigella flexneri 2a str. 2457T]

MTESTSRRPAYARLLDRAVRILAVRDHSEQELRRKLAAPIMGKNGPEEIDATAEDYERVIAWCHEHGYLD

DSRFVARFIASRSRKGYGPARIRQELNQKGISREATEKAMRECDIDWCALARDQATRKYGEPLPTVFSEK

VKIQRFLLYRGYLMEDIQDIWRNFAD

>gi|30064054|ref|NP_838225.1| hypothetical protein S2903 [Shigella flexneri 2a str. 2457T]

MSEALSLFSLFASSFLSATLLPGNSEVVLVAMLLSGISHPWVLVLTATMGNSLGGLTNVILGRFFPLRKT

SRWQEKATGWLKRYGAVTLLLSWMPVVGDLLCLLAGWMRISWGPVIFFLCLGKALRYVAVAAATVQGMMW

WH

>gi|30064053|ref|NP_838224.1| glutamate--cysteine ligase [Shigella flexneri 2a str. 2457T]

MIPDVSQALAWLEKHPQALKGIQRGLERETLRVNADGTLATTGHPEALGSALTHKWITTDFAEALLEFIT
PVDGDIEHMLTFMRDLHRYTARNMGDERMWPLSMPCYIAEGQDIELAQYGTSNTGRFKTLYREGLKNRYG
ALMQTISGVHYNFSLPMAFWQAKCGDISGADAKEKISAGYFRVIRNYYRFGWVIPYLFGASPAICSSFLQ
GKPTSLPFEKTECGMYYLPYATSLRLSDLGYTNKSQSNLGITFNDLYEYVAGLKQAIKTPSEEYAKIGIE
KDGKRLQINSNVLQIENELYAPIRPKRVTRSGESPSDALLRGGIEYIEVRSLDINPFSPIGVDEQQVRFL
DLFMVWCALADAPEMSSSELACTRVNWNRVILEGRKPGLTLGIGCETAQFPLLQVGKDLFRDLKRVAQTL
DSINGGEAYQKVCDELVACFDNPDLTFSARILRSMIDTGIGGTGKAFAEAYRNLLREEPLEILREEDFVA
EREASERRQQEMETADTEPFAVWLEKHA

>gi|30064052|ref|NP_838223.1| S-ribosylhomocysteinase [Shigella flexneri 2a str. 2457T]
MPLLDSFTVDHTRMEAPAVRVAKTMNTPHGDAITVFDLRFCVPNKEVMPERGIHTLEHLFAGFMRNHLNG
NGVEIIDISPMGCRTGFYMSLIGTPDEQRVADAWKAAMEDVLKVQDQNQIPELNVYQCGTYQMHSLQEAQ
DIARSILEREVRINSNEELALPKEKLQELHI

>gi|30064051|ref|NP_838222.1| multidrug resistant protein emrB [Shigella flexneri 2a str. 2457T]
MQQQKPLEGAQLVIMTIALSLATFMQVLNSTIANVAIPTIAGNLGSSLSQGTWVITSFGVANAISIPLTG
WLAKRVGEVKLFLWSTIAFAIASWACGVSSSLNMLIFFRVIQGIVAGPLIPLSQSLLLNNYPPAKRSIAL
ALWSMTVIVAPICGPILGGYISDNYHWGWIFFINVPIGVAVVLMTLQTLRGRETRTERRRIDAVGLALLV
IGIGSLQIMLDRGKELDCFSSQEIIILTVVAVVAICFLIVWELTDDNPIVDLSLFKSRNFTIGCLCISLA
YMLYFGAIVLLPQLLQEVYGYTATWAGLASAPVGIIPVILSPIIGRFAHKLDMRRLVTFSFIMYAVCFYW
RAYTFEPGMDFCASAWPQFIQGFAVVCFFMPLTTITLSGLPPERLAAASSLSNFTRTLAGSIGTSITTTM
WTNRESMHHAQLTESVNPFNPNAQAMYSQLEGLGMTQQQASGWIAQQITNQGLIISANEIFWMSAGIFLV
LLGLVWFAKPPFGAGGGGGGAH

>gi|30064050|ref|NP_838221.1| transcriptional repressor MprA [Shigella flexneri 2a str. 2457T]

MDSSFTPIEQMLKFRASRHEDFPYQEILLTRLCMHMQSKLLENRNKMLKAQGINETLFMALITLESQENH

SIQPSELSCALGSSRTNATRIADELEKRGWIERRKSDNDRRCLHLQLTEKGHEFLREVLPPQHNCLHQLW

SALSTTEKDQLEQITRKLLSRLDQMEQDGVVLEAMS

>gi|30064049|ref|NP_838220.1| hypothetical protein S2897 [Shigella flexneri 2a str. 2457T]

MSYEVLLLGLLVGAANYCFRYLPLRLRVGNARPTKRGAVGILLDTIGIASICALLVVSTAPEVMHDTRRF

VPTLVGFAVLGASFYKTRSIIIPTLLSALAYGLAWKVMAII

>gi|30064048|ref|NP_838219.1| hypothetical protein S2896 [Shigella flexneri 2a str. 2457T]

MESPTPQPAPGSATFMEGCKDSLPIVISYIPVAFAFGLNATRLGFSPLESVFFSCIIYAGASQFVITAML

AAGSSLWVAALSVMAMDVRHVLYGPSLRSRIIQRLQKSKTALWAFGLTDEVFAAATAKLVRNNRRWSENW

MIGIAFSSWSSWVFGTVIGAFSGSGLLQGYPAVEAALGFMLPALFMSFLLASFQRKQSLCVTAALVGALA

GVTLFSIPVAILAGIVCGCLTALIQAFWQGAPDEL

>gi|30064047|ref|NP_838218.1| hypothetical protein S2894 [Shigella flexneri 2a str. 2457T]

MTKPNHELSPALIVLMSIATGLAVASNYYAQPLLDTIARNFSLSASSAGFIVTAAQLGYAAGLLFLVPLG

DMFERRRLIVSMTLLAAGGMLITASSQSLAMMILGTALTGLFSVVAQILVPLAATLASPDKRGKVVGTIM

SGLLLGILLARTVAGLLANLGGWRTVFWVASVLMALMALALWRGLPQMKSETHLNYPQLLGSVFSMFISD

KILRTRALLGCMTFANFSILWTSMAFLLAAPPFNYSDGDIGLFGLAGAAGALGARPAGGFADKGKSHHNF

RSASAVTFMAGNLVWSHFRTGVDYRHSGAGPHRTGRAYH

>gi|30064046|ref|NP_838217.1| glycine betaine transporter periplasmic subunit [Shigella flexneri 2a str. 2457T]

MRHSVLFATAFATLISTQTFAADLPGKGITVNPVQSTITEETFQTLLVSRALEKLGYTVNKPSEVDYNVG

YTSLASGDATFTAVNWTPLHDNMYEAAGGDKKFYREGVFVNGAAQGYLIDKKTADQYKITNIAQLKDPKI

AKLFDTNGDGKADLTGCNPGWGCEGAINHQLAAYELTNTVTHNQGNYAAMMADTISRYKEGKPVFYYTWT

PYWVSNELKPGKDVVWLQVPFSALPGDKNADTKLPNGANYGFPVSTMHIVANKAWTEKNPAAAKLFAIMQ

LPVADINAQNAIMHDGKASEGDIQGHVDGWIKAHQQQFDGWVNEALAAQK

>gi|30064045|ref|NP_838216.1| glycine betaine transporter membrane protein [Shigella flexneri 2a str. 2457T]

MADQNNPWDTTPAADSAAQSADAWGTPTTAPADGGGADWLTSTPAPNVEHFNILDPFHKTLIPLDSWVTE

GIDWVVTHFRPVFQGVRVPVDYILNGFQQLLLGMPAPVAIIVFALITWQISGVGMGVATLVSLIAIGAIG

AWSQAMVTLALVLTALLFCIVIGLPLGIWLARSPRAAKIIRPLLDAMQTTPAFVYLVPIVMLFGIGNVPG

VVVTIIFALPPIIRLTILGINQVPADLIEASRSFGASPRQMLFKVQLPLAMPTIMAGVNQTLMLALSMVV

IASMIAVGGLGQMVLRGIGRLDMGLATVGGVGIVILAIILDRLTQAVGRDSRSRGNRRWYTTGPVGLLTR

PFIK

>gi|30064043|ref|NP_838214.1| ribonucleotide-diphosphate reductase subunit beta [Shigella flexneri 2a str. 2457T]

MKLSRISAINWNKISDDKDLEVWNRLTSNFWLPEKVPLSNDIPAWQTLTVVEQQLTMRVFTGLTLLDTLQ

NVIGAPSLMPDALTPHEEAVLSNISFMEAVHARSYSSIFSTLCQTKDVDAAYAWSEENAPLQRKAQIIQQ

HYRGDDPLKKKIASVFLESFLFYSGFWLPMYFSSRGKLTNTADLIRLIIRDEAVHGYYIGYKYQKNMEKI

SLGQREELKSFAFDLLLELYDNELQYTDELYAETPWADDVKAFLCYNTNKALMNLGYEPLFPAEMAEVNP

AILAALSPNADENHDFFSGSGSSYVMGKAVETEDEDWNF

>gi|30064041|ref|NP_838212.1| ribonucleotide reductase stimulatory protein [Shigella flexneri 2a str. 2457T]

MSQLVYFSSSSENTQRFIERLGLPAVRIPLNERERIQVDEPYILIVPSYGGGGTAGAVPRQVIRFLNDEH

NRALLRGVIASGNRNFGEAYGRAGDVIARKCGVPWLYRFELMGTQSDIENVRKGVTEFWQRQPQNA

>gi|30064039|ref|NP_838210.1| hypothetical protein S2885 [Shigella flexneri 2a str. 2457T]

MYLRPDEVARVLEKVGFTVDVVTQKAYGYRRGENYVYVNREARMGRTALVIHPTLKERSSTLAEPASDIK

TCDHYQQFPLYLAGERHEHYGIPHGFSSRVALERYLNGLFGEAS

>gi|30064038|ref|NP_838209.1| hypothetical protein S2884 [Shigella flexneri 2a str. 2457T]

MFSPQSRLRHAVADTFAMVVYCSVVNMCIEVFLSGMSFEQSFYSRLVAIPVNILIAWPYGMYRDLFMRAA

RKVSPSGWIKNLADILAYVTFQSPVYVAILLVVGADWHQIMAAVSSNIVVSMLMGAVYGYFLDYCRRLFK

VSRYQQVKA

>gi|30064037|ref|NP_838208.1| DNA binding protein, nucleoid-associated [Shigella flexneri 2a str. 2457T]

MSVMLQSLNNIRTLRAMAREFSIDVLEEMLEKFRVVTKERREEEEQQQRELAERQEKISTWLELMKADGI

NPEELLGNSSAAAPRAGKKRQPRPAKYKFTDVNGETKTWTGQGRTPKPIAQALAEGKSLDDFLI

>gi|30064036|ref|NP_838207.1| hypothetical protein S2882 [Shigella flexneri 2a str. 2457T]

MALTTISPHDAQELIARGAKLIDIRDADEYLREHIPEADLAPLSVLEQSGLPPKLRREQIIFHCQAGKRT

SNNADKLAAIAAPAEIFLLEDGIDGWKRAGLPVAVNKSQPLPLMRQVQIAAGGLILNGVVLGYTVNSGFF

LLSGFVGAGLLFAGISGFCGMARLLDKMPWNQRA

>gi|30064031|ref|NP_838202.1| LysM domain/BON superfamily protein [Shigella flexneri 2a str. 2457T]

MGLFNFVKDAGEKLWDAVTGQHDKDDQAKKVQEHLSKTGIPDADKVNIQIADGKATVTGDGLSQEAKEKI

LVAVGNISGIASVDDQVKTATPATASQFYTVKSGDTLSAISKQVYGNANLYNKIFEANKPMLKSPDKIYP

GQVLRIPEE

>gi|30064030|ref|NP_838201.1| DNA-binding transcriptional regulator CsiR [Shigella flexneri 2a str. 2457T]

MTITSLDGYRWLKNDIIRGNFQPDEKLRMSLLTSRYALGVGPLQEALSQLVAERLVTVVNQKGYRVASMS

EQELLDIFDARANMEAMLVSLAIARGGDEWEADVLAKAHLLSKLEACDASEKMLDEWDLRHQAFHTAIVA

GCGSHYLLQMRERLFDLAARYRFIWLRRTVLSVEMLEDKHDQHQTLTAAVLPRDTARASELMRQHLLTPI

PIIQQAMAGN

>gi|30064019|ref|NP_838190.1| integrase [Shigella flexneri 2a str. 2457T]

MARQTKPLSVKEIESAKPKEADYVLYDGDGLELLIKSSGSKIWQFRYIRPVTKTRAKKSIGPYPSVTLAD

ARNYRAESRSLLAKQIDPQEHQQEQLRSSLEAKTNTFQLVAERWWNVKKASVTEDYAEDIWRSLERDVFP

AIGDVSVTDIKAHTLVQAVQPVQARGALETVRRLCQRINEVMIYAQNTGLIDAVPSVNIGKAFEKPQKKN

MPSIRPDQLSQLMQTMRTASISLSTRCLFMWQLLTITRPAEAAEARWEEVDIEAQEWKIPAARMKMNRDH

TVPLSDEAIAVLEMMKPLSGNREFIFPSRIKPNQPMNSQTVNASLKRAGFGGVLVSHGLRSIASTALNEQ

GFPPDAIEAALAHVDKNEVRRAYNRSDYLEQRRPMMQWWANFVMAADRGSMIEGGIKGMKLVG

>gi|30064018|ref|NP_838189.1| SsrA-binding protein [Shigella flexneri 2a str. 2457T]

MTKKKAHKPGSATIALNKRARHEYFIEEEFEAGLALQGWEVKSLRAGKANISDSYVLLRDGEAFLFGANI

TPMAVASTHVVCDPTRTRKLLLNQRELDSLYGRVNREGYTVVALSLYWKNAWCKVKIGVAKGKKQHDKRS

DIKEREWQVDKARIMKNAHR

>gi|30064014|ref|NP_838185.1| recombination and repair protein [Shigella flexneri 2a str. 2457T]

MLAQLTISNFAIVRELEIDFHSGMTVITGETGAGKSIAIDALGLCLGGRAEADMVRTGAARADLCARFSL

KDTPAALRWLEENQLEDGHECLLRRVISSDGRSRGFINGTAVPLSQLRELGQLLIQIHGQHAHQLLTKPE

HQKFLLDGYANETSLLQEMTARYQLWHQSCRDLAHHQQLSQERAARAELLQYQLKELNEFNPQPGEFEQI

DEEYKRLANSGQLLTTSQNALALMADGEDANLQSQLYTAKQLVSELIGMDSKLSGVLDMLEEATIQIAEA

SDELRHYCDRLDLDPNRLFELEQRISKQISLARKHHVSPEALPQYYQSLLEEQQQLDDQADSQETLALAV

TKHHQQALETARALHQQRQQYAEELAQLITDSMHALSMPHGQFTIDVKYDEHHLGADGADRIEFRVTTNP

GQPMQPIAKVASGGELSRIALAIQVITARKMETPALIFDEVDVGISGPTAAVVGKLLRQLGESTQVMCVT

HLPQVAGCGHQHYFVSKETDGAMTETHMQSLDKKARLQELARLLGGSEVTRNTLANAKELLAA

>gi|30064010|ref|NP_838181.1| hypothetical protein S2848 [Shigella flexneri 2a str. 2457T]

MQRLEQRSPDAILLLFLIAQTVDITMPVFALLALVAYSVSLALIVPGLLQKNGGWRRMAIISAVIALVCH

AIALEARILPDGDSGQNLSLLNVGSLVSLVICTVMTIVASRNRGWLLLPIVYAFALINLALATFMPNEYI

THLEATPGMLMHIGLSLFSYATLIIAALYALQLAWIDYQLKNKKLAFNQEMPPLMSIERKMFHITQIGVV

LLTLTLCTGLFYMHNLFSMENIDKAVLSIVAWFVYIVLLWGHYHEGWRGRRVVWFNVAGAVILTLAYFGS

RIVQQLIS

>gi|30064007|ref|NP_838178.1| 16S rRNA-processing protein RimM [Shigella flexneri 2a str. 2457T]

MSKQLTAQAPVDPIVLGKMGSSYGIRGWLRVFSSTEDAESIFDYQPWFIQKAGQWQQVQLESWKHHNQDM

IIKLKGVDDRDAANLLTNCEIVVDSSQLPQLEEGDYYWKDLMGCQVVTTEGYDLGKVVDMMETGSNDVLV

IKANLKDAFGIKERLVPFLDGQVIKKVDLTTRSIEVDWDPGF

>gi|30064006|ref|NP_838177.1| tRNA (guanine-N(1)-)-methyltransferase [Shigella flexneri 2a str. 2457T]

MWIGIISLFPEMFRAITDYGVTGRAVKNGLLSIQSWSPRDFTHDRHRTVDDRPYGGGPGMLMMVQPLRDA

IHAAKAAAGEGAKVIYLSPQGRKLDQAGVSELATNQKLILVCGRYEGIDERVIQTEIDEEWSIGDYVLSG

GELPAMTLIDSVSRFIPGVLGHEASATEDSFAEGLLDCPHYTRPEVLEGMEVPPVLLSGNHAEIRRWRLK

QSLGRTWLRRPELLENLALTEEQARLLAEFKTEHAQQQHKHDGMA

>gi|30064005|ref|NP_838176.1| 50S ribosomal protein L19 [Shigella flexneri 2a str. 2457T]

MSNIIKQLEQEQMKQDVPSFRPGDTVEVKVWVVEGSKKRLQAFEGVVIAIRNRGLHSAFTVRKISNGEGV

ERVFQTHSPVVDSISVKRRGAVRKAKLYYLRERTGKAARIKERLN

>gi|30064004|ref|NP_838175.1| outer membrane lipoprotein [Shigella flexneri 2a str. 2457T]

MIKHLVAPLIFTSLILTGCQSPQGKFTPEQVAAMQSYGFTESAGDWSLGLSDAILFAKNDYKLLPESQQQ

IQTMAAKLASTGLTHARMDGHTDNYGEDSYNEGLSLKRANVVADAWAIGGQIPRSNLTTQGLGKKYPIAS

NKTAQGRAENRRVAVVITTP

>gi|30064003|ref|NP_838174.1| hypothetical protein S2840 [Shigella flexneri 2a str. 2457T]

MRFSHRLFLLLILLLTGAPILAQEPSDVAKNVRMMVSGIVSYTRWPALSGPPKLCIFSSSRFSTALQENA

ATSLPYLPVIIHTQQEAMISGCNGFYFGNESPTFQMELTEQYPSKALLLIAEQNTECIIGSAFCLIIHNN

DVRFAVNLDASSRSGVKVNPDVLMLARKKNDG

>gi|30064002|ref|NP_838173.1| hypothetical protein S2839 [Shigella flexneri 2a str. 2457T]

MMKKFIAPLLALLVSGCQIDPYTHAPTLTSTDWYDVGMEDAISGSAIKDDDAFSDSQADRGLYLKGYAEG

QKKTCQTDFTYARGLSGKSFPASCNNVENASQLHEVWQKRADENASTIRLN

>gi|30064001|ref|NP_838172.1| phospho-2-dehydro-3-deoxyheptonate aldolase [Shigella flexneri 2a str. 2457T]

MHKDALNNVHITDEQVLMTPEQLKAAFPLSLQQEAQIADSRKTISDIIAGRDPRLLVVCGPCSIHDPETA

LEYARRFKALAAEVSDSLYLVMRVYFEKPRTTVGWKGLINDPHMDGSFDVEAGLQIARKLLLELVNMGLP

LATEALDPNSPQYLGDLFSWSAIGARTTESQTHREMASGLSMPVGFKNGTDGSLATAINAMRAAAQPHCF

VGINQAGQVALLQTQGNPDGHVILRGGKAPNYSPADVAQCEKEMEQAGLRPSLMVDCSHGNSNKDYRRQP

AVAESVVAQIKDGNRSIIGLMIESNIHEGNQSSEQPRSEMKYGVSVTDACISWEMTDALLREIHQDLNGQ

LTARVA

>gi|30064000|ref|NP_838171.1| bifunctional chorismate mutase/prephenate dehydrogenase [Shigella flexneri 2a str. 2457T]

MVAELTALRDQIDEVDKALLNLLAKRLELVAEVGKVKSRFGLPIYVPEREASMLASRRAEAEALGVPPDL

IEDVLRRVMRESYSSENDKGFKTLCPSLRPVVIVGGGGQMGRLFEKMLTLSGYQVRILEQHDWDRAADIV

ADAGMVIVSVPIHVTEQVIGKLPPLPKDCILVDLASVKNGPLQAMLAAHDGPVLGLHPMFGPDSGSLAKQ

VVVWCDGRKPEAYQWFLEQIQVWGARLHRISAVEHDQNMAFIQALRHFATFAYGLHLAEENVQLEQLLAL

SSPIYRLELAMVGRLFAQDPQLYADIIMSSERNLALIKRYYKRFGEAIELLEQGDKQAFIDSFRKVEHWF

GDYAQRFQSESRVLLRQANDNRQ

>gi|30063999|ref|NP_838170.1| bifunctional chorismate mutase/prephenate dehydratase [Shigella flexneri 2a str. 2457T]

MTSENPLLALREKISALDEKLLALLAERRELAVEVGKAKLLSHRPVRDIDRERDLLERLITLGKAHHLDA

HYITRLFQLIIEDSVLTQQALLQQHLNKINPHSARIAFLGPKGSYSHLAARQYAARHFEQFIESGCAKFA

DIFNQVETGQADYAVVPIENTSSGAINDVYDLLQHTSLSIVGEMTLTIDHCLLVSGTTDLSTINTVYSHP

QPFQQCSKFLNRYPHWKIEYTESTSAAMEKVAQAKSPHVAALGSEAGGTLYGLQVLERIEANQRQNFTRF

VVLARKAINVSDQVPAKTTLLMATGQQAGALVEALLVLRNHNLIMTRLESRPIHGNPWEEMFYLDIQANL

ESAEMQKALKELGEITRSMKVLGCYPSENVVPVDPT

>gi|30063997|ref|NP_838168.1| translation inhibitor protein RaiA [Shigella flexneri 2a str. 2457T]

MTMNITSKQMEITPAIRQHVADRLAKLEKWQTHLINPHIILSKEPQGFVADATINTPNGVLVASGKHEDM

YTAINELINKLERQLNKLQHKGEARRAATSVKDANFVEEVEEE

>gi|30063990|ref|NP_838161.1| outer membrane protein assembly complex subunit YfiO [Shigella flexneri 2a str. 2457T]

MTRMKYLVAAATLSLFLAGCSGSKEEVPDNPPNEIYATAQQKLQDGNWRQAITQLEALDNRYPFGPYSQQ

VQLDLIYAYYKNADLPLAQAAIDRFIRLNPTHPNIDYVMYMRGLTNMALDDSALQGFFGVDRSDRDPQHA

RAAFSDFSKLVRGYPNSQYTTDATKRLVFLKDRLAKYEYSVAEYYTERGAWVAVVNRIEGMLRDYPDTQA

TRDALPLMENAYRQMQMNAQAEKVAKIIAANSSNT

>gi|30063988|ref|NP_838159.1| alpha-ketoglutarate transporter [Shigella flexneri 2a str. 2457T]

MAESTVTADSKLTSSDTRRRIWAIVGASSGNLVEWFDFYVYSFCSLYFAHIFFPSGNTTTQLLQTAGVFA

AGFLMRPIGGWLFGRIADKHGRKKSMLLSVCMMCFGSLVIACLPGYETIGTWAPALLLLARLFQGLSVGG

EYGTSATYMSEVAVEGRKGFYASFQYVTLIGGQLLALLVVVVLQHTMEDAALREWGWRIPFALGAVLAVV

ALWLRRQLDETSQQETRALKEAGSLKGLWRNRRAFIMVLGFTAAGSLCFYTFTTYMQKYLVNTAGMHANV

ASGIMTAALFVFMLIQPLIGALSDKIGRRTSMLCFGSLAAIFTVPILSALQNVSSPYAAFGLVMCALLIV

SFYTSISGILKAEMFPAQVRALGVGLSYAVANAIFGGSAEYVALSLKSIGMETAFFWYVTLMAVVAFLVS

LMLHRKGKGMRL

>gi|30063985|ref|NP_838156.1| hypothetical protein S2819 [Shigella flexneri 2a str. 2457T]

MSQRGLEALLRPKSIAVIGASMKPNRAGYLMMRNLLAGGFNGPVLPVTPAWKAVLGVLAWPDIASLPFTP

DLAVLCTNASRNLALLEELGEKGCKTCIILSAPASQHEDLRACALRHNMRLLGPNSLGLLAPWQGLNASF

SPVPIKRGKLAFISQSAAVSNTILDWAQQREMGFSYFIALGDSLDIDVDELLDYLARDSKTSAILLYLEQ

LSDARRFVSAARSASRNKPILVIKSGRSPAAQRLLNTTAGMDPAWDAAIQRAGLLRVQDTHELFSAVETL

SHMRPLRGDRLMIISNGAAPAALALDALWSRNGKLATLSEETCQKLRDALPEHVAVSNPLDLRDDASSEH

YVKTLDILLHSQDFDALMVIHSPSAAAPATESAQVLIEAVKHHPRSKYVSLLTNWCGEHSSQEARRLFSE

AGLPTYRTPEGTITAFMHMVEYRRNQKQLRETPALPSNLTSNTAEAHLLLQQAIAEGATSLDTHEVQPIL

QAYGMNTLPTWIASDSTEAVHIAEQIGYPVALKLRSPDIPHKSEVQGVMLYLRTANEVQQAANAIFDRVK

MAWPQARVHGLLVQSMANRAGAQELRVVVEHDPVFGPLIMLGEGGVEWRPEDQAVVALPPLNMNLARYLV

IQGIKSKKIRARSALRPLDVAGLSQLLVQVSNLIVDCPEIQRLDIHPLLASGSEFTALDVTLDISPFEGD

NESRLAVRPYPHQLEEWVELKNGERCLFRPILPEDEPQLQQFISRVTKEDLYYRYFSEINEFTHEDLANM

TQIDYDREMAFVAVRRIDQTEEILGVTRAISDPDNIDAEFAVLVRSDLKGLGLGRRLMEKLITYTRNHGL

QRLNGITMPNNRGMVALARKLGFNVDIQLEEGIVGLTLNLARREES

>gi|30063980|ref|NP_838151.1| autonomous glycyl radical cofactor GrcA [Shigella flexneri 2a str. 2457T]

MITGIQITKAANADLLNSFWLLDSEKGEARCIVAKAGYAEDEVVAVSKLGDIEYREVPVEVKPEVRVEGG

QHLNVNVLRRETLEDAVKHPEKYPQLTIRVSGYAVRFNSLTPEQQRDVIARTFTESL

>gi|30063979|ref|NP_838150.1| neutral amino-acid efflux protein [Shigella flexneri 2a str. 2457T]

MTPILLSAFWTYTLITAMTPGPNNILALSSATSHGFRQSTRVLAGMSLGFLIVMLLCAGISFSLAVIDPA

AVHLLSWAGAAYIVWLAWKIATSPTKEDGLQAKPISFWASFALQFVNVKIILYGVTALSTFVLPQTQALS

WVVGVSVLLAMIGTFGNVCWALAGHLFQRLFRQYGRQLNIVLALLLVYCAVRIFY

>gi|30063978|ref|NP_838149.1| LYSR-type transcriptional regulator [Shigella flexneri 2a str. 2457T]

MFLYSKFDIFDRFGVMDLRRFITLKTVVEEGSFLRASQKLCCTQSTVTFHIQQLEQEFSVQLFEKIGRRM

CLTREGKKLLPHIYELTRVMDTLREAAKKESDPDGELRVVSGETLLSYRMPQVLQRFRQRAPKVRLSLQA

LNCYVIRDALLNDEADVGVFYRVGNDDALNRRELGEQSLVLVASPQIADVDFTEPGRHNACSFIINEPQC

VFRQIFESTLRQRRITVENTIELISIESIKRCVAANIGVSYLPHFAVEKEIESGELIELPFGEQSQTITA

MCAHYAGKAVSPAMHIFIQCVEECFVVA

>gi|30063976|ref|NP_838147.1| enzyme [Shigella flexneri 2a str. 2457T]

MWGADYRRYGCNLHLFVWISLLGYCSHPVLSPFFLQGFIFMSQSTSVLRRNGFTFKQFFVAHDRCAMKVG

TDGILLGAWAPVAGVKRCLDIGAGSGLLALMLAQRTSDSVIIDAVELESEAAAQAQENINQSPWAERINV

HTADIQQWITQQTVRFDLIISNPPYYQQGVECATPQREQARYTTTLDHPSLLTCAAECITEEGFFCVVLP

EQIGNGFTELALSMGWHLRLRTDVAENEARLPHRVLLAFSPQAGECFSDRLVIRGPDQNYSEAYTALTQA

FYLFM

>gi|30063975|ref|NP_838146.1| RNA polymerase sigma factor RpoE [Shigella flexneri 2a str. 2457T]

MSEQLTDQVLVERVQKGDQKAFNLLVVRYQHKVASLVSRYVPSGDVPDVVQEAFIKAYRALDSFRGDSAF

YTWLYRIAVNTAKNYLVAQGRRPPSSDVDAIEAENFESGGALKEISNPENLMLSEELRQIVFRTIESLPE

DLRMAITLRELDGLSYEEIAAIMDCPVGTVRSRIFRAREAIDNKVQPLIRR

>gi|30063974|ref|NP_838145.1| anti-RNA polymerase sigma factor SigE [Shigella flexneri 2a str. 2457T]

MQKEQLSALMDGETLDSELLNELAHNPEMQKTWESYHLIRDSMRGDTPEVLHFDISSRVMAAIEEEPVRQ

SATLIPEAQPAPHQWQKMPFWQKVRPWAAQLTQMGVAACVSLAVIVGVQHYNGQSETSQQPETPVFNTLP

MMGKASPVSLGVPSEATANNGQQQQVQEQRRRINAMLQDYELQRRLHSEQLQFEQAQTQQAAVQVPGIQT

LGTQSQ

>gi|30063973|ref|NP_838144.1| periplasmic negative regulator of sigmaE [Shigella flexneri 2a str. 2457T]

MKQLWFAMSLVTGSLLFSANASATPASGALLQQMNLASQSLNYELSFISINKQGVESLRYRHARLDNRPL

AQLLQMDGPRREVVQRGNEISYFEPGLEPFTLNGDYIVDSLPSLIYTDFKRLSPYYDFISVGRTRIADRL

CEVIRVVARDGTRYSYIVWMDTESKLPMRVDLLDRDGETLEQFRVIAFNVNQDISSSMQTLAKANLPPLL

SVPVGEKAKFSWTPTWLPQGFSEVSSSRRPLPTMDNMPIESRLYSDGLFSFSVNVNRATPSSTDQMLRTG

RRTVSTSVRDNAEITIVGELPPQTAKRIAENIKFGAAQ

>gi|30063972|ref|NP_838143.1| SoxR reducing system protein RseC [Shigella flexneri 2a str. 2457T]

MIKEWATVVSWQNGQALVSCDVKASCSSCASRAGCGSRVLNKLGPQTTHTIVVPCDEPLVPGQKVELGIA

EGSLLSSALLVYMSPLVGLFLIASLFQLLFASDVAALCGAVLGGIGGFLIARGYSRKFAARAEWQPIILS

VALPPGLVRFETSSEDASQ

>gi|30063970|ref|NP_838141.1| signal peptidase I [Shigella flexneri 2a str. 2457T]

MANMFALILVIATLVTGILWCVDKFFFAPKRRERQAAAQAAAGDSLDKATLKKVAPKPGWLETGASVFPV

LAIVLIVRSFIYEPFQIPSGSMMPTLLIGDFILVEKFAYGIKDPIYQKTLIETGHPKRGDIVVFKYPEDP

KLDYIKRAVGLPGDKVTYDPVSKELTIQPGCSSGQACENALPVTYSNVEPSDFVQTFSRRNGGEATSGFF

EVPKNETKENGIRLSERKETLGDVTHRILTVPIAQDQVGMYYQQPGQQLATWIVPPGQYFMMGDNRDNSA

DSRYWGFVPEANLVGRATAIWMSFDKQEGEWPTGVRLSRIGGIH

>gi|30063967|ref|NP_838138.1| DNA repair protein RecO [Shigella flexneri 2a str. 2457T]

MEGWQRAFVLHSRPWSETSLMLDVFTEESGRVRLVAKGARSKRSTLKGALQPFTPLLLRFGGRGEVKTLR

SAEAVSLALPLSGITLYSGLYINELLSRVLEYETRFSELFFDYLHCIQSLAGDTGTPEPALRRFELALLG

HLGYGVNFTHCAGSGEPVDGTMTYRYREEKGFIASVVIDNKTFTGRQLKALNAREFPDADTLRAAKRFTR

MALKPYLGGKPLKSRELFRQFMPKRTVKTHYE

>gi|30063966|ref|NP_838137.1| pyridoxine 5'-phosphate synthase [Shigella flexneri 2a str. 2457T]

MAELLLGVNIDHIATLRNARGTAYPDPVQAAFIAEQAGADGITVHLREDRRHITDRDVRILRQTLDTRMN

LEMAVTEEMLAIAVETKPHFCCLVPEKRQEVTTEGGLDVAGQREKIRDACKRLADAGIQVSLFIDADEEQ

IKAAAEVGAPFIEIHTGCYADAKTDAEQAQELVRIAKAATFAASLGLKVNAGHGLTYHNVKAIAAIPEMH

ELNIGHAIIGRAVMTGLKDAVAEMKRLMLEARG

>gi|30063965|ref|NP_838136.1| 4'-phosphopantetheinyl transferase [Shigella flexneri 2a str. 2457T]

MAILGLGTDIVEIARIEAVIARSGERLARRVLSDNEWAIWKTHHQPVRFLAKRFAVKEAAAKAFGTGIRN

GLAFNQFEVFNDELGKPRLRLWGEALKLAEKLGVVNMHVTLADERHYACATVIIES

>gi|30063963|ref|NP_838134.1| hypothetical protein S2796 [Shigella flexneri 2a str. 2457T]

MPRTVTHNPDSPNNDDVLAASEKWDACKPPYTSAHMKICVAAAKIILAASGVARCSKYEKENYLRIDFSK

AGKVTFYAEFPKKMGLKGKKLGEWPELVIQLAREKALGMADGGLRAESVHAALEMYRDDPKPK

>gi|30063962|ref|NP_838133.1| hypothetical protein S2795 [Shigella flexneri 2a str. 2457T]

MRIDRIKVTFGEREVFSDVTYNRLVEVLDEWIATRSNNNALELFAELRRFWKFCAPTLCNGRNVAASLPD

DYVSSRVQKPTPTRLFTDIESIARLWLNVAACTSVHQKNAVRFMIITGVRPINVHNLRWDYVYEEAGEIV

YPEGVIGMRGAMKTQKAFRLPITPEIRRIIDEQKAWRDSVPECNRDYVFLQPRDPMQPFSKRSLDKLVKT

YSPDGAVK

>gi|30063959|ref|NP_838130.1| bacteriophage protein [Shigella flexneri 2a str. 2457T]

MYGGSPADIAAAIWRKAPPGIDMNGNTTFTVADKEYDPPYPEYVISWQTLKPVSLHVSVTLKKSDYLPSD

ITRQVQQSVLDAFNGTDGGLRARVASVVSAGRYYAGIYKTDPEHIDILGLTVSRDGSSWTTAVTFGIDEI

PVLDVSDISVKLQEA

>gi|30063958|ref|NP_838129.1| hypothetical protein S2790 [Shigella flexneri 2a str. 2457T]

MQNVAATVLAQYAASPRLNALINSFNAALSPDSFISDFYGLIWNIDTAEKYGLDVWGKIVGVSRWLTVKD

DFNYLGFSESRMDTPVMDDPCPFNQAPFYNGKSDTRTVDLSDAVYRRLILMKAMSNITDCSVPDINLMLR

FMFGKKRRAYVLNNGGLRMSYVFESALSLAELAIIQSSGALPSPPGVYVSVVLKESRNEGQ

>gi|30063957|ref|NP_838128.1| tail fiber protein [Shigella flexneri 2a str. 2457T]

MKASDKPRQLAVPFASTGDKNRIPDKATQQTRESGNAAYDSGFPPVTMTAVSAGGIPPHGKDFNGLMYDI

TAAIRFAQAGGLYTYNAGFAGAIGGYAKGAILAGVATTAVWLNTTDDNLTDPEGSDSAGWVNLLEDPKRI

FLRQKNNLSDLQNKGTARDNLQVYSKEQSDQRYVHREGDKITGELKIRGVNALRIFNEAFGLIFRRSEEC

LHLIPTSEGQGENGDIGPLRPFTINLRTGEISMSHKVSVGGGSQVNGALGIGVQNALGGNSIVLGDNDTG

FKQNGDGILDVYANSQRVFRWICPYISRHLLSLNPLQARCRRYSRGER

>gi|30063949|ref|NP_838120.1| DNA-invertase [Shigella flexneri 2a str. 2457T]

MLIGYVRVSTNDQNTDLQRNALNCAGCELIFEDKISGTKSERPGLKKLLKTLSAGDTLVVWKLDRLGRSM

RHLVVLVEALRERGINFRSLTDSIDTSTPMGRFFFHVMGALAEMERELIVERTKAGLEAARAQGRIGGRR

PKLSPE

>gi|30063943|ref|NP_838114.1| 2-component sensor protein [Shigella flexneri 2a str. 2457T]

MLYLNFPSVSIRLFASDESNTLKRWPVFPRSLRQLVMLAFLLILLPLLVLAWQAWQSLNALSDQAALVNR

TTLIDARRSEAMTNAALEMERSYRQYCVLDDPTLAKVYQSQRKRYSEMLDAHAGVLPDDKLYQALRQDLN

NLAQLQCNNSGPDAAAAARLEAFASANTEMVQATRTVVFSRGQQLQREIAERGQYFGWQSLVLFLVSLVM

VLLFTRMIIGPVKNIERMINRLGEGRSLGNSVSFCGPSELRSVGQRILWLSERLSWLESQRHQFLRHLSH

ELKTPLASMREGTELLADQVVGPLTPEQKEVVSILDSSSCNLQKLIEQLLDYNRKQADSAVELENVELAP

LVETVVSAHSLPARAKMMHTDVDLKATACLAEPMLLMSVLDNLYSNAVHYGAESGNICLRSSLHGARVYI

DVINTGTPIPQEERAMIFEPFFQGSHQRKGAVKGSGLGLSIARDCIRRMQGELYLVDESGQDVCFRIELP

SSKNTK

>gi|30063942|ref|NP_838113.1| hypothetical protein S2774 [Shigella flexneri 2a str. 2457T]

MRHIFQRLLPRRLWLAGLPCLALLGCVQNHNKPAIDTPAEEKIPVYQLADYLSTECSDIWALQGKSTETN

PLYWLRAMDCADRLMPAQSRQQARQYDDGSWQNTFKQGILLADAKITPYERRQLVARIEALSTEIPAQVR

PLYQLWRDGQALQLQLAEERQRYSKLQQSSDSELDTLRQQHHVLQQQLELTTRKLENLTDIERQLSTRKP

AGNFSPDTPHESEKPAPSTHEVTPDEP

>gi|30063941|ref|NP_838112.1| 2-component transcriptional regulator [Shigella flexneri 2a str. 2457T]

MSHKPAHLLLVDDDPGLLKLLGLRLTSEGYSVVTAESGAEGLRVLNREKVDLVISDLRMDEMDGMQLFAE

IQKVQPGMPVIILTAHGSIPDAVAATQQGVFSFLTKPVDKDALYQAIDDALEQSAPATDERWREAIVTRS

PLMLRLLEQARLVAQSDVSVLINGQSGTGKEIFAQAIHNASPRNSKPFIAINCGALPEQLLESELFGHAR

GAFTGAVSNREGLFQAAEGGTLFLDEIGDMPAPLQVKLLRVLQERKVRPLGSNRDIDINVRIISATHRDL

SKAMARGEFREDLYYRLNVVSLKIPALAERTEDIPLLANHLLRQAAERHKPFVRAFSTDAMKRLMTASWP

GNVRQLVNVIEQCVALTSSPVISDALVEQALEGENTALPTFVEARNHFELNYLRKLLQITKGNVTHAARM

AGRNRTEFYKLLSRHELDANDFKE

>gi|30063940|ref|NP_838111.1| nitrogen regulatory protein P-II 1 [Shigella flexneri 2a str. 2457T]

MKKIDAIIKPFKLDDVREALAEVGITGMTVTEVKGFGRQKGHTELYRGAEYMVDFLPKVKIEIVVPDDIV

DTCVDTIIRTAQTGKIGDGKIFVFDVARVIRIRTGEEDDAAI

>gi|30063935|ref|NP_838106.1| transport system permease [Shigella flexneri 2a str. 2457T]

MYASSLPLPQGKSVSLKQFVSRHINEIGLLVVIAILYLVFSLNAPGFISLNNQMNVLRDAATIGIAAWAM

TLIIISGEIDVSVGPMVAFVSVCLAFLLQFEVPLAIACLLVLLLGALMGTLAGVLRGVFNVPSFVATLGL

WSALRGMGLFMTNALPVPIDENEVLDWLGGQFLGVPVSALIMMVLFALFVFISRKTAFGRSVFAVGGNAT

AAQLCGINVRRVRILIFTLSGLLAAVTGILLAARLGSGNAGAANGLEFDVIAAVVVGGTALSGGRGSLFG

TLLGVLVITLIGNGLVLLGINSFFQQVVRGVIIVVAVLANILLTQRSSKAKR

>gi|30063933|ref|NP_838104.1| hypothetical protein S2763 [Shigella flexneri 2a str. 2457T]

MTIYTLSHGSLKLDVSDQGGVIEGFWRDTTPLLRPGKKSGVATDASCFPLVPFANRVSGNRFVWQGREYQ

LQPNVEWDAHYLHGDGWLGEWQCVSHSDDSLCLVYEHRSGVYHYRVSQAFHLTADTLTVTLSVTNQGAET

LPFGTGWHPYFPLSPQTRIQAQASGYWLEREQWLAGEFCEQLPQELDFNQLAPLPRQWVNNGFAGWNGQA

RIEQPQEGYAIIMETTPPAPCYFIFVSDPAFDKGYAFDFFCLEPMSHAPDEHHRPEGGDLIALAPGESTT

SEMSLRVEWL

>gi|30063932|ref|NP_838103.1| hypothetical protein S2762 [Shigella flexneri 2a str. 2457T]

MPGAWRGARVCITFKSHYLAKGLVMNTLRYFDFGAARPVLLLIARIAVVLIFIIFGFPKMMGFDGTVQYM

ASLGAPMPMLAAIIAVVMEVPAAILIVLGFFTRPLAVLFIFYTLGTAVIGHHYWDMTGDAVGPNMINFWK

NVSIAGAFLLLAITGPGAISLDRR

>gi|30063929|ref|NP_838100.1| 3-phenylpropionate dioxygenase subunit beta [Shigella flexneri 2a str. 2457T]

MSAQVSLELHHRISQFLFHEASLLDDWKFRDWLAQLDEEIRYTMRTTVNAQTRDRRKGVQPPTTWIFNDT

KDQLERRIARLETGMAWAEEPPSRTRHLISNCQVSETDIPNVFAVRVNYLLYRAQKERDETFYVGTRFDK

VRRLEDDNWRLLERDIVLDQAVITSHNLSVLF

>gi|30063928|ref|NP_838099.1| large terminal subunit of phenylpropionate dioxygenase [Shigella flexneri 2a str. 2457T]

MTTPSDLNIYQLIDTQNGRVTPRIYTDPDIYQLELERIFGRCWLFLAHESQIPKPGDFFNTYMGEDAVVV

VRQKDGSIKAFLNQCRHRAMRVSYADCGNSRAFTCPYHGWSYGINGELIDVPLEPRAYPQGLCKSHWGLN

EVPCVESYKGLIFGNWDTSAPGLHDYLGDIAWYLDGMLDRREGGTEIVGGVQKWVINCNWKFPAEQFASD

QYHALFSHASAVQVLGAKDDGSDKRLGDGQTARPVWETAKDALQFGQDGHGSGFFFTEKPDANVWVDGAV

SSYYRETYAEAEQRLGEVRALRLAGHNNIFPTLSWLNGTATLRVWHPRGPDQVEVWAFCITDKAASDEVK

AAFENSATRAFGPAGFLEQDDSENWCEIQKLLKGHRARNSKLCLEMGLGQEKRRDDGIPGITNYIFSETA

ARGMYQRWADLLSSESWQEVLDKTAAYQQEVMK

>gi|30063927|ref|NP_838098.1| DNA-binding transcriptional regulator HcaR [Shigella flexneri 2a str. 2457T]

MELRHLRYFVAVAQALNFTRAAEKLHTSQPSLSSQIRDLENCVGVPLLVRDKRKVALTAAGECFLQDALA

ILEQAENAKLRARKIVQEDRQLTIGFVPSAEVNLLPKVLPMFRLRQPDTLIELVSLITTQQEEKIRRGEL

DVGLMRHPVYSPEIDYLELFDEPLVVVLPVDHPLAHEKEITAAQLDGVNFVSTDPAYSGSLAPIVKAWFA

QENSQPNIVQVATNILVTMNLVGMGLGVTLIPGYMNNFNTGQVVFRPIAGNVPSIALLMAWKKGEMKLAL

RDFIAIVQERLASVTA

>gi|30063926|ref|NP_838097.1| 3-phenylpropionic acid transporter [Shigella flexneri 2a str. 2457T]

MVLQSTRWLALGYFTYFFSYGIFLPFWSVWLKGIGLTPETIGLLLGAGLVARFLGSLLIAPRVSDPSRLI

SALRVLALLPLLFAVAFWAGAHVAWLMLVMIGFNLFFSPLVPLTDALANTWQKQFPLDYGKVRLWGSVAF

VIGSALTGKLVTMFDYRVILVLLTLGVASMLLGFLIRPTIQPQGASRQQESTGWSAWLALVRQNWRFLAC

VCLLQGAHAAYYGFSAIYWQAAGYSASAVGYLWSLGVVAEVIIFALSNKLFRRCSARDMLLISAICGVVR

WGIMGATTALPWLIVVQILHCGTFTVCHLAAMRYIAARQVSEVIRLQAVYSAVAMGGSIAIMTVFAGFLY

QYLGHGVFWVMALVALPAMFLRPKVVPSC

>gi|30063925|ref|NP_838096.1| stationary phase inducible protein CsiE [Shigella flexneri 2a str. 2457T]

MMPTLAPPSVLSAPQRRCQILLTLFQPGLTATMATFSELNGVDDDIASLDISETGREILRYHQLTLTTGY

DGSYRVEGTVLNQRLCLFHWLRRGFRLCPSFITSQFTPALKSELKRRGIARNFYDDTNLQALVNLCSRRL

QKRFESRDIHFLCLYLQYCLLQHHAGITPQFNPLQRRWAESCLEFQVAQEIGRHWQRRALQHVPPDEPLF

MALLFSMLRVPDPLRDAHQRDRQLRQSIKRLVNHFRELGNVRFYDEQGLCDQLYIHLAQALNRSLFAIGI

DNTLPEEFARLYPRLVRTTRAALAGFESEYGVHLSDEESGLVAVIFGAWLMQENDLHEKQIILLTGNDSE

REAQIEQQLRELTLLPLNIKHMSVKAFLQTGAPRGAALIIAPYTMPLPLFSPPLIYTDLTLTTHQQEQIR

KMLESA

>gi|30063922|ref|NP_838093.1| ATP synthase subunit beta [Shigella flexneri 2a str. 2457T]

MLQNIRIVLVETSHTGNMGSVARAMKTMGLTNLWLVNPLVKPDSQAIALAAGASDVIGNAHIVDTLDEAL

AGCSLVVGTSARSRTLPWPMLDPRECGLKSVAEAANTPVALVFGRERVGLTNEELQKCHYHVAIAANPEY

SSLNLAMAVQVIAYEVRMAWLATQENGEQVEHEETPYPLVDDLERFYGHLEQTLLATGFIRENHPGQVMN

KLRRLFTRARPESQELNILRGILASIEQQNKGNKAE

>gi|30063921|ref|NP_838092.1| DNA-binding transcriptional regulator IscR [Shigella flexneri 2a str. 2457T]

MRLTSKGRYAVTAMLDVALNSEAGPVPLADISERQGISLSYLEQLFSRLRKNGLVSSVRGPGGGYLLGKD

ASSIAVGEVISAVDESVDATRCQGKGGCQGGDKCLTHALWRDLSDRLTGFLNNITLGELVNNQEVLDVSG

RQHTHDAPRTRTQDAIDVKLRA

>gi|30063912|ref|NP_838083.1| enhanced serine sensitivity protein SseB [Shigella flexneri 2a str. 2457T]

MSETKNELEDLLEKAATEPAHRPAFFRTLLESTVWVPGTAAQGEAVVEDSALDLQHWEKEDGTSVIPFFT

SLEALQQAVEDEQAFVVMPVRTLFEMTLGETLFLNAKLPTGKEFMPREISLLIGEEGNPLSSQEILEGGE

SLILSEVAEPPAQMIDSLTTLFKTIKPVKRAFICSIKENEEAQPNLLIGIEADGDIEEIIQATGSVATDT

LPGDEPIDICQVKKGEKGISHFITEHIAPFYERRWGGFLRDFKQNRII

>gi|30063909|ref|NP_838080.1| hypothetical protein S2735 [Shigella flexneri 2a str. 2457T]

MSEQLVTPENVTTKDGKINLLDLNRQQMREFFKDLGEKTFRADQVMKWMYHYCCDNFDEMTDINKVLRGK

LKEVAEIRAPEVVEEQRSSDGTIKWAIAVGDQRVETVYIPEDDRATLCVSSQVGCALECKFCSTAQQGFN

RNLRVSEIIGQVWRAAKIVGAAKVTGQRPITNVVMMGMGEPLLNLNNVVPAMEIMLDDFGFGLSKRRVTL

STSGVVPALDKLGDMIDVALAISLHAPNDEIRDEIVPINKKYNIETFLAAVRRYLEKSNANQGRVTIEYV

MLDHVNDGTEHAHQLAELLKDTPCKINLIPWNPFPDAPYGRSSNSRIDRFSKVLMSYGFTTIVRKTRGDD

IDAACGQLAGDVIDRTKRTLRKRMQGEAIDIKAV

>gi|30063908|ref|NP_838079.1| hypothetical protein S2734 [Shigella flexneri 2a str. 2457T]

MNTEATHDQNEALTTGARLRNAREQLGLSQQAVAERLCLKVSTVRDIEEDKAPADLASTFLRGYIRSYAR

LVHIPEEELLPGLEKQAPLRAAKVAPMQSFSLGKRRKKRDGWLMTFTWLVLFVVIGLSGAWWWQDHKAQQ

EEITTMADQSSAELSSNSEQGQSVPLNTSTTTDPATTSTPPASVDTTATNTQTPAVTAPAPAVDPQQNAV

VSPSQANVDTAATPVPTAATTPDGAAPLPTDQAGVTTPAADPNALVMNFTADCWLEVTDATGKKLFSGMQ

RKDGNLNLTGQAPYKLKIGAPAAVQIQYQGKPVDLSRFIRTNQVARLTLNAEQSPAQ

>gi|30063907|ref|NP_838078.1| 4-hydroxy-3-methylbut-2-en-1-yl diphosphate synthase [Shigella flexneri 2a str. 2457T]

MHNQAPIQRRKSTRIYVGNVPIGDGAPIAVQSMTNTRTTDVEATVNQIKALERVGADIVRVSVPTMDAAE

AFKLIKQRVNVPLVADIHFDYRIALKVAEYGVDCLRINPGNIGNEERIRMVVDCARDKNIPIRIGVNAGS

LEKDLQEKYGEPTPQALLESAMRHVDHLDRLNFDQFKVSVKASDVFLAVESYRLLAKQIDQPLHLGITEA

GGARSGAVKSAIGLGLLLSEGIGDTLRVSLAADPVEEIKVGFDILKSLRIRSRGINFIACPTCSRQEFDV

IGTVNALEQRLEDIITPMDVSIIGCVVNGPGEALVSTLGVTGGNKKSGLYEDGVRKDRLDNNDMIDQLEA

RIRAKASQLDEARRIDVQQVGKIIT

>gi|30063905|ref|NP_838076.1| hypothetical protein S2731 [Shigella flexneri 2a str. 2457T]

MEIYENENDQVEAVKRFFAENGKALAVGGILGVGALTGWRYWNSHQVDSARSASLAYQNAVTAVSEGKPD

SIPAAEKFAAENKNTYGALASLELAQQFVDKNELEKAAAQLQQGLADTSDENLKAVINLRLARVQVQLKQ

ADAALKTLDTIKGEGWAAIVADLRGEALLSKGDKQGARSAWEAGVKSDVTPALSEMMQMKINNLSI

>gi|30063901|ref|NP_838072.1| exodeoxyribonuclease VII large subunit [Shigella flexneri 2a str. 2457T]

MLPSQSPAIFTVSRLNQTVRLLLEHEMGQVWISGEISNFTQPASGHWYFTLKDDTAQVRCAMFRNSNRRV

TFRPQHGQQVLVRANITLYEPRGDYQIIVESMQPAGEGLLQQKYEQLKAKLQAECLFDQQYKKPLPSPAH

CVGVITSKTGAALHDILHVLKRRDPSLPVIIYPTSVQGDDAPGQIVRAIELANQRNECDVLIVGRGGGSL

EDLWSFNDERVARAIFASRIPVVSAVGHETDVTIADFVADLRAPTPSAAAEVVSRNQQELLRQVQSARQR

LEMAMDYYLANRTRRFTQIHHRLQQQHPQLRLARQQTMLERLQKRMSFALENQLKRAGQQQQRLTQRLNQ

QNPQPKIHRTQTRIQQLEYRLAEILRAQLSATRERFGNAVTHLEAVSPLSTLARGYSVTTATDGNVLKKV

KQVKTGEMLTTRLEDGWIESEVKNIQPVKKSRKKVH

>gi|30063898|ref|NP_838069.1| integrase [Shigella flexneri 2a str. 2457T]

MLTDTKLRNLKPRDKLYKVNDREGLYVAVTPAGSISFRYNYSINGRQETITFGRYGVGGITLAEARELLG

DAKKMVAAGKSPAKEKARDKARVKDAETFGAWAEKWLRGYQMADSTRDMRRSVYERELKPKFSNQKLVEI

THEDLRALADAIVERGAPATAVHVREIVLQVFRWAIERGQKVENPAELVRPTSIARFEPRDRALTPEEIG

LMYQYMERVGTSPTNRAAAKLLLLTMVRKSELTNATWSEINFSEALWTIPKERMKRRNPHLVFLSQQALD

IFIAMKTFAGGSDFVLPSRYDSDAPMSAATLNQVLTLTYKAAQKDGKSLTKFGPHDLRRTASTLLHEAGY

NTDWIEKCLAHEQKGVRAVYNKAEYREQRAAMLQDWADMIDEWTSGGSKG

>gi|30063896|ref|NP_838067.1| DNA helicase [Shigella flexneri 2a str. 2457T]

MALDLMAAFTELPPPIDYVLPNMVAGTVGALVSPGGAGKSMLALQLAAQIAGGPDLLEIGEFPTGQVVYL

PAEDPPAAIHHRLHALGAHLSAAERQAVADGLLIEPLIGKCPNIMAASWFDALKRAAEGRRLMILDTLRR

FHIEEENASGPMAQVVGHMEAIAADTGCSIVFLHHASKSAAMMGSGDQQQASRGSSVLVDNIRWQSYLSG

MTQGEAEILGVDDCQRGYFVRFGVSKANYGAPFQELWFRRHDGGVLKPAVLERQCKVKRRQREEA

>gi|30063895|ref|NP_838066.1| replication protein C [Shigella flexneri 2a str. 2457T]

MKKPKHDLTHVRHDPAHCLAPGLFRSLKRGDRKRCKLDVTYTFGEDESMRFVGFEPLGADDMRLLQGIVA

LGGPNGILLTPEPTSETGRQLRLFLEPRFEAIEQDGLVVRESLTKLLSETGMTDSGDNIKALKASLLRMS

NVTILVTKGRRQAAFHLMSHAFDETDGRLWVALNPRIAEAILGHRPYARIDMAEVRVLQTDPARLMHQRL

CGWIDPGKSGRVELDTLCGYVWPDEANAEAMKKRRQTARKALAELAAVGWVVNEYAKGKWEIKRPGPTAT

APVYRRNVPLLPS

>gi|30063893|ref|NP_838064.1| conjugal transfer protein TrbJ [Shigella flexneri 2a str. 2457T]

MKSKILAAKKTALAVALATGFITTTTAPVQAGIPVIDGGNLAQNIMTAIESVAQTLKQIEQYQTQLQQYE

NQLQNTMAPAAYIWDQAQTTINRLIAAQNTLAYYENQLGSLDRYLAKFQDVAYYRSSPCFNGSGGCTPAE

KAAMEENRRLASESQKKANDALFQTVADQQKALKDDARTLERLQGAAQGATGQLQAIGYANQLASQQANQ

LLQIRTMLTAQHNAEAARIAAELDAEARGDARAEQMRTWTFRPSPADNY

>gi|30063890|ref|NP_838061.1| sodium bile acid symporter family protein [Shigella flexneri 2a str. 2457T]

MSAGAQAIAAKPRQAPMSGFERYLTLWVALCIVAGVALGQGLPDVFQAIGRMEVAQVNLPVGLLIWVMIV

PMLVKIDFGALHQVKEHWRGIGVTLFVNWAVKPFSMALLGWLFIRQVFAPFLPADQLDSYIAGLILLAAA

PCTAMVFVWSRLTNGDPLFTLSQVALNDAIMIVAFAPIVGLLLGMSSISVPWDTLLISVVLYIIVPVILA

QLLRRHLLKQGQAAFERAMQKIGPWSMAALLLTLVLLFAFQGEAIIRQPLVIAMLAVPILIQVFFNSGLA

YWLNKRAGEKHSVACPSALIGASNFFELAVAAAISLFGLHSGAALATVVGVLVEVPVMLLVVRVVNRSKS

WYERG

>gi|30063889|ref|NP_838060.1| arsenate reductase [Shigella flexneri 2a str. 2457T]

MTERIYNVLILCTGNSARSIMAEALINTMGQGRFRAYSAGSHPTGKVNPFAVEKVESVNYPTENLRSKSW

DEYATPDAPKMDFIITVCDNAAGEMCPVWPGQPISAHWGFEDPAAVEGTDAEKRRAFEQTFRHMMNRVRL

FVNLPLKMLDQTAIKRELANIGKTEQEA

>gi|30063887|ref|NP_838058.1| transcriptional regulator [Shigella flexneri 2a str. 2457T]

METINAVAALAAIAQESRLAVFRLLVQAGPAGMAAGKISEAAGIPPSSLSFHLKELAHAGMVTSRQEGRF

VIYEANFSTATNLVAFLTENCCGGQVCNLSCTTEAGKVLA

>gi|30063886|ref|NP_838057.1| resolvase [Shigella flexneri 2a str. 2457T]

MRRTKPVAAPMVARVYLRVSTDAQDLERQEAITTAAKAAGYYVAGIYREKASGARADRPELLRMIGDLQP

GEVVIAEKIDRISRLPLPEAERLVASIQAKGARLAVPGVVDLSDLAAEAQGVAKIVLEAVQIMLFRLALQ

MARDDYEDRRERQRQGIELARQAGRYKGRRADPKRRAQVVALRKSGYSINKTAELAGYSAAQVKRIWAEV

SQAEAKQHGAFVEDALTEADALAAVGQDERQEERA

>gi|30063884|ref|NP_838055.1| transposase [Shigella flexneri 2a str. 2457T]

MPHVAARTASRDRDTGRYQSHRPEQTLLYQIVDEYYPAFAALMAEQGKELPGYVQREFEEFLQCGRLEHG

FLRVRCESCHAEHLVAFSCKRRGFCPSCGARRMAESAALLVDEVLPEQPMRQWVLSFPFQLRFLFASRPE

IMGWVLGIVYRVIATHLVKKAGHTHQVAKTGAVTLIQRFGSALNLNVHFHMLFLDGVYVEQSHGSARFRW
VKAPTSPELTQLTHTIAHRVGRYLERQGLLERDVENSYLASDAVDDDPMTPLLGHSITYRIAVGSQAGRK
VFTLQTLPTSGDPFGDGIGKVAGSSLHAGVAARADERKKLERLCRYISRPAVSEKRLSLTRGGNVRYQLK
TPYRDGTTHVIFEPLDFIARLAALVPKPRVNLTRFHGVFAPNSRHRALVTPAKRGRGNKVRVADEPATPA
QRRASMTWAQRLKRVFNIDIETCSGCGGAMKVIACIEDPIVIKQILDHLKHKAETSGTRALPESRAPPAE
LLLGLFD

>gi|30063883|ref|NP_838054.1| phosphoglucosamine mutase [Shigella flexneri 2a str. 2457T]
MVRKYFGTDGIRGKANEGAMTAETALRVGMAAGRVFRRGDHRHRVVIGKDTRLSGYMLEPALTAGFTSMG
MDVFLFGPLPTTYRKNKRPFHPSPTALDRS

>gi|30063882|ref|NP_838053.1| dihydropteroate synthase [Shigella flexneri 2a str. 2457T]
MNKSLIIFGIVNITSDSFSDGGRYLAPDAAIAQARKLMAEGADVIDLGPASSNPDAAPVSSDTEIARIAP
VLDALKADGIPVSLDSYQPATQAYALSRGVAYLNDIRGFPDAAFYPQLAKSSAKLVVMHSVQDGQADRRE
APAGDIMDHIAAFFDARIAALTGAGIKRNRLVLDPGMGFFLGAAPETSLSVLARFDELRLRFDLPVLLSV
SRKSFLRALTGRGPGDVGAATLAAELAAAAGGADFIRTHEPRPLRDGLAVLAALKETARIR

>gi|30063881|ref|NP_838052.1| hypothetical protein S2702 [Shigella flexneri 2a str. 2457T]
MKKVFLCAILASLSYPAIASSLQDQLSAVAEAEQQGKNEEQRQHDEWVAERNREIQQEKQRRANAQAAAN
KRAATAAANKKARQDKLDAEATADKKRDQSYEDELRSLEIQKQKLALAKEEARVKRENEFIDQELKHKAA
QTDVVQSEADANRNMTEGGRDLMKSVGKAEENKSDSWFN

>gi|30063880|ref|NP_838051.1| outer membrane lipoprotein [Shigella flexneri 2a str. 2457T]
MMKFKKCLLPVAMLASFTLAGCQSNADDHAADVYQTDQLNTKQETKTVNIISILPAKVAVDNSQNKRNAQ
AFGALIGAVAGGVIGHNVGSGSNSGTTAGAVGGGAVGAAAGSMVNDKTLVEGVSLTYKEGTKVYTSTQEG

KECQFTTGLAVVITTTYNETRIQPNTKCPEKS

>gi|30063876|ref|NP_838047.1| exopolyphosphatase [Shigella flexneri 2a str. 2457T]

MPIHDKSPRPQEFAAVDLGSNSFHMVIARVVDGAMQIIGRLKQRVHLADGLGPDNMLSEEAMTRGLNCLS

LFAERLQGFSPASVCIVGTHTLRQALNATDFLKRAEKVIPYPIEIISGNEEARLIFMGVEHTQPEKGRKL

VIDIGGGSTELVIGENFEPILVESRRMGCVSFAQLYFPGGVINKENFQRARMAAAQKLETLTWQFRIQGW

NVAMGASGTIKAAHEVLMEMGEKDGIITPERLEKLVKEILRHRNFASLSLPGLSEERKTVFVPGLAILCG

VFDALAIRELRLSDGALREGVLYEMEGRFRHQDVRSRTASSLANQYHIDSEQARRVLDTTMQMYEQWREQ

QPKLAHPQLEALLRWAAMLHEVGLNINHSGLHRHSAYILQNSDLPGFNQEQQLMMATLVRYHRKAIKLDD

LPRFTLFKKKQFLPLIQLLRLGVLLNNQRQATTTPPTLTLITDDSHWTLRFPHDWFSQNALVLLDLEKEQ

EYWEGVAGWRLKIEEESTPEIAA

>gi|30063875|ref|NP_838046.1| polyphosphate kinase [Shigella flexneri 2a str. 2457T]

MGQEKLYIEKELSWLSFNERVLQEAADKSNPLIERMRFLGIYSNNLDEFYKVRFAELKRRIIISEEQGSN

AHSRHLLGKIQSRVLKADQEFDGLYNELLLEMARNQIFLINERQLSVNQQNWLRHYFKQYLRQHITPILI

NPDTDLVQFLKDDYTYLAVEIIRSDSIRYALLEIPSDKVPRFVNLPPEAPRRRKPMILLDNILRYCLDDI

FKGFFDYDALNAYSMKMTRDAEYDLVHEMEASLMELMSSSLKQRLTAEPVRFVYQRDMPNALVEVLREKL

TISRYDSIVPGGRYHNFKDFINFPNVGKANLVNKPLPRLRHIWFDKAQFRNGFDAIRERDVLLYYPYHTF

EHVLELLRQASFDPSVLAIKINIYRVAKDSRIIDSMIHAAHNGKKVTVVVELQARFDEEANIHWAKRLTE

AGVHVIFSAPGLKIHAKLFLISRKENGEVVRYAHIGTGNFNEKTARLYTDYSLLTADARITNEVRRVFNF

IENPYRPVTFDYLMVSPQNSRRLLYEMVDREIANAQQGLPSGITLKLNNLVDKGLVDRLYAASSSGVPVN

LLVRGMCSLIPNLEGISDNIRAISIVDRYLEHDRVYIFENGGDKKVYLSSADWMTRNIDYRIEVATPLLD

PRLKQRVLDIIDILFSDTVKARYIDKELSNRYVPRGNRRKVRAQLAIYDYIKSLEQPE

>gi|30063870|ref|NP_838041.1| DNA replication initiation factor [Shigella flexneri 2a str. 2457T]

MVNFSRFCEILVEVSLNTPAQLSLPLYLPDDETFASFWPGDNSSLLAALQNVLRQEHSGYIYLWAREGAG

RSHLLHAACAELSQRGDAVGYVPLDKRTWFVPEVLDGMEHLSLVCIDNIECIAGDELWEMAIFDLYNRIL

ESGKTRLLITGDRPPRQLNLGLPDLASRLDWGQIYKLQPLSDEDKLQALQLRARLRGFELPEDVGRFLLK

RLDREMRTLFMTLDQLDRASITAQRKLTIPFVKEILKL

>gi|30063869|ref|NP_838040.1| oxidoreductase [Shigella flexneri 2a str. 2457T]

MTKQVKIYHNPRCSKSRETLNLLKENGVEPEVVLYLETPADAATLRDLLKMLGMNSARELMRQKEDLYKE

LNLADSSLSEEALIQAMVDNPKLMERPIMVANGKARIGRPPEQVLEIVG

>gi|30063867|ref|NP_838038.1| permease [Shigella flexneri 2a str. 2457T]

MLEMLMQWYRRRFSDPEAIALLVILVAGFGIIFFFSGLLAPLLVAIVLAYLLEWPTVRLQSIGCSRRWAT

SIVLVVFVGILLLMAFVVLPIAWQQGIYLIRDMPGMLNKLSDFAATLPRRYPVLMDAGIIDAMAENMRSR

MLTMGDSVVKISLASLVGLLTIAVYLVLVPLMVFFLLKDKEQMLNAVRRVLPRNRGLAGQVWKEMNQQIT

NYIRGKVLEMIVVGIATWLGFLLFGLNYSLLLAVLVGFSVLIPYIGAFVVTIPVVGVALFQFGAGTEFWS

CFAVYLIIQALDGNLLVPVLFSEAVNLHPLVIILSVVIFGGLWGFWGVFFAIPLATLIKAVIHAWPDGQI

AQE

>gi|30063866|ref|NP_838037.1| protein processing element [Shigella flexneri 2a str. 2457T]

MPTIHVSIVSFSNSFAYSGGYMTEECGEIVFWTLRKKFVASSDEMPEHSSQVMYYSLAIGHHVGVIDCLN

VAFRCPLTEYEDWLALVEEEQARRKMLGVMTFGEIVIDASHTALLTRAFAPLADDATSVWQARSIQFIHL

LDEIVLEPAIYLMARKIA

>gi|30063862|ref|NP_838033.1| hydrogenase 4 membrane subunit [Shigella flexneri 2a str. 2457T]

MTGSMIVNNLAGLMMLTSLFVISVKSYRLSCGFYACQSLVLVSIFATLSCLFAAEQLLIWSASAFITKVL

LVPLIMTYAARNIPQNIPEKALFGPAMMALLAALIVLLCAFVVQPVKLPMATGLKPALAVALGHFLLGLL

CIVSQRNILRQIFGYCLMENGSHLVLALLAWRAPELVEIGIATDAIFAVIVMVLLARKIWRTHGTLDVNN

LTALKG

>gi|30063859|ref|NP_838030.1| hydrogenase 4 Fe-S subunit [Shigella flexneri 2a str. 2457T]

MNRFVVAEPLWCTGCNTCLAACSDVHKTQGLQQHPRLALAKTSTITAPVVCHHCEEAPCLQVCPVNAISQ

RDDAIQLNESLCIGCKLCAVVCPFGAISASGSRPVNAHAQYVFQAEGSLKDGEENVPPQHALLRWEPGVQ

TVAVKCDLCDFLPEGPACVRACPNQALRLITDDSLQRQMKEKQRLAASWFANGGESPLSLTQEQH

>gi|30063856|ref|NP_838027.1| lipoprotein [Shigella flexneri 2a str. 2457T]

MAYSVQKSRLAKVAGVSLVLLLAACSSDSRYKRQVSGDEAYLEAAPLAELHAPAGMILPVTSGDYAIPVT

NGSGAVGKALDIRPPAQPLALVSGARTQFTGDTASLLVENGRGNTLWPQVVSVLQAKNYTITQRDDAGQT

LTTDWVQWNRLDEDEQYRGRYQISVKPQGYQQAVTVKLLNLEQAGKPVADAASMQRYSTEMMNVISAGLD

KSATDAANAAQNRASTTMDVQSAADDTGLPMLVVRGPFNVVWQRLPAALEKVGMKVTDSTRSQGNMAVTY

KPLSDSDWQELGASDPGLASGDYKLQVGDLDNRSSLQFIDPKGHTLTQSQNDALVAVFQAAFSK

>gi|30063854|ref|NP_838025.1| hypothetical protein S2668 [Shigella flexneri 2a str. 2457T]

MRWQGRRESDNVEDRRNSSGGPSMGGPGFRLPSGKGGLILLIVVLVAGYYGVDLTGLMTGQPVSQQQSTR

SISPNEDEAAKFTSVIQATTEDTWGQQFEKMGKTYQQPKLVMYRGMTRTGCGAGQSIMGPFYCPADGTVY

IDLSFYDDMKDKLGADGDFAQGYVIAHEVGHHVQKLLGIEPKVRQLQQNATQAEVNRLSVRMELQADCFA

GVWGHSMQQQGVLETGDLEEALNAAQAIGDDRLQQQSQGRVVPDSFTHGTSQQRYSWFKRGFDSGDPAQC

NTFGKSI

>gi|30063852|ref|NP_838023.1| esterase YpfH [Shigella flexneri 2a str. 2457T]

MKHDHFVVQSPDKPAQQLLLLFHGVGDNPVAMGEIGSWFAPLFPDALVVSVGGAEPSGNPAGRQWFSVQG

ITEDNRQARVDAIMPTFIETVRYWQKQSGVGANATALIGFSQGAIMALESIKAEPGLASRVIAFNGRYAS

LPETASTATTIHLIHGGEDPVIDLAHAVAAQEALISAGGDVTLDIVEDLGHAIDNRSMQFALDHLRYTIP

KHYFDQALSGGKPGDDDVIEMM

>gi|30063850|ref|NP_838021.1| hypothetical protein S2664 [Shigella flexneri 2a str. 2457T]

MVTLYGIKNCDTIKKARRWLEANNIDYRFHDYRVDGLDSELLNGFINELGWEALLNTRGTTWRKLDETTR

NKITDAASAAALMTEMPAIIKRPLLCAPGKPMLLGFSDSSYQQFFHEV

>gi|30063846|ref|NP_838017.1| hypothetical protein S2659 [Shigella flexneri 2a str. 2457T]

MRYRIFLLFFFALLPTSLVWAAPAQRAFSDWQVTCNNQNFCVARNTGDHNGLVMTLSRSAGAHTDAVLRI

ERGGLKSPDASEGEIAPRLLLDGEPLALSGDKWRISPWLLVTDDTATITAFLQIIQEGKAITLRDGNQTI

SLSGLKAALLFIDAQQKRVGSETAWIKKGDEPPLSVPPAPALKEVAVVNPTPTPLSLEERNDLLDYGNWR

MNGLRCSLDPLRREVNVTALTDDKALMMISCEAGAYNTIDLAWIVSRKKPLASRPVRLRLPFNNGQETNE

LELMNATFDEKSRELVTLAKGRGLSDCGIQARWRFDGQRFRLVRYAAEPTCDNWHGPDARPTLWITR

>gi|30063842|ref|NP_838013.1| hypothetical protein S2655 [Shigella flexneri 2a str. 2457T]

MPNFVIYSDEKLSRYTKVFHRRRLMDKERIIQEFVPGKQVTLAHLIAHPGEELAKKIGVPDAGAIGIMTL

TPGETAMIAGDLALKAADVHIGFLDRFSGALVIYGSVGAVEEALSQTVSGLGRLLNYTLCEMTKS

>gi|30063841|ref|NP_838012.1| hypothetical protein S2654 [Shigella flexneri 2a str. 2457T]

MKRIAFVGSVGAGKTTLFNALQGNYTLARKTLAVEFNDKGDIDTPGEYFSHPRWYHALITTLQDVDMLIY

VHGANDPESRLPAGLLDIGVSKRQIAVISKTDMPDADVAATRKLLLETGFEEPIFELNSHDPQSVQQLVD

YLASLTKQEEAGEKTHHSE

>gi|30063839|ref|NP_838010.1| iso-IS1 ORF2 [Shigella flexneri 2a str. 2457T]

MAFICELDEQWSYVGSKARQHWLGYAYNTKTGGVLAYTFGPRTDQTCRELLALLTPFNIGMLTSDDWGSY

GREVPKNKHLTGKIFTQRIERNNLTLRTRIKRLGRKTICFSRSVEIHEKVIGAFIEKHMFY

>gi|30063838|ref|NP_838009.1| amino acid antiporter [Shigella flexneri 2a str. 2457T]

MSNANIAPKSKKMTVGTLAIMNITAVVSLRFIPSEAEYGLGAIFYYTFAAIMFLVPVAFVAAELATTYPQ

KGGAFRWIGEAFGARWGFVGMLMTWLQVIPYFPTVLTFGAVSVAFIDVDMGVAESIASNKWYILFFVLFV

YWGAICVALRGVGIFSQVSKWCGIIGTIIPAIILVILGFSYLWFSGKPAVIPLGWGDLFPNIANFQNIVL

AASIFLAYAGMEMNAVHVNDLDNPTKKYPIAITIASIGTIAIFLLSTLGVAFIIPTDKISLTQSLLVAYD

MLFEWAGVPWLGSVMAFMLAIGVLGGVVTWIAGPNTGVLAIAKAGYLPKFFQKTNRHGMGHHLMFVQGII

VSVLSVTFVIMPSVQAAFQILSQLTVMLYLVMYMLMFASAIYLRHSQPDTKRPYRAPALFFWATLGFLGS

FLAFVLSFIPPEQIPTGSPTSYVLYLLVLVALFLLIPILIYAFRKSDWKDPNSDFEPFSWEKRTSN

>gi|30063835|ref|NP_838006.1| hypothetical protein S2646 [Shigella flexneri 2a str. 2457T]

MFGNYISTSPEKIIMLALRIMQGIAKTLAEHVLDLKHSPLSKQAMKRQTLRLWAEYSLGTINKIIDMKSG

PSNQSAEEMEFIRRLILIRRDIHSQLHSVGIDINDGTGD

>gi|30063834|ref|NP_838005.1| hypothetical protein S2645 [Shigella flexneri 2a str. 2457T]

MDAFEVLANKGAELVAQRDKTANEGERALLNKQIKAIRMAQFKLISNEVIETIKPQIPQIVADAIKAAGL

EKRIAGLKTNAEKMEMYKQSGAPNPDEYFSPDVEFMAQVEERLGSCLTEEQRRYFDGVDSSAGIDLNSYF

GREIEHFDAAPSMPEPEPEPEMATDPEAEQRKRVFNAIGIRY

>gi|30063832|ref|NP_838003.1| hypothetical protein S2643 [Shigella flexneri 2a str. 2457T]

MTNIERLERCADIMRRRWIYDPEAGLLFSRETKDVIKGSMTNKGHLVVTVHEGKFKVTLTYQKACYVYTY

GAYDETLYEVHHVNLNKQDNRIKNIRLMPKEKHRRIHKNVRKLIRLGHTQWLQPQLIEQSL

>gi|30063830|ref|NP_838001.1| hypothetical protein S2641 [Shigella flexneri 2a str. 2457T]

MPALDLIRPSVTAMRVIASVNAEFARELKLPPHIRSLGLISADSDDVTYIAADEATKQAMVEVVYGRSLY
AGAAHGPSPTAGEVLIMLGGPNPAEVRAGLDAMVAHIENGAAFQWANDAENTAFLAHVVSRTGSYLSSTA
GITLGDPMAYLVAPPLEATYGIDAALKSADVQLVTYVPPPSETNYSAAFLTGSQAACKAACNAFTDAVLE
IARNPIQRA

>gi|30063829|ref|NP_838000.1| hypothetical protein S2639 [Shigella flexneri 2a str. 2457T]
MINALGLLEVDGMVAAIDAADAMLKAANVRLLSHEVLDPGRLTLVVEGDLAACRAALDAGCAAAMRTGRV
ISRKEIGRPDDDTQWLVTGFNRQPKQPVKEPDAPVIVAESADELLALLTSVCQGMTAGEVAAHFGWPLEK
ARNALEQLFSAGTLRKRSSRYRLKPH

>gi|30063827|ref|NP_837998.1| N-acetylmuramoyl-l-alanine amidase I [Shigella flexneri 2a str. 2457T]
MSTFKPLKTLTSRRQVLKAGLAALTLSGMSQAIAKDEPLKTSNGHSKPKAKKSGGKRVVVLDPGHGGIDT
GAIGRNGSKEKHVVLAIAKNVRSILRNHGIDARLTRSGDTFIPLYDRVEIAHKHGADLFMSIHADGFTNP
KAAGASVFALSNRGASSAMAKYLSERENRADEVAGKKATDKDHLLQQVLFDLVQTDTIKNSLTLGSHILK
KIKPVHKLHSRNTEQAAFVVLKSPSVPSVLVETSFITNPEEERLLGTAAFRQKIATAIAEGVISYFHWFD
NQKAHSKKR

>gi|30063825|ref|NP_837996.1| hypothetical protein S2634 [Shigella flexneri 2a str. 2457T]
MKSTEFHPVHYDAHGRLRLPLLFWLVLLLQARTWVLFVIAGASREQGTALLNLFYPDHDNFWLGLIPGIP
AVLAFLLSGRRASFPRTWHVLYFLLLLAQVVLLCWQPWLWLNGESVSGIGLALVVADIVALIWLLTNRRL
RACFNEEKE

>gi|30063824|ref|NP_837995.1| hypothetical protein S2633 [Shigella flexneri 2a str. 2457T]
MKSLRLMLCAMPLMLTGCSTMSSVNWSAANPWNWFGSSTKVSEQGVGELTASTPLQEQAIADALDGDYRL
RSGMKTTNGNVVRFFEVMKGDNVAMVINGDQGTISRIDVLDSDIPADTGVKIGTPFSDLYSKAFGNCQKA

DGDDNRAVECKAEGSQHISYQFSGEWRGPEGLMPSDDTLKNWKVSKIIWRR

>gi|30063823|ref|NP_837994.1| hypothetical protein S2632 [Shigella flexneri 2a str. 2457T]

MSQVQSGILPEHCRAAIWIEANVKGEVDALRAASKTFADKLATFEAKFPDAHLGAVVAFGNNTWRALSGG

VGAEELKDFPGYGKGLAPTTQFDVLIHILSLRHDVNFSVAQAAMEAFGDCIEVKEEIHGFRWVEERDLSG

FVDGTENPAGEETRREVAVIKDGVDSGGSYVFVQRWEHNLKQLNRMSVHDQEMMIGRTKEANEEIDGDER

PETSHLTRVDLKEDGKGLKIVRQSLPYGTASGTHGLYFCAYCARLHNIEQQLLSMFGDTDGKRDAMLRFT

KPVTGGYYFAPSLDKLMAL

>gi|30063822|ref|NP_837993.1| N-acetylmuramic acid phosphotransfer permease [Shigella flexneri 2a str. 2457T]

MAKEISSELLNTILTRVGGPGNIASCGNCMTRLRLGVHDSSLVDPNIKTLEGVKGVILTSDQVQVVFGPG

KAHRAAKAMSELLGDAPVQDAAEIAAQNKRQLKAKQTSGVQQFLAKFATIFTPLIPGFIAAGLLLGIATL

IATVMHVPADAQGTLPDALNFMKVFSKGLFTFLVILVGYNAAQAFGGTGVNGAIIAALFLLGYNPAATTG

YYAGFHDFFGLPIDPRGNIIGVLIAAWACARIEGMVRRFMPDDLDMLLTSLITLLITATLAYLIIMPLGG

WLFEGMSWLFMHLNSNPFGCAVLAGLFLIAVVFGVHQGFIPVYLALMDSQGFNSLFPILSMAGAGQVGAA

LALYWRAQPHSALRSQVRGAIIPGLLGVGEPLIYGVTLPRMKPFVTACLGGAAGGLFIGLIAWWGLPMGL

NSAFGPSGLVALPLMTSAQGILPAMAVYAGGILVAWVCGFIFTTLFGCRNVNLD

>gi|30063820|ref|NP_837991.1| hypothetical protein S2628 [Shigella flexneri 2a str. 2457T]

MLYLTKIRNAESEFTGNEQKIADFLRARVSELKSVSSRQMAKQLGISQSSIVKFAQKLGAQGFTELRMAL

IGEYSASREKTNATALHLHSSITSDDSLEVIARKLNREKELALEQTCALFDYARLQKIIEVISKAPFIQI

TGLGGSALVGRDLSFKLMKIGYRVACEADTHVQATVSQALKKGDVQIAISYSGSKKEIVLCAEAARKQGA

TVIAITSLADSPLRRLAHFTLDTVSGETEWRSSSMSTRTAQNSVTDLLFVGLVQHQ

>gi|30063818|ref|NP_837989.1| thiosulfate transporter subunit [Shigella flexneri 2a str. 2457T]

MAVNLLKKNSLALVASLLLAGHVQATELLNSSYDVSRELFAALNPPFEQQWAKDNGGDKLTIKQSHAGSS

KQALAILQGLKADVVTYNQVTDVQILHDKGKLIPADWQSRLPNNSSPFYSTMGFLVRKGNPKNIHDWNDL

VRSDVKLIFPNPKTSGNARYTYLAAWGAADKADGGDKAKTEQFMTQFLKNVEVFDTGGRGATTTFAERGL

GDVLISFESEVNNIRKQYEAQGFEVVIPKTNILAEFPVAWVDKNVQANGTEKAAKAYLNWLYSPQAQTII

TDYYYRVNNPEVMDKLKDKFPQTELFRVEDKFGSWPEVMKTHFTSGGELDKLLAAGRK

>gi|30063817|ref|NP_837988.1| sulfate/thiosulfate transporter subunit [Shigella flexneri 2a str. 2457T]

MFAVSSRRVLPGFTLSLGTSLLFVCLILLLPLSALVMQLSEMSWAQYWEVITNPQVVAAYKVTLLSAFVA

SIFNGVFGLLMAWILTRYRFPGRTLLDALMDLPFALPTAVAGLTLASLFSVNGFYGEWLAKFDIKVTYTW

LGIAVAMAFTSIPFVVRTVQPVLEELGPEYEEAAETLGATRWQSFCKVVLPELSPALVAGVALSFTRSLG

EFGAVIFIAGNIAWKTEVTSLMIFVRLQEFDYPAASAIASVILAASLLLLFSINTLQSRFGRRVVGH

>gi|30063813|ref|NP_837984.1| hypothetical protein S2620 [Shigella flexneri 2a str. 2457T]

MKKIICLAITLLMTLPAYAKLTVHEEARINAMLEGLAQKKDLIFVRNGDEHTCDEAVSHLRLKLGNTRNR

IDTAEQFIDKVASSSSITGKPYIVKIPGKSDENAQPFLHALIAQTDKTVPAEGN

>gi|30063811|ref|NP_837982.1| glucose-specific PTS system component [Shigella flexneri 2a str. 2457T]

MGLFDKLKSLVSDDKKDTGTIEIIAPLSGEIVNIEDVPDVVFAEKIVGDGIAIKPTGNKMVAPVDGTIGK

IFETNHAFSIESDSGVELFVHFGIDTVELKGEGFKRIAEEGQRVKVGDTVIEFDLPLLEEKAKSTLTPVV

ISNMDEIKELIKLSGSVTVGETPVIRIKK

>gi|30063810|ref|NP_837981.1| phosphoenolpyruvate-protein phosphotransferase [Shigella flexneri 2a str. 2457T]

MISGILASPGIAFGKALLLKEDEIVIDRKKISADQVDQEVERFLSGRAKASAQLETIKTKAGETFGEEKE

AIFEGHIMLLEDEELEQEIIALIKDKHMTADAAAHEVIEGQASALEELDDEYLKERAADVRDIGKRLLRN

ILGLKIIDLSAIQDEVILVAADLTPSETAQLNLKKVLGFITDAGGRTSHTSIMARSLELPAIVGTGSVTS

QVKNDDYLILDAVNNQVYVNPTNEVIDKMRAVQEQVASEKAELAKLKDLPAITLDGHQVEVCANIGTVRD

VEGAERNGAEGVGLYRTEFLFMDRDALPTEEEQFAAYKAVAEACGSQAVIVRTMDIGGDKELPYMNFPKE

ENPFLGWRAIRIAMDRKEILRDQLRAILRASAFGKLRIMFPMIISVEEVRALRKEIEIYKQELRDEGKAF

DESIEIGVMVETPAAATIARHLAKEVDFFSIGTNDLTQYTLAVDRGNDMISHLYQPMSPSVLNLIKQVID

ASHAEGKWTGMCGELAGDERATLLLLGMGLDEFSMSAISIPRIKKIIRNTNFEDAKVLAEQALAQPTTDE

LMTLVNKFIEEKTIC

>gi|30063807|ref|NP_837978.1| sulfate transport protein CysZ [Shigella flexneri 2a str. 2457T]

MVSSFTSAPRSGFYYFAQGWKLVSQPGIRRFVILPLLVNILLMGGAFWWLFTQLDVWIPTFMSYVPDWLQ

WLSYLLWPLAVISVLLVFGYFFSTIANWIAAPFNGLLAEQLEARLTGATPPDTGIFGIMKDVPRIMKREW

QKFVWYLPRAIVLLILYFIPGIGQTVAPVLWFLFSAWMLAIQYCDYPFDNHKVPFKEMRTALRTRKITNM

QFGALTSLFTMIPLLNLFIMPVAVCGATAMWVDCYRDKHAMWR

>gi|30063803|ref|NP_837974.1| LYSR-type transcriptional regulator [Shigella flexneri 2a str. 2457T]

MNYSLKQLKVFVTVAQEKSFSRAGERIGLSQSAVSHSVKELENHTGVRLLDRTTREVVLTDAGQQLALRL

ERLLDELNSTLRDTGRMGQQLSGKVRVAASQTISAHLIPQCIAESHRRYPDIQFVLHDRPQQWVMESIRQ

GDVDFGIVIDPGPVGDLQCEAILSEPFFLLCHRDSALAVEDYVPWQALQGAKLVLQDYASGSRPLIDAAL

ARNGIQANIVQEIGHPATLFPMVAAGIGISILPALALPLPEGSPLVVKRITPVVERQLMLVRRKNRSLST

AAEALWDVVRDQGNALMAGREGDPLYQI

>gi|30063802|ref|NP_837973.1| hypothetical protein S2609 [Shigella flexneri 2a str. 2457T]

MATISSTSIPSIQTQSSNRASQGSDVASQIARISQQIIKLTQQIKEIVDTSGSAEDKQKQAELIQQQITL

LETQLAQLQKQQAEKAQEKEQRLSLNVSLLNPVEKTTHIDIYI

>gi|30063799|ref|NP_837970.1| hypothetical protein S2603 [Shigella flexneri 2a str. 2457T]

MTPDELARLTGYSRQTINKWVRKEGWTTSPKPGVQGGKARLVHVNEQVREYIRNAERPEGQGEAPALSGD

APLEVLLVTLAKEMTPVEQKQFTSLLLREGIIGLLQRLGIRDSK

>gi|30063794|ref|NP_837965.1| hypothetical protein S2595 [Shigella flexneri 2a str. 2457T]

MFRSLFLAAALMAFTPLAANAGEITLLPSIKLQIGDRDHYGNYWDGGHWRDRDYWHRNYEWRKNRWWRHD

NGYHRGWDKRKAYERGYREGWRDRDDHRGKGRGHGHRH

>gi|30063793|ref|NP_837964.1| hypothetical protein S2594 [Shigella flexneri 2a str. 2457T]

MLHPRARTMLLLSLPAVAIGIASSLILIVVMKIASVLQNLLWQRLPGTLGIAQDSPLWIIGVLTLTGIAV

GLVIRFSQGHAGPDPACEPLIGAPVPPSALPRLIVALILGLAGGVSLGPEHPIMTVNIALAVAIGARLLP

RVNRMEWTILASAGTIGALFGTPVAAALIFSQTLNGSSEVPLWDRLFAPLMAAAAGALTTGLFFHPHFSL

PIAHYGQMEMTDILSGAIVAAIAIAAGMVAVWCLPRLHAMMNQMKNPVLVLGIGGFILGILGVIGGPVSL

FKGLDEMQQMVANQAFSTSDYFLLAVIKLAALVVAAASGFRGGRIFPAVFVGVALGLMLHEHVPAVPAAI

TVSCAILGIVLVVTRDGWLSLFMAAVVVPNTTLLPLLCIVMLPAWLLLAGKPMMMVNRPKQQPPHDNV

>gi|30063792|ref|NP_837963.1| glucokinase [Shigella flexneri 2a str. 2457T]

MTKYALVGDVGGTNARLALCDIASGEISQAKTYSGLDYPSLEAVIRVYLEEHKVEVKDGCIAIACPITGD

WVAMTNHTWAFSIAEMKKNLGFSHLEIINDFTAVSMAIPMLKKEHLIQFGGAEPVEGKPIAVYGAGTGLG

VAHLVHVDKRWVSLPGEGGHVDFAPNSEEEAIILEILRAEIGHVSAERVLSGPGLVNLYRAIVKADNRLP

ENLKPKDITERALADSCTDCRRALSLFCVIMGRFGGNLALNLGTFGGVFIAGGIVPRFLEFFKASGFRAA

FEDKGRFKEYVHDIPVYLIVHDYPGLLGSGAHLRQTLGHIL

>gi|30063791|ref|NP_837962.1| PTS system enzyme IIB component [Shigella flexneri 2a str. 2457T]

MSKKLIALCACPMGLAHTFMAAQVLEEAAVEAGYEVKIETQGADGIQNRLTAQDIAEATIIIHSVAVTPE

DNERFESRDVYEITLQDAIKNAAGIIKEIEEMIASEQQ

>gi|30063790|ref|NP_837961.1| transporter [Shigella flexneri 2a str. 2457T]

MAIKKRSATVVPGASGAAAAVKNPQASKSSFWGELPQHVMSGISRMVPTLIMGGVILAFSQLIAYSWLKI

PAEIGIMDALNSGKFSGFDLSLLKFAWLSQSFGGVLFGFAIPMFAAFVANSIGGKLAFPAGFIGGLMSTQ

PTQLLNFDPSTMQWATSSPVPSTFIGALIISIVAGYLVKWMNQKIQLPDFLLAFKTTFLLPILSAIFVML

AMYYVITPFGGWINGGIRTVLTAAGEKGALMYAMGIAAATAIDLGGPINKAAGFVAFSFTTDHVLPVTAR

SIAIVIPPIGLGLATIIDRRLTGKRLFNAQLYPQGKTAMFLAFMGISEGAIPFALESPITAIPSYMVGAI

VGSTAAVWLGAVQWFPESAIWAWPLVTNLGVYMAGIALGAIITALMVVFLRLMMFRKGKLLIDSL

>gi|30063788|ref|NP_837959.1| PTS system enzyme IIA component, enzyme I [Shigella flexneri 2a str. 2457T]

MLTIQFLCPLPNGLHARPAWELKEQCSQWQSEITFINHRQNAKADAKSSLALIGTGTLFNDSCSLNISGS

DEEQARRVLEEYILVRFIDSDSVQPTQAELTAHPLPRSLSRLNPDLLYGNVLASGVGVGTLTLLQSDSLD

SYRAIPASAQDSTRLEHSLATLAEQLNQQLRERDGESKTILSAHLSLIQDDEFAGNIRRLMTEQHQGLGA

AIISNMEQVCAKLSASASDYLRERVSDIRDISEQLLHITWPELKPRNNLVLEKPTILVAEDLTPSQFLSL

DLKNLAGMILEKTGRTSHTLILARASAIPVLSGLPLDAIARYAGQPAVLDAQCGVLAINPNDAVSGYYQV

AQTLADKRQKQQAQAAAQLAYSRDNKRIDIAANIGTALEAPGAFANGAEGVGLFRTEMLYMDRDSAPDEQ

EQFEAYQQVLLAAGDKPIIFRTMDIGGDKSIPYLNIPQEENPFLGYRAVRIYPEFAGLFRTQLRAILRAA

SFGNAQLMIPMVHSLDQILWVKGEIQKAIVELKRDGLRHAETITLGIMVEVPSVCYIIDHFCDEVDFFSI

GSNDMTQYLYAVDRNNPRVSPLYNPITPSFLRMLQQIVTTAHQRGKWVGICGELGGESRYLPLLLGLGLD

ELSMSSPRIPAVKSQLRQLDSEACRELARQACECRSAQEIEALLTAFTPEEDVRPLLALENIFVDQDFSN

KEQAIQFLCGNLGVNGRTEHPFELEEDVWQREEIVTTGVGFGVAIPHTKSQWIRHSSISIARLAKPVDWQ

SEMGEVELVIMLTLGANEGMNHVKVFSQLARKLVNKNFRQSLFAAQDAQSILTLLETELTF

>gi|30063787|ref|NP_837958.1| ARAC-type regulatory protein [Shigella flexneri 2a str. 2457T]

MKAPGLPADQQFFADLFSGLVLNPQLLGRVWFASQPASLPVGSLCIDFPRLDIVLRGEYGNLLEAKQQRM

VEGEMLFIPARAANLPINNKPVMLLSLVFAPTWLGLSFYDSRTTSLLHPARQTQLPSLQRGEGEAMLTAL

THLSRSPLEQNIIQPLVLSLLHLCRNVVNMPPGNSQPRGDFLYHSICNWV

>gi|30063786|ref|NP_837957.1| 2-component transcriptional regulator [Shigella flexneri 2a str. 2457T]

MKVIIVEDEFLAQQELSWLIKEHSQMEIVGTFDDGLDVLKFLQHNRVDAIFLDINIPSLDGVLLAQNISQ

FAHKPFIVFITAWKEHAVEAFELEAFDYILKPYQESRITGMLQKLEAAWQQQQTSSTPAATVTRENDTIN

LVKDERIIVTPINDIYYAEAHEKMTFVYTRRESYVMPMNITEFCSKLPPSHFFRCHRSFCVNLNKIREIE

PWFNNTYILRLKDLDFEVPVSRSKVKEFRQLMHL

>gi|30063781|ref|NP_837952.1| hypothetical protein S2579 [Shigella flexneri 2a str. 2457T]

MKRLIMATMVTAILASSTVWAADNAPVAAQQQTQQTQKTAAAERISEQGLYAMRDVQVARLALFHGDPEK

AKELTNEASALLSDDSTEWAKFAKPGKKTNLNDDQYIVINASVGISESYVATPEKEAAIKIANEKMAKGD

KKGAMEELRLAGVGVMENQYLMPLKQTRNALADAQKLLDKKQYYEANLALKGAEDGIIVDSEALFVN

>gi|30063777|ref|NP_837948.1| hybrid sensory histidine kinase in two-component regulatory system with EvgA [Shigella flexneri 2a str. 2457T]

MKFLPYFFLLCCGLWSTISFADEDYIEYRGISSNNRVTLDPVRLSNKELRWLASKKNLVIAVHKSQTATL

LHTDSQQQVRGINADYLNLLKRALNIKLTLREYADHQKAMDALAEGEVDIVLSHLVTSPPLNNDIAATKP

LIITFPALVTTLHDSMRPLTSPKPVNIARVANYPPDEVIHQSFPKATIISFTNLYQALASVSAGHNDYFI

GSNIITSSMISRYFTHSLNVVKYYNSPRQYNFFLTRKESVILNEVLNRFVDALTNEVRYEVSQNWLDTGN

LAFLNKPLELTEHEKQWIKQHPNLKVLENPYSPPYSMTDENGSVRGVMGDILNIITLQTGLNFFPITVSH

NIHAGTQLSPGGWDIIPGAIYSEDRENNVLFAEAFITTPYVFVMQKAPDSEQTLKKGMKVAIPYYYELHS

QLKEMYPEVEWIQVDNASAAFHKIKEGELDALVATQLNSRYMIDHYYPNELYHFLIPGVPNASLSFAFPR

GEPELKDIINKALNAIPPSEVLRLTEKWIKMPNVTIDTWDLYSEQFYIVTTLSVLLVGSSLLWGFYLLRS

VRRRKVIQGDLENQISFRKALSDSLPNPTYVVNWQGNVISHNSAFEHYFTADYYKNAMLPLENSDSPFKD

VFSNAHEVTAETKENRTIYTQVFEIDNGIEKRCINHWHTLCNLPASDNAVYICGWQDITETRDLINALEV

EKNKAIKATVAKSQFLATMSHEIRTPISSIMGFLELLSGSGLSKEQRVEAISLAYATGQSLLGLIGEILD

VDKIESGNYQLQPQWVDIPTLVQNTCHSFGAIAASKSIALSCSSTFPDHYLVKIDPQAFKQVLSNLLSNA

LKFTTEGAVKITTSLGHIDDNHAVIKMTIMDSGSGLSQEEQQQLFKRYSQTSAGRQQTGSGLGLMICKEL

IKNMQGDLSLESHPGIGTTFTITIPVEISQQVATVEAKAEQPITLPEKLSILIADDHPTNRLLLKRQLNL

LGYDVDEATDGVQALHKVSMQHYDLLITDVNMPNMDGFELTRKLREQNSSLPIWGLTANAQANEREKGLS

CGMNLCLFKPLTLDVLKTHLSQLHQVAHIAPQYRHLDIEALKNNTANDLQLMQEILMTFQHETHKDLPAA

FHALEAGDNRTFHQCIHRIHGAANILNLQKLINISHQLEITPASDDSKPEILQLLNSVKEHIAELDQEIA

VFCQKND

>gi|30063776|ref|NP_837947.1| DNA-binding transcriptional activator EvgA [Shigella flexneri 2a str. 2457T]

MNAIIIDDHPLAIAAIRNLLIKNDIEILAELTEGGSAVQRVETLKPDIVIIDVDIPGVNGIQVLETLRKR

QYSGIIIIVSAKNDHFYGKHCADAGANGFVSKKEGMNNIIAAIEAAKNGYCYFPFSLNRFVGSLTSDQQK

LDSLSKQEISVMRYILDGKDNNDIAEKMFISNKTVSTYKSRLMEKLECKSLMDLYTFAQRNKIG

>gi|30063775|ref|NP_837946.1| multidrug resistance protein K [Shigella flexneri 2a str. 2457T]

MEQINSNKKQSNRKKYFSLLVIVLFIAFSGAYAYWSMELEDMISTDDAYVTGNADPISAQVSGSVTVVNH

KDTNYVRQGDILVSLDKTDATIALNKAKNNLANIVRQTNKLYLQDKQYSAEVASARIQYQQSLEDYNRRV

PLAKQGVISKETLEHTKDTLISSKAALNAAIQAYKANKALVMNTPLNRQPQVVEAADATKEAWLVLKRTD

IRSPVTGYIAQRSVQVGETVSSGQSLMAVVPARQMWVNANFKETQLTDVRIGQSVNIISDLYGENVVFHG

RVTGINMGTGNAFSLLPAQNATGNWIKIVQRVPVEVSLDPKELMEHPLRIGLSMTATIDTKDEDIAEMPD

LASTVTSMPAYTSKALVIDTSPIEKEISNIISHNGQL

>gi|30063773|ref|NP_837944.1| D-serine dehydratase [Shigella flexneri 2a str. 2457T]

MENAKMNSLIAQYPLVKDLVALKETTWFNPSTTSLAEGLPYVGLTEQDVQDAHARLSRFAPYLAKAFAET

AATGGIIESELVAIPAMQKRLEKEYQQPISGQLLLKKDSHLPISGSIKARGGIYEVLAHAEKLALEAGLL

TLDDDYSKLLSPEFKQFFSQYSIAVGSTGNLGLSIGIMSARIGFKVTVHMSADARAWKKAKLRSHGVTVV

EYEQDYGVAVEEGRKAAQSDPNCFFIDDENSRTLFLGYSVAGQRLKAQFAQQGRIVDADNPLFVYLPCGV

GGGPGGVAFGLKLAFGDHVHCFFAEPTHSPCMLLGVHTGLHDQISVQDIGIDNLTAADGLAVGRASCFVG

RAMERLLDGFYTLSDQTMYDMLGWLAQEEGIRLEPSALAGMAGPQRVCASVSYQQMHGFSAEQLRNATHL

VWATGGGMVPEEEMNQYLAKGR

>gi|30063772|ref|NP_837943.1| sucrose specific repressor [Shigella flexneri 2a str. 2457T]

MDLASDVCFCYSARFTANGLGMASLKDVARLAGVSMMTVSRVMHNAESVRPATRDRVLQAIQTLNYVPDL

SARKMRAQGRKPSTLAVLAQDTATTPFSVDILLAIEQTASEFGWNSFLINIFSEDDAARAARQLLAHRPD

GIIYTTMGLRHITLPESLYGENIVLANCVADDPALPSYIPDDYTAQYESTQHLLAAGYRQPLCFWLPESA

LATGYRRQGFEQAWRDAGRDLAEVKQFHMATGDDHYTDLASLLNAHFKSGKPDFDVLICGNDRAAFVAYQ

VLLAKGVRIPQDVAVMGFDNLVGVGHLFLPPLTTIQLPHDIIGREAALHIIEGREGGRVTRIPCPLLIRC
ST

>gi|30063771|ref|NP_837942.1| hypothetical protein S2470 [Shigella flexneri 2a str. 2457T]

MTQSRLHAAQNALAKLHEHRGNTFYPHFHLAPPAGWMNDPNGLIWFNDRYHAFYQHHPMSEHWGPMHWGH

ATSDDMIHWQHEPIALALGDDNDKDGCFSGSAVDDNGVLSLIYTGHVWLDGAGNDDAIREVQCLATSRDG

IHFEKQGVILTPPEGIMHFRDPKVWREADTWWMVVGAKDPGNTGQILLYRGSSLREWAFDRVLAHADAGE

SYMWECPDFFSLGDQHYLMFSPQGMNAEGYSYRNRFQSGVIPGMWSPGRLFAQSGHFTELDNGHDFYAPQ

SFLAKDGRRIVIGWMDMWESPMPSKREGWAGCMTLARELSESNGKLLQRPVHEAESLRQQHQSVSPRRIS

NKYVLQENAQAVEIQLQWALKNSDAEHYGLQLGTGMRLYIDNQSERLILWRYYPHENLDGYRSIPLPQRD

TLALRIFIDTSSVEVFINDGEAVMSSRIYPQPEERELSLYASHGVAVLQHGALWLLG

>gi|30063768|ref|NP_837939.1| transporter, partial [Shigella flexneri 2a str. 2457T]

MAIHEHIRQDGEKELERDAMALLWSAIAAGLSMGASLLAKGIFHAELEGVPGSFLLENLGYTFGFIIVIM

ARQQLFTENTVTAVLPVMQKPTMSNVGLLMRLWGVVLLGNILGTGIAAWAFEYMPIFNEETRDAFVKIGM

DVMKNTPSEMFANAIISGWLIATMVWMFPAAGAAKIVVIILMTWLIALGDTTHIVVGSVEILYLVFNGTL

HWSDFIWPFALPTLAGNICGGTFIFALMSHAQIRNDMSNKRKAEARQKAERAENIKKNDKNPA

>gi|30063767|ref|NP_837938.1| lipoprotein precursor [Shigella flexneri 2a str. 2457T]

MKLRLSALALGTTLLVGCASSGTDQQGRSDPLEGFNRTMYNFNFNVLDPYIVRPVAVAWRDYVPQPARNG

LSNFTGNLEEPAVMVNYFLQGDPYQGMVHFTRFFLNTILGMGGFIDVAGMANPKLQRTEPHRFGSTLGHY

GVGYGPYVQLPFYGSFTLRDDGGDMADALYPVLSWLTWPMSVGKWTLEGIETRAQLLDSDGLLRQSSDPY

IMVREAYFQRHDFIANGGELKPQENPNAQAIQDDLKDIDSE

>gi|30063766|ref|NP_837937.1| hypothetical protein S2558 [Shigella flexneri 2a str. 2457T]

MLPSISINNTSAAYPESINENNNDEINGLVQEFKNLFNGKEGISTCIKHLLELIKNAIRVNDDPYRSNIN

NSSVTYIDIGSNDTDHITIGIDNQEPIELPANYKDKELVRTIINDNIVEKTHDINNKEMIFSALKEIYDG

DPGFIFDKISHKLRHTVTEFDESGKSEPTDLFTWYGKDKKGDSLAIVIKNKNGNDYLSLGYYDQDDYHIQ

RGIRINGDSLTQYCSENARSASAWFESSKAIMAESFATGSDHQVVNELNGERLREPNEVFKRLGRAIRYN

FQVDDAKFRRDNVKEIISTLFANKVDVDHPENKYKDFKNLEDKVEKRLQNRQTKYQNEINQLSALGVNFD

DI

>gi|30063765|ref|NP_837936.1| long-chain fatty acid outer membrane transporter [Shigella flexneri 2a str. 2457T]

MSQKTLFTKSALAVAVALISTQAWSAGFQLNEFSSSGLGRAYSGEGAIADDAGNVSRNPALITMFDRPTF

SAGAVYIDPDVNISGTSPSGRSLKADNIAPTAWVPNMHFVAPINDQFGWGASITSNYGLATEFNDTYAGG

SVGGTTDLETMNLNLSGAYRLNNAWSFGLGFNAIYARAKIERFAGDLGQLVAGQIMQSPAGQTPQGQALA

ATANGIDSNTKIAHLNGNQWGFGWNAGILYELDKNNRYALTYRSEVKIDFKGNYSSDLNRAFNNYGLPIP

TATGGATQSGYLTLNLPEMWEVSGYNRVDPQWAIHYSLAYTSWSQFQQLKATSTSGDTLFQKHEGFKDAY

RIALGTTYYYDDNWTFRTGIAFDDSPVPAQNRSISIPDQDRFWLSAGTTYAFNKDASVDVGVSYMHGQSV

KINEGPYQFESEGKAWLFGTNFNYAF

>gi|30063764|ref|NP_837935.1| hypothetical protein S2556 [Shigella flexneri 2a str. 2457T]

MHIQRKSTMSKCSADETPVCCCMDVGTIMDNSDCTASYSRVFANRAEAEQTLAALTEKSRSVESEPCKIS

PTFTEESDGVRLDIDFTFACEAEMLIFQLGLR

>gi|30063761|ref|NP_837932.1| phosphohistidine phosphatase [Shigella flexneri 2a str. 2457T]

MQVFIMRHGDAALDSASDSVRPLTTNGCDESRLMANWLKGQKVEIERVLVSPFLRAEQTLEEVGDCLNLP

SSAEVLPELTPCGDVGLVSAYLQALTNEGVASVLVISHLPLVGYLVAELCPGETPPMFTTSAIASVTLDE

SGNGTFNWQMSPCNLKMAKAI

>gi|30063758|ref|NP_837929.1| fimbrial-like protein [Shigella flexneri 2a str. 2457T]

MSKFVKTAIAAAMVMGVFTSTATIAAGNNGTARFYGTIEDSVCSIVPDDHKLEVDMGDIGAEKLKNNGTT

TPKSFQIRLQDCVFDTQETMTTTFTGTVSSANSGNYYTIFNTDTGAAFNNVSLAIGDSLGTSYKSGMGID

QKIVKDTSTNKGKAKQTLNFNAWLVGAADAPDLGNFEANTTFQITYL

>gi|30063757|ref|NP_837928.1| hypothetical protein S2541 [Shigella flexneri 2a str. 2457T]

MKKKTTLSEEDQALFRQLMAGTRKIKQDTIVHRPQRKKISEVPVKRLIQEQADASHYFSDEFQPLLNTEG

PVKYVRPDVSHFEAKKLRRGDYSPELFLDLHGLTQLQAKQELGALIAACRREHVFCACVMHGHGKHILKQ

QTPLWLAQHPHVMAFHQAPKEYGGDAALLVLIEVEEWLPPELP

>gi|30063755|ref|NP_837926.1| chorismate synthase [Shigella flexneri 2a str. 2457T]

MAGNTIGQLFRVTTFGESHGLALGCIVDGVPPGIPLTEADLQHDLDRRRPGTSRYTTQRREPDQVKILSG

VFEGVTTGTSIGLLIENTDQRSQDYSAIKDVFRPGHADYTYEQKYGLRDYRGGGRSSARETAMRVAAGAI

AKKYLAEKFGIEIRGCLTQMGDIPLEIKDWSLVEQNPFFCPDPDKIDALDELMRALKKEGDSIGAKVTVV

ASGVPAGLGEPVFDRLDADIAHALMSINAVKGVEIGDGFDVVALRGSQNRDEITKDGFQSNHAGGIFGGI

SSGQQIIAHMALKPTSSITVPGRTINRFGEEVEMITKGRHDPCVGIRAVPIAEAMLAIVLMDHLLRQRAQ

NADVKTDIPRW

>gi|30063754|ref|NP_837925.1| penicillin-insensitive murein endopeptidase [Shigella flexneri 2a str. 2457T]

MNKTAIALLALLASSASLAATPWQKITQPVPGSAQSIGSFSNGCIVGADTLPIQSEHYQVMRTDQRRYFG

HPDLVMFIQRLSSQVSNLGMGTVLIGDMGMPAGGRFNGGHASHQTGLDVDIFLQLPKTRWTSAQLLRPQA

LDLVSRDGKHVVSTLWKPEIFSLIKLAAQDKDVTRIFVNPAIKQQLCLDAGTDRDWLRKVRPWFQHRAHM

HVRLRCPADSLECEDQPLPPSGDGCGAELQSWFEPPKPGTTKPEKKTPPPLPPSCQALLDEHVI

>gi|30063753|ref|NP_837924.1| hypothetical protein S2538 [Shigella flexneri 2a str. 2457T]

METFNSLFMVSPLLLGVLFFVAMLAGFIDSIAGGGGLLTIPALMAAGMSPANALATNKLQACGGSISATI

YFIRRKVVSLSDQKLNIAMTFVGSMSGALLVQYVQADVLRQILPILVICIGLYFLLMPKLGEEDRQRRMY

GLPFALIAGGCVGFYDGFFGPAAGSFYALAFVMLCGFNLAKATAHAKLLNATSNIGGLLLFILGGKVIWA

TGFVMLVGQFLGARMGSRLVLSKGQKLIRPMIVIVSAVMSAKLLYDSHGQEILHWLGMN

>gi|30063752|ref|NP_837923.1| transporting ATPase [Shigella flexneri 2a str. 2457T]

MNSTHHYEQLIEIFNSCFADEFNTRLIKGDDEPIYLPADAEVPYNRIVFAHGFYASAIHEISHWCIAGKA

RRELVDFGYWYCPDGRDAQTQSQFEDVEVKPQAFDWLFCVAAGYPFNVSCDNLEGDFEPDRVVFQRRVHA

QVMDYLTNGIPERPARFIKALQNYYHTPELTAEQFPWPEALN

>gi|30063748|ref|NP_837919.1| hypothetical protein S2533 [Shigella flexneri 2a str. 2457T]

MTAVSQTETRSSASFSLFRISFAVFLTYMTVGLPLPVIPLFVHHDLGYGNTMVGIAVGIQFLATVLTRGY

AGRLADQYGAKRSALQGMLACGLAGGALLLAAILPVSAPFKFALLVIGRLILGFGESQLLTGALTWGLGI

VGPKHSGKVMSWNGMAIYGALAVGAPLGLLIHSHYGFAALAITTMVLPLLAWACNGTVRKVPALAGERPS

LWSVVGLIWKPGLGLALQGVGFAVIGTFVSLYFASKGWAMAGFTLTAFGGAFVVMRVMFGWMPDRFGGVK

VAIVSLLVETVGLLLLWQAPGAWVALAGAALTGAGCSLIFPALGVEVVKRVPSQVRGTALGGYAAFQDIA

LGVSGPLAGMLATTFGYSSVFLAGAISAVLGIIVTILSFRRG

>gi|30063747|ref|NP_837918.1| flagella biosynthesis regulator [Shigella flexneri 2a str. 2457T]

MIQPISGPPPGQPPGQGDNLPSGAGNQPLSSQQRTSLESLMTKVTSLTQQQRAELWAGIRHDIGLSGDSP

LLSRHFPAAEHNLAQRLLAAQKSHSARQLLAQLGEYLRLGNHRQAVTDYIRHNFGQTPLNQLSPEQLKTI

LTLLQEGKMVIPQPQQREATDRPLLPAEHNALKQLVTKLAAATGEPSKQIWQSMLELSGVKDGELIPAKL

FNHLVTWLQARQTLSQQNTPTLESLQMALKQPLDASELAALSAYIQQKYGLSAQSSLSSAQAEDILNQLY

QRRVKGIDPRDMQPLLNPFPPMMDTLQNMATRPALWILLVAIILMLVWLVR

>gi|30063745|ref|NP_837916.1| semialdehyde dehydrogenase [Shigella flexneri 2a str. 2457T]

MSEGWNIAVLGATGAMGEALLETLAERQFPVGEIYALARNESAGEQLRFGGKTITVQDAAEFDWTQAQLA

FFVAGKEATAAWVEEATNSGCLVIDSSGLFALEPDVPLVVPEVNPFVLTDYRNRNVIAVPDSLTSQLLAA

LKPLIDQGGLSRISVTSLISASAQGKKAVDALAGQSAKLLNGIPIDEEDFFGRQLAFNMLPLLPDSEGSV

REERRIVDEVRKILQDEGLMISASVVQAPVFYGHAQMVNFEALRPLAAEEARDAFAQGEDIVLSEENEFP

TQVGDASGTPHLSVGCVRNDYGMPEQVQFWSVADNVRFGGALMAVKIAEKLVQEYLY

>gi|30063743|ref|NP_837914.1| hypothetical protein S2528 [Shigella flexneri 2a str. 2457T]

MDLIYFLIDFILHIDVHLAELVAEYGVWVYAILFLILFCETGLVVMPFLPGDSLLFVAGALASLETNDLN

VHMMVVLMLIAAIVGDAVNYTIGRLFGEKLFSNPNSKIFRRSYLDKTHQFYEKHGGKTIILARFVPIVRT

FAPFVAGMGHMSYRHFAAYNVIGALLWVLLFTYAGYFFGTIPMVQDNLKLLIVGIIVVSILPGVIEIIRH

KRAAARAAK

>gi|30063739|ref|NP_837910.1| colicin V production protein [Shigella flexneri 2a str. 2457T]

MVWIDYAIIAVIAFSSLVSLIHGFVREALSLVTWGCAFFVASHYYTYLSVWFTGFEDELVRNGIAIAVLF

IATLIVGAIVNFVIGQLVEKTGLSGTDRVLGVCFGALRGVLIVAAILFFLDSFTGVSKSEDWSKSQLIPQ

FSFIIRWFFDYLQSSSSFLPRA

>gi|30063737|ref|NP_837908.1| 3-octaprenyl-4-hydroxybenzoate carboxy-lyase [Shigella flexneri 2a str. 2457T]

MKRLIVGISGASGAIYGVRLLQVLRDVTDIETHLVMSQAARQTLSLETDFSLREVQALADVTLDARDISA

SISSGSFQTLGMVILPCSIKTLSGIVHSYTDGLLTRAADVVLKERRPLVLCVRETPLHLGHLRLMTQAAE

IGAVIMPPVPAFYHRPQSLDDVINQTVNRVLDQFAITLPEDLFARWQGA

>gi|30063736|ref|NP_837907.1| lysine-, arginine-, ornithine-binding periplasmic protein [Shigella flexneri 2a str. 2457T]

MKKSILALSLLVGLSAAASSYAALPETVRIGTDTTYALFSSKDAKGDFVGFDIDLGNEMCKRMQVKCTWV

ASDFDALIPSLKAKKIDAIISSLSITDKRQQEIAFSDKLYAADSRLIAAKGSPIQPTLDSLKGKHVGVLQ

GSTQEAYANETWRSKGVDVVAYANQDLVYSDLAAGRLDAALQDEVAASEGFLKQPAGKDFAFAGSSVKDK

KYFGDGTGVGLRKDYAELTAAFNKALGELRQDGTYDKMAKKYFDFNVYGD

>gi|30063734|ref|NP_837905.1| histidine transport system permease [Shigella flexneri 2a str. 2457T]

MLYGFSGVILQGALVTLELAISSVVLAVIIGLIGAGGKLSQNRLSGLIFEGYTTLIRGVPDLVLMLLIFY

GLQIALNMVTEAMGVGQIDIDPMVAGIITLGFIYGAYFTETFRGAFMAVPKGHIEAATAFGFTRGQVFRR

IMFPAMMRYALPGIGNNWQVILKSTALVSLLGLEDVVKATQLAGKSTWEPFYFAIVCGVIYLVFTTVSNG

VLLFLERRYSVGVKRADL

>gi|30063733|ref|NP_837904.1| histidine transport, membrane protein M [Shigella flexneri 2a str. 2457T]

MIEILHEYWKPLLWTDGYRFTGVAITLWLLILSVVIGGVLALFLAIGRVSSNKYIQFPIWLFTYIFRGTP

LYVQLLVFYSGMYTLEIVKGTEFLNAFFRSGLNCTVLALTLNTCAYTTEIFAGAIRSVPHGEIEAARAYG

FSTFKMYRCIILPSALRIALPAYSNEVILMLHSTALAFTATVPDLLKIARDINAATYQPFTAFGIAAVLY

LIISYVLISLFRRAEKRWLQHVKPSSTH

>gi|30063729|ref|NP_837900.1| D-erythro-7,8-dihydroneopterin triphosphate 2'-epimerase [Shigella flexneri 2a str. 2457T]

MAQPAAIIRIKNLRLRTFIGIKEEEINNRQDIVINVTIHYPADKARTSEDINDALNYRTVTKNIIQHVEN

NRFSLLEKLTQDVLDIAREHHWVTYAEVEIDKLHALRYADSVSMTLSWQR

>gi|30063726|ref|NP_837897.1| phosphodiesterase [Shigella flexneri 2a str. 2457T]

MKLMFASDIHGSLPATERVVELFVQSGAQWLVILGDVLNHGPRNALPEGYAPAKVAERLNEVAHKVIAVR
GNCDSEVDQMMLHFPITAPWQQVLLEKQRLFLTHGHLFGPENLPALNQNDVLVYGHTHLPVAEQRGEIFH
FNPGSVSIPKGGNPAGYGMLDNDVLSVIALNDQSIIAQVAINP

>gi|30063725|ref|NP_837896.1| regulator [Shigella flexneri 2a str. 2457T]

MEQRRLASTEWVDIVNEENEVIAQASREQMRAQCLRHRATYIVVHDGMGKILVQRRTETKDFLPGMLDAT
AGGVVQADEQLLESARREAEEELGIAGVPFAEHGQFYFEDKNCRVWGALFSCVSHGPFALQEDEVSGVCW
LTPEEITARCDEFTPDSLKALALWMKRNAKNEAVETETAE

>gi|30063723|ref|NP_837894.1| phosphate acetyltransferase [Shigella flexneri 2a str. 2457T]

MSRIIMLIPTGTSVGLTSVSLGVIRAMERKGVRLSVFKPIAQPRTGGDAPDQTTTIVRANSSTTTAAEPL
KMSYVEGLLSSNQKDVLMEEIVANYHANTKDAEVVLVEGLVPTRKHQFAQSLNYEIAKTLNAEIVFVMSQ
GTDTPEQLKERIELTRNSFGGAKNTNITGVIVNKLNAPVDEQGRTRPDLSEIFDDSSKAKVNNVDPAKLQ
ESSPLPVLGAVPWSFDLIATRAIDMARHLNATIINEGDINTRRVKSVTFCARSIPHMLEHFRAGSLLVTS
ADRPDVLVAACLAAMNGVEIGALLLTGGYEMDARISKLCERAFATGLPVFMVNTNTWQTSLSLQSFNLEV
PVDDHERIEKVQEYVANYINADWIESLTATSERSRRLSPPAFRYQLTELARKAGKRIVLPEGDEPRTVKA
AAICAERGIATCVLLGNPAEINRVAASQGVELGAGIEIVDPEVVRESYVGRLVELRKNKGMTETVAREQL
EDNVVLGTLMLEQDEVDGLVSGAVHTTANTIRPPLQLIKTAPGSSLVSSVFFMLLPEQVYVYGDCAINPD
PTAEQLAEIAIQSADSAAAFGIEPRVAMLSYSTGTSGAGSDVEKVREATRLAQEKRPDLMIDGPLQYDAA
VMADVAKSKAPNSPVAGRATVFIFPDLNTGNTTYKAVQRSADLISIGPMLQGMRKPVNDLSRGALVDDIV
YTIALTAIQSAQQQ

>gi|30063722|ref|NP_837893.1| acetate kinase [Shigella flexneri 2a str. 2457T]

MSSKLVLVLNCGSSSLKFAIIDAVNGEEYLSGLAECFHLPEARIKWKMDGNKQEAALGAGAAHSEALNFI

VNTILAQKPELSAQLTAIGHRIVHGGEKYTSSVVIDESVIQGIKDAASFAPLHNPAHLIGIEEALKSFPQ

LKDKNVAVFDTAFHQTMPEESYLYALPYNLYKEHGIRRYGAHGTSHFYVTQEAAKMLNKPVEELNIITCH

LGNGGSVSAIRNGKCVDTSMGLTPLEGLVMGTRSGDIDPAIIFHLHDTLGMSVDAINKLLTKESGLLGLT

EVTSDCRYVEDNYATKEDAKRAMDVYCHRLAKYIGAYTALMDGRLDAVAFTGGIGENAAMVRELSLGKLG

VLGFEVDHERNLAARFGKSGFINKEGTRPAVVIPTNEELVIAQDASRLTA

>gi|30063721|ref|NP_837892.1| hypothetical protein S2506 [Shigella flexneri 2a str. 2457T]

MSTPDNRSVNFFSLFRRGQHYSKTWPLEKRLAPVFVENRVIKMTRYAIRFMPPIAVFTLCWQIALGGLLG

PAVATALFALSLPMQGLWWLGKRSVTPLPPAILNWFYEVRGKLQESGQVLAPVEGKPDYQALADTLKRAF

KQLDKTFLDDL

>gi|30063720|ref|NP_837891.1| hypothetical protein S2505 [Shigella flexneri 2a str. 2457T]

MEMTNAQRLILSNQYKMMTMLDPANAERYRRLQTIIERGYGLQMRELDREFGELKEETCRTIIDIMDMYH

ALYVSWSNLQDQQSIDERRVTFLGFDAATEARYLGYVRFMVNVEGRYTHFDAGTHGFNAQTPMWEKYQRM

LNVWHACPRQYHLSANEINQIINA

>gi|30063717|ref|NP_837888.1| hypothetical protein S2502 [Shigella flexneri 2a str. 2457T]

MKQSHFFAHLSRLKLINRWPLMRNVRTENVSEHSLQVAMVAHALAAIKNRKFGGNVNAERIALLAMYHDA

SEVLTGDLPTPVKYFNAQIAQEYKAIEKIAQQKLVDMVPEELRDIFAPLIDEHAYSDEEKSLVKQADALC

AYLKCLEELAAGNNEFLLAKTRLEATLEARRSQEMDYFMEVFVPSFHLSLDEISQDSPL

>gi|30063715|ref|NP_837886.1| LysR family NADH dehydrogenase transcriptional regulator [Shigella flexneri 2a str. 2457T]

MISANRPIINLDLDLLRTFVAVADLNTFAAAAAAVCRTQSAVSQQMQRLEQLVGKELFARHGRNKLLTEH

GIQLLGYARKILRFNDEACSSLMFSNLQGVLTIGASDESADTILPFLLNRVSSVYPKLALDVRVKRNAYM

AEMLESQEVDLMVTTHRPSAFKALNLRTSPTHWYCAAEYVLQKGEPIPLVLLDDPSPFRDMVLATLNKVD

IPWRLAYVASTLPAVRAAVKAGLGVTARPVEMMSPDLRVLSGVDGLPPLPDTEYLLCYDPSSNNELAQVI

YQAMESYHNPWQYSPMSAPEGDDSLLIERDIE

>gi|30063706|ref|NP_837877.1| NADH dehydrogenase subunit J [Shigella flexneri 2a str. 2457T]

MEFAFYICGLIAILATLRVITHTNPVHALLYLIISLLAISGVFFSLGAYFAGALEIIVYAGAIMVLFVFV

VMMLNLGGSEIEQERQWLKPQVWIGPAILSAIMLVVIVYAILGVNDQGIDGTPISAKAVGITLFGPYVLA

VELASMLLLAGLVVAFHVGREERAGEVLSNRKDDSAKRKTEEHA

>gi|30063705|ref|NP_837876.1| NADH dehydrogenase subunit K [Shigella flexneri 2a str. 2457T]

MIPLQHGLILAAILFVLGLTGLVIRRNLLFMLIGLEIMINASALAFVVAGSYWGQTDGQVMYILAISLAA

AEASIGLALLLQLHRRRQNLNIDSVSEMRG

>gi|30063698|ref|NP_837869.1| hypothetical protein S2480 [Shigella flexneri 2a str. 2457T]

MIEWQDLHHSELSVSQLYALLQLRCAVFVVEQNCPYQDIDGDDLTGDNRHILGWKNDELVAYARILKSDD

DLEPVVIGRVIVSEALRGEKVGQQLMSKTLETCTHHWPDKPVYLGAQAHLQNFYQSFGFIPVTEVYEEDG

IPHIGMAREVIQA

>gi|30063697|ref|NP_837868.1| hypothetical protein S2479 [Shigella flexneri 2a str. 2457T]

MSNQFGDTRIDDDLTLLSETLEEVLRSSGDPADQKYVELKAHAEKALDDVKKRVSQASDSYYYRAKQAVY

RADDYVHEKPWQGIGVGAAVGLVLGLLLARR

>gi|30063695|ref|NP_837866.1| 2-succinyl-5-enolpyruvyl-6-hydroxy-3-cyclohexene-1-carboxylate synthase [Shigella flexneri 2a str. 2457T]

MSVSAFNRRWAAVILEALTRHGVRHICIAPGSRSTPLTLAAAENSAFIHHTHFDERGLGHLALGLAKVSK

QPVAVIVTSGTAVANLYPALIEAGLTGEKLILLTADRPPELIDCGANQAIRQPGMFASHPTHSISLPRPT

QDIPARWLVSTIDHALGTLHAGGVHINCPFAEPLYGEMDDTGLSWQQRLGDWWQDDKPWLREAPRLESEK

QRDWFFWRQKRGVVVAGRMSAEEGKKVALWAQTLGWPLIGDVLSQTGQPLPCADLWLGNAKATSELQQAQ

IVVQLGSSLTGKRLLQWQASCEPEEYWIVDDIEGRLDPAHHRGRRLIANIADWLELHPAEKRQPWCVEIP

RLAEQAMQAVIARRDAFGEAQLAHRISDYLPEQGQLFVGNSLVVRLIDALSQLPAGYPVYSNRGASGIDG

LLSTAAGVQRASGKPTLAIVGDLSALYDLNALALLRQVSAPLVLIVVNNNGGQIFSLLPTPQSERERFYL

MPQNVHFEHAAAMFELKYHHPQNWQELETAFADAWRTPTTTVIEMVVNDTDGAQTLQQLLAQVSHL

>gi|30063692|ref|NP_837863.1| O-succinylbenzoate synthase [Shigella flexneri 2a str. 2457T]

MRSAQVYRWQIPMDAGVVLRDRRLKTRDGLYVCLREGEREGWGEISPLPGFSQENWEEAQSVLLAWVNNW

LAGDCELPQMPSVAFGVSCALAELTDTLPQAANYRAAPLCNGDPDDLILKLADMPGEKVAKVKVGLYEAV

RDGMVVNLLLEAIPDLHLRLDANRAWTPLKGQQFAKYVNPDYRHRIAFLEEPCKTRDDSRAFARETGIAI

AWDESLREPDFAFVAEEGVRAVVIKPTLTGSLEKVREQVQAAHALGLTAVISSSIESSLGLTQLARIAAW

LTPDTIPGLDTLDLMQAQQVRRWPGSPLPLVDADVQEQLL

>gi|30063689|ref|NP_837860.1| sucrose-6 phosphate hydrolase [Shigella flexneri 2a str. 2457T]

MIWLTLVFASLLSVAGQLCQKQATCFVAINKRRKHIVLWLGLALACLGLAMMLWLLVLQNVPVGIAYPML

SLNFVWVTLAAVKLWHEPVSPRHWCGVAFIIGGIVILGSTV

>gi|30063688|ref|NP_837859.1| 4-amino-4-deoxy-L-arabinose transferase [Shigella flexneri 2a str. 2457T]

MKSVRYLIGIFAFIACYYLLPISTRLLWQPDETRYAEISREMLASGDWIVPHLLGLRYFEKPIAGYWINS

IGQWLFGANNFGVRAGVIFATLLTAALVTWFTLRLWRDKRLALLATVIYLSLFIVYAIGTYAVLDPFIAF

WLVAGMCSFWLAMQAQTWKGKSAGFLLLGITCGMGVMTKGFLALAVPVLSVLPWVATQKRWKDLFIYGWL

AVISCVLTVLPWGLAIAQREPDFWHYFFWVEHIQRFALDDAQHRAPFWYYVPVIIAGSLPWLGLLPGALY

TGWKNRKHSATVYLLSWTIMPLLFFSVAKGKLPTYILSCFAPLAMLLAHYALLAAKNNPLALRINGWINI

AFGVTGIIATFVISPWGPMNTPVWQTFESYKVFCAWSIFSLWAFFGWYTLTNVEKTWSFAALCPLGLALL

VGFSIPDRVMEGKHPQFFVEMTQESLQPSRYILTDSVGVAAGLAWSLQRDDIIMYRQTGELKYGLNYPDA

KGRFVSGDEFANWLNQHRQEGIITLVLSVDRDEDINSLAIPPADAIVRQERLVLIQYRPK

>gi|30063687|ref|NP_837858.1| hypothetical protein S2468 [Shigella flexneri 2a str. 2457T]

MTKVGLRIDVDAFRGTREGVPRLLEILSKHNIQASIFFSVGPDNMGRHLWRLVKPQFLWKMLRSNAASLY

GWDILLAGTAWPGKEIGHANADIIREAAKHHEVGLHAWDHHAWQARSGNWDRQTMIDDIARGLRTLEEII

GQPVTCSAAAGWRADQKVIEAKEAFHLRYNSDCRGAIPFRPLLESGNPGTAQIPVTLPTWDEVIGRDVKA

EDFNGWLLNRILRDKGTPVYTIHAEVEGCAYQHNFVDLLKRAAQEGVTFCPLSELLSETLPLGQVVRGNI

AGREGWLGCQQIAGSR

>gi|30063683|ref|NP_837854.1| protein induced by aluminum [Shigella flexneri 2a str. 2457T]

MKSKKYFIILLALAAIAGLGTHAAWSSNGLPRIDNKTLARLAQQHPVVVLFRHAERCDRSTNQCLSDKTG

ITVKGTQDARELGNAFSADISDFDLYSSNTVRTIQSATWFSAGKKLTVDKRLLQCGNEIYSAIKDLQSKA

PDKNIVIFTHNHCLTYIAKNKRDAIFKPDYLDGLVMHVEKGKVYLDGEFVNH

>gi|30063682|ref|NP_837853.1| hypothetical protein S2463 [Shigella flexneri 2a str. 2457T]

MADDRGVFPGQWALSGGGVESGERIEEALRREIREELGEQLLLTEITPWTFSDDIRTKTYADGRKEEIYM

IYLIFDCVSANREVKINEEFQDYAWVKPEDLVHYDLNVATRKTLRLKGLL

>gi|30063681|ref|NP_837852.1| hypothetical protein S2462 [Shigella flexneri 2a str. 2457T]

MKKIALAGLAGMLLVSASVNAMSISGQAGKEYTNIGVGFGTESTGLALSGNWTHNDDDGDVAGVGLGLNL

PLGPLMATVGGKGVYTNPNYGDEGYAAAVGGGLQWKIGNSFRLFGEYYYSPDSLSSGIQSYEEANAGARY

TIMRPVSIEAGYRYLNLSGKDGNRDNAVADGLYVGVNASF

>gi|30063679|ref|NP_837850.1| hypothetical protein S2460 [Shigella flexneri 2a str. 2457T]

MSNPPYGERGVGASVARAARWGRIENYMAQVNDSLCLLVQVESKTALDNLDEILDVEGIDGVFIGPADLS

ASLGYPDNAGHPEVQRIIETSIRRIRAAGKAAGFLAVAPDMAQQCLAWGANFVAVGVDTMLYSDALDQRL

AMFKSGKNGPRIKGSY

>gi|30063678|ref|NP_837849.1| hypothetical protein S2459 [Shigella flexneri 2a str. 2457T]

MTESTTSSPHDAVFKTFIFTPETARDFLEIHLPEPLRKLCNLQTLRLEPTSFIEKSLRAYYSDVLWSVET

SDGDGYIYCVIEHQSSAEKNMAFRPMRYATAAMQSHLDKGYDRVPLVVPLLFYHGETSPYPYSLNWLDEF

DDPQLARQLYTEAFPLVDITIVPDDEIMQHRRIALLELIQKHIRDHDLIGMVDRITTLLVRGFTNDSQLQ

TLFNYLLQCGDTSRFTRFIQEIAERSPLQKERLMTIAERLRQEGHQIGWQEGKLVGLQQGKLEGLQEGMH

EQAIKIALRMLEQGIDRDQVLAATQLSEADLAANNH

>gi|30063677|ref|NP_837848.1| sn-glycerol-3-phosphate dehydrogenase subunit C [Shigella flexneri 2a str. 2457T]

MNDTSFENCIKCTVCTTACPVSRVNPGYPGPKQAGPDGERLRLKDGALYDEALKYCINCKRCEVACPSDV

KIGDIIQRARAKYDTTRPSLRNFVLSHTDLMGSVSTPFAPIVNTATSLKPVRQLLDAALKIDHRRTLPKY

SFGTFRRWYRSIAAQQAQYKDQVAFFHGCFVNYNHPQLGKDLIKVLNAMGTGVQLLSKEKCCGVPLIANG

FTDKARKQAITNVESIREAVGVKGIPVIATSSTCTFALRDEYPEVLNVDNKGLRDHIELATRWLWRKLDE

GKTLPLKPLPLKVVYHTPCHMEKMGWTLYTLELLRNIPGLELTVLDSQCCGIAGTYGFKKENYPTSQAIG

APLFRQIEESGADLVVTDCETCKWQIEMSTSLRCEHPITLLAQALA

>gi|30063676|ref|NP_837847.1| anaerobic glycerol-3-phosphate dehydrogenase subunit B [Shigella flexneri 2a str. 2457T]

MRFDTVIMGGGLAGLLCGLQLQKHGLRCAIVTRGQSALHFSSGSLDLLSHLPDGQPVADIHSGLESLRQQ

APAHPYSLLGPQRVLDLACQAQALIAESGAQLQGSVELAHQRITPLGTLRATWLSSPEVPVWPLPAKKIC

VVGISGLMDFQAHLAAASLRELDLSVETAEIELPELDVLRNNATEFRAVNIARFLDNEENWPLLLDALIP

VANTCEMILMPACFGLADDKLWRWLNEKLPCSLMLLPTLPPSVLGIRLQNQLQRQFVRQGGVWMPGDEVK

KVTCKNGVVNEIWTRNHADIPLRPRFAVLASGSFFSGGLVAERNGIREPILGLDVLQTATRGEWYKGDFF

APQPWQQFGVTTDETLRPSQAGQTIENLFAIGSVLGGFDPIAQGCGGGVCAVSALHAAQQIAQRAGGQQ

>gi|30063671|ref|NP_837842.1| hypothetical protein S2452 [Shigella flexneri 2a str. 2457T]

MAVSAKYDEFNHWWATEGDWVEEPNYRRNGMSGVQCVERNGKKLYVKRMTHHLFHSVRYPFGRPTIVREV

AVIKELERAGVIVPKIVFGEAVKIEGEWRALLVTEDMAGFISIADWYARHAVSPYSDEVRQAMLKAVALA

FKKMHSVNRQHGCCYVRHIYVKTEGKAEAGFLDLEKSRRRLRRDKAINHDFRQLEKYLEPIPKADWEQVK

AYYYAM

>gi|30063666|ref|NP_837837.1| insertion sequence 2 OrfA protein [Shigella flexneri 2a str. 2457T]

MIVLILVFRLVIGEQIIDVLGPEKRRRRTTQEKIAIVQQSFEPGMTVSLVARQHGVAASQLFLWRKQYQE

GSLTAVAAGEQVVPASELAAAMKQIKELQRLLGKKTMENELLKEAVEYGRAKKWIAHAPLLPGDGE

>gi|30063663|ref|NP_837834.1| hypothetical protein S2442 [Shigella flexneri 2a str. 2457T]

MRHGLLALICWLCCVVAHSEMLNVEQSRLFRAWFVRIAQEQLRQGPSPRWYQQDCAGLVRFAANETLKVH

DSKWLKSNGLSSQYLPPEMTLTPEQRQLAQNWNQGSGKNGPYVTAINLIQYNSQFIGQDINQALPGDMIF

FDQGDAQHLMVWMGRYVIYHTGSATKTDNGMRAVSLQQLMTWKDTRWIPNDSNPNFIGIYRLNFLAR

>gi|30063660|ref|NP_837831.1| hypothetical protein S2436, partial [Shigella flexneri 2a str. 2457T]

MQVRWREPAKTAQQSNIPVTVERQLYRLIPGEEEMSFTLQPVTSNEIDSDALYLDEITLTSEQDAVLRYG

QVEVPLPPGADVERTTWGISVNKPNAAKQQGQLLEKARNEMGELAYMVPVKELTGTVTFRHLLRFSQKGQ

FVLPPARYVRSYAPAQQSVAAGSEWTGMQVK

>gi|30063659|ref|NP_837830.1| hypothetical protein S2435 [Shigella flexneri 2a str. 2457T]

MNWRRIVWLLALVTLPTLAEETPLQLVLRGAQHDQLYQLSSSGVTKVSALPDSLTTPLGSLWKLYVYAWL

EDTHQPEQPYQCRGNSPEEVYCCQAGESITRDTALVRSCGLYFAPQRLHIGADVWGQYWQQRQAPAWLAS

LTTLKPETSVTVKSLLDSLATLPAQNKAQEVLLDVVLDEAKIGVASMLGSRVRVKTWSWFADDKQEIRQG

GFAGWLTDGTPLWVTGSGTSKTVLTRYATVLNRVLPVPTQVASGQCVEVELFARYPLKKITAEKSTTAVK

PSVLNGRYRVTFTNGNHITFVSHGETTLLSEKGKLKLQSHLDREEYVARVLDREAKSTPPEAAKAMTVAI

RTFLQQNANREGDCLTIPDSSATQRVSASPATTGARTMTAWTQELIYAGDPVHYHGSRATEGTLSWRQAT

AQAGQGERYDQILAFAYPDNSLSRWGAPRSTCQLLPKAKAWLAKKMPQWRRILQGETGYNEPDVFAVCRL

VSGFPYTDRQQKRLFIRNFFTLQDRLDLTHEYLHLAFDGYPTGLDENYIETLTRQLLMD

>gi|30063658|ref|NP_837829.1| hypothetical protein S2434 [Shigella flexneri 2a str. 2457T]

MRKIFLPLLLVALSPVAHSEGVQEVEIDAPLSGWHPAEGEDASFSQSINYPASSVNMADDQNISAQIRGK

IKNYAAAGKVQQGRLVVNGASMPQRIESDGSFARPYIFTEGSNSVQVISPDGQSRQKMQFYSTPGTGTIR

ARLRLVLSWDTDNTDLDLHVVTPDGEHAWYGNTVLKNSGALDMDVTTGYGPEIFAMPAPVHGRYQVYINY

YGGRSETELTTAQLTLITDEGSVNEKQETFIVPMRNAGELTLVKSFDW

>gi|30063657|ref|NP_837828.1| hybrid sensory kinase in two-component regulatory system with RcsB and YojN [Shigella flexneri 2a str. 2457T]

MKYLASFRTTLKASRYMFRALALVLWLLIAFSSVFYIVNALHQRESEIRQEFNLSSDQAQRFIQRTSDVM

KELKYIAENRLSAENGVLSPRGRETQADVPAFEPLFADSDCSAMSNTWRGSLESLAWFMRYWRDNFSAAY

DLNRVFLIGSDNLCMANFGLRDMPVERDTALKALHERINKYRNAPQDDSGSNLYWISEGPRPCVGYFYAL

TPVYLANRLQALLGVEQTIRMENFFLPGTLPMGVTILDENGHTLISLTGPESKIKGDPRWMQERSWFGYT

EGFRELVLKKNLPPSSLSIVYSVPVDKVLERIRMLILNAILLNVLAGAALFTLARMYERRIFIPAESDAL

RLEEHEQFNRKIVASAPVGICILRTADGVNILSNELAHTYLNMLTHEDRQRLTQIICGQQVNFVDVLTSN

NTNLQISFVHSRYRNENVAICVLVDVSSRVKMEESLQEMAQAAEQASQSKSMFLATVSHELRTPLYGIIG

NLDLLQTKELPKGVDRLVTAMNNSSSLLLKIISDILDFSKIESEQLKIEPREFSPREVMNHITANYLPLV

VRKQLGLYCFIEPDVPVALNGDPMRLQQVISNLLSNAIKFTDTGCIVLHVRADGDYLSIRVRDTGVGIPA

KEVVRLFDPFFQVGTGVQRNFQGTGLGLAICEKLISMMDGDISVDSEPGMGSQFTVRIPLYGAQYPQKKG

VEGLSGKRCWLAVRNASLCQFLETSLQRSGIVVTTYEGQEPTPEDVLITDEVVSKKWQGRAVVTFCRRHI

GIPLEKAPGEWVHSVAAPHELPALLARIYLIEMESDDPANALPSTDKAVSDNDDMMILVVDDHPINRRLL

ADQLGSLGYQCKTANDGVDALNVLSKNHIDIVLSDVNMPNMDGYRLTQRIRQLGLTLPVIGVTANALAEE

KQRCLESGMDSCLSKPVTLDVIKQTLTVYAERVRKSRDS

>gi|30063656|ref|NP_837827.1| transcriptional regulator RcsB [Shigella flexneri 2a str. 2457T]

MNNMNVIIADDHPIVLFGIRKSLEQIEWVNVVGEFEDSTALINNLPKLDAHVLITDLSMPGDKYGDGITL

IKYIKRHFPSLSIIVLTMNNNPAILSAVLDLDIEGIVLKQGAPTDLPKALAALQKGKKFTPESVSRLLEK

ISAGGYGDKRLSPKESEVLRLFAEGFLVTEIAKKLNRSIKTISSQKKSAMMKLGVENDIALLNYLSSVTL

SPADKD

>gi|30063655|ref|NP_837826.1| phosphotransfer intermediate protein in two-component regulatory system with RcsBC [Shigella flexneri 2a str. 2457T]

MRQKETTATTRFSLLPGSITRFFLLLIIVLLVTMGVMVQSAVNAWLKDKSYQIVDITHAIQKRVDTWRYV

TRQIYDNIAATTSPSSGEGLQETRLKQDVYYLEKPRRKTEALIFGSHDNSTLEMTQRMSTYLDTLWGAEN

VPWSMYYLNGQDNSLVLISTLPLKDLTSGFKESTVSDIVDSRRAEMLQQANALDERESFSNMRRLAWQNG

HYFTLRTTFNQPGHLATVVAFDLPINDLIPPGMPLDSFRLEPDATATGNNDNEKEGTDSVSIHFNSTKIE

ISSALNSTDMRLVWQVPYGTLLLDTLQNILLPLLLNIGLLALALFGYTTFRHFSSRSTESVPSTAVNNEL

RILRAINEEIVSLLPLGLLVHDQESNRTVISNKIADHLLPHLNLQNITTMAEQHQGIIQATINNELYEIR

MFRSQVAPRTQIFIIRDQDREVLVNKKLKQAQRLYEKNQQGRMTFMKNIGDALKEPAQSLAESAAKLNAP

ESKQLANQADVLVRLVEEIQLANMLADDSWKSETVLFSVQDLIDEVVPSVLPAIKRKGLQLLINNHLKAH

DMRRGDRDTLRRILLLLMQYAVTSTQLGKITLEVDQDESSEDRLTFRILDTGEGVSIHEMDNLHFPFINQ

TQNDRYGKADPLAFWLSDQLARKLGGHLNIKTRDGLGTRYSVHIKMLAADPEVEEEEERLLDDVCVMVDV

TSAEIRNIVTRLLENWGATCITPDERLISQDYDIFLTDNPSNLTASGLLLSDDESGVREIGPGQLCVNFN

MSNAMQEAVLQLIEVQLAQEEVTESPLGGDENAQLHASGYYALFVDTVPDDVKRLYTEAATSDFAALAQT

AHRLKGVFAMLNLVPGKQLCETLEHLIREKDVPGIEKYISDIDSYVKSLL

>gi|30063654|ref|NP_837825.1| outer membrane porin protein C [Shigella flexneri 2a str. 2457T]

MKVKVLSLLVPALLVAGAANAAEVYNKDGNKLDLYGKVDGLHYFSDDKSVDGDQTYMRLGFKGETQVTDQ

LTGYGQWEYQIQGNSAENENNSWTRVAFAGLKFQDVGSFDYGRNYGVVYDVTSWTDVLPEFGGDTYGSDN

FMQQRGNGFATYRSTDFFGLVDGLNFAVQYQGKNGSPEGEGMTNNGREALRQNGDGVGGSITYDYEGFGI

GAAVSSSKRTDDQNFGLNRYDERYIGNGDRAETYTGGLKYDANNIYLAAQYTQTYNATRVGNLGWANKAQ

NFEAVAQYQFDFGLRPSLAYLQSKGKNLGVINGRNYDDEDILKYVDVGATYYFNKNMSTYVDYKINLLDD

NQFTRDAGINTDNIVALGLVYQF

>gi|30063653|ref|NP_837824.1| thiamine biosynthesis lipoprotein ApbE [Shigella flexneri 2a str. 2457T]

MEISFTRVALLVAALFFVGCDQKPQPAKTHATEVTVLEGKTMGTFWRASIPGIDAKRSAELKEKIQTQLD

ADDQLLSTYKKDSALMRFNDSQSLSPWPVSEAMADIVTTSLRIGARTDGAMDITVGPLVNLWGFGPEQQP

VQIPSQEQIDAMKAKTGLQHLTVINQSHQQYLQKDLPDLYVDLSTVGEGYAADHLARLMEQEGISRYLVS

VGGALNSRGMNGEGQPWRVAIQKPTDKENAVQAVVDINGHGISTSGSYRNYYELDGKRLSHVIDPQTGRP

IEHNLVSVTVIAPTALEADAWDTGLMVLGPEKAKEVVRREGLAVYMITKEGDSFKTWMSPQFKSFLVSEK

N

>gi|30063649|ref|NP_837820.1| ecotin [Shigella flexneri 2a str. 2457T]

MKTILPEVLFAAFATTSAWAAESVQPLEKIAPYPQAETGMKRQVIQLTPQEDESTLKVELLIGQTLEVDC

NLHRLGGKLESKTLEGWGYDYYVFDKVSSPVSTMMACPDGKKEKKFVTAYLGDAGMLRYNSKLPIVVYTP

DNVDVKYRVWKAEEKIDNAEVR

>gi|30063648|ref|NP_837819.1| ferredoxin-type protein [Shigella flexneri 2a str. 2457T]

MKIDASRRGILTGCWRKASNGIRPPWSGDESHFLTHCTRCDACINACENNILQRGTGGYPSVNFKNNECS

FCYACAQACPESLFSPRHTRAWDLQFTIGDACLAYQSVECRRCQDSCEPMAIIFRPTLSGIYQAQLNSQL

CNGCGACAASCPVSAITAEYLHAH

>gi|30063646|ref|NP_837817.1| nitrate reductase catalytic subunit [Shigella flexneri 2a str. 2457T]

MKLSRRSFMKANAVAAAAAAAAGLSVPGVARAVVGQQEAIKWDKAPCRFCGTGCGVLVGTQQGRVVACQGD

PDAPVNRGLNCIKGYFLPKIMYGKDRLTQPLLRMKNGKYDKEGEFTPITWDQAFDVMEEKFKTALKEKGP

ESIGMFGSGQWTIWEGYAASKLFKAGFRSNNIDPNARHCMASAVVGFMRTFGMDEPMGCYDDIEQADAFV

LWGANMAEMHPILWSRITNSRLSNQNVTVAVLSTYQHRSFELADNGIIFTPQSDLVILNYIANYIIQNNA

INQDFFSKHVNLRKGATDIGYGLRPTHPLEKAAKNPGSDASEPMSFEDYKAFVAEYTLEKTAEMTGVPKD

QLEQLAQLYADPNKKVISYWTMGFNQHTRGVWANNLVYNLHLLTGKISQPGCGPFSLTGQPSACGTAREV

GTFAHRLPADMVVTNEKHRDICEKKWNIPSGTIPAKIGLHAVAQDRALKDGKLNVYWTMCTNNMQAGPNI

NEERMPGWRDPRNFIIVSDPYPTVSALAADLILPTAMWVEKEGAYGNAERRTQFWRQQVQAPGEAKSDLW

QLVQFSRRFKTEEVWPEELLAKKPELRGKTLYEVLYATPEVSKFPVSELAEDQLNDESRELGFYLQKGLF

EEYAWFGRGHGHDLAPFDDYHKARGLRWPVVNGKETQWRYSEGNDPYVKAGEGYKFYGKPDGKAVIFALP

FEPAAEAPDEEYDLWLSTGRVLEHWHTGSMTRRVPELHRAFPEAVLFIHPLDAKARDLRRGDKVKVVSRR

GEVISIVETRGRNRPPQGLVYMPFFDAAQLVNKLTLDATDPLSKETDFKKCAVKLEKV

>gi|30063645|ref|NP_837816.1| quinol dehydrogenase periplasmic component [Shigella flexneri 2a str. 2457T]

MSRSAKPQNGRRRFLRDVVRTAGGLAAVGVALGLQQQTARASGVRLRPPGAINENAFASACVRCGQCVQA

CPYDTLKLATLASGLSAGTPYFVARDIPCEMCEDIPCAKVCPSGALDREIESIDDARMGLAVLVDQENCL

NFQGLRCDVCYRECPKIDEAITLELERNTRTGKHARFLPTVHSDACTGCGKCEKVCVLEQPAIKVLPLSL

AKGELGHHYRFGWLEGNNGKS

>gi|30063644|ref|NP_837815.1| quinol dehydrogenase membrane component [Shigella flexneri 2a str. 2457T]

MANRKRDAGREALEKKGWWRSHRWLVLRRLCQFFVLGMFLSGPWFGVWILHGNYSSSLLFDTVPLTDPLM

TLQSLASGHLPATVALTGAVIITVLYALAGKRLFCSWVCPLNPITDLANWLRRRFDLNQSATIPRHIRYV

LLVVILVGSALTGTLIWEWINPVSLMGRSLVMGFGSGALLILALFLFDLLVVEHGWCGHICPVGALYGVL

GSKGVITVAASDRQKCNRCMDCFHVCPEPHVLRAPVLDEQSPVQVTSRDCMTCGRCVDVCSEDVFTITTR

WSSGAKS

>gi|30063643|ref|NP_837814.1| citrate reductase cytochrome c-type subunit [Shigella flexneri 2a str. 2457T]

MKSHDLKKALCQWTAMLALVVSGAVWAANGVDFSQSPEVSGTQEGAIRMPKEQDRMPLNYVNQPPMIPHS

VEGYQVTTNTNRCLQCHGVESYRTTGAPRISPTHFMDSDGKVGAEVAPRRYFCLQCHVPQADTAPIVGNT

FTPSKGYGK

>gi|30063642|ref|NP_837813.1| cytochrome c-type protein NapC [Shigella flexneri 2a str. 2457T]

MGNSDRKPGLIKRLWKWWRTPSHLALGTLLLIGFVGGIVFWGGFNTGMEKANTEEFCISCHEMRNTVYQE

YMDSVHYNNRSGVRATCPDCHVPHEFVPKMIRKLKASKELYGKIFGVIDTPQKFEAHRLTMAQNEWRRMK

DNNSQECRNCHNFEYMDTTAQKSVAAKMHDQAVKDGQTCIDCHKGIAHKLPDMREVEPGF

>gi|30063640|ref|NP_837811.1| heme exporter protein B, cytochrome c-type biogenesis protein [Shigella flexneri 2a str. 2457T]

MMFWRIFRLELRVAFRHSAEIANPLWFFLIVITLFPLSIGPEPQLLARIAPGIIWVAALLSSLLALERLF

RDDLQDGSLEQLMLLPLPLPAVVLAKVMAHWMVTGLPLLILSPLVAMLLGMDVYGWQVMALTLLLGTPTL

GFLGAPGVALTVGLKRGGVLLSILVLPLTIPLLIFATAAMDAASMHLPVDGYLAILGALLAGTATLSPFA

TAAALRISIQ

>gi|30063639|ref|NP_837810.1| heme exporter protein C [Shigella flexneri 2a str. 2457T]

MWKTLHQLAIPPRLYQICGWFIPWLAIASVVVLTVGWIWGFGFAPADYQQGNSYRIIYLHVPAAIWSMGI

YASMAVAAFIGLVWQMKMANLAVAAMAPIGAVFTFIALVTGSAWGKPMWGTWWVWDARLTSELVLLFLYV

GVIALWHAFDDRRLAGRAAGILVLIGVVNLPIIHYSVEWWNTLHQGSTRMQQSIDPAMRSPLRWSIFGFL

LLSATLTLMRMRNLILLMEKRRPWVSELILKRGRK

>gi|30063637|ref|NP_837808.1| cytochrome c-type biogenesis protein CcmE [Shigella flexneri 2a str. 2457T]

MNIRRKNRLWIACAVLAGLALTIGLVLYALRSNIDLFYTPGEILYGKRETQQMPEVGQRLRVGGMVMPGS

VQRDPNSLKVTFTIYDAEGSVDVSYEGILPDLFREGQGVVVQGELEKGNHILAKEVLAKHDENYTPPEVE

KAMEANHRRPASVYKDPAS

>gi|30063636|ref|NP_837807.1| cytochrome c-type biogenesis protein [Shigella flexneri 2a str. 2457T]

MMPEIGNGLLCLALGIALLLSVYPLWGVARGDARMMASSRLFAWLLFMSVAGAFLVLVNAFVVNDFTVTY

VASNSNTQLPVWYRVAATWGAHEGSLLLWVLLMSGWTFAVAIFSQRIPLDIVARVLAIMGMVSVGFLLFI

LFTSNPFSRTLPNFPIEGRDLNPLLQDPGLIFHPPLLYMGYVGFSVAFAFAIASLLSGRLDSTYARFTRP

WTLAAWIFLTLGIVLGSAWAYYELGWGGWWFWDPVENASFMPWLVGTALMHSLAVTEQRASFKAWTLLLA

ISAFSLCLLGTFLVRSGVLVSVHAFASDPARGMFILAFMVLVIGGSLLLFAARGHKVRSRVNNALWSRES

LLLANNVLLVAAMLVVLLGTLLPLVHKQLGLGSISIGEPFFNTMFTWLMVPFALLLGVGPLVRWGRDRPR

KIRNLLIIAFISTLVLSLLLPWLFESKVVAMTVLGLAMACWIAVLATAEAALRISRGTKTTFSYWGMVAA

HLGLAVTIVGIAFSQNYSVERDVRMKSGDSVDIHEYRFTFRDVKEVTGPNWRGGVATIGVTREGKPETVL

YAEKRYYNTAGSMMTEAAIDGGITRDLYAALGEELENGAWAVRLYYKPFVRWIWAGGLMMALGGLLCLFD

PRYRKRVSPQKTAPEAV

>gi|30063635|ref|NP_837806.1| disulfide oxidoreductase [Shigella flexneri 2a str. 2457T]

MKRKVLLIPLIIFLAIAAALLWQLARNAEGDDPTNLESALIGKPVPKFRLESLDNPGQFYQADVLTQGKP

VLLNVWATWCPTCRAEHQYLNQLSAQGIRVVGMNYKDDRQKAISWLKELGNPYALSLFDGDGMLGLDLGV

YGAPETFLIDGNGIIRYRHAGDLNPRVWEEEIKPLWEKYSKEAAQ

>gi|30063634|ref|NP_837805.1| subunit of heme lyase [Shigella flexneri 2a str. 2457T]

MRFLLGVLMLMISGSALATIDVLQFKDEAQEQQFRQLTEELRCPKCQNNSIADSNSMIATDLRQKVYELM

QEGKSKKEIVDYMVARYGNFVTYDPPLTPLTVLLWVLPVVAIGIGGWVIYARSRRRVRVVPEAFPEQSVP

EGKRAGYVVYLPGIVVALIVAGVSYYQTGNYQQVKIWQQATAQAPALLDRALDPKADPLNEEEMSRLALG

MRTQLQKNPGDIEGWIMLGRVGMALGNASIATDAYATAYRLDPKNSDAALGYAEALTRSSDPNDNRLGGE

LLRQLVRTDHSNIRVLSMYAFNAFEQQRFGEAVAAWEMMLKLLPANDTRRAVIERSIAQAMQHLSPQESK

>gi|30063633|ref|NP_837804.1| transcriptional regulator NarP [Shigella flexneri 2a str. 2457T]

MPEATPFQVMIVDDHPLMRRGVRQLLELDPGFEVVAEAGEGASAIDLANRLDIDVILLDLNMKGMSGLDT

LNALRRDGVTAQIIILTVSDASSDVFALIDAGADGYLLKDSDPEVLLEAIRTGAKGSKVFSERVNQYLRE

REMFGAEEDPFSVLTERELDVLHELAQGLSNKQIASVLNISEQTVKVHIRNLLRKLNVRSRVAATILFLQ

QRGAQ

>gi|30063632|ref|NP_837803.1| ATP-binding component of a transport system [Shigella flexneri 2a str. 2457T]

MASTVEYGETVDGVILEKDIQLVYGTANNTKINPGGEQHIKEFGVSSNTEINGGYQYIEMNGTAEYSVLN

DGYQIVQMGGAANQTTLNNGVLQVYGPANDTTIKGGRLIVEKDGGAVFAAIEKGGLLEVKEGGFALAVDQ

KTGGAIKTTTRAMEVFGTNRLGQFDIKNGIANNMLLENGGSLRVEENDFAYNTTVDSGGLLEVMDGGTAT

GVDKKAGGKLIVSTNALEVSGTNSKGQFSIKDGVSKNYELDDGSGLIVMEDTQAIDTILDEHATMQSLGK

DTGTKVQANAVYDLGRSDQNGSITYSSKAISENMVINNGRANVWAGTMVNVSVRGNDGILEVMKPQINYA

PAMLVGKVVVSEGASFRTHGAVDTSKADVSLENSAWTIIADITTTNQNTRLNLANLAMSGANVIMMAEPV

TRSSVTASAENFITLTTNTLSGNGNFYMRTDMANHQSDQLNVTGQATGDFKIFVTDTGASPAAGDSLTLV

TTGGGDAAFTLGNAGGVVDIGTYEYTLLDNGNHSWSLAENRAQITPSTTDVLNMAAAQPLVFDAELDTVR

ERLGSVKGVSYDTVMWSSAINTRNNVTTDAGAGFEQTLTGLTLGIDSRFSREESSTIRGLFFGYSHSDIG

FDRGGKGNIDSYTLGAYAGWEHQNGAYVDGVVKVDRFANTIHGKMSNGATAFGDYNSNGAGAHVESGFRW

VDGLWSVRPYLAFTGFTTDGQDYTLSNGMRADVGNTRILRAEAGTAVSYHMDLQNGTTLEPWLKAAVRQE

YADSNQVKVNDDGKFNNDVAGTSGVYQAGIRSSFTPTLSGHLSVSYGNGAGVESPWNTQAGVVWTF

>gi|30063631|ref|NP_837802.1| sulfatase [Shigella flexneri 2a str. 2457T]

MVTHRQRYREKVSQMVSWGHWFALFNILLSLVIGSRYLFIADWPTTLAGRIYSYVSIIGHFSFLVFATYL

LILFPLTFIVGSQRLMRFLSVILATAGMTLLLIDSEVFTRFHLHLNPIVWQLVINPDENEMARDWQLMFI

SVPVILLLELVFATWSWQKLRSLTRRRRFARPLAAFLFIAFIASHVVYIWADANFYRPITMQRANLPLSY

PMTARRFLEKHGLLDAQEYQRRLIEQGNPDAVSVQYPLSELRYRDMGTGQNVLLITVDGLNYSRFEKQMP

ALAGFAEQNISFTRHMSSGNTTDNGIFGLFYGISPSYMDGILSTRTPAALITALNQQGYQLGLFSSDGFT

SPLYRQALLSDFSMPSVRTQSDDQTATQWINWLGRYAQEDNRWFSWVSFNGTNIDDSNQQAFARKYSRAA

GNVDDQIKRVLNALRDSGKLDNTVVIITAGRGIPLSEEEETFDWSHGHLQVPLVIHWPGTPAQRINALTD

HTDLMTTLMQRLLHVSTPASEYSQGQDLFNPQRRHYWVTAADNDTLAITTPKKTLVLNNNGKYRTYNLRG

ERVKDEKPQLSLLLQVLTDEKRFIAN

>gi|30063629|ref|NP_837800.1| nucleoid-associated protein NdpA [Shigella flexneri 2a str. 2457T]

MSLDINQIALHQLIKRDEQNLELVLRDSLLEPTETVVEMVAELHRVYSAKNKAYGLFSEESELAQTLRLQ

RQGEEDFLAFSRAATGRLRDELAKYPFADGGFVLFCHYRYLAVEYLLVAVLSNLSSMRVNENLDINPTHY

LDINHADIVARIDLTEWETNPESTRYLTFLKGRVGRKVADFFMDFLGASEGLNAKAQNRGLLQAVDDFTA

EAQLDKAERQNVRQQVYSYCNEQLQAGEEIELESLSKELAGVSEVSFTEFAAEKGYELEESFPADRSTLR

QLTKFAGSGGGLTINFDAMLLGERIFWDPATDTLTIKGTPPNLRDQLQRRTSGGN

>gi|30063626|ref|NP_837797.1| 16S rRNA pseudouridylate synthase A [Shigella flexneri 2a str. 2457T]

MRLDKFIAQQLGVSRAIAGREIRGNRVTVDGEIVRNAAFKLLPEHDVAYDGNPLAQQHGPRYFMLNKPQG

YVCSTDDPDHPTVLYFLDEPVAWKLHAAGRLDIDTTGLVLMTDDGQWSHRITSPRHHCEKTYLVTLESPV

ADDTAEQFAKGVQLHNEKDLTKPAVLEVITPTQVRLTISEGRYHQVKRMFAAVGNHVVELHRERIGGITL

DADLAPGEYRPLTEEEIASVV

>gi|30063625|ref|NP_837796.1| bicyclomycin/multidrug efflux system [Shigella flexneri 2a str. 2457T]

MTTRQHSSFAIVFILGLLAMLMPLSIDMYLPALPVISAQFGVSAGSTQMTLSTYILGFALGQLIYGPMAD

SFGRKPVVLGGTLVFAAAAVACALAQTIDQLIVMRFFHGLAAAAASVVINALMRDIYPKEEFSRMMSFVM

LVTTIALLMAPIVGGWVLVWLSWHYIFWILALAAILASAMIFFLIKETLPPERRQPFHIRTTIGNFAALF

RHKRVLSYMLASGFSFAGMFSFLSAGPFVYIEINHVAPENFGYYFALNIVFLFVMTIFNSRFVRRIGALN

MFRSGLWIQFIMAAWMVISAPLGLGFWSLVVGVAAFVGCVSMVSSNAMAVILDEFPHMAGTASSLAGTFR

FGIGAIVGALLSLATFNSAWPMIWSIAFCATSSILFCLYASRPKKR

>gi|30063624|ref|NP_837795.1| hypothetical protein S2397 [Shigella flexneri 2a str. 2457T]

MTSLQLSIVHRLPQNYRWSAGFAGSKVEPIPQNGPCGDNSLVALKLLSPDGDNAWSVMYKLSQALSDIEV

PCSVLECEGEPCLFVNRQDEFAATCRLKNFGVAIAEPFSNYNPF

>gi|30063622|ref|NP_837793.1| transport system permease [Shigella flexneri 2a str. 2457T]

MSRLSPVNQARWARFRHNRRGYWSLWIFLVLFGLSLCSELIANDKPLLVRYDGSWYFPLLKNYSESDFGG

PLASQADYQDPWLKQRLENNGWVLWAPIRFGATSINFATDKPFPSPPSRQNWLGTDANGGDVLARILYGT

RISVLFGLMLTLCSSVMGVLAGALQGYYGGKVDLWGQRFIEVWSGMPTLFLIILLSSVVQPNFWWLLAIT

VLFGWMSLVGVVRAEFLRTRNFDYIRAAQALGVSDRSIILRHMLPNAMVATLTFLPFILCSSITTLTSLD

FLGFGLPLGSPSLGELLLQGKNNLQAPWLGITAFLSVAILLSLLIFIGEAVRDAFDPNKAV

>gi|30063621|ref|NP_837792.1| transport system permease [Shigella flexneri 2a str. 2457T]

MGAYLIRRLLLVIPTLWAIITINFFIVQIAPGGPVDQAIAAIEFGNAGVLPGAGGEGVRASHAQTGVGNI

SDSNYRGGRGLDPEVIAEITHRYGFDKPIHERYFKMLWDYIRFDFGDSLFRSASVLTLIKDSLPVSITLG

LWSTLIIYLVSIPLGIRKAVYNGSRFDVWSSAFIIIGYAIPAFLFAILLIVFFAGGSYFDLFPLRGLVSA

NFDSLPWYQKITDYLWHITLPVLATVIGGFAALTMLTKNSFLDEVRKQYVVTARAKGVSEKNILWKHMFR

NAMLLVIAGFPATFISMFFTGSLLIEVMFSLNGLGLLGYEATVSRDYPVMFGTLYIFTLIGLLLNIVSDI

SYTLVDPRIDFEGR

>gi|30063620|ref|NP_837791.1| hypothetical protein S2393 [Shigella flexneri 2a str. 2457T]

MIVRILLLFIALFTFGAQAQAIKESYTFAVLGEPRYAFNFNHFDYVNPAAPKGGQITLSALGTFDNFNRY

ALRGNPGARTEQLYDTLFTTSDDEPGSYYPLIAESARYADDYSWVEVAINPRARFHDGSPITARDVEFTF

QKFLTEGVPQFRLVYKGTTVKAIAPLTVRIELAKPGKEDMLSLFSLPVFPEKYWKDHKLSDPLATPPLAS

GPYHVTSWKMGQNIVYSRVKDYWAANLPVNRGRWNFDTIRYDYYLDDNVAFEAFKAGAFDLRMENDAKNW

ATRYTGKNFDKKYIIKDEQKNESAQDTRWLAFNIQRAVFSDRRVREAITLAFDFEWMNKALFYNAWSRTN

SYFQNTEYAARNYPDAAELVLLAPMKKDLPPEVFTQIYQPPVSKGDGYDRDNLLKADKLLNEAGWVLKGQ

QRVNATTGQPLSFELLLPASSNSQWVLPFQHSLQRLGINMDIRKVDNSQITNRMRNRDYDMMPRLWRAMP

WPSSDLQISWSSEYINSTYNAPGVQSPVIDSLINQIIAAQGNKEKLLPLGRALDRVLTWNYYMLPMWYMA

EDRLAWWDKFSQPAVRPVYSLGIDTWWYDVNKAAKLPSARQQGE

>gi|30063619|ref|NP_837790.1| hypothetical protein S2392 [Shigella flexneri 2a str. 2457T]

MFIRAPNSGRKLLLTCIVAGVMIAILVSCLQFLVAWHKHEVKYDTLIIDVQKYLDTYFADLKSTTDRLQP

LTLDTCQQANPELTARAAFSMNVRTFVLVKDKKTFCSSATGEMDIPLNELIPALDINKNVDMAILPGTPM

VPNKPAIVIWYRNPLLKNSGVFAALNLNLTPSLFYSSRQEDYDGVALIIGNTALSTFSSRLMNVNELTDM

PVRETKIAGIPLTVRLYADDWTWNDVWYAFLLGGMSGTVVGLLCYYLMSVRMRPDREIMTAIKREQFYVA

YQPVVDTQALRVTGLEVLLRWRHPVAGEIPPDAFINFAESQKMIVPLTQHLFELIARDAAELEKVLPVGV

KFGINIAPDHLHSESFKADIQKLLTSLPAHHFQIVLEITERDMLKEQEATQLFAWLHSVGVEIAIDDFGT

GHSALIYLERFTLDYLKIDRGFINAIGTETITSPVLDAVLTLAKRLNMLTVTEGVETPEQARWLSERGVN

FMQGYWISHPLPLDDFVRWLKKPYTPQW

>gi|30063618|ref|NP_837789.1| outer membrane lipoprotein [Shigella flexneri 2a str. 2457T]

MVKSQPILRYILRGIPAIAVAVLLSACSANNTAKNMHPETRAVGSETSSLQASQDEFENLVRNVDVKSRI

MDQYADWKGVRYRLGGSTKKGIDCSGFVQRTFREQFGLELPRSTYEQQEMGKSVSRSNLRTGDLVLFRAG

STGRHVGIYIGNNQFVHASTSSGVIISSMNEPYWKKRYNEARRVLSRS

>gi|30063617|ref|NP_837788.1| hypothetical protein S2390 [Shigella flexneri 2a str. 2457T]

MIKNLPQIVLLNIVGPALFLSWYIPVNHGFWLPIDADIFYFFNQKLVESKAFLWLVALTNNRAFDGCSLL

AMGMLMLSFWLKENAPGRRRIVIMGLVMLLTAVVLNQLGQALIPVKRASPTLTFTDINRVSELLSVPTKD

ASRDSFPGDHGMMLLIFSAFMWRYFGKVAGLIALIIFMVFAFPRVMIGAHWFTDIIVGSMTVILIGLPWV

LLTPLSDRLITFFDKSLPGKNKHFQNK

>gi|30063615|ref|NP_837786.1| oxidoreductase [Shigella flexneri 2a str. 2457T]

MKTIASVTLPHHVHAPRYDRQQLQSRIVHFGFGAFHRAHQALLTDRVLNAQGGDWGICEISLFSGDQLMS

QLRAQNHLYTVLEKGADGNQVIIVGAVHECLNAKLDSLAAIIEKFCEPQVAIVSLTITEKGYCIDPATGA

LDTSNPRIIHDLQTPEEPHSAPGILVEALKRRRERSLTPFTVLSCDNIPDNGHVVKNAVLRMAEKRSPEL

AGWIKEHVSFPGTMVDRIVPAATDESLAEISQHLGVNDPCAISCEPFIQWVVEDNFIAGRPAWEVAGVQM

VNDVLPWEEMKLRMLNGSHSFLAYLGYLSGFAYISDCMQDRVFRYAARTLMLDEQAPTLQIKDVDLTQYA

DKLIARFANPSLKHKTWQIAMDGSQKLPQRMLAGIRIHLGRETDWSLLALGVAGWMRYVSGVDDAGNAID

VRDPLSDKIRELVAGSSSEQRVTALLSLREVFGDDLPDNPHFVQAIEQAWQQIVQFGAHQALLNTLKI

>gi|30063611|ref|NP_837782.1| transport [Shigella flexneri 2a str. 2457T]

MHNSPAVSSAKSFDLTSTAFLIVAFLTGIAGALQTPTLSIFLTDEVHARPAMVGFFFTGSAVIGILVSQF

LAGRSDKRGDRKSLIVFCCLLGVLACTLFAWNRNYFVLLFVGVFLSSFGSTANPQMFALAREHADKTGRE

AVMFSSFLRAQVSLAWVIGPPLAYALAMGFSFTVMYLSAAVAFIVCGVMVWLFLPSMQKELPLATGTVEA

PRRNRRDTLLLFVICTLMWGSNSLYIINMPLFIINELHLPEKLAGVMMGTAAGLEIPTMLIAGYFAKRLG

KRFLMRVAAVGGVCFYAGMLMAHSPAILLGLQLLNAIFIGILGGIGMLYFQDLMPGQAGSATTLYTNTSR

VGWIIAGSVAGIVAEIWNYHAVFWFAMVMIIATLFWVMTPTY

>gi|30063610|ref|NP_837781.1| bifunctional fructose-specific PTS IIA/HPr protein [Shigella flexneri 2a str. 2457T]

MFQLSVQDIHPGEKAGDKEEAIRQVAAALVQAGNVAEGYVNGMLAREQQTSTFLGNGIAIPHGTTDTRDQ

VLKTGVQVFQFPEGVTWGDGQVAYVAIGIAASSDKHLGLLRQLTHVLSDDSVAEQLKSATTAEELRALLM

GEKQSEQLKLDNEMLTLDIVASDLLTLQALNAARLKEARAVDATFVTKAINEQPLNLGQGIWLSDSAEGN

LRSAIAVSRAANAFDVDGETAAMLVSVAMNDDQPIAVLKRLADLLLDNKADRLLKADAATLLALLTSDDA

PTDDVLSAEFVVRNEHGLHARPGTMLVNTIKQFNSDITVTNLDGTGKPANGRSLMKVVALGVKKGHRLRF

TAQGADAEQALKAIGDAIAAGLGEGA

>gi|30063608|ref|NP_837779.1| fructose-specific PTS system IIBC component [Shigella flexneri 2a str. 2457T]

MKTLLIIDANLGQARAYMAKTLLGAAARKAKLEIIDNPNDAEMAIVLGDSIPNDSALNGKNVWLGDISRA

VAHPELFLSEAKGHAKPYTAPVTATAPVAASGPKRVVAVTACPTGVAHTFMAAEAIETEAKKRGWWVKVE

TRGSVGAGNAITPEEVAAADLVIVAADIEVDLAKFAGKPMYRTSTGLALKKTSQELDKAVAEATPYEPAG

KAQTATTEGKKESAGAYRHLLTGVSYMLPMVVAGGLCIALSFAFGIEAFKEPGTLAAALMQIGGGSAFAL

MVPVLAGYIAFSIADRPGLTPGLIGGMLAVSTGSGFIGGIIAGFLAGYIAKLISTQLKLPQSMEALKPIL

IIPLISSLVVGLAMIYLIGKPVAGILEGLTHWLQTMGTANAVLLGAILGGMMCTDMGGPVNKAAYAFGVG

LLSTQTYGPMAAIMAAGMVPPLAMGLATMVARRKFDKAQQEGGKAALVLGLCFISEGAIPFAARDPMRVL

PCCIVGGALTGAISMAIGAKLMAPHGGLFVLLIPGAITPVLGYLVAIIAGTLVAGLAYAFLKRPEVDAVA

KAA

>gi|30063606|ref|NP_837777.1| hypothetical protein S2379 [Shigella flexneri 2a str. 2457T]

MSELKISPELLQISPEVQDALKNKKPVVALESTIISHGMPFPQNAQTAIEVEETIRKQGAVPATIAIIGG

VMKVGLSKEKIELLGREGHNVTKVSRRDLPFVVAAGKNGATTVASTMIIAALAGIKVFATGGIGGVHRGA

EHTFDISADLQELANTNVTVVCAGAKSILDLGLTTEYLETFGVPLIGYQTKALPAFFCRTSSFDVSIRLD

SASEIARAMAVKWQSGLNGGLVVANPIPEQFAMPEESINAAIDQAVAEAEEQGVIGKESTPFLLARVAEL

TSGDSLKSNIQLVFNNAILASEIAKEYQRLAG

>gi|30063604|ref|NP_837775.1| DNA-binding transcriptional activator YeiL [Shigella flexneri 2a str. 2457T]

MSESAFKDCFSTDVSADTRLFHFLARDYIVQEGQQPSWLFYLTRGRARLYATLANGRVSLIDFFAAPCFI

GEIELIDKDHEPRAVQAIEECWCLALPMKHYRPLLLNDTLFLRKLCVTLSHKNYRNIVSLTQNQSFPLVN

RLAAFILLSQEGDLYHEKHTQAAEYLGVSYRHLLYVLAQFIHDGLLIKRKDI

>gi|30063603|ref|NP_837774.1| ribonucleoside hydrolase 2 [Shigella flexneri 2a str. 2457T]

MEKRKIILDCEPGHDDAIAMMMAAKHPAIDLLGITIVAGNQTLDKTLINGLNVCQKLEINVPVYAGMPQP

IMRKQIVADNIHGETGLDGPVFEPLTRQAESTHAVKYIIDTLMASDGDITLVPVGPLSNIAVAMRMQPAI

LPKIREIVLMGGAYGTGNFTPSAEFNIFADPEAARVVFTSGVPLVMMGLDLTNQTVCTPDVIARMERVGG

PAGELFSDIMNFTLKTQFEYYGLAGGPVHDATCIGYLINPDGIKTQDMYVEVDVNSGPCYGRTVCDELGV

LGKPANTKVGITIDTDWFWGLVEECVRGYIKTH

>gi|30063600|ref|NP_837771.1| endonuclease IV [Shigella flexneri 2a str. 2457T]

MKYIGAHVSAAGGLANAAIRAAEIDATAFALFTKNQRQWRAAPLTTQTIDEFKAACEKYHYTSAQILPHD

SYLINLGHPVTEALEKSRDAFIDEMQRCEQLGLSLLNFHPGSHLMQISEEDCLARIAESINIALDKTQGV

TAVIENTAGQGSNLGFKFEHLAAIIDGVEDKSRVGVCIDTCHAFAAGYDLRTPAECEKTFADFARIVGFK

YLRGMHLNDAKSTFGSRVDRHHSLGEGNIGHDAFRWIMQNDRFDGIPLILETINPDIWAEEIAWLKAQQT

EKAVA

>gi|30063599|ref|NP_837770.1| hypothetical protein S2372 [Shigella flexneri 2a str. 2457T]

MTNITLQKQHRTLWHFIPGLALSAVITGVALWGGSIPAVAGAGFSALTLAILLGMVLGNTIYPHIWKSCD

GGVLFAKQYLLRLGIILYGFRLTFSQIADVGISGIIIDVLTLSSTFLLACFLGQKVFGLDKHTSWLIGAG

SSICGAAAVLATEPVVKAEASKVTVAVATVVIFGTVAIFLYPAIYPLMSQWFSPETFGIYIGSTVHEVAQ

VVAAGHAISPDAENAAVISKMLRVMMLAPFLILLAARVKQLSGANSGEKSKITIPWFAILFIVVAIFNSF

HLLPQSVVNMLVTLDTFLLAMAMAALGLTTHVSALKKAGAKPLLMALVLFAWLIVGGGAINYVIQSVIA

>gi|30063598|ref|NP_837769.1| DNA-binding transcriptional regulator [Shigella flexneri 2a str. 2457T]

MHITLRQLEVFAEVLKSGSTTQASVMLALSQSAVSAALTDLEGQLGVQLFDRVGKRLVVNEHGRLLYPRA

LALLEQAVEIEQLFREDNGAIRIYASSTIGNYILPAVIARYRHDYPQLPIELSVGNSQDVMQAVLDFRVD

IGFIEGPCHSTEIISEPWLEDELVVFAAPTSPLARGPVTLEQLAAAPWILRERGSGTRDIVDYLLLSHLP

KFEMAMELGNSEAIKHAVRHGLGISCLSRRVIEDQLQAGTLSEVAVPLPRLMRTLWRIHHRQKHLSNALR

RFLDYCDPANVPR

>gi|30063595|ref|NP_837766.1| hypothetical protein S2366 [Shigella flexneri 2a str. 2457T]

MERNVTLDFVRGVAILGILLLNISAFGLPKAAYLNPAWYDAITPQDAWTWAFLDLIGQVKFLTLFALLFG

AGLQMLLPRGRRWIQSRLTLLVLLGFIHGLLFWDGDILLAYGLVGLGVLLLLGLISDSQTSRAWTPDASA

ILYEKYWKLHGGVEAISNRADGVGNSLLALGAQYGWQLAGMMLIGAALMRSGWLKGQFSLRHYRRTGFVL

VAIGVTINLPAIALQWQLDWAYRWCAFLLQMPRELSAPFQAIGYASLFYGFWPQLSRFKLVLAIACVGRM

ALTNYLLQTLICTTLFYHLGLFMHFDRLELLAFVIPVWLANILFSVIWLRYFRQGPVEWLWRQLTLRAAG

PAISKTSR

>gi|30063594|ref|NP_837765.1| DNA-binding transcriptional regulator GalS [Shigella flexneri 2a str. 2457T]

MITIRDVARQAGVSVATVSRVLNNSTLVSADTREAVMKAVSELDYRPNANAQALATQVSDTIGVVVMDVS

DAFFGALVKAVDLVAQQHQKYVLIGNSYHEAEKERHAIEVLIRQRCNALIVHSKALSDDELAQFMDNIPG

MVLINRVVPGYAHRCVCLDNLSGARMATRMLLNNGHQRIGYLSSSHGIEDDAMRKAGWMSALKEQDIIPP

ESWIGTGTPDMPGGEAAMVELLGRNLQLTAVFAYNDNMAAGALTALKDNGIAIPLHLSIIGFDDIPIARY

TDPQLTTVRYPIASMAKLATELALQGAAGNIDPRASHCFMPTLVRRHSVATRQNAAAITNSTNQAM

>gi|30063593|ref|NP_837764.1| galactose-binding transport protein; receptor for galactose taxis [Shigella flexneri 2a str. 2457T]

MNKKVLTLSAVMASMLFGAAAHAADTRIGVTIYKYDDNFMSVVRKAIEQDAKAAPDVQLLMNDSQNDQSK

QNDQIDVLLAKGVKALAINLVDPAAAGTVIEKARGQNVPVVFFNKEPSRKALDSYDKAYYVGTDSKESGI

IQGDLIAKHWAANQGWDLNKDGQIQFVLLKGEPGHPDAEARTTYVIKELNDKGIKTEQLQLDTAMWDTAQ

AKDKMDAWLSGPNANKIEVVIANNDAMAMGAVEALKAHNKSSIPVFGVDALPEALALVKSGALAGTVLND

ANNQAKATFDLAKNLADGKGAADGTNWKIDNKVVRVPYVGVDKDNLAEFSKK

>gi|30063591|ref|NP_837762.1| beta-methylgalactoside transporter inner membrane component [Shigella flexneri 2a str. 2457T]

MSALNKKSFLTYLKEGGIYVVLLVLLAIIIFQDPTFLSLLNLSNILTQSSVRIIIALGVAGLIVTQGTDL

SAGRQVGLAAVVAATLLQSMDNANKVFPEMATMPIALVILIVCAIGAVIGLINGLIIAYLNVTPFITTLG

TMIIVYGINSLYYDFVGASPISGFDSGFSTFAQGFLALGSFRLSYITFYALIAVAFVWVLWNKTRFGKNI

FAIGGNPEAAKISGVNVGLNLLMIYALSGVFYAFGGMLEAGRIGSATNNLGFMYELDAIAACVVGGVSFS

GGVGTVIGVVTGVIIFTVINYGLTYIGVNPYWQYIIKGAIIIFAVALDSLKYARKK

>gi|30063588|ref|NP_837759.1| hypothetical protein S2358 [Shigella flexneri 2a str. 2457T]

MLKRVFLSLLVLIGLLLLTVLGLDRWMSWKTAPYIYDELQDLPYRQVGVVLGTAKYYRTGVINQYYRYRI

QGAINAYNSGKVNYLLLSGDNALQSYNEPMTMRKDLIAAGVDPSDIVLDYAGFRTLDSIVRTRKVFDTND

FIIITQRFHCERALFIALHMGIQAQCYAVPSPKDMLSVRIREFAARFGTLADLYIFKREPRFLGPLVPIP

AMHQVPEDAQGYPAVTPEQLLELQKKQGK

>gi|30063586|ref|NP_837757.1| hypothetical protein S2356 [Shigella flexneri 2a str. 2457T]

MMANIWWSLPLTLIVFFAARKLAARYKFPLLNPLLVAMVVIIPFLMLTGISYDSYFKGSEVLNDLLQPAV

VALAYPLYEQLHQIRARWKSIITICFIGSVVAMVTGTSVALLMGASPEIAASILPKSVTTPIAMAVGGSI

GGIPAISAVCVIFVGILGAVFGHTLLNAMRIRTKAARGLAMGTASHALGTARCAELDYQEGAFSSLALVL

CGIITSLIAPFLFPIILAVMG

>gi|30063585|ref|NP_837756.1| hypothetical protein S2355 [Shigella flexneri 2a str. 2457T]

MSKTLNIIWQYLRAFVLIYACLYAGIFIASLLPVTIPGSIIGMLILFVLLALQILPAKWVNPGCYVLIRY

MALLFVPIGVGVMQYFDLLRAQFGPVVVSCAVSTLVVFLVVSWSSQLVHGERKVVGQKGSEE

>gi|30063583|ref|NP_837754.1| hypothetical protein S2350 [Shigella flexneri 2a str. 2457T]

MLTGNQRETNWPMDLNTLISQYGYAALVIGSLAEGETVTLLGGVAAHQGLLKFPLVVLSVALGGMIGDQV

LYLCGRRFGGKLLRRFSKHQDKIERAQKLIQRHPYLFVIGTRFMYGFRVIGPTLIGASQLPPKIFLPLNI

LGAFAWALIFTTIGYAGGQVIAPWLHNLDQHLKHWVWLILVVVLVVGVRWWLKRRGKKKPDNQA

>gi|30063582|ref|NP_837753.1| hypothetical protein S2349 [Shigella flexneri 2a str. 2457T]

MSHVWGLFSHPDRKMQVINRENETISHHYTHHVLLMAAIPVICAFIGTTQIGWNFGDGTILKLSWFTGLA

LAVLFYGVMLAGVAVMGRVIWWMARNYPQRPSLAHCMVFAGYVATPLFLSGLVALYPLVWLCALVGTVAL

FYTGYLLYLGIPSFLNINKEEGLSFSSSTLAIGVLVLEVLLTLTVILWGYGYRLF

>gi|30063580|ref|NP_837751.1| D-lactate dehydrogenase [Shigella flexneri 2a str. 2457T]

MSSMTTTDNKAFLNELARLVGHSHLLTDPAKTARYRKGFRSGQGDALAVVFPGSLLELWRVLKACVTADK

IILMQAANTGLTEGSTPNGNDYDRDIVIISTLRLDKLHVLGKGEQVLAYPGTTLYSLEKALKPLGREPHS

VIGSSCIGASVIGGICNNSGGSLVQRGPAYTEMSLFARIDEDGKLTLVNHLGIDLGETPEQILSKLDDDR

IKDDDVRHDGRHAHDYDYVHRVRDIEADTPARYNADPDRLFESSGCAGKLAVFAVRLDTFEAEKNQQVFY

IGTNQPEVLTEIRRHILANFENLPVAGEYMHRDIYDIAEKYGKDTFLMIDKLGTDKMPFFFNLKGRTDAM

LEKVKFFRPHFTDRAMQKFGHLFPSHLPPRMKNWRDKYEHHLLLKMAGDGVGEAKSWLVDYFKQAEGDFF

VCTPEEGSKAFLHRFAAAGAAIRYQAVHSDEVEDILALDIALRRNDTEWYEHLPPEIDSQLVHKLYYGHF

MCYVFHQDYIVKKGVDVHALKEQMLELLQQRGAQYPAEHNVGHLYKAPETLQKFYRENDPTNSMNPGIGK

TSKRKNWQEVE

>gi|30063579|ref|NP_837750.1| beta-D-glucoside glucohydrolase [Shigella flexneri 2a str. 2457T]

MKWLCSVGIAVSLVLQPALADDLFGNHPLTPEARDAFVTDLLKKMTVDEKIGQLRLISVGPDNPKEAIRE

MIKDGQVGAIFNTVTRQDIRAMQDQVMELSRLKIPLFFAYDVLHGQRTVFPISLGLASSFNLDAVKTVGR

VSAYEAADDGLNMTWAPMVDVSRDPRWGRASEGFGEDTYLTSIMGKTMVEAMQGKSPADRYSVMTSVKHF

AAYGAVEGGKEYNTVDMSPQRLFNDYMPPYKAGLDAGSGAVMVALNSLNGTPATSDSWLLKDVLRDQWGF

KGITVSDHGAIKELIKHGTAADPEDAVRVALKSGINMSMSDEYYSKYLPGLIKSGKVTMAELDDAARHVL

NVKYDMGLFNDPYSHLGPKESDPVDTNAESRLHRKEAREVARESLVLLKNRLETLPLKKSATIAVVGPLA

DSKRDVMGSWSAAGVADQSVTVLTGIKNSVGENGKVLYAKGANVTSDKGIIDFLNQYEEAVKVDPRSPQE

MIDEAVQTAKQSDVVVAVVGEAQGMAHEASSRTDITIPQGQRDLIAALKATGKPLVLVLMNGRPLALVKE

DQQADAILETWFAGTEGGNAIADVLFGDYNPSGKLPISFPRSVGQIPVYYSHLNTGRPYNADKPNKYTSR

YFDEANGALYPFGYGLSYTTFTVSDVKLSAPTMKRDGKVTASVQVTNTGKREGATVVQMYLQDVTASMSR

PVKQLKGFEKITLKPGETQTVSFPIDIEALKFWNQQMKYDAEPGKFNVFIGTDSARVKKGEFELL

>gi|30063578|ref|NP_837749.1| transport system permease [Shigella flexneri 2a str. 2457T]

MPLLKLWAGSLVMLAAVSLPLQAASPVKVGSKIDTEGALLGNIILQVLESHGVPTVNKVQLGTTPVGRGA

ITSDELDIYPEYTGNGAFFFKDENDAAWKNAGQGYEKVKKLDAEHNKLIWLTPAPANNTWTIAVRQDVAE

KNKLTSLADLSRYLQEGGTFKLAASAEFIERADALPTFEKAYGFKLGQDQLLSLAGGDTAVTIKAAAQQT

SGVNAAMAYGTDGPVAALGLQTLSDPQGVQPIYAPAPVVRESVLKEYPQMAQWLQPVFASLDAKTLQQLN

ASIAVEGLDAKKVAADYLKQKGWAK

>gi|30063577|ref|NP_837748.1| transport system permease [Shigella flexneri 2a str. 2457T]

MTYLRINPVLALLLLLTAIAAALPFISYAPNRLVSGEGLHLWQLWPQTIWMLVGVGCAWLTACFVPAKKG

SIFALILAQFVFVLLVWGAGKAATQLAQNGSALARTSLGRGFWLAAALALLACSDAIRRISTHPLWRWLL

HMQIAIIPLWLLYSGTLNDLSLMKEYANRQDVFDDALAQHLTLLFGAVLPALVIGVPLGIWCYFSTARQG

AIFSLLNVIQTVPSVALFGLLIAPLAALVTAFPWLGKLGIAGTGMTPALIALVLYALLPLVRGVVVGLNQ

IPRDVLESARAMGMSGAQRFLHVQLPLALPVFLRSLRVVMVQTVGMAVIAALIGAGGFGALVFQGLLSSA

IDLVLLGVIPVIVLAVLTDALFDLLIALLKVKRND

>gi|30063575|ref|NP_837746.1| transport system permease [Shigella flexneri 2a str. 2457T]

MKMLRDPLFWLIALFVALIFWLPYSQPLFAALFPQLPRPVYQQESFAALALAHFWLVGISSLFAVIIGTG

AGIAVTRPWGAEFRPLVETIAAVGQTFPPVAVLAIAVPVIGFGLQPAIIALILYGVLPILQVTLAGLGAI

DPSVTEVAKGMGMSRGQRLRKVELPLAAPVILAGVRTSVIINIGTATIASTVGASTLGTPIIIGLSGFNT

AYVIQGALLVALAAIIADRLFERLVQALSQHAK

>gi|30063573|ref|NP_837744.1| hypothetical protein S2340 [Shigella flexneri 2a str. 2457T]

MSEWASEDINAPSLLTGNPALQPQTGGILNIPANHQRIFTLFFTCRSQTNGDVVDQYAVEDRQQRLESRL

LALKPSAQRNKQMCGECLGGVILTALFDPRSQAM

>gi|30063566|ref|NP_837737.1| phage tail fiber protein [Shigella flexneri 2a str. 2457T]

MNTVADVMAGNDGEYCFHARTGKYGVYLKQDWRNEYNVGDIAVYEDSKPGTLNDFLIAPDEGDLKPDVVK

RFEEMVAQAQQSAGAAAGNAQQTAQDVAAAATARDDAQRFAEKARQDATVTAEDRKATAEDVTSTGANAA

AAGQSAQDAAGYARAAEQAKNDIDAALTGTLKTANHLSETAAAGEKAQQKSRDNLGLKSAATMEAQSDIY

DRTKGRLAIPGAFGFGCAFLPEDVIRFDTKSDFLAWVRNALPGEYSVAGPYGIIIPDTRFEGVLSIRWTD

ARPETTEPRYRAKSLTFYGINGPIYHTRYCYWPISRLTGWVKINITTEDIIYRIVASSVRNRWGDPDIGG

LIIAAYQGEADGDKVIRLVRGQSYRGSRLGPVGISVPGTPTGTYIVSPQFFITGCSEHSLPGSYCALSGV

PDAHVSGAMPGLFIRTSRGMHRGN

>gi|30063562|ref|NP_837733.1| 2-component sensor protein [Shigella flexneri 2a str. 2457T]

MYDFNLVLLLLQQMCVFLVIAWLMSKTPLFIPLMQVTVRLPHKFLCYIVFSIFCIMGTWFGLHIDDSIAN

TRAIGAVMGGLLGGPVVGGLVGLTGGLHRYSMGGMTALSCMISTIVEGLLGGLVHSILIRRGRTDKVFNP

ITAGAVTFVAEMVQMLIILAIARPYEDAVRLVSNIAAPMMVTNTVGAALFMRILLDKRAMFEKYTSAFSA

TALKVAASTEGILRQGFNEVNSMKVAQVLYQELDIGAVAITDREKLLAFTGIGDDHHLPGKPISSTYTLK

AIETGEVVYADGNEVPYRCSLHPQCKLGSTLVIPLRGENQRVMGTIKLYEAKNRLFSSINRTLGEGIAQL

LSAQILAGQYERQKAMLTQSEIKLLHAQVNPHFLFNALNTIKAVIRRDSEQASQLVQYLSTFFRKNLKRP

SEFVTLADEIEHVNAYLQIEKARFQSRLQVNIAIPQELSQQQLPAFTLQPIVENAIKHGTSQLLDTGRVA

ISARREGQHLMLEIEDNAGLYQPVTNASGLGMNLVDKRLRERFGDDYGISVACEPDSYTRITLRLPWRDE

A

>gi|30063561|ref|NP_837732.1| two-component response-regulatory protein YehT [Shigella flexneri 2a str. 2457T]

MIKVLIVDDEPLARENLRIFLQEQSDIEIVGECSNAVEGIGAVHKLRPDVLFLDIQMPRISGLEMVGMLD

PEHRPYIVFLTAFDEYAIKAFEEHAFDYLLKPIDEARLEKTLARLRQERSKQDVSLLPENQQALKFIPCT

GHSRIYLLQMKDVAFVSSRMSGVYVTSHEGKEGFTELTLRTLESRTPLLRCHRQYLVNLAHLQEIRLEDN

GQAELILRNGLTVPVSRRYLKSLKEAIGL

>gi|30063558|ref|NP_837729.1| hypothetical protein S2319 [Shigella flexneri 2a str. 2457T]

MKAFNKLFSLVVASVLVFSLAGCGDKEESKKFSANLNGTEIAITYVYKGDKVLKQSSETKIQFASIGATT

KEDAAKTLEPLSAKYKNIAGVEEKSTYTDTYAQENVTIDMEKVDFKALQGISGINVSAEDAKKGITMAQM

ELVMKAAGFKEVK

>gi|30063555|ref|NP_837726.1| hypothetical protein S2316 [Shigella flexneri 2a str. 2457T]

MNSLRPELLELTPQALTALSNAGFVKRSLKELENGNVPEISHENSALIATFSDGVRTQLANGQALKEAQC

TYGASGMCRHRVMLVLSYQRLCATAQPTGKEEEWDPAIWLEELATLPDATRKRAQALVAKSITIELFCAP

GEIPSARLPMSDVRFYSRSSIRFARCDCIEGTLCEHVVLAVQAFVQAKAQQAELTHLIWQMRSEHVTSSD

DPFASEEGKTCRQYVQQLSQALWLSGISQPLIHYEAAFSRAQQAAERCSWRWVSESLRQLRASVDAFHAR

ASHYHAGECLRQLAALNSRLNCAQEMARSDSVGEVPPVPWRTVVGSGIAGEAKLDHLRLVSLGMRCWQDI

EHYGLRIWFTDPDTGSILHLSRSWPRSEQEDSPAATRRLFSFQAGALAGGQIVSQAAKRSADGELLLATR

NRLSSVVPLSPDAWQMLSAPLRQPGIVALREYLRQRPPACIRPLNQVDNLFILPVAECISLGWDSSRQTL

DAQVISGEGEDNLLTLSLPASASAPYAIERMAALLQQTDDPVCLVSGFVSFVDGQLTLEPQVMMTKTRAW

ALDAETAPVVVSLPSASVLPVPSTAHQLLMRCQALLIQLLHNGWRYQEQSAINQAELLANDLTAVGFYRL

AHVLAQFRNTESEARVEAMNNGVLLCEQLFPMLQQQG

>gi|30063554|ref|NP_837725.1| hypothetical protein S2315 [Shigella flexneri 2a str. 2457T]

MSELNDLLTTRELQRWRLILGEAAETTLCGLDDNARQIDHALEWLYGRDPERLQRGERSSGLGGSNLTTP

EWINSIHTLFPQQVIERLESDAVLRYGIEDVVTNLDVLERMQPSESLLRAVLHTKHLMNPEVLAAARRIV

RQVVEEIMARLAKEVRQAFSGVRDRRRRSFIPLARNFDFKSTLRANLQHWHPQHGKLYIEFPRFNSRIKR

QSEQWQLVLLVDQSGSMVDSVIHSAVMAACLWQLPGIRTHLVAFDTSVVDLTADVADPVELLMKVQLGGG

TNIASAMEYGRQLIEQPAKSVIILVSDFYEGGSSSLLTHQVKKCVQSGIKVLGLAALDSTATPCYDRDTA

QALVNVGAQIAAMTPGELASWLAENLQS

>gi|30063553|ref|NP_837724.1| hypothetical protein S2314 [Shigella flexneri 2a str. 2457T]

MSEPLIVGIRHHSPACARLVKSLIESQRPRYVLIEGPADFNDRVDELFLAHQLPVAIYSYCQYQDGAAPG

RGAWTPFAEFSPEWQALQAARRIQAQTYFIDLPCWAQSEEEDDSPDTQDESQTLLLRDTRMDNSDTLWDH

LFEDESQQTALPSALARYFAQLRGDSPGDALNRQREAFMARWITWAMQQNNGDVLVVCGGWHAPALANMW

RECPQEINKPELSSLADAVTGCYLTPYSEKRLDVLAGYLSGMPAPVWQNWCWQWGLQQAGEQLLKTVLTR

LRQHKLPASTADMAAAHLHAMALAQLRGHTLPLRTDWLDAIAGSLIKEALNAPLPWSYRGVIHPDTDPIL

LTLIDTLAGDGFGKLAPSTPQPPLPKDVTCELERTAISLPAELTLNRFNPNGLAQSQVLHRLAILEIPGI

VRQQGSTLTLAGNGEERWKLTRPLSQHAALIEAACFGATLLEAARHKLEADMLDAGGIGSITTCLSQAAS

AGLASFSQQLLEQLTLLIAQENQFAEMGQALEVLYALWRLDEISGMQGAQILQTTLCAAIDRTLWLCESN

GRPDEKEFHAHLHSWQALCHILRDLHSGVQLPGVSLSAAVALLERHSQAIHVLALDRGATLGALMRLEHP

NASAEAALTMLAQLSPAQSGEALHGLLALARHQLACQPAFIAGFSSHLNQLSDADFTNALPDLRAAMAWL

PPRERGTLAHQVLEHYQLAQLPVSALQMPLHCPPQAIAHHQQLEQQALASLQHWGVFHV

>gi|30063552|ref|NP_837723.1| hypothetical protein S2313 [Shigella flexneri 2a str. 2457T]

MRQTFIARKISFISLIHFRDEYMSPQNNHLQRPPAAVLYADELAKLKQNDNAPCPPGWQLSLPAARAFIL

GDSAQNISRKVVISPSAVERMLVTLATGRGLMLVGEPGTAKSLLSELLATSISGDAGLTIQGGASTTEDQ

IKYGWNYALLINHGPSTEALVPAPLYQGMRDGKIVRFEEITRTPLEVQDCLLGMLSDRVMTVPELTGEAS

QLYAREGFNIIATANTRDRGVNEMSAALKRRFDFETVFPIMDFAQELELVASASARLLAHSGIPHKVPDA

VLELLVRTFRDLRANGEKKTSMDTLTAIMSTAEAVNVAHAVGVRAWFLANRAGEPADLVDCIAGTIVKDN

EEDRARLRRYFEQRVATHKEAHWQAYYQARHRLP

>gi|30063546|ref|NP_837717.1| fimbrial-like protein [Shigella flexneri 2a str. 2457T]

MKRSIIAAAVFSSFFMSAGVFAADVDTGTLTIKGNIAESPCKFEAGGDSVSINMPTVPTTVFEGKAKYST
YDDAVGVTSSMLKISCPKEVAGVKLSLITNDKITGNDKAIASSNDTVGDNSDVLDVSAPFNIESYKTAEG
QYAIPFKAKYLKLTDNSVQSGDVLSSLVMRVAQD

>gi|30063545|ref|NP_837716.1| outer membrane protein [Shigella flexneri 2a str. 2457T]

MLRMTPLASAIVALLLGIEAYAAEETFDTHFMIGGMKDQQVANIRLDDNQPLPGQYDIDIYVNKQWRGKY
EIIVKDNPQETCLSREVIKRLGINSDNFASGKQCLTFEQLVQGGSYSWDIGVFRLDFSVPQAWVEELESG
YVPPENWERGINAFYTSYYVSQYYSDYKASGNNKSTYVRFNSGLNLLEWQLHSDASFSKTNNNPGVWKSN
TLYLERGFAQFLGTLRVGDMYTSSDIFDSVRFSGVRLFRDMQMLPNSKQNFTPRVQGIAQSNALVTIEQN
GFVVYQKEVPPGPFAITDLQLAGGGADLDVSVKEADGSVTTYLVPYAAVPNMLQPGVSKYDFAAGRSHIE
GASKQSDFVQAGYQYGFNNLLTLYGGTMVANNYYAFTLGTGWNTRIGAISVDATKSHSKQDNGDVFDGQS
YQIAYNKFVSQTSTRFGLAAWRYSSRDYRTFNDHVWANNKDNYRRDENDIYDIADYYQNDFGRKNSFSAN
MSQSLPEGWGSVSLSTLWRDYWGRSGSSKDYQLSYSNNWRRISYTLAASQAYGENHHEEKRFNIFISIPC
DWGDDVTTPRRQIYMSNSTTFDDQGFASNNTGLSGTVGSRDQFNYGVNLSHQHQGNETTAGANLTWNAPV
ATVNGSYSQSSTYRQTGASVSGGIVAWSGGVNLANRLSETFAVMNAPGIKDAYVNGQKYRTTNRNGVVVY
DGMTPYRENHLMLDVSQSDSEAELRGNRKIAAPYRGAVVLVNFDTDQRKPWFIKALRADGQPLTFGYEVN
DIHGHNIGVVGQGSQLFIRTNEIPPSVNVAIDKQQGLSCTITFGKEIDESRNYICQ

>gi|30063543|ref|NP_837714.1| hydroxyethylthiazole kinase [Shigella flexneri 2a str. 2457T]

MQVDLLSSAQSAHALHLFHQHSPLVHCMTNDVVQTFTANTLLALGASPAMVIETEEASQFAAIASALLIN

VGTLTQPRAQAMRAAVEQAKSSQTRWTLDPVAVGALDYRRHFCHELLSFKPAAIRGNASEIMALAGIANG

GRGVDTTDAAANAIPAAQTLARETGAIVVVTGEVDYVTDGHRIIGIHGGDPLMTKVVGTGCALSAVVAAC

CALPGDTLENVASACHWMKQAGERAVARSEGPGSFVPHFLDALWQLTQEVQA

>gi|30063542|ref|NP_837713.1| phosphomethylpyrimidine kinase [Shigella flexneri 2a str. 2457T]

MKRINALTIAGTDPSGGAGIQADLKTFSALGAYGCSVITALVAQNTRGVQSVYRIEPDFVAAQLDSVFSD

VRIDTTKIGMLAETDIVEAVAERLQRYQIQNVVLDTVMLAKSGDPLLSPSAVATLRSRLLPQVSLITPNL

PEAAALLDAPHARTEQEMLEQGRSLLAMGCGAVLMKGGHLDDEQSPDWLFTREGEQRFTAPRIMTKNTHG

TGCTLSAALAALRPRHTNWADTVQEAKIWLSSALAQADTLEVGHGIGPVHHFHAWW

>gi|30063541|ref|NP_837712.1| hypothetical protein S2289 [Shigella flexneri 2a str. 2457T]

MQLRITSRKKLTALLCALGLISIVAIYPRQTVNFFYSTAVQITDYIHFYGYRPVKSFAIRIPASYTIHGL

DVSRWQERIDWQRVAKMRDNDIRLQFAFIKATEGEKLVDPYFSRNWQLSRENGLLRGAYHYFSPSVSASV

QARLFLQTVDFSQGDFPAVLDVEERGKLSAKELRKRVSQWLKMVEKRTGKKPIIYSGAVFYHTNLAGYFN

EYPWWVAHYYQRRPDNDGMAWRFWQHSDRGQVDGINGPVDFNVFNGTGMSCRHSLMGLKKRLK

>gi|30063540|ref|NP_837711.1| transcriptional regulator [Shigella flexneri 2a str. 2457T]

MEQAHTQLIAQLNERILAADNTPLYIKFAETVKNAVRSGVLEHGNILPGERDLSQLTGVSRITVRKAMQA

LEEEGVVTRSRGYGTQINNIFEYSLKEARGFSQQVVLRGKKPDTLWVNKRVVKCPEEVAQQLAVEAGSDV

FLLKRIRYVDEEAVSIEESWVPAHLIHDVDAIGISLYDYFRSQHIYPQRTRSRVSARMPDAEFQSHIQLD

SKIPVLVIKQVALDQQQRPIEYSISHCRSDLYVFVCEE

>gi|30063539|ref|NP_837710.1| kinase [Shigella flexneri 2a str. 2457T]

MSGARLHTLLPELTTRQPVMVVGAAVIDVIADAYALPWRGCDIELKQQSVNVGGCALNIAVALKRLGIEA

GNALPLGQGVWAEIIRNRMAKEGLISLIDNAESDNGWCLALVEPDGERTFMSFSGVENQWNRQWLARLTV

APGNLLYFSGYQLASPCGELLVEWLEKLQDVTPFIDFCPRIGDIPDALLARIMACRPLVSLNRQEAEIAA

ERFALSAEITTLGKQWQEKFAAPLIVRLDKEGAWYFSNDASGCIPAFPTQVVDTIGAGDSHAGGVLAGLA

SGLPLADAVLLGNAVASWVVGHRGGDCAPTREELLLAHKNV

>gi|30063538|ref|NP_837709.1| fructose-bisphosphate aldolase [Shigella flexneri 2a str. 2457T]

MTDIAQLLGKDADNLLQHRCMTIPSDQLYLPGHDYVDRVMIDNNRPPAVLRNMQTLYNTGRLAGTGYLSI

LPVDQGVEHSAGASFAANPLYFDPKNIVELAIEAGCNCVASTYGVLVSVSRRYAHRIPFLVKLNHNETLS

YPNTYDQTLYASVEQAFNMGAVAVGATIYFGSEESRRQIEEISAAFERAHELGMVTVLWAYLRNSAFKKD

GVDYHVSADLTGQANHLAATIGADIVKQKMAENNGGYKAINYGYTDDRVYSKLTSENPIDLVRYQLANCY

MGRAGLINSGGAAGGETDLSDAVRTAVINKRAGGMGLILGRKAFKKSMADGVKLINAVQDVYLDSKITIA

>gi|30063537|ref|NP_837708.1| tagatose-bisphosphate aldolase [Shigella flexneri 2a str. 2457T]

MYVVSTKQMLNNAQRGGYAVPAFNIHNLETMQVVVETAANLHAPVIIAGTPGTFTHAGTENLLALVSAMA

KQYHHPLAIHLDHHTKFDDIAQKVRSGVRSVMIDASHLPFAQNISRVKEVVDFCHRFDVSVEAELGQLGG

QEDDVQVNEVDALYTNPAQAREFAEATGIDSLAVAIGTAHGMYASAPVLDFSRLENIRQWVNLPLVLHGA

SGLSTKDIQQTIKLGICKINVATELKNAFLQSLKNYLTEHPEATDPRDYLQSAKSAMRDVVSKVIADCGC
EGRA

>gi|30063536|ref|NP_837707.1| tagatose 6-phosphate kinase 1 [Shigella flexneri 2a str. 2457T]

MKTLIARHKAGEHIGICSVCSAHPLVIEAALAFDRNSTRKVLIEATSNQVNQFGGYTGMTPADFREFVFT

IADKVGFARERIILGGDHLGPNCWQQENADAAMEKSVELVKAYVRAGFSKIHLDASMSCAGDTIPLAPET

VAERAAVLCFAAESVATDCQREQLSYVIGTEVPVPGGEASAIQSVHITHVEDAANTLRTHQKAFIARGLT

EALTRVIAIVVQPGVEFDHSNIIHYQPQEAQALAQWIENTRMVYEAHSTDYQTRTAYWELVRDHFAILKV

GPALTFALREAIFALAQIEQELIAPENRSGCLAVIEEVMLDEPQYWKKYYRTGFNDSLLDIRYSLSDRIR

YYWPHSRIKNSVETMMVNLQGVDIPLGMISQYLPKQFERIQSGELSAMPHQLIMNKIYDVLRAYRYGCAE

>gi|30063535|ref|NP_837706.1| galactitol-specific PTS system component IIA [Shigella flexneri 2a str. 2457T]

MTNLFVRSGISFVDRSEVLTYIGNEMLAKGVVHDTWPQALIAREAEFPTGIMLEQHAIAIPHCEAIHAKS

SAIYLLRPTNKVHFQQADDDNDVAVSLVIALIVENPQQQLKLLRCLFGKLQQPDIVETLITLPETQLKEY

FTKYVLDSDE

>gi|30063534|ref|NP_837705.1| galactitol-specific PTS system component IIB [Shigella flexneri 2a str. 2457T]

MKRKIIVACGGAVATSTMAAEEIKELCQSHNIPVELIQCRVNEIETYMDGVHLICTTARVDRSFGDIPLV

HGMPFVSGVGIEALQNKILTILQG

>gi|30063533|ref|NP_837704.1| PTS system galactitol-specific enzyme IIC [Shigella flexneri 2a str. 2457T]

MFSEVMRYILDLGPTVMLPVVIIIFSKILGMKAGDCFKAGLHIGIGFVGIGLVIGLMLDSIGPAAKAMAE

NFDLNLHVVDVGWPGSSPMTWASQIALVAIPIAILVNVAMLLTRMTRVVNVDIWNIWHMTFTGALLHLAT

GSWMIGMAGVVIHAAFVYKLGDWFARDTRNFFELEGIAIPHGTSAYMGPIAVLVDAIIEKIPGVNRIKFS

ADDIQRKFGPFGEPVTVGFVMGLIIGILAGYDVKGVLQLAVKTAAVMLLMPRMIKPIMDGLTPIAKQARS

RLQAKFGGQEFLIGLDPALLLGHTAVVSASLIFIPLTILIAVCVPGNQVLPFGDLATIGFFVAMAVAVHR

GNLFRTLISGVIIMSITLWIATQTIGLHTQPAANAGALKAGGMVASMDQGGSPITWLLIQVFSPQNIPGF

IIIGAIYLTGIFMTWRRARGFTKQEKAVLAE

>gi|30063529|ref|NP_837700.1| galactitol utilization operon repressor [Shigella flexneri 2a str. 2457T]

MTMNSFERRNKIIQLVNEQGTVLVQDLAGVFAASEATIRADLRFLEQKGVVTRFHGGAAKIMSGNSETET

QEVGFKERFQLASAPKNRIAQAAVKMIHEGMTVILDSGSTTMLIAEGLMTAKNITVITNSLPAAFALSEN

KDITLVVCGGTVRHKTRSMHGSIAERSLQDINADLMFVGADGIDAVNGITTFNEGYSISEAMVTAANKVI

AVLDSSKFNRRGFNQVLPIEKIDIIVTDDAVSEVDKLALQKTRVKLITV

>gi|30063528|ref|NP_837699.1| lipid kinase [Shigella flexneri 2a str. 2457T]

MAEFPASLLILNGKSTDNLPLREAIMLLREEGMTIHVRVTWEKGDAARFVEEARKLGVATVIAGGGDGTI

NEVSTALIQCEGDDIPALGILPLGTANDFATSVGIPEALDKALKLAIAGNAIAIDMAQVNKQTCFINMAT

GGFGTRITTETPEKLKAALGGVSYIIHGLMRMDTLQPDRCEIRGENFHWQGDALVIGIGNGRQAGGGQQL

CPNALINDGLLQLRIFTGDEILPALVSTLKSDEDNPNIIEGASSWFDIQAPHEITFNLDGEPLSGQNFHI

EILPAALRCRLPPDCPLLR

>gi|30063527|ref|NP_837698.1| hypothetical protein S2273 [Shigella flexneri 2a str. 2457T]

MPLLYLNTRECRWYLMGEGEMKKIAAISLISIFLISGCAVHNDETSIGKFGLAYKSNIQRKLDNQYYTEA

EASLARGRISGAENIVKNDAAHFCVTQGKKMQIVDLKTEGAGLHGVARLTFKCGE

>gi|30063526|ref|NP_837697.1| hypothetical protein S2271 [Shigella flexneri 2a str. 2457T]

MFKPELLSPAGTLKNMRYAFAYGADAVYAGQPRYSLRVRNNEFNHENLQLGINEAHALGKKFYVVVNIAP

HNAKLKTFIRDLKPVVEMGPDALIMSDPGLIMLVREHFPEMPIHLSVQANAVNWATVKFWQQMGLTRVIL

SRELSLEEIEEIHNQVPDMEIEIFVHGALCMAYSGRCLLSGYINKRDPNQGTCTNACRWEYNVQEGKEDD

VGNIVHKYEPIPVQNVEPTLGIGAPTDKVFMIEEAQRPGEYMTAFEDEHGTYIMNSKDLRAIAHVERLTK

MGVHSLKIEGRTKSFYYCARTAQVYRKAIDDAAAGKPFDTSLLETLEGLAHRGYTEGFLRRHTHDDYQNY

EYGYSVSDRQQFVGEFTGERKGDLAAVAVKNKFSVGDSLELMTPQGNINFTLEHMENAKGEAMPVAPGDG

YTVWLPVPQDLELNYALLMRNFSGETTRNPHGK

>gi|30063525|ref|NP_837696.1| hypothetical protein S2270 [Shigella flexneri 2a str. 2457T]

MLFSISNFNQGVIMAGWFELSKSSDNQFRFVLKAGNGETILTSELYTSKASAEKGIASVRSNSPQEERYE

KKTASNGKFYFNLKAANHQIIGSSQMYATAQSRETGIASVKANGTSQTVKDNT

>gi|30063524|ref|NP_837695.1| DNA-binding transcriptional regulator BaeR [Shigella flexneri 2a str. 2457T]

MTELPIDENTPRILIVEDEPKLGQLLIDYLRAASYAPTLISHGDQVLAYVRQTPPDLILLDLMLPGTDGL

TLCREIRRFSDVPIVMVTAKIEEIDRLLGLEIGADDYICKPYSPREVVARVKTILRRCKPQRELQQQDAE

SPLIIDEGRFQASWRGKMLDLTPAEFRLLKTLSHEPGKVFSREQLLNHLYDDYRVVTDRTIDSHIKNLRR

KLESLDAEQSFIRAVYGVGYRWEADACRIV

>gi|30063523|ref|NP_837694.1| signal transduction histidine-protein kinase BaeS [Shigella flexneri 2a str. 2457T]

MKFWRPGITGKLFLAIFATCIVLLISMHWAVRISFERGFIDYIKHGNEQRLQLLSDALGEQYAQHGNWRF

LRNNDRFVFQILRSFEHDNSEDKPGPGMPPHGWRTQFWVVDQNNKVLVGPRAPIPPDGTRRPILVNGAEV

GAVIASPVERLTRNTDINFDKQQRQTSWLIVALATLLAALATFLLARGLLAPVKRLVDGTHKLAAGDFTT

RVTPTSEDELGKLAQDFNQLASTLEKNQQMRRDFMADISHELRTPLAVLRGELEAIQDGVRKFTPETVAS

LQAEVGTLTKLVDDLHQLSMSDEGALAYQKAPVDLIPLLEVAGGAFRERFASRGLKLQFSLPDSITVFGD

RDRLMQLFNNLLENSLRYTDSGGSLKISAEQHDKTVRLTFADSAPGVSDDQLQKLFERFYRTEGSRNRAS

GGSGLGLAICLNIVEAHNGRIIAAHSPFGGVSITVELPLERDLQREV

>gi|30063522|ref|NP_837693.1| multidrug efflux system protein MdtE [Shigella flexneri 2a str. 2457T]

MTDLPDSTRWQLWIVAFGFFMQSLDTTIVNTALPSMAQSLGESPLHMHMVIVSYVLTVAVMLPASGWLAD

KVGVRNIFFTAIVLFTLGSLFCALSGTLNELLLARALQGVGGAMMVPVGRLTVMKIVPREQYMAAMTFVT

LPGQVGPLLGPALGGLLVEYASWHWIFLINIPVGIIGAIATLMLMPNYTMQTRRFDLSGFLLLAVGMAVL

TLALDGSKGTGLSPLAIAGLVAVGVVALVLYLLHARNNNRALFSLKLFRTRTFSLGLAGSFAGRIGSGML

PFMTPVFLQIGLGFSPFHAGLMMIPMVLGSMGMKRIVVQVVNRFGYRRVLVATTLGLSLVTLLFMTTALL

GWYYVLPFVLFLQGMVNSTRFSSMNTLTLKDLPDNLASSGNSLLSMIMQLSMSIGVTIAGLLLGLFGSQH

ISVDSGTTQTVFMYTWLSMAFIIALPAFIFARVPNDTHQNVAISRRKRSAQ

>gi|30063521|ref|NP_837692.1| multidrug efflux system subunit MdtC [Shigella flexneri 2a str. 2457T]

MKFFALFIYRPVATILLSVAITLCGILGFRMLPVAPLPQVDFPVIMVSASLPGASPETMASSVATPLERS

LGRIAGVSEMTSSSSLGSTRIILQFDFDRDINGAARDVQAAINAAQSLLPSGMPSRPTYRKANPSDAPIM

ILTLTSDTYSQGELYDFASTQLAPTISQIDGVGDVDVGGSSLPAVRVGLTPQALFNQGVSLDDVRTAISN

ANVRKPQGALEDGTHRWQIQTNDELKTAAEYQPLIIHYNNGGAVRLGDVATVTDSVQDVRNAGMTNAKPA

ILLMIRKLPEANIIQTVDSIRAKLPELQETIPAAIDLQIAQDRSPTIRASLEEVEQTLIISVALVILVVF

LFLRSGRATIIPAVAVPVSLIGTFAAMYLCGFSLNNLSLMALTIATGFVVDDAIVVLENIARHLEAGMKP

LQAALQGTREVGFTVLSMSLSLVAVFLPLLLMGGLPGRLLREFAVTLSVAIGISLLVSLTLTPMMCGWML

KASKPREQKRLRGFGRMLVALQQGYGKSLKWVLNHTRLVGVVLLGTIALNISIPKTFFPEQDTGVLMGGI

QADQSISFQAMRGKLQDFMKIIRDDPAVDNVTGFTGGSRVNSGMMFITLKPRDERSETAQQIIDRLRVKL

AKEPGANLFLMAVQDIRVGGRQSNASYQYTLLSDDLAALREWEPKIRKKLATLPELADVNSDQQDNGAEM

NLVYDRDTMARLGIDVQAANSLLNNAFGQRQISTIYQPMNQYKVVMEVDPRYTQDISALEKMFVINNEGK

AIPLSYFAKWQPANAPLSVNHQGLSAASTISFNLPTGKSLSDASAAIDRAMTQLGVPSTVRGSFAGTAQV

FQETMNSQVILIIAAIATVYIVLGILYESYVHPLTILSTLPSAGVGALLALELFNAPFSLIALIGIMLLI

GIVKKNAIMMVDFALEAQRHGNLTPQEAIFQACLLRFRPIMMTTLAALFGALPLVLSGGDGSELRQPLEI

TIVGGLVMSQLLTLYTTPVVYLFFDRLRLRFSRKPKQTVTE

>gi|30063519|ref|NP_837690.1| hypothetical protein S2262 [Shigella flexneri 2a str. 2457T]

MSEQITFATSDFASNPEPRCPCILLLDVSGSMSGRPINELNTGLVTFRDELLADSLALKRVELGIVTFGP

VHVEQPFTSAANFFPPILFAQGDTPMGAAITKALDMVEERKREYRANGISYYRPWIFLITDGAPIDEWQA

AANKVFRGEEDKRFAFFSIGVQGADMKTLAQISVRQPLPLQGLQFRELFSWLSSSLRSVSRSTPGTEVVL

EAPKGWTSV

>gi|30063518|ref|NP_837689.1| hypothetical protein S2261 [Shigella flexneri 2a str. 2457T]

MSWRLVYASAVGTSHISADLPCQDACQMQVAWLNDQQPLLVMFLADGAGSVSQGGEGAMLAVNEAMAYMS

QKVQGGELGLNDILATDIVLTVRQRLFAEAEAKELAVRDFACTFLGLISSANGTLIMQIGDGGVVVDFGH

GLQLPLTPMVGEYANMTHFITDEDAVSRLETFTSTERVHKVAAFTDGIQRLALNMLDNSPHVPFFTPFFN

GLASATQEQLDLLPELLKQFLSSPAVNERTDDDKTLALALWLP

>gi|30063517|ref|NP_837688.1| 3-methyladenine DNA glycosylase [Shigella flexneri 2a str. 2457T]

MYILNWQPPYDWSWMLGFLAARAVSGVETVADSYYARSLAVGEYRGVVTAIPDIARHTLHINLSAGLEPV

AAECLAKMSRLLDLQCNPQIVNGALGKLGAARPGLRLPGSVDAFEQGVRAILGQLVSVAMAAKLTARVAQ

LYGERLDDFPEYICFPTPQRLAAADPQALKALGMPLKRAEALIHLANAALEGTLPMTIPGDVEQAMKTLQ

TFPGIGRWTANYFALRGWQAKDVFLPDDYLIKQRFLGMTPAQIRRYAERWKPWRSYALLHIWYTEGWQPD

EA

>gi|30063515|ref|NP_837686.1| uridine kinase [Shigella flexneri 2a str. 2457T]

MTDQSHQCVIIGIAGASASGKSLIASTLYRELREQVGDEHIGVIPEDCYYKDQSHLSMEERVKTNYDHPS

AMDHSLLLEHLQALKRGSAIDLPVYSYVEHTRMKETVTVEPKKVIILEGILLLTDARLRDELNFSIFVDT

PLDICLMRRIKRDVNERGRSMDSVMAQYQKTVRPMFLQFIEPSKQYADIIVPRGGKNRIAIDILKAKISQ

FFE

>gi|30063514|ref|NP_837685.1| deoxycytidine triphosphate deaminase [Shigella flexneri 2a str. 2457T]

MRLCDRDIEAWLDEGRLSINPRPPVERINGATVDVRLGNKFRTFRGHTAAFIDLSGPKDEVSAALDRVMS

DEIVLDEGEAFYLHPGELALAVTLESVTLPADLVGWLDGRSSLARLGLMVHVTAHRIDPGWSGCIVLEFY

NSGKLPLALRPGMLIGALSFEPLSGPAARPYNRREDAKYRNQQGAVASRIDKD

>gi|30063513|ref|NP_837684.1| assembly protein [Shigella flexneri 2a str. 2457T]

MRRFLTTLMILLVVLVAGLSALVLLVNPNDFRDYMVKQVAARSGYQLQLDGPLRWHVWPQLSILSGRMSL

TAQGASQPLVRADNMRLDVALLPLLSHQLSVKQVMLKGAVIQLTPQTEAVRSEDAPVAPRDNTLPDLSDD

RGWSFDISSLKVADSVLVFQHEDDEQVTIRNIRLQMEQDPQHRGSFEFSGRVNRDQRDLTISLNGTVDAS

DYPHDLTAAIEQINWQLQGADLPKQGIQGQGSFQSQWQESHKRLSFNQISLTANDSTLSGQAQVTLTEKP

EWQLRLQFPQLNLDNLIPLNETANGENGAAQQGQSQSTLPRPVISSRIDEPAYQGLQGFTADILLQASNV

RWRGMNFTDVATQMTNKSGLLEITQLQGKLNGGKVSLPGTLDATSINPRINFQPRLENVEIGTILKAFNY

PISLTGKMSLAGDFSGADIDADAFRHNWQGQAHVEMTDTRMEGMNFQQMIQQAVERNGGDVKAAENFDNV

TRLDRFTTDLTLKDGVVTLNDMQGQSPVLALTGEGMLNLADQTCDTQFDIRVVGGWNGESKLIDFLKETP

VPLRVYGNWQQLNYSLQVDQLLRKHLQDEAKRRLNDWAERNKDSRNGKDVKKLLEKM

>gi|30063512|ref|NP_837683.1| transporter [Shigella flexneri 2a str. 2457T]

MEWIADPSIWAGLITLIVIELVLGIDNLVFIAILAEKLPPKQRDRARVTGLLLAMLMRLLLLASISWLVT

LTQPLFSFRSFTFSARDLIMLFGGFFLLFKATMELNERLEGKDSNNPTQRKGAKFWGVVTQIVVLDAIFS

LDSVITAVGMVDHLLVMMAAVVIAISLMLMASKPLTQFVNSHPTIVILCLSFLLMIGFSLVAEGFGFVIP

KGYLYAAIGFSVMIEALNQLAIFNRRRFLSANQTLRQRTTEAVMRLLSGQKEDAELDAETASMLVDHGNQ

QIFNPQERRMIERVLNLNQRTVSSIMTSRHDIEHIDLNAPEEEIRQLLERNQHTRLVVTDGDDAEDLLGV

VHVIDLLQQSLRGEPLNLRVLIRQPLVFPETLPLLPALEQFRNARTHFAFVVDEFGSVEGIVTLSDVTET

IAGNLPNEVEEIDARHDIQKNADGSWTANGHMPLEDLVQYVPLPLDEKREYHTIAGLLMEYLQRIPKPGE

EVQVGDYLLKTLQVESHRVQKVQIIPLRKDGEMEYEV

>gi|30063508|ref|NP_837679.1| colanic acid biosynthesis acetyltransferase WcaB [Shigella flexneri 2a str. 2457T]

MLEDLRANSWSLRPCCMVLAYRVAHFCSVWRKKNVLNNLWAAPLLVLYRIITECFFGYEIQAAATIGRRF

TIHHGYAVVINKNVVAGDDFTIRHGVTIGNRGADNMACPHIGNGVELGANVIILGDITLGNNVTVGAGSV

VLDSVPDNALVVGEKARVKVIK

>gi|30063505|ref|NP_837676.1| colanic acid biosynthesis acetyltransferase WcaF [Shigella flexneri 2a str. 2457T]

MQDLSGFSVPKGFRGGNAIKVQLWWAVQATIFAWSPQVLYRWRAFLLRLFGAKIGKNVVIRPSVKITYPW

KLTLGDYAWVGDDVNLYTLGEITIGAHSVISQKSYLCTGSHDHASQHFTINATPIVIGEKCWLATDVFVA

PGVTIGDGTVVGARSSVFKSLPANVVCRGNPAVVIRERVETE

>gi|30063502|ref|NP_837673.1| GDP-mannose mannosyl hydrolase [Shigella flexneri 2a str. 2457T]

MFLRQEDFATVVRSTPLVSLDFIVENSRGEFLLGKRTNRPAQGYWFVPGGRVQKDETLEAAFERLTMAEL

GLRLPITAGQFYGVWQHFYDDNFSGTDFTTHYVVLGFRFRVAEEELLLPDEQHDDYRWLTPDALLASNDV

HANSRAYFLAEKRAGVPGL

>gi|30063501|ref|NP_837672.1| glycosyl transferase [Shigella flexneri 2a str. 2457T]

MKILVYGINYSPELTGIGKYTGEMVEWLAAQGHEVRVITAPPYYPQWQVGENYSAWRYKREEGAATVWRC

PLYVPKQPSTLKRLLHLGSFAVSSFFPLMAQRRWKPDRIIGVVPTLFCTPGMRLLAKLSGARTVLHIQDY

EVDAMLGLGLAGKGKGGKVAQLATAFERSGLHNVDNVSTISRSMMNKAIEKGVAADNVIFFPNWSEIARF

QHVADADVDALRNQLGLPDNKKIILYSGNIGEKQGLENVIEAADLLRDEPLIFAIVGQGGGKARLEKMAQ

QRGLRNMQFFPLQSYDALPALLKMGDCHLVVQKRGAADAVLPSKLTNILAVGGNAVITAEAHTELGQLCE

TFPGIAVCVEPESVEALVAGIRQALLLPKHNTVAREYAERTLDKENVLRQFINDIRG

>gi|30063500|ref|NP_837671.1| mannose-1-phosphate guanyltransferase [Shigella flexneri 2a str. 2457T]

MAQSKLYPVVMAGGSGSRLWPLSRVLYPKQFLCLKGDLTMLQTTICRLNGVECESPVVICNEQHRFIVAE

QLRQLNKLTENIILEPAGRNTAPAIALAALAAKRHSPESDPLMLVLAADHVIADEDAFRAAVRNAMPYAE

AGKLVTFGIVPDLPETGYGYIRRGEVSVGEQDTVAFEVAQFVEKPNLETAQAYVASGEYYWNSGMFLFRA

GRYLEELKKYRPDILDACEKAMSAVDPDLDFIRVDEEAFLACPEESVDYAVMEPTADAVVVPMDAGWSDV

GSWSSLWEISAHTAEGNVCHGDVINHKTENSYVYAESGLVTTVGVKDLVVVQTKDAVLIADRNAVQDVKK

VVEQIKADGRHEHRVHREVYRPWGKYDSIDAGDRYQVKRITVKPGEGLSVQMHHHRAEHWVVVAGTAKVT

IDGDIKLLGENESIYIPLGATHCLENPGKIPLDLIEVRSGSYLEEDDVVRFADRYGRV

>gi|30063498|ref|NP_837669.1| UDP-glucose lipid carrier transferase [Shigella flexneri 2a str. 2457T]

MTNLKKRERAKTNASLISMVQRFSDITIMFAGLWLVCEVSGLSFLYMHLLVALITLVVFQMLGGITDFYR

SWRGVRAATEFALLLQNWTLSVIFSAGLVAFNNDFDTQLKIWLAWYGLTSIGLVVCRSCIRIGAGWLRNH

GYNKRMVAVAGDLAAGQMLMESFRNQPWLGFEVVGVYHDPKPGGVSNDWAGNLQQLVEDAKAGKIHNVYI

AMQMCDGARVKKLVHQLADTTCSVLLIPDVFTFNILHSRLEEMNGVPVVPLYDTPLSGVNRLLKRAEDIV

LATLILLLISPVLCCIALAVKLSSPGPVIFRQTRYGMDGKPIKVWKFRSMKVMENDKVVTQATQNDPRVT

KVGNFLRRTSLDELPQFINVLTGGMSIVGPRPHAVAHNEQYRQLIEGYMLRHKVKPGITGWAQINGWRGE

TDTLEKMEKRVEFDLEYIREWSVWFDIKIVFLTVFKGFVNKAAY

>gi|30063497|ref|NP_837668.1| colanic acid exporter [Shigella flexneri 2a str. 2457T]

MSLREKTISGAKWSAIATVIIIGLGLVQMTVLARIIDNHQFGLLTVSLVIIALADTLSDFGIANSIIQRK

EISHLELTTLYWLNVGLGLVVCVAVFLLSDVIGDVLNNPDLAPLIKTLSLAFVVIPHGQQFRALMQKELE

FNKIGMIETSAVLAGFTFTVVSAHFWPLAMTAILGYLVNSAVRTLLFGYFGRKIYCPGLHFSLASVAPNL

RFGAWLTADSIINYLNTNLSTLVLARILGAGVAGGYNLAYNVAVVPPMKLNPIITRVLFPAFAKIQDDTE

KLRVNFYKLLSIVGIINFPALLGLMVVSNNFVPLVFGEKWNSIIPVLQLLCIVGLLRSVGNPIGSLLMAK

ARVDISFKFNVFKTFLFIPAIVIGGQMAGAIGVTLGFLLVQIINTILSYFVMIKPVLGSSYRQYILSLWL

PFYLSLPTLVVSYALGIVLKGQLALGMLLAVQIAAGVLAFVVMIVLSRHPLVVEVKRQFCRSEKMKMLLR

AG

>gi|30063496|ref|NP_837667.1| pyruvyl transferase [Shigella flexneri 2a str. 2457T]

MKLLILGNHTCGNRGDSAILRGLLDAINILNPHTEVDVMSRYPVSSSWLLNRPVMGDPLFLQMTQHNSAA

GVVGRIKKVLRRRYQHQVLLSRVTDTGKLRNIAIAQGFTDFVRLLSGYDAIIQVGGSFFVDLYGVPQFEH

ALCTFMAKKPLFMIGHSVGPFQDEQFNQLANYVFGHCDALILRESVSLDLMKRRNITTAKVEHGVDTAWL

VDHHTEDFTASYAVQHWLDVAAQQKTVAITLRELAPFDKRLGTTQQAYEKAFAGVVNRILDEGYQVIALS

TCTGIDSYNKDDRMVALNLRQHISDPARYHVVMDELNDLEMGKILGACELTVGTRLHSAIISMNFATPAI

AINYEHKSAGIMQQLGLPEMAIDIRHLLDGSLQAMVADTLGQLPVLNARFNEAVSRERQTGMQMVQSVLE

RIGEVK

>gi|30063494|ref|NP_837665.1| colanic acid biosynthesis protein [Shigella flexneri 2a str. 2457T]

MPFKTLSRRTFLTASSALAFLHTPFARALPARQSVNINDYNPHDWIASFKQAFSEGQTVVVPAGFVCDNI

NTGIFIPPGKTLHILGSLRGNGRGRFVLQDGSQVTGGEGGGMHNITLDVRGSDCTIKGLAMSGFGPVMQI

YIGGKNKRVMRNLTIDNLTVSHANYAILRQGFHNQIIGANITNCKFSDLQGDAIEWNVAINDSDILISDH

VIERINCTNGKINWGIGIGLAGSTYDNNYPEDQAVKNFVVANITGSDCRQLIHVENGKHFVIRNINARNI

TPDFSKKAGIDNATVAIYGCDNFVIDNIEMINSAGMLIGYGVIKGKYLSIPQNFRVNNIQLDNTHLAYKL

RGIQISAGNAVSFVSLTNIEMKRASLELHNKPQHLFMRNINVMQESSVGPALSMNFDMRKDVRGVFMAKK

ETLLSLANVHAVNERGQSSVDIDRINHHIVNVEKINFRLPERRE

>gi|30063489|ref|NP_837660.1| dTDP-6-deoxy-L-mannose-dehydrogenase [Shigella flexneri 2a str. 2457T]

MNVIKTEIPDVLIFEPKVFGDERGFFMESFNQKVFEEAVGRKVEFVQDNHSKSTKGVLRGLHYQLEPYAQ

GKLVRCVVGEVFDVAVDIRKSSPTFGKWVGVNLSAENKRQLWIPEGFAHGFCVLSDEAEFVYKTNNFYSK

MQERGILWSDKSINIEWPVQNPLLSDKDINGQKFVDADYFI

>gi|30063488|ref|NP_837659.1| polysaccharide biosynthesis protein [Shigella flexneri 2a str. 2457T]

MSIIKNSVWNLFGYAIPTLIAIPSLGFLARGLGPEGFGVYTIAIALVGYAGIFDVGLTRSVIREIAIHRD

NHHERTKVISTSTSFLVLFSCFGAFLLLIFSDGIVNYLKISGVEHSDIQLAFKLLAICIPLFILNQLWSA

ILEGDEKFGIVNIQKSISSSCIAGIPAIFVFYSATLSAAVAGLIFARVISILVSAYYVRNDIKISGVHFC

YKTFKRLFFFGGWMTVSNIISPVMVYFDRFIVSNIMGADKVAFYSAPAEVILKLGIIPAAIGRAVFPRLS

NIKDFKEFKRNVNKSLLLMFLICLPVIIIGLLYSGLVLKIWFGENYQINSFNILNVLLIGFFFNALAMIP

FSAIQALGKSKITALIHCAELVPYLALLYFMVEKYGLLGAAISWSIRVILDALLLQWLYTRMCSVYEN

>gi|30063487|ref|NP_837658.1| dTDP-rhamnosyl transferase [Shigella flexneri 2a str. 2457T]

MNSNIYAVIVTYNPELKNLNALITELKEQNCYVVVVDNRTNFTLKDKLADIEKVHLICLGRNEGIAKAQN

IGIRYSLEKGAEKIIFFDQDSRIRNEFIKKLSCYMDNENAKIAGPVFIDRDKSHYYPICNIKKNGLREKI

HVTEGQTPFKSSVTISSGTMVSKEVFEIVGMMDEELFIDYVDTEWCLRCLNYGILVHIIPDIEMVHAIGD

KSVKICGINIPIHSPVRRYYRVRNAFLLLRKNHVPLLLSIREVVFSLIHTTLIIATQKNKIEYMKKHILA

TLDGIRGITGGGRYNA

>gi|30063486|ref|NP_837657.1| dTDP-rhamnosyl transferase [Shigella flexneri 2a str. 2457T]

MATYNGECWIEEQLKSIIEQKDVDISIFISDDLSTDNTLNICEEFQLSYPSIINILPSVNKFGGAGKNFY

RLIKDVDLENYDYICFSDQDDIWYKDKIKNAIDCLVFNNANCYSSNVIAYYPSGRKNLVDKAQSQTQFDY
FFEAAGPGCTYVIKKETLIEFKKFIINNKNAAQDICLHDWFLYSFARTRNYSWYIDRKPTMLYRQHENNQ
VGANISFKAKYKRLGLVRNKWYRKEVTKIANALADDSFVNNQLGKGYIGNLILALSFWKLRRKKADKIYI
LLMLILNIF

>gi|30063485|ref|NP_837656.1| O-antigen polymerase [Shigella flexneri 2a str. 2457T]
MNNINKIFITFLCIELIIGGGGRLLEPLGIFPLRYLLFVFSFILLIFNLVTFNFSITQKCVSLFIWLLLF
PFYGFFVGLLAGNKINDILFDVQPYLFMLSLIYLFTLRYTLKVFSCEIFIKIVNAFALYGSLLYISYIIL
LNFGLLNFNLIYEHLSLTSEFFFRPDGAFFSKSFYFFGVGAIISFVDKKYLKCLIIVLAILLTESRGVLL
FTTLSLLLASFKLHKLYLNTIIIILGSVLFIIMLYMVGSRSEDSDSVRFNDLYFYYKNVDLATFLFGRGF
GSFILDRLRIEIVPLEILQKTGVIGVFISLVPMLLIFLKGYFLNSTKTSLMMSLILFFSITVSITNPFLF
TPMGIFIIGVVVLWVFSIENIQISNNLTSGAK

>gi|30063484|ref|NP_837655.1| glycosyl translocase [Shigella flexneri 2a str. 2457T]
MLKIGKLLTSSFFSYFLIGIVNTALHWGVFYACYNNLAFGQGRSNIVGFICAATFSFFANARCSFKVSAT
KARYFIFIFFMGAMSYLFGVLFDLLALSPIFTLFTFSLFSLVLGYCASKYFIFR

>gi|30063483|ref|NP_837654.1| glycosyl transferase, partial [Shigella flexneri 2a str. 2457T]
MYHSFNIAVIIPCYNEQKAIAKVINDFKTNIPTASIYVFDNNSTDSTAQVAEDAGATVHSVPLKGKGNVV
RRMFSDVDADIYLMVDGDDTYDASSAPEMINHLIKNQLDMVVGCRQENGDQNTYRKGHR

>gi|30063482|ref|NP_837653.1| hypothetical protein S2216, partial [Shigella flexneri 2a str. 2457T]
MLSGYRVFSRRYVKSFPCLSRGFEIETELTIHALELRMKYGEVNTKYGERSEGSVSKLSTWSDGFKILKT
IIKLYSLERPLYFFSIIGVLLAALSIILGLPIIVDYIDTGLVRRFPTAFLTASIMLSSIMAFVCGIILHS
NTTTRREMKALFYLSEKNYKLIM

>gi|30063481|ref|NP_837652.1| hypothetical protein S2215 [Shigella flexneri 2a str. 2457T]

MSLNILIIYFLGMVGQFNKIAIFLIFTVCWVLSIIKRQQFRWLAINNIEFSTLFVILFLVLIFVVTLLSS

LRAPGDWDDTMYHLPLARSLVEHHAIVVEQYLRFPLFPQNADLLMALGLQLGDVRLAQFLANICFFVIAC

GLVGCSWEITKTYYPGIIATILLFTINPLKDHLGYAYIDLTLSLFCCSQYSYIYSLRKQ

>gi|30063480|ref|NP_837651.1| hypothetical protein S2214 [Shigella flexneri 2a str. 2457T]

MIAKKRNIQTFLIFSLATILGGIYWYVRSFWISGDPFSPAGGNIFGHYLWNEIDLQVQTAEQARHGISPA

TLDLQQAINKVGSDVVFYAILSVFTLRNNKILWVFFSIVLTYVLFWFSVTQGDRYLSPIYPLSVLQVAIS

IASLIKDINITNYKLGRYFILVCIIFFRGDRCILNSNIFILNT

>gi|30063479|ref|NP_837650.1| hypothetical protein S2213 [Shigella flexneri 2a str. 2457T]

MNFNSRLESRYSYELMKKASEYSELYGDNLIQLGLEDGIYFYKGMAIGDVFGLARYSDWTISNPECEVIP

QDDLIEKMKSFNSSFIVISKRSYANFNPEKYPKFKVLMDTPNGILIAIK

>gi|30063477|ref|NP_837648.1| regulator of length of O-antigen component of lipopolysaccharide chains [Shigella flexneri 2a str. 2457T]

MRTWKFPSVRVMMRVENNNVSGQNHDPEQIDLIDLLVQLWRGKMTIIISVIVAIALAIGYLAVAKEKWTS

TAIITQPDVGQIAGYNNAMNVIYGQAAPKVSDLQETLIGRFSSAFSALAETLDNQEEPEKLTIEPSVKNQ

QLPLTVSYVGQTAEGAQMKLAQYIQQVDDKVNQELEKDLKDNIALGRKNLQDSLRTQEVVAQEQKDLRIR

QIQEALQYANQAQVTKPQVQQTEDVTQDTLFLLGSEALESMIKHEATRPLVFSPNYYQTRQNLLDIEKLK

FDDLDIHAYRYVMKPTLPIRRDSPKKAITLILAVLLGGMVGAGIVLGRNALRNYNAK

>gi|30063476|ref|NP_837647.1| bifunctional phosphoribosyl-AMP cyclohydrolase/phosphoribosyl-ATP [Shigella flexneri 2a str. 2457T]

MLTEQQRRELDWEKTDGLMPVIVQHAVSGEVLMLGYMNPEALDKTIESGKVTFFSRTKQRLWIKGETSGN

FLNVVSIAPDCDNDTLLVLANPIGPTCHKGTSSCFGNTAHQWLFLYQLEQLLAERKYADPETSYTAKLYA

SGTKRIAQKVGEEGVETALAATVHDRFELTNEASDLMYHLLVLLQDQDLDLTTVIENLHKRHQ

>gi|30063475|ref|NP_837646.1| imidazole glycerol phosphate synthase subunit HisF [Shigella flexneri 2a str. 2457T]

MLAKRIIPCLDVRDGQVVKGVQFRNHEIIGDIVPLAKRYAEEGADELVFYDITASSDGRVVDKSWVSRVA

EVIDIPFCVAGGIKSLEDAAKILSFGADKISINSPALADPTLITRLADRFGVQCIVVGIDTWYDAETGKY

HVNQYTGDESRTRVTQWETLDWVEEVQKRGAGEIVLNMMNQDGVCNGYDLKQLKKVREVCHVPLIASGGA

GTMEHFLEAFRDADVDGALAASVFHKQIINIGELKAYLATQGVEIRIC

>gi|30063474|ref|NP_837645.1| 1-(5-phosphoribosyl)-5-[(5-phosphoribosylamino)methylideneamino] imidazole-4-carboxamide isomerase [Shigella flexneri 2a str. 2457T]

MIIPALDLIDGTVVRLHQGDYGKQRDYGNDPLPRLQDYAAQGAEVLHLVDLTGAKDPAKRQIPLIKTLVA

GVNVPVQVGGGVRSEEDVAALLEAGVARVVVGSTAVKSPDVVKGWFERFGADALVLALDVRIDEQGNKQV

AVSGWQENSGVSLEQLVETYLPVGLKHVLCTDISRDGTLAGSNVSLYEEVCARYPQVAFQSSGGIGDIND

VAALRGTGVRGVIVGRALLEGKFTVKEAIACWQNA

>gi|30063473|ref|NP_837644.1| imidazole glycerol phosphate synthase subunit HisH [Shigella flexneri 2a str. 2457T]

MNVVILDTGCANLNSVKSAIARHGYEPKVSRDPDIVLLADKLFLPGVGTAQAAMDQVRERELFDLIKACT

QPVLGICLGMQLLGRRSEESNGVDLLGIIDEDVPKMTDFGLPLPHMGWNRVYPQAGNRLFQGIEDGAYFY

FVHSYAMPVNPWTIAQCNYGEPFTAAVQKDNFYGVQFHPERSGAAGAKLLKNFLEM

>gi|30063472|ref|NP_837643.1| imidazole glycerol-phosphate dehydratase/histidinol phosphatase [Shigella flexneri 2a str. 2457T]

MSQKYLFIDRDGTLISEPPSDFQVDRFDKLAFEPGVIPELLKLQKAGYKLVMITNQDGLGTQSFPQADFD

GPHNLMMQIFTSQGVQFDEVLICPHLPADECDCRKPKVKLVEGYLAEQAMDRANSYVIGDRATDIQLAEN

MGINGLRYDRETLNWPMIGEQLTKRDRYAHVVRNTKETQIDVQVWLDREGGSKINTGVGFFDHMLDQIAT

HGGFRMEINVKGDLYIDDHHTVEDTGLALGEALKIALGDKRGICRFGFVLPMDECLARCALDISGRPHLE

YKAEFTYQRVGDLSTEMIEHFFRSLSYTMGVTLHLKTKGKNDHHRVESLFKAFGRTLRQAIRVEGDTLPS

SKGVL

>gi|30063470|ref|NP_837641.1| histidinol dehydrogenase [Shigella flexneri 2a str. 2457T]

MSFNTIIDWNSCTAEQQRQLLMRPAISASESITRTVNDILDNVKTRGDEALREYSAKFDKTTVTALKVSA

DEIAAASERLSDELKQAMAVAVKSIETFHTAQKLPPVDVETQLGVRCQQVTRPVASVGLYIPGGSAPLFS

TVLMLATPARIAGCKKVVLCSPPPIADEILYAAQLCDVQDVFNVGGAQAIAALAFGTESVPKVDKIFGPG

NAFVTEAKRQVSQRLDGAAIDMPAGPSEVLVIADSGATPDFVASDLLSQAEHGPDSQVILLTPDADMARR

VAEAVERQLAELPRAETTRQALNASRLIVTKDLAQCVEISNQYGPEHLIIQTRNARELVDGITSAGSVFL

GDWSPESAGDYASGTNHVLPTYGYTATCSSLGLADFQKRMTVQELSKEGFSALASTIETLAAAERLTAHK

NAVTLRVNVLKEQA

>gi|30063469|ref|NP_837640.1| ATP phosphoribosyltransferase [Shigella flexneri 2a str. 2457T]

MTDNTRLRIAMQKSGRLSDDSRELLARCGIKINLHTQRLIAMAENMPIDILRVRDDDIPCLVMDGVVDLG

IIGENVLEEELLNRRAQGEDPRYFTLRRLDFGGCRLSLATPVDEAWDGPLSLNGKRIATSYPHLLKRYLD

QKGISFKSCLLNGSVEVAPRAGLADAICDLVSTGATLEANGLREVEVIYRSKACLIQRDGEMEESKQQLI

DKLLTRIQGVIQARESKYIMMHAPTERLDEVIALLPGAERPTILPLAGDQQRVAMHMVSSETLFWETMEK

LKALGASSILVLPIEKMME

>gi|30063465|ref|NP_837636.1| enzyme of sugar metabolism [Shigella flexneri 2a str. 2457T]

MKKVAIVGLGWLGMPLAMSLSARGWQVTGSKTTQDGVEAARMSGIDSYLLRMEPELVCDSDDLDALMDAD

ALVITLPARRSGPGDEFYLQAVQELVDSALAHRIPRIIFTSSTSVYGDAQGTVKETTPRNPVTNSGRVLE

ELEDWLHNLPGTSVDILRLAGLVGPGRHPGRFFAGKTAPDGEHGVNLVHLEDVIGAITLLLQAPKGGHIY

NICAPAHPARNVFYPQMARLLGLQPPQFRNSLDSGKGKIIDGSRICNELGFEYQYPDPLVMPLE

>gi|30063464|ref|NP_837635.1| LYSR-type transcriptional regulator [Shigella flexneri 2a str. 2457T]

MKPLLDVLMILDAIEKEGSFAAASAKLYKTPSALSYTVHKLESDLNIQLLDRSGHRAKFTRTGKMLLEKG

REVLHTVRELEKQAIKLHEGWENELVIGVDDTFPFSLLAPLIEAFYQHHSVTRLKFINGVLGGSWDALTQ

GRADIIVGAMHEPPSSSEFGFSRLGDLEQVFAVAPHHPLAQEEEPLNRRIIKRYRAIVVGDTAQAGASTA

SQLLDEQEAITVFDFKTKLELQISGLGCGYLPRYLAQRFLDSGALIEKKVVAQTLFEPVWIGWNEQTAGL

ASGWWRDEILANSAIAGVYAKSDDGKSAIQRKIIMTSLSFSCHFPPI

>gi|30063462|ref|NP_837633.1| transport system permease [Shigella flexneri 2a str. 2457T]

MFSMILSGLICGALLGFVMQRGRFCLTGGFRDMYIAKNNRMFYALLIAISAQSVGVFALIQAGLLTYEAG

AFPWLGTVIGGYLFGLGIVLAGGCATGTWYRAGEGLIGSWIALFTYMVMSAVMRSPHASGLNQTLQHYTT

EHNSIAETFNLSVWPLVTVLLVITLWVVMKELKKPKLKVATLPPRRTGIAHILFEKRWHPFVTAVLIGLI

ALLAWPLSEATGRMFGLGVTSPTANILQFLVAGDVKYINWGVFLVLGIFVEAFIAAKASREFRVRAADAQ

TTLRSGLGGVLMGFGASIAGGCSIGNGLVMTAMMTWQGWIGLVFMILGVWTASWLVYVRPQRKARLATAA

AN

>gi|30063460|ref|NP_837631.1| exonuclease I [Shigella flexneri 2a str. 2457T]

MTDTDKQPTFLFHDYETFGTHPALDRPAQFAAIRTDDEFNVIGEPEVFYCKPADDYLPRPGAVLITGITP

QEARAKGENEAAFAARIHSLFTVPKTCILGYNNVRFDDEVTRNVFYRNFYDPYAWSWQHDNSRWDLLDVM

RACYALRPEGINWPENDDGLPSFRLEHLTKANGIEHSNAHDAMADVYATIAMAKLVKTRQPRLFDYLFTH

RNKHKLMALIDVPQMKPLAHVSGMFGAWRGNTSWVAPLAWHPENRNAVIMADLAGDISPLLELDIDTLRE

RLYTAKADLGDNAAVPVKLVHINKCPVLAQANTLRPEDADRLGINRQHCLDNLKILRENPQVREKVVAIF

AEAEPFTPSDNVDAQLYNGFFSDADRAAMKIVLETEPRNLPALDITFVDKRIEKLLFNYRARNFPGTLDY

AEQQRWLEHRHQVFTPEFLQGYADELQMLVQQYADDKEKVALLKALWQYAEEIV

>gi|30063455|ref|NP_837626.1| hypothetical protein S2185, partial [Shigella flexneri 2a str. 2457T]

MYNDVLFPGEDINKPISIAAANRFVNRIRGGMDLGYWRTHDFRRTLVTRLSEMNVEPHVTERMLGHELGG

IMSVYNKHDWIEAQRKAYELHADKLFWHIRSISD

>gi|30063453|ref|NP_837624.1| IS629 orfB [Shigella flexneri 2a str. 2457T]

MPLLDKLREQYGVGPVCSELHIAPSTYYHCQQQRHHPDKRSARAQRDDWLKKEIQRVYDENHKVYGVKSG

VSCYGKVSEWPDALWHVSWRLWDLPVFSGVKRSVRPSAGKPLPQATA

>gi|30063449|ref|NP_837620.1| hypothetical protein S2177 [Shigella flexneri 2a str. 2457T]

MLALTNSGCLNESDSHIIRGIKMETTKPSFQDVLEFVRLFRRKNKLQREIQDVEKKIRDNQKRVLLLDNL

SDYIKPGMSVEAIQGIIASMKGDYEDRVDDYIIKNAELSKERRDISKKLKAMGEMKNGEAK

>gi|30063444|ref|NP_837615.1| cobalamin synthase [Shigella flexneri 2a str. 2457T]

MSKLFWAMLSFITRLPVPRRWSQGLDFEHYSRGIITFPLIGLLLGAISGLVFMVLQAWCGAPLAALFSVL

VLALMTGEFHLDGLADTCDGVFSARSRDRMLEIMRDSRLGTHGGLALIFVVLAKILVLSELALRGEPILA

SLAAACAVSRGTAALLMYRHRYAREEGLGNVFIGKIDGRQTCVTLGLAAIFAAVLLLGMHGVAAMVVTMV

AIFILGQLLKRTLGGQTGDTLGAAIELGELVFLLALL

>gi|30063443|ref|NP_837614.1| nicotinate-nucleotide--dimethylbenzimidazole phosphoribosyltransferase [Shigella flexneri 2a str. 2457T]

MQIIADLLNTIPAINSAAMSRAQRHVDGLFKPVGSLGKLEALAIQLAGMPGVNGIPHVGKKAVLVMCADH

GVWEEGVAISPKEVTAIQAENMTRGTTGVCVLAAQAGANVHVIDVGIDTAEPIPGLINMRVARGSGNIAS

APAMSRRQAEKLLLDVICYTRELAKNGVTLFGVGELGMANTTPAAAIVSTITGRDPEEVVGIGANLPTDK

LANKIDVVRRAITLNQPNPQDGVDVLAKVGGFDLVGIAGVMLGAASCGLPVLLDGFLSYAAALAACQMSP

AIKPYLIPSHLSAEKGARIALSHLGLEPYLNMDMRLGEGSGAALAMSIIEAACAIYNNMGELAASNIVLP

GNTTSDLNS

>gi|30063442|ref|NP_837613.1| hypothetical protein S2168 [Shigella flexneri 2a str. 2457T]

MMRRVNILCSFALLFASQNSLAVTYPLPPEGSRLVGQSLTVTVPDHNTQPLETFAAQYGQGLSNMLEANP

GADVFLPKPGSQLTIPQQLILPATVRKGIVVNVAEMRLYYYPPDSNTVEVFPIGIGQAGRETPRNWVTTV

ERKQEAPTWTPTPNIRREYAKRGESLPAFVPAGPDNPMGLYAIYIGRLYAIHGTNANFGIGLRVSQGCIR

LRNDDIKYLFDNVPVGTRVQIIDQPVKYTTEPDGSKWLEVHEPLSRNRAEYESDRKVPLPVTPSLRAFIN

GQEVDVNRANAALQHRSGMPVQISSGSRQMF

>gi|30063441|ref|NP_837612.1| nitrogen assimilation transcriptional regulator [Shigella flexneri 2a str. 2457T]

MNFRRLKYFVKIVDIGSLTQAAEVLHIAQPALSQQVAILEGELNQQLLIRTKRGVTPTDAGKILYTHARA

ILRQCEQAQLAVHNVGQSLSGQVSIGFAPGTAASSITMPLLQAVRAEFPEVVIYLHENSGAVLNEKLINH

QLDMAVIYEHSPVAGVSSQALLKEDLFLVGTQDCPGQNVDVNAIAQMNLFLPSDYSAVRLRVDEAFSLRR

LTAKVIGEIESIATLTAAIASGMGVAVLPESAARSLCGAVNGWMSRITTPSMSLSLSLNLPARANLSPQA

QAVKELLMSVISSPVMEKRQWQLVS

>gi|30063440|ref|NP_837611.1| transcriptional regulator Cbl [Shigella flexneri 2a str. 2457T]

MNFQQLKIIREAARQDYNLTEVANMLFTSQSGVSRHIRELEDEFGIEIFVRRGKRLLGMTEPGKALLVIA

ERILNEASNVRRLADLFTNDTSGVLTIATTHTQARYSLPEVIKAFRELFPEVRLELIQGSPQEIATLLQN

GEADIGIASERLSNDPQLVAFPWFCWHHSLLVPLDHPLTQITPLTLESIAKWPLITYRQGITGRSRTDDA

FTRKGLPADIVLSAQDSDVIKTYVALGLGIGLVAEQSSGEQEEENLIRLDTRHLFDANTVWLGLKRGQLQ

RNYVWRFLELCNAGLSVEDIKRQVMENSEEEIDYQI

>gi|30063438|ref|NP_837609.1| AMP nucleosidase [Shigella flexneri 2a str. 2457T]

MNNKGSGLTPAQALDKLDALYEQSVVALRNAIGKYITIGELPDENARKQGLFVYPSLTVTWDGSTTNPPK

TRAFGRFTHAGSYTTTITRPTLFRSYLNEQLTLLYQDYGAHISVQPSQHEIPYPYVIDGSELTLDRSMSA

DLTRYFPTTELAQIGDETADGIYHPTEFSPLSHFDARRVDFSLARLRHYTGTPVEHFQPFVLFTNYTRYV

DEFVRWGCSQILDPDSPYIALSCAGGNWITAETEAPEEAISDLAWKKHQMPAWHLITADGQGITLVNIGV

GPSNAKTICDHLAVLRPDIWLMIGHCGGLRESQAIGDYVLAHAYLRDDHVLDAVLPPDIPIPSIAEVQRA

LYDATKLVSGRPGEEVKQRLRTGTVVTTDDRNWELRYSASALRFNLSRAVAIDMESATIAAQGYRFRVPY

GTLLCVSDKPLHGEIKLPGQANRFYEGAISEHLQIGIRAIDLLRAEGDRLHSRKLRTFNEPPFR

>gi|30063433|ref|NP_837604.1| hypothetical protein S2148 [Shigella flexneri 2a str. 2457T]

MQFCSSDEFASKTMIKWPWKVQESAHQTALPWQEALSIPLLTCLTEQEQSKLVALAERFLQQKRLVPLQG

FELNSLRSCRIALLFCLPVLELGLEWLDGFHEVLIYPAPFVVDDEWEDDIGLVHNQRIVQSGQSWQQGPI

VLNWLDIQDSFDASGFNLIIHEVAHKLDTRNGDRASGVPFISLREVAGWEHDLHAAMNNIQEEIELVGEN

AASIDAYAASDPAECFAVLSEYFFSAPELFAPRFPSLWQRFCQFYQQDPLQRLHHANDTDSFSATNVH

>gi|30063428|ref|NP_837599.1| hypothetical protein S2141 [Shigella flexneri 2a str. 2457T]

MDSRPGLRTTSPSSGTRTGRSNTRTLPPDPLLECAGRTRWPFGIREPDEDYPVTIIETEGFLNLVSVSAV

ALNQPFSLFFGAGVVLIPVRFECMRTPPVPAGRGGRVIRIVKFGIVRHLFSLLAQQVPVVQADGWIVNQN

DQKKQNPPKRVKCGCVEDA

>gi|30063426|ref|NP_837597.1| crossover junction endodeoxyribonuclease [Shigella flexneri 2a str. 2457T]

MTERIEFVLPYPPTVNTYWRRRGSTYFVSKAGERYRRDVALIVRQQRLKLNLSGRLAIKIIAEPPDKRRR

DLDNILKAPLDALTHAGLLIDDEQFDEINIVRGQLVPGGRLGIKITELGCA

>gi|30063419|ref|NP_837590.1| sheath protein [Shigella flexneri 2a str. 2457T]

MTISFNTVPSNTLVPLFYAEMNNSAANTAVTSAPALLIGHASNDAAIEVNSLVLMPSADYARQICGAGSQ

LARMVEVYRQTDPFGELYVIAVPEARGAAATVRVTVAGEAEESGTLSLYVGRSRVQVPVVNGDDATAVAT

AIKEAVNGVITLPFTASSDAGVVTLTARHKGLYGNELPVCLNYYGSGGGEILPAGLRVVTEVGAAGSGAP

DLTAAVAAMGDEAFDFIGLPFNDAASINMMMTEMNDSSGRWSYARQLYGHVYTAKLGTLSELVGAGDMHN

QQHITLAGYEKETQSPVDELVASRLAREAVFIRNDPARPTQTGELVGMLPAPKGKRFIMTEQQTLLSHGV

ATAYVEGGTLQIQRSVTTYKKNAYGVADNSYLDSETLHTSAYVLRKLKSVITSKYGRHKLANDGTRFGPG

QAIVTPAVIKGELLATYRQMERAGIVENYDLFKQYLIVERDADNPNRLNTLFPPDYVNQLRVFAVVNQFR

LQYSEESA

>gi|30063418|ref|NP_837589.1| hypothetical protein S2127 [Shigella flexneri 2a str. 2457T]

MAKIAGTCFFKVDGQQLSLTGGIEVPMNTNVRDDIVGMAGDVDYKETWRSPYVKGTFKVPKNFPVDKITT

SDQMTITAELANGMVYVLSAAWLHGEANHNAEEGTADLEFHGEEGGYQ

>gi|30063417|ref|NP_837588.1| bacteriophage protein [Shigella flexneri 2a str. 2457T]

MAMNVTEIVLKKPVTAHNEMLHVLELREPTYDEIEALGFPFIISGEGSIKLDSQVALKYIPLLAGIPCSS

AAQMAKLDIFKTSMQILRFFTQSETGSTSGNDSTMLPGSGN

>gi|30063416|ref|NP_837587.1| tail protein [Shigella flexneri 2a str. 2457T]

MADSFQLKAIITAVDKVSAPLKGMQRQLKGFKKEFASLSLGAAGAGTAVLGALALPVKSAIALESKMADV

RKVVDGLDTPEAFKAMTEQVRDLSTELPMSAEGIAEIVAAGGQAGIARDELMQFTDDAVKMGVAFDTTAE

ESGQMMAQWRTAFKLTQGEVAGLADKINYLGNTGPASAKKISDVVTRIGPLGSVAGVASGEIAAMGATIA

GMGVESEIAATGIKNFMLSLTARDSATKSQKKVLRSLRISPKKLAADMQKDARGAMLHVLDSLAKVPKEK

QAAVLKALFGKESLGAIAPLLTNLDLLRTNFNRVADAQQYGGSMQKEYAARAATTENQLLLLQNQINAIS

STLGETFLPSLNEGIKEMKPFLEEVRTFVRENPEVVKTIAKTGAALLTMGVAIGTLTRITKIMGSVMNMT

PAKGLIALLVGGAYLIIDNWETVGPVVKKVWQEVDQVVRAMGGWEQAVKTIATVSALYIGVKAVASIRAA

TVAQNQWTTAAGKTGLKLKGLGKISLIGGLLELGMMAQEFEKEHPWLVKNFVADALNSGFGLNDKFDEWG

KQFHDFVYDMTGWQMPRGDGYLSPDKRYTPNVSLERNQLLSLASSPATRSELKVTFDNAPPGMRVIDLPK

TGDPFMKITHDVGYSPFKR

>gi|30063410|ref|NP_837581.1| hypothetical protein S2117 [Shigella flexneri 2a str. 2457T]

MAIRLHKLAVALGVFIVSAPAFSHGHHSHGKPLTEVEQKAANGVFDDTNVQNRTLSDWDGVWQSVYPLLQ

SGKLDPVFQKKADADKTKTFAEIKDYYRKGYPTDIEMIGIEDGIVEFHRNNETTSCKYDYDGYKILTYKS

GKKGVRYLFECKDPESKAPKYIQFSDHIIAPRKSSHFHIFMGNDSQQSLLNEMENWPTYYPYQLSSEEVV

EEMMSH

>gi|30063409|ref|NP_837580.1| sulfite oxidase subunit YedZ [Shigella flexneri 2a str. 2457T]

MRLTAKQVIWLKVCLHLAGLLPFLWLVWAINHGGLGADPVKDIQHFTGRTALKFLLAALLITPLARYAKQ

PLLIRTRRLLGLWCFAWATLHLTSYALLELGVNNLALLGKELITRPYLTLGIISWVILLALAFTSTQSMQ

RKLGKHWQQLHNFVYLVAILAPIHYLWSVKIISPQPLIYAGLAVLLLALRYKKLLSLFNRLRKQAHNKLS

L

>gi|30063407|ref|NP_837578.1| transcriptional regulatory protein YedW [Shigella flexneri 2a str. 2457T]

MKILLIEDNQRTQEWVTQGLSEAGYVIDAVSDGRDGLYLALKDDYALIILDIMLPGMDGWQILQTLRTAK

QTPVICLTARDSVDDRVRGLDSGANDYLVKPFSFSELLARVRAQLRQHHALNSTLEISGLKMDSVSQSVS

RDNISITLTRKEFQLLWLLASRAGEIIPRTVIASEIWGINFDSDTNTVDVAIRRLRAKVDDPFPEKLIAT

IRGMGYSFVAVKK

>gi|30063406|ref|NP_837577.1| 2-component sensor protein [Shigella flexneri 2a str. 2457T]

MKRLSITVRLTLLFIFLQSVAGAGIVWTLYNGLASELKWRDDTTLINRTAQIKQLLIDGVNPDTLPVYFN

RMMDVSQDILIIHSDGINKIVNRTNVSDDMLNNIPASETISAAGIYRSIINDTEIDALRINIDEVSPSLT

VTVAKLASARHNMLEQYKINSIIICIVAIILCSVLSPLLIRTGVREIKKLSGVTEALNYNDSRVPVEVNA

LPRELKPLGQALNKMHHALVKDFERLSQFADDLAHELRTPINALLGQNQVTLSQIRSIAEYQKTIAGNIE

ELENISRLTENILFLARADKNNVLVKLDSLSLNKEVENLLDYLEYLSDEKEICFKVKCNQQIFADKILLQ

RMLSNLIVNAIRYSPEKSRIHITSFLDANGSLNIDIASPGTKINEPEKLFRRFWRGDNSRHSVGQGLGLS

LVKAIAELHGGSATYHYLSKHNVFRITLPQRN

>gi|30063405|ref|NP_837576.1| chaperone protein HchA [Shigella flexneri 2a str. 2457T]

MTVQTSKNPQVDIAEDNAFFPSEYSLSQYTSPVSDLDGVDYPKPYRGKHKILVIAADERYLPTDNGKLFS

TGNHPIETLLPLYHLHAAGFEFEVATISGLMTKFEYWAMPHKDEKVMPFFEQHKSLFRNPKKLADVVASL

NADSEYAAIFVPGGHGALIGLPESQDVAAALQWAIKNDRFVISLCHGPAAFLALRHSDNPLNGYSICAFP

DAADKQTPDIGYMPGHLTWYFGEELKKMGMNIINDDITGRVHKDRKRLTGDSPFAANALGKLAAQKCWQL

TRVNPCYIVM

>gi|30063399|ref|NP_837570.1| outer membrane pore protein [Shigella flexneri 2a str. 2457T]

MTLRCLLNPWRFSERILTDRENGEKGDNSQDTSYARVGVKGETQINPEMTGYGQFELDLEASNRHNPDQT

RLAYAGLSYKDFGSFDYSRNVGVAYDAEAFTDMFVEWGGDSWAGTDLFMTNRTNGVATYRNTDFFGMVEG

LNFALQYQGKNEGTGNYKANGDGHGLSATYTIDGFSFAGAYANSDRTDWQSGDGKGERAEVWALSTKYDA

NNVYAAVMYGESHNMNSDDGDVVNKTQNFEAVLQYQFDFGLRPSIGYSYSKALDVAGWICPYISRHLLSL

NPLQARCRRYSRGER

>gi|30063397|ref|NP_837568.1| ISEhe3 orfB [Shigella flexneri 2a str. 2457T]

MLDVHPSGFYAWLQQPHSQRHQADLRLTGQIKQFWLESGCVYGYRKIHLDLRDSGQQCGVNRVWRLMKRV

GIKAQVGYRSPRARKGEASIVSPNRLQRQFNPDAPDERWVTDITYIRTHEGWLYLAVVVDLFSRKIIGWS

MQSRMTKDIVLNALLMAVWWRNPEKQVLVHSDQGSQYTSHEWQSFLKSHGLEGSMSRRGNCHDNAVAESF

FQLLKRERIKKKIYGTREEARSDIFDYIEMFYNSKRRHGSSEQMSPTEYENQYYQRLGSV

>gi|30063395|ref|NP_837566.1| DNA mismatch endonuclease, patch repair protein [Shigella flexneri 2a str. 2457T]

MVDVHDKATRSKNMRAIATRDTAIEKRLASLLTGQGLAFRVQDASLPGRPDFVVDEYRCVIFTHGCFWHH

HHCYLFKVPATRTEFWLEKIGKNVERDRRDISRLQELGWRVLIVWECALRRREKLTDAALTERLEEWICG

EGASAQIDTQGIHLLA

>gi|30063394|ref|NP_837565.1| hypothetical protein S2098 [Shigella flexneri 2a str. 2457T]

MRFRQLLPLFGALFALYIIWGSTYFVIRIGVGSWPPLMMAGVRFQAAGILLLAFLLLRGHKLPPLRPLLN

AALIGLLLLAVGNGMVTVAEHQNVPSGIAAVVIATVPLFTLCFSRLFGIKTRKLEWVGIAIGLAGIIMLN

SGGNLSGNPWGAILILIGSISWAFGSVYGSRITLPVGMMAGAIEMLAAGVVLMIASMIAGEKLTALPSLS

GFLAVGYLALFGSIIAINAYMYLIRNVSPALATSYAYVNPVVAVLLGTGLGGETLSKIEWLALGVIVFAV

VLVTLGKYLFPAKPVVAPVIQDASSE

>gi|30063393|ref|NP_837564.1| hypothetical protein S2097 [Shigella flexneri 2a str. 2457T]

MLLAGSSLLTLLDDIATLLDDISVMGKLAAKKTAGVLGDDLSLNAQQVSGVRANRELPVVWGVAKGSLIN

KVILVPLALIISAFIPWAITPLLMIGGAFLCFEGVEKVLHMLEARKHKEDPAQSQQRLEKLAAQDPLKFE

KDKIKGAIRTDFILSAEIVAITLGIVAEAPLLNQVLVLSGIALVVTVGVYGLVGVIVKIDDLGYWLAEKS

SALMQALGKGLLIIAPWLMKALSIVGTLAMFLVGGGIVVHGIAPLHHAIEHIAGQQSAVVAMILPTVLNL

ILGFIIGGIVVLGVKAVAKMRGQAH

>gi|30063389|ref|NP_837560.1| positive regulator for ctr capsule biosynthesis, positive transcription factor [Shigella flexneri 2a str. 2457T]

MSTIIMDLCSYTRLGLTGYLLSRGVKKREINDIETVDDLAIACDSQRPSVVFINEDCFIHDASNSQRIKH

IINQHPNTLFIVFMAIANVHFDEYLLVRKNLLISSKSIKPESLDDILGDILKKETTITSFLNMPTLSLSR

TESSMLRMWMAGQGTIQISDQMNIKAKTVSSHKGNIKRKIKTHNKQVIYHVVRLTDNVTNGIFVNMR

>gi|30063388|ref|NP_837559.1| flagellar biosynthesis protein FliR [Shigella flexneri 2a str. 2457T]

MMQETSDQWLSWLSLYFWPLLRVLALISTAPILSERSVPKRVKLGLAMMITFAIAPSLPANDVPVFSFFA

LWLAVQQILIGIALGFTMQFAFAAVRTAGEIIGLQMGLSFATFVDPGSHLNMPVLARIMDMLALLLFLTF

NGHLWLISLLVDTFHTLPIGSEPLNSNAFLAPTKAGSLIFLNGLMLALPLITLLLTLNLALGLLNRMAPQ

LSIFVIGFPLTLTVGISLMAALMPLIAPFCEHLFSEIFNLLADIISELPLI

>gi|30063385|ref|NP_837556.1| flagellar motor switch protein FliN [Shigella flexneri 2a str. 2457T]

MSDMNNPADDNNGAMDDLWAEALSEQKSTSEKSAADAVFQQFGGGDVSGTLQDIDLIMDIPVKLTVELGR

TRMTIKELLRLTQGSVVALDGLAGEPLDILINGYLIAQGEVVVVADKYGVRITDIITPSERMRRLSR

>gi|30063384|ref|NP_837555.1| flagellar motor switch protein FliM [Shigella flexneri 2a str. 2457T]

MGDSILSQAEIDALLNGDSEVKDEPTASISGESDIRPYDPNTQRRVVRERLQALEIINERFARHFRMGLF

NLLRRSPDITVGAIRIQPYHEFARNLPVPTNLNLIHLKPLRGTGLVVFSPSLVFIAVDNLFGGDGRFPTK

VEGREFTHTEQRVINRMLKLALEGYSDAWKAINPLEVEYVRSEMQVEFTNITTSPNDIVVNTPFHVEIGN

LTGEFNICLPFSMIEPLRELLVNPPLENSRNEDQNWRDNLVRQVQHSQLELVANFADISLRLSQILKLKP

GDVLPIEKPDRIIAHVDGVPVLTSQYGTLNGQYALRIEHLINPILNSLNEEQPK

>gi|30063383|ref|NP_837554.1| flagellar basal body-associated protein FliL [Shigella flexneri 2a str. 2457T]

MTDYAISKKSKRSLWIPILVFITLAACASAGYSYWHSHQVAADDKAQQRVVPSPVFYALDTFTVNLGDAD

RVLYIGITLRLKDEATRSRLSEYLPEVRSRLLLLFSRQDAAVLATEEGKKNLIAEIKTTLSTPLVAGQPK

QDVTDVLYTAFILR

>gi|30063382|ref|NP_837553.1| flagellar hook-length control protein [Shigella flexneri 2a str. 2457T]

MIRLAPLITADVDTTTLPGGKASDAAQDFLTLLSEALAGETTTDKAAPQLLVATDKPTTKGEPLVSDILA

DAQQADLLIPVDETLPVINDEQSTSTPLTTAQTMTLAAVADKNTTKDEKADDLNEDVTASLSALFAMLPG

FDNTPKVTDAPSTVLPAEKPTLFTKLTSAQLTTAQPDDAPGTPAQPLTPLVAEAQSKAEVISTPSPVTAD

ASPLITPHQTQPLPTVAAPVLSAPLGSHEWQQSLSQHISLFTRQGQQSAELRLHPQDLGEVQISLKVDDN

QAQIQMISPHQHVRAALEAALPVLRTQLAESGIQLGQSNISGESFSGQQQAASQQQQSQRTANHEPLAGE

DDDTLPVPVSLQGRVTGNSGVDIFA

>gi|30063380|ref|NP_837551.1| flagellar assembly protein H [Shigella flexneri 2a str. 2457T]

MSDNLPWKTWTPDDLAPPPAEFVPMVESEETIIEEAEPSLEQQLAQLQMQAHEQGYQAGIAEGRQQGHEQ

GYQEGLAQGLEQGLAEAKAQQAPIHARMQQLVSEFQTTLDALDSVIASRLMQMALEAARQVIGQTPTVDN

SALIKQIQQLLQQEPLFSGKPQLRVHPDDLQRVDDMLGATLSLHGWRLRGDPTLHPGGCKVSADEGDLDA

SVATRWQELCRLAAPGVV

>gi|30063379|ref|NP_837550.1| flagellar motor switch protein G [Shigella flexneri 2a str. 2457T]

MSNLTGTDKSVILLMTIGEDRAAEVFKHLSQREVQTLSAAMANVTQISNKQLTDVLAEFEQEAEQFAALN

INANDYLRSVLVKALGEERAASLLEDILETRDTASGIETLNFMEPQSAADLIRDEHPQIIATILVHLRRA

QAADILALFDERLRHDVMLRIATFGGVQPAALAELTEVLNGLLDGQNLKRSKMGGVRTAAEIINLMKTQQ

EEAVITAVREFDGELAQKIIDEMFLFENLVDVDDRSIQRLLQEVDSESLLIALKGAEQPLREKFLRNMSQ

RAADILRDDLAKRGPVRLSQVENEQKAILLIVRRLAETGEMVIGSGEDTYV

>gi|30063378|ref|NP_837549.1| flagellar hook-basal body protein FliE [Shigella flexneri 2a str. 2457T]

MSAIQGIEGVISQLQATAMSARAQESLPQPTISFAGQLHAALDRISDTQTVARTQAEKFTLGEPGVALND

VMTDMQKASVSMQMGIQVRNKLVAAYQEVMSMQV

>gi|30063377|ref|NP_837548.1| multidrug efflux protein [Shigella flexneri 2a str. 2457T]

MNPYIYLGGAILAEVIGTTLMKFSEGFTRLWPSVGTIICYCASFWLLAQTLAYIPTGIAYAIWSGVGIVL

ISLLSWGFFGQRLDLPAIIGMMLICAGVLVINLLSRSAPH

>gi|30063376|ref|NP_837547.1| kinase inhibitor [Shigella flexneri 2a str. 2457T]

MKKLIVSSVLAFITFSAQAAAFQVTSNEIKTGEQLTTSHVFSGFGCEGGNTSPSLTWSGAPEGTKSFAVT

VYDPDAPTGSGWWHWTVANIPATVTYLPTDAGRRDGTKLPTGAVQGRNDFGYAGFGGACPPKGDKPHHYQ

FKVWALKTDKIPVDSNSSGALVGYMLNANKIATAEITPVYEIK

>gi|30063375|ref|NP_837546.1| regulator [Shigella flexneri 2a str. 2457T]

MLARDKSNLKIEEIRMHKHHEIHRVKPLMPALCRIRQGKKVINWETHSLTVDNNQIILFPCGYEFYIVNY

PEAGLYLAEMLYYPIDLIEKFQKFYAITDQIRNTTGFCLPQNPELIYCWEQLKTSISRGFSTQIQEHLAM

GVLLSLGAHHVNCLLLSDSKQSLISRCYNLMLSEPRTKWTANKVARYLYISVSTLHRRLASEGISFQSIL

DDVRLNNALSCYTNDSKTHQRDCQGKWLQVSFSFY

>gi|30063371|ref|NP_837542.1| inner membrane protein [Shigella flexneri 2a str. 2457T]

MSWQQFKHAWLIKFWAPIPAVIAAGILSTYYFGITGTFWAVTGEFTRWGGQLLQLFGVHAEEWGYFKIIH

LEGSPLTRIDGMMILGMFGGCFAAALWANNVKLRMPRSRIRIMQAIIGGIIAGFGARLAMGCNLAAFFTG

IPQFSLHAWFFAIATAIGSWFGARFTLLPIFRIPVKMQKVSAASPLTQKPDQARRRFRLGMLVFFGMLGW

ALLTAMNQPKLGLAMLFGVGFGLLIERAQICFTSAFRDMWITGRTHMAKAIIIGMAVSAIGIFSYVQLGV

EPKIMWAGPNAVIGGLLFGFGIVLAGGCETGWMYRAVEGQVHYWWVGLGNVIGSTILAYYWDDFAPALAT

DWDKINLLKTFGPMGGLLVTYLLLFAALMLIIGWEKRFFRREAPQTAKEIA

>gi|30063370|ref|NP_837541.1| hypothetical protein S2067 [Shigella flexneri 2a str. 2457T]

MKKLAIAGALMLLAGCAEVENYNNVVKTPAPDWLAGYWQTKGPQRALVSPEAIGSLIVTKEGDTLDCRQW

QRGIAVPGKLTLMSDDLTNVTVKRELYEVERDGNTIEYDGMTMERVDRPTAECAAALDKAPLPTPLP

>gi|30063369|ref|NP_837540.1| alpha-amylase [Shigella flexneri 2a str. 2457T]

MRNPTLLQCFHWYYPEGGKLWPELAERADGFNDIGINMVWLPPAYKGASGGYSVGYDSYDLFDLGEFDQK

GSIPTKYGDKAQLLAAIDALKRNDIAVLLDVVVNHKMGADEKEAIRVQRVNADDRTQIDEEIIECEGWTR

YTFPARAGQYSQFIWDFKCFSGIDHIEHPDEDGIFKIVNDYTGEGWNDQVDDELGNFDYLMGENIDFRNH

AVTEEIKYWARWVMEQTQCDGFRLDAVKHIPAWFYKEWIEHVQEVAPKPLFIVAECWSHEVDKLQTYIDQ

VEGKTMLFDAPLQMKFHEASRMGRDYDMTQIFTGTLVEADPFHAVTLVANHDTQPLQALEAPVEPWFKPL

AYALILLRENGVPSVFYPDLYGAHYEDVGGDGQTYPIDMPIIEQLDELILARQRFAHGVQTLFFDHPNCI

AFSRSGTDEYPGCVVVMSNGDDGEKTIHLGENYGNKTWRDFLGNRQESVVTDENGEATFFCNGGSVSVWV

IEEVI

>gi|30063368|ref|NP_837539.1| flagellar biosynthesis protein FliT [Shigella flexneri 2a str. 2457T]

MNNAPHLYFAWQQLVEKSQLMLRLATEEQWDELIASEMAYVNAVQEIAHLTEEVAPSTTMQEQLRPMLHL

ILDNESKVKQLLQIRMDELAKLVGQSSVQKSVLSAYGDQGGFVLAPQDNLF

>gi|30063367|ref|NP_837538.1| flagellar protein FliS [Shigella flexneri 2a str. 2457T]

MYAAKGTQAYAQIGVESTVMSASQQQLVTMLFDGVLSALVRARLFMQDNNQQGKGVSLSKAINIIENGLR

VSLDEESKDELTQNLIALYSYMVRRLLQANLRNDVSAVEEVEALMRNIADAWKESLLSPSSIQDPV

>gi|30063366|ref|NP_837537.1| flagellar capping protein [Shigella flexneri 2a str. 2457T]

MASISSLGVGSGLDLSSILDSLTAAQKATLTPISNQQSSFTAKLSAYGTLKSALTTFQTANTALSKADLF

SATSTTSSTTAFSATTAGNAIAGKYTISVTHLAQAQTLTTRTTRDDTKTAIATSDSKLTIQQGDDKDPIT

IDISAANSSLSGIRDAINNAKAGVSASIINVGNGEYRLSVTSNDTGLDNAMTLSVSGDDALQSFMGYDAS

ASSNGMEVSVAAQNAQLTVNNVAIENSSNTISDALENITLNLNDVTTGNQTLTITQDTSKVQTAIKDWVN

AYNSLIDTFSSLTKYTAVDAGADSQSSSNGALLGDSTLRTIQTQLKSMLSNTVSSSSYKTLAQIGITTDP

SDGKLELDADKLTAALKKDASGVGALIVGDGKKTGITTTIGSNLTSWLSTTGIIKAATDGVSKTLNKLTK

DYNAASDRIDAQVARYKEQFTQLDVLMTSLNSTSSYLTQQFENNSNSK

>gi|30063365|ref|NP_837536.1| flagellin [Shigella flexneri 2a str. 2457T]

MAQVINTNSLSLITQNNINKNQSALSSSIERLSSGLRINSAKDDAAGQAIANRFTSNIKGLTQAARNAND

GISVAQTTEGALSEINNNLQRIRELTVQASTGTNSDSDLDSIQDEIKSRLDEIDRVSGQTQFNGVNVLAK

DGSMKIQVGANDGQTITIDLKKIDSDTLGLNGFNVNGGGAVANTAASKADLVAANATVVGNKYTVSAGYD

AAKASDLLAGVSDGDTVQATINNGFGTAASATNYKYDSASKSYSFDTTTASAADVQKYLTPGVGDTAKGT

ITIDGSAQDVQISSDGKITASNGDKLYIDTTGRLTKNGSGASLTEASLSTLAANNTKATTIDIGGTSISF

TGNSTTPDTITYSVTGAKVDQAAFDKAVSTSGNNVDFTTAGYSVNGTTGAVTKGVDSVYVDNNEALTTSD

TVDFYLQDDGSVTNGSGKAVYKDADGKLTTDAETKAATTADPLKALDEAISSIDKFRSSLGAVQNRLDSA

VTNLNNTTTNLSEAQSRIQDADYATEVSNMSKAQIIQQAGNSVLAKANQVPQQVLSLLQG

>gi|30063364|ref|NP_837535.1| flagella biosynthesis protein FliZ [Shigella flexneri 2a str. 2457T]

MPHFNFDYQEFLMMVQHLKRRPLSRYLKDFKHSQTHCAHCRKLLDRITLVRDGKIVNKIEISRLDTLLDE

KGWQTEQQSWAALCRFCGDLHCKTQSDFFDIIGFKKFLFEQTEMSPGTVREYVVRLRRLGNHLHEQNISL

DQLQDGFLDEILAPWLPTTSTNNYRIALRKYQHYQRQTCTGLVQKSSSLPASDIY

>gi|30063362|ref|NP_837533.1| transport system permease (former yecC) [Shigella flexneri 2a str. 2457T]

MQESIQLVIDSLPFLLKGAGYTLQLSIGGMFFGLLLGFILALMRLSPIWPVRWLARFYISIFRGTPLIAQ

LFMIYYGLPQFGIELDPIPSAMIGLSLNTAAYAAETLRAAISSIDKGQWEAAASIGMTPWQTMRRAILPQ

AARVALPPLSNSFISLVKDTSLAATIQVPELFRQAQLITSRTLEVFTMYLAASLIYWIMATVLSTLQNHF

ENQLNRQEREPK

>gi|30063360|ref|NP_837531.1| transcriptional regulator of ftsQAZ gene cluster [Shigella flexneri 2a str. 2457T]

MQDTDFFSWRRTMLLRFQRMEAAEEAYHEIELQAQQLEYDYYSLCVRHPVPFTRPKVAFYTNYPESWVSY

YQAKNFLAIDPVLNPENFSQGHLMWNDDLFSEAQPLWEAAHAHGLRRGVTQYLMLPNRALGFLSFSRCST

REIPILSDELQLKMQLLVRESLMALMRLNDEIVMTPEMNFSKREKEILKWTAEGKTSAEIAMILSITG

>gi|30063358|ref|NP_837529.1| response regulator [Shigella flexneri 2a str. 2457T]

MINVLLVDDHELVRAGIRRILEDIKGIKVVGEASCGEDAVKWCRTNAVDVVLMDMSMPGIGGLEATRKIA

RSTADVKIIMLTVHTENPLPAKVMQAGAAGYLSKGAAPQEVVSAIRSVYSGQRYIASDIAQQMALSQIEP

EKTESPFASLSERELQIMLMITKGQKVNEISEQLNLSPKTVNSYRYRMFSKLNIHGDVELTHLAIRHGLC

NAETLSSQ

>gi|30063355|ref|NP_837526.1| hypothetical protein S2047 [Shigella flexneri 2a str. 2457T]

MKTGPLNESELEWLDDILTKYNTDHAILDVAELDGLLTAVLSSPQEIEPEQWLVAVWGGADYVPRWASEK

EMTRFMNLAFQHMADTAERLNEFPEQFEPLFGLREVDGSELTIVEEWCFGYMRGVALSDWSTLPDSLKPA

LEAIALHGTEENFERVEKMSPEAFEESVDAIRLAALDLHAYWMAHPQEKAVQQPIKAEEKPGRNDPCPCG

SGKKFKQCCLH

>gi|30063354|ref|NP_837525.1| tyrosine-specific transport protein [Shigella flexneri 2a str. 2457T]

MKNRTLGSVFIVAGTTIGAGMLAMPLAAAGVGFSVTLILLIGLWALMCYTALLLLEVYQHVPADTGLGTL

AKRYLGRYGQWLTGFSMMFLMYALTAAYISGAGELLASSISDWTGISMSATAGVLLFTFVAGGVVCVGTS

LVDLFNRFLFSAKIIFLVVMLVLLLPHIHKVNLLTLPLQQGLALSAIPVIFTSFGFHGSVPSIVSYMDGN

IRKLRWVFIIGSAIPLVAYIFWQVATLGSIDSTTFMGLLANHAGLNGLLQALREMVASPHVELAVHLFAD

LALATSFLGVALGLFDYLADLFQRSNTVGGRLQTGAITFLPPLAFALFYPRGFVMALGYAGVALAVLALI

IPSLLTWQSRKHNPQAGYRVKGGRPALVVVFLCGIAVIGVQFLIAAGLLPEVG

>gi|30063352|ref|NP_837523.1| ferritin [Shigella flexneri 2a str. 2457T]

MLKPEMIEKLNEQMNLELYSSLLYQQMSAWCSYHTFEGAAAFLRRHAQEEMTHMQRLFDYLTDTGNLPRI

NTVESPFAEYSSLDELFQETYKHEQLITQKINELAHAAMTNQDYPTFNFLQWYVSEQHEEEKLFKSIIDK

LSLAGKSGEGLYFIDKELSTLDTQN

>gi|30063351|ref|NP_837522.1| hypothetical protein S2043 [Shigella flexneri 2a str. 2457T]

MRLLILTLSLITLAGCTVTRQAHVSEVDAATGIVRLVYDQAFLQHAHTDRYVSRGIADRACQQEGYTHAI

PFGQPVGNCSLFAGSLCLNTEFTLSYQCHHSAFPVFL

>gi|30063349|ref|NP_837520.1| ferritin-like protein [Shigella flexneri 2a str. 2457T]

MATAGMLLKLNSQMNREFYASNLYLHLSNWCSEQSLNGTATFLRAQAQSNVTQMMRMFNFMKSVGATPIV

KAIDVPGEKLNSLEELFQKTMEEYEQRSSTLAQLADEAKELNDDSTVNFLRDLEKEQQHDGLLLQTILDE

VRSAKLAGMCPVQTDQHVLNVVSHQLH

>gi|30063347|ref|NP_837518.1| L-arabinose transporter permease [Shigella flexneri 2a str. 2457T]

MMSSVSTSGSGAPKSSFSFGRIWDQYGMLVVFAVLFIACAIFVPNFATFINMKGLGLAISMSGMVACGML

FCLASGDFDLSVASVIACAGVTTAVVINLTESLWIGVAAGLLLGVLCGLVNGFVIAKLKINALITTLATM

QIVRGLAYIISDGKAVGIEDESFFALGYANWFGLPAPIWLTVACLIIFGLLLNKTTFGRNTLAIGGNEEA

ARLAGVPVVRTKIIIFVLSGLVSAIAGIILASRMTSGQPMTSIGYELIVISACVLGGVSLKGGIGKISYV

VAGILILGTVENAMNLLNISPFAQYVVRGLILLAAVIFDRYKQKAKRTV

>gi|30063346|ref|NP_837517.1| trehalose-6-phosphate phosphatase [Shigella flexneri 2a str. 2457T]

MTEPLTETPELSAKYAWFFDLDGTLAEIKPHPDQVVVPDNILQGLQLLATASDGALALISGRSMVELDAL

AKPYRFPLAGVHGAERRDINGKTHIVHLPDAIARDISVQLHTVIAQYPGAELEAKGMAFALHYRQAPQHE

DALMTLAQRITQIWPQMALQQGKCVVEIKPRGTSKGEAIAAFMQEAPFIGRTPVFLGDDLTDESGFAVVN

RLGGMSVKIGTGATQASWRLAGVPDVWSWLEMITAALQQKRENNRSDDYESFSRSI

>gi|30063343|ref|NP_837514.1| transcriptional activator FlhC [Shigella flexneri 2a str. 2457T]

MSEKSIVQEARDIQLAMELITLGARLQMLESETQLSRGRLIKLYKELRGSPPPKGMLPFSTDWFMTWEQN

VHASMFCNAWQFLLKTGLCNGVDAVIKAYRLYLEQCPQAEEGPLLALTRAWTLVRFVESGLLQLSSCNCC

GGNFITHAHQPVGSFACSLCQPPSRAVKRRKLSQNPADIIPQLLDEQRVQAV

>gi|30063342|ref|NP_837513.1| flagellar motor protein MotA [Shigella flexneri 2a str. 2457T]

MLILLGYLVVLGTVFGGYLMTGGSLGALYQPAELVIIAGAGIGSFIVGNNGKAIKGTLKALPLLFRRSKY

TKAMYMDLLALLYRLMAKSRQMGMFSLERDIENPRESEIFASYPRILADSVMLDFIVDYLRLIISGHMNT

FEIEALMDEEIETHESEAEVPANSLALVGDSLPAFGIVAAVMGGVHALGSADRPAAELGALIAHAMVGTF

LGILLAYGFISPLASVLRQKSAETSKMMQCVKVTLLSNLNGYAPPIAVEFGRKTLYSSERPSFIELEEHV

RAVKNPQQQTTTEEA

>gi|30063341|ref|NP_837512.1| flagellar motor protein MotB [Shigella flexneri 2a str. 2457T]

MKNQAHPIIVVKRRKAKSHGAAHGSWKIAYADFMTAMMAFFLVMWLISISSPKELIQIAEYFRTPLATAV

TGGDRISNSESPIPGGGDDYTQSQGEVNKQPNIEELKKRMEQSRLRKLRGDLDQLIESDPKLRALRLHLK

IDLVQEGLRIQIIDSQNRPMFRTGSADVEPYMRDILRAIAPVLNGIPNRISLSGHTDDFPYASGEKGYSN

WELSADRANASRRELMVGGLDSGKVLRVVGMAATMRLSDRGPDDAVNRRISLLVLNKQAEQAILHENAES

QNEPVSALEKPEVAPQVSVPTMPSAEPR

>gi|30063340|ref|NP_837511.1| chemotaxis protein CheA [Shigella flexneri 2a str. 2457T]

MSIDISDFYQTFFDEADELLADMEQHLLVLQPEAPDAEQLNAIFRAAHSIKGGAGTFGFSVLQETTHLME

NLLDEARRGEMQLNTDIINLFLETKDIMQEQLDAYKQSQEPDAASYDYICQALRQLALEAKGETPSAVTR

LSVVAKSEPQDEQSRSQLPRRIILSRLKAGEVDLLEEELGHLTTLNDVVKGADSLSAILPGDIAEDDITA

VLCFVIEADQITFETVEVSPKIATPPVLKLAAEQAPTGRVEREKTTRSSESTSIRVAVEKVDQLINLVGE

LVITQSMLAQRSSELDPVNHGDLITSMGQLQRNARDLQESVMSIRMMPMEYVFSRYPRLVRDLAGKLGKQ

VELTLVGSSTELDKSLIERIIDPLTHLVRNSLDHGIELPEKRLAAGKNSVGNLILSAEHQGGNICIEVTD

DGAGLNRERILAKAASQGLTVSENMSDDEVAMLIFAPGFSTAEQVTDVSGRGVGMDVVKRNIQEMGGHVE

IQSKQGTGTTIRILLPLTLAILDGMSVRVADEVFILPLNAVMESLQPREADLHPLAGGERVLEVRGEYLP

IVELWKVFNVAGAKTEATQGIVVILQSGGRRYALLVDQLIGQHQVVVKNLESNYRKVPGISAATILGDGS

VALIVDVSALQAINREQRMANTAA

>gi|30063339|ref|NP_837510.1| purine-binding chemotaxis protein [Shigella flexneri 2a str. 2457T]

MTGMTNVTKLASEPSGQEFLVFTLGDEEYGIDILKVQEIRGYDQVTRIANTPAFIKGVTNLRGVIVPIVD

LRIKFSQVDVDYNDNTVVIVLNLGQRVVGIVVDGVSDVLSLTAEQIRPAPEFAVTLSTEYLTGLGALGDR

MLILVNIEKLLNSEEMALLDSAASEVA

>gi|30063338|ref|NP_837509.1| methyl-accepting chemotaxis protein II, aspartate sensor receptor [Shigella flexneri 2a str. 2457T]

MINRIRVVTLLVMVLGVFALLQLISGSLFFSSLHHSQKSFVVSNQLREQQGELTSTWDLMLQTRINLSRS

AVRMMMDSSNQQSNAKVELLDSARKTLAQAATHYKKFKSMAPLPEMVATSRNFDEKYKNYYTALTELIDY

LDYGNTGAYFAQPTQGMQNAMGEAFAQYALSSEKLYRDIVTDNADDYRFAQWQLAVIALVVVLILLVAWY

GIRRMLLTPLAKIIAHICEIAGGNLANTLTIDGRSEMGDLAQSVSHMQRSLTDTVTHVREGSDAIYAGTR

EIAAGNTDLSSRTEQQASALEETAASMEQLTATVKQNADNARQASQLAQSASDTAQHGGKVVDGVVKTMH

EIADSSKKIADIISVIDGIAFQTNILALNAAVEAARAGEQGRGFAVVAGEVRNLASRSALAAKEIKALIE

DSVSRIDTGSVLVESAGETMNNIVNAVTRVTDIMGEIASASDEQSRGIDQVALAVSEMDRVTQQNASLVQ

ESAAAAAALEEQASRLTQAVSAFRLAASPLTNKPQTPSRPASEQPPAQPRLRITEQDPNWETF

>gi|30063337|ref|NP_837508.1| methyl-accepting protein IV [Shigella flexneri 2a str. 2457T]

MFNRIRISTTLFLILILCGILQIGSNGMSFWAFRDDLQRLNHGEQSNQQRAALAQTRAVMLQASTALNKA

GTLTALSYPADDIKTLMTTARASLTQSTTLFKSFMAMTAGNEHVRALQKETEKSFARWHNDLEHQATWLE

SNQLSDFLTAPVQGSQNAFDVNFEAWQLEINHVLEAASAQSQRNYQISALVFISMIIVAAIYISSALWWT

RKMIVQPLAIIGSHFDSIAAGNLARPIAVYGRNEITAIFASLKTMQQALRGTVSDVRKGSHEMHIGIAEI

VAGNNDLSSRTEQQAASLAQTAASMEQLTATVGQNADNARQASELAKNAATTAQAGGVQVSTMTHTMQEI

ATSSQKIGDIISVIDGIAFQTNILALNAAVEAARAGEQGRGFAVVAGEVRNLASRSAQAAKEIKGLIEES

VNRVQQGSKLVNNAAATMTDIVSSVTRVNDIMGEIASASEEQRRGIEQVAQAVSQMDQVTQQNSSLVEEA

AVATEQLANQADHLSSRVAVFTLEEHEVARHESAQLQIAPVVS

>gi|30063336|ref|NP_837507.1| chemotaxis methyltransferase CheR [Shigella flexneri 2a str. 2457T]

MTSSLPCGQTSLLLQMTERLALSDAHFRRISQLIYQRAGIVLADHKRDMVYNRLVRRLRSLGLTDFGHYL

NLLESNQHSGEWQAFINSLTTNLTAFFREAHHFPLLADHARRRSGEDRVWSAAASTGEEPYSIAMTLADT

LGTVPGRWKVFASDIDTEVLEKARSGIYRHEELKNLTPQQLQRYFMRGTGPHEGLVRVRQELANYVDFAP

LNLLAKQYTVPGPFDAIFCRNVMIYFDQTTQQEILRRFVPLLKPDGLLFAGHSENFSHLERRFTLRGQTV

YALSKD

>gi|30063335|ref|NP_837506.1| chemotaxis-specific methylesterase [Shigella flexneri 2a str. 2457T]

MSKIRVLSVDDSALMRQIMTEIINSHSDMEMVATAPDPLVARDLIKKFNPDVLTLDVEMPRMDGLDFLEK

LMRLRPMPVVMVSSLTGKGSEVTLRALELGAIDFVTKPQLGIREGMLAYSEMIAEKVRTAAKASLAAHKP

LSAPTTLKAGPLLSSEKLIAIGASTGGTEAIRHVLQPLPLSSPALLITQHMPPGFTRSFADRLNKLCQIG

VKEAEDGERVLPGHAYIAPGDRHMELARSGANYQIKIHDGPAVNRHRPSVDVLFHSVAKQAGRNAVGVIL

TGMGNDGAAGMLAMRQAGAWTLAQNEASCVVFGMPREAINMGGVCEVVDLSQVSQQMLATISAGQAIRI

>gi|30063334|ref|NP_837505.1| chemotaxis regulatory protein CheY [Shigella flexneri 2a str. 2457T]

MADKELKFLVVDDFSTMRRIVRNLLKELGFNNVEEAEDGVDALNKLQAGGYGFVISDWNMPNMDGLELLK

TIRADGAMSALPVLMVTAEAKKENIIAAAQAGASGYVVKPFTAATLEEKLNKIFEKLGM

>gi|30063333|ref|NP_837504.1| chemotaxis regulator CheZ [Shigella flexneri 2a str. 2457T]

MMQPSIKPADEHSAGDIIARIGSLTHMLRDSLRELGLVQAIAEAAEAIPDARDRLYYVVQMTAQAAERAL

NSVEASQPHQDQMEKSAKALTQRWDDWFADPIDLADARELVTDTRQFLADVPAHTSFTNAQLLEIMMAQD

FQDLTGQVIKRMMDVIQEIERQLLMVLLENIPEQESRPKRENQSLLNGPQVDTSKAGVVASQDQVDDLLD

SLGF

>gi|30063331|ref|NP_837502.1| hypothetical protein S2019 [Shigella flexneri 2a str. 2457T]

MNVVSNTQLLEQRIADFFTLSDEHKKARVLLDTLACSCPARIFGGMVRDLGLYGVDGFSSDLDIVIGRSR

EELFQTLAELPVKQLRFNKFGGIRFRYHDFEFDIWNLNETWAFQEKLIFCEDESSLLNEVA

>gi|30063329|ref|NP_837500.1| hypothetical protein S2015 [Shigella flexneri 2a str. 2457T]

MEWQQLLNCNRRKDNLKKTNIAFLHPLASGRQEIERDYDRLLFAAPTRRLADKTQVFPLDQNDSIRTRLT

HSHEVANFARGIGMRLAFEMKEHIFGEIPTGIVVERDVPALLAAIGLAHDLGNPPFGHLKWSTKTGHRVR

VFPVSIFRFVWG

>gi|30063328|ref|NP_837499.1| flagellar biosynthesis protein FlhB [Shigella flexneri 2a str. 2457T]

MSDESDDKTEAPTPHRLEKAREEGQIPRSRELTSLLILLVGVSVIWFGGVSLARRLSGMLSAGLHFDHSI

IKDPNLILGQIILLIREAMLALLPLISGVVLVALISPVMLGGLVFSGKSLQPKFSKLNPLPGIKRIFSAQ

TSAELLKAILKTILVGSVTGFFLWHHWPQMMRLMAESPITAMGNAMDLVGLCALLVVLGVIPMVGFDVFF

QIFSHLKKLRMSRQDIRDEFKQSEGDPHVKGRICQMQRAAARRRMMADVPKADVIVNNPTHYSVALQYDE

NKMSAPKVVAKGAGLVALRIREIGAENNVPTLEAPPLARALYRHAEIGQQIPGQLYAAVAEVLAWVWQLK

RWRLAGGQRPVHLLIFRCRQPWILLTRNRPMSNLAAMLRLPANLKST

>gi|30063327|ref|NP_837498.1| flagellar protein [Shigella flexneri 2a str. 2457T]

MRTLLAILLFPLLVQAAGEGMWQASSVGITLNHRGESMSSAPLSARQPASGLMTLVAWRYQLIGPTPAGL

RVRLCSQSRCVELEGQSGTTVAFSGIAAAEPLRFIWEVPGGG

>gi|30063323|ref|NP_837494.1| hypothetical protein S2006 [Shigella flexneri 2a str. 2457T]

MANWQSIDELQDIASDLPRFTHALDELSRRLGLDITPLTADHISLRCHQNVTAERWRRGFEQCGELLSEK

MINGRPICLFKLHEPVQVAHWQFSNVELPWPGEKRYPHEGWEHIEIVLPGDPETLNARALALLSDEGLSL

PGISVKTSSPKGEHERLPNPTLAVTDGKTTIKFHPWSIEEIVASEQSA

>gi|30063321|ref|NP_837492.1| cytochrome C-type protein [Shigella flexneri 2a str. 2457T]

MRGKKRIGLLFLLIAVVVGGGGLLLAQKALHKTSDTAFCLSCHSMSKPFEEYQGTVHFSNQKGIRAECAD

CHIPKSGMDYLFAKLKASKDIYHEFVSGKIDSDDKFETHRQEMAETVWKELKATDSATCRSCHSFDAMDI

ASQSESAQKMHNKAQKGGETCIDCHKGIAHFPPEIKMDDNAAHELESQAATSVTNGAHIYPFKTSRIGEL

ATVNPGTDLTVVDASGKQPIVLLQGYQMQGSENTLYLAAGQRLALATLSEEGIKALTVNGEWQADEYGNQ

WRQASLQGALTDPALADRKPLWQYAEKLDDTYCAGCHASIAADHYTVNAWPSIAKGMGARTSMSENEPDI

LTRYFQYNAKDITEKQ

>gi|30063320|ref|NP_837491.1| biotin sulfoxide reductase [Shigella flexneri 2a str. 2457T]

MALTRREFIKHSGIAAGALVVTSAAPLPAWAEEKGGKILTAGRWGAMSVEVKDGKIVSSTGALAKTIPNS

LQSTAADQVHTTARIQHPMVRKSYLDNPLQPAKGRGEDTYVQVSWEQALKLIHEQHERIRKANGPSAIFA

GSYGWRSSGVLHKAQTLLQRYMNLAGGYSGHSGDYSTGAAQVIMPHVVGSVEVYEQQASWPLILENSQVV

VLWGMNPLNTLKIAWSSTDEQGLEYFHQLKKSGKPVIAIDPIRSETIEFFGDNATWIAPNMGTDVALMLG

IAHTLMTQGKHDKVFLEKYTTGYPQFEEYLTGKSDNTPKSAVWTAEITGVPEAQIVKLAELMAANRTMLM

AGWGIQRQQYGEQKHWMLVTLAAMLGQIGTPGGGFGFSYHYSNGGNPTRVGGVLPEMSAAIAGQASEAAD

DGGMTAIPVARIVDALENPGGKYQHNGKEQTYPNIKMIWWAGGGNFTHHQDTNRLIKAWQKPEMIVVSEC

YWTAAAKHADIVLPITTSFERNDLTMTGDYSNQHIVPMKQAVAPQFEARNDFDVFADLAELLKPGGKEIY

TEGKDEMAWLKFFYDAAQKGARAQRVTMPMFNAFWQQNKLIEMRRSEKNEQYVRYGDFRADPVKNALGTP

SGKIEIYSKTLEKFGYKDCPAHPTWLAPDEWKGTADEKQLQLLTAHPAHRLHSQLNYAELRKKYAVADRE

PITIHTEDAARFGIANGDLVRVWNKRGQILTGAVVTDGIKKGVVCVHEGAWPDLENGLCKNGSANVLTAD

IPSSQRANACAGNSALVYIEKYTGNAPKLTAFDKPAVQA

>gi|30063319|ref|NP_837490.1| enzyme [Shigella flexneri 2a str. 2457T]

MIDFGNFYSLIAKNHLSHWLETLPAQIANWQREQQHGLFKQWSNTVEFLPEIKPYRLDLLHSVTAESEEP

LSTGQIKRIETLMRNLMPWRKGPFSLYGVNIDTEWRSDWKWDRVLPHLSDLTGRTILDVGCGSGYHMWRM

IGAGAHLAVGIDPTQLFLCQFEAVRKLLGNDQRAHLLPLGIEQLPALKAFDTVFSMGVLYHRRSPLEHLW

QLKDQLVNEGELVLETLVIDGDENTVLVPGDRYAQMRNVYFIPSALALKNWLKKCGFVDIRIVDVCVTTT

EEQRRTEWMVTESLSDFLDPHDPSKTVEGYPAPKRAVLIARKP

>gi|30063318|ref|NP_837489.1| hypothetical protein S2001 [Shigella flexneri 2a str. 2457T]

MSHRDTLFSAPIARLGDWTFDERVAEVFPDMIQRSVPGYSNIISMIGMLAERFVQPGTQVYDLGCSLGAA

TLSVRRNIHHDNCKIIAIDNSPAMIERCRRHIDAYKAPTPVDVIEGDIRDIAIENASMVVLNFTLQFLEP

SERQALLDKIYQGLNPGGALVLSEKFSFEDAKVGELLFNMHHDFKRANGYSELEISQKRSMLENVMLTDS

VETHKARLHKAGFEHSELWFQCFNFGSLVALKAEDAA

>gi|30063317|ref|NP_837488.1| hypothetical protein S2000 [Shigella flexneri 2a str. 2457T]

MVSALYAVLSALLLMKFSFDVVRLRMQYRVAYGDGGFSELQSAICIHGNAVEYIPIAIVLMLFMEMNGAE

TWMVHICGIVLLAGRLMHYYGFHHRLFRWRRSGMSATWCALLLMVLANLWYMPWELVFSLR

>gi|30063313|ref|NP_837484.1| hypothetical protein S1992 [Shigella flexneri 2a str. 2457T]

MIIQSKLIRAALVCAAKNDVRYYLNGLHITPKHIEATNGSVALRMAHGIRTKKNIIVQFEGGVPAKAETT

ELIFSKEPIAVHRDQFQRRLSITGIKLVDGCFPDLDRIIPKKFDRCTHPVLQAGYLSYPEKMFGRERKFI

PVQLRPSGDGQAVRIQFDSIINSMYGNPEFVVMPCRDHGDFNVAQEHPE

>gi|30063312|ref|NP_837483.1| hypothetical protein S1991 [Shigella flexneri 2a str. 2457T]

MNTVTINNKQFPVIEYRGQRVVTLAMIDEVHQRPEGTARAAFNRNREHFISGVDYAELGADVIRTDLPEG

TFSKFAPSGIVLFESGYLMLTKPFNDALAWQVQRELVNSYFRTRTPLTEIEMIAAIAADAVRQQKRLNHV

EEQLETVTEAVETIKRGNMRAGYVGYRQVVAKSGMSDTKCRNLVNAYQIPTDTHEFMTPDGLLSRRAIVE

LEPFMAAFHQMMSEADPRGTRWYHPKMGLFQVIGWEDKA

>gi|30063311|ref|NP_837482.1| serine protease [Shigella flexneri 2a str. 2457T]

MKKVIPALALVLLTTGLVGCDRVEPGNVGIKVNKLGDDKGVGEVVGVGRYWTGWNTEVYIFPTFKQMKTY

DEPFSFQMSDGTTIGYHIGVAYKVDPSKVTTVFQTYRKGVDDITDTDLRQKIADALNRLASKMTTDKFID

GGKSELLDAALKDIQEEMTPIGIQVMSLSYVGKPEYPPTVIDSINAKVTANQKTLQCEQEVKQREAEANM

LRAEAAGQADAIRTKAQAEADAIRLRGEALRQNPNVMELEAINKWNGTLPQYMTSGANTPFIQVK

>gi|30063308|ref|NP_837479.1| Q antiterminator encoded by prophage CP-933P [Shigella flexneri 2a str. 2457T]

MNNQYLQFVREQLIIATADLSGATKGQLEAWQENAMFDTGRYRRKKIRYRDEVTGKMITRDNPPIPGKQS

LAKGASIPLVSQVAFSTSSWRRAVLSLEEHYKAWLLWCYSGSICWEYQITITHWAWEEFKAHSGTRKIAE

KTQERLKKLIWLAAQAVKAELFGGEGYEYQELALLAGVTTKNWSKTFTGHWVVMKHIFHRLDSEALLFVM

RTRSEQKAAFSKQSIAKVD

>gi|30063297|ref|NP_837468.1| Iron transport protein, inner membrane component [Shigella flexneri 2a str. 2457T]

MMALLLEPLQFTFMSHALLISLVVSIPCALLSVFLVLKGWALMGDAMSHAVFPGIVLTWILGLPLATGAF

VAGVFCAVATGYLKDNSRIKQDTVMGIVFSGMFAAGLILYIAIKPDVHLDHILFGDMLGITIGDIIQTVI

IAGLVTLVISVKWRDFLLFSFDYQQAQVSGLHTRWLHYGLLCMVSLTIVATLKAVGIILSISLLIAPGAI

AVLLTQRFHIALLLATGISVIVSMTGVWLSFFIDSAPAPTIVVLFAVVFIMTFTVTSINARTKGNAHTQD

LLSPN

>gi|30063296|ref|NP_837467.1| Iron transport protein [Shigella flexneri 2a str. 2457T]

MNVLLEPFSYEYMLNAMWVSAMVGGLCAFLSCYLMLKGWSLIGDALSHSIVPGVAGAYMLGLPFSLGAFF

SGGLAAGSMLFLNQRTRLKEDAIIGLIFSSFFGLGLFMVSLNPTSVNIQTIVLGNILAIDPADILQLTII

GILSIIVLFFKWKDLMVTFFDENHARAIGLHPGRLKILFFTLLSVSTVAALQTVGAFLVICLVVTPGATA

WLLTDRFPRLLIIAVTIGSVTSFLGAWVSYFLDGATGGIIVVAQTLLFLLAFVFAPTHGLLANRRRAHKA

LEDRS

>gi|30063294|ref|NP_837465.1| Iron transport protein [Shigella flexneri 2a str. 2457T]

MLLGCLALTCSIAFQASATEKFKVITTFTIIADMAKNVAGDAAEVSSITKPGAEIHEYQPTPGDIKRAQG

AQLILANGMNLELWFQRFYQHLNGVPEVIVSSGVTPVGITEGPYEGKPNPHAWMSPDNALIYVDNIRDAL

IKYDPANAQTYQRNADTYKAKITQTLAPLRKQITELPENQRWMVTSEGAFSYLARDLGLKELYLWPINAD

QQGTPQQVRKVVDIVKKNHIPAVFSESTISDKPARQVARETGAHYGGVLYVDSLSTENGPVPTYIDLLKV

TTSTLVQGIKAGKREK

>gi|30063293|ref|NP_837464.1| integrase for prophage CP-933R [Shigella flexneri 2a str. 2457T]

MESEKGQSQNLWKFAVYSGLRHGELAALAWEDVDFEKGIVNVRRNLTILDMFGPPKTNAGIRTVALLQPA

LEALKEQYKLTGHHRKSEITFYHREYGRTEKQKLHFVFMPRVCNEKQKPYYSVSSLGTRWNAAVKRAGIR

RRNPYHTRHTFACWLLTAGANPAFIASQMGHETAQMVYEIYGMWIDDMNDEQIAMLNARLS

>gi|30063288|ref|NP_837459.1| bacteriophage protein [Shigella flexneri 2a str. 2457T]

MGFAGDGKTYTASELAIGLVMLMRQRGIEAGNRPVMFLDTETGSDWVKPRFDAENIELFTAKTRSFVDLL

EAINEAESSGSVMIIDSISHFWTGLCDEYARRRNRKRGLEFSDWAWLKQEWRRFTDRFVNSQAHIIMCGR

AGYEYDFFEGDDGKRQLAKTGIKMKAETETGYEPSIRIQMEKQMNIETGQVWRTARILKDRSTRIDGQVF

SNPTFKNFLPHIESLNLGGEHPGIDTSRDNSELFANDGTPTWLKEKRAKEIALDEIIELLNKHHGGTSND

AKRAKADLLEKTFTSRSWERIKGMDWPTIKAGRNALWIELEGVEYAFPDPQTQNETQQAGYDENIPV

>gi|30063287|ref|NP_837458.1| host-nuclease inhibitor protein Gam [Shigella flexneri 2a str. 2457T]

MSVKLRLPQSAYPIFDRIEEMAWARHYQQIVREEKETELADDLEKGLPQHLFESLCIDHLQRHGANKQAI

SHAFDDDVEFQERMAEHIRYMAETIAHHQVDIDLED

>gi|30063286|ref|NP_837457.1| exodeoxyribonuclease VIII [Shigella flexneri 2a str. 2457T]

MNTDKQVVYPLYYEAKNDKVRKRLGIKGGFYWAEAKKLSIAISRGAVAIDDAGYDEDDFKKPVRVNLPVVD

DLPPEGVFDTEFCNRYEKGGEDGITMVFIASSPSVQDKPASTDNTNVNGEDMTEIEENMLLPVSGQELPI

RWLAQHGSEKPVTHVSRDELQALHIARDEELPAVTALAVSHKTSLLDPLEIRDLHKLVRDTDRVFPNPGN

SSLGLMTAFFEAYMDADYTDRGLLTKEWMKGNRVSRITRTASGANAGGGNLTDRGEGFVHDLTSLARDVA

TGVLARSMDVDIYNLHPAHAKRVEEIIAENKPPFSVFRDKFITMPGGMDYSRAIVVASVKEAPIGIEVIP

AHVTEYLNKVLTETDHANPDPEIVDIACGRSSAPMPQRVTEEGKQDDEEKTQPSGAMADEQATAETMEPD

ATEHHQNTQPLDAQSQVNSVDEKYQELRAELHEARKNIPPKNPVDADKLLAALR

>gi|30063281|ref|NP_837452.1| hypothetical protein S1949 [Shigella flexneri 2a str. 2457T]

MLMSLAQCPGFLFAHREECTVEIKKIINPRYTESGAVDCDVFFDDRDQAVPYTATADDVAPTGQQIWQEL

QSGKWGEIAPFTVTPEMLEAAREARRQEIEAWRTEQEAKPFTFEWNGRTWNADASSVARLSPVVMLAKSV

AAQTHMVWSDADNQQVKLSMPELEELAAAMVQAQVDRNDEIYRRQREMKEELSSLDDLASIRAFDVK

>gi|30063273|ref|NP_837444.1| Holliday junction resolvase [Shigella flexneri 2a str. 2457T]

MAIILGIDPGSRVTGYGVIRQVGRQLSYLGSGCIRTKVDDLPSRLKLIYAGVTEIITQFQPDYFAIEQVF

MAKNADSALKLGQARGVAIVAAVNQELPVFEYAARQVKQTVVGMGSAEKSQVQHMVRTLLKLPANPQADA

ADALAIAITHCHVSQNAMQMSESRLNLTRGRLR

>gi|30063272|ref|NP_837443.1| Holliday junction DNA helicase RuvA [Shigella flexneri 2a str. 2457T]

MIGRLRGIIIEKQPPLVLIEVGGVGYEVHMPMTCFYELPEAGQEAIVFTHFVVREDAQLLYGFNNKQERT

LFKELIKTNGVGPKLALAILSGMSAQQFVNAVEREEVGALVKLPGIGKKTAERLIVEMKDRFKGLHGDLF

TPAADLVLTSPASPATDDAEQEAVAALVALGYKPQEASRMVSKIARPDTSSETLIREALRAAL

>gi|30063270|ref|NP_837441.1| high-affinity zinc transporter membrane component [Shigella flexneri 2a str. 2457T]

MIELLFPGWLAGIMLACAAGPLGSFVVWRRMSYFGDTLAHASLLGVAFGLLLDVNPFYAVIAVTLLLAGG

LVWLEKRPQLAIDTLLGIMAHSALSLGLVVVSLMSNIRVDLMAYLFGDLLAVTPEDLISIAIGVVIVVAI

LFWQWRNLLSMTISPDLAFVDGVKLQQVKLLLMLVTALTIGVAMKFVGALIITSLLIIPAATARRFARTP

EQMAGVAVLVGMVAVTGGLTFSAFYDTPAGPSVVLCAALLFIISMMKKQAS

>gi|30063267|ref|NP_837438.1| hypothetical protein S1932 [Shigella flexneri 2a str. 2457T]

MQQIARSVALAFNNLPRPHRVMLGSLTVLTLAVAVWRPYVYHRDATPIVKTIELEQNEIRSLLPEASEPI

DQAAQEDEAIPQDELDDKIAGEAGVHEYVVSTGDTLSSILNQYGIDMGDITQLAAADKELRNLKIGQQLS

WTLTADGELQRLTWEVSRRETRTYDRTAANGFKMTSEMQQGEWVNNLLKGTVGGSFVASARNAGLTSAEV

SAVIKAMQWQMDFRKLKKGDEFAVLMSREMLDGKREQSQLLGVRLRSEGKDYYAIRAEDGKFYDRNGTGL

AKGFLRFPTAKQFRISSNFNPRRTNPVTGRVAPHRGVDFAMPQGTPVLSVGDGEVVVAKRSGAAGYYVAI

RHGRSYTTRYMHLRKILVKPGQKVKRGDRIALSGNTGRSTGPHLHYEVWINQQAVNPLTAKLPRTEGLTG

SDRREFLAQAKEIVPQLRFD

>gi|30063266|ref|NP_837437.1| lipid A biosynthesis (KDO)2-(lauroyl)-lipid IVA acyltransferase [Shigella flexneri 2a str. 2457T]

METKKNNSEYIPEFDKSFRHPRYWGAWLGVAAMAGIALTPPKFRDPILARLGRFAGRLGKSSRRRALINL

SLCFPERSEAEREAIVDEMFATAPQAMAMMAELAIRGPEKIQPRVGWQGLEIIEEMRRNNEKVIFLVPHG

WAVDIPAMLMASQGQKMAAMFHNQGNPVFDYVWNTVRRRFGGRLHARNDGIKPFIQSVRQGYWGYYLPDQ

DHGPEHSEFVDFFATYKATLPAIGRLMKVCRARVVPLFPIYDGKTHRLTIQVRPPMDDLLEADDHTIERR

MNEEVEIFVGPRPEQYTWILKLLKTRKPGEIQPYKRKDLYPIK

>gi|30063262|ref|NP_837433.1| phosphogluconate dehydratase [Shigella flexneri 2a str. 2457T]

MNPQLLRVTNRIIERSRETRSAYLARIEQAKTSTVHRSQLACGNLAHGFAACQPENKASLKSMLRNNIAI

ITSYNDMLSAHQPYEHYPEIIRKALHEANAVGQVAGGVPAMCDGVTQGQDGMELSLLSREVIAMSAAVGL

SHNMFDGALFLGVCDKIVPGLTMAALSFGHLPAVFVPSGPMASGLPNKEKVRIRQLYAEGKVDRMALLES

EAASYHAPGTCTFYGTANTNQMVVEFMGMQLPGSSFVHPDSPLRDALTAAAARQVTRMTGNGNEWMPIGK

MIDEKVVVNGIVALLATGGSTNHTMHLVAMARAAGIQINWDDFSDLSDVVPLMARLYPNGPADINHFQAA

GGVPVLVRELLKAGLLHEDVNTVAGFGLSRYTLEPWLNNGELDWREGAEKSLDSNVIASFEQPFSHHGGT

KVLSGNLGRAVMKTSAVPVENQVIEAPAVVFESQHDVMPAFEAGLLDRDCVVVVRHQGPKANGMPELHKL

MPPLGVLLDRCFKIALVTDGRLSGASGKVPSAIHVTPEAYDGGLLAKVRDGDIIRVNGQTGELTLLVDEA

ELAAREPHIPDLSASRVGTGRELFSALREKLSGAEQGATCITF

>gi|30063261|ref|NP_837432.1| keto-hydroxyglutarate-aldolase/keto-deoxy-phosphogluconate aldolase [Shigella flexneri 2a str. 2457T]

MKNWKTSAESILTTGPVVPVIVVKKLEHAVPMAKALVAGGVRVLEVTLRTECAVDAIRAIAKEVPEAIVG

AGTVLNPQQLAEVTEAGAQFAISPGLTEPLLKAATEGTIPLIPGISTVSELMLGMDYGLKEFKFFPAEAN

GGVKALQAIAGPFSQVRFCPTGGISPANYRDYLALKSVLCIGGSWLVPADALEAGDYDRITKLAREAVEG

AKL

>gi|30063260|ref|NP_837431.1| phosphoribosylglycinamide formyltransferase 2 [Shigella flexneri 2a str. 2457T]

MTLLGTALRPAATRVMLLGSGELGKEVAIECQRLGVEVIAVDRYADAPAMHVAHRSHVINMLDGDALRRV

VELEKPHYIVPEIEAIATDMLIQLEEEGLNVVPCARATKLTMNREGIRRLAAEELQLPTSTYRFADSKSL

FREAVAAIGYPCIVKPVMSSSGKGQTFIRSAEQLAQAWEYAQQGGRAGAGRVIVEGVVKFDFEITLLTVS

AVDGVHFCAPVGHRQEDGDYRESWQPQQMSPLALERAQEIARKVVLALGGYGLFGVELFVCGDEVIFSEV

SPRPHDTGMVTLISQDLSEFALHVRAFLGLPVGGIRQYGPAASAVILPQLTSQNVTFDNVQNAVGADLQI

RLFGKPEIDGSRHLGVVLATSESVVDAIERAKHAAGQVKVQG

>gi|30063259|ref|NP_837430.1| hypothetical protein S1923 [Shigella flexneri 2a str. 2457T]

MKKRGAFLGRLLVSACASVFAANNETSKSVTFPKCEGLDAAGIAASVKRDYQQNRVARWADDQKIVGQAD
PVAWVSLQDIQGKDDKWSVPLTVRGKSADIHYQVCVDCKAGMAEYQRR

>gi|30063258|ref|NP_837429.1| hypothetical protein S1922 [Shigella flexneri 2a str. 2457T]
MANWLNQLQSLLGQSSSSTSSSADQGLGKLLVSGALGGLAGLLVANKSARKLLTKYGTNALLVGGGAVAG
TVLWNKYKDKIRAAHQDEPQFGAQSTPLDERTERLILALVFAAKSDGHIDANERAAIDQQLREAGVEEQG
RVLIEQAIEQPLDPQRLATGVRNEEEALEIYFLSCAAIDIDHFMERSYLNALGDALKIPQDVREGIERDL
EQQKRTLAE

>gi|30063256|ref|NP_837427.1| exodeoxyribonuclease X [Shigella flexneri 2a str. 2457T]
MLRIIDTETCGLQGGIVEIASVDVIDGKIVNPMSLLVRPDRPISPQAMAIHRITEAMVADKPWIEDVIPH
YYGSEWYVAHNASFDRRVLPEMPGEWICTMKLARRLWPGLKYSNMALYKTRKLNVQTPLGLHHHRALYDC
YITAALLIDIMNTSGWTAEQMADITGRPSLMTTFTFGKYRGKAVSDVAERYPGYLRWLFNNLDSMSPELR
LTLKHYLENT

>gi|30063255|ref|NP_837426.1| hypothetical protein S1919 [Shigella flexneri 2a str. 2457T]
MTIIAGLPVEYNDRFIRGIAVFAPWRKTPGNYHQSHGACLGRRSRTITVVDEQPQGMDMDPTCSLFTTGQ
CLGEPDLLASARRLQFFSHQYSIAVLMANARGNSALWDEYGRLIVRADRGSLLLVGQRSSQGWQGDIIPL
R

>gi|30063253|ref|NP_837424.1| hypothetical protein S1917 [Shigella flexneri 2a str. 2457T]
MASTARSLRYALAILTTSLVTPSVWAHAHLTHQYPAANAQVTAAPQAITLNFSEGVETGFSGAKITGPKN

ENIKTLPAKRNEQDQKQLIVPLADSLKPGTYTVDWHVVSVDGHKTKGHYTFSVK

>gi|30063252|ref|NP_837423.1| hypothetical protein S1915 [Shigella flexneri 2a str. 2457T]

MMKKSILAFLLLTSSAAALAAPQVITVSRFEVGKDKWAFNREEVMLTCRPGNALYVINPSTLVQYPLNDI

AQKEVASGKTNAQPISVIQIDDPNNPGEKMSLAPFIERAEKLC

>gi|30063246|ref|NP_837417.1| transport system permease [Shigella flexneri 2a str. 2457T]

MHSERAPFFLKLAAWGGVVFLHFPILIIAAYAFNTEDAAFSFPPQGLTLRWFSVAAQRSDILDAVTLSLK

VAALATLIALVLGTLAAAALWRRDFFGKNAISLLLLLPIALPGIVTGLALLTAFKTINLEPGFFTIVVGH

ATFCVVVVFNNVIARFRRTSWSLVEASMDLGANGWQTFRYVVLPNLSSALLAGGMLAFALSFDEIIVTTF

TAGHERTLPLWLLNQLGRPRDVPVTNVVALLVMLVTTLPNLGAWWLTREGDNGQ

>gi|30063242|ref|NP_837413.1| hypothetical protein S1902 [Shigella flexneri 2a str. 2457T]

MNQSLTLAFLIAAGIGLVVQNTLMARITQTSSTILIAMLLNSLVGIVLFVSILWFKQGMAGFGELVSSVR

WWTLIPGLLGSFFVFASISGYQNVGAATTIAVLVASQLIGGLVLDIFRSHGVPLRALFGPICGAILLVVG

AWLVARRSF

>gi|30063241|ref|NP_837412.1| resistance protein [Shigella flexneri 2a str. 2457T]

MSIRFARKADCAAIAEIYNHAVLYTAAIWNDQTVDADNRIAWFEARTIAGYPVLVSEEDGVVTGYASFGD

WRSFDGFRHTVEHSVYVHPDHQGKGLGRKLLSRLIDEARDCGKHVMVAGIESQNQASLHLHQSLGFVVTA

QMPQVGTKFGHWLDLTFMQLQLDERTEPDAIG

>gi|30063237|ref|NP_837408.1| DNA-binding transcriptional regulator [Shigella flexneri 2a str. 2457T]

MNSFCHAGRVGQKTREGIKMPGTGKMKHVSLTLQVENDLKHQLSIGALKPGARLITKNLAEQLGMSITPV

REALLRLVSVNALSVAPAQAFTVPEVRQSQLDEINRIRYELELMAVALAVENLSSQDIAELQELLGKLQQ

AQEKGDMEQIINVNRLFRLAIYHRSNMPILCEMIEQLWIRMGPALHYLYEAINPAELRERIENYHLLLAA

LKAKDKEGCKHCLAEIMQQNIAILYQQYNR

>gi|30063236|ref|NP_837407.1| IS91 orf [Shigella flexneri 2a str. 2457T]

MLPRFADIFQQGNRWLNWLEKQPEGSVRPVVIESVTKIMACGTTLMGYTQWCCSSPDCCHTKKVCFRCKS

RSCPHCGVKAGAQWIQYLLSLVPYCPWQHIVFTLPCQYWSLVFHNRWLLAEMSRIAADVILEICHQADVE

PGIFTVIHTWGRDQQWHPHIHLSTTAGGVTSGHTWKNLHFYARKVMSMWRYRITRLLSRKYPDLVMPDAL

AAEGSSKREWNRFLDTHYRRGWNVNVSRVMDNTTHVAVYFGSYLKKPPVPMSRLEHYAGQDEIGLRYNSH

RTKREEYLLMSGDEFMERFSWHVADKGFRMVRYYGFLSPAKRRLLEEVVYIITETVRKTAMQIRWRGMYQ

RLLKVDPLKCILCGSQMRFTGLKRGYRLAEQVLMHEPLGPDAVVRLRAAEGKLRPF

>gi|30063230|ref|NP_837401.1| hypothetical protein S1885 [Shigella flexneri 2a str. 2457T]

MTRQKATLIGLIAIVLWSTMVGLIRGVSEGLGPVGGAAAIYSLSGLLLIFTVGFPRIRQIPKGYLLAGSL

LFVSYEIYLALSLGYAATRHQAIEVGMVNYLWPSLTILFAILFNGQKTNWLIVPGLLLALVGVCWVLGGE

NGLHYDEIINNITTSPLSYFLAFIGAFIWAAYCTVTNKYARGFNGITVFVLLTGACLWIYYFLTPQPEMV

FSTPVMIKLISAAFTLGFAYTAWNVGILHGNVTIMAVGSYFTPVLSSALAAVLLSAPLSFSFWQGALMVC

GGSLLCWLATRRG

>gi|30063227|ref|NP_837398.1| formate dehydrogenase-N subunit gamma [Shigella flexneri 2a str. 2457T]

MSKSKMIVRTKFIDRACHWTVVICFFLVALSGISFFFPTLQWLTQTFGTPQMGRILHPFFGIAIFVALMF

MFVRFVHHNIPDKKDIPWLLNIVEVLKGNEHKVADVGKYNAGQKMMFWSIMSMIFVLLVTGVIIWRPYFA

QYFPMQVVRYSLLIHAAAGIILIHAILIHMYMAFWVKGSIKGMIEGKVSRRWAKKHHPRWYREIEKAEAK

KESEEGI

>gi|30063226|ref|NP_837397.1| hypothetical protein S1881 [Shigella flexneri 2a str. 2457T]

MDYCCVGRNNNDSVPREVTLLRVKTLMKMANHPRPGDIIQESLDELNVSLREFARAMEIAPSTASRLLTG

KAALTPEMAIKLSVVIGSSPQMWLNLQNAWSLAEAEKTVVVSRLRRLVTQ

>gi|30063221|ref|NP_837392.1| osmotically inducible protein [Shigella flexneri 2a str. 2457T]

MTIHKKGQAHWEGDIKRGKGTVSTESGVLNQQPYGFNTRFEGEKGTNPEELIGAAHAACFSMALSLMLGE

AGFTPTSIDTTADVSLDKVDAGFAITKIALKSEVAVPGIDASTFDGIIQKAKAGCPVSQVLKAEITLDYQ

LKS

>gi|30063219|ref|NP_837390.1| ATP-dependent peptide transporter membrane subunit [Shigella flexneri 2a str. 2457T]

MMLSEETSAVRPQKQTRFNGAKLVWMLKGSPLTVTGAVIIVLMRLMMIFSPWLATHDPNAIDLTARLLPP

SAAHWFGTDEVGRDLFSRVLVGSQQSILAGLVVVAIAGMIGSLLGCLSGVLGGRADAIIMRIMDIMLSIP

SLVLTMALAAALGPSLFNAMLAIAIVRIPFYVRLARGQALVVRQYTYVQAAKTFGASRWHLINWHILRNS

LPPLIVQASLDIGSAILMAATLGFIGLGAQQPSAEWGAMVANGRNYVLDQWWYCAFPGAAILLTSVGFNL

FGDGIRDLLDPKAGGKQS

>gi|30063218|ref|NP_837389.1| hypothetical protein S1869 [Shigella flexneri 2a str. 2457T]

MDICSRNKKLTIRRPAILVALALLLCSCKSTPPESMVTPPAGSKPPATTQQSSQPMRGIWLATVSRLDWP

PVSSVNISNPTSRARVQQQAMIDKLDHLQRLGINTVFFQVKPDGTALWPSKILPWSDLMTGKIGENPGYD

PLQFMLDEAHKRGMKVHAWFNPYRVSVNTKPGTIRELNSTLSQQPASVYVQHRDWIRTSGDRFVLDPGIP

EVQDWITSIVAEVVSRYPVDGVQFDDYFYTESPGSRLNDNETYRKYGGAFASKADWRRNNTQQLIAKVSH

TIKSIKPGVEFGVSPAGVWRNRSHDPLGSDTRGAAAYDESYADTRRWVEQGLLDYIAPQIYWPFSRSAAR

YDVLAKWWADVVKPTRTRLYIGIAFYKVGEPSKIEPDWMINGGVPELKKQLDLNDAVPEISGTILFREDY

LNKPQTQQAVSYLQSRWGS

>gi|30063217|ref|NP_837388.1| acid sensitivity protein, transporter [Shigella flexneri 2a str. 2457T]

MATSVQTGKAKQLTLLGFFAITASMVMAVYEYPTFATSGFSLVFFLLLGGILWFIPVGLCAAEMATVDGW

EEGGVFAWVSNTLGPRWGFAAISFGYLQIAIGFIPMLYFVLGALSYILKWPALNEDPITKTIAALIILWA

LALTQFGGTKYTARIAKVGFFAGILLPAFILIALAAIYLHSGAPVAIEMDSKTFFPDFSKVGTLVVFVAF

ILSYMGVEASATHVNEMSNPGRDYPLAMLLLMVAAICLSSVGGLSIAMVIPGNEINLSAGVMQTFTVLMS

HVAPEIEWTVRVISALLLLGVLAEIASWIVGPSRGMYVTAQKNLLPAAFAKMNKNGVPVTLVISQLVITS

IALIILTNTGGGNNMSFLIALALTVVIYLCAYFMLFIGYIVLVLKHPDLKRTFNIPGGKGVKLVVAIVGL

LTSIMAFIVSFLPPDNIQGDSTDMYVELLVVSFLVVLALPFILYAVHDRKGKANTGVTLEPINSQNAPKG

HFFLHPRARSPHYIVMNDKKH

>gi|30063211|ref|NP_837382.1| hypothetical protein S1861 [Shigella flexneri 2a str. 2457T]

MKRVLIPGVILCGADVAQAVDDKNMYMYFFEEMTVYAPVPVPVNGNTHYTSESIERLPTGNGNISDLLRT

NPAVRMDSTQSTSLNQGDIRPEKISIHGASPYQNAYLIDGISATNNLNPANESDASSATNISGMSQGYYL

DVSLLDNVTLYDSFVPVEFGRFNGGVIDAKIKRFNADDSKVKLGYRTTRLDWLTSHIDENNKSAFNQGSS

GSTYFSPDFKKNFYTLSFNQELADNFGVTAGLSRRQSDITRADYVSNDGIVAGRAQYKNVIDTALSKFTW

FASDRFTHDLTLKYTGSSRDYNTSTFPQSDREMGNKSYGLAWDMDTQLAWAKLRTTVGWDHISDYTRHDH

DIWYTELSCTYGDITGRCTRGGLGHISQAVDNYTFKTRLDWQKFAVGDVSHQPYFGAEYIYSDAWTERHN

QSESYVINAAGKKTNHTIYHKGKGSLGIDNYTLYMADHISWRNVSLMPGVRYDYDNYLSNHNISPRFMTE

WDIFADQTSMITAGYNRYYGGNILDMGLRDIRNSWTESVSGNKTLTRYQNLKTPYNDELAMGLQQKIDKN

VIARASEAHDQISKSSRTDSATKTTITEYNNDGKTKTHSFNLSFELAEPLHIRQVDINPQIVFSYIKSKG

NLSLNNGYEESNTGDNQVVYNGNLVSYDSVPVADFNNPLKISLNMDFTHQPSGLVWANTLAWQEARKARI

ILGKTNAQYISEYSDYKQYVDEKLDSSLTWDTRLSWTPQFLKQQNLTISADILNVLDSKTAVDTTNTGVA

TYASGRTFWLDVSMKF

>gi|30063210|ref|NP_837381.1| enzyme [Shigella flexneri 2a str. 2457T]

MHVTAKPSSFQCNLKCDYCFYLEKESQFTHEKWMDDSTLKEFIKQYIEASGNQVYFTWQGGEPTLAGLDF

FRKVIHYQQRYAGQKRIFNTLQTNGILLNNEWCAFLKEHEFLVGISIDGPQELHDRYRRSNSGNGTFAKV

IAAIERLKSYQVEFNTLTVINNVNVHYPLEVYHFLKSIGSKHMQFIELLETGTPNIDFSGHSENTFRIID

FSVPPTAYGKFMSTIFMQWVKNDVGEIFIRQFESFVSRFLGNGHTSCIFQESCKDNLVVESNGDIYECDH

FVYPQYKIGNINKSELKTMNSVQLTAQKKRISAKCQQCAYKPICNGGCPKHRITKVNNETVSYFCEGYKI

LFSTMVPYMNAMVELAKNRVPLYHIMDVAKQMENN

>gi|30063207|ref|NP_837378.1| 3-dehydroquinate dehydratase [Shigella flexneri 2a str. 2457T]

MKTVTVKDLVIGAGAPKIIVSLMAKDIARVKSEALAYREADFDILEWRVDHFADLSNVESVMAAAKILRE

TMPEKPLLFTFRSAKEGGEQAISTEAYIALNRAAIDSGLVDMIDLELFTGDDQVKETVAYAHAHDVKVVM

SNHDFHKTPEAEEIIARLRKMQSFDADIPKIALMPQSTSDVLTLLAATLEMQEQYADRPIITMSMAKTGV

ISRLVGEVFGSAATFGAVKKASAPGQISVNDLRTVLTILHQA

>gi|30063206|ref|NP_837377.1| quinate/shikimate dehydrogenase [Shigella flexneri 2a str. 2457T]

MDVTAKYELIGLMAYPIRHSLSPEMQNKALEKAGLPFTYMAFEVDNDSFPGAIEGLKALKMRGTGVSMPN
KQLACEYVDELTPAAKLVGAINTIVNDDGYLRGYNTDGTGHIRAIKESGFDIKGKTMVLLGAGGASTAIG
AQGAIEGLKEIKLFNRRDEFFDKALAFAQRVNENTDCVVTDTDLADQQAFAEALASADILTNGTKVGMKP
LENESLVNDISLLHPGLLVTECVYNPHMTKLLQQAQQAGCKTIDGYGMLLWQGAEQFTLWTGKDFPLEYV
KQVMGFGA

>gi|30063205|ref|NP_837376.1| amino acid/amine transport protein [Shigella flexneri 2a str. 2457T]

MTSLAEKFSTDNAGIAYLISGIGLGRLISILFFGVISDKFGRRAVILMAVIMYLLFFFGIPACPNLTLAY
GLAVCVGIANSALDTGGYPALMECFPKASGSAVILVKAMVSFGQMFYPMLVSYMLLNNIWYGYGLIIPGI
LFVLITLMLLKSKFPSQLVDASVANELPQMNSKPLVWLEGVSSVLFGVAAFSTFYVIVVWMPKYAMAFAG
MSEAEALKTISYYSMGSLVCVFIFAALLKKMVRPIWANVFNSALATITAAIIYLYPSPLVCNAGAFVIGF
SAAGGILQLGVSVMSEFFPKSKAKVTSIYMMMGGLANFVIPLITGYLSNIGLQYIIVLDFTFALLALITA
IIVFSRYYRVFIIPENDVRFGERKFSTRLNTIKHRG

>gi|30063203|ref|NP_837374.1| hypothetical protein S1851 [Shigella flexneri 2a str. 2457T]

MNAYELQALRHIFAMTIDECATWIAQTGDSESWRQWENGKCAIPDCVVEQLLAMRQQRKKHLHAIIEKIN
NRIGNNTMRFFPDLTAFQQVYPDGNFIDWKIYQSVAAELYAHDLERLC

>gi|30063202|ref|NP_837373.1| inner membrane protein [Shigella flexneri 2a str. 2457T]

MVNVRQPRDVAQILLSVLFLAIMIVACLWIVQPFILGFAWAGTVVIATWPVLLRLQKIMFGRRSLAVLVM
TLLLVMVFIIPIALLVNSIVDGSGPLIKAISSGDMTLPDLAWLNTIPVIGAKLYAGWHNLLDMGGTAIMA
KVRPYIGTTTTWFVGQAAHIGRFMVHCALMLLFSALLYWRGEQVAQGIRHFATRLAGVRGDAAVLLAAQA
IRAVALGVVVTALVQAVLGGIGLAVSGVPYATLLTVLMILSCLVQLGPLPVLIPAIIWLYWTGDTTWGTV
LLVWSGVVGTLDNVIRPMLIRMGADLPLILILSGVIGGLIAFGMIGLFIGPVLLAVSWRLFAAWVEEVPP

PTDQPEEILEELGEIEKPNK

>gi|30063200|ref|NP_837371.1| hypothetical protein S1848 [Shigella flexneri 2a str. 2457T]

MIWKRKITLEALNAMGEGNMVGFLDIRFEHIGDDTLEATMPVDSRTKQPFGLLHGGASVVLAESIGSVAG

YLCTEGEQKVVGLEINANHVRSAREGRVRGICKPLHLGSRHQVWQIEIFDEKGRLCCSSRLTTAIL

>gi|30063197|ref|NP_837368.1| cysteine desulfurase activator complex subunit SufB [Shigella flexneri 2a str. 2457T]

MSRNTEATDDVKTWTGGPLNYKEGFFTQLATDELAKGINEEVVRAISAKRNEPEWMLEFRLNAYRAWLEM

EEPHWLKAHYDKLNYQDYSYYSAPSCGNCDDTCASEPGAVQQTGANAFLSKEVEAAFEQLGVPVREGKEV

AVDAIFDSVSVATTYREKLAEQGIIFCSFGEAIHDHPELVRKYLGTVVPGNDNFFAALNAAVASDGTFIY

VPKGVRCPMELSTYFRINAEKTGQFERTILVADEDSYVSYIEGCSAPVRDSYQLHAAVVEVIIHKNAEVK

YSTVQNWFPGDNNTGGILNFVTKRALCEGENSKMSWTQSETGSAITWKYPSCILRGDNSIGEFYSVALTS

GHQQADTGTKMIHIGKNTKSTIISKGISAGHSQNSYRGLVKIMPTATNARNFTQCDSMLIGANCGAHTFP

YVECRNNSAQLEHEATTSRIGEDQLFYCLQRGISEEDAISMIVNGFCKDVFSELPLEFAVEAQKLLAISL

EHSVG

>gi|30063195|ref|NP_837366.1| cysteine desulfurase activator complex subunit SufD [Shigella flexneri 2a str. 2457T]

MAGLPNSSNALQQWHHLFEAEGTKRSPQAQQHLQQLLRTGLPTRKHENWKYTPLEGLTNSQFVSIAGDIS
PQQRDALALTLDAVRLVFVDGRYVPALSDAIEGSGYEVSINDDRQGLPDAIQAEVFLHLTESLAQSVTHI
AVKRGQRPAKPLLLMHITQGVAGEEVNTAHYRHHLDLAEGAEATVIEHFVSLNDARHFTGARFTINVAAN
AHLQHIKLAFENPVSHHFAHNDLLLADDATAFSHSFLLGGAVLRHNTSTQLNGENSTLRINSLAMPVKNE
VCDTRTWLEHNKGFCNSRQLHKTIVSDKGRAVFNGLINVAQHAIKTDGQMTNNNLLMGKLAEVDTKPQLE
IYADDVKCSHGATVGRIDDEQMFYLRSRGINQQDAQQMIIYAFAAELTEALRDEGLKQQVLARIGQRLPG
GAR

>gi|30063193|ref|NP_837364.1| cysteine desufuration protein SufE [Shigella flexneri 2a str. 2457T]

MALLPDKEKLLRNFLRCANWEEKYLYIIELGQRLPELRAEDGSPQNSIQGCQSQVWIVMRQNAQGIIELQ
GDSDAAIVKGLIAVVFILYDQMTPQDIVNFDVRPWFEKMALTQHLTPSRSQGLEAMIRAIRAKAAALS

>gi|30063192|ref|NP_837363.1| hypothetical protein S1840 [Shigella flexneri 2a str. 2457T]

MKRASLLTLTLIGAFSAIQAAWAVDYPLPPTGSRLVGQNQTYTVQEGDKNLQAIARRFDTAAMLILEANN
TIAPVPKPGTTITIPSQLLLPDAPRQGIIVNLAELRLYYYPPGENIVQVYPIGIGLQGLETPVMETRVGQ
KIPNPTWTPTAGIRQRSLERGIKLPPVVPAGPNNPLGRYALRLAHGNGEYLIHGTSAPDSVGLRVSSGCI
RMNAPDIKALFSSVRTGTPVKVINEPVKYSVEPNGMRYVEVHRPLSAEEQQNVQTMPYTLPAGFTQFKDN
KAVDQKLVDKALYRRAGYPVSVSSGATPAASNAPSVESAQNGEPEQGNMLRATQ

>gi|30063186|ref|NP_837357.1| hypothetical protein S1834 [Shigella flexneri 2a str. 2457T]

MNPVDRPLLDIGLTRLEFLRISGKGLAGLTIAPALLSLLGCKQEDIDSGTVGLINTPKGVLVTQRARCTG

CHRCEISCTNFNDGSVGTFFSRIKIHRNYFFGDNGVGSGGGLYGDLNYTADTCRQCKEPQCMNVCPIGAI

TWQQKEGCITVDHKRCIGCSACTTACPWMMATVNTESKKSSKCVLCGECANACPTGALKIIEWKDITV

>gi|30063184|ref|NP_837355.1| oxidoreductase, Fe-S subunit [Shigella flexneri 2a str. 2457T]

MSWIGWTVAATALGDNQMSFTRRKFVLGMGTVIFFTGSASSLLANTRQEKEVRYAMIHDESRCNGCNICA

RACRKTNHVPAQGSRLSIAHIPVTDNDNETQYHFFRQSCQHCEDAPCIDVCPTGASWRDEQGIVRVEKSQ

CIGCSYCIGACPYLVRYLNPVTKVADKCDFCAESRLAKGFPPICVSACPEHALIFGREDSPEIQAWLQDN

KYYQYQLPGTGKPHLYRRFGQHLIKKENV

>gi|30063183|ref|NP_837354.1| hypothetical protein S1830 [Shigella flexneri 2a str. 2457T]

MNPSQHAEQFQSQLANYVPQFTPEFWPVWLIIAGVLLVGMWLVLGLHALLRARGVKKSATDHGEKVYLYS

KAVRLWHWSNALLFVLLLASGLINHFAMVGATAVKSLVAVHEVCGFLLLACWLGFVLINAVGDNGHHYRI

RRQGWLERAAKQTRFYLFGIMQGEEHPFPATTQSKFNPLQQVAYVGVMYGLLPLLLLTGLLCLYPQAVGD

VFPGVRYWLLQAHFALAFISLFFIFGHLYLCTTGRTPHETFKSMVDGYHRH

>gi|30063182|ref|NP_837353.1| hypothetical protein S1829 [Shigella flexneri 2a str. 2457T]

MIITRADLREWRIGAVMYRWFLRHFPRGGSYADIRHALIEEGYTDWAESLVEYAWKKWLADENFAHQEVS

SMQKLATDPGEIPFCSQFARSDDHARIGCCEDNARIATAGYAAQIASMGYSVRIGSVGFNSHIGSSGERA

RVAVTGNSSRISSAGDSSRIANTGMRVRVCTLGERCHVASNGDLVQIASFGANARIANSGDNVHIIASGE

NSTVVSTGVVDSIILGLGGSAALAYHDGERVRFAVAIEGENNIRTGVRYRLNEQHQFVEC

>gi|30063181|ref|NP_837352.1| hypothetical protein S1827 [Shigella flexneri 2a str. 2457T]

MATLLQLHFAFNGPFGDAMAEQLKPLAESINQEPGFLWKVWTESEKNHEAGGIYLFTDEKSALAYLEKHT

ARLKNLGVEEVVAKVFDVNEPLSQINQAKLA

>gi|30063180|ref|NP_837351.1| hypothetical protein S1824 [Shigella flexneri 2a str. 2457T]

MGSDAKNLMSDGNVQIVKTGEVLGATQLTEGELIVEAGGRAENTVVTGAGWLKVATGGIAKCTQYGNNGT

LSVSDGAIATDIVQSEGGAISLSTLATVNGRHPEGEFSVDQGYACGLLLENGGNLRVLEGHRAEKIILDQ

EGGLLVNGTTSAVVVDEGGELLVYPSGEASNCEINQGGVFMLAGKASDTLLAGGTMNNLGGEDSDTIVEN

GSIYRLGTDGLQLYSSGKTQNLSVNVGGRAEVHAGTLENAVIQGGTVILLSPTSADENFVVEEDRAPVEL

TGSVALLDGASMIIGYGAELQQSTITVQQGGVLILDGSTVKGDSVTFIVGNINLNGGKLWLITGAATHVQ

LKVKRLRGEGAICLQTSAKEISPDFINVKGEVNGDIRVEITDASRQTLCNALKLQPDEDGIGATLQPA

>gi|30063178|ref|NP_837349.1| riboflavin synthase subunit alpha [Shigella flexneri 2a str. 2457T]

MFTGIVQGTAKLVSIDEKPNFRTHVVELPDHMLDGLETGASVAHNGCCLTVTEINGNHVSFDLMKETLRI

TNLGDLKVGDWVNVERAAKFSDEIGGHLMSGHIMTTAEVAKILTSENNRQIWFKVQDSQLMKYILYKGFI

GIDGISLTVGEVTPTRFCVHLIPETLERTTLGKKKLGARVNIEIDPQTQAVVDTVERVLAARENAMNQPG

TEA

>gi|30063177|ref|NP_837348.1| cyclopropane-fatty-acyl-phospholipid synthase [Shigella flexneri 2a str. 2457T]

MSSSCIEEVSVPDDNWYRIANELLSRAGIAINGSAPADIRVKNPDFFKRVLQEGSLGLGESYMDGWWECD

RLDMFFSKVLRAGLENQLPHHFKDTLRIAGARLFNLQSKKRAWIVGKEHYDLGNDLFSRMLDPFMQYSCA

YWKDADNLESAQQAKLKMICEKLQLKPGMRVLDIGCGWGGLAHYMASNYDVSVVGVTISAEQQKMAQERC

KGLDVTILLQDYRDLNDQFDRIVSVGMFEHVGPKNYDTYFAVVDRNLKPEGIFLLHTIGSKKTDLNVDPW

INKYIFPNGCLPSVRQIAQSSEPHFVMEDWHNFGADYDTTLMAWYERFLAAWPEIADNYSERFKRMFTYY

LNACAGAFRARDIQLWQVVFSRGVENGLRVAR

>gi|30063176|ref|NP_837347.1| inner membrane transport protein YdhC [Shigella flexneri 2a str. 2457T]

MQPGKRFLVWLAGLSVLGFLATDMYLPAFAAIQADLQTPASAVSASLSLFLAGFAAAQLLWGPLSDRYGR

KPVLLIGLTIFALGSLGMLWVENAATLLVLRFVQAVGVCAAAVIWQALVTDYYPSQKVNRIFATIMPLVG

LSPALAPLLGSWLLVHFSWQAIFATLFAITVVLILPIFWLKPTTKARNNSQDGLTFTDLLRSKTYRGNVL

IYAACSASFFAWLTGSPFILSEMGYSPAVIGLSYVPQTIAFLIGGYGCRAALQKWQGKQLLPWLLVLFAV

SVIATWAAGFISHVSLVEILIPFCVMAIANGAIYPIVVAQALRPFPHATGRAAALQNTLQLGLCFLASLV

VSWLISISTPLLTTTSVMLSTVVLVALGYMMQRCEEVDCPNHGNAEVAHSESH

>gi|30063175|ref|NP_837346.1| DNA-binding transcriptional regulator [Shigella flexneri 2a str. 2457T]

MWSEYSLEVVDAVARNGSFSAAAQELHRVPSAVSYTVRQLEEWLAVPLFERRHRDVELTAAGAWFLKEGR

SVVKKMQITRQQCQQIANGWRGQLAIAVDNIVRPERTRQMIVDFYRHFDDVELLVFQEVFNGVWDALSDG

RVELAIGATRAIPVGGRYAFRDMGMLSWSCVVASHHPLALMDGPFSDDTLRNWPSLVREDTSRTLPKRIT

WLLDNQKRLVVPDWESSATCISAGLCIGMVPTHFAKPWLNEGKWVALELENPFPDSACCLTWQQNDMSPA

LTWLLEYLSDSETLNKEWLREPEETPATGD

>gi|30063174|ref|NP_837345.1| DNA-binding transcriptional repressor PurR [Shigella flexneri 2a str. 2457T]

MATIKDVAKRANVSTTTVSHVINKTRFVAEETRNAVWAAIKELHYSPSAVARSLKVNHTKSIGLLATSSE

AAYFAEIIEAVEKNCFQKGYTLILGNAWNNLEKQRAYLSMMAQKRVDGLLVMCSEYPEPLLAMLEEYRHI

PMVVMDWGEAKADFTDAVIDNAFEGGYMAGRYLIERGHREIGVIPGPLERNTGAGRLAGFMKAMEEAMIK

VPESWIVQGDFEPESGYRAMQQILSQPHRPTAVFCGGDIMAMGALCAADEMGLRVPQDVSLIGYDNVRNA

RYFTPALTTIHQPKDSLGETAFNMLLDRIVNKREEPQSIEVHPRLIERRSVADGPFRDYRR

>gi|30063173|ref|NP_837344.1| transporter [Shigella flexneri 2a str. 2457T]

MKINYPLLALAIGAFGIGTTEFSPMGLLPVIARGVDVSIPAAGMLISAYAVGVMVGAPLMTLLLSHRARR

SALIFLMAIFTLGNVLSAIAPDYMTLMLSRILTSLNHGAFFGLGSVVAASVVPKHKQASAVATMFMGLTL

ANIGGVPAATWLGETIGWRMSFLATAGLGVISMVSLFFSLPKGGTGARPEVKKELAVLMRPQVLSALLTT

VLGAGAMFTLYTYISPVLQSITHATPVFVTAMLVLIGVGFSIGNYLGGKLADRSVNGTLKGFLLLLMVIM

LAIPFLARNEFGAAISMVVWGAATFAVVPPLQMRVMHVASEAPGLSSSVNIGAFNLGNALGAAAGGAVIS

AGLGYSFVPVTGAIVAGLALLLVFMSARKQPETVCVANS

>gi|30063171|ref|NP_837342.1| lipoprotein [Shigella flexneri 2a str. 2457T]

MARINRISITLCALLFTTLPLTPMAHASKQARESSATTHITKKADKKKSTATTKKTQKASQKTAKKAASK

SMTKSKTASSVKKSSITASKNAKTRSKHAVNKTASASFTEKCTKRKGYKSHCVKVKNAASGTLADAHKAK

VQKATKVAMNKLMQQIGKPYRWGGSSPRTGFDCSGLVYYAYKDLVKIRIPRTANEMYHLRDAGPIERSEL

KNGDLVFFRTQGRGTADHVGVYVGNGKFIQSPRTGQEIQITSLSEDYWQRHYVGARRVMTPKTLR

>gi|30063168|ref|NP_837339.1| ribonuclease T [Shigella flexneri 2a str. 2457T]

MSDNAQLTGLCDRFRGFYPVVIDVETAGFNAKTDALLEIAAITLKMDEQGWLMPDTTLHFHVEPFVGANL

QPEALAFNGIDPNDPDRGAVSEYEALHEIFKVVRKGIKASGCNRAIMVAHNANFDHSFMMAAAERASLKR

NPFHPFATFDTAALAGLALGQTVLSKACQTAGMDFDSTQAHSALYDTERTAVLFCEIVNRWKRLGGWPLP

AAEEV

>gi|30063166|ref|NP_837337.1| N-ethylmaleimide reductase [Shigella flexneri 2a str. 2457T]

MSSEKLYSPLKVGAITAANRIFMAPLTRLRSIEPGDIPTPLMAEYYRQRASAGLIISEATQISAQAKGYA

GAPGIHSPEQIAAWKKITAGVHAENGHMALQLWHTGRISHASLQPGGQAPVAPSALSAGTRTSLRDENGQ

AIRVETSMPRALELEEIPGIVNDFRQAIANAREAAFDLVELHSAHGYLLHQFLSPSSNHRTDQYGGSVEN

RARLVLEVVDAGIEEWGADRIGIRVSPIGTFQNTDNGPNEEADALYLIEQLGKRGIAYLHMSEPDWAGGE

PYTDAFREKVRARFHGPIIGAGAYTVEKAETLIGKGLIDAVAFGRDWIANPDLVARLQLKAELNPQRAES

FYGGGAEGYTDYPTL

>gi|30063165|ref|NP_837336.1| hypothetical protein S1807 [Shigella flexneri 2a str. 2457T]

MNKHTEHDTREHLLATGEQLCLQRGFTGMGLSELLKTAEVPKGSFYHYFRSKEAFGVAMLERHYAAYHQR

LAELLQSGEGNYRDRILAYYQQTLNQFCQHGTISGCLTVKLSAEVCDLSEDMRSAMDKGARGVIALLSQA

LENGRENHCLTFCGEPLQQAQVLYALWLGANLQAKISRSFEPLENALAHVKNIIATPAV

>gi|30063164|ref|NP_837335.1| hypothetical protein S1808 [Shigella flexneri 2a str. 2457T]

MFASIMFGVFVHALECSRPVGLVQAKLLLWTLTASIMVIILSEEGEVAEQLEFFPVQSPCRGICQSDERG

FCRGCFRSRDERFNWNKMSDGEKQEVLRLCRQRLMRKLRANKPASSDEPEQPSLF

>gi|30063161|ref|NP_837332.1| hypothetical protein S1803 [Shigella flexneri 2a str. 2457T]

MVTMSIKTIKYFSTIIVAIVAVLAGWWLWNYYMQSPWTRDGKIRAEQVSITPQVSGRIVELNIKDNQLVN

AGELLLTIDKTPFQIAELNAQAQLAKAQSDLAKANNEANRRRHLSQNFIFAEELDTANLNVKAMQASVNA

AQATLKQTQWQLAQTEIRAPVSGWVTNLTTRIGDYADTGKPLFALVDSHSFYVIGYFEETKLRHIREGAP

AQITLYSDNKTLQGHVSSIGRAIYDQSVESDSSLIPDVKPNVPWVRLAQLVPVRFALDKVPGDVTLVSGT

TCSIAVGQ

>gi|30063158|ref|NP_837329.1| outer membrane protein [Shigella flexneri 2a str. 2457T]

MIKRVLVVSMVGLSLVGCVNNDTLSGDVYTASEAKQVQNVSYGTIVNVRPVQIQGGDDSNVIGAIGGAVL

GGFLGNTVGGGTGRSLATAAGAVAGGVAGQGVQSAMNKTQGVELEIRKDDGNTIMVVQKQGNTRFSPGQR

VVLASNGSQVTVSPR

>gi|30063157|ref|NP_837328.1| anhydro-N-acetylmuramic acid kinase [Shigella flexneri 2a str. 2457T]

MKSGRFIGVMSGTSLDGVDVVLATIDEHRVAQLASLSWPIPVSLKQAVLDICQGQQLTLSQFGQLDTQLG

RLFADAVNALLKEQNLQARDIVAIGCHGQTVWHEPTGVAPHTLQIGDNNQIVARTGITVVGDFRRRDIAL

GGQGAPLVPAFHHALLAHPTERRMVLNIGGIANLSLLIPGQLVGGYDTGPGNMLMDAWIWRQAGKPYDKD

AEWARAGKVILPLLQNMLSDPYFSQPAPKSTGREYFNYGWLERHLRHFPGVDPRDVQATLAELTAVTISE

QVLLSGGCERLMVCGGGSRNPLLMARLAVLLLGTEVTTTDAVGISGDDMEALAFAWLAWRTLAGLPGNLP

SVTGASQETVLGAIFPANS

>gi|30063147|ref|NP_837318.1| electron transport complex RsxE subunit [Shigella flexneri 2a str. 2457T]

MSEIKDVIVQGLWKNNSALVQLLGLCPLLAVTSTATNALGLGLATTLVLTLTNLTISTLRHWTPAEIRIP

IYVMIIASVVSAVQMLINAYAFGLYQSLGIFIPLIVTNCIVVGRAEAFTAKKGPALSALDGFSIGTGATC

AMFVLGSLREIIGNGTLFDGADALLGSWAKVLRVEIFHTDSPFLLAMLPPGAFIGLGLMLAGKYLIDERM

KKRRAEAAAERALPNGETGNV

>gi|30063146|ref|NP_837317.1| electron transport complex protein RnfG [Shigella flexneri 2a str. 2457T]

MLKTIRKHGITLALFAAGSTGLTAAINQMTKTTIAEQASLQQKTLFDQVLPAERYNNALAQSCYLVTAPE

LGKGEHRVYIAKQDDKPVAAVLEATAPDGYSGAIQLLVGADFNGTVLGTRVTEHHETPGLGDKIELRLSD

WLTHFAGKKISGADDAHWAVKKDGGDFDQFTGATITPRAVVNAVKRAGLYAQTLPAQLSQLPACGE

>gi|30063145|ref|NP_837316.1| electron transport complex protein RnfD [Shigella flexneri 2a str. 2457T]

MVFRIASSPYTHNQRQTSRIMLLVLLAAVPGIAAQLRFFGWGTLVQILLASVSALLAEALVLKLRKQSVA

ATLKDNSALLTGLLLAVSIPPLAPWWMVVLGTVFAVIIAKQLYGGLGQNPFNPAMIGYVVLLISFPVQMT

SWLPPHEIAVNIPGFIDAIQVIFSGHTASGGDMNTLRLGIDGISQATPLDTFKTSVRAGHSVEQIMQYPI

YSGILAGAGWQWVNLAWLAGGVWLLWQKAIRWHIPLSFLVTLALCATLGWLFSPETLAAPQIHLLSGATM

LGAFFILTDPVTASTTNRGRLMFGALAGLLVWLIRSFGGYPDGVAFAVLLANITVPLIDYYTRPRVYGHR

KG

>gi|30063144|ref|NP_837315.1| electron transport complex protein RnfC [Shigella flexneri 2a str. 2457T]

MLKLFSAFRKNKIWDFNGGIHPPEMKTQTNGTPLRQVLLAQHFVIPLKQHIGAEGELCVSVGDKVLRGQP

LTRGRGKMLPVHAPTSGTVTAIAPHSTAHPSALAELSVIIDADGEDCWIPRDGWVDYRSRSREELIERIH

QFGVAGLGGAGFPTGVKLQGGGDKIETLIINAAECEPYITADDRLMQDCAAQVVEGIRILAHILQPREIL

IGIEDNKPQAISMLRAVLADSNDISLRVIPTKYPSGGAKQLTYILTGKQVPHGGRSSDIGVLMQNVGTAY

AVKRAVIDGEPITERVVTLTGEAIARPGNVWARLGTPVRHLLNDAGFCPSADQMVIMGGPLMGFTLPWLD

VPVVKITNCLLAPSANELGEPQEEQSCIRCSACADACPVDLLPQQLYWFSKGQQHDKATTHNIADCIECG

ACAWVCPSNIPLVQYFRQEKAEIAAIRQEEKRAAEAKARFEARQARLEREKATRLERHKSAAVQPAAKDK

DAIAAALARVKEKQAQATQPIVIKAGERPDNSAIIAEREARKAQARAKQAELEQTNDAATVAEPRKTAVE

AAIARSKARKLEQQQANAEPEEQVDPRKAAVEAAIARAKARKLEQQQANAEPEEQVDPRKAAVEAAIARA

KARKLEQQQANAEPEEQVDPRKAAVEAAIARAKARKLEQQQANAEPEEQVDPRKAAVEAAIARAKARKLE

QQQANAEPEEQVDPRKAAVAAAIARVQAKKAAQQKVVNED

>gi|30063143|ref|NP_837314.1| electron transport complex protein RnfB [Shigella flexneri 2a str. 2457T]

MNAIWIAVAAVSLLGLAFGAILGYASRRFAVEDDPVVEKIDEILPQSQCGQCGYPGCRPYAEAISCNGEK

INRCAPGGEAVMLKIAELLNVEPQPLDGEAQELTPARMVAVIDENNCIGCTKCIQACPVDAIVGATRVMH

TVMSDLCTGCNLCVDPCPTHCISLQPVAETPDSWKWDLNTIPVRIIPVEHHA

>gi|30063142|ref|NP_837313.1| Na(+)-translocating NADH-quinone reductase subunit E [Shigella flexneri 2a str. 2457T]

MTDYLLLFVGTVLVNNFVLVKFLGLCPFMGVSKKLETAMGMGLATTFVMTLTSICAWLIDTWILIPLNLI

YLRTLAFILVIAVVVQFTEMVVRKTSPVLYRLLGIFLPLITTNCAVLGVALLNINLGHNFLQSALYGFSA

AVGFSLVMVLFAAIRERLAVADVPAPFRGNAIALITAGLMSLAFMGFSGLVKL

>gi|30063141|ref|NP_837312.1| hypothetical protein S1783 [Shigella flexneri 2a str. 2457T]

MTTTTPQRIGGWLLGPLAWLLVALLSTTLALLLYTAALSSPQTFQTLGGQALTTQILWGVSFITAIAMWY

YTLWLTIAFFKRRRCVPKHYIIWLLISVLLAVKAFAFSPVEVGIAVRQLLFTLLATALIVPYFKRSSRVK

ATFVNP

>gi|30063136|ref|NP_837307.1| bifunctional maltose and glucose-specific PTS system components IICB [Shigella flexneri 2a str. 2457T]

MTAKTAPKVTLWEFFQQLGKTFMLPVALLSFCGIMLGIGSSLSSHDVITLIPVLGNPVLQAIFTWMSKIG

SFAFSFLPVMFCIAIPLGLARENKGVAAFAGFVGYAVMNLAVNFWLTNKGILPTTDAAVLKANNIQSILG

IQSIDTGILGAVIAGIIVWMLHERFHNIRLPDALAFFGGTRFVPIISSLVMGLVGLVIPLVWPIFAMGIS

GLGHMINSAGDFGPMLFGTGERLLLPFGLHHILVALIRFTDAGGTQEVCGQTVSGALTIFQAQLSCPTTH

GFSESVTRFLSQGKMPAFLGGLPGAALAMYHCARPENRHKIKGLLISGLIACVVGGTTEPLEFLFLFVAP

VLYVIHALLTGLGFTVMSVLGVTIGNTDGNIIDFVVFGILHGLSTKWYMVPVVAAIWFVVYYVIFRFAIT

RFNLKTPGRDSEVASSIEKAVAGAPGKSGYNVPAILEALGGADNIVSLDNCITRLRLSVEDMSLVNVQAV

KDNRAIGVVQLNQHNLQVVIGPQVQSVKDEMAGLMHTVQA

>gi|30063135|ref|NP_837306.1| DNA-binding transcriptional repressor MalI [Shigella flexneri 2a str. 2457T]

MATAKKITIHDVALAAGVSVSTVSLVLNGKGRISTATGERVNAAIEELGFVRNRQASALRGGQSGVIGLI

VRDLSAPFYAELTAGLTEALEAQGRMVFLLHGGKDGEQLAQRFSLLLNQGVDGVVIAGAAGSSDDLRRMA

EEKAIPVIFASRASYLDDVDTVRPDNMQAAQLLTEHLIRNGHQRIAWLGGQSSSLTRAERVGGYCATLLK

FGLPFHSDWVLECTSSQKQAAEAITALLRHNPTISAVVCYNETIAMGAWFGLLKAGRQSGESGVDRYFEQ

QVSLAAFTDATPTTLDDIPVTWASTPARELGITLADRMMQKITHEETHSRNLIIPARLIAAK

>gi|30063132|ref|NP_837303.1| repressor for uid operon [Shigella flexneri 2a str. 2457T]

MMDNMQTEAQPTRTRILNAAREIFSENGFHSASMKAICKSCAISPGTLYHHFISKEALIQAIILQDQERA

LARFREPIEGIHFVDYMVESIVSLTHEAFGQRALVVEIMAEGMRNPQVAAMLKNKHMTITEFVAQRMRDA

QQKGEISPDINTAMTSRLLLDLTYGVLADIEAEDLAREASFAQGLRAMIGGILTAS

>gi|30063125|ref|NP_837296.1| DNA replication terminus site-binding protein [Shigella flexneri 2a str. 2457T]

MARYDLVDRLNTTFRQMEQELATFAAHLEQHKLLVARVFSLPEVKKEDEHNPLNRIEVKQHLGNDAQSLA

LRHFRHLFIQQQSENRSSKAAVRLPGVLCYQVDNLSQAALVSHIQHINKLKTTFEHIVTVESELPTAARF

EWVHRHLPGLITLNAYRTLTVLHDPATLRFGWANKHIIKNLHRDEVLAQLEKSLKSPRSVAPWTREEWQR

KLEREYQDIAALPQNAKLKIKRPVKVQPIARVWYKGDQKQVQHACPTPLIALINRDNGAGVPDVGELLNY

DADNVQHRYKPQAQPLRLIIPRLHLYVAD

>gi|30063124|ref|NP_837295.1| sensor protein RstB [Shigella flexneri 2a str. 2457T]

MKKLFIQFYLLLFVCFLVMSLLVGLVYKFTAERAGKQSLDDLMNSSLYLMRSELREIPPHDWGKTLKEMD

LNLSFDLRVEPLSKYHLDDISMHRLRGGEIVALDDQYTFLQRIPRSHYVLAVGPVPYLYYLHQMRLLDIA

LIAFIAISLAFPVFIWMRPHWQDMLKLEAAAQRFGDGHLNERIHFDEGSSFERLGIAFNQMADNINALIA

SKKQLIDGIAHELRTPLVRLRYRLEMSDNLSAAESQALNRDISQLEALIEELLTYARLDRPQNELHLSEP

DLPLWLSTHLADIQAVTPDKTVRIKTLMQGHYAALDMRLMERVLDNLLNNALRYCHSTVETSLLLSGNRA

TLIVEDDGPGIAPENREHIFEPFVRLDPSRDRSTGGCGLGLAIVHSIALTMGGTVNCDISELGGARFSFS

WPLWHNIPQFTSA

>gi|30063122|ref|NP_837293.1| DNA-binding transcriptional regulator RstA [Shigella flexneri 2a str. 2457T]

MNTIVFVEDDAEVGSLIAAYLAKHDMQVTVEPRGDQAEETILRENPDLVLLDIMLPGKDGMTICRDLRAK

WSGPIVLLTSLDSDMNHILALEMGACDYILKTTPPAVLLARLRLHLRQNEQATLTKGLQETSLTPYKALH

FGTLTIDPINRVVTLANTEISLSTADFELLWELATHAGQIMDRDALLKNLRGVSYDGLDRSVDVAISRLR

KKLLDNAAEPYRIKTVRNKGYLFAPHAWDSDNKQSDD

>gi|30063121|ref|NP_837292.1| hypothetical protein S1760 [Shigella flexneri 2a str. 2457T]

MGLVIKAALGALVVLLIGVLAKTKNYYIAGLIPLFPTFALIAHYIVASERGIEALRATIIFSMWSIIPYF

VYLVSLWYFTGMMRLPAAFVGSVACWGISAWVLIICWIKLH

>gi|30063119|ref|NP_837290.1| arginine/ornithine antiporter [Shigella flexneri 2a str. 2457T]

MEKKLGLSALTALVLSSMLGAGVFSLPQNMAAVASPAALLIGWGITGAGILLLAFAMLILTRIRPELDGG

IFTYAREGFGELIGFCSAWGYWLCAVIANVSYLVIVFSALSFFTDTPELRLFGDGNTWQSIVGASALLWI

VHFLILRGVQTAASINLVATLAKLLPLGLFVVLAMMMFKLDTFKLDFTGLALGVPVWEQVKNTMLITLWV

FIGVEGAVVVSARARNKRDVGKATLLAVLSALGVYLLVTLLSLGVVARPELAEIRNPSMAGLMVEMMGPW

GEIIIAAGLIVSVCGAYLSWTIMAAEVPFLAATHKAFPRIFARQNAQAAPSASLWLTNICVQICLVLIWL

TGSDYNTLLTIASEMILVPYFLVGAFLLKIATRPLHKAVGVGACIYGLWLLYASGPMHLLLSVVLYAPGL

LVFLYARKTHTHDNVLNRQEMVLIGMLLIASVPATWMLVG

>gi|30063118|ref|NP_837289.1| hypothetical protein S1757 [Shigella flexneri 2a str. 2457T]

MKLKNTLLASALLSATAFSVNAATELTPEQAAAVKPFDRVVVTGRFNAIGEAVKAVSRRADKEGAASFYV

VDTSDFGNSGNWRVVADLYKADAEKAEETSNRVINGVVELPKDQAVLIEPFDTVTVQGFYRSQPEVNDAI

TKAAKAKGAYSFYIVRQIDANQGGNQRITAFIYKKDAKKRIVQSPDVIPADSEAGRAALAAGGEAAKKVE

IPGVATTASPSSEVGRFFETQSSKGGRYTVTLPDGTKVEELNKATAAMMVPFDSIKFSGNYGNMTEVSYQ

VAKRAAKKGAKYYHITRQWQKRGNNLTVSADLYK

>gi|30063115|ref|NP_837286.1| transporter [Shigella flexneri 2a str. 2457T]

MAKPIITLNGLKIVIMLGMLVIILCGIRFAAEIIVPFILALFIAVILNPLVQHMVRWRVPRVLAVSILMT

IIVMAMVLLLAYLGSALNELTRTLPQYRNSIMTPLQALEPLLQRVGIDVSVDQLAHYIDPNAAMTLLTNL

LTQLSNAMSSIFLLLLTVLFMLLEVPQLPGKFQQMMARPVEGMAAIQRAIDSVSHYLVLKTAISIITGLV

AWAMLAALDVRFAFVWGLLAFALNYIPNIGSVLAAIPPIAQVLVFNGFYEALLVLAGYLLINLVFGNILE

PRIMGRGLGLSTLVVFLSLIFWGWLLGPVGMLLSVPLTIIVKIALEQTAGGQSIAVLLSDLNKE

>gi|30063114|ref|NP_837285.1| multidrug efflux system protein MdtJ [Shigella flexneri 2a str. 2457T]

MYIYWILLGLAIATEITGTLSMKWASVSEGNGGFILMLVMISLSYIFLSFAVKKIALGVAYALWEGIGIL

FITLFSVLLFDESLSLMKIAGLTTLVAGIVLIKSGTRKARKPELEVNHGAV

>gi|30063113|ref|NP_837284.1| multidrug efflux system protein MdtI [Shigella flexneri 2a str. 2457T]

MAQFEWVHAAWLALAIVLEIVANVFLKFSDGFRRKIFGLLSLAAVLAAFSALSQAVKGIDLSVVYALWGG

FGIAATLAAGWILFGQRLNRKGWIGLVLLLAGMIMVKLA

>gi|30063112|ref|NP_837283.1| hypothetical protein S1751 [Shigella flexneri 2a str. 2457T]

MRTTIAVVLGAISLTSAFVFADKPDVAKSANDEVSTLFFGHDDRVPVNDTTQSPWDAVGQLETASGNLCT

ATLIAPNLALTAGHCLLTPPKGKADKAVALRFVSNKGLWRYEIHDIEGRVDPTLGKRLKADGDGWIVPPA

AAPWDFGLIVLRNPPSGITPLPLFEGDKAALTAALKSAGRKVTQAGYPEDHLDTLYSHQNCEVTGWAQTS

VMSHQCDTLPGDSGSPLMLHTDDGWQLIGVQSSAPAAKDRWRADNRAISVTGFRDKLDQLSQK

>gi|30063110|ref|NP_837281.1| LYSR-type transcriptional regulator [Shigella flexneri 2a str. 2457T]

MNIELRHLRYFVAVAEELHFGRAAARLNISQPPLSQQIQALEQQIGARLLARTNRSVLLTAAGKQFLADS

RQILSMVDDAAARAERLHQGEAGELRIGFTSSAPFIRAVSDTLSLFRRDYPDVHLQTREMNTREQIAPLI

EGTLDMGLLRNTALPETLEHAVIVHEPLMAMIPYDHPLANNPSVTLAELAELAELAKEPFVFFDPHVGTG

LYDDILGLMRRYHLTPVITQEVGEAMTIIGLVSAGLGVSILPASFKRVQLNEMRWVPIAEEDAVSEMWLV

WPKHHEQSPAARNFRIHLLNALR

>gi|30063108|ref|NP_837279.1| dithiobiotin synthetase [Shigella flexneri 2a str. 2457T]

MLKRFFITGTDTSVGKTVVSRALLQALASQGKRVAGYKPVAKGSKETPEGLRNKDALVLQSVSTIELPYE

AVNPIALSEEESSVAHSCPINYTLISNGLANLTDKVDHVVVEGTGGWRSLMNDLRPLSEWVVQEQLPVLM

VVGIQEGCINHALLTAQAIANDGLPLIGWVANRINPGLAHYAEIIDVLGKKLPAPLIGELPYLPRAEQRE

LGQYIRLAMLRSVLAVDRVTV

>gi|30063106|ref|NP_837277.1| twin-argninine leader-binding protein DmsD [Shigella flexneri 2a str. 2457T]

MTHFSQQDNFSVAARVLGALFYYAPESAEAAPLVAVLTSDGWETQWPLPEASLAPLVTAFQTQCEETHAQ

AWQRLFVGPWALPSPPWGSVWLDRESVLFGDSTLALRQWMREKGIQFEMKQNEPEDHFGSLLLMAAWLAE

NGRQTECEELLAWHLFPWSTRFLDVFIEKAEHPFYRALGELARLTLAQWQSQLLIPVAVKPLFR

>gi|30063105|ref|NP_837276.1| DMSO reductase anchor subunit [Shigella flexneri 2a str. 2457T]

MGNGWHEWPLVIFTVLGQCVVGALIVSGIGWFAAKNDADRQHIVRGMFFLWLLMGIGFIASVMHLGSPLR

AFNSLNRIGASGLSNEIAAGSIFFAVGGLWWLVAVIGKMPQALGKLWLLVSMALGVIFVWMMTCVYQIDT

VPTWHNGYTTLAFFLTVLLSGPILAAAILRAARVTFNTTPFAIISVLALIACAEVIVLQGLSLASIHSSV

QQASALVPDYASLQVWRVVLLCAGLGCWLCPLIRRREPHVAGLVLGLILILVGEMIGRVLFYGLHMTVGM

AIAG

>gi|30063104|ref|NP_837275.1| oxidoreductase, Fe-S subunit [Shigella flexneri 2a str. 2457T]

MTTQYGFFIDSSRCTGCKTCELACKDFKDLGPEVSFRRIYEYAGGDWQEDNGIWHQNVFAYYLSISCNHC

DDPACTKVCPSGAMHKREDGFVVVDEDVCIGCRYCHMACPYGAPQYNAEKGHMTKCDGCYSRVAEGKQPI

CVESCPLRALEFGPIEELRQKHGTLAAVAPLPRAHFTKPNIVIKPNANSRPTGDTTGYLANPEEV

>gi|30063100|ref|NP_837271.1| hypothetical protein S1732 [Shigella flexneri 2a str. 2457T]

MARPIATHDNTFTKAYLQQHCGDLLSFDGQGDLSGWLDDVLTGAGRLNESMASNTKPVSPYLILTQLLTH

DTLTVSAVQESLSRKRVALGEPMVSTRYARYVYATVVSASKSVQYHASKAGS

>gi|30063099|ref|NP_837270.1| hypothetical protein S1731 [Shigella flexneri 2a str. 2457T]

MNTELPVTNRQFLIILLTNAESSVDCCRYNLDLLKLSRWYAQAEDWDYEKIREMYAEPIQSYLQTHTEEE

LKRGLQDIFYFNNPYTCEGDSEEMDWIASRSDADLKHISNAMMQVESKWSRRLQVLTEDYENGTFDKLVP

PDAERLQAKADQEQRFKSFAEALAGGASYDF

>gi|30063098|ref|NP_837269.1| hypothetical protein S1730 [Shigella flexneri 2a str. 2457T]

MTSDNKQEYTIHIFGQDITSQDGLFRLDEIRRVGIQIGKVEDNRSTEVARFMRTKAGRSLTNPENGFVKK

INMGHLGTSWLADPVAAIEFGRWLDLGYGLAVTRAFVSMTSSATVQSAVASAEDIKPETKEFIQRHGTGL

YIAGREVSQAEYSDYQAVTAGRCEWR

>gi|30063095|ref|NP_837266.1| hypothetical protein S1723 [Shigella flexneri 2a str. 2457T]

MKLSTCCAALLLALASPAVLAAPGSCERIQSDISQRIINNGVPESSFTLSIVPNDQVDQPDSQVVGHCAN

DTHKILYNRTTSGNVSAPAQSTQDGAPAEPQ

>gi|30063093|ref|NP_837264.1| hypothetical protein S1720 [Shigella flexneri 2a str. 2457T]

MKITLSKRIGLLAFLLPCALALSTTVHAETNKLVIESGDSAQSRQHAAMEKEQWNDTRNLRQKVNKRTEK

EWDKADAAFDNRDKCEQSANINAYWEPNTLRCLDRRTGRVITP

>gi|30063092|ref|NP_837263.1| hypothetical protein S1719 [Shigella flexneri 2a str. 2457T]

MIKTTLLFFATALCEIIGCFLPWLWLKRNASIWLLLPAGISLALFVWLLTLHPAASGRVYAAYGGVYVCT

ALMWLRVVDGVKLSLYDWTGALIALCGMLIIVAGWGRT

>gi|30063086|ref|NP_837257.1| transport system permease [Shigella flexneri 2a str. 2457T]

MRIRYGWELALAALLVIEIVAFGAINPRMLDLNMLLFSTSDFICIGIVALPLTMVIVSGGIDISFGSTIG

LCAIALGVLFQSGVPMPLAILLTLLLGALCGLINAGLIIYTKVNPLVITLGTLYLFAGSALLLSGMAGAT

GYEGIGGFPMAFTDFANLDVLGLPVPLIIFLICLLVFWLWLHKTHAGRNVFLIGQSPRVALYSAIPVNRT

LCALYAMTGLASAVAAVLLVSYFGSARSDLGASFLMPAITAVVLGGANIYGGSGSIIGTAIAVLLVGYLQ

QGLQMAGVPNQVSSALSGALLIVVVVGRSVSLHRQQIKEWLARRANNPLP

>gi|30063085|ref|NP_837256.1| transport system permease [Shigella flexneri 2a str. 2457T]

MLKFIQNNREITALLAVVLLFVLPGFLDRQYLSVQTLTMVYSSAQILILLAMGATLVMLTRNIDVSVGSI

TGMCAVLLGMLLNAGYSLPVACVATLLLGLLAGFFNGALVAWLKIPAIVATLGTLGLYRGIMLLWTGGKW

IEGLPAELKQLSAPLLLGISAIGWLTIILVAFMAWLLAKTAFGRSFYATGDNLQGARQLGVRTEAIRIVA

FSLNGCMAALAGIVFASQIGFILNQTGTGLEMKAIAACVLGGISLLGGSGAIIGAVLGAWFLTQIDSVLV

LLRIPAWWNDFIAGLVLLAVLVFDGRLRCALERNLRRQKYARFMTPPPSVKPASSGKKREAA

>gi|30063083|ref|NP_837254.1| sorC family transcriptional regulator [Shigella flexneri 2a str. 2457T]

MTINDSAISEQGMCEEEQVARIAWFYYHDGLTQSEISDRLGLTRLKVSRLLEKGHQSGIIRVQINSRFEG

CLEYETQLRRQFSLQHVRVIPGLADADVGGRLGIGAAHMLMSLLQPQQMLAIGFGEATMNTLQRLSGFIS

SQQIRLVTLSGGVGSYMTGIGQLNAACSVNIIPAPLRASSADIARTLKNENCVKDVLLAAQAADVAIVGI

GAVSQQDDATIIRSGYISQGEQLMIGRKGAVGDILGYFFDAKGDVVTDIKIHNELIGLPLSALKTIPVRV

GVAGGENKAEAIAAAMKGGYINALVTDQDTAAAILRS

>gi|30063080|ref|NP_837251.1| aldolase [Shigella flexneri 2a str. 2457T]

MDWGMQSRLSWIFNPKTGKTVMLAFDHGYFQGPTIGLERIDINIAPLFEHADVLMCTRGILRSVVPPATN

KPVVLRASGANSILAELSNEAVALSMDDAVRLNSCAVAAQVYIGSEYEHQSIKNIIQLVDAGMKVGMPTM

AVTGVGKDMVRDQRYFSLATRIAAEMGAQIIKTYYVEKGFERIVAGCPVPIVIAGGKKLPERETLEMCWQ

AIDQGASGVDMGRNIFQSDHPVAMMKAVQAVVHHNETADRAYELYLSEKQ

>gi|30063079|ref|NP_837250.1| autoinducer-2 (AI-2) modifying protein LsrG [Shigella flexneri 2a str. 2457T]

MHVTLVEINVHEDKVDEFIEVFRQNHLGSVQEEGNLRFDVLQDPEVNSRFYIYEAYKDEDTVAFHKTTPH
YKTCVAKLESLMTGPRKKRLFNGLMP

>gi|30063078|ref|NP_837249.1| trans-aconitate 2-methyltransferase [Shigella flexneri 2a str. 2457T]
MSDWNPSLYLHFSAERSRPAVELLARVPLENVEYVADLGCGPGNSTALLHQRWPAARITGIDSSPAMIAE
ARSALPDCQFVEADIRNWQPEQALDLIFANASLQWLPDHYELFPHLVSLLNPQGVLAVQMPDNWLEPTHV
LMREVAWEQNYPDRGREPLAGVHAYYDILSEAGCEVDIWRTTYYHQMPSHQAIIDWVTATGLRPWLQDLT
ESEQQLFLTRYHQMLEEQYPLQENGQILLAFPRLFIVARRTE

>gi|30063077|ref|NP_837248.1| hypothetical protein S1701 [Shigella flexneri 2a str. 2457T]
MIVITFNRATFPRLKITMIVRPQQHWLRRIFVWHGSVLSKISSRLLLNFLFSIAVIFMLPWYTHLGIKFT
LAPFSILGVAIAIFLGFRNNAGYARYVEARKLWGQLMIASRSLLREVKTTLPDSASVREFARLQIAFAHC
LRMTLRKQPQAEVLAHYLKTGDLQRVLASNSPANRILLIMGEWLAVQRRNGQLSDILFISLNDRLNDISA
VLAGCERIAYTPIPFAYTLILHRTVYLFCIMLPFALVVDLHYMTPFISVLISYTFISLDCLAEELEDPFG
TENNDLPLDAICNAIEIDLLQMNDEAEIPAKILPDRHYQLT

>gi|30063076|ref|NP_837247.1| altronate oxidoreductase [Shigella flexneri 2a str. 2457T]
MKTLNRRDFPGAQYPERIIQFGEGNFLRAFVDWQIDLLNEHTDLNSGVVVVRPIETSFPPSLSTQDGLYT
TIIRGLNEKGEAVSDARLIRSVNREISVYSEYDEFLKLAHNPEMRFVFSNTTEAGISYHAGDKFDDAPAV
SYPAKLTRLLFERFSHFNGALDKGWIIIPCELIDYNGDALRELVLRYAQEWALPEAFIQWLDQANSFCST
LVDRIVTGYPRDEVAKLEEELGYHDGFLDTAEHFYLFVIQGPKSLATELRLDKYPLNVLIVDDIKPYKER
KVAILNGAHTALVPVAFQAGLDTVGEAMNDAEICAFVEKAIYEEIIPVLDLPRDELESFASAVTGRFRNP
YIKHQLLSIALNGMTKFRTRILPQLLAGQKANGTLPARLTFALAALIAFYRGERNGETYPVQDDAHWLER
YQQLWSQHRDRVIGTQELVAIVLAEKDHWEQDLTQVPGLVEQVANDLDAILEKGMREAVRPLC

>gi|30063075|ref|NP_837246.1| hypothetical protein S1698 [Shigella flexneri 2a str. 2457T]

MLADWFSEQFSTGVLIVPCMLTLAIPGVLPRFKAEQMMPAIAFIVSVIASVVIGGAGSLAFPLPALIWCA

VRYTPQVTCLLTFVTGAVEIVLVANSVIDISVGSPFSIPEMFSARLGIATMAICPIMVSFSVAAINLLMK

QVALRADFDFLTQVYSRSGLYEALKSPSLKQTQHLTVMLLDIDYFKSINDNYGHECGDKVLSVFARHIQK

IVGDKGLVARMGGEEFAVAVPSVNPVDGLLMAEKIRKGVELQPFTWQQKTLYLTVSIGVGSGRASYRTLT

DDFNKLMVEADTCLYRSKKDGRNRTSTMRYGEEVV

>gi|30063074|ref|NP_837245.1| hypothetical protein S1697 [Shigella flexneri 2a str. 2457T]

MQSLDPLFARLSRSKFRSRFRLGMKERQYCLEKGAPVIEQHAADFVAKRLAPALPANDGKQTPMRGHPVF

IAQHATATCCRGCLAKWHNIPQGVSLSEEQQRYIVAVIYHWLVV

>gi|30063072|ref|NP_837243.1| hypothetical protein S1694 [Shigella flexneri 2a str. 2457T]

MVTPVSICNYISLPDDFPARNIAPQVKEVLKDFIDALSTIICDEEWRTSLNTNSATKKIFNNLDNLSYIQ

RTSFRGNDTLYNEKVQFKLTYPVKNGRHKENIEFQVVINLSPIYLDNFRHDGEINIFCAPNPKPVTMGRV

FQTGVERVLFLFMNDFIEQFPMINLGAPIKRAHTPHIEPLPPDHHTAADYLRQFDLLVLNFISRGNFVIL

PRLWNNSEVHRWFVNKDPNLITAILDITDSELKEDLLQSLMDSLGSNKHVQPEVCICFLSLLAEQESPHF

QDLFLFFANMLLHYHQFMNPNESDLNDVLMPASLSDDKIIKHMARRTLKLFVKNETPPKVTHEDLVKNRP

RSPVRPPIPATAKTPDLPERH

>gi|30063071|ref|NP_837242.1| sugar efflux transporter [Shigella flexneri 2a str. 2457T]

MTTNTVSRKVAWLRVVTLAVAAFIFNTTEFVPVGLLSDIAQSFHMQTAQVGIMLTIYAWVVALMSLPFML

MTSQVERRKLLICLFVVFIASHVLSFLSWSFTVLVISRIGVAFAHAIFWSITASLAIRMAPAGKRAQALS

LIATGTALAMVLGLPLGRIVGQYFGWRMTFFAIGIGALVTLLCLIKLLPLLPSEHSGSLKSLPLLFRRPA

LMSIYLLTVVVVTAHYTAYSYIEPFVQNIAGFSANFATALLLLLGGAGIIGSVIFGKLGNQYASALVSTA

IALLLVCLALLLPAANSEIHLGVLSIFWGIAMMIIGLGMQVKVLALAPDATDVAMALFSGIFNIGIGAGA

LVGNQVSLHWSMSMIGYVGAVPAFAALIWSIIIFRRWPVTLEEQTQ

>gi|30063070|ref|NP_837241.1| multiple drug resistance protein MarC [Shigella flexneri 2a str. 2457T]

MLDLFKAIGLGLVVLLPLANPLTTVALFLGLAGNMNSAERNRQSLMASVYVFAIMMVAYYAGQLVMDTFG

ISIPGLRIAGGLIVAFIGFRMLFPQQKAIDSPEAKSKSEELEDEPSANIAFVPLAMPSTAGPGTIAMIIS

SASTVRQSSTFADWVLMVAPPLIFFLVAVILWGSLRSSGAIMRLVGKGGIEAISRLMGFLLVCMGVQFII

NGILEIIKTYH

>gi|30063068|ref|NP_837239.1| DNA-binding transcriptional activator MarA [Shigella flexneri 2a str. 2457T]

MSRRNTDAITIHSILDWIEDNLESPLSLEKVSERSGYSKWHLQRMFKKETGHSLGQYIRSRKMTEIAQKL

KESNEPILYLAERYGFESQQTLTRTFKNYFDVPPHKYRMTNMQGESRFLHPLNHYNS

>gi|30063065|ref|NP_837236.1| transporter [Shigella flexneri 2a str. 2457T]

MATLPFMTIYLSRQYSLSVDLIGYAMTIALTIGVVFSLGFGILADKFDKKRYMLLAITAFASGFIAIPLV

NNVTLVVLFFALINCAYSVFATVLKAWFADNLSSTSKTKIFSINYTMLNIGWTIGPPLGTLLVMQSINLP

FWLAAICSAFPMLFIQIWVKRSEKIIATETGSVWSPKVLLQDKALLWFTCSGFLASFVSGAFASCISQYV

MVIADGDFAEKVVAVVLPVNAAMVVTLQYSVGRRLNPANIRALMTAGTLCFVIGLVGFIFSGNNLLLWGM

SAAVFTVGEIIYAPGEYMLIDHIAPPGMKASYFSAQSLGWLGAAINPLVSGVVLTSLPPSSLFVILALVI

IAAWVLMLKGIRARPWGQPALC

>gi|30063061|ref|NP_837232.1| competence damage-inducible protein A [Shigella flexneri 2a str. 2457T]

MNINKDKIVQLADTDTIENLTSALSQRLIADQLRLTTAESCTGGKLASALCAAEDTPKFYGAGFVTFTDQ

AKMKILSVSQQSLERYSAVSEKVAAEMATGAIERADADVSIAITGYGGPEGGEDGTPAGTVWFAWHIKGQ

NYTAVMHFAGDCETVLALAVRFALAQLLQLLL

>gi|30063058|ref|NP_837229.1| hypothetical protein S1680 [Shigella flexneri 2a str. 2457T]

MTVETQLNPTQPVNQQIYRILRRDIVHCLIAPGTPLSEKEVSVRFNVSRQPVREAFIKLAENGLIQIRPQ

RGSYVNKISMAQVRNGSFIRQAIECAVARRAASMITESQCYQLEQNLHQQRIAIERKQLDDFFELDDNFH

QLLTQIADCQLAWDTIENLKATVDRVRYMSFDHVSPPEMLLRQHLDIFSALQKRDGDAVERAMTQHLQEI

SESVRQIRQENSDWFSEE

>gi|30063056|ref|NP_837227.1| oxidoreductase [Shigella flexneri 2a str. 2457T]

MGNNLLSAKATLPVYDRNNLAPRIVHLGFGAFHRAHQGVYADILATEHFSDWGYYEVNLIGGEQQIADLQ

QQDNLYTVAEMSADAWTARVVGVVKKALHVQMDGLETVLAAMCEPQIAIVSLTITEKGYFHSPATGQLML

EHPMVAADVQNPHQPKTATGVIVEALARRKAAGLPAFTVMSCDNMPENGHVMRDVVTSYAQAVDVKLAQW

IEDNVTFPSTMVDRIVPAVTEDTLAKIEQLTGVRDPAGVACEPFRQWVIEDNFVAGRPEWEKAGAELVSD

VLPYEEMKLRMLNGSHSFLAYLGYLAGYQHINDCMEDEHYRHAAYGMMLQEQAPTLKVQGVDLQDYANRL

IARYSNPALRHRTWQIAMDGSQKLPQRMLDSVRWHLAHDSKFDLLALGVAGWMRYVGGVDEQGNPIEISD

PLLPVIQKAVQSSAEGKARVQSLLAIKAIFGDDLPDNSLFTAKVTEAYLSLLAHGAKATVAKYSVK

>gi|30063041|ref|NP_837212.1| oxidoreductase [Shigella flexneri 2a str. 2457T]

MKKKIESYQGAAGGWGAVKSVANAVRKQMDIRHDVIAMFDMNKPEGFDCPGCAWPDPKNSASFDICENGA

KAIAWEVTDKQVNASFFAENTVQSLLTWGDHELEAAGRLTQPLKYDAVSDCYKPLSWQQAFDEIGARLQS

YSDPNQVEFYTSGRTSNEAAFLYQLFAREYGSNNFPDCSNMCHEPTSVGLAASIGVGKGTVLLEDFEKCD

LVICIGHNPGTNHPRMLTSLRALVKRGAKMIAINPLQERGLERFTAPQNPFEMLTNSETQLASAYYNVRI

GGDMALLKGMMRLLIERDDAASAAGRPSLLDDEFIQTHTVGFDELRRDVLNSEWKDIERISGLSQTQIAE

LADAYAAAERTIICYGMGITQHEHGTQNVQQLVNLLLMKGNIGKPGAGICPLRGHSNVQGDRTVGITEKP

SAEFLARLGERYGFPPPHAPGHAAIASMQAICTGQARALICMGGNFALAMPDREASAVPLTQLDLAVHVA

TKLNRSHLLTARHSYILPVLGRSEIDMQKSGAQAVTVEDSMSMIHASRGVLKPAGVMLKSECAVVAGIAQ

AALPQSVVAWEYLVEDYDRIRNDIEAVLPEFADYNQRIRHPGGFHLINAAAERRWMTPSGKANFITSKGL

LEDPSSAFNSKLVMATVRSHDQYNTTIYGMDDRYRGVFGQRDVVFMSAKQAKICRVKNGERVNLIALTPD

GKRSSRRMDRLKVVIYPMADRSLVTYFPESNHMLTLDNHDPLSGIPGYKSIPVELEPSN

>gi|30063039|ref|NP_837210.1| transcriptional regulator YdeO [Shigella flexneri 2a str. 2457T]

MSLVCSVIFIHHAFNANILDKDYAFSDGEILMVDNAVRTHFEPYERHFKEIGFNENTIKKYLQCTNIQTV

TVPVPAKFLRASNVPTGLLNEMIAYLNSEERNHHNFSELLLFSCLSIFAACKGFITLLTNGVLSVSGKVR

NIVNMKLAHPWKLKDICDCLYISESLLKKKLKQEQTTFSQILLDARMQHAKNLIRVEGSVNKIAEQCGYA

STSYFIYAFRKHFGNSPKRVSKEYRCQRHTGMNTGNTMSALAI

>gi|30063037|ref|NP_837208.1| ARAC-type regulatory protein [Shigella flexneri 2a str. 2457T]

MYQRCFDNASETLFVAGKTPRLSRFAFSDDPKWESGHHVHDNETELIYVKKGVARFTIDSSLYVAHADDI

VVIERGRLHAVASDVNDPATTCTCALYGFQFQGAEENQLLQPHSCPVIAAGQGKEVIKTLFNELSVILPQ

SKNSQTSSLWDALAYTLAILVMLPTY

>gi|30063031|ref|NP_837202.1| phosphoenolpyruvate synthase [Shigella flexneri 2a str. 2457T]

MSNNGSSPLVLWYNQLGMNDVDRVGGKNASLGEMITNLSGMGVSVPNGFATTADAFNQFLDQSGVNQRIY

ELLDKTDIDDVTQLAKAGAQIRQWIIDTPFQPELENAIREAYAQLSADDENASFAVRSSATAEDMPDASF

AGQQETFLNVQGFDAVLVAVKHVFASLFNDRAISYRVHQGYDHRGVALSAGVQRMVRSDLASSGVMFSID

TESGFDQVVFITSAWGLGEMVVQGAVNPDEFYVHKPTLAANRPAIVRRTMGSKKIRMVYAPTQEHGKQVK

IEDVPQEQRDIFSLTNEEVQELAKQAVQIEKHYGRPMDIEWAKDGHTGKLFIVQARPETVRSRGQVMERY

TLHSQGKIIAEGRAIGHRIGAGPVKVIHDISEMNRIEPGDVLVTDMTDPDWEPIMKKASAIVTNRGGRTC

HAAIIARELGIPAVVGCGDATERMKDGENVTVSCAEGDTGYVYAELLEFSVKSSSVETMPDLPLKVMMNV

GNPDRAFDFACLPSEGVGLARLEFIINRMIGVHPRALLEFDDQEPQLQNEIREMMKGFDSPREFYVGRLT

EGIATLGAAFYPKRVIVRLSDFKSNEYANLVGGERYEPDEENPMLGFRGAGRYVSDSFRDCFALECEAVK

RVRNDMGLTNVEIMIPFVRTVDQAKAVVEELARQGLKRGENGLKIIMMCEIPSNALLAEQFLEYFDGFSI

GSNDMTQLALGLDRDSGVVSELFDERNDAVKALLSMAIRAAKKQGKYVGICGQGPSDHEDFAAWLMEEGI

DSLSLNPDTVVQTWLSLAELKK

>gi|30063030|ref|NP_837201.1| hypothetical protein S1645 [Shigella flexneri 2a str. 2457T]

MDNAVDRHVFYISDGTAITAEVLGHAVMSQFPVTISSITLPFVENESRARAVKDQIDAIYHQTGVRPLVF

YSIVLPEIRAIILQSEGFCQDIVQALVAPLQQEMKLDPTPIAHRTHGLNPNNLNKYDARIAAIDYTLAHD

DGISLRNLDQAQVILLGVSRCGKTPTSLYLAMQFGIRAANYPFIADDMDNLVLPASLKPLQHKLFGLTID

PERLAAIREERRENSRYASLRQCRMEVAEVEALYRKNQIPWINSTNYSVEEIATKILDIMGLSRRMY

>gi|30063029|ref|NP_837200.1| phospho-2-dehydro-3-deoxyheptonate aldolase [Shigella flexneri 2a str. 2457T]

MNRTDELRTARIESLVTPAELALRYPVTPGVATHVTDSRRRIEKILNGEDKRLLVIIGPCSIHDLTAAME

YATRLQSLRNQYQSRLEIVMRTYFEKPRTVVGWKGLISDPDLNGSYRVNHGLELARKLLLQVNELGVPTA

TEFLDMVTGQFIADLISWGAIGARTTESQIHREMASALSCPVGFKNGTDGNTRIAVDAIRAARASHMFLS

PDKNGQMTIYQTSGNPYGHIIMRGGKKPNYHADDIAAACDTLHEFDLPEHLVVDFSHGNCQKQHRRQLEV

CEDICQQIRNGSTEIAGIMAESFLREGTQKIVGGQPLTYGQSITDPCLGWEDTERLVEKLAFAVDTRF

>gi|30063026|ref|NP_837197.1| hypothetical protein S1641 [Shigella flexneri 2a str. 2457T]

MKIFLENLYHSDCYFLPIRDNQQLLVGVELITHFSSEDGTVRIPTSRVIAQLTEEQHWQLFSEQLELLKS

CQHFFIQHKLFAWLNLTPQVATLLLDRDNYAGELLKYPFIELLINENYPHLNEGKDNRDLLSLSQMYPLV

LGNLGAGNSTMKAVFDGLFTRVMLEKGFIQQQITHRSFEPFIRAIQAQISPCCNCIIAGGIDTPEILAQI

TPFDFHALQGCLWPAVPINQITTLVQR

>gi|30063025|ref|NP_837196.1| lipoprotein [Shigella flexneri 2a str. 2457T]

MRFCLILITALFLAGCSHHKAPPPNARLSDSITVIAGLNDQLQSWHGTPYRYGGMTRRGVDCSGFVVVTM

RDRFDLHLPRETKQQASIGTQIDKDELLPGDLVFFKTGSGQNGLHVGIYDTNNQFIHASTSKGVMRSSLD

NVYWQKNFWQARRI

K

>gi|30063022|ref|NP_837193.1| vtamin B12-transporter permease [Shigella flexneri 2a str. 2457T]

MLTLARQQQRQNIRWLLCLSVLMLLALLLSLCAGEQWISPGDWFTPRGELFVWQIRLPRTLAVLLVGAAL

AISGAVMQALFENPLAEPGLLGVSNGAGVGLIAAVLLGQGQLPNWALGLCAIAGALIITLILLRFARRHL

STSRLLLAGVALGIICSALMTWAIYFSTSVDLRQLMYWMMGGFGGVDWRQSWLMLALIPVLLWICCQSRP

MNMLALGEISARQLGLPMWFWRNVLVAATGWMVGVSVALAGAIGFIGLVIPHILRLCGLTDHRVLLPGCA

LAGASALLLADIVARLALAAAELPIGVVTATLGAPVFIWLLLKAGR

>gi|30063015|ref|NP_837186.1| translation initiation factor IF-3 [Shigella flexneri 2a str. 2457T]

MSLREALEKAEEAGVDLVEISPNAEPPVCRIMDYGKFLYEKSKSSKEQKKKQKVIQVKEIKFRPSTDEGD

YQVKLRSLIRFLEEGDKAKITLRFRGREMAHQQIGMEVLNRVKDDLQELAVVESFPTKIEGRQMIMVLAP

KKKQ

>gi|30063011|ref|NP_837182.1| hypothetical protein S1625 [Shigella flexneri 2a str. 2457T]

MLAGGMFASLNAAADDSVFTVMDDPASAKKPFEGNLNAGYLAQSGNTKSSSLTADTTMTWYGQTTAWSLW

GNASNTSSNDERSSEKYAAGGRSRFNLTDYDYLFGQASWLTDRYNGYRERDVLTAGYGRQFLNGPVHSFR

FEFGPGVRYDKYTDNASETQPLGYASGAYAWQLTDNAKFTQGVSVFGAEDTTLNSESALNVAINEHFGLK

VAYNVTWNSEPPESAPEHTDRRTTLSLGYSM

>gi|30063007|ref|NP_837178.1| hypothetical protein S1621 [Shigella flexneri 2a str. 2457T]

MTYQQAGRIAVLKRILGWVIFIPALISTLISLLKFMNTRQENQEGINAVMLDFAHVMIDMMQANTPFLNL

FWYNSPTPNFNGGVNVMFWVIFILIFVGLALQDSGARMSRQARFLREGVEDQLILEKAKGEEGLTREQIE

SRIVVPHHTIFLQFFSLYILPVICIAAGYVFFSLLGFI

>gi|30063005|ref|NP_837176.1| hypothetical protein S1619 [Shigella flexneri 2a str. 2457T]

MTAEGHLLFSIACAVFAKNAELTPVLAQGDWWHIVPSAILTCLLPDIDHPKSFLGQRLKWISKPIARAFG

HRGFTHSLLAVFALLATFYLKVPEGWFIPADALQGMVLGYLSHILADMLTPAGVPLLWPCRWRFRLPILV

PQKGNQLERFICMALFVWSVWMPHSLPENSAVRWSSQMINTLQIQFHRLIKHQVEY

>gi|30062999|ref|NP_837170.1| cryptic phospho-beta-glucosidase; cryptic [Shigella flexneri 2a str. 2457T]

MSQKLKVVTIGGGSSYTPELLEGFIKRYHELPVSELWLVDVEGGKAKLDIIFDLCQRMIDNAGVPMKLYK

TLDCREALKDADFVTTQLRVGQLPARELDERIPLSHGYLGQETNGAGGLFKGLRTIPVIFDIVKDVEELC

PNAWVINFTNPAGMVTEAVYRHTGFKRFIGVCNIPIGMKMFIHDVLMLKDSDDLSIDLFGLNHMVFIKDV

LVNGKSRFAELLDGVASGQLKVSGVKNIFDLPFSEGLIRSLNLLPCSYLLYYFKQKEMLAIEMGEYYKGG

ARAQVVQKVEKQLFELYKNPELKVKPKELEQRGGAYYSDAACEVINAIYNDKQAEHYVNIPHHGHIDNIP

ADWAVEMTCKLGRDGATPHPRITHFDDKVMGLIHTIKGFEIAASNAALSGEFNDVLLALNLSPLVHSDRD

AELLAREMILAHEKWLPNFADCIAELKKAH

>gi|30062996|ref|NP_837167.1| N,N'-diacetylchitobiose-specific PTS system transporter subunit IIA [Shigella flexneri 2a str. 2457T]

MMDLDNIPDTQTEAEELEEVVMGLIINSGQARSLAYAALKQAKQGDFAAAKAMMDQSRMALNEAHLVQTK

LIEGDAGEGKMKVSLVLVHAQDHLMTSMLARELITELIELHEKLKA

>gi|30062995|ref|NP_837166.1| N,N'-diacetylchitobiose-specific PTS system transporter subunit IIB [Shigella flexneri 2a str. 2457T]

MEKKHIYLFCSAGMSISLLVSKMRAQAEKYEVPVIIEAFPETLAGEKGQNADVVLLGPQIAYMLPEIQRL

LPNKPVEVIDSLLYGKVDGLGVLKAAVAAIKKAAAN

>gi|30062994|ref|NP_837165.1| DNA-binding transcriptional activator OsmE [Shigella flexneri 2a str. 2457T]

MNKNMAGILSAAAVLTMLAGCTAYDRTKDQFVQPVVKDVKKGMSRAQVAQIAGKPSSEVSMIHARGTCQT

YILGQRDGKAETYFVALDDTGHVINSGYQTCAEYDTDPQAAK

>gi|30062993|ref|NP_837164.1| NAD synthetase [Shigella flexneri 2a str. 2457T]

MTLQQQIIKALGAKPQINAEEEIRRSVDFLKSYLQTYPFIKSLVLGISGGQDSTLAGKLCQMAINELRQE

TRNESLQFIAVRLPYGVQADEQDCQDAIAFIQPDRVLTVNIKGAVLASEQALREAGIELSDFVRGNEKAR

ERMKAQYSIAGMTSGVVVGTDHAAEAITGFFTKYGDGGTDINPLYRLNKRQGKQLLTALGCPEHLYKKAP

TADLEDDRPSLPDEVALGVTYDNIDDYLEGKNLPEQVARTIENWYLKTEHKRRPPINVFDDFWKK

>gi|30062992|ref|NP_837163.1| nucleotide excision repair endonuclease [Shigella flexneri 2a str. 2457T]

MVRRLTSPRLEFEAAAIYEYPEHLHSFLNDLPTRPGVYLFHGESDTMPLYIGKSVNIRSRVLSHLRTPDE

AAMLRQSRRISWICTAGEIGALLLEARLIKEQQPLFNKRLRRNRQLCALQLNEKRVDVVYAKEVDFSRAP

NLFGLFANRRAALQALQSIADEQKLCYGLLGLEPLSRGRACFRSALKRCAGACCGKESHEEHALRLRQSL

ERLRVVCWPWQGAVALKEQHPEMTQYHIIQNWLWLGAVNSLEEATTLIRTPAGFDHDGYKILCKPLLSGN

YEITELDPANDQRAS

>gi|30062991|ref|NP_837162.1| hypothetical protein S1601 [Shigella flexneri 2a str. 2457T]

MEYFDMRKMSVNLWRNAAGETREICTFPPAKRDFYWRASIASIAANGEFSLFPGMERIVTLLEGGEMLLE

SADRFNHTLKPLQPFAFTADQVVKAKLTAGQMSMDFNIMTRLDVCKAKVRIAERTFTTFGSRGGVVFVIN

GAWQLGDKLLTTDQGVCWFDGRHTLRLLQPQGKLLFSEINWLAGHSPDQVQ

>gi|30062990|ref|NP_837161.1| periplasmic protein [Shigella flexneri 2a str. 2457T]

MRKLTALFVASTLALGAANLAHAADTTTAAPADAKPMMHHKGKFGPHQDMMFKDLNLTDAQKQQIHEIMK

GQRDQMKRPPLEERRAMHDIIASDTFDKAKAEAQIAKMEEQRKANMLAHMETQNKIYNILTPEQKKQFNA

NFEKRLTERPAAKGKMPATAE

>gi|30062989|ref|NP_837160.1| succinylglutamate desuccinylase [Shigella flexneri 2a str. 2457T]

MDNFLALTLTGKKPVITEREINGVRWRWLGDGVLELTPLTPPQGALVISAGIHGNETAPVEMLDALLGAI

SHGEIPLRWRLLVILGNPPALKQGKRYCHSDMNRMFGGRWQLFAESGETCRARELEQCLEDFYDQGKESV

RWHLDLHTAIRGSLHPQFGVLPQRDIPWDEKFLTWLGAAGLEALVFHQEPGGTFTHFSVRHFGALACTLE

LGKALPFGQNDLRQFAVTASAIAALLSGESVGIVRTPPLRYRVVSQITRHSPSFEMHMASDTLNFMPFEK

GTLLAQDGEERFTVTHDVEYVLFPNPLVALGLRAGLMLEKIS

>gi|30062985|ref|NP_837156.1| hypothetical protein S1593 [Shigella flexneri 2a str. 2457T]

MNAERNFLFACLIFALVIYAIHAFGLFDLLTDLPHLQTLIRQSGLFGYSLYILLFINAPLFLLPGSILVI

AGGIVFGPLLGTQLSLIAATLASSCSFLLARWLGRDLLLKYVGHSHTFQAIEKGIARNGIDFLILTRLIP

LFPYNIQNYAYGFTTIAFWPYTLISALTTLPGIVIYTVMASDLANEGITLRFILQLCLAGLALFILIQLA

KLYARHKHVDLSASRRSPLTHPKNEG

>gi|30062984|ref|NP_837155.1| hypothetical protein S1592 [Shigella flexneri 2a str. 2457T]

MKALRCALFYNSVWRAWRCLFSSSSQNSTPDTNMWICLLRAAAHLLTQKMKDRTMSQHYSVSWKKGLAAL

CLLAVAGLSGCDQKENAAAKVEYDGLSNSQPLRVDANNHTVTMLVQINGRFLTDDTRHGIVFKDGSNGHK

SLFMAYAPPKAFYEALKEAGGTPGENMTMDNKETTHVTGSKLDISVNWQGAAKAYSFDEVIVDSNGKKLD

MRFGGNLTAAEEKKTGCLVCLDSCPVGIVSNATYTYGAVEKHGEVKFKGNASVLPADNTLATVTFKITE

>gi|30062983|ref|NP_837154.1| hypothetical protein S1591 [Shigella flexneri 2a str. 2457T]

MKIQSRKIWYYRITLIILLFAMLLAWALLPGVHEFINRSVAAFAAVDQQGIERFIQSYGALAAVVSFLLM

ILQAIAAPLPAFLITFANASLFGAFWGGLLSWTSSMAGAALCFFIARVMGREVVEKLTGKTVLDSMDGFF

TRYGKHTILVCRLLPFVPFDPISYAAGLTSIRFRSFFIATGLGQLPATIVYSWAGSMLTGGTFWFVTELF

ILFALTVVIFMAKKIWLERQKRND

>gi|30062982|ref|NP_837153.1| hypothetical protein S1590 [Shigella flexneri 2a str. 2457T]

MGLPPLSKIPFILRPQAWLHRRHYGEVLSPIRWWGRIPFIFYLVSMFVGWLERKRSPLDPVVRSLVSARI

AQMCLCEFCVDITSMKVAERTGSTDKLLAVADWRQSPLFSDEERLALEYAEAASVTPPTVDDALRTRLAA

HFDAQALTELTALIGLQNLSARFNSAMDIPAQGLCRIPEKRS

>gi|30062979|ref|NP_837150.1| cytochrome oxidase [Shigella flexneri 2a str. 2457T]

MLDRHLHPRIKPLLHQCVRVLDKPGITPDGLTLVGFAIGVLALPFLALGWYLAALVVILLNRLLDGLDGA

LARRRGLTDAGGFLDISLDFLFYALVPFGFILAAPEQNALAGGWLLFAFIGTGSSFLAFAALAAKHQIDN

PGYAHKSFYYLGGLTEGTETILLFVLGCLFPAWFAWFAWIFGALCWMTTFTRVWSGYLTLKSLQRQ

>gi|30062978|ref|NP_837149.1| pyrimidine (deoxy)nucleoside triphosphate pyrophosphohydrolase [Shigella flexneri 2a str. 2457T]

MKMIEVVAAIIERDGKILLAQRPAQSDQAGLWEFAGGKVELDESQQQALVRELNEELDIEATVGEYVASH

QREVSGRIIHLHAWHVPDFHGTLQAHEHQALVWCSPEEALQYPLAPADIPLLEAFMALRAARAAD

>gi|30062972|ref|NP_837143.1| hypothetical protein S1573 [Shigella flexneri 2a str. 2457T]

MDALELLINRRSASRLAEPAPTGEQLQNILRAGMRAPDHKSMQPWHFFVIEGEGCERFSAVLEQGAIAAG

SDDKAIDKARNAPFRAPLIITVVAKCKENHKVPRWEQEMSAGCAVMAMQMAAVAQGFGGIWRSGALTESP

VVREAFGCREQDKIVGFLYLGTPQLKASTSINVPDPTPFVTYF

>gi|30062971|ref|NP_837142.1| protease 4 [Shigella flexneri 2a str. 2457T]

MRTLWRFIAGFFKWTWRLLNFVREMVLNLFFIFLVLVGVGIWMQVSGGDSKETASRGALLLDISGVIVDK

PDSSQRFSKLSRQLLGASSDRLQENSLFDIVNTIRQAKDDRNITGIVMDLKNFAGGDQPSMQYIGKALKE

FRDSGKPVYAVGENYSQGQYYLASFANKIWLSPQGVVDLHGFATNGLYYKSLLDKLKVSTHVFRVGTYKS

AVEPFIRDDMSPAAREADSRWIGELWQNYLNTVAANRQIPAQQVFPGAQGLLEGLTKTGGDTAKYALENK

LVDALASSAEIEKALTKEFGWSKTDKNYRAISYYDYALKTPADTGDSIGVVFANGAIMDGEETQGNVGGD

TTAAQIRDARLDPKVKAIVLRVNSPGGSVTASEVIRAELAAARAAGKSVVVSMGGMAASGGYWISTPANY

IVANPSTLTGSIGIFGVITTVENSLDSIGVHTDGVSTSPLADVSITRALPPEAQQMMQLSIENGYKRFIT

LVADARHSTPEQIDKIAQGHVWTGQDAKANGLVDSLGDFDDAVAKAAELAKVKQWHLEYYVDEPTFFDKV

MDNMSGSVRAMLPDAFQAMLPAPLASVASTVKSESDKLAAFNDPQNRYAFCLTCANVR

>gi|30062967|ref|NP_837138.1| DEOR-type transcriptional regulator [Shigella flexneri 2a str. 2457T]

MAAKDRIQAIKQMVANDKKVTVSNLSGIFQVTEETIRRDLEKLEDEGFLTRTYGGAVLNTAVLTENIHFY

KRASSFYEEKLLIARKALPFIDNKTTMAADSSSTVMELLKLLQDRSDLTLLTNSAEAIHVLAQSEIKVVS

TGGELNKNTLSLQGRITKEIISRYHVDIMVMSCKGLDINSGALDSNEAEAEIKKTMIRQATEVALLVDHS

KFDRKAFVQLADFSHINYIITDKSPGAEWIAFCKDNNIQLVW

>gi|30062964|ref|NP_837135.1| aldolase [Shigella flexneri 2a str. 2457T]

MLADIRYWENDATNKYYAIAHFNVWNAEMLMGVKDAAEEAKSPVIISFSTGFVGNTSFEDFSHMMVSMAQ

KATVPVITHWDHGRSIEIIHNAWTHGMNSLMRDASAFDFEENIRLTKEAVDFFHPLGIPVEAELGHVGNE

TVYEEALAGYHYTDPDQAAEFVERTGCDSLAVAIGNQHGVYTSEPQLNFEVVKRVRDAVSVPLVLHGASG

ISDADIKTAISLGIAKINIHTELCQAAMVAVKENQDQPFLHLEREVRKAVKERALEKIELFGSDGKAE

>gi|30062958|ref|NP_837129.1| hypothetical protein S1558 [Shigella flexneri 2a str. 2457T]

MIKKIFALPVIEQISPVLSRRKLDELDLIVVDHPQVKASFALQGAHLLSWKPAGEEEVLWLSNNTPFKNG

VAIRGGVPVCWPWFGPAAQQGLPAHGFARNLPWTLKSHHEDADGVALTFELTQSEETKKFWPHDFTLLAH

FRVGKTCEIDLESHGEFETTSALHTYFNVGDIAKVSVSGLGDRFIDKVNDAKEDVLTDGIQTFPDRTDRV

YLNPQDCSVINDEALNRIIAVGHQHHLNVVGWNPGPALSVSMGDMPDDGYKTFVCVETAYASETQKVTKE

KPAHLAQSIRVAKR

>gi|30062956|ref|NP_837127.1| hypothetical protein S1556 [Shigella flexneri 2a str. 2457T]

MTKLKLLALGVLIATSAGVAHAEGKFSLGAGVGVVEHPYKDYDTDVYPVPVINYEGDNFWFRGLGGGYYL

WNDATDKLSITAYWSPLYFKAKDSGDHQMRHLDDRKSTMMAGLSYAHFTQYGYLRTTLAGDTLDNSNGIV

WDMAWLYRYTNGGLTVTPGIGVQWNSENQNEYYYGVSRKESARSGLRGYNPNDSWSPYLELSASYNFLGD

WSVYGTARYTRLSDEVTDSPMVDKSWTGLISTGITYKF

>gi|30062955|ref|NP_837126.1| hypothetical protein S1555 [Shigella flexneri 2a str. 2457T]

MNIFDHYRQRYEAAKDEEFTLQEFLTTCRQDRSAYANAAERLLMAIGEPVMVDTAQDPRLSRLFSNRVIA

RYPAFEEFYGMEDAIEQIVSYLKHAAQGLEEKKQILYLLGPVGGGKSSLAERLKSLMQLVPIYVLSANGE

RSPVNDHPFCLFNPQEDAQILEKEYGIPRRYLGTIMSPWAAKRLHEFGGDITKFRVVKVWPSILQQIAIA

KTEPGDENNQDISALVGKVDIRKLEHYAQNDPDAYGYSGALCRANQGIMEFVEMFKAPIKVLHPLLTATQ

EGNYNGTEGISALPFNGIILAHSNKSEWVTFRNNKNNEAFLDRVYIVKVPYCLRISEEIKIYEKLLNHSE

LTHAPCAPGTLETLSRFSILSRLKEPENSSIYSKMRVYDGESLKDTDPKAKSYQEYRDYAGVDEGMNGLS

TRFAFKILSRVFNFDHVEVAANPVHLFYVLEQQIEVYWQ

>gi|30062953|ref|NP_837124.1| hypothetical protein S1552 [Shigella flexneri 2a str. 2457T]

MTEMAKGSVTHQQLIALLSQEGANFRVVTHEAVGKCEAVSEIRGTALGQGAKALVCKVKGNGVNQHVLAI

LAADQQADLSQLASHIGGLRASLASPAEVDELTGCVFGAIPPFSFHPKLKLVADPLLFERFDEIAFNAGM

LDKSVILKTADYLRIAQPELVNFRRTA

>gi|30062951|ref|NP_837122.1| hypothetical protein S1550 [Shigella flexneri 2a str. 2457T]

MFDVTLLILLGLAALGFISHNTTVAVSILVLIIVRVTPLSTFFPWIEKQGLSIGIIILTIGVMAPIASGT

LPPSTLIHSFLNWKSLVAIAVGVIVSWLGGRGVTLMGSQPQLVAGLLVGTVLGVALFRGVPVGPLIAAGL

VSLIVGKQ

>gi|30062950|ref|NP_837121.1| amino acid/amine transport protein [Shigella flexneri 2a str. 2457T]

MTCSTSLSGKNRIVLIAGILMIATTLRVTFTGAAPLLDTIRSAYSLTTAQTGLLTTLPLLAFALISPLAA

PVARRFGMERSLFAALLLICAGIAIRSLPSPYLLFGGTAVIGGGIALGNVLLPGLIKRDFPHSVARLTGA

YSLTMGAAAALGSAMVVPLALNGFGWQGALFMLMCFPLLALFLWLPQWRSQQHANLSTSRALHTRGIWRS

PLAWQVTLFLGINSLVYYVIIGWLPAILISHGYSEAQAGSLHGLLQLATAAPGLLIALFLHHVKDQRGIA

AFVALMCAVGAVGLCFMPAHAITWTLLFGFGSGATMILGLTFIGLRQVLRIRRRHSRGWHNPSGICWQPV

GRR

>gi|30062949|ref|NP_837120.1| hypothetical protein S1547 [Shigella flexneri 2a str. 2457T]

MKGRTNTMNIQCKRVYDPAEQSDGYRVLVDRLWPRGIKKTDLALDEWDKEITPSTELRKAFHGEVVDFAT

FREQYRAELAQHEQEGKRLADIAKKQPLTLLYAAKNTTQNHALVLADWLRSL

>gi|30062948|ref|NP_837119.1| hypothetical protein S1546 [Shigella flexneri 2a str. 2457T]

MKIISFVLPCLLVLAGCSTPSQPEAPKPPQIGMANPASVYCQQKGGTLIPVQTAQGVSNNCKLPGGETID

EWALWRRDHPAGEK

>gi|30062947|ref|NP_837118.1| hypothetical protein S1545 [Shigella flexneri 2a str. 2457T]

MSDQIIARVSQSLAKEQSLESLVRQLLEMLEMVTDMESTYLTKVDVEARLQHIMFARNSQKMHIPENFTV

LWDYSLCKRAIDENCFFSDEVPDRWGDCIAARNLGITTFLSTPIHLPDGSFYGTLCAASSEKRQWSERAE

QVLQLFAGLIAQYIQKEALVEQLHEANAALIAQSYTDSLTGLPNRRAIFENLTTLFSLARHLNHKIMIAF

IDLDNFKLINDRFGHNSGDLFLIQVGERLNTLQQNGEVIGRLGGDEFLVVSLNNENADISSLRERIQQQI

RGEYHLGDVDLYYPGASLGIVEVDPETTDADSALHAADIAMYQEKKHKQKTPFVAHPALHS

>gi|30062944|ref|NP_837115.1| repressor protein [Shigella flexneri 2a str. 2457T]

MPPEIMRFKERLEEAMNGESSRAFAAKCGLSDGVIRNYLSGKTYPSLDRLAQIAYATGRPIEWFIQSQSM

PMVEHKNNQENKNSQPDRSTLKTKLIAIVELLEDKELHSAIELFRKKGFEALMPEIFSSDDSIQADLQGI

SQQTLQTAKMLESLPTDTRKKILSKYGIYEQEGLVAPSQEPQDVKKAV

>gi|30062943|ref|NP_837114.1| diogenase beta subunit [Shigella flexneri 2a str. 2457T]

MSDYQMFEVQVSQVEPLTEQVKRFTLVATDGKPLPAFTGGSHILVQMSDGDNQYSNAYSLLSSPHNTSSY
QIAVRLEENSRGGSRFLHQQVKVGDRLTISTPNNLFALIPSARKHLFIAGGIGITPFLSHMAELQHSDID
WQLHYCSRNPESCAFRAELVQHPQAEKVHLHHSSTGTRLELARLLADIEPGTHVYTCGPEALIEAVRSEA
ARLDIAADTLHFEQFAIEDKTGDAFTLVLARSGKEFVVPEEMTILQVIENNKAAKVECLCREGVCGTCET
AILEGEADHRDQYFSDEERASQQSMLICCSRAKGKRLVLDL

>gi|30062940|ref|NP_837111.1| outer membrane protein [Shigella flexneri 2a str. 2457T]

MAVQKNVIKGILAGTFALMLSGCVTVPDAIKGSSTTPQQDLVRVMSAPQLYVGQEARFGGKVVAVQNQQG
KTRLEIATVPLDSGARPTLGEPSRGRIYADVNGFLDPVDFRGQLVTVVGPITGAVDGKIGNTPYKFMVMQ
VTGYKRWHLTQQVIMPPQPIDPWFYGGRGWPYGYGGWGWYNPGPARVQTVVTE

>gi|30062939|ref|NP_837110.1| hypothetical protein S1536 [Shigella flexneri 2a str. 2457T]

MRILAIDTATEACSVALWNDGTVNAHFELCPREHTQRILPMVQDILTTSGTSLTDINALAYGRGPGSFTG
VRIGIGIAQGLALGAELPMIGVSTLMTMAQGAWRKNGATRVLSAIDARMGEVYWAEYQRDENGIWHGEET
EAVLKPELVHERMQQLSGEWVTVGTGWQAWPDLGKESGLVLRDGEVLLPAAEDMLPIACQMFAEGKTVAV
EHAEPVYLRNNVAWKKLPGKE

>gi|30062938|ref|NP_837109.1| enzyme [Shigella flexneri 2a str. 2457T]

MTDDFAPDGQLAKAIPGFKPREPQRQMAVAVTQAIEKGQPLVVEAGTGTGKTYAYLAPALRAKKKVIIST
GSKALQDQLYSRDLPTVSKALKYTGNVALLKGRSNYLCLERLEQQALAGGDLPVQILSDVILLRSWSNQT
VDGDISTCVSVAEDSQAWPLVTSTNDNCLGSDCPMYKDCFVVKARKKAMDADVVVVNHHLFLADMVVKES
GFGELIPEADVMIFDEAHQLPDIASQYFGQSLSSRQLLDLAKDITIAYRTELKDTQQLQKCADRLAQSAQ

DFRLQLGEPGYRGNLRELLANPQIQRAFLLLDDTLELCYDVAKLSLGRSALLDAAFERATLYRTRLKRLK

EINQPGYSYWYECTSRHFTLALTPLSVADKFKELMAQKPGSWIFTSATLSVNDDLHHFTSRLGIEQAESL

LLPSPFDYSRQALLCVPRNLPQTNQPGSARQLAAMLRPIIEANNGRCFMLCTSHAMMRDLAEQFRATMTL

PVLLQGETSKGQLLQQFVSAGNALLVATSSFWEGVDVRGDTLSLVIIDKLPFTSPDDPLLKARMEDCRLR

GGDPFDEVQLPDAVITLKQGVGRLIRDADDRGVLVICDYRLVMRPYGATFLASLPPAPRTRDIARAVRFL

AIPSSR

>gi|30062937|ref|NP_837108.1| hypothetical protein S1534 [Shigella flexneri 2a str. 2457T]

MAVFAARKWKYNEDKMMTIVRIDAEARWSDVVIHNNTLYYTGVPENLDADAFEQTANTLAQIDAVLEKQG

SNKSSILDATIFLADKNDFAAMNKAWDAWVVAGHAPVRCTVQAGLMNPKYKVEIKIVAAV

>gi|30062936|ref|NP_837107.1| hypothetical protein S1533 [Shigella flexneri 2a str. 2457T]

MQIKVIYSLIDNMVNFKDKNMPAVIDKALDFIGAMDVSAPTPSSMNESTAKGIFKYLKELGVPASAADIT

ARADQEGWNPGFTEKMVGWAKKMETGERSVIKNPEYFSTYMQEELKALV

>gi|30062934|ref|NP_837105.1| para-aminobenzoate synthase component I [Shigella flexneri 2a str. 2457T]

MKTLSPTVITLPWRQDAAEFYFSRLSHLPWAMLLHSGYADHPYSRFDIVVADPICTLTTLGKETVVSESE

KRTTTTDDPLQVLQQVLDRADIRPTHNEDLPFQGGALGLFGYDLGRRFESLPEIAEQDIVLPDMAVGIYD

WALIVDHQRHTVSLLSHNDVNARRAWLESQQFSPQEDFTLTSDWQSNMTHEQYGEKFRQVQEYLHSGDCY

QVNLAQRFHATYSGDEWQAFLQLNQANRAPFSAFLRLEQGAILSLSPERFILCDNSEIQTRPIKGTLPRL

PDPQEDSKQAEKLANSAKDRAENLMIVDLMRNDIGRVAVAGSVKVPELFVVEPFPAVHHLVSTITARLPE

QLHASDLLRAAFPGGSITGAPKVRAMEIIDELEPQRRNAWCGSIGYLSFCGNMDTSITIRTLTAINGQIY

CSAGGGIVADSQEEAEYQETFDKVNKILRQLEK

>gi|30062931|ref|NP_837102.1| hypothetical protein S1528 [Shigella flexneri 2a str. 2457T]

MQKAQRIIKTYRRNRMIVCTICALVTLASTLSVRFISQRNLNQQRVVQFANHAVEELDKVLLPLQAGSKV

LLPLIGLPCSVAHLPLRKQAAKLQTVRSIGLVQDGTLYCSSIFGYRNVPVVDILAELPAPQPLLRLTIDR

ALIKGSPVLIQWTPAAGSSNAGVIEMINIDLLTAMLLEPQLPQISSASLTVDKRHLLYGNGLVDSLPQPE

DNENYQVSSQSFPFTINVNGPGATALAWHYLPTQLPLAVLLSLLVGYIAWLATAYRMSFSREINLGLAQH

EFELFCQPLLNARSQQCIGVEILLRWNNPRQGWISPDVFIPIAEEHHLIVPLTHYVMAETIRQHHVFPMS

SQFHVGINVAPSHFRRGVLIKDLNQYWFSAHPIQQLILEITERDALLDVDYRIARELHRKNVKLAIDDFG

TGNSSFSWLETLRPDVLKIDKSFTAAIGSDAVNSTVTDIIIALGQRLNIELVAEGVETQEQAKYLRRHGV

HILQGYLYAQPMPLRDFPKWLAGSQPPPARHNGHITPIMSLR

>gi|30062930|ref|NP_837101.1| transporter [Shigella flexneri 2a str. 2457T]

MEFLMDPSIWAGLLTLVVLEIVLGIDNLVFIAILADKLPPKQRDKARLLGLSLALIMRLGLLSLISWMVT

LTKPLFTVMDFSFSGRDLIMLFGGIFLLFKATTELHERLENRDHDSGHGKGYASFWVVVTQIVILDAVFS

LDAVITAVGMVNHLPVMMAAVVIAMAVMLLASKPLTRFVNQHPTVVVLCLSFLLMIGLSLVAEGFGFHIP

KGYLYAAIGFSIIIEVFNQIARRNFIRHQSTLPLRARTADAILRLMGGKRQANVQHDADNPMPIPEGAFA

EEERYMINGVLTLASRSLRGIMTPRGEISWVDANLGVDEIREQLLSSPHSLFPVCRGELDEIIGIVRAKE

LLVALEEGVDVAAIASASPAIIVPETLDPINLLGVLRRARGSFVIVTNEFGVVQGLVTPLDVLEAIAGEF

PDADETPEIITDGNGWLVKGGTDLHALQQALDVEHLADDDDIATVAGLVISANGHIPRVGDVIDVGPLHI

TIFEANDYRVDLVRIVKEQPAHDEDE

>gi|30062929|ref|NP_837100.1| PTS enzyme IIAB [Shigella flexneri 2a str. 2457T]

MTIAIVIGTHGWAAEQLLKTAEMLLGEQENVGWIDFVPGENAETLIEKYNAQLAKLDTTKGVLFLVDTWG

GSPFNAASRIVVDKEHYEVIAGVNIPMLVETLMARDDDPSFDELVALAVETGREGVKALKAKPVEKAAPA

PAAAAPKAAPTPAKPMGPNDYMVIGLARIDDRLIHGQVATRWTKETNVSRIIVVSDEVAADTVRKTLLTQ

VAPPGVTAHVVDVAKMIRVYNNPKYAGERVMLLFTNPTDVERLVEGGVKITSVNVGGMAFRQGKTQVNNA

VSVDEKDIEAFKKLNARGIELEVRKVSTDPKLKMMDLISKIDK

>gi|30062928|ref|NP_837099.1| PTS enzyme IIC [Shigella flexneri 2a str. 2457T]

MEITTLQIVLVFIVACIAGMGSILDEFQFHRPLIACTLVGIVLGDMKTGIIIGGTLEMIALGWMNIGAAV

APDAALASIISTILVIAGHQSIGAGIALAIPLAAAGQVLTIIVRTITVAFQHAADKAADNGNLTAISWIH

VSSLFLQAMRVAIPAVIVALSVGTSEVQNMLNAIPEVVTNGLNIAGGMIVVVGYAMVINMMRAGYLMPFF

YLGFVTAAFTNFNLVALGVIGTVMAVLYIQLSPKYNRVAGAPAQAAGNNDLDNELD

>gi|30062927|ref|NP_837098.1| mannose-specific PTS system protein IID [Shigella flexneri 2a str. 2457T]

MSEMVDTTQTTTEKKLTQSDIRGVFLRSNLFQGSWNFERMQALGFCFSMVPAIRRLYPENNEARKQAIRR

HLEFFNTQPFVAAPILGVTLALEEQRANGAEIDDGAINGIKVGLMGPLAGVGDPIFWGTVRPVFAALGAG

IAMSGSLLGPLLFFILFNLVRLATRYYGVAYGYSKGIDIVKDMGGGFLQKLTEGASILGLFVMGALVNKW

THVNIPLVVSTITGQDGQTRVTTVQTILDQLMPGLVPLLLTFACMWLLRKKVNPLWIIVGFFVIGIAGYA

CGLLGL

>gi|30062926|ref|NP_837097.1| hypothetical protein S1523 [Shigella flexneri 2a str. 2457T]

MTITDLVLILFIAALLAFAIYDQFIMPRRNGPTLLAIPLLRRGRIDSVIFVGLIVILIYNNVTNHGALIT

TWLLSALALMGFYIFWIRIPKIIFKQKGFFFANVWIEYSRIKAMNLSEDGVLVMQLEQRRLLIRVRNIDD

LEKIYKLLVSGNAANLLI

>gi|30062923|ref|NP_837094.1| hypothetical protein S1520 [Shigella flexneri 2a str. 2457T]

MFAGGDDVFYGYPGQDVVMNITATVLLAFGMSMDAFAASIGKGAPLHKPKFSEALRTGLIFGAVETLTPL

IGWGMGMLASRFVLEWNHWIAFVLLIFLGGRMIIEGFRGADDEDEEPRRRHGFWLLVTTAIATSLDAMAV

GVGLAFLQVNIIATALAIGCATLIMSTLGMMVGRFIGSIIGKKAEILGGLVLIGIGVQILWTHFHG

>gi|30062922|ref|NP_837093.1| 23S rRNA methyltransferase A [Shigella flexneri 2a str. 2457T]

MSFSCPLCHQPLSREKNSYICPQRHQFDMAKEGYVNLLPVQHKRSRDPGDSAEMMQARRAFLDAGHYQPL

RDAIVAQLRERLDEKATAVLDIGCGEGYYTHAFADALPEITTFGLDVSKVAIKAAAKRYPQVTLCVASSH

RLPFSDTSMDAIIRIYAPCKAEELARVVKPGGWVITATPGPRHLMELKGLIYNEVHLHAPHAEQLEGFTL

QQSAELCYPMRLRGDEAVALLQMTPFAWRAKPEVWQTLAAKEVFDCQTDFNIHLWQRSY

>gi|30062917|ref|NP_837088.1| regulator [Shigella flexneri 2a str. 2457T]

MANADLDKQPDSVSSVLKVFGILQALGEEREIGITELSQRVMMSKSTVYRFLQTMKTLGYVAQEGESEKY

SLTLKLFELGARALQNVDLIRSADIQMREISRLTKETIHLGALDEDSIVYIHKIDSMYNLRMYSRIGRRN

PLYSTAIGKVLLAWRDRDEVKQILEGVEYKRSTERTITSTEALLPVLDQVREQGYGEDNEEQEEGLQCIA

VPVFDRFGVVIAGLSISFPTLRFSEERLQEYVAMLHTAARKISAQMGYHDYPF

>gi|30062916|ref|NP_837087.1| transporter [Shigella flexneri 2a str. 2457T]

MVAIHLLPVSYNSATSTVNISARIIPLLIIHQRYKIPMPKVQADGLPLPQRYGAILTIVIGISMAVLDGA

IANVALPTIATDLHATPASSIWVVNAYQIAIVISLLSFSFLGDMFGYRRIYKCGLVVFLLSSLFCALSDS

LQMLTLARVIQGFGGAALMSVNTALIRLIYPQRFLGRGMGINSFIVAVSSAAGPTIAAAILSIASWKWLF

LINVPLGIIALLLAMRFLPPNGSRASKPRFDLPSAVMNALTFGLLITALSGFAQGQSLTLIAAELVVMVV

VGIFFIRRQLSLPVPLLPVDLLRIPLFSLSICTSVCSFCAQMLAMVSLPFYLQTVLGRSEVETGLLLTPW

PLATMVMAPLAGYLIERVHAGLLGALGLFIMATGLFSLVLLPASPADINIIWPMILCGAGFGLFQSPNNH

TIITSAPRERSGGASGMLGTARLLGQSSGAALVALMLNQFGDNGTHVSLMAAAILAVIAACVSGLRITQP

RARA

>gi|30062912|ref|NP_837083.1| hypothetical protein S1509 [Shigella flexneri 2a str. 2457T]

MNKTEFYADLNRDFNALMAGETSFLATLANTSALLYERLTDVNWAGFYLLEDDTLVLGPFQGKIACVRIP

VGRGVCGTAVARNQVQRIEDVHAFDGHIACDAASNSEIVLPLVVKNQIIGVLDIDSTVFARFTDEDEQGL

RQLVAQLEKVLATTDYKKFFASVAG

>gi|30062911|ref|NP_837082.1| hypothetical protein S1508 [Shigella flexneri 2a str. 2457T]

MALNTPQITPTKKITVRAIGEELPRGDYQRCPQCDMLFSLPEINSHQSAYCPRCQAKIRDGRDWSLTRLA

AMAFTMLLLMPFAWGEPLLHIWLLGIRIDANVMQGIWQMTKQGDAITGSMVFFCVIGAPLILVSSIAYLW

FGNRLGMNLRPVLLMLERLKEWVMLDIYLVGIGVASIKVQDYAHIQAGVGLFSFVALVILTTVTLSHLNV

EELWERYYPQRPATRRDEKLRVCLGCHFTGYPDQRGLCPRCHIPLRLRRRHSLQKCWAALLASIVLLLPA

NLLPISIIYLNGGRQEDTILSGIMSLASSNIAVAGIVFIASILVPFTKVIVMFTLLLSIHFKCQQGLRTR

ILLLRMVTWIGRWSILDLFVISLTMSLINRDQILAFTMGPAAFYFGAAVILTILAVEWLDSRLLWDAHES

GNARFDD

>gi|30062904|ref|NP_837075.1| IS91 orf [Shigella flexneri 2a str. 2457T]

MNRATLRAVHAAGADTGKNGRKLPLRAAGYFRIPFVINTYKPEDQLFVSGIHKEGNIQVCAALNGIVKII

KAQNFSVFFVKKIIRMKFHNIAGKFASLWCGFAFTGFS

>gi|30062902|ref|NP_837073.1| hypothetical protein S1495 [Shigella flexneri 2a str. 2457T]

MRTTSFAKVAALCGLLALSGCASKITQPDKYNNYSDLKETTSATGKPVLRWVDPSFDQSKYDSIVWNPIT

YYPVPKPSTQVGQKVLDKILNYTNTEMKEAIAQRKPVVTTAGPRSLIFRGAITGVDTSKEGLQFYEVVPV

ALVVAGTQMATGHRTMDTRLYFEGELIDAATNKPVIKVVRQGEGKDLNNESTPMAFENIKQVIDDMATDA

TMFDVNKK

>gi|30062901|ref|NP_837072.1| tellurite resistance protein TehB [Shigella flexneri 2a str. 2457T]

MIIRDENYFTDKYELTRTHSEVLEAVKVVKPGKTLDLGCGNGRNSLYLAANGYDVDAWDKNAMSIANVER

IKSIENLDNLHTRVVDLNNLTFDGQYDFILSTVVLMFLEAKTIPGLIANMQRCTKPGGYNLIVAAMDTAD

YPCTVGFTFAFKEGELRRYYEGWERVKYNEDVGELHRTDANGNRIKLRFATMLARKK

>gi|30062900|ref|NP_837071.1| potassium-tellurite ethidium and proflavin transporter [Shigella flexneri 2a str. 2457T]

MQSDKVLNLPAGYFGIVLGTIGMGFAWRYASQVWQVSHWLGDGLVILAMIIWGLLTSAFITRLIRFPHSV

LAEVRHPVLSSFVSLFPATTMLVAIGFVPWFRPLAVCMFSFGVVVQLAYAAWQTAGLWRGSHPEEATTPG

LYLPTVANNFISAMACGALGYTDAGLVFLGAGVFSWLSLEPVILQRLRSSGELPTALRTSLGIQLAPALV

ACSAWLSVNGGEGDTLAKMLFGYGLLQLLFMLRLMPWYLSQPFNASFWSFSFGVSALATTGLHLGSGSDN

GFFHTLAVPLFIFTNFIIAILLIRTFALLMQGKLLVRTERAVLMKAEDKE

>gi|30062899|ref|NP_837070.1| hypothetical protein S1492 [Shigella flexneri 2a str. 2457T]

MRKYRLSEEQRAFSYQEDGTKKSVLLRQIIAISDFNDVIAGTAGGWIDRETVLAQEGNCWIYDQNAIAFG

GTVISGNTRITGTSVLWGEVYATDNVWIDNSEISQGAYISDSVTIHDSLVYVQCRIFGHALIDQHSMIVA

AQGLTPDHQLLLQIYDRARVSASRIVHQAQIYGDAVVRYAFIEHRAEVFDFASVEGNEENNVWLCDCAKV

YGHAQVKAGIEEDAIPTIHYSSQVAEYAIVEGNCVLKHHVLIGGNAVVRGGPILLDEHVVIQGESRITGA

VIIENHVELTDHAVVEAFDGDTVHVRGPKVINGEERITRTPLAGLL

>gi|30062898|ref|NP_837069.1| ribosomal-protein-L7/L12-serine acetyltransferase [Shigella flexneri 2a str. 2457T]

MTETIKVSESLELHAVAESHVTPLYQLICKNKTWLQQSLNWPQFVQSEEDTRKTVQGNVMLHQRGYAKMF

MIFKEDELIGVISFNRIEPLNKTAEIGYWLDESHQGQGIISQALQTLIHHYAQSGELRRFVIKCRVDNPQ

SNQVALRNGFILEGCLKQAEFLNDAYDDVNLYARIIDS

>gi|30062895|ref|NP_837066.1| glucan biosynthesis protein D [Shigella flexneri 2a str. 2457T]

MDRRRFIKGSMAMAAVCGTSGIASLFSQAAFAADSDIADGQTQRFDFSILQSMAHDLAQTAWRGAPRPLP

DTLATMTPQAYNSIQYDAEKSLWHNVENRQLDAQFFHMGMGFRRRVRMFSVDPATHLAREIHFRPELFKY

NDAGVDTKQLEGQSDLGFAGFRVFKAPELARRDVVSFLGASYFRAVDDTYQYGLSARGLAIDTYTDSKEE

FPDFTAFWFDTVKPGATTFTVYALLDSASITGAYKFTIHCEKSQVIMDVENHLYARKDIKQLGISPMTSM

FSCGTNERRMCDTIHPQIHDSDRLSMWRGNGEWICRPLNNPQKLQFNAYTDNNPKGFGLLQLDRDFSHYQ

DIMGWYNKRPSLWVEPRNKWGKGTIGLMEIPTTGETLDNIVCFWQPEKAVKAGDEFAFQYRLYWSAQPPV

HCPLARVMATRTGMGGFPEGWAPGEHYPEKWARRFAVDFVGGDLKAAAPKGIEPVITLSSGEAKQIEILY

IEPIDGYRIQFDWYPTSDSTDPVDMRMYLRCQGDAISETWLYQYFPPAPDKRQYVDDRVMS

>gi|30062894|ref|NP_837065.1| hypothetical protein S1487 [Shigella flexneri 2a str. 2457T]

MANSITADEIREQFSQAMSAMYQQEVPQYGTLLELVADVNLAVLENNPQLHEKMVNADELARLNVERHGA

IRVGTAQELATLRRMFAIMGMYPVSYYDLSQAGVPVHSTAFRPIDDASLARNPFRVFTSLLRLELIENEI

LRQKAAEILRQRDIFTPRCRQLLEEYEQQGGFNETQAQEFVQEALETFRWHQLATVDEETYRALHNEHRL

IADVVCFPGCHINHLTPRTLDIDRVQSMMPECGIEPKILIEGPPRREVPILLRQTSFKALEETVLFAGQK

QGTHTARFGEIEQRGVALTPKGRQLYDDLLRNAGTGQDNLTHQMHLQETFRTFPDSEFLMRQQGLAWFRY

RLTPSGEAHRQAIHPGDDPQPLIERGWVVAQPITYEDFLPVSAAGIFQSNLGNETQTRSHGNASREAFEQ

ALGCPVLDEFQLYQEAEERSKRRCGLL

>gi|30062893|ref|NP_837064.1| LYSR-type transcriptional regulator [Shigella flexneri 2a str. 2457T]

MISPRKHQYYPFCCNIIALIVNNILQSSYHNQTTSLVSDTASFLTSLMEKNSLFSQRIRLRHLHTFVAVA

QQGTLGRAAETLNLSQPALSNTLNELEQLTGARLFERGRQGAQLTLPGEQFLTHAVRVLDAINTAGQSLH

RKEGLNNDVVRVGALPTAALGILPSVIGQFHQQQKETTLQVATMSNPMILAGLKTGEIDIGIGRMSDPEL

MTGLNYELLFLESLKLVVRPNHPLLQENVTLSRVLEWPVVVSPEGTAPRQHSDALVQSQGCKIPSGCIET

LSTSLSRQLTVEYDYVWFVPSGAVKDDLRHATLVALPVPGHGAGEPIGILTRVDATFSSGCQLMINAIRK

SMPF

>gi|30062892|ref|NP_837063.1| methyl-accepting chemotaxis protein III, ribose sensor receptor [Shigella flexneri 2a str. 2457T]

MNTTPSQRLGFLHHIRLVPLFACILGGILVLFALSSALAGYFLWQADRDQRDVTAEIEIRTGLANSSDFL

RSARINMIQAGAASRIAEMEAMKRNIAQAESEIKQSQQGYRAYQNRPVKTPADEAFDTELNQRFQAYITG

MQPMLKYAKNGMFEAIINHESEQIRPLDNAYTDILNKAVKIRSTRANQLAELAHQRTRLGGMFMIGAFVL

ALVMTLITFMVLRRIVIRPLQHAAQRIEKIASGDLTMNDEPAGRNEIGRLSRHLQQMQHSLGMTVGTVRQ

GAEEIYRGTSEISAGNADLSSRTEEQAAAIEQTAASMEQLTATVKQNADNAHHASKLAQEASIKASDGGQ

TVSGVVKTMGAISTSSKKISEITAVINSIAFQTNILALNAAVEAARAGEQGRGFAVVASEVRTLASRSAQ

AAKEIEGLISESVRLIDLGSDEVATAGKTMSTIVDAVASVTHIMQEIAAASDEQSRGITQVSQAISEMDK

VTQQNASLVEEASAAAVSLEEQAARLTEAVNVFRLNKHSVSAEPRGAGEPVSFATV

>gi|30062884|ref|NP_837055.1| azoreductase [Shigella flexneri 2a str. 2457T]

MSKVLVLKSSILAGYSQSNQLSDYFVEQWSEKHSADEITVRDLAANPIPVLDGELVGALRPSDAPLTPRQ

QEALALSDELIAELKAHDVIVIAAPMYNFNISTQLKNYFDLVARAGVTFRYTENGPEGLVTGKKAIVITS

RGGIHKDGPTDLVTPYLSTFLGFIGITDVKFVFAEGIAYGPEMAAKAQSDAKAAIDSIVAA

>gi|30062880|ref|NP_837051.1| phosphatidate cytidylyltransferase [Shigella flexneri 2a str. 2457T]

MISGPRWMTLTFFTLISFLALKEYCTLISVHFSRWLYWVIPLNYLLIGFNCFELFLLFIPLTGFLILATW

RVFVGDPSGFQHTVSAIFWGWIMTVFALSHAAWLLMLPTINIQGGALLVLFLLALTESNDIAQYLWGKSC

GRRKVVPKVSPGKTLEGLLGGVITTMIASLIIGPLLTPLNTLQALLAGLLIGISGFCGDVVMSAIKRDIG

VKDSGKLLLGHGGLLDRIDSLIFTAPVFFYFIRYCCY

>gi|30062879|ref|NP_837050.1| hypothetical protein S1464 [Shigella flexneri 2a str. 2457T]

MTLYQIKPLFQSLLRPTMFWLYKHHVTANHITLTALALSLFTGLLLVLVAQPILFLLLPIVLFIRMALNA

LDGMLARECNQQTRLGAILNETGDVISDIALYLPFLFLPESNASLVILMLFCTILTEFCGLLAQTINGVR

SYAGPFGKSDRALIFGLWGLAVAIYPQWMQWNNLLWSIASILLICTAINRCRSVLLMSAER

>gi|30062874|ref|NP_837045.1| hypothetical protein S1458 [Shigella flexneri 2a str. 2457T]

MLGKYKAVLALLLLIILVPLTLLMTLGLWVPTLADIWLPLGTRIALDESPRITRKGLIIPDLRYLVGDCQ

LAHITNASLSHPSRWLLNVGTVELDSACLAKLPQTEQSPAAPKTLAQWQSMLPNTWINIDKLIFSPWQEW

QGKLSLALTSDIQQLRYQGEKVKFQGQLKGQQLTVSELDVVAFENQPPVKLVGEFTMPLVPDGLPVSGHA

TATLNLPQEPSLVDAELDWQENSGQLIVLARDNGDPLLDLPWQITRQQLTVSDGRWSWLYAGFPLSGRLG

VKVDNWQAGLENALVSGRLNVLTQGQAGKGNAVLNFGPGKLSMDNSQLPLQLTGEAKQADLILYARLPAQ

LSGSLSDPTLTFEPGALLRSKGRVIDSLDIDEIRWPLAGVKVTQRGVDGRLQAILQAHENELGDFVLHMD

GLANDFLPDAGRWQWRYWGKGSFTPMNATWDVAGKGEWHDSTITLTDLSTGFDQLQYGTMTVEKPRLILD

KPVVWVRDAQHPSFSGALSLDAGQTLFTGGSVLPPSTLKFSVDGRNPTYFLFKGDLHAGEIGPVRVNGRW

DGIRLRGNAWWPKQSLTVFQPLVPPDWKMNLRDGELYAQVAFSAAPEQGFRAGGHGVLKGGSAWMPDNQV

NGVDFVLPFRFADGAWHLGTRGPVTLRIAEVINLVTAKNITADLQGRYPWTEEEPLLLTDVSVDVLGGNV

LMKQLRMPQHDPALLRLNNLSSSELVSAVNPKQFAMSGAFSGALPLWLNNEKWIVKDGWLANSGLMTLRL

DKDTADAVVKDNMTAGSAINWLRYMEISRSSTKINLDNLGLLTMQANITGTSRVDGKSGTVNLNYHHEEN

IFTLWRSLRFGDNLQAWLEQNARLPGNDCPQGKECEEKQ

>gi|30062872|ref|NP_837043.1| hypothetical protein S1456 [Shigella flexneri 2a str. 2457T]

MMKKTLLLCAFLVGLVSSNVMALTLDEARTQGRVGETFYGYLVALKTDAETEKLVTEINAERKASYQQRA

KQNNVSVDDIAKLAGQKLVARAKPGEYVQGINGKWVRKF

>gi|30062869|ref|NP_837040.1| oxidoreductase, Fe-S subunit [Shigella flexneri 2a str. 2457T]

MITIDGNGAVASVTFRISEVIAIYPITPSSTMAEQADAWAGNGLKNVWGDTPRVVEMQSEAGAIATVHGA

LQTGALSTSFTSSQGLLLMIPTLYKLAGELTPFVLHVAARTVATHALSIFGDHSDVMAVRQTGCAMLCAA

NVQEAQDFALISHIATLKSRVPFIHFFDGFRTSHEINKIVPLADDTILDLMPQAEIDAHRARALNPEHPV

IRGTSANPDTYFQSREATNPWYNAVYDHVEQAMNDFAAATGRQYQPFEYYGHPQAERVIILMGSAIGTCE

EVVDELLTRGEKVGVLKVRLYRPFSAKHLLQALPGSVRSVAVLDRTKEPGAQAEPLYLDVMTALAEAFNN

GERETLPRVIGGRYGLSSKEFGPDCVLAVFAELNAAKPKARFTVGIYDDVTNLSLPLPENTLPNSAKLEA

LFYGLGSDGSVSATKNNIKIIGNSTPWYAQGYFVYDSKKAGGLTVSHLRVSEQPIRSAYLISQADFVGCH

QLQFIDKYQMAERLKPSGIFLLNTPYSADEVWSRLPQEVQAVLNQKKARFYVINAAKIARECGLAARINT

VMQMAFFHLTQILPGDSALAELQGAIAKSYSSKGQDLVERNWQALALARESVEEVPLQPVNPHSANRPPV

VSDAAPDFVKTVTAAMLAGLGDALPVSALPPDGTWPMGTTRWEKRNIAEEIPIWKEELCTQCNHCVAACP

HSAIRAKVVPPEAMENAPASLHSLDVKSRDMRGQKYVLQVAPEDCTGCNLCVEVCPAKDRQNPEIKAINM

MSRQEHVEEEKINYDFFLNLPEIDRSKLERIDIRTSQLITPLFEYSGACPGCGETPYIKLLTQLYGDRML

IANATGCSSIYGGNLPSTPYTTDANGRGPAWANSLFEDNAEFGLGFRLTVDQHRVRVLRLLDQFADKIPA

ELLTALKSDATPEVRREQVAALRQQLNDVAEAHELLRDADALVEKSIWLIGGDGWAYDIGFGGLDHVLSL

TENVNILVLDTQCYSNTGGQASKATPLGAVTKFGEHGKRKARKDLGVSMMMYGHVYVAQISLGAQLNQTV

KAIQEAEAYPGPSLIIAYSPCEEHGYDLALSHDQMRQLTATGFWPLYRFDPRRADEGKLPLALDSRPPSE

ALEETLLHEQRFRRLNSQQPEVAEQLWKDAAANLQKRYDFLAQMAGKAEKSNTD

>gi|30062868|ref|NP_837039.1| filament protein [Shigella flexneri 2a str. 2457T]

MNSVITQKVSSGVTLYADTKTGGFMNRTILVPIDISDSELTQRVISHVEAEAKIDDAEVHFLTVIPSLPY

YASLGLAYSAELPAMDDLKAEAKSQLEEIIKKFKLPTDRVHVHVEEGSPKDRILELAKKIPAHMIIIASH

RPDITTYLLGSNAAAVVRHAECSVLVVR

>gi|30062865|ref|NP_837036.1| zinc transporter [Shigella flexneri 2a str. 2457T]

MEAIKGSDVNVPDAVFAWMLDGRGGVKPLENTDVIDEAHPCWLHLNYVHHDSAQWLATTPLLPNNVRDAL

AGESTRPRVSRFGEGTLITLRCINGSTDERPDQLVAMRVYMDGRLIVSTRQRKVLALDDVVSDLEEGTGP

TDCGGWLVDVCDALTDHSSEFIEQLHDKIIDLEDNLLDQQIPPRGFLALLRKQLIVMRRYMAPQRDVYAR

LASERLPWMSDDQRRRMQDIADRLGRGLDEIDACIARTGVMADEIAQVMQENLARRTYTMSLMAMVFLPS

TFLTGLFGVNLGGIPGGGWQFGFSIFCILLVVLIGGVALWLHRSKWL

>gi|30062861|ref|NP_837032.1| secretion protein [Shigella flexneri 2a str. 2457T]

MIISKKQLIGVVAIGILLTGVVFFIWRVSKGRFIQTTDDAYIGGNITTVASKVSGYISAIEVRDNQSVKK

GDIILRLDDRDYRANVARLEAKIKSSKANLEGIQATITMQQSIIQSASETWQAVKHEEQKRLRDTERYEK

LAQSAAISQQIIDNARFDYQQVAAKERKAANDFLVEKQRLAVLSAQEENVRASIEEVQAALTQALLDLEY

TLVHV

>gi|30062858|ref|NP_837029.1| fumarate/nitrate reduction transcriptional regulator [Shigella flexneri 2a str. 2457T]

MIPEKRIIRRIQSGGCAIHCQDCSISQLCIPFTLNEHELDQLDNIIERKKPIQKGQTLFKAGDELKSLYA

IRSGTIKSYTITEQGDEQITGFHLAGDLVGFDAIGSGHHPSFAQALETSMVCEIPFETLDDLSGKMPNLR

QQMMRLMSGEIKGDQDMILLLSKKNAEERLAAFIYNLSRRFAQRGFSPREFRLTMTRGDIGNYLGLTVET

ISRLLGRFQKSGMLAVKGKYITIENNDALAQLAGHTRNVA

>gi|30062857|ref|NP_837028.1| universal stress protein UspE [Shigella flexneri 2a str. 2457T]

MAMYQNMLVVIDPNQDDQPALRRAVYLHQRIGGKIKAFLPIYDFSYEMTTLLSPDERTAMRQGVISQRTA

WIHEQAKYYLNAGVPIEIKVVWHNRPFEAIIQEVISGGHDLVLKMAHQHDRLEAVIFTPTDWHLLRKCPS

PVWMVKDQPWPEGGKALVAVNLASEEPYHNALNEKLVKETIELAEQVNHTEVHLVGAYPVTPINIAIELP

EFDPSVYNDAIRGQHLLAMKALRQKFGINENMTHVEKGLPEEVIPDLAEHLQAGIVVLGTVGRTGISAAF

LGNTAEQVIDHLRCDLLVIKPDQYQTPVELDDEEDD

>gi|30062851|ref|NP_837022.1| hypothetical protein S1425 [Shigella flexneri 2a str. 2457T]

MIAELFTNNALNLVIIFGSCAALILMSFWFRRGNRKRKGFLFHAVQFLIYTIIISAVGSISNYVIENYKL

KFITPGVIDFICTSLIAVILTIKLFLLINQFEKQQIKKGRDITSARIMSRIIKITIIVVLVLLYGEHFGM

SLSGLLTFGGIGGLAVGMAGKDILSNFFSGIMLYFDRPFSIGDWIRSPDRNIEGTVAEIGWRITKITTFD

NRPLYVPNSLFSSISVENPGRMTNRRITTTIGLRYEDAAKVGVIVEAVREMLKNHPAIDQRQTLLVYFNQ

FADSSLNIMVYCFTKTTVWAEWLAAQQDVYLKIIDIVQSHGADFAFPSQTLYMDNITPPEQGR

>gi|30062850|ref|NP_837021.1| transport periplasmic protein [Shigella flexneri 2a str. 2457T]

MSVRGKLMKHSVSVTCCALLVSSISLSYAAEVPSGTVLAEKQELVRHIKDEPASLDPAKAVGLPEIQVIR

DLFEGLVNQNEKGEIVPGVATQWKSNDNRIWTFTLRDNAKWADGTPVTAQDFVYSWQRLVDPKTLSPFAW

FAALAGINNAQAIIDGKATPDQLGVTAVDAHTLKIQLDKPLPWFVNLTANFAFFPVQKANVESGKEWTKP
GNLIGNGAYVLKERVVNEKLVVVPNTHYWDNAKTVLQKVTFLPINQESAATKRYLAGDIDITESFPKNMY
QKLLKDIPGQVYTPPQLGTYYYAFNTQKGPTADQRVRLALSMTIDRRLMTEKVLGTGEKPAWHFIPDVTA
GFTPEPSPFEQMSQEELNAQAKTLLSAAGYGPQKPLKLTLLYNTSENHQKIAIAVASMWKKNLGVDVKLQ
NQEWKTYIDSRNTGNFDVIRASWVGDYNEPSTFLTLLTSMHSGNISRFNNPAYDKVLAQASTENTVKARN
ADYNAAEKILMEQAPIAPIYQYTNGRLIKPWLKGYPINNPEDVAYSRTMYIVKH

>gi|30062847|ref|NP_837018.1| LYSR-type transcriptional regulator [Shigella flexneri 2a str. 2457T]
MKREEIADLMAFVVVAEERSFTRAAARLSMVQSALSQIVRRIEERLGLRLLTRTTRSVVPTEAGEHLLSV
LGPILHDIDSALTSLSDLQNRPSGTIRITTVEHAAKTILLPAMRTFLKSHPEIDIQLTIDYGLTDVVSER
FDAGVLRCTGNSGYYHLFFF

>gi|30062844|ref|NP_837015.1| murein peptide amidase A [Shigella flexneri 2a str. 2457T]
MTVTRSRAERGAFPPGTEHYGRSLLGAPLIWFPAPAASRESGLILAGTHGDENSSVVTLSCALRTLTPSL
RRHHVVLCVNPDGCQLGLRANANGVDLNRNFPAANWKEGETVYRWNSAAEERDVVLLTGDKPGSEPETQA
LCQLIHRIQPAWVVSFHDPLACIEDPRHNELGEWLAQSFELPLVTSVGYETPGSFGSWCADLNLHCITAE
FPPISSDEASEKYLFAMANLLRWHPKDAIRPS

>gi|30062843|ref|NP_837014.1| muconate cycloisomerase I [Shigella flexneri 2a str. 2457T]
MRIVKVFEEAWPLHTPFVIARGSRSEARVVVVELEEEGIKGTGECTPYPRYGESDASVMAQIMSVVPQLE
KGLTREELQKILPAGAARNALDCALWDLAARKQQQSLADLIGITLPETVITAQTVVIGTPDQMANSASTL
WQAGAKLLKVKLDNHLISERMVAIRTAVPDATLIVDANESWRAEGLAARCQLLADLGVAMLEQPLPAQDD
AALENFIHPLPICADESCHTRSNLKALKGRYEMVNIKLDKTGGLTEALALATEARAQGFSLMLGCMLCTS
RAISAALPLVPQVSFADLDGPTWLAVDVEPALQFTTGELHL

>gi|30062842|ref|NP_837013.1| thiol peroxidase [Shigella flexneri 2a str. 2457T]

MSQTVHFQGNPVTVANSIPQAGSKAQTFTLVAKDLSDVTLGQFAGKRKVLNIFPSIDTGVCAASVRKFNQ

LATEIDNTVVLCISADLPFAQSRFCGAEGLNNVITLSTFRNAEFLQAYGVAIADGPLKGLAARAVVVIDE

NDNVIFSQLVDEITTEPDYEAALAVLKA

>gi|30062841|ref|NP_837012.1| DNA-binding transcriptional regulator TyrR [Shigella flexneri 2a str. 2457T]

MRLEVFCEDRLGLTRELLDLLVLRGIDLRGIEIDPIGRIYLNFAELEFESFSSLMAEIRRIAGVTDVRTV

PWMPSEREHLALSALLEALPEPVLSVDMKSKVDMANPASCQLFGQKLDRLRNHTAAQLINGFNFLRWLES

EPQDSHNEHVVINGQNFLMEITPVYLQDENDQHVLTGAVVMLRSTIRMGRQLQNVAAQDVSAFSQIVAVS

PKMKHVVEQAQKLAMLSAPLLITGDTGTGKDLFAYACHQASPRAGKPYLALNCASIPEDAVESELFGHAP

EGKKGFFEQANGGSVLLDEIGEMSPRMQAKLLRFLNDGTFRRVGEDHEVHVDVRVICATQKNLVELVQKG

VFREDLYYRLNVLTLNLPPLRDCPQDIMPLTELFVARFADEQGVPRPKLAADLNTVLTRYAWPGNVRQLK

NAIYRALTQLDGYELRPQDILLPDYDAATVAVGEDAMEGSLDEITSRFERSVLTQLYRNYPSTRKLAKRL

GVSHTAIANKLREYGLSQKKNEE

>gi|30062840|ref|NP_837011.1| hypothetical protein S1411 [Shigella flexneri 2a str. 2457T]

MTEPLKPRIDFDGPLEVEQNPKFRAQQTFDENQAQNFAPATLDEAQEEEGQVEAVMDAALRPKRSLWRKM

VMGGLALFGASVVGQGIQWTMNAWQTQDWVALGGCAAGALIIGAGVGSVVTEWRRLWRLRQRAHERDEAR

DLLHSHGTGKGRAFCEKLAQQAGIDQSHPALQRWYASIHETQNDREVVSLYAHLVQPVLDAQARREISRS

AAESTLMIAVSPLALVDMAFIAWRNLRLINRIATLYGIELGYYSRLRLFKLVLLNIAFAGASELVREVGM

DWMSQDLAARLSTRAAQGIGAGLLTVRLGIKAMELCRPLPWIDDDKPRLGDFRRQLIGQVKETLQKGKTP

>gi|30062839|ref|NP_837010.1| enzyme [Shigella flexneri 2a str. 2457T]

MKRLKNELNALVNRGVDRHLRLAVTGLSRSGKTAFITAMVNQLLNIHAGARLPLLSAVREERLLGVKRIP

QRDFGIPRFTYDEGLAQLYGDPPAWPTPTRGVSEIRLALRFKSNDSLLRHFKDTSTLYLEIVDYPGEWLL

DLPMLAQDYLSWSRQMTGLLNGQRGEWSAKWRMMCEGLDPLAPADENRLADIAAVWTDYLHHCKQQGLHF

IQPGRFVLPGDMAGAPALQFFPWPDVDAWDESKLAQADKHTNAGMLRERFNYYCEKVVKGFYKNHFLRFD

RQIVLVDCLQPLNSGPQAFNDMRLALTQLMQSFHYGQRTLFRRLFSPVIDKLLFAAMKADHVTVDQHANM

VSLLQQLIQDAWQNAAFERISMDCLGLASVQATTSGIIDVNGEKIPALRGNRLSDGALLTVYPGEVPARL

PGQAFWDKQGFQFEAFRPQVMDVDKPLPHIRLDAALEFLIGDKLR

>gi|30062838|ref|NP_837009.1| LACI-type transcriptional regulator [Shigella flexneri 2a str. 2457T]

MSPTIYDIARVAGVSKSTVSRVLNKQTNISPEAREKVLRAIEELQYQPNKLARALTSSGFDAIMVISTRS

TKTTAGNPFFSEVLHAITAKAEEEGFDVILQTSHNPAEDLQKCESKIKQKMIKGIIMLSSPADESFFAQL

DKYDIPVVVIGKVEGQYAHVYSVDTDNFGDSIALTDALIESGHQNIACLHAPLDVHVSVDRVNGYKQSLS

AHNIAVRDKWIVDGGYTHETALQAARQLLSQSPLPEAVFATDSLKLMSIYRAAAEKNIAIPQQLAVVGYS

NETLSFILTPAPGGIDVPTQELGQQSCELLFRLISGKPSPQNITVATHMTLK

>gi|30062832|ref|NP_837003.1| transport system permease [Shigella flexneri 2a str. 2457T]

MATNKRTLSRIGFYCGLALFLIITLFPFFVMLMTSFKSAKEAISLHPTLLPQQWTLEHYVDIFNPMIFPF

VDYFRNSMVVSVVSSVVAVFLGILGAYALSRLRFKGRMTINASFYTVYMFSGILLVVPLFKIITALGIYD

TEMALIITMVTQTLPTAVFMLKSYFDTIPDEIEEAAMMDGLNRLQIIFRITVPLAMSGLISVFVYCFMVA

WNDYLFASIFLSSASNFTLPVGLNALFSTPDYIWGRMMAASLVTALPVVIMYALLERFIKSGLTAGGVKG

>gi|30062831|ref|NP_837002.1| binding-protein dependent transport protein [Shigella flexneri 2a str. 2457T]

MNRLFSGRSDMPFALLLLAPSLLLLGGLVAWPMVSNIEISFLRLPLNPNIESTFVGVSNYVRILSDPGFW

HSLWMTVWYTALVVAGSTVLGLAVAMFFNREFRLRKTARSLVILSYVTPSISLVFAWKYMFNNGYGIVNY

LGVDLLHLYEQAPLWFDNPGSSFVLVVLFAIWRYFPYAFISFLAILQTIDKSLYEAAEMDGANAWQRFRI

VTLPAIMPVLATVVTLRTIWMFYMFADVYLLTTKVDILGVYLYKTAFAFNDLGKAAAISVVLFIIIFAVI

LLTRKRVNLNGNK

>gi|30062828|ref|NP_836999.1| thiosulfate:cyanide sulfurtransferase [Shigella flexneri 2a str. 2457T]

MAIFIHRRTLTCLKKGLLALALVFSLPVFAAEHWIDVRVPEQYQQEHVQGAINIPLKEVKERIATAVPDK

NDTVKVYCNAGRQSGQAKELLSEMGYTHVENAGGLKDIAMPKVKG

>gi|30062826|ref|NP_836997.1| DNA-binding transcriptional activator PspC [Shigella flexneri 2a str. 2457T]

MAGINLNKKLWRIPQQGMVRGVCAGIANYFDVPVKLVRILVVLSIFFGLALFTLVAYIILSFALDPMPDN

MAFGEQLPSSSELLDEVDRELAASETRLREMERYVTSDTFTLRSRFRQL

>gi|30062824|ref|NP_836995.1| phage shock protein PspA [Shigella flexneri 2a str. 2457T]

MGIFSRFADIVNANINALLEKAEDPQKLVRLMIQEMEDTLVEVRSTSARALAEKKQLTRRIEQASAREVE

WQEKAELALLKEREDLARAALIEKQKLTDLIKSLEHEVTLVDDTLARMKKEIGELENKLSETRARQQALM

LRHQAANSSRDVRRQLDSGKLDEAMARFESFERRIDQMEAEAESHSFGKQKSLDDQFAELKADDAISEQL

AQLKAKMKQDNQ

>gi|30062819|ref|NP_836990.1| DNA-binding transcriptional repressor PuuR [Shigella flexneri 2a str. 2457T]

MSDEGLAPGKRLSEIRQQQGLSQRRAAELSGLTHSAISTIEQDKVSPAISTLQKLLKVYGLSLSEFFSEP

EKPDEPQVVINQDDLIEMGSQGVSMKLVHNGNPNRTLAMIFETYQPGTTTGERIKHQGEEIGTVLEGEIV

LTINGQDYHLVAGQSYAINTGIPHSFSNTSAGICRIISAHTPTTF

>gi|30062818|ref|NP_836989.1| gamma-glutamyl-gamma-aminobutyrate hydrolase [Shigella flexneri 2a str. 2457T]

MRLSMENIMNNPVIGVVMCRNRLKGHATQTLQEKYLNAIIHAGGLPIALPHALAEPSLLEQLLPKLDGIY

LPSSPSNVQPHLYGENGDEPDADPGRDLLSMAIINAALERRIPIFAICRGLQELVVATGGSLHRKLCEQP

ELLEHREDPELPVEQQYAPSHEVQVEEGGLLSALLPECSNFWVNSLHGQGAKVVSPRLRVEARSPDGLVE

AVSVINHPFALGVQWHPEWNSSEYALSRILFEGFITAWQHHIAEKQRL

>gi|30062815|ref|NP_836986.1| peptide transport periplasmic protein [Shigella flexneri 2a str. 2457T]

MRQVLSSLLVIAGLVSGQAIAAPESPPHADIRDSGFVYCVSGQVNTFNPSKASSGLIVDTLAAQFYDRLL

DVDPYTYRLMPELAESWEVLDNGATYRFHLRRDVPFQKTDWFTPTRKMNADDVVFTFQRIFDRNNPWHNV

NGSNFPYFDSLQFADNVKSVRKLDNHTVEFRLAQPDASFLWHLATHYASVMSAEYARKLEKEDRQEQLDR

QPVGTGPYQLSEYRAGQFIRLQRHDDFWRGKPLMPQVVVDLGSGGTGRLSKLLTGECDVLAWPAASQLSI

LRDDPRLRLTLRPGMNVAYLAFNTAKPPLNNPAVRHALALAINNQRLMQSIYYGTAETAAFILPRASWAY

DNEAKITEYNPAKSREQLKALGLENLTLKLWVPTRSQAWNPSPLKTAELIQADMAQVGVKVVIVPVEGRF

QEARLMDMSHDLTLSGWATDSNDPDSFFRPLLSCAAIHSQTNLAHWCNPKFDSVLRKALSSQQLAARIEA

YDEAQSILAQELPILPLASSLRLQAYRYDIKGLVLSPFGNASFAGVYREKQDEVKKP

>gi|30062814|ref|NP_836985.1| peptide transport permease [Shigella flexneri 2a str. 2457T]

MIIFTLRRILLLIVTLFLLTFVGFSLSYFTPHAPLQGASLWNAWVFWFNGLIHWDFGVSSINGQPIAEQL

KELFPATIELCILAFGFALIVGIPVGMIAGITRHKWQDNLINAIALLGFSIPVFWLALLLTLFCSLTLGW

LPVSGRFDLLYEVKPITGFALIDAWLSDSPWRDEMIMSAIRHMILPVITLSVAPTTEVIRLMRISTIEVY
DQNYVKAAATRGLSRFTILRRHVLHNALPPVIPRLGLQFSTMLTLAMITEMVFSWPGLGRWLINAIRQQD
YAAISAGVMVCGSLVIIVNVISDILGAMANPLKHKEWYALR

>gi|30062813|ref|NP_836984.1| peptide transport permease [Shigella flexneri 2a str. 2457T]
MPYDSVYSEKRPPGTLRTAWRKFYSDASAMVGLYGCAGLAVLCIFGGWFAPYGIDQQFLGYQLLPPSWSR
YGEVSFFLGTDDLGRDVLSRLLSGAAPTVGGAFVVTLAATICGLVLGTFAGATHGLRSAVLNHILDTLLA
IPSLLLAIIVVAFAGPSLSHAMFAVWLALLPRMVRSIYSMVHDELEKEYVIAARLDGASTLNILWFAVMP
NITAGLVTEITRALSMAILDIAALGFLDLGAQLPSPEWGAMLGDALELIYVAPWTVMLPGAAIMISVLLV
NLLGDGVRRAIIAGVE

>gi|30062810|ref|NP_836981.1| hypothetical protein S1376 [Shigella flexneri 2a str. 2457T]
MMDKIKSNARDLRRNLTLQERKLWRYLRSRRFGDFKFRRQHPVGSYILDFACCSARVVVELDGGQHDLAV
AYDTRRTSWLESQGWTVLRFWNNEIDCNEEAVLEIILQELNRRSPSP

>gi|30062808|ref|NP_836979.1| oxidoreductase [Shigella flexneri 2a str. 2457T]
MEQRHITGKSHWYHETQSSTTEYDVLPLVPEAAKVSDPFLLDVILDEETLAPFLSWLVPARVLAVELFPD
QLTVTRSQTFTAYERLSTALTVAQVCGVQRLCNYYSARLTPLPGPDSSRESNHRLAQITQYARQLASSPS
IIDNRSRQHLNDVGLTVWDCVIINQIIGFIGFQARTIATFQAYLGHPVRWLPGLAIQNYADASLFADESI
RWRSSYEVEKLPEEHTKSSTAELCQLAETLSLHPISLSLLEKLLNSTRVNAQPDNQLAALLCARINGSPA
CFSTCMDSSNEYKKISTLLRKGENEINRWADRHSVERATVQAIQWLTRAPDRFSAAQFSPLLEHEKSSTQ
IINLLVWSGLCGWINRLKIALGETY

>gi|30062806|ref|NP_836977.1| DEOR-type transcriptional regulator [Shigella flexneri 2a str. 2457T]

MNSRQQTILQMVIDQGQVSVTDLAKATGVSEVTIRQDLNTLEKLSYLRRAHGFAVSLDSDDVETRMMSNY

TLKRELAEFAASLVQPGETIFIENGSSNALLARTLGEQKKNVTIITVSSYIAHLLKDAPCEVILLGGVYQ

KKSESMVGPLTRQCIQQVHFSKAFIGIDGWQPETGFTGRDMMRTDVVNAVLEKECEAIVLTDSSKFGAVH

SYSIGPVERFNRVITDSKIRASDLMHLEHSKLTVHVVDI

>gi|30062804|ref|NP_836975.1| translation initiation factor Sui1 [Shigella flexneri 2a str. 2457T]

MMSDSNSRLVYSTETGRIDEPKAAPVRPKGDGAVRIQRQTSGRKGKGVCLITGIDLDDAELTKLAAELKK

KCGCGGAVKDGVIEIQGDKRDLLKSLLEAKGMKVKLAGG

>gi|30062803|ref|NP_836974.1| orotidine 5'-phosphate decarboxylase [Shigella flexneri 2a str. 2457T]

MTLTASSSSRAVTNSPVVVALDYHNCDDALSFVDKIDPRDCRLKVGKEMFTLFGPQFVRELQQRGFDIFL

DLKFHDIPNTAAHAVAAAADLGVWMVNVHASGGARMMTAAREALVPFGKDAPLLIAVTVLTSMEASDLAD

LGVTLSPADYAERLAALTQKCDLDGVVCSAQEAVRFKQVFGQEFKLVTPGIRPQGSEAGDQRRIMTPEQA

LAAGVDYMVIGRPVTQSVDPAQTLKAINASLQRSA

>gi|30062802|ref|NP_836973.1| tetratricopeptide repeat protein [Shigella flexneri 2a str. 2457T]

MLELLFLLLPVAAAYGWYMGRRSAQQNKQDEANRLSRDYVAGVNFLLSNQQDKAVDLFLDMLKEDTGTVE

AHLTLGNLFRSRGEVDRAIRIHQTLMESASLTYEQRLLAIQQLGRDYMAAGLYDRAEDMFNQLTDETDFR

IGALQQLLQIYQATSEWQKAIDVAERLVKLGKDKQRVEIAHFYCELALQHMASDDLDRAMTLLKKGAAAD

KNSARVSIMMGRVFMAKGEYAKAVESLQRVISQDRELVSETLEMLQTCYQQLGKTAEWAEFLQRAVEENT

GADAELMLADIIEARDGSEAAQVYITRQLQRHPTMRVFHKLMDYHLNEAEEGRAKESLMVLRDMVGEKVR

SKPRYSCQKCGFTAYTLYWHCPSCRAWSTIKPIRGLDGL

>gi|30062801|ref|NP_836972.1| hypothetical protein S1366 [Shigella flexneri 2a str. 2457T]

MKYLLIFLLVLAIFVISVTLGAQNDQQVTFNYLLAQGEYRISTLLAVLFAAGFAIGWLICGLFWLRVRVS

LARAERKIKRLENQLSPATDVAVVPHSSAAKE

>gi|30062800|ref|NP_836971.1| phosphatidylglycerophosphatase B [Shigella flexneri 2a str. 2457T]

MRSIARRTAVGAALLLVMPVAVWISGWRWQPGEQSWLLKAAFWVTETVTQPWGVITHLILFGWFLWCLRF

RIKAAFVLFAILAAAILVGQGVKSWIKDKVQEPRPFVIWLEKTHHIPVDEFYTLKRAERGNLVKEQLAEE

KNIPQYLRSHWQKETGFAFPSGHTMFAASWALLAVGLLWPRRRTLTIAILLVWATGVMGSRLLLGMHWPR

DLVVATLISWALVAVATWLAQRICGPLTPPAEENREIAQREQES

>gi|30062799|ref|NP_836970.1| GTP cyclohydrolase II [Shigella flexneri 2a str. 2457T]

MQLKRVAEAKLPTPWGDFLMVGFEELATGHDHVALVYGDISGHTPVLARVHSECLTGDALFSLRCDCGFQ

LEAALTQIAEEGRGILLYHRQEGRNIGLLNKIRAYALQDQGYDTVEANHQLGFAADERDFTLCADMFKLL

GVNEVRLLTNNPKKVEILTEAGINIVERVPLIVGRNPNNEHYLDTKAEKMGHLLNK

>gi|30062797|ref|NP_836968.1| transcriptional regulator CysB [Shigella flexneri 2a str. 2457T]

MKLQQLRYIVEVVNHNLNVSSTAEGLYTSQPGISKQVRMLEDELGIQIFSRSGKHLTQVTPAGQEIIRIA

REVLSKVDAIKSVAGEHTWPDKGSLYIATTHTQARYALPNVIKGFIERYPRVSLHMHQGPPTQIADAVSK

GNADFAIATEALHLYEDLVMLPCYHWNRAIVVTPDHPLAGKKAITIEELAQYPLVTYTFGFTGRSELDTA

FNRAGLTPRIVFTATDADVIKTYVRLGLGVGVIASMAVDPVADPDLVRVDAHDIFSHSTTKIGFRRSTFL

RSYMYDFIQRFAPHLTRDVVDAAVALRSNEEIEVMFKDIKLPEK

>gi|30062794|ref|NP_836965.1| periplasmic protease [Shigella flexneri 2a str. 2457T]

MELLSEYGLFLAKIVTVVLAIAAIAAIIVNVAQRNKRQRGELRVNNLSEQYKEMKEELAAALMDSHQQKQ

WHKAQKKKHKQEAKAAKAKAKLGEVATDSKPRVWVLDFKGSMDAHEVNSLREEITAVLAAFKPQDQVVLR

LESPGGMVHGYGLAASQLQRLRDKNIPLTVTVDKVAASGGYMMACVADKIVSAPFAIVGSIGVVAQMPNF

NRFLKSKDIDIELHTAGQYKRTLTLLGENTEEGREKFREELNETHQLFKDFVKRMRPSLDIEQVATGEHW

YGQQAVEKGLVDEINTSDEVILSLMEGREVVNVRYMQRKRLIDRFTGSAAESADRLLLRWWQRGQKPLM

>gi|30062792|ref|NP_836963.1| cob(I)yrinic acid a,c-diamide adenosyltransferase [Shigella flexneri 2a str. 2457T]

MSDERYQQRQQRVKEKVDARVAQAQDERGIIIVFTGNGKGKTTAAFGTATRAVGHGKKVGVVQFIKGTWP

NGERNLLEPHGVEFQVMATGFTWDTQNRESDTAACREVWQHAKRMLADSSLDMVLLDELTYMVAYDYLPL

EEVVQALNERPHQQTVIITGRGCHRDILELADTVSELRPIRHAFDAGVKAQIGIDY

>gi|30062791|ref|NP_836962.1| 23S rRNA pseudouridylate synthase B [Shigella flexneri 2a str. 2457T]

MSEKLQKVLARAGHGSRREIESIIEAGRVSVDGKIAKLGDRVEVTPGLKIRIDGHLISVRESAEQICRVL

AYYKPEGELCTRNDPEGRPTVFDRLPKLRGARWIAVGRLDVNTCGLLLFTTDGELANRLMHPSREVEREY

AVRVFGQVDDAKLRDLSRGVQLEDGPAAFKTIKFSGGEGINQWYNVTLTEGRNREVRRLWEAVGVQVSRL

IRVRYGDIPLPKGLPRGGWTELDLAQTNYLRELVELPPETSSKVAVEKDRRRMKANQIRRAVKRHSQVSG

SRRSGGRNNG

>gi|30062789|ref|NP_836960.1| enzyme [Shigella flexneri 2a str. 2457T]

MSDTNYAVIYDLHSHTTASDGCLTPEALVHRAVEMRVGTLAITDHDTTAAIAPAREEISRSGLALNLIPG

VEISTVWENHEIHIVGLNIDITHPLMCEFLAQQTERRNQRAQQIAERLEKAQIPGALEGAQRLAQGGAVT

RGHFARFLVECGKASSMADVFKKYLARGKTGYVPPQWCTIEQAIDVIHHSGGKAVLAHPGRYNLSAKWLK

RLVAHFAEHHGDAMEVAQCQQSPNERTQLATLARQHHLWASQGSDFHQPCPWIELGRKLWLPAGVEGVWQ

LWEQPQNTTEREL

>gi|30062787|ref|NP_836958.1| anthranilate synthase component I [Shigella flexneri 2a str. 2457T]

MQTQKPTLELLTCEGAYRDNPTALFHQLCGNRPATLLLESADIDSKDDLKSLLLIDSALRITALGDTVTI

QALSGNGEALLALLDNALPAGVESEQSPNCRVLRFPPVSPLLDEDARLCSLSVFDAFRLLQNLLNVPKEE

REAMFFGGLFSYDLVAGFEDLPQLSAENNCPDFCFYLAETLMVIDHQKKSTRIQASLFAPNEEEKQRLTA

RLNELRQQLTEAAPPLPVVSVPHMRCECNQSDEEFGGVVRLLQKAIRAGEIFQVVPSRRFSLPCPSPLAA

YYVLKKSNPSPYMFFMQDNDFTLFGASPESSLKYDATSRQIEIYPIAGTRPRGRRADGSLDRDLDSRIEL

EMRTDHKELSEHLMLVDLARNDLARICTPGSRYIADLTKVDRYSYVMHLVSRVVGELRHDLDALHAYRAC

MNMGTLSGAPKVRAMQLIAEAEGRRRGSYGGAVGYFTAHGDLDTCIVIRSALVENGIATVQAGAGVVLDS

VPQSEADETRNKARAVLRAIATAHHAQETF

>gi|30062785|ref|NP_836956.1| bifunctional indole-3-glycerol phosphate synthase/phosphoribosylanthranilate isomerase [Shigella flexneri 2a str. 2457T]

MMQTVLAKIVADKAIWVETRKQQQPLASFQNEVQPSTRHFYDALQGARTAFILECKKASPSKGVIRDDFD

PARIAAIYKHYASAISVLTDEKYFQGSFDFLLIVSQIAPQPILCKDFIIDPYQIYLARYYQADACLLMLS

VLDDDQYRQLAAVAHSLEMGVLTEVSNEEELERAIALGAKVVGINNRDLRDLSIDLNRTRELAPKLGHNV

TVISESGINTYAQVRELSHFANGFLIGSALMAHDDLHAAVRRVLLGENKVCGLTRGQDAKAAYDAGAIYG

GLIFVATSPRCVNVEQAQEVMAAAPLQYVGVFRNHDIADVVDKAKVLSLAAVQLHGNEDQLYIDTLREAL

PAHVAIWKALSVGETLPAREFQHVDKYVLDNGQGGSGQRFDWSLLNGQSLGNVLLAGGLGADNCVEAAQT

GCAGLDFNSAVESQPGIKDARLLASVFQTLRAY

>gi|30062784|ref|NP_836955.1| tryptophan synthase subunit beta [Shigella flexneri 2a str. 2457T]

MTTLLNPYFGEFGGMYVPQILMPALRQLEEAFVSAQKDPEFQAQFNDLLKNYAGRPTALTKCQNITAGTN

TTLYLKREDLLHGGAHKTNQVLGQALLAKRMGKTEIIAETGAGQHGVASALASALLGLKCRIYMGAKDVE

RQSPNVFRMRLMGAEVIPVHSGSATLKDACNEALRDWSGSYETAHYMLGTAAGPHPYPTIVREFQRMIGE

ETKAQILEREGRLPDAVIACVGGGSNAIGMFADFINETNVGLIGVEPGGHGIETGEHGAPLKHGRVGIYF

GMKAPMMQTEDGQIEESYSISAGLDFPSVGPQHAYLNSTGRADYVSITDDEALEAFKTLCLHEGIIPALE

SSHALAHALKMMRENPDKEQLLVVNLSGRGDKDIFTVHDILKARGEI

>gi|30062783|ref|NP_836954.1| tryptophan synthase subunit alpha [Shigella flexneri 2a str. 2457T]

MERYESLFAQLKERKEGAFVPFVTLGDPGIEQSLKIIDTLIEAGADALELGIPFSDPLADGPTIQNATLR

AFAAGVTPAQCFEMLALIRQKHPTIPIGLLMYANLVFNKGIDEFYAQCEKVGVDSVLVADVPVEESAPFR

QAALRHNVAPIFICPPNADDDLLRQIASYGRGYTYLLSRAGVTGAENRAALPLNHLVAKLKEYNAAPPLQ

GFGISAPDQVKAAIDAGAAGAISGSAIVKIIEQHINEPEKMLAALKVFVQPMKAATRS

>gi|30062781|ref|NP_836952.1| structural protein [Shigella flexneri 2a str. 2457T]

MNMKTIEDVFIQLLSDTYSAEKQLTRALAKLARATSNEKLSQAFHAHLEETHGQIERIDQVVESESNLKI

KRMKCVAMEGLIEEANEVIESTEKNEVRDAALIAAAQKVEHYEIASYGTLATLAEQLGYRKAAKLLKETL

EEEKATDIKLTDLALNNVNKKAENKA

>gi|30062780|ref|NP_836951.1| hypothetical protein S1346 [Shigella flexneri 2a str. 2457T]

MNRIEHYHDWLRDAHAMEKQAESMLESMASRIDNYPELRARIEQHLSETKNQIVQLETILDRNDISRSVI

KDSMSKMAALGQSIGGIFPSDEIVKGSIGGYVFEQFEITCYTSLLAAAKNAGDTVSIPIIEAILNEEKQM

ADWLIQHIPQTTEKFLIRSETDGVEAKK

>gi|30062779|ref|NP_836950.1| outer membrane protein W [Shigella flexneri 2a str. 2457T]

MKKLTVAALAVTTLLSGSAFAHEAGEFFMRAGSATVRPTEGAGGTLGSLGGFSVTNNTQLGLTFTYMATD

NIGVELLAATPFRHKIGTRATGDIATVHHLPPTLMAQWYFGDASSKFRPYVGAGINYTTFFDNGFNDHGK

EAGLSDLSLKDSWGAAGQVGVDYLINRDWLVNMSVWYMDIDTTANYKLGGAQQHDSVRLDPWVFMFSAGY

RF

>gi|30062778|ref|NP_836949.1| hypothetical protein S1344 [Shigella flexneri 2a str. 2457T]

MSITAQSVYRDTGNFFRNQFMTILLVSLLCAFITVVLGHVFSPSDAQLAQLNDGVPVSGSSGLFDLVQNM

SPEQQQILLQASAASTFSELIGNAILAGGVILIIQLVSAGQRVSALRAIGASAPILPKLFILIFLTTLLV

QIGIMLVVVPGIIMAILLALAPVMLVQDKMGIFASMRSSMRLTWANMRLVAPAVLSWLLAKTLLLLFASS

FAALTPEIGAVLANTLSNLISAILLIYLFRLYMLIRQ

>gi|30062777|ref|NP_836948.1| intracellular septation protein A [Shigella flexneri 2a str. 2457T]

MKQFLDFLPLVVFFAFYKIYDIYAATAALIVATAIVLIYSWVRFRKVEKMALITFVLVVVFGGLTLFFHN

DEFIKWKVTVIYALFAGALLVSQWVMKKPLIQRMLSKELTLPQPVWSKLNLAWAVFFILCGLANIYIAFW

LPQNIWVNFKVFGLTALTLIFTLLSGIYIYRHMPQEDKS

>gi|30062770|ref|NP_836941.1| cardiolipin synthetase [Shigella flexneri 2a str. 2457T]

MTTVYTLVSWLAILGYWLLIAGVTLRILMKRRAVPSAMAWLLIIYILPLVGIIAYLAVGELHLGKRRAER

ARAMWPSTAKWLNDLKACKHIFAEENSSVAAPLFKLCERRQGIAGVKGNQLQLMTESDDVMQALIRDIQL

ARHNIEMVFYIWQPGGMADQVAESLMAAARRGIHCRLMLDSAGSVAFFRSPWPELMRNAGIEVVEALKVN

LMRVFLRRMDLRQHRKMIMIDNYIAYTGSMNMVDPRYFKQDAGVGQWIDLMARMEGPIATAMGIIYSCDW

EIETGKRILPPPPDVNIMPFEQASGHTIHTIASGPGFPEDLIHQALLTAAYSAHEYLIMTTPYFVPSDDL

LHAICTAAQRGVDVSIILPRKNDSMLVGWASRAFFTELLAAGVKIYQFEGGLLHTKSVLVDGELSLVGTV

NLDMRSLWLNFEITLAIDDKGFGADLAAVQDDYISRSRLLDARLWLKRPLWQRVAERLFYFFSPLL

>gi|30062766|ref|NP_836937.1| oligopeptide transport permease [Shigella flexneri 2a str. 2457T]

MMLSKKNSETLENFSEKLEVEGRSLWQDARRRFMHNRAAVASLIVLVLIALFVILAPMLSQFAYDDTDWA

MMSSAPDMESGHYFGTDSSGRDLLVRVAIGGRISLMVGVAAALVAVVVGTLYGSLSGYLGGKVDSVMMRL

LEILNSFPFMFFVILLVTFFGQNILLIFVAIGMVSWLDMARIVRGQTLSLKRKEFIEAAQVGGVSTPGIV

IRHIVPNVLGVVVVYASLLVPSMILFESFLSFLGLGTQEPLSSWGALLSDGANSMEVSPWLLLFPAGFLV

VTLFCFNFIGDGLRDALDPKDR

>gi|30062765|ref|NP_836936.1| oligopeptide transporter permease [Shigella flexneri 2a str. 2457T]

MLKFILRRCLEAIPTLFILITISFFMMRLAPGSPFTGERTLPPEVMANIEAKYHLNDPIMTQYFSYLKQL

AHGDFGPSFKYKDYSVNDLVASSFPVSAKLGAAAFFLAVILGVSAGVIAALKQNTKWDYTVMGLAMTGVV

IPSFVVAPLLVMIFAIILHWLPGGGWNGGALKFMILPMVALSLAYIASIARITRGSMIEVLHSNFIRTAR

AKGLPMRRIILRHALKPALLPVLSYMGPAFVGIITGSMVIETIYGLPGIGQLFVNGALNRDYSLVLSLTI

LVGALTILFNAIVDVLYAVIDPKIRY

>gi|30062764|ref|NP_836935.1| periplasmic oligopeptide binding protein [Shigella flexneri 2a str. 2457T]

MTNITKRSLVAAGVLAALMAGNVALAADVPAGVTLAEKQTLVRNNGSEVQSLDPHKIEGVPESNISRDLF

EGLLVSDLDGHPAPGVAESWDNKDAKVWTFHLRKDAKWSDGTPVTAQDFVYSWQRSVDPNTASPYASYLQ

YGHIAGIDEILEGKKPITDLGVKAIDDHTLEVTLSEPVPYFYKLLVHPSTSPVPKAAIEKFGEKWTQPGN

IVTNGAYTLKDWVVNERIVLERSPTYWNNAKTVINQVTYLPIASEVTDVNRYRSGEIDMTYNNMPIELFQ

KLKKEIPDEVHVDPYLCTYYYEINNQKPPFNDVRVRTALKLGMDRDIIVNKVKAQGDMPAYGYTPPYTDG

AKLTQPEWFGWSQEKRNEEAKKLLAEAGYTADKPLTINLRYNTSDLHKKLAIAASSLWKKNIGVNVKLVN

QEWKTFLDTRHQGTFDVARAGWCADYNEPTSFLNTMLSNSSMNTAHYKSPAFDSIMAETLKVTDEAQRTA

LYTKAEQQLDKDSAIVPVYYYVNARLVKPWVGGYTGKDPLDNTYTRNMYIVKH

>gi|30062761|ref|NP_836932.1| hypothetical protein S1327 [Shigella flexneri 2a str. 2457T]

MIQTLFDFPVYFKFFIGLFALVNPVGIIPVFISMTSYQTAAARNKTNLTANLSVAIILWISLFLGDTILQ

LFGISIDSFRIAGGILVVTIAMSMISGKLGEDKQNKQEKSETAVRESIGVVPLALPLMAGPGAISSTIVW

GTRYHSISYLFGFFVAIALFALCCWGLFRMAPWLVRVLRQTGINVITRIMGLLLMALGIEFIVTGIKGIF

PGLLN

>gi|30062758|ref|NP_836929.1| thymidine kinase [Shigella flexneri 2a str. 2457T]

MAQLYFYYSAMNAGKSTALLQSSYNYQERGMRTVVYTAEIDDRFGAGKVSSRIGLSSPAKLFNQNSSLFD

EIRAEHEQQAIHCVLVDECQFLTRQQVYELSEVVDQLDIPVLCYGLRTDFRGELFIGSQYLLAWSDKLVE

LKTICFCGRKASMVLRLDQAGRPYNEGEQVVIGGNERYVSVCRKHYKEALQVGSLTAIQERHRHD

>gi|30062757|ref|NP_836928.1| global DNA-binding transcriptional dual regulator H-NS [Shigella flexneri 2a str. 2457T]

MSEALKILNNIRTLRAQARECTLETLEEMLEKLEVVVNERREEESAAAAEVEERTRKLQQYREMLIADGI

DPNELLNSLAAVKSGTKAKRAQRPAKYSYVDENGETKTWTGQGRTPAVIKKAMDEQGKSLDDFLIKQ

>gi|30062756|ref|NP_836927.1| UTP--glucose-1-phosphate uridylyltransferase subunit GalU [Shigella flexneri 2a str. 2457T]

MAAINTKVKKAVIPVAGLGTRMLPATKAIPKEMLPLVDKPLIQYVVNECIAAGITEIVLVTHSSKNSIEN

HFDTSFELEAMLEKRVKRQLLDEVQSICPPHVTIMQVRQGLAKGLGHAVLCAHPVVGDEPVAVILPDVIL

DEYESDLSQDNLAEMIRRFDETGHSQIMVEPVADVTAYGVVDCKGVELAPGESVPMVGVVEKPKADVAPS

NLAIVGRYVLSADIWPLLAKTPPGAGDEIQLTDAIDMLIEKETVEAYHMKGKSHDCGNKLGYMQAFVEYG

IRHNTLGTEFKAWLEEEMGIKK

>gi|30062755|ref|NP_836926.1| response regulator of RpoS [Shigella flexneri 2a str. 2457T]

MTQPLVGKQILIVEDEQVFRSLLDSWFSSLGATTVLAADGVDALELLGGFTPDLMICDIAMPRMNGLKLL

EHIRNRGDQTPVLVISATENMADIAKALRLGVEDVLLKPVKDLNRLREMVFACLYPSMFNSRVEEEERLF

RDWDAMVDNPAAAAKLLQELQPPVQQVISHCRVNYRQLVAADKPGLVLDIAALSENDLAFYCLDVTRAGH

NGVLAALLLRALFNGLLQEQLAHQNQRLPELGALLKQVNHLLRQANLPGQFPLLVGYYHRELKNLILVSA

GLNATLNTGEHQVQISNGVPLGTLGNAYLNQLSQRCDAWQCQIWGTGGRLRLMLSAE

>gi|30062751|ref|NP_836922.1| nitrate reductase 1, cytochrome b(NR), gamma subunit [Shigella flexneri 2a str. 2457T]

MQFLNMFFFDIYPYIAGAVFLIGSWLRYDYGQYTWRAASSQMLDRKGMNLASNLFHIGILGIFVGHFFGM

LTPHWMYEAWLPIEVKQKMAMFAGGASGVLCLIGGVLLLKRRMFSPRVRATTTGADILILSLLVIQCALG

LLTIPFSAQHMDGSEMMKLVGWAQSVVTFHGGASQHLDGVAFIFRLHLVLGMTLFLLFPFSRLVHIWSVP

VEYLTRKYQLVRARH

>gi|30062750|ref|NP_836921.1| nitrate reductase 1, delta subunit [Shigella flexneri 2a str. 2457T]

MIELVIVSRLLEYPDAALWQHQQEMFEAIAASKNLSKEDAHALGIFLRDLTAMDPLDAQVQYSELFDRGR

ATSLLLFEHVHGESRDRGQAMVDLLAQYEQHGLQLNSRELPDHLPLYLEYLSQLPQSEAVEGLKDIAPIL

ALLSARLQQRESRYAVMFDLLLKLANTAIDSDKVAEKIADEARDDTPQALDAVWEEEQVKFFADKGCGDS

AITAHQRRFAGAVAPQYLNITTGGQH

>gi|30062749|ref|NP_836920.1| nitrate reductase 1, beta subunit [Shigella flexneri 2a str. 2457T]

MKIRSQVGMVLNLDKCIGCHTCSVTCKNVWTSREGVEYAWFNNVETKPGQGFPTDWENQEKYKGGWIRKI

NGKLQPRMGNRAMLLGKIFANPHLPGIDDYYEPFDFDYQNLHTAPEGSKSQPIARPRSLITGERMAKIEK

GPNWEDDLGGEFDKLAKDKNFDNIQKAMYSQFENTFMMYLPRLCEHCLNPACVATCPSGAIYKREEDGIV

LIDQDKCRGWRMCITGCPYKKIYFNWKSGKSEKCIFCYPRIEAGQPTVCSETCVGRIRYLGVLLYDADAI

ERAASTENEKDLYQRQLDVFLDPNDPKVIEQAIKDGIPLSVIEAAQQSPVYKMAMEWKLALPLHPEYRTL

PMVWYVPPLSPIQSAADAGELGSNGILPDVESLRIPVQYLANLLTAGDTKPVLRALKRMLAMRHYKRAET

VDGKVDTRALEEVGLTEAQAQEMYRYLAIANYEDRFVVPSSHRELAREAFPEKNGCGFTFGDGCHGSDTK

FNLFNSRRIDAIDVTSKTEPHP

>gi|30062748|ref|NP_836919.1| nitrate reductase 1 alpha subunit [Shigella flexneri 2a str. 2457T]

MSKFLDRFRYFKQKGETFADGHGQLLNTNRDWEDGYRQRWQHDKIVRSTHGVNCTGSCSWKIYVKNGLVT

WETQQTDYPRTRPDLPNHEPRGCPRGASYSWYLYSANRLKYPMMRKRLMKMWREAKALHSDPVEAWASII

EDADKAKSFKQARGRGGFVRSSWQEVNELIAASNVYTIKNYGPDRVAGFSPIPAMSMVSYASGARYLSLI

GGTCLSFYDWYCDLPPASPQTWGEQTDVPESADWYNSSYIIAWGSNVPQTRTPDAHFFTEVRYKGTKTVA

VTPDYAEIAKLCDLWLAPKQGTDAAMALAMGHVMLREFHLDNPSQYFTDYVRRYTDMPMLVMLEERDGYY

AAGRMLRAADLVDALGQENNPEWKTVAFNTNGEMVAPNGSIGFRWGEKGKWNLEQRDGKTGEETELQLSL

LGSQDEIAEVGFPYFGGDGTEHFNKVELENVLLHKLPVKRLQLADGSTALVTTVYDLTLANYGLERGLND

VNCATSYDDVKAYTPAWAEQITGVSRSQIIRIAREFADNADKTHGRSMIIVGAGLNHWYHLDMNYRGLIN

MLIFCGCVGQSGGGWAHYVGQEKLRPQTGWQPLAFALDWQRPARHMNSTSYFYNHSSQWRYETVTAEELL

SPMADKSRYTGHLIDFNVRAERMGWLPSAPQLGTNPLTIAGEAEKAGMNPVDYTVKSLKEGSIRFAAEQP

ENGKNHPRNLFIWRSNLLGSSGKGHEFMLKYLLGTEHGIQGKDLGQQGGVKPEEVDWQDNGLEGKLDLVV

TLDFRLSSTCLYSDIILPTATWYEKDDMNTSDMHPFIHPLSAAVDPAWEAKSDWEIYKAIAKKFSEVCVG

YLGKETDIVTLPIQHDSAAELAQPLDVKDWKKGECDLIPGKTAPHIMVVERDYPATYERFTSIGPLMEKI

GNGGKGIAWNTQSEMDLLRKLNYTKAEGPAKGQPMLNTAIDAAEMILTLAPETNGQVAVKAWAALSEFTG

RDHTHLALNKEDEKIRFRDIQAQPRKIISSPTWSGLEDEHVSYNAGYTNVHELIPWRTLSGRQQLYQDHQ

WMRDFGESLLVYRPPIDTRSVKEVIGQKSNGNPEKALNFLTPHQKWGIHSTYSDNLLMLTLGRGGPVVWL

SEADAKDLGIADNDWIEVFNSNGALTARAVVSQRVPAGMTMMYHAQERIVNLPGSEITQQRGGIHNSVTR

ITPKPTHMIGGYAHLAYGFNYYGTVGSNRDEFVVVRKMKNIDWLDGEGNDQVQESVK

>gi|30062747|ref|NP_836918.1| nitrate transport protein nark [Shigella flexneri 2a str. 2457T]

MSHSSASERATGAVITDWRPEDPAFWQQRGQRIASRNLWISVPCLLLAFCVWMLFSAVAVNLPKVGFNFT

TDQLFMLTALPSVSGALLRVPYSFMVPIFGGRRWTAFSTGILIIPCVWLGFAVQDTSTPYSVFIIISLLC

GFAGANFASSMANISFFFPKQKQGGALGLNGGLGNMGVSVMQLVAPLVVSLSIFAVFGSQGVKQPDGTEL

YLANASWIWVPFLAIFTIAAWFGMNDLATSKASIKEQLPVLKRGHLWIMSLLYLATFGSFIGFSAGFAML

SKTQFPDVQILQYAFFGPFIGALARSAGGALSDRLGGTRVTLVNFILMAIFSGLLFLTLPTDGQGGSFMA

FFAVFLALFLTAGLGSGSTFQMISVIFRKLTMDRVKAEGGSDERAMREAATGTAAALGFISAIGAIGGFF

IPKAFGSSLALTGSPVGAMKVFLIFYIACVVITWAVYGRHSKK

>gi|30062746|ref|NP_836917.1| nitrate/nitrite sensor protein NarX [Shigella flexneri 2a str. 2457T]

MLKRCLSPLTLVNQVALIVLLSTAIGLAGMAVSGWLVQGVQGSAHAINKAGSLRMQSYRLLAAVPLSEKD

KPLIKEMEQTAFSAELTRAAERDGQLAQLQGLQDYWRNELIPALMRAQNRETVSADVSQFVAGLDQLVSG

FDRTTEMRIETVVLVHRVMAVFMALLLVFTIIWLRARLLQPWRQLLAMASAVSHRDFTQRANISGRNEMA
MLGTALNNMSAELAESYAVLEQRVQEKTAGLEHKNQILSFLWQANRRLHSRAPLCERLSPVLNGLQNLTL
LRDIELRVYDTDDEENHQEFTCQPDMTCDDKGCQLCPRGVLPVGDRGTTLKWRLADSHTQYGILLATLPQ
GRHLSHDQQQLVDTLVEQLTATLALDRHQERQQQLIVMEERATIARELHDSIAQSLSCMKMQVSCLQMQG
DALPESSRELLSQIRNELNASWAQLRELLTTFRLQLTEPGLRPALEASCEEYSAKFGFPVKLDYQLPPRL
VPSHQAIHLLQIAREALSNALKHSQASEVVVTVAQNDNQVKLTVQDNGCGVPENAIRSNHYGMIIMRDRA
QSLRGDCRVRRRESGGTEVVVTFIPEKTFTDVQGDTHE

>gi|30062745|ref|NP_836916.1| transcriptional regulator NarL [Shigella flexneri 2a str. 2457T]
MSNQEPATILLIDDHPMLRTGVKQLISMAPDITVVGEASNGEQGIELAESLDPDLILLDLNMPGMNGLET
LDKLREKSLSGRIVVFSVSNHEEDVVTALKRGADGYLLKDMEPEDLLKALHQAAAGEMVLSEALTPVLAA
SLRANRATTERDVNQLTPRERDILKLIAQGLPNKMIARRLDITESTVKVHVKHMLKKMKLKSRVEAAVWV
HQERIF

>gi|30062744|ref|NP_836915.1| hypothetical protein S1307 [Shigella flexneri 2a str. 2457T]
MAPENHDGEKHFAEIVKDFGETSMNDNGLDTGEQAKAFAWGKVRDALSQQVNQHVESWLSPWGNASVDVK
VDNEGHFTGSRGSWFVPLQDNDRYLTWSQLGLTQQDNGLVSNVGVGQRWARGNWLVGYNTFYDNLQDENL
QRAGFGAEAWGEYLRLSANFYQPFAAWHEQTATQEQRMARGYDLTARMRMPFYQHLNTSVSLEQYFGDRV
DLFNSGTGYHNPVALSLGLNYTPVPLVTVTAQHKQGESGENQNNLGLNLNYRFGVPLKKQLSAGEVAESQ
SLRGSRYDNPQRNNLPTLEYRQRKTLTVFLATPPWDLKPGETVPLKLQIRSRYGIRQLIWQGDTQILSLT
PGAQANSAEGWTLIMPDWQNGEGASNHWRLSVVVEDNQGQRVSSNEITLTLVEPFDALSNDELRWEP

>gi|30062743|ref|NP_836914.1| hypothetical protein S1306 [Shigella flexneri 2a str. 2457T]
MQKIVIVANGAPYGSESLFNSLRLAIALREQESNLDLRLFLMSDAVTAGLRGQKPGEGYNIQQMLEILTA
QNVPVKLCKTCTDGRGISTLPLIDGVEIGTLVELAQWTLSADKVLTF

>gi|30062740|ref|NP_836911.1| calcium/sodium:proton antiporter [Shigella flexneri 2a str. 2457T]

MSNAQEAVKTRHKETSLIFPVLVLVVLFLWGSSQTLPVVIAINLLALIGILSSAFSVVRHADVLAHRLGE

PYGSLILSLSVVILEVSLISALMATGDAAPTLMRDTLYSIIMIVTGGLVGFSLLLGGRKFATQYMNLFGI

KQYLIALFPLAIIVLVFPMALPAANFSTGQALLVALISAAMYGVFLLIQAKTHQSLFVYEHEDDSDDDDP

HHGKTSAHSSLWHAIWLIIHLIAVIAVTKMNASSLETLLDSMNAPVAFTGFLVALLILSPEGLGALKAVL

NNQVQRAMNLFFGSVLATISLTVPVVTLIAFMTGNELQFALGAPEMVVMVASLVLCHISFSTGRTNVLNG

AAHLALFAAYLMTIFA

>gi|30062739|ref|NP_836910.1| 2-dehydro-3-deoxyphosphooctonate aldolase [Shigella flexneri 2a str. 2457T]

MKQKVVSIGDINVANDLPFVLFGGMNVLESRDLAMRICEHYVTVTQKLGIPYVFKASFDKANRSSIHSYR

GPGLEEGMKIFQELKQTFGVKIITDVHEPSQAQPVADVVDVIQLPAFLARQTDLVEAMAKTGAVINVKKP

QFVSPGQMGNIVDKFKEGGNEKVILCDRGANFGYDNLVVDMLGFSIMKKVSGNSPVIFDVTHALQCRDPF

GAASGGRRAQVAELARAGMAVGLAGLFIEAHPDPEHAKCDGPSALPLAKLEPFLKQMKAIDDLVKGFEEL

DTSK

>gi|30062737|ref|NP_836908.1| transcriptional regulator [Shigella flexneri 2a str. 2457T]

MTSFSTLLSVHLISIALSVGLLTLRFWLRYQKHPQAFARWTRIVPPVVDTVLLLSGIALMAKAHILPFSG

QAQWLTEKLFGVIIYIVLGFIALDYRRMHSQQARIIAFPLALVVLYIIIKLATTKVPLLG

>gi|30062734|ref|NP_836905.1| glutamyl-tRNA reductase [Shigella flexneri 2a str. 2457T]

MTLLALGINHKTAPVSLRERVSFSPDKLDQALDSLLAQPMVQGGVVLSTCNRTELYLSVEEQDNLQEALI

RWLCDYHNLNEEDLRKSLYWHQDNDAVSHLMRVASGLDSLVLGEPQILGQVKKAFVDSQKGHMKASELER

MFQKSFSVAKRVRTETDIGASAVSVAFAACTLARQIFESLSTVTVLLVGAGETIELVARHLREHKVQKMI

IANRTRERAQILADEVGAEVIALSEIDERLREADIIISSTASPLPIIGKGMVERALKSRRNQPMLLVDIA

VPRDVEPEVGKLANAYLYSVDDLQSIISHNLAQRKAAAVEAETIVAQETSEFMAWLRAQSASETIREYRS

QAEHVRDELTAKALAALEQGGDAQAIMQDLAWKLTNRLIHAPTKSLQQAARDGDNERLNILRDSLGLE

>gi|30062733|ref|NP_836904.1| outer membrane lipoprotein LolB [Shigella flexneri 2a str. 2457T]

MPLPDFRLIRLLPLAALVLTACSVTTPKGPGKSPDSPQWRQHQQDVRNLNQYQTRGAFAYISDQQKVYAR

FFWQQTGQDRYRLLLTNPLGSTELELNAQPGNVQLVDNKGQRYTADDAEEMIGKLTGMPIPLNSLRQWIL

GLPGDATDYKLDDQYRLSEITYSQNGKNWKVVYGGYDTKTQPAMPANMELTDGGQRIKLKMDNWIVK

>gi|30062732|ref|NP_836903.1| 4-diphosphocytidyl-2-C-methyl-D-erythritol kinase [Shigella flexneri 2a str. 2457T]

MRTQWPSPAKLNLFLYITGQRADGYHTLQTLFQFLDYGDTISIELRDDGDIRLLTPVEGVEHEDNLIVRA

ARLLMKTAADSGRLPTGSGANISIDKRLPMGGGLGGGSSNAATVLVALNHLWQCGLSMDELAEMGLTLGA

DVPVFVRGHAAFAEGVGEILTPVDPPEKWYLVAHPGVSIPTPVIFKDPELPRNTPKRSIETLLKCEFSND

CEVIARKRFREVDAVLSWLLEYAPSRLTGTGACVFAEFDTESEARQVLEQAPEWLNGFVAKGVNLSPLHR

AML

>gi|30062726|ref|NP_836897.1| adhesion and penetration protein [Shigella flexneri 2a str. 2457T]

MGIKQHNGNTKADRLAELKIRSPSIQLIKFGAIGLNAIIFSPLLIAADTGSQYGTNITINDGDRITGDTA

DPSGNLYGVMTPAGNTPGNINLGNDVTVNVNDASGYAKGIIIQGKNSSLTANRLTVDVVGQTSAIGINLI

GDYTHADLGTGSTIKSNDDGIIIGHSSTLTATQFTIENSNGIGLTINDYGTSVDLGSGSKIKTDGSTGVY

IGGLNGNNANGAARFTATDLTIDVQGYSAMGINVQKNSVVDLGTNSTIKTNGDNAHGLWSFGQVSANALT

VDVTGAAANGVEVRGGTTTIGADSHISSAQGGGLVTSSSDATINFSGTAAQRNSIFSGGSYGASAQTATA

VINMQNTDITVDRNGSLALGLWALSGGRITGDSLAITGAAGARGIYAMTNSQIDLTSDLVIDMSTPDQMA

IATQHDDGYAASRINASGRMLINGSVLSKGGLINLDMHPGSVWTGSSLSDNVNGGKLDVAMNNSVWNVTS

NSNLDTLALSHSTVDFASHGSTAGTFTTLNVENLSGNSTFIMRADVVGEGNGVNNRGDLLNISGSSAGNH

VLAIRNQGSEATTGNEVLTVVKTTDGAASFSASSQVELGGYLYDVRKNGTNWELYASGTVPEPTPNPEPT

PAPAQPPIVNPDPTPEPAPTPKPTTTADAGGNYLNVGYLLNYVENRTLMQRMGDLRNQSKDGNIWLRSYG

GSLDSFASGKLSGFDMGYSGIQFGGDKRLSDVMPLYVGLYIDSTHASPDYSGGDGTARSDYMGMYASYMA

QNGFYSDLVIKASRQKNSFHVLDSQNNGVNANGTANGMSISLEAGQRFNLSPTGYGFYIEPQTQLTYSHQ

NEMAMKASNGLNIHLNHYESLLGRASMILGYDITAGNSQLNVYVKTGAIREFSGDTEYLLNDSREKYSFK

GNGWNNGVGVSAQYNKQHTFYLEADYTQGNLFDQKQVNGGYRFSF

>gi|30062725|ref|NP_836896.1| DNA-binding transcriptional regulator DhaR [Shigella flexneri 2a str. 2457T]

MRDMSGAFNNDGRGISPLIATSWERCNKLMKRETWNVPHQAQGVTFASIYRRKKAMLTLGQAALEDAWEY

MAPRECALFILDETACILSRNGDPQTLQQLSALGFNDGTYCAEGIIGTCALSLAAISGQAVKTMADQHFK

QALWNWAFCATPLFDSKGRLTGTIALACPVEQTTAADLPLTLAIAREVGNLLLTDSLLAETNRHLNQLNA

LLESMDDGVISWDEQGNLQFINAQAARVLRLDATASQGRAITELLTLPAVLQQAIKLAHPLKHVEATFES

QHQFIDAVITLKPIIETQGTSFILLLHPVEQMRQLMTSQLGKVSHTFAHMPQDDPQTRRLIHFGRQAARS

SFPVLLCGEEGVGKALLSQAIHNESERAAGPYIAVNCELYGDAALAEEFIGGDRTDNENGRLSRLELAHG

GTLFLEKIEYLAVELQSALLQVIKQGVITRLDARRLIPIDVKVIATTTADLAMLVEQNRFSRQLYYALHA

FEITIPPLRMRRGSIPALVNNKLRSLEKRFSTRLKIDDDALARLVSCAWPGNDFELYSVIENLALSSDNG

RIRVSDLPEHLFTEQATDDVSATRLSTSLSFAEVEKEAIINAAQVTGGRIQEMSALLGIGRTTLWRKMKQ

HGIDAGQFKRRV

>gi|30062722|ref|NP_836893.1| dihydroxyacetone kinase subunit M [Shigella flexneri 2a str. 2457T]

MVNLVIVSHSSRLGEGVGELARQMLMSDSCKIAIAAGIDDPQNPIGTDAVKVMEAIESVADADHVLVMMD

MGSALLSAETALELLAPEIAAKVRLCAAPLVEGTLAATVSAASGADIDKVIFDAMHALEAKREQLGLPSS

DTEISDTCPPYDEEARSLAVVIKNRNGLHVRPASRLVYTLSTFNADMLLEKNGKCVTPESINQIALLQVR

YNDTLRLIAKGPEAEEALIAFRHLAEDNFGETEEVAPPTLRPVLPVSGKAFYYQPVLCTVQAKSTLTAEE

EQDRLRQAIDFTLLDLMTLTAKAEASGLDDIAAIFSGHHTLLGDPELLAAASELLQHEHCTAEYAWQQVL

KELSQQYQQLDDEYLQARYIDVDDLLHRTLVHLTQTKEELPQFNSPTILLAENIYPSTVLQLDPAVVKGI

CLSAGSPVSHSALIARELGIGWICQQGEKLYAIQPEETLTLDVKTQRFNRQG

>gi|30062718|ref|NP_836889.1| ABC transporter ATP-binding protein [Shigella flexneri 2a str. 2457T]

MPITRRTFAQALASTLLLQSLPSFSQTVNRFASQSLPEAQNITRIVSAGAPADLLLLAVAPEKMVGFSSF

DFARQALIPLPEHIRQLPRLGRLAGRASTLSLEGLMALHPDLVVDCGNTDETLISQARQVSEQTQIPWLL

LNGKLAQSAEQLTTLGKTLGEEHRAAEQANLASHFVGEAQAFATSPAANLRFYAARGPRGLETGLQGSLH

TEAAELLGLHNVAQIADRHGLTQVSMENLLRWQPDIILVQEAVTADFIRRDPLWQGVKAVAEQRILFLSG

LPFGWLDAPPGINRLLGLRRLHAWLDPAINRQFKSDMQHYAQLFWHCSLSDADYQKLVAS

>gi|30062717|ref|NP_836888.1| iron compound ABC transporter permease [Shigella flexneri 2a str. 2457T]

MRIVNGCILLLAAISITFAAVSGAYHLDMQQLLALILRQENESVQEQIVFWQIRVPRILAALLLGAALAG

AGTTYQGMLRNPLVSPDILGVSAGAGLGACAAILWGLPIVYIQLYAFCGGLMVVAGVWLITRRVTRHDPI

LTLVLVGIALGTLCGAGISLIKTLADPYTQLPSITFWLLGGLSTVTLRDLCYAAPIILIGSLPLFFLRWR

MNLLTLSDDEARSLGLNVTRLRFGLIVCATLITASTVAIAGIIGWIGLVVPHIARLLTGHNHQQLLPMAM

CTGAILLLLTDTLARSIGTTEIPLGILTAFVGAPFFLLLLLRGGRQ

>gi|30062715|ref|NP_836886.1| hypothetical protein S1277 [Shigella flexneri 2a str. 2457T]

MLIDDIDFADLYLQQLKLAHRTEKTPDHWDQRAEKMAENCASPTDSYLQQLIAKIDLQGAQTLFDMGCGP

GTVSLALADKLTTVYGVDYSQGMLNVAARRAAALKADNVHWIQRAWEEDWSDLPRCDIAVASRSTLVADM

RHAMSKLNNQARLRVYTTHLVSTSFVSPAIQRAAGREVIELPNYIFALNVLYQMGIYAHVDFIRGQNCQQ

DNSTWERFEQNVSWSLGALNDDERERLYHWYQQQDARALAPASRDWALIWWDSVPQEAL

>gi|30062712|ref|NP_836883.1| hypothetical protein S1272 [Shigella flexneri 2a str. 2457T]

MSHYHEQFLKQNPLAVLGVLRDLHKAAIPLRLSWNGGQLISKILAITPDKLVLDFGSQAEDNIAVLKAQH

ITITAETQGAKVEFTVEQLQQSEYLQLPAFITVPPPTLWFVQRRRYFRISAPLHPPYFCQTKLADNSTLR

FRLYDLSLGGMGALLETAKPAELQEGMRFAQIEVNMGQWGVFHFDAQLISTSERKVIDGKNETITTPRLS

FRFLNVSPTVERQLQRIIFSLEREAREKADKVRD

>gi|30062711|ref|NP_836882.1| murein transglycosylase E [Shigella flexneri 2a str. 2457T]

MKQRHGNSLLTTETKCISCRLGVPLSPQRRFQAIRIEEVKLRWFAFLIVLLAGCSSKHDYTNPPWNAKVP

VQRAMQWMPISQKAGAAWGVDPQLITAIIAIESGGNPNAVSKSNAIGLMQLKASTSGRDVYRRMGWSGEP

TTSELKNPERNISMGAAYLNILETGPLAGIEDPKVLQYALVVSYANGAGALLRTFSSDRKKAISKINDLD

ADEFLDHVARNHPAPQAPRYIYKLEQALDAM

>gi|30062710|ref|NP_836881.1| L,D-carboxypeptidase A [Shigella flexneri 2a str. 2457T]

MSLFHLIAPSGYSIKQHAALRGIQRLTDAGHQVNNVEVIARRCERFAGTETERLEDLNSLARLTTPNTIV

LAVRGGYGASRLLADIDWQALVARQQHDPLLICGHSDFTAIQCGLLAQGNVITFSGPMLVANFGADELNA

FTEHHFWLALRNETFTIEWQGEGPTCQTEGTLWGGNLAMLISLIGTPWMPKIENGILVLEDINVHPFRVE

RMLLQLYHAGILPRQKAIILGSFSGSTPNDYDAGYNLESVYAFLRSRLSIPLITGLDFGHEQRTVTLPLG

AHAILNNTREGAQLTISGHPVLKM

>gi|30062707|ref|NP_836878.1| alanine racemase [Shigella flexneri 2a str. 2457T]

MTRPIQASLDLQALKQNLSIVRQAAPHARVWSVVKANAYGHGIERIWSALGATDGFALLNLEEAITLRER

GWKGPILMLEGFFHAQDLEIYDQHRLTTCVHSNWQLKALQNARLKAPLDIYLKVNSGMNRLGFQSDRVLT

VWQQLRAMANVGEMTLMSHFAEAEHPDGISGAMARIEQAAEGLECRRSLSNSAATLWHPEAHFDWVRPGI

ILYGASPSGQWRDIANTGLRPVMTLSSEIIGVQTLKAGERVGYGGRYTARDEQRIGIVAAGYADGYPRHA

PTGAPVLVDGVRTMTVGTVSMDMLAVDLTPCPQAGIGTPVELWGKEIKIDDVAAAAGTVGYELMCALALR

VPVVTV

>gi|30062706|ref|NP_836877.1| D-amino acid dehydrogenase small subunit [Shigella flexneri 2a str. 2457T]

MRVVILGSGVVGVASAWYLNQAGHEVTVIDREPGAALETSAANAGQISPGYAAPWAAPGVPLKAIKWMFQ

RHAPLAVRLDGTQFQLKWMWQMLRNCDTSHYMENKGRMVRLAEYSRDCLKALRAETNIQYEGRQGGTLQL

FRTEQQYENATRDIAVLEDAGVPYQLLESSRLAEVDPALAEVAHKLTGGLQLPNDETGDCQLFTQNLARM

AEQAGVKFRFNTPVDQLLCDGEQIYGVKFGDEVIKADAYVMAFGSYSTAMLKGIVDIPVYPLKGYSLTIP

IAQEDGAPVSTILDETYKIAITRFDNRIRVGGMAEIVGFNTELLQPRRETLEMVVRDLYPRGGHVEQATF

WTGLRPMTPDGTPVVGSTRFKNLWLNTGHGTLGWTMACGSGQLLSDLLSGRTPAIPYEDLSVARYSRGFT

PSRPGHLHGAHS

>gi|30062705|ref|NP_836876.1| SpoVR family protein [Shigella flexneri 2a str. 2457T]

MATIDSMNKDTTRLSDGPDWTFDLLDVYLAEIDRVAKLYRLDTYPHQIEVITSEQMMDAYSSVGMPINYP

HWSFGKKFIETERLYKHGQQGLAYEIVINSNPCIAYLMEENTITMQALVMAHACYGHNSFFKNNCLFRSW

TDASSIVDYLIFARKYITECEERYGVDEVERLLDSCHALMNYGVDRYKRPQKISLQEEKARQKSREEYLQ

SQVNMLWRTLPKREEEKTVAEARRYPSEPQENLLYFMEKNAPLLESWQREILRIVRKVSQYFYPQKQTQV

MNEGCATFWHYTILNHLYDEGKVTERFMLEFLHSHTNVVFQPPYNSPWYSGINPYALGFAMFQDIKRICQ

SPTEEDKYWFPDIAGSDWLETLHFAMRDFKDESFISQFLSPKVMRDFRFFTVLDDDRHNYLEISAIHNEE

GYREIRNRLSSQYNLSNLEPNIQIWNVDLRGDRSLTLRYIPHNRAPLDRGRKEVLKHVHRLWGFDVMLEQ

QNEDGSIELLERCPPRMGNL

>gi|30062704|ref|NP_836875.1| fatty acid metabolism regulator [Shigella flexneri 2a str. 2457T]

MVIKAQSPAGFAEEYIIESIWNNRFPPGTILPAERELSELIGVTRTTLREVLQRLARDGWLTIQHGKPTK

VNNFWETSGLNILETLARLDHESVPQLIDNLLSVRTNISTIFIRTAFRQHPDKAQEVLATANEVADHADA

FAELDYNIFRGLAFASGNPIYGLILNGMKGLYTRIGRHYFANPEARSLALGFYHKLSALCSEGAHDQVYE

TVRRYGHESGEIWHRMQKNLPGDLAIQGR

>gi|30062703|ref|NP_836874.1| sodium/proton antiporter [Shigella flexneri 2a str. 2457T]

MEISWGRALWRNFLGQSPDWYKLALIIFLIVNPLIFLISPFVAGWLLVAEFIFTLAMALKCYPLLPGGLL

AIEAVFIGMTSAEHVREEVAANLEVLLLLMFMVAGIYFMKQLLLFIFTRLLLSIRSKMLLSLSFCVAAAF

LSAFLDALTVVAVVISVAVGFYGIYHRVASSRTEDTDLQDDSHIDKHYNVVLEQFRGFLRSLMMHAGVGT

ALGGVMTMVGEPQNLIIAKAAGWHFGDFFLRMSPVTVPVLICGLLTCLLVEKLRWFGYGETLPEKVREVL

QQFDDQSRHQRTRQDKIRLIVQAIIGVWLVTALALHLAEVGLIGLSVIILATSLTGVTDEHAIGKAFTES

LPFTALLTVFFSVVAVIIDQQLFSPIIQFVLQASEHAQLSLFYIFNGLLSSISDNVFVGTIYINEAKAAM

ESGAITLKQYELLAVAINTGTNLPSVATPNGQAAFLFLLTSALAPLIRLSYGRMVWMALPYTLVLTLVGL

LCVEFTLAPVTEWFMQMGWIATL

>gi|30062702|ref|NP_836873.1| disulfide bond formation protein B [Shigella flexneri 2a str. 2457T]

MLRFLNQCSQGRGAWLLMAFTALALELTALWFQHVMLLKPCVLCIYERCALFGVLGAALIGAIAPKTPLR

YVAMVIWLYSAFRGVQLTYEHTMLQLYPSPFATCDFMVRFPEWLPLDKWVPQVFVASGDCAERQWDFLGM

EMPQWLLGIFIAYLIVAVLVVISQPFKAKKRDLFGR

>gi|30062700|ref|NP_836871.1| DNA polymerase V subunit UmuD [Shigella flexneri 2a str. 2457T]

MLFIKPADLREIVTFPLFSDLVQCGFPSPAADYVEQRIDLNQLLIQHPSATYFVKASGDSMIDGGISDGD

LLIVDSAITASHGDIVIAAVDGEFTVKKLQLRPTVQLIPMNSAYSPITISSEDTLDVFGVVIHVVKAMR

>gi|30062697|ref|NP_836868.1| hypothetical protein S1256 [Shigella flexneri 2a str. 2457T]

MPKPGILKSKSMFCAIYRSSKRDQTYLYVEKKDDFSRVPEELMKGFGQPQLAMILPLDGRKKLVNADIEK

VKLALTEQGYYLQLPPPPEDLLKQHLSVMGQKTDDTNK

>gi|30062693|ref|NP_836864.1| septum formation inhibitor [Shigella flexneri 2a str. 2457T]

MSNTPIELKGSSFTLSVVHLHEAEPKVIHQALKDKIAQAPTFLKHAPVVLNVSALEDPVNWSAMHKAVSA

TGLRVIGVSGCKDAQLKAEIEKMGLPILTEGKEKAPRPAPAPQAPAQNTMPVTKTRLIDTPVRSGQRIYA

PQCDLIVTSHVSAGAELIADGNIHVYGMMRGRALAGASGDRETQIFCTNLMAELVSIAGEYWLSDQIPAE

FYGKAARLQLVENALTVQPLN

>gi|30062688|ref|NP_836859.1| hypothetical protein S1244 [Shigella flexneri 2a str. 2457T]

MMMKKFALLAGLFVFAPMTWAQDYNIKNGLPSETYITCAEANEMAKTDSAQVAEIVAVMGNASVASRDLK

IEQSPELSAKVVEKLNQVCAKDPQMLLITAIDDTMRAIGKK

>gi|30062687|ref|NP_836858.1| hypothetical protein S1242 [Shigella flexneri 2a str. 2457T]

MQGKNTIVTTGDYSIGLLSQTSGNLNTDTIIRVNSDGSVTPSFSDGDDTFIVTAGNHAVGVLACASPGSA

CACVSSLDEESTADTGSNENNAIAKLDMAKGEITTHGTESYAAYANGTVVKAGDTLDYTNASVTLTDVDI

TTHGDNAHAIAARQGTVSFNQGEIYTTGPDAAIAKIYNGGTVTLKNTSAVAHQGSGIVLESSINGQEATV

DILSGSSLRSANEILYHKDETSNVTITDSEVSSAADVFINNIKGHLTVDATNSKITGSANISTDDNTHTY

LSLSDNSTWDIKADSTVSNLTVDNSTVYISRADGRDVEPTRLTITENYVGNNGVLHLRTELDDDNSATDK

VVINGNTSGTTRVKVTNAGGSGAYTLNGIEIISVEGESNGEFIKDSRIFAGAYEYSLTRGNTEATNKNWY

LTNFQATSGGETNSGGSSAPTVAPTPVLRPEAGSYVANLAAANTLFVMRLNDRAGETRYIDPVTEQERSS

RLWLRQIGGHNAWRDSNGQLRTTSHRYVSQLGGDLLTGGFTDSDSWRLGVMAGYARDYNLTHSSVSDYRS

KGSVRGYSAGLYATWFADDISKKGAYIDSWAQYSWFKNSVKGDELAYESYSAKGATVSLEAGYGFALNKS

FGLEAAKYTWIFQPQAQAIWMGVDHNAHTEANGSRIENDANNNIQTRLGFRTFIRTQEKNSGPHGDDFEP

FVEMNWIHNSKDFAVSMNGVKVEQDGVSNLGEIKLGVNGNLNPAASVWGNVGVQLGDNGYNDTAVMVGLK

YKF

>gi|30062686|ref|NP_836857.1| hypothetical protein S1241 [Shigella flexneri 2a str. 2457T]

MRNTLIPILVAICLFITGVAILNIQLWYSAKAEYLAGARYAANNINHILEEASQATQTAVNIAGKECDLE

EQYQLGTEAALKPHLRTIIILKQGIVWCTSLPGNRVLLSRIPVVPDSNLLLAPAIDTVNRLPILLYQSQF

ADTRILVTISDQHIRGALNVPLKGVRYVLRVADDIIGPTADVMTLNGHYPYTEKVHSTKYHFTIIFNPPP

LFSFYRLIDKGFGILIFILLIASAAAFLLDRYFNKSATPEEILRRAINNGEIVPFYQPVVNGREGTLRGV

EVLARWKQPHGGYISPAAFIPLAEKSGLIVPLTQSLMNQVARQMNAIASKLPEGFHIGINFSASHIISPT

FVDECLNFRDSFTRRDLNLVLEVTEREPLNVDESLVQRLNILHENGFVIALDDFGTGYSGLSYLHDLHID

YIKIDHSFVGRVNADPESTRILDCVLDLARKLSISIVAEGVETKEQLGYLNQNNITFQQGYYFYKPVTYI

DLVKIILSKPKVKVVVE

>gi|30062683|ref|NP_836854.1| isocitrate dehydrogenase [Shigella flexneri 2a str. 2457T]

MESKVVVPAQGKKITLQNGKLNVPENPIIPYIEGDGIGVDVTPAMLKVVDAAVEKAYKGERKISWMEIYT

GEKSTQVYGQDVWLPAETLDLIREYRVAIKGPLTTPVGGGIRSLNVALRQELDLYICLRPVRYYQGTPSP

VKHPELTDMVIFRENSEDIYAGIEWKADSADAEKVIKFLREEMGVKKIRFPEHCGIGIKPCSEEGTKRLV

RAAIEYAIANDRDSVTLVHKGNIMKFTEGAFKDWGYQLAREEFGGELIDGGPWLKVKNPNTGKEIVIKDV

IADAFLQQILLRPAEYDVIACMNLNGDYISDALAAQVGGIGIAPGANIGDECALFEATHGTAPKYAGQDK

VNPGSIILSAEMMLRHMGWTEAADLIVKGMEGAINAKTVTYDFERLMEGAKLLKCSEFGDAIIKNM

>gi|30062679|ref|NP_836850.1| hypothetical protein S1234 [Shigella flexneri 2a str. 2457T]

MAKNYYDITLALAGICQSARLVQQLAHQGHCDADALHVSLNSIIDMNPSSTLAVFGGSEANLRVGLETLL

GVLNASSCQGLNAELTRYTLSLMVLERKLSSAKGALDTLGNRINGLQRQLEHFDLQSETLMSAMAAIYVD

VISPLGPRIQVTGSPAVLQSPQVQAKVRATLLAGIRAAVLWHQVGGGRLQLMFSRNRLTTQAKQILAHLT

PEL

>gi|30062677|ref|NP_836848.1| DNA-binding transcriptional regulator PhoP [Shigella flexneri 2a str. 2457T]

MRVLVVEDNALLRHHLKVQIQDAGHQVDDAEDAKEADYYLNEHLPDIAIVDLGLPDEDGLSLIRRWRSND

VSLPILVLTARESWQDKVEVLSAGADDYVTKPFHIEEVMARMQALMRRNSGLASQVISLPPFQVDLSRRE

LSINDEVIKLTAFEYTIMETLIRNNGKVVSKDSLMLQLYPDAELRESHTIDVLMGRLRKKIQAQYPQEVI

TTVRGQGYLFELR

>gi|30062676|ref|NP_836847.1| sensor protein PhoQ [Shigella flexneri 2a str. 2457T]

MKKLLRLFFPLSLRVRFLLATAAVVLVLSLAYGMVALIGYSVSFDKTTFRLLRGESNLFYTLAQWENNKL

HVELPENIDKQSPTMTLIYDENGQLLWAQRDVPWLMKMIQPDWLKSNGFHEIEADVNDTSLLLSGDHSIQ

QQLQEVREDDDDAEMTHSVAVNVYPATSRMPKLTIVVVDTIPVELKSSYMVWSWFIYVLSANLLLVIPLL

WVAAWWSLRPIEALAKEVRELEEHNRELLNPATTRELTSLVRNLNRLLKSERERYDKYRTTLTDLTHSLK

TPLAVLQSTLRSLRSEKMSVSDAEPVMLEQISRISQQIGYYLHRASMRGGTLLSRELHPVAPLLDNLTSA

LNKVYQRKGVNISLDISPEISFVGEQNDFVEVMGNVLDNACKYCLEFVEISARQTDEHLYIVVEDDGPGI

PLSKREVIFDRGQRVDTLRPGQGVGLAVAREITEQYEGKIVAGESMLGGARMEVIFGRQHSAPKDE

>gi|30062674|ref|NP_836845.1| terminase of prophage CP-933C [Shigella flexneri 2a str. 2457T]

MRGRPIELEPWQQFAFACILGFKVKATGRRKYTSAFIEVPRKNAKSTTAAILANWFLIMENGQQDIYTAA

VSRDQARIVFDNARQMCLLSRPLRRRVNIQAHKVIHPKSNSLLKPLAAKAATIEGTNPSLSIVDEYHLHP

DNGVYSALELGMGARPEGLLFAITTSGSNVVSACKQHYDYCCQILDGEEVNDSIFVLIYELDDENEVDDP

AMWIKANPNIDVSVDREKLASTIQKARGIPSQWVEMMTKRFNIWCQGTTPWMGNGAWAECAGTFTEEDLH

GQECYAGLDLSSTSDISSVCYAFPVGKTIMLISRHYLPEFQLQNPANKNRAVYRQWAKAGWIRTTPGDCI

DYDRIRDDIMQDAEKFNIRLVGFDTWNATHLRTQLQGAGFEVEPFPQTYLRFSPAAKSFEVFVNRRVIVH

RGDPVLSWSMSNVVMQSDANANIKPNKKKSPNKIDPSVAALMAFGTFQAEHEDFAFDISDSHRQKLEEFS

GV

>gi|30062673|ref|NP_836844.1| hypothetical protein S1227 [Shigella flexneri 2a str. 2457T]

MKQWREKSRQLAERGDLTPADWSNLELYCVNYSIYRKAVADLAARGFSIVNSQGGESRNPALSAKSDAER

VMIKMASLLGFDPISRRKNPPETEEEDELDRLE

>gi|30062670|ref|NP_836841.1| head-tail adaptor [Shigella flexneri 2a str. 2457T]

MNIGRLRDRITIQTLKQTRDITGEILETWEDGHTLWASVNMVSSKEAISSGAELAIGTVRIWIRYRKDIN

ATSRIKVSTGPLAGRVLNIIGQPLPDAARTRLEILCREGAEK

>gi|30062668|ref|NP_836839.1| head maturation protease of prophage CP-933C [Shigella flexneri 2a str. 2457T]

MGEKIMKSMEIRSSEITTSGAGTLTGYVVRWDKLSELLWGEFYEKFQRGAFTEWLAAGNDVRGLYEHDHS

MLLGRTRSGTLKLEEDETGLRFELTPPDTSTGRDVIELVKRGDISGMSFGFRSRKDVWDTTTDPCVRTVL

VAELYEITVTSVPAYPDSGVELARRSLYEQHPEKMPRADNRRWWADLAGV

>gi|30062665|ref|NP_836836.1| hypothetical protein S1217 [Shigella flexneri 2a str. 2457T]

MSEPKFGEKFYKHNGRITILQISAATPGWWVETDEGSSPVASWELCVLSYPDRDVYQDILPVISTDKGMK

PVDIKNMGFQCVMLTEKMMEEMKKNNAGSLH

>gi|30062663|ref|NP_836834.1| bacteriophage P4 DNA primase [Shigella flexneri 2a str. 2457T]

MKLAPNVKKQPRGIKHKDTEVIIFAGSDAWSHAKQWQEQDGPASGDNVPPVWLGPNQLAELDALKIVPDG

KKRVRLYQAGELDLVETKKIGQKLAAADIQDANFYPEGMHVQKCENWRRYLNAERENIAAGLTMPEQKNT

QLAQMADSERAQLLAERFDGVCVHQESEIVHVWRGGVWCPVSTMELSREMVVIYSEHRATFSKRVINNAV

EALKVIAEPMGEPSGDLLPFANGALDLKTGEFSPHTPENWITTHNGIEYTPPAPGENIRDNALNFHKWLE

HAAGKDQRKMMRICAALYMIMANRYDWQMFIEATGDGGSGKSTFTHIASLLAGKQNTVSAEMTSLDDAGG

RAQVVGSRLIVLADQPKYTGEGTGIKKITGGDPVEINPKYEKRFTAVIRAVVLATNNNPMIFTERAGGVA

RRRVIFRFDNIVSEAEKDRELPEKIAAEIPVIIRRLLANFTDSEKARALLLEQRDGDEALAIKQQTDPVI

EFCQFLNFLEEARGLMMGGGGDSVKYTTRNSLYRVYLAFMAYAGRSKPLNVNDFGKAMKPAAKVYGHEYI

TRKVKGVTQTNAITTDECDAFL

>gi|30062661|ref|NP_836832.1| hypothetical protein S1212 [Shigella flexneri 2a str. 2457T]

MCSDTLHTINTKSKAPHCRSSNGASNQQRKLGAVMVAVNHIPHLVHTQTAFVWRFLALSAGESQIIHVTA

WTEREARSRCPSGCVAVFAARIRQGETYAQ

>gi|30062660|ref|NP_836831.1| integrase of prophage CP-933C [Shigella flexneri 2a str. 2457T]

MSRALNKLSDTQLRKINGTPAQKTAFLNDGGNLSVRHSTSGLLTWYFTYRAGTGRGAPPERIKLGNYPDL

SLKSAREKAAQCRAWLAEGKNPRHELNYTVQEALKPVTVGDALTYWLESYAKENRVDYAALKKRLNNHVI

QHIGAMPLDKCELRHWLACFDQVAKRTPVTAGFLLQTCKQALKFCRRRRYAISNVLDDMSVADVGKKPDI

SERVLSTKELGELLQALDKKIFSPYYIALIRLLIVFGCRTVELRLSEISEWDFTEMLWTVPKEHSKTKVA

IFRPIPEAILPFVTQLVEQNRHTGLLLGEVKQETSVSQYGRLAHRRLNHPHWSLHDIRRTFTTMLNDLGV

DPHVVEQLTGHQMPGMQRVYNHSRYLDAKRNALDMWTERLGILAGTHENVTTLPVARRK

>gi|30062658|ref|NP_836829.1| peptidase T [Shigella flexneri 2a str. 2457T]

MDKLLERFLNYVSLDTQSKAGVRQVPSTEGQWKLLHLLKEQLEEMGLINVTLSEKGTLMATLPANVPGDI

PAIGFISHVDTSPDCSGKNVNPQIVENYRGGDIALGIGDEVLSPVMFPVLHQLLGQTLITTDGKTLLGAD

DKAGIAEIMTALAVLQQKNIPHGDIRVAFTPDEEVGKGAKHFDVDAFDARWAYTVDGGGVGELEFENFNA

ASVNIKIVGNNVHPGTAKGVMVNALSLAARIHAEVPADESPEMTEGYEGFYHLASMKGTVERADMHYIIR

DFDRKQFEARKRKMMEIAKKVGKGLHPDCYIELVIEDSYYNMREKVVEHPHILDIAQQAMRDCDIEPELK

PIRGGTDGAQLSFMGLPCPNLFTGGYNYHGKHEFVILEGMEKAVQVIVRIAELTAQRK

>gi|30062655|ref|NP_836826.1| spermidine/putrescine ABC transporter [Shigella flexneri 2a str. 2457T]

MIGRLLRGGFMTAIYAYLYIPIIILIVNSFNSSRFGINWQGFTTKWYSLLMNNDSLLQAAQHSLTMAVFS

ATFATLIGSLTAVALYRYRFRGKPFVSGMLFVVMMSPDIVMAISLLVLFMLLGIQLGFWSLLFSHITFCL

PFVVVTVYSRLKGFDVRMLEAAKDLGASEFTILRKIILPLAMPAVAAGWVLSFTLSMDDVVVSSFVTGPS

YEILPLKIYSMVKVGVSPEVNALATILLVLSLVMVIASQLIARDKTKGNTGDVK

>gi|30062654|ref|NP_836825.1| spermidine/putrescine ABC transporter substrate-binding protein [Shigella flexneri 2a str. 2457T]

MKKWSRHLLAAGALALGMSAAHADDNNTLYFYNWTEYVPPGLLEQFTKETGIKVIYSTYESNETMYAKLK

TYKDGAYDLVVPSTYYVDKMRKEGMIQKIDKSKLTNFSNLDPDMLNKPFDPNNDYSIPYIWGATAIGVNG

DAVDPKSVTSWADLWKPEYKGSLLLTDDAREVFQMALRKLGYSGNTTDPKEIEAAYNELKKLMPNVAAFN

SDNPANPYMEGEVNLGMIWNGSAFVARQAGTPIDVVWPKEGGIFWMDSLAIPANAKNKEGALKLINFLLR

PDVAKQVAETIGYPTPNLAARKLLSPEVANDKTLYPDAETIKNGEWQNDVGAASSIYEEYYQKLKAGR

>gi|30062651|ref|NP_836822.1| outer membrane-specific lipoprotein transporter subunit LolE [Shigella flexneri 2a str. 2457T]

MAMPLSLLIGLRFSRGRRRGGMVSLISVISTIGIALGVAVLIVGLSAMNGFERELNNRILAVVPHGEIEA

VNQPWTNWQEALDNVQKVPGIAAAAPYINFTGLVESGANLRAIQVKGVNPQQEQRLSALPSFVQGDAWRN

FKAGEQQIIIGKGVADALKVKQGDWVSIMIPNSNPEHKLMQPKRVRLHIAGILQLSGQLDHSFAMIPLAD

AQQYLDMGSSVSGIALKMTDVFNANKLVRDAGEVTNSYVYIKSWIGTYGYMYRDIQMIRAIMYLAMVLVI

GVACFNIVSTLVMAVKDKSGDIAVLRTLGAKDGLIRAIFVWYGLLAGLFGSLCGVVIGVVVSLQLTPIIE

WIEKLIGHQFLSSDIYFIDFLPSELHWLDVFYVLVTALLLSLLASWYPARRASNIDPARVLSGQ

>gi|30062649|ref|NP_836820.1| outer membrane-specific lipoprotein transporter subunit LolC [Shigella flexneri 2a str. 2457T]

MYQPVALFIGLRYMRGRAVDRFGRFVSWLSTIGITLGVMALVTVLSVMNGFERELQNNILGLMPQAILSS

EHGSLNPQQLPETAVKLDGVNRVAPITTGDVVLQSARSVAVGVMLGIDPAQKDPLTPYLVNVKQTDLEPG

KYNVILGEQLASQLGVNRGDQIRVMVPSASQFTPMGRIPSQRLFNVIGTFAANSEVDGYEMLVNIEDASR

LMRYPAANITGWRLWLDEPLRVDSLSQQKLPEGSKWQDWRDRKGELFQAVRMEKNMMGLLLSLIVAVAAF

NIITSLGLMVMEKQGEVAILQTQGLTPRQIMMVFMVQGASAGIIGAILGAALGALLASQLNNLMPIIGVL

LDGAALPVAIEPLQVIVIALVAMAIALLSTLYPSWRAAATQPAEALRYE

>gi|30062648|ref|NP_836819.1| hypothetical protein S1199 [Shigella flexneri 2a str. 2457T]

MKQKELWINQIKGLCICLVVIYHSVITFYPHMTTFQHPLSEVLSKCWIYFNLYLAPFRMPVFFFISGYLI

RRYIDSVPWGNCLDKRIWNIFWVLALWGVVQWLALSALNQWLAPERDLSNASNAAYADSTGEFLHGMITA

STSLWYLYALIVYFVVCKIFSRLALPLFALFVLLSVAVNFVPTPWWGMNSVIRNLPYYSLGAWFGATIMT

CVKEVPLRRHLLMASLLTVLAVGAWLFTISLLLSLVSIVVIMKLFYQYEQRFGMRSTSLLNVIGSNTIAI

YTTHRILVEIFSLTLLAQMNAARWSPQVELTLLLVYPFVSLFICTVAGLLVRKLSQRAFSDLLFSPPSLP

AAVSYSR

>gi|30062646|ref|NP_836817.1| hypothetical protein S1197 [Shigella flexneri 2a str. 2457T]

MMIKTRFSRWLTFFTFAAAVALALPAKANTWPLPPASSRLVGENKFHVVENDGGSLEAIAKKYNVGFLAL

LQANPGVDPYVPRAGSVLTIPLQTLLPDAPREGIVINIAELRLYYYPPGKNSVTVYPIGIGQLGGDTLTP

TMVTTVSDKRANPTWTPTANIRARYKAQGIELPAVVPAGPDNPMGHHAIRLAAYGGVYLLHGTNADFGIG

MRVSSGCIRLRDDDIKTLFSQVTPGTKVNIINTPIKVSAEPNGARLVEVHQPLSEKIDDDPQLLPITLNS

AMQSFKDAAQTDAEVMQHVMDVRSGMPVDVRRHQVSPQTL

>gi|30062644|ref|NP_836815.1| hypothetical protein S1195 [Shigella flexneri 2a str. 2457T]

MGSGLVNGGDYFYNNLSFTVTRYNGIMATDSTQCVKKSRGRPKVFDRDAALDKAMKLFWQHGYEATSLAD

LVEATGAKAPTLYAEFTNKEGLFRAVLDRYIDRFAAKHEAQLFCEEKSVESALADYFAAIANCFTSKDTP

AGCFMINNCTTLSPDSGDIANTLKSRHAMQERTLQQFLCQRQARGEIPTHCDVTHLAEFLNCIIQGMSIS

AREGASLEKLMQIAGTTLRLWPELVK

>gi|30062643|ref|NP_836814.1| hypothetical protein S1194 [Shigella flexneri 2a str. 2457T]

MNKSMLAGIGIGVAAALGVAAVASLNVFERGPQYAQVVSATPIKETVKTPRQECRNVTVTHRRPVQDENR

ITGSVLGAVAGGVIGHQFGGGRGKDVATVVGALGGGYAGNQIQGSLQESDTYTTTQQRCKTVYDKSEKML

GYDVTYKIGDQQGKIRMDRDPGTQIPLDSNGQLILNNKV

>gi|30062642|ref|NP_836813.1| respiratory NADH dehydrogenase [Shigella flexneri 2a str. 2457T]

MTTPLKKIVIVGGGAGGLEMATQLGHKLGRKKKAKITLVDRNHSHLWKPLLHEVATGSLDEGVDALSYLA
HARNHGFQFQLGSVIDIDREAKTITIAELRDEKGELLVPERKIAYDTLVMALGSTSNDFNTPGVKENCIF
LDNPHQARRFHQEMLNLFLKYSANLGANGKVNIAIVGGGATGVELSAELHNAIKQLHSYGYKGLTNEALN
VTLVEAGERILPALPPRISAAAHNELTKLGVRVLTQTMVTSADEGGLHTKDGEYIEADLMVWAAGIKAPD
FLKDIGGLETNRINQLVVEPTLQTTRDPDIYAIGDCASCPRPEGGFVPPRAQAAHQMATCAMNNILAQMN
GKPLKNYQYKDHGSLVSLSNFSTVGSLMGNLTRGSMMIEGRIARFVYISLYRMHQIALHGYFKTGLMMLV
GSINRVIRPRLKLH

>gi|30062641|ref|NP_836812.1| hypothetical protein S1192 [Shigella flexneri 2a str. 2457T]
MIIYLHGFDSNSPGNHEKVLQLQFIDPDVRLISYSTRHPKHDMQHLLKEVDKMLQLNVDERPLICGVGLG
GYWAERIGFLCDIRQVIFNPNLFPYENMEGKIDRPEEYADIATKCVTNFREKNRDRCLVILSRNDEALNS
QRTSEELHHYYEIVWDEEQTHKFKNISPHLQRIKAFKTLG

>gi|30062640|ref|NP_836811.1| beta-hexosaminidase [Shigella flexneri 2a str. 2457T]
MGPVMLDVEGYELDAEEREILAHPLVGGLILFTRNYHDPAQLRELVRQIRAASRNHLVVAVDQEGGRVQR
FREGFTRLPAAQSFAALLGMEEGGKLAQEAGWLMASEMIAMDIDISFAPVLDVGHISAAIGERSYHADPQ
KALAIASRFIDGMHEAGMKTTGKHFPGHGAVTADSHKETPCDPRPQAEIRAKDMSVFSSLIRENKLDAIM
PAHVIYSDVDPRPASGSPYWLKTVLRQELGFDGVIFSDDLSMEGAAIMGSYAERGQASLDAGCDMILVCN
NRKGAVSVLDNLSPIKAERVTRLYHKGSFSRQELMDSARWKAISTRLNQLHERWQEEKVGH

>gi|30062639|ref|NP_836810.1| thiamine kinase [Shigella flexneri 2a str. 2457T]
MPFRSNNPITRDELLSRFFPQFHPVTTFNSGLSGGSFLIEHQGQRFVVRQPHDPDAPQSAFLRQYRALSQ
LPASIAPKPHLYLRDWMVVDYLPGAVKTYLPDTNELAGLLYYLHQQPRFGWRITLLPLLELYWQQSDPAR
RTVGWLRMLKRLRKAREPRPLRLSPLHMDVHAGNLVHSASGLKLIDWEYAGDGDIALELAAVWVENTEQH
RQLVNDYATRAKIYPAQLWRQVRRWFPWLLMLKGGWFEYRWRQTGDQQFIRLADDTWRQLLIKQ

>gi|30062638|ref|NP_836809.1| hypothetical protein S1189 [Shigella flexneri 2a str. 2457T]

MTKMSRYALITALAMFLAGCVGQREPAPVEEVKPAPEQPAEPQQPVPTVPSVPTIPQQPGPIEHEDRTAP

PAPHIRHYDWNGAMQPMVSKMLGADGVTAGSVLLVDSVNNRTNGSLNAAEATETLRNALANNGKFTLVSA

QQLSMAKQQLGLSPQDSLGTRSKAIGIARNVGAHYVLYSCASGNVNAPTLQMQLMLVQTGEIIWSGKGAV

SQQ

>gi|30062637|ref|NP_836808.1| hypothetical protein S1188 [Shigella flexneri 2a str. 2457T]

MRKGCFGLVSLVLLLLVGCRSHPEIPVNDEQSLVMESSLLAAGISAEKPVLSTSDIQPSASSTIYNERQE

PVTVHYRFYWYDARGLEMHPLERPRSVTIPAHSAVTLYGSANFLGAHKVRLYLYL

>gi|30062635|ref|NP_836806.1| glucose-specific PTS system IIBC components [Shigella flexneri 2a str. 2457T]

MFKNAFANLQKVGKSLMLPVSVLPIAGILLGVGSANFSWLPAVVSHVMAEAGGSVFANMPLIFAIGVALG

FTNNDGVSALAAVVAYGIMVKTMAVVAPLVLHLPAEEIASKHLADTGVLGGIISGAIAAYMFNRFYRIKL

PEYLGFFAGKRFVPIISGLAAIFTGVVLSFIWPPIGSAIQTFSQWAAYQNPVVAFGIYGFIERCLVPFGL

HHIWNVPFQMQIGEYTNAAGQVFHGDIPRYMAGDPTAGKLSGGFLFKMYGLPAAAIAIWHSAKPENRAKV

GGIMISAALTSFLTGITEPIEFFFMFVAPILYIIHAILAGLAFPICILLGMRDGTSFSHGLIDFIVLSGN

SSKLWLLPIVGIGYAIVYYTIFRVLIKALDLKTPGREDATEDAKATGTSEMAPALVAAFGGKENITNLDA

CITRLRVSVADVSKVDQAGLKKLGAAGVVVAGSGVQAIFGTKSDNLKTEMDEYIRNH

>gi|30062633|ref|NP_836804.1| DNA polymerase III subunit delta' [Shigella flexneri 2a str. 2457T]

MRWYPWLRPDFEKLVASYQAGRGHHALLIQALPGMGDDALIYALSRYLLCQQPQGHKSCGHCRGCQLMQA

GTHPDYYTLAPEKGKNTLGIDAVREVTEKLNEHARLGGAKVVWVTDAALLTDAAANALLKTLEEPPAETW

FFLATREPERLLATLRSRCRLHYLAPPPEQYAVTWLSREVTMSQDALLAALRLSAGSPGAALALFQGDNW

QARETLCQALAYSVPSGDWYSLLAALNHEQAPARLHWLATLLMDALKRHHGAAQVTNVDVPGLVAELANH

LSPSRLQAILGDVCHIREQLMSVTGINRELLITDLLLRIEHYLQPGVVLPVPHL

>gi|30062631|ref|NP_836802.1| hypothetical protein S1181 [Shigella flexneri 2a str. 2457T]

MKKVLLIILLLLVVLGIAAGVGVWKVRHLADSKLLIKEETIFTLKPGTGRLALGEQLYADKIINRPRVFQ

WLLRIEPDLSHFKAGTYRFTPQMTVREMLKLLESGKEAQFPLRLVEGMRLSDYLKQLREAPYIKHTLSDD

KYATVAQALELENPEWIEGWFWPDTLMYTANTTDVALLKRVHKKMVKAVDSAWEGRADGLPYKDKNQLVT

MASIIEKETAVASERDQVASVFINRLRIGMRLQTDPTVIYGMGERYNGKLSRADLETPTAYNTYTITGLP

PGAIATPGADSLKAAAHPAKTPYLYFVADGKGGHTFNTNLASHNKSVQDYLKVLKEKNAQ

>gi|30062630|ref|NP_836801.1| 4-amino-4-deoxychorismate lyase [Shigella flexneri 2a str. 2457T]

MFLINGYKQESLAVSDRATQFGDGCFTTARVIDGKVSLLSAHILRLQDACQRLMISCDFWPQLEQEMKTL

AAEQQNGVLKVVISRGSGGRGYSTLNSGPATRILSVTAYPAHYDRLRNEGMTLALSPVRLGRNPHLAGIK

HLNRLEQVLIRSHLEQTNADEALVLDSEGWVTECCAANLFWRKGNVVYTPRLDQAGVNGIMRQFCIRLLA

QSSYQLVEVQASLEEALQADEMVICNALMPVMPVRACGDVTFSSATLYEYLAPLCERPN

>gi|30062625|ref|NP_836796.1| 3-oxoacyl-ACP synthase [Shigella flexneri 2a str. 2457T]

MYTKIIGTGSYLPEQVRTNADLEKMVDTSDEWIVTRTGIRERHIAAPNETVSTMGFEAATRAIEMAGIEK

DQIGLIVVATTSATHAFPSAACQIQSMLDIKGCPAFDVAAACAGFTYALSVADQYVKSGAVKYALVVGSD

VLARTCDPTDRGTIIIFGDGAGAAVLAASEEPGIISTHLHADGSYGELLTLPNADRVNPENSIHLTMAGN

EVFKVAVTELAHIVDETLAANNLDRSQLDWLVPHQANLRIISATAKKLGMSMDNVVVTLDRHGNTSAASV

PCALDEAVRDGRIKPGQLVLLEAFGGGFTWGSALVRF

>gi|30062622|ref|NP_836793.1| hypothetical protein S1172 [Shigella flexneri 2a str. 2457T]

MQKVKLPLTLDPVRTAQKRLDYQGIYTPDQVERVAESVVSVDSDVECSMSFAIDNQRLAVLNGDAKVTVT

LECQRCGKPFTHQVYTTYCFSPVRSDEQAEALPEAYEPIEVNEFGEIDLLAMVEDEIILALPVVPVHDSE

HCEVSEADMVFGELPEEVQKPNPFAVLASLKRK

>gi|30062619|ref|NP_836790.1| hypothetical protein S1169 [Shigella flexneri 2a str. 2457T]

MSSRVANLTVIVSSKRRRVSVARFSCGKTAQLSKKQTGYYSPEIFPSTGKDCNPQPANCLKDQYVLRHCC

VDDRSGKMGYSVKFLVLTRMDTETASLFHCKPCYSKMTFTIYHPLTHSFFTSCW

>gi|30062617|ref|NP_836788.1| flagellar rod assembly protein/muramidase FlgJ [Shigella flexneri 2a str. 2457T]

MISDSKLLASAAWDAQSLNELKAKASEDPAANIRPVARQVEGMFVQMMLKSMRDALPKDGLFSSEHTRLY

TSMYDQQIAQQMTAGKGLGLAEMMVKQMTPEQPLPEESTPAAPMKFPLETVVRYQNQTLSQLVQKAVPRN

YDDSLPGDSRAFLAQLSLPAQLASQQSGVPHHLILAQAALESGWGQRQIRRENGEPSYNLFGVKASGNWK

GQVTEITTTEYENGEAKKVKAKFRVYSSYLEALSDYVGLLTRNPRYAAVTTAASAEQGAQVLQDAGYATD

PHYARKLTNMIQQMKSISDKVSKTYSMNIDNLF

>gi|30062616|ref|NP_836787.1| flagellar basal body P-ring protein [Shigella flexneri 2a str. 2457T]

MIKFLSALILLLVTTAAQAERIRDLTSVQGVRQNSLIGYGLVVGLDGTGDQTTQTPFTTQTLNNMLSQLG

ITVPTGTNMQLKNVAAVMVTASLPPFGRQGQTIDVVVSSMGNAKSLRGGTLLMTPLKGVDSQVYALAQGN

ILVGGAGASAGGSSVQVNQLNGGRITNGAVIERELPSQFGVGNTLNLQLNDEDFSMAQQIADTINRVRGY

GSATALDARTIQVRVPSGNSSQVRFLADIQNMQVNVTPQDAKVVINSRTGSVVMNREVTLDSCAVAQGNL

SVTVNRQANVSQPDTPFGGGQTVVTPQTQIDLRQSGGSLQSVRSSASLNNVVRALNALGATPMDLMSILQ

SMQSAGCLRAKLEII

>gi|30062615|ref|NP_836786.1| flagellar basal body L-ring protein [Shigella flexneri 2a str. 2457T]

MQKNAAHTYAISSLLVLSLTGCAWIPSTPLVQGATSAQPVPGPTPVANGSIFQSAQPINYGYQPLFEDRR

PRNIGDTLTIVLQENVSASKSSSANASRDGKTNFGFDTVPRYLQGLFGNARADVEASGGNTFNGKGGANA

SNTFSGTLTVTVDQVLVNGNLHVVGEKQIAINQGTEFIRFSGVVNPRTISGSNTVPSTQVADARIEYVGN

GYINEAQNMGWLQRFFLNLSPM

>gi|30062614|ref|NP_836785.1| flagellar basal body rod protein FlgG [Shigella flexneri 2a str. 2457T]

MISSLWIAKTGLDAQQTNMDVIANNLANVSTNGFKRQRAVFEDLLYQTIRQPGAQSSEQTTLPSGLQIGT

GVRPVATERLHSQGNLSQTNNSKDVAIKGQGFFQVMLPDGSSAYIRDGSFQVDQNGQLVTAGGFQVQPAI

TIPANALSITIGRDGVVSVTQQGQAAPVQVGQLNLTTFMNDTGLESIGENLYTETQSSGAPNESTPGLNG

AGLLYQGYVETSNVNVAEELVNMIQVQRAYEINSKAVSTTDQMLQKLTQL

>gi|30062613|ref|NP_836784.1| flagellar hook protein FlgE [Shigella flexneri 2a str. 2457T]

MAFSQAVSGLNAAATNLDVIGNNIANSATYGFKSGTASFADMFAGSKVGLGVKVAGITQDFTDGTTTNTG

RGLDVAISQNGFFRLVDSNGSVFYSRNGQFKLDENRNLLNTQGLQLTGYPVTGTPPTIQQGANPTNISIP

NTLMAAKTTTTASMQINLNSSDPLPTVTPFSASNADSYNKKGSVTVFDSQGNAHDMSVYFVKTGDNNWQV

YTQDSSDPNSIAKTATTLEFNANGTLVDGAMANNIATGAINGAEPATFSLSFLNSMQQNTGANNIVATTQ

NGYKPGDLVSYQINDDGTVVGNNSNEQTQLLGQIVLANFANNEGLASEGDNVWSATQSSGVALLGTAGTG

NFGTLTNGALEASNVDLSKELVNMIVAQRNYKSNAQTIKTQDQILNTRVNLR

>gi|30062612|ref|NP_836783.1| flagellar basal body rod modification protein [Shigella flexneri 2a str. 2457T]

MSIAVTTTDPTNTGVSTTSSSSLTGSNAADLQSSFLTLLVAQLKNQDPTNPMENNELTSQLAQISTVSGI

EKLNTTLGSISGQIDNSQSLQASNLIGHGVMIPGTTVLAGTGSEEGAVTTTTPFGVELQQAADKVTATIT

DKNGAVVRTIDIGELTAGVHSFTWDGSLTDGSTAPNGSYNVAISASNGGTQLVAQPLQFALVQGVIRGNS

GNTLDLGTYGTTTLDEVRQII

>gi|30062611|ref|NP_836782.1| flagellar basal body rod protein FlgB [Shigella flexneri 2a str. 2457T]

MLDKLDAALRFQQEALNLRAQRQEVLAANIANADTPGYQARDIDFASELKKVMQRGRDATSVVALTMTST

QHIPAQALTPPTAELQYRIPDQPSLDGNTVDMDRERTQFADNSLQYQMSLSALSGQIKGMMNVLQSGN

>gi|30062610|ref|NP_836781.1| flagellar basal body P-ring biosynthesis protein FlgA [Shigella flexneri 2a str. 2457T]

MLTIKRSVAIIAILFSPLSTASNLTSQLHNFFSAQLAGVSDEVRVSIRTAPNLLPPCEQPLLSMSNNSRL

WGNVNVLARCGNDKRYLQVNVQATGNYVVAAMPIVRGGKLEAGNVKLKRGRLDTLPPRTVLDINQLVDAV

SLRDLSPDQPIQLTQFRQAWRVKAGQRVNVIASGDGFSANAEGQALNNAAVAQNARVRMVSEQVVSGVVD

ADGNILINL

>gi|30062608|ref|NP_836779.1| flagella synthesis protein FlgN [Shigella flexneri 2a str. 2457T]

MTRLAEILDQMSAVLNDLKTVMDQEQQHLSMGQINGSQLQWITEQKSSLLATLDYLEQLRKKEPNTANSV

DISQRWQEITVKTQQLRQMNQHNGWLLEGQIERNQQALEMLKPHQEPTLYGANGQTSTPHRGSKKISI

>gi|30062607|ref|NP_836778.1| virulence factor [Shigella flexneri 2a str. 2457T]

MNLLKSLAAVSSMTMFSRVLGFARDAIVARIFGAGMATDAFFVAFKLPNLLRRIFAEGAFSQAFVPILAE

YKSKQGEDATRVFVSYVSGLLTLALAVVTVAGMLAAPWVIMVTAPGFADTADKFALTSQLLKITFPYILL

ISLASLVGAILNTWNRFSIPAFAPTLLNISMIGFALFAAPYFNPPVLALAWAVTVGGILQLVYQLPHLKK

IGMLVLPRINFHDAGAMRVVKQMGPAILGVSVSQISLIINTIFASFLASGSVSWMYYADRLMEFPSGVLG

VALGTILLPSLSKSFASGNHDEYNRLMDWGLRLCFLLALPSAVALGILSGPLTVSLFQYGKFTAFDALMT

QRALIAYSVGLIGLIVVKVLAPGFYSRQDIKTPVKIAIVTLILTQLMNLAFIGPLKHAGLSLSIGLAACL

NASLLYWQLRKQKIFTPQPGWMAFLLRLVVAVLVMSGVLLGMLHIMPEWSLGTMPWRLLRLMAVVLAGIA

AYFAALAVLGFKVKEFARRTV

>gi|30062606|ref|NP_836777.1| virulence factor [Shigella flexneri 2a str. 2457T]

MKKLRIGVVGLGGIAQKAWLPVLAAASDWTLQGAWSPTRAKALPICDSWRIPYADSLSSLAASCDAVFVH

SSTASHFDVVSTLLNAGVHVCVDKPLAENLRDAERLVELAARKKLTLMVGFNRRFAPLYGELKTQLATAA

SLRMDKHRSNSVGPHDLYFTLLDDYLHVVDTALWLSGGKASLDGGTLLTNDAGEMLFAEHHFSAGPLQIT

TCMHRRAGSQRETVQAVTDGALIDITDMREWREERGQGVVNKPIPGWQSTLEQRGFVGCARHFIECV

>gi|30062605|ref|NP_836776.1| hypothetical protein S1151 [Shigella flexneri 2a str. 2457T]

MKYQLTALEARVIGCLLEKQVTTPEQYPLSVNGVVTACNQKTNREPVMNLSESEVQEQLDNLVKRHYLRT

VSGFGNRVTKYEQRFCNSEFGDLKLSAAEVALITTLLLRGAQTPGELRSRAARMYEFSDMAEVESTLEQL

ANREDGPFVVRLAREPGKRESRYMHLFSGEVEDQPAVTAMSNMVDGDLQTRVEALEIEVAELKQRLDSLL

AHLGD

>gi|30062604|ref|NP_836775.1| ribosomal-protein-S5-alanine N-acetyltransferase [Shigella flexneri 2a str. 2457T]

MFGYRSNVPKVRLTTDRLVVRLVHDRDAWRLADYYAENRHFLKPWEPVRDESHCYPSGWQARLGMINEFH

KQGSAFYFGLFDPDEKEIIGVANFSNVVRGSFHACYLGYSIGQKWQGKGLMFEALTAAIRYMQRTQHIHR

IMANYMPHNKRSGDLLARLGFEKEGYAKDYLLIDGQWRDHVLTALTTPDWTPGR

>gi|30062603|ref|NP_836774.1| multidrug resistance protein MdtH [Shigella flexneri 2a str. 2457T]

MSRVSQARNLGKYFLLIDNMLVVLGFFVVFPLISIRFVDQMGWAAVMVGIALGLRQFIQQGLGIFGGAIA

DRFGAKPMIVTGMLMRAAGFATMGIAHEPWLLWFSCLLSGLGGTLFDPPRSALVVKLIRPQQRGRFFSLL

MMQDSASAVIGALLGSWLLQYDFRLVCATGAVLFVLCAAFNAWLLPAWKLSTVRTPVREGMTRVMRDKRF

VTYVLTLAGYYMLAVQVMLMLPIMVNDVAGAPSAVKWMYAIEACLSLTLLYPIARWSEKHFRLEHRLMAG

LLIMSLSMMPVGMVSGLQQLFTLICLFYIGSIIAEPARETLSASLADARARGSYMGFSRLGLAIGGAIGY

IGGGWLFDLGKSAHQPELPWMMLGIIGIFTFLALGWQFSQKRAARRLLERDA

>gi|30062602|ref|NP_836773.1| glutaredoxin [Shigella flexneri 2a str. 2457T]

MKLYIYDHCPYCLKARMIFGLKNIPVELHVLLNDDAETPTRMVGQKQVPILQKDDSRYMPESMDIVHYVD

KLDGKPLLTGKRSPAIEEWLRKVNGYVNKLLLPRFAKSAFDEFSTPAARKYFVDKKEASAGNFADLLAHS

DGLIKNISDDLRALDKLIVKPNAVNGELSEDDIQLFPLLRNLTLVAGINWPSRVANYRDNMAKQTQINLL

SSMAI

>gi|30062601|ref|NP_836772.1| hypothetical protein S1147 [Shigella flexneri 2a str. 2457T]

MNKFLFAAALIVSGLLVGCNQLTQYTITEQEINQSLAKHNNFSKDIGLPGVADAHIVLTNLTSQIGREEP

NKVTLTGDANLDMNSLFGSQKATMKLKLKALPVFDKEKGAIFLKEMEVVDATVQPEKMQTVMQTLLPYLN

QALRNYFNQQPAYVLREDGSQGEAMAKKLAKGIEVKPGEIVIPFTD

>gi|30062600|ref|NP_836771.1| dihydroorotase [Shigella flexneri 2a str. 2457T]

MTAPSQVLKIRRPDDWHLHLRDGDMLKTVVPYTSEIYGRAIVMPNLAPPVTTVEAAVAYRQRILDAVPAG

HDFTPLMTCYLTDSLDPNELERGFNEAVFTAAKLYPANATTNSSHGVTSIDAIMPVLERMEKIGMPLLVH

GEVTHADIDIFDREARFIESVMEPLRQRLTALKVVFEHITTKDAADYVRDGNERLAATITPQHLMFNRNH

MLVGGVRPHLYCLPILKRNIHQQALRELVASGFNRVFLGTDSAPHARHRKESSCGCAGCFNAPTALGSYA

TVFEEMNALQHFEAFCSVNGPQFYGLPVNDTFIELVREEQQVAESIALTDDTLVPFLAGETVRWSVKQ

>gi|30062595|ref|NP_836766.1| cytochrome [Shigella flexneri 2a str. 2457T]

MSFTNTPERYGVISAAFHWLSAIIVYGMFALGLWMVTLSYYDGWYHKAPELHKSIGILLMMGLVIRVLWR

VISPPPGPLPSYSPMTRLAAKAGHLALCLLLFAIGISGYLISTADGKQISVFGWFDVPATLADAGAQADF

AGALHFWLAWSVVVLSVMHGFMALKHHFIDKDDTLKRMLGKSSSDYGV

>gi|30062594|ref|NP_836765.1| hypothetical protein S1140 [Shigella flexneri 2a str. 2457T]

MKKSLLGLTFASLMFSAGSAVAADYKIDKEGQHAFVNFRIQHLGYSWLYGTFKDFDGTFTFDEKNPAADK

VNVTINTTSVDTNHAERDKHLRSADFLNTTKYPQATFTSTSVKKDGDELDITGDLTLNGVTKPVTLEAKL

IGQGDDPWGGKRAGFEAEGKIKLKDFNIKTDLGPASQEVDLIISVEGVQQK

>gi|30062592|ref|NP_836763.1| lipid A biosynthesis lauroyl acyltransferase [Shigella flexneri 2a str. 2457T]

MTNLPKFSTALLHPRYWLTWLGIGVLWLVVQLPYPIIYRLGCGLGKLALRFMKRRAKIVHRNLELCFPEM

SEQERRKMVVKNFESVGMGLMETGMAWFWPDRRIARWTEVIGMEHIRDVQAQKRGILLVGIHFLTLELGA

RQFGMQEPGIGVYRPNDNPLIDWLQTWGRLRSNKSMLDRKDLKGMIKALKKGEVVWYAPDHDYGPRSSVF

VPLFAVEQAATTTGTWMLARMSGACLVPFVPRRKPDGKGYQLIMLPPECSPPLDDAETTAAWMNKVVEKC

IMMAPEQYMWLHRRFKTRPEGVPSRY

>gi|30062591|ref|NP_836762.1| hypothetical protein S1136 [Shigella flexneri 2a str. 2457T]

MRRAISYIRFSSERQLKGDSVRRQSKLVTDWLDKNPEFYLDSSLSFKDLGKSAFSGKHLKGGLGDFLTAI

EKGLVKAGDTLLIESLDRLSRQDIDIASELLRRILRAGVDVVTLSDGEHYTRESLKDPLALIKSILIMQR

AHEESLRKSERVQAAWNRKKELISEGIKVSRRCPAWLRLNDDRRTFTIIPDKVEVVKRAFDLRLQGLSFW

AITRTLNDEGHLSLNQYTPKQKGWSDTAVKKLLRNRAVIGCFTPAGREEVQGYYPAIISESLFYRVQQLN

TGQYGRASVSSNPLSVNLFRGIIKCEVYWQ

>gi|30062577|ref|NP_836748.1| hypothetical protein S4815 [Shigella flexneri 2a str. 2457T]

MTLPVFITVIADHDKPQPSGCLLDNLSSAAVKTAGVLISFQAVLSAYQRVMEVGLQRASAERSIDFVFSK

DANQVKDFTKSLAQTTGLDIAELQSQFAGFGASARESLGIQGSEELFRNMIGYSRLMGRSEEEIKRALTA

LSQMAGKGQIMAEELKGQLAEAVPGMVQVFAKATGKTEQELFDAMKKGALKSADTLQKVTQELNKQITAK

GGWKAISESTQAQLGNLKNSWNTTLDSIFRGSENGLQDFTRSLTNLLNALGGSGKSLGESLGSLMTSMSH

GVDSLTDISYRVRAFFDEVTLAYRPLNDTQRKIVDGLSDGLIDGLKMLAVAVTAQKAANVAGGLLNLANA

VTSFGDAVNKAKAGKAGSSGGALNKVGRVVLYAELVNFALDNLNPEAAKNPNLHQSGIGTETNFNPEYKG

DSSKPIVLSLTGLMVQLIKLLTSKNR

>gi|30062576|ref|NP_836747.1| hypothetical protein S4816 [Shigella flexneri 2a str. 2457T]

MVCNYRYKNRQCHCLSGGYMARSAKPRKRKPASQRSKLPRYVVKLHEDDFFDEEDAEVLRFDSFDDAVEC

CADLNIPFFVDAGNKKLVFWFVRVDDEGYPEIARCTEREFATILAGISAGGMYCPECGTVHWPDGVAPPF

>gi|30062574|ref|NP_836745.1| glucosyltransferase MdoH [Shigella flexneri 2a str. 2457T]

MNKTTEYIDAMPIAASEKAALPKTDIRAVHQALDAEHRTWAREDDSPQGSVKARLEQAWPDSLADGQLIK

DDEGRDQLKAMPEAKRSSMFPDPWRTNPVGRFWDRLRGRDVTPRYLARLTKEEQESEQKWRTVGTIRRYI

LLILTLAQTVVATWYMKTILPYQGWALINPMDMVGQDLWVSFMQLLPYMLQTGILILFAVLFCWVSAGFW

TALMGFLQLLIGRDKYSISASTVGDEPLNPEHRTALIMPICNEDVNRVFAGLRATWESVKATGNAKHFDV

YILSDSYNPDICVAEQKAWMELIAEVGGEGQIFYRRRRRRVKRKSGNIDDFCRRWGSQYSYMVVLDADSV

MTGDCLCGLVRLMEANPNAGIIQSSPKASGMDTLYARCQQFATRVYGPLFTAGLHFWQLGESHYWGHNAI

IRVKPFIEHCALAPLPGEGSFAGSILSHDFVEAALMRRAGWGVWIAYDLPGSYEELPPNLLDELKRDRRW

CHGNLMNFRLFLVKGMHPVHRAVFLTGVMSYLSAPLWFMFLALSTALQVVHALTEPQYFLQPRQLFPVWP

QWRPELAIALFASTMVLLFLPKLLSILLIWCKGTKEYGGFWRVTLSLLLEVLFSVLLAPVRMLFHTVFVV

SAFLGWEVVWNSPQRDDDSTSWGEAFKRHGSQLLLGLVWAVGMAWLDLRFLFWLAPIVFSLILSPFVSVI

SSRATVGLRTKRWKLFLIPEEYSPPQVLVDTDRFLEMNRQRSLDDGFMHAVFNPSFNALATAMATARHRA

SKVLEIARDRHVEQALNETPEKLNRDRRLVLLSDPVTMARLHFRVWNSPERYSSWVSYYEGIKLNPLALR

KPDAASQ

>gi|30062573|ref|NP_836744.1| glucan biosynthesis protein G [Shigella flexneri 2a str. 2457T]

MMKMRWLSAAVMLTLYTSSSWAFSIDDVAKQAQSLAGKGYEAPKSNLPSVFRDMKYADYQQIQFNHDKAY

WNNLKTPFKLEFYHQGMYFDTPVKINEVTATAVKRIKYSPDYFTFGDVQHDKDTVKDLGFAGFKVLYPIN

SKDKNDEIVSMLGASYFRVIGAGQVYGLSARGLAIDTALPSGEEFPRFKEFWIERPKPTDKRLTIYALLD

SPRATGAYKFVVMPGRDTVVDVQSKIYLRDKVGKLGVAPLTSMFLFGPNQPSPANNYRPELHDSNGLSIH

AGNGEWIWRPLNNPKHLAVSSFSMENPRGFGLLQRGRDFSRFEDLDDRYDLRPSAWVTPKGEWGKGSVEL

VEIPTNDETNDNIVAYWTPDQLPEPGKEMNFKYTITFSRDEDKLHAPDNAWVQQTRRSTGDVKQSNLIRQ

PDGTIAFVVDFTGAEMKKLPEDTPVTAQTSIGDNGEIVESTVRYNPVTKGWRLVMRVKVKDAKKITEMRA

ALVNADQTLSETWSYQLPANE

>gi|30062572|ref|NP_836743.1| glucans biosynthesis protein [Shigella flexneri 2a str. 2457T]

MNPVPAQREYFLDSIRAWLMLLGIPFHISLIYSSHTWHVNSAEPSLWLTLFNDFIHSFRMQVFFVISGYF

SYMLFLRYPLKKWWKVRVERVGIPMLTAIPLLTLPQFIMLQYVKGKAESWPGLSLYDKYNTLAWELISHL

WFLLVLVVMTTLCVWIFKRIRNNLENSDKTNKKFSMVKLSVIFLCLGIGYAVIRRTIFIVYPPILSNGMF

NFIVMQTLFYLPFFILGALAFIFPHLKALFTTPSRGCTLAAALAFVAYLLNQRYGSGDAWMYETESVITM

VLGLWMVNVVFSFGHRLLNFQSARVTYFVNASLFIYLVHHPLTLFFGAYITPHITSNWLGFLCGLIFVVG

IAIILYEIHLRIPLLKFLFSGKPVVKRENDKAPAR

>gi|30062571|ref|NP_836742.1| synthase [Shigella flexneri 2a str. 2457T]

MMKKTPTSTKDSLPNKEMNDLPRLASAVLPLCSQHPGQCGLFPLEKSLDAFAARYRLAEMAEHTLDVQYY

IWQDDMSGRLLFSALLAAAKRGVRVRLLLDDNNTPGLDDILRLLDSHPRIEVRLFNPFSFRLLRPLGYIT

DFSRLNRRMHNKSFTVDGVVTLVGGRNIGDAYFGAGEEPLFSDLDVMAIGPVVEDVADDFARYWYCKSVS

PLQQVLDVPEGEMADRIELPASWHNDAMTHRYLRKMESSPFINHLVDGTLPLIWAKTRLLSDDPAKGEGK

AKRHSLLPQRLFDIMGSPSERIDIISSYFVPTRAGVAQLLRMVRKGVKIAILTNSLAANDVAVVHAGYAR

WRKKLLRYGVELYELKPTREQSSTLHDRGITGNSGASLHAKTFSIDGKTVFIGSFNFDPRSTLLNTEMGF

VIESETLAQLIDKRFIQSQYDAAWQLRLDRWGRINWVDRHAKKEVILKKEPATSFWKRVMVRLASILPVE

WLL

>gi|30062569|ref|NP_836740.1| hypothetical protein S1114 [Shigella flexneri 2a str. 2457T]

MFRPFLDSLMLGSLFFPFIAIAGSTAQGGVIHFYGQIVEPACDVSTQSSPVEMNCPQNGSIPGKTYSSKA

LMSGNVKNAQIASVKVQYLDKQKKLAVMNIEYN

>gi|30062568|ref|NP_836739.1| autoagglutination protein [Shigella flexneri 2a str. 2457T]

MNALLLLAALSSQITFNTTQQGDMYTIIPEVTLTQSCLCRVQILSLREGSSGQSQTKQEKTLSLPANQPI

ALTKLSLNISPEDRVKIVVTVSDGQALHLSQQWTPSSEKS

>gi|30062565|ref|NP_836736.1| curlin minor subunit [Shigella flexneri 2a str. 2457T]

MKNKLLFMMLTILGAPGIAAAAGYDLANSEYNFAVNELSKSSFNQAAIIGQAGTNNSAQLRQGGSKLLAV

VAQEGSSNRAKIDQTGDYNLAYIDQAGSANDASISQGAYGNTAMIIQKGSGNKANITQYGTQKTAVVVQR

QSQMAIRVTQR

>gi|30062564|ref|NP_836735.1| DNA-binding transcriptional regulator CsgD [Shigella flexneri 2a str. 2457T]

MFNEVHSIHGHTLLLITKPSLQATALLQHLKQSLAITGKLHNIQRSLDDISSGSIILLDMMEADKKLIHY

WQDTLSRKNNNIKILLLNTPEDYPYRDIENWPHINGVFYAMEDQERVVNGLQGVLRGECYFTQKLASYLI

THSGNYRYNSTESALLTHREKEILNKLRIGASNNEIARSLFISENTVKTHLYNLFKKIAVKNRTQAVSWA

NDNLRR

>gi|30062563|ref|NP_836734.1| curli assembly protein CsgE [Shigella flexneri 2a str. 2457T]

MKRYLRWIVAAEFLFAAGNLHAVEVEVPGLLTDHTVSSIGHDFYRAFSDKWESDYTGNLTINERPSARWG

SWITITVNQDVIFQTFLFPLKRDSEKTVVFALIQTEEALNRRQINQALLSTDDLAHDEF

>gi|30062562|ref|NP_836733.1| curli assembly protein CsgF [Shigella flexneri 2a str. 2457T]

MRVKHAVVLLMLISPLSWAGTMTFQFRNPNFGGNPNNGAFLLNSAQAQNSYKDPSYNDDFGIETPSALDN

FTQAIQSQILGGLLSNINTGKPGRMVTNDYIVDIANRDGQLQLNVTDRKTGQTSTIQVSGLQNNSTDF

>gi|30062561|ref|NP_836732.1| hypothetical protein S1103 [Shigella flexneri 2a str. 2457T]

MAAFSAIMRGMNILLSIAITTGILSGIWGWVAVSLGLLSWAGFLGCTAYFACPQGGLKGLAISAATLLSG

VVWAMVIIYGSALAPHLEILGYVITGIVAFLMCIQAKQLLLSFVPGTFIGACATFAGQGDWKLVLPSLAL

GLVFGYAMKNSGLWLAARSAKTAHREQEIKNKA

>gi|30062560|ref|NP_836731.1| oxidoreductase component [Shigella flexneri 2a str. 2457T]

MNEFSILCRVLGSLYYRQPQDPLLVPLFTLIREGKLAANWPLEQDELLTRLQKSCDMTQVSADYNALFIG

DECAVPPYRSAWVEDATEAEVRAFLSERGMPLADTPADHIGTLLLAASWLEDQSTEDESEALETLFSEYL

LPWCGAFLGKVEAHATTPFWRTMAPLTRDAISAMWDELEEDSEE

>gi|30062559|ref|NP_836730.1| hydrolase [Shigella flexneri 2a str. 2457T]

MYPVDLHMHTVASTHAYSTLSDYIAQAKQKGIKLFAITDHGPDMEDAPHHWHFINMRIWPRVVDGVGILR

GIEANIKNVDGEIDCSGKMFDSLDLIIAGFHEPVFAPHDKATNTQAMISTIASGNVHIISHPGNPKYEID

VKAVAEAAAKHQVALEINNSSFLHSRKGSEDNCRAVAAAVRDAGGWVALGSDSHTAFTMGEFEECLKILD

AVDFPLERILNVSPRRLLNFLESRGMAPIAEFADL

>gi|30062558|ref|NP_836729.1| hypothetical protein S1096 [Shigella flexneri 2a str. 2457T]

MIPDYLTFIRFQDKRNLIYIYAIGLILIGFYWKNAGFTFPSEDIGVVSGILALVLYNFIFDLKAYWAYKC

VTKNIDFSWFKKKQNHKIELFLTQPLVAGFLSLIMLSAMSWGLYQLLPSLYALFLISLLGPLVIFLLFRM

IRTSYVKQVAISVAKKVKYKSLTRYVLLSVCISTVVNLLTISPLRNSDSFVTEGQWLTFKSIIALLILCG

VVLAINLFFLRFSKRPAFLGRFFLQEIDLFFSSENTLSTFFAKPLWLRLFILRVIEMMWITLVSVLATLV

EWRIWFEAYFLLCYVPCLIYYFFHCRFLWHNDFMMACDMYFRWGHFNK

>gi|30062555|ref|NP_836726.1| hypothetical protein S1091 [Shigella flexneri 2a str. 2457T]

MQYKDENGVNEPSRRRLLKGIGALAGSCPVAHAQKTQSAPGTLSPDARNEKQPFYGEHQAGILTPQQAAM

MLVAFDVLASDKADLERLFRLLTQRFAFLSQGGAAPETPNPRLPPLDSGILGGYIAPDNLTITLSVGHSL

FDERFGLAPQMPKKLQKMTRFPNDSLDAALCHGDVLLQICANTQDTVNHALRDIIKHTPDLLSVRWKREG

FISDHAARSKGKETPINLLGFKDGTANPDSQNDKLMQKVVWVTADQQEPAWTIGGSYQAVRLIQFRVEFW

DRTPLKEQQTIFGRDKQTGAPLGMQHEHDVPDYASDPEGKVIALDSHIRLANPRTAESESSLMLRRGYSY

SLGVTNSGQLDMGLLFVCYQHDLEKGFLTVQKRLNGEALEEYVKPIGGGYFFSLPGVKDANDYLGRALLQ

V

>gi|30062554|ref|NP_836725.1| hypothetical protein S1090 [Shigella flexneri 2a str. 2457T]

MTINFRRNALQLSVAALFSSAFMANAADVPQVKVTVTDKQCEPMTITVNAGKTQFIIQNHSQKALEWEIL

KGVMVVEERENIAPGFSQKMTANLQPGEYDMTCGLLTNPKGKLIVKGEATADAAQSDALLSLGGAITAYK

AYVMAETTQLVTDTKAFTDAIKAGDIEKAKALYAPTRQHYERIEPIAELFSDLDGSIDAREDDYEQKAAD

PKFTGFHRLEKALFGDNTTKGMDQYAEQLYTDVVDLQKRISELAFPPSKVVGGAAGLIEEVAASKISGEE

DRYSHTDLWDFQANVEGSQKIVDLLRPQLQKANPELLAKVDANFKKVDTILAKYRTKDGFETYDKLTDAD

RNALKGPITALAEDLAQLRGVLGLD

>gi|30062553|ref|NP_836724.1| cytochrome [Shigella flexneri 2a str. 2457T]

MTSRSRQAAYSGIFINETTGEFPQKEQELFEGIVAVIAVVILTWMVFWMRKVSRNVKVQLEQAVDSALQR

GNHHGWALVMMVFFAVAREGLESVFFLLAAFQQDVGIWPPLGAMLGLATAVVLGFLLYWGGIRLNLGAFF

KWTSLFILFVAAGLAAGAIRAFHEAGLWNHFQEIAFDMSAVLSTHSLFGTLMEGIFGYQEAPSVSEVAVW

FIYLIPALVAFALPPRAGATASRSA

>gi|30062550|ref|NP_836721.1| tet operon regulator [Shigella flexneri 2a str. 2457T]

MTQGAVKTTGKRSRAVSAKKKAILSAALDTFSQFGFHGTRLEQIAELAGVSKTNLLYYFPSKEALYIAVL

RQILDIWLAPLKAFREDFAPLAAIKEYIRLKLEVSRDYPQASRLFCMEMLAGAPLLMDELTGDLKALIDE

KSALIAGWVKSGKLAPIDPQHLIFMIWASTQHYADFAPQVEAVTGATLRDEVFFNQTVENVQRIIIEGIR

PR

>gi|30062549|ref|NP_836720.1| hypothetical protein S1085 [Shigella flexneri 2a str. 2457T]

MQDAAPRLTFTLRDEERLMMKIGVFVPIGNNGWLISTHAPQYMPTFELNKAIAQKAEHYHFDFALSMIKL

RGFGGKTEFWDHNLESFTLMAGLAAVTSRIQIYATAATLTLPPAIVARMAATIDSISGGRFGVNLVTGWQ

KPEYEQMGIWPGDDYFSRRYDYLTEYVQVLRDLWGTGKSDFKGDFFTMNDCRVSPQPSIPMKVICAGQSD
AGMAFSAQYADFNFCFGKGVNTPTAFAPTAARMKQATEQTGRDVGSYVLFMVIADETDDATRAKWEHYKA
GADEEALSWLTEQSQKDTRSGTDTNVRQMADPTSAVNINMGTLVGSYASVARMLDEVASVPGAEGVLLTF
DDFLSGIETFGERIQPLMQCRAHLSALTQEVA

>gi|30062548|ref|NP_836719.1| synthetase [Shigella flexneri 2a str. 2457T]
MPRPSLCADSGGGMMTTLTARPEAITFDPQQSAQIVVDMQNAYATPGGYLDLAGFDVSTTRPVIANIQTA
VTAARAAGMLIIWFQNGWDEQYVEAGGPGSPNFHKSNALKTMRKQPQLQGKLLAKGSWDYQLVDELVPQP
GDIVLPKPRYSGFFNTPLDSILRSRGIRHLVFTGIATNVCVESTLRDGFFLEHFGVVLEDATHQAGPEFA
QKAALFNIETFFGWVSDVETFCDALSPTSFARIA

>gi|30062545|ref|NP_836716.1| hypothetical protein S1081 [Shigella flexneri 2a str. 2457T]
MNEAVSPGALSTLFTDARTHNGWRETPVSDETLQELYALMKWGPTSANCSPARIVFIRTAEGKERLRPAL
SSGNLQKTLTAPVTAIVAWDSEFYERLPLLFPHGDARSWFTSSPQLAEETAFRNSSMQAAYLIVACRALG
LDTGPMSGFDRQHVDDAFFAGSTLKSNLLINIGYGDSSKLFARLPRLSFEEACGLL

>gi|30062541|ref|NP_836712.1| TrpR binding protein WrbA [Shigella flexneri 2a str. 2457T]
MAKVLVLYYSMYGHIETMARAVAEGASKVDGAEVVVKRVPETMPPQLFEKAGGKTQTAPVATPQELADYD
AIIFGTPTRFGNMSGQMRTFLDQTGGLWASGALYGKLASVFSSTGTGGGQEQTITSTWTTLAHHGMVIVP
IGYAAQELFDVSQVRGGTPYGATTIAGGDGSRQPSQEELSIARYQGEYVAGLAVKLNG

>gi|30062539|ref|NP_836710.1| glucose-1-phosphatase/inositol phosphatase [Shigella flexneri 2a str. 2457T]

MNKTLIAATVAGIVLLASNAQAQTVPEGYQLQQVLMMSRHNLRAPLANNGSVLEQSTPNKWPEWDVPGGQ

LTTKGGVLEVYMGHYMREWLAEQGIVKSGECPPPDTVYAYANSLQRTVATAQFFITGAFPGCDIPVHHQE

KMGTMDPTFNPMITDDSAAFSEQAVAAMEKELSKLQLTDSYQLLEKIVNYKDSPACKEKQQCSLVDGKNT

FSAKYQQEPGVSGPLKVGNSLVDAFTLQYYEGFPMDQVAWGEIKSDQQWKVLSKLKNGYQDSLFTSPEVA

RNVAKPLVSYIDKALVTDRASAPKITVLVGHDSNIASLLTALDFKPYQLHDQNERTPIGGKIVFQRWHDS

KANRDLMKIEYVYQSAEQLRNADALTLQAPAQRVTLELSGCPIDANGFCPMDKFDSVLNEAVK

>gi|30062538|ref|NP_836709.1| hypothetical protein S1074 [Shigella flexneri 2a str. 2457T]

MGSNIHGISCTANNYLKQAWNDIKNEYEKNQTYSITLFENTLVCFMRLYNELRRKVNEEDTPCLECESLE

KEFEEMQNDNDLSLFMRTLRTNDTQIYSGVSGGITYTIQYVQDVDIVRVSLPGRGSESITDFKGYYWYGF

MEYIENINACDDVFSEYCLDNENMSIQPEQINMPGISDLDTGIDLSGISFIQSEINKTYGLKYAPVDGDG

YCLLRAILVLKEHEYSWALGSHKTQKQVYEEFIKIVDKQTIEALVDTAFYNLREDVKTLFGVDLQSDNKI

QGVGSFMSWSFLFFKKQFIDSCLNDKKCILHLPEFIFNDNKNLLALDTDTSDRIKAVKNFLAVLSDSICS

LFIVNSNVASISLGNESFSTDEDLEYGYLTNTGNHYDVYLPPELFSQAYKLKNKEMNAQLDYLNRYAT

>gi|30062536|ref|NP_836707.1| chaperone-modulator protein CbpM [Shigella flexneri 2a str. 2457T]

MANVTVTFTITEFCLHTGISEEELNEIVGLGVVEPREIQETTWVFDDHAAIVVQRAVRLRHELALDWPGI

AVALTLMDDIAHLKQENRLLRQRLSRFVAHP

>gi|30062533|ref|NP_836704.1| trimethylamine N-oxide reductase, cytochrome c-type subunit [Shigella flexneri 2a str. 2457T]

MRKLWNALRRPSARWSVLALVAIGIVIGIALIVLPHVGIKVTSTTEFCVSCHSMQPVYEEYKQSVHFQNA

SGVRAECHDCHIPPDIPGMVKRKLEASNDIYQTFIAHSIDTPEKFEAKRAELAEREWARMKENNSATCRS

CHNYDAMDHARQHPEAARQMKVAAKDNQSCIDCHKGIAHQLPDMSSGFRKQFDELRASANDSGDTLYSID

IKPIYAAKGDKEASGSLLPASEVKVLKRDGDWLQIEITGWTESAGRQRVLTQFPGKRIFVASIRGDVQQQ

VKTLEKTTVADTNTEWSKLQATAWMKKGDMVNDIKPIWAYADSLYNGTCNECHGAPEIAHFDANGWIGTL

NGMIGFTSLDKREERTLLKYLQMNASDTAGKAHGDKKEEK

>gi|30062532|ref|NP_836703.1| DNA-binding transcriptional regulator TorR [Shigella flexneri 2a str. 2457T]

MPHHIVIVEDKPVTQARLQSYFTQEGYTVSVTASGAGLREIMQNQPVDLILLDINLPDENGLMLTRALRE

RSTVGIILVTGRSDRIDRIVGLEMGADDYVTKPLELRELVVRVKNLLWRIDLARQAQPHTQDYCYRFAGY

CLNVSRHTLERDGEPIKLTRAEYEMLVAFVTNPGEILSRERLLRMLSARRVENPDLRTVDVLIRRLRHKL

SADLLVTQHGEGYFLAADVC

>gi|30062531|ref|NP_836702.1| TMAO reductase system periplasmic protein TorT [Shigella flexneri 2a str. 2457T]

MRVLLFLLLSLFMLSAFSADNLLRWHDAQHFTVQASMPLKAKRVWKLCALYPSLKDSYWLSLNYGMQEAA

RRYGVDLKVLEAGGYSQLATQQAQIDQCKQWGAEAILLGSSTTSFPDLQKQVANLPVIELVNALDDPQVK

SRVGVPWFQMGYQPGRYLVQWAHGKPLNVLLMPGPDNAGGSKEMVEGFRAAIAGSPVRIVDIALGDNDIE

IQRNLLQEMLERHPEIDVVAGTAIAAEAAMGEGRNLKTPLTVVSFYLSHQVYRGLKRGRVIMAASDQMVW

QGELAVEQAIRQLQGQSVSDNVSPPILVLTPKNADREHIRRSLSPGGFRPVYFYQHTSAAKK

>gi|30062530|ref|NP_836701.1| hypothetical protein S1062 [Shigella flexneri 2a str. 2457T]

MAEKKRTRWQRRPGTTGGKLPWNDWRNATTWRKATQFLLLAMNIYIAITFWYWVRYYETAGSTTFVARPG

GIEGWLPIAGLMNLKYSLATGQLPSVHAAAMLLLVAFIVISLLLKKAFCSWLCPVGTLSELIGDLGNKLF

GRQCVLPRWLDIPLRGVKYLLLSLFLYIALLMPAQAIHYFMLSPYSVVMDVKMLDFFRHMGTATLISVTV

LLIASLFIRHAWCRYLCPYGALMGVVSLLSPFKIRRNAESCIDCGKCAKKCPSRIPVDKLIQVRTVECTG

CMTCVESCPVASTLTFSLQKPAANKKAFALSGWLMTLLVLGIMFAVIGYAMYAGVWQSPVPEELYRRLIP

QAPMIGH

>gi|30062524|ref|NP_836695.1| hypothetical protein S1056 [Shigella flexneri 2a str. 2457T]

MNKGKVMKHKLSAILMAFMLTTPAAFAAPEAANGTEATTGTTGTTTTTTGATTTAATTSGVAAGAVGTAT

VVGVATAVGVATLAVVAANDSGDGGSHNTSTTTSTTR

>gi|30062523|ref|NP_836694.1| regulator [Shigella flexneri 2a str. 2457T]

MRPLILSIFALFLAGCTHSQQSMVDTFRASLFDNQDITVAAQQIQALPYSTMYLRLNEGQRIFVVLGYIE

QEQSKWLSQDNAMLVTHNGRLLKTVKLNNNLLEVTNPGRAPLRNALAIKDGSRWTRDILWSEDNHFRSAT

LSSTFSFAGLETLNIAGHNVLCNVWQEEVTSTLPEKQWQNTFWVDSATGQVRQSRQMLGAGVIPVEMTFL

KPAP

>gi|30062522|ref|NP_836693.1| hypothetical protein S1054 [Shigella flexneri 2a str. 2457T]

MNKLQSYFIASVLYVMTPHAFAQGTVTIYLPSEQQTLSVGPVENVVQLVTQPQLRDRLWWPGALLTDSAA

KAKALKDYQHVMAQLASWEAEADDDVAATIKSVRQQLLNLNITGRLPVKLDPDFVRVDENSNPPLVGDYT

LYTVQRPVTITLLGAVSGAGQLPWQAGRSVTDYLQDHPRLAGADKNNVMVITPEGETVVAPVALWNKRHV

EPPPGSQLWLGFSAHVLPEKYADLNDQIVSVLTQRVPD

>gi|30062518|ref|NP_836689.1| phosphoanhydride phosphorylase [Shigella flexneri 2a str. 2457T]

MKAILIPFLSLLIPLTPQSAFAQSEPELKLESMVIVSRHGVRAPTKATQLMQDVTPDAWPTWPVKLGWLT

PRGGELIAYLGHYQRQRLVADGLLAKKGCPQSAQVAIIADVDERTRKTGEAFTAGLAPDCAITVHTQADT

SSPDPLFNPLKTGVCQLDNANVTDAILCRAGGSIADFTGHRQTVFRELERVLNFPQSNLCLNREKQDESC

SLTQALPSELKVSADNVSLTGAVSLASMLTEIFLLQQAQGMPEPGWGRITDSHQWNTLLSLHNAQFYLLQ

RTPEVARSRATPLLDLIMAALTPHPPQKQAYGVTLPTSVLFIAGHDTNLANLGGALELNWTLPGQPDNTP

PGGELVFERWRRLSDNSQWIQVSLVFQTLQQMRDKTPLSLNTPPGEVKLTLAGCEERNAQGMCSLAGFTQ

IVNEARIPACSL

>gi|30062517|ref|NP_836688.1| third cytochrome oxidase, subunit II [Shigella flexneri 2a str. 2457T]

MFDYETLRFIWWLLIGVILVVFMISDGFDMGIGCLLPLVARNDDERRIVINSVGAHWEGNQVWLILAGGA

LFAAWPRVYAAAFSGFYVAMILVLCSLFFRPLAFDYRGKIADARWRKMWDAGLVIGSLVPPVVFGIAFGN

LLLGVPFAFTPQLRVEYLGSFWQLLTPFPLLCGLLSLGMVILQGGVWLQLKTVGVIHLRSQLATKRAALL

VMLCFLLAGYWLWVGIDGFVLLAQDANGPSHPLMKLVAVLPGAWMNNFVESPVLWIFPLLGFFCPLLTVM

AIYRGRPGWGFLMASLMQFGVIFTAGITLFPFVMPSSVSPISSLTLWDSTSSQLTLSIMLVIVLIFLPIV

LLYTLWSYYKMWGRMTTETLRRNKNELY

>gi|30062515|ref|NP_836686.1| hydrogenase-1 operon protein hyaF [Shigella flexneri 2a str. 2457T]

MSETFFHLLGPGTQPNDDSFSMNPLPITCQVNDEPSMAALEQCAHSPQVIALLNELQHQLSERQPPLGEV

LAVDLLNLNADDRHFINTLLGEGEVSVRIQQADDSKSEIQEAIFCGLWRVRRRRGEKLLEDKLEAGCAPL

ALWQAATQNLLPTDSLLPPPIDGLMNGLPLAHELLAHVRNPDAQPHSINLTQLPISEADRLFLSRLCGPG

NIQIRTIGYGESYINATGLRHVWHLRCTDTLKGPLLESYEICPIPEVVLAAPEDLVDSAQRLSEVCQWLA

EAAPT

>gi|30062514|ref|NP_836685.1| hydrogenase-1 operon protein HyaE [Shigella flexneri 2a str. 2457T]

MSNDTPFDALWQRMLARGWTPVSESRLDDWLTQAPDGVVLLSSDPKRTPEVSDNPVMIGELLHEFPDYTW

QVAIADLEQSEAIGDRFGAFRFPATLVFTGGNYRGVLNGIHPWAELINLMRGLVEPQQERAS

>gi|30062513|ref|NP_836684.1| hydrogenase 1 maturation protease [Shigella flexneri 2a str. 2457T]

MSEQHVVVMGLGNLLWADEGFGVRVAERLYAHYHWPEYVEIVDGGTQGLNLLGYVESASHLLILDAIDYG

LEPGTLRTYAGERIPAYLSAKKMSLHQNSFSEVLALADIRGHLPAHIALVGLQPAMLDDYGGSLSELARE

QLPAAEQSALAQLAAWGSVPQPANESRCLNYDCLSMENYEGVRLRQYRMTQEEQG

>gi|30062512|ref|NP_836683.1| hydrogenase 1 b-type cytochrome subunit [Shigella flexneri 2a str. 2457T]

MQQKSDNVVSHYVFEAPVRIWHWLTVLCMAVLMVTGYFIGKPLPSVSGEATYLFYMGYIRLIHFSAGMIF

TVVLLMRIYWAFVGNRYSRELFIVPVWRKSWWQGVWYEIRWYLFLAKRPSADIGHNPIAQAAMFGYFLMS

VFMIITGFALYSEHSQYAIFAPFRYVVEFFYWTGGNSMDIHSWHRLGMWLIGAFVIGHVYMALREDIMSD

DTVISTMVNGYRSHKFGKISNKERS

>gi|30062511|ref|NP_836682.1| hydrogenase 1 large subunit [Shigella flexneri 2a str. 2457T]

MSTQYETQGYTINNAGRRLVVDPITRIEGHMRCEVNINDQNVITNAVSCGTMFRGLEIILQGRDPRDAWA

FVERICGVCTGVHALASVYAIEDAIGIKVPDNANIIRNIMLATLWCHDHLVHFYQLAGMDWIDVLDALKA

DPRKTSELAQSLSSWPKSSPGYFFDVQNRLKKFVEGGQLGIFRNGYWGHPQYKLPPEANLMGFAHYLEAL

DFQREIVKIHAVFGGKNPHPNWIVGGMPCAINIDESGAVGAVNMERLNLVQSIITRTADFINNVMIPDAL

AIGQFNKPWSEIGTGLSDKCVLSYGAFPDIANDFGEKSLLMPGGAVINGDFNNVLPVDLVDPQQVQEFVD

HAWYRYPNDQVGRHPFDGITDPWYNPGDVKGSDTNIQQLNEQERYSWIKAPRWRGNAMEVGPLARTLIAY

HKGDAATVESVDRMMSALNLPLSGIQSTLGRILCRAHEAQWAAGKLQYFFDKLMTNLKNGNLATASTEKW

EPATWPTECRGVGFTEAPRGALGHWAAIRDGKIDLYQCVVPTTWNASPRDPKGQIGAYEAALMNTKMAIP

EQPLEILRTLHSFDPCLACSTHVLGDDGSELISVQVR

>gi|30062510|ref|NP_836681.1| hydrogenase-1 small subunit [Shigella flexneri 2a str. 2457T]

MNNEETFYQAMRRQGVTRRSFLKYCSLAATSLGLGAGMAPKIAWALENKPRIPVVWIHGLECTCCTESFI

RSAHPLAKDVILSLISLDYDDTLMAAAGTQAEEVFEDIITQYNGKYILAVEGNPPLGEQGMFCISSGRPF

IEKLKRAAAGASAIIAWGTCASWGCVQAARPNPTQATPIDKVITDKPIIKVPGCPPIPDVMSAIITYMVT

FDRLPDVDRMGRPLMFYGQRIHDKCYRRAHFDAGEFVQSWDDDAARKGYCLYKMGCKGPTTYNACSSTRW

NDGVSFPIQSGHGCLGCAENGFWDRGSFYSRVVDIPQMGTHSTADTVGLTALGVVAAAVGVHAVASAVDQ

RRRHNQQPTETEHQPGNEDKQA

>gi|30062506|ref|NP_836677.1| oxidoreductase [Shigella flexneri 2a str. 2457T]

MEGKASLGETIDIVDHQGKWLARGAYSPASQIRARVWTFDPSESIDIAFFSRRLQQAQKWRDWLAQKDGL

DSYRLIAGESDGLPGITIDRFGNFLVLQLLSAGAEYQRAALISALQTLYPECAIYDRSDVAVRKKEGMEL

TQGLVTGELPPALLPIEEHGMKLLVDIQHGHKTGYYLDQRDSRLATRRYVENKRVLNCFSYTGGFAVSAL

MGGCSQVVSVDTSQEALDIARQNVELNKLDLSKAEFVRDDVFKLLRTYRDRGEKFDVIVMDPPKFVENKS

QLMGACRGYKDINMLAIQLLNEGGILLTFSCSGLMTSDLFQKIIADAAIDAGRDVQFIEQFRQAADHPVI

ATYPEGLYLKGFACRVM

>gi|30062505|ref|NP_836676.1| hypothetical protein S1034 [Shigella flexneri 2a str. 2457T]

MWNFTLISKVKISREVTMIASKFGIGQQVRHSLLGYLGVVVDIDPVYSLSEPSPDELAVNDELRAAPWYH

VVMEDDNGLPVHTYLAEAQLSSELQDEHPEQPSMDELAQTIRKQLQAPRLRN

>gi|30062504|ref|NP_836675.1| hypothetical protein S1033 [Shigella flexneri 2a str. 2457T]

MVGMSALSYTLLNSLEEIMKETDIAGILTSTHTIALVGASDKPDRPSYRVMKYLLDQGYHVIPVSPKVAG

KTLLGQQGYGTLADVPEKVDMVDVFRNSEAAWGVAQEAIAIGAKTLWMQLGVINEQAAVLARDAGLNVVM

DRCPAIEIPRLGLAK

>gi|30062503|ref|NP_836674.1| hypothetical protein S1032 [Shigella flexneri 2a str. 2457T]

MKTGIVTTLIALCLPVSVFATTLRLSTDVDLLVLDGKKVSSSLLRGADSIELDNGPHQLVFRVEKTIHLS

NSEERLYISPPLVVSFNTQLINQVNFRLPRLENEREANHFDAAPRLELLDGDATPIPVKLDILAITSTAK

TIDYEVEVERYNKSAKRASLPQFATMMADDSTLLSGVSELDAIPPQSQVLTEQRLKYWFKLADPQTRNTF

LQWAEKQPSS

>gi|30062501|ref|NP_836672.1| DNA helicase IV [Shigella flexneri 2a str. 2457T]

MELKATTLGKRLAQHPYDRAVILNAGIKVSGDRHEYLIPFNQLLAIHCKRGLVWGELEFVLPDEKVVRLH

GTEWGETQRFYHHLDAHWRRWSGEMSEIASGVLRQQLDLIATRTGENKWLTREQTSGVQQQIRQALSALP

LPVNRLEEFDNCREAWRKCQAWLKDIESARLQHNQAYTEAMLTEYADFFRQVESSPLNPAQARAVVNGEH

SLLVLAGAGSGKTSVLVARAGWLLARGEASPEQILLLAFGRKAAEEMDERIRERLHTEDITARTFHALAL

HIIQQGSKKVPIVSKLENDTAARHELFIAEWRKQCSEKKAQAKGWRQWLTEEMQWSVPEGNFWDDEKLQR

RLASRLDRWVSLMRMHGGAQAEMIASAPEEIRDLFSKRIKLMAPLLKAWKGALKAENAVDFSGLIHQAIV

ILEKGRFISPWKHILVDEFQDISPQRAALLAALRKQNSQTTLFAVGDDWQAIYRFSGAQMSLTTAFHENF

GEGDRCDLDTTYRFNSRIGEVANRFIQQNPGQLKKPLNSLTNGDKKAVTLLDESQLDALLDKLSGYAKPE

ERILILARYHHMRPASLEKAATRWPKLQIDFMTIHASKGQQADYVIIVGLQEGSGGFPAAARESIMEEAL

LPPVEDFPDAEERRLMYVALTRARHRVWALFNKENPSPFVEILKNLDVPVARKP

>gi|30062500|ref|NP_836671.1| hypothetical protein S1029 [Shigella flexneri 2a str. 2457T]

MRTVLNILNFVLGGFATTLGWLLATLVSIVLIFTLPLTRSCWEITKLSLVPYGNEAIHVDELNPAGKNVL

LNTGGTVLNIFWLIFFGWWLCLMHIATGIAQCISIIGIPVGIANFKIAAIALWPVGRRVVSVETAQAARE

ANARRRFE

>gi|30062499|ref|NP_836670.1| hypothetical protein S1028 [Shigella flexneri 2a str. 2457T]

MAFMLSPLLKRYTWNSAWLYYARIFIALCGTTAFPWWLGDVKLTIPLTLGMVAAALTDLDDRLAGRLRNL

IITLFCFFIASASVELLFPWPWLFAIGLTLSTSGFILLGGLGQRYATIAFGALLIAIYTMLGTSLYEHWY

QQPMYLLAGAVWYNVLTLIGHLLFPVRPLQDNLARCYEQLARYLELKSRMFDPDIEDESQAPLYDLALAN

GQLMATLNQTKLSLLTRLRGDRGQRGTRRTLHYYFVAQDIHERASSSHIQYQTLREHFRHSDVLFRFQRL

MSMQGQACQQLSRCILLRQSYQHDPHFERAFTHIDAALERIRDNGAPADLLKTLGFLLNNLRAIDAQLAT

IESEQAQALPRNNDENELADDSPHGLSDIWLRLSRHFTPESALFRHAVRMSLVLCFGYAIIQITGMHHGY

WILLTSLFVCQPNYNATRHRLKLRIIGTLVGIAIGIPVLWFVPSLEGQLVLLVITGVLFFAFRNVQYAHA

TMFITLLVLLCFNLLGEGFEVALPRVIDTLIGCAIAWAAVSYIWPDWKFRNLPRMLERATEANCRYLDAI

LEQYHQGRDNRLAYRIARRDAHNRDAELASVVSNMSSEPNVTPQIREAAFRLLCLNHTFTSYISALGAHR

EQLTNPEILAFLDDAVCYVDDALHHQPADEERVNQALAGLKQRMQQLEPRADSKEPLVVQQVGLLIALLP

EIGRLQRQITVPTSGRTGVFH

>gi|30062496|ref|NP_836667.1| hypothetical protein S1025 [Shigella flexneri 2a str. 2457T]

MKSLSYKRIYKSQEYLATLGTIEYRSLFGSYSLTVDDTVFAMVSDGELYLRACEQSAQYCVKHPPVWLTY

KKCGRSVTLNYYRVDESLWRNQLKLVRLSKYSLDAALKEKSTRNTRERLKDLPNMSFHLEAILGEVGRLA

P

>gi|30062495|ref|NP_836666.1| SOS cell division inhibitor [Shigella flexneri 2a str. 2457T]

MYTSGYAHRSSSFSSAASKIARVSTENTTAGLISEVVYREDQPMMTQLLLLPLLQQLGQQSRWQLWLTPQ

QKLSREWVQASGLPLTKVMQISQLSPCHTVESMVRALRTGNYSVVIGWLTDDLTEEEHAELVDAANEGNA

MGFIMRPVSASSHTTRQLSGLKIHSNLYH

>gi|30062494|ref|NP_836665.1| outer membrane protein A [Shigella flexneri 2a str. 2457T]

MKKTAIAIAVALAGFATVAQAAPKDNTWYTGAKLGWSQYHDTGFIPNNGPTHENQLGAGAFGGYQVNPYV

GFEMGYDWLGRMPYKGDNINGAYKAQGVQLTAKLGYPITDDLDIYTRLGGMVWRADTKANVPGGASFKDH

DTGVSPVFAGGVEYAITPEIATRLEYQWTNNIGDANTIGTRPDNGLLSLGVSYRFGQGEAAPVVAPAPAP

EVQTKHFTLKSDVLFNFNKATLKPEGQAALDQLYSQLSNLDPKDGSVVVLGYTDRIGSDAYNQGLSERRA

QSVVDYLISKGIPADKISARGMGESNPVTGNTCDNVKQRAALIDCLAPDRRVEIEVKGIKDVVTQPQA

>gi|30062493|ref|NP_836664.1| hypothetical protein S1022 [Shigella flexneri 2a str. 2457T]

MKYQQLENLESGWKWKYLVKKHREGELITRYIEASAAQEAVDVLLSLENEPVLVNGWIDKHMNPELVNRM

KQTIRARRKRHFNAEHQHTRKKSIDLEFIVWQRLAGLAQRRGKTLSETIVQLIEDAENKEKYANKMSSLK

QDLQALLGKE

>gi|30062492|ref|NP_836663.1| ATP-dependent protease [Shigella flexneri 2a str. 2457T]

MTITKLAWRDLVPDTDSYQEIFAQPHLIDENDPLFSDTQPRLQFALEQLLHTRASSSFMLAKAPEESEYL

NLIADAARTLQSDAGQLVGDHYEVSGHSIRLRHAVSADDNFATLTQVVAADWVEAEQLFGCLRQFNGDIT

LQPGLVHQANGGILIISLRTLLAQPLLWMRLKNIVNRERFDWVAFDESRPLPVSVPSMPLKLKVILVGER

ESLADFQEMEPELSEQAIYSEFEDTLQIVDAESVSQWCRWVTFTARHNHLPAPGADAWPVLIREAARYTG

EQETLPLSPQWILRQCKEVASLCDGDTFSGEQLNLMLQQREWREGFLAERMQDEILQEQILIETEGERIG

QINALSVIEFPGHPRAFGEPSRISCVVHIGDGEFTDIERKAELGGNIHAKGMMIMQAFLMSELQLEQQIP

FSASLTFEQSYSEVDGDSASMAELCALISALADVPVNQSIAITGSVDQFGRVQPVGGLNEKIEGFFAICQ

QRELTGKQGVIIPTANVRHLSLHSELVKAVEEGKFTIWAVDDVTDALPLLLNLVWDGEGQTTLMQTIQER

IAQASQQEGRHRFPWPLRWLNWFIPN

>gi|30062491|ref|NP_836662.1| 3-hydroxydecanoyl-ACP dehydratase [Shigella flexneri 2a str. 2457T]

MVDKRESYTKEDLLASGHGELFGAKGPQLPAPNMLMIDRVVKMTETGGNFDKGYVEAELDINPDLWFFGC

HFIGDPVMPGCLGLDAMWQLVGFYLGWLGGEGKGRALGVGEVKFTGQVLPTAKKVTYRIHFKRIVNRRLI

MGLADGEVLVDCRLIYTASDLKVGLFQDTSAF

>gi|30062489|ref|NP_836660.1| hypothetical protein S1018 [Shigella flexneri 2a str. 2457T]

MKKWLVTIAALWLAGCSSGEINKNYYQLPVVQSGTQSTASQGNRLLWVEQVTVPDYLAGNGVVYQTSDVK

YVIANNNLWASPLDQQLRNTLVANLSTQLPGWVVASQPLGSAQDTLNVTVTEFNGRYDGKVIVSGEWLLN

HQGQLIKRPFRLEGVQTQDGYDEMVKVLAGVWSQEAASIAQEIKRLP

>gi|30062488|ref|NP_836659.1| paraquat-inducible protein B [Shigella flexneri 2a str. 2457T]

MESNNGEAKIQKVKNWSPVWIFPIVTALIGAWVLFYHYSHQGPEVTLITANAEGIEGGKTTIKSRSVDVG

VVESATLADDLTHVEIKARLNSGMEKLLHKDTVFWVVKPQIGREGISGLGTLLSGVYIELQPGAKGSKMD

KYDLLDSPPLAPPDAKGIRVILDSKKAGQLSPGDPVLFRGYRVGSVETSTFDTQKRNISYQLFINAPYDR

LVTSNVRFWKDSGIAVDLTSAGMRVEMGSLTTLLSGGVSFDVPEGLDLGQPVAPKTAFVLYDDQKSIQDS

LYTDHIDYLMFFKDSVRGLQPGAPVEFRGIRLGTVSKVPFFAPNMRQTFNDDYRIPVLIRIEPERLKMQL

GENADVVEHLGELLKRGLRGSLKTGNLVTGALYVDLDFYPNTPAITGIREFNGYQIIPTVSGGLAQIQQR

LMEALDKINKLPLNPMIEQATSTLSESQRTMKNLQTTLDSMNKILASQSMQQLPTDMQSTLRELNRSMQG

FQPGSAAYNKMVADMQRLDQVLRELQPVLKTLNEKSNALVFEAKDKKDPEPKRAKQ

>gi|30062487|ref|NP_836658.1| paraquat-inducible protein A [Shigella flexneri 2a str. 2457T]

MCEHHHAAKHILCSQCDMLVALPRLEHGQKAACPRCGTTLTVAWDAPRQRPTAYALAALFMLLLSNLFPF

VNMNVAGVTSEITLLEIPGVLFSEDYASLGTFFLLFVQLVPAFCLITILLLVNRAELPVRLKEQLARVLF

QLKTWGMAEIFLAGVLVSFVKLMAYGSIGVGSSFLPWCLFCVLQLRAFQCVDRRWLWDDIAPMPELRQPL

KPGVTGIRQGLRSCSCCTAILPADEPVCPRCSTKGYVRRRNSLQWTLALLVTSIMLYLPANILPIMVTDL

LGSKMPSTILAGVILLWSEGSYPVAAVIFLASIMVPTLKMIAIAWLCWDAKGHGKRDSERMHLIYEVVEF

VGRWSMIDVFVIAVLSALVRMGGLMSIYPAMGALMFALVVIMTMFSAMTFDPRLSWDRQPESEHEES

>gi|30062485|ref|NP_836656.1| 23S rRNA m(2)G2445 methyltransferase [Shigella flexneri 2a str. 2457T]

MNSLFASTARGLEELLKTELENLGAVECQVVQGGVHFKGDTRLVYQSLMWSRLASRIMLPLGECKVYSDL

DLYLGVQAINWTEMFNPGATFAVHFSGLNDTIRNSQYGAMKVKDAIVDAFTRKNLPRPNVDRDAPDIRVN

VWLHKETASIALDLSGDGLHLRGYRDRAGIAPIKETLAAAIVMRSGWQSGTPLLDPMCGSGTLLIEAAML

ATDRAPGLHRGRWGFSGWAQHDEAIWQEVKAEAQTRARKGLAEYSSHFYGSDSDARVIQRARTNARLAGI

GELITFEVKDVAQLTNPLPKGPYGTVLSNPPYGERLDSEPALIALHSLLGRIMKNQFGGWNLSLFSASPD

LLSCLQLRADKQYKAKNGPLDCVQKNYHVAESTPDSKPAMVAEDYANRLRKNLKKFEKWARQEGIECYRL

YDADLPEYNVAVDRYADWVVVQEYAPPKTIDAHKARQRLFDIIAATISVLGIAPNKLVLKTRERHKGKNQ

YQKLGEKGEFLEVTEYNAHLWVNLTDYLDTGLFLDHRIARRMLGQMSKGKDFLNLFSYTGSATVHAGLGG

ARSTTTVDMSRTYLEWAERNLRLNGLTRRAHRLIQADCLAWLREANEQFDLIFIDPPTFSNSKRMEDAFD

VQRDHLALMKDLKRLLRAGGTIMFSNNKRGFRMDLDGLAKLGLKAQEITQKTLSQDFARNRQIHNCWLIT

AA

>gi|30062483|ref|NP_836654.1| hypothetical protein S1012 [Shigella flexneri 2a str. 2457T]

MLLRFYRVGERQMRIKPDDNWRWYYDEEHDRMMLDLANGMLFRSRFARKMLTPDAFSPAGFCVDDAALYF

SFEEKCRDFNLSKDQKAELVLNALVAIRYLKPQMPKSWHFVSHGEMWVPMPGDAACVWLSDTHEQVNLLV

VESGENAALCLLAQPCVVIAGRAMQLGDAIKIMNDRLKPQVNVDSFSLEQAV

>gi|30062481|ref|NP_836652.1| putativi pili assembly chaperone [Shigella flexneri 2a str. 2457T]

MPARHLYFIMTNTWNRLALLIFAVLSLLVAGELQAGVVVGGTRFIFPADRESISILLTNTSQESWLINSK

INRPTRWAGGEASTVPALLLAAPPLILLKPGTTGTLRLLRTESDILPVDRETLFELSIASVPSGKVENQS

VKVAMRSVFKLFWRPEGLPGDPLEAYQQLRWTRNSQGVQLTNPTPYYINLIQVSVNGKALSNAGVVPPKS
QRQTNWCQVIAPCHVAWRTINDYGGLSAKKEQNLP

>gi|30062480|ref|NP_836651.1| fimbrial-like protein [Shigella flexneri 2a str. 2457T]
MRRRFSILIWFTSEGVMLKRIIWILFLLGLTGGCELFAHDGTVNISGSFRRNTCVLAQDSKQINVQLGDV
SLTRFFHGNYGPEKSFIINLQDCGTDVSTVDVTFSGTPDGVQSEMLSIESGTDAASGLAIAILDDAKILI
PLNQASKDYSLHSGKVPLTFYAQLRPVNSDVQSGKVNASATFVLHYD

>gi|30062479|ref|NP_836650.1| fimbrial-like protein [Shigella flexneri 2a str. 2457T]
MKKKTIYQCVILFFSLLNIHVGMAGPEQVSMHIYGNVVDQGCDVATKSALQNIHIGDFNISDFQAANTVS
TAADLNIDITGCAAGITGADVLFSGEADTLAPTLLKLTDTGGSGGMATGIAVQILDAQSQQEIPLNQVQP
LTPLKAGDNTLKYQLRYKSTKAGATGGNATAVLYFDLVYQ

>gi|30062478|ref|NP_836649.1| fimbrial protein [Shigella flexneri 2a str. 2457T]
MQIIFGEKCVLLLRLFFAAVLMLWCAQTAAYIGQCHTTQGNPYIGVNFGVKTLDEEENTAGVVKDKFYQW
NESNDYYVSCDCDKDNVRSGRWAFAADSPLVYLGDNWYKINDYLAAKVLLQVKGSSPTAVPFENVGTGAD
TRWHICDPGGQRLGGQGASGNSGSFSLKILQPFVGSVVIPPMALARLFECYNIPAGDSCTTTGTPVLVYY
LSGTINSLGSCSVNAGETIEVDLGDVFAANFRVVGHKPLGARTAELAIPVRCNTGNAGLVNVNLSLTATS
DPSYPQAIKTSRPGVGVVVTDSQHNIISPAGGTLPLSIPDDADSIRE

>gi|30062477|ref|NP_836648.1| outer membrane protein [Shigella flexneri 2a str. 2457T]
MYRTHRQHSLLSSGGVPSFIGGLVVFVSAAFNAQAETWFDPAFFKDDPSMVADLSRFEKGQKITPGVYRV
DIVLNQTIVDTRNVNFVEITPEKGIAACLTTESLDAMGVNTDAFPAFKQLDKQVCVPLAEIIPDASVTFN
VNKLRLEISVPQIAIKSNARGYVPPERWDEGINALLLGYSFSGANSIHSSADSDSGDSYFLNLNSGVNLG
PWRLRNNSTWSRSSGQTAEWKNLSSYLQRAVIPLKGELTVGDDYTAGDFFDSVSFRGVQLASDDNMLPDS

LKGFAPVVRGIAKSNAQITIKQNGYTIYQTYVSPGAFEISDLYSTSSSGDLLVEIKEADGSVNSYSVPFS

SVPLLQRQGRIKYAVTLAKYRTNSNEQQESKFAQTTLQWGGPWGTTWYGGGQYAEYYRAAMFGLGFNLGD

FGAISFDATQAKSTLADQSEHKGQSYRFLYAKTLNQLGTNFQLMGYRYSTSGFYTLSDTMYKHMDGYEFN

DGDDEDTPMWSRYYNLFYTKRGKLQVNISQQLGEYGSFYLSGSQQTYWHTDQQDRLLQFGYNTQIKDLSL

GVSWNYSKSRGQPDADQVFALNFSLPLNLLLPRSNDSYTRKKNYAWMTSNTSIDNEGHITQNLGLTETLL

DDGNLSYSVQQGYNSEGKTANGSASMDYKGAFADARVGYNYSDNGSQQQLNYALSGSLVAHSQGITLGQS

LGETNVLIAAPGAENTRVANSTGLKTDWRGYTVVPYATSYRENRIALDAASLKRNVDLENAVVNVVPTKG

ALVLAEFNAHAGARVLMKTSKQGIPLRFGAIATLDGIQTNSGIIDDDGSLYMSGLPAQGAITVRWGEAPD

QICHISYQLTEQQINSAITRMDAICR

>gi|30062474|ref|NP_836645.1| IS1 orfB, A [Shigella flexneri 2a str. 2457T]

MASISIRCPSCSATEGVVRNGKSTAGHQRYLCSHCRKTWQLQFTYTASQPGTHQKIIDMAMNGVGCRASA

RIMGVGLYTVLRHLKNSAESVTSRIQPGSDVIVCAEMDEHWGYVGAKSRQRWLFYAYDRIRRTVVAHVFG

ERTLATLERLLSLLSAFEVVVWMTDGCPLYESRLKGKLHVISKRYTQRIERHNLNLRQHLARLVRKSLSF

SKSVELHDKAIGHYLNIKHYQ

>gi|30062473|ref|NP_836644.1| NAD(P)H-dependent FMN reductase [Shigella flexneri 2a str. 2457T]

MRVITLAGSPRFPSRSSSLLEYAREKLNGLDVEVYHWNLQNFAPEDLLYARFDSPALKTFTEQLQQADGL

IVATPVYKAAYSGALKTLLDLLPERALQGKVVLPLATGGTVAHLLAVDYALKPVLSALKAQEILHGVFAD

DSQLIDYHHRPQFTPNLQTHLDTALETFWQALHRRDVQVPDLLSLRGNAHA

>gi|30062471|ref|NP_836642.1| alkanesulfonate monooxygenase [Shigella flexneri 2a str. 2457T]

MSLNMFWFLPTHGDGHYLGTEEGSRPVDHGYLQQIAQAADRLGYTGVLIPTGRSCEDAWLVAASMIPVTQ

RLKFLVALRPSVTSPTVAARQAATLDRLSNGRALFNLVTGSDPQELAGDGVFLDHSERYEASAEFTQVWR

RLLLGETVNFNGKHIHVRGAKLLFPPIQQPYPPLYFGGSSDVAQELAAEQVDLYLTWGEPPELVKEKIEQ

VRAKAAAYGRKIRFGIRLHVIVRETNDEAWQAAERLISHLDDETIAKAQAAFARTDSVGQQRMAALHNGK

RDNLEISPNLWAGVGLVRGGAGTALVGDGPTVAARINEYAALGIDSFVLSGYPHLEEAYRVGELLFPHLD

VAIPEIPQPQPLNPQGEAVANDFIPRKVAQS

>gi|30062466|ref|NP_836637.1| nicotinate phosphoribosyltransferase [Shigella flexneri 2a str. 2457T]

MTQFASPVLHSLLDTDAYKLHMQQAVFHHYYDVHVAAEFRCRGDDLLGIYADAIREQIQAMQHLRLQDDE

YQWLSALPFFKADYLNWLREFRFNPEQVTVSNDNGKLDIRLSGPWREVILWEVPLLAVISEMVHRYRSPQ

ADVAQALDTLESKLVDFSALTAGLDMSRFHLMDFGTRRRFSREVQETIVKRLQQESWFVGTSNYDLARRL

SLTPMGTQAHEWFQAHQQISPDLANSQRAALAAWLEEYPDQLGIALTDCITMDAFLRDFGVEFASRYQGL

RHDSGDPVEWGEKAIAHYEKLGIDPQSKTLVFSDNLDLRKAVELYRHFSSRVQLSFGIGTRLTCDIPQVK

PLNIVIKLVECNGKPVAKLSDSPGKTICHDKAFVRALRKAFDLPHIKKAS

>gi|30062464|ref|NP_836635.1| outer membrane protein F [Shigella flexneri 2a str. 2457T]

MMKRNILAVIVPALLVAGTANAAEIYNKDGNKVDLYGKTVGLHYFSKGNGENSYGGNGDMTYARLGFKGE

TQINSDLTGYGQWEYNFQGNNSEGADAQTGNKTRLAFAGLKYADVGSFDYGRNYGVVYDALGYTDMLPEF

GGDTAYSDDFFVGRVGGVATYRNSNFFGLVDGLNFAVQYLGKNERDTARRSNGDGVGGSISYEYEGFGIV

GAYGAADRTNLQEAQPLGNGKKAEQWATGLKYDANNIYLAANYGETRNATPITNKFTNTSGFANKTQDVL

LVAQYQFDFGLRPSIAYTKSKAKDVEGIGDVDLVNYFEVGATYYFNKNMSTYVDYIINQIDSDNKLGVGS

DDTVAVGIVYQF

>gi|30062461|ref|NP_836632.1| hypothetical protein S0987 [Shigella flexneri 2a str. 2457T]

MDKFDANRRKLLALGGVALGAAILPTPAFATLSTPRPRILTLNNLHTGESIKAEFFDGRGYIQEELAKLN

HFFRDYRANKIKSIDPGLFDQLYRLQGLLGTRKPVQLISGYRSIDTNNELRARSRGVAKKSYHTKGQAMD

FHIEGIALSNIRKAALSMRAGGVGYYPRSNFVHIDTGPARHW

>gi|30062460|ref|NP_836631.1| hypothetical protein S0986 [Shigella flexneri 2a str. 2457T]

MLLNMMCGRRLSAISLCLAVTFAPLFNAQADEPEVIPGDSPVAVSEQGEALPQAQATAIMAGIQPLPEGA

AEKARTQIESQLPAGYKPVYLNQLQLLYAARDMQPMWENRDAVKAFQQQLAEVAIAGFQPQFNKWVELLT

DPGVNGMARDVVLSDAMMGYLHFIANIPIKGTRWLYSSKPYALSTPPLSVINQWQLALDKGQLPTFVAGL

APQHPQYAAMHESLLVLLSDTKPWPQLTGKATLRPGQWSNDVPALREILQRTGMLDGGPKITLPGDDTPT

DAVVSPSAVTVETAETKPMDKQTTSRSKPAPAVRAAYDNELVEAVKRFQAWQGLGADGAIGPATRDWLNV

TPAQRAGVLALNIQRLRLLPTELSTGIMVNIPAYSLLYYQNGNQVLDSRVIVGRPDRKTPMMSSALNNVV

VNPPWNVPPTLARKDILPKVRNDPGYLESHGYTVMRGWNSREAIDPWEVDWSTITASNLPFRFQQAPGPR

NSLGRYKFNMPSSEAIYLHDTPNHNLFKRDTRALSSGCVRVNKASDLANMLLQDAGWNDKRISDALKQGD

TRYVNIRQSIPVNLYYLTAFVGADDRTQYRTDIYNYDLPARSSSQIVSKAEQLIR

>gi|30062458|ref|NP_836629.1| cell division protein MukB [Shigella flexneri 2a str. 2457T]

MIERGKFRSLTLINWNGFFARTFDLDELVTTLSGGNGAGKSTTMAAFVTALIPDLTLLHFRNTTEAGATS

GSRDKGLHGKLKAGVCYSMLDTINSRHQRVVVGVRLQQVAGRDRKVDIKPFAIQGLPMSVQPTQLVTETL

NERQARVLPLNELKDKLEAMEGVQFKQFNSITDYHSLMFDLGIIARRLRSASDRSKFYRLIEASLYGGIS

SAITRSLRDYLLPENSGVRKAFQDMEAALRENRMTLEAIRVTQSDRDLFKHLISEATNYVAADYMRHANE

RRVHLDKALEFRRELHTSRQQLAAEQYKHVDMARELAEHNGAEGDLEADYQAASDHLNLVQTALRQQEKI

ERYEVDLDELQIRLEEQNEVVAEAIERQEENEARAEAAELEVDELKSQLADYQQALDVQQTRAIQYNQAI

AALNRAKELCHLPDLTADSAAEWLETFQAKELEATEKMLSLEQKMSMAQTAHSQFEQAYQLVVAINGPLA

RNEAWDVARELLREGVDQRHLAEQVQPLRMRLSELEQRLREQQEAERLLADFCKRQGKNFDIDELEALHQ

ELEARIASLSDSVSNAREERMALRQEQEQLQSRIQSLMQRAPVWLAAQNSLNQLSEQCGEEFSSSQDVTE

YLQQLLEREREAIVERDEVGARKNAVDEEIERLSQPGGSEDQRLNALAERFGGVLLSEIYDDVSLEDAPY

FSALYGPSRHAIVVPDLSQVTEHLEGLTDCPEDLYLIEGDPQSFDDSVFSVDELEKAVVVKIADRQWRYS

RFPEVPLFGRAARESRIESLHAEREVLSERFATLSFDVQKTQRLHQAFSRFIGSHLAVAFESDPEAEIRQ

LNSRRVELERALSNHENDNQQQRIQFEQAKEGVTALNRILPRLNLLADDSLADRVDEIRERLDEAQEAAR

FVQQFGNQLAKLEPIVSVLQSDPEQFEQLKEDYAYSQQMQRDARQQAFALTEVVQRRAHFSYSDSAEMLS

GNSDLNEKLRERLEQAEAERTRAREALRGHAAQLSQYNQVLASLKSSYDTKKELLNDLQRELQDIGVRAD

SGAEERARIRRDELHAQLSNNRSRRNQLEKALTFCEAEMDNLTRKLRKLERDYFEMREQVVTAKAGWCAV

MRMVKDNGVERRLHRRELAYLSADDLRSMSDKALGALRLAVADNEHLRDVLRMSEDPKRPERKIQFFVAV

YQHLRERIRQDIIRTDDPVEAIEQMEIELSRLTEELTSREQKLAISSRSVANIIRKTIQREQNRIRMLNQ

GLQNVSFGQVNSVRLNVNVRETHAMLLDVLSEQHEQHQDLFNSNRLTFSEALAKLYQRLNPQIDMGQRTP

QTIGEELLDYRNYLEMEVEVNRGSDGWLRAESGALSTGEAIGTGMSILVMVVQSWEDESRRLRGKDISPC

RLLFLDEAARLDARSIATLFELCERLQMQLIIAAPENISPEKGTTYKLVRKVFQNTEHVHVVGLRGFAPQ

LPETLLGRDEAPSQAS

>gi|30062457|ref|NP_836628.1| condesin subunit E [Shigella flexneri 2a str. 2457T]

MPVKLAQALANPLFPALDSALRSGRHIGLDELDNHAFLMDFQEYLEEFYARYNVELIRAPEGFFYLRPRS

TTLIPRSVLSELDMMVGKILCYLYLSPERLANEGIFTQQELYDELLTLADEAKLLKLVNNRSTGSDVDRQ

KLQEKVRSSLNRLRRLGMVWFMGHDSSKFRITESVFRFGADVRAGDDPREAQRRLIRDGEAMPIENHLQL

NDETEESQPDSGEEE

>gi|30062456|ref|NP_836627.1| condesin subunit F [Shigella flexneri 2a str. 2457T]

MSEFSQTVPELVAWARKNDFSISLPVDRLSFLLAVATLNGERLDGEMSEGELVDAFRHVSDAFEQTSETI

GVRANNAINDMVRQRLLNRFTSEQAEGNAIYRLTPLGIGITDYYIRQREFSTLRLSMQLSIVAGELKRAA

DAAEEGGDEFHWHRNVYAPLKYSVAEIFDSIDLTQRLMDEQQQQVKDDIAQLLNKDWRAAISSCELLLSE

TSGTLRELQDTLEAAGDKLQANLLRIQDATMTHDDLHFVDRLVFDLQSKLDRIISWGQQSIDLWIGYDRH
VHKFIRTAIDMDKNRVFAQRLRQSVQTYFDEPWALTYANADRLLDMRDEEMALRDEEVTGELPEDLEYEE
FNEIREQLAAIIEEQLAVYKTRQVPLDLGLVVREYLSQYPRARHFDVARIVIDQAVRLGVAQADFTGLPA
KWQPINDYGAKVQAHVIDKY

>gi|30062455|ref|NP_836626.1| metallothionein SmtA [Shigella flexneri 2a str. 2457T]
MQDRNFDDIAEKFSRNIYGTTKGQLRQAILWQDLDRVLAEMGPQKLRVLDAGGGEGQTAIKMAERGHQVI
LCDLSAQMIDRAKQAAEAKGVSDNMQFIHCAAQDVASHLETPVDLILFHAVLEWVADPRSVLQTLWSVLR
PGGVLSLMFYNAHGLLMHNMVAGNFDYVQAGMPKKKKRTLSPDYPRDPAQVYLWLEEAGWQIMGKTGVRV
FHDYLREKYQQRDCYEALLELETRYCRQEPYITLGRYIHVTARKPQSKDKV

>gi|30062454|ref|NP_836625.1| hypothetical protein S0980 [Shigella flexneri 2a str. 2457T]
MLFTLKKVIGNMLLPLPLMLLIIGAGLALLWFSRFQKTGKIFISIGWLALLLLSLQPVADRLLRPIESTY
PTWNNSQKVDYIVVLGGGYTWNPQWAPSSNLINNSLPRLNEGIRLWRENPGSKLIFTGGVAKTNTVSTAE
VGARVAQSLGVPREQIITLDLPKDTEEEAAAVKQAIGDAPFLLVTSASHLPRAMIFFQQEGLNPLPAPAN
QLAIDSPLNPWERAIPSPVWLMHSDRVGYETLGRIWQWLKGPSGEPRQE

>gi|30062453|ref|NP_836624.1| hypothetical protein S0979 [Shigella flexneri 2a str. 2457T]
MEQLRAELSHLLGEKLSRIECVNEKADTALWALYDSQGNPMPLMARSFSTPGKARQLAWKTTMLARSGTV
RMPTIYGVMTHEEHPGPDVLLLERMRGVSVEAPARTPERWEQLKDQIVEALLAWHRQDSRGCVGAVDNTQ
ENFWPSWYRQHVEVLWTTLNQFNNTGLTMQDKRILFRTRECLPALFEGFNDNCVLIHGNFCLRSMLKDSR
SDQLLAMVGPGLMLWAPREYELFRLMDNSLAEDLLWSYLQRAPVAESFIWRRWLYVLWDEVAQLVNTGRF
SRRNFDLASKSLLPWLA

>gi|30062450|ref|NP_836621.1| hypothetical protein S0976 [Shigella flexneri 2a str. 2457T]

MSLPHLSLADARNLHLAAQGLLNKPRRRASLEDIPATISRMSLLQIDTINIVARSPYLVLFSRLGNYPAQ

WLDESLARGELMEYWAHEACFMPRSDFRLIRHRMLAPEKMGWKYKDAWMQEHEAEIAQLIQHIHDKGPVR

SADFEHPRKGASGWWEWKPHKRHLEGLFTAGKVMVIERRNFQRVYDLTHRVMPDWDDERDLVSQTEAEII

MLDNSARSLGIFREQWLADYYRLKRPALAAWREARAEQQQIIAVHVEKLGNLWLHADLLPLLERALAGKL

TATHSAVLSPFDPVVWDRKRAEQLFDFSYRLECYTPAPKRQYGYFVLPLLHRGQLVGRMDAKMHRQTGIL

EVISLWLQEGIKPTTMLQKGLRQAITDFASWQQATRVTLGRCPQGLFTDCRTGWEIDPVA

>gi|30062449|ref|NP_836620.1| tetraacyldisaccharide 4'-kinase [Shigella flexneri 2a str. 2457T]

MIEKIWSGESPLWRLLLPLSWLYGLVSGAIRLCYKLKLKRAWRAPVPVVVVGNLTAGGNGKTPVVVWLVE

QLQQRGIRVGVVSRGYGGKAESYPLLLSADTTTAQAGDEPVLIYQRTDAPVAVSPVRSDAVKAILAQHPD

VQIIVTDDGLQHYRLARDVEIVVIDGVRRFGNGWWLPAGPMRERAGRLKSVDAVIVNGGVPRSGEIPMHL

LPGQAVNLRTGTRCDVAQLEHVVAMAGIGHPPRFFATLKMCGVQPEKCVPLADHQSLNHADVSALVSAGQ

TLVMTEKDAVKCRAFAEENWWYLPVDAQLSGDEPAKLLTQLTSLASGN

>gi|30062448|ref|NP_836619.1| lipid transporter ATP-binding/permease [Shigella flexneri 2a str. 2457T]

MHNDKDLSTWQTFRRLWPTIAPFKAGLIVAGVALILNAASDTFMLSLLKPLLDDGFGKTDRSVLVWMPLV

VIGLMILRGITSYVSSYCISWVSGKVVMTMRRRLFGHMMGMPVSFFDKQSTGTLLSRITYDSEQVASSSS

GALITVVREGASIIGLFIMMFYYSWQLSIILIVLAPIVSIAIRVVSKRFRNISKNMQNTMGQVTTSAEQM

LKGHKEVLIFGGQEVETKRFDKVSNRMRLQGMKMVSASSISDPIIQLIASLALAFVLYAASFPSVMDNLT

AGTITVVFSSMIALMRPLKSLTNVNAQFQRGMAACQTLFTILDSEQEKDEGKRVIERATGDVEFRNVTFT

YPGRDVPALRNINLKIPAGKTVALVGRSGSGKSTIASLITRFYDIDEGEILMDGHDLREYTLASLRNQVA

LVSQNVHLFNDTVANNIAYARTEQYSREQIEEAARMAYAMDFINKMDNGLDTVIGENGVLLSGGQRQRIA

IARALLRDSPILILDEATSALDTESERAIQAALDELQKNRTSLVIAHRLSTIEKADEIVVVEDGVIVERG

THNDLLEHRGVYAQLHKMQFGQ

>gi|30062445|ref|NP_836616.1| cytidylate kinase [Shigella flexneri 2a str. 2457T]

MTAIAPVITIDGPSGAGKGTLCKAMAEALQWHLLDSGAIYRVLALAALHHHVDVASEDALVPLASHLDVR

FVSTNGNLEVILEGEDVSGEIRTQEVANAASQVAAFPRVREALLRRQRAFRELPGLIADGRDMGTVVFPD

APVKIFLDASSEERAHRRMLQLQEKGFSVNFERLLAEIKERDDRDRNRAVAPLVPAADALVLDSTTLSIE

QLIEKALQYARQKLALA

>gi|30062442|ref|NP_836613.1| 3-phosphoshikimate 1-carboxyvinyltransferase [Shigella flexneri 2a str. 2457T]

MESLTLQPIARVDGTINLPGSKSVSNRALLLAALAHGKTVLTNLLDSDDVRHMLNALTALGLSYTLSADR

TRCEIIGNGGPLHAEGALELFLGNAGTAMRPLAAALCLDSNDIVLTGEPRMKERPIGHLVDALRLGGAKI

TYLEQENYPPLRLQGGFTGGNVDVDGSVSSQFLTALLMTAPLAPEDTVIRIKGDLVSKPYIDITLNLMKT

FGVEIENQHYQQFVVKGGQSYQSPGTYLVEGDASSASYFLAAAAIKGGTVKVTGIGRNSMQGDIRFADVL

EKMGATICWGDDYISCTRGELNAIDMDMNHIPDAAMTIATAALFAKGTTTLRNIYNWRVKETDRLFAMAT

ELRKVGAEVEEGHDYIRITPPEKLNFAEIATYNDHRMAMCFSLVALSDTPVTILDPKCTAKTFPDYFEQL

ARISQAA

>gi|30062440|ref|NP_836611.1| hypothetical protein S0964 [Shigella flexneri 2a str. 2457T]

MTQTFIPGKDAALEDSIARFQQKLSDLGFQIEEASWLNPVPNVWSVHIRDKECALCFTNGKGATKKAALA

SALGEYFERLSTNYFFADFWLGETIANGPFVHYPNEKWFPLSENDDVPEGLLDDRLRAFYDPENELTGSM

LIDLQSGNEDRGICGLPFTRQSDNQTVYIPMNIIGNLYVSNGMSAGNTRNEARVQGLSEVFERYVKNRII

AESISLPEIPADVLARYPAVVEAIETLETEGFPIFAYDGSLGGQYPVICVVLFNPANGTCFASFGAHPDF

GVALERTVTELLQGRGLKDLDVFTPPTFDDEEVAEHTNLETHFIDSSGLISWDLFKQDADYPFVDWNFSG

TTEEEFATLMAIFNKEDKEVYIADYEHLGVYACRIIVPGMSDIYPAEDLWLANNSMGSHLRETILSLPGS

EWEKEDYLNLIEQLDEEGFDDFTRVRELLGLATGSDNGWYTLRIGELKAMLALAGGDLEQALVWTEWTME

FNSSVFSPERANYYRCLQTLLLLAQEEDRQPLQYLNAFVRMYGADAVEAASAAMSGEAAFYGLQPVDSDL

HAFAAHQSLLKAYEKLQRAKAAFWAK

>gi|30062439|ref|NP_836610.1| formate transporter [Shigella flexneri 2a str. 2457T]

MKADNPFDLLLPAAMAKVAEEAGVYKATKHPLKTFYLAITAGVFISIAFVFYITATTGTGTMPFGMAKLV

GGICFSLGLILCVVCGADLFTSTVLIVVAKASGRITWCQLAKNWLNVYFGNLVGALLFVLLMWLSGEYMT

ANGQWGLNVLQTADHKVHHTFIEAVCLGILANLMVCLAVWMSYSGRSLMDKAFIMVLPVAMFVASGFEHS

IANMFMIPMGIVIRDFASPEFWTAVGSAPENFSHLTVMNFITDNLIPVTIGNIIGGGLLVGLTYWVIYLR

ENDHH

>gi|30062437|ref|NP_836608.1| pyruvate formate lyase-activating enzyme 1 [Shigella flexneri 2a str. 2457T]

MSVIGRIHSFESCGTVDGPGIRFITFFQGCLMRCLYCHNRDTWDTHGGKEVTVEDLMKEVVTYRHFMNAS

GGGVTASGGEAILQAEFVRDWFRACKKEGIHTCLDTNGFVRRYDPVIDELLEVTDLVMLDLKQMNDEIHQ

NLVGVSNHRTLEFAKYLANKNVKVWIRYVVVPGWSDDDDSAHRLGEFTRDMGNVEKIELLPYHELGKHKW

VAMGEEYKLDGVKPPKKETMERVKGILEQYGHKVMF

>gi|30062428|ref|NP_836599.1| IS629 orfB [Shigella flexneri 2a str. 2457T]

MPLLDKLREQYRVGPLCSELHIAPSTYYHCQQQRHHPDKRSARAQRDDWLKKEIQRVYDENHKVYGVRKV

WRQLLREGIRVARCTVARLMEVMGLAGVLRGKKVRTTISRKAVAAGDRVNRQFVAERPDQLWVADFTYVS

TWQGFVYVAFIIDVFAGYIVGWRVSSSMETTFVLDALEQALWARRPSGTVHHSDKGSQYVSLAYTQRLKE

AGLLASTGSTGDSYDNAMAESINGLYKAEVIHRKSWKNRAEVELATLTWVDWYNNRRLLERLGHIPPAEA

EKAYYASIGNDDLAA

>gi|30062423|ref|NP_836594.1| lysozyme protein R of prophage CP-933K [Shigella flexneri 2a str. 2457T]

MSPSLRKAVAVAIGGGAVAIASVLITGPGGNDGLEGVSYIPYKDIIGVWTVCHGHTGKDIMPGKTYTEAE

CKALLNKDLVTVARQINPYIKVDIPETTVSAPSATSCENCPVWQQSRPSILMDTNDEFPDNKRYSLLPFL

FA

>gi|30062420|ref|NP_836591.1| integrase encoded by prophage CP-933K; partial, partial [Shigella flexneri 2a str. 2457T]

MSKIKAIRRGLPDAPLEDITTKEIAAMLNGYIDEGKAASAKLIRSTLSDAFREAIAEGHITTNPVAATRA

AKSEVRRSRLTADEYLKIYQAAESSPCWLRLAMELAVVTGQRVGDLCEMKWSDIVDGYLYVEQSKTGVKI

AIPTTLHVDALGISMKETLDKCKEILGGETIIASTRREPLSSGTVSRYFMRARKASGLSFEGDPPTFHEL

RSLSARLYEKQISDKFAQHLLGHKSDTMASQYRDDRGREWDKIEIK

>gi|30062419|ref|NP_836590.1| pectinesterase [Shigella flexneri 2a str. 2457T]

MNTFSVSRLALALAFGVTLTACSSTPPDQRPSDQTAPGTSSRPILSAKEALNFDAQHYFASLTPGAAAWN

PSPITLPAQPDFVVGPAGTQGVTHTTIQAAVDAAIIKRTNKRQYIAVMPGEYQGTVYVPAAPGGITLYGT

GEKPIDVKIGLSLDGGMSPADWRHDVNPRGKYMPGKPAWYMYDSCQSKRSDSIGVLCSAVFWSQNNGLQL

QNLTIENTLGDSVDAGNHPAVALRTDGDQVQINNVNILGRQNTFFVTNSGVQNRLETNRQPRTLVTNSYI

EGDVDIVSGRGAVVFDNTEFRVVNSRTQQEAYVFAPATLSNIYYGFLAVNSRFNASGDGVAQLGRSLDVD

ANTNGQVVIRDSAINEGFNTAKPWADAVISNRPFAGNTGSVDDNDEIQRNLNDTNYNRMWEYNNRGVGSK

VVAEEKK

>gi|30062418|ref|NP_836589.1| enzyme [Shigella flexneri 2a str. 2457T]

MIKLSEKGVFLASNNEIIAEEHFTGEIKKEEAKKGTIAWSILSSHNTSGNMDKLKIKFDSLASHDITFVG

IVQTAKASGMERFPLPYVLTNCHNSLCAVGGTINGDDHVFGLSAAQRYGGIFVPPHIAVIHQYMREMMAG

GGKMILGSDSHTRYGALGTMAVGEGGGELVKQLLNDTWDIDYPGVVAVHLTGKPAPYVGPQDVALAIIGA

VFKNGYVKNKVMEFVGPGVSALSTDFRNSVDVMTTETTCLSSVWQTDEEVHNWLALHGRGQDYCQLNPQP

MAYYDGCISVDLSAIKPMIALPFHPSNVYEIDTLNQNLTDILREIEIESERVAHGKAKLSLLDKVENGRL

KVQQGIIAGCSGGNYENVIAAANALRGQSCGNDTFSLAVYPSSQPVFMDLAKKGVVADLIGAGAIIRTAF

CGPCFGAGDTPINNGLSIRHTTRNFPNREGSKPANGQMSAVALMDARSIAATAANGGYLTSASELDCWDN

VPEYAFDVTPYKNRVYQGFVKGATQQPLIYGPNIKDWPELGALTDNIVLKVCSKILDEVTTTDELIPSGE

TSSYRSNPIGLAGNVSELTEVFARIKQIAGQEHIDPLQTEIGSMVYAVKPGDGSAREQAASCQRVIGGLA

NIAEEYATKRYRSNVINWGMLPLQMAEVPTFEVGDYIYIPGIKAALDNPGTTFKGYVIHEDAPVTEITLY

MESLTAEEREIIKAGSLINFNKNRQM

>gi|30062415|ref|NP_836586.1| 6-phosphogluconolactonase [Shigella flexneri 2a str. 2457T]

MKQTVYIASPESQQIHVWNLNHEGALTLTQVVDVPGQVQPMVVSPDKRYLYVGVRPEFRVLAYRIAPDDG

ALTFAAESVLPGSPTHISTDHQGQFVFVGSYNAGNVSVTRLEDGLPVGVVDVVEGLDGCHSANISPDNRT

LWVPALKQDRICLFTVSDDGHLVAQDPAEVTTVEGAGPRHMVFHPNEQYAYCVNELNSSVDVWELKDPHG

NIECVQTLDMMPENFSDTRWAADIHITPDGRHLYACDRTASLITVFSVSEDGSVLSKEGFQPTETQPRGF

NVDYSGKYLIAAGQKSHHISVYEIVGEQGLLHEKGRYAVGQGPMWVVVNAH

>gi|30062410|ref|NP_836581.1| exodeoxyribonuclease VIII of prophage CP-933R [Shigella flexneri 2a str. 2457T]

MSTKPLFLLRKAKKSSGEPDVVLWASDDFESTSTTLDYLLVKSGKKLSNYFKAVATNFPVVNDLPPEGEI

DFTWSERYQLSKDSMTWELKPGAAPDDVHHQDNAQETKELAGGQEENAQADAHEDCQDCEVSVATLRFTQ

RLLHIFTYAAGDLKYLHHATREQRKHITALEMDQENSYVQNLLLAIRGMAEPTTLDNAALLRLTDAIKAE

VYWQ

>gi|30062400|ref|NP_836571.1| bacteriophage protein [Shigella flexneri 2a str. 2457T]

MKIRHEHIESVLLALAAEKGQAWVANAITEEYLRQGGGELSLVPGKDWNNQQNIYHRWLKGETKAQREKI

QKLIPAVLAILPRELRHRLCIFDTLERRALLAAQEALSTAIDAHDDAVQAVYRKAHFSGGGSPGDSVVVH

>gi|30062399|ref|NP_836570.1| bacteriophage protein [Shigella flexneri 2a str. 2457T]

MASNWIKLEVITPDKPEIFRLAEILNIDPDAALGKVIRFWAWADQQMIDGNADCNARGVTKSAIDRITFM

SGFADALIQVGWLVENDGGLSLPNFERHNGKSSKKRAVTNERVTKIRELKRKGNAASVTQTDQKALPVEE

EEEDLNTDLPLNPPRQKRASKKFEPEAIELPDWLPETLWHEWVRFRQALRKPIRTEQGDTGTGKIPSAGF

YT

>gi|30062398|ref|NP_836569.1| bacteriophage protein [Shigella flexneri 2a str. 2457T]

MAKPFTPEQREELKTRIVELVHQDGRVTIRQLSDETGISRASVGRLCIELVASGDVYNSGYGLFPSEQAR

KDWQSARKKLSRVKVRKPVVVDPDLIWSLPDGEIRRYDRRLNIICRECRKSEAMQRVLAFYQGNFQEAIL

>gi|30062397|ref|NP_836568.1| hypothetical protein S0913 [Shigella flexneri 2a str. 2457T]

MEALQASEIDYTIFFYNPNIHPQKEYLIRKDENIRFAEQHGVPFIDADYDTDNWFERAKGMEWEPERGIR

CTMCFDMRFERTALYAAENGFSVISSSLGISRWKNMQQVNDCGRRAVAHYPGMVYWDYNWRKQGGSSRMI

EISKREKFYQQEYCGCVYSLRDTNLHRKSQGRPLIKIGQLHYGKEEKE

>gi|30062385|ref|NP_836556.1| MFS family transporter protein [Shigella flexneri 2a str. 2457T]

MSTYTRPVMLLLSGLLLLTLAIAVLNTLVPLWLAQEHMSTWQVGVVSSSYFTGNLVGTLLTGYVIKRIGF

NRSYYLSSFIFAAGCAGLGLMIGFWSWLAWRFVAGVGCAMIWVVVESALMCSGTSRNRGRLLAAYMMVYY

VGTFLGQLLVSKVSTELMSVLPWVTGLTLAGILPLLFTRVLNQQAENHDSTSITAMLKLRQARLGVNGCI

ISGIVLGSLYGLMPLYLNHKGVSNASIGFWMAVLVSAGILGQWPIGRLADKFGRLLVLRVQVFVVILGSI

AMLSQAAMAPALFILGAAGFTLYPVAMAWACEKVEHHQLVAMNQALLLSYTVGSLLGPSFSAMLMQNFSD

NLLFIMIASVSFIYLLMLLRNAGHTPKPVAHV

>gi|30062384|ref|NP_836555.1| hypothetical protein S0897 [Shigella flexneri 2a str. 2457T]

MTKPYVRLDKNDAAVLLVDHQAGLLSLVRDIEPDKFKNNVLALGDLAKYFNLPTILTTSFETGPNGPLVL

ELKAQFPDAPYIARPGNINAWDNEDFVKAVKATGKKQLIIAGVVTEVCVAFPALSAIEEGFDVFVVTDAS

GTFNEITRHSAWDRMSQAGAQLMTWFGVACELHRDWRNDIEGLATLFSNHIPDYRNLMTSYDTLTKQK

>gi|30062383|ref|NP_836554.1| anaerobic dimethyl sulfoxide reductase subunit C [Shigella flexneri 2a str. 2457T]

MGSGWHEWPLMIFTVFGQCVAGGFIVLALALLKGDLRAEAQQRVIACMFGLWVLMGIGFIASMLHLGSPM

RAFNSLNRVGASALSNEIASGSIFFAVGGIGWLLAMLKKLSPALRTLWLVVTMVLGVVFVWMMVRVYNSI

DTVPTWYSIWTPMGWAMRLLPAISVLALVVSGVMSVMQGTELATIHSSVQQAAALVPDYGALMSWRIVLL

AVALCLWIAPQLKGYQPAVPLLSVSFILLLAGELIGRGVFYGLHMTVGMAVAS

>gi|30062381|ref|NP_836552.1| anaerobic dimethyl sulfoxide reductase subunit A [Shigella flexneri 2a str. 2457T]

MASSALTLPFSRIAHAVDSAIPTKSDEKVIWSACTVNCGSRCPLRMHVVDGEIKYVETDNTGDDNYDGLH

QVRACLRGRSMRRRVYNPDRLKYPMKRVGARGEGKFERISWEEAYDIIATNMQRLIKEYGNESIYLNYGT

GTLGGTMTRSWPPGNTLVARLMNCCGGYLNHYGDYSSAQIAEGLNHTYGGWADGNSPSDIENSKLVVLFG

NNPGETRMSGGGVTYYLEQARQKSNARMIIIDPRYTDTGAGREDEWIPIRPGTDAALVNGLAYVMITKNL

VDQAFLDKYCVGYDEKTLPASAPKNGHYKAYILGEGPDGVAKTPEWASQITGVPADKIIKLAREIGSTKP

AFISQGWGPQRHANGEIATRAISMLAILTGNVGINGGNSGAREGSYSLPFVRMPTLENPIQTSISMFMWT

DAIERGPEMTALRDGVRGKDKLDVPIKMIWNYAGNCLINQHSEINRTHEILQDDKKCELIVVIDCHMTSS

AKYADILLPDCTASEQMDFALDASCGNMSYVIFNDQVIKPRFECKTIYEMTSELAKRLGVEQQFTEGRTQ

EEWMRHLYAQSREAIPELPTFEEFRKQGIFKKRDPQGHHVAYKAFREDPQANPLTTPSGKIEIYSQALAD

IAATWELPEGDVIDPLPIYTPGFESYQDPLNKQYPLQLTGFHYKSRVHSTYGNVDVLKAACRQEMWINPL

DAQKRGIHNGDKVRIFNDRGEVHIEAKVTPRMMPGVVALGEGAWYDPDAKRVDKGGCINVLTTQRPSPLA

KGNPSHTNLVQVEKV

>gi|30062378|ref|NP_836549.1| outer-membrane lipoprotein carrier protein [Shigella flexneri 2a str. 2457T]

MMKKIAITCALLSSLVASSVWADAASDLKSRLDKVSSFHASFTQKVTDGSGAAVQEGQGDLWVKRPNLFN

WHMTQPDESILVSDGKTLWFYNPFVEQATATWLKDATGNTPFMLIARNQSSDWQQYNIKQNGDDFVLTPK

ASNGNLKQFTINVGRDGTIHQFSAVEQDDQRSSYQLKSQQNGAVDAAKFTFTPPQGVTVDDQRK

>gi|30062377|ref|NP_836548.1| DNA translocase FtsK [Shigella flexneri 2a str. 2457T]

MSQEYTEDKEVTLTKLSSGRRLLEALLILIVLFAVWLMAALLSFNPSDPSWSQTAWHEPIHNLGGMPGAW

LADTLFFIFGVMAYTIPVIIVGGCWFAWRHQSSDEYIDYFAVSLRIIGVLALILTSCGLAAINADDIWYF

ASGGVIGSLLSTTLQPLLHSSGGTIALLCVWAAGLTLFTGWSWVTIAEKLGGWILNILTFASNRTRRDDT

WVDEDEYEDDEEYEDENHGKQHESRRARILRGALARRKRLAEKFINPMGRQTDAALFSGKRMDDDEEITY

TARGVAADPDDVLFSGNRATQPEYDEYDPLLNSAPITEPVAVAAAATTATQSWAAPVEPVTQTPPVASVD

VPPSQPTVAWQPVPGPQTGEPVIAPAPEGYPQQSQYAQPAVQYNEPLQQPVQPQQPYYAPAAEQPAQQPY

YAPAPEQPVAGNAWQAEEQQSTFAPLSTYQTEQTYQQPAAQEPLYQQPQPVEQQPVVEPEPVVEETKPAR

PPLYYFEEVEEKRAREREQLAAWYQPIPEPVKEPEPIKSSLKAPSVAAVPPVEAAAAVSPLASGVKKATL

ATGAAATVAAPVFSLANSGGPRPQVKEGIGPQLPRPKRIRVPTRRELASYGIKLPSQRAAEEKAREAQRN

QYDSGDQYNDDEIDAMQQDELARQFAQTQQQRYGEQYQHDVPVNAEDADAAAEAELARQFAQTQQQRYSG

EQPAGANPFSLDDFEFSPMKALLDDGPHEPLFTPIVEPVQQPQQPVAPQQQYQQPQQPVAPQQQYQQPQQ

QVAPQPQYQQPQQPVAPQQQYQQPQQPVAPQPQYQQPQQPVAPQPQYQQPQQPVAPQPQDTLLHPLLMRN

GDSRPLHKPTTPLPSLDLLTPPPSEVEPVDTFALEQMARLVEARLADFRIKADVVNYSPGPVITRFELNL

APGVKAARISNLSRDLARSLSTVAVRVVEVIPGKPYVGLELPNKKRQTVYLREVLDNAKFRDNPSPLTVV

LGKDIAGEPVVADLAKMPHLLVAGTTGSGKSVGVNAMILSMLYKAQPEDVRFIMIDPKMLELSVYEGIPH

LLTEVVTDMKDAANALRWCVNEMERRYKLMSALGVRNLAGYNEKIAEADRMMRPIPDPYWKPGDSMDAQH

PVLKKEPYIVVLVDEFADLMMTVGKKVEELIARLAQKARAAGIHLVLATQRPSVDVITGLIKANIPTRIA

FTVSSKIDSRTILDQAGAESLLGMGDMLYSGPNSTLPVRVHGAFVRDQEVHAVVQDWKARGRPQYVDGIT

SDSESEGGAGGFDGAEELDPLFDQAVQFVTEKRKASISGVQRQFRIGYNRAARIIEQMEAQGIVSEQGHN

GNREVLAPPPFD

>gi|30062376|ref|NP_836547.1| leucine-responsive transcriptional regulator [Shigella flexneri 2a str. 2457T]

MVDSKKRPGKDLDRIDRNILNELQKDGRISNVELSKRVGLSPTPCLERVRRLERQGFIQGYTALLNPHYL

DASLLVFVEITLNRGAPDVFEQFNTAVQKLEEIQECHLVSGDFDYLLKTRVPDMSAYRKLLGETLLRLPG

VNDTRTYVVMEEVKQSNRLVIKTR

>gi|30062372|ref|NP_836543.1| leucyl/phenylalanyl-tRNA--protein transferase [Shigella flexneri 2a str. 2457T]

MRLVQLSRHSIAFPSPEGALREPNGLLALGGDLSPARLLMAYQRGIFPWFSPGDPILWWSPDPRAVLWPE

SLHISRSMKRFHKRSPYRVTMNYAFGQVIEGCASDREEGTWITRGVVEAYHRLHELGHAHSIEVWREDEL

VGGMYGVAQGTLFCGESMFSRMENASKTALLVFCEEFIGHGGKLIDCQVLNDHTASLGACEIPRRDYLNY

LNQMRLGRLPNNFWVPRCLFSPQE

>gi|30062369|ref|NP_836540.1| ATP-dependent Clp protease adaptor protein ClpS [Shigella flexneri 2a str. 2457T]

MGKTNDWLDFDQLAEEKVRDALKPPSMYKVILVNDDYTPMEFVIDVLQKFFSYDVERATQLMLAVHYQGK

AICGVFTAEVAETKVAMVNKYARENEHPLLCTLEKA

>gi|30062366|ref|NP_836537.1| macrolide transporter subunit MacA [Shigella flexneri 2a str. 2457T]

MKKRKTVKKRYVIALVIVIAGLITLWRILNAPVPTYQTLIVRPGDLQQSVLATGKLDALRKVDVGAQVSG

QLKTLSVAIGDKVKKDQLLGVIDPEQAENQIKEVEATLMELRAQRQQAEAELKLARVTYSRQQRLAQTQA

VSLQDLDTAATEMAVKQAQIGTIDAQIKRNQASLDTAKTNLDYTRIVAPMAGEVTQITTLQGQTVIAAQQ

APNILTLADMSTMLVKAQVSEADVIHLKPGQKAWFTVLGDPLTRYEGQIKDVLPTPEKVNDAIFYYARFE

VPNPNGLLRLDMTAQVHIQLTDVKNVLTIPLSALGDPVGDNRYKVKLLRNGETREREVTIGARNDTDVEI

VKGLEAGDEVVIGEAKPGAAQ

>gi|30062365|ref|NP_836536.1| enzyme [Shigella flexneri 2a str. 2457T]

MVKSTSCITIDFMNMSQLTERTFTPSESLSSLSLFLSLARGQCRPGKFWHRCSFRQKFLLRSLIMPRLSV

EWMNELSHWPNLNVLLTRQPRLPVRLHRPYLAANLSRKQLLEALRYHYALLRGCMSAEEFSLYLNTPGLQ

LAKLEGKNGEQFTLELTMMISMDKEGDSTILFRNSEGIPLAEITFTLCEYQGKRTMFIGGLQGAKWEIPH

QEIQNATKACHGLFPKRLVMEAACLFAQRLQVEQIIAVSNETHIYRSLRYRDKEGKIHADYNAFWESVGG

VCDAERHYRLPAQIARKEIAEIASKKRAEYRRRYEMLDAIQPQMATMFRG

>gi|30062364|ref|NP_836535.1| hypothetical protein S0876 [Shigella flexneri 2a str. 2457T]

MILERVEIVGFRGINRLSLMLEQNNVLIGENAWGKSSLLDALTLLLSPESDLYHFERDDFWFPPGDINGR

EHHLHIILTFRESLPGRHRVRRYRPLEACWTPCTDGYHRIFYRLEGESAEDGSVMTLRSFLDKDGHPIDV

EDINDQARHLVRLMPVLRLRDARFMRRIRNGTVPNVPNVEVTARQLDFLARELSSHPQNLSDGQIRQGLS

AMVQLLEHYFSEQGAGQARYRLMRRRASNEQRSWRYLDIINRMIDRPGGRSYRVILLGLFATLLQAKGTL

RLDKDARPLLLIEDPETRLHPIMLSVAWHLLNLLPLQRIATTNSGELLSLTPVEHVCRLVRESSRVAAWR

LGPSGLSTEDSRRISFHIRFNRPSSLFARCWLLVEGETETWVINELARQCGHHFDAEGIKVIEFAQSGLK

PLVKFARRMGIEWHVLVDGDEAGKKYAATVRSLLNNDREAEREHLTALPALDMEHFMYRQGFSDVFHRVA

QIPENVPMNLRKIISKAIHRSSKPDLAIEVAMEAGRRGVDSVPTLLKKMFSRVLWLARGRAD

>gi|30062358|ref|NP_836529.1| surface protein [Shigella flexneri 2a str. 2457T]

MCHRAFRLLLCKDWIFMFSGLLIILVPLIVGYLIPLRQQAALKAINQLLSWMVYLILFFMGISLAFLDNL

ASNLLAILHYSAVSITVILLCNIAALMWLERGLPWRNHHQQEKLPSRIAMALESLKLCGVVVIGFAIGLS

GLAFLQHATEASEYTLILLLFLVGIQLRNNGMTLKQIVLNRRGMIVAVVVVVSSLIGGLINAFILDLPIN

TALAMASGFGWYSLSGILLTESFGPVIGSAAFFNDLARELIAIMLIPGLIRRSRSTALGLCGATSMDFTL

PVLQRTGGLDMVPAAIVHGFILSLLVPILIAFFSA

>gi|30062354|ref|NP_836525.1| L-threonine aldolase [Shigella flexneri 2a str. 2457T]

MIDLRSDTVTRPSRAMLEAMMAAPVGDDVYGDDPTVNALQDYAAELSGKEAAIFLPTGTQANLVALLSHC

ERGEEYIVGQAAHNYLFEAGGAAVLGSIQPQPIDAAADGTLPLDKVAMKIKPDDIHFARTKLLSLENTHN

GKVLPREYLKEAWEFTRERNLALHVDGARIFNAVVAYGCELKEITQYCDSFTICLSKGLGTPVGSLLVGN

RDYIKRAIRWRKMAGGGMRQSGILAAAGMYALENNVARLQEDHDNAAWMAEQLREAGADVMRQDTNMLFV

RVGEENAAALGEYMKARNVLINASPIVRLVTHLDVSREQLAEVAAHWRAFLAR

>gi|30062353|ref|NP_836524.1| dTDP-glucose enzyme [Shigella flexneri 2a str. 2457T]

MPQRILVLGASGYIGQHLVRTLSQQGHQILAAARHVDRLAKLQLANVSCHKVDLSWPDNLPALLQDIDTV

YFLVHSMGEGGDFIAQERQLALNVRDALREVPVKQLIFLSSLQAPPHEQSDHLRARQATADILREANVPV

TELRAGIIVGAGSAAFEVMRDMVYNLPVLTPPRWVRSRTTPIALENLLHYLVALLDHPANEHRIFEAAGP

EVLSYQQQFEHFMAVSGKRRWLIPIPLPTRWISVWFLNVITSVPPTTARALIQGLKHDLLADDTALRALI

PQRLIAFDDAVRSTLKEEEKLVNSSDWGYDAQAFARWRPEYGYFAKQAGFTVKTSASLAALWLVVNQIGG

KERYFFGNILWQTRALMDRAIGHNLAKGRPEREYLQTGDAVDSWKVIVVEPKKQLTLLFGMKAPGLGRLC

FTLEDKGDYRTIDVRAFWHPHGMPGLFYWLLMIPAHLFIFRGMAKQIARLAEQSTD

>gi|30062351|ref|NP_836522.1| regulator [Shigella flexneri 2a str. 2457T]

MRRVFWLVAAALLLAGCAGEKGIVEKEGYQLDTRHQAQAAYPRIKVLVIHYTADDFDSSLATLTDKQVSS

HYLIPAVPPRYNGKPRIWQLVPEQELAWHAGISAWRGATRLNDTSIGIELENRGWQKSSGVKYFAPFEQA

QIQALIPLAKDIIARYHIKPENVVAHADIAPQRKDDPGPLFPWQQLAQQGIGAWPDAQRVNFYLAGRAPH

TPVDTASLLELLARYGYDVKPDMTPREQRRVIMAFQMHFRPTLYNGEADAETQAIAEALLEKYGQD

>gi|30062349|ref|NP_836520.1| hypothetical protein S4814 [Shigella flexneri 2a str. 2457T]

MINNISDQASSFPGTQLNQSDNFLDSLREFFAILNPSRKGELSTWDTIYLHLILAINADSDLIKNDVLLA

ENIPSANYQFNTFFSNTFEIDVKKYLGKSEDNEVEIKAGNERISIGIRNVSNGKLERQQFLFPLDYENKL

QEQLDKYFTIESHPLLYRYTIGSKIANVIFEKLYSRIDFNKEQYISFIKDAFIHFYDYSRRYAISENIDK

DAVTNNIALMSTFYDSDNTSGEVLNNDFTEEESFETALDVEHAIVLGFADDNFETKPVHYQDLLTRFSAF

QDTVFNLFPEMHSSHYHDICSVSVDMTKGTQCMIHLMVNEEVFMSLPVPVATMVREDASNLVNLKTLLND

GCFIKYSHFNDVALIKQNISNLYLSHTVVNESILKKCCFENGSLGDVKITNSNVINSAFKNISFRSVKIN

NVNTHSLKFINCNFFNVDMIRVNLSKCLFHECSMHGVKIKPWLPVKWTKELISDYLYGCLLSLYSICARD

IYNMNAGNNVKVAADAFLEIIFSLKNKYCIKLLSAQDRAFIYEFARMIFAYINDKSIEILLLSCFAAADQ

KAIQRYRPQSQDGEDFRSHLQYKLPLSAH

>gi|30062347|ref|NP_836518.1| lipoprotein [Shigella flexneri 2a str. 2457T]

MRYRSLSLLIPCALLLSACTTVTPAYKDNGPRTGSCVQGGPDSVAQQFYDYRIQHRSNDITALRPYLSDK

LATQLSDASRDNSHRELLSSDPFSSRTTLPDSAHVASASTIPNRDARNIPLRVDLKQGDQGWQDEVLMIQ

EGQCWVIDDVRYLGGSVHATAGTLRQSIENR

>gi|30062345|ref|NP_836516.1| arginine 3rd transport system periplasmic binding protein [Shigella flexneri 2a str. 2457T]

MKKVLIAALIAGFSLSATAAETIRFATEAPYPPFESIDANNQIVGFDVDLAQALCKEIDATCTFSNQAFD

SLIPSLKFRRVEAVMAGMDITPEREKQVLFTTPYYDNSALFVGQQGKYTSVDQLKGKKVGVQNGTTHQKF

IMDKHPEITTVPYDSYQNAKLDLQNGRIDSVFGDTAVVTEWLKDNPKLAAVGDKVTDKDYFGTGLGIAVR

QGNTELQQKLNTALEKVKKDGTYETIYNKWFQK

>gi|30062344|ref|NP_836515.1| arginine transporter permease subunit ArtQ [Shigella flexneri 2a str. 2457T]

MNEFFPLASAAGMTVGLAVCALIVGLALAMFFAVWESAKWRPVAWAGSALVTILRGLPEILVVLFIYFGS

SQLLLTLSDGFTINLGFVQIPVQMDIENFDVSPFLCGVIALSLLYAAYASQTLRGALKAVPVGQWESGQA

LGLSKSAIFFRLVMPQMWRHALPGLGNQWLVLLKDTALVSLISVNDLMLQTKSIATRTQEPFTWYIVAAA

IYLVITLLSQYILKRIDLRATRFERRPS

>gi|30062343|ref|NP_836514.1| arginine transporter permease subunit ArtM [Shigella flexneri 2a str. 2457T]

MFEYLPELMKGLHTSLTLTVASLIVALILALIFTIILTLKTPVLVWLVRGYITLFTGTPLLVQIFLIYYG

PGQFPTLQEYPALWHLLSEPWLCALIALSLNSAAYTTQLFYGAIRAIPEGQWQSCSALGMSKKDTLAILL

PYAFKRSLSSYSNEVVLVFKSTSLAYTITLMEVMGYSQLLYGRTYDVMVFGAAGIIYLVVNGLLTLMMRL

IERKALAFERRN

>gi|30062341|ref|NP_836512.1| 23S rRNA methyluridine methyltransferase [Shigella flexneri 2a str. 2457T]

MQCALYDAGRCRSCQWITQPIPEQLSAKTADLKNLLADFPVEEWCAPVSGPEQGFRNKAKMVVSGSVEKT

LLGMLHRDGTPEDLCDCPLYPASFAPVFAALKPFIARAGLTPYNVARKRGELKYILLTESQSDGGMMLRF

VLRSDTKLAQLRKALPWLHEQLPQLKVITVNIQPVHMAIMEGETEIYLTEQQALAERFNDVPLWIRPQSF

FQTNPAVASQLYATARDWVRQLPVKHMWDLFCGVGGFGLHCATPDMQLTGIEIAPEAIACAKQSAAELGL

TRLQFQALDSTQFATAQGEVPELVLVNPPRRGIGKPLCDYLSTMAPRFIIYSSCNAQTMAKDIRELPGYR

IERVQLFDMFPHTAHYEVLTLLVKQ

>gi|30062340|ref|NP_836511.1| hypothetical protein S0854 [Shigella flexneri 2a str. 2457T]

MEDETLGFFKKTSSSHARLNVPALVQVAALAIIMIRGLDVLMIFNTLGVRGIGEFIHRSVQTWSLTLVFL

SSLVLVFIEIWCAFSLVKGRRWARWLYLLTQITAASYLWAASLGYGYPELFSIPGESKREIFHSLMLQKL

PDMLILMLLFVPSTSRRFFQLQ

>gi|30062339|ref|NP_836510.1| putrescine transporter subunit: membrane component of ABC superfamily [Shigella flexneri 2a str. 2457T]

MNNLPVVRSPWRIVILLLGFTFLYAPMLMLVIYSFNSSKLVTVWGGWSTRWYGELLRDDAMMSAVGSSLT

IAACAATAAAILGTIAAVVLVRFGRFRGSNGFAFMITAPLVMPDVITGLSLLLLFVALAHAIGWPADRGM

LTIWLAHVTFCTAYVAVVISSRLRELDRSIEEAAMDLGATPLKVFFVITLPMIMPAIISGWLLAFTLSLD

DLVIASFVSGPGATTLPMLVFSSVRMGVNPEINALATLILGAVGIVGFIAWYLMARAEKQRIRDIQRARR

G

>gi|30062338|ref|NP_836509.1| putrescine transporter subunit: membrane component of ABC superfamily [Shigella flexneri 2a str. 2457T]

MNTLEPAAQSKPPGGFKLWLSQLQMKHGRKLVIALPYIWLILLFLLPFLIVFKISLAEMARAIPPYTELM

EWADGQLSITLNLGNFLQLTDDPLYFDAYLQSLQVAAISTFCCLLIGYPLAWAVAHSKPSTRNILLLLVI

LPSWTSFLIRVYAWMGILKNNGVLNNFLLWLGVIDQPLTILHTNLAVYIGIVYAYVPFMVLPIYTALIRI

DYSLVEAALDLGARPLKTFFTVIVPLTKGGIIAGSMLVFIPAVGEFVIPELLGGPDSIMIGRVLWQEFFN

NRDWPVASAVAIIMLLLLIVPIMWFHKHQQKSVGEHG

>gi|30062336|ref|NP_836507.1| putrescine transporter subunit: periplasmic-binding component of ABC superfamily [Shigella flexneri 2a str. 2457T]

MTALNKKWLSGLVAGALMAVSVGTLAAEQKTLHIYNWSDYIAPDTVANFEKETGIKVVYDVFDSNEVLEG

KLMAGSTGFDLVVPSASFLERQLTAGVFQPLDKSKLPEWKNLDPELLKLVAKHDPDNKFAMPYMWATTGI

GYNVDKVKAVLGENAPVDSWDLILKPENLEKLKSCGVSFLDAPEEVFATVLNYLGKDPNSTKADDYTGPA

TDLLLKLRPNIRYFHSSQYINDLANGDICVAIGWAGDVWQASNRAKEAKNGVNVSFSIPKEGAMAFFDVF

AMPADAKNKDEAYQFLNYLLRPDVVAHISDHVFYVNANKAATPLVSAEVRENPGIYPPADVRAKLFTLKV

QDPKIDRVRTRAWTKVKSGK

>gi|30062335|ref|NP_836506.1| sensory transduction regulator [Shigella flexneri 2a str. 2457T]

MTSLVVPGLDTLRQWLDDLGMSFFECDNCQALHLPHMQNFDGVFDAKIDLIDNTILFSAMAEVRPSAVLP
LAADLSAINASSLTVKAFLDMQDDNLPKLVVCQSLSVMQGVTYEQFAWFVRQSEEQISMVILEANAHQLL
LPTDDEGQNNVTENYFLH

>gi|30062333|ref|NP_836504.1| nitroreductase A [Shigella flexneri 2a str. 2457T]
MTPTIELICGHRSIRHFTDEPISEAQCEAIINSARATSSSSFLQCSSIIRITDKALREELVTLTGGQKHV
AQAAEFWVFCADFNRHLQICPDAQLGLAEQLLLGVVDTAMMAQNALTAAESLGLGGVYIGGLRNNIEAVT
KLLKLPQHVLPLFGLCLGWPADNPDLKPRLPASILVHENSYQPLDKDALAQYDEQLAEYYLTRGSNNRWD
TWSDHIRRTIIKESRPFILDYLHKQGWATR

>gi|30062330|ref|NP_836501.1| hypothetical protein S0844 [Shigella flexneri 2a str. 2457T]
MKHKQRWAGAICCFVLFIVVCLFLATHMKGAFRAAGHPEIGLLFFILPGAVASFFSQRREVLKPLFGAML
AAPCSMLIMRLFFSPTRSFWQELAWLLSAVFWCALGALCFLFISSLFKPQHRKNQ

>gi|30062329|ref|NP_836500.1| hypothetical protein S0843 [Shigella flexneri 2a str. 2457T]
MNINVAELLNGNYILLLFVVLALGLCLGKLRLGSIQLGNSIGVLVVSLLLGQQHFSINTDALNLGFMLFI
FCVGVEAGPNFFSIFFRDGKNYLMLALVMVGSALVIALGLGKLFGWDIGLTAGMLAGSMTSTPVLVGAGD
TLHHSGMESRQLSLALDNLSLGYALTYLIGLVSLIVGARYLPKLQHQDLQTSAQQIARERGLDTDANRKV
YLPVIRAYRVGPELVAWTDGKNLRELGIYRQTGCYIERIRRNGILANPDGDAVLQMGDEIALVGYPDAHA
RLDPSFRNGKEVFDRDLLDMRIVTEEVVVKNHNAVGKRLAQLKLTDHGCFLNRVIRSQIEMPIDDNVVLN
KGDVLQVSGDARRVKTIADRIGFISIHSQVTDLLAFCAFFVIGLMIGMITFQFSTFSFGMGNAAGLLFAG
IMLGFMRANHPTFGYIPQGALSMVKEFGLMVFMAGVGLSAGSGINNGLGAIGGQMLIAGLIVSLVPVVIC

FLFGAYVLRMNRALLFGAMMGARTCAPAMEIISDTARSNIPALGYAGTYAIANVLLTLAGTIIVMVWPGL

G

>gi|30062328|ref|NP_836499.1| DEOR-type transcriptional regulator [Shigella flexneri 2a str. 2457T]

MRRANDPQRREKIIQATLEAVKLYGIHAVTHRKIATLAGVPLGSMTYYFSGIDELLLEAFSSFTEIMSRQ

YQAFFSDVSDAQGACQAITDMIYSSQVATPDNMELMYQLYALASRKPLLKTVMQNWMQRSQQTLEQWFEP

GTARALDAFIEGMTLHFVTDRKPLSREEILRMVERVAG

>gi|30062327|ref|NP_836498.1| DEOR-type transcriptional regulator [Shigella flexneri 2a str. 2457T]

MTVNSSRNALKRRTWALFMFFFLPGLLMASWATRTPAIRDILSVSIAEMGGVLFGLSIGSMSGILCSAWL

VKRFGTRNVILVTMSCALIGMMILSLALWLTSPLLFAVGLGVFGASFGSAEVAINVEGAAVEREMNKTVL

PMMHGFYSLGTLAGAGVGMALTAFGVPATVHILLAALVGIAPIYIAIQAIPDGTGKNAADGTQHGEKGIP

FYRDIQLLLIGVVVLAMAFAEGSANDWLPLLMVDGHGFSPTSGSLIYAGFTLGMTVGRFTGGWFIDSYSR

VAVVRASALMGALGIGLIIFVDSAWVAGVSVVLWGLGASLGFPLTISAASDTGPDAPTRVSVVATTGYLA

FLVGPPLLGYLGEHYGLRSAMLVVLALVILAAIVAKAVAKPDTKTQTAMENS

>gi|30062324|ref|NP_836495.1| chloramphenicol resistance pump Cmr [Shigella flexneri 2a str. 2457T]

MQNKLASGARLGRQALLFPLCLVLYEFSTYIGNDMIQPGMLAVVEQYQAGIDWVPTSMTAYLAGGMFLQW

LLGPLSDRIGRRPVMLAGVVWFIVTCLAILLAQNIEQFTLLRFLQGISFCFIGAVGYAAIQESFEEAVCI

KITALMANVALIAPLLGPLVGAAWIHVLPWEGMFVLFAALAAISFFGLQRAMPETAMRIGEKLSLKELGR

DYKLVLKNGRFVAGALGFVSLPLLAWIAQSPIIIITGEQLSSYEYGLLQVPIFGALIAGNLLLARLTSRR

TVRSLIIMGGWPIMIGLLVAAAATVISSHAYLWMTAGLSIYAFGIGLANAGLVRLTLFASDMSKGTVSAA

MGMLQMLIFTVGIEISKHAWLNGGNGLFNLFNLVNGILWLSLMVIFLKDKQMGNSHEG

>gi|30062323|ref|NP_836494.1| undecaprenyl pyrophosphate phosphatase [Shigella flexneri 2a str. 2457T]

MLENLNLSLFSLINATPDSAPWMISLAIFIAKDLITVVPLLAAVLWLWGLTAQRQLVIKIAIALAVSLFV

SWTMGHLFPHDRPFVENIGYNFLHHAADDSFPSDHGTVIFTFALAFLCWHRLWSGSLLMVLAVVIAWSRV

YLGVHWPLDMLGGLLAGMIGCLSAQIIWQAMGHKLYQRLQSWYRVCFALPIRKGWVRD

>gi|30062322|ref|NP_836493.1| DNA-binding transcriptional repressor DeoR [Shigella flexneri 2a str. 2457T]

METRREERIGQLLQELKRSDKLHLKDAAALLGVSEMTIRRDLNNHSAPVVLLGGYIVLEPRSASHYLLSD

QKSRLVEEKRRAAKLAATLVEPDQTLFFDCGTTTPWIIEAIDNEIPFTAVCYSLNTFLALKEKPHCRAFL

CGGEFHASNAIFKPIDFQQTLNNFCPDIAFYSAAGVHVSKGATCFNLEELPVKHWAMSMAQKHVLVVDHS

KFGKVRPARMGDLKRFDIVVSDCCPEDEYVKYAQTHRIKLMY

>gi|30062318|ref|NP_836489.1| biofilm formation regulatory protein BssR [Shigella flexneri 2a str. 2457T]

MFVDRQRIDLLNRLIDARVDLAAYVQLRKAKGYMSVSESNHLRDNFFKLNRELHDKSLRLNLHLDQEEWS

ALHHAEEALATAAVCLMSGHHDCPTVITVNADKLENCLMSLTLSIQSLQKHAMLEKA

>gi|30062316|ref|NP_836487.1| hypothetical protein S0827 [Shigella flexneri 2a str. 2457T]

MSRINKFVLTVSLLIFIMISAVACGIYTQMVKERVYSLKQSVIDTAFAVANIAEYRRSVAIDLINTLNPT

EEQLLVGLRTAYADSVSPSYLYDVGPYLISSDECIQVKEFEKNYCADIMQVVKYRHVKNTGFISFDGKTF

VYYLYPVTHNRSLIFLLGLERFSLLSKSLVMDSENLMFSLFKNGKPVTGDEYNAKNAIFTVSEAMEHFAY

LPTGLYVFAYKKDVYLRVCTLIIFFAALVAVILGASCLYLVRRVINRGIVEKEAIINNHFERVLDGGLFF

SAADVKKLYSMYNSAFLDDLTKAMGRKSFDEDLKALPEKGGYLCLFDVDKFKNINDTFGHLLGDEVLMKV

VKILKSQIPVDKGKVYRFGGDEFAVIYTGGTLEELLSILKEIVHFQVGSINLSTSIGVAHSNECTTVERL

KMLADERLYKSKKNGRAQISWQ

>gi|30062315|ref|NP_836486.1| transport system permease [Shigella flexneri 2a str. 2457T]

MRLFNWRRQAVLNAMPLVKPDQVRTPWHEFWRRFRRQHMAMTAALFVILLIVVAIFARWIAPYDAENYFD

YDNLNNGPSLQHWFGVDSLGRDIFSRVLVGAQISLAAGVFAVFIGVAIGTLLGLLAGYYEGWWDRLIMRI

CDVLFAFPGILLAIAVVAVLGSGIANVIIAVAIFSIPAFARLVRGNTLVLKQQTFIESARSIGASDMTIL

LRHILPGTVSSIVVFFTMRIGTSIISAASLSFLGLGAQPPTPEWGAMLNEARADMVIAPHVAVFPALAIF

LTVLAFNLLGDGLRDALDPKIKG

>gi|30062314|ref|NP_836485.1| transport system permease [Shigella flexneri 2a str. 2457T]

MLYYVIKRLLGLIPTLFIVSVLVFLFVHMLPGDPARLIAGPEADAQVIELVRQQLGLDQPLYHQFWHYIS

NAVQGDFGLSMVSRRPVADEIASRFMPTLWLTITSMVWAVIFGMAAGIIAAVWRNRWPDRLSMTIAVSGI

SFPAFALGMFLIQVFSVELGWLPTVGADSWQHYILPSLTLGAAVAAVMARFTRASFVDVLSEDYMRTARA

KGVSETWVVLKHGLRNAMIPVVTMMGLQFGFLLGGSIVVEKVFNWPGLGRLLVDSVEMRDYPVIQAEILL

FSLEFILINLVVDVLYAAINPAIRYK

>gi|30062313|ref|NP_836484.1| transporter [Shigella flexneri 2a str. 2457T]

MARAVHRSGLVALGIATALMASCAFAAKDVVVAVGSNFTTLDPYDANDTLSQAVAKSFYQGLFGLDKEMK

LKNVLAESYTVSDDGITYTVKLREGIKFQDGTDFNAVAVKANLDRASDPANHLKRYNLYKNIAKTEAIDP

TTVKITLKQPFSAFINILAHPATAMISPTALEKYGKEIGFHPVGTGPYELDTWNQTDFVKVKKFAGYWQP

GLPKLDSITWRPVADNNTRAAMLQTGEAQFAFPIPYEQATLLEKNKNIELMASPSIMQRYISMNVTQKPF

DNPKVREALNYAINRPALVKVAFAGYATPATGVVPPSIAYAQSYKPWPYDPVKARELLKEAGYPNGFSTT

LWSSHNHSTAQKVLQFTQQQLAQVGIKAQVTAMDAGQRAAEVEGKGQKESGVRMFYTGWSASTGEADWAL

SPLFASQNWPPTLFNTAFYSNKQVDDFLAQALKTNDPAEKTRLYKAAQDIIWQESPWIPLVVEKLVSAHS

KNLTGFWIMPDTGFSFEDADLQ

>gi|30062307|ref|NP_836478.1| pyruvate formate-lyase 2 activating enzyme [Shigella flexneri 2a str. 2457T]

MFERNREATMIFNIQRYSTHDGPGIRTVVFLKGCSLGCRWCQNPESRARTQDLLYDARLCLEGCELCAKA

APEVIERALNGLLIHREKLTPEHLTALTDCCPTQALTVCGEVKSVEEIMATVLRDKPFYDRSGGGLTLSG

GEPFMQPEMATALLQACHEAGIHTAVETCLHVPWKYIAPSLPYIDLFLADLKHVADAPFKQWTDGNAARV

LDNLKKLAAAGKKIIIRVPLIQGFNADETSVKAITDFAADELHVGEIHFLPYHTLGINKYHLLNLPYDAP

EKPLDAPELLDFAQQYACQKGLTATLRG

>gi|30062306|ref|NP_836477.1| formate acetyltransferase [Shigella flexneri 2a str. 2457T]

MTTLKLDTLSDRIKAHKNALVHIVKPPVCTERAQHYTEMYQQHLDKPIPVRRALALAHHLANRTIWIKHD

ELIIGNQASEVRAAPIFPEYTVSWIEKEIDDLADRPGAGFAVSEENKRVLHEVCPWWRGQTVQDRCYGMF

TDEQKGLLATGIIKAEGNMTSGDAHLAVNFPLLLEKGLDGLREKVAERRSRINLTVLEDLHGEQFLKAID

IMLVAVSEHIERFAALARAMAATETRESRRDELLAMAENCDLIAHQPPQTFWQALQLCYFIQLILQIESN

GHSVSFGRMDQYLYPYYRRDVELNQTLDREHAIELLHSCWLKLLEVNKIRSGSHSKASAGSPLYQNVTIG

GQNLVDGQPMDAVNPLSYAILESCGRLRSTQPNLSVRYHAGMSNDFLDACVHVIRCGFGMPAFNNDEIVI

PEFIKLGIEPQDAYDYAAIGCIETAVGGKWGYRCTGMSFINFARVMLAALEGGRDATSGKVFLPQEKALS

AGNFNNFDEVMDAWDTQIRYYTRKSIEIEYVVDTMLEENVHDILCSALVDDCIERAKSIKQGGAKYDWVS

GLQVGIANLGNSLAAVKKLVFEQGAIGQQQLAAALADDFDGLTHEQLRQRLINGAPKYGNDDDTVDTLLA

RAYQTYIDELKQYHNPRYGRGPVGGNYYAGTSSISANVPFGAQTMATPDGRKAHTPLAEGASPASGTDHL

GPTAVIGSVGKLPTAAILGGVLLNQKLNPATLENESDKQKLMILLRTFFEVHKGWHIQYNIVSRETLLEA

KKHPDQYRDLVVRVAGYSAFFTALSPDAQDDIIARTEHML

>gi|30062305|ref|NP_836476.1| hypothetical protein S0815 [Shigella flexneri 2a str. 2457T]

MSVKVIVTDMDGTFLNDAKTYNQPRFMAQYQELKKRGIKFVVASGNQYYQLISFFPELKDEISFVAENGA

LVYEHGKQLFHGELTRHESRIVIGELLKDKQLNFVACGLQSAYVSENAPEAFVALMAKHYHRLKPVKDYQ

EIDDVLFKFSLNLPDEQIPLVIDKLHIALDGIMKPVTSGFGFIDLIIPGLHKANGISRLLKRWDLSPQNV

VAIGDSGNDAEMLKMARYSFAMGNAAENIKQIARYATDDNNHEGALNVIQAVLDNTSPFNS

>gi|30062304|ref|NP_836475.1| hypothetical protein S0814 [Shigella flexneri 2a str. 2457T]

MASTFTSDTLPADHKAAIRQMKHALRAQLGDVQQIFNQLSDDIATRVAEINALKAQGDAVWPVLSYADIK

AGHVTAEQREQIKRRGCAVIKGHFPREQALGWDQSMLDYLNRNRFDEVYKGPGDNFFGTLSASRPEIYPI

YWSQAQMQARQSEEMANAQSFLNRLWTFESDGKQWFNPDVSVIYPDRIRRLPPGTTSKGLGAHTDSGALE

RWLLPAYQHVFANVFNGNLAKYDPWHAAHRTEVEEYTVDNTTKCSVFRTFQGWTALSDMLPGQGLLHVVP

IPEAMAYVLLRPLLDDVPEDELCGVAPGRVLPVSEQWHPLLIEALTSIPKLEAGDSVWWHCDVIHSVAPV

ENQQGWGNVMYIPAAPMCEKNLAYAHKVKAALEKGASPGDFPREDYETNWEGRFTLADLNIHGKRALGMD

V

>gi|30062300|ref|NP_836471.1| manganese transport regulator MntR [Shigella flexneri 2a str. 2457T]

MSRRAGTPIAKKVTQLVNVEEHVEGFRQVREAHRRELIDDYVELISDLIREVGEARQVDMAARLGVSQPT

VAKMLKRLATMGLIEMIPWRGVFLTAEGEKLAQESRERHQIVENFLLVLGVSPEIARRDAEGMEHHVSEE

TLDAFRLFTQKHGAK

>gi|30062299|ref|NP_836470.1| enzyme [Shigella flexneri 2a str. 2457T]

MNLTLKESLVTRSRVFSPWTAFYFLQSLLINLGLGYPFSLLYTAAFTAILLLLWRTLPRVQKVLVGVSSL

VAACYFPFAQAYGAPNFNTLLALHSTNMEESTEILTIFPWYSYLVGLFIFALGVIAIRRKKENEKARWNT

FDSLCLVFSVATFFVAPVQNLAWGGVFKLKDTGYPVFRFAKDVIVNNNEVIEEQERMAKLSGMKDTWTVT

AVKPKYQTYVVVIGESARRDALGAFGGHWDNTPFASSVNGLIFADYIAASGSTQKSLGLTLNRVVDGKPQ

FQDNFVTLANRAGFQTWWFSNQGQIGEYDTAIASIAKRADEVYFLKEGNFEADKNTKDEALLDMTAQVLA

QEHSQPQLIVLHLMGSHPQACDRTQGKYETFVQSKETSCYLYTMTQTDDLLRKLYDQLRNSGSSFSLVYF

SDHGLAFKERGKDVQYLAHDDKYQQNFQVPFMVISSGDKAHRVIKARRSANDFLGFFSQWTGIKAKEINI

KYPFISEKKAGPIYITNFQLQKVDYNHLGTDIFDPKP

>gi|30062298|ref|NP_836469.1| outer membrane protein X [Shigella flexneri 2a str. 2457T]

MKKIACLSALAAVLAFTAGTSVAATSTVTGGYAQSDAQGQMNKMGGFNLKYRYEEDNSPLGVIGSFTYTE

KSRTASSGDYNKNQYYGITAGPAYRINDWASIYGVVGVGYGKFQTTEYPTYKHDTSDYGFSYGAGLQFNP

MENVALDFSYEQSRIRSVDVGTWIAGVGYRF

>gi|30062297|ref|NP_836468.1| threonine and homoserine efflux system [Shigella flexneri 2a str. 2457T]

MPGSLRKMPVWLPIVILLVAMASIQGGASLAKSLFPLVSAPGVTALRLALGTLILIAFFKPWRLRFAKEQ

RLPLLFYGVSLGGMNYLFYLSIQTVPLGIAVALEFTGPLAVALFSSRRPVDFVWVVLAVLGLWFLLPLGQ

DVSHVDLTGCALALGAGACWAIYILSGQRAGAEHGPATVAIGSLIAALIFVPIGALQAGEALWHWSVIPL

GLAVVILSTALPYSLEMIALTRLPTRTFGTLMSMEPALAAVSGMIFLGETLTPIQLLALGAIIAASMGST

LTVRKESKIKELDIN

>gi|30062296|ref|NP_836467.1| DNA starvation/stationary phase protection protein Dps [Shigella flexneri 2a str. 2457T]

MSTAKLVKSKATNLLYTRNDVSDSEKKATVELLNRQVIQFIDLSLITKQAHWNMRGANFIAVHEMLDGFR

TALIDHLDTMAERAVQLGGVALGTTQVINSKTPLKSYPLDIHNVQDHLKELADRYAIVANDVRKAIGEAK

DDDTADILTAASRDLDKFLWFIESNIE

>gi|30062294|ref|NP_836465.1| glutamine ABC transporter permease [Shigella flexneri 2a str. 2457T]

MQFDWSAIWPAIPLLIEGAKMTLWISVLGLAGGLVIGLLAGFARTFGGWIANHVALVFIEVIRGTPIVVQ

VMFIYFALPMAFNDLRIDPFTAAVVTIMINSGAYIAEITRGAVLSIHKGFREAGLALGLSRWETIRYVIL

PLALRRMLPPLGNQWIISIKDTSLFIVIGVAELTRQGQEIIAGNFRALEIWSAVAVFYLIITLVLSFILR

RLERRMKIL

>gi|30062291|ref|NP_836462.1| hypothetical protein S0799 [Shigella flexneri 2a str. 2457T]

MHVIDLSYFVKIYRSKNKHFSVNFLRHLHRAGDLHLSGTTRCIYEDVIMKKCLTLLIATVLSGISLTAYA

AQPMSNLDSGQLRPAGTVSATGASNLSDLEDKLAEKAREQGAKGYVINSAGGNDQMLGTATIYK

>gi|30062286|ref|NP_836457.1| hypothetical protein S0792 [Shigella flexneri 2a str. 2457T]

MESGHRFDAQTLHSFIQAVFRQMGSEEQEAKLVADHLIAANLAGHDSHGIGMIPSYVRSWSQGHLQINHH

AKIVKEAGAAVTLDGDRAFGQVAAHEAMALGIEKAHQHGIAAVALHNSHHIGRIGYWAEQCSAAGFVSIH

FVSVVGIPMVAPFHGRDSRFGTNPFCVVFPRKDNFPLLLDYATSAIAFGKTRVAWHKGVPVPPGCLIDVN

GVPTTNPAVMQESPLGSLLTFAEHKGYALAAMCEILGGALSGGKTTHQETLQTSPDAILNCMTTIIINPE

LFGAPDCNAQTEAFAEWVKASPHDDDKPILLPGEWEVNTRRERQEQGIPLDAGSWQAICDAARQIGMSEE

TLQAFCQQLAS

>gi|30062285|ref|NP_836456.1| glycosyl transferase family protein [Shigella flexneri 2a str. 2457T]

MDYRKIIKEIGRGKNHARDLDRDTARGLYAHMLNGEVPDLELGGVLIALRIKGEGEAEMLGFYEAMQNHT

IKLTPPAGKPMPIVIPSYNGARKQANLTPLLAILLHKLGFPVVVHGVSEDPTRVLTETIFELMGITPTLH

GGQAQAKLDEHQPVFMPVGAFCPPLEKQLAMRWRMGVRNSAHTLAKLATPFAEGEALCLSSVSHPEYIGR
VAKFFSDIGGWALLMHGTEGEVYANPQRCPQINLIDREGMRVLYEKQDPAGSELLPQAKDPETTAQWTER
CLAGSEAIPESLKIQMACCLVATGEAATISDGLARVNQAF

>gi|30062284|ref|NP_836455.1| ATP-dependent DNA helicase DinG [Shigella flexneri 2a str. 2457T]
MALTAALKAQIAAWYKALQEQIPDFIPRAPQRQMIADVAKTLAGEEGRHLAIEAPTGVGKTLSYLIPGIA
IAREEQKTLVVSTANVALQDQIYSKDLPLLKKIIPDLKFTAAFGRGRYVCPRNLTALASTEPTQQDLLAF
LDDELTPNNQEEQKRCAKLKGDLDTYKWDGLRDHTDIAIDDDLWRRLSTDKASCLNRNCYYYRECPFFVT
RREIQEAEVVVANHALVMAAMESEAVLPDPKNLLLVLDEGHHLPDVARDALEMSAEITVPWYRLQLDLFT
KLVATCMEQFRPKTIPPLAIPERLNAHCEELYELIASLNNILNLYMPAGQEAEHRFAMGELPDELLEICQ
RLAKLTEMLRGLAELFLNDLSEKTGSHDIVRLHRLILQMNRALGMFEVQSKLWRLASLAQSSGAPVTKWA
TREEREGQLHLWFHCVGIRVSDQLERLLWRSIPHIIVTSATLRSLNSFSRLQEMSGLKEKAGDRFVALDS
PFNHCEQGKIVIPRMRFEPSIDNEEQHIAEMAAFFREQVESKKYLGMLVLFASGRAMQRFLDYVTDLRLM
LLVQGDQPRYRLVELHRKRVANGERSVLVGLQSFAEGLDLKGDLLSQVHIHKIAFPPIDSPVVITEGEWL
KSLNRYPFEVQSLPSASFNLIQQVGRLIRSHGCWGEVVIYDKRLLTKNYGKRLLDALPVFPIEQPEVPEG
IVKKKEKTKSPRRRRR

>gi|30062283|ref|NP_836454.1| hypothetical protein S0789 [Shigella flexneri 2a str. 2457T]
MPVRAQRIQHVMQDTIINFYSTSDDYGDFSNFAARPIKVDGNTWPTSEHYFQAQKFLDEKYREEIRRVSS
PMVAARMGRNRSKPLRKNWESVKEQVMRKALRAKFEQHAELRVLLLATAPAKLVEHTENDAYWGDGGNGK
GKNRLGYLLMELREQLAIEK

>gi|30062281|ref|NP_836452.1| DNA-binding transcriptional regulator [Shigella flexneri 2a str. 2457T]
MDCAMNNPAMTIKGEQAKKQLIAAALAQFGEYGMNATTREIAAQAGQNIAAITYYFGSKEDLYLACAQWI

ADFIGEQFRPHAEEAERLFAQPQPDRAAIRELILRACRNMIKLLTQDDTVNLSKFISREQLSPTAAYHLV
HEQVISPLHSHLTRLIAAWTGCDANDTRMILHTHALIGEILAFRLGKETILLRTGWTAFDEEKTELINQT
VTCHIDLILQGLSQRSL

>gi|30062280|ref|NP_836451.1| hypothetical protein S0786 [Shigella flexneri 2a str. 2457T]
MMKKPVVIGLAVVVLAAVVAGGYWWYQSRQDNGLTLYGNVDIRTVNLSFRVGGRVESLAVDEGDAIKAGQ
VLGELDHKPYEIALMQAKAGVSVAQAQYDLMLAGYRDEEIAQAAAAVKQAQAAYDYAQNFYNRQQGLWKS
RTISANDLENARSSRDQAQATLKSAQDKLRQYRSGNREQDIAQAKASLEQAQAQLAQAELNLQDSTLIAP
SDGTLLTRAVEPGTVLNEGGTVFTVSLTRPVWVRAYVDERNLDQAQPGRKVLLYTDGRPDKPYHGQIGFV
SPTAEFTPKTVETPDLRTDLVYRLRIVVTDADDALRQGMPVTVQFGDEAGHE

>gi|30062278|ref|NP_836449.1| hypothetical protein S0784 [Shigella flexneri 2a str. 2457T]
MSNPILSWHRVRALCVKETRQIVRDPSSWLIAVVIPLLLLFIFGYGINLDSSKLRVGILLEQRSEAALDF
THTMTGSPYIDATISDNRQELIAKMQSGKIRGLVVIPVDFAEQMERANATAPIQVITDGSEPNTANFVQG
YVEGIWQIWQMQRAEDNGQTFEPLIDVQTRYWFNPAAISQHFIIPGAVTIIMTVIGAILTSLVVAREWER
GTMEALLSTEITRTELLLCKLIPYYFLGMLAMLLCMLVSVFILGVPYRGSLLILFFISSLFLLSTLGMGL
LISTITRNQFNAAQVALNAAFLPSIMLSGFIFQIDSMPAVIRAVTYIIPARYFVSTLQSLFLAGNIPVVL
VVNVLFLIASAVMFIGLTWLKTKRRLD

>gi|30062277|ref|NP_836448.1| hypothetical protein S0783 [Shigella flexneri 2a str. 2457T]
MFHRLWTLIRKELQSLLREPQTRAILILPVLIQVILFPFAATLEVTNATIAIYDEDNGEHSVELTQRFAR
ASAFTHVLLLKSPQEIRPTIDTQKALLLVRFPADFSRKLDTFQTAPLQLILDGRNSNSAQIAANYLQQIV
KNYQQELLEGKPKPNNSELVVRNWYNPNLDYKWFVVPSLIAMITTIGVMIVTSLSVAREREQGTLDQLLV

SPLTTWQIFIGKAVPALIVATFQATIVLAIGIWAYQIPFAGSLALFYFTMVIYGLSLVGFGLLISSLCST

QQQAFIGVFVFMMPAILLSGYVSPVENMPVWLQNLTWINPIRHFTDITKQIYLKDASLDIVWNSLWPLLV

ITATTGSAAYAMFRRKVM

>gi|30062276|ref|NP_836447.1| hypothetical protein S0782 [Shigella flexneri 2a str. 2457T]

MKWQQRVRVATGLSCWQIMLHLLVVALLVVGWMSKTLVHVGVGLCALYCVTVVMMLVFQRHPEQRWREVA

DVLEELTTTWYFGAALIVLWLLSRVLENNFLLAIAGLAILAGPAVVSLLAKDKKLHHLTSKHRVRR

>gi|30062275|ref|NP_836446.1| hypothetical protein S0781 [Shigella flexneri 2a str. 2457T]

MPDQTQQFSFKVLTINIHKGFTAFNRRFILPELRDAVRTVSADIVCLQEVMGAHEVHPLHVENWPDTSHY

EFLADTMWSDFAYGRNAVYPEGHHGNAVLSRYPIEHYENRDVSVDGAEKRGVLYCRIVPPMTGKAIHVMC

VHLGLREAHRQAQLAMLAEWVNELPDGEPVLVAGDFNDWRQKANHPLKVQAGLDEIFTRAHGRPARTFPV

QFPLLRLDRIYVKNASASAPTALPLRTWRHLSDHAPLSAEIHL

>gi|30062274|ref|NP_836445.1| cardiolipin synthase 2 [Shigella flexneri 2a str. 2457T]

MKCSWREGNKIQLLENGEQYYPAVFKAIGEAQERIILETFIWFEDNVGKQLHAALLAAAQRGVKAEVLLD

GYGSPDLSDEFVNELTAAGVVFRYYDPRPRLFGMRTNVFRRMHRKIVVIDARIAFIGGLNYSAEHMSSYG

PEAKQDYAVRLEGPIVEDILQFELENLPGQSAARRWWRRHHKAEENRQPGEAQVLLVWRDNEEHRDDIER

HYLKMLTQAQREVIIANAYFFPGYRFLHALRKAARRGVRIKLIIQGEPDMPIVRVGARLLYNYLVKGGVQ

VFEYRRRPLHGKVALMDDHWATVGSSNLDPLSLSLNLEANVIIHDRHFNQTLRDNLNGIIAADCQQVDET

MLPKRTWWNLTKSVLAFHFLRHFPALVGWLPAHTPRLTQVDPPAQPTMETQDRVETENTGVNP

>gi|30062273|ref|NP_836444.1| hypothetical protein S0779 [Shigella flexneri 2a str. 2457T]

MSKSHPRWRLAKKILTWLFFIAVIVLLVVYAKKVDWEEVWKVIRDYNRVALLSAVGLVVVSYLIYGCYDL

LARFYCGHKLAKRQVMLVSFICYAFNLTLSTWVGGIGMRYRLYSRLGLPGSTITRISSLSITTNWLGYIL

LAGIIFTAGVVELPDYWYVDQTTLRILGIGLLMIIAVYLWFCAFAKHRHMTIKGQKLVLPSWKFALAQML

ISSVNWMVMGAIIWLLLGQSVNYFFVLGVLLVSSIAGVIVHIPAGIGVLEAVFIALLAGEHTSKGSIIAA

LLAYRVLYYFIPLLLALICYLLLESQAKKLRAKNEAAM

>gi|30062272|ref|NP_836443.1| hypothetical protein S0778 [Shigella flexneri 2a str. 2457T]

MESYSQNSNKLDFQHEARILNGIWLITALGLVATAGLAWGAKYLEITATKYDSPPMYVAIGLLLLCMYGL

SKDINKINAAIAGVIYLFLLSLVAIVVASLVPVYAIIIVFSTAGAMFLISMLAGLLFNVDPGSHRFIIMM

TLTGLALVIIVNAALMSERPIWVISCLMIVLWPGIISHGRNKLLELAGKCHSEELWSPVRCAFTGALTLY

YYFIGFFGILAAIAITLVWQRHTRFFH

>gi|30062265|ref|NP_836436.1| structural protein [Shigella flexneri 2a str. 2457T]

MRNRTLADLDRVVALGGGHGLGRVLSSLSSLGSRLTGIVTTTDNGGSTGRIRRSEGGIAWGDMRNCLNQL

ITEPNVASAMFEYRFGGNGELSGHNLGNLMLKALDHLSVRPLEAINLIRNLLKVDTHLIPMSEHPVDLMA

IDDQGHEVYGEVNIDQLTTPIQELLLTPNVPATREAVHAINEADLIIIGPGSFYTSLMPILLLKEIAQAL

RRTPAPMVYIGNLGRELSLPAANLKLESKLAIMEQYVGKKVIDAVIVGPKVDVSAVKERIVIQEVLEASD

IPYRHDRQLLHNALEKALQALG

>gi|30062263|ref|NP_836434.1| dithiobiotin synthetase [Shigella flexneri 2a str. 2457T]

MSKRYFVTGTDTEVGKTVASCALLQAAKAVGYRTAGYKPVASGSEKTPEGLRNSDALALQHNSSLQLDYA

TVNPYTFAESTSPHIISAQEGRPIESLVMSAGLRALEQQADWVLVEGAGGWFTPLSDTFTFADWVTQEQL

PVILVVGVKLGCINHAMLTAQAIQHAGLTLAGWVANDVTPPGKRHAEYMTTLTRMIPAPLLGEIPWLAEN

PENAATGKYINLALL

>gi|30062260|ref|NP_836431.1| biotin synthase [Shigella flexneri 2a str. 2457T]

MAHRPRWTLSQVTELFEKPLLDLLFEAQQVHRQHFEPRQVQVSTLLSIKTGACPEDCKYCPQSSRYKTGL

EAERLMEVEQVLESARKAKAAGSTRFCMGAAWKNPHERDMPYLEQMVQGVKAMGLEACMTLGTLSESQAQ

RLANAGLDYYNHNLDTSPEFYGNIITTRTYQERLDTLEKVRDAGIKVCSGGIVGLGETVKDRAGLLLQLA

NLPTPPESVPINMLVKVKGTPLADNDDVDAFDFIRTIAVARIMMPTSYVRLSAGREQMNEQTQAMCFMAG

ANSIFYGCKLLTTPNPEEDKDLQLFRKLGLNPQQTAVLAGDNEQQQRLGQALMTPDTDEYYNAAAL

>gi|30062258|ref|NP_836429.1| kinase inhibitor protein [Shigella flexneri 2a str. 2457T]

MKLISNDLRDGDKLPHRHVFNGMGYDGDNISPHLAWDDVPAGTKSFVVTCYDPDAPTGSGWWHWVVVNLP

ADTRVLPQGFGSGLVAMPDGVLQTRTDFGKTGYDGAAPPKGETHRYIFTVHALDVERIDVDEGASGAMVG

FNVHFHSLASASITAMFS

>gi|30062254|ref|NP_836425.1| membrane protein precursor [Shigella flexneri 2a str. 2457T]

MRKLYAAILSAAICLAVSGAPAWASEHRSTLSAGYLHASTNAPGSDDLNGINVKYRYEFTDTLGLVTSFS

YANAEDEQKTHYSDTRWHEDSVRNRWFSVMAGLSVRVNEWFSAYAMAGVAYSRVSTFSGDYLRVTDNKGK

THDVLTGSDDNRHSNTSLAWGAGVQFNPTESVAIDLAYEGSGSGDWRTDGFIVGVGYKF

>gi|30062252|ref|NP_836423.1| tail component [Shigella flexneri 2a str. 2457T]

MKTGAEAIRALATQLPAFRQKLSDGWYQVRIAGRDAGETELSARLNEPLANGAVIHIVPRLVGAKSGGVF

QAVLGAAVMAVAIWMPGVGIMASNLLFSLGASMTLGGVAQMLAPKARTPRTQTTDNGKQNTCFSSLDNMV

AQGNVLPVLYGEMRVGSRVVSQEISTADEGDGGQVVVIGR

>gi|30062250|ref|NP_836421.1| minor tail protein [Shigella flexneri 2a str. 2457T]

MQDIRQETLNECTRAEQSARVELWEIDLTEVGGERYFFCNEQNEKGEPVTWQGRQYQAYPIQGSGFELNG

RGCAARPTLTVSNLHGMVTGMAEDLQSLVGGTVVRRKVYARFLDAVNFVNGNSDADPEQEVISRWRIEQC

SELSAVSASFVLATPTETDGAVFPGRIMLANTCMWTYRSDECGYTGRAVADEFDKPTTDIRKDKCSKCMR

GCELRNNTGNFGGFLSINKLSQ

>gi|30062249|ref|NP_836420.1| minor tail protein [Shigella flexneri 2a str. 2457T]

METFHWKVRPDMNVVSEPKVVTVKLGDGYEQRRAAGLNNQLSTYSVTIRVRKGEHPSLKAFLERHGGVRA

FQWTPPYDWKLIRVVCRKWSASVGALWVTVTADFEQVVN

>gi|30062248|ref|NP_836419.1| tail length tape measure protein precursor [Shigella flexneri 2a str. 2457T]

MDQIANLVIDLGIDAAEFKNEIPRIKNLLNGAASDAERSSARMQRFMERQTQAARQTMQAASSAATAASA

HAQTVEKNARAHERMAREVEQTRLRVDALNQKMREEQAQARALAEAQDKAAAAFYRQIDSVKQAGAGLQE

LQRIQQQIRQARNSGGVGQQDYLALISEITAKTRALTQAEEQATRQKAAFIRQLKEQATRQNLSSSELLR

ARAAQLGVSSAAEVYIRKMERAGKATHSLGLKSAAARRELGVLISQMARGNFGALRGSGITLANRAGWTG

ALMSPKGMMTGGVIGGLVAAVLGLGKAWHDGRKEGEKFNRQLALTGHYAGVTVGQLWKLSRAISGNGITQ

HAAAGALAQVVGSGAFHGNDIGMVAKAAAQMERSVGQSVSDTINQFKRLKDDPVNAAKALDNALHFLNAT

QLEQIRVLGEQGRSSDAARIAMSALAEETGKRTSDIDNNLNALGSTLQTLSDWWKQFWDAAMNIGREDSL

DAQIDALQEKIQRAKKYPWTNASTQVEYDQQRLNDLQEKKRRKDLQDAKAQAERNYQEQQKRRNAENAAL

NRMNETEAARHQREIARINAMQYADQAVRDAAIQRENERYEKAIKKNTRATRNDEATRLLLQYSQQQAQV

EGQIAAARQSAGIATERMTEAHKQLLALQQRISDLDGKKLTADEKSVLARKNELIQALTLLDVKQQELQK

QTALNDLRKKTVQLTSQLADKERALREQHNLDIATAGMGDKQRQRYQAQLRIRQEYRQQLQQLENDSRQK

GTYGTEDYRRAEEVLKGSLKRQLNENKRYWQELEVAQGDWKNGAMRAFQNFTADADNAAGTAEQMFTVAF

SSAGNALATFCTTGKLNFKSFTSSLLSDMARIMAQMAMMQAVKGVGSLFGFTTNADGGVYQSADLSRYSG

TVVNRPTFFAFAKGAGVMGEAGPEAILPLRRGADGKLGVVADTGGSGMVMFAPQYNIEINNDGTNGQIGP

AALKVVYDLGKKAAADFMQQQARDGGRLSGAYR

>gi|30062246|ref|NP_836417.1| tail component of prophage CP-933K [Shigella flexneri 2a str. 2457T]

MFLKQDTFNYEKQSVVLSELSGLQRIEYLTFVQQRTAKFDAQEGELPEAERQIAFLRMGMDINAWLVSRS

LWNAEQSQDVETLCASIMTTWSYDALGAGAERVLSLSGMGTIENAGDDDHEALTPEKS

>gi|30062245|ref|NP_836416.1| tail component of prophage CP-933K [Shigella flexneri 2a str. 2457T]

MTTPNPLAKTKGAGTTFWMYTGNGDAFANPLSDTDWLRLAMVKDLQPGEMTADAEDDTYLDDEDADWKTT

TQGQKSVGDTSATLACRPGDSGQKKLVQLFDSGEVCAFRIKYPNGTVDVFRGWLSSLGKTIASKDVMTRT

VKISGVGRPYLAEEGTETVGVTGLTVAPASVSVKAGATTTLTFTVKPDGASDKAISVHSSDPQTASVTLS

GLVATVKGVKQGSVSIVGMTSDGEFVAVAAVTVSAP

>gi|30062244|ref|NP_836415.1| tail component of prophage CP-933K [Shigella flexneri 2a str. 2457T]

MNRHTQIRQSVLARLREQCGDSATFFDGLPAFIDAQELPAVAVWLSDAQYTGKMTDEDDWQAVLHIAVFI

RAQAPDSELDMWMESTIFPALNDVPALSGLIDTLNPLGFNYQRDNEMATWAMAEITYQITYTN

>gi|30062242|ref|NP_836413.1| tail attachment protein [Shigella flexneri 2a str. 2457T]

MDGFDNLFDAALAGVNEVILRDMGISAVITSGELEGTHLTGVFDDPESISFVAGGIRLEDSSPCLFVKTA

DISQLRRQDTLTIGDDSFFVDRITPDDGGCCYIRLRRGSPAPQNNARMRRYDEGT

>gi|30062241|ref|NP_836412.1| DNA-packaging protein [Shigella flexneri 2a str. 2457T]

MATKEENLKRLCELAERLGREPDVSGSAADIAQRVAELEEELGDAGEVAEPDTYSPSKDDPTAHKGESVP

EQPGIVTGDETGQVTVVALATLHTKCNGSVIFVSPGTSFRVSAEVAASMAAGGLAKRQ

>gi|30062240|ref|NP_836411.1| major capsid protein [Shigella flexneri 2a str. 2457T]

MGLFTTRQLLGYTEQKVKFRALFLELFFRRTVNFHTEEVMLDKITGKTPVAAYVSPVVEGKVLRHRGGET

RVLRPGYVKPKHEFNYQQAVERLPGEDPAQLNGPAYRRLRIITDNLKQEEHAIVQVEEMQAVNAVLYGKY

TMEGEQFETVEVDFGRSVGNNITQAGGTEWSAQDRDTFDPTHDLDAYCDFASGTINIAIMDGKVWRLLNG

FKLFREKLDTRRGSNSQLETAVKDLGAVVSFKGYYGDLAIVVAKTSYVAEDGTEKRYLPDGTLVLGNTAA

EGIRCYGAIQDAQALSEGVVASSRYPKHWLTVGDPAREFTMTQSAPLMVLPDPDEFVVVQVK

>gi|30062239|ref|NP_836410.1| capsid protein small subunit [Shigella flexneri 2a str. 2457T]

MVTKTITEQRAEVRIFAGNDPAHTATGCSGISSATPALTPLMLDEDTGKLVVWDGQKAGHVVGILVLPLE

GTETVLTYYKSGTFATEAIHWPERVDTHKKANAFAGSALSHAALP

>gi|30062238|ref|NP_836409.1| head-tail preconnector gp5 [Shigella flexneri 2a str. 2457T]

MRRNLSHIIAAAFNEPLLLEPAYARVFFCALGREMGAASLSVPQQQVQLDAPGMLAETDEYMAGGKRPAR

VYRVVNGIAVLPVTGTLVHRLGGMRPFSGMTGYDGIVACLQQAMADTQVRGVLLDIDSSGGQATGAFDCA

DMIYRLRQLKPVWALCNDTACSAAMLLASACSRRLVTQTSRIGSIGVMMGHVSYAGHLAQAGVDITLIYA

GSHKVDGNQFESLPAKVRQDMQQRIDAARRMFAEKVAMYTGLSVDAVMGTEAAVFEGQSGIEAELADELI

NASDAISVMAATLNTHDTGGTMPQLTATEAAAQENQRVMGILTCQEAKGREQLATMLAGQQGMSVEQARA

ILAVAAPQQSGVSAQSEADRIMACEEAKGREQLAATLAAMPEMTVEKARPILAASPQANAGPSLRDQIMA

LDEAKGAEAQAEKLAACPGMTVENARAVLAAASGRAEPVSASTTAMFEHFMANHSPAAVQGGVPQTSADG

DADVKMLMAMP

>gi|30062237|ref|NP_836408.1| head-tail preconnector gp5, partial [Shigella flexneri 2a str. 2457T]

MLKFRTFFPGDALNLQTAQDSDNGFSALEQALLRYIAAGLGVSYEQLSRDYSKVSYSSARASANESWRYF

MGRRKFIAARLATQMFSCWLEEALLRGIIRPPRARFDFYQARSAWSRAEWISSGRMAIDGLKEVQESVMR

IEAGLSTYEKELALMGEDYQDIFRQQVRESAEREKAGLSRPVWIAQAYQQQIAESRRPEEETTSRET

>gi|30062236|ref|NP_836407.1| head-tail preconnector gp5 [Shigella flexneri 2a str. 2457T]

MKRTPVLIDVNGVPLRESLSYNGGGAGFGGQMAEWLPPAQSADAALLPALRLGNARADDLVRNNGIAANA

VALHKDHIVGHMFLISYRLNWRWLGMRETTAKSFVGEVEAAWSEYAEGMFGEIDVEGKRTFTEFIREGVG

VHAFNGEIFVQPVWDTETTQLFRTRFKAVSPKRVDTPGHGMGNHFLRAGVEVDRYGRAIAYHICEDDFPF

SGAGRWERIPRELPTGRPAMLHIFEPVEDGQTRGANQFYSVMERMKMLDSLQATQLQSAIVKAMYAATIE

SELDTEKAFEYIAGAPQGQQDNPLINILEKFTRWYDTNNVTLGGVKIPHLFSWRCAEPADSAGFRQWIFG

A

>gi|30062234|ref|NP_836405.1| DNA packaging protein of prophage CP-933R; terminase large subunit [Shigella flexneri 2a str. 2457T]

MNISEQQLSNMMVAVTIALQPLVRVLPVTAVEWADQNYYLPKESSYGEGEWKTLQFQVAIMNCMGNDEIR

TVNLIKSARVGYTKMVLGVIGYFIEHKSRNTLLFQPTDSAAEDFMKSHVEATIRDVPCLKKLSPWLGRKH

RDNTLTLKRFSSGVGFWCLGGAAAKNYREKSVDVVCYDELSSFEPDVEKEGSPTLLGDKRIEGSVWPKSI

RGSTPKIKGSCQIEKAANESAHFMRFYVPCPHCGEEQYLKFGDDATSFGLKWEKGKPETVYYLCEHNGCV

IRQSELDQTGGRWICDNTGMWTCDGLTFFSASGNEIPPPRSITFHIWTAYSPFTTRVQIVCDWLDALKDP

NGVKTFVNTTLGETWEEAVGEKLDHQVLMDKVVPYTATVPVRVVYLTAGIDSQRNRFEMYVWGWAPGEEA

FLVDKIIIMGRPDEEETLLRVDAAINKKYRHADGTEMTISRVCWDIGGIDGEIVYQRSKKHGIFRVLPVK

GASVYGKPVITMPKTRNQRGVYLCEVGTDTAKEILYARMKADPTPVNEATSYAIRFPDNPEIFSQTEAQQ

LVAEELVEKWEKGKMRLLWDNKKRRNEALDCLVYAYAALRVSVQRWQLDLAVQLHPARMQLHNRPLKNWQ

RSCPEE

>gi|30062231|ref|NP_836402.1| endopeptidase [Shigella flexneri 2a str. 2457T]

MSRVTAIISALVICIIVCLSWAVNHYRDNAITYKEQRDKNAGELKLANATITDMQQRQRDADALDAKYTK

ELADAKAENDALRRKLDNGGRVLVKGKCPVPSSAETSSASGMGNDATVELSPVAGRNVLGIRDG

>gi|30062230|ref|NP_836401.1| endolysin R of prophage CP-933V [Shigella flexneri 2a str. 2457T]

MSAKIKYGLSAAVLALIAAGASAPQILDQFLNEKEGNHTTAYRDGSGIWTICRGATMVDGKPVIPGMKLS

KEKCDQVNAIERDKALAWVERNIKVPLTEPQKAGIASFCPYNIGPGKCFPSTFYKRLNAGDRKGACEAIR

WWIKDVGRDCRIRSNNCYGQVIRRDQESALACWGIDQ

>gi|30062229|ref|NP_836400.1| hypothetical protein S0732 [Shigella flexneri 2a str. 2457T]

MKHEEMNQRFNHLENEITELNKKLSALVSSEDENKRRDEHYAAIYDYCHKVAHETFMKFLQEKFLPAALS

EKEAAYLRPEYVITVNSAGEEEHKSDFIASAPDKDQEPHRPFRVSCEEGEFVVYENGKPVRASHHHCLKI

INLAIRCLKDENTRVMKRIGRCMGYLQVAAEIEALASGADMDAAVREALLRDFNTPPLRKSLMTGSSRG

>gi|30062227|ref|NP_836398.1| Q protein [Shigella flexneri 2a str. 2457T]

MRDIQQVLERWGAWVANNHEDVTWSSIAAGFKGLIPSKVKSRPQCCDDDAMVICGCMARLNRNNNDLHDL

LVDYYVFGMTFMTLARKHGCSDGYIGKKLQKAEGVVEGMLMMLGVRLEMDKYIEHP

>gi|30062224|ref|NP_836395.1| helicase [Shigella flexneri 2a str. 2457T]

MTTPVWRNDDLEGAVIGAFFLRGADPEVMDILATLPADVFSVRAYQDIYTGICRQARVSGVIDPVLLCNE
MPELAPVITDTGRKTWVKSSLEHYVAALRRNAALRDAEKTLNEALQKLRDAHTCEAAEDALKDAQNMMVT
LSTGKGVIQPVHIDDVLPEVVERVECRNQGLEKSRTLMTGIDELDAKTGGMEPGDLVFIAARPSMGKTEL
ALDIIDKVTEQGHGVLLFTMEMANIQIGERMVSAAGGMPVSRLKSVAHFEDEDWTRFSQGVGRMTGRNIW
MVDQANLAIDEICATTKHHLIKYPETALVVVDYLGLIKTRTTGRHDLAVGEISKGLKGLAKSGGFPLIAL
SQLSRGVESRPNKRPMNSDLKNSGEIEADADIILMLYRDEVYNPDTQARGIAEINITKQRNGSLGTIYRR
FYNGHFLPVDQESARVLSTPMKPGNPRRYSNKRTDSSKMERFF

>gi|30062223|ref|NP_836394.1| replication protein DnaC [Shigella flexneri 2a str. 2457T]
MMTFNLREQQKRLQARMDELRAEIAFAQKGEKPWPYRSCLMREGRGYCEKHGEYHTHILVWSDRNGEDRE
EISCCPDCLIAEANDLTMELSSIKAEELTDNAGIALRFRDCEFDNYLEVNPGAARNLAACRRYAENWPDM
LENGTSLVMTGSCGTGKNHLAVAMAKHIIRNYLASVEITDVMRLTRAVKNCWRNDSEKTADEVIERYASM
DLLIIDEVGVQFGSAAEMAILQEIINARYESILPTILISNLSPEELWAFISPRIADRITDGGRNWLSFNW
PSYRSRIRGVAA

>gi|30062219|ref|NP_836390.1| bacteriophage protein [Shigella flexneri 2a str. 2457T]
MRPSELSDLLWAQVDRVAPHLLPNGKIEGHEWVAGNVNGDKGNSLKVNLIGKKKWADFAEGDGGDMLDLW
MACRGINLHQAMQEAKAFLGIKDDDHHFDARREKKFSRPDRKKIARYVTRTESHLEYLQSRGISPEVVKR
YEVVSGKVWNGERELDALVLPYKRDGELLQVKRISTERPDGKKVIMAEGDCEPCLFGWQALDAGVRVVVL
CEGEIDCMSYAQYGISALSVPFGGGKGAKQQWIEFEYHNLDRFEEIFISMDVDDVGREAAREIVSRLGEH
RCRLVTLPYKDINECLMNGVTEDEIWQYIGTASYFDPEELYSAREFYQDTINAFYGKQQYLFNPPWESLA
DKFQFREAELTLVNGVHGHGKACPLNEPILLADGTWTTHGNVKIGDQVASVDGNPSTVTGIFPQGVRDVY
RVTFEDGRYVDCAGDHLWEVTSRGFTKGEKRRVIDTFGLKRLSETKRHKNGVRIPEITGDFGDHSEPLAW

VIGSLLGDGSLSNGSVKFSNVEPYMIERMKAELPDYNFSGDGKDWLISTARGQVNPLMETLRGYGLMGCT

AKNKFIPRVFFSANKSTRIGMLCGLLETDGYVEKDGTLVFSSASEELRNEVVNKNWPPS

>gi|30062206|ref|NP_836377.1| bacteriophage protein [Shigella flexneri 2a str. 2457T]

MAKPDWEAIESAYRAGVLSLRDIGEKYGVTEGAIRKRAKKLDWARSGGTQVCKNGTQKRKVRTSRKPAIT

GLTQKSTQLKTESTPDTKPIRGMRTDPPTNPFQPGNQQALKHGGYARRLLLKDEVIEDAKALTLEDELFR

LRANNLVAAENIGRWLVSLEDANGDQERKMLIENISAAEKAMMRNTVRIESIVGTLATVGKIFADTAYRK

AATDKVSLEADRLRRDAGIDDGNGERDLNDFYSDIQTDAESGFT

>gi|30062202|ref|NP_836373.1| bacteriophage protein [Shigella flexneri 2a str. 2457T]

MSGNIGANPAIIKLSWIEAAVDAHKTLNFEPSGRKRIGFDVADSGTDKCANVYRHGSVVFWADEWKAKED

ELLKSCQRTYQAALEREADIVYDSIGVGASAGAKFSEINADRKSENAYARRVNYQRFNAGAGVHEPDDEY

NGIPNKDFFANLKAQAWWLTVSEIRLTPLTTENSILWMS

>gi|30062200|ref|NP_836371.1| bacteriophage protein [Shigella flexneri 2a str. 2457T]

MARNKQALRRTVQATADGYENFIARVGMQTPNQHSASTYRANFTSRNRMLVEWSYRSSWIIGEAVDAIPD

DMTRKGIRITSEIDAKDRGILESQLDELQIWDALNDVLKWSRLYGGAVGFIMIEGQAPMTPLRPETIGKG

KFKGILPLDRWMVDPALTRRIKDMGPDLGKPEFYDVVTTATGIPAWRIHHSRLIRFDGVTLPFQQKMTEN

EWGMSVVERIWDRLTAFDSATVGAAQLVYKAHLRTYSVEKLRELIALGGPAYEALLKNIDLIRQFQSNEG

MTLMDSRDKFETHQYSFSGLDDILSQFAEQISGAVGIPLVRLFGQSPKGFSTGDADLANYYDRISSLQER

RLRLPVRRILDIMHRSELGKPLPDDFTFEFNPLWQMSDVDRSTVALNTTNAISTALGDGLMTLKAAMTDL

RENSDVTGIGASITNEDIENAEDEAPPGIGEPDDEPQEPSGGNPVSNQPTQDSEGGRRHRKWSLRWFK

>gi|30062199|ref|NP_836370.1| bacteriophage protein [Shigella flexneri 2a str. 2457T]

MGQVMQSIIAEQVKYIKSLPLEAADRVYDIQNKAIEAVVTGGRAEQFAKEIASTGDVAKSRADLIARTEL

GRATGALDMARAIAIGSDGYIWRTADDGDVRDSHDHMKGKFVRWDSPPTLDGMTGHAGELPNCRCYKEIV

FVRVPFAMKRAA

>gi|30062198|ref|NP_836369.1| bacteriophage protein [Shigella flexneri 2a str. 2457T]

MKYFFETRLGETRYRLADGSLLCKDVPIGRTGKQLYGADDLPKLKPDKFVEIVVTRSPEQVFHPATLASF

EGMSITILHPEDENGNVRLVNPENWKVLAVGHLQNVRRGTGEQSDLMLADLIVKDESAIQLIEDGLREVS

CGYDAEYEQTEPGKAEQVDITGNHVALVPKGRAGNRCAIGDRDTMANQKKNWWNRMRAAIKTGDADTMNE

LVESAPASVTGDEGDLPQGVNLNINLSPQQPLPDKAPEMGGDPTGDSDDDLKTLLKALLAKLERNATGDN

DNKPDDNPTGDGEDDEEETTITGDSAWRAEVIVPGIDLSRKMKPTAFKREVLASADKTLVRQIVGDADIR

KLPKQSVDMAFNAVSEVAKGRNTRATTGDAQRLNMGMTSIASLNKQNAEFWANRKG

>gi|30062197|ref|NP_836368.1| bacteriophage protein [Shigella flexneri 2a str. 2457T]

MNNVFLYRMPVGIAGAVSRPQDLTVEPVVLKSDNAFAAYGLAGKYDDDGFFVPLADGDTADKVKGIYVRP

YPTTSQPDMVRQVGTGKNFPGDAMKRGYVTVNLGSDFDASTIKKGDLH

>gi|30062195|ref|NP_836366.1| bacteriophage protein [Shigella flexneri 2a str. 2457T]

MITFDQATVDSSGAFLIGELERLDQTLNLPLVGYTWSRDIQLREDASIADDISSWTNTSFAAAGTGANPN

GKNWVGKDSTAIAGVNVDIGKSGNPLNLWGMELGWTVIELQAAQQVGRPIDTQKYDGMQLKWQMDNDEQV

YVGDSALNLKGLVTLDGVPVNNAAKTWATSTPDEIRASINQVLSDAWAASGYSVVPRDLLIPPEQFALLS

SIIVSSAGNQSLLTNLQTNTISYHQNGVPLNIRAVKWLKGRGVGNKDRIVAYTNDKKYVRYPLVPLQSVP
VQYRGLYQIVTYYGKLGAVEPVYKETISYVDGI

>gi|30062191|ref|NP_836362.1| phage transposase [Shigella flexneri 2a str. 2457T]
MLQQGKFKTSQGCFEIARPTLEAHDYDREALWSKWDKASDSQRSLAEKWLPSIQATDEMLNQGISTKTAF
ATVAGHYQVSASTLRDKYYQVQKFAKPDWAAALVDGRGASRRNVHKSEFDEDAWQFLIADYLRPEKPAFR
KCYERLELAAREHGWSIPSRATAFRRIQQLDEAMVVACREGEHALMHLIPAQQRTVEHLDAMQWINGDGY
LHNVFVRWFNGDVIRPKTWFWQDVKTRKILGWRCDVSENIDSIRLSFMDVVTRYSIPEDFHITIDNTRGA
ANKWLTGGAPNRYRFKVKEDDPKGLFLLMGAKMHWTSVVAGKGWGQAKPVERAFGVGGLEEYVDKHPALA
GAYTGPNPQAKPDNYGDHAVDAELFLKTFAEGVAMFNARTGRETEMCGGKLSFDDVFEREYARTIVRKPT
EEQKRMLLLPAEAVNVSRKGEFTLKVGGSLKGAKNVYYNMALMNAGVKKVVVRFDPQQLHSTVY

>gi|30062190|ref|NP_836361.1| repressor protein [Shigella flexneri 2a str. 2457T]
MKSANEINQWMTPKQITELDGMPGTIQGVHKRAKKEGWPKRSQEGRRGPGVEYIPAIPAMQKIAIEEMNA
DTLKMFSLLINKLGENEVREIELNMAKYGLSGLMQERPSISVDTLLATLGIDRQTLQTALALHKLPPETR
QEILSMYGVHKQEEPVAPLLEPQDAKKAV

>gi|30062189|ref|NP_836360.1| C4-dicarboxylate transporter DcuC [Shigella flexneri 2a str. 2457T]
MLTFIELLIGVVVIVGVARYIIKGYSATGVLFVGGLLLLIISAIMGHKVLPSSQASTGYSATDIVEYVKI
LLMSRGGDLGMMIMMLCGFAAYMTHIGANDMVVKLASKPLQYINSPYLLMIAAYFVACLMSLAVSSATGL
GVLLMATLFPVMVNVGISRGAAAAICASPAAIILAPTSGDVVLAAQASEMSLIDFAFKTTLPISIAAIIG
MAIAHFFWQRYLDKKEHISHEMLDVSEITTTAPAFYAILPFTPIIGVLIFDGKWGPQLHIITILVICMLI
ASILEFLRSFNTQKVFSGLEVAYRGMADAFANVVMLLVAAGVFAQGLSTIGFIQSLISIATSFGSASIIL

MLVLVILTMLAAVTTGSGNAPFYAFVEMIPKLAHSSGINPAYLTIPMLQASNLGRTLSPVSGVVVAVAGM

AKISPFEVVKRTSVPVLVGLVIVIVATELMVPGTAAAVTGK

>gi|30062188|ref|NP_836359.1| palmitoyl transferase [Shigella flexneri 2a str. 2457T]

MNVSKYVAIFSFVFIQLISVGKVFANADERMTTFRENIAQTWQQPEHYDLYIPAITWHARFAYDKEKTDR

YNERPWGGGFGLSRWDEKGNWHGLYAMAFKDSWNKWEPIAGYGWESTWRPLADENFHLGLGFTAGVTARD

NWNYIPLPVLLPLASVGYGPVTFQMTYIPGTYNNGNVYFAWMRFQF

>gi|30062186|ref|NP_836357.1| camphor resistance protein CrcB [Shigella flexneri 2a str. 2457T]

MLQLLLAVFIGGGTGSVARWLLSMRFNPLHQAIPLGTLAANLIGAFIIGMGFAWFSRMTNIDPVWKVLIT

TGFCGGLTTFSTFSAEVVFLLQEGRFGWALLNVFVNLLGSFAMTALAFWLFSASTVH

>gi|30062179|ref|NP_836350.1| D-alanyl-D-alanine carboxypeptidase [Shigella flexneri 2a str. 2457T]

MNTIFSARIMKRLALTTALCTAFISAAHADDLNIKTMIPGVPQIDAESYILIDYNSGKVLAEQNADVRRD

PASLTKMMTSYVIGQAMKAGKFKETDLVTIGNDAWATGNPVFKGSSLMFLKPGMQVPVSQLIRGINLQSG

NDACVAMADFAAGSQDAFVGLMNSYVNALGLKNTHFQTVHGLDADGQYSSARDMALIGQALIRDVPNEYS

IYKEKEFTFNGIRQLNRNGLLWDNSLNVDGIKTGHTDKAGYNLVASATEGQMRLISAVMGGRTFKGREAE

SKKLLTWGFRFFETVNPLKVGKEFASEPVWFSDSDRASLGVDKDVYLTIPRGRMKDLKASYVLNSSELHA

PLQKNQVVGTINFQLDGKTIEQRPLVVLQEIPEGNFFGKIIDYIKLMFHHWFG

>gi|30062178|ref|NP_836349.1| rare lipoprotein A [Shigella flexneri 2a str. 2457T]

MRKQWLGICIAAGMLAACTSDDGQQQTVSVPQPAVCNGPIVEISGADPRFEPLNATANQDYQRDGKSYKI

VQDPSRFSQAGLAAIYDAEPGSNLTASGEAFDPTKLTAAHPTLPIPSYARITNLANGRMIVVRINDRGPY

GNDRVISLSRAAADRLNTSNNTKVRIDPIIVAQDGSLSGPGMACTTVAKQTYALPAPPDLSGGAGTSSVS

GPQGDILPVSNSTLKSEDPTGAPVTSSGFLGAPTTLAPGVLEGSEPTPAPQPVVTASSTTPATSPAMVTP

QAASQSASGNFMVQVGAVSDQARAQQYQQQLGQKFGVPGRVTQNGAVWRIQLGPFASKAEASTLQQRLQT

EAQLQSFITTAQ

>gi|30062177|ref|NP_836348.1| cell wall shape-determining protein [Shigella flexneri 2a str. 2457T]

MTDNPNKKTFWDKVHLDPTMLLILLALLVYSALVIWSASGQDIGMMERKIGQIAMGLVIMVVMAQIPPRV

YEGWAPYLYIICIILLVAVDAFGAISKGAQRWLDLGIVRFQPSEIAKIAVPLMVARFINRDVCPPSLKNT

GIALVLIFMPTLLVAAQPDLGTSILVALSGLFVLFLSGLSWRLIGVAVVLVAAFIPILWFFLMHDYQRQR

VMMLLDPESDPLGAGYHIIQSKIAIGSGGLRGKGWLHGTQSQLEFLPERHTDFIFAVLAEELGLVGILIL

LALYILLIMRGLWIAARAQTTFGRVMAGGLMLILFVYVFVNIGMVSGILPVVGVPLPLVSYGGSALIVLM

AGFGIVMSIHTHRKMLSKSV

>gi|30062176|ref|NP_836347.1| penicillin-binding protein 2 [Shigella flexneri 2a str. 2457T]

MKLQNSFRDYTAESALFVRRALVAFLGILLLTGVLIANLYNLQIVRFTDYQTRSNENRIKLVPIAPSRGI

IYDRNGIPLALNRTIYQIEMMPEKVDNVQQTLDALRSVVDLTDDDIAAFRKERARSHRFTSIPVKTNLTE

VQVARFAVNQYRFPGVEVKGYKRRYYPYGSALTHVIGYVSKINDKDVERLNNDGKLANYAATHDIGKLGI

ERYYEDVLHGQTGYEEVEVNNRGRVIRQLKEVPPQAGHDIYLTLDLKLQQYIETLLAGSRAAVVVTEPRT

GGVLALVSTPSYDPNLFVDGISSKDYSALLNDPNTPLVNRATQGVYPPASTVKPYVAVSALSAGVITRNT

TLFDPGWWQLPGSEKRYRDWKKWGHGRLNVTRSLEESADTFFYQVAYDMGIDRLSEWMGKFGYGHYTGID

LAEERSGNMPTREWKQKRFKKPWYQGDTIPVGIGQGYWTATPIQMSKALMILINDGIVKVPHLLMSTAED

GKQVPWVQPHEPPVGDIHSGYWELAKDGMYGVANRPNGTANKYFASAPYKIAAKSGTAQVFGLKANETYN

AHKIAERLRDHKLMTAFAPYNNPQVAVAMILENGGAGPAVGTLMRQILDHIMLGDNNTDLPAENPAVAAA

EDH

>gi|30062175|ref|NP_836346.1| rRNA large subunit methyltransferase [Shigella flexneri 2a str. 2457T]

MKLQLVAVGTKMPDWVQTGFTEYLRRFPKDMPFELIEIPAGKRGKNADIKRILDKEGELMLAAAGKNRIV

TLDIPGKPWDTPQLAAELERWKLDGRDVSLLIGGPEGLSPACKAAAEQSWSLSALTLPHPLVRVLVAESL

YRAWSITTNHPYHRE

>gi|30062171|ref|NP_836342.1| DNA polymerase III subunit delta [Shigella flexneri 2a str. 2457T]

MIRLYPEQLRAQLNEGLRAAYLLLGNDPLLLQESQDAVRQVAAAQGFEEHHTFSIDPNTDWNAIFSLCQA

MSLFASRQTLLLLLPENGPNAAINEQLLTLTGLLHDDLLLIVRGNKLSKAQENAAWFTALANRSVQVTCQ

TPEQAQLPRWVAVRAKQLNLELDDAANQVLCYCYEGNLLALAQALERLSLLWPDGKLTLPRVEQAVNDAA

HFTPFHWVDALLMGKSKRALHILQQLRLEGSEPVILLRTLQRELLLLVNLKRQSAHTPLRALFDKHRVWQ

NRRGMMGEALNRLSQPQLRQAVQLLTRTELTLKQDYGQSVWAELEGLSLLLCHKPLADVFIDG

>gi|30062170|ref|NP_836341.1| LPS-assembly lipoprotein RlpB [Shigella flexneri 2a str. 2457T]

MRYLATLLLSLAVLITAGCGWHLRDTTQVPSTMKVMILDSGDPNGPLSRAVRNQLRLNGVELLDKETTRK

DVPSLRLGKVSIAKDTASVFRNGQTAEYQMIMTVNATVLIPGRDIYPISAKVFRSFFDNPQMALAKDNEQ

DMIVKEMYDRAAEQLIRKLPSIRAADIRSDEEQTSTTTDTPATPARVSTMLGN

>gi|30062168|ref|NP_836339.1| hypothetical protein S0660 [Shigella flexneri 2a str. 2457T]

MNKVAQYYRELVASLNERLRNGERDIDALVEQARERVIKTGELTRTEIDELTRAVRRDLEEFAMSYEESL

KEESDSVFMRVIKESLWQELADITDKTQLEWREIFQDLNHHGVYHSGEVVGLGNLVCEKCHFHLPIYTPE

VLTLCPKCGYDQFQRRPFEP

>gi|30062166|ref|NP_836337.1| hypothetical protein S0658 [Shigella flexneri 2a str. 2457T]

MDMGSQKILFALSTPMEIRNECCLPSHSSPKMYLGTCFFDLSSSWGIDDRDDLLRIIHRMIENGHAARLA

GFYHRWFRYSPCEWRDYLAELNEQGQAYAQFVASTAECCGEGGIKAWDYVRMGFLSRMGVLNNWLSEEES

LWIQSRIHLRALRYYSNWRQYFAGYTFGRQYWQSPEDDHLPLLREFLARKEYDDSGNDMFYQLFASDDAY

YPTLSWQPLADYPTCPETLKDMSDL

>gi|30062165|ref|NP_836336.1| enzyme [Shigella flexneri 2a str. 2457T]

MKNCWKILDIEETTDVDIIRRAYLALLPSFHPETDPQGFKQLRQAYEEALRIAQSPAKSVWQPEEYEVAE

HEILLAFRALLASDSERFLPSAWQRFIQQLNYCSMEEIDELRWSLCTIAMNTAHLSFECVVLLAERLRWL

QEENVGEIDEEELESFLYAIAKGNVFNFQTILHLPVAVQNDTIDFYQMFARIWSSHPEWLTLYLAQHRAV

IIPDDAKLHRNLLRWYSASRLGIPELLDYARSWREAEPDNEDARYYEYAQRVYCGEGESLLAELCYYWRE

YPSTQADALMLQWCRQHRVYYYPLVVMMIEARDLVNDQGKPLLYVPGDSARTRFHLYEILSDEKLSALGR

SLVEMVLHKGRKPRISLTRDTEHPLWPLYLVAKQLVQASQPTEESLMPIVSRLDAEDRCPLEALIIRRLL

IQAANFTGQETVEPEPQPQPMPVDDGGLGCLGVIKIIFYIFIFAGLIGKILHLFG

>gi|30062163|ref|NP_836334.1| tRNA ligase [Shigella flexneri 2a str. 2457T]

MNKEEQYLLFALSAPMEILNQSCKPAHDSPKMYTGIKEFDLSSSWGINNRDDLIQTIYQMTDDGHANDLA

GLYLTWQRSSPEEWKALIAGGSERGLIYTQFVAQTAMCCGEGGIKAWDYVRMGFLSRAGVLNNWLTEEES

LWLQSRVYARAHHYYHSWMHYFSAYSLGRLYWQSSQCEDNASLREALTLYKYDSAGSRMFEELAAGSDRF

YATLPWQPLTVQPECPVTLKDVSDL

>gi|30062162|ref|NP_836333.1| hypothetical protein S0654 [Shigella flexneri 2a str. 2457T]

MKTCWQILEIESTTQIDIIRQAYLARLPLCHPETDPQGFKALRQAYEEALRLAVNPVEEADDEEKDATAE

HEILRAFRTLLDSESDRFQPSAWQKFIQQLNTWNMEDVDQLRWPLCAIAIEARYLSLNCASLLAERLNWH

SFNDSEGMDEEEREAFLEAIQAGDCFDFLSLLEYPVALQNQTVEYYFALERCCRYHPDYVTAFLAMEGPW

FIPDDAKLHRKLLRWYSSVQTGMVELIPVAKQWQAEEPESEDARYYQCAQRLYCGEGESLLADLGAYWES

YPSTQADNLLLQWSKRHCPDYFALLVMVIEARSMVDAQGQPLKYVPGESARTRLLWAEILHSGKLSPLGQ

SFIESLFFKRKAWAWWKSRVGSETEEDSPLLDLYRVAEQVVLEAFPKQEMLARLNTRLEGGDAHPLEAII

TRMLLTKVKLEPEDEDVDEPTPENHEEKNDEGEKPQSITSIIKISLTVLVIGYALGKIAMLFS

>gi|30062161|ref|NP_836332.1| ribonucleoside hydrolase 1 [Shigella flexneri 2a str. 2457T]

MALPILLDCDPGHDDAIAIVLALASPELDVKAITSSAGNQTPEKTLRNVLRMLTLLNRTDIPVAGGAVKP

LMRELIIADNVHGESGLDGPALPEPTFAPQNCTAVELMAKTLRESAEPVTIVSTGPQTNVALLLNSHPEL

HSKIARIVIMGGAMGLGNWTPAAEFNIYVDPEAAEIVFQSGIPVVMAGLDVTHKAQIHVEDTERFRAIGN

PVSTIVAELLDFFLEYHKDEKWGFVGAPLHDPCTIAWLLKPELFTTVERWVGVETQGKYTQGMTVVDYYY

LTGNKPNATVIVDVDRQGFVDLLADRLKFYA

>gi|30062159|ref|NP_836330.1| glutamate/aspartate transport system permease [Shigella flexneri 2a str. 2457T]

MYEFDWSSIVPSLPYLLDGLVITLKITVTAVVIGILWGTMLAVMRLSSFAPVSWFAKAYVNVFRSIPLVM

VLLWFYLIVPGFLQNVLGLSPKNDIRLISAMVAFSMFEAAYYSEIIRAGIQSISRGQSSAALALGMTHWQ

SMKLIILPQAFRAMVPLLLTQGIVLFQDTSLVYVLSLADFFRTASTIGERDGTQVEMILFAGFVYFVISL

SASLLVSYLKRRTA

>gi|30062158|ref|NP_836329.1| glutamate/aspartate transport system permease [Shigella flexneri 2a str. 2457T]

MSIDWNWGIFLQQAPFGNTTYLGWVWSGFQVTIALSICAWIIAFLVGSFFGILRTVPNRFLSGLGTLYVE

LFRNVPLIVQFFTWYLVIPELLPEKIGMWFKAELDPNIQFFLSSMLCLGLFTAARVCEQVRAAIQSLPRG

QKNAALAMGLTLPQTYRYVLLPNAYRVIVPPMTSEMMNLVKNSAIASTIGLVDMAAQAGKLLDYSAHAWE

SFTAITLAYVLINAFIMLVMTLVERKVRLPGNMGGK

>gi|30062157|ref|NP_836328.1| glutamate and aspartate transporter subunit [Shigella flexneri 2a str. 2457T]

MQLRKPATAILALALSAGLAQADDAAPAAGSTLDKIAKNGVIVVGHRESSVPFSYYDNQQKVVGYSQDYS

NAIVEAVKKKLNKPDLQVKLIPITSQNRIPLLQNGTFDFECGSTTNNVERQKQAAFSDTIFVVGTRLLTK

KGGDIKDFADLKGKAVVVTSGTTSEVLLNKLNEEQKMNMRIISAKDHGDSFRTLESGRAVAFMMDDALLA

GERAKAKKPDNWDIVGKPQSQEAYGCMLRKDDPQFKKLMDDTIAQVQTSGEAEKWFDKWFKNPIPPKNLN

MNFELSDEMKALFKEPNDKALN

>gi|30062156|ref|NP_836327.1| apolipoprotein N-acyltransferase [Shigella flexneri 2a str. 2457T]

MDFASLIERQRIRLLLALLFGACGTLAFSPYDVWPAAIISLMGLQALTFNRRPLQSAAIGFCWGFGLFGS

GINWVYYVSIATFGGMPGPVNIFLVVLLAAYLSLYTGLFAGVLSRLWPKTTWLRVAIATPALWQVTEFLRG

WVLTGFPWLQFGYSQIDGPLKGLAPLMGVEAINFLLMMVSGLLALALVKRNWRPLVVAVVLFALPFPLRY

IQWFTPQPEKTIQVSMVQGDIPQSLKWDEGQLLNTLKIYYNATAPLMGKSSLIIWPESAITDLEINQQPF

LKALDGELRDKGSSLVTGIVDARLNKQNRYDTYNTIITLGKGAPYSYESADRYNKNHLVPFGEFVPLESI

LRPLAPFFDLPMSSFSRGPYIQPPLSANGIELTAAICYEIILAEQVRDNFRPDTDYLLTISNDAWFGKSI

GPWQHFQMARMRALELARPLLRSTNNGITAVIGPQGEIQAMIPQFAREVLTTNVTPTTGLTPYARTGNWP

LWVLTALFGFAAVLMSLRQRRK

>gi|30062154|ref|NP_836325.1| metalloprotease [Shigella flexneri 2a str. 2457T]

MSQVILDLQLACEDNSGLPEESQFQTWLNAVIPQFQEESEVTIRVVDTAESHSLNLTYRGKDKPTNVLSF

PFEVPPGMEMSLLGDLVICRQVVEKEAQEQGKPLEAHWAHMVVHGSLHLLGYDHIEDDEAEEMEALETEI

MLALGYEDPYIAEKE

>gi|30062153|ref|NP_836324.1| ATP-binding protein in pho regulon [Shigella flexneri 2a str. 2457T]

MFSFRRHTDKRNGLNIDTREITLEPADNARLLSLCGPFDDNIKQLERRLGIEINRRDNHFKLTGRPICVT

AAADILRSLYVDTAPMRGQIQDIEPEQIHLAIKEARVLEQSAESVPEYGKAVNIKTKRGVIKPRTPNQAQ

YIANILDHDITFGVGPAGTGKTYLAVAAAVDALERQEIRRILLTRPAVEAGEKLGFLPGDLSQKVDPYLR

PLYDALFEMLGFEKVEKLIERNVIEVAPLAYMRGRTLNDAFIILDESQNTTIEQMKMFLTRIGFNSKAVI

TGDVTQIDLPRNTKSGLRHAIEVLADVEEISFNFFHSEDVVRHPVVARIVNAYEAWEEAEQKRKAALAAE

RKREEQEQK

>gi|30062146|ref|NP_836317.1| N-acetyl glucosamine specific PTS system components IIABC [Shigella flexneri 2a str. 2457T]

MNILGFFQRLGRALQLPIAVLPVAALLLRFGQPDLLNVAFIAQAGGAIFDNLALIFAIGVASSWSKDSAG

AAALAGAVGYFVLTKAMVTINPEINMGVLAGIITGLVGGAAYNRWSDIKLPDFLSFFGGKRFVPIATGFF

CLVLAAIFGYVWPPVQHAIHAGGEWIVSAGALGSGIFGFINRLLIPTGLHQVLNTIAWFQIGEFTNAAGT

VFHGDINRFYAGDGTAGMFMSGFFPIMMFGLPGAALAMYFAAPKERRPMVGGMLLSVAVTAFLTGVTEPL

EFLFMFLAPLLYLLHALLTGISLFVATLLGIHAGFSFSAGAIDYALMYNLPAASQNVWMLLVMGVIFFAI

YFVVFSLVIRMFNLKTPGREDKEDEIVTEEANSNTEEGLTQLATNYIAAVGGTDNLKAIDACITRLRLTV

ADSARVNDTMCKRLGASGVVKLNKQTIQVIVGAKAESIGDAMKKVVARGPVAAASAEATPATAAPVAKPQ
AVPNAVSIAELVSPITGDVVALDQVPDEAFASKAVGDGVAVKPTDKIVVSPAAGTIVKIFNTNHAFCLET
EKGAEIVVHMGIDTVALEGKGFKRLVEEGAQVSAGQPILEMDLDYLNANARSMISPVVCSNIDDFSGLLI
KAQGHVVAGQTPLYEIKK

>gi|30062144|ref|NP_836315.1| hypothetical protein S0623 [Shigella flexneri 2a str. 2457T]
MRTFSGKRSTLALAIAGVTAMSGFMAMPEARAEGFIDDSTLTGGIYYWQRERDRKDVTDGDKYKTNLSHS
TWNANLDFQSGYAADMFGLDIAAFTAIEMAENGDSSHPNEIAFSKSNKAYDEDWSGDKSGISLYKAAAKF
KYGPVWARAGYIQPTGQTLLAPHWSFMPGTYQGAEAGANFDYGDAGALSFSYMWTNEYKAPWHLEMDEFY
QNDKTTKVDYLHSIGAKYDFKNNFVLEAAFGQAEGYIDQYFAKASYKFDIAGSPLTTSYQFYGTRDKVDD
RSVNDLYDGTAWLQALTFGYRAADVVDLRLEGTWIKADSQQGYFLQRMTPTYASSNGRLDIWWDNRSDFN
ANGEKAVFFSAMYDLKNWNLPGFAIGASYVYAWDAKPATWQSNPDAYYDKNRTIEESAYSLDAVYTIQDG
RAKGTMFKLHFTEYDNHSDIPSWGGGYGNIFQDERDVKFMVIAPFTIF

>gi|30062143|ref|NP_836314.1| hypothetical protein S0622 [Shigella flexneri 2a str. 2457T]
MKKLILIAMMASGLVACAQSTAPQEDSRLKEAYSACINTAQGSPEKIEACQSVLNVLKKEKQHQQFADQE
SVRVLDYQQCLRATQTGHDQAVKADCDKVWQEIRSNNK

>gi|30062142|ref|NP_836313.1| ferric uptake regulator [Shigella flexneri 2a str. 2457T]
MTDNNTALKKAGLKVTLPRLKILEVLQEPDNHHVSAEDLYKRLIDMGEEIGLATVYRVLNQFDDAGIVTR
HNFEGGKSVFELTQQHHHDHLICLDCGKVIEFSDDSIEARQREIAAKHGIRLTNHSLYLYGHCAEGDCRE
DEHAHEGK

>gi|30062141|ref|NP_836312.1| flavodoxin FldA [Shigella flexneri 2a str. 2457T]

MAITGIFFGSDTGNTENIAKMIQKQLGKDVADVHDIAKSSKEDLEAYDILLLGIPTWYYGEAQCDWDDFF

PTLEEIDFNGKLVALFGCGDQEDYAEYFCDALGTIRDIIEPRGATIVGHWPTAGYHFEASKGLADDDHFV

GLAIDEDRQPELTAERVEKWVKQISEELHLDEILNA

>gi|30062140|ref|NP_836311.1| LexA regulated protein [Shigella flexneri 2a str. 2457T]

MYYGALSIRAEAWLIVSPEVTKIMAKEQTDRTTLDLFAHERRPGRPKTNPLSRDEQLRINKRNQLKRDKV

RGLKRVELKLNAEAVEALNELAESRNMSRSELIEEMLMQQLAALRSQGIV

>gi|30062138|ref|NP_836309.1| replication initiation regulator SeqA [Shigella flexneri 2a str. 2457T]

MKTIEVDDELYSYIASHTKHIGESASDILRRMLKFSAASQPAAPVTKEVRVASPAIVEAKPVKTIKDKVR

AMRELLLSDEYAEQKRAVNRFMLLLSTLYSLDAQAFAEATESLHGRTRVYFAADEQTLLKNGNQTKPKHV

PGTPYWVITNTNTGRKCSMIEHIMQSMQFPAELIEKVCGTI

>gi|30062135|ref|NP_836306.1| potassium-transporting ATPase subunit C [Shigella flexneri 2a str. 2457T]

MSGLRPALSTFLFLLLITGGVYPLLTTALGQWWYPWQANGSLIREGDTVRGSALIGQNFTGNGYFHGRPS

ATAEMPYNPQASGGSNLAVSNPELDKQIAARVAALRAANPDASTNVPVELVTASASGLDNNITPQAAAWQ

IPRIAKARNLSVEQLTQLIAKYSQQPLVKYIGQPVVNIVELNLALDKLDE

>gi|30062134|ref|NP_836305.1| potassium-transporting ATPase subunit A [Shigella flexneri 2a str. 2457T]

MAAQGFLLIATFLLVLMVLARPLGSGLARLINDIPLPGTAGVERILFRLPGVSDHEMNWKQYLCAILGLN
MLGLAVLFFMLLGQHYLPLNPQQLPGLSWDLALNTAVSFVTNTNWQSYSGETTLSYFSQMAGLTVQNFLS
AASGIAVIFAFIRAFTRQSMSTLGNAWVDLLRITLWVLVPVALLIALFFIQQGAQQNFLPYQAVNTVEGA
QQLLPMGPVASQEAIKMLGTNGGGFFNANSSHPFENPTALTNFVQMLAIFLIPTALCFAFGEVTGDRRQG
RMLLWAMSVIFVICVGVVMWAEVQGNPHLLALGADSSINMEGKESRFGVLVSSLFAVVTTAASCGAVIAM
HDSFTALGGMVPMWLMQIGEVVFGGVGSGLYGMMLFVLLAVFIAGLMIGRTPEYLGKKIDVREMKLTALA
ILVTPTLVLMGAALAMMTDAGRSAMLNPSPHGFSEVLYAVSSAANNNGSAFAGLSANSPFWNCLLAFCMF
VGRFGVIIPVMAIAGSLVSKKSQPASSGTLPTHGPLFVGLLIGTVLLVGALTFIPALALGPVAEYLS

>gi|30062130|ref|NP_836301.1| Rhs-family protein [Shigella flexneri 2a str. 2457T]
MEEAFWAARQGDALLHTSFMADVLGAVLEVAANVVIDALIVGTTSLLATSVGITMGCTAVVLTGFVAGVA
MVYTGVTEKVSEACSALANMLFPPQIEGYILTGSGDTWINSKPAARAAATAASRQDIEAQEAQAKAEQEE
AQRQEDARTFRDVAGEYLEMAGAIALAVNPVTGPVLMSRALHDQLSTEEGRSEMVDGVTHFVSELWQPTV
ASAAPGSTPTPDDKIDCHKHPSSLTQFLAQKKAAFLDAPLGTALNLLNPGGMLETAFQGVNAVIGSISNL
FKGDDEPPAAEYIAEGARDVRINSQPATRSGARCTCEARVVNEPGNGAFVSPDVRIGGPLLVVRDIRSGR
SQITLVATVALMFLRPGKMLSKIACFAASVGMSMLTQKATSALPHPVNAATGAKYLADDDDFDFSLPGHF
PLDWQRVYSSRDERTEGMFGQGWSVMYEVSLVCTPGSADENCMTFVSGMGRRLDMEAVLPGGGFYSPGEG
LAVRRGEQGHWLISSDDGQCFLFEADPHHPQRQRLKMLGDRNSNCLNLYYDDRGRIVEISGEQQRPCIRL
YYELAAHPRRVTQIYQHFPETAPLLLRRYSYDEAGHLNGVYDSTGHLLREFAYDENHCMTLHRQPGGEGY
YYQWGWYEGPDDAAWRVTGHHTDSGEQYRLAWDLAQRRLCVTDGLGRTRYHQWDAQNQVTAYQDEAGQVT
TFRWSDEERLLLGMTDPQGGKWRYVYDLQGHITETHDPLGRVEQAQWHPVWHQPETEVDAAGNLLGRRAG
ERATAENSVVPFNRLMSYRGVHYRYDEFGRAVEKEGRSGTQSYRYDAEHRMVEVTTARGTYRYVYDALGR
RTEKQHISPDGKPYNRTKFLWDGMRLAQESRPEGTGSLYIYRDQGSYEPLARVDKAGKEGPNRILYFHTD

VNGAPEEMTDSDGKIVWETGYQVWGNTIQEKDHGGVEQNLRYQGQYLDRETGLHYNLHRYYDPDVGRFMV

TDPIGLRGGLNLYSYAPNPLKYADPLGLTPCAVSNQKANRLLDSSETKVTVRSRSDAEQLFMDRYLGHNY

KNMTGESGPSTKNLMEYLTENKTKAGSYHWDDIKDPSVTKPSYRVSGHGPGNPDGDLPHLQVHQHGGSVR

HIFFPWET

>gi|30062128|ref|NP_836299.1| hypothetical protein S0603 [Shigella flexneri 2a str. 2457T]

MNLQRFDDSTLIRIFALHELHRLKEHGLTRGALLDYHSRYKLVFLAHSQPEYRKLGPFVADIHQWQNLDD

FYNQYRQRVIVLLSHPANPRDHTNVLMHVQGYFRPHIDSTERQQLAALIDSYRRGEQPLLAPLMRIKHYM

ALYPDAWLSGQRYFELWPRVINLRHSGVL

>gi|30062125|ref|NP_836296.1| hydrolase-oxidase [Shigella flexneri 2a str. 2457T]

MKNTELEQLINEKLNSAAISDYAPNGLQVEGKETVQKIVTGVTASQALLDEAVRLGADAVIVHHGYFWKG

ESPVIRGMKRNRLKTLLANDINLYGWHLPLDAHPELGNNAQLAALLGITVMGEIEPLVPWGELTMPVPGM

ELASWIEARLGRKPLWCGDTGPEVVQRVAWCTGGGQSFIDSAARFGVDAFITGEVSEQTIHSAREQGLHF

YAAGHHATERGGIRALSEWLNENTDLDVTFIDIPNPA

>gi|30062124|ref|NP_836295.1| carboxylase [Shigella flexneri 2a str. 2457T]

MQRARCYLIGETAVVLELEPPVTLASQKRIWRLAQRLVDMPNVVEAIPGMNNITVILRNPESLALDAIER

LQRWWEESEALEPESRFIEIPVVYGGAGGPDLAVVAAYCGLSEKQVVELHSSVEYVVWFLGFQPGFPYLG

SLPEQLHTPRRAEPRLLVPAGSVGIGGPQTGVYPLATPGGWQLIGHTSLSLFDPARDEPILLRPGDSVRF

VPQKEGIC

>gi|30062123|ref|NP_836294.1| LamB/YcsF family protein [Shigella flexneri 2a str. 2457T]

MKIDLNADLGEGCASDAELLTLVSSANIACGFHAGDAQTMQAFVREAIKNGVAIGAHPSFPDRENFGRSA

MQLPPETVYAQTLYQIGALATIARAQGGVMRHVKPHGMLYNQAAKEAQLADAIARAVYACDPALVLVGLA

GSELIRAGKQYGLTTREEVFADRGYQADGSLVPRSQPGALIENEEQALAQTLEMVQHGRVKSITGEWATV

AAQTVCLHGDGEHALAFARRLRATFAKKGIVVAA

>gi|30062122|ref|NP_836293.1| endonuclease VIII [Shigella flexneri 2a str. 2457T]

MPEGPEIRRAADNLEAAIKGKPLTDVWFAFPQLKTYQSQLIGQHVTHVETRGKALLTHFPNGLTLYSHNQ

LYGVWRVVDTGEEPQTTRVLRVKLQTADKTILLYSASDIEMLRPEQLTTHPFLQRVGPDVLDPNLTPEVV

KERLLSPRFRNRQFAGLLLDQAFLAGLGNYLRVEILWQVGLTGNHKAKDLNAAQLDALAHALLEIPRFSY

ATRGQVDENKHHGALFRFKVFHRDGELCERCGGIIEKTTLSSRPFYWCPGCQH

>gi|30062121|ref|NP_836292.1| transporter [Shigella flexneri 2a str. 2457T]

MLYRYTKEKIMRERRMPVLQWGMLCVLSLLLSIGFLAVHLPAALLLGPMIAGIIFSMRGITLQLPRSAFL

AAQAILGCMIAQNLTGSILTTLAANWPIVLAILLVTLLSSAIVGWLLVRYSSLPGNTGAWGSSPGGAAAM

VAMAQDYGADIRLVAFMQYLRVLFVAGAAVLVTRMMLGDNAEAVNQQIVWFPPVSINLLLTILLAVVAGT

AGCMLRLPSGTMLIPMLAGAVLQSGQLITIELPEWLLAMAYMAIGWRIGLGFDKQILLRALRPLPQILLS

IFALLAICAGMAWGLTRFMHIDFMTAYLATSPGGLDTVAVIAAGSNADMALIMAMQTLRLFSILLTGPAI

ARFISTYAPKRSA

>gi|30062120|ref|NP_836291.1| chaperone [Shigella flexneri 2a str. 2457T]

MTFMKGLPLLLLVASLCSHAALQPDRTRIVFNANDKATSLRVDNRSDKLPYLAYSWLENEKGEKSDDLLV

ALPPIQRLEPKATTQVRIVKQASTTKLPGDRETLFFYNMREIPPAPEKNSDHAVLQVAIQSRIKVFWRPA

ALRKKAGEKVELQLQVSQQGNQLTLKNPTAYYLTIAYLGRNEKGVLPGFKTVMVAPFSTVNTNTGNYSGS

QFYLGYMDDYGALRMTTLNCSGQCHLQAVEAKK

>gi|30062119|ref|NP_836290.1| outer membrane protein [Shigella flexneri 2a str. 2457T]

MDTVNIYRLSFVSCLVVAMPCALAVEFNLNVLDKSMRDRIDISLLKEKGVIAPGEYFVSVAVNNNQISNE

QKINWHKNDDKTIPCINDLLVDKFGLKPEVRQSLPLINQCVDFSSRPEMLFNFDQANQQLNISIPQAWLA

WHSENWTPPSTWKEGVAGVLMDYNLFASSYRPQDGSSSTNLNAYGTAGINTGAWRLRSDYQLNHTDSDDN

HEQSGEISRTYLFRPLPQLGSKLTLGETDFSPNIFDGFSYTGAALASDDRMLPWELRGYAPQISGIAQTN

ATVTISQSGRVIYQKKVPPGPFIIDDLNQSVQGTLDVKVTEEDGRVNNFQVSAASTPFLTRQGQVRYKLA

AGQPRPSMSHQTENETFFSNEVSWGMLSNTSLYSGLLLSGDDYHSAAMGIGQNMLWLGALSFDVTWASSH

FDTQQDERGLSYRFNYSKQVDATNSTISLAAYRFSDRHFHSYANYLDHKYNDSDAQDEKQTISLSVGQPI

TPLNLNLYANLLHQTWWNADASTTANITAGFNVDIGDWRDISISTSFNTTHYEDKDRDNQIYLSISLPFG

NGGRVGYDMQNSSHSTTHRMSWNDTLDERNSWGMSAGLQSDRPDNGAQVSGNYQHLSSAGEWDISGTYAA

NDYSSVSSSWSGSFTATQYGAAFHRRSSTNEPRLMVSTDGVADIPVQGNLDYTNHFGIAVVPLISSYQPS

TVAVNMNDLPDGVTVAENVIKETWIEGAIGYKSLASRSGKDVNVIIRNASGQFPPLGADIRQDDSGISVG

MVGEEGHAWLSGVAENQKFTVVWGDSQHCSLHLPEHMEDTANRLILPCH

>gi|30062118|ref|NP_836289.1| fimbrial-like protein [Shigella flexneri 2a str. 2457T]

MFKGQKTLAALAVSLLFTAPVYAADEGSGEIHFKGEVIEAPCEIHQDDIDKEVELGQVTTSHINQSHHSD

AVAVDLLLVNCDLENSSNGSGGKISKVAVTFDSSAKTTGADPILNNTSTGEATGVGVRLMNKDQSNIVLG

TATPDIDLAPTSSEQTLNFFAWMEQIDQATPVTPGAVTANATYVLDYK

>gi|30062115|ref|NP_836286.1| succinate dehydrogenase cytochrome b556 small membrane subunit [Shigella flexneri 2a str. 2457T]

MVSNASALGRNGVHDFILVRATAIVLTLYIIYMVGFFATSGELTYEVWIGFFASAFTKVFTLLALFSILI

HAWIGMWQVLTDYVKPLALRLMLQLVIVVALVVYVIYGFVVVWGV

>gi|30062107|ref|NP_836278.1| DNA-binding transcriptional repressor MngR [Shigella flexneri 2a str. 2457T]

MGHKPLYRQIADRIREQIARGELKPGDALPTESALQTEFGVSRVTVRQALRQLVEQQILESIQGSGTYVK

EERVNYDIFQLTSFDEKLSDRHVDTHSEVLIFEVIPADDFLQQQLQITVQDRVWHVKRVRYRKQKPMALE

ETWMPLALFPDLTWQVMENSKYHFIEEVKKMVIDRSEQEIIPLMPTEEMSRLLNISQTKPILEKVSRGYL

VDGRVFEYSRNAFNTDDYKFTLIAQPKIIAISTKRPSYDGQPCGNRQPFAIPVEGSGEGYFLLQIFISEQ

FSVLHRYPPGCERFSRLRLPEQRISHPQSLSHRQ

>gi|30062106|ref|NP_836277.1| 2-O-a-mannosyl-D-glycerate specific PTS transporten components IIABC [Shigella flexneri 2a str. 2457T]

MVLFCRAHWRDYKNDQVRIMMNLTTLTHRDALCLNARFTSREEAIHALTQRLAALGKISSTEQFLKEVYR

RESLGPTALSEGLAVPHGKTAAVKEAAFAVATLSEPLQWEGVDGPEAVDLVVLLAIPLNEAGTTHMQLLT

ALTTRLADDEIRARIQSATTPDELLSALDDKGGTQPSASFSNAPTIVCVTACPAGIAHTYMAAEYLEKAG

RKLGVNVYVEKQGANGIEGRLTADQLNSATACIFAAEVAIKESERFNGIPALSVPVAEPIRHAEALIQQS

LTLERGDETRTLQQDTQPVKSVKTELKQALLSGISFAVPLIVAGGTVLAVAVLLSQIFGLQDLFNEENSW

LWMYRKLGGGLLGILMVPVLAAYTAYSLADKPALAPGFAAGLAANMIGSGFLGAVVGGLIAGYLIRWMKN

HLRLSSKFNGFLTFYLYPVLGTLGAGSLMLFVVGEPVAWINNSLTAWLNGLSGSNALLLGAILGFMCSFD

LGGPVNKAAYAFCLGAMANGVYGPYAIFASVKMVSAFTVTASTMLAPRLFKEFEIETGKSTWLLGLAGIT

EGAIPMAIEDPLRVIGSFVLGSMVTGAIVGAMNIGLSTPGAGIFSLFLLHDNGAGGVMAAIGWFGAALVG

AAISTAILLIWRRHAVKHGNYLTDGVMP

>gi|30062104|ref|NP_836275.1| cytochrome d terminal oxidase, polypeptide subunit I [Shigella flexneri 2a str. 2457T]

MMLDIVELSRLQFALTAMYHFLFVPLTLGMAFLLAIMETVYVLSGKQIYKDMTKFWGKLFGINFALGVAT

GLTMEFQFGTNWSYYSHYVGDIFGAPLAIEGLMAFFLESTFVGLFFFGWDRLGKVQHMCVTWLVALGSNL

SALWILVANGWMQNPIASDFNFETMRMEMVSFSELVLNPVAQVKFVHTVASGYVTGAMFILGISAWYMLK

GRDFAFAKRSFAIAASFGMAAVLSVIVLGDESGYEMGDVQKTKLAAIEAEWETQPAPAAFTLFGIPDQEE

ETNKFAIQIPYALGIIATRSVDTPVIGLKELMVQHEERIRNGMKAYSLLEQLRSGSTDQTVRDQFNSMKK

DLGYGLLLKRYTPNVADATEAQIQQATKDSIPRVAPLYFAFRIMVACGFLLLAIIALSFWSVIRNRIGEK

KWLLRAALYGIPLPWIAVEAGWFVAEYGRQPWAIGEVLPTAVANSSLTAGDLIFSMVLICGLYTLFLVAE

LFLMFKFARLGPSSLKTGRYHFEQSSTTTQPAR

>gi|30062103|ref|NP_836274.1| cytochrome d terminal oxidase polypeptide subunit II [Shigella flexneri 2a str. 2457T]

MIDYEVLRFIWWLLVGVLLIGFAVTDGFDMGVGMLTRFLGRNDTERRIMINSIAPHWDGNQVWLITAGGA

LFAAWPMVYAAAFSGFYVAMILVLASLFFRPVGFDYRSKIEETRWRNMWDWGIFIGSFVPPLVIGVAFGN

LLQGVPFNVDEYLRLYYTGNFFQLLNPFGLLAGVVSVGMIITQGATYLQMRTVGELHLRTRATAQVAALV

TLVCFALAGVWVMYGIDGYVVKSTMDHYAASNPLNKEVVREAGAWLVNFNNTPILWAIPALGVVLLLLTI

LTARMDKAAWAFVFSSLTLACIILTAGIAMFPFVMPSSTMMNASLTMWDATSSQLTLNVMTWVAVVLVPI

ILLYTAWCYWKMFGRITKEDIERNTHSLY

>gi|30062101|ref|NP_836272.1| acyl-CoA thioester hydrolase YbgC [Shigella flexneri 2a str. 2457T]

MNTTLFRWPVRVYYEDTDAGGVVYHASYVAFYERARTEMLRHHHFSQQALMAERVAFVVRKMTVEYYAPA

RLDDMLEIQTEITSMRGTSLVFTQRIVNAENTLLNEAEVLVVCVDPLKMKPRALPKSIVAEFKQ

>gi|30062100|ref|NP_836271.1| colicin uptake protein TolQ [Shigella flexneri 2a str. 2457T]

MTDMNILDLFLKASLLVKLIMLILIGFSIASWAIIIQRTRILNAAAREAEAFEDKFWSGIELSRLYQESQ

GKRDNLTGSEQIFYSGFKEFVRLHRANSHAPEAVVEGASRAMRISMNRELENLETHIPFLGTVGSISPYI

GLFGTVWGIMHAFIALGAVKQATLQMVAPGIAEALIATAIGLFAAIPAVMAYNRLNQRVNKLELNYDNFM

EEFTAILHRQAFTVSESNKG

>gi|30062099|ref|NP_836270.1| colicin uptake protein TolR [Shigella flexneri 2a str. 2457T]

MARARGRGRRDLKSEINIVPLLDVLLVLLLIFMATAPIITQSVEVDLPDATESQAVSSNDNPPVIVEVSG

IGQYTVVVEKDRLERLPPEQVVAEVSSRFKANPKTVFLIGGAKDVPYDEIIKALNLLHSAGVKSVGLMTQ

PI

>gi|30062098|ref|NP_836269.1| cell envelope integrity inner membrane protein TolA [Shigella flexneri 2a str. 2457T]

MSKATEQNDKLKRAIIISAVLHVILFAALIWSSFDENIEASAGGGGGSSIDAVMVDSGAVVEQYKRMQSQ

ESSAKRSDEQRKMKEQQAAEELREKQAAEQERLKQLEKERLAAQEQKKQAEEAAKQAELKQKQAEVAAAK

AAADAKAAEEAAKKAAADAKKKAEAEAAKAAAEAQKKAEVAAAALKKKAEAAEAAAAEARKKAATEAAEK

AKAEAEKKAAAEKAAADKKAAAEKAAADKKAAEKAAADKAAADKKAAAEKAAADKKAAAAKAAAEKAAAA

KAAAEADDIFGELSSGKNAPKTGGGAKGNNASPAGSGNTKNNGASGADINNYAGQIKSAIESKFYDASSY

AGKTCTLRIKLAPDGMLLDIKPEGGDPALCQAALAAAKLAKIPKPPSQAVYEVFKNAPLDFKP

>gi|30062097|ref|NP_836268.1| translocation protein TolB [Shigella flexneri 2a str. 2457T]

MKQALRVAFGFLILWASVLHAEVRIVIDSGVDSGRPIGVVPFQWAGPGAAPEDIGGIVAADLRNSGKFNP

LDRARLPQQPGSAQEVQPAAWSALGIDAVVVGQVTPNPDGSYNVAYQLVDTGGAPGTVLAQNSYKVNKQW

LRYAGHTASDEVFEKLTGIKGAFRTRIAYVVQTNGGQFPYELRVSDYDGYNQFVVHRSPQPLMSPAWSPD

GSKLAYVTFESGRSALVIQTLANGAVRQVASFPRHNGAPAFSPDGSKLAFALSKTGSLNLYVMDLASGQI

RQVTDGRSNNTEPTWFPDSQNLAFTSDQAGRPQVYKVNINGGAPQRITWEGSQNQDADVSSDGKFMVMVS

SNGGQQHIAKQDLATGGVQVLSSTFLDETPSLAPNGTMVIYSSSQGMGSVLNLVSTDGRFKARLPATDGQ

VKFPAWSPYL

>gi|30062096|ref|NP_836267.1| peptidoglycan-associated outer membrane lipoprotein [Shigella flexneri 2a str. 2457T]

MQLNKVLKGLMIALPVMAIAACSSNKNASNDGSEGMLGAGTGMDANGGNGNMSSEEQARLQMQQLQQNNI

VYFDLDKYDIRSDFAQMLDAHANFLRSNPSYKVTVEGHADERGTPEYNISLGERRANAVKMYLQGKGVSA

DQISIVSYGKEKPAVLGHDEAAYSKNRRAVLVY

>gi|30062095|ref|NP_836266.1| tol-pal system protein YbgF [Shigella flexneri 2a str. 2457T]

MSSNFRHQLLSLSLLVGIAAPWAAFAQAPISSVSSGSVEDRVTQLERISNAHSQLLTQLQQQLSDNQSDI

DSLRGQIQENQYQLNQVVERQKQILLQIDSLSSGGAAAQSTSSDQSGATASTTPTADAGTANAGAPVKSG

DANTDYNAAIALVQDKSRQDDAMVAFQNFIKNYPDSTYLPNANYWLGQLNYNKGKKDDAAYYFASVVKNY

PKSPKAADAMFKVGVIMQDKGDTAKAKAVYQQVISKYPGTDGAKQAQKRLNAM

>gi|30062094|ref|NP_836265.1| quinolinate synthetase [Shigella flexneri 2a str. 2457T]

MSVMFDPDTAIYPFPPKPTPLSIDEKAYYREKIKRLLKERNAVMVAHYYTDPEIQQLAEETGGCISDSLE

MARFGAKHPASTLLVAGVRFMGETAKILSPEKTILMPTLQVECSLDLGCPVEEFNAFYDAHPDRTVVVYA

NTSAAVKARADWVVTSSIAVELIDHLDSLGEKIIWAPDKHLGRYVQKQTGGDILCWQGACIVHDEFKTQA

LTRLQEEYPDAAILVHPESPQAIVDMADAVGSTSQLIAAAKALPHQRLIVATDRGIFYKMQQAVPDKELL

EAPTAGEGATCRSCAHCPWMAMNDLQAIAEALEQEGSNHEVHVDERLRERALVPLNRMLDFAATLRG

>gi|30062093|ref|NP_836264.1| PnuC protein [Shigella flexneri 2a str. 2457T]

MDFFSVQNILVHIPIGAGGYDLSWIEAVGTIAGLLCIGLASLEKISNYFFGLINVTLFGIIFFQIQLYAS

LLLQVFFFAANIYGWYAWSRQTSQNEAELKIRWLPLPKALSWLAVCVVSIGLMTVFINPVFAFLTRVAVM

IMQALGLQVVMPELQPDAFPFWDSCMMVLSIVAMILMTRKYVENWLLWVIINVISVVIFALQGVYAMSLE

YIILTFIALNGSRMWINSARERGSRALSH

>gi|30062091|ref|NP_836262.1| homeobox protein [Shigella flexneri 2a str. 2457T]

MKMTKLATLFLTATLSLASGAALAADSGAQTNNGQANAAADAGQVAPDARENVAPNNVDNNGVNTGSGGT

MLHSDGSSMNNDGMTKDEEHKNTMCKDGRCPDINKKVQTGDGINNDVDTKTDGTTQ

>gi|30062090|ref|NP_836261.1| phospho-2-dehydro-3-deoxyheptonate aldolase [Shigella flexneri 2a str. 2457T]

MNYQNDDLRIKEIKELLPPVALLEKFPATENAANTVAHARKAIHKILKGNDDRLLVVIGPCSIHDPVAAK

EYATRLLALREELKDELEIVMRVYFEKPRTTVGWKGLINDPHMDNSFQINDGLRIARKLLLDINDSGLPA

AGEFLDMITPQYLADLMSWGAIGARTTESQVHRELASGLSCPVGFKNGTDGTIKVAIDAINAAGAPHCFL

SVTKWGHSAIVNTSGNGDCHIILRGGKEPNYSAKHVAEVKEGLNKAGLPAQVMIDFSHANSSKQFKKQMD

VCADVCQQIAGGEKAIIGVMVESHLVEGNQSLDSGEPLAYGKSITDACIGWEDTDALLRQLVNAVKARRG

>gi|30062083|ref|NP_836254.1| DNA-binding transcriptional regulator ModE [Shigella flexneri 2a str. 2457T]

MQAEILLTLKLQQKLFADPRRISLLKHIALSGSISQGAKDAGISYKSAWDAINEMNQLSEHILVERATGG

KGGGGAVLTRYGQRLIQLYDLLAQIQQKAFDVLSDDDALPLNSLLAAISRFSLQTSARNQWFGTITARDH

DDVQQHVDVLLADGKTRLKVAITAQSGARLGLDEGKEVLILLKAPWVGITQDEAVAQNADNQLPGIISHI

ERGAEQCEVLMALPDGQTLCATVPVNEATSLQQGQNVTAYFNADSVIIATLC

>gi|30062081|ref|NP_836252.1| molybdate transporter periplasmic protein [Shigella flexneri 2a str. 2457T]

MARKWLNLFAGAALSFAVAGNALADEGKITVFAAASLTNAMQDIATQYKKEKGVDVVSSFASSSTLARQI

EAGAPADLFISADQKWMDYAVDKKAIDTATRQTLLGNSLVVVAPKASEQKDFTIDSKTNWTSLLNGGRLA

VGDPEHVPAGIYAKEALQKLGAWDTLSPKLAPAEDVRGALALVERNEAPLGIVYGSDAVASKGVKVVATF

PEDSHKKVEYPVAVVEGHNNATVKAFYDYLKGPQAAEIFKRYGFTTK

>gi|30062080|ref|NP_836251.1| molybdate ABC transporter permease [Shigella flexneri 2a str. 2457T]

MILTDPEWQAVLLSLKVSSLAVLFSLPFGIFFAWLLVRCTFPGKALLDSVLHLPLVLPPVVVGYLLLVSM

GRRGFIGERLYDWFGITFAFSWRGAVLAAAVMSFPLMVRAIRLALEGVDVKLEQAARTLGAGRWRVFFTI

TLPLTLPGIIVGTVLAFARSLGEFGATITFVSNIPSETRTIPSAMYTLIQTPGGESGAARLCIISIALAM

ISLLISEWLARISRERAGR

>gi|30062078|ref|NP_836249.1| phosphotransferase [Shigella flexneri 2a str. 2457T]

MTTRVIALDLDGTLLTPKKTLLPSSIEALARAREAGYQLIIVTGRHHVAIHPFYQALALDTPAICCNGTY

LYDYHAKTVLEADPMPVNKALQLIEMLNEHHIHGLMYVDDAMVYEHPTGHVIRTSNWAQTLPPEQRPTFT

QVASLAETTQQVNAVWKFALTHDDLPQLQHFGKHVEHELGLECEWSWHDQVDIAHGGNSKGKRLTKWVEA

QGWSMENVVAFGDNFNDISMLEAAGTGVAMGNADDAVKARANIVIGDNTTDSIAQFIYSHLI

>gi|30062075|ref|NP_836246.1| citrate lyase synthetase (citrate (pro-3S)-lyase ligase [Shigella flexneri 2a str. 2457T]

MKIKGYQFATLNNIRCKSITTCTFRILFIMFGNDIFTRVKRSENKKMAEIAQFLHENDLSVDTTVEVFIT

VTRDEKLIACGGIAGNIIKCVAISESVRGEGLALTLATELINLAYERHSTHLFIYTKTEYEALFRQCGFS

TLTCVPGVMVLMENSATRLKRYAESLKKFRHPGNKIGCIVMNANPFTNGHRYLIQQAAAQCDWLHLFLVK

EDSSRFPYEDRLDLVLKGTADIPRLTVHRGSEYIISRATFPCYFIKEQSVINHCYTEIDLKIFRQYLAPA

LGVTHRFVGTEPFCRVTAQYNQDMRYWLETPTISAAPIELVEIERLRYQEMPISASRVRQLLAKNDLTAI

APLVPAVTLHYLQNLLEHSRQDAAARQKTPA

>gi|30062072|ref|NP_836243.1| citrate lyase subunit alpha [Shigella flexneri 2a str. 2457T]

MTQKIEQSQRQERVAAWNRRTECDLAAFQNSPKQTYQAEKARDRKLCANLEEAIRRSGLQDGMTVSFHHA

FRGGDLTVNMVMDVIAKMGFKNLTLASSSLSDCHAPLVEHIRQGVVTRIYTSGLRGPLAEEISRGLLAEP

VQIHSHGGRVHLVQSGELNIDVAFLGVPSCDEFGNANGYTGKACCGSLGYAMVDADNAKQVVMLTEELLP

YPHNPASIEQDQVDLIVKVNRVGDAAKIGAGTTRMTTNPRELLIARSAADVIVNSGYFKEGFSMQTGTGG

ASLAVTRFLEDKMRSRDIRADFALGGITATMVDLHEKGLIRKLLDVQSFDSHAAQSLARNPNHIEISANQ

YANWGSKGASVDRLDVVVLSALEIDRQFNVNVLTGSDGVLRGASGGHCDTAIASALSIIVAPLVRGRIPT

LVDNVLTCITPGSSVDILVTDHGIAVNPARPELAERLQEAGIKVVSIEWLRERARLLTGEPQPIEFTDRV

VAVVRYRDGSVIDVVHQVKE

>gi|30062071|ref|NP_836242.1| 2-(5''-triphosphoribosyl)-3'-dephosphocoenzyme-A synthase [Shigella flexneri 2a str. 2457T]

MHLLPELASHHAVSIPELLVSRDERQARQHAWLKHHPVPLVSFTVVAPGPIKDSEVTRRIFNHRVTALRA

LATKQGWQIQEQAALVSASGPEGMLSIAAPARDLKLATIELEHSHPLGRLWDIDVLTPEGEILSRRDYSL

RLAAACCASKAQPSARVEKPIN

>gi|30062070|ref|NP_836241.1| triphosphoribosyl-dephospho-CoA synthase [Shigella flexneri 2a str. 2457T]

MSMPATSTKTTKLATSLIDEYALLGWRAMLTEVNLSPKPGLVDRINCGAHKDMALEDFHRSALAIQGWLP

RFIEFGACSAEMAPEAVLHGLRPIGMACEGDMFRATAGVNTHKGSIFSLGLLCAAIGRLLQLNQPVTPTT

VCSTAASFCRGLTDRELRTNNSRLTAGQRLYQQLGLTGARGEAEAGYPLVINHALPHYLTLLDQGLDPEL

ALLDTLLLLMATNGDTNVASRGGEGGLRWLQREAQTLLQKGGIRTPTDLDYLRQFDRECIERNLSPGGSA

DLLILTCFLAQI

>gi|30062069|ref|NP_836240.1| ribonuclease I [Shigella flexneri 2a str. 2457T]

MSSTPIMKAFCRNAALLAVSLLPLSSANAVALQAKQYGDFDRYVLALSWQTGFCQSQHDRNRNERDECRL

QTETTNKTDFLTVHGLWPGLPKSVAARGVDERRWMRFGCATRPIPNLPEARASRMCSSPETGLSLETAAK

LSEVMPGAGGRSCLERYEYAKHGACFGFDPDAYFGTMVRLNQEIKESEAGKFLADNYGKTVSRRDFDAAF

AKSWGKENVKAVKLTCQGNPAYLTEIQISIKADAINAPLSANSFLPQPHPGNCGKTFVIDKAGY

>gi|30062068|ref|NP_836239.1| nucleoside diphosphate kinase regulator [Shigella flexneri 2a str. 2457T]

MSRPTIIINDLDAERIDILLEQPAYAGLPIADALNAELDRAQMCSPEEMPHDVVTMNSRVKFRNLSDGEV

RVRTLVYPAKMTDSNTQLSVMAPVGAALLGLRVGDSIHWELPGGVATHLEVLELEYQPEAAGDYLL

>gi|30062067|ref|NP_836238.1| hypothetical protein S0533 [Shigella flexneri 2a str. 2457T]

MLLPWLFPQTKRNNFAAKVNSENQEKLLILRKEDASASSGQSELSLVQMGLKQVVASSFWLVFEFQHFKV

GCNAARKFPVNGIANAQPQQCGTYRSHNGKLSIAIGHFCRIHQRAHTHFAIAKIAEFNPAVHCHHVVWHL

FWRTHLGAIQLCV

>gi|30062065|ref|NP_836236.1| hypothetical protein S0531 [Shigella flexneri 2a str. 2457T]

MYKAIIMPVDVFEMELSDKAVRHAEFLAQDDGVIHLLHVLPGSASLSLHRFAADVRRFEEHLQHEAEERL

QTMVSHFTIDPSRIKQHVRFGSVRDEVNELAEELGADVVVIGSRNPSISTHLLGSNASSVIRHANLPVLV

VR

>gi|30062056|ref|NP_836227.1| hypothetical protein S0519 [Shigella flexneri 2a str. 2457T]

MPHNPIRVVVGPANYFSHPGSFNHLHNFFTDEQLSRAVWIYGKRAIAAAQTKLPPAFGLPGAKHILFRGH

CSESDVQQLAAESGDDRSVVIGVGGGALLDTAKALARRLGLPFVAAPTIAATCAAWTPLSVWYNDAGQAL

HYEIFDDANFMVLVEPEIILNAPQQYLLAGIGDTLAKWYEAVVLAPQPETLPLTVRLGINNAQAIRDVLL

NSSEQALADQQNQQLTQSFCDVVDAIIAGGGMVGGLGDRFTRVAAAHAVHNGLTVLPQTEKFLHGTKVAY

GILVQSALLGQDDVLAQLTGAYQRFHLPTTLAELEVDINNQAEIDKVIAHTLRPVESIHYLPVTLTPDTL

RAAFEKVESFKA

>gi|30062054|ref|NP_836225.1| hypothetical protein S0517 [Shigella flexneri 2a str. 2457T]

MIWKRHLTLDELNATSDNTMVAHLGIVYTLLGDDVLEAEMPVDTRTHQPFGLLHGGASAALAETLGSMAG

FMMTRDGQCVVGTELNATHHRPVSEGKVRGVCQPLHLGRQNQSWEIVVFDEQGRRCCTCRLGTAVLG

>gi|30062052|ref|NP_836223.1| 2,3-dihydro-2,3-dihydroxybenzoate synthetase [Shigella flexneri 2a str. 2457T]

MAIPKLQAYALPESHDIPQNKVDWAFELQRAALLIHDMQDYFVSFWGENCPMMEQVIANIAALRDYCKQH

NIPVYYTAQPKEQSDEDRALLNDMWGPGLTRSPEQQKVVDRLTPDADDTVLVKWRYSAFHRSPLEQMLKE

SGRNQLIITGVYAHIGCMTTATDAFMRDIKPFMVADALADFSRDEHLMSLKYVAGRSGRVVMTEELLPAP

IPASKAALREVILPLLDESDEPFDDDNLIDYGLDSVRMMALAARWRKVHGDIDFVMLAKNPTIDAWWKLL

SREVK

>gi|30062050|ref|NP_836221.1| iron-enterobactin transporter periplasmic binding protein [Shigella flexneri 2a str. 2457T]

MRLAPLYRNALLLTGLLLSGIAVVQAADWPRQITDSRGTHTLESQPQRIVSTSVTLTGSLLAIDAPVIAS

GATTPNNRVADDQGFLRQWSKVAKERKLQRLYIGEPSAEAVAAQMPDLILISATGGDSALALYDQLSTIA

PTLIINYDDKSWQSLLTQLGEITGHEKQAAERIAQFDKQLAAAKEQIKLPPQPVTAIVYTAAAHSANLWT

PESAQGQMLEQLGFTLAKLPAGLNASQSQGKRHDIIQLGGENLAAGLNGESLFLFAGDQKDADAIYANPL

LAHLPAVQNKQVYALGTETFRLDYYSAMQVLERLKALF

>gi|30062049|ref|NP_836220.1| enterobactin exporter EntS [Shigella flexneri 2a str. 2457T]

MNKQSWLLNLSLLKTHPAFRAVFLARFISIVSLGLLGVAVPVQIQMMTHSTWQVGLSVTLTGGAMFVGLM

VGGVLADRYERKKVILLARGTCGIGFIGLCLNALLPEPSLLAIYLLGLWDGFFASLGVTALLAATSALVG

RENLMQAGAITMLTVRLGSVISPMIGGLLLATGGVAWNYGLAAAGTFITLLPLLSLPELPPPPQPLEHPL

KSLLAGFRFLLASPLLGGLLTMASAVLVLYPALADNWQMSAAQIGFLYAAIPLGAAIGALTSGKLAHSAR

PGLLMLLSTLGSFLAIGLFGLMPMWILGVVCLALFGWLSAVSSLLQYTMLQTQTPEAMLGRINGLWTAQN

VTGDAIGAALLGGLGAMMTPVASASASGFGLLIIGVLLLLVLVELRRFRQTPPQVTASDS

>gi|30062048|ref|NP_836219.1| iron-enterobactin transporter membrane protein [Shigella flexneri 2a str. 2457T]

MEVRISGSVAVTRAIAVPGLLLLLIIATALSLLIGAKSLPASVVLEAFSGTCQSADCTIVLDARLPRTLA

GLLAGGALGLAGALMQTLTRNPLADPGLLGVNAGASFAIVLGAALFGYSSAQEQLAMAFAGALVASLIVA

FTGSQGGGQLSPVRLTLAGVALAAVLEGLTSGIALLNPDVYDQLRFWQAGSLDIRNLHTLKVVLIPVLIA

GATALLLSRALNSLSLGSDTATALGSRVARTQLIGLLAITVLCGSATAVVGPIAFIGLMMPHMARWLVGA

DHRWSLPVTLLATPALLLFADIIGRVIVPGELRVSVVSAFIGAPVLIFLVRRKTRGGA

>gi|30062047|ref|NP_836218.1| iron-enterobactin transporter permease [Shigella flexneri 2a str. 2457T]

MIYVSRRLLITCLLLISACVVAGIWGLRSGAVTLEISQVFAALMGDAPRSMTMVVTEWRLPRVLMALLIG

AALGVSGAIFQSLMRNPLGSPDVMGFNTGAWSGVLVAMVLFGQDLTAIALAAMVGGIITSLLVWLLAWRN

GIDTFRLIIIGIGVRAMLVAFNTWLLLKASLETALTAGLWNAGSLNGLTWAKTSPSAPIIILMLIAATLL

VRRMRLLEMGDDTACALGVSVERSRLLMMLVAVVLTAAATALAGPISFIALVAPHIARRISGTARWGLTQ

AALCGALLLLAAVLCAQQLFMPYQLPVGVVTVSLGGIYLIVLLIQESRKK

>gi|30062041|ref|NP_836212.1| outer membrane receptor FepA [Shigella flexneri 2a str. 2457T]

MNKKIHSLALLVNLGIYGVAQAQEPTDTPVSHDDTIVVTAAEQNLQAPGVSTITADEIRKNPVARDVSEI

IRTMPGVNLTGNSTSGQRGNNRQIDIRGMGPENTLILIDGKPVSSRNSVRQGWRGERDTRGDTSWVPPEM

IERIEVLRGPAAARYGNGAAGGVVNIITKKGSGEWHGSWDAYFNAPEHKEEGATKRTNFSLTGPLGDEFS

FRLYGNLDKTQADAWDINQGHQSARAGTYATTLPAGREGVINKDINGVVRWDFAPLQSLELEAGYSRQGN

LYAGDTQNTNSDAYTRSKYGDETNRLYRQNYALTWNGGWDNGVTTSNWVQYEHTRNSRIPEGLAGGTEGK

FNEKATQDFVDIDLDDVMLHSEVNLPIDFLVNQTLTLGTEWNQQRMKDLSSNTQALTGTNTGGAIDGVSA

TDRSPYSKAEIFSLFAENNMELTDSTIVTPGLRFDHHSIVGNNWSPALNISQGLGDDFTLKIGIARAYKA

PSLYQTNPNYILYSKGQGCYASAGGCYLQGNDDLKVETSINKEIGLEFKRDGWLAGVTWFRNDYRNKIEA

GYVAVGQNAVGTDLYQWDNVPKAVVEGLEGSLNVPVSETVMWTNNITYMLKSENKTTGDRLSIIPEYTLN

STLSWQAREDLSMQTTFTWYGKQQPKKYNYKGQPAVGPETKEISPYSIVGLSATWDVTKNVSLTGGVDNL

FDKRLWRAGNAQTTGDLAGANYIAGAGAYTYNEPGRTWYMSINTHF

>gi|30062040|ref|NP_836211.1| phosphopantetheinyltransferase component of enterobactin synthase multienzyme complex [Shigella flexneri 2a str. 2457T]

MVDMKTTHTSLPFAGHTLHFVEFDPANFCEQDLLWLPHYAQLQHAGRKRKTEHLAGRIAAVYALREYGYK

CVPAIGELRQPVWPAEVYGSISHCGATALAVVSRQPIGVDIEEIFSAQTATELTDNIITPAEHERLADCG

LAFSLALTLAFSAKESAFKASEIQTDAGFLDYQIISWNKQQVIIHRENEMFAVHWQIKEKIVITLCQHD

>gi|30062035|ref|NP_836206.1| carboxylate-amine ligase [Shigella flexneri 2a str. 2457T]

MPLPDFHVSEPFTLGIELEMQVVNPPGYDLSQDSSMLIDAVKNKITAGEVKHDITESMLELATDVCRDIN

QAAGQFSAMQKVVLQAAADHHLEICGGGTPPFQKWQRQEVCDNERYQRTLENFGYLIQQATVFGQHVHVG

CASGDDAIYLLHGLSRFVPHFIALSAASPYMQGTDTRFAPSRPNIFSAFPDNGPMPWVSNWQQFEALFRC

LSYTTMIDSIKDLHWDIRPSPHFGTVEVRVMDTPLTLSHAVNMAGLIQATAHWLLTERPFKHQEKDYLLY

KFNRFQACRYGLEGVITDPHTGDRRPLTEDTLRLLEKIAPSAHKIGASSAIEALHRQVVSGLNEAQLMRD

FVADGGSLIGLVKKHCEIWAGD

>gi|30062033|ref|NP_836204.1| hypothetical protein S0494 [Shigella flexneri 2a str. 2457T]

MDKQSLHETAKRLALELPFVELCWPFGPEFDVFKIGGKIFMLSSELRGVPFINLKSDPQKSLLNQQIYPS

IKPGYHMNKKHWISVYSGEEISEALLRDLINDSWNLVVDGLAKRDQKRVRPG

>gi|30062032|ref|NP_836203.1| dihydropteridine reductase [Shigella flexneri 2a str. 2457T]

MDIISVALKRHSTKAFDASKKLTPEQAEQIKTLLQYSPSSTNSQPWHFIVASTEEGKARVAKSAAGNYVF

NKRKILDASHVVVFCAKTAMDDAWLKLVVDQEDADGRFATPEAKAANDKGRKFFAYMHRKDLHDDAEWMA

KQVYLNVGNFLLGVAALGLDAVPIEGFDAAILDAEFGLKEKGYTSLVVVPVGHHSVEDFNATLPKSRLPQ

NTTLTEV

>gi|30062031|ref|NP_836202.1| transporter [Shigella flexneri 2a str. 2457T]

MQDLISQVEDLAGIEIDHTTSMVMIFGIIFLTAVVVHIILHWVVLRTFEKRAIASSRLWLQIITQNKLFH

RLAFTLQGIIVNIQAVFWLQKGTEAADILTTCAQLWIMMYALLSVFSLLDVILNLAQKFPAASQLPLKGI

FQGIKLIGAILVGILMISLLIGQSPAILISGLGAMAAVLMLVFKDPILGLVAGIQLSANDMLKLGDWLEM

PKYGADGAVIDIGLTTVKVRNWDNTITTIPTWSLVSDSFKNWSGMSASGGRRIKRSISIDVTSIRFLDED

EMQRLNKAHLLKPYLTSRHQEINEWNRQQGSTESVLNLRRMTNIGTFRAYLNEYLRNHPRIRKDMTLMVR

QLAPGDNGLPLEIYAFTNTVVWLEYESIQADIFDHIFAIVEEFGLRLHQSPTGNDIRSLAGAFKQ

>gi|30062029|ref|NP_836200.1| inner membrane component for iron transport [Shigella flexneri 2a str. 2457T]

MIEWIIRRSVANRFLVLMGALFLSIWGTWTIINTPVDALPDLSDVQVIIKTSYPGQAPQIVENQVTYPLT

TTMLSVPGAKTVRGFSQFGDSYVYVIFEDGTDPYWARSRVLEYLNQVQGKLPAGVSAELGPDATGVGWIY

EYALVDRSGKHDLADLRSLQDWFLKYELKTIPDVAEVASVGGVVKEYQVVIDPQRLAQYGISLAEVKSAL

DASNQEAGGSSIELAEAEYMVRASGYLQTLDDFNHIVLKASENGVPVYLRDVAKVQIGPKMRRGIAELNG

EGEVAGGVVILRSGKNAREVIAAVKDKLETLKSSLPEGVEIVTTYDRSQLIDRAIDNLSGKLLEEFIVVA

VVCAPFLWHVRSALVAIISLPLGLCIAFIVMPFQGLNANIMSLGGIAIAVGAMVDAAIVMIENAHKRLEE

WQHQHPDATLDNKTRWQVITDASVEVGPALFISLLIITLSFIPIFTLEGQEGRLFGPLAFTKTYAMAGAA

LLAIVVIPILMGYWIRGKIPPESSNPLNRFLIRVYHPLLLKVLHWPKTTLLVAALSVLTVLWPLNKVGGE

FLPQINEGDLLYMPSTLPGISAAEAASMLQKTDKLIMSVPEVARVFGKTGKAETATDSAPLEMVETTIQL

KPQDQWRPGMTMDKIIEELDNTVRLPGLANLWVPPIRNRIDMLSTGIKSPIGIKVSGTVLADIDAMAEQI

EEVARTVPGVASALAERLEGGRYINVEINREKAARYGMTVADVQLFVTSAVGGAMVGETVEGIARYPINL

RYPQSWRDSPQALRQLPILTPMKQQITLADVADVKVSTGPSMLKTENARPTSWIYIDARDRDMVSVVHDL

QKAIAEKVQLKPGTSVAFSGQFELLERANHKLKLMVPMTLMIIFVLLYLAFRRVGEALLIISSVPFALVG

GIWLLWWMGFHLSVATGTGFIALAGVAAEFGVVMLMYLRHAIEAEPSLNNPQTFSEQKLDEALYHGAVLR

VRPKAMTVAVIIAGLLPILWGTGAGSEVMSRIAAPMIGGMITAPLLSLFIIPAAYKLMWLHRHRVRN

>gi|30062023|ref|NP_836194.1| periplasmic copper-binding protein [Shigella flexneri 2a str. 2457T]

MKKALQVAMFSLFTVIGFNAQANEHHHETMSEAQPQVISATGVVKGVDLESKKITIHHDPIAAVNWPEMT

MRFTITPQTKMSEIKTGDKVAFNFVQQGNLSLLQDIKVSQ

>gi|30062022|ref|NP_836193.1| copper/silver efflux system outer membrane protein CusC [Shigella flexneri 2a str. 2457T]

MSPCKLLPFCVALALTGCSLAPDYQRPVMPVPQQFSLSQNGLVNAADNYQNVGWRTFFVDNQVKTLISEA

LVNNRDLRMATLKVQEARAQYRLTDADRYPQLNGEGSGSWSGNLKGDSATTREFSTGLNASFDLDFFGRL

KNMSEAERQNYLATEEAQRAVHILLVSNVAQSYFNQQLAYAQLQIAEETLRNYQQSYAFVEKQLLTGSSN

VLALEQARGVIESTRSDIAKRQGELAQANNALQLLLGSYGKLPQAQTVNSGSLQSVKLPAGLSSQILLQR

PDIMEAEHALMAANANIGAARAAFFPSISLTSGISTASSDLSSLFNASSGMWNFIPKIEIPIFNAGRNQA

NLDIAEIRQQQSVVNYEQKIQNAFKEVADALALRQSLDDQISAQQRYLASLQITLQRARALYQHGAVSYL

EVLDAERSLFATRQTLLDLNYARQVNEIYLYTALGGGWQQ

>gi|30062021|ref|NP_836192.1| DNA-binding transcriptional activator CusR [Shigella flexneri 2a str. 2457T]

MKLLIVEDEKKTGEYLTKGLTEAGFVVDLADNGLNGYHLAMTGDYDLIILDIMLPDVNGWDIVRMLRSAN

KGMPILLLTALGTIEHRVKGLELGADDYLVKPFAFAELLARVRTLLRRGAAVIIESQFQVADLMVDLVSR

KVTRSGTRITLTSKEFTLLEFFLRHQGEVLPRSLIASQVWDMNFDSDTNAIDVAVKLLRGKIDNDFEPKL

IQTVRGVGYMLEVPDGQ

>gi|30062020|ref|NP_836191.1| sensor kinase CusS [Shigella flexneri 2a str. 2457T]

MVSKPFQRPFSLATRLTFFISLATIAAFFAFAWIIIHSVKVHFAEQDINDLKEISATLERVLNHPDETQA

RRLMTLEDIVSGYSNVLISLADSHGKTVYHSPGAPDIREFARDAIPDKDARGGEVYLLSGPTIMMPGHGH

GHMEHSNWRMINLPVGPLVDGKPIYTLYIALSIDFHLHYINDLMNKLIMTASVISILIVFIVLLAVHKGH

APIRSVSRQIQNITSKDLDVRLDPQTVPIELEQLVLSFNHMIERIEDVFTRQSNFSADIAHEIRTPITNL

ITQTEIALSQSRSQKELEDVLYSNLEELTRMAKMVSDMLFLAQADNNQLIPEKKMLNLADEVGKVFDFFE

ALAEDRGVELRFVGDKRQVAGDPLMLRRALSNLLSNALRYTPTRETIVVRCQTVDHQVQVSVENPGTPIA

PEHLPRLFDRFYRVDPSRQRKGEGSGIGLAIVKSIVVAHKGTVAVTSDARGTRFVITLPA

>gi|30062019|ref|NP_836190.1| hypothetical protein S0476 [Shigella flexneri 2a str. 2457T]

MRKFIFVLLTLLLVSPFSFAMKGIIWQPQNRDSQVTDTQWQGLMSQLRFQGFDTLVLQWTRYGDAFTQPE

QRTLLFKRAAAAQQTGLKLIVGLNADPEFFMHQKQSSAALESYLNRLLAADLQQARLWSAAPGITPDGWY

ISAEIDDLNWRSEAARQPLLTWLNNAQRLISDVSAKPVYISSFFAGNMSPDGYRQLLEQVKATGVNVWVQ

DGSGVDKLTAEQRERYLQASADCQSSAPASGIVYELFVAGKGKTFTAKPKPDAEIASLLAKRSSCGKDML

YFSLRYLPVAHGILEY

>gi|30062018|ref|NP_836189.1| envelope protein [Shigella flexneri 2a str. 2457T]

MQLSSSEPCVVILTEKEVEVSVNNHATFTLPKNYLAAFACNNNVIELSTLNHVLITHINRNIINDYLLFL

NKKLTCVKPWSRLATPVIACHSRTPEVFRLATNHSKQQSSKPCEAELTRALLFTVLSNFLEQSRFIALLM

YILRSSVRDSVCRIIQSDIQHYWNLRIVASSLCLSPSLLKKKLKNENTSYSQIVTECRMRYAVQMLLMDN

KNITQVAQLCGYSSTSYFISVFKAFYSLTPLNYLAKQRQKVMW

>gi|30062015|ref|NP_836186.1| chaperone [Shigella flexneri 2a str. 2457T]

MMTKIKLLMLMIFYLIISASAHAAGGIALGATRIIYPADAKQTAVWIKNSHTNELFLVNSWIENSSGVKE

KSFIITPPLFVSEPKSENTLRIIYTGPPLAADRESLFWMNVKTIPSVDKNALNGRNVLQLAILSRMKLFL

RPIQLQELPAEAPDTLKFSRSGNYINVHNPSPFYVTLVNLQVGSQKLGNAMAAPRVSSQIPLPSGVQGKL

KFQTVNDYGSVTPVREVNLN

>gi|30062014|ref|NP_836185.1| fimbrial-like protein [Shigella flexneri 2a str. 2457T]

MESINEIEGIYMKLRFISSALAAALFAATGSYAAVVDGGTIHFEGELVNAACSVNTDSADQVVTLGQYRT

DIFNAVGNTSALIPFTIQLNDCDPVVAANAAVAFSGQADAINDNLLAIVSSTNTTTATGVGIEILDNTSA

ILKPDGNSFSTNQNLIPGTNVLHFSARYKGTGTSASAGQANADATFIMRYE

>gi|30062011|ref|NP_836182.1| hypothetical protein S0466 [Shigella flexneri 2a str. 2457T]

MPTVITHAAVPLCIGLGLGSKGLLFAGIILAMLPDADVLSFKFGVAYGNVFGHRGFTHSLVFAFVVPLLC

VLIGRRWFRAGLIRCWLFLTVSLLSHSLLDSVTTGGKGVGWLWPWSDERFFAPWQVIKVAPFALSRYTTA

YGHQVIISELMWVWLPGMLLMGMLWWRRR

>gi|30062008|ref|NP_836179.1| UDP-2,3-diacylglucosamine hydrolase [Shigella flexneri 2a str. 2457T]

MATLFIADLHLCVEEPAITAGFLRFLAGEARKADALYILGDLFEAWIGDDDPNPLHRQMAAAIKAVSDSG

VPCYFIHGNRDFLLGKRFARESGMTLLPEEKVLELYGRRVLIMHGDTLCTDDAGYQAFRAKVHKPWLQML

FLALPLFVRKRIAARMRANSKEANSSKSLAIMDVNQNAVVSAMEKHQVQWLIHGHTHRPAVHELIANQQP

AFRVVLGAWHTEGSMVKVTADDVELIHFPF

>gi|30062006|ref|NP_836177.1| phosphoribosylaminoimidazole carboxylase ATPase subunit [Shigella flexneri 2a str. 2457T]

MKQVCVLGNGQLGRMLRQAGEPLGIAVWPVGLDAEPAAVPFQQSVITAEIERWPETALTRELARHPAFVN

RDVFPIIADRLTQKQLFDKLHLPTAPWQLLADRSEWPAVFDRLGELAIVKRRTGGYDGRGQWSLRANETE

QLPAECYGECIVEQGINFSGEVSLVGARGFDGSTVFYPLTHNLHQDGILRTSVAFPQANAQQQAQAEEML

SAIMQELGYVGVMAMECFVTPQGLLINELAPRVHNSGHWTQNGASISQFELHLRAITDLPLPQPVVNNPS

VMINLIGSDVNYDWLKLPLVHLHWYDKEVRPGRKVGHLNLTDSDTSRLTATLEALIPLLPPEYASGVIWA

QSKFS

>gi|30062005|ref|NP_836176.1| carbamate kinase [Shigella flexneri 2a str. 2457T]

MKTLVVALGGNALLQRGEALTAENQYRNIASAVPALARLARSYRLAIVHGNGPQVGLLALQNLAWKEVEP

YPLDVLVAESQGMIGYMLAQSLSAQPQMPPVTTVLTRIEVSPDDPAFLQPEKFIGPVYQPEEQEALEAAY

GWQMKRDGKYLRRVVASPQPRKILDSEAIELLLKEGHVVICSGGGGVPVTEDGAGSEAVIDKDLAAALLA

EQINADGLVILTDADAVYENWGTPQQRAIRHATPDELAPFAKADGSMGPKVTAVSGYVRSRSKPAWIGAL

SRIEETLAGEAGTCISL

>gi|30062003|ref|NP_836174.1| hypothetical protein S0457 [Shigella flexneri 2a str. 2457T]

MGYLNNVTGYREDLLANRAIVKHGNFALLTPDGLIKNIIPDFENCDATILSTPKLGASFVDYLVTLHQNG

GNQQGFGGEGIETFLYVISGNITAKAEGKTFALSEGGYLYCPPGSLMTFVNAQAEDSQIFLYKRRYVPVE

GHAPWLFSGNASELERIHYEGMDDVILLDFLPKELGFDMNMHILSFAPGASHGYIETHVQEHGAYILSGQ

GVYNLGNAANLLI

>gi|30061998|ref|NP_836169.1| DNA-binding transcriptional repressor AllR [Shigella flexneri 2a str. 2457T]

MTEVRRRGRPGQAEPVAQKGAQALERGIAILQYLEKSGGSSSVSDISLNLDLPLSTTFRLLKVLQAADFV

YQDSQLGWWHIGLGVFNVGAAYIHNRDVLSVAGPFMRRLMLLSGETVNVAIRNGNEAVLIGQLECKSMVR

MCAPLGSRLPLHASGAGKALLYPLAEEELMSIILQTGLQQFTPTTLVDMPTLLKDLEQARELGYTVDKEE

HVVGLNCIASAIYDDVGRVVAAISISGPSSRLTEDRFVSQGELVRDTARDISTALGLKAHP

>gi|30061997|ref|NP_836168.1| ureidoglycolate hydrolase [Shigella flexneri 2a str. 2457T]

MKLQVLPLSQEAFSAYGDVIETQQRDFFHINNGLVERYHDLALVEILEQDRTLISINRAQPANLPLTIHE

LERHPLGTQAFIPMKGEVFVVVVALGDDKPDLSTLRAFITNGEQGVNYHRNVWHHPLFAWQRVTDFLTID

RGGSDNCDVESIPEQELCFA

>gi|30061996|ref|NP_836167.1| DNA-binding transcriptional activator AllS [Shigella flexneri 2a str. 2457T]

MFDPETLRTFIAVAETGSFSKAAERLCKTTATISYRIKLLEENTGVALFFRTTRSVTLTAAGEHLLSQAR

DWLSWLESMPSELQQVNDGVERQVNIVINNLLYNPQAVAQLLAWLNERYPFTQFHISRQIYMGVWDSLLY

EGFSLAIGVTGTEALANTFSLDPLGSVQWRFVMAADHPLANVEEPLTEAQLRRFPAVNIEDSAGTLTKRV

AWRLPGQKEIIVPDMETKIAAHLAGVGIGFLPKSLCQSMIDNQQLVSRVIPTMRPPSPLSLAWRKFGSCK

AVEDIVTLFTQHRPEISGFLEIFGTPRS

>gi|30061995|ref|NP_836166.1| tRNA 2-selenouridine synthase [Shigella flexneri 2a str. 2457T]

MQERHTEQDYRALLIADTPIIDVRAPIEFEQGAMPAAINLPLMDNDERASVGTCYKQQGSDAALALGHKL

VAGEIRQQRMYAWRAACLQNPQGILCCARGGQRSHIVQRWLHEAGIDYPLVEGGYKALRQTAIQATIELA

QKPIVLIGGCTGSGKTLLVQQQPNGVDLEGLARHRGSAFGRTLQPQLSQASFENLLAAEMLKTDARQDLR

LWVLEDESRMIGSNHLPECLRERMTQAAIAVVEDPFEIRLERLNEEYFLRMHHDFTHAYGDEQGWQEYCE

YLHHGLSAIKRRLGLQRYNELAAQLDTALTTQLTTGSTDGHLAWLVPLLKEYYDPMYRYQLEKKAEKVVF

RGEWAEVAVWVKAQ

>gi|30061991|ref|NP_836162.1| metal resistance protein [Shigella flexneri 2a str. 2457T]

MPEKCRKHAMNSHNITNESLALALMLVVVAILISHKEKLALEKDILWSVGRAIIQLIIVGYVLKYIFSVD

DASLTLLMVLFICFNAAWNAQKRSKYIAKAFISSFIAITVGAGITLAVLILSGAIEFIPMQVIPVAGMIA

GNAMVAVGLCYNNLGQRVISEQQQIQEKLSLGATPKQASAILIRDSIRAALIPTVDSAKTVGLVSLPGMM

SGLIFAGIDPVKAIKYQIMVTFMLLSTASLSTIIACYLTYRKFYNSRHQLVVTQLKKK

>gi|30061987|ref|NP_836158.1| DNA-binding transcriptional regulator CueR [Shigella flexneri 2a str. 2457T]

MNISDVAKITGLTSKAIRFYEEKGLVTPPMRSENGYRTYTQQHLNELTLLRQARQVGFNLEESGELVNLF

NDPQRHSADVKRRTLEKVAEIERHIEELQSMRDQLLALANACPGDDSADCPIIENLSGCCHHRAG

>gi|30061983|ref|NP_836154.1| hypothetical protein S0435 [Shigella flexneri 2a str. 2457T]

MIQYVLASLFTGKQQLKTMKQATRKPTTPGDILLYEYLEPLDLKINELAELLHVHRNRVSALINNNRKLT

TEMAFRLAKVFDTTVDFRLNLQAAVDLWEVENNMRTQEELGRIETVAEYLARREERAKKVA

>gi|30061982|ref|NP_836153.1| ligase [Shigella flexneri 2a str. 2457T]

MDLLYRVKTLWAALRGNHYTWPAIDITLPGNRHFHLIGSIHMGSHDMAPLPTRLLKKLKNADALIVEADV

STSDTPFANLPACEALEERISEEQLQNLQHISQEMGISPSLFSTQPLWQIAMVLQATQAQKLGLRAEYGI

DYQLLQAAKQQHKPVIELEGAENQIAMLLQLPDKGLALLDDTLTHWHTNARLLQQMMSWWLNAPPQNNDI

TLPNTFSQSLYDVLMHQRNLAWRDKLRAMPPGRYVVAVGALHLYGEGNLPQMLR

>gi|30061981|ref|NP_836152.1| hypothetical protein S0433 [Shigella flexneri 2a str. 2457T]

MTPAVKLLEKNKISFQIHTYEHDPAETNFGDEVVKKLGLNPDQVYKTLLVAVNGDMKHLAVAVTPVAGQL

DLKKVAKALGAKKVEMADPMVAQRSTGYLVGGISPLGQKKRLPTIIDAPAQEFATIYVSGGKRGLDIELA

AGDLAKILDAKFADIARRD

>gi|30061979|ref|NP_836150.1| fosmidomycin resistance protein [Shigella flexneri 2a str. 2457T]

MAMSEQTQPVAGAAASTTKARTSFGILGAISLSHLLNDMIQSLILAIYPLLQSEFSLTFMQIGMITLTFQ

LASSLLQPVVGYWTDKYPMPWSLPIGMCFTLSGLVLLALAGSFGAVLLAAALVGTGSSVFHPESSRVARM

ASGGRHGLAQSIFQVGGNFGSSLGPLLAAVIIAPYGKGNVAWFVLAALLAIVVLAQISRWYSAQHRMNKG

KPKATIINPLPRNKVVLAVSILLILIFSKYFYMASISSYYTFYLMQKFGLSIQNAQLHLFAFLFAVAAGT

VIGGPVGDKIGRKYVIWGSILGVAPFTLILPYARLHWTGVLTVIIGFILASAFSAILVYAQELLPGRIGM

VSGLFFGFAFGMGGLGAAVLGLIADHTSIELVYKICAFLPLLGMLTIFLPDNRHKD

>gi|30061977|ref|NP_836148.1| inosine-guanosine kinase [Shigella flexneri 2a str. 2457T]

MKFPGKRKSKHYFPVNARDPLLQQFQPENETSAAWVVGIDQTLVDIEAKVDDEFIERYGLSAGHSLVIED

DVAEVLYQELKQKNLITHQFAGGTIGNTMHNYSVLADDRSVLLGVMCSNIEIGSYAYRYLCNTSSRTDLN

YLQGVDGPIGRCFTLIGESGERTFAISPGHMNQLRAESIPEDVIAGASALVLTSYLVRCKPGEPMPEATM

KAIEYAKKYNVPVVLTLGTKFVIAENPQWWQQFLKDHVSILAMNEDEAEALTGESDPLLASDKALDWVEL

VLCTAGPIGLYMAGFTEDEAKRKTQHPLLPGAIAEFNQYEFSRAMRHKDCQNPLRVYSHIAPYMGGPEKI

MNTNGAGDGALAALLHDITANSYHRSNVPNSSKHKFTWLTYSSLAQVCKYANRVSYQVLNQHSPRLTRGL

PEREDSQEESYWDR

>gi|30061972|ref|NP_836143.1| recombination protein RecR [Shigella flexneri 2a str. 2457T]

MQTSPLLTQLMEALRCLPGVGPKSAQRMAFTLLQRDRSGGMRLAQALTRAMSEIGHCADCRTFTEQEVCN

ICSNPRRQENGQICVVESPADIYAIEQTGQFSGRYFVLMGHLSPLDGIGPDDIGLDRLEQRLAEEKITEV

ILATNPTVEGEATANYIAELCAQYDVEASRIAHGVPVGGELEMVDGTTLSHSLAGRHKIRF

>gi|30061971|ref|NP_836142.1| hypothetical protein S0423 [Shigella flexneri 2a str. 2457T]

MFGKGGLGNLMKQAQQMQEKMQKMQEEIAQLEVTGESGAGLVKVTINGAHNCRRVEIDPSLLEDDKEMLE

DLVAAAFNDAARRIEETQKEKMASVSSGMQLPPGFKMPF

>gi|30061968|ref|NP_836139.1| hypothetical protein S0420 [Shigella flexneri 2a str. 2457T]

MQRIILIIIGWLAVVLGTLGVVLPVLPTTPFILLAAWCFARSSPRFHAWLLYRSWFGSYLRFWQKHHAMP

RGVKPRAILLILLTFAISLWFVQMPWVRIMLLVILACLLFYMWRIPVIDEKQEKH

>gi|30061967|ref|NP_836138.1| primosomal replication protein N'' [Shigella flexneri 2a str. 2457T]

MKTALLLEKLEGQLATLRQRCAPVSQFATLSARFDRHLFQTRATTLQSCLDEAGDNLAALRHAVEQQQLP

QVAWLAEHLAAQLEAIAREASAWSLREWDSAPPKISRWQRKRIQHQDFERRLREMVAERRARLARVTDLV

EQQTLHREVKAYEARLARCRHALEKIENRLARLTR

>gi|30061965|ref|NP_836136.1| potassium efflux protein KefA [Shigella flexneri 2a str. 2457T]

MTMFQYYKRSRHFVFSAFIAFVFVLLCQNTAFARASSNGDLPTKADLQAQLDSLNKQKDLSAQDKLVQQD

LTDTLATLDKIDRIKEETVQLRQKVAEAPEKMRQATAALTALSDVDNDEETRKILSTLSLRQLETRVAQA

LDDLQNAQNDLASYNSQLVSLQTQPERVQNAMYNASQQLQQIRSRLDGTDVGETALRPSQKVLMQAQQAL

LNAEIDQQRKSLEGNTVLQDTLQKQRDYVTANSARLEHQLQLLQEAVNSKRLTLTEKTAQEAVSPDEAAR

IQANPLVKQELEINQQLSQRLITATENGNQLMQQNIKVKNWLERALQSERNIKEQIAVLKGSLLLSRILY

QQQQTLPSADELENMTNRIADLRLEQFEVNQQRDALFQSDAFVNKLEEGHTNEVNSEVHDALLQVVDMRR

ELLDQLNKQLGNQLMMAINLQINQQQLMSVSKNLKSILTQQIFWVNSNRPMDWDWIKAFPQSLKDEFKSM

KITVNWEKAWPAVFIAFLAGLPLLLIAGLIHWRLGWLKAYQQKLASAVGSLRNDSQLNTPKAILIDLIRA

LPVCLIILAVGLILLTMQLNISELLWSFSKKLAIFWLVFGLCWKVLEKNGVAVRHFGMPEQQTSHWRRQI

VRISLALLPIHFWSVVAELSPLHLMDDVLGQAMIFFNLLLIAFLVWPMCRESWRDKESHTMRLVTITVLS

IIPIALMVLTATGYFYTTLRLSGRWIETVYLVIIWNLLYQTVLRGLSVAARRIAWRRALARRQNLVKEGA

EGAEPPEEPTIALEQVNQQTLRITMLLMFALFGVMFWAIWSDLITVFSYLDSITLWHYNGTEAGAAVVKN

VTMGSLLFAIIASMVAWALIRNLPGLLEVLVLSRLNMRQGASYAITTILNYIIIAVGAMTVFGSLGVSWD

KLQWLAAALSVGLGFGLQEIFGNFVSGLIILFERPVRIGDTVTIGSFSGTVSKIRIRATTITDFDRKEVI

IPNKAFVTERLINWSLTDTTTRLVIRLGVAYGSDLEKVRKVLLKAATEHPRVMHEPMPEVFFTAFGASTL

DHELRLYVRELRDRSRTVDELNRTIDQLCRENDINIAFNQLEVHLHNEKGDEVTEVKRDYKGDDPTPAVG

>gi|30061964|ref|NP_836135.1| DNA-binding transcriptional repressor AcrR [Shigella flexneri 2a str. 2457T]

MARKTKQEAQETRQHILDVALRLFSQQGVSSTSLGEIAKAAGVTRGAIYWHFKDKSDLFSEIWELSESNI

GELELEYQAKFPGDPLSVLREILIHVLESTVTEERRRLLMEIIFHKCEFVGEMAVVQQAQRNLCLESYDR

IEQTLKHCIEAKMLPADLMTRRAAIIMRGYISGLMENWLFAPQSFDLKKEARDYVAILLEMYLLCPTLRN

PATNE

>gi|30061963|ref|NP_836134.1| acriflavin resistance protein AcrA precursor [Shigella flexneri 2a str. 2457T]

MNKNRGFTPLAVVLMLSGSLALTGCDDKQAQQGGQQMPAVGVVTVKTEPLQITTELPGRTSAYRIAEVRP

QVSGIILKRNFKEGSDIEAGVSLYQIDPATYQATYDSAKGDLAKAQAAANIAQLTVNRYQKLLGTQYISK

QGYDQALADAQQANAAVTAAKAAVETARINLAYTKVTSPISGRIGKSNVTEGALVQNGQATVLATVQQLD

PIYVDVTQSSNDFLRLKQELANGTLKQENGKAKVSLITSDGIKFPQDGTLEFSDVTVDQTTGSITLRAIF

PNPDHTLLPGMFVRARLEEGLNPNAILVPQQGVTRTPRGDATVLVVGADDKVETRPIVASQAIGDKWLVT

EGLKAGDRVVISGLQKVRPGVQVKAQEVTADNNQQAASGAQPEQSKS

>gi|30061962|ref|NP_836133.1| acriflavine resistance protein [Shigella flexneri 2a str. 2457T]

MPNFFIDRPIFAWVIAIIIMLAGGLAILKLPVAQYPTIAPPAVTISASYPGADAKTVQDTVTQVIEQNMN

GIDNLMYMSSNSDSTGTVQITLTFESGTDADIAQVQVQNKLQLAMPLLPQEVQQQGVSVEKSSSSFLMVV

GVINTDGTMTQEDISDYVAANMKDAISRTSGVGDVQLFGSQYAMRIWMNPNELNKFQLTPVDVITAIKAQ

NAQVAAGQLGGTPPVKGQQLNASIIAQTRLTSTEEFGKILLKVNQDGSRVLLRDVAKIELGGENYDIIAE

FNGQPASGLGIKLATGANALDTAAAIRAELAKMEPFFPSGLKIVYPYDTTPFVKISIHEVVKTLVEAIIL

VFLVMYLFLQNFRATLIPTIAVPVVLLGTFAVLAAFGFSINTLTMFGMVLAIGLLVDDAIVVVENVERVM

AEEGLPPKEATRKSMGQIQGALVGIAMVLSAVFVPMAFFGGSTGAIYRQFSITIVSAMALSVLVALILTP

ALCATMLKPIAKGDHGEGKKGFFGWLNRMFEKSTHHYTDSVGGILRSTGRYLVLYLIIVVGMAYLFVRLP

SSFLPDEDQGVFMTMVQLPAGATQERTQKVLNEVTHYYLTKEKNNVESVFAVNGFGFAGRGQNTGIAFVS

LKDWADRPGEENKVEAITMRATRAFSQIKDAMVFAFNLPAIVELGTATGFDFELIDQAGLGHEKLTQARN

QLLAEAAKHPDMLTSVRPNGLEDTPQFKIDIDQEKAQALGVSINDINTTLGAAWGGSYVNDFIDRGRVKK

VYVMSEAKYRMLPDDIGDWYVRAADGQMVPFSAFSSSRWEYGSPRLERYNGLPSMEILGQAAPGKSTGEA

MELMEQLASKLPTGVGYDWTGMSYQERLSGNQAPSLYAISLIVVFLCLAALYESWSTPFSVMLVVPLGVI

GALLAATFRGLTNDVYFQVGLLTTIGLSAKNAILIVEFAKDLMDKEGKGLIEATLDAVRMRLRPILMTSL

AFILGVMPLVISTGAGSGAQNAVGTGVMGGMVTATVLAIFFVPVFFVVVRRRFSRKNEDIEHSHTVDHH

>gi|30061961|ref|NP_836132.1| hypothetical protein S0413 [Shigella flexneri 2a str. 2457T]

MDEYSPKRHDIAQLKFLCETLYHDCLANLEESNHGWVNDPTSAINLQLNELIEHIATFALNYKIKYNEDN

KLIEQIDEYLDDTFMLFSSYGINMQALQKWRKSGNRLFRCFVNATKENPASLSC

>gi|30061959|ref|NP_836130.1| hypothetical protein S0410 [Shigella flexneri 2a str. 2457T]

MTEIQRLLTETIESLNTREKRDNKPRFSISFIRKHPGLFIGMYVAFFATLAVMLQSETLSGSVWLLVVLF

ILLNGFFFFDVYPRYRYEDIDVLDFRVCYNGEWYNTRFVPAALVEAILNSPRVADVHKEQLQKMIVRKGE

LSFYDIFTLARAESTS

>gi|30061957|ref|NP_836128.1| hypothetical protein S0408 [Shigella flexneri 2a str. 2457T]

MKYVDGFVVAVPADKKDAYREMAAKAAPLFKEFGALRIVECWASDVPDGKVTDFRMAVKAEENEEVVFSW

IEYPSKEVRDAANQKMMSDPRMKEFGESMPFDGKRMIYGGFESIIDE

>gi|30061954|ref|NP_836125.1| hypothetical protein S0404 [Shigella flexneri 2a str. 2457T]

MKLVHMASGLAVAIALAACADKSADIQTPAPAANTSISATQQPAIQQPNVSGTVWIRQKVALPPDAVLTV

TLSDASLADAPSKVLAQKAVRTEGKQSPFSFVLPFNPADVQPNARILLSAAITVNDKLVFITDTVQPVIN

QGGTKADLTLVPVQQTAVPVQASGGATTTVPSTSPTQVNPSSAVPAPTQY

>gi|30061948|ref|NP_836119.1| LRP-like transcriptional regulator [Shigella flexneri 2a str. 2457T]

MVFKPHRINCRDFTFKIELKEKKILRGRAMLDKIDRKLLALLQQDCTLSLQALAEAVNLTTTPCWKRLKR

LEDDGILIGKVALLDPEKIGLGLTAFVLIKTQHHSSEWYCRFVTVVTEMPEVLGVWRMAGEYDYLMRVQV

ADMKRYDEFYKRLVNSVPGLSDVTSSFAMEQIKYTTSLPIE

>gi|30061947|ref|NP_836118.1| hypothetical protein S0397 [Shigella flexneri 2a str. 2457T]

MEKEMARLVAFDMDGTLLMPDHHLGEKTLSTLARLRERDITLTFATGRHALEMQHILGALSLDAYLITGN

GTRVHSLEGELLHRDDLPADVAELVLYQQWDTRASMHIFNDDGWFTGKESPALLQVFVYSGFRYQIIDVK

KMPLGSVTKICFCGDHDDLTRLQIQLYEALGERAHLCFSATDCLEVLPVGCNKGAALTVRTQHLGLSLRD

CMAFGDAMNDREMLGSVGSGFIMGNAMPQLRAELPHLPVIGHCRNQAVSHYLTHWLDYPHLPYSPE

>gi|30061946|ref|NP_836117.1| queuosine biosynthesis protein QueC [Shigella flexneri 2a str. 2457T]

MKRAVVVFSGGQDSTTCLVQALQQYDEVHCVTFDYGQRHRAEIDVARELALKLGARAHKVLDVTLLNELA

VSSLTRDSIPVPDYEPEADGIPNTFVPGRNILFLTLAAIYAYQVKAEAVITGVCETDFSGYPDCRDEFVK

ALNHAVSLGMAKDIRFETPLMWIDKAETWALADYYGKLDLVRNETLTCYNGIKGDGCGHCAACNLRANGL

NHYLADKPTVMAAMKQKTGLR

>gi|30061945|ref|NP_836116.1| hypothetical protein S0394 [Shigella flexneri 2a str. 2457T]

MQTQIKVRGYHLDVYQHVNNARYLEFLEEARWDGVENSDSFQWMTAHNIAFVVVNININYRRPAVLSDLL

TITSQLQQLNGKSGILNQVITLEPEGQVVADALITFVCIDLKTQKALALEGELREKLEQMVK

>gi|30061943|ref|NP_836114.1| peptidyl-prolyl cis-trans isomerase (rotamase D) [Shigella flexneri 2a str. 2457T]

MMDSLRTAANSLVLKIIFGIIIVSFILTGVSGYLIGGGNNYAAKVNDQEISRGQFENAFNSERNRMQQQL

GDQYSELAANEGYMKTLRQQVLNRLIDEALLDQYARELKLGISDEQVKQAIFATPAFQVDGKFDNSRYNG

ILNQMGMTADQYAQALRNQLTTQQLINGVAGTDFMLKGETDELAALVAQQRVVREATIDVNALAAKQPVT

EQEIASYYEQNKNNFMTPEQFRVSYIKLVAATMQQPVSDADIQSYYDQHQDQFTQPQRTRYSIIQTKTED

EAKAVLDELNKGGDFAALAKEKSADIISARNGGDMGWLEDATIPDELKNAGLKEKGQLSGVIKSSVGFLI

VRLDDIQPAKVKSLDEVRDDIAAKVKHEKALDAYYALQQKVSDAASNDTESLAGAEQAAGVKATQTGWFS

KDNLPEELNFKPVADAIFNGGLVGENGASGINSDIITVDGDRAFVLRVSEHKPEAVKPLADVQEQVKALV

QHNKAEQQAKVDAEKLLVDLKAGKGAEAMQAAGLKFGEPKTLSRSGRDPISQAAFALPLPAKDKPSYGMA

TDMQGNVVLLALDEVKQGSMPEDQKKAMVQGITQNNAQIVFEALMSNLRKEAKIKIGDALEQQ

>gi|30061938|ref|NP_836109.1| trigger factor [Shigella flexneri 2a str. 2457T]

MQVSVETTQGLGRRVTITIAADSIETAVKSELVNVAKKVRIDGFRKGKVPMNIVAQRYGASVRQDVLGDL

MSRNFIDAIIKEKINPAGAPTYVPGEYKLGEDFTYSVEFEVYPEVELQGLEAIEVEKPIVEVTDADVDGM

LDTLRKQQATWKEKDGAVEAEDRVTIDFTGSVDGEEFEGGKASDFVLAMGQGRMIPGFEDGIKGHKAGEE

FTIDVTFPEEYHAENLKGKAAKFAINLKKVEERELPELTAEFIKRFGVEDGSVEGLRAEVRKNMERELKS

AIRNRVKSQAIEGLVKANDIDVPAALIDSEIDVLRRQAAQRFGGNEKQALELPRELFEEQAKRRVVVGLL

LGEVIRTNELKADEERVKGLIEEMASAYEDPKEVIEFYSKNKELMDNMRNVALEEQAVEAVLAKAKVTEK

ETTFNELMNQQA

>gi|30061928|ref|NP_836099.1| cytochrome o ubiquinol oxidase subunit IV [Shigella flexneri 2a str. 2457T]

MSHSTDHSGASHGSVKTYMTGFILSIILTVIPFWMVMTGAASPAVILGTILAMAVVQVLVHLVCFLHMNT

KSDEGWNMTAFVFTVLIIAILVVGSIWIMWNLNYNMMMH

>gi|30061926|ref|NP_836097.1| hypothetical protein S0373 [Shigella flexneri 2a str. 2457T]

MPGKTAALYDVDKTLKNARVELKTSPDAKNKLREAAQAVGVDLSAFILSAAMERAESVLDNQRRRELSNQ

SWELMNQLIAEPAQPTLALKALMKRKNSDGRQA

>gi|30061921|ref|NP_836092.1| hypothetical protein S4809 [Shigella flexneri 2a str. 2457T]

MKAKSTLLLMILSVFYSHQVLSSEPTQPDVYSVVEKKLNSALPLEENTTFRSQAEWFNLQYELLVSGYPE

RAYNLLSKLETESKNAKYYLDLTASRVAEEEAPETALTFLSKIGLNKPATLTSFESYINSWIKSKQPEKA

MQLLSLDENALNYYLPTVLVAYQIVPDKAISIYNSVYGDNVVIPYQQVTMLLTVAEGYQTKGDIKNAIHY

ADKALNMFDKAIAVDSSRESHYSDEYLKLMNIYAANGNKEKAVVLSQRLHKAITESQSNYLSTLEGILLF

YRNNDMQQDYQNSLSNYIVFIDKIFAFSPSTRSELSLINLLNSLNEVELMRKRLAFLMTAPEYACYNSQY

CYEDKVSALKILYRHHDNELMDKYFTTLLKEISNQPLEDWDIIISNTGEKFSEVGLTKYAQHLASFAEEF

YIKEQKSHSESEMRGLFSSLAELYSFGNDTASANRVFHQHVPAFNTDHMIQYFINAKEWNNARELLIKEE

MLGMHNLILLENICMQKNAECMAHITFTLNKLTTQPAITKIDSVGNEQLYQIGKIYHKLEIKPEPEQQVL

IQSLYDRASGSASATQ

>gi|30061920|ref|NP_836091.1| nucleotide-binding protein [Shigella flexneri 2a str. 2457T]

MPSFDIVSEVDLQEARNAVDNASREVESRFDFRNVEASFELNDASKTIKVLSESDFQVNQLLDILRAKLL

KRGIEGSSLDVPENIVHSGKTWFVEAKLKQGIESATQKKIVKMIKDSKLKVQAQIQGDEIRVTGKSRDDL

QAVMAMVRGGDLGQPFQFKNFRD

>gi|30061919|ref|NP_836090.1| 2-dehydropantoate 2-reductase [Shigella flexneri 2a str. 2457T]

MKITVLGCGALGQLWLTALCKQGHEVQGWLRVPQPYCSVNLVETDGSIFNESLTANDPDFLATSDLLLVT

LKAWQVSDAVKSLASTLPVTTPILLIHNGMGTIEELQNIQQPLLMGTTTHAARRDGNVIIHVANGITHIG

PARQQDGDYSYLADILQTVLPDVAWHNNIRAELWRKLAVNCVINPLTAIWNCPNGELRHHPQEIMQICEE

VAAVIEREGHHTSAEDLRDYVMQVIDATAENISSMLQDIRALRHTEIDYINGFLLRRARAHGIAVPENTR

LFEMVKRKESEYERIGTGLPRPW

>gi|30061917|ref|NP_836088.1| thiamine biosynthesis protein ThiI [Shigella flexneri 2a str. 2457T]

MKFIIKLFPEITIKSQSVRLRFIKILTGNIRNVLKHYDETLAVVRHWDNIEVRAKDENQRLTIRDALTRI

PGIHHILEVEDVPFTDMHDIFEKALVQYRDQLEGKTFCVRVKRRGKHDFSSIDVERYVGGGLNQHIESAR

VKLTNPDVTVHLEVEDDRLLLIKGRYEGIGGFPIGTQEDVLSLISGGFDSGVSSYMLMRRGCRVHYCFFN

LGGAAHEIGVRQVAHYLWNRFGSSHRVRFVAINFEPVVGEILEKIDDGQMGVILKRMMVRAASKVAERYG

VQALVTGEALGQVSSQTLTNLRLIDNVSDTLILRPLISYDKEHIINLARQIGTEDFARTMPEYCGVISKS

PTVKAVKSKIEAEEEKFDFSILDKVVEEANNVDIREIAQQTEQEVVEVETVNGFGPNDVILDIRSVDEQE

DKPLKVEGIDVVSLPFYKLSTKFGDLDQNKTWLLWCERGVMSRLQALYLREQGFNNVKVYRP

>gi|30061913|ref|NP_836084.1| phosphatidylglycerophosphatase A [Shigella flexneri 2a str. 2457T]

MTILSRHKDVAKSRLKMSNPWHLLAVGFGSGLSPIVPGSMGSLAAIPFWYLMTFLPWQLYSLVVMLGICI

GVYLCHQTAKDMGVHDHGSIVWDEFIGMWITLMALPTNDWQWVAAGFVIFRILDMWKPWPIRWFDRNVHG

GMGIMIDDIVAGVISAGILYFIGHHWPLGILS

>gi|30061912|ref|NP_836083.1| thiamine monophosphate kinase [Shigella flexneri 2a str. 2457T]

MACGEFSLIARYFDRVRSSRLDVELGIGDDCALLNIPEKQTLAISTDTLVAGNHFLPDIDPADLAYKALA

VNLSDLAAMGADPAWLTLALTLPDVDEAWLESFSDSLFDLLNYYDMQLIGGDTTRGPLSMTLGIHGFVPM

GRALTRSGAKPGDWIYVTGTPGDSAAGLAILQNRLQVADAKDADYLIKRHLRPSPRILQGQALRDLANSA

IDLSDGLISDLGHIVKASDCGARIDLALLPFSDALSRHVEPEQALRWALSGGEDYELCFTVPELNRGALD

VALGHLGVPFTCIGQMTADIEGLCFIRDGEPVTFDWKGYDHFVTP

>gi|30061911|ref|NP_836082.1| transcription antitermination protein NusB [Shigella flexneri 2a str. 2457T]

MKPAARRRARECAVQALYSWQLSQNDIADVEYQFLAEQDVKDVDVLYFRELLAGVATNTAYLDGLMKPYL

SRLLEELGQVEKAVLRIALYELSKRSDVPYKVAINEAIELAKSFGAEDSHKFVNGVLDKAAPVIRPNKK

>gi|30061910|ref|NP_836081.1| 6,7-dimethyl-8-ribityllumazine synthase [Shigella flexneri 2a str. 2457T]

MNIIEANVATPDARVAITIARFNNFINDSLLEGAIDALKRIGQVKDENITVVWVPGAYELPLAAGALAKT

GKYDAVIALGTVIRGGTAHFEYVAGGASNGLAHVAQDSEIPVAFGVLTTESIEQAIERAGTKAGNKGAEA

ALTALEMINVLKAIKA

>gi|30061909|ref|NP_836080.1| bifunctional diaminohydroxyphosphoribosylaminopyrimidine deaminase/5-amino-6-(5-phosphoribosylamino)uracil reductase [Shigella flexneri 2a str. 2457T]

MQDEYYMARALKLAQRGRFTTHPNPNVGCVIVKDGEIVGEGYHQRAGEPHAEVHALRMAGEKAKGATAYV

TLEPCSHHGRTPPCCDALIAAGVARVVAAMQDPNPQVAGRGLYRLQQAGIDVSHGLMMSEAEQLNKGFLK

RMRTGFPYIQLKLGASLDGRTAMASGESQWITSPQARRDVQRQRAQSHAILTSSATVLADDPALTVRWSE

LDEQTQALYPQQNLRQPVRIVIDSQNRVTPEHRIVQQPGETWFARTQEDSREWPETVRTLLIPEHKGHLD

LVVLMMQLGKQQINSIWVEAGPTLAGVLLQAGLVDELIVYIAPKLLGSDARGLCSLPGLEKLADAPQFKF

KEIRHVGPDVCLHLVGA

>gi|30061908|ref|NP_836079.1| transcriptional regulator NrdR [Shigella flexneri 2a str. 2457T]

MHCPFCFAVDTKVIDSRLVGEGSSVRRRRQCLVCNERFTTFEVAELVMPRVVKSNDVREPFNEEKLRSGM

LRALEKRPVSSDYVEMAINHIKSQLRATGEREVPSKMIGNLVMEQLKKLDKVAYIRFASVYRSFEDIKEF

GEEIARLED

>gi|30061906|ref|NP_836077.1| outer membrane protein Tsx [Shigella flexneri 2a str. 2457T]

MKKTLLAAGAVLALSSSFTVNAAENDKPQYLSDWWHQSVNVVGSYHTRFGPQIRNDTYLEYEAFAKKDWF

DFYGYADAPVFFGGNSDAKGIWNHGSPLFMEIEPRFSIDKLTNTDLSFGPFKEWYFANNYIYDMGRNKDG

RQSTWYMGLGTDIDTGLPMSLSMNVYAKYQWQNYGAANENEWDGYRFKIKYFVPITDLWGGQLSYIGFTN

FDWGSDLGDDSGNAINGIKTRTNNSIASSHILALNYDHWHYSVVARYWHDGGQWNDDAELNFGNGNFNVR

STGWGGYLVVGYNF

>gi|30061905|ref|NP_836076.1| hypothetical protein S0355 [Shigella flexneri 2a str. 2457T]

MAIIPKNYARLESGYREKALKIYPWVCGRCSREFVYSNLRELTVHHIDHDHTNNPEDGSNWELLCLYCHD

HEHSKYTEADQYGTTVIAGEDAQKDVGEAKYNPFADLKAMMNKKK

>gi|30061904|ref|NP_836075.1| preprotein translocase subunit SecF [Shigella flexneri 2a str. 2457T]

MAQEYTVEQLNHGRKVYDFMRWDYWAFGISGLLLIAAIVIMGVRGFNWGLDFTGGTVIEITLEKPAEIDV

MRDALQKAGFEEPMLQNFGSSHDIMVRMPPAEGETGGQVLGSQVLKVINESTNQNAAVKRIEFVGPSVGA

DLAQTGAMALMAALLSILVYVGFRFEWRLAAGVVIALTHDVIITLGILSLFHIEIDLTIVASLMSVIGYS

LNDSIVVSDRIRENFRKIRRGTPYEIFNVSLTQTLHRTLITSGTTLMVILMLYLFGGPVLEGFSLTMLIG

VSIGTASSIYVASALALKLGMKREHMLQQKVEKEGADQPSILP

>gi|30061903|ref|NP_836074.1| preprotein translocase subunit SecD [Shigella flexneri 2a str. 2457T]

MLNRYPLWKYVMLIVVIVIGLLYALPNLFGEDPAVQITGARGVAASEQTLIQVQKTLQEEKITAKSVALE

EGAILARFDSTDTQLRAREALMGVMGDKYVVALNLAPATPRWLAAIHAEPMKLGLDLRGGVHFLMEVDMD

TALGKLQEQNIDSLRSDLREKGIPYTTVRKENNYGLSITFRDAKARDEAIAYLSKRHPDLVISSQGSNQL

RAVMSDARLSEAREYAVQQNINILRNRVNQLGVAEPVVQRQGADRIVVELPGIQDTARAKEILGATATLE

FRLVNTNVDQAAAASGRVPGDSEVKQTREGQPVVLYKRVILTGDHITDSTSSQDEYNQPQVNISLDSAGG

NIMSNFTKDNIGKPMATLFVEYKDSGKKDANGRAVLVKQEEVINIANIQSRLGNSFRITGINNPNEARQL

SLLLRAGALIAPIQIVEERTIGPTLGMQNIEQGLEACLAGLLVSILFMIIFYKKFGLIATSALIANLILI

VGIMSLLPGATLSMPGIAGIVLTLAVAVDANVLINERIKEELSNGRTVQQAIDEGYRGAFSSIFDANITT

LIKVIILYAVGTGAIKGFAITTGIGVATSMFTAIVGTRAIVNLLYGGKRVKKLSI

>gi|30061902|ref|NP_836073.1| preprotein translocase subunit YajC [Shigella flexneri 2a str. 2457T]

MSFFISDAVAATGAPAQGSPMSLILMLVVFGLIFYFMILRPQQKRTKEHKKLMDSIAKGDEVLTNGGLVG

RVTKVAENGYIAIALNDTTEVVIKRDFVAAVLPKGTMKAL

>gi|30061900|ref|NP_836071.1| S-adenosylmethionine:tRNA ribosyltransferase-isomerase [Shigella flexneri 2a str. 2457T]

MRVTDFSFELPESLIAHYPMPERSSCRLLSLDGPTGALTHGTFTDLLDKLNPGDLLVFNNTRVIPARLFG

RKASGGKIEVLVERMLDDKRILAHIRASKAPKPGAELLLGDDESINATMTARHGALFEVEFNDERSVLDI

LNSIGHMPLPPYIDRPDEDADRELYQTVYSEKPGAVAAPTAGLHFDEPLLEKLRAKGVEMAFVTLHVGAG

TFQPVRVDTIEDHIMHSEYAEVPQDVVDAVLAAKARGNRVIAVGTTSVRSLESAAQAAKNDLIEPFFDDT

QIFIYPGFQYKVVDALVTNFHLPESTLIMLVSAFAGYQHTMNAYKAAVEEKYRFFSYGDAMFITYNPQAI

NERVGE

>gi|30061897|ref|NP_836068.1| branched-chain amino acid aminotransferase [Shigella flexneri 2a str. 2457T]

MTHQLRSRDIIALGFMTFALFVGAGNIIFPPMVGLQAGEHVWTAAFGFLITAVGLPVLTVVALAKVGGGV

DSLSTPIGKVAGVLLATVCYLAVGPLFATPRTATVSFEVGIAPLTGDSALPLFIYSLVYFAIVILVSLYP

GKLLDTVGNFLAPLKIIALVILSVVAIVWPAGSVSTATEAYQNAAFSNGFVNGYLTMDTLGAMVFGIVIV

NAARSRGVTEARLLTRYTVWAGLMAGVGLTLLYLALFCLGSDSASLVDQSANGAAILHAYVQHTFGGGGS

FLLAALIFIACLVTAVGLTCACAEFFAQYVPLSYRTLVFILGGFSMVVSNLGLSQLIQISVPVLTAIYPP

CIALVVLSFTRSWWHNSSRVIAPPMFISLLFGILDGIKASAFSDILPSWAQRLPLAEQGLAWLMPTVVMV

VLAIIWDRAAGRQVTSSAH

>gi|30061896|ref|NP_836067.1| phosphate regulon sensor protein [Shigella flexneri 2a str. 2457T]

MLERLSWKRLVLELLLCCLPAFILGAFFGYLPWFLLASVTGLLIWHFWNLLRLSWWLWVDRSMTPPPGRG

SWEPLLYGLHQMQLRNKKRRRELGNLIKRFRSGAESLPDAVVLTTEEGGIFWCNGLAQQILGLRWPEDNG

QNILNLLRYPEFTQYLKTRDFSRPLNLVLNTGRHLEIRVMPYTHKQLLMVARDVTQMHQLEGARRNFFAN

VSHELRTPLTVLQGYLEMMDEQPLEGAVREKALHTMREQTQRMEGLVKQLLTLSKIEAAPTQLLNEKVDV

PMMLRVVEREAQTLSQKKQTFTFEIDNGLKVSGNEDQLRSAISNLVYNAVNHTPEGTHITVRWQRVPHGA

EFSVEDNGPGIAPEHIPRLTERFYRVDKARSRQTGGSGLGLAIVKHAVNHHESRLNIESTVGKGTRFSFV

IPERLIAKNSD

>gi|30061895|ref|NP_836066.1| transcriptional regulator PhoB [Shigella flexneri 2a str. 2457T]

MARRILVVEDEAPIREMVCFVLEQNGFQPVEAEDYDSAVNQLNEPWPDLILLDWMLPGGSGIQFIKHLKR

ESMTRDIPVVMLTARGEEEDRVRGLETGADDYITKPFSPKELVARIKAVMRRISPMAVEEVIKMQGLSLN

PTSHRVMAGEEPLEMGPTEFKLLHFFMTHPERVYSREQLLNHVWGTNVYVEDRTVDVHIRRLRKALEPGG

HDRMVQTVRGTGYRFSTRF

>gi|30061894|ref|NP_836065.1| exonuclease subunit SbcD [Shigella flexneri 2a str. 2457T]

MRILHTSDWHLGQNFYSKSREAEHQAFLDWLLETAQTHQVDAIIVAGDVFDTGSPPSYARTLYNRFVVNL

QQTGCHLVVLAGNHDSVATLNESRDIMAFLNTTVVASAGHAPQILPRRDGTPGAVLCPIPFLRPRDIISS

QAGLNGIEKQQHLLAAITDYYQQHYADACKLRGDQPLPIIATGHLTTVGSSKSDAVRDIYIGTLDAFPAQ

NFPPADYIALGHIHRAQIIGGMEHVRYCGSPIPLSFDECGKSKYVHLVTFSNGKLESVENLNVPVTQPMA

VLKGDLASITAQLEQWRDVSQEPPVWLDIEITTDEYLHDIQRKIQALTESLPVEVLLVRRSREQRERVLA

SQQRETLSELSVEEVFNRRLALEDLDESQQQRLQHLFTTTLRTLAGEHEA

>gi|30061893|ref|NP_836064.1| exonuclease subunit SbcC [Shigella flexneri 2a str. 2457T]

MKILSLRLKNLNSLKGEWKIDFTREPFASNGLFAITGPTGAGKTTLLDAICLALYHETPRLSNVSQSQND

LMTRDTAECLAEVEFEVKGEAYRAFWSQNRARNQPDGNLQVPRVELARCADGKILADKVKDKLELTATLT

GLDYGRFTRSMLLSQGQFAAFLNAKPKERAELLEELTGTEIYGQISAMVFEQHKSARTELEKLQAQASGV

TLLTPEQVQSLTASLQVLTDEEKQLITAQQQEQQSLNWLTRQDELQQEASRRQQALQQALAEEEKAQPQL

AALSLAQPARNLRPHWERIAEHSAALAHIRQQIEEVNTRLQSTMALRASIRHHAAKQSAELQQQQQSLNT

WLQEHDRFRQWNNELAGWRAQFSQQTSDREHLRQWQQQLTHAEQKLNALAAITLTLTADEVATALAQHAE

QRPLRQRLVALHGQIVPQQKRLAQLMVTIQNVTLEQTQRNVALNEMRQRYKEKTQQLADVKTICEQEARI

KTLEAQRAQLQAGQPCPLCGSTSHPAVEAYQALEPGVNQSRLLALENEVKKLGEEGAALRGQLDALTKQL

QRDENEAQSLRQDEQALTQQWQAVTASLNITLQPQDDIQPWLDAQDEHERQLRLLSQRHELQGQIAAHNQ

QIIQYQQQIEQRQQQLLTALAGYALTLPQEDEEESWLATRQQEAQSWQQRQNELTALQNRIQQLTPILET

LPQSDDLPHSEETVALDNWRQVHEQCLALHSQQQTLQQQDVLAAQSLQKAQAQFDTALQASVFDDQQAFL

AALMDEQTLTQLEQLKQNLENQRRQAQTLVTQTAETLAQHQQHRPDGLALTVTVEQIQQELAQTHQKLRE

NTTSQGEIRQQLKQDADNRQQQQTLMQQIAQMTQQVEDWGYLNSLIGSKEGDKFRKFAQGLTLDNLVHLA

NQQLTRLHVRYLLQRKASEALEVEVVDTWQADAVRDTRTLSGGESFLVSLALALALSDLVSHKTRIDSLF

LDEGFGTLDSETLDTALDALDALNASGKTIGVISHVEAMKERIPVQIKVKKINGLGYSKLESTFAVK

>gi|30061892|ref|NP_836063.1| MFS transport protein AraJ [Shigella flexneri 2a str. 2457T]

MKKVILSLALGTFGLGMAEFGIMSVLTELAHNVGISIPAAGHMISYYALVVVVGAPIIALFSSRYSLKHI

LLFLVALCVIGNAMFTLSSSYLMLAIGRLVSGFPHGAFFGVGAIVLSKIIKPGKVTAAVAGMVSGMTVAN

LLGIPLGTYLSQECWRYTFLLIAVFNIAVMASVYFWVPDIRDEAKGNLREQFHFLRSPAPWLIFAATMFG

NAGVFAWFSYVKPYMMFISGFSETAMTFIMMLVGLGMVLGNMLSGRISGRYSPLRIAAVTDFIIVLALLM

LFFCGGMKTTSLIFAFICCAGLFALSAPLQILLLQNAKGGELLGAAGGQIAFNLGSAVGAYCGGMMLTLG

LAYNYVALPAALLSFAAMSSLLLYGRYKRQQAADSPVLAKPLG

>gi|30061890|ref|NP_836061.1| recombination associated protein [Shigella flexneri 2a str. 2457T]

MLWFKNLMVYRLSREISLRAEEMEKQLASMAFTPCGSQDMAKMGWVPPMGSHSDALTHVANGQIVICARK

EEKILPSPVIKQALEAKIAKLEAEQARKLKKTEKDSLKDEVLHSLLPRAFSRFSQTMMWLDTVNGLIMVD

CASAKKAEDTLALLRKSLGSLPVVPLSMENPIELTLTEWVRSGSAAQGFQLLDEAELKSLLEDGGVIRAK

KQDLTSEEITNHIEAGKVVTKLALDWQQRIQFVMCDDGSLKRLKFCDELRDQNEDIDREDFAQRFDADFI

LMTGELAALIQNLIEGLGGEAQR

>gi|30061889|ref|NP_836060.1| hypothetical protein S0337 [Shigella flexneri 2a str. 2457T]

MTQRPWSKLQREIYDLLTPTINLQIHCTRYPMRSQNGGSTDLPRYWITLDKNVIWDYPKDFIAGNAGVRN

FHGETCWYPYLTDICSISDLLREYIDTPKAELLTKQFTSDKWGLVNILRAADRRIGMRRLDQLRRKTHNI

AALKIIARRSE

>gi|30061886|ref|NP_836057.1| hypothetical protein S0334 [Shigella flexneri 2a str. 2457T]

MSASLAILTIGIVPMQEVLPLLTEYIDEDNISHHSLLGKLSREEVMAEYAPEAGEDTILTLLNDNQLAHV

SRRKVERDLQGVVEVLDNQGYDVILLMSTANISSMTARNTIFLEPSRILPPLVSSIVEDHQVGVIVPVEE

MLPVQAQKWQILQKSPVFSLGNPIHDSEQKIIDAGKELLAKGADVIMLDCLGFHQRHRDLLQKQLDVPVL

LSNVLIARLAAELLV

>gi|30061881|ref|NP_836052.1| diguanylate cyclase AdrA [Shigella flexneri 2a str. 2457T]

MFPKIMNDENFFKKAAAHGEEPPLTPQNEHQRSGLRFARRVRLPRAVGLAGMFLPIASTLVSHPPPGWWW

LVLVGWAFVWPHLAWQIASRAVDPLSREIYNLKTDAVLAGMWVGVMGVNVLPSTAMLMILCLNLMGAGGP

RLFVAGLVLMVVSCLVTLELTGITVSFNSAPLEWWLSLPIIVVYPLMFGWVSYQTATKLAEHKRRLQVMS

TRDGMTGVYNRRHWETMLRNEFDNCRRHNRDATLLIIDIDHFKSINDTWGHDVGDEAIVALTRQLQITLR

GSDVIGRFGGDEFAVIMSGTPAESAITAMLRVREGLNTLRLPNTPQVTLRISVGVAPLNPQMSHYREWLK

SADLALYKAKKAGRNRTEVAA

>gi|30061877|ref|NP_836048.1| hypothetical protein S4804 [Shigella flexneri 2a str. 2457T]

MHMIEINSLLLITSVILMSLLAVGLFDKISPINLVEHGRNNQIDGMRGFLAIFVLIHHAAIWNGYLSSGV

WEAPSSNLLANLGQVGVSFFFMITGYLFFSKIISGDQDWTRLYVSRLLRLTPMFIVSLCLIFIIVGFKSG

WRMQVSTEELFVSIMKWLPFTALGMPNINDVKDSFTINAAVTWTLVYEWFFYFSLPVISALIKRKVSIYM

VMISAISLFVFILFFSKIHIASFLFGLLAFLLNKSKIVNGIAKAKVTPIIITAIMVFEMTYFKTTYAPLP

LILCGITFIIIASGCDLYGILRLNITRKLGETTYSVYLLHGIFLYCLMTWIIPNNYTENTFIILVSTTAF

LITFTSCLTFKLIETPFIKLTKQTTTLVKELIPTLTNNNQ

>gi|30061876|ref|NP_836047.1| phage integrase [Shigella flexneri 2a str. 2457T]

MSLFRRGEIWYASFTLPNGKRFKQSLGTKDKRQATELHDKLKAEAWRVSKLGEIPDITFEEACVRWLEEK

AHKKSLDDDKSRIGFWLQHFAGMQLRDITESKIYSAMQKMTNRRHEENWRLRAEACRKKGKPVPEYTPKP

ASVATKATHLSFIKALLRAAEREWKMLDKAPIIKVPQPKNKRIRWLEPHEAQRLIDECPEPLKSVVEFAL

ATGLRRSNIINLEWQQIDMQRRVAWINPEESKSNRAIGVALNDTACRVLKKQIGNHHRWVFVYKESCTKP

DGTKAPTVRKMRYDANTAWKAALRRAGIDDFRFHDLRHTWASWLVQAGVPLSVLQEMGGWESIEMVRRYA

HLAPNHLTEHARQIDSILNPSVPNSSQSKNKEGTNDV

>gi|30061872|ref|NP_836043.1| flippase [Shigella flexneri 2a str. 2457T]

MLKLFVKYTSIGVLNTLIHWVVFGVCIYAAHTSQALANFTGFVVAVSFSFFANARFTFKALTTAMRYMLY

VGFMGILSVIVGWAADKCSLPPIVTLITFSAISLVCGFVYSKFIVFRDAK

>gi|30061870|ref|NP_836041.1| glucosyl tranferase II [Shigella flexneri 2a str. 2457T]

MIKINLFKNANLLAFISCFAISIYCYWGWLYDGTLNIDGEFTNNFYQTITLGRWFHTFLRHYFLPEPFSL

YITPLIALSFIIISAFIICRSLKLESYELLIGMLVFITFPQISYQLEFLNQADTVGIAFLLAAISAIIFH

SQKNRIVIFSGIVLSILSMAIYQTFVTYIIAFVIGLQINSIIRNEKNIRESFYSSCLSLSLIALSTLIYL

LLTKAIKHYFSLESNEYISNYIQNASDIKWLVKSAIDNIYNFYNNPPTGLNLYKWLLIPLLILMFTLTYK

LKTRSIYLISSIIFIYILPVIFIVVVGSGAPPRLFVLMPIVAVILFSCLSNFRSIKYLNCMFFLFIIFNG

VSTSKNLFLNDTLARQKDISLAKEISYTSQTKGISLNGKYIYIYGSNDSGNMLSMSADTFGKSFFWWDGG

NYFRMVAFMNYYGICNCKPANKEQIEKIYPIVKSLPSWPNPDSIAEINGLVIIKLSEKKGWLPFNI

>gi|30061869|ref|NP_836040.1| hypothetical protein S4808 [Shigella flexneri 2a str. 2457T]

MPARKVCQTFFRNALASTHQYRQNAIIDSAAALVGGASLSLTSIGRHLPGPARVKDKIKRVDCLLGNKRL

HNDIPLIFKNITSMMTNKLSWCVIAVDWSGYPFQEYHVLRASLLCDGRSIPLMSQVFPSKKNNEAVEIAF

LDALSGAISPRTRVVIVTDAGFQSAWFRHIKSQGWDFIGRIRGVVKFRLDSDKDKWLDIKMCRGSSEAKY

LGTGTLARKKRSQCEGHFYLYKHSPKGRKSRRARGRPGLPTTEKEQKAAGREPWLIFSNTAEFNAKKIMK

LYSRRMQIEQNFRDEKSERFGFGLRASRSRRGERFLVLSLLVTLASIVLWLLGYHFENKGFHLKYQANSL

KKRRVLSFLTLAENVLRFNPELLRRAQPEKILCRLASTYRSMVLAY

>gi|30061868|ref|NP_836039.1| hypothetical protein S4802 [Shigella flexneri 2a str. 2457T]

MYNNKIDSNRKIRHDWVDALKFLGIFAIYLGHLGLGAGKLYPFVFSYHVPLFFFAAGFFTIKKNDLSVFD

YIKSKFYRLMIPYFTFAFSILIINTINSGETIDYIYNHIYDIIYGVRNNQFVGTIWFINCLFVIIAIDAI

FREIVKNNIVILIISLLSFMLSQTVLNHNPLLDPQWFWNIDSAMAYWWLLPLGRCMFLELTRDRFFGKSK

IGFIVFSITAIMSAYQLLNQKPLLFKIISIFNADIISSSYIQAINTIITTVGLIIFNIFIAKIICGNDFI

VRAGRNTLNICGMEYITKLFIPMALAIIGFSVTIPNPICAIIYTCICVYVSDKIGHWLSRTVGGPFLIK

>gi|30061867|ref|NP_836038.1| phage tail fibre protein [Shigella flexneri 2a str. 2457T]

MSRTLDLILLCRPVQDTVHLLMRITLQWDINKMSYFYSASTNGFYSTEFHGTNIPDDAVEISESEWKTLI

NAQSVTKMITCGENGHPVIVDRPSPTPEQLALINDEKKSALIAEATNVIAPLQDAVDLGMATDDETKLYW

HGKNIGCCLCVLM

>gi|30061866|ref|NP_836037.1| hypothetical protein S0319 [Shigella flexneri 2a str. 2457T]

MHRIDTKTAKKDKFGAGKNGFTRGNPQTGTPATDLDDDYFDMLQEELCSVVEASGASLEKGRHDQLLTAL

RALLLSRKNPFGDIKSDGTVKTALENLGLGEAAKRNVGTGANQIPDMSLFASINTVTAAAQKFPSGLILQ

CGQLNGAPNVSSTYGMRFPMTFSRVIAVVVTLNVTGAAGQPTVSATSVQNTGFNITVSPGSGYGSSADAY

YIAMGY

>gi|30061857|ref|NP_836028.1| DNA-binding transcriptional regulator Crl [Shigella flexneri 2a str. 2457T]

MTLPSGHPKSRLIKKFTALGPYIREGKCEDNRFFFDCLAVCVNVKPAPEVREFWGWWMELEAQESRFTYS

YQFGLFDKAGDWKSVPVKDTEVVERLEHTLREFHEKLRELLTTLNLKLEPANDFRDEPVKLTA

>gi|30061856|ref|NP_836027.1| fermentation/respiration switch protein [Shigella flexneri 2a str. 2457T]

MTQANLSETLFKPRFKHPETSTLVRRFNHGAQPPVQSALDGKTIPHWYRMINRLMWIWRGIDPREILDVQ

ARIVMSDAERTDDDLYDTVIGYRGGNWIYEWATQAMVWQQKACTEEDPQLSGRHWLHAATLYNIAAYPHL

KGDDLAEQAQALSNRAYEEAAQRLPGTMRQMEFTVPGGAPITGFLHMPKGDGPFPTVLMCGGLDAMQTDY

YSLYERYFAPRGIAMLTIDMPSVGFSSKWKLTQDSSLLHQHVLKALPNVPWVDHTRVAAFGFRFGANVAV

RLAYLESPRLKAVACLGPVVHTLLSDFKCQQQVPEMYLDVLASRLGMHDASDEALRVELNRYSLKVQGLL

GRRCPTPMLSGYWKNDPFSPEEDSRLITSSSADGKLLEIPFNPVYRNFDKGLQEITGWIEKRLC

>gi|30061855|ref|NP_836026.1| xanthine-guanine phosphoribosyltransferase [Shigella flexneri 2a str. 2457T]

MSEKYIVTWDMLQIHARKLASRLMPSEQWKGIIAVSRGGLVPGALLARELGIRHVDTVCISSYDHDNQRE

LKVLKRAEGDGEGFIVIDDLVDTGGTAVAIREMYPKAHFVTIFAKPAGRPLVDDYVVDIPQDTWIEQPWD

MGVVFVPPISGR

>gi|30061854|ref|NP_836025.1| aminoacyl-histidine dipeptidase [Shigella flexneri 2a str. 2457T]

MSELSQLSPQPLWDIFAKICSIPHPSYHEEQLAEYIVGWAKEKGFHVERDQVGNILIRQPATAGMENRKP

VVLQAHLDMVPQKNNDTVHDFTKDPIQPYIDGEWVKARGTTLGADNGIGMASALAVLADENVVHGPLEVL

LTMTEEAGMDGAFGLQSNWLQADILINTDSEEEGEIYMGCAGGIDFTSNLHLDREAVPAGFETFKLTLKG

LKGGHSGGEIHVGLGNANKLLVRFLAGHAEELDLRLIDFNGGTLRNAIPREAFATIAVAADKVDALKSLV

NTYQEILKNELAEKEKNLALLLDSVANDKAALIAKSRDTFIRLLNATPNGVIRNSDVAKGVVETSLNVGV

VTMTDNNVEIHCLIRSLIDSGKDYVVSMLDSLGKLAGAKTEAKGAYPGWQPDANSPVMHLVRETYQRLFN

KTPNIQIIHAGLECGLFKKPYPEMDMVSIGPTITGPHSPDEQVHIESVGHYWTLLTELLKEIPAK

>gi|30061851|ref|NP_836022.1| hypothetical protein S0303 [Shigella flexneri 2a str. 2457T]

MNNIQIRDYQPGDFQQLCAIFIRAVTMTVSQHYSLQQIAAWAQIDESRWKEKLAKSQVRVAVINAKLVGF

ITCVEHYIDMLFVDPEYTRRGVASALLKPFIKSESELTVDASITAKPFFERYGFQTVKQQRVECRGAWFI

NFYMRYKPQH

>gi|30061850|ref|NP_836021.1| toxin YafO [Shigella flexneri 2a str. 2457T]

MRVFKTKLIRLQLTAEELDALTADFISYKRDGVLPDIFGRDALYDDSFTWPLIKFERVAHIHLANVNNPF

PPQLRQFSRTNDEAHLVYCQGAFDEQAWLLIAILKPEPHKLARDNNQMHKIGKMAEAFRMRF

>gi|30061847|ref|NP_836018.1| hypothetical protein S0298 [Shigella flexneri 2a str. 2457T]

MTTIKLIVNSVSKSERESIIAALHGQSIFNGGGLSPLNKISPSHPPKPATVAVPEETEKKARDVNEKTAL

LKKKSATELGELATSINTIARDAHMEANLEMEIVPQGLRVLIKDDQNRNMFERGSAQIMPFFKTLLVELA

PVFDSLYNKIIITGHTDAMAYKNNIYNNWNLSGDRALSARRVLEEAGMPEDKVMQVSAMADQMLLDAKNP

QSAGNRRIEIMVLTKSASDTLYQYFGQHGDKVVQPLVQKLDKQQVLSQRMR

>gi|30061846|ref|NP_836017.1| flagellar biosynthetic protein FlhA [Shigella flexneri 2a str. 2457T]

MLSRSDLLTLLTINFIVVTKEAERISEVSARFTLDAMPGKQMAIDADLNAGLINQAQAQTRRKDVASEAD

FYGAMDGASKFVRGDAIAGMMILAINLSGGVCIGIFKYNLSADAAFQQYVLMTIGDGLVAQIPSLLLSTA
AAIIVTRVSDNGDIAHDVRHQLLASPSVLYTATGIMFVLAVVPGMPHLPFLLFSALLGFTGWRMSKRPQA
AKAEEKSLETLTRTITETSEQQVSWETIPLIEPISLSLGYKLVALVDKAQGNPLTQRIRGVRQVISDGNG
VLLPEIRIRENFRLKPSQYAIFINGIEADEADIPADKLMALPSSETYGEIDGVLGNDPAYGIPVTWIQPA
QKAKALNMGYQVIDSASVIATHVNKIVRSYIPDLFNYDDITQLHNRLSSMAPRLAEDLSAALNYSQLLKV
YRALLTEGVSLRDIVTIATVLVASSTVTKDHILLAADVRLALRRSITHPFVRKQELTVYTLNNELENLLT
NVVNQAQQAGKVMLDSVPVDPNMLNQFQSTMPQVKEQMKAAGKDPVLLVPPQLRPLLPRYARLFAPGLHV
LSYNEVPDELELKIMGALM

>gi|30061845|ref|NP_836016.1| hypothetical protein S0297 [Shigella flexneri 2a str. 2457T]
MSEYRRYYIKGGTWFFTVNLRNRRSHLLTTQFQTLRNAIINVKRDRPFEINAWVVLPEHMHCIWTLPESD
DDFSSRWREIKKQFTHACGLKNIWQPRFWEHAIRNTKDYRHHVDYIYINPEKHGWVKQVSDWPFSTFHRD
VARGLYPIDWAGDITDLSAGERIIL

>gi|30061844|ref|NP_836015.1| lipoprotein [Shigella flexneri 2a str. 2457T]
MRFAPFTRQRHHNREQIKQHPEWFPAPLKASDRRWQALAENNHFLSSDHLHNITEVAIHRLEQQLGKPYV
WGGTRPDQGFDCSGLVFYAYNKILEAKLPRTANEMYHYRRATIVANNDLRRGDLLFFHIHSREIADHMGG
YLGDGQFIESLRTGETIRVSRLAEPFWQDHFLGARRILTEEMIL

>gi|30061843|ref|NP_836014.1| hypothetical protein S0295 [Shigella flexneri 2a str. 2457T]
MRKIALILAMLLIPCVSFAGLLGSSSSTTPVSKEYKQQLMGSPVYIQIFKEERTLDLYVKMGEQYQLLDS
YKICKYSGGLGPKQRQGDFKSPEGFYSVQRNQLKPDSRYYKAINIGFPNAYDRAHGYEGKYLMIHGDCVS
IGCYAMTNQGIDEIFQFVTGALVFGQPSVQVSIYPFRMTDANMKRHKYSNFKDFWEQLKPGYDYFEQTRK
PPTVSVANGRYVVSKPLSHEVVQPQLASNYTLPEAK

>gi|30061842|ref|NP_836013.1| amidotransferase [Shigella flexneri 2a str. 2457T]

MCELLGMSANVPTDICFSFTGLVQRGGGTGPHKDGWGITFYEGKGCRTFKDPQPSFNSPIAKLVQDYPIK

SCSVVAHIRQANRGEVALENTHPFTRELWGRNWTYAHNGQLTGYKSLETGNFRPVGETDSEKAFCWLLHK

LTQRYPRTPGNMAAVFKYIASLADELRAKGVFNMLLSDGRYVMAYCSTNLHWITRRAPFGVATLLDQDVE

IDFSSQTTPNDVVTVIATQPLTGNETWQKIMPGEWRLFCLGERVV

>gi|30061841|ref|NP_836012.1| phosphoheptose isomerase [Shigella flexneri 2a str. 2457T]

MYQDLIRNELNEAAETLANFLKDDANIHAIQRAAVLLADSFKAGGKVLSCGNGGSHCDAMHFAEELTGRY

RENRPGYPAIAISDVSHISCVGNDFGFNDIFSRYVEAVGREGDVLLGISTSGNSANVIKAIAAAREKGMK

VITLTGKDGGKMAGTADIEIRVPHFGYADRIQEIHIKVIHILIQLIEKEMVK

>gi|30061839|ref|NP_836010.1| C-lysozyme inhibitor [Shigella flexneri 2a str. 2457T]

MGRISSGGMMFKAITTVAALVIATSAMAQDDLTISSLAKGETTKAAFNQMVQGHKLPAWVMKGGTYTPAQ

TVTLGDETYQVMSACKPHDCGSQRIAVMWSEKSNQMTGLFSAIDEKTSQEKLTWLNVNDALSIDGKTVLF

AALTGSLENHPDGFNFK

>gi|30061837|ref|NP_836008.1| Rhs-family protein [Shigella flexneri 2a str. 2457T]

MTARDYIWNADGEVGGINDKLRGCLVFSYDRSGWLTSRTGQMYDHDHYYYDKAGNLLTDEYQGAVMDNRL

PGYGRDRYRYNEWGELTERRDQQLEWNAQGQLTRVISSNSETRYQYDALGRRISKATSNLHTDRGERSRT

TFVWEGFRLLQETTWQGKRTYLYDAEQPYTPVAAITGRGESQKIWYYHTDLTGTVHEVTAPDGTLVWAGY

QAGFGENRGDISNSGAYFEQPQRLPGQYFDEETGLHYNLFRYYAPECGRFVSQDPIGLNGGLNLYAYAPN

PLGWIDPLGLNSLGIDSNNIFRGDGNYKGGGIGQPKSGTISAQDIIDHL

>gi|30061836|ref|NP_836007.1| Rhs-family protein [Shigella flexneri 2a str. 2457T]

MIEDGSPNVFLGGGTQTVLEISPEIPDWLRQVVDVLYVVAGMLGGLAGAWRQAAKMGSKFGTKCAAKFIG

GEMVGMAISDTVMGLFSNPVDVTTGQKILLPETDFTLPGRLPVTCSRFYASHLETEGLLGRGWRLNWEIN

LREDETYITFIGVQGRELSYPKEMLIPGHQIFDPEEQFYLSRLHDGCYVLHYTDCSYYVFDEFDDHGVAP

LLFMETPYRQRIAFGRENGRLVRVASSSGHHLLLHRTMTQAGERLSHIELLKGGRPGNLVEYRYDDNGQL

TGVVNRAGVTVRQFAYENGLMTEHRNATGFTCTYHWEEIEGFPRVVEHTTSDGEDYRFHYDFAGGQTVVT

GRPEQKWQWWFDEETYVTAHRTPGGGMYRFTYNENHFPVAVELPGERRVTLEYDTLSRVVKETDPAGRVT

QTQWNGSFAEITRRALDDDHVWKADYNEHGQVIRETDPEGRVTRYGYDDQGLPETVCHPGKQQDRYTWNA

LGLLSSHRRITGSVQSWQYTQRGMLARHTDEEKRETRWQYTPEGLVASLSNGNGAQYRFSYDGDGRLTGE

QRPDGLIRMFALNADGFPVIIPTQGTEGGVRNEQQERDALGRLLRSDTQHSTRTFSYNRLDQITEVTLTP

TEEGERLHHMQADTVRFAYDRSGWLTAEHSVHGSIKYRRDALGNPTDITLPDGQHLSHLYYGSGHLLQTA

LDGITVSEYERDSLHRQVIRTQGKLATFSGYNADNRLSWQRSLPGGSQNPQQAVLPRRRTTA

>gi|30061835|ref|NP_836006.1| Rhs-family protein [Shigella flexneri 2a str. 2457T]

MYEAARVDDPIYHTSALAGFLIGAIIGIAIIAVASFAFFTCGFLAGLILSFLADQIASGVLQLGEAIGRS

IHSTVGKILTGSDNVSTNSRPAARAVLSTVICEDHSPEIRIAQGSGNIYINSQLPVRMTTLSVMQ

>gi|30061832|ref|NP_836003.1| Rhs-family protein [Shigella flexneri 2a str. 2457T]

MSFVSTGNKPVGNGGPVITTPPIAGESGGMSTGSAVTDVSGAAEEMAEQAAADLFGALPEPSGLVKAAVA

AAQAAAAAAGISDMAGAVQDAAASLAAGAPGAHNVTVSGSAVPPQMLLFAGMNGSEGLGNLFSYTVQLKT

LDALNLGYVSPAANLPLQLMVGKDLCVSIELDGGGKRYISGLVTAARVVGHESRSVTYELRIEPWLKLLT

HTSDYKAFQNKTVVDILDEVLDEYSFPVEKRLVENYPPRAWQVQYGETDFDFIQRLMQEWGIYWWFEHSE

NSHTLVLVDAINGHKACPDSPLVEWHQEGLKLDKEFIHTITASERLRTGKWVMDDFDFMKPRSLLKSTVA

SPRDTGHAEYEHYEWPGDYFTTGEGEMLTRIRMEAQRSPGSRAHGAGHIRTLMTGYTFTLMNHPTAEINQ

EYLLVQTTLFLRDNAQHSGQDQHFTYVTTFELHPTSEVYRPQRTLSKPHTKGPQSAIVTGPAGQEIWTDK
YGRVKVQFGWDRYGKNDENSSCWIRVSYPWAGKGFGMIQIPRIGQEVLVDFKNGDPDLPIIVGRTYNQDT
MPPWGLPGMASQSGIFSHSLQGGPTNGNMLRFDDKTGAEEVKFHAEKDLNTTVKNNETHTVMVDRIKTIV
KNETISVGEDRNATITKNDGLSVKLAQTINVGTTYRLDVGDQFTLRCGNAALVLHKDGSIEFCGKQLLLH
TSDVMQLIGSGIDMNPDGGTAMTADDIAPLPTPDVSE

>gi|30061829|ref|NP_836000.1| dehydrogenase subunit [Shigella flexneri 2a str. 2457T]
MNVNFFVTCIGDALKSRMARDSVLLLEKLGCRVNFPEKQGCCGQPAINSGYIKEAIPGMKNLIAALEDND
DPIISPAGSCTYAIKSYPTYLADEPEWASRAEKVAARMQDLTSFIVNKLGVVDVGASLQGRAVYHPSCSL
ARKLGVKDEPLTLLKNVRGLELLTFAEQDTCCGFGGTFSVKMAEISGEMVKEKVVHLMEVRPEYLIGADV
SCLLNISGRLQREGQKVKVMHIAEVLMSR

>gi|30061828|ref|NP_835999.1| hypothetical protein S0280 [Shigella flexneri 2a str. 2457T]
MSIKTSNTDFKTRIRQQIEDPIMRKAVASAQQRIGANRQKMVDELGHWEEWRDRAAQIRDHVLSNLDAYL
YQLSEKVTQNGGHVYFAKTKEDATRYILQVAQRKNARKVVKSKSMVTEEIGVNHVLQDAGIQVIETDLGE
YILQLDQDPPSHVVVPAIHKDRHQIRRVLHERLGYEGPETPEAMTLFIRQKIREDFLSAEIGITGCNFAV
AETGSVCLVTNEGNARMCTTLPKTHIAVMGMERIAPTFAEVDVLITMLARSAVGARLTGYNTWLTGPREA
GHVDGPEEFHLVIVDNGRSEVLASEFRDVLRCIRCGACMNTCPAYRHIGGHGYGSIYPGPIGAVISPLLG
GYKDFKDLPYACSLCTACDSVCPVRIPLSKLILRHRRVMAEKGITAKAEQRAIKMFAYANSHPGLWKVGM
MAGAHAASWFINGGKTPLKFGAISDWMEARDLPEADGESFRSWFKKHQAQEKKNG

>gi|30061825|ref|NP_835996.1| hypothetical protein S0277 [Shigella flexneri 2a str. 2457T]
MSEQIKQDIDLIEILFHLKKKIRVILFIMAICMAMVLLFLYINKDNTKVIYSLKINQTTPGILVSCDSNN
NFACQTTMTEDVIQRITTFFQTSPDIKNREIRLEWSGDKRDLPTAEEEISRVQASIIKWYASEYHNGRQV

LDEIQTPSAINSELYTKMIYLTRNWSLYPNGDGCVTISSPEIKNKYPAAICLALGFFLSIVISVMFCLVK

KMVDEYQQNSGQ

>gi|30061822|ref|NP_835993.1| taurine transporter substrate binding subunit [Shigella flexneri 2a str. 2457T]

MNVTVAYQTSAEPAKVAQADNTFAKESGASVDWRKFDSGASIVRALASGDVQIGNLGSSPLAVAASQQVP

IEVFLLASKLGNSEALVVKKTISKPEDLIGKRIAVPFISTTHYSLLAALKHWGIKPGLVEIVNLQPPAII

AAWQRGDIDGAYVWAPAVNALEKDGKVLTDSEQVGQWGAPTLDVWVVRKDFAEKHPEVVKAFAKSAIDAQ

QPYIANPDAWLKQPENISKLARLSGVPEGDVPGLVKGNTYLTPQQQTAELTGPVNKAIIDTAQFLKEQGK

VPAVANDYSQYVTSRFVQ

>gi|30061820|ref|NP_835991.1| taurine transporter subunit [Shigella flexneri 2a str. 2457T]

MSVLINEKLHSRRLKWRWPLSRQVTLSIGTLAVLLTVWWTVAALQLISPLFLPPPQQVLAKLLTIAGPQG

FMDATLWQHLAASLTRIVLALLAAVVIGIPVGIAMGLSPTVRGILDPVIELYRPVPPLAYLPLMVIWFGI

GETSKILLIYLAIFAPVAMSALAGVKSAQQVRIRAAQSLGASRAQVLWFVILPGALPEILTGLRIGLGVG

WSTLVAAELIAATRGLGFMVQSAGEFLATDVVLAGIAVIAIIAFLLELGLRALQRRLTPWHGEVQ

>gi|30061819|ref|NP_835990.1| taurine dioxygenase [Shigella flexneri 2a str. 2457T]

MSERLSITPLGPYIGAQISGADLTRPLSDNQFEQLYHAVLRHQVVFLRDQAITPQQQRALAQRFGELHIH

PVYPHAEGVDEIIVLDTHNDNPPDNDNWHTDVTFIETPPAGAILAAKELPSTGGDTLWTSGIAAYEALSV

PFRQLLSWLRAEHDFRKSFPEYKYRKTEEEHQRWREAVAKNPPLLHPVVRTHPVSGKQALFVNEGFTTRI

VDVSEKESEALLGFLFAHITKPEFQVRWRWQPNDIAIWDNRVTQHYANADYLPQRRIMHRATILGDKPFY

RAG

>gi|30061810|ref|NP_835981.1| transporter [Shigella flexneri 2a str. 2457T]

MFKSFFPKPGTFFLSAFVWALIAVIFWQAGGGDWVARITGASGQIPISAARFWSLDFLIFYAYYIVCVGL

FAFFWFIYSPHRWQYWSILGTALIIFVTWFLVEVGVAVNAWYAPFYDLIQTALSSPHKVTIEQFYREVGV

FLGIALIAVVISVLNNFFVSHYVFRWRTAMNEYYMANWQQLRHIEGAAQRVQEDTMRFASTLENMGVSFI

NAIMTLIAFLPVLVTLSAHVPELPIIGHIPYGLVIAAIVWSLMGTGLLAVVGIKLPGLEFKNQRVEAAYR

KELVYGEDDATRATPPTVRELFSAVRKNYFRLYFHYMYFNIARILYLQVDNVFGLFLLFPSIVAGTITLG

LMTQITNVFGQVRGAFQYLINSWTTLVELMSIYKRLRSFEHELDGDKIQEVTHTLS

>gi|30061809|ref|NP_835980.1| hypothetical protein S0257 [Shigella flexneri 2a str. 2457T]

MSRVNHLSSLSLLAVLVLAGCSSQAPQPLKKGEKAIDVASVVRQKMPASVKDRDAWAKDLATTFESQGLA

PTLENVYSVLAVAQQESNYQADPAVPGLSKIAWQEIDRRAERMHIPAFLVHTALKIKSPNGKSYSERLDS

VRTEKQLSAIFDDLISMVPMGQTLFGSLNPVRTGGPMQVSIAFAEQHTKGYPWKMDGTVRQEVFSRRGGL

WFGTYHLLNYPASYSAPIYRFADFNAGWYASRNAAFQNAVSKASGVKLALDGDLIRYDSKEPGKTELATR

KLAGKLGMSDSEIRRQLEKGDSFSFEETALYKKVYQLAEAKTGKSLPREMLPGIQLESPKITRNLTTAWF

AKRVDERRARCMKQ

>gi|30061808|ref|NP_835979.1| hypothetical protein S0256 [Shigella flexneri 2a str. 2457T]

MADFTLSKSLFSGKYRNASSTPGNIAYALFVLFCFWAGAQLLNLLVHAPGVYERLMQVQETGRPRVEIGL

GVGTIFGLIPFLAGCLIFAVVALWLHWRHRRQ

>gi|30061807|ref|NP_835978.1| hypothetical protein S0255 [Shigella flexneri 2a str. 2457T]

MNLRCESQNGNLKLNLLIITKMLSTPQTVVTEYARMLFFVNGAAMNLPVKIRRDWHYYAFAIGLIFILNG

VVGVLGFEAKGWQTYAVGLVTWVISFWLAGLIIRRRDEETENAQ

>gi|30061806|ref|NP_835977.1| D-alanyl-alanine synthetase A [Shigella flexneri 2a str. 2457T]

MEKLRVGIVFGGKSAEHEVSLQSAKNIVDAIDKSRFDVVLLGIDKQGQWHVSDASNYLLNADDPAHIALR

PSATSLAQVPGKHEHQLIDAQNGQPLPTVDVIFPIVHGTLGEDGSLQGMLRVANLPFVGSDVLASAACMD

KDVTKRLLRDAGLNIAPFITLTRANRHNISFAEVESKLGLPLFVKPANQGSSVGVSKVTSEEQYAIAVDL

AFEFDHKVIVEQGIKGREIECAVLGNDNPQASTCGEIVLTSDFYAYDTKYIDEDGAKVVVPAAIAPEIND

KIRAIAVQAYQTLGCAGMARVDVFLTPENEVVINEINTLPGFTNISMYPKLWQASGLGYTDLITRLIELA

LERHAADNALKTTM

>gi|30061803|ref|NP_835974.1| hypothetical protein S0251 [Shigella flexneri 2a str. 2457T]

MKRDGAMKITLLVTLLFGLVFLTTVGAAERTLTPQQQRMTSCNQQATAQALKGDARKTYMSDCLKNSKSA

PGEKSLTPQQQKMRECNNQATQQALKGDDRNKFMSACLKKAA

>gi|30061800|ref|NP_835971.1| DNA transfer protein [Shigella flexneri 2a str. 2457T]

MANYRDLLEQAGARYGVPEGLMTALGAKESSYNPAAVSSAGAVGLTQVMPGTWRDMGYTDEQMQNPEYQA

DAGARYLAKMYQQFGNWRDSLQAYHDGPGNVMKAKRGEYTPGPEGRGYVDDRFAQWAGDPVTDSTVEQRA

TSAKVHPQQDPNNPFAQLEVQSSEQVSASGVQSDPNNPFAQIEQQAASQQPPQPVSSVAPKPVQQQTVNQ

ANNAPTREEPSLMQQAGDWLTGGQSAGQIAEQAGRGLVNIPFDVLQGGASLINAISQGLGGPKVLDDVYR
PVDRPTDPYAQAGESIGGYLVPGAGVAGNMVIGSLADAANQRGDFAENAAINAGLSIATHGLINGVTRGV
RGASNIISGNKTSAQRATTAPTETSPFSGDAAAATNPAVHAAEARVAQGVPMTPATKNPEEVVRTVAAQK
RPNLASSLDELDINPQAEVLESAERLNVDSLLPSHFSGNEQYKAVEQAIKSRAGSALQVQENEAIRQLAQ
GAGEIIDRVSGAKDALGMSDKFIDTVNGRMSALMKRSDQLYRNVEKAMPAGAKIDAPSTRSMLKQVAEDL
GGMKNLDPIEKRVEVA

>gi|30061799|ref|NP_835970.1| prophage DNA injection protein [Shigella flexneri 2a str. 2457T]
MATWQGTNGGLLAGIGGVNSNAPSVNDIGNTLQLIRQNNDIERSGANNVGLTALQGLSGIAGVFQQEKQA
QRQKEFQQAYANAYASGDRGALRQLATQYPDQIESVRKGMGFIDEEQRNSIGTLAAGARLASSSPEAMQS
WLQNNAGELARVGVNPHDVAQMYQQNPRQFGEFVDHLGMNSLGPEKYFDLQDKMQGRQVTMRGQDLDSQT
AARNQAITMRGQDIQANLGQQRINLDAETNRINNENKRLDRMLSAETNDLKRQEIQSRIAANNQQLQQKQ
QALNDGYKDGINTLTTSMFTLNDIVSSPSLKSITGLRGVIPNVPGSQAADTQARLDTFKSQAYLTAVQAM
RGMGALSDAEGKKLDQAVGSLQNSQSEESFRRNAGVILNTLNQKRNEAVGKYVQQNGIKRVEAPQASIDY
LKQHPELSIDFINRYGYLPSLGQ

>gi|30061798|ref|NP_835969.1| DNA transfer protein [Shigella flexneri 2a str. 2457T]
MLYAFKLGRKLRGEEPYCPEKGGKGGSSDKSAKYAAEAQKYAADLQNQQWQTIMKNLAPFTPLAEQYVNQ
LQNLSSLEGQGQALNQYYNSKQYKDLAGQARYQSLAAAEATGGLGSTATSNQLATIAPTLGQSWLSNQMS
NYNNLANVGLGALQGQANAGQTYANNMSSIAQQSAALAAANANKPSSLQTAISGGTSGAIAGAGLASLLG
TSTPWGAGIGAGIGLLGSLF

>gi|30061797|ref|NP_835968.1| head assembly protein [Shigella flexneri 2a str. 2457T]
MITFKPTRNIDLIEAVGNHPDIIAGSNNGDGYDYKPECRYFEVNVHGQFGGIVYYQEIQPLTLDCHAMYL
PEIRGFSKEIGLAFWRYILTNTTVQCVTSFAARKFRHGQMYCAMIGLKRVGTIKKYFKGVDDVTFYSATR

EELIDFLNHGR

>gi|30061796|ref|NP_835967.1| packaged DNA stabilization protein [Shigella flexneri 2a str. 2457T]

MADSNLNVPVIIQATRLDTSVLPRNIFSQSYLLYVIAQGTDVGNVANKANEAGQGAYDAQVRNDEQDVIL

ADHEQRISAAEATLVNHEERIRQAESTLQDHETRIAQNESDISSLDTRVQSLESQVSDHETRIDALEYAT

TRKKSEVVYSGVSVTIPTAPTNLVSLLKTLTPSSGSLAPFFDTVNNKMVVFNENKTLLFKLSIVGTWPSG

TANRSMQLTFSGSVPDTLVSSRNAATTTDNILLATFFSVDKDGFLATNGSTLTIQSNGAAFTATTIKIIA

EQ

>gi|30061795|ref|NP_835966.1| packaged DNA stabilization protein [Shigella flexneri 2a str. 2457T]

MPIQQLPLMKGVGKDFRNADYIDYLPVNMLATPKEILNSSGYLRSFPGIAKRSDVNGVSRGVEYNMAQNA

VYRVCGGKLYKGESEVGDVAGSGRVSMAHGRTSQAVGVNGQLVEYRYDGTVKTVSNWPTDSGFTQYELGS

VRDITRLRGRYAWSKDGTDSWFITDLEDESHPDRYSAQYRAESQPDGIIGIGTWRDFIVCFGSSTIEYFS

LTGATTVGAALYVAQPSLMVQKGIAGTYCKTPFADSYAFISNPATGAPSVYIIDSGQVSPIASASIEKIL

RSYTADELADGVMESLRFDAHELLIIHLPRHVLVYDASSIANGPQWCVLKTGLYDDVYRAIDFVYEGNQI

TCGDKLESVTGKLQFDISSQYDKQQEHLLFTPLFKADNARVFDLEVESSTGVAQYADRLFLSATTDCINY

GREQMIEQNEPFVYDKRVLWKRVGRIRKNVGFKLRVITKSPVTLSGAQIRIE

>gi|30061794|ref|NP_835965.1| DNA stabilization protein [Shigella flexneri 2a str. 2457T]

MNLTTKGDLVLAALRKLGVASNATLTDVEPQSMEDGVNDLEMMMAEWLGGDASPGINVGYIFADADVAPD

PGDEHGLSNNAINAVIFNLACRIAPDYALEASAKLITTARYGKERLVKLSAMDRAKAAKCKSGYPNRMPV

GSGNQLAKWNGWNYFHRKEPCDNGSE

>gi|30061792|ref|NP_835963.1| coat protein [Shigella flexneri 2a str. 2457T]

MALNEGQLVTYALDEIIGTVQNLTPMASKVTKYPPPAESMQRSSNTVWMPVEQEAPTQTGWDLTGNATGI

LELSVKCNMGDPDNDFFELRADDLRDERSYRRRIQASAKKLANNIESAIAKQATEMGSLVVHDTRAIGPS

TGLPGWDFVSDAERLMFSRELNRDMGISYFLNPDDYRKAGRNLVDGDIFGRVPEEAYRNGTIQRQIAGFD

EILRSPKLPAVTKSTATGVTVSGAQKFKPQAYTLDTDGNKENVDNRVATVTVSSTTGFKRGDKISFIGVK

FLSQMAKNVLTDDATFSITRVIDGTHIEITPKPIALDDASLTKEEKAYANVNTSLADTTPVNVLNVATTT

ANVFWADDSIRLLSQPIPVTHELFAGMKTSSFSIPGIGVNGIFATQGDINTLSGKCRIAVWYSACAVRPE

AIGVGLPNQTA

>gi|30061791|ref|NP_835962.1| scaffolding protein [Shigella flexneri 2a str. 2457T]

MDQTTDIQASEELTLPGNHAAASADGLVVDNANDSAGQEEGFEIVRKDDEKPKQDPATNAEFARRRIERK

RQRELEQQMEAVKRGELPEHLRVNPELPKQPDPNDYLSEDALAKYDYDQSRALAAFQQANSEWQIKAMDA

RSQAVAEQGRKTQEFTQQSAQYVEAARKHYEAAEKLNIPDYQEKEDAFMQLVPPAVGADIMRLFPEKSAA

LMYHLGANPEKTRQLLAMDGQSALIELTRLSERLTLKPRAKPVSEAPLPDEPIQGHAVAANISAIEKQME

AAANKGDVETYRKLKAQLNKGIR

>gi|30061790|ref|NP_835961.1| packaging glycoprotein [Shigella flexneri 2a str. 2457T]

MADENRLNSILCKFDADWMASDEARTEATNDLYFSRVSQWDDWLSNYTTLQYRGQFDVVRPEVRKLVAEM

RRNPVDVLFRPKDGANPDAADVLMGMYRTDMRHNTAKIAVNVGVREQIESGVGAWRLVTQYEDNDPTSNN

QVIRRLPIHEACSHVIWDANSKQMDKSDAKHCTVINALSRNGWKEFAEDYGIDPDTLPSFQNPNDTWLFP

WVSNDVVYVAEYYEVEEEKEKVFIYRDPLTGEPVSYYQQDIKDVIDDLANRGFIKVAERKVKRRRVYKSI

ITCTQILKDREKIAGEHIPIVPVYGEWSFAGDKECYEGVVRLTKDGQRLRNMIMSFNADIVARSPKKKPT

FFPEQIEGYEYMYGGNDDYPYYLQNRTDENGNDLPIGPISYMENPEVPQANAYMLEAATNAVKEVASLGV

DAQAANGQVAFDTVNQLNMRADLETYVFQDNLATAMRRDGEIYASMVNDIYDVPRHVTLTLEDGSEKDVQ

LYAQVVDYQSGNVVTLNDIRGRYECYTDVGPSFQSMKEQNRAEIQELLTKVPQGTPEFQMLMLQYFTLLD

GKGVEMMREYANKQLVMMGLKKPETPEEMEMVQQAQQQPQQPSAEQIQAQGILLQGQAELLKAENQQAQI

QVEAAKVEAQNQLNAAKIAEIFNNMDLDKQAELREYLRLVGQFQQQRSKDARANAELLLKDADQTHSQRM

DFANLMRQVQIPSGGVAETPQ

>gi|30061786|ref|NP_835957.1| IS911 orfA [Shigella flexneri 2a str. 2457T]

MTRWVKQLRDERQGKTPKASPITPEQIEIRELRKKLQRIEMENEILKRLRALDVRLPEQFSIIGKLRAHY

PVVTLCHVFGVHRSSYRYWKNRPEKPDGRRAVLRSQVLELHGISHGSAGARSIATMATRRGYQMGRWLAG

RLMKELGLVSCQQPTHRYKRGGHEHVAIPNYLERQFAVTEPNQVWCGDVTYIWTGKRWAYLAVVLDLFAR

KPVGWAMSFSPDSRLTMKALEMAWETRGKPGGVMFHSDQGSHYTSRQFRQLLWRYQIRQSMSRRGNCWDN

SPMERFFRSLKNEWMPVVGYVSFSEAAHAITDYIVGYYSALRPHEYNGGLPPNESENRYWKNSNSVASFC

>gi|30061785|ref|NP_835956.1| hypothetical protein S0233, partial [Shigella flexneri 2a str. 2457T]

MTTSNTLSDYAVLVPERLEAILDRAAQRAGHEDISELLGCTVCSYAGDDTGIYLLPKRFASISFRSTKDA

KTVDVKVTRNSNTAGYDLELISVVDVLATGSVKVKAGEFDVEKDASFPLIHVIRFTTPRVTINPE

>gi|30061784|ref|NP_835955.1| hypothetical protein S0232 [Shigella flexneri 2a str. 2457T]

MMNPCGYSYGYTLIYLWSSPLVTLERVTEVLTLRQWPQSSRLERGQGGRGTDLCLLDTYRDFCEIFLIQG

HNLPPLAPTDRKVKTCKHQSNSYCGHTADCYYLSLPVEHLADQVDTEDELKQLL

>gi|30061782|ref|NP_835953.1| hypothetical protein S0230 [Shigella flexneri 2a str. 2457T]

MTQKYELIVKGIRNFENKVTVTLALRDKKRFDGEIFDLDISLDRVEGAALEFYEAAARRSIRQVFLDVAA

GLCEGDELLPETRPCSEARYTIKINSSDNSITGC

>gi|30061775|ref|NP_835946.1| hypothetical protein S0219, partial [Shigella flexneri 2a str. 2457T]

MKPNITWGLQKIQAHGIADNTIYATEGTQIIFTVTSPWIPVFKVNDDVKIALTLTRHEEAWWIINQSTEY

CCTVNDQIVEPHHRMRLNEGDLIEWGLSS

>gi|30061774|ref|NP_835945.1| hypothetical protein S0218 [Shigella flexneri 2a str. 2457T]

MLSPEQDLNRHADPFPFNEEPSSQDLDSLRDLVTEAETLQDMVTGGLSIDAILNVLDATGEDETIWPVEK

TPPDILHLLSPEYAPETAHNTVLPDLTRKEHRIIGIDSHYRINPAQHGEIYHDKQ

>gi|30061773|ref|NP_835944.1| hypothetical protein S0217 [Shigella flexneri 2a str. 2457T]

MTNNNELPITLSALLRDYSVVEGIQMAEQQVRMHPAQASRRHSLFQLLCVAGDWSRALQQIQLCARMDAN

YTREAQVFGELIRCEIYRHACFQGEQRPGVILPPPAWMEDLLTALACNARGEAQEADAHRSRALEAITDT

SGQWNGGAFDWISDSDSRTGPVLELIAGGAYIWLPFSQICSLKSPRPAHLIDLIWKPVNVTLNNGDTHSA

>gi|30061772|ref|NP_835943.1| hypothetical protein S0215 [Shigella flexneri 2a str. 2457T]

MSASRQRPGNHLLPTLFDRLCDDAPNQKRDHGISVSPVQIKEIIRRDLSFLLNTVSHEDDIDAARYPYAA

ASVLNYGLPPLAGSFLYEHKWDDIRRAILRTITRFEPRLKASTLQIIPLQDERRQSGHNTLQFEIRGEIL

TQPYPTAFRVRSALDMEQSRITFF

>gi|30061771|ref|NP_835942.1| outer membrane usher protein [Shigella flexneri 2a str. 2457T]

MDPRLLEYYNRELSYLRETGAEFAARHPKVAARLGMQGTDIADPYVERMVEAFSFLTARTQLKIDAEFPR

FTQRLLEVVSPNYVTPTPSMSVAQLHPDTEEGDLAKGFTVPRDTAFFSAIPEGESTACQFRSSQDVTLWP

LAIEEARLTAAPPDMPALHRYLPANIHVAGALRITLRTFGELTFSQLAGLDRLPFYLCGEERTASHLLEL

LHTSAIAPLAGIPGHFDGALDVNLQQPVMYEGLEPDQGLLPLAWNVFHGHNLLHEYFACPERFYFFTPTG

LSAGLQKIDGGVAEIVILLNRLPPDWLIHQTNAAQFSLFCTPVINLFPRVTARIDVTHSTTEQHLVVDRT

HPLDYEVFSVQEVEGLETDTTRKMAFRPLYHTRNNDEGNHGRYFSLRREPRRLSENARRYGTRTPYTGSE

VFLSLVDQYEAPYPENLRHITITAMVTNRDLPCLIARNGRDDLTVDAAIPVAGVGLIRPPRSPQPPMAER

EMAWRLIRQLSFNYLPLADLDHRTGGQALRDLLNLFIPAHDSPQSRQVRSLIGCKTTPVTRRLPGSGLLV

YGRGVSCELTVDEEGFSGISPYLFGLVLEHYIARHVSINTFSQMTLHSMQRGKIMTWPVRAGQRGSV

>gi|30061770|ref|NP_835941.1| periplasmic chaperone of fimbral assembly machinery [Shigella flexneri 2a str. 2457T]

MSNDFTQAQAPPWRYGFLNLMRRVDVQLCTVPAGNTWQPRMEKFRLGQTPALTFAPREIASVGWQEGRLH

ISLYSLVLWGPNGPLPLHYTELARNRTESRR

>gi|30061768|ref|NP_835939.1| hypothetical protein S0211 [Shigella flexneri 2a str. 2457T]

MEIFLSDPISAECPCGPDLEYDPEYLLLFTRAAPREEAQYGDFVSTPENINWAELERDAHRLLMRSKDIR

ILVVLLRCRIQQAGARGLSEALTLLETLCSTYPDAIHPQLLATEDITAEDAAVARSNALAALLDHEGVMA

DIRGITLSNNAAMRLQVRDVERSLSALRPADALAPESVRQQLADLEARGTLPLDAFRQAAETTERLQRHA

RETLNDQAPDFSRLTQLLALLPGAVQSTTPEILPQPQAEQPENATIVHTEQMQAEQIALPVHIAEEISPM

TGAEPRQIRDRNDALERLRVIRRWFEHSEPSSPTIPLLRQAERLVGKRVSEVINEIPVELLEKWDALE

>gi|30061767|ref|NP_835938.1| DNA polymerase III subunit epsilon [Shigella flexneri 2a str. 2457T]

MSTAITRQIVLDTETTGMNQIGAHYEGHKIIEIGAVEVVNRRLTGNNFHVYLKPDRLVDPEAFGVHGIAD

EFLLDKPTFAEVADEFMDYIRGAELVIHNAAFDIGFMDYEFSLLKRDIPKTNTFCKVTDSLAVARKMFPG

KRNSLDALCARYEIDNSKRTLHGALLDAQILAEVYLAMTGGQTSMAFAMEGETQQQQGEATIQRIVRQAS

KLRVVFATDEELAAHEARLDLVQKKGGSCLWRA

>gi|30061765|ref|NP_835936.1| hypothetical protein S0207 [Shigella flexneri 2a str. 2457T]

MMKPARVPQTVVAPDCWGDLPWGELYRKALERQLNPWFTKMYGFHLLKIGNLSAEINCEACAVSHQVNVS

AQGMPVQVQADPLHLPFADKSVDICLLAHTLPWCTDPHRLLREADRVLIDDGWLVISGFNPISLMGLRKL

VPVLRKTSPYNSRMFTLMRQLDWLSLLNFEVLHASRFHVLPWNKHGGKLLNAHIPALGCLQLIVARKRTI

PLTLNPMKQSKNKPRIRQAVGATRQCRKPQA

>gi|30061763|ref|NP_835934.1| membrane-bound lytic murein transglycosylase D [Shigella flexneri 2a str. 2457T]

MKAKAILLASVLLVGCQSTGNVQQHAQSLSAAGQGEAAKFTSQARWMDDGTSIAPDGDLWAFIGDELKMG

IPENDRIREQKQKYLRNKSYLHDVTLRAEPYMYWIAGQVKKRNMPMELVLLPIVESAFDPHATSGANAAG

IWQIIPSTGRNYGLKQTRNYDARRDVVASTTAALNMMQRLNKMFDDDWLLTVAAYNSGEGRVMKAIKTNK

ARGKSTDFWSLPLPQETKQYVPKMLALSDILKNSKRYGVRLPTTDESRALARVHLSSPVEMAKVADMAGI

SVSKLKTFNAGVKGSTLGASGPQYVMVPKKHADQLRESLASGEIAAVQSTLVANNTPLNSRVYTVRSGDT

LSSIASRLGVSTKDLQQWNKLRGSKLKPGQSLTIGAQRLANNSDSITYRVRKGDSLSSIAKRHGVNIKDV

MRWNSDTANLQPGDKLTLFVKNNNMPDS

>gi|30061761|ref|NP_835932.1| hypothetical protein S0203 [Shigella flexneri 2a str. 2457T]

MRKNTYAMRYVAGQPAERILPPGSFASIGQALPPGEPLSTEERIRILVWNIYKQQRAEWLSVLKNYGKDA

HLVLLQEAQTTPELVQFATANYLAADQVPAFVLPQHPSGVMTLSAAHPVYCCPLREREPILRLAKSALVT

VYPLPDTRLLMVVNIHAVNFSLGVDVYSKQLLPIGDQIAHHSGPVIMAGDFNAWSRRRMNALYRFAREMS

LRQVRFTDDQRRRAFGRPLDFVFYRGLNVSEASVLVTRASDHNPLLVEFSPGKPDK

>gi|30061760|ref|NP_835931.1| transcriptional regulator LYSR-type [Shigella flexneri 2a str. 2457T]

MKATSEELAIFVSVVESGSFSRAAEQLGQANSAVSRAVKKLEMKLGVSLLNRTTRQLSLTEEGERYFRRV

QSILQEMAAAESEIMETRNTPRGLLRIDAATPVVLHFLMPLIKPFRERYPEVTLSLVSSETIINLIERKV

DVAIRAGTLTDSSLRARPLFNSYRKIIASPDYISRYGKPETIDDLKQHVCLGFTEPASLNTWPIACSDGQ

LHEVKYGLSSNSGETLKQLCLSGNGIACLSDYMIDKEIARGELVELMADKVLPVEMPFSAVYYSDRAVST

RIRAFIDFLSEHVKTAPGGVMPIS

>gi|30061757|ref|NP_835928.1| D,D-heptose 1,7-bisphosphate phosphatase [Shigella flexneri 2a str. 2457T]

MAKSVPAIFLDRDGTINVDHGYVHEIDNFEFIDGVIDAMRELKKMGFALVVVTNQSGIARGKFTEAQFET

LTEWMDWSLADRDVDLDGIYYCPHHPQGSVEEFRQVCDCRKPHPGMLLSARDYLHIDMAASYMVGDKLED

MQAAVAANVGTKVLVRTGKPITPEAENAADWVLNSLADLPQAIKKQQKPAQ

>gi|30061755|ref|NP_835926.1| DL-methionine transporter permease subunit [Shigella flexneri 2a str. 2457T]

MSEPMMWLLVRGVWETLAMTFVSGFFGFVIGLPVGVLLYVTRPGQIIANAKLYRTVSAIVNIFRSIPFII

LLVWMIPFTRVIVGTSIGLQAAIVPLTVGAAPFIARMVENALLEIPTGLIEASRAMGATPMQIVCKVLLP

EALPGLVNAATITLITLVGYSAMGGAVGAGGLGQIGYQYGYIGYNATVMNTVLVLLVILVYLIQFAGDRI

VRAVTRK

>gi|30061754|ref|NP_835925.1| DL-methionine transporter substrate-binding subunit [Shigella flexneri 2a str. 2457T]

MAFKFKTFAAVGALIGSLALVGCGQDEKDPNHIKVGVIVGAEQQVAEVAQKVAKDKYGLDVELVTFNDYV

LPNEALSKGDIDANAFQHKPYLDQQLKDRGYKLVAVGNTFVYPIAGYSKKIKSLDELQDGSQVAVPNDPT

NLGRSLLLLQKVGLIKLKDGVGLLPTVLDVVENPKNLKIVELEAPQLPRSLDDAQIALAVINTTYASQIG

LTPAKDGIFVEDKDSPYVNLIVTREDNKDAENVKKFVQAYQSDEVYEAANKVFNGGAVKGW

>gi|30061753|ref|NP_835924.1| outer membrane lipoprotein [Shigella flexneri 2a str. 2457T]

MRALPICLVALMLSGCSMLSRSPVEPVQSTAPQPKAEPAKPKAPRATPVRIYTNAEELVGKPFRDLGEVS

GDSCQASNQDSPPSIPTARKQMQINASKMKANAVLLHSCEVTSGTPGCYRQAVCIGSALNITAK

>gi|30061750|ref|NP_835921.1| hypothetical protein S0186 [Shigella flexneri 2a str. 2457T]

MDKPKAYCRLLLPSFLLLSACTVDISQPDPSATAVDAEAKTWAVKFQHQSSFTEQSIKEITAPDLKPGDL

LFSSSLGVTSFGIRVFSTSSVSHVAIFLGDNNVAEATGAGVQIVSLKKAMKHSDKLFVLRVPDLTPQQAT

DITAFANKIKDSGYNYRGIVEFIPFMVTRQMCSLNPFSEDFRQQCVSDLAKAQLSSVCEGDKKSWFCSEF

VTDAFAKSGHPLTLAQSGWISPADLMHMRIGDVSAFKPETQLQYVGHLKPGIYIKAGRFVGLTQ

>gi|30061749|ref|NP_835920.1| lipoprotein involved with copper homeostasis and adhesion [Shigella flexneri 2a str. 2457T]

MVKKAIVTAMAVISLFTLMGCNNRAEVDTLSPAQAAELKPMPQSWRGVLPCADCEGIEISLFLEKDGTWV

MNERYLGAREEPSSFASYGTWARTADKLVLTDSKGEKSYYRAKGDALEMLDREGNPIESQFNYTLEPAQS

SLPMTPMTLRGMYFYMADAATFTDCATGKRFMVANNAELERSYLAARGHSEKPVLLSVEGHFTLEANPDT

GAPTKVLAPDTAGKFYPNKDCSSLGQ

>gi|30061747|ref|NP_835918.1| hypothetical protein S0183 [Shigella flexneri 2a str. 2457T]

MALKATIYKATVNVADLDRNQFLDASLTLARHPSETQERMMLRLLAWLKYADERLQFTRGLCADDEPEAW

LRNDHLGIDLWIELGLPDERRIKKACTQAAEVALFTYNSRAAQIWWQQNQSKCVQFANLSVWYLDDEQLA

KVSAFADRTMTLQATIQDGVIWLSDDKNNLEVNLTAWQQPS

>gi|30061745|ref|NP_835916.1| tRNA(Ile)-lysidine synthetase [Shigella flexneri 2a str. 2457T]

MTLTLNRQLLTSRQILVAFSGGLDSTVLLHQLVQWRTENPGVTLRAIHVHHGLSANADAWVTHCENVCQQ

WQVPLVVERVQLAQEGLGIEAQARQARYQAFARTLLPGEVLVTAQHLDDQCETFLLALKRGSGPAGLSAM

AEVSEFAGTRLIRPLLARTRGELEQWALAHGLRWIEDESNQDDSYDRNFLRLRVVPLLQQRWPHFAEATA

RSATLCAEQESLLDELLADDLAHCQTSQGTLQIAPMLAMSDARRAAIIRRWLAGQNAPMPSRDALVRIWQ

EVALAREDASPCLRLGAFEIRRYQSQLWWIKSVTGQSETIVLWQTWLQPLELPAGLGTVQLTAGGDIRPP

RADEAVSVRFKAPGLLHIVGRNGGRKLKKIWQELGVPPWLRDTTPLLFYGETLIAAAGVFVTQEGVAEGE

NGVSFVWQKTLS

>gi|30061743|ref|NP_835914.1| lysine decarboxylase 2 [Shigella flexneri 2a str. 2457T]

MNIIAIMGPHGVFYKDEPIKELESALVAQGFQIIWPQNSVDLLKFIEHNPRICGVIFDWDEYSLDLCSDI

NQLNEYLPLYAFINTHSTMDVSVQDMRMALWFFEYALGQAEDIAIRMRQYTNEYLDNITPPFTKALFTYV

KERKYTFCTPGHMGGTAYQKSPVGCLFYDFFGGNTLKADVSISVTELGSLLDHTGPHLEAEEYIARTFGA

EQSYIVTNGISTSNKIVGMYAAPSGSTLLIDRNCHKSLAHLLMMNDVVPVWLKPTRNALGILGGIPRREF

TRDSIEEKVAATTQAQWPVHAVITNSTYDGLLYNTDWIKQTLDVPSIHFDSAWVPYTHFHPIYQGKSGMS

GERVAGKVFFETQSTHKMLAALSQASLIHIKGEYDEEAFNEAFMMHTTTSPSYPIVASVKTAAAMLRGNP

GKRLINRSVERALHFRKEVQRLREESDGWFFDIWQPPQVDEAECRPVAPGEQWHGFNDADADHMFLDPVK

VTILTPGMDEQGNMSEEGIPAALVAKFLDERGIVVEKTGPYNLLFLFSIGIDKTKAMGLLRGLTEFKRSY

DLNLRIKNMLPDLYAEDPDFYRNMRIQDLAQGIHKLIRKHDLSGLMLRAFDTLPEMIMTPHQAWQRQIKG

EVETIALEQLVGRVSANMILPYPPGVPLLMPGEMLTKESRTVLDFLLMLCSVGQHYPGFETDIHGAKQDE

DGVYRVRVLKMAG

>gi|30061741|ref|NP_835912.1| DNA polymerase III subunit alpha [Shigella flexneri 2a str. 2457T]

MSEPRFVHLRVHSDYSMIDGLAKTAPLVKKAAALGMPALAITDFTNLCGLVKFYGAGHGAGIKPIVGADF

NVQCDLLGDELTHLTVLAANNTGYQNLALLISKAYQRGYGAAGPIIDRDWLIELNEGLILLSGGRMGDVG

RSLLRGNSALVDECVAFYEEHFPDRYFLELIRTGRPDEESYLHAAVELAEARGLPVVATNDVRFIDSSDF

DAHEIRVAIHDGFTLDDPKRPRNYSPQQYMRSEEEMCELFADIPEALANTVEIAKRCNVTVRLGEYFLPQ

FPTGDMSTEDYLVKRAKEGLEERLAFLFPDEEERLKRRPEYDERLETELQVINQMGFPGYFLIVMEFIQW

SKDNGVPVGPGRGSGAGSLVAYALKITDLDPLEFDLLFERFLNPERVSMPDFDVDFCMEKRDQVIEHVAD

MYGRDAVSQIITFGTMAAKAVIRDVGRVLGHPYGFVDRISKLIPPDPGMTLAKAFEAEPQLPEIYEADEE

VKALIDMARKLEGVTRNAGKHAGGVVIAPTKITDFAPLYCDEEGKHPVTQFDKSDVEYAGLVKFDFLGLR

TLTIINWALEMINKRRAKNGEPPLDIAAIPLDDKKSFDMLQRSETTAVFQLESRGMKDLIKRLQPDCFED

MIALVALFRPGPLQSGMVDNFIDRKHGREEISYPDVQWQHESLKSVLEPTYGIILYQEQVMQIAQVLSGY

TLGGADMLRRAMGKKKPEEMAKQRSVFAEGAEKNGINAELAMKIFDLVEKFAGYGFNKSHSAAYALVSYQ

TLWLKAHYPAEFMAAVMTADMDNTEKVVGLVDECWRMGLKILPPDINSGLYHFHVNDDGEIVYGIGAIKG

VGEGPIEAIIEARNKGGYFRELFDLCARTDTKKLNRRVLEKLIMSGAFDRLGPHRAALMNSLGDALKAAD

QHAKAEAIGQADMFGVLAEEPEQIEQSYASCQPWPEQVVLDGERETLGLYLTGHPINQYLKEIERYVGGV

RLKDMHPTERGKVITAAGLVVAARVMVTKRGNRIGICTLDDRSGRLEVMLFTDALDKYQQLLEKDRILIV

SGQVSFDDFSGGLKMTAREVMDIDEAREKYARGLAISLTDRQIDDQLLNRLRQSLEPHRSGTIPVHLYYQ

RADARARLRFGATWRVSPSDRLLNDLRGLIGSEQVELEFD

>gi|30061739|ref|NP_835910.1| lipid-A-disaccharide synthase [Shigella flexneri 2a str. 2457T]

MTDQRPLTIALVAGETSGDILGAGLIRALKERVPNARFVGVAGPRMQAEGCEAWYEMEELAVMGIVEVLG

RLRRLLHIRADLTKRFGELKPDVFVGIDAPDFNITLEGNLKKQGIKTIHYVSPSVWAWRQKRVFKIGRAT

DLVLAFLPFEKAFYDKYNVPCRFIGHTMADAMPLDPDKNGARDVLGIPYDAHCLALLPGSRGAEVEMLSA

DFLKTAQLLRQTYPDLEIVVPLVNAKRREQFERIKAEVAPDLSVHLLDGMGREAMVASDAALLASGTAAL

ECMLAKCPMVVGYRMKPFTFWLAKRLVKTDYVSLPNLLAGRELVKELLQEECEPQKLAAALLPLLANGKT

SHAMHDTFRELHQQIRCNADEQAAQAVLELAQ

>gi|30061738|ref|NP_835909.1| UDP-N-acetylglucosamine acyltransferase [Shigella flexneri 2a str. 2457T]

MIDKSAFVHPTAIVEEGASIGANAHIGPFCIVGPHVEIGEGTVLKSHVVVNGHTKIGRDNEIYQFASIGE

VNQDLKYAGEPTRVEIGDRNRIRESVTIHRGTVQGGGLTKVGSDNLLMINAHIAHDCTVGNRCILANNAT

LAGHVSVDDFAIIGGMTAVHQFCIIGAHVMVGGCSGVAQDVPPYVIAQGNHATPFGVNIEGLKRRGFSRE

AITAIRNAYKLIYRSGKTLDEVKPEIAELAETYPEVKAFTDFFARSTRGLIR

>gi|30061737|ref|NP_835908.1| (3R)-hydroxymyristoyl-ACP dehydratase [Shigella flexneri 2a str. 2457T]

MTTNTHTLQIEEILELLPHRFPFLLVDRVLDFEEGRFLRAVKNVSVNEPFFQGHFPGKPIFPGVLILEAM

AQATGILAFKSVGKLEPGELYYFAGIDEARFKRPVVPGDQMIMEVTFEKTRRGLTRFKGVALVDGKVVCE

ATMMCARSREA

>gi|30061736|ref|NP_835907.1| UDP-3-O-[3-hydroxymyristoyl] glucosamine N-acyltransferase [Shigella flexneri 2a str. 2457T]

MPSIRLADLAQQLDAELHGDGDIVITGVASMQSAQTGHITFMVNPKYREHLGLCQASAVVMTQDDLPFAK

SAALVVKNPYLTYARMAQILDTTPQPAQNIAPSAVIDATAKLGNNVSIGANAVIESGVELGDNVIIGAGC

FVGKNSKIGAGSRLWANVTIYHEIQIGQNCLIQSGTVVGADGFGYANDRGNWVKIPQIGRVIIGDRVEIG

ACTTIDRGALDDTVIGNGVIIDNQCQIAHNVVIGDNTAVAGGVIMAGSLKIGRYCMIGGASVINGHMEIC

DKVTVTGMGMVMRPITEPGVYSSGIPLQPNKVWRKTAALVMNIDDMSKRLKSLERKVNQQD

>gi|30061735|ref|NP_835906.1| periplasmic chaperone [Shigella flexneri 2a str. 2457T]

MKKWLLAAGLGLALATSAQAADKIAIVNMGSLFQQVAQKTGVSNTLENEFKGRASELQRMETDLQAKMKK

LQSMKAGSDRTKLEKDVMAQRQTFAQKAQAFEQDRARRSNEERGKLVTRIQTAVKSVANSQDIDLVVDAN

AVAYNSSDVKDITADVLKQVK

>gi|30061733|ref|NP_835904.1| zinc metallopeptidase RseP [Shigella flexneri 2a str. 2457T]

MLSFLWDLASFIVALGVLITVHEFGHFWVARRCGVRVERFSIGFGKALWRRTDKLGTEYVIALIPLGGYV

KMLDERAEPVVPELRHHAFNNKSVGQRAAIIAAGPVANFIFAIFAYWLVFIIGVPGVRPVVGEIAANSIA

AEAQIAPGTELKAVDGIETPDWDAVRLQLVDKIGDESTTITVAPFDSDQRRDVKLDLRHWAFEPDKEDPV

SSLGIRPRGPQIEPVLENVQPNSAASKAGLQAGDRIVKVDGQPLTQWVTFVMLVRDNPGKSLALEIERQG

SPLSLTLIPESKPGNGKAIGFVGIEPKVIPLPDEYKVVRQYGPFNAIVEATDKTWQLMKLTVSMLGKLIT

GDVKLNNLSGPISIAKGAGMTAELGVVYYLPFLALISVNLGIINLFPLPVLDGGHLLFLAIEKIKGGPVS

ERVQDFCYRIGSILLVLLMGLALFNDFSRL

>gi|30061730|ref|NP_835901.1| 1-deoxy-D-xylulose 5-phosphate reductoisomerase [Shigella flexneri 2a str. 2457T]

MKQLTILGSTGSIGCSTLDVVRHNPEHFRVVALVAGKNVTRMVEQCLEFSPRYAVMDDEASAKLLKTMLQ

QQGSRTEVLSGQQAACDMAALEEVDQVMAAIVGAAGLLPTLAAIRAGKTILLANKESLVTCGRLFMDAVK

QSKAQLLPVDSEHNAIFQSLPQPIQHNLGYADLEQNGVVSILLTGSGGPFRETPLRDLATMTPDQACRHP

NWSMGRKISVDSATMMNKGLEYIEARWLFNASASQMEVLIHPQSVIHSMVRYQDGSVLAQLGEPDMRTPI

AHTMAWPNRVNSGVKPLDFCKLSALTFAAPDYERYPCLKLAMEAFEQGQAATTALNAANEITVAAFLAQQ

IRFTDIAALNLSVLEKMDMREPQCVDDVLSVDANASEVARKEVMRLAS

>gi|30061728|ref|NP_835899.1| uridylate kinase [Shigella flexneri 2a str. 2457T]

MATNAKPVYKRILLKLSGEALQGTEGFGIDASILDRMAQEIKELVELGIQVGVVIGGGNLFRGAGLAKAG

MNRVVGDHMGMLATVMNGLAMRDALHRAYVNARLMSAIPLNGVCDSYSWAEAISLLRNNRVVILSAGTGN

PFFTTDSAACLRGIEIEADVVLKATKVDGVFTADPAKDPTATMYEQLTYSEVLEKELKVMDLAAFTLARD

HKLPIRVFNMNKPGALRRVVMGEKEGTLITE

>gi|30061724|ref|NP_835895.1| PII uridylyl-transferase [Shigella flexneri 2a str. 2457T]

MNTLPEQYANTALSTLPGQPQNPCAWPRDELTVCGIKAHIDTFQRWLGDAFDNGISAEQLIEARTEFIDQ

LLQRLWIEAGFSQIADLALVAVGGYGRGELHPLSDIDLLILSRKKLPDDQAQKVGELLTLLWDVKLEVGH

SVRTLEECMLEGLSDLTVATNLIESRLLIGDVALFLELQKHIFSEGFWPSDKFYAAKVEEQNQRHQRYHG

TSYNLEPDIKSSPGGLRDIHTLQWVARRHFGATSLDEMVGFGFLTSAERAELNECLHILWRIRFALHLVV

SRYDNRLLFDRQLSVAQRLNYSGEGNEPVERMMKDYFRVTRRVSELNQMLLQLFDEAILALPADEKPRPL

DDEFQLRGTLIDLRDETLFMRQPEAILRMFYTMVRNSAITGIYSTTLRQLRHARRHLQQPLCNIPQARKL

FLSILRHPGAVRRGLLPMHRHSVLGAYMPQWSHIVGQMQFDLFHAYTVDEHTIRVMLKLESFASEETRQR

HPLCVDVWPRLPSTELIFIAALFHDIAKGRGGDHSILGAQDVVHFAELHGLNSRETQLVAWLVRQHLLMS

VTAQRRDIQDPEVIKQFAEEVQTENRLRYLVCLTVADICATNETLWNSWKQSLLRELYFATEKQLRRGMQ

NTPDMRERVRHHQLQALALLRMDNIDEEALHQIWSRCRANYFVRHSPNQLAWHARHLLQHDLSKPLVLLS

PQATRGGTEIFIWSPDRPYLFAAVCAELDRRNLSVHDAQIFTTRDGMAMDTFIVLEPDGSPLSADRHEVI

RFGLEQVLTQSSWQPPQPRRQPAKLRHFTVETEVTFLPTHTDRKSFLELIALDQPGLLARVGKIFADLGI

SLHGARITTIGERVEDLFIIATADRRALNNELQQEVHQRLTEALNPNDKG

>gi|30061723|ref|NP_835894.1| 2,3,4,5-tetrahydropyridine-2,6-carboxylate N-succinyltransferase [Shigella flexneri 2a str. 2457T]

MQQLQNIIETAFERRAEITPANADTVTREAVNQVIALLDSGALRVAEKIDGQWVTHQWLKKAVLLSFRIN

DNQVIEGAESRYFDKVPMKFADYDEARFQKEGFRVVPPAAVRQGAFIARNTVLMPSYVNIGAYVDEGTMV

DTWATVGSCAQIGKNVHLSGGVGIGGVLEPLQANPTIIEDNCFIGARSEVVEGVIVEEGSVISMGVYIGQ

STRIYDRETGEIHYGRVPAGSVVVSGNLPSKDGKYSLYCAVIVKKVDAKTRGKVGINELLRTID

>gi|30061722|ref|NP_835893.1| hypothetical protein S0158 [Shigella flexneri 2a str. 2457T]

MYDNLKSLGITNPEEIDRYSLRQEANNDILKIYFQKDKGEFFAKSVKFKYPRQRKTVVADGVGQGYKEVQ

EISPNLRYIIDELDQICQRDRSEVDLKRKILDDLRHLESVVTNKISEIEADLEKLTRK

>gi|30061720|ref|NP_835891.1| deoxyguanosinetriphosphate triphosphohydrolase [Shigella flexneri 2a str. 2457T]

MAQIDFRKKINWHRRYRSPQGVKTEHEILRIFESDRGRIINSPAIRRLQQKTQVFPLERNAAVRTRLTHS

MEVQQVGRYIAKEILSRLKELKLLEAYGLDELTGPFESIVEMSCLMHDIGNPPFGHFGEAAINDWFRQRL

HPEDAESQPLTDDRCSVAALRLRDGEEPLNELRRKIRQDLCHFEGNAQGIRLVHTLMRMNLTWAQVGGIL

KYTRPAWWRGETPETHHYLMKKPGYYLSEEAYIARLRKELNLALYSRFPLTWIMEAADDISYCVADLEDA

VEKRIFTVEQLYHHLHEAWGQHEKGSLFSLVVENAWEKSRSNSLSRSTEDQFFMYLRVNTLNKLVPYAAQ

RFIDNLPAIFAGTFNHALLEDASECSDLLKLYKNVAVKHVFSHPDVEQLELQGYRVISGLLEIYRPLLSL

SLSDFTELVEKERVKRFPIESRLFHKLSTRHRLAYVEAVSKLPSDSPEFPLWEYYYRCRLLQDYISGMTD

LYAWDEYRRLMAVEQ

>gi|30061719|ref|NP_835890.1| 5'-methylthioadenosine/S-adenosylhomocysteine nucleosidase [Shigella flexneri 2a str. 2457T]

MKIGIIGAMEEEVTLLRDKIENRQTISLGGCEIYTGQLNGTEVALLKSGIGKVAAALGATLLLEHCKPDV

IINTGSAGGLAPTLKVGDIVVSDEARYHDADVTAFGYEYGQLPGCPAGFKADDKLIAAAEACIAELNLNA

VRGLIVSGDAFINGSVGLAKIRHNFPQAIAVEMEATAIAHVCHNFNVPFVVVRAISDVADQQSHLSFDEF

LAVAAKQSSLMVESLVQKLAHG

>gi|30061718|ref|NP_835889.1| vitamin B12-transporter protein BtuF [Shigella flexneri 2a str. 2457T]

MAKSLFRALVALSFLAPLWLNAAPRVITLSPANTELAFAAGITPVGVSSYSDYPPQAQKIEQVSTWQGMN

LERIVALKPDLVIAWRGGNAERQVDQLASLGIKVMWVDATSIEQIANALRQLAPWSPQPDKAEQAAQSLL

DQYVQLKAQYADKPKKRVFLQFGINPPFTSGKESIQNQVLEVCGGENIFKDSRVPWPQVSREQVLARSPQ

AIVITGGPDQIPKIKQYWGEQLKIPVIPLTSDWFERASPRIILAAQQLCNALSQVD

>gi|30061717|ref|NP_835888.1| hypothetical protein S0152 [Shigella flexneri 2a str. 2457T]

MLVYWLDIVGTAVFAIYGVLLAGKLRMDPFGVLVLGVVTAVGDGTIRDMALDHGPVFWVKDPTDLVVAMV

TSMLTIVLVRQPRRLPKWMLPVLDAVGLAVFVGISVNKAFNAEAGPLIAVCMGVITGVGGGIIRDVLVRE

IPMILRTEIYATACIIGGIVHATAYYTFSVPLETASMMGMVVTLLIRLAAIRWHLKLPTFALDENGR

>gi|30061713|ref|NP_835884.1| iron-hydroxamate transporter permease subunit [Shigella flexneri 2a str. 2457T]

MSKRIALFPALLLALLVIVATALTWMNFSQALPRSQWAQAAWSPDIDVIEQMIFHYSLLPRLAISLLVGA

GLGLVGVLFQQVLRNPLAEPTTLGVATGAQLGITVTTLWAIPSAMASQFAALAGACVVGLIVFGVAWGKR

LSPVTLIFAGLVVSLYCGAINQLLVIFHHDQLQSMFLWSTGTLTQTDWGGVERLWPQLLGGVMLTLLLLR

PLTLMGLDDGVARNLGLALSLARLAALSLAIVISALLVNAVGIIGFIGLFAPLLAKMLGARRLLPRLMLA

SLIGALILWLSDQIILWLTRVWMEVSTGSVTALIGAPLLLWLLPRLRSISAPDMKVNDRVATERQHVLAF

ALAGGVLLLMAVVVALSFGRDAHGWTWASGALLEDLMPWRWPRIMAALFAGVMLAVAGCIIQRLTGNPMA

SPEVLGISSGAAFGVVLMLFLVPGNAFGWLLPAGSLGAAVTLLIIMIAAGRGGFSPHRMLLAGMALSTAF

TMLLMMLQASGDPRMAQVLTWISGSTYNATDAQVWRTGIVMVILLAITPLCRRWLTILPLGGDTARAVGM

ALTPTRIALLLLAACLTATATMTIGPLSFVGLMAPHIARMMGFRRTMPHIVISALVGGLLLVFADWCGRM

VLFPFQIPAGLLSTFIGAPYFIYLLRKQSR

>gi|30061712|ref|NP_835883.1| iron-hydroxamate transporter substrate-binding subunit [Shigella flexneri 2a str. 2457T]

MSGLPLISRRRLLTAMALSPLLWQMNTAHAAAIDPNRIVALEWLPVELLLALGIVPYGVADTINYRLWVS

EPPLPDSVIDVGLRTEPNLELLTEMKPSFMVWSAGYGPSSEMLARIAPGRGFNFSDGKHPLAMARKSLTE

MADLLNLQSAAETHLAQYEDFIRSMKPRFVKRGARPLLLTTLIDPRHMLVFGPNSLFQEILDEYGIPNAW

QGETNFWGSTAVSIDRLAAYKDVDVLCFDHDNSKDMDALMATPLWQAMPFVRTGRFQRVPAVWFYGATLS

AMHFVRVLDNAIGGKA

>gi|30061710|ref|NP_835881.1| ferrichrome outer membrane transporter [Shigella flexneri 2a str. 2457T]

MARSKTAQPKHSLRKIAVVVATAVSGMSVYAQAAVEPKEDTITVTAAPAPQESAWGPAATIAARQSATGT

KTDTPIQKVPQSISVVTAEEMALHQPKSVKEALSYTPGVSVGTRGASNTYDHLIIRGFAAEGQSQNNYLN

GLKLQGNFYNDAVIDPYMLERAEIMRGPVSVLYGKSSPGGLLNMVSKRPTTEPLKEVQFKAGTDSLFQTG

FDFSDALDDDGVYSYRLTGLARSANAQQKGSEEQRYAIAPAFTWRPDDKTNFTFLSYFQNEPETGYYGWL

PKEGTVEPLPNGKRLPTDFNEGAKNNTYSRNEKMVGYSFDHEFNDTFTVRQNLRFAENKTSQNSVYGYGV

CSDPANAYSKQCAALAPADKGHYLARKYVVDDEKLQNFSVDTQLQSKFATGDIDHTLLTGVDFMRMRNDI

NAWFGYDDSVPLFNLYNPVNPDFDFNAKDPANSGPYRILNKQKQTGVYVQDQAQWDKVLVTLGGRYDWAD

QESLNRVAGTTDKRDDKQFTWRGGVNYLFDNGVTPYFSYSESFEPSSQVGKDGNIFAPSKGKQYEVGVKY

VPEDRPIVVTGAVYNLTKTNNLMADPEGSFFSVEGGEIRARGVEIEAKAALSASVNVVGSYTYTDAEYTT

DTTYKGNTPAQVPKHMASLWADYTFFDGPLSGLTLGTGGRYTGSSYGDPANSFKVGSYTVVDALVRYDLA

RVGMAGSNVALHVNNLFDREYVASCFNTYGCFWGAERQVVATATFRF

>gi|30061709|ref|NP_835880.1| penicillin-binding protein 1b [Shigella flexneri 2a str. 2457T]

MAGNDREPIGRKGKPTRPVKQKVSRRRYEDDDDYDDYDDYEDEEPMPRKGKGKGKGRKPRGKRGWLWLLL

KLAIVFAVLIAIYGVYLDQKIRSRIDGKVWQLPAAVYGRMVNLEPDMTISKNEMVKLLEATQYRQVSKMT

RPGEFTVQANSIEMIRRPFDFPDSKEGQVRARLTFDGDHLATIVNMENNRQFGFFRLDPRLITMISSPNG

EQRLFVPRSGFPDLLVDTLLATEDRHFYEHDGISLYSIGRAVLANLTAGRTVQGASTLTQQLVKNLFLSS

ERSYWRKANEAYMALIMDARYSKDRILELYMNEVYLGQSGDNEIRGFPLASLYYFGRPVEELSLDQQALL

VGMVKGASIYNPWRNPKLALERRNLVLRLLQQQQIIDQELYDMLSARPLGVQPRGGVISPQPAFMQLVRQ

ELQAKLGDKVKDLSGVKIFTTFDSVAQDAAEKAAVEGIPALKKQRKLSDLETAIVVVDRFSGEVRAMVGG

SEPQFAGYNRAMQARRSIGSLAKPATYLTALSQPKIYRLNTWIADAPIALRQPNGQVWSPQNDDRRYSES

GRVMLVDALTRSMNVPTVNLGMALGLPAVTETWIKLGVPKDQLHPVPAMLLGALNLTPIEVAQAFQTIAS

GGNRAPLSALRSVIAEDGKVLYQSFPQAERAVPAQAAYLTLWTMQQVVQRGTGRQLGAKYPNLHLAGKTG

TTNNNVDTWFAGIDGSTVTITWVGRDNNQPTKLYGASGAMSIYQRYLANQTPTPLNLVPPEDIADMGVDY

DGNFVCSGGMRVLPVWTSDPQSLCQQSEMQQQPSGNPFDQSSQPQQQPQQQPAQQEQKDSDGVAGWIKDM

FGSN

>gi|30061707|ref|NP_835878.1| hypothetical protein S0142 [Shigella flexneri 2a str. 2457T]

MPHMSEPQRLFFAIDLPAEIREQIIHWRATHFPPEAGRPVAADNLHLTLAFLGEVSAEKEKALSLLAGRI

RQPGFTLTLDDAGQWLRSRVVWLGMRQPPRGLIQLANMLRSQAARSGCFQSNRPFHPHITLLRDASEAVT

FPPPGFNWSYAVTEFTLYASSFARGRTRYTPLKRWALTQ

>gi|30061706|ref|NP_835877.1| sugar fermentation stimulation protein A [Shigella flexneri 2a str. 2457T]

MEFSPPLQRATLIQRYKRFLADVITPDGRELTLHCPNTGAMTGCATPGDTVWYSTSDNTKRKYPHTWELT

QSQSGAFICVNTLWANRLTKEAILNESISELSGYSSLKSEVKYGSERSRIDFMLQADSRPDCYIEVKSVT

LAENEQGYFPDAVTERGQKHLRELMSVAAEGQRAVIFFAVLHSAITRFSPARHIDEKYAQLLSEAQQRGV

EILAYKAEISAEGMALKKSLPVTL

>gi|30061705|ref|NP_835876.1| DnaK transcriptional regulator DksA [Shigella flexneri 2a str. 2457T]

MQEGQNRKTSSLSILAIAGVEPYQEKPGEEYMNEAQLAHFRRILEAWRNQLRDEVDRTVTHMQDEAANFP

DPVDRAAQEEEFSLELRNRDRERKLIKKIEKTLKKVEDEDFGYCESCGVEIGIRRLEARPTADLCIDCKT

LAEIREKQMAG

>gi|30061702|ref|NP_835873.1| 2-amino-4-hydroxy-6-hydroxymethyldihydropteridine pyrophosphokinase [Shigella flexneri 2a str. 2457T]

MTVAYIAIGSNLASPLEQVNAALKALGDIPESRILAVSSFYRTPPLGPQDQPDYLNAAVALETSPAPEEL

LNHTQRIELQQGRVRKAERWGPRTLDLDIMLFGNEVINTERLTVPHYDMKNRGFMLWPLFEIAPELAFPD

GETLREVLHTRAFDKLSKW

>gi|30061699|ref|NP_835870.1| 3-methyl-2-oxobutanoate hydroxymethyltransferase [Shigella flexneri 2a str. 2457T]

MKPTTIASLQKYKQDKKRFATITAYDYSFAKLFADEGLNVMLVGDSLGMTVQGHDSTLPVTVADIAYHTA

AVRRGAPNCLLLADLPFMAYATPEQAFENAATVMRAGANMVKIEGGEWLVETVKMLTERAVPVCGHLGLT

PQSVNIFGGYKVQGRGNEASDRLLSDALALEAAGAQLLVLECVPVELAKRITEALAIPVIGIGAGNVTDG

QILVMHDAFGITGGHIPKFAKNFLAETGDIRAAVRQYMAEVESGVYPGEEHSFH

>gi|30061698|ref|NP_835869.1| pantoate--beta-alanine ligase [Shigella flexneri 2a str. 2457T]

MLIIETLPLLRQQIRRLRMEGKRVALVPTMGNLHNGHMKLVDEAKARADVVVVSIFVNPMQFDRPEDLAR

YPRTLQEDCEKLNKRKVDLVFAPSVKEIYPNGTETHTYVDVPGLSTMLEGASRPGHFRGVSTIVSKLFNL

VQPDIACFGEKDFQQLALIRKMVADMGFDIEIVGVPIMRAKDGLALSSRNGYLTAEQRKIAPGLYKVLSS

IADKLQAGERDLDEIITIAGQELNEKGFRADDIQIRDADTLLEVSETSKRAVILVAAWLGDARLIDNKMV

ELA

>gi|30061697|ref|NP_835868.1| hypothetical protein S0131 [Shigella flexneri 2a str. 2457T]

MDAPSTTPHDAVFKQFLMHAETARDFLEIHLPVELRELCDLNTLHLESGSFIEECLKGHSTDVLYSMQMQ

GNPGYLHVVIEHQSKPDKKMAFRMMRYSIAAMHRHLEAGHDKLPLVVPILFYQGEATPYPLSMCWFDMFY
SPELARRVYNSPFPLVDITITPDDEIMQHRRIAILELLQKHIRQRDLMLLLEQLVTLIDEGYTSGSQLVA
MQNYMLQRGHTEQADLFYGVLRDRETGGKSMMTLAQWFEEKGIEKGIQQGRQEERQEFALRLLSKGMSRE
DVAEMANLPLAEIDKVINLI

>gi|30061696|ref|NP_835867.1| aspartate alpha-decarboxylase [Shigella flexneri 2a str. 2457T]
MIRTMLQGKLHRVKVTHADLHYEGSCAIDQDFLDAAGILENEAIDIWNVTNGKRFSTYAIAAERGSRIIS
VNGAAAHCASVGDIVIIASFVTMPDEEARTWRPNVAYFEGDNEMKRTAKAIPVQVA

>gi|30061695|ref|NP_835866.1| hypothetical protein S0129 [Shigella flexneri 2a str. 2457T]
MYKQAVILLLMLFTASVSAALPARYMQTIENAAVWAQISDKMVTVGNIRAGQIIAVEPTAASYYAFNFGF
GKGFIDKGHLEPVQGRQKVEDGLGDLNKPLSNQNLITWKDTPVYNAPSVGSAPFGVLADNLRYPILHKLK
DRLNQTWYQIRIGDRLAYISALDAQPDNGLPVLTYHHILRDEENTRFRHTSTTTSVRAFNNQMAWLRDRG
YATLSMAQLEGYVKNTINLPARAVVITFDDGLKSVSRYAYPVLKQYGMKATVFIVTSRIKRHPQKWNPKS
LQFMSVSELNEIRDVFDFQSHTHFLHRVDGYRRPILLSRSEHNILFDFARSRRALAQFNPHVWYLSYPFG
GFNDKAVKAANDAGFHLAVTTMKGKVKPGDNPLLLKRLYILRTDSLEKMSRLVSNQPQG

>gi|30061694|ref|NP_835865.1| PTS enzyme II B component [Shigella flexneri 2a str. 2457T]
MLGWVITCHDDRAQEILDALEKKHGALLQCRAVNFWRGLSSNMLSRMMCDALHEADSGEGVIFLTDIAGA
PPYRVASLLSHKHSRCEVISGVTLPLIEQMMACRETMTSSEFRECIVELGGPEVSSLWHQQQKNPPFVLK
HNLYEY

>gi|30061693|ref|NP_835864.1| hypothetical protein S0127 [Shigella flexneri 2a str. 2457T]
MMHLYWVALKSIWAKEIHRFMRIWVQTLVPPVITMTLYFIIFGNLIGSRIGDMHGFSYMQFIVPGLIMMS
VITNAYANVASSFFGAKFQRNIEELLVAPVPTHVIIAGYVGGGVARGLFVGILVTAISLFFVPFQVHSWV

FVALTLVLTAVLFSLAGLLNGVFAKTFDDISLVPTFVLTPLTYLGGVFYSLTLLPPFWQGLSHLNPIVYM

ISGFRYGFLGINDVPLVTTFGVLVVFIVAFYLICWSLIQRGRGLRS

>gi|30061691|ref|NP_835862.1| carbonic anhydrase [Shigella flexneri 2a str. 2457T]

MKDIDTLISNNALWSKMLVEEDPGFFEKLAQAQKPRFLWIGCSDSRVPAERLTGLEPGELFVHRNVANLV

IHTDLNCLSVVQYAVDVLEVEHIIICGHYGCGGVQAAVENPELGLINNWLLHIRDIWFKHSSLLGEMPQE

RRLDTLCELNVMEQVYNLGHSTIMQSAWKRGQKVTIHGWAYGIHDGLLRDLDVTATNRETLEQRYRHGIS

NLKLKHANHK

>gi|30061689|ref|NP_835860.1| glucose dehydrogenase [Shigella flexneri 2a str. 2457T]

MAINNTASRRLLVTLTALFAALCGLYLLIGGGWLVAIGGSWYYPIAGLVMLGVAWMLWRSKRAALWLYAA

LLLGTMIWGVWEVGFDFWALTPRSDILVFFGIWLILPFVWRRLVIPASGAVAALVVALLISGGILTWAGF

NDPQEINGTLSADATPAEAISPVADQDWPAYGRNQEGQRFSPLKQIHADNVHKLKEAWVFRTGDVKQPND

PGEITNEVTPIKVGDTLYLCTAHQRLFALDAASGKEKWHYDPELKTNESFQHVTCRGVSYHEAKAETASP

EVMADCPRRIILPVNDGRLIAINAENGKLCETFANKGVLNLQSNMPDTKPGLYEPTSPPIITDKTIVMAG

SVTDNFSTRETSGVIRGFDVNTGELLWAFDPGAKDPNAIPSDEHTFTFNSPNSWAPAAYDAKLDLVYLPM

GVTTPDIWGGNRTPEQERYASSILALNATTGKLAWSYQTVHHDLWDMDLPAQPTLADITVNGQKVPIIYA

PAKTGNIFVLDRRNGELVVPAPEKPVPQGAAKGDYVTPTQPFSELSFRPTKDLSGADMWGATMFDQLVCR

VMFHQMRYEGIFTPPSEQGTLVFPGNLGMFEWGGISVDPNREVAIANPMALPFVSKLLPRGPGNPMEQPK

DAKGTGTESGIQPQYGVPYGVTLNPFLSPFGLPCKQPAWGYISALDLKTNEVVWKKRIGTPQDSMPFPMP

VPVPFNMGMPMLGGPISTAGNVLFIAATADNYLRAYNMSNGEKLWQGRLPAGGQATPMTYEVNGKQYVVI

SAGGHGSFGTKMGDYIVAYALPDDVK

>gi|30061688|ref|NP_835859.1| multicopper oxidase [Shigella flexneri 2a str. 2457T]

MQRRDFLKYSVALGVASALPLWSRAVFAAERPTLPIPDLLTTDARNRIQLTIGAGQSTFGEKTATTWGYN

GNLLGPAVKLQRGKAVTVDIYNQLAEETTLHWHGLEVPGDVDGGPQGIIPPGGKRSVTLNVDQPAATCWF

HPHQHGKTGRQVAMGLAGLVVIEDDEILKLMLPKQWGIDDVPVIVQDKKFSADGQIDYQLDVMTAAVGWF

GDTLLTNGAIYPQHAAPRGWLRLRLLNGCNARSLNFATSDNRPLYVIASDGGLLPEPVKVSELPVLMGER

FEVLVEVNDNKPFDLVTLPVSQMGMAIAPFDKPHPVMRIQPIAISASGALPDTLSSLPALPSLEGLTVRK

LQLSMDPMLDMMGMQMLMEKYGDQAMAGMDHSQMMGHMGHGNMNHMNHGGKFDFHHANKINGQAFD
MNKP

MFAAAKGQYERWVISGVGDMMLHPFHIHGTQFRILSENGKPPAAHRAGWKDTVKVEGNVSEVLVKFNHNA

PKEHAYMAHCHLLEHEDTGMMLGFTV

>gi|30061687|ref|NP_835858.1| hypothetical protein S0121 [Shigella flexneri 2a str. 2457T]

MSIVLPLTGRSSRRHNLIDNNGRRLARSVLTFIFFKPLVEAMKTFFRTVLFGSLIAVCANSYALSESEAE

DMADLTAVFVFLKNDCGYQNLSNGQIRRALVFFAQQNQWDLSNYDTFDMKALGEDSYRDLSGIGIPVAKK

CKALARDSLSLLAYVK

>gi|30061685|ref|NP_835856.1| S-adenosylmethionine decarboxylase [Shigella flexneri 2a str. 2457T]

MKKLKLHGFNNLTKSLSFCIYDICYAKTAEERDGYIAYIDELYNANRLTEILSETCSIIGANILNIARQD

YEPQGASVTILVSEEPVDPKLIDKTEHPGPLPETVVAHLDKSHICVHTYPESHPEGGLCTFRADIEVSTC

GVISPLKALNYLIHQLESDIVTIDYRVRGFTRDINGMKHFIDHEINSIQNFMSDDMKALYDMVDVNVYQE

NIFHTKMLLKEFDLKHYMFHTKPEDLTDSERQEITAALWKEMREIYYGRNMPAV

>gi|30061683|ref|NP_835854.1| bifunctional aconitate hydratase 2/2-methylisocitrate dehydratase [Shigella flexneri 2a str. 2457T]

MLEEYRKHVAERAAEGIAPKPLDANQMAALVELLKNPPAGEEEFLLDLLTNRVPPGVDEAAYVKAGFLAA

IAKGEAKSPLLTPEKAIELLGTMQGGYNIHPLIDALDDAKLAPIAAKALSHTLLMFDNFYDVEEKAKAGN

EYAKQVMQSWADAEWFLNRPALAEKLTVTVFKVTGETNTDDLSPAPDAWSRPDIPLHALAMLKNAREGIE
PDQPGVVGPIKQIEALQQKGFPLAYVGDVVGTGSSRKSATNSVLWFMGDDIPHVPNKRGGGLCLGGKIAP
IFFNTMEDAGALPIEVNVSNLNMGDVIDVYPYKGEVRNHETGELLATFELKTDVLIDEVRAGGRIPLIIG
RGLTTKAREALGLPHSDVFRQAKDVAESDRGFSLAQKMVGRACGVKGIRPGAYCEPKMTSVGSQDTTGPM
TRDELKDLACLGFSADLVMQSFCHTAAYPKPVDVNTHHTLPDFIMNRGGVSLRPGDGVIHSWLNRMLLPD
TVGTGGDSHTRFPIGISFPAGSGLVAFAAATGVMPLDMPESVLVRFKGKMQPGITLRDLVHAIPLYAIKQ
GLLTVEKKGKKNIFSGRILEIEGLPDLKVEQAFELTDASAERSAAGCTIKLNKEPIIEYLSSNIVLLKWM
IAEGYGDRRTLERRIQGMEKWLANPELLEADADAEYAAVIDIDLAEIKEPILCAPNDPDDARPLSAVQGE
KIDEVFIGSCMTNIGHFRAAGKLLDAHKGQLPTRLWVAPPTRMDAAQLTEEGYYSVFGKSGARIEIPGCS
LCMGNQARVADGATVVSTSTRNFPNRLGTGANVFLASAELAAVAALIGKLPTPEEYQTYVAQVDKTAVDT
YRYLNFNQLSQYTEKADGVIFQTAV

>gi|30061679|ref|NP_835850.1| transcriptional regulator PdhR [Shigella flexneri 2a str. 2457T]
MAYSKIRQPKLSDVIEQQLEFLILEGTLRPGEKLPPERELAKQFDVSRPSLREAIQRLEAKGLLLRRQGG
GTFVQSSLWQSFSDPLVELLSDHPESQYDLLETRHALEGIAAYYAALRSTDEDKERIRELHHAIELAQQS
GDLDAESNAVLQYQIAVTEAAHNVVLLHLLRCMEPMLAQNVRQNFELLYSRREMLPLVSSHRTRIFEAIM
VGKPEEAREASHRHLAFIEEILLDRSREESRRERSLRRLEQRKN

>gi|30061677|ref|NP_835848.1| regulatory protein AmpE [Shigella flexneri 2a str. 2457T]
MTLFTTLLVLIFERLFKLGEHWQLDHRLEAFFRRVKHFSLGRTLGMTIIAMGVTFLLLRALQGVLFNVPT
LLVWLLIGLLCIGAGKVRLHYHAYLTAASRNDSHARATMAGELTMIHGVPAGCDEREYLRELQNALLWIN
FRFYLAPLFWLIVGGTWGPVTLMGYAFLRAWQYWLARYQTPHHRLQSGIDAVLHVLDWVPVRLAGVVYAL
IGHGEKALPAWFASLGDFHTSQYQVLTRLAQFSLAREPHVDKVETPKAAVSMAKKTSFVVVVVIALLTIY
GALV

>gi|30061676|ref|NP_835847.1| N-acetyl-anhydromuranmyl-L-alanine amidase [Shigella flexneri 2a str. 2457T]

MLLEQGWLVGARRVPSPHYDCRLDDETPTLLVVHNISLPPGEFGGPWIDALFTGTIDPQAHPFFAEIAHL

RVSAHCLIRRDGEIVQYVPFDKRAWHAGVSQYQGRERCNDFSIGIELEGTDTLAYTDAQYQQLAAVTRAL

IDRYPDIANNMTGHCDIAPDRKTDPGPAFDWARFRALVSKETT

>gi|30061674|ref|NP_835845.1| major pilin subunit [Shigella flexneri 2a str. 2457T]

MDKQRGFTLIELMVVIGIIAILSSIGIPAYQNYLRKAALTDMLQTFVPYRTAVELCALEHGGLDTCDGGS

NGIPSPTTTRYVSAMSVAKGVVSLTGQESLNGLSVVMTPGWDNANGVTGWARNCNIQSDSALQQACEDVF

RFDDAN

>gi|30061673|ref|NP_835844.1| type IV pilin biogenesis protein [Shigella flexneri 2a str. 2457T]

MASKQLWRWHGITGDGNAQDGMLWAESRTLLLMALQQQMVTPLSLKRIAINSAQWRGDKSAEVIHQLATL

LKAGLTLSEGLALLAEQHPSKQWQALLQSLAHDLEQGIAFSNALLPWSEAFPPLYQAMIRTGELTGKLDE

CCFELARQQKSQRQLTDKVKSALRYPIIILAMAIMVVVAMLHFVLPEFAAIYKTFNTPLPALTQGIMTLA

DFSGEWGWLLVLFGFLLAIANKLLMRRPTWLIARQKLLLRIPIMGSLMRGQKLTQIFTILALTQSAGITF

LQGVESVRETMRCPYWVQLLTQIQHDISNGHPIWLALKNAGEFSPLCLQLVRTGEASGSLDLMLDNLAHH

HRDNTMALADNLAALLEPALLIITGGIIGTLVVAMYLPIFHLGDAMSGMG

>gi|30061669|ref|NP_835840.1| hypothetical protein S0101 [Shigella flexneri 2a str. 2457T]

MQTQVLFEHPLNEKMRTWLRIEFLIQQLTVNLPIVDHAGALHFFRNVSELLDVFERAEVRTELLKELNRQ

QRKLQTWIGVPGVDQSRIEALIQQLKAAVSVLISAPRIGQFLREDRLIALVRQRLSIPGGCCSFDLPTLH

IWLHLPQAQRDSQVETWIASLNPLTQALTMVLDLIRQSAPFRKQTSLNGFYQDNGGDADLLRLNLSLDSQ

LYPQISGHKSRFAIRFMPLDTENGQVPERLDFELACC

>gi|30061666|ref|NP_835837.1| nucleoside triphosphate pyrophosphohydrolase [Shigella flexneri 2a str. 2457T]

MKKLQIAVGIIRNENNEIFITRRAADAHMANKLEFPGGKIEMGETPEQAVVRELQEEVGITPQHFSLFEK

LEYEFPDRHITLWFWLVESWEGVPWGKEGQPGEWMSLVGLNADDFPPANEPVIAKLKRL

>gi|30061665|ref|NP_835836.1| preprotein translocase subunit SecA [Shigella flexneri 2a str. 2457T]

MLIKLLTKVFGSRNDRTLRRMRKVVNIINAMEPEMEKLSDEELKGKTAEFRARLEKGEVLENLIPEAFAV

VREASKRVFGMRHFDVQLLGGMVLNERCIAEMRTGEGKTLTATLPAYLNALTGKGVHVVTVNDYLAQRDA

ENNRPLFEFLGLTVGINLPGMPAPAKREAYAADITYGTNNEYGFDYLRDNMAFSPEERVQRKLHYALVDE

VDSILIDEARTPLIISGPAEDSSEMYKRVNKIIPHLIRQEKEDSETFQGEGHFSVDEKSRQVNLTERGLV

LIEELLVKEGIMDEGESLYSPANIMLMHHVTAALRAHALFTRDVDYIVKDGEVIIVDEHTGRTMQGRRWS

DGLHQAVEAKEGVQIQNENQTLASITFQNYFRLYEKLAGMTGTADTEAFEFSSIYKLDTVVVPTNRPMIR

KDLPDLVYMTEAEKIQAIIEDIKERTAKGQPVLVGTISIEKSELVSNELTKAGIKHNVLNAKFHANEAAI

VAQAGYPAAVTIATNMAGRGTDIVLGGSWQAEVAALENPTAEQIEKIKADWQVRHDAVLEAGGLHIIGTE

RHESRRIDNQLRGRSGRQGDAGSSRFYLSMEDALMRIFASDRVSGMMRKLGMKPGEAIEHPWVTKAIANA

QRKVESRNFDIRKQLLEYDDVANDQRRAIYSQRNELLDVSDVSETINSIREDVFKATIDAYIPPQSLEEM

WDIPGLQERLKNDFDLDLPITEWLDKEPELHEETLRERILAQSIEVYQRKEEVVGAEMMRHFEKGVMLQT

LDSLWKEHLAAMDYLRQGIHLRGYAQKDPKQEYKRESFSMFAAMLESLKYEVISTLSKVQVRMPEEVEEL

EQQRRMEAERLAQMQQLSHQDDDSAAAAALAAQTGERKVGRNDPCPCGSGKKYKQCHGRLQ

>gi|30061663|ref|NP_835834.1| UDP-3-O-[3-hydroxymyristoyl] N-acetylglucosamine deacetylase [Shigella flexneri 2a str. 2457T]

MIKQRTLKRIVQATGVGLHTGKKVTLTLRPAPANTGVIYRRTDLNPPVDFPADAKSVRDTMLCTCLVNEH

DVRISTVEHLNAALAGLGIDNIVIEVNAPEIPIMDGSAAPFVYLLLDAGIDELNCAKKFVRIKETVRVED

GDKWAEFKPYNGFSLDFTIDFNHPAIDSSNQRYAMNFSADAFMRQISRARTFGFMRDIEYLQSRGLCLGG

SFDCAIVVDDYRVLNEDGLRFEDEFVRHKMLDAIGDLFMCGHNIIGAFTAYKSGHALNNKLLQAVLAKQE

AWEYVTFQDDAELPLAFKAPSAVLA

>gi|30061662|ref|NP_835833.1| cell division protein FtsZ [Shigella flexneri 2a str. 2457T]

MFEPMELTNDAVIKVIGVGGGGGNAVEHMVRERIEGVEFFAVNTDAQALRKTAVGQTIQIGSGITKGLGA

GANPEVGRNAADEDRDALRAALEGADMVFIAAGMGGGTGTGAAPVVAEVAKDLGILTVAVVTKPFNFEGK

KRMAFAEQGITELSKHVDSLITIPNDKLLKVLGRGISLLDAFGAANDVLKGAVQGIAELITRPGLMNVDF

ADVRTVMSEMGYAMMGSGVASGEDRAEEAAEMAISSPLLEDIDLSGARGVLVNITAGFDLRLDEFETVGN

TIRAFASDNATVVIGTSLDPDMNDELRVTVVATGIGMDKRPEITLVTNKQVQQPVMDRYQQHGMAPLTQE

QKPVAKVVNDNAPQTAKEPDYLDIPAFLRKQAD

>gi|30061661|ref|NP_835832.1| cell division protein FtsA [Shigella flexneri 2a str. 2457T]

MIKATDRKLVVGLEIGTAKVAALVGEVLPDGMVNIIGVGSCPSRGMDKGGVNDLESVVKCVQRAIDQAEL

MADCQISSVYLALSGKHISCQNEIGMVPISEEEVTQEDVENVVHTAKSVRVRDEHRVLHVIPQEYAIDYQ

EGIKNPVGLSGVRMQAKVHLITCHNDMAKNIVKAVERCGLKVDQLIFAGLASSYSVLTEDERELGVCVVD

IGGGTMDIAVYTGGALRHTKVIPYAGNVVTSDIAYAFGTPPSDAEAIKVRHGCALGSIVGKDESVEVPSV

GGRPPRSLQRQTLAEVIEPRYTELLNLVNEEILQLQEKLRQQGVKHHLAAGIVLTGGAAQIEGLAACAQR

VFHTQVRIGAPLNITGLTDYAQEPYYSTAVGLLHYGKESHLNGEAEVEKRVTASVGSWIKRLNSWLRKEF

>gi|30061660|ref|NP_835831.1| cell division protein FtsQ [Shigella flexneri 2a str. 2457T]

MSQAALNTRNSEEEVSSRRNNGTRLAGILFLLTVLTTVLVSGWVVLSWMEDAQRLPLSKLVLTGERHYTR

NDDIRQSILALGEPGTFMTQDVNIIQTQIEQRLPWIKQVSVRKQWPDELKIHLVEYVPIARWNDQHMVDA

EGNTFSVPPDRTSKQVLPMLYGPEGSANEVLQGYREMGQMLAKDRFTLKEAAMTARRSWQLTLNNDIKLN

LGRGDTMKRLVRFVELYPVLQQQAQTDGKRISYVDLRYDSGAAVGWAPLPPEESTQQQNQAQAEQQ

>gi|30061659|ref|NP_835830.1| D-alanine--D-alanine ligase [Shigella flexneri 2a str. 2457T]

MTDKIAVLLGGTSAEREVSLNSGAAVLAGLREGGIDAYPVDPKEVDVTQLKSMGFQKVFIALHGPGGEDG

TLQGMLELMGLPYTGSGVMASALSIDKLRSKLLWQGAGLPVAPWVALTRAEFEKGLSDKQLAEISALGLP

VIVKPSREGSSVGMSKVVAENALQDALRLAFQHDEEVLIEKWLSGPEFTVAILGEEILPSIRIQPSGTFY

DYEAKYLSDETQYFCPAGLEASQEANLQALVLKAWTTLGCKGWGRIDVMLDSDGQFYLLEANTSPGMTSH

SLVPMAARQAGMSFSQLVVRILELAD

>gi|30061658|ref|NP_835829.1| UDP-N-acetylmuramate--L-alanine ligase [Shigella flexneri 2a str. 2457T]

MNTQQLAKLRSIVPEMRRVRHIHFVGIGGAGMGGIAEVLANEGYQISGSDLAPNPVTQQLLNLGATIYFN

HRPENVRDASVVVVSSAISADNPEIVAAHEARIPVIRRAEMLAELMRFRHGIAIAGTHGKTTTTAMVSSI

YAEAGLDPTFVNGGLVKAAGVHARLGHGRYLIAEADESDASFLHLQPMVAIVTNIEADHMDTYQGDFENL

KQTFINFLHNLPFYGRAVMCVDDPVIRELLPRVGRQTTTYGFSEDADVRVEDYQQIGPQGHFTLLRQDKE

PMCVTLNAPGRHNALNAAAAVAVATEEGIDDEAILRALESFQGTGRRFDFLGEFPLEPVNGKSGTAMLVD

DYGHHPTEVDATIKAARAGWPDKNLVMLFQPHRFTRTRDLYDDFANVLTQVDTLLMLEVYPAGEAPIPGA

DSRSLCRTIRGRGKIDPILVPDPAQVAEMLAPVLTGNDLILVQGAGNIGKIARSLAEIKLKPQTPEEEQH

D

>gi|30061657|ref|NP_835828.1| undecaprenyldiphospho-muramoylpentapeptide beta-N-acetylglucosaminyltransferase [Shigella flexneri 2a str. 2457T]

MSGQGKRLMVMAGGTGGHVFPGLAVAHYLMAQGWQVRWLGTADRMEADLVPKHGIEIDFIRISGLRGKGI

KALIAAPLRIFNAWRQARAIMKAYKPDVVLGMGGYVSGPGGLAAWSLGIPVVLHEQNGIAGLTNKWLAKI

ATKVMQAFPGAFPNAEVVGNPVRTDVLALPLPQQRLAGREGPVRVLVVGGSQGARILNQTMPQVAAKLGD

SVTIWHQSGKGSQQSVEQAYAEAGQPQHKVTEFIDDMAAAYAWADVVVCRSGALTVSEIAAAGLPALFVP

FQHKDRQQYWNALPLEKAGAAKIIEQPQLSVDAVANTLAGWSRETLLTMAERARAASIPDATERVANEVS

RAARA

>gi|30061656|ref|NP_835827.1| cell division protein FtsW [Shigella flexneri 2a str. 2457T]

MRLSLPRLKMPRLPGFSILVWISTALKGWVMGSREKDTDSLIMYDRTLLWLTFGLAAIGFIMVTSASMPI

GQRLTNDPFFFAKRDGVYLILAFILAIITLRLPMEFWQRYSATMLLGSIILLMIVLVVGSAVKGASRWID

LGLLRIQPAELTKLSLFCYIANYLVRKGDEVRNNLRGFLKPMGVILVLAVLLLAQPDLGTVVVLFVTTLA

MLFLAGAKLWQFIAIIGMGISAVVLLILAEPYRIRRVTAFWNPWEDPFGSGYQLTQSLMAFGRGELWGQG

LGNSVQKLEYLPEAHTDFIFAIIGEELGYVGVVLALLMVFFVAFRAMSIGRKALEIDHRFSGFLACSIGI

WFSFQALVNVGAAAGMLPTKGLTLPLISYGGSSLLIMSTAIMMLLRIDYETRLEKAQAFVRGSR

>gi|30061655|ref|NP_835826.1| UDP-N-acetylmuramoyl-L-alanyl-D-glutamate synthetase [Shigella flexneri 2a str. 2457T]

MADYQGKNVVIIGLGLTGLSCVDFFLARGVTPRVMDTRMTPPGLDKLPEAVERHTGSLNDEWLMAADLIV

ASPGIALAHPSLSAAADAGIEIVGDIELFCREAQAPIVAITGSNGKSTVTTLVGEMAKAAGVNVGVGGNI

GLPALMLLDDECELYVLELSSFQLETTSSLQAVAATILNVTEDHMDRYPFGLQQYRAAKLRIYENAKVCV

VNADDALTMPIRGADERCVSFGVNMGDCHLNHQQGETWLRVKGEKVLNVKEMKLSGQHNYTNALVALALA

DAAGLPRASSLKALTTFTGLPHRFEVVLEHNGVRWINDSKATNVGSTEAALNGLHVDGTLHLLLGGDGKS

ADFSPLVRYLNGDNVRLYCFGRDGAQLAALRPEVAEQTETMEQAMRLLAPRVQPGDMVLLSPACASLDQF

KNFEQRGNEFARLAKELG

>gi|30061654|ref|NP_835825.1| phospho-N-acetylmuramoyl-pentapeptide-transferase [Shigella flexneri 2a str. 2457T]

MLVWLAEHLVKYYSGFNVFSYLTFRAIVSLLTALFISLWMGPRMIAHLQKLSFGQVVRNDGPESHFSKRG

TPTMGGIMILTAIVISVLLWAYPSNPYVWCVLVVLVGYGVIGFVDDYRKVVRKDTKGLIARWKYFWMSVI

ALGVAFALYLAGKDTPATQLVVPFFKDVMPQLGLFYILLAYFVIVGTGNAVNLTDGLDGLAIMPTVFVAG

GFALVAWATGNMNFASYLHIPYLRHAGELVIVCTAIVGAGLGFLWFNTYPAQVFMGDVGSLALGGALGII

AVLLRQEFLLVIMGGVFVVETLSVILQVGSFKLRGQRIFRMAPIHHHYELKGWPEPRVIVRFWIISLMLV

LIGLATLKVR

>gi|30061653|ref|NP_835824.1| UDP-N-acetylmuramoyl-tripeptide--D-alanyl-D-alanine ligase [Shigella flexneri 2a str. 2457T]

MISVTLSQLTDILNGELQGADITLDAVTTDTRKLTPGCLFVALKGERFDAHDFADQAKAGGAGALLVSRP

LDIDLPQLIVKDTRLAFGELAAWVRQQVPALVVALTGSSGKTSVKEMTAAILSQCGNTLYTAGNLNNDIG

VPMTLLRLTPEYDYAVIELGANHQGEIAWTVSLTRPEAALVNNLAAAHLEGFGSLAGVAKAKGEIFSGLP

ENGIAIMNADNNDWLNWQSVIGSRKVWRFSPNAANSDFTATNIHVTSHGMEFTLQTPTGSVDVLLPLPGR

HNIANALAAAALSMSVGATLDAIKAGLANLKAVPGRLFPIQLAENQLLLDDSYNANVGSMTAAVQVLAEM

PGYRVLVVGDMAELGAESEACHVQVGEAAKAAGIDRVLSMGKQSHAISTASGVGEHFADKTALITRLKSL

IAEQQVITILVKGSRSAAMEEVVRALQENGTC

>gi|30061652|ref|NP_835823.1| UDP-N-acetylmuramoylalanyl-D-glutamate--2,6-diaminopimelate ligase [Shigella flexneri 2a str. 2457T]

MADRNLRDLLAPWVPDAPSRALREMTLDSRVAAAGDLFVAVVGHQADGRRYIPQAIAQGVAAIIAEAKDE

ATDGEIREMHGVPVIYLSQLNERLSALAGRFYHEPSDNLRLVGVTGTNGKTTTTQLLAQWSQLLGETSAV

MGTVGNGLLGKVIPTENTTGSAVDVQHELAGLVDQGATFCAMEVSSHGLVQHRVAALKFPASVFTNLSRD

HLDYHGDMEHYEAAKWLLYSEHHCGQAIINADDEVGRRWLAKLPDAVAVSMEDHINPNCHGRWLKATEVN

YHDSGATIRFSSSWGDGEIESHLMGAFNVSNLLLALATLLALGYPLADLLKTAARLQPVCGRMEVFTAPG

KPTVVVDYAHTPDALEKALQAARLHCAGKLWCVFGCGGDRDKGKRPLMGAIAEEFADVAVVTDDNPRTEE

PRAIINDILAGMLDAGHAKVMEGRAEAVTCAVMQAKENDVVLVAGKGHEDYQIVGNQRLDYSDRVTVARL

LGGIA

>gi|30061651|ref|NP_835822.1| penicillin-binding protein 3; peptidoglycan synthetase [Shigella flexneri 2a str. 2457T]

MKAAAKTQKPKRQEEHANFISWRFALLCGCILLALAFLLGRVAWLQVISPDMLVKEGDMRSLRVQQVSTS

RGMITDRSGRPLAVSVPVKAIWADPKEVHDAGGISVGDRWKALANALNIPLDQLSARINANPKGRFIYLA

RQVNSDMADYIKKLKLPGIHLREESRRYYPSGEVTAHLIGFTNVDSQGIEGVEKSFDKWLTGQPGERIVR

KDRYGRVIEDISSTDSQAAHNLALSIDERLQALVYRELNNAVAFNKAESGSAVLVDVNTGEVLAMANSPS

YNPNNLSGTPKEAMRNRTITDVFEPGSTVKPMVVMTALQRGVVRENSVLNTIPYRINGHEIKDVARYSEL

TLTGVLQKSSNVGVSKLALTMPSSALVDTYSRFGLGKATNLGLVGERSGLYPQKQRWSDIERATFSFGYG

LMVTPLQLARVYATIGSYGIYRPLSITKVDPPVPGERVFPESIVRTVVHMMESVALPGGGGVKAAIKGYR

IAIKTGTAKKVGPDGRYINKYIAYTAGVAPASQPRFALVVVINDPQAGKYYGGAVSAPVFGAIMGGVLRT

MNIEPDALTTGDKNEFVINQGEGTGGRS

>gi|30061650|ref|NP_835821.1| cell division protein FtsL [Shigella flexneri 2a str. 2457T]

MISRVTEALSKVKGSMGSHERHALPGVIGDDLLRFGKLPLCLFICIILTAVTVVTTAHHTRLLTAQREQL

VLERDALDIEWRNLILEENALGDHSRVERIATEKLQMQHVDPSQENIVVQK

>gi|30061648|ref|NP_835819.1| cell division protein MraZ [Shigella flexneri 2a str. 2457T]

MFRGATLVNLDSKGRLSVPTRYREQLLENAAGQMVCTIDIHHPCLLLYPLPEWEIIEQKLSRLLSMNPVE

RRVQRLLLGHASECQMDGAGRLLIAPVLRQHAGLTKEVMLVGQFNKFELWDETTWHQQVKEDIDAEQLAT

GDLSERLQDLSL

>gi|30061645|ref|NP_835816.1| DNA-binding transcriptional regulator FruR [Shigella flexneri 2a str. 2457T]

MKLDEIARLAGVSRTTASYVINGKAKQYRVSDKTVEKVMAVVREHNYHPNAVAAGLRAGRTRSIGLVIPD

LENTSYTRIANYLERQARQRGYQLLIACSEDQPDNEMRCIEHLLQRQVDAIIVSTSLPPEHPFYQRWAND

PFPIVALDRALDREHFTSVVGADQDDAEMLAEELRKFPAETVLYLGALPELSVSFLREQGFRTAWKDDPR

EVHFLYANSYEREAAAQLFEKWLETHPMPQALFTTSFALLQGVMDVTLRRDGKLPSDLAIATFGDNELLD

FLQCPVLAVAQRHRDVAERVLEIVLASLDEPRKPKPGLTRIKRNLYRRGVLSRS

>gi|30061643|ref|NP_835814.1| acetolactate synthase 3 regulatory subunit [Shigella flexneri 2a str. 2457T]

MRRILSVLLENESGALSRVIGLFSQRGYNIESLTVAPTDDPTLSRMTIQTVGDEKVLEQIEKQLHKLVDV

LRVSELGQGAHVEREIMLVKIQASGYGRDEVKRNTEIFRGQIIDVTPSLYTVQLAGTSDKLDAFLASIRD

VAKIVEVARSGVVGLSRGDKIMR

>gi|30061635|ref|NP_835806.1| transporter [Shigella flexneri 2a str. 2457T]

MIWIMTMARRMNGVYAAFMLVAFMMGVAGALQAPTLSLFLSREVGAQPFWIGLFYTVNAIAGIGVSLWLA

KRSDSQGDRRKLIIFCCLMAIGNALLFAFNRHYLTLITCGVLLASLANTAMPQLFALAREYADNSAREVV

MFSSVMRAQLSLAWVIGPPLAFMLALNYGFTVMFSIAAGIFTLSLVLIAFMLLSVARVELPSENALSMQG

DWQDSNVRMLFVASTLMWTCNTMYIIDMPLWISSDLGLPDKLAGFLMGTAAGLEIPAMILAGYYVKRYGK

RRMMVIAVVAGVLFYTGLIFFHSRMALMTLQLFNAVFIGIVAGIGMLWFQDLMPGRAGAATTLFTNTIST

GVILAGVIQGAIAQSWGHFAVYWVIAIISVVALFLTAKVKDV

>gi|30061634|ref|NP_835805.1| transcriptional regulator SgrR [Shigella flexneri 2a str. 2457T]

MPSARLQQQFIRLWQCCEGKSQDTTLNELAALLSCSRRHMRTLLNTMQDRGWLTWEAEVGRGKRSRLTFL

YTGLALQQQRAEDLLEQDRIDQLVQLVGDKATVRQMLVSHLGRSFRQGRHILRVLYYRPLRNLLPGSALR

RSETHIARQIFSSLTRINEENGELEADIAHHWQQISPLHWRFFLRPGVHFHHGRELEMDDVIASLKRINT

LPLYSHITDIVSPTPWTLDIHLTQPDRWLPLLLGQVPAMILPCEWETLSNFASHPIGTGPYAVIRNSTNQ

LKIQAFDDFFGYRALIDEVNVWVLPEIADEPAGGLMLKGPQGEKKEIESRLEEGCYYLLFDSRTHRGANQ

QVRDWVSYVLSPTNLVYFAEEQYQQLWFPAYGLLPRWHHARTIKSEKPAGLESLTLTFYQDHSEHRVIAG

IMQQILASHQVTLEIKEISYDQWHEGEIESDIWLNSANFTLPLDFSLFAHLCEVPLLQHCIPIDWQADAA

RWRNGEMNLANWCQQLVASKAMVPLIHHWLIIQGQRSMRGLRMNTLGWFDFKSAWFAPPDP

>gi|30061633|ref|NP_835804.1| thiamine transporter substrate binding subunit [Shigella flexneri 2a str. 2457T]

MLKKCLPLLLLCTAPVFAKPVLTVYTYDSFAADWGPGPKIKKAFEANCNCELKLVALEDGVSLLNRLRME

GKNSKADVVLGLDNNLLDAASKTGLFAKSGVAADAVNVPGGWNNDTFVPFDYGYFAFVYDKNKLKNPPQS

LKELVESDQNWRVIYQDPRTSTPGLGLLLWMQKVYGDDAPQAWQKLAKKTVTVTKGWSEAYGLFLKGESD

LVLSYTTSPAYHILEEKKDNYAAANFSEGHYLQVEVAARTAASKQPELAQKFLQFMVSPAFQNAIPTGNW

MYPVANVTLPAGFEQLTKPATTLEFTPAEVAAQRQAWISEWQRAVSR

>gi|30061632|ref|NP_835803.1| thiamine transporter membrane protein [Shigella flexneri 2a str. 2457T]

MATRRQPLIPGWLIPGVSAATLVVAVALAAFLALWWNAPQGNWVAVWQDSYLWHVVRFSFWQAFLSALLS

VVPAIFLARALYRRRFPGRLALLRLCAMTLILPVLVAVFGILSVYGRQGWLASLCQSLGLEWTFSPYGLQ

GILLAHVFFNLPMASRLLLQALENIPGEQRQLAAQLGMRGWHFFRFVEWPWLRRQIPPVAALIFMLCFAS

FATVLSLGGGPQATTIELAIYQALSYDYDPARAAMLALLQMVCCLGLVLLSQRLSKAIAPGTTLLQGWRD

PDDRLHSRICDTVLIVLALLLLLPPLLAVIVDGVNRQLPEVLAQPVLWQALWTSLRIALAAGVLCVVLTM

MLLWSSRELRARQKMLAGQALEMSGMLILAMPGIVLATGFFLLLNNTIGLPQSADGIVIFTNALMAIPYA

LKVLENPMRDITARYSMLCQSLGIEGWSRLKVVELRALKRPLAQALAFACVLSIGDFGVVALFGNDDFRT

LPFYLYQQIGSYRSQDGAVTALILLLLCFLLFTVIEKLPGRNVKTD

>gi|30061630|ref|NP_835801.1| hypothetical protein S0062 [Shigella flexneri 2a str. 2457T]

MQALLEHFITQSTVYSLMAVVLVAFLESLALVGLILPGTVLMAGLGALIGSGELSFWHAWLAGIVGCLLG

DWISFWLGWRFKKPLHRWSFLKKNKALLDKTEHALHQHSMFTILVGRFVGPTRPLVPMVAGMLDLPVAKF

ITPNIIGCLLWPPFYFLPGILAGAAIDIPAGMQSGEFKWLLLATAVFLWVGGWLCWRLWRSGKATDRLSH

YLSRGRLLWLTPLISAIGVVALVVLIRHPLMPVYIDILRKVVGV

>gi|30061629|ref|NP_835800.1| DNA-binding transcriptional regulator AraC [Shigella flexneri 2a str. 2457T]

MAEAQNDPLLPGYSFNAHLVAGLTPIEANGYLDFFIDRPLGMKGYILNLTIRGQGVVKNQGREFVCRPGD

ILLFPPGEIHHYGCHPEAREWYHQWVYFRPRAYWHEWLNWPSIFANTGFFRPDEAHQPYFSDLFGQIINA

GQGEGRYSELLAMNLLEQLLLRRMEAINESLHPPMDNRVREACQYISDHLADSNFDIASVAQHVCLSPSR

LSHLFRQQLGISVLSWREDQRISQAKLLLSTTRMPIATVGRNVGFDDQLYFSRVFKKCTGASPSEFRAGC

EEKVNDVAVKLS

>gi|30061627|ref|NP_835798.1| L-arabinose isomerase [Shigella flexneri 2a str. 2457T]

MAIFDNYEVWFVIGSQHLYGPETLRQVTQHAEHVVNALNTEAKLPYKLVLKPLGTTPDEITAICRDANYD

DRCAGLVVWLHTFSPAKMWINGLTMLNKPLLQFHTQFNAALPWDSIDMDFMNLNQTAHGGREFGFIGARM

RQQHAVVTGHWQDKQAHERIGSWMRQAVSKQDTRHLKVCRFGDNMREVAVTDGDKVAAQIKFGFSVNTWA

VGYLVQVVNSISDGDVNALVDEYESCYTMTPATQIHGEKRQNVLEAARIELGMKRFLEQGGFHAFTTTFE

DLHGLKQLPGLPVQRLMQQGYGFAGEGDWKTAALLRIMKVMSTGLQGGTSFMEDYTYHFEKGNDLVLGSH

MLEVCPSIAVEEKPILDVQHLGIGGKDDPARLIFNTQTGPAIVASLIDLGDRYRLLVNCIDTVKTPHSLP

KLPVANALWKAQPDLPTASEAWILAGGAHHTVFSHALNLNDMRQFAEMHDIEITVIDNDTRLPAFKDALR

WNEVYYGFRR

>gi|30061622|ref|NP_835793.1| Dna-J like membrane chaperone protein [Shigella flexneri 2a str. 2457T]

MQYWGKIIGVAVALLMGGGFWGVVLGLLIGHMFDKARSRKMAWFANQRERQALFFATTFEVMGHLTKSKG

RVTEADIHIASQLMDRMNLHGASRTAAQNAFRVGKSDNYPLREKMRQFRSVCFGRFDLIRMFLEIQIQAA

FADGSLHPNERAVLYVIAEELGISRAQFDQFLRMMQGGAQFGGGYQQQSGGGNWQQAQRGPTLEDACNVL

GVKPTDDATTIKRAYRKLMSEHHPDKLVAKGLPPEMMEMAKQKAQEIQQAYELIKQQKGFK

>gi|30061621|ref|NP_835792.1| organic solvent tolerance protein [Shigella flexneri 2a str. 2457T]

MKKRIPTLLATMIATALYSQQGLAADLASQCMLGVPSYDRPLVQGDTNDLPVTINADHAKGDYPDDAVFT

GSVDIMQGNSRLQADEVQLHQKEAPGQPEPVRTVDALGNVHYDDNQVILKGPKGWANLNTKDTNVWEGDY

QMVGRQGRGKADLMKQRGENRYTILDNGSFTSCLPGSDTWSVVGSEIIHDREEQVAEIWNARFKVGPVPI

FYSPYLQLPVGDKRRSGFLIPNAKYTTTNYFEFYLPYYWNIAPNMDATITPHYMHRRGNIMWENEFRYLS

QAGAGLMELDYLPSDKVYEDEHPNDDSSRRWLFYWNHSGVMDQVWRFNVDYTKVSDPSYFNDFDNKYGSS

TDGYATQKFSVGYAVQNFNATVSTKQFQVFSEQNTSSYSAEPQLDVNYYQNDVGPFDTRIYGQAVHFVNT

RDDMPEATRVHLEPTINLPLSNNWGSINTEAKFLATHYQQTNLDWYNSRNTTKLDESVNRVMPQFKVDGK

MVFERDMEMLAPGYTQTLEPRAQYLYVPYRDQSDIYNYDSSLLQSDYSGLFRDRTYGGLDRIASANQVTT

GVTSRIYDDAAVERFNISVGQIYYFTESRTGDDNITWENDDKTGSLVWAGDTYWRISERWGLRGGIQYDT

RLDNVATSNSSIEYRRDEDRLVQLNYHYASPEYIQATLPKYYSTAEQYKNGISQVGAVASRPIADRWSIV

GAYYYDTNANKQADSMLGVQYSSCCYAIRVGYERKLNGWDNDKQHAVYDNAIGFNIELRGLSSNYGLGTQ

EMLRSNILPYQNTL

>gi|30061619|ref|NP_835790.1| 4-hydroxythreonine-4-phosphate dehydrogenase [Shigella flexneri 2a str. 2457T]

MVKTQRVVITPGEPAGIGPDLVVQLAQREWPVELVVCADATLLTDRAAMLGLPLTLRPYSPNSPAQPQTT

GTLTLLPVALRESVTAGQLAIENGHYVVETLARACDGCLNGEFAALITGPVHKGVINDASIPFTGHTEFF

EERSQAKKVVMMLATEELRVALATTHLPLRDIADAITPALLHEVIAILHHDLRTKFGIAEPRILVCGLNP

HAGEGGHMGTEEIDTIIPVLDELRAQGMKLNGPLPADTLFQPKYLDNADAVLAMYHDQGLPVLKYQGFGR

GVNITLGLPFIRTSVDHGTALELAGRGEADVGSFITALNLAIKMIVNTQ

>gi|30061616|ref|NP_835787.1| diadenosine tetraphosphatase [Shigella flexneri 2a str. 2457T]

MATYLIGDVHGCYDELIALLHKVEFTPGKDTLWLTGDLVARGPGSLDVLRYVKSLGDSVRLVLGNHDLHL

LAVFAGISRNKPKDRLTPLLEAPDADELLNWLRRQPLLQIDEEKKLVMAHAGITPQWDLQTAKECARDVE

AVLSSDSYPFFLDAMYGDMPNNWSPELRGLGRLRFITNAFTRMRFCFPNGQLDMYSKESPEEAPAPLKPW

FAIPGPVAEEYSIAFGHWASLEGKGTPEGIYALDTGCCWGGSLTCLRWEDKQYFVQPSNRHKDLGEAAAS

>gi|30061608|ref|NP_835779.1| L-carnitine/gamma-butyrobetaine antiporter [Shigella flexneri 2a str. 2457T]

MKNEKRKTGIEPKVFFPPLIIVGILCWLTVRDLDAANVVINAVFSYVTNVWGWAFEWYMVVMLFGWFWLV

FGPYAKKRLGNEPPEFSTASWIFMMFASCTSAAVLFWGSIEIYYYISTPPFGLEPNSTGAKELGLAYSLF

HWGPLPWATYSFLSVAFAYFFFVRKMEVIRPSSTLVPLAGEKHAKGLFGTIVDNFYLVALIFAMGTSLGL

ATPLVTECMQWLFGIPHTLQLDAIIITCWIILNAICVACGLQKGVRIASDVRSYLSFLMLGWVFIVSGAS

FIMNYFTDSVGMLLMYLPRMLFYTDPIAKGGFPQGWTVFYWAWWVIYAIQMSIFLARISRGRTVRELCFG

MVMGLTASTWILWTVLGSNTLLLMDKNIINIPNLIEQYGVARAIIETWAALPLSTATMWGFFILCFIATV

TLVNACSYTLAMSTCREVRDGEEPPLLVRIGWSILVGIIGIVLLALGGLKPIQTAIIAGGCPLFFVNIMV

TLSFIKDAKQNWKD

>gi|30061603|ref|NP_835774.1| carnitine operon protein CaiE [Shigella flexneri 2a str. 2457T]

MERTLTTVSYYAFEGLIPVVHPTAFVHPSAVLIGDVIVGAGVYIGPLASLRGDYGRLIVQAGANIQDGCI

MHGYCDTDTIVGENGHIGHGAILHGCVIGRDALVGMNSVIMDGAVIGEESIVAAMSFVKAGFSGEKRQLL

MGTPARAVRSVSDDELHWKRLNTKEYQDLVGRCHASLHETQPLRQMEENRPRLQGTTDVTPKR

>gi|30061599|ref|NP_835770.1| dihydrodipicolinate reductase [Shigella flexneri 2a str. 2457T]

MHDANIRVAIAGAGGRMGRQLIQAALALEGVQLGAALEREGSSLLGSDAGELAGAGKTGVTVQSSLDAIK

DDFDVFIDFTRPEGTLNHLAFCRQHGKGMVIGTTGFDEAGKQAIRDAAADIAIVFAANFSVGVNVMLKLL

EKAAKVMGDYTDIEIIEAHHRHKVDAPSGTALAMGEAIAHALDKDLKDCAVYSREGHTGERVPGTIGFAT

VRAGDIVGEHTAMFADIGERLEITHKASSRMTFANGAVRSALWLSGKESGLFDMRDVLDLNNL

>gi|30061598|ref|NP_835769.1| ribonucleoside hydrolase RihC [Shigella flexneri 2a str. 2457T]

MRLPIFLDTDPGIDDAVAIAAAIFAPELDLQLMTTVAGNVSVEKTTRNALQLLHFWNAEIPLAQGAAVPL

VRAPRDAASVHGESGMAGYDFVEHNRKPLGIPAFLAIRDALMRAPEPVTLVAIGPLTNIALLLLQCPECK

PYIRRLVIMGGSAGRGNCTPNAEFNIAADPEAAACVFRSGIEIVMCGLDVTNQAILTPDYLATLPELNRT

GKMLHALFSHYRSGSMQSGLRMHDLCAIAWLVRPDLFTLKPCFVAVETQGEFTSGTTVVDIDGCLGKPAN

VQVALDLNVKGFQQWVAEVLALVP

>gi|30061597|ref|NP_835768.1| 4-hydroxy-3-methylbut-2-enyl diphosphate reductase [Shigella flexneri 2a str. 2457T]

MQILLANPRGFCAGVDRAISIVENALAIYGAPIYVRHEVVHNRYVVDSLRERGAIFIEQISEVPDGAILI

FSAHGVSQAVRNEAKSRDLTVFDATCPLVTKVHMEVARASRRGEESILIGHAGHPEVEGTMGQYSNPEGG

MYLVESPDDVWKLTVKNEEKLSFMTQTTLSVDDTSDVIDALRKRFPKIVGPRKDDICYATTNRQEAVRAL

AEQAEVVLVVGSKNSSNSNRLAELAQRMGKHAFLIDDAKDIQEEWVKEVKCVGVTAGASAPDILVQNVVA

RLQQLGGGEAIPLEGREENIVFEVPKELRVDIREVD

>gi|30061595|ref|NP_835766.1| lipoprotein signal peptidase [Shigella flexneri 2a str. 2457T]

MSQSICSTGLRWLWLVVVVLIIDLGSKYLILQNFALGDTVPLFPSLNLHYARNYGAAFSFLADSGGWQRW

FFAGIAIGISVLLAVMMYRSKATQKLNNIAYALIIGGALGNLFDRLWHGFVVDMIDFYVGDWHFATFNLA

DTAICVGAALIVLEGFLPSKAKKQ

>gi|30061593|ref|NP_835764.1| bifunctional riboflavin kinase/FMN adenylyltransferase [Shigella flexneri 2a str. 2457T]

MKLIRGIHNLSQAPQEGCVLTIGNFDGVHRGHRALLQGLQEEGRKRNLPVMVMLFEPQPLELFATDKAPA

RLTRLREKLRYLAECGVDYVLCVRFDRRFAALTAQNFISDLLVKHLRVKFLAVGDDFRFGAGREGDFLLL

QKAGMEYGFDITSTQTFCEGGVRISSTAVRQALADDNLALAESLLGHPFAISGRVVHGDELGRTIGFPTA

NVPLRRQVSPVKGVYAVEVLGLGEKPLPGVANIGTRPTVAGIRQQLEVHLLDVAMDLYGRHIQVVLRKKI

RNEQRFASLDELKAQIARDELTAREFFGLTKPA

>gi|30061588|ref|NP_835759.1| transcriptional activator NhaR [Shigella flexneri 2a str. 2457T]

MSHINYNHLYYFWHVYKEGSVVGAAEALYLTPQTITGQIRALEERLQGKLFKRKGRGLEPSELGELVYRY

ADKMFTLSQEMLDIVNYRKESNLLFDVGVADALSKRLVSSVLNAAVVEGEPIHLRCFESTHEMLLEQLSQ

HKLDMIISDCPIDSTQQEGLFSVRIGECGVSFWCTNPPPEKPFPACLEERRLLIPGRRSMLGRKLLNWFN

SQGLNVEILGEFDDAALMKAFGAMHNAIFVAPTLYAYDFYADKTVVEIGRVENVMEEYHAIFAERMIQHP

AVQRICNTDYSALFSPAVR

>gi|30061587|ref|NP_835758.1| pH-dependent sodium/proton antiporter [Shigella flexneri 2a str. 2457T]

MKHLHRFFSSDASGGIILIIAAILAMIMANSGATSGWYHDFLETPVQLRVGSLEINKNMLLWINDALMAV

FFLLVGLEVKRELMQGSLASLRQAAFPVIAAIGGMIVPALLYLAFNYADPITREGWAIPAATDIAFALGV

LALLGSRVPLVLKIFLMALAIIDDLGAIIIIALFYTNDLSMASLGVAAVAIAVLAVLNLCGVRRTGVYIL

VGVVLWTAVLKSGVHATLAGVIVGFFIPLKEKHGRSPAKRLEHVLHPWVAYLILPLFAFANAGVSLQGVT

LDGLTSILPLGIIAGLLIGKPLGISLFCWLALRLKLAHLPEGTTYQQIMVVGILCGIGFTMSIFIASLAF

GSVDPELINWAKLGILVGSISSAVIGYSWLRVRLRPSV

>gi|30061583|ref|NP_835754.1| hypothetical protein S0013 [Shigella flexneri 2a str. 2457T]

MKSVFTISASLAISLMLCCTAQANDHKLLGVIAMPRNETNDLALKLPVCRIVKRIQLSADHGDLQLSGAS

VYFKAARSASQSLNIPSEIKEGQTTDWININSDNDNKRCVSKITFSGHTVNSSDMATLKIIGDD

>gi|30061581|ref|NP_835752.1| hypothetical protein S0012 [Shigella flexneri 2a str. 2457T]

MNVNYLNDSDLDFLQHCSEEQLANFARLLTHNEKGKTRLSSILMRNELFKSMEGHPEQHRRNWQLIAGEL

QHFGGDSIANKLRGHGKLYRAILLDVSKRLKLKADKEMSTFEIEQQLLEQFLRNTWKKMDEEHKQEFLHA

VDARVNELEELLPLLMKDKLLAKGVSHLLSSQLTRILRTHAAMSVLGHGLLRGAGLGGPVGAALNGVKAV

SGSTYRVTIPAVLQIACLRRMVSATQV

>gi|30061580|ref|NP_835751.1| hypothetical protein S0010 [Shigella flexneri 2a str. 2457T]

MGNTKLANPAPLGLMGFGMTTILLNLHNVGYFALDGIILAMGIFYGGIAQIFAGLLEYKKGNTFGLTAFT

SYGSFWLTLVAILLMPKLGLTDAPNAQFLGVYLGLWGVFTLFMFFGTLKGARVLQFVFFSLTVLFALLAI

GNIAGNAAIIHFAGWIGLICGASAIYLAMGEVLNEQFGRTVLPIGESH

>gi|30061577|ref|NP_835748.1| hypothetical protein S0006 [Shigella flexneri 2a str. 2457T]

MLILISPAKTLDYQSPLTTTRYTLPELLDNSQQLIHEARKLTPPQISTLMRISDKLAGINAARFHDWQPD

FTPANARQAILAFKGDVYTGLQAETFSEDDFDFAQQHLRMLSGLYGVLRPLDLMQPYRLEMGIRLENARG

KDLYQFWGDIITNKLNEALAAQGDNVVINLASDEYFKSVKPKKLNAEIIKPVFLDEKNGKFKIISFYAKK

ARGLMSRFIIENRLTKPEQLTGFNSEGYFFDEDSSSNGELVFKRYEQR

>gi|30061574|ref|NP_835745.1| homoserine kinase [Shigella flexneri 2a str. 2457T]

MVKVYAPASSANMSVGFDVLGAAVAPVDGALLGDVVTVEAAETFSLNNLGRFADKLPSEPRENIVYQCWE

RFCQELGKQIPVAMTLEKNMPIGSGLGSSACSVVAALMAMNEHCGKPLNDTRLLALMGELEGRISGSIHY

DNVAPCFLGGMQLMIEENDIISQQVPGFDEWLWVLAYPGIKVSTAEARAILPAQYRRQDCIAHGRHLAGF

IHACYSRQPELAAKLMKDVIAEPYRERLLPGFRQARQAVAEIGAVASGISGSGPTLFALCDKPDTAQRVA

DWLGKNYLQNQEGFVHICRLDTAGARVLEN

>gi|30061573|ref|NP_835744.1| bifunctional aspartokinase I/homeserine dehydrogenase I [Shigella flexneri 2a str. 2457T]

MRVLKFGGTSVANAERFLRVADILESNARQGQVATVLSAPAKITNHLVAMIEKTISGQDALPNISDAERI

FAELLTGLAAAQPGFPLAQLKTFVDQEFAQIKHVLHGISLLGQCPDSINAALICRGEKMSIAIMAGVLEA

RGHNVTVIDPVEKLLAVGHYLESTVDIAESTRRIAASRIPADHMVLMAGFTAGNEKGELVVLGRNGSDYS

AAVLAACLRADCCEIWTDVDGVYTCDPRQVPDARLLKSMSYQEAMELSYFGAKVLHPRTITPIAQFQIPC

LIKNTGNPQAPGTLIGASRDEDELPVKGISNLNNMAMFSVSGPGMKGMVGMAARVFAAMSRARISVVLIT

QSSSEYSISFCVPQSDCVRAERAMQEEFYLELKEGLLEPLAVTERLAIISVVGDGMRTLRGISAKFFAAL

ARANINIVAIAQGSSERSISVVVNNDDATTGVRVTHQMLFNTDQVIEVFVIGVGGVGGALLEQLKRQQSW

LKNKHIDLRVCGVANSKALLTSVHGLNLENWQEELAQAKEPFNLGRLIRLVKEYHLLNPVIVDCTSSQAV

ADQYADFLREGFHVVTPNKKANTSSMDYYHQLRYAAEKSRRKFLYDTNVGAGLPVIENLQNLLNAGDELV

KFSGILSGSLSYIFGKLDEGMSFSEATTLAREMGYTEPDPRDDLSGMDVARKLLILARETGRELELADIE

IEPVLPAEFNAEGDVAAFMANLSQLDDLFAARVAKARDEGKVLRYVGNIDEDGVCRVKIAEVDGNDPLFK

VKNGENALAFYSHYYQPLPLVLRGYGAGNDVTAAGVFADLLRTLSWKLGV

>gi|30043893|gb|AAP19612.1| soluble lytic murein transglycosylase [Shigella flexneri 2a str. 2457T]

MYLHLEDALVEKAKQVTWRLLAAGVCLLTVSSVARADSLDEQRSRYAQIKQAWDNRQMDVVEQMMPGLKD

YPLYPYLEYRQITDDLMNQPAVTVTNFVRANPTLPPARTLQSRFVNELARREDWRGLLAFSPEKPGTTEA

QCNYYYAKWNTGQSEEAWQGAKELWLTGKSQPNACDKLFSVWRASGKQDPLAYLERIRLAMKAGNTGLVT

VLAGQMPADYQTIASAIISLANNPNTVLTFARTTGATDFTRQMAAVAFASVARQDAENARLMIPSLAQAQ

QLNEDQIQELRDIVAWRLMGNDVTDEQAKWRDDAIMRSQSTSLIERRVRMALGTGDRRGLNTWLARLPME

AKEKDEWRYWQADLLLERGREAEAKEILHQLIQQRGFYPMVAAQRIGEEYELKIDKAPQNVDSTLTQGPE

MARVRELMYWNLDNTARSEWANLVKSKSKTEQAQLARYAFNNQWWDLSVQATIAGKLWDHLEERFPLAYN

DLFKRYTSGKEIPQSYAMAIARQESAWNPKVKSPVGASGLMQIMPGTATHTVKMFSIPGYSSPGQLLDPE

TNINIGTSYLQYVYQQFGNNRIFSSAAYNAGPGRVRTWLGNSAGRIDAVAFVESIPFSETRGYVKNVLAY

DAYYRYFMGDKPTLMSATEWGRRY

>gi|30043891|gb|AAP19610.1| probable nadAB transcriptional regulator [Shigella flexneri 2a str. 2457T]

MRDTEGDMSSFDYLKTAIKQQGCTLQQVADASGMTKGYLSQLLNAKIKSPSAQKLEALHRFLGLEFPRQK

KTIGVVFGKFYPLHTGHIYLIQRACSQVDELHIIMGFDDTRDRALFEDSAMSQQPTVPDRLRWLLQTFKY

QKNIRIHAFNEEGMEPYPHGWDVWSNGIKKFMAEKGIQPDLIYTSEEADAPQYMEHLGIETVLVDPKRTF

MSISGAQIRENPFRYWEYIPTEVKPFFVRTVAILGGESSGKSTLVNKLANIFNTTSAWEYGRDYVFSHLG

GDEIALQYSDYDKIALGHAQYIDFAVKYANKVAFIDTDFVTTQAFCKKYEGREHPFVQALIDEYRFDLVI

LLENNTPWVADGLRSLGSSVDRKEFQNLLVEMLEENNIEFVRVEEDDYDSRFLRCVELVREMMGEQG

>gi|30043828|gb|AAP19547.1| hypothetical protein S4618 [Shigella flexneri 2a str. 2457T]

MHNIPGVRNTRLPLLQEIVMEILYNIFTVFFNQVMTNAPLLLGIVTCLGYILLRKSVSVIIKGTIKTIIG

FMLLQAGSGILTSTFKPVVAKMSEVYGINGAISDTYASMMATIDRMGDAYSWVGYAVLLALALNICYVLL

RRITGIRTIMLTGHIMFQQAGLIAVTLFIFGYSMWTTIICTAILVSLYWGITSNMMYKPTQEVTDGCGFS

IGHQQQFASLIAYKVAPFLGKKEESVEDLKLPGWLNIFHDNIVSTAIVMTIFFGAILLSFGIDTVQAMAG

KVHWTVYILQTGFSFAVAIFIITQGVRMFVAELSEAFNGISQRLIPGAVLAIDCAAIYSFAPNAVVWGFM

WGTIGQLIAVGILVACGSSILIIPGFIPMFFSNATIGVFANHFGGWRAALKICLVMGMIEIFGCVWVVKL

TGMSAWMGMADWSILAPPMMQGFFSIGIAFMAVIIVIALAYMFFAGRALRAEEDAEKQLAEQSA

>gi|30043824|gb|AAP19543.1| hypothetical protein S4614 [Shigella flexneri 2a str. 2457T]

MWVTLSSRFHSKIAYQVTIREHSPKVKLIRHYTMVSRKRNSVIYRFASLLLVLMLSACSALQGTPQPAPP

VTDHPQEIRRDQTQGLQRIGSVSTMVRGSPDDALAEIRAKAVAAKADYYVVVMVDETIVTGQWYSQAILY

RK

>gi|30043573|gb|AAP19293.1| transcription elongation factor and transcript cleavage factor [Shigella flexneri 2a str. 2457T]

MRIIKQTKGINEMKTPLVTREGYEKLKQELNYLWREERPEVTKKVTWAASLGDRSENADYQYNKKRLREI

DRRVRYLTKCLENLKIVDYSPQQEGKVFFGAWVEIENDDGVTHRFRIVGYDEIFGRKDYISIDSPMARAL

LKKEVGDLAVVNTPAGEANWYVNAIEYVKP

>gi|30043564|gb|AAP19284.1| hypothetical protein S4330 [Shigella flexneri 2a str. 2457T]

MLFIRYCSLLTYKDEHMLTVPGLCWLCRMPLALGHWGICSVCSRATRTDKTLCPQCGLPATHSHLPCGRC

LQKPPPWQRLVTVADYAPPLSPLIHQLKFSRRSEIASALSRLLLLEVLHARRTTGLQLPDRIISVPLWQR

RHWRRGFNQSDLLCQPLSRWLHCQWDSEAVTRTRATATQHFLSARLRKRNLKNAFRLELPVQGRHMVIVD

DVVTTGSTVAEIAQLLLRNGAATVQVWCLCRTL

>gi|30043513|gb|AAP19233.1| hypothetical protein S4273 [Shigella flexneri 2a str. 2457T]

MIRWMNEPLWPFIERKKSMRNLVKYVGIGLLVMGLAACDDKDTNATAQGSVAESNATGNPVNLLDGKLSF

SLPADMTDQSGKLGTQANNMHVWSDATGQKAVIVIMGDDPKEDLAVLAKRLEDQQRSRDPQLQVVTNKAI

ELKGHKMQQLDSIISAKGQTAYSSVILGNVGNQLLTMQITLPADDQQKAQTTAENIINTLVIQ

>gi|30043512|gb|AAP19232.1| putative transport [Shigella flexneri 2a str. 2457T]

MVKMKHCCKNVVILMPEPVAEPALNGLRLNLRIVSIVMFNFASYLTIGLPLAVLPGYVHDVMGFSAFWAG

LVISLQYFATLLSRPHAGRYADLLGPKKIVVFGLCGCFLSGLGYLTAGLTASLPVISLLLLCLGRVILGI

GQSFAGTGSTLWGVGVVGSLHIGRVISWNGIVTYGAMAMGAPLGVVFYHWGGLQALALIIMGVALVAILL

AIPRPTVKASKGKPLPFRAVLGRVWLYGMALALASAGFGVIATFITLFYDAKGWDGAAFALTLFSCAFVG

TRLLFPNGINRIGGLNVAMICFSVEIIGLLLVGVATMPWMAKIGVLLAGAGFSLVFPALGVVAVKAVPQQ

NQGAALATYTVFMDLSLGVTGPLAGLVMSWAGVPVIYLAAAGLVAIALLLTWRLKKRPPEHVPEAASSS

>gi|30043437|gb|AAP19157.1| hypothetical protein S4189 [Shigella flexneri 2a str. 2457T]

MLSPVCPGFVCMRYIKSITQQKLSFLLAIYIGLFMNGAVFYRRFGSYAHDFTVWKGVSAVVELAATVLVT

FFLLRLLSLFGRRSWRILASLVVLFSAGASYYMTFLNVVIGYGIIASVMTTDIDLSKEVVGLNFILWLIA

VSALPLILIWNNRCRYTLLRQLRTPGQRIRSLVVVVLAGIMVWAPIRLLDIQQKKVERATGVDLPSYGGV

VANSYLPSNWLSALGLYAWARVDESSDNNSLLNPAKKFTYQAPQNVDDTYVVFIIGETTRWDHMGIFGYE

RNTTPKLAQEKNLAAFRGYSCDTATKLSLRCMFVRQGGAEDNPQRTLKEQNIFAVLKQLGFSSDLYAMQS

EMWFYSNTMADNIAYREQIGAEPRNRGKPVDDMLLVDEMQQSLGRNPDGKHLIILHTKGSHFNYTQRYPR

SFAQWKPECIGVDSGCTKAQMINSYDNSVTYVDHFISSVIDQVRDKKAIVFYAADHGESINEREHLHGTP

RELAPPEQFRVPMMVWMSDKYLENPANAQAFAQLKKEADMKVPRRHVELYDTIMGCLGYTSPDGGINENN

NWCHIPQTKVAAAN

>gi|30043344|gb|AAP19065.1| putative transcriptional regulator [Shigella flexneri 2a str. 2457T]

MPPGKCLFSGVFCNMAEKQTAKRNRREEILQSLALMLESSDGSQRITTAKLAASVGVSEAALYRHFPSKT

RMFDSLIEFIEDSLITRINLILKDEKDTTARLRLIVLLLLGFGERNPGLTRILTGHALMFEQDRLQGRIN

QLFERIEAQLRQVLREKRMREGEGYTTDETLLASQILAFCEGMLSRFVRSEFKYRPTDDFDARWPLIAAQ
LQ

>gi|30043259|gb|AAP18980.1| hypothetical protein S3995 [Shigella flexneri 2a str. 2457T]

MMMLFSARDRYNSSGQHPRSTDEFIMIRNVLLAFMICSGMTLLGGCSSVMSHTGGKEGTYPGTRASATMI

GDDETNWGTKSLAILDMPFTAVMDTLLLPWDVFRKDSSVRSRVEKSEANAQATNAVIPPARMPDN

>gi|30043236|gb|AAP18957.1| putative transport protein [Shigella flexneri 2a str. 2457T]

MDKTFTPHPVIFALFRFDLNSSSSNTVKVAKTLNSSHISMLKWSAFPLKHATGNTMSEFIAENRGADAIT
RPNWSAVFSVAFCVACLIIVEFLPVSLLTPMAQDLGISEGVAGQSVTVTAFVAMFASLFITQTIQATDRR
YVVILFAVLLTLSCLLVSFANSFSLLLIGRACLGLALGGFWAMSASLTMRLVPPRTVPKALSVIFGAVSI
ALVIAAPLGSFLGELIGWRNVFNAAAVMGVLCIFWIIKSLPSLPGEPSHQKQNTFRLLQRPGVMAGMIAI
FMSFAGQFAFFTYIRPVYMNLAGFGVDGLTLVLLSFGIASFIGTSLSSFILKRSVKLALAGAPLILAVSA
LVLTLWGSDKIVATGVAIIWGLTFALVPVGWSTWITRSLADQAEKAGSIQVAVIQLANTCGAAIGGYALD
NIGLTSPLMLSGTLMLLTALLVTAKVKMKKS

>gi|30043217|gb|AAP18938.1| membrane-bound ATP synthase [Shigella flexneri 2a str. 2457T]
MKNVMSVSLVSRNVARKLLLVQLLVVIASGLLFSLKDPFWGVSAISGGLAVFLPNVLFMIFAWRHQAHTP
AKGRVAWTFAFGEAFKVLAMLVLLVVALAVLKAVFLPLIVTWVLVLVVQILAPAVINNKG

>gi|30043210|gb|AAP18931.1| putative 2-component regulator [Shigella flexneri 2a str. 2457T]
MTKGRHFIMAHPHLLAERISRLSSSLEKGLYERSHAIRLCLLAALSGESVFLLGPPGIAKSLIARRLKFA
FQNARAFEYLMTRFSTPEEVFGPLSIQALKDEGRYERLTSGYLPEAEIVFLDEIWKAGPAILNTLLTAIN
ERQFRNGAHVEKIPMRLLVAASNELPEADSSLEALYDRMLIRLWLDKVQDKANFRSMLTSQQDENDNPVP
DALQVTDEEYERWQKEIGEITLPDHVFELIFILRQQLDKLPDAPYVSDRRWKKAIRLLQASAFFSGRSAV
APVDLILLKDCLWYDAQSLNLIQQQIDVLMTGHAWQQQGMLTRLGAIVQRHLQLQQQQSDKTALTLIRLG
GIFSRRQQYQLPVNVTASTLTLLLQKPLKLHDMEVVHISFERSALEQWLSKGGEIRGKLNGIGFAQKLNL
EVDSTQHLVVRDVSLQGSTLALPGSSAEGLPGEIKQQLEELESDWRKQHALFSEQQKCLFIPGDWLGRIE
DSLQDVGAQIRQAQQC

>gi|30043155|gb|AAP18877.1| diaminopimelate epimerase [Shigella flexneri 2a str. 2457T]
MMQFSKMHGLGNDFMVVDAVTQNVFFSPELIRRLADRHLGVGFDQLLVVEPPYDPELDFHYRIFNADGSE
VAQCGNGARCFARFVRLKGLTNKRDIRVSTANGRMVLTVTDDDLVRVNMGEPNFEPSAVPFRANKAEKTY

IMRAAEQTILCGVVSMGNPHCVIQVDDVDTAAVETLGPVLESHERFPERANIGFMQVVKREHIRLRVYER

GAGETQACGSGACAAVAVGIQQGLLDEEVRVELPGGRLDIAWKGPGHPLYMTGPAVHVYDGFIHL

>gi|30043143|gb|AAP18865.1| hypothetical protein S3857 [Shigella flexneri 2a str. 2457T]

MKCKDDMSAVLTAEQALKLVGEMFVYHMPFNRALGMELERYEKEFAQLAFKNQPMMVGNWAQSILHGGVI

ASALDVAAGLVCVGSTLTRHETISEDELRQRLSRMGTIDLRVDYLRPGRGERFTATSSLLRAGNKVAVAR

VELHNEEQLYIASATATYMVG

>gi|30042987|gb|AAP18709.1| thiamin biosynthesis protein, thiazole moiety [Shigella flexneri 2a str. 2457T]

MSSGRNISCRMATRSCFFRLLQGVEMLRIADKTFDSHLFTGTGKFASSQLMVEAIRASGSQLVTLAMKRV

DLRQHNDAILEPLIAAGVALLPNTSGAKTAEEAIFAAHLAREALGTNWLKLEIHPDARWLLPDPIETLKA

AETLVQQGFVVLPYCGADPVLCKRLEEVGCAAVMPLGAPIGSNQGLETRAMLEIIIQQATVPVVVDAGIG

VPSHATQALEMGADAVLVNTAIAVADDPVNMAKAFRLAVEVGLLARQSGPGSRSYFAHATSPLTGFLEAS

A

>gi|30042976|gb|AAP18698.1| hypothetical protein S3661 [Shigella flexneri 2a str. 2457T]

MSKNDSLPAAGESFLLVYHARLPVISAFHRWHGRCNTRSKTTTGGLTMKRNTKIALVMMALSAMAMGSTS

AFAHGGHGMWQQNAAPLTSEQQTAWQKIHNDFYAQSSALQQQLVTKRYEYNALLAANPPDSSKINAVAKE

MENLRQSLDELRVKRDIAMAEAGIPRGAGMGMGYGGCGGGGHMGMGHW

>gi|30042968|gb|AAP18690.1| repressor of aceBA operon [Shigella flexneri 2a str. 2457T]

MKIISTIQKKEAVMVAPIPAKRGRKPAVATAPATGQVQSLTRGLKLLEWIAESNGSVALTELAQQAGLPN

STTHRLLTTMQQQGFVRQVGELGHWAIGAHAFMVGSSFLQSRNLLAIVHPILRNLMEESGETVNMAVLDQ

SDHEAIIIDQVQCTHLMRMSAPIGGKLPMHASGAGKAFLAQLSEEQVTKLLHRKGLHAYTHATLVSPVHL

KEDLAQTRKRGYSFDDEEHALGLRCLAACIFDEHREPFAAISISGPISRITDDRVTEFGAMVIKAAKEVT

LAYGGMR

>gi|30042898|gb|AAP18621.1| hypothetical protein S3575 [Shigella flexneri 2a str. 2457T]

MKMPIKRVLTLTCWNTRSNWWRISVNFSPKSSQIHHALRTVAGRFAVKSIDYFWHDSCNASKRFHIWESI

MLELLFVIGFFVMLMVTGVSLLGIIAALVVATAIMFLGGMLALMIKLLPWLLLAIAVVWVIKAIKAPKMP

KYQRYDRWRY

>gi|30042895|gb|AAP18618.1| putative regulator [Shigella flexneri 2a str. 2457T]

MFFLEHGKVRTFLTPTLRCPMEKTTTQELLAQAEKICAQRNVRLTPQRLEVLRLMSLQDGAISAYDLLDL

LREAEPQAKPPTVYRALDFLLEQGFVHKVESTNSYVLCHLFDQPTHTSAMFICDRCGAVKEECAEGVEDI

MHTLAAKMGFALRHNVIEAHGLCAACVEVEACRHPEQCQHDHSVQVKKKPR

>gi|30042893|gb|AAP18616.1| DNA-damage-inducible protein F [Shigella flexneri 2a str. 2457T]

MPLGVAVCFSSLFIRLVCMAFLTSSDKALWHLALPMIFSNITVPLLGLVDTAVIGHLDSPVYLGGVAVGA

TATSFLFMLLLFLRMSTTGLTAQAYGAKNPQALARALVQPLLLALGAGALIALLRTPIIDLALHIVGGSE

AVLEQARRFLEIRWLSAPASLANLVLLGWLLGVQYARAPVILLVVGNILNIVLDVWLVMGLHMNVQGAAL

ATVIAEYATLLIGLLMVRKILKLRGISGEMLKTAWRGNFRRLLALNRDIMLRSLLLQLCFGAITVLGARL

GSDIIAVNAVLMTLLTFTAYALDGFAYAVEAHSGQAYGARDGSQLLDVWRAACRQSGIVALLFSVVYLLA

GEHIIALLTSLTQIQQLADRYLIWQVILPLVGVWCYLLDGMFIGATRAAEMRNSMAVAAAGFALTLLTLP

WLGNHGLWLALTVFLALRGLSLAAIWRRHWRNGTWFAAT

>gi|30042808|gb|AAP18531.1| glutamate synthase, large subunit [Shigella flexneri 2a str. 2457T]

MRGARMTRKPRRHALSVPVRSGSEVGFPQSLGEVHDMLYDKSLERDNCGFGLIAHIEGEPSHKVVRTAIH

ALARMQHRGAILADGKTGDGCGLLLQKPDRFFRIVAQERGWRLAKNYAVGMLFLNKDPELAAAARRIVEE

ELQRETLSIVGWRDVPTNEGVLGEIALSSLPRIEQIFVNAPAGWRPRDMERRLFIARRRIEKRLEADKDF

YVCSLSNLVNIYKGLCMPADLPRFYLDLADLRLESAICLFHQRFSTNTVPRWPLAQPFRYLAHNGEINTI

TGNRQWARARTYKFQTPLIPDLHDAAPFVNETGSDSSSMDNMLELLLAGGMDIIRAMRLLVPPAWQNNPD

MDPELRAFFDFNSMHMEPWDGPAGIVMSDGRFAACNLDRNGLRPARYVITKDKLITCASEVGIWDYQPDE

VVEKGRVGPGELMVIDTRSGRILHSAETDDDLKSRHPYKEWMEKNVRRLVPFEDLPDEEVGSRELDDDTL

ASYQKQFNYSAEELDSVIRVLGENGQEAVGSMGDDTPFAVLSSQPRIIYDYFRQQFAQVTNPPIDPLREA

HVMSLATSIGREMNVFCEAEGQAHRLSFKSPILLYSDFKQLTTMKEEHYRADTLDITFDVTKTTLEATVK

ELCDKAEKMVRSGTVLLVLSDRNIAKDRLPVPAPMAVGAIQTRLVDQSLRCDANIIVETASARDPHHFAV

LLGFGATAIYPYLAYETLGRLVDTHAIAKDYRTVMLNYRNGINKGLYKIMSKMGISTIASYRCSKLFEAV

GLHDDVVGLCFQGAVSRIGGASFEDFQQDLLNLSKRAWLARKPISQGGLLKYVHGGEYHAYNPDVVRTLQ

QAVQSGEYSDYQEYAKLVNERPATTLRDLLAITPGENAVNIADVEPASELFKRFDTAAMSIGALSPEAHE

ALAEAMNSIGGNSNSGEGGEDPARYGTNKVSRIKQVASGRFGVTPAYLVNADVIQIKVAQGAKPGEGGQL

PGDKVTPYIAKLRYSVPGVTLISPPPHHDIYSIEDLAQLIFDLKQVNPKAMISVKLVSEPGVGTIATGVA

KAYADLITIAGYDGGTGASPLSSVKYAGCPWELGLVETQQALVANGLRHKIRLQVDGGLKTGVDIIKAAI

LGAESFGFGTGPMVALGCKYLRICHLNNCATGVATQDDKLRKNHYHGLPFKVTNYFEFIARETRELMAQL

GVTRLVDLIGRTDLLKELDGFTAKQQKLALSKLLETAEPHAGKSLYCTENNPPFDNGLLNAQLLQQAKPF

VDERQSKTFWFDIRNTDRSVGASLSGYIAQTHGDQGLAADPIKAYFNGTAGQSFGVWNAGGVELYLTGDA

NDYVGKGMAGGLIAIRPPVGSAFRSHEASIIGNTCLYGATGGRLYAAGRAGERFGVRNSGAITVVEGIGD

NGCEYMTGGIVCILGKTGVNFGAGMTGGFAYVLDESGDFRKRVNPELVEVLSVDDLAIHEEHLRGLITEH

VQHTGSQRGEEILANWSTFATKFALVKPKSSDVKALLGHRSRSAAELRVQAQ

>gi|30042774|gb|AAP18497.1| 7,8-dihydropteroate synthase [Shigella flexneri 2a str. 2457T]

MLRGFFLSIHTRDNIMKLFAQGTSLDLSHPHVMGILNVTPDSFSDGGTHNSLIDAVKHANLMINAGATII

DVGGESTRPGAAEVSVEEELQRVIPVVEAIAQRFEVWISVDTSKPEVIRESAKVGAHIINDIRSLSEPGA

LEAAAETGLPVCLMHMQGNPKTMQEAPKYDDVFAEVNRYFIEQIARCEQAGIAKEKLLLDPGFGFGKNLS
HNYSLLARLAEFHHFNLPLLVGMSRKSMIGQLLNVGPSERLSGSLACAVIAAMQGAHIIRVHDVKETVEA
MRVVEATLSAKENKRYE

>gi|30042769|gb|AAP18492.1| hypothetical protein S3428 [Shigella flexneri 2a str. 2457T]
MGLSTLEQKLTEMITAPVEALGFELVGIEFIRGRTSTLRIYIDSEDGINVDDCADVSHQVSAVLDVEDPI
TVAYNLEVSSPGLDRPLFTAEHYARFVGEEVTLVLRMAVQNRRKWQGVIKAVDGEMITVTVEGKDEVFAL
SNIQKANLVPHF

>gi|30042723|gb|AAP18446.1| hypothetical protein S3376 [Shigella flexneri 2a str. 2457T]
MAYCNPGLESRPNKRNALRRHVVTGIGMKIVIAPDSYKESLSASEVAQAIEKGFREIFPDAQYVSIPVAD
GGEGTVEAMIAATQGSERHAWVTGPLGEKVNASWGISGDGKTAFIEMAAASGLELVPAEKRDPLVTTSRG
TGELILQALESGATNIIIGIGGSATNDGGAGMVQALGAKLCDANGNEIGFGGGSLNTLNDIDISGLDPRL
KDCVIRVACDVTNPLVGDNGASRIFGPQKGASEAMIVELDNNLSHYADVIKKALHVDVKDVPGAGAAGGM
GAALMAFLGAELKSGIEIVTTALNLEEHIHDCTLVITGEGRIDSQSIHGKVPIGVANVAKKYHKPVIGIA
GSLTDDVGVVHQHGIDAVFSVLTSIGTLDEAFRGAYDNICRASRNIAATLAIGMRNTG

>gi|30042644|gb|AAP18368.1| hypothetical protein S3282 [Shigella flexneri 2a str. 2457T]
MGIYHWSRKTKMKRTKSIRHASFRKNWSARHLTPVALAVATVFMLAGCEKSDETVSLYQNADDCSAANPG
KSAECTTAYNNALKEAERTAPKYATREDCVAEFGEGQCQQAPAQAGMAPENQAQAQQSSGSFWMPLMAGY
MMGRLMGGGAGFAQQPLFSSKNPASPAYGKYTDATGKNYGAAQPGRTMTVPKTAMAPKPATTTTVTRGGF
GESVAKQSTMQRSATGTSSRSMGG

>gi|30042537|gb|AAP18261.1| hypothetical protein S3159 [Shigella flexneri 2a str. 2457T]

MGLNVREGEIMAKNRSRRLRKKMHIDEFQELGFSVAWRFPEGTSEEQIDKIVDDFINEVIEPNKLAFDGS

GYLAWEGLICMQEIGKCTEEHQAIVRKWLEECKLDEVRTSELFDVWWD

>gi|30042521|gb|AAP18245.1| hypothetical protein S3143 [Shigella flexneri 2a str. 2457T]

MTAPFFPRILGAVAFLNQETEPLTMNLQHHFLIAMPALQDPIFRRSVVYICEHNTNGAMGIIVNKPLENL

KIEGILEKLKITPEPRDESIRLDKPVMLGGPLAEDRGFILHTPPSNFASSIRISDNTVMTTSRDVLETLG

TDKQPSDVLVALGYASWEKGQLEQEILDNAWLTAPADLNILFKTPIADRWREAAKLIGVDILTMPGVAGH

A

>gi|30042324|gb|AAP18049.1| putative 2-component transcriptional regulator [Shigella flexneri 2a str. 2457T]

MSSQNDYQSNGRYDNIYSQIDSEAKMSFSVDVLANIAIELQRGIGHQDRFQRLITTLRQVLECDASALLR

YDSRQFIPLAIDGLAKDVLGRRFALEGHPRLEAIARAGDVVRFPADSELPDPYDGLIPGQESLKVHACVG

LPLFVGQNLIGALTLDGMQPDQFDVFSDEELRLIAALAAGALSNALLIEQLESQNMLPGDATPFEAVKQT

QMIGLSPGMTQLKKEIEIVAASDLNVLISGETGTGKELVAKAIHEASPRAVNPLVYLNCAALPESVAESE

LFGHVKGAFTGAISNRSGKFEMADNGTLFLDEIGELSLALQAKLLRVLQYGDIQRVGDDRSLRVDVRVLA

ATNRDLREEVLAGRFRADLFHRLSVFPLSVPPLRERGDDVILLAGYFCEQCRLRQGLSRVVLSAGARNLL

QHYSFPGNVRELEHAIHRAVVLARATRNGDEVILEAQHFAFPEVTLPPPEAAAVPVVKQNLREATEAFQR

ETIRQALAQNHHNWAACARMLETDVANLHRLAKRLGLKD

>gi|30042282|gb|AAP18007.1| hypothetical protein S2871 [Shigella flexneri 2a str. 2457T]

MSLLCAFFRNVDIFRLWLRNEHRHVTLHLIRGSLLMNALTAVQNNAVDSGQDYSGFTLIPSAQSPRLLEL

TFTEQTTKQFLEQVAEWPVQALEYKSFLRFRVGKILDDLCANQLQPLLLKTLLNRAEGALLINAVGIDDV

AQADEMVKLATAVAHLIGRSNFDAMSGQYYARFVVKNVDNSDSYLRQPHRVMELHNDGTYVEEITDYVLM

MKIDDWEHLDHYFRHPLARRPMRFAAPPSKNVSKDVFHPVFDVDQQGRPVMRYIDQFVQPKDFEEGVWLS

ELSDAIETSKGILSVPVPVGKFLLINNLFWLHGRDRFTPHPDLRRELMRQRGYFAYATHHYQTHQ

>gi|30042204|gb|AAP17929.1| hypothetical protein S2780 [Shigella flexneri 2a str. 2457T]

MEYKIHSRVSHIACCRHYFIARKDMNGLLRIRQRYQGLAQSDKKLADYLLLQPDTARHLSSQQLANEAGV

SQSSVVKFAQKLGYKGFPALKLALSEALASQPESPSVPIHNQIRGDDPLRLVGEKLIKENTAAMYATLNV

NSEEKLHECVTMLRSARRIILTGIGASGLVAQNFAWKLMKIGFNAAALRDMHALLATVQASSPDDLLLAI

SYTGVRRELNLAADEMLRVGGKVLAITGFTPNALQQRASHCLYTIAEEQATNSASISACHAQGMLTDLLF

IALIQQDLELAPERIRHSEALVKKLV

>gi|30042053|gb|AAP17779.1| hypothetical protein S2640 [Shigella flexneri 2a str. 2457T]

MSTLLSLIGVFRHTNNKFIRIAKQTGASLLPIFHPMMLSPGGANPDVDCITKAAKSRDKKPPLCSEGWKF

GGAKRDRTADLLHAMQALSQLSYSPTMRLRTKFAGCKIWWS

>gi|30042050|gb|AAP17776.1| hypothetical protein S2596 [Shigella flexneri 2a str. 2457T]

MKSPYCHRSNYKLAIAIITVITGTVVTGEIVITGIAIMNGAKTAGGVMIMAIIVAGISVKHMSVAIVKAG

AIVTIIAEKAADMGTAIKRVLQWSTMPDATLARLIRPTNRLFNA

>gi|30042039|gb|AAP17765.1| putative heat shock protein [Shigella flexneri 2a str. 2457T]

MSVPLSTWNLLRYNNSYLQKVTMFPQCKFSREFLHPRYWLTWFGLGVLWLWVQLPYPVLCFLGTRIGAMA

RPFLKRRESIARKNLELCFPQHSAEEREKMIAENFRSLGMALVETGMAWFWPDSRVRKWFDVEGLDNLKR

AQMQNRGVMVVGVHFMSLELGGRVMGLCQPMMATYRPHNNQLMEWVQTRGRMRSNKAMIGRNNLRGIVGA

LKKGEAVWFAPDQDYGRKGSSFAPFFAVENVATTNGTYVLSRLSGAAMLTVTMVRKADYSGYRLFITPEM

EGYPTDENQAAAYMNKIIEKEIMRAPEQYLWIHRRFKTRPVGESSLYI

>gi|30042005|gb|AAP17731.1| putative peptidase [Shigella flexneri 2a str. 2457T]

MRSLTHIAKIEPPPVRRVTYVKHYSIQPANLEFNAEGTPVSRDFDDVYFSNDNGLEETRYVFLGGNQLEV

RFPEHPHPLFVIAESGFGTGLNFLTLWKAFDQFREAHPQAQLQRLHFISFEKFPLTRADLALAHQHWPEL

APWAEQLQAQWPMPLPGCHRLLLDESRVTLDLWFGDINELTSQLDDSLNQKVDAWFLDGFAPAKNPDMWT

QNLFNAMARLARPGGTLATFTSAGFVRRGLQEAGFTMQKRKGFGRKREMLCGVMEQTLPLPCSTPWFNRT

GSSKREVAIIGGGIASALLSLALLRRGWQVALYCADEAPALGASGNRQGALYPLLSKHDEALNRFFSNAF

TFARRFYDQLPVKFDHDWCGVTQLGWDEKSQHKIAQMLSMDLPAELAVAVEANAVEQITGVATNCSGITY

PQGGWLCPAELTRNVLELAQQQGLQIYYQYQLQNLSRKDDCWLLNFAGDQQATHSVVVLANGHQISRFSQ

TSTLPVYSVAGQVSHIPTTPELAELKQVLCYDGYLTPQNPANQHHCIGASYHRGSEDTAYSEDDQQQNRQ

RLIDCFPQAQWAKEVDVSDKEARCGVRCATRDHLPMVGNVPDYEATLVEYASLAEQKDEAVSAPVFDDLF

MFAALGSRGLCSAPLCAEILAAQMSDEPIPMDASTLAALNPNRLWVRKLLKGKAVKAG

>gi|30041979|gb|AAP17705.1| putative S-transferase [Shigella flexneri 2a str. 2457T]

MQGNICAMSAITESKPTRRWAMPDTLVIIFFVAILTSLATWVVPVGMFDSQEVQYQVDGQTKTRKVVDPH

SFRILTNEAGEPEYHRVQLFTTGDERPGLMNFPFEGLTSGSKYGTAVGIIMFMLVIGGAFGIVMRTGTID

NGILALIRHTRGNEILFIPALFILFSLGGAVFGMGEEAVAFAIIIAPLMVRLGYDSITTVLVTYIATQIG

FASSWMNPFCVVVAQGIAGVPVLSGSGLRIVVWVIATLIGLIFTMVYASRVKKNPLLSRVHESDRFFREK

QADVEQRPFTFGDWLVLIVLTAVMVWVIWGVIVNAWFIPEIASQFFTMGLVIGIIGVVFRLNGMTVNTMA

SSFTEGARMMIAPALLVGFAKGILLLVGNGEAGNASVLNTILNSIANAISGLDNAVAAWFMLLFQAVFNF

FVTSGSGQAALTMPLLAPLGDLVGVNRQVTVLAFQFGDGFSHIIYPTSASLMATLGVCRVDFRNWLKVGA

TLLGLLFIMSSVVVIGAQLMGYH

>gi|30041945|gb|AAP17671.1| putative transport/receptor protein [Shigella flexneri 2a str. 2457T]

MSETGNLLCGDKQTAQTYRAVAGTGAGLSWSCHDALAAGLAERTGRHCLPDVKSEFCLGDAGCSKTVART

GIAASLVWGGVHYWWHCDPREYGVMCLIWGLFSVIIASVAQLSLGFAASHLPPMTHLWDFIAALLAFGLD

ARILLLGLLGYLLSVFCWYKTLHKLALSKAYALLSMSYVLVWIASMVLPGWEGTFSLKALLGVACIMSGL

MLIFLPTTKQRY

>gi|30041867|gb|AAP17594.1| putative elongation factor [Shigella flexneri 2a str. 2457T]

MQGVLAGLPSGIPGIGLEIEGAVQHAPQPGRHSIRCSLKVNHRIDERAQYHLFCTSKSANTTCPKGPSRV

LCRDIFDIFDYRNFSMPRANEIKKGMVLNYNGKLLLAKDIDIQSPTARGAATLYKMRFSDVRTGLKVEER

FKGDDIVDTVTLTRRYVDFSYVDGNEYVFMDKEDYTPYTFTKDQIEEELLFMPEGGMPDMQVLTWDGQLL

ALELPQTVDLEIVETAPGIKGASASARNKPATLSTGLVIQVPEYLSPGEKIRIHIEERRYMGRAD

>gi|30041762|gb|AAP17489.1| hypothetical protein S2248 [Shigella flexneri 2a str. 2457T]

MGAGIERRAGIRMTEKVKQHAAPVTGSDEIDIGRLVGTVIEARWWVIGITVVFALCAVVYTFFATPIYSA

DALVQIEQNSGNSLVQDIGSALANKPPASDAEIQLIRSRLVLGKTVDDLDLDIAVSKNTFPIFGAGWDRL

MGRQNETVKVTTFNRPKEMADQVFTLNVLDDKNYTLSSDGGFSARGQAGQMLKKEGVTLMVEAIHARPGS

EFTVTKYSTLGMINQLQNSLTVTENGKDAGVLSLTYTGEDREQIRDILNSIARNYQEQNIERKSAEASKS

LAFLAQQLPEVRSRLDVAENKLNAFRQDKDSVDLPLEAKAVLDSMVNIDAQLNELTFKEAEISKLYTKVH

PAYRTLLEKRQALEEEKAKLNGRVTAMPKNQQEIVRLTRDVESGQQVYMQLLNKEQELKITEASTVGDVR

IIDPAITQPGVLKPKKGLIILGAIILGLMLSIVGVLLRSLFNRGIESPQVLEEHGISVYASIPLSEWQKA

RDSVKTIKGVKRYKQSQLLAVGNPTDLAIEAIRSLRTSLHFAMMQAQNNVLMMTGVSPSIGKTFVCANLA

AVISQTNKRVLLIDCDMRKGYTHELLGTNNVNGLSEILIGQGDITTAAKPTSIAKFDLIPRGQVPPNPSE

LLMSERFAELVNWASKNYDLVLIDTPPILAVTDAAIVGRHVGTTLMVARYAVNTLKEVETSLSRFEQNGI

PVKGVILNSIFRRASAYQDYGYYEYEYKSDAK

>gi|30041615|gb|AAP17343.1| putative 1-aminocyclopropane-1-carboxylate deaminase [Shigella flexneri 2a str. 2457T]

MTKKGAFTSAFFIYAFSACIIRKSQQHTSEAVMPLHNLTRFPRLEFIGAPTPLEYLPRFSDYLGREIFIK

RDEVTPMAMGGNKLRKLEFLAADALREGADTLITAGAIQSNHVRQTAAVAAKLGLHCVALLENPIGTTAE

NYLTNGNRLLLDLFNTQIEMCDALTDPNAQLEELATRVEAQGFRPYVIPVGGSNALGALGYVESALEIAQ

QCEGAVNISSVVVASGSAGTHAGLAVGLEHLMPESELIGVTVSRSVADQLPKVVNLQQAIAKELELTASA

EILLWDDYFAPGYGVPNDEGMEAVKLLARLEGILLDPVYTGKAMAGLIDGISQKRFKDEGPILFIHTGGA

PALFAYHPHV

>gi|30041602|gb|AAP17330.1| hypothetical protein S2042 [Shigella flexneri 2a str. 2457T]

MSCHTSCIDHTSALIHCADEVFEKPLPHLFEAAVFLMGFTLWGKLEFNQRDGFLDCPHGDRVCGSGMMSK

VDILQLTGHFFTNFNGRDYIENIQNLFDNQLASYHIRNQFLIGIQRLGHRKLLIAF

>gi|30041520|gb|AAP17248.1| putative adhesin [Shigella flexneri 2a str. 2457T]

MIGRIMLHKKTLLFAALSAALWGGATQAADAAVVASLKPVGFIASAIADGVTETEVLLPDGASEHDYSLR

PSDVKRLQNADLVVWVGPEMEAFMQKPVSKLPEAKQVTIAQLEDVKPLLMKSIHGDDDDHDHAEKSDEDH

HHGDFNMHLWLSPEIARATAVAIHGKLVELMPQSRAKLDANLKDFEAQLASTETQVGNELAPLKGKGYFV

FHDAYGYFEKQFGLTPLGHFTVNPEIQPGAQRLHEIRTQLVEQKATCVFAEPQFRPAVVESVARGTSVRM

GTLDPLGTNIKLGKTSYSEFLSQLANQYASCLKGD

>gi|30041408|gb|AAP17137.1| transcriptional regulator [Shigella flexneri 2a str. 2457T]

MKLESPLGSDLARLVRIWRALIDHRLKPLELTQTHWVTLHNIHQLPPDQSQIQLAKAIGIEQPSLVRTLD
QLEEKGLISRQTCASDRRAKRIKLTEKAEPLISEMEAVINKTRAEILHGISAEELEQLITLIAKLEHNII
ELQAKG

>gi|30041360|gb|AAP17089.1| acid shock protein [Shigella flexneri 2a str. 2457T]
MKKQIEGMTMKKVLALVVAAAMGLSSAAFAAETATTPAPTATTTKAAPAKTTHHKKQHKAAPAQKAQAAK
KHHKNTKAEQKAPEQKAQAAKKHAGKHGHQQPAKPAAQPAA

>gi|30041343|gb|AAP17072.1| hypothetical protein S1722 [Shigella flexneri 2a str. 2457T]
MIENHLYSLVTVVKYKLLPCLLAIFLTGCDRTEVTLSFTPEMASFSNEFDFDPLRGPVKDFTQTLMDEQG
EVTKRVSGTLSEEGCFDSLELLDLENNTVVALVLDANYYRDAETLEKRVRLQGKCQLAELPSAGVSWETD
DNGFVIKASSKQMQMEYRYDDQGYPLGKTTKSNDKTLSVSATPSTDPIKKLDYTAVTLLNNQRVGNVKQS
CEYDSHANPVDCQLIIVDEGVKPAVERVYTIKNTIDYY

>gi|30041218|gb|AAP16947.1| hypothetical protein S1570 [Shigella flexneri 2a str. 2457T]
MIKETAMPHRALLLVDLQNDFCAGGALAVPEGDSTVEVANRLIDWCQSRGEAVIASQDWHPANHGSFASQ
HGVEPYTPGQLGGLRQTFWPEHCVQNSEGAQLHPLLNQKEIAAVFHKGENPLVDSYSAFFDNGRRQKTAL
DDWLRDHEIDELIVMGLATDYCVKFTVLDALQLGYKVNVITDGCRGVNIQPQDSAHAFMEMSAAGATLYT
LADWEETQG

>gi|30041136|gb|AAP16866.1| cytochrome b(561) [Shigella flexneri 2a str. 2457T]

MLTENYVKAGNVMENKYSRLQISIHWLVFLLVIAAYCAMEFRGFFPRSDRPLINMIHVSCGISILVLMVV

RLLLRLKYPTPPIIPKPKPMMTGLAHLGHLVIYLLFIALPVIGLVMMYNRGNPWFAFGLTMPYASEANFE

RVDSLKSWHETLANLGYFVIGLHAAAALAHHYFWKDNTLLRMMPRKRS

>gi|30041071|gb|AAP16801.1| psp operon transcriptional activator [Shigella flexneri 2a str. 2457T]

MANFIMAEYKDNLLGEANSFLEVLEQVSHLAPLDKPVLIIGERGTGKELIASRLHYLSSRWQGPFISLNC

AALNENLLDSELFGHEAGAFTGAQKRHPGRFERADGGTLFLDELATAPMMVQEKLLRVIEYGELERVGGS

QPLQVNVRLVCATNADLPAMVNEGTFRADLLDRLAFDVVQLPPLRERESDIMLMAEHFAIQMCREIKLPL

FPGFTERARETLLNYRWPGNIRELKNVVERSVYRHGTSDYPLDDIIIDPFKRRPPEEAIAVSENTSLPTL

PLDLREFQMQQEKELLQLSLQQGKYNQKRAAELLGLTYHQFRALLKKHQI

>gi|30041022|gb|AAP16752.1| hypothetical protein S1340 [Shigella flexneri 2a str. 2457T]

MHDLNKDIIFPLQIFALSNNVLYNFPEQGVVPVLYVIYAQDKADSLEKRLSVRPAHLARLQLLHDEGRLL

TAGPMPAVDSNDPGAAGFTGSTVIAEFESLEAAQAWADADPYVAAGVYEHVSVKPFKKVF

>gi|30041017|gb|AAP16747.1| hypothetical protein S1335 [Shigella flexneri 2a str. 2457T]

MSPSDCFTSSARCCKTSPTEVKQVVTMDMDLNNRLTEDETLEQAYDIFLELAADNLDPADVLLFNLQFEE

RGGAELFDPAEDWQEHVDFDLNPDFFAEVVIGLADSEDGEISDVFARILLCREKDHKLCHIIWRE

>gi|30040947|gb|AAP16677.1| hypothetical protein S1258 [Shigella flexneri 2a str. 2457T]

MAEHLMSDVPFWQSKTLDEMSDAEWESLCDGCGQCCLHKLMDEDTDEIYFTNVACRQLNIKTCQCRNYER

RFEFEPDCIKLTRENLPTFEWLPMTCAYRLLAEGKDLPAWHPLLTGSKAAMHGERISVRHIAVKESEVID
WQDHILNKPDWAQ

>gi|30040754|gb|AAP16485.1| putative sulfite reductase [Shigella flexneri 2a str. 2457T]
MRGFVFCYTAASLIKVVIIMLIFEGKEIETDTEGYLKESSQWSEPLAVVIAENEGISLSPEHWEVVRFVR
DFYLEFNTSPAIRMLVKAMANKFGEEKGNSRYLYRLFPKGPAKQATKIAGLPKPVKCI

>gi|30040748|gb|AAP16479.1| methylglyoxal synthase [Shigella flexneri 2a str. 2457T]
MYIMELTTRTLPARKHIALVAHDHCKQMLMSWVERHQPLLEQHVLYATGTTGNLISRATGMNVNAMLSGP
MGGDQQVGALISEGKIDVLIFFWDPLNAVPHDPDVKALLRLATVWNIPVATNVATADFIIQSPHFNDAVD
ILIPDYQRYLADRLK

>gi|30040718|gb|AAP16449.1| hypothetical protein S1000 [Shigella flexneri 2a str. 2457T]
MFRFLTFCLCEVMPMRNIIKLALAGLLSVSTFAVAAESSPEALRIGYQKGSIGMVLAKSHQLLEKRYPQS
KISWVEFPAGPQMLEALNVGSIDLGSTGDIPPIFAQAAGADLVYVGVEPPKPKAEVILVAENSPIKTVAD
LKGHKVAFQKGSSSHNLLLRALRQAGLKFTDIQPTYLTPADARAAFQQGNVDAWAIWDPYYSAALLQGGV
RVLKDGTDLNQTGSFYLAARPYAEKNGAFIQGVLATFSEADALTRSQREQSIALLAKTMGLPAPVIASYL
DHRPPTTIKPVNAEVAALQQQTADLFYENRLVPKKVDIRQRIWQPTQLEGKQL

>gi|30040602|gb|AAP16334.1| putative prismane HCP protein [Shigella flexneri 2a str. 2457T]
MIMFCVQCEQTIRTPAENGCSYAQGMCGKTAETSDLQDLLIAALQGLSAWAVKAREYGIINHDVDSFAPR
AFFSTLTNVNFDSPRIVGYAREAIALREALKAQCLAVDANARVDNPMADLQLVSDDLGELQRQAAEFTPN
KDKAAIGENILGLRLLCLYGLKGAAAYMEHAHVLGQYDNDIYAQYHKIMAWLGTWPADMNALLECSMEIG

QMNFKVMSILDAGETGKYGHPTPTQVNVKATAGKCILISGHDLKDLYNLLEQTEGTGVNVYTHGEMLPAH

GYPELRKFKHLVGNYGSGWQNQQVEFARFPGPIVMTSNCIIDPTVGAYDDRIWTRSIVGWPGVRHLDGED

FSAVIAQAQQMAGFPYSEIPHLITVGFGRQTLLGAADTLIDLVSREKLRHIFLLGGCDGARGERHYFTDF

ATSVPDDCLILTLACGKYRFNKLEFGDIEGLPRLVDAGQCNDAYSAIILAVTLAEKLGCGVNDLPLSLVL

SWFEQKAIVILLTLLSLGVKNIVTGPTAPGFLTPDLLAVLNEKFGLRSITTVEEDMKQLLNA

>gi|30040553|gb|AAP16285.1| putative transaldolase [Shigella flexneri 2a str. 2457T]

MRQLFREFSRDSLHSDKKYILRMVMELYLDTSDVVAVKALSRIFPLAGVTTNPSIIAAGKKPLDVVLPQL

HEAMGGQGRLFAQVMATTAEGMVNDALKLRSIIADIVVKVPVTAEGLAAIKMLKAEGIPTLGTAVYGAAQ

GLLSALAGAEYVAPYVNRIDAQGGSGIQTVTDLHQLLKMHAPQAKVLAASFKTPRQALDCLLAGCESITL

PLDVAQQMISYPAVEAAVTKFEQDWQGAFGRTSI

>gi|30040534|gb|AAP16266.1| putative enzyme [Shigella flexneri 2a str. 2457T]

MWGAGPTFWRTCMMYHIPGVLSPQDVAHFREQLEQAEWVDGRVTTGAQGAQVKNNQQVDTRSALYAALQN

EVLNAVNQHALFFAAALPRTLSTPLFNRYQNNETYGFHVDGAVRSHPQNGWMRTDLSATLFLSDPQSYDG

GELVVNDTFGQHRVKLPAGDLVLYPSSSLHCVTPVTRGVRVASFMWIQSMIRDDKKRAMLFELDKNIQSL

KSRYGENEEILSLLNLYHNLLREWSEI

>gi|30040361|gb|AAP16093.1| succinate dehydrogenase, cytochrome b556 [Shigella flexneri 2a str. 2457T]

MWALFMIRNVKKQRPVNLDLQTIRFPITAIASILHRVSGVITFVAVGILQWLLGTSLSSPEGFEQASAIM

GSFFVKFIMWGILTALAYHVVVGIRHMMMDFGYLEETFEAGKRSAKISFVITVVLSLLAGVLVW

>gi|30040306|gb|AAP16039.1| thiol:disulfide interchange protein [Shigella flexneri 2a str. 2457T]

MTVIGYAFYSTFALTEKNKLMLKKILLLALLPAIAFAEELPSPVKAIEKQGITIIKTFDAPGGMKGYLGK
YQDMGVTIYLTPDGKHAISGYMYNEKGENLSNTLIEKEIYAPAGREMWQRMEQSHWLLDGKKDAPVIVYV
FSDPFCPYCKQFWQQARPWVDSGKVQLRTLLVGVIKPESPATAAAILASKDPAKTWQQYEASGCKLKLNV
PANVSTEQMKVLSDNEKLMDDLGANVTPAIYYMSKENTLQQAVGLPDQKTLNIIMGNK

>gi|30040180|gb|AAP15913.1| putative polymerase/proteinase [Shigella flexneri 2a str. 2457T]
MMNLHALPTSPRRWQCYDGGNFYQTRLRVSESPNMFKKILFPLVALFMLAGCAKPPTTIEVSPTITLPQQ
DPSLMGVTVSINGADQRTDQALAKVTRDNQIVTLTASRALRFLLQEVLEKQMTARGYMVGPNGPVNLQII
VSQLYADVSQGNVRYNIATKADIAIIATAQNGNKMTKNYRASYNVEGAFQASNKNIADAVNSVLTDTIAD
MSQDTSIHEFIKQNAR

>gi|30040151|gb|AAP15884.1| hypothetical protein S0357 [Shigella flexneri 2a str. 2457T]
MLSLLLPSTIRLIATRKGRPMNTNVFRLLLLGSLFSLSACVQQSEVRQMKHSVSTLNQEMTQLNQETVKI
TQQNRLNAKSSSGVYLLPGAKTPARLESQIGTLRMSLVNITPDTDGTTLTLRIQGESNDPLPAFSGTVEY
GQIQGTIDNFQEINVQNQLINAPASVLAPSDVDIPLQLKGISVDQLGFVRIHDIQPVMQ

>gi|30040127|gb|AAP15860.1| hypothetical protein S0331 [Shigella flexneri 2a str. 2457T]
MPHSCREIHCFDNRWQKHKQNYAGRQKRDTIEDYLTKDDFMTIWVDADACPNVIKEILYRAAERMQMPLV
LVANQSLRVPPSRFIRTLRVAAGFDVADNEIVRQCEAGDLVITADIPLAAEAIEKGAAALNSRGERYTPA
TIRERLTMRDFMDTLRASGIQTGGPDSLSQRDRQAFAAELEKWWLEVQRSRG

>gi|30040069|gb|AAP15803.1| putative transporter [Shigella flexneri 2a str. 2457T]

MRQAGLSMAAKHHSNLARLATGWKHAIFLKLTERVSVVGLRNIRRRRKRMDNRSEFLNNVAQALGRPLRL

EPQAEDAPLNNYANERLTQLNQQQRCDAFIQFASDVMLTRCELTSEAKAAEAAIRLCKELGDQSVVISGD

TRLEELGISERLQQECNAVVWDPAKGAENISQAEQAKVGVVYAEYGLTESGGVVLFSAAERGRSLSLLPE

SSLFILRKSTILPRVAQLAEKLHQKAQAGERMPSCINIISGPSSTADIELIKVVGVHGPVKAVYLIIEDC

>gi|30039926|gb|AAP15660.1| hypothetical protein S0118 [Shigella flexneri 2a str. 2457T]

MARCDFGALPGAEEHTMDYEFLRDITGVVKVRMSMGHEVVGHWFNEEVKENLALLDEVEQAAHALKGSER

SWQRAGHEYTLWMDAEEVMVRANQLEFAGDEMEEGMNYYDEESLSLCGVEDFLQVVAAYRNFVQQK

>gi|30039906|gb|AAP15640.1| hypothetical protein S0096 [Shigella flexneri 2a str. 2457T]

MLWTSGFNDKICALNTFEFDRDGNNVSGILTRWRQFGKRYFWPHLLLGMVAASLGLPALSNAAEPNAPAK

ATTRNHEPSAKVNFGQLALLEANTRRPNSNYSVDYWHQHAIRTVIRHLSFAMAPQTLPVAEESLPLQAQH

LALLDTLSALLTQEGTPSEKGYRIDYAHFTPQAKFSTPVWISQAQGIRAGPQRLT

>gi|30039883|gb|AAP15617.1| probable transcriptional activator for leuABCD operon [Shigella flexneri 2a str. 2457T]

MTHSTAMDSVFIRTRIFMFSEFYSFCFFLFYMHDKSYSSGLFLCIPIRERELSVTVELSMPEVQTDHSET

AELSKPQLRMVDLNLLTVFDAVMQEQNITRAAHVLGMSQPAVSNAVARLKVMFNDELFVRYGRGIQPTAR

AFQLFGSVRQALQLVQNELPGSGFEPASSERVFHLCVCSPLDSILTSQIYNHIEQIAPNIHVMFKSSLNQ

NTEHQLRYQETEFVISYEDFHRPEFTSVPLFKDEMVLVASKNHPTIKGPLLKHDVYNEQHAAVSLDRFAS

FSQPWYDTVDKQASIAYQGMAMMSVLSVVSQTHLVAIAPRWLAEEFAESLELQVLPLPLKQNSRTCYLSW

HEAAGRDKGHQWMEEQLVSICKR

>gi|30039844|gb|AAP15578.1| transcriptional regulator of cai operon [Shigella flexneri 2a str. 2457T]

MKLHTIDISTILIWPCLIASKRVIARLLICETGVRMCEGYVEKPLYLLIAEWMMAENRWVIAREISIHFD

IEHSKAVNTLTYILSEVTEISCEVKMIPNKLEGRGCQCQRLVKVVDIDEQIYARLRNNSREKLVGVRKTP

RIPAVPLTELNREQKWQMMLSKSMRR

>gi|30039824|gb|AAP15558.1| positive regulator for sigma 32 heat shock promoters [Shigella flexneri 2a str. 2457T]

MLPLTALTPFNAAPTGPPSPAPRSKPCPSTLIAAWVRKMRVSWLESKCDTPFANNLSFISSGSSSSSSFT

LASTACRNSCLCSSSIFFQVLRRNCSSNCCSISNVDISLSAFSFNRFETSSKMARYNLPCPRSLLAILSP

PKCCNSPAISCQLRRCCSGCPSIDLNSSLRISMLERRVLPFSLWVSNRAKFANCSSLQC

>gi|66361325|pdb|1Z67|A Chain A, Structure Of Homeodomain-like Protein Of Unknown Function S4005 From Shigella Flexneri

SNAXGLFDEVVGAFLKGDAGKYQAILSWVEEQGGIQVLLEKLQSGGLGAILSTWLSNQQRNQSVSGEQLE

SALGTNAVSDLGQKLGVDTSTASSLLAEQLPKIIDALSPQGEVSAQANNDLLSAGXELLKGKLFR

>gi|168988956|pdb|3C87|B Chain B, Crystal Structure Of The Enterobactin Esterase Fes From Shigella Flexneri In The Presence Of Enterobactin

SNAXTALKVGSESWWQSKHGPEWQRLNDEXFEVTFWWRDPQGSEEYSTIKRVWVYITGVTDHHQNSQPQS

XQRIAGTDVWQWTTQLNANWRGSYCFIPTERDDIFSAPSPDRLELREGWRKLLPQAIADPLNPQSWKGGL

GHAVSALEXPQAPLQPGWDCPQAPEIPAKEIIWKSERLKNSRRVWIFTTGDVTAEERPLAVLLDGEFWAQ

SXPVWPVLTSLTHRQQLPPAVYVLIDAIDTTHRAHELPCNADFWLAVQQELLPLVKVIAPFSDRADRTVV

AGQSFGGLSALYAGLHWPERFGCVLSQSGSYWWPHRGGQQEGVLLEKLKAGEVSAEGLRIVLEAGIREPX

IXRANQALYAQLHPIKESIFWRQVDGGHDALCWRGGLXQGLIDLWQPLFHDRS

>gi|146387127|pdb|2PDO|H Chain H, Crystal Structure Of The Putative Acetyltransferase Of Gnat Family From Shigella Flexneri

SNAXEIRVFRQEDFEEVITLWERCDLLRPWNDPEXDIERKXNHDVSLFLVAEVNGEVVGTVXGGYDGHRG

SAYYLGVHPEFRGRGIANALLNRLEKKLIARGCPKIQINVPEDNDXVLGXYERLGYEHADVLSLGKRLIE

DEEY

>gi|334359334|pdb|3QVO|A Chain A, Structure Of A Rossmann-Fold Nad(P)-Binding Family Protein From Shigella Flexneri.

XGSSHHHHHHSSGRENLYFQGHXKNVLILGAGGQIARHVINQLADKQTIKQTLFARQPAKIHKPYPTNSQ

IIXGDVLNHAALKQAXQGQDIVYANLTGEDLDIQANSVIAAXKACDVKRLIFVLSLGIYDEVPGKFVEWN

NAVIGEPLKPFRRAADAIEASGLEYTILRPAWLTDEDIIDYELTSRNEPFKGTIVSRKSVAALITDIIDK

PEKHIGENIGINQPGTDGDKPFFXGS

>gi|296278506|pdb|3M92|B Chain B, The Structure Of Ycin, An Unchracterized Protein From Shigella Flexneri.

XGSSHHHHHHSSGRENLYFQGHXNKETQPIDRETLLKEANKIIREHEDTLAGIEATGVTQRNGVLVFTGD

YFLDEQGLPTAKSTAVFNXFKHLAHVLSEKYHLVDGS

>gi|168177394|pdb|3C8G|D Chain D, Crystal Structure Of A Possible Transciptional Regulator Yggd From Shigella Flexneri 2a Str. 2457t

SNAXATLTEDDVLEQLDAQDNLFSFXXTAHSILLQGIRQFLPSLFVDNDEEIVEYAVXPLLAQSGPLDDI

DVALRLIYALGXXDKWLYADITHFSQYWHYLNEQDETPGFADDITWDFISNVNSITRNATLYDALKAXXF

ADFAVWSEARFSGXVKTALTLAVTTTLKELTP

>gi|90109756|pdb|2FZV|D Chain D, Crystal Structure Of An Apo Form Of A Flavin-Binding Protein From Shigella Flexneri

MHHHHHHSSGVDLGTENLYFQSNAMRLRHLSDPDSLPALDKSFAIERPALGLAPDAPPVRILLLYGSLRA

RSFSRLAVEEAARLLQFFGAETRIFDPSDLPLPDQVQSDDHPAVKELRALSEWSEGQVWCSPERHGQITS

VMKAQIDHLPLEMAGIRPTQGRTLAVMQVSGGSQSFNAVNTLRLLGRWMRMFTIPNQSSIAKAFQEFDAA

GRMKPSPYYDRIADVMEELVRFTALVRPHREALTDRYSERKAAGHVIDEATDLSSIAIAPQPLPESETS

>gi|60594515|pdb|1Y2I|E Chain E, Crystal Structure Of Mcsg Target Apc27401 From Shigella Flexneri

MHHHHHHSSGVDLGTENLYFQSNAXQFSTTPTLEGLTIVEYCGVVTGEAILGANIFRDFFAGIRDIVGGR

SGAYEKELRKAREIAFEELGSQARALGADAVVGIDIDYETVGQNGSXLXVSVSGTAVKTRRNI

>gi|62738666|pdb|1YLO|F Chain F, Crystal Structure Of Protein Of Unknown Function (Possible Aminopeptidase) S2589 From Shigella Flexneri 2a Str. 2457t

SNAXDLSLLKALSEADAIASSEQEVRQILLEEAARLQKEVRFDGLGSVLIRLNESTGPKVXICAHXDEVG

FXVRSISREGAIDVLPVGNVRXAARQLQPVRITTREECKIPGLLDGDRQGNDVSAXRVDIGARTYDEVXQ

AGIRPGDRVTFDTTFQVLPHQRVXGKAFDDRLSCYLLVTLLRELHDAELPAEVWLVASSSEEVGLRGGQT

ATRAVSPDVAIVLDTACWAKNFDYGAANHRQIGNGPXLVLSDKSLIAPPKLTAWIETVAAEIGVPLQADX

FSNGGTDGGAVHLTGTGVPTLVXGPATRHGHCAASIADCRDILQXEQLLSALIQRLTRETVVQLTDFR

>gi|313651521|gb|EFS15917.1| hypothetical protein SF2457T_0176 [Shigella flexneri 2a str. 2457T]

MQWISTTSQKLDERLYRVCVWVKKYHEDAINRVVLTVDLSKARHDPIEDQVRAELLCGHYFLMRKENNAK

SAPADQNFEFLPDGRNLSWQTSTPGLQHLLLNSNVPESLRPLADYIQMRLAKLTMVPMSGTIMKAALEDS

ISWVKVDLRYYWQYELVNSHLGPVQVTHKALVRFGRLAKHDENASAIRMLRQRLSSPFIQEFEMPGEELK

RKQKIMNTVDVKMLFHTHYPGQKLLLARYANGWVLVDCFLFHHIKPKKKAKNKTKAGKSETQNIKSTGSE

DHGTGHYGVHRVVTLTGSGVDQ

>gi|313651520|gb|EFS15916.1| hypothetical protein SF2457T_0175 [Shigella flexneri 2a str. 2457T]

MTVGTVTRHEERLKEVNPIEVSTISDASVDEIIRVIQDFCIDDIDVLSRNSTKVTTKYPGLIIVPEGAEQ

LSGIICDINDAKSDFASAMKRVNSEKHIRFKEVHRKLPGLVTAHSTRKILFVEEQLKKVTFAWRLNRNQV

KTNASDLIAMLDKRRLVAVKSPVTTDLTVVANIDRAKALLERKVLRDGEGYRLCRTNTFPVPIAHLFTYR

PEGKERGSSKYAETDYKVVKASLPIFGVGQKPTIKRLQDWVPAQDEALSNGRRSNHSYTELVPGADLGIF

IMRKV

>gi|313651518|gb|EFS15914.1| hypothetical protein SF2457T_0173 [Shigella flexneri 2a str. 2457T]

MVFWIIISFMFLSAMFMTGHLLNKVVLQPVWLCIWSLPWLGFLLKVRVTEMDKGLVNTLQLGDLPVFRWS

RSVVPGTWSVDRINGRYRLNRIGTDGLTVHYPWRLYYQPLFLTPDKQISDINGREHERKF

>gi|313651517|gb|EFS15913.1| hypothetical protein SF2457T_0172 [Shigella flexneri 2a str. 2457T]

MPLLCWVFTSPFSNWTDKFFTGTEVPEGSLPGLEQAPEAIFRFVLNDEGFDVGFDAVGMDLCCFSIPLST

MPTKNLDDEETLSRLTGDVIHGVLLSLPEYIEMPDRLVYQLTDEVMAFNSHCGNGILHGWTTAQELWRNE

ILPRTTILMQQTSVIH

>gi|313651515|gb|EFS15911.1| hypothetical protein SF2457T_0170 [Shigella flexneri 2a str. 2457T]

MTNSTDNQNYVRAVLAGIGIDFDETEMFISVSHCQSDEVSFTCSISASELRESAGHYVDTLNDTQLAGLD

ADALKKRLVYFLEVFDLVSGQYLDISGKHFATSRFEYDDVCSEILSNSADSAQPGGYDREEYKRLMEVDG

QVLIARFALEKFWDTHFIGLINYVSDEITSGLYEVYRTFSDINMAGYTFSEYSYTRRITDEFSLHISLKE

DDFEEQLTDCYMDETTLPSGKVVLRRNNESIIGIYEGYASKSYFPMVANVRVLDTDGEVVTELYQGVNVS

ELAGGRIKIHDRQELISEVFANLREFIPASEGKIFDAA

>gi|313651514|gb|EFS15910.1| hypothetical protein SF2457T_0169 [Shigella flexneri 2a str. 2457T]

MFNESDDKNFVSAMLKCQLGLNISQEDITIYDKENHFEQLSFKANVALDDLLFYLDLYISELIKHNAPYS

ETEVLRTKIKYFLKVYEKSGFQNIRIRGYHNAHSTIDIVDIASLILAGSVPESEHDSIDPVLRKEIYQNR

MSVEGKVLIARFALKQFFHSDFGDFILEFEKSISKCLNTSLQIIKSVKNSFNRLGQYQYQRRVKDELTLH

LDLNTDEYPACMPDLYIGFKESEGTTGVYRDDEKIIRLYTGVSSGKDVPVMMTVRFTGCDGSVLSESSHG

TFCSVGPTGRVQVCDKVALVQEAVEELRDVV

>gi|313651513|gb|EFS15909.1| hypothetical protein SF2457T_0168 [Shigella flexneri 2a str. 2457T]

MKAIYKIIEAMLVKMGFEGAVIQSVNLETGVIQSLVVKADIPVRTLMPHLRRYLRDCEPLWGHDVDYHPI

AQYCSGAALLKAGPVTLETFRFEVSVSTAHLRLVDVEPDCLNEGVNREKLEYVYYRSSNADKDLILGMVE

KGHNFINHLHELLRSRLSTALPLIFAVTEKIAESSVPQVVTSFFSVDHQINAQIVVEPVPVSVADQIISA

DWNVCSVSGADKFLNHVNRYLSGRVMKRRIYAVIEACDGGSLSLNANLGCFTSPLLVTEYKMVQSHSSSL

YRKAINNSLKQVNSYAPEHSDDLLSM

>gi|313651512|gb|EFS15908.1| hypothetical protein SF2457T_0167 [Shigella flexneri 2a str. 2457T]

MKIIEKIINAFLISRKHSVKVSNVICLSGTDGKFTGICCDADVSFDFLYSYAPAYSSTFLDIPFPGFEDQ

DIADCVKCQLDVVKNKRNRSFLIDHIRFPVSSREGFTLTRGDSYDVTECEYNKERLLHLTRQGRFCEDYL

TFKDGLSSFFSFVNFELHEIVKEGIRLALDVLNKITSDQPDRLIKDFKYHDCFGSYNVQIFSKGLPVDIL

ETMIAPDRLLSNLSGSRQIMKNASRYLKGFALSNRVYMKYGWRLADVDTSDYAKVMSDREMHARDDLKAF

CSVFFKECFASGKYEYEAYLSERQHAIRNGY

>gi|313651511|gb|EFS15907.1| hypothetical protein SF2457T_0166 [Shigella flexneri 2a str. 2457T]

MKLATDLVNAVLIKNGIVPTSINELPVTSLVKPLTQVHLSLYLKTTHLSQFVPDYVESLKDCPVSYDIGL

ELQQCINSAELLCEDMESLEFISVWIELYTCNSKQQRFEKSNFSFRTEDIELAKMLSCSHEILISQAACF

WLLYFEDFMKYVRNRLCALLRQVLILIAGLRNVYLQRNEDQLIKRVKHGNEMLEVTLKAVALCSMERYLK

SYASLRTEAAASGFIKCIQSYISTGWRHGYIVCQYSKNGRSLGKSLPLFIKETSEHNIQKRVNRLISSTI

SGRNKDLGLYDF

>gi|313651509|gb|EFS15905.1| hypothetical protein SF2457T_0164 [Shigella flexneri 2a str. 2457T]

MPDKREQMTYASQAVKRTPHEVTDHFIKMVHARIAEVSGWRYVFDRIPAFKDACDKAPGQVPCPFSGVGK

SKFRFRKKDLFTGCAIHNDFPVNAFCDGIDVLAEYYKLSKTQTCKKILTDFFGMDLYAPLTDADLESERR

YKSTVRATETLDSDEVEKRGRKLEVIYHYTGEIKPESPVWVYLRNRGLNRVLSNLPKDLGLNKRLYYMDK

SLEKPTIYPGMIAIYRDTRGRPLTIHRTFVELNGDKAHVENPKLMMKPPADMTGGSIQLYDPHFNPGTRT

WTLGVAEGIENALSVTEATSTPCWAASSAWCLENVEVPDSLLPPPGVKVIQFYIWADKDLVNTKGTSPGM

ESAKRLQERMKEFFAKRYPTSELTIKVFEPDFDIPVGKKGVDWNDVLKLTGPDGFPVKWAPECLAQL

>gi|313651507|gb|EFS15903.1| von Willebrand factor type A domain protein [Shigella flexneri 2a str. 2457T]

MKFDLKSSPRHIQRLTNIASVISGINGIYVIIDNKVSTPYFNTQNNVCVLPNGDYSDERFVKLIEGFICH

EAGHGRYTEHEVYREAFVGELINADGFISIDDDLKADFQNLKQKQKAYARACRLKGLINLFDDVQMEEKT

GIDYQEAKKRLAVTYALMVEAGRMTVDISSTPQNPVQFIEMYLLNTLRVNVLQQEGHKETLDPFFDYAKK

ILAPVTSEVDEIIHQALSCKSTQNCDSLARKTLALLERLRDEAKEKQQEEEQSKDPHDDTDESSGSENEP

DTEPNGDSQGEGDGDKEGQGDDSGDGKAPAEGNGLPDQSGGDEQNAENSNGEPDGESEGDESATPGSDDA

DSTPSLNDSSNGNSSDGESNFSPEQWDMLAKLLDDFLNSDEESEDFHEVLAKEISVIAASVSDEVKAEFG

ASEWDVPDLNIDLNVYNEALNISQTLGADLSVLQQVKMRGQFKTRDRGLSFDINRLIQSPMGVRDVFRSQ

SESKNRGHVGLVIVRDISGSMSLEHRYIHAIKTDLALTLAIEAISKMHVANVIYPFVDKDFEVIKTFDEN

AEEKLSKFSLGCKGNNTPTGSALNAALELLLESQFDRKIVFLITDGYPTESAYTINDVFSVAESNGIEIA

GVGIKTDVLMGFNEGTFVNVDDISLLPNEVSKLVHQILSN

>gi|313651506|gb|EFS15902.1| hypothetical protein SF2457T_0161 [Shigella flexneri 2a str. 2457T]

MTFNTSKLNKVSAKEISENFIKAYPDLQEGAVITKIEISGCQGSRRTDKIELSYGGDDITDQKSVSKSEV

RWIDIKALSFKSTITSRISTLCSRLCIRYSNMWILPVERMEEFLEEAQEIEREFQAGIQNVVDNYDTHIQ

AEKDRSPLMSDLIDQLKLTKDDFVKSFRFNLAHFIPFSPISVEGDETQDFYQEQLITDLADEAMKVYTKI

SKNDNLRSSTIDRLKQMQDKIISFMFIHKEAVVLAEAIKHIMNNLPKGAISSPRDVAVLKQWFYFMSDAS

MLKRIISGEQKVTDWLDSITRSFNIDSQVTAANSLNTAEDDVFSSANVFREDPVEDLENKAVNAPEPAVG

PDENVSIQASGSNRAFDCELELTGW

>gi|313651504|gb|EFS15900.1| hypothetical protein SF2457T_0159 [Shigella flexneri 2a str. 2457T]

MKQKIRPTAFCSKQMDFFSIFDFEEEEKEIITIVESPLDYINDAIAAQQSIQSEDKHSGPELEPSVSEDV

ALPGNCPKLLKNQILHDISFAAETDYLFTLEEYSAELGGLVFDWAPEDVYQLYVVAMEESLENVRHLVIT

KSLYSTDEFGNITVNPLLEAETRWYMSKSFELTCATHGIDAIEFRSELKKSLYEYTHSYGGENAELARYH

QDKEVILHDCKEEMGWDIFFEQDYLLQENKLAMKWTDRDIMDVYSKVFKSTINLFEKLVVNNKLTLRDTF

GWVIVNPTFERQFEWIESEVFEIVGTHLGYNVAAIRTQMATACRMTFH

>gi|313651503|gb|EFS15899.1| hypothetical protein SF2457T_0158 [Shigella flexneri 2a str. 2457T]

MSKSKARSKALLIAFADLIPDMDKVVNKKLLDSLNVYSGHDNDLIVIMNEDGPTIIELNSLKSVSMLAQK

LSAFSTYYHVEMQQILVNPIDFEKAYTLLKEAPAIPMFKTLADLDKFLNEEFEKYGLNTFLDVDNLDYSL

AKSRELKNDQLVAWVSEIIEKREKLALRNRFNEVTKAHYETVDAMYAAVRPLMKELGFPDELMLHTFSEL

SVFDSKGWDYAIKSKIEFLTKREEQCLDYQMKADKRQATVDELLAQINNAKTVKAPRSFGQLFGFSVIAM

MTFMFIVNKFI

>gi|313651501|gb|EFS15897.1| uvrD/REP helicase family protein [Shigella flexneri 2a str. 2457T]

MGKPTDEQRVIIENANANNMVIAAPGSGKSFTMIEAVISILRQFPYAKVGMVTFTRAATNSLAEKLKRRL

SKKDQDRVLVNTFHGFIRMQLDMVNWKGKMLISSAQRSVIHRALKESGAPFRYPDAEFAIDAIGREMDTD

IISVRHTRQQIHLFNTYQAICQKDHVADLNALSRFVVGQMYSGKMQPLNLTHLVVDEVQDTDSIQYAWIS

LHTRAGVNTSIVGDDDQAIYSFRASGGVKIFQQFEKQFRPNIFYLNTCFRCEPEILKVAGALIEKNVYRY

AKDLRSAKGGGGKVHFRSYVDMDEQIQGILNLINQDPIGWAILSRGNAHLDQLESLIEQPVLRYGGKSFW

DEKETSDVLHLMAFFRHSNDVRLMKRVLALFGENEEVLDQTALSMKGRKVTFGELNIPNESSLETRTLHS

NFTRFTQETREKVEIEKRFANLIKWMELSSIKMRTQKGSPSLSRIALDTCKQWAEKTGWQNMINRAAAMC

LGPKKKDEEYTPDKVVLSTLHGSKGLEWKNVIIMSCNADQIPSKRSVGQEAIEEERRLLFVGFTRAEQQL

HVMWYGDPSFFLSECAEDKLKEAAKSRTESPLTE

>gi|313651500|gb|EFS15896.1| lipoprotein [Shigella flexneri 2a str. 2457T]

MKNKRFVSLVSALVASASLVGCAGNAGQSPNSAQHSDTHGLYWHANAKDSVAKNAYSLAGFDVSFNEETV

QPGGDADNFLSKLLISGSMGYVTGGLSGLSIMSLGSLYSSSDAEYIQVNQYVVFVPNPKKLPYNDESLVR

AGAAYVYNHTKESQVMLGFNPSKQSAALASCKIDRAVINKWSTCELTSKPAPDVMPTNSMYSFQAIRPAT

GTEIPQLNLPAGEYSVIRYVFIPFKGNESSNNFSGIIFRSDSPKFTMPGGAAASINGKDYYLFSGEYGQK

GFPEKTLKSK

>gi|313651499|gb|EFS15895.1| mating pair stabilization TraN family protein [Shigella flexneri 2a str. 2457T]

MKHRKIISSAIIASMIANTMSWAFYVSLINIALTPKIFAADAIFDQLENNFNLANPNANRNATTSAQDIV

EKYKNADSGENLSGKISEKYVGKAESTNLNVGKYGTPNSNESVMSNAVSDGKSIGKAVQLPSMSGGTINS

NYTKEGAKLLSRDSSGNIGISNNPNTTAGTKTSTGELFSSEQKHSDVQFNAGGRYGDENGFINDIKNRKS

QLFEAQSYDGVAYRTLVNANKENPASTIKPNDPMFNAGRNEIGNAVAGTGNWLQNCNTETSKQTITTHYP

DYKEFYCNSPKKDNFNSCTITRDFSVPVYISGGNGDMSMCGDNCVRVWFGRRDDNYWNDGVYDNSLTLKF

HPDAKLATAKIINAEWDDHMRVTLDGTQIFAHIDGAYRSSNYPSPQGQWELKKSWKLDKVYDITEQVKTS

VYQEQDREVTMASRVWVGGGGEGYFEVEMTFENMKLEDKHIQEPAGCFDAVQTPNSFCRFDRFVNMDVGT

KRLPESVLKMATPLYKGDTGYLTWKTNLEGYFCDPLAKEKLCSYDAKGNIMKDPTGKDLCYNYDDIKNMP

DACSTYKNDAACVLDNQTCAEGWFDEGTNTCYMYEQKYTCDRGKDVVREVESQTNSCVGMIPCSGGTCET

GPKEENKDFGKVAAYSNMVQYMQGEAKCEDPNDPNSCSVFEGKAEWCGRSVGFVNGLAKTDCCEKPQGAA

GSLEAIMLAGSMIRNTNWTRVNAQLVNWTGGESGTWASMANSVGEWTASAGKTVGQMWNNVTSSITSVYE

NVAGNLGRTVGSSAAGESGQLAKETMSSFGLGKLKQMAMEKAYDLLPDTVRDFVFKNVATTGGEIVFSAA

VQNFMLALNVIGWIYTAYQVTKMLLEMLVACDQKEMEASIHKNQKSCFTLDTNRCVKYLNLGFTKKCVKK

ATDMCCYNSMLSRVIMQQAYPQLGIDPVTSNCVGLSIGQIQKLDFDKIDLTEWINDAVQVGEVPDQYATF

SESSITANLPFKNENYQLPSERTKDAMGGEENMIKARQENAQAIKEENVDCSYLPRPAICEVGSTYVDPI

TGKEIPKY

>gi|313651498|gb|EFS15894.1| traU family protein [Shigella flexneri 2a str. 2457T]

MSVPVKKLKKLLLEILIAVMLSLSLPAMTMAADTGVPGAMCQSAGVWQGLIKNICWSCIFPMRIMGIGAA

PEGAAPSRPGCYCTDQNGIPEIGWQLSFFQPVKIVEVVKSPWCSPFLEGTMLQKSQFDIGKSNTNQPMTA

TEAGFYDVHLWEFPIMTMLKLLVIGECTAEPYVDASLTYISEVDPMWESDLLTLVLNPEAVVFANPIASM

VCAADCVAVTAGKDNLAAYFCAGCDGNLYPLTGHIYANDDAVRTSSLITQRLLTKLHRQGMLMRTMGADA

MCEKTWEYFTPRSQYRLSMLYPTPEANGPDCCHRLGDSVHDWSTLKGGRKKIGIDNYVYMLWRYNDCCVR

YIPN

>gi|313651496|gb|EFS15892.1| putative type-F conjugative transfer system pilin assembly protein [Shigella flexneri 2a str. 2457T]

MRSFELKTTIKPLASCLAFYALISTAVASPQYQQVADDIARQAQVLGAQIPVPHASEGPLPSGSLDSPDT

KKYIRQAEAMKKNGDLSQQTNRGYVPGMNADSVQAVIDHTQAIRAQSNNSEAVNDIIRRRDEIQGNSQLS

ESALKSVENKPEVMRSQSSNIEKMFGSSGITAADFERKIDSSREEVLSTENGITIFASFSLPDYVLEDLL

RTASEHKARVVFNGLKKGTTRLPETQAAINQLVVKGKFDSPLITIDPDAFNQYQITQVPTIISREQSRFA

KMVGSFNVDFFQRELARKPDQDLFPIAGTTYPVEEKSIIKELEERAQKYDWDGAKKRAVADTWKNQYMVN

LPPAQEHKEWLIDPTIRVTQDVKDKQGRVIASAGELINPLARFPQNLTMIIFDPMNPGQLEWAEKQYRQR

LGSGQVMPMFTRIKQENGWDHLNDLREKFNGKVFKVNEQIIARFQIKNTPALISTEKEKFRITQFSEAEI

RGIGTKIAAEEN

>gi|313651495|gb|EFS15891.1| hypothetical protein SF2457T_0150 [Shigella flexneri 2a str. 2457T]

MRKSGLCLTLLFSLIASIKSVQAEAIMISGKLQADMPAVTFNPGPGDFVAFVNNNTITASGTGNACNVTV

DDRATSSVDNLVCFFEWLPNTSGFTANGFNLTGIPNAAGELKLPYKISYFSGSERQKVEVVNGEYTVNAL

VPVKPTITGLKSSMNGVVQDGYTLKSYLKDEAIRNVTVSVEPRNYVQYISIGSGSACEVAIGGNGCTIDV

GSIKVSDSDLLMGSREITVTANSKNNYFAPPEAQKLTLTWDYRPPVVDHTLWNFTDEAKTISIGGQEIYT

GARTVAVAVKVPQEETEGEWWLPTAMTLTMNPDGVFKPTTKVTLEDGTVIDFKQSWATPLRRTLQPVSGP

QKVGDEYLYIFDLTDLTNGSYAASFTVENNSKNTSTYNEPESKLMLSDNPTLMVLKDGAALAKRAPVYFL

NEIIVAAFQGQAGVADIKAVTIDNKLVELTPTNHKGIYYLPVGDDLEVNADHEITVIAENLYGKIVTFNT

TFTYQPTGFTLKNFEKDITLYSRVRQYTDLMSQTAGDKCTLFTTEENALAYLEWYGDKSDITACYPQWNN

VPDGLEFYFKGRTPGLTGFFNKTGDNLLDYQVYMINGKGSKAVSSRNRHVLITQLPYNPVLTYKKNKVLT

GINDNTALAYTTGGEAARLLAKVVPADVTVYVSQNGGESVKTTFKNRSSNNDATTFVQRLKVASAPLWTK

NVFDIAVEYSKEPTMRTVDTLNVYTVPDFNIRAAMEIEDKKTATTMELPLKVSVGRYNNSTRKSSFSRTT

MGDWDVTIYAQKSVYGKDPETGRYKTTYERTPLTEALPVNDAGVVETKIKIENMDLGNVRLVGIAKVRSP

FADFELTRETSAVSIRVYKGEELEGNLSKSLIIGRIPLSTLVNFKSASTANSDALAPTEWQESSDNGQTW

TMLQDMTGKRSISIRKTEVGKWLYRAKMTNKFTSKESYSDVLTVVTYKQPKLSIDVAEILEGDDLPVTLL

DNDEPIPQGTAEVMWSEDKVNWVQGDTTYNVSAADTLPPTIYARMRYLDSDELAEESSWKETSARLTVAK

PKRLSVSVSGISSVEVGSKVTLDGKYTNPNSRFKNGNDVIEEWTAPDGQKIRGANLSITLTEQMLDKQGY

AAFEYSAWLADSKDSTISTRKVSVKSWVYKFPEMKVSTKLKYTMAPSTLHVALSGIKDGGYPGVTYSREW

IYDKDNISVLKDDGDTKEFAIAKPGKYTLVIVFRDNRNNEQRIENTFVVDEQTPMTVEMTPKFSNKFMRA

PLDVTLRSNIKLAHSADSIDTVTYKVNGEVVPGSKNYWAQLISGLTEKKYEVTLDVVSKMGQRGTASVDF

EVVKNAAPQCALSYTESNMSWSFTNKCIDTDGKLVRYEWYINDELRNVFGSTATLSKNLNRGKQDIRVVA

YDDSGDSATQRTTVYGPDEGASKSVDSVETSQ

>gi|313651493|gb|EFS15889.1| orfB [Shigella flexneri 2a str. 2457T]

MTKKSEKENDRIQISAFWLSERQSPYAYNFLKKNALTHRGEQISLIRSAITTGLVLNNLFPELSSFINGL

NERLTAADLNRFFNDEFNKDKLNNENLKEQISFMLDSKFNELFSMINGCDFSKATFSTTSQSDVSETLKA

ERVRDQRLELIPKELTVNPKPETLEILTPIESAKKNDSGLNTSHGSAAVSASQSENTVLSEHTQIQGVTR

KPKKKANANLANLAK

>gi|313651492|gb|EFS15888.1| stbA family protein [Shigella flexneri 2a str. 2457T]

MTELAKTKLAVAESNSTELETLKVVIDDGSKAAKLVCTNNQGDLVPLLTQNSFVADFRVSHDGLIPFNYL

IDGLQRFSHHSESSNALETTDVAHQYDEISRLNVHHALHSSGLMPQDVHLYVTLPLSQFYTALGETNNEN

IQRKKDNLMKPVERYIDGKRVSFNVVSVTVFPESLPAVTRADEIESIESFESSLVIDLGGTTLDVASITG

QLEQISSVRGFDRIGCSIVYDEIRRYLDSAKLNASNAYIHHLVDNRENKASLKVSPEDLDGVFNAVNGAV

AQLQEKVIKAVTQVEERPHNVFLVGGGSYLIEPAVRKHFDKSKIIMVDNPQFALSLAIADTVFA

>gi|313651491|gb|EFS15887.1| trhO [Shigella flexneri 2a str. 2457T]

MASEHVQTIDLTNFSGRVFFLADPHGHYSTLCNLIHSISEPDEELIVFSTGNLFDYGPEPMELMTAINTG

IFDGRSVRVFSAAGAGEEMLKKLLPINKDRRTYYPSTFLNERWCARGGRWHKQINRNYLEEEICKLLSTQ

LATVMKVLFKGNISIGICPSDYTDIRQGFNNTYNALLAFNQANVNIFQSQFLYGMDHAVRPVNVSDVNLV

VLGRNPVNSIRKAHGLPLTNLPVLIGNCLHINTGSLYMSEISSPALLAPGIPPTTVPAITLVELILASTP

TLICHQMILNKNGIYTQNTAPLNLDSNDNNIEATL

>gi|313651490|gb|EFS15886.1| hypothetical protein SF2457T_0145 [Shigella flexneri 2a str. 2457T]

MLAFLGPSNITNASTKTPIPSSQLSDAKTGVNYEFITIAANYVNGMWLVDGRQRPVIKTSMTKRNYLQIE

NDSASTPLNLVIPKIEFSIIAKNGVFISKFISLDEDASGKRILWLEPGSSMTISFVNDLSSTPLQALVSV

TKQKKEVIAIFGPDQGKKFTLPLPSDSQQSSIDYVPSPEIALRINAPKKLQPAIAIPEDASASLLPSHTY

KIRAAEKVRTFKFSSFVWVGKRNNLLGGSDYFTKELKIYPGENFDISTIQKTTMRTEND

>gi|313651489|gb|EFS15885.1| parB family protein [Shigella flexneri 2a str. 2457T]

MSDEQHIGNDKSRYINAPKRTEVIHRSGLQGLKGQPRLKKLFTLHNGRKLEAEHIIVPAEKVELETTVHP

LNPRNQEALSVNAVRDILKDIEARGVDTEGVAVKRNGVYLLIEGSRRRFCCIQSAKELPLWVLPDDVNAD

DINSIISATQTSRRFSYREVGLKYLREMEEHGFVTNEELANYHGISHVSVSKRVQAAKINSNLIALFPDY

EAIPNSYYNRLFRLQKYIEKNLFSLEEVVENTREEIRDLDISDIAEAQKTVMEKITTVVEKLDIKPPSKG

WDTRELATFTNKDKYARISKSSSGRKIRFEFNRMNRELIDEIEKFIKSKLSEMNN

>gi|313651488|gb|EFS15884.1| parA [Shigella flexneri 2a str. 2457T]

MNLIDKIALVGQRMKSEQISLKESLLVSSRVSVSDDSVDGVDRLIYNHCLNKKNLSDFFGKSRVTFNKIL

ADLEEKELVGAPIYQNKNHLYTRWDVQKIMDALNYPRYSDYYCSRTIVTQNHKGGTGKSTTSGVLAVAAA

LDLHLNARILLIEWDPQGSIGSGMIQSVAEDDVFLTAIDAILGVYEEDSDYRKYLDLGYSEEQIIEGMPF

STHLPNLDVITAFPTDARFKDKYWQCSREERTELLLRFKEVILPVLKSKYDLIIIDTPPEDSPITWAADE

AADGILVAVSPREYDYASTTDFMLTISERFKQSPSKGENLSWFKVLAVNVDDKSPYEKIVLDKLVRTVQE

LFMSANIKNSEAFKAAASRGRTVLDIKKSEELCSPKQLDVAEESVMAVYQQFINEIKSFSVKQGGNV

>gi|313651483|gb|EFS15879.1| hypothetical protein SF2457T_0138 [Shigella flexneri 2a str. 2457T]

MEGSWLRTVAFNPLNRQTMEESWACPMEFNPLNRQTMEESWSCPVAFNPLNRQTMEESWSCPVVFNPLNR

QTMGGSWSGTVAFNPLIGRPWRDYCRVPGHLTTYSSVHDGMLVA

>gi|313651482|gb|EFS15878.1| plasmid transfer protein [Shigella flexneri 2a str. 2457T]

MRSANVYNKELSRHQLGGFIPVYDQIPGTHYFLLDGNRLGFMFICSPSPGVFDNQQDVLTELFKMDFPTD

TICQTSLTALPDLILHLSAWSAVRGGRMEGHDKLKGDLLTAYQLDYYDRSLNEPLKPDHDKLMLRDFQVW

ISFSIPLKSALPSEIEKTRIDALYSDLISKLNTVGLFPHKVGAENWLYCMDKLLHPGKTSRWSEGHVEAS

TMRRLNEQINVPGRKYTVTENHFSSTTQSNDISEHRYFKQLSVVKFPEFVNFGCMYELVVNWLNGRKTIF

SPFMITQTVHFADPLKLSRENVRYKAITNKQASIPTVLTFCPRLKDMDNDYMTITRELEDGARLLHSYLT

FTVMGNSAVDVQSAADQLKSFYLESRVNVADDSYIVFPSFVSSLPMCNDPKTILELDRFEVVSNTGAAHM

TPIFGPWKGNTDRPVLNLVSREGQLLGLDIFKTSASYNMVVGATSGAGKSFWVAYIINNYLGAGPRSNNL

IHYRDTFEGFKNNSYDAFDPDGAQIFVVDVGRSYQGISEQYTNSQFIDFGKKPDFTLNPFAFLTDVTVGE

RVFDEAPVFNDDSNNHDDDKDKVAQTIMVLNQLKIMASEKGNIDDFQQSVMLQLISEEYNESRKVGRTGS

ITGFARRCSNHEDKRIKDIGDQLGQWCEGGIYGNRFTENLPPINFDSRFIVLELEELKGTPHLQTVVLMS

IIQAAQHAMFIKKDGRRRLFILDEAWEYIRPDNASGSSNHSNQFFSSFLEAAWRRFRKTNCAGICITQSF

EDYFTSSVGRALTANSPWKIIMKQEKESIEAMKVNNYFSTTDAEYERMKNIRTVKRAFSEMLVRFENFQE

ICRLYVDRKMELCFTTDSTDRGKLWEIQSRLDCSYGEAIEILYEQEVASKSAA

>gi|313651481|gb|EFS15877.1| traV family protein [Shigella flexneri 2a str. 2457T]

MNIKKVIFSALVVGSSSYLAGCTIGSAESECPGIEKGVICKGPREVMELTNNRDDLSGLGEEEANSGKGK

SAVNDSQYPAQLTPPGAVQYPKSSVLVNKPVTYTTTEVKPVGQLPVMYDQTLKMGAPTSTIGPRPISGNP

VNSNVRMTTSYNSNGSSVNPFLHPSAEVVKKTTQVVTPAPAPRYVAPNSDINASKDLYSINNGQPVNPTL

SSGQVQQYRTQGYKQAVVAPEPLAVLQQGRVMRITFAPYTDDNDALNLPGFVYVNVKPQTWIAGKNSTSN

PARIVPLEVQDAARENMQQQQRATKAVSSNGIVRQL

>gi|313651480|gb|EFS15876.1| htdT [Shigella flexneri 2a str. 2457T]

MKKLLLSLLISITSACPPLTLAVENVLNNRDAAALMEKAKEGGVSTKPLDGVLAKMKSYKPKDAIYIPTG

GLYLFQDERSQLMAVTTDGRYTITGGSVMDILQRKSVLSVEDIRKSFFINLDDAPFPLETVASIPLGNPK

LKRQAAIFITLDCDGCQDLIKKFYDEREKYRVDIVLIPSPGEPKQELRQLWCSKEKGKVNDLDILRWLMG

NKADIEKRLLTKEEAEACPAEPLVASLMLAGIYKLQGVPSVVRQDGLAGNGIPKDFDYWLKQSVEPLLKN

PFDTN

>gi|313651479|gb|EFS15875.1| htdV [Shigella flexneri 2a str. 2457T]

MENNNDNESPKKSESADSLNIDAAKPVGGIRTTKKVTLEIDIEKTLKYLMVAGLAVLIVIYGYKGGRFVY

DNFKAMSQPAYQIAVLDMQTLRKVFNKQNPVLDSQQSKTNFENYFKALMKVYRERGYLVIDASLAVTIPE

NVEIVSYIDLGGSFGEVQSLPGEPKESEIR

>gi|313651478|gb|EFS15874.1| traB pilus assembly family protein [Shigella flexneri 2a str. 2457T]

MDIKKAWENKTVRISVIGAALMVLIVVISQSIFTTPVKKEKKTQKKDMQTGFLIDDSQMNKLSNEESQKT

YNEMVRQNRIDQNAAKADRDKAEKAQQENKAQIASLASQVQQLTTQLTEMQTSRNGNRNLDAGGPRNNVN

EQAPAKPYQLNPNAAVNGVSSGYAPISPTRNSPMRTITQSSIKTNGTDGVIQVMPISENRIREGREVVAG

SEKAPTRTIRGDGTAPVDSKARHAARKDEMFLPATSIITGVLITGLEAPTSLSSKAEPMPVTMRIKKDII

MPNNYTMDLRDCNLLGSAVGDLASQRAYIRATSISCVNSKGKAFDVIVEAYAVSENDGKNGIRGNLISRN

GNAIAGSAFAGGLSALAGSLSPSKVSSLNIDPNSTAQYQSPNIGALGALAGAGAAQGGLNRLVDYYTSIA

EQQWPIVEISPGRPITFVVQKGATIPTNLTSR

>gi|313651477|gb|EFS15873.1| trhK [Shigella flexneri 2a str. 2457T]

MTSKKVFHCTAITAVILLATSNVVFAEDYQLPATVNNPVVMPVGADEFQNGVKNAIIKDTGTTTETVQAT

KPTTALPSLSPASSASPAVNEITGALSNNPTLAGYQQQVKSGNFDSYGRPAGKEPQQNAQSTGSPSKADE

LYVEARNRYKEVQRVNVPPGGNIVLPVSRGLQNRISTSFKNASVSTSTPADEASIFVNGGDVFISTNTDK

PIGIMLSEDSVPESTYNLTLVPLDVPGAMISVTTSLSPTMQAKRETSLDKQNYDEMLARSQSEELTPSDP

RQDDHKQRIIDLLTPVALGEVPSGFSLQEDRLSRIPSSEQSPCNFNMYAKLGQRLVGSRELIDVVLVKND

KPYGQVVADQQCITEGVVASALFDKAFLQPGEETELYIVRDKLFKERQTRVTTRPSLIKR

>gi|313651476|gb|EFS15872.1| trhE [Shigella flexneri 2a str. 2457T]

MKLLSRLKIPFIKNSSNGDFDNKDETSQKGGEEVKGSFLDSKARFSKEIEGSEIGITYSALIKRDEKLLR

VNTVAMIIIGVLVVKNQFLTDPVTIVLPPNMTEEVKVVGNKASESYKTQWALFFSTLIGNINPTNIGFVT

TTILDALSPDLQAKTRESLQQQTNIMQARGVEQSFKPIDMYYDTKNDMVYVWGTKSTRLINVPDKTESSK

WTFEWVLGMKNGRPRIAYVNQYSGTPNIKKITINGKEQLATLDNPPPSIGN

>gi|313651474|gb|EFS15870.1| repFIB replication protein A [Shigella flexneri 2a str. 2457T]

MSDNNEVTHPFDVTNTETGKTYQLSPNSSKSVQPIALLRLSVFTPVGTKEKRYRNFEVDASDELSSMELA

RSEGYDDIRITGLKLSMSTDFKCWLGCIMAFSKYGFASDKITLSFNEFAKMCGISSTNINKRTRSRFQEA

LANLASVVISFRDSKTERFTVTHLVQKAVIDPKKDTVELVGDPSMWELYRYDHKTLLSLQVLSVLAKKEA

AQSLYIYFEAMPAGTLFVSMKRLRERLLLTTPVRTQNQIIRKAMLELKSIGYLEYQEVKKGRDIQFQIFK

RSPKLALAKHS

>gi|313651473|gb|EFS15869.1| hypothetical protein SF2457T_0128 [Shigella flexneri 2a str. 2457T]

MNHCDYQIIEISPKALFIESGFNPREAVIGELCYEQEPVKSTIVSIKQAYKEGRRVDLIKVVKKDSNYVV

RQGHCRYRALSLALAEGADIQLISVVVMNYKNKSEEYLENLDGNRNNGLNPVALAYALAEALKLGSTVES

LAMRYQRSSTAIRNQLKILDMPLELQRLISLNLIKKTLAIEIMLKYSNDHAKVLAHLESCSLLVPSTEPE

PVLSKTSSVQTSSNDEKLDNPETLPVHVIPKEIKTKSITRSSLGIKRLSHKKTERLKTEFIGLIDTVRPA

LNDSNKLTELIFDGQHSNLFTNISSTSITKTTESVKITLDEAKLQLILSLLNDENTAIIIPKSKVTELHE

IRDLLTSE

>gi|313651471|gb|EFS15867.1| hypothetical protein SF2457T_0126 [Shigella flexneri 2a str. 2457T]

MKHSLFFTQDGYLLPSSVKPIYGKDGHYYAVITRECFDCGGAGKIANAVCVTCNGSKTSPATEEKLVLKE

CIEFLETIPELRCNPDLEAWKRRHSEQLALLQADNSKFAASIAQQLNNNLFLTVRQLESFYSTYQPVSEA

LSENAGFPIEHSFNIGDVVSFDIIFTEVRAAFSKGSHSYFFTFTGKKQNDGFALTGNVQDHILLGHSYHL

SGKVRKIHYFDSVPYALLTVFKLTA

>gi|313651470|gb|EFS15866.1| hypothetical protein SF2457T_0125 [Shigella flexneri 2a str. 2457T]

MQNDLGLGFIRKSARTSAKIISRKVTFPLDDLDNAKLTLTGLQCHPSCRFPFYQLSMLIHNQLAEHGVIS

ADENTPFTVSISIHEWLSRARVAFIDYHGLRKSDKTVMITRAHLEELLTYLSTTLAVYVPYSKKRLNFSF

LTSFTLVKTSQSYTLTFPGVLRPFLMLLGGLIQEFMTEKLLKRRNAQFVIFDYLRKSRRHSHKITDIVND

LQLHTVNVRIMNVLTQLADQGLISFICEGKRGERSIEELQFIPYASRDHSEVLSFEEWISPIG

>gi|313651469|gb|EFS15865.1| DNA adenine methylase family protein [Shigella flexneri 2a str. 2457T]

MPFLSKHFPHDKSRRWVEPFIGGGAVFLNMFATEALLADSNPDLINLYRNIQRNKPAFIREVQLLAERHF

EEEDYYVLRNTFNSTSFDDAPLQRAAIFYAMNRLGYNGLCRYNLKRKFSVPWGKRYQFSLDIQKVDYLSF

RLSSVELKTADFGQTLEFAGGGDQIYCDPPYDKISKTSFVSYDGIPFDKSAHVKLADMLVDANRKGASVA

ISNSMTPFTLELYEERGFDIHTHNAYRSVGSQSKSRKKEIEILAVLR

>gi|313651468|gb|EFS15864.1| DNA-binding plasmid partitioning protein [Shigella flexneri 2a str. 2457T]

MKQFRDANPDLCNEVTEQKAWAISPTVVQITEGFNSREMGMGEAYYQLPEVADHLNNIKNAYIRGDYVDP

IRVLIKDGIPYVRQGHCRLKAALMAVAEGHSNLTILCIEMKADEISCELTTIDGNRGLALSPVALGESYR

RLQALGGWSLDRIAEREGKSNTTIAALIRLTGCCVYLKKLIHADAISYVAVLSLIDEFGETEAVARIEKM

IADLEKADSSGIQIKKTPRGQIRVVPSDFKPARVPPVLATKAVEGVKLITTSLLQKLGDIELPEVTDSSE

DEEITFTLNRSMLEMLKNLQSEITESENKQLRRAENRQARLKGEKVTYKTKKNDKNGGEPQSETPPAQA

>gi|313651467|gb|EFS15863.1| DNA restriction methylase [Shigella flexneri 2a str. 2457T]

MSAQFLKCGNQKLYSYLSGYNHLISEADASYAENALRSEYWKRVMALTDVLSVMSDEKKKEWDKQFTADR

YHLPPQEIPDFTLDAVVTTVVALLNDRHQFIKDRVYAVFQSLSRQHKTNKAFGFSTRMITSGVCEGVKDK

WVKLKVEFKESGLMPLSELRVICSYFRGETVKPVYDTKKMVENMVGHVGFRNWISLDGNSIRFRVYKNGS

MHIDVHPDIAERLNNILAAIVPLALPAERVAHTKATLQEFPALKRCIDFHSRMQLAELDFTQNEKEWSSW

TSLGYAGENADKTRQVNADVLRFLGARVTSYHVTFDYDPTEVMRFIGHMGAMPDIKSHQFYPSSARLSEY
VASIIAAGEGERLLEPNIGHGDLLQCFPKSVNVTGIELDTLNCLVSRAKGYETTEADFLAWSAANQIQKF
DCVVMNPPFADNRAKLHLLAAASHLVPGGRLAAVLPLSLQGIDNLLGEGFRTEWMDTFDNEFDNTAVSVR
VLYAERF

>gi|313651465|gb|EFS15861.1| exported protein [Shigella flexneri 2a str. 2457T]

MRKILTGCIIATAIASVYPAAAQIFSYSYTDTNQLAKTLKPATQSYLNPASTMTLNLISGLDRYERVSVT
RDSDKKVMYSAVTGKISVADRIVAADGTEYYGKDLALPALGEGAFTVINDTLDLQQAVVSSSTYHFTIDT
TAPKYTSIYPSQNAGYEMVLSGPVWGLGRGGSGQFSIFADGVDDANGIDKIRLVIKRGNGTVVSDNTLSY
DTAAKRAFYPWIKDSVTQPGMPTSDLNEEFTFNFIITDKAGNTLNIPPQRFLFDDQIGEYTPFAVHDSRV
STSVVPGISSGYVPFVRGLSVLENPYRMVIRIPRTNWKPYRNGGITILNNYGGAKVISEDTTYVYVEVKL
PQGALDINYYRPVNTYQWSGGDLTQYASWLNWDPASVKSPAWGSPAIERQMSDGTWFNSVNWKYFKASDM
PVNLTQIRFNVQARPYDQKITGGATCNIPAGSTSCTVALTQAITNGTTGYLHSGYEVRSTTESTFFMPIW
ENVVWHTLGPSVTGYDYNDTTNILQVYINQPGDGSYFDTVKLSRVWLSDKSRNNADINVTGTLTGRNTAT
GNYTYEFNLKLIPEGSYNVQLNAQDSFNNTGSLDYKTVTVDNTPPSISISYETKPITSSVTVYGLENIRI
QLADTLTKPSLTRMTLRGGPVSDAVELSWVNLGNNLYAPNYPKIFPSLNDGETYTLTVQAKDEMNNVKES
AVEFSYLPNNLVRLENLKTISVSTALKTSDDTPLAVLYASQLRKKDGSIATGKQDAILTVRKDAAFGVTV
NGVSAAPGESKNVQLDLGLGDSRSFPIFPSTSGVVGTSEFMLNIEELK

>gi|313651461|gb|EFS15857.1| hypothetical protein SF2457T_0116 [Shigella flexneri 2a str. 2457T]

MIAFSAYVKSWSLLYTLLAASVSLTVMISLPFIVSPLQHSKFIALRILRLLVITLWTIGLVGVFFSVVER

LVWVNADSYPAWLAKELDSPAITDGENFIKATESFEKICGKNKGYLSVVTKHNGIFMRCDDSLSFDSWWK
GVYRLKTPETR

>gi|313651460|gb|EFS15856.1| replication protein repA [Shigella flexneri 2a str. 2457T]
MSSNNNKLSLVIEESLKDSGEVIQLFPTLKQTIQPKVLLRLGVFVPTLKSTDKGGRKSHHIDATEPLSRL
SIVEGEGYSQIQIYGPRLDMDTDFKSWCGIVYALSNRPLDSEGQLELNFPEFAKFCGFDTKRIDKQLKER
FDASLTRIARTNISFIKKIPGTDDQITINMHLVESTYYDSSTGKIIIKPNSKLNTLYRVDGKTRLYLKTL
QKLARKESAQALYLYLAELPSTFYRIGFDRLRGRLQLSSHLGNQNATVKKALGQLKEIGFLEYSIDKENG
DYVLNILKRNMKP

>gi|313651458|gb|EFS15854.1| exported domain protein [Shigella flexneri 2a str. 2457T]
MAMLGVSVPASAQIYESIFTDTNGIEVHAPSTRLLLNPASPVTLTLIAGLDRYVNVKITSATGTQLLNTT
TTRTSVSDRLKAGDGSEFYGKKVTLPVLGEGRSVVQVSILDLNQSVVATYSYNWLVDTTPLSGNALTGNA
AYGSTAGSVWKMGLEATGQFDFTSTNVTDVNGIEKGMFYVYHSNGSLFSTTQM

>gi|313651457|gb|EFS15853.1| hypothetical protein SF2457T_0112 [Shigella flexneri 2a str. 2457T]
MKFCRRPLIVAVYTALLFPLYSQAQLYNYSYTDTRGTKVNAEPTREYLNPGSAITLNLISGLDRYERVTV
TRSSDNVQMFQSVTGLVTVADRITAADGSEYYGKSMTLPVLGEGTFSLKSETLDNKSNVVATTSYSFSID
ITPPVLPDPMQWIRAGFQYGSLDIFGDRTATQAISLSNVSDARSGLDKAEWFAIDSSGVRRTVNAQLNAL
TGVVQALAAVAAGSTVAPVSPAYYTVGFRVYDKAGNWTETSHRSGIDRTIPSYIQHQVRNAKTGVWEDYV
PGMTIFENPVKFRYKRLKSDSTNFNGTDFGWVDQITNSDNTYFYTEATVSYPETYMYYELHTKAGLNVNL
QYKTLKFTPGNGVAEAPRYLSGQYHFRDANTWTSSDIVRFTGPDCVDLGRVNVEPRSYVQTATIVGIGSC
NVSVGESSCQVSMNYCKTSGKGYVPYQIHIRGTGSYANLGGLQSHFYTYWDMNAPVINSIVSSPLDKTVV
MLVTDSDVTNDWTNYMWATNIFNLTLKDTSNKVSTLSPKNVTVLNYQLKQVTFDYSALPSGKYTATGAAT
DTYNNTAELTLSNTILHDVTPPVVDISFEGKQADGALVKGLENLVITLADDMTKASLKSITLSGGPTSDK

VSVGWYSQGTDKYGLNYPRIFPATDDADSYQLTAVAVDEAGNTTTKVSRFRYVPNNLIEFNTLKTLAVGM

GLKTSDNQPLAYLRTNSIRKKDGSLITGAQTGTLTVRKDAAFAVSMNGVAVVPGDSKDITIDFGQGDGVL

IPIFPATSGNVGESSFMIELPQIQ

>gi|313651456|gb|EFS15852.1| hypothetical protein SF2457T_0111 [Shigella flexneri 2a str. 2457T]

MQSQFLLLGVIWLPPALAMAELERLLKGYDADHSRLLENVLETAHQQFLKFCEPQKREGGGVENHVECPR

QDDALQCLRFMSTRYALAVDLCAAYVCSRYGR

>gi|313651455|gb|EFS15851.1| hypothetical protein SF2457T_0110 [Shigella flexneri 2a str. 2457T]

MKNTVIPTVTENEMGEVITRHSAYGLVSVSRTSTTGQRLYASDLSHKEVVTMTFSESEQIERDGVIRHRL

AEGRRRSPLLQVSLSPAQWATMITSFGMSDGVPCTINSLIRGDYERQPEIGYIESTRERYERQIREAAER

EMAKLHEKLEVLRLLAVKGKAGKRELDEAYQSLLSVINNLPVNLAFTNQLIQESMVNIVSHGKAELEATA

MGVAARLGMKEMSSLASLEEKK

>gi|313651452|gb|EFS15848.1| hypothetical protein SF2457T_0107 [Shigella flexneri 2a str. 2457T]

MAKLISTRELATIITALLVKPELLGELDSPEKHRALMEDLGRVVAEHCGGNVTEIALPETDGTAAEGALQ

NGQPVRYLETKESSPYLIVHPDASLPSVTQNVWMHADHDGWEEHFNGETDALTPEMSEQFRQQVYALLTP

ESHTTSSEMRLTLQDWQLGEAEIPEEDCQPYQVKVLAENKNVQCEVTNETGTTCFGLILEIDRGVPTLHI

DTGSDSLLHIHAAHDGLVLTPDAPSHRFEDAPVDRFSYNSPSLLVPGV

>gi|313651451|gb|EFS15847.1| hypothetical protein SF2457T_0106 [Shigella flexneri 2a str. 2457T]

MPKYLLPAELAEIVCGLLVQPALLNELKTPATHKAFMQDIGELVAKHCGGQIDGVIMPEVRDEDSQADYL

TSPDETPYIVVSPGQDMHSLTDNVWRHHARDGWLDHIKGSVEESPDELPTHKECAVRRYTLQRLLLQEET

SHANLPVQVVRMCEYSTDDVEQTDSSAEFNVTMALGGHAMLAVKDTDSRLVMALTLLIENGVPSVHVNAG
DTDPSVLHIRAAHGGLVLSTDSLNHDFSISPSDQYTYGRPGLLLKAAY

>gi|313651449|gb|EFS15845.1| hypothetical protein SF2457T_0104 [Shigella flexneri 2a str. 2457T]
MDKYFDRSGMAIDNAKIKCIDSVKGTGEYIYRVTCNKCNGRGERNHFYKSRCIACNATGYSLVTTRTCYT
LTALYRIYPEAARKISAAQAAERQRAVQSKTSAFNLWCQNHQELVDAITQQDGENSFLNSLKSTLSRKFP
LSDKQLTVAARILGM

>gi|313651448|gb|EFS15844.1| hypothetical protein SF2457T_0103 [Shigella flexneri 2a str. 2457T]
MDAMFYIQASTKQQASFIFDFLAPRLNAHCQLIEPLNETIIPSLSRTTLSLQPWSQNSQTRTSEMQMVTH
GLTLSGAVMVEHSRQIVDFCDNLGLRLDAPTGKPGWTELINADCEQLNSPRLYPVLIQPGTEGQFQIYVP
APAVIDADTVIASVIDECFSDVTSYCLAGTPFPMTEKPVIRMEAHLSDAHTRVFELTFISYNLALSATVY
DKNLRQVLMLCEDFGVTLQGRAGASCHAYMRNEYRRLAHVGVYSRSGAENDVRAYSSDKEETLIRQLMTQ
ATAGSALIPARHGKQFNGVLMNANPSSATTGFIYLSPEFPQGQFMMTAEVVKSSCTPRGISYIETVDGYR
FIVTAYDTKAMHSLTEFLAVNEDYFTPYRWE

>gi|313651447|gb|EFS15843.1| hypothetical protein SF2457T_0102 [Shigella flexneri 2a str. 2457T]
MNKLDIISSNLDFIFLLNAAGYSASDVVFKRQGASTDFNAGFWTFIAEQKDRYIEVSCSLSKPTAEGTQH
VDVCIDMFENPINDEISRTLDTKTYATNYKYIERVPFTETVKKHLPPALLILFQTSDCIIQGKDA

>gi|313651446|gb|EFS15842.1| hypothetical protein SF2457T_0101 [Shigella flexneri 2a str. 2457T]
MSIQELDFTTCDLFSILDSLNYSREDINGLTPAIFRNGELSRISLSAVQPTRSAEISISQRVSSTTGLRC
YMLLIAQHEDRIHSEALSPDAEAQYQVNHFIRKMAEVTDASTKSVKKCSQVMKKVIKEQQFKANASFGHS

FIKG

>gi|313651445|gb|EFS15841.1| hypothetical protein SF2457T_0100 [Shigella flexneri 2a str. 2457T]

MSLAPDAGATLNKPLTSREAQMSENYFIVKIVCRNGEYEHSSVKLVASDTEANASQTALLNECRDEVEAL

NFEDGGVYDLGGEFFYQVRSCQPVPPEDAEILLRYL

>gi|313651444|gb|EFS15840.1| hypothetical protein SF2457T_0099 [Shigella flexneri 2a str. 2457T]

MKSIFAALPVKDGKSGIWTLDSFVITPEKAQTLKLRAEYTKDQDEYIPAGVYRRLCCDNEVVMSNTPMEI

RTCAAFIERATGCVLINGLGLGMVLHAILQKPDVTHVTVIEKSQDVINLVAESFAHDPRVEIIHADALKY

CPPAGVTYNVVWHDIWPDFSSSNLDEMEALEHKYLRLCDWQDSWGKDQCEAALIRFAALESSLESLMQKG

K

>gi|313651443|gb|EFS15839.1| RES domain protein [Shigella flexneri 2a str. 2457T]

MKSWNSVGTALVYLSETASLTMLETLVHINAPQLLDSYTLLSIDVPDDQIQAFDLNLLPPDWASEDAPAE

LALYGDNWAESGSSVALRIPSALSQVEFNYLLNPAHPAIFDLMKTVQKIPYKFDTRLK

>gi|313651442|gb|EFS15838.1| hypothetical protein SF2457T_0097 [Shigella flexneri 2a str. 2457T]

MESEQLITKITQTLKRPDGSEVRIVVDQAFGSGLTPSLGVYVLRRPTTADNWQLCKTAPHKDWRTMSVDE

YQKHGRSEMLRYVSIGEILRLSAAIGKPMSYVDTCPGLQG

>gi|313651440|gb|EFS15836.1| hypothetical protein SF2457T_0095 [Shigella flexneri 2a str. 2457T]

MANNYTQASFTILCSQEQAQMALDAIAYVTDTDVAEGEHLLSKPVSECSLTELLVLGIIQNHPECDPFEP

TFGQSESPEDNYELELKAEITGKGLAICHDESINLDHAIAVTTAVLSVFNLPEMVTINAAFVCDRPRENE

FGGATIVVTKDTHQYEEGFNFSRLMNEAHDAGVQYALVKVNQYNHEYTYTQCYLMSCKKSESAYDVARHR
LASDESTPDNPGQDGVIILSEEDNTSMALHSVTELMPVVYESLSKLLPSLDELCPVTA

>gi|313651439|gb|EFS15835.1| hypothetical protein SF2457T_0094 [Shigella flexneri 2a str. 2457T]
MTNKILILSASCINEFASDVPQSVFFSIDTSLAKRIRELAAYVKENDLYAAEFFFYDCHWSETLEEEIEV
LMSQAAFIESDSSGQETMLRDVMPSCRTETTSIRVMKESFHLTAVPRHCGDDMMLNTPLIPLAELESNAT
VLITQQYS

>gi|313651438|gb|EFS15834.1| hypothetical protein SF2457T_0093 [Shigella flexneri 2a str. 2457T]
MYIEMKETFLELPSEIQFSEAANAAILTQDKWGDVWQTLKTDADLNYTDAGEPVSLASRVTTATSAIYHA
ITEGWTMCVGYSGGKDSHSLLHLFLMALIRAVRNGSNVSQHHFIQMSDTLIENPEMHHQATQVLSQLRAF
IAEQELPLTVLVARPSMTQSWVGRILTGRGLPTWTNSSTRQCATDLKRSPLRQAKARYLKNAPASVRNKV
CLMLGSRDDESARRADNILRMGGQADSVSLTEHGGELYPVKEWRAQDIWSFLMACGSLARFPLPSFLPDN
FSLATLYKDATGECIWTPEKSTVRSSACGARYGCSLCCAVGIDHSMETLLRTDPEKYGYQAGLNRLQRFL
TKIQYDWSLRDYIGRKVFEGGYVRLQPNVFSSSLTERLFHICCSLDYVEAQRAAAVHEKLLSGEIEDTAH
NRRMSEPQFRLVQEANVLHIDFLWSLHCFNPRPFRAIELYRQVWEGGALDLLDDEPAMQPVPRTPMPAPR
WMKLPEGRIGTSFDGLSDPSSEIAYFDGREDERASRSLMSSGEGLNIVAFEEEDELTVDEDTASWIIWHE
YDGLRQRVSDGEFTPVAAAQYLLRYGAVRISRGKGAVYHRLAQRGQAYSRLGINDQTPLPSLLASRRFKI
LTDSDYRLLVARKLRGQRQKLRFWCCVAACVALHTYNKTPLGGWITTQLETEQQLHLARTGDELKARTLD
AVLTLCNLRISSQQPEERLYYRAVRKRFLETLAGCISPEREGTLREVIWELRMLSGPQSSKKTGFRHVDE
CRERTAALLRPLLKRMVRLLA

>gi|313651435|gb|EFS15831.1| hypothetical protein SF2457T_0090 [Shigella flexneri 2a str. 2457T]

MFLHLVPKILHPMGNLCTLDSVSVPELSLRLTGNDLVAMRPYPNKQYLVGMLKGRRALNGFLVKSPRAFE

EFTMVSVWNIEGFGKITHTLKTFVEDTDYDLVSHDVLLAQGSYRAQASEQCRVHPVYKNIAPVHIEPKME

SLLSTEPNFENDVCETHSWGMLVRSRDEGFKAMTMPSARLQESVALRGDRQPQLEQAIVITG

>gi|313651431|gb|EFS15827.1| putative membrane protein [Shigella flexneri 2a str. 2457T]

MFFILAILIWGFTGLTFKDAILFTSVGLSISVVALSALLVVALTFVMAAMYAAHELGALGKMIISQVVLV

GFWNLIFSVIPDGKLISFFGVNVWQSGSYLVYFMTVLVAVFAAYIALQKQR

>gi|313651429|gb|EFS15825.1| hypothetical protein SF2457T_0084 [Shigella flexneri 2a str. 2457T]

MTTLTFEIAGVKKLLEELRSAERFNATIEQLFEPSNYPGGTPLNEEGKTEVEMNQTGGIFWPSSKHIDPA

RLTPQILLVKDHGVYLITNASLDGTPVSRDTVVYARGMNPSVDDEWYDEAEEALGGDDSSVSIPVAWFEL

ALKKKFNAFSIKVSPTKITLVNG

>gi|313651424|gb|EFS15820.1| hypothetical protein SF2457T_0079 [Shigella flexneri 2a str. 2457T]

MNKPLVSFAELSGNAINVARQSVIDMEMDATREKIGKARSLFHSGIHRAVNGYPLIQSAANQLAVIKRLL

GDTKYLDACITENLCMFSPEGYLYLFMQRRFINEPVA

>gi|313651421|gb|EFS15817.1| hypothetical protein SF2457T_0076 [Shigella flexneri 2a str. 2457T]

MNQTETGPSGFTETTVDEERIAIRVMIGDVVGRYRKQVPPDRVQKATFEVNGGLLMALLLALLMLVCIIQ

RLATGDRLMSFTEEPDVRDIQFTLMLVIEFALGMVVWFLIATGEFSRPWLYCLTPAKPVWLFDEVEDADL

VLLSGNPFIKRWLHKEILKIRRLTYTRLDEELEKIAHYPDSLRHSSVRAHRLSLIAGTKTD

>gi|313651419|gb|EFS15815.1| hypothetical protein SF2457T_0074 [Shigella flexneri 2a str. 2457T]

MSTVENDVKTANNDISLRTYLERTGNGARKSNGLVINPAVVEEEEGFNTRTAGIGESYYSLPDVVAHLNS

LKESYKIDPLSVQAIVVQILNGRPVLRQGACRIRAVAMANIELADEGRELITLIRCEEFRGSRASAEQFT

LDGNSNLALSVVAEALSIKRMVEDAEEPKTFAELAKRRGKTEQHLRQMVRVLDLPEALQAMLVKGEVSMY

VALDEYLYSGDEAVNNITKAIDVYGKATAKTLKFVKAGNIDAAMKPKAAIVPDANATPRKPP

>gi|313651418|gb|EFS15814.1| hypothetical protein SF2457T_0073 [Shigella flexneri 2a str. 2457T]

MTEQTSGEGTNDETTPPQLVDGSAGTNPEPTPERTPAPQMKDPVKTPEPKAVSVSSVLNKKTVTTIADTA

IPLFSRIAEEAKQRAAKNLSDDTYTLVLTNDEMNAICEAHGDLTQYLRKHAEKQNKGQA

>gi|313651414|gb|EFS15810.1| hypothetical protein SF2457T_0069 [Shigella flexneri 2a str. 2457T]

MFEFDRRKQHETADLIFEALTFSPTPELELAKFVRLKSVRRIAQFDDGEVRYGVHVELDEAVNINSFRIF

KDDNTGGFDFYLFSVLGNNAELVQINLDASNIHLQDIFERSTGLCLSY

>gi|313651413|gb|EFS15809.1| hypothetical protein SF2457T_0068 [Shigella flexneri 2a str. 2457T]

MKTLNIQVNDIAAISALGGSMLADIIAAQYKVIDDTKDASEMAMPCIAYGQIPANIGPGVFINRVYSMDS
QSLSASQRYELFNGLATQAFTFFFQSGWYGAFRVVSFRDNGPAGLLNIGIVHDKVLNEFCDDFPRANVSE
TGFNCGMTFDQIRMFISDLAYQMPLYNNQDHFVKLAKHLAA

>gi|313651410|gb|EFS15806.1| hypothetical protein SF2457T_0065 [Shigella flexneri 2a str. 2457T]
MNTFLYVLVGWIIVLYLVNKVREKYSKPKTVNILVKRNGVYQEVEAVVMPRSHNSDVGTGKTGNEAAPQW
LNKEDDISELEYYSGGSLLKAARDFEAQQMEQKF

>gi|313651409|gb|EFS15805.1| hypothetical protein SF2457T_0064 [Shigella flexneri 2a str. 2457T]
MTTANVTTTTYRSDDIVPFRRPQGDLDNRYMPQAYALVRSWASKPAEYGAGVLATYRQPVVNLGYKIKGT
RVVLILVPIECEPVGVKMTDAALWPSLSIGEAMRIMQEAWQNIPELNP

>gi|313651408|gb|EFS15804.1| nuclease-related domain protein [Shigella flexneri 2a str. 2457T]
MDAILMVWRVIGISIIVLVLIVLYCLMGFVSSAIAERRTARQIEIYLPDDAEGLLSDLTLPVNATGTTQI
DHVLIASHGLYVIEQKNYAGKLYGKLEESHWRKWKSSGTLKLQNPFRQNYGHIRAIQSTLRASELECINV
VIINGPCKFEGEKPDWLCMGMEEFVRKVTERRQLNVFKPEAVSFIRGELKLKRKPPGLYTDLNHIHNVTT
RYKTTMRLEQRITYNLLRFSRLILSKMFRSAK

>gi|313651407|gb|EFS15803.1| peptidase family S49 family protein [Shigella flexneri 2a str. 2457T]
MFKRNNKKSPANIKESLDENADRFYKMFRIHTTSKVVISLLALAAAGVSAFNIYDKYQESEGRMDHIAVV
RISGEMGTGSEVGDGSVIATALAKAYNNPHAKAVIIEAESGGGGPSDAIIIYRQINALRSHQQKIERVSH
AESTSKPRNTSDHSASRSQADSNAELSKRNTLDVLSAGSGHFISDMENSYKPIIVSVKGICASACYYAVS
PADAIYADSSALTGSIGVRMDHWNLSRVMDTVGVKNEPLTAGEFKDALDPYHPLSDATRDFMQKQILDAM
HEQFIADVEQGRGKKLLSRPEADAVALYSGRVWTTQQAIRYGLIDGDLTPVEVRSRLSQMYSTTIFKTYN

EPQRSLRSALGMLMSLSTNVETLAGTTSRILDSVQATSYPTVR

>gi|313651406|gb|EFS15802.1| traJ [Shigella flexneri 2a str. 2457T]

MMLYLIYSLGFQNTDELKQQITQEVNASRSLISNDRWLSVIANSEATLSTLINDYKLIDYLNTILIPDTS

TPARGMNYVAEKMTSVNYTVAKNLPLLIYQSIFRWNLILGWLILFSPYLIAMLADGMYQWKLKRYVFGNV

TVQFYRIWFRAFWIIGALTFIYLSMPNMSLFNNIAQLFPPVALLILGIALNRLWSNFQKLM

>gi|313651405|gb|EFS15801.1| hypothetical protein SF2457T_0060 [Shigella flexneri 2a str. 2457T]

MDEQITLTQNQIFSASLKVSKSRSLVKRRMQSLGLKFTESQDVRNRLAGIEKGALKCVGQFCHDNDAESL

SAMAIILSELFLLQGELSTSNEYGDHETSYWTVAQGPCDEWVQSLLASENGRRTFNSFRITFDNSEERRS

LVEKNAKMLGSYLLPYFVNFTNAASAFITLPNSITFKQVQRNKPLIHPETTLSHILTIEDSAFLSRIKFK

LISAIDRLPDPSGQYANMFNHIMDRALLTHLNREQIDSPCVCKKVISTYADTMLTLPIFNTTITGNYRHW

TPWGINFVEFSRQAAKEKSCVYVPEPGQIHWKSPEHKELAEYSLINQIIPQQYHWLLGVPTIWRSHYRDH

SKRLDLFKEWRDANGCG

>gi|313651404|gb|EFS15800.1| membrane protein [Shigella flexneri 2a str. 2457T]

MGFRSDGVNIAPIILYTAVFLLLVCLYRCKTAHPYLMAHWRVFQRQIMFISLKSLRTINKSNFFSNERKY

RQLVQEYKKTNRPVPDRKTYFCNGFEWGPEHADRAYQIANLSSDKREIALPFVLSPIARHFETMARSMGG

NNAIFAVDRRAPIFVTEDNWFGHTLITGNVGTGKTVLQRLLSISMLHLGHVIVVIDPKNDAEWRQSLMDE

ASELGLPFYKFHPAQPSSSVCIDVCNAYTNVSDLTSRLLSLVSVPGEVNPFVQYAEALVSTVITGLSYTD

KKPSIYLIHKNMKSHMSVVNLTIKVMECCFARHYGPDVWMEKVKYASNDTLQVRFKRLTEWFNAHFLNYE

GAEPIEWIDTVGRLVDYSMSDPEHMSKMTAGIMPLFSRLTEQPLNELLSPSPNTLTSREIVTSDGMFSTG

GVLYISLDGLSNPESARAISQLIMSDLTSCAGSRYNADDGDMSSHSRISIFVDEAHSAINNSMINLLAQG

RAAQIALFICTQTISDFIAAANAETANRITGLCNNYISLRVNDTPTQTLVVENFGKSPISTNMVTYTTGS

ETTLPHNNFSGSISERKQTTLEESIPKELLGQVPKFHIVARLQDGRKVVGQIPIAVSEKAMKPNTTLLEM

FLKPAGKVTLRQNVGLSYLNKYLRKLH

>gi|313651403|gb|EFS15799.1| helicase family protein [Shigella flexneri 2a str. 2457T]

MNFRALFLSMQRVFGIFSRRENDVSELMMKDAANFSPFAQIIGEQKYTVPDHPNPEVLKFIEYPTRPAGI

QTFNEQSILSLYRDKLHSISMMLAISDGDIREDAYTFTNLVLKPLIEYIRWIHLLPASENHHHNGIGGLL

SHSLEVAMISLKNANHSELRPIGYQDEEVVRRKVYLYAAFICGLVHDAGKVYDLDIVSLNLSETLTWAPS

SQSLLDWARENNVVEYEIHWRKRIHNQHNIWSSVFLERILDPVCMSFLDRVKKERVYAKMVTALNVYNDG

NDFLSKCVRTSDYYSTGTDLNVLRDPIMGLRSNDAAARAIGTIKHNFTSININNYKTKPMHIIIVNGEVY

LNENAFLDFVLSDFAAHKFNFPQGDAGKTVLVESLVQRGYVEPYDDERVVHYFIPGTYSENEIASIFRNG

IGKLEFYNLLKLRWIGLIFDSYKIPDSVPGLFSVNANKDFIYIDEQKTVTEYRRPVPGRESVTRVTDTVN

DAIENTPQYGLQLVNGPDADSNNIISSENTESITDSLEESGADISNEIFETQVVTAIDTAETVNADEPEQ

VEEHDDRSQIHLVEQLHEMLLSAPLPHHAVINIDSVPYLDLDAAIALIPGIDEAAFCNGPFFQLTYRDGS

LDGMWIVRDVNNLRLIQLGDNCAGMQVSTSEPRNTSSLKSLFDTSMYQPLDIPEAPSVNEAASPPQTPLE

LPQPRLNAPVAEEASSVAEQTNAHSEPDSVIATEYEQYGHLLEETLDSDGEAYSDLIASDSTEAEYPATD

PQSSDFAQLPRETALSVAPGDLDYSEGAIKPPAPDATGKETILTSPEPAEDVRETVAAVEKASHLSPALA

RLFAVSTHAEKKHEKTQEPSPVKEVKNPTSSTTVKAPISIEPPGAEEKEAVEEFTLLNDGEVTELEYVEI

ATMLHQILTKLSGSFKRKRKNRFMVLTQNTFYLTQSCIEKYGTQLNAPELFNQLPQYQVTSGAVVNTKCI

AFNIPTLVAASDRAKVDIELIINKLKEVGNL

>gi|313651402|gb|EFS15798.1| hypothetical protein SF2457T_0057 [Shigella flexneri 2a str. 2457T]

MTDVTMNESEHKVPDVNTRSPHASDNSRRVVQSKNEHYGMATVTITPGTPDFNRFLTARNRSVIRGFDDV

SIAISSLFRTVNAVKHPELVQAIQDWFNELHEENNLMKNNLERHIATIHIDESDPFFSSTEFSPFRFEPV

QLNFNNQNTMRFYKHIFEMNNLLTQMHKFNSLGQLPVSDYNVMAHNIIRSLNMYIERVKKTLNVSRRAKG

AYSPDEFIEKVKQYKSVQAYIAAELSGKRR

>gi|313651400|gb|EFS15796.1| trhR [Shigella flexneri 2a str. 2457T]

MSESDNITRNLLGKLLRTEISIIRSYRAFLMLLPLHGSSKYQTGSPLLQRRLFGNGFGAMIDNAFEVETR

PGSFLVPRSLSKEISWDKFFVAVVDGDTNVIREYDSEDTDFGIYNEGEKVTLLSGQEEFYNPRKIQQLRS

KCVDIQNDYLMQVFFMSMLAPEFVSIFFGLKPATVEAIKDVGMSSLKLINDVVLFPRTIPFTPGLGDDSA

TLKSKVFAWAYEISADIRLGKISDDLMELLRYDTMFTCHRQDVFNTLANKVLLKDY

>gi|313651399|gb|EFS15795.1| pilus assembly protein [Shigella flexneri 2a str. 2457T]

MTRLTLIPSKMLVLLSLFITGVHANEEQALIKDTPFVSGQAFKKGFFWYDDPTRKTEEEITETKPPVASS

TQPEQEEKIELNSKWLKDNMPQLLTKAMDNPTPENLSRFYTAQRLMLDIGTRFSDKSKDYFLKNPMMSEK

RRQPVEKVALDAHRTVVEKNQQSVMKDIFTKSGLFFFFQSTCQFCHEESQILQFMENYYSVDILPVSMDG

RPLQNGLFQDFSVPNAQIIDQFKIREVPTIFLVSKDGSSAQRISEGMITAEELKNTIILAAKGMKLIDDA

SFQSTLDIKRQYTIGEDGVITVNKSEMDSDPFLLQRIMDKKLEGYDMPTADPVNYLNVGGSLGGAYAR

>gi|313651398|gb|EFS15794.1| traH family protein [Shigella flexneri 2a str. 2457T]

MRADFHVCAKKLLLSLAVCTAMIAPNAVADNAMRNIFNGMMTTTSPASFETATRTGVVGGSFSYRTTNVN

TNLVSMSFPKASVGCNGIDVFLGSFSMINGDQLVQVARGIAQGAAIYAFNVAVSAICADCAATINDIQNK

LQALNKFAKDSCNATYSFLTENVGPPSQFANAVSSGPASMLGTLNGLLPDFGSSMTKSPETVTSQVKAKN

PEEFAEKFSGNLFYMSFMDIDKGSMNIGGVTELSGYRLAEQLMSLVGTVIINWDAKGEKAGMEVRPSTMT

VSDFIMGPPAGGSIKMLKCSPAPDPASARKSQCLVMSEVTDGGFKGLKDTISDLLIQVQTKAISDVRLSD

DELRIISYIGIPTILDSLQTFDVPEGYAYIQDISAIAATSLVINMLRQVEAKISAMNIPSESLSGRRQDL

TRLSENLTNQVKAAYELTRSQVGNSSDVISAWDDRRLKRKAFMESLRGTGN

>gi|313651397|gb|EFS15793.1| putative membrane protein [Shigella flexneri 2a str. 2457T]

MDYNIYTVGDIEFVWSALNGIALIFSQYTGVKAFLTTAAVLAGASLFYKTWLWLLNPTKAEVPIFSWILG
LILFSMAIVRVDVTIESVKSGEVRNVDGIPIFIAAMGTVTTNLSQGLLKDYKTAFDPLAPIDFAATTLDD
DITLGPMIRFVKFLQWGGDSQGYCSAFPEPASGLGAMNVCATVQSLAYNCLKGTQNSSAKIAGKETIFND
IFSANIVDSMERINQAMKGSLKNASANIVGVNSSKSATCEEVWSTVKQVTSTPEARQTIALIGQTNGILA
PDEAGGAASGASFTDVMASANGMYGKAIGAYDATLSLFIMNELRNGASKYKTPLGLASDMQLFEASLKRT
NTMASQGQLWLQLSGAAIAFLEMFAYMVAPFALLMLLALGGNGVAAAAKYLQLILFVNMWPITAVMVNAY
VKKVATADLDTWSTLNSQSNAVTWMGLPGLAETYSSYLSVASALYALIPVLTLFLMTQSIHPMMNAMKGV
TPPAPVDSSHLTPKVWDAPNSGKSSFGDVNRTALTSTGQGFSGGGAMDSSNFRLGTWNAGSSIASSQGQG
AAFTSSAMSAASNTFQQAFNQMSEVGRTGQSGQQFTSNLQSLKSFTDQAGANIANSVGTKHGINSQQLAG
VVSNLLLNGSLGLGGEEKLGKGGGGGLAQKVMDSLGIKAGGQVGSGITKSNSGNESLLTDLSKAITTQLT
ENSGLTDQMTKAAAQINSDQFAQTNAFKEAATKMNQSTQTMAENLSTSLSTNASANTGMTLDSRQAINLD
RFSDSIRNKNFSDEDVRNFARKNGLDENAFMEKFNSYNDTFKASTQLGSQLQRTDALVATTRDFSEQKIA
IDTARGETAESNKQDLRETSSLLKGLVADFGGNAQQMLPITNQLDRITGDASGINTIAQAQDRTPEHVNT
SSVMSADRVADVGQSMDAQAKAGLVQNQQNANQHVGGQTESGVTPYNVDKISKEDIQGIHQGNVNKSYSE
ENQNVLSSLEKNTPVVTNETVGRVQGAGVDVQHANQTFDQLQAGSGGKHATSGMGTISDKLNNMYGDNQI
RGISGNLPNYFGRIANDPTKKPDDKRAELAAQAVFTYGASTMATGAEKEQLKADTQNILSELSHYNVRWG
MNDLQNIHEGFNMHNKASGSLDSVVRANLGEGGSGGGLVGQRTQTTTDLLVGNKIESNTERGVIGSTLLS
GQESVARGLDVIGAKPVNDMLSGMGILTTPSSLANDASNPQNMPSSLLGKVGNHLQMSDGVSAVTSRYKD
MGGDGIKAYETASQNSERAIRQQLTDDPRFGPDKADAFVSFMKSNLNNTNEPYQSRIEKAESWLNENKK

>gi|313651396|gb|EFS15792.1| transglycosylase SLT domain protein [Shigella flexneri 2a str. 2457T]

MFIPPVDDVKPIPVPVEIYTQCITDASRFFGIDAELVFTLFDNEGGKVGTFSRNKNGTYDIGPMQINSSN
LPEIRDHFPSVTWRVLAYDACASFWVGTWWLYRKIVDRKGNVFEGIADYNSKTPKVRATYIFNFMIKYNR
RIQRRNGMDELYQWTQPKPQYNGHIVKNLPE

>gi|313651395|gb|EFS15791.1| dsbC [Shigella flexneri 2a str. 2457T]

MNAQTANTDVLPDAALKKLREVGLSIEHIEPSPVKDIYTVISREGVSYVSKDGDYIFTGSLFHVNGKDVE

NTTEQAILKGVREFATKTKSIEYKSANEKYRLAVFTDITCGFCQKLHQDLQSYLDAGISIKFLAFPRAGL

NSVVAGNMAKIWCAAKPNDALDAAMSPTSTIPEGRPDEACLDTIKSHFQVASTIPLQGTPTMVTLSGKPQ

LFTGWMSPENLVTRMGAEQK

>gi|313651394|gb|EFS15790.1| hypothetical protein SF2457T_0049 [Shigella flexneri 2a str. 2457T]

MKSAILTTLMLVAVGSQPVFAAKCQFGACDADTVASSEILVSAAQTDKDAVLKKLNDATSRSDVMSKRFE

SYLSNDRLKVNTDSTKKGPSNEIAKPRDELAPVRQLKAIRPELVQTPIFQPIVAFYERLA

>gi|313651392|gb|EFS15788.1| lipoprotein [Shigella flexneri 2a str. 2457T]

MFAARFASGLTGKGFNISSHQPEYILRFSYSKTQENLQYSELPVTGITGYVIEKKTTRKDKHGQTETDYD

YKPVSGIVGTETVSQRHFWRRLDVEVYPAGKNAQQVLKVSMQSNAPIPSDSVAYSAMIDALTGNLDAPLR

SGNYVASVPWN

>gi|313651391|gb|EFS15787.1| DSBA-like thioredoxin domain protein [Shigella flexneri 2a str. 2457T]

MIKLNYLKPVASVLISAVIATAGSVYITSKYLKPDVPGNDEIGRVAASYLVKNPQYLLEAGKALEKENTN

ASLERIIPYAPALFETKETPNIGPDNAAVAVIEFFDYQCHFCMQVAPVVESVLSQSTDVKFFFKEFPIFA

GSKPVSAMGAATGLHVYQTFGAEAYRKYHNNLMTSAYVFFNNQRAFTLNDLDMVVNKSGFNSSFGDREKS

RYENVISGNMQLGEALGINGTPGFIIMNMQKPDAATTSFIPGAVDEATLKYAIQKARGG

>gi|313651387|gb|EFS15783.1| hypothetical protein SF2457T_0042 [Shigella flexneri 2a str. 2457T]

MAIDTDDNDPVDAEACEKYLAQLRELEAYRAYRTTAAIDWFFDQATRAIHGELWLAACTTFLNGIETSLR

VTMKLKASQAQPQAPTPLVDLSDMATLSNALLRRAHQAGMPVTLLAFPDEQDLLTKIADGAPKLPYAEIV

RVRHNLCHGNILEHIITASDGMGEPVRLFTPECMRDLAQTLSAVSKVWIAGLHQYWCDNNLSMP

>gi|313651386|gb|EFS15782.1| hypothetical protein SF2457T_0041 [Shigella flexneri 2a str. 2457T]

MLPAVRNAEDVAVTMALGTVQNALSQGISEAQVSLMAKVAEREGKIAGAVANIITSRTGAITRALEKMHP

YYAELAAAKLAGTQEGIEQASKIQAGVLTLELFVGKNVTVVDVAEGEPADSSIPLTLCQKKLVVDEEFSA

WSDIDEHFDFRSLSAFLKALKDNQGLVEQIFPTERCVLVMVTTRRFIDYGDTWANAENNEKNSIVFLMVR

NGQNIKQIYSPVESHLGASRLFPSEDEQQGHFRGFDGTTIKFEDVAYTDRLKAHDLMALHYRRLLIMLFG

LDQRLALFGQFYPQHEKANFLNLSFQERYFHFLHDEDGTGLLASPDSQTLDEFISEKNSYIQPGSRLLCN

WRELMTPRTAPGAVKEDSGYSGYSFIADPEDNVSAAVVYRQGDTLAVDVAVKRFSTDKMFNCKVNVSAYS

PWRRGEAELAYLCLDAVGPEELTRFIQQRKFRSNHLFYIRFFKAAIKFLEQERENELPHRQYLLAAMQDS

GMHLPGNIQELIHQCIASWKTGNRGVSLAVGMSTEKGRQALLNQLYRLTQGASEMVSVIQEHVAASGSQL

LRAGVNSSGNYVAFIAPKPYECDNRLEAHAWVHRVVYATGKRNIRETGRSWVSMLERSASEITLWEDEEQ

SRKWYSVTPVFSSWTEKQKLFEMCEKGAGLLKGAMGTPDDEEYGDLLEMWGDAYIACNASGEHVTTPDMF

LPVGLIKSRKSLKLIALGTCSTEHWMYARAGDDDSRELLLELYTSWYEYPDKARARLLSMAEKNSGLRFV

LLEGKSRKLERFAVKRPEEIVNWHAGNLKEIPTMLNDQWACHMAMVSQNDKVYLTPDLLDDDGLPNFNGV

TRQVPGEGYQPVNVYEFESDYFNAVYDADGKKVALCHWYDVTDNSYSAEELIGNMPHNAFKFLQYRLDNI

EQAEAFIKYRNRNYQPRENADWPEPPEGVKRYVIRARS

>gi|313651380|gb|EFS15776.1| integrase core domain protein [Shigella flexneri 2a str. 2457T]

MDEVNRLKIIQDVIDRRLTTQMAAQRLGISDRQCRRLLARYREDGPLGMTSRRRGKSSNNQLPQGLAAYA

LNIIRERYNDFGPTLACEKLAEVHGVHISKETVRKLMTQASLWVPRKQRAPKIQQPRYRRACAGELIQID

GCDHHWFENRGPKCTALVYVDDATSRLMQLRFVKSESTFTYFEATRGYIEMHGKPLALYSDKASVFRINN

KNATGGEGETQFGRAMHELNIQTICAETSAAKGRVERAHLTLQDRLVKELRLQGISTMESANAFAEEFMN

DYNRRFSKAPRQEFDVHRELDVDDDLDMVFTWREARRVSKSLTVQYDKVLYLIEDNEFSRRAIGKYIDVW

HYPDGHKELRLNGVSLPYSTYDKLSEIDQGAIVDNKRLGRALEMAQLVQAERDNNRSQSVPSGDGPSRRR

KAPTTKKSQRSLDQDDMFNALVKLQTRAEEIFGERNQK

>gi|313651379|gb|EFS15775.1| hypothetical protein SF2457T_0034 [Shigella flexneri 2a str. 2457T]

MITFSMDESGYTGYDLLHKEQKFQGASSILINHDDASRLIKEYFPKLQADELKYSSLKRRDSNRKPLFEL

QKHLLSNYPCITCVGDKRFLLILMFIDYAVEPFYYDSGINLYEDGGNFSMASMVYYVGPAYYGSAFDDVL

LQFQNAMKEKTLDSVSLLISKIRRLDWQRLKEFFGPLAFNHPDCIDAIMTDGISTDASFTILNALISRTE

MMSSGEYAIEHDRSKNLLTYNERLNFLINCDKEGEFKHSEIATISFPLNLKKVYQIDSKESPSVQLCDVL

IGACIESVYQLMDSKVLNQNSVLSLYQDSQLIHFIPDIDFEGQKKFRKGSQSEEYLTFIQNEIYSSKL

>gi|313651378|gb|EFS15774.1| ATPase involved in DNA repair [Shigella flexneri 2a str. 2457T]

MVQVLNTTGLNYQLEKTITEAEERIILISPYLKLSNRIKELIEDKNRLKVDIRIVYGKSELNSKEYEWLT

NLPFVRLSFCKNLHAKLYANENQCIVCSLNLYDFSQINNHEIGVLIIKEQEKDAYTSSLNEAQRLIRIST

DNIQPLTTDKETTGSKESEKTNKESSDFTSAFHEEPLTNQNGLITDLNTNSKLTSTNLAKNHKTDLLSLY

KKLIEAGYLMDVEGKYILTDAGIAAGAEAKPNRYKKGENYFLWPDNLAL

>gi|313651377|gb|EFS15773.1| hypothetical protein SF2457T_0032 [Shigella flexneri 2a str. 2457T]

MTRQHPLLKIALNSDGHLSWPILPGETHRCPTCLRHVTTGPDGDLVHSMEEGGNVCIPSASVVISKAIIE

MLSAGERLYVNPIKNGNKTLAPSFIFLHTDQQLRPFINSDYQPAGASWRSDKGFRLGLFFMNERASKINK

DQFDFIAVIDPLTFQSEFDATWSRGEFDDPLKALRYSIVSENLSSVWVKWPVKNLQPRKETVMADYWYDY

YSPHSPAQQAACTATVTGIDGNVSGETIYMLQIALERSTVEYQLVKSLGVILLLDNTGNLVSEAQPHFDI

IIKACISGVRDFVLQNPRTLGFENKVA

>gi|313651375|gb|EFS15771.1| hypothetical protein SF2457T_0030 [Shigella flexneri 2a str. 2457T]

MGQKFAVFIAYDDEPNTKRYSAEFQTQDEYVKGWQSALKKAHHTSGQKSVISCGCRGKGAKRLYVRSLPN

SDTFILIKAANTGTEHDPSCVFFDLDARHTGLKGYASNVVRINNEGTMSIRLGIGMTEKDPPEKSEVPSL

PQIQRPEGGQASMTLSGLLSLLWTEAGLNVWYPNMAGKRNDSLVRYRMLEAAKQIRSGRACIGDHLFIGV

ADSKSKVASEQVQLLSSAELSDKRLLLLSVLPRYDAEKHEKPLKFLPMRNFGGMPLTFFNSDGHWDSVKR

RFPLEYAAWKNGGKVVVFALTSPVSVTSRGISARAHQIVLMLVSDNWIPLDSSYEAIVSRKLDAEHRHYV

KPMRYDASASDVFPDFYLLDTRSDKPFPMEVFGMSTPAYLARKELKKAYYNQEYGAYGWWHWDATEKTEY

QELPHFPEARR

>gi|313651373|gb|EFS15769.1| ATP/GTP-binding protein [Shigella flexneri 2a str. 2457T]

MTDNVNLMNGDEQTASSLSEETSKTPPLSPELLKNSPYSSIAKVKYFTEIEQICLDKSISTENLDQFFKA

HWLRDKMGGSFARAQEMLAAYKQYVDEVPEEARAIEIPDQIKDAFSDFTAFITWYFRLSYTAIQSDSVKI

AKAEITQLRQRNAEILEELSQSKEQATVLNNEKVNLINLLEQQRELSSKLEDSLQEAEETLAGTQSELQH

AQNEIQLLQQTVGTLNQQLSERKQELASQQEYQKQLNDENKAQQVELTALKSQNDHLQRTVSDLKVSVSQ

LEQDLSSVQTHSSELSSSLAEKDTTLTLVRSELSTAKGENDQLRAEAQRLADESLVAKKVQTDQTEELQQ

LRNQMISMEATLNAEKTIAESLRGTIKQLTEAMTGAVAIKPKSSGASKPRNKKTT

>gi|313651371|gb|EFS15767.1| hypothetical protein SF2457T_0026 [Shigella flexneri 2a str. 2457T]

MSMNSQPELKLSTRTEQLASSRDAAMQKFLDGMTLIAEASAICGFSLFNSKIMAPNAFGLPASLAASIEE

GRQQIDRKTWNNLFEETGIDRFWNHNQRAEFRESLRNAPPIASLTVIRSTLRQAVAMRSITLAEGFVDLL

CQLDRRYKTNA

>gi|313651370|gb|EFS15766.1| hypothetical protein SF2457T_0025 [Shigella flexneri 2a str. 2457T]

MAASRITHLITSCTKGKHSQCGSMPELSIRSGQTPEEAMSSWAATIKRSQSASPVPALSLYAGNHWSTAK

EILRTTENLELWVISAGLGFLNSRDLVDAYEATFHDLPFSHRQWWRELTNTFGKERTAKSIETLMAARPF

DDYVIAASPVYIEATEDDILAGASKLNNHIAQLTVVTSGEYSGPLESYLIRSESRMMRQLSSNMVCLNIK

LAQYIIGSRRYS

>gi|313651367|gb|EFS15763.1| hypothetical protein SF2457T_0022 [Shigella flexneri 2a str. 2457T]

MCVERPDGAFDIYTGRFIQFHQIDNSRRKIVEISDPDLVGMTYSIPMVFNGGKFSGQGVTCLSLPAVTLI

KVTFPSRTYQEGQEQITMSSPDQALFSEDVRKNCAGTCVLTGVRGRQRTEAAHIKPRHAGGDPDVTNGIL

LRSDIHTLFDNWHFSIDPDSMKARFSPDVLSVDKDLLQLEGKQIDFSRLQMPINIEHLRHHWNKFFNRHV

NPVIKNALNKKINNYEKRPVSSNIHY

>gi|313651366|gb|EFS15762.1| putative lipoprotein [Shigella flexneri 2a str. 2457T]

MQNCLSGYLKATIVILSAGLTGCDLANGTAESGVYQKYTNGPFKEPTIVMSPGWRMDDHGREALIFGSSH

CPDVNGNNTSEGGCVLIEEHSATVAVTVVDATNQSRRQETWTIERKKDRVIVKRPDNSFVMPWGK

>gi|313651365|gb|EFS15761.1| hypothetical protein SF2457T_0020 [Shigella flexneri 2a str. 2457T]

MREPRPRHTLQVVCVPSLNIQDLGLTSFHSWLLSKGYDTTNARNNRTCWAREGGWHLKRCRHLATGEDHF

WFIAFNGTGGDIFPVKTQQDYRAAYRKLEAYGYAPAVIEQLNTGVAYNLAYSRTPLKRPESATSELKRKP
DVNIQGEPCERFVTERSSAAQNKFKTLLIENFAGRCAVTGWVNGGVLVAAHIEHGTRYNPSNGILLTPTM
HALFDADLMGIDPSTLTVHFKPGIEAGELFEGKTLNPLVYDLDTDRLAARWADFIGAE

>gi|313651363|gb|EFS15759.1| yadA [Shigella flexneri 2a str. 2457T]
MVSPMEKTQSPAERFYESIYYSDESLEEDYLAELRNFSSDHWDTALRAARLSAAVKRFKTSEMLRFILEF
VVPENAQEDAPDLTPLAAKRLCNSLFGRSGSQSILVYVFGQAGRVHRSATCSPKTIEAIAALYRSDAERY
WNSTLATIERVKHTYRAKIRNS

>gi|313651362|gb|EFS15758.1| hypothetical protein SF2457T_0017 [Shigella flexneri 2a str. 2457T]
MEAIRGKIIEIIQEKQLLGFPHLLAGYDGGTVPLDEVSGSGSMFGATADLFVKVNDLCVAVFLDIDRSVA
GKLTFDHLHASEMVAALRIDLPDIEYEISQVQLGRRDGTYSECIEKIIIDATESREWLYHPMMQELGTEP
LKVYEGREPAEAGPLLQRLSDCPLKLPDNLPQSMNVLIRMQQATIVCLCRLTVRASDIAQTDEFPLFMRY
FRLHCLDTSEHVNYEKLLNWQDMITKSKEGQLLTVEERDYLQEIVRIGLYNHRLKSVCLTACEDSRVGES
EGPNNYSLL

>gi|313651358|gb|EFS15754.1| phage integrase family protein [Shigella flexneri 2a str. 2457T]
MNSKPVTRQFEDSDLHQELVTFEVPNNDLKELIFYFSHMKYNTAKTYLQWLRSWNEWYQANAGKEGNEAW
PASSLPVTEPPLLAYLDYLQGSLSHSSIKGCLHALNSIHRKALDRPGIITSKVKSILASLEQAEAREQKV
TRQATPFLVSDLKALIKAHGTTQSVRKLRDLCIIWTGFETLLRSAELRRIRMQDLVLNEQTGSFTLTVYR
TKSTVSTLLTYHLTPHLTATLIRLMDMVKRDQQSHPKDYLFQAVNYQDSGYMPPGWGLRSKGNEINTLLK

NHNMPYRPTRPPIGKNGKPIIVDDEGMLSKNTLLRAFEAFWDELHPQEAGTRCWTGHSVRVGGAIELANA
GYTHLQIMEMGNWSNPEMVSRYIRNIDAGKKAMTKFMREALDE

>gi|313651357|gb|EFS15753.1| inner membrane protein [Shigella flexneri 2a str. 2457T]
MELGIMVCIVLLLSLAAVWISNKTVGLLEIHYFKRPLSMEYAAWLRVMCAGLLFIFIQMVPAFTFLYTLK
LVALLNLGVQTMKLNGVYKFKRTGMMPPKDAPHDLGRFNPFRKK

>gi|313651353|gb|EFS15750.1| initiator Replication family protein [Shigella flexneri 2a str. 2457T]
MAEIAVINHKKRKNSPRIVQSNELTEAAYSLSRDQKRLLYLFVHQIRKSDGSLQEHDGICEIHVAKYAET
FGLTSAEASKDIRQALKGFAGKEVVFYRPEEDAGDEKGYESFPWFIKRAHSPSRGLYSVHINPYLIPFFI
GLQNRFTQFRLSETKEITNPYAMRLYESLCQYRKPDGSGVVSLKIDWIMERYQLPQSYQRMPDFRRRFLK
ASVDEINSRTPMRLSYIEKKKGRQTTHIVFSFRDITSMTIE

>gi|313651349|gb|EFS15747.1| uncharacterized yfaL domain protein [Shigella flexneri 2a str. 2457T]
MTNNASGGAVFLQQGAEFSLLPENETGMTLFANNTVTGEYNNGGAIFAKENSTLNLTDVIFSGNVAGGYG
GAIYSSGTNDTGAVDLRVTNAINNIANDGKGGAIYTINNDVYLSDDVFNNNQAYTSTSYSDGDGGAIDVT
DNNSDSKHPSGYTIINNTAFTNNTAEGYGGAIYTNSATAPYLIDISVDDSYSQNGGVLVDENNSAAGYGD
GPSTAAGGFMYLGLSEVTFDIADGKTLVIGNTENDGAVDSIAGTGLITKTGSGDLVLNADNNDFTGEMQI
ENGEVTLGRSNSLMNVGDTHCQDDPQDCYGLTIGSIDQYQNQAELNVGSTQQTFVHALTGFQNGTLNIDA
GGNVTVNQGSFAGIIEGAGQLTIAQNGSYVLAGAQSMALTGDIVVDDGAVLSLEGDAADLTALQDDPQSI
VLNGGVLDLSDFSTWQSGTSYNDGLEVSGSSGTVIGSQDVVDLAGGDNLHIGGDGKDGVYVVVDASDGQV
SLANNNSYLGTTQIASGTLMVSDNSQLGDTHYNRQVIFTDK

>gi|313651348|gb|EFS15746.1| uncharacterized yfaL domain protein [Shigella flexneri 2a str. 2457T]
MRADGEVAVDAGVDTQWGALMADSSGQHQDEGSTFTKTGAGTLELTASGTTQSAVRVEEGTLKGDVADIF

PYASSLWVGDGATFVTGADQDIQSIDATSSGTIDISDGTVLRLTGQDTSVALNASLFNGDGTLVNATDGV

TLTGELNTNLETDSLTYLADVTVNGDLTNTSGAVSLQNGVAGDTLTVNGDYTGGGTLLFDSELNGDDSVS

DQLVMNGNTAGNTTVVVNSITGIGEPTSTGIKVVDFAADPTQFQNNAQFSLAGSGYVNMGAYDYTLVEDN

NDWYLRSQEVTPPSPPEPDPTPDPDPTPDPDPTPDPAPTPAYQPVLNAKVGGYLNNLRAANQAFMMERRD

HAGGDGQTLNLRVIGGRYHYTAAGQLAQQEDTSTVQLSGGLFSGRWGTDGEWMLGAVGGYSDNQGDSRSN

MTGTRADNQNHGYAVGLTSSWYQHGNQKQGAWLDSWLQYAWFNNDVSEHEDGVDRYHSSGIIASLEAGYQ

WLPGRGVVIEPQAQVIYQGVQQDDFTAANHARVSQSQGDDIQTRLGLHSEWRTAVGVTPTLDLNYYHDPH

ATEIEEDGSTISDDAVKLRGEIKVGVTGNISQRVSLRGSVAWQKGSDDFAQTAGFLSMTVKW

>gi|313651345|gb|EFS15743.1| pufY' protein [Shigella flexneri 2a str. 2457T]

MSGEKKAKGWRFYGLVGFGAIALLSAGVWALQYAGSGPEKTLSPLVVHNNLQIDLNEPDLFLDSDSLSQL

PKDLLTIPFLHDVLSEDFVFYYQNHADRLGIEGSIRRIVYEHDLTLKDKLFSSLLDQPAQAALWHDKQGH

LSHYMVLIQRSGLSKLLEPLLFAATSDS

>gi|313651344|gb|EFS15742.1| uncharacterized protein yfaA [Shigella flexneri 2a str. 2457T]

MRYNGNNALMFATYQDKMLVFSSTDMLFKDDQQDTEATAIASDLLSGKKRWQASFGLEERTAEKTPVRLR

IVVSARLLGFGYQRLMPSFAGVRFEMGNDGWHSFVALNDESASVDASFDFTPVWNSMPAGASFCVAVPYS

HGIAEEMLSHISQENDKLNGALDGAAGLCWYEDSKLQTPLFVGQFDGTAEQAQLPGKLFTQNIGAHESKA

PEGVLPVSQTQQGEAQIWCREVSSRYGQYPKAQAAQPDQLMSDYFFRVSLAMQNKTLLFSLDDTLVNNAL

QTLNKTRPAMVDVIPTDGIVPLYINPQGVAKLLRNETLTSLPKNLEPVFYNAAQTLLMPKLDALSQQPRY

VMKLAQMEPGVAWQWLPITWQPL

>gi|313651342|gb|EFS15740.1| alpha-2-macroglobulin family protein [Shigella flexneri 2a str. 2457T]

MGTGLAKADDSLPSSNYAPPAGGTFFLLADSSFSSSEEAKVRLEAPGRDYRRYQMEEYGGVDVRLYRIPD

PMAFLRQQKNLHRIVVQPQYLGDGLNNTLTWLWDNWYGKSRRVMQRTFSSQSRQNVTQALPELHLGNAII

KPSRYVQNNQFSPLKKYPLVEQFRYPLWQAKPVEPQQGVKLEGASSNFISPQPGNIYIPLGQQEPGLYLV

EAMVGGYRATTVVFVSDTVALSKVSGNELLVWTAGKKQGEAKPGSEILWTEGLGVMTRGVTDDSGTLQLQ

HISPERSYILGKDAEGGVFVSENFFYESEIYNTRLYIFTDRPLYRAGDRVDVKVMGREFRDPLHSSPIVS

APAKLSVLDANGSLLQTVDVTLDARNGGQGSFRLPENAVAGGYELRLAYRNQVYSSSFRVANYIKPHFEI

GLALDKKEFKTGEAVSGKLQLLYPDGEPVKNARVQLSLRAQQLSMVGNDLRYAGRFPVSLEGSETVSDAS

GHVALNLPAADKPSRYLLTVSASDGAAYRVTTTKEILIERGLAHYSLSTAAQYSNSGESVVFRYAALESS

KQVPVTYEWLRLEDRTNHSGELPSGGKSFTVNFAKPGNYNLTLRDKDGLILAGLSHAVSGKGSTAHTGTV

DIVADKTLYQPGETAKMLITFPEPIDEALLTLERDRVEQQSLLSHPANWLTLQRLNDTLYEARVPVSNSF

APNITFSVLYTRNGQYSFQNAGIKVAVPQLDIRVKTDKTHYQPGELVNVE

>gi|313651339|gb|EFS15737.1| alpha-2-macroglobulin family domain protein [Shigella flexneri 2a str. 2457T]

MTTSELTSSLKGKPVSAQLTVGVVDEMIYALQPEIAPNIGKFFYPLGRNNVRTSSSLSFISYDQALSSEP

VAPGATNRSERRVKMLERPRREEVDTAAWMPSLTTDKQGKAYFTFLMPDSLTRWRITARGMNGDGLVGQG

RAYLRSEKNLYMKWSMPTVYRVGDKPAAGLFIFSQQDNEPVALVTKFAGAEMRQTLTLHKGANYISLTQN

IQQSGLLSAELQQNGQVQDSISTKLSFVDNSWPVEQQKNVMLGGGENALMLPEQASNIRLQSSETPQEIF

RNNLDALVDEPWGGVINTGSRLIPLSLAWRSLADHQSAAANDIRQMIQDNRLRLMQLAGPGARFTWWGED

GNGDAFLTAWAWYADWQASQALGVTQQPEYWQHMLDSYAEQADNMPLLHRALVLAWAQEMNLPCKTLLKG

GDEAIARRGTKDEDFSEEDIRDINDSLILDTPESPLADAVANVLTMTLLKKAQLKSTVMPQVQQYAWDKA

ANSNQPLAHTVVLLNSGGDATQTAAILMV

>gi|313651323|gb|EFS15721.1| malate:quinone oxidoreductase domain protein [Shigella flexneri 2a str. 2457T]

MELNYTPQNADGSISIEKAVAINEAFQISRQFWAHQVERGVLRTPRSFINTVPHMSFVWGEDNVNFLRAR

YAALQQSSLFRGMRYSEDHAQIKEWAPLVMEGRDPQQKLALM

>gi|313651322|gb|EFS15720.1| malate:quinone oxidoreductase [Shigella flexneri 2a str. 2457T]

MNYGEITRQLIASLQKKSNFSLQLSSEVRALKRNDDNTWTVTVADLKNGTAQNIRAKFVFIGAGGAALKL

LQESGIPEAKDYAGFPVGGQFLVSENPDVVNRHLAKVYGKASVGAPPMSVPHIDTRVLDGKRVVLFGPFA

TFSTKFLKNGSLWDLMSSTTTSNVMPMMHVGLDNFDLVKYLVSQVMLSEEDRFEALKEYYPQAKKEDWRL

WQAGQRVQIIKRDAEKGGVLRLGTEVVSDQQGTIAALLGASPGASTAAPIMLNLLEKVFGDRVSSPQWQA

TLKAIVPSYGRKLNGDVAATERELQYTSEVLGLKYDKPQAADSTPKPQLKPQPVQKEVADIAL

>gi|313651264|gb|EFS15662.1| colicin I receptor domain protein [Shigella flexneri 2a str. 2457T]

MVVTASSVEQNLKDAPASISVITQEDLQRKPVQNLKDVLKEVPGVQLTNEGDNRKGVSIRGLDSSYTLIL

VDGKRVNSRNAVFRHNDFDLNWIPVDSIERIEVVRGPMSSLYGSDALGGVVNIITKKIGQKWSGTVTVDT

TIQEHRDRGDTYNGQFFTSGPLIDGVLGMKAYGSLAKREKDDPQNSTTTDTGETPRIEGFSSRDGNVEFA

>gi|313651263|gb|EFS15661.1| colicin I receptor domain protein [Shigella flexneri 2a str. 2457T]

MPLTAINQFLTVGGEWRHDKLSDAVNLTGGTSSKTSASQYALFVEDEWRIFEPLALTTGVRMDDHETYGE

HWSPRAYMVYNATDTVTVKGGWATAFKAPSLLQLSPDWTSNSCRGACKIVGSPDLKPETSESWELGLYYM

GEEGWLEGGESSVIVFRNDVKDRISISRTSDVNAAPGYQNFVGFETGANGRRIPVFSYYSVNKARIQGVE

TELKIPFNDEWKLSINYTYNDGRDVSNGENKPLSDLPFHTANGTLDWKPLALEDWSFYVSGHYTGQKRAD

SATAKTPGGYTIWNTGAAWQVTKDVKLRAGVLNLGDKDLSRDDYSYNEDGRRYFMAVDYRF

>gi|313651244|gb|EFS15642.1| outer membrane efflux family protein [Shigella flexneri 2a str. 2457T]

MSAEGLMGPFALNDPAAGTTGPWYTNGTFGLTAGWHLDIWGKNRAEVTARLGTVKARAAEREQTRQLLAG

SVARLYWEWQTQAALNTVLQQIEKEQNTIIATDRQLYQNGITSSVDGVETDINASKTRQQLNDVAGKMKI

IEARLNALTNHQTKSLKLKPVALPKVASQLPDELGYSLLARRADLQAAHWYVESSLSTIDAAKAAFYPDI

NLMAFLQQDALHLSDLFRHSAQQMGVTAGLTLPIFDSGRLNANLDIAKAESNLSIASYNKAVVEAVNDVA

RAASQVQTLAEKNQHQAQIERDALRVVGLAQARFNAGIIAGSRVSEARIPALRERANGLLLQGQWLDASI

RLTGALGGGYKR

>gi|313651221|gb|EFS15619.1| caudovirales tail fibre assembly family protein [Shigella flexneri 2a str. 2457T]

MLHLKNITAGNPKTAEQYQMTKRYSVTWLFSEDGKNWYEELKNFASDTIKIAYTGDGRVVWVGKDVTGIE

PRNASVIEVPDITANRRITAPGYWFYRNDEFVFDYRLKAEDERDALLAQVSARTGEWEEDLLLGLISDED

KEKLKACRIYAKSLQAMDFSTITDKSSYNAIEWPASPEGSS

>gi|313651216|gb|EFS15614.1| uncharacterized protein ybcY [Shigella flexneri 2a str. 2457T]

MDLNEASLNAASTRAGESKIKHKISHDVFEPYPAALHGQFDSISMFYLLHCLPGNISTKSCVIRNAAQAL

TDDGTLYGATILGDGVVHNSFGQKLMRIYNQKGIFSNTKDSEEGLTHILSEHFENVKTKVQGTVVMFSAS

GKK

>gi|313651200|gb|EFS15599.1| histidine kinase-, DNA gyrase B-, and HSP90-like ATPase family protein [Shigella flexneri 2a str. 2457T]

MYIRLDKPENPQSTALALDIANKTLPLFRHVNSESLRKVCEIIRDDIHADAVAITNTDHVLAYVGVGEHN

YQNGDDFISPTTRQAMNYGKIIIKNNDEAHRTPEIHSMLVIPLWEKGVVTGTLKIYYCHAHQITSSLQEM

AVGLSQIISTQLEVSRAEQLREMANKAEIRALQSKINPHFLFNALNAISSSIRLNPDTARQLIFNLSRYL

RYNIELKDDEQIDIKKELYQIKDYIAIEQARFGDKLTVIYDIDEEVNCCIPSLLIQPLVENAIVHGIQPC

KGKGVVTISVAECGNRVRIAVRDTGHGIVPKVIERVEANEMPGNKIGLLNVHHRVKLLYGEGLHIRRLEP

GTEIAFYIPNQRTPVASQATLLL

>gi|313651192|gb|EFS15591.1| membrane transport family protein [Shigella flexneri 2a str. 2457T]

MLTFFIGDLLPIIVIMLLGYFSGRRETFSEDQARAFNKLVLNYALPAALFVSITRANREMIFADTRLTLV

SLVVIVGCFFFSWFGCYKFFKRTHAEAAVCALIAGSPTIGFLGFAVLDPIYGDSVSTGLVVAIISIIVNA

ITIPIGLYLLNPSSGADGKKNSNLSALISAAKEPVVWAPVLATILVLVGVKIPAAWDPTFNLIAKANSGV

AVFAAGLTLAAHKFEFSAEIAYNTFLKLILMPLALLLVGMACHLNSEHLQMMVLAGALPPAFSGIIIASR

FNVYTRTGTASLAVSVLGFVVTAPLWIYVSRLVS

>gi|313651179|gb|EFS15578.1| putative prophage CPS-53 integrase domain protein [Shigella flexneri 2a str. 2457T]

MLTVKQIEAAKPKEKPYRLLDGNGLYLYVPVSGKKVWQLRYKIDGKEKILTVGKYPLMTLQEARDKAWTA

RKDISAGIDPVKAKKASSNNNSFSAIYKEWYEHKKQVWSVGYATELAKMFDDDILPIIGGLEIQDIEPMQ

LLEVIRRFEDRGAIEVVNKNWPPS

>gi|313651165|gb|EFS15564.1| outer membrane usher papC domain protein [Shigella flexneri 2a str. 2457T]

MPDHSLFRLRILPWCIALAMSGSYSSVWAEDDIQFDSRFLELKGDTKIDMKRFSSQGYVEPGKYNLQVQL

NKQPLAEEYDIYWYAGEDDASKSYACLTPELVAQFGLKEDVAKNLQWSHDAKCLKSGQLEGMEIKADLSQ

SALVISLPQAYLEYTYPDRDPPSRWDDGISGIVADYSINAQTRHEENGGDDSNEISGNGTVGVNLGPWRM

RADWQTNYQHTRSNDDDEFSGDETQKNGSGVATMPGGRYHH

>gi|313651164|gb|EFS15563.1| outer membrane usher papC domain protein [Shigella flexneri 2a str. 2457T]

MLPPNLRGYAPDISGVAHTTAKVTVSQMGRVIYETQVPAGPFRIQDLGDSVSGTLHIRIEEQNGQVQEYD

ISTASMPYLTRPGQVRYKIMMGRPQEWGHHVEGEFFSGAEASWGIANGWSLYGGALGDENYQSAALGVGR

DLSTFGAVAFDVTHSHTKLDKDTAYGKGSLDGNSFRVSYSKDFDQLNSRVTFAGYRFSEENFMTMSEYLD

ASDSGMVRTGNDKEMYTATYNQNFRDAGVSVYLNYTRHTYWDREEQTNYNIMLSHYFNMGSIRNVSISMT

GYRYEYDNQADKGMYISLSMPWGDNSTVSYNGTMAVGRTAVRSVISAVSMTRLTIS

>gi|313651163|gb|EFS15562.1| outer membrane usher papC domain protein [Shigella flexneri 2a str. 2457T]

MNVGTSDKHTSVDGYYSHDGSLAQVDLSANYHEGQYTSAGLSLQGGATLTAHGGALHRTQNMGGTRLLID

ADGVADVPVEGNGAAVYTNMFGKAVVSDVNNYYRNQAYIDLNKLPENAEATQSVVQATLTEGAIGYRKFA

VISGQKAMAVLRLQDGSHPPFGAEVKNDNEQTVGLVDDDGNVYLAGVKPGEHMSVFWSGVAHCDINLPDP

LPADLFNGLLLPCQHKGNVAPVVPDDIKPVIQEQTQQVTPTNPPVSVSANQ

>gi|313651162|gb|EFS15561.1| chaperone protein pmfD [Shigella flexneri 2a str. 2457T]

MSDLLCSAKLGATTLALLLSAASLSAQASVTPDRTRLIFNESDKSISVTLRNNDPKLPYLAQSWIEDEKG

NKISSPLTVLPPVQRIDSMMNGQVKVQGMPDINKLPADRESLFYFNVREIPPKSNKANTLQIALQTRIKL

FWRPKALENVSMKNP

>gi|313651160|gb|EFS15559.1| putative minor fimbrial subunit [Shigella flexneri 2a str. 2457T]

MNLRVLVDAPPPCTVNGAAVEFGNVFINKINRVDYKRPIDYSLVCNNLAMDDLRLQMQATTVVINGETVI

STGIPGFGIRVQKSSDHTILDLTSGSWLPFNFSSGVPVLEAVPVKQSGTTLAAAEFNASATIVVDYQ

>gi|313651159|gb|EFS15558.1| fimbrial subunit [Shigella flexneri 2a str. 2457T]

MKRISLILLWGFCSMALSNVSFHGYLVQPPNCTISNAQTIEITFQDVLIDDINGSNYEQTVPYSITCDTA

VRDPLMEMTLSWSGTPSDFDNAAVSSNITGLGIQLKQAGQSFTINTPLVVNETDLPVLTAVPVKKSGVIL

PEADFEAWATLQVDYQ

>gi|313651158|gb|EFS15557.1| fimbrial family protein [Shigella flexneri 2a str. 2457T]

MKKKRTLFFISSLMLLGSGTTIAGDNLHFTGNLISKSCTPVINGSQLAEVHFPAIAASDLMNLGQSERVP

LVFQLKDCHSSTLFNVKVTLTGTEDSALPGFLAFDSSSSASGAGIGIETAAGTSVPINNTTGVTLPLNQG

NNSLNFNTWLQAKSGRDVTSGDFSATVTATFEYF

>gi|313651157|gb|EFS15556.1| uncharacterized protein yfcO [Shigella flexneri 2a str. 2457T]

MKKWTIIFASLMLLVLSVVGASKSYAGDKLMSASFDSVRVYYAMDKVTGAVASSVFNVTVITPKEVAYGK

YDSFAYKGDTLRVISWSGSGSAPTLVLTDFDTINNSNCPGIDTKIFRCAYMTFKITVASDDYGCPWIASF

YSYTDLPGFGSYTAPTVHNTICPTIPVASYDISWSENYVSHNKALRIQSTGSTVTTTLSTYLMEGGRLCD

GSNFSDNDGRGAYCRAVSELLTFTSYGCDKSTVTVTPTRHPVTDKVLHDIVVNVNTSSGQPIDSTCRFQY

VLNELKFVVLL

>gi|313651058|gb|EFS15457.1| integrase core domain protein [Shigella flexneri 2a str. 2457T]

MDGQKVDSARALIARGWGVSLVSRCLRVSRAQLHVILRRTDDWMDGRRSRHTDDTDVLLRIHHVIGELPT

YGYRRVWALLRRQAELDGMPAINAKRVYRLMRQNALLLERKPAVPPSKRAHTGRVAVKESNQRWCSDGFE

FCCDNGERLRVTFALDCCDREALHWAVTTGGFNSETVQDVMLGAVERRFGNDLPSSPVEWLTDNGSCYRA

NETRQFARMLGLEPKNTAVRSPESNGIAESFVKTIKRDYISIMPKPDGLTAAKNLAEAFEHYNEWHPHSA

LGYRSPREYLRQRACNGLSDNRCLEI

>gi|313651051|gb|EFS15451.1| transglycosylase SLT domain protein [Shigella flexneri 2a str. 2457T]

MKKLKINYLFIGILALLLAVALWPSIPWFGKADNRIAAIQARGELRVSTIHTPLTYNEINGKPFGLDYEL

AKQFADYLGVKLKVTVRQNISQLFDDLDNGNADLLAAGLVYNSERVKNYQPGPTYYSVSQQLVYKVGQYR

PRTLGNLTAEQLTVAPGHVVVNDLQTLKETKFPELSWKVDDKKGSAELMEDVIEGKLDYTIADSVAISLF

QRVHPELAVALDITDEQPVTWFSPLDGDNTLSAALLDFFNEMNEDGTLARIEEKYLGHGDDFDYVDTRTF

LRAVDAVLPQLKPLFEKYAEEIDWRLLAAIAYQESHWDAQATSPTGVRGMMMLTKNTAQSLGITDRTDAE

QSISGGVRYLQDMMSKVPESVPENERIWFALAAYNMGYAHMLDARALTAKTKGNPDSWADVKQRLPLLSQ

KPYYSKLTYGYARGHEAYAYVENIRKYQISLVGYLQEKEKQATEAAMQLAQDYPAVSPTELGKEKFPFLS

FLSQSSSNYLTHSPSLLFSRKGSEEKQN

>gi|313651040|gb|EFS15440.1| tetratricopeptide repeat family protein [Shigella flexneri 2a str. 2457T]

MWLENDYIKVMILPELGGRVHRAWDKVKQRDFVYHNEVIKPALVGMLGPWISGGIEFNWPQHHRPTTFMP

VDFTLEAHEDGAQTVWVGETEPMHGLQVMTGFTLRPDRAALEIASRVYNGNATPRHFLWWVNPAVKGGEG

HQSVFPPDVTAVFDHGKRAVSAFPIATGTYYKVDYSAGVDISRYKNVPVPTSYMAEKSQYDFVGAWCHDE

DGGLLHVANHHIAPGKKQWSWGHSEFGQAWDKSLTDNNGPYIELMTGIFADNQPDFTWLDAYEEKRFEQY

FLPYHSLGMVQNASRDAVIKLQRSERGIEWGLYAISPLNGYRLAIREIGKCNALLDDAVALTPATAIQGV

LHGINPDRLTIELSDADGNIVLSYQEHQPQELPLPDVAKAPLAAQDITSTDEAWFIGQHLEQYHHASRSP

FDYYLRGVALDPLDYRCNLALAMLEYNRADFPQAVAYASQALKRAHALNKKPQCGQASLIRASAYERQGQ

YQQAEEDFWRAVWSGNSKAGGYYGLARLAARNDNFDAGLDFCQQSLRACPTNQEVLCLHNLLLVLSGRQD

NARLQREKLLRDYPLNATLWWLNWFDGRSESALAQWRGLCQGRDVNALMTAGQLINWGMPTLAAEMLNAL

DCQRTLPLYLQASLLPKAERGELVAKAIDVFPQFVRFPNTLEEVAALESIEECWFARHLLACFYYNKRSY

NKAIALWQRCVEMSPEFADGWRGLAIHAWNKKHDYELAARYLDNAYQLAPQDARLLFERDLLDKLSGATP

EKRLARLENNLEIALKRDDMTAELLNLWHLTGQADKAADILATRKFHPWEGGEGKVTSQFILNQLLRAWQ

HLDAREPQQASELLHAALYYPENLSEGRLPGQTDNDIWFWQAICANAQGDETEAMRCLRLAATGDRTINI

HSYYNDQPVDYLFWQGMALRLLGEQQTAQQLFSEMKQWAQEMAKTSIEADFFAVSQPDLLSLYGDLQQQH

KEKCLMVAMLASAGLGEVAQYESARAELTAINPAWPKAALFTTVMPFIFNYVH

>gi|313651039|gb|EFS15439.1| ABC transporter periplasmic-binding protein yphF [Shigella flexneri 2a str. 2457T]

MPKKMRTTRNLLLMATLLGSALFARAADKEMTIGAIYLDTQGYYAGVRQGVQDAAKDSSVQVQLIETNAQ

GDISKESTFVDTLVARNVDAIILSAVSENGSSRTVRRASEAGIPVICYNTCINQKGVDKYVSAYLVGDPL

EFGKNWVTLPPIILLPIKLTSRKLPSSIAKPLKFVCSDVKDLKKY

>gi|313651038|gb|EFS15438.1| ABC transporter periplasmic-binding yphF domain protein [Shigella flexneri 2a str. 2457T]

MQRRKGFEEVLKSRVPGAQIVANQEGTVLDKAISVGEKLIISTPDLNAIMGESGGATLGAVKAVRNQNQA

GKIAVFGSDMTTEIAQELENNQVLKAVVDISGKKMGNAVFAQTLKVINKQADGEKVIQVPIDLYTKTEDG

KQWLATHVDGLP

>gi|313651006|gb|EFS15406.1| penicillin-binding 1C domain protein [Shigella flexneri 2a str. 2457T]

MLDNLIEARYLEALINYEDRWFWKHPGVNPFSVARAAWQDLTSGRVISGGSTLTMQVARLLDPHPKTFGG
KILQLWRALQLEWHLSKREILILYLNRAPFGGTLQGIGAASWAYLGKSPANLSYSEAAMLAVLPQVPSRL
RPDRWPERAEAARNKVLERMAAQGVWSREQVKESREEPIWLAPRQMPQLAPLFSRMMLGKSKSDKIVTTL
DAGLQRRLEELAQNWKGRLPPRSSLAMIVVNHTDMRVRGWVGSVDLNDDSRFGHVDMVNAIRSPGSVLKP
FVYGLALDEGLIHPASLLQDVPRHR

>gi|313651005|gb|EFS15405.1| penicillin-binding domain protein [Shigella flexneri 2a str. 2457T]

MSEALVRSLNLPAVQVLEAYGPKRFAAKLRNVGLPLYLPNGAAPNLSLILGGAGAKLEDMAAAYTAFARH
GKAGKLRLQPDDPLLERPLMSSGAAWIIRRIMADEAQPLPDSALPRVAPLAWKTGTSYGYRDAWAIGVNA
RYVIGIWTGRPDGTPVVGQFGFASAVPLLNQVNNILLSRSANLPEDPRPNSVTRGVICWPGGQSLPEGDG
NCRRRLATWLLDGSQPPTLLLSEQEGINGIRFPIWLDDKGQRVAADCPQAKQEMVNVWPLPLEPWLPASE
RRAVRLPPASTICPPYGHDAQLPLQLTGVRDGAIIKRLPGAAEATLPLQSSGGAGERWWFLNGEPLTERG
RNVTLHLTDKGDYQLLVMDDVGQIATVKFVMQ

>gi|313650984|gb|EFS15384.1| P-type conjugative transfer protein TrbL [Shigella flexneri 2a str. 2457T]

MVAMRKKIALGGLMMVATMVLAEPAMAQELSSSGVMNDVLKRFHDAAATWGPAIESAASRLFWTLVVISM
VWTFGMMALRKADIGEFFAEFVRFTIFTGFFWWLLTNANQGMNIAGTIVQSLQTLGAQAGGLSNSNLGPS
SILDLGFELYNRTVQATSELGWRQMATALVMELMALAVLFVLALIAVNLLLLLASAWILLYAGVFFLGFG
GSRWTSDMAINYYKTVLGLAAQLMAMVLLVAIGKEFINHYYTQISENMASQELAVMLVISVILLFLVNKV
PPMISGLVSGGGIGAAGGIGNFGAGAAVGAAVTAASMATGGAALAGKAVMGAAAGAAGGASALQAAFQKA
SASMETGGDMSSMGSVVSSGGNGGGEAGTAGSSPFAQAAGFGDSGSSSSGGGFAKAAKLATGTASELAKG

VGSQVKQGFQERVSETTGGKLAASIRESMEPKEASQSGQFEGNSLGADSGPDSNEVRS

>gi|313650967|gb|EFS15367.1| MASE1 family protein [Shigella flexneri 2a str. 2457T]

MKLNATYIKIRDKWWGLPLFLPSLILPIFAHINTFAHISSGEVFLFYLPLALMISMMMFFSWAALPGITL

GIFVRKYAELGFYETLSLTANFIIIIILCWGGYRVFTPRRNNVSHGDTRLISQRIFWQIVFPATLFLILF

QFAAFVGLLASRENLVGVMPFNLGTLINYQALLVGNLIGVPLCYFIIRVVRNPFYLRSYYSQLKQQVDAK

VTKKEFALWLLALGALLLLLCMPLNEKSTIFSTNYTLSLLLPLMMWGAMRYGYKLISLLWAVVLMISIHS

YQNYIPIYPGYTTQLTITSSSYLVFSFIVNYMAVLATRQRAVVRRIQRLAYVDPVVHLPNVRALNRALRD

APWSALCYLRIPGMEMLVKNYGIMLRIQYEVYWQ

>gi|313650964|gb|EFS15364.1| EAL domain protein [Shigella flexneri 2a str. 2457T]

MSVKLRLPQYKQKLSHWLSPLLEPGEDVYQLSGNDLALRLNTESHQERITALDSHLKQFRFFWDGMPMQP

QIGVSYCYVRSPVNHIYLLLGELNTVAELSIVTNAPENMQRCGAMYLQRELKDKVAMMNRLQQALEHNHF

FLMAQPITGMRGDVYHEILLRMKGENDELIGPGSFLPVAHEFGLSSSIDMWVIEHTLQFMAENRAKMPAH

RFAINLSPTSVCQARFPVEVSQLLAKYQIEAWQLIFEVTESNALTNVKQAQITLQHLQELGCQIAIDDFG

TGYASYARLKNVNADLLKIDGSFIRNIVSNSLDYQIVASICHLARMKKMRVVAEYVENEEIREAVLSLGI

DYMQGYLIGKPQPLIDTLNEIEPIRESA

>gi|313650952|gb|EFS15352.1| putative formate transporter [Shigella flexneri 2a str. 2457T]

MGGLCFTLGFILLAVCGTSLFTSSVMTVMAKSRGVISWRTWLINALLVACGNLAGIACFSLLIWFSGLVM

SENAMWGVAILHCAEGKMHHTFTESVSLGIMCNLMVCLALWMSYCGRSLCDKIVAMILSITLFVASGFEH

CIANLFVIPFAIAIRHFAPTSFWQLAHSSADNFPALTVSHFITANLLPVMLGNIIGGAVLVSICYRAIYL

RQES

>gi|313650949|gb|EFS15349.1| hydrogenase-4 transcriptional activator [Shigella flexneri 2a str. 2457T]

MAMSDEAMFAPPQGITIEAVNGMLAERLAQKHGKASLLRAFIPLPPPFSPVQLIELHVLKSNFYYRYHDD

GSDVTATTEYQGEMVDYSRYAVLLGSSGMAELRFIRTHGSRFTPQDCTLFNWLARIITPVLQSWLNDEAQ

QVALRLLEKDRDHHRVLVDITNAVLSHLDLDDLIADVAREIHHFFGLASVSMVLGDHRKNEKFSLWCSDL

SASHCACLPHNMPGDSVLLTQTLQTRQPTLTHRADDLFLWQRDPLLLLLASNGCESALLIPLTFGNHTPG

ALLLAHTSSTLFSEENCQLLQHIADRIAIAVGNADACRRMTDLQESLQQENHQLSEQLLSNLGVGDIIYQ

SQAMEDLLQQVDIVAKSDSTVLICGETGTGKEVIARAIHQLSPRHDKPLVKINCAAIPASLLESELFGHD

KGAFTGAINTHRGRFEIADGGTLFLDEIGDLPLELQPKLLRVLQEREIERLGGSRTIPVNVRVIAATNRD

LWQMVEDRQFRSDLFYRLNVFPLELPPLRDRPEDIPLLAKHFTQKMARHMNRSIDAIPTEALRQLMSWDW

PGNVRELENVIERAVLLTRGNSLNLHLNVRQSRLLPTLNEDSALRSSMA

>gi|313650938|gb|EFS15338.1| hydrogenase-4 component B domain protein [Shigella flexneri 2a str. 2457T]

MDALQLLTWSLILYLFASLASLFLLGLDRLAIKLSGITSLVGGVIGIISGITQLHAGVTLVAHFATPFDF

ADLTLRMDSLSAFMVLVISLLVVVCSLYSLTYMREYEGKGAAAMGFFMNLFIASMVALLVMDNAFWFIVL

FEMMSLSSWFLVIARQDKTSINAGMLYFFIAHAGSVLIMIAFLLMGRESGSLDFASFARFHFLRGWRRRC

FCWPFSVLARKPG

>gi|313650935|gb|EFS15335.1| glycine cleavage system transcriptional repressor [Shigella flexneri 2a str. 2457T]

MTLSSQHYLVITALGADRPGIVNTITRHVSSCGCNIEDSRLAMLGEEFTFIMLLSGSWNAITLIESTLPL

KGAELDLLIVMKRTTARPRPPMPASVWVQVDVADSPHLIERFTALFDAHHMNIAELVSRTQPAENERAAQ

LHIQITAHSPASADAANIEQAFKALCTELNAQGSINVVNYSQHDEQDGVK

>gi|313650924|gb|EFS15324.1| nitrate/nitrite sensor narQ domain protein [Shigella flexneri 2a str. 2457T]

MQCSQALNTSQIDVHCFRHILQIVRDNEAAEYLELNVGDNWRISEGKPNPELPMQILPVTMQETVYGELH

WQNSHVSSSEPLLNSVSSMLGRGLYFNQAQKHFQQLLLMEERATIARELHDSLAQVLSYLRIQLTLLKRS

IPEDNATAQSIMADFSQALNDAYRQLRELLTTFRLTLQQADLPSALREMLDTLQNQTSAKLTLDCRLPTL

ALDAQMQVHLLQIIREAVLNAMKHANASEIAVSCVTAPDGNHTVYIRDNGIGIGEPKEPEGHYGLNIMRE

RAERLGGTLTFSQPSGGGTLVSISFRSAEGEGKGNTPAEV

>gi|313650923|gb|EFS15323.1| nitrate/nitrite sensor narQ domain protein [Shigella flexneri 2a str. 2457T]

MIVKRPVSASLARAFFYIVLLSILSTGIALLTLASSLRDAEAINIAGSLRMQSYRLGYDLQSGSPQLNAH

RQLFQQALHSPVLTNLNVWYVPEAVKTRYAHLNANWLEMNNRLSKGDLPWYQANINNYVNQIDLFVLALQ

HYAERKMLLVVAISLAGGIGIFTLVFFTLRRIRHQVVAPLNQLVTASQRIEHGQFDSPPLDTSLPNELGL

LAKTFNQMSSELHKLYLSLEASVEEKTRDLHETKRRLEVLYQCSVPRR

>gi|313650912|gb|EFS15312.1| ethanolamine utilization eutA domain protein [Shigella flexneri 2a str. 2457T]

MMKEKQSLGDFVVASAGPHLESVIAGHGAGAQTLSEQRLCRVLNIDIGGGTANYALFDAGKISGTACLNV
GGRLLETDSQGRVVYAHKPGQMIVDECFGAGTDARSLTGAQLVQVTRRMAALIVEVIDGTLSPLAQALMQ
TGLLPAGVTPEIITLSGGVGECYRHQPANPFCFADIGPLLATALHDHPRLREMNVQFPAQTVRSTVIGAG
AHTLSLSGSTIWLEGVQLPLRNLPVAIPIDETDLVSAWQQALLQLDLDPKTDVYVLALPASLPVRYAAVL
TVINALVDFVARFPNPHPLLVVAGQDFGKALGMLLRPQLQQLPLAVIDEVIVRAGDYIDIGTPLFGGSVV
PVTVKSLAFPS

>gi|313650908|gb|EFS15308.1| glutamate racemase [Shigella flexneri 2a str. 2457T]
MFSAESPIGVIDSGVGGLTSVKEIINLLPGEDIIYCGDNMNAPYGNRSADDIISLTKKMLTFLQSRNVKL
VAVACNTISSTLESEEYSGYAKSFPFPILSIIEPAVEDVIRQQYKNVGIIATEFTIKTGCHKELIKKLNS
TINVFGEPSKNLAMLIEEGNLNAPAILNDIKKHVNHLISLHPVNEIILGCTHYPIVQNFFEAVAPDIKFI
NPAHDQAISIKNHLGQLNLLNSSNIGTLQINTSGSMEIYKTVLGELSITKPHTFSIRQF

>gi|313650905|gb|EFS15305.1| glutamate decarboxylase [Shigella flexneri 2a str. 2457T]
MKNKSGNSCYREAVDIHSSLELSFILHKYHLRVDEKKSRNVFSAIRDELILDGDSRQNLATFCQTWLDDE
IRELLNLAIDKNMIYKDEYPQMAEPEGRCVRMLSDSWSFPDSRHTLGCSTIGSSEAAMLGRLALKWQWCK
KREVQGKSTEPDLRSCANMLA

>gi|313650899|gb|EFS15299.1| hypothetical protein SF2457T_0525 [Shigella flexneri 2a str. 2457T]

MRISEQQRQILDYLAKNESITARQAADLVYGGNVTRVQVDAARRSLMTMVSNGLIRKQGRAFVAERHINH

AYSQDVEELRRQILWVFSKQGQEYFLSEHPNFAAKELTVSGLSCALDYLVGEGCVTYKQLWHALNTLVED

GLLIATRENIEYWIMPRSGRRNHARYISGNRYRTPDFNPTQEDRDRAEMMCREQEKRIMDKLFSMQ

>gi|313650894|gb|EFS15294.1| HTH-type transcriptional regulator eutR [Shigella flexneri 2a str. 2457T]

MLSEDVITCQANFLHNPDRVLHMLRSQSALEVKEQHKAALWGFVQQALATFCENPENLHQPAVRKVLGDN

LLMAMGAMLEEAQPMVTAESISHQSYRRLLSRAREYVLENMSEPVTVLDLCNQLHVSRRTLQNAFHAILG

IGPNAWLKRIRLNVVRRELISPWSQSTTVKDAAMQWGFWHLGQFATDYQQLFAEKPSLTLHQRMREWG

>gi|313650880|gb|EFS15280.1| sulfate ABC transporter, permease protein CysW [Shigella flexneri 2a str. 2457T]

MAEVTQLKRYDARPINWGKWFLIGIGMLVSAFILLVPMIYIFVQAFSKGLMPVLQNLADPDMLHAIWLTV

MIALIAVPVNLVFGILLAWLVTRFNFPGRQLLLTLLDIPFAVSPVVAGLVYLLFYGSNGPLGGWLDEHNL

QIMFSWPGMVLVTIFVTCPFVVRELVPVMLSQGSQEDEAAILLGASGWQMFRRVTLPNIRWALLYGVVLT

NARAIGEFGAVSVVSGSIRGETLSLPLQIELLEQDYNTVGSFTAAALLTLMAIITLFLKSMLQWRLENQE

KRAQQEEHHEH

>gi|313650875|gb|EFS15275.1| WGR domain protein [Shigella flexneri 2a str. 2457T]

MKKCFIYHDEKSNKFWWIDYEGDSLAVNYGKVGSIGKFHTKEFDNEEQCLKEASKLIAAKMKKGYQETPD

FNFMDLYYFDDEEIGLHLKTSHPNFQCHFTDPLYMCCWDEESPFGSDEGADALNELENSLRSGLC

>gi|313650867|gb|EFS15267.1| cell division protein ZipA [Shigella flexneri 2a str. 2457T]

MMQDLRLILIIVGAIAIIALLVHGFWTSRKERSSMFRDRPLKRMKSKRDDDSYDEDVEDDEGVGEVRVHR

VNHAPANAQEHEAARPSPQHQYLPPYASAQPRQPVQQPPEAQVPPQHAPRPAQPVQQPAYQPQPEQPLQQ

PVSPQVAPAPQPVHSAPQPAQQAFQPAEPVAAPQPEPVAEPAPVMDKPKRKEAVIIMNVAAHHGSELNGE

LLLNSIQQAGFIFGDMNIYHRHLSPDGSGPALFSLANMVKPGTFDPEMKDFTTPGVTIFMQVPSYGDELQ

NFKLMLQSAQHIADEVGGVVLDDQRRMMTPQKLREYQDIIREVKDANA

>gi|313650859|gb|EFS15259.1| hypothetical protein SF2457T_0482 [Shigella flexneri 2a str. 2457T]

MKRLRNKMTTEELAECLGVAKQTVNRWTREKGWKTEKFPGVKGGRARLILVDTQVCEFIQNTPAFHNTPM

LMEAEERIAEYAPGARAPAYRQIINAIDNMTDIEQEKVAQFLSREGIRNFLARLDIDESA

>gi|313650857|gb|EFS15257.1| MASE1 family protein [Shigella flexneri 2a str. 2457T]

MFVEHNLIKNIKIFTLAFTLTVVLIQLSRFISPLAIIHSSYIFLAWMPLCVMLSILFIFGWRGVVPVLCG

MFCTNLWNFHLSFLQTAVMLGSQTFVVLCACAILRWQLGTRWRYGLTSRYVWQRLFWLGLVTPIGIKCSM

YLVGSFFDFPLKISTFFGDADAIFTVVDLLSLFTAVLIYNMLFYYLTRIYLKLAGSVKRAATSVVHTL

>gi|313650856|gb|EFS15256.1| EAL domain protein [Shigella flexneri 2a str. 2457T]

MLLLLLCTPYENDFIAGYLVPVFFIIFTLGVGNLRYPFLNLTWAVSTLCLLNYNQNFLQGVETEYSLAFI

LAVLISFSVCLLYMVRIYHRSEWLNRRWHLQALTDPLTLLPNFRALEQAPEQEAGKSFCCLRIDNLEFMS

RHYGLMMRVHCIRSICRTLLPLMQENEKLYQLPGSELLLVLSGPETEGRLQHMVNILNSRQIHWNNTGLD

MGYGAAWGRFDGNQETLQPLLGQLSWLAEQSCAHHHVLALDSREEMVSGQTTKQVLLLNTIRTALDQGDL

LLYAQPIRNKEGEGYDEILARLKYDGGIMTPDKFLPLIAQFNLSARFDLQVLESLLKWLATHPCDKKGPR

FSVNLMPLTLLQKNIAGRIIRLFKRYHISPQAVILEITEEQAFSNAESSMYNIEQLHKFGFRIAIDDFGT

GYANYERLKRLQADIIKIDGVFVKDIVTNTLDAMIVRSITDLAKAKSLSVVAEFVETQQQQALLHKLGVQ

YLQGYLIGRPQPLAD

>gi|313650847|gb|EFS15248.1| DNA internalization-related competence protein ComEC/Rec2 [Shigella flexneri 2a str. 2457T]

MKLKVRAVHGQLNDGGFDSQRYAIAQHQPLTGRFLQASVIEPNCSLRAQYLASLQTTLQPYPWNAVILGL

GMGERLSVPKEIKNIMRDTGTAHLMAISGLHIAFAALLAAGLIRSGQIFLPGRWIHWQIPLIGGICCAAF

YAWLTGMQPPALRTVVALATWGMLKLSGRQWSGWDVWICCLAAILLMDPVAILSQSLWLSAAAVAALIFW

YQWFPCPEWQLPPVLRAVVSLIHLQLGITLLLMPMQIVIFHGISLTSFIANLFAIPLVTFITVPLILAAM

VVHFSGPLILEQGLWFLADRSLALLFWGLKSLPEGWINIVECWQWLSFSPWFLLVVWRLNAWRTLPAMCV

AGGLLMCWPLWQKPRPDEWQLYMLDVGQGLAMVIARNGKAILYDTGLA

>gi|313650846|gb|EFS15247.1| uncharacterized ycaI domain protein [Shigella flexneri 2a str. 2457T]

MTQDWPEGDSGQQLIIPWLHWHNLEPEGVILSHEHLDHRGGLDSILHIWPMLWIRSPLNWEHHQPCVRGE

AWQWQGLRFSAHWPLQGSNDKGNNHSCVVKVDDGTNSILLTGDIEAPAEQKMLSRYWQQVQATLLQVPHH

GSNTSSSLPLIQRVNGKVALASASRYNAWRLPSNKVKHRYQQKGYQWLDTPHQGQVTVNFSAQGWRISSL

REQILPRWYHQWFGVPVDNG

>gi|313650823|gb|EFS15224.1| putative aliphatic sulfonates transport permease protein ssuC [Shigella flexneri 2a str. 2457T]

MITDVWKYRGKSRQHLAISSWRALIGFSIGGSLGLILGLISGLSRWGERLLDTSIQMLRNVPHLALIPLV

ILWFAIDESAKIFLVALGTLFPIYINTWHGIRNIDRGLVEMARSYGLSGIPLFIHVILPDALPSIMVGVR

FALGLMWLTLIVAETISANSGIGYLAMNAREFLQTDVVVVAIILYALLGKLADVSAQLLERLWLRWNPAY

HLKEATI

>gi|313650768|gb|EFS15169.1| tyrosine-protein kinase etk [Shigella flexneri 2a str. 2457T]

MIPNSSPESAPEIQLLQSRMILGKTIAELNLRDIVEQKYFPIVGRGWARLTKEKLGELAISWMHIPQLNG

QDQQLTLTVGENGHYTLEGEEFTVNGMVGQRLEKDGVALTIADIKAKPGTQFVLSQRTELEAINALQETF

TVSERSKESGMLELTMTGDDPQLITRILNSIANNYLQQNIARQAAQDSQSLEFLQRQLPEVRSELDQAEE

KLNVYRQQRDSVDLNLEAKAVLEQIVNVDNQLNELTFREAEISQLYKKDHPTYRALLEKRQTLEQERKRL

NKRVSAMPSTQQEVLRLSRDVEAGRAVYLQLLNRQQELSISKSSAIGNVRIIDPAVTQPQPVKPKKALNV

VLGFILGLFISVSAVLARAMLRRGVEAPEQLEEHGISVYSTIPMSEWLDKRTRLRKKNLFSNQQRHRTKN

IPFLAVDNPADSAVEAVRALRTSLHFAMMETENNILMITGATPDSGKTFVSSTLAAVIAQSDQKVLFIDA

DLRRGYSHNLFTVSNEHGLSEYLAGKDELNKVIQHFGKGDFDVITRGQVPPNPSELLMRDRMRQLLEWAN

DHYDLVIVDTPPMLAVSNAAVVGRSVGTSLLVARFGLNTAKEVSLSMQRLEQAGVNIKGAILNGVIKRAS

TAYSYGYNYYGYSYSEKE

>gi|313650753|gb|EFS15154.1| TMAO reductase sytem sensor TorS [Shigella flexneri 2a str. 2457T]

MLQALREQGFDTTAIEQQEQEISRSLRQQGELVGRRLQLRQQQRQLSQQIAAADEIARLEQGQANNATTS

AGATQAGIYDLIEQDQRQAAESALDRLIDIDLEYVNQMNELRLSALRVQQMVMNLGLEQIQKNAPTLEKQ

LNNAVKILQRRQIRIEDPGVRAQVATTLTTVSQYSDLLALFQQDSEISNHLQTLAQNNIAQFAQFSSEVS

QLVDTIELHNQHGLAHLEKASARGQYSLLLLGMVSLCALIMILWRVVYRSFTRPLAEQTQALQRLLDGDI

DSPFPETAGVRELDTIGRLMDAFRSSVHALNRHREQLAAQVKARTAELQELVIEHRQARAEAEKASQAKS

AFLTAMSHEIRTPLYGILGTAQLLADNPALNAQRDDLQAITDSGESLLTILNDILDYSAIEAGGKNVSVS

DEPFEPRPLLESTLQLMSGRVKGRPIRLATAIADDVPSALMGDPRRIRQVITNLLSNALRFTDEGHIILR

SRTDGEQWLVEVEDSGCGIDPAKLAEIFQPFVQVSGKRGGTGLGLTISSRLAQAMGGELSATSTPEVGSC

FCLRLPLRVATAPVPKTANQAVRLDGLRLLLIEDNPLTQRITVEMLNTSGAQVVAIGNAAQALETLQNSE

PFAAALVDFDLPDIDGITLARQLAQQYPSLVLIGFSAHVIDETLRQRTSSLFRGIIPKPVPREVLGQLLA

HYLQLQANNDLPLDVSQLNEDAQLMGTEKIHEWLALFKQHALPLLDEIDIARASQDSEKIKRAAHQLKSS

CSSLGMRSASQLCAQLEQQPLSAPLPHEEITRSVAALEA

>gi|313650748|gb|EFS15149.1| trimethylamine-N-oxide reductase 1 domain protein [Shigella flexneri 2a str. 2457T]

MNNNDLFQASRRRFLAQLGGLTVAGMLGPSLLTPRRATAAQAATEAVISKEGILTGSHWGAIRAMVKDGR

FVAAKPFELDKYPSKMIAGLPDHVHNAARIRYPMVRVDWLRKRHLSDTSQRGDNRFVRVSWDEALDMFYE

ELERVQKTHGPSALLTASGWQSTGMFHNASGMLAKAIALHGNSVGTGGDYSTGAAQVILPRVVGSMEVYE

QQTSWPLVLQNSKTIVLWGSDLLKNQQANWWCPDHDVYEYYEQLKAKVAAGEVEVISIDPVVTSTHEYLG

REHVKHIAVNPQTDVPLQLALAHTLYSENLYDKNFLANYCVGFEQFLPYLLGEKDGQPKDAAWAEKLTGI

DAETIRGLARQMAANRTQIIAGWCVQRMQHGEQWAWMIVVLAAMLGQIGLPGGGFGWHYNGAGTPGRKGV

ILSGFSGSTSIPPVHDNSDYKGYSSTIPIARFIDAILEPGKVINWNGKSVKLPPLKMCIFAGTNPFHRHQ

QINRIIEGWRKLETVIAIDNQWTSTCRFADIVLPATTQFERNDLDQYGNHSNRGIIAMKQVVPP

>gi|313650747|gb|EFS15148.1| trimethylamine-N-oxide reductase 1 domain protein [Shigella flexneri 2a str. 2457T]

MGWLKRIWQEGVQQGKGRGVHLPAFDDFWNNKEYVEFDHPQMFVRHQAFREDPDLEPLGTPSGLIEIYSK

TIADMNYDDCQGHPMWFEKIERSHGGPGSQKYPLHLQSVHPDFRLHSQLCESETLRQQYTVAGKEPVFIN

PQDASARGIRNGDVVRVFNARGQVLAGAVVSDRYAPGVARIHEGAWYDPDKGGEPGALCKYGNPNVLTID

IGTSQLAQATSAHTTLVEIEKYNGTVEQVTAFNGPVEMVAQCEYVPASQVKS

>gi|313650743|gb|EFS15144.1| chaperone torD domain protein [Shigella flexneri 2a str. 2457T]

MAALPATLTVRDDARLELAADFCGLFLMTDKQAALPYASAYKQDEQEIKRLLVEAGMETSGNFNEPADHL

AIYLELLSYLHFSLGEGTVPARRIDSLRQKTLTALRQWLPEFAARCRQYDSFGFYAALSQLLLVLVECDH

QNR

>gi|313650735|gb|EFS15136.1| 4-hydroxyphenylacetate 3-monooxygenase reductase component [Shigella flexneri 2a str. 2457T]

MNIVDQQTFRDAMSCMGAAVNIITTDGPAGRAGFTASAVCSVTDTPPTLLVCLNRGASVWPVFNENRTLC

VNTLSAGQEPLSNLFGGKTPMEHRFAAARWQTGVTGCPQLEEALVSFDCRISQVVSVGTHDILFCAIEAI

HRHTTPYGLVWFDRSYHALMRPAC

>gi|313650721|gb|EFS15122.1| phoH-like family protein [Shigella flexneri 2a str. 2457T]

MGRQKAVIKARREAKRVLRRDSRSHKQREEESVTSLVQMSGVEAIGMARDSRDTSPILARNEAQLHYLQA

IESKQLIFATGEAGCGKTWISAAKAAEALIHKDVDRIIVTRPVLQADEDLGFLPGDIAEKFAPYFRPVYD

LLVRRLGASFMQYCLRPEIGKVETAPFAYMRGRTFENAVVILDEAQNVTAAQMKMFLTRLGENVTVIVNG

DITQCDLPRGVCSGLSDALERFEEDEMVGIVRFGKEDCVRSALCQRTLHAYS

>gi|313650715|gb|EFS15116.1| putative dehydrogenase [Shigella flexneri 2a str. 2457T]

MDIIFYHPTFDTQWWIEALRKAIPQARVRAWKSGDNDSADYVLVWHPPVEMLAGRDLKAVFALGAGVDSI

LSKLQAHPEMLNPSVPLFRLEDTGMGEQMQEYAVSQVLHWFRRFDDYRIQQNSSHWQPLPEYHREDFTIG

ILGAGVLGCKVAQSLQT

>gi|313650710|gb|EFS15111.1| curli production assembly/transport component CsgG family protein [Shigella flexneri 2a str. 2457T]

MVEGSIIGYESNVKSGGVGARYFGIGADTQYQLDQIAVNLRVVNVSTGEILSSVNTSKTILSYEVQAGVF

RFIDYQRLLEGEVGYTSNEPVMLCLMSAIETGVIFLINDGIDRGLWDL

>gi|313650666|gb|EFS15068.1| copper resistance D family protein [Shigella flexneri 2a str. 2457T]

MLAFTWIALRFIHFTSLMLVFGFAMYGAWLAPLMIRRLLTKRSLRLQQHAAVWSLISATAMLAVQGGLMG

TGWTDVFSPNIWQAVLQTQFGGVWLWQIVLALVTLIVALMQPRNMPRLLFMLTTAQFILLAGVGYATLNE

GVTAKIHQTNHAIHLICAAAWFGGLLPVLWCMQLIKGRWRH

>gi|313650651|gb|EFS15053.1| helix-turn-helix domain, rpiR family protein [Shigella flexneri 2a str. 2457T]

MNMLEKIQSQLEHLSKSERKVAEVILASPDNAIHSSIAALALEANVSEPTVNRFCRSMDTRGFPDFKLHL

AQSLANGTPYVNRNVNEDDSVESYTGKIFESAMATLDHVRHSLDKSAINRTVDLLTQAKKIAFFGLGSSA

AVAHDAMNKFFRFNVPVVYSDDIVLQRMSCMNCSDGDVVVLISHTGRTKNLVELAQLARENDAMVIALTS

AGTPLAREATLAITLDVPEDTDIYMPMVSRLAQLTVIDVLATGFTLRRGAKFRDNLKRVKEALKESRFDK

QLLNLSDDR

>gi|313650642|gb|EFS15044.1| hypothetical protein SF2457T_0887 [Shigella flexneri 2a str. 2457T]

MNINYPAEYEIGDIVFTCIGAALFGQISAASNCWSNHVGIIIGHNGEDFLVAESRVPLSTITTLSRFIKR

SSNQRYAIKRLDAGLTEQQKQRIVEQVPSRLRKLYHTGFKYESSRQFCSKFVFDIYKEALCIPVGEIETF

GQLLNSNPNAKLTFWKFWFLGSIPWERKTVTPASLWHHPGLVLIHAEGVETPQPELTEAV

>gi|313650624|gb|EFS15026.1| tail assembly protein K [Shigella flexneri 2a str. 2457T]

MTQTESAILAHARRCAPAESCGFVVRTPEGDRYLPSENISGEPEERFRMAPEDWLRAQMQGEIVALVHSH

PGGLPWLSEADRRLQVQSDLPWWLVCRGTIHKFRCVPHLTGRRFEHGVTDCYTLFRDAYHLAGIEMPDFH

RGDDWWRHGQNLYLDNLEATGLYQVPLSSAQPGDVLLCCFGSSVPNHAAIYCGDGELLHHIPEQLSKRER

YTDKWQRRTHSLWRHREWHASAVYGDLQRIWPPHRPSCRKRGLKPSAHWPHSSRRFVRN

>gi|313650616|gb|EFS15018.1| phage tail assembly protein T [Shigella flexneri 2a str. 2457T]

MRLAREFRRADWRRMLSDMSATELGEWGDYFRMQSFSDVWMDAQFASLKALIVRMVSGSSDAAVADFSLL

PEENGIPERTDEELMHLGEGISGGVRYGPDSQPGH

>gi|313650602|gb|EFS15005.1| PAP2 superfamily protein [Shigella flexneri 2a str. 2457T]

MLQGAGWLLLLAPFFFFTYGSLNQFTAVQDLNSHDIPSQVFGWETAIPFLPWTIVPYWSLDLLYGFSLFV

CSSTFEQRRLVHRLILATVMACCGFFLYPLKFSFIRPEVSGVTGWLFSQLELFDLPYNQSPSLHIILCWL

LWRHFRQHLAVRWRKVCGGWFLLIAISTLTTWQHHFIDVITGLAVGMLIDWMVPVDRRWNYQKLDQRRIK

IALPY

>gi|313650598|gb|EFS15001.1| uncharacterized protein ydbD [Shigella flexneri 2a str. 2457T]

MPCFTAMRAEIALMSGSAFAVTHHAFSSGAGRTSDGNGSHASTLKSPSYTKSVSWQHYQDKQQVEQAINS

SVNLVPFGLSASDWKVHRGDLVVEGNIESNQKLIVLGNLTVKGNISTFSLSNPWVILGNVTATNIVTDSP

LLITGSINASGLVFIDSYYDNPSTIKGSINARGVFINDIIAPVVASSTNSEFMVRASDKNDTENVKKALM

IINPDAYYWGLINDEDALKEIFKRSNIRMAGNVCNQMKKEALFRPKPSPELVQELQMLDEGNVAAFEGRD

IATFDLAIMRTLPRLKGISANLRKQLINSNDEQTIESMARYMPDNEILELTDQQLGYQPVVLGLLDREPL

SVEIMTRMSRLPDGVGPLNLALRENLPLDIVMTLAKRDWDMIIQELYKDAWLLPESIIDGYIRSDDSSIR

QVGAGGQLTYNQAMQLANDSSNNVVTSLAFKLAEMKHHGQLLRMTPQESDKVAAYLYQKFENDDDLIRVL

FLALPDNLQFNFVKRMEKKSPAYFCCRDMQVIHSDAALQRLLTRFNDPEGWSNLAKNQYLSTSMKQKIWQ

RALSHRKNNPKADSDAYETSADMILSELISHGEVDDQMLLNAAALIRLEDWDFLESALVSWDNLPAVVLK

ELQQNTPRNDIWAKFFLRQENSSRAQVDEALRVYYALDPDALAQLDVLAKQPDRIWWSTLAKSNLTFFKF

GALSNRHTPSAALAAEIDPEWWIVAMNNPRFPVDVLKARLKRDPLLALELVNPELDLVRQLALNGKTRAI

REQAMRKLDELY

>gi|313650596|gb|EFS14999.1| heat shock hslJ domain protein [Shigella flexneri 2a str. 2457T]

MKKVAAFVALSLLMAGCVSNDKIAVTPEQLQHHRFVLESVNGKPVTSDKNPPEISFGEKMMISGSMCNRF

SGEGKLSNGELTAKGLAMTRMMCANPQLNELDNTVMTPTY

>gi|313650579|gb|EFS14982.1| uncharacterized ydaM domain protein [Shigella flexneri 2a str. 2457T]

MTWLFCDRIATKIDKNNGISMITHNFNTLDLLTSPVWIVSPFEEQLIYANSAARLLMQDLTFSQLRTGPY

SVSSQKELPKYLSDLQNQHDIIEILTVQRKEEETALSCRLVLRELTETEPVIIFEGIEAPATLGLKASRS

ANYQRKKQGFYARFFLTNSAPMLLIDPSRDGQIVDANLAALNFYGYNHETMCQKHT

>gi|313650578|gb|EFS14981.1| uncharacterized ydaM domain protein [Shigella flexneri 2a str. 2457T]

MHEISHLPGGHNPLNFVHKLADGSTRHVQTYAGPIEIYGDKLMLCIVHDITEQKRLEEQLEHAAHHDAMT

GLLNRRQFYHITEPGQMQHLAIAQDYSLLLIDTDRFKHINDLYGHSKGDEVLCALARPLESCARKGDLVF

RWGGEEFVLLLPRTPLDTALSLAETIRVSVAKVSISGLPRFTVSIGVAHHEGNESIDELFKRVDDALYRA

KNDGRNRVLAA

>gi|313650577|gb|EFS14980.1| smr domain protein [Shigella flexneri 2a str. 2457T]

MLDKLRSGKYPQQASLNLLRQPVEECRKMVFSFIQQALADGLRNVLIIHGKGREDKSHANIVRSYVARWL

TEFDDVQAYCTALPHHGGSGACYVALRKTAQAKQENWERHAKRSR

>gi|313650575|gb|EFS14978.1| drug resistance MFS transporter [Shigella flexneri 2a str. 2457T]

MSMRKHIAFASMCMGLFIAQLDIQIVSSSLNEIGGGLSAGKDEMAWLQTSYLIAEIIVIPLSGWLSRVFS

TRWLFTLSAGIFTLMSIACGLAWNIQIMILFRALQGAAGASMIPLVFTMAFIYYQGKELGLAAAVVSALA

SLSPTLGPTLGGWLTDNLDWRWLFYINILPGIYLVLSIPFLVNFDKPDLSLLKVADYPSIILLAMTLGCL

EYTLEEGARWGWLDDNTILLTSVLALVSFILFAARTLTISNPIMDLHAFKDKNFTLGCFFSFSGGVGIFS

TVYLIPVFLGQIRGLNAEEIGFAVCTTGIFQLFSVPFYFWLSKKINLRWLLMAGLGGFVFSMYLFTPITH

EWGWQELLFPQAIRGISQQFAMAPIVTLTLGGIPKERLKLASGVFNLTRNLGGAIGIALCGSILNNRTNF

HFSRMGEKMVSVPHTVNDFISRSALFFNRSRSDQTSEILASTKLLSQLMLREAQTMAFSVPFC

>gi|313650574|gb|EFS14977.1| RND family drug transporter [Shigella flexneri 2a str. 2457T]

MRQQQDEQGRFSICSRQAAVVRAPIDGIVANRSAHTGSWVEGGTSLVSLVPVSELWVDANYKENQIAGMK

PGMKAEIRADILKGEVFHGHIESLSPATGASFSLIPIENATGNFTKIVQRVPVRIAFDDAKELKQLLRPG

LSVTVSVDER

>gi|313650573|gb|EFS14976.1| transposase [Shigella flexneri 2a str. 2457T]

MLSAFTPRPLKRLFTANQCWTSFLDAGGLRDIEVEAVTKMLACGTRILGVKEYICDKPECPHVRYVTNSC

GSRACPSCGKKATDLWIATQLNRLPDCDWVHLVFTLPDTQWPVFESNRWLLNDVCRLAVENLLYAARKRG

QEPGIFCAIHTYGRRLNWHPHVHVSVTCGGLNKHGQWKKLSFLKDAMRSRWMWNMRQRLLKAWSEGLAMP

ESLSHITTESQRRSLVLKAGGKYWHVYMSKKTAGGRNTARYLGRYLKKPPIAASRLAHYNGGASLSFRYL

DHETENGDGTLTQRELVARLKQHIPEKFFKMVRYFGFLANRVCGEKLPQVYRALGMDKPEPVAKVCYAQM

VKQFLSRDPFECVLCGGRMVYRRAIAGLNVEGLKKNARDISLLRYMPA

>gi|313650571|gb|EFS14974.1| bacterial regulatory helix-turn-helix s, AraC family protein [Shigella flexneri 2a str. 2457T]

MTITLQPKASPGHHIIGLDSEHLNGGTVPWHKHLYAQLLYPAEGVVRVWAGESVWLVHASSALWLPPQMP

HKFVATGNVLLKTVLVSEAESETLGTVCFMTGISPLLRELLIAINQLPPSQSTTDKQQLRFSALETLILQ

EIKMGVKMSLELPWPNDERLQQLCENLLNNQGYLPTLDNLADKINVSSRTLMRLFVKETGLTFRHWVQQM

HVISAVTLLDDGYSLTKIAHRLGYASAESFGNMFKRRTGYSPGKFTRRLTMHNYAITRQMI

>gi|313650552|gb|EFS14956.1| hypothetical protein SF2457T_0983 [Shigella flexneri 2a str. 2457T]

MDIAHQFWHIPHEKIWIEDQSTNCGENARFSITLLNQAVERVHTAIVVQDPTMQRRTMATFRRMTGDNPD

APRWLSYPGFVPQLGNNADSVIFINQLQGLWPVERYLSLLTGELPRLHDDSDGYGPRGRDFIVHVDFPAE

VIHAWQTLKHDAVLIEAMESRSLR

>gi|313650530|gb|EFS14936.1| streptogramin A acetyltransferase [Shigella flexneri 2a str. 2457T]

MDNKIFLNHKGKKSYKERNLFLFSKGDKITIEDNVIAEEYSTMPVKNFSSVGAFSFPTCHFSGNIRIGRF

CSIASNVKIMGGNHPLNRFTTHMMTYNGEFDKFAMSEFERSWTLKPFITKPENPIIGNDVWIGNDVVLKG

GIAIGDGAVIAANSVVTKDVPPYAIVAGVPAKIIRFRFDSNVIDELLRIKWWNYNYSDLPDNNKCDDINY

FVEEMNRLISNGNIQERDYKKFNLSEVFRGL

>gi|313650527|gb|EFS14933.1| binding--dependent transport systems inner membrane component domain protein [Shigella flexneri 2a str. 2457T]

MILPVQAALERLPPSLLQASADLGARPRQTFRYVVLPLAIPGIAAGSIFTFSLTLGDFIVPQLVGPPGYF

IGNMVYSQQGAIGNMPMAAAFTLVPIILIALYLAFVKRLGAFDAL

>gi|313650506|gb|EFS14913.1| hypothetical protein SF2457T_1164 [Shigella flexneri 2a str. 2457T]

MENFIDTLENWRDQKQIISVICKDGIKIDDSIITSFKASKDVGISNGLRIQLTFQEINFKAIVGQTDVSA

ATGRTSTTNDGGATSKKNTGNTTTSLGSPMLTCKELFSYSASELSDEALKARVTCSKSVSVKNGESTFTA

SANE

>gi|313650472|gb|EFS14879.1| flagellar basal-body rod protein flgF [Shigella flexneri 2a str. 2457T]

MDHAIYTAMGAASQTLNQQAVTASNLANASTPGFRAQLNALRAVPVEGLSLPTRTLVTASTPGADMTPGK

MDYTSRPLDVALQQDGWPCRPLTAAKGIRVMAAFRLIPPGN

>gi|313650471|gb|EFS14878.1| flagellar basal-body rod protein flgF [Shigella flexneri 2a str. 2457T]

MIGEAGPIAVPEGAEITIAADGTISALNPGDPANTVAPVGRLKLVKATGSEVQRGDDGIFRLSAETQATR

GPVLQADPTLRVMSGVLEGSNVNAVAAMSDMIASARRFEMQMKVISSVDDNAGRANQLLSMS

>gi|313650466|gb|EFS14873.1| flagellar hook-associated 1 domain protein [Shigella flexneri 2a str. 2457T]

MSSLINNAMSGLNAAQAALNTASNNISSYNVAGYTRQTTIMAQANSTLGAGGWVGNGVYVSGVQREYDTF

ITNQLRAAQTQSSGLTARYEQMSKIDNMLSTSTSSLATQMQDFFTSLQTLVSNAEDPAARQALIGKSEGL

VNQFKTTDQYLRDQDKQVNIAIGASVDQINNYAKQIASLNDQISRLTGVGAGASPNNLLDQRDQLVSELN

QIVGVEVSVQDGGTYNITMPMVTHWFREVRRGNWRQFLPAPTLLVRLSLTLMGRQAILRSRRNY

>gi|313650465|gb|EFS14872.1| flagellar hook-associated 1 domain protein [Shigella flexneri 2a str. 2457T]

MVLATDYKISFDNNQWQVTRLASNTTFTATPDANGKVAFDGLELTFTGTPAVNDSFTLKPVSDAIVNMDV

LITDEAKIAMASEEDAGDSDNRNGQALLDLQSNSKTVGGAKSFNDAYASLVSDIGNKTATLKTSSTTQGN

VVTQLSNQQQSISGVNLDEEYGNLQRFQQYYLANAQVLQTANAIFDALINIR

>gi|313650464|gb|EFS14871.1| flagellar hook-associated 3 domain protein [Shigella flexneri 2a str. 2457T]

MRFSTQMMYQQNMRGITNSQAEWMKYGEQMSTGKRVVNPSDDPIAASQAVVLSQAQAQNSQYTLARTFAT

QKVSLEESVLSQVTTAIQNAQEKIVYASNGTLSDDDRASLATDIQGLRDQLLNLANTTDGNGRYI

>gi|313650463|gb|EFS14870.1| flagellar hook-associated protein 3 [Shigella flexneri 2a str. 2457T]

MGATYKTETAPFSEANGDYVGGTESIKQQVDASRSMVIGHTGDKIFDSITSNAVAEPDGSASETNLFAML

DSAIAALKTPVADSEADKEIAAAALDKTNRGLKNSLNNVLTVRAELGTQLNKLESLDSLGSDRALGQTQQ

MSDLVDVDWNATISSYIMQQTALQASYKAFTDMQGLSLFQLNK

>gi|313650445|gb|EFS14852.1| fhuE receptor domain protein [Shigella flexneri 2a str. 2457T]

MKSLYAATRVTLADPLHLILGARYTNWRVDTLTYSMEKNHTTPYAGLVFDINDNWSTYASYTSIFQPQND

RDSSGKYLAPITGNNYELGLKSDWMNSRLTTTLAIFRIEQDNVAQSTGTPIPGSNGETAYKAVDGTVSKG

VEFELNGAITDNWQLTFGATRYLAEDNEGNAVNPNLPRTTVKMFTSYRLPVMPELTVGGGVNWQNRVYTD

TVTPYGTFRAEQGSYALVDLFTRYQVTKNFSLQGNVNNLFDKTYDTNMEGSIVYGAPRNFSITGTYQF

>gi|313650444|gb|EFS14851.1| fhuE receptor domain protein [Shigella flexneri 2a str. 2457T]

MIVEGSATAPDDGENDYSVTSTSAGTKMQMTQHDIPQSVTIVSQQRMEDQQLQTLGEVMENTLGISKSQA

DSDRALYYSRGFQIDNYMVDGIPTYFESRWNLGDALSDMALFERVEVVRGATGLMTGTGNPSAAINMVRK

HATCREFKGDVSAEYGSWNKERYVADLQSPLTEDGKIRARIVGGYQNNDSWLDRYNSEKTFFSGIVDADL

GDLTTLSAGYEYQRIDVNSPTWGGLPRWNTDGSSNSYDRARSTAPDWAYNDKEINKVFMTLKQRFADTWQ

ATLNATHSEVEFDSKMMYVDAYVNKADGMLVGPYSNYGPGFDHVGGTGWNSGKRKVDALDLFADGSYELF

GRQHNLMFGGSYSKQNNRSVHGPTSSRMKLAVSTTLMAISHKPTGHHRAWRRTIPHI

>gi|313650416|gb|EFS14823.1| hypothetical protein SF2457T_1074 [Shigella flexneri 2a str. 2457T]

MKIEYTPERGRGFVRPGETGKPQNWGFSGIKKAAPKWSRLSEQITRCAVCVCDPKHKHGDDSRYQAGGQC

NQSGSVRCHNCEVLSFDEYSLRYFSRAKEHGANRSDSCSFFLRRLFRAGDVFSVDSSLVIVCACKAMRRS

TSHHGADSFYPLGSMPRCCNSLRIIRFIQAARESSPSCCCAFSIFSRKSGSKRNWNGGLPRLSFLCVDTL

ITPQVMYLCVVTHYTQSTQKAKPRTVGAVTGLLTNNVN

>gi|313650405|gb|EFS14812.1| phage capsid family protein [Shigella flexneri 2a str. 2457T]

MQRIIELRQQKTAIKNQMRDMLENAEKENRSLNDAEGAKFDELRAKAESLDKDISRLEAIADEERSKPGK

SSQTTDPAELRNYILTGETRALSTGVPADGGYTVIPELNTEIMRMLADESTMRRICTVKKISSNEFKQLV

SAGGATVNHGEEGKAREQTSTPQINEVSIKLYPVYAYPRTTQEIVDFSDVDILSWLTGEIGDTFTETEES

DLVVGDGDKKAKGFLSVPRAEKNDKERDFGTLQVIKPSESLAWTSADPLIDLKFALRKKYRKNAV

>gi|313650404|gb|EFS14811.1| phage capsid family protein [Shigella flexneri 2a str. 2457T]

MVNSTTAAKLQKVKNANGDYIWRDRLQAGDPDTLLGLPVEYLEFMPDNVIALGDFKRGYYIVDHETGVRT

RPDNLTEPGFIKIFTQKYLGGGVVDSNAIKILALPEDDD

>gi|313650402|gb|EFS14809.1| phage portal protein, HK97 family [Shigella flexneri 2a str. 2457T]

MWPFRRKKEQRSMTLDEFMALAGTSNTGAGEYVSSGTAESLPAVMNAVTVISDAVATMPCYLYLVRNEKG

KEAREWLDSHPVDHILNERPNAWQTPYQFKRMMIRYCLLSGNAYAVIQWGRDGFPAALHPYPPQSVNVEQ

TGDHNWRYCITDAYTGNIRNYLPWEVLHLRYSTDDGFMGRSPVTICRESLGLGLAQQRHGASVMRDGMMA

AGVITSGEWLDGVKGKQALAALERYKGARNAGKTPILEGGMSYQQLGMSNQDAEWLASRRFTIEDIARMF

NVSPIFLQEYSNSTYSNFSEASRAFLTMTMRPWLANFEQQIKNALLVASPVPGIRYQVEFDSADLLRATP

GERFATYERGIKSDVMCPNEAREREGLSPRDGGDEFS

>gi|313650353|gb|EFS14762.1| HTH-type transcriptional regulator dicA [Shigella flexneri 2a str. 2457T]

MKNETFGARLLHRRKKLKLSQAALGKLVKVAHVTISQWERDETQPAGKRLFALSQALQCSPTWLLFGDED

KQPGEPIPDNQPAILTEDQKELLQLFDALPESEQKALLSEMRARVENFNKLFEELLKARKRSANK

>gi|313650346|gb|EFS14755.1| hypothetical protein SF2457T_1173 [Shigella flexneri 2a str. 2457T]

MEIKPEDELSNIVLFPIKEDDPRNQVNFLYKPSERPYCHHASVRVDEKERQVRCKICGAVVEPFDWMLSV

AKRETRLADDVRLLRQEEQERRRNIEKLIQIERNAKARIRRATKSRTE

>gi|313650297|gb|EFS14709.1| catecholate siderophore receptor fiu domain protein [Shigella flexneri 2a str. 2457T]

MSNKILTNQTNLTSTFYTGSIGHDVSTGVEFTRETQTNYGVNPVTLPAVNIYHPDSRIHPGGLTRNGANA

NGQTDTFAIYAFDTLQITRDFELNGGIRLDNYHTEYDSATACGGSGRGAITCPAGVAKGSPVTTVDTAKS

GNLVNWKAGALYHLTENGNVYINYAVSQQPPGGNNFALAQSGSGNSANRTDFKPQKANTSEIGTKWQVLD

KRLLLTAALFRTDIENEVEQNDDGTYSQYGKKRVEGYEISVAGNITPAWQVIGGYTQQKATIKNGKDVAQ

DGSSSLPYTPEHAFTLWSQYQATDDISVGAGARYIGSIHKGSDGAVGTPAFTEGYWVADAKLGYRVNRNL

DFQLNVYNLFDTDYVASINKSGYRYHPGEPRTFLLTANMHF

>gi|313650296|gb|EFS14708.1| catecholate siderophore receptor fiu domain protein [Shigella flexneri 2a str. 2457T]

MGAFFAGENGNSTTGDAIYMRGADTSNSIYIDGIRDIGSVSRDTFNTEQVEVIKGPSGTDYGRSAPTGSI

NMISKQPRNDSGIDASASIGSAWFRRGTLDVNQVIGDTTAVRLNVMGEKTHDAGRDKVKNERYGVAPSVA

FGLGTANRLYLNYLHVTQHNTPDGGIPTIGLPGYSAPSAGAAALNHSGKVDTHNFYGTDSDYDDSTTDTA

TMRFEHDINDNTTIRNTTRWSRVKQDYLMTAIMGGASNITQPTSDVNSWT

>gi|313650294|gb|EFS14706.1| catecholate siderophore receptor fiu domain protein [Shigella flexneri 2a str. 2457T]

MENNRNFPARQFHSLTFFAGLCIGITPVAQALAAEGQANADDTLVVEASTPSLYAPQQSADPKFSRPVAD

TTRTMTVISEQVIKDQGATNLTDALKNVPGVGNASQLSN

>gi|313650291|gb|EFS14703.1| mechanosensitive ion channel family protein [Shigella flexneri 2a str. 2457T]

MLDNDTSRKELIDQLRTVAATPPAEPVPKIVPPTLVEEQTVLQKVTEVSRHYGEALSARFGQLYRNITGS

PHKPFNPQTFSNALSHFSMLAVLVFGFYWLIRLCALPLYRKMGQWARQKNRERSNWLQLPAMIIGAFIID

LLLLALTLFVGQVLSDNLNAGSRTIAFQQSLFLNAFALIEFFKAVLRLIFCPNVAELRPFTIQDESARYW

GRRLSWLSSLIGYGLIVAVPIISNQVNVQIGALANVIIMLCMTVWALYLIFRNKKEITQHMLNFAEHSLA

FFSLFIRAFALVWHWLASAYFIMLFFFSLFDPGNSLKFMMGATVRSLAIIGIAAFVSGMFSRWLAKTITL

SPHTQRNYPELQKRLNGWLSAALKTARILTVCVAVMLLLSAWGLFDFWNWLQNGAGQKTVDILIRIALIL

FFSAVGWTVLASLIENRLASDIHGRPLPSARTRTLLTLFRNALAVIISTITIMIVLSEIGVNIAPLLAGA

GALGLAISFGSQTLVKDIITGVFIQFENGMNTGDLVTIGPLTGTVERMSIRSVGVRQDTGAYHIIPWSSI

TTFANFVRGIGSVVANYDVDRHEDADKANQALKDAVTELMENEEIRGLIIGEPNFAGIVGLSNTAFTLRV

SFTTLPLKQWTVRFALDSQVKKHFDLAGVRAPVQTYQVLPAPGATPAEPLPPGEPTL

>gi|313650266|gb|EFS14678.1| uncharacterized protein yliE [Shigella flexneri 2a str. 2457T]

MLSLYEKIRLIILFLLAALSFIGLFFMINYQLVSERAVKRADSRFELIQKNVGYFFKDIERSALTLKDSL

YLLKNTEEIQRAVILKMEMMPFLDSVGLVLDDNKYYLFSRRANDKIVVYHQEQVNGPLVDESGRVIFADF

NPSKRPWSVASDDSNNSWNPAYNCFDRPGKKCISFTLRINGKDHDLLAVDKIHVDLNWRYLNEYLDQISA

NDEVLFLKQGHEIIAKNQLAREKLIIYNSEGNYNIIDSVDTEYIAKTSAVPNNALFEIYFYYPGGNLLNA

SDKLFYLPFAFIIIVLLVVYFMTTRVFRRQFSEMTELVNTLAFLPDSTDQIEALKIREGDAKEIISIKNS

IAEMKDAEIERSNKLLSLISYDQESGFIKNMAIIESNNNQYLAVGIIKLCGLEAVEAVFGVDERNKIVRK

LCQRIAEKYAQCCDIVTFNADLYLLLCRENVQTFTRKIAMVNDFDSSFGYRNLRIHKSAICEPLQGENAW

SYAEKLKLAISSIRDHMFSEFIFCDDAKLNEIEENIWIARNIRHAMEIGELFLVYQPIVDINTRAILSAE

ALCRWVSAERGIISPLKFITIAEDIGFINELGYQIIKTAMG

>gi|313650265|gb|EFS14677.1| uncharacterized yliE domain protein [Shigella flexneri 2a str. 2457T]

MKENGLKANSLCVEITETVIERINEHFYLNIEQLRKQGVRISIDDFGTGLSNLKRFYEINPDSIKVDSQF

TGDIFGTAGKIVRIIFDLARYNRIPVIAEGVESEDVARELIKLGCVQAQGYLYQKPMPFSAWDKSGKLVK

E

>gi|313650258|gb|EFS14670.1| D-alanyl-D-alanine carboxypeptidase dacC [Shigella flexneri 2a str. 2457T]

MTQYSSLLRGLAAGSAFLFLFAPTAFAAEQTVEAPSVDARAWILMDYASGKVLAEGNADEKLDPASLTKI

MTSYVVGQALKADKIKLTDMVTVGKDAWATGNPALRGSSVMFLKPGDQVSVADLNKGVIIQSGNDACIAL

ADYVAGSQESFIGLMNGYAKKLGLTNTTFQTVHGLDAPGQFSTARDMALLGKALIHDVPEEYAIHKEKEF

TFNKIRQPNRNRLLWSSNLNVDGMKTGTTAGAGYNLVASATQGDMRLISVVLGAKTDRIRFNESEKLLTW

GFRFFETVTPIKPDATFVTQRVWFGDKIEVNLGAGEAGSVR

>gi|313650250|gb|EFS14662.1| cof-like hydrolase family protein [Shigella flexneri 2a str. 2457T]

MSIKLIAVDMDGTFLSDQKTYNRERFMAQYQQMKAQGIRFVVASGNQYYQLISFFPEIANEIAFVAENGG

WVMSEGKDVFNGELSKDAFATVVEHLLTRPEVEIIACGKNSAYTLKKYDDAMKTVAEMYYHRLEYVDNFD

NLEDIFFKFGLNLSDELIPQVQKALHEAIGDIMVPVHTGNGSIDLIIPGVHKANGLRQLQKLWGIDDSEV

VVFGDGGNDIEMLRQAGFSFAMENAGSAVVAAAKYRAGYNNREGVLDVIDKVLKHEAPFDQ

>gi|313650185|gb|EFS14598.1| HD domain protein [Shigella flexneri 2a str. 2457T]

MKNHHQHQDAAHDLCHFRRVWATAQKLAADDDVDMLVILTACYFHDIVSLAKNHPQRQRSSILAAEETRR

LLREEFVQFPAEKIEAVCHAIAAHSFSAQIAPLTTEAKIVQDADRLEALGAIGLARVFAVSGALSLALFD

GEARCRTGGGE

>gi|313650178|gb|EFS14591.1| cellulose synthesis regulatory protein [Shigella flexneri 2a str. 2457T]

MQYNVDKLIFLRNGMREALVAPLDFTSLRNAVTEFEQHRDEHAWQIELNRRRTLPVNGVSDALVSEGNFL

SRENESLDNEITAALEVGYLLRLAHNSSSMVEQAMYVSRAGFYVSTQPTLFTRNVPTRYYGYVTQPWFIG

HSQRENRHRAVRWFTSQPEHASNTEPQVTVSVPVDSNNYWYGVLGMSIPVRTMQQFLRNAIDKNLDGEYQ

LYDSKLRFLTSSNPDHPTGNIFDPRELALLAQAMEHDTRGGIRMDSRYVSWERLDHFDGVLARVHTLSEG

VRGDFGSISIALTLLWALFTTMLLLSWYVIRRMVSNMYVLQSSLQWQAWHDTLTRLYNRGALFEKARPLA

KLCQTHQHPFSVIQVDLDHFKAINDRFGHQAGDRVLSHAAGLISSSLRAQDVAGRVGGEEFCVILPGANL

TQAAEVAERIRLKLNEKEMLIAKSTTIRISASLGVSSSEETGDYDFEQLQSLADRRLYLAKQAGRNRVFA

SDNA

>gi|313650177|gb|EFS14590.1| putative mannosyl-3-phosphoglycerate phosphatase [Shigella flexneri 2a str. 2457T]

MAQFIARLNELGLQFMQGARFWHVLDASAGKDQAANWIIATYQQLSGRRPTTLGLGDGPNDAPLLEVMDY

AVIVKGLNREGVHLHDEDPARVWRTQREGPEGWREGLDHFFSAR

>gi|313650176|gb|EFS14589.1| putative mannosyl-3-phosphoglycerate phosphatase [Shigella flexneri 2a str. 2457T]

MFSIQQPLLVFSDLDGTLLDSHSYDWQPAAPWLSRLHEANIPVILCSSKTLAEMLYLQKMLGLQGLPLIA

ENGAVIQLAEQWQDIDGFPRIISGISHGEICQVLNTLREKEHFKFTTFDDVDDATIAEWTGLSRS

>gi|313650170|gb|EFS14583.1| flagellar biosynthetic protein FliP [Shigella flexneri 2a str. 2457T]

MQTLVFITSLTFIPAILLMMTSFTRIIIVFGLLRNALGTPSAPPNQVLLGLALFLTFFIMSPVIDKIYVD

AYQPFSEEKISMQEALEKGAQPLREFMLRQTREADLGLFARLANTGPLQGPEAVPMRILLPAYVTSELKT

AFQIGFTIFIPFLIIDLVIASVLMALGMMMVPPATIALPFKLMLFVLVDGWQLLVGSLAQSFYS

>gi|313650164|gb|EFS14577.1| flagellar export protein FliJ [Shigella flexneri 2a str. 2457T]

MAEEQLKMLIDYQNEYRNNLNSDMSAGMTSNRWINYQQFIQTLEKAITQHRQQLNQWTQKVDIALNSWRE

KKQRLQAWQTLQERQSTAALLAENRLDQKKMDEFAQRAAMRKPE

>gi|313650159|gb|EFS14572.1| flagellar M-ring domain protein [Shigella flexneri 2a str. 2457T]

MSNQPAPANNAPISTPPTNQNNRQQQASTTSNSGPRSTQRNETSNYEVDRTIRHTKMNVGDVQRLSVAVV

VNYKTLPDGKPLPLSNEQMKQIEALTREAMGFSEKRGDSLNVVNSPFNSSDESGGALPFWQQQVFIDQLL

AAGRWLLVAGTAGGVVAVAESGTPAANTSRRGGEDCAATGAGPRGSGRCGGSPSEQRRTTPTTAR

>gi|313650158|gb|EFS14571.1| flagellar M-ring domain protein [Shigella flexneri 2a str. 2457T]

MNATAAQTKSLEWLNRLRANPKIPLIVTGSAAVAVMVALILWAKAPDYRTLFSNLSDQDGGAIVSQLTQM

NIPYRFSEASGAIEVPADKVHELRLAQQGLPKGGAVGFELLDQEKFGISQFSEQVNYQRALEGELSRTIE

TIGPVKGARVHLAMPKPSLFVREQKSPSASVTVNLLPGRALDEGQISAIVHLVSSAVAGLPPGNVTLVDQ

GGHLLTQSNTSGRDLNDAQLKYASDVEGRIQQRIEAILSPIVGNGNIHA

>gi|313650149|gb|EFS14562.1| uncharacterized protein yedK [Shigella flexneri 2a str. 2457T]

MCGRFAQSQTREDYLALLAEDIERDIPYDPEPIGRYNVAPGTKVLLLSERDEHLHLDPVFWGYAPGWWDK

PPLINARVETASTSRMFKPLWQHGRAICFADGWFEWKKEGDKKSLILSIALTDNLFLWPR

>gi|313650139|gb|EFS14552.1| RNA polymerase sigma factor for flagellar operon [Shigella flexneri 2a str. 2457T]

MLDELRSRDWVPRSVRRNAREVAQAIGQLEQELGRNATETEVAERLGIDIADYRQMLLDTNNSQLFSYDE

WREEHGDSIELVTDDHQRENPLQQLLDSNLRQRVMEAIETLPEREKLVLTLYYQEELNLKEIGAVLEVGE

SRVSQLHSQAIKRLRTKLGKL

>gi|313650116|gb|EFS14529.1| L-arabinose-binding periplasmic domain protein [Shigella flexneri 2a str. 2457T]

MHKFTKALAAIGLAAVMSQSAMAENLKLGFLVKQPEEPWFQTEWKFADKAGKDLGFEVIKIAVPDGEKTL

NAIDSLAASGAKGFVICTPDPKLGSAIVAKARGYDMKVIAVDDQFVNAKGKPMDTVPLVMMAATKIGERQ

G

>gi|313650115|gb|EFS14528.1| L-arabinose-binding periplasmic protein [Shigella flexneri 2a str. 2457T]

MQKRGWDVKESAVMAITANELDTARRRTTGSMDALKAAGFPEKQIYQVPTKSNDIPGAFDAANSMLVQHP

EVKHWLIVGMNDSTVLGGVRATEGQGFKAADIIGIGINGVDAVSELSKAQATGFYGSLLPSPDVHGYKSS

EMLYNWVAKDVETPKFTEVTDVVLITRDNFKEELEKKGLGGK

>gi|313650087|gb|EFS14500.1| flagellar biosynthesis protein flhA [Shigella flexneri 2a str. 2457T]

MMVLPLPAFILDLLFTFNIALSIMVLLVAMFTQRTLEFAAFPTILLFTMLLRLALNVASTRIILMEGHTG

AAAAGKVVEAFGHFLVGGNFAIGIVVFVILVIINFMVITKGAGRIAEVGARFVLDGMPGKQMAIDADLNA

GLIGEDEAKKRRSEVTQEADFYGSMDGASKFVRGDAIAGILIMVINVVGGLLVGVLQLA

>gi|313650086|gb|EFS14499.1| flagellar biosynthesis protein flhA [Shigella flexneri 2a str. 2457T]

MGHAAESYTLLTIGDGLVAQIPALVISTAAGGIVTRVSTDQDVGEQMVNQLFSNPSVMLLSAAVLGLLGL

VPGMPNLVFLLFTAGLLGLAWWIRGREQKAPAEPKPVKMAENNTVVEATWNDVQLEDSLGMEVGYRLIPM

VDFQQDGELLGRIRSIRKKFAQEMGFLPPVVHIRDNMDLQPARYRILMKGVEIGSGDAYPGRWLAINPGT

AAGTLPGEATVDPAFGLNAIWIESALKEQAQIQGYTVVEASTVVATHLNHLISQHAAELFGRQEAQQLLD

RVAQEMPKLTEDLVPGVVTLTTLHKVLQNLLDEKVPIRDMRTILETLAEHAPIQSDPHELSAVVRVALGR

AITQQWFPGKDEVHVIGLDTPLERLLLQALQGGGGLEPGLADRLLAQTQEALSRQEMLGAPPVLLVNHAL

RPLLSRFLRRSLPQLVVLSNLELSDNRHIRMTATIGGK

>gi|313650081|gb|EFS14494.1| hypothetical protein SF2457T_1388 [Shigella flexneri 2a str. 2457T]

MIKSCLRQEYNLVDAQLNKAYGEAYRYIEQVPRTGAKKLDTEQLNLLKKSQRAWLDFRDKECELILSNED

VQDLSDPYSESEWLSCMIIQTNTRTRQLQLYRNSEDFYPSPLTRG

>gi|313650072|gb|EFS14485.1| uncharacterized yecE domain protein [Shigella flexneri 2a str. 2457T]

MLILDSRPVHASRPHSEAIRDAQRKKPKVPVHAVLTATNPLIRFIGSDDMTQNRELFQVWLQKLAQWHQT

TTPYLFLHTPDIAQAPELVHTLWEDLRKTLPEIGAVPAIPQQSSLF

>gi|313650057|gb|EFS14470.1| insertion element IS110 uncharacterized 43.6 kDa protein [Shigella flexneri 2a str. 2457T]

MTESSDYESVQVFIGVDVGKDTHHAVAINRSGKRLFDKALPNDENKLRSLISDLKQHGQILLVVDQPATI

GALPVAVARSEGVLVGYLPGLAMRRIADLHAGEAKTDARDAAIIAEAARTLPHALRTLKLADEQIAELSM

LCGFDDDLAAQTTQASNRILGLLTQIHPAPERVLGPRLEHPAVLDLLQRYPSPEKLASLGEKKLAAQLCK

LAPRLGKRLAADIAQALAEQTVVVPGTNAAAVVLPRLALQLITLRKQRDEVALEVEQRVLAHPLYPVLTS

MPGVGVRTAARLLTEVACRAFASAAHLAAYAGLAPVTRRSGSPIRGEHPSRRGNKALKRALFLSAFDRAQ

GSALQGVTTPAKMSQGKRHNQALIALARRRCDVLFAMMRDGTFYTPQGS

>gi|313650056|gb|EFS14469.1| DNA packaging protein FI [Shigella flexneri 2a str. 2457T]

MAKKDDNLKRLRELAASLGREPDISGSAADIAQRVAELEEELANMDDTDIRDKSAHPENALTGHENEVIS

AQPETVIQNMDDLVTVVALVTLHTDALHATRDEPLAFVPPGMAFRVFAGVAAGMTERGLARMQ

>gi|313650055|gb|EFS14468.1| tail attachment protein [Shigella flexneri 2a str. 2457T]

MADFDNLFDVALDLADKAIIRNMGIRAVITSGRLKGIMISGVFDDPENISLVAGGVRIEDSLPSLFVKTA

DILRLCRNDSLMIGRESFCVDRITPDDGGCSYIRLRREGLPGNVRAGRYYEGA

>gi|313650054|gb|EFS14467.1| minor tail protein Z [Shigella flexneri 2a str. 2457T]

MKGLENAIRNLNSLDTRMVPQASAWAINRVAQKAVSVATRQVAGNTVAGDNQVKGIPLKLVRQRVRVFKA

SPSGKMTARIRVNRGNLPAIKLNTTRRRAGEGLRVGKYFFRGAFVQQLANGRWHVLRCLPEARFATGHDH

QGRLRKNRLPVEVVKIPLSGPLTQAFEDARDRIIAAEMPKQLGYALKQQLRLHLSK

>gi|313650001|gb|EFS14419.1| uncharacterized protein yjiC [Shigella flexneri 2a str. 2457T]

MSMPLSNAANNQFNTNNHFLHHPKVDSELTRRYEYARLDTENIYLLPLARGNNHNYDGKSVVEIRKLNIG

KQSWPFNYVTDTCREFNGITTTGRMLYRNLKITSALDEIYGGICKKAHAATELAEGLRLNLFMKSPFDPV

EDYTVHEITLGPGCNVPGYAGTTIGYISTLPASQAKRWTNEQPRIDIYIDQIITVSGVANSSGFALAALL

NANIEMGNDPIIGIEAYPGTAEIHSKMGYNVIPGDEDAPLKRMTLQPSSLPELFELKNGEWNYIGK

>gi|313649993|gb|EFS14411.1| outer membrane usher fimD domain protein [Shigella flexneri 2a str. 2457T]

MGDGYTQGDIFDGINFRGAQLASDDNMLPDSQRGFAPVIHGIARGTAQVTIKQNGYGIYNSTVPPGPFTI

NDIYAAGNSGDLQVTIKEADGSTQIFTVPYSSVPLLQREGHTRYSITAGEYRSGNAQQEKPRFFQSTLLH

GLPAGWTIYGGTQLADRYRAFNFGIGKNMEALGALSVDMTQANSTLPDDSQHDGQSVRFLYNKSLNESGT

NIQLVGYRYSTSGYFNFADTTYSRMNGYNIETQDGVIQVKPKFTDYYNLAYNKRGKLQLTVTQQLGRTST

LYLSGSHQTYWGTSNVDEQFQAGLNTAFEDINWTLSYSLTKNAWQKGRDQMLALNVNIPFSHWLRSDSKS

QWRHASASYSMSHDLNGRMTNLAGVYGTLLEDNNLSYSVQTGYAGGGDGNSGSTGYATLNYRGGYGNANI

GYSHSDDIKQLYYGVSGGVLAHANGVTLGQPLNDTVVLVKAPGAKDAKVENQTGVRTDWRGYAVLPYATE

YRENRVALDTNTLADNVDLDNAVANVVPTRGAIVRAEFKARVGIKLLMTLTHNNKPLPFGAMVTSESSQS

SGIVADNGQVYLSGMPLAGKVQVKWGEEENAHCVANYQLPPESQQQLLTQLSAECR

>gi|313649991|gb|EFS14409.1| outer membrane usher fimD domain protein [Shigella flexneri 2a str. 2457T]

MAGFFVRLVVACAFAAQAPLSSADLYFNLRFLADDPQAVADLSRFENGQELPPGTYRVDIYLNNGYMATR

DVTFNTGDSEQGIVPCLTRAQLASMGLNTASVAGMNLLADDACVPLTTMVQDATAHLDVGQQRLNLTIPQ

AFMSNRARGYIPPELWDPGINAGLLNYNFSGNSVQNRVMLPTY

>gi|313649985|gb|EFS14403.1| porin family protein [Shigella flexneri 2a str. 2457T]

MKKAKILSGVLLLCFSSPLISQAATLDVRGGYRSGSHAYETRLKVSEGWQNGWWASMESNTWNTIHDNKK

ENAALNDVQVEVNYAIKLDDQWTVRPGMLTHFSSNGTRYGPYVKLSWDATKDLNFGIRYRYDWKAYRQQD

LSGDMSRDNVHRWDGYVTYHINSDFTFAWQTTLYSKQNDYRYANHKKWATENAFVLQYHMTPDITPYIEY

DYLDRQGVYNGRDNLSENSYRIGVSFKL

>gi|313649983|gb|EFS14401.1| hypothetical protein SF2457T_1519 [Shigella flexneri 2a str. 2457T]

MAYGEGLPLPDREDAPHSRIKQLARFAHTHPGGPPCHFNDIIPLTHCPHDVQDMQGYHHPLATNHQTQYG

TVGQALHIARKLLPFIPDNAGILIVPCCRGGSAFTAGSEGTYSERHGASHDACRWGTDTPLYQDLVSRTR

AALAKNPQNKFLGVCWMQGEFDLMTSDYASHPQHFNHMVEAFRRDLKQYHSQLNNITDAPWFCGDTTWYW

KENFPHSYEAIYGNYQNNVLANIIFVDFQQQGERGLTNAPDEDPDDLSTGYYGSAYRSPENWTTALRSSH

FSTAARRGIISDRFVEAILQFWRER

>gi|313649977|gb|EFS14395.1| transposase family protein [Shigella flexneri 2a str. 2457T]

MSRKTQRYSKEFKAEAVRTVLENQLSISEGASRLSLPEGTLGQWVTAARKGLGTPGSRTVAELESEILQL

RKALNEARLERDILKSNSVFCTGVAEKYALIEQWRQQFPIEAMCQVFGVSRSGYYNWVQHEPSDRKQSDE

RLKLEIKVAHIRTRETYGTRRLQTELAENG

>gi|313649974|gb|EFS14393.1| hypothetical protein SF2457T_1570 [Shigella flexneri 2a str. 2457T]

MISNDILRSVRYILKANNNDLVRILALDNVEATAEQIAVWLRKEDEEGFQRCPDIVLSSFLNGLIYEKRG

KDESAPALEPERRINNNIVLKKLRIAFSLKTDDILVMLPTY

>gi|313649952|gb|EFS14372.1| phage integrase family domain protein [Shigella flexneri 2a str. 2457T]

MAISDTKLRTIYGKPYSGPQEVADADGLSVRISPKGVIQFQYRYRWHGKPNRLGLGRYPSLSLKDARQIT

ADLRKLYFSGTDPRTYFEEKVENSMTVAQCLDYWFDNYVSTTLREKTQALYRSTVMKRMHDAFPNRPASS

ITVKQWVDLLTEEEKDNPRRARQVLSQLRSAISWCMRRQLIDSCAIMSIQPRDFGSRAEVGDRVLSYHEL

AKIWLAIERSRASTSNKLLHQMLMLWGARLSELRLATKTEFDLLDNVWTVPKEHSKMGNVIRRPIFEQM

>gi|313649945|gb|EFS14365.1| hypothetical protein SF2457T_1697 [Shigella flexneri 2a str. 2457T]

MKLIWSEESWDDYLYWQETDKRIVKKINELIKDTRRTPFEGKGKPEPLKHNLSGFWSRRITEEHRLVYAV

TDDSLLIAACRYHY

>gi|313649933|gb|EFS14353.1| UDP-glucose 6-dehydrogenase domain protein [Shigella flexneri 2a str. 2457T]

MKSGSDNFRASSIQGIMKRIKAKGVEVIIYEPVMKEDSFFNSRLERDLATFKQQADVIISNRMAEELKDV

ADKVYTRDLFGSD

>gi|313649907|gb|EFS14327.1| transposase [Shigella flexneri 2a str. 2457T]

MPCFTAMRAEIAQMSGSAFAVTHHAFSSGAGRTSDGNGSHASTLKSPSYTKSVSWQHYLCADAKKVQRQI

LHVPGFWAELSWHLRQRTTGKTKALYNKV

>gi|313649905|gb|EFS14325.1| putative colanic acid polymerase domain protein [Shigella flexneri 2a str. 2457T]

MSTSIRICSYLLLPLIYLLVNVKIAQLGESFPITIVTFLPVLLLLFLERISIKKLMIALGIGAGLTAFNY

LFGQSLDAGKYVTSTMLFVYIVIIIGMVWSIRFKTISPHNHRKILRFFYLVVGLVVALAAVEMAQIILTG

GSSIMESISKYLIYSNSYVLNFIKFGGKRTTALYFEPAFFALALISIWLSIKQFGIKTPKTDALILAGII

LSGSFSGVMTFILFYLLEWAFQYLNKEAIKKKLPLALISLAVFRVGVVIAFPYISTRLGDLGTG

>gi|313649904|gb|EFS14324.1| putative colanic acid biosynthesis glycosyl transferase wcaC domain protein [Shigella flexneri 2a str. 2457T]

MLALGCQFISPSQHVADAFNSLYGPGRCRIINNGIDMATEAILADLPPVRETQGKPKIAVVAHDLRYDGK

TNQQLVREMMALGDKIELHTFGKFSPFTAGNVVNHGFETDKRKLMSALNQMDALVFSSRVDNYPLILCEA

LSIGVPVIATHSDAAREVLQKSGGKTVSEEEVLQLVQLSKPEIAQAIFGTTLAEFSQRSRAAYSGQQMLE

EYVNFYQNL

>gi|313649903|gb|EFS14323.1| putative colanic acid biosynthesis glycosyl transferase wcaC domain protein [Shigella flexneri 2a str. 2457T]

MNILQFNVRLAEGGAAGVALDLHQRALQQGLASHFVYGYGKGGKESVSHQNYPQVIKHTPRMTAMANIAL

FRLFNRDLFGNFNELYRTITRTPGPVVLHFHVLHSYWLNLNSVVRFCEKVKNHKPDVTLVWTLHDHWSVT

GRCAFTDGCEGWKTGCQKCPTLNNYPPVKIDRAHQLVAGKRQLFPRDAGAGLSVYFPQPACG

>gi|313649901|gb|EFS14321.1| putative colanic acid biosynthesis glycosyl transferase wcaA domain protein [Shigella flexneri 2a str. 2457T]

MFDTELKAAQDYDIFLRMVVEYGEPWKVEEATQILHINHGEMQITSSPKKFSGYFHFYRKHKDKFDRASK

KYQLFTLYQIRNKRMTWRTLLTLLSVRNGKRLADGIRGR

>gi|313649900|gb|EFS14320.1| putative colanic acid biosynthesis glycosyl transferase wcaA [Shigella flexneri 2a str. 2457T]

MKDNPLISIYMPTWNRQQLAIRAIKSVLRQDYSNWEMIIVDDCSTSWEQLQQYVTALNDPRITYIHNDIN

SGACAVRNQAIMLAQGEYITGIDDDDEWTPNRLSVFLAHKQQLVTHAFLYANDYVC

>gi|313649890|gb|EFS14310.1| uncharacterized yegE domain protein [Shigella flexneri 2a str. 2457T]

MIWVLSESIGALALVPLGLLFKPHYLLRHRNPRLLFESLLTLAITLTLSWLSMLYLPWPFTFIIVLLMWS

AVRLPRMEAFLIFLTTVMMVSLMMAADPSLLATPRTYLMSHMPWLPFLLILLPANIMTMVMYAFRAERKH

ISESETRFRNAMEYSAIGMALVGTEGQWLQSNKALCQFLGYSQEELRGLTFQQLTWPEDLNKDLQQVEKL

ISGEINTYSMEKRYYNRNGDVVWALLAVSLVRHTDGTPLYFIAQIEDINELKRTEQVNQQLMERITLANE

AGGIGIWEWELKPNIFSWDKRMFELYEIPPHIKPNWQVWYECVLPEDRQHAEKVIRCNHARPLSWNFALP

>gi|313649889|gb|EFS14309.1| uncharacterized yegE domain protein [Shigella flexneri 2a str. 2457T]

MKDGIRHIRALANRVLNKEGEVERLLGINMDMTEVKQLNEALFQEKERLHITLDSIGEAVVCIDMAMKIT

FMNPAAEKMSGWTQEEALGVPLLTVLHITFGDNGPLMENIYSADTSRSAIEQDVVLHCRNGGSYDVHYSI

TPLSTLDGSNIGSVLVIQDVTESRKMLRQLSYSASHDALTHLANRASFEKQLRILLQTVNSTHQRHALVF

IDLDRFKAVNDSAGHAAGDALLRELASLMLSMLRSSDVLARLGGDEFGLLLPDCNIESARFIATRIISAV

NDYHFIWEGRVHRVGASAGITLIDDNNHQAAEVMSQADIACYASKNGGRGRVTVYEPQQAAAHSERAAMS

LDEQWRMIKENQLMMIAHGVASPRIPEARNLWLISLKLWSCEGEIIDEQKFRRSFSDPALSHALDRRVFH

EFFQQAAKAVASKGISIALPLSVAGLSSATLVNDLLEQLENSPLPPRLLHLIIPAEAILDHAESVQKLRL

AGCRIVLSQVGRDLQIFNSLKANMADYLLLDGELCANVQGNLMDEMLITIIQGHAQRLGMKTIAGPVVLP

LVMDTLSGIGVDLIYGDVIADAQPLDLLVNSSYFAIN

>gi|313649886|gb|EFS14306.1| uncharacterized chaperone yegD domain protein [Shigella flexneri 2a str. 2457T]

MALLQKVWRQRLSYRLVRSAEECKIALSSVAETRASLPFISNELATLISQRGLESALSQPLARILEQVQL

ALDNAQEKPDVIYLTGGSARSPLIKKALAEQLPGIPIAGGDDFGSVTAGLAHWAEVVFR

>gi|313649884|gb|EFS14304.1| uncharacterized protein yegI [Shigella flexneri 2a str. 2457T]

MKPTLYTATGECVTPGRELGKGGEGAVYDINEFVDSVAKIYHTPPPALKQDKLAFMAATADAQLLNYVAW

PQATLHGGRGGKVIGFMMPKVSGKEPIHMIYSPAHRRQRYPHCAWDFLLYVARNIASSFATVHEHGHVVG

DVNQNSFMVGRDSKVVLIDSDSFQINANGTLHLCEVGVSHFTPPELQTLSSFVGFERTENHGNFGLALLI

FHVLFGGRHPYSGVPLISDAGNALETDITHFRYAYASDNQRRGLKPPPRSIPLSMLPSDVEAMFQQAFTE

SGVATGRPTAKAWVAALDSLRQQLKKCPVSAMHVYPAHLTDCPWCTLDNQGVIYFIDLGEEVITTGGDFV

LAKVWAMVMASVAPPALQLSLPDHFQPTGRPLPLGLLRREYIILLEIALSALSLLLCGLQAEPRYIILVP

VLAAIWIIGSLTSKAYKAEVQQRREAFNRAKMDYDHLVRQIQQVGGLEGFIAKRTMLEKMKDEILGLPEE

EKLALAALHDTARERQK

>gi|313649880|gb|EFS14300.1| efflux transporter, RND family, MFP subunit [Shigella flexneri 2a str. 2457T]

MLDNLIGARYLTGLGTITAANTVTVRSRVDGQLMALHFQEGQQVKAGDLLAEIDPSQFKVALAQTQGQLA

KDKATLANARRDLARYQQLAKTNLVSRQELDAQQALVSETEGTIKADEASVASAQLQLDWSRITAPVDGR

VGLKQVDVGNQISSGDTTGIVVITQTHPIDLVFTLPESDIATVVQAQKAGKPLMVEAWDRTNSKKLSEGT

LLSLDNQIDATTGTIKVKARFNNQDDALFPNQFVNARMLVDTEQNAVVIPTAALQMGNEGHFVWVLNSEN

KVSKHLVTPGIQDSQKVVIRAGISAGDRVVTDGIDRLTEGAKVEVVEAQSATTPEEKATSREYAKKGARS

>gi|313649879|gb|EFS14299.1| multidrug resistance protein mdtB [Shigella flexneri 2a str. 2457T]

MRPVATTLLMVAILLAGIIGYRALPVSALPEVDYPTIQVVTLYPGASPDVMTSAVTAPLERQFGQMSGLK

QMSSQSSGGASVITLQFQLTLPLNVAEQEVQAAINAATNLLPSDLPNPPVYSKVNPADPPIMTLAVTSTA

MPMTQVEDMVETRVAQKISQISGVGLVTLSGGQRPAVRVKLNAQAIAALGLTSETVRTAITGANVNSAKG

SLDGPSRAVTLSANDQMQSAEEYRQLIIAYQNGAPIRLGDVATVEQGAENSWLGAWANKEQAIVMNVQRQ

PGANIISTADSIRQMLPQLTESLPKSVKVTVLSDRTTNIRASVDDTQFELMMAIALVVMIIYLFLRNIPA

TIIPGVAVPLSLIGTFAVMVFLDFSINNLTLMALTIATGFVVDDAIVVIENISRYIEKGEKPLAAALKGA

GEIGFTIISLTFSLIAVLIPLLFMGDIVGRLFREFAITLAVAILISAVVSLTLTPMMCARMLSQESLRKQ

NRFSRASEKMFDRIIAAYGRGLAKVLNHPWLTLSVALSTLLLSVLLWVFIPKGFFPVQDNGIIQGTLQAP

QSSSFANMAQRQRQVADVILQDPAVQSLTSFVGVDGTNPSLNSARLQINLKPLDERDDRVQKVIARLQTA

VDKVPGVDLFLQPTQDLTIDTQVSRTQYQFTLQATSLDALSTWVPQLMEKLQQLPQLSDVSSDWQDKGLV

AYVNVDRDSASRLGISMADVDNALYNAFGQRLISTIYTQANQYRVVLEHNTENTPGLAALDTIRLTSSDG

GVVPLSSIAKIEQRFAPLSINHLDQFPVTTISFNVPDNYSLGDAVQAIMDTEKTLNLPVDITTQF

>gi|313649850|gb|EFS14270.1| nickel/cobalt efflux system rcnA domain protein [Shigella flexneri 2a str. 2457T]

MTEFTTLLQQGNAWFFIPSAILLGALHGLEPGHSKTMMAAFIIAIKGTIKQAVMLGLAATISHTAVVWLI

AFGGMVISKRFTAQSAEPWLQLISAVIIIGTAFWMFWRT

>gi|313649849|gb|EFS14269.1| nickel/cobalt efflux system rcnA [Shigella flexneri 2a str. 2457T]

MHGHDYEHHHHHHDHEHHHDQGHHHHHEHGEYQDAHARAHANDIKRRFDGREVINWQILLFGLTGGFIPC

PAAITVLLICIQLKALTLGATLVVSFSIGLALTLVTVGVGAAISVQQVAKRWSGFNTLAKRAPYFSSLLI

GLVGVYMGVHGFMGIMR

>gi|313649847|gb|EFS14267.1| uncharacterized protein yehA [Shigella flexneri 2a str. 2457T]

MEIRIMLFILMMMVMPVSYAACYSELSVQHNLVVQGNFALTQTQIATYEHNFNDSSCVSTNTITPMSPSD

IIVGLYNDTIKLNLHFEWTNKNNITLSNNQSSFTSGYSVTVTPAASNAKVNISAGGGGSVMINGVATLSS

ASSSTRGSAAVQFLLCLLGGKSWGACVNSYRNALAQNAGVYSFNLTLSYNPITTTCKQDDLLITLDSIPV

LQLPATGNKATINSKKRDIILRCKNLLGQQNQTSRKMQVYLSSSDLLTNSNTILKGAEDNA

>gi|313649845|gb|EFS14265.1| gram-negative pili assembly chaperone [Shigella flexneri 2a str. 2457T]

MVLPAHAGIVIYGTRIIYPAENKEVMVQLMNQGNRSSLLQAWIDDGDTSLPPEKIQVPFMLTPPVAKIGA

NSGQQVKIKIMPNKLPTNKESIFYLNVLYIPPNSPEQEGKNALKFAMQNRIKLFYRPAGIAPVNKATFKK

LLVNRSGNGLVIKNDSANWVTISDVKANNVKVNYETIMIAPLESQSVNIKSNNANNWYLTIINDHGNYIS

DKI

>gi|313649835|gb|EFS14255.1| putative molybdate metabolism regulator domain protein [Shigella flexneri 2a str. 2457T]

MVVSAIASTPQYVEQAKDNSLQPSQTVTDSLKVADLSTIIQEQPSFVAETRAADKNTDAVLPWLAKDIAV

VFPPEVVHTTLSHRRFPGVPVQQADKLTQLRRLACSVSQRDNKTATFDFSTCSLEWQNTVAQAISQIDGL

KTTQLPSPVMAVLTALEMKCTRYKVREDVMDQIVQEGSLEYATDVIIHLQQIDIKWDGNAANLLI

>gi|313649832|gb|EFS14252.1| putative molybdate metabolism regulator domain protein [Shigella flexneri 2a str. 2457T]

MEQYSRFELRLRLRKHLSLAEESLWQKCAQKLIAAIPHIPEWRQPLIALLLPEKPEIAHEIAQHLLGQKKLP

SLEWLKIVATDEHILASLEKYHEPYAIFDDYYCGAIWSATVLQEQGVAALPRFAPYAASDYCADVLRHIN

HPFALTLLIRVAGHTKRCHDRMTKACAAFPHAALAALAELLVQKEENSWRIMLMTMFISQPALAEQVIPW

LSTPAVAVLKSCQHQLTQPSNHASADLLPAIVVSPPWISKKKKSPIPVLDLAPLGIEPICYLTEEISNQL

LAKYIWYSKHITVSHEESTTNLLARMGFQRRIAGTYIKAPEAVVEAWLNEDYSTLLSEFKVFHSPTGHYW

QLGILTTLPLEAGESSKSMECPYSISTYRYRIRHVTFWTQRFTWFS

>gi|313649831|gb|EFS14251.1| putative molybdate metabolism regulator domain protein [Shigella flexneri 2a str. 2457T]

MLHFGLKGLPGLVNSITRYPQEALPITNYFAASELAPAVARAFNKLKTLRENARTWLLKYPEHALTGLLP

AALGKAGEAQDNARAALRMLTENGHQPLLQEIARRYNQSEVTDAVNALLALDPLDNHPTKIPTLPTFYQP

SIWTRPVLKANAQSLPDNALLHLGEMLRFPQEEALYPGLLQVKDACTADSLAEFAWDLFTAWQTAGAPSK

ESWAFTALGVLGNDDTARKLTPLIRAWPGESQHKRATVGLDILAAIGSDIALMQLNGIAQKLKFKALQER

AKEKIADIAESRELTVAELEDRLAPDLGLDDNGSLLLDFGPRQFTVSFDETLKPFVRDVSGSRLKDLPKP

NKSDYESQANDAVNRYKLLKKDARTVAAQQVARLESAMCLRRRWSPENFQLFLVVHPLVRHLTRRLIWGV

YSADNQLLACFRVAEDNSYSTADDDLFTLPEGDISIGIPHVLEISPTDAVAFGQLFADYELLPPFRQLDR

NSYALTEAERNASELTRWAGRKCPSGRVMGLANKGWIKGEPQDGGWMIKPLGRWSLIMEIDEGFAVGISP

AELSAEQLLSKLWLWEGKAESYGWGSNSTQEAQFSVLDAITASELINDIEALFE

>gi|313649830|gb|EFS14250.1| putative molybdate metabolism regulator [Shigella flexneri 2a str. 2457T]

MDKELPWLADNAQLELKYKKGKTPLSHRNWPGEPVPVITESIIQTLGDELLQKAEKKKNIVWRYENFSPE

WQSAITQAINLIGEHKPSVPARTMAALAFIAQNDNQQLLDEIVQQEGLEYATEVVIARQFIARCYESDPL

VVTLQYQNEDYGYGYRSETYNEFDLRLRKHLSLAEESCWQRCADKLIAALPGITKVRRPFIALILPEKPE

IANELVSLECPRTHFHSKEWLKVVATDPKAVRKLERYWSQDIFSDREASYMSHENHFGYAACAALLREQG

LAVVPRLAIYAHKEDCGSLLVQINHPQVIRTLLLVADKNKPSLQRVAKYSKNFPHATLAALAELLALKEP

PARPGYPIIEDKKLPAQQKARDEYWRTLLQTLMASQPQLAAEVMPWLSTQARAVLNSYLSAPPKPVIDST

DNSQMPEILVSPPWRGRKKKTSIRHFSLPDLTLAPQTYRSPNKHWTPEQQTAIDHFSAMSFDERLATRGA

DIFLRELGFDVHSLEFNKRLLNGQLDAFDAQQLQEYTSIGLLRTYHAHQAKSKQYREDATNALLAQDSIA

LMGGWRTFQHGFYYGNEQRWGIWNLYLIAQMPREMAVSCWQQIVEADFHYTGVEYLLSVLGTDALPGLNT

AFARHPKEIFPLLIHFGATELALPIARVWHLFAGQRNLARQWILQWPEHTATALIPLVFTKSSDKSEAAL

LALRLLYEHGHGEWLQTVANRWQRTDLWPALEQLLKQSPIEIYPARIPKAPDFWHPQMWSRPRLITNNQP

VTDDALEIIGEMLRFTQGGRFYSGLEQLKTFCQPQTLAAFAWDLFTAWQQAGAPAKDNWAFLALSLFGDE

STARDLTTHILAWPQEGKSARAVSGMNILTLMNNDMALMQLHHISQRAKSRPLRDNAAEFLQVVAENRGL

SQEELADRLVPTLGLNDPQALSFDFGPRQFTVRFDENLNPVIFDQQNVRQKSVPRLRADDDQLKAPEALA

RLKGLKKDATQVSKNLLPRLEAALRTTRRWSLADFHSLFVNHPFTRLVTQRLIWGVIRQMNRVVYSTPFV

WPQRGSSAMRKMSQLTCLRTL

>gi|313649829|gb|EFS14249.1| putative molybdate metabolism regulator domain protein [Shigella flexneri 2a str. 2457T]

MTAEMRSEFAQLFADYEIMPPFRQLSRRTVLLTPDESTSNSLTRWEGKSATVGQLMGMRYKGWESGYEDA

FVYDLGEYRLVLKFSPGFNHYNVDSKALMSFRSLRVYRDNKSVTFAELDVFDLSEALSAPDVIFH

>gi|313649787|gb|EFS14210.1| hypothetical protein SF2457T_1760 [Shigella flexneri 2a str. 2457T]

MRVLLRPVLVPELGLVVLKPGRESMPVFHNTRVLVEPEPKSMRNLPSGVVPAVRQPLVEDKTLLPFFSNA

RVIRAAGGAGALSDWLLRHIKSCQWPHGDYHHSETVIHRYGTGAMVLCWHCDNQLRDQTSESLEQLAHQN

LSAWMIDVIGHAISGTQERELSLAELSWWAVRNQVADALPEAVLRRSLGLRAEKIRSMYRESDIVPGEQT

ATSILKQRTKNLAPLPHAHQQQNPPQEETVVSIAVDPESPAQYLQRQKPQREEMPVYTRWVKTQKCMTCG

NQADDPHHIIGHGLGGMGTKADDLFVIPLCRKCHNELHAGVKDFEEKHGSQLLLLIRFLMHARNSGVLKW

KA

>gi|313649785|gb|EFS14208.1| ycgB [Shigella flexneri 2a str. 2457T]

MKMTELTKEQLIEEAKLKIAITKCHPNSGMARVEGELFKIARASLEAEPIAWRYRYVKKGVMDSQGELWV

GDWKYVPKKEDCNDRPNYEIQALFTAPPVPVTSEELVKAVHFYEQLKRENPPASGNQINGLTMSVKRPAN

>gi|313649784|gb|EFS14207.1| phage integrase family protein [Shigella flexneri 2a str. 2457T]

MGRRRKNPEHEKLPPNVYPNKYSYVWKPTSRESVTLTAIKDGLAALWKKYEETVNNRDRAMTFGRLWEKF

LASAYYSDLSPRTQKDYLQHQKKLLAVFGKVPADSIKPEHIRRYMDKRGEQSKTQANHEKSSMSRVYSWG

YERGYVKANPCAGVSKFKAKNRERYVTDKEYQAVLSVAPLPVFIAMEIAYLCAARVSDVLSLKWEQIGND

GIFIQQGKTGKKQIKAWSPRLQAAIEKAKQLPTSAYVISNQYGNRYMYKGFNEMWVEARNRAGKISGILT

DFTFHDLKAKGISDYEGSSRDKQLFSGHKTEGQVLIYDRKVKVSPTLDVPLPENIPRKYSK

>gi|313649777|gb|EFS14200.1| shikimate transporter domain protein [Shigella flexneri 2a str. 2457T]

MAKFSVPLQVLGALWVRNGMEESAEFEQQQHNQAAAKKRIPVIEALLRHPGAFLKIIALRLCELLTMYIV

TAFALNYSTQNMGLPRELFLNIGLLVGGLSCLTIPCFAWLADRFGRRRVYITGALIGTLSAFPFFMALEA

QSIFWIVFFSIMLANIAHDMVVCVQQPMFTEM

>gi|313649770|gb|EFS14193.1| uncharacterized transporter yeeO domain protein [Shigella flexneri 2a str. 2457T]

MGLTISRYIGAVAILWVLAIGFNPALRISLKSYFKPLNFSIIWEVMGIGIPASVESVLFTSGRLLTQMFV

AGMGTSVIAGNFIAFSIAALINLPGSALGSASTIITGRRLGVGQIAQAEIQLRHVFWLSTLGLTAIAWLT

APFAGVMASFYTQDPQVKHVVVILI

>gi|313649769|gb|EFS14192.1| uncharacterized transporter yeeO domain protein [Shigella flexneri 2a str. 2457T]

MGVLSTFLVSWLGKDAMAGVGLADSFNMVIMAFFAAIDLGTTVVVAFSLGKRDRRRARVATRQSLVIMTL

FAVLLATLIHHFGEQIIDFVAGDATTEVKALALTYLELTVLSYPAAAITLIGSGALRGAGNTKIPLLING

SLNILNIIISGILIYGLFSWSGLGWV

>gi|313649763|gb|EFS14186.1| bifunctional adenosylcobalamin biosynthesis protein cobU [Shigella flexneri 2a str. 2457T]

MVVSAIASTPHSSQVLYIATSQILDDEMAARIEHHRQSRPEHWRTVERWQHLDELIHADINPNEAVLLEC

VTTMVTNLLFDYGGDKDPDEWDYQAMEQAINAEIQSLIAACQRCPAKVVLVTNEVGMGIVPESRLARHFR

DIAGRVNQQLAAAANEVWLVVSGIGVKIK

>gi|313649757|gb|EFS14180.1| reverse transcriptase family protein [Shigella flexneri 2a str. 2457T]

MQRKLATWAATDPSLRIQRLLRLITQPEWLAEAARITLSSKGAHTPGVDGVNKTMLQARLAVELQILRDE

LLSGHYQPLPARRVYIPKSNGKLRPLGIPALRDRIVQRAMLMAMEPIWESDFHTLSYGFRPERSVHHAIR

TVKLQLTDCGETRGRWVIEGDLSSYFDTVHHRLLMKAVRRRISDARFMTLLWKTIKAGHIDVGLFRAASE

GVPQGGVISPLLSNIMLNEFDQYLHERYLSGKARKDRWYWNNSIQRGRSTAVRENWQWKPAVAYCRYADD

FVLIVKGTKAQAEAIREECRGVLEGSLKLRLNMDKTKITHVNDGFIFLGHRIIRKRSRYGEMRVVSTIPQ

EKARNFAASLTALLSGNYSESKVDMAEQLNRKLKGWAMFYQFVDFKAKVFSYIDRVVFWKLAHWLARKYR

TGIASLMRWWCKSPKPGQSKTWVLFGKTNHGKLSGEILYRLVGQGKKLFRWRLPEGNPYLRTETRNTYTS

RFTEVAMAFASI

>gi|313649753|gb|EFS14176.1| inner membrane yeeA domain protein [Shigella flexneri 2a str. 2457T]

MGAIVARQDNFHKTSLMGRLRHFVLLNAQKLCDTQHMMQQILLSLVHALYEGNPQPVFANTEKLNDAVEE

LRQLLDNHHDLKVVETPIYGYVWLNMETAHQLELLSNLICRALRK

>gi|313649726|gb|EFS14150.1| bacterial regulatory helix-turn-helix , lysR family protein [Shigella flexneri 2a str. 2457T]

MDSNNQIEPCLSRKSSEGKPQIFTTLRNIDLNLLTIFEAVYVHKGIVNAAKVLNLTPSAISQSIQKLRVI

FPDPLFIRKGQGVTPTAFAMHLHEYISQGLESILGALDIEGSYDKQRTITIATTPSVGALVLPVIYRAIK

THYPQLLLRNPPISDAENQLSQFQTDLIIDNMFCTNRTVQHHVLFTDNMVLICRKGNPLLSLEDDRETID

NAAHVLLLPEGQNFSGLRQRVQEMFPDRQINFTSYNILTIAALVANSDMLAIIPSRFYNLFSRCWPLEKL

PFPSLNEEQIDFSIHYNKFILRDPILHGVIDVIRNAF

>gi|313649670|gb|EFS14094.1| KDP operon transcriptional regulatory protein kdpE [Shigella flexneri 2a str. 2457T]

MTNVLIVEDEQAIRRFLRTALEGDGMRVFEAETLQRGLLEAATRKPDLIILDLGLPDGDGIEFIRDLRQW

SPVPVIVLSARSEESDKIAALDAGADDYLSKPFGIGELQARLRVALRRHSATPPPPRPIRW

>gi|313649669|gb|EFS14093.1| sensor kdpD domain protein [Shigella flexneri 2a str. 2457T]

MYVETPALHRLPEKKRRAILSALRLAQELGAETATLSDPAEEKAVVRYAREHNLGKIILGRPASRRWWRR

ETFADRLARIAPDLDQVLVALDEPPARTINNAPDSRSFKDKWRVQIQGCVVAAALCAVITLIAMQWLMAF

DAANLVMLYLLGVVVALFYGRWPSVVATVINVVSFDLFFIAPRGTLAVSDVQYLLTFAVMLTVGLVIGNL

TAGVRYQARVARYREQRTRHLYEMSKALAVGRSPQDIAATSEQFIASTFHARSQVLLPDDNGKLQPLTHP

QGITPWDDAIAQWSFDKGLPAGAGTDTLPGVPYQILPLKSGEKTYGLVVVEPGNLRQLMIPEQQRLLETF

TLLVANALERLTLTASEEQARMASEREQIRNALLAALSHDLRTPLTVLFGQAEILTLDLASEGSPHARQA

SEIRQHVLNTTRLVNNLLDMARIQSGGFNLKKEWLTLEEVVGSALQMPEPGLSSPINLSLPEPLTLIHVD

GPLFERVLINLLENAVKYAGAQAEIGIDAHVEGENLQLDVWDNGPGLPPGQEQTIFDKFARGNKESAVPG

VGLGLAICRAIVDVHGGTITAFNRPEGGACFRVTLPQQTAPELEDFHEDM

>gi|313649668|gb|EFS14092.1| sensor kdpD domain protein [Shigella flexneri 2a str. 2457T]

MNNEPLRPDPDRLLEQTAAPHRGKLKVFFSACAGVGKTWAMLAEAQRLRAQGLDIVVGVVETHGRKDTAA

MLEGLAVLPLKRQAYRGRHISEFDLDAALARRPALILMDELAHSNAPGSRHPKRWQDIEELLEAGIDVFT

TVNVQHLESLNDVVSGVTGIQVRETVPDPFFDAADDVVLVDLPPDDLRQRLKEGKVYIAGQAERAIEHFF

RKGNLIALRELALRRTADRVDEQMRAWRGHPGEEKVWHTRDAILLCIGHNTGSENWSARRRG

>gi|313649652|gb|EFS14076.1| allophanate hydrolase subunit 2 family protein [Shigella flexneri 2a str. 2457T]

MLKIIRAGMYTTVQDGGRHGFCQSGISHCGALDMPALRIANLLVGNDANAPALEITLGQLTVEFETDGWF

ALTGAGCEARLDDNAVWTGWRLPMKAGQRLTLKRPQHGMRSYLAVAGGIDVPPVMGSCSTDLKVGIGGLE

GRLLKDGD

>gi|313649651|gb|EFS14075.1| allophanate hydrolase subunit 2 family protein [Shigella flexneri 2a str. 2457T]

MEAQGVKQLLWGNRIRALPGPEYHEFDRASQDAFWRSPWQLSSQSNRMGYRLQGQILKRITDRELLSHGL

LPGVVQVPHNGQPIVLMNDAQTTGGYPRIACIIEADMYHLAQIPLGQPIHFVQCSLEEALKARQDQQRYF

EQLAWRLHNEN

>gi|313649647|gb|EFS14071.1| uncharacterized ybgO domain protein [Shigella flexneri 2a str. 2457T]

MIDFGKFNLLDIRRHTMSKTFSIKTTKSQNDQCTDGFKVSSSFYTEETLVEEDKALLIGNGLKLRLLDEN

ASPYTFNKYAEYADFTSDMLVYEKTYTAELSSIAGTPIEAGPFDTVVLFKINYN

>gi|313649597|gb|EFS14021.1| phage integrase family protein [Shigella flexneri 2a str. 2457T]

MPCFTAMRAEIALMSGSAFAVTHHAFSSGAGRTSDGNGSHASTLKSPSYTKSVSWQHYRLAEEDPASATR

PFRTEVKRSRLLIDEYLLIRKIADVQNEWFGLCMDLALVTGQRKGDLAAMRWEDIRDSRLYVEQQKTGAK

IRISLPTTISRLNLTLADVLDNLKKINGKNEKLLGGKTARTIAAQFRIARDTSGLKWEGDPPPFHEIRSL

SGRLHSAEKGSDFTQALLGHRSSSMTDKYRDGRGREWKDI

>gi|313649590|gb|EFS14014.1| UPF0190 protein yedY [Shigella flexneri 2a str. 2457T]

MAKPLTLDHDDLTRRFPLEERIYRMRCVEAWSMVVPWIGFPLHKLLALAEPTSNAKYVAFETIYAPEQMP

GQQDRFIGGGLKYPYVEGLRLDEAMHPLTLMTVGVYGKALPPQNGAPVRLIVPWKYGFKGIKSIVSIKLT

RERPPTTWNLAAPDEYGFYANVNPHVDHPRWSQATERFIGSGGILDVQRQPTLLFNGYADQVASLYRGLD

LRENF

>gi|313649580|gb|EFS14005.1| hypothetical protein SF2457T_1962 [Shigella flexneri 2a str. 2457T]

MTSTLTSKPTLNPALRSFWTMRARNKVLYGGRSSSKSWDAAGIAIFLSNKYTLRFCCARQIQNKIEESVY

TLLKIQIDRFGLRHRFRILNNKIINRVTGSEFVFYGLWRNIEEIKSLEGIDMLWLEEAHALTEYQWKILE

PTIRKEGSECWFIFNPGLVTDFVWRNFVVDPPEGTLIRKINYDENPFLSDTMLKVIDAARRRDPDGFKHV

YEGVPESDDDAAIRLAP

>gi|313649572|gb|EFS13999.1| beta-lactamase family protein [Shigella flexneri 2a str. 2457T]

MSIQHFRVALIPFFAAFCLPVFAHPETLVKVKDAEDQLGARVGYIELDLNSGKILESFRPEERFPMMSTF

KVLLCGAVLSRIDAGQEQLGRRIHYSQNDLVEYSPVTEKHLTDGMTVRELCSAAITMSDNTAANLLLTTI

GGPKELTAFLHNMGDHVTRLDRWEPELNEAIPNDERDTTMPVAMATTLRKLLTGELLTLASRQQLIDWME

ADKVAGPLLRSALPAGWFIADKSGAGERGSRGIIAALGPDGKPSRIVVIYTTGSQATMDERNRQIAEIGA

SLIKHW

>gi|313649558|gb|EFS13988.1| host specificity protein J [Shigella flexneri 2a str. 2457T]

MEREEWVKASSKGHTPREAKDNLKSTQLLSVIDAISEGPVDGPVDGLKSVLLNGTPVLDSEGKTNFSGVT

VVFRAGEQEQTPPEGFESSGSETVLGTEVKYDTPITRTITSANIDRLRLTFGVQALVETTSKGDRNPSEV

RLLVQIQRNGGWVTEKDITIKGKTTSQYLASVVVGNLPPRPFNIRMRRMTPDSTTDQLQNKTLWSSYTEI

IDVKQCYPNTALVGVQVDSEQFGNQQVSRNYHLRGRILQVPSNYNPQTRQYSGIWDGTLKPAYSNNMAWC

LWDMLTHPRYGMGKRLGAADVDKWALYVIGQYCDQSVPDGFGGTEPRITCNAYLTTQRKAWDVLSDFCSA

MRCMPVWNGQTLTFVQDRPSDKVWTYNRSNVVMPDDGAPFRYSFSALKDRHNVVEVNWIDPDNGHETATE

LVEDTQAIVRYGRNVTKMDAFGCTSRGQAHRAGLWLIKTELLETQTVDFSVGAEGLRHVPGDVIEICDDD

YAGISTGGRVLAVNSQTRTLTLDREITLPSSGTTLISLVDGNGNPVSVEVQSVTDGVKVKVSRVPDGVAE

YSVWGLKLPTLRQRLFRCVSIRENDDGTYAITAVQHVPEKEAIVDNGAHFDGDRRGTVNGVTPPAVQHLT

AEVTADSGEYQVLARWDTPKVVKGVSFLLRLTVAADDGSERLVSTARTTETTYRFRQLALGNYSLTVRAV

NARGQQGDPASVSFRIAAPAAPVTIELIPGYFQITVVPKLAVYDPTVQFEFWFSEKRIADIRQVETSARY

LGTALYWIAASINIRPGHDYYFYVRSVNTVGKSAFVEAVGRASDDAEGYLSFYKGLINKTHLGKELWTQI

DNGQLAPDLTEIRTSITNVSNEITQTVNKKLENQSAAIQQIQKVQVDTNNNLNSMWAVKLQQMKDGRLYI

AGIGAGIENTPAGMQSQVLLAADRIAMINPANGNTKPMFVGQGDQIFMNDVFLKRLTAPTITSGGNPPAF

SLTPGGRLTAKNADISGNVNANSGTLNNVTINKNCRVLGKLSANQIEGDLVKTVGKPFPRDSRAPERWPS

GTITVRVYDDQPFDRQIVIPAVAFRGAKHERKNNNIYSSCRLIVKKNGAEIYNRTTLDNTLIYTGVIDMP

AGHGHMTLEFSVSAWLVNGWYPTASISDLLVVVMKKSTAGISIS

>gi|313649531|gb|EFS13962.1| X-Pro dipeptidyl-peptidase family protein [Shigella flexneri 2a str. 2457T]

MMNNKVSFTNSNNPTISLSAVIYFPPKFDETRQYPAIVVSHPGGGVKEQTAGTYAEKLAEKGFVTIAYDA

SYQGESGGEPRQLENPYIRTEDVSAVIDYLTTLSYVDNTRIGAMGICAGAGYTANAAIQDRRIKAIGTVS

AVNIGSMFRNGWENNVKSIDALPHIEAGSNARTSDISSEEYAIMPLAPMKEADAPNEELRQAWEYYHTPR

AQYPTAPGYATLRSLNQIITYDAYHMAEVYLTQPMQIVAGSQAGSKWMSDDLYDRASSQDKRYHIVEGAN

HMDLYDGKAYVAEAISVLAPFFEETL

>gi|313649528|gb|EFS13959.1| alpha/beta superfamily hydrolase domain protein [Shigella flexneri 2a str. 2457T]

MSITGVNIGRLFREGFSNYDPIGVLNAMASQRAKEARGGELQINELLPASLDAAKAHGLTERDVYEATDY

YKTPRGQQPGGATKMLFSHAQKTLAWDAFAFTEVLLTQPVMVVVGEKVGAFGAYRDGLEVYGRAMVSQDR

QLVSLPDFSHYELYDKPEAVQEALAKVIPFFNTHLG

>gi|313649518|gb|EFS13949.1| outer membrane protein G [Shigella flexneri 2a str. 2457T]

MACAQAEEKSDWHFNIGAMYEIENVEGYGEDMDGLAEPSVYFNAANGPWRIALAYYQEGPVDYSAGKRGT

RFDRPELEVHYQFLESDDFSFGLTGGFRNYGYHYVDEPGKDTANMQRWKIAPDWDVKLTDDLRFNGWLSM

YKFANDLNTTGYADTRVETETGLQYTFNETVALRVNYYLERGFNMDDSRNNGEFSTQEIRAYLPLTLGNH

SVTPYTRIGLDR

>gi|313649510|gb|EFS13941.1| putative transient receptor potential locus [Shigella flexneri 2a str. 2457T]

MKIGTQNQAFFPENIRERFRYIKEMGFDGFEIDGKLLVNNIEEVKAAIKETGLPVTTACGGYDGWIGDFI

EERRLNGLKQIERILEALAEVGGFRQRGACLPSAYRR

>gi|313649506|gb|EFS13937.1| extracellular solute-binding family 1 domain protein [Shigella flexneri 2a str. 2457T]

MMQALTYYRDLAANTMPGSNDIMEVKDAFMNGTAPMAIYSTYILPAVIKEGDPKNVGFVVPTEKNSAVYG

MLTSLTITAGQKTEAAEKFVTFMEQADNIADWVMMSPGAALPVNKAVVTTATWKDNDVIKALGELPNQLI

GELPNIQVFGAVGDKNFTRMGDVTGSGVVSSMVHNVTVGKADLPGTLQASQKKLDELIEQH

>gi|313649505|gb|EFS13936.1| bacterial extracellular solute-binding family protein [Shigella flexneri 2a str. 2457T]

MIKSKIVLLSALVSCALISGCKEENKTHVSIEFMHSSVEQERQAVISKLIARFEKENPGITVKQVPVEED

AHNTKVITLSRSGSLPEVIETSHDYAKVMDKEQLIDRKAVATVISNVGEGAFYDGVLRIVRTEDGSAWTG

VPVSAWIGGIWYRKDVLAKAGLEEPKNWQQLLDVAQKLNDPANKKYGIALPTAESVLTEQSFSQFALSN

>gi|313649504|gb|EFS13935.1| putative sucrose phosphorylase domain protein [Shigella flexneri 2a str. 2457T]

MNVTYMNALSRRESSDEERCTRFILAHAILLSFPGVPAIYIQSILGSRNDYAGVEKLGYNRAINRKKYHS

EEITRELNDEATLRHAVYHELSRLITLRRSHNEFHPDNNFTIDTVNSSVMRIQRSNADGNCLTGLFNVSK

NIQHVNITDLHGRDLISEVDILGNEITLRPWQVMWIK

>gi|313649477|gb|EFS13908.1| gmr domain protein [Shigella flexneri 2a str. 2457T]

MQLSAEQAARIREMTVITSSLMMSLTVDESDLSVHLVGRKINKREWAGNASAWHDTPAVARDLSHGLSFA

EQVVSEAHSAIVILDSRGNIQRFNRLCEDYTGLKEHDVIG

>gi|313649476|gb|EFS13907.1| gmr domain protein [Shigella flexneri 2a str. 2457T]

MSRREAAASRRNNRVFFRSGNAYEVELWIPTRKGQRLFLFRNKFVHSGSGKNEIFLICSGTDITEERRTQ

ERLRILANTDSITGLPNRNAMQELIDHAINQADNNKVGVVYLDLDNFKKVNDAYGHLFGDQLLRDVSLAI

LSCLEHDQVLARPGGDEFLVLASNTSQSALEAMASRILTRLRLPFRIGLIEVYTSCSVGISLSPEHGSDS

ATIIRHADTAMYTAKEGGRGQFCVFTPEMNQRVFEYLWLDTNLRKALENDQLVIENHLAWRSAQSGSTST

LAVT

>gi|313649475|gb|EFS13906.1| gmr domain protein [Shigella flexneri 2a str. 2457T]

MDFISYAEESGLIVPLGRWVILDVVRQVAKWRDKGINLRVAVNISARQLADQTIFTALKQVLQELNFEYC

PIDVELTESCLIENDELALSVIQQFSQLGAQVHLDDFGTGYSSLSQLARFPIDAIKLDQVFVRDIHKQPV

SQSLVRAIVAVAQALNLQVIAEGVESAKEDAFLTKNGINERQGFLFAKPMPAVAFERWYKRYLKRA

>gi|313649443|gb|EFS13874.1| protein tonB [Shigella flexneri 2a str. 2457T]

MIMTSITLDLPRRFPWPTLLSVCIHGAVVAGLLYTSVHQVIELPAPAQPISVTMVAPADLEPPQAVQPPP

EPVVEPEPEPEPIPEPPKEAPVVIEKPKPKPKPKPKPVKKVQEQPKRDVKPVESRPASPFENTAPARPTS

STATAATSKPVTSVASGPRALSRNQPQYPARAQALRIEGQVKVKFDVTPDGRVDNVQILSAKPANMFERE

VKNAMRRWRYEPGKPGSGIVVNILFKINGTTEIQ

>gi|313649377|gb|EFS13808.1| TonB dependent receptor protein [Shigella flexneri 2a str. 2457T]

MHASFAASSDLGYLQVSGSQLKQDFLGLPHGVNNDIAGKHGKMINSSADDKRGIVKLGFTPRENDEYTLT

YIKQDGEKDNPPYSGNCGQKSRYWQWPEYDKESFYYQGATQLNDRFTLKSRLYRDTFENTLMMYNSLADL

KNKKGSYSHYSDYSDGAGLQLAADVRENDLLSFAVNWKDDVHREKGAPHAAYDRYEDRTWSLASEYQWAA

ADNVDVVAGISYDWRDSVEAKKHEKDGSITHYDDNNQSAFNWQVMGKYHFANEDTLALSYYDRTRFPTLK

ERYTTSKPAYNQIAIVNPQLKPERARGVDLTWNGAFTRDWGFEVSVYYNRVSDAILSHNINADTIQNQNS

GTVDYSGLDAGIKGKISNILDVGLSYALIHADAKRKDIGKITDLPTQTMTAWMTLKPWEPLSVTLSEEAR

SSSYSNSDGSQKAAGFAVTHIRADYTLGHGFSVNASVNNLFDTQYAYSEGFIEEGRNFWAGVEYTF

>gi|313649360|gb|EFS13791.1| hemolysin E, chromosomal domain protein [Shigella flexneri 2a str. 2457T]

MTEIVADKTVEVVKNAIETADGALDLYNKYLDQVIPWQTFDETIKELSRFKQEYSQAASVLVGDIKTLLM

DSQDKYFEATQTVYEWCGVATQLLAAYILLFDEYNEKKASAPH

>gi|313649346|gb|EFS13778.1| hypothetical protein SF2457T_2217 [Shigella flexneri 2a str. 2457T]

MIYPTNTGKSGEHLRLTTLESVWIQGKLRMWGRWSYIGGGKTGNMFNQLLASKKLTKTAVNEALRRMKKA

GIEKPELEAFLREMINGKQKTWLAHCTDAEALCIDRVISEVLAEHPGLISVLRQRYEGRGMTKRKMAELL

NDAHPEWCFRTCCSRVDVWLNLAEYMLYLPMRDAFSSGDLKTVC

>gi|313649344|gb|EFS13776.1| hypothetical protein SF2457T_2215 [Shigella flexneri 2a str. 2457T]

MRNYAFHKRFFKLLQLGFDYWTPVGGAITPRERKLVSGFVDYLCESVGREHTPALSDAAEQYLNTVATCR

TRDTALLKSFDAFREWVTIQAGFYTEHIYPDGSRGRRAKSIAFANMDETEFQQVYKSVLNVLWNWILFRK

FSSPEEVENVAAQLLEFA

>gi|313649343|gb|EFS13775.1| putative transposase [Shigella flexneri 2a str. 2457T]

MPCFTAMRAEIALMSGSAFAVTHHAFSSGAGRTSDGNGSHASTLKSPSYTKSVSWQHYPVSIGINSGILH

NGLNTHSTAPAENYDGAYTTGQTNLFRITSPGDAGELRFFASNQNR

>gi|313649339|gb|EFS13771.1| hypothetical protein SF2457T_2210 [Shigella flexneri 2a str. 2457T]

MINRTKLEHILEYARQQKCIGQLCKIPPGDMVEIVEMAMRKAGNSPVTLAGWISCSDAVPAEYCDVILLD

DLGNVFPGSWDKVFCPTRGGNKMAFVDKDGVEVESSTHWMPLPEPPQEVNQ

>gi|313649338|gb|EFS13770.1| hypothetical protein SF2457T_2209 [Shigella flexneri 2a str. 2457T]

MTTITKERLQWLANISGRDDIDDIDGGEIRELALIALASLEAEPVAECIVEDGGMCIDGFGEYVGHSLPD

GMHELYAAPRMRQPDGWKAVAVAWKVTFTQVDQESNTFTAIYFYKAEVERWVRLHKACDFRADITPLYAA

PAVPVAVNDDMAYAFHHALSDSSLGSDEIEEIKTGLRAAFANVTIQPEPIVPDEIGPNDSNTFDYVDGWN

ACRAAMLKGDK

>gi|313649337|gb|EFS13769.1| HNH endonuclease family protein [Shigella flexneri 2a str. 2457T]

MALTKKQRAELRMKFGGRCAYCGCELGEKWHADHVKPVIRFDGNMLHQERDDISNMVPECHPCNLHKHCS

SLEDYRRIISDGRREFLASGKGKALVRMGLVEMKSDPVVFWFEKYQEGATA

>gi|313649333|gb|EFS13765.1| DNA replication protein dnaC [Shigella flexneri 2a str. 2457T]

MKNIATGGVLERIRRLTPPHVTAPFRTVAEWREWQLAEGQKRCEEINRLNRQLRVEKILNRSGIQPLHRK

CSFANYRAQNDGQRHALSQAKSIADELMTGCTNFVFSGKPGTGKNHLAAAIGNRLMAKGRSVIIVTVSDV

MSVLHESYDNGKSGEKFLQELCGVDLLVLDEIGMQRDTKNEQVVLNQIVDRRTASLRGVGMLTNINHAAM

NTLLGERVMDRMVMNGGRWVNFNWESWRPNVSHSRVVK

>gi|313649332|gb|EFS13764.1| uncharacterized ydaU domain protein [Shigella flexneri 2a str. 2457T]

MAGVLEGRLSGGTAAEFCNSAVVALQAAGLDVCREYPVPERGDGCGGRIDIVVTDRNGVRCGIELDRNSP

RQKSLLKIGAVETGICVLRRSDIARHTEQGILVIGGAVRQKKFDPLSVDLPDWLSETLWHEWVQFRQALR

KPIRTELGANGAIRELEKFRQQGFTPEQVIRHSIANEYQGLFAPKGVRPETLLRQVNTVSLPDSAIPPGF

RG

>gi|313649295|gb|EFS13728.1| phage anti-repressor domain protein [Shigella flexneri 2a str. 2457T]

MAQLYGTEPVRIRQNHHENKVRFVEGKHFFKVVGNDLKELRVALNYSQNPVSPKARSLILWTERGAARHG

KMLETDQAWDVFEKLEDCYFSQRDPSTPVSCQKSYDTRVLCYQRGGVTVSTIQLRDDDIVISLESWLELA

RANGWFVVRRDKLVERLMQL

>gi|313649288|gb|EFS13722.1| protein fimH [Shigella flexneri 2a str. 2457T]

MPLPLKLYITPVGAAGGVVIKAGEVIARIHMYKIATLGSGNPRNFTWNIISNNSVVMPTGGCTVDSRNVT

VDLPDFPGSAEIPLGVYCSSEQKLSFYLSGATTDSSRQVFANTAPDATKASGVGVTLMRNGKILATGENV

SLGTVNKSKVPLGLSATYGQTGNKVSAGTVQSVIGVTFIYE

>gi|313649258|gb|EFS13692.1| ankyrin repeat B domain protein [Shigella flexneri 2a str. 2457T]

MATGQTQQLITLFKQLPILPEKEIIEIITAQNSVGTPALFLAMMNGHTDNVKIFMQEIQSLVDNHIIHED

NLVKLLQTKSANETPGLYISMLYGFDEIIDIFLNTLTTPIALRAFKQKTGDEYFSHENT

>gi|313649238|gb|EFS13672.1| PTS system, cellobiose-specific IIC component [Shigella flexneri 2a str. 2457T]

MAPFFIGMALAEERKVDALAAGLLSVAAFMTVTPYSVGEAYAVGANWLGGANIISGIIIGLVVAEMFTFI

VRRNWVIKLPDSVPASVSRSFSALIPGFIILSVMGIIAWALNTWGTNFHQIIMDTISTPLASLGSVVGWA

YVIFVPLLWFFGIHGALALTALDNGIMTPWALENIATYQQYGSVEAALAAGKTFHIWAKPMLDSFIFLGG

SGATLGLILAIFIASRRADYRQVAKLALPSGIFQINEPILFGLPIIMNPVMFIPFVLVQPILAAITLAAY

YMGIIPPVTNIAPWTMPTGLGAFFNTNGSVAALLVALFNLGIATLIYLPFVVVANKAQNAIDKEESEEDI

ANP

>gi|313649230|gb|EFS13664.1| N-succinylarginine dihydrolase domain protein [Shigella flexneri 2a str. 2457T]

MAIEVPATQVSVSDAVSTYLFNSQLLSRDDGSMMLVLPQECREHAGVWGYLNELLAADNPISELKVFDLR

ESMANGGGPACLRLRVVLTEEERRAVNPAVMMNDTLFNALNDWVDRYYRDRLTAADLADPQLLREGREAL

DVLSQLLNLGSVYPFQREGGGNG

>gi|313649229|gb|EFS13663.1| N-succinylarginine dihydrolase [Shigella flexneri 2a str. 2457T]

MNAWEVNFDGLVGLTHHYAGLSFGNEASTRHRFQVSNPRLAAKQGLLKMKALADAGFPQAVIPPHERPFI

PVLRQLGFSGSDEQVLEKVARQAPHWLSSVSSASPMWVANAATIAPSADTLDGKVHLTVANLNNKFHRSL

EAPVTESLLKAIFKDEEKFSVHSALPQVALLGDEGAANHNRLGGHYGEPGMLLFVYGREEGNDTRPSRYP

ARQTREASEAVARLNQVNPQQVIFAQQNPDVIDQSVFHNDVIAVSNRQVLFCHQQAFARQSQLLARG

>gi|313649227|gb|EFS13661.1| arginine N-succinyltransferase [Shigella flexneri 2a str. 2457T]

MMVIRPVERSDVSALMQLASKTGGGLTSLPANEATLSARIERAIKTWQGELPKSEQGYVFVLEDSETGTV

AGICAIEVAVGLNDPWYNYRVGTLVHASKELNVYNALPTLFLSNDHTGSSELCTLFLDPDWRKEGNGYLL

SKSRFMFMAAFRDKFNDKVVAEMRGVIDEHGYSPFWQSLGKRFFSMDFSRADFLCGTGQKAFIAELMPKH

PIYTHFLSQEAQDVIGQVHPQTAPARAVLEKEGFRYRNYIDIFDGGPTLECDIDRVRAIRKSRLVEVAEG

QPAQGDFPACLVANENYHHFRVVLARTDPATERLILTAAQLDALKCHAGDRVRLVRLCAEEKTA

>gi|313649219|gb|EFS13653.1| extracellular solute-binding family 1 domain protein [Shigella flexneri 2a str. 2457T]

MRHCGWLLGLLSLFSLATHASDWQEIKNEAKGQTVWFNAWGGDTAINRYLDWVSGEMKTHYAINLKIVRL

ADAADAVKRIQTEAAAGRKTGGSVDLLWVNGENFRTLKE

>gi|313649216|gb|EFS13650.1| ynjB domain protein [Shigella flexneri 2a str. 2457T]

MWREGKDFPPSPARMDALLKAGTLRLSLTFNPAHAQQKIASGDLPASSYSFGFREGMIGNVHFVTIPANA

NASAAAKVVANFLLSPDAQLRKADPVVWGDPSVLDPQKLPDGQREILQSRMPQDLPPVLAEPHAGWVNAL

EQEWLRRYGTH

>gi|313649215|gb|EFS13649.1| inner membrane ABC transporter permease ynjC domain protein [Shigella flexneri 2a str. 2457T]

MVAVIYAPLIPAALTLISPALSLTRWQALFADPQLPQALLATLVSTTIAAVGALLIALLVIVALWPGPKW

QRMCARLPWLLAIPHVAFATSALLLFADGGLLYDYFPYFTPPMDRFGSVWASPLQ

>gi|313649214|gb|EFS13648.1| inner membrane ABC transporter permease ynjC domain protein [Shigella flexneri 2a str. 2457T]

MDLSGSIERKTAVAAGHCAGFAGLQPLAMAMLAIVAWSLSVVDVAIILGPGNPPTLAVISWQWLTQGDAD

QQTKGALASLLLMLLLAAYVLAQLAAHYSPRRWHSQACHTFITRHYAGEFFTLNRCAVCGSAGDPRGSVD

DQ

>gi|313649213|gb|EFS13647.1| inner membrane ABC transporter permease ynjC domain protein [Shigella flexneri 2a str. 2457T]

MVLLAILADQSTINSEALINSLTMGLVATFIALLLLLLWLEWGAQRRQLWLWLPILLPALPLVAGQYTLA

LWLNLDGSWTAVVWGHLLWVMPWMLFILQPAWQRIDSRLILIAQTLGWSRAKIFFYVKCPLMLRPVLIAF

AVGFSVSIAQYMPTLWLGAGRFPTLTTEAVALSSGGSNGILAAQALWQLLLPLIIFALTALVAKWVGYVR

QGIR

>gi|313649211|gb|EFS13645.1| putative thiosulfate sulfurtransferase ynjE domain protein [Shigella flexneri 2a str. 2457T]

MKRVSQMTALAMALGLACASSWAAELAKPLTLDQLQQQNGKAIDTRPSAFYNGWPQTLNGPSGHEPAALN

LSASWLDKMSTEQLNAWIKQHNLKADAPVALYGNGKDVDAVKTRLQKAGFTHISILSDALSEPSRLQKLP

HFEQLVYPQWLHDLQQGKEVTAKPVGDWKVIEAA

>gi|313649203|gb|EFS13637.1| inner membrane protein ynjI [Shigella flexneri 2a str. 2457T]

MKKVLLQNHPGSEKYSFNGWGIFNSNFERVIKENKAMLLCKWGFYLTCVVAVMFVFAAITSNGLNERGLI

TAGCSFLYLLIMMGLIVRAGFKAKKEQLHYYQAKGIEPLSIEKLQALQLIAPYRFYHKQWSETLEFWPRK

PEPGKDTFQYHVLPFDSIDIISKRRESLENQWGIEDSESYCALMEHFLSGDHGANTFKANMEEAPEQVIA

LLNKFAVFPSDYISDCANNRSGKSSAKLIWAAELSWMISISSTAFQNGTIEEELAWHYIMLASRKAHELF

ESEEDYQKNSLMGFLYWHICCYRRKLTDAELEACYRYDKQFWEHYSKKCRWPIRNVPWGASSVKYS

>gi|313649179|gb|EFS13613.1| GGDEF domain protein [Shigella flexneri 2a str. 2457T]

MSVKLRLPHAAEQSEVSPEQRRAICEAFESANNTHGLNLTAHKYPGLRGTLQTASTDCDTIVEAAALLPA

FDQAVEGNRHQDDYGSGLGMAEEKFHYYLDLNDRYVYFYEPVNVEYFAMNNWSFLQSGSIGIDRKDIEKV

FTGRTVLSSIYQDQRTKQNVMSLLTPVYVAGQLKGIVLLDINKNNLRNIFYTHDRPLLWRFLNVTLTDTD

SGRDIIINQSEDNLFQYVSYVHDLPGGIRVSLSIDILYFITSSRKSVLFWILTALILLNMVRMHFRLYQN

VSRENISDAMTGLYNRKILTPELEQRLQKLVQSGSSVMFIAIDMDKLKQINDTLGHQEGDLAITLHDAYK

ASDERLYVNKQNKNSRS

>gi|313649176|gb|EFS13610.1| bacterial regulatory helix-turn-helix s, AraC family protein [Shigella flexneri 2a str. 2457T]

MTRRLIQVLFDELPQQPQQQLHLPVSSHPKIRTMVEMMAKGPVEWGALGQWAGFFAMSERNLARLIVKET

GLSFRQWRQQLQLIMALQGLVKGDTVQKVAHTLGYDSTTAFITMFKKGLGQTPGRYIAGLTTVSPQSAKP

DPRQ

>gi|313649166|gb|EFS13600.1| rieske domain protein [Shigella flexneri 2a str. 2457T]

MNQYTGLGAKVLEACPEVHNLKLAARFTTRTPANWKNIVDNYLECYHCGPAHLGFSDSVQVDRYWHTMHG

NWTLQYGFAKPSEQSFKFEEGTDAAFHGFWLWPCTMLNVTPIKGMMTVIYEFPVDSETTLQNYDIYFTNE

ELTDEQKSLIEWYRDVFHPEDLRLVESVQKGLKSRGYRGQGRIMADSSGSGISEHGIAHFHNLLAQVFKD

>gi|313649137|gb|EFS13571.1| peptidase family M48 family protein [Shigella flexneri 2a str. 2457T]

MMRIALFLLTNLAVMVVFGLVLSLTGIQSSSVQGLMIMALLFGFGGSFVSLLMSKWMALRSVGGEVIEQP

RNERERWLVNTVATQARQAGIAMPQVAIYHAPDINAFATGARRDASLVAVSTGLLQNMSPDEAEAVIAHE

ISHIANGDMVTMTLIQGVVNTFVIFISRILAQLAAGFMGGNRDEGEESNGNPLIYFAVATVLELVFGILA

SIITMWFSRHREFHADAGSAKLVGREKMIAALQRLKTSYEPQEATSMMAFCINGKSKSLSELFMTHPPLD

KRIEALRTGEYLK

>gi|313649135|gb|EFS13569.1| proP effector [Shigella flexneri 2a str. 2457T]

MENQPKLNSSKEVIAFLAERFPHCFSAEGEARPLKIGIFQDLVDRVAGEMNLSKTQLRSALRLYTSSWRY

LYGVKPGATRVDLDGNPCGELDEQHVEHARKQLEEAKARVQAQRAEQQAKKREAAAAAGEKEDAPRRERK

PRPTTPRRKEGAERKPRAQKPVEKAPKTVKAPREEQHTPVSDISALTVGQALKVKAGQNAMDATVLEITK

DGVRVQLNSGMSLIVRAEHLVF

>gi|313649132|gb|EFS13566.1| uncharacterized yebT domain protein [Shigella flexneri 2a str. 2457T]

MSQETPASTTEAQIKNKRRISPFWLLPFIALMIAGWLIWDSYQDRGNTVTIDFMSADGIVPGRTPVRYQG

VEVGTVQDISLSDDLRKIEVKVSIKSDMKDALREETQFWLMTPKASLAGVSGLDALVGGNYIGMMPGKGK

EQDHFVALDTQPKYRLDNGDLMIHLQAPALGSLNSGSLVYFRKIPVGKVYDYAINPNKQGVVIDVLIERR

FTDLVKKGSRF

>gi|313649131|gb|EFS13565.1| uncharacterized yebT domain protein [Shigella flexneri 2a str. 2457T]

MKLESLAALVNGAIAFDSPEESKPAEAEDTFGLYEDLAHSQRGVIIKLELPSGAGLTADSTPLMYQGLEV

GQLTKLDLNPGGKVTGEMTVDPCFVKIPASNYATRNYPLAMLISAPC

>gi|313649130|gb|EFS13564.1| uncharacterized yebT domain protein [Shigella flexneri 2a str. 2457T]

MPGDGEPRKEFVVVPGEKALLQEPDVLTLTLTAPESYGIDAGQPLILHGVQVGQVIDRKLTSKGVTFTVA

IEPQHRELVKGDSKFVVNSRVDVKVGLDGVEFLGASASEWINGGIRILPGDKGEMKASYPLYANLEKALE

NSLSDLPTTTMSLSAETLPDVQAGSVVLYRKFEVGEVITVRPRANAFDIDLHIKPEYRNLLTSNSVFWAE

GGAKVQLNGSGLTVQASPLSRALKGAISFDNLSGASASQRKGDKRILYASETAARAVGRQITLHAFDAGK

LAVGMPIRYLGIDIGQIQTLDLITARNEVQAKAVLYPEYVQTFARGGTRFSVVTPQISAAGVEHLDTILQ

PYINVEPGRGNPRRDFELEEATITDSRYLDGLSIIVEAPEAGSLGIGTPVLFRGLEVGTVTGMTLGTLSD

RVMIAMRISKRYQHLVRNNSVFWLASGYSLDFGLTGGVVKTGTFNQFIRGGIAFATPPGTPLAPKAQEGK

HFLLQESEPKEWREWGTALPK

>gi|313649122|gb|EFS13556.1| serine/threonine-phosphatase 1 domain protein [Shigella flexneri 2a str. 2457T]

MKQPAPVYQRIAGHQWRHIWLSGDIHGCLEQLRRKLWHCRFDPWRDLLISVGDVIDRGPQSLRCLQLLEQ

HWVRAVRGNHEQMAMDALAFQQMSLWLMNGGDWFIALADNQQKQAKTALEKCQHLPFILEVHSRTGKHVI

AHADYPDDV

>gi|313649112|gb|EFS13547.1| uncharacterized protein yneA [Shigella flexneri 2a str. 2457T]

MCPALKRAMQRGVRVLTWDSDTKPECRSYYINQGTPAQLGGMLVDMAARQVNKDKAKVAFFYSSPTVTDQ

NQWVKEAKAKIAKEHPGWEIVTTQFGYNDATKSLQTAEGILKAYSDLDAIIAPDANALPAAAQAAENLKN

DKVAIVGFSTPNVMRPYVERGTVKEFGLWDVVQQGKISVYVADVLLKKGSMKTGDKLDIQGVGQVEVSPN

SVQGYDYEADGNGIVLLPERVIFNKENIGKYDF

>gi|313649075|gb|EFS13510.1| inner membrane metabolite transport ydfJ domain protein [Shigella flexneri 2a str. 2457T]

MLVTLRIIQGLGAGAEISGAGTMLAEYAPKGKRGIISSFVAMGTNCGTLSATAIWAFMFFILSKEELLAW

GWRIPFLASVVVMVFAIWLRMNLKESPVFEKVNDSNQPTAKPAPAGSMFQSKSFWLATGLRFGQAGNSGL

IQTFLAGYLVQTLLFNKAIPTDALMISSILGFMTIPFLGWLSDKIGRRIPYIIMNTSAIVLAWPMLSIIV

DKSYAPSTIMVALIVIHNCAVLGLFALENITMAEMFGCKNRFTRMAISKEIGGLIASGFGPILAGIFCTM

TESWYPIAIMIMAYSVIGLISALKMPEVKDRDLSALEDAAEDQPRVVRAAQPSRSL

>gi|313649065|gb|EFS13500.1| antitermination family protein [Shigella flexneri 2a str. 2457T]

MKLEDLPKYYSPKSPGLTDASASTSKDALSITDVMAAQGMTQNRAEMGFSAFLGKMGISMNDRARATELL

ADYALSRCDRVAALRKLPAEIKPVVMRIMASYAFEDYARSAASKKQCPCCYGEKFIESIVFTNKIQYPDG

KPPVWAKCTKGVYPFYWEEWKKVREVVKVACPECGGKGEVSTACKDCRGRGVAIHREESVKRGMPVIRDC

QRCGGRGYERLPSTEAFNAICEVTNQITRASWEKTVKKFYDALVTRFDIEEAWAERQLKKVTR

>gi|313649052|gb|EFS13488.1| bme18 [Shigella flexneri 2a str. 2457T]

MVGLLSLAYLTGTWSSPKAGTMTTRYGRGPVMLFSTGVMPFGLLMTLFSSLWLIFAGMLLFSAGFFAAHS

VASSWIGPRAKRAKGQASSLYLFSYYLGSSIAGTLGGVFWHNYGWNGVGAFIALMLVIALLVGTRLHRRL

HA

>gi|313649042|gb|EFS13478.1| putative dimethyl sulfoxide reductase chain ynfF [Shigella flexneri 2a str. 2457T]

MKIHTTEALMKAEISHRSLMKTSALGSLALASSAFTLPFSQMVRAAEAPVEEKTVWSSCTVNCGSRCLLR

LHVKDDTVYWVESDTTGDDVYGNHQVRACLRGRSIRRRMNHPDRLKYPMKRVGERGEGKFERISWDEALD

TISDNLRRILKDYGNEAVHVLYGTGVDGGNITNSNVPYRLMNSCGGFLSRYGSYSTAQISAAMSYMFGAN

DGNSPDDIANTKLVVMFGNNPAETRMSGGGVTYYVEQARERSNARMIVIDPRYNDTAAGREDEWLPIRPG

TDGALACAIAWVLITENMVDQPFLDKYCVGYDEKTLPANAPRNAHYKAYILGEGPDGIAKTPEWAAKITS

IPAEKIIQLAREIGSAKPAYICQGWGPQRHSNGEQTSRAIAMLSVLTGNVGINGGNSGVREGSWDLGVEW

FPMLENPVKTQISVFTWTDAIDHGTKMTATRDGVRGKEKLDVPIKFLWCYASNTLINQHGDINHTHEVLQ

DDSKCEMIVGIDHFMTASAKYCDILLPDLMPTEQEDLISHESAGNMGYVILAQPATSAKFERKPIYWMLS

EVAKPKRVPTPATAILMCYSRPAHKRCGSTPLMLRHAVSVMAIPCGYLTIMEKC

>gi|313649040|gb|EFS13476.1| hypothetical protein SF2457T_2511 [Shigella flexneri 2a str. 2457T]

MTDINHDNTVYPSSSFSGSPYRYQQVYTSSPDTRLTQINLLSVAKPQLVRRANGRYTIRFRLKGQTTPFL

SVSTRSTDRRVATMRQRELAATAKAFMLDRPEVSLQELTEHLRSMAEQFLTDASDDYWNGLEVATLVDEK

SNLKELAATQALSLDQQKGIRLALEVLTAAQQRVDTGDTSGLIKLIDDNNLTDDSTIGDSTSILNNEQGD

RPAVFTQERQSSVVFSSLVSSLLAEKVQTLKTSSYKDLSSSLNTVSRFLPEDMDLMSRSGWLAVRDSMLA

SEVRPSTINKLLTKAKMCLDYGLMNGQLEGRNPIERMKLTKDIDSKRRAFTDEELERLLVRVESE

>gi|313649023|gb|EFS13459.1| putative transposase [Shigella flexneri 2a str. 2457T]

MPCFTAMRAEIALMSGSAFAVTHHAFSSGAGRTSDGNGSHASTLKSPSYTKSVSWQHYRVIEPRLPTRCQ

SSPPSLVHRYIRDERFLILISFTIRYFHHFRKQTYFY

>gi|313649008|gb|EFS13445.1| ipaH-like protein [Shigella flexneri 2a str. 2457T]

MTNINTACVKNNASYQLNNALPNKETISSNFCERLEQWGNKSVNNGEERAIAVERIKEAYNSNMASLDLS

YLDLSELPPIPSTVNTLNLENNCLTCLDLADNASLVNINLSVNKINTITFPKEPKLENIYINHNNLESLD

LKNQHSLVNLEAQNNSLKK

>gi|313648992|gb|EFS13429.1| D-alanyl-D-alanine dipeptidase domain protein [Shigella flexneri 2a str. 2457T]

MSDTTELVDLAVIFPDLEIELKYACVDNITGKAIYQQARCLLHKDAITALAKSISIAQLSGLQLVIYDAY

RPQQAQAMLWQACPDPQYVVDVTVGSNHSRGTALDLTLRDDVEVVNDAKRGNVRRTPTKTNAI

>gi|313648991|gb|EFS13428.1| putative enzyme domain protein [Shigella flexneri 2a str. 2457T]

MNLSALHFRSNQLPNQVSDAMQAWGIDGHQLTVEITESMMMEHDTEIFKRIQILRDMGVGLSVDDFGTGF

SGLSRLVSLPVTEIKIDKSFVDRCLTEKRILALLEAITSIGQSLNLTVVAEGVETKEQFEMLRKIHCRVI

QGYFFSRPLPAEEIPGWMSSVLPLKI

>gi|313648990|gb|EFS13427.1| GGDEF domain protein [Shigella flexneri 2a str. 2457T]

MYFKRMKDEWTGLVEQADPLIRAKAAEIALAHAHYLSIEFYRIVRIDPHAEEFLSNEQVERQLKSAMERW

IINVLSAQVDDVERLIQIQHTVAEVHARIGIPVEIVEMGFRVLKKILYPVIFSSDYSAAEKLQVYHFSIN

SIDIAMEVMTRAFTFSDSSASKEDENYRIFSLLENAEEEKERQIASILSWEIDIIYKVLLDSDLGSSLPL

SQADFGLWFNHKGRHYFSGIAEVGHISRLIQDFDGIFNQTMRNTRILNNRSLRVKFLLQIRNTVSQIITL

LRELFEEVSRHEVGMDVLTKLLNRRFLPTIFKREIAHANRTGTPLSVLIIDVDKFKEINDTWGHNTGDEI

LRKVSFLSQKRLVKSKILGAGSSRKLAVS

>gi|313648972|gb|EFS13409.1| major Facilitator Superfamily protein [Shigella flexneri 2a str. 2457T]

MKNPYYPTALGLYFNYLVHGMGVILMSLNMASLETLWQTNAAGVSIVISSLGIGRLSVLLFAGLLSDRFG

RRPFIMLGMCCYMAFFFDILQTNNIIIAYVFGFLAGMANSFLDAGTYPSLMEAFPRSPGTANILIKAFVS

SGQFLLPLIISLLVWAELWFGWSFMIAAGIMFINALFLYRCTFPPHPGRRLPVIKKTTSSTEHRCSIIDL

ASYTLYGYISMATFYLVSQWLAQYGQFVAGMSYTMSIKLLSIYTVGSLLCVFITAPLIRNTVRPTTLLML

YTFISFIALFTVCLHPTFYVVIIFAFVIGFTSAGGVVQIGLTLMAERFPYAKGKATGIYYSTGSIATFTI

PLITAHLSQRSIADIMWFDTAIGFLLALFIGLRSRKKTRHHSLKENVAPGG

>gi|313648956|gb|EFS13393.1| putative transposase [Shigella flexneri 2a str. 2457T]

MPCFTAMRAEIALMSGSAFAVTHHAFSSGAGRTSDGNGSHASTLKSPSYTKSVSWQHYLIVGMINRYKEW

EIGQKRMNCTEKCAELSVKLCWKCAIWGRNQSILLSQAYYVQH

>gi|313648953|gb|EFS13390.1| aldehyde ferredoxin oxidoreductase, domains 2 & 3 family protein [Shigella flexneri 2a str. 2457T]

MKFAGYDVIIIEGKAKSPVWLKIKDDKVSLEKADFLWGKGTRATTEEICRLTSPETCVAAIGQAGENLIP

LSGMLNSRNHSGGAGTGAIMGSKNLKAIAVEGTKGVNIADRQEMKRLNDYMMTELIGANNNHVVPSTPQS

WAEYSDPKSRWTARKGLFWGAAEGGPIETGEIPPGNQNTVGFRTYKSVFDLGPAAEKYTVKMSGCHSCPI

RCMTQMNIPRVKEFGVPSTGGNTCVANFVHTTIFPNGPKDFEDKDDGRVIGNLVGLNLFDDYGLWCNYGQ

LHRDFTYCYSKGVFKRVLPAEEYAEIRWDQLEAGDVNFIKDFYYRLAHRVGELSHLADGSYAIAERWNLG

KEYWGYAKNKLWSPFGYPVHHANEASAQVGSIVKCMFNRDCMTHTHINFIGSGLPLKLQREVAKELFGSE

DAYDETKNYTPINDAKIKYAKWSLLRVCLHNAVTLCNWVWPMTVSPLKSRNYRGDLALEAKFFKAITGEE

MTQEKLDLAAERIFTLHRAYTVKPMQTKDMRNEHDLICSWVFDKDPQIPVFTEGTDKMDRDDMHASLTMF

YKEMGWDPQLGCPTRETLQRLGLEDIAADLAAHNLLPA

>gi|313648952|gb|EFS13389.1| uncharacterized protein ydhW [Shigella flexneri 2a str. 2457T]

MGKMNHQDELPLAKVSEVDEAKRQWLQGMRHPVDTVTEPEPAEILAEFIRQHSAAGQLVARTVFLSPPYS

VAEEELSVLLESIKQNGDYADIACMTGSQDDYYYSTQAMSENYAAMSLQVVEQDICRAIAHAVRFECQTY

PRPYKVAMLMQAPYYFQEAQIEAAIAAMDVAPEYADIRQVESSTAVLYLFSERFMTYGKAYGLCEWFEVE

QFQNP

>gi|313648948|gb|EFS13385.1| uncharacterized protein ydhS [Shigella flexneri 2a str. 2457T]

MKKIAIVGAGPTGIYTLFSLLQQQTPLSISIFEQADEAGVGMPYSDEENSKMMLANIASIEIPPINCTYL

EWLQKQEASHLQRYGVKKETLHDRQFLPRILLGEYFRDQFLRLVDQARQQKFAVAVYESCQVTDLQITNA

GVMLATNQDLPSETFDLAVIATGHVWPDEEEATRTYFPSPWSGLMEAKVDACNVGIMGTSLSGLDAAMAV

AIQHGSFIEDDKQHVVFHRDNASEKLNITLMSRTGILPEADFYCPIPYEPLHIVTDQALNAEIQKGEEGL

LDRVFRLIVEEIKFADPDWSQRIALESLNVDSFAQAWFAERKQRDPFDWAEKNLQEVERNKREKHTVPWR

YVILRLHEAVQEIVPHLNEHDHKRFSKGLARVFIDNYAAIPSESIRRLLALREAGI

>gi|313648934|gb|EFS13371.1| transposase IS200 like family protein [Shigella flexneri 2a str. 2457T]

MDGEPDHVHLLINYPPKLAISSLVNSLKGISNRLLRRDRPDIAVRYYYKGVLWSPGYFASSCEGAPISVI

RQYIEQQQTPGQVENRALYPRPEGRGFTAHRIKD

>gi|313648932|gb|EFS13369.1| transposase, IS605 OrfB family [Shigella flexneri 2a str. 2457T]

MKRLQAFKFQLRPGGQQEREMRRFAGACRFVFNRALALQNENHEAGNKYIPYGKMASWLVEWKNATETQW

LKDSPSQPLQQSLKDLERAYKNFFQKRAAFPRFKKRGQNAAFRYPQDVKLDQENSRIFLPKLGWMRYRNS

RQVTGVVKNVTVSQSCGTWYISIQTESEVSTPVHPSASMIGLDAGVAKLATLSDGTVFEPVNSFQKNQKT

LARLQRQLSRKVKFSNNWQKQKRKIQRLHSCIANIRRDYLHKVTTTVSKNHAMIVIEDLKVSNMSKSAAG

TVSQPGRNVRAKSGLNRSILDQGWYEMRRQLEYKQLWSGGQVLAVPPAYTSQRCACCGHTAKENRLSQSK

FRCQVCGYTANADVNGARNILAAGHAVLACGEMVQSGRSLKQEPTEMIQATA

>gi|313648924|gb|EFS13361.1| fusaric acid resistance conserved region family protein [Shigella flexneri 2a str. 2457T]

MVNITEASQLWDIAQARVCEVIVGILCGGMMMMILPSSSDATALLTALKNMHARLLEHASLLWQPETNDA

IRAAHEGVIGQILTMNLLRIQAFWSHYRFRQQNARLNALLHQQLRMTSVISSLRRMLLNWPSPPGATREI

LEQLLTALASSQTDAYTVARIIAPLRPTNVADYRHVAFWQRLRYFCRLYLQSSQELHRLQSGVDDHARLA

RTSALARHTDNAEAMWSGLRTFCTLMMIGAWSIASQWDAGANALTLAAISCVLYSAVAAPFKSLSLLMRT

LVLLSLFSFVVKFGLMVQISDLWQFLLFLFPLLATMQLLKLQMPKFAALWGQLIVFMGSFIAVTNPPVYD

FADFLNYNLAKIVGVALAWLAFAILRPGSDARKSRRHIRALRRDFVDQLSRHPTLSESEFESLTYHHVSQ

LSNSQDALARRWLLRWGVVLLNCSHVVWQLRDWESRSDPLSRVRDNCISLLRGVMSERGVQQKSLAATLE

ELQRICDSLARHHQPAARELAAIVWRLYCSLSQLEQAPPQGTLAS

>gi|313648888|gb|EFS13325.1| glucuronide transporter [Shigella flexneri 2a str. 2457T]

MGLGLCYSLVNIPYGSLATAMTQQPQSRARLGAARGIAASLTFVCLAFLIGPSIKNSSPEEMVSVYHFWT

IVLVIAGMVLYFICFKSTRENVVRIVAQPSLKISLQTLKRNRPLFMLCIGALCVLISTFAVSASSLFYVR

YVLNDTGLFTVLVLVQNLVGTVASAPLVPGMVARIGKKNTFLIGALLGTCGYLLFFWVSVWSLPVALVAL

AIASIGQGVTMTVMWALEADTVEYGEYLTGVRIEGLTYSLFSFTRKCGQAIGGSIPAFILGLSGYIANQV

>gi|313648885|gb|EFS13322.1| hypothetical protein SF2457T_2545 [Shigella flexneri 2a str. 2457T]

MNKSLVAVGVIVALGVVWTGGAWYTGKKIETHLEDMVAQANAQLKLTAPESNLEVSYQNYHRGVFSSQLQ

LLVKPIAGKENPWIKSGQSVIFNESVDHGPFPLAQLKKLNLIPSMASIQTTLVNNEVSKPLFDMAKGETP

FEINSRIGYSGDSSSDISLNPLNYEQKDEKVAFSGGEFQLNADRDGKAISLSGEAQSGRIDAVNEYNQKV

QLTFNNLKTDGSSTLGSFGERVGNQKLSLEKMTISVEGKELALLEGMEISGKSDLVNDGKTINSQLDYSL

NSLKVQNQDLGSGKLTLKVGQIDGEAWHQFSQQYNAQTQALLAQPEIANNPELYQEKVTEAFFSALPLML

KGDPVITIAPLSWKNSQGESALNLSLFLKDPATTKEAPQTLAQEVDRSVKSLDAKLTIPVDMATELMTQV

AKLEGYQEDQAKKLAKQQVEGASAMGQMFRLTTLQDNTITTSLQYANGQITLNGQKMSLEDFVGMFAMPA

LNVPAVPAIPQQ

>gi|313648856|gb|EFS13295.1| phthalate permease family domain protein [Shigella flexneri 2a str. 2457T]

MDVDVSTSVAGNKPQRIRRIQTVTLVLLFMAGIVNFLDRSSLSVAGEAIRGELGLSATEFGVLLSAFSLS

YGFSQLPSGILLDRFGPRIVLGAGLIFWSLMQALTGMVNSFSHFILMRIGLGGNAANLLI

>gi|313648840|gb|EFS13279.1| multiphosphoryl transfer 2 domain protein [Shigella flexneri 2a str. 2457T]

MALIVEFICELPNGVHARPASHVETLCNTFSSQIEWHNLRTDRKGNAKSALALIGTDTLVGDNCQLLISG

ADEQEGHQRLSQWLRDEFPHCDAPLAEVKSDELEPLPVSLTNLNPQIIRARTVCSGSAGGILTPISSLDL

NALSNLPAAKDVDAEQSALENGLTLVLKNIEFRLLDSDGATSAILEAHRSLAGDTSLHEHLLAGVSAGLS

CAEAIVASANHFCEEFARSSSRYLQERALDVRDVCFQLLQQIYGEQRFPAPGKLTQSAICMADELTPSQF

LELDKNHLKGLLLKSGGTTSHTVILARSFNIPTLVGVDIDALTPWQHQTIYIDGNAGAIVVEPGEAVARY

YQQEARVQDALREQQRV

>gi|313648839|gb|EFS13278.1| multiphosphoryl transfer 2 domain protein [Shigella flexneri 2a str. 2457T]

MEAQAAFGNGAEGVGLFRTEMLYMDRTSAPGESELYNIFCQALESANGRSIIVRTMDIGGDKPVDYLNIP

AEANPFLGYRAVRIYEEYASLFTTQLRSILRASAHGSLKIMIPMISSMEEILWVKEKLAEAKQQLRNEHI

PFDEKIQLGIMLEVPSVMFIIDQCCEEIDFFSIGSNDLTQYLLAVDRDNAKVTRHYNSLNPAFLRALDYD

VQAVHRQGKWIGLCGELGAKGSVLPLLVGLGLDELSMSAPSIPAAKARMAQLDSRECRKLLNQAMACRTS

LEVEHLLAQFRMTQQDAPLVTAECITLESDWRSKEEVLKGMTDNLLLAGRCRYPRKLEADLWAREAVFST

GLGFSFAIPHSKSEHIEQSTISVARLQAPVRWGDDEAQFIIMLTLNKHAAGDQHMRIFSRLARRIMHEEF

RNALVNAASADAIASLLQHELEL

>gi|313648836|gb|EFS13275.1| glycerol dehydrogenase [Shigella flexneri 2a str. 2457T]

MDRIIQSPGKYIQGADVINRLGEYLKPLAERWLVVGDKFVLVFAQSTVEKSFKDAGLVVEITPFGGECSQ

NEIDRLRGIAETAQCGAILGIGGGKTLDTAKALAHFMGVPVAIAPTIASTDAPCSALSVIYTDEGEFDRY

LLLPNNPNMVIVDTKIVAGAPARLLAAGIGDALATWFEARACSRSGATTMAGGKCTQAALALAELCYNTL

LEEGEKAMLAAEQHVVTPALERVIEANTYLSGVGFESGGWRRLTRCITA

>gi|313648833|gb|EFS13272.1| hypothetical protein SF2457T_2723 [Shigella flexneri 2a str. 2457T]

MVIRALRSQKVDLQKLVHEDMAKNFAEYPQKWKLKRPDSNIDHRRVPNLETWFSRHNKTRPISKNAGDYQ

AGDIVSWRLDNGLAHIGVASDGFARDGTPLVIHNIGAGAQEEDVLFSWRMVGHYRYFVK

>gi|313648832|gb|EFS13271.1| hypothetical protein SF2457T_2722 [Shigella flexneri 2a str. 2457T]

MKSAMLAILSGVSWGASAIVAKRLYARHPRVDLLSLTSWQMLYAALVMSVVALLVPQREIDWQPTVFWAL

AYSAILATALAWSLWLFVLKNLPASIASLSTLAVPVCGVLFSWWLLGENPGAVEGSGIVLIVLALALVSR

KKKEAVSVKSI

>gi|313648831|gb|EFS13270.1| hypothetical protein SF2457T_2721 [Shigella flexneri 2a str. 2457T]

MSAAGKSNPLAISGLVVLTLIWSYSWIFMKQVTSYIGAFDFTALRCIFGALVLFIVLLLRGRGMRPTPFK

YTLAIALLQTCGMVGLAQWALVSGGAGKVAILSYTMPFWVVIFAALFLGERLRRGQYFAILIAAFGLFLV

L

>gi|313648793|gb|EFS13233.1| hypothetical protein SF2457T_2940 [Shigella flexneri 2a str. 2457T]

MFDVLMYLFETYIHTEAELRVDQDKLEQDLTDAGFEREDIYNALLWLEKLADYQEGLAEPMQLASDPLSM

RIYTPEECERLDASCRGFLLFLEQIQVLNLETREMVIERVLALDNAEFELDDLKWVILMVLFNIPGCENA

YQQMEELLFEVNEGMLH

>gi|313648766|gb|EFS13206.1| uncharacterized yjbI domain protein [Shigella flexneri 2a str. 2457T]

MIGHITNSGGNNSLMKHDVVQGNNIVDLDLLRNFNGVTGLNRDYFIYISDIFLNIKQRNEKNHAINMLRE

VSISNDTISVKFYRNEKIECACNFLMDKDAQGYIDLSDFDLTNCHFKGDVISKVSFLSSNLQHVTFECKE

IENCNFTKATVDNVIFKCRRLHNVIFLKTSGECVDFSQNILDTVDFSQSQLGHSNFRECQIRNSNFDNCY

LYASHFTRAEFLSAKEISFIKSNLTAVMFDHVRMSTGNF

>gi|313648765|gb|EFS13205.1| uncharacterized protein yjbI [Shigella flexneri 2a str. 2457T]

MELTIDYSDIFGNEDLDGYINNIIKMIDTLPDNAMILKSVLAVKLVMQLKILNIVNKNFIENMKKTFSHC

PYIKDPIIRSYIHSGEDDKFDNFMRQHRFSKVNFDTQQMIHFINRFNMNKGLVDKNNNFFIQLIDQALRS

TDDMIKANAWYLYKEWIRSDDVSPIFIETEEKLRTFNTNKLTRNDNIFILFSSVDDGPVMVVSSQRLHDM

LNPTKDTNWNSTCIYKSRHKMLPINLTQETLFSSKSHGKYALFPIFTASWRATRIKNKGI

>gi|313648747|gb|EFS13187.1| uncharacterized protein yjbS [Shigella flexneri 2a str. 2457T]

MNISYVNSNKTTSLPVELDALNNKDISYAKDFFLYIETQLKIAKDFCRPGEEVSSSIASKVFHAFIDLVN

KIRGKKDFMYICTLCCFAEEVKGDYSHYRTFLFDIGNQYKVKLTQSGKKSSL

>gi|313648739|gb|EFS13179.1| putative transposase [Shigella flexneri 2a str. 2457T]

MPCFTAMRAEIALMSGSAFAVTHHAFSSGAGRTSDGNGSHASTLKSPSYTKSVSWQHYPAGKYQSGICRN

YLYRPFCSLRHAVLAGIFLIYLRRAHGKYAEKSTSQ

>gi|313648733|gb|EFS13173.1| formate dehydrogenase, alpha subunit [Shigella flexneri 2a str. 2457T]

MSNAINEIDNTDLVFVFGYNPADSHPIVANHVINAKRNGAKIIVCDPRKIETARIADMHIALKNGSNIAL

LNAMGHVIIEENLYDKAFVASRTEGFEEYRKIVEGYTPESVEDITGVSASEIRQAARMYAQAKSAAILWG

MGVTQFYQGVETVRSLTSLAMLTSNLGKPHAGVNPVRGQNNVQGACDMGALPDTYPGYQYVKDPANREKF

AKAWGVESLPAHTGYRISELPHRAAHGEVRAAYIMGEDPLQTDAELSAVRKAFEDLELVIVQDIFITKTA

SAADVILPSTSWGEHEGVFTAADRGFQRFFKAVEPKWDLKTDWQIISEIATRMGYPMHYNNTQEIWDELR

HLCPDFYGATYEKMGELGFIQWPCRDTSDADQGTSYLFKEKFDTPNGLAQFFTCDWVAPIDKLTDEYPMV

LSTVREVGHYSCRSMTGNCAALAALADEPGYAQINTEDAKRLGIEDEALV

>gi|313648709|gb|EFS13149.1| EAL domain protein [Shigella flexneri 2a str. 2457T]

MTYMQRGNYVAVINPLFWSEVMSDDPTLQWGVYDTVMKTFFSLSKEASAATFSPLIHLKDLTVQRNGYLY

ATIYSTKRPIAAIVATSYQRLITHFYNHLIFALPAGILGSLVLLLLWLRIRQNYLSPKRKLQRALEKHQL

CLYYQPIIDIKTEKCIGAEALLRWPGEQGQIMNPAEFIPLAEKEGMIEQITDYVIDNVFRDLGAYLATHA

DRYVSINLSASDFHTSRLIARINQKTEQYAVRPQQIKFEVTEHAFLDVEKMTPINSGFPPGWLRSGN

>gi|313648708|gb|EFS13148.1| hypothetical protein SF2457T_2855 [Shigella flexneri 2a str. 2457T]

MSHRARHQLLALPGIIFLVLFPIILSLWIAFLWAKSEVNNQLRTFAQLALDKSELVIRQADLVSDAAERY

QGQVCTPAHQKRMLNIIRGYLYINEFVITIFYAHR

>gi|313648707|gb|EFS13147.1| transposase [Shigella flexneri 2a str. 2457T]

MPCFTAMRAEIALMSGSAFAVTHHAFSSGAGRTSDGNGSHASTLKSPSYTKSVSWQHYRGFYVSGQNHWP

LWRCLIHLFYHHRLDVVPDSGDVRHCDAKQPHCEPTGGNNAQTAADHRKPDIIRPTKTDVECAGQKHHYG
TKKLPAKKNHASGEGSHQTIPLKRADALDSAALFTGGVVHSGYRGACRTQK

>gi|313648699|gb|EFS13139.1| uncharacterized protein yjcZ [Shigella flexneri 2a str. 2457T]
MTKTLLDGPGRVLESVHPRFLVDLAQGDDARHPQAHQQQFRERLMQELLSRVQLQTWTNGGMLNAPLSLR
LTMVEKLASMLDPGHLALTQIAQHLALLQKMDHRQHSAFPELPQQIAALYEWFSARCRWKEKALTQRGLL
VQAGDQSEQIFTRWRAGAYNAWSLPGRCFIVLEELRWGAFGDACRLGSPQAVVLLLGDLREKATQHLAES
INAAPTTRHYYHQWFASSTVPTGGDHADFLSWLGKWTTADKQPVCWSVTQRWQTVALGMPRLCSAQRLVG
AMVEEIFSVNLA

>gi|313648691|gb|EFS13131.1| biodegradative arginine decarboxylase [Shigella flexneri 2a str. 2457T]
MMKVLIVESEFLHQDTWVGNAVERLADALSQQNVTVIKSTSFDDGFAILSSNEAIDCLMFSYQMEHPDEH
QNVRQLIGKLHERQQNVPVFLLGDREKALAAMDRDLLELVDEFAWILEDTADFIAGRAVAAMTRYRQQLL
PPLFSALMKYSDIHEYSWAAPGHQGGVGFTKTPAGRFYHDYYGENLFRTDMGIERTSLGSLLDHTGAFGE
SEKYAARVFGADRSWSVVVGTSGSNRTIMQACMTDNDVVVVDRNCHKSIEQGLMLTGAKPVYMVPSRNRY
GIIGPIYPQEMQPETLQKKISESPLTKDKAGQKPSYCVVTNCTYDGVCYNAKEAQDLLEKTSDRLHFDEA
WYGYARFNPIYADHYAMRGEPGDHNGPTVFATHSIHKLLNALSQASYIHVREGRGAINFSRFNQAYMMHA
TTSPLYAICASNDVAVSMMDGNSGLSLTQEVIDEAVDFRQAMARLYKEFTADGSWFFKPWNKEVVTDPQT
GKTYDFADAPTKLLTTVQDCWVMHPGESWHGFKDIPDNWSMLDPIKVSILAPGMGEDGELEETGVPAALV
TAWLGRHGIVPTRTTDFQIMFLFSMGVTRGKWGTLVNTLCSFKHHYDANTPLAQVMPELVEQYPDTYANM
GIHDLGDTMFAWLKENNPGARLNEAYSGLPVAEITPREAYNAIVDNNVELVSIENLPGRIAANSVIPYPP
GIPMLLSGENFGDKNSPQVSYLRSLQSWDHHFPRFEHETEGTEIIDGIYHVMCVKA

>gi|313648666|gb|EFS13106.1| ankyrin repeat protein A [Shigella flexneri 2a str. 2457T]

MLFPGAPQNRIVYRHIAAQYINDIYQNVDYKPHQDDYSSAEKFLTHFNKKCKNQTLALISSRPEGRCVAA

CGDFGLVMKAYFDKMESNGISVMAAILLVDNHALTVRLRIKNTTEGCTHYVVSVYDPNVTNDKIRIMSES

KEDIKHYSLMDFMNVDYSLLKWSNDHVINQSVAIIPALPKEQLLMLKGTVDEITPPLSPATMNLLMAIGQ

NHQLTQLMIQLQKMPELHRTEMLTAYNSINLPGLYLAINYGNADIVETIFNSLSETGYEGLLSKKNLMHI

LEAKDKNGFSGLFLAISRKDKNVVTSILNVLPKLAATHHLDNEQVYKFLSAKNRTSSHVLYHVMANGDAD

MLKIFLVALPLLIRTCHLTKEQVLDLLKAKDFYGCPRLYLAMQNGHSDIVKVILEALPCLAQEINISASD

IVDLLTAKSLARDTGLFMAMQRGHMNVINTIFNALPTLFNTFKFDKKI

>gi|313648665|gb|EFS13105.1| ankyrin repeat A domain protein [Shigella flexneri 2a str. 2457T]

MFSAIQHKQQNVVETVYLALSDHARLFGFTAEDIMDFWQHKAPQKYSAFELAFEFGHRVIAELILNTLNK

MAESFGFTDNPRYIAEKNYMEALLKKASPHTVR

>gi|313648664|gb|EFS13104.1| isocitrate dehydrogenase kinase/phosphatase domain protein [Shigella flexneri 2a str. 2457T]

MEGQQLRDAIEEYGNAIRQLAAANIFPGDMLFKNFGVTRHGRVVFYDYDEICYMTEVNFRDIPPPRYPED

ELASEPWYSVSPGDVFPEEFRHWLCADPRIGPLFEEMHADLFRADYWRALQNRIREGHVEDVYAYRRRQR

FSVRYGEMLF

>gi|313648663|gb|EFS13103.1| isocitrate dehydrogenase kinase/phosphatase [Shigella flexneri 2a str. 2457T]

MPRGLELLIAQTILQGFDAQYGRFLEVTSGAQQRFEQADWHAVQQAMKNRIHLYDHHVGLVVEQLRCITN

GQSTDAAFLLRVKEHYTRLLPDYPRFEIAESFFNSVYCRLFDHRSLTPERLFIFSSQPERRFRTIPRPLA

KDFHPDHGWESLLMRVISDLPLRLRWQNKSRDIHYIIRHLTETLGTDNLAESHLQVANELFYRNKAAWLV

GKLITPSGTLPFLLPIHQTDDGELFIDTCLTTTAEASIVFGFARSYFMVYAPLPAALVEWLREILPGKTT

AELYMAIGCQKHAKTESYREYLVYLQGCNEQFIEAPGIRGMVMLVFTLPGFDRVFKVIKDKFAPQKEMSA

AHVRACYQLVKEHDRVGRMADTQEFENFVLEKRHISPVLMELLLQEAAEKITDLGEQIVIRHLYNRAADG

AAQYLAGTSGRSAVARRH

>gi|313648661|gb|EFS13101.1| malate synthase A domain protein [Shigella flexneri 2a str. 2457T]

MLIETLPAVFQMDEILHALRDHIVGLNCGRWDYIFSYIKTLKNYPDRVLPDRQAVTMDKPFLNAYSRLLI

KTCHKRGAFAMGGMAAFIPSKDEERNNQVLNKVKADKALEANNGHDGTWIAHPGLADTAMAVFNDILGSR

KNQLEVLREQDAPITADQLLAPCDGERTEEGMRANIRVAVQYIEAWISGNGCVPIYGLMEDAATAEISRT

SIWQWIHHQKTLSNGKPVTKALFRQMLGEEMKVIASELGEERFSQGRFDDAARLMEQITTSDELIDFLTL

PGYRLLA

>gi|313648660|gb|EFS13100.1| malate synthase A domain protein [Shigella flexneri 2a str. 2457T]

MTEQATTTDELAFTRPYGEQEKQILTAEAVEFLTELVTHFTPQRNKLLAARIQQQQDIDNGTLPDFISET

ASIRDADWKIRGIPADLEDRRVEITGPVERKMVINALNANVKVFMADFEDSLAPDWNKVIDGQINLRDAV

NGTISYTNEAGKIYQLKPNPAVLICRVRGLHLPEKHVTWRGEAIPGSLFDFALYFFHNYQALLAKGSGPY

FYLPKTQS

>gi|313648622|gb|EFS13063.1| general L-amino acid transport system permease aapQ domain protein [Shigella flexneri 2a str. 2457T]

MAIVLSVGLFRFNKTYQIKTGQLRRSWPIAAVLIIGLPLLAQWLFGAALHWDVPALQGFNFRGGMVLIPE

LAALTLALSVYTSAFIAEIIRAGIQAVPYGQHEAARSLGLPNPVTLRQVIIPQALRVIIPPLTSQYLNIV

KNSSLAAVIGYPDMVSLFAGTVLNQTGQAIETIAMTMSVYLIISLTISLLMNIYNRRIAIVER

>gi|313648616|gb|EFS13058.1| uncharacterized outer membrane usher yraJ domain protein [Shigella flexneri 2a str. 2457T]

MTSQKDRPTQHEMRLDGSLLDDGRLSYSLEQSLDDDNNHNSSLNASYRSPYGTFSAGYSYGNDSSQYNYG

VTGGVVIHPHGVTLSQYLGNAFALIDANGASGVRIQNYPGIATDPFGYAVVPYLTTYQENRLSVDTTQLP

DNVDLEQTTQFVVPNRGAMVAARFNANIGYRVLVTVSDRNGKPLPFGALASNDDTGQQSIVDEGGILYLS

GISSKSQSWTVRWGNQADQQCQFAFSTPDSEPTTSVLQGTAQCH

>gi|313648585|gb|EFS13027.1| UPF0141 inner membrane yhbX domain protein [Shigella flexneri 2a str. 2457T]

MLIVGESVRVDNMSLYGYTRSTTPQVEAQRKQIKLFNQAISGAPYTALSVPLSLTADSVLSHDIHNYPDN

IINMANQAGFQTFWLSSQSAFRQNGTAVTSIAMRAMETVYVRGFDELLLPHLSQALQQNTQQKKLIVLHL

NGSHEPACSAYPQSSAVFQPQDDQDACYDNSIHYTDSLLGQVFELLKDRRASVMYFADHGLERDPTKKNV

YFHGGREASQQAYHVPMFIWYSPVLGDGVDRTTENNIFSTAYNNYLINAWMGVTKPEQPQTLEEVIAHYK

GDSRVVDANHDVFDYVMLRKEFTEDKQGNPTPEGQG

>gi|313648584|gb|EFS13026.1| UPF0141 inner membrane yhbX domain protein [Shigella flexneri 2a str. 2457T]

MLFFVLTILVVKRISSLPLRLLVAAPFVLLTAADMSISLYSWCTFGTTFNDGFAISVLQSDPDEVVKMLG

MYIPYLCAFAFLSLLFLAVIIKYDVSLPTKKVTGILLLIVISGSLFSACQFAYKDAKNKKAFSPYILAS

>gi|313648548|gb|EFS12990.1| monofunctional biosynthetic peptidoglycan transglycosylase [Shigella flexneri 2a str. 2457T]

MSKSRLTVFSFVRRFLLRLMVVLAVFWGGGIALFSVAPVPFSAVMVERQVSAWLHGNFRYVAHSDWVSMD

QISPWMGLAVIAAEDQKFPEHWGFDVASIEKALAHNERNENRIRGASTISQQTAKISFYGMGVAGYEKGW

KLD

>gi|313648514|gb|EFS12956.1| HTH-type transcriptional activator aaeR [Shigella flexneri 2a str. 2457T]

MERLKRMSVFAKVVEFGSFTAAARQLQMSVSSISQTVSKLEDELQVKLLNRSTRSIGLTEAGRIYYQGCR

RMLHEVQDVHEQLYAFNNTPIGTLRIGCSSTMAQNVLAGLTAKMLKEYPGLSANLVTGIPAPDLIADGLD

VVIRVGALQDSSLFSRRLGAMPMVVCAAKSYLTQYGIPEKPADLSSHSWLEYSVRPDNEFELIAPEGIST

RLIPQGRFVTNDPMTLVRWLTAGAGIAYVPLMWVINEINRGELEILLPRYQSDPRPVYALYTEKDKLPLK

VQVVINSLTDYFVEVGKLFQEMHGRGKEK

>gi|313648512|gb|EFS12954.1| uncharacterized protein yhdP [Shigella flexneri 2a str. 2457T]

MRRLPGILLLTGAALVVIAALLVSGLRIALPHLNAWRPEILNKIESATGMPVEASQLSASWQNFGPTLEA

HDIRAELKDGGEFSVKRVTLALDVWQSLLHMRWQFRDLTFWQLRFRTNTPITSGGSDDSLEASHISDLFL

RQFDHFDLRDSEVSFLTPSGQRAELAIPQLTWLNDPRRHRAEGLVSLSSLTGQHGVMQVRMDLRDDEGLL

SNGRVWLQADDIDLKPWLGKWMQDNIALETAQFSLEGWMTIDKGDVTGGDVWLKQGGASWLGEKQTHTLS

VDNLTAHITRENPGWQFSIPDTRITMDGKPWPSGALTLAWIPEQDVGGKDNKRSDELRIRASNLELAGLE

GIRPLAAKLSPALGDVWRSTQPSGKINTLALDIPLQAADKTRFQASWSDLAWKQWKLLPGAEHFSGTLSG

SVENGLLTASMKQAKMPYETVFRAPLEIADGQATISWLNNDKGFQLDGRNIDVKAKAVHARGGFRYLQPA

NDEPWLGILAGISTDDGSQAWRYFPENLMGKDLVDYLSGAIQGGEADNATLVYGGNPQLFPYKHNEGQFE

VLVPLRNAKFAFQPDWPALTNLDIELDFINDGLWMKTDGVNLGGVRASNLTAVIPDYSKEKLLIDADIKG

PGKAVGPYFDETPLKDSLGATLQELQLDGDVNARLHLDIPLNGELVTAKGEVTLRNNSLFIKPLDSILKN

LSGKFSFINGDLQSEPLTASWFNQPLNVDFSTKEGAKAYQVAVNLNGNWQPAKTGVLPEAVNEALSGSVA

WDGKVGIDLPYHAGATYNVELNGDLNNVSSHLPSPLAKPAGEPLAVNVKVDGNLNSFELTGQAGADNHFN

SRWLLGQKLTLDRAIWAADSKTLPPLPEQSGVELNMPPMNGAEWLALFQKGAAESVGGAASFPQHITLRT

PMLSLGNQQWNNLSIVSQPTANGTLVEAQGREINATLAMRNNAPWLANIKYLYYNPSVAKTRGDSTPSSP

FPTTERINFRGWPDAQIRCTECWFWGQKFGRIDSDITISGDTLTLTNGLIDTGFSRLTADGEWVNNPGNE

RTSLKGKLRGQKIDAAAEFFGVTTPIRQSSFNVDYDLHWRKAPWQPDEATLNGIIHTQLGKGEITEINTG

HAGQLLRLLSVDALMRKLRFDFRDTFGEGFYFDSIRSTAWIKDGVMHTDDTLVDGLEADIAMKGSVNLVR

RDLNMEAVVAPEISATVGVAAAFAVNPIVGAAVFAASKVLGPLWSKVSILRYHISGPLDDPQINEVLRQP

RKEKAQ

>gi|313648508|gb|EFS12950.1| rod shape-determining protein MreC [Shigella flexneri 2a str. 2457T]

MKPIFSRGPSLQIRLILAVLVALGIIIADSRLGTFSQIRTYMDTAVSPFYFVSNAPRELLDGISQTLASR

DQLELENRALRQELLLKNSELLMLGQYKQENARLRELLGSPLRQDEQKMVTQVISTVNDPYSDQVVIDKG

SVNGVYEGQPVISDKGVVGQVVAVAKLTSRVLLICDATHALPIQVLRNDIRVIAAGNGCTDDLQLEHLPA

NTDIRVGDVLVTSGLGGRFPEGYPVAVVSSVKLDTQRAYTVIQARPTAGLQRLRYLLLLWGADRNGANPM

TPEEVHRVANERLMQMMPQVLPSPDAMGPKLPEPATGIAQPTPQQPATGNAATAPVAPTQPAANRSPQRA

TPPQSGAQPPARAPGGQ

>gi|313648493|gb|EFS12935.1| putative transposase [Shigella flexneri 2a str. 2457T]

MPCFTAMRAEIALMSGSAFAVTHHAFSSGAGRTSDGNGSHASTLKSPSYTKSVSWQHYPFSHHCGALFLL

FPVYTQLIATRNRAQKLALLLLIRGWPQTGKRRFPNVL

>gi|313648491|gb|EFS12933.1| general L-amino acid transport system permease aapQ domain protein [Shigella flexneri 2a str. 2457T]

MPHRRSTVKGSLSFANPTVRAWLFQILAVVAVVGIVGWLFQNTVTNLNNRGITSGFAFLDRGAGFGIVQH

LIDYQQGDTYGRVFIVGLLNTLLVSALCIVFASVLGFFIGLARLSDNWLLRKLSTIYIEIFRNIANLLLV

LCRVAQFARTTPSCERV

>gi|313648479|gb|EFS12922.1| uncharacterized HTH-type transcriptional regulator ybhD domain protein [Shigella flexneri 2a str. 2457T]

MIGVRSSSGNRLLIEQQLADKPWKLDWFYEVRHLSTSLGLVEAGLGISALPGLAMPHAPYSSIIGIPLVE

PVIRRTLGIIRRKDAVLSPAAERFFSLLINLWTDDKDNLWTNIVERQRHALQEIS

>gi|313648478|gb|EFS12921.1| uncharacterized HTH-type transcriptional regulator ybhD [Shigella flexneri 2a str. 2457T]

MKHELSSMKAFVILAESSSFNNAAKLLNITQPALTRRIKKMEEDLHIQLFERTTRKVTLTKAGKRLLPEA

RELIKKFDETLFNIRDMNAYHRGMVTLACIPTAVFYFLPLAIGKFNELYPNIKVRILEQGTNNCMESVLC

NESDFGINMNNVTNSSIDFTPLVNEPFVLACRRDHPLAKK

>gi|313648477|gb|EFS12920.1| hypothetical protein SF2457T_3203 [Shigella flexneri 2a str. 2457T]

MKKIPCVMMRGGTSRGAFLLAEHLPEDQTQRDKILMAIMGSGNALEIDGIGGGNPLTSKVAIISRSSDPR

ADVDYLFAQVIVHEQRVDTTPNCGNMLSGVGAFAIENGLIAATSPVTRVRIRNVNTGTFIEADVQTPNGV

VEYEGSARIDGVPGTAAPVALTFLNAAGTKTGKVFPTDNQIDYFDDVPVTCIDMAMPVVIIPAEYLGKTG

YELPAELDADKALLARIESIRLQAGKAMGLGDVSNMVIPKPVLISPAQKGGAINVRYFMPHSCHRALAIT

GAIAISSSCALEGTVTRQIVPSVGYGNINIEHPSGALDVHLSNEGQDATTLRASVIRTTRKIFSGEVYLP

>gi|313648476|gb|EFS12919.1| inner membrane protein ybhI [Shigella flexneri 2a str. 2457T]

MNKKSLWKLILILAIPCIIGFMPAPAGLSELAWVLFGIYLAAIVGLVIKPFPEPVVLLIAVAASMVVVGN

LSDGAFKTTAVLSGYSSGTTWLVFSAFTLSAAFVTTGLGKLIAYLLIGKIGNTMLGLGYVTVFLDLVLAP

ATPSNTARAGGIVLPIINSVAVALGSEPEKSPRRVGHYLMMSIYMVTKTTSYMFFTAMAGNILALKMIND

ILHLQISWGGWALAAGLPGIIMLLVTPLVIYTMYPPEIKKVDNKTIAKAGLAELGPMKIREKMLLGVFVL

ALLGWIFSKSLGVDESTVAIVVMATMLLLGIVTWEDVVKNKGGWNTLIWYGGIIGLSSLLSKVKFFEWLA

EVFKNNLAFDGHGNVAFFVIIFLSIIVRYFFASGSAYIVAMLPVFAMLANVSGAPLMLTALALLFSNSYG

GMVTHYGGAAGPVIFGVGYNDIKSWWLVGAVLTILTFLVHITLGVWWWNMLIGWNML

>gi|313648461|gb|EFS12904.1| uncharacterized HTH-type transcriptional regulator ycaN [Shigella flexneri 2a str. 2457T]

MESLPKIRLFNRTTRSVSLTEAGSNLYERLRPAFDEIQIMLDEMNDFRLTPTGTLKINAARVAARIFLMP

LLVGFTREYPDIKVELTTDDSLIDIVQQGFDAGVRLSGIVEKDMISVAIGPPVKLCVAATPEYFARYGKP

RHPHDLLNHQCVVFRYPSGKPFHWQFAKELEIAVAGNIILDDVDAELEAVLMGAGIGYLLYEQIKEYLDT

GRLECVLEDWITERPGFQVYYPNRQYMSCGLRAFLDYVKTGQICQSQRHRPQ

>gi|313648460|gb|EFS12903.1| amino acid permease family domain protein [Shigella flexneri 2a str. 2457T]

MPFYFRKECPLNSGYLTLVLVAILIMLPTLGIGDMNNLYKWLLNLNSVVMPLRYLWVFVAFIAVVRLAQK

YKPEYVFIRNKPLAMTVGIWCFAFTACACLTGIFPKMEAFTAEWTFQLALNVATPFVLVGLGLIFPLLAR

KANSK

>gi|313648441|gb|EFS12884.1| siderophore-interacting family protein [Shigella flexneri 2a str. 2457T]

MPALRRRLETLSKLAVKPQVSALVSVRDNACQDYLAHLDGFNIEWLAHDEQAVDARLAQMQIPADDYFIW

ITGEGKVVKNLSRRFEAEQYDPQRVRAAAYWHAK

>gi|313648440|gb|EFS12883.1| siderophore-interacting FAD-binding domain protein [Shigella flexneri 2a str. 2457T]

MNNTPRYPQRVRNDLRFRELTVLRVERISAGFQRIVLGGEALDGFTSRGFDDHSKLFFPQPDAHFVPPTV

TEESIVWPEGPRPPSRDYTPLYDELRHELAIDFFIHDGGVASGWAINLRWQVRAVRWWCRKITRISCMSA

MNPECPHCAAAWKR

>gi|313648438|gb|EFS12881.1| aerotaxis receptor domain protein [Shigella flexneri 2a str. 2457T]

MEAARAGEQGKGFAVVAGEVRHLASRSANAANDIRKLIDASADKVQSGSQQVHAAGRTMEDIVAQVKNVT

QLIAQISHSTLEQADGLSSLTRAVDELNLITQKNAELVEESAQVSAMVKHRASRLEDAVTVLH

>gi|313648437|gb|EFS12880.1| aerotaxis receptor [Shigella flexneri 2a str. 2457T]

MSSHPYVTQQNTPLADDTTLMSTTDLQSYITHANDTFVQVSGFTLQELQGQPHNMVRHPDMPKAAFADMW

FTLKKGEPWSGIVKNRRKNGDHYWVRANAVPMVREGKISGYMSIRTRATDEEIAAVEPLYKALNSGRTSK

RIHKGLVVRKGWLGKLPSLPLRWRTRGVMTLMFILLAAMLWFVAAPVVTYFLCVLVVLLASACFEWQIVR

PIENVARQALKVATGERNSVEHLNRSDELGLTLRAVGQLGLMCRWLINDVSSQVSSVRNGSETLAKGTDE

LNEHTQQTVDNVQQTVATMNQMAASVKQNSATASAADKLSITASNAAVQGGEAMTTVIKTMDDIADST

>gi|313648428|gb|EFS12871.1| uncharacterized ygjJ domain protein [Shigella flexneri 2a str. 2457T]

MKLITAPCRALLALPFCYAFSAAGEEARPAEHDDTKTPAITSTSSPSFRFYGELGLGGYMDLEGENKHKY

SDGTYIEGGLEMKYGSWFGLIYGEGWTVQADHDGNA

>gi|313648427|gb|EFS12870.1| uncharacterized protein ygjJ [Shigella flexneri 2a str. 2457T]

MPDHSWGGFEGGINRFYGGYRTNDGTEIMLSLRQDSSLDDLQWWGDFTPDLGYVIPNTRDIMTALKVQNL

SGNFRYSVTATPAGHHDESKAWLHFGKYDRYDDKYTYPAMDERLHPV

>gi|313648426|gb|EFS12869.1| uncharacterized ygjJ domain protein [Shigella flexneri 2a str. 2457T]

MNGYIQYDLAEGITWMNGLEITDGTGQLYLTGLFTPNFAARAWHHTGRADGLDVPGSESGMMVSAMYEAL

KGVYLSTAYTYAKHRPDHADDETTSFMQFGIWYEYGGGRFATAFDSRFYMKNASNDPSDQIFLMQYFYW

>gi|313648411|gb|EFS12854.1| cblD like pilus biogenesis initiator family protein [Shigella flexneri 2a str. 2457T]

MRNRLIAAILGLFGTLTGVQAAPDVTSEITYDLASGRADYYFWKDEASAGNNGYMWYECSYPDLQQTCTA

NGNISTVQIYLTEQRSGMRWPVKLKGFKTAIVSSDEAPLGCKGGKGLQTNLKDSNRSSCTEDGQHYYIYD

TKFLTLYLEQTEMKNLPIGGVWKGKVKLHSNSPAQDYFANITLNTLDPNHIDVFFPEFAHATPRVQLDLH

PTGSVNGSNYAQDLTMLDMCLYDGFNGNAISYEIMLKDEGRPAAGRRDGYFSIYRQGGTTTDEGERIDYR

VKMYNPETGGQIDVRNNENMVWNSINLKRVRPVVLPGIRYAVMCVPTPLTLAVDKFSVMDKQAGYYMGKL

SVIFTPSLPTIN

>gi|313648410|gb|EFS12853.1| outer membrane fimbrial user protein [Shigella flexneri 2a str. 2457T]

MPCFTAMRAEIALMSGSAFAVTHHAFSSGAGRTSDGNGSHASTLKSPSYTKSVSWQHYRNFNGYDADDVS

DSNTDVSDLYYRYDFLRRYYVQAGRMDNRTLFNAQGGNFTFNFLPLGAIDGMRIGSTLSYLNQAQSQQGT

PVMVLLSRNSRVDAYRNEQLLGSFYLNSGSQFIDTSSFPPGSYSVALKVYENNQLTRTELVPFTKTGGLT

DGNAQWFLQAGKTTSQVSDDESSAYQLGVRLPLHPQYELYAGLANADDVSAFELGNNWTADLGRVGNLAI
SASVFRNDDGGKGDMQQANWSNPGWPTLGFYRTNSDGDACTTDSRESYNALSCYESISATVSLNFVGWNM
MLGYTCTQNNTDDSLRWDKQQSFENNYLRQTTAQSISETVQLSASRAIVMRDWILSTSVGVFHRNDNGGD
NDDNGLYLSFSLSDTPTMDSNNNSHSTNVPTDYRYSEQDGDQTSWQLSHTFYNDSFSHKELGVTVGGLNT
DTINSAVNGRWDGQYGNVYATVSDSYDRKNHDHLSAFTGTYSSTLAVSRYGVNLGASGTDDLLGAVLVDV
KGFSEQDEESQDLQLEARVAGSRTLQLGQSDSVLFPYPGFQSGFVEVNDSSQGNQQGTTNIINGAGNREL
MLLPGKLRYREVSASFNYNYIGRLLLPAAVKKFPIVGLNSAMLLVAEDGGFTLEINGSEKELYLLSGQQF
LKCPLSVVKKRASIRYSGDVTCSVVTYSQLPESIQVQAQLKQPKLRGNVQTAQREVAP

>gi|313648405|gb|EFS12848.1| hypothetical protein SF2457T_3130 [Shigella flexneri 2a str. 2457T]
MKYRIALAVSLFALSAGSYATTLCQEKEQNILKEISYAEKHQNQNRIDGLNKALSEVRANCSDSQLRADH
HKKIAKQKDEVAERQQDLAEAKQKGDADRTETGRSAGRAEKAGSARLLTHNSHYLLTWRKLCRKNTLRNI
CVLS

>gi|313648401|gb|EFS12844.1| hypothetical protein SF2457T_3126 [Shigella flexneri 2a str. 2457T]
MILSIDSNDANSAPLHKKTISSLSGAVESMMKKLEDVGVLVAHILMPILFITAGWGKITAYSGTQQYMEA
MGVPGFMLLTAFLFHSNFAEGVNSLMFMKNLTISGGFLLLAITGPGAYSIGRLLNKKW

>gi|313648395|gb|EFS12838.1| hypothetical protein SF2457T_3120 [Shigella flexneri 2a str. 2457T]
MFDSTLNPLWQRYILAVQEEVKPALGCTEPISLALAAAVAAAELEGPVERVEAWVSPNLMKNGLGVTVPG
TGMVGLPIAAALGALGGNANAGLEVLKDATAQAIADAKALLAAGKVSVKIQEPCNEILFSRAKVWNGEKW
ACVTIVGGHTNIVHIETHNGVVFTHQACVAEGEQESPLTVLSRTTLAEILKFVNEVPFAAIRFILDSAKL
NCALSQEGLSGKWGLHIGATLEKQCERGLLAKDLSSSIVIRTSAASDARMGGATLPAMSNSGSGNQGITA
IMPVVVVAEHFGADDERLARALMLSHLSAIYIHNQLPRLSALCAATTAAMGAAAGMAWLVDGRYETISMA
ISSMIGDVSGMICDGASNSCAMKVSTSASAAWKAVLMALDDTAVTGNEGIVAHDVEQSIANLCALASHSM

QQTDRQIIEIMASKAR

>gi|313648385|gb|EFS12828.1| uncharacterized protein yhaC [Shigella flexneri 2a str. 2457T]

MNVIFSCGTGDIVDLSSLDLRNVDLDYYDFTDKHMANTILNHFKLDSTNFTNANMFQVNFVSSTQNATIS

WDYLLKITPVLISISDMYSEEKIKFVESCLNELGDITEEQLKIMRFAIIKSIPRATLTDKLENELTKEIY

KSSSKINNCLNRIKLPEMKEFSSKKTYDYIDTIIEDYENIKENAYLVVPQINYTMDLNIEDSSSEELIPS

RKTKILRTMALRSGNITHMKHITQRINILPERTIRIITTF

>gi|313648382|gb|EFS12825.1| 2-dehydro-3-deoxyglucarate aldolase [Shigella flexneri 2a str. 2457T]

MPTNEPEIIKRLLDIGFYNFLIPFVETKEEAEQAVASTRYPPEGIRGVSVSHRANMFGTVADYFAQSNKN

ITILVQIESQQGVDNVDAIAATEGVDGIFVGPSDLAAALGHLGNASHPDVQKAIQHIFNRASAHGKPSGI

LAPVEADARRYLEWGATFVAVGSDLGVFRSATHKLADTFKK

>gi|313648351|gb|EFS12795.1| glucitol/sorbitol permease IIC component [Shigella flexneri 2a str. 2457T]

MVTGILPLLISLQVIMNALINFIGQHRIERFAQRCAGNPVSRYLLLPCIGTFVFCNPMTLSLGRFMPEKY

KPSYYAAASYSCHSMNGLFPHINPANCLFILALPAV

>gi|313648338|gb|EFS12782.1| multidrug resistance A domain protein [Shigella flexneri 2a str. 2457T]

MTGYVSRRAVQPGAQISPTTPLMAVVPATNMWVDANFKETQIANMRIGQPVTITTDIYGDDVKYTGKVVG

LDMGTGSAFSLLPAQNATGNWIKVVQRLPVRIELDQKQLEQYPLRIGLSTLVSVNTTNRDGQVLANKVRS

TPVAVSTAREISLAPVNKLIDDIVKANAG

>gi|313648337|gb|EFS12781.1| multidrug resistance A domain protein [Shigella flexneri 2a str. 2457T]

MVLRHFEETDDAYVAGNQIQIMSQVSGSVTKVWADNTDFVKEGDVLVTLDPTDARQAFEKAKTALASSVR

QTHQLMINSKQLQANIEVQKIALAKAQSDYNRRVPLGNANLIGREELQHARDAVTSAQAQLDVAIQQYNA

NQAMILGTKLEDQPAVQQAATEVRNA

>gi|313648304|gb|EFS12748.1| alpha amylase, catalytic domain protein [Shigella flexneri 2a str. 2457T]

MFSIKPGPGNLPIDNPTLLSWNITDGDLNSKFNTLEYLNCITNIINACGVYPQDLKDREIISTFHAEKVI

NDLLKNDYKISLSPDTTYRELNKAAQSSITAPDRIGEGKTWVYQRDTMVERGDNSGVHQYGPAEHFTHII

SDKPSPKDKYVAYAINIPDYELAADVYNINVTSPSGQQETFKILINPEHLRQTLERKSLTAVQKSQCEII

TPKKPGEAILHAFNATYQQIRENMSEFARSHYGYIQIPPVTTFRADGPETPEEEKGYWFHAYQPEDLCTI

HNPMGDLQDFIALVKDAKKFGIDIIPDYTFNFMGIGGSGKNDLDYPSADIRAKISKDIESGIPGYWQGQV

LIPFIKDPVTKERKQIHPEDIHLTAKDFEASKDNISKDEWKNLHALKEKSLNGMPKTTPKSDQVIMLQNQ

YVREMRKYGVRGLRYDAAKHSKHEQIERSITPPLKNYNERLHNTNLFNPKYHEKTVMNYMEYLVTCQLNE

EQMSSLLYERDDLSAIDFSLLMKTIKAFSFGGDLLTLASKPGSTISSIPSERRILININHDFPNNGNLFN

DFLFNHQQDEQLAMAYMAALPFSRPLVYWDGQVLKSTTEIKNYDGPTRVGGEAWLNKGCSTYQQLYNEFH

ALYIDKAGIWSAFEGVFATKNVLAFSRGDSVNINHSPHDGLVIINKGNEEVEGTWPNKLQPGIYKNMGSN

SVNIIINNTRKIIPPCKVFTLRGGSLNINIPRRSALLLGKTGEPPNYLYL

>gi|313648282|gb|EFS12726.1| inner membrane protein yfiN [Shigella flexneri 2a str. 2457T]

MHNGLVEALKNITDVVHDVRSNRNFSRRVSEERIAEFHRFALDFNSLLDEMEEWQLRLQAKNAQLLRTAL

HDPLTGLANRAAFRSGINTLMNNSDARKTSALLFLDGDNFKYINDTWGHATGDRVLIEIAKRLAEFGGLR

HKAYRLGGDEFAMVLYDVQSESEVQQICSALTQIFNLPFDLHNGHQTTMTLSIGYAMTIEHASAEKLQEL

ADHNMYQAKHQRAEKLVR

>gi|313648229|gb|EFS12673.1| phage regulatory , Rha family protein [Shigella flexneri 2a str. 2457T]

MLNVAIENQNGWNYSAPAPHKTGAGIATPTMTTAHNRAQAVFLCVKHSHIQIMVGRAGQPQGWPVSVVTG

CSNPVRLTTHEIATSGGESFKLTIEAAIMATILTLSHPDATIENGRAVTTSVAVAEFFRKMHKNVIQKIE

TLECSPEFNRLNFKPVTYTDAKGEKRPMYQITKNGFVFLVMGFTGKKAAAFKEAYIAEFDRMEAELRQNN

TPPADKMIPGDGRTLVVHFDKFGNVEFTETVPDGALVCTLETFRFYLEKQGWTLVNRGAIKNMTVEQLLS

LK

>gi|313648213|gb|EFS12658.1| serine/threonine-protein phosphatase 2 [Shigella flexneri 2a str. 2457T]

MQQEAIVLLLKFHHLPHIIEITNDIIKYVIAHADYPGDEYLFGKEIAESELLWPVYRVQKSLNGELQQIN

GADYFIFGHMMFDNIQTFANQIYIDTGSPKSGRLSFYKIR

>gi|313648210|gb|EFS12655.1| putative transposase [Shigella flexneri 2a str. 2457T]

MPCFTAMRAEIALMSGSAFAVTHHAFSSGAGRTSDGNGSHASTLKSPSYTKSVSWQHYLNLKGHYDSKEE

GFANRYNFAYLLHKHHAKAAKQHLSRNQHQELAVRFFP

>gi|313648206|gb|EFS12651.1| hydrogenase expression/formation protein HypE [Shigella flexneri 2a str. 2457T]

MNNIQLAHGSGGQAMQQLINSLFMEAFANPWLAEQEDQARLDLAQLVAEGDRLAFSTDSYVIDPLFFPGG

NIGKLAICGTANDVAGSGAIPRYLSCGFILEEGLPMETLKAVVTSMAETARTAGIAIVTGDTKVVQRGAA

DKLFINTAGMGAIPANIHWGAQTLTAGDVLLVSGTLGDHGATILNLREQLGLDGELVSDCAVLTPLIQTL

RDIPGVKALRDATRGGVNAVVHEFAAACGCGIEISESALPVKPAVRGVCELLGLDALNFANEGKLVIAVE

RNAAEQVLAALHSHPLGKDAALIGEVVERKGVRLAGLYGVKRTLDLPHAEPLPRIC

>gi|313648184|gb|EFS12630.1| RNA polymerase sigma factor rpoD [Shigella flexneri 2a str. 2457T]

MEQNPQSQLKLLVTRGKEQGYLTYAEVNDHLPEDIVDSDQIEDIIQMINDMGIQVMEEAPDADDLMLAEN

TADEDAAEAAAQVLSSVESEIGRTTDPVRMYMREMGTVELLTREGEIDIAKRIEDGINQVQCSVAEYPEA

ITYLLEQYDRVEAEEARLSDLITGFVDPNAEEDLAPTATHVGSELSQEDLDDDEDEDEEDGDDDSADDDN

SIDPELAREKFAELRAQYVVTRDTIKAKGRSHAAAQEEILKLSEVFKQFRLVPKQFDYLVNSMRVMMDRV

RTQERLIMKLCVEQCKMPKKNFITLFTGNETSDTWFNAAIAMNKPWSEKLHDVSEEVHRALQKLQQIEEE

TGLTIEQVKDINRRMSIGEAKARRAKKEMVEANLRLVISIAKKYTNRGLQFLDLIQEGNIGLMKAVDKFE

YRRGYKFSTYATWWIRQAITRSIADQARTIRIPVHMIETINKLNRISRQMLQEMGREPTPEELAERMLMP

EDKIRKVLKIAKEPISMETPIGDDEDSHLGDFIEDTTLELPLDSATTESLRAATHDVLAGLTAREAKVLR

MRFGIDMNTDHTLEEVGKQFDVTRERIRQIEAKALRKLRHPSRSEVLRSFLDD

>gi|313648166|gb|EFS12612.1| inner membrane yqiK domain protein [Shigella flexneri 2a str. 2457T]

MTLEQEQQVKTRTAEQNARIAAFEAERRREAEQTRILAERQIQETEIEREQAVRSRKVEAEREVRIKEIE

QQQVTEIANQTKSIAIAAKSEQQSQAEARANLALAEAVSAQQNVETTRQTAEADRAKQVALIAAAQDAET

KAVELTVRAKAEKEAAEMQAAAIVELAEATHKKGLAEAEAQRALNDAINVLSDEQTSLKFKLALLQALPA

VIEKSVEPMKSIDGIKIIQVDGLNSGSAAGDANTGNVGGGNLAEQALSAALSYRTQAPLIDSLLNEIGVS

GGSLAALTSPLISTTPVAENVK

>gi|313648148|gb|EFS12594.1| outer membrane protein [Shigella flexneri 2a str. 2457T]

MQMKKLLPILIGLSLSGFSSLSQAENLMQVYQQARLSNPELRKSAADRDAAFEKINEARSPLLPQLGLGA

DYTYSNGYRDANGINSNATSASLQLTQSIFDMSKWRALTLQEKAAGIQDVTYQTDQQTLILNTATAYFNV

LNAIDVLSYTQAQKEAIYRQLDQTTQRFNVGLVAITDVQNARAQYDTVLANEVTARNNLDNAVEQLRQIT

GNYYPELAALNVENFKTDKPQPVNALLKEAEKRNLSLLQARLSQDLEREQIRQAQDGHLPTLDLTASTGI

SDTSYSGSKTRGAAGTQYDDSNMGQNKVGLSFSLPIYQGGMVNSQVKQAQYNFVGASEQLESAHRSVVQT

VRSSFNNINASISSINAYKQAVVSAQSSLDAMEAGYSVGTRTIVDVLDATTTLYNAKQELANARYNYLIN

QLNIKSALGTLNEQDLLALNNALSKPVSTNPENVAPQTPEQNAIADGYAPDSPAPVVQQTSARTTTSNGH

NPFRN

>gi|313648133|gb|EFS12579.1| radical SAM superfamily protein [Shigella flexneri 2a str. 2457T]

MSSISLIQPDRDLFSWPQYWAACFGPAPFLPMSREEMDQLGWDSCDIILVTGDAYVDHPSFGMAICGRML

EAQGFRVGIIAQPDWSSKDDFMRLGKPNLFFGVTAGNMDSMINRYTADRRLRHDDAYTPDNVAGKRPDRA

TLVYTQRCKEAWKDVPVILGGIEASLRRTAHYDYWSDTVRRSVLVDSKADMLMFGNGERPLVEVAHRLAM

GEPISEIRDVRNTAIIVKEALPGWSGVDSTRLDTLGKIDPIPHPYGEDLPCADNKPVAPKKQEAKAVIVQ

PPRQKPWEKTYVLLPSFEKVKGDKVLYAHASRILHHETNPGCARALMQKHGDRYVWINPPAIPLSTEEMD

SVFALPYKRVPHPAYGNARIPAYEMIRFSVNIMRGCFGGCSFCSITEHEGRIIQSRSEDSIINEIEAIRD

TVPGFTGVISDLGGPTANMYMLRCKSPRAEQTCRRLSCVYPDICPHMDTNHEPTINLYRRARDLKGIKKI

LIASGVRYDIAVEDPRYIKELATHHVGGYLKIAPEHTEEGPLSKMMKPGMGSYDRFKELFDTYSKQAGKE

QYLIPYFISAHPGTRDEDMVNLALWLKKHRFRLDQVQNFYPSPLANSTTMYYTGKKPLAKIGYKSEGVFV

PKGDKQRRLHKALLRYHDPANWPLIRQALEAMGKKHLIGSRRDCLVPAPTIEEMREARRQNRNTRPALTK

HTPMATQRQTPATAKKASSTQSRPVNAGAKKRPKAAVGR

>gi|313648091|gb|EFS12537.1| yghO protein [Shigella flexneri 2a str. 2457T]

MECDLLMIKIEKVINKNDLKAFIAFPSSLYPDDPNWIPPLFIERNEHLSAKNPGTDHIIWQAWVAKKAGQ

IVGRITAQIDTLHRERYGEDTGHFGMIDAIDDPQDFAALFGAAEAWLKSQGASKISGPFSLNINQESGLL

IEGFDTPPCAMMPHGKPWYAAHIEQLGYHKGIDLLAWWMQRTDLTFSPALKKLMDQVRKKVTIRCINRQR

FAEEMQILREIFNSGWQHNWGFVPFTEHEFATMGDQLKYLVPDDMIYIAEIDSAPCAFIVACLTSTRRLP

I

>gi|313648085|gb|EFS12531.1| glycolate oxidase iron-sulfur subunit [Shigella flexneri 2a str. 2457T]

MLEGNEVTLKTQEHLDRCLTCRNCETTCPSGVRYHNLLDIGRDIVEQKVKRPLPERMLREGLRQVVPRPA

VFRALTQVGLVLRPFLPEQVRAKLPAETVKAKPRPPLRHKRRVLMLEGCAQPTLSPNTNAATARVLDRLG

ISVMSANEAGCCGAVDYHLNAQEKGLARARNNIDAWWPAIEAGAEAILQTASGCGAFVKEYGQMLKNDAL

YADKARQVSELAVDLVELLREEPLEKLAIRGDKKLAFHCPCTLQHAQKLNGEVEKVLLRLGFTLTDVPDS

HLCCGSAGTYALTHPDLARQLRDNKMNALESGKPEMIVTANIGCQTHLASAGRTSVRHWIEIVEQALEKE

>gi|313648076|gb|EFS12522.1| type IV leader peptidase family protein [Shigella flexneri 2a str. 2457T]

MAAIWLLRVDLLFVALFLIFISWGDILLRIISHRYLLVLTVLIFLALILQHQKPNLVAAGSVLLVGFFLF

SAGIIGGGDVKLLTVLSLAIDEHELANFLVAMTFCGALVVLAGLLFFRKSIRENGVPYAVPISLAFLLTY

PVFPLFC

>gi|313648059|gb|EFS12505.1| aec68 [Shigella flexneri 2a str. 2457T]

MTMLQIVGALILLIAGFAILRLLFRALTSTASALAGFILLCLFGPALLAGYITERITRLFHIRWLAGVFL

TIAGMIISFMWGLDGKHIALEAHTFDSVKFILTTALAAGLLALPVQIRTIQQNGLTPEDISKEINGYYCC

FYTAFFLMACSAYAPLIALQFDISPSLMWWGGLLYWLAALVTLLWAASQIQALKRLTSAISQTLEEQPVL

NSKSWLSSLQNDYSLPETLTERIWLTLISQRISRGELREFELADGNWLLNNAWYERNMAGFNEQLKENLS

FTPDELKTLFRNRLNLSPEANDDFLDRCLDGGDWYPFSEGRRFVSFHHVDELRICASCGLTEVHHAPENH

KPAPEWYCSSLCHETETLCQDIYERSYTGFISDATANGLILMKLPETWSTNEKMFASGGQGHGFAAERGN

HIVDRVRLKNARILGDNNARNGADRLVSGTEIQTKYCSTAARSVGAAFDGQNGQYRYMGNHGPMQLEVPR

DQYAGAVETMKNKIREGKVPGVTDPAEASRLIRRGHLTYTQARNITRFGTIESVTYDIAEGSVVSLAAGG

ISFALTASVFWLSTGDRDAALQTAAVQAGKTFTRTLAVYVTTQQLHRLTVVQGMLKHIDFSTASPTVRQA

LQKGTGAGNISALNKVMKGTLVTSLALVAVTTSPDMIKMLRGRISGAQFIRNLAVASSGVAGGAVGSVAG

GILFSPLGPFGALTGRVVGGVLGGMIASAVSGKIAGALVEEDRVKILAMIQEQVTWLAGSFLLTGHEIEN

LNANLARVIDQNALEIIFAAGIQQRAATNMLIKPLVVSIIRQRPVMEYDASHLGKMVNRLEEAFPPELPA

>gi|313648058|gb|EFS12504.1| antigen 43 [Shigella flexneri 2a str. 2457T]

MKRHLNTCYRLVWNHITGAFVVASELARAQGKRGGVAVALSLAAVTSLPVLAADIVVHPGETVNGGTLVN

HDNQFVSGTADGVTVSTGLELGPDSDENTGGQWIKAGGTGRNTTVTANGRQIVQAGGTASDTVIRDGGGQ

SLNGLAVNTTLDNRGEQWVHGGGKAAGTIINQDGYQTIKHGGLATGTIVNTGAEGGPESENVSSGQMVGG

TAESTTINKNGRQVIWSSGMARDTLIYAGGDQTVHGEAHNTRLEGGNQYVHNGGTATETLINRDGWQVIK

EGGTAAHTTINQKGKLQVNAGGKASDVTQNTGGALVTSTAATVTGTNRLGAFSVVEGKADNVVLENGGRL

DVLTGHTATNTRVDDGGTLDVRNGGTATTVSMGNGGVLLADSGAAVSGTRSDGKAFSIGGGQADALMLEK

GSSFTLNAGDTATDTTVNGGLFTARGGTLAGTTTLNNGAILTLSGKTVNNDTLTIREGDALLQGGALTGN

GSVEKSGSGTLTVSNTTLTQKAVNLNEGTLTLNDSTVTTDVIAQRGTALKLTGSTVLNGAIDPTNVTLAS

GATWNIPDNATVQSVVDDLSHAGQIHFTSTRTGKFVPATLKVKNLNGQNGTISLRVRPDMAQNNADRLVI

DGGRATGKTILNLVNAGNSASGLATSGKGIQVVEAINGATTEEGAFIQGNKLQAGAFNYSLNRDSDESWY

LRSENAYRAEVPLYASMLTQAMDYDRILAGSRSHQTGVSGENNSVRLSIQGGHLGHDNNGGIARGATPES

SGSYGFVRLEGDLLRTEVAGMSVTAGVYGAAGHSSVDVKDDDGSRAGTVRDDAGSLGGYLNLIHNASGLW

ADIVAQGTRHSMKASSDNNDFRVRGWGWLGSLETGLPFSITDNLMLEPQLQYTWQGLSLDDGQDNASYVK

FGHGSAQHVRAGFRLGSHHDMNFGKGTSSRDTLRGSAKHSVRELPVNWWVQPSVIRTFSSRGDMSMGTAA

AGSNMTFSPSQNGTSLDLQAGLEARVRENITLGVQASYAHSINGSSAEGYNSQATLNVTF

>gi|313648046|gb|EFS12492.1| hypothetical protein SF2457T_3646 [Shigella flexneri 2a str. 2457T]

MDEYCHTHKLSRSKVINALLSATAPVLNDINCYYQLAGELQSRLLNGVYQRDLPRKRNVVSAEKYCLEIW

ENKLFTRRILEFDSSNGVLYALKHKRHYRRDKMIGRVESRCIKDICEYQMQLSGEKTKYACFIYIERTIY

NHDNPSDKIPVKAAVGNAVILLAKDVIYNEYFFDLRQSFFVSVTDLMVSGAKGIPETQTYPDVYCWIPLF

SINSGVLITPVYKIDPLKPVTVKKPDKITVVCNYRE

>gi|313648033|gb|EFS12479.1| ISL3 family transposase domain protein [Shigella flexneri 2a str. 2457T]

MVARGFTGSETIVRDAVAKWRKGWNPPVTTAVRLPSVSRVSRWLMPWRITRDEENYASRFISLMCEKEPE

LKIAQQLALEFYRILKTQNKSQLSSWFTRVHESGSAEFRRVAAGMEADAAAICEAISSRWSNGVVEGHVN

RLKKMLKRQMYGRAGFELLRQRVMSPLA

>gi|313648032|gb|EFS12478.1| ISSfl4 ORF3 [Shigella flexneri 2a str. 2457T]

MKTQSRHSELAKAFAYALNQWPALTYYANDGWVEIDNNIAENALRAVSLGRKNFLFFGSDHGGERGALLY

SLIGTCKLNDVDPESYLRHVLGVIADWPVNRVSELLPWRIALPAE

>gi|313648015|gb|EFS12461.1| uncharacterized protein yggN [Shigella flexneri 2a str. 2457T]

MMRKMLLAAALSVTAMTAHADYQCSVTPRDDVIVSPQTVQVKGENGNLVITPDGNVMYNGKQYSLNAAQR

EQAKDYQAELRSTLPWIDEGAKSRVEKARIALDKIIVQEMGDSSKMRSRLTKLDAQLKEQMNRIIETRSD

GLTFHYKAIDQVRAEGQQLVNQAMGGILQDSINEMGAKAVLKSGGNPLQNVLGSLGGLQSSIQTEWKKQE

KDFQQFGKDVCSRVVTLEDSRKALVGNLK

>gi|313647975|gb|EFS12421.1| uncharacterized protein yggC [Shigella flexneri 2a str. 2457T]

MKIELTVNGLNVQAQYHDEEIERVHKLLLRMLAALQTVNPQRRTVVFLCAPPGTGKSTLTTFWEYLAQQD

PELPAIQALPMDGFHHYNSWLDAHQLRPFKGAPETFDVAKLAENLRQVVEGDCTWPQYDRQKHDPVEDAL

HVTAPLVIVEGNWLLLDDEKWCQLAQFCDFSIFINAPAAALRERLVGRKLAGGVSLADAEAFYDRTDGPN

VRRGLEESLPANLTLMMTATGEYRLVD

>gi|313647962|gb|EFS12408.1| succinyl-CoA:coenzyme A transferase [Shigella flexneri 2a str. 2457T]

MIIELNHYHDPRVAELADIVIPGAPPRRNSVSIFHAMDRVGTRYVQIDPKKIVAVVETNLPDAGNMLDKQ

NPMCQQIADNVVTFLLQEMAHGRIPPEFLPLQSGVGNINNAVMARLGENPEIPPFMMYSEVLQESVVHLL

ETGKISGASASSLTISADSLRKIYDNMDYFASRIVLRPQEISNNPEIIRRLGVIALNVGLEFDIYGHANS

THVAGVDLMNGIGGSGDFERNAYLSIFMAPSIAKEGKISTVVPMCSHVDHSEHSVKVIITEQGIADLRGL

SPLQRARTIIDNCAHPMYRDYLHRYLENAPGGHIHHDLSHVFDLHRNLIATGSMLG

>gi|313647961|gb|EFS12407.1| acetyl-CoA hydrolase/transferase family protein [Shigella flexneri 2a str. 2457T]

METQWTRMTADEAAEIIQHNDMVAFSGFTPAGSPKALPAAIARRANEQHEAKKPYQIRLLTGASISAAAD

DVLSDADAVSWRAPYQTSSSLRKKINQGAVSFVDLHLSEVAQMVNYGFFGDIDVAVIEASALAPDGRVWL

TSGIGNAPT

>gi|313647913|gb|EFS12359.1| uncharacterized ygeQ domain protein [Shigella flexneri 2a str. 2457T]

MKGKSALTLLLAGIFSCGTCQATEAEVTSESVFNILNSRGAATDKSYLSLNPDKYPNYRLLIHSAKLKNE

IKSHYTKDETQGLLTLTENTRKLTLTEKPWGTFILASTFEDDKTAAETHYDAVWLRDSLWGYMALVSDQG

NSVAAKKVLLTLWGYMSTPDQIKRMQDIISNPKRLDGILILPT

>gi|313647840|gb|EFS12286.1| hypothetical protein SF2457T_3435 [Shigella flexneri 2a str. 2457T]

MESLPFVAAQPTIVPQLQQQVTGLTSSLNSQEKKELTHKLESIFNNTQVQIAVLIVSTTKDETIEQYATR

VFDNWRLGDAKRNDGILIVVAWSDRTVRIQVGYGLEEKVTDALAGDIIRSNMIPAFKQQKLAKGLELAII

ALNNQLTSQHQYPANPSESESTSSTDHYYFAIFWVFAVMFFPFWFFHQGSNFCRACKSGVCISAIHLLDL

FLFSDKTFSIAVFSFFFTFTIFMVFTCLCVLQKRASGRSYHSDNSGSAGGSSGGASGRW

>gi|313647829|gb|EFS12275.1| glycerol-3-phosphate responsive antiterminator family protein [Shigella flexneri 2a str. 2457T]

MIAAVKDNASLQLAIDSECQFISVLYGNICTISNIVKKIKNAGKYAFIHVDLLEGASNKEVVIQFLKLVT

EADGIISTKASMLKAARAEVFFCIHHLFIVDSISFHNIDK

>gi|313647821|gb|EFS12267.1| CRISPR-associated helicase Cas3 domain protein [Shigella flexneri 2a str. 2457T]

MTFFDFWGKNRRGEKEGIDDYHLLCWHLLDVAAMGYLMVKRNCFGLADYFRQLGISDKEQAARFFAWLLC

WHDIGKFARSFQQLYLPPEFKIQEGARKNYEKISHSTLGYWLWNHYLSECQELLPSSSLSPRKLRRVIEM

WMPVTTGHHGRPPDRMDELDNFLPEDKAAARDFLLEIKPLFPRIEIPTFWDDDEGVELIKQLSWYISATV

VLADWTGSSTRFFPRVAQAMDIEDYWQKALVQAQNALTVFPPQAETAPFTGINTLFPFIENTTPLQQKVL

DLDINQPGPQLFILEDVTGAGKTEAALILAHRLMAAGNAQGLFFGLPTMATANAMYDRLVKTWLAFYSPE

VFPAPAEINRRNTVRFSRK

>gi|313647807|gb|EFS12253.1| RNA polymerase sigma factor rpoS [Shigella flexneri 2a str. 2457T]

MSQNTLKVHDLNEDAEFDENGVEVFDEKALVEEEPSDNDLAEEELLSQGATQRVLDATQLYLGEIGYSPL

LTAEEEVYFARRALRGDVASRRRMIESNLRLVVKIARRYGNRGLALLDLIEEGNLGLIRAVEKFDPERGF

RFSTYATWWIRQTIERAIMNQTRTIRLPIHIVKELNVYLRTARELSHKLDHEPSAEEIAEQLDKPVDDVS

RMLRLNERITSVDTPWVVIPKKRCWTSWPMKKRTVRKIPRKMTI

>gi|313647795|gb|EFS12242.1| D-ribose-binding periplasmic domain protein [Shigella flexneri 2a str. 2457T]

MNMKKLATLVSAVALSATVSANAMAKDTIALVVSTLNNPFFVSLKDGAQKEADKLGYNLVVLDSQNNPAK

ELANVQDLTVRGTKILLINPTDSDAVGNAVKMAN

>gi|313647794|gb|EFS12241.1| D-ribose-binding periplasmic protein [Shigella flexneri 2a str. 2457T]

MSHIASDNVLGGKIAGDYIAKKAGEGAKVIELQGIAGTSAARERGEGFQLAVAAHKFNVLASQPADFDRT

KGLNVMQNLLTAHPDVQAVFAQNDEMALGALRALQTAGKSDVMVVGFDGTPDGEKAVNDGKLAATIAQLP

DQIGAKGVETADKVLKGEKVQAKYPVDLKLVVKQ

>gi|313647791|gb|EFS12238.1| drug resistance MFS transporter [Shigella flexneri 2a str. 2457T]

MSDKKKRSMSGLPWIAAMAFFMQALDATILNTALPAIAHSLNRSPLAMQSAIISYTLTVAMLIPVSGWLA

DRFGTRRIFTLAVSLFTLGSLACALSNSLPQLVVFRVIQGIGDAMMMPVARPALLRAYPRNELLPVLNFV

AMPGLVGPILGPVLGGVLVTWATWHWIFLINIPIGIAGLLYARKHMPNFTTARRRFDITGFLLFGLSLVL

FSSGIELFGEKIVASWIALTVIVTSIGLLLLYILHARRTPNPLISLDLFKTRTFSIGIVGNIATRLGTGC

VPFLMPLMLQVGFGYQAFIAGCMMAPTALGSIIAKSMVTQVLRRLGYRHTLVGITVIIGLMIAQFSLQSP

AMAIWMLILPLFILGMAMSTQFTAMNTITLADLTDDNASSGNSVLAVTQQLSISLGVAVSAAVLRIYEGM

EGTTTVEQFHYTFITMGIITVASAAMFMLLKTTDGNNLIKRQRKSMPNRVPSESE

>gi|313647790|gb|EFS12237.1| bacterial regulatory s, gntR family protein [Shigella flexneri 2a str. 2457T]

MPLSAQQLAAQKNLSYVLAEKLAQRILKGEYEPGTILPGEIELGEQFGVSRTAVREAVKTLTAKGMVLPR

PRIGTRVMPQSNWNFLDQELLTWWITEENFHQVIDHFLVMRICLEPQACLLAATVGTAEQKAHLNTLMAE

MAALKENFRRERWIEVDMAWHEHIYEMSANPFLTSFASLFHSVYHTYFTSITSDTVIKLDLHQAIVDAII

QSDGDAAFKACQALLRSPDK

>gi|313647787|gb|EFS12234.1| competence comM domain protein [Shigella flexneri 2a str. 2457T]

MPGESSATVKQRVMAARERQFKRQNKLNAWLDSPEIRQFCKLESEDAMWLEETLIHLGLSIRAWQRLLKV

ARTIADIDQSDIITRQHLQEAVSYRAIDRLLIHLQKLLT

>gi|313647786|gb|EFS12233.1| competence protein comM [Shigella flexneri 2a str. 2457T]

MSLSIVHTRAALEVNAPPITVEVHISKGLPGLTMVGLTETTVKEARDRVRSAIINSGYEYPAKKITINLA

PADLPKEGGRYDLPIAIALLAASEQLTANKLDEYELVGELALTGALRGVPGAISSATEAIKSGRKIIVAK

DNEDEVGLINGEGCLIADHLQAVCAFLEGKNTLERPKPTDAVSRALQHDLSDVVGQEQGKRGLEITAAGG

HNLLLIGPPGTGKTMLASRINGLLPDLSNEEALESAAILSLVNAESVQKQWRQRPFRSPHHSASLTAMVG

GGAIPGPGEISLAHNGVLFLDELPEFERRTLDALREPIESGQIHLSRTRAKITYPARFQLVAAMNPSPTG

HYQGNHNRCTPEQTLRYLTGDPITTPRHFE

>gi|313647749|gb|EFS12197.1| fimbrial family protein [Shigella flexneri 2a str. 2457T]

MNAPFEYHANNPSGNTKYNCNRIEPLSISSGAKAIVYFYIKKTFAGKLIIPETKIVTLYGTISRDTPVDY

SQPMADVYIRGDITAPQSCEINNLKPVCFDFKEIPAADFSSVVGSAVTTHKITKTVTIECENLGILNTDD

ISTSFYATEPNTDNSMVVTSNSNVGIKIYDKNNKEIKVNGGELPTDMDKSTVYGEKSGSVTFSAAPASLT

GARPAPGQFTATATITVEIVR

>gi|313647747|gb|EFS12195.1| phosphate ABC transporter, permease protein PstC [Shigella flexneri 2a str. 2457T]

MAATKPAFNPPGKKGDIIFSVLVKLAALIVLLMLGGIIVSLIISSWPSIQKFGLAFLWTKEWDAPNDIYG

ALVPIYGTLVTSFIALLIAVPVSFGIALFLTELAPGWLKRPLGIAIELLAAIPSIVYGMWGLFIFAPLFA

VYFQEPVGNIMSNIPIVGALFSGPAFGIGILAAGVILAIMIIPYIAAVMRDVFEQTPVMMKESAYGIGCT

TWEVIWRIVLPFTKNGVIGGIMLGLGRALGETMAVTFIIGNTYQLDSASLYMPGNSITSALANEFAEAES

GLHVAALMELGLILFVITFIVLAASKFMIMRLAKNEGAR

>gi|313647733|gb|EFS12181.1| hypothetical protein SF2457T_3945 [Shigella flexneri 2a str. 2457T]

MCIADGSAAEKFAGSFQDDASIEGVEFEYDEEDEFAGIKNTYPDEMLKELVERTPGYHGWQQEFWLAHCG

DFCAFIGYVGWNDIKDRLDEFANLEEDCENFGIRNSDLAKCLQKGGDCQGYLFRCLHCGKLRLWGDFS

>gi|313647723|gb|EFS12171.1| HTH-type transcriptional regulator yidZ domain protein [Shigella flexneri 2a str. 2457T]

MPFYFRKECPLNSGYLRALDNVLQELGRERTIAMSLPEFEQSLFMAAQPDNLLLATAPRYCQYYNQLHQL

PLVALPLPFDESQQKKLEVPFTLLWHKRNSHNPKIVWLRETIKNLYASMA

>gi|313647720|gb|EFS12168.1| HTH-type transcriptional regulator yidZ domain protein [Shigella flexneri 2a str. 2457T]

MKKSITTLDLNLLLCLQLLMQERSVTKAAKRMNVTPSAVSKSLAKLRAWFDDPLFVNSPLGLSPTQLMVS

MEQNLAEWMQMSNLLLDKPHHQTPRGLKFELAAESPLMMIMLNALSKRIYQRYPQATIKLRNWDYDSLDA

ITRGEVDIGFSGRESHPRSRELLSSLPLAIDYEVLFSDVPCVWLRKDHPALHETWNLDTFLRYPHISICW

EQSDTWVRCTGNSGHYHLFFF

>gi|313647704|gb|EFS12152.1| lipoproteinC [Shigella flexneri 2a str. 2457T]

MKLNFKGFFKAAGLFPLALMLSGCISYALVSHTAKGSSGKYQSQSDTITGLSQAKDSNGTKGYVFVGESL

DYLITDGADDIVKMLNDPALNRHNIQVADDARFVLNAGKKKFTGTISLYYYWNNEEEKALATHYGFACGV

QHCTRSLENLKGTIHEKNKNMDYSKVMAFYHPFKVRFYEYYSPRGIPDGVSAALLPVTVTLDIITAPLQF

LVVYAVNQ

>gi|313647701|gb|EFS12149.1| KDPG and KHG aldolase family protein [Shigella flexneri 2a str. 2457T]

MQWQTKLPLIAILRGITPDEALAHVGAVIDAGFDAVEIPLNSPQWEQSIPAIVDAYGDKALIGAGTVLKP

EQVDALARMGCQLIVTPNIHSEVIRRAVGYGMTVCPGCATATEAFTALEAGAQALKIFPSSAFGPQYIKA

LKAVLPPDIAVFAVGGVTPENLAQWIDAGCVGAGLGSDLYRAGQSVERTAQQAAAFVKAYREAVQ

>gi|313647689|gb|EFS12137.1| PTS system maltose-specific EIICB component [Shigella flexneri 2a str. 2457T]

MLSQIQRFGGAMFTPVLLFPFAGIVVGLAILLQNPMFVGESLTDPNSLFAQIVHIIEEGGWTVFRNMPLI

FAVGLPIGLAKQAQGRACLAVMVSFLTWNYFINAMGMTWGSYFGVDFTQDAVAGSGLTMMAGIKTLDTSI

IGAIIISGIVTALHNRLFDKKLPVFLGIFQGTSYVVIIAFLVMIPCAWLTLLGWPKVQMGIESLQAFLRS

AGALGVWVYTFLERILIPTGLHHFIYGPFIFGPAAVEGGIQMYWAQHLQEFSLSAEPLKSLFPEGGFALH

GNSKIFGAVGISLAMYFTAAPENRVKVAGLLIPATLTAMLVGITEPLEFTFLFISPLLFAVHAVLAASMS

TVMYLFGVVGNMGGGLIDQVLPQNWIPMFSNHADMMLTQIAIGLCFTLLYFVVFRTLILQFNMCTPGRED

AEVKLYSKAEYKASRGQTTAAEPKKELDQAAGILQALGGVGNISSINNCATRLRIALHDMSQTLDDEVFK

KLGAHGVFRSGDAIQVIIGLHVSQLREQLDSLINSHQSAENVAITEAV

>gi|313647688|gb|EFS12136.1| putative 6-phospho-alpha-glucosidase [Shigella flexneri 2a str. 2457T]

MTKFSVVVAGGGSTFTPGIVLMLLANQDRFPLRALKFYDNDGARQEVIAEACKVILKEKAPDIAFSYTTD

PEVAFSDVDFVMAHIRVGKYPMRELDEKIPLRHGVVGQETCGPGGIAYGMRSIGGVLELVDYMEKYSPNA

>gi|313647661|gb|EFS12109.1| serine protease sat domain protein [Shigella flexneri 2a str. 2457T]

MKETESSYNKKFNSDYKSNNQQTSFDQPDWKTGVFKFDTLHLNNADFSISRNANVEGNISANKSAITIGD

KNAYIDNLAGKNITNNGFDFKQTISTNLSIGETKFTGGITAHNSQIAIGDQAVVTLNGATFLNNTPISID

KGAKVIAQNSMFTTKGIDISGELTMMGIPEQNSKTVTPGLHYAADGFRLSGGNANFIARNMASVTGNIYA

DDAATITLGQPETETPTISSAYQAWAETLLYGFDTAYRGAITAPKATVSMNNAIWHLNSQSSINRLETKD

SMVRFTGDNGKFTTLTVDNLTIDDSAFVLRANLAQADQ

>gi|313647660|gb|EFS12108.1| putative serine protease [Shigella flexneri 2a str. 2457T]

MIYKNDKTFRNLEIFGDSGSGAYLYDNKLEKWVLVGTTHGIASVNGDQLTWITKYNDKLVSELKDTYSHK

INLNGNNVTIKNTDITLHQNNADTTGTQEKITKDKDIVFTNGGNVLFKDNLDFGSGGIIFDEGHEYNING

QRFTFKGAGIDIGKESIVNWNALYSSDDVLHKIGPGTLNVQKKQGANIKIGEGNVILNEEGTFNNIYLAS

GNGKVILNKDNSLGNDQYAGIFFTKRGGTLDLNGHNQTFTRIAATDDGTTITNSDTKKEAVLAINNEDSY

IYHGNINGNIKLTHNINSQDKKTNAKLILDGSVNKKNDVEVSNASLTM

>gi|313647618|gb|EFS12068.1| cellulose synthase operon C domain protein [Shigella flexneri 2a str. 2457T]

MDIAAGDKAAARSQLAKLPATDNTSLNTQRRVALAQAQLGDTAAAQQTFNKLIPQAKSQPPSMESAMVLR

DGAKFEAQAGDPKQALETYKDAMVASGVTTTRPQDNDTFTRLTRNDEKDDWLKRGVRSDAADLYRQQDLN

VTLEHDYWGSSGTGGYSDLKAHTTMLQVDAPYSDGRMFFRSDFVNMNVGSFSTNADGKWDDNWGTCTLQD

CSGNRSQSDSGASVAVGWRNDVWSWDIGTTPMGFNVVDVVGGISYSDDIGPLGYTVNAHRRPISSSLLAF

GGQKDSPSNTGKKWGGVRADGVGLSLSYDKGEVNGVWASLSGDQLTGKNVEDNWRVRWMTGYYYKVINQN

NRRVTIGLNNMIWHYDKDLSGYSLGQGGYYSPQEYLSFAIPVMWRERTENWSWELGASGSWSHSRTKTMP

RYPLMNLIPTDWQEEAARQSNDGGSSQGFGYTARALLERRVTSNWFVGTAIDIQQVKDYAPSHFLLYVRY

SAAGWQGDMDLPPQPLIPYADW

>gi|313647617|gb|EFS12067.1| cellulose synthase operon C domain protein [Shigella flexneri 2a str. 2457T]

MDSGNTNAVRGLANIYRQQSPEKAEAFIASLSASQRRSIDDIERSLQNDRLAQQAEVLENQGKWAQAAAL

QRQRLALGPGSVWITYRLSQDLWQAGQRSQADTLMRNLAQQKPNNPEQVYAYGLYLSGHNQDRAALAHIN

SLPRAQWNSNIQELVNRLQSDQVLETANRLRESGKEAEAEAMLRQQPPSTRIDLTLADWA

>gi|313647615|gb|EFS12065.1| cyclic di-GMP-binding protein [Shigella flexneri 2a str. 2457T]

MKRKLFWICAVAMGMSAFPSFMTQATPATQPLINAEPAVAAQTEQNPQVGQVMSGEQGADAPIVAQNGPS

RDVKLTFAQIAPPPGSMVLRGINPNGSIEFGMRSDEVVTKAMLNFEYTPSPSLLPVQSQLKVYLNDELMG

VLPVTKEQLGKKTLAQMPINPLFITDFNRVRLEFVGHYQDVCENPASTTLWLDVGRSSGLDLTYQTLNVK

NDLSHFPVPFFDPRDNRTNTLPMVFAGAPDVGLQQASAIVASWFGSRSGWRGQNFPVLYNQLPDRNAIVF

ATNDKRPDFLRDHPAVKAPVIEMINHPQNPYVKLLVVFGRDDKDLLQAAKGIAQGNILFRGESVVVNEVK

PLLPRKPYDAPNWVRTDRPVTFGELKTYEEQLQSSGLEPAAINVSLNLPPDLYLMRSTGIDMDINYRYTM

PPVKDSSRMDISLNNQFLQSFNLSSKQEANRLLLRIPVLQGLLDGKTDVSIPALKLGATNQLRFDFEYMN

PMPGGSVDNCITFQPVQNHVVIGDDSTIDFSKYYHFIPMPDLRAFANAGFPFSRMADLSQTITVMPKAPN

EAQMETLLNTVGFIGAQTGFPAINLTVTDDGSTIQGKDADIMIIGGIPDKLKDDKQIDLLVQATESWVKT

PMRQTPFPGIVPDESDRAAETQSTLTSFGAMAAVIGFQSPYNDQRSVIALLADSPRGYEMLNDAVNDSGK

RATMFGSVAVIRESGINSLRVGDVYYVGHLPWFERLWYALANHPILLAVLATISVILLAWVLWRLLRIIS

RRRLNPDNE

>gi|313647614|gb|EFS12064.1| cellulose synthase catalytic subunit [UDP-forming] domain protein [Shigella flexneri 2a str. 2457T]

MFHFLSGIPRLIFLTAPLAFLLLHAYIIYAPALMIALFVLPHMIHASLTNSKIQGKYRHSFWSEIYETVL

AWYIAPPTLVALINPHKGKFNVTAKGGLVEEEYVDWVISRPYIFLVLLNLVGVAVGIWRYFYGPPTEMLT

VVVSMVWVFYNLIVLGGAVAVSVESKQVRRSNRVEMTMPAAIAREDGHLFSCTVQDFSDGGLGIKINGQA

QILEGQKVNLLLKRGQQEYVFPTQVARVMGNEVGLKLMPLTTQQHIDFVQCTFARADTWALWQDSYPEDK

PLESLLDILKLGFRGYRHLAEFAPSSVKGIFRVLTSLVSWVVSFIPRRPERSETAQPSDQALAQQ

>gi|313647613|gb|EFS12063.1| cellulose synthase catalytic subunit [UDP-forming] domain protein [Shigella flexneri 2a str. 2457T]

MSAGARRLILGIIVTFSLILALICVTQPFNPLAQFIFLMLLWGVALIVRRMPGRFSALMLIVLSLTVSCR

YIWWRYTSTLNWDDPVSLVCGLILLFAETYAWIVLVLGYFQVVWPLNRQPVPLPKDMSLWPSVDIFVPTY

NEDLNVVKNTIYASLGIDWPKDKLNIWILDDGGREEFRQFAQNVGVKYIARTTHEHAKAGNINNALKYAK

GEFVSIFDCDHVPTRSFLQMTMGWFLKEKQLAMMQTPHHFFSPDPFERNLGRFRKTPNEGTLFYGLVQDG

NDMWDATFFCGSCAVIRRKPLDEIGGIAVETVTEDAHTSLRLHRRGYTSAYMRIPQAAGLATESLSAHIG

QRIRWARGMV

>gi|313647612|gb|EFS12062.1| cellulose synthase catalytic subunit [UDP-forming] domain protein [Shigella flexneri 2a str. 2457T]

MSILTRWLLIPPVNARLIGRYRDYRRHGASAFSATLGCFWMILAWIFIPLEHPRWQRIRAEHKNLYTHIN

ASRPRPLDPVRYLIQTCWLLIGASRKETPKPRSGHFQVCKIFVDVTING

>gi|313647583|gb|EFS12033.1| putative transposase [Shigella flexneri 2a str. 2457T]

MPCFTAMRAEIALMSGSAFAVTHHAFSSGAGRTSDGNGSHASTLKSPSYTKSVSWQHYLRLEVVNHYFTT

DDGYRIISARFGVPRTQVRTWVALYEKHGEKGLIPKPKGVSADPELRIKVVKAVIEQHMSLNQAAAHFML

AGSGSVARWLKVYEERGEAGLRALKISTKRNIAISVDPEKAASALELSKDRRIEDLERQVRFLETRLMYL

KKLKALAHPTKK

>gi|313647565|gb|EFS12015.1| xylose isomerase domain protein [Shigella flexneri 2a str. 2457T]

MLIEPKPQEPTKHQYDYDAATVYGFLKQFGLEKEIKLNIEANHATLAGHSFHHEIATAIALGLFGSVDAN

RGDAQLGWDTDQFPNSVEENALVMYEILKAGGFTTGGLNFDAKVRRQSTDKYDLFYGHIGAMDTMALALK

IAARMIEDGELDKRIAQRYSGWNSELGQQILKGQMSLADLAKYAQEHNLSPVHQSGRQEQLENLVNHYLF

DK

>gi|313647564|gb|EFS12014.1| xylose isomerase domain protein [Shigella flexneri 2a str. 2457T]

MQAYFDQLDRVRYEGSKSSNPLAFRHYNPDELVLGKRMEEHLRFAACYWHTFCWNGADMFGVGAFNRPWQ

QPGEALALAKRKADVAFEFFHKLHVPFYCFHDVDVSPEGASLKEYINNFAQMVDVLAAKQEESGVKLLWG

TANCFTNPRYGAGAATNPDPEVFSWAATQVVTAMEATHKLGGENYVLWGGREGYETLLNTDLRQEREQLG

RFMQMVVEHKHKIGF

>gi|313647560|gb|EFS12010.1| xylose operon regulatory domain protein [Shigella flexneri 2a str. 2457T]

MFTKRHRITLLFNANKAYDRQVVEGVGEYLQASQSEWDIFIEEDFRARIDKIKDWLGDVVIADFDDKQIE

QALADVDVPIVGVGGSYHLAESYPPVHYIATDNYALVESAFLHLKEKGVNRFAFYGLPESSGKRWATERE

YAFRQLVAEEKYRGVVYQGLETAPENWQHAQNRLAD

>gi|313647559|gb|EFS12009.1| xylose operon regulatory domain protein [Shigella flexneri 2a str. 2457T]

MIGIDNEELTRYLSRVALSSVAQGARQMGYQAAKLLHRLLDKEEMPLQRILVPPVRVIERRSTDYRSLTD

PAVIQAMHYIRNHACKGIKVDQVLDAVGISRSNLEKRFKEEVGETIHAMIHAEKLEKARSLLISTTLSIN

EISQMCGYPSLQYFYSVFKKAYDTTPKEYRDVNSEVML

>gi|313647549|gb|EFS11999.1| 2,3-diketo-L-gulonate TRAP transporter small permease yiaM domain protein [Shigella flexneri 2a str. 2457T]

MAFMDNAHVQVTFLVEKLSPAWQRRVALVTHSLILFICGALAWGATLKTIQDWSDYSPILGLPIGLMYAA

CLPTSLVIAFFELRHLYQLITRSNSLTSPPQGA

>gi|313647528|gb|EFS11978.1| hypothetical protein SF2457T_4057 [Shigella flexneri 2a str. 2457T]

MNLKKIFFSAVTVSVLCALTGCDYIEEGKPESSLLKQQEEHNNKIVLLEKQQAQLKSQLETIQKQQTGII

NSTKTLTHVIKSVKDQQNTFIFTEFNPAKTKYFILNNGSVALAGRVLSIDATENGSVIHISLVNLLSTPI

SNIGFNATWGGEKPVDAKEFARWQQLLFNTSMKSTLKLLPGQWQDINLTLKGVSPNNLGYLKLAINMENI

QFDNLPSAENRQKRSKK

>gi|313647525|gb|EFS11975.1| hemagglutinin family protein [Shigella flexneri 2a str. 2457T]

MATNTTNIANNTSNIATNTTNISNLTETVTNLGEDALKWDKDNGVFTAAHGTETTSKITNVKDGDLTTGS

TDAVNGSQLKTTNDAVATNTTNIATNTTNISNLTETVTNLGEDALKWDKDNGVFTAAHGNNTASKITNIL

DGTVTATSSDAINGSQLYDLSSNIATYFGGNASVNTDGVFTGPTYKIGETNYYNVGDALAAINSSFSTSL

GDALLWDATAGKFSAKHGTNGDASVITDVADGEISDSSSDAVNGSQLHGVSSYVVDALGGGAEVNADGTI

TAPTYTIANADYDNVGDALNAIDTTPDDALLWDADAGENGAFSAAHGKDKTASVITNVANGAISAASSDA

INGSQLYTTNKYIADALGGDAEVNADGTITAPTYTIANAEYNNVGDALDALDDNALLWDKTANGGAGAYN

ASHDGKASIITNVANGSISEDSTDAVNGSQLNATNMMIEQNTQIINQLAGNTDATYIEENGAGINYVRTN

DNDLAFNDASASGVGATAVGYNAVASGASSVAIGQNSSSTVDTGIALGSSSVSSRVIAKGSRDTSVTENG

VVIGYDTTDGELLGALSIGDDGKYRQIINVADGSEAHDAVTVRQLQNAIGAVATTPTKYFHANSTAEDSL

AVGEDSLAMGAKTVVNGNAGIGIGLNTLVLADAINGIAIGSNARANHANSIAMGNGSQTTRGAQTGYTAY

NMDAPQNSVGEFSVGSEDGQRQITNVAAGSADTDAVNVGQLKVTDERVAQNTQSITNLNNQVTNLDTRVT

NIENGIGDIVTTGSTKYFKTNTDGVDANAQGKDSVAIGSGSIAAADNSVALGTGSVAEEENTISVGSSTN

QRRITNVAASVNATDAVNVSQLKSSEAGGVRYDTKADGSIDYSNITLGGGNGSTTRISNVSAGVNNNDAV

NYAQLKQSAQETKQYTDQRMVEMDNKLSKTESKLSGGIASAMAMTGLPQAYTPGASMASIGGGTYNGESA

VALGVSMVSANGRWVYKLQGSTNSQGEYSAALGAGIQW

>gi|313647507|gb|EFS11957.1| htrL protein [Shigella flexneri 2a str. 2457T]

MKSSTTIITAYFDIGRGDWTANKGFREKLARSVDVYFSYFERLAALENEMIIFTSPDLKPRIEAIRNGKP

TTVIVIDIKKKFRYIRSRIEKIQKDESFTNRLEPRQLKNPEYWSPEYVLVCNLKAYFVNKAINMGLVKTP

LVAWIDFGYCRKPNVTRGLKIWDFPFDENKMHLFTIKKGLTVTSQQQAFDFMIGNHVYIIGGAIVGSQHK

WKEFYKLVLESQKITLNNNIVDDDQGIFVMCYYKRSDLFNLNYLGRGKWFDLFRCFRSNTLGAKMQALRI

FLSRK

>gi|313647468|gb|EFS11918.1| sugar (Glycoside-Pentoside-Hexuronide) transporter family protein [Shigella flexneri 2a str. 2457T]

MGGVITNDPTQRISLQSWRFVLATAGGMLSTVLMMPLVNLIGGDNKPLGFQGGIVVLSVVAFMMLAFCFF

TTKERVEAPPTTTSMREDLRDIWQNDQWRIVGLLTIFNILAVCVRGGAMMYYVTWILGTPEVFVAFLTTY

CVGNLIGSALAKPLTDWKCKVTIFWWTNALLAVISLAMFFVPMQASITMFVFIFVIGVLHQLVTPIQWVM

MFDTVDYGEWCNGKRLTGISFAGTLFVLKLGLAFGGALIGWMLAYGGYDAAEKAQNSATISIIIALFTIV

PAICYLLSAIIAKRYYSLTTHNLKTVMEQLAQGKRRCQQQFTSQEVQN

>gi|313647442|gb|EFS11892.1| putative serine protease [Shigella flexneri 2a str. 2457T]

MNKIYSLKYSAATGGLIAVSELAKRVSGKTNRKLVATMLSLAVAGTVNAANIDISNVWARDYLDLAQNKG

IFQPGATDVTITLKNGDKFSFHNLSIPDFSGAAASGAATAIGGSYSVTVAHNKKNPQAAETQVYAQSSYK

VVDRRNSNDFEIQRLNKFVVETVGATPAETNPTTYSDALERYGIVTSDGSKKIIGFRAGSGGTSFINGES

KISTNSAYSHDLLSASLFEVTQWDSLDLPLYFQTSVIT

>gi|313647438|gb|EFS11889.1| chaperone protein aggD [Shigella flexneri 2a str. 2457T]

MNADLSVVEMVFIIMKNFNVLTKVVFCLSFLSSAFPTYAADDVKVTQNTSVFKLKLSATRLIYEQGSDGA

AITVTNPQDWPMLVQSKVFEEDKVTAAPFIVTPPLFRLNPGQQNKMWVVQTAPALQDREKLHWLCATGIT

PKKSDVWAEKDKEKIAENVNLNIQVNVHTCIKLITRPSGIEGKMVNASEKLSWKKQGDKIVAVNQSPFYI

NLSSLQVGGSKIEKLDYIAPFSQHEFVMPKTSGQDIKWSVITDEGGESRLFISHLQS

>gi|313647437|gb|EFS11888.1| bacterial regulatory s, luxR family protein [Shigella flexneri 2a str. 2457T]

MVNDLTYKKLESTNINCNILSPENTTWTNVTVIGLDLFAICGVCSLLKRVGGVKLQVFTSLPTDMNVLTD

SSIVIWVKMRHDGLPELAGHIVKVCRRYRLMRQLVISDAIPEGMTNDIGPLSRVWLTHGSAKVDILLSSL

RKVLNAHCLPGPLFIKRLGRVQWRVLLLRAKGISTRKIAEICGIGLKTVSAHESAIRERLGTRNKSEYVW

LVRNAQLIQQAIPALGRDASRYERCNNMRTKQKV

>gi|313647436|gb|EFS11887.1| O-Antigen Polymerase family protein [Shigella flexneri 2a str. 2457T]

MPFYFRKECPLNSGYLNFPYGIFQQRNVLASWLATGCGVALYLALTARTRSLTLICLLSLYPLSTALVLT

QSRVGLLGLFCVIVLAALADRPRWRGRPLSGLLRLIFVASLLCWCVGISNYAMPGETTADFTHSSSNDQR

IRILKGAISLIQQRPLTGWGLGSFEATFPQALEDVGVTNIESDTATHPHNELLFVTAEGGIVALFGFLLL

SGVWLWPCICRLKMTNNNAHHQCEKWFIALMGLPLVIHMMTEYPLYTSVPHLMLLLLLFRIGMPENVMNQ

VRIPKTVRLGILPVIVLIMTAALVILVAGFDAQRELTRAEADMNEGFIPRLPEHSWKYLTQAERLERDQH

LLIANKPGFVRDTESMSLFTVWGNKWLAIHNDADVSAAMIFIAHRRGDYLTAEKIRKRAARVFVNDERFT

RGGN

>gi|313647412|gb|EFS11863.1| permease family protein [Shigella flexneri 2a str. 2457T]

MFVFLAVELSLLFIVISAGVSLIRQKVPDHKIQQMMGARKGRGYLLAALLGAVTPFCSCSTIPMLRGLLS

AKAGFGPTLTFLFVSPLLNPIIVGLMWMTFGWKVTLLYAIIAAGVSVLASIILDSLGFERHIIASKSSSA

NCCAPAKTSPGTTYTPIKVSCCSPAAKAIENPVVTCCSTKAVVSINPIKLATKDALQQFKDVLPYLLLGV

LIGSFIYGFIPSAWIAAHAGADNPFAIPLSAVVGIPLYIRAEAVIPLASVLMTKGMGLGALMALIIGSAG

ASLTEVILLKSMFRMPMIAAFLTVILGMAILMGYLTQLLF

>gi|313647411|gb|EFS11862.1| hypothetical protein SF2457T_4176 [Shigella flexneri 2a str. 2457T]

MSIDFTPGIINTYHGDIYNCTTNTDNVKTPDTPKWPCDNWEEQQPINSTFSGEGYNPEQFDLAQQQLQQI

NACHTNTTYTNADYSKVVVQLVSLINNIETISSTPLTQQTQSILNQIHNIRYEKDKDSVCQIIVVVNPEP

NNPIITKILVEEGIPERFSVQTVSSDNKNFAGQRADLPTDIRDIQSLYLKMAKLYIEHSKNENRMEALAG

CDFIDFNMTGQDMSKLVLTLSKFYFEDLLNINFTDANLFNTIFSHKENPIPKLHKYEQHLDKQINGLFST

LLTINDNSLRAKAEIASRIIDFLEAKVVNLSFDDILKYKQEFKKICYKQLQEFTTPSLYNKIQKWATMSK

NEFIEFHYETLQPEKISYPFYLKRDLPNEKDINYGVEIEIPSGKRIRLSNHYQNIIP

>gi|313647408|gb|EFS11859.1| uncharacterized protein yhiD [Shigella flexneri 2a str. 2457T]

MVIGSGMYELGIYGSVMTLLVLEVFHQLTFRLMNKNYHLQLTLVNGNTVSMLDWFKQQKIKTDLVSLQEN

EDHEVVAIDIQLHATTLIEDLLRLLKGMAGVKGVSIS

>gi|313647407|gb|EFS11858.1| mgtC family protein [Shigella flexneri 2a str. 2457T]

MTAEFIIRLILAAIACGAIGMERQMRGKGAGLRTHVLIGMGSALFMIVSKYGFADVLSLDHVGLDPSRIA

AQVVTGVGFIGAGNILVRNQNIVGLTTAADIWVTAA

>gi|313647402|gb|EFS11853.1| multidrug resistance protein MdtE [Shigella flexneri 2a str. 2457T]

MLTACDDKSAENAAAMTPEVGVVTLSPGSVNVLSELPGRTVPYEVAEIRPQVGGIIIKRNFIEGDKVNQG

DSLYQIDPAPLQAELNSAKGSLAKALSTASNARITFNRQASLLKTNYVSRQDYDTARTQLNEAEANVTVA

KAAVEQATINLQYANVTSPITGVSGKSSVTVGALVTANQADSLVTVQRLDPIYVDLTQSVQDFLRMKEEV

ASGQIKQVQGSTPVQLNLENGKRYSQTGTLKFSDPTVDETTGSVTLRAIFPNPNGDLLPGMYVTALVDEG

SRQNVFMTR

>gi|313647337|gb|EFS11789.1| putative nitrite transporter [Shigella flexneri 2a str. 2457T]

MFTDTINKCAANAARIARLSANNPLGFWVSSAMAGAYVGLGIILIFTLGNLLDPSVRPLVMGATFGIALT

LVIIAGSELFTGHTMFLTFGVKAGSISHGQMWAILPQTWLGNLVGSVFVAMLYSWGGGSLLPVDTSIVHS

VALAKTTAPAMVLFFKGALCNWLVCLAIWMALRTEGAAKFIAIWWCLLAFIASGYEHSIANMTLFALSWF

GNHSEAYTLAGIGHNLLWVTLGNTLSGAVFMGLGYWYATPKANRPVADKFNQTETAAG

>gi|313647321|gb|EFS11773.1| hypothetical protein SF2457T_4321 [Shigella flexneri 2a str. 2457T]

MRRTFIKKEGVVITTLARYLLGEKCGNRLKTIDELATECRSSVGLTQAALKTLESSGAIRIERRGRNGSY

LVEMDNKALLSHVDINNVVCAMPLPYTRLYEGLASGLKAQFDGIPFYYAHMRGADIRVECLLNGVYDMAV

VSRLAAESYLSQNNLCIALELGPHTYVGEHQLICRKGESGNVKRVGLDSRSADQKIMTDVFFGDSDVERV

DLSYHESLQRIVKGDVDAVIWNVVAENELTMLGLEATPLTDDPRFLQATEAVVLTRVDDYPMQQLLRAVV

DKHALLAHQQRVVSGEQEPSY

>gi|313647280|gb|EFS11732.1| HTH-type transcriptional regulator malT domain protein [Shigella flexneri 2a str. 2457T]

MLIPSKLSRPVRLDHTVVRERLLAKLSGANNFRLALITSPAGYGKTTLISQWAAGKNDIGWYSLDEGDNQ

QERFASYLIAAVQQATNGHCAICETMEQKRQYASLTSLFAQLFIELAEWHSPLYLVIDDYHLITNPVIHE

SMRFFIRHQPENLTLVVLSRNLPQLGIANLRVRDQLLEIGSQQLAFTHQEAKQFFDCRLSSPIEAAESSR

ICDDVSGWATAL

>gi|313647279|gb|EFS11731.1| HTH-type transcriptional regulator malT [Shigella flexneri 2a str. 2457T]

MDNVDLATRHFLLKSAILRSMNDALITRVTGEENGQMRLEEIERQGLFLQRMDDTGEWFCYHPLFGNFLR

QRCQWELAAELPEIHRAAAESWMAQGFPSEAIHHALAAGDALMLRDILLNHAWSLFNHSELSLLEESLKA

LPWDSLLENPQLVLLQAWLMQSQHRYGEVNTLLARAEHEIKDIREGTMHAEFNALRAQVAINDGNQDEAE

RLAKLALEELPPGWFYSRIVATSVLGEVLHCKGELTRSLALVQQTEQMARQHDVWHYALWSLIQQSEILF

AQGFLQTAWETQEKAFQLINEQHLEQLPMHEFLVRIRAQLLWAWARLDEAEASARSGIEVLSSYQPQQQL

QCLAMLIQCSLARGDLDNARSQLNRLENLLGNGKYHSDWISNANKVRVIYWQMTGDKAAAANWLRHTAKP

EFANNHFLQGQWRNIARAQILLGEFEPAEIVLEELNENARSLRLMSDLNRNLLLLNQLYWQAGRKSDAQR

VLLDALKLANRTGFISHFVIEGEAMAQQLRQLIQLNTLPELEQHRAQRILREINQHHRHKFAHFDENFVE

RLLNHPEVPELIRTSPLTQREWQVLGLIYSGYSNEQIAGELEVAATTIKTHIRNLYQKLGVAHRQAAVQH

AQKLLKMMGYGV

>gi|313647274|gb|EFS11726.1| rhomboid family protein [Shigella flexneri 2a str. 2457T]

MLMITSFANPRVAQAFVDYMATQGVILTIQQHNQSDVWLADESQAERVRAELARFLENPADPRYLAASWL

AGHTGSGLHYRRYPFFAALRERAGPVTWVMMIACVVVFIAMQILGDQEVMLWLAWPFDPTLKFEFWRYFT

HALMHFSLMHILFNLLWWWYLGGAVEKRLGSGKLIVITLISALLSGYMQQKFSGPWFGGLSGVVYALMGY

VWLRGERDPQSGIYLQRGLIIFALIWIVAGWFDLFGMSMANGAHIAGLAVGLAMAFVDSLNARKRK

>gi|313647260|gb|EFS11712.1| HTH-type transcriptional regulator gntR [Shigella flexneri 2a str. 2457T]

MKKKRPVLQDVADRVGVTKMTVSRFLRNPEQVSVALRGKIAAALDELGYIPNRAPDILSNATSRAIGVLL

PSLTNQVFAEVLRGIESVTDAHGYQTMLAHYGYKPEMEQERLESMLSWNIDGLILTERTHTPRTLKMIEV

AGIPVVELMDSQSPCLDIAVGFDNFEAARQMTTAIIARGHRHIAYLGARLDERTIIKQKGYEQAMLDAGL

VPYSVMVEQSSSYSSGIELIRQARREYPQLDGVFCTNDDLAVGAAFECQRLGLKVPDDMAIAGFHGHDIG

QVMEPRLASVLTPRERMGSIGAERLLARIRGESVTPKMLDLGFTLSPGGSI

>gi|313647214|gb|EFS11666.1| uncharacterized protein yhiL [Shigella flexneri 2a str. 2457T]

MKAIDNIFKYIPSNHKDKHSDKVNNHQHHSKVDKTHRSKIVEIDKLDNDSQIDNDFGMHIIYFLQHGHWR

VNEHSLQMEKIWFYNSEPSIDIQEYNSFADNTTNTFIFTIIPDNNHVIKLSSPITVKIECKDGYYFINFS

GDKSDIIYKVNGLSIIPENFFTLLSGNFKADWRWDVSKETFPKDKFDSYVKSVFSKIDFYKQCGVINPQN

ANTAYFGDTDGRVGAVLYSLLVSGHIGIREKGWSLLCELLKHEDMASSAYIHKNNKLLYNLLNTRDMILN

ELHQHVFLKDDAITPCIFLGDHTGDRFSTIFGDKYILTLLNSMRNMEGNKDSRINKNVIVLAGNHEINFN

GNYTARLENYKLSAGDTYNLIKTLDVCNYNSETKVLTSHHGIIRNEENQCYCLGALQVPFNQMKDPIDPE

ELANICNKKHKQHMDDHLLHLIRSNAIAPTPVYENYFNNTTAFRPKPEDIFKCGQTLKKTKQKFGHYGLG

VDQHQKIDNYTMGLNSWKIAPNERGDKKGVPGLSCFQPQ

>gi|313647213|gb|EFS11665.1| inner membrane protein yhiM [Shigella flexneri 2a str. 2457T]

MCWKVQGNVTGHVLISLAAICLALFTTAFIIISQLTRGVNTFYNTLFPIIGYAGSIITMIWGWALLAGND

VMADEFVAGHVIFGVGMIAACVSTVAASSGHFLLIPKNAAGSKSDGTPVQAYSSLIGNCLIAVPVLLTLL

GFIWSITLLHSADITPHYVALG

>gi|313647208|gb|EFS11660.1| fimbrial Usher family protein [Shigella flexneri 2a str. 2457T]

MSITTNRYASGYATLTEAVSAQDERNRKRDKNSHDGSTISLSQPLGNIGNLNFNTTRYNSSRGTGNTRST

SLSYSTVWRGITFSINWAKNDLLTSHKWKVDRKLSVGISVPLSLGDENQIYASSQMSRSGEQGNNYQVSL

SGQNSGGVWWDVATNITNAHQSQPKSTMNIVQVGKNGSYGQFSSHYSSSENMKQLGANLSGGILITRDGL

TFGQNVDGTLALIEAPGATGVNVNGWPGLSTDFRGYAILPVQPYRRDDVILDEKTIGKNYDLPQTSQLVV

PTAGAVVPATLAVKSGDKGLVTLKQKEGKPIPFGAVISYSKDTENMAGIVGEDGIAYVSGLSAEGEFNVK

WGYSKDQSCIAKYQLPAKKSASGLYQIAATCL

>gi|313647198|gb|EFS11651.1| L-rhamnose-proton symporter [Shigella flexneri 2a str. 2457T]

MAFDRRGQCSLFLRSVQKSKKWSWETMWSVGGIVSWIILPWAISALLLPDFWAYYSSFSLSTLLPVFLFG

AMWGIGNINYGLTMRYLGMSMGIGIAIGITLIVGTLMTPIINGNFDVLINTEGGRMTLLGVLVALIGVGI

VTRAGQLKERKMGIKAEEFNLKKGLVLAVMCGIFSAGMSFAMNAAKPMHEAAAALGVDPLYVALPSYVVI

MGGGAIINLGFCFIRLAKVKDLSLKADFSLAKPLIIHNVLLSALGGLMWYLQFFFYA

>gi|313647183|gb|EFS11637.1| hypothetical protein SF2457T_4432 [Shigella flexneri 2a str. 2457T]

MSSNNEYDLFKNRETSLWKRKVPESAALAMTGTVYKLFGNRLLNARNQLDKNLLEMAMQMVYGKDMVTKS

GDILIDIQLNKDGSLQSKHYTLDVGEVINSYNIDPLCECFKY

>gi|313647169|gb|EFS11623.1| fructose-like PTS system EIIBC component [Shigella flexneri 2a str. 2457T]

MAMESSLRIVAITNCPAGIAHTYMVAEALEQKARSLGHTIKVETQGSSGVENRLSSEEIAAADYVILATG

RGLSGDDRARFAGKKVYEIAISLALKNIDQIFSELPTNSQLFAADSGVKLGKQEVQSGSVMSHLMAGVSA

ALPFVIGGGILVALANMLVQFGLPYTDMSKGAPSFTWVVESIGYLGFTFMIPIMGAYIASSIADKPAFAP

AFLVCYLANDKALLGTQSGAGFLGAVVLGLAIGYFVFWFRKVRLGKALQPLLGSMLIPFVTLLVFGVLTY

YVIGPVMSDLMGGLLHFLNTIPPSMKFAAAFLVGAMLAFDMGGPINKTAWFFCFSLLEKHIYDWYAIVGV

VALMPPVAAGLATFIAPKLFTRQEKEAASSAIVVGATVATEPAIPYALAAPLPMITANTLAGGITGVLVI

AFGIKRLAPGLGIFDPLIGLMSPVGSFYLVLAIGLALNISFIIVLKGLWLRRKAKAAQQELVHEH

>gi|313647151|gb|EFS11606.1| hypothetical protein SF2457T_4524 [Shigella flexneri 2a str. 2457T]

MHYRNVVCLLSCSLFLSSAWGCRLSAPEHNIYQKQGKGVVYLRPYKETNLSLPDVNYKSLRRLPNLLIDP

TTLDEWDKEPPLTDLTTDYLYEGAQAWYPHYSWHSDGRNILYAGEVVQNPPGTPPVDVASFKAWGDFAAD

KHSLYFEGKRTDDNGGGNSLDIKTLHQVEFRPPWDPDLLGLILRDANFLYINGHRLADPESFRVLAQKSW

DQRGKFSTTFNPCIAVPFGPWDTLARTRTKILLNGEQLDADPDTFSVVRWMPGSLLTWRDKNGLQRKVLD

KENLAWDEDLTKHCLDFSLLEKKVFWRKGPACKQEELPGLDPEQFQPISDAVAQHQDSLYTIIETESGNR

KLEIVKLDDPNLIINKRFNAGKRHGYLLTRAEG

>gi|313647132|gb|EFS11587.1| threonine efflux protein [Shigella flexneri 2a str. 2457T]

MLMLFLTVAMVHIVALMSPGPDFFFVSQTAVSRSRKEAMMGVLGITCGVMVWAGIALLGLHLIIEKMAWL

HTLIMVGGGLYLCWMGYQMLRGALKKEAVSAPAPQVELAKSGRSFLKGLLTNLANPKAIIYFGSVFSLFV

GDNVGTTERWGIFALIIVETLAWFTVVASLFALPQMRRGYQRLAKWIDGFAGALFAGFGIHLIISR

>gi|313647131|gb|EFS11586.1| homoserine/Threonine efflux family protein [Shigella flexneri 2a str. 2457T]

MTLEWWFAYLLTSIILSLSPGSGAINTMTTSLNHGYRGAVASIAGLQTGLAIHIVLVGVGLGTLFSRSVI

AFEVLKWAGAAYLIWLGIQQWRAAGAIDLKSLASTQSRRHLFQRAVFVNLTNPKSIVFLAALFPQFIMPQ

QPQLMQYIVLGVTTIVVDIIVMIGYATLAQRIALWIKGPKQMKALNKIFGSLFMLVGALLASARHA

>

>gi|313647054|gb|EFS11510.1| ferric enterobactin transport fepE domain protein [Shigella flexneri 2a str. 2457T]

MSSLNIKQGSDAHFSEYPLASPSNNEIDLLNLIEVLWRAKKTVMAVVFAFACAGLLISFILPQKWTSSAV

ITPAEAIQWQDLEKTFTKLRVLDLDVNIDRGGAFNLFIKKFQSVSLLEEYLRSSPYVMD

>gi|313647053|gb|EFS11509.1| ferric enterobactin transport protein fepE [Shigella flexneri 2a str. 2457T]

MHRAIVALSEKMKAVDDNASKKKDEPSLYTSWTLSFTAPTSKEAQTVLSGYIDYISALVVKESIENVRNK

LEIKTQFEKEKLAQDRIKTKNQLDANIQRLNYSLDIANAAGIKKPVYSNGQAVKDDPDFSISLGADGIER

KLEIEKAVTDVAELNGELRNRQYLVEQLTKANVNDVNFTPFKYQLSPSLPVKKDGPGKAIIVILSALIGG

MVACGSVLLRYAMASRKQDAMMADHLV

>gi|313647046|gb|EFS11502.1| putative isochorismate synthase [Shigella flexneri 2a str. 2457T]

MERQAIPEQTTFEQMGARAAALTATPQVDKVVLSRLIDITTDAAIDSGVLLERLIAQNPVSYNFHVPLAD

GGVLLGASPELLLRKDGERFSSIPLAGSARRQPDEVLDREAGNRLLASEKDRHEHELGTQAMKEVLRERS

SELHVPSSPQLITTPTLWHLATPFEGKANSQENALTLACLLHPTPALSGFPHQAATQVIAELEPFDRELF

GGIVGWCDSEGNGEWVVTIRCAKLRENQVRLFAGAGIVPASSPLGEWRETGVKLSTMLNVFGLH

>gi|313647037|gb|EFS11493.1| ibrB domain protein [Shigella flexneri 2a str. 2457T]

MPNDYNPNNVAPPEKKLLQKSIEIDGFTQPIVVTHTDKNAMEIVDGFHRHEIGKGSSSLKLRLKGYLPVT

CLEGTRNQRIAATIRHNRARGRHQITAMSEIVRELSQLGWDDNKIGKELGMDSDEVLRLKQINGLQELFA

DRQYSRAWTVK

>gi|313647033|gb|EFS11489.1| ibrA domain protein [Shigella flexneri 2a str. 2457T]

MYQAGVPLRHMRICEPFGPEQRQGLWLCHVIEPDRWAAMCARVSGVKSGGIYAGHDNHFYGHRKILKPEH

LDWQEYALLLLNSMPEKTAEHYRNKIAIYLHWYQKKGIEVPQTQQGDIGAKDIPSWRRICKVLLNNDYWC

RALSFSPTKAKNYQRYNERIKGKRQEWGILCNND

>gi|313647032|gb|EFS11488.1| ibrA domain protein [Shigella flexneri 2a str. 2457T]

MYTDVIEEFYWVALPLTTQNSLSQYQPEWQCWEPDVEWVRQPPQDAITAPDFFCFYQPGMTFEQFVREFA

EWFSQKRPAAMMIGIRADESYNRFVAIASLNKQRFADDKPWTTAAPGGHSWYIYPIYDWKVADIWT

>gi|313647029|gb|EFS11485.1| bacterial regulatory helix-turn-helix , lysR family protein [Shigella flexneri 2a str. 2457T]

MANLYDLKKFDLNLLVIFECIYQHLSISKAAESLYITPSAVSQSLQRLRAQFNDPLFIRSGKGIAPTTTG

LNLHHHLEKNLKGLEQTINIVNKSELKKNFIIYGPQLISCSNNSMLIRCLRQDASVEIECHDILMSAENA

EELLVHRKADLVITQMPVISRSVICMPLHTIRNTLICSNKHPRITDNSTYEQIMAEEFTQLIAKSAGVDD

IQMEIDEKFMNRKISFRGSSLLTIINSIAVTDLLGIVPYELYNYHRDFLNLKEIKLEHPLPSIKLYISYN

KSSLNNLVFSRFIDRLNESF

>gi|313647019|gb|EFS11475.1| citrate carrier domain protein [Shigella flexneri 2a str. 2457T]

MGALTRREWTLIGLVLLSLGLWVFGSEVINATAVGLLAVSLMLALHVVPWKDITRYNSAWNTLVNLATLV

VMANGLTRSGFIDWFAGTMSTRLEGFSPNATVIVLVLVFYFAHYLFASLSAHTATMLPVILAVGKGIPGV

PMEQLCILLVLSIGIMGCLTPYATGPGVIIYGCGYVKSKDYWRLGAIFGVIYISMLLLVGWPILAMWN

>gi|313647018|gb|EFS11474.1| citrate carrier domain protein [Shigella flexneri 2a str. 2457T]

MSLAKDNIWKLLAPLVVMGVMFLIPVPDGMPPQAWHYFAVFVAMIVGMILEPIPATAISFIAVTICVIGS

NYLLFDAKELADPAFNAQKQALKWGLAGFSSTTVWLVFGAFIFALGYEVSGLGRRIALFLVKFMDKRTLT

LGYAIVIIDILLAPFTPSNTARTGGTVFPVIKNLPPLFKSFPNDPSARRIGGYLMWMMVISTSLSSSMFV

TGADQTCWVWSSSAKLPVSRLAGCSGSSASCRLGLSCLSLRRGFPTCCTNRKSHTVKKWQPGRVMN

>gi|313646980|gb|EFS11437.1| acyl carrier protein phosphodiesterase [Shigella flexneri 2a str. 2457T]

MTDNLPEVREAQEWFRSETRRVAPITLDVMWDHFLSRHWSQLSPDFPLQEFVCYAREQVMTILPDSPPRF

INLNNYLWSEQWLVRYRDMDFIQNVLNGMASRRPRLDALRDSWYDLDAHYDALETRFWQFYPRMMAQASH

KAL

>gi|313646953|gb|EFS11410.1| acetyltransferase, GNAT family [Shigella flexneri 2a str. 2457T]

MYALRASLKKSVRNSDCAAKALIDRQSGELIGICTFTAYSLERQRVSGVLQGSQPSEIGVVRLVMLGGAR

KYQKRGFGQDLLCDFFEHVKKIHPALPIKGVYLDADPAAINFYARLGFVQLSATPNAFGAVPMFLAIQHI

LAA

>gi|313646945|gb|EFS11402.1| COX Aromatic Rich Motif family protein [Shigella flexneri 2a str. 2457T]

MTSNSVMNSFFIPRLGSQIYAMAGMQTRLHLIANEPGTYDGISASYSGPGFSGMKFKAIATPDRAEFDQW

VAKAKQSPNTMSDMAAFEKLAAPSEYNQVEYFSNVKPDLFADVINKFMAHGKSMDMTQPEGEHSAHEGME

GMDMSHAESAH

>gi|313646928|gb|EFS11385.1| bacterial extracellular solute-binding s, family 5 Middle family protein [Shigella flexneri 2a str. 2457T]

MRQAQEAGWLEWQAQSGRGKRGQLRFLVTPESLRNAMMEQALETGKQQDVLELAQLAPGELRTLLQPFMG

GQWQNDTPTLRIPYYRPLEPLQPGFLPGRAEQHLAGQIFSGLTRFDNNTQRPIGDLAHHWETSTDRLRWD

FYLRSTLHWHNGDAVKASHLHQRLLMLLQLPALDQLFISVKRIEVTHPQCLTFFLHRPDYWLAHRLASYC

SHLAHPQFPLIGTGPFRLTQFTAELVRLESHDYYHLRHPLLKAVEYWITPPLFEKDLGTSCRHPVQITIG

KPEELQRVSQVSSGISLGFCYLTLRKSPRLSLWQARKVISIIHQSGLLQTLEVGENLITASHALLPGWTI

PHWQVPDEVKLPKTLTLVYHLPIELHTMAERLQATLAAEGCELTIIFHNAKNWDDTTLLAHADLMMGDRL

IGEAPEYTLEQWLRCDPLWPHVFDAPAYAHLQSTLDAVQVMPDEENRFNALKAVFSQLMADATLTPLFNY

HYRISAPPGVNGVRLTPRGWFEFTEAWLPAPSQ

>gi|313646914|gb|EFS11371.1| EAL domain protein [Shigella flexneri 2a str. 2457T]

MLVRTRHLVGLISGVLILSVLLPVGLSIWLAHQQVETSFIEELDTYSSRVAIRANKVATQGKDALQELER

WQGAACSEAHLMEMRRVSYSYRYIQEVVYIDNNVPQCSSLEHESPPDTFPELGKISKDGYRVWLTSHNDL

GIIRYMVAMGTAHYVVMIDPASFIDVIPYSSWQIDAAIIGNAHNVVITSSDEIAQGIITRLQKTPGEHIE

NNGIIYDILPFLEMNISIITWASTKMLQKGWHRQVFIWLPLGLVIGLLAAMFVLRILRRIQSPHHRLQDA

IENRDICVHYQPIVSLANGKIVGAEALARWPQTDGSWLSPDSFIPLAQQTGLSEPLTLLIIRSVFEDMGD

CLRQHPQQHISINLESTVLTSEKIPQLLREMINHYQVNPRQIALELTEREFADPKTSAPIISRYREAGHE

IYLDDFGTGYSSLSYLQDLDVDILKIDKSFVDALEYKNVTPHIIEMAKTLKLKMVAEGIETSKQEEWLRQ

HGVHYGQGWLYSKALPKEDFLRWAEQHL

>gi|313646912|gb|EFS11369.1| maltose O-acetyltransferase [Shigella flexneri 2a str. 2457T]

MTEAYIEPTFRCDYGYNIFLGNNFFANFDCVMLDVCPIRIGDNCMLAPGVHIYTATHPIDPVARNSGAEL

GKPVTIGNNVWIGGRAVINPGVTIGDNVVVASGAVVTKGVPDNVVVGGNPARIIKKL

>gi|313646882|gb|EFS11339.1| nfeD-like family protein [Shigella flexneri 2a str. 2457T]

MMELMVVHPHIFWLSLGGLLLAAEMLGGNGYLLWSGVAAVITGLVVWLVPLGWEWQGVMFAILTLLAAWL

WWKWLSRRVREQKHSDSHLNQRGQQLIGRRFVLESPLVNGRGHMRVGDSSWPVSASEDLGAGTHVEVIAI

EGITLHIRAVSS

>gi|313646875|gb|EFS11332.1| arylesterase [Shigella flexneri 2a str. 2457T]

MNFNNVFRWHLPFLFLVLLTFRAAAADTLLILGDSLSAGYRMSASAAWPALLNDKWQSKTSVVNASISGD

TSQQGLARLPALLKQHQPRWVLVELGAMMVCVVFSHSKPSKRCARFCRMSKPPTLNHC

>gi|313646873|gb|EFS11330.1| permease family protein [Shigella flexneri 2a str. 2457T]

MIARWFWREWRSPSLLIVWLALSLAVACVLALGNISDRMEKGLSQQSREFMAGDRALRSSREVPQAWLEE

AQKRGLKVGKQLTFATMTFAGDTPQLANVKAVDDIYPMYGGLQTNPPGLKPQAGSVLLAPRLMALLNLKT

GDTIDVGDATLRIAGEVIQEPDSGFNPFQMAPRLMMNLADVDKTGAVQPGSRVTWRYKFGGNENQLDGYE

KWLLPQLKPEQRWYGLEQDEGALGRSMERSQQFLLLSALLTLLLAVAAVAVAMNHYCRSRYDLVAILKTL

GAGRAQLRKLIVGQWLMVLTLSAVTGGAIGLLFENVLMVLLKPVLPAALPPASLWPWLWALGTMTVISLL

VGLRPYRLLLATQPLRVLRNDVVANVWPLKFYLPIVSVVVVLLLAGLMGGSMLLWAVLAGAVVLALLCGV

LGWMLLNVLRRMTLKSLPLRLAVSRLLRQPWSTLSQLSAFSLSFMLLALLLVLRGDLLDRWQQQLPPESP

NYFLINIATEQVAPLKAFLAEHHIVPESFYPVVRARLTAINDKPTEGNEDEALNRELNLTWQNTRPDHNP

IVAGNWPPKADEVSMEEGLAKRLNVALGDTVTFMGDTQEFRAKVTSLRKVDWESLRPNFYFIFPEGALDG

QPQSWLTSFRWENGNGMLTQLNRQFPTISLLDIGAILKQVGQVLEQVSRALEVMVVLVTACGMLLLLAQV

QVGMRQRHQELVVWRTLGAGKKLLRTTLWCDVRHAWVCFRPGGRNWCGNGTGSVAGESV

>gi|313646861|gb|EFS11318.1| uncharacterized ylbF domain protein [Shigella flexneri 2a str. 2457T]

MAQGIRLGRFTVKQPQRYCLLRITPPSHPQPLAAAWMQRAEETGLFGPLAMAASDPLPAELRQFRHCFQA

ALNGVKTDWRHWLGKGPGLTPSHDDTLSGMLLAAWYYGALDARAGRPFFACSDNLQLVTTAVSVSYLRYA

AQGYFASPLLHFVHALSCPKRTAVAIDSLLALGHTSGADTLLGFWLGQQLLQGKP

>gi|313646849|gb|EFS11306.1| outer membrane usher sfmD domain protein [Shigella flexneri 2a str. 2457T]

MKIPTTTDIPQRYTWCLAGICYSSLAILPSFLSYAESYFNPAFLLENGTSVADLSRFERGNHQPAGVYRV

DLWRNDEFIGSQDIVFESTTENTGDKSGGLMPCFNQVLLERIGLNSSAFPELAQQQNNKCINLLKAVPDA

TINFDFAAMRLNITIPQIALLSSAHGVMTPTY

>gi|313646844|gb|EFS11301.1| bacteriophage N4 adsorption A domain protein [Shigella flexneri 2a str. 2457T]

MRSGAMSTANNNVGGAAPGKSYRSYGQLEAEYRIGRNMLLEGDLLSVYSRVFADTGENGVMMPVKNPMSG

TGLRWKPLRDQIFFLAVEQQLPLNGQNGASDTMLRASASFFNGGKYSDEWHPNGSGWFAQNLYLDAAQYI

RQDIQAWTADYRVSWHQKVANGQTIEPYAHVQDNGYRDKGTQGAQLGGVGVRWNIWTGETHYDAWPHKVS

LGVEYQHTFKAINQRNGERNNAFLTIGVHW

>gi|313646843|gb|EFS11300.1| bacteriophage N4 adsorption A domain protein [Shigella flexneri 2a str. 2457T]

MLLAGQLDDRILALQSQGIFTDPQSYITYATALAYRGEKARLQHYLIENKPLFTTGAQEKSWLYLLSKYS

ANPVQALANYTVQFADNRQYVVGATLPVLLKEGQYDAAQKLLATLPANEMLEERYAVSVATRNKAESLRL

ARLLYQQEPTNLPRLDQLTWQLMQNEQSREAADLLLQRYPFQGDARVSQTLMARLASLLESHPYLATPAK

VAILSKPLPLAEQRQWQSQLPGIADNCPAIVRLLGDMSPSYDAAAWNRLAKCYRDTLPGVALYAWLQAEQ

RQPSAWQHRAVAYQAYQVEDYATALAAWQKISLHDMSNDDLLAAANTAQAAGNGAARDRWLQQAEQRELG

NNAQYWWLHARRYIPAQPELALNDLTRSINIAPSANSYVARATIYRQRHNVPAAVSDLRAALELEPNNSN

TQAALGYALWDSGDIAQSREMLEQAHKGLPDDPALIRQLAYVNQRLDDMPATQHYARLVIDDIDNQA

>gi|313646842|gb|EFS11299.1| bacteriophage N4 adsorption A domain protein [Shigella flexneri 2a str. 2457T]

MKENNLNRVIGWSGLLLTSLLSTSALADNIGTSAEELGLSDYRHFVIYPRLDKALKAQKNNDEATAIREF

EYIHQQVPDNIPLTLYLAEAYRHFGHDDRARLLLEDQLKRHPGDARLERSLAAIPVEVKSVTTVEELLAQ

QKAGDAAPTLRCRSEVGQNALRLAQLPVARAQLNDATFAASPEGKTLRTDLLQRAIYLKQ

>gi|313646841|gb|EFS11298.1| bacteriophage N4 adsorption B domain protein [Shigella flexneri 2a str. 2457T]

MLYNAVQHQWLTEQQTGEIWRQYVPHQFLFAEILTTLGHINRSAINVLLLRHERSSLPLGKFLVTEGVIS

QETLDRVLTIQRELQVSMQSLLLKAGLNTEQVAQLESENEGE

>gi|313646840|gb|EFS11297.1| putative bacteriophage N4 adsorption protein B [Shigella flexneri 2a str. 2457T]

MDWLLDVFATWLYGLKVIAITLAVIMFISGLDDFFIDVVYWVRRIKRKLSVYRRYPRMSYRELYKPDEKP

LAIMVPAWNETGVIGNMAELAATTLDYENYHIFVGTYPNDPDTQRDVDEVCARFPNVHKVVCARPGPTSK

ADCLNNVLDAITQFERSANFAFAGFILHDAEDVISPMELRLFNYLVERKDLIQIPVYPFEREWTHFTSMT

YIDEFSELHGKDVPVREALAGQVPSAGVGTCFSRRAVTALLADGNGIAFDVQSLTEDYDIGFRLKEKGMT

EIFVRFPVVDEAKEREQRKFLQHARTSNMICVREYFPDTFSTAVRQKSRWIIGIVFQGFKTHKWTSSLTL

NYFLWRDRKGAISNFVSFLAMLVMIQLLLLLAYESLWPDAWHFLSIFSGSAWLMTLLWLNFGLMVNRIVQ

RVIFVTGYYGLTQGLLSVLRLFWGNLINFMANWRVLKQVLQHGDPRRVAWDKTTHDFPSVTGDTRSLRPL

GQILLENQVITEEQLDTALRNRVEGLRLGGSMLMQGLISAEQLAQALAEQNGVAWESIDAWQIPSSLIAE

MPASVALHYAVLPLRLENDELIVGSEDGIDPVSLAALTRKVGRKVRYVIVLR

>gi|313646834|gb|EFS11291.1| cation efflux system cusB domain protein [Shigella flexneri 2a str. 2457T]

MKKIALIIGSMIAGGIISAAGFTWFDQAEPHAEKMPTAERKILFWYDPMYPNTRFDKPGKSPFMDMDLVP

KYADEESSASGVRIDPTQTQNLGVKTATVTRGPLTFAQSFPTNVSYNEYQYAIVQARAAGFIDKVYPLTV

GDKVQKGTPLLDLTIPDWVEAQSEYLLLRETGGTATQTEGILERLRLAGMPEADIRHLIATQKIQTRFTL

KAPIDGVITAFDLRAGMNIAKDNVVAKEEGRLLICVCHCLALSTVVN

>gi|313646826|gb|EFS11283.1| cation efflux system cusB domain protein [Shigella flexneri 2a str. 2457T]

MDPVWVTAAIPESIAWLVKDASQFTLTVPARPDKTLTIRKWTLLPGVDAATRTLQLRLEVDNADEALKPG

MNAWLQLNTASEPMLLIPSQALIDTGNEQRVITVDADGRFVPKRVAVFQASQGVTALRSGLAEGEKVVSG

GLFLIDSEANISGALERMRSESATHAH

>gi|313646779|gb|EFS11238.1| carbohydrate diacid regulator domain protein [Shigella flexneri 2a str. 2457T]

MAGWHLDTKMAQDIVARTMRIIDTNINVMDARGRIIGSGDRERIGELHEGALLVLSQGRVVDIDDAVARH

LHGVRQGINLPLRLEGEIVGVIGLTGEPENLRKYGELVCMTAEMMLEQSRLMHLLAQDSRLREELVMNLI

QAEENTPALLNGRNGWGSISINREWWLLLRSTAVSLAWTAQWRSYNNCKTR

>gi|313646778|gb|EFS11237.1| carbohydrate diacid regulator domain protein [Shigella flexneri 2a str. 2457T]

MAELQQLQNALTTPERNNLVAIVSLTEMVVLKPALNSFGRWDAEDHRKRVEQLITRMKEYGQLRFRVSLG

NYFTGPGSIARSYRTAKTTMVVGKQRMPESRCYFYQDLMLPVLLDSLRGDWQANELARPLARLKAMDNNG

LLRRTLAAWFRHNVQPLATSKALFIHRNTLEYRLNRISELTGLDLGNFDDRLLLYVALQLDEER

>gi|313646707|gb|EFS11166.1| DNA packaging gp2 domain protein [Shigella flexneri 2a str. 2457T]

MWFDEEPPYSIYGEGLTRTNKYGQFSILTFTPLMGMSDVVTKFLKNPSKSQKVVNMTIYDAEHYTDEQKE

QIIASYPEHEREARARGIPTMGSGRIFQIPEETIKCQPFECPDHFYVIGGMDFGWDHPQAQVQLWWDKEA

DTIYVSRVWKAKEKTAVQAWGAVKSWAHKVPTAWPHDGNQHEKGGGEQLKGQYADAGFMMLQEHATWPDG

GNAVEPGITELRDMMLDGRFKVFNTCEPFFEEFRLYHRDENGKIVKLNDDVLSAVRYAYMMRRFAKMMRD

IKKPKEKKIPAPIRPIARRT

>gi|313646683|gb|EFS11142.1| uncharacterized protein yaiV [Shigella flexneri 2a str. 2457T]

MLSVVKPLQEFGKLDKCLSRYGTRFEFNNEKQVIFSSDVNNEDTFVILEGVISLRREENVLIGITQAPYI

MGLADGLMKNDIPYKLISEGNCTGYHLPAKQTRATLLSMIDWNEELRSRIGVMNYIHQRTRISRSVVAEV

LAALRKGGYIEMNKGKLVAINRLPSEY

>gi|313646682|gb|EFS11141.1| uncharacterized protein yaiT [Shigella flexneri 2a str. 2457T]

MSYSHFNNDLSATMSNSTYVDGSTNSDAWGFGLKTGYDFKLGDAGYVTPYGSISGLFQSGDDYQLSNDMK

VDGQPYDSMRYELGVDAGYTFTYSEDQALTPYFKLAYVYDDSNNDNDVNGDSIDNGTEGSAVRVGLGTQF

SFTKNFSAYTDANYLGGGDVDQDWSANVGVKYTW

>gi|313646677|gb|EFS11136.1| outer membrane autotransporter barrel domain protein [Shigella flexneri 2a str. 2457T]

MITDVWKYRGKSTDCADRADDYYHDRLALTVDNSTIDDNYEHYTYNGTYNNAADTHVVDVYNIGTAITLD

QEVDLSITNNSHVAGITLTQGYEWEDIDDNTVSTGVNSSEVFNNTITVKDSTVTSGSWTDEGTTGWFGNT

GNASDYSGKSNFVTVDTDGDGVADSTIASWDDVALAVVAHPNVDNAMQT

>gi|313646672|gb|EFS11131.1| outer membrane autotransporter barrel domain protein [Shigella flexneri 2a str. 2457T]

MHSWKKKLVVSQLALACTLAITSQANAATNDISGQTYNTFHHYNDATYADDVYYDGYVGWNNYAADSYYN

GDIYPVINNATVNGVISTYYLDDGISTNTNANSLTIKNSTIHGMIYSVCMTTD

>gi|313646656|gb|EFS11115.1| hypothetical protein SF2457T_4844 [Shigella flexneri 2a str. 2457T]

MRAIYLSVQQAWNGKITYSVSGESEFAKKFQGKALPFDVRIISASQNEDWLVIATKVLPGADLRTYVDFK

NSTVHVDSAGLEKVAKCINCNNTLQVNIPHEAGHVLGYLDDDYDSSSPYVGDISGLMNVGMELWERYLKN

ATITLNIIMPETKFTLLNVTK

>gi|313646511|gb|EFS10972.1| type IV pilus assembly tapB domain protein [Shigella flexneri 2a str. 2457T]

MNIPQLTALCLRYQGVLLDASEEVVHVAVVDAPSHELLDALHFATTKRIEITCWTRQQMEGHASRTQQTL

PVAVQEKHQPKAELLTRTLQSALEQRASDIHIEPADNAYRIRLRIDGVLHPLPDVSPDAGVALTARLKVL

GNLDIAEHRLPQDGQFTVELAGNTVSFRIATLPCRGGEKVVLRLLQQVSQALDVNTLGMQPLQLADFAHA

LQQPQGLVLVTGPTGSGKTVTWCCPRYTPIPPLKRWYVYSKWGSPAGCYHRRLRW

>gi|313646501|gb|EFS10962.1| uncharacterized yacH domain protein [Shigella flexneri 2a str. 2457T]

MAWRHNPNYRNGVPYHDQDMAKRFHQTDVNGGMSATQLPAPTRDSQRQAAASQFQQRTHAAPVITRDTQR

QAAAQRFNEAEHYGSYDDFRDFSRRQPLTQQQKDAARQRYQSASPEQRQAVREKMQTNPQIQQRREAARQ

RIQSASPEQRQAVREKMQTNPQNQQRREAARERIQSATPEQRQVFKEKVQQRPLNQQQRDNARQRIQSAS

PEQRQVFREKVQESRPQRLNDSNHTVRLNNEQRSAVRERLSERGARRLER

>gi|313646499|gb|EFS10960.1| uncharacterized yacH domain protein [Shigella flexneri 2a str. 2457T]

MTLPFKPHVLALICSAGLCAASAGLYIKSRTVEAPVEPQSTQLAVSDAAAVTLPATVSAPPVIPAVVKSA

FSTAQIDQWVAPVALYPDALLSQVLMASTYPTNVAQAVQWSHDNPLKQGDAAIQAVSDQPWDASVKSLVA

FPQLMALMGENPQWVQNLGDAFLAQPQDVMDSVQRLRQLAQQTGSLKSSTEQKVITTTKKAVPVKQTVTA

PVIPSNTVLTANPVITEPATTVISIEPANPDVVYIPNYNPTVVYGNWANTAYPPVYLPPPAGEPFVDSFV

RGFGYSMGVATTYALFSSID

>gi|313646480|gb|EFS10941.1| uncharacterized fimbrial-like protein yadC [Shigella flexneri 2a str. 2457T]

MPCFTAMRAEIALMSGSAFAVTHHAFSSGAGRTSDGNGSHASTLKSPSYTKSVSWQHYPTKVGTENGQLL

GNTLTGNDAAKGVGVLIEGLATSKNPLMTLKPNDSNSVYKDYDPRGKDDTTGGVYPDQDTGITYPLHFQA

TLQQDGTIPIEAGEFKATSTFQVTYP

>gi|313646468|gb|EFS10930.1| IS222, transposase ORFA [Shigella flexneri 2a str. 2457T]

MDRAVRMVKWHTEFGHLNRGDMLTSEQHRCSNEKRNFSAEFKRESAQLVVDQKYTVADAAKAMDVGLSTM

TRWVKQLRDERQGKTPKASPITPEQIEIRELRKKLQRIEMENEILKKATALLMSDSLNSSR

>gi|313646451|gb|EFS10913.1| hypothetical protein SF2457T_5366 [Shigella flexneri 2a str. 2457T]

MGKISDLNYSQHITLADNFKQKNEALDTWYVGMNDFARIAGGQNSRSNILSPRAFLEFLAKIFTLGYVDF

SKRSNEAGRNMMAHIEFSSYSKDTDGNEKMKFYMNNPEGERADLSKVKIEITLASASTKGIREGHTVIIF

KQSDGSTNRYEGKSFERKDDSSLHLITNKVLACYQREANKKIARLLNNHQKLNNIQELNDSQELNNSQKL

NNSQELNNSQVSCKGSVDSTITDLLEKALNKG

>gi|313646428|gb|EFS10890.1| inner membrane ABC transporter permease protein yjfF [Shigella flexneri 2a str. 2457T]

MIKRNLPLMITIGVFVLGYLYCLTQFPGFASTRVICNILTDNAFLGIIAVGMTFVILSGGIDLSVGSVIA

FTGVFLAKVVGDFGLSPLLAFPLVLVMGCAFGAFMGLLIDALKIPAFIITLAGMFFLRGVSYLVSEESIP

INHPIYDTLSSLAWKIPGGGRLSAMGLLMLAVVVIGIFLAHCTRFGNQVYAIGGNATSANLMGISTRSTT

IRIYMLSTGLATLAGIVFSIYTQAGYALAGVGVELDAIASVVIGGTLLSGGVGTVLGTLFGVAIQGLIQT

YINFDGTLSS

>gi|313646425|gb|EFS10887.1| sugar transporter subunit: periplasmic-binding component of ABC superfamily [Shigella flexneri 2a str. 2457T]

MDAIFIAPVVATGWEPVLKEAKDAEIPVFLLDRSIDVKDKSLYMTTVTADNILEGKLIGDWLVKEVNGKP

CNVVELQGTVGASVAIDRKKGFAEAIKNAPNIKIIRSQSGDFTRSKGKEVMESFIKAENNGKNICMVYAH

NDDMVIGAIQAIKEAGLKPGKDILTGSIDGVPDIYKAMMDGEANASVELTPNMAGPAFDALEKYKKDGTM

PEKLTLTKSTLYLPDTAKEELEKKKNMGY

>gi|313646413|gb|EFS10875.1| hypothetical protein SF2457T_5328 [Shigella flexneri 2a str. 2457T]

MLPRIRHNNFIGAVELFVKSSHTKTHSNDFFNNIQHAFKKKDWVSNYDSLLTLRESFRCATQIDKNSYQV

LSSKNETVNAMDNFLISFSLKDNGAEYTITLRGSGFEYEEIPITINEYNSFMDFKNREFPLEQNRRLYAC

DILQKKQSDIPKRIKGYIRQAFGDVSFGYALLEDVVSKLKRGKFELQIPGGGIKECDGWYIYEKIIDDNF

AIVIESLGFALKIYGGDERFRNGSSVVLEDEDYSLIYNFLVNAGCQQVELAEQVDAIVSANLAADSNITK

EKICEKYKSTIEAFKKEQLALPVLVRCKNSET

>gi|313646403|gb|EFS10865.1| bacterial regulatory s, tetR family protein [Shigella flexneri 2a str. 2457T]

MYIDTSNVQSLKEKLLLCAVNEFAEYGYEGARVDNIVKAAGCSKQTVYHHFGNKENLFIEVLEYTWNDIR

QKEKALDFSDLPPQKAIEKIIDFTWDYYIANPWFLKIVHSENQSKGVHYAKSQRLLEINHAHLQLMESLL

DEGKKYNIFKPGIDPLQVNINIAALGGYYLINQHTLGLVYHISMVSPQALEARRKVIKETILSWLLVDPS

STAHE

>gi|313646366|gb|EFS10828.1| amino acid permease-associated region domain protein [Shigella flexneri 2a str. 2457T]

MMAEMGAAYRKEEGGIYSWMNNSVGPRFAFIGTFMWFSSYIIWMVSTSAKVWVPFSTFLYGSDMTQHWRI

AGLEPTQVVGLLAVAWMILVTVVASKGINKIARITAVGGIAVMCLNLVLLLVSITILLLNGGHFAQDINF

LASPNPGYQSGLAMLSFVVFAIFAYGGIEAVGGLVDKTENPEKNFAKGIVFAAIVISIGYSLAIFLWGVS

TNWQQVLSNGSVNLGNITYVLMKSLGVMLGNALHLSPEASLSLGVWFARITGLSMFLAYTGAFAIHR

>gi|313646365|gb|EFS10827.1| amino acid permease family domain protein [Shigella flexneri 2a str. 2457T]

MTRLNAMGMPSIAMWMQCGLVTVFILLVSFGGGTASAFFNKLTLMANVSMTLPYLFLALAFPFFKARQDL

DRPFVIFKTRMSAMIATVVVVLVVTFANVFTIIQPVVEAGDWDSTLWMIGGPVFFSLLAMAIYQNYCSRM

ANKPELALD

>gi|313646339|gb|EFS10801.1| aidB domain protein [Shigella flexneri 2a str. 2457T]

MHWQTHTIFNQPIPLNNSNLYLSDGALCEAVTREGAGWDSDFLASIGQQLGTAESLELGRLANVNPPELL

RYDAQGRRLDDVRFHPAWHLLMQALCTNRVHNLAWEEDARSGAFVARAARFMLHAQVEAGSLCPVTMTFA

ATPLLLQMLPTPFQDWTTPLLSDRYDSHLLPGGQKRGLLIGMGMTEKQGGSDVMSNTTRAERLEDGSYRL

VGHKWFFSVPQSDAHLVLAQTAGGLSCFFVPRFLPDGQRNAIRLERLKDKLGNRSNASCEVEFQDAIGWL

LGQEGEGIRLILKMGG

>gi|313646336|gb|EFS10798.1| aidB domain protein [Shigella flexneri 2a str. 2457T]

MCLDVLRVLNKQAGVYDLLSEAFVEVKGQDRYFDRAVRRLQQQLRKPAEELGREITHQLFLLGCGAQMLK

YASPPMAQAWCQVMLDTRGGVRLSEQIQNDLLLRATGGVCV

>gi|313646316|gb|EFS10778.1| hypothetical protein SF2457T_5226 [Shigella flexneri 2a str. 2457T]

MTDTHSIAQPLEAEVSPANNRQLTVSYASRYPDYSRIPAITLKGQWLEAAGFATGTAVDVKVMEGCIVLT

AQPAAAEESELMQSLRKVCKLSARKQRQVQEFIGVITGKQKVA

>gi|313646314|gb|EFS10776.1| RNA methylase family UPF0020 family protein [Shigella flexneri 2a str. 2457T]

MSISSVIKSLQDIMRKDAGVDGDAQRLGQLSWLLFLKIFDAQEEALELEQDNYQYPIPQRYLWRSWAANA

QGITGDSLLEFVNDDLFPALKNLTAPIDKNPRGYVVKQAFSDAYNYMKNGTLLRQVINKLNEIDFTSASE

RHLFGDIYEQILKDLQSAGNAGEFYTPRAVTRFMVDRVDPKLGESIMDPACGTGGFLACAFDHVKNKYVK

SVADHQTLQQQIHGVEKKQLPHLLATTNMLLHGIEVPVQIRHDNTLNKPLSSWDEQLDVIVTNPPFGGTE

EDGIEKNFPAEMQTRETADLFLQLIVEVLAKNGRAAVVLPDGTLFGEGVKTKIKKLLTEECNLHTIVRLP

NGVFNPYTGIKTNLLFFTKGQPTKEIWFYEHPYPAGVKNYSKTKPMKFEEFQAEIDWWGNEADGFASRVE

NEQAWKVSIDDVIARNFNLDIKNPHQAETVSHDPDELLAQYAKQQAEIQTLRNQLRDILGAALSGKEVN

>gi|313646313|gb|EFS10775.1| type III restriction enzyme, res subunit [Shigella flexneri 2a str. 2457T]

MAELNLSNLTEADIITKCVMPAILNAGWDNTTQIRQEVKLRDGKVIVRGKVAARRTVKSADIVLYHKPGI

PLAVIEAKANKHEIGKGMQQGIEYARLLDVPFVFATNGDGFIFRDATAAEGECLEKQITLDDFPSPAELW

QKFCLWKGYTQAQLPVITQDYYDDGSGKSPRYYQLQAINKTIEAVSNGQNRVLLVMATGTGKTYTAFQII

WRLWKSKNKKRILFLADRNILVDQTKNNDFQPFGTAMTKVSGRTIDPAYEIHLALYQAITGPEEDQKAFK

QVAPDFFDLIVIDECHRGSASEDSAWREILDYFSSATQIGLTATPKETHEVSSTDYFGDPVYVYSLKEGI

EDGFLAPYKVVRVDIDVDLQGWRPTKGQTDLNGEVIDDRIYNQKDFDRTMVIDERTELVARTITDYLKRT

NPMDKTIVFCNDIDHAERMRRALVNLNPQQVKKNDKYVMKITGDDEIGKAQLDNFINPKKPYPVIATTSE

LMTTGVDAKTCKLVVLDQNIQSMTKFKQIIGRGTRIDERYGKLWFTILDFKKATELFADERFDGIPEKVM

DTTPEDIADPESDFEEKLEEISEHDDEQVTGVDEPPAPPYQVTDTDDVGPFPEEDEKKIRKFHVNGVAVG

VIAQRVQYYDADGKLVTESFKDYTRKTLLKEYASLDDFTRKWQDADRKEAIIHELEQQGIIWEVLAEEVG

KDLDPFDMLCHVVYGQPPLTRKERAENVHKRNYFTKYSEAAQAVLDNLLDKYADAGVQEIESIQVLKLKP

FDSMGTLPEIIKTGFGDRNGYNQALSELENEIYQLPPRSA

>gi|313646310|gb|EFS10772.1| hypothetical protein SF2457T_5220 [Shigella flexneri 2a str. 2457T]

MAFMNRMARTEDFNLDDFSQFITAFEKVYMHGWLKKQIKSQREMVCYSALVAINNDMPFDSVINQINQHA

DNSGFIAALDEDLYEPRPNQVNLIKAILLRLDMEQQDESVIKTYTGRITIEHILPQALVNEYWINRFQPQ

EHVYWLHKIGNLTLISGSKNSETQHYDFIKKKSIYEKLNSKSSFYLTKDVCNSSEWGLAELKMRHEKMKT

QLKKLWLV

>gi|313646304|gb|EFS10766.1| 4-hydroxyphenylacetate 3-monooxygenase oxygenase component [Shigella flexneri 2a str. 2457T]

MKDVYIKLEKETDAGIIVSGAKVVATNSALTHYNMIGFGSAQVMGENPDFALMFVAPMDADGVKLISRAS

YEMVAGATGSPYDYPLSSRFDENDAILVMDNVLIPWENVLIYRDFDRCRRWTMEGGFARMYPLQACVRLA

VKLDFITALLKKSLECTGTLEFRGVQADLGEVVAWRNTFWALSDSMCSEATPWVNGAYLPDHAALQTYRV

LAPMAYAKIKNIIERNVTSGLIYLPSSARDLNNPQIDQYLAKYVRGSNGMDHVQRIKILKLMWDAIGSEF

GGRHELYEINYSGSQDEIRLQCLRQAQSSGNMDKMMAMVDRCLSEYDQNGWTVPHLHNNDDINMLDKLLK

>gi|313646303|gb|EFS10765.1| 4-hydroxyphenylacetate 3-monooxygenase oxygenase component domain protein [Shigella flexneri 2a str. 2457T]

MKPEDFRASTQRPFTGEEYLKSLQDGREIYIYGERVKDVTTHPAFRNAAASVAQLYDALHKPEMQDSLCW

NTDTGSGGYTHKFFRVAKSADDLRQQRDAIAEWSRLSYGWMGRTPDYKAAFGCALGANPGFYGQFEQNAR

NWYTRIQETGLYFNHAIVNPPIVICRPIK

>gi|313646289|gb|EFS10751.1| bacterial regulatory s, gntR family protein [Shigella flexneri 2a str. 2457T]

MSRSQNLRHNVINQVIDDMARGHIPSPLPSQSALAEMYNISRTTVRHILSQLRECGVLTQVGNDYVIARK

PDHDDGFACTTASMSEQNKVFEQAFFTMINQRQLRPRETFSELQLARAAGVSPVVVREYLLKFGRYNLIQ

SEKRGQWSMKQFDQSYAEQLFELREMLETHSLQHFLNLPDHDPRWLQAKTMLERHRLLRDNIGNSFRMFS

QLDRDFHSLLLSAADNIFFDQSLEIISVIFHFHYQWDESDLKQRNIIAVDEHMTILSALICRSDLDATLA

LRNHLNSAKQSMIRSINENTRYAH

>gi|313646281|gb|EFS10743.1| hypothetical protein SF2457T_5191 [Shigella flexneri 2a str. 2457T]

MQTEQQRAVTRLCIQCGLFLLQHGAESALVDELSSRLGRALGMDSVESSISSNAIVLTTIKDGQCLTSTR

KNHDRGINMHVVTEVQHIVILAEHHLLDYKGVEKRFSQIQPLRYPRWLVALMVGLSCACFCKLNNGGWDG

AVITFFASTTAMYIRQLLAQRHLHPQINFCLTAFAATTISGLLLQLPTFSNTPTIAMAASVLLLVPGFPL

INAVADMFKGHINTGLARWAIASLLTLATCVGVVMALTIWGLRGWV

>gi|313646278|gb|EFS10740.1| bacterial regulatory s, luxR family protein [Shigella flexneri 2a str. 2457T]

MESLPGLVIADLAGESEDPRSVCEHYYSLISQYREIHWVFMVSRSWYSQAVELLMCPTATLLSDVEPIEN

LVKTVRSGNTHAERISAMLTSPAMTETHDFSYRSVILTLSERKVLRLLGKGWGINQIASLLKKSNKTISA

QKNSAMRRLAIHSNAEMYAWINSAQGARELNLPSVYGDAAEWNTAELRREMSHS

>gi|313646248|gb|EFS10710.1| hypothetical protein SF2457T_5155 [Shigella flexneri 2a str. 2457T]

MKRTQRQDHRLVIDAGASEFPGGKHADRPFLTTNLINTGITRLTGPSASPSRTFSRITAGNGKVAERASP

RWLAFSITTQLKVLSPSMPASIATQKSASGSKRRAFATRFRAPARVSSLPKGCSGTPLSTATDAIWQDPS

PKIS

>gi|313646232|gb|EFS10694.1| amino-acid carrier alsT domain protein [Shigella flexneri 2a str. 2457T]

MVILFAFSSIVANYIYAENNLFFLRLNNPKAIWCLRICTFATVIGGTLLSLPLMWQLADIIMACMAITNL

TAILLLSPVVHTIASDYLRQRKLGVRPVFDPLRYPDIGRQLSPDAWDDVSQE

>gi|313646231|gb|EFS10693.1| amino acid carrier family protein [Shigella flexneri 2a str. 2457T]

MPVFFSFINSVLWGSVMIYLLFGAGCWFTFRTGFVQFRYIRQFGKSLKNSIHPQPGGLTSFQSLCTSLAA

RVGSGNLAGVALAITAGGPGAVFWMWVAAFIGMATSFAECSLAQLYKERDVNGQFRGGPAWYMARGLGMR

WMGVLFAVFLLIAYGIIFSGVQANAVARALSFSFDFPPLVTGIILAVFALLAITRGLHGVARLMQGFVPL

MAIIWVLTSLVICVINIGQLPHVIWSIFESAFGWQEAAGGAAGYTLSQAITNGFQRSMFSNEVGMGSTPN

AAAAAASWPPHPAAQGIVQMIGIFIDTLVICTASAMLILLAGNGTTYMPLEGIQLIQKAMRVLMGS
