# Supplementary material for: Finding Potential Therapeutic Targets against Shigella flexneri through Proteome Exploration
Source: Front Microbiol. 2016 Nov 22;7:1817. doi: 10.3389/fmicb.2016.01817 (PMC5118456; doi:10.3389/fmicb.2016.01817)
Supplement: Supplementary file 11 [file DataSheet6.PDF]

>gi|161486506|ref|NP_836827.2| spermidine/putrescine ABC transporter [Shigella flexneri 2a str. 2457T]

MKNTSKFQNVVIVTIVGWLVLFVFLPNLMIIGTSFLTRDDASFVKMVFTLDNYTRLLDPLYFEVLLHSLN

MALIATLACLVLGYPFAWFLAKLPHKVRPLLLFLLIVPFWTNSLIRIYGLKIFLSTKGYLNEFLLWLGVI

DTPIRIMFTPSAVIIGLVYILLPFMVMPLYSSIEKLDKPLLEAARDLGASKLQTFIRIIIPLTMPGIIAG

CLLVMLPAMGLFYVSDLMGGAKNLLIGNVIKVQFLNIRDWPFGAATSITLTIVMGLMLLVYWRASRLLNK

KVELE

>gi|161486375|ref|NP_839574.2| 30S ribosomal protein S4 [Shigella flexneri 2a str. 2457T]

MARYLGPKLKLSRREGTDLFLKSGVRAIDTKCKIEQAPGQHGARKPRLSDYGVQLREKQKVRRIYGVLER

QFRNYYKEAARLKGNTGENLLALLEGRLDNVVYRMGFGATRAEARQLVSHKAIMVNGRVVNIASYQVSPN

DVVSIREKAKKQSRVKAALELAEQREKPTWLEVDAGKMEGTFKRKPERSDLSADINEHLIVELYSK

>gi|30065597|ref|NP_839768.1| methyl-accepting chemotaxis protein I, serine sensor receptor [Shigella flexneri 2a str. 2457T]

MLKRIKIVTSLLLVLAVFGLLQLTSGGLFFNALKNDKENFTVLQTIRQQQPTLNGSWVALLQTRNTLNRA

GIRYMMDQNNIGSGSTVAELMQSASISLKQAEKNWADYEALPRDPRQSTAAAAEIKRNYDIYHNALAELI

QLLGAGKINEFFDQPTQGYQDGFEKQYVAYMEQNDRLYDIAVSDNNASYSQAMWILVGVMIVVLAVIFAV

WFGIKASLVAPMNRLIDSIRHIAGGDLVKPIEVDGSNEMGQLAESLRHMQGELMRTVGDVRNGANAIYSG

ASEIATGNNDLSSRTEQQAASLEETAASMEQLTATVKQNAENARQASHLALSASETAQRGGKVVDNVVQT

MRDISTSSQKIADIISVIDGITFQTNILALNAAVEAARAGEQGRGFAVVAGEVRNLAQRSAQAVREIKSL

IEDSVGKVDVGSTLVESAGETMAEIVSAVTRVTDIMGEIASASDEQSRGIDQVGLAVAEMDRVTQQNAAL

VEESAAAAAALEEQASRLTEAVAVFRIQQQQQQRETSAVVKTVTPATPRKMAVADSGENWETF

>gi|30065585|ref|NP_839756.1| carbon starvation protein [Shigella flexneri 2a str. 2457T]

MPGFTMDTKKLFKHIPWVILGIIGAFCLAVVALRRGEHVSALWIVVASVSVYLVAYRYYSLYIAQKVMKL

DPTRATPAVINNDGLNYVPTNRYVLFGHHFAAIAGAGPLVGPVLAAQMGYLPGTLWLLAGVVLAGAVQDF

MVLFISSRRNGASLGEMIKEEMGPVPGTIALFGCFLIMIIILAVLALIVVKALAESPWGVFTVCSTVPIA

LFMGIYMRFIRPGRVGEVSVIGIVLLVASIYFGGVIAHDPYWGPALTFKDTTITFALIGYAFVSALLPVW

LILAPRDYLATFLKIGVIVGLALGIVVLNPELKMPAMTQYIDGTGPLWKGALFPFLFITIACGAVSGFHA

LISSGTTPKLLANETDARFIGYGAMLMESFVAIMALVAASIIEPGLYFAMNTPPAGLGITMPNLHEMGGE

NAPIIMAQLKDVTAHAAATVSSWGFVISPEQILQTAKDIGEPSVLNRAGGAPTLAVGIAHVFHKVLPMAD

MGFWYHFGILFEALFILTALDAGTRSGRFMLQDLLGNFIPFLKKTDSLVAGIIGTAGCVGLWGYLLYQGV

VDPLGGVKSLWPLFGISNQMLAAVALVLGTVVLIKMKRTQYIWVTVVPAVWLLICTTWALGLKLFSTNPQ

MEGFFYMASQYKEKIANGTDLTAQQIANMNHIVVNNYTNAGLSILFLIVVYSIIFYGFKTWLAVRNSDKR

TDKETPYVPIPEGGVKISSHH

>gi|30065429|ref|NP_839600.1| periplasmic chaperone [Shigella flexneri 2a str. 2457T]

MSNKNVNVRKSQEITFCLLAGILMFMAMMVAGRAEAGVALGATRVIYPAGQKQVQLAVTNNDENSTYLIQ

SWVENADGVKDGRFIVTPPLFAMKGKKENTLRILDATNNQLPQDRESLFWMNVKAIPSMDKSKLTENMLQ

LAIISRIKLYYRPAKLALPPDQAAEKLRFRRSANSLTLINPTPYYLTVTELNAGTRVLENALVPPMGEST

VKLPSDAGSNITYRTINDYGALTPKMTGVME

>gi|30065404|ref|NP_839575.1| DNA-directed RNA polymerase subunit alpha [Shigella flexneri 2a str. 2457T]

MQGSVTEFLKPRLVDIEQVSSTHAKVTLEPLERGFGHTLGNALRRILLSSMPGCAVTEVEIDGVLHEYST

KEGVQEDILEILLNLKGLAVRVQGKDEVILTLNKSGIGPVTAADITHDGDVEIVKPQHVICHLTDENASI

SMRIKVQRGRGYVPASTRIHSEEDERPIGRLLVDACYSPVERIAYNVEAARVEQRTDLDKLVIEMETNGT

IDPEEAIRRAATILAEQLEAFVDLRDVRQPEVKEEKPEFDPILLRPVDDLELTVRSANCLKAEAIHYIGD

LVQRTEVELLKTPNLGKKSLTEIKDVLASRGLSLGMRLENWPPASIADE

>gi|30065350|ref|NP_839521.1| hypothetical protein S4380 [Shigella flexneri 2a str. 2457T]

MTNSNRIKLTWISFLSYALTGALVIVTGMVMGNIADYFNLPVSSMSNTFTFLNAGILISIFLNAWLMEIV

PLKTQLRFGFLLMVLAVAGLMFSHSLALFSTAMFILGVVSGITMSIGTFLITQMYEGRQRGSRLLFTDSF

FSMAGMIFPMIAAFLLARSIEWYWVYACIGLVYVAIFILTFGCEFPALGKRAPKTDAPVEKEKWGIGVLF

LSVAALCYILGQLGFISWVPEYAKGLGMSLNDAGTLVSNFWMSYMVGMWAFSFILRFFDLQRILTVLAGL

AAILMYVFNTGTPVHMAWSILALGFFSSAIYTTIITLGSQQTKVPSPKLVNFVLTCGTIGTMLTFVVTGT

IVEHSGPQAALLTANGLYAVVFVMCFLLGFVSRHRQHNTLTSH

>gi|30064994|ref|NP_839165.1| transcriptional regulator [Shigella flexneri 2a str. 2457T]

MIYKSIAERLRIRLNSADFTLNSLLPGEKKLAEEFAVSRMTIRKAIDLLVAWGLVVRRHGSGTYLVRKDV

LHQTASLTGLVEVLKRQGKTVTSQVLIFEIMPAPPAIASQLRIQINEQIYFSRRVRFVEGKPLMLEDSYM

PVKLFRNLSLQHLEGSKFEYIEQECGILIGGNYESLMPVLADRLLARQMKVAEHTPLLRITSLSYSESGE

FLNYSVMFRNASEY

>gi|30064893|ref|NP_839064.1| site-specific tyrosine recombinase XerC [Shigella flexneri 2a str. 2457T]

MTDLHTDVERYLRYLSVERQLSPITLLNYQRQLEAIINFASENGLQSWQQCDAAMVRNFAVRSRRKGLGA

ASLALRLSALRSFFDWLVSQNELKANPAKGVSAPKAPRHLPKNIDVDDMNRLLDIDINDPLAVRDRAMLE

VMYGAGLRLSELVGLDIKHLDLESGEVWVMGKGSKERRLPIGRNAVAWIEHWLDLRDLFGSEDDALFLSK

LGKRISARNVQKRFAEWGIKQGLNNHVHPHKLRHSFATHMLESSGDLRGVQELLGHANLSTTQIYTHLDF

QHLASVYDAAHPRAKRGK

>gi|30064772|ref|NP_838943.1| primosome assembly protein PriA [Shigella flexneri 2a str. 2457T]

MPVAHVALPVPLPRTFDYLLPEGMTVKAGCRVRVPFGKQQERIGIVVSVSDASELPLTELKAVVEVLDGE

PVFTHSVWRLLLWAADYYHHPIGDVLFHALPILLRQGRPAANAPMWYWFATEQGQAVDLNSLKRSPKQQQ

ALAALRQGKIWRDQVATLEFNDAALQALRKKGLCDLASETPEFSDWRTNYAVSGERLRLNTEQATAVGAI

HSAADTFSAWLLAGVTGSGKTEVYLSVLENVLAQGKQALVMVPEIGLTPQTIARFRERFNAPVEVLHSGL

NDSERLSAWLKAKNGEAAIVIGTRSALFTPFKNLGVIVIDEEHDSSYKQQEGWRYHARDLAVYRAHSEQI

PIILGSATPALETLCNVQQKKYRLLRLTRRAGNARPAIQHVLDLKGQKVQAGLAPALITRMRQHLQANNQ

VILFLNRRGFAPALLCHDCGWIAECPRCDHYYTLHQAQQHLRCHHCDSQRPVPRQCPSCGSTHLVPVGLG

TEQLEQTLAPLFPDVPISRIDRDTTSRKGALEQQLAEVHRGGARILIGTQMLAKGHHFPDVTLVALLDVD

GALFSADFRSAERFAQLYTQVAGRAGRAGKQGEVVLQTHHPEHPLLQTLLYKGYDAFAEQALAERRMMQL

PPWTSHVIVRAEDHNNQHAPLFLQQLRNLILSSPLADDKLWVLGPVPALAPKRGGRWRWQILLQHPSRVR

LQHIISGTLALINTIPDSRKVKWVLDVDPIEG

>gi|30064723|ref|NP_838894.1| anti-RNA polymerase sigma 70 factor [Shigella flexneri 2a str. 2457T]

MLNQLDNLTERVRGSNKLVDRWLHVRKHLLVAYYNLVGIKPGKESYMRLNEKALDDFCQSLVDYLSAGHF

SIYERILHKLEGNGQLARAAKIWPQLEANTQQIMDYYDSSLETAIDHDNYLEFQQVLSDIGEALEARFVL

EDKLILLVLDAARVKHPA

>gi|30064701|ref|NP_838872.1| sorbose-permease PTS system IIB component [Shigella flexneri 2a str. 2457T]

MNITLARIDDRLIHGQVTTVWSKVANAQRIIICNDEVYNDEVRRTLLRQAAPPGMKVNVVNIEKAVAVYH

NPQYQDETVFYLFTRPQDALAMVRQGVKIGTLNIGGMAWRPGKKQLTKAVSLDDDDINAFHELNNLGVIL

DLRVVASDPSINIIDKINEQLIAN

>gi|30064551|ref|NP_838722.1| IS600 orf [Shigella flexneri 2a str. 2457T]

MAHIRTRETYGTRRLQTELAENGIIVGRDRLARLRKELRLRCKQKRKFRATTNPNHNLPVAPNLLNQTFA

PTAPNQVWVADLTYVATQEGWLYLAGIKDVYTCEIVGYAMGERMTKELTGKALFMALRSQRPPAGLIHHS

DRGSQYCAYDYRVIQEQSGLKTSMSRKGNCYDNAPMESFWGTLKNESLSHYRFNNRDEAISVIREYIEIF

YNRQRRHSRLGNISPAAFRENIIRWLLKKRTNGSVRYCQYTSKVAMIYIEQLELIHKSGDVLYPVKITRK

SSGKTAFHLVPFGLNKTHDLLEVEDASEAIRLVIDERHSIRCSTLTATITNKKGKRIKRTGIYSIKGVNI

KEYNVR

>gi|30064535|ref|NP_838706.1| D-arabinose 5-phosphate isomerase [Shigella flexneri 2a str. 2457T]

MSHVELQPGFDFQQAGKEVLAIERECLAELDQYINQNFTLACEKMFWCKGKVVVMGMGKSGHIGRKMAAT

FASTGTPSFFVHPSEAAHGDLGMVTPQDVVIAISNSGESSEITALIPVLKRLHVPLICITGRPESSMARA

ADVHLCVKVAKEACPLGLAPTSSTTATLVMGDALAVALLKARGFTAEDFALSHPGGALGRKLLLRVNDIM

HTGDEIPHVKKTASLRDALLEVTRKNLGMTVICDDNMMIEGIFTDGDLRRVFDMGVDVRQLSIADVMTPG

GIRVRPGILAVEALNLMQSRHITSVMVADGDHLLGVLHMHDLLRAGVV

>gi|30064457|ref|NP_838628.1| formate acetyltransferase 3 [Shigella flexneri 2a str. 2457T]

MKVDIDTSDKLYADAWLGFKGTDWKSEINVRDFIQHNYTPYEGDESFLAEATPATTELWEKVMEGIRIEN

ATHAPVDFDTNIATTITAHDAGYINQPLEKIVGLQTDAPLKRALHPFGGINMIKSSFHAYGREMDSEFEY

LFTDLRKTHNQGVFDVYSPDMLRCRKSGVLTGLPDGYGRGRIIGDYRRVALYGISYLVRERELQFADLQS

RLEKGEDLEATIRLREELAEHRHALLQIQEMAAKYGFDISRPAQNAQEAVQWLYFAYLAAVKSQNGGAMS

LGRTASFLDIYIERDFKAGVLNEQQAQELIDHFIMKIRMVRFLRTPEFDSLFSGDPIWATEVIGGMGLDG

RTLVTKNSFRYLHTLHTMGPAPEPNLTILWSEELPIAFKKYAAQVSIVTSSLQYENDDLMRTDFNSDDYA

IACCVSPMVIGKQMQFFGARANLAKTLLYAINGGVDEKLKIQVGPKTAPLMDDVLDYDKVMDSLDHFMDW

LAVQYISALNIIHYMHDKYSYEASLMALHDRDVYRTMACGIAGLSVATDSLSAIKYARVKPIRDENGLAV

DFEIDGEYPQYGNNDERVDSIACDLVERFMKKIKALPTYRNAVPTQSILTITSNVVYGQKTGNTPDGRRA

GTPFAPGANPMHGRDRKGAVASLTSVAKLPFTYAKDGISYTFSIVPAALGKEDPVRKTNLVGLLDGYFHH

EADVEGGQHLNVNVMNREMLLDAIEHPEKYPNLTIRVSGYAVRFNALTREQQQDVISRTFTQAL

>gi|30063781|ref|NP_837952.1| hypothetical protein S2579 [Shigella flexneri 2a str. 2457T]

MKRLIMATMVTAILASSTVWAADNAPVAAQQQTQQTQKTAAAERISEQGLYAMRDVQVARLALFHGDPEK

AKELTNEASALLSDDSTEWAKFAKPGKKTNLNDDQYIVINASVGISESYVATPEKEAAIKIANEKMAKGD

KKGAMEELRLAGVGVMENQYLMPLKQTRNALADAQKLLDKKQYYEANLALKGAEDGIIVDSEALFVN

>gi|30063512|ref|NP_837683.1| transporter [Shigella flexneri 2a str. 2457T]

MEWIADPSIWAGLITLIVIELVLGIDNLVFIAILAEKLPPKQRDRARVTGLLLAMLMRLLLLASISWLVT

LTQPLFSFRSFTFSARDLIMLFGGFFLLFKATMELNERLEGKDSNNPTQRKGAKFWGVVTQIVVLDAIFS

LDSVITAVGMVDHLLVMMAAVVIAISLMLMASKPLTQFVNSHPTIVILCLSFLLMIGFSLVAEGFGFVIP

KGYLYAAIGFSVMIEALNQLAIFNRRRFLSANQTLRQRTTEAVMRLLSGQKEDAELDAETASMLVDHGNQ

QIFNPQERRMIERVLNLNQRTVSSIMTSRHDIEHIDLNAPEEEIRQLLERNQHTRLVVTDGDDAEDLLGV

VHVIDLLQQSLRGEPLNLRVLIRQPLVFPETLPLLPALEQFRNARTHFAFVVDEFGSVEGIVTLSDVTET

IAGNLPNEVEEIDARHDIQKNADGSWTANGHMPLEDLVQYVPLPLDEKREYHTIAGLLMEYLQRIPKPGE

EVQVGDYLLKTLQVESHRVQKVQIIPLRKDGEMEYEV

>gi|30063508|ref|NP_837679.1| colanic acid biosynthesis acetyltransferase WcaB [Shigella flexneri 2a str. 2457T]

MLEDLRANSWSLRPCCMVLAYRVAHFCSVWRKKNVLNNLWAAPLLVLYRIITECFFGYEIQAAATIGRRF

TIHHGYAVVINKNVVAGDDFTIRHGVTIGNRGADNMACPHIGNGVELGANVIILGDITLGNNVTVGAGSV

VLDSVPDNALVVGEKARVKVIK

>gi|30063505|ref|NP_837676.1| colanic acid biosynthesis acetyltransferase WcaF [Shigella flexneri 2a str. 2457T]

MQDLSGFSVPKGFRGGNAIKVQLWWAVQATIFAWSPQVLYRWRAFLLRLFGAKIGKNVVIRPSVKITYPW

KLTLGDYAWVGDDVNLYTLGEITIGAHSVISQKSYLCTGSHDHASQHFTINATPIVIGEKCWLATDVFVA

PGVTIGDGTVVGARSSVFKSLPANVVCRGNPAVVIRERVETE

>gi|30063433|ref|NP_837604.1| hypothetical protein S2148 [Shigella flexneri 2a str. 2457T]

MQFCSSDEFASKTMIKWPWKVQESAHQTALPWQEALSIPLLTCLTEQEQSKLVALAERFLQQKRLVPLQG

FELNSLRSCRIALLFCLPVLELGLEWLDGFHEVLIYPAPFVVDDEWEDDIGLVHNQRIVQSGQSWQQGPI

VLNWLDIQDSFDASGFNLIIHEVAHKLDTRNGDRASGVPFISLREVAGWEHDLHAAMNNIQEEIELVGEN

AASIDAYAASDPAECFAVLSEYFFSAPELFAPRFPSLWQRFCQFYQQDPLQRLHHANDTDSFSATNVH

>gi|30063426|ref|NP_837597.1| crossover junction endodeoxyribonuclease [Shigella flexneri 2a str. 2457T]

MTERIEFVLPYPPTVNTYWRRRGSTYFVSKAGERYRRDVALIVRQQRLKLNLSGRLAIKIIAEPPDKRRR

DLDNILKAPLDALTHAGLLIDDEQFDEINIVRGQLVPGGRLGIKITELGCA

>gi|30063273|ref|NP_837444.1| Holliday junction resolvase [Shigella flexneri 2a str. 2457T]

MAIILGIDPGSRVTGYGVIRQVGRQLSYLGSGCIRTKVDDLPSRLKLIYAGVTEIITQFQPDYFAIEQVF

MAKNADSALKLGQARGVAIVAAVNQELPVFEYAARQVKQTVVGMGSAEKSQVQHMVRTLLKLPANPQADA

ADALAIAITHCHVSQNAMQMSESRLNLTRGRLR

>gi|30063272|ref|NP_837443.1| Holliday junction DNA helicase RuvA [Shigella flexneri 2a str. 2457T]

MIGRLRGIIIEKQPPLVLIEVGGVGYEVHMPMTCFYELPEAGQEAIVFTHFVVREDAQLLYGFNNKQERT

LFKELIKTNGVGPKLALAILSGMSAQQFVNAVEREEVGALVKLPGIGKKTAERLIVEMKDRFKGLHGDLF

TPAADLVLTSPASPATDDAEQEAVAALVALGYKPQEASRMVSKIARPDTSSETLIREALRAAL

>gi|30063267|ref|NP_837438.1| hypothetical protein S1932 [Shigella flexneri 2a str. 2457T]

MQQIARSVALAFNNLPRPHRVMLGSLTVLTLAVAVWRPYVYHRDATPIVKTIELEQNEIRSLLPEASEPI

DQAAQEDEAIPQDELDDKIAGEAGVHEYVVSTGDTLSSILNQYGIDMGDITQLAAADKELRNLKIGQQLS

WTLTADGELQRLTWEVSRRETRTYDRTAANGFKMTSEMQQGEWVNNLLKGTVGGSFVASARNAGLTSAEV

SAVIKAMQWQMDFRKLKKGDEFAVLMSREMLDGKREQSQLLGVRLRSEGKDYYAIRAEDGKFYDRNGTGL

AKGFLRFPTAKQFRISSNFNPRRTNPVTGRVAPHRGVDFAMPQGTPVLSVGDGEVVVAKRSGAAGYYVAI

RHGRSYTTRYMHLRKILVKPGQKVKRGDRIALSGNTGRSTGPHLHYEVWINQQAVNPLTAKLPRTEGLTG

SDRREFLAQAKEIVPQLRFD

>gi|30063205|ref|NP_837376.1| amino acid/amine transport protein [Shigella flexneri 2a str. 2457T]

MTSLAEKFSTDNAGIAYLISGIGLGRLISILFFGVISDKFGRRAVILMAVIMYLLFFFGIPACPNLTLAY

GLAVCVGIANSALDTGGYPALMECFPKASGSAVILVKAMVSFGQMFYPMLVSYMLLNNIWYGYGLIIPGI

LFVLITLMLLKSKFPSQLVDASVANELPQMNSKPLVWLEGVSSVLFGVAAFSTFYVIVVWMPKYAMAFAG

MSEAEALKTISYYSMGSLVCVFIFAALLKKMVRPIWANVFNSALATITAAIIYLYPSPLVCNAGAFVIGF

SAAGGILQLGVSVMSEFFPKSKAKVTSIYMMMGGLANFVIPLITGYLSNIGLQYIIVLDFTFALLALITA

IIVFSRYYRVFIIPENDVRFGERKFSTRLNTIKHRG

>gi|30062777|ref|NP_836948.1| intracellular septation protein A [Shigella flexneri 2a str. 2457T]

MKQFLDFLPLVVFFAFYKIYDIYAATAALIVATAIVLIYSWVRFRKVEKMALITFVLVVVFGGLTLFFHN

DEFIKWKVTVIYALFAGALLVSQWVMKKPLIQRMLSKELTLPQPVWSKLNLAWAVFFILCGLANIYIAFW

LPQNIWVNFKVFGLTALTLIFTLLSGIYIYRHMPQEDKS

>gi|30062766|ref|NP_836937.1| oligopeptide transport permease [Shigella flexneri 2a str. 2457T]

MMLSKKNSETLENFSEKLEVEGRSLWQDARRRFMHNRAAVASLIVLVLIALFVILAPMLSQFAYDDTDWA

MMSSAPDMESGHYFGTDSSGRDLLVRVAIGGRISLMVGVAAALVAVVVGTLYGSLSGYLGGKVDSVMMRL

LEILNSFPFMFFVILLVTFFGQNILLIFVAIGMVSWLDMARIVRGQTLSLKRKEFIEAAQVGGVSTPGIV

IRHIVPNVLGVVVVYASLLVPSMILFESFLSFLGLGTQEPLSSWGALLSDGANSMEVSPWLLLFPAGFLV

VTLFCFNFIGDGLRDALDPKDR

>gi|30062510|ref|NP_836681.1| hydrogenase-1 small subunit [Shigella flexneri 2a str. 2457T]

MNNEETFYQAMRRQGVTRRSFLKYCSLAATSLGLGAGMAPKIAWALENKPRIPVVWIHGLECTCCTESFI

RSAHPLAKDVILSLISLDYDDTLMAAAGTQAEEVFEDIITQYNGKYILAVEGNPPLGEQGMFCISSGRPF

IEKLKRAAAGASAIIAWGTCASWGCVQAARPNPTQATPIDKVITDKPIIKVPGCPPIPDVMSAIITYMVT

FDRLPDVDRMGRPLMFYGQRIHDKCYRRAHFDAGEFVQSWDDDAARKGYCLYKMGCKGPTTYNACSSTRW

NDGVSFPIQSGHGCLGCAENGFWDRGSFYSRVVDIPQMGTHSTADTVGLTALGVVAAAVGVHAVASAVDQ

RRRHNQQPTETEHQPGNEDKQA

>gi|30062504|ref|NP_836675.1| hypothetical protein S1033 [Shigella flexneri 2a str. 2457T]

MVGMSALSYTLLNSLEEIMKETDIAGILTSTHTIALVGASDKPDRPSYRVMKYLLDQGYHVIPVSPKVAG

KTLLGQQGYGTLADVPEKVDMVDVFRNSEAAWGVAQEAIAIGAKTLWMQLGVINEQAAVLARDAGLNVVM

DRCPAIEIPRLGLAK

>gi|30062501|ref|NP_836672.1| DNA helicase IV [Shigella flexneri 2a str. 2457T]

MELKATTLGKRLAQHPYDRAVILNAGIKVSGDRHEYLIPFNQLLAIHCKRGLVWGELEFVLPDEKVVRLH

GTEWGETQRFYHHLDAHWRRWSGEMSEIASGVLRQQLDLIATRTGENKWLTREQTSGVQQQIRQALSALP

LPVNRLEEFDNCREAWRKCQAWLKDIESARLQHNQAYTEAMLTEYADFFRQVESSPLNPAQARAVVNGEH

SLLVLAGAGSGKTSVLVARAGWLLARGEASPEQILLLAFGRKAAEEMDERIRERLHTEDITARTFHALAL

HIIQQGSKKVPIVSKLENDTAARHELFIAEWRKQCSEKKAQAKGWRQWLTEEMQWSVPEGNFWDDEKLQR

RLASRLDRWVSLMRMHGGAQAEMIASAPEEIRDLFSKRIKLMAPLLKAWKGALKAENAVDFSGLIHQAIV

ILEKGRFISPWKHILVDEFQDISPQRAALLAALRKQNSQTTLFAVGDDWQAIYRFSGAQMSLTTAFHENF

GEGDRCDLDTTYRFNSRIGEVANRFIQQNPGQLKKPLNSLTNGDKKAVTLLDESQLDALLDKLSGYAKPE

ERILILARYHHMRPASLEKAATRWPKLQIDFMTIHASKGQQADYVIIVGLQEGSGGFPAAARESIMEEAL

LPPVEDFPDAEERRLMYVALTRARHRVWALFNKENPSPFVEILKNLDVPVARKP

>gi|30062294|ref|NP_836465.1| glutamine ABC transporter permease [Shigella flexneri 2a str. 2457T]

MQFDWSAIWPAIPLLIEGAKMTLWISVLGLAGGLVIGLLAGFARTFGGWIANHVALVFIEVIRGTPIVVQ

VMFIYFALPMAFNDLRIDPFTAAVVTIMINSGAYIAEITRGAVLSIHKGFREAGLALGLSRWETIRYVIL

PLALRRMLPPLGNQWIISIKDTSLFIVIGVAELTRQGQEIIAGNFRALEIWSAVAVFYLIITLVLSFILR

RLERRMKIL

>gi|30062107|ref|NP_836278.1| DNA-binding transcriptional repressor MngR [Shigella flexneri 2a str. 2457T]

MGHKPLYRQIADRIREQIARGELKPGDALPTESALQTEFGVSRVTVRQALRQLVEQQILESIQGSGTYVK

EERVNYDIFQLTSFDEKLSDRHVDTHSEVLIFEVIPADDFLQQQLQITVQDRVWHVKRVRYRKQKPMALE

ETWMPLALFPDLTWQVMENSKYHFIEEVKKMVIDRSEQEIIPLMPTEEMSRLLNISQTKPILEKVSRGYL

VDGRVFEYSRNAFNTDDYKFTLIAQPKIIAISTKRPSYDGQPCGNRQPFAIPVEGSGEGYFLLQIFISEQ

FSVLHRYPPGCERFSRLRLPEQRISHPQSLSHRQ

>gi|30061705|ref|NP_835876.1| DnaK transcriptional regulator DksA [Shigella flexneri 2a str. 2457T]

MQEGQNRKTSSLSILAIAGVEPYQEKPGEEYMNEAQLAHFRRILEAWRNQLRDEVDRTVTHMQDEAANFP

DPVDRAAQEEEFSLELRNRDRERKLIKKIEKTLKKVEDEDFGYCESCGVEIGIRRLEARPTADLCIDCKT

LAEIREKQMAG

>gi|30061702|ref|NP_835873.1| 2-amino-4-hydroxy-6-hydroxymethyldihydropteridine pyrophosphokinase [Shigella flexneri 2a str. 2457T]

MTVAYIAIGSNLASPLEQVNAALKALGDIPESRILAVSSFYRTPPLGPQDQPDYLNAAVALETSPAPEEL

LNHTQRIELQQGRVRKAERWGPRTLDLDIMLFGNEVINTERLTVPHYDMKNRGFMLWPLFEIAPELAFPD

GETLREVLHTRAFDKLSKW

>gi|30061599|ref|NP_835770.1| dihydrodipicolinate reductase [Shigella flexneri 2a str. 2457T]

MHDANIRVAIAGAGGRMGRQLIQAALALEGVQLGAALEREGSSLLGSDAGELAGAGKTGVTVQSSLDAIK

DDFDVFIDFTRPEGTLNHLAFCRQHGKGMVIGTTGFDEAGKQAIRDAAADIAIVFAANFSVGVNVMLKLL

EKAAKVMGDYTDIEIIEAHHRHKVDAPSGTALAMGEAIAHALDKDLKDCAVYSREGHTGERVPGTIGFAT

VRAGDIVGEHTAMFADIGERLEITHKASSRMTFANGAVRSALWLSGKESGLFDMRDVLDLNNL

>gi|30061597|ref|NP_835768.1| 4-hydroxy-3-methylbut-2-enyl diphosphate reductase [Shigella flexneri 2a str. 2457T]

MQILLANPRGFCAGVDRAISIVENALAIYGAPIYVRHEVVHNRYVVDSLRERGAIFIEQISEVPDGAILI

FSAHGVSQAVRNEAKSRDLTVFDATCPLVTKVHMEVARASRRGEESILIGHAGHPEVEGTMGQYSNPEGG

MYLVESPDDVWKLTVKNEEKLSFMTQTTLSVDDTSDVIDALRKRFPKIVGPRKDDICYATTNRQEAVRAL

AEQAEVVLVVGSKNSSNSNRLAELAQRMGKHAFLIDDAKDIQEEWVKEVKCVGVTAGASAPDILVQNVVA

RLQQLGGGEAIPLEGREENIVFEVPKELRVDIREVD

>gi|30043828|gb|AAP19547.1| hypothetical protein S4618 [Shigella flexneri 2a str. 2457T]

MHNIPGVRNTRLPLLQEIVMEILYNIFTVFFNQVMTNAPLLLGIVTCLGYILLRKSVSVIIKGTIKTIIG

FMLLQAGSGILTSTFKPVVAKMSEVYGINGAISDTYASMMATIDRMGDAYSWVGYAVLLALALNICYVLL

RRITGIRTIMLTGHIMFQQAGLIAVTLFIFGYSMWTTIICTAILVSLYWGITSNMMYKPTQEVTDGCGFS

IGHQQQFASLIAYKVAPFLGKKEESVEDLKLPGWLNIFHDNIVSTAIVMTIFFGAILLSFGIDTVQAMAG

KVHWTVYILQTGFSFAVAIFIITQGVRMFVAELSEAFNGISQRLIPGAVLAIDCAAIYSFAPNAVVWGFM

WGTIGQLIAVGILVACGSSILIIPGFIPMFFSNATIGVFANHFGGWRAALKICLVMGMIEIFGCVWVVKL

TGMSAWMGMADWSILAPPMMQGFFSIGIAFMAVIIVIALAYMFFAGRALRAEEDAEKQLAEQSA

>gi|30043573|gb|AAP19293.1| transcription elongation factor and transcript cleavage factor [Shigella flexneri 2a str. 2457T]

MRIIKQTKGINEMKTPLVTREGYEKLKQELNYLWREERPEVTKKVTWAASLGDRSENADYQYNKKRLREI

DRRVRYLTKCLENLKIVDYSPQQEGKVFFGAWVEIENDDGVTHRFRIVGYDEIFGRKDYISIDSPMARAL

LKKEVGDLAVVNTPAGEANWYVNAIEYVKP

>gi|30042898|gb|AAP18621.1| hypothetical protein S3575 [Shigella flexneri 2a str. 2457T]

MKMPIKRVLTLTCWNTRSNWWRISVNFSPKSSQIHHALRTVAGRFAVKSIDYFWHDSCNASKRFHIWESI

MLELLFVIGFFVMLMVTGVSLLGIIAALVVATAIMFLGGMLALMIKLLPWLLLAIAVVWVIKAIKAPKMP

KYQRYDRWRY

>gi|30042774|gb|AAP18497.1| 7,8-dihydropteroate synthase [Shigella flexneri 2a str. 2457T]

MLRGFFLSIHTRDNIMKLFAQGTSLDLSHPHVMGILNVTPDSFSDGGTHNSLIDAVKHANLMINAGATII

DVGGESTRPGAAEVSVEEELQRVIPVVEAIAQRFEVWISVDTSKPEVIRESAKVGAHIINDIRSLSEPGA

LEAAAETGLPVCLMHMQGNPKTMQEAPKYDDVFAEVNRYFIEQIARCEQAGIAKEKLLLDPGFGFGKNLS

HNYSLLARLAEFHHFNLPLLVGMSRKSMIGQLLNVGPSERLSGSLACAVIAAMQGAHIIRVHDVKETVEA

MRVVEATLSAKENKRYE

>gi|30040947|gb|AAP16677.1| hypothetical protein S1258 [Shigella flexneri 2a str. 2457T]

MAEHLMSDVPFWQSKTLDEMSDAEWESLCDGCGQCCLHKLMDEDTDEIYFTNVACRQLNIKTCQCRNYER

RFEFEPDCIKLTRENLPTFEWLPMTCAYRLLAEGKDLPAWHPLLTGSKAAMHGERISVRHIAVKESEVID

WQDHILNKPDWAQ

>gi|313651501|gb|EFS15897.1| uvrD/REP helicase family protein [Shigella flexneri 2a str. 2457T]

MGKPTDEQRVIIENANANNMVIAAPGSGKSFTMIEAVISILRQFPYAKVGMVTFTRAATNSLAEKLKRRL

SKKDQDRVLVNTFHGFIRMQLDMVNWKGKMLISSAQRSVIHRALKESGAPFRYPDAEFAIDAIGREMDTD

IISVRHTRQQIHLFNTYQAICQKDHVADLNALSRFVVGQMYSGKMQPLNLTHLVVDEVQDTDSIQYAWIS

LHTRAGVNTSIVGDDDQAIYSFRASGGVKIFQQFEKQFRPNIFYLNTCFRCEPEILKVAGALIEKNVYRY

AKDLRSAKGGGGKVHFRSYVDMDEQIQGILNLINQDPIGWAILSRGNAHLDQLESLIEQPVLRYGGKSFW

DEKETSDVLHLMAFFRHSNDVRLMKRVLALFGENEEVLDQTALSMKGRKVTFGELNIPNESSLETRTLHS

NFTRFTQETREKVEIEKRFANLIKWMELSSIKMRTQKGSPSLSRIALDTCKQWAEKTGWQNMINRAAAMC

LGPKKKDEEYTPDKVVLSTLHGSKGLEWKNVIIMSCNADQIPSKRSVGQEAIEEERRLLFVGFTRAEQQL

HVMWYGDPSFFLSECAEDKLKEAAKSRTESPLTE

>gi|313651469|gb|EFS15865.1| DNA adenine methylase family protein [Shigella flexneri 2a str. 2457T]

MPFLSKHFPHDKSRRWVEPFIGGGAVFLNMFATEALLADSNPDLINLYRNIQRNKPAFIREVQLLAERHF

EEEDYYVLRNTFNSTSFDDAPLQRAAIFYAMNRLGYNGLCRYNLKRKFSVPWGKRYQFSLDIQKVDYLSF

RLSSVELKTADFGQTLEFAGGGDQIYCDPPYDKISKTSFVSYDGIPFDKSAHVKLADMLVDANRKGASVA

ISNSMTPFTLELYEERGFDIHTHNAYRSVGSQSKSRKKEIEILAVLR

>gi|313651164|gb|EFS15563.1| outer membrane usher papC domain protein [Shigella flexneri 2a str. 2457T]

MLPPNLRGYAPDISGVAHTTAKVTVSQMGRVIYETQVPAGPFRIQDLGDSVSGTLHIRIEEQNGQVQEYD

ISTASMPYLTRPGQVRYKIMMGRPQEWGHHVEGEFFSGAEASWGIANGWSLYGGALGDENYQSAALGVGR

DLSTFGAVAFDVTHSHTKLDKDTAYGKGSLDGNSFRVSYSKDFDQLNSRVTFAGYRFSEENFMTMSEYLD

ASDSGMVRTGNDKEMYTATYNQNFRDAGVSVYLNYTRHTYWDREEQTNYNIMLSHYFNMGSIRNVSISMT

GYRYEYDNQADKGMYISLSMPWGDNSTVSYNGTMAVGRTAVRSVISAVSMTRLTIS

>gi|313651162|gb|EFS15561.1| chaperone protein pmfD [Shigella flexneri 2a str. 2457T]

MSDLLCSAKLGATTLALLLSAASLSAQASVTPDRTRLIFNESDKSISVTLRNNDPKLPYLAQSWIEDEKG

NKISSPLTVLPPVQRIDSMMNGQVKVQGMPDINKLPADRESLFYFNVREIPPKSNKANTLQIALQTRIKL

FWRPKALENVSMKNP

>gi|313651039|gb|EFS15439.1| ABC transporter periplasmic-binding protein yphF [Shigella flexneri 2a str. 2457T]

MPKKMRTTRNLLLMATLLGSALFARAADKEMTIGAIYLDTQGYYAGVRQGVQDAAKDSSVQVQLIETNAQ

GDISKESTFVDTLVARNVDAIILSAVSENGSSRTVRRASEAGIPVICYNTCINQKGVDKYVSAYLVGDPL

EFGKNWVTLPPIILLPIKLTSRKLPSSIAKPLKFVCSDVKDLKKY

>gi|313651006|gb|EFS15406.1| penicillin-binding 1C domain protein [Shigella flexneri 2a str. 2457T]

MLDNLIEARYLEALINYEDRWFWKHPGVNPFSVARAAWQDLTSGRVISGGSTLTMQVARLLDPHPKTFGG

KILQLWRALQLEWHLSKREILILYLNRAPFGGTLQGIGAASWAYLGKSPANLSYSEAAMLAVLPQVPSRL

RPDRWPERAEAARNKVLERMAAQGVWSREQVKESREEPIWLAPRQMPQLAPLFSRMMLGKSKSDKIVTTL

DAGLQRRLEELAQNWKGRLPPRSSLAMIVVNHTDMRVRGWVGSVDLNDDSRFGHVDMVNAIRSPGSVLKP

FVYGLALDEGLIHPASLLQDVPRHR

>gi|313650743|gb|EFS15144.1| chaperone torD domain protein [Shigella flexneri 2a str. 2457T]

MAALPATLTVRDDARLELAADFCGLFLMTDKQAALPYASAYKQDEQEIKRLLVEAGMETSGNFNEPADHL

AIYLELLSYLHFSLGEGTVPARRIDSLRQKTLTALRQWLPEFAARCRQYDSFGFYAALSQLLLVLVECDH

QNR

>gi|313650721|gb|EFS15122.1| phoH-like family protein [Shigella flexneri 2a str. 2457T]

MGRQKAVIKARREAKRVLRRDSRSHKQREEESVTSLVQMSGVEAIGMARDSRDTSPILARNEAQLHYLQA

IESKQLIFATGEAGCGKTWISAAKAAEALIHKDVDRIIVTRPVLQADEDLGFLPGDIAEKFAPYFRPVYD

LLVRRLGASFMQYCLRPEIGKVETAPFAYMRGRTFENAVVILDEAQNVTAAQMKMFLTRLGENVTVIVNG

DITQCDLPRGVCSGLSDALERFEEDEMVGIVRFGKEDCVRSALCQRTLHAYS

>gi|313650575|gb|EFS14978.1| drug resistance MFS transporter [Shigella flexneri 2a str. 2457T]

MSMRKHIAFASMCMGLFIAQLDIQIVSSSLNEIGGGLSAGKDEMAWLQTSYLIAEIIVIPLSGWLSRVFS

TRWLFTLSAGIFTLMSIACGLAWNIQIMILFRALQGAAGASMIPLVFTMAFIYYQGKELGLAAAVVSALA

SLSPTLGPTLGGWLTDNLDWRWLFYINILPGIYLVLSIPFLVNFDKPDLSLLKVADYPSIILLAMTLGCL

EYTLEEGARWGWLDDNTILLTSVLALVSFILFAARTLTISNPIMDLHAFKDKNFTLGCFFSFSGGVGIFS

TVYLIPVFLGQIRGLNAEEIGFAVCTTGIFQLFSVPFYFWLSKKINLRWLLMAGLGGFVFSMYLFTPITH

EWGWQELLFPQAIRGISQQFAMAPIVTLTLGGIPKERLKLASGVFNLTRNLGGAIGIALCGSILNNRTNF

HFSRMGEKMVSVPHTVNDFISRSALFFNRSRSDQTSEILASTKLLSQLMLREAQTMAFSVPFC

>gi|313650527|gb|EFS14933.1| binding--dependent transport systems inner membrane component domain protein [Shigella flexneri 2a str. 2457T]

MILPVQAALERLPPSLLQASADLGARPRQTFRYVVLPLAIPGIAAGSIFTFSLTLGDFIVPQLVGPPGYF

IGNMVYSQQGAIGNMPMAAAFTLVPIILIALYLAFVKRLGAFDAL

>gi|313650353|gb|EFS14762.1| HTH-type transcriptional regulator dicA [Shigella flexneri 2a str. 2457T]

MKNETFGARLLHRRKKLKLSQAALGKLVKVAHVTISQWERDETQPAGKRLFALSQALQCSPTWLLFGDED

KQPGEPIPDNQPAILTEDQKELLQLFDALPESEQKALLSEMRARVENFNKLFEELLKARKRSANK

>gi|313650164|gb|EFS14577.1| flagellar export protein FliJ [Shigella flexneri 2a str. 2457T]

MAEEQLKMLIDYQNEYRNNLNSDMSAGMTSNRWINYQQFIQTLEKAITQHRQQLNQWTQKVDIALNSWRE

KKQRLQAWQTLQERQSTAALLAENRLDQKKMDEFAQRAAMRKPE

>gi|313649443|gb|EFS13874.1| protein tonB [Shigella flexneri 2a str. 2457T]

MIMTSITLDLPRRFPWPTLLSVCIHGAVVAGLLYTSVHQVIELPAPAQPISVTMVAPADLEPPQAVQPPP

EPVVEPEPEPEPIPEPPKEAPVVIEKPKPKPKPKPVKKVQEQPKRDVKPVESRPASPFENTAPARPTS

STATAATSKPVTSVASGPRALSRNQPQYPARAQALRIEGQVKVKFDVTPDGRVDNVQILSAKPANMFERE

VKNAMRRWRYEPGKPGSGIVVNILFKINGTTEIQ

>gi|313649227|gb|EFS13661.1| arginine N-succinyltransferase [Shigella flexneri 2a str. 2457T]

MMVIRPVERSDVSALMQLASKTGGGLTSLPANEATLSARIERAIKTWQGELPKSEQGYVFVLEDSETGTV

AGICAIEVAVGLNDPWYNYRVGTLVHASKELNVYNALPTLFLSNDHTGSSELCTLFLDPDWRKEGNGYLL

SKSRFMFMAAFRDKFNDKVVAEMRGVIDEHGYSPFWQSLGKRFFSMDFSRADFLCGTGQKAFIAELMPKH

PIYTHFLSQEAQDVIGQVHPQTAPARAVLEKEGFRYRNYIDIFDGGPTLECDIDRVRAIRKSRLVEVAEG

QPAQGDFPACLVANENYHHFRVVLARTDPATERLILTAAQLDALKCHAGDRVRLVRLCAEEKTA

>gi|313648972|gb|EFS13409.1| major Facilitator Superfamily protein [Shigella flexneri 2a str. 2457T]

MKNPYYPTALGLYFNYLVHGMGVILMSLNMASLETLWQTNAAGVSIVISSLGIGRLSVLLFAGLLSDRFG

RRPFIMLGMCCYMAFFFDILQTNNIIIAYVFGFLAGMANSFLDAGTYPSLMEAFPRSPGTANILIKAFVS

SGQFLLPLIISLLVWAELWFGWSFMIAAGIMFINALFLYRCTFPPHPGRRLPVIKKTTSSTEHRCSIIDL

ASYTLYGYISMATFYLVSQWLAQYGQFVAGMSYTMSIKLLSIYTVGSLLCVFITAPLIRNTVRPTTLLML

YTFISFIALFTVCLHPTFYVVIIFAFVIGFTSAGGVVQIGLTLMAERFPYAKGKATGIYYSTGSIATFTI

PLITAHLSQRSIADIMWFDTAIGFLLALFIGLRSRKKTRHHSLKENVAPGG

>gi|313648888|gb|EFS13325.1| glucuronide transporter [Shigella flexneri 2a str. 2457T]

MGLGLCYSLVNIPYGSLATAMTQQPQSRARLGAARGIAASLTFVCLAFLIGPSIKNSSPEEMVSVYHFWT

IVLVIAGMVLYFICFKSTRENVVRIVAQPSLKISLQTLKRNRPLFMLCIGALCVLISTFAVSASSLFYVR

YVLNDTGLFTVLVLVQNLVGTVASAPLVPGMVARIGKKNTFLIGALLGTCGYLLFFWVSVWSLPVALVAL

AIASIGQGVTMTVMWALEADTVEYGEYLTGVRIEGLTYSLFSFTRKCGQAIGGSIPAFILGLSGYIANQV

>gi|313648508|gb|EFS12950.1| rod shape-determining protein MreC [Shigella flexneri 2a str. 2457T]

MKPIFSRGPSLQIRLILAVLVALGIIIADSRLGTFSQIRTYMDTAVSPFYFVSNAPRELLDGISQTLASR

DQLELENRALRQELLLKNSELLMLGQYKQENARLRELLGSPLRQDEQKMVTQVISTVNDPYSDQVVIDKG

SVNGVYEGQPVISDKGVVGQVVAVAKLTSRVLLICDATHALPIQVLRNDIRVIAAGNGCTDDLQLEHLPA

NTDIRVGDVLVTSGLGGRFPEGYPVAVVSSVKLDTQRAYTVIQARPTAGLQRLRYLLLLWGADRNGANPM

TPEEVHRVANERLMQMMPQVLPSPDAMGPKLPEPATGIAQPTPQQPATGNAATAPVAPTQPAANRSPQRA

TPPQSGAQPPARAPGGQ

>gi|313647807|gb|EFS12253.1| RNA polymerase sigma factor rpoS [Shigella flexneri 2a str. 2457T]

MSQNTLKVHDLNEDAEFDENGVEVFDEKALVEEEPSDNDLAEEELLSQGATQRVLDATQLYLGEIGYSPL

LTAEEEVYFARRALRGDVASRRRMIESNLRLVVKIARRYGNRGLALLDLIEEGNLGLIRAVEKFDPERGF

RFSTYATWWIRQTIERAIMNQTRTIRLPIHIVKELNVYLRTARELSHKLDHEPSAEEIAEQLDKPVDDVS

RMLRLNERITSVDTPWVVIPKKRCWTSWPMKKRTVRKIPRKMTI

>gi|313647791|gb|EFS12238.1| drug resistance MFS transporter [Shigella flexneri 2a str. 2457T]

MSDKKKRSMSGLPWIAAMAFFMQALDATILNTALPAIAHSLNRSPLAMQSAIISYTLTVAMLIPVSGWLA

DRFGTRRIFTLAVSLFTLGSLACALSNSLPQLVVFRVIQGIGDAMMMPVARPALLRAYPRNELLPVLNFV

AMPGLVGPILGPVLGGVLVTWATWHWIFLINIPIGIAGLLYARKHMPNFTTARRRFDITGFLLFGLSLVL

FSSGIELFGEKIVASWIALTVIVTSIGLLLLYILHARRTPNPLISLDLFKTRTFSIGIVGNIATRLGTGC

VPFLMPLMLQVGFGYQAFIAGCMMAPTALGSIIAKSMVTQVLRRLGYRHTLVGITVIIGLMIAQFSLQSP

AMAIWMLILPLFILGMAMSTQFTAMNTITLADLTDDNASSGNSVLAVTQQLSISLGVAVSAAVLRIYEGM

EGTTTVEQFHYTFITMGIITVASAAMFMLLKTTDGNNLIKRQRKSMPNRVPSESE

>gi|313647260|gb|EFS11712.1| HTH-type transcriptional regulator gntR [Shigella flexneri 2a str. 2457T]

MKKKRPVLQDVADRVGVTKMTVSRFLRNPEQVSVALRGKIAAALDELGYIPNRAPDILSNATSRAIGVLL

PSLTNQVFAEVLRGIESVTDAHGYQTMLAHYGYKPEMEQERLESMLSWNIDGLILTERTHTPRTLKMIEV

AGIPVVELMDSQSPCLDIAVGFDNFEAARQMTTAIIARGHRHIAYLGARLDERTIIKQKGYEQAMLDAGL

VPYSVMVEQSSSYSSGIELIRQARREYPQLDGVFCTNDDLAVGAAFECQRLGLKVPDDMAIAGFHGHDIG

QVMEPRLASVLTPRERMGSIGAERLLARIRGESVTPKMLDLGFTLSPGGSI

>gi|313647169|gb|EFS11623.1| fructose-like PTS system EIIBC component [Shigella flexneri 2a str. 2457T]

MAMESSLRIVAITNCPAGIAHTYMVAEALEQKARSLGHTIKVETQGSSGVENRLSSEEIAAADYVILATG

RGLSGDDRARFAGKKVYEIAISLALKNIDQIFSELPTNSQLFAADSGVKLGKQEVQSGSVMSHLMAGVSA

ALPFVIGGGILVALANMLVQFGLPYTDMSKGAPSFTWVVESIGYLGFTFMIPIMGAYIASSIADKPAFAP

AFLVCYLANDKALLGTQSGAGFLGAVVLGLAIGYFVFWFRKVRLGKALQPLLGSMLIPFVTLLVFGVLTY

YVIGPVMSDLMGGLLHFLNTIPPSMKFAAAFLVGAMLAFDMGGPINKTAWFFCFSLLEKHIYDWYAIVGV

VALMPPVAAGLATFIAPKLFTRQEKEAASSAIVVGATVATEPAIPYALAAPLPMITANTLAGGITGVLVI

AFGIKRLAPGLGIFDPLIGLMSPVGSFYLVLAIGLALNISFIIVLKGLWLRRKAKAAQQELVHEH

>gi|313646928|gb|EFS11385.1| bacterial extracellular solute-binding s, family 5 Middle family protein [Shigella flexneri 2a str. 2457T]

MRQAQEAGWLEWQAQSGRGKRGQLRFLVTPESLRNAMMEQALETGKQQDVLELAQLAPGELRTLLQPFMG

GQWQNDTPTLRIPYYRPLEPLQPGFLPGRAEQHLAGQIFSGLTRFDNNTQRPIGDLAHHWETSTDRLRWD

FYLRSTLHWHNGDAVKASHLHQRLLMLLQLPALDQLFISVKRIEVTHPQCLTFFLHRPDYWLAHRLASYC

SHLAHPQFPLIGTGPFRLTQFTAELVRLESHDYYHLRHPLLKAVEYWITPPLFEKDLGTSCRHPVQITIG

KPEELQRVSQVSSGISLGFCYLTLRKSPRLSLWQARKVISIIHQSGLLQTLEVGENLITASHALLPGWTI

PHWQVPDEVKLPKTLTLVYHLPIELHTMAERLQATLAAEGCELTIIFHNAKNWDDTTLLAHADLMMGDRL

IGEAPEYTLEQWLRCDPLWPHVFDAPAYAHLQSTLDAVQVMPDEENRFNALKAVFSQLMADATLTPLFNY

HYRISAPPGVNGVRLTPRGWFEFTEAWLPAPSQ

>gi|313646849|gb|EFS11306.1| outer membrane usher sfmD domain protein [Shigella flexneri 2a str. 2457T]

MKIPTTTDIPQRYTWCLAGICYSSLAILPSFLSYAESYFNPAFLLENGTSVADLSRFERGNHQPAGVYRV

DLWRNDEFIGSQDIVFESTTENTGDKSGGLMPCFNQVLLERIGLNSSAFPELAQQQNNKCINLLKAVPDA

TINFDFAAMRLNITIPQIALLSSAHGVMTPTY

>gi|313646468|gb|EFS10930.1| IS222, transposase ORFA [Shigella flexneri 2a str. 2457T]

MDRAVRMVKWHTEFGHLNRGDMLTSEQHRCSNEKRNFSAEFKRESAQLVVDQKYTVADAAKAMDVGLSTM

TRWVKQLRDERQGKTPKASPITPEQIEIRELRKKLQRIEMENEILKKATALLMSDSLNSSR

>gi|313646413|gb|EFS10875.1| hypothetical protein SF2457T_5328 [Shigella flexneri 2a str. 2457T]

MLPRIRHNNFIGAVELFVKSSHTKTHSNDFFNNIQHAFKKKDWVSNYDSLLTLRESFRCATQIDKNSYQV

LSSKNETVNAMDNFLISFSLKDNGAEYTITLRGSGFEYEEIPITINEYNSFMDFKNREFPLEQNRRLYAC

DILQKKQSDIPKRIKGYIRQAFGDVSFGYALLEDVVSKLKRGKFELQIPGGGIKECDGWYIYEKIIDDNF

AIVIESLGFALKIYGGDERFRNGSSVVLEDEDYSLIYNFLVNAGCQQVELAEQVDAIVSANLAADSNITK

EKICEKYKSTIEAFKKEQLALPVLVRCKNSET

>gi|313646231|gb|EFS10693.1| amino acid carrier family protein [Shigella flexneri 2a str. 2457T]

MPVFFSFINSVLWGSVMIYLLFGAGCWFTFRTGFVQFRYIRQFGKSLKNSIHPQPGGLTSFQSLCTSLAA

RVGSGNLAGVALAITAGGPGAVFWMWVAAFIGMATSFAECSLAQLYKERDVNGQFRGGPAWYMARGLGMR

WMGVLFAVFLLIAYGIIFSGVQANAVARALSFSFDFPPLVTGIILAVFALLAITRGLHGVARLMQGFVPL

MAIIWVLTSLVICVINIGQLPHVIWSIFESAFGWQEAAGGAAGYTLSQAITNGFQRSMFSNEVGMGSTPN

AAAAAASWPPHPAAQGIVQMIGIFIDTLVICTASAMLILLAGNGTTYMPLEGIQLIQKAMRVLMGS
