# Supplementary material for: Finding Potential Therapeutic Targets against Shigella flexneri through Proteome Exploration
Source: Front Microbiol. 2016 Nov 22;7:1817. doi: 10.3389/fmicb.2016.01817 (PMC5118456; doi:10.3389/fmicb.2016.01817)
Supplement: Supplementary file 13 [file DataSheet8.PDF]

>gi|30065350|ref|NP_839521.1| hypothetical protein S4380 [Shigella flexneri 2a str. 2457T]

MTNSNRIKLTWISFLSYALTGALVIVTGMVMGNIADYFNLPVSSMSNTFTFLNAGILISIFLNAWLMEIV

PLKTQLRFGFLLMVLAVAGLMFSHSLALFSTAMFILGVVSGITMSIGTFLITQMYEGRQRGSRLLFTDSF

FSMAGMIFPMIAAFLLARSIEWYWVYACIGLVYVAIFILTFGCEFPALGKRAPKTDAPVEKEKWGIGVLF

LSVAALCYILGQLGFISWVPEYAKGLGMSLNDAGTLVSNFWMSYMVGMWAFSFILRFFDLQRILTVLAGL

AAILMYVFNTGTPVHMAWSILALGFFSSAIYTTIITLGSQQTKVPSPKLVNFVLTCGTIGTMLTFVVTGT

IVEHSGPQAALLTANGLYAVVFVMCFLLGFVSRHRQHNTLTSH

>gi|30063433|ref|NP_837604.1| hypothetical protein S2148 [Shigella flexneri 2a str. 2457T]

MQFCSSDEFASKTMIKWPWKVQESAHQTALPWQEALSIPLLTCLTEQEQSKLVALAERFLQQKRLVPLQG

FELNSLRSCRIALLFCLPVLELGLEWLDGFHEVLIYPAPFVVDDEWEDDIGLVHNQRIVQSGQSWQQGPI

VLNWLDIQDSFDASGFNLIIHEVAHKLDTRNGDRASGVPFISLREVAGWEHDLHAAMNNIQEEIELVGEN

AASIDAYAASDPAECFAVLSEYFFSAPELFAPRFPSLWQRFCQFYQQDPLQRLHHANDTDSFSATNVH

>gi|30063267|ref|NP_837438.1| hypothetical protein S1932 [Shigella flexneri 2a str. 2457T]

MQQIARSVALAFNNLPRPHRVMLGSLTVLTLAVAVWRPYVYHRDATPIVKTIELEQNEIRSLLPEASEPI

DQAAQEDEAIPQDELDDKIAGEAGVHEYVVSTGDTLSSILNQYGIDMGDITQLAAADKELRNLKIGQQLS

WTLTADGELQRLTWEVSRRETRTYDRTAANGFKMTSEMQQGEWVNNLLKGTVGGSFVASARNAGLTSAEV

SAVIKAMQWQMDFRKLKKGDEFAVLMSREMLDGKREQSQLLGVRLRSEGKDYYAIRAEDGKFYDRNGTGL

AKGFLRFPTAKQFRISSNFNPRRTNPVTGRVAPHRGVDFAMPQGTPVLSVGDGEVVVAKRSGAAGYYVAI

RHGRSYTTRYMHLRKILVKPGQKVKRGDRIALSGNTGRSTGPHLHYEVWINQQAVNPLTAKLPRTEGLTG

SDRREFLAQAKEIVPQLRFD

>gi|30062504|ref|NP_836675.1| hypothetical protein S1033 [Shigella flexneri 2a str. 2457T]

MVGMSALSYTLLNSLEEIMKETDIAGILTSTHTIALVGASDKPDRPSYRVMKYLLDQGYHVIPVSPKVAG
KTLLGQQGYGTLADVPEKVDMVDVFRNSEAAWGVAQEAIAIGAKTLWMQLGVINEQAAVLARDAGLNVVM
DRCPAIEIPRLGLAK

>gi|30043828|gb|AAP19547.1| hypothetical protein S4618 [Shigella flexneri 2a str. 2457T]
MHNIPGVRNTRLPLLQEIVMEILYNIFTVFFNQVMTNAPLLLGIVTCLGYILLRKSVSVIIKGTIKTIIG
FMLLQAGSGILTSTFKPVVAKMSEVYGINGAISDTYASMMATIDRMGDAYSWVGYAVLLALALNICYVLL
RRITGIRTIMLTGHIMFQQAGLIAVTLFIFGYSMWTTIICTAILVSLYWGITSNMMYKPTQEVTDGCGFS
IGHQQQFASLIAYKVAPFLGKKEESVEDLKLPGWLNIFHDNIVSTAIVMTIFFGAILLSFGIDTVQAMAG
KVHWTVYILQTGFSFAVAIFIITQGVRMFVAELSEAFNGISQRLIPGAVLAIDCAAIYSFAPNAVVWGFM
WGTIGQLIAVGILVACGSSILIIPGFIPMFFSNATIGVFANHFGGWRAALKICLVMGMIEIFGCVWVVKL
TGMSAWMGMADWSILAPPMMQGFFSIGIAFMAVIIVIALAYMFFAGRALRAEEDAEKQLAEQSA

>gi|30040947|gb|AAP16677.1| hypothetical protein S1258 [Shigella flexneri 2a str. 2457T]
MAEHLMSDVPFWQSKTLDEMSDAEWESLCDGCGQCCLHKLMDEDTDEIYFTNVACRQLNIKTCQCRNYER
RFEFEPDCIKLTRENLPTFEWLPMTCAYRLLAEGKDLPAWHPLLTGSKAAMHGERISVRHIAVKESEVID
WQDHILNKPDWAQ
