# Supplementary material for: Finding Potential Therapeutic Targets against Shigella flexneri through Proteome Exploration
Source: Front Microbiol. 2016 Nov 22;7:1817. doi: 10.3389/fmicb.2016.01817 (PMC5118456; doi:10.3389/fmicb.2016.01817)
Supplement: Supplementary file 14 [file DataSheet9.PDF]

>gi|30061702|ref|NP_835873.1| 2-amino-4-hydroxy-6-hydroxymethyldihydropteridine pyrophosphokinase [Shigella flexneri 2a str. 2457T]

MTVAYIAIGSNLASPLEQVNAALKALGDIPESRILAVSSFYRTPPLGPQDQPDYLNAAVALETSPAPEEL

LNHTQRIELQQGRVRKAERWGPRTLDLDIMLFGNEVINTERLTVPHYDMKNRGFMLWPLFEIAPELAFPD

GETLREVLHTRAFDKLSKW

>gi|30061599|ref|NP_835770.1| dihydrodipicolinate reductase [Shigella flexneri 2a str. 2457T]

MHDANIRVAIAGAGGRMGRQLIQAALALEGVQLGAALEREGSSLLGSDAGELAGAGKTGVTVQSSLDAIK

DDFDVFIDFTRPEGTLNHLAFCRQHGKGMVIGTTGFDEAGKQAIRDAAADIAIVFAANFSVGVNVMLKLL

EKAAKVMGDYTDIEIIEAHHRHKVDAPSGTALAMGEAIAHALDKDLKDCAVYSREGHTGERVPGTIGFAT

VRAGDIVGEHTAMFADIGERLEITHKASSRMTFANGAVRSALWLSGKESGLFDMRDVLDLNNL

>gi|30042774|gb|AAP18497.1| 7,8-dihydropteroate synthase [Shigella flexneri 2a str. 2457T]

MLRGFFLSIHTRDNIMKLFAQGTSLDLSHPHVMGILNVTPDSFSDGGTHNSLIDAVKHANLMINAGATII

DVGGESTRPGAAEVSVEEELQRVIPVVEAIAQRFEVWISVDTSKPEVIRESAKVGAHIINDIRSLSEPGA

LEAAAETGLPVCLMHMQGNPKTMQEAPKYDDVFAEVNRYFIEQIARCEQAGIAKEKLLLDPGFGFGKNLS

HNYSLLARLAEFHHFNLPLLVGMSRKSMIGQLLNVGPSERLSGSLACAVIAAMQGAHIIRVHDVKETVEA

MRVVEATLSAKENKRYE

>gi|313651006|gb|EFS15406.1| penicillin-binding 1C domain protein [Shigella flexneri 2a str. 2457T]

MLDNLIEARYLEALINYEDRWFWKHPGVNPFSVARAAWQDLTSGRVISGGSTLTMQVARLLDPHPKTFGG

KILQLWRALQLEWHLSKREILILYLNRAPFGGTLQGIGAASWAYLGKSPANLSYSEAAMLAVLPQVPSRL

RPDRWPERAEAARNKVLERMAAQGVWSREQVKESREEPIWLAPRQMPQLAPLFSRMMLGKSKSDKIVTTL

DAGLQRRLEELAQNWKGRLPPRSSLAMIVVNHTDMRVRGWVGSVDLNDDSRFGHVDMVNAIRSPGSVLKP

FVYGLALDEGLIHPASLLQDVPRHR

>gi|313649443|gb|EFS13874.1| protein tonB [Shigella flexneri 2a str. 2457T]

MIMTSITLDLPRRFPWPTLLSVCIHGAVVAGLLYTSVHQVIELPAPAQPISVTMVAPADLEPPQAVQPPP

EPVVEPEPEPEPIPEPPKEAPVVIEKPKPKPKPKPKPVKKVQEQPKRDVKPVESRPASPFENTAPARPTS

STATAATSKPVTSVASGPRALSRNQPQYPARAQALRIEGQVKVKFDVTPDGRVDNVQILSAKPANMFERE

VKNAMRRWRYEPGKPGSGIVVNILFKINGTTEIQ

>gi|313647260|gb|EFS11712.1| HTH-type transcriptional regulator gntR [Shigella flexneri 2a str. 2457T]

MKKKRPVLQDVADRVGVTKMTVSRFLRNPEQVSVALRGKIAAALDELGYIPNRAPDILSNATSRAIGVLL

PSLTNQVFAEVLRGIESVTDAHGYQTMLAHYGYKPEMEQERLESMLSWNIDGLILTERTHTPRTLKMIEV

AGIPVVELMDSQSPCLDIAVGFDNFEAARQMTTAIIARGHRHIAYLGARLDERTIIKQKGYEQAMLDAGL

VPYSVMVEQSSSYSSGIELIRQARREYPQLDGVFCTNDDLAVGAAFECQRLGLKVPDDMAIAGFHGHDIG

QVMEPRLASVLTPRERMGSIGAERLLARIRGESVTPKMLDLGFTLSPGGSI
