# Supplementary material for: Finding Potential Therapeutic Targets against Shigella flexneri through Proteome Exploration
Source: Front Microbiol. 2016 Nov 22;7:1817. doi: 10.3389/fmicb.2016.01817 (PMC5118456; doi:10.3389/fmicb.2016.01817)
Supplement: Supplementary file 15 [file Image1.PDF]

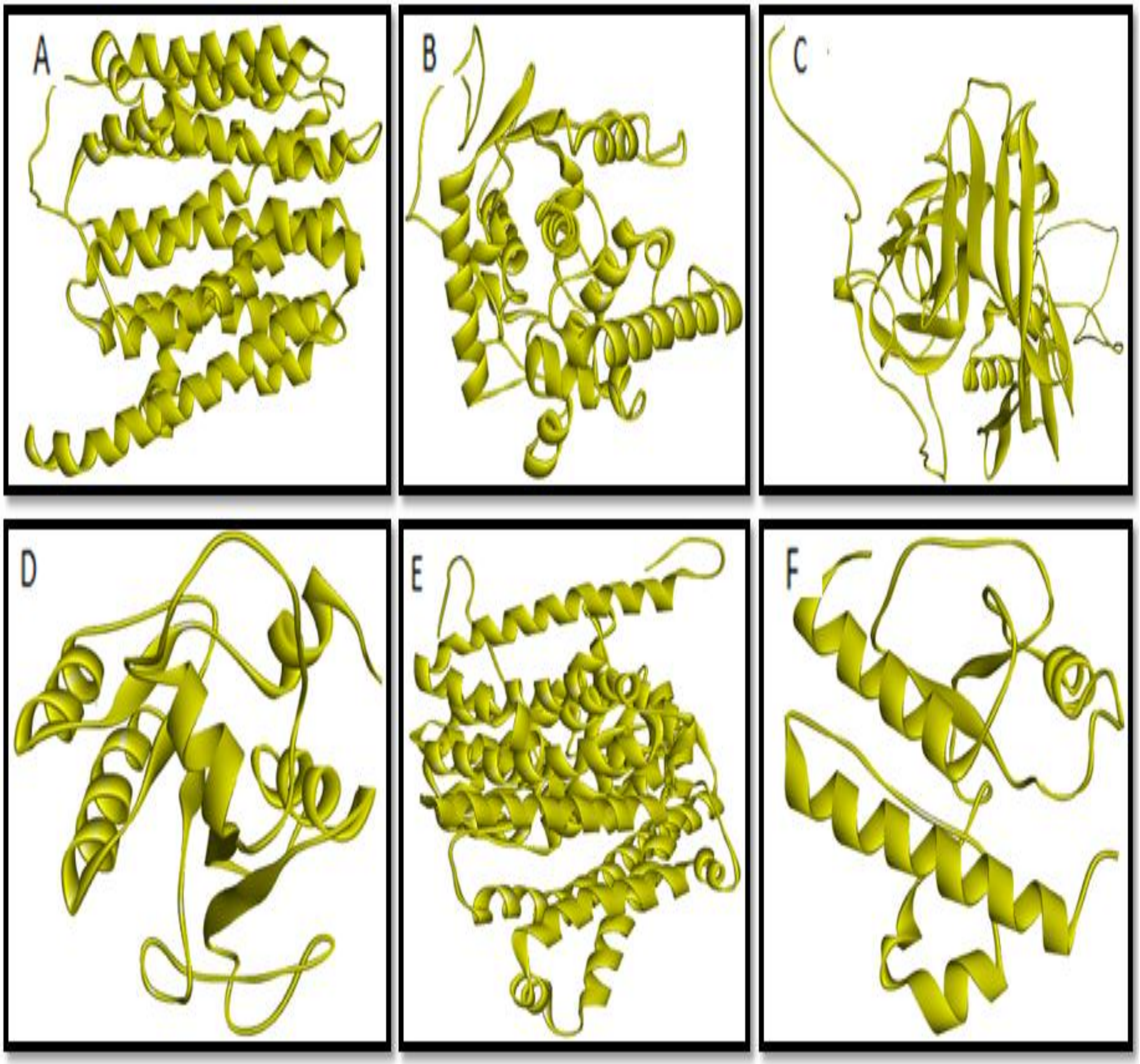

Supplementary Fig, S1: **Predicted 3D model of Hypothetical Proteins**. A. NP_839521.1 (Template 3O7Q_A); B. NP_837604.1 (Template 3KHI_A); C. NP_837438.1 (Template 4RNY_A); D. NP_836675.1 (Template 2D5A_A); E. AAP19547.1 (Template 4RP9_A); F. AAP16677.1 (Template 5EG1_A)
