# Supplementary material for: Finding Potential Therapeutic Targets against Shigella flexneri through Proteome Exploration
Source: Front Microbiol. 2016 Nov 22;7:1817. doi: 10.3389/fmicb.2016.01817 (PMC5118456; doi:10.3389/fmicb.2016.01817)
Supplement: Supplementary file 17 [file Image3.PDF]

Overall model quality

Z-Score: **-4.26**

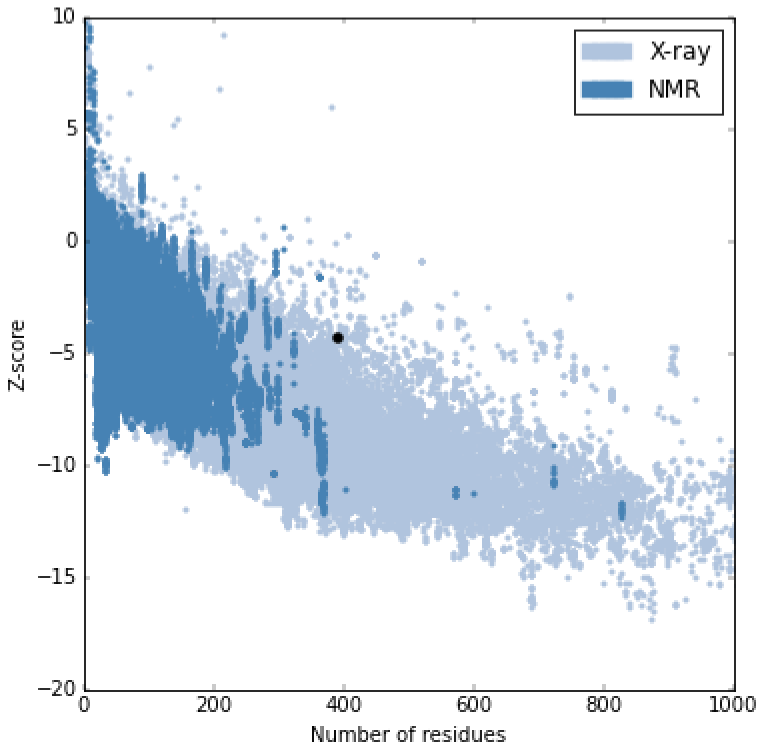

Supplementary Figure, S3_1: Model quality assessment of predicted model (NP_839521.1).

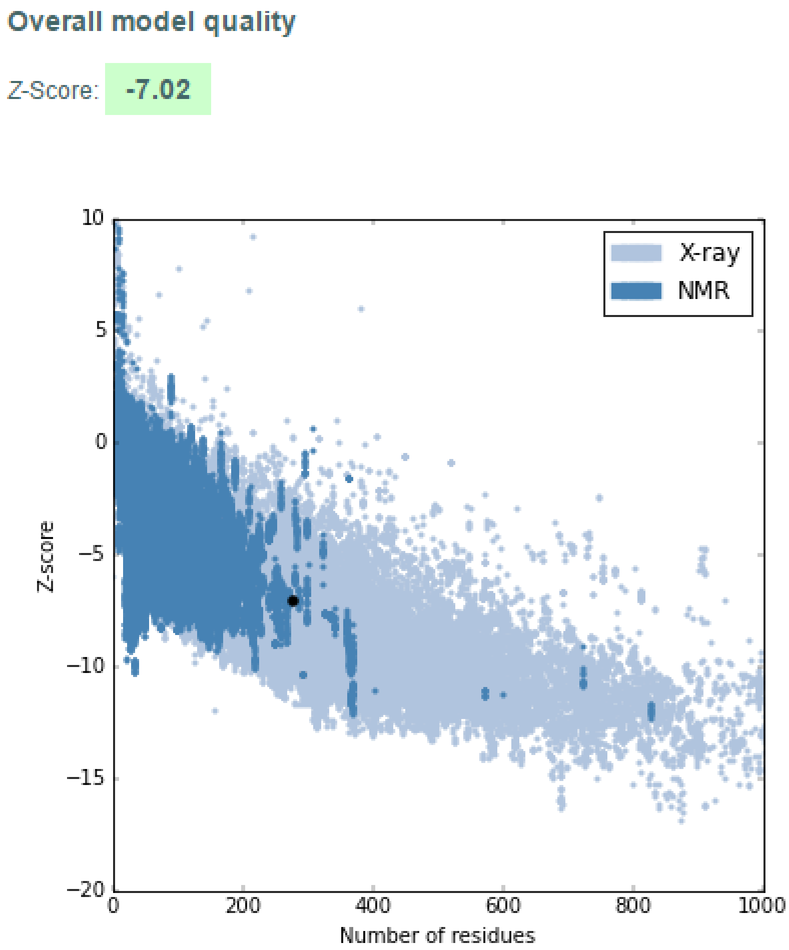

Supplementary Figure, S3_2: Quality assessment of predicted model (NP_837604.1).

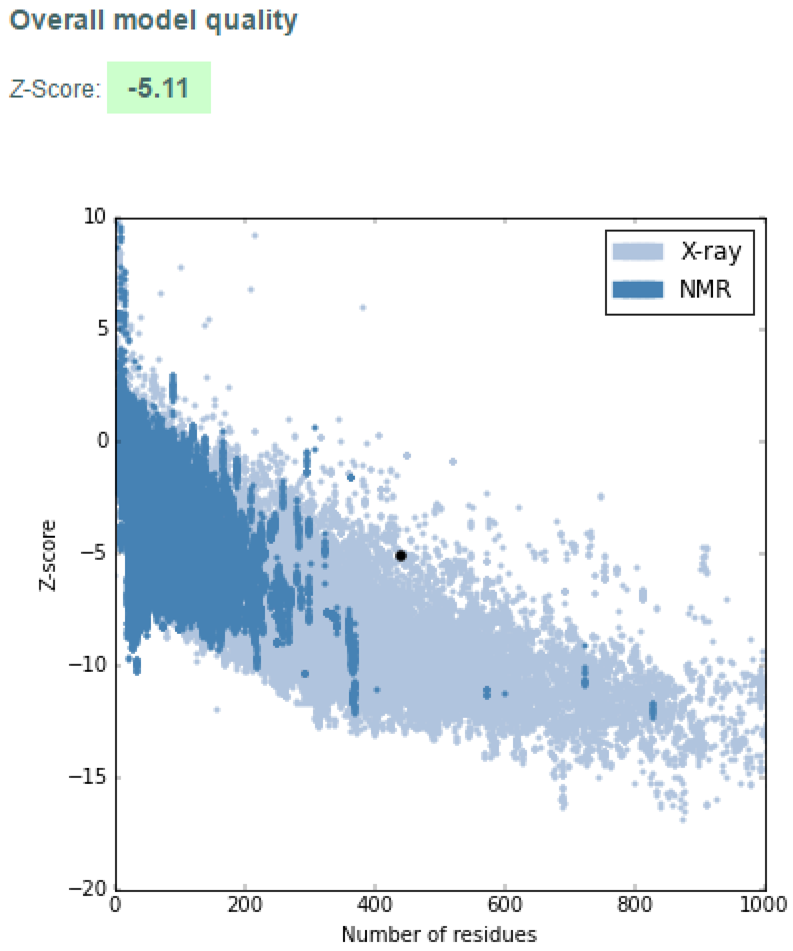

Supplementary Figure, S3_3: Quality model assessment of predicted model (NP_837438.1)

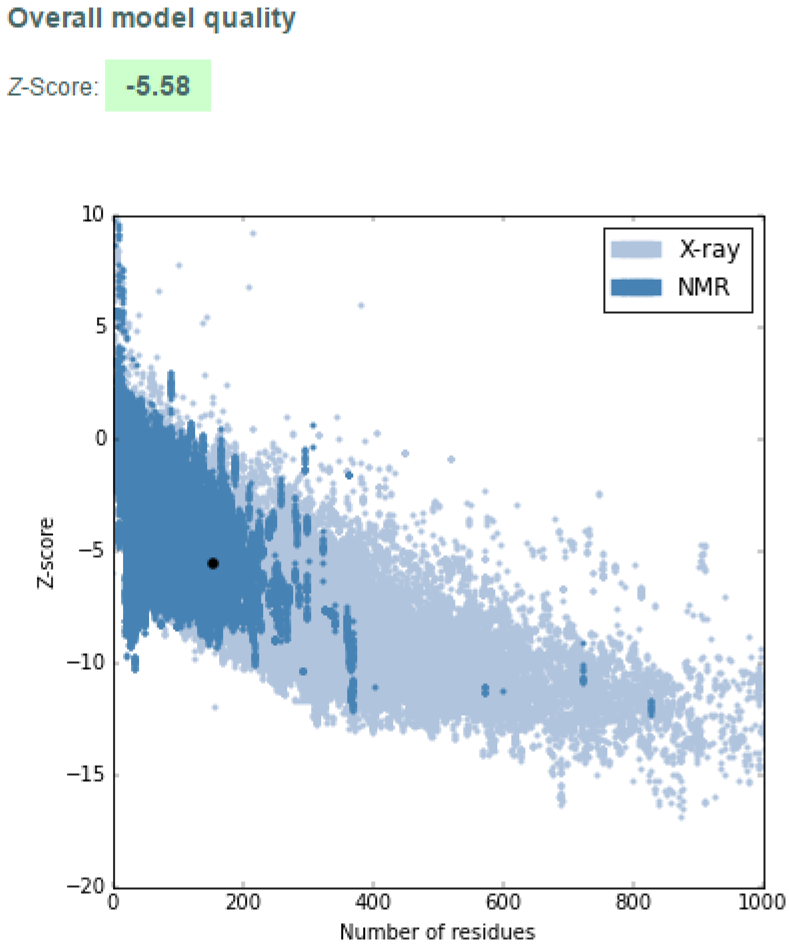

Supplementary Figure, S3_4: Quality assessment of predicted model (NP_836675.1).

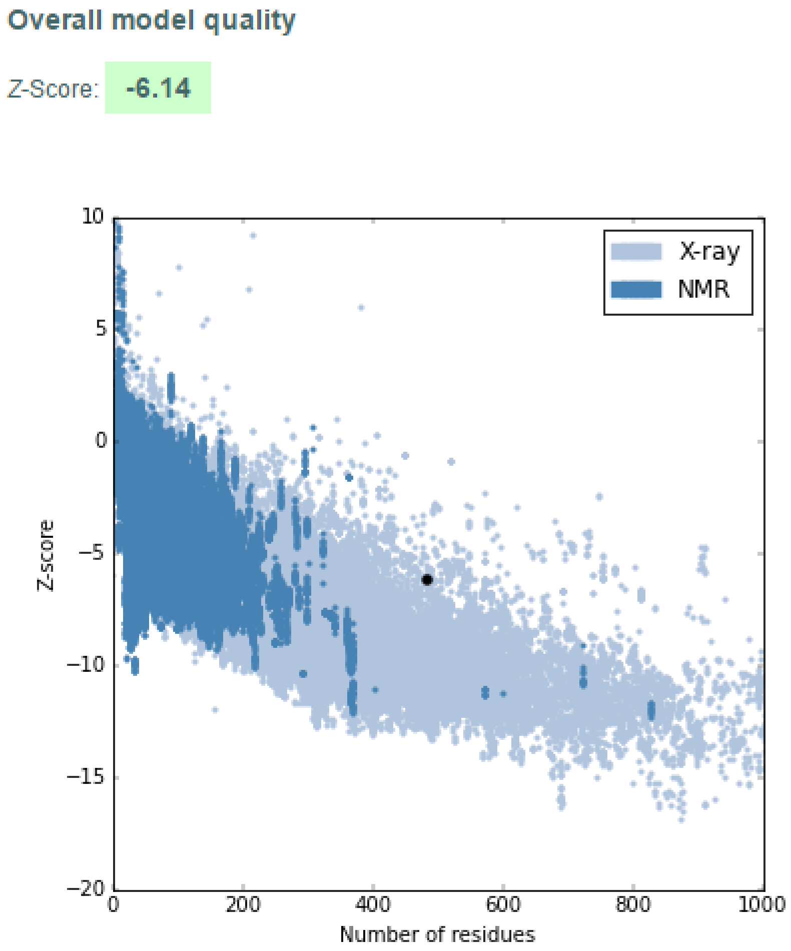

Supplementary Figure, S3_5: Quality assessment of predicted model (AAP19547.1).

Overall model quality

Z-Score: **-0.23**

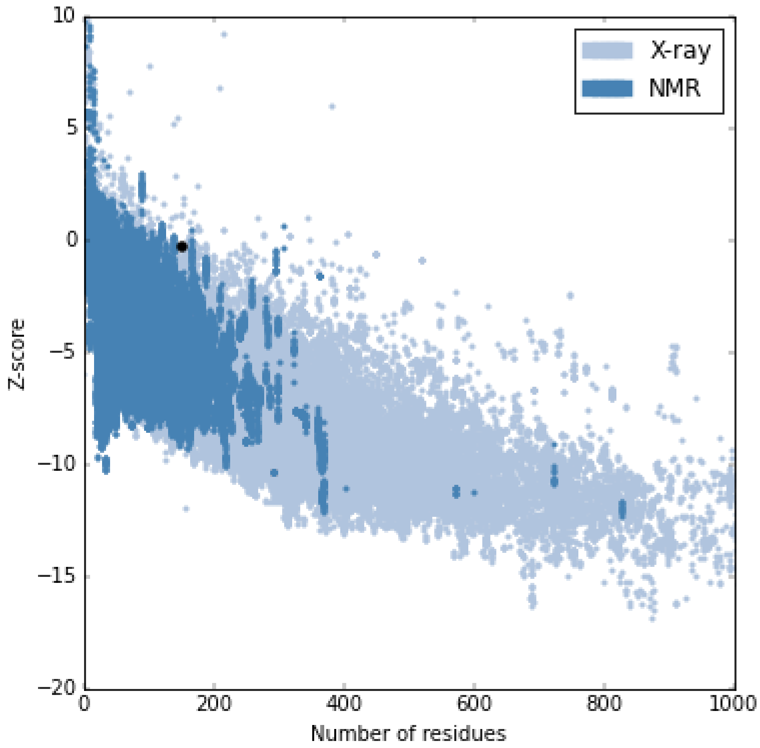

Supplementary Figure, S3_6: Quality assessment of predicted model (AAP16677.1).
