# Supplementary figures and images for: Finding Potential Therapeutic Targets against Shigella flexneri through Proteome Exploration
Source: Front Microbiol. 2016 Nov 22;7:1817. doi: 10.3389/fmicb.2016.01817 (PMC5118456; doi:10.3389/fmicb.2016.01817)

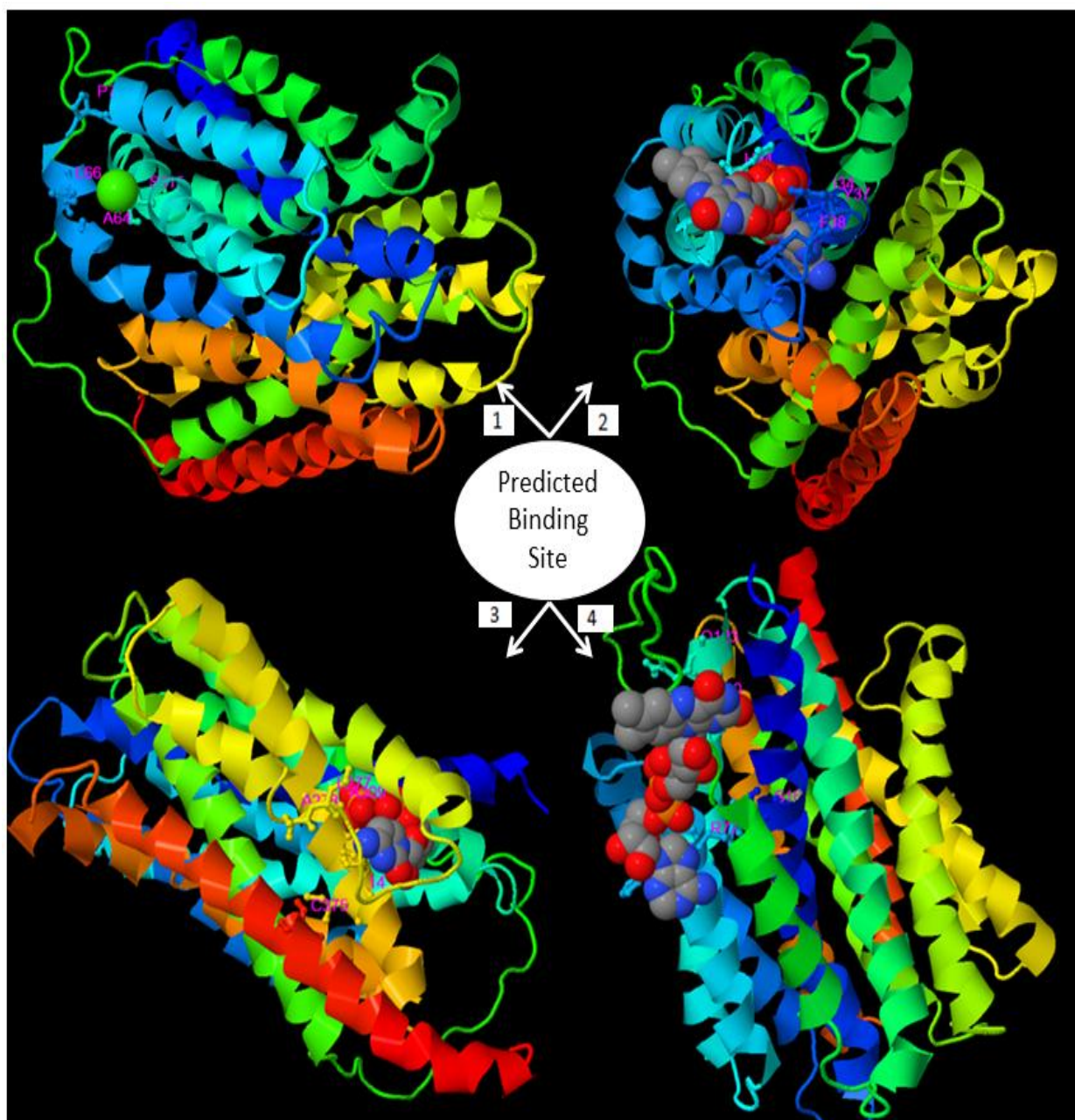

**Supplementary Fig, S4:** predicted Binding site of model (NP\_839521.1).

Supplement: Supplementary file 18 [file Image4.PDF]

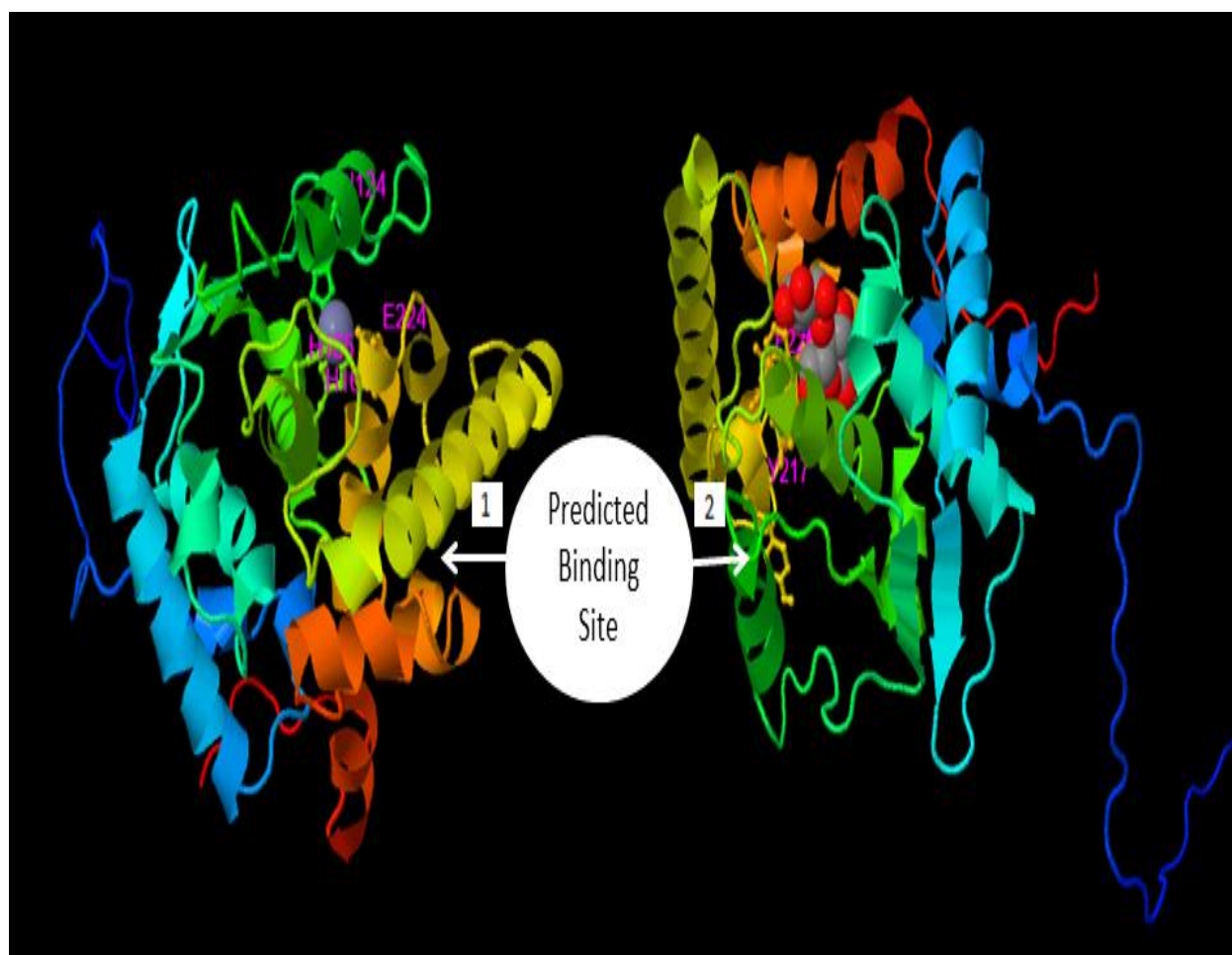

**Supplementary Fig, S5:** predicted Binding site of model (NP\_83704.1).

Supplement: Supplementary file 19 [file Image5.PDF]

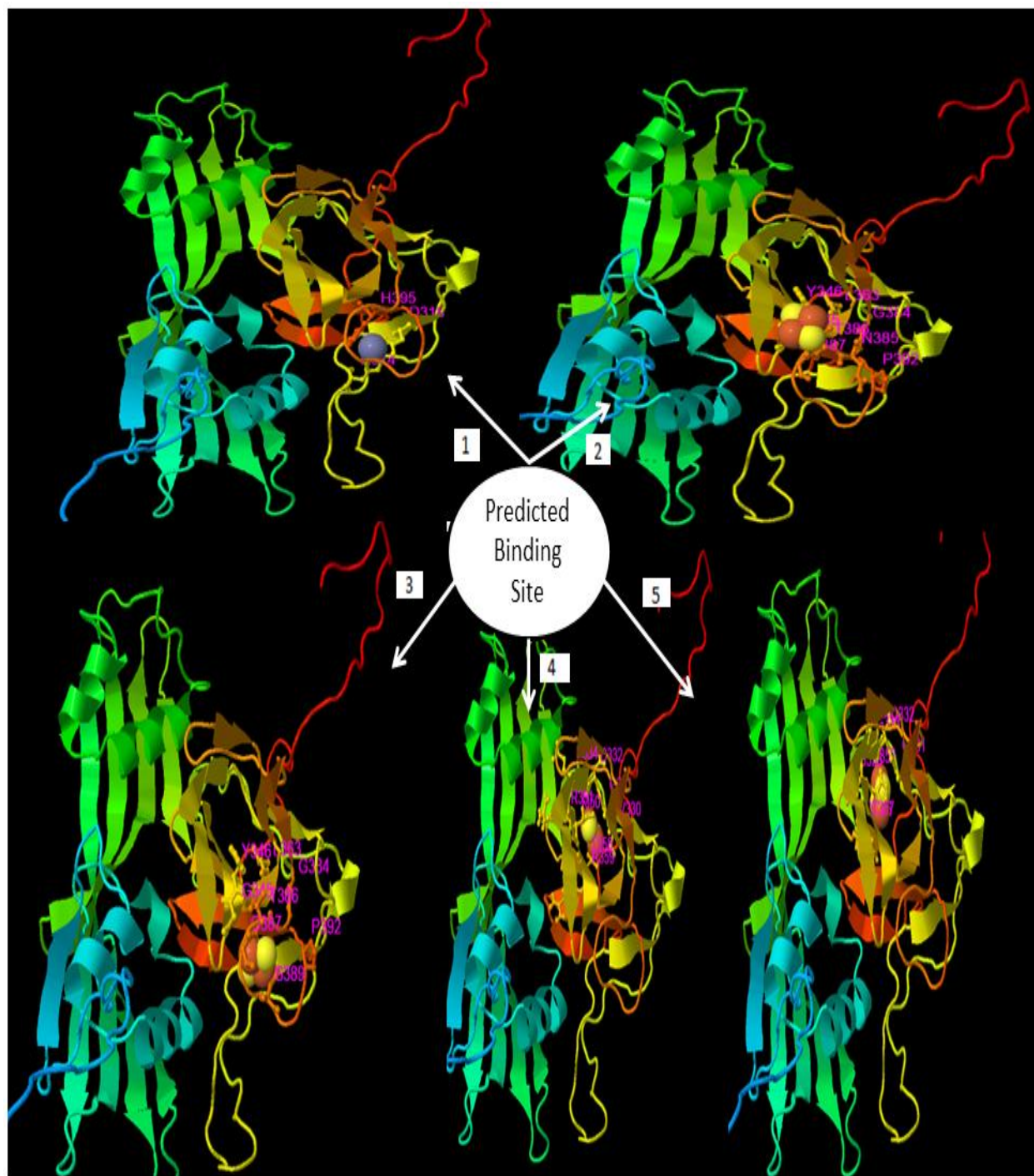

**Supplementary Fig, S6:** predicted Binding site of model (NP\_837438.1).

Supplement: Supplementary file 20 [file Image6.PDF]

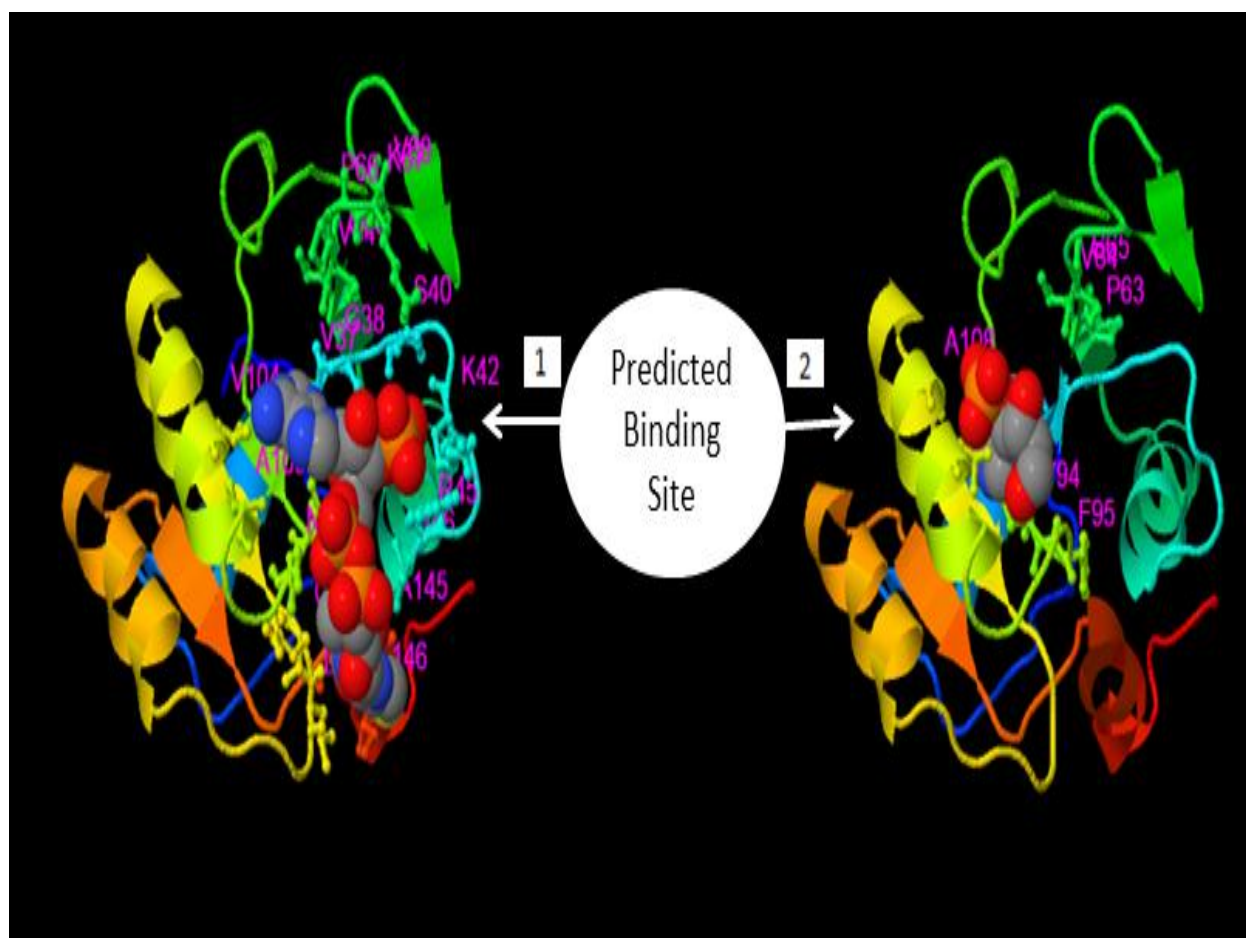

**Supplementary Fig, S7:** predicted Binding site of model (NP\_836675.1).

Supplement: Supplementary file 21 [file Image7.PDF]

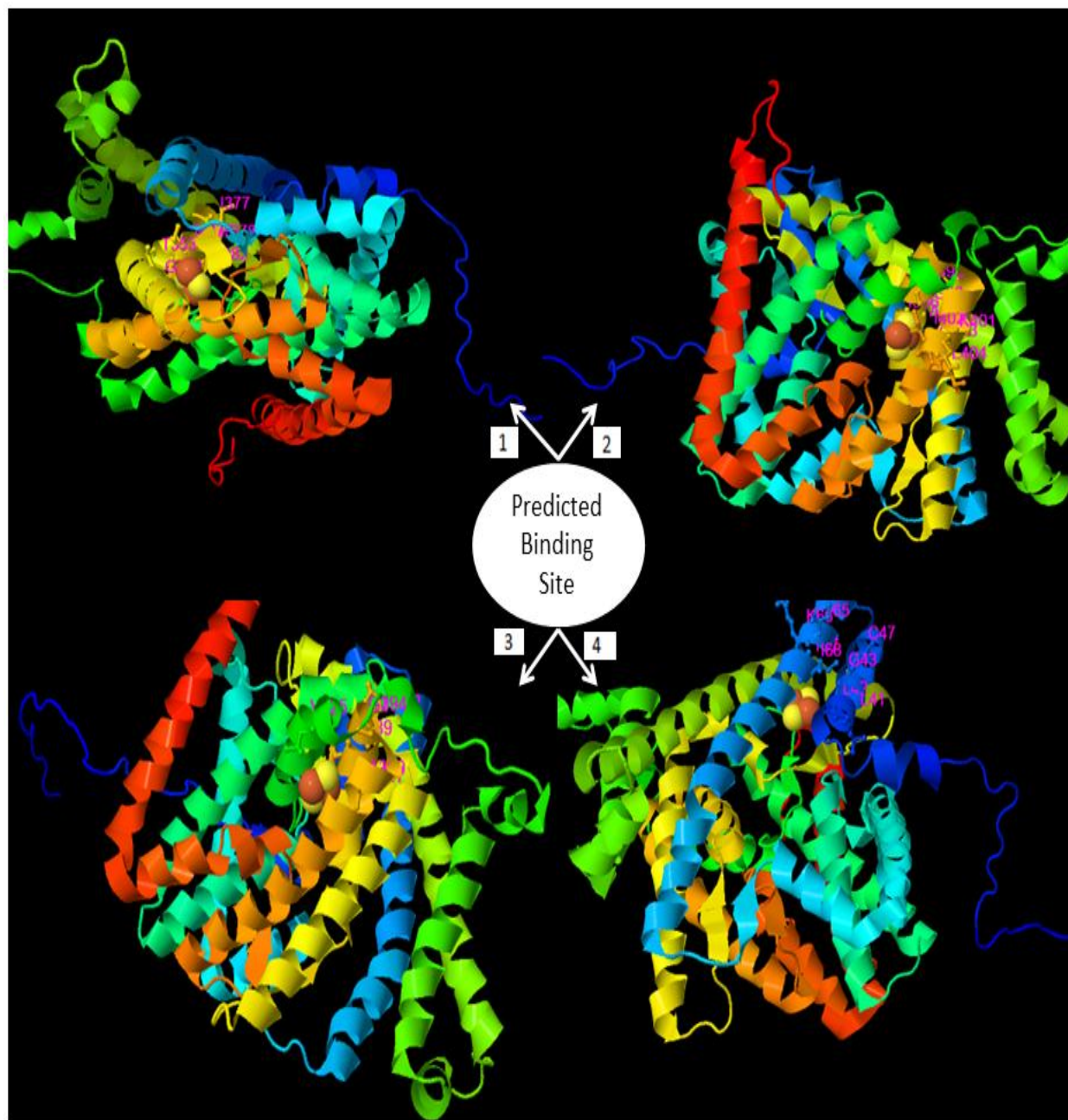

**Supplementary Fig, S8:** predicted Binding site of model (AAP19547.1).

Supplement: Supplementary file 22 [file Image8.PDF]

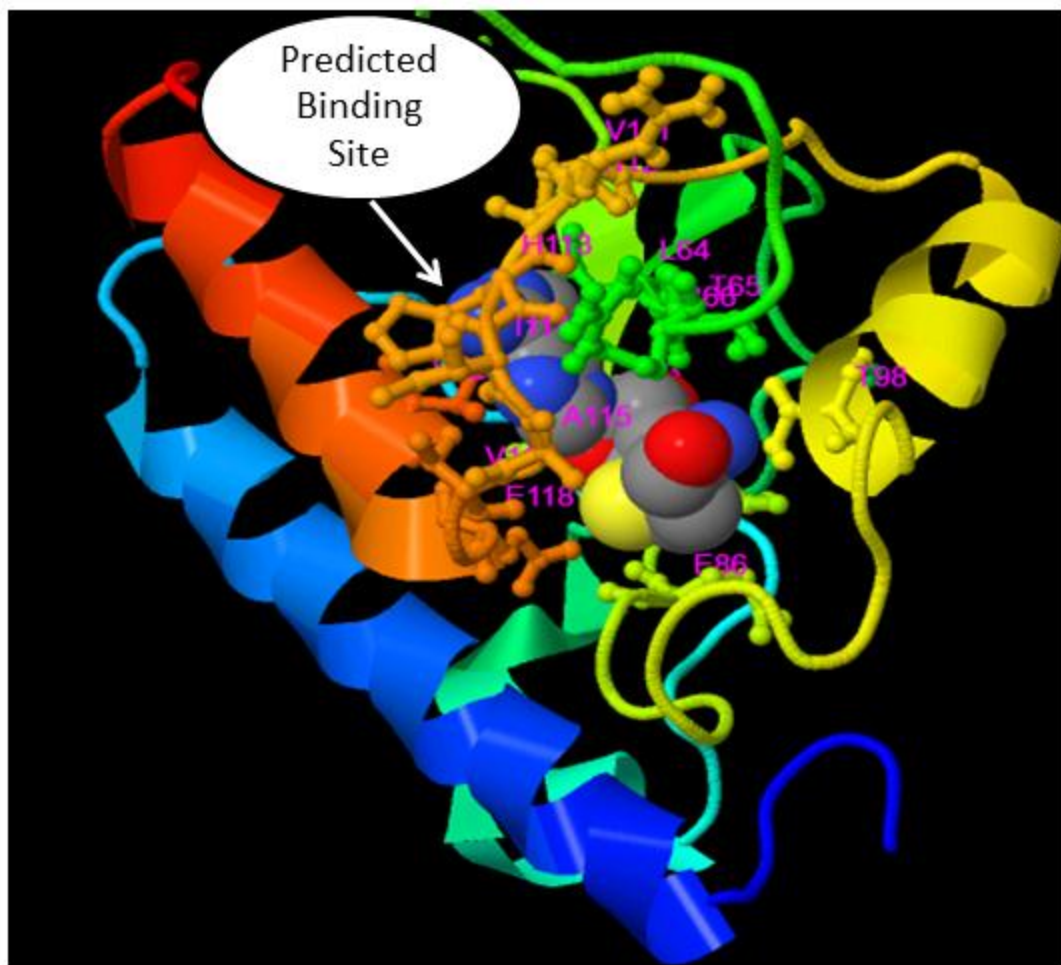

**Supplementary Fig, S9:** Predicted Binding site of model (AAP16677.1).

Supplement: Supplementary file 23 [file Image9.PDF]
