# Supplementary material for: Finding Potential Therapeutic Targets against Shigella flexneri through Proteome Exploration
Source: Front Microbiol. 2016 Nov 22;7:1817. doi: 10.3389/fmicb.2016.01817 (PMC5118456; doi:10.3389/fmicb.2016.01817)
Supplement: Supplementary file 6 [file DataSheet1.PDF]

>gi|559111466|tpe|CDI36422.1| TPA: proteolysis tag peptide encoded by tmRNA Esche_coli_K12, partial [Shigella flexneri 2a str. 2457T]

ANDENYALAAICDAKGNSFADAMKACGFTLPNAPLTPRQAIKAFVELHIEQGCVLESNGQSIGVVNAIVGQRRYTVTLN
GESNHAGTTPMGYRRDTVYAFSRICHQSIEKAKKMGDPLVLTFGKVEPRPNTVNVVPGKTTFTIDCRHTDAAVLRDFT
QQLENDMRAICDEMDIGIDIDLWMDEEPVPMNKELVATLTELCEREKLNYRVMHSGAGHDAQIFAP

>gi|559111464|tpe|CDI36421.1| TPA: proteolysis tag peptide encoded by tmRNA Esche_coli_K12, partial [Shigella flexneri 2a str. 2457T]

ANDENYALAAIACGEITHSRQAIEETLPWLSSFGADPTGGMTRLLYSPEWLETQQQFKKRMAASGL

ETRFDEVGNLYGRLSGTEYPQEVVLSGSHIDTVVNGGNLDGQFGALAAWLAIDWLKTQYGAPLRTVEVVA

MAEEEGSRFPYVFWGSKNIFGLANPDDVRNICDAKGNSFADAMKACGFTLPNAPLTPRQAIKAFVELHIE

QGCVLESNGQSIGVVNAIVGQRRYTVTLNGESNHAGTTPMGYRRDTVYAFSRICHQSIEKAKKMGDPLVL

TFGKVEPRPNTVNVVPGK

>gi|229089135|ref|NP_836175.2| allantoate amidohydrolase [Shigella flexneri 2a str. 2457T]

MVVHCRIIHRITHRIACGEITHSRQAIEETLPWLSSFGADPTGGMTRLLYSPEWLETQQQFKKRMAASGL

ETRFDEVGNLYGRLSGTEYPQEVVLSGSHIDTVVNGGNLDGQFGALAAWLAIDWLKTQYGAPLRTVEVVA

MAEEEGSRFPYVFWGSKNIFGLANPDDVRNICDAKGNSFADAMKACGFTLPNAPLTPRQAIKAFVELHIE

QGCVLESNGQSIGVVNAIVGQRRYTVTLNGESNHAGTTPMGYRRDTVYAFSRICHQSIEKAKKMGDPLVL

TFGKVEPRPNTVNVVPGKTTFTIDCRHTDAAVLRDFTQQLENDMRAICDEMDIGIDIDLWMDEEPVPMNK

ELVATLTELCEREKLNYRVMHSGAGHDAQIFAPRVPTCMIFIPSINGISHNPAERTNITDLAEGVKTLAL

MLYQLAWQE

>gi|229089134|ref|NP_838753.2| rod shape-determining protein MreB [Shigella flexneri 2a str. 2457T]

MLKKFRGMFSNDLSIDLGTANTLIYVKGQGIVLNEPSVVAIRQDRAGSPKSVAAVGHDAKQMLGRTPGNI

AAIRPMKDGVIADFFVTEKMLQHFIKQVHSNSFMRPSPRVLVCVPVGATQVERRAIRESAQGAGAREVFL

IEEPMAAAIGAGLPVSEATGSMVVDIGGGTTEVAVISLNGVVYSSSVRIGGDRFDEAIINYVRRNYGSLI

GEATAERIKHEIGSAYPGDEVREIEVRGRNLAEGVPRGFTLNSNEILEALQEPLTGIVSAVMVALEQCPP

ELASDISERGMVLTGGGALLRNLDRLLMEETGIPVVVAEDPLTCVARGGGKALEMIDMHGGDLFSEE

>gi|229089133|ref|NP_839120.2| regulatory ATPase RavA [Shigella flexneri 2a str. 2457T]

MAHPHLLAERISRLSSSLEKGLYERSHAIRLCLLAALSGESVFLLGPPGIAKSLIARRLKFAFQNARAFE

YLMTRFSTPEEVFGPLSIQALKDEGRYERLTSGYLPEAEIVFLDEIWKAGPAILNTLLTAINERQFRNGA

HVEKIPMRLLVAASNELPEADSSLEALYDRMLIRLWLDKVQDKANFRSMLTSQQDENDNPVPDALQVTDE

EYERWQKEIGEITLPDHVFELIFILRQQLDKLPDAPYVSDRRWKKAIRLLQASAFFSGRSAVAPVDLILL

KDCLWYDAQSLNLIQQQIDVLMTGHAWQQQGMLTRLGAIVQRHLQLQQQQSDKTALTLIRLGGIFSRRQQ

YQLPVNVTASTLTLLLQKPLKLHDMEVVHISFERSALEQWLSKGGEIRGKLNGIGFAQKLNLEVDSTQHL

VVRDVSLQGSTLALPGSSAEGLPGEIKQQLEELESDWRKQHALFSEQQKCLFIPGDWLGRIEDSLQDVGA

QIRQAQQC

>gi|229089132|ref|NP_839340.2| outer membrane lipoprotein [Shigella flexneri 2a str. 2457T]

MFILLAAVVSGALAVSGCTTNPYTGEREAGKSAIGAGLGSLVGAGIGALSSSKKDRGKGALIGAAAGAAL

GGGVGYYMDVQEAKLRDKMRGTGVSVTRSGDNIILNMPNNVTFDSSSAPLKPAGANTLTGVAMVLKEYPK

TAVNVIGYTDSTGGHDLNMRLSQQRADSVASALITQGVDASRIRTQGLGPANPIASNSTAEGKAQNRRVE

ITLSPL

>gi|229089131|ref|NP_839360.2| phosphodiesterase [Shigella flexneri 2a str. 2457T]

MAMVAAVVLVFVFIFCTVLLFHLVQQNRYNTATQLESIARSVREPLSSAILKGDIPEAEAILASIKPAGV

VSRADVVLPNQFQALRKSFIPERPVPVMVTRLFELPVQISLGVYSLERPANPQPIAYLVLQADSFRMYKF

VMSTLSTLVTIYLLLSLILTVAISWCINRLILHPLRNIARELNAIPAQELVGHQLALPRLHQDDEIGMLV

RSYNLNQQLLQRHYEEQNENAMRFPVSDLPNKALLMEMLEQVVARKQTTALMIITCETLRDTAGVLKEAQ

REILLLTLVEKLKSVLSPRMILAQISGYDFAVIANGVQEPWHAITLGQQVLTIMSERLPIERIQLRPHCS

IGVAMFYGDLTAEQLYSRAISAAFTARHKGKNQIQFFDPQQMEAAQKRLTEESDILNALENHQFAIWLQP

QVEMTSGKLVSAEVLLRIQQPDGSWDLPDGLIDRIECCGLMVTVGHWVLEESCRLLAAWQERGIMLPLSV

NLSALQLMHPNMVADMLELLTRYRIQPGTLILEVTESRRIDDPHAAVAILRPLRNAGVRVALDDFGMGYA

GLRQLQHMKSLPIDVLKIDKMFVEGLPEDSSMIAAIIMLAQSLNLQMIAEGVETEAQRDWLAKAGVGIAQ

GFLFARPLPIEIFEESYLEEK

>gi|229089130|ref|NP_839492.3| peptidoglycan synthetase [Shigella flexneri 2a str. 2457T]

MKFVKYFLILAVCCILLGAGSIYGLYRYIEPQLPDVVTLKDVRLQIPMQIYSADGELIAQYGEKRRIPVT

LDQIPPEMVKAFIATEDSRFYEHHGVDPVGIFRAASVALFSGHASQGASTITQQLARNFFLSPERTMMRK

IKEVFLAIRIEQLLTKDEILELYLNKIYLGYRAYGVGAAAQVYFGKTVDQLTLNEMAVIAGLPKAPSTFN

PLYSMDRAVARRNVVLSRMLDEGYITQQQFDQTRTEAINANYHAPEIAFSAPYLSEMVRQEMYNRYGESA

YEDGYRIYTTITRKVQQAAQQAVRNNVLDYDMRHGYRGPANVLWKVGESAWDNNKITDTLKALPTYGPLL

PAAVTSANPQGATAMLADGSTVALSMEGVRWARPYRSDTQQGPTPRKVTDVLQTGQQIWVRQVGDAWWLA

QVPEVNSALVSINPQNGAVMALVGGFDFNQSKFNRATQALRQVGSNIKPFLYTAAMDKGLTLASMLNDVP

ISRWDAGAGSDWQPKNSPPQYAGPIRLRQGLGQSKNVVMVRAMRAMGVDYAAEYLQRFGFPAQNIVHTES

LALGSASFTPMQVARGYAVMANGGFLVDPWFISKIENDQGGVIFEAKPKVACPECDIPVIYGDTQKSNVL

ENNDVEDVAISREQQNVSVPMPQLEQANQALVAKTGAQEYAPHVINTPLAFLIKSALNTNIFGEPGWQGT

GWRAGRDLQRRDIGGKTGTTNSSKDAWFSGYGPGVVTSVWIGFDDHRRNLGHTTASGAIKDQISGYEGGA

KSAQPAWDAYMKAVLEGVPEQPLTPPPGIVTVNIDRSTGQLANGGNSREEYFIEGTQPTQQAVHEVGTTI

IDNGEAQELF

>gi|229089129|ref|NP_839775.2| hypothetical protein S4664 [Shigella flexneri 2a str. 2457T]

MGVIEFLLALAQDMILAAIPAVGFAMVFNVPVRALRWCALLGSIGHGSRMILMTSGLNIEWSTFMASMLV

GTIGIQWSRWYLAHPKVFTVAAVIPMFPGISAYTAMISSVKISQLGYSEPLMITLLTNFLTASSIVGALS

IGLSIPGLWLYRKRPRV

>gi|161486555|ref|NP_835762.2| 30S ribosomal protein S20 [Shigella flexneri 2a str. 2457T]

MANIKSAKKRAIQSEKARKHNASRRSMMRTFIKKVYAAIEAGDKAAAQKAFNEMQPIVDRQAAKGLIHKN

KAARHKANLTAQINKLA

>gi|161486554|ref|NP_835773.2| DNA-binding transcriptional activator CaiF [Shigella flexneri 2a str. 2457T]

MCEGYVEKPLYLLIAEWMMAENRWVIAREISIHFDIEHSKAVNTLTYILSEVTEISCEVKMIPNKLEGRG

CQCQRLVKVVDIDEQIYARLRNNSREKLVGVRKTPRIPAVPLTELNREQKWQMMLSKSMRR

>gi|161486553|ref|NP_835780.2| electron transfer flavoprotein FixA [Shigella flexneri 2a str. 2457T]

MKIITCYKCVPDEQDIAVNNADGSLDFSKADAKISQYDLNAIEAACQLKQQAAEAQVTALSVGGKALTNA

KGRKDVLSRGPDELIVVIDDQFEQALPQQTASALAAAAQKAGFDLILCGDGSSDLYAQQVGLLVGEILNI

PAVNGVSKIISLTADTLTVERELEDETETLSIPLPAVVAVSTDINSPQIPSMKAILGAAKKPVQVWSAAD

IGFNAVDAWSEQQVAAPKQRERQRIVIEGDGEEQIAAFAENLRKVI

>gi|161486552|ref|NP_835809.2| 3-isopropylmalate dehydrogenase [Shigella flexneri 2a str. 2457T]

MSKNYHIAVLPGDGIGPEVMTQALKVLNAVRNRFAMRITTSHYDVGGAAIDNHGQPLPPATVEGCEQADA

VLFGSVGGPKWEHLPPDQQPERGALLPLRKHFKLFSNLRPAKLYQGLEAFCPLRADIAANGFDIQCVREL

TGGIYFGQPKGREGSGQYEKAFDTEVYHRFEIERIARIAFESARKRRHKVTSIDKANVLQSSILWREIVN

EIATEYPDVELAHMYIDNATMQLIKDPSQFDVLLCSNLFGDILSDECAMITGSMGMLPSASLNEQGFGLY

EPAGGSAPDIAGKNIANPIAQILSLALLLRYSLDADDAASAIERAINRALEEGIRTGDLARGAAAVSTDE

MGDIIARYVAEGV

>gi|161486551|ref|NP_835812.2| leucine transcriptional activator [Shigella flexneri 2a str. 2457T]

MPEVQTDHSETAELSKPQLRMVDLNLLTVFDAVMQEQNITRAAHVLGMSQPAVSNAVARLKVMFNDELFV

RYGRGIQPTARAFQLFGSVRQALQLVQNELPGSGFEPASSERVFHLCVCSPLDSILTSQIYNHIEQIAPN

IHVMFKSSLNQNTEHQLRYQETEFVISYEDFHRPEFTSVPLFKDEMVLVASKNHPTIKGPLLKHDVYNEQ

HAAVSLDRFASFSQPWYDTVDKQASIAYQGMAMMSVLSVVSQTHLVAIAPRWLAEEFAESLELQVLPLPL

KQNSRTCYLSWHEAAGRDKGHQWMEEQLVSICKR

>gi|161486550|ref|NP_835813.2| acetolactate synthase 3 catalytic subunit [Shigella flexneri 2a str. 2457T]

MEMLSGAEMVVRSLIDQGVKQVFGYPGGAVLDIYDALHTVGGIDHVLVRHEQAAVHMADGLARATGEVGV

VLVTSGPGATNAITGIATAYMDSIPLVVLSGQVATSLIGYDAFQECDMVGISRPVVKHSFLVKQTEDIPQ

VLKKAFWLAASGRPGPVVVDLPKDILNPANKLPYVCPESVSMRSYNPTTTGHKGQIKRALQTLVAAKKPV

VYVGGGAIMAGCHQQLKETVEALNLPVVSSLMGMGAFPATHRQALGMLGMHGTYEANMTMHNADVIFAVG

VRFDDRTTNNLAKYCPNATVLHIDIDPTSISKTVTADIPIVGDARQVLEQMLELLSQESAHQPLDEIRDW

WQQIEQWRALQCLKYDTHSEKIKPQAVIETLWRLTNGDAYVTSDVGQHQMFAALYYPFDKPRRWINSGGL

GTMGFGLPAALGVKMALPEETVVCVTGDGSIQMNIQELSTALQYELPVLVVNLNNRYLGMVKQWQDMIYS

GRHSQSYMQSLPDFVRLAEAYGHVGIQISHPHELESKLSEALEQVRNNRLVFVDVTVDGSEHVYPMQIRG

GGMDEMWLSKTERT

>gi|161486549|ref|NP_835835.2| SecA regulator SecM [Shigella flexneri 2a str. 2457T]

MSGILTRWRQFGKRYFWPHLLLGMVAASLGLPALSNAAEPNAPAKATTRNHEPSAKVNFGQLALLEANTR

RPNSNYSVDYWHQHAIRTVIRHLSFAMAPQTLPVAEESLPLQAQHLALLDTLSALLTQEGTPSEKGYRID

YAHFTPQAKFSTPVWISQAQGIRAGPQRLT

>gi|161486548|ref|NP_835855.2| hypothetical protein S0118 [Shigella flexneri 2a str. 2457T]

MDYEFLRDITGVVKVRMSMGHEVVGHWFNEEVKENLALLDEVEQAAHALKGSERSWQRAGHEYTLWMDAE

EVMVRANQLEFAGDEMEEGMNYYDEESLSLCGVEDFLQVVAAYRNFVQQK

>gi|161486547|ref|NP_835861.2| hypoxanthine-guanine phosphoribosyltransferase [Shigella flexneri 2a str. 2457T]

MKHTVEVMIPEAEIKARIAELGRQITERYKDSGSDMVLVGLLRGSFMFMADLCREVQVSHEVDFMTASSY

GSGMSTTRDVKILKDLDEDIRGKDVLIVEDIIDSGNTLSKVREILSLREPKSLAICTLLDKPSRREVNVP

VEFIGFSIPDEFVVGYGIDYAQRYRHLPYIGKVILLDE

>gi|161486546|ref|NP_835874.2| poly(A) polymerase I [Shigella flexneri 2a str. 2457T]

MTLPRCTIFTRVANFCRKVLSREESEAEQAVARPQVTVIPREQHAISRKDISENALKVMYRLNKAGYEAW

LVGGGVRDLLLGKKPKDFDVTTNATPEQVRKLFRNCRLVGRRFRLAHVMFGPEIIEVATFRGHHEGNVSD

RTTSQRGQNGMLLRDNIFGSIEEDAQRRDFTINSLYYSVADFTVRDYVGGMKDLKDGVIRLIGNPETRYR

EDPVRMLRAVRFAAKLGMRISPETAEPIPRLATLLNDIPPARLFEESLKLLQAGYGYETYKLLCEYHLFQ

PLFPTITRYFTENGDSPMERIIEQVLKNTDTRIHNDMRVNPAFLFAAMFWYPLLETAQKIAQESGLTYHD

AFALAMNDVLDEACRSLAIPKRLTTLTRDIWQLQLRMSRRQGKRAWKLLEHPKFRAAYDLLALRAEVERN

AELQRLVKWWGEFQVSAPPDQKGMLNELDEEPSPRRRTRRPRKRAPRREGTT

>gi|161486545|ref|NP_835875.2| glutamyl-Q tRNA(Asp) synthetase [Shigella flexneri 2a str. 2457T]

MTDTQYIGRFAPSPSGELHFGSLIAALGSYLQARARQGRWLVRIEDIDPPREVPGAAETILRQLEHYGLH

WDGDVLWQSQRHDAYREALAWLHEQGLSYYCTCTRARIQSIGGIYDGHCRVLHHGPDNAAVRIRQQHPVT

QFTDLLRGIIHADEKLAREDFIIHRRDGLFAYNLAVVVDDHFQGVTEIVRGADLIEPTVRQIPLYQLFGW

KVPDYIHLPLALNPQGAKLSKQNHAPALPKGDPRPVLIAALQFLGQQAEAHWQDFSVEQILQSAVKNWML

TAVPESAIVNSTFSNASC

>gi|161486544|ref|NP_835903.2| CDP-diglyceride synthase [Shigella flexneri 2a str. 2457T]

MLKYRLISAFVLIPVVIAALFLLPPVGFAIVTLVVCMLAAWEWGQLSGFTTRSQRVWLAVLCGLLLALML

FLLPEYHRNIHQPLVEISLWASLGWWIVALLLVLFYPGSAAIWRNSKTLRLIFGVLTIVPFFWGMLALRA

WHYDENHYSGAIWLLYVMILVWGADSGAYMFGKLFGKHKLAPKVSPGKTWQGFIGGLATAAVISWGYGMW

ANLDVAPVTLLICSIVAALASVLGDLTESMFKREAGIKDSGHLIPGHGGILDRIDSLTAAVPVFACLLLL

VFRTL

>gi|161486543|ref|NP_835915.2| hypothetical protein S0180 [Shigella flexneri 2a str. 2457T]

MLGLKQVHHIAIIATDYAVSKAFYCDILGFTLQSEVYREARDSWKGDLALNGQYVIELFSFPFPPERPSR

PEACGLRHLAFSVDDIDAAVAHLESHNVKCEAIRVDPYTQKRFTFFNDPDGLPLELYEQ

>gi|161486542|ref|NP_835986.2| insertion element IS2 transposase InsD [Shigella flexneri 2a str. 2457T]

MDSARALIARGWGVSLVSRCLRVSRAQLHVILRRTDDWMDGRRSRHTDDTDVLLRIHHVIGELPTYGYRR

VWALLRRQAELDGMPAINAKRVYRLMRQNALLLERKPAVPPSKRAHTGRVAVKESNQRWCSDGFEFCCDN

GERLRVTFALDCCDREALHWAVTTGGFNSETVQDVMLGAVERRFGNDLPSSPVEWLTDNGSCYRANETRQ

FARMLGLEPKNTAVRSPESNGIAESFVKTIKRDYISIMPKPDGLTAAKNLAEAFEHYNEWHPHSALGYRS

PREYLRQRACNGLSDNRCLEI

>gi|161486541|ref|NP_835987.2| insertion sequence 2 OrfA protein [Shigella flexneri 2a str. 2457T]

MIDVLGPEKRRRRTTQEKIAIVQQSFEPGMTVSLVARQHGVAASQLFLWRKQYQEGSLTAVAAGEQVVPA

SELAAAMKQIKELQRLLGKKTMENELLKEAVEYGRAKKWIAHAPLLPGDGE

>gi|161486540|ref|NP_835998.2| transporter [Shigella flexneri 2a str. 2457T]

MDNRSEFLNNVAQALGRPLRLEPQAEDAPLNNYANERLTQLNQQQRCDAFIQFASDVMLTRCELTSEAKA

AEAAIRLCKELGDQSVVISGDTRLEELGISERLQQECNAVVWDPAKGAENISQAEQAKVGVVYAEYGLTE

SGGVVLFSAAERGRSLSLLPESSLFILRKSTILPRVAQLAEKLHQKAQAGERMPSCINIISGPSSTADIE

LIKVVGVHGPVKAVYLIIEDC

>gi|161486539|ref|NP_836011.2| acyl-CoA dehydrogenase [Shigella flexneri 2a str. 2457T]

MMILSILATVVLLGALFYHRVSLFISSLILLAWTAALGVAGLWSAWVLVPLTIILVPFNFAPMRKSMISA

PVFRGFRKVMPPMSRTEKEAIDAGTTWWEGDLFQGKPDWKKLHNYPQPRLTAEEQAFLDGPVEEACRMAN

DFQITHELADLPPELWAYLKEHRFFAMIIKKEYGGLEFSAYAQSRVLQKLSGVSGILAITVGVPNSLGPG

ELLQHYGTDEQKDHYLPRLARGQEIPCFALTSPEAGSDAGAIPDTGIVCMGEWQGQQVLGMRLTWNKRYI

TLAPIATVLGLAFKLSDPEKLLGGAEDLGITCALIPTTTPGVEIGRRHFPLNVPFQNGPTRGKDVFVPID

YIIGGPKMAGQGWRMLVECLSVGRGITLPSNSTGGVKSVALATGAYSHIRRQFKISIGKMEGIEEPLARI

AGNAYVMDAAASLITYGIMLGEKPAVLSAIVKYHCTHRGQQSIIDAMDITGGKGIMLGQSNFLARAYQGA

PIAITVEGANILTRSMMIFGQGAIRCHPYVLEEMEAAKNNDVNAFDKLLFKHIGHVGSNKVRSFWLGLTR

GLTSSTPTGDATKRYYQHLNRLSANLALLSDVSMALLGGSLKRRERISARLGDILSQLYLASAVLKRYDD

EGRNEADLPLVHWGVQDALYQAEQAMDNLLQNFPNRVVAGLLNVVIFPTGRHYLAPSDKLDHKMAKILQV

PNATRSRIGRGQYLTPSEHNPVGLLEEALVDVIAADPIHQRICKELGKNLPFTRLDELAHNALTKGLIDK

DEAAILVKAEESRLRSINVDDFDPEELATKPVKLPEKVRKVEAA

>gi|161486538|ref|NP_836054.2| hypothetical protein S0331 [Shigella flexneri 2a str. 2457T]

MTIWVDADACPNVIKEILYRAAERMQMPLVLVANQSLRVPPSRFIRTLRVAAGFDVADNEIVRQCEAGDL

VITADIPLAAEAIEKGAAALNSRGERYTPATIRERLTMRDFMDTLRASGIQTGGPDSLSQRDRQAFAAEL

EKWWLEVQRSRG

>gi|161486537|ref|NP_836062.2| fructokinase [Shigella flexneri 2a str. 2457T]

MRIGIDLGGTKTEVIALGDAGEQLYRHRLPTPRDDYRQTIETIATLVDMAEQATGQRGTVGMGIPGSISP

YTGVVKNVNSTWLNGQPFDKDLSARLQREVRLANDANCLAVSEAVDGAAAGAQTVFAVIIGTGCGAGVAF

NGRTHIGGNGTAGEWGHNPLPWMDEDELRYREEVPCYCGKQGCIETFISGTGFATDYRRLSGHALKGSEI

IRLVEESDPVAELALRRYELRLAKSLAHVVNILDPDVIVLGGGMSNVDRLYQTVGQLIKQFVFGGECETP

VSKAKHGDSSGVRGAAW

>gi|161486536|ref|NP_836078.2| hypothetical protein S0357 [Shigella flexneri 2a str. 2457T]

MNTNVFRLLLLGSLFSLSACVQQSEVRQMKHSVSTLNQEMTQLNQETVKITQQNRLNAKSSSGVYLLPGA

KTPARLESQIGTLRMSLVNITPDTDGTTLTLRIQGESNDPLPAFSGTVEYGQIQGTIDNFQEINVQNQLI

NAPASVLAPSDVDIPLQLKGISVDQLGFVRIHDIQPVMQ

>gi|161486535|ref|NP_836107.2| hypothetical protein S0385 [Shigella flexneri 2a str. 2457T]

MFKKILFPLVALFMLAGCAKPPTTIEVSPTITLPQQDPSLMGVTVSINGADQRTDQALAKVTRDNQIVTL

TASRALRFLLQEVLEKQMTARGYMVGPNGPVNLQIIVSQLYADVSQGNVRYNIATKADIAIIATAQNGNK

MTKNYRASYNVEGAFQASNKNIADAVNSVLTDTIADMSQDTSIHEFIKQNAR

>gi|161486534|ref|NP_836108.2| transcriptional regulator BolA [Shigella flexneri 2a str. 2457T]

MMIRERIEEKLRAAFQPVFLEVVDESYRHNVPAGSESHFKVVLVSDRFTGERFLNRHRMIYSTLAEELST

TVHALALHTYTIKEWEGLQDTVFASPPCRGAGSIA

>gi|161486533|ref|NP_836183.2| hypothetical protein S0467 [Shigella flexneri 2a str. 2457T]

MATFSLGKHPHVELCDLLKLEGWSESGAQAKIAIAEGQVKVDGAVETRKRCKIVAGQTVSFAGHSVQVVA

>gi|161486532|ref|NP_836233.2| disulfide isomerase/thiol-disulfide oxidase [Shigella flexneri 2a str. 2457T]

MLKKILLLALLPAIAFAEELPSPVKAIEKQGITIIKTFDAPGGMKGYLGKYQDMGVTIYLTPDGKHAISG

YMYNEKGENLSNTLIEKEIYAPAGREMWQRMEQSHWLLDGKKDAPVIVYVFSDPFCPYCKQFWQQARPWV

DSGKVQLRTLLVGVIKPESPATAAAILASKDPAKTWQQYEASGCKLKLNVPANVSTEQMKVLSDNEKLMD

DLGANVTPAIYYMSKENTLQQAVGLPDQKTLNIIMGNK

>gi|161486531|ref|NP_836287.2| succinate dehydrogenase cytochrome b556 large membrane subunit [Shigella flexneri 2a str. 2457T]

MIRNVKKQRPVNLDLQTIRFPITAIASILHRVSGVITFVAVGILQWLLGTSLSSPEGFEQASAIMGSFFV

KFIMWGILTALAYHVVVGIRHMMMDFGYLEETFEAGKRSAKISFVITVVLSLLAGVLVW

>gi|161486530|ref|NP_836352.2| lipoyltransferase [Shigella flexneri 2a str. 2457T]

MYQDKILVRQLGLQPYEPISRAMHEFTDTRDNSTLDEIWLVEHYPVFTQGQAGKAEHILMPGDIPVIQSD

RGGQVTYHGPGQQVMYVLLNLKRRKLGVRELVTLLEQTVVNTLAELGIEAHPRADAPGVYVGEKKICSLG

LRIRRGCSFHGLALNVNMDLSPFLRINPCGYAGMEMAKISQWKPEATTNNIAPRLLENILALLNNPDFEY

ITA

>gi|161486529|ref|NP_836374.2| insertion element IS2 transposase InsD [Shigella flexneri 2a str. 2457T]

MDSARALIARGWGVSLVSRCLRVSRAQLHVILRRTDDWMDGRRSRHTDDTDVLLRIHHVIGELPTYGYRR

VWALLRRQAELDGMPAINAKRVYRLMRQNALLLERKPAVPPSKRAHTGRVAVKESNQRWCSDGFEFCCDN

GERLRVTFALDCCDREALHWAGTTGGFNSETVQDVMLGAVERRFGNDLPSSPVEWLTDNGSCYRANETRQ

FARMLGLEPKNTAVRSPESNGIAESFVKTIKRDYISIMPKPDGLTAAKNLAEAFEHYNEWHPHSALGYRS

PREYLRQRACNGLSDNRCLEI

>gi|161486528|ref|NP_836375.2| insertion sequence 2 OrfA protein [Shigella flexneri 2a str. 2457T]

MIDVLGPEKRRRRTTQEKIAIVQQSFEPGMTVSLVARQHGVAASQLFLWRKQYQEGSLTAVAAGEQVVPA

SELAAAMKQIKELQRLLGKKTMENELLKEAVEYGRAKKWIAHAPLLPGDGE

>gi|161486527|ref|NP_836460.2| hydroxylase [Shigella flexneri 2a str. 2457T]

MMYHIPGVLSPQDVAHFREQLEQAEWVDGRVTTGAQGAQVKNNQQVDTRSALYAALQNEVLNAVNQHALF

FAAALPRTLSTPLFNRYQNNETYGFHVDGAVRSHPQNGWMRTDLSATLFLSDPQSYDGGELVVNDTFGQH

RVKLPAGDLVLYPSSSLHCVTPVTRGVRVASFMWIQSMIRDDKKRAMLFELDKNIQSLKSRYGENEEILS

LLNLYHNLLREWSEI

>gi|161486526|ref|NP_836479.2| fructose-6-phosphate aldolase [Shigella flexneri 2a str. 2457T]

MELYLDTSDVVAVKALSRIFPLAGVTTNPSIIAAGKKPLDVVLPQLHEAMGGQGRLFAQVMATTAEGMVN

DALKLRSIIADIVVKVPVTAEGLAAIKMLKAEGIPTLGTAVYGAAQGLLSALAGAEYVAPYVNRIDAQGG

SGIQTVTDLHQLLKMHAPQAKVLAASFKTPRQALDCLLAGCESITLPLDVAQQMISYPAVEAAVTKFEQD

WQGAFGRTSI

>gi|161486525|ref|NP_836508.2| putrescine transporter ATP-binding subunit [Shigella flexneri 2a str. 2457T]

MNDAIPRPQAKTRKALTPLLEIRNLTKSYDGQHAVDDVSLTIYKGEIFALLGASGCGKSTLLRMLAGFEQ

PSAGQIMLDGVDLSQVPPYLRPINMMFQSYALFPHMTVEQNIAFGLKQDKLPKAEISSRVNEMLGLVHMQ

EFAKRKPHQLSGGQRQRVALARSLAKRPKLLLLDEPMGALDKKLRDRMQLEVVDILERVGVTCVMVTHDQ

EEAMTMAGRIAIMNRGKFVQIGEPEEIYEHPTTRYSAEFIGSVNVFEGVLKERQEDGLVLDSPGLVHPLK

VDADASVVDNVPVHVALRPEKIMLCEEPPANGCNFAVGEVIHIAYLGDLSVYHVRLKSGQMISAQLQNAH

RHRKGLPTWGDEVRLCWEVDSCVVLTV

>gi|161486524|ref|NP_836528.2| hydroxylamine reductase [Shigella flexneri 2a str. 2457T]

MFCVQCEQTIRTPAENGCSYAQGMCGKTAETSDLQDLLIAALQGLSAWAVKAREYGIINHDVDSFAPRAF

FSTLTNVNFDSPRIVGYAREAIALREALKAQCLAVDANARVDNPMADLQLVSDDLGELQRQAAEFTPNKD

KAAIGENILGLRLLCLYGLKGAAAYMEHAHVLGQYDNDIYAQYHKIMAWLGTWPADMNALLECSMEIGQM

NFKVMSILDAGETGKYGHPTPTQVNVKATAGKCILISGHDLKDLYNLLEQTEGTGVNVYTHGEMLPAHGY

PELRKFKHLVGNYGSGWQNQQVEFARFPGPIVMTSNCIIDPTVGAYDDRIWTRSIVGWPGVRHLDGEDFS

AVIAQAQQMAGFPYSEIPHLITVGFGRQTLLGAADTLIDLVSREKLRHIFLLGGCDGARGERHYFTDFAT

SVPDDCLILTLACGKYRFNKLEFGDIEGLPRLVDAGQCNDAYSAIILAVTLAEKLGCGVNDLPLSLVLSW

FEQKAIVILLTLLSLGVKNIVTGPTAPGFLTPDLLAVLNEKFGLRSITTVEEDMKQLLNA

>gi|161486523|ref|NP_836530.2| insertion element IS2 transposase InsD [Shigella flexneri 2a str. 2457T]

MDSARALIARGWGVSLVSRCLRVSRAQLHVILRRTDDWMDGRRSRHTDDTDVLLRIHHVIGELPTYGYRR

VWALLRRQAELDGMPAINAKRVYRIMRQNALLLERKPAVPPSKRAHTGRVAVKESNQRWCSDGFEFCCDN

GERLRVTFALDCCDREALHWAVTTGGFNSETVQDVMLGAVERRFGNDLPSSPVEWLTDNGSCYRANETRQ

FARMLGLEPKNTAVRSPESNGIAESFVKTIKRDYISIMPKPDGLTAAKNLAEAFEHYNEWHPHSALGYRS

PREYLRQRACNGLSDNRCLEI

>gi|161486522|ref|NP_836531.2| insertion sequence 2 OrfA protein [Shigella flexneri 2a str. 2457T]

MIDVLGPEKRRRRTTQEKIAIVQQSFEPGMTVSLVARQHGVAASQLFLWRKQYQEGSLTAVAAGEQVVPA

SELAAAMKQIKELQRLLGKKTMENELLKEAVEYGRAKKWIAHAPLLPGDGE

>gi|161486521|ref|NP_836575.2| insertion element IS2 transposase InsD [Shigella flexneri 2a str. 2457T]

MDSARALIARGWGVSLVSRCLRVSRAQLHVILRRTDDWMDGRRSRHTDDTDVLLRIHHVIGELPTYGYRR

VWALLRRQAELDGMPAINAKRVYRLMRQNALLLERKPAVPPSKRAHTGRVAVKESNQRWCSDGFEFCCDN

GERLRVTFALDCCDREALHWAVTTGGFNSETVQDVMLGAVERRFGNDLPSSPVEWLTDNGSCYRANETRQ

FARMLGLEPKNTAVRSPESNGIAESFVKTIKRDYISIMPKPDGLTAAKNLAEAFEHYNEWHPHSALGYRS

PREYLRQRACNGLSDNRCLEI

>gi|161486520|ref|NP_836576.2| insertion sequence 2 OrfA protein [Shigella flexneri 2a str. 2457T]

MIDVLGPEKRRRRTTQEKIAIVQQSFEPGMTVSLVARQHGVAASQLFLWRKQYQEGSLTAVAAGEQVVPA

SELAAAMKQIKELQRLLGKKTMENELLKEAVEYGRAKKWIAHAPLLPGDGE

>gi|161486519|ref|NP_836640.2| insertion element IS2 transposase InsD [Shigella flexneri 2a str. 2457T]

MDSARALIARGWGVSLVSRCLRVSRAQLHVILRRTDDWMDGRRSRHTDDTDVLLRIHHVIGELPTYGYRR

VWALLRRQAELDGMPAINAKRVYRIMRQNALLLERKPAVPPSKRAHTGRVAVKESNQRWCSDGFEFCCDN

GERLRVTFALDCCDREALHWAVTTGGFNSETVQDVMLGAVERRFGNDLPSSPVEWLTDNGSCYRANETRQ

FARMLGLEPKNTAVRSPESNGIAESFVKTIKRDYISIMPKPDGLTAAKNLAEAFEHYNEWHPHSALGYRS

PREYLRQRACNGLSDNRCLEI

>gi|161486518|ref|NP_836641.2| insertion sequence 2 OrfA protein [Shigella flexneri 2a str. 2457T]

MIDVLGPEKRRRRTTQEKIAIVQQSFEPGMTVSLVARQHGVAASQLFLWRKQYQEGSLTAVAAGEQVVPA

SELAAAMKQIKELQRLLGKKTMENELLKEAVEYGRAKKWIAHAPLLPGDGE

>gi|161486517|ref|NP_836643.2| alkanesulfonate transporter substrate-binding subunit [Shigella flexneri 2a str. 2457T]

MRNIIKLALAGLLSVSTFAVAAESSPEALRIGYQKGSIGMVLAKSHQLLEKRYPQSKISWVEFPAGPQML

EALNVGSIDLGSTGDIPPIFAQAAGADLVYVGVEPPKPKAEVILVAENSPIKTVADLKGHKVAFQKGSSS

HNLLLRALRQAGLKFTDIQPTYLTPADARAAFQQGNVDAWAIWDPYYSAALLQGGVRVLKDGTDLNQTGS

FYLAARPYAEKNGAFIQGVLATFSEADALTRSQREQSIALLAKTMGLPAPVIASYLDHRPPTTIKPVNAE

VAALQQQTADLFYENRLVPKKVDIRQRIWQPTQLEGKQL

>gi|161486516|ref|NP_836668.2| insertion sequence 2 OrfA protein [Shigella flexneri 2a str. 2457T]

MIDVLGPEKRRRRTTQEKIAIVQQSFEPGMTVSLVARQHGVAASQLFLWRKQYQEGSLTAVAAGEQVVPA

SELAAAMKQIKELQRLLGKKTMENELLKEAVEYGRAKKWIAHAPLLPGDGE

>gi|161486515|ref|NP_836673.2| methylglyoxal synthase [Shigella flexneri 2a str. 2457T]

MELTTRTLPARKHIALVAHDHCKQMLMSWVERHQPLLEQHVLYATGTTGNLISRATGMNVNAMLSGPMGG

DQQVGALISEGKIDVLIFFWDPLNAVPHDPDVKALLRLATVWNIPVATNVATADFIIQSPHFNDAVDILI

PDYQRYLADRLK

>gi|161486514|ref|NP_836679.2| sulfur transfer protein TusE [Shigella flexneri 2a str. 2457T]

MLIFEGKEIETDTEGYLKESSQWSEPLAVVIAENEGISLSPEHWEVVRFVRDFYLEFNTSPAIRMLVKAM

ANKFGEEKGNSRYLYRLFPKGPAKQATKIAGLPKPVKCI

>gi|161486513|ref|NP_836690.2| phosphotyrosine-protein phosphatase [Shigella flexneri 2a str. 2457T]

MAQLKFNSILVVCTGNICRSPIGERLLRKRLPGVKVKSAGVHGLVKHPADATAADVAANHGVSLEGHAGR

KLTAEMARNYDLILAMESEHIAQVTAIAPEVRGKTMLFGQWLEQKEIPDPYRKSQDAFEHVYSMLERASQ

EWAKRLSR

>gi|161486512|ref|NP_836755.2| insertion sequence 2 OrfA protein [Shigella flexneri 2a str. 2457T]

MIDVLGPEKRRRRTTQEKIAIVQQSFEPGMTVSLVARQHGVAASQLFLWRKQYQEGSLTAVAAGEQVVPA

SELAAAMKQIKELQRLLGKKTMENELLKEAVEYGRAKKWIAHAPLLPGDGE

>gi|161486511|ref|NP_836756.2| insertion element IS2 transposase InsD [Shigella flexneri 2a str. 2457T]

MDSARALIARGWGVSLVSRCLRVSRAQLHVILRRTDDWMDGRRSRHTDDTDVLLRIHHVIGELPTYGYRR

VWALLRRQAELDGMPAINAKRVYRLMRQNALLLERKPAVPPSKRAHTGRVAVKESNQRWCSDGFEFCCDN

GERLRVTFALDCCDREALHWAVTTGGFNSETVQDVMLGAVERRFGNDLPSSPVEWLTDNGSCYRANETRQ

FARMLGLEPKNTAVRSPESNGIAESFVKTIKRDYISIMPKPDGLTAAKNLAEAFEHYNEWHPHSALGYRS

PREYLRQRACNGLSDNRCLEI

>gi|161486510|ref|NP_836769.2| biofilm formation regulatory protein BssS [Shigella flexneri 2a str. 2457T]

MEKNNEVIQTHPLVGWDISTVDSYDALMLRLHYQTPNKSEQEGTEVGQTLWLTTDVARQFISILEAGIAK

IESGDFPVNEYRRH

>gi|161486509|ref|NP_836789.2| ribonuclease E [Shigella flexneri 2a str. 2457T]

MKRMLINATQQEELRVALVDGQRLYDLDIESPGHEQKKANIYKGKITRIEPSLEAAFVDYGAERHGFLPL

KEIAREYFPANYSAHGRPNIKDVLREGQEVIVQIDKEERGNKGAALTTFISLAGSYLVLMPNNPRAGGIS

RRIEGDDRTELKEALASLELPEGMGLIVRTAGVGKSAEALQWDLSFRLKHWEAIKKAAESRPAPFLIHQE

SNVIVRAFRDYLRQDIGEILIDNPKVLELARQHIAALGRPDFSSKIKLYTGEIPLFSHYQIESQIESAFQ

REVRLPSGGSIVIDSTEALTAIDINSARATRGGDIEETAFNTNLEAADEIARQLRLRDLGGLIVIDFIDM

TPVRHQRAVENRLREAVRQDRARIQISHISRFGLLEMSRQRLSPSLGESSHHVCPRCSGTGTVRDNESLS

LSILRLIEEEALKENTQEVHAIVPVPIASYLLNEKRSAVNAIETRQDGVRCVIVPNDQMETPHYHVLRVR

KGEETPTLSYMLPKLHEEAMALPSEEEFAERKRPEQPALATFAMPDVPPAPTPAEPAATVVAPAPKAATA

TPAAPAQPGLLSRFFGALKALFSGGEEAKPTEQPTPKAEAKPERQQDRRKPRQSNRRDRNERRDTRSERT

EGSDNREENRRNRRQAQQQTAETRESRQQAEVTEKARTTDEQQAPRRERSRRRNDDKRQAQQEAKALNVE

EQSVQETEQEERVRPVQPRRKQRQLNQKVRYEQSVAEEAVVAPVVEETAAAEPIVQEAPAPRTELVKVPL

PVVAQTAPEQQEENNADNRDNGGMPRRSRRSPRHLRVSGQRRRRYRDERYPTQSPMPLTVACASPELASG

KVWIRYPIVRPQDVQVEEQREQEEVQVQPMVTEVPVAAAVEPVVSAPVVEEMAEVVEAPVPVAEPQPEVV

ETTHPEVIAAAVTEQPQVITESDVAVAQEVAEHAEPMVEPQEETADADIEEVAETAEVVVAEPEVVAQPA

APVVAEVAAEVETVAAVEPEITVEHNHATAPMTRAPAPEYVPEAPRHSDWQRPTFAFEGKGAAGGHTATH

HASAAPARPQPVE

>gi|161486508|ref|NP_836792.2| Maf-like protein [Shigella flexneri 2a str. 2457T]

MPKLILASTSPWRRALLEKLQISFECAAPEVDETPRSDESPRQLVLRLAQEKSQSLASRYPDHLIIGSDQ

VCVLDGEITGKPLTEENARLQLRKASGNIVTFYTGLALFNSANGHLQTEVEPFDVHFRHLSEAEIDNYVR

KEHPLHCAGSFKSEGFGITLFERLEGRDPNTLVGLPLIALCQMLRREGKNPLMG

>gi|161486507|ref|NP_836795.2| glycerol-3-phosphate acyltransferase PlsX [Shigella flexneri 2a str. 2457T]

MTRLTLALDVMGGDFGPSVTVPAALQALNSNSQLTLLLVGNPDAITPLLAKADFEQRSRLQIIPAQSVIA

SDARPSQAIRASRGSSMRMALELVKEGRAQACVSAGNTGALMGLAKLLLKPLEGIERPALVTVLPHQQKG

KTVVLDLGANVDCDSTMLVQFAIMGSVLAEEVVEIPNPRVALLNIGEEEVKGLDSIRDASAVLKTIPSIN

YIGYLEANELLTGKTDVLVCDGFTGNVTLKTMEGVVRMFLSLLKSQGEGKKRSWWLLLLKRWLQKSLTRR

FSHLNPDQYNGACLLGLRGTVIKSHGAANQRAFAVAIEQAVQAVQRQVPQRIAARLESVYPAGFELLDGG

KSGTLR

>gi|161486506|ref|NP_836827.2| spermidine/putrescine ABC transporter [Shigella flexneri 2a str. 2457T]

MKNTSKFQNVVIVTIVGWLVLFVFLPNLMIIGTSFLTRDDASFVKMVFTLDNYTRLLDPLYFEVLLHSLN

MALIATLACLVLGYPFAWFLAKLPHKVRPLLLFLLIVPFWTNSLIRIYGLKIFLSTKGYLNEFLLWLGVI

DTPIRIMFTPSAVIIGLVYILLPFMVMPLYSSIEKLDKPLLEAARDLGASKLQTFIRIIIPLTMPGIIAG

CLLVMLPAMGLFYVSDLMGGAKNLLIGNVIKVQFLNIRDWPFGAATSITLTIVMGLMLLVYWRASRLLNK

KVELE

>gi|161486505|ref|NP_836851.2| tRNA-specific 2-thiouridylase MnmA [Shigella flexneri 2a str. 2457T]

MSETAKKVIVGMSGGVDSSVSAWLLQQQGYQVEGLFMKNWEEDDGEEYCTAAADLADAQAVCDKLGIELH

TVNFAAEYWDNVFELFLAEYKAGRTPNPDILCNKEIKFKAFLEFAAEDLGADYIATGHYVRRADVDGKSR

LLRGLDSNKDQSYFLYTLSHEQIAQSLFPVGELEKPQVRKIAEDLGLVTAKKKDSTGICFIGERKFREFL

GRYLPAQPGKIITVDGDEIGEHQGLMYHTLGQRKGLGIGGTKEGTEEPWYVVDKDVENNILVVAQGHEHP

RLMSVGLIAQQLHWVDREPFTGTMRCTVKTRYRQTDIPCTVKALDDDRIKVIFDEPVAAVTPGQSAVFYN

GEVCLGGGIIEQRLPLPV

>gi|161486504|ref|NP_836865.2| insertion element IS2 transposase InsD [Shigella flexneri 2a str. 2457T]

MDSARALIARGWGVSLVSRCLRVSRAQLHVILRRTDDWMDGRRSRHTDDTDVLLRIHHVIGELPTYGYRR

VWALLRRQAELDGMPAINAKRVYRLMRQNALLLERKPAVPPSKRAHTGRVAVKESNQRWCSDGFEFCCDN

GERLRVTFVLDCCDREALHWAGTTGGFNSETVQDVMLGAVERRFGNDLPSSPVEWLTDNGSCYRANETRQ

FARMLGLEPKNTAVRSPESNGIAESFVKTIKRDYISIMPKPDGLTAAKNLAEAFEHYNEWHPHSALGYRS

PREYLRQRACNGLSDNRCLEI

>gi|161486503|ref|NP_836870.2| hypothetical protein S1258 [Shigella flexneri 2a str. 2457T]

MSDVPFWQSKTLDEMSDAEWESLCDGCGQCCLHKLMDEDTDEIYFTNVACRQLNIKTCQCRNYERRFEFE

PDCIKLTRENLPTFEWLPMTCAYRLLAEGKDLPAWHPLLTGSKAAMHGERISVRHIAVKESEVIDWQDHI

LNKPDWAQ

>gi|161486502|ref|NP_836880.2| potassium/proton antiporter [Shigella flexneri 2a str. 2457T]

MDATTIISLFILGSILVTSSILLSSFSSRLGIPILVIFLAIGMLAGVDGVGGIPFDNYPFAYMVSNLALA

IILLDGGMRTQASSFRVALGPALSLATLGVLITSGLTGMMAAWLFNLDLIEGLLIGAIVGSTDAAAVFSL

LGGKGLNERVGSTLEIESGSNDPMAVFLTITLIAMIQQHESSVSWMFVVDILQQFGLGIVIGLGGGYLLL

QMINRIALPAGLYPLLALSGGILIFALTTALEGSGILAVYLCGFLLGNRPIRNRYGILQNFDGLAWLAQI

AMFLVLGLLVNPSDLLPIAIPALILSAWMIFFARPLSVFAGLLPFRGFNLRERVFISWVGLRGAVPIILA

VFPMMAGLENARLFFNVAFFVVLVSLLLQGTSLSWAAKKAKVVVPPVGRPVSRVGLDIHPENPWEQFVYQ

LSADKWCVGAALRDLHMPKETRIAALFRDNQLLHPTGSTRLREGDVLCVIGRERDLPALGKLFSQSPPVA

LDQRFFGDFILEASAKYADVALIYGLEDGREYRDKQQTLGEIVQQLLGAAPVVGDQVEFAGMIWTVAEKE

DNEVLKIGVRVAEEEAES

>gi|161486501|ref|NP_836924.2| hypothetical protein S1319 [Shigella flexneri 2a str. 2457T]

MSQLCPCGSAVEYSLCCHPYVSGEKVAPDPEHLMRSRYCAFVMQDADYLIKTWHPSCGAAALRAELMTGF

AHTEWLGLTVFEHCWQDADNIGFVSFVARFTEGGKTGAIIERSRFLKENGQWYYIDGTRPQFGRNDPCPC

GSGKKFKKCCGQ

>gi|161486500|ref|NP_836940.2| dsDNA-mimic protein [Shigella flexneri 2a str. 2457T]

MDMDLNNRLTEDETLEQAYDIFLELAADNLDPADVLLFNLQFEERGGAELFDPAEDWQEHVDFDLNPDFF

AEVVIGLADSEDGEISDVFARILLCREKDHKLCHIIWRE

>gi|161486499|ref|NP_836945.2| YciI-like protein [Shigella flexneri 2a str. 2457T]

MLYVIYAQDKADSLEKRLSVRPAHLARLQLLHDEGRLLTAGPMPAVDSNDPGAAGFTGSTVIAEFESLEA

AQAWADADPYVAAGVYEHVSVKPFKKVF

>gi|161486498|ref|NP_836994.2| phage shock protein operon transcriptional activator [Shigella flexneri 2a str. 2457T]

MAEYKDNLLGEANSFLEVLEQVSHLAPLDKPVLIIGERGTGKELIASRLHYLSSRWQGPFISLNCAALNE

NLLDSELFGHEAGAFTGAQKRHPGRFERADGGTLFLDELATAPMMVQEKLLRVIEYGELERVGGSQPLQV

NVRLVCATNADLPAMVNEGTFRADLLDRLAFDVVQLPPLRERESDIMLMAEHFAIQMCREIKLPLFPGFT

ERARETLLNYRWPGNIRELKNVVERSVYRHGTSDYPLDDIIIDPFKRRPPEEAIAVSENTSLPTLPLDLR

EFQMQQEKELLQLSLQQGKYNQKRAAELLGLTYHQFRALLKKHQI

>gi|161486497|ref|NP_837059.2| cytochrome b561 [Shigella flexneri 2a str. 2457T]

MENKYSRLQISIHWLVFLLVIAAYCAMEFRGFFPRSDRPLINMIHVSCGISILVLMVVRLLLRLKYPTPP

IIPKPKPMMTGLAHLGHLVIYLLFIALPVIGLVMMYNRGNPWFAFGLTMPYASEANFERVDSLKSWHETL

ANLGYFVIGLHAAAALAHHYFWKDNTLLRMMPRKRS

>gi|161486496|ref|NP_837085.2| carboxy-terminal protease [Shigella flexneri 2a str. 2457T]

MNMFFRLTALAGLLAIAGQTFAVEDITRADQIPVLKEETQHATVSERVTSRFTRSHYRQFDLDQAFSAKI

FDRYLNLLDYSHNVLLASDVEQFAKKKTELGDELRSGKLDVFYDLYNLAQKRRFERYQYALSVLEKPMDF

TGNDTYNLDRSKAPWPKNEAELNAQWDSKVKFDELSLKLTGKTDKEIRETLTRRYKFAIRRLAQTNSEDV

FSLAMTAFAREIDPHTNYLSPRNTEQFNTEMSLSLEGIGAVLQMDDDYTVINSMVAGGPAAKSKAISVGD

KIVGVGQTGKPMVDVIGWRLDDVVALIKGPKGSKVRLEILPAGKGTKTRTVTLTRERIRLEDRAVKMSVK

TVGKEKVGVLDIPGFYVGLTDDVKVQLQKLEKQNVSSVIIDLRSNGGGALTEAVSLSGLFIPAGPIVQVR

DNNGKVREDSDTDGQVFYKGPLVVLVDRFSASASEIFAAAMQDYGRALVVGEPTFGKGTVQQYRSLNRIY

DQMLRPEWPALGSVQYTIQKFYRVNGGSTQRKGVTPDIIMPTGNEETETGEKFEDNALPWDSIDAATYVK

SGDLTAFEPELLKEHNARIAKDPEFQNIMKDIARFNAMKDKRNIVSLNYAVREKENNEDDATRLARLNER

FKREGKPELKKLDDLSKDYQEPDPYLDETVNIALDLAKLEKARPAEQPAPVK

>gi|161486495|ref|NP_837140.2| nicotinamidase/pyrazinamidase [Shigella flexneri 2a str. 2457T]

MPHRALLLVDLQNDFCAGGALAVPEGDSTVEVANRLIDWCQSRGEAVIASQDWHPANHGSFASQHGVEPY

TPGQLGGLRQTFWPEHCVQNSEGAQLHPLLNQKEIAAVFHKGENPLVDSYSAFFDNGRRQKTALDDWLRD

HEIDELIVMGLATDYCVKFTVLDALQLGYKVNVITDGCRGVNIQPQDSAHAFMEMSAAGATLYTLADWEE

TQG

>gi|161486494|ref|NP_837146.2| insertion sequence 2 OrfA protein [Shigella flexneri 2a str. 2457T]

MIDVLGPEKRRRRTTQEKIAIVQQSFEPGMTVSLVARQHGVAASQLFLWRKQYQEGSLTAVAAGEQVVPA

SELAAAMKQIKELQRLLGKKTMENELLKEAVEYGRAKKWIAHAPLLPGDGE

>gi|161486493|ref|NP_837147.2| insertion element IS2 transposase InsD [Shigella flexneri 2a str. 2457T]

MDSARALIARGWGVSLVSRCLRVSRAQLHVILRRTDDWMDGRRSRHTDDTDVLLRIHHVIGELPTYGYRR

VWALLRRQAELDGMPAINAKRVYRLMRQNALLLERKPAVPPSKRAHTGRVAVKESNQRWCSDGFEFCCDN

GERLRVTFALDCCDREALHWAVTTGGFNSETVQDVMLGAVERRFGNDLPSSPVEWLTDNGSCYRANETRQ

FARMLGLEPKNTAVRSPESNGIAESFVKTIKRDYISIMPKPDGLTAAKNLAEAFEHYNEWHPHSALGYRS

PREYLRQRACNGLSDNRCLEI

>gi|161486492|ref|NP_837237.2| O-acetylserine/cysteine export protein [Shigella flexneri 2a str. 2457T]

MSRKDGVLALLVVVVWGLNFVVIKVGLHNMPPLMLAGLRFMLVAFPAIFFVARPKVPLNLLLGYGLTISF

AQFAFLFCAINFGMPAGLASLVLQAQAFFTIVLGAFTFGERLHGKQLAGIALAIFGVLVLIEDSLNGQHV

AMLGFMLTLAAAFSWACGNIFNKKIMSHSTRPAVMSLVIWSALIPIIPFFVVSLILDGSASMIHSLVTID

MTTILSLMYLAFVATIVGYGIWGTLLGRYETWRVAPLSLLVPVVGLASAALLLDERLTGLQFLGAVLIMT

GLYINVFGLRWRKAVKVGS

>gi|161486491|ref|NP_837240.2| DNA-binding transcriptional repressor MarR [Shigella flexneri 2a str. 2457T]

MKSTSDLFNEIIPLGRLIHMVNQKKDRLLNEYLSPLDITAAQFKVLCSIRCAACITPVELKKVLSVDLGA

LTRMLDRLVCKGWVERLPNPNDKRGVLVKLTTSGAAICEQCHQLVGQDLHQELTKNLTADEVATLEHLLK

KVLP

>gi|161486490|ref|NP_837252.2| insertion element IS2 transposase InsD [Shigella flexneri 2a str. 2457T]

MDSARALIARGWGVSLVSRCLRVSRAQLHVILRRTDDWMDGRRSRHTDDTDVLLRIHHVIGELPTYGYRR

VWALLRRQAELDGMPAINAKRVYRLMRQNALLLERKPAVPPSKRAHTGRVAVKESNQRWCSDGFEFCCDN

GERLRVTFALDCCDREVLHWAVTTGGFNSETVQDVMLGAVERRFGNDLPSSPVEWLTDNGSCYRANETRQ

FARMLGLEPKNTAVRSPESNGIAESFVKTIKRDYISIMPKPDGLTAAKNLAEAFEHYNEWHPHSALGYRS

PREYLRQRACNGLSDNRCLEI

>gi|161486489|ref|NP_837253.2| insertion sequence 2 OrfA protein [Shigella flexneri 2a str. 2457T]

MIDVLGPEKRRRRTTQEKIAIVQQSFEPGMTVSLVARQHGVAASQLFLWRKQYQEGSLTAVAAGEQVVPA

SELAAAMKQIKELQRLLGKKTMENELLKEAVEYGRAKKWIAHAPLLPGDGE

>gi|161486488|ref|NP_837258.2| insertion sequence 2 OrfA protein [Shigella flexneri 2a str. 2457T]

MIDVLGPEKRRRRTTQEKIAIVQQSFEPGMTVSLVARQHGVAASQLFLWRKQYQEGSLTAVAAGEQVVPA

SELAAAMKQIKELQRLLGKKTMENELLKEAVEYGRAKKWIAHAPLLPGDGE

>gi|161486487|ref|NP_837259.2| insertion element IS2 transposase InsD [Shigella flexneri 2a str. 2457T]

MDSARALIARGWGVSLVSRCLRVSRAQLHVILRRTDDWMDGRRSRHTDDTDVLLRIHHVIGELPTYGYRR

VWALLRRQAELDGMPAINAKRVYRLMRQNALLLERKPAVPPSKRAHTGRVAVKESNQRWCSDGFEFCCDN

GERLRVTFALDCCDREALHWAVTTGGFNSETVQDIMLGAVERRFGNDLPSSPVEWLTDNGSCYRANETRQ

FARMLGLEPKNTAVRSPESNGIAESFVKTIKRDYISIMPKPDGLTAAKNLAEAFEHYNEWHPHSALGYRS

PREYLRQRACNGLSDNRCLEI

>gi|161486486|ref|NP_837265.2| hypothetical protein S1722 [Shigella flexneri 2a str. 2457T]

MKYKLLPCLLAIFLTGCDRTEVTLSFTPEMASFSNEFDFDPLRGPVKDFTQTLMDEQGEVTKRVSGTLSE

EGCFDSLELLDLENNTVVALVLDANYYRDAETLEKRVRLQGKCQLAELPSAGVSWETDDNGFVIKASSKQ

MQMEYRYDDQGYPLGKTTKSNDKTLSVSATPSTDPIKKLDYTAVTLLNNQRVGNVKQSCEYDSHANPVDC

QLIIVDEGVKPAVERVYTIKNTIDYY

>gi|161486485|ref|NP_837278.2| voltage-gated ClC-type chloride channel ClcB [Shigella flexneri 2a str. 2457T]

MFRRLLIATVVGILAVFAVAGFRHAMLLLEWLFLNNDSGSLVNAATNLSSWRRLLTPALGGLAAGLLLMG

WQKFTQQRPHAPTDYMEALQTDGQFDYAASLVKSLASLLVVTSGSAIGREGAMILLAALAASCFAQRFTP

RQEWKLWIACGAAAGMAAAYRAPLAGSLFIAEVLFGTMMLASLGPVIISAIVAWLVSNLINHSDALLYNV

QLSVTVQARDYALIISTGVLAGLCGPLLLTLMNACHRGFVSLKLAPPWQLALGGLIVGLLSLFTPAVWGN

GYSTVQSFLTAPPLLMIIAGIFLCKLCAVLASSGSGAPGGVFTPTLFIGLAIGMLYGRSLGLWFPDGEEI

TLLLGLTGMATLLAATTHAPIMSTLMICEMTGEYQLLPGLLIACVIASVISRTLHRDSIYRQHTAQHS

>gi|161486484|ref|NP_837282.2| acid shock protein precursor [Shigella flexneri 2a str. 2457T]

MKKVLALVVAAAMGLSSAAFAAETATTPAPTATTTKAAPAKTTHHKKQHKAAPAQKAQAAKKHHKNTKAE

QKAPEQKAQAAKKHAGKHGHQQPAKPAAQPAA

>gi|161486483|ref|NP_837301.2| outer membrane porin protein [Shigella flexneri 2a str. 2457T]

MRKIVAMAVICLTAASGLTSAYAAQLADDEAGLRIRLKNELRRADKPSAGAGRDIYAWVQGGLLDFNSGY

YSNIVGVEGGAYYVYKLGARADMSTRWYLDGDKSFGFALGAVKIKPSENSLLKLGRFGTDYSYGSLPYRI

PLMVGSSQRTLPTVSEGALGYWALTPNIDLWGMWRSRVFLWTDSTTGIRDEGVYNSQTGKYDKHRARSFL

AASWYDDTSRYSLGASVQKDVSNQIQSILEKSIPLDPNYTLKGELLGFYAQLEGLSRNTSQPNETALVSG

QLTWNAPWGSVFGSGGYLRHAMNGAVVDTDIGYPFSLSLDRNREGMQSWQLGANYRVTPQFTLTFAPIVT

RGYESSKRDVRIEGAGILGGMNYRVSEGPLQGMNFFLAADKGREKRDGSTLGDRLNYWDVKMSIQYDFML

K

>gi|161486482|ref|NP_837310.2| oxidoreductase [Shigella flexneri 2a str. 2457T]

MSDNIRVGLIGYGYASKTFHAPLIAGTPGLELAVISSSDETKVKADWPTVTVVSEPKHLFNDPNIDLIVI

PTPNDTHFPLAKAALEAGKHVVVDKPFTVTLSQARELDALAKSLGRVLSVFHNRRWDSDFLTLKGLLAEG

VLGEVAYFESHFDRFRPQVRDRWREQGGPGSGIWYDLAPHLLDQAITLFGLPVSMTVDLAQLRPGAQSTD

YFHAILSYPQRRVILHGTMLAAAESARYIVHGSRGSYVKYGLDPQEERLKNGERLPQEDWGYDMRDGVLT

RVEGEERVEETLLTVPGNYPAYYAAIRDALNGDGENPVPASQAIQVMELIELGIESAKHRATLCLA

>gi|161486481|ref|NP_837326.2| insertion element IS2 transposase InsD [Shigella flexneri 2a str. 2457T]

MDSARALIARGWGVSLVSRCLRVSRAQLHVILRRTDDWMDGRRSRHTDDTDVLLRIHHVIGELPTYGYRR

VWALLRRQAELDGMPAINAKRVYRLMRQNALLLERKPAVPPSKRAHTGRVAVKESNQRWCSDGFEFCCDN

GERLRVTFALDCCDREALHWAVTTGGFNSETVQDVMLGAVERRFGNDLPSSPVEWLTDNGSCYRANETRQ

FARMLGLEPKNTAVRSPESNGIAESFVKTIKRDYISIMPKPDGLTAAKNLAEAFEHYNEWHPHSALGYRS

PREYLRQRACNGLSDNRCLEI

>gi|161486480|ref|NP_837327.2| insertion sequence 2 OrfA protein [Shigella flexneri 2a str. 2457T]

MIDVLGPEKRRRRTTQEKIAIVQQSFEPGMTVSLVARQHGVAASQLFLWRKQYQEGSLTAVAAGEQVVPA

SELAAAMKQIKELQRLLGKKTMENELLKEAVEYGRAKKWIAHAPLLPGDGE

>gi|161486479|ref|NP_837330.2| transcriptional regulator SlyA [Shigella flexneri 2a str. 2457T]

MESPLGSDLARLVRIWRALIDHRLKPLELTQTHWVTLHNIHQLPPDQSQIQLAKAIGIEQPSLVRTLDQL

EEKGLISRQTCASDRRAKRIKLTEKAEPLISEMEAVINKTRAEILHGISAEELEQLITLIAKLEHNIIEL

QAKG

>gi|161486478|ref|NP_837410.2| insertion element IS2 transposase InsD [Shigella flexneri 2a str. 2457T]

MDSARALIARGWGVSLVSRCLRVSRAQLHVILRRTDDWMDGRRSRHTDDTDVLLRIHHVIGELPTYGYRR

VWALLRRQAELDGMPAINAKRVYRLMRQNALLLERKPAVPPSKRAHTGRVAVKESNQRWCSDGFEFCCDN

GERLRVTFALDCCDREALHWAVTTGGFNSETVQDVMLGAVERRFGNDLPSSPVEWLTDNGSCYRANETRQ

FARMLGLEPKNTAVRSPESNGIAESFVKTIKRDYISIMPKPDGLTAAKNLAEAFEHYNEWHPHSALGYRS

PREYLRQRACNGLSDNRCLEI

>gi|161486477|ref|NP_837411.2| insertion sequence 2 OrfA protein [Shigella flexneri 2a str. 2457T]

MIDVLGPEKRRRRTTQEKIAIVQQSFEPGMTVSLVARQHGVAASQLFLWRKQYQEGSLTAVAAGEQVVPA

SELAAAMKQIKELQRLLGKKTMENELLKEAVEYGRAKKWIAHAPLLPGDGE

>gi|161486476|ref|NP_837439.2| high-affinity zinc transporter periplasmic component [Shigella flexneri 2a str. 2457T]

MLHKKTLLFAALSAALWGGATQAADAAVVASLKPVGFIASAIADGVTETEVLLPDGASEHDYSLRPSDVK

RLQNADLVVWVGPEMEAFMQKPVSKLPEAKQVTIAQLEDVKPLLMKSIHGDDDDHDHAEKSDEDHHHGDF

NMHLWLSPEIARATAVAIHGKLVELMPQSRAKLDANLKDFEAQLASTETQVGNELAPLKGKGYFVFHDAY

GYFEKQFGLTPLGHFTVNPEIQPGAQRLHEIRTQLVEQKATCVFAEPQFRPAVVESVARGTSVRMGTLDP

LGTNIKLGKTSYSEFLSQLANQYASCLKGD

>gi|161486475|ref|NP_837455.2| insertion element IS2 transposase InsD [Shigella flexneri 2a str. 2457T]

MDSARALIARGWGVSLVSRCLRVSRAQLHVILRRTDDWMDGRRSRHTDDTDVLLRIHHVIGELPTYGYRR

VWALLRRQAELDGMPAINAKRVYRLMRQNALLLERKPAVPPSKRAHTGRVAVKESNQRWCSDGFEFCCDN

GERLRVTFALDCCDREALHWAVTTGGFNSETVQDVMLGAVERRFGNDLPSSPVEWLTDNGSCYRANETRQ

FARMLGLEPKNTAVRSPESNGIAESFVKTIKRDYISIMPKPDGLTAAKNLAEAFEHYNEWHPHSALGYRS

PREYLRQRACNGLSDNRCLEI

>gi|161486474|ref|NP_837528.2| excinuclease ABC subunit C [Shigella flexneri 2a str. 2457T]

MSDQFDAKAFLKTVTSQPGVYRMYDAGGTVIYVGKAKDLKKRLSSYFRSNLASRKTEALVAQIQQIDVTV

THTETEALLLEHNYIKLYQPRYNVLLRDDKSYPFIFLSGDTHPRLAMHRGAKHAKGEYFGPFPNGYAVRE

TLALLQKIFPIRQCENSVYRNRSRPCLQYQIGRCLGPCVEGLVSEEEYAQQVEYVRLFLSGKDDQVLTQL

ISRMETASQNLEFEEAACIRDQIQAVRRVTEKQFVSNTGDDLDVIGVAFDAGMACVHVLFIRQGKVLGSR

SYFPKVPGGTELSEVVETFVGQFYLQGSQMRTLPGEILLDFNLSDKTLLADSLSELAGRKINVQTKPRGD

RARYLKLARTNAATALTSKLSQQSTVHQRLTALASVLKLPEVKRMECFDISHTMGEQTVASCVVFDANGP

LRAEYRRYNITGITPGDDYAAMNQVLRRRYGKAIDDSKIPDVILIDGGKGQLAQAKNVFAELDVSWDKNH

PLLLGVAKGADRKAGLETLFFEPEGEGFSLPPDSPALHVIQHIRDESHDHAIGGHRKKRAKVKNTSSLET

IEGVGPKRRQMLLKYMGGLQGLRNASVEEIAKVPGISQGLAEKIFWSLKH

>gi|161486473|ref|NP_837534.2| D-cysteine desulfhydrase [Shigella flexneri 2a str. 2457T]

MPLHNLTRFPRLEFIGAPTPLEYLPRFSDYLGREIFIKRDEVTPMAMGGNKLRKLEFLAADALREGADTL

ITAGAIQSNHVRQTAAVAAKLGLHCVALLENPIGTTAENYLTNGNRLLLDLFNTQIEMCDALTDPNAQLE

ELATRVEAQGFRPYVIPVGGSNALGALGYVESALEIAQQCEGAVNISSVVVASGSAGTHAGLAVGLEHLM

PESELIGVTVSRSVADQLPKVVNLQQAIAKELELTASAEILLWDDYFAPGYGVPNDEGMEAVKLLARLEG

ILLDPVYTGKAMAGLIDGISQKRFKDEGPILFIHTGGAPALFAYHPHV

>gi|161486472|ref|NP_837557.2| flagellar biosynthesis protein FliO [Shigella flexneri 2a str. 2457T]

MNNHATVQSSAPVSAEPLLQVSGALIAIIALILAAAWLVKRLGFAPKRTGVNGLKISASASLGARERVVV

VDVEDARLVLGVTAGQINLLHKLPPSAPTEEIPQTDFQSVMKNLLKRGGRS

>gi|161486471|ref|NP_837571.2| insertion element IS2 transposase InsD [Shigella flexneri 2a str. 2457T]

MDSARALIARGWGVSLVSRCLRVSRAQLHVILRRTDDWKDGRRSRHSDDTDVLLRIHHVIGELPTYGYRR

VWALLRRQAELDGMPAINAKRIYRLMRQNALLLERKLAVPPSKRAHTGRVAVKESNQRWCSDGFEFRCDN

GEKLRVTFALDCCDRKALHWAVTTGGFNSETVQDVMLGAVERRFGNDLPSSPVEWLTDNGSCYRANETRQ

FARMLGLEPKNTAVRSPESNGIAESFVKTIKRDYISIMPKPDGLTAAKNLAEAFEHYNEWHPHSALGYRS

PREYLRQRACNGLSDNRCLEI

>gi|161486470|ref|NP_837572.2| insertion sequence 2 OrfA protein [Shigella flexneri 2a str. 2457T]

MIDVLGPEKRRRRTTQEKIAIVQQSFEPGMTVSLVARQHGVAASQLFLWRKQYQEGSLTAVAAGEQVVPA

SELAAAMKQIKELQRLLGKKTMENELLKEAVEYGRAKKWIAHAPLLPGDGE

>gi|161486469|ref|NP_837680.2| tyrosine kinase [Shigella flexneri 2a str. 2457T]

MTEKVKQHAAPVTGSDEIDIGRLVGTVIEARWWVIGITVVFALCAVVYTFFATPIYSADALVQIEQNSGN

SLVQDIGSALANKPPASDAEIQLIRSRLVLGKTVDDLDLDIAVSKNTFPIFGAGWDRLMGRQNETVKVTT

FNRPKEMADQVFTLNVLDDKNYTLSSDGGFSARGQAGQMLKKEGVTLMVEAIHARPGSEFTVTKYSTLGM

INQLQNSLTVTENGKDAGVLSLTYTGEDREQIRDILNSIARNYQEQNIERKSAEASKSLAFLAQQLPEVR

SRLDVAENKLNAFRQDKDSVDLPLEAKAVLDSMVNIDAQLNELTFKEAEISKLYTKVHPAYRTLLEKRQA

LEEEKAKLNGRVTAMPKNQQEIVRLTRDVESGQQVYMQLLNKEQELKITEASTVGDVRIIDPAITQPGVL

KPKKGLIILGAIILGLMLSIVGVLLRSLFNRGIESPQVLEEHGISVYASIPLSEWQKARDSVKTIKGVKR

YKQSQLLAVGNPTDLAIEAIRSLRTSLHFAMMQAQNNVLMMTGVSPSIGKTFVCANLAAVISQTNKRVLL

IDCDMRKGYTHELLGTNNVNGLSEILIGQGDITTAAKPTSIAKFDLIPRGQVPPNPSELLMSERFAELVN

WASKNYDLVLIDTPPILAVTDAAIVGRHVGTTLMVARYAVNTLKEVETSLSRFEQNGIPVKGVILNSIFR

RASAYQDYGYYEYEYKSDAK

>gi|161486468|ref|NP_837752.2| D-alanyl-D-alanine endopeptidase [Shigella flexneri 2a str. 2457T]

MLIMPKFRVSLFSLALMLAVPFAPQAVAKTAAATTASQPEIASGSAMIVDLNTNKVIYSNHPDLVRPIAS

ISKLMTAMVVLDARLPLDEKLKVDISQTPEMKGVYSRVRLNSEISRKDMLLLALMSSENRAAASLAHHYP

GGYKAFIKAMNAKAKSLGMNNTRFVEPTGLSVHNVSTARDLTKLLIASKQYPLIGQLSTTREDMATFSNP

TYTLPFRNTNHLVYRDNWNIQLTKTGFTNAAGHCLVMRTVINNKPVALVVMDAFGKYTHFADASRLRTWI

ETGKVMPVPAAALSYKKQKAAQMAAAGQTAQND

>gi|161486467|ref|NP_837785.2| elongation factor P [Shigella flexneri 2a str. 2457T]

MPRANEIKKGMVLNYNGKLLLAKDIDIQSPTARGAATLYKMRFSDVRTGLKVEERFKGDDIVDTVTLTRR

YVDFSYVDGNEYVFMDKEDYTPYTFTKDQIEEELLFMPEGGMPDMQVLTWDGQLLALELPQTVDLEIVET

APGIKGASASARNKPATLSTGLVIQVPEYLSPGEKIRIHIEERRYMGRAD

>gi|161486466|ref|NP_837838.2| insertion element IS2 transposase InsD [Shigella flexneri 2a str. 2457T]

MDSARALIARGWGVSLVSRCLRVSRAQLHVILRRTDDWMDGRRSRHTDDTDVLLRIHHVIGELPTYGYRR

VWALLRRQAELDGMPAINAKRVYRLMRQNALLLERKPAVPPSKRAHTGRVAVKESNQRWCSDGFEFCCDN

GERLRVTFALDCCDREALHWAVTTGGFNSETVQDVMLGAVERRFGNDLPSSPVEWLTDNGSCYRANETRQ

FARMLGLEPKNTAVRSPESNGIAESFVKTIKRDYISIMPKPDGLTAAKNLAEAFEHYNEWHPHSALGYRS

PREYLRQRACNGLSDNRCLEI

>gi|161486465|ref|NP_837840.2| ribonucleotide-diphosphate reductase subunit beta [Shigella flexneri 2a str. 2457T]

MAYTTFSQTKNDQLKEPMFFGQPVNVARYDQQKYDIFEKLIEKQLSFFWRPEEVDVSRDRIDYQALPEHE

KHIFISNLKYQTLLDSIQGRSPNVALLPLISIPELETWVETWAFSETIHSRSYTHIIRNIVNDPSVVFDD

IVTNEQIQKRAEGISSYYDELIEMTSYWHLLGEGTHTVNGKTVTVSLRELKKKLYLCLMSVNALEAIRFY

VSFACSFAFAERELMEGNAKIIRLIARDEALHLTGTQHMLNLLRSGADDPEMAEIAEECKQECYDLFVQA

AQQEKDWADYLFRDGSMIGLNKDILCQYVEYITNIRMQAVGLDLPFQTRSNPIPWINTWLVSDNVQVAPQ

EVEVSSYLVGQIDAEVDTDDLSNFQL

>gi|161486464|ref|NP_837855.2| UDP-4-amino-4-deoxy-L-arabinose--oxoglutarate aminotransferase [Shigella flexneri 2a str. 2457T]

MSEFLPFSRPAMGVEELAAVKEVLESGWITTGPKNQALEQAFCQLTGNQHAIAVSSATAGMHITLMALEI

GKGDEVITPSLTWVSTLNMISLLGATPVMVDVDRDTLMVTPEAIESAITPRTKAIIPVHYAGAPADIDAI

RAIGERYGIAVIEDAAHAVGTYYKGRHIGAKGTAIFSFHAIKNITCAEGGLIVTDNENLARQLRMLKFHG

LGVDAYDRQTWGRAPQAEVLTPGYKYNLTDINAAIALTQLVKLEHLNTRRREIAQQYQQALAALPFQPLS

LPAWPHVHAWHLFIIRVDEQRCGISRDALMEALKERGIGTGLHFRAAHTQKYYRERFPTLSLPNTEWNSE

RICSLPLFPDMTTADADRVITALQQLAGQ

>gi|161486463|ref|NP_837861.2| hypothetical protein S2471 [Shigella flexneri 2a str. 2457T]

MCLIWGLFSVIIASVAQLSLGFAASHLPPMTHLWDFIAALLAFGLDARILLLGLLGYLLSVFCWYKTLHK

LALSKAYALLSMSYVLVWIASMVLPGWEGTFSLKALLGVACIMSGLMLIFLPTTKQRY

>gi|161486462|ref|NP_837867.2| menaquinone-specific isochorismate synthase [Shigella flexneri 2a str. 2457T]

MQSLTTALENLLRHLSQEIPATPGIRVIDIPFPLKDAFDALSWLASQQTYPQFYWQQRNGDEEAAVLGAI

TRFTSLDQAQRFLRQHPEHADLRIWGLNAFDPSQGNLLLPRLEWRRCGGKATLRLTLFSESSLQHDAIQA

KEFIATLVSIKPLPGLHLTTTREQHWPDKTGWTQLIELATKTIAEGELDKVVLARATDLHFASPVNAAAM

MAASRRLNLNCYHFYMAFDGENAFLGSSPERLWRRRDKALRTEALAGTVANHPDDKQAQQLGEWLMADDK

NQRENMLVVEDICQRLQADTQTLDVLPPQVLRLRKVQHLRRCIWTSLNKADDVICLHQLQPTAAVAGLPR

DLARQFIARHEPFTREWYAGSAGYLSLQQSEFCVSLRSAKISGNVVRLYAGAGIVRGSDPEQEWQEIDNK

AAGLRTLLQME

>gi|161486461|ref|NP_837870.2| ribonuclease Z [Shigella flexneri 2a str. 2457T]

MELIFLGTSAGVPTRTRNVTAILLNLQHPTQSGLWLFDCGEGTQHQLLHTAFNPGKLDKIFISHLHGDHL

FGLPGLLCSRSMSGIIQPLTIYGPQGIREFVETALRISGSWTDYPLEIVEIGAGEILDDGLRKVTAYPLE

HPLECYGYRIEEHDKPGALNAQALKAAGVPPGPLFQELKAGKTITLEDGRQINGADYLAAPVPGKALAIF

GDTGPCDAALDLAKGVDVMVHEATLDITMEAKANSRGHSSTRQAATLAREAGVGKLIITHVSSRYDDKGC

QHLLRECRSIFPATELANDFTVFNV

>gi|161486460|ref|NP_837873.2| NADH dehydrogenase subunit N [Shigella flexneri 2a str. 2457T]

MTITPQNLIALLPLLIVGLTVVVVMLSIAWRRNHFLNATLSVIGLNAALVSLWFVGQAGAMDVTPLMRVD

GFAMLYTGLVLLASLATCTFAYPWLEGYNDNKDEFYLLVLIAALGGILLANANHLASLFLGIELISLPLF

GLVGYAFRQKRSLEASIKYTILSAAASSFLLFGMALVYAQSGDLSFVALGKNLGDGMLNEPLLLAGFGMM

IVGLGFKLSLVPFHLWTPDVYQGAPAPVSTFLATASKIAIFGVVMRLFLYAPVGDSEAIRVVLAIIAFAS

IIFGNLMALSQTNIKRLLGYSSISHLGYLLVALIALQTGEMSMEAVGVYLAGYLFSSLGAFGVVSLMSSP

YRGPDADSLFSYRGLFWHRPILAAVMTVMMLSLAGIPMTLGFIGKFYVLAVGVQAHLWWLVGAVVVGSAI

GLYYYLRVAVSLYLHAPEQPGRDAPSNWQYSAGGIVVLISALLVLVLGVWPQPLISIVRLAMPLM

>gi|161486459|ref|NP_837895.2| hypothetical protein S2509 [Shigella flexneri 2a str. 2457T]

MSAITESKPTRRWAMPDTLVIIFFVAILTSLATWVVPVGMFDSQEVQYQVDGQTKTRKVVDPHSFRILTN

EAGEPEYHRVQLFTTGDERPGLMNFPFEGLTSGSKYGTAVGIIMFMLVIGGAFGIVMRTGTIDNGILALI

RHTRGNEILFIPALFILFSLGGAVFGMGEEAVAFAIIIAPLMVRLGYDSITTVLVTYIATQIGFASSWMN

PFCVVVAQGIAGVPVLSGSGLRIVVWVIATLIGLIFTMVYASRVKKNPLLSRVHESDRFFREKQADVEQR

PFTFGDWLVLIVLTAVMVWVIWGVIVNAWFIPEIASQFFTMGLVIGIIGVVFRLNGMTVNTMASSFTEGA

RMMIAPALLVGFAKGILLLVGNGEAGNASVLNTILNSIANAISGLDNAVAAWFMLLFQAVFNFFVTSGSG

QAALTMPLLAPLGDLVGVNRQVTVLAFQFGDGFSHIIYPTSASLMATLGVCRVDFRNWLKVGATLLGLLF

IMSSVVVIGAQLMGYH

>gi|161486458|ref|NP_837911.2| hypothetical protein S2525 [Shigella flexneri 2a str. 2457T]

MASKFQNRLVGTIVLVALGVIVLPGLLDGQKKHYQDEFAAIPLVPKAGDRDEPDMMPAATQALPTQPPEG

AAEEVRAGDAAAPSLDPATIAANNTEFEPEPAPVAPPKPKPVEPPKPKVEVPPAPKPEPKPVVEEKAAPT

GKAYVVQLGALKNADKVNEIVGKLRGAGYRVYTSPSTPVQGKITRILVGPDASKDKLKGSLGELKQLSGL

SGVVMGYTPN

>gi|161486457|ref|NP_837921.2| 5-methylaminomethyl-2-thiouridine methyltransferase [Shigella flexneri 2a str. 2457T]

MKHYSIQPANLEFNAEGTPVSRDFDDVYFSNDNGLEETRYVFLGGNQLEVRFPEHPHPLFVIAESGFGTG

LNFLTLWKAFDQFREAHPQAQLQRLHFISFEKFPLTRADLALAHQHWPELAPWAEQLQAQWPMPLPGCHR

LLLDESRVTLDLWFGDINELTSQLDDSLNQKVDAWFLDGFAPAKNPDMWTQNLFNAMARLARPGGTLATF

TSAGFVRRGLQEAGFTMQKRKGFGRKREMLCGVMEQTLPLPCSTPWFNRTGSSKREVAIIGGGIASALLS

LALLRRGWQVALYCADEAPALGASGNRQGALYPLLSKHDEALNRFFSNAFTFARRFYDQLPVKFDHDWCG

VTQLGWDEKSQHKIAQMLSMDLPAELAVAVEANAVEQITGVATNCSGITYPQGGWLCPAELTRNVLELAQ

QQGLQIYYQYQLQNLSRKDDCWLLNFAGDQQATHSVVVLANGHQISRFSQTSTLPVYSVAGQVSHIPTTP

ELAELKQVLCYDGYLTPQNPANQHHCIGASYHRGSEDTAYSEDDQQQNRQRLIDCFPQAQWAKEVDVSDK

EARCGVRCATRDHLPMVGNVPDYEATLVEYASLAEQKDEAVSAPVFDDLFMFAALGSRGLCSAPLCAEIL

AAQMSDEPIPMDASTLAALNPNRLWVRKLLKGKAVKAG

>gi|161486456|ref|NP_837949.2| hypothetical protein S2575 [Shigella flexneri 2a str. 2457T]

MSFHLRLFSRHKQMTNNESKGPFEGLLVIDMTHVLNGPFGTQLLYNMGARVIKVEPPGHGDDTRTFGPYV

DGQSLYYSFINHGKESVVLDLKNDHDKSIFINMLKQADVLAENFRLGTMEKLGFSWERLQEINPRLIYAS

SSGFGHTGPLKDAPAYDTIIQAMSGIMMETGYPDAPPVRVGTSLADLCGGVYLFSGIVSALYGREKSQRG

AHVDIAMFDATLSFLEHGLMAYIATGKSPQRLGNRHPYMAPFDVFDTQDKPITICCGNDKLFSALCQALE

LTELVNDPRFSSNILRVQNQAILKQYIERTLKTQAAEVWLAKIHEVGVPVAPLLSVAEAINLPQTQARNM

LIEAGGIMMPGNPIKISGCADPHVMPGAATLDQHGEQIRQEFSS

>gi|161486455|ref|NP_837955.2| lipid A biosynthesis palmitoleoyl acyltransferase [Shigella flexneri 2a str. 2457T]

MFPQCKFSREFLHPRYWLTWFGLGVLWLWVQLPYPVLCFLGTRIGAMARPFLKRRESIARKNLELCFPQH

SAEEREKMIAENFRSLGMALVETGMAWFWPDSRVRKWFDVEGLDNLKRAQMQNRGVMVVGVHFMSLELGG

RVMGLCQPMMATYRPHNNQLMEWVQTRGRMRSNKAMIGRNNLRGIVGALKKGEAVWFAPDQDYGRKGSSF

APFFAVENVATTNGTYVLSRLSGAAMLTVTMVRKADYSGYRLFITPEMEGYPTDENQAAAYMNKIIEKEI

MRAPEQYLWIHRRFKTRPVGESSLYI

>gi|161486454|ref|NP_838118.2| hypothetical protein S2779 [Shigella flexneri 2a str. 2457T]

MATHERRVVFFDLDGTLHQQDMFGSFLRYLLRRQPLNALLVLPLLPIIAIALLIKGRAARWPMSLLLWGC

TFGHSEARLQTLQADFVRWFRDNVTAFPLVQERLTTYLLSSDADIWLITGSPQPLVEAVYFDTPWLPRVN

LIASQIQRGYGGWVLTMRCLGHEKVAQLERKIGTPLRLYSGYSDSNQDNPLLYFCQHRWRVTPRGELQQL

E

>gi|161486453|ref|NP_838119.2| DNA-binding transcriptional regulator [Shigella flexneri 2a str. 2457T]

MNGLLRIRQRYQGLAQSDKKLADYLLLQPDTARHLSSQQLANEAGVSQSSVVKFAQKLGYKGFPALKLAL

SEALASQPESPSVPIHNQIRGDDPLRLVGEKLIKENTAAMYATLNVNSEEKLHECVTMLRSARRIILTGI

GASGLVAQNFAWKLMKIGFNAAALRDMHALLATVQASSPDDLLLAISYTGVRRELNLAADEMLRVGGKVL

AITGFTPNALQQRASHCLYTIAEEQATNSASISACHAQGMLTDLLFIALIQQDLELAPERIRHSEALVKK

LV

>gi|161486452|ref|NP_838126.2| insertion sequence 2 OrfA protein [Shigella flexneri 2a str. 2457T]

MIDVLGPEKRRRRTTQEKIAIVQQSFEPGMTVSLVARQHGVAASQLFLWRKQYQEGSLTAVAAGEQVIPA

SELAAAMKQIKELQRLLGKKTMENELLKEAVEYGRAKKWIAHAPLLPGDGE

>gi|161486451|ref|NP_838127.2| insertion element IS2 transposase InsD [Shigella flexneri 2a str. 2457T]

MDSARALIARGWGVSLVSRCLRVSRAQLHVILRRTDDWMDGRRSRHTDDTDVLLRIHHVIGELPTYGYRR

VWALLRRQAELDGMPAINAKRVYRLMRQNALLLERKPAVPPSKRAHTGRVAVKESNQRWCSDGFEFCCDN

GERLRVTFALDCCDREALHWAVTTGGFNSETVQDVMLGAVERRFGNDLPSSPVEWLTDNGSCYRANETRQ

FARMLGLEPKNTAVRSPESNGIAESFVKTIKRDYISIMPKPDGLTAAKNLAEAFEHYNEWHPHSALGYRS

PREYLRQRACNGLSDNRCLEI

>gi|161486450|ref|NP_838197.2| hypothetical protein S2871 [Shigella flexneri 2a str. 2457T]

MNALTAVQNNAVDSGQDYSGFTLIPSAQSPRLLELTFTEQTTKQFLEQVAEWPVQALEYKSFLRFRVGKI

LDDLCANQLQPLLLKTLLNRAEGALLINAVGIDDVAQADEMVKLATAVAHLIGRSNFDAMSGQYYARFVV

KNVDNSDSYLRQPHRVMELHNDGTYVEEITDYVLMMKIDDWEHLDHYFRHPLARRPMRFAAPPSKNVSKD

VFHPVFDVDQQGRPVMRYIDQFVQPKDFEEGVWLSELSDAIETSKGILSVPVPVGKFLLINNLFWLHGRD

RFTPHPDLRRELMRQRGYFAYATHHYQTHQ

>gi|161486449|ref|NP_838204.2| insertion element IS2 transposase InsD [Shigella flexneri 2a str. 2457T]

MDSARALIARGWGVSLVSRCLRVSRAQLHVILRRTDDWMDGRRSRHTDDTDVLLRIHHVIGELPTYGYRR

VWALLRRQAELDGMPAINAKRVYRLMRQNALLLERKPAVPPSKRAHTGRVAVKESNQRWCSDGFEFCCDN

GERLRVTFALDCCDREALHWAVTTGGFNSETVQDVMLGAVERRFGNDLPSSPVEWLTDNGSCYRANETRQ

FARMLGLEPKNTAVRSPESNGIAESFVKTIKRDYISIMPKPDGLTAAKNLAEAFEHYNEWHPHSALGYRS

PREYLRQRACNGLSDNRCLEI

>gi|161486448|ref|NP_838205.2| insertion sequence 2 OrfA protein [Shigella flexneri 2a str. 2457T]

MIDVLGPEKRRRRTTQEKIAIVQQSFEPGMTVSLVARQHGVAASQLFLWRKQYQEGSLTAVAAGEQVVPA

SELAAAMKQIKELQRLLGKKTMENELLKEAVEYGRAKKWIAHAPLLPGDGE

>gi|161486447|ref|NP_838238.2| D-arabinose 5-phosphate isomerase [Shigella flexneri 2a str. 2457T]

MSEALLNTGRQTLMLELQEASRLPERLGDDFVRAANIILHCEGKVVVSGIGKSGHIGKKIAATLASTGTP

AFFVHPAEALHGDLGMIESRDVMLFISYSGGAKELDLIIPRLEDKSIALLAMTGKPTSPLGLAAKAVLDI

SVEREACPMHLAPTSSTVNTLMMGDALAMAVMQARGFNEEDFARSHPAGALGARLLNKVHHLMRRDDAIP

QVALTASVMDAMLELSRTGLGLVAVCDAQQQVQGVFTDGDLRRWLVGGGALTTPVNEAMTTGGTTLQAQS

RAIDAKEVLMKRKITAAPVVDENGKLTGAINLQDFYQAGII

>gi|161486446|ref|NP_838239.2| anaerobic nitric oxide reductase transcriptional regulator [Shigella flexneri 2a str. 2457T]

MSFSVDVLANIAIELQRGIGHQDRFQRLITTLRQVLECDASALLRYDSRQFIPLAIDGLAKDVLGRRFAL

EGHPRLEAIARAGDVVRFPADSELPDPYDGLIPGQESLKVHACVGLPLFVGQNLIGALTLDGMQPDQFDV

FSDEELRLIAALAAGALSNALLIEQLESQNMLPGDATPFEAVKQTQMIGLSPGMTQLKKEIEIVAASDLN

VLISGETGTGKELVAKAIHEASPRAVNPLVYLNCAALPESVAESELFGHVKGAFTGAISNRSGKFEMADN

GTLFLDEIGELSLALQAKLLRVLQYGDIQRVGDDRSLRVDVRVLAATNRDLREEVLAGRFRADLFHRLSV

FPLSVPPLRERGDDVILLAGYFCEQCRLRQGLSRVVLSAGARNLLQHYSFPGNVRELEHAIHRAVVLARA

TRNGDEVILEAQHFAFPEVTLPPPEAAAVPVVKQNLREATEAFQRETIRQALAQNHHNWAACARMLETDV

ANLHRLAKRLGLKD

>gi|161486445|ref|NP_838337.2| hypothetical protein S3032 [Shigella flexneri 2a str. 2457T]

MNREKGVSSLALVLMLLVLGSLLLQGMSQQDRSFASRVSMESQSLRRQAIVQSALAWGKMHSWQTQPAVQ

CSQYAGTDAQVCLRLLADNEALLIAGYEGVSLWRTGEVIDGKIVFSPRGWSDFCPLKEGALCQLP

>gi|161486444|ref|NP_838399.2| methylmalonyl-CoA decarboxylase [Shigella flexneri 2a str. 2457T]

MSYQYVNVATINKVAVIEFNYGRKLNALSKVFIDDLMQALSDLNRPEIRCIILRAPSGSKVFSAGHDIHE

LPSGGRDPLSYDDPLRQITRMIQKFPKPIISMVEGSVWGGAFEMIMSSDLIIAASTSTFSMTPVNLGVPY

NLVGIHNLTRDAGFHIVKELIFTASPITAQRALAVGILNHVVEVEELEDFTLQMAHHISEKAPLAIAVIK

EELRVLGEAHTMNSDEFERIQGMRRAVYDSEDYQEGMNAFLEKRKPNFVGH

>gi|161486443|ref|NP_838435.2| hypothetical protein S3143 [Shigella flexneri 2a str. 2457T]

MNLQHHFLIAMPALQDPIFRRSVVYICEHNTNGAMGIIVNKPLENLKIEGILEKLKITPEPRDESIRLDK

PVMLGGPLAEDRGFILHTPPSNFASSIRISDNTVMTTSRDVLETLGTDKQPSDVLVALGYASWEKGQLEQ

EILDNAWLTAPADLNILFKTPIADRWREAAKLIGVDILTMPGVAGHA

>gi|161486442|ref|NP_838440.2| hypothetical protein S3148 [Shigella flexneri 2a str. 2457T]

MSAVTVNDDGLVLRLYIQPKASRDSIVGLHGDEVKVAITAPPVDGQANSHLVKFLGKQFRVAKSQVVIEK

GELGRHKQIKIINPQQIPPEIAALIN

>gi|161486441|ref|NP_838451.2| hypothetical protein S3159 [Shigella flexneri 2a str. 2457T]

MAKNRSRRLRKKMHIDEFQELGFSVAWRFPEGTSEEQIDKIVDDFINEVIEPNKLAFDGSGYLAWEGLIC

MQEIGKCTEEHQAIVRKWLEECKLDEVRTSELFDVWWD

>gi|161486440|ref|NP_838471.2| insertion sequence 2 OrfA protein [Shigella flexneri 2a str. 2457T]

MIDVLGPEKRRRRTTQEKIAIVQQSFEPGMTVSLVARQHGVAASQLFLWRKQYQEGSLTAVAAGEQVVPA

SELAAAMKQIKELQRLLGKKTMENELLKEAVEYGRAKKWIAHAPLLPGDGE

>gi|161486439|ref|NP_838472.2| insertion element IS2 transposase InsD [Shigella flexneri 2a str. 2457T]

MDSARALIARGWGVSLVSRCLRVSRAQLHVILRRTDDWMDGRRSRHTDDTDVLLRIHHVIGELPTYGYRR

VWALLRRQAELDGMPAINAKRVYRLMRQNALLLERKPAVPPSKRAHTGRVAVKESNQRWCSDGFEFCCDN

GERLRVTFALDCCDREALHWAVTTGGFNSETVQDVMLGAVECRFGNDLPSSPVEWLTDNGSCYRANETRQ

FARMLGLEPKNTAVRSPESNGIAESFVKTIKRDYISIMPKPDGLTAAKNLAEAFEHYNEWHPHSALGYRS

PREYLRQRACNGLSDNRCLEI

>gi|161486438|ref|NP_838537.2| 2,5-diketo-D-gluconate reductase A [Shigella flexneri 2a str. 2457T]

MANPTVIKLQDGNVMPQLGLGVWQASNEEVITAIQKALEVGYRSIDTAAAYKNEEGVGKALKNASVNREE

LFITTKLWNDDHKRPREALLDSLKKLQLDYIDLYLMHWPVPAIDHYVEAWKGMIELQKEGLIKSIGVCNF

QIHHLQRLIDETGVTPVINQIELHPLMQQRQLHAWNATHKIQTESWSPLAQGGKGVFDQKVIRDLADKYG

KTPAQIVIRWHLDSGLVVIPKSVTPSRIAENFDVWDFRLDKDELGKIAKLDQGKRLGPDPDQFGG

>gi|161486437|ref|NP_838558.2| hypothetical protein S3282 [Shigella flexneri 2a str. 2457T]

MKRTKSIRHASFRKNWSARHLTPVALAVATVFMLAGCEKSDETVSLYQNADDCSAANPGKSAECTTAYNN

ALKEAERTAPKYATREDCVAEFGEGQCQQAPAQAGMAPENQAQAQQSSGSFWMPLMAGYMMGRLMGGGAG

FAQQPLFSSKNPASPAYGKYTDATGKNYGAAQPGRTMTVPKTAMAPKPATTTTVTRGGFGESVAKQSTMQ

RSATGTSSRSMGG

>gi|161486436|ref|NP_838564.2| insertion sequence 2 OrfA protein [Shigella flexneri 2a str. 2457T]

MIDVLGPEKRRRRTTQEKIAIVQQSFEPGMTVSLVARQHGVAASQLFLWRKQYQEGSLTAVAAGEQVVPA

SELAAAMKQIKELQRLLGKKTMKNELLKEAVEYGRAKKWIAHAPLLPGDGE

>gi|161486435|ref|NP_838565.2| insertion sequence 2 OrfB protein [Shigella flexneri 2a str. 2457T]

MDSARALIARGWGVSLVSRCLRVSRAQLHVILRRTDDWMDGRRSRHTDDTDVLLRIHHVIGELPTYGYRR

VWALLRRQAELDGMPAINAKRVYRLMRQNALLLERKPAVPPSKRAHTGRVAVKESNQRWCSDGFEFCCDN

GERLRVTFALDCCDREALHWAVTTGGFNSETVQDVMLGAVERRFGNDLPSSPVEWLTDNGSCYRANETRQ

FARMLGLEPKNTAVRSPESNGIAESFVKTIKRDYISIMPKPDGLTAAKNLAEAFEHYNEWHPHSALGYRS

PRG

>gi|161486434|ref|NP_838589.2| putrescine--2-oxoglutarate aminotransferase [Shigella flexneri 2a str. 2457T]

MIREPPEHILNRLPSSASALACSAHALNLIEKRTLDHEEMKALNREVIEYFKEHVNPGFLEYRKSVTAGG

DYGAVEWQAGGLNTLVDTQGQEFIDCLGGFGIFNVGHRNPVVVSAVQNQLAKQPLHSQELLDPLRAMLAK

TLAALTPGKLKYSFFCNSGTESVEAALKLAKAYQSPRGKFTFIATSGVFHGKSLGALSATAKSTFRKPFM

PLLPGFRHVPFGNIEAMRTALNECKKTGDDVAAVILEPIQGEGGVILPPPGYLTAVRKLCDEFGALMILD

EVQTGMGRTGKMFACEHENVQPDILCLAKALGGGVMPIGATIATEEVFSVLFDNPFLHTTTFGGNPLACA

AALATINVLLEQNLPAQAEQKGDMLLDGFRQLAREYPDLVQEARGKGMLMAIEFVDNEIGYNFASEMFRQ

RVLVAGTLNNAKTIRIEPPLTLTIEQCELVIKAARKALAAMRVSVEEA

>gi|161486433|ref|NP_838629.2| propionate/acetate kinase [Shigella flexneri 2a str. 2457T]

MRKEMNEFPVVLVINCGSSSIKFSVLDASDCEVLMSGIADGINSENAFLSVNGGEPAPLAHHSYEGALKA

IAFELEKRNLNDSVALIGHRIAHGGSIFTESAIITDEVIDNIRRVSPLAPLHNYANLSGIESAQQLFPGV

TQVAVFDTSFHQTMAPEAYLYGLPWKYYEELGVRRYGFHGTSHRYVSQRAHSLLNLAEDDSGLVVAHLGN

GASICAVRNGQSVDTSMGMTPLEGLMMGTRSGDVDFGAMSWVASQTNQSLGDLERVVNKESGLLGISGLS

SDLRVLEKAWHEGHERAQLAIKTFVHRIARHIAGHAASLRRLDGIIFTGGIGENSSLIRRLVMEHLAVLG

VEIDTEMNNRSNSFGERIVSSENAHVICVVIPTNEEKMIALDAIHLGKVNAPAEFA

>gi|161486432|ref|NP_838635.2| glycerate kinase [Shigella flexneri 2a str. 2457T]

MKIVIAPDSYKESLSASEVAQAIEKGFREIFPDAQYVSIPVADGGEGTVEAMIAATQGSERHAWVTGPLG

EKVNASWGISGDGKTAFIEMAAASGLELVPAEKRDPLVTTSRGTGELILQALESGATNIIIGIGGSATND

GGAGMVQALGAKLCDANGNEIGFGGGSLNTLNDIDISGLDPRLKDCVIRVACDVTNPLVGDNGASRIFGP

QKGASEAMIVELDNNLSHYADVIKKALHVDVKDVPGAGAAGGMGAALMAFLGAELKSGIEIVTTALNLEE

HIHDCTLVITGEGRIDSQSIHGKVPIGVANVAKKYHKPVIGIAGSLTDDVGVVHQHGIDAVFSVLTSIGT

LDEAFRGAYDNICRASRNIAATLAIGMRNTG

>gi|161486431|ref|NP_838636.2| tartronate semialdehyde reductase [Shigella flexneri 2a str. 2457T]

MTMKVGFIGLGIMGKPMSKNLLKAGYSLVVADRNPEAIADVIAAGAETASTAKAIAEQCDVIITMLPNSP

HVKEVALGENGIIEGAKPGTVLIDMSSIAPLASREISEALKAKGIDMLDAPVSGGEPKAIDGTLSVMVGG

DKAIFDKYYDLMKAMAGSVVHTGAIGAGNVTKLANQVIVALNIAAMSEALTLATKAGVNPDLVYQAIRGG

LAGSTVLDAKAPMVMDRNFKPGFRIDLHIKDLANALDTSHGVGAQLPLTAAVMEMMQALRADGLGTADHS

ALVCYYEKLAKVEVTR

>gi|161486430|ref|NP_838655.2| insertion sequence 2 OrfA protein [Shigella flexneri 2a str. 2457T]

MIDVLGPEKRRRRTTQEKIAIVQQSFEPGMTVSLVARQHGVAASQLFLWRKQYQEGSLTAVAAGEQVVPA

SELAAAMKQIKELQRLLGKKTMENELLKEAVEYGRAKKWIAHAPLLPGDGE

>gi|161486429|ref|NP_838673.2| ATP-dependent RNA helicase DeaD [Shigella flexneri 2a str. 2457T]

MAEFETTFADLGLKAPILEALNDLGYEKPSPIQAECIPHLLNGRDVLGMAQTGSGKTAAFSLPLLQNLDP

ELKAPQILVLAPTRELAVQVAEAMTDFSKHMRGVNVVALYGGQRYDVQLRALRQGPQIVVGTPGRLLDHL

KRGTLDLSKLSGLVLDEADEMLRMGFIEDVETIMAQIPEGHQTALFSATMPEAIRRITRRFMKEPQEVRI

QSSVTTRPDISQSYWTVWGMRKNEALVRFLEAEDFDAAIIFVRTKNATLEVAEALERNGYNSAALNGDMN

QALREQTLERLKDGRLDILIATDVAARGLDVERISLVVNYDIPMDSESYVHRIGRTGRAGRAGRALLFVE

NRERRLLRNIERTMKLTIPEVELPNAELLGKRRLEKFAAKVQQQLESSDLDQYRALLSKIQPTAEGEELD

LETLAAALLKMAQGERTLIVPPDAPMRPKREFRDRDDRGPRDRNDRGPRGDREDRPRRERRDVGDMQLYR

IEVGRDDGVEVRHIVGAIANEGDISSRYIGNIKLFASHSTIELPKGMPGEVLQHFTRTRILNKPMNMQLL

GDAQPHTGGERRGGGRGFGGERREGGRNFSGERREGGRGDGRRFSGERREGRAPRRDDSTGRRRFGGDA

>gi|161486428|ref|NP_838681.2| hypothetical protein S3428 [Shigella flexneri 2a str. 2457T]

MSTLEQKLTEMITAPVEALGFELVGIEFIRGRTSTLRIYIDSEDGINVDDCADVSHQVSAVLDVEDPITV

AYNLEVSSPGLDRPLFTAEHYARFVGEEVTLVLRMAVQNRRKWQGVIKAVDGEMITVTVEGKDEVFALSN

IQKANLVPHF

>gi|161486427|ref|NP_838686.2| dihydropteroate synthase [Shigella flexneri 2a str. 2457T]

MKLFAQGTSLDLSHPHVMGILNVTPDSFSDGGTHNSLIDAVKHANLMINAGATIIDVGGESTRPGAAEVS

VEEELQRVIPVVEAIAQRFEVWISVDTSKPEVIRESAKVGAHIINDIRSLSEPGALEAAAETGLPVCLMH

MQGNPKTMQEAPKYDDVFAEVNRYFIEQIARCEQAGIAKEKLLLDPGFGFGKNLSHNYSLLARLAEFHHF

NLPLLVGMSRKSMIGQLLNVGPSERLSGSLACAVIAAMQGAHIIRVHDVKETVEAMRVVEATLSAKENKR

YE

>gi|161486426|ref|NP_838720.2| glutamate synthase [Shigella flexneri 2a str. 2457T]

MTRKPRRHALSVPVRSGSEVGFPQSLGEVHDMLYDKSLERDNCGFGLIAHIEGEPSHKVVRTAIHALARM

QHRGAILADGKTGDGCGLLLQKPDRFFRIVAQERGWRLAKNYAVGMLFLNKDPELAAAARRIVEEELQRE

TLSIVGWRDVPTNEGVLGEIALSSLPRIEQIFVNAPAGWRPRDMERRLFIARRRIEKRLEADKDFYVCSL

SNLVNIYKGLCMPADLPRFYLDLADLRLESAICLFHQRFSTNTVPRWPLAQPFRYLAHNGEINTITGNRQ

WARARTYKFQTPLIPDLHDAAPFVNETGSDSSSMDNMLELLLAGGMDIIRAMRLLVPPAWQNNPDMDPEL

RAFFDFNSMHMEPWDGPAGIVMSDGRFAACNLDRNGLRPARYVITKDKLITCASEVGIWDYQPDEVVEKG

RVGPGELMVIDTRSGRILHSAETDDDLKSRHPYKEWMEKNVRRLVPFEDLPDEEVGSRELDDDTLASYQK

QFNYSAEELDSVIRVLGENGQEAVGSMGDDTPFAVLSSQPRIIYDYFRQQFAQVTNPPIDPLREAHVMSL
ATSIGREMNVFCEAEGQAHRLSFKSPILLYSDFKQLTTMKEEHYRADTLDITFDVTKTTLEATVKELCDK
AEKMVRSGTVLLVLSDRNIAKDRLPVPAPMAVGAIQTRLVDQSLRCDANIIVETASARDPHHFAVLLGFG
ATAIYPYLAYETLGRLVDTHAIAKDYRTVMLNYRNGINKGLYKIMSKMGISTIASYRCSKLFEAVGLHDD
VVGLCFQGAVSRIGGASFEDFQQDLLNLSKRAWLARKPISQGGLLKYVHGGEYHAYNPDVVRTLQQAVQS
GEYSDYQEYAKLVNERPATTLRDLLAITPGENAVNIADVEPASELFKRFDTAAMSIGALSPEAHEALAEA
MNSIGGNSNSGEGGEDPARYGTNKVSRIKQVASGRFGVTPAYLVNADVIQIKVAQGAKPGEGGQLPGDKV
TPYIAKLRYSVPGVTLISPPPHHDIYSIEDLAQLIFDLKQVNPKAMISVKLVSEPGVGTIATGVAKAYAD
LITIAGYDGGTGASPLSSVKYAGCPWELGLVETQQALVANGLRHKIRLQVDGGLKTGVDIIKAAILGAES
FGFGTGPMVALGCKYLRICHLNNCATGVATQDDKLRKNHYHGLPFKVTNYFEFIARETRELMAQLGVTRL
VDLIGRTDLLKELDGFTAKQQKLALSKLLETAEPHAGKSLYCTENNPPFDNGLLNAQLLQQAKPFVDERQ
SKTFWFDIRNTDRSVGASLSGYIAQTHGDQGLAADPIKAYFNGTAGQSFGVWNAGGVELYLTGDANDYVG
KGMAGGLIAIRPPVGSAFRSHEASIIGNTCLYGATGGRLYAAGRAGERFGVRNSGAITVVEGIGDNGCEY
MTGGIVCILGKTGVNFGAGMTGGFAYVLDESGDFRKRVNPELVEVLSVDDLAIHEEHLRGLITEHVQHTG
SQRGEEILANWSTFATKFALVKPKSSDVKALLGHRSRSAAELRVQAQ

>gi|161486425|ref|NP_838724.2| N-acetylmannosamine kinase [Shigella flexneri 2a str. 2457T]
MTILAIDIGGTKLAAALIGADGQIRDRRELPTPASQTPEALRDALSALVSPLQAHAQRVAIASTGIIRDG
SLLALNPHNLGGLLHFPLVKTLEQLTNLPTIAINDAQAAAWAEYQALEGDITDMVFITVSTGVGGGVVSG
GKLLTGPGGLAGHIGHTLADPHGPVCGCGRTGCVEAIASGRGIAAAAQGELAGADARTIFTRAGQGDEQA
QQLIHRSARTLARLIADIKATTDCQCVVVGGSVGLAEGYLALVEMYLAQEPAAFHVDLLAAHYRHDAGLL
GAALLAQGEKL

>gi|161486424|ref|NP_838726.2| sialic acid transporter [Shigella flexneri 2a str. 2457T]
MSTTTQNIPWYRHLNRAQWRAFSAAWLGYLLDGFDFVLIALVLTEVQGEFGLTTVQAASLISAAFISRWF

GGLMLGAMGDRYGRRLAMVTSIVLFSAGTLACGFAPGYITMFIARLVIGMGMAGEYGSSATYVIESWPKH

LRNKASGFLISGFSVGAVVAAQVYSLVVPVWGWRALFFIGILPIIFALWLRKNIPEAEDWKEKHAGKAPV

RTMVDILYRGEHRIANIVMTLAAATALWFCFAGNLQNAAIVAVLGLLCAAIFISFMVQSTGKRWPTGVML

MVVVLFAFLYSWPIQALLPTYLKTDLAYNPHTVANVLFFSGFGAAVGCCVGGFLGDWLGTRKAYVCSLLA

SQLLIIPVFAIGGANVWVLGLLLFFQQMLGQGIAGILPKLIGGYFDTDQRAAGLGFTYNVGALGGALAPI

IGALIAQRLDLGTALASLSFSLTFVVILLIGLDMPSRVQRWLRPEALRTHDAIDGKPFSGAVPFGSAKND

LVKTKS

>gi|161486423|ref|NP_838745.2| hypothetical protein S3497 [Shigella flexneri 2a str. 2457T]

MSLFPVIVVFGLSFPPIFFELLLSLAIFWLVRRVLVPTGIYDFVWHPALFNTALYCCLFYLISRLFV

>gi|161486422|ref|NP_838802.2| glycerol-3-phosphate acyltransferase [Shigella flexneri 2a str. 2457T]

MSGWPRIYYKLLNLPLSILVKSKSIPADPAPELGLDTSRPIMYVLPYNSKADLLTLRAQCLAHDLPDPLE

PLEIDGTLLPRYVFIHGGPRVFTYYTPKEESIKLFHDYLDLHRSNPNLDVQMVPVSVMFGRAPGREKGEV

NPPLRMLNGVQKFFAVLWLGRDSFVRFSPSVSLRRMADEHGTDKTIAQKLARVARMHFARQRLAAVGPRL

PARQDLFNKLLASRAIAKAVEDEARSKKISHEKAQQNAIALMEEIAANFSYEMIRLTDRILGFTWNRFYQ

GINVHNAERVRQLAHDGHELVYVPCHRSHMDYLLLSYVLYHQGLVPPHIAAGINLNFWPAGPIFRRLGAF

FIRRTFKGNKLYSTVFREYLGELFSRGYSVEYFVEGGRSRTGRLLDPKTGTLSMTIQAMLRGGTRPITLI

PIYIGYEHVMEVGTYAKELRGATKEKESLPQMLRGLSKLRNLGQGYVNFGEPMPLMTYLNQHVPDWRESI

DPIEAVRPAWLTPTVNNIAADLMVRINNAGAANAMNLCCTALLASRQRSLTREQLTEQLNCYLDLMRNVP

YSTDSTVPSASASELIDHALQMNKFEVEKDTIGDIIILPREQAVLMTYYRNNIAHMLVLPSLMAAIVTQH

RHISRDVLMEHVNVLYPMLKAELFLRWDRDELPDVIDALANEMQRQGLITLQDDELHINPAHSRTLQLLA

AGARETLQRYAITFWLLSANPSINRGTLEKESRTVAQRLSVLHGINAPEFFDKAVFSSLVLTLRDEGYIS

DSGDAEPAETMKVYQLLAELITSDVRLTIESATQGEG

>gi|161486421|ref|NP_838805.2| DNA-damage-inducible SOS response protein [Shigella flexneri 2a str. 2457T]

MAFLTSSDKALWHLALPMIFSNITVPLLGLVDTAVIGHLDSPVYLGGVAVGATATSFLFMLLLFLRMSTT

GLTAQAYGAKNPQALARALVQPLLLALGAGALIALLRTPIIDLALHIVGGSEAVLEQARRFLEIRWLSAP

ASLANLVLLGWLLGVQYARAPVILLVVGNILNIVLDVWLVMGLHMNVQGAALATVIAEYATLLIGLLMVR

KILKLRGISGEMLKTAWRGNFRRLLALNRDIMLRSLLLQLCFGAITVLGARLGSDIIAVNAVLMTLLTFT

AYALDGFAYAVEAHSGQAYGARDGSQLLDVWRAACRQSGIVALLFSVVYLLAGEHIIALLTSLTQIQQLA

DRYLIWQVILPLVGVWCYLLDGMFIGATRAAEMRNSMAVAAAGFALTLLTLPWLGNHGLWLALTVFLALR

GLSLAAIWRRHWRNGTWFAAT

>gi|161486420|ref|NP_838807.2| zinc uptake transcriptional repressor [Shigella flexneri 2a str. 2457T]

MEKTTTQELLAQAEKICAQRNVRLTPQRLEVLRLMSLQDGAISAYDLLDLLREAEPQAKPPTVYRALDFL

LEQGFVHKVESTNSYVLCHLFDQPTHTSAMFICDRCGAVKEECAEGVEDIMHTLAAKMGFALRHNVIEAH

GLCAACVEVEACRHPEQCQHDHSVQVKKKPR

>gi|161486419|ref|NP_838810.2| phage shock protein G [Shigella flexneri 2a str. 2457T]

MLELLFVIGFFVMLMVTGVSLLGIIAALVVATAIMFLGGMLALMIKLLPWLLLAIAVVWVIKAIKAPKMP

KYQRYDRWRY

>gi|161486418|ref|NP_838840.2| multidrug efflux system protein MdtO [Shigella flexneri 2a str. 2457T]

MSALNSLPLPVVRLLAFFHEELSERRPGRVPQIVQLWVGCLLVILISMTFEIPFVALSLAVLFYGIQSNA

FYTKFVAILFVVATVLEIGSLFLIYKWSYGEPLIRLIIAGPILMGCMFLMRTHRLGLVFFAVAIYGQTFP

AMLDYPEVVVRLTLWCIVVGLYPTLLMTLIGVLWFPNRAITQMHQALNDRLDDAISHLTDSLAPLPETRI

EREALALQKLNVFCLADDANWRTQSAWWQSCVATVTYIYSTLNRYDPTSFADSQAIIEFRQKLASEINKL

QHAVAEGQCWQSDWRLSESEAVAARECNLENICQTLLQLGQMNPNTPPTPAAKPPSMVADAFTNPDYIRY

AVKTLLACLICYTFYSGVDWEGIHTCMLTCVIVANPNVGSSYQKMVLRFGGAFCGAILALLFTLLVMPWL

DNIVELLFVLAPIFLLGAWIATSSERSSYIGTQMVVTFALATLENVFGPVYDLVEIRDRALGIIIGTVVS

AMIYTFVWPESEARTLPQKLAGALGMLSKVMRIPRQQEVTALRTYLQIRIGLHAAFNACEEMCQRVALER

QLDSEERALLIERSQTVIRQGRDILHAWDATWNSAQALDNALQPDRAGQFADALEKYAAGLATALSRSPQ

ITLEETPTSQAILPTLLKQEQHVCQLFARLPDWTAPALTPATEQAQGATQ

>gi|161486417|ref|NP_838866.2| insertion sequence 2 OrfA protein [Shigella flexneri 2a str. 2457T]

MIDVLGPEKRRRRTTQEKIAIVQQSFEPGMTVSLVARQHGVAASQLFLWRKQYQEGSLTAVAAGEQVVPA

SELAAAMKQIKELQRLLGKKTMENELLKEAVEYGRAKKWIAHAPLLPGDGE

>gi|161486416|ref|NP_838867.2| insertion element IS2 transposase InsD [Shigella flexneri 2a str. 2457T]

MDSARALIARGWGVSLVSRCLRVSRAQLHVILRRTDDWMDGRRSRHTDDTDVLLRIHHVIGELPTYGYRR

VWALLRRQAELDGMPAINAKRVYRLMRQNALLLERKPAVPPSKRAHTGRVAVKESNQRWCSDGFEFCCDN

GERLRVTFALDCCDREALHWAVTTGGFNSETVQDVMLGAVERRFGNDLPSSPVEWLTDNGSCYRANETRQ

FARMLGLEPKNTAVRSPESNGIAESFVKTIKRDYISIMPKPDGLTAAKNLAEAFEHYNEWHPHSALGYRS

PREYLRQRACNGLSDNRCLEI

>gi|161486415|ref|NP_838879.2| IclR family transcriptional regulator [Shigella flexneri 2a str. 2457T]

MVAPIPAKRGRKPAVATAPATGQVQSLTRGLKLLEWIAESNGSVALTELAQQAGLPNSTTHRLLTTMQQQ

GFVRQVGELGHWAIGAHAFMVGSSFLQSRNLLAIVHPILRNLMEESGETVNMAVLDQSDHEAIIIDQVQC

THLMRMSAPIGGKLPMHASGAGKAFLAQLSEEQVTKLLHRKGLHAYTHATLVSPVHLKEDLAQTRKRGYS

FDDEEHALGLRCLAACIFDEHREPFAAISISGPISRITDDRVTEFGAMVIKAAKEVTLAYGGMR

>gi|161486414|ref|NP_838887.2| zinc resistance protein [Shigella flexneri 2a str. 2457T]

MKRNTKIALVMMALSAMAMGSTSAFAHGGHGMWQQNAAPLTSEQQTAWQKIHNDFYAQSSALQQQLVTKR

YEYNALLAANPPDSSKINAVAKEMENLRQSLDELRVKRDIAMAEAGIPRGAGMGMGYGGCGGGGHMGMGH

W

>gi|161486413|ref|NP_838898.2| thiazole synthase [Shigella flexneri 2a str. 2457T]

MLRIADKTFDSHLFTGTGKFASSQLMVEAIRASGSQLVTLAMKRVDLRQHNDAILEPLIAAGVALLPNTS

GAKTAEEAIFAAHLAREALGTNWLKLEIHPDARWLLPDPIETLKAAETLVQQGFVVLPYCGADPVLCKRL

EEVGCAAVMPLGAPIGSNQGLETRAMLEIIIQQATVPVVVDAGIGVPSHATQALEMGADAVLVNTAIAVA

DDPVNMAKAFRLAVEVGLLARQSGPGSRSYFAHATSPLTGFLEASA

>gi|161486412|ref|NP_838913.2| glutamate racemase [Shigella flexneri 2a str. 2457T]

MATKLQDGNTPCLAATPSEPRPTVLVFDSGVGGLSVYDEIRHLLPDLHYIYAFDNVAFPYGEKSEAFIVE

RVVAIVTAVQERYPLALAVVACNTASTVSLPALREKFDFPVVGVVPAIKPAARLTANGIVGLLATRGTVK

RSYTHELIARFANECQIEMLGSAEMVELAEAKLHGEDVSLDALKRILRPWLRMKEPPDTVVLGCTHFPLL

QEELLQVLPEGTRLVDSGAAIARRTAWLLEHEAPDAKSADANIAFCMAMTPEAEQLLPVLQRYGFETLEK

LAVLG

>gi|161486411|ref|NP_838918.2| soluble pyridine nucleotide transhydrogenase [Shigella flexneri 2a str. 2457T]

MPHSYDYDAIVIGSGPGGEGAAMGLVKQGARVAVIERYQNVGGGCTHWGTIPSKALRHAVSRIIEFNQNP

LYSDHSRLLRSSFADILNHADNVINQQTRMRQGFYERNHCEILQGNARFVDEHTLALDCPDGSVETLTAE

KFVIACGSRPYHPTDVDFTHPRIYDSDSILSMHHEPRHVLIYGAGVIGCEYASIFRGMDVKVDLINTRDR

LLAFLDQEMSDSLSYHFWNSGVVIRHNEEYEKIESCDDGVIMHLKSGKKLKADCLLYANGRTGNTDSLAL

QNIGLETDSRGQLKVNSMYQTAQPHVYAVGDVIGYPSLASAAYDQGRIAAQALVKGEATAHLIEDIPTGI

YTIPEISSVGKTEQQLTAMKVPYEVGRAQFKHLARAQIVGMNVGTLKILFHRETKEILGIHCFGERAAEI

IHIGQAIMEQKGGGNTIEYFVNTTFNYPTMAEAYRVAALNGLNRLF

>gi|161486410|ref|NP_838963.2| periplasmic repressor CpxP [Shigella flexneri 2a str. 2457T]

MRIVTAAVMASTLAVSSLSHAAEVGSGDNWHPGEELTQRSTQSHMFDGISLTEHQRQQMRDLMQQARHEQ

PPVNVSELETMHRLVTAENFDENAVRAQAEKMANEQIARQVEMAKVRNQMYRLLTPEQQAVLNEKHQQRM

EQLRDVTQWQKSSSLKLLSSSNSRSQ

>gi|161486409|ref|NP_838967.2| insertion sequence 2 OrfA protein [Shigella flexneri 2a str. 2457T]

MIDVLGPEKRRRRTTQEKIAIVQQSFEPGMTVSLVARQHGVAASQLFLWRKQYQEGSLTAVAAGEQVVPA

SELAAAMKQIKELQRLLGKKTMENELLKEAVEYGRAKKWIAHAPLLPGDGE

>gi|161486408|ref|NP_838968.2| insertion element IS2 transposase InsD [Shigella flexneri 2a str. 2457T]

MDSARALIARGWGVSLVSRCLRVSRAQLHVILRRTDDWMDGRRSRHTDDTDVLLRIHHVIGELPTYGYRR

VWALLRRQAELDGMPAINAKRVYRLMRQNALLLERKPAVPPSKRAHTGRVAVKESNQRWCSDGFEFCCDN

GERLRVTFALDCCDREVLHWAVTTGGFNSETVQDVMLGAVERRFGNDLPSSPVEWLTDNGSCYRANETRQ

FARMLGLEPKNTAVRSPESNGIAESFVKTIKRDYISIMPKPDGLTAAKNLAEAFEHYNEWHPHSALGYRS

PREYLRQRACNGLSDNRCLEI

>gi|161486407|ref|NP_839017.2| ribosome biogenesis GTP-binding protein YsxC [Shigella flexneri 2a str. 2457T]

MTNLNYQQTHFVMSAPDIRHLPSDTGIEVAFAGRSNAGKSSALNTLTNQKSLARTSKTPGRTQLINLFEV

ADGKRLVDLPGYGYAEVPEEMKRKWQRALGEYLEKRQSLQGLVVLMDIRHPLKDLDQQMIEWAVDSNIAV

LVLLTKADKLASGARKALLNMVREAVLAFNGDVQVETFSSLKKQGVDKLRQKLDTWFSEMQPVEETQDGE

>gi|161486406|ref|NP_839054.2| hypothetical protein S3857 [Shigella flexneri 2a str. 2457T]

MSAVLTAEQALKLVGEMFVYHMPFNRALGMELERYEKEFAQLAFKNQPMMVGNWAQSILHGGVIASALDV

AAGLVCVGSTLTRHETISEDELRQRLSRMGTIDLRVDYLRPGRGERFTATSSLLRAGNKVAVARVELHNE

EQLYIASATATYMVG

>gi|161486405|ref|NP_839066.2| diaminopimelate epimerase [Shigella flexneri 2a str. 2457T]

MQFSKMHGLGNDFMVVDAVTQNVFFSPELIRRLADRHLGVGFDQLLVVEPPYDPELDFHYRIFNADGSEV

AQCGNGARCFARFVRLKGLTNKRDIRVSTANGRMVLTVTDDDLVRVNMGEPNFEPSAVPFRANKAEKTYI

MRAAEQTILCGVVSMGNPHCVIQVDDVDTAAVETLGPVLESHERFPERANIGFMQVVKREHIRLRVYERG

AGETQACGSGACAAVAVGIQQGLLDEEVRVELPGGRLDIAWKGPGHPLYMTGPAVHVYDGFIHL

>gi|161486404|ref|NP_839077.2| insertion sequence 2 OrfA protein [Shigella flexneri 2a str. 2457T]

MIDVLGPEKRRRRTTQEKIAIVQQSFEPGMTVSLVARQHGVAASQLFLWRKQYQEGSLTAVAAGEQVVPA

SELAAAMKQIKELQRLLGKKTMENELLKEAVEYGRAKKWIAHAPLLPGDGE

>gi|161486403|ref|NP_839078.2| insertion element IS2 transposase InsD [Shigella flexneri 2a str. 2457T]

MDSARALIARGWGVSLVSRCLRVSRAQLHVILRRTDDWMDGRRSRHTDDTDVLLRIHHVIGELPTYGYRR

VWALLRRQAELDGMPAINAKRVYRLMRQNALLLERKPAVPPSKRAHTGRVAVKESNQRWCSDGFEFCCDN

GERLRVTFALDCCDREALHWAVTTGGFNSETVQDVMLGAVERRFGNDLPSSSVEWLTDNGSCYRANETRQ

FARMLGLEPKNTAVRSPESNGIAESFVKTIKRDYISIMPKPDGLTAAKNLAEAFEHYNEWHPHSALGYRS

PREYLRQRACNGLSDNRCLEI

>gi|161486402|ref|NP_839121.2| hypothetical protein S3943 [Shigella flexneri 2a str. 2457T]

MLTLDTLNVMLAVSEEGLIEEMIIALLASPQLAVFFEKFPRLKAAITDDVPRWREALRSRLKDARVPPEL

TEEVMCYQQSQLLSTPQFIVQLPQILDLLHRLNSPWAEQARQLVDANSTITSALHTLFLQRWRLSLIVQA

TTLNQQLLEEEREQLLSEVQERMTLSGQLEPILADNNTAAGRLWDMSAGQLKRGDYQFIVKYGEFLNEQP

ELKRLAEQLGRSREAKSIPRNDAQMETFRTMVREPATVPEQVDGLQQSDDILRLLPPELATLGITELEYE

FYRRLVEKQLLTYRLHGESWREKVFERPVVHKDYDEQPRGPFIVCVDTSGSMGGFNEQCAKAFCLALMRI

ALAENRRCYIMLFSTEIVRYELSGPQGIEQAIRFLSQQFRGGTDLASCFRAIMERLQSREWFDADAVVIS

DFIAQRLPDDVTSKVKELQRVHQHRFHAVAMSAHGKPGIMRIFDHIWRFDTGMRSRLLRRWRR

>gi|161486401|ref|NP_839127.2| F0F1 ATP synthase subunit I [Shigella flexneri 2a str. 2457T]

MSVSLVSRNVARKLLLVQLLVVIASGLLFSLKDPFWGVSAISGGLAVFLPNVLFMIFAWRHQAHTPAKGR

VAWTFAFGEAFKVLAMLVLLVVALAVLKAVFLPLIVTWVLVLVVQILAPAVINNKG

>gi|161486400|ref|NP_839140.2| insertion element IS2 transposase InsD [Shigella flexneri 2a str. 2457T]

MDSARALIARGWGVSLVSRCLRVSRAQLHVILRRTDDWMDGRRSRHTDDTDVLLRIHHVIGELPTYGYRR

VWALLRRQAELDGMPAINAKRVYRLMRQNALLLERKPAVPPSKRAHTGRVAVKESNQRWCSDGFEFCCDN

GERLRVTFALDCCDREALHWAVTTGGFNSETVQDVMLGAVERRFGNDLPSSPVEWLTDNGSCYRANETRQ

FARMLGLEPKNTAVRSPESNGIAESFVKTIKRDYISIMPKPDGLTAAKNLAEAFEHYNEWHPHSALGYRS

PREYLRQRACNGLSDNRCLEI

>gi|161486399|ref|NP_839141.2| insertion sequence 2 OrfA protein [Shigella flexneri 2a str. 2457T]

MIDVLGPEKRRRRTTQEKIAIVQQSFEPGMTVSLVARQHGVAASQLFLWRKQYQEGSLTAVAAGEQVVPA

SELAAAMKQIKELQRLLGKKTMENELLKEAVEYGRAKKWIAHAPLLPGDGE

>gi|161486398|ref|NP_839146.2| ribonucleoside transporter [Shigella flexneri 2a str. 2457T]

MSEFIAENRGADAITRPNWSAVFSVAFCVACLIIVEFLPVSLLTPMAQDLGISEGVAGQSVTVTAFVAMF

ASLFITQTIQATDRRYVVILFAVLLTLSCLLVSFANSFSLLLIGRACLGLALGGFWAMSASLTMRLVPPR

TVPKALSVIFGAVSIALVIAAPLGSFLGELIGWRNVFNAAAVMGVLCIFWIIKSLPSLPGEPSHQKQNTF

RLLQRPGVMAGMIAIFMSFAGQFAFFTYIRPVYMNLAGFGVDGLTLVLLSFGIASFIGTSLSSFILKRSV

KLALAGAPLILAVSALVLTLWGSDKIVATGVAIIWGLTFALVPVGWSTWITRSLADQAEKAGSIQVAVIQ

LANTCGAAIGGYALDNIGLTSPLMLSGTLMLLTALLVTAKVKMKKS

>gi|161486397|ref|NP_839169.2| hypothetical protein S3995 [Shigella flexneri 2a str. 2457T]

MIRNVLLAFMICSGMTLLGGCSSVMSHTGGKEGTYPGTRASATMIGDDETNWGTKSLAILDMPFTAVMDT

LLLPWDVFRKDSSVRSRVEKSEANAQATNAVIPPARMPDN

>gi|161486396|ref|NP_839217.2| insertion sequence 2 OrfA protein [Shigella flexneri 2a str. 2457T]

MIDVLGPEKRRRRTTQEKIAIVQQSFEPGMTVSLVARQHGVAASQLFLWRKQYQEGSLTAVAAGEQVVPA

SELAAAMKQIKELQRLLGKKTMKNELLKEAVEYGRAKKWIAHAPLLPGDGE

>gi|161486395|ref|NP_839218.2| insertion element IS2 transposase InsD [Shigella flexneri 2a str. 2457T]

MDSARALIARGWGVSLVSRCLRVSRAQLHVILRRTDDWMDGRRSRHTDDTDVLLRIHHVIGELPTYGYRR

VWALLRRQAELDGMPAINAKRVYRLMRQNALLLERKPAVPPSKRAHTGRVAVKESNQRWCSDGFEFCCDN

GERLRVTFALDCCDREALHWAVTTGGFNSETVQDVMLGAVERRFGNDLPSSPVEWLTDNGSCYRANETRQ

FARMLGLEPKNTAVRSPESNGIAESFVKTIKRDYISIMPKPDGLTAAKNLAEAFEHYNEWHPHSALGYRS

PREYLRQRACNGLSDNRCLEI

>gi|161486394|ref|NP_839229.2| insertion sequence 2 OrfA protein [Shigella flexneri 2a str. 2457T]

MIDVLGPEKRRRRTTQEKIAIVQQSFEPGMTVSLVARQHGVAASQLFLWRKQYQEGSLTAVAAGEQVVPA

SELAAAMKQIKELQRLLGKKTMENELLKEAVEYGRAKKWIAHAPLLPGDGE

>gi|161486393|ref|NP_839230.2| insertion element IS2 transposase InsD [Shigella flexneri 2a str. 2457T]

MDSARALIARGWGVSLVSRCLRVSRAQLHVILRRTDDWMDGRRSRHTDDTDVLLRIHHVIGELPTYGYRR

VWALLRRQAELDGMPAINAKRVYRIMRQNALLLERKPAVPPSKRAHTGRVAVKESNQRWCSDGFEFCCDN

GERLRVTFALDCCDREALHWAVTTGGFNSETVQDVMLGAVERRFGNDLPSSPVEWLTDNGSCYRANETRQ

FARMLGLEPKNTAVRSPESNGIAESFVKTIKRDYISIMPKPDGLTAAKNLAEAFEHYNEWHPHSALGYRS

PREYLRQRACNGLSDNRCLEI

>gi|161486392|ref|NP_839254.2| nucleoid occlusion protein [Shigella flexneri 2a str. 2457T]

MAEKQTAKRNRREEILQSLALMLESSDGSQRITTAKLAASVGVSEAALYRHFPSKTRMFDSLIEFIEDSL

ITRINLILKDEKDTTARLRLIVLLLLGFGERNPGLTRILTGHALMFEQDRLQGRINQLFERIEAQLRQVL

REKRMREGEGYTTDETLLASQILAFCEGMLSRFVRSEFKYRPTDDFDARWPLIAAQLQ

>gi|161486391|ref|NP_839256.2| bifunctional phosphopantothenoylcysteine decarboxylase/phosphopantothenate synthase [Shigella flexneri 2a str. 2457T]

MSLAGKKIVLGVSGGIAAYKTPELVRRLRDRGADVRVAMTEAAKAFITPLSLQAVSGYPVSDSLLDPAAE

AAMGHIELGKWADLVILAPATADLIARVAAGMANDLVSTICLATPAPVAVLPAMNQQMYRAAATQHNLEV

LASRGLLIWGPDSGSQACGDIGPGRMLDPLTIVDMAVAHFSPVNDLKHLNIMITAGPTREPLDPVRYISN

HSSGKMGFAIAAAAARRGANVTLVSGPVSLPTPPFVKRVDVMTALEMEAAVNASVQQQNIFIGCAAVADY

RAATVAPEKIKKQATQGDELTIKMVKNPDIVAGVAALKDHRPYVVGFAAETNNVEEYARQKRIRKNLDLI

CANDVSQPTQGFNSDNNALHLFWQDGDKVLPLERKELLGQLLLDEIVTRYDEKNRR

>gi|161486390|ref|NP_839257.2| DNA repair protein RadC [Shigella flexneri 2a str. 2457T]

MKVKNNAQLLMPREKMLKFGISALTDVELLALFLRTGTRGKDVLTLAKEMLENFGSLYGLLTSEYEQFSG

VHGIGVAKFAQLKGIAELARRYYNVRMREESPLLSPEMTREFLQSQLTGEEREIFMVIFLDSQHRVITHS

RLFSGTLNHVEVHPREIIREAIKINASALILAHNHPSGCAEPSKADKLITERIIKSCQFMDLRVLDHIVI

GRGEYVSFAERGWI

>gi|161486389|ref|NP_839282.2| hypothetical protein S4116 [Shigella flexneri 2a str. 2457T]

MRGKAINTMTRAVKPRRFAIRPIIYASVLSAGVLLCAFSAHADERDQLKSIQADIAAKERAVRQKQQQRA

SLLAQLKKQEEAISEATRKLRETQNTLNQLNKQIDEMNASIAKLEQQKAAQERSLAAQLDAAFRQGEHTG

IQLILSGEESQRGQRLQAYFGYLNQARQETIAQLKQTREEVTMQHAELEEKQSEQQTLLYEQRAQQAKLT

QALNERKKTLAGLESSIQQGQQQLSELRANESRLRNSIARAEAAAKARAEREAREAQAVRERQKEATRKG

TTYKPTESEKSLMSRTGGLGAPRGQAFWPVRGPTLHRYGEQLQGELRWKGMVIGASEGTEVKAIADGRVI

LADWLQGYGLVVVVEHGKGDMSLYGYNQSALVSVGSQVRAGQPIALVGSSGGQGRPSLYFEIRRQGQAVN

PQPWLGR

>gi|161486388|ref|NP_839346.2| phosphoethanolamine transferase [Shigella flexneri 2a str. 2457T]

MRYIKSITQQKLSFLLAIYIGLFMNGAVFYRRFGSYAHDFTVWKGVSAVVELAATVLVTFFLLRLLSLFG

RRSWRILASLVVLFSAGASYYMTFLNVVIGYGIIASVMTTDIDLSKEVVGLNFILWLIAVSALPLILIWN

NRCRYTLLRQLRTPGQRIRSLVVVVLAGIMVWAPIRLLDIQQKKVERATGVDLPSYGGVVANSYLPSNWL

SALGLYAWARVDESSDNNSLLNPAKKFTYQAPQNVDDTYVVFIIGETTRWDHMGIFGYERNTTPKLAQEK

NLAAFRGYSCDTATKLSLRCMFVRQGGAEDNPQRTLKEQNIFAVLKQLGFSSDLYAMQSEMWFYSNTMAD

NIAYREQIGAEPRNRGKPVDDMLLVDEMQQSLGRNPDGKHLIILHTKGSHFNYTQRYPRSFAQWKPECIG

VDSGCTKAQMINSYDNSVTYVDHFISSVIDQVRDKKAIVFYAADHGESINEREHLHGTPRELAPPEQFRV

PMMVWMSDKYLENPANAQAFAQLKKEADMKVPRRHVELYDTIMGCLGYTSPDGGINENNNWCHIPQTKVA

AAN

>gi|161486387|ref|NP_839402.2| insertion element IS2 transposase InsD [Shigella flexneri 2a str. 2457T]

MDSARALIARGWGVSLVSRCLRVSRAQLHVILRRTDDWMDGRRSRHTDDTDVLLRIHHVIGELPTYGYRR

VWALLRRQAELDGMPAINAKRVYRLMRQNALLLERKPAVPPSKRAHTGRVAVKESNQRWCSDGFEFCCDN

GERLRVTFALDCCDREALHWAVTTGGFNSETVQDVMLGAVERRFGNDLPSSPVEWLTDNGSCYRANETRQ

FARMLGLEPKNTAVRSPESNGIAESFVKTIKRDYISIMPKPDGLTAAKNLAEAFEHYNEWHPHSALGYRS

PREYLRQRACNGLSDNRCLEI

>gi|161486386|ref|NP_839403.2| insertion sequence 2 OrfA protein [Shigella flexneri 2a str. 2457T]

MIDVLGPEKRRRRTTQEKIAIVQQSFEPGMTVSLVARQHGVAASQLFLWRKQYQEGSLTAVAAGEQVVPA

SELAAAMKQIKELQRLLGKKTMENELLKEAVEYGRAKKWIAHAPLLPGDGE

>gi|161486385|ref|NP_839421.2| major facilitator superfamily transporter [Shigella flexneri 2a str. 2457T]

MPEPVAEPALNGLRLNLRIVSIVMFNFASYLTIGLPLAVLPGYVHDVMGFSAFWAGLVISLQYFATLLSR

PHAGRYADLLGPKKIVVFGLCGCFLSGLGYLTAGLTASLPVISLLLLCLGRVILGIGQSFAGTGSTLWGV

GVVGSLHIGRVISWNGIVTYGAMAMGAPLGVVFYHWGGLQALALIIMGVALVAILLAIPRPTVKASKGKP

LPFRAVLGRVWLYGMALALASAGFGVIATFITLFYDAKGWDGAAFALTLFSCAFVGTRLLFPNGINRIGG

LNVAMICFSVEIIGLLLVGVATMPWMAKIGVLLAGAGFSLVFPALGVVAVKAVPQQNQGAALATYTVFMD

LSLGVTGPLAGLVMSWAGVPVIYLAAAGLVAIALLLTWRLKKRPPEHVPEAASSS

>gi|161486384|ref|NP_839422.2| hypothetical protein S4273 [Shigella flexneri 2a str. 2457T]

MRNLVKYVGIGLLVMGLAACDDKDTNATAQGSVAESNATGNPVNLLDGKLSFSLPADMTDQSGKLGTQAN

NMHVWSDATGQKAVIVIMGDDPKEDLAVLAKRLEDQQRSRDPQLQVVTNKAIELKGHKMQQLDSIISAKG

QTAYSSVILGNVGNQLLTMQITLPADDQQKAQTTAENIINTLVIQ

>gi|161486383|ref|NP_839451.2| gluconate kinase [Shigella flexneri 2a str. 2457T]

MSTTNHDHHIYVLMGVSGSGKSAVASEVAHQLHAAFLDGDFLHPRRNIEKMASGEPLNDDDRKPWLQALN

DSAFAMQRTNKVSLIACSALKKHYRDLLREGNPNLSFIYLKGDFDVIESRLKARKGHFFKTQMLVTQFET

LQEPGADETDVLVVDIDQPLEGVVASTIEVIKKGK

>gi|161486382|ref|NP_839473.2| gluconate periplasmic binding protein [Shigella flexneri 2a str. 2457T]

MLTVPGLCWLCRMPLALGHWGICSVCSRATRTDKTLCPQCGLPATHSHLPCGRCLQKPPPWQRLVTVADY

APPLSPLIHQLKFSRRSEIASALSRLLLLEVLHARRTTGLQLPDRIISVPLWQRRHWRRGFNQSDLLCQP

LSRWLHCQWDSEAVTRTRATATQHFLSARLRKRNLKNAFRLELPVQGRHMVIVDDVVTTGSTVAEIAQLL

LRNGAATVQVWCLCRTL

>gi|161486381|ref|NP_839475.2| insertion element IS2 transposase InsD [Shigella flexneri 2a str. 2457T]

MDSARALIARGWGVSLVSRCLRVSRAQLHVILRRTDDWMDGRRSRHTDDTDVLLRIHHVIGELPTYGYRR

VWALLRRQAELDGMPAINAKRVYRLMRQNALLLERKPAVPPSKRAHTGRVAVKESNQRWCSDGFEFCCDN

GERLRVTFALDCCDREALHWAVTTGGFNSETVQDVMLGAVERRFGNDLPSSPVEWLTDNGSCYRANETRQ

FARMLGLEPKNTAVRSPESNGIAESFVKTIKRDYISIMPKPDGLTAAKNLAEAFEHYNEWHPHSALGYRS

PREYLRQRACNGLSDNRCLEI

>gi|161486380|ref|NP_839476.2| insertion sequence 2 OrfA protein [Shigella flexneri 2a str. 2457T]

MIDVLGPEKRRRRTTQEKIAIVQQSFEPGMTVSLVARQHGVAASQLFLWRKQYQEGSLTAVAAGEQVVPA

SELAAAMKQIKELQRLLGKKTMENELLKEAVEYGRAKKWIAHAPLLPGDGE

>gi|161486379|ref|NP_839482.2| transcription elongation factor GreB [Shigella flexneri 2a str. 2457T]

MKTPLVTREGYEKLKQELNYLWREERPEVTKKVTWAASLGDRSENADYQYNKKRLREIDRRVRYLTKCLE

NLKIVDYSPQQEGKVFFGAWVEIENDDGVTHRFRIVGYDEIFGRKDYISIDSPMARALLKKEVGDLAVVN

TPAGEANWYVNAIEYVKP

>gi|161486377|ref|NP_839497.2| shikimate kinase I [Shigella flexneri 2a str. 2457T]

MAEKRNIFLVGPMGAGKSTIGRQLAQQLNMEFYDSDQEIEKRTGADVGWVFDLEGEEGFRDREEKVINEL

TEKQGIVLATGGGSVKSRETRNRLSARGVVVYLETTIEKQLARTQRDKKRPLLHVETPPREVLEALANER

NPLYEEIADVTIRTDDQSAKVVANQIIHMLESN

>gi|161486376|ref|NP_839515.2| fructoselysine-6-P-deglycase [Shigella flexneri 2a str. 2457T]

MLSEENKMLDIDKSTVDFLVTENMVQEVEKVLSHDVPLVHAIVEEMVKRDIDRIYFVACGSPLNAAQTAK

HLADRFSDLQVYAISGWEFCDNTPYRLDDRCAVIGVSDYGKTEEVIKALELGRACGALTAAFTKRADSPI

TSAAEFSIDYQADCIWEIHLLLCYSVVLEMITRLAPNAEIGKIKNDLKQLPNALGHLVRTWEEKGRQLGE

LASQWPMIYTVAAGPLRPLGYKEGIVTLMEFTWTHGCVIESGEFRHGPLEIVEPGVPFLFLLGNDESRHT

TERAINFVKQRTDNVIVIDYAEISQGLHPWLAPFLMFVPMEWLCYYLSIYKDHNPDERRYYGGLVEY

>gi|161486375|ref|NP_839574.2| 30S ribosomal protein S4 [Shigella flexneri 2a str. 2457T]

MARYLGPKLKLSRREGTDLFLKSGVRAIDTKCKIEQAPGQHGARKPRLSDYGVQLREKQKVRRIYGVLER

QFRNYYKEAARLKGNTGENLLALLEGRLDNVVYRMGFGATRAEARQLVSHKAIMVNGRVVNIASYQVSPN

DVVSIREKAKKQSRVKAALELAEQREKPTWLEVDAGKMEGTFKRKPERSDLSADINEHLIVELYSK

>gi|161486374|ref|NP_839581.2| insertion sequence 2 OrfA protein [Shigella flexneri 2a str. 2457T]

MIDVLGPEKRRRRTTQEKIAIVQQSFEPGMTVSLVARQHGVAASQLFLWRKQYQEGSLTAVAAGEQVVPA

SELAAAMKQIKELQRLLGKKTMENELLKEAVEYGRAKKWIAHAPLLPGDGE

>gi|161486373|ref|NP_839582.2| insertion element IS2 transposase InsD [Shigella flexneri 2a str. 2457T]

MDSARALIARGWGVSLVSRCLRVSRAQLHVILRRTDDWMDGRRSRHTDDTDVLLRIHHVIGELPTYGYRR

VWALLRRQAELDGMPAINAKRVYRLMRQNALLLERKPAVPPSKRAHTGRVAVKESNQRWCSDGFEFCCDN

GERLRVTFALDCCDREALHWAVTTGGFNSETVQDVMLGAVERRFGNDLPSSPVEWLTDNGSCYRANETRQ

FARMLGLEPKNTAVRSPESNGIAESFVKTIKRDYISIMPKPDGLTAAKNLAEAFEHYNEWHPHSALGYRS

PREYLRQRACNGLSDNRCLEI

>gi|161486372|ref|NP_839583.2| RNA 2'-phosphotransferase-like protein [Shigella flexneri 2a str. 2457T]

MAKYNEKELADTSKFLSFVLRHKPEAIGIVLDREGWADINKLILCAQKAGKRLTRALLDTVVATSDKKRF

SYSSDGRCIRAVQGHSTSQVAISFAEKTPPQFLYHGTASRFLDEIKKQGLIAGDRHYVHLSADEATARKV

GARHGSPVVLTVKAQEMAKRGIPFWQAENGVWLTSTVAVEFLEWSFMPTGVQKQ

>gi|161486371|ref|NP_839686.2| FxsA [Shigella flexneri 2a str. 2457T]

MRWLPFIAIFLYVYIEISIFIQVAHVLGVLLTLVLVIFTSVIGMSLVRNQGFKNFVLMQQKMAAGENPAA

EMIKSVSLIIAGLLLLLPGFFTDFLGLLLLLPPVQKHLTVKLMPHLRFSRMPGGGFSAGTGGGNTFDGEY

QRKDDDRDRLDHKDDRQD

>gi|161486370|ref|NP_839707.2| ribosome-associated GTPase [Shigella flexneri 2a str. 2457T]

MSKNKLSKGQQRRVNANHQRRLKTSKEKPDYDDNLFGEPDEGIVISRFGMHADVESADGDVHRCNIRRTI

RSLVTGDRVVWRPGKPAAEGVNVKGIVEAVHERTSVLTRPDFYDGVKPIAANIDQIVIVSAILPELSLNI

IDRYLVACETLQIEPIIVLNKIDLLDDEGMAFVNEQMDIYRNIGYRVLMVSSHTQDGLKPLEEALTGRIS

IFAGQSGVGKSSLLNALLGLQKEILTNDVSDNSGLGQHTTTAARLYHFPHGGDVIDSPGVREFGLWHLEP

EQITQGFVEFHDYLGLCKYRDCKHDTDPGCAIREAVEEGKIAETRFENYHRILESMAQVKTRKNFSDTDD

>gi|161486369|ref|NP_839708.2| oligoribonuclease [Shigella flexneri 2a str. 2457T]

MSANENNLIWIDLEMTGLDPERDRIIEIATLVTDANLNILAEGPTIAVHQSDEQLALMDDWNVRTHTASG

LVERVKASTMGDREAELATLEFLKQWVPAGKSPICGNSIGQDRRFLFKYMPELEVYFHYRYLDVSTLKEL

ARRWKPEILDGFTKQGTHQAMDDIRESVAELAYYREHFIKL

>gi|161486368|ref|NP_839722.2| exoribonuclease R [Shigella flexneri 2a str. 2457T]

MSQDPFQEREAEKYANPIPSREFILEHLTKREKPASRDELAVELHIEGEEQLEGLRRRLRAMERDGQLVF

TRRQCYALPERLDLVKGTVIGHRDGYGFLRVEGRKDDLYLSSEQMKTCIHGDQVLAQPLGADRKGRREAR

IVRVLVPKTSQIVGRYFTEAGVGFVVPDDSRLSFDILIPPDQIMGARMGFVVVVELTQRPTRRTKAVGKI

VEVLGDNMGTGMAVDIALRTHEIPYIWPQAVEQQVAGLKEEVPEEAKAGRVDLRDLPLVTIDGEDARDFD

DAVYCEKKRGGGWRLWVAIADVSYYVRPPTPLDREARNRGTSVYFPSQVIPMLPEVLSNGLCSLNPQVDR

LCMVCEMTVSSKGRLTGYKFYEAVMSSHARLTYTKVWHILQGDQDLREQYAPLVKHLEELHNLYKVLDKA

REERGGISFESEEAKFIFNAERRIERIEQTQRNDAHKLIEECMILANISAARFVEKAKEPALFRIHDKPS

TEAITSFRSVLAELGLELPGGNKPEPRDYAELLESVADRPDAEMLQTMLLRSMKQAIYDPENRGHFGLAL

QSYAHFTSPIRRYPDLTLHRAIKYLLAKEQGHQGNTTETGGYHYSMEEMLQLGQHCSMAERRADEATRDV

ADWLKCDFMLDQVGNVFKGVISSVTGFGFFVRLDDLFIDGLVHVSSLDNDYYRFDQVGQRLMGESSGQTY

RLGDRVEVRVEAVNMDERKIDFSLISSERAPRNVGKTAREKAKKGDAGKKGGKRCQVGKKVNFEPDSAFR

GEKKTKPKAAKKDARKAKKPSAKTQKIAAATKAKRAAKKKVAE

>gi|161486367|ref|NP_839731.2| hypothetical protein S4614 [Shigella flexneri 2a str. 2457T]

MVSRKRNSVIYRFASLLLVLMLSACSALQGTPQPAPPVTDHPQEIRRDQTQGLQRIGSVSTMVRGSPDDA

LAEIRAKAVAAKADYYVVVMVDETIVTGQWYSQAILYRK

>gi|161486366|ref|NP_839735.2| ascorbate-specific PTS system enzyme IIC [Shigella flexneri 2a str. 2457T]

MEILYNIFTVFFNQVMTNAPLLLGIVTCLGYILLRKSVSVIIKGTIKTIIGFMLLQAGSGILTSTFKPVV

AKMSEVYGINGAISDTYASMMATIDRMGDAYSWVGYAVLLALALNICYVLLRRITGIRTIMLTGHIMFQQ

AGLIAVTLFIFGYSMWTTIICTAILVSLYWGITSNMMYKPTQEVTDGCGFSIGHQQQFASLIAYKVAPFL

GKKEESVEDLKLPGWLNIFHDNIVSTAIVMTIFFGAILLSFGIDTVQAMAGKVHWTVYILQTGFSFAVAI

FIITQGVRMFVAELSEAFNGISQRLIPGAVLAIDCAAIYSFAPNAVVWGFMWGTIGQLIAVGILVACGSS

ILIIPGFIPMFFSNATIGVFANHFGGWRAALKICLVMGMIEIFGCVWVVKLTGMSAWMGMADWSILAPPM

MQGFFSIGIAFMAVIIVIALAYMFFAGRALRAEEDAEKQLAEQSA

>gi|161486365|ref|NP_839771.2| phosphoglycerol transferase I [Shigella flexneri 2a str. 2457T]

MSELLSFALFLASVLIYAWKAGRNTWWFAATLTVLGLFVVLNITLFASDYFTGDGINDAVLYTLTNSLTG

AGVSKYILPGIGIVLGLTAVFGALGWILRRRRHHPHHFGYSLLALLLALGSVDASPAFRQITELVKSQSR

DGDPDFAAYYKEPSKTIPDPKLNLVYIYGESLERTYFDNEAFPDLTPELGALKNEGLDFSHTQQLPGTDY

TIAGMVASQCGIPLFAPFEGNASASVSSFFPQNICLGDILKNSGYQNYFVQGANLRFAGKDVFLKSHGFD

HLYGSEELKSVVADPHYRNDWGFYDDTVLDEAWKKFEELSRSGQRFSLFTLTVDTHHPDGFISRTCNRKK

YDFDGKPNQSFSAVSCSQENIATFINKIKASPWFKDTVIVVSSDHLAMNNTAWKYLNKQDRNNLFFVIRG

DKPQQKTLAVKRNTMDNGATVLDILGGDNYLGLGRSSLSGQSMSEIFLNIKEKTLAWKPDIIRLWKFPKE

MKEFTIDQQKNMIAFSGSHFRLPLLLRVSDKRVEPLPESEYSAPLRFQLADFAPRDNFVWVDSCYKMAQL

WAPELALSTDWCVSQGQLGGQQIVQHVDKTTWKSKTAFKDTVIDMARYKGNVDTLKIVDNDIRYKADSFI

FNVAGAPEEVKQFSGISRPESWGRWSNAQLGDEVKIEYKHPLPKKFDLVITAKAYGNNASRPIPVRVGNE

EQTLVLGNEVTTTTLHFDNPTDADTLVIVPPEPVSTNEGNILGHSPRKLGIGMVEIKVVEREG

>gi|161486364|ref|NP_839778.2| DNA-binding transcriptional activator BglJ [Shigella flexneri 2a str. 2457T]

MSSIGIESLFRKFAGNPYKLHTYTSQESFQDAMSRISFAAVIFSFSAMRSERREGLSCLTELAIKFPRTR

RLVIVDDDIEARLIGSLSPSPLDGVLSKASTLEIFHQELFLSLNGVRQATDRLNNQWYINQSRTLSPTER

EILRFMSRGYSMTQIAEQLKRNIKTIRAHKFNVMSKLGVSSDAGLLEAADILLCMRHCEASNVLHPY

>gi|161486363|ref|NP_839798.2| nicotinamide-nucleotide adenylyltransferase [Shigella flexneri 2a str. 2457T]

MSSFDYLKTAIKQQGCTLQQVADASGMTKGYLSQLLNAKIKSPSAQKLEALHRFLGLEFPRQKKTIGVVF

GKFYPLHTGHIYLIQRACSQVDELHIIMGFDDTRDRALFEDSAMSQQPTVPDRLRWLLQTFKYQKNIRIH

AFNEEGMEPYPHGWDVWSNGIKKFMAEKGIQPDLIYTSEEADAPQYMEHLGIETVLVDPKRTFMSISGAQ

IRENPFRYWEYIPTEVKPFFVRTVAILGGESSGKSTLVNKLANIFNTTSAWEYGRDYVFSHLGGDEIALQ

YSDYDKIALGHAQYIDFAVKYANKVAFIDTDFVTTQAFCKKYEGREHPFVQALIDEYRFDLVILLENNTP

WVADGLRSLGSSVDRKEFQNLLVEMLEENNIEFVRVEEDDYDSRFLRCVELVREMMGEQG

>gi|161486362|ref|NP_839800.2| lytic murein transglycosylase [Shigella flexneri 2a str. 2457T]

MEKAKQVTWRLLAAGVCLLTVSSVARADSLDEQRSRYAQIKQAWDNRQMDVVEQMMPGLKDYPLYPYLEY

RQITDDLMNQPAVTVTNFVRANPTLPPARTLQSRFVNELARREDWRGLLAFSPEKPGTTEAQCNYYYAKW

NTGQSEEAWQGAKELWLTGKSQPNACDKLFSVWRASGKQDPLAYLERIRLAMKAGNTGLVTVLAGQMPAD

YQTIASAIISLANNPNTVLTFARTTGATDFTRQMAAVAFASVARQDAENARLMIPSLAQAQQLNEDQIQE

LRDIVAWRLMGNDVTDEQAKWRDDAIMRSQSTSLIERRVRMALGTGDRRGLNTWLARLPMEAKEKDEWRY

WQADLLLERGREAEAKEILHQLIQQRGFYPMVAAQRIGEEYELKIDKAPQNVDSTLTQGPEMARVRELMY

WNLDNTARSEWANLVKSKSKTEQAQLARYAFNNQWWDLSVQATIAGKLWDHLEERFPLAYNDLFKRYTSG

KEIPQSYAMAIARQESAWNPKVKSPVGASGLMQIMPGTATHTVKMFSIPGYSSPGQLLDPETNINIGTSY

LQYVYQQFGNNRIFSSAAYNAGPGRVRTWLGNSAGRIDAVAFVESIPFSETRGYVKNVLAYDAYYRYFMG

DKPTLMSATEWGRRY

>gi|146133915|ref|NP_836102.2| ISSfl3 orfC, partial [Shigella flexneri 2a str. 2457T]

MKVLAPGNGKTKTGRLWVYVRDDRNAGSSLPAAVWXAYSXDRXXXXPQJHLAXXXGRKPDRQ

>gi|30065639|ref|NP_839810.1| RNA methyltransferase [Shigella flexneri 2a str. 2457T]

MRITIILVAPARAENIGAAARAMKTMGFSELRIVDSQAHLEPATRWVAHGSGDIIDNIKVFPTLAESLHD

VDFTVATTARSRAKYHYYATPVELVPLLEEKSSWMSHAALVFGREDSGLTNEELALADVLTGVPMVADYP

SLNLGQAVKVYCYQLATLIQQPAKSDTTADQHQLQALRERVMALLTTLAVADDIKLVDWLQQRLGLLEQR

DTAMLHRLLHDIEKNITK

>gi|30065638|ref|NP_839809.1| hypothetical protein S4705 [Shigella flexneri 2a str. 2457T]

MTKVRNCVLDALSINVNNIISLVVGTFPQDPTVSKTAVILTILTAT

>gi|30065637|ref|NP_839808.1| two-component response regulator [Shigella flexneri 2a str. 2457T]

MQTPHILIVEDELVTRNTLKSIFEAEGYDVFEATDGAEMHQILSEYDINLVIMDINLPGKNGLLLARELR

EQANVALMFLTGRDNEVDKILGLEIGADDYITKPFNPRELTIRARNLLSRTMNLGTVSEERRSVESYKFN

GWELDINSRSLIGPDGEQYKLPRSEFRAMLHFCENPGKIQSRAELLKKMTGRELKPHDRTVDVTIRRIRK

HFESTPDTPEIIATIHGEGYRFCGDLED

>gi|30065636|ref|NP_839807.1| hypothetical protein S4703 [Shigella flexneri 2a str. 2457T]

MLKSPLFWKMTTLFGAVLLLLIPIMLIRQVIVERADYRSDVEDAIRQSTSGPQKLVGPLIAIPVTELYTV

QEEDKTVERKRSFIHFWLPESLMVDGNQNVEERKIGIYTGQVWHSDLTLKADFDVSRLSELNAPNIILGK

PFIVISVGDARGIGVVKAPEVNGTALTIEPGTGLEQGGQGVHIPLPEGDWRKQNLQLNMALNLSGTGDLS

VVPAGRNSEMTLTSNWPHPSFLGDFLPAKREASESGFQAQWQSSWFANNLGERFASGNDTGWENFPAFSV

AVTTPADQYQLTDRATKYAILLIALTFMAFFVFETLTAQRLHPMQYLLVGLSLVMFYLLLLALSEHIGFT

VAWIIASLIGALMNGIYLQAVLKGWRNSMLFTLALLLLDGVMWGLLNSADSALLLGTSVLVVALAGMMFV

TRNIDWYAFSLPKMKASKEVTTDDELRIWK

>gi|30065635|ref|NP_839806.1| sensory histidine kinase CreC [Shigella flexneri 2a str. 2457T]

MRIGMRLLLGYFLLVAVAAWFVLAIFVKEVKPGVRRATEGTLIDTATLLAELARPDLLSGDPTHGQLAQA

FNQLQHRPFRANIGGINKVRNEYHVYMTDAHGKVLFDSANKAVGQDYSRWNDVWLTLRGQYGARSTLQNP

ADPESSVMYVAAPIMGGSRLIGVLSVGKPNAAMAPVIKRSERRILWASAILLGIALVIGAGMVWWINRSI

ARLTRYADSVTDNKPVPLPDLGSSELRKLAQALESMRVKLEGKNYIEQYVYALTHELKSPLAAIRGAAEI

LREGPPPEVVARFTDNILTQNARMQALVETLLRQARLENRQEVVLTAVDVAALFRRVSEARTVQLAEKNI

TLHVMPTEVNVAAEPALLDQALGNLLDNAIDFTPESGCITLSAEVDQEHVTLKVLDTGSGIPDYALSRIF

ERFYSLPRANGQKSSGLGLAFVSEVARLFNGEVTLRNVQEGGVLASLRLHRHFT

>gi|30065634|ref|NP_839805.1| DNA-binding response regulator CreB [Shigella flexneri 2a str. 2457T]

MQRETVWLVEDEQGIADTLVYMLQQEGFAVEVFERGLPVLDKARQQVPDVMILDVGLPDISGFELCRQLL

ALHPALPVLFLTARSEEVDRLLGLEIGADDYVAKPFSPREVCARVRTLLRRVKKFSTPSPVIRIGHFELN

EPAAQISWFDTPLTLTRYEFLLLKTLLKSPGRVWSRQQLMDSVWEDAQDTYDRTVDIHIKTLRAKLRAIN

PDLSPINTHRGMGYSLRGL

>gi|30065633|ref|NP_839804.1| hypothetical protein S4700 [Shigella flexneri 2a str. 2457T]

MKYKHLILSLSLIMLGPLAHAEEIGSVDTVFKMIGPDHKIVVEAFDDPDVKNVTCYVSRAKTGGIKGGLG

LAEDTSDAAISCQQVGPIELSDRIKNGKAQGEVVFKKRTSLVFKSLQVVRFYDAKRNALAYLAYSDKVVE

GSPKNAISAVPVMPWRQ

>gi|30065632|ref|NP_839803.1| right origin-binding protein [Shigella flexneri 2a str. 2457T]

MDQAGIIRDLLIWLEGHLDQPLSLDNVAAKAGYSKWHLQRMFKDVTGHAIGAYIRARRLSKSAVALRLTA

RPILDIALQYRFDSQQTFTRAFKKQFAQTPALYRRSPEWSAFGIRPPLRLGEFTMPEHKFVTLEDTPLIG

VTQSYSCSLEQISDFRHEMRYQFWHDFLGNAPTIPPVLYGLNETRPSQDKDDEQEVFYTTALAQDQADGY

VLTGHPVMLQGGEYVMFTYEGLGTGVQEFILTVYGTCMPMLNLTRRKGQDIERYYPAEDAKAGDRPINLR

CELLIPIRR

>gi|30065631|ref|NP_839802.1| phosphoglycerate mutase [Shigella flexneri 2a str. 2457T]

MLQVYLVRHGETQWNAERRIQGQSDSPLTAKGEQQAMQVATRAKELGITHIISSDLGRTRRTAEIIAQAC

GCDIIFDSRLRELNMGVLEKRHIDSLTEEEENWRRQLVNGTVDGRIPEGESMQELSDRVNAALESCRDLP

QGSRPLLVSHGIALGCLVSTILGLPAWAERRLRLRNCSISRVDYQESLWLASGWVVETAGDISHLDAPAL

DELQR

>gi|30065630|ref|NP_839801.1| Trp operon repressor [Shigella flexneri 2a str. 2457T]

MAQQSPYSAAMAEQRHQEWLRFVDLLKNAYQNDLHLPLLNLMLTPDEREALGTRVRIVEELLSGEMSQRE

LKNELGAGIATITRGSNSLKAAPVELRQWLEEVLLKSD

>gi|30065628|ref|NP_839799.1| ABC transporter ATP-binding protein [Shigella flexneri 2a str. 2457T]

MAQFVYTMHRVGKVVPPKRHILKNISLSFFPGAKIGVLGLNGAGKSTLLRIMAGIDKDIEGEARPQPDIK

IGYLPQEPQLNPEHTVRESIEEAVSEVVNALKRLDEVYALYADPDADFDKLAAEQGRLEEIIQAHDGHNL

NVQLERAADALRLPDWDAKIANLSGGERRRVALCRLLLEKPDMLLLDEPTNHLDAESVAWLERFLHDFEG

TVVAITHDRYFLDNVAGWILELDRGEGIPWEGNYSSWLEQKDQRLAQEASQEAARRKSIEKELEWVRQGT

KGRQSKGKARLARFEELNSTEYQKRNETNELFIPPGPRLGDKVLEVSNLRKSYGDRLLIDDLSFSIPKGA

IVGIIGPNGAGKSTLFRMISGQEQPDSGTITLGETVKLASVDQFRDSMDNSKTVWEEVSGGLDIMKIGNT

EMPSRAYVGRFNFKGVDQGKRVGELSGGERGRLHLAKLLQVGGNMLLLDEPTNDLDIETLRALENALLEF

PGCAMVISHDRWFLDRIATHILDYQDEGKVEFFEGNFTEYEEYKKRTLGADALEPKRIKYKRIAK

>gi|30065626|ref|NP_839797.1| DNA repair protein RadA [Shigella flexneri 2a str. 2457T]

MAKAPKRAFVCNECGADYPRWQGQCSACHAWNTITEVRLAASPTVARNERLSGYAGSAGVAKVQKLSDIS

LEELPRFSTGFKEFDRVLGGGVVPGSAILIGGNPGAGKSTLLLQTLCKLAQQMKTLYVTGEESLQQVAMR

AHRLGLPTDNLNMLSETSIEQICLIAEEEQPKLMVIDSIQVMHMADVQSSPGSVAQVRETAAYLTRFAKT

RGVAIVMVGHVTKDGSLAGPKVLEHCIDCSVLLDGDADSRFRTLRSHKNRFGAVNELGVFAMTEQGLREV

SNPSAIFLSRGDEVTSGSSVMVVWEGTRPLLVEIQALVDHSIMANPRRVAVGLEQNRLAILLAVLHRHGG

LQMADQDVFVNVVGGVKVTETSADLALLLAMVSSLRDRPLPQDLVVFGEVGLAGEIRPVPSGQERISEAA

KHGFRRAIVPAANVPKKAPEGMQIFGVKKLSDALSVFDDL

>gi|30065625|ref|NP_839796.1| phosphoserine phosphatase [Shigella flexneri 2a str. 2457T]

MPNITWCDLPEDVSLWPGLPLSLSGDEVMPLDYHAGRSGWLLYGRGLDKQRLTQYQSKLGAAMVIVAAWC

VEDYQVIRLAGSLTARATRLAHEAQLDVAPLGKIPHLRTPGLLVMDMDSTAIQIECIDEIAKLAGTGEMV

AEVTERAMRGELDFTASLRSRVATLKGADANILQQVRENLPLMPGLTQLVLKLETLGWKVAIASGGFTFF

AEYLRDKLRLTAVVANELEIMDGKFTGNVIGDIVDAQYKAKTLTRLAQEYEIPLAQTVAIGDGANDLPMI

KAAGLGIAYHAKPKVNEKAEVTIRHADLMGVFCILSGSLNQK

>gi|30065624|ref|NP_839795.1| hypothetical protein S4690 [Shigella flexneri 2a str. 2457T]

MARTKLKFRLHRAVIVLFCLALLVALMQGASWFSQNHQRQRNPQLEELARTLARQVTLNVAPLMRTDSPD

EKRIQAILDQLTDESRILDAGVYDEQGDLIARSGESVEVRDRLALDGKKAGGYFNQQIVEPIAGKNGPLG

YLRLTLDTHTLATEAQQVDNTTNILRLMLLLSLAIGVVLTRTLLQGKRTRWQQSPFLLTASKPVPEEEES

EKKE

>gi|30065623|ref|NP_839794.1| hypothetical protein S4688 [Shigella flexneri 2a str. 2457T]

MIDLENQEREIINLMFSQRISWLAAVRIRHKLSLAEVSKMLGISINSLKQIEKTERLSSNIKSKMAEIYG

CPPELLICPSWMTAEHK

>gi|30065622|ref|NP_839793.1| purine nucleoside phosphorylase [Shigella flexneri 2a str. 2457T]

MATPHINAEMGDFADVVLMPGDPLRAKYIAETFLEDAREVNNVRGMLGFTGTYKGRKISVMGHGMGIPSC

SIYTKELITDFGVKKIIRVGSCGAVLPHVKLRDVVIGMGACTDSKVNRIRFKDHDFAAIADFDMVRNAVD

AAKALGVDARVGNLFSADLFYSPDGEMFDVMEKYGILGVEMEAAGIYGVAAEFGAKALTICTVSDHIRTH

EQTTAAERQTTFNDMIKIALESVLLGDKE

>gi|30065621|ref|NP_839792.1| phosphopentomutase [Shigella flexneri 2a str. 2457T]

MKRAFIMVLDSFGIGATEDAERFGDVGADTLGHIAEACAKGEADNGRKGPLNLPNLTRLGLAKAHEGSTG

FIPAGMDGNAEVIGAYAWAHEMSSGKDTPSGHWEIAGVPVLFEWGYFSDHENSFPQELLDKLVERANLPG

YLGNCHSSGTVILDQLGEEHMKTGKPIFYTSADSVFQIACHEETFGLDKLYELCEIAREELTNGGYNIGR

VIARPFIGDKAGNFQRTGNRHDLAVEPPAPTVLQKLVDEKHGQVVSVGKIADIYANCGITKKVKATGLDA

LFDATIKEMKEAGDNTIVFTNFVDFDSSWGHRRDVAGYAAGLELFDRRLPELMSLLRDDDILILTADHGC

DPTWTGTDHTREHIPVLVYGPKVKPGSLGHRETFADIGQTLAKYFGTSDMEYGKAMF

>gi|30065620|ref|NP_839791.1| thymidine phosphorylase [Shigella flexneri 2a str. 2457T]

MFLAQEIIRKKRDGHALSDEEIRFFINGIRDNTISEGQIAALAMTIFFHDMTMPERVSLTMAMRDSGTVL

DWKSLHLNGPIVDKHSTGGVGDVTSLMLGPMVAACGGYIPMISGRGLGHTGGTLDKLESIPGFDIFPDDN

RFREIIKDVGVAIIGQTSSLAPADKRFYATRDITATVDSIPLITASILAKKLAEGLDALVMDVKVGSGAF

MPTYELSEALAEAIVGVANGAGVRTTALLTDMNQVLASSAGNAVEVREAVQFLTGEYRNPRLFDVTMALC

VEMLISGKLAKDDAEARAKLQAVLDNGKAAEVFGRMVAAQKGPTDFVENYAKYLPTAMLTKAVYADTEGF

VSEMDTRALGMAVVAMGGGRRQASDTIDYSVGFTDMARLGDQVDGQRPLAVILAKDENSWQEAAKAVKAA

IKLADNAPESTPTVYRRISE

>gi|30065619|ref|NP_839790.1| deoxyribose-phosphate aldolase [Shigella flexneri 2a str. 2457T]

MTDLKASSLRALKLMDLTTLNDDDTDEKVIALCHQAKTPVGNTAAICIYPRFIPIARKTLKEQGTPEIRI

ATVTNFPHGNDDIEIALAETRAAIAYGADEVDVVFPYRALMAGDEQVGFDLVKACKEACAAANVLLKVII

ETGELKDEALIRKASEISIKAGADFIKTSTGKVAVNATPESARIMMEVIRDMGVEKTVGFKPAGGVRTAE

DAQKYLAIADELFGADWADARHYRFGASSLLASLLKALGHGDGKSASSY

>gi|30065618|ref|NP_839789.1| hypothetical protein S4683 [Shigella flexneri 2a str. 2457T]

MPTSHENALQQRCQQIVTSPVLSPEQKRHFLALEAENNLPYPQLPAEARRALDEGVICDMFEGHAPYKPR

YVLPDYARFLANGSEWLELEGAKDLDDALSLLTILYHHVPSVTSMPVYLGQLDALLQPYVRILTQNEIDV

RIKRFWRYLDRTLPDAFMHANIGPSDSPITRAILRADAQVSPNLTFIYDPEITPDDLLLEVAKNICECSK

PHIANGPVHDKIFTKGGYGIVSCYNSLPLAGGGSTLVRLNLKAIAERSESLDDFFTRTLPHYCQQQIAII

DARCEFLYQQSHFFENSFLVKEGLINPERFVPMFGMYGLAEAVNLLCEKEGIAARYGKEAAANEVGYRIS

AQLAEFVANTPVKYGWQKRAMLHAQSGISSDIGTTPGARLPYGDEPDPITHLQTVAPHHAYYYSGISDIL

TLDETIKRNPQALVQLCLGAFKAGMREFTANVSGNDLVRVTGYMVRLSDLEKYRAEGSRTNTTWLGEEAA

RNTRILERQPRVISHEQQMRFSQ

>gi|30065617|ref|NP_839788.1| activating enzyme [Shigella flexneri 2a str. 2457T]

MNSRCALVSKIIPFSCVDGPGSRLALFLQGCNLRCKNCHNPWTMGRCNDCGECVPQCPHQALQIVDGKVV

WNAVVCEQCDTCLKMCPQHATPMAQSMSVDEVLSHVRKAVLFIEGITVSGGEATTQLPFVVALFTAIKND

PQLRHLTCLVDSNGMLSETGWEKLLPVCDGAMLDLKAWGSECHQQLTGRDNQQIKRSIYLLAERGKLAEL

RLLMIPGQVDYLQHIEELAAFIKGLGDVPVRLNAFHAHGVYGEAQSWASATPEDVEPLADALKVRGVSRL

IFPALYL

>gi|30065616|ref|NP_839787.1| hypothetical protein S4681 [Shigella flexneri 2a str. 2457T]

MQALAENYQPLYAVLGLHPGMLEKHSDVSLEQLQQALERRPAKVVAVGEIGLDLFGDDPQFERQQWLLDE

QLKLAKRYDLPVILHSRRTHDKLAMHLKRHDLPRAGVVHGFSGSQQQAERFVQLGYKIGVGGTITYPRAS

KTRDVIAKLPLASLLLETDAPDMPLNGFQGQPNRPEQAARVFAVLCELRPEPADEIAEVLLNNTYAVFNV

RG

>gi|30065615|ref|NP_839786.1| hypothetical protein S4680 [Shigella flexneri 2a str. 2457T]

MGQRIPVTLGNIAPLSLRPFQPGRIALVCEGGGQRGIFTAGVLDEFMRAQFNPFDLYLGTSAGAQNLSAF

ICNQPGYARKVIMRYTTKREFFDPLRFVRGGNLIDLDWLVEATASQMPLQMDTAARLFDSGKSFYMCACR

QDDYAPNYFLPTKQNWLDVIRASSAIPGFYRSGVSLEGINYLDGGISDAIPVKEAARQGAKTLVVIRTVP

SQMYYTPQWFKRMERWLGDSSLQPLVNLVQHHETSYRDIQQFIEKPPGKLRISEIYPPKPLHSIALGSRI

PALREDYKLGRLCGRYFLATVGKLLTEKAPLTRHLVPVVTPESIVIPPAPVANDTLVAEVSDAPQANDPT

FNNEDLA

>gi|30065614|ref|NP_839785.1| periplasmic protein [Shigella flexneri 2a str. 2457T]

MTMTRLKISKTLLAVMLTSAVATGSAYAENNAQTTNESAGQKVDSSMNKVGNFMDDSAITAKVKAALVDH

DNIKSTDISVKTDQKVVTLSGFVESQAQAEEAVKVAKGVEGVTSVSDKLHVRDAKEGSVKGYAGDTATTS

EIKAKLLADDIVPSRKVKVETTDGVVQLSGTVDSQAQSDRAESIAKAVDGVKSVKNDLKTK

>gi|30065613|ref|NP_839784.1| peptide chain release factor 3 [Shigella flexneri 2a str. 2457T]

MTLSPYLKEVAKRRTFAIISHPDAGKTTITEKVLLFGQAIQTAGTVKGRGSNQHAKSDWMEMEKQRGISI

TTSVMQFPYHDCLVNLLDTPGHEDFSEDTYRTLTAVDCCLMVIDAAKGVEDRTRKLMEVTRLRDTPILTF

MNKLDRDIRDPMELLDEVENELKIGCAPITWPIGCGKLFKGVYHLYKDETYLYQSGKGHTIQEVRIVKGL

NNPDLDAAVGEDLAQQLRDELELVKGASNEFDKELFLAGEITPVFFGTALGNFGVDHMLNGLVEWAPAPM

PRQTDTRTVEASEDKFTGFVFKIQANMDPKHRDRVAFMRVVSGKYEKGMKLRQVRTAKDVVISDALTFMA

GDRSHVEEAYPGDILGLHNHGTIQIGDTFTQGEMMKFTGIPNFAPELFRRIRLKDPLKQKQLLKGLVQLS

EEGAVQVFRPISNNDLIVGAVGVLQFDVVVARLKSEYNVEAVYESVNVATARWVECADAKKFEEFKRKNE

SQLALDGGDNLAYIATSMVNLRLAQERYPDVQFHQTREH

>gi|30065612|ref|NP_839783.1| nucleotidase [Shigella flexneri 2a str. 2457T]

MKWDWIFFDADETLFTFDSFTGLQRMFLDYSVTFTAEDFQDYQAVNKPLWVDYQNGAITSLQLQHGRFES

WAERLKVEAGLLNDAFINAMAEICTPLPGAVSLLNAIRSNAKIGIITNGFSALQQVRLERTGLRDYFDLL

VISEEVGVAKPNKKIFDYALEQAGNPDRSRVLMVGDTAESDILGGINAGLATCWLNAHNREQPEGIAPTW

TVSSLHELEQLLCKH

>gi|30065611|ref|NP_839782.1| ribosomal-protein-alanine N-acetyltransferase [Shigella flexneri 2a str. 2457T]

MNTISSLETTDLPAAYHIEQRAHAFPWSEKTFASNQGERYLNFQLTQNGKMAAFAITQVVLDEATLFNIA

VDPDYQRQGLGRALLEHLIDELEKRGVATLWLEVRASNAAAIALYESLGFNEATIRRNYYPTTDGREDAI

IMALPISM

>gi|30065610|ref|NP_839781.1| DNA polymerase III subunit psi [Shigella flexneri 2a str. 2457T]

MTSRRDWQLQQLGITQWSLRRPGALQGEIAIASPAHVRLVMVANDLPALTDPLVSDVLRALTVSPDQVLQ

LTPEKIAMLPQGSRCNSWRLGTDEPLSLEGAQVASPALTELRANPTARAALWQQICTYEHDFFPRND

>gi|30065609|ref|NP_839780.1| 16S ribosomal RNA m2G1207 methyltransferase [Shigella flexneri 2a str. 2457T]

MSAFTPASEVLLRHSDDFEQSRILFAGDLQDDLPARLDTAASRAHTQQFHHWQVLSCQMGDNARFSLVAT

ANDVADCDTLIYYWPKNKPEAQFQLMNLLSLLPVGTDIFVVGENRSGVRSAEQMLADYAPLNKVDSARRC

GLYFGRLEKQPVFDADKFWGEYSVDGLTVKTLPGVFSRDGLDVGSQLLLSTLTPHTKGKVLDVGCGAGVL

SVAFARHSPKIRLTLCDVSAPAVEASRATLAANGVEGEVFASNVFSEVKGCFDMIISNPPFHDGMQTSLD

AAQTLIRGAVRHLNSGGELRIVANAFLPYPDVLDETFGFHEVIAQTGRFKVYRAIMTRQAKKG

>gi|30065608|ref|NP_839779.1| ferric iron reductase involved in ferric hydroximate transport [Shigella flexneri 2a str. 2457T]

MAYRSAPLYEDVIWRTHLQPQDAGLAQAVRAMIAKHREHLLEFIRLDEPAPLNAMTLAQWSSPNALSSLL

AVYSDHIYRNQPTMIRENKPLISLWAQWYIGLMVPPLMLALLTQEKALDVSPEHFHAEFHETGRVACFWV

DVCEDKNATPHSPQQRMETLISQALVPVVQALEATGEINGKLIWSNTGYLINWYLTEMKQLLGEATVESL

RHALFFEKTLTNGEDNPLWRTVVLRDGLLVRRTCCQRYRLPDVQQCGYCTLK

>gi|30065606|ref|NP_839777.1| IS1 orfB [Shigella flexneri 2a str. 2457T]

MSRQCTHYGRWPQHGFTSLKKLRPQSVTSRIQPGSDVIVCAEMDEHWGYVGAKSRQRWLFYAYDRIRRTV

VAHVFGERTLATLERLLSLLSAFEVVVWMTDGWPLYESRLKGKLHVISKRYTQRIERHNLNLRQHLARLG

RKSLSFSKSVELHDKVIGHYLNIKHYQ

>gi|30065605|ref|NP_839776.1| IS1 orfA [Shigella flexneri 2a str. 2457T]

MASISIRCPSCSATEGVVRNGKSTAGHQRYLCSHCRKTWQLQFTYTASQPGTHQKIIDMAMNGVGCRASA

RIMGVGLNTVLRHLKNSGRSR

>gi|30065603|ref|NP_839774.1| primosomal protein DnaI [Shigella flexneri 2a str. 2457T]

MSSRVLTPDVVGIDALVHDHQTVLAKAEGGVVAVFANNAPAFYAVTPARLAELLALEEKLARPGSDVALD

DQLYQEPQAAPVAVPMGKFAMYPDWQPDADFIRLAALWGVALREPVTTEELASFIAYWQAEGKVFHHVQW

QQKLARSLQIGRASNGGLPKRDVNTVSEPDSQIPPGFRG

>gi|30065602|ref|NP_839773.1| DNA replication protein DnaC [Shigella flexneri 2a str. 2457T]

MKNVGDLMQRLQKMMPAHIKPAFKTGEELLAWQKEQGAIRSAALERENRAMKMQRTFNRSGIRPLHQNCS

FENYRVECEGQMNALSKARQYVEEFDGNIASFIFSGKPGTGKNHLAAAICNELLLRGKSVLIITVADIMS

AMKDTFRNSGTSEEQLLNDLSNVDLLVIDEIGVQTESKYEKVIINQIVDRRSSSKRPTGMLTNSNMEEMT

KLLGERVMDRMRLGNSLWVIFNWDSYRSRVTGKEY

>gi|30065601|ref|NP_839772.1| hypothetical protein S4661 [Shigella flexneri 2a str. 2457T]

MMMKTIKHLLCCAIAASALISTGVHAASWKDALSSAASELGNQNSTTQEGGWSLASLTNLLSSGNQALSA

DNMNNAAGILQYCAKQKLASVTDAENIKNQVLEKLGLNSEEQKEDTNYLDGIQGLLKTKDGQQLNLDNIG

TTPLAEKVKTKACDLVLKQGLNFIS

>gi|30065599|ref|NP_839770.1| hypothetical protein S4658 [Shigella flexneri 2a str. 2457T]

MYNISRTTVRHILSQLRECGVLTQVGNDYVIARKPDHDDGFACTTASMSEQNKVFEQAFFTMINQRQLRP

RETFSELQLARAAGVSPVVVREYLLKFGRYNLIQSEKRGQWSMKQFDQSYAEQLFELREMLETHSLQHFL

NLPDHDPRWLQAKTMLERHRLLRDNIGNSFRMFSQLDRDFHSLLLSAADNIFFDQSLEIISVIFHFHYQW

DESDLKQRNIIAVDEHMTILSALICRSDLDATLALRNHLNSAKQSMIRSINENTRYAH

>gi|30065598|ref|NP_839769.1| transporter [Shigella flexneri 2a str. 2457T]

MEKENITLDPRSSFTPSSSADIPVPPDGLVQRSTRIKRIQTTAMLLLFFAAVINYLDRSSLSVANLTIRE

ELGLSATEIGALLSVFSLAYGIAQLPCGPLLDRKGPRLMLGLGMFFWSLFQAMSGMVHSFTQFVLVRIGM

GIGEAPMNPCGVKVINDWFNIKERGRPMGFFNAASTIGVAVSPPILAAMMLVMGWRGMFITIGVLGIFLA

IGWYMLYRNREHVELTAVEQAYLNAGSVNARRDPLNFAEWRSLFRNRTMWGMMLGFSGINYTAWLYLAWL

PGYLQTAYNLDLKSTGLMAAIPFLFGAAGMLVNGYVTDWLVKGGMAPIKSRKICIITGMFCSAAFTLIVP

QATTSMMAVLLIGMALFCIHFAGTSCWGLIHVAVASRMTASVGSIQNFASFICASFAPIITGFIVDTTHS

FRLALIICGCVTAAGALAYIFLVRQPINDPRKD

>gi|30065597|ref|NP_839768.1| methyl-accepting chemotaxis protein I, serine sensor receptor [Shigella flexneri 2a str. 2457T]

MLKRIKIVTSLLLVLAVFGLLQLTSGGLFFNALKNDKENFTVLQTIRQQQPTLNGSWVALLQTRNTLNRA

GIRYMMDQNNIGSGSTVAELMQSASISLKQAEKNWADYEALPRDPRQSTAAAAEIKRNYDIYHNALAELI

QLLGAGKINEFFDQPTQGYQDGFEKQYVAYMEQNDRLYDIAVSDNNASYSQAMWILVGVMIVVLAVIFAV

WFGIKASLVAPMNRLIDSIRHIAGGDLVKPIEVDGSNEMGQLAESLRHMQGELMRTVGDVRNGANAIYSG

ASEIATGNNDLSSRTEQQAASLEETAASMEQLTATVKQNAENARQASHLALSASETAQRGGKVVDNVVQT

MRDISTSSQKIADIISVIDGITFQTNILALNAAVEAARAGEQGRGFAVVAGEVRNLAQRSAQAVREIKSL

IEDSVGKVDVGSTLVESAGETMAEIVSAVTRVTDIMGEIASASDEQSRGIDQVGLAVAEMDRVTQQNAAL

VEESAAAAAALEEQASRLTEAVAVFRIQQQQQQQRETSAVVKTVTPATPRKMAVADSGENWETF

>gi|30065596|ref|NP_839767.1| regulator of the 4HPA-hydroxylase operon [Shigella flexneri 2a str. 2457T]

MHDSLTIALLQAREAAMSYFRPIVKRHNLTEQQWRIVRILAESPSMDFHDLAYRACILRPSLTGILTRME

RDGLVLRLKPINDQRKLYISLTKEGQALYNRAQTQIEEAYRQIEAQFTAEKMQQLTHLLEEFIALGNSRQ

EDIPGDNE

>gi|30065595|ref|NP_839766.1| 5-oxo-1,2,5-tricarboxilic-3-penten acid decarboxilase/isomerase [Shigella flexneri 2a str. 2457T]

MKGTIFAVALNHRSQLDAWQEAFQQSPYKAPPKTAVWFIKPRNTVIGCGEPIPFPQGEKVLSGATVALIV

GKTATKVREEDAAEYIAGYALDNDVSLPEESFYRPAIKAKCRDGFCPIGETVALSNVDNLTIYTEINGRP

ADHWNTADLQRNAAQLLSALSEFATLNPGDAILLGTPQARVEIQPSDRVRVLAEGFPPLENPVVDEREVT

TRKSFPTLPHPHGTLFALGLNYADHASELEFKPPEEPLVFLKAPNTLTGDNQTSVRPNNIEYMHYEAELV

VVIGKQARNVSEADAMDYVAGYTVCNDYAIRDYLENYYRPNLRVKSRDGLTPMLSTIVPKEAIPDPHNLT

LRTFVNGELRQQGTTADLIFSVPFLIAYLSEFMTLNPGDMIATGTPKGLSDVVPGDEVVVEVEGVGRLVN

RIVSEETAK

>gi|30065594|ref|NP_839765.1| 5-carboxy-2-hydroxymuconate semialdehyde dehydrogenase [Shigella flexneri 2a str. 2457T]

MKKVNHWINGKNVAGNDYFQTTNPATGEVLADVASGGEAEINQAVAAAKEAFPKWADLPMKERARLMRRL

GDLIDQNVPEIAAMETADTGLPIHQTKNVLIPRASHNFEFFAEVCQQMNGKTYPVDDKMLNYTLVQPVGV

CALASPWNVPFMTATWKVAPCLALGNTAVLKMSELSPLTADRLGELALEAGIPAGVLNVVQGYGATAGDA

LVRHHDVRAVSFTGGTATGRNIMKNAGLKKYSMELGGKSPVLIFEDADIERALDAALFTIFSINGERCTA

GSRIFIQQSIYPEFVKRFAERANRLRVGDPNDPNTQVRALISQQHWEKVSGYIRLGIEEGATLLAGGPDK

PSDLPAHLKGGNFLRPTVLADVDNRMRVAQEEIFGPVACLLPFKDEAEGLRLANDVEYGLASYIWTQDVS

KVLRLARGIEAGMVFVNTQNVRDLRQPFGGVKASGTGREGGEYSFEVFAEMKNVCISMGDHPIPKWGV

>gi|30065593|ref|NP_839764.1| homoprotocatechuate dyoxygenase [Shigella flexneri 2a str. 2457T]

MGKLALAAKITHVPSMYLSELPGKNHGCRQGAIDGHKEISKRCREMGVDTIIVFDTHWLVNSAYHINCAD

HFEGVYTSNELPHFIRDMTYNYEGNPELGQLIAEEALKLGVRAKAHNIPSLKLEYGTLVPMRYMNEDKHF

KVVSISAFCTVHDFADSRKLGEAILKAIEQYDGTVAVLASGSLSHRFIDDQRAEEGMNSYTREFDRQMDE

RVVKLWREGQFKEFCNMLPEYADYCYGEGNMHDTVMLLGMLGWDKYDGKVEFITELFPSSGTGQVNAVFP

LPA

>gi|30065592|ref|NP_839763.1| 5-carboxymethyl-2-hydroxymuconate delta-isomerase [Shigella flexneri 2a str. 2457T]

MPHFIVECTENIREEARLPELFASVNTALAATGIFPLGGIRSRAHWIDTWQMADGKHDYAFVHMTLKIGS

GRSLESRQEVGEMLFDLIKTHFASLMESRYLALSFEIAELHPTLNFKQNNVHALFK

>gi|30065591|ref|NP_839762.1| 2-oxo-hepta-3-ene-1,7-dioic acid hydratase [Shigella flexneri 2a str. 2457T]

MFDKHTHTLIAQRLDQAEKQREQIRAISLDYPEITIEDAYAVQREWVRLKIAESRTLKGHKIGLTSKAMQ

ASSQISEPDYGALLDDMFFHDGSDIPTDRFIVPRIEVELAFVLAKPLRGPNCTLFDVYNATDYVIPALEL

IDARCHNIDPETQRPRKVFDTISDNAANAGVILGGRPIKPDELDLRWISALMYRNGVIEETGVAAGVLNH

PANGVAWLANKLAPYDVQLETGQIILGGSFTLPVPARKGDTFHVDYGNMGSISCRFV

>gi|30065590|ref|NP_839761.1| 2,4-dihydroxyhept-2-ene-1,7-dioic acid aldolase [Shigella flexneri 2a str. 2457T]

MENSFKAALKAGRPQIGLWLGLSSSYSAELLAGPGFDWLLIDGEHAPNNVQTVLTQLQAIAPYPSQPVVR

PSWNDPVQIKQLLDVGTQTLLVPMVQNADEARKAVRATRYPPAGIRGVGSALARASRWNRIPDYLQKAND

QMCVLVQIETREAMKNLPQILDVEGVDGVFIGPADLSADMGYAGNPQHPEVQAAIEQAIVQIREAGKAPG

ILIANEQLAKRYLELGALFVAVGVDTTLLARAAEALAARFGNSSSISMAKQNNNSVY

>gi|30065589|ref|NP_839760.1| 4-hydroxyphenylacetate permease [Shigella flexneri 2a str. 2457T]

MTTSTLQYKDNKTVEIEHRVINKLFRRLIIFLFILFVFSFLDRINIGFAGLTMGKDLGLTSTMFGLAATL

FYVTYVMCGIPSNMMLAKIGARRWIAGIMVVWGIASTCTMFATSPETLYLLRMLVGIAEAGFLPGILVYL

TWWFPAYHRARANALFMIAMPVTMMLGLILSGYILALDGLWNLKGWQWLFLLEGLPSVILGVVTWFYLND

TPDQATWLDENEKQALKTMILREQESAFAHASVPRSTLREVLTPAIMLYTLAYFCLTNTLSAINIWTPQI

LQSFNTGSSNIMIGLLAAIPQFCTIFGMIWWSRRSDRLKERKKHTILPYLFAAAGWLLASATDHSLIQLS

SIIMASMGSFTAMTIFWTTPDQVISLQSRAVAMAVINAVGNVGSAVSPLLIGILKDATGSFSSGLWFVAG

LLVLGALVMTRIPMGSREHSLSSELIAPKPQ

>gi|30065588|ref|NP_839759.1| 4-hydroxyphenylacetate 3-monooxygenase operon regulatory protein [Shigella flexneri 2a str. 2457T]

MSSEAIFEHGYENIDLVREYDARYAGEDVHYETFARLANFFGRDMRPHWHDRYFQLHYLVTGRITLQLDE

HFYSVRAPLFVLTPPSVPHTFFTNPDSDGHVLTVRQELIWPLVEKLWPGSGDAAMLQGICLSLEGMEHTL

AALNHYWPIISLEFQQNKQGREILLQSLAQSIFTILLREAPPNDISTCSVRGEMCLFQKFNRLIDENYRQ

HLMVPEYATMLGMSESRLTELCRRFANQSPKRLIFERVSREARRLLLYSEQSINQIALDLGYKDPAYFAR

FFNRMMGCSPTQFRGR

>gi|30065587|ref|NP_839758.1| hypothetical protein S4646 [Shigella flexneri 2a str. 2457T]

MATALIIGNKTWLIDAGYGASLQLVKNNIPLRQIDNILLTHLHSDHILDYPSVLMNAWASGLQDKTIHVF

GPPGTRAMTAASWQVFDRDITLRMEEEGKPDPRKLVTAQDVGEGVVYKDDTVTVTALKVPHSPFPDGEAF

AYRFDTQGKRIVFSGDTSYFPPLATFSEGADILVHEAVHEPSVAKLADSIGNGKTLAKAIASHHTTIEDV

GKIANEAKVKKLILSHLVPITVPDETWKQEAQKTIQVLSSLVMKI

>gi|30065586|ref|NP_839757.1| component C of the 4HPA-hydroxylase [Shigella flexneri 2a str. 2457T]

MQLDEQRLRFHDAMASLSAAVNIITTEGDAGQCGITATAVCSVTDTPPSLMVCINANSAMNPVFQGNGKL

CVNVLNHEQELMARHFAGMTGMAMEERFSLSCWQKGPLAQPVLKGSLASLEGEIRDVQAIGTHLVYLVEI

KNIILSAEGHGLIYFKRRFHPVMLEMEAAI

>gi|30065585|ref|NP_839756.1| carbon starvation protein [Shigella flexneri 2a str. 2457T]

MPGFTMDTKKLFKHIPWVILGIIGAFCLAVVALRRGEHVSALWIVVASVSVYLVAYRYYSLYIAQKVMKL

DPTRATPAVINNDGLNYVPTNRYVLFGHHFAAIAGAGPLVGPVLAAQMGYLPGTLWLLAGVVLAGAVQDF

MVLFISSRRNGASLGEMIKEEMGPVPGTIALFGCFLIMIIILAVLALIVVKALAESPWGVFTVCSTVPIA

LFMGIYMRFIRPGRVGEVSVIGIVLLVASIYFGGVIAHDPYWGPALTFKDTTITFALIGYAFVSALLPVW

LILAPRDYLATFLKIGVIVGLALGIVVLNPELKMPAMTQYIDGTGPLWKGALFPFLFITIACGAVSGFHA

LISSGTTPKLLANETDARFIGYGAMLMESFVAIMALVAASIIEPGLYFAMNTPPAGLGITMPNLHEMGGE

NAPIIMAQLKDVTAHAAATVSSWGFVISPEQILQTAKDIGEPSVLNRAGGAPTLAVGIAHVFHKVLPMAD

MGFWYHFGILFEALFILTALDAGTRSGRFMLQDLLGNFIPFLKKTDSLVAGIIGTAGCVGLWGYLLYQGV

VDPLGGVKSLWPLFGISNQMLAAVALVLGTVVLIKMKRTQYIWVTVVPAVWLLICTTWALGLKLFSTNPQ

MEGFFYMASQYKEKIANGTDLTAQQIANMNHIVVNNYTNAGLSILFLIVVYSIIFYGFKTWLAVRNSDKR

TDKETPYVPIPEGGVKISSHH

>gi|30065584|ref|NP_839755.1| hypothetical protein S4641 [Shigella flexneri 2a str. 2457T]

MFGNLGQAKKYLGQAAKMLIGIPDYDNYVEHMKTNHPDKPYMSYEEFFRERQNARYGGDGKGGMRCC

>gi|30065583|ref|NP_839754.1| GTP-binding protein YjiA [Shigella flexneri 2a str. 2457T]

MIENEFGEVSVDDQLIGDRATQIKTLTNGCICCSRSNELEDALLDLLDNLDKGNIQFDRLVIECTGMADP

GPIIQTFFSHEILCQLYLLDGVIALVDAVHADEQMNQFTIAQSQVGYADRILLTKTDVAGEAEKLRERLA

RINARAPVYTVTHGDIDLGLLFNTNGFMLEENVVSTKPRFHFIADKQNDISSIVVELDYPVDISEVSRVM

ENLLLESADKLLRYKGMLWIDGEPNRLLFQGVQRLYSADWDRPWGDEKPHSTMVFIGIQLPEEEIRAAFA

GLRK

>gi|30065582|ref|NP_839753.1| IS1 orfB [Shigella flexneri 2a str. 2457T]

MSRQCTHYGRWPQHGFTSLKKLRPQSVTSRIQPGSDVIVCAEMDEHWGYVGAKSRQRWLFYAYDRIRRTV

VAHVFGERTLATLERLLSLLSAFEVVVWMTDGWPLYESRLKGKLHVISKRYTQRIERHNLNLRQHLARLV

RKSLSFSKSVELHDKVIGHYLNIKHYQ

>gi|30065581|ref|NP_839752.1| IS1 orfA [Shigella flexneri 2a str. 2457T]

MASISIRCPSCSATEGVVRNGKSTAGHQRYLCSHCRKTWQLQFTYTASQPGTHQKIIDMAMNGVGCRASA

RIMGVGLNTVLRHLKNSGRSR

>gi|30065580|ref|NP_839751.1| hypothetical protein S4635 [Shigella flexneri 2a str. 2457T]

MSVEKLIVDHMETWTSALQTRSTAGRGSSGKIDLYGIKKLRELILELAVRGKLVPQDPNDEPASDLLKRI

AAEKAELVKQGKIKKQKPLPEISEEEKPFELPEGWEWVHLPDIYCSISESSRKIKSSEILPEGKYPVIEQ

SQEFISGYCNNECLLIKLNNPVIVFGDHTRNIKFIDFDFVVGADGVKILSPILICERFFFWQLRSFKLDV

RGYARHFKVLNSCLFALPPIAEQERIVEKVSSLMSLCDQLEQQSLTSLDAHQQLVETLLGTLTDSQNTAE

LAENWARISEHFDTLFTTEASVDALKQTILQLAVMGKLVPQDPNDEPASELLKRIAQEKAQLVKEGKIQK

PLPPISDEEKPFELPEGWEWCRIGNIVNIKSELVSPKDYLNLYQVAPDIIEKGTGRVISKRTVKESGVKG

PNSRFYKGQIVYSKIRPSLSKVFLAEYNGLCSADMYPLDCYINPNYLLKYILSIPFLMQVKKAENRIKMP

KLNSDSFYNIIVAIPPYNEQQAIFDKINSIEAVCNGLISYIGIYHKTQLHLADALTDAAIN

>gi|30065579|ref|NP_839750.1| hypothetical protein S4633 [Shigella flexneri 2a str. 2457T]

MLFDHLRDEVMRLDAGITQEVLKLYIAFKAETNFVDVVPQKSRLRLSLNMQFHELVDPKGIAKDVTNVGR

WGNGDVEIGFSDLAQLPYIMGLIRQAFEKQMESALV

>gi|30065578|ref|NP_839749.1| IS600 orfA [Shigella flexneri 2a str. 2457T]

MSRKTQRYSKEFKAEAVRTVLENQLSISEGASRLSLPEGTLGQWVTAARKGLGTPGSRTVAELESEILQL

RKALNEARLERDILKKATAYFAQESLKNTR

>gi|30065577|ref|NP_839748.1| IS600 orfB [Shigella flexneri 2a str. 2457T]

MAHIRTRETYGTRRLQTELAENGIIVGRDRLARLRKELRLRCKQKRKFRATTNPNHNLPVAPNLLNQTFA

PTAPNQVWVADLTYVATQEGWLYLAGIKDVYTCEIVGYAMGERMTKELTGKALFMALRSQRPPAGLIHHS

DRGSQYCAYDYRVIQEQFGLKTSMSRKGNCYDNAPMESFWGTLKNESLSHYRFNNRDEAISVIREYIEIF

YNRQRRHSRLGNISPAAFREKYHQMAA

>gi|30065576|ref|NP_839747.1| ISEhe3 orfB [Shigella flexneri 2a str. 2457T]

MLDVHPSGFYAWLQQPHSQRHQADLRLTGQIKQFWLESGCVYGYRKIHLDLRDSGQQCGVNRVWRLMKRV

GIKAQVGYRSPRARKGEASIVSPNRLQRQFNPDAPDERWVTDITYIRTHEGWLYLAVVVDLFSRKIIGWS

MQSRMTKDIVLNALLMAVWRRNPEKQVLVHSDQGSQYTSHEWQSFLKSHGLEG

>gi|30065575|ref|NP_839746.1| ISEhe3 orfA [Shigella flexneri 2a str. 2457T]

MSGKRYPEEFKTEAVKQVVDRGYSVASVATRLDITTHSLYAWIKKYGPDSSTNKEQSDAQAEIRRLQKEL

KRVTDERDILKKAAAYFAKLSD

>gi|30065574|ref|NP_839745.1| 50S ribosomal protein L9 [Shigella flexneri 2a str. 2457T]

MQVILLDKVANLGSLGDQVNVKAGYARNFLVPQGKAVPATKKNIEFFEARRAELEAKLAEVLAAANARAE

KINALETVTIASKAGDEGKLFGSIGTRDIADAVTAAGVEVAKSEVRLPNGVLRTTGEHEVSFQVHSEVFA

KVIVNVVAE

>gi|30065573|ref|NP_839744.1| 30S ribosomal protein S18 [Shigella flexneri 2a str. 2457T]

MARYFRRRKFCRFTAEGVQEIDYKDIATLKNYITESGKIVPSRITGTRAKYQRQLARAIKRARYLSLLPY

TDRHQ

>gi|30065572|ref|NP_839743.1| primosomal replication protein N [Shigella flexneri 2a str. 2457T]

MTNRLVLSGTVCRTPLRKVSPSGIPHCQFVLEHRSVQEEAGFHRQAWCQMPVIVSGHENQAITHSITVGS

RITVQGFISCHKAKNGLSKMVLHAEQIELIDSGD

>gi|30065571|ref|NP_839742.1| 30S ribosomal protein S6 [Shigella flexneri 2a str. 2457T]

MRHYEIVFMVHPDQSEQVPGMIERYTAAITGAEGKIHRLEDWGRRQLAYPINKLHKAHYVLMNVEAPQEV

IDELETTFRFNDAVIRSMVMRTKHAVTEASPMVKAKDERRERRDDFANETADDAEAGDSEE

>gi|30065570|ref|NP_839741.1| hypothetical protein S4624 [Shigella flexneri 2a str. 2457T]

MFSRVLALLAVLLLSANTWAAIEINNHQARNMDDVQSLGVIYINHNFATESEARQALNEETDAQGATYYH

VILMREPGSNGNMHASADIYR

>gi|30065569|ref|NP_839740.1| L-ribulose-5-phosphate 4-epimerase [Shigella flexneri 2a str. 2457T]

MQKLKQQVFEANMDLPRYGLVTFTWGNVSAIDRERGLVVIKPSGVAYETMKADDMVVVDMSGKVVEGEYR

PSSDTATHLELYRRYPSLGGIVHTHSTHATAWAQAGLAIPALGTTHADYFFGDIPCTRGLSEEEVQGEYE

LNTGKVIIETLGNAEPLHTPGIVVYQHGPFAWGKDAHDAVHNAVVMEEVAKMAWIARSINPQLNHIDSYL

MNKHFMRKHGPNAYYGQK

>gi|30065568|ref|NP_839739.1| L-xylulose 5-phosphate 3-epimerase [Shigella flexneri 2a str. 2457T]

MLSKQIPLGIYEKALPAGECWLERLRLAKTLGFDFVEMSVDETNERLSRLDWSREQRLALVNAIVETGVR

VPSMCLSAHRRFPLGSEDDAVRAQGLEIMRKAIQFAQDVGIRVIQLAGYDVYYQEANNETRRRFRDGLKE

SVEMASRAQVTLAMEIMDYPLMNSISKALGYAHYLNNPWFQLYPDIGNLSAWDNDVQMELQAGIGHIVAV

HVKDTKPGVFKNVPFGEGVVDFERCFETLKQSGYCGPYLIEMWSETAEDPAAEVVKACDWVKARMAKAGM

VEAA

>gi|30065567|ref|NP_839738.1| 3-keto-L-gulonate-6-phosphate decarboxylase [Shigella flexneri 2a str. 2457T]

MSLPMLQVALDNQTMDSAYETTRLIAEEVDIIEVGTILCVGEGVRAVRDLKALYPHKIVLADAKIADAGK

ILSRMCFEANADWVTVICCADINTAKGALDVAKEFNGDVQIELTGYWTWEQAQQWRDAGIQQVVYHRSRD

AQAAGVAWGEADITAIKRLSDMGFKVTVTGGLALEDLPLFKGIPIHVFIAGRSIRDAASPVEAARQFKRS

IAELWG

>gi|30065566|ref|NP_839737.1| L-ascorbate-specific enzyme IIA component of PTS [Shigella flexneri 2a str. 2457T]

MKLHDSLAENKSIRLQAEAETWQDAVKIGVDLLVAADVVEPRYYQAILDAVEQHGPYFVLAPGLAMPHGR

PEEGVKKTGFALVTLKKPLEFNHEDNDPVDILITMAAVDANTHQEVGIMQIVNLFEDEENFDRLRACRTE

QEVLDLIDRTNAAA

>gi|30065565|ref|NP_839736.1| L-ascorbate-specific enzyme IIB component of PTS [Shigella flexneri 2a str. 2457T]

MTVRILAVCGNGQGSSMIMKMKVDQFLTQSNIDHTVNSCAVGEYKSELSGADIIIASTHIAGEITVTGNK

YVVGVRNMLSPADFGPKLLEVIKAHFPQDVK

>gi|30065563|ref|NP_839734.1| L-ascorbate 6-phosphate lactonase [Shigella flexneri 2a str. 2457T]

MAMSKVKSITRESWILSTFPEWGSWLNEEIEQEQVAPGTFAMWWLGCTGIWLKSEGGTNVCVDFWCGTGK

QSHGNPLMKQGHQMQRMAGVKKLQPNLRTTPFVLDPFAIRQIDAVLATHDHNDHIDVNVAAAVMQNCADD

VPFIGPKTCVDLWIGWGVPKERCIVVKPGDVVKVKDIEIHALDAFDRTALITLPADQKAAGVLPDGMDDR

AVNYLFKTPGGSLYHSGDSHYSNYYAKHGNEHQIDVALGSYGENPRGITDKMTSADMLRMGEALNAKVVI

PFHHDIWSNFQADPQEIRVLWEIKKDRLKYGFKPFIWQVGGKFTWPLDKDNFEYHYPRGFDDCFTIEPDL

PFKSFL

>gi|30065562|ref|NP_839733.1| transcriptional repressor UlaR [Shigella flexneri 2a str. 2457T]

MTEAQRHQILLEMLAQLGFVTVEKVVERLGISPATARRDINKLGESGKLKKVRNGAEAITQQRPRWTPMN

LHQAQNHDEKVRIAKAASQLVNPGESVVINCGSTAFLLGREMCGKPVQIITNYLPLANYLIDQEHDSVII

MGGQYNKSQSITLSPQGSENSLYAGHWMFTSGKGLTAEGLYKTDMLTAMAEQKMLSVVGKLVVLVDSSKI

GERAGMLFSRADQIDMLITGKNANPEILQQLEAQGVSILRV

>gi|30065561|ref|NP_839732.1| esterase [Shigella flexneri 2a str. 2457T]

MIEIESRELADIPVLHAYPVGQKDTPLPCVIFYHGFTSSSLVYSYFAVALAQAGLRVIMPDAPDHGSRFS

GDEARRLNQFWQILLQSMQEFTTLRAAIAEENWLLDDRLAVGGASMGAMTALGITARHPTVKCTASMMGS

GYFTSLARSLFPPLIPETAAQQNEFNKIVAPLAEWEATNHLEQLGDRPLLLWHGLDDDVVPADESLRLQQ

ALSETGRDKLLTCSWQPGVRHRITPEALDAAVTFFRQHL

>gi|30065559|ref|NP_839730.1| hypothetical protein S4613 [Shigella flexneri 2a str. 2457T]

MGKRKMELTMKQLLASPSLQLVTYPASATAQSAEFASADCVTGLNEIGQISVSNISGDPQDVERIVALKA

DEQGASWYRIITMYEDQQPDNWRVQAILYA

>gi|30065558|ref|NP_839729.1| IS1 orfA [Shigella flexneri 2a str. 2457T]

MASISIRCPSCSATEGVVRNGKSTAGHQRYLCSHCRKTWQLQFTYTASQPGKHQKIIDMAMNGVGCRASA

RIMGVGLNTVLRHLKNSGRSR

>gi|30065557|ref|NP_839728.1| IS1 orfB [Shigella flexneri 2a str. 2457T]

MSRQCTHYGRWPQHGFTSLKKLRPQSVTSRIQPGSDVIVCAEMDEHWGYVGAKSRQRWLFYAYDRIRRTV

VAHVFGERTLATLERLLSLLSAFEVVVWMTDGWPLYESRLKGKLHVISKRYTQRIERHNLNLRQHLARLV

RKSLSFSKSVELHDKVIGHYLNIKHYQ

>gi|30065556|ref|NP_839727.1| synthetase/amidase [Shigella flexneri 2a str. 2457T]

MLRHNVPVRRDLDQIAANNGFDFHIIDNEIYWDESRAYRFTLRQIEEQIEKPTAELHQMCLEVVDRAVKD

EEILTQLAIPPLYWDVIAESWRARDPSLYGRMDFAWCGNAPVKLLEYNADTPTSLYESAYFQWLWLEDAR

RSGIIPRDADQYNAIQERLISRFSELYSRELFYFCCCQDTDEDRSTVLYLQDCAQQAGQESRFIYIEDLG

LGVGGVLTDLDDNVIQRAFKLYPLEWMMRDDNGPLLRKRREQWVEPLWKSILSNKGLMPLLWRFFPGHPN

LLASWFEGEKSQIAAGESYVRKPIYSREGGNVTIFDGQNNVVDHADGDYADEPMIYQAFQPLPRFGDSYT

LIGSWIVDDEACGMGIREDNTLITKDTSRFVPHYIAG

>gi|30065555|ref|NP_839726.1| hypothetical protein S4607 [Shigella flexneri 2a str. 2457T]

MARKRKSRNNSKIGHGAISRIGRPNNPFEPRRNRYAQKYLTLALMGGAAFFVLKGCSDSSDVDNDGDGTF

YATVQDCIDDGNNADICARGWNNAKAAFYADVPKNMTQQNCQSKYENCYYDNVEQSWIPVVSGFLLSRVI

RKDRDEPFVYNSGGSSFASRPVWRNTSGDYSWRFGSGKKESYSSGGFTTRKASTVSRGGYGRSSSARGHW

GG

>gi|30065554|ref|NP_839725.1| IS1 orfB [Shigella flexneri 2a str. 2457T]

MSRQCTHYGRWPQHGFTSLKKLRPQSVTSRIQPGSDVIVCAEMDEHWGYVGAKSRQRWLFYAYDRIRRTV

VAHVFGERTLATLERLLSLLSAFEVVVWMTDGCPLYESRLKGKLHVISKRYTQRIERHNLNLRQHLARLV

RKSLSFSKSVELHDKVIGHYLNIKHYQ

>gi|30065553|ref|NP_839724.1| IS1 orfA [Shigella flexneri 2a str. 2457T]

MASISIRCPSCSATEGVVRNGKSTAGHQRYLCSHCRKTWQLQFTYTASQPGTHQKIIDMAMNGVGCRASA

RIMGVGLNTVLRHLKNSGRSR

>gi|30065552|ref|NP_839723.1| 23S rRNA (guanosine-2'-O-)-methyltransferase [Shigella flexneri 2a str. 2457T]

MSEMIYGIHAVQALLERAPERFQEVFILKGREDKRLLPLIHALESQGVVIQLANRQYLDEKSDGAVHQGI

IARVKPGRQYQENDLPDLIASLDQPFLLILDGVTDPHNLGACLRSADAAGVHAVIVPKDRSAQLNATAKK

VACGAAESVPLIRVTNLARTMRMLQEENIWIVGTAGEADHTLYQSKMTGRLALVMGAEGEGMRRLTREHC

DELISIPMAGSVSSLNVSVATGICLFEAVRQRS

>gi|30065550|ref|NP_839721.1| transcriptional repressor NsrR [Shigella flexneri 2a str. 2457T]

MQLTSFTDYGLRALIYMASLPEGRMTSISEVTDVYGVSRNHMVKIINQLSRAGYVTAVRGKNGGIRLGKP

ASAIRIGDVVRELEPLSLVNCSSEFCHITPACRLKQALSKAVQSFLTELDNYTLADLVEENQPLYKLLLV

E

>gi|30065549|ref|NP_839720.1| adenylosuccinate synthetase [Shigella flexneri 2a str. 2457T]

MGNNVVVLGTQWGDEGKGKIVDLLTERAKYVVRYQGGHNAGHTLVINGEKTVLHLIPSGILRENVTSIIG

NGVVLSPAALMKEMKELEDRGIPVRERLLLSEACPLILDYHVALDNAREKARGAKAIGTTGRGIGPAYED
KVARRGLRVGDLFDKETFAEKLKEVMEYHNFQLVNYYKAEAVDYQKVLDDTMAVADILTSMVVDVSDLLD
QARQRGDFVMFEGAQGTLLDIDHGTYPYVTSSNTTAGGVATGSGLGPRYVDYVLGILKAYSTRVGAGPFP
AELFDETGEFLCKQGNEFGATTGRRRRTGWLDTVAVRRAVQLNSLSGFCLTKLDVLDGLKEVKLCVAYRM
PDGREVTTTPLAADDWKGVEPIYETMPGWSESTFGVKDRSGLPQAALNYIKRIEELTGVPIDIISTGPDR
TETMILRDPFDA

>gi|30065548|ref|NP_839719.1| hypothetical protein S4599 [Shigella flexneri 2a str. 2457T]
MNSTIWLALALVLVLEGLGPMLYPKAWKKMISAMTNLPDNILRRFGGGLVVAGVVVYYMLRKTIG

>gi|30065547|ref|NP_839718.1| FtsH protease regulator HflC [Shigella flexneri 2a str. 2457T]
MRKSVIAIIIIVLVVLYMSVFVVKEGERGITLRFGKVLRDDDNKPLVYEPGLHFKIPFIETVKMLDARIQ
TMDNQADRFVTKEKKDLIVDSYIKWRISDFSRYYLATGGGDISQAEVLLKRKFSDRLRSEIGRLDVKDIV
TDSRGRLTLEVRDALNSGSAGTEDEVTTPAADNAIAEAAERVTAETKGKVPVINPNSMAALGIEVVDVRI
KQINLPTEVSEAIYNRMRAEREAVARRHRSQGQEEAEKLRATADYEVTRTLAEAERQGRIMRGEGDAEAA
KLFADAFSKDPDFYAFIRSLRAYENSFSGNQDVMVMSPDSDFFRYMKTPTSATR

>gi|30065546|ref|NP_839717.1| FtsH protease regulator HflK [Shigella flexneri 2a str. 2457T]
MAWNQPGNNGQDRDPWGSSKPGGNSEGNGNKGGRDQGPPDLDDIFRKLSKKLGGLGGGKGTGSGGGSSSQ
GPRPQLGGRVVTIAAAAIVIIWAASGFYTIKEAERGVVTRFGKFSHLVEPGLNWKPTFIDEVKPVNVEAV
RELAASGVMLTSDENVVRVEMNVQYRVTNPEKYLYSVTSPDDSLRQATDSALRGVIGKYTMDRILTEGRT
VIRSDTQRELEETIRPYDMGITLLDVNFQAARPPEEVKAAFDDAIAARENEQQYIREAEAYSNEVQPRAN
GQAQRILEEARAYKAQTILEAQGEVARFAKLLPEYKAAPEITRERLYIETMEKVLGNTRKVLVNDKGGNL
MVLPLDQMLKGGNAPAAKSDNGASNLLRLPPASSSTTSGASNTSSTSQGDIMDQRRANAQRNDYQRQGE

>gi|30065545|ref|NP_839716.1| GTPase HflX [Shigella flexneri 2a str. 2457T]

MFDRYDAGEQAVLVHIYFTQDKDMEDLQEFESLVSSAGVEALQVITGSRKAPHPKYFVGEGKAVEIAEAV

KAMGASVVLFDHALSPAQERNLERLCECRVIDRTGLILDIFAQRARTHEGKLQVELAQLRHLATRLVRGW

THLERQKGGIGLRGPGETQLETDRRLLRNRIVQIQSRLERVEKQREQGRQSRIKADVPTVSLVGYTNAGK

STLFNRITEARVYAADQLFATLDPTLRRIDVADVGETVLADTVGFIRHLPHDLVAAFKATLQETRQATLL

LHVIDAADVRVQENIEAVNTVLEEIDAHEIPTLLVMNKIDMLEDFEPRIDRDEENKPIRVWLSAQTGAGI

PQLFQALTERLSGEVAQHTLRLPPQEGRLRSRFYQLQAIEKEWMEEDGSVSLQVRMPIVDWRRLCKQEPA

LIDYLI

>gi|30065544|ref|NP_839715.1| RNA-binding protein Hfq [Shigella flexneri 2a str. 2457T]

MAKGQSLQDPFLNALRRERVPVSIYLVNGIKLQGQIESFDQFVILLKNTVSQMVYKHAISTVVPSRPVSH

HSNNAGGGTSSNYHHGSSAQNTSAQQDSEETE

>gi|30065543|ref|NP_839714.1| tRNA delta(2)-isopentenylpyrophosphate transferase [Shigella flexneri 2a str. 2457T]

MSDISKASLPKAIFLMGPTASGKTALAIELRKILPVELISVDSVLIYKGMDIGTAKPNAEELLAAPHRLL

DIRDPSQAYSAADFRRDALAEMADITAAGRIPLLVGGTMLYFKALLEGLSPLPSADPEVRARIEQQAAEQ

GWESLHRQLQEVDPVAAARIHPNDPQRLSRALEVFFISGKTLTELTQTSGDALPYQVHQFAIAPASRELL

HQRIEQRFHQMLASGFEAEVRALFARGDLHTDLPSIRCVGYRQMWSYLEGEISYDEMVYRGVCATRQLAK

RQITWLRGWEGVHWLDSEKPEQARDEVLQVVGAIAG

>gi|30065542|ref|NP_839713.1| DNA mismatch repair protein [Shigella flexneri 2a str. 2457T]

MPIQVLPPQLANQIAAGEVVERPASVVKELVENSLDAGATRIDIDIERGGAKLIRIRDNGCGIKKDELAL

ALARHATSKIASLDDLEAIISLGFRGEALASISSVSRLTLTSRTAEQQEAWQAYAEGRDMDVTVKPAAHP

VGTTLEVLDLFYNTPARRKFLRTEKTEFSHIDEIIRRIALARFDVTINLSHNGKIVRQYRAVPEGGQKER

RLGAICGTAFLEQALAIEWQHGDLTLRGWVADPNHTTPALAEIQYCYVNGRMMRDRLINHAIRQACEDKL

GADQQPAFVLYLEIDPHQVDVNVHPAKHEVRFHQSRLVHDFIYQGVLSVLQQQLETPLPLDDEPQPAPRA

IPENRVAAGRNHFAEPAAREPVAPRYSPAPASGSRPAAPWPNAQPGYQKQQGEVYRQLLQTPAPMQKPKA

PEPQEPALAANSQSFGRVLTIVHSDCALLERDGNISLLSLPVAERWLRQAQLTPGEAPVCAQPLLIPLRL

KVSGEEKSALEKAQSALAELGIDFQSDAQHVTIRAVPLPLRQQNLQILIPELIGYLAKQSVFEPGNIAQW

IARNLMSEHAQWSMAQAITLLADVERLCPQLVKTPPGGLLQSVDLHPAIKALKDE

>gi|30065541|ref|NP_839712.1| N-acetylmuramoyl-l-alanine amidase II [Shigella flexneri 2a str. 2457T]

MMYRIRNWLVATLLLLCTPVGAATLSDIQVSNGNQQARITLSFIGDPDYAFSHQSKRTVALDIKQTGMIQ

GLPLLFSGNNLVKAIRSGTPKDAQTLRLVVDLTENGKTEAVKRQNGSNYTVVFTINADVPPPPPPPPVVA

KRVETPAVVAPRVSEPARNPFKTESNRTTGVISSNTVTRPAARATANTGDKIIIAIDAGHGGQDPGAIGP

GGTREKNVTIAIARKLRTLLNDDPMFKGVLTRDGDYFISVMGRSDVAHKQNANFLVSIHADAAPNRSATG

ASVWVLSNRRANSEMASWLEQHEKLSELLGGAGDVLANSQSDPYLSQAVLDLQFGHSQRVGYDVATSMIS

QLQRIGEIHKRRPEHASLGVLRSPDIPSVLVETGFISNNSEERLLASDDYQQQLAEAIYKGLRNYFLAHP

MQSAPQGATAQTASTVTTPDRTLPN

>gi|30065540|ref|NP_839711.1| ATPase [Shigella flexneri 2a str. 2457T]

MMNRVIPLPDEQATLDLGERVAKACDGATVIYLYGDLGAGKTTFSRGFLQALGHQGNVKSPTYTLVEPYT

LDNLMVYHFDLYRLADPEELEFMGIRDYFANDAICLVEWPQQGTGVLPDPDVEIHIDYQAQGREARVSAV

SSAGELLLARLAG

>gi|30065539|ref|NP_839710.1| hypothetical protein S4589 [Shigella flexneri 2a str. 2457T]

MTDHTMKKNPVSIPHTVWYADDIRRGEREAADVLGLTLYELMLRAGEAAFQVCRSAYPDARHWLVLCGHG

NNGGDGYVVARLAKAVGIEVTLLAQESDKPLPEEAALAREAWLNAGGEIHASNIVWPESVDLIVDALLGT

GLQQAPRESISQLIDHANSHPAPIVAVDIPSGLLAETGATPGAVINADHTITFIALKLGLLTGKARDVTG

QLHFDSLGLDSWLAGQETKIQRFSAEQLSHWLKPRRPTSHKGDHGRLVIIGGDHGTAGAIRMTGEAALRA

GAGLVRVLTRSENIAPLLTARPELMVHELTMDSLTESLEWADVVVIGPGLGQQEWGKKALQKVENFRKPM

LWDADALNLLAINPDKRHNRVITPHPSEAARLLGCSVAEIESDRLHCAKRLVQRYGGVAVLKGAGTVVAA

HPDALGIIDAGNAGMAIGGKGDVLSGIIGALLGQKLSPYDAACAGCVAHGAAADVLAARFGTRGMLATDL

FSTLQRIVNPEVTDKNHDESSNSAP

>gi|30065538|ref|NP_839709.1| hypothetical protein S4590 [Shigella flexneri 2a str. 2457T]

MSEPLDLNQLAQKIKQWGLELGFQQVGITDTDLSESEPKLQAWLDKQYHGEMDWMARHGMLRARPHELLP

GTLRVISVRMNYLPANAAFASTLKNPKLGYVSRYALGRDYHKLLRNRLKKLGEMIQQHCVSLNFRPFVDS

APILERPLAEKAGLGWTGKHSLILNREAGSFFFLGELLVDIPLPVDQPVEEGCGKCVACMTICPTGAIVE

PYTVDARRCISYLTIELEGALPEELRPLMGNRIYGCDDCQLICPWNRYSQLTTEDDFSPRKPLHAPELIE

LFAWSEEKFLKVTEGSAIRRIGHLRWLRNIAVALGNAPWDETILTALESRKGEHPLLDEHIAWAIAQQIE

RRNACIVEVQLPKKQRLVRVIEKGLPRDA

>gi|30065535|ref|NP_839706.1| phosphatidylserine decarboxylase [Shigella flexneri 2a str. 2457T]

MLNSFKLSLQYILPKLWLTRLAGWGASKRAGWLTKLVIDLFVKYYKVDMKEAQKPDTASYRTFNEFFVRP

LRDEVRPIDTDPNVLVMPADGVISQLGKIEEDKILQAKGHNYSLEALLAGNYLMADLFRNGTFVTTYLSP

RDYHRVHMPCNGILREMIYVPGDLFSVNHLTAQNVPNLFARNERVICLFDTEFGPMAQILVGATIVGSIE

TVWAGTITPPREGIIKRWTWPAGENDGSVALLKGQEMGRFKLGSTVINLFAPGKVNLVEQLESLSVTKIG

QPLAVSTETFVTPDAEPAPLPAEEIEAEHDASPLVDDKKDQV

>gi|30065534|ref|NP_839705.1| hypothetical protein S4581 [Shigella flexneri 2a str. 2457T]

MRLIITFLMAWCLSWGAYAATAPDSKQITQELEQAKAAKPAQPEVVEALQSALNALEERKGSLERIKQYQ

QVIDNYPKLSATLRAQLNNIRDEPRSVSPGMSTDALNQEILQVSSQLLDKSRQAQQEQERAREIADSLNQ

LPQQQTDARRQLNEIERRLGTLTGNTPLNQAQNFALQSDSARLKALVDELELAQLSANNRQELARLRSEL

AEKESQQLDAYLQALRNQLNSQRQLEAERALESTELLAENSADLPKDIVAQFKINRELSAALNQQAQRME

LVASQQRQAASQTLQVRQALNTLREQSQWLGSSNLLGEALRAQVARLPEMPKPQQLDTEMAQLRVQRLRY

EDLLNKQPLLRQIHQADGQPLTAEQNRILEAQLRTQRELLNSLLQGGDTLLLELTKLKVSNRQLEDALKE

VNEATHRYLFWASDVRPMTIAWPLEIAQDLRRLISLDTFSQLGKASVMMLTSKETILPLFGALILVGCSI

YSRRYFTRFLERSAAKVGKVTQDHFWLTLRTLFWSILVASPLPVLWMTLGYGLREAWPYPLAVAIGDGVT

ATVPLLWVVMICATFARPNGLFIAHFGWPRERVSRGMRYYLMSIGLIMALMMFDNLDDREFSGSLGRLCF

ILICGALAVVTLSLKKAGIPLYLNKEGSGDNITNHMLWNMMIGAPLVAILASAVGYLATAQALLARLETS

VAIWFLLLVVYHVIRRWMLIQRRRLAFDRAKHRRAEMLAQRARGEEEAHHHSSPEGAIEVDESEVDLDAI

SAQSLRLVRSILMLIALLSVIVLWSEIHSAFGFLENISLWDVTSTVQGVESLEPITLGAVLIAILVFIIT

TQLVRNLPALLELAILQHLDLTPGTGYAITTITKYLLMLIGGLVGFSMIGIEWSKLQWLVAALGVGLGFG

LQEIFANFISGLIILFEKPIRIGDTVTIRDLTGSVTKINTRATTISDWDRKEIIVPNKAFITEQFINWSL

SDSVTRVVLTIPAPADANSEEVTEILLTAARRCSLVIDNPAPEVFLVDLQQGIQIFELRIYAAEMGHRMP

LRHEIHQLILAGFHAHGIDMPFPPFQMRLESLNGKQTGRTLTSAGKGRQAGSL

>gi|30065533|ref|NP_839704.1| IS1 orfA [Shigella flexneri 2a str. 2457T]

MASISIRCPSCSATEGVVRNGKSTAGHQRYLCSHCRKTWQLQFTYTASQPDTHQKIIDMAMNGVGCRASA

RIMGVGLNTVLRHLKNSGRSR

>gi|30065532|ref|NP_839703.1| lysyl-tRNA synthetase [Shigella flexneri 2a str. 2457T]

MSETASWQPSASIPNLLKRAAIMAEIRRFFADRGVLEVETPCMSQATVTDIHLVPFETRFVGPGHSQGMN

LWLMTSPEYHMKRLLVAGCGPVFQLCRSFRNEEMGRYHNPEFTMLEWYRPHYDMYRLMNEVDDLLQQVLD

CPAAESLSYQQAFLRYLEIDPLSADKTQLREVAAKLDLSNVADTEEDRDTLLQLLFTFGVEPNIGKEKPT

FVYHFPASQASLAQISTEDHRVAERFEVYYKGIELANGFHELTDAREQQQRFEQDNRKRAARGLPQHPID

QNLIEALKVGMPDCSGVALGVDRLVMLALGAETLAEVIAFSVDRA

>gi|30065531|ref|NP_839702.1| fumarate reductase flavoprotein subunit [Shigella flexneri 2a str. 2457T]

MQTFQADLAIVGAGGAGLRAAIAAAQANPNAKIALISKVYPMRSHTVAAEGGSAAVAQDHDSFEYHFHDT

VAGGDWLCEQDVVDYFVHHCPTEMTQLELWGCPWSRRPDGSVNVRRFGGMKIERTWFAADKTGFHMLHTL

FQTSLQFPQIQRFDEHFVLDILVDDGHVRGLVAINMMEGTLVQIRANAVVMATGGAGRVYRYNTNGGIVT

GDGMGMALSHGVPLRDMEFVQYHPTGLPGSGILMTEGCRGEGGILVNKNGYRYLQDYGMGPETPLGEPKN

KYMELGPRDKVSQAFWHEWRKGNTISTPRGDVVYLDLRHLGEKKLHERLPFICELAKAYVGVDPVKEPIP

VRPTAHYTMGGIETDQNCETRIKGLFAVGECSSVGLHGANRLGSNSLAELVVFGRLAGEQATERAATAGN

GNEAAIEAQAAGVEQRLKDLVNQDGGENWAKIRDEMGLAMEEGCGIYRTPELMQKTIDKLAELQERFKRV

RITDTSSVFNTDLLYTIELSHGLNVAECMAHSAMARKESRGAHQRLDEGCTERDDVNFLKHTLAFRDADG

TTRLEYSDVKITTLPPAKRVYGGEADAADKAEAANNKEKANG

>gi|30065530|ref|NP_839701.1| fumarate reductase iron-sulfur subunit [Shigella flexneri 2a str. 2457T]

MAEMKNLKIEVVRYNPEVDTAPHSAFYEVPYDATTSLLDALGYIKDNLAPDLSYRWSCRMAICGSCGMMV

NNVPKLACKTFLRDYTDGMKVEALANFPIERDLVVDMTHFIESLEAIKPYIIGNSRTADQGTNIQTPAQM

AKYHQFSGCINCGLCYAACPQFGLNPEFIGPAAITLAHRYNEDSRDHGKKERMAQLNSQNGVWSCTFVGY

CSEVCPKHVDPAAAIQQGKVESSKDFLIATLKPR

>gi|30065529|ref|NP_839700.1| fumarate reductase subunit C [Shigella flexneri 2a str. 2457T]

MTTKRKPYVRPMTSTWWKKLPFYRFYMLREGTAVPAVWFSIELIFALFALKNGPEAWAGFVDFLQNPVIV

IINLITLTAALLHTKTWFELAPKAANIIVKDEKMGPEPIIKSLWAVTVVATIVILFVALYW

>gi|30065528|ref|NP_839699.1| fumarate reductase subunit D [Shigella flexneri 2a str. 2457T]

MINPNPKRSDEPVFWGLFGAGGMWSAIIAPVMILLVGILLPLGLFPGDALSYERVLAFAQSFIGRVFLFL

MIVLPLWCGLHRMHHAMHDLKIHVPAGKWVFYGLAAILTVVTLIGVVTI

>gi|30065527|ref|NP_839698.1| beta-lactamase [Shigella flexneri 2a str. 2457T]

MFKTTLCALLITASYSTFAAPQQINDIVHRTITPLIEQQKIPGMAVAVIYQGKPYYFTWGYADIAKKQPV

TQQTLFELGSVSKTFTGVLGGDAIARGEIKLSDPATKYWPELTAKQWNGITLLHLATYTAGGLPLQVPDE

VKSSSDLLRFYQNWQPAWAPGTQRLYANSSIGLFGALAVKPSGLSFEQAMQTRVFQPLKLNHTWINVPPP

EEKNYAWGYREGKAVHVSPGALDAEAYGVKSTIEDMARWVQSNLPPLDINEKTLQQGIQLAQSRYWQTGD

MYQGLGWEMLDWPVNPDSIINGSDNKIALAARPVKAIKPPTPAVRASWVHKTGATGGFGSYVAFIPEKEL

GIVMLANKNYPNPARVAAACQILNALQ

>gi|30065526|ref|NP_839697.1| outer membrane lipoprotein Blc [Shigella flexneri 2a str. 2457T]

MRLLPLVAAATAAFLVVACSFPTPPRGVTVVNNFDAKRYLGTWYEIARFDHRFERGLEKVTATYSLRDDG

GLNVINKGYNPDRGMWQQSEGKAYFTGAPTRAALKVSFFGPFYGGYNVIALDREYRHALVCGPDRDYLWI

LSRTPTISDEVKQEMLAVATREGFDVSKFIWVQQPGS

>gi|30065525|ref|NP_839696.1| suppresser of groEL [Shigella flexneri 2a str. 2457T]

MPFVFSAIVIKVIVEIPLPPGKISVQPSALQDDLQTPLFTGDGPKFPEPDMSWIILVIAGLLEVVWAVGL

KYTHGFSRLTPSVITVTAMIVSMALLAWAMKSLPVGTAYAVWTGIGAVGAAITGIVLLGESANPMRLASL

ALIVLGIIGLKLSTH

>gi|30065524|ref|NP_839695.1| elongation factor P [Shigella flexneri 2a str. 2457T]

MATYYSNDFRAGLKIMLDGEPYAVEASEFVKPGKGQAFARVKLRRLLTGTRVEKTFKSTDSAEGADVVDM

NLTYLYNDGEFWHFMNNETFEQLSADAKAIGDNAKWLLDQAECIVTLWNGQPISVTPPNFVELEIVDTDP

GLKGDTAGTGGKPATLSTGAVVKVPLFVQIGEVIKVDTRSGEYVSRVK

>gi|30065523|ref|NP_839694.1| hypothetical protein S4569 [Shigella flexneri 2a str. 2457T]

MAHIVTLNTPSREDWLTQLADVVTDPDELLRLLNIDADEKLLAGRSAKKLFALRVPRSFIDRMEKGNPDD

PLLRQVLTSQDEFVVASGFSTDPLEEQHSVVPGLLHKYHNRALLLVKGGCAVNCRYCFRRHFPYAENQGN

KRNWQTALEYVAAHPELDEMIFSGGDPLMAKEHELDWLLTQLEAIPHIKRLRIHSRLPIVIPARITEALV
ERFARSTLQILLVNHINHANEVDETFRQAMAKLRRVGVTLLNQSVLLRGVNDNAQTLANLSNALFDAGVM
PYYLHVLDKVQGAAHFMVSDDEARQIMRELLTLVSGYLVPKLAREIGGEPSKTPLDLQLRQQ

>gi|30065522|ref|NP_839693.1| IS1 orfB [Shigella flexneri 2a str. 2457T]
MSRQCTHYGRWPQHGFTSLKKLRPQSVTSRIQPGSDVIVCAEMDEHWGYVGAKSRQRWLFYAYDRIRRTV
VAHVFGERTLATLERLLSLLSAFEVVVWMTDGWPLYESRLKGKLHVISKRYTQRIERHNLNLRQHLARLV
RKSLSFSKSVELHDKVIGHYLNIKHYQ

>gi|30065521|ref|NP_839692.1| IS1 orfA [Shigella flexneri 2a str. 2457T]
MASISIRCPSCSATEGVVRNGKSTAGHQRYLCSHCRKTWQLQFTYTASQPGTHQKIIDMAMNGVGCRASA
RIMGVGLNTVLRHLKNSGRSR

>gi|30065520|ref|NP_839691.1| hypothetical protein S4566 [Shigella flexneri 2a str. 2457T]
MAISIKGVNTGVIRKSNNFIALALKIKEPRNKESLFFMSVMELRDLLIALESRLHQKHKLDAAARLQYEQ
ARDKVIKKMAENIPEILVDELKNADINRRVNTLELTDNQGENLTFVLTLHDGSKCELVVNELQIEMLARA
IIHAINNAEMRELALRITSLLDFLPLYDVDCQDNGNLEYDTYSQPEWKHNLFDHYLAVLYRFKDESGKEQ
FSGAVVKTREATPGKEIEAITRRMLDFSPRLKKLAGVPCQVYVRTVAANNAQPLTQDQCLRALHHLRVQS
TSKTAPQAK

>gi|30065519|ref|NP_839690.1| hypothetical protein S4565 [Shigella flexneri 2a str. 2457T]
MASSSLIMGNNMHVKYLAGIVGAALLMAGCSSSNELSAAGQSVRIVDEQPGAECQLIGTATGKQSNWLSG
QHGEEGGSMRGAANDLRNQAAAMGGNVIYGISSPSQGMLSSFVPTDSQIIGQVYKCPN

>gi|30065518|ref|NP_839689.1| chaperonin GroEL [Shigella flexneri 2a str. 2457T]

MAAKDVKFGNDARVKMLRGVNVLADAVKVTLGPKGRNVVLDKSFGAPTITKDGVSVAREIELEDKFENMG

AQMVKEVASKANDAAGDGTTTATVLAQAIITEGLKAVAAGMNPMDLKRGIDKAVTAAVEELKALSVPCSD

SKAIAQVGTISANSDETVGKLIAEAMDKVGKEGVITVEDGTGLQDELDVVEGMQFDRGYLSPYFINKPET

GAVELESPFILLADKKISNIREMLPVLEAVAKAGKPLLIIAEDVEGEALATLVVNTMRGIVKVAAVKAPG

FGDRRKAMLQDIATLTGGTVISEEIGMELEKATLEDLGQAKRVVINKDTTTIIDGVGEEAAIQGRVAQIR

QQIEEATSDYDREKLQERVAKLAGGVAVIKVGAATEVEMKEKKARVEDALHATRAAVEEGVVAGGGVALI

RVASKLADLRGQNEDQNVGIKVALRAMEAPLRQIVLNCGEEPSVVANTVKGGDGNYGYNAATEEYGNMID

MGILDPTKVTRSALQYAASVAGLMITTECMVTDLPKNDAADLGAAGGMGGMGGMGGMM

>gi|30065517|ref|NP_839688.1| co-chaperonin GroES [Shigella flexneri 2a str. 2457T]

MNIRPLHDRVIVKRKEVETKSAGGIVLTGSAAAKSTRGEVLAVGNGRILENGEVKPLDVKVGDIVIFNDG

YGVKSEKIDNEEVLIMSESDILAIVEA

>gi|30065516|ref|NP_839687.1| inner membrane protein YjeH [Shigella flexneri 2a str. 2457T]

MSGLKQELGLAQGIGLLSTSLLGTGVFAVPALAALVAGNNSLWAWPVLIILVFPIAIVFAILGRHYPSAG

GVAHFVGMAFGSRLERVTGWLFLSVIPVGLPAALQIAAGFGQAMFGWHSWQLLLAELGTLALVWYIGTLG

ASSSANLQTVIAGLIVALIVAIWWAGDIKPASIPFPAPGNIELTGLFAALSVMFWCFVGLEAFAHLASEF

KNPERDFPRALMIGLLLAGLVYWGCTVVVLHFDAYGEQMAAAASLPKIVVQLFGVGALWIACVIGYLACF

ASLNIYIQSFARLVWSQAQHNPDHYLARLSSRHIPNNALNAVLGCCVVSTLVIHALEINLDALIIYANGI

FIMIYLLCMLAGCKLLQGRYRLLAVVGGLLCVLLLAMVGWKSLYALIMLVGLWLFLPKRKTPENGITT

>gi|30065514|ref|NP_839685.1| aspartate ammonia-lyase [Shigella flexneri 2a str. 2457T]

MCLKQIIGSLKKKVHMSNNIRIEEDLLGTREVPADAYYGVHTLRAIENFYISNNKISDIPEFVRGMVMVK

KAAAMANKELQTIPKSVANAIIAACDEVLNNGKCMDQFPVDVYQGGAGTSVNMNTNEVLANIGLELMGHQ

KGEYQYLNPNDHVNKCQSTNDAYPTGFRIAVYSSLIKLVDAINQLREGFERKAVEFQDILKMGRTQLQDA

VPMTLGQEFRAFSILLKEEVKNIQRTAELLLEVNLGATAIGTGLNTPKEYSPLAVKKLAEVTGFPCVPAE

DLIEATSDCGAYVMVHGALKRLAVKMSKICNDLRLLSSGPRAGLNEINLPELQAGSSIMPAKVNPVVPEV

VNQVCFKVIGNDTTVTMAAEAGQLQLNVMEPVIGQAMFESVHILTNACYNLLEKCINGITANKEVCEGYV

YNSIGIVTYLNPFIGHHNGDIVGKICAETGKSVREVVLERGLLTEAELDDIFSVQNLMHPAYKAKRYTDE

SEQ

>gi|30065513|ref|NP_839684.1| anaerobic C4-dicarboxylate transporter [Shigella flexneri 2a str. 2457T]

MLVVELIIVLLAIFLGARLGGIGIGFAGGLGVLVLAAIGVKPGNIPFDVISIIMAVIAAISAMQVAGGLD

YLVHQTEKLLRRNPKYITILAPIVTYFLTIFAGTGNISLATLPVIAEVAKEQGVKPCRPLSTAVVSAQIA

ITASPISAAVVYMSSVMEGHGISYLHLLSVVIPSTLLAVLVMSFLVTMLFNSKLSDDPIYRKRLEEGLVE

LRGEKQIEIKSGAKTSVWLFLLGVVGVVIYAIINSPSMGLVEKPLMNTTNAILIIMLSVATLTTVICKVD

TDNILNSSTFKAGMSACICILGVAWLGDTFVSNNIDWIKDTAGEVIQGHPWLLAVIFFFASALLYSQAAT

AKALMPMALALNVSPLTAVASFAAVSGLFILPTYPTLVAAVQMDDTGTTRIGKFVFNHPFFIPGTLGVAL

AVCFGFVLGSFML

>gi|30065512|ref|NP_839683.1| divalent-cation tolerance protein CutA [Shigella flexneri 2a str. 2457T]

MLDEKSSNTTSVVVLCTAPDEATAQDLAAKVLAEKLAACATLIPGATSLYYWEGKLEQEYEVQMILKTTV

SHQQALLECLKSHHPYQTPELLVLPVTHGDTDYLSWLNASLR

>gi|30065511|ref|NP_839682.1| thiol:disulfide interchange protein precursor [Shigella flexneri 2a str. 2457T]

MAQRIFTLILLLLCSTSVFAGLFDAPGRSQFVPADQAFAFDFQQNQHDLNLTWQIKDGYYLYRKQIRITPE

HAKIADEQLPQGVWHEDEFYGKSEIYRDRLTLPVTINQASAGATLTVTYQGCADAGFCYPPETKTVPLSE

VVANNAASQPVSVSQQEQHTAQLPFSALWALLIGIGIAFTPCVLPMYPLISGIVLGGKQRLSTARALLLT

FIYVQGMALTYTALGLVVAAAGLQFQAALQHPYVLIGLAIVFTLLAMSMFGLFTLQLPSSLQTRLTLMSN

RQQGGSPSGVFVMGAIAGLICSPCTTAPLSAILLYIAQSGNMWPGGGTLYLYALGMGLPLMLITVFGNRL
LPKSGPWMEQVKTAFGFVILALPVFLLERVIGDVWGLRLWSALGVAFFGWAFITSLQAKRGWMRVVQIIL
LAAALVSVRPLQDWAFGATHTAQTQTHLNFTQIKTVDELNQALVEAKGKPVMLDLYADWCVACKEFEKYT
FSDPQVQKALADTVLLQANVTANDAQDVALLKHLNVLGLPTILFFDGQGQEHPQARVTGFMDAETFSAHL
RDRQP

>gi|30065510|ref|NP_839681.1| transcriptional regulator [Shigella flexneri 2a str. 2457T]
MQREDVLGEALKLLELQGIANTTLEMVAERVDYPLDELRRFWPDKEAILYDALRYLSQQIDVWRRQLMLD
ETQTAEQKLLARYQALSECVKNNRYPGCLFIAACTFYPDPGHPIHQLADQQKSAAYDFTHELLTTLEVDD
PAMVAKQMELVLEGCLSRMLVNRSQADVDTAHRLAEDILRFARCRQGGALT

>gi|30065509|ref|NP_839680.1| IS1 orfB [Shigella flexneri 2a str. 2457T]
MSRQCTHYGRWPQHGFTSLKKLRPQSVTSRIQPGSDVIVCAEMDEHWGYVGAKSRQRWLFYAYDRIRRTV
VAHVFGERTLATLERLLSLLSAFEVVVWMTDGWPLYESRLKGKLHVISKRYTQRIERHNLNLRQHLARLV
RKSLSFSKSVELHDKVIGHYLNIKHYQ

>gi|30065508|ref|NP_839679.1| IS1 orfA [Shigella flexneri 2a str. 2457T]
MASISIRCPSCSATEGVVRNGKSTAGHQRYLCSHCRKTWQLQFTYTASQPGKHQKIIDMAMNGVGCRASA
RIMGVGLNTVLRHLKNSGRSR

>gi|30065507|ref|NP_839678.1| transport of lysine/cadaverine [Shigella flexneri 2a str. 2457T]
MSSAKKIGLFACTGVVAGNMMGSGIALLPANLASIGGIAIWGWIISIIGAMSLAYVYARLATKNPQQGGP
IAYAGEISPAFGFQTGVLYYHANWIGNLAIGITAVSYLSTFFPVLNDPVPAGIACIAIVWVFTFVNMLGG
TWVSRLTTIGLVLVLIPVVMTAIVGWHWFDAATYAANWNTADTTDGHAIIKSILLCLWAFVGVESAAVST
GMVKNPKRTVPLATMLGTGLAGIVYIAATQVLSGMYPSSVMAASGAPFAISASTILGNWAAPLVSAFTAF

ACLTSLGSWMMLVGQAGVRAANDGNFPKVYGEVDSNGIPKKGLLLAAVKMTALMILITLMNSAGGKASDL

>gi|30065506|ref|NP_839677.1| IS600 orfB [Shigella flexneri 2a str. 2457T]

MCQVFGVSRSGYYDRVQHAPSDRKQSDERLKLEIKVAHIRTRETYGTRRLQTELAENGIIVGRDRLARLR

KELRLRCKQKRKFRATTNPNHNLPVAPNLLNQTFAPTAPNQVWVADLTYVATQEGWLYLAGIKDVYTCEI

VGYAMGERMTKELTGKALFMALRSQRPPAGLIHHSDRGSQYCAYDYRVIQEQSGLKTSMSRKGNCYDNAP

MESFWGTLKNESLSHYRFNNRDEAISVIREYIEIFYNRQRRHSRLGNISPAAFREKYHQMAA

>gi|30065505|ref|NP_839676.1| IS1 orfA [Shigella flexneri 2a str. 2457T]

MASISIRCPSCSATEGVVRNGKSTAGHQRYLCSHCRKTWQLQFTYTASQPGTHQKIIDMAMNGVGCRASA

RIMGVGLNTVLRHLKNSGRSR

>gi|30065504|ref|NP_839675.1| IS1 orfB [Shigella flexneri 2a str. 2457T]

MSRQCTHYGRWPQHGFTSLKKLRPQSVTSRIQPGSDVIVCAEMDEHWGYVGAKSRQRWLFYAYDRIRRTV

VAHVFGERTLATLERLLSLLSAFEVVVWMTDGWPLYESRLKGKLHVISKRYTQRIERHNLNLRQHLARLG

RKSLSFSKSVELHDKVIGHYLNIKHYQ

>gi|30065503|ref|NP_839674.1| hypothetical protein S4548 [Shigella flexneri 2a str. 2457T]

MRDLLQRPDLFSINTATLGYKTPLPAIIDACAARGIGAIAPWRRELQGEDLQQITRQLAASNMSVSGLCR

STYYTAPTLAERKLAIDDNRRALDDAAVLNAACYMQVVGGLPTGTKDLYEAREQVKQGIRQLLPHSKDVG

VPIALEPLHPMTAADRSCLCTLRQALDWCDELDPDGEFGLGVAVDVYHVWWDPDLASQILRAGKRILAFH

VSDWLMPTTDLVNDRGMPGDGVINIPSIRRLVENAGFNGAIELEIFSPYWWQKDINSTGNDSNLLIVFYV

QIMPDDLVMQLHRF

>gi|30065502|ref|NP_839673.1| 3-ketoacyl-ACP reductase [Shigella flexneri 2a str. 2457T]

MKSTRPVAVITGATRGIGKGCAQELARGGFNLLINDRPDADSVEKLHITQQECLAEGVEVICFPADVGDL

SLHEEMLDAAQNQWGRLDCLLNNAGISVKKRGDLLDLEPDSFDQNIAINTRAPFFLAQAFSKRLLAQPKP

EAELPHRSIIFVSSINAIMLAMNRGEYTIAKTAVSAAARLFAARLCNEQIGVYEVRPGLIKTDMTIPATA

YYDELIAKGLVPWGRWGYPADIASTVRAMAEGKLIYTCGQAVAIDGGLSMPRF

>gi|30065501|ref|NP_839672.1| hypothetical protein S4546 [Shigella flexneri 2a str. 2457T]

MHSENQSKGVHYAKSQRLLEINHAHLQLMESLLDEGKKYNIFKPGIDPLQVNINIAALGGYYLINQHTLG

LVYHISMVSPQALEARRKVIKETILSWLLVDPSSTAHE

>gi|30065500|ref|NP_839671.1| hypothetical protein S4545 [Shigella flexneri 2a str. 2457T]

MIEEQDVEQEETMPGRFELKPTLEKVWHAPDNFRFMDPLPPMHRRGIIIAAIVLVVGFLLPSDDTPNASV

VTREAQLDIQSQSQPPTEEQPQTTQTQPFQPDSGIDNQWRSYRVEPGKTMAQLFRDHGLPATDVYAMAQV

EGAGKPLSNLQNGQMVKIRQNASGVVTGLTIDTGNNQQVLFTRQPDGSFIRAR

>gi|30065499|ref|NP_839670.1| D-alanine/D-serine/glycine permease [Shigella flexneri 2a str. 2457T]

MVDQVKVVADDQAPAEQSLRRNLTNRHVQLIAIGGAIGTGLFMGSGKTISLAGPSIIFVYMIIGFMLFFV

MRAMGELLLSNLEYKSFSDFASDLLGPWAGYFTGWTYWFCWVVTGMADVVAITAYAQFWFPDLSDWVASL

AVIVLLLTLNLATVKMFGEMEFWFAMIKIVAIVSLIVVGLVMVAMHFQSPTGVEASFAHLWNDGGWFPKG

LSGFFAGFQIAVFAFVGIELVGTTAAETKDPEKSLPRAINSIPIRIIMFYVFALIVIMSVTPWSSVVPEK

SPFVELFVLVGLPAAASVINFVVLTSAASSANSGVFSTSRMLFGLAQEGVAPKAFAKLSKRAVPAKGLTF

SCICLLGGVVMLYVNPSVIGAFTMITTVSAILFMFVWTIILCSYLVYRKQRPHLHEKSIYKMPLGKLMCW

VCMAFFVFVVVLLTLEDDTRQALLVTPLWFIALGLGWLFIGKKRAAELRK

>gi|30065498|ref|NP_839669.1| iron-sulfur cluster repair di-iron protein [Shigella flexneri 2a str. 2457T]

MAYRDQPLGELALSIPRASALFRKYDMDYCCGGKQTLARAAARKELDVEVIEAELAKLAEQPIEKDWRSA

PLAEIIDHIIVRYHDRHREQLPELILQATKVERVHADKPSVPKGLTKYLTMLHEELSSHMMKEEQILFPM

IKQGMGSQAMGPISVMESEHDEAGELLEVIKHTTNNVTPPPEACTTWKAMYNGINELIDDLMDHISLENN

VLFPRALAGE

>gi|30065497|ref|NP_839668.1| transmembrane subunit [Shigella flexneri 2a str. 2457T]

MISGVLYALLAGLMWGLIFVGPLIVPEYPAMLQSMGRYLALGLIALPIAWLGRVRLRQLARRDWLTALML

TMMGNLIYYFCLASAIQRTGAPVSTMIIGTLPVVIPVFANLLYSQRDGKLAWGKLAPALICIGIGLASVN

IAELNHGLPDFDWARYTSGIVLALVSVVCWAWYALRNARWLRENPDKHPMMWATAQALVTLPVSLIGYLV

ACYWLNIQTPDFSLPFGPRPLVFISLMVAIAVLCSWVGALCWNVASQRLPTVILGPLIVFETLAGLLYTF

LLRQQMPPLMTLSGIALLVIGVVIAVRAKPEKPLTESVSES

>gi|30065496|ref|NP_839667.1| oxidoreductase [Shigella flexneri 2a str. 2457T]

MIAITGATGQLGHYIIESLMKTVPASQIVAIVRNPAKAQALAAQGITVRQADYSDEAALTSALQGVEKLL

LISSSEVGQRAPQHRNVINAAKAAGVKFIAYTSLLHADTSPLGLADEHIETEKMLADSGIVYTLLRNGWY

SENYLASAPAALEHGVFIGAAGDGKIASATRADYATAAARVISEAGHEGKVYELAGDSAWTLTQLAAELT

KQSGKQVTYQNLSEADFAAALKSVGLPDGLADMLADSDVGASKGGLFDNSKTLSKLIGCPTTTLAESVSH

LFNVNN

>gi|30065495|ref|NP_839666.1| hypothetical protein S4540 [Shigella flexneri 2a str. 2457T]

MGSLHRFVLCLNTLTPKLTFSKYVQKGKYEMSQVSLSQQLKEGNLFAEQCPSREVLKHVTSRWGVLILVA

LREGTHRFSDLRRKIGGVSEKMLAQSLQALEQDGFLNRIAYPVVPPHVEYSLTPLGEQVSEKVAALADWI

ELNLPEVLAVRDERAA

>gi|30065494|ref|NP_839665.1| bifunctional 2',3'-cyclic nucleotide 2'-phosphodiesterase/3'-nucleotidase periplasmic precursor protein [Shigella flexneri 2a str. 2457T]

MIKFSATLLATLIAASVNAATVDLRIMETTDLHSNMMDFDYYKDTATEKFGLVRTASLINDARNEVKNSV

LVDNGDLIQGSPLADYMSAKGLKAGDVHPVYKALNTLDYTVGTLGNHEFNYGLDYLKNALAGAKFPYVNA

NVIDARTKQPMFTPYLIKDTEVVDKDGKKQTLKIGYIGVVPPQIMGWDKANLSGKVTVNDITETVRKYVP

EMREKGADVIVVLAHSGLSADPYKVMAENSVYYLSEIPGVNAIMFGHAHAVFPGKDFADIEGADITKGTL

NGVPAVMPGMWGDHLGVVDLQLSNDSGKWQVTQAKAEARPIYDIANKKSLAAEDSKLVETLKADHDATRQ

FVSKPIGKSADNMYSYLALVQDDPTVQVVNNAQKAYVEHYIQGDPDLAKLPVLSAAAPFKVGGRKNDPAS

YVEVEKGQLTFRNAADLYLYPNTLIVVKASGKEVKEWLECSAGQFNQIAPNSTKPQSLINWDGFRTYNFD

VIDGVNYQIDVTQPARYDGECQMINANAERIKNLTFNGKPIDPNAMFLVATNNYRAYGGKFAGTGDSHIA

FASPDENRSVLAAWIADESKRAGEIHPAADNNWRLAPIAADKKLDIRFETSPSDKAAAFIKEKGQYPMNK

VATDDIGFAIYQVDLSK

>gi|30065493|ref|NP_839664.1| adenosine-3'(2'),5'-bisphosphate nucleotidase [Shigella flexneri 2a str. 2457T]

MLDQVCQLARNAGDAIMQVYDGTKPMDVVSKADNSPVTAADIAAHTVIMDGLRTLAPDIPVLSEEDPPGW

EVRQHWQRYWLVDPLDGTKEFIKRNGEFTVNIALIDHGKPILGVVYAPVMNVMYSAAEGKAWKEECGVRK

LIQVRDARPPLVVISRSHADAELKEYLQQLGEHQTTSIGSSLKFCLVAEGQAQLYPRFGPTNIWDTAAGH

AVAAAAGAHVHDWQGKPLDYTPRESFLNPGFRVSIY

>gi|30065492|ref|NP_839663.1| hypothetical protein S4535 [Shigella flexneri 2a str. 2457T]

MTLRKILALTCLLLPMMASTHQFEIGQRVPPIGITDRGELVLDKDQFSYKTWNSAQLVGKVRVLQHIAGR

TSAKEKNATLIEAIKSAKLPHDRYQTTTIVNTDDAIPGSGMFVRSSLESNKKLYPWSQFIVDSNGVARGA

WQLDEESSAVVVLDKDGRVQWAKDGALTPEEVQQVMDLLQKLLK

>gi|30065491|ref|NP_839662.1| hypothetical protein S4534 [Shigella flexneri 2a str. 2457T]

MAPHRRNTQQEITMKIFQRYNPLQVAKYVKILFRGRLYIKDIGAFEFDKGKILIPKVKDKLHLSVMSEVN

RQVMRLQTEMA

>gi|30065490|ref|NP_839661.1| transporter [Shigella flexneri 2a str. 2457T]

MLNSILVILCLIAVSAFFSMSEISLAASRKIKLKLLADEGNINAQRVLNMQENPGMFFTVVQIGLNAVAI

LGGIVGDAAFSPAFHSLFSRYMSAELSEQLSFILSFSLVTGMFILFADLTPKRIGMIAPEAVALRIINPM

RFCLYVCTPLVWFFNGLANMIFRIFKLPMVRKDDITSDDIYAVVEAGALAGVLRKQEHELIENVFELESR

TVPSSMTPRENVIWFDLHEDEQSLKNKVAEHPHSKFLVCNEDIDHIIGYVDSKDLLNRVLANQSLALNSG

VQIRNTLIVPDTLTLSEALESFKTAGEDFAVIMNEYALVVGIITLNDVMTTLMGDLVGQGLEEQIVARDE

NSWLIDGGTPIDDVMRVLDIDEFPQSGNYETIGGFMMFMLRKIPKRTDSVKFAGYKFEVVDIDNYRIDQL

LVTRIDSKATALSPKLPDAKDKEESVA

>gi|30065489|ref|NP_839660.1| methionine sulfoxide reductase A [Shigella flexneri 2a str. 2457T]

MSLFDKKHLVSPADALPGRNTPMPVATLHAVNGHSMTNVPDGMEIAIFAMGCFWGVERLFWQLPGVYSTA

AGYTGGYTPNPTYREVCSGDTGHAEAVRIVYDPSVISYEQLLQVFWENHDPAQGMRQGNDHGTQYRSAIY

PLTPEQDAAARASLERFQAAMLAADDDRHITTEIANATPFYYAEDDHQQYLHKNPYGYCGIGGIGVCLPP

EA

>gi|30065488|ref|NP_839659.1| hypothetical protein S4531 [Shigella flexneri 2a str. 2457T]

MRYIRQLCCVSLLCLSGSAVAANVRLQVEGLSGQLEKNVRAQLSTIESDEVTPDRRFRARVDDAIREGLK

ALGYYQPTIEFDLRPPPKKGRQVLIAKVTPGVPVLIGGTDVVLRGGARTDKDYLKLLDTRPAIGTVLNQG

DYENFKKSLTSIALRKGYFDSEFTKAQLGIALGLHKAFWDIDYNSGERYRFGHVTFEGSQIRDEYLQNLV

PFKEGDEYESKDLAELNRRLSATGWFNSVVVAPQFDKARETKVLPLTGVVSPRTENTIETGVGYSTDVGP

RVKATWKKPWMNSYGHSLTTSTSISAPEQTLDFSYKMPLLKNPLEQYYLVQGGFKRTDLNDTESDSTTLV

ASRYWDLSSGWQRAINLRWSLDHFTQGEITNTTMLFYPGVMISRTRSRGGLMPTWGDSQRYSIDYSNTAW

GSDVDFSVFQAQHVWIRTLYDRHRFVTRGTLGWIETGDFDKVPPDLRFFVGGDRSIRGYKYKSIAPKYAN

GDLKGASKLITGSLEYQYNVTGKWWGAVFVDSGEAVSDIRRSDFKTGTGVGVRWESPVGPIKLDFAVPVA

DKDEHGLQFYIGLGPEL

>gi|30065487|ref|NP_839658.1| hypothetical protein S4530 [Shigella flexneri 2a str. 2457T]

MSLWKKISLGVVIVILLLLGSVAFLVGTTSGLHLVFKAADRWVPGLDIGKVTGGWRDLTLSDVRYEQLGV

AVKAGNLHLGVGLECLWNSSVCINDLALKDIQVNIDSKKMPPSEQVEEEEDSGPLDLSTPYPITLTRVAL

DNVNIKIDDTTVSVMDFTSGLNWQEKTLTLKPTSLKGLLIALPKVVEVAQEEVVEPKIENPQPEEKPLGE

TLKDLFSRPVLPEMTDVHLPLNLNIEEFKGEQLRVTGDTDITVRTMLLKVSSIDGNTKLDALDIDSNQGI

VNASGTAQLSDNWPVDITLNSTLNVEPLKGEKVKLKVGGALREQLEIGVNLSGPVDMDLRAQTRLAEAGL

PLNVEVNSKQLYWPFTGEKQYQADDLKLKLTGKMTDYTLSMRTAVKGQEIPPATITLDAKGNEQQVNLDK

LTVAALEGKTELKALLDWQQAISWRGELTLNGINTAKEFPEWPSKLNGLIKTRGSLYGGTWQMEVPELKL

TGNVKQNKVNVDGTLKGNSYMQWMIPGLHLELGPNSAEVKGELGVKDLNLDATINAPGLDNALPGLGGTA

KGLVKVRGTVEAPQLLADITARGLRWQELSVAQVRVEGDIKSTDQIAGKLDVRVEQISQPDVNINLVTLN

AKGSEKQHELQLRIQGEPVSGQLNLAGSFDRKEERWKGTLSNTRFQTPVGPWSLTRDIALDYRNKEQKIS

IGPHCWLNPNAELCVPQTIDAGAEGRAVVNLNRFDLAMLKPFMPETTQASGIFTGKADVAWDTTKEGLPQ

GSITLSGRNVQVTQTVNDAALPVAFQTLNLTAELRNNRAELGWTIRLTNNGQFDGQVQVTDPQGRRNLGG

NVNIRNFNLAMINPIFTRGEKAAGMVSANLRLGGDVQSPQLFGQLQVTGVDIDGNFMPFDMQPSQLAVNF

NGMRSTLAGTVRTQQGEIYLNGDADWSQIENWRARVTAKGSKVRITVPPMVRMDVSPDVVFEATPNLFTL

DGRVDVPWARIVVHDLPESAVGVSSDVVMLNDNLQPEEPKTASIPINSNLIVHVGNNVRIDAFGLKARLT

GDLNVVQDKQGLGLNGQINIPEGRFHAYGQDLIVRKGELLFSGPPDQPYLNIEAIRNPDATEDDVIAGVR

VTGLADEPKAEIFSDPAMSQQAALSYLLRGQGLESDQSDSAAMTSMLIGLGVAQSGQIVGKIGETFGVSN

LALDTLGVGDSSQVVVSGYVLPGLQVKYGVGIFDSIATLTLRYRLMPKLYLEAVSGVDQALDLLYQFEF

>gi|30065486|ref|NP_839657.1| hypothetical protein S4529 [Shigella flexneri 2a str. 2457T]

MRIFVYGSLRHKQGNSHWMTNAQLLGDFSIDNYQLYSLGHYPGAVPGNGTVHGEVYRIDNATLAELDALR

TRGGEYARQLIQTPYGSAWMYVYQRPVDGLKLIESGDWLDRDK

>gi|30065485|ref|NP_839656.1| hypothetical protein S4528 [Shigella flexneri 2a str. 2457T]

MITYPCLTSRRFQLALIHRRVVDKRTSMHSHTASESTGARIHRPWCARHQVRPAWRCQYDKLHRVPFRSP

ELRLDSGPGYTTGSYRY

>gi|30065484|ref|NP_839655.1| inorganic pyrophosphatase [Shigella flexneri 2a str. 2457T]

MSLLNVPAGKDLPEDIYVVIEIPANADPIKYEIDKESGALFVDRFMSTAMFYPCNYGYINHTLSLDGDPV

DVLVPTPYPLQPGSVIRCRPVAVLKMTDEAGEDAKLVAVPHSKLSKEYDHIKDVNDLPELLKAQIAHFFE

HYKDLEKGKWVKVEGWENAEAAKAEIVASFERAKNK

>gi|30065483|ref|NP_839654.1| ATP-binding component of a transport system [Shigella flexneri 2a str. 2457T]

MTTDQHQEILRTEGLSKFFPGVKALDNVDFSLRRGEIMALLGENGAGKSTLIKALTGVYHADRGTIWLEG

QTISPKNTAHAQQLGIGTVYQEVNLLPNMSVADNLFIGREPKRFGLLRRKEMEKRATELMASYGFSLDVR

EPLNRFSVAMQQIVAICRAIDLSAKVLILDEPTASLDTQEMELLFGLMRQLRDRGVSLIFVTHFLDQVYQ

VSDRITVLRNGSFVGCRETRELPQIELVKMMLGRELDTHALQRAGRTLLSDKPVAAFKNYGKKGTIAPFD

LEVRPGEIVGLAGLLGSGRTETAEVIFGIKPADSGTALIKGKPQTLRSPHQASVLGIGFCPEDRKTDGII

AAASVRENIILALQAQRGWLRPISRKEQQEIAERFIRQLGIRTPSTEQPIEFLSGGNQQKVLLSRWLLTR

PQFLILDELTRGIDVGAHAEIIRLIETLCADGLALLVISSELEELVGYADRVIIMRDRKQVAEIPLAQLS

VPAIMNAIAA

>gi|30065482|ref|NP_839653.1| transport system permease [Shigella flexneri 2a str. 2457T]

MMPQSLPDTTPPKRRFHWPTGMPQLAALLLVLLVDSLVAPHFWQVVLQDGRLFGSPIDILNRAAPVALLA

IGITLVIATGGIDLSVGAVMAIAGATTAAMTVAGFSLPIVLLSALGTGILAGLWNGILVAILKIQPFVAT

LILMVAGRGVAQLITAGQIVTFNSPDLSWFGSGSLLFLPTPVIIAVLTLLLFWLLTRKTALGMFIEVVGI

NIRAAKNAGVNTRIIVMLTYVLSGLCAAIAGIIVAADIRGADANNAGLWLELDAILAVVIGGGSLMGGRF

NLLLSVVGALIIQGMNTGILLSGFPPEMNQVVKAVVVLCVLIVQSQRFISLIKGVRSHDKT

>gi|30065481|ref|NP_839652.1| IS4 orf [Shigella flexneri 2a str. 2457T]

MHIGQALDLVSRYDSLRNPLTSLGDYLDPELISRCLAESGTVTLRKRRLPLEMMVWCIVGMVLERKEPLH

QIVNRLDIMLPGNRPFVAPSAVIQARQRLGSEAVRRVFTKTAQLWHNATPHPHWCGLTLLAIDGVFWRTP

DTPENDAAFPRQTHAGNPALYPQVKMVCQMELTSHLLTAAAFGTMKNSENELAEQLIEQTGDNTLTLMDK

GYYSLGLLNGWSLAGEHRHWMIPLRKGAQYEELRKLGKGDHLVKLKTSPQARKKWPGLGNEVTARLLTVT

RKGKVCHLLTSMTDAMRFPGGEMADLYSNRWEIELGYREIKQTMQLSRLTLRSKKPELVEQELWGVLLAY

NLVRYQMIKMAEHLKGYWPNQLSFSESCGMVMRMLMTLQGASPGRIPELMRDLASMGQLVKLPTRRGRAF

PRVVKERPWKYPTAPKKSQSVA

>gi|30065480|ref|NP_839651.1| fructose-1,6-bisphosphatase [Shigella flexneri 2a str. 2457T]

MKTLGEFIVEKQHEFSHATGELTALLSAIKLGAKIIHRDINKAGLVDILGASGAENVQGEVQQKLDLFAN

EKLKAALKARDIVAGIASEEEDEIVVFEGCEHAKYVVLMDPLDGSSNIDVNVSVGTIFSIYRRVTPVGTP

VTEEDFLQPGNKQVAAGYVVYGSSTMLVYTTGCGVHAFTYDPSLGVFCLCQERMRFPEKGKTYSINEGNY

IKFPNGVKKYIKFCQEEDKSTNRPYTSRYIGSLVADFHRNLLKGGIYLYPSTASHPDGKLRLLYECNPMA

FLAEQAGGKASDGKERILDIIPETLHQRRSFFVGNDHMVEDVERFIREFPDA

>gi|30065479|ref|NP_839650.1| ligase [Shigella flexneri 2a str. 2457T]

MRIHILGICGTFMGGLAMLARQLGHEVTGSDANVYPPMSTLLEKQGIELIQGYDASQLEPQPDLVIIGNA

MTRGNPCVEAVLEKNIPYMSGPQWLHDFVLRDRWVLAVAGTHGKTTTAGMATWILEQCGYKPGFVIGGVP

GNFEVSARLGESDFFVIEADEYDCAFFDKRSKFVHYCPRTLILNNLEFDHADIFDDLKAIQKQFHHLVRI

VPGQGRIIWPENDINLKQTMAMGCWSEQELVGEQGHWQAKKLTTDASEWEVLLDGEKVGEVKWSLVGEHN

MHNGLMAIAAARHVGVAPADAANALGSFINARRRLELRGEANGVTVYDDFAHHPTAILATLAALRGKVGG

TARIIAVLEPRSNTMKMGICKDDLAPSLGRADEVFLLQPAHIPWQVAEVAEACVQPAHWSGDVDTLADMV

VKTAQPGDHILVMSNGGFGGIHQKLLDGLAKKAEAAQ

>gi|30065478|ref|NP_839649.1| hypothetical protein S4518 [Shigella flexneri 2a str. 2457T]

MTKQPEDWLDDVPGDDIEDEDDEIIWVSKSEIKRDAEELKRLGAEIVDLGKNALDKIPLDADLRPAIELA

QRIKMEGRRRQLQLIGKMLRQRDVEPIRQALDKLKNRHNQQVVLFHKLENLRDRLIDQGDDAIAEVLNLW

PDADRQQLRTLIRNAKKEKEGNKPPKSARQIFQYLRELAENEG

>gi|30065477|ref|NP_839648.1| peptidase PmbA [Shigella flexneri 2a str. 2457T]

MALAMKVISQVEAQRKILEEAVSTALELASGKSDGAEVAVSKTTGISVSTRYGEVENVEFNSDGALGITV

YHQNRKGSASSTDLSPQAIARTVQAALDIARYTSPDPCAGVADKELLAFDAPNLDLFHPAEVSPDEAIEL

AARAEQAALQADKRITNTEGGSFNSHYGVKVFGNSHGMLQGYCSTRHSLFSCVIAEENGDMERDYAYTIG

RAMSDLQTPEWVGADCARRTLSRLSPRKLSTMKAPVIFANEVATGLFGHLVGAIAGGSVYRKSTFLLDSL

GKQILPDWLTIEEHPHLLKGLASTPFDSEGVRTERRDIIKDGILTQWLLTSYSARKLGLKSTGHAGGIHN

WRIAGQGLSFEQMLKEMGTGLVVTELMGQGVSAITGDYSRGAAGFWVENGEIQYPVSEITIAGNLKDMWR

NIVTVGNDIETRSNIQCGSVLLPEMKIAGQ

>gi|30065476|ref|NP_839647.1| anaerobic ribonucleotide reductase-activating protein [Shigella flexneri 2a str. 2457T]

MNYHQYYPVDIVNGPGTRCTLFVSGCVHECPGCYNKSTWRVNSGQPFTKAMEDKIIKDLNDTRIKRQGIS

LSGGDPLHPQNVPDILKLVKRIRAECPGKDIWVWTGYKLDELNAAQMQVVDLINVLVDGKFVQDLKDPSL

IWRGSSNQVVHHLR

>gi|30065475|ref|NP_839646.1| anaerobic ribonucleoside triphosphate reductase [Shigella flexneri 2a str. 2457T]

MTPHVMKRDGCKVPFKSERIKEAILRAAKAAEVDDADYCATVAAVVNEQMQGRNQVDINEIQTAVENQLM

SGPYKQLARAYIEYRHDRDIEREKRGRLNQEIRGLVEQTNASLLNENANKDSKVIPTQRDLLAGIVAKHY

ARQHLLPRDVVQAHERGDIHYHDLDYSPFFPMFNCMLIDLKGMLTQGFKMGNAEIEPPKSISTATAVTAQ

IIAQVASHIYGGTTINRIDEVLAPFVTASYNKHRKTAEEWNIPDAEGYANSRTIKECYDAFQSLEYEVNT

LHTANGQTPFVTFGFGLGTSWESRLIQESILRNRIAGLGKNRKTAVFPKLVFAIRDGLNHKKGDPNYDIK

QLALECASKRMYPDILNYDQVVNVTGSFKTPMGCRSFLGVWENENGEQIHDGRNNLGVISLNLPRIALEA

KGDEATFWKLLDERLVLARKALMTRIARLEGVKARVAPILYMEGACGVRLNADDDVSEIFKNGRASISLG

YIGIHETINALFSGEHVYDNEKLRAKGIAIVERLRQAVDQWKEETGYGFSLYSTPSENLCDRFCRLDTAE

FGVVPGVTDKGYYTNSFHLDVEKKVNPYDKIDFEAPYPPLANGGFICYGEYPNIQHNLKALEDVWDYSYQ

HVPYYGTNTPIDECYECGFTGEFECTSKGFTCPKCGNHDASRVSVTRRVCGYLGSPDARPFNAGKQEEVK

RRVKHLGNGQIG

>gi|30065474|ref|NP_839645.1| trehalose-6-phosphate hydrolase [Shigella flexneri 2a str. 2457T]

MTNLPHWWQNGVIYQIYPKSFQDTTGSGTGDLRGVIQRLDYLHKLGVDAIWLTPFYVSPQVDNGYDVANY

TAIDPTYGTLDDFDELVTQAKSRGIRIILDMVFNHTSTQHAWFREALNKESPYRQFYIWRDGEPETPPNN

WRSKFGGSAWRWHAESEQYYLHLFAPEQADLNWENPAVRAELKKVCEFWADRGVDGLRLDVVNLISKDPR

FPEDLDGDGRRFYTDGPRAHEFLHEMNRDVFTPRGLMTVGEMSSTSLEHCQRYAALTGSELSMTFNFHHL

KVDYPGGEKWTLAKPDFVALKTLFRHWQQGMHNVAWNALFWCNHDQPRIVSRFGDEGEYRVPAAKMLAMV

LHGMQGTPYIYQGEEIGMTNPHFTRITDYRDVESLNMFAELRNDGRDADELLAILASKSRDNSRTPMQWS

NGDNAGFTAGEPWIGLGDNYQQINVEAALADDSSVFYTYQKLIALRKQEAILTWGNYQDLLPNSPVLWCY

RREWKGQTLLVIANLSREIQPWQPGQMRGNWQLVMHNYEEASPQPCAMNLRPFEAVWWLQK

>gi|30065473|ref|NP_839644.1| trehalose(maltose)-specific PTS system components IIBC [Shigella flexneri 2a str. 2457T]

MSKINQTDIDRLIELVGGRGNIATVSHCITRLRFVLNQPANARPKEIEQLPMVKGCFTNAGQFQVVIGTN

VGDYYQALIASTGQAQVDKEQVKKAARQNMKWHEQLISHFAEIFFPLLPALISGGLILGFRNVIGDLPMS

NGQTLAQMYPSLQTIYDFLWLIGEAIFFYLPVGICWSAVKKMGGTPILGIVLGVTLVSPQLMNAYLLGQQ

LPEVWNFGMFSIAKVGYQAQVIPALLAGLALGVIETRLKRIVPDYLYLVVVPVCSLILAVFLAHALIGPF

GRMIGDGVAFAVRHLMTGSFAPIGAALFGFLYAPLVITGVHQTTLAIDLQMIQSMGGTPVWPLIALSNIA

QGSAVIGIIISSRKHNEREISVPAAISAWLGVTEPAMYGINLKYRFPMLCAMIGSGLAGLLCGLNGVMAN
GIGVGGLPGILSIQPSYWQVFALAMAIAIIIPIVLTSFIYQRKYRLGTLDIV

>gi|30065472|ref|NP_839643.1| trehalose repressor [Shigella flexneri 2a str. 2457T]
MQNRLTIKDIARLSGVGKSTVSRVLNNESGVSQRTRERVEAVMNQHGFSPSRSARAMRGQSDKVVAIIVT
RLDSLSENLAVQTMLPAFYEQGYDPIMMENQFSPQLVAEHLGVLKRRNIDGVVLFGFTGITEEMLAHWQS
SLVLLARDAKGFASVCYDDEGAIKILMQRLYDQGHRNISYLGVPHSDVTTGKRRHEAYLAFCKAHKLHPV
AALPGLAMKQGYENVAKVITPETTALLCATDTLALGASKYLQEQRIDTLQLASVGNTPLMKFLHPEIVTV
DPGYAEAGRQAACQLIAQVTGRSEPQQIIIPATLS

>gi|30065471|ref|NP_839642.1| magnesium-transporting ATPase MgtA [Shigella flexneri 2a str. 2457T]
MFKEIFTRFIRHLPSRLVHRDPLPGAQQTVNTAVPPSLSAHCLKMAVMPEEELWKTFDTHPEGLNQAEVE
SAREQHGDNKLPAQQPSPWWVHLWVCYRNPFNILLTILGAISYATEDLFAAGVIALMVAISTLLNFIQEA
RSTKAADALKAMVSNTATVLRVINDKGENGWLEIPIDQLVPGDIIKLAAGDMIPADLRILQARDLFVAQA
SLTGESLPVEKAATTRQPEHSNPLECDTLCFMGTTVVSGTAQAMVIATGANTWFGQLAGRVSEQESEPNA
FQQGISRVSMLLIRFMLVMAPVVLLINGYTKGDWWEAALFALSVAVGLTPEMLPMIVTSTLARGAVKLSK
QKVIVKHLDAIQNFGAMDILCTDKTGTLTQDKIVLENHTDISGKTSERVLHSAWLNSHYQTGLKNLLDTA
VLEGTDEESARSLASRWQKIDEIPFDFERRRMSVVVAENTEHHQLVCKGALQEILNVCSQVRHNGEIVPL
DDTMLRKIKRVTDTLNRQGLRVVAVATKYLPAREGDYQRADESDLILEGYIAFLDPPKETTAPALKALKA
SGITVKILTGDSELVAAKVCHEVGLDAGEVVIGSDIETLSDDELANLAQRTTLFARLTPMHKERIVTLLK
REGHVVGFMGDGINDAPALRAADIGISVDGAVDIAREAADIILLEKSLMVLEEGVIEGRRTFANMLKYIK
LTASSNFGNVFSVLVASAFLPFLPMLPLHLLIQNLLYDVSQVAIPFDNVDDEQIQKPQRWNPADLGRFMV
FFGPISSIFDILTFCLMWWVFHANTPETQTLFQSGWFVVGLLSQTLIVHMIRTRRVPFIQSCASWPLMIM
TVIVMIVGIALPFSPLASYLQLQALPLSYFPWLVAILAGYMTLTQLVKGFYSRRYGWQ

>gi|30065470|ref|NP_839641.1| hypothetical protein S4509 [Shigella flexneri 2a str. 2457T]

MSQTFYRCNKGEIMSKTIATENAPAAIGPYVQGVDLGNMIITSGQIPVNPKTGEVPADVAAQARQSLDNV

KAIVEAAGLKVGDIVKTTVFVKDLNDFATVNATYEAFFTEHNATFPARSCVEVARLPKDVKIEIEAIAVR

R

>gi|30065469|ref|NP_839640.1| aspartate carbamoyltransferase regulatory subunit [Shigella flexneri 2a str. 2457T]

MTHDNKLQVEAIKRGTVIDHIPAQIGFKLLSLFKLTETDQRITIGLNLPSGEMGRKDLIKIENTFLSEEQ

VDQLALYAPQATVNRIDNYEVVGKSRPSLPERIDNVLVCPNSNCISHAEPVSSSFAVRKRANDIALKCKY

CEKEFSHNVVLAN

>gi|30065468|ref|NP_839639.1| aspartate carbamoyltransferase catalytic subunit [Shigella flexneri 2a str. 2457T]

MANPLYQKHIISINDLSRDDLNLVLATAAKLKANPQPELLKHKVIASCFFEASTRTRLSFETSMHRLGAS

VVGFSDSANTSLGKKGETLADTISVISTYVDAIVMRHPQEGAARLATEFSGNVPVLNAGDGSNQHPTQTL

LDLFTIQETQGRLDNLHVAMVGDLKYGRTVHSLTQALAKFDGNRFYFIAPDALAMPQYILDMLDEKGIAW

SLHSSIEEVMAEVDILYMTRVQKERLDPSEYANVKAQFVLRASDLHNAKANMKVLHPLPRVDEIATDVDK

TPHAWYFQQAGNGIFARQALLALVLNRDLVL

>gi|30065467|ref|NP_839638.1| pyrBI operon leader peptide [Shigella flexneri 2a str. 2457T]

MVQCVRHFVLPRLKKDAGLPFFFPLITHSQPLNRGAFFCLGVRR

>gi|30065466|ref|NP_839637.1| hypothetical protein S4506 [Shigella flexneri 2a str. 2457T]

MSNTLNHTSSRQIVRHYTHSQKRCKHLMQYFVSANGLFELKVKIYAFLFSMILEGKCRSVSIIADISCFF

LFHFHAIRNAFYSIHPTYRAEGENERLTLLLIAQGYALSL

>gi|30065465|ref|NP_839636.1| hypothetical protein S4504 [Shigella flexneri 2a str. 2457T]

MVERTAVFPAGRHSLYAEHRYSAAIRSGDLLFVSGQVGSREDGTPEPDFQQQVRLAFDNLHATLAAAGCT

FDDIIDVTSFHTDPEKQSEDIMTVKNEIFSAPPYPNWTAVGVTWLAGFDFEIKVIARIPEL

>gi|30065464|ref|NP_839635.1| oxidoreductase [Shigella flexneri 2a str. 2457T]

MGAFTGKTVLILGGSRGIGAAIVRRFVTDGANVRFTYAGSKDAAERLAQETGATAVFTDSADRDAVIDVV

RKSGALDILVVNAGIGVFGDALELNADDIDRLFKINIHAPYHASVEAARQMPEGGRILIIGSVNGDRMPV

AGMAAYAASKSALQGMARGLARDFGPRGITINVVQPGPIDTDVNPANGPMRDMLHSFMAIKRHGQPEEVA

GMVAWLAGPEASFVTGAMHTIDGAFGA

>gi|30065463|ref|NP_839634.1| hypothetical protein S4502 [Shigella flexneri 2a str. 2457T]

MSLKLLIILVLTPRVSTRLLAVRLGYLAVYSMNMSVRKLFRLPIFFVMIVR

>gi|30065462|ref|NP_839633.1| hypothetical protein S4501, partial [Shigella flexneri 2a str. 2457T]

MVLEGIHSHDPQARDIAIQYYHAAETTIYDYIARRHPQSAQCVTDFMSTVMSGLSAKAREGHSIEQLCAT

AALAGEAIKTLLKE

>gi|30065461|ref|NP_839632.1| hypothetical protein S4500 [Shigella flexneri 2a str. 2457T]

MIIGNIHNLQPWLPQELRQAIEHIKAHVTAETPKGKHDIEGNRLFYLISEDMTEPYEARRAEYHARYLDI

QIVLKGQEGMTFSTQPAGAPDTDWLADKDIAFLPEGVDEKTVILNEGDFVVFYPGEVHKPLCAVGAPAQV

RKAVVKMLMA

>gi|30065460|ref|NP_839631.1| IS1 orfB [Shigella flexneri 2a str. 2457T]

MSRQCTHYGRWPQHGFTSLKKLRPQSVTSRIQPGSDVIVCAEMDEHWGYVGAKSRQRWLFYAYDRIRRTV

VAHVFGERTLATLERLLSLLSAFEVVVWMTDGWPLYESRLKGKLHVISKRYTQRIERHNLNLRQHLARLV

RKSLSFSKSVELHDKVIGHYLNIKHYQ

>gi|30065459|ref|NP_839630.1| IS1 orfA [Shigella flexneri 2a str. 2457T]

MASISIRCPSCSATEGVVRNGKSTAGHQRYLCSHCRKTWQLQFTYTDSQPGTHQKIIDMAMNGVGCRASA

RIMGVGLNTVLRHLKNSGRSR

>gi|30065458|ref|NP_839629.1| ornithine carbamoyltransferase subunit I [Shigella flexneri 2a str. 2457T]

MSGFYHKHFLKLLDFTPAELNSLLQLAAKLKADKKSGKEEAKLTGKNIALIFEKDSTRTRCSFEVAAYDQ

GARVTYLGPSGSQIGHKESIKDTARVLGRMYDGIQYRGYGQEIVETLAEYAGVPVWNGLTNEFHPTQLLA

DLLTMQEHLPGKAFNEMTLVYAGDARNNMGNSMLEAAALTGLDLRLVAPQACWPEAALVTECRALAQQNG

GNITLTEDVAKGVEGADFIYTDVWVSMGEAKEKWAERIALLRDYQVNSKMMQLTGNPEVKFLHCLPAFHD

DQTTLGKKMAEEFGLHGGMEVTDEVFESAASIVFDQAENRMHTIKAVMVATLSKLNN

>gi|30065457|ref|NP_839628.1| hypothetical protein S4495 [Shigella flexneri 2a str. 2457T]

MANPEQLEEQREETRLIIEELLEDGSDPDALYTIEHHLSADDLETLEKAAVEAFKLGYEVTDPEELEVED

GDIVICCDILSECALNADLIDAQVEQLMTLAEKFDVEYDGWGTYFEDPNGEDGDDEDFVDEDDDGVRH

>gi|30065456|ref|NP_839627.1| hypothetical protein S4493 [Shigella flexneri 2a str. 2457T]

MAQVINEMDVPSHSFVFHGTGERYFLICVVNVLLTIITLGIYLPWALMKCKRYLYANMEVNGQRFSYGIT

GGNVFVSCLVFVFFYFAILMTVSADMPLVGCVLTLLLLVLLIFMAAKGLRYQALMTSLNGVRFSFNCSLK

GFWWVTFFLPILMAIGMGTVFFISTKMLHANSSSSVIISVVLMAIVGIVSIGIFNGTLYSLVMSFLWSNT

SFGIHRFKVKLDTAYCIKYAILAFLALLPFLAVAGYIIFDQILNEYDSSGYANDDIENLQQFMEMQRKMI

IAQLIYYFGIAVSTSYLTVSLRNHFMSNLSLNDGRIRFRSTLTYHGMLYRMCALVVISGITGGLAYPLLK

IWMIDWQAKNTYLLGDLDDLPLINKEEQPDKGFLASISRGVMPSLPFL

>gi|30065455|ref|NP_839626.1| valyl-tRNA synthetase [Shigella flexneri 2a str. 2457T]

MEKTYNPQDIEQPLYEHWEKQGYFKPNGDESQESFCIMIPPPNVTGSLHMGHAFQQTIMDTMIRYQRMQG

KNTLWQVGTDHAGIATQMVVERKIAAEEGKTRHDYGREAFIDKIWEWKAESGGTITRQMRRLGNSVDWER

ERFTMDEGLSNAVKEVFVRLYKEDLIYRGKRLVNWDPKLRTAISDLEVENRESKGSMWHIRYPLADGAKT

ADGKDYLVVATTRPETLLGDTGVAVNPEDPRYKDLIGKYVILPLVNRRIPIVGDEHADMEKGTGCVKITP

AHDFNDYEVGKRHALPMINILTFDGDIRESAQVFDTKGNESDVYSSEIPAEFQKLERFAARKAVVAAVDA

LGLLEEIKPHDLTVPYGDRGGVVIEPMLTDQWYVRADVLAKPAVEAVENGDIQFVPKQYENMYFSWMRDI

QDWCISRQLWWGHRIPAWYDEAGNVYVGRNEEEVRKENNLGANVALRQDEDVLDTWFSSALWTFSTLGWP

ENTDALRQFHPTSVMVSGFDIIFFWIARMIMMTMHFIKDENGKPQVPFHTVYMTGLIRDDEGQKMSKSKG

NVIDPLDMVDGISLPELLEKRTGNMMQPQLADKIRKRTEKQFPNGIEPHGTDALRFTLAALASTGRDINW

DMKRLEGYRNFCNKLWNASRFVLMNTEGQDCGFNGGEMTLSLADRWILAEFNQTIKAYREALDSFRFDIA

AGILYEFTWNQFCDWYLELTKPVMNGGTEAELRGTRHTLVTVLEGLLRLAHPIIPFITETIWQRVKVLCG

ITADTIMLQPFPQYDASQVDEAALADTEWLKQAIVAVRNIRAEMNIAPGKPLELLLRGCSADAERRVNEN

RGFLQTLARLESITVLPADDKGPVSVTKIIDGAELLIPMAGLINKEDELARLAKEVAKIEGEISRIENKL

ANEGFVARAPEAVIAKEREKLEGYAEAKAKLIEQQAVIAAL

>gi|30065454|ref|NP_839625.1| DNA polymerase III subunit chi [Shigella flexneri 2a str. 2457T]

MKNATFYLLDNDTTVDGLSAVEQLVCEIAAERWRSGKRVLIACEDEKQAYRLDEALWARPAESFVPHNLA

GEGPRGSAPVEIAWPQKRSSSPRDILISLRTSFADFATAFTEVVDFVPYEDSLKQLARERYKAYRVAGFN

LNTATWK

>gi|30065453|ref|NP_839624.1| leucyl aminopeptidase [Shigella flexneri 2a str. 2457T]

MEFSVKSGSPEKQRSACIVVGVFEPRRLSPIAEQLDKISDGYISALLRRGELEGKPGQTLLLHHVPNVLS

ERILLIGCGKERELDERQYKQVIQKTINTLNDTGSMEAVCFLTELHVKGRNNYWKVRQAVETAKETLYSF

DQLKTNKSEPRRPLRKMVFNVPTRRELTSGERAIQHGQAIAAGIKAAKDLGNMPPNICNAAYLASQARQL

ADSYSKNVITRVIGEQQMKELGMHSYLAVGQGSQNESLMSVIEYKGNASEDARPIVLVGKGLTFDSGGIS
IKPSEGMDEMKYDMCGAAAVYGVMRMVAELQLPINVIGVLAGCENMPGGRAYRPGDVLTTMSGQTVEVLN
TDAEGRLVLCDVLTYVERFEPEAVIDVATLTGACVIALGHHITGLMTNHNPLAHELIAASEQSGDRAWRL
PLGDEYQEQLESNFADMANIGGRPGGAITAGCFLSRFTRKYNWAHLDIAGTAWRSGKAKGATGRPVALLA
QFLLNRAGFNGEE

>gi|30065452|ref|NP_839623.1| hypothetical protein S4489 [Shigella flexneri 2a str. 2457T]
MIIIRYLVRETLKSQLAILFILLLIFFCQKLVRILGAAVDGDIPANLVLSLLGLGVPEMAQLILPLSLFL
GLLMTLGKLYTESEITVMHACGLSKAVLVKAAMILAVFTAIVAAVNVMWAGPWSSRHQDEVLAEAKANPG
MAALAQGQFQQATNGSSVLFIESVDGSDFKDVFLAQIRPKGNARPSVVVADSGHLTQLRDGSQVVTLNQG
TRFEGTALLRDFRITDFQDYQAIIGHQAVALDPNDTDQMDMRTLWNTDTDRARAELNWRITLVFTVFMMA
LMVVPLSVVNPRQGRVLSMLPAMLLYLLFFLIQTSLKSNGGKGKLDPTLWMWTVNLIYLALAIVLNLWDT
VPVRRLRASFSRKGAV

>gi|30065451|ref|NP_839622.1| hypothetical protein S4488 [Shigella flexneri 2a str. 2457T]
MQPFGVLDRYIGKTIFTTIMMTLFMLVSLSGIIKFVDQLKKAGQGSYDALGAGMYTLLSVPKDVQIFFPM
AALLGALLGLGMLAQRSELVVMQASGFTRMQVALSVMKTAIPLVLLTMAIGEWVAPQGEQMARNYRAQAM
YGGSLLSTQQGLWAKDGNNFVYIERVKGDEVLGGISIYAFNENRRLQSVRYAATAKFDPEHKVWRLSQVD
ESDLTNPKQITGSQTVSGTWKTDLTPDKLGVVALDPDALSISGLHNYVKYLKSSGQDAGRYQLNMWSKIF
QPLSVAVMMLMALSFIFGPLRSVPMGVRVVTGISFGFVFYVLDQIFGPLTLVYGIPPIIGALLPSASFFL
ISLWLLMRKS

>gi|30065450|ref|NP_839621.1| hypothetical protein S4487 [Shigella flexneri 2a str. 2457T]
MSEPLLIARTPDTELFLLPGMANRHGLITGATGTGKTVTLQKLAESLSEIGVPVFMADVKGDLTGIAQAG
TASEKLLTRLKNIGVNDWQPHANPVVVWDIFGEKGHTVRATVSDLGPLLLARLLNLNDVQSGVLNIIFRI

ADDQGLLLLDFKDLRAITQYIGDNAKSFQNQYGNISSASVGAIQRGLLSLEQQGAAHFFGEPMLDIKDWM
RTDANGKGVINILSAEKLYQMPKLYAASLLWMLSELYEQLPEAGDLEKPKLVFFFDEAHLLFNDAPQVLL
DKIEQVIRLIRSKGVGVWFVSQNPSDIPDNVFGQLGNRVQHALRAFTPKDQKAVKTAAQTMRANPAFDTE
KAIQELGTGEALISFLDAKGSPSVVERAMVIAPCSRMGPVTEDERNGLINHSPVYGKYEDEVDRESAYEM
LQKGFQASTEQQNNPPVKGKEVAVDDGILGGLKDILFGTTGPRGGKKDGVVQTMAKSAARQVTNQIVRGD
VGEFAGGEKKISQT

>gi|30065449|ref|NP_839620.1| oxidoreductase [Shigella flexneri 2a str. 2457T]
MLYTSQTTPEKAPKMSMIKSYAAKEAGGELEVYEYDPGELKPQDVEVQVDYCGICHSDLSMIDNEWGFSQ
YPLVAGHEVIGRVVALGSAAQDKGLQVGQRVGIGWTARSCGHCDACISGNQINCEQGAVPTIMNRGGFAE
KLRADWQWVIPLPENIDIESAGPLLCGGITVFKPLLMHHITATSRVGVIGIGGLGHIAIKLLHAMGCEVT
AFSSNPAKEQEVLAMGADKVVNSRDPQALKALSGQFDLIINTVNVSLDWQPYFEALTYGGNFHTVGAVLT
PLSVPAFTLIAGDRSISGSATGTPYELRKLMRFAARSKVAPTTELFPMSKINDAIQHVRDGKARYRVVLK
ADF

>gi|30065448|ref|NP_839619.1| P4-type integrase [Shigella flexneri 2a str. 2457T]
MALSDVKVRSAKPEAKAYKLTDGEGMVLLVHPNGSKYWRLRYRFGGKEKMLALGKYPEVSLADARARRDE
ARKLLANGVDPSENKKAVKVEQEQEAITFEVVARDWHASNQKWSASHSARVLKSLEDNLFTAIGKRNIAE
LKTRDLLVPIKAVESSGRLEVAARLQQRTTAIMRFAVQSGLIDYNPAQEIAGAVATAKRQHRAALELNRI
PELLHRIDHYSGRPLTRLAVELTLLVFIRSSELRFARWSEIDFETAMWTIPGEREQLEGVKHSQRGSKMR
TPHLVPLSRQALSILEKIKSMSGNRELIFVGDHDPRKPMSENTVNKALRVMGYDTKVEVCGHGFRTMACS
SLIESGLWSRDAVERQMSHQERSSVRAAYIHKAEHLGERRLMFEVVNKNWPPS

>gi|30065447|ref|NP_839618.1| IS911 orfB, partial [Shigella flexneri 2a str. 2457T]
MKELGLVSCQQPTHRYKRGGHEHVAIPNYLERQFAVTEPNQVWCGDVTYIWTGKRWAYLAVVLDLFARKP

VGWAMSFSPDSRLTMKALEMAWETRGKPGGVMFHSDQGSHYTSRQFRQLLWRYQIRQSMSRRGNCWDNSP
MERFFRSLKNEWMPVVGYVSFSEAAHAITDYIVGYYSALRPHEYNGGLPPNESENRYWKNSNSVASFC

>gi|30065446|ref|NP_839617.1| IS911 orfA [Shigella flexneri 2a str. 2457T]
MKKRNFSAEFKRESAQLVVDQKYTVADAAKAMDVGLSTMTRWVKQLRDERQGKTPKASPITPEQIEIREL
RKKLQRIEMENEILKKATALLMSDSLNSSR

>gi|30065445|ref|NP_839616.1| IS1 orfB [Shigella flexneri 2a str. 2457T]
MSRQCTHYGRWPQHGFTSLKKLRPQSVTSRIQPGSDVIVCAEMDEHWGYVGAKSRQRWLFYAYDRIRRTV
VAHVFGERTLATLERLLSLLSAFEVVVWMTDGWPLYESRLKGKLHVISKRYTQRIERHNLNLRQHLARLV
RKSLSFSKSVELHDKVIGHYLNIKHYQ

>gi|30065444|ref|NP_839615.1| IS1 orfA [Shigella flexneri 2a str. 2457T]
MASISIRCPSCSATEGVVRNGKSTAGHQRYLCSHCRKTWQLQFTYTASQPGTHQKIIDMAMNGVGCRASA
RIMGVGLNTVLRHLKNSGRSR

>gi|30065443|ref|NP_839614.1| IS600 orfB [Shigella flexneri 2a str. 2457T]
MAHIRTRETYGTRRLQTELAENGIIVGRDRLARLRKELRLRCKQKRKFRATTNPNHNLPVAPNLLNQTFA
PTAPNQVWVADLTYVATQEGWLYLAGIKDVYTCEIVGYAMGERMTKELTGKALFMALRSQRPPAGLIHHS
DRGSQYCAYDYRVIQEQSGLKTSMSRKGNCYDNAPMESFWGTLKNESLSHYRFNNRDEAISVIREYIEIF
YNRQRRHSRLGNISPAAFREKYHQMAA

>gi|30065442|ref|NP_839613.1| IS600 orfA [Shigella flexneri 2a str. 2457T]
MSRKTQRYSKEFKAEAVRTVLENQLSISEGASRLSLPEGTLGQWVTAARKGLGTPGSRTVAELESEILQL
RKALNEARLERDILKKATAYFAQESLKNTR

>gi|30065441|ref|NP_839612.1| ISEhe3a orf [Shigella flexneri 2a str. 2457T]

MSGKRYPEEFKTEAVKQVVDRGYSVASVATRLDITTHSLYAWIKKYGPDSSTNKEQSDAQAEIRRLQKEL

KRVTDERDILKKAAAYFAKLSD

>gi|30065440|ref|NP_839611.1| hypothetical protein S4474 [Shigella flexneri 2a str. 2457T]

MWAFARRALNVEDILCLPIQDCDKSHIWLLVKDDQRLE

>gi|30065439|ref|NP_839610.1| DeoR family transcriptional regulator [Shigella flexneri 2a str. 2457T]

MLEVSVMTIRRDLHQEDEPLPLTLLGGYIVMVNKPAPSMPVIHDVPKNHRDDLPIAILAAGMVNENDLIF

FDNGQEIPLVISMIPDAITFTGICYSHRVFVALNEKPNVTAILCGGTYRARSDAFYDASNSSPLDSLNPR

KIFISASGVHNHFGVSWFNPEDLATKRKVMNRGLRKILLARHALFDEVASASLAPISAFDVLISDRPLPA

DYVTHCQNGSVKIITPDSEDE

>gi|30065438|ref|NP_839609.1| IS1 orfA [Shigella flexneri 2a str. 2457T]

MASISIRCPSCSATEGVVRNGKSTAGHLRYLCSHCRKTWQLQFTYTASQPGTHQKIIDMAMNGVGCRASA

RIMGVGLNTVLRHLKNSGRSR

>gi|30065437|ref|NP_839608.1| IS1 orfB [Shigella flexneri 2a str. 2457T]

MSRQCTHYGRWPQHGFTSLKKLRPQSVTSRIQPGSDVIVCAEMDEHWGYVGAKSRQRWLFYAYDRIRRTV

VAHVFGERTLATLERLLSLLSAFEVVVWMTDGWPLYESCLKGKLHVISKRYTQRIERHNLNLRQHLARLV

RKSLSFSKSVELHDKVIGHYLNIKHYQ

>gi|30065436|ref|NP_839607.1| hypothetical protein S4470 [Shigella flexneri 2a str. 2457T]

MQGYHHPLATNHQTQYGTVGQALHIARKLLPFIPDNAGILIVPCCRGGSAFTAGSEGTYSERHGASHDAC

RWGTDTPLYQDLVSRTRAALAKNPQNKFLGVCWMQGEFDLMTSDYASHPQHFNHMVEAFRRDLKQYHSQL
NNITDAPWFCGDTTWYWKENFPHSYEAIYGNYQNNVLANIIFVDFQQQGERGLTNAPDEDPDDLSTGYYG
SAYRSPENWTTALRSSHFSTAARRGIISDRFVEAILQFWRER

>gi|30065435|ref|NP_839606.1| hypothetical protein S4469 [Shigella flexneri 2a str. 2457T]
MTTLTARVFTTAEIIYRKTVIALVCHLNCSRQETVTMNKTIMALAIMMASFAANASVLPETPVPFKSGTG
AIDNDTVYIGLGSAGTAWYKLDTQAKDKKWTALAAFPGGPREQATSAFIDGNLYVFGGIGKNSEGLTQVF
NDVHKYNPKTNSWVKLMSHAPMGMAGHVTFVHNGKAYVTGGVNQNIFNGYFEDLNEAGKDSTAIDKINAH
YFDKKAEDYFFNKFLLSFDPSTQQWSYAGESPWYGTAGAAVVNKGDKTWLINGEAKPGLRTDAVFELDFT
GNNLKWNKLDPVSSPDGVAGGFAGISNDSLIFAGGAGFKGSRENYQNGKNYAHEGLKKSYSTDIHLWHNG
KWDKSGELSQGRAYGVSLPWNNSLLIIGGETAGGKAVTDSVLISVKDNKVTVQN

>gi|30065434|ref|NP_839605.1| hypothetical protein S4468 [Shigella flexneri 2a str. 2457T]
MESNTWNTIHDNKKENAALNDVQVEVNYAIKLDDQWTVRPGMLTHFSSNGTRYGPYVKLSWDATKDLNFG
IRYRYDWKAYRQQDLSGDMSRDNVHRWDGYVTYHINSDFTFAWQTTLYSKQNDYRYANHKKWATENAFVL
QYHMTPDITPYIEYDYLDRQGVYNGRDNLSENSYRIGVSFKL

>gi|30065433|ref|NP_839604.1| recombinase; regulator for fimA [Shigella flexneri 2a str. 2457T]
MRNKADNKKRNFLTHSEIESLLKAANTGPHATRNYCLILLCFIHGFRASEICRLRISDIDLKAKCIYIHR
LKKGFSTTHPLLNKEVQALKNWLSIRTSYPHAESEWVFLSRKGNPLSRQQFYHIISTSGGNAGLSLEIHP
HMLRYSCGFALANMGIDTRLI

>gi|30065432|ref|NP_839603.1| tyrosine recombinase [Shigella flexneri 2a str. 2457T]
MSKRRYLTGKEVQAMMQAVCYGATGARDYCLILLAYRHGMRISELLDLHYQDLDLNEGRINIRRLKNGFS
TVHPLRFDEREAVERWTQERANWKGADRTDAIFISRRGSRLSRQQAYRIIRDAGIEAGTVTQTHPHMLRH

ACGYELAERGADTRLIQDYLGHRNIRHTVRYTASNAARFAGLWERNNLINEKLKREEV

>gi|30065431|ref|NP_839602.1| major type 1 subunit fimbrin (pilin) [Shigella flexneri 2a str. 2457T]

MKIKTLAIVVLSALSLSSAAALADTTTVNGGTIHFKGEVVNAACAVDAGSVDQTVQLGQVRTASLKQAGA

TSSAVGFNIQLNDCDTTVATKAAVAFLGTAIDATRTDVLALQSSAAGSATNVGVQILDRTGNALTLDGAT

FSAQTTLNNGTNTIPFQARYYAIGEATPGAANADATFKVQYQ

>gi|30065430|ref|NP_839601.1| fimbrial protein [Shigella flexneri 2a str. 2457T]

MRMQPSRFSINNLPRFKDVITGRDAHPCAIKITMKRKRLFLLASLLPMFALAGNKWNTTLPGGNMQFQGV

IIAETCRIEAGDKQMTVNMGQISSNRFHAAGEDSAPVPFVIHLRECSTVVSERVGVAFHGVADGKNPDVL

SVGEGPGIASNIGVALFDDEGNLVPINRPPANWKRLYSGSTSLHFIAKYRATGRRVTGGIANAQAWFSLT

YQ

>gi|30065429|ref|NP_839600.1| periplasmic chaperone [Shigella flexneri 2a str. 2457T]

MSNKNVNVRKSQEITFCLLAGILMFMAMMVAGRAEAGVALGATRVIYPAGQKQVQLAVTNNDENSTYLIQ

SWVENADGVKDGRFIVTPPLFAMKGKKENTLRILDATNNQLPQDRESLFWMNVKAIPSMDKSKLTENMLQ

LAIISRIKLYYRPAKLALPPDQAAEKLRFRRSANSLTLINPTPYYLTVTELNAGTRVLENALVPPMGEST

VKLPSDAGSNITYRTINDYGALTPKMTGVME

>gi|30065428|ref|NP_839599.1| IS1 orfA [Shigella flexneri 2a str. 2457T]

MASISIRCPSCSATEGVVRNGKSTAGHQRYLCSHCRKTWQLQFTYTASQPGKHQKIIDMAMNGVGCRASA

RIMGVGLNTVLRHLKNSGRSR

>gi|30065427|ref|NP_839598.1| IS1 orfB [Shigella flexneri 2a str. 2457T]

MSRQCTHYGRWPQHGFTSLKKLRPQSVTSRIQPGSDVIVCAEMDEHWGYVGAKSRQRWLFYAYDRIRRTV

VAHVFGERTLATLERLLSLLSAFEVVVWMTDGCPLYESRLKGKLHVISKRYTQRIERHNLNLRQHLARLV

RKSLSFSKSVELHDKVIGHYLNIKHYQ

>gi|30065426|ref|NP_839597.1| minor fimbrial subunit, precursor polypeptide [Shigella flexneri 2a str. 2457T]

MRNKPFYLLCAFLWLAVSRVLAADSTITIRGYVRDNGCSVAAESTNFTVDLMENAAKQFNNIGATTPVVP

FRILLSPCGNAVSAVKVGFTGVADSHNANLLALENTVSAAAGLGIQLLNEQQNQIPLNAPSSAISWTTLT

PGKPNTLNFYARLMATQVPVTAGHINATATFTLEYQ

>gi|30065425|ref|NP_839596.1| minor fimbrial subunit, precursor polypeptide [Shigella flexneri 2a str. 2457T]

MKWCKRGYVLAAMLALASATIQAADVTITVNGKVVAKPCTVSTTNATVDLGDLYSFSLMSAGAASAWHDV

ALELTNCPVGTSRVTASFSGAADSTGYYKNQGTAQNIQLELQDDSGNTLNTGATKTVQVDDSSQSAHFPL

QVRALTVNGGATQGTIQAVISITYTYS

>gi|30065424|ref|NP_839595.1| minor fimbrial subunit, D-mannose specific adhesin [Shigella flexneri 2a str. 2457T]

MKRAITLFAVLLMGWSVNAWSFACKTANGTAIPIGGGSANVYVNLAPVVNVGQNLVVDLSTQIFCHNDYP

ETITDYVTLQRGSAYGGVLSNFSGTVKYSGSSYPFPTTSETPRVVYNSRTDKPWPVALYLTPVSSAGGVA

IKAGSLIAVLILRQTNNYNSDDFQFVWNIYANNDVVVPTGGCDVSARDVTVTLPDYPGSVPIPLTVYCAK

SQNLGYYLSGTTADAGNSIFTNTASFSPAQGVGVQLTRNGTIIPANNTVSLGAVGTSAVSLGLTANYART

GGQVTAGNVQSIIGVTFVYQ

>gi|30065423|ref|NP_839594.1| fructuronate transporter [Shigella flexneri 2a str. 2457T]

MHVLNILWVVFGIGLMLILNLKFKINSMVALLVAALSVGMLAGMDLMSLLHTMKAGFGNTLGELAIIVVF

GAVIGKLMVDSGAAHQIAHTLLARLGLRYVQLSVIIIGLIFGLAMFYEVAFIMLAPLVIVIAAEAKIPFL

KLAIPAVAAATTAHSLFPPQPGPVALVNAYGADMGMVYIYGVLVTIPSVICAGLILPKFLGNLERPTPSF

LKADQPVDMNNLPSFGVSILVPLIPAIIMISTTIANIWLVKDTPAWEVVNFIGSSPIAMFIAMVVAFVLF

GTARGHDMQWVMNAFESAVKSIAMVILIIGAGGVLKQTIIDTGIGDTIGMLMSHGNISPYIMAWLITVLI

RLATGQGVVSAMTAAGIISAAILDPATGQLVGVNPALLVLATAAGSNTLTHINDASFWLFKGYFDLSVKD

TLKTWGLLELVNSVVGLIIVLIISMVA

>gi|30065422|ref|NP_839593.1| mannonate dehydratase [Shigella flexneri 2a str. 2457T]

MEQTWRWYGPNDPVSLADVRQAGATGVVTALHHIPNGEVWSVEEILKRKAIVEDAGLVWSVVESVPIHED

IKTHTGNYEQWIANYQQTLRNLAQCGIRTVCYNFMPVLDWTRTDLEYVLPDGSKALRFDQIEFAAFEMHI

LKRPGAEADYTEEEIAQAAERFATMSDEDKARLTRNIIAGLPGAEEGYTLDQFRKHLELYKDIDKAKLRE

NFAVFLKAIIPVAEEVGVRMAVHPDDPPRPILGLPRIVSTIEDMQWMVDTVNSMANGFTMCTGSYGVRAD

NDLVDMIKQFGPRIYFTHLRSTMREDNPKTFHEAAHLNGDVDMYKVVKAIVEEEHRRKAEGKEDLIPMRP

DHGHQMLDDLKKKTNPGYSAIGRLKGLAEVRGVELAIQRAFFSR

>gi|30065421|ref|NP_839592.1| D-mannonate oxidoreductase [Shigella flexneri 2a str. 2457T]

MTTIVDSNLPVARPSWDHSRLESRIVHLGCGAFHRAHQALYTHHLLESTDSDWGICEVNLMPGNDRMLIE

NLKKQQLLYTVAEKGAESTELKIIGSMKEALHPEIDGCEGILNAMARPQTAIVSLTVTEKGYCADAASGQ

LDLNNPLIKHDLENPTAPKSAIGYIVEALRLRREKGLKAFTVMSCDNVRENGHVAKVAVLGLAQARDPQL

AAWIEENVTFPCTMVDRIVPAATPETLQEIADQLGVYDPCAIACEPFRQWVIEDNFVNGRPDWDKVGTQF

VADVVPFEMMKLRMLNGSHSFLAYLGYLGGYETIADTMTNPDYRKAAFALMMQEQAPTLSMPEGTDLNAY

ATLLIERFSNPSLRHRTWQIAMDGSQKLPQRLLDPVRLHLQNGGSWRHLALGVAGWMGYTQGVDEQGNAI

DVVDPMLAEFQKINAQYQGADRVKALLGLSGIFADDLPQNADFVGAVTSAYQQLCERGARECVAAL

>gi|30065420|ref|NP_839591.1| DNA-binding transcriptional repressor UxuR [Shigella flexneri 2a str. 2457T]

MKSVTSAQRPYQEVGAMIHDLIIKTPYNPGERLPPEREIAEMLDVTRTVVREALIMLEIKGLVEVRRGAG

IYVLDSSGSHNTDSPDANVCNDAGPFELLQARQLLESNIAEFAALQATREDIVKMRQALQLEERELASSA

PGSSESGDMQFHLAIAEATHNSMLVELFRQSWQWRENNPMWIQLHSHLDDSLYRKEWLGDHKQILAALIK
KDARAAKLAMWQHLENVKQRLLEFSNVDDIYFDGYLFDSWPLDKVDA

>gi|30065419|ref|NP_839590.1| IS1 orfA [Shigella flexneri 2a str. 2457T]
MASISIRCPSCSATEGVVRNGKSTAGHQRYLCSHCRKTWQLQFTYTASQPGTHQKIIDMAMNGVGCRASA
RIMGVGLNTVLRHLKNSGRSR

>gi|30065418|ref|NP_839589.1| IS1 orfB [Shigella flexneri 2a str. 2457T]
MSRQRTHYGRWPQHGFTSLKKLRPQSVTSRIQPGSDVIVCAEMDEHWGYVGAKSRQRWLFYAYDRIRRTV
VAHVFGERTLATLERLLSLLSAFEVVVWMTDGWPLYESRLKGKLHVISKRYTQRIERHNLNLRQHLARLV
RKSLSFSKSVELHDKVIGHYLNIKHYQ

>gi|30065417|ref|NP_839588.1| hypothetical protein S4448 [Shigella flexneri 2a str. 2457T]
MRKMMRQSLQAVLPEISGNKTSLLRKSVCSDLLTLFNSPHSALPSLLVSGMPEWQVHNPSDKHLQSWYCR
QLRSALLFHEPRIAALQVNLKEAYCHTLAISLEIMLYHDDEPLTFDLVWDNGGWRSATLENVS

>gi|30065416|ref|NP_839587.1| DNA-binding transcriptional regulator [Shigella flexneri 2a str. 2457T]
MDDCGAVLHNIETKWLYDFLTLEKCRNFSQAAVSRNVSQPAFSRRIRALEQAIGVELFNRQVTPLQLSEQ
GKIFHSQIRHLLQQLESNLAELRGGSDYAQRKIKIAAAHSLSLGLLPSIISQMPPLFTWAIEAIDVDEAV
DKLREGQSDCIFSFHDEDLLEAPFDHIRLFESQLFPVCASDEHGEALFNLAQPHFPLLNYSRNSYMVRLI
NRTLTRHSELSFSTFFVSSMSELLKQVALDGCGIAWLPEYAIQQEIRSGQLVVLNRDEQVIPIQAYAYRM
NTRMNPVAERFWRELRELEIVLS

>gi|30065415|ref|NP_839586.1| isoaspartyl dipeptidase [Shigella flexneri 2a str. 2457T]
MIDYTAAGFTLLQGAHLYAPEDRGICDVLVANGKIIAVASNIPSDIVPNCTVVDLSGQILCPGFIDQHVH

LIGGGGEAGPTTRTPEVALSRLTEAGVTSVVGLLGTDSISRHPESLLAKTRALNEEGISAWMLTGAYHVP

SRTITGSVEKDVAIIDRVIGVKCAISDHRSAAPDVYHLANMAAESRVGGLLGGKPGVTVFHMGDSKKALK

PIYDLLENCDVPISKLLPTHVNRNVPLFEQALEFARKGGTIDITSSIDEPVAPAEGIARAVQAGIPLARV

TLSSDGNGSQPFFDDEGNLTHIGVAGFETLLETVQVLVKDYDFSISDALRPLTSSVAGFLNMSGKGEILP

GNDADLLVMTPELRIEQVYARGKLMVKDGKACVKGTFETA

>gi|30065414|ref|NP_839585.1| hypothetical protein S4445 [Shigella flexneri 2a str. 2457T]

MTTQVRKNVMDMFIDGARRGFTIATTNLLPNVVMAFVIIQALKITGLLDWVGHICEPVMALWGLPGEAAT

VLLAALMSMGGAVGVAASLATADALTGHDVTVLLPAMYLMGNPVQNVGRCLGTAEVNAKYYPHIITVCVI

NALLSIWVMQLIV

>gi|30065413|ref|NP_839584.1| hypothetical protein S4444 [Shigella flexneri 2a str. 2457T]

MGIVMTQQGDAVAGELATEKVGIKGYLAFFLTIIFFPGVFSGTDSWWRVFDFSVLNGSFGQLPGANGATT

SFRGVGGAGAKDGFLFALELAPSVILSLGIISITDGLGGLRAAQQLMTPVLKPLLGIPGICSLALIANLQ

NTDAAAGMTKELAQEGEITERDKVIFAAYQTSGSAIITNYFSSGVAVFAFLGTSVIVPLAVILVFKFVGA

NILRVWLNFEERRNPTQGAQA

>gi|30065409|ref|NP_839580.1| ISSfl3 orfD [Shigella flexneri 2a str. 2457T]

MEHIPLYRQSEIYARQGVELSRNTMVRWVSEMADKLRPLYIALNDYVLEAGKVHADDTPVKVLAPGNGKT

KTGRLWVYVRDDRNAGSSLPAAVWFAYSADRKGEHPQLHLAKYQGVLQADAYAGYNVLYETGRVKEAGCL

AHARRKIHDEDVRRPTEMTQEALRRIAELYDIEAEIRGSPAEERLAVRKARSVQLMQSLYDWIQLQRKTL

SKHAEMAKAFDYILNHWNALNEFCRDGWVEIDNNIGENALRSVAVGRKNYLFFGSDKGGESAAIIYSLLV

TCKQNEVEPEDWLREVIEKLNDWPSNQVHKLLPWNFSSVK

>gi|30065408|ref|NP_839579.1| ISSfl3 orfC [Shigella flexneri 2a str. 2457T]

MNNELPDDIELLKAMLRKQQSRLRQYACQVAGYEQEIERLKAQLDRLRRMLFGQSSEKKRHKLENQIRQA

EKRLSELENRLNTARNLLEDASSVTDSPDTSPPSENPIASKPESPGRKSSRKPLPAELPRETHRLLPAET

SCPACGGVLKEMGETISEQLDIINTAFKVIETIRPKLACSRCDVIGTTSS

>gi|30065407|ref|NP_839578.1| ISSfl3 orfB [Shigella flexneri 2a str. 2457T]

MITLPTGTKIWIIAGITDMRCGFNGLASKVQNTLKDDPFSGHIFVFRGRSGKMVKILWADRDGLCLFTKR

LAGDPGRESAPDASSVIHATGGDRVATSQTDRTAWHPDITRDKTRE

>gi|30065406|ref|NP_839577.1| ISSfl3 orfA [Shigella flexneri 2a str. 2457T]

MNSQTTKDIPCFRSYLPDALRLRFEDKLTIRAIAQRLGLSHSTIHTLFQRFLASGIAWPLPDSVSFAQLD

AILYANRKKELTEPQIREGSWRKERRTSYSREFKVRLAKQALQPGAVVARIAREHDINDNLLFKWKSQYE

DGLLSDDDIQECMPVPVALTDTPEPTRPVTNPFWRNKHDERPEGAPGNVPRCELHLKSGVVKLFDPLTPE

LLRALIREMKGGIR

>gi|30065405|ref|NP_839576.1| 50S ribosomal protein L17 [Shigella flexneri 2a str. 2457T]

MRHRKSGRQLNRNSSHRQAMFRNMAGSLVRHEIIKTTLPKAKELRRVVEPLITLAKTDSVANRRLAFART

RDNEIVAKLFNELGPRFASRAGGYTRILKCGFRAGDNAPMAYIELVDRSEKAEAAAE

>gi|30065404|ref|NP_839575.1| DNA-directed RNA polymerase subunit alpha [Shigella flexneri 2a str. 2457T]

MQGSVTEFLKPRLVDIEQVSSTHAKVTLEPLERGFGHTLGNALRRILLSSMPGCAVTEVEIDGVLHEYST

KEGVQEDILEILLNLKGLAVRVQGKDEVILTLNKSGIGPVTAADITHDGDVEIVKPQHVICHLTDENASI

SMRIKVQRGRGYVPASTRIHSEEDERPIGRLLVDACYSPVERIAYNVEAARVEQRTDLDKLVIEMETNGT

IDPEEAIRRAATILAEQLEAFVDLRDVRQPEVKEEKPEFDPILLRPVDDLELTVRSANCLKAEAIHYIGD

LVQRTEVELLKTPNLGKKSLTEIKDVLASRGLSLGMRLENWPPASIADE

>gi|30065402|ref|NP_839573.1| 30S ribosomal protein S11 [Shigella flexneri 2a str. 2457T]

MAKAPIRARKRVRKQVSDGVAHIHASFNNTIVTITDRQGNALGWATAGGSGFRGSRKSTPFAAQVAAERC

ADAVKEYGIKNLEVMVKGPGPGRESTIRALNAAGFRITNITDVTPIPHNGCRPPKKRRV

>gi|30065401|ref|NP_839572.1| 30S ribosomal protein S13 [Shigella flexneri 2a str. 2457T]

MARIAGINIPDHKHAVIALTSIYGVGKTRSKAILAAAGIAEDVKISELSEGQIDTLRDEVAKFVVEGDLR

REISMSIKRLMDLGCYRGLRHRRGLPVRGQRTKTNARTRKGPRKPIKK

>gi|30065400|ref|NP_839571.1| 50S ribosomal protein L36 [Shigella flexneri 2a str. 2457T]

MKVRASVKKLCRNCKIVKRDGVIRVICSAEPKHKQRQG

>gi|30065399|ref|NP_839570.1| preprotein translocase subunit SecY [Shigella flexneri 2a str. 2457T]

MAKQPGLDFQSAKGGLGELKRRLLFVIGALIVFRIGSFIPIPGIDAAVLAKLLEQQRGTIIEMFNMFSGG

ALSRASIFALGIMPYISASIIIQLLTVVHPTLAEIKKEGESGRRKISQYTRYGTLVLAIFQSIGIATGLP

NMPGMQGLVINPGFAFYFTAVVSLVTGTMFLMWLGEQITERGIGNGISIIIFAGIVAGLPPAIAHTIEQA

RQGDLHFLVLLLVAVLVFAVTFFVVFVERGQRRIVVNYAKRQQGRRVYAAQSTHLPLKVNMAGVIPAIFA

SSIILFPATIASWFGGGTGWNWLTTISLYLQPGQPLYVLLYASAIIFFCFFYTALVFNPRETADNLKKSG

AFVPGIRPGEQTAKYIDKVMTRLTLVGALYITFICLIPEFMRDAMKVPFYFGGTSLLIVVVVIMDFMAQV

QTLMMSSQYESALKKANLKGYGR

>gi|30065398|ref|NP_839569.1| 50S ribosomal protein L15 [Shigella flexneri 2a str. 2457T]

MRLNTLSPAEGSKKAGKRLGRGIGSGLGKTGGRGHKGQKSRSGGGVRRGFEGGQMPLYRRLPKFGFTSRK

AAITAEVRLSDLAKVEGGVVDLNTLKAANIIGIQIEFAKVILAGEVTTPVTVRGLRVTKGARAAIEAAGG

KIEE

>gi|30065397|ref|NP_839568.1| 50S ribosomal protein L30 [Shigella flexneri 2a str. 2457T]

MAKTIKITQTRSAIGRLPKHKATLLGLGLRRIGHTVEREDTPAIRGMINAVSFMVKVEE

>gi|30065396|ref|NP_839567.1| 30S ribosomal protein S5 [Shigella flexneri 2a str. 2457T]

MAHIEKQAGELQEKLIAVNRVSKTVKGGRIFSFTALTVVGDGNGRVGFGYGKAREVPAAIQKAMEKARRN

MINVALNNGTLQHPVKGVHTGSRVFMQPASEGTGIIAGGAMRAVLEVAGVHNVLAKAYGSTNPINVVRAT

IDGLENMNSPEMVAAKRGKSVEEILGK

>gi|30065395|ref|NP_839566.1| 50S ribosomal protein L18 [Shigella flexneri 2a str. 2457T]

MDKKSARIRRATRARRKLQELGATRLVVHRTPRHIYAQVIAPNGSEVLVAASTVEKAIAEQLKYTGNKDA

AAAVGKAVAERALEKGIKDVSFDRSGFQYHGRVQALADAAREAGLQF

>gi|30065394|ref|NP_839565.1| 50S ribosomal protein L6 [Shigella flexneri 2a str. 2457T]

MSRVAKAPVVVPAGVDVKINGQVITIKGKNGELTRTLNDAVEVKHADNTLTFGPRDGYADGWAQAGTARA

LLNSMVIGVTEGFTKKLQLVGVGYRAAVKGNVINLSLGFSHPVDHQLPAGITAECPTQTEIVLKGADKQV

IGQVAADLRAYRRPEPYKGKGVRYADEVVRTKEAKKK

>gi|30065393|ref|NP_839564.1| 30S ribosomal protein S8 [Shigella flexneri 2a str. 2457T]

MSMQDPIADMLTRIRNGQAANKAAVTMPSSKLKVAIANVLKEEGFIEDFKVEGDTKPELELTLKYFQGKA

VVESIQRVSRPGLRIYKRKDELPKVMAGLGIAVVSTSKGVMTDRAARQAGLGGEIICYVA

>gi|30065392|ref|NP_839563.1| 30S ribosomal protein S14 [Shigella flexneri 2a str. 2457T]

MAKQSMKAREVKRVALADKYFAKRAELKAIISDVNASDEDRWNAVLKLQTLPRDSSPSRQRNRCRQTGRP

HGFLRKFGLSRIKVREAAMRGEIPGLKKASW

>gi|30065391|ref|NP_839562.1| 50S ribosomal protein L5 [Shigella flexneri 2a str. 2457T]

MAKLHDYYKDEVVKKLMTEFNYNSVMQVPRVEKITLNMGVGEAIADKKLLDNAAADLAAISGQKPLITKA

RKSVAGFKIRQGYPIGCKVTLRGERMWEFFERLITIAVPRIRDFRGLSAKSFDGRGNYSMGVREQIIFPE

IDYDKVDRVRGLDITITTTAKSDEEGRALLAAFDFPFRK

>gi|30065390|ref|NP_839561.1| 50S ribosomal protein L24 [Shigella flexneri 2a str. 2457T]

MAAKIRRDDEVIVLTGKDKGKRGKVKNVLSSGKVIIEGINLVKKHQKPVPALNQPGGIVEKEAAIQVSNV

AIFNAATGKADRVGFRFEDGKKVRFFKSNSETIK

>gi|30065389|ref|NP_839560.1| 50S ribosomal protein L14 [Shigella flexneri 2a str. 2457T]

MIQEQTMLNVADNSGARRVMCIKVLGGSHRRYAGVGDIIKITIKEAIPRGKVKKGDVLKAVVVRTKKGVR

RPDGSVIRFDGNACVLLNNNSEQPIGTRIFGPVTRELRSEKFMKIISLAPEVL

>gi|30065388|ref|NP_839559.1| 30S ribosomal protein S17 [Shigella flexneri 2a str. 2457T]

MTDKIRTLQGRVVSDKMEKSIVVAIERFVKHPIYGKFIKRTTKLHVHDENNECGIGDVVEIRECRPLSKT

KSWTLVRVVEKAVL

>gi|30065387|ref|NP_839558.1| 50S ribosomal protein L29 [Shigella flexneri 2a str. 2457T]

MKAKELREKSIEELNTELLNLLREQFNLRMQAASGQLQQSHLLKQVRRDVARVKTLLNEKAGA

>gi|30065386|ref|NP_839557.1| 50S ribosomal protein L16 [Shigella flexneri 2a str. 2457T]

MLQPKRTKFRKMHKGRNRGLAQGTDVSFGSFGLKAVGRGRLTARQIEAARRAMTRAVKRQGKIWIRVFPD

KPITEKPLAVRMGKGKGNVEYWVALIQPGKVLYEMDGVPEELAREAFKLAAAKLPIKTTFVTKTVM

>gi|30065385|ref|NP_839556.1| 30S ribosomal protein S3 [Shigella flexneri 2a str. 2457T]

MGQKVHPNGIRLGIVKPWNSTWFANTKEFADNLDSDFKVRQYLTKELAKASVSRIVIERPAKSIRVTIHT

ARPGIVIGKKGEDVEKLRKVVADIAGVPAQINIAEVRKPELDAKLVADSITSQLERRVMFRRAMKRAVQN

AMRLGAKGIKVEVSGRLGGAEIARTEWYREGRVPLHTLRADIDYNTSEAHTTYGVIGVKVWIFKGEILGG

MAAVEQPEKPAAQPKKQQRKGRK

>gi|30065384|ref|NP_839555.1| 50S ribosomal protein L22 [Shigella flexneri 2a str. 2457T]

METIAKHRHARSSAQKVRLVADLIRGKKVSQALDILTYTNKKAAVQVKKVLESAIANAEHNDGADIDDLK

VTKIFVDEGPSMKRIMPRAKGRADRILKRTSHITVVVSDR

>gi|30065383|ref|NP_839554.1| 30S ribosomal protein S19 [Shigella flexneri 2a str. 2457T]

MPRSLKKGPFIDLHLLKKVEKAVESGDKKPLRTWSRRSTIFPNMIGLTIAVHNGRQHVPVFVTDEMVGHK

LGEFAPTRTYRGHAADKKAKKK

>gi|30065382|ref|NP_839553.1| 50S ribosomal protein L2 [Shigella flexneri 2a str. 2457T]

MAVVKCKPTSPGRRHVVKVVNPELHKGKPFAPLLEKNSKSGGRNNNGRITTRHIGGGHKQAYRIVDFKRN

KDGIPAVVERLEYDPNRSANIALVLYKDGERRYILAPKGLKAGDQIQSGVDAAIKPGNTLPMRNIPVGST

VHNVEMKPGKGGQLARSAGTYVQIVARDGAYVTLRLRSGEMRKVEADCRATLGEVGNAEHMLRVLGKAGA

ARWRGVRPTVRGTAMNPVDHPHGGGEGRNFGKHPVTPWGVQTKGKKTRSNKRTDKFIVRRRSK

>gi|30065381|ref|NP_839552.1| 50S ribosomal protein L23 [Shigella flexneri 2a str. 2457T]

MIREERLLKVLRAPHVSEKASTAMEKSNTIVLKVAKDATKAEIKAAVQKLFEVEVEVVNTLVVKGKVKRH

GQRIGRRSDWKKAYVTLKEGQNLDFVGGAE

>gi|30065380|ref|NP_839551.1| 50S ribosomal protein L4 [Shigella flexneri 2a str. 2457T]

MELVLKDAQSALTVSETTFGRDFNEALVHQVVVAYAAGARQGTRAQKTRAEVTGSGKKPWRQKGTGRARS

GSIKSPIWRSGGVTFAARPQDHSQKVNKKMYRGALKSILSELVRQDRLIVVEKFSVEAPKTKLLAQKLKD

MALEDVLIITGELDENLFLAARNLQKVDVRDATGIDPVSLIAFDKVVMTADAVKQVEEMLA

>gi|30065379|ref|NP_839550.1| 50S ribosomal protein L3 [Shigella flexneri 2a str. 2457T]

MIGLVGKKVGMTRIFTEDGVSIPVTVIEVEANRVTQVKDLANDGYRAIQVTTGAKKANRVTKPEAGHFAK

AGVEAGRGLWEFRLAEGEEFTVGQSISVELFADVKKVDVTGTSKGKGFAGTVKRWNFRTQDATHGNSLSH

RVPGSIGQNQTPGKVFKGKKMAGQMGNERVTVQSLDVVRVDAERNLLLVKGAVPGATGSDLIVKPAVKA

>gi|30065378|ref|NP_839549.1| 30S ribosomal protein S10 [Shigella flexneri 2a str. 2457T]

MQNQRIRIRLKAFDHRLIDQATAEIVETAKRTGAQVRGPIPLPTRKERFTVLISPHVNKDARDQYEIRTH

LRLVDIVEPTEKTVDALMRLDLAAGVDVQISLG

>gi|30065377|ref|NP_839548.1| bacterioferritin [Shigella flexneri 2a str. 2457T]

MKGDVKIINYLNKLLGNELVAINQYFLHARMFKNWGLMRLNDVEYHESIDEMKHADKYIERILFLEGIPN

LQDLGKLGIGEDVEEMLQSDLRLELEGAKDLREAIAYADSVHDYVSRDMMIEILADEEGHIDWLETELDL

IGKIGLQNYLQSQIKVKD

>gi|30065376|ref|NP_839547.1| bacterioferritin-associated ferredoxin [Shigella flexneri 2a str. 2457T]

MYVCLCNGVSDKKIRQAVRQFHPQSFQQLRKFIPVGNQCGKCIRAAREVMQDELMQMPEFKEIA

>gi|30065375|ref|NP_839546.1| elongation factor Tu [Shigella flexneri 2a str. 2457T]

MSKEKFERTKPHVNVGTIGHVDHGKTTLTAAITTVLAKTYGGAARAFDQIDNAPEEKARGITINTSHVEY

DTPTRHYAHVDCPGHADYVKNMITGAAQMDGAILVVAATDGPMPQTREHILLGRQVGVPYIIVFLNKCDM

VDDEELLELVEMEVRELLSQYDFPGDDTPIVRGSALKALEGDAEWEAKILELAGFLDSYIPEPERAIDKP

FLLPIEDVFSISGRGTVVTGRVERGIIKVGEEVEIVGIKETQKSTCTGVEMFRKLLDEGRAGENVGVLLR

GIKREEIERGQVLAKPGTIKPHTKFESEVYILSKDEGGRHTPFFKGYRPQFYFRTTDVTGTIELPEGVEM

VMPGDNIKMVVTLIHPIAMDDGLRFAIREGGRTVGAGVVAKVLG

>gi|30065374|ref|NP_839545.1| elongation factor EF-2 [Shigella flexneri 2a str. 2457T]

MDWMEQEQERGITITSAATTAFWSGMAKQYEPHRINIIDTPGHVDFTIEVERSMRVLDGAVMVYCAVGGV

QPQSETVWRQANKYKVPRIAFVNKMDRVGANFLKVVNQIKTRLGANPVPLQLAIGAEEHFTGVVDLVKMK

AINWNDADQGVTFEYEDIPADMVELANEWHQNLIESAAEASEELMEKYLGGEELTEAEIKGALRQRVLNN

EIILVTCGSAFKNKGVQAMLDAVIDYLPSPVDVPAINGILDDGKDTPAERHASDDEPFSALAFKIATDPF

VGNLTFFRVYSGVVNSGDTVLNSVKAARERFGRIVQMHANKREEIKEVRAGDIAAAIGLKDVTTGDTLCD

PDAPIILERMEFPEPVISIAVEPKTKADQEKMGLALGRLAKEDPSFRVWTDEESNQTIIAGMGELHLDII

VDRMKREFNVEANVGKPQVAYRETIRQKVTDVEGKHAKQSGGRGQYGHVVIDMYPLEPGSNPKGYEFIND

IKGGVIPGEYIPAVDKGIQEQLKAGPLAGYPVVDMGIRLHFGSYHDVDSSELAFKLAASIAFKEGFKKAK

PVLLEPIMKVEVETPEENTGDVIGDLSRRRGMLKGQESEVTGVKIHAEVPLSEMFGYATQLRSLTKGRAS

YTMEFLKYDEAPSNVAQAVIEARGK

>gi|30065373|ref|NP_839544.1| 30S ribosomal protein S7 [Shigella flexneri 2a str. 2457T]

MPRRRVIGQRKILPDPKFGSELLAKFVNILMVDGKKSTAESIVYSALETLAQRSGKSELEAFEVALENVR

PTVEVKSRRVGGSTYQVPVEVRPVRRNALAMRWIVEAARKRGDKSMALRLANELSDAAENKGTAVKKRED

VHRMAEANKAFAHYRW

>gi|30065372|ref|NP_839543.1| 30S ribosomal protein S12 [Shigella flexneri 2a str. 2457T]

MATVNQLVRKPRARKVAKSNVPALEACPQKRGVCTRVYTTTPKKPNSALRKVCRVRLTNGFEVTSYIGGE

GHNLQEHSVILIRGGRVKDLPGVRYHTVRGALDCSGVKDRKQARSKYGVKRPKA

>gi|30065371|ref|NP_839542.1| sulfur transfer complex subunit TusB [Shigella flexneri 2a str. 2457T]

MLHTLHRSPWLTDFAALLRLLSEGDELLLLQDGVTAAVDGNRYLESLRNAPIKVYALNEDLIARGLTGQI

LNDIIPIDYTDFVRLTVKHSSQMAW

>gi|30065370|ref|NP_839541.1| sulfur relay protein TusC [Shigella flexneri 2a str. 2457T]

MKRIAFVFSTAPHGTAAGREGLDALLATSALTDDLAVFFIADGVFQLLSGQKPDAVLARDYIATFKLLSL

YDIEQCWVCAASLRERGLDPQTPFVVEATPLEADALRRELANYDVILRF

>gi|30065369|ref|NP_839540.1| sulfur transfer complex subunit TusD [Shigella flexneri 2a str. 2457T]

MRFAIVVTGPAYGTQQASSAFQFAQALIAEGHKLSSVFFYREGVYNANQLTSPASDEFDLVRGWQQLNAQ

HGVALNICVAAALRRGIVDETEAGRLGLASSNLQPGFTLSGLGALAEASLTCDRVVQF

>gi|30065368|ref|NP_839539.1| hypothetical protein S4398 [Shigella flexneri 2a str. 2457T]

MSRSLLTNETSELDLLDQRPFDQTDFDILKSYEAVVDGLAMLIGSHCEIVLHSLQDLKCSAIRIANGEHT

GRKIGSPITDLALRMLHDMTGADSSVSKCYFTRAKSGVLMKSLTIAIRNREQRVIGLLCINMNLDVPFSQ

IMSTFVPPETPDVGSSVNFASSVEDLVTQTLEFTIEEVNADRNVSNNAKNRQIVLNLYEKGIFDIKDAIN

QVADRLNISKHTVYLYIRQFKSGDFQGQDK

>gi|30065367|ref|NP_839538.1| FKBP-type peptidyl-prolyl cis-trans isomerase [Shigella flexneri 2a str. 2457T]

MKSLFKVTLLATTMAVALHAPITFAAEAAKPATTADSKASFKNDDQKSAYALGASLGRYMENSLKEQEKL

GIKLDKDQLIAGVQDAFADKSKLSDQEIEQTLQAFEARVKSSAQAKMEKDAADNEAKGKEYREKFAKEKG

VKTSSTGLVYQVVEAGKGEAPKDSDTVVVNYKGTLIDGKEFDNSYTRGEPLSFRLDGVIPGWTEGLKNIK

KGGKIKLVIPPELAYGKAGVPGIPPNSTLVFDVELLDVKPAPKADAKPEADAKAADSAKK

>gi|30065366|ref|NP_839537.1| hypothetical protein S4396 [Shigella flexneri 2a str. 2457T]

MQDLSLEARLAELESRLAFQEITIEELNVTVAAHEMEMAKLRDHLRLLTEKLKASQPSNIASQAEETPPP

HY

>gi|30065365|ref|NP_839536.1| FKBP-type peptidyl-prolyl cis-trans isomerase [Shigella flexneri 2a str. 2457T]

MKVAKDLVVSLAYQVRTEDGVLVDESPVSAPLDYLHGHGSLISGLETALEGHEVGDKFDVAVGANDAYGQ

YDENLVQRVPKDVFMGVDELQVGMRFLAETDQGPVPVEITAVEDDHVVVDGNHMLAGQNLKFNVEVVAIR

EATEEELAHGHVHGAHDHHHDHDHDGCCGGHGHDHGHEHGGEGCCGGKGNGGCGCH

>gi|30065364|ref|NP_839535.1| glutathione-regulated potassium-efflux system protein KefB [Shigella flexneri 2a str. 2457T]

MLAGIAIGPWGLGFISDVDEILHFSELGVVFLMFIIGLELNPSKLWQLRRSIFGVGAAQVLLSAALLAGL

LMLTDFAWQAAVVGGIGFAMSSTAMALQLMREKGMNRSESGQLGFSVLLFQDLAVIPALALVPLLAGSAD

EHFDWMKIGMKVLEFVGMLIGGRYLLRPVFRFIAASGVREVFTAATLLLVLGSALFMDALGLSMALGTFI

AGVLLAESEYRHELETAIDPFKGLLLGLFFISVGMSLNLGVLYTHLLWVVISVVVLVAVKILVLYLLARL

YGVRSSERMQFAGVLSQGGEFAFVLFSTASSQRLFQGDQMAPLLMKLVDKWLSRQFNGPEEEDEKPWVND

DKPQVIVVGFGRFGQVIGRLLMANKMRITVLERDISAVNLMRKYGYKVYYGDATQVDLLRSAGAEAAESI

VITCNEPEDTMKLVEICQQHFPHLHILARARGRVEAHELLQAGVTQFSRETFSSALELGRKTLVTLGMHP

HQAQRAQLHFRRLDMRMLRELIPMHADTVQISRAREARRELEEIFQREMQQERRQLDGWDEFE

>gi|30065363|ref|NP_839534.1| glutathione-regulated potassium-efflux system ancillary protein KefG [Shigella flexneri 2a str. 2457T]

MSQPAKVLLLYAHPESQDSVANRVLLKPATQLSNVTVHDLYAHYPDFFIDIPREQALLREHEVIVFQHPL

YTYSCPALLKEWLDRVLSRGFASGPGGNQLAGKYWRNVITTGEPESAYRYDALNRYPMSDVLRPFELAAG

MCRMHWLSPIIIYWARRQSAKELASHARAYGDWLANPLSPGGR

>gi|30065362|ref|NP_839533.1| ABC transporter ATP-binding protein [Shigella flexneri 2a str. 2457T]

MIVFSSLQIRRGVRVLLDNATATINPGQKVGLVGKNGCGKSTLLALLKNEISADGGSYTFPGSWQLAWVN

QETPALPQAALEYVIDGDREYRQLEAQLHDANERNDGHAIATIHSKLDAIDAWSIRSRAASLLHGLGFSN

EQLERPVSDFSGGWRMRLNLAQALICRSDLLLLDEPTNHLDLDAVIWLEKWLKSYQGTLILISHDRDFLD

PIVDKIIHIEQQSMFEYTGNYSSFEVQRATRLAQQQAMYESQQERVAHLQSYIDRFRAKATKAKQAQSRI

KMLERMELIAPAHVDNPFRFSFRAPESLPNPLLKMEKVSAGYGDRIILDSIKLNLVPGSRIGLLGRNGAG

KSTLIKLLAGELAPVSGEIGLAKGIKLGYFAQHQLEYLRADESPIQHLARLAPQELEQKLRDYLGGFGFL

GDKVTEETRRFSGGEKARLVLALIVWQRPNLLLLDEPTNHLDLDMRQALTEALIEFEGALVVVSHDRHLL

RSTTDDLYLVHDRKVEPFDGDLEDYQQWLSDVQKQENQTDEAPKENANSAQARKDQKRREAELRAQTQPL

RKEIARLEKEMEKLNAQLAQAEEKLGDSELYDQSRKAELTACLQQQASAKSGLEECEMAWLEAQEQLEQM

LLEGQSN

>gi|30065361|ref|NP_839532.1| hydrolase [Shigella flexneri 2a str. 2457T]

MAQITTTDANEFSSSAEFTPMRGFSNCHLQTMLPRLFRRQVKFTPYWQRLELPDGDFVDLAWSEDPAQAK

HKPRLVVFHGLEGSLNSPYPHGLVEAAQKRGWLGVVMHFRGCSGEPNRMHRIYHSGETEDASWFLRWLQR

EFGHAPTAAVGYSLGGNMLACLLAKEGNDLPVDAAVIVSAPFMLEACSYHMEKGFSRVYQRYLLNLLKAN

AARKLAAYPGTLPINLAQLKSVRRIREFDDLITARIHGYADAIDYYRQCSAMPMLNRIAKPTLIIHAKDD

PFMDHQVIPKPESLPSQVEYQLTEHGGHVGFIGGTLLHPQMWLESRIPDWLTTYLEAKSC

>gi|30065360|ref|NP_839531.1| hypothetical protein S4390 [Shigella flexneri 2a str. 2457T]

MLIPWQDLSPETLENLIESFVLREGTDYGEHERTLEQKVADVKRQLQCGEAVLVWSELHETVNIMPRSQF

RE

>gi|30065359|ref|NP_839530.1| phosphoribulokinase [Shigella flexneri 2a str. 2457T]

MSAKHPVIAVTGSSGAGTTTTSLAFRKIFAQLNLHAAEVEGDSFHRYTRPEMDMAIRKARDAGRHISYFG

PEANDFGLLEQTFIEYGQSGKGKSRKYLHTYDEAVPWNQVPGTFTPWQPLPEPTDVLFYEGLHGGVVTPQ

HNVAQHVDLLVGVVPIVNLEWIQKLIRDTSERGHSREAVMDSVVRSMEDYINYITPQFSRTHLNFQRVPT

VDTSNPFAAKGIPSLDESFVVIHFRNLEGIDFPWLLAMLQGSFISHINTLVVPGGKMGLAMELIMLPLVQ

RLMEGKKIE

>gi|30065358|ref|NP_839529.1| hypothetical protein S4388 [Shigella flexneri 2a str. 2457T]

MQARVKWVEGLTFLGESASGHQILMDGNSGDKAPSPMEMVLMAAGGCSAIDVVSILQKGRQDVVDCEVKL

TSERREEAPRLFTHINLHFIVTGRDLKDAAVARAVDLSAEKYCSVALMLEKAVNITHSYEVVAA

>gi|30065357|ref|NP_839528.1| cAMP-regulatory protein [Shigella flexneri 2a str. 2457T]

MVLGKPQTDPTLEWFLSHCHIHKYPSKSTLIHQGEKAETLYYIVKGSVAVLIKDEEGKEMILSYLNQGDF

IGELGLFEEGQERSAWVRAKTACEVAEISYKKFRQLIQVNPDILMRLSAQMARRLQVTSEKVGNLAFLDV

TGRIAQTLLNLAKQPDAMTHPDGMQIKITRQEIGQIVGCSRETVGRILKMLEDQNLISAHGKTIVVYGTR

>gi|30065356|ref|NP_839527.1| hypothetical protein S4386 [Shigella flexneri 2a str. 2457T]

MWRRLIYHPDINYALRQTLVLCLPVAVGLMLGELRFGLLFSLVPACCNIAGLDTPHKRFFKHLIIGASLF

ATCSLLTQLLLVKDVPLPFLLTGLTLVLGVTAELGPLHAKLLPASLLAAIFTLSLAGYMPVWEPLLIYAL

GTLWYGLFNWFWFWIWREQPLRESLSLLYRELADYCEAKYSLLTQHTDPEKALPPLLVRQQKAVDLITQC

YQQMHMLSAQNNTDYKRMLRIFQEALDLQEHISVSLHQPEEVQKLVERSHAEEVIRWNAQTVAARLRVLA

DDILYHRLPTRFTMEKQIGALEKIARQHPDNPVGQFCYWHFSRIARVLRTQKPLYARDLLADKQRRMPLL

PALKSYLSLKSPALRNAGRLSVMLSVASLMGTVLHLPKSYWILMTVLLVTQNGYGATRLRIVNRSVGTVV

GLIIAGVALHFKIPESYTLTLMLITTLASYLILRKNYGWATVGFTITAVYTLQLLWLNGEQYILPRLIDT

IIGCLIAFGGTVWLWPQWQSGLLRKNAHDALEAYQEAIRLILSEDPQPTPLAWQRMRVNQAHNTLYNSLN

QAMQEPAFNSHYLADMKLWVTHSQFIVEHINAMTTLAREHRALPPELAQEYLQSCEIAIQRCQQRLEYDE

PGSSGDANIMDAPEMQPHEGAAGTLEQHLQRVIGHLNTMHTISSMAWRQRPHHGIWLSRKLRDSKA

>gi|30065355|ref|NP_839526.1| bifunctional N-succinyldiaminopimelate-aminotransferase/acetylornithine transaminase protein [Shigella flexneri 2a str. 2457T]

MAIEQTAITRATFDEVILPIYAPAEFIPVKGQGSRIWDQQGKEYVDFAGGIAVTALGHCHPALVNALKTQ

GETLWHISNVFTNEPALRLGRKLIEATFAERVVFMNSGTEANETAFKLARHYACVRHSPFKTKIIAFHNA

FHGRSLFTVSVGGQPKYSDGFGPKPADIIHVPFNDLHAVKAVMDDHTCAVVVEPIQGEGGVTAATPEFLQ

GLRELCDQHQALLVFDEVQCGMGRTGDLFAYRYYGVTPDILTSAKALGGGFPISAMLTTAEIASAFHPGS

HGSTYGGNPLACAVAGAAFDIINTPEVLEGIQAKRQRFVDHLQKIDQQYDVFSDIRGMGLLIGAELKPHY

KGRARDFLYAGAEVGVMVLNAGPDVMRLAPSLVVEDADIDEGMQRFAHAVAKVVGA

>gi|30065354|ref|NP_839525.1| para-aminobenzoate synthase component II [Shigella flexneri 2a str. 2457T]

MILLIDNYDSFTWNLYQYFCELGADVLVKRNDALTLADIDALKPQKIVISPGPCTPDEAGISLDVIRHYA

GRLPILGVCLGHQAMAQAFGGKVVRAAKVMHGKTSPITHSGEGVFRGLANPLTVTRYHSLVVEPDSLPAC

FDVTAWSETREIMGIRHRQWDLEGVQFHPESILSEQGHQLLANFLHL

>gi|30065353|ref|NP_839524.1| cell filamentation protein Fic [Shigella flexneri 2a str. 2457T]

MSDKFGEGRDLYLYPGLDIMRNRLNIRQQQRLEQAAYEMTALRAATIELGPLVRGLPHLRTIHRQLYQDI

FDWAGQLREVDIYQGDTPFCHFAYIEKEGNALMQDLEEEGYLVGLEKAKFVERLAHYYCEINVLHPFRVG

SGLAQRIFFEQLAIHAGYQLSWQGIEKEAWNQANQSGAMGDLTALQMIFSKVVSEAGESE

>gi|30065352|ref|NP_839523.1| hypothetical protein S4382 [Shigella flexneri 2a str. 2457T]

MKKLTDKQKSRLWELQRNRNFQASRRLEGVEMPLVTLTAAEALARLEELRRHYER

>gi|30065351|ref|NP_839522.1| peptidyl-prolyl cis-trans isomerase A (rotamase A) [Shigella flexneri 2a str. 2457T]

MFKSTLAAMAAVFALSALSALSPAAMAAKGDPHVLLTTSAGNIELELDKQKAPVSVQNFVDYVNSGFYNN

TTFHRVIPGFMIQGGGFTEQMQQKKPNPPIKNEADNGLRNTRGTIAMARTADKDSATSQFFINVADNAFL

DHGQRDFGYAVFGKVVKGMDVADKISQVPTHDVGPYQNVPSKPVVILSAKVLP

>gi|30065350|ref|NP_839521.1| hypothetical protein S4380 [Shigella flexneri 2a str. 2457T]

MTNSNRIKLTWISFLSYALTGALVIVTGMVMGNIADYFNLPVSSMSNTFTFLNAGILISIFLNAWLMEIV

PLKTQLRFGFLLMVLAVAGLMFSHSLALFSTAMFILGVVSGITMSIGTFLITQMYEGRQRGSRLLFTDSF

FSMAGMIFPMIAAFLLARSIEWYWVYACIGLVYVAIFILTFGCEFPALGKRAPKTDAPVEKEKWGIGVLF

LSVAALCYILGQLGFISWVPEYAKGLGMSLNDAGTLVSNFWMSYMVGMWAFSFILRFFDLQRILTVLAGL

AAILMYVFNTGTPVHMAWSILALGFFSSAIYTTIITLGSQQTKVPSPKLVNFVLTCGTIGTMLTFVVTGT

IVEHSGPQAALLTANGLYAVVFVMCFLLGFVSRHRQHNTLTSH

>gi|30065349|ref|NP_839520.1| nitrite reductase (NAD(P)H) subunit [Shigella flexneri 2a str. 2457T]

MSKVRLAIIGNGMVGHRFIEDLLDKSDAANFDITVFCEEPRIAYDRVHLSSYFSHHTAEELSLVREGFYE

KHGIKVLVGERAITINRQEKVIHSSAGRTVFYDKLIMATGSYPWIPPIKGSDTQDCFVYRTIEDLNAIES

CARRSKRGAVVGGGLLGLEAAGALKNLGIETHVIEFAPMLMAEQLDQMGGEQLRRKIESMGVRVHTSKNT

LEIVQEGVEARKTMRFADGSELEVDFIVFSTGIRPRDKLATQCGLDVAPRGGIVINDSCQTSDPDIYAIG

ECASWNNRVFGLVAPGYKMAQVAVDHILGSENAFEGADLSAKLKLLGVDVGGIGDAHGRTPGARSYVYLD

ESKEIYKRLIVSEDNKTLLGAVLVGDTSDYGNLLQLVLNAIELPENPDSLILPAHSGSGKPSIGVDKLPD

SAQICSCFDVTKGDLIAAINKGCHTVAALKAETKAGTGCGGCIPLVTQVLNAELAKQGIEVNNNLCEHFA

YSRQELFHLIRVEGIKTFEELLAKHGKGYGCEVCKPTVGSLLASCWNEYILKPEHTPLQDSNDNFLANIQ

KDGTYSVIPRSPGGEITPEGLMAVGRIAREFNLYTKITGSQRLAMFGAQKDDLPEIWRQLIEAGFETGHA

YAKALRMAKTCVGSTWCRYGVGDSVGLGVELENRYKGIRTPHKMKFGVSGCTRECSEAQGKDVGIIATEK

GWNLYVCGNGGMKPRHADLLAADIDRETLIKYLDRFMMFYIRTADKLTRTAPWLENLEGGIDYLKAVIID

DKLGLNAHLEEEMARLREAVVCEWTETVNTPSAQTRFKHFINSDKRDPNVQMVPEREQHRPATPYERIPV

TLVEDNA

>gi|30065348|ref|NP_839519.1| nitrite reductase small subunit [Shigella flexneri 2a str. 2457T]

MSQWKDICKIDDILPETGVCALLGDKQVAIFRPYHSDQVFAISNIDPFFESSVLSRGLIAEHQGELWVAS
PLKKQRFRLSDGLCMEDEQFSVKHYEARVKDGVVQLRG

>gi|30065347|ref|NP_839518.1| nitrite reductase, NirC protein, partial [Shigella flexneri 2a str. 2457T]
MFLTFGVKAGSISHGQMWAILPQTWLGNLVGSVFVAMLYSWGGGSLLPVDTSIVHSVALAKTTAPAMVLF
FKGALCNWLVCLAIWMALRTEGAAKFIAIWWCLLAFIASGYEHSIANMTLFALSWFGNHSEAYTLAGIGH
NLLWVTLGNTLSGAVFMGLGYWYATPKANRPVADKFNQTETAAG

>gi|30065346|ref|NP_839517.1| siroheme synthase [Shigella flexneri 2a str. 2457T]
MDHLPIFCQLRDRDCLIVGGGDVAERKARLLLDAGARLTVNALAFIPQFTAWADAGMLTLVEGPFDESLL
DTCWLAIAATDDDTLNQRVSEAAEARRIFCNVVDAPKAASFIMPSIIDRSPLMVAVSSGGTSPVLARLLR
EKLESLLPLHLGQVAKYAGQLRGRVKQQFATMSERRRFWEKLFVNDRLAQSLANNDQKAITETTEQLINE
PLDHRGEVVLVGAGPGDAGLLTLKGLQQIQQADVVVYDRLVSDDIMNLVRRDADRVFVGKRAGYHCVPQE
EINQILLREAQKGKRVVRLKGGDPFIFGRGGEELETLCNAGIPFSVVPGITAASGCSAYSGIPLTHRDYA
QSVRLITGHLKTGGELDWENLAAEKQTLVFYMGLNQAATIQQKLIEHGMPGEMPVAIVENGTAVTQRVID
GTLTQLGELAQQMNSPSLIIIGRVVGLRDKLNWFSNH

>gi|30065345|ref|NP_839516.1| hypothetical protein S4375 [Shigella flexneri 2a str. 2457T]
MNKFIKVALVGAVLATLTACTGHIENRDKNCSYDYLLHPAISISKIIGGCGPTAQ

>gi|30065343|ref|NP_839514.1| fructoselysine 3-epimerase [Shigella flexneri 2a str. 2457T]
MKTGMFTCGHQRLPIEHAFRDASELGYDGIEIWGGRPHAFAPDLKAGGIKQIKALAQTYQMPIIGYTPET
NGYPYNMMLGDEHMRRESLDMIKLAIDMAKEMNAGYTLISAAHAGYLTPPNVIWGRLAENLSELCEYAEN
IGMDLILEPLTPYESNVVCNANDVLHALALVPSPRLFSMVDICAPYVQAEPVMSYFDKLGDKLRHLHIVD
SDGASDTHYIPGEGKMPLRELMRDIIDRGYEGYSTVELVTMYMNEPRLYARQALERFRALLPEDER

>gi|30065342|ref|NP_839513.1| fructoselysine 6-kinase [Shigella flexneri 2a str. 2457T]

MKTLATIGDNCVDIYPQLNKAFSGGNAVNVAVYCTRYGIQPGCITWVGDDDYGTKLKQDLARMGVDISHV

HTKHGVTAQTQVELHDNDRVFGDYTEGVMADFALSEEDYAWLAQYDIVHAAIWGHAEDAFPQLHAAGKLT

AFDFSDKWDSPLWQTLVPHLDFAFASAPQEDEALRLKMKAIVARGAGTVIVTLGENGSIAWDGAQFWRQA

PEPVTVIDTMGAGDSFIAGFLCGWSAGMTLPQAMAQGTACAAKTIQYHGAW

>gi|30065341|ref|NP_839512.1| DNA-binding transcriptional regulator FrlR [Shigella flexneri 2a str. 2457T]

MLLLAGKRPYNPSNSFFISGSIMSATDRYSHQLLYATVRQRLLDDIAQGVYQAGQQIPTENELCTQYNVS

RITIRKAISDLVADGVLIRWQGKGTFVQSQKVENALLTVSGFTDFGVSQGKSTKEKVIEQERVSAAPFCE

KLNIPGNSEVFHLCRVMYLDKEPLFIDSSWIPLSRYPDFDEIYVEGSSTYQLFQERFDTRVVSDKKTIDI

FAATRPQAKWLKCELGEPLFRISKIAFDQNDKPVHVSELFCRANRITLTIDNKRH

>gi|30065340|ref|NP_839511.1| hypothetical protein S4368 [Shigella flexneri 2a str. 2457T]

MKTFPLQSLTLAEAQQKQFALVDTICRHFPGSEFLAGGDLGLTPSLNQPRITQRVEQVLADAFHAQAAAL

VQGAGTGAIRAGLAALLKPGQRLLVHDAPVYPTTRVIIEQMGLTLITADFNDLSALKQVVDEQQPDAALV

QHTRQQPQDSYVLADVLATLRAAGVPALTDDNYAVMKVARIGCECGANVSTFSCFKLFGPEGVGAVVGDA

DVISRIRATLYSGGSQIQGSQALEVLRGLVFAPLIHAVKAGVSERLLALLNGGAVAEVKSAVIANAQSKV

LIVEFHQLIAARVLEEAQKRGALPYPVGAESKYEIPPLFYRLSGTFRQANPQLEHCAIRINPNRSGEETV

LRILRESIASI

>gi|30065339|ref|NP_839510.1| transport system permease [Shigella flexneri 2a str. 2457T]

MDLYIQIIVVACLTGMTSLLAHRSAAVFHDGIHPILPQLIEGYMNRREAGSIAFGLSIGFVASVGISFTL

KTGLLNAWLLFLPTDILGVLAINSLMAFGLGAIWGVLILTCLLPVNQLLTALPVDVLGSLGELSSPVVSA

FALFPLVAIFYQFGWKQSLVAAVVVLMTRVVVVRYFPHLNPESIEIFIGMVMLLGIAITHDLRHRDENDI

DASGLSVFEERTSRIIKNLPYIAIVGALIAAVASMKIFAGSEVSIFTLEKAYSAGVTPEQSQTLINQAAL
AEFMRGLGFVPLIATTALATGVYAVAGFTFVYAVGYLSPNPMVAAVLGAVVISAEVLLLRSIGKWLGRYP
SVRNASDNIRNAMNMLMEVALLVGSIFAAIKMAGYTGFSIAVAIYFLNESLGRPVQKMAAPVVAVMITGI
LLNVLYWLGLFVPA

>gi|30065338|ref|NP_839509.1| hypothetical protein S4366 [Shigella flexneri 2a str. 2457T]
MKIPLNFSNKDRLMKKIGVAGLQREQIKKTIEATAPGCFEVFIHNDMEAAMKVKSGQLDYYIGACNTGAG
AALSIAIAVIGYNKSCTIAKPGIKAKDEHIAKMIAEGKVAFGLSVEHVEHAIPMLINHLK

>gi|30065337|ref|NP_839508.1| hydrolase [Shigella flexneri 2a str. 2457T]
MSFDPTGYTLAHEHLHIDLSGFKNNVDCRLDQYAFICQEMNDLMTRGVRNVIEMTNRYMGRNAQFMLDVM
RETGINVVACTGYYQDAFFPEHVVTRSVQELAQEMVDEIEQGIDGTELKAGIIAEIGSSEGKITPLEEKV
FIAAALAHNQTGRPISTHTSFSTMGLEQLALLQAHGVDLSRVTVGHCDLKDNLDNILKMIDLGAYVQFDT
IGKNSYYPDEKRIAMLHALRDRGLLNRVMLSMDITRRSHLKANGGYGYDYLLTTFIPQLRQSGFSQADVD
VMLRENPSQFFQ

>gi|30065336|ref|NP_839507.1| mutase [Shigella flexneri 2a str. 2457T]
MARFVVLVIDSFGVGAMKDVTLVRPQDAGANTCGHILSQLPHLQLPTLEKLGLINTLGYAPGDMQPSDSA
TWGVAELQHEGGDTFMGHQEILGTRPLPPLRMPFRDVIDRVEQALVSAGWQVERRGDDLQFLSVNQAVAI
GDNLEADLGQVYNITANLSVISFDDAIKIGRIVRDQVQVGRVITFGGLLTNSQRILDAAESKEGRFIGIN
APRSGAYDNGFQVVHMGYGVDEKVQVPQKLYEAGVPTVLVGKVADIVSNPYGVSWQNLVDSQRIMDITLD
EFNTHPTAFICINIQETDLAGHAEDVARYAERLQVVDRNLARLVEAMQPDDCLVVMADHGNDPTIGHSHH
TREVVPVLVYQQGLVATQLGVRTTLSDVGATVCEFFRAPPPQNGRSFLSSLRFAGDTL

>gi|30065335|ref|NP_839506.1| hypothetical protein S4363 [Shigella flexneri 2a str. 2457T]

MFVEALKRQNPALISAALSLWQQGKIAPDSWVIDVDQILENGKRLIETARLYGIELYLMTKQFGRNPWLA

EKLLALGYSGIVAVDYKEARVMRRAGLPVAHQGHLVQIPCHQVADAVEQGTDIITVFTLDKAREVSAAAV

KAGRVQPVLLKVYSDDDFLYPGQESGFVQHSLHEVIAEIKKLPGLHLAGLTHFPCLLWDEAAGKVLPTPN

LHTLVQARDQLAKSGIAIEQLNVPSATSCTSLPLLAEYGATHAEPGHALTGTIPANQQGDQPERIAMLWL

SEISHHFRGDSYCYGGGYYRRGHAQHALVFTPENQKITETNLKTVDDSSIDYTLPLAGEYPVSSAVVLCF

RTQIFVTRSDVVLVSGIQRGEPEIVGRYDSLGNPLEA

>gi|30065334|ref|NP_839505.1| hypothetical protein S4362 [Shigella flexneri 2a str. 2457T]

MGNRNPVIKRKASDMETRLNLLCEAGVIDKDVCKGMMQVVNVLEKECHLPVRSEQGTMAMTHMASALMRS

RRGEEIEPLDNELLAELAQSSHWQAVVQLHQELLKEFALEVNPCEEGYFLANLYGLWMAANEEV

>gi|30065333|ref|NP_839504.1| hypothetical protein S4361 [Shigella flexneri 2a str. 2457T]

MDNKALLSHVDINNVVCAMPLPYTRLYEGLASGLKAQFDGIPFYYAHMRGADIRVECLLNGVYDMAVVSR

LAAESYLSQNNLCIALELGPHTYVGEHQLICRKGESGNVKRVGLDSRSADQKIMTDVFFGDSDVERVDLS

YHESLQRIVKGDVDAVIWNVVAENELTMLGLEATPLTDDPRFLQATEAVVLTRVDDYPMQQLLRAVVDKH

ALLAHQQRVVSGEQEPSY

>gi|30065332|ref|NP_839503.1| tryptophanyl-tRNA synthetase [Shigella flexneri 2a str. 2457T]

MTKPIVFSGAQPSGELTIGNYMGALRQWVNMQDDYHCIYCIVDQHAITVRQDAQKLRKATLDTLALYLAC

GIDPEKSTIFVQSHVPEHAQLGWALNCYTYFGELSRMTQFKDKSARYAENINAGLFGYPVLMAADILLYQ

TNLVPVGEDQKQHLELSRDIAQRFNALYGEIFKVPEPFIPKSGARVMSLLEPTKKMSKSDDNRNNVIGLL

EDPKSVVKKIKRAVTDSDEPPVVRYDVQNKAGVSNLLDILSAVTGQSIPELEKQFEGKMYGHLKGEVADA

VSGMLTELQERYHRFRNDEAFLQQVMKDGAEKASVHASRTLKAVYEAIGFVAKP

>gi|30065331|ref|NP_839502.1| phosphoglycolate phosphatase [Shigella flexneri 2a str. 2457T]

MNKFEDIRGVAFDLDGTLVDSAPGLAAAVDMALYALELPVAGEERVITWIGNGADVLMERALTWARQERA

TLRKTMGKPPVDDDIPAEEQVRILRKLFDRYYGEVAEEGTFLFPHVADTLGALQAKGLPLGLVTNKPTPF

VAPLLEALDIAKYFSVVIGGDDVQNKKPHPDPLLLVAERMGIAPQQMLFVGDSRNDIQAAKAAGCPSVGL

TYGYNYGEAIDLSQPDVIYQSINDLLPALGLPHSENQESKND

>gi|30065330|ref|NP_839501.1| ribulose-phosphate 3-epimerase [Shigella flexneri 2a str. 2457T]

MKQYLIAPSILSADFARLGEDTAKALAAGADVVHFDVMDNHYVPNLTIGPMVLKSLRNYGITAPIDVHLM

VKPVDRIVPDFAAAGASIITFHPEASEHVDRTLQLIKENGCKAGLVFNPATPLSYLDYVMDKLDVILLMS

VNPGFGGQSFIPQTLDKLREVRRRIDESGFDIRLEVDGGVKVNNIGEIAAAGADMFVAGSAIFDQPDYKK

VIDEMRSELAKVSHE

>gi|30065329|ref|NP_839500.1| DNA adenine methylase [Shigella flexneri 2a str. 2457T]

MKKNRAFLKWAGGKYPLLDDIKRHLPKGECLVEPFVGAGSVFLNTDFSRYILADINSDLISLYNIVKMRT

DEYVQAARELFVPETNCAEVYYQFREEFNKSQDPFRRAVLFLYLNRYGYNGLCRYNLRGEFNVPFGRYKK

PYFPEAELYHFAEKAQNAFFYCESYADSMERADDASVVYCDPPYAPLSATANFTAYHTNSFTLEQQAHLA

EIAEGLVERHIPVLISNHDTMLTREWYQRAKLHVVKVRRSISSNGGTRKKVDELLALYKPGVVSPAKK

>gi|30065328|ref|NP_839499.1| hypothetical protein S4356 [Shigella flexneri 2a str. 2457T]

MDEFKPEDELKPDPSDRRTGRSRQSSERSERTERGEPQINFDDIELDDTDDRRPTRAQKERNEEPEIEEE

IDESEDETVDEERVERRPRKRKKAASKPASRQYMMMGVGILVLLLLIIGIGSALKAPSTTSSDQTASGEK

SIDLAGNATDQANGVQPAPGTTSAENTQQDVSLPPISSTPTQGQTPVATDGQQRVEVQGDLNNALTQPQN

QQQLNNVAVNSTLPTEPATVAPVRNGNASRDTAKTQTAERPSTTRPVRQQAVIEPKKPQATVKTEPKPVA

QTPKRTEPEPAAPVASTKAPAATSTPAPKETATTAPVQTASPAQTTATPAAGGKTAGNVGSLKSAPSSHY

TLQLSSSSNYDNLNGWAKKENLKNYVVYETTRNGQPWYVLVSGVYASKEEAKKAVSTLPADVQAKNPWAK

PLRQVQADLK

>gi|30065327|ref|NP_839498.1| 3-dehydroquinate synthase [Shigella flexneri 2a str. 2457T]

MERIVVTLGERSYPITIASGLFNEPASFLPLKSGEQVMLVTSETLAPLYLDKVRGVLEQAGVNVDSVILP

DGEQYKSLAVLDTVFTALLQKPHGRDTTLVALGGGVVGDLTGFAAASYQRGVRFIQVPTTLLSQVDSSVG

GKTAVNHPLGKNMIGAFYQPASVVVDLDCLKTLPPRELASGLAEVIKYGIILDGAFFNWLEENLDALLRL

DGPAMAYCIRRCCELKAEVVAADERETGLRALLNLGHTFGHAIEAEMGYGNWLHGEAVAAGMVMAARTSE

RLGQFSSAETQRIITLLTRAGLPVNGPREMSAQAYLPHMLRDKKVLAGEMRLILPLAIGKSEVRSGVSHE

LVLNAIADCQSA

>gi|30065325|ref|NP_839496.1| outer membrane porin HofQ [Shigella flexneri 2a str. 2457T]

MKQWIAALLLMLIPGVQAAKPQKVTLMVDDVPVAQVLQALAEQEKLNLVVSPDVSGTVSLHLTDVPWKQA

LQTVVKSAGLITRQEGNILSVHSIAWQNDNIARQEAEQARAQANLPLENRSITLQYADAGELAKAGEKLL

SAKGSMTVDKRTNRLLLRDNKTALSALEQWVAQMDLPVGQVELSAHIVTINEKSLRELGVKWTLADAQHA

GGVGQVTTLGSDLSVATATTHVGFNIGRINGRLLDLELSALEQKQQLDIIASPRLLASHLQPASIKQESE

IPYQVSSGESGATSVEFKEAVLGMEVTPTVLQKGRIRLKLHISQNVPGQVLQQADGEVLAIDKQEIETQV

EVKSGETLALGGIFTRKNKSGQDSVPLLGDIPWFGQLFRHDGKEDERRELVVFITPRLVSSE

>gi|30065324|ref|NP_839495.1| hypothetical protein S4351 [Shigella flexneri 2a str. 2457T]

MNMFFDWWFATSPRLRQFCWAFWLLMLVTLIFLSSTHHEERDALIRLRASHHQQWAALYRLVDTTPFSEE

KTLPFSPLDFQLSGAQLVSWHPSAQGGELALKTLWEAVPSAFTRLAERNVSVSRFSLSVEGDDLLFTLQL

EMPHEG

>gi|30065323|ref|NP_839494.1| hypothetical protein S4350 [Shigella flexneri 2a str. 2457T]

MNPPINFLPWRQQRRTAFLRFWLLMFVAPLLLAVGITLILRLTGSAEARIDAVLLQAEQQLARSLQITKP

RLLEQQQLREQRSQRQRQRQFTRDWQSALEALAALLPEHAWLTTISWQQGTLEIKGLTTSITALNALETS

LRQDASFHLNQRGATQQDAQGRWQFEYQLTRKVSDEHVL

>gi|30065322|ref|NP_839493.1| hypothetical protein S4349 [Shigella flexneri 2a str. 2457T]

MTGDKEIITMTFKIWQIGLHLQQQEAAAVAIVRGAKECFLQRWWRLPLAHDIIKDGRIVDAQQLAKTLLP

WSRELPQRHHIMLAFPASRTLQRSFPRPSMSLGEREQTAWLSGTMARELDMDPDSLRFDYSEDSLSPAYN

VTAAQSKELATLLTLAERLRVHVSAITPDASALQRFLPFLPSHQQCLAWRDNEQWLWATRYSWGRKLAVG

MTSAKELAAALSVDPDSVAICGEGGFDPWEAVSVRQPPLPPPGGDFAIALGLALGKAY

>gi|30065320|ref|NP_839491.1| ADP-ribose diphosphatase NudE [Shigella flexneri 2a str. 2457T]

MNKSLQKPTILNVETVARSRLFTVESVDLEFSNGVRRVYERMRPTNREAVMIVPIVDDHLILIREYAVGT

ESYELGFSKGLIDPGESVYEAANRELKEEVGFGANDLTFLKKLSMAPSYFSSKMNIVVAQDLYPESLEGD

EPEPLPQVRWPLAHMMDLLEDPDFNEARNVSALFLVREWLKGQGRV

>gi|30065319|ref|NP_839490.1| dehydrogenase [Shigella flexneri 2a str. 2457T]

MSTIVIFLAALLACSLLAGWLIKVRSRRRQLPWTNAFADAQTRKLTPEERSAVENYLESLTQVLQVPGPT

EASAAPISLALNAESNNVMMLTHAITRYGISTDDPNKWRYYLDSVEVHLPPFWEQYINDENTVELIHTDS

LPLVISLNGHTLQEYMQETRGYALQPVPSTQASIRGEESEQIELLNIRKETHEEYALSRPRGLREALLIV

ASFLMFFFCLITPDVFVPWLAGGALLLLGAGLWGLFAPPAKSSLREIHCLRGTPRRWGLFGENDQEQINN

ISLGIIDLVYPAHWQPYIAQDLGQQTDIDIYLDRHVVRQGRYLSLHDEVKNFPLQHWLRSTIIAAGSLLV

LFMLLFWIPLDMPLKFTLSWMKGAQTIEATSVKQLADAGVRVGDTLRISGTGMCNIRTSGTWSAKTNSPF

LPFDCSQIIWNDARSLPLPESELVNKATALTEAVNRQLHPKPEDESRVSASLRSAIQKSGMVLLDDFGDI

VLKTADLCSAKDDCVRLKNALVNLGNSKDWDALVKRANAGKLDGVNVLLRPVSAESLDNLVATSTAPFIT

HETARAAQSLNSPAPGGFLIVSDEGSDFVDQPWPSASLYDYPPQEQWNAFQKLAQMLMHTPFNAEGIVTK

IFTDANGTQHIGLHPIPDRSGLWRYLSTTLLLLTMLGSAIYNGVQAWRRYQRHRTRMMEIQAYYESCLNP

QLITPSESLIE

>gi|30065318|ref|NP_839489.1| phosphatase [Shigella flexneri 2a str. 2457T]

MLPCRANCFTLEISLMHINIAWQDVDTVLLDMDGTLLDLAFDNYFWQKLVPETWGAKNGVTPQEAMEYMR

QQYHDVQHTLNWYCLDYWSEQLGLDICAMTTEMGPRAVLREDTIPFLEALKASGKQRILLTNAHPHNLAV

KLEHTGLDAHLDLLLSTHTFGYPKEDQRLWHAVAEATGLKAERTLFIDDSEAILDAAAQFGIRYCLGVTN

PDSGIAEKQYQRHPSLNDYRRLIPSLM

>gi|30065317|ref|NP_839488.1| ribosome-associated heat shock protein Hsp15 [Shigella flexneri 2a str. 2457T]

MKEKPAVEVRLDKWLWAARFYKTRALAREMIEGGKVHYNGQRSKPSKIVELNATLTLRQGNDERTVIVKA

ITEQRRPASEAALLYEETAESVEKREKMAMARKLNALTMPHPDRRPDKKERRDLLRFKHGDSE

>gi|30065316|ref|NP_839487.1| Hsp33-like chaperonin [Shigella flexneri 2a str. 2457T]

MPQHDQLHRYLFENFAVRGELVTVSETLQQILENHDYPQPVKNVLAELLVATSLLTATLKFDGDITVQLQ

GDGPMNLAVINGNNNQQMRGVARVQGEIPENADLKTLVGNGYVVITITPSEGERYQGVVGLEGDTLAACL

EDYFMRSEQLPTRLFIRTGDVDGKPAAGGMLLQVMPAQNTQQDDFDHLATLTETIKTEELLTLPANEVLW

RLYHEEEVTVYDPQDVEFKCTCSRERCADALKTLPDEEVDSILAEDGEIDMHCDYCGNHYLFNAMDIAEI

RNNASPADP

>gi|30065315|ref|NP_839486.1| transporter [Shigella flexneri 2a str. 2457T]

MDNVELSPATRWGMIATGLLQGMIATGLLQGLVCYLLIAWLAGKNHSWIVYGVPATVAFSSVLLFSVISF

KQKRLWGWLALVFIATTGMSGWLKWQTDGMNPWRAEKAIWDFGCYLLLMAMLLLPWIQQSLRIRNGSSRY

SYFYQSVWHNVLILLVIFLSNGLTWLVLLLWGELFKLVGITFFNTLFFATDWFMYLTFGLVTALAVILAR

TQSRLIDSIQKLFTLIATGLLPLVSLLTLMFIITLPFTGLSAISRHISAAGLLLTLAFLQLILMAIVRDP

QKASLPWTGPLRCLIKTALLVAPLYVFVATWGLWLRVAQYGWTVDRLQGALAELVLLVWSLGYFVSIVWR

NGQNPLVLQGKVNLAVSLLVLVILVLLNSPVLDSMRISVNSHMARYQSGKNTPDQVTIYMLEQSGRYGRA

ALESLKSDAGFMKDPKRARDLLMALDGEQHLQEQVSEKVFAENVLIAPGSVKPDATFWSALIQDRYNVMT

CIEKDACVLVEQDLNSDGQAERILFAFNDDRVIVYGFDSDRKEWDALDMSLLPNEITKEKLLTAAKDGKL

GTRPKAWRDLTVDGETLEINLSK

>gi|30065314|ref|NP_839485.1| phosphoenolpyruvate carboxykinase [Shigella flexneri 2a str. 2457T]

MRVNNGLPPQELEAYGISDVHDIVYNPSYDLLYQEELDPSLTGYERGVLTNLGAVAVDTGIFTGRSPKDK

YIVRDDTTRDTFWWADKGKGKNDNKPLSPETWQHLKGLVTRQLSGKRLFVVDAFCGANPDTRLSVRFITE

VAWQAHFVKNMFIRPSDEELAGFKPDFIVMNGAKCTNPQWKEQGLNSENFVAFNLTERMQLIGGTWYGGE

MKKGMFSMMNYLLPLKGIASMHCSANVGEKGDVAVFFGLSGTGKTTLSTDPKRRLIGDDEHGWDDDGVFN

FEGGCYAKTIKLSKEAEPEIYNAIRRDALLENVTVREDGTIDFDDGSKTENTRVSYPIYHIDNIVKPVSK

AGHATKVIFLTADAFGVLPPVSRLTADQTQYHFLSGFTAKLAGTERGITEPTPTFSACFGAAFLSLHPTQ

YAEVLVKRMQAAGAQAYLVNTGWNGTGKRISIKDTRAIIDAILNGSLDNAETFTLPMFNLAIPTELPGVD

TKILDPRNTYASPEQWQEKAETLAKLFIDNFDKYTDTPAGAALVAAGPKL

>gi|30065313|ref|NP_839484.1| osmolarity sensor protein [Shigella flexneri 2a str. 2457T]

MRRLRFSPRSSFARTLLLIVTLLFASLVTTYLVVLNFAILPSLQQFNKVLAYEVRMLMTDKLQLEDGTQL

VVPPAFRREIYRELGISLYSNEAAEEAGLRWAQHYEFLSHQMAQQLGGPTEVRVEVNKSSPVVWLKTWLS

PNIWVRVPLTEIHQGDFSPLFRYTLAIMLLAIGGAWLFIRIQNRPLVDLEHAALQVGKGIIPPPLREYGA

SEVRSVTRAFNHMAAGVKQLADDRTLLMAGVSHDLRTPLTRIRLATEMMSEQDGYLAESINKDIEECNAI

IEQFIDYLRTGQEMPMEMADLNAVLGEVIAAESGYEREIETALYPGSIEVKMHPLSIKRAVANMVVNAAR

YGNGWIKVSSGTEPNRAWFQVEDDGPGIAPEQRKHLFQPFVRGDSARTISGTGLGLAIVQRIVDNHNGML

ELGTSERGGLSIRAWLPVPVTRAQGTTKEG

>gi|30065312|ref|NP_839483.1| osmolarity response regulator [Shigella flexneri 2a str. 2457T]

MQENYKILVVDDDMRLRALLERYLTEQGFQVRSVANAEQMDRLLTRESFHLMVLDLMLPGEDGLSICRRL

RSQSNPMPIIMVTAKGEEVDRIVGLEIGADDYIPKPFNPRELLARIRAVLRRQANELPGAPSQEEAVIAF

GKFKLNLGTREMFREDEPMPLTSGEFAVLKALVSHPREPLSRDKLMNLARGREYSAMERSIDVQISRLRR

MVEEDPAHPRYIQTVWGLGYVFVPDGSKA

>gi|30065310|ref|NP_839481.1| hypothetical protein S4337 [Shigella flexneri 2a str. 2457T]

MPFIARYRKEITGGLDDTQLRNLETRLSYLRELEERRQAILKSISEQGKLTDDLAKAINATLSKTELEDL

YLPYKPKRRTRGQIAIEAGLEPLADLLWSDPSHTPEVAAAQYVDADKGVADTKAALDGARYILMERFAED

AALLAKVRDYLWKNAHLVSTVVSGKEEEGAKFRDYFDHHEPLSTVPSHRALAMFRGRNEGVLQLSLNADP

QFDEPPKESYCEQIIMDHLGLRLNNAPADSWRKSVVSWTWRIKVLMHLETELMGTVRERAEDEAINVFAR

NLHDLLMAAPAGLRATMGLDPGLRTGVKVAVVDATGKLVATDTIYPHTGQAAKAAMTVAALCEKHNVELV

AIGNGTASRETERFYLDVQKQFPKVTAQKVIVSEAGASVYSASELAAQEFPDLDVSLRGAVSIARRLQDP

LAELVKIDPKSIGVGQYQHDVSQTQLARKLDAVVEDCVNAVGVDLNTASVPLLTRVAGLTRMMAQNIVAW

RDENGQFQNRQQLLKVSRLGPKAFEQCAGFLRINHGDNPLDASTVHPEAYPVVERILAATQQALKDLMGN

SSELRNLKASDFTDEKFGVPTVTDIIKELEKPGRDPRPEFKTAQFADGVETMNDLQPGMILEGAVTNVTN

FGAFVDIGVHQDGLVHISSLSNKFVEDPHTVVKAGDIVKVKVLEVDLQRKRIALTMRLDEQPGETNSRRG

GGNERPQNNRPAAKPRGREAQPAGNSAMMDALAAAMGKKR

>gi|30065309|ref|NP_839480.1| ferrous iron transport protein A [Shigella flexneri 2a str. 2457T]

MQYTPDTAWKITGFSREISPAYRQKLLSLGMLPGSSFDVVRVAPLGDPIHIETRRVSLVLRKKDLALLEV

EAVSC

>gi|30065308|ref|NP_839479.1| ferrous iron transport protein B [Shigella flexneri 2a str. 2457T]

MKKLTIGLIGNPNSGKTTLFNQLTGARQRVGNWAGVTVERKEGQFSTTDHQVTLVDLPGTYSLTTISSQT

SLDEQIACHYILSGDADLLINVVDASNLERNLYLTLQLLELGIPCIVALNMLDIAEKQNIRIEIDALSAR

LGCPVIPLVSTRGRGIEALKLAIDRYKANENVELVHYAQPLLNEADSLAKVMPSDIPLKQRRWLGLQMLE

GDIYSRAYAGEASQHLDATLARLRNEMDDPALHIADARYQCIAAICDVVSNTLTAEPSRFTTAVDKIVLN

RFLGLPIFLFVMYLMFLLAINIGGALQPLFNVGSVALFVHGIQWIGYTLHFPDWLTIFLAQGLGGGINTV

LPLVPQIGMMYLFLSFLEDSGYMARAAFVMDRLMQALGLPGKSFVPLIVGFGCNVPSVMGARTLDAPRER

LMTIMMAPFMSCGARLAIFAVFAAAFFGQNGALAVFSLYMLGIVMAVLTGLMLKYTIMRGEATPFVMELP

VYHVPHVKSLIIQTWQRLKGFVLRAGKVIIIVSIFLSAFNSFSLSGKIVDNINDSALASVSRVITPVFKP

IGVHEDNWQATVGLFTGAMAKEVVVGTLNTLYTAENIQDEEFNPAEFNLGEELFSAVDETWQSLKDTFSL

SVLMNPIEASKGNGEMGTGAMGVMDQKFGSAAAAYSYLIFVLLYVPCISVMGAIARESSRGWMGFSILWG

LNIAYSLATLFYQVASYSQHPTYSLVCILAVILFNIVAIGLLRRARSRVDIELLATRKSVSSCCAASTTG

DCH

>gi|30065307|ref|NP_839478.1| hypothetical protein S4334 [Shigella flexneri 2a str. 2457T]

MASLIQVRDLLALRGRMEATQISQTLNTPQPMINAMLQQLESMGKAVRIQEEPDGCLSGSCKSCPEGKAC

LREWWALR

>gi|30065306|ref|NP_839477.1| hypothetical protein S4333 [Shigella flexneri 2a str. 2457T]

MSKKQSSTPHDALFKLFLRQPETARDFLAFHLPAPIHALCDMKTLKLESRSFIDDDLRESYSDVLWSVKT

EQGPGYIYCLIEHQSTSNKLIAFRMMRYAIAAMQNHLDAGYKTLPMVVPLLFYHGIESPYPYSLCWLDCF

ADPKLARQLYASAFPLIDVTVMPDDEIMQHRRMALLELIQKHIRQRDLMGLVEQMACLLSSGYANDRQIK

GLFNYILQTGDAVRFNDFIDGVAERSPKHKESLMTIAERLRQEGEQSKALHIAKIMLESGVPLADIMRFT

GLSEEELAAASRLAP

>gi|30065303|ref|NP_839474.1| carboxylesterase BioH [Shigella flexneri 2a str. 2457T]

MNNIWWQTKGQGNVHLVLLHGWGLNAEVWRCIDEELSSHFTLHLVDLPGFGRSRGFGALSLADMAEAVLQ

QAPDKAIWLGWSLGGLVASQIALTHPERVQALVTVASSPCFSARDEWPGIKPDVLAGFQQQLSDDFQRTV

ERFLALQTMGTETARQDARALKKTVLALPMPEVDVLNGGLEILKTVDLRQPLQNVSMPFLRLYGYLDGLV

PRKVVPMLDKLWPHSESYIFAKAAHAPFISHPVEFCHLLVALKQRVLVVSES

>gi|30065301|ref|NP_839472.1| DNA uptake protein [Shigella flexneri 2a str. 2457T]

MIRISDAAQAHFAKLLANQEEGTQIRVFVINPGTPNAECGVSYCPPDAVEATDTALKFDLLTAYVDELSA

PYLEDAEIDFVTDQLGSQLTLKAPNAKMRKVADDAPLMERVEYMLQSQINPQLAGHGGRVSLMEITEDGY

AILQFGGGCNGCSMVDVTLKEGIEKQLLNEFPELKGVRDLTEHQRGEHSYY

>gi|30065300|ref|NP_839471.1| high-affinity transport permease for gluconate [Shigella flexneri 2a str. 2457T]

MPLVIVAIGVILLLLLLMIRFKMNGFIALVLVALAVGLMQGMPLDKVIGSIKAGVGGTLGSLALIMGFGAM

LGKMLADCGGAQRIATTLIAKFGKKHIQWAVVLTGFTVGFALFYEVGFVLMLPLVFTIAASANIPLLYVG

VPMAAALSVTHGFLPPHPGPTAIATIFNADMGKTLLYGTILAIPTVILAGPVYARVLKGIDKPIPEGLYS

AKTFSEEEMPSFGVSVWTSLVPVVLMAMRAIAEMILPKGHAFLPVAEFLGDPVMATLIAVLIAMFTFGLN

RGRSMDQINDTLVSSIKIIAMMLLIIGGGGAFKQVLVDSGVDKYIASMMHETNISPLLMAWSIAAVLRIA

LGSATVAAITAGGIAAPLIATTGVSPELMVIAVGSGSVIFSHVNDPGFWLFKEYFNLTIGETIKSWSMLE

TIISVCGLVGCLLLNMVI

>gi|30065299|ref|NP_839470.1| 4-alpha-glucanotransferase [Shigella flexneri 2a str. 2457T]

MESKRLDNAALAAGISPNYINAHGKPQSISAETKRRLLDAMHQRTATKVAVTPVPNVMVYTSGKKMPMVV

EGSGEYSWQLTTEEGTQYKGHVTGGKAFNLPTKLPEGYHTLTLTQDDQRAHCRVIVAPKRCYEPQALLNK

QKLWGACVQLYTLRSEKNWGIGDFGDLKAMLVDVAKRGGSFIGLNPIHALYPANPESASPYSPSSRRWLN

VIYIDVNAVEDFHLSEEAQAWWQLPTTQQTLQQARDADWVDYSTVTALKMTALRMAWKGFAQRDDEQMAA

FRQFVAEQGDSLFWQAAFDALHAQQVKEDEMRWGWPAWPEMYKNVDSPEVRQFCEEHRDDVDFYLWLQWL

AYSQFAACWEISQGYEMPIGLYRDLAVGVAEGGAETWCDRELYCLKASVGAPPDILGPLGQNWGLPPMDP

HIITARAYEPFIELLRANMQNCGALRIDHVMSMLRLWWIPYGETADQGAYVHYPVDDLLSILALESKRHR

CMVIGEDLGTVPVEIVGKLRSSGVYSYKVLYFENDHEKTFRAPKAYPEQSMAVAATHDLPTLRGYWESGD

LTLGKTLGLYPDEVVLRGLYQDRELAKQGLLDALHKYGCLPKRAGHKASLMSMTPTLNRGLQRYIADSNS

ALLGLQPEDWLDMAEPVNIPGTSYQYKNWRRKLSATLESMFADDGVNKLLKDLDRRRRAAAKKK

>gi|30065298|ref|NP_839469.1| maltodextrin phosphorylase [Shigella flexneri 2a str. 2457T]

MSQPIFNDKQFQEVLSRQWQRYGLNSAAEMTPRQWWLAVSEALAEMLRAQPFAKPVANQRHVNYISMEFL

IGRLTGNNLLNLGWYQDVQDSLKAYDINLTDLLEEEIDPALGNGGLGRLAACFLDSMATVGQSATGYGLN

YQYGLFRQSFVDGKQVEAPDDWHRSNYPWFRHNEALDVQVGIGGKVMKDGRWEPEFTITGQAWDLPVVGY

RNGVAQPLRLWQATHAHPFDLTKFNDGDFLRAEQQGINAEKLTKVLYPNDNHTAGKKLRLMQQYFQCACS

VADILRRHHLAGRKLHELADYEVIQLNDTHPTIAIPELLRVLTDEHQMSWDDAWAITSKTFAYTNHTLMP

EALERWDVKLVKGLLPRHMQIINEINTRFKTLVEKTWPGDEKVWAKLAVVHDKQVHMANLCVVGGFAVNG

VAALHSDLVVKDLFPEYHQLWPNKFHNVTNGITPRRWIKQCNPALAALLDKSLQKEWANDLDQLINLEKF

ADDAKFRQQYREIKQANKVCLAEFVKVRTGIEINPQAIFDIQIKRLHEYKRQHLNLLHILALYKEIRENL

QADRVPRVFLFGAKAAPGYYLAKNIIFAINKVADVINNDPLVGDKLKVVFLPDYCVSAAEKLIPAADISE

QISTAGKEASGTGNMKLALNGALTVGTLDGANVEIAEKVGEENIFIFGHTVEQVKAILAKGYDPVKWRKK

DKVLDAVLKELESGKYSDGDKHAFDQMLHSIGKQGGDPYLVMADFAAYVEAQKQVDVLYRDQEAWTRAAI

LNTARCGMFSSDRSIRDYQARIWQAKR

>gi|30065297|ref|NP_839468.1| RNA 3'-terminal-phosphate cyclase, partial [Shigella flexneri 2a str. 2457T]

MLEPLLAKIGIHQQTTLLRHGFYPAGGGVVATEVSPVASFSTLQLGERGNIVQMRGEVLLAGVPRHVAER

EIATLAGIFSLHEQNIHNLPRDQGPGNTVSLEVESENITERFFVVGEKRVSAEVVAAQLVKEVKRYLASP

AAVGEYLADQLVLPMALAGAGEFTVAHPSCHLLTNIAVVERFLPVRFGLIETDGVTRVSIE

>gi|30065296|ref|NP_839467.1| hypothetical protein S4321 [Shigella flexneri 2a str. 2457T]

MNYELLTTENAPVKMWTKGVPVEADARQQLINTAKMPFIFKHIAVMPDVHLGKGSTIGSVIPTKGAIIPA

AVGVDIGCGMNALRTALTAADLPENLAELRQAIETAVPHGRTTGRCKRDKGAWENPPFNVDAKWAELEAG

YQWLTQKYPRFLNTNNYKHLGTLGTGNHFIEICLDESEQVWIMLHSGSRGIGNAIGTYFIDLAQKEMQDQ

LETLPSRDLAYFMEGTEYFDDYLRAVAWAQLFASLNRDAMMENVVTALQSITQKTVRQPQTLAMEEINCH

HNYVQKEQLFGEEIYVTRKGAVSARAGQYGIIPGSMGAKSFIVRGLGNEESFCSCSHGAGRVMSRTKAKK

LFSVEDQIRATAHVECRKDAEVIDEIPMAYKDIDAVMAAQSDLVEVIYTLRQVVCVKG

>gi|30065295|ref|NP_839466.1| 2-component regulator [Shigella flexneri 2a str. 2457T]

MRKTVAFGFVGTVLDYAGRGSQRWSKWRPSLCIYQQESLVIDRLELLHDARSRSLFETLKRDIASVSPET

EVVGVEIELHNPWDFEEVYACLHDFARGYEFQPEKEDYLIHITTGTHVAQICWFLLAEARYLPARLIQSS

TPRKKEQPRGPGEVTIIDLDLSRYNAIASRFAEERQQTLDFLKSGIATRNPHFNRMIEQIEKVAIKSRAP

ILLNGPTGAGKSFLARRIFELKQARHQFSGAFVEVNCATLRGDTAMSTLFGHVKGAFTGARESREGLLRS

ANGGMLFLDEIGELGADEQAMLLKAIEEKTFYPFGSDRQVSSDFQLIAGTVRDLRQLVAEGKFREDLYAR

INLWTFTLPGLRQRQEDIEPNLDYEVERHATLTGDSVRFNTEARRAWLAFATSPQATWRGNFRELSASVT

RMATFATSGRITLDVVEDEINRLRYNWQESRPSALTALLGAEAENIDLFDRMQLEHVIAICRQAKSLSAA

GRQLFDVSRQGKASVNDADRLRKYLARFGLTWEAVQDQHSSS

>gi|30065294|ref|NP_839465.1| DNA-binding transcriptional repressor GlpR [Shigella flexneri 2a str. 2457T]

MKQTQRHNGIIELVKQQGYVSTEELVEHFSVSPQTIRRDLNELAEQNLILRHHGGAALPSSSVNTPWHDR

KATQTEEKERIARKVAEQIPNGSTLFIDIGTTPEAVAHALLNHSNLRIVTNNLNVANTLMVKEDFRIILA

GGELRSRDGGIIGEATLDFISQFRLDFGILGISGIDSDGSLLEFDYHEVRTKRAIIENSRHVMLVVDHSK

FGRNAMVNMGSISMVDAVYTDAPPPVSVMQVLTDHHIQLELC

>gi|30065293|ref|NP_839464.1| intramembrane serine protease GlpG [Shigella flexneri 2a str. 2457T]

MITSFANPRVAQAFVDYMATQGVILTIQQHNQSDVWLADESQAERVRAELARFLENPADPRYLAASWLAG

HTGSGLHYRRYPFFAALRERAGPVTWVMMIACVVVFIAMQILGDQEVMLWLAWPFDPTLKFEFWRYFTHA

LMHFSLMHILFNLLWWWYLGGAVEKRLGSGKLIVITLISALLSGYMQQKFSGPWFGGLSGVVYALMGYVW

LRGERDPQSGIYLQRGLIIFALIWIVAGWFDLFGMSMANGAHIAGLAVGLAMAFVDSLNARKRK

>gi|30065292|ref|NP_839463.1| thiosulfate sulfurtransferase [Shigella flexneri 2a str. 2457T]

MDQFECINVADAHQKLQEKEAVLVDIRDPQSFAMGHAVQAFHLTNDTLGAFMRDNDFDTPVMVMCYHGNS

SKGAAQYLLQQGYDVVYSIDGGFEAWQRQFPAEVAYGA

>gi|30065291|ref|NP_839462.1| glycerol-3-phosphate dehydrogenase [Shigella flexneri 2a str. 2457T]

MAASMVLVSRADAAGRGLSVLMLEAQDLACATSSASSKLIHGGLRYLEHYEFRLVSEALAEREVLLKMAP

HIAFPMRFRLPHRPHLRPAWMIRIGLFMYDHLGKRTSLPGSTGLRFGANSVLKPEIKRGFEYSDCWVDDA

RLVLANAQMVVRKGGEVLTRTRATSARRENGLWIVEAEDIDTGKKYSWQARGLVNATGPWVKQFFDEGMH

LPSPYGIRLIKGSHIVVPRVHTQKQAYILQNEDKRIVFVIPWMDEFSIIGTTDVEYKGDPKAVKIEESEI

NYLLKVYNAHFKKQLSRDDIVWTYSGVRPLCDDESDSPQAITRDYTLDIHDENGKAPLLSVFGGKLTTYR

KLAEHALEKLTPYYQGIGPAWTKESVLPGGAIEGDRDDYAARLRRRYPFLTESLARHYTRTYGSNSELLL

GNAGTVSDLGEDFGHEFYEAELKYLVDHEWVRRTDDALWRRTKQGMWLNADQQSRVSQWLVEYTQQKLSL

AS

>gi|30065290|ref|NP_839461.1| IS4 orf [Shigella flexneri 2a str. 2457T]

MTDAMRFPGGEMADLYSHRWEIELGYREIKQTMQLSRLTLRSKKPELVEQELWGVLLAYNLVRYQMIKMA

EHLKGYWPNQLSFSESCGMVMRMLMTLQGASPGRIPELMRDLASMGQLVKLPTRRGRAFPRVVKERPWKY

PTAPKKSQSVA

>gi|30065289|ref|NP_839460.1| IS4 orf [Shigella flexneri 2a str. 2457T]

MHIGQALDLVSRYDSLRNPLTSLGDYLDPELISRCLAESGTVTLRKRRLPLEMMVWCIVGMALERKEPLH

QIVNRLDIMLPGNRPFVAPSAVIQARQRLGSEAVRRVFTKTAQLWHNATPHPHWCGLTLLAIDGVFWRTP

DTPENDAAFPRQTHAGNPALYPQVKMVCQMELTSHLLTAAAFGTMKNSENELAEQLIEQTGDNTLTLMDK

GYYSLGLLNGWSLAGEHRHWMIPLRKGAQYEELRKLGKGDHLVKLKTSPQARKKWPGLGNEVTARLLTVR

AKEKSAIC

>gi|30065288|ref|NP_839459.1| glycogen phosphorylase [Shigella flexneri 2a str. 2457T]

MNAPFTYSSPTLSVEALKHSIAYKLMFTIGKDPVVANKHEWLNATLFAVRDRLVERWLRSNRAQLSQETR

QVYYLSMEFLIGRTLSNAMLSLGIYEDVQGALEAMGLNLEELIDEENDPGLGNGGLGRLAACFLDSLATL

GLPGRGYGIRYDYGMFKQNIVNGSQKESPDYWLEYGNPWEFKRHNTRYKVRFGGRIQQEGKKTRWIETEE

ILGVAYDQIIPGYDTDATNTLRLWSAQASSEINLGKFNQGDYFAAVEDKNHSENVSRVLYPDDSTYSGRE

LRLRQEYFLVSSTIQDILSRHYQLHKTYDNLADKIAIHLNDTHPVLSIPEMMRLLIDEHQFSWDDAFEVC

CQVFSYTNHTLMSEALETWPVDMLGKILPRHLQIIFEINDYFLKTLQEQYPNDTDLLGRASIIDESNGRR

VRMAWLAVVVSHKVNGVSELHSNLMVQSLFADFAKIFPGRFTNVTNGVTPRRWLAVANPSLSAVLDEHLG

RNWRTDLSLLNELQQHCDFPMVNHAVHQAKLENKKRLAEYIAQQLNVVVNPKALFDVQIKRIHEYKRQLM

NVLHVITRYNRIKADPDAKWVPRVNIFGGKAASAYYMAKHIIHLINDVAKVINNDPQIGDKLKVVFIPNY

SVSLAQLIIPAADLSEQISLAGTEASGTSNMKFALNGALTIGTLDGANVEMLDHVGADNIFIFGNTAEEV

EELRRQGYKPREYYEKDEELHQVLTQIGSGVFSPEDPGRYRDLVDSLINFGDHYQVLADYRSYVDCQDKV

DELYELQEEWTAKAMLNIANMGYFSSDRTIKEYADHIWHIDPVRL

>gi|30065287|ref|NP_839458.1| glycogen synthase [Shigella flexneri 2a str. 2457T]

MQVLHVCSEMFPLLKTGGLADVIGALPAAQIADGVDARVLLPAFPDIRRGVTDAQVVSRRDTFAGHITLL

FGHYNGVGIYLIDAPHLYDRPGSPYHDTNLFAYTDNVLRFALLGWVGAEMASGLDPFWRPDVVHVHDWHA

GLAPAYLAARGRPAKSVFTVHNLAYQGMFYAHHMNDIQLPWSFFNIHGLEFNGQISFLKAGLYYADHITA

VSPTYAREITEPQFAYGMEGLLQQRHREGRLSGVLNGVDEKIWSPETDLLLASRYTRDTLEDKAENKSQL

QIAMGLKVDDKVPLFAVVSRLTSQKGLDLVLEALPGLLEQGGQLALLGAGDPVLQEGFLAAAAEYPGQVG

VQIGYHEAFSHRIMGGADVILVPSRFEPCGLTQLYGLKYGTLPLVRRTGGLADTVSDCSLENLADGVASG

FVFEDSNAWSLLRAIRRAFVLWSRPSLWRFVQRQAMAMDFSWQVAAKSYRELYYRLK

>gi|30065286|ref|NP_839457.1| glucose-1-phosphate adenylyltransferase [Shigella flexneri 2a str. 2457T]

MVSLEKNDHLMLARQLPLKSVALILAGGRGTRLKDLTNKRAKPAVHFGGKFRIIDFALSNCINSGIRRMG

VITQYQSHTLVQHIQRGWSFFNEEMNEFVDLLPAQQRMKGENWYRGTADAVTQNLDIIRRYKAEYVVILA

GDHIYKQDYSRMLIDHVEKGARCTVACMPVPIEEASAFGVMAVDENDKIIEFVEKPANPPSMPNDPSKSL

ASMGIYVFDADYLYELLEEDDRDENSSHDFGKDLIPKITEAGLAYAHPFPLSCVQSDPDAEPYWRDVGTL

EAYWKANLDLASVVPELDMYDRNWPIRTYNESLPPAKFVQDRSGSHGMTLNSLVSGGCVISGSVVVQSVL

FSRVRVNSFCNIDSAVLLPEVWVGRSCRLRRCVIDRACVIPEGMVIGENAEEDARRFYRSEEGIVLVTRE

MLRKLGHKQER

>gi|30065285|ref|NP_839456.1| glycogen debranching enzyme [Shigella flexneri 2a str. 2457T]

MTQLAIGKPAPLGAHYDGQGVNFTLFSAHAERVELCVFDANGQEHRYDLPGNSGDIWHGYLPDARPGLRY

GYRVHGPWQPAEGHRFNPAKLLIDPCARQIDGEFKDNPLLHAGHNEPDYRDNAAIAPKCVVVVDHYDWED

DAPPRTPWGSTIIYEAHVKGLTYLHPEIPVEIRGTYKALGHPVMINYLKQLGITALELLPVAQFASEPRL

QRMGLSNYWGYNPVAMFALHPAYACSPETALDEFRDAIKALHKAGIEVILDIVLNHSAELDLDGPLFSLR

GIDNRSYYWIREDGDYHNWTGCGNTLNLSHPAVVDYASACLRYWVETCHVDGFRFDLAAVMGRTPEFRQD

APLFTAIQNCPVLSQVKLIAEPWDIAPSGYQVGNFPPLFAEWNDHFRDAARRFWLHYDLPLGAFAGRFAA

SSDVFKRNGRLPSAAINLVTAHDGFTLRDCVCFNHKHNEANGEENRDGTNNNYSNNHGKEGLGGTLDLVE

RRRDSIHALLTTLLLSQGTPMLLAGDEHGHSQHGNNNAYCQDNQLNWLDWSQASSGLTAFTAALIHLRKR

IPALVENRWWEEGDGNVRWLNRYAQPLSTDEWQNGPKQLQILLSDRFLIAINATLEVTEIVLPAGEWHAI

PPFAGEDNPVITVVWQGPAHGLCVFQR

>gi|30065284|ref|NP_839455.1| glycogen branching enzyme [Shigella flexneri 2a str. 2457T]

MSDRIDRDVINALIAGHFADPFSVLGMHKTTAGLEVRALLPDATDVWVIEPKTGRKLAKLECLDSRGFFS

GVIPRRKNFFRYQLAVVWHGQQNLIDDPYRFGPLIQEMDAWLLSEGTHLRPYETLGAHADTMDGVTGTRF

SVWAPNARRVSVVGQFNYWDGRRHPMRLRKESGIWELFIPGAHNGQLYKYEMIDANGNLRLKSDPYAFEA

QMRPETASLICGLPEKVVQTEERKKANQFDAPISIYEVHLGSWRRHTDNNFWLSYRELADQLVPYAKWMG

FTHLELLPINEHPFDGSWGYQPTGLYAPTRRFGTRDDFRYFIDAAHAAGLNVILDWVPGHFPTDDFALAE

FDGTNLYEHSDPREGYHQDWNTLIYNYGRREVSNFLVGNALYWIERFGIDALRVDAVASMIYRDYSRKEG

EWIPNEFGGRENLEAIEFLRNTNRILGEQVSGAVTMAEESTDFPGVSRPQDMGGLGFWYKWNLGWMHDTL

DYMKLDPIYRQYHHDKLTFGMLYNYTENFVLPLSHDEVVHGKKSILDRMPGDAWQKFANLRAYYGWMWAF

PGKKLLFMGNEFAQGREWNHDASLDWHLLEGGDNWHHGVQRLVRDLNLTYRHHKAMHELDFDPYGFEWLV

VDDKERSVLIFVRRDKEGNEIIVASNFTPVPRHDYRFGINQPGKWREILNTDSIHYHGSNAGNGGTVHSD

EIASHGRQHSLSLTLPPLATIWLVREAE

>gi|30065283|ref|NP_839454.1| aspartate-semialdehyde dehydrogenase [Shigella flexneri 2a str. 2457T]

MKNVGFIGWRGMVGSVLMQRMVEERDFDAIRPVFFSTSQLGQAAPSFGGTTGTLQDAFDLEALKALDIIV

TCQGGDYTNEIYPKLRESGWQGYWIDAASSLRMKDDAIIILDPVNQDVITDGLNNGIRTFVGGNCTVSLM

LMSLGGLFANDLVDWVSVATYQAASGGGARHMRELLTQMGHLYGHVADELATPSSAILDIERKVTTLTRS

GELPVDNFGVPLAGSLIPWIDKQLDNGQSREEWKGQAETNKILNTSSVIPVDGLCVRVGALRCHSQAFTI

KLKKDVSIPTVEELLAAHNPWAKVVPNDREITMRELTPAAVTGTLTTPVGRLRKLNMGPEFLSAFTVGDQ

LLWGAAEPLRRMLRQLA

>gi|30065282|ref|NP_839453.1| dITP- and XTP- hydrolase [Shigella flexneri 2a str. 2457T]

MNEIISAAVLLILIMDPLGNLPIFMSVLKHTEPKRRRAIMVRELLIALLVMLVFLFAGEKILAFLSLRAE

TVSISGGIILFLIAIKMIFPSASGNSSGLPAGEEPFIVPLAIPLVAGPTILATLMLLSHQYPNQMGHLVI

ALLLAWGGTFVILLQSSLFLRLLGEKGVNALERLMGLILVMMATQMFLDGIRMWMKG

>gi|30065281|ref|NP_839452.1| low affinity gluconate transporter [Shigella flexneri 2a str. 2457T]

MKARMHAFLALMVVSMGAGLFSGMPLDKIAATMEKGMGGTLGFLAVVVALGAMFGKILYETGAVDQIAVK

MLKSFGHSRAHYAIGLAGLVCALPLFFEVAIVLLISVAFSMARHTGTNLVKLVIPLFAGVAAAAAFLVPG

PAPMLLASQMNADFGWMILIGLCAAIPGMIIAGPLWGNFISRYVELHIPDDISEPHLGEGKMPSFGFSLS

LILLPLVLVGLKTIAARFVPEGSTAYEWFEFIGHPFTAILVACLVAIYGLAMRQGMPKDKVMEICGHALQ

PAGIILLVIGAGGVFKQVLVDSGVGPALGEALTGMGLPIAITCFVLAAAVRIIQGAATVACLTAVGLVMP

VIEQLNYSGAQMAALSICIAGGSIVVSHVNDAGFWLFGKFTGATEAETLKTWTMMETILGTVGAIVGMIA

FQLLS

>gi|30065279|ref|NP_839450.1| regulator of gluconate (gnt) operon [Shigella flexneri 2a str. 2457T]

MTVSRFLRNPEQVSVALRGKIAAALDELGYIPNRAPDILSNATSRAIGVLLPSLTNQVFAEVLRGIESVT

DAHGYQTMLAHYGYKPEMEQERLESMLSWNIDGLILTERTHTPRTLKMIEVAGIPVVELMDSQSPCLDIA

VGFDNFEAARQMTTAIIARGHRHIAYLGARLDERTIIKQKGYEQAMLDAGLVPYSVMVEQSSSYSSGIEL

IRQARREYPQLDGVFCTNDDLAVGAAFECQRLGLKVPDDMAIAGFHGHDIGQVMEPRLASVLTPRERMGS

IGAERLLARIRGESVTPKMLDLGFTLSPGGSI

>gi|30065278|ref|NP_839449.1| hypothetical protein S4301 [Shigella flexneri 2a str. 2457T]

MIYLRKANERGHANHGWLDSWHTFSCANYYDPNFMGFSALRVINDDVIEAGQGFGTHPHKDMEILTYVLE

GTVEHQDSMGNKEQVPAGEFQIMSAGTGIRHSEYNPSSTERLHLYQIWIMPEENGITPRYEQRRFDAVQG

KQLVLSPDARDGSLKVHQDMELYRWALLKDEQSVHQIAAERRVWIQVVKGNVTINGVKASTSDGLAIWDE

QAISIHADSDSEVLLFDLPPV

>gi|30065277|ref|NP_839448.1| dehydrogenase [Shigella flexneri 2a str. 2457T]

MVINCAFIGFGKSTTRYHLPYVLNRKDSWHVAHIFRRHAKPEEQAPIYSHIHFTSDLDEVLNDPDVKLVV

VCTHADSHFEYAKRALEAGKNVLVEKTFTPTLAQAKELFALAKSKGLTVTPYQNRRFDSCFLTAKKAIES

GKLGEIVEVESHFDYYRPVAETKPGLPQDGAFYGLGVHTMDQIISLFGRPDHVAYDIRSLRNKANPDDTF

EAQLFYGDLKAIVKTSHLVKIDYPKFIVHGKKGSFIKYGIDQQETSLKANIMPGEPGFAADDSVGVLEYV

NDEGVTVREEMKPEMGDYGRVYDALYQTITHGAPNYVKESEVLTNLEILERGFEQASPSTVTLAN

>gi|30065276|ref|NP_839447.1| acetyltransferase YhhY [Shigella flexneri 2a str. 2457T]

MSEIVIRHAETRDYEAIRQIHAQPEVYCNTLQVPHPSDHMWQERLADRPGIKQLVACIDGDVVGHLTIDV

QQRPRRSHVADFGICVDSRWKNRGVASALMREMIEMCDNWLRVDRIELTVFVDNAPAIKVYKKFGFEIEG

TGKKYALRNGEYVDAYYMARVK

>gi|30065275|ref|NP_839446.1| gamma-glutamyltranspeptidase [Shigella flexneri 2a str. 2457T]

MIKPTFLRRVAIAALLSGSCFSAAAAPPAPPVSYGVEEDVFHPVRAKQGMVASVDATATQVGVDILKEGG

NAVDAAVAVGYALAVTHPQAGNLGGGGFMLIRSKNGNTTAIDFREMAPAKATRDMFLDDQGNPDSKKSLT

SHLASGTPGTVAGFSLALDKYGTMPLNKVVQPAFKLARDGFIVNDALADDLKTYGSEVLPNHENSKAIFW

KEGEPLKKGDTLVQANLAKSLEMIAENGPDEFYKGTIAEQIAQEMQKNGGLITKEDLAAYKAVERTPISG

DYRGYQVYSMPPPSSGGIHIVQILNILENFDMKKYGFGSADAMQIMAEAEKYAYADRSEYLGDPDFVKVP

WQALTNKAYAKSIADQIDIKKAKPSSEIRPGKLAPYESNQTTHYSVVDKDGNAVAVTYTLNTTFGTGIVA

GESGILLNNQMDDFSAKPGVPNVYGLVGGDANAVGPNKRPLSSMSPTIVVKDGKTWLVTGSPGGSRIITT

VLQMVVNSIDYGMNVAEATNAPRFHHQWLPDELRVEKGFSPDTLKLLEAKGQKVALKEAMGSTQSIMVGP

DGELYGASDPRSVDDLTAGY

>gi|30065274|ref|NP_839445.1| hypothetical protein S4297 [Shigella flexneri 2a str. 2457T]

MKRLLILTALLPFVGFAQPINTLNNPNQPGYQIPSQQRMQTQMQTQQIQQKGMLNQQLKTQTQLQQQHLE

NQINNNSQRVLQSQPGERNPARQQMLPNTNGGMLNSNRNPDSSLNQQHMLPERRNGDMLNQPSTPQPDIP

LKTIGP

>gi|30065273|ref|NP_839444.1| glycerophosphodiester phosphodiesterase [Shigella flexneri 2a str. 2457T]

MSNWPYPRIVAHRGGGKLAPENTLAAIDVGAKYGHKMIEFDAKLSKDGEIFLLHDDNLERTSNGWGVAGE

LNWQDLLRVDAGSWYSKAFKGEPLPLLSQVAERCREHGMMANIEIKPTTGTGPLTGKMVALAARQLWAGM

TPPLLSSFEIDALEAAQQAAPELPRGLLLDEWRDDWRELTARLGCVSIHLNHKLLDKARVMQLKDAGLRI

LVYTVNKPQHAAELLRWGVDCICTDAIDVIGPNFTAQ

>gi|30065272|ref|NP_839443.1| glycerol-3-phosphate transporter membrane protein [Shigella flexneri 2a str. 2457T]

MIENRPWLTIFSHTMLILGIAVILFPLYVAFVAATLDKQAVYAAPMTLIPGTHLLENIHNIWVNGVGTNS

APFWRMLLNSFVMAFSITLGKITVSMLSAFAIVWFRFPLRNLFFWMIFITLMLPVEVRIFPTVEVIANLK

MLDSYAGLTLPLMASATATFLFRQFFMTLPDELVEAARIDGASPMRFFCDIVFPLSKTNLAALFVITFIY

GWNQYLWPLLIITDVDLGTTVAGIKGMIATGEGTTEWNSVMAAMLLTLIPPVVIVLVMQRAFVRGLVDSE

K

>gi|30065271|ref|NP_839442.1| glycerol-3-phosphate transporter permease [Shigella flexneri 2a str. 2457T]

MSSSRPVFRSRWLPYLLVAPQLIITVIFFIWPAGEALWYSLQSVDPFGFSSQFVGLDNFVTLFHDSYYLD

SFWTTIKFSTFVTVSGLLVSLFFAALVEYIVRGSRFYQTLMLLPYAVAPAVAAVLWIFLFNPGRGLITHF

LAEFGYDWNHAQNSGQAMFLVVFASVWKQISYNFLFFYAALQSIPRSLIEAAAIDGAGPIRRFFKIALPL

IAPVSFFLLVVNLVYAFFDTFPVIDAATSGGPVQATTTLIYKIYREGFTGLDLASSAAQSMVLMFLVIVL

TVVQFRYVESKVRYQ

>gi|30065270|ref|NP_839441.1| glycerol-3-phosphate transporter periplasmic binding protein [Shigella flexneri 2a str. 2457T]

MKPLHYTASALALGLALMGNAQAVTTIPFWHSMEGELGKEVDSLAQRFNAENPDYKIVPTYKGNYEQNLS

AGIAVFRTGNAPAILQVYEVGTATMMASKAIKPVYDVFKEAGIQFDESQFVPTVSGYYSDSKTGHLLSQP

FNSSTSVLYYNKDAFKKAGLDPEQPPKTWQDLADYSAKLKASGIKCGYASGWQGWIQLENFSAWNGLPFA

SKNNGFDGTDAVLEFNKPEQVKHIAMLEEMNKKGDFSYVGRKDESTEKFYNGDCAMTTASSGSLANIREY

AKFNYGVGMMPYDADAKDAPQNAIIGGASLWVMQGKDKETYTGVAKFLDFLAKPENAAEWHQKTGYLPIT

KAAYDLTREQGFYEKNPGADTATRQMLNKPPLPFTKGLRLGNMPQIRVIVDEELESVWTGKKTPQQALDT

AVERGNQLLRRFEKSTKS

>gi|30065269|ref|NP_839440.1| leucine/isoleucine/valine transporter ATP-binding subunit [Shigella flexneri 2a str. 2457T]

MEKVMLSFDKVSAHYGKIQALHEVSLHINQGEIVTLIGANGAGKTTLLGTLCGDPRATSGRIVFDDKDIT

DWQTAKIMREAVAIVPEGRRVFSRMTVEENLAMGGFFAERDQFQERIKWVYELFPRLHERRVQRAGTMSG

GEQQMLAIGRALMSNPRLLLLDEPSLGLAPIIIQQIFDTIEQLREQGMTIFLVEQNANQALKLADRGYVL

ENGHVVLSDTGDALLANEAVRSAYLGG

>gi|30065268|ref|NP_839439.1| leucine/isoleucine/valine transporter ATP-binding subunit [Shigella flexneri 2a str. 2457T]

MSQPLLSVNGLMMRFGGLLAVNNVNLELYPQEIVSLIGPNGAGKTTVFNCLTGFYKPTGGTILLRDQHLE

GLPGQQIARMGVVRTFQHVRLFREMTVIENLLVAQHQQLKTGLFSGLLKTPSFRRAQSEALDRAATWLER

IGLLEHANRQASNLAYGDQRRLEIARCMVTQPEILMLDEPAAGLNPKETKELDELIAELRNHHNTTILLI

EHDMKLVMGISDRIYVVNQGTPLANGTPEQIRNNPDVIRAYLGEA

>gi|30065267|ref|NP_839438.1| leucine/isoleucine/valine transporter permease subunit [Shigella flexneri 2a str. 2457T]

MKPMHIAMALLSAAMFFVLAGVFMGVQLELDGTKLVVDTASDIRWQWMFIGTAVVFFFQLLRPAFQKGLK

SVSGPKFILPAIDGSTVKQKLFLVALLVLVVAWPFMVSRGTVDIATLTMIYIILGLGLNVVVGLSGLLVL

GYGGFYAIGAYTFALLNHYYGLGFWTCLPIAGLMAAAAGFLLGFPVLRLRGDYLAIVTLGFGEIVRILLL

NNTEITGGPNGISQIPKPTLFGLEFSRTAREGGWDTFSNFFGLKYDPSDRVIFLYLVALLLVVLSLFVIN

RLLRMPLGRAWEALREDEIACRSLGLSPRRIKLTAFTISAAFAGFAGTLFAARQGFVSPESFTFAESAFV

LAIVVLGGMGSQFAVILAAVLLVVSRELMRDFNEYSMLMLGGLMVLMMIWRPQGLLPMTRPQLKLKNGAA

KGEQA

>gi|30065266|ref|NP_839437.1| branched-chain amino acid ABC transporter permease [Shigella flexneri 2a str. 2457T]

MSEQFLYFLQQMFNGVTLGSTYALIAIGYTMVYGIIGMINFAHGEVYMIGSYVSFMIIAALMMMGIDTGW

LLVAAGFVGAIVIASAYGWSIERVAYRPVRNSKRLIALISAIGMSIFLQNYVSLTEGSRDVALPSLFNGQ

WVVGHSENFSASITTMQAVIWIVTFLAMLALTIFIRYSRMGRACRACAEDLKMASLLGINTDRVIALTFV

IGAAMAAVAGVLLGQFYCVINPYIGFMAGMKAFTAAVLGGIGSIPGAMIGGLILGIAEALSSAYLSTEYK

DVVSFALLILVLLVMPTGILGRPEVEKV

>gi|30065265|ref|NP_839436.1| high-affinity leucine-specific transport protein, periplasmic binding protein [Shigella flexneri 2a str. 2457T]

MKRNAKTIIAGMIALAISHTAMADDIKVAVVGAMSGPIAQWGDMEFNGARQAIKDINVKGGIKGDKLVGV

EYDDACDPKQAVAVANKIVNDGIKYVIGHLCSSSTQPASDIYEDEGILMISPGATNPELTQRGYQHIMRT

AGLDSSQGPTAAKYILETVKPQRIAIIHDKQQYGEGLARSVQDGLKAANANVVFFDGITAGEKDFSALIA

RLKKENIDFVYYGGYYPEMGQMLRQARSVGLKTQFMGPEGVGNASLSNIAGDAAEGMLVTMPKRYDQDPA

NQGIVDALKADKKDPSGPYVWITYAAVQSLATALERTGSDEPLALVKDLKANGANTVIGPLNWDEKGDLK

GFDFGVFQWHADGSSTAAK

>gi|30065264|ref|NP_839435.1| hypothetical protein S4286 [Shigella flexneri 2a str. 2457T]

MKLTIIRLENFSDQDRIDLQKIWPEYSPSSLQVDDNHRIYAARFNERLLAAVRVTLSGTEGALDSLRVRE

VTRRRGVGQYLLEEVLRNNPGVSCWWMADAGVEDRGVMTAFMQALGFTAQQGGWEKR

>gi|30065263|ref|NP_839434.1| high-affinity amino acid transport protein, periplasmic binding protein [Shigella flexneri 2a str. 2457T]

MLNNNITTHVITRRMGILRMNIKGKALLAGCIALAFSNMALAEDIKVAVVGAMSGPVAQYGDQEFTGAEQ

AVADINAKGGIKGNKLQIVKYDDACDPKQAVAVANKVVNDGIKYVIGHLCSSSTQPASDIYEDEGILMIT

PAATAPELTARGYQLILRTTGLDSDQGPTAAKYILEKVKPQRIAIVHDKQQYGEGLARAVQDGLKKGNAN

VVFFDGITAGEKDFSTLVARLKKENIDFVYYGGYHPEMGQILRQARAAGLKTQFMGPEGVANVSLSNIAG

ESAEGLLVTKPKNYDQVPANKPIVDAIKAKKQDPSGAFVWTTYAALQSLQAGLNQSDDPAEIAKYLKANS
VDTVMGPLTWDEKGDLKGFEFGVFDWHANGTATDAK

>gi|30065262|ref|NP_839433.1| RNA polymerase factor sigma-32 [Shigella flexneri 2a str. 2457T]
MTDKMQSLALAPVGNLDSYIRAANAWPMLSADEERALAEKLHYHGDLEAAKTLILSHLRFVVHIARNYAG
YGLPQADLIQEGNIGLMKAVRRFNPEVGVRLVSFAVHWIKAEIHEYVLRNWRIVKVATTKAQRKLFFNLR
KTKQRLGWFNQDEVEMVARELGVTSKDVREMESRMAAQDMTFDLSSDDDSDSQPMAPVLYLQDKSSNFAD
GIEDDNWEEQAANRLTDAMHGLDERSQDIIRARWLDEDNKSTLQELADRYGVSAERVRQLEKNAMKKLRA
AIEA

>gi|30065261|ref|NP_839432.1| cell division protein FtsX [Shigella flexneri 2a str. 2457T]
MNKRDAINHIRQFGGRLDRFRKSVGGSGDGGRNAPKRAKSSPKPVNRKTNVFNEQVRYAFHGALQDLKSK
PFATFLTVMVIAISLTLPSVCYMVYKNVNQAATQYYPSPQITVYLQKTLDDDAAAGVVAQLQAEQGVEKV
NYLSREDALGEFRNWSGFGGALDMLEENPLPAVAVVIPKLDFQGTESLNTLRDRITQINGIDEVRMDDSW
FARLAALTGLVGRVSAMIGVLMVAAVFLVIGNSVRLSIFARRDSINVQKLIGATDGFILRPFLYGGALLG
FSGALLSLILSEILVLRLSSAVAEVAQVFGTKFDINGLSFDECLLLLLVCSMIGWVAAWLATVQHLRHFT
PE

>gi|30065260|ref|NP_839431.1| cell division protein FtsE [Shigella flexneri 2a str. 2457T]
MIRFEHVSKAYLGGRQALQGVTFHMQPGEMAFLTGHSGAGKSTLLKLICGIERPSAGKIWFSGHDITRLK
NREVPFLRRQIGMIFQDHHLLMDRTVYDNVAIPLIIAGASGDDIRRRVSAALDKVGLLDKAKNFPIQLSG
GEQQRVGIARAVVNKPAVLLADEPTGNLDDALSEGILRLFEEFNRVGVTVLMATHDINLISRRSYRMLTL
SDGHLHGGVGHE

>gi|30065259|ref|NP_839430.1| cell division protein FtsY [Shigella flexneri 2a str. 2457T]

MAKEKRRGFFSWLGFGQKEQTPEKETEVQNEQPVVEEIVQAQEPAKASEQAVEEQPQAHTEAEAETFAAD
VVEVTEQVVESEKAQPEAEVVAQPEPVVEETPEPVAIEREELPLPEDVNAEEVSPEEWQAEAETVEIVEA
AEEEAAKEEITDEELEAQALAAEAAEEAVMVVPPAEEEQPVAEIAQEQEKPTKEGFFARLKRSLLKTKEN
LGSGFISLFRGKKIDDDLFEELEEQLLIADVGVETTRKIITNLTEGASRKQLRDAEALYGLLKEEMGEIL
AKVDEPLNVEDKTPFVILMVGVNGVGKTTTIGKLARQFEQQGKSVMLAAGDTFRAAAVEQLQVWGQRNNI
PVIAQHTGADSASVIFDAIQAAKARNIDVLIADTAGRLQNKSHLMEELKKIVRVMKKLDVEAPHEVMLTI
DASTGQNAVSQAKLFHEAVGLTGITLTKLDGTAKGGVIFSVADQFGIPIRYIGVGERIEDLRPFKADDFI
EALFARED

>gi|30065258|ref|NP_839429.1| 16S rRNA m(2)G966-methyltransferase [Shigella flexneri 2a str. 2457T]
MKKPNHSGSGQIRIIGGQWRGRKLPVPDSPGLRPTTDRVRETLFNWLAPVIVDAQCLDCFAGSGALGLEA
LSRYAAGATLIEMDRVVSQQLIKNLATLKAGNARVVNSNAMSFLAQKGTPHNIVFVDPPFRRGLLEETIN
LLEDNGWLADEALIYVESEVENGLPTVPANWSLHREKVAGQVAYRLYQHEAQGESDAD

>gi|30065257|ref|NP_839428.1| hypothetical protein S4279 [Shigella flexneri 2a str. 2457T]
MLINIGRLLMLCVWGFLILNLVHPFPRPLNIFVNVALIFTVLMHGMQLALLKSTLPKDGPQMTTAEKVRI
FLFGVFELLVWQKKFKVKK

>gi|30065256|ref|NP_839427.1| receptor [Shigella flexneri 2a str. 2457T]
MSKPPLFFIVIIGLIVVAASFRFMQQRREKADNDMAPLQQKLVVVSNKREKPINDRRSRQQEVTPAGTSM
RYEASFKPQSGGMEQTFRLDAQQYHALTVGDKGTLSYKGTRFVSFVGEQ

>gi|30065255|ref|NP_839426.1| enzyme [Shigella flexneri 2a str. 2457T]
MLWSFIAVCLSAWLSVDASYRGPTWQRWVFKPLTLLLLLLLAWQAPMFDAISYLVLAGLCASLLGDALTL
LPRQRLMYAIGAFFLSHLLYTIYFASQMTLSFFWPLPLVLLVLGALLLAIIWTRLEEYRWPICTFIGMTL

VMVWLAGELWFFRPTAPALSAFVGASLLFISNFVWLGSHYRRRFRADNAIAAACYFAGHFLIVRSLYL

>gi|30065254|ref|NP_839425.1| zinc/cadmium/mercury/lead-transporting ATPase [Shigella flexneri 2a str. 2457T]

MSTPDNHGKKAPQFAAFKPLTTVQNANDCCCDGACSSSPTLSENVSGTRYSWKVSGMDCAACARKVENAV

RQLAGVNQVQVLFATEKLVVDADNDIRAQVESAVQKAGYSLRDEQAADEPQASRLKENLPLITLIVMMAI

SWGLEQFNHPFGQLAFIATTLVGLYPIARQALRLIKSGSYFAIETLMSVAAIGALFIGATAEAAMVLLLF

LIGERLEGWAASRARQGVSALMALKPETATRLRNGEREEVAINSLRPGDVIEVAAGGRLPADGKLLSPFA

SFDESALTGESIPVERATGDKVPAGATSVDRLVTLEVLSEPGASAIDRILKLIEEAEERRAPIERFIDRF

SRIYTPAIMAVALLVTLVPPLLFAASWQEWIYKGLTLLLIGCPCALVISTPAAITSGLAAAARRGALIKG

GAALEQLGRVTQVAFDKTGTLTVGKPRVTAIHPATGISESELLTLAAAVEQGATHPLAQAIVREAQVAEL

AIPTAESQRALVGSGIEAQVNGERVLICAAGKHPADAFAGLINELESAGQTVVLVVRNDDVLGIIALQDT

LRADATTAISELNALGVKGVILTGDNPRAAAAIAGELGLEFKAGLLPEDKVKAVTKLNQHAPLAMVGDGI

NDAPAMKAAAIGIAMGSGTDVALETADAALTHNHLRGLVQMIELARATHANIRQNITIALGLKGIFLVTT

LLGMTGLWLAVLADTGATVLVTANALRLLRRR

>gi|30065253|ref|NP_839424.1| sulfur transfer protein SirA [Shigella flexneri 2a str. 2457T]

MTDLFSSPDHTLDALGLRCPEPVMMVRKTVRNMQPGETLLIIADDPATTRDIPGFCTFMEHELVAKETDG

LPYRYLIRKGG

>gi|30065252|ref|NP_839423.1| hypothetical protein S4274 [Shigella flexneri 2a str. 2457T]

MNVFSQTQRYKALFWLSLFHLLVITSSNYLVQLPVSIFGFHTTWGAFSFPFIFLATDLTVRIFGAPLARR

IIFAVMIPALLISYVISSLFYMGSWQGFGALAHFNLFVARIATASFMAYALGQILDVHVFNRLRQSRRWW

LAPTASTLFGNVSDTLAFFFIAFWRSPDAFMAEHWMEIALVDYCFKVLISIVFFLPMYGVLLNMLLKRLA

DKSEINALQAS

>gi|30065249|ref|NP_839420.1| hypothetical protein S4271 [Shigella flexneri 2a str. 2457T]

METPQPDKTGMHILLKLASLVVILAGIHAAADIIVQLLLALFFAIVLNPLVTWFIRRGVQRPVAITIVVV

VMLIALTALVGVLAASFNEFISMLPKFNKELTRKLFKLQEMLPFLNLHMSPERMLQRMDSEKVVTFTTAL

MTGLSGAMASVLLLVMTVVFMLFEVRHVPYKMRFALNNPQIHIAGLHRALKGVSHYLALKTLLSLWTGVI

VWLGLALMGVQFALMWAVLAFLLNYVPNIGAVISAVPPMIQVLLFNGVYECILVGALFLVVHMVIGNILE

PRMMGHRLGMSTMVVFLSLLIWGWLLGPVGMLLSVPLTSVCKIWMETTKGGSKLAILLGPGRPKSRLPG

>gi|30065248|ref|NP_839419.1| holo-(acyl carrier protein) synthase 2 [Shigella flexneri 2a str. 2457T]

MYRIVLGKVSTLSAAPLPPGLREQAPQGPRRERWLAGRTLLSHTLSPLPEIIYGEQGKPAFAPETPLWFN

LSHSGDDIALLLSDEGEVGCDIEVIRPRVNWRWLANAVFSLGEHAEMDAVHPDQQLEMFWRIWTRKEAIV

KQRGGSAWQIVSVDSTYHSSLSVSHCQLENLSLAICTPTPFTLTADSVQWIDSVN

>gi|30065247|ref|NP_839418.1| periplasmic binding protein for nickel [Shigella flexneri 2a str. 2457T]

MLSTLRRTLFALLACASFIVHAAAPDEITTAWPVNVGPLNPHLYTPNQMFAQSMVYEPLVKYQADGSVIP

WLAKSWTHSEDGKTWTFTLRDDVKFSNGEPFDAEAAAENFRAVLDNRQRHAWLELANQIVDVKALNKTEL

QITLKSAYYPFLQELALPRPFRFIAPSQFKNHETMNGIKAPIGTGPWILQESKLNQYDVFVRNENYWGEK

PAIKKITFNVIPDPTTRAVAFETGDIDLLYGNEGLLPLDTFARFSQNPAYHTQLSQPIETVMLALNTAKA

PTNELAVREALNYAVNKKSLIDNALYGTQQVADTLFAPSVPYANLGLKPRQYDPQKAKELLEKAGWTLPA

GKDIREKNGQPLRIELSFIGTDALSKSMAEIIQADMRQIGADVSLIGEEESSIYARQRDGRFGMIFHRTW

GAPYDPHAFLSSMRVPSHADFQAQQGLADKPLIDKEIGEVLATHDETQRQALYRDILTRLHDEAVYLPIS

YISMMVVSKPELGNIPYAPIATEIPFEQIKPVKP

>gi|30065246|ref|NP_839417.1| nickel transporter permease NikB [Shigella flexneri 2a str. 2457T]

MLRYVLRRFLLLIPMVLAASVIIFLMLRLGTGDPALDYLRLSNLPPTPEMLASTRTMLGLDQPLYVQYGT

WLWKALHLDFGISFASQRPVLDDMLNFLPATLELAGAALVLILLTSVPLGIWAARHRDRLPDFAVRFIAF

LGVSMPNFWLAFLLVMAFSVYLQWLPAMGYGSWQHIILPAVSIAFMSLAINARLLRASMLDVAGQRHVTW

ARLRGLNDKQTERRHILRNASLPMITAVGMHIGELIGGTMIIENIFAWPGVGRYAVSAIFNRDYPVIQCF

TLMMVVVFVVCNLIVDLLNAALDPRIRRHEGAHA

>gi|30065245|ref|NP_839416.1| nickel transporter permease NikC [Shigella flexneri 2a str. 2457T]

MNFFLSSRWSVRLALIIIALLALIALTSQWWQPYDPQAIDLPSCLLSPDAQHWLGTDHLGRDIFSRLMAA

TRVSLGSVMACLLLVLTLGLVIGGSAGLIGGHVDQATMRVADMFMTFPTSILSFFMVGVLGTGLTNVIIA

IALSHWAWYARMVRSLVISLRQREFVLASRLSGAGHVRVFVDHLAGAVIPSLLVLATLDIGHMMLHVAGM

SFLGLGVTAPTAEWGVMINDARQYIWTQPLQMFWPGLALFISVMAFNLVGDALRDHLDPHLVTEHAH

>gi|30065244|ref|NP_839415.1| nickel transporter ATP-binding protein NikD [Shigella flexneri 2a str. 2457T]

MPQQIELRNITLQAAQPLVHGVSLTLQRGRVLALVGGSGSGKSLTCAATLGILPAGVRQTAGEILADGKP

VSPYALRGIKIATIMQNPRSAFNPLHTMHTHARETCLALGKPADDATLTAAIEAVGLENAARVLKLYPFE

MSGGMLQRMMIAMAVLCESPFIIADEPTTDLDVVAQARILDLLESIMQKQAPGMLLVTHDMGVVARLADD

VAVMSQGKIVEQGDVETLFNAPKHTVTRSLVSAHLALYGMELAS

>gi|30065243|ref|NP_839414.1| nickel transporter ATP-binding protein NikE [Shigella flexneri 2a str. 2457T]

MTLLNVCGLSHHYAHGGFSGKHQHQAVLNNVSLTLKSGETVALLGRSGCGKSTLARLLVGLESPSQGNIS

WHGESLAKLNRAQRKAFRRDIQMVFQDSISAVNPRKTVREILREPMRHLLSLKKSEQLARASEMLKAVDL

DDSVLDKRPPQLSGGQLQLVCLARALAVEPKLLILDEAVSNLDLVLQAGVIRLLKKLQQQFGTACLFITH

DLRLVERFCQRVMVMDNGQIAETQAVGDKLTFSSDAGRVLQNAVLPAFPVRRRTSEKV

>gi|30065242|ref|NP_839413.1| nickel responsive regulator [Shigella flexneri 2a str. 2457T]

MQRVTITLDDDLLETLDSLSQRRGYNNRSEAIRDILRSALAQEATQQHGTQGFAVLSYVYEHEKRDLASR

IVSTQHHHHDLSVATLHVHINHDDCLEIAVLKGDMGDVQHFADDVIAQRGVRHGHLQCLPKED

>gi|30065241|ref|NP_839412.1| hypothetical protein S4263 [Shigella flexneri 2a str. 2457T]

MIKLKTPNSMEIAEQPAVITYVPELNAFRGKFLGLSGYCDFVSDSIQGLQKEGELSLREYLEDCKAAGIE

PYARTEKIKTFTLRYPESLSERLNNAAAQQQVSVNTYIIETLNERLNHL

>gi|30065240|ref|NP_839411.1| transporter [Shigella flexneri 2a str. 2457T]

MRHLRNIFNLGIKELRSLLGDKAMLTLIVFSFTVSVYSSATVTPGSLNLAPIAIADMDQSQLSNRIVNSF

YRPWFLPPEMITADEMDAGLDAGRYTFAINIPPNFQRDVLAGRQPDIQVNVDATRMSQAFTGNGYIQNII

NGEVNSFVARYRDNSEPLVSLETRMRFNPNLDPAWFGGVMAIINNITMLAIVLTGSALIREREHGTVEHL

LVMPITPFEIMMAKIWSMGLVVLVVSGLSLVLMVKGVLGVPIEGSIPLFMLGVALSLFATTSIGIFMGTI

ARSMPQLGLLVILVLLPLQMLSGGSTPRESMPQMVQDIMLTMPTTHFVSLAQAILYRGAGFEIVWPQFLT

LMAIGGAFFTIALLRFRKTIGTMA

>gi|30065239|ref|NP_839410.1| ATP-binding component of a transport system [Shigella flexneri 2a str. 2457T]

MTHLELVPVPPVAQLAGVSQHYGKTVALNNITLDIPARCMVGLIGPDGVGKSSLLSLISGARVIEQGNVV

VLGGDMHDPKHRRDVCPRIAWMPQGLGKNLYHTLSVYENVDFFARLFGHDKAEREVRINELLTSTGLAPF

RDRPAGKLSGGMKQKLGLCCALIHDPELLILDEPTTGVDPLSRAQFWDLIDSIRQRQSNMSVLVATAYME

EAERFDWLVAMNAGEVLATGSAEELRQQTQSATLEEAFINLLPQAQRQAHQAVVIPPYQPENAEIAIEAR

DLTMRFGSFVAVDHVNFRIPRGEIFGFLGSNGCGKSTTMKMLTGLLPASEGEAWLFGQPVDPKDIDTRRR

VGYMSQAFSLYNELTVRQNLELHARLFHIPEAEIPARVAEMSERFKLNDVEDVLPESLPLGIRQRLSLAV

AVIHRPEMLILDEPTSGVDPVARDMFWQLMVDLSRQDKVTIFISTHFMNEAERCDRISLMHAGKVLASGT

PQELVEKRGAASLEEAFIAYLQEAAGQSNEAEAPPVVHDTTHAPRQGFSLRRLFSYSRREALELRRDPVR

STLALMGTVILMLIMGYGISMDVENLRFAVLDRDQTVSSQAWTLNLSGSRYFIEQPPLTSYDELDRRMRA

GDITVAIEIPPNFGRDIARGTPVELGVWIDGAMPSRAETVKGYVQAMHQSWLQDVASRQSTPASQSGLMN

IETRYRYNPDVKSLPAIVPAVIPLLLMMIPSMLSALSVVREKELGSIINLYVTPTTRSEFLLGKQLPYIA
LGMLNFFLLCGLSVFVFGVPHKGSFLTLTLAALLYIIIATGMGLLISTFMKSQIAAIFGTAIITLIPATQ
FSGMIDPVASLEGPGRWIGEVYPTSHFLTIARGTFSKALDLTDLWQLFIPLLIAIPLVMGLSILLLKKQE
G

>gi|30065238|ref|NP_839409.1| hypothetical protein S4260 [Shigella flexneri 2a str. 2457T]
MDKSKRHLAWWVVGALAVAAVVAWWLLRPAGVPEGFAVSNGRIEATEVDIASKIAGRIDTILVKEGQFVR
EGEVLAKMDTRVLQEQRLEAIAQIKEAQSAVAAAQALLEQRQSETRAAQSLVNQRQAELDSVAKRHTRSR
SLAQRGAISAQQLDDDRAAAESARAALESAKAQVSASKAAIEAARTNIIQAQTRVEAAQATERRIAADID
DSELKAPRDGRVQYRVAEPGEVLAAGGRVLNMVDLSDVYMTFFLPTEQAGTLKLGGEARLILDAAPDLRI
PATISFVASVAQFTPKTVETSDERLKLMFRVKARIPPELLQQHLEYVKTGLPGVAWVRVNEELPWPDDLV
VRLPQ

>gi|30065237|ref|NP_839408.1| hypothetical protein S4259 [Shigella flexneri 2a str. 2457T]
MKIGTVAGTNGSTTTIATNDMVQEHVTNFTKELFGYIANGIGDDISSIARTMLGEVVEKIDDWQIERFQQ
SIQDDKISFTIQTNHSEKYSMLSGMRAHILRRNNCYQFIVTINSKNYGCPLDNTDINWCSIVYLLNNMTV
NDNANDVAVTESYKPIWNWEISQYNVFDIKFETIIKPQFADRTYFSNCSPVDPTSTRPTYFGDTDGSVGA
VLYALFATGHLGIMAEGENFLSQLLNIEDEVLNVLLRENFNEQLDTNVNTIISILNRRDNVLESLQPYLV
INKDAVTPCTFLGDQTGDRFSNICGDQFIIDLLKRIMSINENVHVLAGNHETNCNGNYMQNFTRMKPLDE
DTYDGIKDYPVCFYDPKYKIMANHHGITFDDQRKRYIIGPITVSIDEMTNALDPVELAEIINKKHHAIIN
GKKFKTSRAISCRSFNRYFSVSTDYRPKLEALLACSQMLGINQVVAHNGNGGRERIGETGTVLGLNARDS
KHAGRMFSMHNCQINPGAGPEITTPWKSYQHEKNRNGLMPLIRRRTMLQL

>gi|30065236|ref|NP_839407.1| hypothetical protein S4838 [Shigella flexneri 2a str. 2457T]
MTKLMQFVQRCYYMTNKKMYFILILVFTLLQVCFFALWKARDGSTTSLECTSTLTRNAKTDHSLYYSANL

SVILKKDGSGSFTIVGLTDEDTPRKFSHSYFFTYKIDSNGRISGNAKAKVSGLENQIKDENFRLNFLDAS

LTGKGNARLSKFNNVYIFSIPGLIINTCAPI

>gi|30065235|ref|NP_839406.1| hypothetical protein S4837 [Shigella flexneri 2a str. 2457T]

MERRCFFIINDAIIFDPNKCSLSNVNTQDEINFPATAGCILAVLLESNGGAVDRNSIIDSVLERFSFDLS

NNTLNQYISLLRKSFKNIGVEENVILTIPKVGFYISENITISKQSYDDSHLQEDVVEKVERKRSIPLFRI

LISLVVVIVIIEMFAVAFKDTYSITDYPLVKSGKIDTCDLYLSENLNEIGLAKSQIEAAEISKKHLPCVP

GANFIYDVNSMHYLLGEGQRYLSRCIKSSNDETYAICSEVLLYD

>gi|30065234|ref|NP_839405.1| hypothetical protein S4836 [Shigella flexneri 2a str. 2457T]

MLSKITQELLKMNNVLLMRPDDSEFMIPDDWSGLNAKGITYEQDNRVTESFLPFKSFYCSPVCTTVELIS

QNNCFAMGIRYLLEKKRDLRLNHFKAFSHKCETKPDVLIMTGCTVADTYSIFSRIVEENIMKTMTPIVII

TGHHSEDLCSLASKLGGVKIISPTEKIAKIENAINDAFNSKPKKIRFLTDILSPQQIETMKMVAQGRSVN

EISAAMNISIKTVYAHKVRAINKLGIKKKVYEALFYKALIQFN

>gi|30065233|ref|NP_839404.1| hypothetical protein S4255 [Shigella flexneri 2a str. 2457T]

MKKTLLAIMLAGTAFASQAGTLVSQGTEASANLTLTKPIVVNNTIQPVKGVYSGTLTAWTPLATGIVGAS

DGQSHDYAVTFPDDIYAESSTSADAVISGDNNPDHKLKVSLTTLEQDPPSAASEEIGGKRYMMLKNTGTG

GAYRVVSHMKEQVVEPDSYTIRTQAYIYAE

>gi|30065230|ref|NP_839401.1| fimbrial protein remnant [Shigella flexneri 2a str. 2457T]

MFYRLSLILIMALLAGQLSAQEWSFDSSQLEGNVSADTVAMFNQGEQLPGNYRVEIYLNGEKVDVGEFPF

HRPESPEEKELVPCLTVDDLIHYGIKIDKSSSDTDNKKNQCFKWNSIEGLKVNYDFDSQRVQITVPQLYL

QDKKSSLAPVSLWNEGVAAFRMVYQTNIDISKQNDNQSTTRNSRYGRFTPGFNLGAWRFRSSVTWSKELG

QSERWQRGYMWFERGINAIKSRLTLGESYTSSEVFDSIPFRGGMLATDDAMTPPEDSYYTPVVHGIAQSE

AQVIIKQNGQIIFTRSVPPGPFALDNLPTLAVGGELDVTVRESNGEEQYFSVPFQTPAIALHEGYFKYSV

MGGNIKKKV

>gi|30065229|ref|NP_839400.1| chaperone [Shigella flexneri 2a str. 2457T]

MLVQSKVFEEDKVTAAPFIVTPPLFRLNPGQQNKMWVVQTAPALQDREKLHWLCATGITPKKSDVWAEKD

KEKIAENVNLNIQVNVHTCIKLITRPSGIEGKMVNASEKLSWKKQGDKIVAVNQSPFYINLSSLQVGGSK

IEKLDYIAPFSQHEFVMPKTSGQDIKWSVITDEGGESRLFISHLQS

>gi|30065228|ref|NP_839399.1| hypothetical protein S4249 [Shigella flexneri 2a str. 2457T]

MLAALADRPRWRGRPLSGLLRLIFVASLLCWCVGISNYAMPGETTADFTHSSSNDQRIRILKGAISLIQQ

RPLTGWGLGSFEATFPQALEDVGVTNIESDTATHPHNELLFVTAEGGIVALFGFLLLSGVWLWPCICRLK

MTNNNAHHQCEKWFIALMGLPLVIHMMTEYPLYTSVPHLMLLLLLFRIGMPENVMNQVRIPKTVRLGILP

VIVLIMTAALVILVAGFDAQRELTRAEADMNEGFIPRLPEHSWKYLTQAERLERDQHLLIANKPGFVRDT

ESMSLFTVWGNKWLAIHNDADVSAAMIFIAHRRGDYLTAEKIRKRAARVFVNDERFTRGGN

>gi|30065227|ref|NP_839398.1| IS600 orfA [Shigella flexneri 2a str. 2457T]

MSRKTQRYSKEFKAEAVRTVLENQLSISEGASRLSLPEGTLGQWVTAARKGLGTPGSRTVAELESEILQL

RKALNEARLERDILKKATAYFAQESLKNR

>gi|30065226|ref|NP_839397.1| IS1 orfB [Shigella flexneri 2a str. 2457T]

MSRQCTHYGRWPQHGFTSLKKLRPQSVTSRIQPGSDVIVCAEMDEHWGYVGAKSRQRWLFYAYDRIRRTV

VAHVFGERTLATLERLLSLLSAFEVVVWMTDGWPLYESRLKGKLHVISKRYTQRIERHNLNLRQHLARLG

RKSLSFSKSVELHDKVIGHYLNIKHYQ

>gi|30065225|ref|NP_839396.1| IS1 orfA [Shigella flexneri 2a str. 2457T]

MAFISIRCPSCSATEGVVRNGKSTAGHQRYLCSHCRKTWQLQFTYTASQPGTHQKIIDMAMNGVGCRASA

RIMGVGLNTVLRHLKNSGRSR

>gi|30065224|ref|NP_839395.1| hypothetical protein S4245 [Shigella flexneri 2a str. 2457T]

MERFDAIIIGAGAAGMFCSALAGQVGRRVLLIDNGKILGRKILMSGGGRCNFTNLYVEPGAYLSQNPHFC

KSALARFTQWDFIDLVNKHGIAWHEKTLGQLFCDDSAQQIVDMLVDECEKGNVTFRLRSEVLSVAKDETG

FTLDLNGMTVGCEKLVIATGGLSMPGLGASPFGYKIAEQFGLNVLPTRAGLVPFTLHKPLLEELQVLAGV

AVPSVITAENGTVFRENLLFTHRGLSGPAVLQISSYWQPGEFVSINLLPDVDLETFLNEQRNAHPNQSLK

NTLAVHLPKRLVERLQQLGQIPDVSLKQLNVCDQQALISTLTDWRVQPNGTEGYRTAEVTLGGVDTNELS

SRTMEARKVPGLYFIGEVMDVTGWLGGYNFQWAWSSAWACAQDLASIVK

>gi|30065223|ref|NP_839394.1| low-affinity phosphate transport protein [Shigella flexneri 2a str. 2457T]

MLHLFAGLDLHTGLLLLLALAFVLFYEAINGFHDTANAVATVIYTRAMRSQLAVVMAAVFNFLGVLLGGL

SVAYAIVHMLPTDLLLNMGSSHGLAMVFSMLLAAIIWNLGTWYFGLPASSSHTLIGAIIGIGLTNALMTG

TSVVDALNIPKVLSIFGSLIVSPIVGLVFAGGLIFLLRRYWSGTKKRARIHLTPAEREKKDGKKKPPFWT

RIALILSAIGVAFSHGANDGQKGIGLVMLVLIGVAPAGFVVNMNATGYEITRTRDAINNVEAYFEQHPAL

LKQATGADQLVPAPEAGATQPAEFHCHPSNTINALNRLKGMLTTDVESYDKLSLDQRSQMRRIMLCVSDT

IDKVVKMPGVSADDQRLLKKLKSDMLSTIEYAPVWIIMAVALALGIGTMIGWRRVATTIGEKIGKKGMTY

AQGMSAQMTAAVSIGLASYTGMPVSTTHVLSSSVAGTMVVDGGGLQRKTVTSILMAWVFTLPAAVLLSGG

LYWFSLQFL

>gi|30065222|ref|NP_839393.1| universal stress protein UspB [Shigella flexneri 2a str. 2457T]

MISTVALFWALCVVCIVNMARYFSSLRALLVVLRNCDPLLYQYVDGGGFFTSHGQPNKQVRLVWYIYAQR

YRDHHDDEFIRRCERVRRQFILTSALCGLVVVSLIALMIWH

>gi|30065221|ref|NP_839392.1| universal stress protein [Shigella flexneri 2a str. 2457T]

MAYKHILIAVDLSPESKVLVEKAVSMARPYNAKVSLIHVDVNYSDLYTGLIDVNLGDMQKRISEETHHAL

TELSTNAGYPITETLSGSGDLGQVLVDAIKKYDMDLVVCGHHQDFWSKLMSSARQLINTVHVDMLIVPLR

DEEE

>gi|30065220|ref|NP_839391.1| methyltransferase [Shigella flexneri 2a str. 2457T]

MKICLIDETGAGDGALSVLAARWGLEHDEDNLMALVLTPEHLELRKRDEPKLGGIFVDFVGGAMAHRRKF

GGGRGEAVAKAVGIKGDYLPDVVDATAGLGRDAFVLASVGCRVRMLERNPVVAALLDDGLARGYADAEIG

GWLQERLQLIHASSLTALTDITPRPQVVYLDPMFPHKQKSALVKKEMRVFQSLVGPDLDADGLLEPARLL

ATKRVVVKRPDYAPPLANVATPNAVVTKGHRFDIYAGTPV

>gi|30065219|ref|NP_839390.1| oligopeptidase A [Shigella flexneri 2a str. 2457T]

MTNPLLTPFELPPFSKILPEHVVPAVTKALNDCRENVERVVAQGAPYTWENLCQPLAEVDDVLGRIFSPV

SHLNSVKNSPELREAYEQTLPLLSEYSTWVGQHEGLYKAYRDLRDGDHYATLNTAQKKAVDNALRDFELS

GIGLPKEKQQRYGEIATRLSELGNQYSNNVLDATMGWTKLVTDEAGLAGMPESALAAAKAQAEAKELEGY

LLTLDIPSYLPVMTYCDNQALREEMYRAYSTRASDQGPNAGKWDNSKVMEEILALRHELAQLLGFENYAF

KSLATKMAENPQQVLDFLTDLAKRARPQGEKELEQLRAFAKAEFGVDELQPWDIAYYSEKQKQHLYSISD

EQLRPYFPENKAVNGLFEVVKRIYGITAKERKDVDVWHPDVRFFELYDENNELRGSFYLDLYARENKRGG

AWMDGCVGQMRKADGSLQKPVAYLTCNFNRPVNGKPALFTHDEVITLFHEFGHGLHHMLTRIETAGVSGI

SGVPWDAVELPSQFMENWCWEPEALAFISGHYETGEPLPKELLDKMLAAKNYQAALFILRQLEFGLFDFR

LHAEFRPDQGAKILETLAEIKKLVAVVPSPSWGRFPHAFSHIFAGGYAAGYYSYLWADVLAADAFSRFEE

EGIFNRETGQSFLDNILSRGGSEEPMDLFKRFRGREPQLDAMLEHYGIKG

>gi|30065218|ref|NP_839389.1| hypothetical protein S4238 [Shigella flexneri 2a str. 2457T]

MLSYRHSFHAGNHADVLKHTVQSLIIESLKEKDKPFLYLDTHAGAGRYQLGSEHAERTGEYLEGIARIWQ

QDDLPAELEAYINVVKHFNRSGQLRYYPGSPLIARQLLREQDSLQLTELHPSDYPLLRSGFQKDSRARVE

KADGFQQLKAKLPPVSRRGLILIDPPYEMKTDYQAVVSGIAEGYKRFATGTYALWYPVVLRQQIKRMIHD

LEATGIRKILQIELAVLPDSDRRGMTASGMIVINPPWKLEQQMNNVLSWLHSKLVPAGTGHATVSWIVPE

>gi|30065217|ref|NP_839388.1| glutathione reductase [Shigella flexneri 2a str. 2457T]

MTKHYDYIAIGGGSGGIASINRAAMYGQKCALIEAKELGGTCVNVGCVPKKVMWHAAQIREAIHMYGPDY

GFDTTINKFNWETLIASRTAYIDRIHTSYENVLGKNNVDVIKGFARFVDAKTLEVNGETITADHILIATG

GRPSHPDIPGVEYGIDSDGFFALPALPERVAVVGAGYIAVELAGVINGLGAKTHLFVRKHAPLRSFDPMI

SETLVEVMNAEGPQLHTNAIPKAVVKNADGSLTLELEDGRSETVDCLIWAIGREPANDNINLEAAGVKTN

EKGYIVVDKYQNTNIEGIYAVGDNTGAVELTPVAVAAGRRLSERLFNNKPDEHLDYSNIPTVVFSHPPIG

TVGLTEPQAREQYGDDQVKVYKSSFTAMYTAVTTHRQPCRMKLVCVGPEEKIVGIHGIGFGMDEMLQGFA

VALKMGATKKDFDNTVAIHPTAAEEFVTMR

>gi|30065216|ref|NP_839387.1| IS1 orfB [Shigella flexneri 2a str. 2457T]

MSRQCTHYGRWPQHGFTSLKKLRPQSVTSRIQPGSDVIVCAEMDEHWGYVGAKSRQRWLFYAYDRIRRTV

VAHVFGERTLATLERLLSLLSAFEVVVWMTDGWPLYESRLKGKLHVISKRYTQRIERHNLNLRQHLARLG

RKSLSFSKSVELHDKVIGHYLNIKHYQ

>gi|30065215|ref|NP_839386.1| IS1 orfB [Shigella flexneri 2a str. 2457T]

MASISIRCPSCSATEGVVRNGKSTAGHQRYLCSHCRKTWQLQFTYTASQPGTHQKIIDMAMNGVGCRASA

RIMGVGLNTVLRHLKNSGRSR

>gi|30065214|ref|NP_839385.1| arsenate reductase [Shigella flexneri 2a str. 2457T]

MSNITIYHNPACGTSRNTLEMIRNSGTEPTIIHYLETPPTRDELVKLIADMGISVRALLRKNVEPYEELG

LAEDKFTDDRLIDFMLQHPILINRPIVVTPLGTRLCRPSEVVLEILPDAQKGAFSKEDGEKVVDEAGKRL

K

>gi|30065213|ref|NP_839384.1| arsenical pump membrane protein [Shigella flexneri 2a str. 2457T]

MLLAGAIFVLTIVLVIWQPKGLGIGWSATLGAVLALVTGVVHPGDIPVVWNIVWNATAAFIAVIIISLLL

DESGFFEWAALHVSRWGNGRGRLLFTWIVLLGAAVAALFANDGAALILTPIVIAMLLALGFSKGTTLAFV

MAAGFIADTASLPLIVSNLVNIVSADFFGLGFREYASVMVPVDIAAIVATLVMLHLYFRKDIPQNYDMAL

LKSPAEAIKDPATFKTGWVVLLLLLVGFFVLEPLGIPVSAIAAVGALILFVVAKRGHAINTGKVLRGAPW

QIVIFSLGMYLVVYGLRNAGLTEYLSGVLNVLADNGLWAATLGTGFLTAFLSSIMNNMPTVLVGALSIDG

STASGVIKEAMVYANVIGCDLGPKITPIGSLATLLWLHVLSQKNMTISWGYYFRTGIIMTLPVLFVTLAA

LALRLSFTL

>gi|30065212|ref|NP_839383.1| DNA-binding transcriptional repressor ArsR [Shigella flexneri 2a str. 2457T]

MLPIQLFKILADETRLGIVLLLSELGELCVCDLCTALDQSQPKISRHLALLRESGLLLDRKQGKWVHYRL

SPHIPAWAAKIIDEAWRCEQEKIQAIVRNLARQNCSGDSKNICS

>gi|30065211|ref|NP_839382.1| IS1 orfA [Shigella flexneri 2a str. 2457T]

MASISIRCPSCSATEGVVRNGKSTAGHQRYLCSHCRKTWQLQFTYTASQPGTHQKIIDMAMNGVGCRASA

RIMGVGLNTVLRHLKNSGRSR

>gi|30065210|ref|NP_839381.1| IS1 orfB [Shigella flexneri 2a str. 2457T]

MSRQCTHYGRWPQHGFTSLKKLRPQSVTSRIQPGSDVIVCAEMDEHWGYVGAKSRQRWLFYAYDRIRRTV

VAHVFGERTLATLERLLSLLSAFEVVVWMTDGWPLYESRLKGKLHVISKRYTQRIERHNLNLRQHLARLV

RKSLSFSKSVELHDKVIGHYLNIKHYQ

>gi|30065209|ref|NP_839380.1| hypothetical protein S4229 [Shigella flexneri 2a str. 2457T]

MFLAVELSLLFIVISAGVSLIRQKVPDHKIQQMMGARKGRGYLLAALLGAVTPFCSCSTIPMLRGLLSAK

AGFGPTLTFLFVSPLLNPIIVGLMWMTFGWKVTLLYAIIAAGVSVLASIILDSLGFERHIIASKSSSANC

CAPAKTSPGTTYTPIKVSCCSPAAKAIENPVVTCCSTKAVVSINPIKLATKDALQQFKDVLPYLLLGVLI

GSFIYGFIPSAWIAAHAGADNPFAIPLSAVVGIPLYIRAEAVIPLASVLMTKGMGLGALMALIIGSAGAS

LTEVILLKSMFRMPMIAAFLTVILGMAILMGYLTQLLF

>gi|30065208|ref|NP_839379.1| outer membrane protein induced after carbon starvation [Shigella flexneri 2a str. 2457T]

MVLIFVDKDSNMNMTKGALILSLSFLLAACSSIPQNIKGNNQPDIQKSFVAVHNQPGLYVGQQARFGGKV

INVINGKTDTLLEIAVLPLDSYAKPDIEANYQGRLLARQSGFLDPVNYRNHFVTILGTIQGEQPGFINKV

PYNFLEVNMQGIQVWHLREVVNTTYNLWDYGYGAFWPEPGWGAPYYTNAVSQVTPELVK

>gi|30065207|ref|NP_839378.1| hypothetical protein S4226 [Shigella flexneri 2a str. 2457T]

MFLIITRDTMFFTAMKNILSKGNVVHIQNEEEIDVMLHQNAFVIIDTLMNNVFHSNFLTQIERLKPVHVI

VFSPFNIKRCLGKVPVTFVPRTITIIDFVALINGSYCSVPEANVSLSRKQHQVLSCIANQMTTEDILEKL

KISLKTFYCHKHNIMMILNLKRINELVRHQHIDYLV

>gi|30065206|ref|NP_839377.1| acid-resistance protein [Shigella flexneri 2a str. 2457T]

MGYKMNISSLRKAFIFMGAVAALSLVNAQSALAANESAKDMTCQEFIDLNPKAMTPVAWWMLHEETVYKG

GDTVTLNETDLTQIPKVIEYCKKNPQKNLYTFKNQASNDLPN

>gi|30065205|ref|NP_839376.1| acid-resistance protein [Shigella flexneri 2a str. 2457T]

MKKVLGVILGGLLLLPVVSNAADAQKAADNKKPVNSWTCEDFLAVDESFQPTAVGFAEALNNKDKPEDAV

LDVQGIATVTPAIVQACTQDKQANFKDKVKGEWDKIKKDM

>gi|30065204|ref|NP_839375.1| acid-resistance membrane protein [Shigella flexneri 2a str. 2457T]

MLYIDKATILKFDLEMLKKHRRAIQFIAVLLFIVGLLCISFPFVSGDILSTVVGALLICSGIALIVGLFS

NRSHNFWPVLSGFLVAVAYLLIGYFFIRAPELGIFAIAAFIAGLFCVAGVIRLMSWYRQRSMKGSWLQLV

IGVLDIVIAWIFLGATPMVSVTLVSTLVGIELIFSAASLFSFASLFVKQQ

>gi|30065203|ref|NP_839374.1| hypothetical protein S4221 [Shigella flexneri 2a str. 2457T]

MIFLMTKDSFLLQGFWQLKDNHEMIKINSLSEIKKVGNKPFKVIIDTYHNHILDEEAIKFLEKLDAERII

VLAPYHISKLKAKAPIYFVSRKESIKNLLEITYGKHLPHKNSQLCFSHNQFKIMQLILKNKNESNITSTL

NISQQTLKIQKFNIMYKLKLRRMSDIVTLGITSYF

>gi|30065202|ref|NP_839373.1| IS1 orfB [Shigella flexneri 2a str. 2457T]

MSRQCTHYGRWPQHGFTSLKKLRPQSVTSRIQPGSDVIVCAEMDEHWGYVGAKSRQRWLFYAYDRIRRTV

VAHVFGERTLATLERLLSLLSAFEVVVWMTDGWPLYESRLKGKLHVISKRYTQRIERHNLNLRQHLARLV

RKSLSFSKSVELHDKVIGHYLNIKHYQ

>gi|30065201|ref|NP_839372.1| IS1 orfA [Shigella flexneri 2a str. 2457T]

MASISIRCPSCSATEGVVRNGKSTAGHQRYLCSHCRKTWQLQFTYTASQPGTHQKIIDMAMNGVGCRASA

RIMGVGLNTVLRHLKNSGRSR

>gi|30065200|ref|NP_839371.1| trehalase [Shigella flexneri 2a str. 2457T]

MLNQKIQNPNPDELMIEVDLCYELDPYELKLDEMIEAEPEPEMIEGLPASDALTPADRYLELFEHVQSAK

IFPDSKTFPDCAPKMDPLDILIRYRKVRRHRDFDLRKFVENHFWLPEVYSSEYVSDPQNSLKEHIDQLWP

VLTREPQDHIPWSSLLALPQSYIVPGGRFSETYYWDSYFTMLGLAESGREDLLKCMADNFAWMIENYGHI

PNGNRTYYLSRSQPPVFALMVELFEEDGVRGARRYLDHLKMEYAFWMDGAESLIPNQAYRHVVRMPDGSL

LNRYWDDRDTPRDESWLEDVETAKHSGRPPNEVYRDLRAGAASGWDYSSRWLRDTGRLASIRTTQFIPID

LNAFLFKLESAIANISALKGEKETEALFRQKASARRDAVNRYLWDDENGIYRDYDWRREQLALFSAAAIV

PLYVGMANHEQADRLANAVRSRLLTPGGILASEYETGEQWDKPNGWAPLQWMAIQGFKMYGDDLLGDEIA
RSWLKTVNQFYLEQHKMIEKYHIADGVPREGGGGEYPLQDGFGWTNGVVRRLIGLYGEP

>gi|30065199|ref|NP_839370.1| IS4 orf [Shigella flexneri 2a str. 2457T]
MHIGQALDLVSRYDSLRNPLTSLGDYLDPELISRCLAESGTVTLRKRRLPLEMMVWCIVGMALERKEPLH
QIVNRLDIMLPGNRPFVAPSAVIQARQRLGSEAVRRVFTKTAQLWHNATPHPHWCGLTLLAIDGVFWRTP
DTPENDAAFPRQTHAGNPALYPQVKMVCQMELTSHLLTAAAFGTMKNSENELAEQLIEQTGDNTLTLMDK
GYYSLGLLNGWSLAGEHRHWMIPLRKGAQYEELRKLGKGDHLVKLKTSPQARKKWPGLGNEVTARLLTVT
RKGKVCHLLTSMTDAMRFPGGEMADLYSHRWEIELGYREIKQTMQLSRLTLRSKKPELVEQELWGVLLAY
NLVRYQMIKMAEHLKGYWPNQLSFSESCGMVMRMLMTLQGASPGRIPELMRDLASMGQLVKLPTRRGRAF
PRVVKERPWKYPTAPKKSQSVA

>gi|30065198|ref|NP_839369.1| regulator [Shigella flexneri 2a str. 2457T]
MQIVMFDRQSIFIHGIKISLQQRIPGVSIQGVSQADELWQKLESYPEALVMLDGDQDGEFCYWLLQKTVV
QFPEVKVLITATDCNKRWLQEVIHFNVLAIVPRDSTVETFALAVNSAAMGMMFLPGDWRTTPEKDIKDLK
SLSARQREILTMLAAGESNKEIGRALNISTGTVKAHLESLYRRLEVKNRTQAAMMLNISS

>gi|30065197|ref|NP_839368.1| transcriptional regulator LYSR-type [Shigella flexneri 2a str. 2457T]
MLEKTINNAICALLFRCEQQSVKEMDKIHAMQLFIKVAELESFSRAADFFALPKGSVSRQIQALEHQLGT
QLLQRTTRRVKLTPEGMTYYQRAKDVLSNLSELDGLFQQDATSISGKLRIDIPSGIAKNLLLPRLSEFLY
QHPGIELELSSHNRPVDILHDGFDCVIRTGALPEDGVIARPLGKLTMVNCASPHYLTRFGYPQSPDDLTS
HAIVRYTPHLGVHPLGFEVASVNGVQWFKSGGMLTVNSSENYLAAGLAGLGIIQIPRIAVREALRAGRLI
EVLPGYRAEPLSLSLVYPQRRELSRRVNLFMQWLAGVMKEHLD

>gi|30065196|ref|NP_839367.1| hypothetical protein S4213 [Shigella flexneri 2a str. 2457T]

MTQENEIKRPTQDLEHEPIKQLDNSEKGGKVSQALETVTTTAEKVQRQPVIAHLIRATERFNDRLGNQFG
AAITYFSFLSMIPILMVSFAAGGFVLASHPMLLQDIFDKILQNISDPTLAATLKNTINTAVQQRTTVGLV
GLAVALYSGINWMGNLREAIRAQSRDVWERSPQDQEKFWVKYLRDFISLIGLLIALIVTLSITSVAGSAQ
QMIISALHLNSIEWLKPTWRLIGLAISIFANYLLFFWIFWRLPRHRPRKKALIRGTFLAAIGFEVIKIVM
TYTLPSLMKSPSGAAFGSVLGLMAFFYFFARLTLFCAAWIATAEYKDDPRMPGKTQP

>gi|30065195|ref|NP_839366.1| transporter [Shigella flexneri 2a str. 2457T]
MQATATTLDHEQEYTPINSRNKVLVASLIGTAIEFFDFYIYATAAVIVFPHIFFPQGDPTAATLQSLATF
AIAFVARPIGSAVFGHFGDRVGRKATLVASLLTMGISTVVIGLLPGYATIGIFAPLLLALARFGQGLGLG
GEWGGAALLATENAPPRKRALYGSFPQLGAPIGFFFANGTFLLLSWLLTDEQFMSWGWRVPFIFSAVLVI
IGLYVRVSLHESPVFEKVAKAKKQVKIPLGTLLTKHVRVTVLGTFIMLATYTLFYIMTVYSMTFSTAAAP
VGLGLPRNEVLWMLMMAVIGFGVMVPVAGLLADAFGRRKSMVIITTLIILFALFAFNPLLGSGNPILVFA
FLLLGLSLMGLTFGPMGALLPELFPTEVRYTGASFSYNVSSILGASVAPYIAAWLQANYGLGAVGLYLAA
MAGLTLIALLLTHETRHQSL

>gi|30065194|ref|NP_839365.1| hypothetical protein S4211 [Shigella flexneri 2a str. 2457T]
MSKAGKITAAISGAFLLLIVVAIILIATFDWNRLKPTINQKVSAELNRPFAIRGNLGVVWERQKQETGWR
SWVPWPHVHAEDIILGNPPDIPEVTMVHLPRVEATLAPLALLTKTVWLPWIKLEKPDARLIRLSEKNNNW
TFNLANDDNKDANAKPSAWSFRLDNILFDQGRIAIDDKVSKADLEIFVDPLGKPLPFSEVTGSKGKADKE
KVGDYIFGLKAQGRYNGEPLTGTGKIGGMLALRGEGTPFPVQADFRSGNTRVAFDGVVNDPMKMGGVDLR
LKFSGDSLGDLYELTGVLLPDTPPFETDGRLVAKIDTEKSSVFDYRGFNGRIGDSDIHGSLVYTTGKPRP
KLEGDVESRQLRLADLGPLIGVDSGKGAEKSKRSEQKKGEKSVQPAGKVLPYDRFETDKWDVMDADVRFK
GRRIEHGSSLPISDLSTHIILKNADLRLQPLKFGMAGGSIAANIHLEGDKKPMQGRADIQARRLKLKELM
PDVELMQKTLGEMNGDAELRGSGNSVAALLGNSNGNLKLLMNDGLVSRNLMEIVGLNVGNYIVGAIFGDD
EVRVNCAAANLNIANGVARPQIFAFDTENALINVTGTASFASEQLDLTIDPESKGIRIITLRSPLYVRGT

FKNPQAGVKAGPLIARGAVAAALATLVTPAAALLALISPSEGEANQCRTILSQMKK

>gi|30065193|ref|NP_839364.1| EAL domain-containing protein [Shigella flexneri 2a str. 2457T]

MIRQVIQRISNPEASIESLQERRFWLQCERAYTWQPIYQTCGRLMAVELLTVVTHPLNPSQRLPPDRYFT

EITVSHRMEVVKEQIDLLAQKADFFVEHGLLASVNIDGPTLIALRQQPKILRQIERLPWLRFELVEHIRL

PKDSTFASMCEFGPLWLDDFGTGMANFSALSEVRYDYIKIARELFVMLRQSPEGRTLFSQLLHLMNRYCR

GVIVEGVETPEEWRDVQNSPAFAAQGWFLSRPAPMETLNTAVLAL

>gi|30065192|ref|NP_839363.1| ketodeoxygluconokinase [Shigella flexneri 2a str. 2457T]

MFKSDAARTRQRLSARKGRTLPPGRGKFPHSTTESFRNTFWLKKIMEHCFNMVDQQTTTAQTNANFLQIR

FTTMSKKIAVIGECMIELSEKGADVKRGFGGDTLNTSVYIARQVDPAALTVHYVTALGTDSFSQQMLDAW

HGENVDTSLTQRMENRLPGLYYIETDSTGERTFYYWRNEAAAKFWLASEQSAAICEELANFDYLYLSGIS

LAILSPTSREKLLSLLRECRAKGGKVIFDNNYRPRLWASKEETQQVYQQMLECTDIAFLTLDDEDALWGQ

QPVEDVIARTHNAGVKEVVVKRGADSCLVSIAGEALVDVPAVKLPKEKVIDTTAAGDSFSAGYLAVRLTG

GSAENAAKRGHLTASTVIQYRGAIIPREAMPA

>gi|30065191|ref|NP_839362.1| hypothetical protein S4208 [Shigella flexneri 2a str. 2457T]

MQGIKIRLLAGGLLMMATAGYVQADALQPDPAWQQGTLSNGLQWQVLTTPQRPSDRVEIRLLVNTGSLAE

STQQSGYSHAIPRIALTQSGGLDAAQARSLWQQGIDPKRPMPPVIVSYDTTLFNLSLPNNRNDLLKEALS

YLANATGKLTITPETINHALQSQDMVATWPADTKEGWWRYRLKGSTLLGHDPADPLKQPVEAEKIKDFYQ

KWYTPDAMTLLVVGNVDARSVVDQINKTFGELKGKRETPAPVPTLSPLRAEAVSIMTDAVRQDRLSIMWD

TPWQPIRESAALLRYWRADLAREALFWHVQQALSASDSKDIGLGFDCRVLYLRAQCAINIESPNDKLNSN

LNLVARELAKVRDKGLPEEEFNALVAQKKLELQKLFAAYARADTDILMGQRMRSLQNQVVDIAPEQYQKL

RQDFLNSLTVEMLNQDLRQQLSNDMALILLQPKGEPEFNMKALQAAWDQIMAPSTAAATTSVATDDVHPE

VTDIPPAQ

>gi|30065190|ref|NP_839361.1| C4-dicarboxylate transporter DctA [Shigella flexneri 2a str. 2457T]

MKTSLFKSLYFQVLTAIAIGILLGHFYPEIGEQMKPLGDGFVKLIKMIIAPVIFCTVVTGIAGMESMKAV

GRTGAVALLYFEIVSTIALIIGLIIVNVVQPGAGMNVDPATLDAKAVAVYADQAKDQGIVAFIMDVIPAS

VIGAFASGNILQVLLFAVLFGFALHRLGSKGQLIFNVIESFSQVIFGIINMIMRLAPIGALGAMAFTIGK

YGVGTLVQLGQLIICFYITCILFVVLVLGSIAKATGFSIFKFIRYIREELLIVLGTSSSESALPRMLDKM

EKLGCRKSVVGLVIPTGYSFNLDGTSIYLTMAAVFIAQATNSQMDIVHQITLLIVLLLSSKGAAGVTGSG

FIVLAATLSAVGHLPVAGLALILGIDRFMSEARALTNLVGNGVATIVVAKWVKELDHKKLDDVLNNRAPD

GKTHELSS

>gi|30065188|ref|NP_839359.1| endo-1,4-D-glucanase [Shigella flexneri 2a str. 2457T]

MNVLRSGIVTMLLLAAFSVQAACTWPAWEQFKKDYISQEGRVIDPSDARKITTSEGQSYGMFFALAANDR

AAFDNILDWTQNNLAQGSLKERLPAWLWGKKENSKWEVLDSNSASDGDVWMAWSLLEAGRLWKEQRYTDI

GSALLKRIAREEVVTVPGLGSMLLPGKVGFAEDNSWRFNPSYMPPTLAQYFTRFGAPWTTLRETNQHLLL

ETAPKGFSPDWVRYEKDKGWQLKAEKTLISSYDAIRVYMWVGMMPDSDPQKARMLNRFKPMATFTEKNGY

PPEKVDVATGKAQGKGPVGFSAAMLPFLQNRDAQAVQRQRVADNFPGSDTYYNYVLTLFGQGWDQHRFRF

STKGELLPDWGQECANSH

>gi|30065187|ref|NP_839358.1| cellulose synthase regulator protein [Shigella flexneri 2a str. 2457T]

MAMGMSAFPSFMTQATPATQPLINAEPAVAAQTEQNPQVGQVMSGEQGADAPIVAQNGPSRDVKLTFAQI

APPPGSMVLRGINPNGSIEFGMRSDEVVTKAMLNFEYTPSPSLLPVQSQLKVYLNDELMGVLPVTKEQLG

KKTLAQMPINPLFITDFNRVRLEFVGHYQDVCENPASTTLWLDVGRSSGLDLTYQTLNVKNDLSHFPVPF

FDPRDNRTNTLPMVFAGAPDVGLQQASAIVASWFGSRSGWRGQNFPVLYNQLPDRNAIVFATNDKRPDFL

RDHPAVKAPVIEMINHPQNPYVKLLVVFGRDDKDLLQAAKGIAQGNILFRGESVVVNEVKPLLPRKPYDA

PNWVRTDRPVTFGELKTYEEQLQSSGLEPAAINVSLNLPPDLYLMRSTGIDMDINYRYTMPPVKDSSRMD

ISLNNQFLQSFNLSSKQEANRLLLRIPVLQGLLDGKTDVSIPALKLGATNQLRFDFEYMNPMPGGSVDNC
ITFQPVQNHVVIGDDSTIDFSKYYHFIPMPDLRAFANAGFPFSRMADLSQTITVMPKAPNEAQMETLLNT
VGFIGAQTGFPAINLTVTDDGSTIQGKDADIMIIGGIPDKLKDDKQIDLLVQATESWVKTPMRQTPFPGI
VPDESDRAAETQSTLTSFGAMAAVIGFQSPYNDQRSVIALLADSPRGYEMLNDAVNDSGKRATMFGSVAV
IRESGINSLRVGDVYYVGHLPWFERLWYALANHPILLAVLATISVILLAWVLWRLLRIISRRRLNPDNE

>gi|30065186|ref|NP_839357.1| cell division protein [Shigella flexneri 2a str. 2457T]
MAVLGLQGVRGGVGTTTITAALAWSLQMLGENVLVVDACPDNLLRLSFNVDFTHRQGWARAMLDGQDWRD
AGLRYTSQLDLLPFGQLSIEEQENPQHWQTRLSDICSGLQQLKASGRYQWILIDLPRDASQITHQLLSLC
EHSLAIVNVDANCHIRLHQQALPDGAHILINDFRIGSQVQDDIYQLWLQSQRRLLPMLIHRDEAMAECLA
AKQPVGEYRSDALAAEEILTLANWCLLNYSGLKTPVGSAS

>gi|30065185|ref|NP_839356.1| hypothetical protein S4200 [Shigella flexneri 2a str. 2457T]
MNNNEPDTLPDPAIGYIFQNDIVALKQAFSLPDIDYADISQREQLAAALKRWPLLAEFAQQK

>gi|30065184|ref|NP_839355.1| protease [Shigella flexneri 2a str. 2457T]
MRDIVDPVFSIGISSLWDELRHMPAGGVWWFNVDRHEDAISLANQTIASQAETAHVAVISMDSDPAKIFQ
LDDSQGPEKIKLFSMLNHEKGLYYLARDLQCSIDPHNYLFILVCANNAWQNIPAERLRSWLDKMNKWSRL
NHCSLLVINPGNNNDKQFSLLLEEYRSLFGLASLRFQGDQHLLDIAFWCNEKGVSARQQLSVQQQNGIWT
LVQSEEAEIQPRSDEKRILSNVAVLEGAPPLSEHWQLFNNNEVLFNEARTAQAATVVFSLQQNAQIEPLA
RSIHTLRRQRGSAMKILVRENTASLRATDERLLLACGANMVIPWNAPLSRCLTMIESVQGQKFSRYVPED
ITTLLSMTQPLKLRGFQKWDVFCNAVNNMMNNPLLPAHGKGVLVALRPVPGIRVEQALTLCRPNRTGDIM
TIGGNRLVLFLSFCRINDLDTALNHIFPLPTGDIFSNRMVWFEDDQISAELVQMRLLAPEQWGMPLPLTQ
SSKPVINAEHDGRHWRRIPEPMRLLDDAVERSS

>gi|30065183|ref|NP_839354.1| hypothetical protein S4198 [Shigella flexneri 2a str. 2457T]

MMTISDIIEIIIVCALIFFPLGYLARHSLRRIRDTLRLFFAKPRYVKPAGTLRRTEKARATKK

>gi|30065182|ref|NP_839353.1| hypothetical protein S4197 [Shigella flexneri 2a str. 2457T]

MTQFTQNTAMPSSLWQYWRGLSGWNFYFLVKFGLLWAGYLNFHPLLNLVFAAFLLMPIPRYSLHRLRHWI

ALPIGFALFWHDIWLPGPESIMSQGSQVAGFSTDYLIDLVTRFINWQMIGAIFVLLVAWLFLSQWIRITV

FVVAILLWLNVLTLAGPSFSLWPAGQPTTTVTTTGGNAAATVAATGGAPVVGDMPAQTAPPTTANLNAWL

NNFYNAEAKRKSTFPSSLPADAQPFELLVINICSLSWSDIEAAGLMSHPLWSHFDIDFKNFNSATSYSGP

AAIRLLRASCGQTSHTNLYQPANNDCYLFDNLSKLGFTQHLMMGHNGQFGGFLKEVRENGGMQTELMDQT

NLPVILLGFDGSPVYDDTAVLNRWLDVTEKDKNSRSATFYNTLPLHDGNHYPGVSKTADYKARAQKFFDE

LDAFFTELEKSGRKVMVVVVPEHGGALKGDRMQVSGLRDIPSPSITDVPVGVKFFGMKAPHQGAPIVIDQ

PSSFLAISDLVVRVLDGKIFTEDNVDWKKLTSGLPQTAPVSENSNAVVIQYQDKPYVRLNGGDWVPYPQ

>gi|30065181|ref|NP_839352.1| transporter protein [Shigella flexneri 2a str. 2457T]

MQHNTLSKHNQKLPFTRYDFGWVLLCIGMAIGAGTVLMPVQIGLKGIWVFITAAIIAYPVTWVVQDIYLK

TLSESDSCNDYTDIISHYLGKNWGIFLGVIYFLMIIHGIFIYSLSVVFDSASYLKTFGLTDADLSQSLLY

KVAIFAVLIAIASGGERLLFKISGPMVVVKVGIIVVFGFAMIPHWNFANITAFPQASVFFRDVLLTIPFC

FFSAVFIQVLNPMNIAYRKREADKVLATRLALRTHRISYITLIAVILFFAFSFTFSISHEEAVSAFEQNI

SALALAAQVIPGHIIHITSTVLNIFAVLTAFFGIYLGFHEAIKGIILNLLSRIIDTKKINSRMLTLAICA

FIVITLTIWVSFRVSVLVFFQLGSPLYGIVSCLIPFFLIYKVAQLEKLRGFKAWLILLYGILLCLSPLLK

LIE

>gi|30065180|ref|NP_839351.1| dipeptide transporter ATP-binding subunit [Shigella flexneri 2a str. 2457T]

MSTQEATSQQPLLQAIDLKKHYPVKKGMFVPERLVKALDGVSFNLERGKTLAVVGESGCGKSTLGRLLTM

IETPTGGELYYQGQDLLKHDPQAQKLRRQKIQIVFQNPYGSLNPRKKVGQILEEPLLINTSLSKEQRREK

ALSMMAKVGLKTEHYDRYPHMFSGGQRQRIAIARGLMLDPDVVIADEPVSALDVSVRAQVLNLMMDLQQE

LGLSYVFISHDLSVVEHIADEVMVMYLGRCVEKGTKDQIFNNPRHPYTQALLSATPRLNPDDRRERIKLT

GELPSPLNPPPGCAFNARCRRRFGPCTQLQPQLKDYGGQLVACFAVDQDENPQR

>gi|30065179|ref|NP_839350.1| dipeptide transporter ATP-binding subunit [Shigella flexneri 2a str. 2457T]

MALLNVDKLSVHFGDESAPFRAVDRISYSVKQGEVVGIVGESGSGKSVSSLAIMGLIDYPGRVMAEKLEF

NGQDLQRISEKERRNLVGAEVAMIFQDPMTSLNPCYTVGFQIMEAIKVHQGGNKSTRRQRAIDLLNQVGI

PDPASRLDVYPHQLSGGMSQRVMIAMAIACRPKLLIADEPTTALDVTIQAQIIELLLELQQKENMALVLI

THDLALVAEAAHKIIVMYAGQVVETGDAHAIFHAPRHPYTQALLRALPEFAQDKERLASLPGVVPGKYDR

PNGCLLNPRCPYATDRCRAEEPALNMLADGRQSKCHYPLDDAGRPTL

>gi|30065178|ref|NP_839349.1| dipeptide transporter [Shigella flexneri 2a str. 2457T]

MSQVTENKVISAPVPMTPLQEFWHYFKRNKGAVVGLVYVAIVLFIAIFANWIAPYNPADQFRDALLAPPA

WQEGGSMAHLLGTDDVGRDVLSRLMYGARLSLLVGCLVVVLSLIMGVILGLIAGYFGGLVDNIIMRVVDI

MLALPSLLLALVLVAIFGPSIGNAALALTFVALPHYVRLTRAAVLVEVNRDYVTASRVAGAGAMRQMFIN

IFPNCLAPLIVQASLGFSNAILDMAALGFLGMGAQPPTPEWGTMLSDVLQFAQSAWWVVTFPGLAILLTV

LAFNLMGDGLRDALDPKLKQ

>gi|30065177|ref|NP_839348.1| dipeptide transporter permease DppB [Shigella flexneri 2a str. 2457T]

MLQFILRRLGLVIPTFIGITLLTFAFVHMIPGDPVMIMAGERGISPERHVQLLAELGLDKPMWQQYLHYI

WGVMHGDLGISMKSRIPVWEEFVPRFQATLELGVCAMIFATAVGIPVGVLAAVKRGSIFDHTAVGLALTG

YSMPIFWWGMMLIMLVSVHWNLTPVSGRVSDMVFLDDSNPLTGFMLIDTAIWGEDGNFIDAVAHMILPAI

VLGTIPLAVIVRMTRSSMLEVLGEDYIRTARAKGLTRMRVIIVHALRNAMLPVVTVIGLQVGTLLAGAIL

TETIFSWPGLGRWLIDALQRRDYPVVQGGVLLVATMIILVNLLVDLLYGVVNPRIRHKK

>gi|30065176|ref|NP_839347.1| dipeptide transport protein [Shigella flexneri 2a str. 2457T]

MRISLKKSGMLKLGLSLVAMTVAASVQAKTLVYCSEGSPEGFNPQLFTSGTTYDASSVPLYNRLVEFKIG

TTEVIPGLAEKWEVSEDGKTYTFHLRKGVKWHDNKEFKPTREMNADDVVFSFDRQKNAQNPYHKVSGGSY

EYFEGMGLPELISEVKKVDDNTVQFVLTRPEAPFLADLAMDFASILSKEYADAMMKAGTPEKLDLNPIGT

GPFQLQQYQKDSRIRYKAFDGYWGTKPQIDTLVFSITPDASVRYAKLQKNECQVMPYPNPADIARMKQDK

SINLMEMPGLNVGYLSYNVQKKPLDDVKVRQALTYAVNKDAIIKAVYQGAGVSAKNLIPPTMWGYNDDVQ

DYTYDPEKAKALLKEAGLEKGFSIDLWAMPVQRPYNPNARRMAEMIQADWAKVGVQAKIVTYEWGEYLKR

AKDGEHQTVMMGWTGDNGDPDNFFATLFSCAASEQGSNYSKWCYKPFEDLIQPARATDDHNKRVELYKQA

QVVMHDQAPALIIAHSTVFEPVRKEVKGYVVDPLGKHHFENVSIE

>gi|30065174|ref|NP_839345.1| resistance protein [Shigella flexneri 2a str. 2457T]

MTPSNYQRTRWLTLIGTIITQFALGSVYTRSLFNGALSAKLDAPVSQVAFSFGLLSLGLAISSSVAGKLQ

ERFGVKRVTVASGILLGLGFFLTAHSNNLMMLWLSAGVLVGLADGAGYLLTLSNCVKWFPERKGLISAFA

IGSYGLGSLGFKFIDTQLLETVGLEKTFVIWGAIALVMIVFGATLMKDAPKQEVKTSNGVVEKDYTLAES

MRKPQYWMLAVMFLTACMSGLYVIGVAKDIAQSLAHLDVVSAANAVTVISIANLSGRLVLGILSDKIARI

RVITIGQVISLVGMAALLFAPLNAVTFFAAIACVAFNFGGTITVFPSLVSEFFGLNNLAKNYGVIYLGFG

IGSIFGSIIASLFGGFYVTFYVIFALLILSLALSTTIRQPEQKMLREAHGSL

>gi|30065173|ref|NP_839344.1| lipase [Shigella flexneri 2a str. 2457T]

MIIKKSGGRWQLSLLASVVISAFFLNTAYAWQQEYIVDTQPGHSTERYTWDSDHQPDYNDILSQRIQSSQ

RALGLEVNLAEETPVDVTSSMSMGWNFPLYEQVTTGPVAALHYDGTTTSMYNEFGDSTTTLTDPLWHASV

SSLGWRVDSRLGDLRPWAQISYNQQFGENIWKAQSGLSRMTATNQNGNWLDVTVGADMLLNQNIAAYAAL

TQAENTTNNSDYLYTMGVSARF

>gi|30065172|ref|NP_839343.1| 3-methyladenine DNA glycosylase [Shigella flexneri 2a str. 2457T]

MERCGWVSQGPLYIAYHDNEWGVPETDSKKLFEMICFEGQQAGLSWITVLKKRENYRAYFHQFDPVKVAA
MQEEDVERLVQDAGIIRHRGKIQAIIGNARAYLQMEQNGEPFPDFVWSFVNHQPQVTQATTLSEIPTSTS
ASDALSKALKKRGFKFVGTTICYSFMQACGLVNDHVVGCCCYLGNKP

>gi|30065171|ref|NP_839342.1| hypothetical protein S4185 [Shigella flexneri 2a str. 2457T]
MIREAQRSELPAILELWLESTTWGHPFIKANYWRDCIPLVRDAYLANAQNWVWEEDGKLLGFVSIMEGRF
LAAMFVAPKAVRRGIGKALMQYVQQRYPHLMLEVYQKNQPAIDFYRAQGFHIVDCAWQDETQLPTWIMSW
PVVQTL

>gi|30065170|ref|NP_839341.1| biotin sulfoxide reductase [Shigella flexneri 2a str. 2457T]
MLVETDGETVFSSRGALATGMENSLQSAVRDQVHSNTRVRFPMVRKGFLASPENPQGIRGQDEFVRVSWD
EALDLIHQQHKRIREAYGPASIFAGSYGWRSNGVLHKASTLLQRYMALAGGYTGHLGDYSTGAAQAIMPY
VVGGSEVYQQQTSWPLVLEHSDVVVLWSANPLNTLKIAWNASDEQGLSYFSALRDSGKKLICIDPMRSET
VDFFGDKMEWVAPHMGTDVALMLGIAHTLVENGWHDEAFLARCTTGYAVFASYLLGESDGIAKNAEWAAE
ICGVGAAKIRELAAIFHQNTTMLMAGWGMQRQQFGEQKHWMIVTLAAMLGQIGTPGGGFGLSYHFANGGN
PTRRSAVLSSMQGSLPGGCDAVDKIPVARIVEALENPGGAYQHNGMNRHFPDIRFIWWAGGANFTHHQDT
NRLIRAWQKPELVVISECFWTAAAKHADIVLPATTSFERNDLTMTGDYSNQHLVPMKQVVPPRYEARNDF
DVFAELSERWEKGGYARFTEGKSQLQWLETFYNVARQRGASQQVELPPFAEFWQANQLIEMPENPDSERF
IRFADFCRDPLAHPLKTASGKIEIFSQRIADYGYPDCPGHPMWLEPDEWQGNAEPEQLQVLSAHPAHRLH
SQLNYSSLRELYAVANREPVTIHPDDAQARGITEGDMVRVWNSRGQILAGAVISEGIKPGVICIHEGAWP
DLDLTADGICKNGAVNVLTKDLPSSRLGNGCAGNTTLAWLEKYNGPELTLTAFEPPASS

>gi|30065168|ref|NP_839339.1| 2-hydroxyacid dehydrogenase [Shigella flexneri 2a str. 2457T]
MKPSVILYKALPDDLLQRLQAHFTVHQVANLSPQTVVQNAAIFAEAEGLLGSNENVDAALLEKMPKLRAT
STISVGYDNFDVDALTARKILLMHTPTVLTETVADTLMALVLSTARRVVEVAERVKAGEWTASIGPDWYG

TDVHHKTLGIVGMGRIGMALAQRVHFGFNMPILYNARRHHKEAEERFNARYCDLDTLLQESDFVCLILPL

TDETHHLFGAEQFAKMKSSAIFINAGRGPVVDENALIAALQKGEIHAAGLDVFEQEPLSVDSPLLSMANV

VAVPHIGSATHETRYGMAACAVDNLIDALQGKVEKNCVNPHVAD

>gi|30065167|ref|NP_839338.1| hypothetical protein S4181 [Shigella flexneri 2a str. 2457T]

MILPGRLRRKGILQACPGLSLSRQTRVCRCALFLGERSKKMATGKSCSRWFAPLAALLMVVSLSGCFDKE

GDQRKAFIDFLQNTVMRSGERLPTLTADQKKQFGPFVSDYAILYGYSQQVNQAMDSGLRPVVDSVNAIRV

PQDYVTQSGPLREMNGSLGVLAQQLQNAKLQADAAHSALKQSDDLKPVFDQAFTKVVTTPADALQPLIPA

AQTFTQQLVMVGDYIAQQGTQVSFVANGIQFPTSQQASEYNKLIAPLPAQHQAFNQAWTTAVTATQ

>gi|30065166|ref|NP_839337.1| hypothetical protein S4180, partial [Shigella flexneri 2a str. 2457T]

MEYKDPMHELLSSLEQIVFKDETQKITLTHRTTSCTEIEQL

>gi|30065165|ref|NP_839336.1| major cold shock protein [Shigella flexneri 2a str. 2457T]

MSGKMTGIVKWFNADKGFGFITPDDGSKDVFVHFSAIQNDGYKSLDEGQKVSFTIESGAKGPAAGNVTSL

>gi|30065164|ref|NP_839335.1| IS150 transposase [Shigella flexneri 2a str. 2457T]

MKVLNELRQFYPLDELLRAAEIPRSTFYYHLKALSKPDKYADVKKRIGEIYHENRGRYGYRRVTLSLHRE

GKQINHKAVQRLMGTLSLKAAIKVKRYRSYRGEVGQTAPNVLQRDFKATRPNEKWVTDVTEFAVNGRKLY

LSPVIDLFNNEVISYSLSERPVMNMVENMLDQAFKKLNPHEHPVLHSDQGWQYRMRRYQNILKEHGIKQS

MSRKGNCLDNAVVECFFGTLKSECFYLDEFSNISELKDAVTEYIEYYNSRRISLKLKGLTPIEYRNQTYM

PRV

>gi|30065163|ref|NP_839334.1| IS1 orfA [Shigella flexneri 2a str. 2457T]

MASISIRCPSCSATEGVVRNGKSTAGHQRYLCSHCRKTWQLQFTYTASQPGTHQKIIDMAMNGVGCRASA

RIMGVGLNTVLRHLKNSGRSR

>gi|30065162|ref|NP_839333.1| IS1 orfB [Shigella flexneri 2a str. 2457T]

MSRQCTHYGRWPQHGFTSLKKLRPQSVTSRIQPGSDVIVCAEMDEHWGYVGAKSRQRWLFYAYDRIRRTV

VAHVFGERTLATLERLLSLLSAFEVVVWMTDGWPLYESRLKGKLHVISKRYTQRIERHNLNLRQHLARLV

RKSLSFSKSVELHDKVIGHYLNIKHYQ

>gi|30065161|ref|NP_839332.1| cytochrome C peroxidase [Shigella flexneri 2a str. 2457T]

MKMVSRITAIGLAGVAICYLGLSGYVWYHDNKRSKQADVQASAVSENNKVLGFLREKGCDYCHTPSAELP

AYYYIPGAKQLMDYDIKLGYKSFNLEAVRAALLADKPVSQSDLNKIEWVMQYETMPPTRYTALHWAGKVS

DEERAEILAWIAKQRAEYYASNDTAPEHRNEPVQPIPQKLPTDAQKVALGFALYHDPRLSADSTISCAHC

HTLNAGGVDGRKTSIGVGGAVGPINAPTVFNSVFNVEQFWDGRAATLQDQAGGPPLNPIEMASKSWDEII

AKLEKDPQLKAQFLEVYPQGFSGENITDAIAEFEKTLITPDSPFDKWLRGDENALTAQQKKGYQLFKDNK

CATCHGGIILGGRSFEPLGLKKDFNFGEITAADIGRMNVTKEERDKLRQKVPGLRNVALTAPYFHRGDVP

TLDGAVKLMLRYQVGKELPQEDVDDIVAFLHSLNGVYTPYMQDKQ

>gi|30065160|ref|NP_839331.1| glutamate decarboxylase [Shigella flexneri 2a str. 2457T]

MDQKLLTDFRSELLDSRFGAKAISTIAESKRFPLHEMRDDVAFQIINDELYLDGNARQNLATFCQTWDDE

NVHKLMDLSINKNWIDKEEYPQSAAIDLRCVNMVADLWHAPAPKNGQAVGTNTIGSSEACMLGGMAMKWR

WRKRMEAAGKPTDKPNLVCGPVQICWHKFARYWDVELREIPMRPGQLFMDPKRMIEACDENTIGVVPTFG

VTYTGNYEFPQPLHDALDKFQADTGIDIDMHIDAASGGFLAPFVAPDIVWDFRLPRVKSISASGHKFGLA

PLGCGWVIWRDEEALPQELVFNVDYLGGQIGTFAINFSRPAGQVIAQYYEFLRLGREGYTKVQNASYQVA

AYLADEIAKQGPYEFICTGRPDEGIPAVCFKLKDGEDPGYTLYDLSERLRLRGWQVPAFTLGGEATDIVV

MRIMCRRGFEMDFAELLLEDYKASLKYLSDHPKLQGIAQQNSFKHT

>gi|30065159|ref|NP_839330.1| DNA-binding transcriptional regulator GadX [Shigella flexneri 2a str. 2457T]

MQSLHGNCLIAYARHKYILTMVNGEYRYFNGGDLVFADASQIRVDKCVENFVLVSRDTLSLFLPMLKEEA

LNLHAHKKISSLLVHHCSRDIPVFQEVAQLSQNKNLRYAEMLRKRALIFALLSVFLEDEHFIPLLLNVLQ

PNMRTRVCTVINNNIAHEWTLARIASELLMSPSLLKKKLREEETSYSQLLTECRMQRALQLIVIHGFSIK

RVAVSCGYHSVSYFIYVFRNYYGMTPTEYQERSAQGLPNRDSAASIVAQGNFYGTNRSAEGIRL

>gi|30065158|ref|NP_839329.1| ARAC-type regulatory protein [Shigella flexneri 2a str. 2457T]

MTHVCSVILIRRSFDIYHEQQKISLHNESILLLEKNLADDFAFCSPDTRRLDIDELTVCHYLQNIRQLPR

NLGLHSKDRLLINQSPPMPLVTAIFDSFNESGVNSPILSNMLYLSCLSMFSHKKELIPLLFNSISTVSGK

VERLISFDIAKRWYLRDIAERMYTSESLIKKKLQDENTCFSKILLASRMSMARRLLELRQIPLHTIAEKC

GYSSTSYFINTFRQYYGVTPHQFAQHSPGTFS

>gi|30065157|ref|NP_839328.1| hypothetical protein S4169 [Shigella flexneri 2a str. 2457T]

MIFKNILNNFARFILLVPQEGVTHNAQGKATALILDKDDVVQLREIEASKAIGDQWVVTSGLQAGDRVIV

SGLQRIRPGIKARAISSSQENASTESKQ

>gi|30065156|ref|NP_839327.1| IS1 orfA [Shigella flexneri 2a str. 2457T]

MASISIRCPSCSATEGVVRNGKSTAGHQRYLCSHCRKTWQLQFTYTASQPGTHQKIIDMAMNGVGCRASA

RIMGVGLNTVLRHLKNSGRSR

>gi|30065155|ref|NP_839326.1| IS1 orfB [Shigella flexneri 2a str. 2457T]

MSCQCTHYGRWPQHGFTSLKKLRPQSVTSRIQPGSDVIVCAEMDEHWGYVGAKSRQRWLFYAYDRIRRTV

VAHVFGERTLATLERLLSLLSAFEVVVWMTDGWPLYESRLKGKLHVISKRYTQRIERHNLNLRQHLARLV

RKSLSFSKSVELHDKVIGHYLNIKHYQ

>gi|30065154|ref|NP_839325.1| glycyl-tRNA synthetase subunit beta [Shigella flexneri 2a str. 2457T]

MSEKTFLVEIGTEELPPKALRSLAESFAANFTAELDNAGLAHGTVQWFAAPRRLALKVANLAEAQPDREI

EKRGPAIAQAFDAEGKPSKAAEGWARGCGITVDQAERLTTDKGEWLLYRAHVKGESTEALLPNMVATSLA

KLPIPKLMRWGASDVHFVRPVHTVTLLLGDKVIPATILGIQSDRVIRGHRFMGEPEFTIDNADQYPEILR

ERGKVIADYEERKAKIKADAEEAARKIGGNADLSESLLEEVASLVEWPVVLTAKFEEKFLAVPAEALVYT

MKGDQKYFPVYANDGKLLPNFIFVANIESKDPQQIISGNEKVVRPRLADAEFFFNTDRKKRLEDNLPRLQ

TVLFQQQLGTLRDKTDRIQALAGWIAEQIGADVNHATRAGLLSKCDLMTNMVFEFTDTQGVMGMHYARHD

GEAEDVAVALNEQYQPRFAGDDLPSNPVACALAIADKMDTLAGIFGIGQHPKGDKDPFALRRAALGVLRI

IVEKNLNLDLQTLTEEAVRLYGDKLTNANVVDDVIDFMLGRFRAWYQDEGYTVDTIQAVLARRPTRPADF

DARMKAVSHFRTLEAAAALAAANKRVSNILAKSDEVLSDRVNASTLKEPEEIKLAMQVVVLRDKLEPYFA

EGRYQDALVELAELREPVDAFFDKVMVMVDDKELRLNRLTMLEKLRELFLRVADISLLQ

>gi|30065153|ref|NP_839324.1| glycyl-tRNA synthetase subunit alpha [Shigella flexneri 2a str. 2457T]

MQKFDTRTFQGLILTLQDYWARQGCTIVQPLDMEVGAGTSHPMTCLRALGPEPMAAAYVQPSRRPTDGRY

GENPNRLQHYYQFQVVIKPSPDNIQELYLGSLKELGMDPTIHDIRFVEDNWENPTLGAWGLGWEVWLNGM

EVTQFTYFQQVGGLECKPVTGEITYGLERLAMYIQGVDSVYDLVWSDGPLGKTTYGDVFHQNEVEQSTYN

FEYADVDFLFTCFEQYEKEAQQLLALENPLPLPAYERILKAAHSFNLLDARKAISVTERQRYILRIRTLT

KAVAEAYYASREALGFPMCNKDK

>gi|30065152|ref|NP_839323.1| hypothetical protein S4164 [Shigella flexneri 2a str. 2457T]

MQPKIYWIDNLRGIACLMVVMIHTTTWYVTNAHSVSPVTWDIANVLNSASRVSVPLFFMISGYLFFGERS

AQPRHFLRIGLCLIFYSAIALLYIALFTSINMELALKNLLQKPVFYHLWFFFAIAVIYLVSPLIQVKNVG

GKMLLVLMVVIGIIANPNTVPQKIDGFEWLPINLYINGDTFYYILYGMLGRAIGMMDTQHKALSWVSAAL

FATGVFIISRGTLYELQWRGNFADTWYLYCGPMVFICAIALLTLVKNTLDTRTIRGLGLISRHSLGIYGF

HALIIHALRTRGIELKNWPILDIIWIFCATLAASLLLSMLVQRIDRNRLVS

>gi|30065151|ref|NP_839322.1| hypothetical protein S4163 [Shigella flexneri 2a str. 2457T]

MDNKISTYSPAFSIVSWIALVGGIVTYLLGLWNAEMQLNEKGYYFAVLVLGLFSAASYQKTVRDKYEGIP

TTSIYYMTCLTVFIISVALLMVGLWNATLLLSEKGFYGLAFFLSLFGAVAVQKNIRDAGINPPKETQVTQ

EEYSE

>gi|30065150|ref|NP_839321.1| hypothetical protein S4162 [Shigella flexneri 2a str. 2457T]

MKTSKTVAKLLFVVGALVYLVGLWISCPLLSGKGYFLGVLMTATFGNYAYLRAEKLGQLDNFFTHICQLV

ALITIGLLFIGVLNAPINAYEMVIYPIAFFVCLFGQMRLFRSV

>gi|30065149|ref|NP_839320.1| xylulokinase [Shigella flexneri 2a str. 2457T]

MYIGIDLGTSGVKVILLNEQGEVVASQTEKLTVSRPHPLWSEQDPEQWWQATDRAMKALGDQHSLQDVKA

LGIAGQMHGATLLDAQQRVLRPAILWNDGRCAQECTLLEARVPQSRVITGNLMMPGFTAPKLLWVQRHEP

EIFRQIDKVLLPKDYLRLRMTGEFASDMSDAAGTMWLDVAKRDWSDVMLQACDLSRDQMPALYEGSEITG

ALLPEVAKAWGMATVPVVAGGGDNAAGAVGVGMVDANQAMLSLGTSGVYFAVSEGFLSKPESAVHSFCHA

LPQRWHLMSVMLSAASCLDWAAKLTGLCNVPALIAAAQQADESAEPVWFLPYLSGERTPHNNPQAKGVFF

GLTHQHGPNELARAVLEGVGYALADGMDVVHACGIKPQSVTLIGGGARSEYWRQMLADISGQQLDYRTGG

DVGPALGAARLAQIAANPEKSLIELLPQLPLEQSHLPDAQRYAAYQPRRETFRRLYQQLLPLMA

>gi|30065148|ref|NP_839319.1| D-xylose transporter subunit XylF [Shigella flexneri 2a str. 2457T]

MKIKNILLTLCTSLLLTNVAAHAKEVKIGMAIDDLRLERWQKDRDIFVKKAESLGAKVFVQSANGNEETQ

MSQIENMINRGVDVLVIIPYNGQVLSNVVKKAKQEGIKVLAYDRMINDADIDFYISFDNEKVGELQAKAL

VDIVPQGNYFLMGGSPVDNNAKLFRAGQMKVLKPYVDSGKIKVVGDQWVDGWLPENALKIMENALTANNN

KIDAVVASNDATAGGAIQALSAQGLSGKVAISGQDADLAGIKRIAAGTQTMTVYKPITLLANTAAEIAVE

LGNGQEPKADTSLNNGLKDVPSRLLTPIDVNKNNIKDTVIKDGFHKESEL

>gi|30065147|ref|NP_839318.1| xylose transporter ATP-binding subunit [Shigella flexneri 2a str. 2457T]

MPYLLEMKNITKTFGSVKAIDNVSLRLNAGEIVSLCGENGSGKSTLMKVLCGIYPHGSYEGEIIFAGEEI

QASHIRDTERKGIAIIHQELALVKELTVLENIFLGNEITHNGIMDYDLMTLRYQKLLAQVSLSISPDTRV

GDLGLGQQQLVEIAKALNKQVRLLILDEPTASLTEQETSVLLDIIRDLQQHGIACIYISHKLNEVKAISD

TICVIRDGQHIGTRDAAGMSEDDIITMMVERELTAIYPNEPHTTGDEILRIEHLTAWHPVNRHIKRVNDV

SFSLKRGEILGIAGLVGAGRTETIQCLFGVWPGQWEGKIYIDGKQVDIRNCQQAITQGIAMVPEDRKRDG

IVPVMAVGKNITLAALNKFTGGISQLDDAAEQKCILESIQQLKVKTSSPDLAIGRLSGGNQQKAILARCL

LLNPRILILDEPTRGIDIGAKYEIYKLINQLVQQGIAVIVISSELPEVLGLSDRVLVMHEGKLKANLINH

NLTQEQVMEAALRSEHHVEKQSV

>gi|30065146|ref|NP_839317.1| xylose transporter membrane component [Shigella flexneri 2a str. 2457T]

MSKSNPSEVKLAVPTSGGFSGLKSLNLQVFVMIAAIIAIMLFFTWTTDGAYLSARNVSNLLRQTAITGIL

AVGMVFVIISAEIDLSVGSMMGLLGGVAAICDVWLGWPLPLTIIVTLVLGLLLGAWNGWWVAYRKVPSFI

VTLAGMLAFRGILIGITNGTTVSPTSAAMSQIGQSYLPASTGFIIGALGLMAFVGWQWRGRMRRQALGLQ

SPASTAVVGRQALTAIIVLGAIWLLNDYRGVPTPVLLLTLLLLGGMFMATRTAFGRRIYAIGGNLEAARL

SGINVERTKLAVFAINGLMVAIAGLILSSRLGAGSPSAGNIAELDAIAACVIGGTSLAGGVGSVAGAVMG

AFIMASLDNGMSMMDVPTFWQYIVKGAILLLAVWMDSATKRRS

>gi|30065145|ref|NP_839316.1| hypothetical protein S4155 [Shigella flexneri 2a str. 2457T]

MILTPIRRYGAMILMLLTLVFSSEVLAKTHTTTASQKSHLTKASNKQVSSKQEYSRNSAKSSSLPDLRKY

PSGTPRKKAFLRTVMPYITSQNAAITAERNWLISKQYQGQWAPAERARLKDIAKRYKVKWSGNTRKIPWN

TLLERVDIIPTSMVATMAAAESGWGTSKLARNNNNLFGMKCMKGRCTNAPGKVKGYSQFSSVKESVSAYV

TNLNTHPAYSSFRKSRAQLRKADQEVTATAMIHKLKGYSTKGKSYNNYLFAMYQDNQRLIAAHM

>gi|30065144|ref|NP_839315.1| periplasmic alpha-amylase precursor [Shigella flexneri 2a str. 2457T]

MKLAACFLTLLPGFAVAASWTSPGFPAFSEQGTGTFVSHAQLPKGTRPLTLNFDQQCWQPADAIKLNQML

SLQPCSNTPPQWRLFRDGKYTLQIDTRSGTPTLMISIQNAAEPVANLVRECPKWDGLPLTLDVSATFPEG

AAVRDYYSQQIAIVKNGQITLQPAATSNGLLLLERAETDTSAPFDWHNATVYFVLTDRFENGDPSNDQSY

GRHKDGMAEIGTFHGGDLRGLTNKLDYLQQLGVNALWISAPFEQIHGWVGGGTKGDFPHYAYHGYYTQDW

TNLDANMGNEADLRTLVDSAHQRGIRILFDVVMNHTSYATLADMQEYQFGALYLSGDEVKKTLGERWSDW

KPAAGQTWHSFNDYINFSDKTGWDKWWGKNWIRTDIGDYDNPGFDDLTMSLAFLPDIKTESTTASGLPVF

YKNKTDTHAKVIEGFTPRDYLTHWLSQWVRDYGIDGFRVDTAKHVELPAWQQLKTEASSALREWKKTNPD

KALDDKPFWMTGEAWGHGVMQSDYYRHGFDAMINFDYQEQAAKAVDCLAQMDTTWQQMAEKLQGFNVLSY

LSSHDTRLFREGGDKAAELLLLAPGAVQIFYGDESSRPFGPTGSDPLQGTRSDMNWQDVSGKSAANVAHW

QKISQFRARHPAIGAGKQTTLSLKQGYGFVREHGDDKVLVIWAGQQ

>gi|30065143|ref|NP_839314.1| valine--pyruvate transaminase [Shigella flexneri 2a str. 2457T]

MTFSLFGDKFTRHSGITLLMEDLNDGLRTPGAIMLGGGNPAQIPEMQDYFQTLLTDMLESGKATDALCNY

DGPQGKTELLTLLAGMLREKLGWDIEPQNIALTNGSQSAFFYLFNLFAGRRADGRVKKVLFPLAPEYIGY

ADAGLEEDLFVSARPNIELLPEGQFKYHVDFEHLHIGEETGMICVSRPTNPTGNVITDEELLKLDALANQ

HGIPLVIDNAYGVPFPGIIFSEGRPLWNPNIVLCMSLSKLGLPGSRCGIIIANEKIITAITNMNGIISLA

PGGIGPAMMCEMIKRNDLLRLSETVIKPFYYQRVQETIAIIRRYLPENRCLIHKPEGAIFLWLWFKDLPI

TTEQLYQRLKARGVLMVPGHNFFPGLDKPWPHTHQCMRMNYVPEPEKIEAGVKILAEEIERAWAESH

>gi|30065142|ref|NP_839313.1| hypothetical protein S4152 [Shigella flexneri 2a str. 2457T]

MNRFIIADATKCIGCRTCEVACAVSHQENQDCAALSPDEFISRIRVIKDHSWTTPVACHQCEDAPCANVC

PVDAISREHGHIFVEQTRCIGCKSCMLACPFGAMEVVSSRKKARAIKCDLCWHRETGPACVEACPTKALQ

CMDVEKVQRHRLQQQPV

>gi|30065141|ref|NP_839312.1| regulator [Shigella flexneri 2a str. 2457T]

MGKEVMGKKENEMAQEKERPAGSQSLFRGLMLIEILSNYPNGCPLAHLSELAGLNKSTVHRLLQGLQSCG

YVTTAPAAGSYRLTTKFIAVGQKALSSLNIIHIAAPHLEALNIATGETINFSSREDDHAILIYKLEPTTG

MLRTRAYIGQHMPLYCSAMGKIYMAFGHPDYVKSYWENHQHEIQPLTRNTITELPAMFDELAHIRESGAA

MDREENELGVSCIAVPVFDIHGRVPYAVSISLSTSRLKQVGEKNLLKPLRETAQAISNELGFTVRDDLGA

IT

>gi|30065140|ref|NP_839311.1| 2,3-diketo-L-gulonate reductase [Shigella flexneri 2a str. 2457T]

MKVTFEQLKAAFNRVLISRGVDSETADACAEMFARTTESGVYSHGVNRFPRFIQQLENGDIIPDAQPKRI

TSLGAIEQWDAQRSIGNLTAKKMMDRAIELAADHGIGLVALRNANHWMRGGSYGWQAAEKGYIGICWTNS

IAVMPPWGAKECRIGTNPLIVAIPSTPITMVDMSMSMFSYGMLEVNRLAGRQLPVDGGFDDEGNLTKEPG

VIEKNRRILPMGYWKGSGMSIVLDMIATLLSDGASVAEVTQDNSDEYGISQIFIAIEVDKLIDGPTRDAK

LQRIMDYVTTAERADENQAIRLPGHEFTTLLAENRRNGITVDDSVWAKIQAL

>gi|30065139|ref|NP_839310.1| IS1 orfA [Shigella flexneri 2a str. 2457T]

MASISIRCPSCSATEGVVRNGKSTAEHQRYLCSHCRKTWQLQFTYTASQPGTHQKIIDMAMNGVGCRASA

RIMGVGLNTVLRHLKNSGRSR

>gi|30065138|ref|NP_839309.1| IS1 orfB [Shigella flexneri 2a str. 2457T]

MSRQCTHYGRWPQHGFTSLKKLRPQSVTSRIQPGSDVIVCAEMDEHWGYVGAKSRQRWLFYAYDRIRRTV

VAHVFGERTLATLERLLSLLSAFEVVVWMTDGWPLYESRLKGKLHVISKRYTQRIERHNLNLRQHLARLG

RKSLSFSKSVELHDKVIGHYLNIKHYQ

>gi|30065137|ref|NP_839308.1| hypothetical protein S4145 [Shigella flexneri 2a str. 2457T]

MAVLIFLGCLLGGIAIGLPIAWALLLCGAALMFWLDMFDVQIMAQTLVNGADSFSLLAIPFFVLAGEIMN

AGGLLKRIVDLPMKLVGHKPGGLGYVGVLAAMIMASLSGSAVADTAAVAALLVPMMRSANYPVNRAAGLI

ASGGIIAPIIPPSIPFIIFGVSSGLSISKLFMAGIAPGIMMGATLMLTWWRQASRLNLPRQQKATMQEIW

HSFVSGIWALFLPVIIIGGFRSGLFTPTEAGAVAAFYALFVATVIYREMTFATLWHVLIGAAKTTSVVMF

LVASAQVSAWLITIAELPMMVSDLLQPLVDSPRLLFIVIMVAILIVGMVMDLTPTVLILTPVLMPLVKEA

GIDPIYFGVMFIINCSIGLITPPIGNVLNVISGVAKLKFDDAVRGVFPYVLVLYSLLVVFVFIPDLIILP

LKWMVMLPTY

>gi|30065136|ref|NP_839307.1| IS1 orfA [Shigella flexneri 2a str. 2457T]

MASISIRCPSCSATEGVVRNGKSTAGHQRYLCSHCRKTWQLQFTYTASQPGTHQKIIDMAMNGVGCRASA

RIMGVGLNTVLRHLKNSGRSR

>gi|30065135|ref|NP_839306.1| IS1 orfB [Shigella flexneri 2a str. 2457T]

MSCQCTHYGRWPQHGFTSLKKLRPQSVTSRIQPGSDVIVCAEMDEHWGYVGAKSRQRWLFYAYDRIRRTV

VAHVFGERTLATLERLLSLLSAFEVVVWMTDGWPLYESRLKGKLHVISKRYTQRIERHNLNLRQHLARLG

RKSLSFSKSVELHDKVIGHYLNIKHYQ

>gi|30065134|ref|NP_839305.1| aldehyde dehydrogenase B (lactaldehyde dehydrogenase) [Shigella flexneri 2a str. 2457T]

MIAVVKLLPTGEDFASHVYPCYTSHRKETIMTNNPPSAQIKPGEYGFPIKLKARYDNFIGGEWVAPADGE

YYQNLTPVTGQLLCEVASSGKRDIDLALDAAHKVKDKWAHTSVQDRAAILFKIADRMEQNLELLATAETW

DNGKPIRETSAADVPLAIDHFRYFASCIRAQEGGISEVDSETVAYHFHEPLGVVGQIIPWNFPLLMASWK

MAPALAAGNCVVLKPARLTPLSVLLLMEIVGDLLPPGVVNVVNGAGGEIGEYLATSKRIAKVAFTGSTEV

GQQIMQYATQNIIPVTLELGGKSPNIFFADVMDEEDAFFDKALEGFALFAFNQGEVCTCPSRALVQESIY

ERFMERAIRRVESIRSGNPLDSVTQMGAQVSHGQLETILNYIDIGKKEGADVLTGGRRKLLEGELKDGYY

LEPTILFGQNNMRVFQEEIFGPVLAVTTFKTMEEALELANDTQYGLGAGVWSRNGNLAYKMGRGIQAGRV

WTNCYHAYPAHAAFGGYKQSGIGRETHKMMLEHYQQTKCLLVSYSDKPLGLF

>gi|30065133|ref|NP_839304.1| alcohol dehydrogenase [Shigella flexneri 2a str. 2457T]

MASSTFFIPSVNVIGADSLTDAMNMMADYGFTRTLIVTDNMLTKLGMAGDVQKALEERNIFSVIYDGTQP

NPTTENVAAGLKLLKENNCDSVISLGGGSPHDCAKGIALVAANGGDIRDYEGVDRSAKPQLPMIAINTTA

GTASEMTRFCIITDEARHNKMAIVDKHVTPLLSVNDSSLMIGMPKSLTAATGMDALTHAIEAYVSIAATP

ITDACALKAVTMIAENLPLAVEDGSNAKAREAMAYAQFLAGMAFNNASLGYVHAMAHQLGGFYNLPHGVC

NAVLLPHVQVFNSKVAAARLRDCAAAMGVNVTGKNDAEGAEACINAIRELAKKVDIPAGLRDLNVKEEDF

AVLATNALKDACGFTNPIQATHEEIVVIYRAAM

>gi|30065132|ref|NP_839303.1| selenocysteinyl-tRNA-specific translation factor [Shigella flexneri 2a str. 2457T]

MIIATAGHVDHGKTTLLQAITGVNADRLPEEKKRGMTIDLGYAYWPQPDGRVPGFIDVPGHEKFLSNMLA

GVGGIDHALLVVACDDGVMAQTREHLAILQLTGNPMLTVALTKADRVDEARVDEVERQVKEVLREYGFAE

AKLFITAATEGRGIDALREHLLQLPEREHASQHSFRLAIDRAFTVKGAGLVVTGTALSGEVKVGDSLWLT

GVNKPMRVRALHAQNQPTETANAGQRIALDIAGDAEKEQINRGDWLLADVPPEPFTRVIVELQTHAPLTQ

WQPLHIHHAASHVTGRVSLLEDNLAELVFDTPLWLADNDRLVLRDISARNTLAGARVVMLNPPRRGKRKP

EYLQWLASLARAQNDSEALAVHLQRGAVNLPDFAWARQLNGEGMRELLQQPGYIQAGYSLLNAPVAARWQ

RKILDTLATYHEQHRDEPGPGRERLRRMALPMEDEALVLLLIEKMRESGDILSHHGWLHLPDHKAGFSEE

QQAIWQKAEPLFGDEPWWVRDLAKETGTDEQAMRLTLRQAAQQGIITAIVKDRYYRNDRIVEFANMIRDL

DQECGSTCAADFRDRLGVGRKLAIQILEYFDRIGFTRRRGNDHLLRDALLFPEK

>gi|30065131|ref|NP_839302.1| selenocysteine synthase [Shigella flexneri 2a str. 2457T]

MTTETRSLYSQLPAIDRLLRDSSFLSLRDTYGHTRVVELLRQMLDEAREVIRGSQTLPAWCENWAQEVDA

RLTKEAQSALRPVINLTGTVLHTNLGRALQAEAAVEAVAQAMRSPVTLEYDLDDAGRGHRDRALAQLLCR

ITGAEDACIVNNNAAAVLLMLAATASGKEVVVSRGELVEIGGAFRIPDVMRQAGCTLHEVGTTNRTHAND

YRQAVNENTALLMKVHTSNYSIQGFTKAIDEAELVALGKELDVPVVTDLGSGSLVDLSQYGLPKEPMPQE

LIAAGVSLVSFSGDKLLGGPQAGIIVGKKEMIARLQSHPLKRALRADKMTLAALEATLRLYLHPEALSEK

LPTLRLLTRSAEVIQIQAQRLQAPLVAHYGAEFAVQVMPCLSQIGSGSLPVDRLPSAALTFTPHDGRGSH

LESLAARWRELPVPVIGRIYDGRLWLDLRCLEDEQRFLEMLLK

>gi|30065130|ref|NP_839301.1| glutathione S-transferase [Shigella flexneri 2a str. 2457T]

MKLVGSYTSPFVRKLFILLLEKGITFEFINELPYNADNGVAQFNPLGKVPVLVTEEGECWFDSPIIAEYI

ELMNVAPAMLPRDPLESLRVRKIEALADGIMDAGLVSVREQARPAAQQSEDELLRQREKINRSLDVLEGY

LVDGTLKTDTVNLATIAIACAVGYLNFRRVAPGWCVDRPHLVKLVENLFSRESFARTEPPKA

>gi|30065129|ref|NP_839300.1| hypothetical protein S4137 [Shigella flexneri 2a str. 2457T]

MDLLIVLTYVALATAALGGVFLVSGLILLMNYNHPYTFTAQKAVIAIPITPQVTGIVTEVTDKNNQLIQK

GEVLFKLDPVRYQARVDRLQADLMTATHNIKTLRAQLTEAQANTTQVSAERDRLFKNYQRYLKGSQAAVN

PFSERDIDDARQNFLAQDALVKGSVAEQAQIQSQLDSMVNGEQSQIVSLRAQLTEAKYNLEQTVIRAPSN

GYVTQVLIRPGTYAAALPLRPVMVFIPEQKRQIVAQFRQNSLLRLKPGDDAEVVFNALPGQVFHGKLTSI

LPVVPGGSYQAQGVLQSLTVVPGTDGVLGTIELDPNDDIDALPDGIYAQVAVYSDHFSHVSVMRKVLLRM

TSWMHYLYLDH

>gi|30065128|ref|NP_839299.1| hypothetical protein S4136 [Shigella flexneri 2a str. 2457T]

MFLNYFALGVLIFVFLVIFYGIIAIHDIPYLIAKKRNHPHADAIHTAGWVSLFTLHVIWPFLWIWATLYQ

PERGWGMQSHVASQEKATEPEIAALSDRISRLEHQLAAEKKTDYSTFPEI

>gi|30065127|ref|NP_839298.1| PTS system, mannitol-specific enzyme IIABC components [Shigella flexneri 2a str. 2457T]

MSSDIKIKVQSFGRFLSNMVMPNIGAFIAWGIITALFIPTGWLPNETLAKLVGPMITYLLPLLIGYTGGK

LVGGKRGGVVGAITTMGVIVGADMPMFLGSMIAGPLGGWCIKHFDRWVDGKIKSGFEMLVNNFSAGIIGM

ILAILAFLGIGPIVEALSKMLAAGVNFMVVHDMLPLASIFVEPAKILFLNNAINHGIFSPLGIQQSHELG

KSIFFLIEANPGPGMGVLLAYMFFGRGSAKQSAGGAAIIHFLGGIHEIYFPYVLMNPRLILAVILGGMTG

VFTLTILGGGLVSPASPGSILAVLAMTPKGAYFANIAGVCAAMAVSFVVSAILLKTSKVKEEDDIEAATR

RMQDMKAESKGTSPLSAGDVTNDLSHVRKIIVACDAGMGSSAMGAGVLRKKIQDAGLSQISVTNSAINNL

PPDVDLVITHRDLTERAMRQVPQAQHISLTNFLDSGLYTSLTERLVAAQRHTENEVKVKDSLKDSFDDSS

ANLFKLGAENIFLGRKAATKEEAIRFAGEQLVKGGYVEPEYVQAMLDREKLTPTYLGESIAVPHGTVEAK

DRVLKTGVVFCQYPEGVRFGEEEDDIARLVIGIAARNNEHIQVITSLTNALDDESVIERLAHTTSVDEVL

ELLAGRK

>gi|30065126|ref|NP_839297.1| mannitol-1-phosphate 5-dehydrogenase [Shigella flexneri 2a str. 2457T]

MKALHFGAGNIGRGFIGKLLADAGIQLTFADVNQVVLDALNARHSYQVHVVGETEQVDTVSGVNAVSSIG

DDVVDLIAQVDLVTTAVGPVVLERIAPAIAKGLVKRKEQGNESPLNIIACENMVRGTTQLKGHVMNALPE

DAKAWVEEHVGFVDSAVDRIVPPSASATNDPLEVTVETFSEWIVDKTQFKGALPNIPGMELTDNLMAFVE

RKLFTLNTGHAITAYLGKLAGHQTIRDAILDEKIRAVVKGAMEESGAVLIKRYGFDADKHAAYIQKILGR

FENPYLKDDVERVGRQPLRKLSAGDRLIKPLLGTLEYSLPHKNLIQGIAGAMHFRSEDDPQAQELAALIA

DKGPQAALAQISGLDANSEVVSEAVTAYKAMQ

>gi|30065125|ref|NP_839296.1| mannitol repressor protein [Shigella flexneri 2a str. 2457T]

MVDQAQDTLRPNNRLSDMQATMEQTQAVENRVLERLNAGKTVRSFLITAVELLTEAVNLLVLQVFRKDDY

AVKYAVEPLLDGDGPLGDLSVRLKLIYGLGVINRQEYEDAELLMALREELNHDGNEYAFTDDEILGPFGE

LHCVAALPPPPQFEPADSSLYAMQIQRYQQAVRSTMVLSLTELISKISLKKAFQK

>gi|30065124|ref|NP_839295.1| hypothetical protein S4132 [Shigella flexneri 2a str. 2457T]

MKEVEKNEIKRLSDRLDAIRHQQADLSLVEAADKYAELEKEKATLEAEIARLREVHSQKLSKEAQKLMKM

PFQRAITKKEQADMGKLKKSVRGLVVVHPMTALGREMGLEEMTGFSKTTF

>gi|30065123|ref|NP_839294.1| IS1 orfB [Shigella flexneri 2a str. 2457T]

MSCQCTHYGRWPQHGFTSLKKLRPQSVTSRIQPGSDVIVCAEMDEHWGYVGAKSRQRWLFYAYDRIRRTV

VAHVFGERTLATLERLLSLLSAFEVVVWMTDGWPLYESRLKGKLHVISKRYTQRIERHNLNLRQHLARLV

RKSLSFSKSVELHDKVIGHYLNIKHYQ

>gi|30065122|ref|NP_839293.1| IS1 orfA [Shigella flexneri 2a str. 2457T]

MASISIRCPSCSATEGVVRNGKSTAGHQRYLCSHCRKTWQLQFTYTASQPGTHQKIIDMAMNGVGCRASA

RIMGVGLNTVLRHLKNSGRSR

>gi|30065121|ref|NP_839292.1| L-lactate permease [Shigella flexneri 2a str. 2457T]

MNLWQQNYDPAGNIWLSSLIASLPILFFFFALIKLKLKGYVAASWTVAIALAVALLFYKMPVANALASVV

YGFFYGLWPIAWIIIAAVFVYKISVKTGQFDIIRSSILSITPDQRLQMLIVGFCFGAFLEGAAGFGAPVA

ITAALLVGLGFKPLYAAGLCLIVNTAPVAFGAMGIPILVAGQVTGIDSFEIGQMVGRQLPFMTIIVLFWI

MAIMDGWRGIKETWPAVVVAGGSFAIAQYLSSNFIGPELPDIISSLVSLLCLTLFLKRWQPVRVFRFGDL

GASQVDMTLAHTGYTAGQVLRAWTPFLFLTATVTLWSIPPFKALFASGGALYEWVINIPVPYLNKLVARM

PPVVSEATAYAAVFKFDWFSATGTAILFAALLSIVWLKMKPSDAISTFGSTLKELALPIYSIGMVLAFAF

ISNYSGLSSTLALALAHTGHAFTFFSPFLGWLGVFLTGSDTSSNALFAALQATAAQQIGVSDLLLVAANT

TGGVTGKMISPQSIAIACAAVGLVGKESDLFRFTVKHSLIFTCMVGVITTLQAYVLTWMIP

>gi|30065120|ref|NP_839291.1| DNA-binding transcriptional repressor LldR [Shigella flexneri 2a str. 2457T]

MIVLPRRLSDEVADRVRALIDEKNLEAGMKLPAERQLAMQLGVSRNSLREALAKLVSEGVLLSRRGGGTF

IRWRHDTWSEQNIVQPLKTLMADDPDYSFDILEARYAIEASTAWHAAMRATPGDKEKIQLCFEATLSEDP

DIASQADVRFHLAIAEASHNIVLLQTMRGFFDVLQSSVKHSRQRMYLVPPVFSQLTEQHQAVIDAIFAGD

ADGARKAMMAHLSFVHTTMKRFDEDQARHARITRLPGEHNEHSREKNA

>gi|30065119|ref|NP_839290.1| L-lactate dehydrogenase [Shigella flexneri 2a str. 2457T]

MIISAASDYRAAAQRILPPFLFHYMDGGAYSEYTLRRNVEDLSEVALRQRILKNMSDLSLETTLFNEKLS

MPVALAPVGLCGMYARRGEVQAAKAADAHGIPFTLSTVSVCPIEEVAPAIKRPMWFQLYVLRDRGFMRNA

LERAKAAGCSTLVFTVDMPTPGARYRDAHSGMSGPNAAMRRYLQAVTHPQWAWDVGLNGRPHDLGNISAY

LGKPTGLEDYIGWLGNNFDPSISWKDLEWIRDFWDGPMVIKGILDPEDARDAVRFGADGIVVSNHGGRQL

DGVLSSARALPAIADAVKGDIAILADSGIRNGLDVVRMIALGADTVLLGRAFLYALATAGQAGVANLLNL

IEKEMKVAMTLTGAKSISEITQDSLVQGLGKELPTALAPMAKGNAA

>gi|30065118|ref|NP_839289.1| tRNA/rRNA methyltransferase YibK [Shigella flexneri 2a str. 2457T]

MLNIVLYEPEIPPNTGNIIRLCANTGFRLHIIEPMGFAWDDKRLRRAGLDYHEFTAVTRHHDYRAFLEAE

NPQRLFALTTKGTPAHSAVSYQDGDYLMFGPETRGLPASILDALPAEQKIRIPMVPDSRSMNLSNAVSVV

VYEAWRQLGYPGALLRD

>gi|30065117|ref|NP_839288.1| serine acetyltransferase [Shigella flexneri 2a str. 2457T]

MSCEELEIVWNNIKAEARTLADCEPMLASFYHATLLKHENLGSALSYMLANKLSSPIMPAIAIREVVEEA

YAADPEMIASAACDIQAVRTRDPAVDKYSTPLLYLKGFHALQAYRIGHWLWNQGRRALAIFLQNQVSVTF

QVDIHPAAKIGRGIMLDHATGIVVGETAVIENDVSILQSVTLGGTGKSGGDRHPKIREGVMIGAGAKILG

NIEVGRGAKIGAGSVVLQPVPPHTTAAGVPARIVGKPDSDKPSMDMDQHFNGINHTFEYGDGI

>gi|30065116|ref|NP_839287.1| NAD(P)H-dependent glycerol-3-phosphate dehydrogenase [Shigella flexneri 2a str. 2457T]

MNQRNASMTVIGAGSYGTALAITLARNGHEVVLWGHDPEHIATLERDRCNAAFLPDVPFPDTLHLESDLA

TALAASRNILVVVPSHVFGEVLRQIKPLMRPDARLVWATKGLEAETGRLLQDVAREALGDQIPLAVISGP

TFAKELAAGLPTAISLASTDQTFADDLQQLLHCGKSFRVYSNPDFIGVQLGGAVKNVIAIGAGMSDGIGF

GANARTALITRGLAEMSRLGAALGADPATFMGMAGLGDLVLTCTDNQSRNRRFGMMLGQGMDVQSAQEKI

GQVVEGYRNTKEVRELAHRFGVEMPITEEIYQVLYCGKNAREAALTLLGRARKDERSSH

>gi|30065115|ref|NP_839286.1| preprotein translocase subunit SecB [Shigella flexneri 2a str. 2457T]

MSEQNNTEMTFQIQRIYTKDISFEAPNAPHVFQKDWQPEVKLDLDTASSQLADDVYEVVLRVTVTASLGE

ETAFLCEVQQGGIFSIAGIEGTQMAHCLGAYCPNILFPYARECITSMVSRGTFPQLNLAPVNFDALFMNY

LQQQAGEGTEEHQDA

>gi|30065114|ref|NP_839285.1| glutaredoxin 3 [Shigella flexneri 2a str. 2457T]

MANVEIYTKETCPYCHRAKALLSSKGVSFQELPIDGNAAKREEMIKRSGCTTVPQIFIDAQHIGGCDDLY

ALDARGGLDPLLK

>gi|30065113|ref|NP_839284.1| hypothetical protein S4118 [Shigella flexneri 2a str. 2457T]

MQEIMQFVGRHPILSIAWIALLVAVLVTTFKSLTSKVKVITRGEATRLINKEDAVVVDLRQRDDFRKGHI

AGSINLLPSEIKANNVGELEKHKDKSVIVVDGSGMQCQEPANALTKAGFAQVFVLKEGVAGWAGENLPLV

RGK

>gi|30065112|ref|NP_839283.1| phosphoglyceromutase [Shigella flexneri 2a str. 2457T]

MSVSKKPMVLVILDGYGYREEQQDNAIFSAKTPVMDALWANRPHTLIDASGLEVGLPDRQMGNSEVGHVN

LGAGRIVYQDLTRLDVEIKDRAFFANPVLAGAVHKAKNAGKAVHIMGLLSAGGVHSHEDHIMAMVELAAE

RGAEKIYLHAFLDGRDTPPRSAESSLKKFEEKFAALGKGRVASIIGRYYAMDRDNRWDRVEKAYDLLTLA

QGEFQADTAVAGLQAAYARDENDEFVKATVIRAEGQPDAAMEDGDALIFMNFRADRAREITRAFVNADFD

GFARKKVVNVDFVMLTEYAADIKTAVAYPPASLVNTFGEWMAKNDKTQLRISETEKYAHVTFFFNGGVEE

SFKGEDRILINSPKVATYDLQPEMSSAELTEKLVAAIKSGKYDTIICNYPNGDMVGHTGVMEAAVKAVEA

LDHCVEEVAKAVESVGGQLLITADHGNAEQMRDPATGQAHTAHTNLPVPLIYVGDKNVKAVAGGKLSDIA

PTMLSLMGMEIPQEMTGKPLFIVE

>gi|30065110|ref|NP_839281.1| hypothetical protein S4115 [Shigella flexneri 2a str. 2457T]

MLAMPSAISVAVLPDSPHAREMATKAHNSGHEVLIHLPMAPLSKQPLEKNTLRPEMSSDEIERIIRSAVN

NVPYAVGINNHMGSKMTSNLFGMQKVMQALERYNLYFLDSVTIGNTQAMRAAQGTGVKVIKRKVFLDDSQ

NEADIRVQFNRAIDLARRNGSTIAIGHPHPSTVRVLQQMVYNLPPDITLVKASSLLNEPQVDTSTPPKNA

VPDAPRNPFRGVKLCKPKKPIEPVYANRFFEVLSESISQSTLIVYFQHQWQGWGKQPEAAKFNASAN

>gi|30065109|ref|NP_839280.1| regulator [Shigella flexneri 2a str. 2457T]

MYKRLITNVRSVKVGYQALLWSFRLWQWRDKTRSHHRITRSAFNLR

>gi|30065108|ref|NP_839279.1| IS1 orfB [Shigella flexneri 2a str. 2457T]

MSRQCTHYGRWPQHGFTSLKKLRPQSVTSRIQPGSDVIVCAEMDEHWGYVGAKSRQRWLFYAYDRIRRTV

VAHVFGERTLATLERLLSLLSAFEVVVWMTDGWPLYESRLKGKLHVISKRYTQRIERHNLNLRQHLARLV

RKSLSFSKSVELHDKVIGHYLNIKHYQ

>gi|30065107|ref|NP_839278.1| IS1 orfA [Shigella flexneri 2a str. 2457T]

MASISIRCPSCSATEGVVRNGKSTAGHQRYLCSHCRKTWQLQFTYTASQPGTHQKIIDMAMNGVGCRASA

RIMGVGLNTVLRHLKNSGRSR

>gi|30065106|ref|NP_839277.1| L-threonine 3-dehydrogenase [Shigella flexneri 2a str. 2457T]

MKALSKLKAEEGIWMTDVPVPELGHNDLLIKIRKTAICGTDVHIYNWDEWSQKTIPVPMVVGHEYVGEVV

GIGQEVKGFKIGDRVSGEGHITCGHCRNCRGGRTHLCRNTIGVGVNRPGCFAEYLVIPAFNAFKIPDNIS

DDLASIFDPFGNAVHTALSFDLVGEDVLVSGAGPIGIMAAAVAKHVGARNVVITDVNEYRLELARKMGIT

RAVNVAKENLNDVMAELGMTEGFDVGLEMSGAPPAFRTMLDTMNHGGRIAMLGIPPSDMSIDWTKVIFKG

LFIKGIYGREMFETWYKMAALIQSGLDLSPIITHRFSIDDFQKGFDAMRSGQSGKVILSWD

>gi|30065105|ref|NP_839276.1| 2-amino-3-ketobutyrate coenzyme A ligase [Shigella flexneri 2a str. 2457T]

MRGEFYQQLTNDLETARAEGLFKEERIITSAQQADITVADGSHVINFCANNYLGLANHPDLIAAAKAGMD

SHGFGMASVRFICGTQDSHKELEQKLAAFLGMEDAILYSSCFDANGGLFETLLGAEDAIISDALNHASII

DGVRLCKAKRYRYANNDMQELEARLKEAREAGARHVLIATDGVFSMDGVIANLKGVCDLADKYDALVMVD

DSHAVGFVGENGRGSHEYCDVMGRVDIITGTLGKALGGASGGYTAARKEVVEWLRQRSRPYLFSNSLAPA

IVAASIKVLEMVEAGSELRDRLWANARQFREQMSAAGFTLAGADHAIIPVMLGDAVVAQKFARELQKEGI

YVTGFFYPVVPKGQARIRTQMSAAHTPEQITRAVEAFTRIGKQLGVIA

>gi|30065104|ref|NP_839275.1| ADP-L-glycero-D-manno-heptose-6-epimerase [Shigella flexneri 2a str. 2457T]

MIIVTGGAGFIGSNIVKALNDKGITDILVVDNLKDGTKFVNLVDLDIADYMDKEDFLIQIMAGEEFGDVE

AIFHEGACSSTTEWDGKYMMDNNYQYSKELLHYCLEREIPFLYASSAATYGGRTSDFIESREYEKPLNVY

GYSKFLFDEYVRQILPEANSQIVGFRYFNVYGPREGHKGSMASVAFHLNTQLNNGESPKLFEGSENFKRD

FVYVGDVADVNLWFLENGVSGIFNLGTGRAESFQAVADATLAYHKKGQIEYIPFPDKLKGRYQAFTQADL

TNLRAAGYDKPFKTVAEGVTEYMAWLNRDA

>gi|30065103|ref|NP_839274.1| ADP-heptose--LPS heptosyltransferase [Shigella flexneri 2a str. 2457T]

MKILVIGPSWVGDMMMSQSLYRTLQARYPQAIIDVMAPAWCRPLLSRMPEVNEAIPMPLGHGALEIGERR

KLGHSLREKRYDRAYVLPNSFKSALVPFFAGIPHRTGWRGEMRYGLLNDVRVLDKEAWPLMVERYVALAY

DKGIMRTAQDLPQPLLWPQLQVSEGEKSYTCNQFSLSSERPMIGFCPGAEFGPAKRWPHYHYAELAKQLI

DEGYQVVLFGSAKDHEAGNEILAALNTEQQAWCRNLAGETQLDQAVILIAACKAIVTNDSGLMHVAAALN

RPLVALYGPSSPDFTPPLSHKARVIRLITGYHKVRKGDAAEGYHQSLIDITPQRVLEELNALLLQEEA

>gi|30065102|ref|NP_839273.1| ADP-heptose--LPS heptosyltransferase [Shigella flexneri 2a str. 2457T]

MGDVLHTLPALTDAQQAIPGIKFDWVVEEGFAQIPSWHAAVERVIPVAIRRWRKAWFSAPIKAERKAFRE

ALQAENYDAVIDAQGLVKSAALVTRLAHGVKHGLDWQTAREPLASLFYNCKHHIAKQQHAVERTRELFAK

SLGYSKPQTQGDYAIAQHFLTNLPTDAGEYAVFLHATTRDDKHWPEEHWRELIGLLADSGIRIKLPWGAP

HEEERAKRLAEGFAYVEVLPKMSLEGVARVLAGAKFVVSVDTGLSHLTAALDRPNITVYGPTDPGLIGGY

GKNQVECRSTSMSLADLPAQTVFQNLNLEIITNKLTSEIR

>gi|30065101|ref|NP_839272.1| lipid A-core, surface polymer ligase [Shigella flexneri 2a str. 2457T]

MTSTLFFSLEKKNWIAYWNRALVFLFITTYFLGGITRYKHLIVILMTITTIVYLCKRPKHYLSLFKTFLF

GSVAILTIAALLSLLQSPDAGASMKEVFKAIIENTLLCTIAIPVILRDEKREDVEKIVFFSFISALGLRC

FSELITYYKDYQQGIMPFADYRHRSISDSMVFLFPALLNLWLIKSAKYRISFVVLSVIFIFLILGTLSRG

AWLSVLVIGLIWILMFKQWKLLLVGVMVSIIALSVIFTHKEMTAKLTYKLQQTNSSYRYANGTQGSALDL

ILENPVIGYGYGNVAYKDVYNKRVIDYPEWTFRQSIGPHNFALFIWFGTGLLGLVSLMMLYCAILKECIK

NGVKNKYRSPYNAYYIILLSFIGYFVIRGNVEQIEPNLLGVYAGLLLAMKNK

>gi|30065100|ref|NP_839271.1| lipopolysaccharide 1,2-N- acetylglucosaminetransferase [Shigella flexneri 2a str. 2457T]

MVDKIIFTVTPIFSIPPRGAAAVETWMYQVAQRTNFPNRIVCIKNPGYSNYTFVNDNCSIHRVGFSRIYK

RLFQKWTRLDPLPYSQRILNIAHDFPITKESVIVVHNSMKLYTQIRKLAPQARVVIHMHNAFEPKLLEQN

VKMIVPSLYLKKYYQSYLANADIEIVPNGIDLETYQSNFQPITRSELNISPEEKIIFYAGRIVPDKGILL

LMQSFEKLAAAHKNLKLVVIGDYTEMSKSDKGAYQRNVREIAKRLKDRCIMLGSIPPEKMYCYYPLADLV

VIPSQFQEPFCMVAIEAMGAGKPVLVSTRGGMTEFVKENTTGFHLKEPMTADSISSDILKTLANPELTAV

AKQGQDFVFDHYSWDGVTQRFEEVIHNWFE

>gi|30065099|ref|NP_839270.1| UDP-glucose:(galactosyl) LPS alpha1,2-glucosyltransferase [Shigella flexneri 2a str. 2457T]

MDFKHLTQFKDIIELDKRPVKLDERETFNVSWGIDENYQVGAAISIASILEKNKQNKFTFHIIADYLDKE

YIELLSQLATKYQTVIKLYHIDSEPLKALPQSNIWPVSIYYRLLSFDYFSARLDSLLYLDADIVCKGSLN

ELIALEFKDEYGAVVIDVDAMQSKSAERLCNEDFNGSYFNSGVMYINLREWLKQRLTEKFFDLLSDESII

KKLKYPDQDILNLMFLHHAKILPRKYNCIYTIKSEFEEKNSEYYTRFINDDTVFIHYTGITKPWHDWANY

ASADYFRNIYNISPWRNIPYKKAVKKHEHKEKYKHLLYQKKFLDGVFTAIKYNVMKG

>gi|30065098|ref|NP_839269.1| lipopolysaccharide core biosynthesis protein [Shigella flexneri 2a str. 2457T]

MIYNKTINGLKVFIKDNDPFYEQVLNDFLTCRVKTLKVFRSIDDTKVILIDTARGPLVLKVYAPKHKMTE

RFLKSCIKKDYYENLIYQTDRVRGEGIQSINDYFLLAERKTLNFAHYYIMLIEYIEGVGLNEYLEISEDL

KDQLSESIKELHQHGMVSGDPHKGNFIVSEKGLRLIDLSGKKTTAVLKAKDRIDLERHYNIKNELKDFGY

TYLIFKKKIKKVIRDVKVKLGLKSK

>gi|30065097|ref|NP_839268.1| UDP-D-galactose:(glucosyl)lipopolysaccharide- alpha-1,3-D-galactosyltransferase [Shigella flexneri 2a str. 2457T]

MSQLNDSDIILFEYNFHYQNIRSKNTLDIAFGIDRNFLFGCGVAIASILLNNREISCEFHVFTDYISDKD

KLYFSDLAKQYNSRINIYVINCDKLKSLPSTKNWTYATYFRFIIADYFYHKHEKILYLDADIACKGSIKE

LLDYQFSTNEIAAVVAERDIEWWQNRASVLTTPQLASGYFNAGFLLINIDEWNLNNISSKAIEMLRDPDW

VSKITHLDQDVLNVLLNGKVKFISEKYNTRYSINYELKDKVDNPVNDDTVFIHYVGPTKPWHEWADYPVS

RSFLIAKAASPWSKEDLLKPVNSNQYRYCAKHKFKQKHYMAGIFNYLKYYKEKCF

>gi|30065096|ref|NP_839267.1| lipopolysaccharide core biosynthesis protein [Shigella flexneri 2a str. 2457T]

MVWMVELKEPFATLWRGKDPFEEVKTLQGEVFRELETRRTLRFEMAGKSYFLKWHRGTTLKEIIKNLLSL

RMPVLGADREWNAIHRLRDVGVDTMYGVAFGEKGMNPLTRTSFIITEDLTPTISLEDYCADWATNPPDVR

VKRMLIKRVATMVRDMHAAGINHRDCYICHFLLHLPFSGKEEELKISVIDLHRAQLRTRVPGRWRDKDLI

GLYFSSMNIGLTQRDIWRFMKVYFAAPLKDILKQEQGLLSQAEAKATKIRERTIRKSL

>gi|30065095|ref|NP_839266.1| LPS alpha1,3-glucosyltransferase [Shigella flexneri 2a str. 2457T]

MIVAFCLYKYFPFGGLQRDFMRIAQTVAARGHHVRVYTQSWEGECPDVFEVIKVPVKSHTNHGRNAEYFA

WVQKHLREHPVDKVVGFNKMPGLDVYYAADVCYAEKVAQEKGFFYRLTSRYRHYAAFERATFEQGKPTQL

LMLTDKQIADFQKHYQTEAERFHILPPGIYPDRKYSQQPANSREIFRKKNGITEQQYLLLQVGSDFTRKG

ADRSIEALASLPDSLRHNTLLYVVGQDKPRKFEALAEKRGVRSNVHFFSGRNDVSELMAAADLLLHPAYQ

EAAGIVLLEAITAGLPVLTTAVCGYAHYIVDANCGEAIAEPFRQETLNEILRKALTQSSLRQAWAENARH

YADTQDLYSLPEKAADIITGGLDG

>gi|30065094|ref|NP_839265.1| lipopolysaccharide core biosynthesis protein [Shigella flexneri 2a str. 2457T]

MDKPFRRILLIKMRFHGDMLLTTPVISSLKKNYPDAKIDVLLYQDTIPILSENPEINALYGIKNKKAKAS

EKIANFFHLIKVLRANKYDLIVNLTDQWMVAILVRLLNARVKISQDYHHRQSAFWRKSFTHLVPLQGGNV

VESNLSVLTPLGLDSLVKQTTMSYPPASWKRMRRELDHAGVGQNYVVIQPTARQIFKCWDNAKFSAVIDA

LHARGYEVVLTSGPDKDDLACVNEIAQGCQTPPVTALAGKVTFPELGALIDHAQLFIGVDSAPAHIAAAV

NTPLISLFGATDHIFWRPWSNNMIQFWAGDYREMPTRDQRDRNEMYLSVIPAADVIAAVDKLLPSSTTGT

SL

>gi|30065093|ref|NP_839264.1| IS1 orfB [Shigella flexneri 2a str. 2457T]

MSRQCTHYGRWPQHGFTSLKKLRPQSVTSRIQPGSDVIVCAEMDEHWGYVGAKSRQRWLFYAYDRIRRTV

VAHVFGERTLATLERLLSLLSAFEVVVWMTDGWPLYESRLKGKLHVISKRYTQRIERHNLNLRQHLARLV

RKPLSFSKSVELHDKVIGHYLNIKHYQ

>gi|30065092|ref|NP_839263.1| IS1 orfA [Shigella flexneri 2a str. 2457T]

MASISIRCPSCSATEGVVRNGKSTAGHQRYLCSHCRKTWQLQFTYTASQPGTHQKIIDMAMNGVGCRASA

RIMGVGLNTVLRHLKNSGRSR

>gi|30065091|ref|NP_839262.1| 3-deoxy-D-manno-octulosonic-acid transferase [Shigella flexneri 2a str. 2457T]

MLELLYTALLYLIQPLIWIRLWVRGRKAPAYRKRWGERYGFYRHPLKPGGIMLHSVSVGETLAAIPLVRA

LRHRYPDLPITVTTMTPTGSERVQSAFGKDVQHVYLPYDLPDALNRFLNKVDPKLVLIMETELWPNLIAA

LHKRKIPLVIANARLSARSAAGYAKLGKFVRRLLRRITLIAAQNEEDGARFVALGAKNNQVTVTGSLKFD

ISVTPQLAAKAVTLRRQWAPHRPVWIATSTHEGEESVVIAAHQALLQQFPNLLLILVPRHPERFPDAINL

VRQAGLSYITRSSGEVPSTSTQVVVGDTMGELMLLYGIADLAFVGGSLVERGGHNPLEAAAHAIPVLMGP

HTFNFKDICARLEQASGLITVTDATTLAKEVSSLLTDAVYRSFYGRHAVEVLYQNQGALQRLLQLLEPYL

PPKTH

>gi|30065090|ref|NP_839261.1| phosphopantetheine adenylyltransferase [Shigella flexneri 2a str. 2457T]

MQKRAIYPGTFDPITNGHIDIVTRATQMFDHVILAIAASPSKKPMFTLEERVALAQQATAHLGNVEVVGF

SDLMANFARNQHATVLIRGLRAVADFEYEMQLAHMNRHLMPELESVFLMPSKEWSFISSSLVKEVARHQG

DVTHFLPENVHQALMAKLA

>gi|30065089|ref|NP_839260.1| formamidopyrimidine-DNA glycosylase [Shigella flexneri 2a str. 2457T]

MPELPEVETSRRGIEPHLVGATILHAVVRNGRLRWPVSEEIYRLSDQPVLSVQRRAKYLLLELPEGWIII

HLGMSGSLRILPEELPPEKHDHVDLVMSNGKVLRYTDPRRFGAWLWTKELEGHNVLAHLGPEPLSDDFNG

EYLHQKCAKKKTAIKPWLMDNKLVVGVGNIYASESLFAAGIHPDRLASSLSLAECELLARVIKAVLLRSI

EQGGTTLKDFLQSDGKPGYFAQELQVYGRKGEPCRVCGTPIVATKHAQRATFYCRQCQK

>gi|30065088|ref|NP_839259.1| 50S ribosomal protein L33 [Shigella flexneri 2a str. 2457T]

MAKGIREKIKLVSSAGTGHFYTTTKNKRTKPEKLELKKFDPVVRQHVIYKEAKIK

>gi|30065087|ref|NP_839258.1| 50S ribosomal protein L28 [Shigella flexneri 2a str. 2457T]

MSRVCQVTGKRPVTGNNRSHALNATKRRFLPNLHSHRFWVESEKRFVTLRVSAKGMRVIDKKGIDTVLAE

LRARGEKY

>gi|30065084|ref|NP_839255.1| deoxyuridine 5'-triphosphate nucleotidohydrolase [Shigella flexneri 2a str. 2457T]

MMKKIDVKILDPRVRKEFPLPTYATSGSAGLDLRACLDDAVELAPGDTTLVPTGLAIHIADPSLAAMMLP

RSGLGHKHGIVLGNLVGLIDSDYQGQLMISVWNRGQDSFTIQPGERIAQMIFVPVVQAEFNLVEDFDATD

RGEGGFGHSGRQ

>gi|30065082|ref|NP_839253.1| orotate phosphoribosyltransferase [Shigella flexneri 2a str. 2457T]

MKSYQRQFIEFALSKQVLKFGEFTLKSGRKSPYFFNAGLFNTGRDLALLGRFYAEALVDSGIEFDLLFGP

AYKGIPIATTTAVALAEHHDLDLPYCFNRKEAKDHGEGGNLVGSALQGRVMLVDDVITAGTAIRESMEII

QANGATLAGVLISLDRQERGRGEISAIQEVERDYNCKVISIITLKDLIAYLEEKPEMAEHLAAVKAYREE

FGV

>gi|30065081|ref|NP_839252.1| ribonuclease PH [Shigella flexneri 2a str. 2457T]

MRPAGRSNNQVRPVTLTRNYTKHAEGSVLVEFGDTKVLCTASIEEGVPRFLKGQGQGWITAEYGMLPRST

HTRNAREAAKGKQGGRTMEIQRLIARALRAAVDLKALGEFTITLDCDVLQADGGTRTASITGACVALADA

LQKLVENGKLKTNPMKGMVAAVSVGIVNGEAICDLEYVEDSAAETDMNVVMTEDGRIIEVQGTAEGEPFT

HEELLTLLALARGGIESIVATQKAALAN

>gi|30065080|ref|NP_839251.1| hypothetical protein S4085 [Shigella flexneri 2a str. 2457T]

MIRSMTAYARREIKGEWGSATWEMRSVNQRYLETYFRLPEQFRSLEPVVRERIRSRLTRGKVECTLRYEP

DVSAQGELILNEKLAKQLVTAANWVKMKSDEGEINPVDILRWPGVMAAQEQDLDAIAAEILAALDGTLDD

FIVARETEGQALKALIEQRLEGVTAEVVKVRAHMPEILQWQRERLVAKLEDAQVQLENNRLEQELVLLAQ

RIDVAEELDRLEAHVKETYNILKKKEAVGRRLDFMMQEFNRESNTLASKSINAEVTNSAIELKVLIEQMR

EQIQNIE

>gi|30065079|ref|NP_839250.1| DNA-damage-inducible protein D [Shigella flexneri 2a str. 2457T]

MNEHHQPFEEIKLINANGAEQWSARQLGKLLGYSEYRHFIPVLTRAKEACENSGHTIDDHFEEILDMVKI

GSNAKRALKDIVLSRYACYLVVQNGDPAKPVIAAGQTYFTIQTRRQELADDEAFKQLREDEKRLFLRNEL

KEHNKQLVEAAQQAGVATATDFAIFQNHGYQGLYGGLDQKAIHQLKGLKKSQKILDHMGSTELAANLFRA

TQTEEKLKRDSVNSKQQANTTHFDVGSKVRQTIQELGGTIPEELPTPQVSIKQLENSVKITEKK

>gi|30065078|ref|NP_839249.1| hypothetical protein S4083 [Shigella flexneri 2a str. 2457T]

MTMRTNRYPARQIFRERTMLLHILYLVGITAEAMTGALAAGRRRMDTFGVIIIATATAIGGGSVRDILLG

HYPLGWVKHPEYVIIVATAAVLTTIVAPVMPYLRKVFLVLDALGLVVFSIIGAQVALDMGHGPIIAVVAA

VTTGVFGGVLRDMFCKRIPLVFQKELYAGVSFASAVLYIALQHYVSNHDVVIISTLVFGFFARLLALRLK

LGLPVFYYSHEGH

>gi|30065077|ref|NP_839248.1| NAD-dependent DNA ligase LigB [Shigella flexneri 2a str. 2457T]

MKVWMAILISILCWQSSVWAVCPAWSPARAQEEISRLQQQIKQWDDDYWKEGKSEVEDGVYDQLSARLTQ

WQRCFVSEPRDVMMPPLNGAVMHPVAHTGVRKMADKNALSLWMRERSDLWVQPKVDGVAVTLVYRDGKLN

KAISRGNGLKGEDWTQKVSLISAVLQTVSGPLANSTLQGEIFLQREGHIQQQMGGINARAKVAGLMMRQG

NSDTLNSLAVFVWAWPDGPQLMTDRLKELATAGFTLTQRYTRAVKNADEVARVRNEWWKAKLPFVTDGVV

VRGAKEPESRHWLPGQAEWLVAWKYQPVAQVAEVKAIQFAVGKSGKISVVASLAPVMLDDKKVQRVNIGS

VRRWQEWDIAPGDQILVSLAGQGIPRIDDVVWRGAERTKPTPPENRFNPLTCYFASDVCQEQFISRLVWL

GSKQVLGLDGIGEAGWRALHQTHRFEHIFSWLLLTPEQLQNTPGIAKSKSAQLWHQFNLARNQPFTRWVM

AMGIPLTRAALNASDERSWSQLLFSTEQFWQQLPGTGSGRARQVIEWKENAQIKKLGSWLAAQQITGFEP

>gi|30065076|ref|NP_839247.1| guanylate kinase [Shigella flexneri 2a str. 2457T]

MAQGTLYIVSAPSGAGKSSLIQALLKTQPLYDTQVSVSHTTRQPRPGEVHGEHYFFVNHDEFKEMISRDA

FLEHAEVFGNYYGTSREAIEQVLATGVDVFLDIDWQGAQQIRQKMPHARSIFILPPSKIELDRRLRGRGQ

DSEEVIAKRMAQAVAEMSHYAEYDYLIVNDDFDTALTDLKTIIRAERLRMSRQKQRHDALISKLLAD

>gi|30065075|ref|NP_839246.1| DNA-directed RNA polymerase subunit omega [Shigella flexneri 2a str. 2457T]

MARVTVQDAVEKIGNRFDLVLVAARRARQMQVGGKDPLVPEENDKTTVIALREIEEGLINNQILDVRERQ

EQQELEAAELQAVTAIAEGRR

>gi|30065074|ref|NP_839245.1| bifunctional (p)ppGpp synthetase II/ guanosine-3',5'-bis pyrophosphate 3'-pyrophosphohydrolase [Shigella flexneri 2a str. 2457T]

MYLFESLNQLIQTYLPEDQIKRLRQAYLVARDAHEGQTRSSGEPYITHPVAVACILAEMKLDYETLMAAL

LHDVIEDTPATYQDMEQLFGKSVAELVEGVSKLDKLKFRDKKEAQAENFRKMIMAMVQDIRVILIKLADR

THNMRTLGSLRPDKRRRIARETLEIYSPLAHRLGIHHIKTELEELGFEALYPNRYRVIKEVVKAARGNRK

EMIQKILSEIEGRLQEAGIPCRVSGREKHLYSIYCKMVLKEQRFHSIMDIYAFRVIVNDSDTCYRVLGQM

HSLYKPRPGRVKDYIAIPKANGYQSLHTSMIGPHGVPVEVQIRTEDMDQMAEMGVAAHWAYKEHGETSTT

AQIRAQRWMQSLLELQQSAGSSFEFIESVKSDLFPDEIYVFTPEGRIVELPAGATPVDFAYAVHTDIGHA

CVGARVDRQPYPLSQPLTSGQTVEIITAPGARPNAAWLNFVVSSKARAKIRQLLKNLKRDDSVSLGRRLL

NHALGGSRKLNEIPQENIQRELDRMKLATLDDLLAEIGLGNAMSVVVAKNLQHGDASIPPATQSHGHLPI

KGADGVLITFAKCCRPIPGDPIIAHVSPGKGLVIHHESCRNIRGYQKEPEKFMAVEWDKETAQEFITEIK

VEMFNHQGALANLTAAINTTTSNIQSLNTEEKDGRVYSAFIRLTARDRVHLANIMRKIRVMPDVIKVTRN

RN

>gi|30065073|ref|NP_839244.1| tRNA guanosine-2'-O-methyltransferase [Shigella flexneri 2a str. 2457T]

MNPTRYARICEMLARRQPDLTVCMEQVHKPHNVSAIIRTADAVGVHEVHAVWPGSRMRTMASAAAGSNSW

VQVKTHRTIGDAVAHLKGQGMQILATHLSDNAVDFREIDYTRPTCILMGQEKTGITQEALALADQDIIIP

MIGMVQSLNVSVASALILYEAQRQRQNAGMYLRENSMLPEAEQQRLLFEGGYPVLAKVAKRKGLPYPHVN
QQGEIEADADWWATMQAAG

>gi|30065072|ref|NP_839243.1| ATP-dependent DNA helicase RecG [Shigella flexneri 2a str. 2457T]
MKGRLLDAVPLSSLTGVGAALSNKLAKINLHTVQDLLLHLPLRYEDRTHLYPIGELLPGVYATVEGEVLN
CNISFGGRRMMTCQISDGSGILTMRFFNFSAAMKNSLATGRRVLAYGEAKRGKYGAEMIHPEYRVQGDLS
TPELQETLTPVYPTTEGVKQATLRKLTDQALDLLDTCAIEELLPPELSQGMMTLPEALRTLHRPPPTLQL
SDLETGQHPAQRRLILEELLAHNLSMLALRAGAQRFHAQPLSANDTLKNKLLAALPFKPTGAQARVVAEI
EHDMALDVPMMRLVQGDVGSGKTLVAALAALRAIAHGKQVALMAPTELLAEQHANNFRNWFAPLGIEVGW
LAGKQKGKARLSQQEAIASGQVQMIVGTHAIFQEQVQFNGLALVIIDEQHRFGVHQRLALWEKGQQQGFH
PHQLIMTATPIPRTLAMTAYADLDTSVIDELPPGRTPVTTVAIPDTRRTDIIDRVRHACITEGRQAYWVC
TLIEESELLEAQAAEATWEELKLALPELNVGLVHGRMKPAEKQAVMASFKQGELHLLVATTVIEVGVDVP
NASLMIIENPERLGLAQLHQLRGRVGRGAVASHCVLLYKTPLSKTAQIRLQVLRDSNDGFVIAQKDLEIR
GPGELLGTRQTGNAEFKVADLLRDQAMIPEVQRLARHIHERYPQQAKALIERWMPETERYSNA

>gi|30065071|ref|NP_839242.1| glutamate transport protein [Shigella flexneri 2a str. 2457T]
MFHLDTLATLVAATLTLLLGRKLVHSVSFLKKYTIPEPVAGGLLVALALLVLKKSMGWEVNFDMSLRDPL
MLAFFATIGLNANIASLRAGGRVVGIFLIVVVGLLVMQNAIGIGMASLLGLDPLMGLLAGSITLSGGHGT
GAAWSKLFIERYGFTNATEVAMACATFGLVLGGLIGGPVARYLVKHSTTPNGIPDDQEVPTAFEKPDVGR
MITSLVLIETIALIAICLTVGKIVAQLLAGTAFELPTFVCVLFVGVILSNGLSIMGFYRVFERAVSVLGN
VSLSLFLAMALMGLKLWELASLALPMLAILVVQTIFMALYAIFVTWRMMGKNYDAAVLAAGHCGFGLGAT
PTAIANMQAITERFGPSHMAFLVVPMVGAFFIDIVNALVIKLYLMLPIFAG

>gi|30065070|ref|NP_839241.1| transporter [Shigella flexneri 2a str. 2457T]
MSVSTLESENAQPVAQTQNSELIYRLEDRPPLPQTLFAACQHLLAMFVAVITPALLICQALGLPAQDTQH

IISMSLFASGVASIIQIKAWGPVGSGLLSIQGTSFNFVAPLIMGGTALKTGGADVPTMMAALFGTLMLAS

CTEMVISRVLHLARRIITPLVSGVVVMIIGLSLIQVGLTSIGGGYAAMSDNTFGAPKNLLLAGVVLALII

LLNRQRNPYLRVASLVIAMAAGYALAWFMGMLPESNEPMTQELIMVPTPLYYGLGIEWSLLLPLMLVFMI

TSLETIGDITATSDVSEQPVSGPLYMKRLKGGVLANGLNSFVSAVFNTFPNSCFGQNNGVIQLTGVASRY

VGFVVALMLIVLGLFPAVSGFVQHIPEPVLGGATLVMFGTIAASGVRIVSREPLNRRAILIIALSLAVGL

GVSQQPLILQFAPEWLKNLLSSGIAAGGITAIVLNLIFPPEKQ

>gi|30065069|ref|NP_839240.1| hypothetical protein S4074 [Shigella flexneri 2a str. 2457T]

MKFIGKLLLYILIALLVVIAGLYFLLQTRWGAEHISAWVSENSDYHLAFGAMDHRFSAPSHIVLENVTFG

RDGQPAPLVAKSVDIALSSRQLTEPRHVDTILLENGTLNLTDQTAPLPFKADRLQLRDMAFNSPNSEWKL

SAQRVNGGVVPWSPEAGKVLGTKAQIQFSAGSLSLNDVPATNVLIEGSIDNDRVTLTNLGADIARGTLTG

NAQRNADGSWQVENLRMADIRLQSEKSLTDFFAPLRSVPSLQIGRLEVIDARLQGPDWAVTDLDLSLRNM

TFSKDDWQTQEGKLSMNASEFIYGSLHLFDPIINAEFSPQGVALRQFTSRWEGGMVRTSGNWLRDGKTLI

LDDAAIAGLEYTLPKNWQQLWMETTPGWLNSLQLKRFSASRNLIIDIDPDFPWQLTALDGYGANLTLVTD

HKWGVWSGSANLNAAAATFNRVDVRRPSLALTANSSTVNISELSAFTEKGILEATASVSQTPQRQTHISL

NGRGVPVNILQQWGWPELPLTGDGNIQLTASGDIQANAPLKPTVSGQLHAVNAAKQQVTQTMNAGVVSSG

EVTSTEPVR

>gi|30065068|ref|NP_839239.1| alpha-xylosidase [Shigella flexneri 2a str. 2457T]

MKISDGNWLIQPGLNLIHPLQVFEVEQQDNEMVVYAAPRDVRERTWQLDTPLFTLRFFSPQEGIVGVRIE

HFQGALNNGPHYPLNILQDVKVTIENTERYAEFKSGNLSARVSKGEFWSLDFLRNGERITGSQVKNNGYV

QDTNNQRNYMFERLDLGVGETVYGLGERFTALVRNGQTVETWNRDGGTSTEQAYKNIPFYMTNRGYGVLV

NHPQCVSFEVGSEKVSKVQFSVESEYLEYFVIDGPTPKAVLDRYTRFTGRPALPPPWSFGLWLTTSFTTN

YDEATVNSFIDGMAERNLPLHVFHFDCFWMKAFQWCDFEWDPLTFPDPEGMIRRLKAKGLKICVWINPYI

GQKSPVFKELQEKGYLLKRPDGSLWQWDKWQPGLAIYDFTNPDACKWYADKLKGLVAMGVDCFKTDFGER

IPIDVQWFDGSDSQKMHNHYAYIYNELVWNVLKDTVGEEEAVLFARSASVGAQKFPVHWGGDCYANYESM

AESLRGGLSIGLSGFGFWSHDIGGFENTAPAHVYKRWCAFGLLSSHSRLHGSKSYRVPWAYDDESCDVVR

FFTQLKCRMMPYLYREAARANARGTPMMRAMMMEFPDDPACDYLDRQYMLGDNVMVAPVFTEAGDVQFYL

PEGRWTHLWHNDELDGSRWHKQQHGFLSLPVYVRDNTLLALGNNDQRPDYVWHEGTAFHLFNLQDGHEAV

CEVPAADGSVIFTLKAARTGNTITVTGAGEAKNWTLCLRNVVKVNGLQGGSQAESEQGLVVTPQGNALTI

TL

>gi|30065067|ref|NP_839238.1| integrase [Shigella flexneri 2a str. 2457T]

MNSSKAGGCHGINDIKVKTAKPKDKPYKLADGGGMYLLINTNGSKYWRMKYRFAGKEKMLSIGVYPDVTL

ADAREKRSEARKLLAAGGDPGEAKKEEKIAQQMSLKNTFEAIAREWHQLKADRWSLRYRDEIIDTFEKDI

FPYIGKRPIAEIKPMKLLEALRKMEKRGALEKMRKVRQRCGEVFRYAIVTGRADYNPAPDLASALATPKK

VHFPFLTANELPHFLNDLAGYTGSIITKTATQIIMLTGVRTQELRFARWEDIDFETKLWEIPAEVMKMKR

PHIVPLSEQVIMLFKQLEPISKHHPLVFIGRNDPRKPISKESINQVIELLGYKGRLTGHGFRHTMSTILH

EQGFNSAWIEMQLAHVDKNSIRGTYNHALYLDGRREMMQWYADYIDSLSSRES

>gi|30065066|ref|NP_839237.1| hypothetical protein S4834 [Shigella flexneri 2a str. 2457T]

MNDRLCFEVHDNKGYFVFPDTWFGPLLGEFEEVLDAYDADEISETSYINKLRRLAQREPDFIDIHAHLAY

AFLEQNAPRKALNAALKGLAAGNRIIPESFCGEIIWMHPENRPYLRALYAAILANVHLQRHQDAVMLTDK

ILAYNPEDNQGARWLLGSELLRTGDHERAFSVLKEHADEFSPYWYELGLLHFLNGEHVKAATAFRHGFAT

NTYIAEMLCGNLHPFPLAVWHDFSGSLDTAEDYYATYSPLWGQYPEALLFVNWLYNHSSVLHERSEIIKC

AEMLIQEDDFEICESILRQQEHLWKRIDKTLSEEIVQKCRNMNGEYIWPWILPFSAAGMKHSSIQYQ

>gi|30065065|ref|NP_839236.1| hypothetical protein S4833 [Shigella flexneri 2a str. 2457T]

MDENALRFASYWRNSLADAESGKGCFERKDAQNFTHWHGIAAGRLDEAIVNKFFKGEKDDVETVNVILRP

KVYFRLLQHGKDRSAGTPDIVTPIVTPALLSREGFLYPTPATSIPRDLLEPLPKGAFSIGEIGQYDKYKT

THTDPTHVMWTRP

>gi|30065064|ref|NP_839235.1| IS3 orfB [Shigella flexneri 2a str. 2457T]

MKYVFIENHRAEFSIKAMCRVLRVARSGWYVWLRRRHQMSLRQQFRLTCDAAVHKAFFEAKQRYGAPRLA

DELPEFNIKTIAASLRRQGLRAKASRKFSPVSYRAHGLPVLENLLEQDFSASGPKPEVGG

>gi|30065063|ref|NP_839234.1| IS3 orfA [Shigella flexneri 2a str. 2457T]

MTKPVSISKKPRKQHTPEFRNEALKLAERIGVAAAARELSLYESQLYAWRSKQQQQMSSSERESELAAEN

VRLKRQLAEQAEELSILQKAATYFAKRLK

>gi|30065062|ref|NP_839233.1| hypothetical protein S4831 [Shigella flexneri 2a str. 2457T]

MMLFSIIIMLLSPKITHANEKVICQNSASFVTKEFICGTILVSLILSSLFCYISISPSIFMHTYGLSTFS

YSIIFSFSVLFFIIGNQLSKIEKLSNLWFLLPNSQFYRITNKWFKNRICRIYYRFYSNNKRWYNQFPVSN

RKRQRCWSRNYFRSWFTNNFITVNSRNRHCRK

>gi|30065061|ref|NP_839232.1| IS629 orfB [Shigella flexneri 2a str. 2457T]

MARCTVARLMAVMGLAGVLRGKKVRTTISRKAVAAGDRVNRQFVAERPDQLWVADFTYVSTWRGFVYVAF

IIDVFAGYIVGWRVSSSMETTFVLDALEQALWARRPSGTVHHSDKGSQYVSLVYTQRLKEAGLLASTGST

GDSYDNAMAESINGLYKAEVIHRKSWKNRAEVELATLTWVDWYNNRRLLERLGHIPPAEAEKAYYASIGN

DDLAA

>gi|30065060|ref|NP_839231.1| IS629 orfA [Shigella flexneri 2a str. 2457T]

MTKNTRFSPEVRQRAVRMVLESQGEYDSQWATICSIAPKIGCTPETLRVRVRQHERDTGGGDGGLTTAER

QRLKELERENRELRRSNDILRQASAYFAKAEFDRLWKK

>gi|30065057|ref|NP_839228.1| colV-immunity protein [Shigella flexneri 2a str. 2457T]

MKRWFKYPVTFDLHYTVSHRLIFAPARFLFLLVVTILNAYDHSILAPVWLCFNSAFRADKQLLAGILLVL

MSVPLFTSADFHIPSNRAYSLSLDYSIVRLKFFLNNNRSTAGRSSPFFITHCTDNHNLISRN

>gi|30065056|ref|NP_839227.1| IS1 orfB [Shigella flexneri 2a str. 2457T]

MSRQCTHYGRWPQHGFTSLKKLRPQSVTSRIQPGSDVIVCAEMDEHWGYVGAKSRQRWLFYAYDRIRRTV

VAHVFGERTLATLERLLSLLSAFEVVVWMTDGWPLYESRLKGKLHVISKRYTQRIERHNLNLRQHLARLV

RKSLSFSKSVELHDKVIGHYLNIKHYQ

>gi|30065055|ref|NP_839226.1| IS1 orfA [Shigella flexneri 2a str. 2457T]

MASISIRCPSCSATEGVVRNGKSTAGHQRYLCSHCRKTWQLQFTYTASQPGTHQKIIDMAMNGVGCRASA

RIMGVGLNTVLRHLKNSGRSR

>gi|30065054|ref|NP_839225.1| hypothetical protein S4829 [Shigella flexneri 2a str. 2457T]

MPCFTAMRAEIALMSGSAFAVTHHAFSSGAGRTSDGNGSHASTLKSPSYTKSVSWQHYPWSETKAAYRCF

SNDKVSANKIMTPHKENITERAQQFKRVLVLQDTTELNYSGQKEKQGVGPKKHKDERNLFLHPQLVISES

GVCLGVYDDYQWFRDELKTSKNTRQEICNDLLHKKHVSEKETWRWVEGYNKATELARQCPDTHVLSISDR

EGDFYDLFERAAQTPGIKADWLVRMKFNNRATLNINGKRDHRLLHERIMEITPQQLVEFTIPDGRGQQAR

TLPDMLSSDAGPLSYPLIYSSKVMIISLFDSTVMPDKYRHNGK

>gi|30065053|ref|NP_839224.1| membrane transport protein [Shigella flexneri 2a str. 2457T]

MSPGIEDTPQKPLSCWPLAFSAGLLGIGQNGLLVVLPVLVIQTNLSLSVWAALLMLGSMLFLPSSPWWGK

QISRTGSKPVVLWALGGYGISFTLLGLGSVLMATSAITTAVGLGILIIARIAYGLTVSAMVPACQVWALQ

RAGEGNRMAALATISSGLSCGRLFGPLCAAAMLAIHPLAPLGLLMAAPVLALLMLLRLPGTPPQPTPECK

SVSLKRDCLPYLLCAILLAAAVSMMQLGLSPALTRQFATDTTAISQQVAWLLGLSAVAALIAQFGVLRPQ

RLTPVALLLSAGVLMSGGLAIMLSEQLWLFYPGCAVLSFGAALATPAYQLLLNDKLADGAGAGWLATSHT

LGYGLCALLVPLVSKTGVAIALIMAALFATILFTIVSVFIWHYRTIK

>gi|30065052|ref|NP_839223.1| siderophore biosynthesis protein [Shigella flexneri 2a str. 2457T]

MRIIIDIIVIILLCGSCLTMTLPSEKPATDVAAQCFLNALIRETTDWKLTEYPPDELLIPLDEQKSLHFR

VAYFSPTQHHRFAFPARLVTASGSYPVDFTTLSRLIIDKLRHQLFLPVPLCETFHQRVLESHVHTQQAID

ARHDWAALREKALNFGEAEQALLTGHAFHPAPKSHEPFNRREAERYLPDMAPHFPLRWFSVDKTQIAGES

LHLNLQQRLTRFAAENAPQLLNELSDNQWLFPLHPWQGEYLLQQGWCQALVAKGLIKDLGEAGTSWLPTT

SSRSLYCATSRDMIKFSLSVRLTNSIRTLSVKEVKRGMRLARLAQTDGWQMLQVRFPTFRVMQEDGWAGL

LDLNGNIMQESLFALRENLLVDQPKSQTNVLVSLTQAAPDGGDSLLVSAVKRLSDRLGITVQQAAHAWVD

AYCQQVLKPLFTAEADYGLVLLAHQQNILVQMLGDLPVGFIYRDCQGSAFMPHATDWLDSIGEAQAENIF

THEQLLRYFPYYLLVNSTFAVTAALGAAGLDSESNLMARVRASLAEVRDQVTHKTCLNYVLESPYWNVKG

NFFCYLNDHNENTIVDPSVIYFDFANPLQAQEV

>gi|30065051|ref|NP_839222.1| siderophore biosynthesis protein [Shigella flexneri 2a str. 2457T]

MSEANIIHSRYGLRCEKLDKPLNLGWGLDNSAVLHCPGELPTGWLCDALDQIFIAAPQLSAVALPWAEWR

EEPQALTLFGQVKSDIIHRTAFWQLPLWLSSPANRASGEMVFDAEREIYFPQRPPRPQGEVYRRYDPRIR

RMLSFRIADPVSDAERFTRWMNDPRVEYFWEQSGSLEVQTAYLERQLTGKHAFPLIGCFDDRPFSYFEIY

WAAEDRIGRHYSWQPFDRGLHLLVGEQQWRGAHYVQSWLRGLTHYLLLDEPRTQRTVLEPRTDNQRLFRH

LEPAGYRTIKEFDFPHKRSRMVMADRHHFFTEVGL

>gi|30065050|ref|NP_839221.1| siderophore biosynthesis protein [Shigella flexneri 2a str. 2457T]

MNHKDWDFVNRRLVAKMLSEMEYEQVFHAESQGDDHYCINLPGAQWRFIAERGIWGWLWIDAQTLRCTDE

PVLAQTLLMQLKPVLSMSDATVAEHMQDLYATLLGDLQLLKARRGLSASDLINLDADRLQCLLSGHPKFV

FNKGRRGWGKEALERYAPEYTNTFRLHWLAVKREHMIWRCDNDLDIQQLLTAAMDPQEFTRFSQVWQENG

LDHNWLPLPVHPWQWQQKIATDFIADFAEGRMVSLGEFGDQWLAQQSLRTLTNASRRGGLDIKLPLTIYN
TSCYRGIPGRYIAAGPLASRWLQQVFATDATLVQSGAVILGEPAAGYVSHEGYAALARAPYRYQEMLGVI
WRENPCRWLKPDESPVLMATLMECDENNQPLAGAYIDRSGLDAETWLTQLFRVVVVPLYHLLCRYGVALI
AHGQNITLAMKEGVPQRVLLKDFQGDMRLVKEAFPEMDSLPQEVRDVTSRLSADYLIHDLQTGHFVTVLR
FISPLMVRLGVPERRFYQLLAAVLSDYMNKHPQMAERFALFSLFRPQIIRVVLNPVKLTWPDLDGGSRML
PNYLENLQNPLWLVTQEYES

>gi|30065049|ref|NP_839220.1| lysine:N6-hydroxylase [Shigella flexneri 2a str. 2457T]
MKKSVDFIGVGTGPFNLSIAALSHQIEELNCLFFDEHPHFSWHPGMLVPDCHMQTVFLKDLVSAVAPTNP
YSFVNYLVKHKKFYRFLTSRLRTVSREEFSDYLRWAAEDMNNLYFSHTVENIDFDKKSRLFLVQTSRGEY
FAHNICLGTGKQPYLPPCVKHVTQSCFHASEMNLRRPDLSGKRITVVGGGQSGADLFLNALRGEWGEAAE
INWVSRRNNFNALDEAAFADEYFTPEYISGFSGLKEDIRHQLLDEQKMTSDGITADSLLTIYRELYHRFE
VLRKPRNIRLLPSRSVTTLESSGPGWRLLMEHHLDQGRESLESDVVIFATGYRSALPQILPSLMPLITMH
DKNTFKVRDDFTLEWSGPKENNIFAVNASMQTHGIAEPQLSLMAWRSARILNRVLGRDLFDLSMPPALIQ
WRSGSRKKPQPEAASLTHYTANIQE

>gi|30065048|ref|NP_839219.1| ferric siderophore receptor [Shigella flexneri 2a str. 2457T]
MRKKYMPRALGPLLLVVLSPAVAQQNDDNEIIVSASRSNRTVAEMAQTTWVIENAELEQQIQGGKELKDA
LAQLIPGLDVSSQSRTNYGMNMRGRPLVVLIDGVRLNSSRSDSRQLDSVDPFNIDHIEVISGATALYGGG
STGGLINIVTKKGQPETMMEFEAGTKSGFNSSKDHDERIAGAVSGGNDHISGRLSVAYQKFGGWFDGNGD
ATLLDNTQTGLQHSNRLDIMGTGTLNIDESRQLQLITQYYKSQGDDNYGLNLGKGFSAISGSSTPYVSKG
LNSDRIPGTERHLISLQYSDSDFLGQELVGQVYYRDESLRYYPFPTVNANKQATAFSSSQQDTDQYGMKL
TLNSQLMDGWQITWGLDAEHERFTSNQMFFDLAQASASGGLNNHKIYTTGRYPSYDITNLAAFLQSSYDI
NDIFTVSGGVRYQYTENRVDDFIDYTQQQKIATGKAISADAIPGGSVDYDNFLFNAGLLMHITERQQAWF
NFSQGVALPDPGKYYGRGIYGAAVNGHLPLTKSVNVSDSKLEGVKVDSYELGWRFIGDNLRTQIAAYYSL

SNKSVERNKDLTISVKDDRRRIYGVEGAVDYLIPDTDWSTGVNFNVLKTESKVNGQWQKYDVKESSPSKA

TAYINWAPEPWSLRVQSTTSFDVSDAEGNDINGYTTVDFISSWQLPVGTLSFSVENLFDRDYTTVWGQRA

PLYYSPGYGPASLYDYKGRGRTFGLNYSVLF

>gi|30065045|ref|NP_839216.1| long polar fimbriae [Shigella flexneri 2a str. 2457T]

MMTTRIVVGLTAGTCLIFSQNLMAEVSVFNPALLEINHQSGVDIRQFNRANLMPPGVYSVDIFINGKMFE

RQDVTFVQDNPDADLHACFIAIKKTLSSFGIKVDALKSFNDVDETVCLDPAPRIEGSSWQFDSDKLQLNI

SIHQIYMDAMAYDYISPTRWDEGINALTINYDFSGSHTLRSDYGSQETDTSYLNLRNGLNIGPWRLRNYS

TLNTSDGRAEYNSISTWIQRDIAALRSQIMIGDTWTASDIFDSTQIRGARLYTDNDMLPASQNGFAPVVR

GIAKSNATVIIRQNGYVIYQSAVPQGAFEITDLNTASTGGDLDVTIKEEDGSEQRFTQPYASLAILKREG

LTDVDVSVGELRDEDGFTPDVLQAQILHGFSHGITLYGGMQAAENYGSAALGVGKDLGALGAISFDVTHA

RANFSHDDTETGQSYRFLYSKLFDDTDTSLRLVGYRYSTEGYYTLNEWASRRNSPEDFWETGNRRSRVEG

TLTQSLGRDYGNLYLTLSRQQYWHTDDVERLMQFGYSSSWKRLSWNVSWSYSNTARQGTGNNHASDNTSE

QIYMLSLSVPLSGWWGNSYATYSVSQNDNSGSSHQLGLSGTALERNNLSWNLMQSYNSHDDEVGGNMSLT

YDGSYGTVNGSYNYSQNSQRLNYGIRGGILAHSEGVTLSQELGETIALVKAPGAAGLEIDNMRGAATDWR

GYTVKTQLNPYDENRVAISDNYFSKSNIELDNTVVTMVPTRGAVVKAEFVTHVGYRVLFRVLNANGKPVP

FGAIAAIQDASLADSGIVGDRGELYLSGLPEKGQVTLSWGENASTKCIFNYSFSTPESESGLIEQGVTCH

>gi|30065044|ref|NP_839215.1| IS1 orfA [Shigella flexneri 2a str. 2457T]

MASISIRCPSCSATEGVVRNGKSTAGHQRYLCSHCRKTWQLQFTYTASQPGTHQKIIDMAMNGVGCRASA

RIMGVGLNTVLRHLKNSGRSR

>gi|30065043|ref|NP_839214.1| phosphate ABC transporter substrate-binding protein [Shigella flexneri 2a str. 2457T]

MKVMRTTVATVVAATLSMSAFSVFAEASLTGAGATFPAPVYAKWADTYQKETGNKVNYQGIGSSGGVKQI

IANTVDFGASDAPLSDEKLAQEGLFQFPTVIGGVVLAVNIPGLKSGELVLDGKTLGDIYLGKIKKWDDEA

IAKLNPGLKLPSQNIAVVRRADGSGTSFVFTSYLAKVNEEWKNNVGTGSTVKWPIGLGGKGNDGIAAFVQ

RLPGAIGYVEYAYAKQNNLAYTKLISADGKPVSPTEENFANAAKGADWSKTFAQDLTNQKGEDAWPITST

TFILIHKDQKKPEQGTEVLKFFDWAYKTGAKQANDLDYASLPDSVVEQVRAAWKTNIKDSSGKPLY

>gi|30065042|ref|NP_839213.1| phosphate transporter permease subunit PstC [Shigella flexneri 2a str. 2457T]

MAATKPAFNPPGKKGDIIFSVLVKLAALIVLLMLGGIIVSLIISSWPSIQKFGLAFLWTKEWDAPNDIYG

ALVPIYGTLVTSFIALLIAVPVSFGIALFLTELAPGWLKRPLGIAIELLAAIPSIVYGMWGLFIFAPLFA

VYFQEPVGNIMSNIPIVGALFSGPAFGIGILAAGVILAIMIIPYIAAVMRDVFEQTPVMMKESAYGIGCT

TWEVIWRIVLPFTKNGVIGGIMLGLGRALGETMAVTFIIGNTYQLDSASLYMPGNSITSALANEFAEAES

GLHVAALMELGLILFVITFIVLA

>gi|30065041|ref|NP_839212.1| phosphate transporter permease subunit PtsA [Shigella flexneri 2a str. 2457T]

MAMVEMQTTAALAESRRKMQARRRLKNRIALTLSMATMAFGLFWLIWILMSTITRGIDGMSLALFTEMTP

PPNTEGGGLANALAGSGLLILWATVFGTPLGIMAGIYLAEYGRKSWLAEVIRFINDILLSAPSIVVGLFV

YTIVVAQMEHFSGWAGVIALALLQVPIVIRTTENMLKLVPDSLREAAYALGTPKWKMISAITLKASVSGI

MTGILLAIARIAGETAPLLFTALSNQFWSTDMMQPIANLPVTIFKFAMSPFAEWQQLAWAGVLIITLCVL

LLNILARVVFAKNKHG

>gi|30065040|ref|NP_839211.1| phosphate transporter subunit [Shigella flexneri 2a str. 2457T]

MSMVETAPSKIQVRNLNFYYGKFHALKNINLDIAKNQVTAFIGPSGCGKSTLLRTFNKMFELYPEQRAEG

EILLDGDNILTNSQDIALLRAKVGMVFQKPTPFPMSIYDNIAFGVRLFEKLSRADMDERVQWALTKAALW

NETKDKLHQSGYSLSGGQQQRLCIARGIAIRPEVLLLDEPCSALDPISTGRIEELITELKQDYTVVIVTH

NMQQAARCSDHTAFMYLGELIEFSNTDDLFTKPAKKQTEDYITGRYG

>gi|30065039|ref|NP_839210.1| transcriptional regulator PhoU [Shigella flexneri 2a str. 2457T]

MDSLNLNKHISGQFNAELESIRTQVMTMGGMVEQQLSDAITAMHNQDSDLAKRVIEGDKNVNMMEVAIDE

ACVRIIAKRQPTASDLRLVMVISKTIAELERIGDVADKICRTALEKFSQQHQPLLVSLESLGRHTIQMLH

DVLDAFARMDIDEAVRIYREDKKVDQEYEGIVRQLMTYMMEDSRTIPSVLTALFCARSIERIGDRCQNIC

EFIFYYVKGQDFRHVGGDELDKLLAGKDSDK

>gi|30065038|ref|NP_839209.1| transcriptional antiterminator BglG [Shigella flexneri 2a str. 2457T]

MNMQITKILNNNVVVVIDDQQREKVVMGRGIGFQKRAGERINSSGIEKEYALSSHELNGRLSELLSHIPL

EVMATCDRLISLAQERLGKLQDSIYISLTDHCQFAIKRFQQNVLLWDIQRLYPKEFQLGEEALTIIDKRL

GVQLPKDEVGFIAMHLVSAQMSGNMEDVAGVTQLMREMLQLIKFQFSLNYQEESLSYQRLVTHLKFLSWR

ILEHASINDSDESLQQAVKQNYPQAWQCAERIAIFIGLQYQRKISPAEIMFLAINIERVRKEH

>gi|30065037|ref|NP_839208.1| beta-glucoside-specific PTS system components IIABC [Shigella flexneri 2a str. 2457T]

MTELARKIVAGVGGADNIVSLMHCATRLRFKLKDESKAQAEVLKKTPGIIMVVESGGQFQVVIGNHVADV

FLAVNSVAGLGEKAQQAPENDEKGNLLNRFVYVISGIFTPLIGLMAATGILKGMLALALTFQWTTEQSGT

YLILFSASDALFGFFPIILGYTAGKRFGGNPFTAMVIGGALVHPLILTAFENGQKADAPGLDFLGIPVTL

LNYSSSVIPIIFSAWLCSILERRLNAWLPSAIKNFFTPLLCLMVITPVTFLLVGQLSTWISEMIAAGYLW

LYQAVPAFAGAVMGGFWQIFVMFGLHRGLVPLCINNFTVLGYDTMIPLLMPAIMAQVGAALGVFLCERDA

QKKVVAGSAALTGLFGITEPAVYGVNLPRKYPFVIACISGALGATIIGYAQTKVYSFGLPSIFTFMQTIP

STGIDFTVWASVIGGVIAIGCAFVGTLMLHFITSKRQPAQGAPQEKTPEVITPPEQGGICSPMTGEIVPL

IHVADTTFASGLLGKGIAILPSVGEVRSPVAGRIASLFATLHAIGIESDDGVEILIHVGIDTVKLDGKFF

SAHVNVGDKVNTGDRLISFDIPAIREAGFDLTTPVLISNSDDFTDVLPHGTAQISAGEPLLSIIR

>gi|30065036|ref|NP_839207.1| phospho-beta-glucosidase B cryptic [Shigella flexneri 2a str. 2457T]

MKAFPETFLWGGATAANQVEGAWLEDGKGITTSDLQPHGVMGKMEPRILGKENIKDVAIDFYYRYPEDIA

LFAEMGFTCLRISIAWARIFPQGDEAEPNEAGLAFYDRLFDEMAQAGIKPLVTLSHYEMLYGLVKNYGGW

ANRAVIDHFEHYARTVFTRYQHKVALWLTFNEINMSLHAPFTGVGLAEESGEAEVYQAIHHQLVASARAV

KAYHSLIPEAKIGNMLLGGLVYPLTCQPQDMLQAMEENRRWMFFGDVQARGQYPGYMQRFFRDHNITIEM

TESDAEDLKHTVDFISFSYYMTGCVSHDESINKNAQGNILNMIPNPHLKSSEWGWQIDPVGLRVMLPTY

>gi|30065035|ref|NP_839206.1| IS1 orfA [Shigella flexneri 2a str. 2457T]

MASISIRCPSCSATEGVVRNGKSTAGHQRYLCSHCRKTWQLQFTYTASQPGTHQKIIDMAMNGVGCRASA

RIMGVGLNTVLRHLKNSGRSR

>gi|30065034|ref|NP_839205.1| IS1 orfB [Shigella flexneri 2a str. 2457T]

MSRQCTHYGRWPQHGFTSLKKLRPQSVTSRIQPGSDVIVCAEMDEHWGYVGAKSRQRWLFYAYDRIRRTV

VAHVFGERTLATLERLLSLLSAFEVVVWMTDGWPLYESRLKGKLHVISKRYTQRIERHNLNLRQHLARLG

RKSLSFSKSVELHDKVIGHYLNIKHYQ

>gi|30065033|ref|NP_839204.1| receptor protein [Shigella flexneri 2a str. 2457T]

MAPLAFSAQSLAESLTVEQRLELLEKALRETQSELKKYKDEEKKKYTPATVNRSVSTNDQGYAANPFPTS

SAAKPDAVLVKNEEKNASETGSIYSSMTLKDFSKFVKDEIGFSYNGYYRSGWGTASHGSPKSWAIGSLGR

FGNEYSGWFDLQLKQRVYNENGKRVDAVVMIDGNVGQQYSTGWFGDNAGGENFMQFSDMYVTTKGFLPFA

PEADFWVGKHGAPKIEIQMLDWKTQRTDAAAGVGLENWKVGPGKIDIALVREDVDDYDRSLQNKQQINTH

TIDLRYKDIPLWDKATLMVSGRYVTANESASEKDNQDNNGYYDWKDTWMFGTSLTQKFDKGGFNEFSFLV

ANNSIARNFGRYAGASPFTTFNGRYYGDHTGGTAVRLTSQGEAYIGDHFIVANAIVYSFGNNIYSYETGA

HSDFESIRAVVRPAYIWDQYNQTGVELGYFTQQNKDANSNKFNESGYKTTLFHTFKVNTSMLTSRLEIRF

YATYIKALENELDGFTFEDNKDAQFAVGAQAEIWW

>gi|30065032|ref|NP_839203.1| xylanase [Shigella flexneri 2a str. 2457T]

MNIKIAALTLAIASGISAQWAIAADMPASPAPTIPVKQYVTQVNADNSVTFRYFAPGAKNVSVVVGVPVP

DNIHPMTKDEAGVWSWRTPILKGNLYEYFFNVDGVRSIDTGTAMTKPQRQVNSSMILVPGSYLDTRSVAH

GDLIAITYHSNALQSERQMYVWTPPGYTGMGEPLPVLYFYHGFGDTGRSAIDQGRIPQIMDNLLAEGKIK

PMLVVIPDTETDAKGIIPEDFVPQERRKVFYPLNAKAADRELMNDIIPLISKRFSVRKDADGRALAGLSQ

GGYQALVSGMNHLESFGWLATFSGVTTTTVPDEGVAARLNDPAAINQQLRNFTVVVGDKDVVTGKDIAGL

KTELEQKKIKFDYQEYPGLNHEMDVWRPAYAAFVQKFKIRH

>gi|30065031|ref|NP_839202.1| 6-phosphogluconolactonase [Shigella flexneri 2a str. 2457T]

MKLIITEDYQEMSRVAAHHLLGYMSKTRRVNLAITAGSTPKGMYEYLTTLVKGKPWYDNCYFYNFDEIPF

RGKEGEGVTITNLRNLFFTPAGIKEENIQKLTIDNYREHDQKLAREGGLDLVVLGLGADGHFCGNLPNTT

HFHEQTVEFPIQGEMVDIVAHGELGGDFSLVPDSYVTMGPKSIMAAKNLLIIVSGAGKAQALKNVLQGPV

TEDVPASVLQLHPSLMVIADKAAAAELAFG

>gi|30065030|ref|NP_839201.1| ISSfl3 orfC [Shigella flexneri 2a str. 2457T]

MNNELPDDIELLKAMLRKQQSRLRQYACQVAGYEQEIERLKAQLDRLRRMLFGQSSEKKRHKLENQIRQA

EKRLSELENRLNTARNLLEDASSVTDSPDTSPPSENPIASKPESPGRKSSRKPLPAELPRETHRLLPAET

SCPACGGVLKEMGETISEQLDIINTAFKVIETIRPKLACSRCDVIVQAPLPPKPIERGYASAGLLARILV

SKYMEHIPLYRQSEIYARQGVELSRNTMVRWVSEMADKLRPLYIALNDYVLEAGKVHADDTPVKVLAPGN

GKTKTGRLWVYVRDDRNAGSSLPAAVWFAYSADRKGEHPQLHLAKYQGVLQADAYAGYNVLYETGRVKEA

GCLAHARRKIHDEDVRRPTEMTQEALRRIAELYDIEAEIRGSPAEERLAVRKARSVQLMQSLYDWIQLQR

KTLSKHAKMAKAFDYILNHWNALNEFCRDGRVEIDNNIGENALRSVAVGRKNYLFFGSDKGGESAAIIYS

LLVTCKQNEVEPEDWLREVIEKLNDWPSNQVHELLPWNFSSVK

>gi|30065029|ref|NP_839200.1| ISSfl3 orfB [Shigella flexneri 2a str. 2457T]

MITLPTGTKIWIIAGITDMRCGFNGLASKVQNTLKDDPFSGHIFVFRGRSGKMVKILWADRDGLCLFTKR

LAGDPGRESAPDASSVIHATGGDRVATSQTDRTAWHPDITRDKTRE

>gi|30065028|ref|NP_839199.1| ISSfl3 orfA [Shigella flexneri 2a str. 2457T]

MNSQTTKDIPCFRSYLPDALRLRFEDKLTIRAIAQRLGLSHSTIHTLFQRFLASGIAWPLPDSVSFAQLD

AILYANRKKELTEPQIREGSWRKERRTSYSREFKVRLAKQALQPGAVVARIAREHDINNNLLFKWKSQYE

DGLLSDDDIQECMPVPVALTDTPEPTRPVTNPFWRNKHDERPEGAPGNVPRCELHLKSGVVKLFDPLTPE

LLRALIREMKGGIR

>gi|30065027|ref|NP_839198.1| inner membrane protein [Shigella flexneri 2a str. 2457T]

MSVSRRVIHHGLYFAVLGPLIGVLFLVLYIFFAKEPLVLLVIIQVLPLFLLLSITTGAIPALLTGVMVAC

LPEKIGSQKNYRCLAGGIGGVVITEIYCAVIVHIKGMASSELFENILSGDSLVVRIIPALLAGVVMSRII

TRLPGLDISCPETDSLS

>gi|30065026|ref|NP_839197.1| 6-phosphogluconate phosphatase [Shigella flexneri 2a str. 2457T]

MSRIEAVFFDCDGTLVDSEVICSRAYVTMFQEFGITLDPEEVFKRFKGVKLYEIIDIVSLEHGVTLAKTE

AEHVYRAEVARLFDSAIEGAGALLSAITAPMCVVSNGPNNKMQHSMGKLNMLHYFPDKLFSGYDIQRWKP

DPALMFHAAKAMNVNVENCILVDDSVAGAQSGIDAGMEVFYFCADPHNKPIVHPKVTTFTHLSQLPELWK

ARGWDITA

>gi|30065025|ref|NP_839196.1| membrane / transport protein [Shigella flexneri 2a str. 2457T]

MSHQHTTQTSGQRMLERVFKLREHGTTARTEVIAGFTTFLTMVYIVFVNPQILGVAGMDTSAVFVTTCLI

AAFGSIMMGLFANLPVALAPAMGLNAFFAFVVVQAMGLPWQIGMGAIFWGAVGLLLLTIFRVRYWMIANI

PVSLRVGITSGIGLFIGMMGLKNAGVIVANSETLVSIGNLTSHSVLLGILGFFIIAILASRNIHAAVLVS

IVVTTLLGWMLGDVHYNGIVSAPPSVMTVVGHVDLAGSFNLGLAGVIFSFMLVNLFDSSGTLIGVTDKAG

LADEKGKFPRMKQALYVDSISSVTGSFIGTSSVTAYIESSSGVSVGGRTGLTAVVVGLLFLLVIFLSPLA

GMVPDYAAAGALIYVGVLMTSSLARVNWQDLTESVPAFITAVMMPFSFSITEGIALGFISYCVMKIGTGR

LRDLSPCVIIVALLFILKIVFIDAH

>gi|30065024|ref|NP_839195.1| hypothetical protein S4025 [Shigella flexneri 2a str. 2457T]

MSEKLQVVTLLGSLRKGSFNGMVARTLPKIAPASMEVNALPSIADIPLYDADVQQEEGFPATVEALAEQI

RQADGVVIVTPEYNYSVPGGLKNAIDWLSRLPDQPLAGKPVLIQTSSMGVIGGARCQYHLRQILVFLDAM

VMNKPEFMGGVIQNKVDPQTGEVIDQGTLDHLTGQLTAFGEFIQRVKI

>gi|30065023|ref|NP_839194.1| hypothetical protein S4024 [Shigella flexneri 2a str. 2457T]

MATHFARGILTEGHLISVRLPSQCHQEARNIPPHRQSRFLASRGLLAELMFMLYGIGELPEIVTLPKGKP

VFSDKNLPSFSISYAGNMVGVALTTEGECGLDMELQRATRGFHSPHAPDNHTFSSNESLWISKQNDPNEA

RAQLITLRRSVLKLTGDVLNDDPRDLQLLPIAGRLKCAHVNHVEALCDAEDVLVWSVAVTPAIEKLSVWE

LDGKHSWKSLPDIHSRANNPTSRMMRFAQLSTVKSFSPN

>gi|30065022|ref|NP_839193.1| IS600 orfA [Shigella flexneri 2a str. 2457T]

MSRKTQRYSKEFKAEAVRTVLENQLSISEGASRLSLPEGTLGQWVTAARKGLGTPGSRTLAELESEILQL

RKALNEARLERDILKKATAYFAQESLKNTR

>gi|30065021|ref|NP_839192.1| IS600 orfB [Shigella flexneri 2a str. 2457T]

MAHIRTRETYGTRRLQTELAENGIIVGRDRLARLRKELRLRCKQKRKFRATTNPNHNLPVAPNLLNQTFA

PTAPNQVWVADLTYVATQEGWLYLAGIKDVYTCEIVGYAMGERMTKELTGKALFMALRSQRPPAGLIHHS

DRGSQYCAYDYRVIQEQFGLKTSMSRKGNCYDNAPMESFWGTLKNESLSHYRFNNRDEAISVIREYIEIF

YNRQRRHSRLGNISPAAFREKYHQMAA

>gi|30065020|ref|NP_839191.1| multidrug efflux system protein MdtL [Shigella flexneri 2a str. 2457T]

MSRFLICSFALVLLYPAGIDMYLVGLPRIAADLNASEAQLHIAFSVYLAGMAAAMLFAGKVADRSGRKPV

AIPGAALFIIASVFCSLAETSTLFLAGRFLQGLGAGCCYVVAFAILRDTLDDRRRAKVLSLLNGITCIIP

VLAPVLGHLIMLKFPWQSLFWAMAMMGIAVLMLSLFILKETRPASPAASDKPRENSESLLNRFFLSRVVI

TTLSVSVILTFVNTSPVLLMEIMGFERGEYATIMALTAGVSMTFSFSTPFALGIFKPRTLMITSQVLFLA

AGITLAVSPSHAVSLFGITLICAGFSVGFGVAMSQALGPFSLRAGVASSTLGIAQVCGSSLWIWLAAVVG

IGAWNMLIGILIACSIVSLLLIMFVAPGRPVAAHEEIHHHA

>gi|30065019|ref|NP_839190.1| tryptophan permease TnaB [Shigella flexneri 2a str. 2457T]

MTDQAEKKHSAFWGVMVIAGTVIGGGMFALPVDLAGAWFFWGAFILIIAWFSMLHSGLLLLEANLNYPVG

SSFNTITKDLIGNTWNIISGITVAFVLYILTYAYISANGAIISETISMNLGYHANPRIVGICTAIFVASV

LWISSLAASRITSLFLGLKIISFVIVFGSFFFQVDYSILRDATSTTAGTSYFPYIFMALPVCLASFGFHG

NIPSLIICYGKRKDKLIKSVVFGSLLALVIYLFWLYCTMGNIPRESFKAIISSGGNIDSLVKSFLGTKQH

GIIEFCLLVFSNLAVASSFFGVTLGLFDYLADLFKIDNSHGGRFKTVLLTFLPPALLYLIFPNGFIYGIG

GAGLCATIWAVIIPAVLAIKARKKFPNQMFTVWGGNLIPAIVILFGITVILCWFGNVFNVLPKFG

>gi|30065018|ref|NP_839189.1| tryptophanase [Shigella flexneri 2a str. 2457T]

MENFKHLPEPFRIRVIEPVKRTTRAYREEAIIKSGMNPFLLDSEDVFIDLLTDSGTGAVTQSMQAAMMRG

DEAYSGSRSYYALAESVKNIFGYQYTIPTHQGRGAEQIYIPVLIKKREQEKGLDRSKMVAFSNYFFDTTQ

GHSQINGCTVRNVYIKEAFDTGVRYDFKGNFDLEGLERGIEEVGPNNVPYIVATITSNSAGGQPVSLANL

KAMYSIAKKYDIPVVMDSARFAENAYFIKQREAEYKDWTIEQITRETYKYADMLAMSAKKDAMVPMGGLL

CMKDDSFFDVYTECRTLCVVQEGFPTYGGLEGGAMERLAVGLYDGMNLDWLAYRIAQVQYLVDGLEEIGV

VCQQAGGHAAFVDAGKLLPHIPADQFPAQALACELYKVAGIRAVEIGSFLLGRDPKTGKQLPCPAELLRL

TIPRATYTQTHMDFIIEAFKHVKENAANIKGLTFTYEPKVLRHFTAKLKEV

>gi|30065017|ref|NP_839188.1| tryptophanase leader peptide [Shigella flexneri 2a str. 2457T]

MNILHVCVTSKWFNIDNKIVDHRP

>gi|30065016|ref|NP_839187.1| IS1 orfA [Shigella flexneri 2a str. 2457T]

MASISIRCPSCSATEGVVRNGKSTAGHQRYLCSHCRKTWQLQFTYTASQPGTHQKIIDMAMNGVGCRASA

RIMGVGLNTVLRHLKNSGRSR

>gi|30065015|ref|NP_839186.1| IS1 orfB [Shigella flexneri 2a str. 2457T]

MSRQCTHYGRWPQHGFTSLKKLRPQSVTSRIQPGSDVIVCAEMDEHWGYVGAKSRQRWLFYAYDRIRRTV

VAHVFGERTLATLERLLSLLSAFEVVVWMTDGWPLYESRLKGKLHVISKRYTQRIERHNLNLRQHLARLG

RKSLSFSKSVELHDKVIGHYLNIKHYQ

>gi|30065014|ref|NP_839185.1| tRNA modification GTPase TrmE [Shigella flexneri 2a str. 2457T]

MSDNDTIVAQATPPGRGGVGILRISGFKAREVAETVLGKLPKPRYADYLPFKDADGSVLDQGIALWFPGP

NSFTGEDVLELQGHGGPVILDLLLKRILTIPGLRIARPGEFSERAFLNDKLDLAQAEAIADLIDASSEQA

ARSALNSLQGAFSARVNHLVEALTHLRIYVEAAIDFPDEEIDFLSDGKIEAQLNDVMADLDAVRAEARQG

SLLREGMKVVIAGRPNAGKSSLLNALAGREAAIVTDIAGTTRDVLREHIHIDGMPLHIIDTAGLREASDE

VERIGIERAWQEIEQADRVLFMVDGTTTDAVDPAEIWPEFIARLPAKLPITVVRNKADITGETLGMSEVN

GHALIRLSARTGEGVDVLRNHLKQSMGFDTNMEGGFLARRRHLQALEQAAEHLQQGKAQLLGAWAGELLA

EELRLAQQNLSEITGEFTSDDLLGRIFSSFCIGK

>gi|30065013|ref|NP_839184.1| inner membrane protein translocase component YidC [Shigella flexneri 2a str. 2457T]

MDSQRNLLVIALLFVSFMIWQAWEQDKNPQPQAQQTTQTTTTAAGSAADQGVPASGQGKLISVKTDVLDL

TINTRGGDVEQALLPAYPKELNSTQPFQLLETSPQFIYQAQSGLTGRDGPDNPANGPRPLYNVEKDAYVL

AEGQNELQVPMTYTDAAGNTFTKTFVLKRGDYAVNVNYNVQNAGEKPLEISSFGQLKQSITLPPHLDTGS

SNFALHTFRGAAYSTPDEKYEKYKFDTIADNENLNISSKGGWVAMLQQYFATAWIPHNDGTNNFYTANLG

NGIVAIGYKSQPVLVQPGQTGAMNSTLWVGPEIQDKMAAVAPHLDLTVDYGWLWFISQPLFKLLKWIHSF

VGNWGFSIIIITFIVRGIMYPLTKAQYTSMAKMRMLQPKIQAMRERLGDDKQRISQEMMALYKAEKVNPL

GGCFPLLIQMPIFLALYYMLMGSVELRQAPFALWIHDLSAQDPYYILPILMGVTMLFIQKMSPTTVTDPM

QQKIMTFMPVIFTVFFLWFPSGLVLYYIVSNLVTIIQQQLIYRGLEKRGLHSREKKKS

>gi|30065012|ref|NP_839183.1| ribonuclease P [Shigella flexneri 2a str. 2457T]

MVKLAFPRELRLLTPSQFTFVFQQPQRAGTPQITILGRLNSLGHPRIGLTVAKKNVRRAHERNRIKRLTR

ESFRLRQHELPAMDFVVVAKKGVADLDNRALSEALEKLWRRHCRLARGS

>gi|30065011|ref|NP_839182.1| 50S ribosomal protein L34 [Shigella flexneri 2a str. 2457T]

MKRTFQPSVLKRNRSHGFRARMATKNGRQVLARRRAKGRARLTVSK

>gi|30065010|ref|NP_839181.1| chromosomal replication initiation protein [Shigella flexneri 2a str. 2457T]

MSLSLWQQCLARLQDELPATEFSMWIRPLQAELSDNTLALYAPNRFVLDWVRDKYLNNINGLLTSFCGAD

APQLRFEVGTKPVTQTPQAAVTSNVAAPAQVAQTQPQRAAPSTRSGWDNVPAPAEPTYRSNVNVKHTFDN

FVEGKSNQLARAAACQVADNPGGAYNPLFLYGGTGLGKTHLLHAVGNGIMARKPNAKVVYMHSERFVQDM

VKALQNNAIEEFKRYYRSVDALLIDDIQFFANKERSQEEFFHTFNALLEGNQQIILTSDRYPKEINGVED

RLKSRFGWGLTVAIEPPELETRVAILMKKADENDIRLPGEVAFFIAKRLRSNVRELEGALNRVIANANFT

GRAITIDFVREALRDLLALQEKLVTIDNIQKTVAEYYKIKVADLLSKRRSRSVARPRQMAMALAKELTNH

SLPEIGDAFGGRDHTTVLHACRKIEQLREESHDIKEDFSNLIRTLSS

>gi|30065009|ref|NP_839180.1| DNA polymerase III subunit beta [Shigella flexneri 2a str. 2457T]

MKFTVEREHLLKPLQQVSGPLGGRPTLPILGNLLLQVADGTLSLTGTDLEMEMVARVALVQPHEPGATTV

PARKFFDICRGLPEGAEIAVQLEGERMLVRSGRSRFSLSTLPAADFPNLDDWQSEVEFTLPQATMKRLIE

ATQFSMAHQDVRYYLNGMLFETEGEELRTVATDGHRLAVCSMPIGQSLPSHSVIVPRKGVIELMRMLDGG

DNLLRVQIGSNNIRAHVGDFIFTSKLVDGRFPDYRRVLPKNPDKHLEAGCDLLKQAFARAAILSNEKFRG

VRLYVSENQLKITANNPEQEEAEEILDVTYSGAEMEIGFNVSYVLDVLNALKCENVRMMLTDSVSSVQIE

DAASQSAAYVVMPMRL

>gi|30065008|ref|NP_839179.1| recombination protein F [Shigella flexneri 2a str. 2457T]

MSLTRLLIRDFRNIETADLALSPGFNFLVGANGSGKTSVLEAIYTLGHGRAFRSLQIGRVIRHEQEAFVL

HGRLQGEERETAIGLTKDKQGDSKVRIDGTDGHKVAELAHLMPMQLITPEGFTLLNGGPKYRRAFLDWGC

FHNEPGFFTAWSNLKRLLKQRNAALRQVTRYEQLRPWDKELIPLAEQISTWRAEYSAGIAADMDDTCKQF

LPEFSLTFSFQRGWEKETEYAEVLERNFERDRQLTYTAHGPHKADLRIRADGAPVEDTLSRGQLKLLMCA

LRLAQGEFLTRESGRRCLYLIDDFASELDDERRGLLASRLKATQSQVFVSAISAEHVIDMSDENSKMFTV

EKGKITD

>gi|30065007|ref|NP_839178.1| DNA gyrase subunit B [Shigella flexneri 2a str. 2457T]

MSNSYDSSSIKVLKGLDAVRKRPGMYIGDTDDGTGLHHMVFEVVDNAIDEALAGHCKEIIVTIHADNSVS

VQDDGRGIPTGIHPEEGVSAAEVIMTVLHAGGKFDDNSYKVSGGLHGVGVSVVNALSQKLELVIQREGKI

HRQIYEHGVPQAPLAVTGETEKTGTMVRFWPSLETFTNVTEFEYEILAKRLRELSFLNSGVSIRLRDKRD

GKEDHFHYEGGIKAFVEYLNKNKTPIHPNIFYFSTEKDGIGVEVALQWNDGFQENIYCFTNNIPQRDGGT

HLAGFRAAMTRTLNAYMDKEGYSKKAKVSATGDDAREGLIAVVSVKVPDPKFSSQTKDKLVSSEVKSAVE

QQMNELLAEYLLENPTDAKIVVGKIIDAARAREAARRAREMTRRKGALDLAGLPGKLADCQERDPALSEL

YLVEGDSAGGSAKQGRNRKNQAILPLKGKILNVEKARFDKMLSSQEVATLITALGCGIGRDEYNPDKLRY

HSIIIMTDADVDGSHIRTLLLTFFYRQMPEIVERGHVYIAQPPLYKVKKGKQEQYIKDDEAMDQYQISIA

LDGATLHTNASAPALAGEALEKLVSEYNATQKMINRMERRYPKAMLKELIYQPTLTEADLSDEQTVTRWV

NALVSELNDKEQHGSQWKFDVHTNAEQNLFEPIVRVRTHGVDTDYPLDHEFITGGEYRRICTLGEKLRGL

LEEDAFIERGERRQPVASFEQALDWLVKESRRGLSIQRYKGLGEMNPEQLWETTMDPESRRMLRVTVKDA

IAADQLFTTLMGDAVEPRRAFIEENALKAANIDI

>gi|30065006|ref|NP_839177.1| hypothetical protein S4005 [Shigella flexneri 2a str. 2457T]

MGLFDEVVGAFLKGDAGKYQAILSWVEEQGGIQVLLEKLQSGGLGAILSTWLSNQQRNQSVSGEQLESAL

GTNAVSDLGQKLGVDTSTASSLLAEQLPKIIDALSPQGEVSAQANNDLLSAGMELLKGKLFR

>gi|30065005|ref|NP_839176.1| sugar phosphatase [Shigella flexneri 2a str. 2457T]

MAIKLIAIDMDGTLLLPDHTISPAVKNAIAAARARGVNVVLTTGRPYAGVHNYLKELHMEQPGDYCITYN

GALVQKAADGSTVAQTALSYDDYRFLEKLSREVGSHFHALDRTTLYTANRDISYYTVHESFVATIPLVFC

EAEKMDPNTQFLKVMMIDEPAILDQAIARIPQEVKEKYTVLKSAPYFLEILDKRVNKGTGVKSLADVLGI

KPEEIMAIGDQENDIAMIEYAGVGVAMDNAIPSVKEVANFVTKSNLEDGVAFAIEKYVLN

>gi|30065003|ref|NP_839174.1| regulator protein for dgo operon [Shigella flexneri 2a str. 2457T]

MTLNKTDRIVITLGKQIVHGKYVPGSPLPAEAELCEEFATSRNIIREVFRSLMAKRLIEMKRYRGAFVAP

RNQWNYLDTDVLQWVLENDYDPRLISAMSEVRNLVEPAIARWAAERATSSDLAQIESALNEMIANNQDRE

AFNEADIRYHEAVLQSVHNPVLQQLSIAISSLQRAVFERTWMGDEANMPQTLQEHKALFDAIRHQDGDAA

EQAALTMIASSTRRLKEIT

>gi|30065002|ref|NP_839173.1| 2-oxo-3-deoxygalactonate kinase [Shigella flexneri 2a str. 2457T]

MTARYIAIDWGSTNLRAWLYQGDHCLESRQSEAGVTRLNGKSPAAVLAEVTTDWREENTPVVMAGMVGSN

VGWKVAPYLSVPARFSSIGEQLTSVGDNIWIIPGLCVSHDDNHNVMRGEETQLIGARALAPSSLYVMPGT

HCKWVQADSQQINDFRTVMTGELHHLLLNHSLIGAGLPPQENSADAFAAGLERGLNAPAILPQLFEVRAS

HVLGTLPREQVSEFLSGLLIGAEVASMRDYVTHQHAITLVAGTSLTARYQQAFQAMGCDVAAVAGDTAFQ

AGIRSIAHAVAN

>gi|30065001|ref|NP_839172.1| D-galactonate transport protein [Shigella flexneri 2a str. 2457T]

MVSGFAMPKIWRKLAMDIPVNAAKPGRRRYLTLVMIFITVVICYVDRANLAVASAHIQEEFGITKAEMGY
VFSAFAWLYTLCQIPGGWFLDRVGSRVTYFIAIFGWSVATLFQGFATGLMSLIGLRAITGIFEAPAFPTN
NRMVTSWFPEHERASAVGFYTSGQFVGLAFLTPLLIWIQEMLSWHWVFIVTGGIGIIWSLIWFKVYQPPR
LTKGISKAELDYIRDGGGLVDGDAPVKKEARQPLTAKDWKLVFHRKLIGVYLGQFAVASALWFFLTWFPN
YLTQEKGITALKAGFMTTVPFLAAFVGVLLSGWVADLLVRKGFSLGFARKTPIICGLLISTCIMGANYTN
DPMMIMCLMALAFFGNGFASITWSLVSSLAPMRLIGLTGGVFNFAGGLGGITVPLVVGYLAQGYGFAPAL
VYISAVALIGALSYILLVGDVKRVG

>gi|30065000|ref|NP_839171.1| oxidoreductase [Shigella flexneri 2a str. 2457T]
MEHFDVAIIGLGPAGSALARKLAGKMQVIALDKKHQCGTEGFSKPCGGLLAPDAQRSFIRDGLTLPVDVI
ANPQIFSVKTVDVAASLTRNYQRSYININRHAFDLWMKSLIPASVEVYHDSLCRKIWREDDKWHVIFRAD
GWEQHITARYLVGADGANSMVRRHLYPDHQIRKYVAIQQWFAEKHPVPFYSCIFDNAITDCYSWSISKDG
YFIFGGAYPMKDGQTRFTTLKEKMSAFQFQFGKAVKSEKCTVLFPSRWQDFVCGKDNAFLIGEAAGFISA
SSLEGISYALDSAEILRAVFLKQPEKSNAAYWRATHKLRLKLFGKIVKSRCLTAPALRKWIMRSGVAHIP
QLKDYPTRFTSPTSRM

>gi|30064999|ref|NP_839170.1| hypothetical protein S3996 [Shigella flexneri 2a str. 2457T]
MMAGTVLYQDRAMKQITFAPRNHLLTNTNTNTNTNTNTWTPDSQWLVFDVRPSGASFTGETIERVNIHTG
EVEVIYRASQGAHVGVVTVHPKSEKYVFIHGPENPDETWHYDFHHRRGVIAEGGKVSNLDAMDITAPYTP
GALRGGSHVHVFSPNGERGSFTYNDHVMHQLDPALDLRNVGVAAPFGPVNVQKQHPREYSGSHWCVLVSK
TTPTPQPGSDEINRAYEEGWVGNHALAFIGDTLSPKGEKVPELFIVELPQDEASWKSAGDAPLSGTETTL
PAPPRGVVQRRLTFTHHRAYPGLVNVPRHWVRCNPQGTQIAFLMRDDNGIVQLWLISPQGGEPRKLTHNK
TDIQSAFNWHPSGEWLGFVLDNRIVCAHAQSGEVEYLTENHANPPSADAVVFSPDGQWLAWMEGGQLWIT
ETDR

>gi|30064997|ref|NP_839168.1| heat shock protein IbpA [Shigella flexneri 2a str. 2457T]

MRNFDLSPLYRSAIGFDRLFNHLENNQSQSNGGYPPYNVELVDENHYRIAIAVAGFAESELEITAQDNLL

VVKGAHADEQKERTYLYQGIAERNFERKFQLAENIHVRGANLVNGLLYIDLERVIPEAKKPRRIEIN

>gi|30064996|ref|NP_839167.1| heat shock chaperone IbpB [Shigella flexneri 2a str. 2457T]

MRNFDLSPLMRQWIGFDKLANALQNAGESQSFPPYNIEKSDDNHYRITLALAGFRQEDLEIQLEGTRLSV

KGTPEQPKEEKKWLHQGLMNQPFSLSFTLAENMEVSGATFVNGLLHIDLIRNEPEPIAAQRIAISERPAL

NS

>gi|30064995|ref|NP_839166.1| hypothetical protein S3992 [Shigella flexneri 2a str. 2457T]

MSDIALTVSILALVAVVGLFIGNVKFRGIGLGIGGVLFGGIIVGHFVSQAGMTLSSDMLHVIQEFGLILF

VYTIGIQVGPGFFASLRVSGLRLNLFAVLIVIIGGLVTAILHKLFDIPLPVVLGIFSGAVTNTPALGAGQ

QILRDLGTPMEMVDQMGMSYAMAYPFGICGILFTMWMLRVIFRVNVETEAQQHESSRTNGGALIKTINIR

VENPNLHDLAIKDVPILNGDKIICSRLKREETLKVPSPDTIIQLGDLLHLVGQPADLHNAQLVIGQEVDT

SLSTKGTDLRVERVVVTNENVLGKRIRDLHFKERYDVVISRLNRAGVELVASGDISLQFGDILNLVGRPS

AIDAVANVLGNAQQKLQQVQMLPVFIGIGLGVLLGSIPVFVPGFPAALKLGLAGGPLIMALILGRIGSIG

KLYWFMPPSANLALRELGIVLFLSVVGLKSGGDFVNTLVNGEGLSWIGYGALITAVPLITVGILARMLAK

MNYLTMCGMLAGSMTDPPALAFANNLHPTSGAAALSYATVYPLVMFLRIITPQLLAVLFWSIG

>gi|30064994|ref|NP_839165.1| transcriptional regulator [Shigella flexneri 2a str. 2457T]

MIYKSIAERLRIRLNSADFTLNSLLPGEKKLAEEFAVSRMTIRKAIDLLVAWGLVVRRHGSGTYLVRKDV

LHQTASLTGLVEVLKRQGKTVTSQVLIFEIMPAPPAIASQLRIQINEQIYFSRRVRFVEGKPLMLEDSYM

PVKLFRNLSLQHLEGSKFEYIEQECGILIGGNYESLMPVLADRLLARQMKVAEHTPLLRITSLSYSESGE

FLNYSVMFRNASEY

>gi|30064993|ref|NP_839164.1| arbutin specific enzyme IIB component of PTS, partial [Shigella flexneri 2a str. 2457T]

MFSNHADMMLTQIAIGLCFTLLYFVVFRTLILQFNMCTPGREDAEVKLYSKAEYKASRGQTTAAEPKKEL

DQAAGILQALGGVGNISSINNCATRLRIALHDMSQTLDDEVFKKLGAHGVFRSGDAIQVIIGLHVSQLRE

QLDSLINSHQSAENVAITEAV

>gi|30064992|ref|NP_839163.1| ARAC-type regulatory protein [Shigella flexneri 2a str. 2457T]

MNGKLQSSDVKNETPYNIPLLINENVISSGISLISLWHTYADEHYRVIWPRDKKKPLIANSWVAVYTVQG

CGKILLKNGEQITLHSNCIIFLKPMDIHSYHCEGLVWEQYWMEFTPTSMMDIPVGQQSVIYNGEIYNQEL

TEVAELITSPEAIKNNLAVAFLTKIIYQWICLMSADGKKDPQRRQIEKLIATLHASLQQRWSVADMAATI

PCSEAWLRRLFLRYTGKTPKEYYLDARLDLALSLLKQQGNSVGEVADTLNFFDSFHFSKAFKHKFGYAPS

AVLKNTDQHPTDASPHN

>gi|30064991|ref|NP_839162.1| symporter YidK [Shigella flexneri 2a str. 2457T]

MNSLQILSFVGFTLLVAVITWWKVRKTDTGSQQGYFLAGRSLKAPVIAASLMLTNLSTEQLVGLSGQAYK

SGMSVMGWEVTSAVTLIFLALIFLPRYLKRGIATIPDFLEERYDKTTRIIIDFCFLIATGVCFLPIVLYS

GALALNSLFHVGESLQISHGAAIWLLVILLGLAGILYAVIGGLRAMAVADSINGIGLVIGGLMVPVFGLI

AIGKGSFMQGIEQITTVHAEKLNSVGGPTDPLPIGAAFTGLILVNTFYWCTNQGIVQRTLASKSLAEGQK

GALLTAVLKMLDPLVLVLPGLIAFHLYQDLPKADMAYPTLVNNVLPVPLEGFFGAVLFGAVISTFNGFLN

SASTLFSMGIYRRIINQNAEPQQLVTVGRKFGFFIAIVSVLVAPWIANAPQGLYSWMKQLNGIYNVPLVT

IIIMGFFFPRIPALAAKVAMGIGIISYITINYLVKFDFHFLYVLACTFCINVVVMLVIGFIKPRATPFTF

KDAFAVDMKPWKNVKIASIGILFAMIGVYVGLAEFGGYGTRWLAMISYFIAAVVIVYLIFDSWWHRHDPA

VTFTPDAKDSL

>gi|30064990|ref|NP_839161.1| hypothetical protein S3984 [Shigella flexneri 2a str. 2457T]

MGIIAQNKISSLGMLFGAIALMMGIIHFSFGPFSAPPPTLESIVADKTAEIKRGLLAGIKGEKITTVEKK

EDMDIDKILDQSGIALAIAALLCAFIGGMRKENRWGIRGALVFGIGIVCSILLIFLIFSFLTGGSLV

>gi|30064989|ref|NP_839160.1| hypothetical protein S3983 [Shigella flexneri 2a str. 2457T]

MKISRLGEAPDYRFSLANERTFLAWIRTALGFLAAGVGLDQLAPDFATPVIRELLALLLCLFSGGLAMYG

YLRWLRNEKAMRLKEDLPYTNSLLIISLILMVVAVIVMGLVLYAG

>gi|30064988|ref|NP_839159.1| hypothetical protein S3982 [Shigella flexneri 2a str. 2457T]

MPDSRKARRIADPGLQPERTSLAWFRTMLGYGALMALAIKHNWHQAGMLFWISIGILAIVALILWHYTRN

RNLMDVTNSDFSQFHVVRDKFLISLAVLSLAILFAVTHIHQLIVFIERVA

>gi|30064987|ref|NP_839158.1| transcriptional regulator [Shigella flexneri 2a str. 2457T]

MTGSQVIDAEEDRHKLVVEYKDALQPADFYHNFKQRGIRSVQLIPYLEFDDRGDLTAASVTAELWGKFLI

ALFECWVRADISRISIELFDATLQKWCGSENPQPRRDCQACDWHRLCPHARQETPDSVLCAGYQAFYSYS

APHMRVMRDLIKQHRSPMELMTMLR

>gi|30064986|ref|NP_839157.1| multidrug resistance protein D [Shigella flexneri 2a str. 2457T]

MLVLLVAVGQMAQTIYIPAIADMARDLNVREGAVQSVMGAYLLTYGVSQLFYGPISDRVGRRPVILVGMS

IFMLATLVAVTTSSLTVLIAASAMQGMGTGVGGVMARTLPRDLYERTQLRHANSLLNMGILVSPLLAPLI

GGLLDTMWNWRACYLFLLVLCAGVTFSMARWMPETRPVDAPRTRLLTSYKTLFGNSGFNCYLLMLIGGLA

GIAAFEACSGVLMGAVLGLSSMTVSILFILPIPAAFFGAWFAGRPNKRFSTLMWQSVICCLLAGLLMWIP

DWFGVMNVWTLLVPAALFFFGAGMLFPLATSGAMEPFPFLAGTAGALVGGLQNIGSGVLASLSAMLPQTG

QGSLGLLMTLMGLLIVLCWLPLATRMSHQGQPV

>gi|30064985|ref|NP_839156.1| ilvB operon leader peptide [Shigella flexneri 2a str. 2457T]

MTTSMFNAKLLPTAPSAAVVVVRVVVVVGNAP

>gi|30064984|ref|NP_839155.1| acetolactate synthase catalytic subunit [Shigella flexneri 2a str. 2457T]

MASSGTTSTRKRFTGAEFIVHFLEQQGIKIVTGIPGGSILPVYDALSQSTQIRHILARHEQGAGFIAQGM

ARTDGKPAVCMACSGPGATNLVTAIADARLDSIPLICITGQVPASMIGTDAFQEVDTYGISIPITKHNYL

VRHIEELPQVMSDAFRIAQSGRPSPVWIDIPKDVQTAVFEIEAQPAVAEKAVAPAFSEESIRDAATMINA

AKRPVLYLGGGVINAPARVRELAEKAQLPTTMTLMALGMLPKAHPLSLGMLGMHGVRSTNYILQEADLLI

VLGARFDDRAIGKTEQFCPNAKIIHVDIDRAELGKIKQPHVAIQADVDDVLAQLIPRVEAQPRAEWHQLV

ADLQREFPCPIPKACDPLSHYGLINAVAACVDDNAIITTDVGQHQMWTAQAYPLNRPRQWLTSGGLGTMG

FGLPAAIGAALANPDRKVLCFSGDGSLMMNIQEMATASENQLDVKIILMNNEALGLVHQQQSLFYEQGVF

AATYPGKINFMQIAAGFGLETCDLNNEADPQAALQEIINRPGPALIHVRIDAEEKVYPMVPPGAANTEMV

GE

>gi|30064983|ref|NP_839154.1| acetolactate synthase 1 regulatory subunit [Shigella flexneri 2a str. 2457T]

MQNTTHNNVILELTVRNHPGVMTHVCGLFARRAFNVEGILCLPIQDSDKSHIWLLVNDDQRLEQMISQIA

KLEDVVKVQRNQSDPTMFNKIAVFFQ

>gi|30064982|ref|NP_839153.1| DNA-binding transcriptional activator UhpA [Shigella flexneri 2a str. 2457T]

MITVALIDDHLIVRSGFAQLLGLEPDLQVVAEFGSGREALAGLPGRGVQVCICDISMPDISGLELLSQLP

KGMATIMLSVHDSPALVEQALNAGARGFLSKRCSPDELIAAVHTVATGGCYLTPDIAIKLASGRQDPLTK

RERQVAEKLAQGMAVKEIAAELGLSPKTVHVHRANLMEKLGVSNDVELARRMFDGW

>gi|30064981|ref|NP_839152.1| sensory histidine kinase UhpB [Shigella flexneri 2a str. 2457T]

MKTLFSRLITVIACFFIFSAAWFCLWSISLHLVERPDMAVLLFPFGLRLGLMLQCPRGYWPVLLGAEWLL

IYWLTQAVGLTHFPLLMIGSLLTLLPVALISRYRHQRDWRTLLLQGAALTAAALLQSLPWLWHGKESWNA

LLLTLTGGLTLAPICLVFWHYLANNTWLPLGPSLVSQPINWRGRHLVWYLLLFVISLWLQLGLPDELSRF

TPFCLALPIIALAWHYGWQGALIATLMNAIALIASQTWRDHLVDLLLSLLVQSLTGLLLGAGIQRLRELN

QSLQKELARNQHLAERLLETEESVRRDVARELHDDIGQTITAIRTQAGIVQRLAADNASVKQSGQLIEQL

SLGVYDAVRRLLGRLRPRQLDDLTLEQAIRSLMREMELEGRGIVSHLEWRIDESALSENQRVTLFRVCQE

GLNNIVKHADASAVTLQGWQQDERLMLVIEDDGSGLPPGSGQQGFGLTGMRERVTALGGTLHISCLHGTR

VSVSLPQRYV

>gi|30064980|ref|NP_839151.1| regulatory protein UhpC [Shigella flexneri 2a str. 2457T]

MLPFLKAPADAPLMTDKHEIDARYRYWRRHILLTIWLGYALFYFTRKSFNAAVPEILANGVLSRSDIGLL

ATLFYITYGVSKFVSGIVSDRSNARYFMGIGLIATGIMNILFGFSTSLWAFAVLWVLNAFFQGWGSPVCA

RLLTAWYSRTERGGWWALWNTAHNVGGALIPIVMAASALHYGWRAGMMIAGCMAIVVGIFLCWRLRDRPQ

ALGLPAVGEWRHDALEIAQQQEGAGLTRKEILTKYVLLNPYIWLLSFCYVLVYVVRAAINDWGNLYMSET

LGVDLVTANTAVTMFELGGFIGALVAGWGSDKLFNGNRGPMNLIFAAGILLSVGSLWLMPFASYVMQATC

FFTIGFFVFGPQMLIGMAAAECSHKEAAGAATGFVGLFAYLGASLAGWPLAKVLDTWHWSGFFVVISIAA

GISALLLLPFLNAQTPREA

>gi|30064979|ref|NP_839150.1| sugar phosphate antiporter [Shigella flexneri 2a str. 2457T]

MLAFLNQVRKPTLDLPLEVRRKMWFKPFMQSYLVVFIGYLTMYLIRKNFNIAQNDMISTYGLSMTQLGMI

GLGFSITYGVGKTLVSYYADGKNTKQFLPFMLILSAICMLGFSASMGSGSVSLFLMIAFYALSGFFQSTG

GSCSYSTITKWTPRRKRGTFLGFWNISHNLGGAGAAGVALFGANYLFDGHVIGMFIFPSIIALIVGFIGL

RYGSDSPESYGLGKAEELFGEEISEEDKETESTDMTKWQIFVEYVLKNKVIWLLCFANIFLYVVRIGIDQ

WSTVYAFQELKLSKAVAIQGFTLFEAGALVGTLLWGWLSDLANGRRGLVACIALALIIATLGVYQHASNE

YIYLASLFALGFLVFGPQLLIGVAAVGFVPKKAIGAADGIKGTFAYLIGDSFAKLGLGMIADGTPVFGLT

GWAGTFAALDIAAIGCICLMAIVAVMEERKIRREKKIQQLTVA

>gi|30064978|ref|NP_839149.1| cryptic adenine deaminase [Shigella flexneri 2a str. 2457T]

MNNSINHKFHHISRAEYQELLAVSRGDAVADYIIDNVSILDLINAGEISGPIVIKGRYIAGVGAEYADTP

ALQRIDARGATAVPGFIDAHLHIESSMMTPVTFETATLPRGLTTVICDPHEIVNVMGEAGFAWFARCAEQ

ARQNQYLQVSSCVPALEGCDVNGASFTLEQMLAWRDHPQVTGLAEMMDYPGVINGQNALLDKLDAFRYLT

LDGHCPGLGGKELNAYIAAGIENCHESYQLEEGRRKLQLGMSLMIREGSAASNLNALAPLINEFNSPQCM

LCTDDRNPWEIAHEGHIDALIRRLIEQHNVPLHVAYRVASWSTARHFGLNHLGLLAPGKQADIVLLSDAR

KVTVQQVLVKGEPIDAQTLQAEESARLALSAPPYGNTIARQPVSASDFALQFTPGKRYRVIDVIHNELIT

HSRSSVYSENGFDRDDVCFIAVLERYGQRLAPACGLLGGFGLNEGALAATVSHDSHNIVVIGRSAEEMAL

AVNQVIQDGGGLCVVRNGQVQSHLPLPIAGLMSTDTAQSLAEQIDALKAAARECGPLPDEPFIQMAFLSL

PVIPALKLTSQGLFDGEKFAFTTLEVTE

>gi|30064977|ref|NP_839148.1| hypothetical protein S3971 [Shigella flexneri 2a str. 2457T]

MIIITEPLLSFVLQKQGIKSPPMDKKMNNDNTDYVSNESGTLSRLFKLPQHGTTVRTELIAGMTTFLTMV

YIVFVNPQILGAAQMDPKVVFVTTCLIAGIGSIAMGIFANLPVALAPAMGLNAFFAFVVVGAMGISWQTG

MGAIFWGAIGLFLLTLFRIRYWMISNIPLSLRIGITSGIGLFIALMGLKNTGVIVANKDTLVMIGDLSSH

GVLLGILGFFIITVLSSRHFHAAVLVSIVVTSCCGLFFGDVHFSGVYSIPPDISGVIGEVDLSGALTLEL

AGIIFSFMLINLFDSSGTLIGVTDKAGLIDSNGKFPNMNKALYVDSVSSVAGAFIGTSSVTAYIESTSGV

AVGGRTGLTAVVVGVMFLLVMFFSPLVAMVPPYATAGALIFVGVLMTSSLARVNWDDFTESVPAFITTVM

MPFTFSITEGIALGFMSYCIMKVCTGRWRDLNLCVVVVAALFALKIILVD

>gi|30064976|ref|NP_839147.1| hypothetical protein S3970 [Shigella flexneri 2a str. 2457T]

MSSDYAGELMIWIMLATLAVVFVVGFRVLTSGARKAIRRLSDRLNIDVVPVESMVDQMGKSAGDEFLRYL

HRPDESHLQNAAQVLLIWQIVIVDDSEQNLLQWHRILQKARLSAPITDAQVRLALGFLRETEPEMQDINA

FQMRYNAFFQPAEGVHWLH

>gi|30064974|ref|NP_839145.1| transporter [Shigella flexneri 2a str. 2457T]

MKPTTLLLIFTFFAMPGIVYAESPFSSLQSAKEKTTVLQDLRKICTPHASLSDEAWEKLMLSDENNKQHI

REAIVAMERNNQSNYWEALGKVECPDM

>gi|30064973|ref|NP_839144.1| IS600 orfA [Shigella flexneri 2a str. 2457T]

MSRKTQRYSKEFKAEAVRTVLENQLSISEGASRLSLPEGTLGQWVTAARKGLGTPGSRTLAELESEILQL

RKALNEARLERDILKKATAYFAQESLKNTR

>gi|30064972|ref|NP_839143.1| IS600 orfB [Shigella flexneri 2a str. 2457T]

MAHIRTRETYGTRRLQTELAENGIIVGRDRLARLRKELRLRCKQKRKFRATTNPNHNLPVAPNLLNQTFA

PTAPNQVWVADLTYVATQEGWLYLAGIKDVYTCEIVGYAMGERMTKELTGKALFMALRSQRPPAGLIHHS

DRGSQYCAYDYRVIQEQFGLKTSMSRKGNCYDNAPMESFWGTLKNESLSHYRFNNRDEAISVIREYIEIF

YNRQRRHSRLGNISPAAFREKYHQMAA

>gi|30064971|ref|NP_839142.1| secreted autotransporter toxin, partial [Shigella flexneri 2a str. 2457T]

MFTTKGIDISGELTMMGIPEQNSKTVTPGLHYAADGFRLSGGNANFIARNMASVTGNIYADDAATITLGQ

PETETPTISSAYQAWAETLLYGFDTAYRGAITAPKATVSMNNAIWHLNSQSSINRLETKDSMVRFTGDNG

KFTTLTVDNLTIDDSAFVLRANLAQADQ

>gi|30064968|ref|NP_839139.1| major fimbrial subunit [Shigella flexneri 2a str. 2457T]

MKRNIIGGAFTLASLMLAGHALAEDGVVNFVGEIVDTTCEVTSDTADQIVPLGKVSKNAFSGVGSLASPQ

KFSIKLENCPATYTQAAVRFDGTEAPGGDGDLKVGTPLTAGNPGDFTGTGQAIAATGVGIRIFNQSDNSQ

VKLYNDSAYTAIDAEGKAEMKFIARYVATNATVTAGTANADSQFTVEYKK

>gi|30064967|ref|NP_839138.1| IS4 orf [Shigella flexneri 2a str. 2457T]

MHIGQALDLVSRYDSLRNPLTSLGDYLDPELISRCLAESGTVTLRKRRLPLEMMVWCIVGMALERKEPLH

QIVNRLDIMLPGNRPFVAPSAVIQARQRLGSEAVRRVFTKTAQLWHNATPHPHWCGLTLLAIDGVFWRTP

DTPENDAAFPRQTHAGNPALYPQVKMVCQMELTSHLLTAAAFGTMKNSENELAEQLIEQTGDNTLTLMDK

GYYSLGLLNAWSLAGEHRHWMIPLRKGAQYEELRKLGKGDHLVKLKTSPQARKKWPGLGNEVTARLLTVT

RKGKVCHLLTSMTDAMRFPGGEMADLYSHRWEIELGYREIKQTMQLSRLTLRSKKPELVEQELWGVLLAY

NLVRYQMIKMAEHLKGYWPNQLSFSESCGMVMRMLMTLQGASPGRIPELMSDLASMGQLVKLPTRRGRAF

PRVVKERPWKYPTAPKKSQSVA

>gi|30064966|ref|NP_839137.1| glucosamine--fructose-6-phosphate aminotransferase [Shigella flexneri 2a str. 2457T]

MCGIVGAIAQRDVAEILLEGLRRLEYRGYDSAGLAVVDAEGHMTRLRRLGKVQMLAQAAEEHPLHGGTGI

AHTRWATHGEPSEANAHPHVSEHIVVVHNGIIENHEPLREELKARGYTFVSETDTEVIAHLVNWELKQGG

TLREAVLRAIPQLRGAYGTVIMDSRHPDTLLAARSGSPLVIGLGMGENFIASDQLALLPVTRRFIFLEEG

DIAEITRRSVNIFDKTGAEVKRQDIESNLQYDAGDKGIYRHYMQKEIYEQPNAIKNTLTGRISHGQVDLS

ELGPNADELLSKVEHIQILACGTSYNSGMVSRYWFESLAGIPCDVEIASEFRYRKSAVRRNSLMITLSQS

GETADTLAGLRLSKELGYLGSLAICNVPGSSLVRESDLALMTNAGTEIGVASTKAFTTQLTVLLMLVAKL

SRLKGLDASIEHDIVHGLQALPSRIEQMLSQDKRIEALAEDFSDKHHALFLGRGDQYPIALEGALKLKEI

SYIHAEAYAAGELKHGPLALIDADMPVIVVAPNNELLEKLKSNIEEVRARGGQLYVFADQDAGFVSSDNM

HIIEMPHVEEVIAPIFYTVPLQLLAYHVALIKGTDVDQPRNLAKSVTVE

>gi|30064965|ref|NP_839136.1| bifunctional N-acetylglucosamine-1-phosphate uridyltransferase/glucosamine-1-phosphate acetyltransferase [Shigella flexneri 2a str. 2457T]

MLNNAMSVVILAAGKGTRMYSDLPKVLHTLAGKAMVQHVIDAANELGAAHVHLVYGHGGDLLKQALKDDN

LNWVLQAEQLGTGHAMQQAAPFFADDEDILMLYGDVPLISVETLQRLRDAKPQGGIGLLTVKLDDPTGYG

RITRENGKVTGIVEHKDATDEQRQIQEINTGILIANGADMKRWLAKLTNNNAQGEYYITDIIALAYQEGR

EIVAVHPQRLSEVEGVNNRLQLSRLERVYQSEQAEKLLLAGVMLRDPARFDLRGTLTHGRDVEIDTNVII

EGNVTLGHRVKIGTGCVIKNSVIGDDCEISPYTVVEDANLAAACTIGPFARLRPGAELLEGAHVGNFVEM

KKARLGKGTKAGHLTYLGDAEIGDNVNIGAGTITCNYDGANKFKTIIGDDVFVGSDTQLVAPVTVGKGAT

IAAGTTVTRNVGENALAISRVPQTQKEGWRRPVKKK

>gi|30064964|ref|NP_839135.1| F0F1 ATP synthase subunit epsilon [Shigella flexneri 2a str. 2457T]

MTYHLDVVSAEQQMFSGLVEKIQVTGSEGELGIYPGHAPLLTAIKPGMIRIVKQHGHEEFIYLSGGILEV

QPGNVTVLADTAIRGQDLDEARAMEAKRKAEEHISSSHGDVDYAQASAELAKAIAQLRVIELTKKAM

>gi|30064963|ref|NP_839134.1| F0F1 ATP synthase subunit beta [Shigella flexneri 2a str. 2457T]

MATGKIVQVIGAVVDVEFPQDAVPRVYDALEVQNGNERLVLEVQQQLGGGIVRTIAMGSSDGLRRGLDVK

DLEHPIEVPVGKATLGRIMNVLGEPVDMKGEIGEEERWAIHRAAPSYEELSNSQELLETGIKVIDLMCPF

AKGGKVGLFGGAGVGKTVNMMELIRNIAIEHSGYSVFAGVGERTREGNDFYHEMTDSNVIDKVSLVYGQM

NEPPGNRLRVALTGLTMAEKFRDEGRDVLLFVDNIYRYTLAGTEVSALLGRMPSAVGYQPTLAEEMGVLQ

ERITSTKTGSITSVQAVYVPADDLTDPSPATTFAHLDATVVLSRQIASLGIYPAVDPLDSTSRQLDPLVV

GQEHYDTARGVQSILQRYQELKDIIAILGMDELSEEDKLVVARARKIQRFLSQPFFVAEVFTGSPGKYVS

LKDTIRGFKGIMEGEYDHLPEQAFYMVGSIEEAVEKAKKL

>gi|30064962|ref|NP_839133.1| F0F1 ATP synthase subunit gamma [Shigella flexneri 2a str. 2457T]

MAGAKEIRSKIASVQNTQKITKAMEMVAASKMRKSQDRMAASRPYAETMRKVIGHLAHGNLEYKHPYLED

RDVKRVGYLVVSTDRGLCGGLNINLFKKLLAEMKTWTDKGVQCDLAMIGSKGVSFFNSVGGNVVAQVTGM

GDNPSLSELIGPVKVMLQAYDEGRLDKLYIVSNKFINTMSQVPTISQLLPLPASDDDDLKHKSWDYLYEP

DPKALLDTLLRRYVESQVYQGVVENLASEQAARMVAMKAATDNGGSLIKELQLVYNKARQASITQELTEI

VSGAAAV

>gi|30064961|ref|NP_839132.1| F0F1 ATP synthase subunit alpha [Shigella flexneri 2a str. 2457T]

MQLNSTEISELIKQRIAQFNVVSEAHNEGTIVSVSDGVIRIHGLADCMQGEMISLPGNRYAIALNLERDS

VGAVVMGPYADLAEGMKVKCTGRILEVPVGRGLLGRVVNTLGAPIDGKGPLDHDGFSAVEAIAPGVIERQ

SVDQPVQTGYKAVDSMIPIGRGQRELIIGDRQTGKTALAIDAIINQRDSGIKCIYVAIGQKASTISNVVR

KLEEHGALANTIVVVATASESAALQYLAPYAGCAMGEYFRDRGEDALIIYDDLSKQAVAYRQISLLLRRP

PGREAFPGDVFYLHSRLLERAARVNAEYVEAFTKGEVKGKTGSLTALPIIETQAGDVSAFVPTNVISITD

GQIFLETNLFNAGIRPAVNPGISVSRVGGAAQTKIMKKLSGGIRTALAQYRELAAFSQFASDLDDATRKQ

LDHGQKVTELLKQKQYAPMSVAQQSLVLFAAERGYLADVELSKIGSFEAALLAYVDRDHAPLMQEINQTG

GYNDEIEGKLKGILDSFKATQSW

>gi|30064960|ref|NP_839131.1| F0F1 ATP synthase subunit delta [Shigella flexneri 2a str. 2457T]

MSEFITVARPYAKAAFDFAVEHQSVERWQDMLTFAAEVTKNEQMAELLSGALAPETLAESFIAVCGEQLD

ENGQNLIRVMAENGRLNALPDVLEQFIHLRAVSEATAEVDVISAAALSEQQLAKISAAMEKRLSRKVKLN

CKIDKSVMAGVIIRSGDMVIDGSVRGRLERLADVLQS

>gi|30064959|ref|NP_839130.1| F0F1 ATP synthase subunit B [Shigella flexneri 2a str. 2457T]

MNLNATILGQAIAFVLFVLFCMKYVWPPLMAAIEKRQKEIADGLASAERAHKDLDLAKASATDQLKKAKA

EAQVIIEQANKRRSQILDEAKAEAEQERTKIVAQAQAEIEAERKRAREELRKQVAILAVAGAEKIIERSV

DEAANSDIVDKLVAEL

>gi|30064958|ref|NP_839129.1| F0F1 ATP synthase subunit C [Shigella flexneri 2a str. 2457T]

MENLNMDLLYMAAAVMMGLAAIGAAIGIGILGGKFLEGAARQPDLIPLLRTQFFIVMGLVDAIPMIAVGL

GLYVMFAVA

>gi|30064957|ref|NP_839128.1| F0F1 ATP synthase subunit A [Shigella flexneri 2a str. 2457T]

MASENMTPQDYIGHHLNNLQLDLRTFSLVDPQNPPATFWTINIDSMFFSVVLGLLFLVLFRSVAKKATSG

VPGKFQTAIELVIGFVNGSVKDMYHGKSKLIAPLALTIFVWVFLMNLMDLLPIDLLPYIAEHVLGLPALR

VVPSADVNVTLSMALGVFILILFYNIKMKGIGGFTKELTLQPFNHWAFIPVNLILEGVSLLSKPVSLGLR

LFGNMYAGELIFILIAGLLPWWSQWILNVPWAIFHILIITLQAFIFMVLTIVYLSMASEEH

>gi|30064955|ref|NP_839126.1| 16S rRNA methyltransferase GidB [Shigella flexneri 2a str. 2457T]

MLNKLSLLLKDAGISLTDHQKNQLIAYVNMLHKWNKAYNLTSVRDPNEMLVRHILDSIVVAPYLQGERFI

DVGTGPGLPGIPLSIVRPEAHFTLLDSLGKRVRFLRQVQHELKLENIEPVQSRVEEFPSEPPFDGVISRA

FASLNDMVSWCHHLPGEQGRFYALKGQMPEDEIALLPEEYQVESVVKLQVPALDGERHLVVIKANKI

>gi|30064954|ref|NP_839125.1| tRNA uridine 5-carboxymethylaminomethyl modification enzyme GidA [Shigella flexneri 2a str. 2457T]

MFYPDPFDVIIIGGGHAGTEAAMAAARMGQQTLLLTHNIDTLGQMSCNPAIGGIGKGHLVKEVDALGGLM

AKAIDQAGIQFRILNASKGPAVRATRAQADRVRYRQAVRTALENQPNLMIFQQAVEDLIVENDRVVGAVT

QMGLKFRAKAVVLTVGTFLDGKIHIGLDNYSGGRAGDPPSIPLSRRLRELPLRVGRLKTGTPPRIDARTI

DFSVLAQQHGDNPMPVFSFMGNASLHPQQVPCYITHTNEKTHDVIRSNLDRSPMYAGVIEGVGPRYCPSI

EDKVMRFADRNQHQIFLEPEGLTSNEIYPNGISTSLPFDVQMQIVRSMQGMENAKIVRPGYAIEYDFFDP

RDLKPTLESKFIQGLFFAGQINGTTGYEEAAAQGLLAGLNAARLSADKEGWAPARSQAYLGVLVDDLCTL

GTKEPYRMFTSRAEYRLMLREDNADLRLTEIGRELGLVDDERWARFNEKLENIERERQRLKSTWVTPSAE

AAAEVNAHLTAPLSREASGEDLLRRPEMTYEKLTTLTPFAPALTDEQAAEQVEIQVKYEGYIARQQDEIE

KQLRNENTLLPATLDYRQISGLSNEVIAKLNDHKPASIGQASRISGVTPAAISILLVWLKKQGMLRRSA

>gi|30064953|ref|NP_839124.1| flavodoxin [Shigella flexneri 2a str. 2457T]

MADITLISGSTLGGAEYVAEHLAEKLEEVGFTTETLHGPLLEDLSASGIWLVISSTHGAGDIPDNLSPFY

EALQEQKPDLSAVRFGTIGIGSREYDTFCGAIDKLEAELKNSGAKQTGETLKINILDHDIPEDPAEEWLG

SWINLLK

>gi|30064952|ref|NP_839123.1| DNA-binding transcriptional regulator AsnC [Shigella flexneri 2a str. 2457T]

MENYLIDNLDRGILEALMGNARTAYAELAKQFGVSPGTIHVRVEKMKQAGIITGARIDVSPKQLGYDVGC

FIGIILKSAKDYPSALAKLESLDEVTEAYYTTGHYSIFIKVMCRSIDALQHVLINKIQTIDEIQSTETLI

VLQNPIMRTIKP

>gi|30064951|ref|NP_839122.1| asparagine synthetase AsnA [Shigella flexneri 2a str. 2457T]

MKTAYIAKQRQISFVKSHFSRQLEERLGLIEVQAPILSRVGDGTQDNLSGCEKAVQVKVKALPDAQFEVV

HSLAKWKRQTLGQHDFSAGEGLYTHMKALRPDEDRLSPLHSVYVDQWDWERVMGDGERQLSTLKSTVEAI

WAGIKATEAAVNEEFGLAPFLPDQIHFVHSQELLSRYPDLDAKGRERAIAKDLGAVFLVGIGGKLSDGHR

HDVRAPDYDDWSTPSELGHAGLNGDILVWNPVLEDAFELSSMGIRVDADTLKHQLALTGDEDRLQLEWHQ

ALLRGEMPQTIGGGIGQSRLTMLLLQLPHIGQVQCGVWPAAVRESVPSLL

>gi|30064948|ref|NP_839119.1| IS4 orf [Shigella flexneri 2a str. 2457T]

MHIGQALDLVSRYDSLRNPLTSLGDYLDPELISRCLAESGTVTLRKRRLPLEMMVWCIVGMALERKEPLH

QIVNRLDIMLPGNRPFVAPSAVIQARQRLGSEAVRRVFTKTAQLWHNATPHPHWCGLTLLAIDGVFWRTP

DTPENDAAFPRQTHAGNPALYPQVKMVCQMELTSHLLTAAAFGTMKNSENELAEQLIEQTGDNTLTLMDK

GYYSLGLLNGWSLAGEHRHWMIPLRKGAQYEELRKLGKGDHLVKLKTSPQARKKWPGLGNEVTARLLTVT

RKGKVCHLLTSMTDAMRFPGGEMADLYSNRWEIELGYREIKQTMQLSRLTLRSKKPELVEQELWGVLLAY

NLVRYQMIKMAEHLKGYWPNQLSFSESCGMVMRMLMTLQGASPGRIPELMRDLASMGQLVKLPTRRGRAF

PRVVKERPWKYPTAPKKSQSVA

>gi|30064947|ref|NP_839118.1| potassium transport protein Kup [Shigella flexneri 2a str. 2457T]

MSTDNKQSLPAITLAAIGVVYGDIGTSPLYTLRECLSGQFGFGVERDAVFGFLSLIFWLLIFVVSIKYLT

FVMRADNAGEGGILTLMSLAGRNTSARTTSMLVIMGLIGGSFFYGEVVITPAISVMSAIEGLEIVAPQLD

TWIVPLSIIVLTLLFMIQKHGTAMVGKLFAPIMLTWFLILAGLGLRSIIANPEVLHALNPMWAVHFFLEY

KTVSFIALGAVVLSITGGEVLYADMGHFGKFSIRLAWFTVVLPSLTLNYFGQGALLLKNPEAIKNPFFLL
APDWALIPLLIIAALATVIASQAVISGVFSLTRQAVRLGYLSPMRIIHTSEMESGQIYIPFVNWMLYVAV
VIVIVSFEHSSNLAAAYGIAVTGTMVLTSILSTTVARQNWHWNKYFVALILIAFLCVDIPLFTANLDKLL
SGGWLPLSLGTVMFIVMTTWKSERFRLLRRMHEHGNSLEAMIASLEKSPPVRVPGTAVYMSRAINVIPFA
LMHNLKHNKVLHERVILLTLRTEDAPYVHNVRRVQIEQLSPTFWRVVASYGWRETPNVEEVFHRCGLEGL
SCRMMETSFFMSHESLILGKRPWYLRLRGKLYLLLQRNALRAPDQFEIPPNRVIELGTQVEI

>gi|30064946|ref|NP_839117.1| D-ribose pyranase [Shigella flexneri 2a str. 2457T]
MKKGTVLNSDISSVISRLGHTDTLVVCDAGLPIPKSTTRIDMALTQGVPSFMQVLGVVTNEMQVEAAIIA
EEIKQHNPQLHETLLTHLEQLQKHQGNTIEIRYTTHEQFKQQTAESQAVIRSGECSPYANIILCAGVTF

>gi|30064945|ref|NP_839116.1| IS1 orfA [Shigella flexneri 2a str. 2457T]
MASISIRCPSCSATEGVVRNGKSTAGHQRYLCSHCRKTWQLQFTYTASQPGTHQKIIDMAMNGVGCRASA
RIMGVGLNTVLRHLKNSGRSR

>gi|30064944|ref|NP_839115.1| IS1 orfB [Shigella flexneri 2a str. 2457T]
MSRQCTHYGRWPQHGFTSLKKLRPQSVTSRIQPGSDVIVCAEMDEHWGYVGAKSRQRWLFYAYDRIRRTV
VAHVFGERTLATLERLLSLLSAFEVVVWMTDGWPLYESRLKGKLHVISKRYTQRIERHNLNLRQHLARLG
RKSLSFSKSVELHDKVIGHYLNIKHYQ

>gi|30064943|ref|NP_839114.1| ribose ABC transporter permease [Shigella flexneri 2a str. 2457T]
MITQTVSGRRYFTKAWLMEQKSLIALLVLIAIVSTLSPNFFTINNLFNILQQTSVNAIMAVGMTLVILTS
GIDLSVGSLLALTGAVAASIVGIEVNALVAVAAALTLGAAIGAVTGVIVAKGRVQAFIATLVMMLLLRGV
TMVYTNGSPVNTGFTENADLFGWFGIGRPLGVPTPVWIMGIVFLAAWYMLHHTRLGRYIYALGGNEAATR
LSGINVNKIKIIVYSLCGLLASLAGIIEVARLSSAQPTAGTGYELDAIAAVVLGGTSLAGGKGRIVGTLI

GALILGFLNNGLNLLGVSSYYQMIVKAVVILLAVLVDNKKQ

>gi|30064942|ref|NP_839113.1| ribokinase [Shigella flexneri 2a str. 2457T]

MDIPNMQNAGSLVVLGSINADHILNLQSFPTPGETVTGNHYQVAFGGKGANQAVAAGRSGANIAFIACTG

DDSIGESVRQQLATDNIDITPVSVIKGESTGVALIFVNGEGENVIGIHAGANAALSPSLVEAQRERIANA

SALLMQLESPLESVMAAAKIAHQNKTIVALNPAPARELPDELLALVDIITPNETEAEKLTGIRVENDEDA

AKAAQVLHEKGIRTVLITLGSRGVWASVNGEGQRVPGFRVQAVDTIAAGDTFNGALITALLEEKPLPEAI

RFAHAAAAIAVTRKGAQPSVPWREEIDAFLDRQR

>gi|30064941|ref|NP_839112.1| transcriptional repressor RbsR [Shigella flexneri 2a str. 2457T]

MKDVARLAGVSTSTVSHVINKDRFVSEAITAKVEAAIKELNYAPSALARSLKLNQTHTIGMLITASTNPF

YSELVRGVERSCFERGYSLVLCNTEGDEQRMNRNLETLMQKRVDGLLLLCTETHQPSREIMQRYPTVPTV

MMDWAPFDGDSDLIQDNSLLGGDLATQYLIDKGHTRIACITGPLDKTPARLRLEGYRAAMKRAGLNIPDG

YEVTGDFEFNGGFDAMRQLLSHPLRPQAVFTGNDAMAVGVYQALYQAELQVPQDIAVIGYDDIELASFMT

PPLTTIHQPKDELGELAIDVLIHRITQPTLQQQRLQLTPILMERGSA

>gi|30064940|ref|NP_839111.1| transporter [Shigella flexneri 2a str. 2457T]

MSGLPWIAAMAFFMQALDATILNTALPAIAHSLNRSPLAMQSAIISYTLTVAMLIPVSGWLADRFGTRRI

FTLAVSLFTLGSLACALSNSLPQLVVFRVIQGIGDAMMMPVARPALLRAYPRNELLPVLNFVAMPGLVGP

ILGPVLGGVLVTWATWHWIFLINIPIGIAGLLYARKHMPNFTTARRRFDITGFLLFGLSLVLFSSGIELF

GEKIVASWIALTVIVTSIGLLLLYILHARRTPNPLISLDLFKTRTFSIGIVGNIATRLGTGCVPFLMPLM

LQVGFGYQAFIAGCMMAPTALGSIIAKSMVTQVLRRLGYRHTLVGITVIIGLMIAQFSLQSPAMAIWMLI

LPLFILGMAMSTQFTAMNTITLADLTDDNASSGNSVLAVTQQLSISLGVAVSAAVLRIYEGMEGTTTVEQ

FHYTFITMGIITVASAAMFMLLKTTDGNNLIKRQRKSMPNRVPSESE

>gi|30064939|ref|NP_839110.1| hypothetical protein S3929 [Shigella flexneri 2a str. 2457T]

MSRTAVREAVKTLTAKGMVLPRPRIGTRVMPQSNWNFLDQELLTWWITEENFHQVIDHFLVMRICLEPQA

CLLAATVGTAEQKAHLNTLMAEMAALKENFRRERWIEVDMAWHEHIYEMSANPFLTSFASLFHSVYHTYF

TSITSDTVIKLDLHQAIVDAIIQSDGDAAFKACQALLRSPDK

>gi|30064938|ref|NP_839109.1| transcriptional regulator HdfR [Shigella flexneri 2a str. 2457T]

MDTELLKTFLEVSRTRHFGRAAESLYLTQSAVSFRIRQLENQLGVNLFTRHRNNIRLTAAGEKLLPYAET

LMSTWQAARKEVAHTSRHNEFSIGASASLWECMLNQWLGRLYQNQDAHTGLQFEARIAQRQSLVKQLHER

QLDLLITTEAPKMDEFSSQLLGYFTLALYTSAPSKLKGDLNYLRLEWGPDFQQHEAGLIGADEVPILTTS

SAELAQQQIAMLNGCTWLPVSWARKKGGLHTVVDSTTLSRPLYAIWLQNSDKNALIRDLLKINVLDEVY

>gi|30064937|ref|NP_839108.1| hypothetical protein S3920 [Shigella flexneri 2a str. 2457T]

MAESFTTTNRYFDNKHYPRGFSRHGDFTIKEAQLLERHGYAFNELDLGKREPVTEEEKLFVAVCRGEREP

VTEAERVWSKYMTRIKRPKRFHTLSGGKPQVEGAEDYTDSDD

>gi|30064936|ref|NP_839107.1| ilvG operon leader peptide [Shigella flexneri 2a str. 2457T]

MTALLRVISLVVISVVVIIIPPCGAALGRGKA

>gi|30064935|ref|NP_839106.1| acetolactate synthase 2 catalytic subunit [Shigella flexneri 2a str. 2457T]

MNGAQWVVHALRAQGVNTVFGYPGGAIMPVYDALYDGGVEHLLCRHEQGAAMAAIGYARATGKTGVCIAT

SGPGATNLITGLADALLDSIPVVAITGQVSAPFIGTDAFQEVDVLGLSLACTKHSFLVQSLEELPRIMAE

AFDVASSGRPGPVLVDIPKDIQLASGDLEPWFTTVENEVTFPHAEVEQARQMLAKAQKPMLYVGGGVGMA

QAVPALREFLAATKMPATCTLKGLGAVEADYPYYLGMLGMHGTKAANFAVQECDLLIAVGARFDDRVTGK

LNTFAPHASVIHMDIDPAEMNKLRQAHVALQGDLNALLPALQQPLNINDWQQHCAQLRDEHSWRYDHPGD

AIYAPLLLKQLSDRKPADCVVTTDVGQHQMWAAQHIAHTRPENFITSSGLGTMGFGLPAAVGAQVARPND

TVVCISGDGSFMMNVQELGTVKRKQLPLKIVLLDNQRLGMVRQWQQLFFQERYSETTLTDNPDFLMLASA

FGIPGQHITRKDQVEAALDTMLNSDGPYLLHVSIDELENVWPLVPPGASNSEMLEKLS

>gi|30064934|ref|NP_839105.1| acetolactate synthase 2 regulatory subunit [Shigella flexneri 2a str. 2457T]

MQHQVNVSARFNPETLERVLRVVRHRGFHVCSMNMAAASDAQNINIELTVASPRSVDLLFSQLNKLVDVA

HVAICQSTTTSQQIRA

>gi|30064933|ref|NP_839104.1| branched-chain amino acid aminotransferase [Shigella flexneri 2a str. 2457T]

MTTKKADYIWFNGEMVRWEDAKVHVMSHALHYGTSVFEGIRCYDSHKGPVVFRHREHMQRLHDSAKIYRF

PVSQSIDELMEACRDVIRKNNLTSAYIRPLIFVGDVGMGVNPPAGYSTDVIIAAFPWGAYLGAEALEQGI

DAMVSSWNRAAPNTIPTAAKAGGNYLSSLLVGSEARRHGYQEGIALDVNGYISEGAGENLFEVKDGVLFT

PPFTSSALPGITRDAIIKLAKELGIEVREQVLSRESLYLADEVFMSGTAAEITPVRSVDGIQIGEGRCGP

VTKRIQQAFFGLFTGETEDKWGWLDQVNQ

>gi|30064932|ref|NP_839103.1| dihydroxy-acid dehydratase [Shigella flexneri 2a str. 2457T]

MPKYRSATTTHGRNMAGARALWRATGMTDADFGKPIIAVVNSFTQFVPGHVHLRDLGKLVAEQIEAAGGV

AKEFNTIAVDDGIAMGHGGMLYSLPSRELIADSVEYMVNAHCADAMVCISNCDKITPGMLMASLRLNIPV

IFVSGGPMEAGKTKLSDQIIKLDLVDAMIQGADPKVSDSQSDQVERSACPTCGSCSGMFTANSMNCLTEA

LGLSQPGNGSLLATHADRKQLFLNAGKRIVELTKRYYEQNDESALPRNIASKAAFENAMTLDIAMGGSTN

TVLHLLAAAQEAEIDFTMSDIDKLSRKVPQLCKVAPSTQKYHMEDVHRAGGVIGILGELDRAGLLNRDVK

NVLGLTLPQTLEQYDVMLTQDDAVKNMFRAGPAGIRTTQAFSQDCRWDSLDDDRANGCIRSLEHAYSKDG

GLAVLYGNFAENGCIVKTAGVDDSILKFTGPAKVYESQDDAVEAILGGKVVAGDVVVIRYEGPKGGPGMQ

EMLYPTSFLKSMGLGKACALITDGRFSGGTSGLSIGHVSPEAASGGSIGLIEDGDLIAIDIPNRGIQLQV

SDAELAARREAQEARGDKAWTPKNRERQVSFALCAYASLATSADKGAVRDKSKLGG

>gi|30064931|ref|NP_839102.1| threonine dehydratase [Shigella flexneri 2a str. 2457T]

MADSQPLSGAPEGAEYLRAVLRAPVYEAAQVTPLQKMEKLSSRLDNVILVKREDRQPVHSFKLRGAYAMM

AGLTEEQKAHGVITASAGNHAQGVAFSSARLGVKALIVMPTATADIKVDAVRGFGGEVLLHGANFDEAKA

KAIELSQQQGFTWVPPFDHPMVIAGQGTLALELLQQDAHLDRVFVPVGGGGLAAGVAVLIKQLMPQIKVI

AVEAEDSACLKAALDAGHPVDLPRVGLFAEGVAVKRIGDETFRLCQEYLDDIITVDSDAICAAMKDLFED

VRAVAEPSGALALAGMKKYIAQHNIRGERLAHILSGANVNFHGLRYVSERCELGEQREALLAVTIPEEKG

SFLKFCQLLGGRSVTEFNYRFADAKNACIFVGVRLSRGLEERKEILQMLNDGGYSVVDLSDDEMAKLHVR

YMVGGRPSHPLQERLYSFEFPESPGALLRFLNTLGTHWNISLFHYRSHGTDYGRVLAAFELGDHEPDFET

RLNELGYDCHDETNNPAFRFFLAG

>gi|30064930|ref|NP_839101.1| DNA-binding transcriptional regulator IlvY [Shigella flexneri 2a str. 2457T]

MDLRDLKTFLHLAESRHFGRSARAMHVSPSTLSRQIQRLEEDLGQPLFVRDNRTVTLTEAGEELRVFAQQ

TLLQYQQLRHTIDQQGPLLYGELHIFCSVTAAYSHLPPILDRFRAEHPSVEIKLTTGDAADAMEKVVTGE

ADLAIAGKPETLPGAVAFSMLENLAVVLIAPALPCPVRNQVSVEKPDWSTVPFIMADQGPVRRRIELWFR

RNKISNPMIYATVGGHEAMVSMVALGCGVALLPEVVLENSPEPVRNRVMILERSDEKTPFELGVCAQKKR

LHEPLIEAFWKILPNH

>gi|30064929|ref|NP_839100.1| ketol-acid reductoisomerase [Shigella flexneri 2a str. 2457T]

MANYFNTLNLRQQLAQLGKCRFMGRDEFADGASYLQGKKVVIVGCGAQGLNQGLNMRDSGLDISYALRKE

AIAEKRASWRKATENGFKVGTYEELIPQADLVVNLTPDKQHSDVVRTVQPLMKDGAALGYSHGFNIVEVG

EQIRKDITVVMVAPKCPGTEVREEYKRGFGVPTLIAVHPENDPKGEGMAIAKAWAAATGGHRAGVLESSF

VAEVKSDLMGEQTILCGMLQAGSLLCFDKLVEEGTDPAYAEKLIQFGWETITEALKQGGITLMMDRLSNP

AKLRAYALSEQLKEIMAPLFQKHMDDIISGEFSSGMMADWANDDKKLLTWREETGKTAFETAPQYEGKIG

EQEYFDKGVLMIAMVKAGVELAFETMVDSGIIEESAYYESLHELPLIANTIVRKRLYEMNVVISDTAEYG

NYLFSYACVPLLKPFMAELQPGDLGKAIPEGAVDNAQLRDVNEAIRSHAIEQVGKKLRGYMTDMKRIAVA

G

>gi|30064928|ref|NP_839099.1| peptidyl-prolyl cis-trans isomerase C (rotamase C) [Shigella flexneri 2a str. 2457T]

MAKTAAALHILVKEEKLALDLLVQIKNGADFGKLAKKHSICPSGKRGGDLGEFRQGQMVPAFDKVVFSCP

VLEPTGPLHTQFGYHIIKVLYRN

>gi|30064927|ref|NP_839098.1| ATP-dependent DNA helicase Rep [Shigella flexneri 2a str. 2457T]

MRLNPGQQQAVEFVTGPCLVLAGAGSGKTRVITNKIAHLIRGCGYQARHIAAVTFTNKAAREMKERVGQT

LGRKEAHGLMISTFHTLGLDIIKREYAALGMKANFSLFDDTDQLALLKELTEGLIEDDKVLLQQLISTIS

NWKNDLKTPSQAAASAIGERDRIFAHCYGLYDAHLKACNVLDFDDLILLPTLLLQRNEEVRERWQNKIRY

LLVDEYQDTNTSQYELVKLLVGSRARFTVVGDDDQSIYSWRGARPQNLVLLSQDFPALKVIKLEQNYRSS

GRILKAANILIANNPHVFEKRLFSELGYGTELKVLSANNEEHEAERVTGELIAHHFVNKTQYKDYAILYR

GNYQSRVFEKFLMQNRIPYKISGGTSFFSRPEIKDLLAYLRVLTNPDDDSAFLRIVNTPKREIGPATLKK

LGEWAMTRNKSMFTASFDMGLSQTLSGRGYEALTRFTHWLAEIQRLAEREPIAAVRDLIHGMDYESWLYE

TSPSTKAAEMRMKNVNQLFSWMTEMLEGSELDEPMTLTQVVTRFTLRDMMERGESEEELDQVQLMTLHAS

KGLEFPYVYMVGMEEGFLPHQSSIDEDNIDEERRLAYVGITRAQKELTFTLCKERRQYGELVRPEPSRFL

LELPQDDLIWEQERKVVSAEERMQKGQSHLANLKAMMAAKRGK

>gi|30064926|ref|NP_839097.1| guanosine pentaphosphate phosphohydrolase [Shigella flexneri 2a str. 2457T]

MGSTSSLYAAIDLGSNSFHMLVVREVAGSIQTLTRIKRKVRLAAGLNSENDLSNEAMERGWQCLRLFAER

LQDIPPSQIRVVATATLRLAVNAGDFIAKAQEILGCPVQVISGEEEARLIYQGVAHTTGGADQRLVVDIG

GASTELVTGTGAQTTSLFSLSMGCVTWLERYFADRNLGQENFDAAEKAAREVLRPVADELRYHGWKVCVG

ASGTVQALQEIMMAQGMDERITLEKLQQLKQRAIHCGRLEELEIDGLTLERALVFPSGLAILIAIFTELN

IQCMTLAGGALREGLVYGMLHLAVEQDIRSRTLRNIQRRFMIDIDQAQRVAKVAANFFDQVENEWHLEAI

SRDLLISACQLHEIGLSVDFKQAPQHAAYLVRNLDLPGFTPAQKKLLATLLLNQTNPVDLSSLHQQNAVP

PRVAEQLCRLLRLAIIFASRRRDDLVPEMTLQANHELLTLTLPQGWLTQHPLGKEIIAQENQWQSYVHWP

LEVH

>gi|30064925|ref|NP_839096.1| ATP-dependent RNA helicase RhlB [Shigella flexneri 2a str. 2457T]

MSKTHLTEQKFSDFALHPKVVEALEKKGFHNCTPIQALALPLTLAGRDVAGQAQTGTGKTMAFLTSTFHY

LLSHPAIADRKVNQPRALIMAPTRELAVQIHADAEPLAEATGLKLGLAYGGDGYDKQLKVLESGVDILIG

TTGRLIDYAKQNHINLGAIQVVVLDEADRMYDLGFIKDIRWLFRRMPPANQRLNMLFSATLSYRVRELAF

EQMNNAEYIEVEPEQKTGHRIKEELFYPSNEEKMRLLQTLIEEEWPDRAIIFANTKHRCEEIWGHLAADG

HRVGLLTGDVAQKKRLRILDEFTRGDLDILVATDVAARGLHIPAVTHVFNYDLPDDCEDYVHRIGRTGRA

GASGHSISLACEEYALNLPAIETYIGHSIPVSKYNPDALMTDLPKPLRLTRPRTGNGPRRTGAPRNRRRS

G

>gi|30064924|ref|NP_839095.1| thioredoxin [Shigella flexneri 2a str. 2457T]

MLHQQRNQHARLIPVELYMSDKIIHLTDDSFDTDVLKADGAILVDFWAEWCGPCKMIAPILDEIADEYQG

KLTVAKLNIDQNPGTAPKYGIRGIPTLLLFKNGEVAATKVGALSKGQLKEFLDANLA

>gi|30064923|ref|NP_839094.1| rho operon leader peptide [Shigella flexneri 2a str. 2457T]

MRSEQISGSSLNPSCRFSSAYSPVTRQRKDMSR

>gi|30064922|ref|NP_839093.1| transcription termination factor Rho [Shigella flexneri 2a str. 2457T]

MNLTELKNTPVSELITLGENMGLENLARMRKQDIIFAILKQHAKSGEDIFGDGVLEILQDGFGFLRSADS

SYLAGPDDIYVSPSQIRRFNLRTGDTISGKIRPPKEGERYFALLKVNEVNFDKPENARNKILFENLTPLH

ANSRLRMERGNGSTEDLTARVLDLASPIGRGQRGLIVAPPKAGKTMLLQNIAQSIAYNHPDCVLMVLLID

ERPEEVTEMQRLVKGEVVASTFDEPASRHVQVAEMVIEKAKRLVEHKKDVIILLDSITRLARAYNTVVPA

SGKVLTGGVDANALHRPKRFFGAARNVEEGGSLTIIATALIDTGSKMDEVIYEEFKGTGNMELHLSRKIA

EKRVFPAIDYNRSGTRKEELLTTQEELQKMWILRKIIHPMGEIDAMEFLINKLAMTKTNDDFFEMMKRS

>gi|30064921|ref|NP_839092.1| UDP-GlcNAc:undecaprenylphosphate GlcNAc-1-phosphate transferase [Shigella flexneri 2a str. 2457T]

MNLLTVSTDLISIFLFTTLFLFFARKVAKKVGLVDKPNFRKRHQGLIPLVGGISVYAGICFTFGIVDYYI

PHASLYLACAGVLVFIGALDDRFDISVKIRATIQAAVGIVMMVFGKLYLSSLGYIFGSWEMVLGPFGYFL

TLFAVWAAINAFNMVDGIDGLLGGLSCVSFAAIGMILWFDGQTSLAIWCFAMIAAILPYIMLNLGILGRR

YKVFMGDAGSTLIGFTVIWILLETTQGKTHPISPVTALWIIAIPLMDMVAIMYRRLRKGMSPFSPDRQHI

HHLIMRAGFTSRQAFVLITLAAALLASIGVLAEYSHFVPEWVMLVLFLLAFFLYGYCIKRAWKVARFIKR

VKRRLRRNRGGSPNLTK

>gi|30064920|ref|NP_839091.1| lipopolysaccharide biosynthesis protein WzzE [Shigella flexneri 2a str. 2457T]

MTQPMPGKPAEDAENELDIRGLFRTLWAGKLWIIGMGLAFALIALAYTFFARQEWSSTAITDRPTVNMLG

GYYSQQQFLRNLDVRSNMASADQPSVMDEAYKEFVMQLASWDTRREFWLQTDYYKQRMVGNSKADAALLD

EMINNIQFIPGDFTRAVNDSVKLIAETAPDANNLLRQYVAFASQRAASHLNDELKGAWAARTIQMKAQVK

RQEEVAKAIYDRRMNSIEQALKIAEQHNISRSATDVPAEELPDSEMFLLGRPMLQARLENLQAVGPAFDL

DYDQNRAMLNTLNVGPTLDPRFQTYRYLRTPEEPVKRDSPRRAFLMIMWGIVGGLIGAGVALTRRCSK

>gi|30064919|ref|NP_839090.1| UDP-N-acetyl glucosamine-2-epimerase [Shigella flexneri 2a str. 2457T]

MSAKALAAYSKRIDVKVLTVFGTRPEAIKMAPLVHALAKDPFFEAKVCVTAQHREMLDQVLKLFSIVPDY

DLNIMQPGQGLTEITCRILEGLKPILAEFKPDVVLVHGDTTTTLATSLAAFYQRIPVGHVEAGLRTGALY

SPWPEEANRTLTGHLAMYHFSPTETSRQNLLRENVADSRIFITGNTVIDALLWVRDQVMSSDTLRSELAA

NYPFIAPDKKMILVTGHRRESFGRGFEEICHALADIATTHQDIQIVYPVHLNPNVREPVNRILGHVKNVI

LIDPQEYLPFVWLMNHAWLILTDSGGIQEEAPSLGKPVLVMRDTTERPEAVTAGTVRLVGTDKQRIVEEV

TRLLKDENEYQAMSRAHNPYGDGQACSRILEALKNNRISL

>gi|30064918|ref|NP_839089.1| UDP-N-acetyl-D-mannosamine dehydrogenase [Shigella flexneri 2a str. 2457T]

MSFATISVIGLGYIGLPTAAAFASRQKQVIGVDINQHAVDTINRGEIHIVEPDLASVVKTAVEGGFLRAS

TTPVEADAWLIAVPTPFKGDHEPDMTYVESAARSIAPVLKKGALVILESTSPVGSTEKMAEWLAEMRPDL

TFPQQVGEQADVNIAYCPERVLPGQVMVELIKNDRVIGGMTPVCSARASELYKIFLEGECVVTNSRTAEM

CKLTENSFRDVNIAFANELSLICADQGINVWELIRLANRHPRVNILQPGPGVGGHCIAVDPWFIVAQNPQ

QARLIRTAREVNDHKPFWVIDQVKAAVADCLAATDKRASELKIACFGLAFKPNIDDLRESPAMEIAELIA

QWHSGETLVVEPNIHQLPKKLTGLCTLAQLDEALATADVLVMLVDHSQFKVINGDNVHQQYVVDAKGVWR

>gi|30064917|ref|NP_839088.1| dTDP-glucose 4,6-dehydratase [Shigella flexneri 2a str. 2457T]

MRKILITGGAGFIGSALVRYIINETSDAVVVVDKLTYAGNLMSLAPVAQSERFAFEKVDICDRAELARVF

TEHQPDCVMHLAAESHVDRSIDGPAAFIETNIVGTYTLLEAARAYWNTLTEDKKSAFRFHHISTDEVYGD

LHSTDDFFTETTPYAPSSPYSASKASSDHLVRAWLRTYGLPTLITNCSNNYGPYHFPEKLIPLMILNALA

GKPLPVYGNGQQIRDWLYVEDHARALYCVATTGKVGETYNIGGHNERKNLDVVETICELLEELASNKPHG

VAYYRDLITFVADRPGHDLRYAIDASKIARELGWLPQETFESGMRKTVQWYLANESWWKQVQDGSYQGER

LGLKG

>gi|30064916|ref|NP_839087.1| glucose-1-phosphate thymidylyltransferase [Shigella flexneri 2a str. 2457T]

MKGIILAGGSGTRLHPITRGVSKQLLPIYDKPMIYYPLSVLMLAGIREILIITTPEDKGYFQRLLGDGSE

FGIQLEYAEQPSPDGLAQAFIIGETFLNGEPSCLVLGDNIFFGQGFSPKLRHVAARTEGATVFGYQVMDP

ERFGVVEFDDNFRAISLEEKPKQPKSNWAVTGLYFYDSKVVEYAKQVKPSERGELEITSINQMYLEAGNL

TVELLGRGFAWLDTGTHDSLIEASTFVQTVEKRQGFKIACLEEIAWRNGWLDDEGVKRAASSLAKTGYGQ

YLLELLRARPRQY

>gi|30064915|ref|NP_839086.1| TDP-fucosamine acetyltransferase [Shigella flexneri 2a str. 2457T]

MQAKIAASNTGELDALQQLGFSLVEGEVDLALPVNNVSDSGAVVAQETDIPALRQLASAAFAQSRFRAPW

YAPDASGRFYAQWIENAVRGTFDHQCLILRAASGDIRGYVSLRELNATDARIGLLAGRGAGAELMQTALN

WAYARGKTTLRVATQMGNTAALKRYIQSGANVESTAYWLYR

>gi|30064914|ref|NP_839085.1| TDP-4-oxo-6-deoxy-D-glucose transaminase [Shigella flexneri 2a str. 2457T]

MIPFNAPPVVGTELDYMQSAMGSGKLCGDGGFTRRCQQWLEQRFGSAKVLLTPSCTASLEMAALLLDIQP

GDEVIMPSYTFVSTANAFVLRGAKIVFVDVRPDTMNIDETLIEAAITDKTRVIVPVHYAGVACEMDTIMA

LAKKHNLFVVEDAAQGVMSTYKGRALGTIGHIGCFSFHETKNYTAGGEGGATLINDKALIERAEIIREKG

TNRSQFFRGQVDKYTWRDIGSSYLMSDLQAAYLWAQLEAADRINQQRLALWQNYYDTLAPLAKAGRIELP

SIPDGCVQNAHMFYIKLRDIDDRSALINFLKEAEIMAVFHYIPLHGCPAGEHFGEFHGEDRYTTKESERL

LRLPLFYNLSPVNQRTVIATLLNYFS

>gi|30064913|ref|NP_839084.1| cytochrome [Shigella flexneri 2a str. 2457T]

MSLAKASLWTAASTLVKIGAGLLVGKLLAVSFGPAGLGLAANFRQLITVLGVLAGAGIFNGVTKYVAQYH

DNPQQLRRVVGTSSAMVLGFSTLMALVFVLAAAPISQGLFGNTDYQGLVRLVALVQMGIAWGNLLLALMK

GFRDAAGNALSLIVGSLIGVLAYYVSYRLGGYEGALLGLALIPALVVIPAAIMLIKRGVIPLSYLKPSWD

NGLAGQLSKFTLMALITSVTLPVAYIMMRKLLAAQYSWDEVGIWQGVSSISDAYLQFITASFSVYLLPTL

SRLTEKRDITREVVKSLKFVLPAVAAASFTVWLLRDFAIWLLLSNKFTAMRDLFAWQLVGDVLKVGAYVF

GYLVIAKASLRFYILAEVSQFTLLMVFAHWLIPAHGALGAAQAYMATYIVYFSLCCGVFLLWRRRA

>gi|30064912|ref|NP_839083.1| 4-alpha-L-fucosyltransferase [Shigella flexneri 2a str. 2457T]

MTVLIHVLGSDIPHHNRTVLRFFNDALAATSEHAREFMVVGKDDGLSDSCPALSVQFFPGKKSLAEAVIA

KAKANRQQRFFFHGQFNPTLWLALLSGGIKPSQFFWHIWGADLYELSSGLRYKLFYPLRRLAQKRVGCVF

ATRGDLSFFAKTHPKVRGELLYFPTRMDPSLNTMANDRQREGKMTILVGNSGDRSNEHVAALRAVHQQFG

DTVKVVVPMGYPPNNEAYIEEVRQAGLELFSEENLQILSEKLEFDAYLALLRQCDLGYFIFARQQGIGTL

CLLIQAGIPCVLNRENPFWQDMTEQHLPVLFTTDDLNEDIVREAQRQLASADKNTIAFFSPNYLQGWQRA

LAIAAGEVA

>gi|30064911|ref|NP_839082.1| common antigen polymerase [Shigella flexneri 2a str. 2457T]

MSLLQFSGLFVVWLLCTLFIATLTWFEFRRVRFNFNVFFSLLFLLTFFFGFPLTSVLVFRFDVGVAPPEI

LLQVLLSAGCFYAVYYVTYKTRLRKRVADVPRRPLFTMNRVETNLTWVILMGIALVSVGIFFMHNGFLLF

RLNSYSQIFSSEVSGVALKRFFYFFIPAMLVVYFLRQDSKAWLFFLVSTVAFGLLTYMIVGGTRANIIIA

FAIFLFIGIIRGWISLWMLAAAGVLGIVGMFWLALKRYGMNVSGDEAFYTFLYLTRDTFSPWENLALLLQ

NYDNIDFQGLAPIVRDFYVFIPSWLWPGRPSMVLNSANYFTWEVLNNHSGLAISPTLIGSLVVMGGALFI

PLGAIVVGLIIKWFDWLYELGNRETNRYKAAILHSFCFGAIFNMIVLAREGLDSFVSRVVFFIVVFGACL

MIAKLLYWLFESAGLIHKRTKSSLRTQVEG

>gi|30064910|ref|NP_839081.1| UDP-N-acetyl-D-mannosaminuronic acid transferase [Shigella flexneri 2a str. 2457T]

MNNNTTAPTYTLRGLQLIGWRDMQHALDYLFADGQLKQGTLVAINAEKMLTIEDNAEVRELINAAEFKYA

DGISIVRSVRKKYPQAQVSRVAGADLWEELMARAGKEGTPVFLVGGKPEVLAQTEAKLRNQWNVNIVGSQ

DGYFKPEQRQALFERIHASGAQIVTVAMGSPKQEIFMRDCRLVHPDALYMGVGGTYDVFTGHVKRAPKIW

QTLGLEWLYRLLSQPSRIKRQLRLLRYLRWHYTGNL

>gi|30064909|ref|NP_839080.1| transporter [Shigella flexneri 2a str. 2457T]

MADNKPELQRGLEARHIELIALGGTIGVGLFMGAASTLKWAGPSVLLAYIIAGLFVFFIMRSMGEMLFLE

PVTGSFAVYAHRYMSPFFGYLTAWSYWFMWMAVGISEITAIGVYVQFWSPEMAQWIPALIAVALVALANL

AAVRLYGEIEFWFAMIKVTTIIVMIVIGLGVIFFGFGNGGQSIGFSNLTEHGGFFAGGWKGFLTALCIVV

ASYQGVELIGITAGEAKNPQVTLRSAVGKVLWRILIFYVGAIFVIVTIFPWNEIGSNGSPFVLTFAKIGI

TAAAGIINFVVLTAALSGCNSGMYSCGRMLYALAKNRQLPAAMAKVSRHGVPVAGVAVSIAILLIGSCLN

YIIPNPQRVFVYVYSASVLPGMVPWFVILISQLRFRRAHKAAIASHPFRSILFPWANYVTMAFLICVLIG

MYFNEDTRMSLFVGIIFMLAVTAIYKVFGLNRHGKAHKLEE

>gi|30064908|ref|NP_839079.1| arylsulfatase regulator [Shigella flexneri 2a str. 2457T]

MLQQVPTRAFHVIAKPSGSDCNLNCDYCFYLEKQSLYREKPVTHMDDDTLEAYIRHYIAASELQNEVAFT

WQGGEPTLLGLEFYRRAVALQAKYGAGRKISNSFQTNGVLLDDEWCAFLAENNFLVGLSLDGPAEIHNQY

RVTKGGRPTHKLVMRALTLLQKHHVDYNVLVCVNRTSAQQPLQVYDFLCDAGVEFIQFIPVVERLADETA

VRDGLKLHAPGDIQGELTEWSVRPDEFGEFLVAIFDHWIKRDVGKIFVMNIEWAFANFVGAPGAVCHHQP

TCGRSVIVEHNGDVYACDHYVYPQYRLGNMHQQTIAEMIDSPQQQVFGEDKFKQLPAQCRSCNVLKACWG

GCPKHRFMLDASGKPGLNYLCAGYQRYFRHLPPYLKAMADLLAHGRPASDIMQAHLMVVNK

>gi|30064905|ref|NP_839076.1| protoheme IX biogenesis protein [Shigella flexneri 2a str. 2457T]

MLKVLLLFVLLIAGIVVGPMIAGHQGYVLIQTDNYNIETSVTGLAIILILAMVVLFAIEWLLRRIFRTGA

HTRGWFVGRKRRRARKQTEQALLKLAEGDYQQVEKLMAKNADHAEQPVVNYLLAAEAAQQRGDEARANQH

LERAAELAGNDTIPVEITRVRLQLARNENHAARHGVDKLLEVTPRHPEVLRLAEQAYIRTGAWSSLLDII

PSMAKAHVGDEEHRAMLEQQAWIGLMDQARADNGSEGLRNWWKNQSRKTRHQVALQVAMAEHLIECDDHD

TAQQIIIDGLKRQYDDRLLLPIPRLKTNNPEQLEKVLRQQIKNVGDRPLLWSTLGQSLMKHGEWQEASLA

FRAALKQRPDAYDYAWLADALDRLHKPEEAAAMRRDGLMLTLQNNPPQ

>gi|30064904|ref|NP_839075.1| uroporphyrinogen III C-methyltransferase [Shigella flexneri 2a str. 2457T]

MTEQEKTSAVVEETREAVDTTSQPVATEKKSKNNTALILSAVAIAIALAAGVGLYGWGKQQAVNQTATSD

ALANQLTALQKAQESQKAELEGIIKQQAAQLKQANRQQETLAKQLDEVQQKIATISGSDAKTWLLAQADF

LVKLAGRKLWSDQDVTTSAALLKSADASLADMNDPSLITVRRAITDDIASLSAVSQVDYDGIILKLNQLS

NQVDNLRLADNDSDGSPMDSDGEELSSSISEWRINLQKSWQNFMDNFITIRRRDDTAVPLLAPNQDIYLR

ENIRSRLLVAAQAVPRHQEETYRQALENVSTWVRAYYDTDDATTKAFLDEVDQLSQQNISMDLPETLQSQ

AMLEKLMQTRVRNLLAQPAAGTTEAKPAPAPAPAPAPQGDTPAAAPQGE

>gi|30064903|ref|NP_839074.1| uroporphyrinogen-III synthase [Shigella flexneri 2a str. 2457T]

MSILVTRPSPAGEELVSRLRTLGQVAWHFPLIEFSPGRQLPQLADQLAALGESDLLFALSQHAVAFAQSQ

LHQQDRKWPQLPTYFAIGRTTALALHTVSGQKILYPQDREISEVLLQLPELQNIAGKRALILRGNGGREL

IGDTLTVRGAEVTFCECYQRCAIHYDGAEEAMRWQSREVTMVVVTSGEMLQQLWSLIPQWYREHWLLHCR

LLVVSERLAKLARELGWQDIKVADNADNDALLRALQ

>gi|30064902|ref|NP_839073.1| porphobilinogen deaminase [Shigella flexneri 2a str. 2457T]

MLDNVLRIATRQSPLALWQAHYVKDKLMASHPGLVVELVPMVTRGDVILDTPLAKVGGKGLFVKELEVAL

LENRADIAVHSMKDVPVEFPQGLGLVTICEREDPRDAFVSNNYDSLDALPAGSIVGTSSLRRQCQLAERR

PDLIIRSLRGNVGTRLSKLDNGEYDAIILAVAGLKRLGLESRIRAALPPEISLPAVGQGAVGIECRLDDT

RTRELLAALNHHETALRVTAERAMNTRLEGGCQVPIGSYAELIDGEIWLCALVGAPDGSQIIRGERRGAP

QDAEQMGISLAEELLNNGAREILAEVYNGEAPA

>gi|30064901|ref|NP_839072.1| adenylate cyclase [Shigella flexneri 2a str. 2457T]

MYLYIETLKQRLDAINQLRVDRALAAMGPAFQQVYSLLPTLLHYHHPRMPGYLDGNVPKGICLYTPDETQ

RHYLNELELYRGMSVQDPPKGELPITGVYTMGSTSSVGQSCSSDLDIWVCHQSWLDSEERQLLQRKCSLL

ESWAASLGVEVSFFLIDENRFRHNESGSLGGEDCGSTQHILLLDEFYRTAVRLAGKRILWNMVPCDEEEH

YDDYVMTLYAQGVLTPNEWLDLGGLSSLSAEEYFGASLWQLYKSIDSPYKAVLKTLLLEAYSWEYPNPRL

LAKDIKQRLHDGEIVSFGLDPYCMMLERVTEYLTAIEDFTRLDLVRRCFYLKVCEKLSRERACVGWRRAV

LSQLVSEWGWDEARLAMLDNRANWKIDQVREAHNELLDAMMQSYRNLIRFARRNNLSVSASPQDIGVLTR

KLYAAFEALPGKVTLVNPQISPDLSEPNLTFIYVPPGRANRSGWYLYNRAPNIESIISHQPLEYNRYLNK

LVAWAWFNGLLTSRTRLYIKGNGIVDLPKLQEMVADVSHHFPLRLPAPTPKALYSPCEIRHLAIIVNLEY

DPTAAFRNQVVHFDFRKLDVFSFGENQNCLVGSVDLLYRNSWNEVRTLHFNGEQSMIEALKTILGKMHQD

AAPPDSVEVFCYSQHLRGLIRTRVQQLVSECIELRLSSTRQETGRFKALRVSGQTWGLFFERLNVSVQKL

ENAIEFYGAISHNKLHGLSVQVETNHVKLPAVVDGFASEGIIQFFFEETQDENGFNIYILDESNRVEVYH

HCEGSKEELVRDVSRFYSSSHDRFTYGSSFINFNLPQFYQIVKVDGREQVIPFRTKSIGNLPPANQDHDT

PLLQQYFS

>gi|30064900|ref|NP_839071.1| frataxin-like protein [Shigella flexneri 2a str. 2457T]

MNDSEFHRLADQLWLTIEERLDDRDGDSDIDCEINGGVLTITFENGSKIIINRQEPLHQVWLATKQGGYH

FDLKGDEWICDRSGETFWDLLEQAATQQAGETVSFR

>gi|30064899|ref|NP_839070.1| IS1 orfA [Shigella flexneri 2a str. 2457T]

MASISIRCPSCSATEGVVRNGKSTAGHQRYLCSHCRKTWQLQFTYTASQPGTHQKIIDMAMNGVGCRASA

RIMGVGLNTVLRHLKNSGRSR

>gi|30064898|ref|NP_839069.1| IS1 orfB [Shigella flexneri 2a str. 2457T]

MSRQRTHYGRWPQHGFTSLKKLRPQSVTSRIQPGSDVIVCAEMDEHWGYVGAKSRQRWLFYAYDRIRRTV

VAHVFGERTLATLERLLSLLSAFEVVVWMTDGWPLYESRLKGKLHVISKRYTQRIERHNLNLRQHLARPV

RKSLSFSKSVELHDKVIGHYLNIKHYQ

>gi|30064897|ref|NP_839068.1| hypothetical protein S3871 [Shigella flexneri 2a str. 2457T]

MVYLRPYKETNLSLPDVNYKSLRRLPNLLIDPTTLDEWDKEPPLTDLTTDYLYEGAQAWYPHYSWHSDGR

NILYAGEVVQNPPGTPPVDVASFKAWGDFAADKHSLYFEGKRTDDNGGGNSLDIKTLHQVEFRPPWDPDL

LGLILRDANFLYINGHRLADPESFRVLAQKSWDQRGKFSTTFNPCIAVPFGPWDTLARTRTKILLNGEQL

DADPDTFSVVRWMPGSLLTWRDKNGLQRKVLDKENLAWDEDLTKHCLDFSLLEKKVFWRKGPACKQEELP

GLDPEQFQPISDAVAQHQDSLYTIIETESGNRKLEIVKLDDPNLIINKRFNAGKRHGYLLTRAEG

>gi|30064896|ref|NP_839067.1| outer membrane lipoprotein [Shigella flexneri 2a str. 2457T]

MKNVFKALTVLLTLFSLTGCGLKGPLYFPPADKNAPPPTKPVETQTQSTVPDKNDRATGDGPSQVNY

>gi|30064894|ref|NP_839065.1| hypothetical protein S3868 [Shigella flexneri 2a str. 2457T]

MKQPGEELQETLTELDDRAVVDYLIKNPEFFIRNARAVEAIRVPHPVRGTVSLVEWHMARARNHIHVLEE

NMALLMEQAIANEGLFYRLLYLQRSLTAASSLDDMLMRFHRWARDLGLAGASLRLFPDRWRLGAPSNHTH

LALSRQSFEPLRIQRLGQEQHYLGPLNGPELLVVLPEAKAVGSVAMSMLGSDADLGVVLFTSRDASHYQQ

GQGTQLLHEIALMLPELLERWIERV

>gi|30064893|ref|NP_839064.1| site-specific tyrosine recombinase XerC [Shigella flexneri 2a str. 2457T]

MTDLHTDVERYLRYLSVERQLSPITLLNYQRQLEAIINFASENGLQSWQQCDAAMVRNFAVRSRRKGLGA

ASLALRLSALRSFFDWLVSQNELKANPAKGVSAPKAPRHLPKNIDVDDMNRLLDIDINDPLAVRDRAMLE

VMYGAGLRLSELVGLDIKHLDLESGEVWVMGKGSKERRLPIGRNAVAWIEHWLDLRDLFGSEDDALFLSK

LGKRISARNVQKRFAEWGIKQGLNNHVHPHKLRHSFATHMLESSGDLRGVQELLGHANLSTTQIYTHLDF

QHLASVYDAAHPRAKRGK

>gi|30064892|ref|NP_839063.1| flavin mononucleotide phosphatase [Shigella flexneri 2a str. 2457T]

MRFYRPLGRISALTFDLDDTLYDNRPVILRTEREALTFVQNYHPALRSFQNEDLQRLRQAVREAEPEIYH

DVTRWRFRSIEQAMLDAGLSAEEASAGAHAAMINFAKWRSRIDVPQQTHDTLKQLAKKWPLVAITNGNAQ

PELFGLGDYFEFVLRAGPHGRSKPFSDMYFLAAEKLNVPIGEILHVGDDLTTDVGGAIRSGMQACWIRPE

NGDLMQTWDSRLLPHLEISRLASLTSLI

>gi|30064891|ref|NP_839062.1| DNA-dependent helicase II [Shigella flexneri 2a str. 2457T]

MDVSYLLDSLNDKQREAVAAPRSNLLVLAGAGSGKTRVLVHRIAWLMSVENCSPYSIMAVTFTNKAAAEM

RHRIGQLMGTSQGGMWVGTFHGLAHRLLRAHHMDANLPQDFQILDSEDQLRLLKRLIKAMNLDEKQWPPR

QAMWYINSQKDEGLRPHHIQSYGNPVEQTWQKVYQAYQEACDRAGLVDFAELLLRAHELWLNKPHILQHY

RERFTNILVDEFQDTNNIQYAWIRLLAGDTGKVMIVGDDDQSIYGWRGAQVENIQRFLNDFPGAEIIRLE

QNYRSTSNILSAANALIENNNGRLGKKLWTDGADGEPISLYCAFNELDEARFVVNRIKTWQDNGGALAEC

AILYRSNAQSRVLEEALLQASMPYRIYGGMRFFERQEIKDALSYLRLIANRNDDAAFERVVNTPTRGIGD

RTLDVVRQTSRDRQLTLWQACRELLQEKALAGRAASALQRFMELIDALAQETADMPLHVQTDRVIKDSGL

RTMYEQEKGEKGQTRIENLEELVTATRQFSYNEEDEDLMPLQAFLSHAALEAGEGQADTWQDAVQLMTMH

SAKGLEFPQVFIVGMEEGMFPSQMSLDEGGRLEEERRLAYVGVTRAMQKLTLTYAETRRLYGKEVYHRPS

RFIGELPEECVEEVRLRATVSRPVSHQRMGTPMVENDSGYKLGQRVRHAKFGEGTIVNMEGSGEHSRLQV

AFQGQGIKWLVAAYARLESV

>gi|30064890|ref|NP_839061.1| hypothetical protein S3864 [Shigella flexneri 2a str. 2457T]

MQSGPMLMENGVINPRIHPNVASRKIRNGVGINKHGNAVFLLSQQATNFYDFACYAKAKLNVEQLLYLGG

TISHMYMKGGAIPWQRYPFVTMISVERKG

>gi|30064889|ref|NP_839060.1| IS4 orf [Shigella flexneri 2a str. 2457T]

MHIGQALDLVSRYDSLRNPLTSLGDYLDPELISRCLAESGTVTLRKRRLPLEMMVWCIVGMALERKEPLH

QIVNRLDIMLPGNRPFVAPSAVIQARQRLGSEAVRRVFTKTAQLWHNATPHPHWCGLTLLAIDGVFWRTP

DTPENDAAFPRQTHAGNPALYPQVKMVCQMELTSHLLTAAAFGTMKNSENELAEQLIEQTGDNTLTLMDK

GYYSLGLLNGWSLAGEHRHWMIPLRKGAQYEELRKLGKGDHLVKLKTSPQARKKWPGLGNEVTARLLTVT

RKGKVCHLLTSMTDAMRFPGGEMADLYSNRWEIELGYREIKQTMQLSRLTLRSKKPELVEQELWGVLLAY

NLVRYQMIKMAEHLKGYWPNQLSFSESCGMVMRMLMTLQGASPGRIPELMSDLASMGQLVKLPTRRGRAF

PRVVKERPWKYPTAPKKSQSVA

>gi|30064888|ref|NP_839059.1| hypothetical protein S3862 [Shigella flexneri 2a str. 2457T]

MAHRLLIGKGMITLNLKRIFLTLTLLPLFAVAADDCALSDPTLTVQAYTVNPQTERVKMYWQKANGEAWG

TLHALLADINSQGQVQMAMNGGIYDESYAPLGLYIENGQQKV

>gi|30064887|ref|NP_839058.1| magnesium/nickel/cobalt transporter CorA [Shigella flexneri 2a str. 2457T]

MLSAFQLENNRLTRLEVEESQPLVNAVWIDLVEPDDDERLRVQSELGQSLATRPELEDIEASARFFEDDD

GLHIHSFFFFEDAEDHAGNSTVAFTIRDGRLFTLRERELPAFRLYRMRARSQSMVDGNAYELLLDLFETK

IEQLADEIENIYSDLEQLSRVIMEGHQGDEYDEALSTLAELEDIGWKVRLCLMDTQRALNFLVRKARLPG

GQLEQAREILRDIESLLPHNESLFQKVNFLMQAAMGFINIEQNRIIKIFSVVSVVFLPPTLVASSYGMNF

EFMPELKWSFGYPGAIIFMILAGLAPYLYFKRKNWL

>gi|30064886|ref|NP_839057.1| hypothetical protein S3860 [Shigella flexneri 2a str. 2457T]

MSKEYMNDDSLSEKWKYRFNFYDQHGFPGFWGATPEYKAAFKALKVRQRLTIQMNFIAFFCSWIYLFVLG

LWKKAIIVLLLGILSLFVGALIGVNILGIAVAAYVAVNTNKWFYEKEVKGLNTWSL

>gi|30064885|ref|NP_839056.1| hypothetical protein S3859 [Shigella flexneri 2a str. 2457T]

MLTGDSHKDVKFMLRMFIPTSNGKISRHRYIFLFILINFIFAFLIIFFNDGEAGFLVIVSTIALHYLVIN

MNCQRLRDSGFIYIKIYVFGTLAVYIISIITMIAEDFACSGNGSMIFLICYFSTFSMLMLAPTDSSKQ

>gi|30064884|ref|NP_839055.1| hypothetical protein S3858 [Shigella flexneri 2a str. 2457T]

MDAKQTRQGVLLALAAYFIWGIAPAYFKLIYYVPADEILTHRVIWSFFFMVVLMSICRQWSYLKTLIQTP

QKIFMLAVSAVLIGGNWLLFIWAVNNHHMLEASLGYFINPLVNIVLGMIFLGERFRRMQWLAVILAICGV

LVQLWTFGSLPIIALGLAFSFAFYGLVRKKIAVEAQTGMLIETMWLLPVAAIYLFAIADSSTSHMGQNPM

SLNLLLIAAGIVTTVPLLCFTAAATRLRLSTLGFFQYIGPTLMFLLAVTFYGEKPGADKMVTFTFIWVAL

AIFVMDAIYTQRRTSK

>gi|30064882|ref|NP_839053.1| phospholipase A [Shigella flexneri 2a str. 2457T]

MRTLQGWLLPVFMLPMAVYAQEATVKEVHDAPAVRGSIIANMLQEHDNPFTLYPYDTNYLIYTQTSDLNK

EAIASYDWAENARKDEVKFQLSLAFPLWRGILGPNSVLGASYTQKSWWQLSNSEESSPFRETNYEPQLFL

GFATDYRFAGWTLRDVEMGYNHDSNGRSDPTSRSWNRLYTRLMAENGNWLVEVKPWYVVGNTDDNPDITK

YMGYYQLKIGYHLGDAVLSAKGQYNWNTGYGGAELGLSYPITKHVRLYTQVYSGYGESLIDYNFNQTRVG

VGVMLNDLF

>gi|30064881|ref|NP_839052.1| ATP-dependent DNA helicase RecQ [Shigella flexneri 2a str. 2457T]

MNVAQAEVLNLESGAKQVLQETFGYQQFRPGQEEIIDTVLSGRDCLVVMPTGGGKSLCYQIPALLLNGLT

VVVSPLISLMKDQVDQLQANGVAAACLNSTQTREQQLEVMTGCRTGQIRLLYIAPERLMLDNFLEHLAHW

NPVLLAVDEAHCISQWGHDFRPEYAALGQLRQRFPTLPFMALTATADDTTRQDIVRLLGLNDPLIQISSF

DRPNIRYMLMEKFKPLDQLMRYVQEQRGKSGIIYCNSRAKVEDTAARLQSRGISAAAYHAGLENNVRADV

QEKFQRDDLQIVVATVAFGMGINKPNVRFVVHFDIPRNIESYYQETGRAGRDGLPAEAMLFYDPADMAWL

RRCLEEKPQGQLQDIERHKLNAMGAFAEAQTCRRLVLLNYFGEGRQEPCGNCDICLDPPKQYDGSTDAQI

ALSTIGRVNQRFGMGYVVEVIRGANNQRIRDYGHDKLKVYGMGRDKSHEHWVSVIRQLIHLGLVTQNIAQ

HSALQLTEAARPVLRGESSLQLAVPRIVALKPKAMQKSFGGNYDRKLFAKLRKLRKSIADESNVPPYVVF

NDATLIEMAEQMPITASEMLSVNGVGMRKLERFGKPFMALIRAHVDGDDEE

>gi|30064880|ref|NP_839051.1| hypothetical protein S3854 [Shigella flexneri 2a str. 2457T]

MGYQMLRGALKKEAVSAPAPQVELAKSGRSFLKGLLTNLANPKAIIYFGSVFSLFVGDNVGTTERWGIFA

LIIVETLAWFTVVASLFALPQMRRGYQRLAKWIDGFAGALFAGFGIHLIISR

>gi|30064879|ref|NP_839050.1| hypothetical protein S3853, partial [Shigella flexneri 2a str. 2457T]

MIAFEVLKWAGAAYLIWLGIQQWRAAGAIDLKSLASTQSRRHLFQRAVFVNLTNPKSIVFLAALFPQFIM

PQQPQLMQYIVLGVTTIVVDIIVMIGYATLAQRIALWIKGPKQMKALNKIFGSLFMLVGALLASARHA

>gi|30064878|ref|NP_839049.1| lysophospholipase L2 [Shigella flexneri 2a str. 2457T]

MFQQQKDWETRENAFAAFTMGPLTDFWRQRDEAEFTGVDDIPVRFVRFRAQHHDRVVVICPGRIESYVKY

AELAYDLFHLGFDVLIIDHRGQGRSGRLLADPHLGHVNRFNDYVDDLAAFWQQEVQPGPWRKRYILAHSM

GGAISTLFLQRHPGVCDAIALTAPMFGIVIRMPSFMARQILNWAEAHPRFRDGYAIGTGRWRALPFAINV

LTHSRQRYRRNLRFYADDPTIRVGGPTYHWVRESILAGEQVLAGAGDDATQTLLLQAEEERVVDNRMHDR

FCELRTAAGHPVEGGRPLVIKGAYHEILFEKDAMRSVALHAIVDFFNRHNSPSGNRSTEV

>gi|30064877|ref|NP_839048.1| sugar phosphatase [Shigella flexneri 2a str. 2457T]

MYQVVASDLDGTLLSPDHTLSPYAKETLKLLTARGINFVFATGRHHVDVGQIRDNLEIKSYMITSNGARV

HDLDGNLIFAHNLDRDIASDLFGVVNDNPDIITNVYRDDEWFMNRHRPEEMRFFKEAVFKYALYEPGLLE

PEGISKVFFTCDSHEQLLPLEQAINARWGDRVNVSFSTLTCLEVMAGGVSKGHALEAVAKKLGYSLKDCI

AFGDGMNDAEMLSMAGKGCIMGSAHQRLKDLHPELEVIGTNADDAVPHYLRKLYLS

>gi|30064876|ref|NP_839047.1| hypothetical protein S3850 [Shigella flexneri 2a str. 2457T]

MALLIITTILWAFSFSFYGEYLAGHVDSYFAVLVRVGLAALVFLPFLRTRSNSLKTVGLYMLVGAMQLGV

MYMLSFRAYLYLTVSELLLFTVLTPLYITLIYDIMSKRRLRWGYAFSALLAVIGAGIIRYDQVTDHFWTG

LLLVQLSNITFAIGMVGYKRLMETRPMPQYNAFAWFYLGAFLVAVIAWFLLGNAQKMPQTTLQWGILVFL

GVVASGIGYFMWNYGATQVDAGTLGIMNNMHVPAGLLVNLAIWHQQPHWPTFITGALVILASLWVHRKWV

APRSSQTADDRRRDCALSE

>gi|30064875|ref|NP_839046.1| regulator for metE and metH [Shigella flexneri 2a str. 2457T]

MIEVKHLKTLQALRNCGSLAAAAATLHQTQSALSHQFSDLEQRLGFRLFVRKSQPLRFTPQGEILLQLAN

QVLPQISQALQACNEPQQTRLRIAIECHSCIQWLTPALGNFHKNWPQVEMDFKSGVTFDPQPALQQGELD

LVMTSDILPRSGLHYSPMFDYEVRLVLAPDHPLAAKTRITPEDLASETLLIYPVQRSRLDVWRHFLQPAG

VSPSLKSVDNTLLLIQMVAARMGIAALPHWVVESFERQGLVVTKTLGEGLWSRLYAAVRDGEQRQPVTEA

FIRSARNHACDHLPFVKSAERPTYDAPTVRPGSPARL

>gi|30064874|ref|NP_839045.1| 5-methyltetrahydropteroyltriglutamate/homocysteine S-methyltransferase [Shigella flexneri 2a str. 2457T]

MTILNHTLGFPRVGLRRELKKAQESYWAGNSTREELLTVGRELRARHWDQQKQAGIDLLPVGDFAWYDHV

LTTSLLLGNVPPRHQNKDGSVDIDTLFRIGRGRAPTGEPAAAAEMTKWFNTNYHYMVPEFVKGQQFKLTW

TQLLEEVDEALALSHNVKPVLLGPVTYLWLGKVKGEQFDRLSLLNDILPVYQQVLAELAKRGIEWVQIDE

PALVLELPQAWLDAYKPAYDALQGQVKLLLTTYFEGVTPNLDTITALPVQGLHVDLVHGKDNVVELHKRL

PSDWLLSAGLINGRNVWRADLTEKYAQIKDIVGKRDLWVASSCSLLHSPIDLSVETRLDAEVKSWFAFAL

QKCHELALLRDALNSGDTAALAEWSAPIQARRHSTRVHNPAVEKRLAAITAQDSQRANVYEVRAEAQRAR

FKLPAWPTTTIGSFPQTTEIRTLRLDFKKGNLDANNYRTGIAEHIKQAIVEQERLGLDVLVHGEAERNDM

VEYFGEHLDGFVFTQNGWVQSYGSRCVKPPIVIGDISRPAPITVEWAKYAQSLTDKPVKGMLTGPVTILC

WSFPREDVSRETIAKQIALALRDEVADLEAAGIGIIQIDEPALREGLPLRRSDWDAYLQWGVEAFRINAA

VAKDDTQIHTHMCYCEFNDIMDSIAALDADVITIETSRSDMELLESFEEFDYPNEIGPGVYDIHSPNVPS

VEWIEALLKKAAKRIPAERLWVNPDCGLKTRGWPETRAALANMVQAAQNLRRG

>gi|30064873|ref|NP_839044.1| hypothetical protein S3847 [Shigella flexneri 2a str. 2457T]

MNIAAVAGSPTGDMIEIGQHLRVRHFCYQTAKQRGDIGKIIRIAFAEVKFRCDSQIPLQRQTATNVADMF

MHAKNFLHYNDHWQWAIALLWSGMVSRHVIPL

>gi|30064872|ref|NP_839043.1| uridine phosphorylase [Shigella flexneri 2a str. 2457T]

MSKSDVFHLGLTKNDLQGATLAIVPGDPDRVEKIAALMDKPVKLASHREFTTWRAELDGKPVIVCSTGIG

GPSTSIAVEELAQLGIRTFLRIGTTGAIQPHINVGDVLVTTASVRLDGASLHFAPLEFPAVADFECTTAL

VEAAKSIGATTHVGVTVSSDTFYPGQERYDTYSGRVVRHFKGSMEEWQAMGVMNYEMESATLLTMCASQG

LRAGMVAGVIVNRTQQEIPNAETMKQTESHAVKIVVEAARRLL

>gi|30064871|ref|NP_839042.1| DNA recombination protein RmuC [Shigella flexneri 2a str. 2457T]

MVYAVIALVGVAIGWLFASYQHAQQKAEQLAEREEMVAELSAAKQQITQSEHWRAECELLNNEVRSLQSI

NTSLEADLREVTTRMEAAQQHADDKIRQMINSEQRLSEQFENLANRIFEHSNRRVDEQNRQSLNSLLSPL

REQLDGFRRQVQDSFGKEAQERHTLTHEIRNLQQLNAQMAQEAINLTRALKGDNKTQGNWGEVVLTRVLE

ASGLREGYEYETQVSIENDARSRMQPDVIVRLPQGKDVVIDAKMTLVAYERYFNAEDDYTRESALQEHIA

SVRNHIRLLGRKDYQQLPGLRTLDYVLMFIPVEPAFLLALDRQPELITEALKNNIMLVSPTTLLVALRTI

ANLWRYEHQSRNAQQIADRASKLYDKMRLFIDDMSAIGQSLDKAQDNYRQAMKKLSSGRGNVLAQAEAFR

GLGVEIKREINPDLAEQAVSQDEEYRLRSVPEQPNDEAYQRDDEYNQQSR

>gi|30064870|ref|NP_839041.1| ubiquinone/menaquinone biosynthesis methyltransferase [Shigella flexneri 2a str. 2457T]

MVDKSQETTHFGFQTVAKEQKADMVAHVFHSVASKYDVMNDLMSFGIHRLWKRFTIDCSGVRRGQTVLDL

AGGTGDLTAKFSRLVGETGKVVLADINESMLKMGREKLRNIGVIGNVEYVQANAEALPFPDNTFDCITIS

FGLRNVTDKDKALRSMYRVLKPGGRLLVLEFSKPIIEPLSKAYDAYSFHVLPRIGSLVANDADSYRYLAE

SIRMHPDQDTLKAMMQDAGFESVDYYNLTAGVVALHRGYKF

>gi|30064869|ref|NP_839040.1| hypothetical protein S3842 [Shigella flexneri 2a str. 2457T]

MPFKPLVTAGIESLLNTFLYRSPALKTARSRLLGKVLRVEVKGFSTSLILVFSERQVDVLGEWAGDADCT

VIAYASVLPKLRDRQQLTALIRSGELEVQGDIQVVQNFVALADLAEFDPAELLAPYTGDIAAEGISKAMR

GGAKFLHHGIKRQQRYVAEAITEEWRMAPGPLEVAWFAEETAAVERAVDALTKRLEKLEAK

>gi|30064868|ref|NP_839039.1| ubiquinone biosynthesis protein UbiB [Shigella flexneri 2a str. 2457T]

MTPGEVRRLYFIIRTFLSYGLDELIPKMRITLPLRLWRYSLFWMPNRHKDKLLGERLRLALQELGPVWIK

FGQMLSTRRDLFPPHIADQLALLQDKVAPFDGKLAKQQIEAAMGGLPVEAWFDDFEIKPLASASIAQVHT

ARLKSNGKEVVIKVIRPDILPVIKADLKLIYRLARWVPRLLPDGRRLRPTEVVREYEKTLIDELNLLRES

ANAIQLRRNFEDSPMLYIPEVYPDYCSEGMMVMERIYGIPVSDVAALEKNGTNMKLLAERGVQVFFTQVF

RDSFFHADMHPGNIFVSYEHPENPKYIGIDCGIVGSLNKEDKRYLAENFIAFFNRDYRKVAELHVDSGWV

PPDTNVEEFEFAIRTVCEPIFEKPLAEISFGHVLLNLFNTARRFNMEVQPQLVLLQKTLLYVEGVGRQLY

PQLDLWKTAKPFLESWIKDQVGIPALVRAFKEKAPFWVEKMPELPELVYDSLRQGKYLQHSVDKIARELQ

SNHVRQGQSRYFLGIGATLVLSGTFLLVSRPEWGLMPGWLMAGGLIAWFVGWRKTR

>gi|30064867|ref|NP_839038.1| twin arginine translocase A [Shigella flexneri 2a str. 2457T]

MRLCLIIIYHRGTCMGGISIWQLLIIAVIVVLLFGTKKLGSIGSDLGASIKGFKKAMSDDEPKQDKTSQD

ADFTAKTIADKQADTNQEQAKTEDAKRHDKEQV

>gi|30064866|ref|NP_839037.1| sec-independent translocase [Shigella flexneri 2a str. 2457T]

MFDIGFSELLLVFIIGLVVLGPQRLPVAVKTVAGWIRALRSLATTVQNELTQELKLQEFQDSLKKVEKAS

LTNLTPELKASMDELRQAAESMKRSYVANDPEKASDEAHTIHNPVVKDNEAAHEGVTPAAAQTQASSPEQ

KPETTPEPVVKPAADAEPKTAAPSPSSSDKP

>gi|30064865|ref|NP_839036.1| twin-arginine protein translocation system subunit TatC [Shigella flexneri 2a str. 2457T]

MSVEDTQPLITHLIELRKRLLNCIIAVIVIFLCLVYFANDIYHLVSAPLIKQLPQGSTMIATDVASPFFT

PIKLTFMVSLILSAPVILYQVWAFIAPALYKHERRLVVPLLVSSSLLFYIGMAFAYFVVFPLAFGFLANT

APEGVQVSTDIASYLSFVMALFMAFGVSFEVPVAIVLLCWMGITSSEDLRKKRPYVLVGAFVVGMLLTPP

DVFSQTLLAIPMYCLFEIGVFFSRFYVGKGRNREEENDAEAESEKTEE

>gi|30064864|ref|NP_839035.1| DNase TatD [Shigella flexneri 2a str. 2457T]

MFDIGVNLTSSQFAKDRDDVVARAFDAGVNGLLITGTNLRESQQAQKLARQYSSCWSTAGVHPHDSSQWQ

AATEEAIIELAAQPEVVAIGECGLDFNRNFSTPEEQERAFVAQLRIAAELNMPVFMHCRDAHERFMTLLE

PWLDKLPGAVLHCFTGTREEMQACVARGIYIGITGWVCDERRGLELRELLPLIPAEKLLIETDAPYLLPR

DLTPKPSSRRNEPAHLPHILQRIAHWRGEDAAWLAATTDANVKTLFGIAF

>gi|30064863|ref|NP_839034.1| transcriptional activator RfaH [Shigella flexneri 2a str. 2457T]

MQSWYLLYCKRGQLQRAQEHLERQAVNCLAPMITLEKIVRGKRTAVSEPLFPNYLFVEFDPEVIHTTTIS

ATRGVSHFVRFGASPAIVPSAVIHQLSVYKPKDIVDPAPPYPGDKVIITEGAFEGFQAIFTEPDGEARSM

LLLNLINKEIKHSVKNTEFRKL

>gi|30064862|ref|NP_839033.1| 3-octaprenyl-4-hydroxybenzoate carboxy-lyase [Shigella flexneri 2a str. 2457T]

MDAMKYNDLRDFLTLLEQQGELKRITLPVDPHLEITEIADRTLRAGGPALLFENPKGYSMPVLCNLFGTP

KRVAMGMGQEDVSALREVGKLLAFLKEPEPPKGFRDLFDKLPQFKQVLNMPTKRLRGAPCQQKIVSGDDV

DLNRIPIMTCWPEDAAPLITWGLTVTRGPHKERQNLGIYRQQLIGKNKLIMRWLSHRGGALDYQEWCAAH

PGERFPVSVALGADPATILGAVTPVPDTLSEYAFAGLLRGTKTEVVKCISNDLEVPASAEIVLEGYIDPG

EMAPEGPYGDHTGYYNEVDNFPVFTVTHITQREDAIYHSTYTGRPPDEPAVLGVALNEVFVPILQKQFPE

IVDFYLPPEGCSYRLAVVTIKKQYAGHAKRVMMGVWSFLRQFMYTKFVIVCDDDVNARDWNDVIWAITTR

MDPARDTVLVENTPIDYLDFASPVSGLGSKMGLDATNKWPGETQREWGRPIKKDPDVVAHIDAIWDELAI

FNNGKSA

>gi|30064861|ref|NP_839032.1| FMN reductase [Shigella flexneri 2a str. 2457T]

MTTLSCKVTSVAAITDTVYRVRIVPDAAFSFRAGQYLMVVMDERDKRPFSMASTPDEKGFIELHIGASEI

NLYAKAVMDRILKDHQIVVDLPHGEAWLRDDEERPMILIAGGTGFSYARSILLTALARNPNRDITIYWGG

REEQHLYDLCELEALSLKHLGLQVVPVVEQPEAGWRGRTGTVLTAVLQDHGTLAEHDIYIAGRFEMAKIA

RDLFCSERNAREDRLFGDAFAFI

>gi|30064860|ref|NP_839031.1| 3-ketoacyl-CoA thiolase [Shigella flexneri 2a str. 2457T]

MEQVVIVDAIRTPMGRSKGGAFRNVRAEDLSAHLMRSLLARNPALEAAALDDIYWGCVQQTLEQGFNIAR

NAALLAEVPHSVPAVTVNRLCGSSMQALHDAARMIMTGDAQACLVGGVEHIGHVPMSHGVDFHPGLSRNV

AKAAGMMGLTAEMLARMHGISREMQDAFAARSHARAWAATQSAAFKNEIIPTGGHDADGVLKQFNYDEVI

RPETTVEALATLRPAFDPVNGTVTAGTSSALSDGAAAMLVMSESRAHELGLKPRARVRSMAVVGCDPSIM

GYGPVPASKLALKKAGLSASDIGVFEMNEAFAAQILPCIKDLGLMEQIDEKINLNGGAIALGHPLGCSGA

RISTTLLNLMERKDVQFGLATMCIGLGQGIATVFERV

>gi|30064859|ref|NP_839030.1| multifunctional fatty acid oxidation complex subunit alpha [Shigella flexneri 2a str. 2457T]

MLYKGDTLYLDWLEDGIAELVFDAPGSVNKLDTATVASLGEAIGVLEQQSDLKGLLLRSNKAAFIVGADI

TEFLSLFLVPEEQLSQWLHFANSVFNRLEDLPVPTIAAVNGYALGGGCECVLATDYRLATPDLRIGLPET

KLGIMPGFGGSVRMPRMLGADSALEIIAAGKDVGADQALKIGLVDGVVKAEKLIEGAMAILRQAINGDLD

WKAKRQPKQEPLKLSKIEATMSFTIAKGMVAQTAGKHYPAPITAVKTIEAAARFGREEALNLENKSFVPL

AHTNEARALVGIFLNDQYVKGKAKKLTKDVETPKQAAVLGAGIMGGGIAYQSAWKGVPVVMKDINDKSLT

LGMTEAAKLLNKQLERGKIDGLKLAGVISTIHPTLDYAGFDRVDIVVEAVVENPKVKKAVLAETEQKVRQ

DTVLASNTSTIPISELANALERPENFCGMHFFNPVHRMPLVEIIRGEKSSDETIAKVVAWASKMGKTPIV

VNDCPGFFVNRVLFPYFAGFSQLLRDGADFRKIDKVMEKQFGWPMGPAYLLDVVGIDTAHHAQAVMAAGF

PQRMQKDYRDAIDALFDANRFGQKNGLGFWRYKEDSKGKPKKEEDAAVEDLLAEVSQPKRDFSEEEIIAR

MMIPMVNEVVRCLEEGIIATPAEADMALVYGLGFPPFHGGAFRWLDTLGSAKYLDMAQQYQHLGPLYEVP

EGLRNKARHNEPYYPPVEPARPVGDLKTA

>gi|30064858|ref|NP_839029.1| proline dipeptidase [Shigella flexneri 2a str. 2457T]

MESLASLYKNHIATLQERTRDALARFKLDALLIHSGELFNVFLDDHPYPFKVNPQFKAWVPVTQVPNCWL

LVDGVNKPKLWFYLPVDYWHNVEPLPTSFWTEDVEVIALPKADGIGSLLPAARGNIGYIGPVPERALQLG

IEASNINPKGVIDYQHYYRSFKTEYELACMREAQKMAVNGHRAAEEAFRSGMSEFDINIAYLTATGHRDT

DVPYSNIVALNEHAAVLHYTKLDHQAPEEMRSFLLDAGAEYNGYAADLTRTWSAKSDNDYAQLVKDVNDE

QLALIATMKAGVSYVDYHIQFHQRIAKLLRKHQIITDMSEEAMVENDLTGPFMPHGIGHPLGLQVHDVAG

FMQDDSGTHLAAPAKYPYLRYTRILQPGMVLTIEPGIYFIESLLAPWREGQFSKHFNWQKIEALKPFGGI

RIEDNVVIHENNVENMTRDLKLA

>gi|30064857|ref|NP_839028.1| hypothetical protein S3828 [Shigella flexneri 2a str. 2457T]

MESWLIPAAPVTVVEEIKKSRFITMLAHTDGVEAAKAFVESVRAEHPDARHHCVAWVAGAPDDSQQLGFS

DDGEPAGTAGKPMLAQLMGSGVGEITAVVVRYYGGILLGTGGLVKAYGGGVNQALRQLTTQRKTPLTEYT

LQCEYSQLTGIEALLGQCDGKIINSDYQAFVLLRVALPAAKVAEFSAKLADFSRGSLQLLAIEE

>gi|30064856|ref|NP_839027.1| potassium transporter [Shigella flexneri 2a str. 2457T]

MHFRAITRIVGLLVILFSGTMIIPGLVALIYRDGAGRAFTQTFFVALAIGSMLWWPNRKEKGELKSREGF

LIVVLFWTVLGSVGALPFIFSESPNLTITDAFFESFSGLTTTGATTLVGLDSLPHAILFYRQMLQWFGGM

GIIVLAVAILPILGVGGMQLYRAEMPGPLKDNKMRPRIAETAKTLWLIYVLLTVACALALWFAGMDAFDA

IGHSFATIAIGGFSTHDASIGYFDSPTINTIIAIFLLISGCNYGLHFSLLSGRSLKVYWRDPEFRMFIGV

QFTLVVICTLVLWFHNVYSSALMTINQAFFQVVSMATTAGFTTDSIARWPLFLPVLLLCSAFIGGCAGST

GGGLKVIRILLLFKQGNRELKRLVHPNAVYSIKLGNRALPERILEAVWGFFSAYALVFIVSMLAIIATGV

DDFSAFASVVATLNNLGPGLGVVADNFTSMNPVAKWILIANMLFGRLEVFTLLVLFTPTFWRE

>gi|30064855|ref|NP_839026.1| protoporphyrinogen oxidase [Shigella flexneri 2a str. 2457T]

MKTLILFSTRDGQTREIASYLASELKELGIQTDVANVHRIEEPQWENYDRVVIGASIRYGHYHSAFQEFV

KKHATRLNSMPSAFYSVNLVARKPEKRTPQTNSYARKFLMNSQWRPDRCAVIAGALRYPRYRWYDRFMLK

LIMKMSGGETDTRKEVVYTDWEQVANFAREIAHLTDKPTLK

>gi|30064854|ref|NP_839025.1| molybdopterin-guanine dinucleotide biosynthesis protein B [Shigella flexneri 2a str. 2457T]

MAGKTMIPLLAFAAWSGTGKTTLLKKLIPALCARGIRPGLIKHTHHDMDVDKPGKDSYELRKAGAVQTIV

ASQQRWALMTETPDEEELDLHFLASRMDTSKLDLILVEGFKHEEIAKIVLFRDGAGHRPEELVIDRHVIA

VASDVPLNLDVALLDINDVEGLADFVVEWMQKQNG

>gi|30064853|ref|NP_839024.1| molybdopterin-guanine dinucleotide biosynthesis protein A [Shigella flexneri 2a str. 2457T]

MNLMTTITGVVLAGGKARRMGGVDKGLLDLNGKPLWQHVADALKTQLSHVVVNANRHQEIYQAGGLKVIE

DSLADYPGPLAGMLSVMQQEAGEWFLFCPCDTPYIPHDLAARLNHQRKDAPVVWVHDGERDHPTIALVNR

AIEPLLLEYLQAGERRVMAFMRLAGGHAVDFSDHKDAFVNVNTPEELARWQEKR

>gi|30064852|ref|NP_839023.1| hypothetical protein S3818 [Shigella flexneri 2a str. 2457T]

MKCKRLNEVIELLQPAWQKEPDFNLLQFLQKLAKESGFDGELADLTDDILIYHLKMRDSAKDAVIPGLQK

DYEEDFKTALLRARGVIKE

>gi|30064851|ref|NP_839022.1| serine/threonine protein kinase [Shigella flexneri 2a str. 2457T]

MNNSAFTFQTLHPDTIMDALFKQGIRVDSGLTPLNSYENRVYQFQDEERRRFVVKFYRPERWTADQILEE

HQFALQLVNDEVPVAAPVAFNGQTLLNHQGFYFAVFPSVGGRQFEADNIDQMEAVGRYLGRMHQTGRKQL

FIHRPTIGLNEYLIEPRKLFEDATLIPSGLKAAFLKATDELIAAVTAHWREDFTVLRLHGDCHAGNILWR

DGPMFVDLDDARNGPAIQDLWMLLNGDKAQQRMQLETIIEAYEEFSEFDTAEIGLIEPLRAMRLVYYLAW

LMRHWADPAFPKNFPWLTGEDYWLRQTATFIEQAKVLQEPPLQLTPMY

>gi|30064850|ref|NP_839021.1| periplasmic protein disulfide isomerase I [Shigella flexneri 2a str. 2457T]

MKKIWLALAGLVLAFSASAAQYEDGKQYTTLEKPVAGAPQVLEFFSFFCPHCYQFEEVLHISDNVKKKLP

EGVKMTKYHVNFMGGDLGKDLTQAWAVAMALGVEDKVTVPLFEGVQKTQTIRSASDIRDVFINAGIKGEE

YDAAWNSFVVKSLVAQQEKAAADVQLRGVPAMFVNGKYQLNPQGMDTSNMDVFVQQYADTVKYLSEEK

>gi|30064849|ref|NP_839020.1| hypothetical protein S3815 [Shigella flexneri 2a str. 2457T]

MDIQSFAVLSGNIYMIRKSATGVIVALAVIWGGGTWYTGTQIQPGIEKFIKDFNDAKKKGEHAYDMTLSY

KNFDKGFFNSRFQMQMTFDNGAPDLNIKPGQKVVFDVDVEHGPLPITMLMHGNVIPALAAAKVNLVNNEL

TQPLFIAAKNKSPVEATLRFAFGGSFSTTLDVAPAEYGKFSFGEGQFTFNGDGSSLSNLDIEGKVEDIVL

QLSPMNKVTAKSFTIDSLARLEEKKFPVGESESKFNQINIINHGEDVAQIDAFVAKTRLDRVKDKDYINV

NLTYELDKLTKGNQQLGSGEWSLIAESIDPSAVRQFIIQYNIAMQKQLAAHPELANDEVALQEVNAALFK

EYLPLLQKSEPTIKQPVRWKNALGELNANLDISIADPAKSSSSTNKDIKSLNFDVKLPLNVVTETAKQLN

LSEGMDAEKAQKQADKQISGMMTLGQMFQLITIDNNTASLQLRYTPGKVVFNGQEMSEEEFMSRAGRFVH

>gi|30064848|ref|NP_839019.1| acyltransferase [Shigella flexneri 2a str. 2457T]

MANLLNKFIMTRILAAITLLLSIVLTILVTIFCSVPIIIAGIVKLLLPVPVIWRKVSRFCDFMMYCWCEG

LAVLLHLNPHLQWEVHGLEGLSKKNWYLLICNHRSWADIVVLCVLFRKHIPMNKYFLKQQLAWVPFLGLA

CWALDMPFMKRYSRAYLLRHPERRGQDVETTRRSCEKFRLHPTTIVNFVEGSRFTQEKHQQTHSTFQNLL

PPKAAGIAMALNVLGKQFDKLLNVTLCYPDNNRQPFFDMLSGKLTRIVVHVDLQPIADELHGDYINDKSF

KRHFQQWLNSLWQEKDRLLTSLMSSQRQNK

>gi|30064847|ref|NP_839018.1| DNA polymerase I [Shigella flexneri 2a str. 2457T]

MVQIPQNPLILVDGSSYLYRAYHAFPPLTNSAGEPTGAMYGVLNMLRSLIMQYKPTHAAVVFDAKGKTFR

DELFEHYKSHRPPMPDDLRAQIEPLHAMVKAMGLPLLAVSGVEADDVIGTLAREAEKAGRPVLISTGDKD

MAQLVTPNITLINTMTNTILGPEEVVNKYGVPPELIIDFLALMGDSSDNIPGVPGVGEKTAQALLQGLGG

LDTLYAEPEKIAGLSFRGAKTMAAKLEQNKEVAYLSYQLATIKTDVELELTCEQLEVQQPAAEELLGLFK

KYEFKRWTADVEVGKWLQAKGAKTAAKPQETSVADEAPEVTATVISYDNYVTILDEETLKAWIAKLEKAP

VFAFDTETDSLDNISANLVGLSFAIEPGVAAYIPVAHDYLDAPDQISHERALELLKPLLEDEKALKVGQN

LKYDRGILANYGIELRGIAFDTMLESYILNSVAGRHDMDSLAERWLKHKTITFEEIAGKGKNQLTFNQIA

LEEAGRYAAEDADVTLQLHLKMWPDLQKHKGPLNVFENIEMPLVPVLSRIERNGVKIDPKVLHNHSEELT

LRLAELEKKAHEIAGEEFNLSSTKQLQTILFEKQGIKPLKKTPGGAPSTSEEVLEELALDYPLPKVILEY

RGLAKLKSTYTDKLPLMINPKTGRVHTSYHQAVTATGRLSSTDPNLQNIPVRNEEGRRIRQAFIAPEDYV

IVSADYSQIELRIMAHLSRDKGLLTAFAEGKDIHRATAAEVFGLPLETVTSEQRRSAKAINFGLIYGMSA

FGLARQLNIPRKEAQKYMDLYFERYPGVLEYMERTRAQAKELGYVETLDGRRLYLPDIKSSNGARRAAAE

RAAINAPMQGTAADIIKRAMIAVDAWLQAEQPRVRMIMQVHDELVFEVHKDDVDAVAKQIHQLMENCTRL

DVPLLVEVGSGENWDQAH

>gi|30064845|ref|NP_839016.1| hypothetical protein S3810 [Shigella flexneri 2a str. 2457T]

MKPSSSNSRSKGHAKARRKTREELDQEARDRKRQKKRRGHAPGSRAAGGNTTSGSKGQNAPKDPRIGSKT

PIPLGVTEKVTKQHKPKSEKPMLSPQAELELLETDERLDALLERLEAGETLSAEEQSWVDAKLDRIDELM

QKLGLSYDDDEEEEEDEKQEDMMRLLRGN

>gi|30064844|ref|NP_839015.1| coproporphyrinogen III oxidase [Shigella flexneri 2a str. 2457T]

MSVQQIDWDLALIQKYNYSGPRYTSYPTALEFSEDFGEQAFLQAVARYPERPLSLYVHIPFCHKLCYFCG

CNKIVTRQQHKADQYLDALEQEIVHRAPLFAGRHVSQLHWGGGTPTYLNKAQISRLMKLLRENFHFNTDA

EISIEVDPREIELDVLDHLRAEGFNRLSMGVQDFNKEVQRLVNREQDEEFIFALLNHAREIGFTSTNIDL

IYGLPKQTPESFAFTLKRVAELNPDRLSVFNYAHLPTIFAAQRKIKDADLPSPQQKLDILQETIAFLTQS

GYQFIGMDHFARPDDELAVAQREGVLHRNFQGYTTQGDTDLLGMGVSAISMIGDCYAQNQKELKQYYQQV

YEQGNALWRGIALTRDDCIRRDVIKSLICNFRLDYAPLEKQWDLHFADYFAEDLKLLAPLAKDGLVDVDE

KGIQVTAKGRLLIRNICMCFDTYLRQKARMQQFSRVI

>gi|30064843|ref|NP_839014.1| nitrogen regulation protein NR(I) [Shigella flexneri 2a str. 2457T]

MQRGIVWVVDDDSSIRWVLERALAGAGLTCTTFENGAEVLEALASKTPDVLLSDIRMPGMDGLALLKQIK

QRHPMLPVIIMTAHSDLDAAVSAYQQGAFDYLPKPFDIDEAVALVERAISHYQEQQQPRNIQLNGPTTDI

IGEAQAMQDVFRIIGRLSRSSISVLINGESGTGKELVAHALHRHSPRTKAPFIALNMAAIPKDLIESELF

GHEKGAFTGANTIRQGRFEQADGGTLFLDEIGDMPLDVQTRLLRVLADGQFYRVGGYAPVKVDVRIIAAT
HQNLEQRVQEGKFREDLFHRLNVIRVHLPPLRERREDIPRLARHFLQVAARELGVEAKLLHPETEAALTR
LAWPGNVRQLENTCRWLTVMAAGQEVLIQDLPGELFESTVAESTSQMQPDSWATLLAQWADRALRSGHQN
LLSEAQPELERTLLTTALRHTQGHKQEAARLLGWGRNTLTRKLKELGME

>gi|30064842|ref|NP_839013.1| nitrogen regulation protein NR(II) [Shigella flexneri 2a str. 2457T]
MATGTQPDAGQILNSLINSILLIDDNLAIHYANPAAQQLLAQSSRKLFGTPLPELLSYFSLNIELMQESL
EAGQGFTDNEVTLVIDGRSHILSVTAQRMPDGMILLEMAPMDNQRRLSQEQLQHAQQVAARDLVRGLAHE
IKNPLGGLRGAAQLLSKALPDPSLLEYTKVIIEQADRLRNLVDRLLGPQLPGTRVTESIHKVAERVVTLV
SMELPDNVRLIRDYDPSLPELAHDPDQIEQVLLNIVRNALQALGPEGGEIILRTRTAFQLTLHGERYRLA
ARIDVEDNGPGIPPHLQDTLFYPMVSGREGGTGLGLSIARNLIDQHSGKIEFTSWPGHTEFSVYLPIRK

>gi|30064841|ref|NP_839012.1| glutamine synthetase [Shigella flexneri 2a str. 2457T]
MSAEHVLTMLNEHEVKFVDLRFTDTKGKEQHVTIPAHQVNAEFFEEGKMFDGSSIGGWKGINESDMVLMP
DASTAVIDPFFADSTLIIRCDILEPGTLQGYDRDPRSIAKRAEDYLRSTGIADTVLFGPEPEFFLFDDIR
FGSSISGSHVAIDDIEGAWNSSTQYEGGNKGHRPAVKGGYFPVPPVDSAQDIRSEMCLVMEQMGLVVEAH
HHEVATAGQNEVATRFNTMTKKADEIQIYKYVVHNVAHRFGKTATFMPKPMFGDNGSGMHCHMSLSKNGV
NLFAGDKYAGLSEQALYYIGGVIKHAKAINALANPTTNSYKRLVPGYEAPVMLAYSARNRSASIRIPVVS
SPKARRIEVRFPDPAANPYLCFAALLMAGLDGIKNKIHPGEAMDKNLYDLPPEEAKEIPQVAGSLEEALN
ELDLDREFLKAGGVFTDEAIDAYIALRREEDDRVRMTPHPVEFELYYSV

>gi|30064840|ref|NP_839011.1| GTP-binding protein [Shigella flexneri 2a str. 2457T]
MIEKLRNIAIIAHVDHGKTTLVDKLLQQSGTFDSRAETQERVMDSNDLEKERGITILAKNTAIKWNDYRI
NIVDTPGHADFGGEVERVMSMVDSVLLVVDAFDGPMPQTRFVTKKAFAYGLKPIVVINKVDRPGARPDWV
VDQVFDLFVNLDATDEQLDFPIVYASALNGIAGLDHEDMAEDMTPLYQAIVDHVPAPDVDLDGPFQMQIS

QLDYNSYVGVIGIGRIKRGKVKPNQQVTIIDSEGKTRNAKVGKVLGHLGLERIETDLAEAGDIVAITGLG

ELNISDTVCDTQNVEALPALSVDEPTVSMFFCVNTSPFSGKEGKFVTSRQILDRLNKKLVHNVALRVEET

EDADAFRVSGRGELHLSVLIENMRREGFELAVSRPKVIFREIDGRKQEPYENVTLDVEEQHQGSVMQALG

ERKGDLKNMNPDGKGRVRLDYVIPSRGLIGFRSEFMTMTSGTGLLYSTFSHYDDVRPGEVGQRQNGVLIS

NGQGKAVAFALFGLQDRGKLFLGHGAEVYEGQIIGIHSRSNDLTVNCLTGKKLTNMRASGTDEAVVLVPP

IRMTLEQALEFIDDDELVEVTPTSIRIRKRHLTENDRRRANRAPKDD

>gi|30064839|ref|NP_839010.1| transcriptional regulator [Shigella flexneri 2a str. 2457T]

MAENQSTVENAKEKLDRWLKDGITTPGGKLPSERELGELLGIKRMTLRQALLNLEAESKIFRKDRKGWFV

TQPRFNYSPELSASFQRAAIEQGREPSWGFTEKNRTSDIPETLAPLIAVTPSTELYRITGWGALEGHKVF

YHETYINPEVAPGFIEQLENHSFSAVWEKCYQKETVVKKLIFKPVRMPGDISKYLGGSAGMPAILIEKHR

ADQQGNIVQIDIEYWRFEAVDLIINL

>gi|30064838|ref|NP_839009.1| hypothetical protein S3803 [Shigella flexneri 2a str. 2457T]

MVTINNARKILQRVDTLPLYLHAYAFHLNMRLERVLPADLLDIASENNLRGVKIHVLDGERFSLGNMDDK

ELSAFGDKARRLNLDIHIETSASDKASIDEAVAIALKTGATSVRFYPRYEGNLRDVLSIIANDIAYVRET

YQDSGLTFTIEQHEDLKSHELVSLVKESEMESLSLLFDFANMINANEHPIDALKTMAPHITQVHIKDALI

VKEQGGLGHKACISGQGDMPFKALLTHLICLGDDEPQVTAYGLEEEVDYYASAFRFEDEDDNPWIPYRQM

SETPLPENHLLDARLRKEKEDAINQINHVRNVLQQIKQEANHLLNH

>gi|30064837|ref|NP_839008.1| resistance protein, partial [Shigella flexneri 2a str. 2457T]

MLTKKKWALFSLLTLCGGTIYKLPSLKDAFYIPMQEYFHLTNGQIGNAMSVNSFVTTVGFFLSIYFADKL

PRRYTMSFSLIATGLLGVYLTTMPGYWGILFVWALFGVTCDMMNWPVLLKSVSRLGNSEQQGRLFGFFET

GRGIVDTVVAFSALAVFTWFGSGLLGFKAGIWFYSLIVIAVGIIIFFVLNDKEEAPSVEVKKEDGASQNT

SMTSVLRDKTIWLIAFNVFFVYAVYCGLTFFIPFLKNIYLLPVALVGAYGIINQYCLKMIGGPIGGMISD

KILKSPSKYLCYTFIISTAALVLLIMLPHESMPVYLGMACTLGFGAIVFTQRAVFFAPIGEAKIGNAANL

LI

>gi|30064836|ref|NP_839007.1| IS1 orfA [Shigella flexneri 2a str. 2457T]

MASISIRCPSCSATEGVVRNGKSTAGHQRYLCSHCRKTWQLQFTYTASQPGTHQKIIDMAMNGVGCRASA

RIMGVGLNTVLRHLKNSGRSR

>gi|30064835|ref|NP_839006.1| IS1 orfB [Shigella flexneri 2a str. 2457T]

MSRQCTHYGRWPQHGFTSLKKLRPQSVTSRIQPGSDVIVCAEMDEHWGYVGAKSRQRWLFYAYDRIRRTV

VAHVFGERTLATLERLLSLLSAFEVVVWMTDGWPLYESRLKGKLHVISKRYTQRIERHNLNLRQHLARLG

RKSLSFSKSVELHDKVIGHYLNIKHYQ

>gi|30064834|ref|NP_839005.1| permease [Shigella flexneri 2a str. 2457T]

MSDHNPLTLKLNLREKIAYGMGDVGSNLMLCIGTLYLLKLYTDELGMPAYYGGIIFLVAKFFTAFTDMLT

GFLLDSRKNIGPKGKFRPFILYAAVPAALIATLQFIATTFCLPVKTTIATTLFMMFGLSYSLMNCSYGAM

IPAITKNPNERAQLAAYRQGGATIGLLICTVAFIPLQSLFSDSTVGYACAALMFSIGGFIFMMLCYRGVK

EHYVDTAPTGHKASILKSFCAIFRNPPLLVLCIANLCTLAAFNIKLAIQVYYTQYVLNDINLLSWMGFFS

MGCILVGVLLVPVTVKCFGKKQVYLAGMVLWAVGDILNYFWGSNSFTFVMFSCVAFFGTAFVNSLNWALV

PDTVDYGEWKTGIRAEGSVYTGYTFFRKISAALAGFLPGIMLTQIGYVPNIAQSDATLQGLRQLIFIWPC

ALAIIAALTMGFFYTLNEKRFALIIEEINQRKNKEMATEKKTASVTL

>gi|30064833|ref|NP_839004.1| alpha-glucosidase [Shigella flexneri 2a str. 2457T]

MDTPRPQLLDFQFHQNNDSFTLHFQQRLILTHNKDNPCLWIGSGIADIDMFRGNFSIKDKLQEKIALTDA

IVSQSPDGWLIHFSRGSDISATLNISADDQGRLLLELQNDNLNHNRIWLRLAAQPEDHIYGCGEQFSYFD

LRGKPFPLWTSEQGVGRNKQTYVTWQADCKENAGGDYYWTFFPQPTFVSTQKYYCHVDNSCYMNFDFSAP

EYHELALWEDKATLRFECADTYISLLEKLTALLGRQPELPDWIYDGVTLGIQGGTEVCQKKLDTMRNAGV

KVNGIWAQDWSGIRITSFGKRVMWNWKWNSENYPQLDSRIKQWYQEGVQFLAYINPYVASDKDLCEEAAQ

HGYLAKDASGGDYLVEFGEFYGGVVDLTNPEAYAWFKEVIKKNMIELGCGGWMADFGEYLPTDTYLHNGV

SAEIMHNAWPALWAKCNYEALEETGKLGEILFFMRAGSTGSQKYSTMMWAGDQNVDWSLDDGLASVVPAA

LSLAMTGHGLHHSDIGGYTTLFEMKRSKELLLRWCDFSAFTPMMRTHEGNRPGDNWQFDGDAETIAHFAR

MTTVFTTLKPYLKEAVALNAKSGLPVMRPLFLHYEDDAHTYSLKYQYLLGRDILVAPVHEEGRSDWTLYL

PEDNWVHAWTGEAFRGGEVTVNAPIGKPPVFYRADSEWAALFASLNSI

>gi|30064832|ref|NP_839003.1| aldose-1-epimerase [Shigella flexneri 2a str. 2457T]

MQITNMHCSGQTVSLAAGDYHATIVTVGAGLAELTFQGCHLVIPHKPEEMPLAHLGKVLIPWPNRIANGC

YRYQGQEYQLPINEHSSKAAIHGLLAWRDWQISELTTTSVTLTAFLPPSYGYPFMLASQVVYSLNAHTGL

SVEIASQNIGTVAAPYGVGIHPYLTCNLTSVDEYLFQLPANQVYAVDEHVNPTTLHHVDELDLNFTQAKK

IAATKIDHTFKTANDLWEMTITHPQQALSVSLCSDQPWVQVYSGEKLQRQGLAVEPMSCPPNAFNSGIDL

LLLEPGKPHRLFFNIYGQRK

>gi|30064831|ref|NP_839002.1| hypothetical protein S3794 [Shigella flexneri 2a str. 2457T]

MKWFNTLSHNRWLEQETDRIFDFGKNSVVPTGFGWLGNKGQIKEEMGTHLWITARMLHVYSVAAAMGRPG

AYALVDHGIKAMNGALRDKKYGGWYACVNDEGVVDASKQGYQHFFALLGAASAVTTGHPEARKLLDYTIE

IIEKYFWSEEEQMCLESWDEAFSKTEEYRGGNANMHAVEAFLIVYDVTHDKKWLDRAIRVASVIIHDVAR

NNHYRVNEHFDTQWNPLPDYNKDNPAHRFRAFGGTPGHWIEWGRLMLHIHAALEARCEQPPAWLLEDAKG

LFNATVRDAWAPDGADGIVYTVDWEGKPVVRERVRWPIVEAMGTAYALYTVTGDRQYETWYQTWWEYCIK

YLMDYENGSWWQELDADNKVITKVWDGKQDIYHLLHCLVIPRIPLAPGMAPAVAAGLLDINAK

>gi|30064830|ref|NP_839001.1| aldolase [Shigella flexneri 2a str. 2457T]

MNKYTINDITRASGGFAMLAVDQREAMRMMFAAAGAPAPVADSVLTDFKVNAAKALSPYASAILVDQQFC

YRQVVEQNAIAKSCAMIVAADEFIPGNGIPVDSVVIDRKINPLQIKQDGGKALKLLVLWRSDEDAQQRLD
MVKEFNELCHSHGLVSIIEPVVRPPRRGDKFDREQAIIDAAKELGDSGADLYKVEMPLYGKGPQQELLCA
SQRLNDHINMPWVILSSSVDEKLFPRAVRVAMTAGASGFLAGRAVWASVVGLPDNELMLRDVCAPKLQQL
GDIVDEMMAKRR

>gi|30064829|ref|NP_839000.1| dehydrogenase [Shigella flexneri 2a str. 2457T]
MAAIAFIGLGQMGSPMASNLLQQGHQLRVFDVNAEAVRHLVDKGATPAANPAQAAKDAEFIITMLPNGDL
VRNVLFGENGVCEGLSTDALVIDMSTIHPLQTDKLIADMQAKGFSMMDVPVGRTSANAITGTLLLLAGGT
AEQVERATPILMAMGSELINAGGPGMGIRVKLINNYMSIALNALSAEAAVLCEALNLPFDVAVKVMSGTA
AGKGHFTTSWPNKVLSGDLSPAFMIDLAHKDLGIALDVANQLHVPMPLGAASREVYSQARAAGRGRQDWS
AILEQVRVSAGMTAKVKM

>gi|30064828|ref|NP_838999.1| kinase [Shigella flexneri 2a str. 2457T]
MIRVACVGITVMDRIYYVEGLPTESGKYVARNYTEVGGGPAATAAVAAARLGAQVDFIGRVGDDDAGNSL
LAELESWGVNTRYTKRYNQAKSSQSAIMVDTKGERIIINYPSPDLLPDAEWLEEIDFSQWDVVLADVRWH
DGAKKAFTLARQAGVMTVLDGDITPQDISELVALSDHAAFSEPGLARLTGVKEMASALKQAQTLTNGHVY
VTQGSAGCDWLENGGRQHQPAFKVDVVDTTGAGDVFHGALAVALATSGDLAESVRFASGVAALKCTRPGG
RAGIPDCDQTRSFLSLFV

>gi|30064827|ref|NP_838998.1| DEOR-type transcriptional regulator [Shigella flexneri 2a str. 2457T]
MSLTELTGNPRHDQLLMLIAERGYMNIDELANLLDVSTQTVRRDIRKLSEQGLITRHHGGAGRASSVVNT
AFEQREVSQTEKKKAIAEAVADYIPDGSTIFITIGTTVEHVARALLNHNHLRIITNSLRVAHILYHNPRF
EVMVPGGTLRSHNSGIIGPSAASFVADFRADYLVTSVGAIESDGALMEFDVNEANVVKTMMAHARNILLV
ADHTKYHASAAVEIGNVAQVTALFTDELPPAALKSRLQDSQIEIILPQEDA

>gi|30064826|ref|NP_838997.1| phosphatase [Shigella flexneri 2a str. 2457T]

MLYIFDLGNVIVDIDFNRVLGAWSDLTRIPLASLKKSFHMGEAFHQHERGEISDEAFAEALCHEMALPLS

YEQFSHGWQAVFVALRPEVIAIMHKLREQGHRVVVLSNTNRLHTTFWPEEYPEIRDAADHIYLSQDLGMR

KPEARIYQHVLQAEGFSPSDTVFFDDNADNIEGANQLGITSILVKDKTTIPDYFAKVLC

>gi|30064825|ref|NP_838996.1| ribonuclease BN [Shigella flexneri 2a str. 2457T]

MLKTIQDKARHRTRPLWAWLKLLWQRIDEDNMTTLAGNLAYVSLLSLVPLVAVVFALFAAFPMFSDVSIQ

LRHFIFANFLPATGDVIQRYIEQFVANSNKMTAVGACGLIVTALLLMYSIDSALNTIWRSKRARPKIYSF

AVYWMILTLGPLLAGASLAISSYLLSLRWASDLNTVIDNVLRIFPLLLSWISFWLLYSIVPTIRVPNRDA

IVGAFVAALLFEAGKKGFALYITMFPSYQLIYGVLAVIPILFVWVYWTWCIVLLGAEITVTLGEYRKLKQ

AAEQEEDDEP

>gi|30064824|ref|NP_838995.1| D-tyrosyl-tRNA(Tyr) deacylase [Shigella flexneri 2a str. 2457T]

MIALIQRVTRASVTVEGEVTGEIGAGLLVLLGVEKDDDEQKANRLCERVLGYRIFSDAEGKMNLNVQQAG

GSVLVVSQFTLAADTERGMRPSFSKGASPDRAEALYDYFVERCRQQEMNTQTGRFAADMQVSLVNDGPVT

FWLQV

>gi|30064823|ref|NP_838994.1| acetyltransferase [Shigella flexneri 2a str. 2457T]

MSQLPGLSRETRESIAMYHLQVPQTEEELERYYHFRWEMLRKPLHQPKGSERDAWDAMAHHQMVVDEQGN

LVAVGRLYINADNEASIRFMVVHPDVQDKGLGTLMAMTLESVARQEGVKRVTCSAREDAVEFFAKLGFVN

QGEITTPTTTPIRHFLMIKPVATLDDILHRGDWCAQLQQAWYEHIPLSEKMGVRIQQYTGQKFITTMPET

GNQNPHHTLFAGSLFSLATLTGWGLIWLMLRERHLGGTIILADAHIRYSKPISGKPHAVADLGALSGDLD

RLARGRKARVQMQVEIFGDETPGAVFEGTYIVLPAKPFGPYEEGGNEEE

>gi|30064822|ref|NP_838993.1| IS1 orfB [Shigella flexneri 2a str. 2457T]

MSRQCTHYGRWPQHGFTSLKKLRPQSVTSRIQPGSDVIVCAEMDEHWGYVGAKSRQRWLFYAYDRIRRTV

VAHVFGERTLATLERLLSLLSAFEVVVWMTDGWPLYESRLKGKLHVISKRYTQRIERHNLKLRQHLARLG

RKSLSFSKSVELHDKVIGHYLNIKHYQ

>gi|30064821|ref|NP_838992.1| IS1 orfA [Shigella flexneri 2a str. 2457T]

MASISIRCPSCSATEGVVRNGKSTAGHLRYLCSHCRKTWQLQFTYTASQPGTHQKIIDMAMNGVGCRASA

RIMGVGLNTVLRHLKNSGRSR

>gi|30064820|ref|NP_838991.1| hypothetical protein S4828 [Shigella flexneri 2a str. 2457T]

MTGTVYKLFGNRLLNARNQLDKNLLEMAMQMVYGKDMVTKSGDILIDIQLNKDGSLQSKHYTLDVGEVIN

SYNIDPLCECFKY

>gi|30064819|ref|NP_838990.1| hypothetical protein S4827 [Shigella flexneri 2a str. 2457T]

MCERMKGGISMFNHTTKTADNIEHNLQLSFLVGSRPSITNKYSAKDVGPDILALPVNVYHADFKIKINNA

VEKASSIVLCAVQKNLHAARKVLSGIIGNVPKNNKPKTIIFDLRFLSAIKQKVFLGSEKPLFVKHTAMMR

EACKELPQSVEYVPLACEAHSHRSVALAKTVAAPIMATGVGLALMYATSSGPYYAQSLSGSPDIEMIKNR

MAELFNQSDISVRNKYSPAFDKWNELVDKLNHERNTVPFACLTTILATAINETLEGDTNVAVVMGCKSAK

DRTISIVLGNSMLQTLLEKRLADSGEIENLFDQQGYFSCDSLTPEELMMLKDLFDIRVLHVSNKFNVGLQ

GNINTDVLQDSFFKNVDFIHEYNSYTVGMGV

>gi|30064818|ref|NP_838989.1| hypothetical protein S3783 [Shigella flexneri 2a str. 2457T]

MSNICKVVVMAMNTVFLHLSEEAIKRLNKLRGWRKVSRSAILREAVEQYLERQQFPVRKAKGGRQKGEVV

GVDDQCKEHKE

>gi|30064817|ref|NP_838988.1| hypothetical protein S3782 [Shigella flexneri 2a str. 2457T]

MNSLAGIDMGRILLDLSNEVIKQLDDLEVQRNLPRADLLREAVDQYLINQSQTARTSVPGIWQGCEEDGV

EYQRKLREEW

>gi|30064816|ref|NP_838987.1| formate dehydrogenase accessory protein FdhE [Shigella flexneri 2a str. 2457T]

MSIRIIPQDELGSSEKRTADMIPPLLFPRLKNLYNRRAERLRELAENNPLGDYLRFAALIAHAQKVVLYD

HPLEMDLTARIKEASAQGKPPLDIHVLPRDKHWQKLLMALIAELKPEMSGPALAVIENLEKASTQELEDM

ASALFASDFSSVSSDKAPFIWAALSLYWAQMANLIPGKARAEYGEQRQYCPVCGSMPVSSMVQIGTTQGL

RYLHCNLCETEWHVVRVKCSNCEQSGKLHYWSLDDEQAAIKAESCDDCGTYLKILYQEKEPKVEAVADDL

ASLVLDARMEQEGYARSSINPFLFPGEGE

>gi|30064815|ref|NP_838986.1| formate dehydrogenase-O subunit gamma [Shigella flexneri 2a str. 2457T]

MKRRDTIVRYTAPERINHWITAFCFILAAVSGLGFLFPSFNWLMQIMGTPQLARILHPFVGVVMFASFII

MFFRYWHQNLINRDDIFWAKNIRKIVVNEEVGDTGRYNFGQKCVFWAAIIFLVLLLVSGVIIWRPYFAPA

FSIPVIRFALMLHSFAAVALIVVIMVHIYAALWVKGTITAMVEGWVTSAWAKKHHPRWYREVRKTTEKKA

E

>gi|30064814|ref|NP_838985.1| formate dehydrogenase-O, iron-sulfur subunit [Shigella flexneri 2a str. 2457T]

MAYQSQDIIRRSATNGLTPAPQARDFQEEVAKLIDVTTCIGCKACQVACSEWNDIRDTVGNNIGVYDNPN

DLSAKSWTVMRFSEVEQNDKLEWLIRKDGCMHCSDPGCLKACPAEGAIIQYANGIVDFQSEQCIGCGYCI

AGCPFDIPRLNPEDNRVYKCTLCVDRVVVGQEPACVKTCPTGAIHFGTKESMKTLASERVAELKTRGYDN

AGLYDPAGVGGTHVMYVLHHADKPNLYHGLPENPEISETVKFWKGIWKPLAAVGFAATFAASIFHYVGVG

PNRADEEENNLHEEKDEERK

>gi|30064813|ref|NP_838984.1| formate dehydrogenase-O, major subunit [Shigella flexneri 2a str. 2457T]

MQVSRRQFFKICAGGMAGTTAAALGFAPSVALAETRQYKLLRTRETRNTCTYCSVGCGLLMYSLGDGAKN

AKASIFHIEGDPDHPVNRGALCPKGAGLVDFIHSESRLKFPEYRAPGSDKWQQISWEEAFDRIAKLMKED

RDANYIAQNAEGVTVNRWLSTGMLCASASSNETGYLTQKFSRALGMLAVDNQARVUHGPTVASLAPTFGR

GAMTNHWVDIKNANLVVVMGGNAAEAHPVGFRWAMEAKIHNGAKLIVIDPRFTRTAAVADYYAPIRSGTD

IAFLSGVLLYLLNNEKFNHEYTEAYTNASLIVREDYGFEDGLFTGYDAEKRKYDKSSWTYELDENGFAKR

DTTLQHPRCVWNLLKQHVSRYTPDVVENICGTPKDAFLKVCEYIAETSAHDKTASFLYALGWTQHSVGAQ

NIRTMAMIQLLLGNMGMAGGGVNALRGHSNIQGLTDLGLLSQSLPGYMTLPSEKQTDLQTYLTANTPKPL

LEGQVNYWGNYPKFFVSMMKAFFGDKATAENSWGFDWLPKWDKGYDVLQYFEMMKEGKVNGYICQGFNPV

ASFPNKNKVIGCLSKLKFLVTIDPLNTETSNFWQNHGELNEVDSSKIQTEVFRLPSTCFAEENGSIVNSG

RWLQWHWKGADAPGIALTDGEILSGIFLRLRKMYAEQGGANPDQVLNMTWNYAIPHEPKSEEVAMESNGK

ALADITDPATGAVIVKKGQQLSSFAQLRDDGTTSCGCWIFAGSWTPEGNQMARRDNADPSGLGNTLGWAW

AWPLNRRILYNRASADPQGNPWDPKRQLLKWDGTKWTGWDIPDYSAAPPGSGVGPFIMQQEGMGRLFALD

KMAEGPFPEHYEPFETPLGTNPLHPNVISNPAARIFKDDAEALGKADKFPYVGTTYRLTEHFHYWTKHAL

LNAILQPEQFVEIGESLANKLGIAQGDTVKVSSNRGYIKAKAVVTKRIRTLKANGKDIDTIGIPIHWGYE

GVAKKGFIANTLTPFVGDANTQTPEFKSFLVNVEKV

>gi|30064812|ref|NP_838983.1| formate dehydrogenase accessory protein [Shigella flexneri 2a str. 2457T]

MKKTQRKEIENVTNITGVRQIELWRRDDLQHPRLDEVAEEVPVALVYNGISHVVMMASPKDLEYFALGFS

LSEGIIESPRDIFGMDVVPSCNGLEVQIELSSRRFMGLKERRRALAGRTGCGVCGVEQLNDIGKPVQPLP

FTQTFDLNKLDDALRHLNDFQPVGRLTGCTHAAAWMLPSGELVGGHEDVGRHVALDKLLGRRSQEGESWQ

QGAVLVSSRASYEMVQKSAMCGVEILFAVSAATTLAVEVAERCNLTLVGFCKPGRATVYTHPQRLSN

>gi|30064811|ref|NP_838982.1| IS1 orfB [Shigella flexneri 2a str. 2457T]

MSRQCTHYGRWPQHGFTSLKKLRPQSVTSHIQPGSDVIVCAEMDEHWGYVGAKSRQRWLFYAYDRIRRTV

VAHVFGERTLATLERLLSLLSAFEVVVWMTDGWPLYESRLKGKLHVISKRYTQRIERHNLNLRQHLARLG

RKSLSFSKSVELHDKVIGHYLNIKHYQ

>gi|30064810|ref|NP_838981.1| IS1 orfA [Shigella flexneri 2a str. 2457T]

MASISIRCPSCSATEGVVRNGKSTAGHQRYLCSHCRKTWQLQFTYTASQPDTHQKIIDMAMNGVGCRASA

RIMGVGLNTVLRHLKNSGRSR

>gi|30064809|ref|NP_838980.1| frv operon regulatory protein, partial [Shigella flexneri 2a str. 2457T]

MLNERQLKIVDLLEQQPRTPGELAQQTGVSGRTILRDIDYLNFTLNGKARIFASGSAGYQLEIFERRSFF

QLLQKHDNDDRLLALLLLNTFTPRAQLASALNLPETWVAERLPQLKQRYERTCCLASRPGLGHFIDETEE

KLVILLANLLRKDPFLIPLAGITRDNLQHLSTACDNQHRWPLIQGDYLSSLILAIYALRNQLTDEWPQYP

VMLPTY

>gi|30064808|ref|NP_838979.1| fructose-specific phosphotransferase system protein FrvX [Shigella flexneri 2a str. 2457T]

MNIELLQQLCEASAVSGDEQEVRDILINTLEPCVNEITFDGLGSFVARKGNKGPKVAVVGHMDEVGFMVT

HIDESGFLRFTTIGGWWNQSMLNHRVTIRTHKGVKIPGVIGFVAPHALTEKQRQQPLSFDEMFIDIGANS

RDEVEKRGVAMGDFISPEANFACWGEDKVVGKALDNRIGCAMMAELLQTVNNPEITLYGVGSVEEEVGLR

GAQTSAEHIKPDVVIVLDTAVAGDVPGIDNIKYPLKLGQGPGLMLFDKRYFPNQKLVAALKNCAAHNDLP

LQFSTMKTGATDGGRYNVMGGGRPVVALCLPTRYLHANSGMISKADYDALLTLIRGFLTTLTAEKVNAFS

QFRQVD

>gi|30064807|ref|NP_838978.1| PTS transporter components IIBC [Shigella flexneri 2a str. 2457T]

MESSLRIVAITNCPAGIAHTYMVAEALEQKARSLGHTIKVETQGSSGVENRLSSEEIAAADYVILATGRG

LSGDDRARFAGKKVYEIAISLALKNIDQIFSELPTNSQLFAADSGVKLGKQEVQSGSVMSHLMAGVSAAL

PFVIGGGILVALANMLVQFGLPYTDMSKGAPSFTWVVESIGYLGFTFMIPIMGAYIASSIADKPAFAPAF

LVCYLANDKALLGTQSGAGFLGAVVLGLAIGYFVFWFRKVRLGKALQPLLGSMLIPFVTLLVFGVLTYYV

IGPVMSDLMGGLLHFLNTIPPSMKFAAAFLVGAMLAFDMGGPINKTAWFFCFSLLEKHIYDWYAIVGVVA

LMPPVAAGLATFIAPKLFTRQEKEAASSAIVVGATVATEPAIPYALAAPLPMITANTLAGGITGVLVIAF

GIKRLAPGLGIFDPLIGLMSPVGSFYLVLAIGLALNISFIIVLKGLWLRRKAKAAQQELVHEH

>gi|30064806|ref|NP_838977.1| fructose-like phosphotransferase system subunit EIIA [Shigella flexneri 2a str. 2457T]

MAALTASCIDLNIQGNGAYSVLKQLATIALQNGFITDSHQFLQTLLLREKMHSTGFGSGVAVPHGKSACV

KQPFVLFARKAQAIDWKASDGEDVNCWICLGVPQSGEEDQVKIIGTLCRKIIHQEFIHQLQQGDTDQVLA

LLNQTLSS

>gi|30064805|ref|NP_838976.1| hypothetical protein S3770 [Shigella flexneri 2a str. 2457T]

MIRKAFVMQVNPDAHEEYQRRHNPIWPELEAVLKSHGVHNYAIYLDKARNLLFAMVEIESEERWNAVAST

DVCQRWWKYMTDVMPANPDNSPVSSELQEVFYLP

>gi|30064804|ref|NP_838975.1| rhamnulose-1-phosphate aldolase, partial [Shigella flexneri 2a str. 2457T]

MPLLANTPFIVTGSGKFFRNVQLDPAANLGVVKVDSDGAGYHILWGLTNEAVPTSELPAHFLSHCERIKA

TNGKDRVIMHCHATNLIALTYVLENDTAVFTRQLWEGSTECLVVFPDGVGILPWMVPGTDEIGQATAQEM

QKHSLVLWPFHGVFGSGPTLDEAFGLIDTAEKSAQVLVKVYSMGGMKQTISREELIALGKRFGVTPLASA

LAL

>gi|30064803|ref|NP_838974.1| L-rhamnose isomerase [Shigella flexneri 2a str. 2457T]

MTTQLEQAWELAKQRFAAVGIDVEEALRQLDRLPVSMHCWQGDDVSGFENPEGSLTGGIQATGNYPGKAR

NASELRADLEQAMRLIPGPKRLNLHAIYLESDTPVSRDQIKPEHFKNWVEWAKANQLGLDFNPSCFSHPL

SADGFTLSHPDDSIRQFWIDHCKASRRVSAYFGEQLGTPSVMNIWIPDGMKDITVDRLAPRQRLLAALDE

VISEKLNPAHHIDAVESKLFGIGAESYTVGSNEFYMGYATSRQTALCLDAGHFHPTEVISDKISAAMLYV

PQLLLHVSRPVRWDSDHVVLLDDETQAIASEIVRHDLFDRVHIGLDFFDASINRIAAWVIGTRNMKKALL

RALLEPTAELRKLEAAGDYTARLALLEEQKSLPRQAVWEMYCQHHDTPAGSEWLESVRAYEKAILSQRG

>gi|30064802|ref|NP_838973.1| rhamnulokinase [Shigella flexneri 2a str. 2457T]

MTFRNCVAVDLGASSGRVMLARYERECRSLTLREIHRFNNGLHSQNGYVTWDVDSLESAIRLGLNKVCEE

GIRIDSIGIDTWGVDFVLLDQQGQRVGLPVAYRDSRTNGLMPQAQQQLGKRDIYQRSGIQFLPFNTLYQL

RALTEQQPELIPHIAHALLMPDYFSYRLTGKMNWEYTNATTTQLVNINSDDWDESLLAWSGANKAWFGRP

THPGNVIGHWICPQGNEIPVVAVASHDTASAVIASPLNGSRAAYLSSGTWSLMGFESQTPFTNDTALAAN

ITNEGGAEGRYRVLKNIMGLWLLQRVLQERQINDLPALIAATQALPACRFIINPNDDRFINPDEMCSEIQ

AACRETAQPIPGSDAELARCIFDSLALLYADVLHELAQLRGEDFSQLHIVGGGCQNTLLNQLCADACGIR

VIAGPVEASTLGNIGIELMTLDELNNVDDFRQVVSTTANLTTFTPNPDSEIAHYVAQIHSTRQTKELCA

>gi|30064801|ref|NP_838972.1| transcriptional activator RhaS [Shigella flexneri 2a str. 2457T]

MTVLHSVDFFPSCNASVAIEPRLPQADFPEHHHDFHEIVIVEHGTGIHVFNGQPYTITGGTVCFVRDHDR

HLYEHTDNLCLTNVLYRSPDRFQFLAGLNQLLPQELDGQYPSHWRVNHSVLQQVRQLVAQMEQQEGENDL

PSTASREILFMQLLLLLRKSSLQENLENSASRLNLLLAWLEDHFADEVNWDAVADQFSLSLRTLHRQLKQ

QTGLTPQRYLNRLRLMKARHLLRHSEASVTDIAYRCGFSDSNHFSTLFRREFNWSPRDIRQGRDGFLQ

>gi|30064800|ref|NP_838971.1| transcriptional activator RhaR [Shigella flexneri 2a str. 2457T]

MAFCNNANLLNVFVRHIANNQLRSLAEVATVAHQLKLLKDDFFASDQQAVAVADRYPQDVFAEHTHDFCE

LVIVWRGNGLHVLNDRPYRITRGDLFYIHADDKHSYASVNDLVLQNIIYCPERLKLNLDWQGAIPGFSAS

AGQPHWRLGSVGMAQARQVIGQLEHESSQHVPFANEMAELLFGQLVMLLNRHRYTSDSLPPTSSETLLDK

LITRLAASLKSPFALDKFCDEASCSERVLRQQFRQQTGMTINQYLRQVRVCHAQYLLQHSRLLISDISTE

CGFEDSNYFSVVFTRETGMTPSQWRHLNSQKD

>gi|30064799|ref|NP_838970.1| superoxide dismutase [Shigella flexneri 2a str. 2457T]

MSYTLPSLPYAYDALEPHFDKQTMEIHHTKHHQTYVNNANAALESLPEFANLPVEELITKLDQLPADKKT

VLRNNAGGHANHSLFWKGLKKGTTLQGDLKAAIERDFGSVDNFKAEFEKAAASRFGSGWAWLVLKGDKLA

VVSTANQDSPLMGEAISGASGFPILGLDVWEHAYYLKFQNRRPDYIKEFWNVVNWDEAAARFAAKK

>gi|30064798|ref|NP_838969.1| 2-keto-3-deoxygluconate permease [Shigella flexneri 2a str. 2457T]

MQIKRSIEKIPGGMMLVPLFLGALCHTFSPGAGKYFGSFTNGMITGTVPILAVWFFCMGASIKLSATGTV

LRKSGTLVVTKIAVAWVVAAIASRIIPEHGVEVGFFAGLSTLALVAAMDMTNGGLYASIMQQYGTKEEAG

AFVLMSLESGPLMTMIILGTAGIASFEPHVFVGAVLPFLVGFALGNLDPELREFFSKAVQTLIPFFAFAL

GNTIDLTVIAQTGLLGILLGVAVIIVTGIPLIIADKLIGGGDGTAGIAASSSAGAAVATPVLIAEMVPAF

KPMAPAATSLVATAVIVTSILVPILTSIWSRKVKARAAKIEILGTVK

>gi|30064795|ref|NP_838966.1| hypothetical protein S3759 [Shigella flexneri 2a str. 2457T]

MRYPVDVYTGKIQAYPEGKPSAIAKIQVDGELMLTELGLEGDEQAEKKVHGGPDRALCHYPREHYLYWVR

EFPEQAELFVAPAFGENLSTDGLTESNVHMGDIFRWGEALIQVSQPRSPCYKLNYHFDISDIAQLMQNTG

KVGWLYSVIAPGKVSADAPLELVSRVSDVTVQEAAAIAWHMPFDDDQYHRLLSAAGLSKSWTRTMQKRRL

SGKIEDFSRRLWGK

>gi|30064794|ref|NP_838965.1| two-component sensor protein [Shigella flexneri 2a str. 2457T]

MIGSLTARIFAIFWLTLVLVLMLVLMLPKLDSRQMTELLDSEQRQGLMIEQHVEAELANDPPNDLMWWRR

LFRAIDKWAPPGQRLLLVTTEGRVIGAERSEMQIIRNFIGQADNADHPQKKKYGRVELVGPFSVRDGEDN

YQLYLIRPASSSQSDFINLLFDRPLLLLIVTMLVSTPLLLWLAWSLAKPARKLKNAADEVAQGNLRQHPE

LEAGPQEFLAAGASFNQMVTALERMMTSQQRLLSDISHELRTPLTRLQLGTALLRRRSGESKELERIETE

AQRLDSMINDLLVMSRNQQKNALVSETIKANQLWSEVLDNAAFEAEQMGKSLTVNFPPGPWPLYGNPNAL

ESALENIVRNALRYSHTKIEVGFAVDKDGITITVDDDGPGVSPEDREQIFRPFYRTDEARDRESGGTGLG

LAIVETAIQQHRGWVKAEDSPLGGLRLVIWLPLYKRS

>gi|30064793|ref|NP_838964.1| DNA-binding transcriptional regulator CpxR [Shigella flexneri 2a str. 2457T]

MNKILLVDDDRELTSLLKELLEMEGFNVIVAHDGEQALDLLDDSIDLLLLDVMMPKKNGIDTLKALRQTH

QTPVIMLTARGSELDRVLGLELGADDYLPKPFNDRELVARIRAILRRSHWSEQQQNNDNGSPTLEVDALV

LNPGRQEASFDGQTLELTGTEFTLLYLLAQHLGQVVSREHLSQEVLGKRLTPFDRAIDMHISNLRRKLPD

RKDGHPWFKTLRGRGYLMVSAS

>gi|30064791|ref|NP_838962.1| ferrous iron efflux protein F [Shigella flexneri 2a str. 2457T]

MNQSYGRLVSRAAIAATAMASLLLLIKIFAWWYTGSVSILAALVDSLVDIGASLTNLLVVRYSLQPADDN

HSFGHGKAESLAALAQSMFISGSALFLFLTGIQHLVSPTPMTDPGVGVIVTIVALICTIILVSFQRWVVR

RTQSQAVRADMLHYQSDVMMNGAILLALGLSWYGWHRADALFALGIGIYILYSALRMGYEAVQSLLDRAL

PDEERQEIIDIVTSWPGVSGAHDLRTRQSGPTRFIQIHLEMEDSLPLVQAHMVADQVEQAILRRFPGSDV

IIHQDPCSVVPREGKRSMLS

>gi|30064790|ref|NP_838961.1| 6-phosphofructokinase [Shigella flexneri 2a str. 2457T]

MLTSGGDAPGMNAAIRGVVRSALTEGLEVMGIYDGYLGLYEDRMVQLDRYSVSDMINRGGTFLGSARFPE

FRDENIRAVAIENLKKRGIDALVVIGGDGSYMGAMRLTEMGFPCIGLPGTIDNDIKGTDYTIGFFTALST

VVEAIDRLRDTSSSHQRISVVEVMGRYCGDLTLAAAIAGGCEFVVVPEVEFSREDLVNEIKAGIAKGKKH

AIVAITEHMCDVDELAHFIEKETGRETRATVLGHIQRGGSPVPYDRILASRMGAYAIDLLLAGYGGRCVG

IQNEQLVHHDIIDAIENMKRPFKGDWLDCAKKLY

>gi|30064789|ref|NP_838960.1| sulfate transporter subunit [Shigella flexneri 2a str. 2457T]

MNKWGVGLTFLLAATSVMAKDIQLLNVSYDPTRELYEQYNKAFSAHWKQQTGDNVVIRQSHGGSGKQATS

VINGIEADVVTLALAYDVDAIAERGRIDKEWIKRLPDNSAPYTSTIVFLVRKGNPKQIHDWNDLIKPGVS

VITPNPKSSGGARWNYLAAWGYALHHNNNDQAKAQDFVRALYKNVEVLDSGARGSTNTFVERGIGDVLIA

WENEALLAANELGKDKFEIVTPSESILAEPTVSVVDKVVEKKGTKEVAEAYLKYLYSPEGQEIAAKNYYR

PRDAEVAKKYENAFPKLKLFTIDEEFGGWTKAQKEHFANGGTFDQISKR

>gi|30064788|ref|NP_838959.1| CDP-diacylglycerol pyrophosphatase [Shigella flexneri 2a str. 2457T]

MKKAGLLFLVMIVIAVVAAGIGYWKLTGEESDTLRKIVLEECLPNQQQNQNPSPCAEVKPNAGYVVLKDL

NGPLQYLLMPTYRINGTESPLLTDPSTPNFFWLAWQARDFMSKKYGQPVPDRAVSLAINSRTGHTQNHFH

IHISCIRPDVREQLDKNLANISSRWLPLPGGLRGHEYLARRVTESELVQRSPFMMLAEEVPEAREHMGSY

GLAMVRQSDNSFVLLATQRNLLTLNRASAEEIQDHECEILR

>gi|30064787|ref|NP_838958.1| triosephosphate isomerase [Shigella flexneri 2a str. 2457T]

MRHPLVMGNWKLNGSRHMVHELVSNLRKELAGVAGCAVAIAPPEMYIDMAKREAEGSHIMLGAQNVDLNL

SGAFTGETSAAMLKDIGAQYIIIGHSERRTYHKESDELIAKKFAVLKEQGLTPVLCIGETEAENEAGKTE

EVCARQIDAVLKTQGAAAFEGAVIAYEPVWAIGTGKSATPAQAQAVHKFIRDHIAKVDANIAEQVIIQYG

GSVNASNAAELFAQPDIDGALVGGASLKADAFAVIVKAAEAAKQA

>gi|30064786|ref|NP_838957.1| hypothetical protein S3749 [Shigella flexneri 2a str. 2457T]

MKPGCTLFFLLCSALTVTTTAHAQTPDTATTAPYLLAGAPTFDLSISQFREDFNSQNPSLPLNEFRAIDS

SPDKANLTRAASKINENLYASTALERGTLKIKSIQMTWLPIQGPEQKAAKAKAQEYMAAVIRTLTPLMTK

TQSQKKLQSLLTAGKNKRYYTETEGALRYVVADNGEKGLTFAVEPIKLALSESLEGLNK

>gi|30064785|ref|NP_838956.1| hypothetical protein S3747 [Shigella flexneri 2a str. 2457T]

MKDVVDKCSTKGCAIDIGTVIDNDNCTSKFSRFFATREEAESFMTKLKELAAAASSADEGASVAYKIKDL

EGQVELDAAFTFSCQAEMIIFELSLRSLA

>gi|30064784|ref|NP_838955.1| universal stress protein UspD [Shigella flexneri 2a str. 2457T]

MAYKHIGVAISGNEEDALLVNKALELARHNDAHLTLIHIDDGLSELYPGIYFPATEDILQLLKNKSDNKL

YKLTKNIQWPKTKLRIERGEMPETLLEIMQKEQCDLLVCGHHHSFINRLMPAYRGMINKMSADLLIVPFI

DK

>gi|30064783|ref|NP_838954.1| ferredoxin-NADP reductase [Shigella flexneri 2a str. 2457T]

MADWVTGKVTKVQNWTDALFSLTVHAPVLPFTAGQFTKLGLEIDGERVQRAYSYVNSPDNPDLEFYLVTV

PDGKLSPRLAALKPGDEVQVVSEAAGFFVLDEVPDCETLWMLATGTAIGPYLSILQLGKDLDRFKNLVLV

HAARYAADLSYLPLMQELEKRYEGKLRIQTVVSRETAAGSLTGRIPALIESGELESAIGLPMNKETSHVM

LCGNPQMVRDTQQLLKETRQMTKHLRRRPGHMTAEHYW

>gi|30064782|ref|NP_838953.1| fructose 1,6-bisphosphatase II [Shigella flexneri 2a str. 2457T]

MRRELAIEFSRVTESAALAGYKWLGRGDKNTADGAAVNAMRIMLNQVNIDGTIVIGEGEIDEAPMLYIGE

KVGTGRGDAVDIAVDPIEGTRMTAMGQANALAVLAVGDKGCFLNAPDMYMEKLIVGPGAKGTIDLNLPLA

DNLRNVAAALGKPLSELTVTILAKPRHDAVIAEMQQLGVRVFAIPDGDVAASILTCMPDSEVDVLYGIGG

APEGVVSAAVIRALDGDMNGRLLARHDVKGDNEENRRIGEQELARCKAMGIEAGKVLRLGDMARSDNVIF

SATGITKGDLLEGISRKGNIATTETLLIRGKSRTIRRIQSIHYLDRKDPEMQVHIL

>gi|30064781|ref|NP_838952.1| glycerol kinase [Shigella flexneri 2a str. 2457T]

MTEKKYIVALDQGTTSSRAVVMDHDANIISVSQREFEQIYPKPGWVEHDPMEIWATQSSTLVEVLAKADI

SSDQIAAIGITNQRETTIVWEKETGKPIYNAIVWQCRRTAEICEHLKRDGLEDYIRSNTGLVIDPYFSGT

KVKWILDHVEGSHERARRGELLFGTVDTWLIWKMTQGRVHVTDYTNASRTMLFNIHTLDWDDKMLEVLDI

PREMLPEVRRSSEVYGQTNIGGKGGTRIPISGIAGDQQAALFGQLCVKEGMAKNTYGTGCFMLMNTGEKA

VKSENGLLTTIACGPTGEVNYALEGAVFMAGASIQWLRDEMKLINDAYDSEYFATKVQNTNGVYVVPAFT

GLGAPYWDPYARGAIFGLTRGVNANHIIRATLESIAYQTRDVLEAMQADSGIRLHALRVDGGAVANNFLM

QFQSDILGTRVERPEVREVTALGAAYLAGLAVGFWQNLDELQEKAVIEREFRPGIETTERNYRYAGWKKA

VKRAMAWEEHDE

>gi|30064780|ref|NP_838951.1| glycerol diffusion facilitator protein, partial [Shigella flexneri 2a str. 2457T]

MSQTSTLKGQCIAEFLGTGLLIFFGVGCVAALKVAGASFGQWEISVIWGLGVAMAIYLTAGVSGAHLNPA

VTIALWLFACFDKRKVIPFIVSQVAGAFCAAALVYGLYYNLFFDFEQTHHIVRGSVESVDLAGTFSTYPN

PHINFVQAFAVEMVITAILMGLILALTDDGNGVPRGPLAPLLIGLLIAVIGASMGPLTGFAMNPARDFGP

KVFA

>gi|30064779|ref|NP_838950.1| hypothetical protein S3741 [Shigella flexneri 2a str. 2457T]

MTMSLEVFEKLEAKVQQAIDTITLLQMEIEELKEKNNSLSQEVQNAQHQREELERENNHLKEQQNGWQER

LQALLGRMEEV

>gi|30064778|ref|NP_838949.1| ribonuclease activity regulator protein RraA [Shigella flexneri 2a str. 2457T]

MKYDTSELCDIYQEDVNVVEPLFSNFGGRASFGGQIITVKCFEDNGLLYDLLEQNGRGRVLVVDGGGSVR

RALVDAELARLAVQNEWEGLVIYGAVRQVDDLEELDIGIQAMAAIPVGAAGEGIGESDVRVNFGGVTFFS

GDHLYADNTGIILSEDPLDIE

>gi|30064777|ref|NP_838948.1| 1,4-dihydroxy-2-naphthoate octaprenyltransferase [Shigella flexneri 2a str. 2457T]

MTEQQISRTQAWLESLRPKTLPLAFAAIIVGTALAWWQGHFDPLVALLALITAGLLQILSNLANDYGDAV

KGSDKPDRIGPLRGMQKGVITQQKMKRALIITVVLICLSGLALVAVACHTLADFVGFLILGGLSIIAAIT

YTVGNRPYGYIGLGDISVLVFFGWLSVMGSWYLQAHTLIPALILPATACGLLATAVLNINNLRDINSDRE

NGKNTLVVRLGEVNARRYHACLLMGSLVCLALFNLFSLHSLWGWLFLLAAPLLVKQARYVMREMDPVAMR

PMLERTVKGALLTNLLFVLGIFLSQWAA

>gi|30064776|ref|NP_838947.1| ATP-dependent protease ATP-binding subunit HslU [Shigella flexneri 2a str. 2457T]

MSEMTPREIVSELDKHIIGQDNAKRSVAIALRNRWRRMQLNEELRHEVTPKNILMIGPTGVGKTEIARRL

AKLANAPFIKVEATKFTEVGYVGKEVDSIIRDLTDAAVKMVRVQAIEKNRYRAEELAEERILDVLIPPAK

NNWGQTEQQQEPSAARQAFRKKLREGQLDDKEIEIDLAAAPMGVEIMAPPGMEEMTSQLQSMFQNLGGQK

QKARKLKIKDAMKLLIEEEAAKLVNPEELKQDAIDAVEQHGIVFIDEIDKICKRGESSGPDVSREGVQRD

LLPLVEGCTVSTKHGMVKTDHILFIASGAFQIAKPSDLIPELQGRLPIRVELQALTTSDFERILTEPNAS

ITVQYKALMATEGVNIEFTDSGIKRIAEAAWQVNESTENIGARRLHTVLERLMEEISYDASDLSGQNITI

DADYVSKHLDALVADEDLSRFIL

>gi|30064775|ref|NP_838946.1| ATP-dependent protease peptidase subunit [Shigella flexneri 2a str. 2457T]

MTTIVSVRRNGHVVIAGDGQATLGNTVMKGNVKKVRRLYNDKVIAGFAGGTADAFTLFELFERKLEMHQG

HLVKAAVELAKDWRTDRMLRKLEALLAVADETASLIITGNGDVVQPENDLIAIGSGGPYAQAAARALLEN

TELSAREIAEKALDIAGDICIYTNHFHTIEELSYKA

>gi|30064774|ref|NP_838945.1| essential cell division protein FtsN [Shigella flexneri 2a str. 2457T]

MAQRDYVRRSQPAPSRRKKSTSRKKQRNLPAVSPAMVAIAAAVLVTFIGGLYFITHHKKEESETLQSQKV

TGNGLPPKPEERWRYIKELESRQPGVRAPTEPSAGGEVKTPEQLTPEQRQLLEQMQADMRQQPTQLVEVP

WNEQTPEQRQQTLQRQRQAQQLAEQQRLAQQSRTTEQSWQQQTRTSQAAPVQAQPRQSKPASTQQPYQDL

LQTPAHTTAQSKPQQAAPVARAADAPKPTAEKKDERRWMVQCGSFRGAEQAETVRAQLAFEGFDSKITTN

NGWNRVVIGPVKGKENADSTLNRLKMAGHTNCIRLAAGG

>gi|30064773|ref|NP_838944.1| DNA-binding transcriptional regulator CytR [Shigella flexneri 2a str. 2457T]

MKAKKQETAATMKDVALKAKVSTATVSRALMNPDKVSQATRNRVEKAAREVGYLPQPMGRNVKRNESRTI

LVIVPDICDPFFSEIIRGIEVTAANHGYLVLIGDCAHQNQQEKTFIDLIITKQIDGMLLLGSRLPFDASI

EEQRNLPPMVMANEFAPELELPTVHIDNLTAAFDAVNYLYEQGHKRIGCIAGPEEMPLCHYRLQGYVQAL

RRCGIMVDPQYIARGDFTFEAGSKAMQQLLDLPQPPTAVFCHSDVMALGALSQAKRQGLKVPEDLSIIGF

DNIDLTQFCDPPLTTIAQPRYEIGREAMLLLLDQMQGQHVGSGSRLMDCELIIRGSTRALP

>gi|30064772|ref|NP_838943.1| primosome assembly protein PriA [Shigella flexneri 2a str. 2457T]

MPVAHVALPVPLPRTFDYLLPEGMTVKAGCRVRVPFGKQQERIGIVVSVSDASELPLTELKAVVEVLDGE

PVFTHSVWRLLLWAADYYHHPIGDVLFHALPILLRQGRPAANAPMWYWFATEQGQAVDLNSLKRSPKQQQ

ALAALRQGKIWRDQVATLEFNDAALQALRKKGLCDLASETPEFSDWRTNYAVSGERLRLNTEQATAVGAI

HSAADTFSAWLLAGVTGSGKTEVYLSVLENVLAQGKQALVMVPEIGLTPQTIARFRERFNAPVEVLHSGL

NDSERLSAWLKAKNGEAAIVIGTRSALFTPFKNLGVIVIDEEHDSSYKQQEGWRYHARDLAVYRAHSEQI

PIILGSATPALETLCNVQQKKYRLLRLTRRAGNARPAIQHVLDLKGQKVQAGLAPALITRMRQHLQANNQ

VILFLNRRGFAPALLCHDCGWIAECPRCDHYYTLHQAQQHLRCHHCDSQRPVPRQCPSCGSTHLVPVGLG

TEQLEQTLAPLFPDVPISRIDRDTTSRKGALEQQLAEVHRGGARILIGTQMLAKGHHFPDVTLVALLDVD

GALFSADFRSAERFAQLYTQVAGRAGRAGKQGEVVLQTHHPEHPLLQTLLYKGYDAFAEQALAERRMMQL

PPWTSHVIVRAEDHNNQHAPLFLQQLRNLILSSPLADDKLWVLGPVPALAPKRGGRWRWQILLQHPSRVR

LQHIISGTLALINTIPDSRKVKWVLDVDPIEG

>gi|30064771|ref|NP_838942.1| 50S ribosomal protein L31 [Shigella flexneri 2a str. 2457T]

MKKDIHPKYEEITASCSCGNVIKIRSTVGHDLNLDVCSKCHPFFTGKQRDVATGGRVDRFNKRFNIPGSK

>gi|30064770|ref|NP_838941.1| peptidoglycan peptidase [Shigella flexneri 2a str. 2457T]

MKNRLLILSLLVSVPAFAWQPQTGDIIFQISRSSQSKAIQLATHSDYSHTGMLVMRNKKPYVFEAVGPVK

YTPLKQWIAHGEKGNYVVRRVEGGLSVEQQQKLTQTAKRYLGKPYDFSFSWSDDRQYCSEVVWKVYQNAL

GMRVGEQQKLKEFDLSNPLVQAKLKERYGKNIPLEETVVSPQAVFDAPQLTTVAKEWPLFSW

>gi|30064769|ref|NP_838940.1| transcriptional repressor protein MetJ [Shigella flexneri 2a str. 2457T]

MAEWSGEYISPYAEHGKKSEQVKKITVSIPLKVLKILTDERTRRQVNNLRHATNSELLCEAFLHAFTGQP

LPDDADLRKERSDEIPEAAKEIMREMGINPETWEY

>gi|30064768|ref|NP_838939.1| cystathionine gamma-synthase [Shigella flexneri 2a str. 2457T]

MTRKQATIAVRSGLNDDEQYGCVVPPIHLSSTYNFTGFNEPRAHDYSRRGNPTRDVVQRALAELEGGAGA

VLTNTGMSAIHLVTTVFLKPGDLLVAPHDCYGGSYRLFDSLAKRGCYRVLFVDQGDEQALRAALAEKPKL

VLVESPSNPLLRVVDIAKICHLAREVGAVSVVDNTFLSPALQNPLALGADLVLHSCTKYLNGHSDVVAGV

VIAKDPDVVTELAWWANNIGVTGGAFDSYLLLRGLRTLVPRMELAQRNAQAIVKYLQTQPLVKKLYHPSL

PENQGHEIAARQQKGFGAMLSFELDGDEQTLCRFLGGLSLFTLAESLGGVESLISHAATMTHAGMAPEAR

AAAGISETLLRISTGIEDGEDLIADLENGFRAANKG

>gi|30064767|ref|NP_838938.1| bifunctional aspartate kinase II/homoserine dehydrogenase II [Shigella flexneri 2a str. 2457T]

MSVIAQAGAKGRQLHKFGGSSLADVKCYLRVAGIMAEFSQPDDMMVVSAAGSTTNQLINWLKLSQTDRLS

AHQVQQTLRRYQCDLISGLLPAEEADSLISAFVSDLERLAALLDSGINDAVYAEVVGHGEVWSARLMSAV

LNQQGLPAAWLDAREFLRAERAAQPQVDEGLSYPLLQQLLVQHPSKRLVVTGFISRNNAGETVLLGRNGS

DYSATQIGALAGVSRVTIWSDVAGVYSADPRKVKDACLLPLLRLDEASELARLAAPVLHARTLQPVSGSE

IDLQLRCSYTPDQGSTRIERVLASGTGARIVTSHDDVCLIEFQVPASQDFKLAHKEIDQILKRAQVRPLA

VGVHNDRQLLQFCYTSEVADSALKILDEAGLPGELRLRQGLALVAMVGAGVTRNPLHCHRFWQQLKGQPV

EFTWQSDDGISLVAVLRTGPTESLIQGLHQSVFRAEKRIGLVLFGKGNIGSRWLELFAREQSTLSARTGF

EFVLAGVVDSRRSLLSYDGLDASRALAFFNDEAVEQDEESLFLWMRAHPYDDLVVLDVTASQQLADQYLD

FASHGFHVISANKLAGASDSNKYRQIHDAFEKTGRHWLYNATVGAGLPINHTVRDLIDSGDTILSISGIF

SGTLSWLFLQFDGSVPFTELVDQAWQQGLTEPDPRDDLSGKDVMRKLVILAREAGYNIEPDQVRVESLVP

AHCEGGSIDHFFENGDELNEQMVQRLEAAREMGLVLRYVARFDANGKARVGVEAVREDYPLASLLPCDNV

FAIESRWYRDNPLVIRGPGAGRDVTAGAIQSDINRLAQLL

>gi|30064766|ref|NP_838937.1| 5,10-methylenetetrahydrofolate reductase [Shigella flexneri 2a str. 2457T]

MSFFHASQRDALNQSLAEVQGQINVSFEFFPPRTSEMEQTLWNSIDRLSSLKPKFVSVTYGANSGERDRT

HSIIKGIKDRTGLEAAPHLTCIDATPDELRTIARDYWNNGIRHIVALRGDLPPGSGKPEMYASDLVTLLK

EVADFDISVAAYPEVHPEAKSAQADLLNLKRKVDAGANRAITQFFFDVESYLRFRDRCVSAGIDVEIIPG

ILPVSNFKQAKKFADMTNVRIPAWMAQMFDGLDDDAETRKLVGANIAMDMVKILSREGVKDFHFYTLNRA

EMSYAICHTLGVRPGL

>gi|30064765|ref|NP_838936.1| catalase; hydroperoxidase HPI(I) [Shigella flexneri 2a str. 2457T]

MSTSDDIHNTTATGKCPFHQGGHDQSAGAGTTTRDWWPNQLRVDLLNQHSNRSNPLGEDFDYRKEFSKLD

YYGLKKDLKALLTESQPWWPADWGSYAGLFIRMAWHGAGTYRSIDGRGGAGRGQQRFAPLNSWPDNVSLD

KARRLLWPIKQKYGQKISWADLFILAGNVALENSGFRTFGFGAGREDVWEPDLDVNWGDEKAWLTHRHPE

ALAKAPLGATEMGLIYVNPEGPDHSGEPLSAAAAIRATFGNMGMNDEETVALIAGGHTLGKTHGAGPTSN

VGPDPEAAPIEEQGLGWASTYGSGVGADAITSGLEVVWTQTPTQWSNYFFENLFKYEWVQTRSPAGAIQF

EAVDAPEIIPDPFDPSKKRKPTMLVTDLTLRFDPEFEKISRRFLNDPQAFNEAFARAWFKLTHRDMGPKS

RYIGPEVPKEDLIWQDPLPQPIYNPTEQDIIDLKFAIADSGLSVSELVSVAWASASTFRGGDKRGGANGA

RLALMPQRDWDVNAAAVRALPVLEKIQKESGKASLADIIVLAGVVGVEKAASAAGLSIHVPFAPGRVDAR

QDQTDIEMFELLEPIADGFRNYRARLDVSTTESLLIDKAQQLTLTAPEMTALVGGMRVLGANFDGSKNGV

FTDRVGVLSNDFFVNLLDMRYEWKATDESKELFEGRDRETGEVKYTASRADLVFGSNSVLRAVAEVYASS

DAHEKFVKDFVAAWVKVMNLDRFDLL

>gi|30064764|ref|NP_838935.1| fructose-like permease EIIC subunit 2 [Shigella flexneri 2a str. 2457T]

MNELVQILKNTRQHLMTGVSHMIPFVVSGGILLAVSVMLYGKGAVPDAVADPNLKKLFDIGVAGLTLMVP

FLAAYIGYSIAERSALAPCAIGAWVGNSFGAGFFGALIAGIIGGIVVHYLKKIPVHKVLRSVMPIFIIPI

IGTLITAGIMMWGLGEPVGALTNSLTQWLQGMQQGSIVMLAVIMGLMLAFDMGGPVNKVAYAFMLICVAQ

GVYTVVAIAAVGICIPPLGMGLATLIGRKNFSAEERETGKAALVMGCVGVTEGAIPFAAADPLRVIPSIM

VGSVCGAVTAALVGAQCYAGWGGLIVLPVVEGKLGYIAAVAVGAVVTAVCVNVLKSLARKNGSSTDEKED

DLDLDFEIN

>gi|30064763|ref|NP_838934.1| fructose-like phosphotransferase EIIB subunit 2 [Shigella flexneri 2a str. 2457T]

MTKIIAVTACPSGVAHTYMAAEALESAAKAKGWEVKVETQGSIGLENELTAEDVASADMVILTKDIGIKF

EERFAGKTIVRVNISDAVKRADAIMSKIEAHLAQTA

>gi|30064762|ref|NP_838933.1| formate acetyltransferase 2 [Shigella flexneri 2a str. 2457T]

MTNRISRLKTALFANTREISLERALLYTASHRQTEGEPVILRRAKATAYILEHVEISIRDEELIAGNRTV

KPRAGIMSPEMDPYWLLKELDQFPTRPQDRFAISEEDKRIYREELFPYWEKRSMKDFINGQMTDEVKAAT

NTQIFSINQTDKGQGHIIIDYPRLLNHGLGELVAQMQQHCQQQPENHFYQAALLLLEASQKHILRYAELA

ETMAANCTDAQRREELLTIAEISRHNAQHKPQTFWQACQLFWYMNIILQYESNASSLSLGRFDQYMLPFY

QASLTQGEDPAFLKELLESLWVKCNDIVLLRSTSSARYFAGFPTGYTALLGGLTENGRSAVNVLSFLCLD

AYQSVQLPQPNLGVRTNALIDTPFLMKTAETIRLGTGIPQIFNDEVVVPAFLNRGVSLEDARDYSVVGCV

ELSIPGRTYGLHDIAMFNLLKVMEICLHENEGNAALTYEGLLEQIRAKISHYITLMVEGSNICDIGHRDW

APVPLLSSFISDCLEKGRDITDGGARYNFSGVQGIGIANLSDSLHALKGMVFEQQRLSFDELLSVLKANF

ATPEGEKVRARLINRFEKYGNDIDEVDNISAELLRHYCKEVEKYQNPRGGYFTPGSYTVSAHVPLGSVVG

ATPDGRFAGEQLADGGLSPMLGQDAQGPTAVLKSVSKLDNTLLSNGTLLNVKFTPATLEGEAGLRKLADF

LRVFTQLKLQHIQFNVVNADTLREAQQRPQDYAGLVVRVAGYSAFFVELSKEIQDDIIRRTAHQL

>gi|30064761|ref|NP_838932.1| pyruvate formate lyase II activase [Shigella flexneri 2a str. 2457T]

MTSSAGQRISCNVVETRRDDVARIFNIQRYSLNDGEGIRTVVFFKGCPHLCPWCANPESISGKIQTVRRE

AKCLHCAKCLRDADECPSGAFERIGRDISLDALEREVMKDDIFFRTSGGGVTLSGGEVLMQAEFATRFLQ

RLRLWGVSCAIETAGDAPASKLLPLAKLCDEVLFDLKIMDATQARDVVKMNLPRVLENLRLLVSEGVNVI

PRLPLIPGFTLSRENMQQALDVLIPLKIRQIHLLPFHQYGEPKYRLLGKTWSMKEVPAPSSADVTTMREM

AERAGFQVTVGG

>gi|30064760|ref|NP_838931.1| fructose-like phosphotransferase EIIB subunit 3 [Shigella flexneri 2a str. 2457T]

MAYLVAVTACVSGVAHTYMAAERLEKLCQLEKWGVSIETQGALGTENRLADEDIRRADVALLITDIELAG

AERFEHCRYVQCSIYAFLREPQRVMSAVRKVLSAPQQTHLILE

>gi|30064759|ref|NP_838930.1| ARAC-type regulatory protein [Shigella flexneri 2a str. 2457T]

MYHDVSYLLSRLINGPLSLRQIYFASSNGPVPDLAYQVDFPRLEIVLEGEFVDTGAGATLVPGDVLYVAA

GGWNFPQWKTPATTFSVLFGKQQLGFSVVQWDGKQYQNLAKQHVARRGPRIGSFLLQTLNEMQMQPQEQQ

TARLIVASLLSHCRDLLGSQIQTASRSQALFEAIRDYIDERYASALTRESVAQAFYISPNYLSHLFQKTG

AIGFNEYLNHTRLEHAKTLLKGYDLKVKEVAHACGFVDSNYFCRLFRKNTEHSPSEYRRQYHSQLTEKPT

TPE

>gi|30064758|ref|NP_838929.1| hypothetical protein S3714 [Shigella flexneri 2a str. 2457T]

MHSTEVQAKPLSSWKALGWALLYFWFFSTLLQAIIYISGYSGTNGIRDSLLFSSLWLIPVFLFPKRIKII

AAVIGVVLWTASLAALCYYVIYGQEFSQSVLFVMFETNTNEASEYLSQYFSLKIVLIALAYTAVAVLLWT

RLRPVYIPKPWRYVVSFALLYGLILHPIAMNTFIKNKPFEKTLDNLASRMEPAAPWQFLTGYYQYRQQLN

SLTKLLNENNALPPLANFKDESGNEPRTLVLVIGESTQRGRMSLYGYPRETTPELDALHKTDPNLTVFNN

VVTSRPYTIEILQQALTFANEKNPDLYLTQPSLMNMMKQAGYKTFWITNQQTMTARNTMLTVFSRQTDKQ

YYMNQQRTQSAREYDTNVLKPFQDVLNDPAPKKLIIVHLLGTHIKYKYRYPENQGKFDGNTDHVPPGLSA

EELESYNDYDNANLYNDHVVASLIKDFKAADPNGFLVYFSDHGEEVYDTPPHKTQGRNEDNPTRHMYTIP

FLLWTSEKWQATHPRDFSQDVDRKYSLAELIHTWSDLAGLSYDGYDPTRSVVNPQFKETTRWIGNPYKKN

ALIDYDTLPYGDQVGNQ

>gi|30064757|ref|NP_838928.1| phosphoenolpyruvate carboxylase [Shigella flexneri 2a str. 2457T]

MNEQYSALRSNVSMLGKVLGETIKDALGEHILERVETIRKLSKSSRAGNDANRQELLTTLQNLSNDELLP

VARAFSQFLNLANTAEQYHSISPKGEAASNPEVIARTLRKLKNQPELSEDTIKKAVESLSLELVLTAHPT

EITRRTLIHKMVEVNACLKQLDNKDIADYERNQLMRRLRQLIAQSWHTDEIRKLRPSPVDEAKWGFAVVE

NSLWQGVPNYLRELNEQLEENLGYKLPVEFVPVRFTSWMGGDRDGNPNVTADITRHVLLLSRWKATDLFL

KDIQVLVSELSMVEATPELLALVGEEGAAEPYRYLMKNLRSRLMATQAWLEARLKGEELPKPEGLLTQNE

ELWEPLYACYQSLQACGMGIIANGDLLDTLRRVKCFGVPLVRIDIRQESTRHTEALGELTRYLGIGDYES

WSEADKQAFLIRELNSKRPLLPRNWQPSAETREVLDTCQVIAEAPQGSIAAYVISMAKTPSDVLAVHLLL

KEAGIGFAMPVAPLFETLDDLNNANDVMTQLLNIDWYRGLIQGKQMVMIGYSDSAKDAGVMAASWAQYQA

QDALIKTCEKAGIELTLFHGRGGSIGRGGAPAHAALLSQPPGSLKGGLRVTEQGEMIRFKYGLPEITVSS

LSLYTGAILEANLLPPPEPKESWRRIMDELSVISCDLYRGYVRENKDFVPYFRSATPEQELGKLPLGSRP

AKRRPTGGVESLRAIPWIFAWTQNRLMLPAWLGAGTALQKVVEDGKQSELEAMCRDWPFFSTRLGMLEMV

FAKADLWLAEYYDQRLVDKALWPLGKELRNLQEEDIKVVLAIANDSHLMADLPWIAESIQLRNIYTDPLN

VLQAELLHRSRQAEKEGQEPDPRVEQALMVTIAGIAAGMRNTG

>gi|30064756|ref|NP_838927.1| acetylornithine deacetylase [Shigella flexneri 2a str. 2457T]

MKNKLPPFIEIYRALIATPSISATEEALDQSNADLITLLADWFKDLGFNVEVQPVPGTRNKFNMLASYGQ

GAGGLLLAGHTDTVPFDDGRWTRDPFTLTEHDGKLYGLGTADMKGFFAFILDALRDVDVTKLKKPLYILA

TADEETSMAGARYFAETTALRPDCAIIGEPTSLQPVRAHKGHISNAIRIQGQSGHSSDPARGINAIELMH

DAIGHILQLRDNLKERYHYEAFTVPYPTLNLGHIHGGDASNRICACCELHMDIRPQPGMTLNELNGLLND

ALAPVSERWPGRLTVDELHPPIPGYECPPNHQLVEVVEKLLGAKTEVVNYCTEAPFIQTLCPTLVLGPGS

INQAHQPDEYLETRFIKPTRELIIQVIHHFCWH

>gi|30064755|ref|NP_838926.1| N-acetyl-gamma-glutamyl-phosphate reductase [Shigella flexneri 2a str. 2457T]

MLNTLIVGASGYAGAELVTYVNRHPHMNITALTVSAQSNDAGKLISDLHPQLKGIVELPLQPMSDISEFS

PGVDVVFLATAHEVSHDLAPQFLEAGCVVFDLSGAFRVNDATFYEKYYGFTHQYPELLEQAAYGLAEWCG

NKLKEANLIAVPGCYPTAAQLALKPLIDADLLDLNQWPVINATSGVSGAGRKAAISNSFCEVSLQPYGVF

THRHQPEIATHLGADVIFTPHLGNFPRGILETITCRLKSGVTQAQVAQALQQAYAHKPLVRLYDKGVPAL

KNVVGLPFCDIGFAVQGEHLIIVATEDNLLKGAAAQAVQCANIRFGYAETQSLI

>gi|30064754|ref|NP_838925.1| acetylglutamate kinase [Shigella flexneri 2a str. 2457T]

MNPLIIKLGGVLLDSEEALERLFSALVNYRESHQRPLVIVHGGGCVVDELMKGLNLPVKKKNGLRVTPAD

QIDIITGALAGTANKTLLAWAKKHQIAAVGLFLGDGDSVKVTQLDEELGHVGLAQPGSPKLINSLLENGY

LPVVSSIGVTDEGQLMNVNADQAATALAATLGADLILLSDVSGILDGKGQRIAEMTAAKAEQLIEQGIIT

DGMIVKVNAALDAARTLGRPVDIASWRYAEQLPALFNGMPMGTRILA

>gi|30064753|ref|NP_838924.1| argininosuccinate lyase [Shigella flexneri 2a str. 2457T]

MALWGGRFTQAADQRFKQFNDSLRFDYRLAEQDIVGSVAWSKALVTVGVLTAEEQAQLEEALNVLLEDVR

ARPQQILESDAEDIHSWVEGKLIDKVGQLGKKLHTGRSRNDQVATDLKLWCKDTVSELLTANRQLQSALV

ETAQNNQDAVMPGYTHLQRAQPVTFAHWCLAYVEMLARDESRLQDALKRLDVSPLGCGALAGTAYEIDRE

QLAGWLGFASATRNSLDSVSDRDHVLELLSAAAIGMVHLSRFAEDLIFFNTGEAGFVELSDRVTSGSSLM

PQKKNPDALELIRGKCGRVQGALTGMMMTLKGLPLAYNKDMQEDKEGLFDALDTWLDCLHMAALVLDGIQ

VKRPRCQEAAQQGYANATELADYLVAKGVPFREAHHIVGEAVVEAIRQGKPLEDLPLSELQKFSQVIGED

VYPILSLQSCLEKRAAKGGVSPQQVAQAIAFAQARLE

>gi|30064752|ref|NP_838923.1| IS4 orf [Shigella flexneri 2a str. 2457T]

MHIGQALDLVSRYDSLRNPLTSLGDYLDPELISRCLAESGTVTLRKRRLPLEMMVWCIVGMVLERKEPLH

QIVNRLDIMLPGNRPFVAPSAVIQARQRLGSEAVRRVFTKTAQLWHNATPHPHWCGLTLLAIDGVFWRTP

DTPENDAAFPRQTHAGNPALYPQVKMVCQMELTSHLLTAAAFGTMKNSENELAEQLIEQTGDNTLTLMDK

GYYSLGLLNGWSLAGEHRHWMIPLRKGAQYEELRKLGKGDHLVKLKTSPQARKKWPGLGNEVTARLLTVT

RKGKVCHLLTSMTDAMRFPGGEMADLYSNRWEIELGYREIKQTMQLSRLTLRSKKPELVEQELWGVLLAY

NLVRYQMIKMAEHLKGYWPNQLSFSESCGMVMRMLMTLQGASPGRIPELMSDLASMGQLVKLPTRRGRAF

PRVVKERPWKYPTAPKKSQSVA

>gi|30064751|ref|NP_838922.1| sensor protein [Shigella flexneri 2a str. 2457T]

MKIIAADVFVTCPGRNFVTLKITTESGLCGLGDATLNGRELSVASYLKDHLCPQLIGRDASRIEDIWQFF

YKGAYWRRGPVTMSAISAIDMALWDIKAKAANMPLYQLLGGASREGVMVYCHTTGRTIDEVLEDYAKHQQ

MGFKAIRVQCGVPGMQTTYGLAKGKGLAYEPATKGLWPEEQLWSSEKYLDFTPKLFEAVRNKFGFNEHLL

HDMHHRLTPIEAARFGKSIEDYRLFWMEDPTPAENQECFRLIRQHTVTPIAVGEVFNSIWDCKQLIEEQL

IGYIRTTITHAGGITGMRRIADFASLYQVRTGSHGPSDLSPVCHAAALHFDLWVPNFGVQEYMGYSEQML

EVFPYSWRFEEGYMHPGDEPGLGISFDEKLAAKYPYDPEYLPVARLEDGTLLNW

>gi|30064750|ref|NP_838921.1| IS1 orfA [Shigella flexneri 2a str. 2457T]

MASISIRCPSCSATEGVVRNGKSTAGHQRYLCSHCRKTWQLQFTYTASQPGTHQKIIDMAMNGVGCRASA

RIMGVGLNTVLRHLKNSGRSR

>gi|30064749|ref|NP_838920.1| IS1 orfB [Shigella flexneri 2a str. 2457T]

MSRQRTHYGRWPQHGFTSLKKLRPQSVTSRIQPGSDVIVCAEMDEHWGYVGAKSRQRWLFYAYDRIRRTV

VAHVFGERTLATLERLLSLLSAFEVVVWMTDGWPLYESRLKGKLHVISKRYTQRIERHNLNLRQHLARLV

RKSLSFSKSVELHDKVIGHYLNIKHYQ

>gi|30064748|ref|NP_838919.1| DNA-binding transcriptional regulator OxyR [Shigella flexneri 2a str. 2457T]

MNIRDLEYLVALAEHRHFRRAADSCHVSQPTLSGQIRKLEDELGVMLLERTSRKVLFTQAGMLLVDQART

VLREVKVLKEMASQQGETMSGPLHIGLIPTVGPYLLPHIIPMLHQTFPKLEMYLHEAQTHQLLAQLDSGK

LDCVILALVKESEAFIEVPLFDEPMLLAIYEDHPWANRECVLMADLAGEKLLMLEDGHCLRDQAMGFCFE

AGADEDTHFRATSLETLRNMVAAGSGITLLPALAVPPERKRDGVVYLPCIKPEPCRTIGLVYRPGSPLRS

RYEQLAEAIRARMDGHFDKVLKQAV

>gi|30064746|ref|NP_838917.1| DNA-binding transcriptional repressor FabR [Shigella flexneri 2a str. 2457T]

MFILWYSASSTFGKDSDIVMGVRAQQKEKTRRSLVEAAFSQLSAERSFASLSLREVAREAGIAPTSFYRH

FRDVDELGLTMVDESGLMLRQLMRQARQRIAKGGSVIRTSVSTFMEFIGNNPNAFRLLLRERSGTSAAFR

AAVAREIQHFIAELADYLELENHMPRAFTEAQAEAMVTIVFSAGAEALDVGVEQRRQLEERLVLQLRMIS

KGAYYWYRREQEKTAIIPGNVKDE

>gi|30064745|ref|NP_838916.1| hypothetical protein S3698 [Shigella flexneri 2a str. 2457T]

MKQANQDRGTLLLALVAGLSINGTFAALFSSIVPFSVFPIISLVLTVYCLHQRYLNRTMSVGLPGLAAAC

FILGVLLYSTVVRAEYPDIGSNFFPAVLSVIMVFWIGAKMRNRKQEVAE

>gi|30064744|ref|NP_838915.1| tRNA (uracil-5-)-methyltransferase [Shigella flexneri 2a str. 2457T]

MTPEHLPTEQYEAQLAEKVVRLQSMMAPFSDLVPEVFRSPVSHYRMRAEFRIWHDGDDLYHIIFDQQTKS

RIRVDSFPAASELINQLMTAMIAGVRNNPVLRHKLFQIDYLTTLSNQAVVSLLYHKKLDDEWRQEAEALR

DALRAQNLNVHLIGRATKTKIALDQDYIDERLPVAGKEMIYRQVENSFTQPNAAMNIQMLEWALDVTKGS

KGDLLELYCGNGNFSLALARNFDRVLATEIAKPSVAAAQYNIAANHIDNVQIIRMAAEEFTQAMNGVREF

NRLQGIDLKSYQFETIFVDPPRSGLDSETEKMVQAYPRILYISCNPETLCKNLETLSQTHKVERLALFDQ

FPYTHHMECGVLLTAK

>gi|30064743|ref|NP_838914.1| vitamin B12/cobalamin outer membrane transporter [Shigella flexneri 2a str. 2457T]

MIKKASLLTACSVTAFSAWAQDTSPDTLVVTAIRFEQPRSTVLAPTTVVTRQDIDRWQSTSVNDVLRRLP

GVDITQNGGSGQLSSIFIRGTNASHVLVLIDGVRLNLAGVSGSADLSQFPIALVQRIEYIRGPRSAVYGS

DAIGGVVNIITTRDEPGTEISAGWGSNSYQNYDVSTQQQLGDKTRVTLLGDYAHTHGYDVVAYGNTGTQA

QTDNDGFLSKTLYGALEHNFTDAWSGFVRGYGYDNRTNYDAYYSPGSPLLDTRKLYSQSWDAGLRYNGEL

IKSQLITSYSHSKDYNYDPHYGRYDSSATLDEMKQYTVQWANNVIVGHGSIGAGVDWQKQTTTPGTGYVE

DGYDQRNTGIYLTGLQQVGDFTFEGAARSDDNSQFGRHGTWQTSAGWEFIEGYRFIASYGTSYKALNLGQ

LYGFYGNPNLDPEKSKQWEGAFEGLTAGVNWRISGYRNDVSDLIDYDDHTLKYYNEGKARIKGVEATANF

DTGPLTHTVSYDYVDARNAITDTPLLRRAKQQVKYQLDWQLYDFDWGITYQYLGTRYDKDYSSYPYQTVK

MGGVSLWDLAVAYPVTSHLTVRGKIANLFDKDYETVYGYQTAGREYTLSGSYTF

>gi|30064741|ref|NP_838912.1| UDP-N-acetylenolpyruvoylglucosamine reductase [Shigella flexneri 2a str. 2457T]

MNHSLKPWNTFGIDHNAQHIVCAEDEQQLLNAWQHATAEGQPVLILGEGSNVLFLEDYRGTVIINRIKGI

EIHDEPDAWYLHVGAGENWHRLVKYTLQEGMPGLENLALIPGCVGSSPIQNIGAYGVELQRVCAYVDCVE

LATGKQVRLTAKECRFGYRDSIFKHEYQDRFAIVAVGLRLPKEWQPVLTYGDLTRLDPTTVTPQQVFNAV

CHMRTTKLPDPKVNGNAGSFFKNPVVSAETAKALLAQFPTAPNYPQAGGSVKLAAGWLIDQCQLKGMQMG

GAAVHRQQALVLINEDNAKSEDVVQLAHHVRQKVGEKFNVWLEPEVRFIGASGEVSAVETIS

>gi|30064740|ref|NP_838911.1| biotin--protein ligase [Shigella flexneri 2a str. 2457T]

MKDNTVPLKLIALLANGEFHSGEQLGETLGMSRAAINKHIQTLRDWGVDVFTVPGKGYSLPEPIQLLNAE

QILGQLDGGSVAVLPVIDSTNQFLLDRIGELKSGDACVAEYQQAGRGRRGRKWFSPFGANLYLSMFWRLE

QGPAAAIGLSLVIGIVMAEVLRKLGADKVRVKWPNDLYLQDRKLAGILVELTGKTGDAAQIVIGAGINMA

MRRVEESVVNQGWITLQEAGINLDRNTLAAMLIRELRAALELFEQEGLAPYLSRWEKLDNFINRPVKLII

GDKEIFGISRGIDKQGALLLEQDGIIKPWMGGEISLRSAEK

>gi|30064739|ref|NP_838910.1| pantothenate kinase [Shigella flexneri 2a str. 2457T]

MTPYLQFDRNQWAALRDSVPMTLSEDEIARLKGINEDLSLEEVAEIYLPLSRLLNFYISSNLRRQAVLEQ

FLGTNGQRIPYIISIAGSVAVGKSTTARVLQALLSRWPEHRRVELITTDGFLHPNQVLKERGLMKKKGFP

ESYDMHRLVKFVSDLKSGVPNVTAPVYSHLIYDVIPDGDKTVVQPDILILEGLNVLQSGMDYPHDPHHVF

VSDFVDFSIYVDAPEDLLQTWYINRFLKFREGAFTDPDSYFHNYAKLTKEEAIKTAMTLWKEINWLNLKQ

NILPTRERASLILTKSANHVVEEVRLRK

>gi|30064738|ref|NP_838909.1| hypothetical protein S3687 [Shigella flexneri 2a str. 2457T]

METCEFMQLKNCIICFIGHILCYTMKKQPNGGQISTLFQHHCAQQCAPAKP

>gi|30064737|ref|NP_838908.1| elongation factor Tu [Shigella flexneri 2a str. 2457T]

MSKEKFERTKPHVNVGTIGHVDHGKTTLTAAITTVLAKTYGGAARAFDQIDNAPEEKARGITINTSHVEY

DTPTRHYAHVDCPGHADYVKNMITGAAQMDGAILVVAATDGPMPQTREHILLGRQVGVPYIIVFLNKCDM

VDDEELLELVEMEVRELLSQYDFPGDDTPIVRGSALKALEGDAEWEAKILELAGFLDSYIPEPERAIDKP

FLLPIEDVFSISGRGTVVTGRVERGIIKVGEEVEIVGIKETQKSTCTGVEMFRKLLDEGRAGENVGVLLR

GIKREEIERGQVLAKPGTIKPHTKFESEVYILSKDEGGRHTPFFKGYRPQFYFRTTDVTGTIELPEGVEM

VMPGDNIKMVVTLIHPIAMDDGLRFAIREGGRTVGAGVVAKVLS

>gi|30064736|ref|NP_838907.1| preprotein translocase subunit SecE [Shigella flexneri 2a str. 2457T]

MSANTEAQGSGRGLEAMKWVVVVALLLAAIVGNYLYRDIMLPLRALAVVILIAAAGGVALLTTKGKATIA

FAREARTEVRKVIWPTRQETLHTTLIVAAVTAVMSLILWGLDGILVRLVSFITGLRF

>gi|30064735|ref|NP_838906.1| transcription antitermination protein NusG [Shigella flexneri 2a str. 2457T]

MSEAPKKRWYVVQAFSGFEGRVATSLREHIKLHNMEDLFGEVMVPTEEVVEIRGGQRRKSERKFFPGYVL

VQMVMNDASWHLVRSVPRVMGFIGGTSDRPAPISDKEVDAIMNRLQQVGDKPRPKTLFEPGEMVRVNDGP

FADFNGVVEEVDYEKSRLKVSVSIFGRATPVELDFSQVEKA

>gi|30064734|ref|NP_838905.1| 50S ribosomal protein L11 [Shigella flexneri 2a str. 2457T]

MAKKVQAYVKLQVAAGMANPSPPVGPALGQQGVNIMEFCKAFNAKTDSIEKGLPIPVVITVYADRSFTFV

TKTPPAAVLLKKAAGIKSGSGKPNKDKVGKISRAQLQEIAQTKAADMTGADIEAMTRSIEGTARSMGLVV

ED

>gi|30064733|ref|NP_838904.1| 50S ribosomal protein L1 [Shigella flexneri 2a str. 2457T]

MAKLTKRMRVIREKVDATKQYDINEAIALLKELATAKFVESVDVAVNLGIDARKSDQNVRGATVLPHGTG

RSVRVAVFTQGANAEAAKAAGAELVGMEDLADQIKKGEMNFDVVIASPDAMRVVGQLGQVLGPRGLMPNP

KVGTVTPNVAEAVKNAKAGQVRYRNDKNGIIHTTIGKVDFDADKLKENLEALLVALKKAKPTQAKGVYIK

KVSISTTMGAGVAVDQAGLSASVN

>gi|30064732|ref|NP_838903.1| 50S ribosomal protein L10 [Shigella flexneri 2a str. 2457T]

MALNLQDKQAIVAEVSEVAKGALSAVVADSRGVTVDKMTELRKAGREAGVYMRVVRNTLLRRAVEGTPFE

CLKDAFVGPTLIAYSMEHPGAAARLFKEFAKANAKFEVKAAAFEGELIPASQIDRLATLPTYEEAIARLM

ATMKEASAGKLVRTLAAVRDAKEAA

>gi|30064731|ref|NP_838902.1| 50S ribosomal protein L7/L12 [Shigella flexneri 2a str. 2457T]

MSITKDQIIEAVAAMSVMDVVELISAMEEKFGVSAAAAVAVAAGPVEAAEEKTEFDVILKAAGANKVAVI

KAVRGATGLGLKEAKDLVESAPAALKEGVSKDDAEALKKALEEAGAEVEVK

>gi|30064730|ref|NP_838901.1| DNA-directed RNA polymerase subunit beta [Shigella flexneri 2a str. 2457T]

MVYSYTEKKRIRKDFGKRPQVLDVPYLLSIQLDSFQKFIEQDPEGQYGLEAAFRSVFPIQSYSGNSELQY

VSYRLGEPVFDVQECQIRGVTYSAPLRVKLRLVIYEREAPEGTVKDIKEQEVYMGEIPLMTDNGTFVING

TERVIVSQLHRSPGVFFDSDKGKTHSSGKVLYNARIIPYRGSWLDFEFDPKDNLFVRIDRRRKLPATIIL

RALNYTTEQILDLFFEKVIFEIRDNKLQMELVPERLRGETASFDIEANGKVYVEKGRRITARHIRQLEKD

DVKLIEVPVEYIAGKVVAKDYIDESTGELICAANMELSLDLLAKLSQSGHKRIETLFTNDLDHGPYISET

LRVDPTNDRLSALVEIYRMMRPGEPPTREAAESLFENLFFSEDRYDLSAVGRMKFNRSLLREEIEGSGIL

SKDDIIDVMKKLIDIRNGKGEVDDIDHLGNRRIRSVGEMAENQFRVGLVRVERAVKERLSLGDLDTLMPQ

DMINAKPISAAVKEFFGSSQLSQFMDQNNPLSEITHKRRISALGPGGLTRERAGFEVRDVHPTHYGRVCP

IETPEGPNIGLINSLSVYAQTNEYGFLETPYRKVTDGVVTDEIHYLSAIEEGNYVIAQANSNLDEEGHFV

EDLVTCRSKGESSLFSRDQVDYMDVSTQQVVSVGASLIPFLEHDDANRALMGANMQRQAVPTLRADKPLV

GTGMERAVAVDSGVTAVAKRGGVVQYVDASRIVIKVNEDEMYPGEAGIDIYNLTKYTRSNQNTCINQMPC

VSLGEPVERGDVLADGPSTDLGELALGQNMRVAFMPWNGYNFEDSILVSERVVQEDRFTTIHIQELACVS

RDTKLGPEEITADIPNVGEAALSKLDESGIVYIGAEVTGGDILVGKVTPKGETQLTPEEKLLRAIFGEKA

SDVKDSSLRVPNGVSGTVIDVQVFTRDGVEKDKRALEIEEMQLKQAKKDLSEELQILEAGLFSRIRAVLV

AGGVEAEKLDKLPRDRWLELGLTDEEKQNQLEQLAEQYDELKHEFEKKLEAKRRKITQGDDLAPGVLKIV

KVYLAVKRRIQPGDKMAGRHGNKGVISKINPIEDMPYDENGTPVDIVLNPLGVPSRMNIGQILETHLGMA

AKGIGDKINAMLKQQQEVAKLREFIQRAYDLGADVRQKVDLSTFSDEEVMRLAENLRKGMPIATPVFDGA

KEAEIKELLKLGDLPTSGQIRLYDGRTGEQFERPVTVGYMYMLKLNHLVDDKMHARSTGSYSLVTQQPLG

GKAQFGGQRFGEMEVWALEAYGAAYTLQEMLTVKSDDVNGRTKMYKNIVDGNHQMEPGMPESFNVLLKEI

RSLGINIELEDE

>gi|30064729|ref|NP_838900.1| DNA-directed RNA polymerase subunit beta' [Shigella flexneri 2a str. 2457T]

MKDLLKFLKAQTKTEEFDAIKIALASPDMIRSWSFGEVKKPETINYRTFKPERDGLFCARIFGPVKDYEC

LCGKYKRLKHRGVICEKCGVEVTQTKVRRERMGHIELASPTAHIWFLKSLPSRIGLLLDMPLRDIERVLY

FESYVVIEGGMTNLERQQILTEEQYLDALEEFGDEFDAKMGAEAIQALLKSMDLEQECEQLREELNETNS

ETKRKKLTKRIKLLEAFVQSGNKPEWMILTVLPVLPPDLRPLVPLDGGRFATSDLNDLYRRVINRNNRLK

RLLDLAAPDIIVRNEKRMLQEAVDALLDNGRRGRAITGSNKRPLKSLADMIKGKQGRFRQNLLGKRVDYS
GRSVITVGPYLRLHQCGLPKKMALELFKPFIYGKLELRGLATTIKAAKKMVEREEAVVWDILDEVIREHP
VLLNRAPTLHRLGIQAFEPVLIEGKAIQLHPLVCAAYNADFDGDQMAVHVPLTLEAQLEARALMMSTNNI
LSPANGEPIIVPSQDVVLGLYYMTRDCVNAKGEGMVLTGPKEAERLYRSGLASLHARVKVRITEYEKDAN
GELVAKTSLKDTTVGRAILWMIVPKGLPYSIVNQALGKKAISKMLNTCYRILGLKPTVIFADQIMYTGFA
YAARSGASVGIDDMVIPEKKHEIISEAEAEVAEIQEQFQSGLVTAGERYNKVIDIWAAANDRVSKAMMDN
LQTETVINRDGQEEKQVSFNSIYMMADSGARGSAAQIRQLAGMRGLMAKPDGSIIETPITANFREGLNVL
QYFISTHGARKGLADTALKTANSGYLTRRLVDVAQDLVVTEDDCGTHEGIMMTPVIEGGDVKEPLRDRVL
GRVTAEDVLKPGTADILVPRNTLLHEQWCDLLEENSVDAVKVRSVVSCDTDFGVCAHCYGRDLARGHIIN
KGEAIGVIAAQSIGEPGTQLTMRTFHIGGAASRAAAESSIQVKNKGSIKLSNVKSVVNSSGKLVITSRNT
ELKLIDEFGRTKESYKVPYGAVLAKGDGEQVAGGETVANWDPHTMPVITEVSGFVRFTDMIDGQTITRQT
DELTGLSSLVVLDSAERTAGGKDLRPALKIVDAQGNDVLIPGTDMPAQYFLPGKAIVQLEDGVQISSGDT
LARIPQESGGTKDITGGLPRVADLFEARRPKEPAILAEISGIVSFGKETKGKRRLVITPVDGSDPYEEMI
PKWRQLNVFEGERVERGDVISDGPEAPHDILRLRGVHAVTRYIVNEVQDVYRLQGVKINDKHIEVIVRQM
LRKATIVNAGSSDFLEGEQVEYSRVKIANRELEANGKVGATYSRDLLGITKASLATESFISAASFQETTR
VLTEAAVAGKRDELRGLKENVIVGRLIPAGTGYAYHQDRMRRRAAGEAPAAPQVTAEDASASLAELLNAG
LGGSDNE

>gi|30064728|ref|NP_838899.1| thiamine biosynthesis protein ThiH [Shigella flexneri 2a str. 2457T]
MKTFSNRWRQLDWDDIRLRINGKTAADVERALNASQLTRDDMMALLSPAASGYLEQLAQQAQRLTRQRFG
NTVSFYVPLYLSNLCANDCTYCGFSMSNRIKRKTLDEADIARESAAIREMGFEHLLLVTGEHQAKVGMDY
FRRHLPALREQFSSLQMEVQPLAETEYAELKQLGLDGVMVYQETYHEATYTRHHLKGKKQDFFWRLETPD
RLGRAGIDKIGLGALIGLSDNWRVDCYMVAEHLLWLQQHYWQSRYSVSFPRLRPCTGGIEPASIMDERQL
VQTICAFRLLAPEIELSLSTRESPWFRDRVIPLAINNVSAFSKTQPGGYADNHPELEQFSPHDDRRPEAV
AAVLTAQGLQPVWKDWDSYLGRASQRL

>gi|30064726|ref|NP_838897.1| thiamine biosynthesis protein ThiF [Shigella flexneri 2a str. 2457T]

MNDRDFMRYSRQILLDDIALDGQQKLLDSQVLIIGLGGLGTPAALYLAGAGVGTLVLADDDDVHLSNLQR

QILFTTEDINSPKSQVSQQRLTQLNPDIQLTALQQRLTGEALKDAVAQADVVLDCTDNMATRQEINAACV

ALNTPLITASAVGFGGQLMVLTPPWEQGCYRCLWPDNQQPERNCRTAGVVGPVVGVMGTLQALEAIKLLS

GIETPAGELRLFDGKSSQWRSLALRRASGCPVCGGSNADPV

>gi|30064725|ref|NP_838896.1| thiamine-phosphate pyrophosphorylase [Shigella flexneri 2a str. 2457T]

MYQPDFPPVPFRLGLYPVVDSVQWIERLLDAGVRTLQLRIKDRRDEEVEADVVAAIALGRRYNARLFIND

YWRLAIKHQAYGVHLGQEDLQATDLNAIRAAGLRLGVSTHDDMEIDVALAARPSYIALGHVFPTQTKQMP

SAPQGLEQLARHVERLADYPTVAIGGISLARAPAVIATGVGSIAVVSAITQAADWRLATAQLLEIAGVGD

E

>gi|30064724|ref|NP_838895.1| thiamine biosynthesis protein ThiC [Shigella flexneri 2a str. 2457T]

MSATKLTRREQRARAQHFIDTLEGTAFPNSKRIYITGTHPGVRVPMREIQLSPTLIGGSKEQPQYEENEA

IPVYDTSGPYGDPQIAINVQQGLAKLRQPWIDARADTEELTVRSSDYTRTRLADDGLDELRFSGLLTPKR

AKTGRRVTQLHYARQGIITPEMEFIAIRENMGRERIRSEVLRHQHPGMSFGAHLPENITAEFVRDEVAAG

RAIIPANINHPESEPMIIGRNFLVKVNANIGNSAVTSSIEEEVEKLVWSTRWGADTVMDLSTGRYIHETR

EWILRNSPVPIGTVPIYQALEKVNGIAEDLTWEAFRDTLLEQAEQGVDYFTIHAGVLLRYVPMTAKRLTG

IVSRGGSIMAKWCLSHHQENFLYQHFREICEICAAYDVSLSLGDGLRPGSIQDANDEAQFAELHTLGELT

KIAWEYDVQVMIEGPGHVPMQMIRRNMTEELEHCHEAPFYTLGPLTTDIAPGYDHFTSGIGAAMIGWFGC

AMLCYVTPKEHLGLPNKEDVKQGLITYKIAAHAADLAKGHPGAQIRDNAMSKARFEFRWEDQFNLALDPF

TARAYHDETLPQESGKVAHFCSMCGPKFCSMKISQEVRDYAAAQTIEVGMADMSENFRARGGEIYLHKEE

A

>gi|30064723|ref|NP_838894.1| anti-RNA polymerase sigma 70 factor [Shigella flexneri 2a str. 2457T]

MLNQLDNLTERVRGSNKLVDRWLHVRKHLLVAYYNLVGIKPGKESYMRLNEKALDDFCQSLVDYLSAGHF

SIYERILHKLEGNGQLARAAKIWPQLEANTQQIMDYYDSSLETAIDHDNYLEFQQVLSDIGEALEARFVL

EDKLILLVLDAARVKHPA

>gi|30064722|ref|NP_838893.1| NADH pyrophosphatase [Shigella flexneri 2a str. 2457T]

MDRIIEKLDHGWWVVSHEQKLWLPKGELPYGEAANFDLVGQRALQIGEWQGEPVWLIQQQRRHDMGSVRQ

VIDLDVGLFQLAGRGVQLAEFYRSHKYCGYCGHEMYPSKTEWAMLCSHCRERYYPQIAPCIIVAIRRDDS

LLLAQHTRHRNGVHTVLAGFVEVGETLEQAVAREVMEESGIKVKNLRYVTSQPWPFPQSLMTAFMAEYDS

GDIVIDPKELLEANWYRYDDLPLLPPPGTVARRLIEDTVAMCRAEYE

>gi|30064721|ref|NP_838892.1| uroporphyrinogen decarboxylase [Shigella flexneri 2a str. 2457T]

MTELKNDRYLRALLRQPVDVTPVWMMRQAGRYLPEYKATRAQAGDFMSLCKNAELACEVTLQPLRRYPLD

AAILFSDILTVPDAMGLGLYFEAGEGPRFTSPVTCKADVDKLPIPDPEDELGYVMNAVRTIRHELKGEVP

LIGFSGSPWTLATYMVEGGSSKAFTVIKKMMYADPQALHALLDKLAKSVTLYLNAQIKAGAQAVMIFDTW

GGVLTGRDYQQFSLYYMHKIVDGLLRENDGRRVPVTLFTKGGGQWLEAMAETGCDALGLDWTTDIADARR

RVGNKVALQGNMDPSMLYAPPARIEEEVATILAGFGHGEGHVFNLGHGIHQDVPPEHAGVFVEAVHRLSE

QYHR

>gi|30064720|ref|NP_838891.1| endonuclease V [Shigella flexneri 2a str. 2457T]

MDLASLRAQQIELASSVICEDRLDKDPPDLIAGADVGFEQGGEVTRAAMVLLKYPSLELVEYKVARIATT

MPYIPGFLSFREYPALLAAWEMLSQKPDLVFVDGHGISHPRRLGVASHFGLLVDVPTIGVAKKRLCGKFE

PLSSKPGALAPLMDKGEQLAWVWRSKARCNPLFIATGHRVSVDSALAWVQRCMKGYRLPEPTRWADAVAS

ERPAFVRYTANQP

>gi|30064719|ref|NP_838890.1| hypothetical protein S3664 [Shigella flexneri 2a str. 2457T]

MLQNPIHLRLERLESWQHVTFMACLCERMYPNYAMFCQQTGFGDGQIYRRILDLIWETLTVKDAKVNFDS

QLEKFEEAIPSADDFDLYGVYPAIDACVALSELVHSRLSGETLEHAVEVSKTSITTVAMLEMTQAGREMG

DEELKENPAVEQEWDIQWEIFRLLAECEERDIELIKGLRADLRESGESNIGIIFQQ

>gi|30064718|ref|NP_838889.1| transcriptional regulator HU subunit alpha [Shigella flexneri 2a str. 2457T]

MNKTQLIDVIAEKAELSKTQAKAALESTLAAITESLKEGDAVQLVGFGTFKVNHRAERTGRNPQTGKEIK

IAAANVPAFVSGKALKDAVK

>gi|30064717|ref|NP_838888.1| hypothetical protein S3662 [Shigella flexneri 2a str. 2457T]

MNSFNEGVVSPLLSFWRRSLMLAGALFLTACSHNSSLPPFTASGFAEDQGAVRIWRKDSGDNVHLLAVFS

PWRSGDTTTREYRWQGDNLTLININVYSKPPVNIRARFDDRGDLSFMQRESDGEKQQLSNDQIDLYRYRA

DQIRQISDALRQGRVVLRQGRWHAMEQTVTTCEGQTIKPDLDSQAIAHIERRQSRSSVDVSVAWLEAPEG

SQLLLVANSDFCRWQPNEKTF

>gi|30064715|ref|NP_838886.1| sensor protein ZraS [Shigella flexneri 2a str. 2457T]

MRFMQRSKDSLAKWLSAILPVVIVGLVGLFAVTVIRDYGRETAAARQTLLEKGSVLIRALESGSRVGMGM

RMHHAQQQALLEEMAGQPGVRWFAVTDEQGTIVMHSNSGMVGKQLYSPQEMQQLHPGDEEAWRRIDSADG

EPVLEIYRQFQPMFAAGMYRMRHMQQYAATPQAIFIAFDASNIVSAEDREQRNTLIILFALATVLLASVL

SFFWYRRYLRSRQLLQDEMKRKEKLVALGHLAAGVAHEIRNPLSSIKGLAKYFAERAPAGGEAHQLAQVM

AKEADRLNRVVSELLELVKPTHLALQAVDLNTLINHSLQLVSQDANSREIQLRFTANDTLPEIQADPDRL

TQVLLNLYLNAIQAIGQHGVISVTASESGAGVKISVTDSGKGIAADQLEAIFTPYFTTKAEGTGLGLAVV

HNIVEQHGGTIQVASQEGKGSTFTLWLPVNITRKDPQG

>gi|30064714|ref|NP_838885.1| transcriptional regulatory protein ZraR [Shigella flexneri 2a str. 2457T]

MTHDNIDILVVDDDISHCTILQALLRGWGYNVALANSGRQALEQVREQVFDLVLCDVRMAEMDGIATLKE

IKALNPAIPVLIMTAYSSVETAVEALKTGAQDYLIKPLDFDNLQATLEKALAHTHSIDAETPAVTASQFG

MVGKSPAMQHLLSEIALVAPSEATVLIHGDSGTGKELVARAIHASSARSEKPLVTLNCAALNESLLESEL

FGHEKGAFTGADKRREGRFVEADGGTLFLDEIGDISPMMQVRLLRAIQEREVQRVGSNQTISVDVRLIAA

THRDLAAEVNAGRFRQDLYYRLNVVAIEVPSLRQRREDIPLLAGHFLQRFAERNRKAVKGFTPQAMDLLI

HYDWPGNIRELENAVERAVVLLTGEYISERELPLAIASTPIPLGQSQDIQPLVEVEKEVILAALEKTGGN

KTEAARQLGITRKTLLAKLSR

>gi|30064713|ref|NP_838884.1| phosphoribosylamine--glycine ligase [Shigella flexneri 2a str. 2457T]

MKVLVIGNGGREHALAWKAAQSPLVETVFAAPGNAGTALEPALQNVAIGVTDIPALLDFAQNEKIDLTIV

GPEAPLVKGVVDTFRAAGLKIFGPTAGAAQLEGSKAFTKDFLARHKIPTAEYQNFTEVEPALVYLREKGA

PIVIKADGLAAGKGVIVAMTLEEAEAAVHDMLAGNAFGDAGHRIVIEEFLDGEEASFIVMVDGEHVLPMA

TSQDHKRVGDKDNGPNTGGMGAYSPAPVVTDEVHQRTMERIIWPTVKGMAAEGNTYTGFLYAGLMIDKQG

NPKVIEFNCRFGDPETQPIMLRMKSDLVELCLAACEGKLDEKTSEWDERASLSVVMAAGGYPGDYRTGDV

IHGLPLEEVADGKVFHAGTKLADDEQVVTSGGRVLCVTALGHTVAEAQKRAYALMTDIHWDDCFCRKDIG

WRAIEREQN

>gi|30064712|ref|NP_838883.1| bifunctional phosphoribosylaminoimidazolecarboxamide formyltransferase/IMP cyclohydrolase [Shigella flexneri 2a str. 2457T]

MQQRRPVRRALLSVSDKAGIVEFAQALSARGVELLSTGGTARLLAEKGLPVTEVSDYTGFPEMMDGRVKT

LHPKVHGGILGRRGQDDAIMEEHQIQPIDMVVVNLYPFAQTVAREGCSLEDAVENIDIGGPTMVRSAAKN

HKDVAIVVKSSDYDAIIKEMDDNEGSLTLATRFDLAIKAFEHTAAYDSMIANYFGSMVPAYHGESKEAAG

RFPRTLNLNFIKKQDMRYGENSHQQAAFYIEENVKEASVATATQVQGKALSYNNIADTDAALECVKEFAE

PACVIVKHANPCGVAIGNSILDAYDRAYKTDPTSAFGGIIAFNRELDAETAQAIISRQFVEVIIAPSASE

EALKITAAKQNVRVLTCGQWGERVPGLDFKRVNGGLLVQDRDLGMVGAEELRVVTQRQPTEQELRDALFC

WKVAKFVKSNAIVYAKNNMTIGIGAGQMSRVYSAKIAGIKAADEGLEVKGSSMASDAFFPFRDGIDAAAA

AGVTCVIQPGGSIRDDEVIAAADEHGIAMLFTDMRHFRH

>gi|30064711|ref|NP_838882.1| hypothetical protein S3652 [Shigella flexneri 2a str. 2457T]

MVISIRRSRHEEGEELVAIWCRSVDATHDFLSAEYRAELEELVRSFLPEAPLWVAVNEREQPVGFMLLSG

QHMDALFIDPDVCGCGVGRMLVEHALSMAPELTTNVNEQNEQAVGFYKKVGFKVTGRSEVDDLGKPYPLL

NLAYVGE

>gi|30064710|ref|NP_838881.1| homoserine O-succinyltransferase [Shigella flexneri 2a str. 2457T]

MPIRVPDELPAVNFLREENVFVMTTSRASGQEIRPLKVLILNLMPKKIETENQFLRLLSNSPLQVDIQLL

RIDSRESRNTPAEHLNNFYCNFEDIQEQNFDGLIVTGAPLGLVEFNDVAYWPQIKQVLEWSKDHVTSTLF

VCWAVQAALNILYGIPKQTRTDKLSGVYEHHILHPHALLTRGFDDSFLAPHSRYADFPAALIRDYTDLEI

LAETEEGDAYLFASKDKRIAFVTGHPEYDAQTLAQEYLRDVEAGLDPDVPYNYFPHNDPQNKPRASWRSH

GNLLFTNWLNYYVYQITPYDLRHMNPTLD

>gi|30064709|ref|NP_838880.1| isocitrate lyase [Shigella flexneri 2a str. 2457T]

MKTRTQQIEELQKEWTQPRWEGITRPYSAEDVVKLRGSVNPECTLAQLGAAKMWRLLHGESKKGYINNLG

ALTGGQALQQAKAGIEAVYLSGWQVAADANLAASMYPDQSLYPANSVPAVVERINNTFRRADQIQWSVGI

EPGDPRYVDYFLPIVADAEAGFGGVLNAFELMKAMIEAGAAAVHFEDQLASVKKCGHMGGKVLVPTQEAI

QKLVAARLAADVTGVPTLLVARTDADAADLITSDCDPYDSEFITGERTSEGFFRTHAGIEQAISRGLAYA

PYADLVWCETSTPDLELARRFAQAIHAKYPGKLLAYNCSPSFNWQKNLDDKTIASFQQQLSDMGYKFQFI

TLAGIHSMWFNMFDLANAYAQGEGMKHYVEKVQQPEFAAAKDGYTFVSHQQEVGTGYFDKVTTIIQGGTS

SVTALTGSTEESQF

>gi|30064707|ref|NP_838878.1| B12-dependent methionine synthase [Shigella flexneri 2a str. 2457T]

MSSKVEQLRAQLNERILVLDGGMGTMIQSYRLNEADFRGERFADWPCDLKGNNDLLVLSKPEVIAAIHNA

YFEAGADIIETNTFNSTTIAMADYQMESLSAEINFAAAKLARACADEWTARTPEKPRYVAGVLGPTNRTA

SISPDVNDPAFRNITFDQLAAAYRESTKALVEGGADLILIETVFDTLNAKAAVFAVKTEFEALGVELPIM

ISGTITDASGRTLSGQTTEAFYNSLRHAEALTFGLNCALGPDELRQYVQELSRIAECYVTAHPNAGLPNA

FGEYDLDADTMAKQIREWAQAGFLNIVGGCCGTTPQHIAAMSRAVEGLAPRKLPEIPVACRLSGLEPLNI

GDDSLFVNVGERTNVTGSAKFKRLIKEEKYSEALDVARQQVENGAQIIDINMDEGMLDAEAAMVRFLNLI

AGEPDIARVPIMIDSSKWDVIEKGLKCIQGKGIVNSISMKEGVDAFIHHAKLLRRYGAAVVVMAFDEQGQ

ADTRARKIEICRRAYKILTEEVGFPPEDIIFDPNIFAVATGIEEHNNYAQDFIGACEDIKRELPHALISG

GVSNVSFSFRGNDPVREAIHAVFLYYAIRNGMDMGIVNAGQLAIYDDLPAELRDAVEDVILNRRDDGTER

LLELAEKYRGSKADDTANAQQAEWRSWDVNKRLEYSLVKGITEFIEQDTEEARQQATRPIEVIEGPLMDG

MNVVGDLFGEGKMFLPQVVKSARVMKQAVAYLEPFIEASKEQGKTNGKMVIATVKGDVHDIGKNIVGVVL

QCNNYEIVDLGVMVPAEKILRIAKEVNADLIGLSGLITPSLDEMVNVAKEMERQGFAIPLLIGGATTSKA

HTAVKIEQNYSGPTVYVQNASRTVGVVAALLSDTQRDDFVARTCKEYETVRIQHGRKKPRTPPVTLEAAR

DNDFAFDWQAYTPPVAHRLGVQEVEASIETLRNYIDWTPFFMTWSLAGKYPRILEDEVVGVEAQRLFKDA

NDMLDKLSAEKTLNPRGVVGLFPANRVGDDIEIYRDETRTHVINVSHHLRQQTEKTGFANYCLADFVAPK

LSGKADYIGAFAVTGGLEEDALADAFEAQHDDYNKIMVKALADRLAEAFAEYLHERVRKVYWGYAPNENL

SNEELIRENYQGIRPAPGYPACPEHTEKATIWELLEVEKHTGMKLTESFAMWPGASVSGWYFSHPDSKYY

AVAQIQRDQVEDHARRKGMSVSDVERWLAPNLGYDAD

>gi|30064706|ref|NP_838877.1| alpha helix protein [Shigella flexneri 2a str. 2457T]

MLTLLHLLSAVALLVWGTHIVRTGVMRVFGARLRTVLSRSVEKKPLAFCAGIGVTALVQSSNATTMLVTS

FVAQDLVALAPALVIVLGADVGTALMARILTFDLSWLSPLLIFIGVIFFLGRKQSRAGQLGRVGIGLGLI

LLALELIVQAVTPITQANGVQVIFASLTGDILLDALIGAMFAIISYSSLAAVLLTATLTAAGIISFPVAL

CLVIGANLGSGLLAMLNNSAANAAARRVALGSLLFKLVGSLIILPFVHLLAETMGKLSLPKAELVIYFHV

FYNLVRCLVMLPFVDPMARFCKTIIRDEPELDTQLRPKHLDVSALDTPTLALANAARETLRIGDAMEQMM

EGLNKVMHGEPRQEKELRKLADDINVLYTAIKLYLARMPKEELAEEESRRWAEIIEMSLNLEQASDIVER

MGSEIADKSLAARRAFSLDGLKELDALYEQLLSNLKLAMSVFFSGDVTSARRLRRSKHRFRILNRRYSHA

HVDRLHQQNVQSIETSSLHLGLLGDMQRLNSLFCSVAYSVLEQPDEDEGRDEY

>gi|30064705|ref|NP_838876.1| peptidase E [Shigella flexneri 2a str. 2457T]

MELLLLSNSTLPGKAWLEHALPLIAEQLQGRRSAVFIPFAGVTQTWDDYTEKTAAVLAPLGVSVTGIHSV

VDPVAAIENAEIVIVGGGNTFQLLKQCRERGLLAPITDVVKRGALYIGWSAGANLACPTIRTTNDMPIVD

PQGFDALNLFPLQINPHFTNALPEGHKGETREQRIRELLVVAPELTIIGLPEGNWITVSKGHATLGGPNT

TYVFKAGEEAVPLEAGHRF

>gi|30064704|ref|NP_838875.1| L-sorbose-1-P-reductase [Shigella flexneri 2a str. 2457T]

MKTTALRLYGKRDLRLETFDLPEMQEDEILATVVTDSLCLSSWKEANLGENHKKVPDDVTTNPIIIGHEF

CGDILAVGKKWQHKFQPGQRYVIQANLQLPDRPDCPGYSFPWVDGEATHVVIPNEVMEQYCLLAYDGETY

FEGSLVEPLSCVIGAFNANYHLQEGSYNHTMGIRPQGHTLILGGTGPMGLLAIDYALHGPVNPSLLVITD

TDNDKLSYARKHYPSEPQTLIHYLNATDAAFDTLMALSGGHGFDDIFVFVPNEGLVTLASSLLATDGCLN

FFAGPQDKHFSAPINFYDVHYAFTHYVGTSGGNTDDMRAAVKLIEEKKVQAAKVVTHILGLNAAGETTLE

LPAVGGGKKLVYTGKYLPLTSLTQIQDQALAAILARHQGIWSGEVEQYLLAHAEAISHD

>gi|30064703|ref|NP_838874.1| sorbose-permease PTS system IID component [Shigella flexneri 2a str. 2457T]

MEQRKITRSDLVSMFLRSNLQQASFNFERIHGLGFCYDMIPAIKRLYPLKEDQVAALRLHLVFFNTTPAV

CGPVIGAEIDDGTINGIKVGLMGPLAGVGDPLVWGTLRPITAALGASLALSGNILGPLLFFFIFNAVRLA

MKWYGLQLGFRKGVNIVSDMGGNVLQKLTEGASILGLFVMGVLVTKWTSINVPLVVSQTHAADGSTVTMT

VQNILDQLCPGLLALGLTLLMVRLLNKKINPVWLIFALFGLGIIGNALGFLS

>gi|30064702|ref|NP_838873.1| sorbose-permease PTS system IIC component [Shigella flexneri 2a str. 2457T]

MGSVLDEFQTHRPLIACTVIGLILGDLKTGIILGGTLELIALCWMNVGAAQSPDSALASIISAILVIVGQ

QSIATGIAIALPVAAAGQVLTVFARTITVVFQHAADKAAEEARFRTLDILHVSALGVQALRVAIPALIVS

LFVSADMVSNMLNAIPEFVTRGLQIAGGFIVVVGYAMVLRMMGVKYLMPFFFLGFLAGGYLDLSLLAFGG

VGVIMALVYIQLNPQWCKAEPQLQATASTALDQLDD

>gi|30064701|ref|NP_838872.1| sorbose-permease PTS system IIB component [Shigella flexneri 2a str. 2457T]

MNITLARIDDRLIHGQVTTVWSKVANAQRIIICNDEVYNDEVRRTLLRQAAPPGMKVNVVNIEKAVAVYH

NPQYQDETVFYLFTRPQDALAMVRQGVKIGTLNIGGMAWRPGKKQLTKAVSLDDDDINAFHELNNLGVIL

DLRVVASDPSINIIDKINEQLIAN

>gi|30064700|ref|NP_838871.1| sorbose-permease PTS system IIA component [Shigella flexneri 2a str. 2457T]

MVNAIFCAHGKLACAMLESVQMVYGNANVEAVAFVPGENAGDIAVKLEKLVSIHNQDEWLIAVDLQCGSP

WNAAAMLAMRNPRLRVISGLSLPLALELVDNQDSMNVDELCEHLTQIAKQTCVVWKQLATTEEDF

>gi|30064699|ref|NP_838870.1| sorbitol-6-phosphate 2-dehydrogenase [Shigella flexneri 2a str. 2457T]

MQTWLNLQDKIIIVTGGASGIGLAIVEELLAQGANVQMVDIHGGDGQYEGHKGYQFWPTDISSTKEVNHT

VAEIIQRFGRIDGLVNNAGVNFPRLLVDEKAPAGQYELNEAAFEKMVNINQKGVFLMSQAVARQMVKQHD

GVIVNVSSESGLEGSEGQSCYAATKAALNSFTRSWSKELGKHGIRVVGIAPGILEKTGLRTPEYEEALAW

TRNITVEQLREGYTKNAIPIGRAGRLAEVADFVCYLLSERASYITGVTTNIAGGKTRG

>gi|30064698|ref|NP_838869.1| sor-operon regulator [Shigella flexneri 2a str. 2457T]

MENSDDIRLIVKIAQLYYEQDMTQAQIARELGIYRTTISRLLKRGRDQGMVTIAINYDYNENLWLEQQLK

QKFGLKDVVVVSGNDEDEETQLAMMGLHGAQLLDRLLEPGDIVGFSWGRAVSALVENLPQAGQSRQLICV

PIIGGPSGKLESRYHVNTLTYSAAAKLKGESHLADFPALLDNPLIRNGIMQSQHFKTISAYWDNLDIALV

GIGSPAIRDGANWHAFYGGEESDDLNARQVAGDICSRFFDIHGAMVETNMSEKTLSIEMNKLKQARYSIG

IAMSEEKYSGIIGALRGKYINCLVTNSSTAELLLK

>gi|30064697|ref|NP_838868.1| 23S rRNA pseudouridine synthase F [Shigella flexneri 2a str. 2457T]

MLPDSSVRLNKYISESGICSRREADRYIEQGNVFLNGKRATIGDQVKPGDIVKVNGQLIEPREAEDLVLI

ALNKPVGIVSTTEDGERDNIVDFVNHSKRVFPIGRLDKDSQGLIFLTNHGDLVNKILRAGNDHEKEYLVT

VDKPITDEFIRGMGAGVPILGTVTKKCKVKKEAPFVFRITLVQGLNRQIRRMCEHFGYEVKKLERTRIMN

VSLSGIPLGEWRDLTDDELIDLFKLIENSSSEAKPKAKAKPKTAGIKRPVVKMEKTAEKGGRPASNGKRF

TSPGRKKKGR

>gi|30064694|ref|NP_838865.1| sensory histidine kinase DcuS [Shigella flexneri 2a str. 2457T]

MRHSLPYHILRKRPMKLSTTVILMVSAVLFSVLLVVHLIYFSQISDMTRDGLANKALAVARTLADSPEIR

QGLQKKPQESGIQAIAEAVRKRNDLLFIVVTDMQSLRYSHPEAQRIGQPFKGDDILNALNGEENVAINRG

FLAQALRVFTPIYDENHKQIGVVAIGLELSRVTQQINDSRWSIIWSVLFGMLVGLIGTCILVKVLKKILF

GLEPYEISTLFEQRQAMLQSIKEGVVAVDDRGEVTLINDAAQELLNYRKSQDDEKLSTLSHSWSQVVDVS

EVLRDGTPRRDEEITIKDRLLLINTVPVRSNGVIIGAISTFRDKTEVRKLMQRLDGLVNYADALRERSHE

FMNKLHVILGLLHLKSYKQLEDYILKTANNYQEEIGSLLGKIKSPVIAGFLISKINRATDLGHTLILNSE

SQLPDSGSEDQVATLITTLGNLIENALEALGPEPGGEISVTLHYRHGWLHCEVNDDGPGIAPDKIDHIFD

KGVSTKGSERGVGLALVKQQVENLGGSIAVESEPGIFTQFFVQIPWDGERSNR

>gi|30064693|ref|NP_838864.1| DNA-binding transcriptional activator DcuR [Shigella flexneri 2a str. 2457T]

MINVLIIDDDAMVAELNRRYVAQIPGFQCCGTASTLEKAKEIIFNSDAPIDLILLDIYMQKENGLDLLPV

LHNARCKSDVIVISSAADAATIKDSLHYGVVDYLIKPFQASRFEEALTGWRQKKMALEKHQYYDQAELDQ

LIHGSSSNEQDPRRLPKGLTPQTLRTLCQWIDAHQDYEFSTDELANEVNISRVSCRKYLIWLVNCHILFT

SIHYGVTGRPVYRYRIQAEHYSLLKQYCQ

>gi|30064692|ref|NP_838863.1| anaerobic C4-dicarboxylate transporter [Shigella flexneri 2a str. 2457T]

MLFTIQLIIILICLFYGARKGGIALGLLGGIGLVILVFVFHLQPGKPPVDVMLVIIAVVAASATLQASGG

LDVMLQIAEKLLRRNPKYVSIVAPFVTCTLTILCGTGHVVYTILPIIYDVAIKNNIRPERPMAASSIGAQ

MGIIASPVSVAVVSLVAMLGNVTFDGRHLEFLDLLAITIPSTLIGILAIGIFSWFRGKDLDKDEEFQKFI

SVPENREYVYGDTATLLDKKLPKSNWLAMWIFLGAIAVVALLGADSDLRPSFGGKPLSMVLVIQMFMLLT

GALIIILTKTNPASISKNEVFRSGMIAIVAVYGIAWMAETMFGAHMSEIQGVLGEMVKEYPWAYAIVLLL

VSKFVNSQAAALAAIVPVALAIGVDPAYIVASAPACYGYYILPTYPSDLAAIQFDRSGTTHIGRFVINHS

FILPGLIGVSVSCVFGWIFAAMYGFL

>gi|30064691|ref|NP_838862.1| fumarase B, fumarate hydratase class I [Shigella flexneri 2a str. 2457T]

MSNKPFIYQAPFPMGKDNTEYYLLTSDYVSVADFDGETILKVEPEALTLLAQQAFHDASFMLRPAHQKQV

AAILHDPEASENDKYVALQFLRNSEIAAKGVLPTCQDTGTAIIVGKKGQRVWTGGGDEEALSKGVYNTYI

EDNLRYSQNAALDMYKEVNTGTNLPAQIDLYAVDGDEYKFLCVAKGGGSANKTYLYQETKALLTPGKLKN

FLVEKMRTLGTAACPPYHIAFVIGGTSAENNLKTVKLASAHYYDELPTEGNEHGQAFRDVQLEQELLEEA

QKLGLGAQFGGKYFAHDIRVIRLPRHGASCPVGMGVSCSADRNIKAKINREGIWIEKLEHNLGQYIPQEL

RQAGEGEAVKVDLNRPMKEILAQLSQYPVSTRLSLTGTIIVGRDIAHAKLKELIDAGKELPQYIKDHPIY

YAGPAKTPAGYPSGSLGPTTAGRMDSYVDLLQSHGGSMIMLAKGNRSQQVTDACHKHGGFYLGSIGGPAA

VLAQQSIKHLECVAYPELGMEAIWKIEVEDFPAFILVDDKGNDFFQQIVNKQCANCTK

>gi|30064690|ref|NP_838861.1| hypothetical protein S3628 [Shigella flexneri 2a str. 2457T]

MTRTLKPLILNTGALALTLILIYTGISAHDKLTWLLEVTPVIIVVPLLLATARRYPLTLLLYTLIFFHAI

ILMVGGQYTYAKVPVGFEVQEWLGLSRNPYDKLGHFFQGLVPALVAREILVRGMYVRGRKMVAFLVCCVA

LAISAMYELIEWWAALAMGQGADDFLGTQGDQWDTQSDMFCALLGALTTVILLARFHCRQLRRYGLITG

>gi|30064689|ref|NP_838860.1| melibiose:sodium symporter [Shigella flexneri 2a str. 2457T]

MTTKLSYGFGAFGKDFAIGIVYMYLMYYYTDVVGLSVGLVGTLFLVARIWDAINDPIMGWIVNATRSRWG

KFKPWILIGTLANSVILFLLFSAHLFEGTTQIVFVCVTYILWGMTYTIMDIPFWSLVPTITLDKREREQL

VPYPRFFASLAGFVTAGVTLPFVNYVGGGDRGFGFQMFTLVLIAFFIVSTIITLRNVHEVFSSDNQSSAE

GSHLTLKAIVALIYKNDQLSCLLGMALAYNVASNIITGFAIYYFSYVIGDADLFPYYLSYAGAANLVTLV

FFPRLVKSLSRRILWAGASILPVLSCGVLLLMALMSYHNVVLIVIAGILLNVGTALFWVLQVIMVADTVD

YGEYKLHVRCESIAYSVQTMVVKGGSAFAAFFIAVVLGMIGYVPNVEQSTQALLGMQFIMIALPTLFFMV

TLILYFRFYRLNGDTLRRIQIHLLDKYRKVPPEPVHADIPVGAVSDVKA

>gi|30064688|ref|NP_838859.1| alpha-galactosidase [Shigella flexneri 2a str. 2457T]

MMSAPKITFIGAGSTIFVKNILGDVFHREALKTAHIALMDIDPTRLEESHIVVRKLMDSAGASGKITCHT

QQKEALEDADFVVVAFQIGGYEPCTVTDFEVCKRHGLEQTIADTLGPGGIMRALRTIPHLWQICENMTEV

CPDATMLNYVNPMAMNTWAMYARYPYIKQVGLCHSVQGTAEELARDLNIDPATLRYRCAGINHMAFYLEL

ERKTADGSYVNLYPELLAAYEAGQAPKPNIHGNTRCQNIVRYEMFKKLGYFVTESSEHFAEYTPWFIKLG

REDLIERYKVPLDEYPKRCVEQLANWHKELEEYKNASRIDIKPSREYASTIMNAIWTGEPSVIYGNVRND

GLIDNLPQGCCVEVACLVDANGIQPTKVGTLPSHLAALMQTNINVQTLLTEAILTENRDRVYHAAMMDPH

TAAVLGIDEIYALVDDLIAAHGDWLPSWLHR

>gi|30064687|ref|NP_838858.1| DNA-binding transcriptional regulator MelR [Shigella flexneri 2a str. 2457T]

MNTDTFMCSSDEKQTRSPLSLYSEYQRMEIEFRAPHIMPTSHWHGQVEVNVPFDGDVEYLINNEKVNINQ

GHITLFWACTPHQLTDTGTCQSMAIFNLPMHLFLSWPLDKDLINHVTHGMVIKSLATQQLSPFEVRRWQQ

ELNSPNEQIRQLAIDEIGLMLKRFSLSSWEPILVNKTSRTHKNSVSRHAQFYVSQMLGFIAENYDQALTI

NDVAEHVKLNANYAMGIFQRVMQLTMKQYITAMRINHVRALLSDTDKSILDIALTAGFRSSSRFYSTFGK

YVGMSPQQYRKLSQQRRQTFPG

>gi|30064686|ref|NP_838857.1| biodegradative arginine decarboxylase [Shigella flexneri 2a str. 2457T]

MKVLIVESEFLHQDTWVGNAVERLADALSQQNVTVIKSTSFDDGFAILSSNEAIDCLMFSYQMEHPDEHQ

NVRQLIGKLHERQQNVPVFLLGDREKALAAMDRDLLELVDEFAWILEDTADFIAGRAVAAMTRYRQQLLP

PLFSALMKYSDIHEYSWAAPGHQGGVGFTKTPAGRFYHDYYGENLFRTDMGIERTSLGSLLDHTGAFGES

EKYAARVFGADRSWSVVVGTSGSNRTIMQACMTDNDVVVVDRNCHKSIEQGLMLTGAKPVYMVPSRNRYG

IIGPIYPQEMQPETLQKKISESPLTKDKAGQKPSYCVVTNCTYDGVCYNAKEAQDLLEKTSDRLHFDEAW

YGYARFNPIYADHYAMRGEPGDHNGPTVFATHSIHKLLNALSQASYIHVREGRGAINFSRFNQAYMMHAT

TSPLYAICASNDVAVSMMDGNSGLSLTQEVIDEAVDFRQAMARLYKEFTADGSWFFKPWNKEVVTDPQTG

KTYDFADAPTKLLTTVQDCWVMHPGESWHGFKDIPDNWSMLDPIKVSILAPGMGEDGELEETGVPAALVT

AWLGRHGIVPTRTTDFQIMFLFSMGVTRGKWGTLVNTLCSFKHHYDANTPLAQVMPELVEQYPDTYANMG

IHDLGDTMFAWLKENNPGARLNEAYSGLPVAEITPREAYNAIVDNNVELVSIENLPGRIAANSVIPYPPG

IPMLLSGENFGDKNSPQVSYLRSLQSWDHHFPRFEHETEGTEIIDGIYHVMCVKA

>gi|30064685|ref|NP_838856.1| ARAC-type regulatory protein [Shigella flexneri 2a str. 2457T]

MRICSDQPCIVLLTEKDVWIRVNGKEPISLKANHMALLNCENNIIDVSSLNNTLVAHISHDIIKDYLRFL

NKDLSQIPVWQRSATPILTLPCLTPDVFRVAAQHSMMPAETESEKERTRALLFTVLSRFLDSKKFLSLMM

YMLRNCVSDSVYQIIESDIHKDWNLSMVASCLCLSPSLLKKKLKSENTSYSQIITTCRMRYAVNELMMDG

KNISQVSQSCGYNSTSYFISVFKDFYGMTPLHYVSQHRERTVA

>gi|30064684|ref|NP_838855.1| arginine:agmatin antiporter [Shigella flexneri 2a str. 2457T]

MSSDADAHKVGLIPVTLMVSGNIMGSGVFLLPANLASTGGIAIYGWLVTIIGALGLSMVYAKMSFLDPSP

GGSYAYARRCFGPFLGYQTNVLYWLACWIGNIAMVVIGVGYLSYFFPILKDPLVLTITCVVVLWIFVLLN

IVGPKMITRVQAVATVLALIPIVGIAVFGWFWFRGETYMAAWNVSGLGTFGAIQSTLNVTLWSFIGVESA

SVAAGVVKNPKRNVPIATIGGVLIAAVCYVLSTTAIMGMIPNAALRVSASPFGDAARMALGDTAGAIVSF

CAAAGCLGSLGGWTLLAGQTAKAAADDGLFPPIFARVNKAGTPVAGLIIVGILMTIFQLSSISPNATKEF

GLVSSVSVIFTLVPYLYTCAALLLLGHGHFGKARPAYLAVTTIAFLYCIWAVVGSGAKEVMWSFVTLMVI

TAMYALNYNRLHKNPYPLDAPISKD

>gi|30064683|ref|NP_838854.1| cell division protein [Shigella flexneri 2a str. 2457T]

MRTLFDGNTVMLKRLLKRPSLNLLAWLLLAAFYISICLNIAFFKQVLQALPLDSLHNVLVFLSMPVVAFS

VINIVLTLSSFLWLNRPLACLFILIGAAAQYFIMTYGIVIDRSMIANIIDTTPAESYALMTPQMLLTLGF

SGVLAELIACWLKIKPATSRLRSVLFRGANILVSVLLILLVAALFYKDYASLFRNNKELVKSLSPSNSIV

ASWSWYSHQRLANLPLVRIGEDAHRNPLMQNEKRKNLTILIVGETSRAENFSLNGYPRETNPRLAKDNVV

YFPNTASCGTATAVSVPCMFSDMPREHYKEELAQHQEGVLDIIQRAGINVLWNDNDGGCKGACDRVPHQN

VTALNLPDQCINGECYDEVLFHGLEEYINNLQGDGVIVLHTIGSHGPTYYNRYPPQFRKFTPTCDTNEIQ

TCTKEQLVNTYDNTLVYVDYIVDKAINLLKEHQDKFTTSLVYLSDHGESLGENGIYLHGLPYAIAPDSQK

QVPMLLWLSEDYQKRYQVDQNCLQKQAQTQHYSQDNLFSTLLGLTGVETKYYQAADDILQTCRRVSK

>gi|30064682|ref|NP_838853.1| DNA-binding transcriptional regulator BasR [Shigella flexneri 2a str. 2457T]

MKILIVEDDTLLLQGLILAAQTEGYACDGVTTARMAEQSLEDGHYSLVVLDLGLPDEDGLHFLARIRQKK

YTLPVLILTARDTLTDKIAGLDVGADDYLVKPFALEELHARIRALLRRHNNQGESELIVGNLTLNMGRRQ

VWMGGEELILTPKEYALLSRLMLKAGSPVHREILYNDIYNWDNEPSTNTLEVHIHNLRDKVGKARIRTVR

GFGYMLVANEEN

>gi|30064681|ref|NP_838852.1| sensor protein BasS/PmrB [Shigella flexneri 2a str. 2457T]

MNLMRFLRRPISLRQRLILTIGAILLVFELISVFWLWHESTEQIQLFEQALRDNRNNDRHIMREIREAVA

SLIVPGVFMVSLTLFICYQAVRRITRPLAELQKELEARTADNLTPIAIHSATLEIEAVVSALNDLVSRLT

STLDNERLFTADVAHELRTPLAGVRLHLELLAKTHHIDVAPLVARLDQMMESVSQLLQLARAGQSFSSGN

YQHVKLLEDVILPSYDELSTMLDQRQQTLLLPESAADITVQGDATLLRMLLRNLVENAHRYSPQGSNIMI

KLQEDGGAVMAVEDEGPGIDESKCGELSKAFVRMDSRYGGIGLGLSIVSRITQLHHGQFFLQNRQETSGT

RAWVRLKKDQNVANQI

>gi|30064680|ref|NP_838851.1| proline/glycine betaine transporter [Shigella flexneri 2a str. 2457T]

MLKRKKVKPITLRDVTIIDDGKLRKAITAASLGNAMEWFDFGVYGFVAYALGKVFFPGADPSVQMVAALA

TFSVPFLIRPLGGLFFGMLGDKYGRQKILAITIVIMSISTFCIGLIPSYDTIGIWAPILLLICKMAQGFS

VGGEYTGASIFVAEYSPDRKRGFMGSWLDFGSIAGFVLGAGVVVLISTIVGEANFLDWGWRIPFFIALPL

GIIGLYLRHALEETPAFQQHVDKLEQGDREGLQDGPKVSFKEIATKYWRSLLTCIGLVIATNVTYYMLLT

YMPSYLSHNLHYSEDHGVLIIIAIMIGMLFVQPVMGLLSDRFGRRPFVLLGSVALFVLAIPAFILINSNV

IGLIFAGLLMLAVILNCFMGVMASTLPAMFPTHIRYSALAAAFNISVLVAGLTPTLAAWLVESSQNLMMP

AYYLMVVAVIGLITGVTMKETANRPLKGATPAASDIQEAKEILVEHYDNIEQKIDNIDHEIADLQAKRTR

LVQQHPRIDE

>gi|30064679|ref|NP_838850.1| hypothetical protein S3617 [Shigella flexneri 2a str. 2457T]

MLESVHPRFLVDLAQGDDARHPQAHQQQFRERLMQELLSRVQLQTWTNGGMLNAPLSLRLTMVEKLASML

DPGHLALTQIAQHLALLQKMDHRQHSAFPELPQQIAALYEWFSARCRWKEKALTQRGLLVQAGDQSEQIF

TRWRAGAYNAWSLPGRCFIVLEELRWGAFGDACRLGSPQAVVLLLGDLREKATQHLAESINAAPTTRHYY

HQWFASSTVPTGGDHADFLSWLGKWTTADKQPVCWSVTQRWQTVALGMPRLCSAQRLVGAMVEEIFSVNL

A

>gi|30064678|ref|NP_838849.1| hypothetical protein S3616 [Shigella flexneri 2a str. 2457T]

MYTQTLYELSQEAERLLQLSRQQLQLLEKMPLSVPGDDAPQLALPWSQPNIAERHAMLNNELCKISRLEM

VLAIVGTMKAGKSTTINAIVGTEVLPNRNRPMTALPTLIRHTPGQKEPVLHFSHVAPIDCLIQQLQQRLR

DCDIKHLTDVLEIDKDMRALMQRIENGVAFEKYYLGAQPIFHCLKSLNDLVRLAKALDVDFPFSAYAAIE

HIPVIEVEFVHLAGLESYPGQLTLLDTPGPNEAGQPHLQKMLNQQLARASAVLAVLDYTQLKSISDEEVR

EAILAVGQSVPLYVLVNKFDQQDRNSDDADQVRALISGTLMKGCITPQQIFPVSSMWGYLANRARYELAN

NGKLPAPEQQRWVEDFAHAALGRRWRHADLADLEHIRHAADQLWEDSLFAQPIQALLHAAYANASLYALR

SAAHKLLNYAQQAREYLDFRAHGLNVACEQLRQNIHQVEESLQLLQLNQAQVSGEIKHEIELALTSANHF

LRQQQDAVNAQLAALFQDDSEPLSEIRTRCETLLQTAQNTISRDFTLRFAEMESTLCRVLTDVIRPIEQQ

VKMELSESGFRPGFHFPVFHGVVPHFNTRQLFSEVISRQEATDEQSTRLGVVRETFSRWLNQPDWGRGNE

KSPTETVDYSVLQRALSAEVDLYCQQMAKVLAEQVDESVTAGMNTFFAEFASCLTELQTRLRESLALRQQ

NESVVRLMQQQLQQTVMTHSWIYTDAQLLRDDIQTLFTAERY

>gi|30064677|ref|NP_838848.1| hypothetical protein S3615 [Shigella flexneri 2a str. 2457T]

MSLPHCPKCNSEYTYEDNGMYICPECAYEWNDAEPAQESDELIVKDANGNLLADGDSVTIIKDLKVKGSS

SMLKIGTKVKNIRLVEGDHNIDCKIDGFGPMKLKSEFVKKN

>gi|30064676|ref|NP_838847.1| hypothetical protein S3614 [Shigella flexneri 2a str. 2457T]

MPLSPYLSFAGNCSDAIAYYQRTLGAELLYKISFGEMPKSAQDSAENCPSGMQFPDTAIAHANVRIAGSD

IMMSDAIPSGKASYSGFTLVLDSQQVEEGKRWFDNLAANGKIEMAWQETFWAHGFGKVTDKFGVPWMINV

VKQQPTQ

>gi|30064675|ref|NP_838846.1| phosphonate/organophosphate ester transporter subunit [Shigella flexneri 2a str. 2457T]

MQTIIRVEKLAKTFNQHQALHAVDLNIHHSEMVALLGPSGSGKSTLLRHLSGLITGDKSAGSHIELLGRT

VQREGRLARDIRKSRANTGYIFQQFNLVNRLSVLENVLIGALGSTPFWRTCFSWFTGEQKQRALQALTRV

GMVHFAHQRVSTLSGGQQQRVAIARALMQQAKVILADEPIASLDPESARIVMDTLRDINQNDGITVVVTL

HQVDYALRYCERIVALRQGHVFYDGSSQLFDNERFDHLYRSINRIEENAKAA

>gi|30064674|ref|NP_838845.1| periplasmic binding protein component of phosphonate transporter [Shigella flexneri 2a str. 2457T]

MNAKIIASLAFTSMFSLSTLLTPAHAEEQEKALNFGIISTESQQNLKPQWTPFLQDMEKKLGVKVNAFFA

PDYAGIIQGMRFNKVDIAWYGNLSAMEAVDRANGQVFAQTVAADGSPGYWSVLIVNKDSPINNLNDLLAK

RKDLTFGNGDPNSTSGFLVPGYYVFAKNNISASDFKRTVNAGHETNALAVANKQVDVATNNTENLDKLKT

SAPEKLKELKVIWKSPLILGDPIVWRKNLSETTKDKIYDFFMNYGKTPEEKAVLERLGWAPFRASSDLQL

VPIRQLALFKEIQSVKGNKGLNEQDKLAKTTEIQAQLDDLDRLNNALSAMSSVSKAVQ

>gi|30064673|ref|NP_838844.1| membrane channel protein component of phosphonate transporter [Shigella flexneri 2a str. 2457T]

MPDAVQAPYPAYRPEPNMQTITIAPPKRSWFSLLSWAVVLDVLVVSWQGAEMAPLTLIKDGGNMATFAVM

TPTY

>gi|30064672|ref|NP_838843.1| IS1 orfB [Shigella flexneri 2a str. 2457T]

MSRQCTHYGRWPQHGFTSLKKLRPQSVTSRIQPGSDVIVCAEMDEHWGYVGAKSRQRWLFYAYDRIRRTV

VAHVFGERTLATLERLLSLLSAFEVVVWMTDGWPLYESRLKGKLHVISKRYTQHIERHNLNLRQHLARLV

RKSLSFSKSVELHDKVIGHYLNIKHYQ

>gi|30064671|ref|NP_838842.1| IS1 orfA [Shigella flexneri 2a str. 2457T]

MASISIRCPSCSATEGVVRNGKSTAGHQRYLCSHCRKTWQLQFTYTASQPGTHQKIIDMAMNGVGCRASA

RIMGVGLNTVLRHLKNSGRSR

>gi|30064670|ref|NP_838841.1| multidrug resistance protein MdtN [Shigella flexneri 2a str. 2457T]

MESTPKKAPRSKFPALLVVALALVALVFVIWRVDSAPSTNDAYASADTIDVVPEVSGRIVELAVTDNQAV

KQGDLLFRIDPRPYEANLAKAEASLAALDKQIMLTQRSVDAQQFGADSVNATVEKARAAAKQATDTLRRT

EPLLKEGFVSAEDVDRARTAQRAAEADLNAVLLQAQSAASAVSGVDALVAQRAAVEADIALTKLHLEMTT

VRAPFDGRIISLKTSVGQFASAMRPIFTLIDTRHWYVIANFRETDLKNIRSGTPATIRLMSDSGKTFEGK

VDSIGYGVLPDDGGLVLGGLPKVSRSINWVRVAQRFPVKIMVDKPDPEMFRIGASAVANLEPQ

>gi|30064668|ref|NP_838839.1| outer membrane efflux protein MdtP [Shigella flexneri 2a str. 2457T]

MINRQLSRLLLCSILGSTTLISGCALVRKDSAPHQQLKPEQIKLADDIHLASSGWPQAQWWKQLNDPQLD

ALIQRTLSGSHTLAEAKLREEKAQSQADLLDAGSQLQVAALGMLNRQRVSANGFLSPYAMDAPALGMDGP

YYTEATVGLFAGLDLDLWGVHRSAVAAAIGAHNAALAETAAVELSLTTGVAQLYYSMQASYQMLDLLEQT

RDVIDYAVKAHQSKVAHGLEAQVPFHGARAQILAVDKQIAAVKGQITETRESLRALIGAGASDMPEIKPV

ALPRVQTGIPATLSYELLARRPDLQAMRWYVQASLDQVDSARALFYPSFDIKAFFGLDAIHLDTLFKKTS

RQFNFIPGLKLPLFDGGRLNANLEGTRAASNMMIERYNQSVLNAVRDVAVNGTRLQTLNDEREMQAEHVE

ATRFTQRAAEAAYQRGLTSRLQATEARLPVLAEEMSLLMLDSRRVIQSIQLMKSLGGGYQAAPVVEKK

>gi|30064667|ref|NP_838838.1| hypothetical protein S3605 [Shigella flexneri 2a str. 2457T]

MLFLTFFAHANDSEPGSQYLKAAEAGDRRAQYFLADSWFSSGDLSKAEYWAQKAADSGDADACALLAQIK

ITNPVSLDYPQAKVLAEKAAQAGSKEGEVTLAHILVNTQAGKPDYPKAISLLENASEDLENDSAVDAQML

LGLIYANGVGIKADDDKATWYFKRSSAISRTGYSEYWAGMMFLNGEEGFIEKNKQKALHWLNLSCMEGFD

TGCEEFEKLTNG

>gi|30064666|ref|NP_838837.1| glutamate/aspartate:proton symporter [Shigella flexneri 2a str. 2457T]

MKNIKFSLAWQILFAMVLGILLGSYLHYHSDSRDWLVVNLLSPAGDIFIHLIKMIVVPIVISTLVVGIAG

VGDAKQLGRIGAKTIIYFEVITTVAIILGITLANVFQPGAGVDMSQLATVDISKYQSTTEAVQSSSHGIM

GTILSLVPTNIVASMAKGEMLPIIFFSVLFGLGLSSLPATHREPLVTVFRSISETMFKVTHMVMRYAPVG

VFALIAVTVANFGFSSLWPLAKLVLLVHFAILFFALVVLGIVARLCGLSVWILIRILKDELILAYSTASS

ESVLPRIIEKMEAYGAPASITSFVVPTGYSFNLDGSTLYQSIAAIFIAQLYGIDLSIWQEIILVLTLMVT

SKGIAGVAGVSFVVLLATLGSVGIPLEGLAFIAGVDRILDMARTALNVVGNALAVLVIAKWEHKFDRKKA

LAYEREVLGKFDKTADQ

>gi|30064665|ref|NP_838836.1| formate-dependent nitrite reductase complex subunit NrfG [Shigella flexneri 2a str. 2457T]

MKQPQIPVKMLTTLTILMVFLCVGSYLLSPKWQAVRAEYQRQRDPLHQFASQQTPEAQLQALQDKIRANP

QNSEQWALLGEYYLWQNDYSNSLLAYRQALQLRGENAELYAALATVLYYQASQHMTAQTRAMIDKALALD

SNEITALMLLASDAFMQANYAQAIELWQKVMDLNSPRINRTQLVESINMAKLLQRRSD

>gi|30064664|ref|NP_838835.1| formate-dependent nitrite reductase complex subunit NrfF [Shigella flexneri 2a str. 2457T]

MNKGLLTLLLLFTCFARAQVVDTWQFANPQQQQQALNIASQLRCPQCQNQNLLESNAPVAVSMRHQVYSM

VSEGKNEVEIIGWMTERYGDFVRYNPPLTGQTLVLWALPVVLLLLMALILWRVRAKR

>gi|30064663|ref|NP_838834.1| heme lyase subunit NrfE [Shigella flexneri 2a str. 2457T]

MLTPLTAFAGVRLRWPAMMRLTCIGILAQFALLLLAFGVLTYCFLISDFSVIYVAQHSYSLLSWELKLAA

VWGGHEGSLLLWVLLLSAWSALFAWHYRQQSDPLFPLTLAVLSLMLAALLLFVVLWSDPFVRIFPPAIEG

RDLNPMLQHPGLIFHPPLLYLGYGGLMVAASVALASLLRGEFDAASARICWRWALPGWSALTAGIILGSW

WAYCELGWGGWWFWDPVENASLLPWLSATALLHSLSLTRQRGIFRHWSLLLAIVTLMLSLLGTLIVRSGI

LVSVHAFALDNVRAVPLFSLFALISLASLALYGWRARDGGPAVRFSGLSREMLILATLLLFCAVLLIVLV

GTLYPMIYGLLGWGRLSVGAPYFNRATLPFGLLMLVVIVLATFVSGKRVQLPALVAHAGVLLFAAGIVVS

SVSRQEISLNLQPGQQVTLAGYTFRFERLDLQARGNYTSEKAIVALFDHQQRIGELTPERRFYEARRQQM

MEPSIRWNGIHDWYAVMGEKTRADRYAFRLYVQSGVRWIWGGGLLMIAGALLSGWRGKKRDE

>gi|30064662|ref|NP_838833.1| formate-dependent nitrate reductase, transmembrane protein [Shigella flexneri 2a str. 2457T]

MTQTSAFHFESLVWDWPIAIYLFLIGISAGLVTLAVLLRRFYPQAGGADSTLLRTTLIVGPGAVILGLLI

LVFHLTRPWTFWKLMFHYSFTSVMSMGVMLFQLYMVVLVLWLAKIFEHDLLALQQRWLPKLGIVQKVLSL

LTPVHRGLETLMLVLAVLLGAYTGFLLSALKSYPFLNNPSLPVLLLFSGISSGAAVALIAMAIRQRSNPH

STEAHFVHRMEIPVVWGEIFLLVAFFVGLALGDDGKVRALVAAALGGGFWTWWFWLGVAGLGLIVPMLLKP

WVNRSSGIPAVLAACGASLVGVLMLRFFILYAGQLTVA

>gi|30064661|ref|NP_838832.1| formate-dependent nitrite reductase [Shigella flexneri 2a str. 2457T]

MTWSRRQFLTGVGVLAAVSGTAGRVVAKTLNINGVRYGMVHDESLCIGCTACMDACREVNKVPEGVSRLT

IIRSEPQGEFPDVKYRFFRKSCQHCDHAPCVDVCPTGASFRDAASGIVDVNPDLCVGCQYCIAACPYRVR

FIHPVTKTADKCDFCRKTNLQAGKLPACVEACPTKALTFGNLDDPNSEISQLLRQKPTYRYKLALGTKPK

LYRVPFKYGEVSQ

>gi|30064660|ref|NP_838831.1| cytochrome c nitrite reductase pentaheme subunit [Shigella flexneri 2a str. 2457T]

MSVLRSLLTAGVLASGLLWSLNGITATPAVQASGDRYEVTQQRNPDAACLDCHKPDTEGMHGKHASVINP

NNKLPVTCTNCHGQASPQHREGVKDVMRFNEPMYKVGEQNSVCMSCHLPEQLQKAFWPHDVHVTKVACAS

CHSLHPQQDTMQTLSDKGRIKICVDCHSDQRTNPNFNPASVPLLKEQP

>gi|30064659|ref|NP_838830.1| cytochrome c552 [Shigella flexneri 2a str. 2457T]

MTRIKINARRIFSLLIPFFFFTSVHAEQTAAPAKPVTVEAKNETFAPQHHDQYLSWKATSEQSERVDALA

EDPRLVILWAGYPFSRDYNKPRGHAFAVTDVRETLRTGAPKNAEDGPLPMACWSCKSPDVARLIQKDGED

GYFHGKWARGGPEIVNNLGCADCHNTASPEFAKGKPELTLSRPYAARAMEAIGKPFEKAGRFDQQSMVCG

QCHVEYYFDGKNKAVKFPWDDGMKVENMEQYYDKIAFSDWTNSLSKTPMLKAQHPEYETWTAGIHGKNNV

TCIDCHMPKVQNAEGKLYTDHKIGNPFDNFAQTCANCHTQDKAALQKVVAERKQSINDLKIKVEDQLVHA

HFEAKAALDAGATEAEMKPIQDDIRHAQWRWDLAIASHGIHMHAPEEGLRMLGTAMDKAADARTKLARLL

ATKGITHEIQIPDISTKEKAQQAIGLNMEQIKAEKQDFIKTVIPQWEEQARKNGLLSQ

>gi|30064658|ref|NP_838829.1| hypothetical protein S3595 [Shigella flexneri 2a str. 2457T]

MNGTIYQRIEDNAHFRELVEKRQRFATILSIIMLAVYIGFILLIAFAPGWLGTPLNPNTSVTRGIPIGVG

VIVISFVLTGIYIWRANGEFDRLNNEVLHEVQAS

>gi|30064657|ref|NP_838828.1| acetate permease [Shigella flexneri 2a str. 2457T]

MKRVLTALAATLPFAANAADAISGAVERQPTNWQAIIMFLIFVVFTLGITYWASKRVRSRSDYYTAGGNI

TGFQNGLAIAGDYMSAASFLGISALVFTSGYDGLIYSLGFLVGWPIILFLIAERLRNLGRYTSADVASYR

LKQGPIRILSACGSLVVVALYLIAQMVGAGKLIELLFGLNYHIAVVLVGVLMMMYVLFGGMLATTWVQII

KAVLLLFGASFMAFMVMKHVGFSFNNLFSEAMAVHPKGVDIMKPGGLVKDPISALSLGLGLMFGTAGLPH

ILMRFFTVSDAREARKSVFYATGFMGYFYILTFIIGFGAIMLVGANPEYKDAAGHLIGGNNMAAVHLANA

VGGNLFLGFISAVAFATILAVVADLTLAGASAVSHDLYANVFKKGATEREELRVSKITVLILGVIAIILG

VLFENQNIAFMVGLAFAIAASCNFPIILLSMYWSKLTTRGAMLGGWLGLITAVVLMILGPTIWVQILGHE

KAIFPYEYPALFSISVAFLGIWFFSATDNSAEGARERELFRAQFIRSQTGFGVEQGRAH

>gi|30064656|ref|NP_838827.1| hypothetical protein S3593 [Shigella flexneri 2a str. 2457T]

MRYNGLNNMFFPLCQINDNHSITSSSHTKKTKSDNYSKHHKNTLIDNKALSLFKIDDHEKVIGLIQKMKR

IYDSLPSGKITKETDRKIHKYFIDIALYANNKCDDRITRRVYLSKEKDVSIKVVYFINNVAVHNNTIEIP

QIVNGGYDFSHLSLKGIVIKDEDLSNSNFAGCRLQNAIFQDCNMYKTNFYYAIMEKILFDNCILDDSNFA

QIKMADGTLNACSAMHVQFYNAAMNRANIKNTFLDYSNFYMAYMAEVNLYKVIAPYVNLFKADLSFSKLD

LINFEHADLSRVNLNKAILQNINLIDSKLFCTWLTNTFLEMVICTGSNMANVNFNNANLSNCHFNCSILT

KACMFNTRLYRVNFDEASVQGMGISILRGEENIPIDSDTLVTLQKFFEEDCTSHTGMSQTEDNINAVAMK

ITADIMQHAD

>gi|30064655|ref|NP_838826.1| hypothetical protein S3592 [Shigella flexneri 2a str. 2457T]

MEIFFTILIMTLVVSLSGVVTRVMPFQIPLPLMQIAIGALLAWPTFGLHVEFDPELFLVLFIPPLLFADG

WKTPTREFLEHGREIFGLALALVVVTVVGIGFLIYWVVPGIPLIPAFALAAVLSPTDAVALSGIVGEGRI

PKKIMGILQGEALMNDASGLVSLKFAVAVAMGTMIFTVGGATVEFMKVAIGGILAGFVVSWLYGRSLRFL

SRWGGDEPATQIVLLFLLPFASYLIAEHIGVSGILAAVAAGMTITRSGVMRRAPLAMRLRANSTWAMLEF

VFNGMVFLLLGLQLPGILETSLMAAEIDPNVEIWMLFTDIILIYAALMLVRFGWLWTMKKFSNRFLKKKP

MEFGSWTTREILIASFAGVRGAITLAGVLSIPLLLPDGNVFPARYELVFLAAGVILFSLFVGVVMLPILL

QHIEVADHSQQLKEERIARAATAEVAIVAIQKMEERLAADTEENIDNQLLTEVSSRVIGNLRRRADGRND

VESSVQEENLERRFRLAALRSERAELYHLRATREISNETLQKLLHDLDLLEALLIEENQ

>gi|30064654|ref|NP_838825.1| hypothetical protein S3591 [Shigella flexneri 2a str. 2457T]

MSTPSARTGGSLDAWFKISQRGSTVRQEVVAGLTTFLAMVYSVIVVPGMLGKAGFPPAAVFVATCLVAGL

GSIVMGLWANLPLAIGCAISLTAFTAFSLVLGQHISVPVALGAVFLMGVLFTVISATGIRSWILRNLPHG

VAHGTGIGIGLFLLLIAANGVGLVIKNPLDGLPVALGDFATFPVIMSLAGLAVIIGLEKLKVPGGILLTI

IGISIVGLIFDPNVHFSGVFAMPSLSDENGNSLIGSLDIMGALNPVVLPSVLALVMTAVFDATGTIRAVA

GQANLLDKDGQIIDGGKALTTDSMSSVFSGLVGAAPAAVYIESAAGTAAGGKTGLTAITVGVLFLLILFL

SPLSYLVPGYATAPALMYVGLLMLSNVAKIDFADFVDAMAGLVTAVFIVLTCNIVTGIMIGFATLVIGRL

VSGEWRKLNIGTVVIAVALVTFYAGGWAI

>gi|30064653|ref|NP_838824.1| redox-sensing activator of soxS [Shigella flexneri 2a str. 2457T]

MEKKLPRIKALLTPGEVAKRSGVAVSALHFYESKGLITSIRNSGNQRRYKRDVLRYVAIIKIAQRIGIPL

ATIGEAFGVLPEGHTLSAKEWKQLSSQWREELDRRIHTLVALRDELDGCIGCGCLSRSDCPLRNPGDRLG

EEGTGARLLEDEQN

>gi|30064652|ref|NP_838823.1| DNA-binding transcriptional regulator SoxS [Shigella flexneri 2a str. 2457T]

MSHQKIIQDLIAWIDVHIDQPLNIDVVAKKSGYSKWYLQRMFRTVTHQTLGDYIRQRRLLLAAVELRTTE

RPIFDIAMDLGYVSQQTFSRVFRRQFDRTPSDYRHRL

>gi|30064651|ref|NP_838822.1| hypothetical protein S3587 [Shigella flexneri 2a str. 2457T]

MYHASREQRRAVQGIGSFQRNCLMATLTTGVVLLRWQLLSAVMMFLASTLNIRFRRSDYVGLAVISSGLG

VVSACWFAMGLLGITMADITAIWHNIESVMIEEMNQTPPQWPMILT

>gi|30064650|ref|NP_838821.1| IS1 orfA [Shigella flexneri 2a str. 2457T]

MASISIRCPSCSATEGVVRNGKSTAGHQRYLCSHCRKTWQLQFTYTASQPGTHQKIIDMAMNGVGCRASA

RIMGVGLNTVLRHLKNSGRSR

>gi|30064649|ref|NP_838820.1| IS1 orfB [Shigella flexneri 2a str. 2457T]

MSRQCTHYGRWPQHGFTSLKKLRPQSVTSRIQPGSDVIVCAEMDEHWGYVGAKSRQRWLFYAYDRIRRTV

VAHVFGERTLATLERLLSLLSAFEVVVWMTDGWPLYESRLKGKLHVISKRYTQHIERHNLNLRQHLARLV

RKSLSFSKSVELHDKVIGHYLNIKHYQ

>gi|30064648|ref|NP_838819.1| single-stranded DNA-binding protein [Shigella flexneri 2a str. 2457T]

MASRGVNKVILVGNLGQDPEVRYMPNGGAVANITLATSESWRDKATGEMKEQTEWHRVVLFGKLAEVASE

YLRKGSQVYIEGQLRTRKWTDQSGQDRYTTEVVVNVGGTMQMLGGRQGGGAPAGGNIGGGQPQGGWGQPQ

QPQGGNQFSGGAQSRPQQSAPAAPSNEPPMDFDDDIPF

>gi|30064647|ref|NP_838818.1| excinuclease ABC subunit A [Shigella flexneri 2a str. 2457T]

MDKIEVRGARTHNLKNINLVIPRDKLIVVTGLSGSGKSSLAFDTLYAEGQRRYVESLSAYARQFLSLMEK

PDVDHIEGLSPAISIEQKSTSHNPRSTVGTITEIHDYLRLLFARVGEPRCPDHDVPLAAQTVSQMVDNVL

SQPEGKRLMLLAPIIKERKGEHTKTLENLASQGYIRARIDGEVCDLSDPPKLELQKKHTIEVVVDRFKVR

DDLTQRLAESFETALELSGGTAVVADMDDPKAEELLFSANFACPICGYSMRELEPRLFSFNNPAGACPTC

DGLGVQQYFDPDRVIQNPELSLAGGAIRGWDRRNFYYFQMLKSLADHYKFDVEAPWGSLSANVHKVVLYG

SGKENIEFKYMNDRGDTSIRRHPFEGVLHNMERRYKETESSAVREELAKFISNRPCASCEGTRLRREARH

VYVENTPLPAISDMSIGHAMEFFNNLKLAGQRAKIAEKILKEIGDRLKFLVNVGLNYLTLSRSAETLSGG

EAQRIRLASQIGAGLVGVMYVLDEPSIGLHQRDNERLLGTLIHLRDLGNTVIVVEHDEDAIRAADHVIDI

GPGAGVHGGEVVAEGPLEAIMAVPESLTGQYMSGKRKIEVPKKRVPANPEKVLKLTGARGNNLKDVTLTL

PVGLFTCITGVSGSGKSTLINDTLFPIAQRQLNGATIAEPAPYRDIQGLEHFDKVIDIDQSPIGRTPRSN

PATYTGVFTPVRELFAGVPESRARGYTPGRFSFNVRGGRCEACQGDGVIKVEMHFLPDIYVPCDQCKGKR

YNRETLEIKYKGKTIHEVLDMTIEEAREFFDAVPALARKLQTLMDVGLTYIRLGQSATTLSGGEAQRVKL

ARELSKRGTGQTLYILDEPTTGLHFADIQQLLDVLHKLRDQGNTIVVIEHNLDVIKTADWIVDLGPEGGS

GGGEILVSGTPETVAECEASHTARFLKPML

>gi|30064646|ref|NP_838817.1| hypothetical protein S3582 [Shigella flexneri 2a str. 2457T]

MTISELLQYCMAKPSAEQSVHNDWKATQIKVEDVLFAMVKEVENRPAVSLKTSPELAELLRQQHSDVRPS

RHLNKAHWSTVYLDGSLPDSQIYYLVDASYQQAVNLLPEEKRKLLVQL

>gi|30064645|ref|NP_838816.1| hypothetical protein S3581 [Shigella flexneri 2a str. 2457T]

MWYQKTLTLSAKSRGFHLVTDEILNQLADMPRVNIGLLHLLLQHTSASLTLNENCDPTVRHDMECFFLRT

VPDNGNYEHDYEGADDMPSHIKSSMLGTSLVLPVHKGRIQTGTWQGIWLGEHRIHGGSRRIIATLQGE

>gi|30064644|ref|NP_838815.1| acid phosphatase/phosphotransferase [Shigella flexneri 2a str. 2457T]

MRKITQAISAVCLLFALNSSAVALASSPSPLNPGTNVARLAEQAPIHWVSVAQIENSLAGRPPMAVGFDI

DDTVLFSSPGFWRGKKTFSPESEDYLKNPVFWEKMNNGWDEFSIPKEVARQLIDMHVRRGDAIFFVTGRS

PTKTETVSKTLADNFHIPATNMNPVIFAGDKPGQNTKSQWLQDKNIRIFYGDSDNDITAARDVGARGIRI

LRASNSTYKPLPQAGAFGEEVIVNSEY

>gi|30064643|ref|NP_838814.1| aromatic amino acid aminotransferase [Shigella flexneri 2a str. 2457T]

MFQKVDAYAGDPILTLMERFKEDPRSDKVNLSIGLYYNEDGIIPQLKAVAEAEARLNAQPHGASLYLPME

GLNSYRHAIAPLLFGADHPVLQQQRVATIQTLGGSGALKVGADFLKRYFPESGVWVSDPTWENHVAIFAG

AGFEVSTYPWYDEVTNSVRFNDLLATLKTLPARSIVLLHPCCHNPTGADLTNDQWDAVIEILKARELIPF

LDIAYQGFSAGMEEDAYAIRAIASAGLPALVSNSFSKIFSLYGERVGGLSVLCEDAEAAGRVLGQLKATV

RRNYSSPPNFGAQVVAAVLNDEALKASWLAEVEEMRTRILAMRQELVKVLSTEMPERNFDYLLNQRGMFS

YTGLSAAQVDRLREEFGVYLIASGRMCVAGLNTANVQRVAKAFAAVM

>gi|30064642|ref|NP_838813.1| alanine racemase [Shigella flexneri 2a str. 2457T]

MQAATVVINRRALRHNLQRLRELAPASKMVAVVKANAYGHGLLETARTLPDADAFGVARLEEALRLRAGG

ITKPVLLLEGFFDARDLPTISAQHFHTAVHNEEQLAALEEASLDEPVTVWMKLDTGMHRLGVRPEQAEAF

YHRLTQCKNVRQPVNIVSHFARADEPKCGATEKQLAIFNTFCEGKPGQRSIAASGGILLWPQSHFDWVRP

GIILYGVSPLEDRSTGADFGCQPVMSLTSSLIAVREHKAGEPVGYGGTWVSERDTRLGVVAMGYGDGYPR

AAPSGTPVLVNGREVPIVGRVAMDMICVDLGPQAQDKAGDPVILWGEGLPVERIAEMTKVSAYELITRLT

SRVAMKYVD

>gi|30064641|ref|NP_838812.1| replicative DNA helicase [Shigella flexneri 2a str. 2457T]

MAGNKPFNKQQAEPRERDPQVAGLKVPPHSIEAEQSVLGGLMLDNERWDDVAERVVADDFYTRPHRHIFT

EMARLQESGSPIDLITLAESLERQGQLDSVGGFAYLAELSKNTPSAANISAYADIVRERAVVREMISVAN

EIAEAGFDPQGRTSEDLLDLAESRVFKIAESRANKDEGPKNIADVLDATVARIEQLFQQPHDGVTGVNTG

YDDLNKKTAGLQPSDLIIVAARPSMGKTTFAMNLVENAAMLQDKPVLIFSLEMPSEQIMMRSLASLSRVD

QTKIRTGQLDDEDWARISGTMGILLEKRNIYIDDSSGLTPTEVRSRARRIAREHGGIGLIMIDYLQLMRV

PALSDNRTLEIAEISRSLKALAKELNVPVVALSQLNRSLEQRADKRPVNSDLRESGSIEQDADLIMFIYR

DEVYHENSDLKGIAEIIIGKQRNGPIGTVRLTFNGQWSRFDNYAGPQYDDE

>gi|30064640|ref|NP_838811.1| quinone oxidoreductase, NADPH-dependent [Shigella flexneri 2a str. 2457T]

MATRIEFHKHGGPEVLQAVEFTPADPAENEIQVENKAIGINFIDTYIRSGLYPPPSLPSGLGTEAAGIVS

KVGSGVKHIKAGDHVVYAQSALGAYSSVHNINADKAAILPAAISFEQAAASFLKGLTVYYLLRKTYEIKP

DEQFLFHAAAGGIGLIACQWAKALGAKLIGTVGTAQKAQSALKAGAWQVINYREENLVERLKEITGGKKV

RVVYDSVGRDTWERSLDCLQRRGLMVSFGNSSGAVTGVNLGILNQKGSLYVTRPSLQGYITTREELTEAS
NELFSLIASGVIKVDVAEQQKYPLKDAQRAHEILESRATQGSSLLIP

>gi|30064638|ref|NP_838809.1| tRNA-dihydrouridine synthase A [Shigella flexneri 2a str. 2457T]
MQKINQTSAMPEKTDVHWSGRFSVAPMLDWTDRHCRYFLRLLSRNTLLYTEMVTTGAIIHGKGDYLAYSE
EEHPVALQLGGSDPAALAQCAKLAEARGYDEINLNVGCPSDRVQNGMFGACLMGNAQLVADCVKAMRDVV
SIPVTVKTRIGIDDQDSYEFLCDFINTVSGKGECEMFIIHARKAWLSGLSPKENREIPPLDYPRVYQLKR
DFPHLTMSINGGIKSLEEAKAHLQHMDGVMVGREAYQNPGILAAVDREIFGSSDTDADPVAVVRAMYPYI
EHELSQGTYLGHIIRHMLGLFQGIPGARQWRRYLSENAHKAGADINVLEHALKLVADKR

>gi|30064637|ref|NP_838808.1| hypothetical protein S3573 [Shigella flexneri 2a str. 2457T]
MWVNKYTDDCTDEDLNDRDFIASVVDRAIFHFAINSICNPGDNKDAMPIEQCTFDVETKNGLPSTVQLFY
EESKDNEPLANIHFQAIGSGFLTFVNACQEHDDNSLKLFASLLISLSYSSAYADLSETVYINENNESYLK
AQFEKLYQRDMKKYLGEMKRLADGGEMNFDGYLDKMSHLVNEGTLDPDILSKMRDAAPQLISFAKSFDPT
SKEEIKILTDTSKLIYDLFGVKSEK

>gi|30064635|ref|NP_838806.1| stress-response protein [Shigella flexneri 2a str. 2457T]
MNKDEAGGNWKQFKGKVKEQWGKLTDDDMTIIEGKRDQLVGKIQERYGYQKDQAEKEVVDWETRNEYRW

>gi|30064633|ref|NP_838804.1| LexA repressor [Shigella flexneri 2a str. 2457T]
MKALTARQQEVFDLIRDHISQTGMPPTRAEIAQRLGFRSPNAAEEHLKALARKGVIEIVSGASRGIRLLQ
EEEEGLPLVGRVAAGEPLLAQQHIEGHYQVDPSLFKPNADFLLRVSGMSMKDIGIMDGDLLAVHKTQDVR
NGQVVVARIDDEVTVKRLKKQGNKVELLPENSEFKPIVVDLRQQSFTIEGLAVGVIRNGDWL

>gi|30064632|ref|NP_838803.1| diacylglycerol kinase [Shigella flexneri 2a str. 2457T]

MANNTTGFTRIIKAAGYSWKGLRAAWINEAAFRQEGVAVLLAVVIACWLDVDAITRVLLISSVMLVMIVE

ILNSAIEAVVDRIGSEYHELSGRAKDMGSAAVLIAIIVAVITWCILLWSHFG

>gi|30064630|ref|NP_838801.1| 4-hydroxybenzoate octaprenyltransferase [Shigella flexneri 2a str. 2457T]

MEWSLTQNKLLAFHRLMRTDKPIGALLLLWPTLWALWVATPGVPQLWIMAVFVAGVWLMRAAGCVVNDYA

DRKFDGHVKRTANRPLPSGAVTEKEARALFVVLVLISFLLVLTLNTMTILLSIAALALAWVYPFMKRYTH

LPQVVLGAAFGWSIPMAFAAVSESVPLSCWLMFLANILWAVAYDTQYAMVDRDDDVKIGIKSTAILFGQY

DKLIIGILQIGVLALMAIIGELNGLGWGYYWSIVVAGALFVYQQKLIANREREACFKAFMNNNYVGLVLF

LGLAMSYWHF

>gi|30064629|ref|NP_838800.1| chorismate pyruvate lyase [Shigella flexneri 2a str. 2457T]

MSHPALTQLRALRYFKEIPALDPQLLDWLLLEDSMTKRFEQQGKTVSVTMIREGFVEQNEIPEELPLLPK

ESRYWLREILLCADGEPWLAGRTVVPVSTLSGPELALQKLGKTPLGRYLFTSSTLTRDFIEIGRDAGLWG

RRSRLRLSGKPLLLTELFLPASPLY

>gi|30064628|ref|NP_838799.1| maltose regulon periplasmic protein [Shigella flexneri 2a str. 2457T]

MKMNKSLIALCLSAGLLASAPGISLADVNYVPQNTSDAPAIPSAALQQLTWTPVDQSKTQTTQLATGGQQ

LNVPGISGPVAAYSVPANIGELTLTLTSEVNKQTSVFAPNVLILDQNMTPSAFFPSSYFTYQELGVMSAD

RLEGVMRLTPALGQQKLYVLVFTTEKDLQQTTQLLDPAKAYAKGVGNSIPDIPDPVARHTSDGLLKLKVK

TNSSSSVLVGPLFGSSAPAPVTVGNTAAPAVAAPAPVPVKKSEPMLNDTESYFNTAIKNAVAKGDVDKAL

KLLDEAERLGSTSARSTFISSVKGKG

>gi|30064627|ref|NP_838798.1| maltoporin [Shigella flexneri 2a str. 2457T]

MITLRKLPLAVAVAAGVMSAQAMAVDFHGYARSGIGWTGSGGEQQCFQTTGAQSKYRLGNECETYAELKL

GQEVWKEGDKSFYFDTNVAYSVAQQNDWEATDPAFREANVQGKNLIEWLPGSTIWAGKRFYQRHDVHMID

FYYWDISGPGAGLENIDVGFGKLSLAATRSSEAGGSSSFASNNIYDYTNETANDVFDVRLAQMEINPGGT

LELGVDYGRANLRDNYRLVDGASKDGWLFTAEHTQSVLKGFNKFVVQYATDSMTSQGKGLSQGSGVAFDN

EKFAYNINNNGHMLRILDHGAISMGDNWDMMYVGMYQDINWDNDNGTKWWTVGIRPMYKWTPIMSTVMEI

GYDNVESQRTGDKNNQYKITLAQQWQAGDSIWSRPAIRVFATYAKWDEKWGYDYNGDSKVNPNYGKAVPA

DFNGGSFGRGDSDEWTFGAQMEIWW

>gi|30064626|ref|NP_838797.1| maltose/maltodextrin transporter ATP-binding protein [Shigella flexneri 2a str. 2457T]

MASVQLQNVTKAWGEVVVSKDINLDIHEGEFVVFVGPSGCGKSTLLRMIAGLETITSGDLFIGEKRMNDT

PPAERGVGMVFQSYALYPHLSVAENMSFGLKLAGAKKEVINQRVNQVAEVLQLAHLLDRKPKALSGGQRQ

RVAIGRTLVAEPSVFLLDEPLSNLDAALRVQMRIEISRLHKRLGRTMIYVTHDQVEAMTLADKIVVLDAG

RVAQVGKPLELYHYPADRFVAGFIGSPKMNFLPVKVTATAIDQVQVELPMPNRQQVWLPVESRDVQVGAN

MSLGIRPEHLLPSDIADVILEGEVQVVEQLGNETQIYIQIPSIRQNLVYRQNDVVLVEEGATFAIGLPPE

RCHLFREDGTACRRLHKEPGV

>gi|30064625|ref|NP_838796.1| maltose ABC transporter periplasmic protein [Shigella flexneri 2a str. 2457T]

MKIKTGARILALSALTTMMFSASALAKIEEGKLVIWINGDKGYNGLAEVGKKFEKDTGIKVTVEHPDKLE

EKFPQVAATGDGPDIIFWAHDRFGGYAQSGLLAEITPDKAFQDKLYPFTWDAVRYNGKLIAYPIAVEALS

LIYNKDLLPNPPKTWEEIPALDKELKAKGKSALMFNLQEPYFTWPLIAADGGYAFKYENGKYDIKDVGVD

NAGAKAGLTFLVDLIKNKHMNADTDYSIAEAAFNKGETAMTINGPWAWSNIDTSKVNYGVTVLPTFKGQP

SKPFVGVLSTGINAASPNKELAKEFLENYLLTDEGLEAVNKDKPLGAVALKSYEEELAKDPRIAATMENA

QKGEIMPNIPQMSAFWYAVRTAVINAASGRQTVDEALKDAQTRITK

>gi|30064624|ref|NP_838795.1| maltose transporter membrane protein [Shigella flexneri 2a str. 2457T]

MDVIKKKHWWQSDALKWSVLGLLGLLVGYLVVLMYAQGEYLFAITTLILSSAGLYIFANRKAYAWRYVYP

GMAGMGLFVLFPLVCTIAIAFTNYSSTNQLTFERAQEVLLDRSWQAGKTYNFGLYPAGDEWQLALSDGET

GKNYLSDAFKFGGEQKLQLKETTAQPEGERANLRVITQNRQALSDITAILPDGNKVMMSSLRQFSGTQPL

YTLDGDGTLTNNQSGVKYRPNNQIGFYQSITADGNWGDEKLSPGYTVTTGWKNFTRVFTDEGIQKPFLAI

FVWTVVFSLITVFLTVAVGMVLACLVQWEALRGKAVYRVLLILPYAVPSFISILIFKGLFNQSFGEINMM

LSALFGVKPAWFSDPTTARTMLIIVNTWLGYPYMMILCMGLLKAIPDDLYEASAMDGAGPFQNFFKITLP

LLIKPLTPLMIASFAFNFNNFVLIQLLTNGGPDRLGTTTPAGYTDLLVNYTYRIAFEGGGGQDFGLAAAI

ATLIFLLVGALAIVNLKATRMKFD

>gi|30064623|ref|NP_838794.1| maltose transporter permease [Shigella flexneri 2a str. 2457T]

MAMVQPKSQKARLFITHLLLLLFIAAIMFPLLMVVAISLRQGNFATGSLIPEQISWDHWKLALGFSVEQA

DGRITPPPFPVLLWLWNSVKVAGISAIGIVALSTTCAYAFARMRFPGKATLLKGMLIFQMFPAVLSLVAL

YALFDRLGEYIPFIGLNTHGGVIFAYLGGIALHVWTIKGYFETIDSSLEEAAALDGATPWQAFRLVLLPL

SVPILAVVFILSFIAAITEVPVASLLLRDVNSYTLAVGMQQYLNPQNYLWGDFAAAAVMSALPITIVFLL

AQRWLVNGLTAGGVKG

>gi|30064622|ref|NP_838793.1| D-xylose transporter XylE [Shigella flexneri 2a str. 2457T]

MNTQYNSSYIFSITLVATLGGLLFGYDTAVISGTVESLNTVFVAPQNLSESAANSLLGFCVASALIGCII

GGALGGYCSNRFGRRDSLKISAVLFFISGVGSAWPELGFTSINPDNTVPIYLAGYVPEFVIYRIIGGIGV

GLASMLSPMYIAELAPAHIRGKLVSFNQFAIIFGQLLVYCVNYFIARSGDASWLNTDGRRYMFASECIPA

LLFLMLLYTVPESPRWLMSRGKQEQTESILRKIMGNTLATQAVQEIKHSLDHGRKTGGRLLMFGVGVIVI

GVMLSIFQQFVGINVVLYYAPEVFKTLGASTDIALLQTIIVGVINLTFTVLAIMTVDKFGRKPLQIIGAL

GMAIGMFSLGTAFYTQASGIVALLSMLFYVAAFAMSWGPVCWVLLSEIFPNAIRGKALAIAVAAQWLANY

FVSWTFPMMDKNSWLVAHFHNGFSYWIYGCMGVLAALFMWKFVPETKGKTLEELEALWEPETKKTQQTAT

L

>gi|30064621|ref|NP_838792.1| phosphate-starvation-inducible protein PsiE [Shigella flexneri 2a str. 2457T]

MTSLSRPRVEFISTILQTVLNLGLLCLGLILVVFLGKETVHLADVLFAPEQTSKYELVEGLVVYFLYFEF

IALIVKYFQSGFHFPLRYFVYIGITAIVRLIIVDHKSPLDVLIYSAAILLLVITLWLCNSKRLKRE

>gi|30064620|ref|NP_838791.1| hypothetical protein S3555 [Shigella flexneri 2a str. 2457T]

MKKRHLLSLLALGISTACYGETYPAPIGPSQSDFGGVGLLQTPTARMAREGELSLNYRDNDQYRYYSASV

QLFPWLETTLRYTDVRTRQYSSVEAFSGDQTYKDKAFDLKLRLWEESYWLPQVAVGARDIGGTGLFDAEY

LVASKAWGPFDFTLGLGWGYLGTSGNVKNPLCSASDKYCYRDNSYKQAGSIDGSQMFHGPASLFGGVEYQ

TPWQPLRLKLEYEGNNYQQDFAGKLEQKSKFNVGAIYRVTDWADVNLSYERGNTFMFGVTLRTNFNDLRP

SYIDNARPQYQPQPQDAILQHSVVANQLTLLKYNAGLADPQIQAKGDTLYVTGEQVKYRDSREGIIRANR

IVMNDLPDGIKTIRITENRLNMPQVTTETDVASLKNHLAGEPLGHETTLAHKRVEPVVPKSTEQGWYIDK

SRFDFHIDPVLNQSVGGPENFYMYQLGVMGTADLWLTDHLLTTGSLFANLANNYDKFNYTNPPQDSHLPR

VRTHVREYVQNDVYVNNLQANYFQHLGNGFYGQVYGGYLETMFGGAGAEVLYRPLDSNWAFGLDANYVKQ

RDWRSAKDMMKFTDYSVKTGHLTAYWTPSFAQDVLVKASVGQYLAGDKGGTLEIAKRFDSGVVVGGYATI

TNVSKEEYGEGDFTKGVYVSVPLDLFSSGPTRSRAAIGWTPLTRDGGQQLGRKFQLYDMTSDRSVNFR

>gi|30064619|ref|NP_838790.1| hypothetical protein S3554 [Shigella flexneri 2a str. 2457T]

MIKQTIVALLLSVGASSVFAAGTVKVFSNGSSEAKTLTGAEHLIDLVGQPRLANSWWPGAVISEELATAA

ALRQQQALLTRLAEQGADSSTDDAAAINALRQQIQALKVTGRQKINLDPDIVRVAERGNPPLQGNYTLWV

GPPPSTVTLFGLISHPGNQPFTPGRDVASYLSDQSLLSGADRSYAWVVYPDGRTQKAPVAYWNKRHVEPM

PGSIIYVGLADSVWSETPDALNADILQTLTQRIPQ

>gi|30064618|ref|NP_838789.1| hypothetical protein S3553 [Shigella flexneri 2a str. 2457T]

MVILPLWRRVVKRPALILICLLLQACSATTKELGNSLWDSLFGTPGVQLTDDDIQNMPYASQYMQLNGGP

QLFVVLAFAEDGQQKWVTQDQATLVTQHGRLVKTLLGGDNLIEVNNLAADPLIKPAQIVDGATWTRTMGW

TEYQQVRYATARSVFKWDGTDTVKVGSDETLVRVLDEEVSTDQARWHNRYWIDSEGQIRQSEQYLGADYF
PVKTTLIKAAKQ

>gi|30064617|ref|NP_838788.1| hypothetical protein S3552 [Shigella flexneri 2a str. 2457T]
MKKVLYGIFAISALAATSAWAAPVQVGEAAESAATSVSAGSSSATSVSTVSSAVGVALAATGGGDGSNTG
TTTTTTTSTQ

>gi|30064616|ref|NP_838787.1| glucose-6-phosphate isomerase [Shigella flexneri 2a str. 2457T]
MKNINPTQTAAWQALQKHFDEMKDVTIADLFAKDGDRFSKFSATFDDQMLVDYSKNRITEETLAKLQDLA
KECDLAGAIKSMFSGEKINRTENRAVLHVALRNRSNTPILVDGKDVMPEVNAVLEKMKTFSEAIISGEWK
GYTGKAITDVVNIGIGGSDLGPYMVTEALRPYKNHLNMHFVSNVDGTHISEVLKKVNPETTLFLVASKTF
TTQETMTNAHSARDWFLKAAGDEKHVAKHFAALSTNAKAVGEFGIDTANMFEFWDWVGGRYSLWSAIGLS
IVLSIGFDNFVELLSGAHAMDKHFSTTPAEKNLPVLLALIGIWYNNFFGAETEAILPYDQYMHRFAAYFQ
QGNMESNGKYVDRNGNVVDYQTGPIIWGEPGTNGQHAFYQLIHQGTKMVPCDFIAPAITHNPLSDHHQKL
LSNFFAQTEALAFGKSREVVEQEYRDQGKDPATLDYVVPFKVFEGNRPTNSILLREITPFSLGALIALYE
HKIFTQGVILNIFTFDQWGVELGKQLANRILPELKDDKEISSHDSSTNGLINRYKAWRG

>gi|30064615|ref|NP_838786.1| aspartate kinase III [Shigella flexneri 2a str. 2457T]
MSEIVVSKFGGTSVADFDAMNRSADIVLSDANVRLVVLSASAGITNLLVALAEGLEPGERFEKLDAIRNI
QFAILERLRYPNVIREEIERLLENITVLAEAAALATSPALTDELVSHGELMSTLLFVEILRERDVQAQWF
DVRKVMRTNDRFGRAEPDVAALAELAALQLLPRLNEGLVITQGFIGSENKGRTTTLGRGGSDYTAALLAE
ALHASRVDIWTDVPGIYTTDPRVVSAAKRIDEIAFAEAAEMATFGAKVLHPATLLPAVRSDIPVFVGSSK
DPRAGGTLVCNKTENPPLFRALALRRNQTLLTLHSLNMLHSRGFLAEVFGILARHNISVDLITTSEVSVA
LTLDTTGSTSTGDTLLTQSLLMELSALCRVEVEEGLALVALIGNDLSKACGVGKEVFGVLEPFNIRMICY
GASSHNLCFLVPGEDAEQVVQKLHSNLFE

>gi|30064614|ref|NP_838785.1| ISSfl3 orfC [Shigella flexneri 2a str. 2457T]

MNNELPDDIELLKAMLRKQQSRLRQYACQVAGYEQEIERLKAQLDRLRRMLFGQSSEKKRHKLENQIRQA

EKRLSELENRLNTARNLLEDASSVTDSPDTSPPSENPIASKPESPGRKSSRKPLPAELPRETHRLLPAET

SCPACGGVLKEMGETISEQLDIINTAFKVIETIRPKLACSRCDVIVQAPLPPKPIERGYASAGLLARILV

SKYMEHIPLYRQSEIYARQGVELSRNTMVRWVSEMADKLRPLYIALNDYVLEAGKVHADDTPVKVLAPGN

GKTKTGRLWVYVRDDRNAGSSLPAAVWFAYSADRKGEHPQLHLAKYQGVLQADAYAGYNVLYETGRVKEA

GCLAHARRKIHDEDVRRPTEMTQEALRRIAELYDIEAEIRGSPAEERLAVRKARSVQLMQSLYDWIQLQR

KTLSKHAEMAKAFDYILNHWNALNEFCRDGRVEIDNNIGENALRSVAVGRKNYLFFGSDKGGESAAIIYS

LLVTCKQNEVEPEDWLREVIEKLNDWPSNQVHELLPWNFLSVK

>gi|30064613|ref|NP_838784.1| ISSfl3 orfB [Shigella flexneri 2a str. 2457T]

MITLPTGTKIWIIAGITDMRCGFNGLASKVQNTLKDDPFSGHIFVFRGRSGKMVKILWADRDGLCLFTKR

LAGDPGRESAPDASSVIHATGGDRVATSQTDRTAWHPDITRDKTRE

>gi|30064612|ref|NP_838783.1| ISSfl3 orfA [Shigella flexneri 2a str. 2457T]

MNSQTTKDIPCFRSYLPDALRLRFEDKLTIRAIAQRLGLSHSTIHTLFQRFLASGIAWPLPDSVSFAQLD

AILYANRKKELTEPQIREGSWRKERRTSYSREFKVRLAKQALQPGAVVARIAREHDINNNLLFKWKSQYE

DGLLSDDDIQECMPVPVALTDTPEPTRPVTNPFWRNKHDERPEGAPGNVPRCELHLKSGVVKLFDPLTPE

LLRALIREMKGGIR

>gi|30064611|ref|NP_838782.1| potassium transporter peripheral membrane component [Shigella flexneri 2a str. 2457T]

MKIIILGAGQVGGTLAENLVGENNDITVVDTNGERLRTLQDKFDLRVVQGHGSHPRVLREAGADDADMLV

AVTSSDETNMVACQVAYSLFNTPNRIARIRSPDYVRDADKLFHSDAVPIDHLIAPEQLVIDNIYRLIEYP

GALQVVNFAEGKVSLAVVKAYYGGPLIGNALSTMREHMPHIDTRVAAIFRHDRPIRPQGSTIVEAGDEVF

FIAASQHIRAVMSELQRLEKPYKRIMLVGGGNIGAGLARRLEKDYSVKLIERNQQRAAELAEKLQNTIVF
FGDASDQELLAEEHIDQVDLFIAVTNDDEANIMSAMLAKRMGAKKVMVLIQRRAYVDLVQGSVIDIAISP
QQATISALLSHVRKADIVGVSSLRRGVAEAIEAVAHGDESTSRVVGRVIDEIKLPPGTIIGAVVRGNDVM
IANDNLRIEQGDHVIMFLTDKKFITDVERLFQPSPFFL

>gi|30064610|ref|NP_838781.1| 16S rRNA methyltransferase B [Shigella flexneri 2a str. 2457T]
MKKQRNLRSMAAQAVEQVVEQGQSLSNILPPLQQKVSDKDKALLQELCFGVLRTLSQLDWLINKLMARPM
TGKQRTVHYLIMVGLYQLLYTRIPPHAALAETVEGAIAIKRPQLKGLINGVLRQFQRRQEELLAEFNTRD
ARYLHPSWLLKRLQKAYPEQWQSIAEANNQRPPMWLRINRTHHSRDSWLALLDEAGMKGFPHADYPDAVR
LETPAPVHALPGFEDGWVTVQDASAQGCMTWLAPQNGEHILDLCAAPGGKTTHILEVAPEAQVVAVDIDE
QRLSRIYDNLKRLGMKATVKQGDGRYPSQWCGEQQFDRILLDAPCSATGVIRRHPDIKWLRRDRDIPELA
QLQSEILDAIWPHLKSGGTLVYATCSVLPEENSLQIKAFLQRTADAELCETGTPEQPGKQNQPGAEEGDG
FFYAKLIKK

>gi|30064609|ref|NP_838780.1| methionyl-tRNA formyltransferase [Shigella flexneri 2a str. 2457T]
MSESLRIIFAGTPDFAARHLDALLSSGHNVVGVFTQPDRPAGRGKKLMPSPIKVLAEEEGLPVFQPVSLR
PQENQQLVADLQADVMVVVAYGLILPKAVLEMPRLGCINIHGSLLPRWRGAAPIQRSLWAGDAETGVTIM
QMDVGLDTGDMLYKLSCPITAEDTSGTLYDKLAELGPQGLITTLKQLADGTAKPEVQDETLVTYAEKLSK
EEARIDWSLSAAQLERCIRAFNPWPMSWLEIEGQPVKVWKASVIDTATNAAPGTILEANKQGIQVATGDG
ILNLLSLQPAGKKAMSAQDLLNSRREWFVPGNRLV

>gi|30064608|ref|NP_838779.1| peptide deformylase [Shigella flexneri 2a str. 2457T]
MSVLQVLHIPDERLRKVAKPVEEVNAEIQRIVDDMFETMYAEEGIGLAATQVDIHQRIIVIDVSENRDER
LVLINPELLEKSGETGIEEGCLSIPEQRALVPRAEKVKIRALDRDGKPFELEAEGLLAICIQHEMDHLVG
KLFMDYLSPLKQQRIRQKVEKLDRLKARA

>gi|30064607|ref|NP_838778.1| DNA protecting protein DprA [Shigella flexneri 2a str. 2457T]

MVDIEIWLRLMSISSLYGDDMVRIAHWLAKQSHIDAVVLQQTGLTLRQAQRFLSFPRKSIESSLCWLEQP

NHHLIPADSEFYPPQLQATTDYPGALFVEGELHALHSFQLAVVGSRAHSWYGERWGRLFCETLATCGVTI

TSGLARGIDGVVHKAALQVNGVSIAVLGNGLNTIHPRRHARLAASLFEQGGALVSEFPLDVPPLAYNFPR

RNRIISGLSKGVLVVEAALRSGSLVTARCALEQGREVFALPGPIGNPGSEGPHWLIKQGAILVTEPEEIL

ENLQFGLHWLPDAPENSFYSPDQQDVALPFPELLANVGDEVTPVDVVAERAGQPVPEVVTQLLELELAGW

IAAVPGGYVRLRRACHVRRTNVFV

>gi|30064606|ref|NP_838777.1| hypothetical protein S3540 [Shigella flexneri 2a str. 2457T]

MYLFETYIHTEAELRVDQDKLEQDLTDAGFEREDIYNALLWLEKLADYQEGLAEPMQLASDPLSMRIYTP

EECERLDASCRGFLLFLEQIQVLNLETREMVIERVLALDNAEFELDDLKWVILMVLFNIPGCENAYQQME

ELLFEVNEGMLH

>gi|30064605|ref|NP_838776.1| DNA topoisomerase [Shigella flexneri 2a str. 2457T]

MRNNESCPKCGAELVIRSGKHGPFLGCSQYPACDYVRPLKSSADGHIVKVLEGQVCPVCGANLVLRQGRF

GMFIGCSNYPECEHTELIDKPDETAITCPQCRTGHLVQRRSRYGKTFHSCDRYPECQFAINFKPIAGECP

ECHYPLLIEKKTAQGVKHFCASKQCGKPVSAE

>gi|30064604|ref|NP_838775.1| ribosome maturation factor [Shigella flexneri 2a str. 2457T]

MNNNLQGDAIAAAIDVLNEKRVIAYPTEAVFGVGCDPDSETAVMRLLELKQRPVDKGLILIAANYEQLKP

YIDDTMLTDVQRETIFSCWPGPVTFVFPAPATTPRWLTGRFDSLAVRVTDHPLVVALCQAYGKPLVSTSA

NLSGLPPCRTVDEVRAQFGAAFPVVPGETGGRLNPSEIRDALTGELFRQG

>gi|30064603|ref|NP_838774.1| shikimate 5-dehydrogenase [Shigella flexneri 2a str. 2457T]

METYAVFGNPIAHSKSPFIHQQFAQQLNIEHPYGRVLAPINDFINTLNAFFRAGGKGANVTVPFKEEAFA

RADELTERAALAGAVNTLKRLEDGRLLGDNTDGVGLLSDLERLSFIRPGLRILLIGAGGASRGVLLPLLS

LDCAVTITNRTVSRAEELAKLFAHTGSIQALGMDELEGHEFDLIINATSSGISGDIPAIPSSLIHPGIYC

YDMFYQKGKTPFLAWCEQRGSKRNADGLGMLVAQAAHAFLLWHGVLPDVEPVIKQLQEELSA

>gi|30064602|ref|NP_838773.1| hypothetical protein S3536 [Shigella flexneri 2a str. 2457T]

MNQAIQFPDREEWDENKKCVCFPALVNGMQLTCAISGESLAYRFTGDTPEQWLASFRQHRWDLEEEAENL

IQEQSEDDQGWVWLP

>gi|30064601|ref|NP_838772.1| transferase [Shigella flexneri 2a str. 2457T]

MKCFTLSSQRRRIIGIPIFCTSIFLIFFSVCCFFTLFAAIAHKTVLFCILKDLHKANNAPKVISKSFIAE

VSMSDVLRPYRDLFPQIGQRVMIDDSSVVIGDVRLADDVGIWPLVVIRGDVHYVQIGARTNIQDGSMLHV

THKSSYNPDGNPLTIGEDVTVGHKVMLHGCTIGNRVLVGMGSILLDGAIVEDDVMIGAGSLVPQNKRLES

GYLYLGSPVKQIRPLSDEEKAGLRYSANNYVKWKDEYLDQGNQTQP

>gi|30064600|ref|NP_838771.1| ATP-binding component of a transport system [Shigella flexneri 2a str. 2457T]

MSQILLQPANAMITLENVHKWYGQFHVLKNINLTVQPGERIVLCGPSGSGKSTTIRCINHLEEHQQGRIV

VDGIELNEDIRNIERVRQEVGMVFQHFNLFPHLTVLQNCTLAPIWVRKMPKKEAEALAMHYLERVRIAEH

AHKFPGQISGGQQQRVAIARSLCMKPKIMLFDEPTSALDPEMVKEVLDTMIGLAQSGMTMLCVTHEMGFA

RTVADRVIFMDRGEIVEQATPDEFFAHPKSERTRAFLSQVIH

>gi|30064599|ref|NP_838770.1| transport system permease [Shigella flexneri 2a str. 2457T]

MTKVLLSQPSRPASHNSSRAMVWVRKNLFSSWSNSLLTIGCIWLMWELIPPLLNWAFLQANWVGSTRADC

TKAGACWVFIHERFGQFMYGLYPHDQRWRINLALLIGLVSIAPMFWKILPHRGRYIAAWAVIYPLIVWWL

MYGGFFGLERVETRQWGGLTLTLIIASVGIAGALPWGILLALGRRSHMPIVRILSVIFIEFWRGVPLITV

LFMSSVMLPLFMAEGTSIDKLIRALVGVILFQSAYVAEVVRGGLQALPKGQYEAAESLALGYWKTQGLVI

LPQALKLVIPGLVNTIIALFKDTSLVIIIGLFDLFSSVQQATVDPAWLGMSTEGYVFAALIYWIFCFSMS

RYSQHLEKRFNTGRTPH

>gi|30064598|ref|NP_838769.1| IS1 orfA [Shigella flexneri 2a str. 2457T]

MASISIRCPSCSATEGVVRNGKSTAGHQRYLCSHCRKTWQLQFTYTASQPGTHQKIIDMAMNGVGCRASA

RIMGVGLNTVLRHLKNSGRSR

>gi|30064597|ref|NP_838768.1| IS1 orfB [Shigella flexneri 2a str. 2457T]

MSRQCTHYGRWPQHGFTSLKKLRPQSVTSRIQPGSDVIVCAEMDEHWGYVGAKSRQRWLFYAYDRIRRTV

VAHVFGERTLATLERLLSLLSAFEVVVWMTDGWPLYESRLKGKLHVISKRYTQRIERHNLNLRQHLARLV

RKSLSFSKSVELHDKVIGHYLNIKHYQ

>gi|30064596|ref|NP_838767.1| periplasmic binding transport protein [Shigella flexneri 2a str. 2457T]

MQCGISDGLPRFSYADADGKFSGIDVDVCRGVAAAVFGDDTKVKYTPLTAKERFTALQSGEVDLLSRNTT

WTSSRDAGMGMAFTGVTYYDGIGFLTHDKAGLKSAKELDGATVCIQAGTDTELNVADYFKANNMKYTPVT

FDRSDESAKALESGRCDTLASDQSQLYALRIKLSNPAEWIVLPEVISKEPLGPVVRRGDDEWFSIVRWTL

FAMLNAEEMGINSQNVDEKAANPATPDMAHLLGKEGDYGKDLKLDNKWAYNIIKQVSNYSEIFERNVGSE

SPLKIKRGQNNLWNNGGIQYAPPVR

>gi|30064595|ref|NP_838766.1| IS1 orfA [Shigella flexneri 2a str. 2457T]

MASISIRCPSCSATEGVVRNGKSTAGHQRYLCSHCRKTWQLQFTYTASQPGTHQKIIDMAMNGVGCRASA

RIMGVGLNTVLRHLKNSGRSR

>gi|30064594|ref|NP_838765.1| IS1 orfB [Shigella flexneri 2a str. 2457T]

MSRQRTHYGRWPQHGFTSLKKLRPQSVTSRIQPGSDVIVCAEMDEHWGYVGAKSRQRWLFYAYDRIRRTV

VAHVFGERTLATLERLLSLLSAFEVVVWMTDGWPLYESRLKGKLHVISKRYTQRIERHNLNLRQHLARLG

RKSLSFSKSVELHDKVIGHYLNIKHYQ

>gi|30064593|ref|NP_838764.1| methyltransferase [Shigella flexneri 2a str. 2457T]

MRTGCEPTRFGNEAKTIIHGDAFAELKKLPTESVDLLFADPPYNIGKNFDGLIEAWKEDLFIDWLFEVIA

ECHRVLKKQGSMYIMNSTENMPFIDLQCRKLFTIKSRIVWSYDSSGVQAKKHYGSMYEPILMMVKDAKNY

TFNGDAILVEAKTGSQRALIDYRKNPPQPYNHQKVPGNVWDFPRVRYLMDEYENHPTQKPKALLKRIILA

SSNPGDIVLDPFAGSFTTGAVAIASGRKFIGIEINSEYIKMGLRRLDVASHYSAEELAKVKKRKTGNLSK

RSRLSEVDPDLIAK

>gi|30064592|ref|NP_838763.1| DNA-binding protein Fis [Shigella flexneri 2a str. 2457T]

MFEQRVNSDVLTVSTVNSQDQVTQKPLRDSVKQALKNYFAQLNGQDVNDLYELVLAEVEQPLLDMVMQYT

RGNQTRAALMMGINRGTLRKKLKKYGMN

>gi|30064591|ref|NP_838762.1| dehydrogenase [Shigella flexneri 2a str. 2457T]

MSSNPQVWESDKSRLRMVHIDEPGIRTVQIAGSAPKEMADAARINVESGAQIIDINMGCPAKKVNRKLAG

SALLQYPDVVKSILTEVVNAVDVPVTLKIRTGWAPEHRNCEEIAQLAEDCGIQALTIHGRTRACLFNGEA

EYDSIRAVKQKVSIPVIANGDITDPLKARAVLDYTGADALMIGRAAQGRPWIFREIQHYLDTGELLPPLP

LAEVKRLLCAHVRELHDFYGPAKGYRIARKHVSWYLQEHAPNDQFRRTFNAIEDASEQLEALEAYFENFA

>gi|30064590|ref|NP_838761.1| 50S ribosomal protein L11 methyltransferase [Shigella flexneri 2a str. 2457T]

MPWIQLKLNTTGANAEDLSDALMEAGAVSITFQDTHDTPVFEPLPGETRLWGDTDVIGLFDAETDMNDVV

AILENHPLLGAGFAHKIEQLEDKDWEREWMDNFHPMRFGERLWICPSWRDVPDENAVNVMLDPGLAFGTG

THPTTSLCLQWLDSLDLTGKTVIDFGCGSGILAIAALKLGAAKAIGIDIDPQAIQASRDNAERNGVSDRL

ELYLPKDQPEEMKADVVVANILAGPLRELAPLISVLPVSGGLLGLSGILASQAESVCEAYADSFALDPVV

EKEEWCRITGRKN

>gi|30064589|ref|NP_838760.1| sodium/panthothenate symporter [Shigella flexneri 2a str. 2457T]

MQLEVILPLVAYLVVVFGISVYAMRKRSTGTFLNEYFLGSRSMGGIVLAMTLTATYISASSFIGGPGAAY

KYGLGWVLLAMIQLPAVWLSLGILGKKFAILARRYNAVTLNDMLFARYQSRLLVWLASLSLLVAFVGAMT

VQFIGGARLLETAAGIPYETGLLIFGISIALYTAFGGFRASVLNDTMQGLVMLIGTVVLLIGVVHAAGGL

SNAVQTLQTIDPQLVTPQGADDILSPAFMTSFWVLVCFGVIGLPHTAVRCISYKDSKAVHRGIIIGTIIV

AILMFGMHLAGALGRAVIPDLTVPDLVIPTLMVKVLPPFAAGIFLAAPMAAIMSTINAQLLQSSATIIKD

LYLNIRPDQMQNETRLKRMSAVITLVLGALLLLAAWKPPEMIIWLNLLAFGGLEAVFLWPLVLGLYWERA

NAKGALSAMIVGGVLYAVLATLNIQYLGFHPIVPSLLLSLLAFLVGNRFGTSVPQATVLTTDK

>gi|30064588|ref|NP_838759.1| hypothetical protein S3512 [Shigella flexneri 2a str. 2457T]

MDTRFVQAHKEARWALGLTLLYLAVWLVDAYLPGVAPGFTGFPRWFEMACILTPLLFIGLCWAMVKFIYR

DIPLEDDDAA

>gi|30064587|ref|NP_838758.1| acetyl-CoA carboxylase biotin carboxylase subunit [Shigella flexneri 2a str. 2457T]

MLDKIVIANRGEIALRILRACKELGIKTVAVHSSTDRDLKHVLLADETVCIGPAPSVKSYLNIPAIISAA

EITGAVAIHPGYGFLSENANFAEQVERSGFIFIGPKAETIRLMGDKVSAIAAMKKAGVPCVPGSDGPLGD

DMDKNRAIAKRIGYPVIIKASGGGGGRGMRVVRGDAELAQSISMTRAEAKAAFSNDMVYMEKYLENPRHV

EIQVLADGQGNAIYLAERDCSMQRRHQKVVEEAPAPGITPELRRYIGERCAKACVDIGYRGAGTFEFLFE

NGEFYFIEMNTRIQVEHPVTEMITGVDLIKEQLRIAAGQPLSIKQEEVHVRGHAVECRINAEDPNTFLPS

PGKITRFHAPGGFGVRWESHIYAGYTVPPYYDSMIGKLICYGENRDVAIARMKNALQELIIDGIKTNVDL

QIRIMNDENFQHGGTNIHYLEKKLGLQEK

>gi|30064586|ref|NP_838757.1| acetyl-CoA carboxylase biotin carboxyl carrier protein subunit [Shigella flexneri 2a str. 2457T]

MDIRKIKKLIELVEESGISELEISEGEESVRISRAAPAASFPVMQQAYAAPMMQQPAQSNAAAPATVPSM

EAPAAAEISGHIVRSPMVGTFYRTPSPDAKAFIEVGQKVNVGDTLCIVEAMKMMNQIEADKSGTVKAILV

ESGQPVEFDEPLVVIE

>gi|30064585|ref|NP_838756.1| hypothetical protein S3509 [Shigella flexneri 2a str. 2457T]

MVSRNHDFPVWMMLYNFLTKVFDESCPANRFSR

>gi|30064584|ref|NP_838755.1| dehydrogenase [Shigella flexneri 2a str. 2457T]

MQALLLEQQDGKTLASVQTLDERRLPEGDVTVDVHWSSLNYKDALAITGKGKIIRNFPMIPGIDFAGTVR

TSEDPRFHAGQEVLLTGWGVGENHWGGLAEQARVKGDWLVAMPQGLDARKAMIIGTAGFTAMLCVMALED

AGVRPQDGEIVVTGASGGVGSTAVALLHKLGYQVVAVSGRESTHEYLKSLGASGILPRDEFAESRPLEKQ

VWAGAIDTVGDKVLAKVLAQMNYGGCVAACGLAGGFTLPTTVMPFILRNVRLQGVDSVMTPPERRAQAWQ

RLVADLPESFYTQAAKEISLSEAPNFAEAIINNQIQGRTLVKVN

>gi|30064583|ref|NP_838754.1| regulatory protein CsrD [Shigella flexneri 2a str. 2457T]

MRLTTKFSAFVTLLTGLTIFVTLLGCSLSFYNAIQYKFSHRVQAVATAIDTHLVSNDFSALRPQITELMV

SADIVRVDLLHGDKQVYTLARNGSYRPVGTSDLFRELSVPLIKHPGMSLRLVYQDPMGNYFHSLMTTAPL

TGAIGFIILMLFLAVRWLQRQLAGQELLETRATRILNGERGSNVLGTIYEWPPRTSSALDTLLREIQNAR

EQHSRLDTLIRSYAAQDVKTGLNNRLFFDNQLATLLEDQEKVGTHGIVMMIRLPDFNMLSDTWGHSQVEE

QFFTLTNLLSTFMMRYPGALLARYHRSDFAALLPHRTLKEAESIASQLIKAVDTLPNNKMLDRDDMIHIG

ICAWRSGQDTEQVMEHAESATRNAGLQGGNSWAIYDDSLPEKGRGNVRWRTLIEQMLSRGGPRLYQKPAV

TREGQVHHRELMCRIFDGNEEVSSAEYMPMVLQFGLSEEYDRLQISRLIPLLRYWPEENLAIQVTVESLI

RPRFQRWLRDTLMQCEKSQRKRIIIELAEADVGQHISRLQPVIRLVNALGVRVAVNQAGLTLVSTSWIKE

LNVELLKLHPGLVRNIEKRTENQLLVQSLVEACSGTSTHVYATGVRSRSEWQTLIQRGVTGGQGDFFASS

QPLDTNVKKYSQRYSV

>gi|30064581|ref|NP_838752.1| rod shape-determining protein MreC [Shigella flexneri 2a str. 2457T]

MKPIFSRGPSLQIRLILAVLVALGIIIADSRLGTFSQIRTYMDTAVSPFYFVSNAPRELLDGISQTLASR

DQLELENRALRQELLLKNSELLMLGQYKQENARLRELLGSPLRQDEQKMVTQVISTVNDPYSDQVVIDKG

SVNGVYEGQPVISDKGVVGQVVAVAKLTSRVLLICDATHALPIQVLRNDIRVIAAGNGCTDDLQLEHLPA

NTDIRVGDVLVTSGLGGRFPEGYPVAVVSSVKLDTQRAYTVIQARPTAGLQRLRYLLLLWGADRNGANPM

TPEEVHRVANERLMQMMP

>gi|30064580|ref|NP_838751.1| rod shape-determining protein MreD [Shigella flexneri 2a str. 2457T]

MASYRSQGRWVIWLSFLIALLLQIMPWPDNLIVFRPNWVLLILLYWILALPHRVNVGTGFVMGAILDLIS

GSMLGVRVLAMSIIAYLVALKYQLFRNLALWQQALVVMLLSLVVDIIVFWAEFLVINVSFRPEVFWSSVV

NGVLWPWIFLLMRKVRQQFAVQ

>gi|30064579|ref|NP_838750.1| Maf-like protein [Shigella flexneri 2a str. 2457T]

MTSLYLASGSPRRQELLAQLGVTFERIVTGIEEQRQPQESAQQYVVRLAREKAQAGVAQTAQDLPVLGAD

IIVILNGEVLEKPRDAEHAAQMLRKLSGQTHQVMTAVALADSQHILDCLVVTDVTFRTLTDEDIAGYVAS

GEPLDKAGAYGIQGLGGCFVRKINGSYHAVVGLPLVETYELLSNFNALREKRDKHDG

>gi|30064578|ref|NP_838749.1| ribonuclease G [Shigella flexneri 2a str. 2457T]

MTAELLVNVTPSETRVAYIDGGILQEIHIEREARRGIVGNIYKGRVSRVLPGMQAAFVDIGLDKAAFLHA

SDIMPHTECVAGEEQKQFTVRDISELVRQGQDLMVQVVKDPLGTKGARLTTDITLPSRYLVFMPGASHVG

VSQRIESESERERLKKVVAEYCDEQGGFIIRTAAEGVGEAELASDAAYLKRVWTKVMERKKRPQTRYQLY

GELALAQRVLRDFADAELDRIRVDSRLTYEALLEFTSEYIPEMTSKLEHYTGRQPIFDLFDVENEIQRAL

ERKVELKSGGYLIIDQTEAMTTVDINTGAFVGHRNLDDTIFNTNIEATQAIARQLRLRNLGGIIIIDFID

MNNEDHRRRVLHSLEQALSKDRVKTSVNGFSALGLVEMTRKRTRESIEHVLCNECPTCHGRGTVKTVETV
CYEIMREIVRVHHAYDSDRFLVYASPAVAEALKGEESHSLAEVEIFVGKQVKVQIEPLYNQEQFDVVMM

>gi|30064577|ref|NP_838748.1| hypothetical protein S3500 [Shigella flexneri 2a str. 2457T]
MDNLTAHITRENPGWQFSIPDTRITMDGKPWPSGALTLAWIPEQDVGGKDNKRSDELRIRASNLELAGLE
GIRPLAAKLSPALGDVWRSTQPSGKINTLALDIPLQAADKTRFQASWSDLAWKQWKLLPGAEHFSGTLSG
SVENGLLTASMKQAKMPYETVFRAPLEIADGQATISWLNNDKGFQLDGRNIDVKAKAVHARGGFRYLQPA
NDEPWLGILAGISTDDGSQAWRYFPENLMGKDLVDYLSGAIQGGEADNATLVYGGNPQLFPYKHNEGQFE
VLVPLRNAKFAFQPDWPALTNLDIELDFINDGLWMKTDGVNLGGVRASNLTAVIPDYSKEKLLIDADIKG
PGKAVGPYFDETPLKDSLGATLQELQLDGDVNARLHLDIPLNGELVTAKGEVTLRNNSLFIKPLDSILKN
LSGKFSFINGDLQSEPLTASWFNQPLNVDFSTKEGAKAYQVAVNLNGNWQPAKTGVLPEAVNEALSGSVA
WDGKVGIDLPYHAGATYNVELNGDLNNVSSHLPSPLAKPAGEPLAVNVKVDGNLNSFELTGQAGADNHFN
SRWLLGQKLTLDRAIWAADSKTLPPLPEQSGVELNMPPMNGAEWLALFQKGAAESVGGAASFPQHITLRT
PMLSLGNQQWNNLSIVSQPTANGTLVEAQGREINATLAMRNNAPWLANIKYLYYNPSVAKTRGDSTPSSP
FPTTERINFRGWPDAQIRCTECWFWGQKFGRIDSDITISGDTLTLTNGLIDTGFSRLTADGEWVNNPGNE
RTSLKGKLRGQKIDAAAEFFGVTTPIRQSSFNVDYDLHWRKAPWQPDEATLNGIIHTQLGKGEITEINTG
HAGQLLRLLSVDALMRKLRFDFRDTFGEGFYFDSIRSTAWIKDGVMHTDDTLVDGLEADIAMKGSVNLVR
RDLNMEAVVAPEISATVGVAAAFAVNPIVGAAVFAASKVLGPLWSKVSILRYHISGPLDDPQINEVLRQP
RKEKAQ

>gi|30064576|ref|NP_838747.1| protease TldD [Shigella flexneri 2a str. 2457T]
MSLNLVSEQLLAANGLKHQDLFAILGQLAERRLDYGDLYFQSSYHESWVLEDRIIKDGSYNIDQGVGVRA
ISGEKTGFAYADQISLLALEQSAQAARTIVRDSGDGKVQTLGAVEHSPLYTSVDPLQSMSREEKLDILRR
VDKVAREADKRVQEVTASLSGVYELILVAATDGTLAADVRPLVRLSVSVLVEEDGKRERGASGGGGRFGY
EFFLADLDGEVRADAWAKEAVRMALVNLSAVAAPAGTMPVVLGAGWPGVLLHEAVGHGLEGDFNRRGTSV

FSGQVGELVASELCTVVDDGTMVDRRGSVAIDDEGTPGQYNVLIENGILKGYMQDKLNARLMGMTPTGNG

RRESYAHLPMPRMTNTYMLPGKSTPQEIIESVEYGIYAPNFGGGQVDITSGKFVFSTSEAYLIENGKVTK

PVKGATLIGSGIETMQQISMVGNDLKLDNGVGVCGKEGQSLPVGVGQPTLKVDNLTVGGTA

>gi|30064575|ref|NP_838746.1| DNA-binding transcriptional regulator [Shigella flexneri 2a str. 2457T]

MSVFAKVVEFGSFTAAARQLQMSVSSISQTVSKLEDELQVKLLNRSTRSIGLTEAGRIYYQGCRRMLHEV

QDVHEQLYAFNNTPIGTLRIGCSSTMAQNVLAGLTAKMLKEYPGLSANLVTGIPAPDLIADGLDVVIRVG

ALQDSSLFSRRLGAMPMVVCAAKSYLTQYGIPEKPADLSSHSWLEYSVRPDNEFELIAPEGISTRLIPQG

RFVTNDPMTLVRWLTAGAGIAYVPLMWVINEINRGELEILLPRYQSDPRPVYALYTEKDKLPLKVQVVIN

SLTDYFVEVGKLFQEMHGRGKEK

>gi|30064573|ref|NP_838744.1| p-hydroxybenzoic acid efflux subunit AaeA [Shigella flexneri 2a str. 2457T]

MKTLIRKFSRTAITVVLVILAFIAIFNAWVYYTESPWTRDARFSADVVAIAPDVSGLITQVNVHDNQLVK

KGQILFTIDQPRYQKALEEAQADVAYYQVLAQEKRQEAGRRNRLGVQAMSREEIDQANNVLQTVLHQLAK

AQATRDLAKLDLERTVIRAPADGWVTNLNVYTGEFITRGSTAVALVKQNSFYVLAYMEETKLEGVRPGYR

AEITPLGSNKVLKGTVDSVAAGVTNASSTRDDKGMATIDSDLEWVRLAQRVPVRIRLDNQQENIWPAGTT

ATVVVTGKQDRDESQDSFFRKMAHRLREFG

>gi|30064572|ref|NP_838743.1| p-hydroxybenzoic acid efflux subunit AaeB [Shigella flexneri 2a str. 2457T]

MGIFSIANQHIRFAVKLATAIVLALFVGFHFQLETPRWAVLTAAIVAAGPAFAAGGEPYSGAIRYRGFLR

IIGTFIGCIAGLVIIIAMIRAPLLMILVCCIWAGFCTWISSLVRIENSYAWGLAGYTALIIVITIQPEPL

LTPQFAVERCSEIVIGIVCAIMADLLFSPRSIKQEVDRELESLLVAQYQLMQLCIKHGDGEVVDKAWGDL

VRRTTALQGMRSNLNMESSRWARANRRLKAINTLSLTLITQSCETYLIQNTRPELITDTFREFFDTPVET

AQDVHKQLKRLRRVIAWTGERETPVTIYSWVAAATRYQLLKRGVISNTKINATEEEILQGEPEVKVESAE

RHHAMVNFWRTTLSCILGTLFWLWTGWTSGSGAMVMIAVVTSLAMRLPNPRMVAIDFIYGTLAALPLGLL

YFLVIIPNTQQSMLLLCISLAVLGFFLGIEVQKRRLGSMGALASTINIIVLDNPMTFHFSQFLDSALGQI

VGCVLAFTVILLVRDKSRDRTGRVLLNQFVSAAVSAMTTNVARRKENHLPALYQQLFLLMNKFPGDLPKF

RLALTMIIAHQRLRDAPIPVNEDLSAFHRQMRRTADHVISARSDDKRRRYFGQLLEELEIYQEKLRIWQA

PPQVTEPVHRLAGMLHKYQHALTDS

>gi|30064571|ref|NP_838742.1| hypothetical protein S3494 [Shigella flexneri 2a str. 2457T]

MNIYAFDFDEIESQEDFYRDFSQTFGLAKDKVRDLDSLWDVLMNDVLPLPLEIEFVHLGEKTRRRFGALI

LLFDEAEEELEGHLRFNVRH

>gi|30064570|ref|NP_838741.1| hypothetical protein S3493 [Shigella flexneri 2a str. 2457T]

MGHETKAQLKDYVEVKIMKIKTTVAALSVLSVLSFGAFAADSIDAAQAQNREAIGTVSVSGVASSPMDIR

EMLNKKAEEKGATAYQITEARSGDTWHATAELYK

>gi|30064569|ref|NP_838740.1| arginine repressor [Shigella flexneri 2a str. 2457T]

MRSSAKQEELVKAFKALLKEEKFSSQGEIVAALQEQGFDNINQSKVSRMLTKFGAVRTRNAKMEMVYCLP

AELGVPTTSSPLKNLVLDIDYNDAVVVIHTSPGAAQLIARLLDSLGKAEGILGTIAGDDTIFTTPANGFT

VKDLYEAILELFDQEL

>gi|30064568|ref|NP_838739.1| malate dehydrogenase [Shigella flexneri 2a str. 2457T]

MKVAVLGAAGGIGQALALLLKTQLPSGSELSLYDIAPVTPGVAVDLSHIPTAVKIKGFSGEDATPALEGA

DVVLISAGVARKPGMDRSDLFNVNAGIVKNLVQQVAKNCPKACIGIITNPVNTTVAIAAEVLKKAGVYDK

NKLFGVTTLDIIRSNTFVAELKGKQPGEVEVPVIGGHSGVTILPLLSQVPGVSFTEQEVADLTKRIQNAG

TEVVEAKAGGGSATLSMGQAAARFGLSLVRALQGEQGVVECAYVEGDGQYARFFSQPLLLGKNGVEERKS

IGTLSAFEQNALEGMLDTLKKDIALGKEFVNK

>gi|30064567|ref|NP_838738.1| serine endoprotease [Shigella flexneri 2a str. 2457T]

MFVKLLRSVAIGLIVGAILLVAMPSLRSLNPLSTPQFDSTDETPASYNLAVRRAAPAVVNVYNRGLNTNS

HNQLEIRTLGSGVIMDQRGYIITNKHVINDADQIIVALQDGRVFEALLVGSDSLTDLAVLKINATGGLPT

IPINARRVPHIGDVVLAIGNPYNLGQTITQGIISATGRIGLNPTGRQNFLQTDASINHGNSGGALVNSLG

ELMGINTLSFDKSNDGETPEGIGFAIPFQLATKIMDKLIRDGRVIRGYIGIGGREIAPLHAQSGGIDQLQ

GIVVNEVSPDGPAANAGIQVNDLIISVDNKPAISALETMDQVAEIRPGSVIPVVVMRDDKQLTLQVTIQE

YPATN

>gi|30064566|ref|NP_838737.1| cytochrome d ubiquinol oxidase subunit III [Shigella flexneri 2a str. 2457T]

MTWEYALIGLVVGIIIGAVAMRFGNRKLRQQQALQYELEKNKAELDEYREELVSHFARSAELLDTMAHDY

RQLYQHMAKSSSSLLPELSAEANPFRNRLAESEASNDQAPVQVPRDYSEGASGLLRTGAKRD

>gi|30064565|ref|NP_838736.1| hypothetical protein S3487 [Shigella flexneri 2a str. 2457T]

MQSVTPTSQYLKALNEGSHQPDDVQKEAVSRLEIIYQELINSRPPAPRTSGLMARVGKLWGKREDTKHTP

VRGLYMWGGVGRGKTWLMDLFYQSLPGERKQRLHFHRFMLRVHEELTALQGQTDPLEIIADRFKAETDVL

CFDEFFVSDITDAMLLGGLMKALFTRGITLVATSNIPPDELYRNGLQRARFLPAIDAIKQHCDVMNVDAG

VDYRLRTLTQAHLWLSPLNDETRAQMDKLWLALAGVKRENSPTLEINHRPLATMGVENQTLAVSFTTLCV

DARSQHDYIALSRLFHTVMLFDVPVMTRLMESEARRFIALVDEFYERHVKLVVSAEVPLYEIYQGERLKF

EFQRCLSRLQEMQSEEYLKREHLAG

>gi|30064564|ref|NP_838735.1| 50S ribosomal protein L13 [Shigella flexneri 2a str. 2457T]

MKTFTAKPETVKRDWYVVDATGKTLGRLATELARRLRGKHKAEYTPHVDTGDYIIVLNADKVAVTGNKRT

DKVYYHHTGHIGGIKQATFEEMIARRPERVIEIAVKGMLPKGPLGRAMFRKLKVYAGNEHNHAAQQPQVL

DI

>gi|30064563|ref|NP_838734.1| 30S ribosomal protein S9 [Shigella flexneri 2a str. 2457T]

MAENQYYGTGRRKSSAARVFIKPGNGKIVINQRSLEQYFGRETARMVVRQPLELVDMVEKLDLYITVKGG

GISGQAGAIRHGITRALMEYDESLRSELRKAGFVTRDARQVERKKVGLRKARRRPQFSKR

>gi|30064562|ref|NP_838733.1| stringent starvation protein A [Shigella flexneri 2a str. 2457T]

MAVAANKRSVMTLFSGPTDIYSHQVRIVLAEKGVSFEIEHVEKDNPPQDLIDLNPNQSVPTLVDRELTLW

ESRIIMEYLDERFPHPPLMPVYPVARGESRLYMHRIEKDWYTLMNTIINGSASEADAARKQLREELLAIA

PVFGQKPYFLSDEFSLVDCYLAPLLWRLPQLGIEFSGPGAKELKGYMTRVFERDSFLASLTEAEREMRLG

RS

>gi|30064561|ref|NP_838732.1| ClpXP protease specificity-enhancing factor [Shigella flexneri 2a str. 2457T]

MDLSQLTPRRPYLLRAFYEWLLDNQLTPHLVVDVTLPGVQVPMEYARDGQIVLNIAPRAVGNLELANDEV

RFNARFGGIPRQVSVPLAAVLAIYARENGAGTMFEPEAAYDEDTSIMNDEEASADNETVMSVIDGDKPDH

DDDTHPDDEPPQPPRGGRPALRVVK

>gi|30064560|ref|NP_838731.1| cryptic C4-dicarboxylate transporter DcuD [Shigella flexneri 2a str. 2457T]

MFGIIISVIVLITMGYLILKNYKPQVVLAAAGVFLMMCGVWLGFGGVLDPAKSSGYLIVDIYNEILRMLS

NRIAGLGLSIMAVGGYARYMERIGASCAMVSLLSRPLKLIRSPYIILSATYVIGQIMAQFITSASGLGML

LMVTLFPTLVSLGVSRLSAVAVIATTMSIEWGILETNSIFAAQVAGMKIATYFFHYQLPVASCVIISVAI

SHFFVQRAFDKKDKNINHEQAEQKTLDNVPPLYYAILPVMPLILMLGSLFLAHVGLMKSELHLVVVMLLS

LTVTMFVEFFRKHNLRETMDDVQAFFDGMGTQFVNVVTLVVAGEIFAKGLTTIGTVDAVIRGAEHSGLGG

IGVMIIMALVIAICAIVMGSGNAPFMSFASLIPNIAAGLHVPAVVMIMPMHFATTLARAVSPITAVVVVT

SGIAGVSPFAVVKRTAIPMAVGFVVNMIATITLFY

>gi|30064559|ref|NP_838730.1| IS1 orfB [Shigella flexneri 2a str. 2457T]

MSRQCTHYGRWPQHGFTSLKKLRPQSVTSRIQPGSDVIVCAEMDEHWGYVGAKSRQRWLFYAYDRIRRTV

VAHVFGERTLATLERLLSLLSAFEVVVWMTDGWPLYESRLKGKLHVISKRYTQRIERHNLNLRQHLARLG

RKSLSFSKSVELHDKVIGHYLNIKHYQ

>gi|30064558|ref|NP_838729.1| IS1 orfA [Shigella flexneri 2a str. 2457T]

MAFISIRCPSCSATEGVVRNGKSTAGHQRYLCSHCRKTWQLQFTYTASQPGTHQKIIDMAMNGVGCRASA

RIMGVGLNTVLRHLKNSGRSR

>gi|30064557|ref|NP_838728.1| transcriptional regulator NanR [Shigella flexneri 2a str. 2457T]

MNAFDSQTEDSSPAIGRNLRSRPLARKKLSEMVEEELEQMIRRREFGEGEQLPSERELMAFFNVGRPSVR

EALAALKRKGLVQINNGERARVSRPSADTIIGELSGMAKDFLSHPGGIAHFEQLRLFFESSLVRYAAEHA

TDEQIDLLAKALEINSQSLDNNAAFIRSDVDFHRVLAEIPGNPIFMAIHVALLDWLIAARPTVTDQALHE

HNNVSYQQHIAIVDAIRRHDPDEADRALQSHLNSVSATWHAFGQTTNKKK

>gi|30064556|ref|NP_838727.1| N-acetylneuraminate lyase [Shigella flexneri 2a str. 2457T]

MATNLRGVMAALLTPFDQQQALDKASLRRLVQFNIQQGIDGLYVGGSTGEAFVQSLSEREQVLEIVAEEA

KGKIKLIAHVGCVSTAESQQLAASAKRYGFDAVSAVTPFYYPFSFEEHCDHYRAIIDSADGLPMVVYNIP

ALSGVKLTLDQINTLVTLPGVGALKQTSGDLYQMEQIRREHPDLVLYNGYDEIFASGLLAGADGGIGSTY

NIMGWRYQGIVKALKEGDIQTAQKLQTECNKVIDLLIKTGVFRGLKTVLHYMDVVSVPLCRKPFGPVDEK

YLPELKALAQQLMQERG

>gi|30064554|ref|NP_838725.1| N-acetylmannosamine-6-phosphate 2-epimerase [Shigella flexneri 2a str. 2457T]

MSLLAQLDQKIAANGGLIVSCQPVPDSPLDKPEIVAAMALAAEQAGAVAIRIEGVANLQATRAVVSVPII

GIVKRDLEDSPVRITAYIEDVDALAQAGADIIAIDGTDRPRPVPVETLLARIHHHGLLAMTDCSTPEDGL
ACQKLGAEIIGTTLSGYTTPETPEEPDLALVKTLSEAGCRVIAEGRYNTPAQAADAMRHGAWAVTVGSAI
TRLEHICQWYNTAMKKAVL

>gi|30064552|ref|NP_838723.1| hypothetical protein S3474 [Shigella flexneri 2a str. 2457T]
MMMGEVQSLPSAGLHPALQDALTLALAARPQEKAPGRYELQGDNIFMNVMTFNTQSPVEKKAELHEQYID
IQLLLNGEERILFGMAGTARQCEEFHHEDDYQLCSAIENEQTIILKPGMFAVFMPGEPHKPGCVVGEPGE
IKKVVVKVKADLMA

>gi|30064551|ref|NP_838722.1| IS600 orf [Shigella flexneri 2a str. 2457T]
MAHIRTRETYGTRRLQTELAENGIIVGRDRLARLRKELRLRCKQKRKFRATTNPNHNLPVAPNLLNQTFA
PTAPNQVWVADLTYVATQEGWLYLAGIKDVYTCEIVGYAMGERMTKELTGKALFMALRSQRPPAGLIHHS
DRGSQYCAYDYRVIQEQSGLKTSMSRKGNCYDNAPMESFWGTLKNESLSHYRFNNRDEAISVIREYIEIF
YNRQRRHSRLGNISPAAFRENIIRWLLKKRTNGSVRYCQYTSKVAMIYIEQLELIHKSGDVLYPVKITRK
SSGKTAFHLVPFGLNKTHDLLEVEDASEAIRLVIDERHSIRCSTLTATITNKKGKRIKRTGIYSIKGVNI
KEYNVR

>gi|30064550|ref|NP_838721.1| glutamate synthase [Shigella flexneri 2a str. 2457T]
MSQNVYQFIDLQRVDPPKKPLKIRKIEFVEIYEPFSEGQAKAQADRCLSCGNPYCEWKCPVHNYIPNWLK
LANEGRIFEAAELSHQTNTLPEVCGRVCPQDRLCEGSCTLNDEFGAVTIGNIERYINDKAFEMGWRPDMS
GVKQTGKKVAIIGAGPAGLACADVLTRNGVKAVVFDRHPEIGGLLTFGIPAFKLEKEVMTRRREIFTGMG
IEFKLNTEVGRDVQLDDLLSDYDAVFLGVGTYQSMRGGLENEDADGVYAALPFLIANTKQLMGFGETRDE
PFVSMEGKRVVVLGGGDTAMDCVRTSVRQGAKHVTCAYRRDEENMPGSRREVKNAREEGVEFKFNVQPLG
IEVNGNGKVSGVKMVRTEMGEPDAKGRRRAEIVAGSEHIVPADAVIMAFGFRPHNMEWLAKHSVELDSQG
RIIAPEGNDNAFQTSNPKIFAGGDIVRGSDLVVTAIAEGRKAADGIMNWLEV

>gi|30064548|ref|NP_838719.1| hypothetical protein S3469 [Shigella flexneri 2a str. 2457T]

MQLQKLVNMFGGDLTRRYGQKVHKLTLHGGFSCPNRDGTIGRGGCTFCNVASFADEAQQHRSIAEQLAHQ

ANLVNRAKRYLAYFQAYTSTFAEVQVLRSMYQQAVSQANIVGLCVGTRPDCVPDAVLDLLCEYKDQGYEV

WLELGLQTAHDKTLHRINRGHDFACYQRTTQLARQRGLKVCSHLIVGLPGEGQAECLQTLERVVETSVDG

IKLHPLHIVKGSIMAKAWEAGRLNGIELEDYTLTAGEMIRHTPPEVIYHRISASARRPTLLAPLWCENRW

TGMVELDRYLNEHGVQGSALGRPWLPPTE

>gi|30064547|ref|NP_838718.1| aerobic respiration control sensor protein ArcB [Shigella flexneri 2a str. 2457T]

MKQIRLLAQYYVDLMMKLGLVRFSMLLALALVVLAIVVQMAVTMVLHGQVESIDVIRSIFFGLLITPWAV

YFLSVVVEQLEESRQRLSRLVQKLEEMRERDLSLNVQLKDNIAQLNQEIAVREKAEAELQETFGQLKIEI

KEREETQIQLEQQSSFLRSFLDASPDLVFYRNEDKEFSGCNRAMELLTGKSEKQLVHLKPADVYSPEAAA

KVIETDEKVFRHNVSLTYEQWLDYPDGRKACFEIRKVPYYDRVGKRHGLMGFGRDITERKRYQDALERAS

RDKTTFISTISHELRTPLNGIVGLSRILLDTELTAEQEKYLKTIHVSAVTLGNIFNDIIDMDKMERRKVQ

LDNQPVDFTSFLADLENLSALQAQQKGLRFNLEPTLPLPHQVITDGTRLRQILWNLISNAVKFTQQGQVT

VRVRYDEGDMLHFEVEDSGIGIPQDELDKIFAMYYQVKDSHGGKPATGTGIGLAVSRRLAKNMGGDITVT

SEQGKGSTFTLTIHAPSVAEEVDDAFDEDDMPLPALNVLLVEDIELNVIVARSVLEKLGNSVDVAMTGKA

ALEMFKPGEYDLVLLDIQLPDMTGLDISRELTKRYPREDLPPLVALTANVLKDKQEYLNAGMDDVLSKPL

SVPALTAMIKKFWDTQDDEESTVTTEENSKSEALLDIPMLEQYLELVGPKLITDGLAVFEKMMPGYVSVL

ESNLTAQDKKGIVEEGHKIKGAAGSVGLRHLQQLGQQIQSPDLPAWEDNVGEWIEEMKEEWRHDVEVLKA

WVAKATKK

>gi|30064546|ref|NP_838717.1| isoprenoid biosynthesis protein with amidotransferase-like domain [Shigella flexneri 2a str. 2457T]

MKKIGVILSGCGVYDGSEIHEAVLTLLAISRSGAQAVCFAPDKQQVDVINHLTGEAMTETRNVLIEAARI

TRGEIRPLAQADAAELDALIVPGGFGAAKNLSNFASLGSECTVDRELKALAQAMHQAGKPLGFMCIAPAM

LPKIFDFPLRLTIGTDIDTAEVLEEMGAEHVPCPVDDIVVDEDNKIVTTPAYMLAQNIAEAASGIDKLVS

RVLVLAE

>gi|30064545|ref|NP_838716.1| hypothetical protein S3465 [Shigella flexneri 2a str. 2457T]

MIRLSEQSPLGTGRHRKCYAHPEDAQRCIKIVYHRGDGGDKEIRRELKYYAHLGRRLKDWSGIPRYHGTV

ETDCGTGYVYDVIADFDGKPSITLTEFAEQCRYEEDIAQLRQLLKQLKRYLQDNRIVTMSLKPQNILCHR

ISESEVIPVVCDNIGESTLIPLATWSKWCCLRKQERLWKRFIAQPALAIALQKDLQPRESKTLALTSREA

>gi|30064544|ref|NP_838715.1| phosphohistidinoprotein-hexose phosphotransferase component of N-regulated PTS system (Npr) [Shigella flexneri 2a str. 2457T]

MTVKQTVEITNKLGMHARPAMKLFELMQGFDAEVLLRNDEGTEAEANSVIALLMLDSAKGRQIEVEATGP

QEEEALAAVIALFNSGFDED

>gi|30064543|ref|NP_838714.1| hypothetical protein S3463 [Shigella flexneri 2a str. 2457T]

MVLMIVSGRSGSGKSVALRALEDMGFYCVDNLPVVLLPDLARTLADREISAAVSIDVRNMPESPEIFEQA

MSNLPDAFSPQLLFLDADRNTLIRRYSDTRRLHPLSSKNLSLESAIDKESDLLEPLRSRADLIVDTSEMS

VHELAEMLRTRLLGKRERELTMVFESFGFKHGIPIDADYVFDVRFLPNPHWDPKLRPMTGLDKPVAAFLD

RHTEVHNFIYQTRSYLELWLPMLETNNRSYLTVAIGCTGGKHRSVYIAEQLADYFRSRGKNVQSRHRTLE

KRKP

>gi|30064542|ref|NP_838713.1| PTS IIA-like nitrogen-regulatory protein PtsN [Shigella flexneri 2a str. 2457T]

MTNNDTTLQLSSVLNRECTRSRVHCQSKKRALEIISELAAKQLSLPPQVVFEAILTREKMGSTGIGNGIA

IPHGKLEEDTLRAVGVFVQLETPIAFDAIDNQPVDLLFALLVPADQTKTHLHTLSLVAKRLADKTICRRL

RAAQSDEELYQIITDTEGTPDEA

>gi|30064541|ref|NP_838712.1| sigma(54) modulation protein [Shigella flexneri 2a str. 2457T]

MQLNITGNNVEITEALREFVTAKFAKLEQYFDRINQVYVVLKVEKVTHTSDATLHVNGGEIHASAEGQDM

YAAIDGLIDKLARQLTKHKDKLKQH

>gi|30064540|ref|NP_838711.1| RNA polymerase factor sigma-54 [Shigella flexneri 2a str. 2457T]

MKQGLQLRLSQQLAMTPQLQQAIRLLQLSTLELQQELQQALESNPLLEQIDTHEEIDTRETQDSETLDTA

DALEQKEMPEELPLDASWDTIYTAGTPSGTSGDYIDDELPVYQGETTQTLQDYLMWQVELTPFSDTDRAI

ATSIVDAVDETGYLTVPLEDILESMGDEEIDIDEVEAVLKRIQRFDPVGVAAKDLRDCLLIQLSQFDKTT

PWLEEARLIISDHLDLLANHDFRTLMRVTRLKEDVLKEAVNLIQSLDPRPGQSIQTGEPEYVIPDVLVRK

HNGHWTVELNSDSIPRLQINQHYASMCNNARNDGDSQFIRSNLQDAKWLIKSLESRNDTLLRVSRCIVEQ

QQAFFEQGEEYMKPMVLADIAQAVEMHESTISRVTTQKYLHSPRGIFELKYFFSSHVNTEGGGEASSTAI

RALVKKLIAAENPAKPLSDSKLTSLLSEQGIMVARRTVAKYRESLSIPPSNQRKQLV

>gi|30064539|ref|NP_838710.1| ABC transporter ATP-binding protein [Shigella flexneri 2a str. 2457T]

MATLTAKNLAKAYKGRRVVEDVSLTVNSGEIVGLLGPNGAGKTTTFYMVVGIVPRDAGNIIIDDDDISLL

PLHARARRGIGYLPQEASIFRRLSVYDNLMAVLQIRDDLSAEQREDRANELMEEFHIEHLRDSMGQSLSG

GERRRVEIARALAANPKFILLDEPFAGVDPISVIDIKRIIEHLRDSGLGVLITDHNVRETLAVCERAYIV

SQGHLIAHGTPTEILQDEHVKRVYLGEDFRL

>gi|30064538|ref|NP_838709.1| lipopolysaccharide transport periplasmic protein LptA [Shigella flexneri 2a str. 2457T]

MKFKTNKLSLNLVLASSLLAASIPAFAVTGDTDQPIHIESDQQSLDMQGNVVTFTGNVIVTQGTIKINAD

KVVVTRPGGEQGKEVIDGYGKPATFYQMQDNGKPVEGHASQMHYELAKDFVVLTGNAYLQQVDSNIKGDK

ITYLVKEQKMQAFSDKGKRVTTVLVPSQLQDKNNKGQTPAQKKGN

>gi|30064537|ref|NP_838708.1| hypothetical protein S3457 [Shigella flexneri 2a str. 2457T]

MSKARRWVIIVLSLAVLVMIGINMAEKDDTAQVVVNNNDPTYKSEHTDTLVYNPEGALSYRLIAQHVEYY

SDQAVSWFTQPVLTTFDKDKIPTWSVKADKAKLTNDRMLYLYGHVEVNALVPDSQLRRITTDNAQINLVT

QDVTSEDLVTLYGTTFNSSGLKMRGNLRSKNAELIEKVRTSYEIQNKQTQP

>gi|30064536|ref|NP_838707.1| 3-deoxy-D-manno-octulosonate 8-phosphate phosphatase [Shigella flexneri 2a str. 2457T]

MSKAGASLATCYGPVSADVMAKAENIRLLILDVDGVLSDGLIYMGNNGEELKAFNVRDGYGIRCALTSDI

EVAIITGRKAKLVEDRCATLGITHLYQGQSNKLIAFSDLLEKLAIAPENVAYVGDDLIDWPVMEKVGLSV

AVADAHPLLIPRADYVTRIAGGRGAVREVCDLLLLAQGKLDEAKGQSI

>gi|30064535|ref|NP_838706.1| D-arabinose 5-phosphate isomerase [Shigella flexneri 2a str. 2457T]

MSHVELQPGFDFQQAGKEVLAIERECLAELDQYINQNFTLACEKMFWCKGKVVVMGMGKSGHIGRKMAAT

FASTGTPSFFVHPSEAAHGDLGMVTPQDVVIAISNSGESSEITALIPVLKRLHVPLICITGRPESSMARA

ADVHLCVKVAKEACPLGLAPTSSTTATLVMGDALAVALLKARGFTAEDFALSHPGGALGRKLLLRVNDIM

HTGDEIPHVKKTASLRDALLEVTRKNLGMTVICDDNMMIEGIFTDGDLRRVFDMGVDVRQLSIADVMTPG

GIRVRPGILAVEALNLMQSRHITSVMVADGDHLLGVLHMHDLLRAGVV

>gi|30064534|ref|NP_838705.1| calcium/sodium:proton antiporter [Shigella flexneri 2a str. 2457T]

MLLATALLIVGLLLVVYSADRLVFAASILCRTFGIPPLIIGMTVVSIGTSLPEIIVSLAASLHEQRDLAV

GTALGSNIINILLILGLAALVRPFTVHSDVLRRELPLMLLVSVVAGSVLYDGQLSRSDGIFLLFLAVLWL

LFIVKLARQGTDSLTREQLAELPRDGGLPVAFLWLGIALIIMPVATRMVVDNATVLANYFAISELTMALT

AIAIGTSLPELATAIAGVRKGENDIAVGNIIGANIFNIVIVLGLPALITPGEIDPLAYSRDYSVMLLVSI

IFALLCWRRSPQPGRGVGVLLTGGFIVWLAMLYWLSPILVE

>gi|30064533|ref|NP_838704.1| ABC transporter ATP-binding protein [Shigella flexneri 2a str. 2457T]

MEQSVANLVDMRDVSFTRGNRCIFDNISLTVPRGKITAIMGPSGIGKTTLLRLIGGQIAPDHGEILFDGE

NIPAMSRSRLYTVRKRMSMLFQSGALFTDMNVFDNVAYPLREHTQLPAPLLHSTVMMKLEAVGLRGAAKL

MPSELSGGMARRAALARAIALEPDLIMFDEPFVGQDPITMGVLVKLISELNSALGVTCVVVSHDVPEVLS

IADHAWILADKKIVAHGSAQALQTNPDPRVRQFLDGIADGPVPFRYPAGDYHADLLPGS

>gi|30064532|ref|NP_838703.1| integral membrane protein [Shigella flexneri 2a str. 2457T]

MLLNALASLGHKGIKTLRTFGRAGLMLFNALVGKPEFRKHAPLLVRQLYNVGVLSMLIIVVSGVFIGMVL

GLQGYLVLTTYSAETSLGMLVALSLLRELGPVVAALLFAGRAGSALTAEIGLMRATEQLSSMEMMAVDPL

RRVISPRFWAGVISLPLLTVIFVAVGIWGGSLVGVSWKGIDSGFFWSAMQNAVDWRMDLVNCLIKSVVFA

ITVTWISLFNGYDAIPTSAGISRATTRTVVHSSLAVLGLDFVLTALMFGN

>gi|30064531|ref|NP_838702.1| cytoplasmic membrane protein [Shigella flexneri 2a str. 2457T]

MQTKKNEIWVGIFLLAALLAALFVCLKAANVTSIRTESTYTLYATFDNIGGLKARSPVSIGGVVVGRVAD

ITLDPKTYLPRVTLEIEQRYNHIPDTSSLSIRTSGLLGEQYLALNVGFEDPELGTAILKDGDTIQDTKSA

MVLEDLIGQFLYGSKGDDNKNSGDAPAAAPGNNETTEPVGTTK

>gi|30064530|ref|NP_838701.1| hypothetical protein S3450 [Shigella flexneri 2a str. 2457T]

MFKRLMMVALLVIAPLSAATAADQTNPYKLMDEAAQKTFDRLKNEQPQIRANPDYLRTIVDQELLPYVQV

KYAGALVLGQYYKSATPAQREAYFAAFREYLKQAYGQALAMYHGQTYQIAPEQPLGDKTIVPIRVTIIDP

NGRPPVRLDFQWRKNSQTGNWQAYDMIAEGVSMITTKQNEWGTLLRTKGIDGLTAQLKSISQQKITLEEK

K

>gi|30064529|ref|NP_838700.1| hypothetical protein S3449 [Shigella flexneri 2a str. 2457T]

MGNAAAYQRYRRPDCATEIDFSTENHSGREKIMSESLSWMQTGDTLALSGELDQDVLLPLWEMREEAVKG

ITCIDLSRVSRVDTGGLALLLHLIDLAKKQGNNVTLQGVNDKVYTLAKLYNLPADVLPR

>gi|30064528|ref|NP_838699.1| hypothetical protein S3448 [Shigella flexneri 2a str. 2457T]

MIEDPMENNEIQSVLMNALSLQEVHVSGDGSHFQVIAVGELFDGMSRVKKQQTVYGPLMEYIADNRIHAV

SIKAYTPAEWARDRKLNGF

>gi|30064527|ref|NP_838698.1| UDP-N-acetylglucosamine 1-carboxyvinyltransferase [Shigella flexneri 2a str. 2457T]

MDKFRVQGPTKLQGEVTISGAKNAALPILFAALLAEEPVEIQNVPKLKDVDTSMKLLSQLGAKVERNGSV

HIDARDVNVFCAPYDLVKTMRASIWALGPLVARFGQGQVSLPGGCTIGARPVDLHISGLEQLGATIKLEE

GYVKASVDGRLKGAHIVMDKVSVGATVTIMCAATLAEGTTIIENAAREPEIVDTANFLITLGAKISGQGT

DRIVIEGVERLGGGVYRVLPDRIETGTFLVAAAISRGKIICRNAQPDTLDAVLAKLRDAGADIEVGEDWI

SLDMHGKRPKAVNVRTAPHPAFPTDMQAQFTLLNLVAEGTGFITETVFENRFMHVPELSRMGAHAEIESN

TVICHGVEKLSGAQVMATDLRASASLVLAGCIAEGTTVVDRIYHIDRGYERIEDKLRALGANIERVKGE

>gi|30064526|ref|NP_838697.1| DNA-binding transcriptional regulator Nlp [Shigella flexneri 2a str. 2457T]

MESNFIDWHPADIIAGLRKKGTSMAAESRRNGLSSSTLANALSRPWPKGEMIIAKALGTDPWVIWPSRYH

DPQTHEFIDRTQLMRSYTKPKK

>gi|30064525|ref|NP_838696.1| octaprenyl diphosphate synthase [Shigella flexneri 2a str. 2457T]

MNLEKINELTAQDMAGVNAAILEQLNSDVQLINQLGYYIVSGGGKRIRPMIAVLAARAVGYEGNAHVTIA

ALIEFIHTATLLHDDVVDESDMRRGKATANAAFGNAASVLVGDFIYTRAFQMMTSLGSLKVLEVMSEAVN

VIAEGEVLQLMNVNDPDITEENYMRVIYSKTARLFEAAAQCSGILAGCTPEEEKGLQDYGRYLGTAFQLI

DDLLDYNADGEQLGKNVGDDLNEGKPTLPLLHAMHHGTPEQAQMIRTAIEQGNGRHLLEPVLEAMNACGS

LEWTRQRAEEEADKAIAALQVLPDTPWREALIGLAHIAVQRDR

>gi|30064524|ref|NP_838695.1| 50S ribosomal protein L21 [Shigella flexneri 2a str. 2457T]

MYAVFQSGGKQHRVSEGQTVRLEKLDIATGETVEFAEVLMIANGEEVKIGVPFVDGGVIKAEVVAHGRGE
KVKIVKFRRRKHYRKQQGHRQWFTDVKITGISA

>gi|30064523|ref|NP_838694.1| 50S ribosomal protein L27 [Shigella flexneri 2a str. 2457T]

MAHKKAGGSTRNGRDSEAKRLGVKRFGGESVLAGSIIVRQRGTKFHAGANVGCGRDHTLFAKADGKVKFE
VKGPKNRKFISIEAE

>gi|30064522|ref|NP_838693.1| hypothetical protein S3442 [Shigella flexneri 2a str. 2457T]

MKQQAGIGILLALTTAICWGALPIAMKQVLEVMEPPTIVFYRFLMASIGLGAILAVKKRLPPLRVFRKPR
WLILLAVATAGLFGNFILFSSSLQYLSPTASQVIGQLSPVGMMVASVFILKEKMRSTQVVGALMLLSGLV
MFFNTSLVEIFTKLTDYTWGVIFGVGAATVWVSYGVAQKVLLRRLASPQILFLLYTLCTIALFPLAKPGV
IAQLSHWQLACLIFCGLNTLVGYGALAEAMARWQAAQVSAIITLTPLFTLFFSDLLSLAWPDFFARPMLN
LLGYLGAFVVVAGAMYSAIGHRIWGGLRKHTTVVSQPRAGE

>gi|30064521|ref|NP_838692.1| GTPase ObgE [Shigella flexneri 2a str. 2457T]

MKFVDEASILVVAGDGGNGCVSFRREKYIPKGGPDGGDGGDGGDVWMEADENLNTLIDYRFEKSFRAERG
QNGASRDCTGKRGKDVTIKVPVGTRVIDQGTGETMGDMTKHGQRLLVAKGGWHGLGNTRFKSSVNRTPRQ
KTNGTPGDKRELLLELMLLADVGMLGMPNAGKSTFIRAVSAAKPKVADYPFTTLVPSLGVVRMDNEKSFV
VADIPGLIEGAAEGAGLGIRFLKHLERCRVLLHLIDIDPIDGTDPVENARIIISELEKYSQDLAAKPRWL
VFNKIDLLDKAEAEEKAKAIAEALGWEDKYYLISAASGLGVKDLCWDVMTFIIENPVVQAEEAKQPEKVE
FMWDDYHRQQLEEIAEEDDEDWDDDWDEDDEEGVEFIYKR

>gi|30064520|ref|NP_838691.1| D-alanyl-D-alanine carboxypeptidase [Shigella flexneri 2a str. 2457T]

MRFSRFIIGLTSCIAFSVQAANVDEYVTQLPAGANLALMVQKVGASAPAIDYHSQQMALPASTQKVITAL

AALIQLGPDFRFTTTLETKGNVENGVLKGDLVARFGADPTLKRQDIRNMVATLKKSGVNQIDGNVLIDTS

IFASHDKAPGWPWNDMTQCFSAPPAAAIVDRNCFSVSLYSAPKPGDMAFIRVASYYPVTMFSQVRTLPRG

SAEAQYCELDVVPSDLNRFTLTGCLPQRSEPLPLAFAVQDGASYAGAILKDELKQAGITWSGTLLRQTQV

NEPGTVVASKQSAPLHDLLKIMLKKSDNMIADTVFRMIGHARFNVPGTWRAGSDAVRQILRQQAGVDIGN

TIIADGSGLSRHNLIAPATMMQVLQYIAQHDNELNFISMLPLAGYDGSLQYRAGLHQAGVDGKVSAKTGS

LQGVYNLAGFITTASGQRMAFVQYLSGYAVKPADQRNRRIPLVRFESRLYKDIYQNN

>gi|30064519|ref|NP_838690.1| transcription elongation factor GreA [Shigella flexneri 2a str. 2457T]

MQAIPMTLRGAEKLREELDFLKSVRRPEIIAAIAEAREHGDLKENAEYHAAREQQGFCEGRIKDIEAKLS

NAQVIDVTKMPNNGRVIFGATVTVLNLDSDEEQTYRIVGDDEADFKQNLISVNSPIARGLIGKEEDDVVV

IKTPGGEVEFEVIKVEYL

>gi|30064518|ref|NP_838689.1| RNA-binding protein YhbY [Shigella flexneri 2a str. 2457T]

MNLSTKQKQHLKGLAHPLKPVVLLGSNGLTEGVLAEIEQALEHHELIKVKIATEDRETKTLIVEAIVRET

GACNVQVIGKTLVLYRPTKERKISLPR

>gi|30064517|ref|NP_838688.1| 23S rRNA methyltransferase J [Shigella flexneri 2a str. 2457T]

MTGKKRSASSSRWLQEHFSDKYVQQAQKKGLRSRAWFKLDEIQQSDKLFKPGMTVVDLGAAPGGWSQYVV

TQIGGKGRIIACDLLPMDPIVGVDFLQGDFRDELVMKALLERVGDSKVQVVMSDMAPNMSGTPAVDIPRA

MYLVELALEMCRDVLAPGGSFVVKVFQGEGFDEYLREIRSLFTKVKVRKPDSSRARSREVYIVATGRKP

>gi|30064516|ref|NP_838687.1| ATP-dependent metalloprotease [Shigella flexneri 2a str. 2457T]

MAKNLILWLVIAVVLMSVFQSFGPSESNGRKVDYSTFLQEVNNDQVREARINGREINVTKKDSNRYTTYI

PVQDPKLLDNLLTKNVKVVGEPPEEPSLLASIFISWFPMLLLIGVWIFFMRQMQGGGGKGAMSFGKSKAR

MLTEDQIKTTFADVAGCDEAKEEVAELVEYLREPSRFQKLGGKIPKGVLMVGPPGTGKTLLAKAIAGEAK

VPFFTISGSDFVEMFVGVGASRVRDMFEQAKKAAPCIIFIDEIDAVGRQRGAGLGGGHDEREQTLNQMLV
EMDGFEGNEGIIVIAATNRPDVLDPALLRPGRFDRQVVVGLPDVRGREQILKVHMRRVPLAPDIDAAIIA
RGTPGFSGADLANLVNEAALFAARGNKRVVSMVEFEKAKDKIMMGAERRSMVMTEAQKESTAYHEAGHAI
IGRLVPEHDPVHKVTIIPRGRALGVTFFLPEGDAISASRQKLESQISTLYGGRLAEEIIYGPEHVSTGAS
NDIKVATNLARNMVTQWGFSEKLGPLLYAEEEGEVFLGRSVAKAKHMSDETARIIDQEVKALIERNYNRA
RQLLTDNMDILHAMKDALMKYETIDAPQIDDLMARRDVRPPAGWEEPGASNNSGDNGSPKAPRPVDEPRT
PNPGNTMSEQLGDK

>gi|30064514|ref|NP_838685.1| phosphoglucosamine mutase [Shigella flexneri 2a str. 2457T]
MSNRKYFGTDGIRGRVGDAPITPDFVLKLGWAAGKVLARHGSRKIIIGKDTRISGYMLESALEAGLAAAG
LSALFTGPMPTPAVAYLTRTFRAEAGIVISASHNPFYDNGIKFFSIDGTKLPDAVEEAIEAEMEKEISCV
DSAELGKASRIVDAAGRYIEFCKATFPNELSLSELKIVVDCANGATYHIAPNVLRELGANVIAIGCEPNG
VNINAEVGATDVRALQARVLAEKADLGIAFDGDGDRVIMVDHEGNKVDGDQIMYIIAREGLRQGQLRGGA
VGTLMSNMGLELALKQLGIPFARAKVGDRYVLEKMQEKGWRIGAENSGHVILLDKTTTGDGIVAGLQVLA
AMARNHMSLHDLCSGMKMFPQILVNVRYTAGSGDPLEHESVKAVTAEVEAALGNRGRVLLRKSGTEPLIR
VMVEGEDEAQVTEFAHRIADAVKAV

>gi|30064513|ref|NP_838684.1| preprotein translocase subunit SecG [Shigella flexneri 2a str. 2457T]
MYEALLVVFLIVAIGLVGLIMLQQGKGADMGASFGAGASATLFGSSGSGNFMTRMTALLATLFFIISLVL
GNINSNKTNRGSEWENLSAPAKTEQTQPAAPAKPTSDIPN

>gi|30064512|ref|NP_838683.1| alkaline phosphatase I [Shigella flexneri 2a str. 2457T]
MFQPQDDQDACYDNSIHYTDSLLGQVFELLKDRRASVMYFADHGLERDPTKKNVYFHGGREASQQAYHVP
MFIWYSPVLGDGVDRTTENNIFSTAYNNYLINAWMGVTKPEQPQTLEEVIAHYKGDSRVVDANHDVFDYV
MLRKEFTEDKQGNPTPEGQG

>gi|30064511|ref|NP_838682.1| argininosuccinate synthase [Shigella flexneri 2a str. 2457T]

MTTILKHLPVGQRIGIAFSGGLDTSAALLWMRQKGAVPYAYTANLGQPDEEDYDAIPRRAMEYGAENARL

IDCRKQLVAEGIAAIQCGAFHNTTGGLTYFNTTPLGRAVTGTMLVAAMKEDGVNIWGDGSTYKGNDIERF

YRYGLLTNAELQIYKPWLDTDFIDELGGRHEMSEFMIACGFDYKMSVEKAYSTDSNMLGATHEAKDLEYL

NSSVKIVNPIMGVKFWDESVKIPAEEVTVRFEQGHPVALNGKTFSDDVEMMLEANRIGGRHGLGMSDQIE

NRIIEAKSRGIYEAPGMALLHIAYERLLTGIHNEDTIEQYHAHGRQLGRLLYQGRWFDSQALMLRDSLQR

WVASQITGEVTLELRRGNDYSILNTVSENLTYKPERLTMEKGDSVFSPDDRIGQLTMRNLDITDTREKLF

GYAKTGLLSSSATSGLPQVENMENKGQ

>gi|30064509|ref|NP_838680.1| transcription elongation factor NusA [Shigella flexneri 2a str. 2457T]

MNKEILAVVEAVSNEKALPREKIFEALESALATATKKKYEQEIDVRVQIDRKSGDFDTFRRWLVVDEVTQ

PTKEITLEAARYEDESLNLGDYVEDQIESVTFDRITTQTAKQVIVQKVREAERAMVVDQFREHEGEIITG

VVKKVNRDNISLDLGNNAEAVILREDMLPRENFRPGDRVRGVLYSVRPEARGAQLFVTRSKPEMLIELFR

IEVPEIGEEVIEIKAAARDPGSRAKIAVKTNDKRIDPVGACVGMRGARVQAVSTELGGERIDIVLWDDNP

AQFVINAMAPADVASIVVDEDKHTMDIAVEAGNLAQAIGRNGQNVRLASQLSGWELNVMTVDDLQAKHQA

EAHAAIDTFTKYLDIDEDFATVLVEEGFSTLEELAYVPMKELLEIEGLDEPTVEALRERAKNALATIAQA

QEESLGDNKPADDLLNLEGVDRDLAFKLAARGVCTLEDLAEQGIDDLADIEGLTDEKAGALIMAARNICW

FGDEA

>gi|30064508|ref|NP_838679.1| translation initiation factor IF-2 [Shigella flexneri 2a str. 2457T]

MTDVTIKTLAAERQTSVERLVQQFADAGIRKSADDSVSAQEKQTLIDHLNQKNSGPDKLTLQRKTRSTLN

IPGTGGKSKSVQIEVRKKRTFVKRDPQEAERLAAEEQARREAEESAKREAQQKAEREAAEQAKREAAEQA

KREAAEKDKVSNQQDDMTKNAQAEKARREQEAAELKRKAEEEARRKLEEEARRVAEEARRMAEENKWTDN

AEPTEDSSDYHVTTSQHARQAEDESDREVEGGRGRGRNAKAARPKKGNKHAESKADREEARAAVRGGKGG

KRKGSSLQQGFQKPAQAVNRDVVIGETITVGELANKMAVKGSQVIKAMMKLGAMATINQVIDQETAQLVA

EEMGHKVILRRENELEEAVMSDRDTGAAAEPRAPVVTIMGHVDHGKTSLLDYIRSTKVASGEAGGITQHI

GAYHVETENGMITFLDTPGHAAFTSMRARGAQATDIVVLVVAADDGVMPQTIEAIQHAKAAQVPVVVAVN

KIDKPEADPDRVKNELSQYGILPEEWGGESQFVHVSAKAGTGIDELLDAILLQAEVLELKAVRKGMASGA

VIESFLDKGRGPVATVLVREGTLHKGDIVLCGFEYGRVRAMRNELGQEVLEAGPSIPVEILGLSGVPAAG

DEVTVVRDEKKAREVALYRQGKFREVKLARQQKSKLENMFANMTEGEVHEVNIVLKADVQGSVEAISDSL

LKLSTDEVKVKIIGSGVGGITETDATLAAASNAILVGFNVRADASARKVIEAESLDLRYYSVIYNLIDEV

KAAMSGMLSPELKQQIIGLAEVRDVFKSPKFGAIAGCMVTEGVVKRHNPIRVLRDNVVIYEGELESLRRF

KDDVNEVRNGMECGIGVKNYNDVRTGDVIEVFEIIEIQRTIA

>gi|30064507|ref|NP_838678.1| ribosome-binding factor A [Shigella flexneri 2a str. 2457T]

MAKEFGRPQRVAQEMQKEIALILQREIKDPRLGMMTTVSGVEMSRDLAYAKVYVTFLNDKDEDAVKAGIK

ALQEASGFIRSLLGKAMRLRIVPELTFFYDNSLVEGMRMSNLVTSVVKHDEERRVNPDDSKED

>gi|30064506|ref|NP_838677.1| tRNA pseudouridine synthase B [Shigella flexneri 2a str. 2457T]

MSRPRRRGRDINGVLLLDKPQGMSSNDALQKVKRIYNANRAGHTGALDPLATGMLPICLGEATKFSQYLL

DSDKRYRVIARLGQRTDTSDADGQIVEERPVTFSAEQLAAALDTFRGDIEQIPSMYSALKYQGKKLYEYA

RQGIEVPREARPITVYELLFIRHEGNELELEIHCSKGTYIRTIIDDLGEKLGCGAHVIYLRRLAVSKYPV

ERIVTLEHLRELVEQAEQQDIPAAELLDPLLMPMDSPASDYPVVNLPLTSSVYFKNGNPVRTSGAPLEGL

VRVTEGENGKFIGMGEIDDEGRVAPRRLVVEYPA

>gi|30064505|ref|NP_838676.1| 30S ribosomal protein S15 [Shigella flexneri 2a str. 2457T]

MSLSTEATAKIVSEFGRDANDTGSTEVQVALLTAQINHLQGHFAEHKKDHHSRRGLLRMVSQRRKLLDYL

KRKDVARYTQLIERLGLRR

>gi|30064504|ref|NP_838675.1| polynucleotide phosphorylase/polyadenylase [Shigella flexneri 2a str. 2457T]

MRRRSGINTSAVRYCLRKRKDITLLNPIVRKFQYGQHTVTLETGMMARQATAAVMVSMDDTAVFVTVVGQ

KKAKPGQDFFPLTVNYQERTYAAGRIPGSFFRREGRPSEGETLIARLIDRPIRPLFPEGFVNEVQVIATV

VSVNPQVNPDIVAMIGASAALSLSGIPFNGPIGAARVGYINDQYVLNPTQDELKESKLDLVVAGTEAAVL

MVESEAELLSEDQMLGAVVFGHEQQQVVIQNINELVKEAGKPRWDWQPEPVNEALNARVAALAEARLSDA

YRITDKQERYAQVDVIKSETIATLLAEDETLDENELGEILHAIEKNVVRSRVLAGEPRIDGREKDMIRGL

DVRTGVLPRTHGSALFTRGETQALVTATLGTARDAQVLDELMGERTDTFLFHYNFPPYSVGETGMVGSPK

RREIGHGRLAKRGVLAVMPDMDKFPYTVRVVSEITESNGSSSMASVCGASLALMDAGVPIKAAVAGIAMG

LVKEGDNYVVLSDILGDEDHLGDMDFKVAGSREGISALQMDIKIEGITKEIMQVALNQAKGARLHILGVM

EQAINAPRGDISEFAPRIHTIKINPDKIKDVIGKGGSVIRALTEETGTTIEIEDDGTVKIAATDGEKAKH

AIRRIEEITAEIEVGRVYTGKVTRIVDFGAFVAIGGGKEGLVHISQIADKRVEKVTDYLQMGQEVPVKVL

EVDRQGRIRLSIKEATEQSQPAAAPEAPAAEQGE

>gi|30064503|ref|NP_838674.1| lipoprotein NlpI [Shigella flexneri 2a str. 2457T]

MKPFLRWCFVATALTLAGCSNTSWRKSEVLAVPLQPTLQQEVILARMEQILASRALTDDERAQLLYERGV

LYDSLGLRALARNDFSQALAIRPDMPEVFNYLGIYLTQAGNFDAAYEAFDSVLELDPTYNYAHLNRGIAL

YYGGRDKLAQDDLLAFYQDDPNDPFRSLWLYLAEQKLDEKQAKEVLKQHFEKSDKEQWGWNIVEFYLGNI

SEQTLMERLKADATDNTSLAEHLSETNFYLGKYYLSLGDLDSATALFKLAVANNVHNFVEHRYALLELSL

LGQDQDDLAESDQQ

>gi|30064501|ref|NP_838672.1| tryptophan permease [Shigella flexneri 2a str. 2457T]

MATLTTTQTSPSLLGGVVIIGGTIIGAGMFSLPVVMSGAWFFWSMAALIFTWFCMLHSGLMILEANLNYR

IGSSFDTITKDLLGKGWNVVNGISIAFVLYILTYAYISASGSILHHTFAEMSLNVPARAAGFGFALLVAF

VVWLSTKAVSRMTAIVLGAKVITFFLTFGSLLGHVQPATLFNVAESNASYAPYLLMTLPFCLASFGYHGN

VPSLMKYYGKDPKTIVKCLVYGTLMALALYTIWLLATMGNIPRPEFIGIAEKGGNIDVLVQALSGVLNSR

SLDLLLVVFSNFAVASSFLGVTLGLFDYLADLFGFDDSAVGRLKTALLTFAPPVVGGLLFPNGFLYAIGY

AGLAATIWAAIVPALLARASRKRFGSPKFRVWGGKPMIALILVFGVGNALVHILSSFNLLPVYQ

>gi|30064500|ref|NP_838671.1| hypothetical protein S3418 [Shigella flexneri 2a str. 2457T]

MTDKTIAFSLLDLAPIPEGSSAREAFSHSLDLARLAEKRGYHRYWLAEHHNMTGIASAATSVLIGYLAAN

TTTLHLGSGGVMLPNHLPLVIAEQFGTLNTLYPGRIDLGLGRAPGSDQRTMMALRRHMSGDIDNFPRDVA

ELVDWFDARDPNPHVRPVPGYGEKIPVWLLGSSLYSAQLAAQLGLPFAFASHFAPDMLFQALHLYRSNFK

PSARLEKPYAMVCINIIAADSNRDAEFLFTSMQQAFVKLRRGETGQLPPPIQNMDQFWSPSEQYDVQQAL

SMSLVGDKAKVRHGLQSILRETDADEIMVNGQIFDHQARLHSFELAMDVKEELLG

>gi|30064499|ref|NP_838670.1| hypothetical protein S3417 [Shigella flexneri 2a str. 2457T]

MKYSLGPVLWYWPKETLEDFYQQAAASSADVIYLGEAVCSKRRATKVGDCLEMAKSLAGSGKQIVLSTLA

LVQASSELGELKRYVENGEFLIEASDLGVVNMCAERKLPFVAGHALNCYNAVTLKILLKQGMMRWCMPVE

LSRDWLVNLLNQCDELGIRNQFEVEVLSYGHLPLAYSARCFTARSEDRPKDECETCCIKYPNGRNVLSQE

NQQVFVLNGIQTMSGYVYNLGNELASMQGLVDVVRLSPQGTDTFAMLDAFRANENGAVPLPLTANSDCNG

YWRRLAGLELQA

>gi|30064498|ref|NP_838669.1| collagenase [Shigella flexneri 2a str. 2457T]

MELLCPAGNLPALKAAIENGADAVYIGLKDDTNARHFAGLNFTEKKLQEAVSFVHQHRRKLHIAINTFAH

PDGYARWQRAVDMAAQLGADALILADLAMLEYAAERYPHIERHVSVQASATNEEAINFYHRHFDVARVVL

PRVLSIHQVKQLARVTPVPLEVFAFGSLCIMSEGRCYLSSYLTGESPNTVGACSPARFVRWQQTPQGLES

RLNEVLIDRYQDGENAGYPTLCKGRYLVDGERYHALEEPTSLNTLELLPELMAANIASVKIEGRQRSPAY

VSQAAKVWRQAIDRCKADPQNFVPQSAWMETLGSMSEGTQTTLGAYHRKWQ

>gi|30064497|ref|NP_838668.1| hypothetical protein S3415 [Shigella flexneri 2a str. 2457T]

MLDKLRSRIVHLGPSLLSVPVKLTPFALKRQVLEQVLSWQFRQALDDGELEFLEGRWLSIHVRDIDLQWF

TSVVNGKLVVSQNTQADVSFSADASDLLMIAARKQDPDTLFFQRRLVIEGDTELGVYVKNLMDAIELEQM

PKALRMMLLQLADFVEAGMKTAPETKQTSVGEPC

>gi|30064496|ref|NP_838667.1| hypothetical protein S3414 [Shigella flexneri 2a str. 2457T]

MLIRVEIPIDAPGIDALLRRSFESDAEAKLVHDLREDGFLTLGLVATDDEGQVIGYVAFSPVDVQGEDLQ

WVGMAPLAVDEKYRGQGLARQLVYEGLDSLNEFGYAAVVTLGDPALYSRFGFELAAHHDLRCRWPGTESA

FLVHRLADDALNGVTGLVEYHEHFNRF

>gi|30064495|ref|NP_838666.1| GIY-YIG nuclease superfamily protein [Shigella flexneri 2a str. 2457T]

MTPWFLYLIRTADNKLYTGITTDVERRYQQHQSGKGAKALRGKGELTLAFSAPVGDRSLALRAEYRVKQL

TKRQKERLVAEGAGFAELLSSLQTPEIKSD

>gi|30064494|ref|NP_838665.1| hypothetical protein S3412 [Shigella flexneri 2a str. 2457T]

METLIAISRWLAKQHVVTWCVQQEGELWCANAFYLFDAQKVAFYILTEEKTRHAQMSGPQAAVAGTVNGQ

PKTVALIRGVQFKGEIRRLEGEESDLARKAYNRRFPVARMLSAPVWEIRLDEIKFTDNTLGFGKKMIWLR

DSGTEQA

>gi|30064493|ref|NP_838664.1| hypothetical protein S3411 [Shigella flexneri 2a str. 2457T]

MENSPHPMQQRGGSMSKKIAVLITDEFEDSEFTSPADEFRKAGHEVITIEKQAGKTVKGKKGEASVTIDK

SIDEVTPAEFDALLLPGGHSPDYLRGDNRFVTFTRDFVNSGKPVFAICHGPQLLISADVIRGRKLTAVKP

IIIDVKNAGVEFYDQEVVVDKDQLVTSRTPDDLPAFNREALRLLGA

>gi|30064492|ref|NP_838663.1| hypothetical protein S3410 [Shigella flexneri 2a str. 2457T]

MSRGAQGRQFCKGTKMSQVLITGATGLVGGHLLRMLINEPKVNAIAAPTRRPLGDMPGVFNPHDPQLSDA

LAQVTEPIDIVFCCLGTTRREAGSKEAFIHADYTLVVDTALTGRRLGAQHMLVVSAMGANAHSPFFYNRV

KGEMEEALIAQNWPKLTIARPSMLLGDRSKQRMNETLFAPLFRLLPGNWKSIDARDVARVMLAEAMRPEH

EGVTILSSSELRKRAE

>gi|30064491|ref|NP_838662.1| hypothetical protein S3409 [Shigella flexneri 2a str. 2457T]

MTGQSSSQAATPIQWWKPALFFLVVIAGLWYVKWQPYYGKAFTAAETHSIGKSILAQADANPWQAALDYA

MIYFLAVWKAAVLGVILGSLIQVLIPRDWLLRTLGQSRFRGTLLGTLFSLPGMMCTCCAAPVAAGMRRQQ

VSMGGALAFWMGNPVLNPATLVFMGFVLGWGFAAIRLVAGLVMVLLIATLVQKWVRETPQTQAPVEIDIP

EAQSGFFSRWGRALWTLFWSTIPVYILAVLVLGAARVWLFPHADGAVDNSLMWVVAMAVAGCLFVIPTAA

EIPIVQTMMLAGMGTAPALALLMTLPAVSLPSLIMLRKAFPAKALWLTGAMVAVSGVIVGGLALLF

>gi|30064490|ref|NP_838661.1| hypothetical protein S3408 [Shigella flexneri 2a str. 2457T]

MKALSPIAVLISALLLQGCVAAAVVGTAAVGTKAATDPRSVGTQVDDGTLEVRVNSALSKDEQIKKETRI

NVTAYQGKVLLVGQSPNAELSARAKQIAMGVDGANEVYNEIRQGQPIGLGEASNDTWITTKVRSQLLTSD

LVKSSNVKVTTENGEVFLMGLVTEREAKAAADIASRVSGVKRVTTAFTFIK

>gi|30064489|ref|NP_838660.1| DnaA initiator-associating protein DiaA [Shigella flexneri 2a str. 2457T]

MQERIKACFTESIQTQIAAAEALPDAISRAAMTLVQSLLNGNKILCCGNGTSAANAQHFAASMINRFETE

RPSLPAIALNTDNVVLTAIANDRLHDEVYAKQVRALGHAGDVLLAISTRGNSRDIVKAVEAAVTRDMTIV

ALTGYDGGELAGLLGPQDVEIRIPSHRSARIQEMHMLTVNCLCDLIDNTLFPHQDD

>gi|30064488|ref|NP_838659.1| hypothetical protein S3406 [Shigella flexneri 2a str. 2457T]

MATVPTRSGSPRQLTTKQTGDAWEAQARRWLEGKGLRFIAANVNERGGEIDLIMREGLTTVFVEVRYRRS

ALYGGAAASVTRSKQHKLLQTARLWLARHNGSFDTVDCRFDVVAFTGNEVEWIKDAFNDHS

>gi|30064487|ref|NP_838658.1| glycosylase [Shigella flexneri 2a str. 2457T]

MVPSTFSRLKAARCLPVVLAALIFAGCGTHTPDQSTAYMQGTAQADSAFYLQQMQQSSDDTRINWQLLAI

RALVKEGKTGQAVELFNQLPQELNDSQRREKTLLAAEIKLAQKDFAGAQNLLAKITPADLEQNQQARYWQ

AKIDASQGRPSIDLLRALIAQEPLLGAKEKQQNIDATWQALSSMTQEQANTLVINADENILQGWLDLQRV

WFDNRNDPDMMKAGIADWQKRYPNNPGAKMLPTQLVNVKAFKPASTNKIALLLPLNGQAAVFGRTIQQGF

EAAKNIGTQPVAAQVAAAPAADVAEQPQPQTADSVASPAQASVSDLTGDQPAAQPVPVSAPATSTAAVSA

PANPSAELKIYDTSSQPLSQILSQVQQDGASIVVGPLLKNNVEELLKSNTPLNVLALNQPENIENRVNIC

YFALSPEDEARDAARHIRDQGKQAPLVLIPRSALGDRVANAFAQEWQKLGGGTVLQQKFGSTSELRAGVN

GGSGIALTGSPITPRATTDSGMTTNNPTLQTTPTDDQFTNNGGRVDAVYIVATPGEIAFIKPMIAMRNGS

QSGATLYASSRSAQGTAGPDFRLEMEGLQYSEIPMLAGGNLPLMQQALSAVNNDYSLARMYAMGVDAWSL

ANHFSQMRQVQGFEINGNTGSLTANPDCVINRKLSWLQYQQGQVVPAS

>gi|30064486|ref|NP_838657.1| hypothetical protein S3404 [Shigella flexneri 2a str. 2457T]

MKQHQSADNSQGQLYIVPTPIGNLADITQRTLEVLQAVDLIAAEDTRHTGLLLQHFGINARLFALHDHNE

QQKAETLLAKLQEGQNIALVSDAGTPLINDPGYHLVRTCREAGIRVVPLPGPCAAITALSAAGLPSDRFC

YEGFLPAKSKGRRDALKAIEAEPRTLIFYESTHRLLDSLEDIVAVLGESRYVVLARELTKTWETIHGAPV

GELLAWVKEDENRRKGEMVLIVEGHKAQEEDLPADALRTLALLQAELPLKKAAALAAEIHGVKKNALYKY

ALEQQG

>gi|30064485|ref|NP_838656.1| fimbrial protein [Shigella flexneri 2a str. 2457T]

MKRAPLITGLLLISTSCAYASSGGCGADSTSGATNYSSVVDDVTVNQTDNVTGREFTSATLSSTNWQYAC

SCSAGKAVKLVYMVSPVLTTTGHQTGYYKLNDSLDIKTTLQANDIPGLTTDQVVSVNTRFTQIKSSTVYS

AATQTGVCQGDTSRYGPVNIGANTTFTLYVTKPFLGSMTIPKTDIAVIKGAWVDGMGSPSTGDFHDLVKL

SIQGNLTAPQSCKINQGDVIKVNFGFINGQKFTTRNAMPDGFTPVDFDITYDCGDTSKIKNSLQMRIDGT

TGVVDQYNLVARRRSSDNVPDVGIRIENLGGGVANIPFQNGILPVDPSGHGTVNMRAWPVNLVGGELETG

KFQGTATITVMVR

>gi|30064483|ref|NP_838654.1| IS2 orfB [Shigella flexneri 2a str. 2457T]

MTTGGFNSETVQDVMLGAVERRFGNDLPSSPVEWLTDNGSCYRANETRQFARMLGLEPKNTAVRSPESNG

IAESFVKTIKRDYISIMPKPDGLTAAKNLAEAFEHYNEWHPHSALGYRSPREYLRQRACNRLSDNRCLEI

>gi|30064482|ref|NP_838653.1| chaperone [Shigella flexneri 2a str. 2457T]

MSKRTFAVILTLLCSFCIGQALAGGIVLQRTRVIYDASRKEAALPVANKGAETPYLLQSWVDNIDGKSRA

PFIITPPLFRLEASDDSSLRIIKTADNLPENKESLFYINVRAIPAKKKSDDVNANELTLVFKTRVKMFYR

PAHLKGRVNDAWKSLEFKRSDHSLNIYNPTEYYVVFAGLAVDKTDLTSKIEYIAPGEHKQLPLPASGGKN

VKWAAINDYGGSSGTETRPLQ

>gi|30064481|ref|NP_838652.1| IS1 orfA [Shigella flexneri 2a str. 2457T]

MASISIRCPSCSATEGVVRNGKSTAGHQRYLCSHCRKTWQLQFTYTASQPGTHQKIIDMAMNGVGCRASA

RIMGVGLNTVLRHLKNSGRSR

>gi|30064480|ref|NP_838651.1| IS1 ORF2 [Shigella flexneri 2a str. 2457T]

MSRQCTHYGRWPQHGFTSLKKLRPQSVTSRIQPGSDVIVCAEMDEHWGYVGAKSRQRWLFYAYDRIRRTV

VAHVFGERTLATLERLLSLLSAFEVVVWMTDGWPLYESRLKGKLHVISKRYTQRIERHNLNLRQHLARLG

RKLLSFSKSVELHDKVIGHYLNIKHYQ

>gi|30064479|ref|NP_838650.1| galactosamine-6-phosphate isomerase [Shigella flexneri 2a str. 2457T]

MERGTASGGASLLKEFHPVQTLQQVENYTALSERASEYLLAVIRSKPDAVICLATGATPLLTYHYLVEKI

HQQQVDISQLTFVKLDEWVDLPLTMPGTCETFLQQHIVQPLGLREDQLISFRSEEINETECERVTNLIAR

KGGLDLCVLGLGKNGHLGLNEPGESLQPACHISQLDARTQQHEMLKTTGRPVTRGITLGLKDILNAREVL

LLVTGEGKQDATERFLTAKVSTAIPASFLWLHSNFICLINT

>gi|30064478|ref|NP_838649.1| N-acetylgalactosamine-specific PTS system transporter subunit IID [Shigella flexneri 2a str. 2457T]

MGSEISKKDITRLGFRSSLLQASFNYERMQAGGFTWAMLPILKKIYKDDKPGLSAAMKDNLEFINTHPNL

VGFLMGLLISMEEKGENRDTIKGLKVALFGPIAGIGDAIFWFTLLPIMAGICSSFASQGNLLGPILFFAV

YLLIFFLRVGWTHVGYSVGVKAIDKVRENSQMIARSATILGITVIGGLIASYVHINVVTSFAIDSTHSVA

LQQDFFDKVFPNILPMAYTLLMYYFLRVKKAHPVLLIGVTFVLSIVCSAFGIL

>gi|30064477|ref|NP_838648.1| N-acetylgalactosamine-specific PTS system transporter subunit IIC [Shigella flexneri 2a str. 2457T]

MHEITLLQGLSLAALVFVLGIDFWLEALFLFRPIIVCTLTGAILGDIQTGLITGGLTELAFAGLTPAGGV

QPPNPIMAGLMTTVIAWSTGVDAKTAIGLGLPFSLLMQYVILFFYSAFSLFMTKADKCAKEADTAAFSRL

NWTTMLIVASAYAVIAFLCTYLAQGAMQALVKAMPAWLTHGFEVAGGILPAVGFGLLLRVMFKAQYIPYL

IAGFLFVCYIRVSNLLPVAVLGAGFAVYEFFNAKSRQQAQPQPVASKNEEEDYSNGI

>gi|30064476|ref|NP_838647.1| IS1 orfB [Shigella flexneri 2a str. 2457T]

MSRQCTHYGRWPQHGFTSLKKLRPQSVTSRIQPGSDVIVCAEMDEHWGYVGAKSRQRWLFYAYDRIRRTV

VAHVFGERTLATLERLLSLLSAFEVVVWMTDGWPLYESRLKGKLHVISKRYTQRIERHNLNLRQHLARLV

RKSLSFSKSVELHDKVIGHYLNIKHYQ

>gi|30064475|ref|NP_838646.1| IS1 orfA [Shigella flexneri 2a str. 2457T]

MASISIRCPSCSATEGVVRNGKSTAGHQRYLCSHCRKTWQLQFTYTASQPGTHQKIIDMAMNGVGCRASA

RIMGVGLNTVLRHLKNSGRSR

>gi|30064474|ref|NP_838645.1| N-acetylglucosamine-6-phosphate deacetylase [Shigella flexneri 2a str. 2457T]

MTHVLRARRLLTEEGWFDDHQLRVADGVIAAIEPIPVGVTERDAERLCPAYIDTHVHGGAGVDVMDDAPD

VLDKLAMHKAREGVGSWLPTTVTAPLNTIHAALKRIAQRCQRGGPGAQVLGSYLEGPYFTPQNKGAHPPG

NAANLLI

>gi|30064473|ref|NP_838644.1| transport enzyme subunit [Shigella flexneri 2a str. 2457T]

MLSIILTGHGGFASGMEKAMKQILGDQSQFIAIDFPETSSAALLTSQLEEAIAQLDCEDGIVFLTDLLGG

TPFRVASTLAMQKPGCEVITGTNLQLLLEMVLEREGLSGEEFRVQALECGHRGLTSLVDELGRCHEECPV

EEGI

>gi|30064472|ref|NP_838643.1| transport enzyme subunit [Shigella flexneri 2a str. 2457T]

MASNQTTLPNVSENEETLLTGVNENVYEDRSIAAELTKKDINRVAWRSMLLQASFNYERMQASGWLYGLL

PALKKIHTNKRDLARAMKGHMGFFNTHPFLVTFVIGIILAMERSKQDVNSIQSTKIAVGAPLGGIGDAMF

WLTLLPICGGIGASLALQGSILGAVVFIVLFNVVHLGLRFGLAHYAYRMGVAAIPLIKANTKKVGHAASI

VGMTVIGALVATYVRLSTTLEITAGDAVVKLQADVIDKLMPAFLPLVYTLTMFWLVRRG

>gi|30064471|ref|NP_838642.1| N-acetylgalactosameine-specific IIC component 2 [Shigella flexneri 2a str. 2457T]

MEISLLQAFALGIIAFIAGLDMFNGLTHMHRPVVLGPLVGLVLGDLHTGILTGDTLELVWMGLAPLAGAQ

PPNVIIGTIVGTAFAITTGVKPDVAVGVAVPFAVAVQMGITFLFSVMSGVMSRCDRMAENADTRGIERVN

YLALLALGTFYFLCAFLPIYFGAEHAKTIIDVLPQRLIDGLGVAGGIMPAIGFAVLLKIMMKNVYIPYFI

LGFVAATWLKLPVLAIAAAALAMALIDLLRKSPEPTQPAAQKEEFEDGI

>gi|30064470|ref|NP_838641.1| N-acetylgalactosamine-specific PTS system transporter subunit IIB [Shigella flexneri 2a str. 2457T]

MKANKQNKEEHAMPNIVLSRIDERLIHGQVGVQWVGFAGANLVLVANDEVAEDPVQQNLMEMVLAEGIAV

RFWTLQKVIDNIHRAADRQKILLVCKTPADFLTLVKGGVPVNRINVGNMHYANGKQQIAKTVSVDAGDIA

AFNDLKAAGVECFVQGVPTEPAVDLFKLL

>gi|30064469|ref|NP_838640.1| tagatose 6-phosphate kinase 2 [Shigella flexneri 2a str. 2457T]

MKHLTEMVRQHKAGKTNAIYAVCSAHPLVLEAAIRYASANQTPLLIEATSNQVDQFGGYTGMTPADFRGF

VCQLADSLNFPQDALILGGDHLGPNRWQNLPAAQAMANADDLIKSYVAAGFKKIHLDCSMSCQDDPIPLT

DDIVAERAARLAKVAEETCREHFGEADLEYVIGTEVPVPGGAHETLSELAVTTPDAARATLEAHRHAFEK

QGLNAIWPRIIALVVQPGVEFDHTNVIDYQPAKASALSQMVENYETLIFEAHSTDYQTPQSLRQLVIDHF

AILKVGPALTFALREALFSLAAIEEELVPAKACSGLRQVLENVMLDRPEYWQSHYHGDGNARRLARGYSY

SDRVRYYWPDSQIDDAFAHLVRNLADSPIPLPLISQYLPLQYVKVRSGELQPTPRELIINHIQDILAQYH

TACEGQ

>gi|30064468|ref|NP_838639.1| DNA-binding transcriptional regulator AgaR [Shigella flexneri 2a str. 2457T]

MSNTDASGEKRVTGTSERREQIIQRLRQQGSVQVNDLSALYGVSTVTIRNDLAFLEKQGIAVRAYGGALI

CDSTTPSVEPSVEDKSALNTAMKRSVAKAAVELIQPGHRVILDSGTTTFEIARLMRKYTDVIAMTNGMNV

ANALLEAEGVELLMTGGHLRRQSQSFYGDQAEQSLQNYHFDMLFLGVDAIDLERGVSTHNEDEARLSRRM

CEVAERIIVVTDSSKFNRSSLHKIIDTQRIDMIIVDEGIPADSLEGLRKAGVEVILVGDHASSL

>gi|30064467|ref|NP_838638.1| hydrolase [Shigella flexneri 2a str. 2457T]

MANIEIRQETPTAFYIKVHDTDNVAIIVNDNGLKAGTRFPDGLELIEHIPQGHKVALLDIPANGEIIRYG

EVIGYAVRAIPRGSWIDESMVVLPEAPPLHTLPLATKVPEPLPPLEGYTFEGYRNADGSVGTKNLLGITT

SVHCVAGVVDYVVKIIERDLLPKYPNVDGVVGLNHLYGCGVAINAPAAVVPIRTIHNISLNPNFGGEVMV

IGLGCEKLQPERLLTGTDDVQAIPVESASIVSLQDEKHVGFQSMVEDILQVAERHLQKLNQRQRETCPAS

ELVVGMHCGGSDAFSGVTANPAVGYASDLLVRCGATVMFSEVTEVRDAIHLLTPRAVNEEVGKRLLEEME

WYDNYLNMGKTDRSANPSPGNKKGGLANVVEKALGSIAKSGKSAIVEVLSPGQRPTKRGLIYAATPASDF

VCGTQQVASGITVQVFTTGRGTPYGLMAVPVIKMAPRTELANRWFDLMDINAGTIATGEETIEEEGWKLF

HFILDVASGKKKTFSDQWGLHNQLAVFNPAPVT

>gi|30064466|ref|NP_838637.1| transporter [Shigella flexneri 2a str. 2457T]

MILDTVDEKRKGVHTRYLILLIIFIVTAVNYADRATLSIAGTEVAKELQLSAVSMGYIFSAFGWAYLLMQ

IPGGWLLDKFGSKKVYTYSLFFWSLFTFLQGFVDMFPLAWAGISMFFMRFMLGFSEAPSFPANARIVAAW

FPTKERGTASAIFNSAQYFSLALFSPLLGWLTFAWGWEHVFTVMGVICFVLTALWIKLIHNPTDHPRMSE

EELKFISENGAVVDMDHKKPGSAAASGPKLHYIKQLLSNRMMLGVFFGQYFINTITWFFLTWFPIYLVQE

KGMSILKVGLVASIPALCGFAGGVLGGVFSDYLIKRGLSLTLARKLPIVLGMLLASTIILCNYTNNTTLV

VMLMALAFFGKGFGALGWPVISDTAPKEIVGLCGGVFNVFGNVASIVTPLVIGYLVSELHSFNAALVFVG

CSALMAMVCYLFVVGDIKRMELQK

>gi|30064463|ref|NP_838634.1| hypothetical protein S3374 [Shigella flexneri 2a str. 2457T]

MHQKQKLIDAGLKLMATAKMPDALRLSGLRHLCNILNLRAFVGRIRRSRSIRHE

>gi|30064462|ref|NP_838633.1| DNA-binding transcriptional activator TdcR [Shigella flexneri 2a str. 2457T]

MTGITIFYGDNIIRYVVNTKKGLRPYFKQLPDNYQAKFELNLMSKFSNFIINKPFSAINTAARHIFSRYL

LENKHLFYQYFKISNTGIDHLEQLINVNFFSSDRTSFCECNRFP

>gi|30064461|ref|NP_838632.1| DNA-binding transcriptional activator TdcA [Shigella flexneri 2a str. 2457T]

MSTILLPKTQHLVVFQEVIRSGSIGSAAKELGLTQPAVSKIINDIEDYFGVELVVRKNTGVTLTPAGQLL

LSRSESITREMKNMVNEISGMSSEAVVEVSFGFPSLIGFTFMSGMINKFKEVFPKAQVSMYEAQLSSFLP

AIRDGRLDFAIGTLSAEMKLQDLHVEPLFESEFVLVASKSRTCTGTTTLESLKNEQWVLPQTNMGYYSEL

LTTLQRNGISIENIVKTDSVVTIYNLVLNADFLTVIPCDMTSPFGSNQFITIPVEETLPVAQYAAVWSKN

YRIKKAASVLVELAKEYSSYNGCRRRQLIEVG

>gi|30064460|ref|NP_838631.1| threonine dehydratase [Shigella flexneri 2a str. 2457T]

MHITYDLPVAIDDIIEAKQRLAGRIYKTGMPRSNYFSERCKGEIFLKFENMQRTGSFKIRGAFNKLSSLT

DAEKRKGVVACSAGNHAQGVSLSCAMLGIDGKVVMPKGAPKSKVAATCDYSAEVVLHGDNFNDTIAKVSE

IVEMEGRIFIPPYDDPKVIAGQGTIGLEIMEDLYDVDNVIVPIGGGGLIAGIAVAIKSINPTIRVIGVQS

ENVHGMAASFHSGEITTHRTTGTLADGCDVSRPGNLTYEIVRELVDDIVLVSEDEIRNSMIALIQRNKVV

TEGAGALACAALLSGKLDQYIQNRKTVSIISGGNIDLSRVSQITGFVDA

>gi|30064459|ref|NP_838630.1| threonine/serine transporter TdcC [Shigella flexneri 2a str. 2457T]

MSTSDSIVSSQTKQSSWRKSDTTWTLGLFGTAIGAGVLFFPIRAGFGGLIPILLMLVLAYPIAFYCHRAL

ARLCLSGSNPSGNITETVEEHFGKTGGVVITFLYFFAICPLLWIYGVTITNTFMTFWENQLGFAPLNRGF

VALFLLLLMAFVIWFGKDLMVKVMSYLVWPFIASLVLISLSLIPYWNSAVIDQVDLGSLSLTGHDGILIT

VWLGISIMVFSFNFSPIVSSFVVSKREEYEKDFGRDFTERKCSQIISRASMLMVAVVMFFAFSCLFTLSP

ANMAEAKAQNIPVLSYLANHFASMTGTKTTFAITLEYAASIIALVAIFKSFFGHYLGTLEGLNGLILKFG

YKGDKTKVSLGKLNTLSMIFIMGSTWVVAYANPNILDLIEAMGAPIIASLLCLLPMYAIRKAPSLAKYRG

RLDNVFVTVIGLLTILNIVYKLF

>gi|30064457|ref|NP_838628.1| formate acetyltransferase 3 [Shigella flexneri 2a str. 2457T]

MKVDIDTSDKLYADAWLGFKGTDWKSEINVRDFIQHNYTPYEGDESFLAEATPATTELWEKVMEGIRIEN

ATHAPVDFDTNIATTITAHDAGYINQPLEKIVGLQTDAPLKRALHPFGGINMIKSSFHAYGREMDSEFEY

LFTDLRKTHNQGVFDVYSPDMLRCRKSGVLTGLPDGYGRGRIIGDYRRVALYGISYLVRERELQFADLQS

RLEKGEDLEATIRLREELAEHRHALLQIQEMAAKYGFDISRPAQNAQEAVQWLYFAYLAAVKSQNGGAMS

LGRTASFLDIYIERDFKAGVLNEQQAQELIDHFIMKIRMVRFLRTPEFDSLFSGDPIWATEVIGGMGLDG

RTLVTKNSFRYLHTLHTMGPAPEPNLTILWSEELPIAFKKYAAQVSIVTSSLQYENDDLMRTDFNSDDYA

IACCVSPMVIGKQMQFFGARANLAKTLLYAINGGVDEKLKIQVGPKTAPLMDDVLDYDKVMDSLDHFMDW

LAVQYISALNIIHYMHDKYSYEASLMALHDRDVYRTMACGIAGLSVATDSLSAIKYARVKPIRDENGLAV

DFEIDGEYPQYGNNDERVDSIACDLVERFMKKIKALPTYRNAVPTQSILTITSNVVYGQKTGNTPDGRRA

GTPFAPGANPMHGRDRKGAVASLTSVAKLPFTYAKDGISYTFSIVPAALGKEDPVRKTNLVGLLDGYFHH

EADVEGGQHLNVNVMNREMLLDAIEHPEKYPNLTIRVSGYAVRFNALTREQQQDVISRTFTQAL

>gi|30064456|ref|NP_838627.1| hypothetical protein S3365 [Shigella flexneri 2a str. 2457T]

MKKIIETQRAPGAIGPYVQGVDLGSMVFTSGQIPVCPQTGEIPADVQDQARLSLENVKAIVVAAGLSVGD

IIKMTVFITDLNDFATINEVYKQFFDEHQATYPTRSCVQVARLPKDVKLEIEAIAVRSA

>gi|30064455|ref|NP_838626.1| L-serine deaminase [Shigella flexneri 2a str. 2457T]

MISAFDIFKIGIGPSSSHTVGPMNAGKSFIDRLESSGLLTATSHIVVDLYGSLSLTGKGHATDVAIIMGL

AGNSPQDVVIDEIPAFIELVTRSGRLPVASGAHIVDFPVAKNIIFHPEMLPRHENGMRITAWKGQEALLS

KTYYSVGGGFIVEEEHFGLSHDVETSVPYDFHSAGELLKMCDYNGLSISGLMMHNELALRSKAEIDAGFA

RIWQVMHDGIERGMNTEGVLPGPLNVPCRAVALRRQLVSSDNISNDPMNVIDWINMYALAVSEENAAGGR

VVTAPTNGACGIIPAVLAYYDKFRRPVNERSIARYFLAAGAIGALYKMNASISGAEVGCQGEIGVACSMA

AAGLTELLGGSPAQVCNAAEIAMEHNLGLTCDPVAGQVQIPCIERNAINAVKAVNAARMAMRRTSAPRVS

LDKVIETMYETGKDMNDKYRETSRGGLAIKVVCG

>gi|30064454|ref|NP_838625.1| transport system permease [Shigella flexneri 2a str. 2457T]

MEIASNKGVIADASTPAGRAGMSESEWREAIKFDSTDTGWVIMSIGMAIGAGIVFLPVQVGLMGLWVFLL

SSVIGYPAMYLFQRLFINTLAESPECKDYPSVISGYLGKNWGILLGALYFVMLVIWMFVYSTAITNDSAS

YLHTFGVTEGLLSDSPFYGLVLICILVAISSRGEKLLFKISTDMVLTKLLVVAALGVSMVGMWHLYNVGS

LPPLGLLVKNAIITLPFTLTSILFIQTLSPMVISYRSREKSIEVARHKALRAMNIAFGILFVTVFFYAVS

FTLAMGHDEAVKAYEQNISALAIAAQFISGDGAAWVKVVSVILNIFAVMTAFFGVYLGFREATQGIVMNI

LRRKMPAEKINENLVQRGIMIFAILLAWSAIVLNAPVLSFTSICSPIFGMVGCLIPAWLVYKVPALHKYK

GMSLYLIIVTGLLLCVSLFLAFS

>gi|30064453|ref|NP_838624.1| hypothetical protein S3360 [Shigella flexneri 2a str. 2457T]

MFDSTLNPLWQRYILAVQEEVKPALGCTEPISLALAAAVAAAELEGPVERVEAWVSPNLMKNGLGVTVPG

TGMVGLPIAAALGALGGNANAGLEVLKDATAQAIADAKALLAAGKVSVKIQEPCNEILFSRAKVWNGEKW

ACVTIVGGHTNIVHIETHNGVVFTHQACVAEGEQESPLTVLSRTTLAEILKFVNEVPFAAIRFILDSAKL

NCALSQEGLSGKWGLAYWRDAGKTVRARFAGERSLFIHCDSYQRGIRCAYGRRYASGYE

>gi|30064452|ref|NP_838623.1| hypothetical protein S3359, partial [Shigella flexneri 2a str. 2457T]

MIRTSAASDARMGGATLPAMSNSGSGNQGITAIMPVVVVAEHFGADDERLARALMLSHLSAIYIHNQLPR

LSALCAATTAAMGAAAGMAWLVDGRYETISMAISSMIGDVSGMICDGASNSCAMKVSTSASAAWKAVLMA

LDDTAVTGNEGIVAHDVEQSIANLCALASHSMQQTDRQIIEIMASKAR

>gi|30064451|ref|NP_838622.1| hypothetical protein S3358 [Shigella flexneri 2a str. 2457T]

MSKKSTKKRQPAKPVVAKEPARTAKNFGYEEMLSELEAIVADAETRLAEDEATA

>gi|30064450|ref|NP_838621.1| hypothetical protein S3357 [Shigella flexneri 2a str. 2457T]

MITTRTARQCGQADYGWLQARYTFSFGHYFDPKLLGYASLRVLNQEVLAPGAAFQPRTYPKVDILNVILD

GEAEYRDSEGNHVQASAGEALLLSTQPGVSYSEHNLSKDKPLTRMQLWLDTCPQRENPLIQKLALNMDKQ

QLIASPEGAMGSLQLRQQVWLHHIVLDKGESANFQLHGPRAYLQSIHGKFHALTHHEEKAALTCGDGAFI

RDEANITLVADSPLRALLIDLPV

>gi|30064449|ref|NP_838620.1| LYSR-type transcriptional regulator [Shigella flexneri 2a str. 2457T]

MAKERALTLEALRVMDAIDRRGSFAAAADELGRVPSALSYTMQKLEEELDVVLFDRSGHRTKFTNVGRML

LERGRVLLEAADKLTTDAEALARGWETHLTIVTEALVPTPAFFPLIDKLAAKANTQLAIITEVLAGAWER

LEQGRADIVIAPDMHFRSSSEINSRKLYTLMNVYVAAPDHPIHQEPEPLSEVTRVKYRGIAVADTARERP

VLTVQLLDKQPRLTVSTIEDKRQALLAGLGVATMPYPMVEKDIAEGRLRVVSPESTSEIDIIMAWRRDSM

GEAKSWCLREIPKLFAGK

>gi|30064448|ref|NP_838619.1| cytochrome [Shigella flexneri 2a str. 2457T]

MDWYLKVLKNYVGFRGRARRKEYWMFILVNIIFTFVLGLLDKMLGWQRAGGEGILTIIYGILVFLPWWAV

QFRRLHDTDRSAWWALLFLIPFIGWLIIIVFNCQAGTPGENRFGPDPKLEQE

>gi|30064447|ref|NP_838618.1| transferase [Shigella flexneri 2a str. 2457T]

MGQLIDGVWHDTWYDTKSTGGKFQRSASAFRNWLTADGAPGPTGTGGFIAEKDRYHLYVSLACPWAHRTL

IMRKLKGLEPFISVSVVNPLMLENGWTFDDSFPGATGDTLYQHEFLYQLYLHADPHYSGRVTVPVLWDKK

NHTIVSNESAEIIRMFNTAFDALGAKAGDYYPPALQTKIDELNGWIYDTVNNGVYKAGFATSQQAYDEAV

AKVFESLARLEQILGQHRYLTGNQLTEADIRLWTTLVRFDPVYVTHFKCDKHRISDYLNLYGFLRDIYQM

PGIAETVNFDHIRNHYFRSHKTINPTGIISIGPWQDLDEPHGRDVRFG

>gi|30064446|ref|NP_838617.1| hypothetical protein S3351 [Shigella flexneri 2a str. 2457T]

MSSKVERERRKAQLLSQIQQQRLDISASSREWLEATGAYDRRWNMLLSLRSWALVGSRVMAIWTIRHPNM

LVRWARRGFGVWSAWRLVKTTLKQQQLRG

>gi|30064445|ref|NP_838616.1| hypothetical protein S3350 [Shigella flexneri 2a str. 2457T]

MADTHHAQGPGKSVLGIGQRIVSIMVEMVETRLRLAVVELEEEKANLFQLLLMLGLTMLFAAFGLMSLMV

LIIWAVDPQYRLNAMIATTVVLLLLALIGGIWTLRKSRKSTLLRHTRHELANDRQLLEEESREQ

>gi|30064444|ref|NP_838615.1| hypothetical protein S3349 [Shigella flexneri 2a str. 2457T]

MSKEHTTEHLRAELKSLSDTLEEVLSSSGEKSKEELSKIRSKAEQALKQSRYRLGETGDAIAKQTRVAAA

RADEYVRENPWTGVGIGAAIGVVLGVLLSRR

>gi|30064443|ref|NP_838614.1| hypothetical protein S3347 [Shigella flexneri 2a str. 2457T]

MSLRQLAWSGTVLLLVGTLLLAWSAVRQQESTLAIRAVHQGTTMPDGFSIWHHLDAHGIPFKSITPKNDT

LLITFDSSDQSAAAKAVLDRTLPHGYIIAQQDNNSQAMQWLTRLRDNSHRFG

>gi|30064442|ref|NP_838613.1| hypothetical protein S3346 [Shigella flexneri 2a str. 2457T]

MELLTQLLQALWAQDFETLANPSMIGMLYFVLFVILFLENGLLPAAFLPGDSLLVLVGVLIAKGAMGYPQ

TILLLTVAASLGCWVSYIQGRWLGNTRTVQNWLSHLPAHYHQRAHHLFHKHGLSALLIGRFIAFVRTLLP

TIAGLSGLNNARFQFFNWMSGLLWVLILTTLGYMLGKTPVFLKYEDQLMSCLMLLPVVLLVFGLAGSLVV

LWKKKYGNRG

>gi|30064441|ref|NP_838612.1| DNA-binding transcriptional repressor ExuR [Shigella flexneri 2a str. 2457T]

MEITEPRRLYQQLAADLKERIEQGVYLVGDKLPAERFIADEKNVSRTVVREAIIMLEVEGYVEVRKGSGI

HVVSNQPRHQQAADNNMEFANYGPFELLQARQLIESNIAEFAATQVTKQDIMKLMAIQEQARGEQCFRDS

EWDLQFHIQVALATQNSALAAIVEKMWTQRSHNPYWKKLHEHIDSRTVDNWCDDHDQILKALIRKDPHAA

KLAMWQHLENTKIMLFNETSDDFEFNADRYLFAENPVVHLDTATSGSK

>gi|30064440|ref|NP_838611.1| IS1 orfB [Shigella flexneri 2a str. 2457T]

MSRQCTHYGRWPQHGFTSLKKLRPQSVTSRIQPGSDVIVCAEMDEHWGYVGAKSRQRWLFYAYDRIRRTV

VAHVFGERTLATLERLLSLLSAFEVVVWMTDGWPLYESRLKGKLHVISKRYTQRIERHNLNLRQHLARLG

RKSLSFSKSVELHDKVIGHYLNIKHYQ

>gi|30064439|ref|NP_838610.1| IS1 orfA [Shigella flexneri 2a str. 2457T]

MASISIRCPSCSATEGVVRNGKSTAGHQRYLCSHCRKTWQLQFTYTASQPGTHQKIIDMAMNGVGCRASA

RIMGVGLNTVLRHLKNSGRSR

>gi|30064438|ref|NP_838609.1| hypothetical protein S3342 [Shigella flexneri 2a str. 2457T]

MDNRTLFNAQGGNFTFNFLPLGAIDGMRIGSTLSYLNQAQSQQGTPVMVLLSRNSRVDAYRNEQLLGSFY

LNSGSQFIDTSSFPPGSYSVALKVYENNQLTRTELVPFTKTGGLTDGNAQWFLQAGKTTSQVSDDESSAY

QLGVRLPLHPQYELYAGLANADDVSAFELGNNWTADLGRVGNLAISASVFRNDDGGKGDMQQANWSNPGW

PTLGFYRTNSDGDACTTDSRESYNALSCYESISATVSLNFVGWNMMLGYTCTQNNTDDSLRWDKQQSFEN

NYLRQTTAQSISETVQLSASRAIVMRDWILSTSVGVFHRNDNGGDNDDNGLYLSFSLSDTPTMDSNNNSH

STNVPTDYRYSEQDGDQTSWQLSHTFYNDSFSHKELGVTVGGLNTDTINSAVNGRWDGQYGNVYATVSDS

YDRKNHDHLSAFTGTYSSTLAVSRYGVNLGASGTDDLLGAVLVDVKGFSEQDEESQDLQLEARVAGSRTL

QLGQSDSVLFPYPGFQSGFVEVNDSSQGNQQGTTNIINGAGNRELMLLPGKLRYREVSASFNYNYIGRLL

LPAAVKKFPIVGLNSAMLLVAEDGGFTLEINGSEKELYLLSGQQFLKCPLSVVKKRASIRYSGDVTCSVV

TYSQLPESIQVQAQLKQPKLRGNVQTAQREVAP

>gi|30064437|ref|NP_838608.1| hypothetical protein S3341 [Shigella flexneri 2a str. 2457T]

MKNLPIGGVWKGKVKLHSNSPAQDYFANITLNTLDPNHIDVFFPEFAHATPRVQLDLHPTGSVNGSNYAQ

DLTMLDMCLYDGFNGNAISYEIMLKDEGRPAAGRRDGYFSIYRQGGTTTDEGERIDYRVKMYNPETGGQI

DVRNNENMVWNSINLKRVRPVVLPGIRYAVMCVPTPLTLAVDKFSVMDKQAGYYMGKLSVIFTPSLPTIN

>gi|30064436|ref|NP_838607.1| transport protein of hexuronates [Shigella flexneri 2a str. 2457T]

MRKIKGLRWYMIALVTLGTVLGYLTRNTVAAAAPTLMEELNISTQQYSYIIAAYSAAYTVMQPVAGYVLD

VLGTKIGYAMFAVLWAVFCGATALAGSWGGLAVARGAVGAAEAAMIPAGLKASSEWFPAKERSIAVGYFN

VGSSIGAMIAPPLVVWAIVMHSWQMAFIISGALSFIWAMAWLIFYKHPRDQKHLTDEERDYIINGQEAQH

QVSTAKKMSVGQILRNRQFWGIALPRFLAEPAWGTFNAWIPLFMFKVYGFNLKEIAMFAWMPMLFADLGC

ILGGYLPPLFQRWFGVNLIVSRKMVVTLGAVLMIGPGMIGLFTNPYVAIMLLCIGGFAHQALSGALITLS

SDVFGRNEVATANGLTGMSAWLASTLFALVVGALADTIGFSPLFAVLAVFDLLGALVIWTVLQNKPAIEV

AQETHNDPAPQH

>gi|30064435|ref|NP_838606.1| glucuronate isomerase [Shigella flexneri 2a str. 2457T]

MTPFMTEDFLLDTEFARRLYHDYAKDQPIFDYHCHLPPQQIAEDYRFKNLYDIWLKGDHYKWRAMRTNGV

AERLCTGDASDREKFDAWAATVPHTIGNPLYHWTHLELRRPFGITGKLLSPSTADEIWNECNELLAQDNF

SARGIMQQMNVKMVGTTDDPIDSLEHHAEIAKDGSFTIKVLPSWRPDKAFNIEQATFNDYMAKLGEVSDT

DIRRFADLQTALTKRLDHFAAHGCKVSDHALDVVMFAEANEAELDSILARRLAGETLSEHEVAQFKTAVL

VFLGAEYARRGWVQQYHIGALRNNNLRQFKLLGPDVGFDSINDRPMAEELSKLLSKQNEENLLPKTILYC

LNPRDNEVLGTMIGNFQGEGMPGKMQFGSGWWFNDQKDGMERQMTQLAQLGLLSRFVGMLTDSRSFLSYT

RHEYFRRILCQMIGRWVEAGEAPADINLLGEMVKNICFNNARDYFAIELN

>gi|30064434|ref|NP_838605.1| altronate hydrolase [Shigella flexneri 2a str. 2457T]

MQYIKIHALDNVAVALADLAEGTEVSVDNQTVTLRQDVARGHKFALTDIAKGANVIKYGLPIGYALADIA

AGEHVHAHNTRTNLSDLDQYRYQLDFQDLPAQAADPEVQIYRRANGDVGVRNELWILPTVGCVNGIARQI

QNRFLKETNNAEGTDGVFLFSHTYGCSQLGDDHINTRTMLQNMVRHPNAGAVLVIGLGCENNQVAAFRET

LGDIDPERVHFMICQQQDDEIEAGIEHLHQLYNVMRNDKREPGKLSELKFGLECGGSDGLSGITANPMLG

RFSDYVIANGGTTVLTEVPEMFGAEQLLMDHCRDEATFEKLVTMVNDFKQYFIAHDQPIYENPSPGNKAG

GITTLEDKSLGCTQKAGSSVVVDVLRYGERLKTPGLNLLSAPGNDAVATSALAGAGCHMVLFSTGRGTPY

GGFVPTVKIATNSELAAKKKHWIDFDAGQLIHGKAMPQLLEEFIDTIVEFANGKQTCNERNDFRELAIFK

SGVTL

>gi|30064433|ref|NP_838604.1| hypothetical protein S3337 [Shigella flexneri 2a str. 2457T]

MTAYWLAQGVGVIAFLIGITTFFNRDERRFKKQLSVYSAVIGVHFFLLGTYPAGASAILNAIRTLITLRT

RSLWVMAIFIVLTGGIGLAKFHHPVELLPVIGTIVSTWALFRCKGLTMRCVMWFSTCCWVIHNFWAGSIG

GTMIEGSFLLMNGLNIIRFWRMQKRGIDPFKVEKTPPAIDERG

>gi|30064432|ref|NP_838603.1| serine/threonine transporter SstT [Shigella flexneri 2a str. 2457T]

MTTQRSPGLFRRLAHGSLVKQILVGLVLGILLAWISKPAAEAVGLLGTLFVGALKAVAPILVLMLVMASI

ANHQHGQKTNIRPILFLYLLGTFSAALAAVVFSFAFPSTLHLSSSAGDISPPSGIVEVMRGLVMSMVSNP

IDALLKGNYIGILVWAIGLGFALRHGNETTKNLVNDMSNAVTFMVKLVIRFAPFGIFGLVSSTLATTGFS

TLWGYAQLLVVLVGCMLLVALVVNPLLVWWKIRRNPFPLVLLCLRESGVYAFFTRSSAANIPVNMALCEK

LNLDRDTYSVSIPLGATINMAGAAITITVLTLAAVNTLGIPVDLPTALLLSVVASLCACGASGVAGGSLL

LIPLACNMFGISNDIAMQVVAVGFIIGVLQDSCETALNSSTDVLFTAAACQAEDDRLANSALRN

>gi|30064431|ref|NP_838602.1| transporter [Shigella flexneri 2a str. 2457T]

MNTVGTPLLWGGFAVVVAIMLAIDLLLQGRRGAHAMTMKQAAAWSLVWVTLSLLFNAAFWWYLVQTEGRA

VADPQALAFLTGYLIEKSLAVDNVFVWLMLFSYFSVPAALQRRVLVYGVLGAIVLRTIMIFTGSWLISQF

DWILYIFGAFLLFTGVKMALAHEDESGIGDKPLVRWLRGHLRMTDTIDNEHFFVRKNGLLYATPLMLVLI

LVELSDVIFAVDSIPAIFAVTTDPFIVLTSNLFAILGLRAMYFLLAGVAERFSMLKYGLAVILVFIGIKM

LIVDFYHIPIAVSLGVVFGILVMTFIINAWVNYRHDKQRGE

>gi|30064430|ref|NP_838601.1| hypothetical protein S3334 [Shigella flexneri 2a str. 2457T]

MEYPRVMIRFAVIGTNWITRQFVEAAHESGKYKLTAVYSRSLEQAQHFANDFSVEHLFTSLEAMAESDAI

DAVYIASPNSLHFSQTQLFLSHKIHVICEKPLASNLAEVDAAIACARENQVVLFEAFKTACLPNFHLLRQ

ALPKAGKLRKVFFNYCQYSSRYQRYLDGENPNTFNPAFSNGSIMDIGFYCLASAVALFGEPKSVQATASL

LASGVDAHGVVVMDYGDFSVTLQHSKVSDSVLGSEIQGEAGSLVIEKLSECQKVCFVPRGSQMQDLTQPQ

HINTMLYEAELFATLVDEHLVDHPGLAVSRITAKLLTEIRRQTGVIFPADSVKLYLPK

>gi|30064429|ref|NP_838600.1| hypothetical protein S3333 [Shigella flexneri 2a str. 2457T]

MLRAFARLLLRICFSRRTLKIACLLLLVAGATIFIADRVMVNASKQLTWGDVNAVPARNVGLLLGARPGN
RYFTRRIDTAAALYHAGKVKWLLVSGDNGRKNYDEASGMQQALIAKGVPAKVIFCDYAGFSTLDSVVRAK
KVFGENHITIISQEFHNQRTIWLAKQYGIDAIGFNAPDLNMKHGFYTQLREKLARVSAVIDAKILHRQPK
YLGPSVMIGPFSEHGCPAKE

>gi|30064428|ref|NP_838599.1| hypothetical protein S3332 [Shigella flexneri 2a str. 2457T]
MSIPFFSFSWAVMSNLTYLQGYPEQLLSQVRTLINEQRLGDVLAKRYPGTHDYATDKALWQYTQDLKNQF
LRNAPPINKVMYDNKIHVLKNALGLHTAVSRVQGSKLKAKAEIRVATVFRNAPEPFLRMIVVHELAHLKE
KEHNKAFYQLCCHMEPQYHQLEFDTRLWLTQLSLGQDKI

>gi|30064427|ref|NP_838598.1| enzyme [Shigella flexneri 2a str. 2457T]
MSHLDNGFRSLTLQRFPATDDVNPLQAWEAADEYLLQQLDDTEILGPVLILNDAFGALSCALAEHKPYSI
GDSYISELATRENLRLNGIDESSVKFLDSTADYPQQPGVVLIKVPKTLALLEQQLRALRKVVTSDTRIIA
GAKARDIHTSTLELFEKVLGPTTTTLAWKKARLINCTFNEPPLADAPQTVSWKLEGTDWTIHNHANVFSR
TGLDIGARFFMQHLPENLEGEIVDLGCGNGVIGLTLLDKNPQAKVVFVDESPMAVASSRLNVETNMPEAL
DRSEFMINNALSGVEPFRFNAVLCNPPFHQQHALTDNVAWEMFHYARRCLKINGELYIVANRHLDYFHKL
KKIFGNCTTIATNNKFVVLKAVKLGRRR

>gi|30064426|ref|NP_838597.1| hypothetical protein S3330 [Shigella flexneri 2a str. 2457T]
MHLITQKALKDAAEKYPQHKTELVALGNTIAKGYFKKPESLKAVFPSLDNFKYLDKHYVFNVGGNELRVV
AMVFFESQKCYIREVMTHKEYDFFTAVHRTKGKK

>gi|30064425|ref|NP_838596.1| hypothetical protein S3329 [Shigella flexneri 2a str. 2457T]
MIAIADILQAGEKLTAVAPFLAGIQNEEQYTQALELVDHLLLNDPENPLLDLVCAKITAWEESAPEFAEF
NAMAQAMPGGIAVIRTLMDQYGLTLSDLPEIGSKSMVSRVLSGKRKLTLEHAKKLATRFGISPALFID

>gi|30064424|ref|NP_838595.1| NADPH dehydrogenase [Shigella flexneri 2a str. 2457T]

MSYPSLFAPLDLGFTTLKNRVLMGSMHTGLEEYPDGAERLAAFYAERARHGVALIVSGGIAPDLTGVGME

GGAMLNDASQIPHHRTITEAVHQEGGKIALQILHTGRYSYQPHLVAPSALQAPINRFVPHELTHEEILQL

IDDFARCAQLAREAGYDGVEVMGSEGYLINEFLTLRTNQRSDQWGGDYRNRMRFAVEVVRTVRERVGNDF

IIIYRLSMLDLVEGGGTFAETVELAQAIEAAGATIINTGIGWHEARIPTIATPVPRGAFSWVTRKLKGHV

SLPLVTTNRINDPQVADDILSRGDADIVSMARPFLADAELLSKAQSGRADEINTCIGCNQGCLDQIFVGK

VTSCLVNPRACHETKMPILPAVQKKNLAVVGAGPAGLAFAINAAARGHQVTLFDAHSEIGGQFNIAKQIP

GKEEFYETLRYYRRMIEVTGVTLKLNHTVTADQLQAFDETILASGIVPRVPPIDGIDHPKVLSYLDVLRD

KAPVGNKVAIIGCGGIGFDTAMYLSQPGESTSQNIARFCNEWGIDSSLQQAGGLSPQGMQIPRSPRQIVM

LQRKASKPGQGLGKTTGWIHRTTLLSRGVKMIPGVSYQKIDDDGLHVVINGETQVLAVDNVVICAGQEPN

RALAQPLIDSGKTVHLIGGCDVAMELDARRAIAQGTRLALEI

>gi|30064423|ref|NP_838594.1| glycosyl hydrolase [Shigella flexneri 2a str. 2457T]

MKIKTILTPVTCALLISFSAHAANADNYKNVINRTGAPQYMKDYDYDDHQRFNPFFDLGAWHGHLLPDGP

NTMGGFPGVALLTEEYINFMASNFDRLTVWQDGKKVNFTLEAYSIPGALVQKLTAKDVQVEMTLRFATPR

TSLLETKIISNKPLDLVWDGELLEKLEAKEGKPLSDKTIAGEYPDYQRKISATRDGLKVTFGKVRATWDL

LTSGESEYQVHKSLPVQTEINGNRFTSKAHINGSTTLYTTYSHLLTAQEVSKEQMQIRDILARPAFYLTA

SQQRWEEYLKKGLTNPDATPEQTRVAVKAIETLNGNWRSPGGAVNYNTVTPSVTGRWFSGNQTWPWDTWK

QAFAMAHFNPDIAKENIRAVFSWQIQPGDSVRPQDVGFVPDLIAWNLSPERGGDGGNWNERNTKPSLAAW

SVMEVYNVTQDKAWLAEMYPKLVAYHDWWLRNRDHNGNGVPEYGATRDKAHNTESGEMLFTVKKGDKEET

QSGLNNYARVVEKGQYDSLEIPAQVAASWESGRDDAAVFGFIDKEQLDKYVANGGKRSDWTVKFAENRSQ

DGTLLGYSLLQESVDQASYMYSDNHYLAEMATILGKPEEAKRYRQLAQQLADYINTCMFDPTTQFYYDVR

IEDKPLANGCAGKPIVERGKGPEGWSPLFNGAATQANADPVVKVMLDPKEFNTFVPLGTAALTNPAFGAD

IYWRGRVWVDQFWFGLKGMERYGYRDDALKLADTFFRHAKGLTADGPIQENYNPLTGAQQGAPNFSWSAA

HLYMLYNDFFRKQ

>gi|30064422|ref|NP_838593.1| oxidoreductase [Shigella flexneri 2a str. 2457T]

MSDTKRNTIGKFGLLSLTFAAVYSFNNVINNNIELGLASAPMFFLATIFYFIPFCLIIAEFVSLNKNSEA

GVYAWVKSSLGGRWAFITAYTYWFVNLFFFTSLLPRVIAYASYAFLGYEYIMTPVATTVISMVLFAFSTW

VSTNGAKMLGPITSVTSTLMLLLTLSYILLAGTALVGGVQPADPITVDAMIPNFNWAFLGVTTWIFMAAG

GAESVAVYVNDVKGGSKSFVKVIILAGIFIGVLYSVSSVLINVFVSSKELKFTGGSVQVFHGMAAYFGLP

EALMNRFVGLVSFTAMFGSLLMWTATPVKIFFSEIPEGIFGKKTVELNENGVPARAAWIQFLIVIPLMII

PMLGSNTVQDLMNTIINMTAAASMLPPLFIMLAYLNLRAKLDHLPRDFRMGSRRTGIIVVSMLIAIFAVG

FVASTFPTGANILTIIFYNVGGIVIFLGFAWWKYSKYIKGLTAEERHIEATPASNVD

>gi|30064421|ref|NP_838592.1| cryptic beta-D-galactosidase subunit beta [Shigella flexneri 2a str. 2457T]

MRIIDNLEQFRQIYASGKKWQRCVEAIENIDNIQPGVAHSIGDSLTYRVENDSATDALFTGHRRYFEVHY

YLQGQQKIEYAPKETLQVVEYYRDETDREYLKGCGETVEVHEGQIVICDIHEAYRFICNNAVKKVVLKVT

IEDGYFHNK

>gi|30064420|ref|NP_838591.1| cryptic beta-D-galactosidase subunit alpha [Shigella flexneri 2a str. 2457T]

MNRWENIQLTHENRLAPRAYFFSYDSVAQARTFARETSSLFLPLSSQWNFHFFDHPLQVPEAFTSELMAD

WGHITVPAMWQMEGHGKLQYTDEGFPFPIDVPFVPSDNPTGAYQRIFTLSDGWQGKQTLIKFDGVETYFE

VYVNGQYVGFSKGSRLTAEFDISAMVKTGDNLLCVRVMQWADSTYVEDQDMWWSAGIFRDVYLVGKHLTH

INDFTVRTDFDEAYCDATLSCEVVLENLAASPVVTTLEYTLFDGERVVHSSAIDHLAIEKLTSASFAFTV

EQPQQWSAESPYLYHLVMTLKDANGNVLEVVPQRVGFRDIKVRDGLFWINNRYVMLHGVNRHDNDHRKGR

AVGMDRVEKDLQLMKQHNINSVRTAHYPNDPRFYELCDIYGLFVMAETDVESHGFANVGDISRITDDPQW

EKVYVERIVRHIHAQKNHPSIIIWSLGNESGYGCNIRAMYHAAKALDDTRLVHYEEDRDAEVVDIISTMY

TRVPLMNEFGEYPHPKPRIICEYAHAMGNGPGGLTEYQNVFYKHDCIQGHYVWEWCDHGIQAQDDHGNVW

YKFGGDYGDYPNNYNFCLDGLIYSDQTPGSGLKEYKQVIAPVKIHALTRGELKVENKLWFTTLDDYTLHA

EVRAEGETLATQQIKLRDVAPNSEAPLQITLPQLDAREAFLNITVTKDSRTRYSEAGHSIATYQFPLKEN

TAQPVPFAPNNARPLTLEDDRLSCTVRGYNFAITFSKMSGKPTSWQVNGESLLTREPKINFFKPMIDNHK

QEYEGLWQPNHLQIMQEHLRDFAVEQSDGEVLIISRTVIAPPVFDFGMRCTYIWRIAADGQVNVALSGER

YGDYPHIIPCIGFTMGINGEYDQVAYYGRGPGENYADSQQANIIDIWRSTVDAMFENYPFPQNNGNRQHV

RWTALTNRHGNGLLVVPQRPINFSAWHYTQENIHAAQHCNELQRSDDITLNLDHQLLGLGSNSWGSEVLD

SWRVWFRDFSYGFTLLPVSGGEATAQSLASYEFGAGFFSTNLHSENKQ

>gi|30064419|ref|NP_838590.1| DNA-binding transcriptional repressor EbgR [Shigella flexneri 2a str. 2457T]

MATLKDIAIEAGVSLATVSRVLNDDPTLNVKEETKHRILEIAEKLEYKTSSARKLQTGAVNQHHILAIYS

YQQELEINDPYYLAIRHGIETQCEKLAIELTNCYEHNGLPDIKNVTGILIVGKPTPALRAAASALTDNIC

FIDFHEPGSGYDAVDIDLARISKEIIDFYINQGVNRIGFIGGEDEPGKADIREVAFAEYGRLKQVVREED

IWRGGFSSSSGYELAKQMLAREDYPKALFVASDSIAIGVLRAIHERGLNIPQDISLISVNDIPTARFTFP

PLSTVRIHSEMMGSQGVNLVYEKARDGRALPLLVFVPSKLKLRGTTR

>gi|30064417|ref|NP_838588.1| hypothetical protein S3318 [Shigella flexneri 2a str. 2457T]

MSHHHEGCCKHEGQPRHEGCCKGEKSEHEHCGHGHQHEHGQCCGGRHGRGGGRRQRFFGHGELRLVILDI

LSRDDSHGYELIKAIENLTQGNYTPSPGVIYPTLDFLQEQSLITIREEEGGKKQIALTEQGAQWLEENRE

QVEMIEERIKARCVGAALRQNPQMKRALDNFKAVLDLRVNQSDISDAQIKKIIAVIDRAAFDITQLD

>gi|30064416|ref|NP_838587.1| IS4 orf [Shigella flexneri 2a str. 2457T]

MHIGQALDLVSRYDSLRNPLTSLGDYLAPELISRCLAESGTVTLRKRRLPLEMMVWCIVGMALERKEPLH

QIVNRLDIMLPGNRPFVAPSAVIQARQRLGSEAVRRVFTKTAQLWHNATPHPHWCGLTLLAIDGVFWRTP

DTPENDAAFPRQTHAGNPALYPQVKMVCQMELTSHLLTAAAFGTMKNSENELAEQLIEQTGDNTLTLMDK

GYYSLGLLNGWSLAGEHRHWMIPLRKGAQYEELRKLGKGDHLVKLKTSPQARKKWPGLGNEVTARLLTVT

RKGKVCHLLTSMTDAMRFPGGEMADLYSNRWEIELGYREIKQTMQLSRLTLRSKKPELVEQELWGVLLAY

NLVRYQMIKMAEHLKGYWPNQLSFSESCGMVMRMLMTLQGASPGRIPELMRDLASMGQLVKLPTRRGRAF

PRVVKERPWKYPTAPKKSQSVA

>gi|30064415|ref|NP_838586.1| RNA polymerase sigma factor RpoD [Shigella flexneri 2a str. 2457T]

MEQNPQSQLKLLVTRGKEQGYLTYAEVNDHLPEDIVDSDQIEDIIQMINDMGIQVMEEAPDADDLMLAEN

TADEDAAEAAAQVLSSVESEIGRTTDPVRMYMREMGTVELLTREGEIDIAKRIEDGINQVQCSVAEYPEA

ITYLLEQYDRVEAEEARLSDLITGFVDPNAEEDLAPTATHVGSELSQEDLDDDEDEDEEDGDDDSADDDN

SIDPELAREKFAELRAQYVVTRDTIKAKGRSHAAAQEEILKLSEVFKQFRLVPKQFDYLVNSMRVMMDRV

RTQERLIMKLCVEQCKMPKKNFITLFTGNETSDTWFNAAIAMNKPWSEKLHDVSEEVHRALQKLQQIEEE

TGLTIEQVKDINRRMSIGEAKARRAKKEMVEANLRLVISIAKKYTNRGLQFLDLIQEGNIGLMKAVDKFE

YRRGYKFSTYATWWIRQAITRSIADQARTIRIPVHMIETINKLNRISRQMLQEMGREPTPEELAERMLMP

EDKIRKVLKIAKEPISMETPIGDDEDSHLGDFIEDTTLELPLDSATTESLRAATHDVLAGLTAREAKVLR

MRFGIDMNTDHTLEEVGKQFDVTRERIRQIEAKALRKLRHPSRSEVLQ

>gi|30064414|ref|NP_838585.1| DNA primase [Shigella flexneri 2a str. 2457T]

MAGRIPRVFINDLLARTDIVDLIDARVKLKKQGKNFHACCPFHNEKTPSFTVNGEKQFYHCFGCGAHGNA

IDFLMNYDKLEFVETVEELAAMHNLEVPFEAGSGPSQIERHQRQTLYQLMDGLNTFYQQSLQQPVATSAR

QYLEKRGLSHEVIARFAIGFAPPGWDNVLKRFGGNPENRQSLIDAGMLVTNDQGRSYDRFRERVMFPIRD

KRGRVIGFGGRVLGNDTPKYLNSPETDIFHKGRQLYGLYEAQQDNAEPNRLLVVEGYMDVVALAQYGINY

AVASLGTSTTADHIQLLFRATNNVICCYDGDRAGRDAAWRALETALPYMTDGRQLRFMFLPDGEDPDTLV

RKEGKEAFEARMEQAMPLSAFLFNSLMPQVDLSTPDGRARLSTLALPLISQVPGETLRIYLRQELGNKLG

ILDDSQLERLMPKAAESGVSRPVPQLKRTTMRILIGLLVQNPELATLVPPLENLDENKLPGLGLFRELVN

TCLSQPGLTTGQLLEHYRGTNNAATLEKLSMWDDIADKNIAEQTFTDSLNHMFDSLLELRQEELIARERT

HGLSNEERLELWTLNQELAKK

>gi|30064413|ref|NP_838584.1| 30S ribosomal protein S21 [Shigella flexneri 2a str. 2457T]

MPVIKVRENEPFDVALRRFKRSCEKAGVLAEVRRREFYEKPTTERKRAKASAVKRHAKKLARENARRTRL

Y

>gi|30064412|ref|NP_838583.1| DNA-binding/iron metalloprotein/AP endonuclease [Shigella flexneri 2a str. 2457T]

MRVLGIETSCDETGIAIYDDEKGLLANQLYSQVKLHADYGGVVPELASRDHVRKTVPLIQAALKESGLTA

KDIDAVAYTAGPGLVGALLVGATVGRSLAFAWNVPAIPVHHMEGHLLAPMLEDNPPEFPFVALLVSGGHT

QLISVTGIGQYELLGESIDDAAGEAFDKTAKLLGLDYPGGPLLSKMAAQGTAGRFVFPRPMTDRPGLDFS

FSGLKTFAANTIRDNGTDDQTRADIARAFEDAVVDTLMIKCKRALDQTGFKRLVMAGGVSANRTLRAKLA

EMMKKRRGEVFYARPEFCTDNGAMIAYAGMVRFKAGATADLGVSVRPRWPLAELPAA

>gi|30064411|ref|NP_838582.1| hypothetical protein S3309 [Shigella flexneri 2a str. 2457T]

MKPSTEWWRYLAPLAVIAIIALLPVPAGLENHTWLYFAVFTGVIVGLILEPVPGAVVAMVGISIIAILSP

WLLFSPEQLAQPGFKFTAKSLSWAVSGFSNSVIWLIFAAFMFGTGYEKTGLGRRIALILVKKMGHRTLFL

GYAVMFSELILAPVTPSNSARGAGIIYPIIRNLPPLYQSQPNDSSSRSIGSYIMWMGIVADCVTSAIFLT

AMAPNLLLIGLMKSASHATLSWGDWFLGMLPLSILLVLLVPWLAYVLYPPVLKSGDQVPRWAETELQAMG

PLCSREKRMLGLMVGALVLWIFGGDYIDAAMVGYSVVALMLLLRIISWDDIVSNKAAWNVFFWLASLITL

ATGLNNTGFISWFGKLLAGSLSGYSPTMVMVTLIVVFYLLRYFFASATAYTCALAPMMIAAALAMPEIPL

PVFCLMVGAAIGLGSILTPYATGPSPIYYGSGYLPTADYWRLGAIFGLIFLVLLVITGLLWMPVVLL

>gi|30064410|ref|NP_838581.1| L(+)-tartrate dehydratase subunit beta [Shigella flexneri 2a str. 2457T]

MKKILTTPIKAEDLQDIRVGDVIYLTGTLVTCRDVCHRRLIELKRPIPYDLNGKAIFHAGPIVRKNGDKW

EMVSVGPTTSMRMESFEREFIEQTGVKLVVGKGGMGPLTEEGCQKFKALHVIFPAGCAVLAATQVEEIEE

VHWTELGMPESLWVCRVKEFGPLIVSIDTHGNNLIAENKKLFAERRDPIVEEICEHVHYIK

>gi|30064409|ref|NP_838580.1| tartrate dehydratase subunit alpha [Shigella flexneri 2a str. 2457T]

MSESNKQQAVNKLTEIVANFTAMISTRMPDDVVDKLKQLKDAETSSMGKIIYHTMFDNMQKAIDLNRPAC

QDTGEIMFFVKVGSRFPLLGELQSILKQAVEEATVKAPLRHNAVEIFDEVNTGKNTGSGVPWVTWDIIPD

NDDAEIEVYMAGGGCTLPGRSKVLMPSEGYEGVVKFVFENISTLAVNACPPVLVGVGIATSVETAAVLSR

KAILRPIGSRHPNPKAAELELRLEEGLNRLGIGPQGLTGNSSVMGASSRHAAGSCRSHL

>gi|30064408|ref|NP_838579.1| transcriptional activator TtdR [Shigella flexneri 2a str. 2457T]

MLNSWPLAKDLQVLVEIVHSGSFSAAAATLGQTPAFVTKRIQILENTLATTLLNRSARGVALTESGQRCY

EHALEILTQYQRLVDDVTQIKTRPEGMIRIGCSFGFGRSHIAPAITELMRNYPELQVHFELFDRQIDLVQ

DNIDLDIRINDEIPDYYIAHLLTKNKRILCAAPEYLQKYPQPQSLQELSRHDCLVTKERDMTHGIWELGN

GQEKKSVKVSGHLSSNSGEIVLQWALEGKGIMLRSEWDVLPFLESGKLVQVLPEYAQSANIWAVYRELLY

RSMKFVSARNFWRHGASNGWASPMKAIRSCRSTRNHSGSFSRFLLNFVQIFVS

>gi|30064407|ref|NP_838578.1| glycerol-3-phosphate acyltransferase PlsY [Shigella flexneri 2a str. 2457T]

MSAIAPGMILIAYLCGSISSAILVCRLCGLPDPRTSGSGNPGATNVLRIGGKGAAVAVLIFDVLKGMLPV

WGAYELGVSPFWLGLIAIAACLGHIWPVFFGFKGGKGVATAFGAIAPISWDLTGVMAGTWLLTVLLSGYS

SLGAIVSALIAPFYVWWFKPQFTFPVSMLSCLILLRHHDNIQRLWRRQETKIWTKFKRKREKDPE

>gi|30064406|ref|NP_838577.1| bifunctional dihydroneopterin aldolase/dihydroneopterin triphosphate 2'-epimerase [Shigella flexneri 2a str. 2457T]

MDIVFIEQLSVITTIGVYDWEQTIEQKLVFDIEMAWDNRKAAKSDDVADCLSYADIAETVVSHVEGARFA

LVERVAEEVAELLLARFNSPWVRIKLSKPGAVARAANVGVIIERGNNLKENN

>gi|30064405|ref|NP_838576.1| undecaprenyl pyrophosphate phosphatase [Shigella flexneri 2a str. 2457T]

MSDMHSLLIAAILGVVEGLTEFLPVSSTGHMIIVGHLLGFEGDTAKTFEVVIQLGSILAVVVMFWRRLFG

LIGIHFGRPLQHEGESKGRLTLIHILLGMIPAVVLGLLFHDTIKSLFNPINVMYALVVGGLLLIAAECLK

PKEPRAPGLDDMTYRQAFMIGCFQCLALWPGFSRSGATISGGMLMGVSRYAASEFSFLLAVPMMMGATAL

DLYKSWGFLTTGDIPMFAVGFITAFVVALIAIKTFLQLIKRISFIPFAIYRFIVAAAVYVVFF

>gi|30064404|ref|NP_838575.1| multifunctional tRNA nucleotidyl transferase/2'3'-cyclic phosphodiesterase/2'nucleotidase/phosphatase [Shigella flexneri 2a str. 2457T]

MKIYLVGGAVRDALLGLSVKDRDWVVVGSTPQEMLDAGYQQVGRDFPVFLHPQTHEEYALARTERKSGSG

YTGFTCYTAPDVTLEDDLKRRDLTINALAQDDNGEIIDPYNGLGDLQNRLLRHVSPAFGEDPLRVLRVAR

FAARYAHLGFRIADETLTLMREMTHAGELEHLTPERVWKETESALTTRNPQVFFQVLRDCGALRVLFPEI

DALFGVPAPARWHPEIDTGIHTLMTLSMAAMLSPQVDVRFATLCHDLGKGLTPPELWPRHHGHGPAGVKL

VEQLCQRLRVPNEIRDLARLVAEFHDLIRTFPMLNPKTIVKLFDSIDAWRKPQRVEQLALTSEADVRGRT

GFESADYPQGRWLREGWEVAQSVPTKAVVEAGFKGVEIREELTRRRIAAVASWKEQRCPKPD

>gi|30064403|ref|NP_838574.1| signal transduction protein [Shigella flexneri 2a str. 2457T]

MPKLRLIGLTLLALSATAVSHAEETRYVSDELNTWVRSGPGDHYRLVGTVNAGEEVTLLQTDANTNYAQV

KDSSGRTAWIPLKQLSTEPSLRSRVPDLENQVKTLTDKLTNIDNTWNQRTAEMQQKVAQSDSVINGLKEE

NQKLKNELIVAQKKVDAASVQLDDKQRTIIMQWFMYGGGVLGLGLLLGLVLPHLIPSRKRKDRWMN

>gi|30064402|ref|NP_838573.1| hypothetical protein S3300 [Shigella flexneri 2a str. 2457T]

MAQEIELKFIVNHSAVEALRDHLNTLGGEHHDPVQLLNIYYETPDNWLRRHDMGLRIRGENGRYEMTMKV

AGRVTGGLHQRPEYNVALSEPTLDLAQLPTEIWPNGELPADLASRVQSLFSTDFYREKWLVEIDGSRIEI

ALDQGEVKAGEFAEPICELELELLSGDTRAVLKLANQLVSQTGLRQGSLSKAARGYHLAQGNPAREIKPT

TILHVAAKADVEQGLEAALELVLAQWQYHEELWVRGNDAAKEQVLAAISLVRHTLMLFGGIVPRKASTHL

RDLLTQCEATIASAVSAVTAVYSTETAMAKLALTEWLVSKAWQPFLDAKAQGKISDSFKRFADIHLSRHA

AELKSVFCQPLGDRYRDQLPRLMRDIDSILLLAGYYDPVVAQAWLENWQGLRHAIATGQRIEIEHFRNEA

NNQEPFWLHSGKR

>gi|30064401|ref|NP_838572.1| bifunctional glutamine-synthetase adenylyltransferase/deadenyltransferase [Shigella flexneri 2a str. 2457T]

MKPLSSPLQQYWQTVVERLPELLAEESLSAQAKSVLTFSDFVQDSVIAHPEWLTELESQPPQADEWQHYS

VWLQEALSNVSDEAGLMRELRLFRRRIMVRIAWAQTLALVTEESILQQLSYLAETLIVAARDWLYDACCR

EWGTPCNAQGEAQPLLILGMGKLGGGELNFSSDIDLIFAWPEHGCTQGGRRELDNAQFFTRMGQRLIKVL

DQPTQDGFVYRVDMRLRPFGESGPLVLSFAALEDYYQEQGRDWERYAMVKARIMGDSEGVYANELRAMLR

PFVFRRYIDFSVIQSLRNMKGMIAREVRRRGLTDNIKLGAGGIREIEFIVQVFQLIRGGREPLLQSRSLL

PTLSAIAALHLLSENDAEQLRVAYLFLRRLENLLQSINDEQTQTLPSDELNRARLAWAMDFADWPQLTGA

LTAHMTNVRRVFNELIGDDESETQEESLSEQWRELWQDALQEDDTTPVLAHLSEDDRKQVLTMIADFRKE

LDKRTIGPRGRQVLDHLMPHLLSDVCAREDAAVTLSRITALLVGIVTRTTYLELLSEFPAALKHLISLCA

ASPMIASQLARYPLLLDELLDPNTLYQPTATDAYRDELRQYLLRVPEDDEEQQLEALRQFKQAQLLRIAA

ADIAGTLPVMKVSDHLTWLAEAMIDAVVQQAWGQMVARYGKPNHLNEREGRGFAVVGYGKLGGWELGYSS

DLDLIFLHDCPMDAMTDGEREIDGRQFYLRLAQRIMHLFSTRTSSGILYEVDARLRPSGAAGMLVTSAEA

FADYQKNEAWTWEHQALVRARVVYGDPQLTAHFDAVRREIMTLPREGKTLQTEVREMREKMRAHLGNKHR

NRFDIKADEGGITDIEFITQYLVLRYAHEKPKLTRWSDNVRILELLAQNDIMEEQEAMALTRAYTTLRDE

LHHLALQELPGHVSEDCFTAERDLVRASWQKWLVEE

>gi|30064400|ref|NP_838571.1| bifunctional heptose 7-phosphate kinase/heptose 1-phosphate adenylyltransferase [Shigella flexneri 2a str. 2457T]

MKVTLPEFERAGVMVVGDVMLDRYWYGPTSRISPEAPVPVVKVNTIEERPGGAANVAMNIASLGANARLV

GLTGIDDAARALSKSLADVNVKCDFVSVPTHPTITKLRVLSRNQQLIRLDFEEGFEGVDPQPLHERINQA

LSSIGALVLSDYAKGALASVQQMIQLARKAGVPVLIDPKGTDFERYRGATLLTPNLSEFEAVVGKCKTEE

EIVERGMKLIADYELSALLVTRSEQGMSLLQPGKAPLHMPTQAQEVYDVTGAGDTVIGVLAATLAAGNSL

EEACFFANAAAGVVVGKLGTSTVSPIELENAVRGRADTGFGVMTEEELKLAVAAARKRGEKVVMTNGVFD

ILHAGHVFYLANARKLGDRLIVAVNSDASTKRLKGDSRPVNPLEQRMIVLGALEAVDWVVSFEEDTPQRL

IAGILPDLLVKGGDYKPEEIAGSKEVWANGGEVLVLNFEDGCSTTNIIKKIQQDKKG

>gi|30064399|ref|NP_838570.1| IS1 orfA [Shigella flexneri 2a str. 2457T]

MASISIRCPSCSATEGVVRNGKSTAGHQRYLCSHCRKTWQLQFTYTASQPGTHQKIIDMAMNGVGCRASA

RIMGVGLNTVLRHLKTQAAVGNLAHTTGQ

>gi|30064398|ref|NP_838569.1| glycogen synthesis protein GlgS [Shigella flexneri 2a str. 2457T]

MEHSLNSLNNFDFLARSFARMHAEGRPVDILAVTGNMDEEHRTWFCARYAWYCQQMMQARELELEH

>gi|30064397|ref|NP_838568.1| IS4 orf [Shigella flexneri 2a str. 2457T]

MTRYQMIKMAEHLKGYWPNQLSFSESCGMVMRMLMTLQGASPGRIPELMRDLASMGQLVKLPTRRGRAFP

RVVKERPWKYPTAPKKSQSVA

>gi|30064396|ref|NP_838567.1| IS1 orfB [Shigella flexneri 2a str. 2457T]

MSRQCTHYGRWPQHGFTSLKKLRPQSVTSRIQPGSDVIVCAEMDEHWGYVGAKSRQRWLFYAYDRIRRTV

VAHVFGERTLATLERLLSLLSAFEVVVWMTDGWPLYESRLKGKLHVISKRYTQRIERHNLNLRQHLARLG

RKSLSFSKSVELHDKVIGHYLNIKHYQ

>gi|30064395|ref|NP_838566.1| IS1 orfA [Shigella flexneri 2a str. 2457T]

MASISIRCPSCSATEGVVRNGKSTAGHQRYLCSHCRKTWQLQFTYTASQPGTHQKIIDMAMNGVGCRASA

RIMGVGLNTVLRHLKNSGRSR

>gi|30064392|ref|NP_838563.1| hypothetical protein S3287 [Shigella flexneri 2a str. 2457T]

MTYPYRTTMVLNTYQYRETTMIDPKKIEQIARQVHESMPKGIREFGEDVEKKIRQTLQAQLTRLDLVSRE

EFDVQTQVLLRTREKLALLEQRISELENRSTEIKKQPDPETLPPTL

>gi|30064391|ref|NP_838562.1| 3,4-dihydroxy-2-butanone 4-phosphate synthase [Shigella flexneri 2a str. 2457T]

MNQTLLSSFGTPFERVENALAALREGRGVMVLDDEDRENEGDMIFPAETMTVEQMALTIRHGSGIVCLCI

TEDRRKQLDLPMMVENNTSAYGTGFTVTIEAAEGVTTGVSAADRITTVRAAIADGAKPSDLNRPGHVFPL

RAQAGGVLTRGGHTEATIDLMTLAGFKPAGVLCELTNDDGTMARAPECIEFANKHNMALVTIEDLVAYRQ

AHERKAS

>gi|30064390|ref|NP_838561.1| zinc transporter ZupT [Shigella flexneri 2a str. 2457T]

MSVPLILTILAGAATFIGAFLGVLGQKPSNRLLAFSLGFAAGIMLLISLMEMLPAALAAEGMSPVLGYGM

FIFGLLGYFGLDRMLPHAHPQDLMQKSVQLLPKSIKRTAILLTLGISLHNFPEGIATFVTASSNLELGFG

IALAVALHNIPEGLAVAGPVYAATGSKRTAILWAGISGLAEILGGVLAWLILGSMISPVVMAAIMAAVAG

IMVALSVDELMPLAKEIDPNNNPSYGVLCGMSVMGFSLVLLQTAGIG

>gi|30064389|ref|NP_838560.1| hypothetical protein S3284 [Shigella flexneri 2a str. 2457T]

MTPLVKDIIMSSTRMPALFLGHGSPMNVLEDNLYTRSWQKLGMTLPRPQAIVVVSAHWFTRGTGVTAMET

PPTIHDFGGFPQALYDTHYPAPGSPALAQRLVELLAPIPVTLDKEAWGFDHGSWGVLIKMYPDADIPMVQ

LSIDSSKPAAWHFEMGRKLAALRDEGIMLVASGNVVHNLRTVKWHGDSSPYPWATSFNEYVKANLTWQGP

VEQHPLVNYLDHEGGALSNPTPEHYLPLLYVLGVWDGQEPITIPVDGIEMGSLSMLSVQIG

>gi|30064388|ref|NP_838559.1| synthetase/amidase [Shigella flexneri 2a str. 2457T]

MERVSITERPDWREKAHEYGFNFHTMYGEPYWCEDAYYKLTLAQVEKLEEVTAELHQMCLKVVEKVIASD

ELMTKFRIPKHTWSFVRQSWLTHQPSLYSRLDLAWDGTGEPKLLENNADTPTSLYEAAFFQWIWLEDQLN

AGNLPEGSDQFNSLQEKLIDRFVELREQYGFQLLHLTCCRDTVEDRGTIQYLQDCATEAEIATEFLYIDE

IGLGEKGQFTDLQDQVISNLFKLYPWEFMLREMFSTKLEDAGVRWLEPAWKSIISNKALLPLLWEMFPNH

PNLLPAYFAEDDHPQMEKYVVKPIFSREGANVSIIENGKTIEAAEGPYGEEGMIVQQFHPLPKFGDSYML

IGSWLVNDQPAGIGIREDRALITQDMSRFYPHIFVE

>gi|30064386|ref|NP_838557.1| hypothetical protein S3281 [Shigella flexneri 2a str. 2457T]

MTTTGLRPRLNVRQRKDTGYLPHSSPFSLQFRPAILYSDGYLPLVPEDKNETDKIHTPRIVPQKLERTPS

DTSRSRGCHCFYAGWL

>gi|30064385|ref|NP_838556.1| outer membrane channel protein [Shigella flexneri 2a str. 2457T]

MKKLLPILIGLSLSGFSSLSQAENLMQVYQQARLSNPELRKSAADRDAAFEKINEARSPLLPQLGLGADY

TYSNGYRDANGINSNATSASLQLTQSIFDMSKWRALTLQEKAAGIQDVTYQTDQQTLILNTATAYFNVLN

AIDVLSYTQAQKEAIYRQLDQTTQRFNVGLVAITDVQNARAQYDTVLANEVTARNNLDNAVEQLRQITGN

YYPELAALNVENFKTDKPQPVNALLKEAEKRNLSLLQARLSQDLEREQIRQAQDGHLPTLDLTASTGISD

TSYSGSKTRGAAGTQYDDSNMGQNKVGLSFSLPIYQGGMVNSQVKQAQYNFVGASEQLESAHRSVVQTVR

SSFNNINASISSINAYKQAVVSAQSSLDAMEAGYSVGTRTIVDVLDATTTLYNAKQELANARYNYLINQL

NIKSALGTLNEQDLLALNNALSKPVSTNPENVAPQTPEQNAIADGYAPDSPAPVVQQTSARTTTSNGHNP

FRN

>gi|30064384|ref|NP_838555.1| ADP-ribose pyrophosphatase NudF [Shigella flexneri 2a str. 2457T]

MLKPDNLPVTFGKNDVEIIARETLYRGFFSLDLYRFRHRLFNGQMSHEVRREIFERGHAAVLLPFDPVRD

EVVLIEQIRIAAYDTSETPWLLEMVAGMIEEGESVEDVARREAIEEAGLIVKRTKPVLSFLASPGGTSER

SSIMVGEVDATTASGIHGLADENEDIRVHVVSREQAYQWVEEGKIDNAASVIALQWLQLHHQALKNEWA

>gi|30064383|ref|NP_838554.1| hypothetical protein S3278 [Shigella flexneri 2a str. 2457T]

MKRYTPDFPEMMRLCEMNFSQLRRLLPRNDAPGETVSYQVANAQYRLTIVESTRYTTLVTIEQTAPAISY

WSLPSMTVRLYHDAMVAEVCSSQQIFRFKARYDYPNKKLHQRDEKHQINQFLADWLRYCLAHGAMAIPVY

>gi|30064382|ref|NP_838553.1| cyclic 3',5'-adenosine monophosphate phosphodiesterase [Shigella flexneri 2a str. 2457T]

MESLLTLPLAGEARGRILQITDTHLFAQKHEALLGVNTWESYQAVLEAIRPHQHEFDLIVATGDLAQDQS

SAAYQHFAEGIASFRAPCVWLPGNHDFQPAMYSALQDAGISPAKRVFIGEQWQILLLDSQVFGVPHGELS

EFQLEWLERKLADAPERHTLLLLHHHPLPAGCSWLDQHSLRNAGELDTVLAKFPHVKYLLCGHIHQELDL

DWNGRRLLATPSTCVQFKPHCSNFTLDTIAPGWRTLELHADGTLTTEVHRLADTRFQPDTASEGY

>gi|30064381|ref|NP_838552.1| esterase YqiA [Shigella flexneri 2a str. 2457T]

MSTLLYLHGFNSSPRSAKASLLKNWLAEHHPDVEMIIPQLPPYPSDAAELLESIVLEHGGDSLGIVGSSL

GGYYATWLSQCFMLPAVVVNPAVRPFELLTDYLGQNENPYTGQQYVLESRHIYDLKVMQIDPLEAPDLIW

LLQQTGDEVLDYRQAVAYYASCRQTVIEGGNHAFTGFEDYFNPIVDFLGLHHL

>gi|30064380|ref|NP_838551.1| DNA topoisomerase IV subunit B [Shigella flexneri 2a str. 2457T]

MTQTYNADAIEVLTGLEPVRRRPGMYTDTTRPNHLGQEVIDNSVDEALAGHAKRVDVILHADQSLEVIDD

GRGMPVDIHPEEGVPAVELILCRLHAGGKFSNKNYQFSGGLHGVGISVVNALSKRVEVNVRRDGQVYNIA

FENGEKVQDLQVVGTCGKRNTGTSVHFWPDETFFDSPRFSVSRLTHVLKAKAVLCPGVEITFKDEINNTE

QRWCYQDGLNDYLAEAVNGLPTLPEKPFIGNFAGDTEAVDWALLWLPEGGELLTESYVNLIPTMQGGTHV

NGLRQGLLDAMREFCEYRNILPRGVKLSAEDIWDRCAYVLSVKMQDPQFAGQTKERLSSRQCAAFVSGVV

KDAFILWLNQNVQAAELLAEMAISSAQRRMRAAKKVVRKKLTSGPALPGKLADCTAQGLNRTELFLVEGD

SAGGSAKQARDREYQAIMPLKGKILNTWEVSSDEVLASQEVHDISVAIGIDPDSDDLSQLRYGKICILAD

ADSDGLHIATLLCALFVKHFRALVKHGHVYVALPPLYRIDLGKEVYYALTEEEKEGVLEQLKRKKGKPNV

QRFKGLGEMNPMQLRETTLDPNTRRLVQLTIDDEDDQRTDAMMDMLLAKKRSEDRRNWLQEKGDMAEIEV

>gi|30064379|ref|NP_838550.1| hypothetical protein S3274 [Shigella flexneri 2a str. 2457T]

MLTVIAEIRTRPGQHHRQAVLDQFAKIVPTVLKEEGCHGYAPMVDCAAGVSFQSMAPDSIVMIEQWESIA

HLEAHLQTPHMKAYSEAVKGDVLEMNIRILQPGI

>gi|30064378|ref|NP_838549.1| modulator of drug activity B [Shigella flexneri 2a str. 2457T]

MSNILIINGAKKFAHSNGQLNDTLTEVADGTLRDLGHDVRIVRADSDYNVKAEVQNFLWADVVIWQMPGW

WMGAPWTVKKYIDDVFTEGHGTLYASDGRTRKDPSKKYGSGSLVQDKKYMLSLTWNAPMEAFTEKDQFFH

GVGVDGVYLPFHKANQFLGMEPLPTFIANDVIKMPDVPRYTEEYRKHLVEIFG

>gi|30064377|ref|NP_838548.1| hypothetical protein S3272 [Shigella flexneri 2a str. 2457T]

MLKQKIKTIFEALLYIMLTYWLIDSFFAFNKYDWMLESGGNICSIPSVSGEDRILQAMIAAFFLLTPLII

LILRKLFMREMFEFWLYVFSLVICLVCGWWLFWGRFIFCY

>gi|30064376|ref|NP_838547.1| sensor protein QseC [Shigella flexneri 2a str. 2457T]

MKFTQRLSLRVRLTLIFLILASVTWLLSSFVAWKQTTDNVDELFDTQLMLFAKRLSTLDLNEINAADRMA

QTPNKLKHGHVDDDALTFAIFTHDGRMVLNDGDNGEDIPYSYQREGFADGQLVGEDDPWRFVWMTSPDGK

YRIVVGQEWEYREDMALAIVAGQLIPWLVALPIMLIIMMVLLGRELAPLNKLALALRMRDPDSEKPLNAT

GVPSEVRPLVESLNQLFARTHAMMVRERRFTSDAAHELRSPLTALKVQTEVAQLSDDDPQARKKALLQLH

SGIDRATRLVDQLLTLSRLDSLDNLQDVAEIPLEDLLQSSVMDIYHTAQQAKIDVRLTLNAHGIKRTGQP

LLLSLLVRNLLDNGPGVTPEALARIGERFYRPPGQTATGSGLGLSIVQRIAKLHDMNVEFGNAEQGGFEA

KVSW

>gi|30064375|ref|NP_838546.1| DNA-binding transcriptional regulator QseB [Shigella flexneri 2a str. 2457T]

MRILLIEDDMLIGDGIKTGLSKMGFSVDWFTQGRQGKEALYSAPYDAVILDLTLPGMDGRDILREWREKG

QREPVLNLTARDALAERVEGLRLGADDYLCKPFALIEVAARLEALMRRTNGQASNELRHGNVMLDPGKRI

ATLAGEPLTLKPKEFALLELLMRNAGRVLPRKLIEEKLYTWDEEVTSNAVEVHVHHLRRKLGSDFIRTVH
GIGYTLGEK

>gi|30064374|ref|NP_838545.1| hypothetical protein S3269 [Shigella flexneri 2a str. 2457T]
MKKFAAVIAVMALCSAPVMAAEQGGFSGPSVTQSQAGGFQGPNGSVTTVESAKSLRDDTWVTLRGNIVER
ISDDLYVFKDASGTINVDIDHKRWNGVTVTPKDTVEIQGEVDKDWNSVEIDVKQIRKVNP

>gi|30064373|ref|NP_838544.1| hypothetical protein S3268 [Shigella flexneri 2a str. 2457T]
MTNLTLDVNIIDFPSIPVAMLPHRCSPELLNYSVAKFIMWRKETGLSPVNQSQTFGVAWDAPATTAPEAF
RFDICGSVSEPIPDNRYGVSNGELTGGRYAVARHVGELDDISHTIWGIIRHWLPASGEKMRKAPILFHYT
NLAEGVTEQRLETDVYVPLA

>gi|30064372|ref|NP_838543.1| DNA topoisomerase IV subunit A [Shigella flexneri 2a str. 2457T]
MSDMAERLALHEFTENAYLNYSMYVIMDRALPFIGDGLKPVQRRIVYAMSELGLNASAKFKKSARTVGDV
LGKYHPHGDSACYEAMVLMAQPFSYRYPLVDGQGNWGAPDDPKSFAAMRYTESRLSKYSELLLSELGQGT
ADWVPNFDGTLQEPKMLPARLPNILLNGTTGIAVGMATDIPPHNLREVAQAAIALIDQPKTTLDQLLDIV
QGPDYPTEAEIITSRAEIRKIYENGRGSVRMRAVWKKEDGAVVISALPHQVSGARVLEQIAAQMRNKKLP
MVDDLRDESDHENPTRLVIVPRSNRVDMDQVMNHLFATTDLEKSYRINLNMIGLDGRPAVKNLLEILSEW
LVFRRDTVRRRLNYRLEKVLKRLHILEGLLVAFLNIDEVIEIIRNEDEPKPALMSRFGLTETQAEAILEL
KLRHLAKLEEMKIRGEQSELEKERDQLQGILASERKMNNLLKKELQADAQAYGDDRRSPLQEREEAKAMS
EHDMLPSEPVTIVLSQMGWVRSAKGHDIDAPGLNYKAGDSFKAAVKGKSNQPVVFVDSTGRSYAIDPITL
PSARGQGEPLTGKLTLPPGATVDHMLMESDDQKLLMASDAGYGFVCTFNDLVARNRAGKALITLPENAHV
MPPVVIEDASDMLLAITQAGRMLMFPVSDLPQLSKGKGNKIINIPSAEAARGEDGLAQLYVLPPQSTLTI
HVGKRKIKLRPEELQKVTGERGRRGTLMRGLQRIDRVEIDSPRRASSGDSEE

>gi|30064371|ref|NP_838542.1| 1-acyl-sn-glycerol-3-phosphate acyltransferase [Shigella flexneri 2a str. 2457T]

MLYIFRLIITVIYSILVCVFGSIYCLFSPRNPKHVATFGHMFGRLAPLFGLKVECRKPADAESYGNAIYI

ANHQNNYDMVTASNIVQPPTVTVGKKSLLWIPFFGQLYWLTGNLLIDRNNRTKAHGTIAEVVNHFKKRRI

SIWMFPEGTRSRGRGLLPFKTGAFHAAIAAGVPIIPVCVSTTSNKINLNRLHNGLVIVEMLPPIDVSQYG

KDQVRELAAHCRSIMEQKIAELDKEVAEREAAGKV

>gi|30064370|ref|NP_838541.1| repressor protein for FtsI [Shigella flexneri 2a str. 2457T]

MSLSRRQFIQASGIALCAGAVPLKASAAGQQQPLPVPPLLESRRGQPLFMTVQRAHWSFTPGTRASVWGI

NGRYLGPTIRVWKGDDVKLIYSNRLTENVSMTVAGLQVPGPLMGGPARMMSPNADWAPVLPIRQNAATLW

YHANTPNRTAQQVYNGLAGMWLVEDEVSKSLPIPNHYGVDDFPVIIQDKRLDNFGTPEYNEPGSGGFVGD

TLLVNGVQSPYVEVSRGWVRLRLLNASNSRRYQLQMSDGRPLHVISGDQGFLPAPVSVKQLSLAPGERRE

ILVDMSNGDEVSITCGEAASIVDRIRGFFEPSSILVSTLVLTLRPTGLLPLVTDSLPMRLLPTEIMAGSP

IRSRDISLGDDPGINGQLWDVNRIDVTAQQGTWERWTVRADEPQAFHIEGVMFQIRNVNGAMPFPEDRGW

KDTVWVDGQVELLVYFGQPSWAHFPFYFNSQTLEMADRGSIGQLLVNPVP

>gi|30064369|ref|NP_838540.1| hypothetical protein S3263, partial [Shigella flexneri 2a str. 2457T]

MQKHGDRYVWINPPAIPLSTEEMDSVFALPYKRVPHPAYGNARIPAYEMIRFSVNIMRGCFGGCSFCSIT

EHEGRIIQSRSEDSIINEIEAIRDTVPGFTGVISDLGGPTANMYMLRCKSPRAEQTCRRLSCVYPDICPH

MDTNHEPTINLYRRARDLKGIKKILIASGVRYDIAVEDPRYIKELATHHVGGYLKIAPEHTEEGPLSKMM

KPGMGSYDRFKELFDTYSKQAGKEQYLIPYFISAHPGTRDEDMVNLALWLKKHRFRLDQVQNFYPSPLAN

STTMYYTGKKPLAKIGYKSEGVFVPKGDKQRRLHKALLRYHDPANWPLIRQALEAMGKKHLIGSRRDCLV

PAPTIEEMREARRQNRNTRPALTKHTPMATQRQTPATAKKASSTQSRPVNAGAKKRPKAAVGR

>gi|30064368|ref|NP_838539.1| outer membrane lipoprotein [Shigella flexneri 2a str. 2457T]

MKTIFTVGAVVLATCLLSGCVNEQKVNQLASNVQTLNAKIARLEQDMKALRPQIYAAKSEANRANTRLDA

QDYFDCLRCLRMYAE

>gi|30064367|ref|NP_838538.1| hypothetical protein S3261 [Shigella flexneri 2a str. 2457T]

MKIILLFLAALASFTVHAQPPSQTVEQTVRQIYQNYKSDASTPYFGETGERAITSARIQQALTLNDNLTL

PGNIGWLDYDPVCDCQDFGDLVLESVAITQTDVDHADAVVRFRIFKDDKEKTTQTLKMVAENGRWVIDDI

VSNHGSVLQAVNSENEKTLAALASLQKEQPESFVAELFEHIADYSWPWTWVVSDSYRQAVNAFYKTTFKT

ANNPDEDMQIERQFIYDNPICFGEESLFSRVDEIRVLEKTADSARIHVRFTLTNGNNEEQELVLQRREGK

WEIADFIRPNSGSLLKQIEAKTAARLKQ

>gi|30064365|ref|NP_838536.1| oxidoreductase [Shigella flexneri 2a str. 2457T]

MNNFNLHTPTRILFGKGAIAGLREQIPHDARVLITYGGGSVKKTGVLDQVLDALKGMDVLEFGGIEPNPA

YETLMNAVKLVREQKVTFLLAVGGGSVLDGTKFIAAAANYPENIDPWHILQTGGKEIKSAIPMGCVLTLP

ATGSESNAGAVISRKTTGDKQAFHSAHVQPVFAVLDPVYTYTLPPRQVANGVVDAFVHTVEQYVTKPVDA

KIQDRFAEGILLTLIEDGPKALKEPENYDVRANVMWAATQALNGLIGTGVPQDWATHMLGHELTAMHGLD

HAQTLAIVLPALWNEKRETKRAKLLQYAERVWNITEGSDDERIDAAIAATRNFFEQLGVPTHLSDYGLDG

SSIPALLKKLEEHGMTQLGENHDITLDVSRRIYEAAR

>gi|30064364|ref|NP_838535.1| ARAC-type regulatory protein [Shigella flexneri 2a str. 2457T]

MRVGVCRLKLFITCSLCWANMRAENDLPDVFHCDRQRPGSQCSFLPMSCLFLQSAGEMLQNCAQSNCRII

PKKLRDMKREEICRLLADKVNKLKNKENSLSELLPDVRLLYGETPFARTPVMYEPGIIILFSGHKIGYIN

ERVFRYDANEYLLLTVPLPFECETYATSEVPLAGLRLNVDILQLQELLMDIGEDEHFQPSMAASGINSAT

LSEEILCAAERLLDVMERPLDARILGKQIIREILYYVLTGPCGGALLALVSRQTHFSLISRVLKRIENKY

TENLSVEQLAAEANMSVSAFHHNFKSVTSTSPLQYLKNYRLHKARMMIIHDGMKASAAAMRVGYESASQF

SREFKRYFGVTPGEDAARMRAMQGN

>gi|30064363|ref|NP_838534.1| hypothetical protein S3257 [Shigella flexneri 2a str. 2457T]

MAVIQDIIAALWQHDFAALADPHIVSVVYFVMFATLFLENGLLPASFLPGDSLLILAGALIAQGVMDFLP

TIAILTAAASLGCWLSYIQGSWLGNTKTVKGWLAQLPAKYHQRATCMFDRHGLLALLAGRFLAFVRTLLP

TMAGISGLPNRRFQFFNWLSGLLWVSVVTSFGYALSMIPFVKRHEDQVMTFLMILPIALLTAGLLGTLFV

VIKKKYCNA

>gi|30064362|ref|NP_838533.1| cystathionine beta-lyase [Shigella flexneri 2a str. 2457T]

MADKKLDTQLVNAGRSKKYTLGAVNSVIQRASSLVFESMEAKKHATRNRANGELFYGRRGTLTHFSLQQA

MCELEGGAGCALFPCGAAAVANSILAFVEQGDHVLMTNTAYEPSQDFCSKILSKLGVTTSWFDPLIGADI

VKHLQPNTKIVFLESPGSITMEVHDVPAIVAAVRSVVPDAIIMIDNTWAAGVLFKALDFGIDVSIQAATK

YLVGHSDAMIGTAVCNARCWEQLRENAYLMGQMVDADTAYITSRGLRTLGVRLRQHHESSLKVAEWLAEH

PQVARVNHPALPGSKGHEFWKRDFTGSSGLFSFVLKKKLNDEELANYLDNFSLFSMAYSWGGYESLILAN

QPEHIAAIRPQGEIDFSGTLIRLHIGLEDVDDLIADLDAGFARIV

>gi|30064361|ref|NP_838532.1| hypothetical protein S3255 [Shigella flexneri 2a str. 2457T]

MLVTFLLRKRKEKKAKVRQYANSNENDYQFDVVLILLCADFVTCVLEIHSG

>gi|30064360|ref|NP_838531.1| biopolymer transport protein ExbB [Shigella flexneri 2a str. 2457T]

MGNNLMQTDLSVWGMYQYADIVVKCVMIGLILASVVTWAIFFSKSVEFFNQKRRLKREQQLLAEARSLNQ

ANDIAADFGSKSLSLHLLNEAQNELELSEGSDDNEGIKERTSFRLERRVAAVGRQMGRGNGYLATIGAIS

PFVGLFGTVWGIMNSFIGIAQTQTTNLAVVAPGIAEALLATAIGLVAAIPAVVIYNVFARQIGGFKAMLG

DVAAQVLLLQSRDLDLEASAAAHPVRVAQKLRAG

>gi|30064359|ref|NP_838530.1| biopolymer transport protein ExbD [Shigella flexneri 2a str. 2457T]

MAMHLNENLDDNGEMHDINVTPFIDVMLVLLIIFMVAAPLATVDVKVNLPASTSTPQPRPEKPVYLSVKA

DNSMFIGNDPVTDETMITALNALTEGKKDTTIFFRADKTVDYETLMKVMDTLHQAGYLKIGLVGEETAKA

K

>gi|30064358|ref|NP_838529.1| hypothetical protein S3252 [Shigella flexneri 2a str. 2457T]

MNALSGLQTHEDSTCCNRFCRPDERSASGNSTLLRFGGFFADQTYLQVTRLMQRIHYLHQRLVIDGFVRS

EEDGGVFLAFG

>gi|30064357|ref|NP_838528.1| oxidoreductase [Shigella flexneri 2a str. 2457T]

MSHLKDPTTQYYTGEYPKQKQPTPGIQAKMTPIPDCGEKTYVGSGRLKDRKALVTGGDSGIGRAAAIAYA

REGADVAISYLPVEEEDAQDVKKIIEECGRKAVLLPGDLSDEKFARSLVHEAHKALGGLDIMALVAGKQV

AIPDIADLTSEQFQKTFAINVFALFWLTQEAIPLLPKGASVITTSSIQAYQPSPHLLDYAATKAAILNYS

RGLAKQVAEKGIRVNIVAPGPIWTALQISGGQTQDKIPQFGQKTPMKRAGQPAELAPVYVYLASQESSYV

TAEVHGVCGGEHLG

>gi|30064356|ref|NP_838527.1| hypothetical protein S3250 [Shigella flexneri 2a str. 2457T]

MERFLENAMYASRWLLAPVYFGLSLALVALALKFFQEIIHVLPNIFSMAESDLILVLLSLVDMTLVGGLL

VMVMFSGYENFVSQLDISENKEKLKWLGKMDATSLKNKVAASIVAISSIHLLRVFMDAKNVPDNKLMWYV

IIHLTFVLSAFVMGYLDRLTRHNH

>gi|30064355|ref|NP_838526.1| aldo-keto reductase [Shigella flexneri 2a str. 2457T]

MVWLANPERYGQMQYRYCGKSGLRLPALSLGLWHNFGHVNALESQRAILRKAFDLGITHFDLANNYGPPP

GSAEENFGRLLREDFAAYRDELIISTKAGYDMWPGPYGSGGSRKYLLASLDQSLKRMGLEYVDIFYSHRV

DENTPMEETASALAHAVQSGKALYVGISSYSPERTQKMVELLHEWKIPLLIHQPSYNLLNRWVDKSGLLD

TLQNNGVGCIAFTPLAQGLLTGKYLNGIPQDSRMHREGNKVRGLTPKMLTEANLNSLRLLNEMAQQRGQS

MAQMALSWLLKDDRVTSVLIGASRAEQLEENVQALNNLTFSTEELAQIDQHIADGELNLWQASSDK

>gi|30064354|ref|NP_838525.1| hypothetical protein S3247, partial [Shigella flexneri 2a str. 2457T]

MNDFFAAIEFMQRYPQATGKVGITGFCYGGGVSNAAAVAYPELACAVPFYGRQAPTADVAKIEAPLLLHF

AELDTRINEGWPPYEAALKDNNKVYEAYIYPGVNHGFHNDSTPRYDKSAADLAWQRTLKWFDKYLS

>gi|30064353|ref|NP_838524.1| hypothetical protein S3246 [Shigella flexneri 2a str. 2457T]

MNNHFGKGLMAGLKATHADSAVNVTKFCADYKRGFVLGYSHRMYEKTGDRQLSAWEAGILTRRYGLDKEM

VMDFFRENNSCSTLRFFMAGYRLEN

>gi|30064352|ref|NP_838523.1| hydrogenase 2 small subunit [Shigella flexneri 2a str. 2457T]

MTGDNTLIHSHGINRRDFMKLCAALAATMGLSSKAAAEMAESVTNPQRPPVIWIGAQECTGCTESLLRAT

HPTVENLVLETISLEYHEVLSAAFGHQVEENKHNALEKYKGQYVLVVDGSIPLKDNGIYCMVAGEPIVDH

IRKAAEGSAAIIAIGSCSAWGGVAAAGVNPTGAVSLQEVLPGKTVINIPGCPPNPHNFLATVAHIITYGK

PPKLDDKNRPTFAYGRLIHEHCERRPHFDAGRFAKEFGDEGHREGWCLYHLGCKGPETYGNCSTLQFCDV

GGVWPVAIGHPCYGCNEKGIGFHKGIHQLANVENQTPRSQKPDVNAKEGGNVSAGAIGLLGGVVGLVAGV

SVMAVRELGRQQKKDNADSRGE

>gi|30064351|ref|NP_838522.1| hydrogenase 2 protein HybA [Shigella flexneri 2a str. 2457T]

MNRRNFIKAASCGALLTGALPSVSHAAAENRPPIPGSLGMLYDSTLCVGCQACVTKCQDINFPERNPQGE

QTWSNNDKLSPYTNNIIQVWTSGTGVNKDQEENGYAYIKKQCMHCVDPNCVSVCPVSALKKDPKTGIVHY

DKDVCTGCRYCMVACPYNVPKYDYNNPFGALHKCELCNQKGVERLDKGGLPGCVEVCPAGAVIFGTREEL

MAEAKKRLALKPGSEYHYPRQTLKSGDTYLHTVPKYYPHLYGEKEGGGTQVLVLTGVPYENLDLPKLDDL

STGARSENIQHTLYKGMMLPLAVLAGLTVLVRRNTKNDHHDGGDDHES

>gi|30064350|ref|NP_838521.1| hydrogenase 2 b cytochrome subunit [Shigella flexneri 2a str. 2457T]

MSHDPQPLGGKIISKPVMIFGPLIVICMLLIVKRLVFGLGSVSDLNGGFPWGVWIAFDLLIGTGFACGGW

ALAWAVYVFNRGQYHPLVRPALLASLFGYSLGGLSITIDVGRYWNLPYFYIPGHFNVNSVLFETAVCMTI

YIGVMALEFAPALFERLGWKVSLQRLNKVMFFIIALGALLPTMHQSSMGSLMISAGYKVHPLWQSYEMLP

LFSLLTAFIMGFSIVIFEGSLVQAGLRGNGPDEKSLFVKLTNTISVLLAIFIGLRFGELIYRDKLSLAFA

GDFYSVMFWIEVLLMLFPLVVLRVAKLRNDSRMLFLSALSALLGCATWRLTYSLVAFNPGGGYAYFPTWE

ELLISIGFVAIEICAYIVLIRLLPILPPLKQNDHNRHEASKA

>gi|30064349|ref|NP_838520.1| hydrogenase 2 large subunit [Shigella flexneri 2a str. 2457T]

MSQRITIDPVTRIEGHLRIDCEIENGVVSKAWASGTMWRGMEEIVKNRDPRDAWMIVQRICGVCTTTHAL

SSVRAAESALNIDVPVNAQYIRNIILAAHTTHDHIVHFYQLSALDWVDITSALQADPTKASEMLKGVSTW

HLNSPEEFTKVQNKIKDLVASGQLGIFANGYWGHPAMKLPPEVNLIAVAHYLQALECQRDANRVVALLGG

KTPHIQNLAVGGVANPINLDGLGVLNLERLMYIKSFIDKLSDFVEQVYKVDTAVIAAFYPEWLTRGKGAV

NYLSVPEFPTDSKNGSFLFPGGYIENADLSSYRPITSHSDEYLIKGIQESAKHSWYKDEAPQAPWEGTTI

PAYDGWSDDGKYSWVKSPTFYGKTVEVGPLANMLVKLAAGRESTQNKLNEIVAIYQKLTGNTLEVAQLHS

TLGRIIGRTVHCCELQDILQNQYSALITNIGKGDHTTFVKPNIPATGEFKGVGFLEAPRGMLSHWMVIKD

GIISNYQAVVPSTWNSGPRNFNDDVGPYEQSLVGTPVADPNKPLEVVRTIHSFDPCMACAVHVVDADGNE

VVSVKVL

>gi|30064348|ref|NP_838519.1| hydrogenase 2 maturation endopeptidase [Shigella flexneri 2a str. 2457T]

MRILVLGVGNILLTDEAIGVRIVEALEQRYILPDYVEILDGGTAGMELLGDMANRDHLIIADAIVSKKNT

PGTMMILRDEEVPALFTNKISPHQLGLADVLSALRFTGEFPKKLTLVGVIPESLEPHIGLTPTVEAMIEP

ALEQVLAALRESGVEAIPREAIHD

>gi|30064347|ref|NP_838518.1| hydrogenase 2-specific chaperone [Shigella flexneri 2a str. 2457T]

MTEEIAGFQTSPKAQVQAAFEEIARRSMHDLSFLHPSMPVYVSDFTLFEGQWTGCVITPWMLSAVIFPGP

DQLWPLRKVSEKIGLQLPYGTMTFTVGELDGVSQYLSCSLMSPLSHSMSIEEGQRLTDDCARMILSLPVT

NPDVPHAGRRALLFGRRRGENA

>gi|30064346|ref|NP_838517.1| hydrogenase nickel incorporation protein HybF [Shigella flexneri 2a str. 2457T]

MHELSLCQSAVEIIQRQAEQHDVKRVTAVWLEIGALSCVEESAVRFSFEIVCHGTVAQGCDLHIVYKPAQ

AWCWDCSQVVEIHQHDAQCPLCHGERLRVDTGDSLIVKSIEVE

>gi|30064345|ref|NP_838516.1| hydrogenase 2 accessory protein HypG [Shigella flexneri 2a str. 2457T]

MCIGVPGQVLAVGEDIHQLAQVEVCGIKRDVNIALICEGNPADLLGQWVLVHVGFAMSIIDEDEAKATLD

ALRQMDYDITSA

>gi|30064344|ref|NP_838515.1| glutathione S-transferase [Shigella flexneri 2a str. 2457T]

MTCYTASTLPLRRQLTMTDNTYQPAKVWTWDKSAGGAFANINRPFSGPTHEKTLPVGKHPLQLYSLGTPN

GQKVTIMLEELLALGVTGAEYDAWLIRIGDGDQFSSGFVEVNPNSKIPALRDHTHNPPIRVFESGSILLY

LAEKFGYFLPQDLAKRTETLNWLFWLQGAAPFLGGGFGHFYHYAPVKIEYAINRFTMEAKRLLDVLDKQL

AQHKFVAGDEYTIADMAIWPWFGNVVLGGVYDAAEFLDAGSYKHVQRWAKEVGERPAVKRGRIVNRTNGP

LNEQLHERHDASDFETNTEDKRQG

>gi|30064343|ref|NP_838514.1| bifunctional glutathionylspermidine amidase/glutathionylspermidine synthetase [Shigella flexneri 2a str. 2457T]

MSKGTTSQDAPFGTLLGYAPGGVAIYSSDYSSLDPQEYEDDAVFRSYIDDEYMGHKWQCVEFARRFLFLN

YGVVFTDVGMAWEIFSLRFLREVVNDNILPLQAFPNGSPRAPVAGALLIWDKGGEFKDTGHVAIITQLHG

NKVRIAEQNVIHSPLPQGQQWTRELEMVVENGCYTLKDTFDDTTILGWMIQTEDTEYSLPQPEIAGELLK

ISGARLENKGQFDGKWLDEKDPLQNAYVQANGQVINQDPYHYYTITESAEQELIKATNELHLMYLHATDK

VLKDDNLLALFDIPKILWPRLRLSWQRRRHHMITGRMDFCMDERGLKVYEYNADSASCHTEAGLILERWA

EQGYKGNGFNPAEGLINELAGAWKHSRARPFVHIMQDKDIEENYHAQFMEQALHQAGFETRILRGLDELG

WDAAGQLIDGEGRLVNCVWKTWAWETAFDQIREVSDREFAAVPIRTGHPQNEVRLIDVLLRPEVLVFEPL

WTVIPGNKAILPILWSLFPHHRYLLDTDFTVNDELVKTGYAVKPIAGRCGSNIDLVSHHEEVLDKTSGKF

AEQKNIYQQLWCLPKVDGKYIQVCTFTVGGNYGGTCLRGDESLVIKKESDIEPLIVVKK

>gi|30064342|ref|NP_838513.1| low-affinity phosphate transport protein [Shigella flexneri 2a str. 2457T]

MLNLFVGLDIYTGLLLLLALAFVLFYEAINGFHDTANAVATVIYTRAMQPQLAVVMAAFFNFFGVLLGGL

SVAYAIVHMLPIDLLLNMGSTHGLAMVFSMLLAAIIWNLGTWFFGLPASSSHTLIGAIIGIGLTNALLTG

SSVMDALNLREVTKIFSSLIVSPIVGLVIAGGLIFLLRRFWSGTKKRDRIHRIPEDRKKKKGKRKPPFWT

RIALIVSAAGVAFSHGANDGQKGIGLVMLVLVGIAPAGFVVNMNASGYEITRTRDAVTNFEHYLQQHPEL

PQKLIAMEPPLPAASTDGTQVTEFHCHPANTFDAIARVKTMLPGNMESYEPLSVSQRSQLRRIMLCISDT

SAKLAKLPGVSKEDQNLLKKLRSDMLSTIEYAPVWIIMAVALALGIGTMIGWRRVAMTIGEKIGKRGMTY

AQGMAAQMTAAVSIGLASYIGMPVSTTHVLSSAVAGTMVVDGGGLQRKTVTSILMAWVFTLPAAIFLSGG

LYWIALQLI

>gi|30064341|ref|NP_838512.1| hypothetical protein S3234 [Shigella flexneri 2a str. 2457T]

MQSITPPLIAVIGSDASGKSTVCEHLITVVEKYGAAERVHLGKQAGNVGRAVTKLPLMGKSLHKTIERNQ

VKTAKKLPGPVPALVITAFVARRLLRFRHMLACRRRGLIVLTDRYPQDQIPGAYDGTVFPPNVEGGRFVS

WLASQERKAFHWMASHKPDLVIKLNVDLEVACARKPDHKRESLARKIAITPQLTFGGAQLVDIDANQPLE

QVLVDAEKAITDFMTARGYH

>gi|30064340|ref|NP_838511.1| hypothetical protein S3233 [Shigella flexneri 2a str. 2457T]

MNQRNMSIINSTPVRVIAIVGCDGSGKSTLTASLVNELAARMPTEHIYLGQSSGRIGEWISQLPVIGAPC

GCYLRSKAAHVHEKPLTPPGNITALVIYLLSCWRAYKFRKMLCKSQQGYLLITDRYPQVEVPGFRFDGPQ

LAKTTGGNGWIKMLRQRELKLYQWMASYLPVLLIRLGIDEQTAFARKPDHQLAALQEKIAVTPQLTFNGA

KILELDGRQPADEIMQASLRAIHAALS

>gi|30064339|ref|NP_838510.1| hypothetical protein S3232 [Shigella flexneri 2a str. 2457T]

MDALQTQTVNSTTAPQPNYIPGLIAVVGCDGTGKSTLTTDLVKSLQQHWQTERRYLGLLSGEDGDKIKRL

PLVGVWLERRLAAKSSKTQSMKTKSPALWAAVIMYCFSLRRMANLRKVQRLAQSGVLVVSDRFPQAEISG

FYYDGPGIGVERATGKISMFLAQRERSLYQKMAQYRPELIIRLGIDIDTAISRKPDHEYAELQDKIGVMS

TIGYNGTKILEIDSRAPYSEVLEQVQKAVSLVAIVSDRRSLT

>gi|30064338|ref|NP_838509.1| hypothetical protein S3231 [Shigella flexneri 2a str. 2457T]

MAGFNIKHWFADGAFRTIIRNSAWLGSSNVESALLGLLALSCAGKGMTPAMFGVLVIVQSYAKSISDFIK

FQTWQLVVQYGTPALTNNNPQQFRNVVSFSFSLDIVSGAVAIVGGIALLPFLSHSLGLDDQSFWLAALYC

TLIPSMASSTPTGILRAVDRFDLIAVQQATKPFLRAAGSVAAWYFDFGFVGFVIAWYVSNLVGGTMYWWF

AARELRRRNIHNAFKLNLFESARHIKGAWSFVWSTNIAHSIWSARNSCSTVLVGIVLGPAAAGLFKIAMT

FFDAAGTPAGLLGKSFYPEVMRLDPRTTRPWLLGVKSGLLAGGIGILVALAVLIVGKPLISLVFGVKYLE

AYDLIQVMLGAIVISMLGFPQESLLLMAGKQRAFLVAQTIASIGYIVLLFMFCHLFGVLGAAFAYFGGQC

LDVALSLIPTLKAFFQRHSLLYNAAGEKS

>gi|30064337|ref|NP_838508.1| hypothetical protein S3230 [Shigella flexneri 2a str. 2457T]

MMRKYFPLEASERLFVAIEEDDVVDAQVSLPPTIALSCTTEIIHDNYALCLQFWLNGVDRQELLRLVRKQ

AKGDELTADERKQFKYMRARYKHLRFAQRLYLKKHQAGFLFGKTTVFLGRFQDGFRNGKKNIVSYYGNLL

RIYLSSPVWSLVNYSLRHSQLESVSSFIAYRQKQMHTLKEIIAKPRLTGREFHDVRKIISQQVSYYDTLR

SLDPENKEALQISRFLAAINGLMGDKHDDMVADDMENRQSYYAPLALDSDICQRLELLISRFPL

>gi|30064336|ref|NP_838507.1| hypothetical protein S3229 [Shigella flexneri 2a str. 2457T]

MSSRLIIALIIMLLAPGVQAHNFVTGKTVTPVYIQEGGELLLNSDDEIHYQKWNSTQLAGKVRIIQYIAG

RKSAKKKNSLLIKAVEAANFPQDRFQPTTIVNTDDAIFGTGYFVVGKIEKNKRRYPWAQFVIDGNGQGRV

AWRLPEQSSTILVLNKAGQIQWAKDGSLTPEEVDHVIALAQKLINE

>gi|30064335|ref|NP_838506.1| hypothetical protein S3228 [Shigella flexneri 2a str. 2457T]

MKLVEHYIMRGTRRLVLIIVGFLIFIFASYSAQRYLTEAANGTLALDVVLDIVFYKVLIALEMLLPVGLY

VSVGVTLGQMYTDSEITAISAAGGSPGRLYKAVLYLAIPLSIFVTLLSMYGRPWAYAQIYQLEQQSQSEL

DVRQLRAKKFNTNDNGRMILSQTVDQDNNRLTDALIYTSTANRTRIFRAGSVDVVDPSPEKPTVMLHNGT

AYLLDHQGRDDNEQIYRNLQLHLNPLDQSPNVKRKAKSVTELARSAFPADHAELQWRQSRGLTALLMALL

AISLSRVKPRQGRFSTLLPLTLLFIAIFYGGDVCRTLVANGAIPLIPGLWLVPGLMLMGLLILVARDFSL

LQKFSR

>gi|30064334|ref|NP_838505.1| hypothetical protein S3227 [Shigella flexneri 2a str. 2457T]

MNVFSRYLIRHLFLGFAAAAGLLLPLFTTFNLINELDGVSPGGYRWTQAVLVVLMTLPRTLVELSPFIAL

LGGIVGLGQLSKNSELTAIRSTGFSIFRIALVALVAGILWTVSLGAIDEWVASPLQQQALQIKSTATALG

EDDDITGNMLWARRGNEFVTVKSLNEQGQPVGVEIFHYRDDLSLESYIYARSATIEDDKTWVLHGVNHKK

WLNGKETLETLDNLAWQSAFTSMNLEELSMPGNTFSVRQLNHYIHYLQETGQPSSEYHLALWEKLGQPIL

TLAMILLAVPFTFSAPRSPGMGSRLAVGVIVGLLTWISYQIMVNLGLLFALSAPVTALGLPVAFVLVALS

LVYWYDRQH

>gi|30064333|ref|NP_838504.1| class II aminotransferase, partial [Shigella flexneri 2a str. 2457T]

MVLLDADSHASIYDACSLGGAEIIRFRHNDAKDLERRMVRLGERAKEAIIIVEGIYSMLGDVAPLAEIVD

IKRRLGGYLIVDEAHSFGVLGATGRGLAEAVGVEDDVDIIVGTFSKSLASIGGFAVVSEAMEVLRYGSRP

YIFTASPSPSCIATVRSSLRTIATQPELRQKLMDNANHLYDGLQKLGYELSSHISPVVPVIIGSKEEGLR

IWRELISPGVYVNLILPPAAPAGITLLRCSVNAAHSREQIDAIIQAFATLKQ

>gi|30064332|ref|NP_838503.1| class II aminotransferase [Shigella flexneri 2a str. 2457T]

MGLYDKYARLAGERLQFSDNGLTPFGTCIDEVYSATEGRIGNKKVILAGTNNYLGLTFNHDAISEGQAAL

AAQGTGTTGSRMANGSYAPHLALEKEIAEFFNRPTAIVFQPVIPLI

>gi|30064331|ref|NP_838502.1| hypothetical protein S3224 [Shigella flexneri 2a str. 2457T]

MVNREIVMDYILSCLQDLVENGVEIKPDSDLVNDLGLESIKVMDLLMMLEDRFDISIPINILLDVKTPAQ

LMETLLPWLENK

>gi|30064330|ref|NP_838501.1| hypothetical protein S3223 [Shigella flexneri 2a str. 2457T]

MTGATGFIGKYIIDNLLARGFHVRALTRTARAHVNDNLTWVRGSLEDTHSLSELVAGASVVVHCAGQVRG

HKEEIFTRCNVDGSLRLMQAAKESGFCQRFLFISSLAARHPELSWYAKSKYVAEQRLAAMADEITLGVFR

PTAVYGPGDKELKPLFDWMLRGLLPRLGAPDTQLSFLHVTDFAQAVGQWLSAETVQTQTYELCDGVAGGY

DWQRVRQLAAKARCGSVRMVDIPLPVLTGLADISTALSRLAGKEPMLTRSKIRELTHADWSASNNRISED

INWFPGISLEHALRNGLF

>gi|30064329|ref|NP_838500.1| acyl-CoA synthetase [Shigella flexneri 2a str. 2457T]

MRYADFPTLVDALDYAALSRAGMNFYDRRCQLEDQLEYQTLKTRAEAGAKRLLSLNLKKGERVALIAETS

SGFVEAFFACQYAGLVAVPLAIPMGVGQRDSWSAKLQGLLASCQPAAIITGDEWLPLVNAATHNNPELHV

LSHAWFKALPEADVALQRPVPNDIAYLQYTSGSTRFPRGVIITHREVMANLRAISHDGIKLRPSDRCVSW

QPFYHDMGLVGFLLPPVATQLSVDYLRTQDFAMRPLQWLKLISKNRGTVSVAPPFGYELCQRRVNEKDLA

ELDLSCWRVAGIGAEPISAEQLHQFAECFRQVNFDNKTFMPCYGLAENALAVSFSDEASGVVVNEVDRDI

LEYQGKAVAPGAETRAVSTFVNCGKALPEHGIEIRNEAGMPVAERVVGHICISGPSLMSGYFGDQVSQDE

IAATGWLDTGDLGYLLDGYLYVTGRIKDLIIIRGRNIWPQDIEYIAEQEPEIHSGDAIAFVTAQEKIILQ

IQCRISDEERRGQLIHALAARIQSEFGVTAAIDLLPPHSIPRTSSGKPARAEAKKRYQKAYAASLNVQES

LA

>gi|30064328|ref|NP_838499.1| DNA-binding transcriptional regulator GlcC [Shigella flexneri 2a str. 2457T]

MKDERRPICEVVAESIERLIIDGVLKVGQPLPSERRLCEKLGFSRSALREGLTVLRGRGIIETAQGRDSH

VARLNREQDTSPLIHLFSTQPRTLYDLLDVRALLEGESARLAATLGTQADFVVITRCYEKMLAASENHKE

ISLIEHAQLDHAFHLAICQASHNQVLVFTLQSLTDLMFNSVFASVNDLYHRPQQKKQIDRQHARIYNAVL

QRLPHVAQRAARDHVRTVKKNLHDIELEGHHLIRSAVPLEMNKVGM

>gi|30064327|ref|NP_838498.1| hypothetical protein S3217 [Shigella flexneri 2a str. 2457T]

MKTKVILSQQMASAIIAAGQEEAQKNNWSVSIAVADDGGHLLALSRMDDCAPIAAYISQEKARTAALGRR

ETKGYEEMVNNGRTAFVTAPLLTSLEGGVPVVVDGQIIGAVGVSGLTGAQDAQVAKVAAAVLAK

>gi|30064326|ref|NP_838497.1| malate synthase G [Shigella flexneri 2a str. 2457T]

MSQTITQGRLRIDANFKRFVDGEVLPGVELDAAAFWHNVDEIVHDLAPENRQLLAERDRIQAALDEWHRS

NPGPVKDKAAYKSFLRELGYLVPQPERVTVETTGIDSEITSQAGPQLVVPAMNARYALNAANARWGSLYD

ALYGSDIIPQEGAMVSGYDPQRGEQVIAWVRRFLDESLPLENGSYQDVVAFKVVDKQLRIQLKNGKETTL

RTPAQFVGYRGDAAAPTCILLKNNGLHIELQIDANGRIGKDDPAHINDVIVEAAISTILDCEDSVAAVDA

EDKILLYRNLLGLMQGTLQEKMEKNGRQIVRKLNDDRQYTAADGSEISLHGRSLLFIRNVGHLMTIPVIW

DSEGNEIPEGILDGVMTGAIALYDLKVQKNSRTGSVYIVKPKMHGPQEVAFANKLFTRIETMLGMAPNTL

KMGIMDEERRTSLNLRSCIAQARNRVAFINTGFLDRTGDEMHSVMEAGPMLRKNQMKSTPWIKAYERNNV

LSGLFCGLRGKAQIGKGMWAMPDLMADMYSQKGDQLRAGANTAWVPSPTAATLHALHYHHTNVQSVQANI

AQTEFSAEFEPLLDDLLTIPVAENANWSAQEIQQELDNNVQGILGYVVRWVEQGIGCSKVPDIHNVALME

DRATLRISSQHIANWLRHGILTKEQVQASLENMAKVVDQQNAGDPAYRPMAGNFANSCAFKAASDLIFLG

VKQPNGYTEPLLHAWRLREKESH

>gi|30064325|ref|NP_838496.1| permease [Shigella flexneri 2a str. 2457T]

MVTWTQMYMPMGGLGLSALVALIPIIFFFVALAVLRLKGHVAGAITLILSILIAIFAFKMPIDMAFAAAG

YGFIYGLWPIAWIIVAAVFLYKLTVASGQFDIIRSSVISITDDQRLQVLLIGFSFGALLEGAAGFGATVA

FGALGVPILVAGQVTGIDPFHIGAMAGRQLPFLSVLVPFWLVAMMDGWKGVKETWPAALVAGGSFAVTQF

FTSNYIGPELPDITSALVSIVSLALFLKVWRPKNTETAISMGQSAGAMVVNKPSSGGPVPSEYSLGQIIR

AWSPFLILTVLVTIWTMKPFKALFAPGGAFYSLVINFQIPHLHQQVLKAAPIVAQPTPMDAVFKFDPLSA

GGTAIFIAAIISIFILGVGIKKGIGVFAEMLISLKWPILSIGMVLAFAFVTNYSGMSTTLALVLAGTGVM

FPFFSPFLGWLGVFLTGSDTSSNALFGSLQSTTAQQINVSDTLLVAANTSGGVTGKMISPQSIAVACAAT

GMVGRESELFRYTVKHSLIFASVITLLQAYVFTGMLVS

>gi|30064324|ref|NP_838495.1| IS1 orfA [Shigella flexneri 2a str. 2457T]

MASISIRCPSCSATEGVVRNGKSTAGHQRYLCSHCRKTWQLQFTYTASQPGTHQKIIDMAMNGVGCRASA

RIMGVGLNTVLRHLKNSGRSR

>gi|30064323|ref|NP_838494.1| IS1 orfB [Shigella flexneri 2a str. 2457T]

MSRQCTHYGRWPQHGFTSLKKLRPQSVTSRIQPGSDVIVCAEMDEHWGYVGAKSRQRWLFYAYDRIRRTV

VPHVFGERTLATLERLLSLLSAFEVVVWMTDGWPLYESRLKGKLHVISKRYTQRIERHNLNLRQHLARLG

RKSLSFSKSVELHDKVIGHYLNIKHYQ

>gi|30064322|ref|NP_838493.1| hypothetical protein S3212 [Shigella flexneri 2a str. 2457T]

MSDDRFYFSLGQGVKFYPASRRITTSTGNVINLSENGYRFLLLLLQGESDKQHIINEVWREQKGSVSDSS

YYGQIYMLRKALDVAGLSSSLIKTIPRKGVRYLGKVEKHVCENERDSDEDSSSYTNDSEAREVNTDANIP

ENDNSQEFSTEELLPIKHTDKNIEWYRTKQWSVFISVLTVLAVCWLTTLVFLVLLFLNQDK

>gi|30064321|ref|NP_838492.1| hypothetical protein S3211 [Shigella flexneri 2a str. 2457T]

MNTFITKYYGKTKQCFARFAKDERGVTAIEYALIGVAMATLLAFIFGDQNSGFLGAIKDAFDAIAAAIQQ

VTISGTSNP

>gi|30064320|ref|NP_838491.1| IS3 orfB [Shigella flexneri 2a str. 2457T]

MKYVFIEKHQAEFSIKAMCRVLRVARSGWYTWCQRRTRISTRQQFRQHCDSVVLAAFTRSKQRYGAPRLT

DELRAQGYPFNVKTVAASLRRQGLRAKASRKFSPVSYRAHGLPVSENLLEQDFYASGPNQKWPGDITYLR

TDEGWLYLAVVIDLWSRAVIGWSMSPRMTAQLPCDALQMALWRRKRPRNVIVHTDRGGQYCSADYQAQLK

RHNLRGSMSAKGCCYDNACVESFFHSLKVECIHGEHFISREIMRATVFNYIECDYNRWRRHSWCGGLSPE

QFENQNLA

>gi|30064319|ref|NP_838490.1| IS3 orfA [Shigella flexneri 2a str. 2457T]

MTKTVSTSKKPRKQHSPEFRSEALKLAERIGVTAAARELSLYESQLYNWRSKQQNQQTSSERELEMSTEI

ARLKRQLAERDEELAILQKAATYFAKRLK

>gi|30064318|ref|NP_838489.1| hypothetical protein S3207 [Shigella flexneri 2a str. 2457T]

MSEITASRPEVVNGHTDVICSTSIRHILAVRKSTLLQIDTLIRQLAEVSAMTESIRGKTALDWAMKQDFR

CGCWLMEKPETAMKAITRNLDRELWRDLMQRSGMLSLMDAQTRDTWYRSLEYDNFPEISEANILSTFEQL

HQNKDEVFERGVINVFKGLSWDYKTNSPCKFGSKIIVNNLVRWDRWGFHLITGQQTDRLVDLERMLHLFS

GKPIPDNRENITIRLDGHIQSVQGKERYEDEMFIIKYFKKGSAHITFKRLELIDRINDIIARYFPSVLSA

>gi|30064317|ref|NP_838488.1| hypothetical protein S3205 [Shigella flexneri 2a str. 2457T]

MKLALTLEADSVNVQALNMGRIVVDVDGVNLSELINKVSENGYSLRVVDKSDQHATSTPPPLTTLTCIRC

STAHITETDNAWLYSLSHQTNDDGETEWIHFTGSGYLLRTDAWSYPVLRLKRLGLSRTFRRLVVTLTRRY

GVSLIHLDAGAECLPGFPTFDW

>gi|30064316|ref|NP_838487.1| hypothetical protein S3204 [Shigella flexneri 2a str. 2457T]

MNTLPDTHVREASGCPSPITIWQTLLTRLLDQHYGLTLNDTPFADERVIEQHIEAGISLCDAVNFLVEKY
ALVRTDQPGFSAGAPSQLINSIDILRARRATGLMTRDNYRTVNNITRGKHPEAKQ

>gi|30064315|ref|NP_838486.1| structural protein [Shigella flexneri 2a str. 2457T]
MSDTLPGTTPPDDNHDRPWWGLPCTVTPCFGARLVQEGNRLHYLADRAGIRGRFSDVDAYHLDQAFPLLM
KQLELMLTGGELNPRHQHTVTLYAKGLTCEADTLGSCGYVYLAVYPTPAAPATTV

>gi|30064314|ref|NP_838485.1| hypothetical protein S3202 [Shigella flexneri 2a str. 2457T]
MKIITRGEAMRIHRQHPASRLFPFCTGKYRWHGSTDTYTGREVQDIPGVLAVFAERRKDSFGPYVRLMSV
TLN

>gi|30064313|ref|NP_838484.1| RADC family DNA repair protein [Shigella flexneri 2a str. 2457T]
MVAGTMQQLSFLPGEMTTRERSLILRALKTLDRHLHEPGVAFTSTHAAREWLILNMAGLEREEFRVLYLN
NQNQLIAGETLFTGTINRTEVHPREVIKRALYHNAAAVVLAHNHPSGEVPPSKADRLITERLVQALALVD
IRVPDHLIVGGSQVFSFAEHGLL

>gi|30064312|ref|NP_838483.1| hypothetical protein S3200 [Shigella flexneri 2a str. 2457T]
MKTLSQNTTSSACAPETDLQQLVATLVPDEQRISFWPQHFGLIPQWVTLEPRVFGWMDRLCEDYCGGIWN
LYTLNNGGAFMAPEPDDDDDETWVLFNAMNGNRAEMSPEAAGIAACLMTYSHHACRTECYAMTVHYYRLR
DYALQHPECSAIMRIID

>gi|30064311|ref|NP_838482.1| hypothetical protein S3199 [Shigella flexneri 2a str. 2457T]
MQLVSRFGYANQIRRDRPLTHEELMHHVPGIFGEEKHTSRSQNYTYIPTITVLESLQREGFQPFFACQTR
VRDPGRRGYTKHMLRLRRAGEINGEHVPEIILLNSHDGTSSYQMLPGYFRFVCQNGCVCGQSLGEVRVPH
RGNVVEKVIEGAYEVVGVFDRIEEKRDAMQSLVLPPPARQALAQAALTYRYGDEHQPVTTADILTPRRRE

DYGKDLWSTYQTIQENMLKGGISGRSAKGKRIHTRAIHNIDTDIKLNRALWVMAETLLESLR

>gi|30064310|ref|NP_838481.1| hypothetical protein S3197 [Shigella flexneri 2a str. 2457T]

MIHLFKTCMITAFILGLTWSAPLRAQDQRYISIRNTDTIWLPGNICAYQFRLDNGGNGEGFGPLTITLQL

KDKYGQTLVTRKMETEAFGDSNATRTTDAFLETECVENVATTEIIKATEESNGHRVSLPLSVFDPQDYHP

LLITVSGKNVN

>gi|30064309|ref|NP_838480.1| hypothetical protein S3196 [Shigella flexneri 2a str. 2457T]

MLQIVGALILLIAGFAILRLLFRALTSTASALAGFILLCLFGPALLAGYITERITRLFHIRWLAGVFLTI

AGMIISFMWGLDGKHIALEAHTFDSVKFILTTALAAGLLALPVQIRTIQQNGLTPEDISKEINGYYCCFY

TAFFLMACSAYAPLIALQFDISPSLMWWGGLLYWLAALVTLLWAASQIQALKRLTSAISQTLEEQPVLNS

KSWLSSLQNDYSLPETLTERIWLTLISQRISRGELREFELADGNWLLNNAWYERNMAGFNEQLKENLSFT

PDELKTLFRNRLNLSPEANDDFLDRCLDGGDWYPFSEGRRFVSFHHVDELRICASCGLTEVHHAPENHKP

APEWYCSSLCHETETLCQDIYERSYTGFISDATANGLILMKLPETWSTNEKMFASGGQGHGFAAERGNHI

VDRVRLKNARILGDNNARNGADRLVSGTEIQTKYCSTAARSVGAAFDGQNGQYRYMGNHGPMQLEVPRDQ

YAGAVETMKNKIREGKVPGVTDPAEASRLIRRGHLTYTQARNITRFGTIESVTYDIAEGSVVSLAAGGIS

FALTASVFWLSTGDRDAALQTAAVQAGKTFTRTLAVYVTTQQLHRLTVVQGMLKHIDFSTASPTVRQALQ

KGTGAGNISALNKVMKGTLVTSLALVAVTTSPDMIKMLRGRISGAQFIRNLAVASSGVAGGAVGSVAGGI

LFSPLGPFGALTGRVVGGVLGGMIASAVSGKIAGALVEEDRVKILAMIQEQVTWLAGSFLLTGHEIENLN

ANLARVIDQNALEIIFAAGIQQRAATNMLIKPLVVSIIRQRPVMEYDASHLGKMVNRLEEAFPPELPA

>gi|30064308|ref|NP_838479.1| outer membrane fluffing protein, partial [Shigella flexneri 2a str. 2457T]

MEGKADNVVLENGGRLDVLTGHTATNTRVDDGGTLDVRNGGTATTVSMGNGGVLLADSGAAVSGTRSDGK

AFSIGGGQADALMLEKGSSFTLNAGDTATDTTVNGGLFTARGGTLAGTTTLNNGAILTLSGKTVNNDTLT

IREGDALLQGGALTGNGSVEKSGSGTLTVSNTTLTQKAVNLNEGTLTLNDSTVTTDVIAQRGTALKLTGS

TVLNGAIDPTNVTLASGATWNIPDNATVQSVVDDLSHAGQIHFTSTRTGKFVPATLKVKNLNGQNGTISL

RVRPDMAQNNADRLVIDGGRATGKTILNLVNAGNSASGLATSGKGIQVVEAINGATTEEGAFIQGNKLQA

GAFNYSLNRDSDESWYLRSENAYRAEVPLYASMLTQAMDYDRILAGSRSHQTGVSGENNSVRLSIQGGHL

GHDNNGGIARGATPESSGSYGFVRLEGDLLRTEVAGMSVTAGVYGAAGHSSVDVKDDDGSRAGTVRDDAG

SLGGYLNLIHNASGLWADIVAQGTRHSMKASSDNNDFRVRGWGWLGSLETGLPFSITDNLMLEPQLQYTW

QGLSLDDGQDNASYVKFGHGSAQHVRAGFRLGSHHDMNFGKGTSSRDTLRGSAKHSVRELPVNWWVQPSV

IRTFSSRGDMSMGTAAAGSNMTFSPSQNGTSLDLQAGLEARVRENITLGVQASYAHSINGSSAEGYNSQA

TLNVTF

>gi|30064307|ref|NP_838478.1| outer membrane fluffing protein, partial [Shigella flexneri 2a str. 2457T]

MKRHLNTCYRLVWNHITGAFVVASELARAQGKRGGVAVALSLAAVTSLPVLAADIVVHPGETVNGGTLVN

HDNQFVSGTADGVTVSTGLELGPDSDENTGGQWIKAGGTGRNTTVTANGRQIVQAGGTASDTVIRDGGGQ

SLNGLAVNTTLDNRGEQWVHGGGKAAGTIINQDGYQTIKHGGLATGTIVNTGAEGGPESENVSSGQMVGG

TAESTTINKNGRQVIWSSGMARDTLIYAGGDQTVHGEAHNTRLEGGNQYVHNGGTATETLINRDGWQVIK

EGGTAAHTTINQKESCR

>gi|30064306|ref|NP_838477.1| hypothetical protein S3193 [Shigella flexneri 2a str. 2457T]

MNPSDAIEAIEKPLSSLPYSLSRHILEHLRKLTSHEPVIGIMGKSGAGKSSLCNALFQGEVTPVSDVHAG

TREVQRFRLSGHGHSMVITDLPGVGESRDRDAEYEALYRDILPELDLVLWLIKADDRALSVDEYFWRHIL

QCGHQQVLFVVTQADKTEPCHEWDMAGIQPSPAQEQNIREKTEAVFRLFRPVHPVVAVSARTGWELDTLV

SALMTALPDHAASPLMTRLQDELRTESVRSQAREQFTGAVDRIFDTAESVCVASVVRTALRAVRDTVVSV

ARAVWNWIFF

>gi|30064305|ref|NP_838476.1| hypothetical protein S4826 [Shigella flexneri 2a str. 2457T]

MNNEQMLIKCRDPRKCRFYAEYLNHKYPNEFPHFAPDNPQNCIRNVNLFLKEKYCNVSPPPFGTFGTTEE

EALNNEINELEIIAGQAILPEESFLWLKTDELATFFTWTTIYLSKEDHTKFPKINHNSSGIKNGIEVRKI

RTVTDETLNTYKKSNLPDNPSSHNERIKVIISYFDSCHSLINKNAKSIYLEELRQAWLKKKSACQKFKWL

LPDDDEMCQWFWTRLKQKQDEKHPVHNPVTRWFIPSSTKEYYLASLVAFALWKEAPDTIELFRTRINHAW

HQKKQKDKYRSDGKKAINIHIRSNVKEMLDELRTVYGLRTGEFFEMLITEKYLEHKRRK

>gi|30064304|ref|NP_838475.1| hypothetical protein S4825 [Shigella flexneri 2a str. 2457T]

MVVLNTPGKYQYDLRNHLLVNLFPIALDAEITTSNVNKHQLHRSNIPAALSGNSGITDYVPFSAERNAAM

VIWRTAIDSRPAVCNDEEGTPGTL

>gi|30064303|ref|NP_838474.1| hypothetical protein S3191 [Shigella flexneri 2a str. 2457T]

MQQLENRCDLLLIQHQKWMTSVTRLIVAHGMGSPHLHGYHRLTLAHFFLPEKGSVISVAPQGLYQVVNPG

TPPFIPAIQEGLMTSIQTHEIMLLTHFNLGGVLLSELHRLGENRLANRLNSLLRRFDDRDLYHTLIWLCW

YDLMCAHSMQPWTEELKHKSHAELENWAVARKREKRELELMIDEYLLYAC

>gi|30064302|ref|NP_838473.1| hypothetical protein S3190 [Shigella flexneri 2a str. 2457T]

MNTPVSLMDDQMVDMSFITQLTGLTDKWFYKLIKHGAFPPPIKLGRSSRWLKSEVEAWLQARIAQSRP

>gi|30064299|ref|NP_838470.1| hypothetical protein S3187 [Shigella flexneri 2a str. 2457T]

MQTYNTNPAYEMAPQLLEHFNQHLDSLFGVYSKLLPFRMDFAYRKNTLSYRCACRYAMCAEMLQLINEVG

EKLVGYAWVMEYTERKGLHIHFVGYLNGQSHRSSYLVSRLMGDIWRRVTEGNGYYHWCRFNKNYPVNINH

VIHYSDHKAVNDLRYAISYLAKREQKECGIILKCSGLPEKSNRGRPRLDSPLPGICALV

>gi|30064298|ref|NP_838469.1| hypothetical protein S3186 [Shigella flexneri 2a str. 2457T]

MYAKSFLALDGNGRLTGARTAQTAPYDSYTCHLCGSALRYHPQYDTERPWFEHADEGLTEHGHECPYVRP

ERREIRLIKRLQQFVPDTLPVVRKASWHCRQCHHDYYGEQYCTHCQTGRFSEEGGAE

>gi|30064297|ref|NP_838468.1| hypothetical protein S3185 [Shigella flexneri 2a str. 2457T]

MNGIKTGLGITPGEHIISADSALSRNIRQCFCLSCHGRLILQTDAQGAWFEHDLHALSEQQKADCVVLNP

EKSHPYIEDMAMFLSPLPVVLEWHCVMCEQFFHGKKFCEACGTGIYCRAVCTKSVYSYQPDLFEDCGGAS

T

>gi|30064296|ref|NP_838467.1| IS629 orfA [Shigella flexneri 2a str. 2457T]

MTKNTRFSPEVRQRAVRMVLESQSEYDSQWATICSIAPKIGCTPETLRVWVRQHERDTGGGDGGLTTAER

QRLKELERENRELRRSNDILRQASAYFAKAEFDRLWKK

>gi|30064295|ref|NP_838466.1| reverse transcriptase-like protein [Shigella flexneri 2a str. 2457T]

MQRKLATWAATDPSLRIQRLLRLITQPEWLAEAARITLSSKGAHTPGVDGVNKTMLQARLAVELQILRDE

LLSGHYQPLPARRVYIPKSNGKLRPLGIPALRDRIVQRAMLMAMEPIWESDFHTLSYGFRPERSVHHAIR

TVKLQLTDCGETRGRWVIEGDLSSYFDTVHHRLLMKAVRRRISDARFMTLLWKTIKAGHIDVGLFRAAVK

VCHRAVLYRRYYRTSC

>gi|30064294|ref|NP_838465.1| reverse transcriptase-like protein, partial [Shigella flexneri 2a str. 2457T]

MLNEFDQYLHERYLSGKARKDRWYWNNSIQRGRSTAVRENWQWKPAVAYCRYADDFVLIVKGTKAQAEAI

REECRGVLEGSLKLRLNMDKTKITHVNDGFIFLGHRIIRKRSRYGEMRVVSTIPQEKARNFAASLTALLS

GNYSESKVDMAEQLNRKLKGWAMFYQFVDFKAKVFSYIDRVVFWKLAHWLARKYRTGIASLMRWWCKSPK

PGQSKTWVLFGKTNHGKLSGEILYRLVGQGKKLFRWRLPEGNPYLRTETRNTYTSRFTEVAMAFASI

>gi|30064293|ref|NP_838464.1| serine protease precurser [Shigella flexneri 2a str. 2457T]

MQDDFDAPVDFVSGLGPLNWTYDKTSGTGTLSQGSKNWTMHGQKDNDLNAGKNLVFSGQNGAIILKDSVT

QGAGYLEFKDSYTVSAESGKTWTGAGIITDKGTNVTWKVNGVAGDNLHKLGEGTLTINGTGVNPGGLKTG

DGIVVLNQQADTAGNIQAFSSVNLASGRPTVVLGDARQVNPDNISWGYRGGKLDLNGNAVTFTRLQAADY

GAVITNNAQQKSQLLLDLKAQDTNVSEPTIGNISPFGGTGTPGNLYSMILNSQTRFYILKSASYGNTLWG

NSLNDPAQWEFVGMNKNKAVQTVKDRILAGRAKQPVIFHGQLTGNMDVAIPQVPGGRKVIFDGSVNLPEG

TLSQDSGTLIFQGHPVIHASISGSAPVSLNQKDWENRQFTMKTLSLKDADFHLSRNASLNSDIKSDNSHI

TLGSDRAFVDKNDGTGNYVIPEEGTSVPDTVNDRSQYEGNITLNHNSALDIGSRFTGGIDAYDSAVSITS

PDVLLTAPGAFAGSSLTVHDGGHLTALNGLFSDGHIQAGKNGKITLSGTPVKDTANQYAPAVYLTDGYDL

TGDNAALEITRGAHASGDIHASAASTVTIGSDTPAELASAETAASAFAGSLLEGYNAAFNGAITGGRADV

SMHNALWTLGGDSAIHSLTVRNSRISSEGDRTFRTLTVNKLDATGSDFVLRTDLKNADKINVTEKATGSD

NSLNVSFMNNPAQGQALNIPLVTAPAGTSAEMFKAGTRVTGFSRVTPTLHVDTSGGNTKWILDGFKAEAD

KAAAAKADSFMNAGYKNFMTEVNNLNKRMGDLRDTNGDAGAWARIMSGAGSADGGYSDNYTHVQVGFDKK

HELDGVDLFTGVTMTYTDSSADSHAFSGKTKSVGGGLYASALFESGAYIDLIGKYIHHDNDYTGNFASLG

TKHYNTHSWYAGAETGYRYHLTEDTFIEPQAELVYGAVSGKTFRWKDGDMDLSMKNRDFSPLVGRTGVEL

GKTFSGKDWSVTARAGTSWQFDLLNNGETVLRDASGEKRIKGEKDSRMLFNVGMNAQIKDNMRFGLEFEK

SAFGKYNVDNAVNANFRYMF

>gi|30064292|ref|NP_838463.1| hypothetical protein S3177 [Shigella flexneri 2a str. 2457T]

MIYTTNAIESLNSVIRHAIKKRKVFPTDDSVKKVVWLAIQSASQKWTMPLKDWRMAMSRFIIEFGDRLDG

HF

>gi|30064291|ref|NP_838462.1| serine protease [Shigella flexneri 2a str. 2457T]

MNKIYSLKYSHITGGLVAVSELTRKVSVGTSRKKVILGIILSSIYGSYGETAFAAMLDINNIWTRDYLDL

AQNRGEFRPGATNVQLMMKDGKIFHFPELPVPDFSAVSNKGATTSIGGAYSVTATHNGTQHHAITTQSWD

QTAYKASNRVSSGDFSVHRLNKFVVETTGVTESADFSLSPEDAMKRYGVNYNGKEQIIGFRAGAGTTSTI

LNGKQYLFGQNYNPDLLSASLFNLDWKNKSYIYTNRTPFKNSPIFGDSGSGSYLYDKEQQKWVFHGVTST

VGFISSTNIAWTNYSLFNNILVNNLKKNFTNTMQLDGKKQELSSIIKDKDLSVSGGGVLTLKQDTDLGIG

GLIFDKNQTYKVYGKDKSYKGAGIDIDNNTTVEWNVKGVAGDNLHKIGSGTLDVKIAQGNNLKIGNGTVI

LSAEKAFNKIYMAGGKGTVKINAKDALSESGNGEIYFTRNGGTLDLNGYDQSFQKIAATDAGTTVTNSNV

KQSTLSLTNTDAYMYHGNVSGNISINHIINTTQQHNNNANLIFDGSVDIKNDISVRNAQLTLQGHATEHA

IFKEGNNNCPIPFLCQKDYSAAIKDQESTVNKRYNTEYKSNNQIASFSQPDWESRKFNFRKLNLENATLS

IGRDANVKGHIEAKNSQIVLGNKTAYIDMFSGRNITGEGFGFRQQLRSGDSAGESSFNGSLSAQNSKITV

GDKSTVTMTGALSLINTDLIINKGATVTAQGKMYVDKAIELAGTLTLTGTPTENNKYSPAIYMSDGYNMT

EDGATLKAQNYAWVNGNIKSDKKASILFGVDQYKEDNLDKTTHTPLATGLLGGFDTSYTGGIDAPAASAS

MYNTLWRVNGQSALQSLKTRDSLLLFSNIENSGFHTVTVNTLDATNTAVIMRADLSQSVNQSDKLIVKNQ

LTGSNNSLSVDIQKVGNNNSGLNVDLITAPKGSNKEIFKASTQAIGFSNISPVISTKEDQEHTTWTLTGY

KVAENTASSGAAKSYMSGNYKAFLTEVNNLNKRMGDLRDTNGEAGAWARIMSGAGSASGGYSDNYTHVQI

GVDKKHELDGLDLFTGLTMTYTDSHASSNAFSGKTKSVGAGLYASAIFDSGAYIDLISKYVHHDNEYSAT

FAGLGTKDYSSHSLYVGAEAGYRYHVTEDSWIEPQAELVYGAVSGKRFDWQDRGMSVTMKDKDFNPLIGR

TGVDVGKSFSGKDWKVTARAGLGYQFDLFANGETVLRDASGEKRIKGEKDGRILMNVGLNAEIRDNLRFG

LEFEKSAFGKYNVDNAINANFRYSF

>gi|30064290|ref|NP_838461.1| hypothetical protein S4823 [Shigella flexneri 2a str. 2457T]

MVKSHGTVSVDGKVSDADLTYLEEVANSTGQEVDKSRLTSQACARTALITDVGIALATELETAGQKWSLG

FPPKFQRVDLFNYNVLVRNYDSSAFKGDRYHNTKNGINADIGASTDLDDNWTLGLVAQNLISRSIETKEV

NGITETFRIRPQVTAGVSWHNAMFTTAFDVDLTPASGFTSDSNRQFAAIGTEFNAWKWAQLRAGYRQNLA

GNDGSAFTAGVGISPFDVVHLDVAGLIGTDNTYGAVAQFQFTF

>gi|30064289|ref|NP_838460.1| superfamily I DNA helicase [Shigella flexneri 2a str. 2457T]

MDENALGFASYWRNSLADAESGKGSFKRKDAQNFTHWHGIAAGRLDEAIVSKFFEGEKDDVETVDVILRP

KVYFRLLQHGKDRSAGAPDIVTPIVTPALLSREGFLYPTPATSIPRDLLEPLPKGAFSIGEIGQYDKYKT

THTTFSINFDDSVDKTAETDEEREARYAALQQEWRQYLYDSERLLKSVAGDWIEKPEQYELAEHGYIVKT

AQSGGASSHILSLYDHLLVCNKDVPLFNRFASREVHAAESLLAPGAKFSDRLGHSGDKFPLAKAQRDALS

HFLDARHGDILAVNGPPGTGKTTLVLSIIATQWARAALEKSEPPVIIATSTNNQAVTNIIEAFGKDFSQG

SGAMAGRWLPELKSFGAYFPSSSRKAEAAKKYQTEDFFNQVESKEYVEDALLFYLEKAKAAFPGKECSSP

EKVIELLHGQLAAKSEQLIRLNATWQTLSQIRAARELIANDIEQYLDNLNKLLSGQEQKVTLLKSAKTEW

KKYRAGESLIYSLFSWLPAVRNKRQYQIQLFLEDKLGALIAGNQWSDPETIERNIDGLLNSAEREQTTYR

QQIDSAHEIVLKEQQAVQEWQRLAFDLGYEGDEELSFSQADELADTQIRFPAFLLTTHYWEGRWLMDMAS

IDDLQDEKKKKGAKGVTARWQRRMKLTPCVVMTCYMLPGNMQISEHKGQRKFEKSYLYDFADLLIVDEAG

QVLPEVAAASFALAKKALVIGDTEQIPPIWSIAPAIDVGNMLAEKILSGSTQEEITEKYTAIADLGKSAA

SGSVMKIAQFASRYQYDPELARGMYLYEHRRCYDNIIGYCNTLCYHGKLLPKRGREESNLMPAMGYLHID

GKGELASSGSRYNLLEAETIAVWLAENQQNIEAHYGKSLHEVVGIVTPFSAQVSTIKQVLGKQDISTGTN

EKSLTVGTVHSLQGAERAIVIFSPVYSKHEDGGFIDSDNSMLNVAVSRAKDSFLVFGDMDLFEVQPASSP

RGLLAKYLFESEKNALSFDYKERKDLKTAGTKIYTLHGVEQHDNFLNQTFENTSKHITIISPWLTWQRLE

QTGFLDSMIAACSRGINVTIVTDRSYNTEHNDFEKRKEKQQNFKAALEKLNALGIATKLVNRVHSKIVIG

DDGLLCVGSFNWFSATREARYERYDTSMVYCGDNLKGEIEAIYNSLERRQV

>gi|30064288|ref|NP_838459.1| P4-type integrase [Shigella flexneri 2a str. 2457T]

MALTDAKIRAAKPTDKAYKLTDGAGMFLLVHPNGSRYWRLRYRILGKEKTLALGVYPEVSLSEARTKRDE

ARKLISEGIDPCEQKRVKKVVPDLQLSFEHIARRWHASNKQWAQSHSDKVLKSLETHVFPFIGNRDITTL

NTPDLLIPVRAAEAKQIYEIASRLQQRISAVMRYAVQSGIIRYNPALDMAGALTTVKRQHRPALELSRLP

ELLSRIDGYKGQPVTRLAVMLNLLVFIRSSELRYARWSEIDIDNSMWTIPAEREPLPGVKFSHRGSKMRT

PHLVPLSKQVVAILAELQTWAGENGLIFTGAHDPRKPISENTVNKALRVMGYDTTQEVCGHGFRAMACSA

LIESGLWSRDAVERQMSHQERNGVRAAYIHKAEHLEERRLMLQWWADFLDANREKGISPFEYAKINNPLK

>gi|30064287|ref|NP_838458.1| transporter [Shigella flexneri 2a str. 2457T]

MVIGPFINASAVLLGGVLGALLSQRLPERIRVSMTSIFGLASLGIGILLVVKCANLPAMVLATLLGALIG

EICLLEKGVNTAVAKAQNLFRHSRKKPAHESFIQNYVAIIVLFCASGTGIFGAMNEGMTGDPSILIAKSF

LDFFTAMIFACSLGIAVSVISIPLLIIQLTLAWAAALILPLITPSMMADFSAVGGLLLLATGLRICGIKM

FPVVNMLPALLLAMPLSAAWTAWFA

>gi|30064286|ref|NP_838457.1| ornithine decarboxylase [Shigella flexneri 2a str. 2457T]

MGQGFPPCPVFLLPRNGFALMKSMNIAASSELVSRLSSHRRVVALGDTDFTDVAAVVITAADSRSGILAL

LKRTGFHLPVFLYSEHAVELPAGVTAVINGNEQQWLELESAACQYEENLLPPFYDTLTQYVEMGNSTFAC

PGHQHGAFFKKHPAGRHFYDFFGENVFRADMCNADVKLGDLLIHEGSAKDAQKFAAKVFHADKTYFVLNG

TSAANKVVTNALLTRGDLVLFDRNNHKSNHHGALIQAGTTPVYLEASRNPFGFIGGIDAHCFNEEYLRQQ

IRDVAPEKADLPRPFRLTIIQLGTYDGTVYNARQVIDTIGHLCDYILFDSAWVGYEQFISMMADSSPLLL

ELNENDPGIFVTQLVHKQQAGFSQTSQIHKKDNHIRGQARFCPHKRLNNAFMLHASTSPFYPLFAALDVN

AKIHEGESGRRLWAECVELGIESRKAILARCKLFRPFIPPVVDGKLWQDYPTSVLASDRRFFSFEPGAKW

HGFEGYAADQYFVDPCKLLLTTPGINAETGEYSDFGVPATILAHYLRENGIVPEKCDLNSILFLLTPAES

HEKLAQLVAMLAQFEQHIEDDSSLAEVLPSVYNKYPVRYRDYTLRQLCQEMHDLYVSFDVKDLQKAMFRQ

QSFPSVVMNPQDAHSAYIRGEVELVRIRDAEGRIAAEGALPYPPGVLCVVPGEVWGGAVQRYFLALEEGV

NLLPGFSPELQGVYSETDADGMKRLYGYVLK

>gi|30064285|ref|NP_838456.1| permease for nucleosides [Shigella flexneri 2a str. 2457T]

MCFFKHSSASRCEEINMNLKLQLKILSFLQFCLWGSWLTTLGSYMFVTLKFDGASIGAVYSSLGIAAVFM

PALLGIVADKWLSAKWVYAICHTIGAITLFMAAQVTTPEAMFLVILINSFAYMPTLGLINTISYYRLQNA

GMDIVTDFPPIRIWGTIGFIMAMWVVSLSGFELSHMQLYIGAALSAILVLFTLTLPHIPVAKQQANQSWT

TLLGLDAFALFKNKRMAIFFIFSMLLGAELQITNMFGNTFLHSFDKDPMFASSFIVQHASIIMSISQISE

TLFILTIPFFLSRYGIKNVMMISIVAWILRFALFAYGDPTPFGTVLLVLSMIVYGCAFDFFNISGSVFVE

KEVSPAIRASAQGMFLMMTNGFGCILGGIVSGKVVEMYTQNGITDWQTVWLIFAGYSVVLAFAFMAMFKY

KHVRVPTGTQTVSH

>gi|30064284|ref|NP_838455.1| murein transglycosylase C [Shigella flexneri 2a str. 2457T]

MMKKYLALALIAPLLISCSTTKKGDTYNEAWVKDTNGFDILMGQFAHNIENIWGFKEVVIAGPKDYVKYT

DQYQTRSHINFDDGTITIETIAGTEPAAHLRRAIIKTLLMGDDPSSVDLYSDVDDITISKEPFLYGQVVD

NTGQPIRWEGRASNFADYLLKNRLKSRSNGLRIIYSVTINMVPNHLDKRAHKYLGMVRQASRKYGVDESL

ILAIMQTESSFNPYAVSRSDALGLMQVVQNTAGKDVFRSQGKSGTPSRSFLFDPASNIDTGTAYLAMLNN

VYLGGIDNPTSRRYAVITAYNGGAGSVLRVFSNDKIQAANIINTMTPGDVYQTLTTRHPSAESRRYLYKV

NTAQKSYRRR

>gi|30064283|ref|NP_838454.1| hypothetical protein S3162 [Shigella flexneri 2a str. 2457T]

MSRTIFCTFLQREAEGQDFQLYPGELGKRIYNEISKEAWAQWQHKQTMLINEKKLNMMNAEHRKLLEQEM

VNFLFEGKEVHIEGYTPEDKK

>gi|30064282|ref|NP_838453.1| adenine DNA glycosylase [Shigella flexneri 2a str. 2457T]

MQASQFSAQVLDWYDKYGRKTLPWQIDKTPYKVWLSEVMLQQTQVATVIPYFERFMAHFPTVTDLANAPL

DEVLHLWTGLGYYARARNLHKAAQQVATLHGGKFPETFEEVAALPGVGRSTAGAILSLSLGKHFPILDGN

VKRVLARCYAVSGWPGKKEVENKLWSLSEQVTPAVGVERFNQAMMDLGAMICTRSKPKCSLCPLQNGCIA

ATNNSWSLYPGKKPKQTLPERTGYFLLLQHEDEVLLAQRPPSGLWGGLYCFPQFADEESLRHWLAQRQIA

ADNLTQLTAFRHTFSHFHLDIVPMWLPVSSFTGCMDEGNALWYNLAQPPSVGLAAPVERLLQQLRTGAPV

>gi|30064281|ref|NP_838452.1| tRNA (guanine-N(7)-)-methyltransferase [Shigella flexneri 2a str. 2457T]

MKNDVISPEFDENGRPLRRIRSFVRRQGRLTKGQEHALENYWPVMGVEFSEDMLDFPALFGREAPVTLEI

GFGMGASLVAMAKDRPEQDFLGIEVHSPGVGACLASAHEEGLSNLRVMCHDAVEVLHKMIPDNSLRMVQL

FFPDPWHKARHNKRRIVQVPFAELVKSKLQLGGVFHMATDWEPYAEHMLEVMSSIDGYKNLSESNDYVPR

PASRPVTKFEQRGHRLGHGVWDLMFERVK

>gi|30064279|ref|NP_838450.1| hypothetical protein S3158 [Shigella flexneri 2a str. 2457T]

MRKMLLAAALSVTAMTAHADYQCSVTPRDDVIVSPQTVQVKGENGNLVITPDGNVMYNGKQYSLNAAQRE

QAKDYQAELRSTLPWIDEGAKSRVEKARIALDKIIVQEMGDSSKMRSRLTKLDAQLKEQMNRIIETRSDG

LTFHYKAIDQVRAEGQQLVNQAMGGILQDSINEMGAKAVLKSGGNPLQNVLGSLGGLQSSIQTEWKKQEK

DFQQFGKDVCSRVVTLEDSRKALVGNLK

>gi|30064278|ref|NP_838449.1| L-asparaginase II [Shigella flexneri 2a str. 2457T]

MEFFKKTALAALVMGFSGAALALPNITILATGGTIAGGGDSATKSNYTAGKVGVENLVNAVPQLKDIANV

KGEQVVNIGSQDMNDNVWLTLAKKINADCDKTDGFVITHGTDTMEETAYFLDLTVKCDKPVVMVGAMRPS

TSMSADGPFNLYNAVVTAADKASANRGVLVVMNDTVLDGRDVTKTNTTDVATFKSVNYGPLGYIHNGKID

YQRTPARKHTSDTPFDVSKLNELPKVGIVYNYANASDLPAKALVDAGYDGIVSAGVGNGNLYKSVFDTLA

TAAKNGTAVVRSSRVPTGATTQDAEVDDAKYGFVASGTLNPQKARVLLQLALTQTKDPQQIQQIFNQY

>gi|30064277|ref|NP_838448.1| alpha helix chain [Shigella flexneri 2a str. 2457T]

MKKQWIVGTALLMLMTGNAWADGEPPTENILKDQFKKQYHGILKLDAISLKNLDAKGNQATWSAEGDVSS

SDDLYTWVGQLADYELLEQTWTKDKPVKFSAMLTSKGTPASGWSVNFYSFQAAASDRGRVVDDIKTNNKY

LIVNSEDFNYRFSQLESALNTQKNSIPALEKEVKALDKQMVAAQKAADAYWGKDANGKQMTREEAFKKIH

QQRDEFNKQNDSEAFAVKYDKEVYQPAIAACHKQSEECYEVPIQQKRDFDINEQRRQTFLQSQKLSRKLQ

DDWVTLEKGQYPLTMKVSEINSKKVAILMKIDDINQANER

>gi|30064276|ref|NP_838447.1| hypothetical protein S3155 [Shigella flexneri 2a str. 2457T]

MDSYIAITLFGCFFVLVFIGVPISFSIGIATVASMLLMFPWDIAAITVSQRLANGLDNFALLAIPFFIFA

GTLMNSGGIAIRLINLAQVMVGRVPGSLGHVNVLANMMFGSISGSAVAAAAAVGGTLNPIQTKEGYDPAF

STAVNVSSCITGLLIPPSNVLIVFSLTAGGVSVASLFMAGYLPGILMGLAVMIVCGIIAKRRGYPLSERA

TFAQACKAFLDALPSLLLVFIVMGGILGGIFTATEASAIAVVYTFILSVLIYREVKWRHLPKLILESVVT

TSIVLLLIGFSVGMSWAMTNADIPYMISDALMGISDNPLIILLLINIVLLIVGIFMDMTPAVLIFTPIFL

PIAQELGMDPVHFGIMMVANLCIGLLTPPVGSALFVGCSISGVKIQHLIKPLLLFYAALLVALMMITYIP

QISLFIPQLLGLM

>gi|30064275|ref|NP_838446.1| IS1 orfA [Shigella flexneri 2a str. 2457T]

MASISIRCPSCSATEGVVRNGKSTAGHQRYLCSHCRKTWQLQFTYTASQPGTHQKIIDMAMNGVGCRASA

RIMGVGLNTVLRHLKNSGRSR

>gi|30064274|ref|NP_838445.1| hypothetical protein S3153 [Shigella flexneri 2a str. 2457T]

MIFNRLKLAVDRVIATFSVAVMLALVVCVVWQVFSRYVLNQPSTLTDELARFLMIWVGLLGAAYTVGAQR

HLSIDLFALALNKRKQLLLSIVINVLILGFAGSVIVTGGLKLIDKTLATSQVSAAMQIPMGYVYIILPLS

GVVMMFYALCFINQSIQQLKQPAQEAS

>gi|30064273|ref|NP_838444.1| hypothetical protein S3152 [Shigella flexneri 2a str. 2457T]

MDSTKDKGFFALSAYVAGTRSFYAKKPITKPEDLKGLKIRVQPSPTTIKMIELMGGSPTPISFGEVYTAM

QQGVVDGAENNVPSWMQTRHIEIAKVFSEDEHASIPDFLVISTKTWNKLTPEQQQILETAAKKSEAYQQK

LWEKIDADTRAQAKAMGGEIVKVDKAPFRKAVQPLFDDFKKDPKQAALLEKFDNAAQ

>gi|30064272|ref|NP_838443.1| hypothetical protein S3151 [Shigella flexneri 2a str. 2457T]

MKMRSFTRSLVCASLLALVSTGVNAAEKVALKLAHNLERSHVVHQSFEELAKEVKQLSKGNMVIRIYPSS

QMGNARETMELLQNGALDMTKGSASDLESFDNIYAIYNLPFLLSCFW

>gi|30064271|ref|NP_838442.1| coproporphyrinogen III oxidase [Shigella flexneri 2a str. 2457T]

MVKLPPLSLYIHIPWCVQKCPYCDFNSHALKGEVPHDDYVQHLLCDLDNDVAYAQGREVKTIFIGGGTPS

LLSGPAMQTLLDGVRARLPLAADAEITMEANPGTVEADRFVDYQRAGVNRISIGVQSFSEEKLKRLGRIH

GPQEAKRAAKLASGLGLRSFNLDLMHGLPDQSLEEALGDLRQAIELNPPHLSWYQLTIEPNTLFGSRPPV

LPDDDALWDIFEQGHQLLTAAGYQQYETSAYAKPGYQCQHNLNYWRFGDYIGIGCGAHGKVTFPDGRILR

TTKTRHPRGFMQGRYLESQRDVEAADKPFEFFMNRFRLLEAAPRVEFIAYTGLCEDVIRPQLDEAIAQGY

LTECADYWQITEHGKLFLNSLLELFLAE

>gi|30064270|ref|NP_838441.1| deoxyribonucleotide triphosphate pyrophosphatase [Shigella flexneri 2a str. 2457T]

MQKVVLATGNAGKVRELASLLSDFGLDIVAQTDLGVDSAEETGLTFIENAILKARHAAKVTGLPAIADDS

GLAVDALGGAPGIYSARYSGEDATDQKNLQKLLETLKDVPDDQRQARFHCVLVYLRHAEDPTPLVCHGSW

PGVITREPAGTGGFGYDPIFFVPSEGKTAAELTREEKSAISHRGQALKLLLDALRNG

>gi|30064268|ref|NP_838439.1| resistance protein [Shigella flexneri 2a str. 2457T]

MNTLTFLLSTVIELYTMVLLLRIWMQCAHCDFYTPFSQFVVKVAQPIIGPLRRVIPAMGPIDSASLLVAY

ILSFIKAIVLFEVVTFLPIIWIAGLLILLKTIGLLIFWVLLVMAIMSWVSQGRSPIEYVLIQLADPLLRP

IRRLLPAMGGIDFSPMILVLLLYAINMGVAEVLQATGNMLLPGLWMAL

>gi|30064267|ref|NP_838438.1| hypothetical protein S3145 [Shigella flexneri 2a str. 2457T]

MNDIAHNLAQVRDKISAAATRCGRSPEEITLLAVSKTKPASAIAEAIDAGQRQFGENYVQEGVDKIRHFQ

ELGVTGLEWHFIGPLQSNKSRLVAEHFDWCHTIDRLRIATRLNEQRPAELPPLNVLIQINISDENSKSGI

QLAELDELAAAVAELPRLRLRGLMAIPALESEYVRQFEVARQMAVAFAGLKTRYPHIDTLSLGMSDDMEA

AIAAGSTMVRIGTAIFGARDYSKK

>gi|30064266|ref|NP_838437.1| transporter [Shigella flexneri 2a str. 2457T]

MPGYAQYRSFSEDLSMNMEEIVALSVKHNVSDLHLCSAWPARWRIRGRMEAAPFDAPDVEELLREWLDDD

QRAILLENGQLDFAVSLAENQRLRGSAFAQRQGISLALRLLPSHCPQLEQLGAPPVLPELLKSENGLILV

TGATGSGKSTTLAAMVGYLNQHADAHILTLEDPVEYLYASQRCLIQQREIGLHCMTFASGLRAALREDPD

VILLGELRDSETIRLALTAAETGHLVLATLHTRGAAQAVERLVDSFPAQEKDPVRNQLAGSLRAVLSQKL

EVDKQEGRVALFELLINTPAVGNLIREGKTHQLPHVIQTGQKVGMLTFQQSYQQRVGEGRL

>gi|30064265|ref|NP_838436.1| Holliday junction resolvase-like protein [Shigella flexneri 2a str. 2457T]

MSGTLLAFDFGTKSIGVAVGQRITGTARPLPAIKAQDGTPDWNLIERLLKEWQPDEIIVGLPLNMDGTEQ

PLTARARKFANRIHGRFGVEVKLHDERLSTVEARSGLFEQGGYRALNKGKVDSASAVIILESYFEQGY

>gi|30064263|ref|NP_838434.1| glutathione synthetase [Shigella flexneri 2a str. 2457T]

MIKLGIVMDPIANINIKKDSSFAMLLEAQRRGYELHYMEMGDLYLINGEARAHTRTLNVKQNYEEWFSFV

GEQDLPLADLDVILMRKDPPFDTEFIYATYILERAEEKGTLIVNKPQSLRDCNEKLFTAWFSDLTPETLV

TRNXAQLKAFWEKHSDIILKPLDGMGGASIFRVKEGDPNLGVIAETLTEHGTCYCMAQNYLPAIKDGDKR

VLVVDGEPVPYCLARIPQGGETRGNLAAGGRGEPRPLTESDWKIARQIGPTLKEKGLIFVGLDIIGDRLT

EINVTSPTCIREIEAEFPVSITGMLMDAIEARLQQQ

>gi|30064262|ref|NP_838433.1| 16S ribosomal RNA methyltransferase RsmE [Shigella flexneri 2a str. 2457T]

MRIPRIYHPEPLTSHSHIALCEDAANHIGRVLRMGPGQALQLFDGSNQVFDAEITSASKKSVEVKVLEGQ

IDDRESPLHIHLGQVMSRGEKMEFTIQKSIELGVSLITPLFSERCGVKLDSERLNKKLQQWQKIAIAACE

QCGRNRVPEIRPAMDLEAWCAEQDEGLKLNLHPRASNSINTLPLPVERVRLLIGPEGGLSADEIAMTARY

QFTDILLGPRVLRTETTALTAITALQVRFGDLG

>gi|30064261|ref|NP_838432.1| DNA-specific endonuclease I [Shigella flexneri 2a str. 2457T]

MYRYLSIAAVVLSAAFSGPTLAEGINSFSQAKAAAVKVHADAPGTFYCGCKINWQGKKGVVDLQSCGYQV

RKNENRASRVEWEHIVPAWQFGHQRQCWQDGGRKNCAKDPVYRKMESDMHNLQPSVGEVNGDRGNFMYSQ

WNGGEGQYGQCAMKVDFKEKAAEPPARARGAIARTYFYMRDQYNLTLSRQQTQLFNAWNKMYPVTDWECE

RDERIAKVQGNHNPYVQRACQARKS

>gi|30064260|ref|NP_838431.1| hypothetical protein S3139 [Shigella flexneri 2a str. 2457T]

MKTSRLPIAIQQAVMRRLREKLAQANLKLGRNYPEPKLSYTQRGTSAGTAWLESYEIRLNPVLLLENSEA

FIEEVVPHELAHLLVWKHFGRVAPHGKEWKWMMESVLGVPARRTHQFELQSVRRNTFPYRCKCQEHQLTV

RRHNRVVRGEAIYRCVHCGEQLVAK

>gi|30064259|ref|NP_838430.1| major facilitator superfamily galactose-proton symporter [Shigella flexneri 2a str. 2457T]

MTFFVCFLAALAGLLFGLDIGVIAGALPFIADEFQITSHTQEWVVSSMMFGAAVGAVGIGWLSFKLGRKK

SLMIGAILFVAGSLFSAAAPNVEVLILSRVLLGLAVGVASYTAPLYLSEIAPEKIRGSMISMYQLMITIG

ILGAYLSDTAFSYTGAWRWMLGVIIIPAILLLIGVFFLPDSPRWFAAKRRFVDAERVLLRLRDTSAEAKR

ELDEIRESLQVKQSGWALFKENSNFRRAVFLGVLLQVMQQFTGMNVIMYYAPKIFELAGYTNTTEQMWGT

VIVGLTNVLATFIAIGLVDRWGRKPTLTLGFLVMAAGMGVLGTMMHIGIHSPSAQYFAIAMLLMFIVGFA

MSAGPLIWVLCSEIQPLKGRDFGITCSTATNWIANMIVGATFLTMLNTLGNANTFWVYAALNVLFILLTL

WLVPETKHVSLEHIERNLMKGRKLREIGAHD

>gi|30064258|ref|NP_838429.1| S-adenosylmethionine synthetase [Shigella flexneri 2a str. 2457T]

MAKHLFTSESVSEGHPDKIADQISDAVLDAILEQDPKARVACETYVKTGMVLVGGEITTSAWVDIEEITR

NTVREIGYVHSDMGFDANSCAVLSAIGKQSPDINQGVDRADPLEQGAGDQGLMFGYATNETDVLMPAPIT

YAHRLVQRQAEVRKNGTLPWLRPDAKSQVTFQYDDGKIVGIDAVVLSTQHSEEIDQKSLQEAVMEEIIKP

ILPAEWLTSATKFFINPTGRFVIGGPMGDCGLTGRKIIVDTYGGMARHGGGAFSGKDPSKVDRSAAYAAR

YVAKNIVAAGLADRCEIQVSYAIGVAEPTSIMVETFGTEKVPSEQLTLLVREFFDLRPYGLIQMLDLLHP

IYKETAAYGHFGREHFPWEKTDKAQLLRDAAGLK

>gi|30064257|ref|NP_838428.1| hypothetical protein S3136 [Shigella flexneri 2a str. 2457T]

MVADPTTTLQVKNTGSLSVNRYGWINIWMAILGQFFTRFPLFFESCLILLKTWLEIFPDNAGILRIYLLQ

FSAIVGYKTRRAA

>gi|30064256|ref|NP_838427.1| hypothetical protein S3135 [Shigella flexneri 2a str. 2457T]

MGITSAGMQSRDAECGERVFTRTVRQVKQQTTVHYFVSPPRPPVKTNPQAKTLISTRLEVATRKKRRVLF

I

>gi|30064255|ref|NP_838426.1| hypothetical protein S3134 [Shigella flexneri 2a str. 2457T]

MLNNVMKKKPVAQSERQHTLLENPCAYGLLSQFQAATVVNCFTLNKII

>gi|30064254|ref|NP_838425.1| arginine decarboxylase [Shigella flexneri 2a str. 2457T]

MSDDMSMGLPSSAGEHGVLRSMQEVAMSSQEASKMLRTYNIAWWGNNYYDVNELGHISVCPDPDVPEARV

DLAQLVKTREAQGQRLPALFCFPQILQHRLRSINAAFKRARESYGYNGDYFLVYPIKVNQHRRVIESLIH

SGEPLGLEAGSKAELMAVLAHAGMTRSVIVCNGYKDREYIRLALIGEKMGHKVYLVIEKMSEIAIVLDEA

ERLNVVPRLGVRARLASQGSGKWQSSGGEKSKFGLAATQVLQLVETLREAGRLDSLQLLHFHLGSQMANI

RDIATGVRESARFYVELHKLGVNIQCFDVGGGLGVDYEGTRSQSDCSVNYGLNEYANNIIWAIGDACEEN

GLPHPTVITESGRAVTEHHTVLVSNIIGVERNEYTVPTAPVEDAPRALQSMWETWQEMHEPGTRRSLREW

LHDSQMDLHDIHIGYSSGTFSLQERAWAEQLYLSMCHEVQKQLDPQNRAHRPIIDELQERMADKMYVNFS

LFQSMPDAWGIDQLFPVLPLEGLDQVPERRAVLLDITCDSDGAIDHYIDGDGIATTMPMPEYDPENPPML

GFFMVGAYQEILGNMHNLFGDTEAVDVFVFPDGSVEVELSDEGDTVADMLQYVQLDPKTLLTQFRDQVKK

TDLDAELQQQFLEEFEAGLYGYTYLEDE

>gi|30064253|ref|NP_838424.1| IS1 orfA [Shigella flexneri 2a str. 2457T]

MASISIRCPSCSATEGVVRNGKSTAGHQRYLCSHCRKTWQLQFTYTASQPGKHQKIIDMAMNGVGCRASA

RIMGVGLNTVLRHLKNSGRSR

>gi|30064252|ref|NP_838423.1| IS1 orfB [Shigella flexneri 2a str. 2457T]

MSRQCTHYGRWPQHGFTSLKKLRPQSVTSRIQPGSDVIVCAEMDEHWGYVGAKSRQRWLFYAYDRIRRTV

VAHVFGERTLATLERLLSLLSAFEVVVWMTDGCPLYESRLKGKLHVISKRYTQRIERHNLNLRQHLARLV

RKSLSFSKSVELHDKVIGHYLNIKHYQ

>gi|30064251|ref|NP_838422.1| lipoprotein, partial [Shigella flexneri 2a str. 2457T]

MNYQLYVRNYSRNTNYEIVATADGLDVLNGKQGSLNNNGYIVNAGDSLVIKGFRKDKHTEAAFQFANVAD

SYAANSAQGDVRNTGVIGFAAFELQGPAQNALPPCSGQAFPADNNGYAPPPCRK

>gi|30064250|ref|NP_838421.1| agmatinase [Shigella flexneri 2a str. 2457T]

MSTLGHQYDNSLVSNAFGFLRLPMNFQPYDSDADWVITGVPFDMATSGRAGGRHGPAAIRQVSTNLAWEH

NRFPWNFDMRERLNVVDCGDLVYAFGDAREMSEKLQAHAEKLLAAGKRMLSFGGDHFVTLPLLRAHAKHF

GKMALVHFDAHTDTYANGCEFDHGTMFYTAPKEGLIDPNHSVQIGIRTEFDKDNGFTVLDACQVNDRSVD

DVIAQVKQIVGDMPVYLTFDIDCLDPAFAPGTGTPVIGGLTSDRAIKLVRGLKDLNIVGMDVVEVAPAYD

QSEITALAAATLALEMLYIQAAKKGE

>gi|30064249|ref|NP_838420.1| hypothetical protein S3128 [Shigella flexneri 2a str. 2457T]

MLACCAICSPVRCAFPYTETERRFTCKRRIFRIILTLCGRENMKIRALLVAMSVATVLTGCQNMDSNGLL

SSGAEAFQAYSLSDAQVKTLSDQACQEMDSKATIAPANSEYAKRLTTIANALGNNINGQPVNYKVYMAKD

VNAFAMANGCIRVYSGLMDMMTDNEVEAVIGHEMGHVALGHVKKGMQVALGTNAVRVAAASAGGIVGSLS

QSQLGDLGEKLVNSQFSQRQEAEADDYSYDLLHQRGISPAGLATSFEKLAKLEEGRQSSMFDDHPASAER

AQHIRDRISADGIK

>gi|30064248|ref|NP_838419.1| transketolase [Shigella flexneri 2a str. 2457T]

MSSRKELANAIRALSMDAVQKAKSGHPGAPMGMADIAEVLWRDFLKHNPQNPSWADRDRFVLSNGHGSML
IYSLLHLTGYDLPMEELKNFRQLHSKTPGHPEVGYTAGVETTTGPLGQGIANAVGMAIAEKTLAAQFNRP
GHDIVDHYTYAFMGDGCMMEGISHEVCSLAGTLKLGKLIAFYDDNGISIDGHVEGWFTDDTAMRFEAYGW
HVIRDIDGHDAASIKRAVEEARAVTDKPSLLMCKTIIGFGSPNKAGTHDSHGAPLGDAEIALTREQLGWK
YAPFEIPSEIYAQWDAKEAGQAKESAWNEKFAAYAKAYPQEAAEFTRRMKGEMPSDFDAKAKEFIAKLQA
NPAKIASRKASQNAIEAFGPLLPEFLGGSADLAPSNLTLWSGSKAINEDAAGNYIHYGVREFGMTAIANG
ISLHGGFLPYTSTFLMFVEYARNAVRMAALMKQRQVMVYTHDSIGLGEDGPTHQPVEQVASLRVTPNMST
WRPCDQVESAVAWKYGVERQDGPTALILSRQNLAQQERTEEQLANIARGGYVLKDCAGQPELIFIATGSE
VELAVAAYEKLTAEGVKARVVSMPSTDAFDKQDAAYRESVLPKAVTARVAVEAGIADYWYKYVGLNGAIV
GMTTFGESAPAELLFEEFGFTVDNVVAKAKALL

>gi|30064247|ref|NP_838418.1| serine protease [Shigella flexneri 2a str. 2457T]
MKAPVSLTSFRPQKSLAIAIGVLAVVVLPFLSYYTVNEGERGILLRYGKIVKVAEPGLGFKIPFMESVEK
ISTRNQAVVYQGLQAYSRDQQPAQMTVSVSFHIKPSEAGAVYTTYNTIEALKDRLIVRQLPTQLENIFGQ
YTAISAVQDRTKLVQDLQNAMRKAVVGPVVIDGVQIENIDFSDAYEKSIENRMKAEVAIATRKQNLETEK
IQAQIAVTQAQAEADSKLAAAKAEAETIRVRGAAEAETIRLKSAAEAEAIRLRGEALRDNPGLVALTTAE
RWDGKLPDTMIPGSTVPFISTK

>gi|30064246|ref|NP_838417.1| IS1 orfA [Shigella flexneri 2a str. 2457T]
MASISIRCPSCSATEGVVRNGKSTAGHQRYLCSHCRKTWQLQFTYTASQPGKHQKIIDMAMNGVGCRASA
RIMGVGLNTVLRHLKNSGRSR

>gi|30064245|ref|NP_838416.1| IS1 orfB [Shigella flexneri 2a str. 2457T]
MSRQCTHYGRWPQHGFTSLKKLRPQSVTSRIQPGSDVIVCAEMDEHWGYVGAKSRQRWLFYAYDRIRRTV
VAHVFGERTLATLERLLSLLSAFEVVVWMTDGCPLYESRLKGKLHVISKRYTQRIERHNLNLRQHLARLV

RKSLSFSKSVELHDKVIGHYLNIKHYQ

>gi|30064244|ref|NP_838415.1| oxidoreductase, partial [Shigella flexneri 2a str. 2457T]

MGLPSGYSAGYSYEYFGGNATYMIIPEIAINLGCVLPYHGSYFAAASLAEPMCCIIGAYHANYHTTQYVY

EHRMGVKPGGNIALLACAGPMGIGAIDYAINGGIQPSRVVVVDIDDKRLAQVQKLLPVDLAASKGIELVY

VNTKGMSDPIQMLRALTGDVGFDDIFVYAAVPAVVEMADELLAEDGCLNFFAGLTDKNFKVPFNFYNVHY

NSTHVVGTSGGSTDDMKEAIALSATGQLQPSFMVTHIGGLDAVPETVLNLPDIPGGKKLIYNGVTMPLTA

IADFAEKGKTDPLFKELARLVEETHGIWNEQAEKYLLAQFGVDIGEAAQ

>gi|30064243|ref|NP_838414.1| fructose-1,6-bisphosphatase II-like protein [Shigella flexneri 2a str. 2457T]

MMSLAWPLFRVTEQAALAAWPQTGCGDKNKIDGLAVTAMRQALNDVAFRGRVVIGEGEIDHAPMLWIGEE

VGKGDGPEVDIAVDPIEGTRMVAMGQSNALAVMAFAPRDSLLHAPDMYMKKLVVNRLAAGAIDLSLPLAD

NLRNVARALGKPLDKLRMVTLDKPRLSAAIEEATQLGVKVFALPDGDVAASVLTCWQDNPYDVMYTIGGA

PEGVISACAVKALGGDMQAELIDFCQAKGDYTENRQIAEQERKRCKAMGVDVNRVYSLDELVRGNDILFS

ATGVTGGELVNGIQQTANGVRTQTLLIGGADQTCNIIDSLH

>gi|30064242|ref|NP_838413.1| DNA-binding transcriptional regulator [Shigella flexneri 2a str. 2457T]

MATLTEDDVLEQLDAQDNLFSFMKTAHSILLQGIRQFLPSLFVDNDEEIVEYAVKPLLAQSGPLDDIDVA

LRLIYALGKMDKWLYADITHFSQYWHYLNEQDETPGFADDITWDFISNVNSITRNATLYDALKAMKFADF

AVWSEARFSGMVKTALTLAVTTTLKELTP

>gi|30064241|ref|NP_838412.1| ATP-binding protein of ABC transport system [Shigella flexneri 2a str. 2457T]

MLTLNKISYRWPGAATDCLCDISLQLKQGEWLALTGDNGAGKSTLLRVMAGLLTPTAGTVMLQQQAMKNL

KNRQRAAKVGVLFQEAENQLFHSTVADEIAFGLKLQKCPADEITQRTHAALQCCQLADTANSHPLDLHNA

QRRMVAVACLEALSPPLLLLDEPSRDFDENWLSVFESWLEKCRQRGTSVVAISHDAAFTRRHFSRVVRLE

DGVIRNVNPPDDIHP

>gi|30064240|ref|NP_838411.1| ATP-binding protein of ABC transport system [Shigella flexneri 2a str. 2457T]

MVTLEQFRYCPTHSTHPPFCYDFHYVKPGMVAIFGDNGSGKSTLAQLMAGWYPDFLPREITGTGTLLGTP

IGHLPLNEQSATIQLVQQSPYLQLSGCTFSVEEEVAFGPENLCLAEAEIMARIDAALTLTECQPLRHRHP

ATLSGGETQRVIIACAIAMQPKLLILDEAFSRLTPQASEMLLQRLQHWAFERGSLIILFERHRTPFLNYC

QQAWQLQNGALQPLC

>gi|30064239|ref|NP_838410.1| lipoprotein, partial [Shigella flexneri 2a str. 2457T]

MQYVPVQCFIRALFASRLPPGVAYLFAGPLLVVEQLKRQLAIIHEAQRARGVPLDEGWYQRLRAMPALII

PLTHNALNDLAVRGAALDMRAFRIHNRRTTLWAPADSTLQRVARYTMILLMLTEFGAWIWLR

>gi|30064238|ref|NP_838409.1| lipoprotein, partial [Shigella flexneri 2a str. 2457T]

MHPFTSLTLWALAACTTLILPAQTILPIYSAATFFCLIALKATRRRAKYVVWLMFSLGAGLWLVHGGWLP

EWLSGTPRSPERWSHAITLWLLILAIVSTSQL

>gi|30064237|ref|NP_838408.1| inner membrane protein [Shigella flexneri 2a str. 2457T]

MARSHFSSQALVLIVISIAINMIGGQLASMVKLPIFLDSIGTLISAVLLGPVIGMLTGLLTNLLWGLLTD

PIAAAFAPVAMVIGLVSGWLARAGWFRTLPKVVVSGVIITLAVTVVAVPLRTALFGGVTGSGADLFVAWM

HSMGQNLVESVAITVIGANLVDKILTAVIVWLLLRQLPIRTTRHFPAMAAVR

>gi|30064236|ref|NP_838407.1| DNA-binding protein, partial [Shigella flexneri 2a str. 2457T]

MRYAGQEGTTLLNGTFEVISLNGTLEQSGEHLHLCVSDPHGTMLGGHMMPGCTVRTTLELVIGSLEELAF

SRQPCALSGYDELHISPVK

>gi|30064235|ref|NP_838406.1| erythrose 4-phosphate dehydrogenase [Shigella flexneri 2a str. 2457T]

MTVRVAINGFGRIGRNVVRALYESGRRAEITVVAINELADAAGMAHLLKYDTSHGRFAWEVRQERDQLFV

GDDAIRVLHERSLQSLPWRELGVDVVLDCTGVYGSREHGEAHIAAGAKKVLFSHPGSNDLDATVVYGVNQ

DQLRAEHRIVSNASCTTNCIIPVIKLLDDAYGIESGTVTTIHSAMHDQQVIDAYHPDLRRTRAASQSIIP

VDTKLAAGITRFFPQFNDRFEAIAVRVPTINVTAIDLSVTVKKPVKANEVNLLLQKAAQGAFHGIVDYTE

LPLVSVDFNHDPHSAIVDGTQTRVSGAHLIKTLVWCDNEWGFANRMLDTTLAMATVAFR

>gi|30064234|ref|NP_838405.1| phosphoglycerate kinase [Shigella flexneri 2a str. 2457T]

MSVIKMTDLDLAGKRVFIRADLNVPVKDGKVTSDARIRASLPTIELALKQGAKVMVTSHLGRPTEGEYNE

EFSLLPVVNYLKDKLSNPVRLVKDYLDGVDVAEGELVVLENVRFNKGEKKDDETLSKKYAALCDVFVMDA

FGTAHRAQASTHGIGKFADVACAGPLLAAELDALGKALKEPARPMVAIVGGSKVSTKLTVLDSLSKIADQ

LIVGGGIANTFIAAQGHDVGKSLYEADLVDEAKRLLTTCNIPVPSDVRVATEFSETAPATLKSVNDVKAD

EQILDIGDASAQELAEILKNAKTILWNGPVGVFEFPNFRKGTEIVANAIADSEAFSIAGGGDTLAAIDLF

GIADKISYISTGGGAFLEFVEGKVLPAVAMLEERAKK

>gi|30064233|ref|NP_838404.1| fructose-bisphosphate aldolase [Shigella flexneri 2a str. 2457T]

MSKIFDFVKPGVITGDDVQKVFQVAKENNFALPAVNCVGTDSINAVLETAAKVKAPVIVQFSNGGASFIA

GKGVKSDVPQGAAILGAISGAHHVHQMAEHYGVPVILHTDHCAKKLLPWIDGLLDAGEKHFAATGKPLFS

SHMIDLSEESLQENIEICSKYLERMSKIGMTLEIELGCTGGEEDGVDNSHMDASALYTQPEDVDYAYTEL

SKISPRFTIAASFGNVHGVYKPGNVVLTPTILRDSQEYVSKKHNLPHNSLNFVFHGGSGSTAQEIKDSVS

YGVVKMNIDTDTQWATWEGVLNYYKANEAYLQGQLGNPKGEDQPNKKYYDPRVWLRAGQTSMIARLEKAF

QELNAIDVL

>gi|30064232|ref|NP_838403.1| mechanosensitive channel MscS [Shigella flexneri 2a str. 2457T]

MEDLNVVDSINGAGSWLVANQALLLSYAVNIVAALAIIIVGLIIARMISNAVNRLMISRKIDATVADFLS
ALVRYGIIAFTLIAALGRVGVQTASVIAVLGAAGLAVGLALQGSLSNLAAGVLLVMFRPFRAGEYVDLGG
VAGTVLSVQIFSTTMRTADGKIIVIPNGKIIAGNIINFSREPVRRNEFIIGVAYDSDIDQVKQILTNIIQ
SEDRILKDREMTVRLNELGASSINFVVRVWSNSGDLQNVYWDVLERIKREFDAAGISFPYPQMDVNFKRV
KEDKAA

>gi|30064231|ref|NP_838402.1| arginine exporter protein [Shigella flexneri 2a str. 2457T]
MFSYYFQGLALGAAMILPLGPQNAFVMNQGIRRQYHIMIALLCAISDLVLICAGIFGGSALLMQSPWLLA
LVTWGGVVFLLWYGFGAFKTAMSSNIELASAEVLKQGRWKIIATMLAVTWLNPHVYLDTFVVLGSLGGQL
DVEPKRWFALGTISASFLWFFGLAILAAWLAPRLRTAKSQRIINLVVGCVMWFIALQLARDGIAHAQALF
S

>gi|30064230|ref|NP_838401.1| hypothetical protein S3107 [Shigella flexneri 2a str. 2457T]
MKFKVIALAALMGISGMAAQANELPDGPHIVTSGTASVDAVPDIATLAIEVNVAAKDAATAKKQADERVA
QYISFLELNQIAKKDISSANLRTQPDYDYQDGKSILKGYRAVRTVEVTLRQLDKLNSLLDGALKAGLNEI
RSVSLGVAQPDAYKDKARKAAIDNAIHQAQELANGFHRKLGPVYSVRYHVSNYQPSPMVRMMKADAAPVS
AQETYEQAAIQFDDQVDVVFQLEPVDQQPAKTPAAQ

>gi|30064229|ref|NP_838400.1| LYSR-type transcriptional regulator [Shigella flexneri 2a str. 2457T]
MDIFISKKMRNFILLAQTNNIARAAEKIHMTASPFGKSIAALEEQIGYTLFTRKDNNISLNKAGQELYQK
LFPVYQRLSAIDNEIHNSGRRSREIVIGIDNTYPTIIFDQLISLGDKYEGVTAQPVEFSENGVIDNLFDR
QLDFIISPQHVSARVQELENLTISELPPLRLGFLVSRRYEERQEQELLQELPWLQMRFQNRANFEAMIDA
NMRPCGINPTIIYRPYSFMAKISAVERGHFLTVIPHFAWRLVNPATLKYFDAPHRPMYMQEYLYSIRNHR
YTATIFSILLKIVTGQTINPASSRLQLNYGVSRRRG

>gi|30064227|ref|NP_838398.1| arginine/ornithine transport system ATPase [Shigella flexneri 2a str. 2457T]

MINEATLAESIRRLRQGERATLAQAMTLVESRHPRHQALSTQLLDAIMPFCGNALRLGVTGTPGAGKSTF

LEAFGMLLIREGLKVAVIAVDPSSPVTGGSILGDKTRMNDLARAEAAFIRPVPSSGHLGGASQRARELML

LCEAAGYDVVIVETVGIAQSETEVARMVDCFISLQIAGGGDDLQGIKKGLMEVADLLVINKDDGDNHTNV

AIARHMYESALHILRRKYDEWQPRGLTCSALEKRGIDEIWHAIIDFKTALTASGRLQQVRQQQSVEWLRK

QTEEEVLNHLFANEDFDRYYRQTLLAVKNNTLSPRTGLRQLSEFIQTQYFD

>gi|30064226|ref|NP_838397.1| methylmalonyl-CoA mutase [Shigella flexneri 2a str. 2457T]

MSNEQEWQQLANKELSRREKTVDSLVQQTAEGIAIKPLYTEADLDNLEVTGTLPGLPPYVRGPRATMYTA

QPWTIRQYAGFSTAKESNAFYRRNLAAGQKGLSVAFDLATHRGYDSDNPRVAGDVGKAGVAIDTVEDMKV

LFDQIPLDKMSVSMTMNGAVLPVLAFYIVAAEEQGVTPDKLTGTIQNDILKEYLCRNTYIYPPKPSMRII

ADIIAWCSGNMPRFNTISISGYHMGEAGANCVQQVAFTLADGIEYIKAAISAGLKIDDFAPRLSLFFGIG

MDLFMNVAMLRAARYLWSEAVSGFGAQDPKSLALRTHCQTSGWSLTEQDPYNNVIRTTIEALAATLGGTQ

SLHTNAFDEALGLPTDFSARIARNTQIIIQEESELCHTVDPLAGSYYIESLTDQIVKQARAIIQQIDEAG

GMAKAIEAGLPKRMIEEASAREQSLIDQGKRVIVGVNKYKLDHEDETDVLEIDNVMVRNEQIASLERIRA

TRDDAAVTAALNALTHAAQHNENLLAAAVNAARVRATLGEISDALEVAFDRYLVPSQCVTGVIAQSYHQS

EKSASEFDAIVAQTEQFLADNGRRPRILIAKMGQDGHDRGAKVIASAYSDLGFDVDLSPMFSTPEEIARL

AVENDVHVVGASSLAAGHKTLIPELVEALKKWGREDICVVAGGVIPPQDYAFLQERGVAAIYGPGTPMLD

SVRDVLNLISQHHD

>gi|30064225|ref|NP_838396.1| chromosome replication initiation inhibitor protein [Shigella flexneri 2a str. 2457T]

MKRPDYRTLQALDAVIRERGFERAAQKLCITQSAVSQRIKQLENMFGQPLLVRTVPPRPTEQGQKLLALL

RQVELLEEEWLGDEQTGSTPLLLSLAVNADSLATWLLPALAPVLADSPIRLNLQVEDETRTQERLRRGEV

VGAVSIQHQALPSCLVDKLGALDYLFVSSKPFAEKYFPNGVTRSALLKAPVVAFDHLDDMHQAFLQQNFD

LPPGSVPCHIVNSSEAFVQLARQGTTCCMIPHLQIEKELASGELIDLTPGLFQRRMLYWHRFAPESRMMR
KVTDALLDYGHKVLRQD

>gi|30064224|ref|NP_838395.1| ribose-5-phosphate isomerase A [Shigella flexneri 2a str. 2457T]
MTQDELKKAVGWAALQYVQPGTIVGVGTGSTAAHFIDALGTMKGQIEGAVSSSDASTEKLKSLGIHVFDL
NEVDSLGIYVDGADEINGHMQMIKGGGAALTREKIIASVAEKFICIADASKQVDILGKFPLPVEVIPMAR
SAVARQLVKLGGRPEYRQGVVTDNGNVILDVHGMEILDPIAMENAINAIPGVVTVGLFANRGADVALIGT
PDGVKTIVK

>gi|30064223|ref|NP_838394.1| D-3-phosphoglycerate dehydrogenase [Shigella flexneri 2a str. 2457T]
MAKVSLEKDKIKFLLVEGVHQKALESLRAAGYTNIEFHKGALDDEQLKESIRDAHFIGLRSRTHLTEDVI
NAAEKLVAIGCFCIGTNQVDLDAAAKRGIPVFNAPFSNTRSVAELVIGELLLLLRGVPEANAKAHRGVWN
KLAAGSFEARGKKLGIIGYGHIGTQLGILAESLGMYVYFYDIENKLPLGNATQVQHLSDLLNMSDVVSLH
VPENPSTKNMMGAKEISLMKPGSLLINASRGTVVDIPALCDALASKHLAGAAIDVFPTEPATNSDPFTSP
LCEFDNVLLTPHIGGSTQEAQENIGLEVAGKLIKYSDNGSTLSAVNFPEVSLPLHGGRRLMHIHENRPGV
LTALNKIFAEQGVNIAAQYLQTSAQMGYVVIDIEADEDVAEKALQAMKAIPGTIRARLLY

>gi|30064222|ref|NP_838393.1| ligase [Shigella flexneri 2a str. 2457T]
MIRQRRRALTPEQQLEMGQQAATRMMTYPPVVMAHTVAVFLSFDGELDTQPLIEQLWRAGKRVYLPVLHP
FSAGNLLFLNYHPQSELVMNRLKIHEPKLDVRDVLPLSRLDVLITPLVAFDEYGQRLGMGGGFYDRTLQN
WQHYKTQPVGYAHDCQLVEKLPVEEWDIPLPAVVTPSKVWEW

>gi|30064221|ref|NP_838392.1| Z-ring-associated protein [Shigella flexneri 2a str. 2457T]
MSAQPVDIQIFGRSLRVNCPPDQRDALNQAADDLNQRLQDLKERTRVTNTEQLVFIAALNISYELAQEKA
KTRDYAASMEQRIRMLQQTIEQALLEQGRITEKTNQNFE

>gi|30064220|ref|NP_838391.1| hypothetical protein S3094 [Shigella flexneri 2a str. 2457T]

MSIQNEMPGYNEMNQYLNQQGTGLTPAEMHGLISGMICGGNDDSSWLPLLHDLTNEGMAFGHELAQALRK

MHSATSDALQDDGFLFQLYLPDGDDVSVFDRADALAGWVNHFLLGLGVTQPKLDKVTGETGEAIDDLRNI

AQLGYDEDEDQEELEMSLEEIIEYVRVAALLCHDTFTHPQPTAPEVQKPTLH

>gi|30064219|ref|NP_838390.1| proline aminopeptidase P II [Shigella flexneri 2a str. 2457T]

MSEISRQEFQRRRQALVEQMQPGSAALIFAAPEVTRSADSEYPYRQNSDFWYFTGFNEPEAVLVLIKSDD

THNHSVLFNRVRDLTAEIWFGRRLGQDAAPEKLGVDRALAFSEINQQLYQLLNGLDVVYHAQGEYAYADE

IVNSALEKLRKGSRQNLTAPATMIDWRPVVHEMRLFKSPEEIAVLRRAGEITAMAHTRAMEKCRPGMFEY

HLEGEIHHEFNRHGARYPSYNTIVGSGENGCILHYTENESELRDGDLVLIDAGCEYKGYAGDITRTFPVN

GKFTQAQREIYDIVLESLETSLRLYRPGTSILEVTGEVVRIMVSGLVKLGILKGNVDELIAQNAHRPFFM

HGLSHWLGLDVHDVGVYGQDRSRILEPGMVLTVEPGLYIAPDADVPEQYRGIGIRIEDDIVITETGNENL

TASVVKKPEEIEALMAAARKQ

>gi|30064218|ref|NP_838389.1| 2-octaprenyl-6-methoxyphenyl hydroxylase [Shigella flexneri 2a str. 2457T]

MSVIIVGGGMAGATLALAISRLSHGALPVHLIEATAPESHAHPGFDGRAIALAAGTCQQLARIGVWQSLA

DCATAITTVHVSDRGHAGFVTLAAEDYQLAALGQVVELHNVGQRLFALLRKAPGVMLHCPDRVANVARTQ

SHVEVTLESGEMLTGRVLIAADGTHSALATACGVDWQQEPYEQLAVIANVATSVAHEGRAFERFTQHGPL

AMLPMSDGRCSLVWCHPLERREEVLSWSDEKFCRELQSAFGWRLGQITHAGKRSAYPLALTRAARPITHR

TVLVGNAAQTLHPIAGQGFNLGMRDVMSLAETLTQAHERGEDIGDYGILCRYQQRRQSDREATIGVTDSL

VHLFANRWAPLVVGRNIGLMTMELFTPARDVLAQRTLGWVAR

>gi|30064217|ref|NP_838388.1| hypothetical protein S3091 [Shigella flexneri 2a str. 2457T]

MQSVDVAIVGGGMVGLAVACGLQGSGLRVAVLEQRVPEPLAVDALPQLRVSAINAASEKLLTRLGVWQDI

LARRASCYHGMEVWDKDSFGHISFDDQSMGYSHLGHIVENSVIHYALWNKAQQSSDITLLAPTELQQVAW

GENETFLTLKDGSMLTARLVIGADGANSWLRNKADIPLTFWDYQHHALVATIRTEEPHDAVARQVFHGEG

ILAFLPLSDPHLCSIVWSLSPEEAQRMQQASEDEFNRALNIAFDNRLGLCKVESARQVFPLTGRYACQFA

AHRLALVGDAAHTIHPLAGQGVNLGFMDAAELIAELKRLHRQGKDIGQYIYLRRYERSRKHSAALMLAGM

QGFRDLFSGANPAKKLLRDIGLKLADTLPGVKPQLIRQAMGLNDLPEWLR

>gi|30064216|ref|NP_838387.1| glycine cleavage system aminomethyltransferase T [Shigella flexneri 2a str. 2457T]

MAQQTPLYEQHTLCGARMVDFHGWMMPLHYGSQIDEHHAVRTDAGMFDVSHMTIVDLRGSRTREFLRYLL

ANDVAKLTKSGKALYSGMLNASGGVIDDLIVYYFTEDFFRLVVNSATREKDLSWITQHAEPFGIEITVRD

DLSMIAVQGPNAQAKAATLFNDAQRQAVEGMKPFFGVQVGDLFIATTGYTGEAGYEIALPNEKAADFWRA

LVEAGVKPCGLGARDTLRLEAGMNLYGQEMDETISPLAANMGWTIAWEPTDRDFIGREALEVQREHGTEK

LVGLVMTEKGVLRNELPVRFTDAQGNQHEGIITSGTFSPTLGYSIALARVPEGIGETAIVQIRNREMPVK

VTKPVFVRNGKAVA

>gi|30064215|ref|NP_838386.1| glycine cleavage system protein H [Shigella flexneri 2a str. 2457T]

MSNVPAELKYSKEHEWLRKEADGTYTVGITEHAQELLGDMVFVDLPEVGATVSAGDDCAVAESVKAASDI

YAPVSGEIVAVNDALSDSPELVNSEPYAGGWIFKIKASDESELESLLDATAYEALLEDE

>gi|30064214|ref|NP_838385.1| glycine dehydrogenase [Shigella flexneri 2a str. 2457T]

MTQTLSQLENSGAFIERHIGPDAAQQQEMLNAVGAQSLNALTGQIVPKDIQLATPPQVGAPATEYAALAE

LKAIASRNKRFTSYIGMGYTAVQLPPVILRNMLENPGWYTAYTPYQPEVSQGRLEALLNFQQVTLDLTGL

DMASASLLDEATAAAEAMAMAKRVSKLKNANRFFVASNVHPQTLDVVRTRAETFGFEVIVDDAQKVLDHQ

DVFGVLLQQVGTTGEIHDYTALISELKSRKIVVSVAADIMALVLLTAPGKQGADIVFGSAQRFGVPMGYG

GPHAAFFAAKDEYKRSMPGRIIGVSKDAAGNTALRMAMQTREQHIRREKANSNICTSQVLLANIASLYAV

YHGPVGLKRIANRIHRLTDILAAGLQQKGLKLRHAHYFDTLCVEVADKAGVLTRAEAAEINLRSDILNAV

GITLDETTTRENVMQLFSVLLGDNHGLEIDTLDKDVAHDSRSIQPAMLRDDEILTHPVFNRYHSETEMMR

YMHSLERKDLALNQAMIPLGSCTMKLNAAAEMIPITWTEFAELHPFCPPEQAEGYQQMIAQLADWLVKLT

GYDAVCMQPNSGAQGEYAGLLAIRHYHESRNEGHRDICLIPASAHGTNPASAHMAGMQVVVVACDKNGNI

DLTDLRAKAEQAGDNLSCIMVTYPSTHGVYEETIREVCEVVHQFGGQVYLDGANMNAQVGITSPGFIGAD

VSHLNLHKTFCIPHGGGGPGMGPIGVKAHLAPFVPGHSVVQIEGMLTRQGAVSAAPFGSASILPISWMYI

RMMGAEGLKKASQVAILNANYIASRLQDAFPVLYTGRDGRVAHECILDIRPLKEETGISELDIAKRLIDY

GFHAPTMSFPVAGTLMVEPTESESKVELDRFIDAMLAIRAEIDQVKAGVWPLEDNPLVNAPHIQSELVAE

WAHPYSREVAVFPAGVADKYWPTVKRLDDVYGDRNLFCSCVPISEYQ

>gi|30064213|ref|NP_838384.1| hypothetical protein S3087 [Shigella flexneri 2a str. 2457T]

MAIALVTGGSRGIGRATALLLAQEGYTVAVNYQQNLHAAQEVMNLIMQAGGKAFVLQADISDENQVVAMF

TAIDQHDEPLAALVNNAGILFTQCTVENLTAERINRVLSTNVTGYFLCCREAVKRMALKNGGSGGAIVNV

SSVASRLGSPGEYVDYAASKGAIDSLTTGLSLEVAAQGIRVNCVRPGFIYTEMHASGGEPGRVDRVKSNI

PMQRGGQAEEVAQAIVWLLSDKASYVTGSFIDLAGGK

>gi|30064212|ref|NP_838383.1| 6-phospho-beta-glucosidase [Shigella flexneri 2a str. 2457T]

MKKLTLPKDFLWGGAVAAHQVEGGWNKGGKGPSICDVLTCGAHGVPREITKEVLPGKYYPNHEAVDFYGH

YKEDIKLFAEMGFKCFRTSIAWTRIFPKGDEAQPNEEGLKFYDSLFDELLKYNIEPVITLSHFEMPLHLV

QQYGSWTNRKFVDFFVRFAEVVFERYKHKVKYWMTFNEINNQRNWRAPLFGYCCSGVVYTEHDNPEETMY

QVLHHQFVASALAVKAARRINPEMKVGCMLAMVPLYPYSCNPDDVMFAQESMRERYVFTDVQLCGYYPSY

VLNEWERRGFNIKMEDGDLDVLREGTCDYLGFSYYMTNAVKAEGGTGDAISGFEGSVPNPYVKASDWGWQ

IDPVGLRYALCELYERYQRPLFIVENGFGAYDKVEEDGSINDDYRIDYLRAHIEEMKKAVTYDGVDLMGY

TPWGCIDCVSFTTGQYSKRYGFIYVNKHDDGTGDMSRSRKKSFNWYKEVIASNGEKL

>gi|30064211|ref|NP_838382.1| oxidoreductase [Shigella flexneri 2a str. 2457T]

MVQKPLIKQGYSLAEEIANSVSHGIGLVFGIVGLVLLLVQAVDLNASATAITSYSLYGGSMILLFLASTL
YHAIPHQRAKMWLKKFDHCAIYLLIAGTYTPFLLVGLDSPLARGLMIVIWSLALLGILFKLTIAHRFKIL
SLVTYLAMGWLSLVVIYEMAVKLAAGSVTLLAVGGVVYSLGVIFYVCKRIPYNHAIWHGFVLGGSVCHFL
AIYLYIGQA

>gi|30064210|ref|NP_838381.1| global regulator [Shigella flexneri 2a str. 2457T]
MAFTPFPPRQPTASARLPLTLMTLDDWALATITGADSEKYMQGQVTADVSQMAEDQHLLAAHCDAKGKMW
SNLRLFRDGDGFAWIERRSVREPQLTELKKYAVFSKVTIAPDDERVLLGVAGFQARAALANLFSELPSKE
KQVVKEGATTLLWFEHPAERFLIVTDEATANMLTDKLRGEAELNNSQQWLALNIEAGFPVIDAANSGQFI
PQATNLQALGGISFKKGCYTGQEMVARAKFRGANKRALWLLAGSASRLPEAGEDLELKMGENWRRTGTVL
AAVKLEDGQVVVQVVMNNDMEPDSIFRVRDDANTLHIEPLPYSLEE

>gi|30064209|ref|NP_838380.1| hypothetical protein S3082 [Shigella flexneri 2a str. 2457T]
MDINNKARIHWACRRGMRELDISIMPFFEHEYDSLSDDEKRIFIRLLECDDPDLFNWLMNHGKPADAELE
MMVRLIQTRNRERGPVAI

>gi|30064208|ref|NP_838379.1| hypothetical protein S3081 [Shigella flexneri 2a str. 2457T]
MVLWQSDLRVSWRAQWLSLLIHGLVAAVILLMPWPLSYTPLWMVLLSLVVFDCVRSQRCINARQGEIRLL
MDGRLRWQGQEWSIVKAPWMIKSGMMLRLRSDGGKRQHLWLAADSMDEAEWRDLRRILLQQETQR

>gi|30064207|ref|NP_838378.1| flavodoxin FldB [Shigella flexneri 2a str. 2457T]
MNMGLFYGSSTCYTEMAAEKIRDIIGPELVTLHNLKDDSPKLMEQYDVLILGIPTWDFGEIQEDWEAVWD
QLDDLNLEGKIIALYGLGDQLGYGEWFLDALGMLHDKLSTKGVKFVGYWPTEGYEFTSPKPVIADGQLFV
GLALDETNQYDLSDERIQSWCEQILNEMAEHYA

>gi|30064206|ref|NP_838377.1| site-specific tyrosine recombinase XerD [Shigella flexneri 2a str. 2457T]

MKQELARIEQFLDALWLEKNLAENTLNAYRRDLSMMVEWLHHRGLTLATAQSDDLQALLAERLEGGYKAT

SSARLLSAVRRLFQYLYREKFREDDPSAHLASPKLPQRLPKDLSEAQVERLLQAPLIDQPLELRDKAMLE

VLYATGLRVSELVGLTMSDISLRQGVVRVIGKGNKERLVPLGEEAVYWLETYLEHGRPWLLNGVSIDVLF

PSQRAQQMTRQTFWHRIKHYAVLAGIDSEKLSPHVLRHAFATHLLNHGADLRVVQMLLGHSDLSTTQIYT

HVATERLRQLHQQHHPRA

>gi|30064205|ref|NP_838376.1| thiol:disulfide interchange protein DsbC [Shigella flexneri 2a str. 2457T]

MKKGFMLFTLLAAFSGFAQADDAAIQQTLAKMGIKSSDIQPAPVAGMKTVLTNSGVLYITDDGKHIIQGP

MYDVSGTAPVNVTNKMLLKQLNALEKEMIVYKAPQEKHVITVFTDITCGYCHKLHEQMADYNALGITVRY

LAFPRQGLDSDAEKEMKAIWCAKDKNKAFDDVMAGKSVAPASCDVDIADHYALGVQLGVSGTPAVVLSNG

TLVPGYQPPKDMKEFLDEHQKMTSGK

>gi|30064204|ref|NP_838375.1| ssDNA exonuclease RecJ [Shigella flexneri 2a str. 2457T]

MKQQIQLRRREVDETADLPAELPPLLRRLYASRGVRSAQELERSVKGMLPWQQLSGVEKAVEILYNAFRE

GTRIIVVGDFDADGATSTALSVLAMRSLGCSNIDYLVPNRFEDGYGLSPEVVDQAHARGAQLIVTVDNGI

SSHAGVEHARSLGIPVIVTDHHLPGDTLPAAEAIINPNLRDCNFPSKSLAGVGVAFYLMLALRTFLRDQG

WFDERGIAIPNLAELLDLVALGTVADVVPLDANNRILTWQGMSRIRAGKCRPGIKALLEVANRDAQKLAA

SDLGFALGPRLNAAGRLDDMSVGVALLLCDNIGEARVLANELDALNQTRKEIEQGMQVEALTLCEKLERS

RDTLPGGLAMYHPEWHQGVVGILASRIKERFHRPVIAFAPAGDGTLKGSGRSIQGLHMRDALERLDTLYP

GMMLKFGGHAMAAGLSLEEDKFELFQQRFGELVTEWLAPSLLQGEVVSDGPLSPAEMTMEVAQLLRDAGP

WGQMFPEPLFDGHFRLLQQRLVGERHLKVMVEPVGGGPLLDGIAFNVDTALWPDNGVREVQLAYKLDINE

FRGNRSLQIIIDNIWPI

>gi|30064203|ref|NP_838374.1| peptide chain release factor 2 [Shigella flexneri 2a str. 2457T]

MFEINPVNNRIQDLTERSDVLRGYLDYDAKKERLEEVNAELEQPDVWNEPERAQALGKERSSLEAVVDTL

DQMKQGLEDVSGLLELAVEADDEETFNEAVAELDALEEKLAQLEFRRMFSGEYDSADCYLDIQAGSGGTE

AQDWASMLERMYLRWAESRGFKTEIIEESEGEVAGIKSVTIKISGDYAYGWLRTETGVHRLVRKSPFDSG

GRRHTSFSSAFVYPEVDDDIDIEINPADLRIDVYRASGAGGQHVNRTESAVRITHIPTGIVTQCQNDRSQ

HKNKDQAMKQMKAKLYELEMQKKNAEKQAMEDNKSDIGWGSQIRSYVLDDSRIKDLRTGVETRNTQAVLD

GSLDQFIEASLKAGL

>gi|30064202|ref|NP_838373.1| lysyl-tRNA synthetase [Shigella flexneri 2a str. 2457T]

MSEQHAQGADAVVDLNNELKTRREKLANLREQGIAFPNDFRRDHTSDQLHAEFDGKENEELEALNIEVAV

AGRMMTRRIMGKASFVTLQDVGGRIQLYVARDDLPEGVYNEQFKKWDLGDILGAKGKLFKTKTGELSIHC

TELRLLTKALRPLPDKFHGLQDQEARYRQRYLDLISNDESRNTFKVRSQILSGIRQFMVNRGFMEVETPM

MQVIPGGAAARPFITHHNALDLDMYLRIAPELYLKRLVVGGFERVFEINRNFRNEGISVRHNPEFTMMEL

YMAYADYKDLIELTESLFRTLAQDILGKTEVTYGDVTLDFGKPFEKLTMREAIKKYRPETDMADLDNFDS

AKAIAESIGIHVEKSWGLGRIVTEIFEEVAEAHLIQPTFITEYPAEVSPLARRNDINPEITDRFEFFIGG

REIGNGFSELNDAEDQAQRFLDQVAAKDAGDDEAMFYDEDYVTALEHGLPPTAGLGIGIDRMVMLFTNSH

TIRDVILFPAMRPVK

>gi|30064201|ref|NP_838372.1| isopentenyl-diphosphate delta-isomerase [Shigella flexneri 2a str. 2457T]

MQTEHVILLNAQGVPTGTLEKYAAHTADTLLHLAFSSWLFNAKGQLLVTRRALSKKAWPGVWTNSVCGHP

QLGESSEDAVIRRCRYELGVEITPPESIYPDFRYRATDPRGIVENEVCPVFAARTTSALQINDDEVMDYQ

WCDLADVLRGIDATPWAFSPWMVMQATNREARIRLSAFTQLK

>gi|30064200|ref|NP_838371.1| permease [Shigella flexneri 2a str. 2457T]

MCLDSFINVNGCFLFWRAKGETLMSAIDSQLPSSSGQDRPTDEVDRILSPGKLIILGLQHVLVMYAGAVA

VPLMIGDRLGLSKDAIAMLISSDLFCCGIVTLLQCIGIGRFMGIRLPVIMSVTFAAVTPMIAIGMNPDIG

LLGIFGATIAAGFITTLLAPLIGRLMPLFPPLVTGVVITSIGLSIIQVGIDWAAGGKGNPQYGNPVYLGI

SFAVLIFILLITRYAKGFMSNVAVLLGIVFGFLLSWMMNEVNLSGLHDASWFAIVTPMSFGMPIFDPVSI

LTMTAVLIIVFIESMGMFLALGEIVGRKLSSHDIICGLRVDGVGTMIGGTFNSFPHTSFSQNVGLVSVMR

VHSRWVCISSGIILILFGMVPKMAVLVASIPQFVLGGAGLVMFGMVLATGIRILSRCNYTTNRYNLYIVA

ISLGVGMTPTLSHDFFSKLPAVLQPLLHSGIMLATLSAVVLNVFFNGYQHHADLVKESVSDKDLEVRTVR

MWLLMRKLKKNEHGE

>gi|30064199|ref|NP_838370.1| IS1 orfB [Shigella flexneri 2a str. 2457T]

MSRQCTHYGRWPQHGFTSLKKLRPQSVTSRIQPGSDVIVCAEMDEHWGYVGAKSRQRWLFYAYDRIRGTV

VAHVFGERTLATLERLLSLLSAFEVVVWMTDGWPLYESRLKGKLHVISKRYTQRIERHNLNLRQHLARLG

RKSLSFSKSVELHDKVIGHYLNIKHYQ

>gi|30064198|ref|NP_838369.1| IS1 orfA [Shigella flexneri 2a str. 2457T]

MASISIRCPSCSATEGVVRNGKSTAGHQRYLCSHCRKTWQLQFTYTASQPGKHQKIIDMAMNGVGCRASA

RIMGVGLNTVLRHLKNSGRSR

>gi|30064197|ref|NP_838368.1| xanthine dehydrogenase subunit XdhC [Shigella flexneri 2a str. 2457T]

MNHSETITTECTINGMPFQLHAAPGTPLSELLREQGLLSVKQGCCVGECGACTVLVDGTAIDSCLYLAAW

AEGKEIRTLEGEAKGGKLSHVQQAYAKSGTVQCGFCTPGLIMATTAMLAKPREKPLTITEIRRGLAGNLC

RCTGYQMIVNTVLDCEKTK

>gi|30064196|ref|NP_838367.1| xanthine dehydrogenase subunit XdhB [Shigella flexneri 2a str. 2457T]

MFDFASYHRAATLADAINLLADNPQAKLLAGGTDVLIQLHHHNDRYRHIVDIHNLAELRGITLAEDGSLR

IGSATTFTQLIEDPITQRHLPALCAAATFIAGPQIRNVATYGGNICNGATSADSATPTLIYDAKLEIHSP

RGVRFVPINGFHTGPGKVSLEHDEILVAFHFPPQPKEHAGSAHFKYAMRDAMDISTIGCAAHCRLDNGNF
SELRLAFGVAAPTPIRCQHAEQTAQNAPLNLQTLEAISESVLQDVAPRSSWRASKEFRLHLIQTMTKKVI
SEAVAAAGGKLQ

>gi|30064195|ref|NP_838366.1| xanthine dehydrogenase subunit XdhA [Shigella flexneri 2a str. 2457T]
MEAREATATGESCMRVDAIAKVTGRARYTDDYVMAGMCYAKYVRSPIAHGYAVSINDEQARSLPGVLAIF
TWEDVPDIPFATAGHAWTLDENKRDTADRALLTRHVRHHGDAVAIVVARDELTAEKAAQLVSIEWQELPV
ITTPEAALTEDAAPIHNGGNLLKQSTMSTGNVQQTIDAADYQVQGHYQTPVIQHCHMESVTSLAWMEDDS
RITIVSSTQIPHIVRRVVGQALDIPWSCVRVIKPFVGGGFGNKQDVLEEPMAAFLTSKLGGIPVKVSLSR
EECFLATRTRHAFTIDGQMGVNRDGTLKGYSLDVLSNTGAYASHGHSIASAGGNKVAYLYPRCAYAYSSK
TCYTNLPSAGAMRGYGAPQVVFAVESMLDDAATALGIDPVEIRLRNAAREGDANPLTGKRICSAGLPECL
EKGRKIFEWEKRRAECQNQQGNLRRGVGVACFSYTSNTWPVGVEIAGARLLMNQDGTINVQSGATEIGQG
ADTVFSQMVAETVGVPVSDVRVISTQDTDVTPFDPGAFASRQSYVAAPALRSAALLLKEKIIAHAAVMLH
QSAMNLTLIKGHIVLVERPEEPLMSLKDLAMDAFYHPERGGQLSAESSIKTTTNPPAFGCTFVDLTVDIA
LCKVTINRILNVHDSGHILNPLLAEGQVHGGMGMGIGWALFEEMIIDAKSGVVRNPNLLDYKMPTMPDLP
QLESAFVEINEPQSAYGHKSLGEPPIIPVAAAIRNAVKMATGVAINTLPLTPKRLYEEFHLAGLI

>gi|30064194|ref|NP_838365.1| lipoprotein [Shigella flexneri 2a str. 2457T]
MFFKRGKILSAGRLNKKSLGIVMFLSVGLLLAGCSGSKSSDTGTYSGSVYTVKRGDTLYRISRTTGTSVK
ELARLNGISPPYTIEVGQKLKLGGAKSSSSTRKSTAKSTTKTASVTPSSAVPKSSWPPVGQRCWLWPTTG
KVIMPYSTADGGNKGIDISAPRGTPIYAAGAGKVVYVGNQLRGYGNLIMIKHSEDYITAYAHNDTMLVNN
GQSVKAGQKIATMGSTDAASVRLHFQIRYRATAIDPLRYLPPQGSKPKC

>gi|30064193|ref|NP_838364.1| integrase [Shigella flexneri 2a str. 2457T]
MLKSRTYLYQRNGVFYIRLRMKTTSRLTASLPSHNRYKLASVSLRTKDRRTAMAHSRHIKSALKAIHADN

PNASYEELREHLKTIVEWELSVSRDDLNDPESYQLYVDQYDDIKSNLREAVATERLTVDQHRYINDVIGV

LKACQDRLNGDSSGLLSYLEPETGSLRPSVSLSVLAEPEVPEPKALTLASLIEQYEQENAQNWKPATLSE

NRASHSTLIEIFDYLDIQDVGKATRADMLRVREVLQQLPKNRKQRFKSMPLSDLLNRESKTDCLDVVTIN

NKYLIKMAAVFKWAVRNDLIAKNLTEGLELKVPQRKASDARDAFSPEQVGQLLVAAKAYSQKTSGKPYHY

YVTALAAITGARLNEVAQLQVKDVRTTEAGTVFIHINEDDSSLPGKSIKNAHSDRCVPLVDGAYGFVLAD

FMSLVEDRRKTEGDNAMVFNGLKLMKNGYGEQVSKWFNRTLLPKVLADRSGLAFHSFRHTVATQLKQHGV

ELAYAQAIMGHSSGSITYDRYAKEVEVETLKEKLAESLSVKKIDGK

>gi|30064192|ref|NP_838363.1| hypothetical protein S3063 [Shigella flexneri 2a str. 2457T]

MASEDWQREHHCRTGIIFTAFAIEAMFIFYRKQVDPGYDKTQKECRKTMHKNTLKLCGINNYMGTKPYQI

IKECLEVRDAIAHGDSYTSSFNFSADHLDNQDDIAKVSWR

>gi|30064191|ref|NP_838362.1| hypothetical protein S3062 [Shigella flexneri 2a str. 2457T]

MSQQEISIINLDQLVSMTSVEIAELTGKEHKHVLRDIRNMVEELNGAKTEHCSTLSSELNGSKFGLVGEE

VYKDAKGESRTMYRLDRKHTFILVAGYSVHLRAKCYDHIQTLERRVLQLEDQKKRAAIQSANRRGVTWGD

YCKTYGLPAQKLMTALLQHRGLFRKNPISNEWSVNPKYSDCFRIIKPSDQKFSAGGYNFRFNAKGLEVFG

KPEMVDKMRGILIAFTGTDQQKQEHLLKLAQSGKVEGI

>gi|30064190|ref|NP_838361.1| hypothetical protein S3060 [Shigella flexneri 2a str. 2457T]

MMFNNNNWKLSVTDINLYENTVSLDGQSYPLSLAIKTLIPGYLSGLPSTSREAMELLEALAEAGVTIGNF

FSNDLMTAYQRRQMNKRAEAERIAKELASQKERTREMFMTEDEWQKELQRREQVKAERRTYGENLRSATH

SAGRSRAAIVADLESGGNWMDSL

>gi|30064189|ref|NP_838360.1| hypothetical protein S3059 [Shigella flexneri 2a str. 2457T]

MNMTKNAFQFGIEPVRITDTDNIQVNEGLPTNADPQVYALQLAKTVKAMLNGVLKDAQDNIPFPVEVLPT

RNSLPTPIIAHTLADRSVLVPVRGGKRPEVVTAPSGTEITVEPIEQAILVSHQTKLWDQKSTTGFTQGTL

QQDALNICDNVIRTINSKMVDVLESSKLLKTVELPALTGSLTAKADAIMDALYENTESSFGSEVSDYGII

AHESHLKALSRLAAKQGFGGEDAIVDMLGTDVAYYNGEDRGVFMMAKRFTALSFGCFRHDGESITVVLSR

DGDSQSHDLEILGKVFVVAEAATTIKMGTGSATAVLPVVKRLSFTKEAN

>gi|30064188|ref|NP_838359.1| IS3 orfB [Shigella flexneri 2a str. 2457T]

MKYVFIEKHQAEFSIKAMCRVLRVARSGWYTWCQRRTRISTRQQFRQHCDSVVLAAFTRSKQRYGAPRLT

DELRAQGYPFNVKTVAASLRRQGLRAKASRKFSPVSYRAHGLPVSENLLEQDFYASGPNQKWPGDITYLR

TDEGWLYLAVVIDLWSRAVIGWSMSPRMTAQLPCDALQMALWRRKRPRNVIVHTDRGGQYCSADYQAQLK

RHNLRGSMSAKGCCYDNACVESFFHSLKVECIHGEHFISREIMRATVFNYIECDYNRWRRHSWCGGLSPE

QFENQNLA

>gi|30064187|ref|NP_838358.1| IS3 orfA [Shigella flexneri 2a str. 2457T]

MTKTVSTSKKPRKQHSPEFRSEALKLAERIGVTAAARELSLYESQLYNWRSKQQNQQTSSERELEMSTEI

ARLKRQLAERDEELAILQKAATYFAKRLK

>gi|30064186|ref|NP_838357.1| transporter protein [Shigella flexneri 2a str. 2457T]

MVLFITALVAWPLTYWPHKALCQFILSSKTSAGEGITGAVTHYYGKKIGNLITTLYFIAFFVVVLIYAVA

ITNSLTEQLAKHMVIDLRIRMLVSLGVVLILNLIFLMGRHATIRVMGFLVFPLIAYFLFLSIYLVGSWQP

DLLTTQVEFNQNTLHQIWISIPVMVFAFSHTPIISTFAIDRREKYGEHAMDKCKKIMKVAYLIICISVLF

FVFSCLLSIPSSYIEAAKEEGVTILSALSMLPNAPAWLSISGIIVAVVAMSKSFLGTYFGVIEGATEVVK

TTLQQVGVKKSRAFNHALSIMLVSLITFIVCCINPNAISMIYAISGPLIAMILFIMPTLSTYLIPALKPW

RSIGNLITLIVGILCVSVMFFS

>gi|30064185|ref|NP_838356.1| acetyl-CoA acetyltransferase [Shigella flexneri 2a str. 2457T]

MKDVVIVGALRTPIGCFRGALAGHSAVELGSLVVKALIERTGVPAYAVDEVILGQVLTAGAGQNPARQSA

IKGGLPNSVSAITINDVCGSGLKALHLATQAIQCGEADIVIAGGQENMSRAPHVLTDSRTGEQLGNSQLV

DSLVHDGLWDAFNDYHIGVTAENLAREYGISRQLQDAYALSSQQKARAAIDAGRFKDEIVPVMIQSNGQT

LVVDTDEQPRTDASAEGLARLNPSFDSLGSVTAGNASSINDGAAAVMMMSEAKARALNLPVLARIRAFAS

VGVDPALMGIAPVYATRRCLERVGWQLADVDLIEANEAFAAQALSVGKMLEWDERRVNVNGGAIALGHPI

GASGCRILVSLVHEMVKRNARKGLATLCIGGGQGVALTIERDE

>gi|30064184|ref|NP_838355.1| 5-keto-4-deoxyuronate isomerase [Shigella flexneri 2a str. 2457T]

MDVRQSIHGAHAKTLDTQGLRNEFLVEKVFVADEYTMVYSHIDRIIVGGIMPITKTVSVGGEVGKQLGVS

YFLERRELGVINIGGAGTITVDGQCYEIGHRDALYVGKGAKEVVFASIDTATPAKFYYNCAPAHTTYPTK

KVTPDEVSPVTLGDNLTSNRRTINKYFVPDVLETCQLSMGLTELAPGNLWNTMPCHTHERRMEVYFYFNM

DDDACVFHMMGQPQETRHIVMHNEQAVISPSWSIHSGVGTKAYTFIWGMVGENQVFNDMDHVGVKDLR

>gi|30064183|ref|NP_838354.1| 2-deoxy-D-gluconate 3-dehydrogenase [Shigella flexneri 2a str. 2457T]

MILSAFSLQGKVAVVTGCDTGLGQGMALGLAQAGCDIVGINIVEPTETIEQVTALGRRFLSLTADLRKID

GIPGLLDRAVAEFGHIDILVNNAGLIRREDALEFSEKDWDDVMNLNIKSVFFMSQAAAKHFIAQGNGGKI

INIASMLSFQGGIRVPSYTASKSGVMGVTRLMANEWAKHNINVNAIAPGYMATNNTQQLRADEQRSAEIL

DRIPAGRWGLPSDLMGPVVFLASSASDYVNGYTIAVDGGWLAR

>gi|30064182|ref|NP_838353.1| low-affinity L-arabinose transport system proton symport protein [Shigella flexneri 2a str. 2457T]

MVTINTESALTPRSLRDTRRMNMFVSVAAAVAGLLFGLDIGVIAGALPFITDHFVLTSRLQEWVVSSMML

GAAIGALFNGWLSFRLGRKYSLMAGAILFVLGSIGSAFATSVEMLIAARVVLGIAVGIASYTAPLYLSEM

ASENVRGKMISMYQLMVTLGIVLAFLSDTAFSYSGNWRAMLGVLALPAVLLIILVVFLPNSPRWLAEKGR

HIEAEEVLRMLRDTSEKAREELNEIRESLKLKQGGWALFKINRNVRRAVFLGMLLQAMQQFTGMNIIMYY

APRIFKMAGFTTTEQQMIATLVVGLTFMFATFIAVFTVDKAGRKPALKIGFSVMALGTLVLGYCLMQFDN

GTASSGLSWLSVGMTMMCIAGYAMSAAPVVWILCSEIQPLKCRDFGITCSTTTNWVSNMIIGAAFLTLLD

SIGAAGTFWLYTALNIAFVGITFWLIPETKNVTLEHIERKLMAGEKLRNIGV

>gi|30064181|ref|NP_838352.1| racemase [Shigella flexneri 2a str. 2457T]

MKTIGLLGGMSWESTIPYYRLINEGIKQRLGGLHSAQMLLHSVDFHEIEECQRRGEWDKTGDILAEAALG

LQRAGAEGIVLCTNTMHKVADAIESRCTLPFLHIADATGRAITGAGMTRVALLGTRYTMEQDFYRGRLTE

QFSINCLIPEADERAKINQIIFEELCLGQFTEASRAYYAQVIARLAEQGAQGVIFGCTEIGLLVPEERSV

LPVFDTAAIHAEDAVAFMLS

>gi|30064180|ref|NP_838351.1| DNA-binding transcriptional regulator LysR [Shigella flexneri 2a str. 2457T]

MAAVNLRHIEIFHAVMTAGSLTEAAHLLHTSQPTVSRELARFEKVIGLKLFERVRGRLHPTVQGLRLFEE

VQRSWYGLDRIVSAAESLREFRQGELSIACLPVFSQSFLPQLLQPFLARYPDVSLNIVPQESPLLEEWLS

AQRHDLGLTETLHTPTGTERTELLSLDEVCVLPPGHPLAVKKVLTPDDFQGENYISLSRTDSYRQLLDQL

FTEHQVKRRMIVETHSAASVCAMVRAGVGISVVNPLTALDYAASGLVVRRFSIAVPFTVSLIRPLHRPSS

ALVQAFSGHLQAGLPKLVTSLDAILSSATTA

>gi|30064179|ref|NP_838350.1| diaminopimelate decarboxylase [Shigella flexneri 2a str. 2457T]

MPHSLFSTDTDLTAENLLRLPAEFGCPVWVYDAQIIRRQIAALKQFEVVRFAQKACSNIHILRLMREQGV

KVDSVSLGEIERALAAGYNPQTHPDDIVFTADVIDQATLERVSELQIPVNAGSVDTLDQLGQVSPGHRVW

LRVNPGFGHGHSQKTNTGGENSKHGIWYTDLPAALDVIQRHHLQLVGIHMHIGSGVDYAHLEQVCGAMVR

QVIEFGQDLQAISAGGGLSIPYQQGEEAVDTEHYYGLWNAAREQIARHLGHPVKLEIEPGRFLVAQSGVL

ITQVRSVKQMGSRHFVLVDAGFNDLMRPAMYGSYHHISALAADGRSLEHAPTVETVVAGPLCESGDVFTQ

QEGGNVETRALPEVKAGDYLVLHDTGAYGASMSSNYNSRPLLPEVLFDNGQARLIRRRQTIEELLALELL

>gi|30064178|ref|NP_838349.1| DNA-binding transcriptional regulator GalR [Shigella flexneri 2a str. 2457T]

MATIKDVARLAGVSVATVSRVINNSPKASEASRLAVHSAMESLSYHPNANARALAQQTTETVGLIVGDVS

DPFFGAMVKAVEQVAYHTGNFLLIGNGYHNEQKERQAIEQLIRHRCAALVVHAKMIPDADLASLMKQMPG

MVLINRILPGFENRCIALDDRYGAWLATRHLIQQGHTRIGYLCSNHSISDAEDRLQGYYDALAESGIPAN

DRLVTFGEPDESGGEQAMTELLGRGRNFTAVACYNDSMAAGAMGVLNDNGIDVPGEISLIGFDDVLVSRY

VRPRLTTVRYPIVTMATQAAELALALADNRPLPEITNVFSPTLVRRHSVSTPSLEASHHATSD

>gi|30064177|ref|NP_838348.1| bifunctional acyl-[acyl carrier protein] synthetase/2-acylglycerophosphoethanolamine acyltransferase [Shigella flexneri 2a str. 2457T]

MLFSFFRNLCRVLYRVRVTGDTQALKGERVLITPNHVSFIDGILLGLFLPVRPVFAVYTSISQQWYMRWL

KSFIDFVPLDPTQPMAIKHLVRLVEQGRPVVIFPEGRITTTGSLMKIYDGAGFVAAKSGATVIPVRIEGA

ELTHFSRLKGLVKRRLFPQITLHILPPTQVAMPDAPRARDRRKIAGEMLHQIMMEARMAVRPRETLYESL

LSAMYRFGAGKKCVEDVNFTPDSYRKLLTKTLFVGRILEKYSVEGERIGLMLPNAGISAAVIFGAIARRR

IPAMMNYTAGVKGLTSAITAAEIKTIFTSRQFLDKGKLWHLPEQLTQVRWVYLEDLKADVTTADKVWIFA

HLLMPRLAQVKQQPEEEALILFTSGSEGHPKGVVHSHKSILANVEQIKTIADFTTNDRFMSALPLFHSFG

LTVGLFTPLLTGAEVFLYPSPLHYRIVPDLVYDRSCTVLFGTSTFLGHYARFANPYDFYRLRYVVAGAEK

LQESTKQLWQDKFGLRILEGYGVTECAPVVSINVPMAAKPGTVGRILPGMDARLLSVPGIEEGGRLQLKG

PNIMNGYLRVEKPGVLEVPTAENVRGEMERGWYDTGDIVRFDEQGFVQIQGRAKRFAKIAGEMVSLEMVE

QLALGVSPDKVHATAIKSDASKGEALVLFTTDNELTRDKLQQYAREHGVPELAVPRDIRYLKQMPLLGSG

KPDFVTLKSWVDEAEQHDE

>gi|30064176|ref|NP_838347.1| lysophospholipid transporter LplT [Shigella flexneri 2a str. 2457T]

MSESVHTNTSLWSKGMKAVIVAQFLSAFGDNALLFATLALLKAQFYPEWSQPILQMVFVGAYILLAPFVG

QVADSFAKGRVMMFANGLKLLGAASICFGINPFLGYTLVGVGAAACSPAKYGILGELTTGSKLVKANGLM

EASAIAAILLGSVAGGVLADWHVLVALAACALAYGGAVVANIYIPKLAARPGQSWNLINMTRSFLNACTS

LWCNGETRFSLVGTSLFWGAGVTLRFLLVLWVPVALGITDNATPTYLNAMVAIGIVVGAGAAAKLVTLET
VSRCMPAGILIGVVVPIFSLQHELLPAYALLMLIGVLGGFFVVPLNALLQERGKKSVGAGNAIAVQNLGE
NSAMLLMLGIYSLAVMVGIPVVPIGIGFGALFALAITALWIWQRRH

>gi|30064175|ref|NP_838346.1| aldo-keto reductase [Shigella flexneri 2a str. 2457T]
MQYHRIPHSSLEVSTLGLGTMTFGEQNSEADAHAQLDYAVAQGINLIDVAEMYPVPPRPETQGLTETYVG
NWLAKHGSREKLIIASKVSGPSRNNDKGIRPDQALDRKNIREALHDSLKRLQTDYLDLYQVHWPQRPTNC
FGKLGYSWTDSAPAVSLLDTLDALAEYQRAGKIRYIGVSNETAFGVMRYLHLADKHDLPRIVTIQNPYSL
LNRSFEVGLAEVSQYEGVELLAYSCLGFGTLTGKYLNGAKPAGARNTLFSRFTRYSGEQTQKAVAAYVDI
ARRHGLDPAQMALAFVRRQPFVASTLLGATTMDQLKTNIESLHLELSEDVLAEIEAVHQVYTYPAP

>gi|30064174|ref|NP_838345.1| hypothetical protein S3041 [Shigella flexneri 2a str. 2457T]
MKKWAVIISAVGLAFAVSGCSSDYVMATKDGRMILTDGKPEIDDDTGLVSYHDQQGNAMQINRDDVSQII
ER

>gi|30064173|ref|NP_838344.1| transporter [Shigella flexneri 2a str. 2457T]
MLFAWITDPNAWLALGTLTLLEIVLGIDNIIFLSLVVAKLPTAQRAHARRLGLAGAMVMRLALLASISWV
TRLTNPLFTIFSQEISARDLILLLGGLFLIWKASKEIHESIEGEEEGLKTRVSSFLGAIVQIMLLDIIFS
LDSVITAVGLSDHLFIMMAAVVIAVGVMMFAARSIGDFVERHPSVKMLALSFLILVGFTLILESFDIHVP
KGYIYFAMFFSIAVESLNLIRNKKNPL

>gi|30064172|ref|NP_838343.1| DNA mismatch repair protein [Shigella flexneri 2a str. 2457T]
MSQPRPLLSPPETEEQLLAQAQQLSGYTLGELAALDGLVTPENLKRDKGWIGVLLEIWLGASAGSKPEQD
FAALGVELKTIPVDSLGRPLETTFVCVARLTGNSGVTWETSHVRHKLKRVLWIPVEGERSIPLAQRRVGS
PLLWSPNEEEDRQLREDWEELMDMIVLGQVERITARHGEYLQIRPKAANAKALTEAIGARGERILTLPRG

FYLKKNFTSALLARHFLIQ

>gi|30064171|ref|NP_838342.1| dinucleoside polyphosphate hydrolase [Shigella flexneri 2a str. 2457T]

MIDDDGYRPNVGIVICNRQGQVMWARRFGQHSWQFPQGGINPGESAEQAMYRELFEEVGLSRKDVRILAS

TRNWLRYKLPKRLVRWDTKPVCIGQKQKWFLLQLVSGDAEINMQTSSTPEFDGWRWVSYWYPVRQVVSFK

RDVYRRVMKEFASVVMSLQENTPKPQNASAYRRKRG

>gi|30064170|ref|NP_838341.1| fused phosphoenolpyruvate-protein phosphotransferase PtsP/GAF domain [Shigella flexneri 2a str. 2457T]

MLTRLREIVEKVASAPRLNEALNILVTDICLAMDTEVCSVYLADHDRRCYYLMATRGLKKPRGRTVTLAF

DEGIVGLVGRLAEPINLADAQKHPSFKYIPSVKEERFRAFLGVPIIQRRQLLGVLVVQQRELRQYDESEE

SFLVTLATQMAAILSQSQLTALFGQYRQTRIRALPAAPGVAIAEGWQDATLPLMEQVYQASTLDPALERE

RLTGALEEAANEFRRYSKRFAAGAQKETAAIFDLYSHLLSDTRLRRELFAEVDKGSVAEWAVKTVIEKFA

EQFAALSDNYLKERAGDLRALGQRLLFHLDDANQGPNAWPERFILVADELSATTLAELPQDRLVGVVVRD

GAANSHAAIMVRALGIPTVMGADIQPSVLHRRTLIVDGYRGELLVDPEPVLLQEYQRLISEEIELSRLAE

DDVNLPAQLKSGERIKVMLNAGLSPEHEEKLGSCIDGIGLYRTEIPFMLQSGFPSEEEQVAQYQGMLQMF

NDKPVTLRTLDVGADKQLPYMPISEENPCLGWRGIRITLDQPEIFLIQVRAMLRANAATGNLNILLPMVT

SLDEVDEARRLIERAGREVEEMIGYEIPKPRIGIMLEVPSMVFMLPHLAKRVDFISVGTNDLTQYILAVD

RNNTRVANIYDSLHPAMLRALAMIAREAEIHGIDLRLCGEMAGDPMCVAILIGLGYRHLSMNGRSVARVK

YLLRRIDFAEAENLAQRSLEAQLATEVRHQVAAFMERRGMGGLIRGGL

>gi|30064169|ref|NP_838340.1| prolipoprotein diacylglyceryl transferase [Shigella flexneri 2a str. 2457T]

MTSSYLHFPEFDPVIFSIGPVALHWYGLMYLVGFIFAMWLATRRANRPGSGWTKNEVENLLYAGFLGVFL

GGRIGYVLFYNFPQFMADPLYLFRVWDGGMSFHGGLIGVIVVMIIFARRTKRSFFQVSDFIAPLIPFGLG

AGRLGNFINGELWGRVDPNFPFAMLFPGSRTEDILLLQTNPQWQSIFDTYGVLPRHPSQLYELLLEGVVL

FIILNLYIRKPRPMGAVSGLFLIGYGAFRIIVEFFRQPDAQFTGAWVQYISMGQILSIPMIVAGVIMMVW
AYRRSPQQHVS

>gi|30064168|ref|NP_838339.1| thymidylate synthase [Shigella flexneri 2a str. 2457T]
MKQYLELMQKVLDEGTQKNDRTGTGTLSIFGHQMRFNLQDGFPLVTTKRCHLRSIIHELLWFLQGDTNIA
YLHENNVTIWDEWADENGDLGPVYGKQWRAWPTPDGRHIDQITTVLNQLKNDPDSRRIIVSAWNVGELDK
MALAPCHAFFQFYVADGKLSCQLYQRSCDVFLGLPFNIASYALLVHMMAQQCDLEVGDFVWTGGDTHLYS
NHMDQTHLQLSREPRPLPKLIIKRKPESIFDYRFEDFEIEGYDPHPGVKAPVAI

>gi|30064167|ref|NP_838338.1| hypothetical protein S3033 [Shigella flexneri 2a str. 2457T]
MPVKEQGFSLLEVLIAMAISSVLLLGAARFLPALQRESLTSTRKLALEDEIWLRVFTVAKHLQRAGYCHG
SCTGEGLEIVGQGDCIIVQWDANSNGIWDREPVKESDQIGFRLKEHVLETLRGATSCEGKGWDKVTNPDA
IIIDTFQVVRQDVSGFSPVLTVNMRAASKSEPQTVVDASYSVTGFNL

>gi|30064165|ref|NP_838336.1| hypothetical protein S3031 [Shigella flexneri 2a str. 2457T]
MSASLKNQQGFSLPEVMVAMVLMVLMVLMVLMVMIVTALSGIQRTSMNSLASRNQYQQLWRHGWQQTQLR
AISPPANWQVNRMQTSQAGCVSISVTLVSPGGREGEMTRLHCPNCQ

>gi|30064164|ref|NP_838335.1| exonuclease V subunit gamma [Shigella flexneri 2a str. 2457T]
MLRVYHSNRLDVLEALMEFIVERERLDDPFEPEMILVQSTGMAQWLQMTLSQKFGIAANIDFPLPASFIW
DMFVRVLPEIPKESAFNKQSMSWKLMTLLPQLLEREDFTLLRHYLTDDSDKRKLFQLSSKAADLFDQYLV
YRPDWLAQWETGHLVEGLGEAQAWQAPLWKALVEYTHELGQPRWHRANLYQRFIETLESATTCPPGLPSR
VFICGISALPPVYLQALQALGKHIEIHLLFTNPCRYYWGDIKDPAYLAKLLTRQRRHSFEDRELPLFRDS
ENAGQLFNSDGEQDVGNSLLASWGKLGRDYIYLLSDLESSQELDAFVDVTPDNLLHNIQSDILELENRAV
AGVNIEEFSRSDNKRPLDPLDSSITFHVCHSPQREVEVLHDRLLAMLEEAPTLTPRDIIVMVADIDSYSP

FIQAVFGSAPADRYLPYAISDRRARQSHPVLEAFISLLSLPDSRFVSEDVLALLDVPVLAARFDITEEGL

RYLRQWVNESGIRWGIDDDNVRELELPATGQHTWRFGLTRMLLGYAMESAQGEWQSVLPYDESSGLIAEL

VGHLASLLMQLNIWRRGLAQERPLEEWLPVCRDMLNAFFLPDAETEAAMTLIEQQWQAIIAEGLGAQYGD

AVPLSLLRDELAQRLDQERISQRFLAGPVNICTLMPMRSIPFKVVCLLGMNDGVYPRQLAPLGFDLMSQK

PKRGDRSRRDDDRYLFLEALISAQQKLYISYIGRSIQDNSERFPSVLVQELIDYIGQSHYLPGDEALNCD

ESEARVKAHLTCLHTRMPFDPQNYQPGERQSYAREWLPAASQAGKAHSEFVQPLPFTLPETVPLETLQRF

WAHPVRAFFQMRLQVNFHTEDSEIPDTEPFILEGLSRYQINQQLLNALVEQDDAERLFRRFRAAGDLPYG

AFGEIFWETQCQEMQQLADRVIACRQPGQSMEIDLACNGVQITGWLPQVQPDGLLRWRPSLLSVAQGMQL

WLEHLVYCASGGNGESRLFLRKDGEWRFPPLAAEQALHYLSQLIEGYREGMSAPLLVLPESGGAWLKTCY

DAQNDAMLDDDSTLQKARTKFLQAYEGNMMVRGEGDDIWYQRLWRQLTPETMETIVEQSQRFLLPLFRFN

QS

>gi|30064163|ref|NP_838334.1| protease III [Shigella flexneri 2a str. 2457T]

MPRSTWFKALLLFVALWAPLSQAETGWQPIQETIRKSDKDNRQYQAIRLDNGMVVLLVSDPQAVKSLSAL

VVPVGSLEDPEAYQGLAHYLEHMSLMGSKKYPQADSLAEYLKMHGGSHNASTAPYRTAFYLEVENDALPG

AVDRLADAIAEPLLDKKYAERERNAVNAELTMARTRDGMRMAQVSAETINPAHPGSKFSGGNLETLSDKP

GNPVQQALKDFHEKYYSANLMKAVIYSNKPLPELAKMAADTFGRVPNKESKKPEITVPVVTDAQKGIIIH

YVPALPRKVLRVEFRIDNNSAKFRSKTDELITYLIGNRSPGTLSDWLQKQGLVEGISANSDPIVNGNSGV

LAISASLTDKGLANRDQVVAAIFSYLNLLREKGIDKQYFDELANVLDIDFRYPSITRDMDYVEWLADTMI

RVPVEHTLDAVNIADRYDAKAVKERLAMMTPQNARIWYISPKEPHNKTAYFVDAPYQVDKISAQTFADWQ

KKAADIALSLPELNPYIPDDFSLIKSEKKYDHPELIVDESNLRVVYAPSRYFASEPKADVSLILRNPKAM

DSARNQVMFALNDYLAGLALDQLSNQASVGGISFSTNANNGLMINANGYTQRLPQLFQALLEGYFSYTAT

EEQLEQAKSWYNQMMDSAEKGKAFEQAIMPAQMLSQVPYFSRDERRKILPSITLKEVLAYRDALKSGARP

EFMVIGNMTEAQATTLARDVQKQLGADGSEWCRNKDVVVDKKQSVIFEKAGNSTDSALAAVFVPTGYDEY

TSSAYSSLLGQIVQPWFYNQLRTEEQLGYAVFAFPMSVGRQWGMGFLLQSNDKQPSFLWERYKAFFPTAE

AKLRAMKPDEFAQIQQAVITQMLQAPQTLGEEASKLSKDFDRGNMRFDSRDKIVAQIKLLTPQKLADFFH

QAVVEPQGMAILSQISGSQNGKAEYVHPEGWKVWENVSALQQTMPLMSEKNE

>gi|30064162|ref|NP_838333.1| exonuclease V subunit beta [Shigella flexneri 2a str. 2457T]

MSDVAETLDPLRLPLQGERLIEASAGTGKTFTIAALYLRLLLGLGGSAAFPRPLIVEELLVVTFTEAATA

ELRGRIRSNIHELRIACLRETTDNPLYERLLEEIDDKAQAAQWLLLAERQMDEAAVFTIHGFCQRMLNLN

AFESGMLFEQQLIEDESLLRYQACADFWRRHCYPLPREIAQVVFETWKGPQALLRDINRYLQGEPPVIKA

PPPDDETLASRHAQIVARIDTVKQQWRDAVGELDALIESSGIDRRKFNRSNQAKWIEKISAWAEEETNSY

QLPESLEKFSQRFLEDRTKAGGETPRHPLFEAIDQLLAEPLSIRDLLITRALAEIRETVAREKRRRGELG

FDDMLSRLDSALRSESGEVLAAAIRTRFPVAMIDEFQDTDPQQYRIFRRIWHHQPETALLLIGDPKQAIY

AFRGADIFTYMKARSEVHAHYTLDTNWRSAPGMVNSVNKLFSQTDDTFMFREIPFIPVKSAGKNQALRFV

FKGETQPAMKMWLMEGESCGVGDYQSTMAQVCAAQIRDWLQAGQRGEALLMNGDDARPVRASDISVLVRS

RQEAAQVRDALTLLEIPSVYLSNRDSVFETLEAQEMLWLLQAVMTPERENTLRSALATSMMGLNALDIET

LNNDEHAWDAVVEEFDGYRQIWRKRGVMPMLRALMSARNIAENLLATAGGERRLTDILHISELLQEAGTQ

LESEHALVRWLSQHILEPDSNASSQQMRLESDKHLVQIVTIHKSKGLEYPLVWLPFITNFRVQDQAFYHD

RHSFEAVLDLNAAPESVDLAEVERLAEDLRLLYVALTRSVWHCSLGVAPLVRRRGDKKGDTDVHQSALGR

LLQKGEPQDAAGLRTCIEALCDDDIAWQTAQTGDNQPWQVNDALTAELNARTLQRLPGDNWRVTSYSGLQ

QRGHGIAQDLMPRLDVDAAGVVSVVEEPTLTPHQFPRGASPGTFLHSLFEDLDFTQPVDPNWVQEKLELG

GFESQWEPVLTEWITAVLQAPLNETGVSLSQLSDRDKQVEMEFYLPISEPLIASQLDALIRQFDPLSAGC

PPLEFMQVRGMLKGFIDLVFRHEGRYYLLDYKSNWLGEDSSAYTQQAMAAAMQAHRYDLQYQLYTLALHR

YLRHRIADYDYDLHFGGVIYLFLRGVDKEHPQQGIYTTRPNAGLIALMDEMFAGMTLEEA

>gi|30064161|ref|NP_838332.1| exonuclease V subunit alpha [Shigella flexneri 2a str. 2457T]

MKLQKQLLEAVEHKQLRPLDVQFALTVAGDEHPAVTLAAALLSHDAGEGHVCLPLSRLENNEASNPLLAT

CVSEIGELQNWEECLLASQAVSRGDEPTPMILCGDRLYLNRMWCNERTVARFFNEVNHTIEVDEALLAQT

LDKLFPVSDEINWQKVAAAVALTRRISVISGGPGTGKTTTVAKLLAALIQMADGERCRIRLAAPTGKAAA

RLTESLGKALRQLPLTDEQKKRIPEDASTLHRLLGAQPGSQRLRHHAGNPLHLDVLVVDEASMIDLPMMS

RLIDALPDHARVIFLGDRDQLASVEAGAVLGDICAYANAGFTAERARQLSRLTGTHVPAGTGTEAASLRD

SLCLLQKSYRFGSDSGIGQLAAAINRGDKTAVKTVFQQDFTDIEKRLLQSGEDYIAMLEEALAGYGRYLD

LLQARAEPDLIIQAFNEYQLLCALREGPFGVAGLNERIEQFMQQKRKIHRHPHSRWYEGRPVMIARNDSA

LGLFNGDIGIALDRGQGTRVWFAMPDGNIKSVQPSRLPEHETTWAMTVHKSQGSEFDHAALILPSQRTPV

VTRELVYTAVTRARRRLSLYADERILSAAIATRTERRSGLAALFSSRE

>gi|30064160|ref|NP_838331.1| N-acetylglutamate synthase [Shigella flexneri 2a str. 2457T]

MVKERKTELVEGFRHSVPYINTHRGKTFVIMLGGEAIEHENFSSIVNDIGLLHSLGIRLVVVYGARPQID

ANLAAHHHEPLYHKNIRVTDAKTLELVKQAAGTLQLDITARLSMSLNNTPLQGAHINVVSGNFIIAQPLG

VDDGVDYCHSGRIRRIDEDALHRQLESGAIVLMGPVAVSVTGESFNLTSEEIATQLAIKLKAEKMIGFCS

SQGVTNDDGDIVSELFPNEAQARVEAQEEKGDYNSGTVRFLRGAVKACRSGVRRCHLISYQEDGALLQEL

FSRDGIGTQIVMESAEQIRRATINDIGGILELIRPLEQQGILVRRSREQLEMEIDKFTIIQRDNTTIACA

ALYPFPEEKIGEMACVAVHPDYRSSSRGEVLLERIAAQAKQSGLSKLFVLTTRSIHWFQERGFTPVDIDL

LPESKKQLYNYQRKSKVLMADLG

>gi|30064159|ref|NP_838330.1| amidase [Shigella flexneri 2a str. 2457T]

MTDYASFAKVSGQISRLLVTQLRFLLLGRGMSGSNTAISRRRLLQGAGAMWLLSVSQVSLAAVSQVVAVR

VWPASSYTRVTVESNRQLKYKQFALSNPERVVVDIEDVNLNSVLKGMAAQIRADDPFIKSARVGQFDPQT

VRMVFELKQNVKPQLFALAPVAGFKERLVMDLYPANAQDMQDPLLALLEDYNKGDLEKQVPPAQSGPQPG

KAGRDRPIVIMLDPGHGGEDSGAVGKYKTREKDVVLQIARRLRSLIEEEGNMKVYMTRNEDIFIPLQVRV

AKAQKQRADLFVSIHADAFTSRQPSGSSVFALSTKGATSTAAKYLAQTQNASDLIGGVSKSGDRYVDHTM

FDMVQSLTIADSLKFGKAVLNKLGKINKLHKNQVEQAGFAVLKAPDIPSILVETAFISNVEEERKLKTAT

FQQEVAESILAGIKAYFADGATLARRG

>gi|30064158|ref|NP_838329.1| murein transglycosylase A [Shigella flexneri 2a str. 2457T]

MKGRWVKYLLMGTVVAMLAACSSKPTDRGQQYKDGKFTQPFSLVNQPDAVGAPINAGDFAEQINHIRNSS

PRLYGNQSNVYNAVQEWLRAGGDTRNMRQFGIDAWQMEGADNYGNVQFTGYYTPVIQARHTRQGEFQYPI

YRMPPKRGRLPSRAEIYAGALGDKYILAYSNSLMDNFIMDVQGSGYIDFGDGSPLNFFSYAGKNGHAYRS

IGKVLIDRGEVKKEDMSMQAIRHWGETHSEAEVRELLEQNPSFVFFKPQSFAPVKGASAVPLVGRASVAS

DRSIIPPGTTLLAEVPLLDNNGKFNGQYELRLMVALDVGGAIKGQHFDIYQGIGPEAGHRAGWYNHYGRV

WVLKTAPGAGNVFSG

>gi|30064157|ref|NP_838328.1| enzyme [Shigella flexneri 2a str. 2457T]

MSVVISDAWRQRFGGTARLYGEKALQLFADAHICVVGIGGVGSWAAEALARTGIGAITLIDMDDVCVTNT

NRQIHALRDNVGLAKAEVMAERIRQINPECRVTVVDDFVTPDNVAQYMSAGYSYVIDAIDSVRPKAALIA

YCRRNKIPLVTTGGAGGQIDPTQIQVTDLAKTIQDPLAAKLRERLKSDFGVVKNSKGKLGVDCVFSTEAL

VYPQSDGTVCAMKATAEGPKRMDCASGFGAATMVTATFGFVAVSHALKKMMAKAARQG

>gi|30064156|ref|NP_838327.1| hypothetical protein S3020 [Shigella flexneri 2a str. 2457T]

MTNPQFAGHPFGTTVTAETLRNTFAPLSQWEDKYRQLIMLGKQLPALPDELKAQAKEIAGCENRVWLGYT

VAENGKMHFFGNSEGRIVRGLLAVLLTAVEGKTAAELQAQSPLALFDELGLRAQLSASRSQGLNALSEAI

IAATKQV

>gi|30064155|ref|NP_838326.1| cysteine sulfinate desulfinase [Shigella flexneri 2a str. 2457T]

MNVFNPAQFRAQFPALQDAGVYLDSAATALKPEAVVEATRQFYSLSAGNVHRSQFAEAQRLTARYEAARE

KVAQLLNAPDDKTIVWTRGTTESINMVAQCYARPRLQPGDEIIVSVAEHHANLVPWLMVAQQTRAKVVKL

PLNAQRLPDVDLLPELITPRSRILALGQMSNVTGGCPDLARAITFAHSAGMVVMVDGAQGTVHFPADVQQ

LDIDFYAFSGHKLYGPTGIGVLYGKSELLEAMSPWLGGGKMIHEVSFDGFTTQSAPWKLEAGTPNVAGVI

GLSAALEWLADYDINQAESWSRSLATLAEEALAKRPGFRSFRCQDSSLLAFDFAGVHHSDMVTLLAEYGI

ALRAGQHCAQPLLAELGVTGTLRASFAPYNTKSDVDALVNAVDRALELLVD

>gi|30064154|ref|NP_838325.1| hypothetical protein S3018 [Shigella flexneri 2a str. 2457T]

MMKKTAAIISACMLTFALSACSGSNYVMHTNDGRTIVSDGKPQTDNDIGMISYKDANGNKQQINRTDVKE

MVELEQ

>gi|30064153|ref|NP_838324.1| DNA-binding transcriptional activator GcvA [Shigella flexneri 2a str. 2457T]

MSKRLPPLNALRVFDAAARHLSFTRAAEELFVTQAAVSHQIKSLEDFLGLKLFRRRNRSLLLTEEGQSYF

LDIKEIFSQLTEATRKLQARSAKGALTVSLLPSFAIHWLVPRLSSFNSAYPGIDVRIQAVDRQEDKLADD

VDVAIFYGRGNWPGLRVEKLYAEYLLPVCSPLLLTGEKPLKTPEDLAKHTLLHDASRRDWQTYTRQLGFN

HINVQQGPIFSHSAMVLQAAIHGQGVALANNVMAQSEIEAGRLVCPFNDVLVSKNAFYLVCHDSQAELGK

IAAFRQWILAKAAAEQEKFRFRYEQ

>gi|30064152|ref|NP_838323.1| hypothetical protein S3016 [Shigella flexneri 2a str. 2457T]

MTSRFMLIFAAISGFIFVALGAFGAHVLSKTMGAVEMGWIQTGLEYQAFHTLAILGLAVAMQRRISIWFY

WSSVFLALGTVLFSGSLYCLALSHLRLWAFVTPVGGVSFLAGWALMLVGAIRLKRKGVSHE

>gi|30064151|ref|NP_838322.1| RNA 2'-O-ribose methyltransferase [Shigella flexneri 2a str. 2457T]

MNKVVLLCRPGFEKECAAEITDKAGQREIFGFARVKENAGYVIYECYQPDDGDKLIRELPFSSLIFARQW

FVVGELLQHLPPEDRITPIVGMLQGVVEKGGELRVEVADTNESKELLKFCRKFTVPLRAALRDAGVLANY

ETPKRPVVHVFFIAPGCCYTGYSYSNNNSPFYMGIPRLKFPAEAPSRSTLKLEEAFHVFIPADEWDERLA

NGMWAVDLGACPGGWTYQLVKRNMWVYSVDNGPMAQSLMDTGQVTWLREDGFKFRPTRSNISWMVCDMVE

KPAKVAALMAQWLVNGWCRETIFNLKLPMKKRYEEVSHNLAYIQAQLDEHGINAQIQARQLYHDREEVTV

HVRRIWAAVGGRRDER

>gi|30064150|ref|NP_838321.1| DNA-binding transcriptional activator FucR [Shigella flexneri 2a str. 2457T]

MKAARQQAIVDLLLNHTSLTTEALSEQLKVSKETIRRDLNELQTQGKILRNHGRAKYIHRQNQDSGDPFH

IRLKSHYAHKADIAREALAWIEEGMVIALDASSTCWYLARQLPDINIQVFTNSHPICHELGKRERIQLIS

SGGTLERKYGCYVNPSLISQLKSLEIDLFIFSCEGIDSSGALWDSNAINADYKSMLLKRAAQSLLLIDKS

KFNRSGEARIGHLDEVTHIISDERQVATSLVTA

>gi|30064149|ref|NP_838320.1| protein of fucose operon [Shigella flexneri 2a str. 2457T]

MLKTISPLISPELLKVLAEMGHGDEIIFSDAHFPAHSMEPQVIRADGLLVSDLLQAIIPLFELDSYAPPL

VMMAAVEGDTLDPEVERRYRNALSLQAPCPDIIRINRFAFYERAQKAFAIVITGERAKYGNILLKKGVTP

>gi|30064148|ref|NP_838319.1| L-fucose isomerase [Shigella flexneri 2a str. 2457T]

MKKISLPKIGIRPVIDGRRMGVRESLEEQTMNMAKATAALLTEKLRHACGAAVECVISDTCIAGMAEAAA

CEEKFSSQNVGLTITVTPCWCYGSETIDMDPTRPKAIWGFNGTERPGAVYLAAALAAHSQKGIPAFSIYG

HDVQDADDTSIPADVEEKLLRFARAGLAVASMKGKSYLSLGGVSMGIAGSIVDHNFFESWLGMKVQAVDM

TELRRRIDQKIYDEAELEMALAWADKNFRYGEDENNKQYQRNAEQSRAVLRESLLMAMCIRDMMQGNSKL

ADIGRVEESLGYNAIAAGFQGQRHWTDQYPNGDTAEAILNSSFDWNGVREPFVVATENDSLNGVAMLMGH

QLTGTAQVFADVRTYWSPEAIERVTGHKLDGLAEHGIIHLINSGSAALDGSCKQRDSEGNPTMKPHWEIS

QQEADACLAATEWCPAIHEYFRGGGYSSRFLTEGGVPFTMTRVNIIKGLGPVLQIAEGWSVELPKDVHDI

LNKRTNSTWPTTWFAPRLTGKGPFTDVYSVMANWGANHGVLTIGHVGADFITLASMLRIPVCMHNVEETK

VYRPSAWAAHGMDIEGQDYRACQNYGPLYKR

>gi|30064147|ref|NP_838318.1| L-fucose transporter [Shigella flexneri 2a str. 2457T]

MGNTSIQTQSYRAVDKDAGQSRSYIIPFALLCSLFFLWAVANNLNDILLPQFQQAFTLTNFQAGLIQSAF

YFGYFIIPIPAGILMKKLSYKAGIITGLFLYALGAALFWPAAEIMNYTLFLVGLFIIAAGLGCLETAANP

FVTVLGPESSSHFRLNLAQTFNSFGAIIAVVFGQSLILSNVPHQSQDVLDKMSPEQLSAYKHSLVLSVQT

PYMIIVAIVLLVALLIMLTKFPALQSDNHSDAKQGSFSASLSRLARIRHWRWAVLAQFCYVGAQTACWSY

LIRYAVEEILGMTAGFAANYLTGTMVCFFIGRFTGTWLISRFAPHKVLAAYALIAMALCLISAFAGGHVG

LIALTLCSAFMSIQYPTIFSLGIKNLGQDTKYGSSFIVMTIIGGGIVTPVMGFVSDAAGNIPTAELIPAL

CFAVIFIFARFRSQTATN

>gi|30064146|ref|NP_838317.1| L-fuculose phosphate aldolase [Shigella flexneri 2a str. 2457T]

MERNKLARQIIDTCLEMTRLGLNQGTAGNVSVRYQDGMLITPTGIPYEKLTESHIVFIDGNGKHEEGKLP

SSEWRFHMAAYQSRPDANAVVHNHAVHCTAVSILNRSIPAIHYMIAAAGGNSIPCAPYATFGTRELSEHV

ALALKNRKATLLQHHGLIACEVNLEKALWLAHEVEVLAQLYLTTLAITDPVPVLSDEEIAVVLEKFKTYG

LRIEE

>gi|30064145|ref|NP_838316.1| L-1,2-propanediol oxidoreductase [Shigella flexneri 2a str. 2457T]

MANRMILNETAWFGRGAVGALTDEVKRRGYQKALIVTDKTLVQCGVVAKVTDKMDAAGLAWAIYDGVVPN

PTITVVKEGLGVFQNSGADYLIAIGGGSPQDTCKAIGIISNNPEFADVRSLEGLSPTNKPSVPILAIPTT

AGTAAEVTINYVITDEEKRRKFVCVDPHDIPQVAFIDADMMDGMPPALKAATGVDALTHAIEGYITRGAW

ALTDALHIKAIEIIAGALRGSVAGDKDAGEEMALGQYVAGMGFSNVGLGLVHGMAHPLGAFYNTPHGVAN

AILLPHVMRYNADFTGEKYRDIARVMGVKVEGMSLEGARNAAVEAVFALNRDVGIPPHLRDVGVRKEDIS

ALAQAALDDVCTGGNPREATLEDIVELYHTAW

>gi|30064144|ref|NP_838315.1| exonuclease IX [Shigella flexneri 2a str. 2457T]

MRSLFLFSQPAIACSGIECYPYRLIFKGVIVAVHLLIVDALNLIRRIHAVQGSPCVETCQHALDQLIMHS

QPTHAVAVFDDENRSSGWRHQRLPDYKADRPPMPEELHDEMPALRAAFEQRGVPCWSASGNEADDLAATL

AVKVTQAGHQATIVSTDKGYCQLLSPTLRIRDYFQKRWLDAPFIDKEFGVQPQQLPDYWGLAGISSSKVP

GVAGIGPKSATQLLVEFQSLEGIYENLDAVAEKWRKKLETHKEMAFLCRDIARLQTDLHIDGNLQQLRLV

R

>gi|30064143|ref|NP_838314.1| L-serine dehydratase (deaminase), L-SD2 [Shigella flexneri 2a str. 2457T]

MISVFDIFKIGIGPSSSHTVGPMKAGKQFTDDLIARNLLKDVTRVVVDVYGSLSLTGKGHHTDIAIIMGL

AGNLPDTVDIDSIPGFIQDVNTHGRLMLANGQHEVEFPVDQCMNFHADNLSLHENGMRITALAGDKVVYS

QTYYSIGGGFIVDEEHFGQQDSAPVEVPYPYSSAADLQKHCQETGLSLSGLMMKNELALHSKEELEQHLA

NVWEVMRGGIERGISTEGVLPGKLRVPRRAAALRRMLVSQDKTTTDPMAVVDWINMFALAVNEENAAGGR

VVTAPTNGACGIIPAVLAYYDKFIREVNANSLARYLLVASAIGSLYKMNASISGAEVGCQGEVGVACSMA

AAGLAELLGASPAQVCIAAEIAMEHNLGLTCDPVAGQVQVPCIERNAIAAVKAVNAARMALRRTSEPRVC

LDKVIETMYETGKDMNAKYRETSRGGLAMKIVACD

>gi|30064142|ref|NP_838313.1| serine transporter [Shigella flexneri 2a str. 2457T]

METTQTSTIASKDSRSAWRKTDTMWMLGLYGTAIGAGVLFLPINAGVGGMIPLIIMAILAFPMTFFAHRG

LTRFVLSGKNPGEDITEVVEEHFGIGAGKLITLLYFFAIYPILLVYSVAITNTVESFMSHQLGMTPPPRA

ILSLILIVGMMTIVRFGEQMIVKAMSILVFPFVGVLMLLALYLIPQWNGAALETLSLDTASATGNGLWMT

LWLAIPVMVFSFNHSPIISSFAVAKREEYGDMAEQKCSKILAFAHIMMVLTVMFFVFSCVLSLTPADLAA

AKEQNISILSYLANHFNAPVIAWMAPIIAIIAITKSFLGHYLGAREGFNGMVIKSLRGKGKSIEINKLNR

ITALFMLVTTWIVATLNPSILGMIETLGGPIIAMILFLMPMYAIQKVPAMRKYSGHISNVFVVVMGLIAI

SAIFYSLFS

>gi|30064141|ref|NP_838312.1| IS4 orf [Shigella flexneri 2a str. 2457T]

MHIGQALDLVSRYDSLRNPLTSLGDYLDPELISRCLAESGTVTLRKRRLPLEMMVWCIVGMALERKEPLH

QIVNRLDIMLPGNRPFVAPSAVIQARQRLGSEAVRRVFTKTAQLWHNATPHPHWCGLTLLAIDGVFWRTP

DTPENDAAFPRQTHAGNPALYPQVKMVCQMELTSHLLTAAAFGTMKNSENELAEQLIEQTGDNTLTLMDK

GYYSLGLLNGWSLAGEHRHWMIPLRKGAQYEELRKLGKGDHLVKLKTSPQARKKWPGLGNEVTARLLTVT

RKGKVCHLLTSMTDAMRFPGEEMADLYSNRWEIELGYREIKQTMQLSRLTLRSKKPELVEQELWGVLLAY
NLVRYQMIKMAEHLKGYWPNQLSFSESCGMVMRMLMTLQGASPGRIPELMRDLASMGQLVKLPTRRGRAF
PRVVKERPWKYPTAPKKRPVRLLN

>gi|30064140|ref|NP_838311.1| hypothetical protein S3003 [Shigella flexneri 2a str. 2457T]
MITHISPLGSMDMLSQLEVDMLKRTASSDLYQLFRNCSLAVLNSGSLTDNSKELLSRFENFDINVLRRER
GVKLELINPPEEAFVDGRIIRALQANLFAVLRDILFVYGQIHNTVRFPNLNLDNSVHITNLVFSILRNAR
ALHVGEAPNMVVCWGGHSINENEYLYARRVGNQLGLRELNICTGCGPGAMEAPMKGAAVGHAQQRYKDSR
FIGMTEPSIIAAEPPNPLVNELIIMPDIEKRLEAFVRIAHGIIIFPGGVGTAEELLYLLGILMNPANKDQ
VLPLILTGPKESADYFRVLDEFVVHTLGENARRHYRIIIDDAAEVARQMKKSMPLVKENRRDTGDAYSFN
WSMRIAPDLQMPFEPSHENMANLKLYPDQPVEVLAADLRRAFSGIVAGNVKEVGIRAIEEFGPYKINGDK
EIMRRMDDLLQGFVAQHRMKLPGSAYIPCYEICT

>gi|30064139|ref|NP_838310.1| 7-cyano-7-deazaguanine reductase [Shigella flexneri 2a str. 2457T]
MSSYANHQALAGLTLGKSTDYRDTYDASLLQGVPRSLNRDPLGLKADNLPFHGTDIWTLYELSWLNAKGL
PQVAVGHVELDYTSVNLIESKSFKLYLNSFNQTRFNNWDEVRQTLERDLSTCAQGKISVALYRLDELEGQ
PIGHFNGTCIDDQDITIDNYEFTTDYLENATSGEKVVEETLVSHLLKSNCLITHQPDWGSIQIQYRGRQI
DREKLLRYLVSFRHHNEFHEQCVERIFNDLLRFCQPEKLSVYARYTRRGGLDINPWRSNSDFVPSTTRLV
RQ

>gi|30064138|ref|NP_838309.1| SecY interacting protein Syd [Shigella flexneri 2a str. 2457T]
MDDLTAQALKDFTARYCDAWHEEHKSWPLSEELYGVPSPCIISTTEDAVYWQPQPFTGEQNVNAVERAFD
IVIQPTIHTFYTTQFAGDMHAQFGDIKLTLLQTWSEDDFRRVQENLIGHLVTQKRLKLPPTLFIATLEEE
LEVISVCNLSGEVCKETLGTRKRTNLASNLAEFLNQLKPLL

>gi|30064137|ref|NP_838308.1| hypothetical protein S2999 [Shigella flexneri 2a str. 2457T]

MTTHDRVRLQLQALEALLREHQHWRNDEPQPHQFNSTQPFFMDTMEPLEWLQWGLIPRMHDLLNNNQPLP

GAFAVAPYYEMALATDHPQRALILAELEKLDALFADDAS

>gi|30064136|ref|NP_838307.1| tRNA pseudouridine synthase C [Shigella flexneri 2a str. 2457T]

MLEILYQDEWLVAVNKPSGWLVHRSWLDRDEKVVVMQTVRDQIGQHVFTAHRLDRPTSGVLLMGLSSEAG

RLLAQQFEQHQIQKRYHAIVRGWLMEEAVLDYPLVEELDKIADKFAREDKGPQPAVTHYRGLATVEMPVA

TGRYPTTRYGLVELEPKTGRKHQLRRHLAHLRHPIIGDSKHGDLRQNRSGAEHFGLQRLMLHASQLSLTH

PFTGEPLTIHAGLDDTWMQALSQFGWRGLLPENERVEFSAPSGQDGEISS

>gi|30064135|ref|NP_838306.1| flavodoxin [Shigella flexneri 2a str. 2457T]

MAEIGIFVGTMYGNSLLVAEEAEAILTAQGHKATVFEDPELSDWLPYQDKYVLVVTSTTGQGDLPDSIVP

LFQGIKDSLGFQPNLRYGVIALGDSSYVNFCNGGKQFDALLQEQSAQRVGEMLLIDASENPEPETESNPW

VEQWGTLLS

>gi|30064134|ref|NP_838305.1| transporter [Shigella flexneri 2a str. 2457T]

MSSLSQAASSVEKRTNARYWIVVMLFIVTSFNYGDRATLSIAGSEMAKDIGLDPVGMGYVFSAFSWAYVI

GQIPGGWLLDRFGSKRVYFWSIFIWSMFTLLQGFVDIFSGFGIIVALFTLRFLVGLAEAPSFPGNSRIVA

AWFPVQERGTAVSIFNSAQYFATVIFAPIMGWLTHEVGWSHVFFFMGGLGIVISFIWLKVIHEPNQHPGV

NQKELEYIAAGGALINMDQQSTKVKVPFSVKWGQIKQLLGSRMMIGVYIGQYCINALTYFFITWFPVYLV

QARGMSILKAGFVASVPAVCGFIGGVLGGIISDWLMRRTGSLNIARKTPIVMGMLLSMVMVFCNYVNVEW

MIIGFMALAFFGKGIGALGWAVMADTAPKEISGLSGGLFNMFGNISGIVTPIAIGYIVGTTGSFNGALIY

VGVHALIAVLSYLVLVGDIKRIELKPVAGQ

>gi|30064133|ref|NP_838304.1| glucarate dehydratase [Shigella flexneri 2a str. 2457T]

MATQSSPVITDMKVIPVAGHDSMLLNIGGAHNAYFTRNIVVLTDNAGHTGIGEAPGGEVIYQTLVDAIPM

VLGQEVARLNKVVQQVHKGNQAADFDTFGKGAWTFELRVNAVAALEAALLDLLGKALNVPVCELLGPGKQ

RETITVLGYLFYIGDRTKTDLPYLENTPGNHEWYQLRHQKAMNSEAVVRLAEASQDRYGFKDFKLKGGVL

PGEQEIDTVRALKKSFPDARITVDPNGAWLLDEAISLCKGLNDVLTYAEDPCGAEQGFSGREVMAEFRRA

TGLPVATNMIATNWREMGHAVMLNAVDIPLADPHFWTLSGAVRVAQLCDDWGLTWGCHSNNHFDISLAIF

THVGAAAPGNPTAIDTHWIWQEGDCRLTKNPLEIKNGKIAVPDAPGLGVELDWEQVQKAHEAYKRLPCGA

RNDAGPMQYLIPGWTFDRKRPVFGRH

>gi|30064132|ref|NP_838303.1| glucarate dehydratase [Shigella flexneri 2a str. 2457T]

MSSQFTTPVVTEMQVIPVAGHDSMLMNLSGAHAPFFTRNIVIIKDNSGHTGVGEIPGGEKIRKTLEDAIP

LVVGKTLGEYKNVLTLVRNTFADRDASGRGLQTFDLRTTIHVVTGIEAAMLDLLGQHLGVNVASLLGDGQ

QRSEVEMLGYLFFVGNRKATPLPYQSQPDDSCDWYRLRHEEAMTPDAVVRLAEAAYEKYGFNDFKLKGGV

LAGEEEAESIVALAQRFPQARITLDPNGAWSLNEAIKIGKYLKGSLAYAEDPCGAEQGFSGREVMAEFRR

ATGLPTATNMIATDWRQMGHTLSLQSVDIPLADPHFWTMQGSVRVAQMCHEFGLTWGSHSNNHFDISLAM

FTHVAAAAPGKITAIDTHWIWQEGNQRLTKEPFEIKGGLVQVPEKPGLGVEIDMDQVMKAHELYQKHGLG

ARDDAMGMQYLIPGWTFDNKRPCMVR

>gi|30064131|ref|NP_838302.1| hybrid sensory histidine kinase BarA [Shigella flexneri 2a str. 2457T]

MTNYSLRARMMILILAPTVLIGLLLSIFFVVHRYNDLQRQLEDAGASIIEPLAVSTEYGMSLQNRESIGQ

LISVLHRRHSDIVRAISVYDENNRLFVTSNFHLDPSSMQLGSNVPFPRQLTVTRDGDIMILRTPIISESY

SPDESPSSDAKNSQNMLGYIALELDLKSVRLQQYKEIFISCVMMLFCIGIALIFGWRLMRDVTGPIRNMV

NTVDRIRRGQLDSRVEGFMLGELDMLKNGINSMAMSLAAYHEEMQHNIDQATSDLRETLEQMEIQNVELD

LAKKRAQEAARIKSEFLANMSHELRTPLNGVIGFTRLTLKTELTPTQRDHLNTIERSANNLLAIINDVLD

FSKLEAGKLILESIPFPLRSTLDEVVTLLAHSSHDKGLELTLNIKSDVPDNVIGDPLRLQQIITNLVGNA

IKFTENGNIDILVEKRALSNTKVQIEVQIRDTGIGIPERDQSRLFQAFRQADASISRRHGGTGLGLVITQ

KLVNEMGGDISFHSQPNRGSTFWFHINLDLNPNIIIEGPSIQCLAGKRLAYVEPNSAAAQCTLDILSETP

LEVVYSPTFSALPPAHYDMMLLGIAVTFREPLTMQHERLAKAVSMTDFLMLALPCHAQVNAEKLKQDGIG

ACLLKPLTPTRLLPALTEFCHHKQNTLLPVTDESKLAMTVMAVDDNPANLKLIGALLEDMVQHVELCDSG

HQAVERAKQMPFDLILMDIQMPDMDGIRACELIHQLPHQRQTPVIAVTAHAMAGQKEKLLGAGMSDYLAK

PIEEERLHNLLLRYKPGSGISSRVVTPEVNEIVVNPNATLDWQLALRQAAGKTDLARDMLQMLLDFLPEV

RNKVEEQLAGENPEGLVDLIHKLHGSCGYSGVPRMKNLCQLIEQQLRSGTKEEDLEPELLELLDEMDNVA

REASKILG

>gi|30064130|ref|NP_838301.1| 23S rRNA 5-methyluridine methyltransferase [Shigella flexneri 2a str. 2457T]

MAQFYSAKRRTTTRQIITVSVNDLDSFGQGVARHNGKTLFIPGLLPQENAEVAVTEDKKQYARAKVVRRL

SDSPERETPRCPHFGVCGGCQQQHASVDLQQRSKSAALARLMKHDVSEVIADVPWGYRRRARLSLNYLPK

TQQLQMGFRKAGSSDIVDVKQCPILAPQLEALLPKVRACLGSLQAMRHLGHVELVQATSGTLMILRHTAP

LSSADREKLERFSHSEGLDLYLAPDSEILETVSGEMPWYDSNGLRLTFSPRDFIQVNAGVNQKMVARALE

WLDVQPEDRVLDLFCGMGNFTLPLATQAASVVGVEGVPALVEKGQQNARLNGLQNVTFYHENLEEDVTKQ

PWAKNGFDKVLLDPARAGAAGVMQQIIKLEPIRIVYVSCNPATLARDSEALLKAGYTIARLAMLDMFPHT

GHLESMVLFSRVK

>gi|30064129|ref|NP_838300.1| GDP/GTP pyrophosphokinase [Shigella flexneri 2a str. 2457T]

MVAVRSAHINKAGEFDPEKWIASLGITSQKSCECLAETWAYCLQQTQGHPDASLLLWRGVEMVEILSTLS

MDIDTLRAALLFPLADANVVSEDVLRESVGKSVVNLIHGVRDMAAIRQLKATHTDSVSSEQVDNVRRMLL

AMVDDFRCVVIKLAERIAHLREVKDAPEDERVLAAKECTNIYAPLANRLGIGQLKWELEDYCFRYLHPTE

YKRIAKLLHERRLDREHYIEEFVGHLRAEMKAEGVKAEVYGRPKHIYSIWRKMQKKNLAFDELFDVRAVR

IVAERLQDCYAALGIVHTHYRHLPDEFDDYVANPKPNGYQSIHTVVLGPGGKTVEIQIRTKQMHEDAELG

VAAHWKYKEGAAAGGARSGHEDRIAWLRKLIAWQEEMADSGEMLDEVRSQVFDDRVYVFTPKGDVVDLPA

GSTPLDFAYHIHSDVGHRCIGAKIGGRIVPFTYQLQMGDQIEIITQKQPNPSRDWLNPNLGYVTTSRGRS

KIHAWFRKQDRDKNILAGRQILDDELEHLGISLKEAEKHLLPRYNFNDVDELLAAIGGGDIRLNQMVNFL

QSQFNKPSAEEQDAAALKQLQQKSYTPQNRSKDNGRVVVEGVGNLMHHIARCCQPIPGDEIVGFITQGRG

ISVHRADCEQLAELRSHAPERIVDAVWGESYSAGYSLVVRVVANDRSGLLRDITTILANEKVNVLGVASR

SDTKQQLATIDMTIEIYNLQVLGRVLGKLNQVPDVIDARRLHGS

>gi|30064128|ref|NP_838299.1| nucleoside triphosphate pyrophosphohydrolase [Shigella flexneri 2a str. 2457T]

MNQIDRLLTIMQRLRDPENGCPWDKEQTFATIAPYTLEETYEVLDAIAREDFDDLRGELGDLLFQVVFYA

QMAQEEGRFDFNDICAAISDKLERRHPHVFADSSAENSSEVLARWEQIKTEERAQKAQHSALDDIPRSLP

ALMRAQKIQKRCANVGFDWTTLGPVVDKVYEEIDEVMYEARQAVVDQAKLEEEMGDLLFATVNLARHLGT

KAEIALQKVNEKFERRFREVERIVAARGLEMTGVDLETMEEVWQQVKRQEIDL

>gi|30064127|ref|NP_838298.1| CTP synthetase [Shigella flexneri 2a str. 2457T]

MTTNYIFVTGGVVSSLGKGIAAASLAAILEARGLNVTIMKLDPYINVDPGTMSPIQHGEVFVTEDGAETD

LDLGHYERFIRTKMSRRNNFTTGRIYSDVLRKERRGDYLGATVQVIPHITNAIKERVLEGGEGHDVVLVE

IGGTVGDIESLPFLEAIRQMAVEIGREHTLFMHLTLVPYMAASGEVKTKPTQHSVKELLSIGIQPDILIC

RSDRAVPANERAKIALFCNVPEKAVISLKDVDSIYKIPGLLKSQGLDDYICKRFSLNCPEANLSEWEQVI

FEEANPVSEVTIGMVGKYIELPDAYKSVIEALKHGGLKNRVSVNIKLIDSQDVETRGVEILKGLDAILVP

GGFGYRGVEGMITTARFARENNIPYLGICLGMQVALIDYARHVANMENANSTEFVPDCKYPVVALITEWR

DENGNVEVRSEKSDLGGTMRLGAQQCQLVDDSLVRQLYNAPTIVERHRHRYEVNNMLLKQIEDAGLRVAG

RSGDDQLVEIIEVPNHPWFVACQFHPEFTSTPRDGHPLFAGFVKAASEFQKRQAK

>gi|30064126|ref|NP_838297.1| phosphopyruvate hydratase [Shigella flexneri 2a str. 2457T]

MSKIVKIIGREIIDSRGNPTVEAEVHLEGGFVGMAAAPSGASTGSREALELRDGDKSRFLGKGVTKAVAA

VNGPIAQALIGKDAKDQAGIDKIMIDLDGTENKSKFGANAILAVSLANAKAAAAAKGMPLYEHIAELNGT

PGKYSMPVPMMNIINGGEHADNNVDIQEFMIQPVGAKTVKEAIRMGSEVFHHLAKVLKAKGMNTAVGDEG

GYAPNLGSNAEALAVIAEAVKAAGYELGKDITLAMDCAASEFYKDGKYVLAGEGNKAFTSEEFTHFLEEL

TKQYPIVSIEDGLDESDWDGFAYQTKVLGDKIQLVGDDLFVTNTKILKEGIEKGIANSILIKFNQIGSLT

ETLAAIKMAKDAGYTAVISHRSGETEDATIADLAVGTAAGQIKTGSMSRSDRVAKYNQLIRIEEALGEKA

PYNGRKEIKGQA

>gi|30064125|ref|NP_838296.1| IS1 orfB [Shigella flexneri 2a str. 2457T]

MSRQCTHYGRWPQHGFTSLKKLRPQSVTSRIQPGSDVIVCAEMDEHWGYVGAKSRQRWLFYAYDRIRRTV

VAHVFGERTLATLERLLSLLSAFEVVVWMTDGWPLYESRLKGKLHVISKRYTQRIERHNLNLRQHLARLV

RKSLSFSKSVELHDKVIGHYLNIKHYQ

>gi|30064124|ref|NP_838295.1| IS1 orfA [Shigella flexneri 2a str. 2457T]

MASISIRCPSCSATEGVVRNGKSTAGHQRYLCSHCRKTWQLQFTYTASQPGTHQKIIDMAMNGVGCRASA

RIMGVGLNTVLRHLKNSGRSR

>gi|30064123|ref|NP_838294.1| hypothetical protein S2984 [Shigella flexneri 2a str. 2457T]

MQYPINEMFQTLQGEGYFTGVPAIFIRLQGCPVGCAWCDTKHTWEKLEDREVSLFSILAKTKESDKWGAA

SSEDLLAVIGRQGYTARHVVITGGEPCIHDLLPLTDLLEKNGFSCQIETSGTHEVRCTPNTWVTVSPKLN

MRGGYEVLSQALERANEIKHPVGRVRDIEALDELLATLTDDKPRVIALQPISQKDDATRLCIETCIARNW

RLSMQTHKYLNIA

>gi|30064122|ref|NP_838293.1| IS1 orfB [Shigella flexneri 2a str. 2457T]

MSRQCTHYGRWPQHGFTSLKKLRPQSVTSRIQPGSDVIVCAEMDEHWGYVGAKSRQRWLFYAYDRIRRTV

VAHVFGERTLATLERLLSLLSAFEVVVWMTDGWPLYESRLKGKLHVISKRYTQRIERHNLNLRQHLARLG

RKSLSFSKSVELHDKVIGHYLNIKHYQ

>gi|30064121|ref|NP_838292.1| IS1 orfA [Shigella flexneri 2a str. 2457T]

MASISIRCPSCSATEGVVRNGKSTAGHQRYLCSHCRKTWQLQFTYTASQPGTHQKIIDMAMNGVGCRASA

RIMGVGLNTVLRHLKNSGRSR

>gi|30064120|ref|NP_838291.1| hypothetical protein S2981 [Shigella flexneri 2a str. 2457T]

MKIFKYTPENNLFYGYILEDMKTGFNILREIMVEGYRPSIARLYDAEDGTQHFTHFADGKCVLIFMAEGN

PRIAKATGEGIAEIVARYPQCQRVDSKLIETWFNNLNWGPDKVAAERVQILKTGNMGFTTEVSGCWSCIH

EIYESVINRIRTEFPHADDITMLGGHSSHSYQNGTNMYFVYDYNVVDCKPEEEIDKYHNPLNKIICEETI

RLGGSMVHHHGIGKHRVHWSKLEHGSAWALLEGLKKQFDPNGIMNTGTIYPIEK

>gi|30064119|ref|NP_838290.1| transporter [Shigella flexneri 2a str. 2457T]

MTGRCLFGFSGEKPFLLPDNEGVKMNTSPVRMDDLPLNRFHCRIATLTFGAHLTDGYVLGVIGYAIIQLT

PAMQLTPFMAGMIGGSALLGLFLGSLVLGWISDHIGRQKIFTFSFLLITLASFLQFFATTPEHLIGLRIL

IGIGLGGDYSVGHTLLAEFSPRRHRGILLGAFSVVWTVGYVLASIAGHHFISENPEAWRWLLASAALPAL

LITLLRWGTPESPRWLLCQGRFAEAHAIVHRYFGPHVLLGDEVVTATHKHIKTLFSSRYWRRTAFNSVFF

VCLVIPWFVIYTWLPTIAQTIGLEDALTASLMLNALLIVGALLGLVLTHLLAHRKFLLGSFLLLAATLVV

MACLPSGSSLTLLLFVLFSTTISAVSNLVGILPAESFPTDIRSLGVGFATAMSRLGAAVSTGLLPWVLAQ

WGMQVTLLLLATVLLVGFVVTWLWAPETKALPLVAAGNVGGANEHSVSV

>gi|30064118|ref|NP_838289.1| transporter [Shigella flexneri 2a str. 2457T]

MNILLAFKAEPDAGMLAEKEWQAAAQGNSGPDVSLLRSLLGADEQAAAALLLAQRKNGTPMSLTALSMGD

ERALHWLRYLMALGFEEAVLLETAADLRFAPEFVARHIAEWQRQNPLDLIITGCQSSEGQNGQTPFLLAE

MLGWPCFTQVERFTLDALFITLEQRTEHGLRCCRVRLPAVIAVRQCGEVALPVPGMRQRMAAGKAEIIRK

TVAAEMPAMQCLQLARAEQRRGATLIDGQTVAEKAQKLWQDYLRQRMQP

>gi|30064117|ref|NP_838288.1| flavoprotein [Shigella flexneri 2a str. 2457T]

MAGLFTPKDAAMNIAIVTINQENAAIASWLAAQDFSGCTLAHWQIEPQPVVAEQVLDAMVEQWQRTPADV

VLFPPGTFGDELSTRLAWRLHGASICQVTSLDIPTVSMRKSHWGNALTATLQTEKRPLCLSLARQAGAAK

NATLPSGMQQLNIVPGALPDWLVSTEDLKNVSRDPLAEARRVLVVGQGGEADNQEIVMLAEKLGAEVGYS

RARVMNGGVDAEKVIGISGHLLAPEVCIVVGASGAAALMAGVRNSKFMVAINHDASAAVFSQADIGVVDD

WKVVLEALVTNIHAEYQ

>gi|30064116|ref|NP_838287.1| hypothetical protein S2976 [Shigella flexneri 2a str. 2457T]

MASGGRCYQESAMSVARNLWRVADAPHIVPADSVERQMVQRLINACPVGLFSLTQEGDLRIDYRGCLECG

TCRLLCDESTLQQWRYPPSGFGITYRFG

>gi|30064115|ref|NP_838286.1| hypothetical protein S2975 [Shigella flexneri 2a str. 2457T]

MLCSGYLGHDMEDDCDIIIIGAGIAGTACALRCARADLSVLLLERAEIPGSKNLSGRRLYTHALAELLPQ

FHLTAPLERRITHESLSLLTPDGATTFSSLQPGGESWSVLRARFDPWLVAEAEKEGVECIPGATVDALYE

ENGRVCGVICGDDILRARYVVLAEGANSVLAERHGLVTRPAGEAMALGIKEVLSLETSAIEERFHLENNE

GAALLFSGGICDDLPGGAFLYTNQQTLSLGIVCPLSSLTQSRVPASELLTRFKAHPAVRTLIKNTESLEY

GAHLVPEGGLHSMPVQYAGNGWLLVGDALRSCVNTGISVRGMDMALTGAQAAAQTLISACQHREPQNLFA

LYHHNVERSLLWDVLQRYQHVPALLQRPGRYRAWPALMQDISRDLWDQGDKPVPPLRQLFWHHLRRHGLW

HLAGDVIRSLRCL

>gi|30064114|ref|NP_838285.1| 6-pyruvoyl tetrahydrobiopterin synthase [Shigella flexneri 2a str. 2457T]

MSTTLFKDFTFEAAHRLPHVPEGHKCGRLHGHSFMVRLEITGEVDPHTGWIIDFAELKAAFKPTYERLDH

HYLNDIPGLENPTSEVLAKWIWDQVKPVVPLLSAVMVKETCTAGCIYRGE

>gi|30064113|ref|NP_838284.1| sulfite reductase subunit alpha [Shigella flexneri 2a str. 2457T]

MTTQVPPSALLPLNPEQLARLQAATTDLTPTQLAWVSGYFWGVLNQQPVALAVTPAPAAEMPGITIISAS

QTGNARRVAEALRDDLLAAKLNVKLVNAGDYKFKQIASEKLLIVVTSTQGEGEPPEEAVALHKFLFSKKA

PKLENTAFAVFSLGDSSYEFFCQSGKDFDSKLAELGGERLLDRVDSDVEYQAAASEWRARVVDALKSRAP

VAAPSQSVATGAVNEIHTSPYSKDAPLVASLSVNQKITGRNSEKDVRHIEIDLGDSGLRYQPGDALGVWY

QNDPALVKELVELLWLTGDEPVTVEGKTLPLNEALQWHFELTVNTANIVENYATLTRSETLLPLVGDKAK

LQHYAATTLIVDMVRFSPAQLDAEALINLLRPLTPRLYSIASSQAEVENEVHVTVGVVRYDVEGRARAGG

ASSFLADRVEEEGEVRVFIEHNDNFRLPANLETPVIMIGPGTGIAPFRAFMQQRAADEAPGKNWLFFGNP

HFTEDFLYQVEWQRYVKEGVLTRIDLAWSRDQKEKVYVQDKLREQGAELWRWINDGAHIYVCGDANRMAK

DVEQALLEVIAEFGGMDTEAADEFLSELRVERRYQRDVY

>gi|30064112|ref|NP_838283.1| sulfite reductase subunit beta [Shigella flexneri 2a str. 2457T]

MSEKHPGPLVVEGKLTDAERMKLESNYLRGTIAEDLNDGLTGGFKGDNFLLIRFHGMYQQDDRDIRAERA

EQKLEPRHAMLLRCRLPGGVITTKQWQAIDKFAGENTIYGSIRLTNRQTFQFHGILKKNVKPVHQMLHSV

GLDALATANDMNRNVLCTSNPYESQLHAEAYEWAKKISEHLLPRTRAYAEIWLDQEKVATTDEEPILGQT

YLPRKFKTTVVIPPQNDIDLHANDMNFVAIAENGKLVGFNLLVGGGLSIEHGNKKTYARTASEFGYLPLE

HTLAVAEAVVTTQRDWGNRTDRKNAKTKYTLERVGVETFKAEVERRAGIKFEPIRPYEFTGRGDRIGWVK

GIDDNWHLTLFIENGRILDYPGRPLKTGLLEIAKIHKGDFRITANQNLIIAGVPESEKAKIEKIAKESGL

MNAVTPQRENSMACVSFPTCPLAMAEAERFLPSFIDNIDNLMVKHGVSDEHIVMRVTGCPNGCGRAMLAE

VGLVGKAPGRYNLHLGGNRIGTRIPRMYKENITEPEILASLDELIGRWAKEREAGEGFGDFTVRAGIIRP

VLDPARDLWD

>gi|30064111|ref|NP_838282.1| phosphoadenosine phosphosulfate reductase [Shigella flexneri 2a str. 2457T]

MSKLDLNALNELPKVDRILALAETNAELEKLDAEGRVAWALDNLPGEYVLSSSFGIQAAVSLHLVNQIHP

DIPVILTDTGYLFPETYRFIDELTDKLKLNLKVYRATESAAWQEARYGKLWEQGVEGIEKYNDINKVEPM

NRALKELNAQTWFAGLRREQSGSRANLPVLAIQRGVFKVLPIIDWDNRTIYQYLQKHGLKYHPLWDEGYL

SVGDTHTTRKWEPGMAEEETRFFGLKRECGLHEG

>gi|30064110|ref|NP_838281.1| alkaline phosphatase isozyme conversion aminopeptidase [Shigella flexneri 2a str. 2457T]

MFSALRHRTAALALGVCFILPVHASSPKPGDFANTQARHIATFFPGRMTGTPAEMLSADYIRQQFQQMGY

RSDIRTFNSRYIYTARDNRKNWHNVTGSTVIAAHEGKAPQQIIIMAHLDTYAPLSDADADANLGGLTLQG

IDDNAAGLGVMLELAERLKNTPTEYGIRFVATSGEEEGKLGAENLLKRMSDTEKKNTLLVINLDNLIVGD

KLYFNSGVKTPEAVRKLTRDRALAIARSHGIAATTNPGLNKNYPKGTGCCNDAEIFDKAGIAVLSVEATN

WNLGNKDGYQQRAKTPAFPAGNSWHDVRLDNQQHIDKALPGRIERRCRDVMRIMLPLVKELAKAS

>gi|30064109|ref|NP_838280.1| sulfate adenylyltransferase subunit 2 [Shigella flexneri 2a str. 2457T]

MAQKRLTHLRQLEAESIHIIREVAAEFSNPVMLYSIGKDSSVMLHLARKAFYPGTLPFPLLHVDTGWKFR

EMYEFRDRTAKAYGCELLVHKNPEGVAMGINPFVHGSAKHTDIMKTEGLKQALNKYGFDAAFGGARRDEE

KSRAKERIYSFRDRFHRWDPKNQRPELWHNYNGQINKGESIRVFPLSNWTEQDIWQYIWLENIDIVPLYL

AAERPVLERDGMLMMIDDNRIDLQPGEVIKKRMVRFRTLGCWPLTGAVESNAQTLPEIIEEMLVSTTSER

QGRVIDRDQAGSMELKKRQGYF

>gi|30064108|ref|NP_838279.1| sulfate adenylyltransferase subunit 1 [Shigella flexneri 2a str. 2457T]

MNTVLAQQIANEGGVEAWMIAQQHKSLLRFLTCGSVDDGKSTLIGRLLHDTRQIYEDQLSSLHNDSKRHG

TQGEKLDLALLVDGLQAEREQGITIDVAYRYFSTEKRKFIIADTPGHEQYTRNMATGASTCELAILLIDA

RKGVLDQTRRHSFISTLLGIKHLVVAINKMDLVDYSEETFTRIREDYLTFAGQLPGNLDIRFVPLSALEG

DNVASQSESMPWYSGLTLLEVLETVEIQRVVDAQPMRFPVQYVNRPNLDFRGYAGMLASGRVEVGQRVKV

LPSGVESNVARIVTFDGDREEVFAGEAITLVLTDEIDISRGDLLLAADEALPAVQSASVDVVWMAEQPLS

PGQSYDIKIAGKKTRARVDGIRYQVDINNLTQREVENLPLNGIGLVDLTFDEPLVLDRYQQNPVTGGLIF

IDRLSNVTVGAGMVHEPVSQATAASSEFSAFELELNALVRRHFPHWGARDLLGDK

>gi|30064107|ref|NP_838278.1| adenylylsulfate kinase [Shigella flexneri 2a str. 2457T]

MALHDENVVWHSHPVTVQQRELHHGHRGVVLWFTGLSGSGKSTVAGALEEALHKLSVSTYLLDGDNVRHG

LCSDLGFSDADRKENIRRVGEVANLMVEAGLVVLTAFISPHRAERQMVRERVGEGRFIEVFVDTPLAICE

ARDPKGLYKKARAGELRNFTGIDSVYEAPESAEIHLNGEQLVTNLVQQLLDLLRQNDIIRS

>gi|30064106|ref|NP_838277.1| hypothetical protein S2965 [Shigella flexneri 2a str. 2457T]

MRNSHNITLTNNDSLTEDEETTWSLPGAVVGFISWLFALAMPMLIYGSNTLFFFLYTWPFFLALMPVAVV

VGIALHSLMDGKLRYSIVFTLVTVGIMFGALFMWLLG

>gi|30064105|ref|NP_838276.1| cell division protein FtsB [Shigella flexneri 2a str. 2457T]

MGKLTLLLLAILVWLQYSLWFGKNGIHDYTRVNDDVAALQATNAKLKARNDQLFAEIDDLNGGQEALEER

ARNELSMTRPGETFYRLVPDASKRAQSAGQNNR

>gi|30064104|ref|NP_838275.1| 2-C-methyl-D-erythritol 4-phosphate cytidylyltransferase [Shigella flexneri 2a str. 2457T]

MATTHLDVCAVVPAAGFGRRMQTECPKQYLSIGNQTILEHSVHALLAHPRVTRVVIAISPGDSRFAQLPL

ANHPQITVVDGGDERADSVLAGLKAAGDVQWVLVHDAARPCLHQDDLARLLALSETSRTGGILAAPVRDT

MKRAEPGKNAIAHTVDRNGLWHALTPQFFPRELLHDCLTRALNEGATITDEASALEYCGFHPQLVEGRAD

NIKVTRPEDLALAEFYLTRTIHQENT

>gi|30064103|ref|NP_838274.1| 2-C-methyl-D-erythritol 2,4-cyclodiphosphate synthase [Shigella flexneri 2a str. 2457T]

MRIGHGFDVHAFGGEGPIIIGGVRIPYEKGLLAHSDGDVALHALTDALLGAAALGDIGKLFPDTDPAFKG

ADSRELLREAWRRIQAKGYTLGNVDVTIIAQAPKMLPHIPQMRVFIAEDLGCHMDDVNVKATTTEKLGFT

GRGEGIACEAVALLIKATK

>gi|30064102|ref|NP_838273.1| tRNA pseudouridine synthase D [Shigella flexneri 2a str. 2457T]

MIEFDNLTYLHGKPQGTGLLKANPEDFVVVEDLGFEPDGEGEHILVRILKNGCNTRFVADALAKFLKIHS

REVSFAGQKDKHAVTEQWLCARVPGKEMPDLSAFQLEGCQVLEYARHKRKLRLGALKGNAFTLVLREVSN

RDDVEQRLIDICVKGVPNYFGAQRFGIGGSNLQGALRWAQTNTPVRDRNKRSFWLSAARSALFNQIVAER

LKKADVNQVVDGDALQLAGRGSWFVATTEELAELQRRVNDKELMITAALPGSGEWGTQREALAFEQAAVA

AETELQALLVREKVEAARRAMLLYPQQLSWNWWDDVTVEIRFWLPAGSFATSVVRELINTTGDYAHIAE

>gi|30064101|ref|NP_838272.1| stationary phase survival protein SurE [Shigella flexneri 2a str. 2457T]

MRILLSNYDGVHAPGIQTLAKALREFADVQVVAPDRNRSGASNSLTLESSLRTFTFENGDIAVQMGTPTD

CVYLGVNALMRPRPDIVVSGINAGPNLGDDVIYSGTVAAAMEGRHLGFPALAVSLDGHKHYDTAAAVTCS

ILRALCKEPLRTGRILNINVQDLPLDQIKGIRVTRCGTRHPADQVIPQQDPRGNTLYWIGPPGGKCDAGP

GTDFAAVDEGYVSITPLHVDLTAHSAQDVVSDWLNSVGVGTQW

>gi|30064100|ref|NP_838271.1| protein-L-isoaspartate O-methyltransferase [Shigella flexneri 2a str. 2457T]

MVSRRVQALLDQLRAQGIQDEQVLNALAAVPREKFVDEAFEQKAWDNIALPIGQGQTISQPYMVARMTEL

LELTPQSRVLEIGTGSGYQTAILAHLVQHVCSVERIKGLQWQARRRLKNLDLHNVSTRHGDGWQGWQARA

PFDAIIVTAAPPEIPTALMTQLDEGGILVLPVGEEHQYLKRVRRRGGEFIIDTVEAVRFVPLVKGELA

>gi|30064099|ref|NP_838270.1| lipoprotein NlpD [Shigella flexneri 2a str. 2457T]

MSAGSPKFTVRRIAALSLVSLWLAGCSDTSNPPAPVSSVNGNAPANTNSGMLITPPPKMGTTSTAQQPQI

QPVQQPQIQATQQPQIQPVQPVAQQPVQMENGRIVYNRQYGNIPKGSYSGSTYTVKKGDTLFYIAWITGN

DFRDLAQRNNIQAPYALNVGQTLQVGNASGTPITGGNAITQADAAEQGVVIKPAQNSTVAVASQPTITYS

ESSGEQSANKMLPNNKPTATTVTAPVTVPTASTTEPTVSSTSTSTPISTWRWPTEGKVIETFGASEGGNK

GIDIAGSKGQAIIATADGRVVYAGNALRGYGNLIIIKHNDDYLSAYAHNDTMLVREQQEVKAGQKIATMG

STGTSSTRLHFEIRYKGKSVNPLRYLPQR

>gi|30064098|ref|NP_838269.1| transcriptional regulator [Shigella flexneri 2a str. 2457T]

MALRNKAFHQLRQLFQQHTARWQHELPDLTKPQYAVMRAIADKPGIEQVALIEAAVSTKATLAEMLARME

NRGLVRREHDVADKRRRFVWLTAEGEKLLAAAIPIGDSVDEEFLGRLSGEEQELFMQLVRKMMST

>gi|30064097|ref|NP_838268.1| phenylacrylic acid decarboxylase-like protein [Shigella flexneri 2a str. 2457T]

MKLIVGMTGATGAPLGVALLQALREMPNVETHLVMSKWAKTTIELETPYSARDVAALADFSHNPADQAAI

ISSGSFRTDGMIVIPCSMKTLAGIRAGYADGLVGRAADVVLKEGRKLVLVPREMPLSTIHLENMLALSRM

GVAMVPPMPAFYNHPETVDDIVHHVVARVLDQFGLEHPHARRWQGLPQARNFSQENE

>gi|30064096|ref|NP_838267.1| 4-hydroxybenzoate decarboxylase, partial [Shigella flexneri 2a str. 2457T]

MAFDDLRSFLQALDDHGQLLKISEEVNAEPDLAAAANATGRIGDGAPALWFDNIRGFTDARVAMNTIGSW

QNHAISLGLPPNTPVKKQIDEFIRRWDNFPIAPERRANPAWAQNTVDGEEINLFDILPLFRLNDGDGGFY

LDKACVVSRDPLDPDNFGKQNVGIYRMEVKGKRKLGLQPVPMHDIALHLHKAEERGEDLPIAITLGNDPI

ITLMGATPLKYDQSEYEMAGALRESPYPIATAPLTGFDVPWGSEVILEGVIESRKREIEGPFGEFTGHYS

GGRNMTVVRIDKVSYRSKPIFESLYLGMPWTEIDYL

>gi|30064095|ref|NP_838266.1| regulatory protein [Shigella flexneri 2a str. 2457T]

MRKAPQGSFPADGKEPIIERIFRLVERYPSRNAAARAWGINEGTLKNYYNRRDYAPIPRPYQLKKIAESE

NVTLEWLMTGEGDVELKDSKEPKKENADNVDAQILMFLSFLKPQEKQQVADVLGRKGAEQLLILLDEHIQ

ELHALVGVRRAIALSLGNLPDEKVREIYALSEAKGDHLNLPQKAASA

>gi|30064094|ref|NP_838265.1| DNA-binding protein [Shigella flexneri 2a str. 2457T]

MRQREDSDRDWPSLKIQLALKDNGWSSFRELSLHAGYAQDSLKAVVRTPNKKYERVISDAIGVPPEIIWP

SRYRNDGFIRKAS

>gi|30064093|ref|NP_838264.1| phage transposase [Shigella flexneri 2a str. 2457T]

MQSYVTVNDLLGVPGMPATTKGIRQALQRFSRDLGDVSRRREGTKAIEYHIDCLPEITRKALRERYVEQL

VATENNVSEVKAVTRKTRNPDAVQAIEAYRGSPQLMEERLNALTENQRWVSEARAALVVEVLKLESAGNP

GRLKAINFLVEKARKGELPERLQQAAVNANAKRGANRTISRDPLYQWVLKYNQSQNAAERLLLLAPGKRD

EIKPEEISWLPEFLAQYRQVNGRPMSEAYEDFVAEWQRRHADEPYMLEVMPSYDVVRYAMKKLPEVVKQK

GRVTGSEYRQLEGFTRRDWTAMPVNYVWIGDGHGMKLKCAHPIHGRPFSPEVTFVIDGGTRFVVGWSLDL

AENVFAVAGAIQHGIRNHGKPFLYYSDNGSGETADMLDKEVVGILPRLGIKHPTGIAGNPQGRGIIERLN

RTLPMRIARRYRTYFGKGADRESLRVLNRDLRSAFNALQQDKPLNDRQKAAMRELPSWAELIEAIREGVE

WYNNRPHSELPMKPNGRHYSPTEFRKKRQAEEDTEIEWLSDLELRDMFRPMVERPVRRCEIQWLNNIYYA

PELRDEHGRKVLISYDIHDAERITVRRKDGSFICEAIWNGNKRAAFAVSAEYHKQQQRIKGMRKRAEEKI

RDAEDEGIQILEHKQAEPWLSNVYRPVGNVVAVQQPEYEEEHDEEFERDFRLGMQKLFAMQEEDDPLA

>gi|30064092|ref|NP_838263.1| IS600 orfB [Shigella flexneri 2a str. 2457T]

MAHIRTRETYGTRRLQTELAENGIIVGRDRLARLRKELRLRCKQKRKFRATTNPNHNLPVAPNLLNQTFA

PTAPNQVWVADLTYVATQEGWLYLAGIKDVYTCEIVGYAMGERMTKELTGKALFMALRSQRPPAGLIHHS

DRGSQYCAYDYRVIQEQFGLKTSMSRKGNCYDNAPMESFWGTLKNESLSHYRFNNRDEAISVIREYIEIF

YNRQRRHSRLGNISPAAFREKYHQMTA

>gi|30064091|ref|NP_838262.1| IS600 orfA [Shigella flexneri 2a str. 2457T]

MSRKTQRYSKEFKAEAVRTVLENQLSISEGASRLSLPEGTLGQWVTAARKGLGTPGSRTVAELESEILQL

RKALNEARLERDILKKATAYFAQESLKNTR

>gi|30064090|ref|NP_838261.1| DEOR-type transcriptional regulator [Shigella flexneri 2a str. 2457T]

MSAGQRKEQKLIPVERRQIILEMVAEKGIVSIAELTDRINVSHMTIRRDLQKLEQQGAVVLVSGGVQSPG
RVAHEPSHQVKTALAMTQKAAIGKLAASLVLPGSCIYLDAGTTTLAIAQHLIHMESLTVVTNDFVIANYL
LDNSNCTIIHTGGAVCRENRSCVGEAAATMLCSLMIDQAFISASSWSVRGISTPAEDKVTVKRAIASASR
QRVLVCDATKYGQVATWLALPLSEFDQIITDDGLPESASRALAKQDLSLLVAKNE

>gi|30064089|ref|NP_838260.1| DNA mismatch repair protein MutS [Shigella flexneri 2a str. 2457T]
MSAIENFDAHTPMMQQYLKLKAQHPEILLFYRMGDFYELFYDDAKRASQLLDISLTKRGASAGEPIPMAG
IPYHAVENYLAKLVNQGESVAICEQIGDPATSKGPVERKVVRIVTPGTISDEALLQERQDNLLAAIWQDS
KGFGYATLDISSGRFRLSEPADRETMAAELQRSNPAELLYAEDFAEMSLIEGRRGLRRRPLWEFEIDTAR
QQLNLQFGTRDLVGFGVENAPRGLCAAGCLLQYAKDTQRTTLPHIRSITMEREQDSIIMDAATRRNLEIT
QNLAGGAENTLASVLDCTVTPMGSRMLKRWLHMPVRDTRVLLERQQTIGALQDFTAELQPVLRQVGDLER
ILARLALRTARPRDLARMRHAFQQLPELRAQLETVDSAPVQALREKMGEFAELRDLLERAIIDTPPVLVR
DGGVIASGYNEELDEWRALADGATDYLERLEVRERERTGLDTLKVGFNAVHGYYIQISRGQSHLAPINYM
RRQTLKNAERYIIPELKEYEDKVLTSKGKALALEKQLYEELFDLLLPHLEALQQSASALAELDVLVNLAE
RAYTLNYTCPTFIDKPGIRITEGRHPVVEQVLNEPFIANPLNLSPQRRMLIITGPNMGGKSTYMRQTALI
ALMAYIGSYVPAQKVEIGPIDRIFTRVGAADDLASGRSTFMVEMTETANILHNATEYSLVLMDEIGRGTS
TYDGLSLAWACAENLANKIKALTLFATHYFELTQLPEKMEGVANVHLDALEHGDTIAFMHSVQDGAASKS
YGLAVAALAGVPKEVIKRARQKLRELESISPNAAATQVDGTQMSLLSVPEETSPAVEALENLDPDSLTPR
QALEWIYRLKSLV

>gi|30064088|ref|NP_838259.1| IS1 orfA [Shigella flexneri 2a str. 2457T]
MASISIRCPSCSATEGVVRNGKSTAGHQRYLCSHCRKTWQLQFTYTASQPGTHQKIIDMAMNGVGCRASA
RIMGVGLNTVLRHLKNSGRSR

>gi|30064087|ref|NP_838258.1| IS1 orfB [Shigella flexneri 2a str. 2457T]

MSRQCTHYGRWPQHGFTSLKKLRPQSVTSRIQPGSDVIVCAEMDEHWGYVGAKSRQRWLFYAYDRIRRTV

VAHVFGERTLATLERLLSLLSAFEVVVWMTDGWPLYESRLKGKLHVISKRYTQRIERHNLNLRQHLARLV

RKSLSFSKSVELHDKVIGHYLNIKHYQ

>gi|30064086|ref|NP_838257.1| hypothetical protein S2941 [Shigella flexneri 2a str. 2457T]

MSGKRISREKLTIKKMIDLYQAKCPQASAEPEHYEALFVYAQKRLDKCVFGEEKPACKQCPVHCYQPAKR

EEMKQIMRWAGPRMLWRHPILTVRHLIDDKRPVPELPEKYRPKKPRE

>gi|30064085|ref|NP_838256.1| formate hydrogen-lyase transcriptional activator for fdhF, hyc and hyp operons [Shigella flexneri 2a str. 2457T]

MSYTPMSDLGQQGLFDITRTLLQQPDLASLCEALSQLVKRSALADNAAIVLWQAQTQRASYYASREKDTP

IKYEDETVLAHGPVRSILSRPDTLHCSYEEFCETWPQLAAGGLYPKFGHYCLMPLAAGGHIFGGCEFIRY

DERPWSEKEFNRLQTFTQIVSVVTEQIQSRVVNNVDYELLCRERDNFRILVAITNAVLSRLDMDELVSEV

AKEIHYYFDIDDISIVLRSHRKNKLNIYSTHYLDKQHPAHEQSEVDEAGTLTERVFKSKEMLLINLYERD

DLAPYERMLFDTWGNQIQTLCLLPLMSGDTMLGVLKLAQCEEKVFTTTNLNLLRQIAERVAIAVDNALAY

QEIHRLKERLVDENLALTEQLNNVDSEFGEIIGRSEAMYSVLKQVEMVAQSDSTVLILGETGSGKELIAR

AIHNLSGRNNRRMVKMNCAAMPAGLLESDLFGHERGAFTGANAQRIGRFELADKSSLFLDEVGDMPLELQ

PKLLRVLQEQEFERLGSNKIIQTDVRLIAATNRDLKKMVADREFRSDLYYRLNVFPIHLPPLRERPEDIP

LLAKAFTFKIARRLGRNIDSIPAETLRTLSNMEWPGNVRELENVIERAVLLTRGNVLQLSLPDIALPEPE

TPPAATVVAQEGEDEYQLIVRVLKETNSVVAGPKGAAQRLGLKRTTLLSRMKRLGIDKSALI

>gi|30064084|ref|NP_838255.1| hypE product [Shigella flexneri 2a str. 2457T]

MQQLINSLFMEAFANPWLAEQEDQARLDLAQLVAEGDRLAFSTDSYVIDPLFFPGGNIGKLAICGTANDV

AGSGAIPRYLSCGFILEEGLPMETLKAVVTSMAETARTAGIAIVTGDTKVVQRGAADKLFINTAGMGAIP

ANIHWGAQTLTAGDVLLVSGTLGDHGATILNLREQLGLDGELVSDCAVLTPLIQTLRDIPGVKALRDATR

GGVNAVVHEFAAACGCGIEISESALPVKPAVRGVCELLGLDALNFANEGKLVIAVERNAAEQVLAALHSH

PLGKDAALIGEVVERKGVRLAGLYGVKRTLDLPHAEPLPRIC

>gi|30064083|ref|NP_838254.1| hydrogenase isoenzyme HypD [Shigella flexneri 2a str. 2457T]

MRFVDEYRAPEQVMQLIEHLRERASHLSYTAERPLRIMEVCGGHTHAIFKFGLDQLLPENVEFIHGPGCP

VCVLPMGRIDTCVEIASHPEVIFCTFGDAMRVPGKQGSLLQAKARGADVRIVYSPMDALKLAQENPTRKV

VFFGLGFETTMPTTAITLQQAKARDVQNFYFFCQHITLIPTLRSLLEQPDNGIDAFLAPGHVSMVIGTDA

YNFIASDFHRPLVVAGFEPLDLLQGVVMLVEQKIAAHSKVENQYRRVVPDAGNLLAQQAIADVFCVNGDS

EWRGLGVIESSGVHLTPDYQRFDAEAHFRPAPQQVCDDPRARCGEVLTGKCKPHQCPLFGNTCNPQTAFG

ALMVSSEGACAAWYQYRQQESEA

>gi|30064082|ref|NP_838253.1| hydrogenase assembly chaperone [Shigella flexneri 2a str. 2457T]

MCIGVPGQIRTIDGNQAKVDVCGIQRDVDLTLVGSCDENGQPRVGQWVLVHVGFAMSVINEAEARDTLDA

LQNMFDVEPDVGALLYGEEK

>gi|30064081|ref|NP_838252.1| hydrogenase nickel incorporation protein HypB [Shigella flexneri 2a str. 2457T]

MCTTCGCGEGNLYIEGDEHNPHSAFRSAPFAPAARPQMKITGIKAPEFTPSQTEEGDLHYGHGEAGTHTP

GMSQRRMLEVEIDVLDKNNRLAERNRARFAARKQLVLNLVSSPGSGKTTLLTETLMRLKDSVPCAVIEGD

QQTVNDAARIRATGTPAIQVNTGKGCHLDAQMIADAAPRLPLDDNGILFIENVGNLVCPASFDLGEKHKV

AVLSVTEGEDKPLKYPHMFAAASLMLLNKVDLLPYLNFDVEKCIACAREVNPEIEIILISATSGEGMDQW

LNWLETQRCA

>gi|30064080|ref|NP_838251.1| hydrogenase nickel incorporation protein [Shigella flexneri 2a str. 2457T]

MGGDMHEITLCQRALELIEQQAAKHGAKRVTGVWLKIGAFSCVETSSLAFCFDLVCRGSVAEGCKLHLEE

QEAECWCETCQQYVTLLTQRVRRCPQCHGDMLQIVADDGLQIRRIEIDQE

>gi|30064079|ref|NP_838250.1| formate hydrogenlyase regulatory protein HycA [Shigella flexneri 2a str. 2457T]

MTIWEISEKADYIAQRHRRLQDQWHIYCNSLVQGITLSKARLHHAMSCAPDKELCFVLFEHFRIYVTLAD

GFNSHTIEYYVETKDGEDKQRIAQAQLSIDGMIDGKVNIRDREQVLEHYLEKIAGVYDSLYTAIENNVPV

NLSQLVKGQSPAA

>gi|30064078|ref|NP_838249.1| small subunit of hydrogenase-3, iron-sulfur protein (part of formate hydrogenlyase (FHL) complex) [Shigella flexneri 2a str. 2457T]

MNRFVIADSTLCIGCHTCEAACSETHRQHGLQSMPRLRVMLNEKESAPQLCHHCEDAPCAVVCPVNAITR

VDGAVQLNESLCVSCKLCGIACPFGAIEFSGSRPLDIPANANTPKAPPAPPAPARVSTLLDWVPGIRAIA

VKCDLCSFDEQGPACVRMCPTKALHLVDNTDIARVSKRKRELTFNTDFGDLTLFQQAQSGEAK

>gi|30064077|ref|NP_838248.1| formate hydrogenlyase subunit 3 [Shigella flexneri 2a str. 2457T]

MSPPLFWHFSFQKALSGWIAGIGGAVGSLYTAAAGFTVLTGAVGVSGALSLVSYDVQISPLNAIWLITLG

LCGLFVSLYNIDWHRHAQVKCNGLQINMLMAAAVCAVIASNLGMFVVMAEVMALCAVFLTSNSKEGKLWF

ALRRLGTLLLVIACWLLWQRYGTLDLRLLDMRMQQLPLGSDIWLLGVIGFGLLAGIIPLHGWVPQAHANA

SAPAAALFSTVVMKIGLLGMLTLSLLGGNAPLWWGIALLVLGMITAFVGGLYALMEHNIQRLLAYHTLEN

IGIILLGLGLGLGAGVTGIALEQPALIALGLVGGLYHLLNHSLFKSVLFLGAGSVWFRTGHRDIEKLGGI

GKKMPVISIAMLVGLMAMAALPPLNGFAGEWVIYQSFFKLSNSGAFVARLLGPLLAVGLAITGALAVMCM

AKVYGVTFLGAPRTKEAENATCAPLLMSVSVVALAICCVIGGVAAPWLLPMLSAAVPLPLEPANTTVSQP

MITLLLIACPLLPFIIMAICKGDRLPSRSRGAAWVCGYDHEKSMVITAHGFAMPVKQAFAPVLKLRKWLN

PVSLVPGWQCEGSALLFRRMALVELAVLVVIIVSRGA

>gi|30064076|ref|NP_838247.1| membrane-spanning protein of hydrogenase 3 (part of FHL complex) [Shigella flexneri 2a str. 2457T]

MSVLYPLIQALVLFAVAPLLSGITRVARARLHNRRGPGVLQEYRDIIKLLGRQSVGPDASGWMFRLTPYV

MVGVMLTIATALPVVTVGSPLPQLGDLITLLYLFAIARFFFAISGLDTGSPFTAIGASREAMLGVLVEPM

LLLGLWVAAQVAGSTNISNITDTVYHWPLSQSIPLVLALCACAFATFIEMGKLPFDLAEAEQELQEGPLS

EYSGSGFGVMKWGISLKQLVVLQMFVGVFIPWGQMETFTVGGLLLALVIAIVKLVVGVLVIALFENSMAR

LRLDITPRITWAGFGFAFLAFVSLLAA

>gi|30064075|ref|NP_838246.1| large subunit of hydrogenase 3 (part of FHL complex) [Shigella flexneri 2a str. 2457T]

MSEEKLGQHYLAALNEAFLGVVLDHAWQTKDQLTVTVKVNYLPEVVEFLYYKQGGWLSVLFGNDERKLNG

HYAVYYVLSMEKGTKCWVTVRVEVDANKPEYPSVTPRVPAAVWGEREVRDMYGLIPVGLPDERRLVLPDD

WPDELYPLRKDSMDYRQRPAPTTDAETYEFINELGDKKNNVVPIGPLHVTSDEPGHFRLFVDGENIIDAD

YRLFYVHRGMEKLAETRMGYNEVTFLSDRVCGICGFAHSTAYTTSVENAMGIQVPERAQMIRAILLEVER

LHPHLLNLGLACHFTGFDSGFMQFFRVRETSMKMAEILTGARKTYGLNLIGGIRRDLLKDDMIQTRQLAQ

QMRREVQELVDVLLSTPNMEQRTVGIGRLDPEIARDFSNVGPMVRASGLARDTRADHPFVGYGLLPMEVH

SEQGCDVISRLKVRINEVYTALNMIDYGLDNLPGGPLMVEGFTYIPHRFALGFAEAPRGDDIHWSMTGDN

QKLYRWRCRAATYANWPTLRYMLRGNTVSYVPLIIGSLDPCYSCTDRMTVVDVRKKKSKVVPYKELERYS

IERKNSPLK

>gi|30064074|ref|NP_838245.1| iron-sulfur protein of hydrogenase 3 (part of FHL complex) [Shigella flexneri 2a str. 2457T]

MFTFIKKVIKTGTATSSYPLEPIAQGQSANGAVHCVDCV

>gi|30064073|ref|NP_838244.1| ascBF operon repressor [Shigella flexneri 2a str. 2457T]

MTTMLEVAKRAGVSKATVSRVLSGNGYVSQETKDRVFQAVEESSYRPNLLARNLSAKSTQTLGLVVTNTL

YHGIYFSELLFHAARMAEEKGRQLLLADGKHSAEEERQAIQYLLDLRCDAIMIYPRFLSVDEIDDIIDAH

SQPIMVLNRRLRNNSSHSVWCDHKQTSFNAVAELINAGHQEIAFLTGSMDSPTSIERLAGYKDALSQHGI

ALNEKLIANGKWTPASGAEGVETLLERGAKFSALVASNDDMAIGAIKALHERGVAVPEQVSVIGFDDIAI

APYIVPALSSVKIPVTEMIQEIIGRLIFMLDGGDFSPPKTFSGKLIRRDSLIALSR

>gi|30064072|ref|NP_838243.1| electron transport protein HydN [Shigella flexneri 2a str. 2457T]

MNRFIIADASKCIGCRTCEVACVVSHQENQDCASLTPETFLPRIHVIKGVNISTATVCRQCEDAPCANVC

PNGAISRDKGFVPVMQERCIGCKTCVVACPYGAMEVVVRPVIRNSGAGLNVRADKAEANKCDLCNHREDG

PACMAACPTHALICVDRNKLEQLSAEKRRRTALMF

>gi|30064071|ref|NP_838242.1| transcriptional regulatory protein [Shigella flexneri 2a str. 2457T]

MAKNTSCGVQLRIRGKVQGVGFRPFVWQLAQQLNLHGDVCNDGDGVEVRLLEDPETFLVQLHQHCPPLAR

IDSVEREPFIWSQLPTEFTIRQSTGGTMNTQIVPDAATCPACLAEMNTPGERRYRYPFINCTHCGPRFTI

IRAMPYDRPFTVMAAFPLCPACDKEYRDPLDRRFHAQPVACPECGPHLEWVSHGEHAEQEAALQAAIAQL

KMGNIVAIKGIGGFHLACDARNSNAVATLRERKHRPAKPLAVMLPVAESLPDAARQLLTTPAAPIVLVDK

KYVPELCDDIAPGLNEVGVMLPANPLQHLLLQELQCPLVMTSGNLSGKPPAISNEQALADLQGIADGFLL

HNRDIVQRMDDSVVRESGEMLRRSRGYVPDALVLPPGFKNVPPVLCLGADLKNTFCLVRGEQAVLSQHLG

DLSDDGIQMQWREALRLMQNIYDFTPQYIVHDVHPGYVSSQWASEMNLPTQTVLHHHAHAAACLAEHQWP

LDGGDVIALTLDGIGMGENGALWGGECLRVNYRECEHLGGLPAVALPGGDLAAKQPWRNLLAQCLRFVPE

WQNYPETASVQQQNWSVLARAIERGINAPLASSCGRLFDAVAAALGCAPATLSYEGEAACALEALAASCD

GVTHPVTMPRVDNQLDLATFWQQWLNWQALVNQRAWAFHDALAQGFAALMREQATMRGITTLVFSGGVIH

NRLLRARLAHYLADFTLLFPQSLPAGDGGLSLGQGVIAAARWLAGEVQNG

>gi|30064070|ref|NP_838241.1| nitric oxide reductase [Shigella flexneri 2a str. 2457T]

MSNGIVIIGSGFAARQLVKNIRKQDATIPLTLIAADSIDEYNKPDLSHVISQGQRADDLTRQTAGEFAGQ

FNLRLFPHTWVTDIDAEAHVVKSQNNQWQYDKLVLATGASAFVPPVPGRELMLTLNSQQEYRACETQLRD

ARRVLIVGGGLIGSELAMDFCRAGKAVTLIDNAASILASLMPPEVSSRLQHRLTEMGVHLLLKSQLQGLE

KTDSGILATLDHQRSIEVDAVIAATGLRPETALARRAGLTINRGVCVDSYLQTSNADIYALGDCAEINGQ

VLPFLQPIQLSGMVLAKNLLGNNTPLKLPAMLVKIKTPELPLHLAGETQRQDLRWQINTERQGMVARGVD

DADQLRAFVVSEDRMKEAFVLLKTLPV

>gi|30064069|ref|NP_838240.1| anaerobic nitric oxide reductase flavorubredoxin [Shigella flexneri 2a str. 2457T]

MSIVVKNNIHWVGQRDWEVRDFHGTEYKTLRGSSYNSYLIREEKNVLIDTVDHKFSREFVQNLRNEIDLA

DIDYIVINHAEEDHAGALTELMAQIPDTPIYCTANAIDSINGHHHHPEWNFNVVKTGDTLDIGNGKQLIF

VETPMLHWPDSMMTYLTGDAVLFSNDAFGQHYCDEHLFNDEVDQTELFEQCQRYYANILTPFSRLVTPKI

TEILGFNLPVDMIATSHGVVWRDNPTQIVELYLKWAADYQEDRITIFYDTMSNNTRMMADAIAQGIAETD

PRVAVKIFNVARSDKNEILTNVFRSKGVLVGTSTMNNVMMPKIAGLVEEMTGLRFRNKRASAFGSHGWSG

GAMDRLSTRLQDAGFEMSLSLKAKWRPDQDALELCREHGREIARQWALAPLPQSTVNTVVKEETSATTTA

DLGPRMQCSVCQWIYDPAKGEPMQDVAPGTPWSEVPDNFLCPECSLGKDVFDELASEAK

>gi|30064066|ref|NP_838237.1| DNA-bindng transcriptional repressor SrlR [Shigella flexneri 2a str. 2457T]

MKPRQRQAAILEYLQKQGKCSVEELAQYFDTTGTTIRKDLVILEHAGTVIRTYGGVVLNKEESDPPIDHK

TLINTHKKELIAEAAVSFIHDGDSIILDAGSTVLQIVPLLSRFNNITVMTNSLHIVNALSELDNEQTILM

PGGTFRKKSASFHGQLAENAFEHFTFDKLFMGTDGIDLNAGVTTFNEVYTVSKAMCNAAREVILMADSSK

FGRKSPNVVCSLESVDKLITDAGIDPAFRQALEEKGIDVIITGESNE

>gi|30064065|ref|NP_838236.1| DNA-binding transcriptional activator GutM [Shigella flexneri 2a str. 2457T]

MVSALITVAVIAWCAQLALGGWQISRFNRAFDTLCQQGRVGVDRSSGRFKPRVVVAIALDDQQCIVDTLF

MKGLTVFARPQKIPAITGMHAGDLQPDVIFPHDPLSQNALSLALKLKRG

>gi|30064064|ref|NP_838235.1| sorbitol-6-phosphate dehydrogenase [Shigella flexneri 2a str. 2457T]

MNQVAVVIGGGQTLGAFLCHGLAAEGYRVAVVDIQSDKAANVAQEINAEYGEGMAYGFGADATSEQSVLA

LSRGVDEIFGRVDLLVYSAGIAKAAFISDFQLGDFDRSLQVNLVGYFLCAREFSRLMIRDGIQGRIIQIN

SKSGKVGSKHNSGYSAAKFGGVGLTQSLALDLAEYGITVHSLMLGNLLKSRMFQSLLPQYATKLGIKPDQ

VEQYYIDKVPLKRGCDYQDVLNMLLFYASPKASYCTGQSINVTGGQVMF

>gi|30064063|ref|NP_838234.1| glucitol/sorbitol-specific PTS system component IIA [Shigella flexneri 2a str. 2457T]

MTVIYQTTITRIGASATDALSDQMLITFREGAPADLEEYCFIHCHGELKGALHPGLQFSLGQHRYPVTAV

GSVAEDNLRELGHVTLRFDGLNEAEFPGTVHVAGPVPDDIAPGSVLKFESVKE

>gi|30064062|ref|NP_838233.1| PTS system, glucitol/sorbitol-specific IIB component and second of two IIC components [Shigella flexneri 2a str. 2457T]

MTRIRIEKGTGGWGGPLELEATPGKKIVYITAGTRPAIVDKLAQLTGWQAIDGFKEGEPAEAEIGVAVID

CGGTLRCGIYPKRRIPTINIHSTGKSGPLAQYIVEDIYVSGVKEENITVVGDATPQPSSVGRDYDTSKKI

TEQSDGLLAKVGMGMGSAVAVLFQSGRDTIDTVLKTILPFMAFVSALIGIIMASGLGDWIAHGLAPLASH

PLGLVMLALICSFPLLSPFLGPGAVIAQVIGVLIGVQIGLGNIPPHLALPALFAINAQAACDFIPVGLSL

AEARQDTVRVGVPSVLVSRFLTGAPTVLIAWFVSGFIYQ

>gi|30064061|ref|NP_838232.1| murein hydrolase B [Shigella flexneri 2a str. 2457T]

MFKRRYVTLLPLFVLLAACSSKPKPTETETTTGTPSGGFLLEPQHNVMQMGGDFANNPNAQQFIDKMVNK

HGFDRQQLQEILSQAKRLDSVLRLMDNQAPTTSVKPPSGPNGAWLRYRKKFITPDNVQNGVVFWNQYEDA

LNRAWQVYGVPPEIIVGIIGVETRWGRVMGKTRILDALATLSFNYPRRAEYFSGELETFLLMARDEQDDP

LNLKGSFAGAMGYGQFMPSSYKQYAVDFSGDGHINLWDPVDAIGSVANYFKAHGWVKGDQVAIMANGQAP

GLPNGFKTKYSISQLAAAGLTPQQPLGNHQQASLLRLDVGTGYQYWYGLPNFYTITRYNHSTHYAMAVWQ

LGQAVALARVQ

>gi|30064060|ref|NP_838231.1| competence damage-inducible protein A [Shigella flexneri 2a str. 2457T]

MTDSELMQLSEQVGQALKARGATVTTAESCTGGWVAKVITDIAGSSAWFERGFVTYSNEAKAQMIGVREE

TLAQHGAVSEPVVVEMAIGALKAARADYAVSISGIAGPDGGSEEKPVGTVWFAFATARGEGITRRECFSG

DRDAVRRQATAYALQTLWQQFLQNT

>gi|30064059|ref|NP_838230.1| recombinase A [Shigella flexneri 2a str. 2457T]

MAIDENKQKALAAALGQIEKQFGKGSIMRLGEDRSMDVETISTGSLSLDIALGAGGLPMGRIVEIYGPES

SGKTTLTLQVIAAAQREGKTCAFIDAEHALDPIYARKLGVDIDNLLCSQPDTGEQALEICDALARSGAVD

VIVVDSVAALTPKAEIEGEIGDSHMGLAARMMSQAMRKLAGNLKQSNTLLIFINQIRMKIGVMFGNPETT

TGGNALKFYASVRLDIRRIGAVKEGENVVGSETRVKVVKNKIAAPFKQAEFQILYGEGINFYGELVDLGV

KEKLIEKAGAWYSYKGEKIGQGKANATAWLKDNPETAKEIEKKVRELLLSNPNSTPDFSVDDSEGVAETN

EDF

>gi|30064058|ref|NP_838229.1| recombination regulator RecX [Shigella flexneri 2a str. 2457T]

MTESTSRRPAYARLLDRAVRILAVRDHSEQELRRKLAAPIMGKNGPEEIDATAEDYERVIAWCHEHGYLD

DSRFVARFIASRSRKGYGPARIRQELNQKGISREATEKAMRECDIDWCALARDQATRKYGEPLPTVFSEK

VKIQRFLLYRGYLMEDIQDIWRNFAD

>gi|30064057|ref|NP_838228.1| alanyl-tRNA synthetase [Shigella flexneri 2a str. 2457T]

MSKSTAEIRQAFLDFFHSKGHQVVASSSLVPHNDPTLLFTNAGMNQFKDVFLGLDKRNYSRATTSQRCVR

AGGKHNDLENVGYTARHHTFFEMLGNFSFGDYFKHDAIQFAWELLTSEKWFALPKERLWVTVYESDDEAY

EIWEKEVGIPRERIIRIGDNKGAPYASDNFWQMGDTGPCGPCTEIFYDHGDHIWGGPPGSPEEDGDRYIE

IWNIVFMQFNRQADGTMEPLPKPSVDTGMGLERIAAVLQHVNSNYDIDLFRTLIQAVAKVTGATDLSNKS

LRVIADHIRSCAFLIADGVMPSNENRGYVLRRIIRRAVRHGNMLGAKETFFYKLVGPLIDVMGSAGEDLK

RQQAQVEQVLKTEEEQFARTLERGLALLDEELAKLSGDTLDGETAFRLYDTYGFPVDLTADVCRERNIKV

DEAGFEAAMEEQRRRAREASGFGADYNAMIRVDSASEFKGYDHLELNGKVTALFVDGKAVDAINAGQEAV

VVLDQTPFYAESGGQVGDKGELKGANFSFAVEDTQKYGQAIGHIGKLAAGSLKVGDAVQADIDEARRARI

RLNHSATHLMHAALRQVLGTHVSQKGSLVNDKVLRFDFSHNEAMKPEEIRAVEDLVNAQIRRNLPIETNI

MNLEAAKAKGAMALFGEKYDERVRVLSMGDFSTELCGGTHASRTGDIGLFRIISESGTAAGVRRIEAVTG

EGAITTVHADSDRLSEVAHLLKGDSNNLADKVRSVLERTRQLEKELQQLKEQAAAQESANLSSKAIDVNG

VKLLVSELSGVEPKMLRTMVDDLKNQLGSTIIVLATVAEGKVSLIAGVSKDVTDRVKAGELIGMVAQQVG

GKGGGRPDMAQAGGTDAAALPAALASVKGWVSAKLQ

>gi|30064056|ref|NP_838227.1| carbon storage regulator [Shigella flexneri 2a str. 2457T]

MLILTRRVGETLMIGDEVTVTVLGVKGNQVRIGVNAPKEVSVHREEIYQRIQAEKSQQSSY

>gi|30064055|ref|NP_838226.1| fructose-1-phosphatase [Shigella flexneri 2a str. 2457T]

MYERYAGLIFDMDGTILDTAPTHRKAWREVLGHYGLQYDIQAMIALNGSPTWRIAQAIIELNQADLDPHA

LAREKTEAVRSMLLDSVEPLPLVEVVKSWHGRRPMAVGTGSESAIAEALLAHLGLRRYFDAVVAADHVKH

HKPAPDTFLLCAQRMGVQPTQCVVFEDADFGIQAARAAGMDAVDVRLL

>gi|30064054|ref|NP_838225.1| hypothetical protein S2903 [Shigella flexneri 2a str. 2457T]

MSEALSLFSLFASSFLSATLLPGNSEVVLVAMLLSGISHPWVLVLTATMGNSLGGLTNVILGRFFPLRKT

SRWQEKATGWLKRYGAVTLLLSWMPVVGDLLCLLAGWMRISWGPVIFFLCLGKALRYVAVAAATVQGMMW

WH

>gi|30064053|ref|NP_838224.1| glutamate--cysteine ligase [Shigella flexneri 2a str. 2457T]

MIPDVSQALAWLEKHPQALKGIQRGLERETLRVNADGTLATTGHPEALGSALTHKWITTDFAEALLEFIT

PVDGDIEHMLTFMRDLHRYTARNMGDERMWPLSMPCYIAEGQDIELAQYGTSNTGRFKTLYREGLKNRYG

ALMQTISGVHYNFSLPMAFWQAKCGDISGADAKEKISAGYFRVIRNYYRFGWVIPYLFGASPAICSSFLQ

GKPTSLPFEKTECGMYYLPYATSLRLSDLGYTNKSQSNLGITFNDLYEYVAGLKQAIKTPSEEYAKIGIE

KDGKRLQINSNVLQIENELYAPIRPKRVTRSGESPSDALLRGGIEYIEVRSLDINPFSPIGVDEQQVRFL

DLFMVWCALADAPEMSSSELACTRVNWNRVILEGRKPGLTLGIGCETAQFPLLQVGKDLFRDLKRVAQTL

DSINGGEAYQKVCDELVACFDNPDLTFSARILRSMIDTGIGGTGKAFAEAYRNLLREEPLEILREEDFVA

EREASERRQQEMETADTEPFAVWLEKHA

>gi|30064052|ref|NP_838223.1| S-ribosylhomocysteinase [Shigella flexneri 2a str. 2457T]

MPLLDSFTVDHTRMEAPAVRVAKTMNTPHGDAITVFDLRFCVPNKEVMPERGIHTLEHLFAGFMRNHLNG

NGVEIIDISPMGCRTGFYMSLIGTPDEQRVADAWKAAMEDVLKVQDQNQIPELNVYQCGTYQMHSLQEAQ

DIARSILEREVRINSNEELALPKEKLQELHI

>gi|30064051|ref|NP_838222.1| multidrug resistant protein emrB [Shigella flexneri 2a str. 2457T]

MQQQKPLEGAQLVIMTIALSLATFMQVLNSTIANVAIPTIAGNLGSSLSQGTWVITSFGVANAISIPLTG

WLAKRVGEVKLFLWSTIAFAIASWACGVSSSLNMLIFFRVIQGIVAGPLIPLSQSLLLNNYPPAKRSIAL

ALWSMTVIVAPICGPILGGYISDNYHWGWIFFINVPIGVAVVLMTLQTLRGRETRTERRRIDAVGLALLV

IGIGSLQIMLDRGKELDCFSSQEIIILTVVAVVAICFLIVWELTDDNPIVDLSLFKSRNFTIGCLCISLA

YMLYFGAIVLLPQLLQEVYGYTATWAGLASAPVGIIPVILSPIIGRFAHKLDMRRLVTFSFIMYAVCFYW

RAYTFEPGMDFCASAWPQFIQGFAVVCFFMPLTTITLSGLPPERLAAASSLSNFTRTLAGSIGTSITTTM

WTNRESMHHAQLTESVNPFNPNAQAMYSQLEGLGMTQQQASGWIAQQITNQGLIISANEIFWMSAGIFLV

LLGLVWFAKPPFGAGGGGGGAH

>gi|30064050|ref|NP_838221.1| transcriptional repressor MprA [Shigella flexneri 2a str. 2457T]

MDSSFTPIEQMLKFRASRHEDFPYQEILLTRLCMHMQSKLLENRNKMLKAQGINETLFMALITLESQENH

SIQPSELSCALGSSRTNATRIADELEKRGWIERRKSDNDRRCLHLQLTEKGHEFLREVLPPQHNCLHQLW

SALSTTEKDQLEQITRKLLSRLDQMEQDGVVLEAMS

>gi|30064049|ref|NP_838220.1| hypothetical protein S2897 [Shigella flexneri 2a str. 2457T]

MSYEVLLLGLLVGAANYCFRYLPLRLRVGNARPTKRGAVGILLDTIGIASICALLVVSTAPEVMHDTRRF

VPTLVGFAVLGASFYKTRSIIIPTLLSALAYGLAWKVMAII

>gi|30064048|ref|NP_838219.1| hypothetical protein S2896 [Shigella flexneri 2a str. 2457T]

MESPTPQPAPGSATFMEGCKDSLPIVISYIPVAFAFGLNATRLGFSPLESVFFSCIIYAGASQFVITAML

AAGSSLWVAALSVMAMDVRHVLYGPSLRSRIIQRLQKSKTALWAFGLTDEVFAAATAKLVRNNRRWSENW

MIGIAFSSWSSWVFGTVIGAFSGSGLLQGYPAVEAALGFMLPALFMSFLLASFQRKQSLCVTAALVGALA

GVTLFSIPVAILAGIVCGCLTALIQAFWQGAPDEL

>gi|30064047|ref|NP_838218.1| hypothetical protein S2894 [Shigella flexneri 2a str. 2457T]

MTKPNHELSPALIVLMSIATGLAVASNYYAQPLLDTIARNFSLSASSAGFIVTAAQLGYAAGLLFLVPLG

DMFERRRLIVSMTLLAAGGMLITASSQSLAMMILGTALTGLFSVVAQILVPLAATLASPDKRGKVVGTIM

SGLLLGILLARTVAGLLANLGGWRTVFWVASVLMALMALALWRGLPQMKSETHLNYPQLLGSVFSMFISD

KILRTRALLGCMTFANFSILWTSMAFLLAAPPFNYSDGDIGLFGLAGAAGALGARPAGGFADKGKSHHNF

RSASAVTFMAGNLVWSHFRTGVDYRHSGAGPHRTGRAYH

>gi|30064046|ref|NP_838217.1| glycine betaine transporter periplasmic subunit [Shigella flexneri 2a str. 2457T]

MRHSVLFATAFATLISTQTFAADLPGKGITVNPVQSTITEETFQTLLVSRALEKLGYTVNKPSEVDYNVG

YTSLASGDATFTAVNWTPLHDNMYEAAGGDKKFYREGVFVNGAAQGYLIDKKTADQYKITNIAQLKDPKI

AKLFDTNGDGKADLTGCNPGWGCEGAINHQLAAYELTNTVTHNQGNYAAMMADTISRYKEGKPVFYYTWT

PYWVSNELKPGKDVVWLQVPFSALPGDKNADTKLPNGANYGFPVSTMHIVANKAWTEKNPAAAKLFAIMQ

LPVADINAQNAIMHDGKASEGDIQGHVDGWIKAHQQQFDGWVNEALAAQK

>gi|30064045|ref|NP_838216.1| glycine betaine transporter membrane protein [Shigella flexneri 2a str. 2457T]

MADQNNPWDTTPAADSAAQSADAWGTPTTAPADGGGADWLTSTPAPNVEHFNILDPFHKTLIPLDSWVTE

GIDWVVTHFRPVFQGVRVPVDYILNGFQQLLLGMPAPVAIIVFALITWQISGVGMGVATLVSLIAIGAIG

AWSQAMVTLALVLTALLFCIVIGLPLGIWLARSPRAAKIIRPLLDAMQTTPAFVYLVPIVMLFGIGNVPG

VVVTIIFALPPIIRLTILGINQVPADLIEASRSFGASPRQMLFKVQLPLAMPTIMAGVNQTLMLALSMVV

IASMIAVGGLGQMVLRGIGRLDMGLATVGGVGIVILAIILDRLTQAVGRDSRSRGNRRWYTTGPVGLLTR

PFIK

>gi|30064044|ref|NP_838215.1| glycine betaine transporter ATP-binding subunit [Shigella flexneri 2a str. 2457T]

MAIKLKIKNLYKIFGEHPQRAFKYIEQGLSKEQILEKTGLSLGVKDASLAIEEGEIFVIMGLSGSGKSTM

VRLLNRLIEPTRGQVLIDGVDIAKISDAELREVRRKKIAMVFQSFALMPHMTVLDNTAFGMELAGINAEE

RREKALDALRQVGLENYAHSYPDELSGGMRQRVGLARALAINPDILLMDEAFSALDPLIRTEMQDELVKL

QAKHQRTIVFISHDLDEAMRIGDRIAIMQNGEVVQVGTPDEILNNPANDYVRTFFRGVDISQVFSAKDIA

RRTPNGLIRKTPGFGPRSALKLLQDEDREYGYVIERGNKFVGAVSIDSLKTALTQQQGLDAALIDAPLAV

DAQTPLSELLSHVGQAPCAVPVVDEDQQYVGIISKGMLLRALDREGVNNG

>gi|30064043|ref|NP_838214.1| ribonucleotide-diphosphate reductase subunit beta [Shigella flexneri 2a str. 2457T]

MKLSRISAINWNKISDDKDLEVWNRLTSNFWLPEKVPLSNDIPAWQTLTVVEQQLTMRVFTGLTLLDTLQ

NVIGAPSLMPDALTPHEEAVLSNISFMEAVHARSYSSIFSTLCQTKDVDAAYAWSEENAPLQRKAQIIQQ

HYRGDDPLKKKIASVFLESFLFYSGFWLPMYFSSRGKLTNTADLIRLIIRDEAVHGYYIGYKYQKNMEKI

SLGQREELKSFAFDLLLELYDNELQYTDELYAETPWADDVKAFLCYNTNKALMNLGYEPLFPAEMAEVNP

AILAALSPNADENHDFFSGSGSSYVMGKAVETEDEDWNF

>gi|30064042|ref|NP_838213.1| ribonucleotide-diphosphate reductase subunit alpha [Shigella flexneri 2a str. 2457T]

MDYHALNAMLNLYDSAGRIQFDKDRQAVDAFIATHVRPNSVTFSSQQQRLNWLVNEGYYDESVLNRYSRD

FVITLFAHAHTSGFRFQTFLGAWKFYTSYTLKTFDGKRYLEDFADRVTMVALTLAQGDETLALQLTDEML

SGRFQPATPTFLNCGKQQRGELVSCFLLRIEDNMESIGRAVNSALQLSKRGGGVAFLLSNLREAGAPIKR

IENQSSGVIPVMKMLEDAFSYANQLGARQGAGAVYLHAHHPDILRFLDTKRENADEKIRIKTLSLGVVIP

DITFHLAKENAQMALFSPYDVERVYGKPFADIAISEHYDELVADERIRKKYLNARDFFQRLAEIQFESGY

PYIMYEDTVNRANPIAGRINMSNLCSEILQVNSASEYDENLDYARTGHDISCNLGSLNIAHTMDSPDFSR

TVETAVRGLTAVSDMSHIRSVPSIEAGNAASHAIGLGQMNLHGYLAREGIAYGSPEALDFTNLYFYTITW

HALRTSMLLARERGETFAGFKQSRYASGEYFSQYLQGNWQPKTAKVGELFARSCITLPTREMWAQLRDDV

MRYGIYNQNLQAVPPTGSISYINHATSSIHPIVAKVEIRKEGKTGRVYYPAPFMTNENLALYQDAYEIGA

EKIIDTYAEATRHVDQGLSLTLFFPDTATTRDINKAQIYAWRKGIKTLYYIRLRQMALEGTEIEGCVSCA

L

>gi|30064041|ref|NP_838212.1| ribonucleotide reductase stimulatory protein [Shigella flexneri 2a str. 2457T]

MSQLVYFSSSSENTQRFIERLGLPAVRIPLNERERIQVDEPYILIVPSYGGGGTAGAVPRQVIRFLNDEH

NRALLRGVIASGNRNFGEAYGRAGDVIARKCGVPWLYRFELMGTQSDIENVRKGVTEFWQRQPQNA

>gi|30064040|ref|NP_838211.1| glutaredoxin-like protein [Shigella flexneri 2a str. 2457T]

MRITIYTRNDCVQCHATKRAMENRGFDFEMINVDRVPEAAEALRAQGFRQLPVVIAGDLSWSGFRPDMIN

RLHPAPHAASA

>gi|30064039|ref|NP_838210.1| hypothetical protein S2885 [Shigella flexneri 2a str. 2457T]

MYLRPDEVARVLEKVGFTVDVVTQKAYGYRRGENYVYVNREARMGRTALVIHPTLKERSSTLAEPASDIK

TCDHYQQFPLYLAGERHEHYGIPHGFSSRVALERYLNGLFGEAS

>gi|30064038|ref|NP_838209.1| hypothetical protein S2884 [Shigella flexneri 2a str. 2457T]

MFSPQSRLRHAVADTFAMVVYCSVVNMCIEVFLSGMSFEQSFYSRLVAIPVNILIAWPYGMYRDLFMRAA

RKVSPSGWIKNLADILAYVTFQSPVYVAILLVVGADWHQIMAAVSSNIVVSMLMGAVYGYFLDYCRRLFK

VSRYQQVKA

>gi|30064037|ref|NP_838208.1| DNA binding protein, nucleoid-associated [Shigella flexneri 2a str. 2457T]

MSVMLQSLNNIRTLRAMAREFSIDVLEEMLEKFRVVTKERREEEEQQQRELAERQEKISTWLELMKADGI

NPEELLGNSSAAAPRAGKKRQPRPAKYKFTDVNGETKTWTGQGRTPKPIAQALAEGKSLDDFLI

>gi|30064036|ref|NP_838207.1| hypothetical protein S2882 [Shigella flexneri 2a str. 2457T]

MALTTISPHDAQELIARGAKLIDIRDADEYLREHIPEADLAPLSVLEQSGLPPKLRREQIIFHCQAGKRT

SNNADKLAAIAAPAEIFLLEDGIDGWKRAGLPVAVNKSQPLPLMRQVQIAAGGLILNGVVLGYTVNSGFF

LLSGFVGAGLLFAGISGFCGMARLLDKMPWNQRA

>gi|30064035|ref|NP_838206.1| hypothetical protein S2881 [Shigella flexneri 2a str. 2457T]

MTELAQLQASTEQAAALLKAMSHPKRLLILCMLSGSPGTSAGELTRITGLSASATSQHLARMRDEGLINS

QRDAQRILYSIKNEAVNAIIATLKNVYCP

>gi|30064032|ref|NP_838203.1| hypothetical protein S2878 [Shigella flexneri 2a str. 2457T]

MGFWRIVITIILPPIGVLLGKGFGWAFIINILLTLLGYIPGLIHAFWVQTRD

>gi|30064031|ref|NP_838202.1| LysM domain/BON superfamily protein [Shigella flexneri 2a str. 2457T]

MGLFNFVKDAGEKLWDAVTGQHDKDDQAKKVQEHLSKTGIPDADKVNIQIADGKATVTGDGLSQEAKEKI

LVAVGNISGIASVDDQVKTATPATASQFYTVKSGDTLSAISKQVYGNANLYNKIFEANKPMLKSPDKIYP

GQVLRIPEE

>gi|30064030|ref|NP_838201.1| DNA-binding transcriptional regulator CsiR [Shigella flexneri 2a str. 2457T]

MTITSLDGYRWLKNDIIRGNFQPDEKLRMSLLTSRYALGVGPLQEALSQLVAERLVTVVNQKGYRVASMS

EQELLDIFDARANMEAMLVSLAIARGGDEWEADVLAKAHLLSKLEACDASEKMLDEWDLRHQAFHTAIVA

GCGSHYLLQMRERLFDLAARYRFIWLRRTVLSVEMLEDKHDQHQTLTAAVLPRDTARASELMRQHLLTPI

PIIQQAMAGN

>gi|30064029|ref|NP_838200.1| gamma-aminobutyrate transporter [Shigella flexneri 2a str. 2457T]

MGQSSQPHELGGGLKSRHVTMLSIAGVIGASLFVGSSVAIAEADPAVLLAYLFAGLLVVMIMRMLAEMAV

ATPDTGSFSTYADKAIGRWAGYTIGWLYWWFWVLVIPLEANIAAMILHSWVPGIPIWLFSLVITLALTGS

NLLSVKNYGEFEFWLALCKVIAILAFIFLGAVAISGFYPYAEVSGISRLWDSGGFMPNGFGAVLSAMLIT

MFSFMGAEIVTIAAAESDMPEKHIVRATNSVIWRISIFYLCSIFVVVALIPWNMPGLKAVGSYRSVLELL

NIPHAKLIMDCVILLSVTSCLNSALYTASRMLYSLSRRGDAPAVMGKINRSKTPYVAVLLSTGAAFLTVV

VNYYAPAKVFKFLIDSSGAIALLVYLVIAVSQLRMRKILRAEGSEIRLRMWLDPWLTWLVIGFITFVLVV

MLFRPAQQLEVISTGLLAIGIICTVPIMARWKKLVLWQKTPVHNTR

>gi|30064028|ref|NP_838199.1| 4-aminobutyrate aminotransferase [Shigella flexneri 2a str. 2457T]

MLNTGHLHPKVVAAVEAQLKKLSHTCFQVLAYEPYLELCEIMNQKVPGDFAKKTLLVTTGSEAVENAVKI

ARAATKRSGTIAFSGEYHGRTHYTLALTGKVNPYSAGMGLMPGHVYRALYPCPLHGISEDDAIASIHRIF

KNDAAPEDIAAIMIEPVQGEGGFYAASPAFMQRLRALCDEHGIMLIADEVQSGAGRTGTLFAMEQMGVAP

DLTTFAKSIAGGFPLAGVTGRAEVMDAVAPGGLGGTYAGNPIACVAALEVLKVFEQENLLQKANDLGQKL

KDGLLAIAEKHPEIGDVRGLGSMIAIELFEDGDHSKPDAKLTAEIVARARDKGLILLSCGPYYNVLRILV

PLTIEDAQIRQGLEIIGQCFDEAKQ

>gi|30064027|ref|NP_838198.1| hydroxyglutarate oxidase [Shigella flexneri 2a str. 2457T]

MAISLTPLTTTRRISKRKGIERMYDFVIIGGGIIGMSTAMQLIDVYPDARIALLEKESGPACHQTGHNSG

VIHAGVYYTPGSLKAQFCLAGNRATKAFCDQNGIRYDNCGKMLVATSQLEMERMRALWERTAANGIEREW

INAVELREREPNITGLGGIFVPSSGIVSYREVTAAMAKIFQARGGEIIYNAEVSALSEHKNGVVIRTRQG

GEYEASTLISCSGLMADRLVKMLGLEPGFIICPFRGEYFRLAPEHNQIVNHLIYPIPDPAMPFLGVHLTR

MIDGSVTVGPNAVLAFKREGYRKRDFSFSDTLEILSSSGIRRVLQNHLRSGLGEMKNSLCKSGYLRLVQK

YCPRLSLSDLQPWPAGVRAQAVSPDGKLIDDFLFVTTPRTIHTCNAPSPAATSAIPIGAHIVSKVQTLLA

SQSNPGRTLRAARSVDALHAAFNQ

>gi|30064025|ref|NP_838196.1| hypothetical protein S2870 [Shigella flexneri 2a str. 2457T]

MNINHSPHDGLVIINKGNEEVEGTWPNKLQPGIYKNMGSNSVNIIINNTRKIIPPCKVFTLRGGSLNINI

PRRSALLLGKTGEPPNYLYL

>gi|30064023|ref|NP_838194.1| IS3 orfB [Shigella flexneri 2a str. 2457T]

MVIDLWSRAVIGWSMSPRMTAQPALRCPADGAVAA

>gi|30064022|ref|NP_838193.1| IS3 orfB [Shigella flexneri 2a str. 2457T]

MKYVFIEKHQAEFSIKAMCRVLRVARSGWYTWCQRRTRISTRQQFRQHCDSVVLAAFTRSKQRYGAPRLT

DELRAQGYPFNVKTVAASLRRQGLRAKASRKFSPVSYRAHGLPVSENLLEQDFYCQWPEPEVARRHHVLT

YR

>gi|30064021|ref|NP_838192.1| IS3 orfA [Shigella flexneri 2a str. 2457T]

MTKTVSTSKKPRKQHSPEFRSEALKLAERIGVTAAARELSLYESQLYNWRSKQQNQQTSSERELEMSTEI

ARLKRQLAERDEELAILQKAATYFAKRLK

>gi|30064020|ref|NP_838191.1| IS1 orfA [Shigella flexneri 2a str. 2457T]

MASISIRCPSCSATEGVVRNGKSTAGHQRYLCSHCRKTWQLQFTYTASQPGTHQKIIDMAMNGVGYRASA

RIMGVGLNTVLRHLKNSGRSGNLAHTTRQ

>gi|30064019|ref|NP_838190.1| integrase [Shigella flexneri 2a str. 2457T]

MARQTKPLSVKEIESAKPKEADYVLYDGDGLELLIKSSGSKIWQFRYIRPVTKTRAKKSIGPYPSVTLAD

ARNYRAESRSLLAKQIDPQEHQQEQLRSSLEAKTNTFQLVAERWWNVKKASVTEDYAEDIWRSLERDVFP

AIGDVSVTDIKAHTLVQAVQPVQARGALETVRRLCQRINEVMIYAQNTGLIDAVPSVNIGKAFEKPQKKN

MPSIRPDQLSQLMQTMRTASISLSTRCLFMWQLLTITRPAEAAEARWEEVDIEAQEWKIPAARMKMNRDH

TVPLSDEAIAVLEMMKPLSGNREFIFPSRIKPNQPMNSQTVNASLKRAGFGGVLVSHGLRSIASTALNEQ

GFPPDAIEAALAHVDKNEVRRAYNRSDYLEQRRPMMQWWANFVMAADRGSMIEGGIKGMKLVG

>gi|30064018|ref|NP_838189.1| SsrA-binding protein [Shigella flexneri 2a str. 2457T]

MTKKKAHKPGSATIALNKRARHEYFIEEEFEAGLALQGWEVKSLRAGKANISDSYVLLRDGEAFLFGANI

TPMAVASTHVVCDPTRTRKLLLNQRELDSLYGRVNREGYTVVALSLYWKNAWCKVKIGVAKGKKQHDKRS

DIKEREWQVDKARIMKNAHR

>gi|30064017|ref|NP_838188.1| hypothetical protein S2856 [Shigella flexneri 2a str. 2457T]

MPQISRTALVPYSAEQMYQLVNDVQSYPQFLPGCTGSRILESTPGQMTAAVDVSKAGISKTFTTRNQLTS

NQSILMNLVDGPFKKLIGGWKFTPLSQEACRIEFHLDFEFTNKLIELAFGRVFKELAANMVQAFTVRAKE

VYSAR

>gi|30064016|ref|NP_838187.1| hypothetical protein S2855 [Shigella flexneri 2a str. 2457T]

MLGKIAVEVAYALPEKQYLQRVTLQEGATVEEAIRASGLLELRTDIDLTKNKVGIYSRPAKLSDSVHDGD

RVEIYRPLIADPKELRRQRAEKSANK

>gi|30064015|ref|NP_838186.1| small membrane protein A, partial [Shigella flexneri 2a str. 2457T]

MSDPFGTNTWFYVFRQQPGHEGVTQQTLTLTFNSSGVLTNIDNKPALSGN

>gi|30064014|ref|NP_838185.1| recombination and repair protein [Shigella flexneri 2a str. 2457T]

MLAQLTISNFAIVRELEIDFHSGMTVITGETGAGKSIAIDALGLCLGGRAEADMVRTGAARADLCARFSL

KDTPAALRWLEENQLEDGHECLLRRVISSDGRSRGFINGTAVPLSQLRELGQLLIQIHGQHAHQLLTKPE

HQKFLLDGYANETSLLQEMTARYQLWHQSCRDLAHHQQLSQERAARAELLQYQLKELNEFNPQPGEFEQI

DEEYKRLANSGQLLTTSQNALALMADGEDANLQSQLYTAKQLVSELIGMDSKLSGVLDMLEEATIQIAEA

SDELRHYCDRLDLDPNRLFELEQRISKQISLARKHHVSPEALPQYYQSLLEEQQQLDDQADSQETLALAV

TKHHQQALETARALHQQRQQYAEELAQLITDSMHALSMPHGQFTIDVKYDEHHLGADGADRIEFRVTTNP

GQPMQPIAKVASGGELSRIALAIQVITARKMETPALIFDEVDVGISGPTAAVVGKLLRQLGESTQVMCVT

HLPQVAGCGHQHYFVSKETDGAMTETHMQSLDKKARLQELARLLGGSEVTRNTLANAKELLAA

>gi|30064013|ref|NP_838184.1| inorganic polyphosphate/ATP-NAD kinase [Shigella flexneri 2a str. 2457T]

MNNHFKCIGIVGHPRHPTALTTHEMLYRWLCTKGYEVIVEQQIAHELQLKNVKTGTLAEIGQLADLAVVV

GGDGNMLGAARTLARYDIKVIGINRGNLGFLTDLDPDNAQQQLADVLEGHYISEKRFLLEAQVCQQDCQK

RISTAINEVVLHPGKVAHMIEFEVYIDEIFAFSQRSDGLIISTPTGSTAYSLSAGGPILTPSLDAITLVP

MFPHTLSARPLVINSSSTIRLRFSHRRNDLEISCDSQIALPIQEGEDVLIRRCDYHLNLIHPKDYSYFNT

LSTKLGWSKKLF

>gi|30064012|ref|NP_838183.1| heat shock protein GrpE [Shigella flexneri 2a str. 2457T]

MSSKEQKTPEGQAPEEIIMDQHEEIEAVEPEASAEQVDPRDEKIANLEAQLAEAQTRERDGILRVKAEME

NLRRRTELDIEKAHKFALEKFINELLPVIDSLDRALEVADKANPDMSAMVEGIELTLKSMLDVVRKFGVE

VIAETNVPLDPNVHQAIAMVESDDVAPGNVLGIMQKGYTLNGRTIRAAMVTVAKAKA

>gi|30064011|ref|NP_838182.1| transporter, partial [Shigella flexneri 2a str. 2457T]

MTVDDIMVPRSEIIGIDINDDWKSILRQLSHSPHGRIVLYRDSLDDAISMLRVREAWRLMSEKKEFTKET

MLRAADEIYFVPEGTPLSTQLVKFQRNKKKVGLVVNEYGDIQGLVTVEDILEEIVGDFTTSMSPTLAEEV

TPQNDGSVIIDGTANVREINKAFNWHLPEDDARTVNGVILEALEEIPVAGTRVRIGKYDIDILDVQDNMI

KQVKVFPVKPLRESVAE

>gi|30064010|ref|NP_838181.1| hypothetical protein S2848 [Shigella flexneri 2a str. 2457T]

MQRLEQRSPDAILLLFLIAQTVDITMPVFALLALVAYSVSLALIVPGLLQKNGGWRRMAIISAVIALVCH

AIALEARILPDGDSGQNLSLLNVGSLVSLVICTVMTIVASRNRGWLLLPIVYAFALINLALATFMPNEYI

THLEATPGMLMHIGLSLFSYATLIIAALYALQLAWIDYQLKNKKLAFNQEMPPLMSIERKMFHITQIGVV

LLTLTLCTGLFYMHNLFSMENIDKAVLSIVAWFVYIVLLWGHYHEGWRGRRVVWFNVAGAVILTLAYFGS

RIVQQLIS

>gi|30064009|ref|NP_838180.1| signal recognition particle protein [Shigella flexneri 2a str. 2457T]

MFDNLTDRLSRTLRNISGRGRLTEDNVKDTLREVRMALLEADVALPVVREFINRVKEKAVGHEVNKSLTP

GQEFVKIVRNELVAAMGEENQTLNLAAQPPAVVLMAGLQGAGKTTSVGKLGNFLREKHKKKVLVVSADVY

RPAAIKQLETLAEQVGVDFFPSDVGQKPVDIVNAALKEAKLKFYDVLLVDTAGRLHVDEAMMDEIKQVHA

SINPVETLFVVDAMTGQDAANTAKAFNEALPLTGVVLTKVDGDARGGAALSIRHITGKPIKFLGVGEKTE

ALEPFHPDRIASRILGMGDVLSLIEDIESKVDRAQAEKLASKLKKGDGFDLNDFLEQLRQMKNMGGMASL

MGKLPGMGQIPDNVKSQMDDKVLVRMEAIINSMTMKERAKPEIIKGSRKRRIAAGCGMQVQDVNRLLKQF

DDMQRMMKKMKKGGMAKMMRSMKGMMPPGFPGR

>gi|30064008|ref|NP_838179.1| 30S ribosomal protein S16 [Shigella flexneri 2a str. 2457T]

MVTIRLARHGAKKRPFYQVVVADSRNARNGRFIERVGFFNPIASEKEEGTRLDLDRIAHWVGQGATISDR

VAALIKEVNKAA

>gi|30064007|ref|NP_838178.1| 16S rRNA-processing protein RimM [Shigella flexneri 2a str. 2457T]

MSKQLTAQAPVDPIVLGKMGSSYGIRGWLRVFSSTEDAESIFDYQPWFIQKAGQWQQVQLESWKHHNQDM

IIKLKGVDDRDAANLLTNCEIVVDSSQLPQLEEGDYYWKDLMGCQVVTTEGYDLGKVVDMMETGSNDVLV

IKANLKDAFGIKERLVPFLDGQVIKKVDLTTRSIEVDWDPGF

>gi|30064006|ref|NP_838177.1| tRNA (guanine-N(1)-)-methyltransferase [Shigella flexneri 2a str. 2457T]

MWIGIISLFPEMFRAITDYGVTGRAVKNGLLSIQSWSPRDFTHDRHRTVDDRPYGGGPGMLMMVQPLRDA

IHAAKAAAGEGAKVIYLSPQGRKLDQAGVSELATNQKLILVCGRYEGIDERVIQTEIDEEWSIGDYVLSG

GELPAMTLIDSVSRFIPGVLGHEASATEDSFAEGLLDCPHYTRPEVLEGMEVPPVLLSGNHAEIRRWRLK

QSLGRTWLRRPELLENLALTEEQARLLAEFKTEHAQQQHKHDGMA

>gi|30064005|ref|NP_838176.1| 50S ribosomal protein L19 [Shigella flexneri 2a str. 2457T]

MSNIIKQLEQEQMKQDVPSFRPGDTVEVKVWVVEGSKKRLQAFEGVVIAIRNRGLHSAFTVRKISNGEGV

ERVFQTHSPVVDSISVKRRGAVRKAKLYYLRERTGKAARIKERLN

>gi|30064004|ref|NP_838175.1| outer membrane lipoprotein [Shigella flexneri 2a str. 2457T]

MIKHLVAPLIFTSLILTGCQSPQGKFTPEQVAAMQSYGFTESAGDWSLGLSDAILFAKNDYKLLPESQQQ

IQTMAAKLASTGLTHARMDGHTDNYGEDSYNEGLSLKRANVVADAWAIGGQIPRSNLTTQGLGKKYPIAS

NKTAQGRAENRRVAVVITTP

>gi|30064003|ref|NP_838174.1| hypothetical protein S2840 [Shigella flexneri 2a str. 2457T]

MRFSHRLFLLLILLLTGAPILAQEPSDVAKNVRMMVSGIVSYTRWPALSGPPKLCIFSSSRFSTALQENA

ATSLPYLPVIIHTQQEAMISGCNGFYFGNESPTFQMELTEQYPSKALLLIAEQNTECIIGSAFCLIIHNN

DVRFAVNLDASSRSGVKVNPDVLMLARKKNDG

>gi|30064002|ref|NP_838173.1| hypothetical protein S2839 [Shigella flexneri 2a str. 2457T]

MMKKFIAPLLALLVSGCQIDPYTHAPTLTSTDWYDVGMEDAISGSAIKDDDAFSDSQADRGLYLKGYAEG

QKKTCQTDFTYARGLSGKSFPASCNNVENASQLHEVWQKRADENASTIRLN

>gi|30064001|ref|NP_838172.1| phospho-2-dehydro-3-deoxyheptonate aldolase [Shigella flexneri 2a str. 2457T]

MHKDALNNVHITDEQVLMTPEQLKAAFPLSLQQEAQIADSRKTISDIIAGRDPRLLVVCGPCSIHDPETA

LEYARRFKALAAEVSDSLYLVMRVYFEKPRTTVGWKGLINDPHMDGSFDVEAGLQIARKLLLELVNMGLP

LATEALDPNSPQYLGDLFSWSAIGARTTESQTHREMASGLSMPVGFKNGTDGSLATAINAMRAAAQPHCF

VGINQAGQVALLQTQGNPDGHVILRGGKAPNYSPADVAQCEKEMEQAGLRPSLMVDCSHGNSNKDYRRQP

AVAESVVAQIKDGNRSIIGLMIESNIHEGNQSSEQPRSEMKYGVSVTDACISWEMTDALLREIHQDLNGQ

LTARVA

>gi|30064000|ref|NP_838171.1| bifunctional chorismate mutase/prephenate dehydrogenase [Shigella flexneri 2a str. 2457T]

MVAELTALRDQIDEVDKALLNLLAKRLELVAEVGKVKSRFGLPIYVPEREASMLASRRAEAEALGVPPDL

IEDVLRRVMRESYSSENDKGFKTLCPSLRPVVIVGGGGQMGRLFEKMLTLSGYQVRILEQHDWDRAADIV

ADAGMVIVSVPIHVTEQVIGKLPPLPKDCILVDLASVKNGPLQAMLAAHDGPVLGLHPMFGPDSGSLAKQ

VVVWCDGRKPEAYQWFLEQIQVWGARLHRISAVEHDQNMAFIQALRHFATFAYGLHLAEENVQLEQLLAL

SSPIYRLELAMVGRLFAQDPQLYADIIMSSERNLALIKRYYKRFGEAIELLEQGDKQAFIDSFRKVEHWF

GDYAQRFQSESRVLLRQANDNRQ

>gi|30063999|ref|NP_838170.1| bifunctional chorismate mutase/prephenate dehydratase [Shigella flexneri 2a str. 2457T]

MTSENPLLALREKISALDEKLLALLAERRELAVEVGKAKLLSHRPVRDIDRERDLLERLITLGKAHHLDA

HYITRLFQLIIEDSVLTQQALLQQHLNKINPHSARIAFLGPKGSYSHLAARQYAARHFEQFIESGCAKFA

DIFNQVETGQADYAVVPIENTSSGAINDVYDLLQHTSLSIVGEMTLTIDHCLLVSGTTDLSTINTVYSHP

QPFQQCSKFLNRYPHWKIEYTESTSAAMEKVAQAKSPHVAALGSEAGGTLYGLQVLERIEANQRQNFTRF

VVLARKAINVSDQVPAKTTLLMATGQQAGALVEALLVLRNHNLIMTRLESRPIHGNPWEEMFYLDIQANL

ESAEMQKALKELGEITRSMKVLGCYPSENVVPVDPT

>gi|30063997|ref|NP_838168.1| translation inhibitor protein RaiA [Shigella flexneri 2a str. 2457T]

MTMNITSKQMEITPAIRQHVADRLAKLEKWQTHLINPHIILSKEPQGFVADATINTPNGVLVASGKHEDM

YTAINELINKLERQLNKLQHKGEARRAATSVKDANFVEEVEEE

>gi|30063996|ref|NP_838167.1| hypothetical protein S2834 [Shigella flexneri 2a str. 2457T]

MSCRFFILLVVKLKRFSHYRSHQIWLALRYSSSKKTPLPAISHKKDSLTKSDKIMRFPSHILTSGTVC

>gi|30063995|ref|NP_838166.1| IS4 orf [Shigella flexneri 2a str. 2457T]

MHIGQALDLVSRYDSLRNPLTSLGDYLDPELISRCLAESGTVTLRKRRLPLEMMVWCIVGMALERKEPLH

QIVNRLDIMLPGNRPFVAPVPLFRPASAWEVRLSAACSRKQRSSGITPRRIRTGAA

>gi|30063994|ref|NP_838165.1| IS4 orf, partial [Shigella flexneri 2a str. 2457T]

MFTKTAQLWHNATPHPHWCGLTLLAIDGVFWRTPDTPENDAAFPRQTHAGNPALYPQVKMVCQMELTSHL

LTAAAFGTMKNSENELAEQLIEQTGDNTLTLMDKGYYSLGLLNGWSLAGEHRHWMIPLRKGAQYEELRKL

GKGDHLVKLKTSPQARKKWPGLGNEVTARLLTVTRKGKVCHLLTSMTDAMRFPGGEMADLYSHRWEIELG

YREIKQTMQLSRLTLRSKKPELVEQELWGVLLAYNLVRYQMIKMAEHLKGYWPNQLSFSESCGMVMRMLM

TLQGASPGRIPELMRDLASMGQLVKLPTRRGRAFPRVVKERPWKYPTAPKKSQSVA

>gi|30063993|ref|NP_838164.1| protein disaggregation chaperone [Shigella flexneri 2a str. 2457T]

MRLDRLTNKFQLALADAQSLALGHDNQFIEPLHLMSALLNQEGGSVSPLLTSAGINAGQLRTDINQALNR

LPQVEGTGGDVQPSQDLVRVLNLCDKLAQKRGDNFISSELFVLAALESRGTLADILKATGATTANITQAI

EQMRGGESVNDQGAEDQRQALKKYTIDLTERAEQGKLDPVIGRDEEIRRTIQVLQRRTKNNPVLIGEPGV

GKTAIVEGLAQRIINGEVPEGLKGRRVLALDMGALVAGAKYRGEFEERLKGVLNDLAKQEGNVILFIDEL

HTMVGAGKADGAMDAGNMLKPALARGELHCVGATTLDEYRQYIEKDAALERRFQKVFVAEPSVEDTIAIL

RGLKERYELHHHVQITDPAIVAAATLSHRYIADRQLPDKAIDLIDEAASSIRMQIDSKPEELDRLDRRII

QLKLEQQALMKESDEASKKRLDMLNEELSDKERQYSELEEEWKAEKASLSGTQTIKAELEQAKIAIEQAR

RVGDLARMSELQYGKIPELEKQLEAATQLEGKTMRLLRNKVTDAEIAEVLARWTGIPVSRMMESEREKLL

RMEQELHHRVIGQNEAVDAVSNAIRRSRAGLADPNRPIGSFLFLGPTGVGKTELCKALANFMFDSDEAMV

RIDMSEFMEKHSVSRLVGAPPGYVGYEEGGYLTEAVRRRPYSVILLDEVEKAHPDVFNILLQVLDDGRLT

DGQGRTVDFRNTVVIMTSNLGSDLIQERFGELDYAHMKELVLGVVSHNFRPEFINRIDEVVVFHPLGEQH

IASIAQIQLKRLYKRLEERGYEIHISDEALKLLSENGYDPVYGARPLKRAIQQQIENPLAQQILSGELVP

GKVIRLEVNEDRIVAVQ

>gi|30063992|ref|NP_838163.1| hypothetical protein S2830 [Shigella flexneri 2a str. 2457T]

MSKLIVPQWPLPKGVAACSSTRIGGVSLPPYDSLNLGAHCGDNPDHVEENRKRLFAAGNLPSKPVWLEQV

HGKDVLKLTGEPYASKRADASYSNTPGTVCAVMTADCLPVLFCNRAGTEVAAVHAGWRGLCAGVLEETVS

CFADKPENILAWLGPAIGPRAFEVGAEVREAFMAVDAKASAAFIQHGDKYLADIYQLARQRLANVGVEQI

FGGDRCTYTENETFFSYRRDKTTGRMASFIWLI

>gi|30063991|ref|NP_838162.1| 23S rRNA pseudouridine synthase D [Shigella flexneri 2a str. 2457T]

MAQRVQLTATVSENQLGQRLDQALAEMFPDYSRSRIKEWILDQRVLVNGKVCDKPKEKVLGGEQVAINAE

IEEEARFEPQDIPLDIVYEDEDIIVINKPRDLVVHPGAGNPDGTVLNALLHYYPPIADVPRAGIVHRLDK

DTTGLMVVAKTVPAQTRLVESLQRREITREYEAVAIGHMTAGGTVDEPISRHPTKRTHMAVHPMGKPAVT

HYRIMEHFRVHTRLRLRLETGRTHQIRVHMAHITHPLVGDPVYGGRPRPPKGASEAFISTLRKFDRQALH

ATMLRLYHPISGIEMEWHAPIPQDMVELIEVMRADFEEHKDEVDWL

>gi|30063990|ref|NP_838161.1| outer membrane protein assembly complex subunit YfiO [Shigella flexneri 2a str. 2457T]

MTRMKYLVAAATLSLFLAGCSGSKEEVPDNPPNEIYATAQQKLQDGNWRQAITQLEALDNRYPFGPYSQQ

VQLDLIYAYYKNADLPLAQAAIDRFIRLNPTHPNIDYVMYMRGLTNMALDDSALQGFFGVDRSDRDPQHA

RAAFSDFSKLVRGYPNSQYTTDATKRLVFLKDRLAKYEYSVAEYYTERGAWVAVVNRIEGMLRDYPDTQA

TRDALPLMENAYRQMQMNAQAEKVAKIIAANSSNT

>gi|30063989|ref|NP_838160.1| IS4 orf [Shigella flexneri 2a str. 2457T]

MHIGQALDLVSRYDSLRNPLTSLGDYLDPELISRCLAESGTVTLRKRRLPLEMMVWCIVGMALERKEPLH

QIVNRLDIMLPGNRPFVAPSAVIQARQRLGSEAVRRVFTKTAQLWHNATPHPHWCGLTLLAIDGVFWRTP

DTPENDAAFPRQTHAGNPALYPQVKMVCQMELTSHLLTAAAFGTMKNSENELAEQLIEQTGDNTLTLMDK

GYYSLGLLNGWSLAGEHRHWMIPLRKGAQYEELRKLGKGDHLVKLKTSPQARKKWPGLGNEVTARLLTVT

RKGKVCHLLTSMTDAMRFPGGEMGDLYSNRWEIELGYREIKQTMQLSRLTLRSKKPELVEQELWGVLLAY

NLVRYQMIKMAEHLKGYWPNQLSFSESCGMVMRMLMTLQGASPGRIPELMRDLASMGQLVKLPTRRGRAF

PRVVKERPWKYPTAPKKSQSVA

>gi|30063988|ref|NP_838159.1| alpha-ketoglutarate transporter [Shigella flexneri 2a str. 2457T]

MAESTVTADSKLTSSDTRRRIWAIVGASSGNLVEWFDFYVYSFCSLYFAHIFFPSGNTTTQLLQTAGVFA

AGFLMRPIGGWLFGRIADKHGRKKSMLLSVCMMCFGSLVIACLPGYETIGTWAPALLLLARLFQGLSVGG

EYGTSATYMSEVAVEGRKGFYASFQYVTLIGGQLLALLVVVVLQHTMEDAALREWGWRIPFALGAVLAVV

ALWLRRQLDETSQQETRALKEAGSLKGLWRNRRAFIMVLGFTAAGSLCFYTFTTYMQKYLVNTAGMHANV

ASGIMTAALFVFMLIQPLIGALSDKIGRRTSMLCFGSLAAIFTVPILSALQNVSSPYAAFGLVMCALLIV

SFYTSISGILKAEMFPAQVRALGVGLSYAVANAIFGGSAEYVALSLKSIGMETAFFWYVTLMAVVAFLVS

LMLHRKGKGMRL

>gi|30063987|ref|NP_838158.1| hypothetical protein S2821 [Shigella flexneri 2a str. 2457T]

MDNDSWSGQDKAQHFIASAILSAAGNEYSQHQGMSRDRSAMFGLMFSVSLGASKELWDSRPEGSGWSWKD

LAWDVAGASTGYTVWQLTRH

>gi|30063986|ref|NP_838157.1| phosphatidylserine synthase [Shigella flexneri 2a str. 2457T]

MLSKFKRNKHQQHLAQLPKISQSVDDVDFFYAPADFRETLLEKIASAKQRICIVALYLEQDDGGKGILNA

LYEAKRQRPELDVRVLVDWHRAQRGRIGAAASNTNADWYCRMAQENPGVDVPVYGVPINTREALGVLHFK

GFIIDDSVLYSGASLNDVYLHQHDKYRYDRYHLIRNRKMSDIMFEWVTQNIMNGRGVNRLDDVNRPKSPE

IKNDIRLFRQELRDAAYHFQGDADNDQLSVTPLVGLGKSSLLNKTIFHLMPCAEQKLTICTPYFNLPAIL

VRNIIQLLREGKKVEIIVGDKTANDFYIPEDEPFKIIGALPYLYEINLRRFLSRLQYYVNTDQLVVRLWK

DDDNTYHLKGMWVDDKWMLITGNNLNPRAWRLDLENAILIHDPQLELAPQREKELELIRERTTIVKHYRD

LQSIADYPVKVRKLIRRLRRIRIDRLISRIL

>gi|30063985|ref|NP_838156.1| hypothetical protein S2819 [Shigella flexneri 2a str. 2457T]

MSQRGLEALLRPKSIAVIGASMKPNRAGYLMMRNLLAGGFNGPVLPVTPAWKAVLGVLAWPDIASLPFTP

DLAVLCTNASRNLALLEELGEKGCKTCIILSAPASQHEDLRACALRHNMRLLGPNSLGLLAPWQGLNASF

SPVPIKRGKLAFISQSAAVSNTILDWAQQREMGFSYFIALGDSLDIDVDELLDYLARDSKTSAILLYLEQ

LSDARRFVSAARSASRNKPILVIKSGRSPAAQRLLNTTAGMDPAWDAAIQRAGLLRVQDTHELFSAVETL

SHMRPLRGDRLMIISNGAAPAALALDALWSRNGKLATLSEETCQKLRDALPEHVAVSNPLDLRDDASSEH

YVKTLDILLHSQDFDALMVIHSPSAAAPATESAQVLIEAVKHHPRSKYVSLLTNWCGEHSSQEARRLFSE

AGLPTYRTPEGTITAFMHMVEYRRNQKQLRETPALPSNLTSNTAEAHLLLQQAIAEGATSLDTHEVQPIL

QAYGMNTLPTWIASDSTEAVHIAEQIGYPVALKLRSPDIPHKSEVQGVMLYLRTANEVQQAANAIFDRVK

MAWPQARVHGLLVQSMANRAGAQELRVVVEHDPVFGPLIMLGEGGVEWRPEDQAVVALPPLNMNLARYLV

IQGIKSKKIRARSALRPLDVAGLSQLLVQVSNLIVDCPEIQRLDIHPLLASGSEFTALDVTLDISPFEGD

NESRLAVRPYPHQLEEWVELKNGERCLFRPILPEDEPQLQQFISRVTKEDLYYRYFSEINEFTHEDLANM

TQIDYDREMAFVAVRRIDQTEEILGVTRAISDPDNIDAEFAVLVRSDLKGLGLGRRLMEKLITYTRNHGL

QRLNGITMPNNRGMVALARKLGFNVDIQLEEGIVGLTLNLARREES

>gi|30063984|ref|NP_838155.1| hypothetical protein S2818 [Shigella flexneri 2a str. 2457T]

MAFFRRSLMTENAVLQLRAERIARATRPFLARGNRVRRCQRCLLPEKLCLCSTITPAQAKSRFCLLMFDT

EPMKPSNTGRLIADILPDTVAFQWSRTEPSQDLLDLVQNPDYQPMVVFPASYADEQREVIFTPPAGKPPL

FIMLDGTWPEARKMFRKSPYLDNLPVISVDLSRLSAYRLREAQAAGQYCTAEVAIALLDMAGDTGAAAGL

GEHFTRFKTRYLAGKTQHLGSITAEQLESV

>gi|30063983|ref|NP_838154.1| thioredoxin 2 [Shigella flexneri 2a str. 2457T]

MNTVCTHCQAINRIPDDRIEDAAKCGRCGHDLFDGEVINATGETLDKLLKDDLPVVIDFWAPWCGPCRNF

APIFEDVAQERSGKVRFVKVNTEAERELSSRFGIRSIPTIMIFKNGQVVDMLNGAVPKAPFDSWLNESL

>gi|30063982|ref|NP_838153.1| methyltransferase [Shigella flexneri 2a str. 2457T]

MNDEMKGKSGKVKVMYVRSDDDSDKRTHNPRTGKGGGRPGKSRADGGRRPARDDKQSQPRDRKWEDSPWR

TVSRAPGDETPEKADHGGISGKSFIDPEVLRRQRAEETRVYGENACQALFQSRPEAIVRAWFIQSVTPRF

KEALRWMAANRKAYHVVDEAELTKASGTEHHGGVCFLIKKRNGTTVQQWVIQAGAQDCVLALENESNPHN

LGGMMRSCAHFGVKGVVVQDAALLESGAAIRTAEGGAEHVQPITGDNIVNVLDDFRQAGYTVVTTSSEQG

KPLFKTSLPAKMVLVLGQEYEGLPDAARDPNDLRVKIDGTGNVAGLNISVATGVLLGEWWRQNKA

>gi|30063981|ref|NP_838152.1| uracil-DNA glycosylase [Shigella flexneri 2a str. 2457T]

MANELTWHDVLAEEKQQPYFLNTLQTVASERQSGVTIYPPQKDVFNAFRFTELGDVKVVILGQDPYHGPG

QAHGLAFSVRPGIAIPPSLLNMYKELENTIPGFTRPNHGYLESWARQGVLLLNTVLTVRAGQAHSHASLG

WETFTDKVISLINQHREGVVFLLWGSHAQKKGAIIDKQRHHVLKAPHPSPLSAHRGFFGCNHFVLANQWL

EQRGETPIDWMPVLPAECE

>gi|30063980|ref|NP_838151.1| autonomous glycyl radical cofactor GrcA [Shigella flexneri 2a str. 2457T]

MITGIQITKAANADLLNSFWLLDSEKGEARCIVAKAGYAEDEVVAVSKLGDIEYREVPVEVKPEVRVEGG

QHLNVNVLRRETLEDAVKHPEKYPQLTIRVSGYAVRFNSLTPEQQRDVIARTFTESL

>gi|30063979|ref|NP_838150.1| neutral amino-acid efflux protein [Shigella flexneri 2a str. 2457T]

MTPILLSAFWTYTLITAMTPGPNNILALSSATSHGFRQSTRVLAGMSLGFLIVMLLCAGISFSLAVIDPA

AVHLLSWAGAAYIVWLAWKIATSPTKEDGLQAKPISFWASFALQFVNVKIILYGVTALSTFVLPQTQALS

WVVGVSVLLAMIGTFGNVCWALAGHLFQRLFRQYGRQLNIVLALLLVYCAVRIFY

>gi|30063978|ref|NP_838149.1| LYSR-type transcriptional regulator [Shigella flexneri 2a str. 2457T]

MFLYSKFDIFDRFGVMDLRRFITLKTVVEEGSFLRASQKLCCTQSTVTFHIQQLEQEFSVQLFEKIGRRM

CLTREGKKLLPHIYELTRVMDTLREAAKKESDPDGELRVVSGETLLSYRMPQVLQRFRQRAPKVRLSLQA

LNCYVIRDALLNDEADVGVFYRVGNDDALNRRELGEQSLVLVASPQIADVDFTEPGRHNACSFIINEPQC

VFRQIFESTLRQRRITVENTIELISIESIKRCVAANIGVSYLPHFAVEKEIESGELIELPFGEQSQTITA

MCAHYAGKAVSPAMHIFIQCVEECFVVA

>gi|30063977|ref|NP_838148.1| ATP-dependent RNA helicase SrmB [Shigella flexneri 2a str. 2457T]

MTVTTFSELELDESLLEALQDKGFTRPTAIQAAAIPPALDGRDVLGSAPTGTGKTAAYLLPALQHLLDFP

RKKSGPPRILILTPTRELAMQVADHARELAKHTHLDIATITGGVAYMNHAEVFSENQDIVVATTGRLLQY

IKEENFDCRAVETLILDEADRMLDMGFAQDIEHIAGETRWRKQTLLFSATLEGDAIQDFAERLLEDPVEV

SANPSTRERKKIHQWYYRADDLEHKTALLVHLLKQPEATRSIVFVRKRERVHELANWLREAGINNCYLEG

EMVQGKRNEAIKRLTEGRVNVLVATDVAARGIDIPDVSHVFNFDMPRSGDTYLHRIGRTARAGRKGTAIS

LVEAHDHLLLGKVGRYIEEPIKARVIDELRPKTRAPSEKQTGKPSKKVLAKRAEKKKAKEKEKPRVKKRH

RDTKNIGKRRKPSGTGVPPQTTEE

>gi|30063976|ref|NP_838147.1| enzyme [Shigella flexneri 2a str. 2457T]

MWGADYRRYGCNLHLFVWISLLGYCSHPVLSPFFLQGFIFMSQSTSVLRRNGFTFKQFFVAHDRCAMKVG

TDGILLGAWAPVAGVKRCLDIGAGSGLLALMLAQRTSDSVIIDAVELESEAAAQAQENINQSPWAERINV

HTADIQQWITQQTVRFDLIISNPPYYQQGVECATPQREQARYTTTLDHPSLLTCAAECITEEGFFCVVLP

EQIGNGFTELALSMGWHLRLRTDVAENEARLPHRVLLAFSPQAGECFSDRLVIRGPDQNYSEAYTALTQA

FYLFM

>gi|30063975|ref|NP_838146.1| RNA polymerase sigma factor RpoE [Shigella flexneri 2a str. 2457T]

MSEQLTDQVLVERVQKGDQKAFNLLVVRYQHKVASLVSRYVPSGDVPDVVQEAFIKAYRALDSFRGDSAF

YTWLYRIAVNTAKNYLVAQGRRPPSSDVDAIEAENFESGGALKEISNPENLMLSEELRQIVFRTIESLPE

DLRMAITLRELDGLSYEEIAAIMDCPVGTVRSRIFRAREAIDNKVQPLIRR

>gi|30063974|ref|NP_838145.1| anti-RNA polymerase sigma factor SigE [Shigella flexneri 2a str. 2457T]

MQKEQLSALMDGETLDSELLNELAHNPEMQKTWESYHLIRDSMRGDTPEVLHFDISSRVMAAIEEEPVRQ

SATLIPEAQPAPHQWQKMPFWQKVRPWAAQLTQMGVAACVSLAVIVGVQHYNGQSETSQQPETPVFNTLP

MMGKASPVSLGVPSEATANNGQQQQVQEQRRRINAMLQDYELQRRLHSEQLQFEQAQTQQAAVQVPGIQT

LGTQSQ

>gi|30063973|ref|NP_838144.1| periplasmic negative regulator of sigmaE [Shigella flexneri 2a str. 2457T]

MKQLWFAMSLVTGSLLFSANASATPASGALLQQMNLASQSLNYELSFISINKQGVESLRYRHARLDNRPL

AQLLQMDGPRREVVQRGNEISYFEPGLEPFTLNGDYIVDSLPSLIYTDFKRLSPYYDFISVGRTRIADRL

CEVIRVVARDGTRYSYIVWMDTESKLPMRVDLLDRDGETLEQFRVIAFNVNQDISSSMQTLAKANLPPLL

SVPVGEKAKFSWTPTWLPQGFSEVSSSRRPLPTMDNMPIESRLYSDGLFSFSVNVNRATPSSTDQMLRTG

RRTVSTSVRDNAEITIVGELPPQTAKRIAENIKFGAAQ

>gi|30063972|ref|NP_838143.1| SoxR reducing system protein RseC [Shigella flexneri 2a str. 2457T]

MIKEWATVVSWQNGQALVSCDVKASCSSCASRAGCGSRVLNKLGPQTTHTIVVPCDEPLVPGQKVELGIA

EGSLLSSALLVYMSPLVGLFLIASLFQLLFASDVAALCGAVLGGIGGFLIARGYSRKFAARAEWQPIILS

VALPPGLVRFETSSEDASQ

>gi|30063971|ref|NP_838142.1| GTP-binding protein LepA [Shigella flexneri 2a str. 2457T]

MKNIRNFSIIAHIDHGKSTLSDRIIQICGGLSDREMEAQVLDSMDLERERGITIKAQSVTLDYKASDGET

YQLNFIDTPGHVDFSYEVSRSLAACEGALLVVDAGQGVEAQTLANCYTAMEMDLEVVPVLNKIDLPAADP

ERVAEEIEDIVGIDATDAVRCSAKTGVGVQDVLERLVRDIPPPEGDPEGPLQALIIDSWFDNYLGVVSLI

RIKNGTLRKGDKVKVMSTGQTYNADRLGIFTPKQVDRTELKCGEVGWLVCAIKDIHGAPVGDTLTLARNP

AEKALPGFKKVKPQVYAGLFPVSSDDYEAFRDALGKLSLNDASLFYEPESSSALGFGFRCGFLGLLHMEI

IQERLEREYDLDLITTAPTVVYEVETTSREVIYVDSPSKLPAVNNIYELREPIAECHMLLPQAYLGNVIT

LCVEKRGVQTNMVYHGNQVALTYEIPMAEVVLDFFDRLKSTSRGYASLDYNFKRFQASDMVRVDVLINGE

RVDALALITHRDNSQNRGRELVEKMKDLIPRQQFDIAIQAAIGTHIIARSTVKQLRKNVLAKCYGGDISR

KKKLLQKQKEGKKRMKQIGNVELPQEAFLAILHVGKDNK

>gi|30063970|ref|NP_838141.1| signal peptidase I [Shigella flexneri 2a str. 2457T]

MANMFALILVIATLVTGILWCVDKFFFAPKRRERQAAAQAAAGDSLDKATLKKVAPKPGWLETGASVFPV

LAIVLIVRSFIYEPFQIPSGSMMPTLLIGDFILVEKFAYGIKDPIYQKTLIETGHPKRGDIVVFKYPEDP

KLDYIKRAVGLPGDKVTYDPVSKELTIQPGCSSGQACENALPVTYSNVEPSDFVQTFSRRNGGEATSGFF

EVPKNETKENGIRLSERKETLGDVTHRILTVPIAQDQVGMYYQQPGQQLATWIVPPGQYFMMGDNRDNSA

DSRYWGFVPEANLVGRATAIWMSFDKQEGEWPTGVRLSRIGGIH

>gi|30063969|ref|NP_838140.1| ribonuclease III [Shigella flexneri 2a str. 2457T]

MNPIVINRLQRKLGYTFNHQELLQQALTHRSASSKHNERLEFLGDSILSYVIANALYHRFPRVDEGDMSR

MRATLVRGNTLAELAREFELGECLRLGPGELKSGGFRRESILADTVEALIGGVFLDSDIQTVEKLILNWY

QTRLDEISPGDKQKDPKTRLQEYLQGRHLPLPTYLVVQVRGEAHDQEFTIHCQVSGLSEPVVGTGSSRRK

AEQAAAEQALKKLELE

>gi|30063968|ref|NP_838139.1| GTP-binding protein Era [Shigella flexneri 2a str. 2457T]

MSIDKSYCGFIAIVGRPNVGKSTLLNKLLGQKISITSRKAQTTRHRIVGIHTEGAYQAIYVDTPGLHMEE

KRAINRLMNKAASSSIGDVELVIFVVEGTRWTPDDEMVLNKLREGKAPVILAVNKVDNVQEKADLLPHLQ

FLASQMNFLDIVPISAETGLNVDTIAAIVRKHLPEATHHFPEDYITDRSQRFMASEIIREKLMRFLGAEL

PYSVTVEIERFVSNERGGCDINGLILVEREGQKKMVIGNKGAKIKTIGIEARKDMQEMFEAPVHLELWVK

VKSGWADDERALRSLGYVDDL

>gi|30063967|ref|NP_838138.1| DNA repair protein RecO [Shigella flexneri 2a str. 2457T]

MEGWQRAFVLHSRPWSETSLMLDVFTEESGRVRLVAKGARSKRSTLKGALQPFTPLLLRFGGRGEVKTLR

SAEAVSLALPLSGITLYSGLYINELLSRVLEYETRFSELFFDYLHCIQSLAGDTGTPEPALRRFELALLG

HLGYGVNFTHCAGSGEPVDGTMTYRYREEKGFIASVVIDNKTFTGRQLKALNAREFPDADTLRAAKRFTR

MALKPYLGGKPLKSRELFRQFMPKRTVKTHYE

>gi|30063966|ref|NP_838137.1| pyridoxine 5'-phosphate synthase [Shigella flexneri 2a str. 2457T]

MAELLLGVNIDHIATLRNARGTAYPDPVQAAFIAEQAGADGITVHLREDRRHITDRDVRILRQTLDTRMN

LEMAVTEEMLAIAVETKPHFCCLVPEKRQEVTTEGGLDVAGQREKIRDACKRLADAGIQVSLFIDADEEQ

IKAAAEVGAPFIEIHTGCYADAKTDAEQAQELVRIAKAATFAASLGLKVNAGHGLTYHNVKAIAAIPEMH

ELNIGHAIIGRAVMTGLKDAVAEMKRLMLEARG

>gi|30063965|ref|NP_838136.1| 4'-phosphopantetheinyl transferase [Shigella flexneri 2a str. 2457T]

MAILGLGTDIVEIARIEAVIARSGERLARRVLSDNEWAIWKTHHQPVRFLAKRFAVKEAAAKAFGTGIRN

GLAFNQFEVFNDELGKPRLRLWGEALKLAEKLGVVNMHVTLADERHYACATVIIES

>gi|30063964|ref|NP_838135.1| hypothetical protein S2797 [Shigella flexneri 2a str. 2457T]

MSLLITKRCINCDMCEPECPNEAISMGDHIYEINSDKCTECVGHYETPTCQKVCPIPNTIVKDPAHVETE

EQLWDKFVLMHHADKI

>gi|30063963|ref|NP_838134.1| hypothetical protein S2796 [Shigella flexneri 2a str. 2457T]

MPRTVTHNPDSPNNDDVLAASEKWDACKPPYTSAHMKICVAAAKIILAASGVARCSKYEKENYLRIDFSK

AGKVTFYAEFPKKMGLKGKKLGEWPELVIQLAREKALGMADGGLRAESVHAALEMYRDDPKPK

>gi|30063962|ref|NP_838133.1| hypothetical protein S2795 [Shigella flexneri 2a str. 2457T]

MRIDRIKVTFGEREVFSDVTYNRLVEVLDEWIATRSNNNALELFAELRRFWKFCAPTLCNGRNVAASLPD

DYVSSRVQKPTPTRLFTDIESIARLWLNVAACTSVHQKNAVRFMIITGVRPINVHNLRWDYVYEEAGEIV

YPEGVIGMRGAMKTQKAFRLPITPEIRRIIDEQKAWRDSVPECNRDYVFLQPRDPMQPFSKRSLDKLVKT

YSPDGAVK

>gi|30063961|ref|NP_838132.1| IS600 orfB [Shigella flexneri 2a str. 2457T]

MAHIRTRETYGTRRLQTELAENGIIVGRDRLARLRKELRLRCKQKRKFRATTNPNHNLPVAPNLLNQTFA

PTAPNQVWVADLTYVATQEGWLYLAGIKDVYTCEIVGYAMGERMTKELTGKALFMALRSQRPPAGLIHHS

DRGSQYCAYDYRVIQEQFGLKTSMSRKGNCYDNAPMESFWGTLKNESLSHYRFNNRDEAISVIREYIEIF

YNRQRRHSRLGNISPAAFREKHHQMAA

>gi|30063960|ref|NP_838131.1| IS600 orfA [Shigella flexneri 2a str. 2457T]

MSRKTQRYSKEFKAEAVRTVLENQLSISEGASRLSLPEGTLGQWVTAARKGLGTPGSRTLAELESEILQL

RKALNEARLERDILKKATAYFAQESLKNTR

>gi|30063959|ref|NP_838130.1| bacteriophage protein [Shigella flexneri 2a str. 2457T]

MYGGSPADIAAAIWRKAPPGIDMNGNTTFTVADKEYDPPYPEYVISWQTLKPVSLHVSVTLKKSDYLPSD

ITRQVQQSVLDAFNGTDGGLRARVASVVSAGRYYAGIYKTDPEHIDILGLTVSRDGSSWTTAVTFGIDEI

PVLDVSDISVKLQEA

>gi|30063958|ref|NP_838129.1| hypothetical protein S2790 [Shigella flexneri 2a str. 2457T]

MQNVAATVLAQYAASPRLNALINSFNAALSPDSFISDFYGLIWNIDTAEKYGLDVWGKIVGVSRWLTVKD

DFNYLGFSESRMDTPVMDDPCPFNQAPFYNGKSDTRTVDLSDAVYRRLILMKAMSNITDCSVPDINLMLR

FMFGKKRRAYVLNNGGLRMSYVFESALSLAELAIIQSSGALPSPPGVYVSVVLKESRNEGQ

>gi|30063957|ref|NP_838128.1| tail fiber protein [Shigella flexneri 2a str. 2457T]

MKASDKPRQLAVPFASTGDKNRIPDKATQQTRESGNAAYDSGFPPVTMTAVSAGGIPPHGKDFNGLMYDI

TAAIRFAQAGGLYTYNAGFAGAIGGYAKGAILAGVATTAVWLNTTDDNLTDPEGSDSAGWVNLLEDPKRI

FLRQKNNLSDLQNKGTARDNLQVYSKEQSDQRYVHREGDKITGELKIRGVNALRIFNEAFGLIFRRSEEC

LHLIPTSEGQGENGDIGPLRPFTINLRTGEISMSHKVSVGGGSQVNGALGIGVQNALGGNSIVLGDNDTG

FKQNGDGILDVYANSQRVFRWICPYISRHLLSLNPLQARCRRYSRGER

>gi|30063954|ref|NP_838125.1| tail fiber protein [Shigella flexneri 2a str. 2457T]

MAISDTGRNSADNVDGVYYRPLQKLINGTWYNVASI

>gi|30063953|ref|NP_838124.1| tail fiber assembly protein [Shigella flexneri 2a str. 2457T]

MTKRYSVTWLFSEDGKNWYEELKNFASDTIKIAYTGDGRVVWVGKDVTGIEPRNASVIEVPDITANRRIT

APGYWFYRNDEFVFDYRLKAEDERDALLAQVSARTGEWEEDLLLGLISDEDKEKLKACRIYAKSLQAMDF

STITDKSSYNAIEWPASPEGSS

>gi|30063952|ref|NP_838123.1| tail fiber protein [Shigella flexneri 2a str. 2457T]

MSVVISGALTDGAGIPMSGYHIILKSRVNTPEVVMNTVADVMTGNDGEYCFHARTGKYGVYLKQDWRNEY

NVGDIAVYEDSKPGTLNDFLIAPDEGDLKPDVVKRFEEMVAQAQQSAGAAAGNAQQTAQDVAAAAGYARA

AEQAKNDIDAALTGTLKTANHLSEIAAAGEKAQQKSRDNLGLKSAPTMEAQSDIYDRTKGRLAIPGAFGF

GCAFLPEDVIRFDTKSDFLAWVRNALPGEYSVAGPYGIIIPDTRFEGVLSIRWTDARPETTEPRYRAKSL

TFYGINGPIYHTRYCYWPISRLTGWVKINITTEDIIYRIVASSVRNRWGDPDIGGLIIAAYQGEADGDKV

IRLVRGQSYRGSRLGPVGISVPSTPTGTYIVSPQFFITGCSEHSLPGSYCALSGVPDAHVSGAMPGLFIR

TSRGMHRGN

>gi|30063951|ref|NP_838122.1| hypothetical protein S2783 [Shigella flexneri 2a str. 2457T]

MTFFFDDRDQAVPYTATADDVAPTGQQIWQELQSGKWGEIAPFTVTPEMLEAAREARRQEIEAWRTEQEA

KPFTFEWNGRIWNAGPDSLGRLSPVVMLAKSVTAQTHMAWSDADNQQVKLSMPELEELAESQKTALLTEA

ESVIRQPGRAVRLNRETDESGEARGRASVFPESVRSVVYAM

>gi|30063950|ref|NP_838121.1| invasion plasmid antigen [Shigella flexneri 2a str. 2457T]

MREINMLRNISSCLFPHISTITSPNHYLSEWDDWEKQGLPEEQRTEAVRRLRACLTSKGHKLDLRALALS

SLPVLPACIKKLDVSCNKLTILTDLPENIKELIARDNFLTHISALPHYLITLDVSENQLENLPLLPDTIK

SLSAEYNRLSTLPSLPLNLKKLEVRNNELQTLPSLPSNLKILKVAHNHLTELPPLPRRLQLLFAYSNRLS

NLPNIQENIIMRRFFYFENNQITTIPTNLFRLDPHITIEIANNPLSDQTLLFLIQQTSVPNFNGPQFRIS

LSDQNRLFLRQMLPQNLHSRHIRVITEGGQNFQIPPLPETVAAWFPEADRREVSTQWTSFSTEENSRAFS

AFLDRLSDTVSARNTSGFREQVAAWLEKLSASAELRQQSFTVAADATESCEDRVALTWNNLRKTLLVHQA

SEGLFDNDTGALLSLGREMFRLEILEDIARDKVRTLHFVDEIEVYLAFQTMLAEKLQLSTAVKEMRFYGV

SGVTANDLRTAEAMVRSREENEFTDWFSLWGPWHAVLKRTEADRWAQAEEQKYEMLENEYPQRVADRLKA

SGLSGDADAEREAGAQVMRETEQQIYRQLTDEVLALRLPENGSQLHHS

>gi|30063949|ref|NP_838120.1| DNA-invertase [Shigella flexneri 2a str. 2457T]

MLIGYVRVSTNDQNTDLQRNALNCAGCELIFEDKISGTKSERPGLKKLLKTLSAGDTLVVWKLDRLGRSM

RHLVVLVEALRERGINFRSLTDSIDTSTPMGRFFFHVMGALAEMERELIVERTKAGLEAARAQGRIGGRR

PKLSPE

>gi|30063946|ref|NP_838117.1| tRNA-specific adenosine deaminase [Shigella flexneri 2a str. 2457T]

MRRAFITGVFFLSEVEFSHEYWMRHALTLAKRAWDEREVPVGAVLVHNNRVIGEGWNRPIGRHDPTAHAE

IMALRQGGLVMQNYRLIDATLYVTLEPCVMCAGAMIHSRIGRVVFGARDAKTGAAGSLMDVLHHPGMNHR

VEITEGILADECAALLSDFFRMRRQEIKAQKKAQSSTD

>gi|30063945|ref|NP_838116.1| transglycosylase [Shigella flexneri 2a str. 2457T]

MSTIHTPLTYNEINGKPFGLDYELAKQFADYLGVKLKVTVRQNISQLFDDLDNGNADLLAAGLVYNSERV

KNYQPGPTYYSVSQQLVYKVGQYRPRTLGNLTAEQLTVAPGHVVVNDLQTLKETKFPELSWKVDDKKGSA

ELMEDVIEGKLDYTIADSVAISLFQRVHPELAVALDITDEQPVTWFSPLDGDNTLSAALLDFFNEMNEDG

TLARIEEKYLGHGDDFDYVDTRTFLRAVDAVLPQLKPLFEKYAEEIDWRLLAAIAYQESHWDAQATSPTG

VRGMMMLTKNTAQSLGITDRTDAEQSISGGVRYLQDMMSKVPESVPENERIWFALAAYNMGYAHMLDARA

LTAKTKGNPDSWADVKQRLPLLSQKPYYSKLTYGYARGHEAYAYVENIRKYQISLVGYLQEKEKQATEAA

MQLAQDYPAVSPTELGKEKFPFLSFLSQSSSNYLTHSPSLLFSRKGSEEKQN

>gi|30063944|ref|NP_838115.1| phosphoribosylformylglycinamidine synthase [Shigella flexneri 2a str. 2457T]

MMEILRGSPALSAFRINKLLARFQAARLPVHNIYAEYVHFADLNAPLNDDEHAQLERLLKYGPALASHAP

QGKLLLVTPRPGTISPWSSKATDIAHNCGLQQVNRLERGVAYYIEAGTLTNEQWQQVTAELHDRMMETVF

FDLDDAEQLFAHHQPTPVTSVDLLGQGRQALIDANLRLGLALAEDEIDYLQDAFTKLGRNPNDIELYMFA

QANSEHCRHKIFNADWIIDGEQQPKSLFRMIKNTFETTPDHVLSAYKDNAAVMEGSEVGRYFADHETGRY

NFHQEPAHILMKVETHNHPTAISPWPGAATGSGGEIRDEGATGRGAKPKAGLVGFSVSNLRIPGFEQPWE

EDFGKPERIVTALDIMTEGPLGGAAFNNEFGRPALNGYFRTYEEKVNSHNGEELRGYHKPIMLAGGIGNI

RADHVQKGEINVGAKLVVLGGPAMNIGLGGGAASSMASGQSDADLDFASVQRDNPEMERRCQEVIDRCWQ

LGDANPILFIHDVGAGGLSNAMPELVSDGGRGGKFELRDILSDEPGMSPLEIWCNESQERYVLAVAADQL

PLFDELCKRERAPYAVIGEATEELHLSLHDRHFDNQPIDLPLDVLLGKTPKMTRDVQTLKAKGDALVREG

ITIADAVKRVLHLPTVAEKTFLVTIGDRSVTGMVARDQMVGPWQVPVANCAVTTASLDSYYGEAMAIGER

APVALLDFAASARLAVGEALTNIAATQIGDIKRIKLSANWMAAAGHPGEDAGLYEAVKAVGEELCPALGL

TIPVGKDSMSMKTRWQEGNEEREMTSPLSLVISAFARVEDVRHTITPQLCTEDNALLLIDLGKGNNALGA

TALAQVYRQLGDKPADVRDVVQLKGFYDAIQALVAQRKLLAYHDRSDGGLLVTLAEMAFAGHCGIDADIA

SLGDDRLAALFNEELGAVIQVRAADREAVESVLAQHGLADCVHYVGQAVSGDRFVITANGQTVFSESRTT

LRVWWAETTWQMQRLRDNPECADQEHQAKSNDADPGLNVKLSFDINEDVAAPYIATGARPKVAVLREQGV

NSHVEMAAAFQRGGFDAIDVHMSDLLTGRTGLEDFHALVACGGFSYGDVLGAGEGWAKSILFNDRVRDEF

ATFFHRPQTLALGVCNGCQMMSNLRELIPGSELWPRFVRNTSDRFEARFSLVEVTQSPSLLLQGMVGSQM

PIAVSHGEGRVEVRDAAHLAALESKGLVALRYVDNFGKVTETYPANPNGSPNGITAVTTESGRVTIMMPH

PERVFRTVSNSWHPENWGEDGPWMRIFRNARKQLG

>gi|30063943|ref|NP_838114.1| 2-component sensor protein [Shigella flexneri 2a str. 2457T]

MLYLNFPSVSIRLFASDESNTLKRWPVFPRSLRQLVMLAFLLILLPLLVLAWQAWQSLNALSDQAALVNR

TTLIDARRSEAMTNAALEMERSYRQYCVLDDPTLAKVYQSQRKRYSEMLDAHAGVLPDDKLYQALRQDLN

NLAQLQCNNSGPDAAAAARLEAFASANTEMVQATRTVVFSRGQQLQREIAERGQYFGWQSLVLFLVSLVM

VLLFTRMIIGPVKNIERMINRLGEGRSLGNSVSFCGPSELRSVGQRILWLSERLSWLESQRHQFLRHLSH

ELKTPLASMREGTELLADQVVGPLTPEQKEVVSILDSSSCNLQKLIEQLLDYNRKQADSAVELENVELAP

LVETVVSAHSLPARAKMMHTDVDLKATACLAEPMLLMSVLDNLYSNAVHYGAESGNICLRSSLHGARVYI

DVINTGTPIPQEERAMIFEPFFQGSHQRKGAVKGSGLGLSIARDCIRRMQGELYLVDESGQDVCFRIELP

SSKNTK

>gi|30063942|ref|NP_838113.1| hypothetical protein S2774 [Shigella flexneri 2a str. 2457T]

MRHIFQRLLPRRLWLAGLPCLALLGCVQNHNKPAIDTPAEEKIPVYQLADYLSTECSDIWALQGKSTETN

PLYWLRAMDCADRLMPAQSRQQARQYDDGSWQNTFKQGILLADAKITPYERRQLVARIEALSTEIPAQVR

PLYQLWRDGQALQLQLAEERQRYSKLQQSSDSELDTLRQQHHVLQQQLELTTRKLENLTDIERQLSTRKP

AGNFSPDTPHESEKPAPSTHEVTPDEP

>gi|30063941|ref|NP_838112.1| 2-component transcriptional regulator [Shigella flexneri 2a str. 2457T]

MSHKPAHLLLVDDDPGLLKLLGLRLTSEGYSVVTAESGAEGLRVLNREKVDLVISDLRMDEMDGMQLFAE

IQKVQPGMPVIILTAHGSIPDAVAATQQGVFSFLTKPVDKDALYQAIDDALEQSAPATDERWREAIVTRS

PLMLRLLEQARLVAQSDVSVLINGQSGTGKEIFAQAIHNASPRNSKPFIAINCGALPEQLLESELFGHAR

GAFTGAVSNREGLFQAAEGGTLFLDEIGDMPAPLQVKLLRVLQERKVRPLGSNRDIDINVRIISATHRDL

SKAMARGEFREDLYYRLNVVSLKIPALAERTEDIPLLANHLLRQAAERHKPFVRAFSTDAMKRLMTASWP

GNVRQLVNVIEQCVALTSSPVISDALVEQALEGENTALPTFVEARNHFELNYLRKLLQITKGNVTHAARM

AGRNRTEFYKLLSRHELDANDFKE

>gi|30063940|ref|NP_838111.1| nitrogen regulatory protein P-II 1 [Shigella flexneri 2a str. 2457T]

MKKIDAIIKPFKLDDVREALAEVGITGMTVTEVKGFGRQKGHTELYRGAEYMVDFLPKVKIEIVVPDDIV

DTCVDTIIRTAQTGKIGDGKIFVFDVARVIRIRTGEEDDAAI

>gi|30063939|ref|NP_838110.1| nitric oxide dioxygenase [Shigella flexneri 2a str. 2457T]

MLDAQTIATVKATIPLLVETGPKLTAHFYDRMFTHNPELKEIFNMSNQRNGDQREALFNAIAAYASNIEN

LPALLPAVEKIAQKHTSFQIKPEQYNIVGEHLLATLDEMFSPGQEVLDAWGKAYGVLANVFINREAEIYN

ENASKAGGWEGTRDFRIVAKTPRSALITSFELEPVDGGAVAEYRPGQYLGVWLKPEGFPHQEIRQYSLTR

KPDGKGYRIAVKREEGGQVSNWLHNHANVGDVVKLVAPAGDFFMAVADDTPVTLISTGVGQTPMLAMLDT

LAKAGHTAQVNWFHAAENGEVHAFADEVKELGQSLPRFTAHTWYRQPSEADRAKGQFDSEGLMDLSKLEG

AFSDPTMQFYLCGPVGFMQFTAKQLVDLGVKQENIHYECFGPHKVL

>gi|30063938|ref|NP_838109.1| serine hydroxymethyltransferase [Shigella flexneri 2a str. 2457T]

MLKREMNIADYDAELWQAMEQEKVRQEEHIELIASENYTSPRVMQAQGSQLTNKYAEGYPGKRYYGGCEY

VDIVEQLAIDRAKELFGADYANVQPHSGSQANFAVYTALLEPGDTVLGMNLAHGGHLTHGSPVNFSGKLY

NIVPYGIDATGHIDYADLEKQAKEHKPKMIIGGFSAYSGVVDWAKMREIADSIGAYLFVDMAHVAGLVAA

GVYPNPVPHAHVVTTTTHKTLAGPRGGLILAKGGSEELYKKLNSAVFPGGQGGPLMHVIAGKAVALKEAM

EPEFKTYQQQVAKNAKAMVEVFLERGYKVVSGGTDNHLFLVDLVDKNLTGKEADAALGRANITVNKNSVP

NDPKSPFVTSGIRVGTPAITRRGFKEAEAKELAGWMCDVLDSINDEAVIERIKGKVLDICARYPVYA

>gi|30063937|ref|NP_838108.1| NAGC-like transcriptional regulator [Shigella flexneri 2a str. 2457T]

MRACINNQQIRHHNKCVILELLYRQKRANKSTLARLAQISIPAVSNILQELESEKRVVNIDDESQTRGHS

SGTWLIAPEGDWTLCLNVTPTSIECQVANACLSPKGEFEYLQIDVPTPQALLSEIEKCWHRHRKLWPDRT

INLALAIHGQVDPVTGVSQTMPQAPWTTPVEVKYLLEEKLGIRVLVDNDCVMLALAEKWQNNSQERDFCV

INVDYGIGSSFVINEQIYRGSLYGSGQIGHTIVNPDGVVCDCGRYGSLETVASLSALKKQARVWLKSQPV

NTQLEPEKLTTAQLIAAWQSGEPWITSWVDRSANAIGLSLYNFLNILNINQIWLYGRSCAFGENWLNTII

RQTGFNPFDRDEGPSVKATQIGFGQLSRAQQVLGIGYLYVEAQLRQI

>gi|30063936|ref|NP_838107.1| ATP-binding component of a transport system [Shigella flexneri 2a str. 2457T]

MFTATEAVPVAKVVAGNKRYPGVVALDNVNFTLNKGEVRALLGKNGAGKSTLIRMLTGSERPDSGDIWIG

ETRLEGDEATLTRRAAELGVRAVYQELSLVEGLTVAENLCLGQWPRRNGMIDYLQMAQDAQRCLQALGVD

VSPEQLVSTLSPAQKQLVEIARVMKGEPRVVILDEPTSSLASAEVELVISAVKKMSALGVAVIYVSHRME

EIRRIASYATVMRDGQVAGDVMLENTSTHHIVSLMLGRDHVDIAPVAPQEIVDQAVLEVRALRHKPKLED

ISFTLRRGEVLGIAGLLGAGRSELLKAIVGLETYEQGEIVINGEKIMRPDYGDMLKRGIGYTPENRKEAG

IIPWLGVDENTVLTNRQKISTNGVLQWSTIRRLTEEVMQRMTVKAASSETPIGTLSGGNQQKVVIGRWVY

AASQILLLDEPTRGVDIEAKQQIYRIVRELAAEGKSVVFISSEVEELPLVCDRILLLQHGTFSQEFHSPV

NVDELMSAILSVH

>gi|30063935|ref|NP_838106.1| transport system permease [Shigella flexneri 2a str. 2457T]

MYASSLPLPQGKSVSLKQFVSRHINEIGLLVVIAILYLVFSLNAPGFISLNNQMNVLRDAATIGIAAWAM

TLIIISGEIDVSVGPMVAFVSVCLAFLLQFEVPLAIACLLVLLLGALMGTLAGVLRGVFNVPSFVATLGL

WSALRGMGLFMTNALPVPIDENEVLDWLGGQFLGVPVSALIMMVLFALFVFISRKTAFGRSVFAVGGNAT

AAQLCGINVRRVRILIFTLSGLLAAVTGILLAARLGSGNAGAANGLEFDVIAAVVVGGTALSGGRGSLFG

TLLGVLVITLIGNGLVLLGINSFFQQVVRGVIIVVAVLANILLTQRSSKAKR

>gi|30063934|ref|NP_838105.1| oxidoreductase [Shigella flexneri 2a str. 2457T]

MKINRIVNEGFMKTMLAAYLLGNSTVDLREVAVPTPGINQVLIKMKSSGICGSDVHYIYHQHRATAAAPD

KPLYQGFINGHEPCGQIVAMGQGCRHFKEGDRVLVYHISGCGFCPNCRRGFPISCTGKGKAAYGWQRDGG

HAEYLLAEEKDLILLPDALSYEDGAFISCGVGTAYEGILRGEVSGSDNVLVVGLGPVGMMAMMLAKGRGA

KRIIGVDMLPERLAMAKQLGGMDHGYLATTEGLPQIIAELTHGGADVALDCSGNAAGRLLALQSTADWGR

VVYIGETGKVEFEVSADLMHHQRRIIGSWVTSLFHMEKCAHDLTDWKLWPRNAITHRFSLEQAGDAYVLM

ASGKCGKVVINFPD

>gi|30063933|ref|NP_838104.1| hypothetical protein S2763 [Shigella flexneri 2a str. 2457T]

MTIYTLSHGSLKLDVSDQGGVIEGFWRDTTPLLRPGKKSGVATDASCFPLVPFANRVSGNRFVWQGREYQ

LQPNVEWDAHYLHGDGWLGEWQCVSHSDDSLCLVYEHRSGVYHYRVSQAFHLTADTLTVTLSVTNQGAET

LPFGTGWHPYFPLSPQTRIQAQASGYWLEREQWLAGEFCEQLPQELDFNQLAPLPRQWVNNGFAGWNGQA

RIEQPQEGYAIIMETTPPAPCYFIFVSDPAFDKGYAFDFFCLEPMSHAPDEHHRPEGGDLIALAPGESTT

SEMSLRVEWL

>gi|30063932|ref|NP_838103.1| hypothetical protein S2762 [Shigella flexneri 2a str. 2457T]

MPGAWRGARVCITFKSHYLAKGLVMNTLRYFDFGAARPVLLLIARIAVVLIFIIFGFPKMMGFDGTVQYM

ASLGAPMPMLAAIIAVVMEVPAAILIVLGFFTRPLAVLFIFYTLGTAVIGHHYWDMTGDAVGPNMINFWK

NVSIAGAFLLLAITGPGAISLDRR

>gi|30063931|ref|NP_838102.1| 2,3-dihydroxy-2,3-dihydrophenylpropionate dehydrogenase [Shigella flexneri 2a str. 2457T]

MSDLHNESIFITGGGSGLGLALVERFIEEGAQVATLELSAAKVASLRQRFGEHILAVEGNVTCYADYQRA

VDQILTRSGKLDCFIGNAGIWDHNASLVNTPAETLETGFHELFNVNVLGYLLGAKACAPALIASEGSMIF

TLSNAAWYPGGGGPLYTTSKHAATGLIRQLAYELAPKVRVNGVGPCGMASDLRGPQALGQSETSIMQSLT

PEKIAAILPLQFFPQPADFTGPYVMLASRRNNRALSGVMINADAGLAIRGIRHVAAGLDL

>gi|30063930|ref|NP_838101.1| 3-phenylpropionate dioxygenase ferredoxin subunit [Shigella flexneri 2a str. 2457T]

MNRIYACPVADVPEGEALRIDTSPVIALFNVGGEFYAINDRCSHGNASMSEGYLEDDATVECPLHAASFC

LKTGKALCLPATDPLTTYPVHVEGGDIFIDLPEAQP

>gi|30063929|ref|NP_838100.1| 3-phenylpropionate dioxygenase subunit beta [Shigella flexneri 2a str. 2457T]

MSAQVSLELHHRISQFLFHEASLLDDWKFRDWLAQLDEEIRYTMRTTVNAQTRDRRKGVQPPTTWIFNDT

KDQLERRIARLETGMAWAEEPPSRTRHLISNCQVSETDIPNVFAVRVNYLLYRAQKERDETFYVGTRFDK

VRRLEDDNWRLLERDIVLDQAVITSHNLSVLF

>gi|30063928|ref|NP_838099.1| large terminal subunit of phenylpropionate dioxygenase [Shigella flexneri 2a str. 2457T]

MTTPSDLNIYQLIDTQNGRVTPRIYTDPDIYQLELERIFGRCWLFLAHESQIPKPGDFFNTYMGEDAVVV

VRQKDGSIKAFLNQCRHRAMRVSYADCGNSRAFTCPYHGWSYGINGELIDVPLEPRAYPQGLCKSHWGLN

EVPCVESYKGLIFGNWDTSAPGLHDYLGDIAWYLDGMLDRREGGTEIVGGVQKWVINCNWKFPAEQFASD

QYHALFSHASAVQVLGAKDDGSDKRLGDGQTARPVWETAKDALQFGQDGHGSGFFFTEKPDANVWVDGAV

SSYYRETYAEAEQRLGEVRALRLAGHNNIFPTLSWLNGTATLRVWHPRGPDQVEVWAFCITDKAASDEVK

AAFENSATRAFGPAGFLEQDDSENWCEIQKLLKGHRARNSKLCLEMGLGQEKRRDDGIPGITNYIFSETA

ARGMYQRWADLLSSESWQEVLDKTAAYQQEVMK

>gi|30063927|ref|NP_838098.1| DNA-binding transcriptional regulator HcaR [Shigella flexneri 2a str. 2457T]

MELRHLRYFVAVAQALNFTRAAEKLHTSQPSLSSQIRDLENCVGVPLLVRDKRKVALTAAGECFLQDALA

ILEQAENAKLRARKIVQEDRQLTIGFVPSAEVNLLPKVLPMFRLRQPDTLIELVSLITTQQEEKIRRGEL

DVGLMRHPVYSPEIDYLELFDEPLVVVLPVDHPLAHEKEITAAQLDGVNFVSTDPAYSGSLAPIVKAWFA

QENSQPNIVQVATNILVTMNLVGMGLGVTLIPGYMNNFNTGQVVFRPIAGNVPSIALLMAWKKGEMKLAL

RDFIAIVQERLASVTA

>gi|30063926|ref|NP_838097.1| 3-phenylpropionic acid transporter [Shigella flexneri 2a str. 2457T]

MVLQSTRWLALGYFTYFFSYGIFLPFWSVWLKGIGLTPETIGLLLGAGLVARFLGSLLIAPRVSDPSRLI

SALRVLALLPLLFAVAFWAGAHVAWLMLVMIGFNLFFSPLVPLTDALANTWQKQFPLDYGKVRLWGSVAF

VIGSALTGKLVTMFDYRVILVLLTLGVASMLLGFLIRPTIQPQGASRQQESTGWSAWLALVRQNWRFLAC

VCLLQGAHAAYYGFSAIYWQAAGYSASAVGYLWSLGVVAEVIIFALSNKLFRRCSARDMLLISAICGVVR

WGIMGATTALPWLIVVQILHCGTFTVCHLAAMRYIAARQVSEVIRLQAVYSAVAMGGSIAIMTVFAGFLY

QYLGHGVFWVMALVALPAMFLRPKVVPSC

>gi|30063925|ref|NP_838096.1| stationary phase inducible protein CsiE [Shigella flexneri 2a str. 2457T]

MMPTLAPPSVLSAPQRRCQILLTLFQPGLTATMATFSELNGVDDDIASLDISETGREILRYHQLTLTTGY

DGSYRVEGTVLNQRLCLFHWLRRGFRLCPSFITSQFTPALKSELKRRGIARNFYDDTNLQALVNLCSRRL

QKRFESRDIHFLCLYLQYCLLQHHAGITPQFNPLQRRWAESCLEFQVAQEIGRHWQRRALQHVPPDEPLF

MALLFSMLRVPDPLRDAHQRDRQLRQSIKRLVNHFRELGNVRFYDEQGLCDQLYIHLAQALNRSLFAIGI

DNTLPEEFARLYPRLVRTTRAALAGFESEYGVHLSDEESGLVAVIFGAWLMQENDLHEKQIILLTGNDSE

REAQIEQQLRELTLLPLNIKHMSVKAFLQTGAPRGAALIIAPYTMPLPLFSPPLIYTDLTLTTHQQEQIR

KMLESA

>gi|30063924|ref|NP_838095.1| enzyme [Shigella flexneri 2a str. 2457T]

MALPVNKRVPKILFILFVVAFCVYLVPRVAINFFYYPDDKIYGPDPWSAESVEFTAKDGTRLQGWFIPSS

TGPADNAIATIIHAHGNAGNMSAHWPLVSWLPERNFNVFMFDYRGFGKSKGTPSQAGLLDDTQSAINVVR

HRSDVNPQQLVLFGQSIGGANILAVIGQGDREGIRAVILDSTFASYATIANQMIPGSGYLLDESYSGENY

IASVSPIPLLLIHGKADHVIPWQHSEKLYSLAKEPKRLILIPDGEHIDAFSDRHGNVYREQMVDFILSAL

NPQN

>gi|30063923|ref|NP_838094.1| inositol monophosphatase [Shigella flexneri 2a str. 2457T]

MHPMLNIAVRAARKAGNLIAKNYETPDAVEASQKGSNDFVTNVDKAAEAVIIDTIRKSYPQHTIITEESG

ELEGTDQDVQWVIDPLDGTTNFIKRLPHFAVSIAVRIKGRTEVAVVYDPMRNELFTATRGQGAQLNGYRL

RGSTARDLDGTILATGFPFKAKQYATTYINIVGKLFNECADFRRTGSAALDLAYVAAGRVDGFFEIGLRP

WDFAAGELLVREAGGIVSDFTGGHNYMLTGNIVAGNPRVVKAILANMRDELSDALKH

>gi|30063922|ref|NP_838093.1| ATP synthase subunit beta [Shigella flexneri 2a str. 2457T]

MLQNIRIVLVETSHTGNMGSVARAMKTMGLTNLWLVNPLVKPDSQAIALAAGASDVIGNAHIVDTLDEAL

AGCSLVVGTSARSRTLPWPMLDPRECGLKSVAEAANTPVALVFGRERVGLTNEELQKCHYHVAIAANPEY

SSLNLAMAVQVIAYEVRMAWLATQENGEQVEHEETPYPLVDDLERFYGHLEQTLLATGFIRENHPGQVMN

KLRRLFTRARPESQELNILRGILASIEQQNKGNKAE

>gi|30063921|ref|NP_838092.1| DNA-binding transcriptional regulator IscR [Shigella flexneri 2a str. 2457T]

MRLTSKGRYAVTAMLDVALNSEAGPVPLADISERQGISLSYLEQLFSRLRKNGLVSSVRGPGGGYLLGKD

ASSIAVGEVISAVDESVDATRCQGKGGCQGGDKCLTHALWRDLSDRLTGFLNNITLGELVNNQEVLDVSG

RQHTHDAPRTRTQDAIDVKLRA

>gi|30063920|ref|NP_838091.1| cysteine desulfurase [Shigella flexneri 2a str. 2457T]

MKLPIYLDYSATTPVDPRVAEKMMQFMTMDGTFGNPASRSHRFGWQAEEAVDIARNQIADLVGADPREIV

FTSGATESDNLAIKGAANFYQKKGKHIITSKTEHKAVLDTCRQLEREGFEVTYLAPQRNGIIDLKELEAA

MRDDTILVSIMHVNNEIGVVQDIAAIGEMCRARGIIYHVDATQSVGKLPIDLSQLKVDLMSFSGHKIYGP

KGIGALYVRRKPRVRIEAQMHGGGHERGMRSGTLPVHQIVGMGEAYRIAKEEMATEMERLRGLRNRLWNG

IKDIEEVYLNGDLEHGAPNILNVSFNYVEGESLIMALKDLAVSSGSACTSASLEPSYVLRALGLNDELAH

SSIRFSLGRFTTEEEIDYTIELVRKSIGRLRDLSPLWEMYKQGVDLNSIEWAHH

>gi|30063919|ref|NP_838090.1| scaffold protein [Shigella flexneri 2a str. 2457T]

MAYSEKVIDHYENPRNVGSFDNNDENVGSGMVGAPACGDVMKLQIKVNDEGIIEDARFKTYGCGSAIASS

SLVTEWVKGKSLDEAQAIKNTDIAEELELPPVKIHCSILAEDAIKAAIADYKSKREAK

>gi|30063918|ref|NP_838089.1| iron-sulfur cluster assembly protein [Shigella flexneri 2a str. 2457T]

MSITLSDSAAARVNTFLANRGKGFGLRLGVRTSGCSGMAYVLEFVDEPTPEDIVFEDKGVKVVVDGKSLQ

FLDGTQLDFVKEGLNEGFKFTNPNVKDECGCGESFHV

>gi|30063917|ref|NP_838088.1| co-chaperone HscB [Shigella flexneri 2a str. 2457T]

MDYFTLFGLPARYQLDTQALSLRFQDLQRQYHPDKFASGSLAEQLAAVQQSATINQAWQTLRHPLMRAEY

LLSLHGFDLASEQHTVRDTAFLMEQLELREELDEIEQAKDEARLESFIKRVKKMFDTRHQLMVEQLDNEA

WDAAADTVRKLRFLDKLRSSAEQLEEKLLDF

>gi|30063916|ref|NP_838087.1| chaperone protein HscA [Shigella flexneri 2a str. 2457T]

MALLQISEPGLSAAPHQRRLAAGIDLGTTNSLVATVRSGQAETLADHEGRHLLPSVVHYQQQGHSVGYDA

RTNAALDTANTISSVKRLMGRSLADIQQRYPHLPYQFQASENGLPMIETAAGLLNPVRVSADILKALAAR

ATEALAGELDGVVITVPAYFDDAQRQGTKDAARLAGLHVLRLLNEPTAAAIAYGLDSGQEGVIAVYDLGG

GTFDISILRLSRGVFEVLATGGDSALGGDDFDHLLADYIREQADIPDRSDNRVQRELLDATIAAKIALSD

ADSVTVNVAGWQGEISREQFNELIAPLVKRTLLACRRALKDAGVEADEVLEVVMVGGSTRVPLVRERVGE

FFGRPPLTSIDPDKVVAIGAAIQADILVGNKPDSEMLLLDVIPLSLGLETMGGLVEKVIPRNTTIPVARA

QDFTTFKDGQTAMSIHVMQGERELVQDCRSLARFALRGIPALPAGGAHIRVTFQVDADGLLSVTAMEKST

GVEASIQVKPSYGLTDSEIASMIKDSMSYAEQDVKARMLAEQKVEAARVLESLHGALAADAALLSAAERQ

VIDNAAAHLSEVAQGDDVDAIEQAIKNVDKQTQDFAARRMDQSVRRALKGHSVDEV

>gi|30063915|ref|NP_838086.1| [2FE-2S] ferredoxin, electron carrer protein [Shigella flexneri 2a str. 2457T]

MPKIVILPHQDLCPDGAVLEANSGETILDAALRNGIEIEHACEKSCACTTCHCIVREGFDSLPESSEQED

DMLDKAWGLEPESRLSCQARVTDEDLVVEIPRYTINHAREH

>gi|30063914|ref|NP_838085.1| hypothetical protein S2743 [Shigella flexneri 2a str. 2457T]

MGLKWTDSREIGEALYDAYPDLDPKTVRFTDMHQWICDLEDFDDDPQASNEKILEAILLVWLDEAE

>gi|30063913|ref|NP_838084.1| aminopeptidase B [Shigella flexneri 2a str. 2457T]

MTEAMKITLSTQPADARWGEKATYSINNDGITLHLNGADDLGLIQRAARKIDGLGIKHVQLSGEGWDADR

CWAFWQGYKGPKGTRKVEWPDLDDAQRQELDNRLMIIDWVRDTINAPAEELGPSQLAQRAVDLISNVAGD

RVTYRITKGEDLREQGYMGLHTVGRGSERSPVLLALDYNPTGDKEAPVYACLVGKGITFDSGGYSIKQTA

FMDSMKSDMGGAATVTGALAFAIMRGLNKRVKLFLCCADNLISGNAFKLGDIITYRNGKKVEVMNTDAEG

RLVLADGLIDASAQKPEMIIDAATLTGAAKTALGNDYHALFSFDDALAGRLLASASQENEPFWRLPLAEF

HRSQLPSNFAELNNTGSAAYPAGASTAAGFLSHFVENYQQGWLHIDCSATYRKAPVEQWSAGATGLGVRT

IANLLTA

>gi|30063912|ref|NP_838083.1| enhanced serine sensitivity protein SseB [Shigella flexneri 2a str. 2457T]

MSETKNELEDLLEKAATEPAHRPAFFRTLLESTVWVPGTAAQGEAVVEDSALDLQHWEKEDGTSVIPFFT

SLEALQQAVEDEQAFVVMPVRTLFEMTLGETLFLNAKLPTGKEFMPREISLLIGEEGNPLSSQEILEGGE

SLILSEVAEPPAQMIDSLTTLFKTIKPVKRAFICSIKENEEAQPNLLIGIEADGDIEEIIQATGSVATDT

LPGDEPIDICQVKKGEKGISHFITEHIAPFYERRWGGFLRDFKQNRII

>gi|30063911|ref|NP_838082.1| ISSfl4 orf [Shigella flexneri 2a str. 2457T]

MTESSDYESVQVFIGVDVGKDTHHAVAINRSGKRLFDKALPNDENKLRSLISDLKQHGQILLVVDQPATI

GALPVAVARSEGVLVGYLPGLAMRRIADLHAGEAKTDVRDAAIIAEAARTLPHALRTLKLADEQIAELSM

LCGFDDDLAAQTTQASNRIRGLLTQIHPAPERVLGPRLEHPAVLDLLQRYPSPEKLASLGEKKLAAQLCK

LAPRLGKRLAADIAQALAEQTVVVPGTNAAAVVLPRLALQLITLRKQRDEVALEVEQRVLAHPLYPVLTS

MPGVGVRTAARLLTEVACRAFASAAHLAAYAGLAPVTRRSGSSIRGEHPSRRGNKALKRALFLSAFAALR

DPLSRAYYTRKMSQGKRHNQALIALARRRCDVLFAMMRDGTFYTPQGS

>gi|30063910|ref|NP_838081.1| nucleoside diphosphate kinase [Shigella flexneri 2a str. 2457T]

MAIERTFSIIKPNAVAKNVIGNIFARFEAAGFKIVGTKMLHLTVEQARGFYAEHDGKPFFDGLVEFMTSG

PIVVSVLEGENAVQRHRDLLGATNPANALAGTLRADYADSLTENGTHGSDSVESAAREIAYFFGEGEVCP

RTR

>gi|30063909|ref|NP_838080.1| hypothetical protein S2735 [Shigella flexneri 2a str. 2457T]

MSEQLVTPENVTTKDGKINLLDLNRQQMREFFKDLGEKTFRADQVMKWMYHYCCDNFDEMTDINKVLRGK

LKEVAEIRAPEVVEEQRSSDGTIKWAIAVGDQRVETVYIPEDDRATLCVSSQVGCALECKFCSTAQQGFN

RNLRVSEIIGQVWRAAKIVGAAKVTGQRPITNVVMMGMGEPLLNLNNVVPAMEIMLDDFGFGLSKRRVTL

STSGVVPALDKLGDMIDVALAISLHAPNDEIRDEIVPINKKYNIETFLAAVRRYLEKSNANQGRVTIEYV

MLDHVNDGTEHAHQLAELLKDTPCKINLIPWNPFPDAPYGRSSNSRIDRFSKVLMSYGFTTIVRKTRGDD

IDAACGQLAGDVIDRTKRTLRKRMQGEAIDIKAV

>gi|30063908|ref|NP_838079.1| hypothetical protein S2734 [Shigella flexneri 2a str. 2457T]

MNTEATHDQNEALTTGARLRNAREQLGLSQQAVAERLCLKVSTVRDIEEDKAPADLASTFLRGYIRSYAR

LVHIPEEELLPGLEKQAPLRAAKVAPMQSFSLGKRRKKRDGWLMTFTWLVLFVVIGLSGAWWWQDHKAQQ

EEITTMADQSSAELSSNSEQGQSVPLNTSTTTDPATTSTPPASVDTTATNTQTPAVTAPAPAVDPQQNAV

VSPSQANVDTAATPVPTAATTPDGAAPLPTDQAGVTTPAADPNALVMNFTADCWLEVTDATGKKLFSGMQ

RKDGNLNLTGQAPYKLKIGAPAAVQIQYQGKPVDLSRFIRTNQVARLTLNAEQSPAQ

>gi|30063907|ref|NP_838078.1| 4-hydroxy-3-methylbut-2-en-1-yl diphosphate synthase [Shigella flexneri 2a str. 2457T]

MHNQAPIQRRKSTRIYVGNVPIGDGAPIAVQSMTNTRTTDVEATVNQIKALERVGADIVRVSVPTMDAAE

AFKLIKQRVNVPLVADIHFDYRIALKVAEYGVDCLRINPGNIGNEERIRMVVDCARDKNIPIRIGVNAGS

LEKDLQEKYGEPTPQALLESAMRHVDHLDRLNFDQFKVSVKASDVFLAVESYRLLAKQIDQPLHLGITEA

GGARSGAVKSAIGLGLLLSEGIGDTLRVSLAADPVEEIKVGFDILKSLRIRSRGINFIACPTCSRQEFDV

IGTVNALEQRLEDIITPMDVSIIGCVVNGPGEALVSTLGVTGGNKKSGLYEDGVRKDRLDNNDMIDQLEA

RIRAKASQLDEARRIDVQQVGKIIT

>gi|30063906|ref|NP_838077.1| histidyl-tRNA synthetase [Shigella flexneri 2a str. 2457T]

MAKNIQAIRGMNDYLPGETAIWQRIEGTLKNVLGSYGYSEIRLPIVEQTPLFKRAIGEVTDVVEKEMYTF

EDRNGDSLTLRPEGTAGCVRAGIEHGLLYNQEQRLWYIGPMFRHERPQKGRYRQFHQLGCEVFGLQGPDI

DAELIMLTARWWRALGIFEHVTLELNSIGSLEARANYRDALVAFLEQHKEKLDEDCKRRMYTNPLRVLDS

KNPEVQALLNDAPALGDYLDEESREHFAGLCKLLESAGIAYTVNQRLVRGLDYYNRTVFEWVTNSLGSQG

TVCAGGRYDGLVEQLGGRATPAVGFAMGLERLVLLVQAVNPEFKADPVVDIYLVASGADTQSAAMALAER

LRDELLGVKLMTNHGGGNFKKQFARADKWGARVAVVLGESEVANGTAVVKDLRSGEQTAVAQDSVAAHLR

TLLG

>gi|30063905|ref|NP_838076.1| hypothetical protein S2731 [Shigella flexneri 2a str. 2457T]

MEIYENENDQVEAVKRFFAENGKALAVGGILGVGALTGWRYWNSHQVDSARSASLAYQNAVTAVSEGKPD

SIPAAEKFAAENKNTYGALASLELAQQFVDKNELEKAAAQLQQGLADTSDENLKAVINLRLARVQVQLKQ

ADAALKTLDTIKGEGWAAIVADLRGEALLSKGDKQGARSAWEAGVKSDVTPALSEMMQMKINNLSI

>gi|30063904|ref|NP_838075.1| outer membrane protein assembly complex subunit YfgL [Shigella flexneri 2a str. 2457T]

MQLRKLLLPGLLSVTLLSGCSLFNSEEDVVKMSPLPTVENQFTPTTAWSTSVGSGIGNFYSNLHPALADN

VVYAADRAGLVKALNADDGKEIWSVSLAEKDGWFSKEPALLSGGVTVSGGHVYIGSEKAQVYALNTSDGT

VAWQTKVAGEALSRPVVSDGLVLIHTSNGQLQALNEADGAVKWTVNLDMPSLSLRGESAPATAFGAAVVG

GDNGRVSAVLMEQGQMIWQQRISQATGSTEIDRLSDVDTTPVVVNGVVFALAYNGNLTALDLRSGQIMWK

RELGSVNDFIVDGNRIYLVDQNDRVMALTIDGGVTLWAQSDLLHRLLTSPVLYNGNLVVGDSEGYLHWIN

VEDGRFVAQQKVDSSGFQTEPVAADGKLLIQAKDGTVYSITR

>gi|30063903|ref|NP_838074.1| GTP-binding protein EngA [Shigella flexneri 2a str. 2457T]

MVPVVALVGRPNVGKSTLFNRLTRTRDALVADFPGLTRDRKYGRAEIEGREFICIDTGGIDGTEDGVETR

MAEQSLLAIEEADVVLFMVDARAGLMPADEAIAKHLRSREKPTFLVVNKTDGLDPDQAVVDFYSLGLGEI

YPIAASHGRGVLSLLEHVLLPWMEDLAPQEEVDEDAEYWAQFEAEENGEEEEEDDFDPQSLPIKLAIVGR

PNVGKSTLTNRILGEERVVVYDMPGTTRDSIYIPMERDGREYVLIDTAGVRKRGKITDAVEKFSVIKTLQ

AIEDANVVMLVIDAREGISDQDLSLLGFILNSGRSLVIVVNKWDGLSQEVKEQVKETLDFRLGFIDFARV

HFISALHGSGVGNLFESVREAYDSSTRRVGTSMLTRIMTMAVEDHQPPLVRGRRVKLKYAHAGGYNPPIV

VIHGNQVKDLPDSYKRYLMNYFRKSLDVMGSPIRIQFKEGENPYANKRNTLTPTQMRKRKRLMKHIKKNK

>gi|30063902|ref|NP_838073.1| hypothetical protein S2728 [Shigella flexneri 2a str. 2457T]

MNVEGMATGGIHMELHCPKCQHVLDQDNGHARCPSCGEFIEMKALCPDCHQPLQVLKACGAVDYFCQHGH

GLISKKRVEFVLA

>gi|30063901|ref|NP_838072.1| exodeoxyribonuclease VII large subunit [Shigella flexneri 2a str. 2457T]

MLPSQSPAIFTVSRLNQTVRLLLEHEMGQVWISGEISNFTQPASGHWYFTLKDDTAQVRCAMFRNSNRRV

TFRPQHGQQVLVRANITLYEPRGDYQIIVESMQPAGEGLLQQKYEQLKAKLQAECLFDQQYKKPLPSPAH

CVGVITSKTGAALHDILHVLKRRDPSLPVIIYPTSVQGDDAPGQIVRAIELANQRNECDVLIVGRGGGSL

EDLWSFNDERVARAIFASRIPVVSAVGHETDVTIADFVADLRAPTPSAAAEVVSRNQQELLRQVQSARQR

LEMAMDYYLANRTRRFTQIHHRLQQQHPQLRLARQQTMLERLQKRMSFALENQLKRAGQQQQRLTQRLNQ

QNPQPKIHRTQTRIQQLEYRLAEILRAQLSATRERFGNAVTHLEAVSPLSTLARGYSVTTATDGNVLKKV

KQVKTGEMLTTRLEDGWIESEVKNIQPVKKSRKKVH

>gi|30063900|ref|NP_838071.1| inosine 5'-monophosphate dehydrogenase [Shigella flexneri 2a str. 2457T]

MLRIAKEALTFDDVLLVPAHSTVLPNTADLSTQLTKTIRLNIPMLSAAMDTVTEARLAIALAQEGGIGFI

HKNMSIERQAEEVRRVKKHESGVVTDPQTVLPTTTLREVKELTERNGFAGYPVVTEENELVGIITGRDVR

FVTDLNQPVSVYMTPKERLVTVREGEAREVVLAKMHEKRVEKALVVDDEFHLIGMITVKDFQKAERKPNA

CKDEQGRLRVGAAVGAGAGNEERVDALVAAGVDVLLIDSSHGHSEGVLQRIRETRAKYPDLQIIGGNVAT

AAGARALAEAGCSAVKVGIGPGSICTTRIVTGVGVPQITAVADAVEALEGTGIPVIADGGIRFSGDIAKA

IAAGASAVMVGSMLAGTEESPGEIELYQGRSYKSYRGMGSLGAMSKGSSDRYFQSDNAADKLVPEGIEGR

VAYKGRLKEIIHQQMGGLRSCMGLTGCGTIDELRTKAEFVRISGAGIQESHVHDVTITKESPNYRLGS

>gi|30063899|ref|NP_838070.1| GMP synthase [Shigella flexneri 2a str. 2457T]

MTENIHKHRILILDFGSQYTQLVARRVRELGVYCELWAWDVTEAQIRDFNPSGIILSGGPESTTEENSPR

APQYVFEAGVPIFGVCYGMQTMAMQLGGHVEASNEREFGYAQVEVVNDSALVRGIEDALTADGKPLLDVW

MSHGDKVTAIPSDFVTVASTESCPFAIMANEEKRFYGVQFHPEVTHTRQGMRMLERFVRDICQCEALWTP

AKIIDDAVARIREQVGDDKVILGLSGGVDSSVTAMLLHRAIGKNLTCVFVDNGLLRLNEAEQVLDMFGDH

FGLNIVHVPAEDRFLSALAGENDPEAKRKIIGRVFVEVFDEEALKLEDVKWLAQGTIYPDVIESAASATG

KAHVIKSHHNVGGLPKEMKMGLVEPLKELFKDEVRKIGLELGLPYDMLYRHPFPGPGLGVRVLGEVKKEY

CDLLRRADAIFIEELRKADLYDKVSQAFTVFLPVRSVGVMGDGRKYDWVVSLRAVETIDFMTAHWAHLPY

DFLGRVSNRIINEVNGISRVVYDISGKPPATIEWE

>gi|30063898|ref|NP_838069.1| integrase [Shigella flexneri 2a str. 2457T]

MLTDTKLRNLKPRDKLYKVNDREGLYVAVTPAGSISFRYNYSINGRQETITFGRYGVGGITLAEARELLG

DAKKMVAAGKSPAKEKARDKARVKDAETFGAWAEKWLRGYQMADSTRDMRRSVYERELKPKFSNQKLVEI

THEDLRALADAIVERGAPATAVHVREIVLQVFRWAIERGQKVENPAELVRPTSIARFEPRDRALTPEEIG

LMYQYMERVGTSPTNRAAAKLLLLTMVRKSELTNATWSEINFSEALWTIPKERMKRRNPHLVFLSQQALD

IFIAMKTFAGGSDFVLPSRYDSDAPMSAATLNQVLTLTYKAAQKDGKSLTKFGPHDLRRTASTLLHEAGY

NTDWIEKCLAHEQKGVRAVYNKAEYREQRAAMLQDWADMIDEWTSGGSKG

>gi|30063897|ref|NP_838068.1| hypothetical protein S2722 [Shigella flexneri 2a str. 2457T]

MQNIKTLINRKKLLEMIPLSTRTIYNLEQRGDFPRRIALTSRNVAWDLSEVEEWIEARKSSGDQAARPGP

IEG

>gi|30063896|ref|NP_838067.1| DNA helicase [Shigella flexneri 2a str. 2457T]

MALDLMAAFTELPPPIDYVLPNMVAGTVGALVSPGGAGKSMLALQLAAQIAGGPDLLEIGEFPTGQVVYL

PAEDPPAAIHHRLHALGAHLSAAERQAVADGLLIEPLIGKCPNIMAASWFDALKRAAEGRRLMILDTLRR

FHIEEENASGPMAQVVGHMEAIAADTGCSIVFLHHASKSAAMMGSGDQQQASRGSSVLVDNIRWQSYLSG

MTQGEAEILGVDDCQRGYFVRFGVSKANYGAPFQELWFRRHDGGVLKPAVLERQCKVKRRQREEA

>gi|30063895|ref|NP_838066.1| replication protein C [Shigella flexneri 2a str. 2457T]

MKKPKHDLTHVRHDPAHCLAPGLFRSLKRGDRKRCKLDVTYTFGEDESMRFVGFEPLGADDMRLLQGIVA

LGGPNGILLTPEPTSETGRQLRLFLEPRFEAIEQDGLVVRESLTKLLSETGMTDSGDNIKALKASLLRMS

NVTILVTKGRRQAAFHLMSHAFDETDGRLWVALNPRIAEAILGHRPYARIDMAEVRVLQTDPARLMHQRL

CGWIDPGKSGRVELDTLCGYVWPDEANAEAMKKRRQTARKALAELAAVGWVVNEYAKGKWEIKRPGPTAT

APVYRRNVPLLPS

>gi|30063894|ref|NP_838065.1| stabilization protein [Shigella flexneri 2a str. 2457T]

MANVLIRNLPAEVHRALKVRAALNDRSTDAEIREILTAAVQPLELEEIGREVGLSESDKKTE

>gi|30063893|ref|NP_838064.1| conjugal transfer protein TrbJ [Shigella flexneri 2a str. 2457T]

MKSKILAAKKTALAVALATGFITTTTAPVQAGIPVIDGGNLAQNIMTAIESVAQTLKQIEQYQTQLQQYE

NQLQNTMAPAAYIWDQAQTTINRLIAAQNTLAYYENQLGSLDRYLAKFQDVAYYRSSPCFNGSGGCTPAE

KAAMEENRRLASESQKKANDALFQTVADQQKALKDDARTLERLQGAAQGATGQLQAIGYANQLASQQANQ

LLQIRTMLTAQHNAEAARIAAELDAEARGDARAEQMRTWTFRPSPADNY

>gi|30063892|ref|NP_838063.1| mating pair formation protein, partial [Shigella flexneri 2a str. 2457T]

MIIEVVAMRKKIALGGLMMVATMVLAEPAMAQELSSSGVMNDVLKRFHDAAATWGPAIESAASRLFWTLV

VISMVWTFGMMALRKADIGEFFAEFVRFTIFTGFFWWLLTNANQGMNIAGTIVQSLQTLGAQAGGLSNSN

LGPSSILDLGFELYNRTVQATSELGWRQMATALVMELMALAVLFVLALIAVNLLLLLASAWILLYAGVFF

LGFGGSRWTSDMAINYYKTVLGLAAQLMAMVLLVAIGKEFINHYYTQISENMASQELP

>gi|30063891|ref|NP_838062.1| hypothetical protein S2715, partial [Shigella flexneri 2a str. 2457T]

MATGGAALAGKAVMGAAAGAAGGASALQAAFQKASASMETGGDMSSMGSVVSSGGNGGGEAGTAGSSPFA

QAAGFGDSGSSSSGGGFAKAAKLATGTASELAKGVGSQVKQGFQERVSETTGGKLAASIRESMEPKEASQ

SGQFEGNSLGADSGPDSNEVRS

>gi|30063890|ref|NP_838061.1| sodium bile acid symporter family protein [Shigella flexneri 2a str. 2457T]

MSAGAQAIAAKPRQAPMSGFERYLTLWVALCIVAGVALGQGLPDVFQAIGRMEVAQVNLPVGLLIWVMIV

PMLVKIDFGALHQVKEHWRGIGVTLFVNWAVKPFSMALLGWLFIRQVFAPFLPADQLDSYIAGLILLAAA

PCTAMVFVWSRLTNGDPLFTLSQVALNDAIMIVAFAPIVGLLLGMSSISVPWDTLLISVVLYIIVPVILA

QLLRRHLLKQGQAAFERAMQKIGPWSMAALLLTLVLLFAFQGEAIIRQPLVIAMLAVPILIQVFFNSGLA

YWLNKRAGEKHSVACPSALIGASNFFELAVAAAISLFGLHSGAALATVVGVLVEVPVMLLVVRVVNRSKS

WYERG

>gi|30063889|ref|NP_838060.1| arsenate reductase [Shigella flexneri 2a str. 2457T]

MTERIYNVLILCTGNSARSIMAEALINTMGQGRFRAYSAGSHPTGKVNPFAVEKVESVNYPTENLRSKSW

DEYATPDAPKMDFIITVCDNAAGEMCPVWPGQPISAHWGFEDPAAVEGTDAEKRRAFEQTFRHMMNRVRL

FVNLPLKMLDQTAIKRELANIGKTEQEA

>gi|30063888|ref|NP_838059.1| arsenical resistance protein [Shigella flexneri 2a str. 2457T]

MRLRHLSDPDSLPALDKSFAIERPALGLAPDAPPVRILLLYGSLRARSFSRLAVEEAARLLQFFGAETRI

FDPSDLPLPDQVQSDDHPAVKELRALSEWSEGQVWCSPERHGQITSVMKAQIDHLPLEMAGIRPTQGRTL

AVMQVSGGSQSFNAVNTLRLLGRWMRMFTIPNQSSIAKAFQEFDAAGRMKPSPYYDRIADVMEELVRFTA

LVRPHREALTDRYSERKAAGHVIDEATDLSSIAIAPQPLPESETS

>gi|30063887|ref|NP_838058.1| transcriptional regulator [Shigella flexneri 2a str. 2457T]
METINAVAALAAIAQESRLAVFRLLVQAGPAGMAAGKISEAAGIPPSSLSFHLKELAHAGMVTSRQEGRF
VIYEANFSTATNLVAFLTENCCGGQVCNLSCTTEAGKVLA

>gi|30063886|ref|NP_838057.1| resolvase [Shigella flexneri 2a str. 2457T]
MRRTKPVAAPMVARVYLRVSTDAQDLERQEAITTAAKAAGYYVAGIYREKASGARADRPELLRMIGDLQP
GEVVIAEKIDRISRLPLPEAERLVASIQAKGARLAVPGVVDLSDLAAEAQGVAKIVLEAVQIMLFRLALQ
MARDDYEDRRERQRQGIELARQAGRYKGRRADPKRRAQVVALRKSGYSINKTAELAGYSAAQVKRIWAEV
SQAEAKQHGAFVEDALTEADALAAVGQDERQEERA

>gi|30063885|ref|NP_838056.1| hypothetical protein S2706 [Shigella flexneri 2a str. 2457T]
MKKPNQDDEPFFITEEIAAEMIAGGYEFELPPIPCTIRLRDVLERMTDAELALQPGEIADQERERCRRKP
CSTS

>gi|30063884|ref|NP_838055.1| transposase [Shigella flexneri 2a str. 2457T]
MPHVAARTASRDRDTGRYQSHRPEQTLLYQIVDEYYPAFAALMAEQGKELPGYVQREFEEFLQCGRLEHG
FLRVRCESCHAEHLVAFSCKRRGFCPSCGARRMAESAALLVDEVLPEQPMRQWVLSFPFQLRFLFASRPE
IMGWVLGIVYRVIATHLVKKAGHTHQVAKTGAVTLIQRFGSALNLNVHFHMLFLDGVYVEQSHGSARFRW
VKAPTSPELTQLTHTIAHRVGRYLERQGLLERDVENSYLASDAVDDDPMTPLLGHSITYRIAVGSQAGRK
VFTLQTLPTSGDPFGDGIGKVAGSSLHAGVAARADERKKLERLCRYISRPAVSEKRLSLTRGGNVRYQLK
TPYRDGTTHVIFEPLDFIARLAALVPKPRVNLTRFHGVFAPNSRHRALVTPAKRGRGNKVRVADEPATPA
QRRASMTWAQRLKRVFNIDIETCSGCGGAMKVIACIEDPIVIKQILDHLKHKAETSGTRALPESRAPPAE
LLLGLFD

>gi|30063883|ref|NP_838054.1| phosphoglucosamine mutase [Shigella flexneri 2a str. 2457T]

MVRKYFGTDGIRGKANEGAMTAETALRVGMAAGRVFRRGDHRHRVVIGKDTRLSGYMLEPALTAGFTSMG

MDVFLFGPLPTTYRKNKRPFHPSPTALDRS

>gi|30063882|ref|NP_838053.1| dihydropteroate synthase [Shigella flexneri 2a str. 2457T]

MNKSLIIFGIVNITSDSFSDGGRYLAPDAAIAQARKLMAEGADVIDLGPASSNPDAAPVSSDTEIARIAP

VLDALKADGIPVSLDSYQPATQAYALSRGVAYLNDIRGFPDAAFYPQLAKSSAKLVVMHSVQDGQADRRE

APAGDIMDHIAAFFDARIAALTGAGIKRNRLVLDPGMGFFLGAAPETSLSVLARFDELRLRFDLPVLLSV

SRKSFLRALTGRGPGDVGAATLAAELAAAAGGADFIRTHEPRPLRDGLAVLAALKETARIR

>gi|30063881|ref|NP_838052.1| hypothetical protein S2702 [Shigella flexneri 2a str. 2457T]

MKKVFLCAILASLSYPAIASSLQDQLSAVAEAEQQGKNEEQRQHDEWVAERNREIQQEKQRRANAQAAAN

KRAATAAANKKARQDKLDAEATADKKRDQSYEDELRSLEIQKQKLALAKEEARVKRENEFIDQELKHKAA

QTDVVQSEADANRNMTEGGRDLMKSVGKAEENKSDSWFN

>gi|30063880|ref|NP_838051.1| outer membrane lipoprotein [Shigella flexneri 2a str. 2457T]

MMKFKKCLLPVAMLASFTLAGCQSNADDHAADVYQTDQLNTKQETKTVNIISILPAKVAVDNSQNKRNAQ

AFGALIGAVAGGVIGHNVGSGSNSGTTAGAVGGGAVGAAAGSMVNDKTLVEGVSLTYKEGTKVYTSTQEG

KECQFTTGLAVVITTTYNETRIQPNTKCPEKS

>gi|30063879|ref|NP_838050.1| hypothetical protein S2700 [Shigella flexneri 2a str. 2457T]

MSQATSMRKRHRFNSRMTRIVLLISFIFFFGRFIYSSVGAWQHHQSKKEAQQSTLSVESPVQR

>gi|30063878|ref|NP_838049.1| IS600 orfB [Shigella flexneri 2a str. 2457T]

MAHIRTRETYGTRRLQTELAENGIIVGRDRLARLRKELRLRCKQKRKFRATTNPNHNLPVAPNLLNQTFA

PTAPNQVWVADLTYVATQEGWLYLAGIKDVYTCEIVGYAMGERMTKELTGKALFMALRSQRPPAGLIHHS

DRGSQYCAYDYRVIQEQFGLKTSMSRKGNCYDNAPMESFWGTLKNESLSHYRFNNRDEAISVIREYIEIF

YNRQRRHSRLGNISPAAFREKYHQMAA

>gi|30063877|ref|NP_838048.1| IS600 orfA [Shigella flexneri 2a str. 2457T]

MSRKTQRYSKEFKAEAVRTVLENQLSISEGASRLSLPEGTLGQWVTAARKGLGTPGSRTLAELESEILQL

RKALNEARLERDILKKATAYFAQESLKNTR

>gi|30063876|ref|NP_838047.1| exopolyphosphatase [Shigella flexneri 2a str. 2457T]

MPIHDKSPRPQEFAAVDLGSNSFHMVIARVVDGAMQIIGRLKQRVHLADGLGPDNMLSEEAMTRGLNCLS

LFAERLQGFSPASVCIVGTHTLRQALNATDFLKRAEKVIPYPIEIISGNEEARLIFMGVEHTQPEKGRKL

VIDIGGGSTELVIGENFEPILVESRRMGCVSFAQLYFPGGVINKENFQRARMAAAQKLETLTWQFRIQGW

NVAMGASGTIKAAHEVLMEMGEKDGIITPERLEKLVKEILRHRNFASLSLPGLSEERKTVFVPGLAILCG

VFDALAIRELRLSDGALREGVLYEMEGRFRHQDVRSRTASSLANQYHIDSEQARRVLDTTMQMYEQWREQ

QPKLAHPQLEALLRWAAMLHEVGLNINHSGLHRHSAYILQNSDLPGFNQEQQLMMATLVRYHRKAIKLDD

LPRFTLFKKKQFLPLIQLLRLGVLLNNQRQATTTPPTLTLITDDSHWTLRFPHDWFSQNALVLLDLEKEQ

EYWEGVAGWRLKIEEESTPEIAA

>gi|30063875|ref|NP_838046.1| polyphosphate kinase [Shigella flexneri 2a str. 2457T]

MGQEKLYIEKELSWLSFNERVLQEAADKSNPLIERMRFLGIYSNNLDEFYKVRFAELKRRIIISEEQGSN

AHSRHLLGKIQSRVLKADQEFDGLYNELLLEMARNQIFLINERQLSVNQQNWLRHYFKQYLRQHITPILI

NPDTDLVQFLKDDYTYLAVEIIRSDSIRYALLEIPSDKVPRFVNLPPEAPRRRKPMILLDNILRYCLDDI

FKGFFDYDALNAYSMKMTRDAEYDLVHEMEASLMELMSSSLKQRLTAEPVRFVYQRDMPNALVEVLREKL

TISRYDSIVPGGRYHNFKDFINFPNVGKANLVNKPLPRLRHIWFDKAQFRNGFDAIRERDVLLYYPYHTF

EHVLELLRQASFDPSVLAIKINIYRVAKDSRIIDSMIHAAHNGKKVTVVVELQARFDEEANIHWAKRLTE

AGVHVIFSAPGLKIHAKLFLISRKENGEVVRYAHIGTGNFNEKTARLYTDYSLLTADARITNEVRRVFNF

IENPYRPVTFDYLMVSPQNSRRLLYEMVDREIANAQQGLPSGITLKLNNLVDKGLVDRLYAASSSGVPVN

LLVRGMCSLIPNLEGISDNIRAISIVDRYLEHDRVYIFENGGDKKVYLSSADWMTRNIDYRIEVATPLLD

PRLKQRVLDIIDILFSDTVKARYIDKELSNRYVPRGNRRKVRAQLAIYDYIKSLEQPE

>gi|30063874|ref|NP_838045.1| phosphoribosylglycinamide formyltransferase [Shigella flexneri 2a str. 2457T]

MNIVVLISGNGSNLQAIIDACKTNKIKGTVRAVFSNKADAFGLERARQAGIATHTLIASAFDSREAYDRE

LIHEIDMYAPDVVVLAGFMRILSPAFVSHYAGRLLNIHPSLLPKYPGLHTHRQALENGDEEHGTSVHFVT

DELDGGPVILQAKVPVFAGDTEDDITARVQTQEHAIYPLVISWFADGRLKMHENAAWLDGQRLPPQGYAA

DE

>gi|30063873|ref|NP_838044.1| phosphoribosylaminoimidazole synthetase [Shigella flexneri 2a str. 2457T]

MGTQAVTDKTSLSYKDAGVDIDAGNALVGRIKGVVKKTRRPEVMGGLGGFGALCALPQKYREPVLVSGTD

GVGTKLRLAMDLKRHDTIGIDLVAMCVNDLVVQGAEPLFFLDYYATGKLDVDTASAVISGIAEGCLQSGC

SLVGGETAEMPGMYHGEDYDVAGFCVGVVEKSEIIDGSKVSDGDVLIALGSSGPHSNGYSLVRKILEVSG

CDPQTTELDGKPLADHLLAPTRIYVKSVLELIEKVDVHAIAHLTGGGFWENIPRVLPDNTQAVIDESSWQ

WPEVFNWLQTAGNVERHEMYRTFNCGVGMIIALPAPEVDKALALLNANGENAWKIGIIKASDSEQRVVIE

>gi|30063872|ref|NP_838043.1| uracil phosphoribosyltransferase [Shigella flexneri 2a str. 2457T]

MKIVEVKHPLVKHKLGLMREQDISTKRFRELASEVGSLLTYEATADLETEKVTIEGWNGPVEIDQIKGKK

ITVVPILRAGLGMMDGVLENVPSARISVVGMYRNEETLEPVPYFQKLVSNIDERMALIVDPMLATGGSVI

ATIDLLKKAGCSSIKVLVLVAAPEGIAALEKAHPDVELYTASIDQGLNEHGYIIPGLGDAGDKIFGTK

>gi|30063871|ref|NP_838042.1| uracil transporter [Shigella flexneri 2a str. 2457T]

MTRRAIGVSERPPLLQTIPLSLQHLFAMFGATVLVPVLFHINPATVLLFNGIGTLLYLFICKGKIPAYLG

SSFAFISPVLLLLPLGYEVALGGFIMCGVLFCLVSFIVKKAGTGWLDVLFPPAAMGAIVAVIGLELAGVA

AGMAGLLPAEGQTPNSKTIIISITTLAVTVLGSVLFRGFLAIIPILIGVLVGYALSFAMGIVDTTPIINA

HWFALPTLYTPRFEWFAILTILPAALVVIAEHVGHLVVTANIVKKDLLRDPGLHRSMFANGLSTVISGFF

GSTPNTTYGENIGVMAITRVYSTWVIGGAAIFAILLSCVGKLAAAIQMIPLPVMGGVSLLLYGVIGASGI

RVLIESKVDYNKAQNLILTSVILIIGVSGAKVNIGAAELKGMALATIVGIGLSLIFKLISVLRPEEVVLD

AEDADITDK

>gi|30063870|ref|NP_838041.1| DNA replication initiation factor [Shigella flexneri 2a str. 2457T]

MVNFSRFCEILVEVSLNTPAQLSLPLYLPDDETFASFWPGDNSSLLAALQNVLRQEHSGYIYLWAREGAG

RSHLLHAACAELSQRGDAVGYVPLDKRTWFVPEVLDGMEHLSLVCIDNIECIAGDELWEMAIFDLYNRIL

ESGKTRLLITGDRPPRQLNLGLPDLASRLDWGQIYKLQPLSDEDKLQALQLRARLRGFELPEDVGRFLLK

RLDREMRTLFMTLDQLDRASITAQRKLTIPFVKEILKL

>gi|30063869|ref|NP_838040.1| oxidoreductase [Shigella flexneri 2a str. 2457T]

MTKQVKIYHNPRCSKSRETLNLLKENGVEPEVVLYLETPADAATLRDLLKMLGMNSARELMRQKEDLYKE

LNLADSSLSEEALIQAMVDNPKLMERPIMVANGKARIGRPPEQVLEIVG

>gi|30063868|ref|NP_838039.1| hypothetical protein S2687 [Shigella flexneri 2a str. 2457T]

MFRQLKKNLVATLIAAMTIGQVAPAFADSADTLPDMGTSAGSTLSIGQEMQMGDYYVRQLRGSAPLINDP

LLTQYINSLGMRLVSHANSVKTPFHFFLINNDEINAFAFFGGNVVLHSALFRYSDNESQLASVMAHEISH

VTQRHLARAMEDQQRSAPLTWVGALGSILLAMASPQAGMAALTGTLAGTRQGMISFTQQNEQEADRIGIQ

VLQRSGFDPQAMPTFLEKLLDQARYSSRPPEILLTHPLPESRLADARNRANQMRPMVVQSSEDFYLAKAR

TLGMYNSGRNQLTSDLLDEWAKGNVRQQRAAQYGRALQAMEANKYDEARKTLQPLLAAEPGNAWYLDLAT

DIDLGQNKANEAINRLKNARDLRTNPVLQLNLANAYLQGGQPQEAANILNRYTFNNKDDSNGWDLLAQAE

AALNNRDQELAARAEGYALAGRLDQAISLLSSASSQVKLGSLQQARYDARIDQLRQLQERFKPYTKM

>gi|30063867|ref|NP_838038.1| permease [Shigella flexneri 2a str. 2457T]

MLEMLMQWYRRRFSDPEAIALLVILVAGFGIIFFFSGLLAPLLVAIVLAYLLEWPTVRLQSIGCSRRWAT

SIVLVVFVGILLLMAFVVLPIAWQQGIYLIRDMPGMLNKLSDFAATLPRRYPVLMDAGIIDAMAENMRSR

MLTMGDSVVKISLASLVGLLTIAVYLVLVPLMVFFLLKDKEQMLNAVRRVLPRNRGLAGQVWKEMNQQIT

NYIRGKVLEMIVVGIATWLGFLLFGLNYSLLLAVLVGFSVLIPYIGAFVVTIPVVGVALFQFGAGTEFWS

CFAVYLIIQALDGNLLVPVLFSEAVNLHPLVIILSVVIFGGLWGFWGVFFAIPLATLIKAVIHAWPDGQI

AQE

>gi|30063866|ref|NP_838037.1| protein processing element [Shigella flexneri 2a str. 2457T]

MPTIHVSIVSFSNSFAYSGGYMTEECGEIVFWTLRKKFVASSDEMPEHSSQVMYYSLAIGHHVGVIDCLN

VAFRCPLTEYEDWLALVEEEQARRKMLGVMTFGEIVIDASHTALLTRAFAPLADDATSVWQARSIQFIHL

LDEIVLEPAIYLMARKIA

>gi|30063865|ref|NP_838036.1| hydrogenase 4 Fe-S subunit [Shigella flexneri 2a str. 2457T]

MSPVLTQYVSQPITLDEQTQKMKRHLLQDIRRSAYVYRIDCGGCNACEIEIFAAITPVFDAERFGIKVVS

SPRHADILLFTGAVTRAMRMPALRAYESAPDHKICVSYGACGVGGGIFHDLYSVWGGSDTIVPIDVWIPG

CPPTPAATIHGFAVALGLLQQKIHAVDYRDPTGVTMQPLWPQIPPSQRIAIEREARRLAGYRQGREICDR

LLRHLSDDPTGNRVNTWLRDADDPRLNSIVQQLFRVLRGLHD

>gi|30063864|ref|NP_838035.1| hydrogenase 4 subunit H [Shigella flexneri 2a str. 2457T]

MLKLLKTIMRAGTATVKYPFAPLEVSPGFRGKPDLMPSQCIACGACACACPANALTIQTDDQQNSRTWQL

YLGRCIYCGRCEEVCPTRAIQLTNNFELTVTHKADLYTHATFHLQRCSRCERPFAPQKTVALAAELLAQQ

QNAPQNREMLWAQASVCPECKQRATLINDDTDVPLVAKEQL

>gi|30063863|ref|NP_838034.1| hydrogenase 4 subunit F [Shigella flexneri 2a str. 2457T]

MSYSVMFALLLLTPLLFSLLCFACRKRGLSATCTVTVLHSLGITLLLILALWVVQTAADAGEIFAAGLWL

HIDGLGGLFLAILGVIGFLTGVYSIGYMRHEVAHGELSPVTLCDYYGFFHLFLFTMLLVVTSNNLIVMWA

AIEATTLSSAFLVGIYGQRSSLESAWKYIIICTVGVAFGLFGTVLVYANAASVMPQAEMAIFWSEVLKQS

SLLDPTLMLLAFVFVLIGFGTKTGLFPMHAWLPDAHSEAPSPVSALLSAVLLNCALLVLIRYYIIICQAI

GSDFPNRLLLIFGMLSVAVAAFFILVQRDIKRLLAYSSVENMGLVAVALGIGGPLGIFAALLHTLNHSLA

KTLLFCGSGNVLLKYGTRDLNVVCGMLKIMPFTAVLFGGGALALALAGMPPFNIFLSEFMTVTAGLARNH

LLIIVLLLLLLTLVLAGLVRMAARVLMAKPPQAVNRGDLGWLTTSPMVILLVMMLAMGTHIPQPVIRILA

GASTIVLSGTHDLPAQRSTWHDFLPSGTASVSEKHSER

>gi|30063862|ref|NP_838033.1| hydrogenase 4 membrane subunit [Shigella flexneri 2a str. 2457T]

MTGSMIVNNLAGLMMLTSLFVISVKSYRLSCGFYACQSLVLVSIFATLSCLFAAEQLLIWSASAFITKVL

LVPLIMTYAARNIPQNIPEKALFGPAMMALLAALIVLLCAFVVQPVKLPMATGLKPALAVALGHFLLGLL

CIVSQRNILRQIFGYCLMENGSHLVLALLAWRAPELVEIGIATDAIFAVIVMVLLARKIWRTHGTLDVNN

LTALKG

>gi|30063861|ref|NP_838032.1| hydrogenase 4 subunit D [Shigella flexneri 2a str. 2457T]

MENLALTTLMLPFIGALVVSFSPQRRAAEWGVLFAALTTLCMLSLISAFYQADKVAVTLTLVNVGDVALF

GLVIDRVSTLILFVVVFLGLLVTIYSTGYLTDKNREHPHNGTNRYYAFLLVFIGAMAGLVLSSTLLGQLL

FFEITGGCSWALISYYQSDKAQRSALKALLITHIGSLGLYLAAATLFLQTGTFVLSAMSELHGDARYLVY

GGILFAAWGKSAQLPMQAWLPDAMEAPTPISAYLHAASMVKVGVYIFARAIIDGGNIPHVIGGVGMVMTL

VTILYGFLMYLPQQDMKRLLAWSTITQLGWMFFGLSLSIFGSRLALEGSIAYIVNHAFAKSLFFLVAGAL

SYSCGTRLLPRLRGVLHTLPLPGVGFCVAALAITGVPPFNGFFSKFPLFAAGFALSVEYWILLPAMILLM

IESVASFAWFIRWFGRVVPGKPSEAVADAAPLPGSMRLVLIVLIVMSLISSVIAATWLQ

>gi|30063860|ref|NP_838031.1| hydrogenase 4 membrane subunit [Shigella flexneri 2a str. 2457T]

MRQTLCDGYLVIFALAQAVILLMLTPLFTGISRQIRARMHSRRGPGIWQDYRDIHKLFKRQEVAPTSSGL

MFRLMPWVLISSMLVLAMALPLFITVSPFAGGGDLITLIYLLALFRFFFALSGLDTGSPFAGVGASRELT

LGILVEPMLILSLLVLALIAGSTHIEMISNTLAMGWNSPLTTVLALLACGFACFIEMGKIPFDVAEAEQE

LQEGPLTEYSGAGLALAKWGLGLKQVVMAALFVALFLPFGRAQELSLACLLTSLVVTLLKVLLIFVLASI

AENTLARGRFLLIHHVTWLGFSLAALAWVFWLTGL

>gi|30063859|ref|NP_838030.1| hydrogenase 4 Fe-S subunit [Shigella flexneri 2a str. 2457T]

MNRFVVAEPLWCTGCNTCLAACSDVHKTQGLQQHPRLALAKTSTITAPVVCHHCEEAPCLQVCPVNAISQ

RDDAIQLNESLCIGCKLCAVVCPFGAISASGSRPVNAHAQYVFQAEGSLKDGEENVPPQHALLRWEPGVQ

TVAVKCDLCDFLPEGPACVRACPNQALRLITDDSLQRQMKEKQRLAASWFANGGESPLSLTQEQH

>gi|30063858|ref|NP_838029.1| thioredoxin-dependent thiol peroxidase [Shigella flexneri 2a str. 2457T]

MNPLKAGDIAPKFSLPDQDGEQVNLTDFQGQRVLVYFYPKAMTPGCTVQACGLRDNMDELKKAGVDVLGI

STDKPEKLSRFAEKELLNFTLLSDEDHQVCEQFGVWGEKSFMGKTYDGIHRISFLIDADGKIEHVFDDFK

TSNHHDVVLNWLKEHA

>gi|30063857|ref|NP_838028.1| dihydrodipicolinate synthase [Shigella flexneri 2a str. 2457T]

MFTGSIVAIVTPMDEKGNVCRASLKKLIDYHVASGTSAIVSVGTTGESATLNHDEHADVVMMTLDLADGR

IPVIAGTGANATAEAISLTQRFNDSGIVGCLTVTPYYNRPSQEGLYQHFKAIAEHTDLPQILYNVPSRTG

CDLLPETVGRLAKVKNIIGIKEATGNLTRVNQIKELVSDDFVLLSGDDASALDFMQLGGHGVISVTANVA

ARDMAQMCKLAAEGHFAEARVINQRLMPLHNKLFVEPNPIPVKWACKELGLVATDTLRLPMTPITDSGRE

TVRAALKHAGLL

>gi|30063856|ref|NP_838027.1| lipoprotein [Shigella flexneri 2a str. 2457T]

MAYSVQKSRLAKVAGVSLVLLLAACSSDSRYKRQVSGDEAYLEAAPLAELHAPAGMILPVTSGDYAIPVT

NGSGAVGKALDIRPPAQPLALVSGARTQFTGDTASLLVENGRGNTLWPQVVSVLQAKNYTITQRDDAGQT

LTTDWVQWNRLDEDEQYRGRYQISVKPQGYQQAVTVKLLNLEQAGKPVADAASMQRYSTEMMNVISAGLD

KSATDAANAAQNRASTTMDVQSAADDTGLPMLVVRGPFNVVWQRLPAALEKVGMKVTDSTRSQGNMAVTY

KPLSDSDWQELGASDPGLASGDYKLQVGDLDNRSSLQFIDPKGHTLTQSQNDALVAVFQAAFSK

>gi|30063855|ref|NP_838026.1| phosphoribosylaminoimidazole-succinocarboxamide synthase [Shigella flexneri 2a str. 2457T]

MQKQAELYRGKAKTVYSTENPDLLVLEFRNDTSAGDGARIEQFDRKGMVNNKFNYFIMSKLAEAGIPTQM

ERLLSDTECLVKKLDMVPVECVVRNRAAGSLVKRLGIEEGIELNPPLFDLFLKNDAMHDPMVNESYCETF

GWVSKENLARMKELTYKANDVLKKLFDDAGLILVDFKLEFGLYKGEVVLGDEFSPDGSRLWDKETLEKMD

KDRFRQSLGGLIEAYEAVARRLGVQLD

>gi|30063854|ref|NP_838025.1| hypothetical protein S2668 [Shigella flexneri 2a str. 2457T]

MRWQGRRESDNVEDRRNSSGGPSMGGPGFRLPSGKGGLILLIVVLVAGYYGVDLTGLMTGQPVSQQQSTR

SISPNEDEAAKFTSVIQATTEDTWGQQFEKMGKTYQQPKLVMYRGMTRTGCGAGQSIMGPFYCPADGTVY

IDLSFYDDMKDKLGADGDFAQGYVIAHEVGHHVQKLLGIEPKVRQLQQNATQAEVNRLSVRMELQADCFA

GVWGHSMQQQGVLETGDLEEALNAAQAIGDDRLQQQSQGRVVPDSFTHGTSQQRYSWFKRGFDSGDPAQC

NTFGKSI

>gi|30063853|ref|NP_838024.1| hypothetical protein S2667 [Shigella flexneri 2a str. 2457T]

MAELTALYTLTAQMKREGICRLLVLSGEEGWCFDHALKLRDALPGDWLWISPQLDAENHCSPSALQTLLG

REFRHAVFDARHGFDAAAFAALSGTLKAGSWLVLLLPVWEEWENQPDADSLRWSDCPDPIATPHFVQHLK

RVLTADNDAILWRQNQPFSLAHFTPRTDWHPATGAPQPEQQQLLQQLLTMPPGVAAVTAARGRGKSALAG

QLISRIAGSAIVTAPAKAATDVLAQFAGEKFRFIAPDALLASDEQADWLVVDEAAAIPAPLLHQLVSRFP

RTLLTTTVQGYEGTGRGFLLKFCARFPHLHRFELQQPIRWAQGCPLEKMVSEALVFNDENFTHTPQGNIV

ISAFEQTLWRSEPETPLKVYQLLSGAHYRTSPLDLRRMMDAPGQHFLQAAGENEIAGALWLVDEGGLSQE

LSQAVWAGFRRPRGNLVAQSLAAHGSNPLAATLRGRRVSRIAVHPARQREGTGRQLIAGALQYTHDLDYL

SVSFGYTGELWRFWQRCGFVLVRMGNHREASSGCYTAMALLPMSDAGKQLAEREHYRLRRDAQALAQWNG

EMLPVDPLNDAILSDDDWLELAGFAFAHRPLLTSLGCLLRLLQTSELALPALRGRLQKNASDAQLCTTLK

LSGRKMLLVRQREEAAQALFALNEVRTERLRDRITQWQFFH

>gi|30063852|ref|NP_838023.1| esterase YpfH [Shigella flexneri 2a str. 2457T]

MKHDHFVVQSPDKPAQQLLLLFHGVGDNPVAMGEIGSWFAPLFPDALVVSVGGAEPSGNPAGRQWFSVQG

ITEDNRQARVDAIMPTFIETVRYWQKQSGVGANATALIGFSQGAIMALESIKAEPGLASRVIAFNGRYAS

LPETASTATTIHLIHGGEDPVIDLAHAVAAQEALISAGGDVTLDIVEDLGHAIDNRSMQFALDHLRYTIP

KHYFDQALSGGKPGDDDVIEMM

>gi|30063851|ref|NP_838022.1| succinyl-diaminopimelate desuccinylase [Shigella flexneri 2a str. 2457T]

MSCPVIELTQQLIRRPSLSPDDAGCQALLIERLQAIGFTVERMDFADTQNFWAWRGQGETLAFAGHTDVV

PPGDADRWINPPFEPTIRDGMLFGRGAADMKGSLAAMVVAAERFVAQHPNHTGRLAFLITSDEEASAHNG

TVKVVEALMARNERLDYCLVGEPSSIEVVGDVVKNGRRGSLTCNLTIHGVQGHVAYPHLADNPVHRAAPF

LNELVAIEWDQGNEFFPATSMQIANIQAGTGSNNVIPGELFVQFNFRFSTELTDEMIKAQVLALLEKHQL

RYTVDWWLSGQPFLTARGKLVDAVVNAVEHYNEIKPQLLTTGGTSDGRFIARMGAQVVELGPVNATIHKI

NECVNAADLQLLARMYQRIMEQLVA

>gi|30063850|ref|NP_838021.1| hypothetical protein S2664 [Shigella flexneri 2a str. 2457T]

MVTLYGIKNCDTIKKARRWLEANNIDYRFHDYRVDGLDSELLNGFINELGWEALLNTRGTTWRKLDETTR

NKITDAASAAALMTEMPAIIKRPLLCAPGKPMLLGFSDSSYQQFFHEV

>gi|30063849|ref|NP_838020.1| aminoglycoside/multidrug efflux system [Shigella flexneri 2a str. 2457T]

MANFFIDRPIFAWVLTILLCLTGTLAIFSLPVEQYPDLAPPNVRVTANYPGASAQTLENTVTQVIEQNMT

GLDNLMYMSSQSSGTGQASVTLSFKAGTDPDEAVQQVQNQLQSAMRKLPQAVQNQGVTVRKTGDTNILTI

AFVSTDGSMDKQDIADYVASNIQDPLSRVNGVGDIDAYGSQYSMRIWLDPAKLNSFQMTAKDVTDAIESQ

NAQIAVGQLGGTPSVDKQALNATINAQSLLQTPEQFRDITLRVNQDGSEVRLGDVATVEMGAEKYDYLSR

FNGKPASGLGVKLASSANEMATAELVLNRLDELAQYFPHGLEYKVAYETTSFVKASIEDVVKTLLEAIAL

VFLVMYLFLQNFRATLIPTIAVPVVLMGTFSVLYAFGYSVNTLTMFAMVLAIGLLVDDAIVVVENVERIM

SEEGLTPREATRKSMGQIQGALVGIAMVLSAVFVPMAFFGGTTGAIYRQFSITIVAAMVLSVLVAMLLTP

ALCATLLKPLKKGEHHGQKGFFAWFNQMFNRNAERYEKGVAKILHRSLRWIVIYVLLLGGMVFLFLRLPT

SFLPLEDRGMFTTSVQLPSGSTQQQTLKVVEQIEKYYFTHEKDNIMSVFATVGSGPGGNGQNVARMFIRL

KDWSERDSKTGTSFAIIERATKAFNQIKEARVIASSPPAISGLGSSAGFDMELQDHAGAGHDALMAARNQ

LLALAAENPELTRVRHNGLDDSPQLQIDIDQRKAQALGVDIDDINDTLQTAWGSSYVNDFMDRGRVKKVY

VQAAAPYRMLPDDINLWYVRNKDGGMVPFSAFATSRWETGSPRLERYNGYSAVEIVGEAAPGVSTGTAMD

IMESLVKQLPNGFGLEWTAMSYQERLSGAQAPALYAISLLVVFLCLAALYESWSVPFSVMLVVPLGVIGA

LLATWMRGLENDVYFQVGLLTVIGLSAKNAILIVEFANEMNQKGHDLFEATLHACRQRLRPILMTSLAFI

FGVLPMATSTGAGSGGQHAVGTGVMGGMISATILAIYFVPLFFVLVRRRFPLKPRPE

>gi|30063848|ref|NP_838019.1| oxidoreductase Fe-S binding subunit [Shigella flexneri 2a str. 2457T]

MNRFIMANSQQCLGCHACEIACVMAHNDEQHVLSQHHFHPRITVIKHQQQRSAVTCHHCEDAPCARSCPN

GAISHVDDSIQVNQQKCIGCKSCVVACPFGTMQIVLTPVAAGKVKATAHKCDLCAGRENGPACVENCPAD

ALQLVTDVALSGMAKSRRLRTARQEHQPWHASTAAQEMPVMSKVEQMQATPARGEPDKLAIEARKTGFDE

IYLPFRADQAQREASRCLKFGEHSVCEWTCPLHNHIPQWIELVKAGNIDAAVELSHQTNTLPEITGRVCP

QDRLCEGACTIRDEHGAVTIGNIERYISDQALAKGWRPDLSHVTKVDKRVAIIGAGPAGLACADVLTRNG

VAVTVYDRHPEIGGLLTFGIPSFKLDKSLLARRREIFSAMGIHFELNCEVGKDVSLDLLLEQYDAVFVGV

GTYRSMKAGLPNEDAPGVYDALPFLIANTKQVMGLEELPEEPFINTAGLNVVVLGGGDTAMDCVRTALRH

GASNVTCAYRRDEANMPGSKKEVKNAREEGANFEFNVQPVALELNEQGHVCGIRFLRTRLGEPDAQGRRR

PVPIEGSEFVMPADAVIMAFGFNPHGMPWLESHGVTVDKWGRIIADVESQYRYQTTNPKIFAGGDAVRGA

DLVVTAMAEGRHAAQGIIDWLGVKSVKSH

>gi|30063847|ref|NP_838018.1| hypothetical protein S2660 [Shigella flexneri 2a str. 2457T]

MTQQITLIKDKILSDNYFTLHNITYDLTRKDGEVIRHKREVYDRGNGATILLYNAKKKSVVLIRQFRVAT

WVNGNESGQLIETCAGLLDNDEPEVCIRKEAIEETGYEVGEVRKLFELYMSPGGVTELIHFFIAEYSDNQ

RANAGGGVEDEDIEVLELPFSQALEMIKTGEIRDGKTVLLLNYLQMSHLMD

>gi|30063846|ref|NP_838017.1| hypothetical protein S2659 [Shigella flexneri 2a str. 2457T]

MRYRIFLLFFFALLPTSLVWAAPAQRAFSDWQVTCNNQNFCVARNTGDHNGLVMTLSRSAGAHTDAVLRI

ERGGLKSPDASEGEIAPRLLLDGEPLALSGDKWRISPWLLVTDDTATITAFLQIIQEGKAITLRDGNQTI

SLSGLKAALLFIDAQQKRVGSETAWIKKGDEPPLSVPPAPALKEVAVVNPTPTPLSLEERNDLLDYGNWR

MNGLRCSLDPLRREVNVTALTDDKALMMISCEAGAYNTIDLAWIVSRKKPLASRPVRLRLPFNNGQETNE

LELMNATFDEKSRELVTLAKGRGLSDCGIQARWRFDGQRFRLVRYAAEPTCDNWHGPDARPTLWITR

>gi|30063845|ref|NP_838016.1| transketolase [Shigella flexneri 2a str. 2457T]

MSRKDLANAIRALSMDAVQKANSGHPGAPMGMADIAEVLWNDFLKHNPTDPTWYDRDRFILSNGHASMLL

YSLLHLTGYDLPLEELKNFRQLHSKTPGHPEIGYTPGVETTTGPLGQGLANAVGLAIAERTLAAQFNQPD

HEIVDHFTYVFMGDGCLMEGISHEVCSLAGTLGLGKLIGFYDHNGISIDGETEGWFTDDTAKRFEAYHWH

VIHEIDGHDPQAVKEAILEAQSVKDKPSLIICRTVIGFGSPNKAGKEEAHGAPLGEEEVALARQKLGWHH

PPFEIPKEIYHAWDAREKGEKAQQSWNEKFAAYKKAHPQLAEEFPRRMSGGLPKDWEKTTQKYINELQAN

PAKIATRKASQNTLNAYGPMLPELLGGSADLAPSNLTIWKGSVSLKEDPAGNYIHYGVREFGMTAIANGI

AHHGGFVPYTATFLMFVEYARNAARMAALMKARQIMVYTHDSIGLGEDGPTHQAVEQLASLRLTPNFSTW

RPCDQMEAAVGWKLAVERHNGPTALILSRQNLAQVERTPDQVKEIARGGYVLKDSGGKPDIILIATGSEM

EITLQAAEKLAGEGRNVRVVSLPSTDIFDAQDEEYRESVLPSNVAARVAVEAGIADYWYKYVGLKGAIVG

MTGYGESAPADKLFPFFGFTAENIVAKAHKVLGVKGA

>gi|30063844|ref|NP_838015.1| transaldolase A [Shigella flexneri 2a str. 2457T]

MNELDGIKQFTTVVADSCDIESIRHYHPQDATTNPSLLLKAAGLSQYEHLIDDAIAWGKKNGKTQEQQVV

AACDKLAVNFGAEILKIVPGCVSTEVDARLSFDKEKSIEKARHLVDLYQQQGVEKSRILIKLASTWEGIR

AAEELEKEGINCNLTLLFSFTQARACAEAGVFLISPFVGRIYDWYQARKPMDPYVVEEDPGVKSVRNIYD

YYKQHHYETIVMGASFRRTEQILALTGCDRLTIAPNLLKELQEKVSPVVRKLIPPSQTFPRPAPMSEAEF

RWEHNQDAMAVEKLSEGIRLFAVDQRKLEDLLAAKL

>gi|30063843|ref|NP_838014.1| malic enzyme [Shigella flexneri 2a str. 2457T]

MDDQLKQSALDFHEFPVPGKIQVSPTKPLATQRDLALAYSPGVAAPCLEIEKDPLKAYKYTARGNLVAVI

SNGTAVLGLGNIGALAGKPVMEGKGVLFKKFAGIDVFDIEVDELDPDKFIEVVAALEPTFGGINLEDIKA

PECFYIEQKLRERMNIPVFHDDQHGTAIISTAAILNGLRVVEKNISDVRMVVSGAGAAAIACMNLLVALG

LQKHNIVVCDSKGVIYQGREPNMAETKAAYAVVDDGKRTLDDVIEGADIFLGCSGPKVLTQEMVKKMARA

PMILALANPEPEILPPLAKEVRPDAIICTGRSDYPNQVNNVLCFPFIFRGALDVGATAINEEMKLAAVRA

IAELAHAEQSEVVASAYGVQDLSFGPEYIIPKPFDPRLIVKIAPAVAKAAMESGVATRPIADFDVYIDKL

TEFVYKTNLFMKPIFSQARKAPKRVVLPEGEEARVLHATQELVTLGLAKPILIGRPNVIEMRIQKLGLQI

KAGVDFEIVNNESDPRFKEYWTEYFQIMKRRGVTQEQAQRALISNPTVIGAIMVQRGEADAMICGTVGDY

HEHFSVVKNVFGYRDGVHTAGAMNALLLPSGNTFIADTYVNDEPDAEELAEITLMAAETVRRFGIEPRVA

LLSHSNFGSSDCPSSSKMRQALELVRDRAPDLMIDGEMHGDAALVEAIRNDRMPDSPLKGSANILVMPNM

EAARISYNLLRVSSSEGVTVGPVLMGVAKPVHVLTPIASVRRIVNMVALAVVEAQTQPL

>gi|30063842|ref|NP_838013.1| hypothetical protein S2655 [Shigella flexneri 2a str. 2457T]

MPNFVIYSDEKLSRYTKVFHRRRLMDKERIIQEFVPGKQVTLAHLIAHPGEELAKKIGVPDAGAIGIMTL

TPGETAMIAGDLALKAADVHIGFLDRFSGALVIYGSVGAVEEALSQTVSGLGRLLNYTLCEMTKS

>gi|30063841|ref|NP_838012.1| hypothetical protein S2654 [Shigella flexneri 2a str. 2457T]

MKRIAFVGSVGAGKTTLFNALQGNYTLARKTLAVEFNDKGDIDTPGEYFSHPRWYHALITTLQDVDMLIY

VHGANDPESRLPAGLLDIGVSKRQIAVISKTDMPDADVAATRKLLLETGFEEPIFELNSHDPQSVQQLVD

YLASLTKQEEAGEKTHHSE

>gi|30063840|ref|NP_838011.1| IS1N orfA [Shigella flexneri 2a str. 2457T]

MTSVNIHCPRCQSAQVYRHGQNPKGRDRLRCRDCHRVFQFTYTYQARKPGMKELITEMAFNGAGVRDTAR

TLKIGSNTVIRTLKNSRQSE

>gi|30063839|ref|NP_838010.1| iso-IS1 ORF2 [Shigella flexneri 2a str. 2457T]

MAFICELDEQWSYVGSKARQHWLGYAYNTKTGGVLAYTFGPRTDQTCRELLALLTPFNIGMLTSDDWGSY

GREVPKNKHLTGKIFTQRIERNNLTLRTRIKRLGRKTICFSRSVEIHEKVIGAFIEKHMFY

>gi|30063838|ref|NP_838009.1| amino acid antiporter [Shigella flexneri 2a str. 2457T]

MSNANIAPKSKKMTVGTLAIMNITAVVSLRFIPSEAEYGLGAIFYYTFAAIMFLVPVAFVAAELATTYPQ

KGGAFRWIGEAFGARWGFVGMLMTWLQVIPYFPTVLTFGAVSVAFIDVDMGVAESIASNKWYILFFVLFV

YWGAICVALRGVGIFSQVSKWCGIIGTIIPAIILVILGFSYLWFSGKPAVIPLGWGDLFPNIANFQNIVL

AASIFLAYAGMEMNAVHVNDLDNPTKKYPIAITIASIGTIAIFLLSTLGVAFIIPTDKISLTQSLLVAYD

MLFEWAGVPWLGSVMAFMLAIGVLGGVVTWIAGPNTGVLAIAKAGYLPKFFQKTNRHGMGHHLMFVQGII

VSVLSVTFVIMPSVQAAFQILSQLTVMLYLVMYMLMFASAIYLRHSQPDTKRPYRAPALFFWATLGFLGS

FLAFVLSFIPPEQIPTGSPTSYVLYLLVLVALFLLIPILIYAFRKSDWKDPNSDFEPFSWEKRTSN

>gi|30063837|ref|NP_838008.1| hypothetical protein S2648 [Shigella flexneri 2a str. 2457T]

MAEPEGRCVRMLSDSWSFPDSRHTLGCSTIGSSEAAMLGRLALKWQWCKKREVQGKSTEPDLRSCANMLA

>gi|30063836|ref|NP_838007.1| hypothetical protein S2647 [Shigella flexneri 2a str. 2457T]

MKQDIADRLEILEGQRAEAKQLRKQARRAHRNNEAELLTKYISFTNYCIYECYKEDAEDWLDSLPEQY

>gi|30063835|ref|NP_838006.1| hypothetical protein S2646 [Shigella flexneri 2a str. 2457T]

MFGNYISTSPEKIIMLALRIMQGIAKTLAEHVLDLKHSPLSKQAMKRQTLRLWAEYSLGTINKIIDMKSG

PSNQSAEEMEFIRRLILIRRDIHSQLHSVGIDINDGTGD

>gi|30063834|ref|NP_838005.1| hypothetical protein S2645 [Shigella flexneri 2a str. 2457T]

MDAFEVLANKGAELVAQRDKTANEGERALLNKQIKAIRMAQFKLISNEVIETIKPQIPQIVADAIKAAGL

EKRIAGLKTNAEKMEMYKQSGAPNPDEYFSPDVEFMAQVEERLGSCLTEEQRRYFDGVDSSAGIDLNSYF

GREIEHFDAAPSMPEPEPEMATDPEAEQRKRVFNAIGIRY

>gi|30063833|ref|NP_838004.1| hypothetical protein S2644 [Shigella flexneri 2a str. 2457T]

MHSLDSYFQRTTAPKSAAQERREEFHEKVMRSADYIADKFVETVRPLVDEVADKLQSEMPEDMEGTAKRR

LICELSRRFGVSISAFK

>gi|30063832|ref|NP_838003.1| hypothetical protein S2643 [Shigella flexneri 2a str. 2457T]

MTNIERLERCADIMRRRWIYDPEAGLLFSRETKDVIKGSMTNKGHLVVTVHEGKFKVTLTYQKACYVYTY

GAYDETLYEVHHVNLNKQDNRIKNIRLMPKEKHRRIHKNVRKLIRLGHTQWLQPQLIEQSL

>gi|30063831|ref|NP_838002.1| hypothetical protein S2642 [Shigella flexneri 2a str. 2457T]

MRISEQQRQILDYLAKNESITARQAADLVYGGNVTRVQVDAARRSLMTMVSNGLIRKQGRAFVAERHINH

AYSQDVEELRRQILWVFSKQGQEYFLSEHPNFAAKELTVSGLSCALPGPAVKVALLTSSYGTP

>gi|30063830|ref|NP_838001.1| hypothetical protein S2641 [Shigella flexneri 2a str. 2457T]

MPALDLIRPSVTAMRVIASVNAEFARELKLPPHIRSLGLISADSDDVTYIAADEATKQAMVEVVYGRSLY

AGAAHGPSPTAGEVLIMLGGPNPAEVRAGLDAMVAHIENGAAFQWANDAENTAFLAHVVSRTGSYLSSTA

GITLGDPMAYLVAPPLEATYGIDAALKSADVQLVTYVPPPSETNYSAAFLTGSQAACKAACNAFTDAVLE

IARNPIQRA

>gi|30063829|ref|NP_838000.1| hypothetical protein S2639 [Shigella flexneri 2a str. 2457T]

MINALGLLEVDGMVAAIDAADAMLKAANVRLLSHEVLDPGRLTLVVEGDLAACRAALDAGCAAAMRTGRV

ISRKEIGRPDDDTQWLVTGFNRQPKQPVKEPDAPVIVAESADELLALLTSVCQGMTAGEVAAHFGWPLEK

ARNALEQLFSAGTLRKRSSRYRLKPH

>gi|30063828|ref|NP_837999.1| ARAC-type regulatory protein, partial [Shigella flexneri 2a str. 2457T]

MLRSQSALEVKEQHKAALWGFVQQALATFCENPENLHQPAVRKVLGDNLLMAMGAMLEEAQPMVTAESIS

HQSYRRLLSRAREYVLENMSEPVTVLDLCNQLHVSRRTLQNAFHAILGIGPNAWLKRIRLNVVRRELISP

WSQSTTVKDAAMQWGFWHLGQFATDYQQLFAEKPSLTLHQRMREWG

>gi|30063827|ref|NP_837998.1| N-acetylmuramoyl-l-alanine amidase I [Shigella flexneri 2a str. 2457T]

MSTFKPLKTLTSRRQVLKAGLAALTLSGMSQAIAKDEPLKTSNGHSKPKAKKSGGKRVVVLDPGHGGIDT

GAIGRNGSKEKHVVLAIAKNVRSILRNHGIDARLTRSGDTFIPLYDRVEIAHKHGADLFMSIHADGFTNP

KAAGASVFALSNRGASSAMAKYLSERENRADEVAGKKATDKDHLLQQVLFDLVQTDTIKNSLTLGSHILK

KIKPVHKLHSRNTEQAAFVVLKSPSVPSVLVETSFITNPEEERLLGTAAFRQKIATAIAEGVISYFHWFD

NQKAHSKKR

>gi|30063826|ref|NP_837997.1| acetyltransferase [Shigella flexneri 2a str. 2457T]

MEIRVFRQEDFEEVITLWERCDLLRPWNDPEMDIERKMNHDVSLFLVAEVNGEVVGTVMGGYDGHRGSAY

YLGVHPEFRGRGIANALLNRLEKKLIARGCPKIQINVPEDNDMVLGMYERLGYEHADVLSLGKRLIEDEE

Y

>gi|30063825|ref|NP_837996.1| hypothetical protein S2634 [Shigella flexneri 2a str. 2457T]

MKSTEFHPVHYDAHGRLRLPLLFWLVLLLQARTWVLFVIAGASREQGTALLNLFYPDHDNFWLGLIPGIP

AVLAFLLSGRRASFPRTWHVLYFLLLLAQVVLLCWQPWLWLNGESVSGIGLALVVADIVALIWLLTNRRL

RACFNEEKE

>gi|30063824|ref|NP_837995.1| hypothetical protein S2633 [Shigella flexneri 2a str. 2457T]

MKSLRLMLCAMPLMLTGCSTMSSVNWSAANPWNWFGSSTKVSEQGVGELTASTPLQEQAIADALDGDYRL

RSGMKTTNGNVVRFFEVMKGDNVAMVINGDQGTISRIDVLDSDIPADTGVKIGTPFSDLYSKAFGNCQKA

DGDDNRAVECKAEGSQHISYQFSGEWRGPEGLMPSDDTLKNWKVSKIIWRR

>gi|30063823|ref|NP_837994.1| hypothetical protein S2632 [Shigella flexneri 2a str. 2457T]

MSQVQSGILPEHCRAAIWIEANVKGEVDALRAASKTFADKLATFEAKFPDAHLGAVVAFGNNTWRALSGG

VGAEELKDFPGYGKGLAPTTQFDVLIHILSLRHDVNFSVAQAAMEAFGDCIEVKEEIHGFRWVEERDLSG

FVDGTENPAGEETRREVAVIKDGVDSGGSYVFVQRWEHNLKQLNRMSVHDQEMMIGRTKEANEEIDGDER

PETSHLTRVDLKEDGKGLKIVRQSLPYGTASGTHGLYFCAYCARLHNIEQQLLSMFGDTDGKRDAMLRFT

KPVTGGYYFAPSLDKLMAL

>gi|30063822|ref|NP_837993.1| N-acetylmuramic acid phosphotransfer permease [Shigella flexneri 2a str. 2457T]

MAKEISSELLNTILTRVGGPGNIASCGNCMTRLRLGVHDSSLVDPNIKTLEGVKGVILTSDQVQVVFGPG

KAHRAAKAMSELLGDAPVQDAAEIAAQNKRQLKAKQTSGVQQFLAKFATIFTPLIPGFIAAGLLLGIATL

IATVMHVPADAQGTLPDALNFMKVFSKGLFTFLVILVGYNAAQAFGGTGVNGAIIAALFLLGYNPAATTG

YYAGFHDFFGLPIDPRGNIIGVLIAAWACARIEGMVRRFMPDDLDMLLTSLITLLITATLAYLIIMPLGG

WLFEGMSWLFMHLNSNPFGCAVLAGLFLIAVVFGVHQGFIPVYLALMDSQGFNSLFPILSMAGAGQVGAA

LALYWRAQPHSALRSQVRGAIIPGLLGVGEPLIYGVTLPRMKPFVTACLGGAAGGLFIGLIAWWGLPMGL

NSAFGPSGLVALPLMTSAQGILPAMAVYAGGILVAWVCGFIFTTLFGCRNVNLD

>gi|30063821|ref|NP_837992.1| N-acetylmuramic acid-6-phosphate etherase [Shigella flexneri 2a str. 2457T]

MQLEKMITEGSNTASAEIDRVSTLEMCRIINDEDKTVPPAVDRVLPDIAAAIDVIHAQVSGGGRLIYLGA

GTSGRLGILDASECPPTYGVKPGLVVGLIAGGEYAIQHAVEGAEDSREGGVNDLKNINLTAQDVVVGIAA

SGRTPYVIAGLEYARQLGCRTVGISCNPGSAVSTTAEFAITPIVGAEVVTGSSRMKAGTAQKLVLNMLST

GLMIKSGKVFGNLMVDVVATNEKLHVRQVNIVKNATGCNAEQAEAALIACERNCKTAIVMVLKNLDAAEA

KKRLDQHGGFIRQVLDKE

>gi|30063820|ref|NP_837991.1| hypothetical protein S2628 [Shigella flexneri 2a str. 2457T]

MLYLTKIRNAESEFTGNEQKIADFLRARVSELKSVSSRQMAKQLGISQSSIVKFAQKLGAQGFTELRMAL

IGEYSASREKTNATALHLHSSITSDDSLEVIARKLNREKELALEQTCALFDYARLQKIIEVISKAPFIQI

TGLGGSALVGRDLSFKLMKIGYRVACEADTHVQATVSQALKKGDVQIAISYSGSKKEIVLCAEAARKQGA

TVIAITSLADSPLRRLAHFTLDTVSGETEWRSSSMSTRTAQNSVTDLLFVGLVQHQ

>gi|30063819|ref|NP_837990.1| short chain dehydrogenase [Shigella flexneri 2a str. 2457T]

MGKLTGKTALITGALQGIGEGIARTFARHGANLILLDISPEIEKLADELCGRGHRCTAVVADVRDPASVA

AAIKRAKEKEGRIDILVNNAGVCRLGSFLDMSDEDRDFHIDINIKGVWNVTKAVLPEMIARKDGRIVMMS

SVTGDMVADPGETAYALTKAAIVGLTKSLAVEYAQSGIRVNAICPGYVRTPMAESIARQSNPEDPESVLT

EMAKAIPLCRLADPLEVGELAAFLASDESSYLTGTQNVIDGGSTLPETVSVGI

>gi|30063818|ref|NP_837989.1| thiosulfate transporter subunit [Shigella flexneri 2a str. 2457T]

MAVNLLKKNSLALVASLLLAGHVQATELLNSSYDVSRELFAALNPPFEQQWAKDNGGDKLTIKQSHAGSS

KQALAILQGLKADVVTYNQVTDVQILHDKGKLIPADWQSRLPNNSSPFYSTMGFLVRKGNPKNIHDWNDL

VRSDVKLIFPNPKTSGNARYTYLAAWGAADKADGGDKAKTEQFMTQFLKNVEVFDTGGRGATTTFAERGL

GDVLISFESEVNNIRKQYEAQGFEVVIPKTNILAEFPVAWVDKNVQANGTEKAAKAYLNWLYSPQAQTII

TDYYYRVNNPEVMDKLKDKFPQTELFRVEDKFGSWPEVMKTHFTSGGELDKLLAAGRK

>gi|30063817|ref|NP_837988.1| sulfate/thiosulfate transporter subunit [Shigella flexneri 2a str. 2457T]

MFAVSSRRVLPGFTLSLGTSLLFVCLILLLPLSALVMQLSEMSWAQYWEVITNPQVVAAYKVTLLSAFVA

SIFNGVFGLLMAWILTRYRFPGRTLLDALMDLPFALPTAVAGLTLASLFSVNGFYGEWLAKFDIKVTYTW

LGIAVAMAFTSIPFVVRTVQPVLEELGPEYEEAAETLGATRWQSFCKVVLPELSPALVAGVALSFTRSLG

EFGAVIFIAGNIAWKTEVTSLMIFVRLQEFDYPAASAIASVILAASLLLLFSINTLQSRFGRRVVGH

>gi|30063816|ref|NP_837987.1| sulfate transport system permease W protein, partial [Shigella flexneri 2a str. 2457T]

MFSWPGMVLVTIFVTCPFVVRELVPVMLSQGSQEDEAAILLGASGWQMFRRVTLPNIRWALLYGVVLTNA

RAIGEFGAVSVVSGSIRGETLSLPLQIELLEQDYNTVGSFTAAALLTLMAIITLFLKSMLQWRLENQEKR

AQQEEHHEH

>gi|30063815|ref|NP_837986.1| sulfate/thiosulfate transporter subunit [Shigella flexneri 2a str. 2457T]

MSIEIANIKKSFGRTQVLNDISLDIPSGQMVALLGPSGSGKTTLLRIIAGLEHQTSGHIRFHGTDVSRLH

ARDRKVGFVFQHYALFRHMTVFDNITFGLTVLPRRERPNAAAIKAKVTKLLEMVQLAHLADRYPAQLSGG

QKQRVALARALAVEPQILLLDEPFGALDAQVRKELRRWLRQLHEELKFTSVFVTHDQEEATEVADRVVVM

SQGNIEQADAPNQVWREPATRFVLEFMGEVNRLQGTIRGGQFHVGAHRWPLGYTPAYQGPVDLFLRPWEV

DISRRTSLDSPLPVQVLEASPKGHYTQLVVQPLGWYNEPLTVVMHGDDAPQHGERLFVGLQHARLYNGDE

RIETRDEELALAQSA

>gi|30063814|ref|NP_837985.1| cysteine synthase B [Shigella flexneri 2a str. 2457T]

MSTLEQTIGNTPLVKLQRMGPDNGSEVWLKLEGNNPAGSVKDRAALSMIVEAEKRGEIKPGDVLIEATSG

NTGIALAMIAALKGYRMKLLMPDNMSQERRAAMRAYGAELILVTKEQGMEGARDLALEMANRGEGKLLDQ

FNNPDNPYAHYTTTGPEIWQQTGGRITHFVSSMGTTGTITGVSCFMREQSKPVTIVGLQPEEGSSIPGIR

RWPAEYLPGIFNAALVDEVLDIHQRDAENTMRELAVREGIFCGVSSGGAVAGALRVAKANPGAVVVAIIC

DRGDRYLSTGVFGEEHFSQGAGI

>gi|30063813|ref|NP_837984.1| hypothetical protein S2620 [Shigella flexneri 2a str. 2457T]

MKKIICLAITLLMTLPAYAKLTVHEEARINAMLEGLAQKKDLIFVRNGDEHTCDEAVSHLRLKLGNTRNR

IDTAEQFIDKVASSSSITGKPYIVKIPGKSDENAQPFLHALIAQTDKTVPAEGN

>gi|30063812|ref|NP_837983.1| pyridoxal kinase [Shigella flexneri 2a str. 2457T]

MSSLLLFNDKSRALQADIVAVQSQVVYGSVGNSIAVPAIKQNGLNVFAVPTVLLSNTPHYDTFYGGAIPD

EWFSGYLRALQERDALRQLRAVTTGYMGTASQIKILAEWLTALRKDHPDLLIMVDPVIGDIDSGIYVKPD

LPEAYRQYLLPLAQGITPNIFELEILTGKNCRDLDSAIAAAKSLLSDTLKWVVITSASGNEENQEMQVVV

VSADSVNVISHSRVKTDLKGTGDLFCAQLISGLLKGKALNDAVHRAGLRVLEVMRYTQQHESDELILPPL

AEA

>gi|30063811|ref|NP_837982.1| glucose-specific PTS system component [Shigella flexneri 2a str. 2457T]

MGLFDKLKSLVSDDKKDTGTIEIIAPLSGEIVNIEDVPDVVFAEKIVGDGIAIKPTGNKMVAPVDGTIGK

IFETNHAFSIESDSGVELFVHFGIDTVELKGEGFKRIAEEGQRVKVGDTVIEFDLPLLEEKAKSTLTPVV

ISNMDEIKELIKLSGSVTVGETPVIRIKK

>gi|30063810|ref|NP_837981.1| phosphoenolpyruvate-protein phosphotransferase [Shigella flexneri 2a str. 2457T]

MISGILASPGIAFGKALLLKEDEIVIDRKKISADQVDQEVERFLSGRAKASAQLETIKTKAGETFGEEKE

AIFEGHIMLLEDEELEQEIIALIKDKHMTADAAAHEVIEGQASALEELDDEYLKERAADVRDIGKRLLRN

ILGLKIIDLSAIQDEVILVAADLTPSETAQLNLKKVLGFITDAGGRTSHTSIMARSLELPAIVGTGSVTS

QVKNDDYLILDAVNNQVYVNPTNEVIDKMRAVQEQVASEKAELAKLKDLPAITLDGHQVEVCANIGTVRD

VEGAERNGAEGVGLYRTEFLFMDRDALPTEEEQFAAYKAVAEACGSQAVIVRTMDIGGDKELPYMNFPKE

ENPFLGWRAIRIAMDRKEILRDQLRAILRASAFGKLRIMFPMIISVEEVRALRKEIEIYKQELRDEGKAF

DESIEIGVMVETPAAATIARHLAKEVDFFSIGTNDLTQYTLAVDRGNDMISHLYQPMSPSVLNLIKQVID

ASHAEGKWTGMCGELAGDERATLLLLGMGLDEFSMSAISIPRIKKIIRNTNFEDAKVLAEQALAQPTTDE

LMTLVNKFIEEKTIC

>gi|30063809|ref|NP_837980.1| phosphohistidinoprotein-hexose phosphotransferase component of PTS system (Hpr) [Shigella flexneri 2a str. 2457T]

MFQQEVTITAPNGLHTRPAAQFVKEAKGFTSEITVTSNGKSASAKSLFKLQTLGLTQGTVVTISAEGEDE

QKAVEHLVKLMAELE

>gi|30063808|ref|NP_837979.1| cysteine synthase A [Shigella flexneri 2a str. 2457T]

MSKIFEDNSLTIGHTPLVRLNRIGNGRILAKVESRNPSFSVKCRIGANMIWDAEKRGVLKPGVELVEPTS

GNTGIALAYVAAARGYKLTLTMPETMSIERRKLLKALGANLVLTEGAKGMKGAIQKAEEIVASNPEKYLL

LQQFSNPANPEIHEKTTGPEIWEDTDGQVDVFIAGVGTGGTLTGVSRYIKGTKGKTDLISVAVEPTDSPV

IAQVLAGEEIKPGPHKIQGIGAGFIPANLDLKLVDKVIGITNEEAISTARRLMEEEGILAGISSGAAVAA

ALKLQEDESLTNKNIVVILPSSGERYLSTALFADLFTEKELQQ

>gi|30063807|ref|NP_837978.1| sulfate transport protein CysZ [Shigella flexneri 2a str. 2457T]

MVSSFTSAPRSGFYYFAQGWKLVSQPGIRRFVILPLLVNILLMGGAFWWLFTQLDVWIPTFMSYVPDWLQ

WLSYLLWPLAVISVLLVFGYFFSTIANWIAAPFNGLLAEQLEARLTGATPPDTGIFGIMKDVPRIMKREW

QKFVWYLPRAIVLLILYFIPGIGQTVAPVLWFLFSAWMLAIQYCDYPFDNHKVPFKEMRTALRTRKITNM

QFGALTSLFTMIPLLNLFIMPVAVCGATAMWVDCYRDKHAMWR

>gi|30063806|ref|NP_837977.1| cell division protein ZipA [Shigella flexneri 2a str. 2457T]

MQDLRLILIIVGAIAIIALLVHGFWTSRKERSSMFRDRPLKRMKSKRDDDSYDEDVEDDEGVGEVRVHRV

NHAPANAQEHEAARPSPQHQYLPPYASAQPRQPVQQPPEAQVPPQHAPRPAQPVQQPAYQPQPEQPLQQP

VSPQVAPAPQPVHSAPQPAQQAFQPAEPVAAPQPEPVAEPAPVMDKPKRKEAVIIMNVAAHHGSELNGEL

LLNSIQQAGFIFGDMNIYHRHLSPDGSGPALFSLANMVKPGTFDPEMKDFTTPGVTIFMQVPSYGDELQN

FKLMLQSAQHIADEVGGVVLDDQRRMMTPQKLREYQDIIREVKDANA

>gi|30063805|ref|NP_837976.1| NAD-dependent DNA ligase LigA [Shigella flexneri 2a str. 2457T]

MESIEQQLTELRTTLCHHEYLYHVMDAPEIPDAEYDRLMRELRELETKHPELITPDSPTQRVGAAPLAAF

SQIRHEVPMLSLDNVFDEESFLAFNKRVQDRLKNNEKVTWCCELKLDGLAVSILYENGVLVSAATRGDGT

TGEDITSNVRTIRAIPLKLHGENIPARLEVRGEVFLPQAGFEKINEDARRTGGKVFANPRNAAAGSLRQL

DPRITAKRPLTFFCYGVGVLEGGELPDTHLGRLLQFKKWGVPVSDRVTLCESAEEVLAFYHKVEEDRPTL

GFDIDGVVIKVNSLEQQEQLGFVARAPRWAVAFKFPAQEQMTFVRDVEFQVGRTGAITPVARLEPVHVAG

VLVSNATLHNADEIERLGLRIGDKVVIRRAGDVIPQVVNVVLSERPEDTREVVFPTHCPVCGSDVERVEG

EAVARCTGGLICGAQRKESLKHFVSRRAMDVDGMGDKIIDQLVEKEYVHTPADLFKLTAGKLTGLERMGP

KLAQNVVNALEKAKETTFARFLYALGIREVGEATAAGLAAYFGTLEALEAASIEELQKVPDVGIVVASHV

HNFFAEESNRNVISELLAEGVHWPAPIVINAEEIDSPFAGKTVVLTGSLSQMSRDDAKARLVELGAKVAG

SVSKKTDLVIAGEAAGSKLAKAQELGIEVIDEAEMLRLLGS

>gi|30063804|ref|NP_837975.1| cytochrome oxidase [Shigella flexneri 2a str. 2457T]

MKLFRILDPFTLTLITVVLLASFFPARGDFVPFFENLTTAAIALLFFMHGAKLSREAIIAGGGHWRLHLW

VMCSTFVLFPILGVLFAWWKPVNVDPMLYSGFLYLCILPATVQSAIAFTSMAGGNVAAAVCSASASSLLG

IFLSPLLVGLVMNVHGARGSLEQVGKIMLQLLLPFVLGHLSRPWIGDWVSRNKKWIAKTDQTSILLVVYT

AFSEAVVNGIWHKVGWGSLLFIVVVSCVLLAIVIVVNVFMARRLSFNKADEITIVFCGSKKSLANGIPMA

NILFPTSVIGMMVLPLMIFHQIQLMVCAVLARRYKRQTEQLQAQQESSADKA

>gi|30063803|ref|NP_837974.1| LYSR-type transcriptional regulator [Shigella flexneri 2a str. 2457T]

MNYSLKQLKVFVTVAQEKSFSRAGERIGLSQSAVSHSVKELENHTGVRLLDRTTREVVLTDAGQQLALRL

ERLLDELNSTLRDTGRMGQQLSGKVRVAASQTISAHLIPQCIAESHRRYPDIQFVLHDRPQQWVMESIRQ

GDVDFGIVIDPGPVGDLQCEAILSEPFFLLCHRDSALAVEDYVPWQALQGAKLVLQDYASGSRPLIDAAL

ARNGIQANIVQEIGHPATLFPMVAAGIGISILPALALPLPEGSPLVVKRITPVVERQLMLVRRKNRSLST

AAEALWDVVRDQGNALMAGREGDPLYQI

>gi|30063802|ref|NP_837973.1| hypothetical protein S2609 [Shigella flexneri 2a str. 2457T]

MATISSTSIPSIQTQSSNRASQGSDVASQIARISQQIIKLTQQIKEIVDTSGSAEDKQKQAELIQQQITL

LETQLAQLQKQQAEKAQEKEQRLSLNVSLLNPVEKTTHIDIYI

>gi|30063801|ref|NP_837972.1| glutamyl-tRNA synthetase [Shigella flexneri 2a str. 2457T]

MKIKTRFAPSPTGYLHVGGARTALYSWLFARNHGGEFVLRIEDTDLERSTPEAIEAIMDGMNWLSLEWDE

GPYYQTKRFDRYNAVIDQMLEEGTAYKCYCSKERLEALREEQMAKGEKPRYDGRCRHSHEHHADDEPCVV

RFANPQEGSVVFDDQIRGPIEFSNQELDDLIIRRTDGSPTYNFCVVVDDWDMEITHVIRGEDHINNTPRQ

INILKALKAPVPVYAHVSMINGDDGKKLSKRHGAVSVMQYRDDGYLPEALLNYLVRLGWSHGDQEIFTRE

EMIKYFTLNAVSKSASAFNTDKLLWLNHHYINALPPEYVATHLQWHIEQENIDTRNGPQLADLVKLLGER

CKTLKEMAQSCRYFYEDFAEFDADAAKKHLRPVARQPLEVVRDKLAAITDWTAENVHHAIQATADELEVG

MGKVGMPLRVAVTGAGQSPALDVTVHAIGKTRSIERINKALDFIAERENQQ

>gi|30063800|ref|NP_837971.1| hypothetical protein S2604 [Shigella flexneri 2a str. 2457T]

MTTEELAECLGVAKQTVNRWTREKGWKTEKFPGVKGGRARLILVDTQVCEFIQNTPAFHNTPMLMEAEER

IAEYAPGARAPAYRQIINAIDNMTDIEQEKVAQFLSREGIRNFLARLDIDESA

>gi|30063799|ref|NP_837970.1| hypothetical protein S2603 [Shigella flexneri 2a str. 2457T]

MTPDELARLTGYSRQTINKWVRKEGWTTSPKPGVQGGKARLVHVNEQVREYIRNAERPEGQGEAPALSGD

APLEVLLVTLAKEMTPVEQKQFTSLLLREGIIGLLQRLGIRDSK

>gi|30063797|ref|NP_837968.1| permease of transport system for 3 nucleosides [Shigella flexneri 2a str. 2457T]

MDRVLHFVLALAVVVILALLVSSDRKKIRIRYVIQLLVIEVLLAWFFLNSDVGLGFVKGFSEMFEKLLGF

ANEGTNFVFGSMNDQGLAFFFLKVLCPIVFISALIGILQHIRVLPVIIRAIGFLLSKVNGMGKLESFNAV

SSLILGQSENFIAYKDILGKISRNRMYTMAATAMSTVSMSIVGAYMTMLEPKYVVAALVLNMFSTFIVLS

LINPYRVDASEENIQMSNLHEGQSFFEMLGEYILAGFKVAIIVAAMLIGFIALIAALNALFATVTGWFGY

SISFQGILGYIFYPIAWVIGVPSSEALQVGSIMATKLVSNEFVAMMDLQKIASTLSPRAEGIISVFLVSF

ANFSSIGIIAGAVKGLNEEQGNVVSRFGLKLVYGSTLVSVLSASIAALVL

>gi|30063796|ref|NP_837967.1| manganese transport protein MntH [Shigella flexneri 2a str. 2457T]

MTNYRVESSSGRAARKMRLALMGPAFIAAIGYIDPGNFATNIQAGASFGYQLLWVVVWANLMAMLIQILS

AKLGIATGKNLAEQIRDHYPRPVVWFYWVQAEIIAMATDLAEFIGAAIGFKLILGVSLLQGAVLTGIATF

LILMLQRRGQKPLEKVIGGLLLFVAAAYIVELIFSQPNLAQLGKGMVIPSLPTSEAVFLAAGVLGATIMP

HVIYLHSSLTQHLHGGSRQQRYSATKWDVAIAMTIAGFVNLVMMATAAAAFHFSGHTGVADLDEAYLTLQ

PLLSHAAATVFGLSLVAAGLSSTVVGTLAGQVVMQGFIRFHIPLWVRRTVTMLPSFIVILMGLDPTRILV

MSQVLLSFGIALALVPLLIFTSDSKLMGDLVNSKRVKQTGWVIVVLVVALNIWLLVGTALGL

>gi|30063794|ref|NP_837965.1| hypothetical protein S2595 [Shigella flexneri 2a str. 2457T]

MFRSLFLAAALMAFTPLAANAGEITLLPSIKLQIGDRDHYGNYWDGGHWRDRDYWHRNYEWRKNRWWRHD

NGYHRGWDKRKAYERGYREGWRDRDDHRGKGRGHGHRH

>gi|30063793|ref|NP_837964.1| hypothetical protein S2594 [Shigella flexneri 2a str. 2457T]

MLHPRARTMLLLSLPAVAIGIASSLILIVVMKIASVLQNLLWQRLPGTLGIAQDSPLWIIGVLTLTGIAV

GLVIRFSQGHAGPDPACEPLIGAPVPPSALPRLIVALILGLAGGVSLGPEHPIMTVNIALAVAIGARLLP

RVNRMEWTILASAGTIGALFGTPVAAALIFSQTLNGSSEVPLWDRLFAPLMAAAAGALTTGLFFHPHFSL

PIAHYGQMEMTDILSGAIVAAIAIAAGMVAVWCLPRLHAMMNQMKNPVLVLGIGGFILGILGVIGGPVSL

FKGLDEMQQMVANQAFSTSDYFLLAVIKLAALVVAAASGFRGGRIFPAVFVGVALGLMLHEHVPAVPAAI

TVSCAILGIVLVVTRDGWLSLFMAAVVVPNTTLLPLLCIVMLPAWLLLAGKPMMMVNRPKQQPPHDNV

>gi|30063792|ref|NP_837963.1| glucokinase [Shigella flexneri 2a str. 2457T]

MTKYALVGDVGGTNARLALCDIASGEISQAKTYSGLDYPSLEAVIRVYLEEHKVEVKDGCIAIACPITGD

WVAMTNHTWAFSIAEMKKNLGFSHLEIINDFTAVSMAIPMLKKEHLIQFGGAEPVEGKPIAVYGAGTGLG

VAHLVHVDKRWVSLPGEGGHVDFAPNSEEEAIILEILRAEIGHVSAERVLSGPGLVNLYRAIVKADNRLP

ENLKPKDITERALADSCTDCRRALSLFCVIMGRFGGNLALNLGTFGGVFIAGGIVPRFLEFFKASGFRAA

FEDKGRFKEYVHDIPVYLIVHDYPGLLGSGAHLRQTLGHIL

>gi|30063791|ref|NP_837962.1| PTS system enzyme IIB component [Shigella flexneri 2a str. 2457T]

MSKKLIALCACPMGLAHTFMAAQVLEEAAVEAGYEVKIETQGADGIQNRLTAQDIAEATIIIHSVAVTPE

DNERFESRDVYEITLQDAIKNAAGIIKEIEEMIASEQQ

>gi|30063790|ref|NP_837961.1| transporter [Shigella flexneri 2a str. 2457T]

MAIKKRSATVVPGASGAAAAVKNPQASKSSFWGELPQHVMSGISRMVPTLIMGGVILAFSQLIAYSWLKI

PAEIGIMDALNSGKFSGFDLSLLKFAWLSQSFGGVLFGFAIPMFAAFVANSIGGKLAFPAGFIGGLMSTQ

PTQLLNFDPSTMQWATSSPVPSTFIGALIISIVAGYLVKWMNQKIQLPDFLLAFKTTFLLPILSAIFVML

AMYYVITPFGGWINGGIRTVLTAAGEKGALMYAMGIAAATAIDLGGPINKAAGFVAFSFTTDHVLPVTAR

SIAIVIPPIGLGLATIIDRRLTGKRLFNAQLYPQGKTAMFLAFMGISEGAIPFALESPITAIPSYMVGAI

VGSTAAVWLGAVQWFPESAIWAWPLVTNLGVYMAGIALGAIITALMVVFLRLMMFRKGKLLIDSL

>gi|30063789|ref|NP_837960.1| exoaminopeptidase [Shigella flexneri 2a str. 2457T]

MDLSLLKALSEADAIASSEQEVRQILLEEAARLQKEVRFDGLGSVLIRLNESTGPKVMICAHMDEVGFMV

RSISREGAIDVLPVGNVRMAARQLQPVRITTREECKIPGLLDGDRQGNDVSAMRVDIGARTYDEVMQAGI

RPGDRVTFDTTFQVLPHQRVMGKAFDDRLSCYLLVTLLRELHDAELPAEVWLVASSSEEVGLRGGQTATR

AVSPDVAIVLDTACWAKNFDYGAANHRQIGNGPMLVLSDKSLIAPPKLTAWIETVAAEIGVPLQADMFSN

GGTDGGAVHLTGTGVPTLVMGPATRHGHCAASIADCRDILQMEQLLSALIQRLTRETVVQLTDFR

>gi|30063788|ref|NP_837959.1| PTS system enzyme IIA component, enzyme I [Shigella flexneri 2a str. 2457T]

MLTIQFLCPLPNGLHARPAWELKEQCSQWQSEITFINHRQNAKADAKSSLALIGTGTLFNDSCSLNISGS

DEEQARRVLEEYILVRFIDSDSVQPTQAELTAHPLPRSLSRLNPDLLYGNVLASGVGVGTLTLLQSDSLD

SYRAIPASAQDSTRLEHSLATLAEQLNQQLRERDGESKTILSAHLSLIQDDEFAGNIRRLMTEQHQGLGA

AIISNMEQVCAKLSASASDYLRERVSDIRDISEQLLHITWPELKPRNNLVLEKPTILVAEDLTPSQFLSL

DLKNLAGMILEKTGRTSHTLILARASAIPVLSGLPLDAIARYAGQPAVLDAQCGVLAINPNDAVSGYYQV

AQTLADKRQKQQAQAAAQLAYSRDNKRIDIAANIGTALEAPGAFANGAEGVGLFRTEMLYMDRDSAPDEQ

EQFEAYQQVLLAAGDKPIIFRTMDIGGDKSIPYLNIPQEENPFLGYRAVRIYPEFAGLFRTQLRAILRAA

SFGNAQLMIPMVHSLDQILWVKGEIQKAIVELKRDGLRHAETITLGIMVEVPSVCYIIDHFCDEVDFFSI

GSNDMTQYLYAVDRNNPRVSPLYNPITPSFLRMLQQIVTTAHQRGKWVGICGELGGESRYLPLLLGLGLD

ELSMSSPRIPAVKSQLRQLDSEACRELARQACECRSAQEIEALLTAFTPEEDVRPLLALENIFVDQDFSN

KEQAIQFLCGNLGVNGRTEHPFELEEDVWQREEIVTTGVGFGVAIPHTKSQWIRHSSISIARLAKPVDWQ

SEMGEVELVIMLTLGANEGMNHVKVFSQLARKLVNKNFRQSLFAAQDAQSILTLLETELTF

>gi|30063787|ref|NP_837958.1| ARAC-type regulatory protein [Shigella flexneri 2a str. 2457T]

MKAPGLPADQQFFADLFSGLVLNPQLLGRVWFASQPASLPVGSLCIDFPRLDIVLRGEYGNLLEAKQQRM

VEGEMLFIPARAANLPINNKPVMLLSLVFAPTWLGLSFYDSRTTSLLHPARQTQLPSLQRGEGEAMLTAL

THLSRSPLEQNIIQPLVLSLLHLCRNVVNMPPGNSQPRGDFLYHSICNWV

>gi|30063786|ref|NP_837957.1| 2-component transcriptional regulator [Shigella flexneri 2a str. 2457T]

MKVIIVEDEFLAQQELSWLIKEHSQMEIVGTFDDGLDVLKFLQHNRVDAIFLDINIPSLDGVLLAQNISQ

FAHKPFIVFITAWKEHAVEAFELEAFDYILKPYQESRITGMLQKLEAAWQQQQTSSTPAATVTRENDTIN

LVKDERIIVTPINDIYYAEAHEKMTFVYTRRESYVMPMNITEFCSKLPPSHFFRCHRSFCVNLNKIREIE

PWFNNTYILRLKDLDFEVPVSRSKVKEFRQLMHL

>gi|30063785|ref|NP_837956.1| aminotransferase [Shigella flexneri 2a str. 2457T]

MADTRPERRFTRIDRLPPYVFNITAELKMAARRRGEDIIDFSMGNPDGATPPHIVEKLCTVAQRPDTHGY

STSRGIPRLRRAISRWYQDRYDVEIDPESEAIVTIGSKEGLAHLMLATLDHGDTVLVPNPSYPIHIYGAV

IAGAQVRSVPLVEGVDFFNELERAIRESYPKPKMMILGFPSNPTAQCVELEFFEKVVALAKCYDVLVVHD

LAYADIVYDGWKAPSIMQVPGARDVAVEFFTLSKSYNMAGWRIGFMVGNKTLVSALARIKSYHDYGTFTP

LQVAAIAALEGDQQCVRDIAEQYKRRRDVLVKGLHEAGWMVEMPKASMYVWAKIPEPYAAMGSLEFAKKL

LNEAKVCVSPGIGFGDYGDTHVRFALIENHDRIRQAIRGIKAMFRADGLLPVSSKHIHENAE

>gi|30063783|ref|NP_837954.1| hypothetical protein S2581 [Shigella flexneri 2a str. 2457T]

MIYLWMFLALCIVCVSGYIGQVLNVVSAVSSFFGMVILAALIYYFTMWLTGGNELVTGIFMFLAPACGLM

IRFMVGYGRR

>gi|30063782|ref|NP_837953.1| hypothetical protein S2580 [Shigella flexneri 2a str. 2457T]

MKVNLILFSLFLLVSIMACNVFAFSISGGVSERSYKETEKTSAMTTTHSTKLQPSQAILFKMREDVPPLN

LTEEITPTYPTKANYLIHPVR

>gi|30063781|ref|NP_837952.1| hypothetical protein S2579 [Shigella flexneri 2a str. 2457T]

MKRLIMATMVTAILASSTVWAADNAPVAAQQQTQQTQKTAAAERISEQGLYAMRDVQVARLALFHGDPEK

AKELTNEASALLSDDSTEWAKFAKPGKKTNLNDDQYIVINASVGISESYVATPEKEAAIKIANEKMAKGD

KKGAMEELRLAGVGVMENQYLMPLKQTRNALADAQKLLDKKQYYEANLALKGAEDGIIVDSEALFVN

>gi|30063780|ref|NP_837951.1| formyl-coenzyme A transferase [Shigella flexneri 2a str. 2457T]

MSTPLQGIKVLDFTGVQSGPSCTQMLAWFGADVIKIERPGVGDVTRHQLRDIPDIDALYFTMLNSNKRSI

ELNTKTAEGKEVMEKLIREADILVENFHPGAIDHMGFTWEHIQEINPRLIFGSIKGFDECSPYVNVKAYE

NVAQAAGGAASTTGFWDGPPLVSAAALGDSNTGMHLLIGLLAALLHREKTGRGQRVTMSMQDAVLNLCRV

KLRDQQRLDKLGYLEEYPQYPNGTFGDAVPRGGNAGGGGQPGWILKCKGWETDPNAYIYFTIQEQNWENT

CKAIGKPEWITDPAYSTAHARQPHIFDIFAEIEKYTVTIDKHEAVAYLTQFDIPCAPVLSMKEISLDPSL

RQSGSVVEVEQPLRGKYLTVGCPMKFSAFTPDIKAAPLLGEHTAAVLQELGYSDDEIAAMKQNHAI

>gi|30063779|ref|NP_837950.1| oxalyl-CoA decarboxylase [Shigella flexneri 2a str. 2457T]

MSDQLQMTDGMHIIVEALKQNNIDTIYGVVGIPVTDMARHAQAEGIRYIGFRHEQSAGYAAAASGFLTQK

PGICLTVSAPGFLNGLTALANATVNGFPIIMISGSSDRAIVDLQQGDYEELDQMNAAKPYAKAAFRVNQP

QDLGIALARAIRVSVSGRPGGVYLDLPANVLAATMEKDEALTTIVKVENPSPALLPCPKSVTSAISLLAK

AERPLIILGKGAAYSQADEQLREFIESAQIPFLPMSMAKGILEDTHPLSAAAARSFALANADVVMLVGAR

LNWLLAHGKKGWAADTQFIQLDIEPQEIDSNRPIAVPVVGDIASSMQGMLAELKQNTFTTPLVWRDILNI

HKQQNAQKMHEKLSTDTQPLNYFNALSAVRDVLRENQDIYLVNEGANTLDNARNIIDMYKPRRRLDCGTW

GVMGIGMGYAIGASVTSGSPVVAIEGDSAFGFSGMEIETICRYNLPVTIVIFNNGGIYRGDGVDLSGAGA

PSPTDLLHHARYDKLMDAFRGVGYNVTTTDELRHALTTGIQSRKPTIINVVIDPAAGTESGHITKLNPKQ

VAGN

>gi|30063777|ref|NP_837948.1| hybrid sensory histidine kinase in two-component regulatory system with EvgA [Shigella flexneri 2a str. 2457T]

MKFLPYFFLLCCGLWSTISFADEDYIEYRGISSNNRVTLDPVRLSNKELRWLASKKNLVIAVHKSQTATL

LHTDSQQQVRGINADYLNLLKRALNIKLTLREYADHQKAMDALAEGEVDIVLSHLVTSPPLNNDIAATKP

LIITFPALVTTLHDSMRPLTSPKPVNIARVANYPPDEVIHQSFPKATIISFTNLYQALASVSAGHNDYFI

GSNIITSSMISRYFTHSLNVVKYYNSPRQYNFFLTRKESVILNEVLNRFVDALTNEVRYEVSQNWLDTGN

LAFLNKPLELTEHEKQWIKQHPNLKVLENPYSPPYSMTDENGSVRGVMGDILNIITLQTGLNFFPITVSH

NIHAGTQLSPGGWDIIPGAIYSEDRENNVLFAEAFITTPYVFVMQKAPDSEQTLKKGMKVAIPYYYELHS

QLKEMYPEVEWIQVDNASAAFHKIKEGELDALVATQLNSRYMIDHYYPNELYHFLIPGVPNASLSFAFPR

GEPELKDIINKALNAIPPSEVLRLTEKWIKMPNVTIDTWDLYSEQFYIVTTLSVLLVGSSLLWGFYLLRS

VRRRKVIQGDLENQISFRKALSDSLPNPTYVVNWQGNVISHNSAFEHYFTADYYKNAMLPLENSDSPFKD

VFSNAHEVTAETKENRTIYTQVFEIDNGIEKRCINHWHTLCNLPASDNAVYICGWQDITETRDLINALEV

EKNKAIKATVAKSQFLATMSHEIRTPISSIMGFLELLSGSGLSKEQRVEAISLAYATGQSLLGLIGEILD

VDKIESGNYQLQPQWVDIPTLVQNTCHSFGAIAASKSIALSCSSTFPDHYLVKIDPQAFKQVLSNLLSNA

LKFTTEGAVKITTSLGHIDDNHAVIKMTIMDSGSGLSQEEQQQLFKRYSQTSAGRQQTGSGLGLMICKEL

IKNMQGDLSLESHPGIGTTFTITIPVEISQQVATVEAKAEQPITLPEKLSILIADDHPTNRLLLKRQLNL

LGYDVDEATDGVQALHKVSMQHYDLLITDVNMPNMDGFELTRKLREQNSSLPIWGLTANAQANEREKGLS

CGMNLCLFKPLTLDVLKTHLSQLHQVAHIAPQYRHLDIEALKNNTANDLQLMQEILMTFQHETHKDLPAA

FHALEAGDNRTFHQCIHRIHGAANILNLQKLINISHQLEITPASDDSKPEILQLLNSVKEHIAELDQEIA

VFCQKND

>gi|30063776|ref|NP_837947.1| DNA-binding transcriptional activator EvgA [Shigella flexneri 2a str. 2457T]

MNAIIIDDHPLAIAAIRNLLIKNDIEILAELTEGGSAVQRVETLKPDIVIIDVDIPGVNGIQVLETLRKR

QYSGIIIIVSAKNDHFYGKHCADAGANGFVSKKEGMNNIIAAIEAAKNGYCYFPFSLNRFVGSLTSDQQK

LDSLSKQEISVMRYILDGKDNNDIAEKMFISNKTVSTYKSRLMEKLECKSLMDLYTFAQRNKIG

>gi|30063775|ref|NP_837946.1| multidrug resistance protein K [Shigella flexneri 2a str. 2457T]

MEQINSNKKQSNRKKYFSLLVIVLFIAFSGAYAYWSMELEDMISTDDAYVTGNADPISAQVSGSVTVVNH
KDTNYVRQGDILVSLDKTDATIALNKAKNNLANIVRQTNKLYLQDKQYSAEVASARIQYQQSLEDYNRRV
PLAKQGVISKETLEHTKDTLISSKAALNAAIQAYKANKALVMNTPLNRQPQVVEAADATKEAWLVLKRTD
IRSPVTGYIAQRSVQVGETVSSGQSLMAVVPARQMWVNANFKETQLTDVRIGQSVNIISDLYGENVVFHG
RVTGINMGTGNAFSLLPAQNATGNWIKIVQRVPVEVSLDPKELMEHPLRIGLSMTATIDTKDEDIAEMPD
LASTVTSMPAYTSKALVIDTSPIEKEISNIISHNGQL

>gi|30063774|ref|NP_837945.1| multidrug resistance protein Y [Shigella flexneri 2a str. 2457T]

MAITKSTPAPLTGGTLWCVTIALSLATFMQMLDSTISNVAIPTISGFLGASTDEGTWVITSFGVANAIAI
PVTGRLAQRIGELRLFLLSVTFFSLSSLMCSLSTNLDVLIFFRVVQGLMAGPLIPLSQSLLLRNYPPEKR
TFALALWSMTVIIAPICGPILGGYICDNFSWGWIFLINVPMGIIVLTLCLTLLKGRETETSPVKMNLPRL
TLLVLGVGGLQIMLDKGRDLDWFNSSTIIILTVVSVISLISLVIWESTSENPILDLSLFKSRNFTIGIVS
ITCAYLFYSGAIVLMPQLLQKTMGYNAIWAGLAYAPIGIMPLLISPLIGRYGNKIDMRVLVTFSFLMYAV
CYYWRSVTFMPTIDFTGIILPQFFQGFAVACFFLPLTTISFSGLPDNKFANASSMSNFFRTLSGSVGTSL
TMTLWGRRESLHHSQLTATIDQFNPVFNSSSQIMDKYYGSLSGVLNEINNEITQQSLSISANEIFRMAAI
AFILLTVLVWFAKPPFTAKGVG

>gi|30063773|ref|NP_837944.1| D-serine dehydratase [Shigella flexneri 2a str. 2457T]

MENAKMNSLIAQYPLVKDLVALKETTWFNPSTTSLAEGLPYVGLTEQDVQDAHARLSRFAPYLAKAFAET
AATGGIIESELVAIPAMQKRLEKEYQQPISGQLLLKKDSHLPISGSIKARGGIYEVLAHAEKLALEAGLL
TLDDDYSKLLSPEFKQFFSQYSIAVGSTGNLGLSIGIMSARIGFKVTVHMSADARAWKKAKLRSHGVTVV
EYEQDYGVAVEEGRKAAQSDPNCFFIDDENSRTLFLGYSVAGQRLKAQFAQQGRIVDADNPLFVYLPCGV
GGGPGGVAFGLKLAFGDHVHCFFAEPTHSPCMLLGVHTGLHDQISVQDIGIDNLTAADGLAVGRASCFVG

RAMERLLDGFYTLSDQTMYDMLGWLAQEEGIRLEPSALAGMAGPQRVCASVSYQQMHGFSAEQLRNATHL

VWATGGGMVPEEEMNQYLAKGR

>gi|30063772|ref|NP_837943.1| sucrose specific repressor [Shigella flexneri 2a str. 2457T]

MDLASDVCFCYSARFTANGLGMASLKDVARLAGVSMMTVSRVMHNAESVRPATRDRVLQAIQTLNYVPDL

SARKMRAQGRKPSTLAVLAQDTATTPFSVDILLAIEQTASEFGWNSFLINIFSEDDAARAARQLLAHRPD

GIIYTTMGLRHITLPESLYGENIVLANCVADDPALPSYIPDDYTAQYESTQHLLAAGYRQPLCFWLPESA

LATGYRRQGFEQAWRDAGRDLAEVKQFHMATGDDHYTDLASLLNAHFKSGKPDFDVLICGNDRAAFVAYQ

VLLAKGVRIPQDVAVMGFDNLVGVGHLFLPPLTTIQLPHDIIGREAALHIIEGREGGRVTRIPCPLLIRC

ST

>gi|30063771|ref|NP_837942.1| hypothetical protein S2470 [Shigella flexneri 2a str. 2457T]

MTQSRLHAAQNALAKLHEHRGNTFYPHFHLAPPAGWMNDPNGLIWFNDRYHAFYQHHPMSEHWGPMHWGH

ATSDDMIHWQHEPIALALGDDNDKDGCFSGSAVDDNGVLSLIYTGHVWLDGAGNDDAIREVQCLATSRDG

IHFEKQGVILTPPEGIMHFRDPKVWREADTWWMVVGAKDPGNTGQILLYRGSSLREWAFDRVLAHADAGE

SYMWECPDFFSLGDQHYLMFSPQGMNAEGYSYRNRFQSGVIPGMWSPGRLFAQSGHFTELDNGHDFYAPQ

SFLAKDGRRIVIGWMDMWESPMPSKREGWAGCMTLARELSESNGKLLQRPVHEAESLRQQHQSVSPRRIS

NKYVLQENAQAVEIQLQWALKNSDAEHYGLQLGTGMRLYIDNQSERLILWRYYPHENLDGYRSIPLPQRD

TLALRIFIDTSSVEVFINDGEAVMSSRIYPQPEERELSLYASHGVAVLQHGALWLLG

>gi|30063770|ref|NP_837941.1| IS4 orf [Shigella flexneri 2a str. 2457T]

MHIGQALDLVSRYDSLRNPLTSLGDYLDPELISRCLAESGTVTLRKRRLPLEMMVWCIVGMALERKEPLH

QIVNRLDIMLPGNRPFVAPSAVIQARQRLGSEAVRRVFTKTAQLWHNATPHPHWCGLTLLAIDGVFWRTP

DTPENDAAFPRQTHAGNPALYPQVKMVCQMELKWHTEFGHLNRGDMLTSEQHRCSNEKKKF

>gi|30063769|ref|NP_837940.1| IS911 orfA [Shigella flexneri 2a str. 2457T]

MKKRNFSAEFKRESAQLVVDQKYTVADAAKAMDVGLSTMTRWVKQLRDERQGKTPKASPITPEQIEIREL

RKKLQRIEMENEILKKATALLMSDSLNSSR

>gi|30063768|ref|NP_837939.1| transporter, partial [Shigella flexneri 2a str. 2457T]

MAIHEHIRQDGEKELERDAMALLWSAIAAGLSMGASLLAKGIFHAELEGVPGSFLLENLGYTFGFIIVIM

ARQQLFTENTVTAVLPVMQKPTMSNVGLLMRLWGVVLLGNILGTGIAAWAFEYMPIFNEETRDAFVKIGM

DVMKNTPSEMFANAIISGWLIATMVWMFPAAGAAKIVVIILMTWLIALGDTTHIVVGSVEILYLVFNGTL

HWSDFIWPFALPTLAGNICGGTFIFALMSHAQIRNDMSNKRKAEARQKAERAENIKKNDKNPA

>gi|30063767|ref|NP_837938.1| lipoprotein precursor [Shigella flexneri 2a str. 2457T]

MKLRLSALALGTTLLVGCASSGTDQQGRSDPLEGFNRTMYNFNFNVLDPYIVRPVAVAWRDYVPQPARNG

LSNFTGNLEEPAVMVNYFLQGDPYQGMVHFTRFFLNTILGMGGFIDVAGMANPKLQRTEPHRFGSTLGHY

GVGYGPYVQLPFYGSFTLRDDGGDMADALYPVLSWLTWPMSVGKWTLEGIETRAQLLDSDGLLRQSSDPY

IMVREAYFQRHDFIANGGELKPQENPNAQAIQDDLKDIDSE

>gi|30063766|ref|NP_837937.1| hypothetical protein S2558 [Shigella flexneri 2a str. 2457T]

MLPSISINNTSAAYPESINENNNDEINGLVQEFKNLFNGKEGISTCIKHLLELIKNAIRVNDDPYRSNIN

NSSVTYIDIGSNDTDHITIGIDNQEPIELPANYKDKELVRTIINDNIVEKTHDINNKEMIFSALKEIYDG

DPGFIFDKISHKLRHTVTEFDESGKSEPTDLFTWYGKDKKGDSLAIVIKNKNGNDYLSLGYYDQDDYHIQ

RGIRINGDSLTQYCSENARSASAWFESSKAIMAESFATGSDHQVVNELNGERLREPNEVFKRLGRAIRYN

FQVDDAKFRRDNVKEIISTLFANKVDVDHPENKYKDFKNLEDKVEKRLQNRQTKYQNEINQLSALGVNFD

DI

>gi|30063765|ref|NP_837936.1| long-chain fatty acid outer membrane transporter [Shigella flexneri 2a str. 2457T]

MSQKTLFTKSALAVAVALISTQAWSAGFQLNEFSSSGLGRAYSGEGAIADDAGNVSRNPALITMFDRPTF

SAGAVYIDPDVNISGTSPSGRSLKADNIAPTAWVPNMHFVAPINDQFGWGASITSNYGLATEFNDTYAGG

SVGGTTDLETMNLNLSGAYRLNNAWSFGLGFNAIYARAKIERFAGDLGQLVAGQIMQSPAGQTPQGQALA

ATANGIDSNTKIAHLNGNQWGFGWNAGILYELDKNNRYALTYRSEVKIDFKGNYSSDLNRAFNNYGLPIP

TATGGATQSGYLTLNLPEMWEVSGYNRVDPQWAIHYSLAYTSWSQFQQLKATSTSGDTLFQKHEGFKDAY

RIALGTTYYYDDNWTFRTGIAFDDSPVPAQNRSISIPDQDRFWLSAGTTYAFNKDASVDVGVSYMHGQSV

KINEGPYQFESEGKAWLFGTNFNYAF

>gi|30063764|ref|NP_837935.1| hypothetical protein S2556 [Shigella flexneri 2a str. 2457T]

MHIQRKSTMSKCSADETPVCCCMDVGTIMDNSDCTASYSRVFANRAEAEQTLAALTEKSRSVESEPCKIS

PTFTEESDGVRLDIDFTFACEAEMLIFQLGLR

>gi|30063763|ref|NP_837934.1| 3-ketoacyl-CoA thiolase [Shigella flexneri 2a str. 2457T]

MGQVLPLVTRQGDRIAIVSGLRTPFARQATAFHGIPTVDLGKMVVGELLARSEIPAEVIEQLVFGQVVQM

PEAPNIAREIVLGTGMNVHTDAYSVSRACATSFQAVANVAESLMAGTIRAGIAGGADSSSVLPIGVSKKL

AYVLVDVNKARTMSQRLKLFSRLRLRDLMPVPPAVAEYSTGLRMGDTAEQMAKTYGITREQQDALAHRSH

QRAAQAWSDGKLKEEVMTAFIPPYKQPLVEDNNIRGNSSLADYAKLRPAFDRKHGTVTAANSTPLTDGAA

AVILMTESRAKELGLVPLGYLRSYAFTAIDVWQDMLLGPAWSTPLALERAGLTMSELTLIDMHEAFAAQT

LANIQLLGSERFAREVLGRAHATGEVDDSKFNVLGGSIAYGHPFAATGARMITQTLHELRRRGGGFGLVT

ACAAGGLGAAMVLEAE

>gi|30063762|ref|NP_837933.1| multifunctional fatty acid oxidation complex subunit alpha [Shigella flexneri 2a str. 2457T]

MEMASAFTLNVRLDNIAIITIDVPDEKMNTLKAEFASQVRAIIKQLRENKELRGVVFISAKPDNFIAGAD

INMIGNCKTAQEAEALARQGQQLMAEIHALPIPVIAAIHGACLGGGLELALACHGRVCTDDPKTVLGLPE

VQLGLLPGSGGTQRLPRLIGVSTALEMILTGKQLRAKQALKLGLVDDVVPHSILLEAAVELAKKDRPSSR

PLPVRERILAGPLGRALLFKMVGKKTEHKTQGNYPATERILEVVETGLAQGTSSGYDAEARAFGELAMTP
QSQALRSIFFASTDVKKDPGSDAPPAPLNSVGILGGGLMGGGIAYVTACKAGLPVRIKDINPQGINHALK
YSWDQLEGKVRRRHLKASERDKQLALISGTTDYRGFAHRDLIIEAVFENLELKQQMVAEVEQNCAAHTIF
ASNTSSLPIGDIAAHAARPEQVIGLHFFSPVEKMPLVEIIPHAGTSAQTIATTVKLAKKQGKTPIVVRDK
AGFYVNRILAPYINEAIRMLTEGERVEHIDAALVKFGFPVGPIQLLDEVGIDTGTKIIPVLEAAYGERFS
APANVVSSILNDDRKGRKNGRGFYLYGQKGRKSKKQVDPAIYPLIGAQGQGRLSAPQVAERCVMLMLNEA
VRCVDEQVIRSVRDGDIGAVFGIGFPPFLGGPFRYIDSLGAGEVVAIMQRLATQYGSRFTPCERLVEMGA
RGESFWKTTATDLQ

>gi|30063761|ref|NP_837932.1| phosphohistidine phosphatase [Shigella flexneri 2a str. 2457T]
MQVFIMRHGDAALDSASDSVRPLTTNGCDESRLMANWLKGQKVEIERVLVSPFLRAEQTLEEVGDCLNLP
SSAEVLPELTPCGDVGLVSAYLQALTNEGVASVLVISHLPLVGYLVAELCPGETPPMFTTSAIASVTLDE
SGNGTFNWQMSPCNLKMAKAI

>gi|30063760|ref|NP_837931.1| IS1 orfA [Shigella flexneri 2a str. 2457T]
MASISIRCPSCSATEGVVRNGKSTAGHQRYLCSHCRKTWQLQFTYTASQPGTHQKIIDMAMNGVGCRASA
RIMGVGLNTVLRHLKNSGRSR

>gi|30063759|ref|NP_837930.1| IS1 orfB [Shigella flexneri 2a str. 2457T]
MSRQCTHYGRWPQHGFTSLKKLRPQSVTSRIQPGSDVIVCAEMDEHWGYVGAKSRQRWLFYAYDRIRRTV
VAHVFGERTLATLERLLSLLSAFEVVVWMTDGWPLYESRLKGKLHVISKRYTQRIERHNLNLRQHLARLV
RKSLSFSKSVELHDKVIGHYLNIKHYQ

>gi|30063758|ref|NP_837929.1| fimbrial-like protein [Shigella flexneri 2a str. 2457T]
MSKFVKTAIAAAMVMGVFTSTATIAAGNNGTARFYGTIEDSVCSIVPDDHKLEVDMGDIGAEKLKNNGTT

TPKSFQIRLQDCVFDTQETMTTTFTGTVSSANSGNYYTIFNTDTGAAFNNVSLAIGDSLGTSYKSGMGID

QKIVKDTSTNKGKAKQTLNFNAWLVGAADAPDLGNFEANTTFQITYL

>gi|30063757|ref|NP_837928.1| hypothetical protein S2541 [Shigella flexneri 2a str. 2457T]

MKKKTTLSEEDQALFRQLMAGTRKIKQDTIVHRPQRKKISEVPVKRLIQEQADASHYFSDEFQPLLNTEG

PVKYVRPDVSHFEAKKLRRGDYSPELFLDLHGLTQLQAKQELGALIAACRREHVFCACVMHGHGKHILKQ

QTPLWLAQHPHVMAFHQAPKEYGGDAALLVLIEVEEWLPPELP

>gi|30063756|ref|NP_837927.1| N5-glutamine S-adenosyl-L-methionine-dependent methyltransferase [Shigella flexneri 2a str. 2457T]

MAGISLLLDQAFHRHFADFFTLRSVDNRVLLNLASPRHQLAKQSLVLLAKCCFLFHLSVSISNFPTVYPT

RSTIDRFKRIFPHSPRVNADLSSSSWQTSRRNYCDHALEEYVDKIFVDEAVNELQTIQDILRWSVSRFSA

ANIWYGHGTDNPWDEAVQLVLPSLYLPLDIPEDMRTARLTSSEKHRIVERVIRRVNERIPVAYLTNKAWF

CGHEFYVDERVLVPRSPIGELINNKFAGLISKQPQHILDMCTGSGCIAIACAYAFPEAEVDAVDISPDAL

AVAEQNIEEHGLIHNVIPIRSDLFRDLPKVQYDLIVTNPPYVDAEDMSDLPNEYRHEPELGLASGTDGLK

LTRRILGNAADYLADDGVLICEVGNSMVHLMEQYPDVPFTWLEFDNGGDGVFMLTKEQLIAAREHFAIYK

D

>gi|30063755|ref|NP_837926.1| chorismate synthase [Shigella flexneri 2a str. 2457T]

MAGNTIGQLFRVTTFGESHGLALGCIVDGVPPGIPLTEADLQHDLDRRRPGTSRYTTQRREPDQVKILSG

VFEGVTTGTSIGLLIENTDQRSQDYSAIKDVFRPGHADYTYEQKYGLRDYRGGGRSSARETAMRVAAGAI

AKKYLAEKFGIEIRGCLTQMGDIPLEIKDWSLVEQNPFFCPDPDKIDALDELMRALKKEGDSIGAKVTVV

ASGVPAGLGEPVFDRLDADIAHALMSINAVKGVEIGDGFDVVALRGSQNRDEITKDGFQSNHAGGIFGGI

SSGQQIIAHMALKPTSSITVPGRTINRFGEEVEMITKGRHDPCVGIRAVPIAEAMLAIVLMDHLLRQRAQ

NADVKTDIPRW

>gi|30063754|ref|NP_837925.1| penicillin-insensitive murein endopeptidase [Shigella flexneri 2a str. 2457T]

MNKTAIALLALLASSASLAATPWQKITQPVPGSAQSIGSFSNGCIVGADTLPIQSEHYQVMRTDQRRYFG

HPDLVMFIQRLSSQVSNLGMGTVLIGDMGMPAGGRFNGGHASHQTGLDVDIFLQLPKTRWTSAQLLRPQA

LDLVSRDGKHVVSTLWKPEIFSLIKLAAQDKDVTRIFVNPAIKQQLCLDAGTDRDWLRKVRPWFQHRAHM

HVRLRCPADSLECEDQPLPPSGDGCGAELQSWFEPPKPGTTKPEKKTPPPLPPSCQALLDEHVI

>gi|30063753|ref|NP_837924.1| hypothetical protein S2538 [Shigella flexneri 2a str. 2457T]

METFNSLFMVSPLLLGVLFFVAMLAGFIDSIAGGGGLLTIPALMAAGMSPANALATNKLQACGGSISATI

YFIRRKVVSLSDQKLNIAMTFVGSMSGALLVQYVQADVLRQILPILVICIGLYFLLMPKLGEEDRQRRMY

GLPFALIAGGCVGFYDGFFGPAAGSFYALAFVMLCGFNLAKATAHAKLLNATSNIGGLLLFILGGKVIWA

TGFVMLVGQFLGARMGSRLVLSKGQKLIRPMIVIVSAVMSAKLLYDSHGQEILHWLGMN

>gi|30063752|ref|NP_837923.1| transporting ATPase [Shigella flexneri 2a str. 2457T]

MNSTHHYEQLIEIFNSCFADEFNTRLIKGDDEPIYLPADAEVPYNRIVFAHGFYASAIHEISHWCIAGKA

RRELVDFGYWYCPDGRDAQTQSQFEDVEVKPQAFDWLFCVAAGYPFNVSCDNLEGDFEPDRVVFQRRVHA

QVMDYLTNGIPERPARFIKALQNYYHTPELTAEQFPWPEALN

>gi|30063751|ref|NP_837922.1| hypothetical protein S2536 [Shigella flexneri 2a str. 2457T]

MIAEFESRILALIDGMVDHASDDELFASGYLRGHLTLAIAELESGDDHSAQAVHTTVSQSLEKAIGAGEL

SPRDQALVTDMWENLFQQASQL

>gi|30063749|ref|NP_837920.1| 3-oxoacyl-ACP synthase [Shigella flexneri 2a str. 2457T]

MKRAVITGLGIVSSIGNNQQEVLASLREGRSGITFSQELKDSGMRSHVWGNIKLDTTGLIDRKVVRFMSD

ASIYAFLSMEQAIADAGLSPEAYQNNPRVGLIAGSGGGSPRFQVFGADAMRGPRGLKAVGPYVVTKAMAS

GVSACLATPFKIHGVNYSISSACATSAHCIGNAVEQIQLGKQDIVFAGGGEELCWEMACEFDAMGALSTK

YNDTPEKASRTYDAHRDGFVIAGGGGMVVVEELEHALARGAYIYAEIVGYGATSDGADMVAPSGEGAVRC

MQMAMHGVDTPIDYLNSHGTSTPVGDVKELAAIREVFGDKSPAISATKAMTGHSLGAAGVQEAIYSLLML

EHGFIAPSINIEELDEQAAGLNIVTETTDRELTTVMSNSFGFGGTNATLVMRKLKRLIRSRPE

>gi|30063748|ref|NP_837919.1| hypothetical protein S2533 [Shigella flexneri 2a str. 2457T]

MTAVSQTETRSSASFSLFRISFAVFLTYMTVGLPLPVIPLFVHHDLGYGNTMVGIAVGIQFLATVLTRGY

AGRLADQYGAKRSALQGMLACGLAGGALLLAAILPVSAPFKFALLVIGRLILGFGESQLLTGALTWGLGI

VGPKHSGKVMSWNGMAIYGALAVGAPLGLLIHSHYGFAALAITTMVLPLLAWACNGTVRKVPALAGERPS

LWSVVGLIWKPGLGLALQGVGFAVIGTFVSLYFASKGWAMAGFTLTAFGGAFVVMRVMFGWMPDRFGGVK

VAIVSLLVETVGLLLLWQAPGAWVALAGAALTGAGCSLIFPALGVEVVKRVPSQVRGTALGGYAAFQDIA

LGVSGPLAGMLATTFGYSSVFLAGAISAVLGIIVTILSFRRG

>gi|30063747|ref|NP_837918.1| flagella biosynthesis regulator [Shigella flexneri 2a str. 2457T]

MIQPISGPPPGQPPGQGDNLPSGAGNQPLSSQQRTSLESLMTKVTSLTQQQRAELWAGIRHDIGLSGDSP

LLSRHFPAAEHNLAQRLLAAQKSHSARQLLAQLGEYLRLGNHRQAVTDYIRHNFGQTPLNQLSPEQLKTI

LTLLQEGKMVIPQPQQREATDRPLLPAEHNALKQLVTKLAAATGEPSKQIWQSMLELSGVKDGELIPAKL

FNHLVTWLQARQTLSQQNTPTLESLQMALKQPLDASELAALSAYIQQKYGLSAQSSLSSAQAEDILNQLY

QRRVKGIDPRDMQPLLNPFPPMMDTLQNMATRPALWILLVAIILMLVWLVR

>gi|30063746|ref|NP_837917.1| erythronate-4-phosphate dehydrogenase [Shigella flexneri 2a str. 2457T]

MKILVDENMPYARDLFSRLGEVIAVPGRPIPVAQLADADALMVRSVTKVNESLLAGKPIKFVGTATAGTD

HVDEAWLKQAGIGFSAAPGCNAIAVVEYVFSSLLMLAERDGFSLHDRTVGIVGVGNVGRRLQARLEALGI

KTLLCDPPRADRGDEGDFRSLDELVQRADILTFHTPLFKDGPYKTLHLADEKLIRSLKPGAILINACRGA

VVDNTALLTCLNEGQKLSVVLDVWEGEPELNVELLTKVDIGTPHIAGYTLEGKARGTTQVFEAYSKFIGH

EQHVALDTLLPAPEFGRITLHGPLDQPTLKRLVHLVYDVRRDDAPLRKVAGIPGEFDKLRKNYLERREWS

SLYVICDDASAASLLCKLGFNAVHHPAR

>gi|30063745|ref|NP_837916.1| semialdehyde dehydrogenase [Shigella flexneri 2a str. 2457T]

MSEGWNIAVLGATGAMGEALLETLAERQFPVGEIYALARNESAGEQLRFGGKTITVQDAAEFDWTQAQLA

FFVAGKEATAAWVEEATNSGCLVIDSSGLFALEPDVPLVVPEVNPFVLTDYRNRNVIAVPDSLTSQLLAA

LKPLIDQGGLSRISVTSLISASAQGKKAVDALAGQSAKLLNGIPIDEEDFFGRQLAFNMLPLLPDSEGSV

REERRIVDEVRKILQDEGLMISASVVQAPVFYGHAQMVNFEALRPLAAEEARDAFAQGEDIVLSEENEFP

TQVGDASGTPHLSVGCVRNDYGMPEQVQFWSVADNVRFGGALMAVKIAEKLVQEYLY

>gi|30063744|ref|NP_837915.1| tRNA pseudouridine synthase A [Shigella flexneri 2a str. 2457T]

MSDQQQLPVYKIALGIEYDGSKYYGWQRQNEVRSVQEKLEKALSQVANEPITVFCAGRTDAGVHGTGQVV

HFETTAQRKDAAWTLGVNANLPGDIAVRWVKAVPDDFHARFSATARRYRYIIYNHRLRPAVLSKGVTHFY

EPLDAERMHRAAQCLLGENDFTSFRAVQCQSRTPWRNVMHINVTRHGPYVVVDIKANAFVHHMVRNIVGS

LMEVGAHNQPESWIAELLAAKDRTLAAATAKAEGLYLVAVDYPDRYDLPKPPMGPLFLAD

>gi|30063743|ref|NP_837914.1| hypothetical protein S2528 [Shigella flexneri 2a str. 2457T]

MDLIYFLIDFILHIDVHLAELVAEYGVWVYAILFLILFCETGLVVMPFLPGDSLLFVAGALASLETNDLN

VHMMVVLMLIAAIVGDAVNYTIGRLFGEKLFSNPNSKIFRRSYLDKTHQFYEKHGGKTIILARFVPIVRT

FAPFVAGMGHMSYRHFAAYNVIGALLWVLLFTYAGYFFGTIPMVQDNLKLLIVGIIVVSILPGVIEIIRH

KRAAARAAK

>gi|30063742|ref|NP_837913.1| acetyl-CoA carboxylase subunit beta [Shigella flexneri 2a str. 2457T]

MSWIERIKSNITPTRKASIPEGVWTKCDSCGQVLYRAELERNLEVCPKCDHHMRMTARNRLHSLLDEGSL

VELGSELEPKDELKFRDSKKYKDRLASAQKETGEKDALVVMKGTLYGMPVVAAAFEFAFMGGSMGSVVGA

RFVRAVEQAQEDNCPLICFSASGGARMQEALMSLMQMAKTSAALAKMQERGLPYISVLTDPTMGGVSASF

AMLGDLNIAEPKALIGFAGPRVIEQTVREKLPPRFQRSEFLIEKGAIDMIVRRPEMRLKLASILAKLMNL

PAPNPEAPREGVVVPPVPDQEPEA

>gi|30063741|ref|NP_837912.1| bifunctional folylpolyglutamate synthase/ dihydrofolate synthase [Shigella flexneri 2a str. 2457T]

MIIKRTPQAASPLASWLSYLENLHSKTIDLGLERVSQVAARLGVLKPAPFVFTVAGTNGKGTTCRTLESI

LMAAGYKVGVYSSPHLVRYTERVRVQGQELPESAHTASFAEIESARGDISLTYFEYGTLSALWLFKQAQL

DVVILEVGLGGRLDATNIVDADVAVVTSIALDHTDWLGPDRESIGLEKAGIFRSEKPAIVGEPEMPSTIA

DVAQEKGALLQRRGVEWNYSVTDHDWAFSDAHGTLENLPLPLVPQPNAATALAALRASGLEVSENAIRDG

IASAILPGRFQIVSESPRVIFDVAHNPHAAEYLTGRMKALPKNGRVLAVIGMLHDKDIAGTLAWLKSVVD

DWYCAPLEGPRGATAEQLLEHLGNGKSFDSVAQAWDAAMADAKAEDTVLVCGSFHTVAHVMEVIDARRSG

GK

>gi|30063739|ref|NP_837910.1| colicin V production protein [Shigella flexneri 2a str. 2457T]

MVWIDYAIIAVIAFSSLVSLIHGFVREALSLVTWGCAFFVASHYYTYLSVWFTGFEDELVRNGIAIAVLF

IATLIVGAIVNFVIGQLVEKTGLSGTDRVLGVCFGALRGVLIVAAILFFLDSFTGVSKSEDWSKSQLIPQ

FSFIIRWFFDYLQSSSSFLPRA

>gi|30063738|ref|NP_837909.1| amidophosphoribosyltransferase [Shigella flexneri 2a str. 2457T]

MCGIVGIAGVMPVNQSIYDALTVLQHRGQDAAGIITIDANNCFRLRKANGLVSDVFEARHMQRLQGNMGI

GHVRYPTAGSSSASEAQPFYVNSPYGITLAHNGNLTNAHELRKKLFEEKRRHINTTSDSEILLNIFASEL

DNFRHYPLEADNIFAAIAATNRLIRGAYACVAMIIGHGMVAFRDPNGIRPLVLGKRDIDENRTEYMVASE

SVALDTLGFDFLRDVAPGEAIYITEEGQLFTRQCADNPVSNPCLFEYVYFARPDSFIDKISVYSARVNMG

TKLGEKIAREWEDLDIDVVIPIPETSCDIALEIARILGKPYRQGFVKNRYVGRTFIMPGQQLRRKSVRRK

LNANRAEFRDKNVLLVDDSIVRGTTSEQIIEMAREAGAKKVYLASAAPEIRFPNVYGIDMPSATELIAHG

REVDEIRQIIGADGLIFQDLNDLIDAVRAENPDIQQFECSVFNGVYVTKDVDQGYLDFLDTLRNDDAKAV

QRQNEVENLEMHNEG

>gi|30063737|ref|NP_837908.1| 3-octaprenyl-4-hydroxybenzoate carboxy-lyase [Shigella flexneri 2a str. 2457T]

MKRLIVGISGASGAIYGVRLLQVLRDVTDIETHLVMSQAARQTLSLETDFSLREVQALADVTLDARDISA

SISSGSFQTLGMVILPCSIKTLSGIVHSYTDGLLTRAADVVLKERRPLVLCVRETPLHLGHLRLMTQAAE

IGAVIMPPVPAFYHRPQSLDDVINQTVNRVLDQFAITLPEDLFARWQGA

>gi|30063736|ref|NP_837907.1| lysine-, arginine-, ornithine-binding periplasmic protein [Shigella flexneri 2a str. 2457T]

MKKSILALSLLVGLSAAASSYAALPETVRIGTDTTYALFSSKDAKGDFVGFDIDLGNEMCKRMQVKCTWV

ASDFDALIPSLKAKKIDAIISSLSITDKRQQEIAFSDKLYAADSRLIAAKGSPIQPTLDSLKGKHVGVLQ

GSTQEAYANETWRSKGVDVVAYANQDLVYSDLAAGRLDAALQDEVAASEGFLKQPAGKDFAFAGSSVKDK

KYFGDGTGVGLRKDYAELTAAFNKALGELRQDGTYDKMAKKYFDFNVYGD

>gi|30063735|ref|NP_837906.1| histidine-binding periplasmic protein of high-affinity histidine transport system [Shigella flexneri 2a str. 2457T]

MKKLVLSLSLVLAFSSATAALAAIPQNIRIGTDPTYAPFESKNSQGELVGFDIELAKELCKRINTQCTFV

ENPLDALIPSLKAKKIDAIMSSLSITEKRQQEIAFTDKLYAADSRLVVAKNSDIQPTVESLKGKRVGVLQ

GTTQETFGNEHWAPKGIEIVSYQGQDNIYSDLTAGRIDAAFQDEVAASEGFLKQPVGKDYKFGGPSVKDE

KLFGVGTGMGLRKEDNELREALNKAFAEMRADGTYEKLAKKYFDFDVYGG

>gi|30063734|ref|NP_837905.1| histidine transport system permease [Shigella flexneri 2a str. 2457T]

MLYGFSGVILQGALVTLELAISSVVLAVIIGLIGAGGKLSQNRLSGLIFEGYTTLIRGVPDLVLMLLIFY

GLQIALNMVTEAMGVGQIDIDPMVAGIITLGFIYGAYFTETFRGAFMAVPKGHIEAATAFGFTRGQVFRR

IMFPAMMRYALPGIGNNWQVILKSTALVSLLGLEDVVKATQLAGKSTWEPFYFAIVCGVIYLVFTTVSNG

VLLFLERRYSVGVKRADL

>gi|30063733|ref|NP_837904.1| histidine transport, membrane protein M [Shigella flexneri 2a str. 2457T]

MIEILHEYWKPLLWTDGYRFTGVAITLWLLILSVVIGGVLALFLAIGRVSSNKYIQFPIWLFTYIFRGTP

LYVQLLVFYSGMYTLEIVKGTEFLNAFFRSGLNCTVLALTLNTCAYTTEIFAGAIRSVPHGEIEAARAYG

FSTFKMYRCIILPSALRIALPAYSNEVILMLHSTALAFTATVPDLLKIARDINAATYQPFTAFGIAAVLY

LIISYVLISLFRRAEKRWLQHVKPSSTH

>gi|30063732|ref|NP_837903.1| histidine/lysine/arginine/ornithine transporter subunit [Shigella flexneri 2a str. 2457T]

MSENKLNVIDLHKRYGEHEVLKGVSLQANAGDVISIIGSSGSGKSTFLRCINFLEKPSEGSIVVNGQTIN

LVRDKDGQLKVADKNQLRLLRTRLTMVFQHFNLWSHMTVLENVMEAPIQVLGLSKQEARERAVKYLAKVG

IDERAQGKYPVHLSGGQQQRVSIARALAMEPEVLLFDEPTSALDPELVGEVLRIMQQLAEEGKTMVVVTH

EMGFARHVSTHVIFLHQGKIEEEGAPEQLFGNPQSPRLQQFLKGSLK

>gi|30063731|ref|NP_837902.1| hypothetical protein S2516 [Shigella flexneri 2a str. 2457T]

MTISTTSTPHDAVFKSFLRHPDTARNFINIHLPAPLRKLCDLTTLKLEPNSFIDEDLRQYYSDLLWSVKT

QEGVGYIYVVIEHQSKPEELMAFRMMRYSIAAMQNHLDAGYKELPLVIPMLFYHGCRSPYPYSLCWLDEF

AEPAIARKIYSSAFPLVDITVVPDDEIMQHRKMALLELIQKHIRQRDLLGLVDQIVSLLVTGNTNDRQLK

ALFNYVLQTGDAQRFRAFIGEITERAPQEKEKLMTIADRLREEGAMQGKHEEALRIAQEMLDRGLDRELV

MMVTRLSPDDLIAQSH

>gi|30063730|ref|NP_837901.1| sugar nucleotide epimerase [Shigella flexneri 2a str. 2457T]

MNIVITGGTGLIGRHLIPRLLELGHQITVVTRNPQKASSVLGPRVTLWQGLADQSNLNGVDAVINLAGEP

IADKRWTHKQKERLCQSRWNITQKLVDLINASDTPPSVLISGSATGYYGDLGEVVVTEEEPPHNEFTHKL

CARWEEIACRAQSDKTRVCLLRTGVVLAPDGGILGKMLPPFRLGLGGPIGSGRQYLAWIHIDDMVNGILW

LLDNELRGPFNMVSPYPVRNEQFAHALGHALHRPAILRVPTTAIRLLMGESSVLVLGGQRALPKRLEEAG

FAFRWYDLEEALADVVR

>gi|30063729|ref|NP_837900.1| D-erythro-7,8-dihydroneopterin triphosphate 2'-epimerase [Shigella flexneri 2a str. 2457T]

MAQPAAIIRIKNLRLRTFIGIKEEEINNRQDIVINVTIHYPADKARTSEDINDALNYRTVTKNIIQHVEN

NRFSLLEKLTQDVLDIAREHHWVTYAEVEIDKLHALRYADSVSMTLSWQR

>gi|30063728|ref|NP_837899.1| glutathione S-transferase [Shigella flexneri 2a str. 2457T]

MIDLYFAPTPNGHKITLFLEEAELDYRLIKVDLGKGGQFRPEFLRISPNNKIPAIVDHSPADGGEPLSLF

ESAAILLYLAEKTGLFLSHETRERAATLQWLFWQVGGLGPMLGQNHHFNHAAPQTIPYAIERYQVETQRL

YHVLNKRLENSPWLGGENYSIADIACWPWVNAWTRLRIDLAMYPAVKNWHERIRSRPATGQALLKAQLGD

ERSDS

>gi|30063727|ref|NP_837898.1| hypothetical protein S2512 [Shigella flexneri 2a str. 2457T]

MSKPAITLWSDAHFFSPYVLSAWVALQEKGLSFHIKTIDLDSGEHLQPTWQGYGQTRRVPLLQIDDFELS

ESSAIAEYLEDRFAPPTWERIYPLDLENRARARQIQAWLRSDLMPIREERPTDVVFAGAKKAPLTAEGKA

SAEKLFAMAEHLLALGQPNLFGEWCIADTDLALMINRLVLHGDEVPERLVDYATFQWQRASVQRFIALSA

KQSG

>gi|30063726|ref|NP_837897.1| phosphodiesterase [Shigella flexneri 2a str. 2457T]

MKLMFASDIHGSLPATERVVELFVQSGAQWLVILGDVLNHGPRNALPEGYAPAKVAERLNEVAHKVIAVR

GNCDSEVDQMMLHFPITAPWQQVLLEKQRLFLTHGHLFGPENLPALNQNDVLVYGHTHLPVAEQRGEIFH

FNPGSVSIPKGGNPAGYGMLDNDVLSVIALNDQSIIAQVAINP

>gi|30063725|ref|NP_837896.1| regulator [Shigella flexneri 2a str. 2457T]

MEQRRLASTEWVDIVNEENEVIAQASREQMRAQCLRHRATYIVVHDGMGKILVQRRTETKDFLPGMLDAT
AGGVVQADEQLLESARREAEEELGIAGVPFAEHGQFYFEDKNCRVWGALFSCVSHGPFALQEDEVSGVCW
LTPEEITARCDEFTPDSLKALALWMKRNAKNEAVETETAE

>gi|30063723|ref|NP_837894.1| phosphate acetyltransferase [Shigella flexneri 2a str. 2457T]
MSRIIMLIPTGTSVGLTSVSLGVIRAMERKGVRLSVFKPIAQPRTGGDAPDQTTTIVRANSSTTTAAEPL
KMSYVEGLLSSNQKDVLMEEIVANYHANTKDAEVVLVEGLVPTRKHQFAQSLNYEIAKTLNAEIVFVMSQ
GTDTPEQLKERIELTRNSFGGAKNTNITGVIVNKLNAPVDEQGRTRPDLSEIFDDSSKAKVNNVDPAKLQ
ESSPLPVLGAVPWSFDLIATRAIDMARHLNATIINEGDINTRRVKSVTFCARSIPHMLEHFRAGSLLVTS
ADRPDVLVAACLAAMNGVEIGALLLTGGYEMDARISKLCERAFATGLPVFMVNTNTWQTSLSLQSFNLEV
PVDDHERIEKVQEYVANYINADWIESLTATSERSRRLSPPAFRYQLTELARKAGKRIVLPEGDEPRTVKA
AAICAERGIATCVLLGNPAEINRVAASQGVELGAGIEIVDPEVVRESYVGRLVELRKNKGMTETVAREQL
EDNVVLGTLMLEQDEVDGLVSGAVHTTANTIRPPLQLIKTAPGSSLVSSVFFMLLPEQVYVYGDCAINPD
PTAEQLAEIAIQSADSAAAFGIEPRVAMLSYSTGTSGAGSDVEKVREATRLAQEKRPDLMIDGPLQYDAA
VMADVAKSKAPNSPVAGRATVFIFPDLNTGNTTYKAVQRSADLISIGPMLQGMRKPVNDLSRGALVDDIV
YTIALTAIQSAQQQ

>gi|30063722|ref|NP_837893.1| acetate kinase [Shigella flexneri 2a str. 2457T]
MSSKLVLVLNCGSSSLKFAIIDAVNGEEYLSGLAECFHLPEARIKWKMDGNKQEAALGAGAAHSEALNFI
VNTILAQKPELSAQLTAIGHRIVHGGEKYTSSVVIDESVIQGIKDAASFAPLHNPAHLIGIEEALKSFPQ
LKDKNVAVFDTAFHQTMPEESYLYALPYNLYKEHGIRRYGAHGTSHFYVTQEAAKMLNKPVEELNIITCH
LGNGGSVSAIRNGKCVDTSMGLTPLEGLVMGTRSGDIDPAIIFHLHDTLGMSVDAINKLLTKESGLLGLT
EVTSDCRYVEDNYATKEDAKRAMDVYCHRLAKYIGAYTALMDGRLDAVAFTGGIGENAAMVRELSLGKLG
VLGFEVDHERNLAARFGKSGFINKEGTRPAVVIPTNEELVIAQDASRLTA

>gi|30063721|ref|NP_837892.1| hypothetical protein S2506 [Shigella flexneri 2a str. 2457T]

MSTPDNRSVNFFSLFRRGQHYSKTWPLEKRLAPVFVENRVIKMTRYAIRFMPPIAVFTLCWQIALGGLLG

PAVATALFALSLPMQGLWWLGKRSVTPLPPAILNWFYEVRGKLQESGQVLAPVEGKPDYQALADTLKRAF

KQLDKTFLDDL

>gi|30063720|ref|NP_837891.1| hypothetical protein S2505 [Shigella flexneri 2a str. 2457T]

MEMTNAQRLILSNQYKMMTMLDPANAERYRRLQTIIERGYGLQMRELDREFGELKEETCRTIIDIMDMYH

ALYVSWSNLQDQQSIDERRVTFLGFDAATEARYLGYVRFMVNVEGRYTHFDAGTHGFNAQTPMWEKYQRM

LNVWHACPRQYHLSANEINQIINA

>gi|30063719|ref|NP_837890.1| phosphatase [Shigella flexneri 2a str. 2457T]

MRCKGFLFDLDGTLVDSLPAVERAWSNWARRHGLAPEEVLAFIHGKQAITSLRHFMAGKSEADIAAEFTR

LEQIEATETEGITALPGAIALLNHLNKAGIPWAIVTSGSMPVARARHKIAGLPAPEVFVTAERVKRGKPE

PDAYLLGAQLLGLAPQECVVVEDAPAGVLSGLAAGCHVIAVNAPADTPRLNEVDLVLHSLEQITVTKQPN

GDVIIQ

>gi|30063718|ref|NP_837889.1| transporter [Shigella flexneri 2a str. 2457T]

MNGELIWVLSLLAVAIVLFATGRVRMDAVALFVIVAFALSGTLTVPEVFSGFSDPNVVLIAALFIIGDGL

VRTGVATVMGTWLVKVAGNSEIKMLVLLMLTVAGLGAFMSSTSVVAIFIPVVLSVAMRMQTSPSRLMMPL

SFAGLISGMMTLVATPPNLVVNSELLREGYHGFSFFSVTPIGLVVLVLGILYMLVMRFMLKGDTQTPQRE

GWTRRTFRDLIREYRLTGRARRLAIRPGSPMIGQRLDDLKLRERYGANVIGVERWRRFRRVIVNVNGVSE

FRARDVLLIDMSAADVDLRQFCSEQLLEPMVLRGEYFSDQALDVGMAEISLIPESELIGKSVREIGFRTR

YGLNVVGLKRNGVALEGSLADEPLLLGDIILVVGNWKLIGMLAKQGRDFVALNLPEEVSEASPAHSQAPH

AIFCLVLMVALMLTNEIPNPVAAIIACLLMGKFRCIDAESAYKSIHWPSIILIVGMMPFAVALQKTGGVA

LAVKGLMDIGGGYGPHMMLGCLFVLSAVIGLFISNTATAVLMAPIALAAAKTMGVSPYPFAMVVAMAASA

AFMTPVSSPVNTLVLGPGNYSFSDFVKLGVPFTIIVMAVCVVMIPMLFPF

>gi|30063717|ref|NP_837888.1| hypothetical protein S2502 [Shigella flexneri 2a str. 2457T]

MKQSHFFAHLSRLKLINRWPLMRNVRTENVSEHSLQVAMVAHALAAIKNRKFGGNVNAERIALLAMYHDA

SEVLTGDLPTPVKYFNAQIAQEYKAIEKIAQQKLVDMVPEELRDIFAPLIDEHAYSDEEKSLVKQADALC

AYLKCLEELAAGNNEFLLAKTRLEATLEARRSQEMDYFMEVFVPSFHLSLDEISQDSPL

>gi|30063716|ref|NP_837887.1| aminotransferase AlaT [Shigella flexneri 2a str. 2457T]

MSPIEKSSKLENVCYDIRGPVLKEAKRLEEEGNKVLKLNIGNPAPFGFDAPDEILVDVIRNLPTAQGYCD

SKGLYSARKAIMQHYQARGMRDVTVEDIYIGNGVSELIVQAMQALLNSGDEMLVPAPDYPLWTAAVSLSS

GKAVHYLCDESSDWFPDLDDIRAKITPRTRGIVIINPNNPTGAVYSKELLMEIVEIARQHNLIIFADEIY

DKILYDDAEHHSIAPLAPDLLTITFNGLSKTYRVAGFRQGWMVLNGPKKHAKGYIEGLEMLASMRLCANV

PAQHAIQTALGGYQSISEFITPGGRLYEQRNRAWELINDIPGVSCVKPRGALYMFPKIDAKRFNIHDDQK

MVLDFLLQEKVLLVQGTAFNWPWPDHFRIVTLPRVDDIELSLSKFARFLSGYHQL

>gi|30063715|ref|NP_837886.1| LysR family NADH dehydrogenase transcriptional regulator [Shigella flexneri 2a str. 2457T]

MISANRPIINLDLDLLRTFVAVADLNTFAAAAAAVCRTQSAVSQQMQRLEQLVGKELFARHGRNKLLTEH

GIQLLGYARKILRFNDEACSSLMFSNLQGVLTIGASDESADTILPFLLNRVSSVYPKLALDVRVKRNAYM

AEMLESQEVDLMVTTHRPSAFKALNLRTSPTHWYCAAEYVLQKGEPIPLVLLDDPSPFRDMVLATLNKVD

IPWRLAYVASTLPAVRAAVKAGLGVTARPVEMMSPDLRVLSGVDGLPPLPDTEYLLCYDPSSNNELAQVI

YQAMESYHNPWQYSPMSAPEGDDSLLIERDIE

>gi|30063714|ref|NP_837885.1| NADH dehydrogenase subunit A [Shigella flexneri 2a str. 2457T]

MSMSTSTEVIAHHWAFAIFLIVAIGLCCLMLVGGWFLGGRARARSKNVPFESGIDSVGSARLRLSAKFYL

VAMFFVIFDVEALYLFAWSTSIRESGWVGFVEAAIFIFVLLAGLVYLVRIGALDWTPARSRRERMNPETN

SIANRQR

>gi|30063713|ref|NP_837884.1| NADH dehydrogenase subunit B [Shigella flexneri 2a str. 2457T]

MDYTLTRIDPNGENDRYPLQKQEIVTDPLEQEVNKNVFMGKLNDMVNWGRKNSIWPYNFGLSCCYVEMVT

SFTAVHDVARFGAEVLRASPRQADLMVVAGTCFTKMAPVIQRLYDQMLEPKWVISMGACANSGGMYDIYS

VVQGVDKFIPVDVYIPGCPPRPEAYMQALMLLQESIGKERRPLSWVVGDQGVYRANMQSERERKRGERIA

VTNLRTPDEI

>gi|30063712|ref|NP_837883.1| bifunctional NADH:ubiquinone oxidoreductase subunit C/D [Shigella flexneri 2a str. 2457T]

MVNNMTDLTAQEPAWQTRDHLDDPVIGELRNRFGPDAFTVQATRTGVPVVWIKREQLLEVGDFLKKLPKP

YVMLFDLHGMDERLRTHREGLPAADFSVFYHLISIDRNRDIMLKVALAENDLHVPTFTKLFPNANWYERE

TWDLFGITFDGHPNLRRIMMPQTWKGHPLRKDYPARATEFSPFELTKAKQDLEMEALTFKPEEWGMKRGT

ENEDFMFLNLGPNHPSAHGAFRIVLQLDGEEIVDCVPDIGYHHRGAEKMGERQSWHSYIPYTDRIEYLGG

CVNEMPYVLAVEKLAGITVPDRVNVIRVMLSELFRINSHLLYISTFIQDVGAMTPVFFAFTDRQKIYDLV

EAITGFRMHPAWFRIGGVAHDLPRGWDRLLREFLDWMPKRLASYEKAALQNTILKGRSQGVAAYGAKEAL

EWGTTGAGLRATGIDFDVRKARPYSGYENFDFEIPVGGGVSDCYTRVMLKVEELRQSLRILEQCLNNMPE

GPFKADHPLTTPPPKERTLQHIETLITHFLQVSWGPVMPANESFQMIEATKGINSYYLTSDGSTMSYRTR

IRTPSYAHLQQIPAAIRGSLVSDLIVYLGSIDFVMSDVDR

>gi|30063711|ref|NP_837882.1| NADH dehydrogenase subunit E [Shigella flexneri 2a str. 2457T]

MHENQQPQTEAFELSAAEREAIEHEMHHYEDPRAASIEALKIVQKQRGWVPDGAIHAIADVLGIPASDVE

GVATFYSQIFRQPVGRHVIRYCDSVVCHINGYQGIQAALEKKLNIKPGQTTFDGRFTLLPTCCLGNCDKG

PNMMIDEDTHAHLTPEAIPELLERYK

>gi|30063710|ref|NP_837881.1| NADH dehydrogenase I subunit F [Shigella flexneri 2a str. 2457T]

MKNIIRTPETHPLTWRLRDDKQPVWLDEYRSKNGYEGARKALTGLSPDEIVNQVKDAGLKGRGGAGFSTG

LKWSLMPKDESMNIRYLLCNADEMEPGTYKDRLLMEQLPHLLVEGMLISAFALKAYRGYIFLRGEYIEAA

VNLRRAIAEATEAGLLGKNIMGTGFDFELFVHTGAGRYICGEETALINSLEGRRANPRSKPPFPATSGVW

GKPTCVNNVETLCNVPAILANGVEWYQNISKSKDAGTKLMGFSGRVKNPGLWELPFGTTAREILEDYAGG

MRDGLKFKAWQPGGAGTDFLTEAHLDLPMEFESIGKAGSRLGTALAMAVDHEINMVSLVRNLEEFFARES

CGWCTPCRDGLPWSVKILRALERGEGQPGDIETLEQLCRFLGPGKTFCAHAPGAVEPLQSAIKYFREEFE

AGIKQPFSNTHLINGIQPNLLKERW

>gi|30063709|ref|NP_837880.1| NADH dehydrogenase subunit G [Shigella flexneri 2a str. 2457T]

MATIHVDGKEYEVNGADNLLEACLSLGLDIPYFCWHPALGSVGACRQCAVKQYQNAEDTRGRLVMSCMTP

ASDGTFISIDDEEAKQFRESVVEWLMTNHPHDCPVCEEGGNCHLQDMTVMTGHSFRRYRFTKRTHRNQDL

GPFISHEMNRCIACYRCVRYYKDYADGTDLGVYGAHDNVYFGRPEDGTLESEFSGNLVEICPTGVFTDKT

HSERYNRKWDMQFAPSICQQCSIGCNISPGERYGELRRIENRYNGTVNHYFLCDRGRFGYGYVNLKDRPR

QPVQRRGDDFITLNAEQAMQGAADILRQSKKVIGIGSPRASVESNFALRELVGEENFYTGIAHGEQERLQ

LALKVLREGGIYTPALREIESYDAVLVLGEDVTQTGARVALAVRQAVKGKAREMAAAQKVADWQIAAILN

IGQRAKHPLFVTNVDDTRLDDIAAWTYRAPVEDQARLGFAIAHALDNSAPAVDGIEPELQNKIDVIVQAL

AGAKKPLIISGTNAGSIEVIQAAANVAKALKGRGADVGITMIARSVNSMGLGIMGGGSLEEALTELETGR

ADAVVVLENDLHRYASATRVNAALAKAPLVMVVDHQRTAIMENAHLVLSSASFAESDGTVINNEGRAQRF

FQVYDPAYYDSKTVMLESWRWLHSLHSTLLSREVDWTQLDHVIDAVVAKIPELAGIKDAAPDATFRIRGQ

KLAREPHRYSGRTAMRANISVHEPRQPQDIDTMFTFSMEGNNQPTAHRSQVPFAWAPGWNSPQAWNKFQD

EVGGKLRFGDPGVRLFETSENGLDYFTSVPARFQPQDGKWRIAPYYHLFGSDELSQRAPVFQSRMPQPYI

KLNPADAAKLGVNAGTRVSFSYDGNTVTLPVEIAEGLTAGQVGLPMGMSGIAPVLAGAHLEDLKEAQQ

>gi|30063708|ref|NP_837879.1| NADH dehydrogenase subunit H [Shigella flexneri 2a str. 2457T]

MSWISPELIEILLTILKAVVILLVVVTCGAFMSFGERRLLGLFQNRYGPNRVGWGGSLQLVADMIKMFFK

EDWIPKFSDRVIFTLAPMIAFTSLLLAFAIVPVSPGWVVADLNIGILFFLMMAGLAVYAVLFAGWSSNNK

YSLLGAMRASAQTLSYEVFLGLSLMGVVAQAGSFNMTDIVNSQAHVWNVIPQFFGFITFAIAGVAVCHRH

PFDQPEAEQELADGYHIEYSGMKFGLFFVGEYIGIVTISALMVTLFFGGWQGPLLPPFIWFALKTAFFMM

MFILIRASLPRPRYDRVMSFGWKICLPLTLINLLVTAAVILWQAQ

>gi|30063707|ref|NP_837878.1| NADH dehydrogenase subunit I [Shigella flexneri 2a str. 2457T]

MTLKELLVGFGTQVRSIWMIGLHAFAKRETRMYPEEPVYLPPRYRGRIVLTRDPDGEERCVACNLCAVAC

PVGCISLQKAETKDGRWYPEFFRINFSRCIFCGLCEEACPTTAIQLTPDFEMGEYKRQDLVYEKEDLLIS

GPGKYPEYNFYRMAGMAIDGKDKGEAENEAKPIDVKSLLP

>gi|30063706|ref|NP_837877.1| NADH dehydrogenase subunit J [Shigella flexneri 2a str. 2457T]

MEFAFYICGLIAILATLRVITHTNPVHALLYLIISLLAISGVFFSLGAYFAGALEIIVYAGAIMVLFVFV

VMMLNLGGSEIEQERQWLKPQVWIGPAILSAIMLVVIVYAILGVNDQGIDGTPISAKAVGITLFGPYVLA

VELASMLLLAGLVVAFHVGREERAGEVLSNRKDDSAKRKTEEHA

>gi|30063705|ref|NP_837876.1| NADH dehydrogenase subunit K [Shigella flexneri 2a str. 2457T]

MIPLQHGLILAAILFVLGLTGLVIRRNLLFMLIGLEIMINASALAFVVAGSYWGQTDGQVMYILAISLAA

AEASIGLALLLQLHRRRQNLNIDSVSEMRG

>gi|30063704|ref|NP_837875.1| NADH dehydrogenase subunit L [Shigella flexneri 2a str. 2457T]

MNMLALTIILPLIGFVLLAFSRGRWSENVSAIVGVGSVGLAALVTAFIGVDFFANGEQAYSQPLWTWMSV

GDFNIGFNLVLDGLSLTMLSVVTGVGFLIHMYASWYMRGEEGYSRFFAYTNLFIASMVVLVLADNLLLMY

LGWEGVGLCSYLLIGFYYTDPKNGAAAMKAFVVTRVGDVFLAFALFILYNELGTLNFRDMVELAPAHFAD

GNNMLMWATLMLLGGAVGKSAQLPLQTWLADAMAGPTPVSALIHAATMVTAGVYLIARTHGLFLMTPEVL

HLVGIVGAVTLLLAGFAALVQTDIKRVLAYSTMSQIGYMFLALGVQAWDAAIFHLMTHAFFKALLFLASG

SVILACHHEQNIFKMGGLRKSIPLVYLCFLVGGAALSALPLVTAGFFSKDEILAGAMANGHINLMVAGLV

GAFMTSLYTFRMIFIVFHGKEQIHAHAVKGVTHSLPLIVLLILSTFVGALIVPPLQGVLPQTTELAHGSM

LTLEITSGVVAVVGILLAAWLWLGKRSLVTSIANSAPGRLLSTWWYNAWGFDWLYDKVFVKPFLGIAWLL

KRDPLNSMMNIPAVLSRFAGKGLLLSENGYLRWYVASMSIGAVVVLALLMVLR

>gi|30063703|ref|NP_837874.1| NADH dehydrogenase subunit M [Shigella flexneri 2a str. 2457T]

MLLPWLILIPFIGGFLCWQTERFGVKVPRWIALITMGLTLALSLQLWLQGGYSLTQSAGIPQWQSEFDMP

WIPRFGISIHLAIDGLSLLMVVLTGLLGVLAVLCSWKEIEKYQGFFHLNLMWILGGVIGVFLAIDMFLFF

FFWEMMLVPMYFLIALWGHKASDGKTRITAATKFFIYTQASGLVMLIAILALVFVHYNATGVWTFNYEEL

LNTPMSSGVEYLLMLGFFIAFAVKMPVVPLHGWLPDAHSQAPTAGSVDLAGILLKTAAYGLLRFSLPLFP

NASAEFAPIAMWLGVIGIFYGAWMTFAQTDIKRLIAYTSVSHMGFVLIAIYTGSQLAYQGAVIQMIAHGL

SAAGLFILCGQLYERIHTRDMRMMGGLWSKMKWLPALSLFFAVATLGMPGTGNFVGEFMILFGSFQVVPV

ITVISTFGLVFASVYSLAMLHRAYFGKAKSQIASQELPGMSLRELFMILLLVVLLVLLGFYPQPILDTSH

SAIGNIQQWFVNSVTTTRP

>gi|30063701|ref|NP_837872.1| IS1 orfB [Shigella flexneri 2a str. 2457T]

MSRQCTHYGRWPQHGFTSLKKLRPQSVTSRIQPGSDVIVCAEMDEHWGYVGAKSRQRWLFYAYDRIRRTV

VAHVFGERTLATLERLLSLLSAFEVVVWMTDGWPLYESRLKGKLHVISKRYTQRIERHNLKLRQHLARLG

RKSLSFSKSVELHDKVIGHYLNIKHYQ

>gi|30063700|ref|NP_837871.1| IS1 orfA [Shigella flexneri 2a str. 2457T]

MASISIRCPSCSATEGVVRNGKSTAGHQRYLCSHCRKTWQLQFTYTASQPGTHQKIIDMAMNGVGCRASA

RIMGVGLNTVLRHLKNSGRSR

>gi|30063698|ref|NP_837869.1| hypothetical protein S2480 [Shigella flexneri 2a str. 2457T]

MIEWQDLHHSELSVSQLYALLQLRCAVFVVEQNCPYQDIDGDDLTGDNRHILGWKNDELVAYARILKSDD

DLEPVVIGRVIVSEALRGEKVGQQLMSKTLETCTHHWPDKPVYLGAQAHLQNFYQSFGFIPVTEVYEEDG

IPHIGMAREVIQA

>gi|30063697|ref|NP_837868.1| hypothetical protein S2479 [Shigella flexneri 2a str. 2457T]

MSNQFGDTRIDDDLTLLSETLEEVLRSSGDPADQKYVELKAHAEKALDDVKKRVSQASDSYYYRAKQAVY

RADDYVHEKPWQGIGVGAAVGLVLGLLLARR

>gi|30063695|ref|NP_837866.1| 2-succinyl-5-enolpyruvyl-6-hydroxy-3-cyclohexene-1-carboxylate synthase [Shigella flexneri 2a str. 2457T]

MSVSAFNRRWAAVILEALTRHGVRHICIAPGSRSTPLTLAAAENSAFIHHTHFDERGLGHLALGLAKVSK

QPVAVIVTSGTAVANLYPALIEAGLTGEKLILLTADRPPELIDCGANQAIRQPGMFASHPTHSISLPRPT

QDIPARWLVSTIDHALGTLHAGGVHINCPFAEPLYGEMDDTGLSWQQRLGDWWQDDKPWLREAPRLESEK

QRDWFFWRQKRGVVVAGRMSAEEGKKVALWAQTLGWPLIGDVLSQTGQPLPCADLWLGNAKATSELQQAQ

IVVQLGSSLTGKRLLQWQASCEPEEYWIVDDIEGRLDPAHHRGRRLIANIADWLELHPAEKRQPWCVEIP

RLAEQAMQAVIARRDAFGEAQLAHRISDYLPEQGQLFVGNSLVVRLIDALSQLPAGYPVYSNRGASGIDG

LLSTAAGVQRASGKPTLAIVGDLSALYDLNALALLRQVSAPLVLIVVNNNGGQIFSLLPTPQSERERFYL

MPQNVHFEHAAAMFELKYHHPQNWQELETAFADAWRTPTTTVIEMVVNDTDGAQTLQQLLAQVSHL

>gi|30063694|ref|NP_837865.1| acyl-CoA thioester hydrolase YfbB [Shigella flexneri 2a str. 2457T]

MILHAQAKHGKLGLPWLVFLHGFSGDCHEWQEVGEAFADYSRLYVDLLGHGGSAAISVDGFDDVTDLLRK

TLVSYNILDFWLVGYSLGGRVAMMAACQGLAGLCGVIVEGGHPGLQNAEQRAERQRSDRQWAQRFRTEPL

TAVFADWYQQPVFASLNDDQRRELVALRSNNNGATLAAMLEATSLAVQPDLRANLSARTFAFYYLCGERD

SKFRALAAELAADCHVIPRAGHNAHRENPAGVIASLAQILRF

>gi|30063693|ref|NP_837864.1| naphthoate synthase [Shigella flexneri 2a str. 2457T]

MIYPDEAMPYAPVEWHDCSEGFEDIRYEKSTDGIAKITINRPQVRNAFRPLTVKEMIQALADARYDDNIG

VIILTGAGDKAFCSGGDQKVRGDYGGYKDDSGVHHLNVLDFQRQIRTCPKPVVAMVAGYSIGGGHVLHMM

CDLTIAADNAIFGQTGPKVGSFDGGWGASYMARIVGQKKAREIWFLCRQYDAKQALDMGLVNTVVPLADL

EKETVRWCREMLQNSPMALRCLKAALNADCDGQAGLQELAGNATMLFYMTEEGQEGRNAFNQKRQPDFSK

FKRNP

>gi|30063692|ref|NP_837863.1| O-succinylbenzoate synthase [Shigella flexneri 2a str. 2457T]

MRSAQVYRWQIPMDAGVVLRDRRLKTRDGLYVCLREGEREGWGEISPLPGFSQENWEEAQSVLLAWVNNW

LAGDCELPQMPSVAFGVSCALAELTDTLPQAANYRAAPLCNGDPDDLILKLADMPGEKVAKVKVGLYEAV

RDGMVVNLLLEAIPDLHLRLDANRAWTPLKGQQFAKYVNPDYRHRIAFLEEPCKTRDDSRAFARETGIAI

AWDESLREPDFAFVAEEGVRAVVIKPTLTGSLEKVREQVQAAHALGLTAVISSSIESSLGLTQLARIAAW

LTPDTIPGLDTLDLMQAQQVRRWPGSPLPLVDADVQEQLL

>gi|30063691|ref|NP_837862.1| O-succinylbenzoic acid--CoA ligase [Shigella flexneri 2a str. 2457T]

MIFSDWPWRHWRQVRGEAIALRLNDEQLNWRELCARVDELATGYAVQGVVEGSGVMLRAWNTPQTLLAWL

ALLQCGARVLPVNPQLPQPLLEELLPNLTLQFALVPDGENTFPALTSLHIQLVEGVHAAAWQPTRLCSMT

LTSGSTGLPKAAVHTYQAHLASAQGVLSLIPFGDHDDWLLSLPLFHVSGQGIMWRWLYAGARMTVRDKQP

LEQMLAGCTHASLVPTQLWRLLVNRSSVSLKAVLLGGAAIPVELTEQAREQGIRCFCGYGLTEFASTVCA

KEADGLADVGLPLPGREVKIVNNEVWLRAASMAEGYWRNGQLVSLVNDEGWYATRDRGEMHNGKLTIVGR

LDNLFFSGGEGIQPEEVERVIAAHPAVLQVFIVSVADKEFGHRPVAVVEYDQQTVDLGEWVKDKLARFQQ

PVRWLTLPPELKNGGIKISRQALKEWVQRQQ

>gi|30063689|ref|NP_837860.1| sucrose-6 phosphate hydrolase [Shigella flexneri 2a str. 2457T]

MIWLTLVFASLLSVAGQLCQKQATCFVAINKRRKHIVLWLGLALACLGLAMMLWLLVLQNVPVGIAYPML

SLNFVWVTLAAVKLWHEPVSPRHWCGVAFIIGGIVILGSTV

>gi|30063688|ref|NP_837859.1| 4-amino-4-deoxy-L-arabinose transferase [Shigella flexneri 2a str. 2457T]

MKSVRYLIGIFAFIACYYLLPISTRLLWQPDETRYAEISREMLASGDWIVPHLLGLRYFEKPIAGYWINS

IGQWLFGANNFGVRAGVIFATLLTAALVTWFTLRLWRDKRLALLATVIYLSLFIVYAIGTYAVLDPFIAF

WLVAGMCSFWLAMQAQTWKGKSAGFLLLGITCGMGVMTKGFLALAVPVLSVLPWVATQKRWKDLFIYGWL

AVISCVLTVLPWGLAIAQREPDFWHYFFWVEHIQRFALDDAQHRAPFWYYVPVIIAGSLPWLGLLPGALY

TGWKNRKHSATVYLLSWTIMPLLFFSVAKGKLPTYILSCFAPLAMLLAHYALLAAKNNPLALRINGWINI

AFGVTGIIATFVISPWGPMNTPVWQTFESYKVFCAWSIFSLWAFFGWYTLTNVEKTWSFAALCPLGLALL

VGFSIPDRVMEGKHPQFFVEMTQESLQPSRYILTDSVGVAAGLAWSLQRDDIIMYRQTGELKYGLNYPDA

KGRFVSGDEFANWLNQHRQEGIITLVLSVDRDEDINSLAIPPADAIVRQERLVLIQYRPK

>gi|30063687|ref|NP_837858.1| hypothetical protein S2468 [Shigella flexneri 2a str. 2457T]

MTKVGLRIDVDAFRGTREGVPRLLEILSKHNIQASIFFSVGPDNMGRHLWRLVKPQFLWKMLRSNAASLY

GWDILLAGTAWPGKEIGHANADIIREAAKHHEVGLHAWDHHAWQARSGNWDRQTMIDDIARGLRTLEEII

GQPVTCSAAAGWRADQKVIEAKEAFHLRYNSDCRGAIPFRPLLESGNPGTAQIPVTLPTWDEVIGRDVKA

EDFNGWLLNRILRDKGTPVYTIHAEVEGCAYQHNFVDLLKRAAQEGVTFCPLSELLSETLPLGQVVRGNI

AGREGWLGCQQIAGSR

>gi|30063686|ref|NP_837857.1| hypothetical protein S2467, partial [Shigella flexneri 2a str. 2457T]

MAQLRVAIAPDDIAITLHHKLCHAARQLLEQTLPAIKHGNILEIAQRENEATCFGRRTPDDSFLEWHKPA

SVLHNMVRAVADPWPGAFSYVGNQKFTVWSSRVHPHASKAQPGSVISVAPLLIACGDGALEIVTGQAGDG

ITMQGSQLAQMLGLVQGSRLNSQPACTARRRTRVLILGVNGFIGNHLTERLLREDHYEVYGLDIGSDAIS

RFLNHPHFHFVEGDISIHSEWIEYHVKKCDVVLPLVAIATPIEYTRNPLRVFELDFEENLRIIRYCVKYR

KRIIFPSTSEVYGMCSDKYFDEDHSNLIVGPVNKPRWIYSVSKQLLDRVIWAYGEKEGLQFTLFLPFNWM

GPRLDNLNAARIGSSRAITQLILNLVEGSPIKLIDGGKQKRCFTDIRDGIEALYHIIENAGNRCDGEIIN

IGNPENEASIEELGEMLLASFEKHPLRHHFPPFAGFRVVESSCYYGKGYQDVEHRKPSIRNAHRCLDWEP

KIDMQETIDETLDFFLRTVDLTDKPS

>gi|30063685|ref|NP_837856.1| undecaprenyl phosphate 4-deoxy-4-formamido-L-arabinose transferase [Shigella flexneri 2a str. 2457T]

MFEIHPVKKVSVVIPVYNEQESLPELIRRTTTACESLGKEYEILLIDDGSSDNSAHMLVEASQAENSHIV

SILINRNYGQHSAIMAGFSHVTGDLIITLDADLQNPPEEIPRLVAKADEGYDVVGTVRQNRQDSWFHKTA

SKMINRLIQRTTGKAMGDYGCMLRAYRRHIVDAMLHCHERSTFIPILANIFARRAIEIPVHHAEREFGES

KYSFMRLINLMYDLVTCLTTTPLRMLSLLGSIIAIGGFSIAVLLVILRLTFGPQWAAEGVFMLFAVLFTF

IGAQFIGMGLLGEYIGRIYTDVRARPRYFVQQVIRPSSKENE

>gi|30063683|ref|NP_837854.1| protein induced by aluminum [Shigella flexneri 2a str. 2457T]

MKSKKYFIILLALAAIAGLGTHAAWSSNGLPRIDNKTLARLAQQHPVVVLFRHAERCDRSTNQCLSDKTG

ITVKGTQDARELGNAFSADISDFDLYSSNTVRTIQSATWFSAGKKLTVDKRLLQCGNEIYSAIKDLQSKA

PDKNIVIFTHNHCLTYIAKNKRDAIFKPDYLDGLVMHVEKGKVYLDGEFVNH

>gi|30063682|ref|NP_837853.1| hypothetical protein S2463 [Shigella flexneri 2a str. 2457T]

MADDRGVFPGQWALSGGGVESGERIEEALRREIREELGEQLLLTEITPWTFSDDIRTKTYADGRKEEIYM

IYLIFDCVSANREVKINEEFQDYAWVKPEDLVHYDLNVATRKTLRLKGLL

>gi|30063681|ref|NP_837852.1| hypothetical protein S2462 [Shigella flexneri 2a str. 2457T]

MKKIALAGLAGMLLVSASVNAMSISGQAGKEYTNIGVGFGTESTGLALSGNWTHNDDDGDVAGVGLGLNL

PLGPLMATVGGKGVYTNPNYGDEGYAAAVGGGLQWKIGNSFRLFGEYYYSPDSLSSGIQSYEEANAGARY

TIMRPVSIEAGYRYLNLSGKDGNRDNAVADGLYVGVNASF

>gi|30063680|ref|NP_837851.1| competence damage-inducible protein A [Shigella flexneri 2a str. 2457T]

MLKVEMLSTGDEVLYGQIVDTNAAWLADFFFHQGLPLSRRNTVGDNLDDLVTILRERSQHADVLIVNGGL

GPTSDDLSALAAATAKGEGLVLHEAWLKEMERYFHERGRVMAPSNRKQAELPASAEFINNPVGTACGFAV

QLNRCLMFFTPGVPSEFKVMVEHEILPRLRERFSLPQPPVCLRLTTFGRSESDLAQSLDTLQLPPGVTMG

YRSSMPIIELKLTGPASEQQAMEKLWLDVKRVAGQSVIFEGTEGLPAQISRELQNRQFSLTLSEQFTGGL

LALQLSRAGAPLLACEVIPSQEETLAQTAHWITERRANHFAGLALAVSGFENEHLNFALATPDGTFALRV

RFSTTRYSLAIRQEVCAMMALNMLRRWLNGQDIASEHGWIEVVESMTLSV

>gi|30063679|ref|NP_837850.1| hypothetical protein S2460 [Shigella flexneri 2a str. 2457T]

MSNPPYGERGVGASVARAARWGRIENYMAQVNDSLCLLVQVESKTALDNLDEILDVEGIDGVFIGPADLS

ASLGYPDNAGHPEVQRIIETSIRRIRAAGKAAGFLAVAPDMAQQCLAWGANFVAVGVDTMLYSDALDQRL

AMFKSGKNGPRIKGSY

>gi|30063678|ref|NP_837849.1| hypothetical protein S2459 [Shigella flexneri 2a str. 2457T]

MTESTTSSPHDAVFKTFIFTPETARDFLEIHLPEPLRKLCNLQTLRLEPTSFIEKSLRAYYSDVLWSVET

SDGDGYIYCVIEHQSSAEKNMAFRPMRYATAAMQSHLDKGYDRVPLVVPLLFYHGETSPYPYSLNWLDEF

DDPQLARQLYTEAFPLVDITIVPDDEIMQHRRIALLELIQKHIRDHDLIGMVDRITTLLVRGFTNDSQLQ

TLFNYLLQCGDTSRFTRFIQEIAERSPLQKERLMTIAERLRQEGHQIGWQEGKLVGLQQGKLEGLQEGMH

EQAIKIALRMLEQGIDRDQVLAATQLSEADLAANNH

>gi|30063677|ref|NP_837848.1| sn-glycerol-3-phosphate dehydrogenase subunit C [Shigella flexneri 2a str. 2457T]

MNDTSFENCIKCTVCTTACPVSRVNPGYPGPKQAGPDGERLRLKDGALYDEALKYCINCKRCEVACPSDV

KIGDIIQRARAKYDTTRPSLRNFVLSHTDLMGSVSTPFAPIVNTATSLKPVRQLLDAALKIDHRRTLPKY

SFGTFRRWYRSIAAQQAQYKDQVAFFHGCFVNYNHPQLGKDLIKVLNAMGTGVQLLSKEKCCGVPLIANG

FTDKARKQAITNVESIREAVGVKGIPVIATSSTCTFALRDEYPEVLNVDNKGLRDHIELATRWLWRKLDE

GKTLPLKPLPLKVVYHTPCHMEKMGWTLYTLELLRNIPGLELTVLDSQCCGIAGTYGFKKENYPTSQAIG

APLFRQIEESGADLVVTDCETCKWQIEMSTSLRCEHPITLLAQALA

>gi|30063676|ref|NP_837847.1| anaerobic glycerol-3-phosphate dehydrogenase subunit B [Shigella flexneri 2a str. 2457T]

MRFDTVIMGGGLAGLLCGLQLQKHGLRCAIVTRGQSALHFSSGSLDLLSHLPDGQPVADIHSGLESLRQQ

APAHPYSLLGPQRVLDLACQAQALIAESGAQLQGSVELAHQRITPLGTLRATWLSSPEVPVWPLPAKKIC

VVGISGLMDFQAHLAAASLRELDLSVETAEIELPELDVLRNNATEFRAVNIARFLDNEENWPLLLDALIP

VANTCEMILMPACFGLADDKLWRWLNEKLPCSLMLLPTLPPSVLGIRLQNQLQRQFVRQGGVWMPGDEVK

KVTCKNGVVNEIWTRNHADIPLRPRFAVLASGSFFSGGLVAERNGIREPILGLDVLQTATRGEWYKGDFF

APQPWQQFGVTTDETLRPSQAGQTIENLFAIGSVLGGFDPIAQGCGGGVCAVSALHAAQQIAQRAGGQQ

>gi|30063675|ref|NP_837846.1| sn-glycerol-3-phosphate dehydrogenase subunit A [Shigella flexneri 2a str. 2457T]

MKTRDSQSSDVIIIGGGATGAGIARDCALRGLRVILVERHDIATGATGRNHGLLHSGARYAVTDAESARE

CISENQILKRIARHCVEPTNGLFITLPEDDLSFQATFIRACEEAGISAEAIDPQQARIIEPAVNPALIGA

VKVPDGTVDPFRLTAANMLDAKEHGAVILTAHEVTGLIREGATVCGVRVRNHLTGETQALHAPVVVNAAG

IWGQHIAEYADLRIRMFPAKGSLLIMDHRINQHVINRCRKPSDADILVPGDTISLIGTTSLRIDYNEIDD

NRVTAEEVDILLREGEKLAPVMAKTRILRAYSGVRPLVASDDDPSGRNVSRGIVLLDHAERDGLDGFITI

TGGKLMTYRLMAEWATDAVCRKLGNTRPCTTADLALPGSQEPAEVTLRKVISLPAPLRGSAVYRHGDRTP

AWLSEGRLHRSLVCECEAVTAGEVQYAVENLNVNSLLDLRRRTRVGMGTCQGELCACRAAGLLQRFNVTT

SAQSIEQLSTFLNERWKGVQPIAWGDALRESEFTRWVYQGLCGLEKEQKDAL

>gi|30063674|ref|NP_837845.1| sn-glycerol-3-phosphate transporter [Shigella flexneri 2a str. 2457T]

MLSIFKPAPHKARLPAAEIDPTYRRLRWQIFLGIFFGYAAYYLVRKNFALAMPYLVEQGFSRGDLGFALS

GISIAYGFSKFIMGSVSDRSNPRVFLPAGLILAAAVMLFMGFVPWATSSIAVMFVLLFLCGWFQGMGWPP

CGRTMVHWWSQKERGGIVSVWNCAHNVGGGIPPLLFLLGMAWFNDWHAALYMPAFCAILVALFAFAMMRD

TPQSCGLPPIEEYKNDYPDDYNEKAEQELTAKQIFMQYVLPNKLLWYIAIANVFVYLLRYGILDWSPTYL

KEVKHFALDKSSWAYFFYEYAGIPGTLLCGWMSDKVFRGNRGATGVFFMTLVTIATIVYWMNPAGNPTVD

MICMIVIGFLIYGPVMLIGLHALELAPKKAAGTAAGFTGLFGYLGGSVAASAIVGYTVDFFGWDGGFMVM

IGGSILAVILLIVVMIGEKRRHEQLLQKRNGG

>gi|30063673|ref|NP_837844.1| glycerophosphodiester phosphodiesterase [Shigella flexneri 2a str. 2457T]

MKLKLKNLSMAIMMSTIVMGSSAMAADSNEKIVIAHRGASGYLPEHTLPAKAMAYAQGADYLEQDLVMTK

DDHLVVLHDHYLDRVTDVADRFPDRARKDGRYYAIDFTLDEIKSLKFTEGFDIENGKKVQTYPGRFPMGK

SDFRVHTFEEEIEFVQGLNHSTGKNIGIYPEIKAPWFHHQEGKDIAAKTLEVLKKYGYTGKDDKVYLQCF

DADELKRIKNELEPKMGMELNLVQLIAYTDWNETQQKQPDGSWVNYNYDWMFKPGAMKQVAEYADGIGPD

YHMLIEETSQPGNIKLTGMVQDAQQNKLVVHPYTVRSDKLPEYTPDVNQLYDALYNKAGVNGLFTDFPDK

AVKFLNKE

>gi|30063672|ref|NP_837843.1| hypothetical protein S2453 [Shigella flexneri 2a str. 2457T]

MNFIRQGLGIALQPELTLKSIAGELCSVPHEPTFYRQISLLTKEKPVEGSPLFLLQMCMEQLVAIGKI

>gi|30063671|ref|NP_837842.1| hypothetical protein S2452 [Shigella flexneri 2a str. 2457T]

MAVSAKYDEFNHWWATEGDWVEEPNYRRNGMSGVQCVERNGKKLYVKRMTHHLFHSVRYPFGRPTIVREV

AVIKELERAGVIVPKIVFGEAVKIEGEWRALLVTEDMAGFISIADWYARHAVSPYSDEVRQAMLKAVALA

FKKMHSVNRQHGCCYVRHIYVKTEGKAEAGFLDLEKSRRRLRRDKAINHDFRQLEKYLEPIPKADWEQVK

AYYYAM

>gi|30063670|ref|NP_837841.1| 2Fe-2S ferredoxin [Shigella flexneri 2a str. 2457T]

MARVTLRITGTQLLCQDEHPSLLAALESHNVAVEYQCREGYCGSCRTRLVAGQVDWIAEPLAFIQPGEIL

PCCCRAKGDIEIEL

>gi|30063668|ref|NP_837839.1| ribonucleotide-diphosphate reductase subunit alpha [Shigella flexneri 2a str. 2457T]

MNQNLLVTKRDGSTERINLDKIHRVLDWAAEGLHNVSISQVELRSHIQFYDGIKTSDIHETIIKAAADLI

SRDAPDYQYLAARLAIFHLRKKAYGQFEPPALYDHVVKMVEMGKYDNHLLEDYTEEEFKQMDTFIDHDRD

MTFSYAAVKQLEGKYLVQNRVTGEIYESAQFLYILVAACLFSNYPRETRLQYVKRFYDAVSTFKISLPTP

IMSGVRTPTRQFSSCVLIECGDSLDSINATSSAIVKYVSQRAGIGINAGRIRALGSPIRGGEAFHTGCIP

FYKHFQTAVKSCSQGGVRGGAATLFYPMWHLEVESLLVLKNNRGVEGNRVRHMDYGVQINKLMYTRLLKG

EDITLFSPSDVPGLYDAFFADQEEFERLYTKYEKDDSIRKQRVKAVELFSLMMQERASTGRIYIQNVDHC

NTHSPFDPTIAPVRQSNLCLEIALPTKPLNDVNDENGEIALCTLSAFNLGAINSLDELEELAILAVRALD

ALLDYQDYPIPAAKRGAMGRRTLGIGVINFAYYLAKHGKRYSDGSANNLTHKTFEAIQYYLLKASNELAK

EQGACPWFNETTYAKGILPIDTYKKDLDAIANEPLHYDWEALRESIKTHGLRNSTLSALMPSETSSQISN

ATNGIEPPRGYVSIKASKDGILRQVVPDYEHLHDAYELLWEMPGNDGYLQLVGIMQKFIDQSISANTNYD

PSRFPSGKVPMQQLLKDLLTAYKFGVKTLYYQNTRDGAEDAQDDLVPSIQDDGCESGACKI

>gi|30063666|ref|NP_837837.1| insertion sequence 2 OrfA protein [Shigella flexneri 2a str. 2457T]

MIVLILVFRLVIGEQIIDVLGPEKRRRRTTQEKIAIVQQSFEPGMTVSLVARQHGVAASQLFLWRKQYQE

GSLTAVAAGEQVVPASELAAAMKQIKELQRLLGKKTMENELLKEAVEYGRAKKWIAHAPLLPGDGE

>gi|30063665|ref|NP_837836.1| 3-demethylubiquinone-9 3-methyltransferase [Shigella flexneri 2a str. 2457T]

MNAEKSPENHNVDHEEIAKFEAVASRWWDLEGEFKPLHRINPLRLGYIAERAGGLFGKKVLDVGCGGGIL

AESMAREGATVTGLDMGFEPLQVAKLHALESGIQVDYVQETVEEHAAKHAGQYDVVTCMEMLEHVPDPQS

VVRACAQLVKPGGDVFFSTLNRNGKSWLMAVVGAEYILRMVPKGTHDVKKFIKPAELLGWVDQTSLKERH

ITGLHYNPITNSFKLGPGVDVNYMLHTQNK

>gi|30063664|ref|NP_837835.1| DNA gyrase subunit A [Shigella flexneri 2a str. 2457T]

MSDLAREITPVNIEEELKSSYLDYAMSVIVGRALPDVRDGLKPVHRRVLYAMNVLGNDWNKAYKKSARVV

GDVIGKYHPHGDSAVYDTIVRMAQPFSLRYMLVDGQGNFGSIDGDSAAAMRYTEIRLAKIAHELMADLEK

ETVDFVDNYDGTEKIPDVMPTKIPNLLVNGSSGIAVGMATNIPPHNLTEVINGCLAYIDDEDISIEGLME

HIPGPDFPTAAIINGRRGIEEAYRTGRGKVYIRARAEVEVDAKTGRETIIVHEIPYQVNKARLIEKIAEL

VKEKRVEGISALRDESDKDGMRIVIEVKRDAVGEVVLNNLYSQTQLQVSFGINMVALHHGQPKIMNLKDI

IAAFVRHRREVVTRRTIFELRKARDRAHILEALAVALANIDPIIELIRHAPTPAEAKTALVANPWQLGNV

AAMLERAGDDAARPEWLEPEFGVRDGLYYLTEQQAQAILDLRLQKLTGLEHEKLLDEYKELLDQIAELLR

ILGSADRLMEVIREELELVREQFGDKRRTEITANSADINLEDLITQEDVVVTLSHQGYVKYQPLSEYEAQ

RRGGKGKSAARIKEEDFIDRLLVANTHDHILCFSSRGRVYSMKVYQLPEATRGARGRPIVNLLPLEQDER

ITAILPVTEFEEGVKVFMATANGTVKKTVLTEFNRLRTAGKVAIKLVDGDELIGVDLTSGEDEVMLFSAE

GKVVRFKESSVRAMGCNTTGVRGIRLGEGDKVVSLIVPRGDGAILTATQNGYGKRTAVAEYPTKSRATKG

VISIKVTERNGLVVGAVQVDDCDQIMMITDAGTLVRTRVSEISIVGRNTQGVILIRTAEDENVVGLQRVA

EPVDEEDLDTIDGSAAEGDDEIAPEVDVDDEPEEE

>gi|30063663|ref|NP_837834.1| hypothetical protein S2442 [Shigella flexneri 2a str. 2457T]

MRHGLLALICWLCCVVAHSEMLNVEQSRLFRAWFVRIAQEQLRQGPSPRWYQQDCAGLVRFAANETLKVH

DSKWLKSNGLSSQYLPPEMTLTPEQRQLAQNWNQGSGKNGPYVTAINLIQYNSQFIGQDINQALPGDMIF

FDQGDAQHLMVWMGRYVIYHTGSATKTDNGMRAVSLQQLMTWKDTRWIPNDSNPNFIGIYRLNFLAR

>gi|30063662|ref|NP_837833.1| IS911 orfA [Shigella flexneri 2a str. 2457T]

MKKRNFSAEFKRESAQLVVDQKYTVADAAKAMDVGLSTMTRWVKQLRDERQGKTPKASPITPEQIEIREL

RKKLQRIEMENEILKKATALLMSDSLNSSR

>gi|30063661|ref|NP_837832.1| IS911 orfB, partial [Shigella flexneri 2a str. 2457T]

MKELGLVSCQQPTHRYKRGGHEHVAIPNYLERQFAVTEPNQVWCGDVTYIWTGKRWAYLAVVLDLFARKP

VGWAMSFSPDSRLTMKALEMAWETRGKPGGVMFHSDQGSHYTSRQFRQLLWRYQIRQSMSRRGNCWDNSP

MERFFRSLKNEWMPVVGYVSFSEAAHAITDYIVGYYSALRPHEYNGGLPPNESENRYWKNSNSVASFC

>gi|30063660|ref|NP_837831.1| hypothetical protein S2436, partial [Shigella flexneri 2a str. 2457T]

MQVRWREPAKTAQQSNIPVTVERQLYRLIPGEEEMSFTLQPVTSNEIDSDALYLDEITLTSEQDAVLRYG

QVEVPLPPGADVERTTWGISVNKPNAAKQQGQLLEKARNEMGELAYMVPVKELTGTVTFRHLLRFSQKGQ

FVLPPARYVRSYAPAQQSVAAGSEWTGMQVK

>gi|30063659|ref|NP_837830.1| hypothetical protein S2435 [Shigella flexneri 2a str. 2457T]

MNWRRIVWLLALVTLPTLAEETPLQLVLRGAQHDQLYQLSSSGVTKVSALPDSLTTPLGSLWKLYVYAWL

EDTHQPEQPYQCRGNSPEEVYCCQAGESITRDTALVRSCGLYFAPQRLHIGADVWGQYWQQRQAPAWLAS

LTTLKPETSVTVKSLLDSLATLPAQNKAQEVLLDVVLDEAKIGVASMLGSRVRVKTWSWFADDKQEIRQG

GFAGWLTDGTPLWVTGSGTSKTVLTRYATVLNRVLPVPTQVASGQCVEVELFARYPLKKITAEKSTTAVK

PSVLNGRYRVTFTNGNHITFVSHGETTLLSEKGKLKLQSHLDREEYVARVLDREAKSTPPEAAKAMTVAI

RTFLQQNANREGDCLTIPDSSATQRVSASPATTGARTMTAWTQELIYAGDPVHYHGSRATEGTLSWRQAT

AQAGQGERYDQILAFAYPDNSLSRWGAPRSTCQLLPKAKAWLAKKMPQWRRILQGETGYNEPDVFAVCRL

VSGFPYTDRQQKRLFIRNFFTLQDRLDLTHEYLHLAFDGYPTGLDENYIETLTRQLLMD

>gi|30063658|ref|NP_837829.1| hypothetical protein S2434 [Shigella flexneri 2a str. 2457T]

MRKIFLPLLLVALSPVAHSEGVQEVEIDAPLSGWHPAEGEDASFSQSINYPASSVNMADDQNISAQIRGK

IKNYAAAGKVQQGRLVVNGASMPQRIESDGSFARPYIFTEGSNSVQVISPDGQSRQKMQFYSTPGTGTIR

ARLRLVLSWDTDNTDLDLHVVTPDGEHAWYGNTVLKNSGALDMDVTTGYGPEIFAMPAPVHGRYQVYINY

YGGRSETELTTAQLTLITDEGSVNEKQETFIVPMRNAGELTLVKSFDW

>gi|30063657|ref|NP_837828.1| hybrid sensory kinase in two-component regulatory system with RcsB and YojN [Shigella flexneri 2a str. 2457T]

MKYLASFRTTLKASRYMFRALALVLWLLIAFSSVFYIVNALHQRESEIRQEFNLSSDQAQRFIQRTSDVM

KELKYIAENRLSAENGVLSPRGRETQADVPAFEPLFADSDCSAMSNTWRGSLESLAWFMRYWRDNFSAAY

DLNRVFLIGSDNLCMANFGLRDMPVERDTALKALHERINKYRNAPQDDSGSNLYWISEGPRPCVGYFYAL

TPVYLANRLQALLGVEQTIRMENFFLPGTLPMGVTILDENGHTLISLTGPESKIKGDPRWMQERSWFGYT

EGFRELVLKKNLPPSSLSIVYSVPVDKVLERIRMLILNAILLNVLAGAALFTLARMYERRIFIPAESDAL

RLEEHEQFNRKIVASAPVGICILRTADGVNILSNELAHTYLNMLTHEDRQRLTQIICGQQVNFVDVLTSN

NTNLQISFVHSRYRNENVAICVLVDVSSRVKMEESLQEMAQAAEQASQSKSMFLATVSHELRTPLYGIIG

NLDLLQTKELPKGVDRLVTAMNNSSSLLLKIISDILDFSKIESEQLKIEPREFSPREVMNHITANYLPLV

VRKQLGLYCFIEPDVPVALNGDPMRLQQVISNLLSNAIKFTDTGCIVLHVRADGDYLSIRVRDTGVGIPA

KEVVRLFDPFFQVGTGVQRNFQGTGLGLAICEKLISMMDGDISVDSEPGMGSQFTVRIPLYGAQYPQKKG

VEGLSGKRCWLAVRNASLCQFLETSLQRSGIVVTTYEGQEPTPEDVLITDEVVSKKWQGRAVVTFCRRHI

GIPLEKAPGEWVHSVAAPHELPALLARIYLIEMESDDPANALPSTDKAVSDNDDMMILVVDDHPINRRLL

ADQLGSLGYQCKTANDGVDALNVLSKNHIDIVLSDVNMPNMDGYRLTQRIRQLGLTLPVIGVTANALAEE

KQRCLESGMDSCLSKPVTLDVIKQTLTVYAERVRKSRDS

>gi|30063656|ref|NP_837827.1| transcriptional regulator RcsB [Shigella flexneri 2a str. 2457T]

MNNMNVIIADDHPIVLFGIRKSLEQIEWVNVVGEFEDSTALINNLPKLDAHVLITDLSMPGDKYGDGITL

IKYIKRHFPSLSIIVLTMNNNPAILSAVLDLDIEGIVLKQGAPTDLPKALAALQKGKKFTPESVSRLLEK

ISAGGYGDKRLSPKESEVLRLFAEGFLVTEIAKKLNRSIKTISSQKKSAMMKLGVENDIALLNYLSSVTL

SPADKD

>gi|30063655|ref|NP_837826.1| phosphotransfer intermediate protein in two-component regulatory system with RcsBC [Shigella flexneri 2a str. 2457T]

MRQKETTATTRFSLLPGSITRFFLLLIIVLLVTMGVMVQSAVNAWLKDKSYQIVDITHAIQKRVDTWRYV

TRQIYDNIAATTSPSSGEGLQETRLKQDVYYLEKPRRKTEALIFGSHDNSTLEMTQRMSTYLDTLWGAEN

VPWSMYYLNGQDNSLVLISTLPLKDLTSGFKESTVSDIVDSRRAEMLQQANALDERESFSNMRRLAWQNG

HYFTLRTTFNQPGHLATVVAFDLPINDLIPPGMPLDSFRLEPDATATGNNDNEKEGTDSVSIHFNSTKIE

ISSALNSTDMRLVWQVPYGTLLLDTLQNILLPLLLNIGLLALALFGYTTFRHFSSRSTESVPSTAVNNEL

RILRAINEEIVSLLPLGLLVHDQESNRTVISNKIADHLLPHLNLQNITTMAEQHQGIIQATINNELYEIR

MFRSQVAPRTQIFIIRDQDREVLVNKKLKQAQRLYEKNQQGRMTFMKNIGDALKEPAQSLAESAAKLNAP

ESKQLANQADVLVRLVEEIQLANMLADDSWKSETVLFSVQDLIDEVVPSVLPAIKRKGLQLLINNHLKAH

DMRRGDRDTLRRILLLLMQYAVTSTQLGKITLEVDQDESSEDRLTFRILDTGEGVSIHEMDNLHFPFINQ

TQNDRYGKADPLAFWLSDQLARKLGGHLNIKTRDGLGTRYSVHIKMLAADPEVEEEEERLLDDVCVMVDV

TSAEIRNIVTRLLENWGATCITPDERLISQDYDIFLTDNPSNLTASGLLLSDDESGVREIGPGQLCVNFN

MSNAMQEAVLQLIEVQLAQEEVTESPLGGDENAQLHASGYYALFVDTVPDDVKRLYTEAATSDFAALAQT

AHRLKGVFAMLNLVPGKQLCETLEHLIREKDVPGIEKYISDIDSYVKSLL

>gi|30063654|ref|NP_837825.1| outer membrane porin protein C [Shigella flexneri 2a str. 2457T]

MKVKVLSLLVPALLVAGAANAAEVYNKDGNKLDLYGKVDGLHYFSDDKSVDGDQTYMRLGFKGETQVTDQ

LTGYGQWEYQIQGNSAENENNSWTRVAFAGLKFQDVGSFDYGRNYGVVYDVTSWTDVLPEFGGDTYGSDN

FMQQRGNGFATYRSTDFFGLVDGLNFAVQYQGKNGSPEGEGMTNNGREALRQNGDGVGGSITYDYEGFGI

GAAVSSSKRTDDQNFGLNRYDERYIGNGDRAETYTGGLKYDANNIYLAAQYTQTYNATRVGNLGWANKAQ

NFEAVAQYQFDFGLRPSLAYLQSKGKNLGVINGRNYDDEDILKYVDVGATYYFNKNMSTYVDYKINLLDD

NQFTRDAGINTDNIVALGLVYQF

>gi|30063653|ref|NP_837824.1| thiamine biosynthesis lipoprotein ApbE [Shigella flexneri 2a str. 2457T]

MEISFTRVALLVAALFFVGCDQKPQPAKTHATEVTVLEGKTMGTFWRASIPGIDAKRSAELKEKIQTQLD

ADDQLLSTYKKDSALMRFNDSQSLSPWPVSEAMADIVTTSLRIGARTDGAMDITVGPLVNLWGFGPEQQP

VQIPSQEQIDAMKAKTGLQHLTVINQSHQQYLQKDLPDLYVDLSTVGEGYAADHLARLMEQEGISRYLVS

VGGALNSRGMNGEGQPWRVAIQKPTDKENAVQAVVDINGHGISTSGSYRNYYELDGKRLSHVIDPQTGRP

IEHNLVSVTVIAPTALEADAWDTGLMVLGPEKAKEVVRREGLAVYMITKEGDSFKTWMSPQFKSFLVSEK

N

>gi|30063652|ref|NP_837823.1| O6-methylguanine-DNA methyltransferase; transcription activator/repressor [Shigella flexneri 2a str. 2457T]

MKNATCLTDDQRWQSVLARDPNADGEFVFAVRTTGIFCRPSCRARHALRENVSFYANASEALAAGFRPCK

RCQPDKANPRQHRLDKITHACRLLEQETPVTLEALADQVAMSPFHLHRLFKATTGMTPKAWQQAWRARRL

RESLAKGESVTTSILNAGFPDSSSYYRKADETLGMTAKQFRHGGENLAVRYALADCELGRCLVAESERGI

CAILLGDDDATLISELQQMFPAADNAPADLTFQQHVREVIASLNQRDTPLTLPLDIRGTAFQQQVWQALR

TIPCGETVSYQQLANAIGKPKAVRAVASACAANKLAIVIPCHRVVRGDGTLSGYRWGVSRKAQLLRREAE

NEER

>gi|30063651|ref|NP_837822.1| alkylated DNA repair protein [Shigella flexneri 2a str. 2457T]

MLDLFADAEPWQEPLAAGAVILRRFAFNAAEQLIRDINDVASQSPFRQMVAPGGYTMSVAMTNCGHLGWT

THRQGYLYSPIDPQTNKPWPAMPQSFHDLCQRAATAAGYPDFQPDACLINRYAPGAKLSLHQDKDEPDLR

APIVSVSLGLPAIFQFGGLKRNDPLKRLLLEHGDVVVWGGESRLFYHGIQPLKAGFHPLTTDCRYNLTFR

QAGKKE

>gi|30063650|ref|NP_837821.1| multidrug transporter membrane component/ATP-binding component [Shigella flexneri 2a str. 2457T]

MELLVLVWRQYRWPFISVMALSLASAALGIGLIAFINQRLIETADTSLLVLPEFLGLLLLLMAVTLGSQL

ALTTLGHHFVYRLRSEFIKRILDTHVERIEQLGSASLLAGLTSDVRNITIAFVRLPELGQGIILTIGSSA

YLWMLSGKMLLVTAIWMAITIWGGFVLVARVYKHMATLRETEDKLYTDFQTVLEGRKELTLNRERAEYVF

NNLYIPDAQEYRHHIIRADTFHLSAVNWSNIMMLGAIGLVFWMANSLGWADTNVAATYSLTLLFLRTPLL

SAVGALPTLLTAQVAFNKLNKFALAPFKAEFPRPQAFPNWQTLELRNVTFAYQDNAFSVGPINLTIKRGE

LLFLIGGNGSGKSTLAMLLTGLYQPQSGEILLDGKPVSGEQPEDYRKLFSAVFTDVWLFDQLLGPEGQPA

NPQLVEKWLAQLKMAHKLELSNGRIVNLKLSKGQKKRVALLLALAEERDIILLDEWAVDQDPHFRREFYQ

VLLPLMQEMGKTIFAISHDDHYFIHADRLLEMRNGQLSELTGEERDAASRDAVARTA

>gi|30063649|ref|NP_837820.1| ecotin [Shigella flexneri 2a str. 2457T]

MKTILPEVLFAAFATTSAWAAESVQPLEKIAPYPQAETGMKRQVIQLTPQEDESTLKVELLIGQTLEVDC

NLHRLGGKLESKTLEGWGYDYYVFDKVSSPVSTMMACPDGKKEKKFVTAYLGDAGMLRYNSKLPIVVYTP

DNVDVKYRVWKAEEKIDNAEVR

>gi|30063648|ref|NP_837819.1| ferredoxin-type protein [Shigella flexneri 2a str. 2457T]

MKIDASRRGILTGCWRKASNGIRPPWSGDESHFLTHCTRCDACINACENNILQRGTGGYPSVNFKNNECS

FCYACAQACPESLFSPRHTRAWDLQFTIGDACLAYQSVECRRCQDSCEPMAIIFRPTLSGIYQAQLNSQL

CNGCGACAASCPVSAITAEYLHAH

>gi|30063647|ref|NP_837818.1| assembly protein for periplasmic nitrate reductase [Shigella flexneri 2a str. 2457T]

MHTNWQVCSLVVQAKSERISDISTQLNAFPGCEVAVSDAPSGQLIVVVEAEDSETLIQTIESVRNVEGVL

AVSLVYHQQEEQGEETP

>gi|30063646|ref|NP_837817.1| nitrate reductase catalytic subunit [Shigella flexneri 2a str. 2457T]

MKLSRRSFMKANAVAAAAAAAGLSVPGVARAVVGQQEAIKWDKAPCRFCGTGCGVLVGTQQGRVVACQGD

PDAPVNRGLNCIKGYFLPKIMYGKDRLTQPLLRMKNGKYDKEGEFTPITWDQAFDVMEEKFKTALKEKGP

ESIGMFGSGQWTIWEGYAASKLFKAGFRSNNIDPNARHCMASAVVGFMRTFGMDEPMGCYDDIEQADAFV

LWGANMAEMHPILWSRITNSRLSNQNVTVAVLSTYQHRSFELADNGIIFTPQSDLVILNYIANYIIQNNA

INQDFFSKHVNLRKGATDIGYGLRPTHPLEKAAKNPGSDASEPMSFEDYKAFVAEYTLEKTAEMTGVPKD

QLEQLAQLYADPNKKVISYWTMGFNQHTRGVWANNLVYNLHLLTGKISQPGCGPFSLTGQPSACGTAREV

GTFAHRLPADMVVTNEKHRDICEKKWNIPSGTIPAKIGLHAVAQDRALKDGKLNVYWTMCTNNMQAGPNI

NEERMPGWRDPRNFIIVSDPYPTVSALAADLILPTAMWVEKEGAYGNAERRTQFWRQQVQAPGEAKSDLW

QLVQFSRRFKTEEVWPEELLAKKPELRGKTLYEVLYATPEVSKFPVSELAEDQLNDESRELGFYLQKGLF

EEYAWFGRGHGHDLAPFDDYHKARGLRWPVVNGKETQWRYSEGNDPYVKAGEGYKFYGKPDGKAVIFALP

FEPAAEAPDEEYDLWLSTGRVLEHWHTGSMTRRVPELHRAFPEAVLFIHPLDAKARDLRRGDKVKVVSRR

GEVISIVETRGRNRPPQGLVYMPFFDAAQLVNKLTLDATDPLSKETDFKKCAVKLEKV

>gi|30063645|ref|NP_837816.1| quinol dehydrogenase periplasmic component [Shigella flexneri 2a str. 2457T]

MSRSAKPQNGRRRFLRDVVRTAGGLAAVGVALGLQQQTARASGVRLRPPGAINENAFASACVRCGQCVQA

CPYDTLKLATLASGLSAGTPYFVARDIPCEMCEDIPCAKVCPSGALDREIESIDDARMGLAVLVDQENCL

NFQGLRCDVCYRECPKIDEAITLELERNTRTGKHARFLPTVHSDACTGCGKCEKVCVLEQPAIKVLPLSL

AKGELGHHYRFGWLEGNNGKS

>gi|30063644|ref|NP_837815.1| quinol dehydrogenase membrane component [Shigella flexneri 2a str. 2457T]

MANRKRDAGREALEKKGWWRSHRWLVLRRLCQFFVLGMFLSGPWFGVWILHGNYSSSLLFDTVPLTDPLM

TLQSLASGHLPATVALTGAVIITVLYALAGKRLFCSWVCPLNPITDLANWLRRRFDLNQSATIPRHIRYV

LLVVILVGSALTGTLIWEWINPVSLMGRSLVMGFGSGALLILALFLFDLLVVEHGWCGHICPVGALYGVL

GSKGVITVAASDRQKCNRCMDCFHVCPEPHVLRAPVLDEQSPVQVTSRDCMTCGRCVDVCSEDVFTITTR

WSSGAKS

>gi|30063643|ref|NP_837814.1| citrate reductase cytochrome c-type subunit [Shigella flexneri 2a str. 2457T]

MKSHDLKKALCQWTAMLALVVSGAVWAANGVDFSQSPEVSGTQEGAIRMPKEQDRMPLNYVNQPPMIPHS

VEGYQVTTNTNRCLQCHGVESYRTTGAPRISPTHFMDSDGKVGAEVAPRRYFCLQCHVPQADTAPIVGNT

FTPSKGYGK

>gi|30063642|ref|NP_837813.1| cytochrome c-type protein NapC [Shigella flexneri 2a str. 2457T]

MGNSDRKPGLIKRLWKWWRTPSHLALGTLLLIGFVGGIVFWGGFNTGMEKANTEEFCISCHEMRNTVYQE

YMDSVHYNNRSGVRATCPDCHVPHEFVPKMIRKLKASKELYGKIFGVIDTPQKFEAHRLTMAQNEWRRMK

DNNSQECRNCHNFEYMDTTAQKSVAAKMHDQAVKDGQTCIDCHKGIAHKLPDMREVEPGF

>gi|30063641|ref|NP_837812.1| cytochrome c biogenesis protein CcmA [Shigella flexneri 2a str. 2457T]

MGMLEARELLCERDERTLFSGLSFTLNAGEWVQITGSNGAGKTTLLRLLTGLSRPDAGEVLWQGQPLHQV

RDSYHQTLLWIGHQPGIKTRLTALENLHFYHRDGDAAQCLEALAQAGLAGFEDIPVNQLSAGQQRRVALA

RLWLTRATLWILDEPFTAIDVNGVDRLTQRMAQHTEQGGIVILTTHQPLNVAESKIRRISLTQTRAA

>gi|30063640|ref|NP_837811.1| heme exporter protein B, cytochrome c-type biogenesis protein [Shigella flexneri 2a str. 2457T]

MMFWRIFRLELRVAFRHSAEIANPLWFFLIVITLFPLSIGPEPQLLARIAPGIIWVAALLSSLLALERLF

RDDLQDGSLEQLMLLPLPLPAVVLAKVMAHWMVTGLPLLILSPLVAMLLGMDVYGWQVMALTLLLGTPTL

GFLGAPGVALTVGLKRGGVLLSILVLPLTIPLLIFATAAMDAASMHLPVDGYLAILGALLAGTATLSPFA

TAAALRISIQ

>gi|30063639|ref|NP_837810.1| heme exporter protein C [Shigella flexneri 2a str. 2457T]

MWKTLHQLAIPPRLYQICGWFIPWLAIASVVVLTVGWIWGFGFAPADYQQGNSYRIIYLHVPAAIWSMGI

YASMAVAAFIGLVWQMKMANLAVAAMAPIGAVFTFIALVTGSAWGKPMWGTWWVWDARLTSELVLLFLYV

GVIALWHAFDDRRLAGRAAGILVLIGVVNLPIIHYSVEWWNTLHQGSTRMQQSIDPAMRSPLRWSIFGFL

LLSATLTLMRMRNLILLMEKRRPWVSELILKRGRK

>gi|30063638|ref|NP_837809.1| heme exporter protein C [Shigella flexneri 2a str. 2457T]

MTPAFASWNEFFAMGGYAFFVWLAVVMTVIPLVVLVVHSVMQHRAILRGVAQQRAREARLRAAQQQEAA

>gi|30063637|ref|NP_837808.1| cytochrome c-type biogenesis protein CcmE [Shigella flexneri 2a str. 2457T]

MNIRRKNRLWIACAVLAGLALTIGLVLYALRSNIDLFYTPGEILYGKRETQQMPEVGQRLRVGGMVMPGS

VQRDPNSLKVTFTIYDAEGSVDVSYEGILPDLFREGQGVVVQGELEKGNHILAKEVLAKHDENYTPPEVE

KAMEANHRRPASVYKDPAS

>gi|30063636|ref|NP_837807.1| cytochrome c-type biogenesis protein [Shigella flexneri 2a str. 2457T]

MMPEIGNGLLCLALGIALLLSVYPLWGVARGDARMMASSRLFAWLLFMSVAGAFLVLVNAFVVNDFTVTY

VASNSNTQLPVWYRVAATWGAHEGSLLLWVLLMSGWTFAVAIFSQRIPLDIVARVLAIMGMVSVGFLLFI

LFTSNPFSRTLPNFPIEGRDLNPLLQDPGLIFHPPLLYMGYVGFSVAFAFAIASLLSGRLDSTYARFTRP

WTLAAWIFLTLGIVLGSAWAYYELGWGGWWFWDPVENASFMPWLVGTALMHSLAVTEQRASFKAWTLLLA

ISAFSLCLLGTFLVRSGVLVSVHAFASDPARGMFILAFMVLVIGGSLLLFAARGHKVRSRVNNALWSRES

LLLANNVLLVAAMLVVLLGTLLPLVHKQLGLGSISIGEPFFNTMFTWLMVPFALLLGVGPLVRWGRDRPR

KIRNLLIIAFISTLVLSLLLPWLFESKVVAMTVLGLAMACWIAVLATAEAALRISRGTKTTFSYWGMVAA

HLGLAVTIVGIAFSQNYSVERDVRMKSGDSVDIHEYRFTFRDVKEVTGPNWRGGVATIGVTREGKPETVL

YAEKRYYNTAGSMMTEAAIDGGITRDLYAALGEELENGAWAVRLYYKPFVRWIWAGGLMMALGGLLCLFD

PRYRKRVSPQKTAPEAV

>gi|30063635|ref|NP_837806.1| disulfide oxidoreductase [Shigella flexneri 2a str. 2457T]

MKRKVLLIPLIIFLAIAAALLWQLARNAEGDDPTNLESALIGKPVPKFRLESLDNPGQFYQADVLTQGKP

VLLNVWATWCPTCRAEHQYLNQLSAQGIRVVGMNYKDDRQKAISWLKELGNPYALSLFDGDGMLGLDLGV

YGAPETFLIDGNGIIRYRHAGDLNPRVWEEEIKPLWEKYSKEAAQ

>gi|30063634|ref|NP_837805.1| subunit of heme lyase [Shigella flexneri 2a str. 2457T]

MRFLLGVLMLMISGSALATIDVLQFKDEAQEQQFRQLTEELRCPKCQNNSIADSNSMIATDLRQKVYELM

QEGKSKKEIVDYMVARYGNFVTYDPPLTPLTVLLWVLPVVAIGIGGWVIYARSRRRVRVVPEAFPEQSVP

EGKRAGYVVYLPGIVVALIVAGVSYYQTGNYQQVKIWQQATAQAPALLDRALDPKADPLNEEEMSRLALG

MRTQLQKNPGDIEGWIMLGRVGMALGNASIATDAYATAYRLDPKNSDAALGYAEALTRSSDPNDNRLGGE

LLRQLVRTDHSNIRVLSMYAFNAFEQQRFGEAVAAWEMMLKLLPANDTRRAVIERSIAQAMQHLSPQESK

>gi|30063633|ref|NP_837804.1| transcriptional regulator NarP [Shigella flexneri 2a str. 2457T]

MPEATPFQVMIVDDHPLMRRGVRQLLELDPGFEVVAEAGEGASAIDLANRLDIDVILLDLNMKGMSGLDT

LNALRRDGVTAQIIILTVSDASSDVFALIDAGADGYLLKDSDPEVLLEAIRTGAKGSKVFSERVNQYLRE

REMFGAEEDPFSVLTERELDVLHELAQGLSNKQIASVLNISEQTVKVHIRNLLRKLNVRSRVAATILFLQ

QRGAQ

>gi|30063632|ref|NP_837803.1| ATP-binding component of a transport system [Shigella flexneri 2a str. 2457T]

MASTVEYGETVDGVILEKDIQLVYGTANNTKINPGGEQHIKEFGVSSNTEINGGYQYIEMNGTAEYSVLN

DGYQIVQMGGAANQTTLNNGVLQVYGPANDTTIKGGRLIVEKDGGAVFAAIEKGGLLEVKEGGFALAVDQ

KTGGAIKTTTRAMEVFGTNRLGQFDIKNGIANNMLLENGGSLRVEENDFAYNTTVDSGGLLEVMDGGTAT

GVDKKAGGKLIVSTNALEVSGTNSKGQFSIKDGVSKNYELDDGSGLIVMEDTQAIDTILDEHATMQSLGK

DTGTKVQANAVYDLGRSDQNGSITYSSKAISENMVINNGRANVWAGTMVNVSVRGNDGILEVMKPQINYA

PAMLVGKVVVSEGASFRTHGAVDTSKADVSLENSAWTIIADITTTNQNTRLNLANLAMSGANVIMMAEPV

TRSSVTASAENFITLTTNTLSGNGNFYMRTDMANHQSDQLNVTGQATGDFKIFVTDTGASPAAGDSLTLV

TTGGGDAAFTLGNAGGVVDIGTYEYTLLDNGNHSWSLAENRAQITPSTTDVLNMAAAQPLVFDAELDTVR

ERLGSVKGVSYDTVMWSSAINTRNNVTTDAGAGFEQTLTGLTLGIDSRFSREESSTIRGLFFGYSHSDIG

FDRGGKGNIDSYTLGAYAGWEHQNGAYVDGVVKVDRFANTIHGKMSNGATAFGDYNSNGAGAHVESGFRW

VDGLWSVRPYLAFTGFTTDGQDYTLSNGMRADVGNTRILRAEAGTAVSYHMDLQNGTTLEPWLKAAVRQE

YADSNQVKVNDDGKFNNDVAGTSGVYQAGIRSSFTPTLSGHLSVSYGNGAGVESPWNTQAGVVWTF

>gi|30063631|ref|NP_837802.1| sulfatase [Shigella flexneri 2a str. 2457T]

MVTHRQRYREKVSQMVSWGHWFALFNILLSLVIGSRYLFIADWPTTLAGRIYSYVSIIGHFSFLVFATYL

LILFPLTFIVGSQRLMRFLSVILATAGMTLLLIDSEVFTRFHLHLNPIVWQLVINPDENEMARDWQLMFI

SVPVILLLELVFATWSWQKLRSLTRRRRFARPLAAFLFIAFIASHVVYIWADANFYRPITMQRANLPLSY

PMTARRFLEKHGLLDAQEYQRRLIEQGNPDAVSVQYPLSELRYRDMGTGQNVLLITVDGLNYSRFEKQMP

ALAGFAEQNISFTRHMSSGNTTDNGIFGLFYGISPSYMDGILSTRTPAALITALNQQGYQLGLFSSDGFT

SPLYRQALLSDFSMPSVRTQSDDQTATQWINWLGRYAQEDNRWFSWVSFNGTNIDDSNQQAFARKYSRAA

GNVDDQIKRVLNALRDSGKLDNTVVIITAGRGIPLSEEEETFDWSHGHLQVPLVIHWPGTPAQRINALTD

HTDLMTTLMQRLLHVSTPASEYSQGQDLFNPQRRHYWVTAADNDTLAITTPKKTLVLNNNGKYRTYNLRG

ERVKDEKPQLSLLLQVLTDEKRFIAN

>gi|30063630|ref|NP_837801.1| hypothetical protein S2403 [Shigella flexneri 2a str. 2457T]

MPQISRYSNEQVEQLLAELLNVLEKHKAPTDLSLMVLGNMVTNLINTSIAPAQRQAIANSFASALQSSIN

EDKAH

>gi|30063629|ref|NP_837800.1| nucleoid-associated protein NdpA [Shigella flexneri 2a str. 2457T]

MSLDINQIALHQLIKRDEQNLELVLRDSLLEPTETVVEMVAELHRVYSAKNKAYGLFSEESELAQTLRLQ

RQGEEDFLAFSRAATGRLRDELAKYPFADGGFVLFCHYRYLAVEYLLVAVLSNLSSMRVNENLDINPTHY

LDINHADIVARIDLTEWETNPESTRYLTFLKGRVGRKVADFFMDFLGASEGLNAKAQNRGLLQAVDDFTA

EAQLDKAERQNVRQQVYSYCNEQLQAGEEIELESLSKELAGVSEVSFTEFAAEKGYELEESFPADRSTLR

QLTKFAGSGGGLTINFDAMLLGERIFWDPATDTLTIKGTPPNLRDQLQRRTSGGN

>gi|30063628|ref|NP_837799.1| 50S ribosomal protein L25 [Shigella flexneri 2a str. 2457T]

MFTINAEVRKEQGKGASRRLRAANKFPAIIYGGKEAPLAIELDHDKVMNMQAKAEFYSEVLTIVVDGKEI

KVKAQDVQRHPYKPKLQHIDFVRA

>gi|30063627|ref|NP_837798.1| ATP-dependent helicase [Shigella flexneri 2a str. 2457T]

MIFTLRPYQQEAVDATLNHFRRHKTPAVIVLPTGAGKSLVIAELARLARGRVLVLAHVKELVAQNHAKYQ

ALGLEADIFAAGLKRKESHGKVVFGSVQSVARNLDAFQGEFSLLIVDECHRIGDDEESQYQQILTHLTKV

NPHLRLLGLTATPFRLGKGWIYQFHYHGMVRGDEKALFRDCIYELPLRYMIKHGYLTPPERLDMPVVQYD

FSRLQAQSNGLFSEADLNRELKKQQRITPHIISQIMEFAEKRKGVMIFAATVEHAKEIVGLLPAEDAALI

TGDNPGAERDVLIEDFKAQRFRYLVNVAVLTTGFDAPHVDLIAILRPTESVSLYQQIVGRGLRLAPGKTD

CLILDYAGNPHDLYAPEVGTPKGKSDNVPVQVFCPACGFANTFWGKTTADGTLIEHFGRRCQGWFEDDDG

HREQCDFRFRFKNCPQCNAENDIAARRCRECDTVLVDPDDMLKAALRLKDALVLRCSGMSLQHGHDEKGE

WLKITYYDEDGADVSERFRLQTPAQRTAFEQLFIRPHTRTPGIPLRWITAADILAQQALLRHPDFVVARM

KGQYWQVREKVFDYEGRFRRAHELRG

>gi|30063626|ref|NP_837797.1| 16S rRNA pseudouridylate synthase A [Shigella flexneri 2a str. 2457T]

MRLDKFIAQQLGVSRAIAGREIRGNRVTVDGEIVRNAAFKLLPEHDVAYDGNPLAQQHGPRYFMLNKPQG

YVCSTDDPDHPTVLYFLDEPVAWKLHAAGRLDIDTTGLVLMTDDGQWSHRITSPRHHCEKTYLVTLESPV

ADDTAEQFAKGVQLHNEKDLTKPAVLEVITPTQVRLTISEGRYHQVKRMFAAVGNHVVELHRERIGGITL

DADLAPGEYRPLTEEEIASVV

>gi|30063625|ref|NP_837796.1| bicyclomycin/multidrug efflux system [Shigella flexneri 2a str. 2457T]

MTTRQHSSFAIVFILGLLAMLMPLSIDMYLPALPVISAQFGVSAGSTQMTLSTYILGFALGQLIYGPMAD

SFGRKPVVLGGTLVFAAAAVACALAQTIDQLIVMRFFHGLAAAAASVVINALMRDIYPKEEFSRMMSFVM

LVTTIALLMAPIVGGWVLVWLSWHYIFWILALAAILASAMIFFLIKETLPPERRQPFHIRTTIGNFAALF

RHKRVLSYMLASGFSFAGMFSFLSAGPFVVYIEINHVAPENFGYYFALNIVFLFVMTIFNSRFVRRIGALN

MFRSGLWIQFIMAAWMVISAPLGLGFWSLVVGVAAFVGCVSMVSSNAMAVILDEFPHMAGTASSLAGTFR

FGIGAIVGALLSLATFNSAWPMIWSIAFCATSSILFCLYASRPKKR

>gi|30063624|ref|NP_837795.1| hypothetical protein S2397 [Shigella flexneri 2a str. 2457T]

MTSLQLSIVHRLPQNYRWSAGFAGSKVEPIPQNGPCGDNSLVALKLLSPDGDNAWSVMYKLSQALSDIEV

PCSVLECEGEPCLFVNRQDEFAATCRLKNFGVAIAEPFSNYNPF

>gi|30063623|ref|NP_837794.1| ATP-binding component of a transport system [Shigella flexneri 2a str. 2457T]

MTQTLLAIENLSVGFRHQQTVRTVVNDVSLQIEAGETLALVGESGSGKSVTALSILRLLPSPPVEYLSGD

IRFHGESLLHASDQTLRGVRGNKIAMIFQEPMVSLNPLHTLEKQLYEVLSLHRGMRREAARGEILNCLDR

VGIRQAAKRLTDYPHQLSGGERQRVMIAMALLTRPELLIADEPTTALDVSVQAQILQLLRELQGELNMGM

LFITHNLSIVRKLAHRVAVMQNGRCVEQNNAATLFASPTHPYTQKLLNSEPSGDPVPLPEPASTLLDVEQ

LQVAFPIRKGILKRIVDHNVVVKNISFTLRAGETLGLVGESGSGKSTTGLALLRLITSQGSIVFDGQPLQ

DLNRRQLLPTRHRIQVVFQDPNSSLNPRLNVLQIIEEGLRVHQPTLSAAQREQQVIAVMHEVGLDPETRH

RYPAEFSGGQRQRIAIARALILKPSLIILDEPTSSLDKTVQAQILTLLKSLQQKHQLAYLFISHDLHVVR

ALCHQVIVLRQGEVVEQGPCARVFATPQQEYTRQLLALS

>gi|30063622|ref|NP_837793.1| transport system permease [Shigella flexneri 2a str. 2457T]

MSRLSPVNQARWARFRHNRRGYWSLWIFLVLFGLSLCSELIANDKPLLVRYDGSWYFPLLKNYSESDFGG

PLASQADYQDPWLKQRLENNGWVLWAPIRFGATSINFATDKPFPSPPSRQNWLGTDANGGDVLARILYGT

RISVLFGLMLTLCSSVMGVLAGALQGYYGGKVDLWGQRFIEVWSGMPTLFLIILLSSVVQPNFWWLLAIT

VLFGWMSLVGVVRAEFLRTRNFDYIRAAQALGVSDRSIILRHMLPNAMVATLTFLPFILCSSITTLTSLD

FLGFGLPLGSPSLGELLLQGKNNLQAPWLGITAFLSVAILLSLLIFIGEAVRDAFDPNKAV

>gi|30063621|ref|NP_837792.1| transport system permease [Shigella flexneri 2a str. 2457T]

MGAYLIRRLLLVIPTLWAIITINFFIVQIAPGGPVDQAIAAIEFGNAGVLPGAGGEGVRASHAQTGVGNI
SDSNYRGGRGLDPEVIAEITHRYGFDKPIHERYFKMLWDYIRFDFGDSLFRSASVLTLIKDSLPVSITLG
LWSTLIIYLVSIPLGIRKAVYNGSRFDVWSSAFIIIGYAIPAFLFAILLIVFFAGGSYFDLFPLRGLVSA
NFDSLPWYQKITDYLWHITLPVLATVIGGFAALTMLTKNSFLDEVRKQYVVTARAKGVSEKNILWKHMFR
NAMLLVIAGFPATFISMFFTGSLLIEVMFSLNGLGLLGYEATVSRDYPVMFGTLYIFTLIGLLLNIVSDI
SYTLVDPRIDFEGR

>gi|30063620|ref|NP_837791.1| hypothetical protein S2393 [Shigella flexneri 2a str. 2457T]

MIVRILLLFIALFTFGAQAQAIKESYTFAVLGEPRYAFNFNHFDYVNPAAPKGGQITLSALGTFDNFNRY
ALRGNPGARTEQLYDTLFTTSDDEPGSYYPLIAESARYADDYSWVEVAINPRARFHDGSPITARDVEFTF
QKFLTEGVPQFRLVYKGTTVKAIAPLTVRIELAKPGKEDMLSLFSLPVFPEKYWKDHKLSDPLATPPLAS
GPYHVTSWKMGQNIVYSRVKDYWAANLPVNRGRWNFDTIRYDYYLDDNVAFEAFKAGAFDLRMENDAKNW
ATRYTGKNFDKKYIIKDEQKNESAQDTRWLAFNIQRAVFSDRRVREAITLAFDFEWMNKALFYNAWSRTN
SYFQNTEYAARNYPDAAELVLLAPMKKDLPPEVFTQIYQPPVSKGDGYDRDNLLKADKLLNEAGWVLKGQ
QRVNATTGQPLSFELLLPASSNSQWVLPFQHSLQRLGINMDIRKVDNSQITNRMRNRDYDMMPRLWRAMP
WPSSDLQISWSSEYINSTYNAPGVQSPVIDSLINQIIAAQGNKEKLLPLGRALDRVLTWNYYMLPMWYMA
EDRLAWWDKFSQPAVRPVYSLGIDTWWYDVNKAAKLPSARQQGE

>gi|30063619|ref|NP_837790.1| hypothetical protein S2392 [Shigella flexneri 2a str. 2457T]

MFIRAPNSGRKLLLTCIVAGVMIAILVSCLQFLVAWHKHEVKYDTLIIDVQKYLDTYFADLKSTTDRLQP
LTLDTCQQANPELTARAAFSMNVRTFVLVKDKKTFCSSATGEMDIPLNELIPALDINKNVDMAILPGTPM
VPNKPAIVIWYRNPLLKNSGVFAALNLNLTPSLFYSSRQEDYDGVALIIGNTALSTFSSRLMNVNELTDM
PVRETKIAGIPLTVRLYADDWTWNDVWYAFLLGGMSGTVVGLLCYYLMSVRMRPDREIMTAIKREQFYVA
YQPVVDTQALRVTGLEVLLRWRHPVAGEIPPDAFINFAESQKMIVPLTQHLFELIARDAAELEKVLPVGV

KFGINIAPDHLHSESFKADIQKLLTSLPAHHFQIVLEITERDMLKEQEATQLFAWLHSVGVEIAIDDFGT
GHSALIYLERFTLDYLKIDRGFINAIGTETITSPVLDAVLTLAKRLNMLTVTEGVETPEQARWLSERGVN
FMQGYWISHPLPLDDFVRWLKKPYTPQW

>gi|30063618|ref|NP_837789.1| outer membrane lipoprotein [Shigella flexneri 2a str. 2457T]
MVKSQPILRYILRGIPAIAVAVLLSACSANNTAKNMHPETRAVGSETSSLQASQDEFENLVRNVDVKSRI
MDQYADWKGVRYRLGGSTKKGIDCSGFVQRTFREQFGLELPRSTYEQQEMGKSVSRSNLRTGDLVLFRAG
STGRHVGIYIGNNQFVHASTSSGVIISSMNEPYWKKRYNEARRVLSRS

>gi|30063617|ref|NP_837788.1| hypothetical protein S2390 [Shigella flexneri 2a str. 2457T]
MIKNLPQIVLLNIVGPALFLSWYIPVNHGFWLPIDADIFYFFNQKLVESKAFLWLVALTNNRAFDGCSLL
AMGMLMLSFWLKENAPGRRRIVIMGLVMLLTAVVLNQLGQALIPVKRASPTLTFTDINRVSELLSVPTKD
ASRDSFPGDHGMMLLIFSAFMWRYFGKVAGLIALIIFMVFAFPRVMIGAHWFTDIIVGSMTVILIGLPWV
LLTPLSDRLITFFDKSLPGKNKHFQNK

>gi|30063616|ref|NP_837787.1| hypothetical protein S2389 [Shigella flexneri 2a str. 2457T]
MTRTNLITGFLGSGKTTSILHLLAHKDPNEKWAVLVNEFGEVGIDGALLADSGALLKEIPGGCMCCVNGL
PMQVGLNTLLRQGKPDRLLIEPTGLGHPKQILDLLTAPVYEPWIDLRATLCILDPRLLLDEKSASNENFR
DQLAAADIIVANKSDRATPESEQALQRWWQQNGGDRQLIHSEHGKVDGHLLDLPRRNLAELPASAAHSHQ
HVVKKGLAALSLPEHQRWRRSLNSGQGYQACGWIFDADTVFDTIGILEWARLAPVERVKGVLRIPEGLVR
INRQGDDLHIETQNVAPPDSRIELISSSEADWNALQSALLKLRLATTA

>gi|30063615|ref|NP_837786.1| oxidoreductase [Shigella flexneri 2a str. 2457T]
MKTIASVTLPHHVHAPRYDRQQLQSRIVHFGFGAFHRAHQALLTDRVLNAQGGDWGICEISLFSGDQLMS
QLRAQNHLYTVLEKGADGNQVIIVGAVHECLNAKLDSLAAIIEKFCEPQVAIVSLTITEKGYCIDPATGA

LDTSNPRIIHDLQTPEEPHSAPGILVEALKRRRERSLTPFTVLSCDNIPDNGHVVKNAVLRMAEKRSPEL

AGWIKEHVSFPGTMVDRIVPAATDESLAEISQHLGVNDPCAISCEPFIQWVVEDNFIAGRPAWEVAGVQM

VNDVLPWEEMKLRMLNGSHSFLAYLGYLSGFAYISDCMQDRVFRYAARTLMLDEQAPTLQIKDVDLTQYA

DKLIARFANPSLKHKTWQIAMDGSQKLPQRMLAGIRIHLGRETDWSLLALGVAGWMRYVSGVDDAGNAID

VRDPLSDKIRELVAGSSSEQRVTALLSLREVFGDDLPDNPHFVQAIEQAWQQIVQFGAHQALLNTLKI

>gi|30063613|ref|NP_837784.1| IS1 orfA [Shigella flexneri 2a str. 2457T]

MASISIRCPSCSATEGVVRNGKSTAGHQRYLCSHCRKTWQLQFTYTASQPGTHQKIIDMVMNGVGCRASA

RIMGVGLNTVLRHLKNSGRSR

>gi|30063612|ref|NP_837783.1| IS1 orfB [Shigella flexneri 2a str. 2457T]

MSRQCTHYGRWPQHGFTSLKKLRPQSVTSRIQPGSDVIVCAEMDEHWGYVGAKSRQRWLFYAYDRIRRTV

VAHVFGERTLATLERLLSLLSAFEVVVWMTDGCPLYESRLKGKLHVISKRYTQRIERHNLNLRQHLARLG

RKSLSFSKSVELHDKVIGHYLNIKHYQ

>gi|30063611|ref|NP_837782.1| transport [Shigella flexneri 2a str. 2457T]

MHNSPAVSSAKSFDLTSTAFLIVAFLTGIAGALQTPTLSIFLTDEVHARPAMVGFFFTGSAVIGILVSQF

LAGRSDKRGDRKSLIVFCCLLGVLACTLFAWNRNYFVLLFVGVFLSSFGSTANPQMFALAREHADKTGRE

AVMFSSFLRAQVSLAWVIGPPLAYALAMGFSFTVMYLSAAVAFIVCGVMVWLFLPSMQKELPLATGTVEA

PRRNRRDTLLLFVICTLMWGSNSLYIINMPLFIINELHLPEKLAGVMMGTAAGLEIPTMLIAGYFAKRLG

KRFLMRVAAVGGVCFYAGMLMAHSPAILLGLQLLNAIFIGILGGIGMLYFQDLMPGQAGSATTLYTNTSR

VGWIIAGSVAGIVAEIWNYHAVFWFAMVMIIATLFWVMTPTY

>gi|30063610|ref|NP_837781.1| bifunctional fructose-specific PTS IIA/HPr protein [Shigella flexneri 2a str. 2457T]

MFQLSVQDIHPGEKAGDKEEAIRQVAAALVQAGNVAEGYVNGMLAREQQTSTFLGNGIAIPHGTTDTRDQ

VLKTGVQVFQFPEGVTWGDGQVAYVAIGIAASSDKHLGLLRQLTHVLSDDSVAEQLKSATTAEELRALLM

GEKQSEQLKLDNEMLTLDIVASDLLTLQALNAARLKEARAVDATFVTKAINEQPLNLGQGIWLSDSAEGN

LRSAIAVSRAANAFDVDGETAAMLVSVAMNDDQPIAVLKRLADLLLDNKADRLLKADAATLLALLTSDDA

PTDDVLSAEFVVRNEHGLHARPGTMLVNTIKQFNSDITVTNLDGTGKPANGRSLMKVVALGVKKGHRLRF

TAQGADAEQALKAIGDAIAAGLGEGA

>gi|30063609|ref|NP_837780.1| 1-phosphofructokinase [Shigella flexneri 2a str. 2457T]

MSRRVATITLNPAYDLVGFCPEIERGEVNLVKTTGLHAAGKGINVAKVLKDLGIDVTVGGFLGKDNQDGF

QQLFSELGIANRFQVVQGRTRINVKLTEKDGEVTDFNFSGFEVTPADWERFVTDSLSWLGQFDMVCVSGS

LPSGVSPEAFTDWMTRLRSQCPCIIFDSSREALVAGLKAAPWLVKPNRRELEIWAGRKLPEMKDVIEAAH

ALREQGIAHVVISLGAEGALWVNASGEWIAKPPSVDVVSTVGAGDSMVGGLIYGLLMRESSEHTLRLATA

VAALAVSQSNVGITDRPQLAAMMARVDLQPFN

>gi|30063608|ref|NP_837779.1| fructose-specific PTS system IIBC component [Shigella flexneri 2a str. 2457T]

MKTLLIIDANLGQARAYMAKTLLGAAARKAKLEIIDNPNDAEMAIVLGDSIPNDSALNGKNVWLGDISRA

VAHPELFLSEAKGHAKPYTAPVTATAPVAASGPKRVVAVTACPTGVAHTFMAAEAIETEAKKRGWWVKVE

TRGSVGAGNAITPEEVAAADLVIVAADIEVDLAKFAGKPMYRTSTGLALKKTSQELDKAVAEATPYEPAG

KAQTATTEGKKESAGAYRHLLTGVSYMLPMVVAGGLCIALSFAFGIEAFKEPGTLAAALMQIGGGSAFAL

MVPVLAGYIAFSIADRPGLTPGLIGGMLAVSTGSGFIGGIIAGFLAGYIAKLISTQLKLPQSMEALKPIL

IIPLISSLVVGLAMIYLIGKPVAGILEGLTHWLQTMGTANAVLLGAILGGMMCTDMGGPVNKAAYAFGVG

LLSTQTYGPMAAIMAAGMVPPLAMGLATMVARRKFDKAQQEGGKAALVLGLCFISEGAIPFAARDPMRVL

PCCIVGGALTGAISMAIGAKLMAPHGGLFVLLIPGAITPVLGYLVAIIAGTLVAGLAYAFLKRPEVDAVA

KAA

>gi|30063607|ref|NP_837778.1| hypothetical protein S2380 [Shigella flexneri 2a str. 2457T]

MREKDYVVIIGSANIDVAGYSHESLNYADSNPGKIKFTPGGVGRNIAQNLALLGNKAWLLSAVGSDFYGQ

SLLTQTNQSGVYVDKCLIVPGENTSSYLSLLDNTGEMLVAINDMNISNAITAEYLAQHREFIQRAKVIVA

DCNISEEALAWILDNAANVPVFVDPVSAWKCVKVRDRLNQNHTLKPNRLEAETLSGIALSGRDDVAKVAA

WFHQHGLNRLVLSMGGDGVYYSDISGENGWSAPIKTNVINVTGAGDAMMAGLASCWVDGMPFAESVRFAQ

GCSSMALSCEYTNNPDLSIANVISLVENAECLN

>gi|30063606|ref|NP_837777.1| hypothetical protein S2379 [Shigella flexneri 2a str. 2457T]

MSELKISPELLQISPEVQDALKNKKPVVALESTIISHGMPFPQNAQTAIEVEETIRKQGAVPATIAIIGG

VMKVGLSKEKIELLGREGHNVTKVSRRDLPFVVAAGKNGATTVASTMIIAALAGIKVFATGGIGGVHRGA

EHTFDISADLQELANTNVTVVCAGAKSILDLGLTTEYLETFGVPLIGYQTKALPAFFCRTSSFDVSIRLD

SASEIARAMAVKWQSGLNGGLVVANPIPEQFAMPEESINAAIDQAVAEAEEQGVIGKESTPFLLARVAEL

TSGDSLKSNIQLVFNNAILASEIAKEYQRLAG

>gi|30063605|ref|NP_837776.1| transport system permease [Shigella flexneri 2a str. 2457T]

MDIMRSVVGMVVLLAIAFLLSVNKKSISLRTVGAALLLQIAIGGIMLYFPPGKWAVEQAALGVHKVMSYS

DAGSAFIFGSLVGPKMDVLFDGAGFIFAFRVLPAIIFVTALISLLYYIGVMGLLIRILGSIFQKALNISK

IESFVAVTTIFLGQNEIPAIVKPFIDRVNRNELFTAICSGMASIAGSVMIGYAGMGVPIDYLLAASLMAI

PGGILFARILSPATEPSQVTFENLSFSETPPKSFIEAAASGAMTGLKIAAGVATVVMAFVAIIALINGII

GGIGGWFGFANASLESIFGYVLAPLAWIMGVDWSDANLAGSLIGQKLAINEFVAYLSFSPYLQTGGTLEV

KTVAIISFALCGFANFGSIGVVVGAFSAISPKRAPEIAQLGLRALAAATLSNLMSATIAGFFIGLA

>gi|30063604|ref|NP_837775.1| DNA-binding transcriptional activator YeiL [Shigella flexneri 2a str. 2457T]

MSESAFKDCFSTDVSADTRLFHFLARDYIVQEGQQPSWLFYLTRGRARLYATLANGRVSLIDFFAAPCFI

GEIELIDKDHEPRAVQAIEECWCLALPMKHYRPLLLNDTLFLRKLCVTLSHKNYRNIVSLTQNQSFPLVN

RLAAFILLSQEGDLYHEKHTQAAEYLGVSYRHLLYVLAQFIHDGLLIKRKDI

>gi|30063603|ref|NP_837774.1| ribonucleoside hydrolase 2 [Shigella flexneri 2a str. 2457T]

MEKRKIILDCEPGHDDAIAMMMAAKHPAIDLLGITIVAGNQTLDKTLINGLNVCQKLEINVPVYAGMPQP

IMRKQIVADNIHGETGLDGPVFEPLTRQAESTHAVKYIIDTLMASDGDITLVPVGPLSNIAVAMRMQPAI

LPKIREIVLMGGAYGTGNFTPSAEFNIFADPEAARVVFTSGVPLVMMGLDLTNQTVCTPDVIARMERVGG

PAGELFSDIMNFTLKTQFEYYGLAGGPVHDATCIGYLINPDGIKTQDMYVEVDVNSGPCYGRTVCDELGV

LGKPANTKVGITIDTDWFWGLVEECVRGYIKTH

>gi|30063602|ref|NP_837773.1| transport system permease [Shigella flexneri 2a str. 2457T]

MDVMRSVLGMVVLLAIAFLLSVNKKKISLRTVGAALVLQVVIGGIMLWLPPGRWVAEKVAFGVHKVMAYS

DAGSAFIFGSLVGPKMDTLFDGAGFIFGFRVLPAIIFVTALVSILYYIGVMGILIRILGGIFQKALNISK

IESFVAVTTIFLGQNEIPAIVKPFIDRLNRNELFTAICSGMASIAGSTMIGYAALGVPVEYLLAASLMAI

PGGILFARLLSPATESSQVSFNNLSFTETPPKSIIEADATGAMTGLKIAAGVATVVMAFVAIIALINGII

GGVGGWFGFEHASLESILGYLLAPLAWVMGVDWSDANLAGSLIGQKLAINEFVAYLNFSPYLQTAGTLDA

KTVAIISFALCGFANFGSIGVVVGAFSAVAPHRAPEIAQLGLRALAAATLSNLMSATIAGFFIGLA

>gi|30063601|ref|NP_837772.1| hypothetical protein S2374 [Shigella flexneri 2a str. 2457T]

MNNREKEILAILRRNPLIQQNEIADMLQISRSRVAAHIMDLMRKGRIKDKGYILTEQEYCVVVGTINMDI

RGMADIHYPQAASHPGTIHCSAGGVGRNIAHNLALLGRDVHLLSVIGDDFYGEMLLEETRRAGVNVSGCV

RLHGQSTSTYLAIANRDDQTVLAINDTHLLEQLTPQLLNGSRDLLRHAGVVLADCNLTAEALEWVFTLAD

EIPVFVDTVSEFKAGKIKHWLAHIHTLKPTLPELEILWGQAITSDADRNAAVNALHQQGVQQLFVYLPDE

SVYCSEKDGEQFLLTVPAHTTVDSFGADDGFMAGLVYSFLEGNNFRDSARFAMACAAISRASGSLNNPTL

SADNALSLVPMV

>gi|30063600|ref|NP_837771.1| endonuclease IV [Shigella flexneri 2a str. 2457T]

MKYIGAHVSAAGGLANAAIRAAEIDATAFALFTKNQRQWRAAPLTTQTIDEFKAACEKYHYTSAQILPHD

SYLINLGHPVTEALEKSRDAFIDEMQRCEQLGLSLLNFHPGSHLMQISEEDCLARIAESINIALDKTQGV

TAVIENTAGQGSNLGFKFEHLAAIIDGVEDKSRVGVCIDTCHAFAAGYDLRTPAECEKTFADFARIVGFK

YLRGMHLNDAKSTFGSRVDRHHSLGEGNIGHDAFRWIMQNDRFDGIPLILETINPDIWAEEIAWLKAQQT

EKAVA

>gi|30063599|ref|NP_837770.1| hypothetical protein S2372 [Shigella flexneri 2a str. 2457T]

MTNITLQKQHRTLWHFIPGLALSAVITGVALWGGSIPAVAGAGFSALTLAILLGMVLGNTIYPHIWKSCD

GGVLFAKQYLLRLGIILYGFRLTFSQIADVGISGIIIDVLTLSSTFLLACFLGQKVFGLDKHTSWLIGAG

SSICGAAAVLATEPVVKAEASKVTVAVATVVIFGTVAIFLYPAIYPLMSQWFSPETFGIYIGSTVHEVAQ

VVAAGHAISPDAENAAVISKMLRVMMLAPFLILLAARVKQLSGANSGEKSKITIPWFAILFIVVAIFNSF

HLLPQSVVNMLVTLDTFLLAMAMAALGLTTHVSALKKAGAKPLLMALVLFAWLIVGGGAINYVIQSVIA

>gi|30063598|ref|NP_837769.1| DNA-binding transcriptional regulator [Shigella flexneri 2a str. 2457T]

MHITLRQLEVFAEVLKSGSTTQASVMLALSQSAVSAALTDLEGQLGVQLFDRVGKRLVVNEHGRLLYPRA

LALLEQAVEIEQLFREDNGAIRIYASSTIGNYILPAVIARYRHDYPQLPIELSVGNSQDVMQAVLDFRVD

IGFIEGPCHSTEIISEPWLEDELVVFAAPTSPLARGPVTLEQLAAAPWILRERGSGTRDIVDYLLLSHLP

KFEMAMELGNSEAIKHAVRHGLGISCLSRRVIEDQLQAGTLSEVAVPLPRLMRTLWRIHHRQKHLSNALR

RFLDYCDPANVPR

>gi|30063597|ref|NP_837768.1| lysine transporter [Shigella flexneri 2a str. 2457T]

MGSETKTTEAPGLRRELKARHLTMIAIGGSIGTGLFVASGATISQAGPGGALLSYMLIGLMVYFLMTSLG

ELAAYMPVSGSFATYGQNYVEEGFGFALGWNYWYNWAVTIAVDLVAAQLVMSWWFPDTPGWIWSALFLGV

IFLLNYISVRGFGEAEYWFSLIKVTTVIVFIIVGVLMIIGIFKGAQPAGWSNWTIGEAPFAGGFAAMIGV

AMIVGFSFQGTELIGIAAGESEDPAKNIPRAVRQVFWRILLFYVFAILIISLIIPYTDPSLLRNDVKDIS

VSPFTLVFQHAGLLSAAAVMNAVILTAVLSAGNSGMYASTRMLYTLACDGKAPRIFAKLSRGGVPRNALY

ATTVIAGLCFLTSMFGNQTVYLWLLNTSGMTGFIAWLGIAISHYRFRRGYVLQGHDINDLPYRSGFFPLG

PIFAFILCLIITLGQNYEAFLKDTIDWGGVAATYIGIPLFLIIWFGYKLIKGTHFVRYSEMKFPQNDKK

>gi|30063596|ref|NP_837767.1| GTP cyclohydrolase I [Shigella flexneri 2a str. 2457T]

MPSLSKEAALVHEALVARGLETPLRPPVHEMDNETRKSLIAGHMTEIMQLLNLDLADDSLMETPHRIAKM

YVDEIFSGLDYANFPKITLIENKMKVDEMVTVRDITLTSTCEHHFVTIDGKATVAYIPKDSVIGLSKINR

IVQFFAQRPQVQERLTQQILIALQTLLGTNNVAVSIDAVHYCVKARGIRDATSATTTTSLGGLFKSSQNT

RHEFLRAVRHHN

>gi|30063595|ref|NP_837766.1| hypothetical protein S2366 [Shigella flexneri 2a str. 2457T]

MERNVTLDFVRGVAILGILLLNISAFGLPKAAYLNPAWYDAITPQDAWTWAFLDLIGQVKFLTLFALLFG

AGLQMLLPRGRRWIQSRLTLLVLLGFIHGLLFWDGDILLAYGLVGLGVLLLLGLISDSQTSRAWTPDASA

ILYEKYWKLHGGVEAISNRADGVGNSLLALGAQYGWQLAGMMLIGAALMRSGWLKGQFSLRHYRRTGFVL

VAIGVTINLPAIALQWQLDWAYRWCAFLLQMPRELSAPFQAIGYASLFYGFWPQLSRFKLVLAIACVGRM

ALTNYLLQTLICTTLFYHLGLFMHFDRLELLAFVIPVWLANILFSVIWLRYFRQGPVEWLWRQLTLRAAG

PAISKTSR

>gi|30063594|ref|NP_837765.1| DNA-binding transcriptional regulator GalS [Shigella flexneri 2a str. 2457T]

MITIRDVARQAGVSVATVSRVLNNSTLVSADTREAVMKAVSELDYRPNANAQALATQVSDTIGVVVMDVS

DAFFGALVKAVDLVAQQHQKYVLIGNSYHEAEKERHAIEVLIRQRCNALIVHSKALSDDELAQFMDNIPG

MVLINRVVPGYAHRCVCLDNLSGARMATRMLLNNGHQRIGYLSSSHGIEDDAMRKAGWMSALKEQDIIPP

ESWIGTGTPDMPGGEAAMVELLGRNLQLTAVFAYNDNMAAGALTALKDNGIAIPLHLSIIGFDDIPIARY

TDPQLTTVRYPIASMAKLATELALQGAAGNIDPRASHCFMPTLVRRHSVATRQNAAAITNSTNQAM

>gi|30063593|ref|NP_837764.1| galactose-binding transport protein; receptor for galactose taxis [Shigella flexneri 2a str. 2457T]

MNKKVLTLSAVMASMLFGAAAHAADTRIGVTIYKYDDNFMSVVRKAIEQDAKAAPDVQLLMNDSQNDQSK

QNDQIDVLLAKGVKALAINLVDPAAAGTVIEKARGQNVPVVFFNKEPSRKALDSYDKAYYVGTDSKESGI

IQGDLIAKHWAANQGWDLNKDGQIQFVLLKGEPGHPDAEARTTYVIKELNDKGIKTEQLQLDTAMWDTAQ

AKDKMDAWLSGPNANKIEVVIANNDAMAMGAVEALKAHNKSSIPVFGVDALPEALALVKSGALAGTVLND

ANNQAKATFDLAKNLADGKGAADGTNWKIDNKVVRVPYVGVDKDNLAEFSKK

>gi|30063592|ref|NP_837763.1| galactose/methyl galaxtoside transporter ATP-binding protein [Shigella flexneri 2a str. 2457T]

MVSSTTPSSGEYLLEMSGINKSFPGVKALDNVNLKVRPHSIHALMGENGAGKSTLLKCLFGIYQKDSGTI

LFQGKEIDFHSAKEALENGISMVHQELNLVLQRSVMDNMWLGRYPTKGMFVDQDKMYRETKAIFDELDID

IDPRARVGTLSVSQMQMIEIAKAFSYNAKIVIMDEPTSSLTEKEVNHLFTIIRKLKERGCGIVYISHKME

EIFQLCDEVTVLRDGQWIATEPLAGLTMDKIIAMMVGRSLNQRFPDKENKPGEVILEVRNLTSLRQPSIR

DVSFDLHKGEILGIAGLVGAKRTDIVETLFGIREKSAGTITLHGKQINNHNANEAINHGFALVTEERRST

GIYAYLDIGFNSLISNIRNYKNKVGLLDNSRMKSDTQWVIDSMRVKTPGHRTQIGSLSGGNQQKVIIGRW

LLTQPEILMLDEPTRGIDVGAKFEIYQLIAELAKKGKGIIIISSEMPELLGITDRILVMSNGLVSGIVDT

KTTTQNEILRLASLHL

>gi|30063591|ref|NP_837762.1| beta-methylgalactoside transporter inner membrane component [Shigella flexneri 2a str. 2457T]

MSALNKKSFLTYLKEGGIYVVLLVLLAIIIFQDPTFLSLLNLSNILTQSSVRIIIALGVAGLIVTQGTDL

SAGRQVGLAAVVAATLLQSMDNANKVFPEMATMPIALVILIVCAIGAVIGLINGLIIAYLNVTPFITTLG

TMIIVYGINSLYYDFVGASPISGFDSGFSTFAQGFLALGSFRLSYITFYALIAVAFVWVLWNKTRFGKNI

FAIGGNPEAAKISGVNVGLNLLMIYALSGVFYAFGGMLEAGRIGSATNNLGFMYELDAIAACVVGGVSFS

GGVGTVIGVVTGVIIFTVINYGLTYIGVNPYWQYIIKGAIIIFAVALDSLKYARKK

>gi|30063590|ref|NP_837761.1| oxidoreductase [Shigella flexneri 2a str. 2457T]

MPQQNYLDELTPAFTPLLAIKEASRCLLCHDAPCSQACPAQTDPGKFIRSIYFRNFKGAAETIRENNALG

AVCARVCPTEKLCQSGCTRAGVDAPIDIGRLQRFVTDFEQQTGMEIYQPGTKTLGKVAIIGAGPAGLQAS

VTLTNQGYDVTIYEKEAHPGGWLRNGIPQFRLPQSVLDAEIARIEKMGVTIKCNNEIGKTLTLEQLKAEN

RAVLVTVGLSSGSGLPLFEHSDVEIAVDFLQRVRQAQGDISIPQSALIIGGGDVAMDVASTLKVLGCQAV

TCVAREELDEFPASEKEFASARELGVSIIDGFTPVAVEGNKVTFKHVRLSGELTMAADKIILAVGQHARL

DAFAELEQQRNTIKTQNYQIRDPQVFAAGDIVEGDKTVVYAVKTGKEAAEAIHHYLEGACSC

>gi|30063589|ref|NP_837760.1| hypothetical protein S2359 [Shigella flexneri 2a str. 2457T]

MDVQQFFVVAVFFLIPIFCFREAWKGWRAGAIDKRVKNAPEPVYVWRAKNPGLFFAYMVAYIGFGILSIG

MIVYLIFYR

>gi|30063588|ref|NP_837759.1| hypothetical protein S2358 [Shigella flexneri 2a str. 2457T]

MLKRVFLSLLVLIGLLLLTVLGLDRWMSWKTAPYIYDELQDLPYRQVGVVLGTAKYYRTGVINQYYRYRI

QGAINAYNSGKVNYLLLSGDNALQSYNEPMTMRKDLIAAGVDPSDIVLDYAGFRTLDSIVRTRKVFDTND

FIIITQRFHCERALFIALHMGIQAQCYAVPSPKDMLSVRIREFAARFGTLADLYIFKREPRFLGPLVPIP

AMHQVPEDAQGYPAVTPEQLLELQKKQGK

>gi|30063587|ref|NP_837758.1| cytidine deaminase [Shigella flexneri 2a str. 2457T]

MHPRFQTAFAQLADNLQSALEPILADKYFPALLTGEQVSSLKSATGLDEDALAFALLPLAAACARTPLSN

FNVGAIARGVSGTWYFGANMEFIGATMQQTVHAEQSAISHAWLSGEKALAAITVNYTPCGHCRQFMNELN

SGLDLRIHLPGREAHALRDYLPDAFGPKDLEIKTLLMDEQDHGYALTGDALSQAAIAAANRSHMPYSKSP

SGVALECKDGRIFSGSYAENAAFNPTLPPLQGALILLNLKGYDYPDIQRAVLAEKADAPLIQWDATSATL

KALGCHSIDRVLLA

>gi|30063586|ref|NP_837757.1| hypothetical protein S2356 [Shigella flexneri 2a str. 2457T]

MMANIWWSLPLTLIVFFAARKLAARYKFPLLNPLLVAMVVIIPFLMLTGISYDSYFKGSEVLNDLLQPAV

VALAYPLYEQLHQIRARWKSIITICFIGSVVAMVTGTSVALLMGASPEIAASILPKSVTTPIAMAVGGSI

GGIPAISAVCVIFVGILGAVFGHTLLNAMRIRTKAARGLAMGTASHALGTARCAELDYQEGAFSSLALVL

CGIITSLIAPFLFPIILAVMG

>gi|30063585|ref|NP_837756.1| hypothetical protein S2355 [Shigella flexneri 2a str. 2457T]

MSKTLNIIWQYLRAFVLIYACLYAGIFIASLLPVTIPGSIIGMLILFVLLALQILPAKWVNPGCYVLIRY

MALLFVPIGVGVMQYFDLLRAQFGPVVVSCAVSTLVVFLVVSWSSQLVHGERKVVGQKGSEE

>gi|30063584|ref|NP_837755.1| tRNA-dihydrouridine synthase C [Shigella flexneri 2a str. 2457T]

MRVLLAPMEGVLDSLVRELLTEVNDYDLCITEFVRVVDQLLPVKVFHRICPELQNASRTPSGTLVRVQLL

GQFPQWLAENAARAVELGFWGVDLNCGCPSKTVNGSGGGATLLKDPELIYQGAKAMREAVPAHLPVSVKV

RLGWDSGEKKFEIADAVQQAGATELVVHGRTKEQGYRAEHIDWQAIGEIRQRLNIPVIANGEIWDWQSAQ

QCMAISGCDAVMIGRGALNIPNLSRVVKYNEPRMPWPEVVALLQKYTRLEKQGDTGLYHVARIKQWLSYL

RKEYDEATELFQHVRVLNNSPDIARAIQAIDIEKL

>gi|30063583|ref|NP_837754.1| hypothetical protein S2350 [Shigella flexneri 2a str. 2457T]

MLTGNQRETNWPMDLNTLISQYGYAALVIGSLAEGETVTLLGGVAAHQGLLKFPLVVLSVALGGMIGDQV

LYLCGRRFGGKLLRRFSKHQDKIERAQKLIQRHPYLFVIGTRFMYGFRVIGPTLIGASQLPPKIFLPLNI

LGAFAWALIFTTIGYAGGQVIAPWLHNLDQHLKHWVWLILVVVLVVGVRWWLKRRGKKKPDNQA

>gi|30063582|ref|NP_837753.1| hypothetical protein S2349 [Shigella flexneri 2a str. 2457T]

MSHVWGLFSHPDRKMQVINRENETISHHYTHHVLLMAAIPVICAFIGTTQIGWNFGDGTILKLSWFTGLA

LAVLFYGVMLAGVAVMGRVIWWMARNYPQRPSLAHCMVFAGYVATPLFLSGLVALYPLVWLCALVGTVAL

FYTGYLLYLGIPSFLNINKEEGLSFSSSTLAIGVLVLEVLLTLTVILWGYGYRLF

>gi|30063580|ref|NP_837751.1| D-lactate dehydrogenase [Shigella flexneri 2a str. 2457T]

MSSMTTTDNKAFLNELARLVGHSHLLTDPAKTARYRKGFRSGQGDALAVVFPGSLLELWRVLKACVTADK

IILMQAANTGLTEGSTPNGNDYDRDIVIISTLRLDKLHVLGKGEQVLAYPGTTLYSLEKALKPLGREPHS

VIGSSCIGASVIGGICNNSGGSLVQRGPAYTEMSLFARIDEDGKLTLVNHLGIDLGETPEQILSKLDDDR

IKDDDVRHDGRHAHDYDYVHRVRDIEADTPARYNADPDRLFESSGCAGKLAVFAVRLDTFEAEKNQQVFY

IGTNQPEVLTEIRRHILANFENLPVAGEYMHRDIYDIAEKYGKDTFLMIDKLGTDKMPFFFNLKGRTDAM

LEKVKFFRPHFTDRAMQKFGHLFPSHLPPRMKNWRDKYEHHLLLKMAGDGVGEAKSWLVDYFKQAEGDFF

VCTPEEGSKAFLHRFAAAGAAIRYQAVHSDEVEDILALDIALRRNDTEWYEHLPPEIDSQLVHKLYYGHF

MCYVFHQDYIVKKGVDVHALKEQMLELLQQRGAQYPAEHNVGHLYKAPETLQKFYRENDPTNSMNPGIGK

TSKRKNWQEVE

>gi|30063579|ref|NP_837750.1| beta-D-glucoside glucohydrolase [Shigella flexneri 2a str. 2457T]

MKWLCSVGIAVSLVLQPALADDLFGNHPLTPEARDAFVTDLLKKMTVDEKIGQLRLISVGPDNPKEAIRE

MIKDGQVGAIFNTVTRQDIRAMQDQVMELSRLKIPLFFAYDVLHGQRTVFPISLGLASSFNLDAVKTVGR

VSAYEAADDGLNMTWAPMVDVSRDPRWGRASEGFGEDTYLTSIMGKTMVEAMQGKSPADRYSVMTSVKHF

AAYGAVEGGKEYNTVDMSPQRLFNDYMPPYKAGLDAGSGAVMVALNSLNGTPATSDSWLLKDVLRDQWGF

KGITVSDHGAIKELIKHGTAADPEDAVRVALKSGINMSMSDEYYSKYLPGLIKSGKVTMAELDDAARHVL

NVKYDMGLFNDPYSHLGPKESDPVDTNAESRLHRKEAREVARESLVLLKNRLETLPLKKSATIAVVGPLA

DSKRDVMGSWSAAGVADQSVTVLTGIKNSVGENGKVLYAKGANVTSDKGIIDFLNQYEEAVKVDPRSPQE

MIDEAVQTAKQSDVVVAVVGEAQGMAHEASSRTDITIPQGQRDLIAALKATGKPLVLVLMNGRPLALVKE

DQQADAILETWFAGTEGGNAIADVLFGDYNPSGKLPISFPRSVGQIPVYYSHLNTGRPYNADKPNKYTSR

YFDEANGALYPFGYGLSYTTFTVSDVKLSAPTMKRDGKVTASVQVTNTGKREGATVVQMYLQDVTASMSR

PVKQLKGFEKITLKPGETQTVSFPIDIEALKFWNQQMKYDAEPGKFNVFIGTDSARVKKGEFELL

>gi|30063578|ref|NP_837749.1| transport system permease [Shigella flexneri 2a str. 2457T]

MPLLKLWAGSLVMLAAVSLPLQAASPVKVGSKIDTEGALLGNIILQVLESHGVPTVNKVQLGTTPVGRGA

ITSDELDIYPEYTGNGAFFFKDENDAAWKNAGQGYEKVKKLDAEHNKLIWLTPAPANNTWTIAVRQDVAE

KNKLTSLADLSRYLQEGGTFKLAASAEFIERADALPTFEKAYGFKLGQDQLLSLAGGDTAVTIKAAAQQT

SGVNAAMAYGTDGPVAALGLQTLSDPQGVQPIYAPAPVVRESVLKEYPQMAQWLQPVFASLDAKTLQQLN

ASIAVEGLDAKKVAADYLKQKGWAK

>gi|30063577|ref|NP_837748.1| transport system permease [Shigella flexneri 2a str. 2457T]

MTYLRINPVLALLLLLTAIAAALPFISYAPNRLVSGEGLHLWQLWPQTIWMLVGVGCAWLTACFVPAKKG

SIFALILAQFVFVLLVWGAGKAATQLAQNGSALARTSLGRGFWLAAALALLACSDAIRRISTHPLWRWLL

HMQIAIIPLWLLYSGTLNDLSLMKEYANRQDVFDDALAQHLTLLFGAVLPALVIGVPLGIWCYFSTARQG

AIFSLLNVIQTVPSVALFGLLIAPLAALVTAFPWLGKLGIAGTGMTPALIALVLYALLPLVRGVVVGLNQ

IPRDVLESARAMGMSGAQRFLHVQLPLALPVFLRSLRVVMVQTVGMAVIAALIGAGGFGALVFQGLLSSA

IDLVLLGVIPVIVLAVLTDALFDLLIALLKVKRND

>gi|30063576|ref|NP_837747.1| ATP-binding component of a transport system [Shigella flexneri 2a str. 2457T]

MIEFSHVSKLFGAQKAVNDLNLNFQEGSFSVLIGTSGSGKSTTLKMINRLVEHDSGVIRFAGEEIRSLPV

LELRRRMGYAIQSIGLFPHWSVAQNIATVPQLQKWSRARIDDRIDELMALLGLESNLRERYPHQLSGGQQ

QRVGVARALAADPQVLLMDEPFGALDPVTRGALQQEMTRIHRLLGRTIVLVTHDIDEALRLAEHLVLMDH

GEVVQQGNPLTMLTRPANDFVRQFFGRSELGVHLLSLRSVADYVRREERAEGEALAEEMTLRDALSLFVA

RGCEVLPVVNTQGQPCGTLHFQDLLVEA

>gi|30063575|ref|NP_837746.1| transport system permease [Shigella flexneri 2a str. 2457T]

MKMLRDPLFWLIALFVALIFWLPYSQPLFAALFPQLPRPVYQQESFAALALAHFWLVGISSLFAVIIGTG

AGIAVTRPWGAEFRPLVETIAAVGQTFPPVAVLAIAVPVIGFGLQPAIIALILYGVLPILQVTLAGLGAI

DPSVTEVAKGMGMSRGQRLRKVELPLAAPVILAGVRTSVIINIGTATIASTVGASTLGTPIIIGLSGFNT

AYVIQGALLVALAAIIADRLFERLVQALSQHAK

>gi|30063574|ref|NP_837745.1| hypothetical protein S2341 [Shigella flexneri 2a str. 2457T]

MRIAKIGVIALFLFMALGGIGGVMLAGYTFILRAG

>gi|30063573|ref|NP_837744.1| hypothetical protein S2340 [Shigella flexneri 2a str. 2457T]

MSEWASEDINAPSLLTGNPALQPQTGGILNIPANHQRIFTLFFTCRSQTNGDVVDQYAVEDRQQRLESRL

LALKPSAQRNKQMCGECLGGVILTALFDPRSQAM

>gi|30063572|ref|NP_837743.1| IS629 orfB [Shigella flexneri 2a str. 2457T]

MARCTVARLMAVMGLAGVLRGKKVRTTIGRKAVAAGDRVNRQFVAERPDQLWVADFTYVSTWRGFVYVAF

IIDVFAGYIVGWRVSSSMETTFVLDALEQALWARRPSGTVHHSDKGSQYVSLAYTQRLKEAGLLASTGST

GDSYDNAMAESINGLYKSEVIHRKSWKNRAEVELATLTWVDWYNNRRLLERLGHIPPAEAEKAYYASIGN

DDLAA

>gi|30063571|ref|NP_837742.1| IS629 orfA [Shigella flexneri 2a str. 2457T]

MTKNTRFSPEVRQRAVRMVLESQGEYDSQWATICSIAPKIGCTPETLRVWGRQHERDTGGGDGGLTTAER

QRLKEPERENRELRRSNDILRQASAYFAKAEFDRLWKK

>gi|30063570|ref|NP_837741.1| IS1 B [Shigella flexneri 2a str. 2457T]

MSRQCTHYGRWPQHGFTSLKKLRPQSVTSRIQPGSDVIVCAEMDEHWGYVGAKSRQRWLFYAYDRIRRTV

VAHVFGERTLATLERLLSLLSAFEVVVWMTDGWPLYESRLKGKLHVISKRYTQRIERHNLNLRQHLARLG

RKSLSFSKSVELHDKVIGHYLNIKHYQ

>gi|30063569|ref|NP_837740.1| IS1 orfA [Shigella flexneri 2a str. 2457T]

MASISIRCPSCSATEGVVRNGKSTAGHQRYLCSHCRKTWQLQFTYTASQPGTHQKIIDMAMNGVGCRASA

RIMGVGLNTVLRHLKNSGRSR

>gi|30063568|ref|NP_837739.1| phage tail fiber protein [Shigella flexneri 2a str. 2457T]

MAISDTGRNSADNVDGVYYRPLQKLINGTWYNVASI

>gi|30063567|ref|NP_837738.1| phage tail fiber assembly protein [Shigella flexneri 2a str. 2457T]

MTKRYSVTWLFSEDGKNWYEELKNFASDTIKIAYTGDGRVVWVGKDVTGIEPRNASVIEVPDITANRRIT

APGYWFYRNDEFVFDYRLKAEDERDALLAQVSARTGEWEEDLLLGLISDEDKEKLKACRIYAKSLQAMDF

STITDKSSYNAIEWPASPEGSS

>gi|30063566|ref|NP_837737.1| phage tail fiber protein [Shigella flexneri 2a str. 2457T]

MNTVADVMAGNDGEYCFHARTGKYGVYLKQDWRNEYNVGDIAVYEDSKPGTLNDFLIAPDEGDLKPDVVK

RFEEMVAQAQQSAGAAAGNAQQTAQDVAAAATARDDAQRFAEKARQDATVTAEDRKATAEDVTSTGANAA

AAGQSAQDAAGYARAAEQAKNDIDAALTGTLKTANHLSETAAAGEKAQQKSRDNLGLKSAATMEAQSDIY

DRTKGRLAIPGAFGFGCAFLPEDVIRFDTKSDFLAWVRNALPGEYSVAGPYGIIIPDTRFEGVLSIRWTD

ARPETTEPRYRAKSLTFYGINGPIYHTRYCYWPISRLTGWVKINITTEDIIYRIVASSVRNRWGDPDIGG

LIIAAYQGEADGDKVIRLVRGQSYRGSRLGPVGISVPGTPTGTYIVSPQFFITGCSEHSLPGSYCALSGV

PDAHVSGAMPGLFIRTSRGMHRGN

>gi|30063565|ref|NP_837736.1| hypothetical protein S2331 [Shigella flexneri 2a str. 2457T]

MTFFFDDRDQAVPYTATADDVAPTGQQIWQELQSGKWGEIAPFTVTPEMLEAAREARRQEIEAWRTEQEA

KPFTFEWNGRIWNAGPDSLGRLSPVVMLAKSVTAQTHMAWSDADNQQVKLSMPELEELAESQKTALLTEA

ESVIRQPGRAVRLNRETDESGEARGRASVFPESVRSVVYAM

>gi|30063564|ref|NP_837735.1| hypothetical protein S2329 [Shigella flexneri 2a str. 2457T]

MLPQHSDIEIAWYASIQQEPNGWKTVTTQFYIQGFSEHIAPLQDAVDLEIATEEENSLLEAWKKYRVLLN

RVDTSTAPDIEWPEEPDTM

>gi|30063563|ref|NP_837734.1| DNA-damage-inducible protein [Shigella flexneri 2a str. 2457T]

MRVEICIAKEKITKMPNGAVDALKEELTRRISKRYDDVEVIVKATSNDGLSVTRTADKDSAKTFVQETLK

DTWESADEWFVH

>gi|30063562|ref|NP_837733.1| 2-component sensor protein [Shigella flexneri 2a str. 2457T]

MYDFNLVLLLLQQMCVFLVIAWLMSKTPLFIPLMQVTVRLPHKFLCYIVFSIFCIMGTWFGLHIDDSIAN

TRAIGAVMGGLLGGPVVGGLVGLTGGLHRYSMGGMTALSCMISTIVEGLLGGLVHSILIRRGRTDKVFNP

ITAGAVTFVAEMVQMLIILAIARPYEDAVRLVSNIAAPMMVTNTVGAALFMRILLDKRAMFEKYTSAFSA

TALKVAASTEGILRQGFNEVNSMKVAQVLYQELDIGAVAITDREKLLAFTGIGDDHHLPGKPISSTYTLK

AIETGEVVYADGNEVPYRCSLHPQCKLGSTLVIPLRGENQRVMGTIKLYEAKNRLFSSINRTLGEGIAQL

LSAQILAGQYERQKAMLTQSEIKLLHAQVNPHFLFNALNTIKAVIRRDSEQASQLVQYLSTFFRKNLKRP

SEFVTLADEIEHVNAYLQIEKARFQSRLQVNIAIPQELSQQQLPAFTLQPIVENAIKHGTSQLLDTGRVA

ISARREGQHLMLEIEDNAGLYQPVTNASGLGMNLVDKRLRERFGDDYGISVACEPDSYTRITLRLPWRDE

A

>gi|30063561|ref|NP_837732.1| two-component response-regulatory protein YehT [Shigella flexneri 2a str. 2457T]

MIKVLIVDDEPLARENLRIFLQEQSDIEIVGECSNAVEGIGAVHKLRPDVLFLDIQMPRISGLEMVGMLD

PEHRPYIVFLTAFDEYAIKAFEEHAFDYLLKPIDEARLEKTLARLRQERSKQDVSLLPENQQALKFIPCT

GHSRIYLLQMKDVAFVSSRMSGVYVTSHEGKEGFTELTLRTLESRTPLLRCHRQYLVNLAHLQEIRLEDN

GQAELILRNGLTVPVSRRYLKSLKEAIGL

>gi|30063560|ref|NP_837731.1| IS1 orfA [Shigella flexneri 2a str. 2457T]

MASISIRCPSCSATEGVVRNGKSTAGHQRYLCSHCRKTWQLQFTYIASQPGTHQKIIDMAMNGVGCRASA

RIMGVGLNTVLRHLKNSGRSR

>gi|30063559|ref|NP_837730.1| IS1 orfB [Shigella flexneri 2a str. 2457T]

MSRQCTHYGRWPQHGFTSLKKLRPQSVTSRIQPGSDVIVCAEMDEHWGYVGAKSRQRWLFYAYDRIRRTV

VAHVFGERTLATLERLLSLLSAFEVVVWMTDGCPLYESRLKGKLHVISKRYTQRIERHNLNLRQHLARLG

RKSLSFSKSVELHDKVIGHYLNIKHYQ

>gi|30063558|ref|NP_837729.1| hypothetical protein S2319 [Shigella flexneri 2a str. 2457T]

MKAFNKLFSLVVASVLVFSLAGCGDKEESKKFSANLNGTEIAITYVYKGDKVLKQSSETKIQFASIGATT

KEDAAKTLEPLSAKYKNIAGVEEKSTYTDTYAQENVTIDMEKVDFKALQGISGINVSAEDAKKGITMAQM

ELVMKAAGFKEVK

>gi|30063557|ref|NP_837728.1| IS1 orfA [Shigella flexneri 2a str. 2457T]

MASISIRCPSCSATEGVVRNGKSTAGHQRYLCSHCRKTWQLQFTYTASQPGTHQKIIDMAMNGVGCRASA

RIMGVGLNTVLRHLKNSGRSR

>gi|30063556|ref|NP_837727.1| IS1 orfB [Shigella flexneri 2a str. 2457T]

MSRQCTHYGRWPQHGFTSLKKLRPQSVTSRIQPGSDVIVCAEMDEHWGYVGAKSRQRWLFYAYDRIRRTV

VAHVFGERTLATLERLLSLLSAFEVVVWMTDGCPLYESRLKGKLHVISKRYTQRIERHNLNLRQHLARLG

RKSLSFSKSVELHDKVIGHYLNIKHYQ

>gi|30063555|ref|NP_837726.1| hypothetical protein S2316 [Shigella flexneri 2a str. 2457T]

MNSLRPELLELTPQALTALSNAGFVKRSLKELENGNVPEISHENSALIATFSDGVRTQLANGQALKEAQC

TYGASGMCRHRVMLVLSYQRLCATAQPTGKEEEWDPAIWLEELATLPDATRKRAQALVAKSITIELFCAP

GEIPSARLPMSDVRFYSRSSIRFARCDCIEGTLCEHVVLAVQAFVQAKAQQAELTHLIWQMRSEHVTSSD

DPFASEEGKTCRQYVQQLSQALWLSGISQPLIHYEAAFSRAQQAAERCSWRWVSESLRQLRASVDAFHAR

ASHYHAGECLRQLAALNSRLNCAQEMARSDSVGEVPPVPWRTVVGSGIAGEAKLDHLRLVSLGMRCWQDI

EHYGLRIWFTDPDTGSILHLSRSWPRSEQEDSPAATRRLFSFQAGALAGGQIVSQAAKRSADGELLLATR

NRLSSVVPLSPDAWQMLSAPLRQPGIVALREYLRQRPPACIRPLNQVDNLFILPVAECISLGWDSSRQTL

DAQVISGEGEDNLLTLSLPASASAPYAIERMAALLQQTDDPVCLVSGFVSFVDGQLTLEPQVMMTKTRAW

ALDAETAPVVVSLPSASVLPVPSTAHQLLMRCQALLIQLLHNGWRYQEQSAINQAELLANDLTAVGFYRL

AHVLAQFRNTESEARVEAMNNGVLLCEQLFPMLQQQG

>gi|30063554|ref|NP_837725.1| hypothetical protein S2315 [Shigella flexneri 2a str. 2457T]

MSELNDLLTTRELQRWRLILGEAAETTLCGLDDNARQIDHALEWLYGRDPERLQRGERSSGLGGSNLTTP

EWINSIHTLFPQQVIERLESDAVLRYGIEDVVTNLDVLERMQPSESLLRAVLHTKHLMNPEVLAAARRIV

RQVVEEIMARLAKEVRQAFSGVRDRRRRSFIPLARNFDFKSTLRANLQHWHPQHGKLYIEFPRFNSRIKR

QSEQWQLVLLVDQSGSMVDSVIHSAVMAACLWQLPGIRTHLVAFDTSVVDLTADVADPVELLMKVQLGGG

TNIASAMEYGRQLIEQPAKSVIILVSDFYEGGSSSLLTHQVKKCVQSGIKVLGLAALDSTATPCYDRDTA

QALVNVGAQIAAMTPGELASWLAENLQS

>gi|30063553|ref|NP_837724.1| hypothetical protein S2314 [Shigella flexneri 2a str. 2457T]

MSEPLIVGIRHHSPACARLVKSLIESQRPRYVLIEGPADFNDRVDELFLAHQLPVAIYSYCQYQDGAAPG

RGAWTPFAEFSPEWQALQAARRIQAQTYFIDLPCWAQSEEEDDSPDTQDESQTLLLRDTRMDNSDTLWDH

LFEDESQQTALPSALARYFAQLRGDSPGDALNRQREAFMARWITWAMQQNNGDVLVVCGGWHAPALANMW

RECPQEINKPELSSLADAVTGCYLTPYSEKRLDVLAGYLSGMPAPVWQNWCWQWGLQQAGEQLLKTVLTR

LRQHKLPASTADMAAAHLHAMALAQLRGHTLPLRTDWLDAIAGSLIKEALNAPLPWSYRGVIHPDTDPIL

LTLIDTLAGDGFGKLAPSTPQPPLPKDVTCELERTAISLPAELTLNRFNPNGLAQSQVLHRLAILEIPGI

VRQQGSTLTLAGNGEERWKLTRPLSQHAALIEAACFGATLLEAARHKLEADMLDAGGIGSITTCLSQAAS

AGLASFSQQLLEQLTLLIAQENQFAEMGQALEVLYALWRLDEISGMQGAQILQTTLCAAIDRTLWLCESN

GRPDEKEFHAHLHSWQALCHILRDLHSGVQLPGVSLSAAVALLERHSQAIHVLALDRGATLGALMRLEHP

NASAEAALTMLAQLSPAQSGEALHGLLALARHQLACQPAFIAGFSSHLNQLSDADFTNALPDLRAAMAWL

PPRERGTLAHQVLEHYQLAQLPVSALQMPLHCPPQAIAHHQQLEQQALASLQHWGVFHV

>gi|30063552|ref|NP_837723.1| hypothetical protein S2313 [Shigella flexneri 2a str. 2457T]

MRQTFIARKISFISLIHFRDEYMSPQNNHLQRPPAAVLYADELAKLKQNDNAPCPPGWQLSLPAARAFIL

GDSAQNISRKVVISPSAVERMLVTLATGRGLMLVGEPGTAKSLLSELLATSISGDAGLTIQGGASTTEDQ

IKYGWNYALLINHGPSTEALVPAPLYQGMRDGKIVRFEEITRTPLEVQDCLLGMLSDRVMTVPELTGEAS

QLYAREGFNIIATANTRDRGVNEMSAALKRRFDFETVFPIMDFAQELELVASASARLLAHSGIPHKVPDA

VLELLVRTFRDLRANGEKKTSMDTLTAIMSTAEAVNVAHAVGVRAWFLANRAGEPADLVDCIAGTIVKDN

EEDRARLRRYFEQRVATHKEAHWQAYYQARHRLP

>gi|30063551|ref|NP_837722.1| methionyl-tRNA synthetase [Shigella flexneri 2a str. 2457T]

MTQVAKKILVTCALPYANGSIHLGHMLEHIQADVWVRYQRMRGHEVNFICADDAHGTPIMLKAQQLGITP

EQMIGEMSQEHQTDFAGFNISYDNYHSTHSEENRQLSELIYSRLKENGFIKNRTISQLYDPEKGMFLPDR

FVKGTCPKCKSPDQYGDNCEVCGATYSPTELIEPKSVVSGATPVMRDSEHFFFDLPSFSEMLQAWTRSGA

LQEQVANKMQEWFESGLQQWDISRDAPYFGFEIPNAPGKYFYVWLDAPIGYMGSFKNLCDKRGDSVSFDE

YWKKDSTAELYHFIGKDIVYFHSLFWPAMLEGSNFRKPTNLFVHGYVTVNGAKMSKSRGTFIKASTWLNH

FDADSLRYYYTAKLSSRIDDIDLNLEDFVQRVNADIVNKVVNLASRNAGFINKRFDGVLASELADPELYK

TFTDAAEVIGEAWESREFGKAVREIMALADLANRYVDEQAPWVVAKQEGRDADLQAICSMGINLFRVLMT

YLKPVLPKLTERAEAFLNTELTWDGIQQPLLGHKVNPFKALYNRIDMKQVEALVEASKEEVKATTAPVTG

PLADDPIQETITFDDFAKVDLRVALIENAEFVEGSDKLLRLTLDLGGEKRNVFSGIRSAYPDPQALIGRH

TIMVANLAPRKMRFGISEGMVMAAGPGGKDIFLLSPDAGAKPGHQVK

>gi|30063550|ref|NP_837721.1| ATPase [Shigella flexneri 2a str. 2457T]

MNEQSQAKSPEALRAMVAGTLANFQHPTLKHNLTTLKALHHVAWMDDTLHVELVMPFVWHSAFEELKEQC

SAELLRITGAKAIDWKLSHNIATLKRVKNQPGINGVKNIIAVSSGKGGVGKSSTAVNLALALAAEGAKVG

ILDADIYGPSIPTMLGAENQRPTSPDGTHMAPIMSHGLATNSIGYLVTDDNAMVWRGPMAGKALMQMLQE

TLWPDLDYLVLDMPPGTGDIQLTLAQNIPVTGAVVVTTPQDIALIDAKKGIVMFEKVEVPVLGIVENMSV

HICSNCGHHEPIFGTGGAEKLAEKYHTQLLGQMPLHISLREDLDKGTPTVISRPESEFTAIYRQLADRVA

AQLYWQGEVIPGEISFRAV

>gi|30063549|ref|NP_837720.1| hypothetical protein S2301 [Shigella flexneri 2a str. 2457T]

MNKYWLSGIIFLAYGLASPAVSSETATLTINGRISPPTCSMAMVNSQPQQHCGQLTYNVDTRHQVSSPVK

GVTTEVVVAGSDSKRRIVLNRYD

>gi|30063548|ref|NP_837719.1| IS1 orfA [Shigella flexneri 2a str. 2457T]

MASISIRCPSCSATEGVVRNGKSTAGHQRYLCSHCRKTWQLQFTYTASQPGTHQKIIDMAMNGVGCRASA

RIMGVGLNTVLRHLKNSGRSR

>gi|30063547|ref|NP_837718.1| IS1 orfB [Shigella flexneri 2a str. 2457T]

MSCQCTHYGRWPQHGFTSLKKLRPQSVTSRIQPGSDVIVCAEMDEHWGYVGAKSRQRWLFYAYDRIRRTV

VAHVFGERTLATLERLLSLLSAFEVVVWMTDGWPLYESRLKGKLHVISKRYTQRIERHNLNLRQHLARLG

RKSLSFSKSVELHDKVIGHYLNIKHYQ

>gi|30063546|ref|NP_837717.1| fimbrial-like protein [Shigella flexneri 2a str. 2457T]

MKRSIIAAAVFSSFFMSAGVFAADVDTGTLTIKGNIAESPCKFEAGGDSVSINMPTVPTTVFEGKAKYST
YDDAVGVTSSMLKISCPKEVAGVKLSLITNDKITGNDKAIASSNDTVGDNSDVLDVSAPFNIESYKTAEG
QYAIPFKAKYLKLTDNSVQSGDVLSSLVMRVAQD

>gi|30063545|ref|NP_837716.1| outer membrane protein [Shigella flexneri 2a str. 2457T]
MLRMTPLASAIVALLLGIEAYAAEETFDTHFMIGGMKDQQVANIRLDDNQPLPGQYDIDIYVNKQWRGKY
EIIVKDNPQETCLSREVIKRLGINSDNFASGKQCLTFEQLVQGGSYSWDIGVFRLDFSVPQAWVEELESG
YVPPENWERGINAFYTSYYVSQYYSDYKASGNNKSTYVRFNSGLNLLEWQLHSDASFSKTNNNPGVWKSN
TLYLERGFAQFLGTLRVGDMYTSSDIFDSVRFSGVRLFRDMQMLPNSKQNFTPRVQGIAQSNALVTIEQN
GFVVYQKEVPPGPFAITDLQLAGGGADLDVSVKEADGSVTTYLVPYAAVPNMLQPGVSKYDFAAGRSHIE
GASKQSDFVQAGYQYGFNNLLTLYGGTMVANNYYAFTLGTGWNTRIGAISVDATKSHSKQDNGDVFDGQS
YQIAYNKFVSQTSTRFGLAAWRYSSRDYRTFNDHVWANNKDNYRRDENDIYDIADYYQNDFGRKNSFSAN
MSQSLPEGWGSVSLSTLWRDYWGRSGSSKDYQLSYSNNWRRISYTLAASQAYGENHHEEKRFNIFISIPC
DWGDDVTTPRRQIYMSNSTTFDDQGFASNNTGLSGTVGSRDQFNYGVNLSHQHQGNETTAGANLTWNAPV
ATVNGSYSQSSTYRQTGASVSGGIVAWSGGVNLANRLSETFAVMNAPGIKDAYVNGQKYRTTNRNGVVVY
DGMTPYRENHLMLDVSQSDSEAELRGNRKIAAPYRGAVVLVNFDTDQRKPWFIKALRADGQPLTFGYEVN
DIHGHNIGVVGQGSQLFIRTNEIPPSVNVAIDKQQGLSCTITFGKEIDESRNYICQ

>gi|30063544|ref|NP_837715.1| hypothetical protein S2292 [Shigella flexneri 2a str. 2457T]
MSHTIRDKQKLKARASKIQGQVVALKKMLDEPHECAAVLQQIAAIRGAVNGLMREVIKGHLTEHIVHQGD
ELKREEDLDVVLKVLDSYIK

>gi|30063543|ref|NP_837714.1| hydroxyethylthiazole kinase [Shigella flexneri 2a str. 2457T]
MQVDLLSSAQSAHALHLFHQHSPLVHCMTNDVVQTFTANTLLALGASPAMVIETEEASQFAAIASALLIN
VGTLTQPRAQAMRAAVEQAKSSQTRWTLDPVAVGALDYRRHFCHELLSFKPAAIRGNASEIMALAGIANG

GRGVDTTDAAANAIPAAQTLARETGAIVVVTGEVDYVTDGHRIIGIHGGDPLMTKVVGTGCALSAVVAAC

CALPGDTLENVASACHWMKQAGERAVARSEGPGSFVPHFLDALWQLTQEVQA

>gi|30063542|ref|NP_837713.1| phosphomethylpyrimidine kinase [Shigella flexneri 2a str. 2457T]

MKRINALTIAGTDPSGGAGIQADLKTFSALGAYGCSVITALVAQNTRGVQSVYRIEPDFVAAQLDSVFSD

VRIDTTKIGMLAETDIVEAVAERLQRYQIQNVVLDTVMLAKSGDPLLSPSAVATLRSRLLPQVSLITPNL

PEAAALLDAPHARTEQEMLEQGRSLLAMGCGAVLMKGGHLDDEQSPDWLFTREGEQRFTAPRIMTKNTHG

TGCTLSAALAALRPRHTNWADTVQEAKIWLSSALAQADTLEVGHGIGPVHHFHAWW

>gi|30063541|ref|NP_837712.1| hypothetical protein S2289 [Shigella flexneri 2a str. 2457T]

MQLRITSRKKLTALLCALGLISIVAIYPRQTVNFFYSTAVQITDYIHFYGYRPVKSFAIRIPASYTIHGL

DVSRWQERIDWQRVAKMRDNDIRLQFAFIKATEGEKLVDPYFSRNWQLSRENGLLRGAYHYFSPSVSASV

QARLFLQTVDFSQGDFPAVLDVEERGKLSAKELRKRVSQWLKMVEKRTGKKPIIYSGAVFYHTNLAGYFN

EYPWWVAHYYQRRPDNDGMAWRFWQHSDRGQVDGINGPVDFNVFNGTGMSCRHSLMGLKKRLK

>gi|30063540|ref|NP_837711.1| transcriptional regulator [Shigella flexneri 2a str. 2457T]

MEQAHTQLIAQLNERILAADNTPLYIKFAETVKNAVRSGVLEHGNILPGERDLSQLTGVSRITVRKAMQA

LEEEGVVTRSRGYGTQINNIFEYSLKEARGFSQQVVLRGKKPDTLWVNKRVVKCPEEVAQQLAVEAGSDV

FLLKRIRYVDEEAVSIEESWVPAHLIHDVDAIGISLYDYFRSQHIYPQRTRSRVSARMPDAEFQSHIQLD

SKIPVLVIKQVALDQQQRPIEYSISHCRSDLYVFVCEE

>gi|30063539|ref|NP_837710.1| kinase [Shigella flexneri 2a str. 2457T]

MSGARLHTLLPELTTRQPVMVVGAAVIDVIADAYALPWRGCDIELKQQSVNVGGCALNIAVALKRLGIEA

GNALPLGQGVWAEIIRNRMAKEGLISLIDNAESDNGWCLALVEPDGERTFMSFSGVENQWNRQWLARLTV

APGNLLYFSGYQLASPCGELLVEWLEKLQDVTPFIDFCPRIGDIPDALLARIMACRPLVSLNRQEAEIAA

ERFALSAEITTLGKQWQEKFAAPLIVRLDKEGAWYFSNDASGCIPAFPTQVVDTIGAGDSHAGGVLAGLA

SGLPLADAVLLGNAVASWVVGHRGGDCAPTREELLLAHKNV

>gi|30063538|ref|NP_837709.1| fructose-bisphosphate aldolase [Shigella flexneri 2a str. 2457T]

MTDIAQLLGKDADNLLQHRCMTIPSDQLYLPGHDYVDRVMIDNNRPPAVLRNMQTLYNTGRLAGTGYLSI

LPVDQGVEHSAGASFAANPLYFDPKNIVELAIEAGCNCVASTYGVLVSVSRRYAHRIPFLVKLNHNETLS

YPNTYDQTLYASVEQAFNMGAVAVGATIYFGSEESRRQIEEISAAFERAHELGMVTVLWAYLRNSAFKKD

GVDYHVSADLTGQANHLAATIGADIVKQKMAENNGGYKAINYGYTDDRVYSKLTSENPIDLVRYQLANCY

MGRAGLINSGGAAGGETDLSDAVRTAVINKRAGGMGLILGRKAFKKSMADGVKLINAVQDVYLDSKITIA

>gi|30063537|ref|NP_837708.1| tagatose-bisphosphate aldolase [Shigella flexneri 2a str. 2457T]

MYVVSTKQMLNNAQRGGYAVPAFNIHNLETMQVVVETAANLHAPVIIAGTPGTFTHAGTENLLALVSAMA

KQYHHPLAIHLDHHTKFDDIAQKVRSGVRSVMIDASHLPFAQNISRVKEVVDFCHRFDVSVEAELGQLGG

QEDDVQVNEVDALYTNPAQAREFAEATGIDSLAVAIGTAHGMYASAPVLDFSRLENIRQWVNLPLVLHGA

SGLSTKDIQQTIKLGICKINVATELKNAFLQSLKNYLTEHPEATDPRDYLQSAKSAMRDVVSKVIADCGC

EGRA

>gi|30063536|ref|NP_837707.1| tagatose 6-phosphate kinase 1 [Shigella flexneri 2a str. 2457T]

MKTLIARHKAGEHIGICSVCSAHPLVIEAALAFDRNSTRKVLIEATSNQVNQFGGYTGMTPADFREFVFT

IADKVGFARERIILGGDHLGPNCWQQENADAAMEKSVELVKAYVRAGFSKIHLDASMSCAGDTIPLAPET

VAERAAVLCFAAESVATDCQREQLSYVIGTEVPVPGGEASAIQSVHITHVEDAANTLRTHQKAFIARGLT

EALTRVIAIVVQPGVEFDHSNIIHYQPQEAQALAQWIENTRMVYEAHSTDYQTRTAYWELVRDHFAILKV

GPALTFALREAIFALAQIEQELIAPENRSGCLAVIEEVMLDEPQYWKKYYRTGFNDSLLDIRYSLSDRIR

YYWPHSRIKNSVETMMVNLQGVDIPLGMISQYLPKQFERIQSGELSAMPHQLIMNKIYDVLRAYRYGCAE

>gi|30063535|ref|NP_837706.1| galactitol-specific PTS system component IIA [Shigella flexneri 2a str. 2457T]

MTNLFVRSGISFVDRSEVLTYIGNEMLAKGVVHDTWPQALIAREAEFPTGIMLEQHAIAIPHCEAIHAKS

SAIYLLRPTNKVHFQQADDDNDVAVSLVIALIVENPQQQLKLLRCLFGKLQQPDIVETLITLPETQLKEY

FTKYVLDSDE

>gi|30063534|ref|NP_837705.1| galactitol-specific PTS system component IIB [Shigella flexneri 2a str. 2457T]

MKRKIIVACGGAVATSTMAAEEIKELCQSHNIPVELIQCRVNEIETYMDGVHLICTTARVDRSFGDIPLV

HGMPFVSGVGIEALQNKILTILQG

>gi|30063533|ref|NP_837704.1| PTS system galactitol-specific enzyme IIC [Shigella flexneri 2a str. 2457T]

MFSEVMRYILDLGPTVMLPVVIIIFSKILGMKAGDCFKAGLHIGIGFVGIGLVIGLMLDSIGPAAKAMAE

NFDLNLHVVDVGWPGSSPMTWASQIALVAIPIAILVNVAMLLTRMTRVVNVDIWNIWHMTFTGALLHLAT

GSWMIGMAGVVIHAAFVYKLGDWFARDTRNFFELEGIAIPHGTSAYMGPIAVLVDAIIEKIPGVNRIKFS

ADDIQRKFGPFGEPVTVGFVMGLIIGILAGYDVKGVLQLAVKTAAVMLLMPRMIKPIMDGLTPIAKQARS

RLQAKFGGQEFLIGLDPALLLGHTAVVSASLIFIPLTILIAVCVPGNQVLPFGDLATIGFFVAMAVAVHR

GNLFRTLISGVIIMSITLWIATQTIGLHTQPAANAGALKAGGMVASMDQGGSPITWLLIQVFSPQNIPGF

IIIGAIYLTGIFMTWRRARGFTKQEKAVLAE

>gi|30063532|ref|NP_837703.1| IS1 orfA [Shigella flexneri 2a str. 2457T]

MASISIRCPSCSATEGVVRNGKSTAGHQRYLCSHCRKTWQLQFTYTASQPGTHQKIIDMAMNGVGCRASA

RIMGVGLNTVLRHLKNSGRSR

>gi|30063531|ref|NP_837702.1| IS1 orfB [Shigella flexneri 2a str. 2457T]

MSRQCTHYGRWPQHGFTSLKKLRPQSVTSRIQPGSDVIVCAEMDEHWGYVGAKSRQRWLFYAYDRIRRTV

VAHVFGERTLATLERLLSLLSAFEVVVWMTDGCPLYESRLKGKLHVISKRYTQRIERHNLNLRQHLARLG

RKSLSFSKSVELHDKVIGHYLNIKHYQ

>gi|30063530|ref|NP_837701.1| galactitol-1-phosphate dehydrogenase [Shigella flexneri 2a str. 2457T]

MKSVVNDTDGIVRVAKSVIPEIKHQDEVRVKIASSGLCGSDLPRIFKNGAHYYPITLGHEFSGYIDAVGS

GVDDLHPGDAVACVPLLPCFTCPECLKGFYSQCAKYDFIGSRRDGGFAEYIVVKRKNVFALPTDMPIEDG

AFIEPITVGLHAFHLAQGCENKNVIIIGAGTIGLLAIQCAVALGAKSVTAIDISSEKLALAKSFGAMQTF

NSSEMSAPQMQSVLRELRFNQLILETAGVPQTVELAVEIAGPHAQLALVGTLHQDLHLTSATFGKILRKE

LTVIGSWMNYSSPWPGQEWETASRLLTERKLSLEPLIAHRGSFESFTQAVRDIARNAMQGKVLLIP

>gi|30063529|ref|NP_837700.1| galactitol utilization operon repressor [Shigella flexneri 2a str. 2457T]

MTMNSFERRNKIIQLVNEQGTVLVQDLAGVFAASEATIRADLRFLEQKGVVTRFHGGAAKIMSGNSETET

QEVGFKERFQLASAPKNRIAQAAVKMIHEGMTVILDSGSTTMLIAEGLMTAKNITVITNSLPAAFALSEN

KDITLVVCGGTVRHKTRSMHGSIAERSLQDINADLMFVGADGIDAVNGITTFNEGYSISEAMVTAANKVI

AVLDSSKFNRRGFNQVLPIEKIDIIVTDDAVSEVDKLALQKTRVKLITV

>gi|30063528|ref|NP_837699.1| lipid kinase [Shigella flexneri 2a str. 2457T]

MAEFPASLLILNGKSTDNLPLREAIMLLREEGMTIHVRVTWEKGDAARFVEEARKLGVATVIAGGGDGTI

NEVSTALIQCEGDDIPALGILPLGTANDFATSVGIPEALDKALKLAIAGNAIAIDMAQVNKQTCFINMAT

GGFGTRITTETPEKLKAALGGVSYIIHGLMRMDTLQPDRCEIRGENFHWQGDALVIGIGNGRQAGGGQQL

CPNALINDGLLQLRIFTGDEILPALVSTLKSDEDNPNIIEGASSWFDIQAPHEITFNLDGEPLSGQNFHI

EILPAALRCRLPPDCPLLR

>gi|30063527|ref|NP_837698.1| hypothetical protein S2273 [Shigella flexneri 2a str. 2457T]

MPLLYLNTRECRWYLMGEGEMKKIAAISLISIFLISGCAVHNDETSIGKFGLAYKSNIQRKLDNQYYTEA

EASLARGRISGAENIVKNDAAHFCVTQGKKMQIVDLKTEGAGLHGVARLTFKCGE

>gi|30063526|ref|NP_837697.1| hypothetical protein S2271 [Shigella flexneri 2a str. 2457T]

MFKPELLSPAGTLKNMRYAFAYGADAVYAGQPRYSLRVRNNEFNHENLQLGINEAHALGKKFYVVVNIAP

HNAKLKTFIRDLKPVVEMGPDALIMSDPGLIMLVREHFPEMPIHLSVQANAVNWATVKFWQQMGLTRVIL

SRELSLEEIEEIHNQVPDMEIEIFVHGALCMAYSGRCLLSGYINKRDPNQGTCTNACRWEYNVQEGKEDD

VGNIVHKYEPIPVQNVEPTLGIGAPTDKVFMIEEAQRPGEYMTAFEDEHGTYIMNSKDLRAIAHVERLTK

MGVHSLKIEGRTKSFYYCARTAQVYRKAIDDAAAGKPFDTSLLETLEGLAHRGYTEGFLRRHTHDDYQNY

EYGYSVSDRQQFVGEFTGERKGDLAAVAVKNKFSVGDSLELMTPQGNINFTLEHMENAKGEAMPVAPGDG

YTVWLPVPQDLELNYALLMRNFSGETTRNPHGK

>gi|30063525|ref|NP_837696.1| hypothetical protein S2270 [Shigella flexneri 2a str. 2457T]

MLFSISNFNQGVIMAGWFELSKSSDNQFRFVLKAGNGETILTSELYTSKASAEKGIASVRSNSPQEERYE

KKTASNGKFYFNLKAANHQIIGSSQMYATAQSRETGIASVKANGTSQTVKDNT

>gi|30063524|ref|NP_837695.1| DNA-binding transcriptional regulator BaeR [Shigella flexneri 2a str. 2457T]

MTELPIDENTPRILIVEDEPKLGQLLIDYLRAASYAPTLISHGDQVLAYVRQTPPDLILLDLMLPGTDGL

TLCREIRRFSDVPIVMVTAKIEEIDRLLGLEIGADDYICKPYSPREVVARVKTILRRCKPQRELQQQDAE

SPLIIDEGRFQASWRGKMLDLTPAEFRLLKTLSHEPGKVFSREQLLNHLYDDYRVVTDRTIDSHIKNLRR

KLESLDAEQSFIRAVYGVGYRWEADACRIV

>gi|30063523|ref|NP_837694.1| signal transduction histidine-protein kinase BaeS [Shigella flexneri 2a str. 2457T]

MKFWRPGITGKLFLAIFATCIVLLISMHWAVRISFERGFIDYIKHGNEQRLQLLSDALGEQYAQHGNWRF

LRNNDRFVFQILRSFEHDNSEDKPGPGMPPHGWRTQFWVVDQNNKVLVGPRAPIPPDGTRRPILVNGAEV

GAVIASPVERLTRNTDINFDKQQRQTSWLIVALATLLAALATFLLARGLLAPVKRLVDGTHKLAAGDFTT

RVTPTSEDELGKLAQDFNQLASTLEKNQQMRRDFMADISHELRTPLAVLRGELEAIQDGVRKFTPETVAS

LQAEVGTLTKLVDDLHQLSMSDEGALAYQKAPVDLIPLLEVAGGAFRERFASRGLKLQFSLPDSITVFGD

RDRLMQLFNNLLENSLRYTDSGGSLKISAEQHDKTVRLTFADSAPGVSDDQLQKLFERFYRTEGSRNRAS

GGSGLGLAICLNIVEAHNGRIIAAHSPFGGVSITVELPLERDLQREV

>gi|30063522|ref|NP_837693.1| multidrug efflux system protein MdtE [Shigella flexneri 2a str. 2457T]

MTDLPDSTRWQLWIVAFGFFMQSLDTTIVNTALPSMAQSLGESPLHMHMVIVSYVLTVAVMLPASGWLAD

KVGVRNIFFTAIVLFTLGSLFCALSGTLNELLLARALQGVGGAMMVPVGRLTVMKIVPREQYMAAMTFVT

LPGQVGPLLGPALGGLLVEYASWHWIFLINIPVGIIGAIATLMLMPNYTMQTRRFDLSGFLLLAVGMAVL

TLALDGSKGTGLSPLAIAGLVAVGVVALVLYLLHARNNNRALFSLKLFRTRTFSLGLAGSFAGRIGSGML

PFMTPVFLQIGLGFSPFHAGLMMIPMVLGSMGMKRIVVQVVNRFGYRRVLVATTLGLSLVTLLFMTTALL

GWYYVLPFVLFLQGMVNSTRFSSMNTLTLKDLPDNLASSGNSLLSMIMQLSMSIGVTIAGLLLGLFGSQH

ISVDSGTTQTVFMYTWLSMAFIIALPAFIFARVPNDTHQNVAISRRKRSAQ

>gi|30063521|ref|NP_837692.1| multidrug efflux system subunit MdtC [Shigella flexneri 2a str. 2457T]

MKFFALFIYRPVATILLSVAITLCGILGFRMLPVAPLPQVDFPVIMVSASLPGASPETMASSVATPLERS

LGRIAGVSEMTSSSSLGSTRIILQFDFDRDINGAARDVQAAINAAQSLLPSGMPSRPTYRKANPSDAPIM

ILTLTSDTYSQGELYDFASTQLAPTISQIDGVGDVDVGGSSLPAVRVGLTPQALFNQGVSLDDVRTAISN

ANVRKPQGALEDGTHRWQIQTNDELKTAAEYQPLIIHYNNGGAVRLGDVATVTDSVQDVRNAGMTNAKPA

ILLMIRKLPEANIIQTVDSIRAKLPELQETIPAAIDLQIAQDRSPTIRASLEEVEQTLIISVALVILVVF

LFLRSGRATIIPAVAVPVSLIGTFAAMYLCGFSLNNLSLMALTIATGFVVDDAIVVLENIARHLEAGMKP

LQAALQGTREVGFTVLSMSLSLVAVFLPLLLMGGLPGRLLREFAVTLSVAIGISLLVSLTLTPMMCGWML

KASKPREQKRLRGFGRMLVALQQGYGKSLKWVLNHTRLVGVVLLGTIALNISIPKTFFPEQDTGVLMGGI

QADQSISFQAMRGKLQDFMKIIRDDPAVDNVTGFTGGSRVNSGMMFITLKPRDERSETAQQIIDRLRVKL

AKEPGANLFLMAVQDIRVGGRQSNASYQYTLLSDDLAALREWEPKIRKKLATLPELADVNSDQQDNGAEM

NLVYDRDTMARLGIDVQAANSLLNNAFGQRQISTIYQPMNQYKVVMEVDPRYTQDISALEKMFVINNEGK

AIPLSYFAKWQPANAPLSVNHQGLSAASTISFNLPTGKSLSDASAAIDRAMTQLGVPSTVRGSFAGTAQV

FQETMNSQVILIIAAIATVYIVLGILYESYVHPLTILSTLPSAGVGALLALELFNAPFSLIALIGIMLLI

GIVKKNAIMMVDFALEAQRHGNLTPQEAIFQACLLRFRPIMMTTLAALFGALPLVLSGGDGSELRQPLEI

TIVGGLVMSQLLTLYTTPVVYLFFDRLRLRFSRKPKQTVTE

>gi|30063520|ref|NP_837691.1| ISSfl4 orf [Shigella flexneri 2a str. 2457T]

MTESSDYESVQVFIGVDVGKDTHHAVAINRSGKRLFDKALPNDENKLRSLISDLKQHGQILLVVDQPATI

GALPVAVARSEGVLVGYLPGLAMRRIADLHAGEAKTDARDAAIIAEAARTLPHALRTLKLADEQIAELSM

LCGFDDDLAAQTTQASNRIRGLLTQIHPAPERVLGPRLEHPAVLDLLQRYPSPEKLASLGEKKLAAQLCK

LAPRLGKRLAADIAQALAEQTVVVPGTNAAAVVLPRLALQLITLRKQRDEVALEVEQRVLAHPLYPVLTS

MPGVGVRTAARLLTEVACRAFASAAHLAAYAGLAPVTRRSGSSIRGEHPSRRGNKALKRALFLSAFAALR

DPLSRAYYTRKMSQGKRHNQVLIALARRRCDVLFAMMRDGTFYTPQGS

>gi|30063519|ref|NP_837690.1| hypothetical protein S2262 [Shigella flexneri 2a str. 2457T]

MSEQITFATSDFASNPEPRCPCILLLDVSGSMSGRPINELNTGLVTFRDELLADSLALKRVELGIVTFGP

VHVEQPFTSAANFFPPILFAQGDTPMGAAITKALDMVEERKREYRANGISYYRPWIFLITDGAPIDEWQA

AANKVFRGEEDKRFAFFSIGVQGADMKTLAQISVRQPLPLQGLQFRELFSWLSSSLRSVSRSTPGTEVVL

EAPKGWTSV

>gi|30063518|ref|NP_837689.1| hypothetical protein S2261 [Shigella flexneri 2a str. 2457T]

MSWRLVYASAVGTSHISADLPCQDACQMQVAWLNDQQPLLVMFLADGAGSVSQGGEGAMLAVNEAMAYMS

QKVQGGELGLNDILATDIVLTVRQRLFAEAEAKELAVRDFACTFLGLISSANGTLIMQIGDGGVVVDFGH

GLQLPLTPMVGEYANMTHFITDEDAVSRLETFTSTERVHKVAAFTDGIQRLALNMLDNSPHVPFFTPFFN

GLASATQEQLDLLPELLKQFLSSPAVNERTDDDKTLALALWLP

>gi|30063517|ref|NP_837688.1| 3-methyladenine DNA glycosylase [Shigella flexneri 2a str. 2457T]

MYILNWQPPYDWSWMLGFLAARAVSGVETVADSYYARSLAVGEYRGVVTAIPDIARHTLHINLSAGLEPV

AAECLAKMSRLLDLQCNPQIVNGALGKLGAARPGLRLPGSVDAFEQGVRAILGQLVSVAMAAKLTARVAQ

LYGERLDDFPEYICFPTPQRLAAADPQALKALGMPLKRAEALIHLANAALEGTLPMTIPGDVEQAMKTLQ

TFPGIGRWTANYFALRGWQAKDVFLPDDYLIKQRFLGMTPAQIRRYAERWKPWRSYALLHIWYTEGWQPD

EA

>gi|30063516|ref|NP_837687.1| ISSfl4 orf [Shigella flexneri 2a str. 2457T]

MTESSDYESVQVFIGVDVGKDTHHAVAINRSGKRLFDKALPNDENKLRSLISDLKQHGQILLVVDQPATI

GALPVAVARSEGVLVGYLPGLAMRRIADLHAGEAKTDARDAAIIAEAARTLPHALRTLKLADEQIAELSM

LCGFDDDLAAQTTQASSRIRGLLTQIHPAPERVLGPRLEHPAVLDLLQRYPSPEKLASLGEKKLAAQLCK

LAPRLGKRLAADIAQALAEQTVVVPGTNAAAVVLPRLALQLITLRKQRDEVALEVEQRVLAHPLYPVLTS

MPGVGVRTAARLLTEVACRAFASAAHLAAYAGLAPVTRRSGSSIRGEHPSRRGNKALKRALFLSAFTALR

DPLSRAYYTRKMSQGKRHNQALIALARRRCDVLFAMMRDGTFYTPQGS

>gi|30063515|ref|NP_837686.1| uridine kinase [Shigella flexneri 2a str. 2457T]

MTDQSHQCVIIGIAGASASGKSLIASTLYRELREQVGDEHIGVIPEDCYYKDQSHLSMEERVKTNYDHPS

AMDHSLLLEHLQALKRGSAIDLPVYSYVEHTRMKETVTVEPKKVIILEGILLLTDARLRDELNFSIFVDT

PLDICLMRRIKRDVNERGRSMDSVMAQYQKTVRPMFLQFIEPSKQYADIIVPRGGKNRIAIDILKAKISQ

FFE

>gi|30063514|ref|NP_837685.1| deoxycytidine triphosphate deaminase [Shigella flexneri 2a str. 2457T]

MRLCDRDIEAWLDEGRLSINPRPPVERINGATVDVRLGNKFRTFRGHTAAFIDLSGPKDEVSAALDRVMS

DEIVLDEGEAFYLHPGELALAVTLESVTLPADLVGWLDGRSSLARLGLMVHVTAHRIDPGWSGCIVLEFY

NSGKLPLALRPGMLIGALSFEPLSGPAARPYNRREDAKYRNQQGAVASRIDKD

>gi|30063513|ref|NP_837684.1| assembly protein [Shigella flexneri 2a str. 2457T]

MRRFLTTLMILLVVLVAGLSALVLLVNPNDFRDYMVKQVAARSGYQLQLDGPLRWHVWPQLSILSGRMSL

TAQGASQPLVRADNMRLDVALLPLLSHQLSVKQVMLKGAVIQLTPQTEAVRSEDAPVAPRDNTLPDLSDD

RGWSFDISSLKVADSVLVFQHEDDEQVTIRNIRLQMEQDPQHRGSFEFSGRVNRDQRDLTISLNGTVDAS

DYPHDLTAAIEQINWQLQGADLPKQGIQGQGSFQSQWQESHKRLSFNQISLTANDSTLSGQAQVTLTEKP

EWQLRLQFPQLNLDNLIPLNETANGENGAAQQGQSQSTLPRPVISSRIDEPAYQGLQGFTADILLQASNV

RWRGMNFTDVATQMTNKSGLLEITQLQGKLNGGKVSLPGTLDATSINPRINFQPRLENVEIGTILKAFNY

PISLTGKMSLAGDFSGADIDADAFRHNWQGQAHVEMTDTRMEGMNFQQMIQQAVERNGGDVKAAENFDNV

TRLDRFTTDLTLKDGVVTLNDMQGQSPVLALTGEGMLNLADQTCDTQFDIRVVGGWNGESKLIDFLKETP

VPLRVYGNWQQLNYSLQVDQLLRKHLQDEAKRRLNDWAERNKDSRNGKDVKKLLEKM

>gi|30063512|ref|NP_837683.1| transporter [Shigella flexneri 2a str. 2457T]

MEWIADPSIWAGLITLIVIELVLGIDNLVFIAILAEKLPPKQRDRARVTGLLLAMLMRLLLLASISWLVT

LTQPLFSFRSFTFSARDLIMLFGGFFLLFKATMELNERLEGKDSNNPTQRKGAKFWGVVTQIVVLDAIFS

LDSVITAVGMVDHLLVMMAAVVIAISLMLMASKPLTQFVNSHPTIVILCLSFLLMIGFSLVAEGFGFVIP

KGYLYAAIGFSVMIEALNQLAIFNRRRFLSANQTLRQRTTEAVMRLLSGQKEDAELDAETASMLVDHGNQ

QIFNPQERRMIERVLNLNQRTVSSIMTSRHDIEHIDLNAPEEEIRQLLERNQHTRLVVTDGDDAEDLLGV

VHVIDLLQQSLRGEPLNLRVLIRQPLVFPETLPLLPALEQFRNARTHFAFVVDEFGSVEGIVTLSDVTET

IAGNLPNEVEEIDARHDIQKNADGSWTANGHMPLEDLVQYVPLPLDEKREYHTIAGLLMEYLQRIPKPGE

EVQVGDYLLKTLQVESHRVQKVQIIPLRKDGEMEYEV

>gi|30063511|ref|NP_837682.1| polysaccharide export protein [Shigella flexneri 2a str. 2457T]

MMKSKMKLMPLLVSVTLISGCTVLPGSNMSTMGKDVIKQQDADFDLDKMVNVYPLTPRLIDQLRPRPNVA

RPNMTLESEIANYQYRVGPGDVLNVTVWDHPELTTPAGQYRSSSDTGNWVQPDGTMFYPYIGKVHVVGKT

LAEIRSDITGRLATYIADPQVDVNIAAFRSQKAYISGQVNKSGQQAITNVPLTILDAINAAGGLTDTADW

RNVVLTHNGREERISLQALMQNGDLNQNRLLYPGDILYVPRNDDLKVFVMGEVKKQSTLKMDFSGMTLTE

ALANAEGIDMTTSNASGIFVIRPLKGEGGRNGKIANIYQLDMSDATSLVMATEFRLQPYDVVYVTTAPVS

RWNRLINQLLPTISGVRYMTDTASDIHNW

>gi|30063510|ref|NP_837681.1| tyrosine phosphatase [Shigella flexneri 2a str. 2457T]

MFNNILVVCVGNICRSPMAERLLQRYHPELKVESAGLGALVGKGADPTAISVAAEHQLSLEGHCARQISR

RLCRNYDLILTMDKRHIERLCEMAPEMRGKVMLFGHWDNECEIPDPYHKSRETFAAVYTLLERSARQWAQ

ALNAEQV

>gi|30063508|ref|NP_837679.1| colanic acid biosynthesis acetyltransferase WcaB [Shigella flexneri 2a str. 2457T]

MLEDLRANSWSLRPCCMVLAYRVAHFCSVWRKKNVLNNLWAAPLLVLYRIITECFFGYEIQAAATIGRRF

TIHHGYAVVINKNVVAGDDFTIRHGVTIGNRGADNMACPHIGNGVELGANVIILGDITLGNNVTVGAGSV

VLDSVPDNALVVGEKARVKVIK

>gi|30063507|ref|NP_837678.1| IS1 orfB [Shigella flexneri 2a str. 2457T]

MSRQCTHYGRWPQHGFTSLKKLRPQSVTSRIQPGSDVIVCAEMDEHWGYVGAKSRQRWLFYAYDRIRRTV

VAHVFGERTLATLERLLSLLSAFEVVVWMTDGWPLYESRLKGKLHVISKRYTQRIERHNLNLRQHLARLV

RKSLSFSKSVELHDKVIGHYLNIKHYQ

>gi|30063506|ref|NP_837677.1| IS1 orfA [Shigella flexneri 2a str. 2457T]

MASISIRCPSCSATEGVVRNGKSTAGHLRYLCSHCRKTWQLQFTYTASQPGTHQKIIDMAMNGVGCRASA

RIMGVGLNTVLRHLKNSGRSR

>gi|30063505|ref|NP_837676.1| colanic acid biosynthesis acetyltransferase WcaF [Shigella flexneri 2a str. 2457T]

MQDLSGFSVPKGFRGGNAIKVQLWWAVQATIFAWSPQVLYRWRAFLLRLFGAKIGKNVVIRPSVKITYPW

KLTLGDYAWVGDDVNLYTLGEITIGAHSVISQKSYLCTGSHDHASQHFTINATPIVIGEKCWLATDVFVA

PGVTIGDGTVVGARSSVFKSLPANVVCRGNPAVVIRERVETE

>gi|30063504|ref|NP_837675.1| GDP-D-mannose dehydratase [Shigella flexneri 2a str. 2457T]

MSKVALITGVTGQDGSYLAEFLLEKGYEVHGIKRRASSFNTERVDHIYQDPHTCNPKFHLHYGDLSDTSN

LTRILREVQPDEVYNLGAMSHVAVSFESPEYTADVDAMGTLRLLEAIRFLGLEKKTRFYQASTSELYGLV

QEIPQKETTPFYPRSPYAVAKLYAYWITVNYRESYGMYACNGILFNHESPRRGETFVTRKITRAIANIAQ

GLESCLYLGNMDSLRDWGHAKDYVKMQWMMLQQEQPEDFVIATGVQYSVRQFVEMAAAQLGIKLRFEGTG

VEEKGIVVSVTGHDAPGVKPGDVIIAVDPRYFRPAEVETLLGDPTKAHEKLGWKPEITLREMVSEMVAND

LEAAKKHSLLKSHGYDVAIALES

>gi|30063503|ref|NP_837674.1| nucleotide di-P-sugar epimerase or dehydratase [Shigella flexneri 2a str. 2457T]

MSKQRIFIAGHCGMVGSAIKRQLEQRGDVELVLRTRDELNLLDSRAVHDFFASERIDQVYLAAAKVGGIV

ANNTYPADFIYQNMMIESNIIHAAHQNDVNKLMFLGSSCIYPKLAKQPMAESELLQGTLEPTNEPYAIAK

IAGIKLCESYNRQYGRDYRTVMPTNLYGPHDNFHPSNSHVIPALLRRFHEATAQNAPDVVVWGSGTPMRE

FLHVDDMAAASIHVMELAHEVWLENTQPMLSHINVGTGVDCTIRELAQTIAKVVGYKGRVVFDASKPDGT

PRKLLDVTRLHQLGWYHEISLEAGLASTYQWFLENQDRFRGE

>gi|30063502|ref|NP_837673.1| GDP-mannose mannosyl hydrolase [Shigella flexneri 2a str. 2457T]

MFLRQEDFATVVRSTPLVSLDFIVENSRGEFLLGKRTNRPAQGYWFVPGGRVQKDETLEAAFERLTMAEL

GLRLPITAGQFYGVWQHFYDDNFSGTDFTTHYVVLGFRFRVAEEELLLPDEQHDDYRWLTPDALLASNDV

HANSRAYFLAEKRAGVPGL

>gi|30063501|ref|NP_837672.1| glycosyl transferase [Shigella flexneri 2a str. 2457T]

MKILVYGINYSPELTGIGKYTGEMVEWLAAQGHEVRVITAPPYYPQWQVGENYSAWRYKREEGAATVWRC

PLYVPKQPSTLKRLLHLGSFAVSSFFPLMAQRRWKPDRIIGVVPTLFCTPGMRLLAKLSGARTVLHIQDY

EVDAMLGLGLAGKGKGGKVAQLATAFERSGLHNVDNVSTISRSMMNKAIEKGVAADNVIFFPNWSEIARF

QHVADADVDALRNQLGLPDNKKIILYSGNIGEKQGLENVIEAADLLRDEPLIFAIVGQGGGKARLEKMAQ

QRGLRNMQFFPLQSYDALPALLKMGDCHLVVQKRGAADAVLPSKLTNILAVGGNAVITAEAHTELGQLCE

TFPGIAVCVEPESVEALVAGIRQALLLPKHNTVAREYAERTLDKENVLRQFINDIRG

>gi|30063500|ref|NP_837671.1| mannose-1-phosphate guanyltransferase [Shigella flexneri 2a str. 2457T]

MAQSKLYPVVMAGGSGSRLWPLSRVLYPKQFLCLKGDLTMLQTTICRLNGVECESPVVICNEQHRFIVAE

QLRQLNKLTENIILEPAGRNTAPAIALAALAAKRHSPESDPLMLVLAADHVIADEDAFRAAVRNAMPYAE

AGKLVTFGIVPDLPETGYGYIRRGEVSVGEQDTVAFEVAQFVEKPNLETAQAYVASGEYYWNSGMFLFRA

GRYLEELKKYRPDILDACEKAMSAVDPDLDFIRVDEEAFLACPEESVDYAVMEPTADAVVVPMDAGWSDV

GSWSSLWEISAHTAEGNVCHGDVINHKTENSYVYAESGLVTTVGVKDLVVVQTKDAVLIADRNAVQDVKK

VVEQIKADGRHEHRVHREVYRPWGKYDSIDAGDRYQVKRITVKPGEGLSVQMHHHRAEHWVVVAGTAKVT

IDGDIKLLGENESIYIPLGATHCLENPGKIPLDLIEVRSGSYLEEDDVVRFADRYGRV

>gi|30063499|ref|NP_837670.1| phosphomannomutase [Shigella flexneri 2a str. 2457T]

MKKLTCFKAYDIRGKLGEELNEDIAWRIGRAYGEFLKPKTIVLGGDVRLTSETLKLALAKGLQDAGVDVL

DIGMSGTEEIYFATFHLGVDGGIEVTASHNPMDYNGMKLVREGARTISGDTGLRDVQRLAEANDFPPVDE

TKRGRYQQINLRDAYVDHLFGYINVKNLTPLKLVINSGNGAAGPVVDAIEARFKALGAPVELIKVHNTPD

GNFPNGIPNPLLPECRDDTRNAVIKHGADMGIAFDGDFDRCFLFDEKGQFIEGYYIVGLLAEAFLEKNPG

AKIIHDPRLSWNTIDVVTAAGGTPVMSKTGHAFIKERMRKEDAIYGGEMSAHHYFRDFAYCDSGMIPWLL

VAELVCLKGKTLGELVRDRMAAFPASGEINSKLAQPVEAINRVEQHFSREALAVDRSDGISMTFADWRFN
LRTSNTEPVVRLNVESRGDVPLMEARTRTLLTLLNE

>gi|30063498|ref|NP_837669.1| UDP-glucose lipid carrier transferase [Shigella flexneri 2a str. 2457T]
MTNLKKRERAKTNASLISMVQRFSDITIMFAGLWLVCEVSGLSFLYMHLLVALITLVVFQMLGGITDFYR
SWRGVRAATEFALLLQNWTLSVIFSAGLVAFNNDFDTQLKIWLAWYGLTSIGLVVCRSCIRIGAGWLRNH
GYNKRMVAVAGDLAAGQMLMESFRNQPWLGFEVVGVYHDPKPGGVSNDWAGNLQQLVEDAKAGKIHNVYI
AMQMCDGARVKKLVHQLADTTCSVLLIPDVFTFNILHSRLEEMNGVPVVPLYDTPLSGVNRLLKRAEDIV
LATLILLLISPVLCCIALAVKLSSPGPVIFRQTRYGMDGKPIKVWKFRSMKVMENDKVVTQATQNDPRVT
KVGNFLRRTSLDELPQFINVLTGGMSIVGPRPHAVAHNEQYRQLIEGYMLRHKVKPGITGWAQINGWRGE
TDTLEKMEKRVEFDLEYIREWSVWFDIKIVFLTVFKGFVNKAAY

>gi|30063497|ref|NP_837668.1| colanic acid exporter [Shigella flexneri 2a str. 2457T]
MSLREKTISGAKWSAIATVIIIGLGLVQMTVLARIIDNHQFGLLTVSLVIIALADTLSDFGIANSIIQRK
EISHLELTTLYWLNVGLGLVVCVAVFLLSDVIGDVLNNPDLAPLIKTLSLAFVVIPHGQQFRALMQKELE
FNKIGMIETSAVLAGFTFTVVSAHFWPLAMTAILGYLVNSAVRTLLFGYFGRKIYCPGLHFSLASVAPNL
RFGAWLTADSIINYLNTNLSTLVLARILGAGVAGGYNLAYNVAVVPPMKLNPIITRVLFPAFAKIQDDTE
KLRVNFYKLLSIVGIINFPALLGLMVVSNNFVPLVFGEKWNSIIPVLQLLCIVGLLRSVGNPIGSLLMAK
ARVDISFKFNVFKTFLFIPAIVIGGQMAGAIGVTLGFLLVQIINTILSYFVMIKPVLGSSYRQYILSLWL
PFYLSLPTLVVSYALGIVLKGQLALGMLLAVQIAAGVLAFVVMIVLSRHPLVVEVKRQFCRSEKMKMLLR
AG

>gi|30063496|ref|NP_837667.1| pyruvyl transferase [Shigella flexneri 2a str. 2457T]
MKLLILGNHTCGNRGDSAILRGLLDAINILNPHTEVDVMSRYPVSSSWLLNRPVMGDPLFLQMTQHNSAA
GVVGRIKKVLRRRYQHQVLLSRVTDTGKLRNIAIAQGFTDFVRLLSGYDAIIQVGGSFFVDLYGVPQFEH

ALCTFMAKKPLFMIGHSVGPFQDEQFNQLANYVFGHCDALILRESVSLDLMKRRNITTAKVEHGVDTAWL

VDHHTEDFTASYAVQHWLDVAAQQKTVAITLRELAPFDKRLGTTQQAYEKAFAGVVNRILDEGYQVIALS

TCTGIDSYNKDDRMVALNLRQHISDPARYHVVMDELNDLEMGKILGACELTVGTRLHSAIISMNFATPAI

AINYEHKSAGIMQQLGLPEMAIDIRHLLDGSLQAMVADTLGQLPVLNARFNEAVSRERQTGMQMVQSVLE

RIGEVK

>gi|30063495|ref|NP_837666.1| colanic acid biosynthesis glycosyl transferase [Shigella flexneri 2a str. 2457T]

MKVGFFLLKFPLSSETFVLNQITAFIDMGFEVEIVALQKGDTENTHAAWTKYNLVARTRWLQDEPAGKVA

KLRHRASQTLRGIHRKNTWHALNLKRYGAESRNLILSAICGQVATPFRADVFIAHFGPAGVTAAKLRELG

IIRGKIATIFHGIDISSREVLNHYTPEYQQLFRRGDLMLPISDLWAGRLQKMGCPREKIAVSRMGVDMTR

FSPRPVKAPATPLEIISVARLTEKKGLHVAIEACRQLKEQGVAFRYRILGIGLWERRLRTIIEQYQLEDV

VEMPGFKPSHEVKEMLDDADVFLLPSVTGADGDMEGIPVALMEAMAVGIPVVSTLHSGIPELVEADKSGW

LLPENDARALAQRLAAFSQLDTDELAPVVKRACEKVEHDFNQQVINRELASLLQAL

>gi|30063494|ref|NP_837665.1| colanic acid biosynthesis protein [Shigella flexneri 2a str. 2457T]

MPFKTLSRRTFLTASSALAFLHTPFARALPARQSVNINDYNPHDWIASFKQAFSEGQTVVVPAGFVCDNI

NTGIFIPPGKTLHILGSLRGNGRGRFVLQDGSQVTGGEGGGMHNITLDVRGSDCTIKGLAMSGFGPVMQI

YIGGKNKRVMRNLTIDNLTVSHANYAILRQGFHNQIIGANITNCKFSDLQGDAIEWNVAINDSDILISDH

VIERINCTNGKINWGIGIGLAGSTYDNNYPEDQAVKNFVVANITGSDCRQLIHVENGKHFVIRNINARNI

TPDFSKKAGIDNATVAIYGCDNFVIDNIEMINSAGMLIGYGVIKGKYLSIPQNFRVNNIQLDNTHLAYKL

RGIQISAGNAVSFVSLTNIEMKRASLELHNKPQHLFMRNINVMQESSVGPALSMNFDMRKDVRGVFMAKK

ETLLSLANVHAVNERGQSSVDIDRINHHIVNVEKINFRLPERRE

>gi|30063493|ref|NP_837664.1| UTP--glucose-1-phosphate uridylyltransferase subunit GalF [Shigella flexneri 2a str. 2457T]

MTNLKAVIPVAGLGMHMLPATKAIPKEMLPIVDKPMIQYIVDEIVAAGIKEILLVTHASKNAVENHFDTS

YELESLLEQRVKRQLLAEVQSICPPGVIIMNVRQGEPLGLGHSILCARPAIGDNPFVVVLPDVVIDDASA

DPLRYNLAAMIARFNETGRSQVLAKRMPGDLSEYSVIQTKEPLDREGKVSRIVEFIEKPDQPQTLDSDIM

AVGRYVLSADIWPELERTQPGAWGRIQLTDAIAELAKKQSVDAMLMTGDSYDCGKKMGYMQAFVKYGLRN

LKEGAKFRKGIEKLLSE

>gi|30063492|ref|NP_837663.1| dTDP-glucose 4,6 dehydratase [Shigella flexneri 2a str. 2457T]

MKILVTGGAGFIGSAVVRHIINNTQDSVVNVDKLTYAGNLESLADVSDSERYAFEHADICDAVAMSRIFA

QHQPDAVMHLAAESHVDRSITGPAAFIETNIVGTYVLLEAARNYWSALNDEKKKSFRFHHISTDEVYGDL

PHPDEANNNEALPLFTETTAYAPSSPYSASKASSDHLVRAWKRTYGLPTIVTNCSNNYGPYHFPEKLIPL

VILNALEGKALPIYGKGDQIRDWLYVEDHARALYTVVTEGKAGETYNIGGHNEKKNIDVVLTICDLLDEI

VPKEKSYREQITYVADRPGHDRRYAIDADKISRELGWKPQETFESGIRKTVEWYLANTNWVENVKSGTYQ

SWIEQNYEGRQ

>gi|30063491|ref|NP_837662.1| dTDP-4-dehydrorhamnose reductase [Shigella flexneri 2a str. 2457T]

MNILLFGKTGQVGWELQRALAPLGNLIALDVHSTDYCGDFSNPEGVAETVKKIRPDVIVNAAAHTAVDKA

ESEPNFAQLLNATCVEAIAKAANEVGAWVIHYSTDYVFPGNGDTPWLETDATAPLNVYGETKLAGEKALQ

EHCAKHLIFRTSWVYAGKGNNFAKTMLRLAKEREELAVINDQFGAPTGAELLADCTAHAIRVAANKPEVA

GLYHLVAGGTTTWHDYAALVFEEARRAGINLALNKLNAVPTTAYPTPARRPHNSRLNTEKFQQNFALVLP

DWQVGVKRMLNELFTTTAI

>gi|30063490|ref|NP_837661.1| glucose-1-phosphate thymidylyltransferase [Shigella flexneri 2a str. 2457T]

MKTRKGIILAGGSGTRLYPVTMAVSKQLLPIYDKPMIYYPLSTLMLAGIRDILIISTPQDTPRFQQLLGD

GSQWGLNLQYKVQPSPDGLAQAFIIGEEFIGGDDCALVLGDNIFYGHDLPKLMDTAVNRESGATVFAYHV

NDPERYGVVEFDDNGTAISLEEKPQEPKSNYAVTGLYFYDNDVVEMAKNLKPSARGELEITDINRIYMDQ

GRLSVAMMGRGYAWLDTGTHQSLIEASNFIATIEERQGLKVSCPEEIAHRKGFIDAEQVKVLAEPLKKNA

YGQYLLKMIKGY

>gi|30063489|ref|NP_837660.1| dTDP-6-deoxy-L-mannose-dehydrogenase [Shigella flexneri 2a str. 2457T]

MNVIKTEIPDVLIFEPKVFGDERGFFMESFNQKVFEEAVGRKVEFVQDNHSKSTKGVLRGLHYQLEPYAQ

GKLVRCVVGEVFDVAVDIRKSSPTFGKWVGVNLSAENKRQLWIPEGFAHGFCVLSDEAEFVYKTNNFYSK

MQERGILWSDKSINIEWPVQNPLLSDKDINGQKFVDADYFI

>gi|30063488|ref|NP_837659.1| polysaccharide biosynthesis protein [Shigella flexneri 2a str. 2457T]

MSIIKNSVWNLFGYAIPTLIAIPSLGFLARGLGPEGFGVYTIAIALVGYAGIFDVGLTRSVIREIAIHRD

NHHERTKVISTSTSFLVLFSCFGAFLLLIFSDGIVNYLKISGVEHSDIQLAFKLLAICIPLFILNQLWSA

ILEGDEKFGIVNIQKSISSSCIAGIPAIFVFYSATLSAAVAGLIFARVISILVSAYYVRNDIKISGVHFC

YKTFKRLFFFGGWMTVSNIISPVMVYFDRFIVSNIMGADKVAFYSAPAEVILKLGIIPAAIGRAVFPRLS

NIKDFKEFKRNVNKSLLLMFLICLPVIIIGLLYSGLVLKIWFGENYQINSFNILNVLLIGFFFNALAMIP

FSAIQALGKSKITALIHCAELVPYLALLYFMVEKYGLLGAAISWSIRVILDALLLQWLYTRMCSVYEN

>gi|30063487|ref|NP_837658.1| dTDP-rhamnosyl transferase [Shigella flexneri 2a str. 2457T]

MNSNIYAVIVTYNPELKNLNALITELKEQNCYVVVVDNRTNFTLKDKLADIEKVHLICLGRNEGIAKAQN

IGIRYSLEKGAEKIIFFDQDSRIRNEFIKKLSCYMDNENAKIAGPVFIDRDKSHYYPICNIKKNGLREKI

HVTEGQTPFKSSVTISSGTMVSKEVFEIVGMMDEELFIDYVDTEWCLRCLNYGILVHIIPDIEMVHAIGD

KSVKICGINIPIHSPVRRYYRVRNAFLLLRKNHVPLLLSIREVVFSLIHTTLIIATQKNKIEYMKKHILA

TLDGIRGITGGGRYNA

>gi|30063486|ref|NP_837657.1| dTDP-rhamnosyl transferase [Shigella flexneri 2a str. 2457T]

MATYNGECWIEEQLKSIIEQKDVDISIFISDDLSTDNTLNICEEFQLSYPSIINILPSVNKFGGAGKNFY

RLIKDVDLENYDYICFSDQDDIWYKDKIKNAIDCLVFNNANCYSSNVIAYYPSGRKNLVDKAQSQTQFDY

FFEAAGPGCTYVIKKETLIEFKKFIINNKNAAQDICLHDWFLYSFARTRNYSWYIDRKPTMLYRQHENNQ

VGANISFKAKYKRLGLVRNKWYRKEVTKIANALADDSFVNNQLGKGYIGNLILALSFWKLRRKKADKIYI

LLMLILNIF

>gi|30063485|ref|NP_837656.1| O-antigen polymerase [Shigella flexneri 2a str. 2457T]

MNNINKIFITFLCIELIIGGGGRLLEPLGIFPLRYLLFVFSFILLIFNLVTFNFSITQKCVSLFIWLLLF

PFYGFFVGLLAGNKINDILFDVQPYLFMLSLIYLFTLRYTLKVFSCEIFIKIVNAFALYGSLLYISYIIL

LNFGLLNFNLIYEHLSLTSEFFFRPDGAFFSKSFYFFGVGAIISFVDKKYLKCLIIVLAILLTESRGVLL

FTTLSLLLASFKLHKLYLNTIIIILGSVLFIIMLYMVGSRSEDSDSVRFNDLYFYYKNVDLATFLFGRGF

GSFILDRLRIEIVPLEILQKTGVIGVFISLVPMLLIFLKGYFLNSTKTSLMMSLILFFSITVSITNPFLF

TPMGIFIIGVVVLWVFSIENIQISNNLTSGAK

>gi|30063484|ref|NP_837655.1| glycosyl translocase [Shigella flexneri 2a str. 2457T]

MLKIGKLLTSSFFSYFLIGIVNTALHWGVFYACYNNLAFGQGRSNIVGFICAATFSFFANARCSFKVSAT

KARYFIFIFFMGAMSYLFGVLFDLLALSPIFTLFTFSLFSLVLGYCASKYFIFR

>gi|30063483|ref|NP_837654.1| glycosyl transferase, partial [Shigella flexneri 2a str. 2457T]

MYHSFNIAVIIPCYNEQKAIAKVINDFKTNIPTASIYVFDNNSTDSTAQVAEDAGATVHSVPLKGKGNVV

RRMFSDVDADIYLMVDGDDTYDASSAPEMINHLIKNQLDMVVGCRQENGDQNTYRKGHR

>gi|30063482|ref|NP_837653.1| hypothetical protein S2216, partial [Shigella flexneri 2a str. 2457T]

MLSGYRVFSRRYVKSFPCLSRGFEIETELTIHALELRMKYGEVNTKYGERSEGSVSKLSTWSDGFKILKT

IIKLYSLERPLYFFSIIGVLLAALSIILGLPIIVDYIDTGLVRRFPTAFLTASIMLSSIMAFVCGIILHS

NTTTRREMKALFYLSEKNYKLIM

>gi|30063481|ref|NP_837652.1| hypothetical protein S2215 [Shigella flexneri 2a str. 2457T]

MSLNILIIYFLGMVGQFNKIAIFLIFTVCWVLSIIKRQQFRWLAINNIEFSTLFVILFLVLIFVVTLLSS

LRAPGDWDDTMYHLPLARSLVEHHAIVVEQYLRFPLFPQNADLLMALGLQLGDVRLAQFLANICFFVIAC

GLVGCSWEITKTYYPGIIATILLFTINPLKDHLGYAYIDLTLSLFCCSQYSYIYSLRKQ

>gi|30063480|ref|NP_837651.1| hypothetical protein S2214 [Shigella flexneri 2a str. 2457T]

MIAKKRNIQTFLIFSLATILGGIYWYVRSFWISGDPFSPAGGNIFGHYLWNEIDLQVQTAEQARHGISPA

TLDLQQAINKVGSDVVFYAILSVFTLRNNKILWVFFSIVLTYVLFWFSVTQGDRYLSPIYPLSVLQVAIS

IASLIKDINITNYKLGRYFILVCIIFFRGDRCILNSNIFILNT

>gi|30063479|ref|NP_837650.1| hypothetical protein S2213 [Shigella flexneri 2a str. 2457T]

MNFNSRLESRYSYELMKKASEYSELYGDNLIQLGLEDGIYFYKGMAIGDVFGLARYSDWTISNPECEVIP

QDDLIEKMKSFNSSFIVISKRSYANFNPEKYPKFKVLMDTPNGILIAIK

>gi|30063478|ref|NP_837649.1| 6-phosphogluconate dehydrogenase [Shigella flexneri 2a str. 2457T]

MSKQQIGVVGMAVMGRNLALNIESRGYTVSIFNRSREKTEEVIAENPGKKLAPYYTVKEFVESLETPRRI

LLMVKAGAGTDAAIDSLKPYLDKGDIIIDGGNTFFQDTIRRNRELSAEGFNFIGTGVSGGEEGALKGPSI

MPGGQKEAYELVAPILTKIAAVAEDGEPCVTYIGADGAGHYVKMVHNGIEYGDMQLIAEAYSLLKGGLNL

SNEELAQTFTEWNNGELSSYLIDITKDIFTKKDEDGNYLVDVILDEAANKGTGKWTSQSALDLGEPLSLI

TESVFARYISSLKDQRVAASKVLSGPQAQSAGDKAEFIEKVRSALYLGKIVSYAQGFSQLRAASEEYNWD

LNYGEIAKIFRAGCIIRAQFLQKITDAYAENPQIANLLLAPYFKQIADDYQQALRDVVAYAVQNGIPVPT

FAAAVAYYDSYRAAVLPANLIQAQRDYFGAHTYKRIDKEGVFHTEWLD

>gi|30063477|ref|NP_837648.1| regulator of length of O-antigen component of lipopolysaccharide chains [Shigella flexneri 2a str. 2457T]

MRTWKFPSVRVMMRVENNNVSGQNHDPEQIDLIDLLVQLWRGKMTIIISVIVAIALAIGYLAVAKEKWTS

TAIITQPDVGQIAGYNNAMNVIYGQAAPKVSDLQETLIGRFSSAFSALAETLDNQEEPEKLTIEPSVKNQ

QLPLTVSYVGQTAEGAQMKLAQYIQQVDDKVNQELEKDLKDNIALGRKNLQDSLRTQEVVAQEQKDLRIR

QIQEALQYANQAQVTKPQVQQTEDVTQDTLFLLGSEALESMIKHEATRPLVFSPNYYQTRQNLLDIEKLK

FDDLDIHAYRYVMKPTLPIRRDSPKKAITLILAVLLGGMVGAGIVLGRNALRNYNAK

>gi|30063476|ref|NP_837647.1| bifunctional phosphoribosyl-AMP cyclohydrolase/phosphoribosyl-ATP [Shigella flexneri 2a str. 2457T]

MLTEQQRRELDWEKTDGLMPVIVQHAVSGEVLMLGYMNPEALDKTIESGKVTFFSRTKQRLWIKGETSGN

FLNVVSIAPDCDNDTLLVLANPIGPTCHKGTSSCFGNTAHQWLFLYQLEQLLAERKYADPETSYTAKLYA

SGTKRIAQKVGEEGVETALAATVHDRFELTNEASDLMYHLLVLLQDQDLDLTTVIENLHKRHQ

>gi|30063475|ref|NP_837646.1| imidazole glycerol phosphate synthase subunit HisF [Shigella flexneri 2a str. 2457T]

MLAKRIIPCLDVRDGQVVKGVQFRNHEIIGDIVPLAKRYAEEGADELVFYDITASSDGRVVDKSWVSRVA

EVIDIPFCVAGGIKSLEDAAKILSFGADKISINSPALADPTLITRLADRFGVQCIVVGIDTWYDAETGKY

HVNQYTGDESRTRVTQWETLDWVEEVQKRGAGEIVLNMMNQDGVCNGYDLKQLKKVREVCHVPLIASGGA

GTMEHFLEAFRDADVDGALAASVFHKQIINIGELKAYLATQGVEIRIC

>gi|30063474|ref|NP_837645.1| 1-(5-phosphoribosyl)-5-[(5-phosphoribosylamino)methylideneamino] imidazole-4-carboxamide isomerase [Shigella flexneri 2a str. 2457T]

MIIPALDLIDGTVVRLHQGDYGKQRDYGNDPLPRLQDYAAQGAEVLHLVDLTGAKDPAKRQIPLIKTLVA

GVNVPVQVGGGVRSEEDVAALLEAGVARVVVGSTAVKSPDVVKGWFERFGADALVLALDVRIDEQGNKQV

AVSGWQENSGVSLEQLVETYLPVGLKHVLCTDISRDGTLAGSNVSLYEEVCARYPQVAFQSSGGIGDIND

VAALRGTGVRGVIVGRALLEGKFTVKEAIACWQNA

>gi|30063473|ref|NP_837644.1| imidazole glycerol phosphate synthase subunit HisH [Shigella flexneri 2a str. 2457T]

MNVVILDTGCANLNSVKSAIARHGYEPKVSRDPDIVLLADKLFLPGVGTAQAAMDQVRERELFDLIKACT

QPVLGICLGMQLLGRRSEESNGVDLLGIIDEDVPKMTDFGLPLPHMGWNRVYPQAGNRLFQGIEDGAYFY

FVHSYAMPVNPWTIAQCNYGEPFTAAVQKDNFYGVQFHPERSGAAGAKLLKNFLEM

>gi|30063472|ref|NP_837643.1| imidazole glycerol-phosphate dehydratase/histidinol phosphatase [Shigella flexneri 2a str. 2457T]

MSQKYLFIDRDGTLISEPPSDFQVDRFDKLAFEPGVIPELLKLQKAGYKLVMITNQDGLGTQSFPQADFD

GPHNLMMQIFTSQGVQFDEVLICPHLPADECDCRKPKVKLVEGYLAEQAMDRANSYVIGDRATDIQLAEN

MGINGLRYDRETLNWPMIGEQLTKRDRYAHVVRNTKETQIDVQVWLDREGGSKINTGVGFFDHMLDQIAT

HGGFRMEINVKGDLYIDDHHTVEDTGLALGEALKIALGDKRGICRFGFVLPMDECLARCALDISGRPHLE

YKAEFTYQRVGDLSTEMIEHFFRSLSYTMGVTLHLKTKGKNDHHRVESLFKAFGRTLRQAIRVEGDTLPS

SKGVL

>gi|30063471|ref|NP_837642.1| histidinol-phosphate aminotransferase [Shigella flexneri 2a str. 2457T]

MSTVTITDLARENVRNLTPYQSARRLGGNGDVWLNANEYPTAVEFKLTQQTLNRYPECQPKAVIENYAQY

AGVKPEQVLVSRGADEGIELLIRAFCEPGKDAILYCPPTYGMYSVSAETIGVECRTVPTLENWQLDLQGI

SDKLDGVKVVYVCSPNNPTGQLINPQDFRTLLELTRGKAIVVADEAYIEFCPQASLAGWLAEYPHLAILR

TLSKAFALAGLRCGFTLANEEVINLLMKVIPPYPLSTPVADIAAQALSPQGIVAMSERVAQIITEREYLI

AALKEIPCVEQVFDSETNYILARFKASSAVFKSLWDQGIILRDQNKQPSLSGCLRITVGTREESQRVIDA

LRAEQV

>gi|30063470|ref|NP_837641.1| histidinol dehydrogenase [Shigella flexneri 2a str. 2457T]

MSFNTIIDWNSCTAEQQRQLLMRPAISASESITRTVNDILDNVKTRGDEALREYSAKFDKTTVTALKVSA

DEIAAASERLSDELKQAMAVAVKSIETFHTAQKLPPVDVETQLGVRCQQVTRPVASVGLYIPGGSAPLFS

TVLMLATPARIAGCKKVVLCSPPPIADEILYAAQLCDVQDVFNVGGAQAIAALAFGTESVPKVDKIFGPG

NAFVTEAKRQVSQRLDGAAIDMPAGPSEVLVIADSGATPDFVASDLLSQAEHGPDSQVILLTPDADMARR

VAEAVERQLAELPRAETTRQALNASRLIVTKDLAQCVEISNQYGPEHLIIQTRNARELVDGITSAGSVFL

GDWSPESAGDYASGTNHVLPTYGYTATCSSLGLADFQKRMTVQELSKEGFSALASTIETLAAAERLTAHK

NAVTLRVNVLKEQA

>gi|30063469|ref|NP_837640.1| ATP phosphoribosyltransferase [Shigella flexneri 2a str. 2457T]

MTDNTRLRIAMQKSGRLSDDSRELLARCGIKINLHTQRLIAMAENMPIDILRVRDDDIPCLVMDGVVDLG

IIGENVLEEELLNRRAQGEDPRYFTLRRLDFGGCRLSLATPVDEAWDGPLSLNGKRIATSYPHLLKRYLD

QKGISFKSCLLNGSVEVAPRAGLADAICDLVSTGATLEANGLREVEVIYRSKACLIQRDGEMEESKQQLI

DKLLTRIQGVIQARESKYIMMHAPTERLDEVIALLPGAERPTILPLAGDQQRVAMHMVSSETLFWETMEK

LKALGASSILVLPIEKMME

>gi|30063468|ref|NP_837639.1| antitoxin YefM [Shigella flexneri 2a str. 2457T]

MRTISYSEARQNLSATMMKAVEDHAPILITRQNGEACVLMSLEEYNSLEETAYLLRSPANARRLMDSIDS

LKSGKGTEKDIIEWVMLPTY

>gi|30063467|ref|NP_837638.1| IS1 orfA [Shigella flexneri 2a str. 2457T]

MASISIRCPSCSATEGVVRNGKSTAGHQRYLCSHCRKTWQLQFTYTASQPGTHQKIIDMVMNGVGCRASA

RIMGVGLNTVLRHLKNSGRSR

>gi|30063466|ref|NP_837637.1| IS1 orfB [Shigella flexneri 2a str. 2457T]

MAHVFGERTLATLERLLSLLSAFEVVVWMTDGWPLYESRLKGKLHVISKRYTQRIERHNLNLRQHLARLV

RKSLSFSKSVELHDKVIGHYLNIKHYQ

>gi|30063465|ref|NP_837636.1| enzyme of sugar metabolism [Shigella flexneri 2a str. 2457T]

MKKVAIVGLGWLGMPLAMSLSARGWQVTGSKTTQDGVEAARMSGIDSYLLRMEPELVCDSDDLDALMDAD

ALVITLPARRSGPGDEFYLQAVQELVDSALAHRIPRIIFTSSTSVYGDAQGTVKETTPRNPVTNSGRVLE

ELEDWLHNLPGTSVDILRLAGLVGPGRHPGRFFAGKTAPDGEHGVNLVHLEDVIGAITLLLQAPKGGHIY

NICAPAHPARNVFYPQMARLLGLQPPQFRNSLDSGKGKIIDGSRICNELGFEYQYPDPLVMPLE

>gi|30063464|ref|NP_837635.1| LYSR-type transcriptional regulator [Shigella flexneri 2a str. 2457T]

MKPLLDVLMILDAIEKEGSFAAASAKLYKTPSALSYTVHKLESDLNIQLLDRSGHRAKFTRTGKMLLEKG

REVLHTVRELEKQAIKLHEGWENELVIGVDDTFPFSLLAPLIEAFYQHHSVTRLKFINGVLGGSWDALTQ

GRADIIVGAMHEPPSSSEFGFSRLGDLEQVFAVAPHHPLAQEEEPLNRRIIKRYRAIVVGDTAQAGASTA

SQLLDEQEAITVFDFKTKLELQISGLGCGYLPRYLAQRFLDSGALIEKKVVAQTLFEPVWIGWNEQTAGL

ASGWWRDEILANSAIAGVYAKSDDGKSAIQRKIIMTSLSFSCHFPPI

>gi|30063463|ref|NP_837634.1| amino acid/amine transport protein [Shigella flexneri 2a str. 2457T]

MSHNVTPNTSRVELRKTLTLVPVVMMGLAYMQPMTLFDTFGIVSGLTDGHVPTAYAFALIAILFTALSYG

KLVRRYPSAGSAYTYAQKSISPTVGFMVGWSSLLDYLFAPMINILLAKIYFEALVPSIPSWMFVVALVAF

MTAFNLRSLKSVANVNTVIVVLQVVLIAVILGMVVYGVFEGEGAGTLASTRPFWSGDAHVIPMITGATIL

CFSFTGFDGISNLSEETKDAERVIPRAIFLTALIGGMIFIFATYFLQLYFPDISRFKDPDASQPEIMLYV

AGKAFQVGALIFSTITVLASGMAAHAGVARLMYVMGRDGVFPKSFFGYVHPKWRTPAMNIILVGAIALLA

INFDLVMATALINFGALVAFTFVNLSVISQFWIREKRNKTLKDHFQYLFLPMCGALTVGALWVNLEESSM

VLGLIWAAIGLIYLACVTKSFRNPVPQYEDVA

>gi|30063462|ref|NP_837633.1| transport system permease [Shigella flexneri 2a str. 2457T]

MFSMILSGLICGALLGFVMQRGRFCLTGGFRDMYIAKNNRMFYALLIAISAQSVGVFALIQAGLLTYEAG

AFPWLGTVIGGYLFGLGIVLAGGCATGTWYRAGEGLIGSWIALFTYMVMSAVMRSPHASGLNQTLQHYTT

EHNSIAETFNLSVWPLVTVLLVITLWVVMKELKKPKLKVATLPPRRTGIAHILFEKRWHPFVTAVLIGLI

ALLAWPLSEATGRMFGLGVTSPTANILQFLVAGDVKYINWGVFLVLGIFVEAFIAAKASREFRVRAADAQ

TTLRSGLGGVLMGFGASIAGGCSIGNGLVMTAMMTWQGWIGLVFMILGVWTASWLVYVRPQRKARLATAA

AN

>gi|30063461|ref|NP_837632.1| hypothetical protein S2194 [Shigella flexneri 2a str. 2457T]

MAIKKLDVVTQVCPFPLIEAKAALAEMASGDELVIEFDCTQATEAIPQWAAEEGHAITDYQQIGDAAWSI

TVQKA

>gi|30063460|ref|NP_837631.1| exonuclease I [Shigella flexneri 2a str. 2457T]

MTDTDKQPTFLFHDYETFGTHPALDRPAQFAAIRTDDEFNVIGEPEVFYCKPADDYLPRPGAVLITGITP

QEARAKGENEAAFAARIHSLFTVPKTCILGYNNVRFDDEVTRNVFYRNFYDPYAWSWQHDNSRWDLLDVM

RACYALRPEGINWPENDDGLPSFRLEHLTKANGIEHSNAHDAMADVYATIAMAKLVKTRQPRLFDYLFTH

RNKHKLMALIDVPQMKPLAHVSGMFGAWRGNTSWVAPLAWHPENRNAVIMADLAGDISPLLELDIDTLRE

RLYTAKADLGDNAAVPVKLVHINKCPVLAQANTLRPEDADRLGINRQHCLDNLKILRENPQVREKVVAIF

AEAEPFTPSDNVDAQLYNGFFSDADRAAMKIVLETEPRNLPALDITFVDKRIEKLLFNYRARNFPGTLDY

AEQQRWLEHRHQVFTPEFLQGYADELQMLVQQYADDKEKVALLKALWQYAEEIV

>gi|30063459|ref|NP_837630.1| ISSfl3 orfA, partial [Shigella flexneri 2a str. 2457T]

MPDSVSFAQLDAILYANRKKELTEPQIREGSWRKERRTSYSREFKVRLAKQALQPGAVVARIAREHDIND

NLLFKWKSQYEDGLLSDDDIQECMPVPVALTDTPEPTRPVTNPFWRNKHDERPEGAPGNVPRCELHLKSG

VVKLFDPLTPELLRALIREMKGGIR

>gi|30063458|ref|NP_837629.1| ISSfl3 orfB [Shigella flexneri 2a str. 2457T]

MITLPTGTKIWIIAGITDMRCGFNGLASKVQNTLKDDPFSGHIFVFRGRSGKMVKILWADRDGLCLFAKR

LAGDPGRESAPDASSVIHATGGDRVATSQTDRTAWHPDITRDKTRE

>gi|30063457|ref|NP_837628.1| ISSfl3 orfC [Shigella flexneri 2a str. 2457T]

MNNELPDDIELLKAMLRKQQSRLRQYACQVAGYEQEIERLKAQLDRLRRMLFGQSSEKKRHKLENQIRQA

EKRLSELENRLNTARNLLEDASSVTDSPDTSPPSENPIASKPESPGRKSSRKPLPAELPRETHRLLPAET

SCPACGGVLKEMGETISEQLDIINTAFKVIETIRPKLACSRCDVIGTTSS

>gi|30063456|ref|NP_837627.1| ISSfl3 orfD [Shigella flexneri 2a str. 2457T]

MEIDNNIGENALRSVAVGRKNYLFFGSDKGGESAAIIYSLLVTCKQNEVEPEDWLREVIEKLNDWPSNQV

HKLLPWNFSSVK

>gi|30063455|ref|NP_837626.1| hypothetical protein S2185, partial [Shigella flexneri 2a str. 2457T]

MYNDVLFPGEDINKPISIAAANRFVNRIRGGMDLGYWRTHDFRRTLVTRLSEMNVEPHVTERMLGHELGG

IMSVYNKHDWIEAQRKAYELHADKLFWHIRSISD

>gi|30063454|ref|NP_837625.1| ISSfl3 orfC [Shigella flexneri 2a str. 2457T]

MNNELPDDIELLKAMLRKQQSRLRQYACQVAGYEQEIERLKAQLDRLRRMLFGQSSEKKRHKLENQIRQA

EKRLSELENRLNTARNLLEDASSVTDSPDTSPPSENPIASKPEFPGRKSSRKPLPAELPRETHRLLPAET

SCPACGGVLKEMGETISEQLDIINTAFKVIETIRPKLACSRCDVIVQAPLPPKPIERGYASAGLLARILV

SKYMEHIPLYRQSEIYARQGVELSRNTMVRWVSEMADKLRPLYIALNDYVLEAGKVHADDTPVKVLAPGN

GKTKTGRLWVYVRDDRNAGSSLPAAVWFAYSADRKGEHPQLHLAKYQGVLQADAYAGYNVLYETGRVKEA

GCLAHARRKTHDEDVRRPTEMTQEALRRIAELYDIEAEIRGSPAEERLAVRKARSVQLMQSLYDWIQLQR

KTLSKHAEMAKAFDYILNHWNALNEFCRDGWVEIDNNIGENALRSVAVGRKNYLFFGSDKGGESAAIIYS

LLVTCKQNEVEPEDWLREVIEKLNDWPSNQVHELLPWNFSSVK

>gi|30063453|ref|NP_837624.1| IS629 orfB [Shigella flexneri 2a str. 2457T]

MPLLDKLREQYGVGPVCSELHIAPSTYYHCQQQRHHPDKRSARAQRDDWLKKEIQRVYDENHKVYGVKSG

VSCYGKVSEWPDALWHVSWRLWDLPVFSGVKRSVRPSAGKPLPQATA

>gi|30063452|ref|NP_837623.1| IS629 orfA [Shigella flexneri 2a str. 2457T]

MAERPDQLWVADFTYVSTWRGFVYVAFIIDVFAGYIVGWRVSSSMETTFVLDALEQALWARRPSGTVHHS

DKGSQYVSLAYTQRLKEAGLLASTGSTGDSYDNAMAESINGLYKAEVIHRKSWKNRAEVELATLMWVDWY

NNRRLLERLGHIPPAEAEKAYYASIGNDDLAA

>gi|30063451|ref|NP_837622.1| ISSfl3 orfB [Shigella flexneri 2a str. 2457T]

MITLPTGTKIWIIAGITDMRCGFNGLASKVQNTLKDDPFSGHIFVFRGRSGKMVKILCEPPRVSWRVFYL

>gi|30063450|ref|NP_837621.1| ISSfl3 orfA [Shigella flexneri 2a str. 2457T]

MNSQTTKDIPCFRSYLPDALRLRFEDKLTIRAIAQRLGLSHSTIHTLFQRFLASGIAWPLPDSVSFAQLD

AILYANRKKELTEPQIREGSWRKERRTSYSREFKVRLAKQALQPGAVVARIAREHDINDNLLFKWKSQYE

DGLLSDDDIQECMPVPVALTDTPEPTRPVTNPFWRNKHDERPEGAPGNVPRCELHLKSGVVKLFDPLTPE

LLRALIREMKGGIR

>gi|30063449|ref|NP_837620.1| hypothetical protein S2177 [Shigella flexneri 2a str. 2457T]

MLALTNSGCLNESDSHIIRGIKMETTKPSFQDVLEFVRLFRRKNKLQREIQDVEKKIRDNQKRVLLLDNL

SDYIKPGMSVEAIQGIIASMKGDYEDRVDDYIIKNAELSKERRDISKKLKAMGEMKNGEAK

>gi|30063448|ref|NP_837619.1| IS629 orfB [Shigella flexneri 2a str. 2457T]

MARCTVARLMAVMGLAGVLRGKKVRTTISRKAVAAGDRVNRQFVAERPDQLWVADFTYVSTWRGFVYVAF

IIDVFAGYIVGWRVSSSMETTFVLDALEQALWARRPSGTVHHSDKGSQYVSLAYTQRLKEAGLLASTGST

GDSYDNAMAESINGLYKAEVIHRKSWKNRAEVELATLTWVDWYNNRRLLERLGHIPPAEAEKAYYASIGN

DDLAA

>gi|30063447|ref|NP_837618.1| IS629 orfA [Shigella flexneri 2a str. 2457T]

MTKNTRFSPEVRQRAVRMVLESQGEYDSQWATICSIAPKIGCTPETLRVWGRQHERDTGGGDGGLTTAER

QRLKEPERENRELRRSNDILRQASAYFAKAEFDRLWKK

>gi|30063446|ref|NP_837617.1| IS600 orfA [Shigella flexneri 2a str. 2457T]

MSRKTQRYSKEFKAEAVRTVLENQLSISEGASRLSLPEGTLGQWVTAARKGLGTPGSRTLAELESEILQL

RKALNEARLERDILKKATAYFAQESLKNTR

>gi|30063445|ref|NP_837616.1| IS600 orfB [Shigella flexneri 2a str. 2457T]

MAHIRTRETYGTRRLQTELAENGIIVGRDRLARLRKELRLRCKQKRKFRATTNPNHNLPVAPNLLNQTFA

PTAPNQVWVADLTYVATQEGWLYLAGIKDVYTCEIVGYAMGERMTKELTGKALFMALRSQRPPAGLIHHS

DRGSQYCAYDYRVIQEQSGLKTSMSRKGNCYDNAPMESFWGTLKNESLSHYRFNNRDEAISVIREYIEIF

YNRQRRHSRLGNISPAAFREKYHQMAA

>gi|30063444|ref|NP_837615.1| cobalamin synthase [Shigella flexneri 2a str. 2457T]

MSKLFWAMLSFITRLPVPRRWSQGLDFEHYSRGIITFPLIGLLLGAISGLVFMVLQAWCGAPLAALFSVL

VLALMTGEFHLDGLADTCDGVFSARSRDRMLEIMRDSRLGTHGGLALIFVVLAKILVLSELALRGEPILA

SLAAACAVSRGTAALLMYRHRYAREEGLGNVFIGKIDGRQTCVTLGLAAIFAAVLLLGMHGVAAMVVTMV

AIFILGQLLKRTLGGQTGDTLGAAIELGELVFLLALL

>gi|30063443|ref|NP_837614.1| nicotinate-nucleotide--dimethylbenzimidazole phosphoribosyltransferase [Shigella flexneri 2a str. 2457T]

MQIIADLLNTIPAINSAAMSRAQRHVDGLFKPVGSLGKLEALAIQLAGMPGVNGIPHVGKKAVLVMCADH

GVWEEGVAISPKEVTAIQAENMTRGTTGVCVLAAQAGANVHVIDVGIDTAEPIPGLINMRVARGSGNIAS

APAMSRRQAEKLLLDVICYTRELAKNGVTLFGVGELGMANTTPAAAIVSTITGRDPEEVVGIGANLPTDK

LANKIDVVRRAITLNQPNPQDGVDVLAKVGGFDLVGIAGVMLGAASCGLPVLLDGFLSYAAALAACQMSP

AIKPYLIPSHLSAEKGARIALSHLGLEPYLNMDMRLGEGSGAALAMSIIEAACAIYNNMGELAASNIVLP

GNTTSDLNS

>gi|30063442|ref|NP_837613.1| hypothetical protein S2168 [Shigella flexneri 2a str. 2457T]

MMRRVNILCSFALLFASQNSLAVTYPLPPEGSRLVGQSLTVTVPDHNTQPLETFAAQYGQGLSNMLEANP

GADVFLPKPGSQLTIPQQLILPATVRKGIVVNVAEMRLYYYPPDSNTVEVFPIGIGQAGRETPRNWVTTV

ERKQEAPTWTPTPNIRREYAKRGESLPAFVPAGPDNPMGLYAIYIGRLYAIHGTNANFGIGLRVSQGCIR

LRNDDIKYLFDNVPVGTRVQIIDQPVKYTTEPDGSKWLEVHEPLSRNRAEYESDRKVPLPVTPSLRAFIN

GQEVDVNRANAALQHRSGMPVQISSGSRQMF

>gi|30063441|ref|NP_837612.1| nitrogen assimilation transcriptional regulator [Shigella flexneri 2a str. 2457T]

MNFRRLKYFVKIVDIGSLTQAAEVLHIAQPALSQQVAILEGELNQQLLIRTKRGVTPTDAGKILYTHARA

ILRQCEQAQLAVHNVGQSLSGQVSIGFAPGTAASSITMPLLQAVRAEFPEVVIYLHENSGAVLNEKLINH

QLDMAVIYEHSPVAGVSSQALLKEDLFLVGTQDCPGQNVDVNAIAQMNLFLPSDYSAVRLRVDEAFSLRR

LTAKVIGEIESIATLTAAIASGMGVAVLPESAARSLCGAVNGWMSRITTPSMSLSLSLNLPARANLSPQA

QAVKELLMSVISSPVMEKRQWQLVS

>gi|30063440|ref|NP_837611.1| transcriptional regulator Cbl [Shigella flexneri 2a str. 2457T]

MNFQQLKIIREAARQDYNLTEVANMLFTSQSGVSRHIRELEDEFGIEIFVRRGKRLLGMTEPGKALLVIA

ERILNEASNVRRLADLFTNDTSGVLTIATTHTQARYSLPEVIKAFRELFPEVRLELIQGSPQEIATLLQN

GEADIGIASERLSNDPQLVAFPWFCWHHSLLVPLDHPLTQITPLTLESIAKWPLITYRQGITGRSRTDDA

FTRKGLPADIVLSAQDSDVIKTYVALGLGIGLVAEQSSGEQEEENLIRLDTRHLFDANTVWLGLKRGQLQ

RNYVWRFLELCNAGLSVEDIKRQVMENSEEEIDYQI

>gi|30063439|ref|NP_837610.1| hypothetical protein S2161 [Shigella flexneri 2a str. 2457T]

MGRKWANIVAKKTAKDGATSKIYAKFGVEIYAAAKQGEPDPELNTSLKFVIERAKQAQVPKHVIDKAIDK

AKGGGDETFVQGRYEGFGPNGSMIIAETLTSNVNRTIANVRTIFNKKGGNIGAAGSVSYMFDNTGVIVFK

GTDPDHIFEILLEAEVDVRDVTEEEGNIVIYTEPADLHKGIAALKAAGITEFSTTELEMIAQSEVELSPE

DLEIFEGLVDALEDDDDVQKVYHNVANL

>gi|30063438|ref|NP_837609.1| AMP nucleosidase [Shigella flexneri 2a str. 2457T]

MNNKGSGLTPAQALDKLDALYEQSVVALRNAIGKYITIGELPDENARKQGLFVYPSLTVTWDGSTTNPPK

TRAFGRFTHAGSYTTTITRPTLFRSYLNEQLTLLYQDYGAHISVQPSQHEIPYPYVIDGSELTLDRSMSA

DLTRYFPTTELAQIGDETADGIYHPTEFSPLSHFDARRVDFSLARLRHYTGTPVEHFQPFVLFTNYTRYV

DEFVRWGCSQILDPDSPYIALSCAGGNWITAETEAPEEAISDLAWKKHQMPAWHLITADGQGITLVNIGV

GPSNAKTICDHLAVLRPDIWLMIGHCGGLRESQAIGDYVLAHAYLRDDHVLDAVLPPDIPIPSIAEVQRA

LYDATKLVSGRPGEEVKQRLRTGTVVTTDDRNWELRYSASALRFNLSRAVAIDMESATIAAQGYRFRVPY

GTLLCVSDKPLHGEIKLPGQANRFYEGAISEHLQIGIRAIDLLRAEGDRLHSRKLRTFNEPPFR

>gi|30063437|ref|NP_837608.1| IS600 orfB [Shigella flexneri 2a str. 2457T]

MAHIRTRETYGTRRLQTELAENGIIVGRDRLACLRKELRLRCKQKRKFRATTNPNHNLPVAPNLLNQTFA

PTAPNQVWVADLTYVATQEGWLYLAGIKDVYTCEIVGYAMGERMTKELTGKALFMALRSQRPPAGLIHHS

DRGSQYCAYDYRVIQEQSGLKTSMSRKGNCYDNAPMESFWGTLKNESLSHYRFNNRDEAISVIREYIEIF

YNRQRRHSRLGNISPAAFREKYHQMAA

>gi|30063436|ref|NP_837607.1| IS600 orfA [Shigella flexneri 2a str. 2457T]

MSRKTRRYSKEFKAEAVRTVLENQLSISEDASRLFLPEGTLGQWVTAARKGLGTPGSRTLAELESEILQL

RKALNEARLERDILKKATAYFAQESLKNTR

>gi|30063435|ref|NP_837606.1| IS911 orfA, partial [Shigella flexneri 2a str. 2457T]

MADAAKAMDVGLSTMTRWVKQLRDERQGKTPKASPITPEQIEIRELRKKLQRIEMENEILKKATALLMSD

SLNSSR

>gi|30063434|ref|NP_837605.1| integrase [Shigella flexneri 2a str. 2457T]

MRHAVHQELIDTNPAANLGGVTTPPVRRHYPALPLERLPELLERIGAYHQGRELTRHAVLLMLHVFIRSS

ELRFARWSEIDFTNRVWTIPATREPIIGVRYSGRGAKMRMPHIVPLSEQSIAILKQIKDITGNNELIFPG

DHNPYKPMCENTVNKALRVMGYDTKKDICGHGFRAMACSALMESGLWAKDAVERQMSHQERNTVRMAYIH

KAEHLEARKAMMQWWSDYLEACRESYAPPYTIGKNKFIP

>gi|30063433|ref|NP_837604.1| hypothetical protein S2148 [Shigella flexneri 2a str. 2457T]

MQFCSSDEFASKTMIKWPWKVQESAHQTALPWQEALSIPLLTCLTEQEQSKLVALAERFLQQKRLVPLQG

FELNSLRSCRIALLFCLPVLELGLEWLDGFHEVLIYPAPFVVDDEWEDDIGLVHNQRIVQSGQSWQQGPI

VLNWLDIQDSFDASGFNLIIHEVAHKLDTRNGDRASGVPFISLREVAGWEHDLHAAMNNIQEEIELVGEN

AASIDAYAASDPAECFAVLSEYFFSAPELFAPRFPSLWQRFCQFYQQDPLQRLHHANDTDSFSATNVH

>gi|30063432|ref|NP_837603.1| integrase for prophage CP-933U [Shigella flexneri 2a str. 2457T]

MTFGRLWEKFLASAYYSDLSPRTQKDYLQHQKKLLAVFGKVPADSIKPEHIRRYMDKRGEQSKTQANHEK

SSMSRVYSWGYERGYVKANPCAGVSKFKAKNRERYVTDKEYQAVLSVAPLPVFIAMEIAYLCAARVSDVL

SLKWEQIGNDGIFIQQGKTGKKQIKAWSPRLQAAIEKAKQLPTSAYVISNQYGNRYMYKGFNEMWVEARN

RAGKISGILTDFTFHDLKAKGISDYEGSSRDKQLFSGHKTEGQVLIYDRKVKVSPTLDVPLPENIPRKYS

K

>gi|30063431|ref|NP_837602.1| hypothetical protein S2145, partial [Shigella flexneri 2a str. 2457T]

MENGLPFYTNRSGKPIVSRDLFTCNKTLPPREVEPNFGAI

>gi|30063430|ref|NP_837601.1| hypothetical protein S2144 [Shigella flexneri 2a str. 2457T]

MARVEGELFKIARASLEAEPIAWRYRYVKKGVMDSQGELWVGDWKYVPKKEDCNDRPNYEIQALFTAPPV

PVTSEELVKAVHFYEQLKRENPPASGNQINGLTMSVKRPAN

>gi|30063429|ref|NP_837600.1| cell killing protein of prophage [Shigella flexneri 2a str. 2457T]

MLNTCRLASYVPKGKEKQAMKQQKAMLIALIVICLTVIVTALVTRKDLCEVRIRTGQTEVAVFTAYEPEE

>gi|30063428|ref|NP_837599.1| hypothetical protein S2141 [Shigella flexneri 2a str. 2457T]

MDSRPGLRTTSPSSGTRTGRSNTRTLPPDPLLECAGRTRWPFGIREPDEDYPVTIIETEGFLNLVSVSAV

ALNQPFSLFFGAGVVLIPVRFECMRTPPVPAGRGGRVIRIVKFGIVRHLFSLLAQQVPVVQADGWIVNQN

DQKKQNPPKRVKCGCVEDA

>gi|30063427|ref|NP_837598.1| hypothetical protein S2142 [Shigella flexneri 2a str. 2457T]

MPVFHNTRVLVEPEPKSMRNLPSGVVPAVRQPLVEDKTLLPFFSNARVIRAAGGAGALSDWLLRHIKSCQ

WPHGDYHHSETVIHRYGTGAMVLCWHCDNQLRDQTSESLEQLAHQNLSAWMIDVIGHAISGTQERELSLA

ELSWWAVRNQVADALPEAVLRRSLGLRAEKIRSMYRESDIVPGEQTATSILKQRTKNLAPLPHAHQQQNP

PQEETVVSIAVDPESPAQYLQRQKPQREEMPVYTRWVKTQKCMTCGNQADDPHHIIGHGLGGMGTKADDL

FVIPLCRKCHNELHAGVKDFEEKHGSQLLLLIRFLMHARNSGVLKWKA

>gi|30063426|ref|NP_837597.1| crossover junction endodeoxyribonuclease [Shigella flexneri 2a str. 2457T]

MTERIEFVLPYPPTVNTYWRRRGSTYFVSKAGERYRRDVALIVRQQRLKLNLSGRLAIKIIAEPPDKRRR

DLDNILKAPLDALTHAGLLIDDEQFDEINIVRGQLVPGGRLGIKITELGCA

>gi|30063425|ref|NP_837596.1| Q antiterminator of prophage [Shigella flexneri 2a str. 2457T]

MNNQYLQFVREQLIIATADLSGATKGQLEAWQENAMFDTGRYRRKKIRYRDEVTGKMITRDNPPIPGKQS

LAKGTSIPLVSPVEFSTSSWRRAVLSLEEHYKAWLLWCYSGSICWEYQIAITQWAWNEFNAQSGTRKIAG

KTQERLKKLIWLAAQAVKAELFGGEGYEYQELALLAGVTTKNWSKTFTRHWVAMKHIFHRLDSEALLFVM

RTRSKKRRHFQSKVLQK

>gi|30063424|ref|NP_837595.1| ISSfl3 orfA, partial [Shigella flexneri 2a str. 2457T]

MPDSVSFAQLDAILYANRKKELTEPQIREGSWRKERRTSYSREFKVRLAKQALQPGAVVARIAREHDIND

NLLFKWKSQYEDGLLSDDDIQECMPVPVALTDTPEPTRPVTNPFWRNKHDERPEGAPGNVPRCELHLKSG

VVKLFDPLTPELLRALIREMKGGIR

>gi|30063423|ref|NP_837594.1| ISSfl3 orfB [Shigella flexneri 2a str. 2457T]

MITLPTGTKIWIIAGITDMRCGFNGLASKVQNTLKDDPFSGHIFVFRGRSGKMVKILWADRDGLCLFTKR

LAGDPGRESAPDASSVIHATGGDRVATSQTDRTAWHPDITRDKTRE

>gi|30063422|ref|NP_837593.1| IS600 orfA [Shigella flexneri 2a str. 2457T]

MSRKTQRYSKEFKAEAVRTVLENQLSISEGASRLSLPEGTLGQWVTAARKGLGTPGSRTVAELESEILQL

RKALNEARLERDILKKATAYFAQESLKNTR

>gi|30063421|ref|NP_837592.1| IS600 orfB [Shigella flexneri 2a str. 2457T]

MAHIRTRETYGTRRLQTELAENGIIVGRDRLARLRKELRLRCKQKRKFRATTNPNHNLPVAPNLLNQTFA

PTAPNQVWVADLTYVATQEGWLYLAGIKDVYTCEIVGYAMGERMTKELTGKALFMALRSQRPPAGLIHHS

DRGSQYCAYDYRVIQEQSGLKTSMSRKGNCYDNAPMESFWGTLKNESLSHYRFNNRDEAISVIREYIEIF

YNRQRRHSRLGNISPAAFREKYHQMAA

>gi|30063420|ref|NP_837591.1| hypothetical protein S2129 [Shigella flexneri 2a str. 2457T]

MPDPQGGEIVYVGGTLLDLNRYELYYQFDFTAKYEITEEDTRQAEDVNALPDLSLLSIDVDYIDPGTGPD

GDIEHHLEMRFPQN

>gi|30063419|ref|NP_837590.1| sheath protein [Shigella flexneri 2a str. 2457T]

MTISFNTVPSNTLVPLFYAEMNNSAANTAVTSAPALLIGHASNDAAIEVNSLVLMPSADYARQICGAGSQ

LARMVEVYRQTDPFGELYVIAVPEARGAAATVRVTVAGEAEESGTLSLYVGRSRVQVPVVNGDDATAVAT

AIKEAVNGVITLPFTASSDAGVVTLTARHKGLYGNELPVCLNYYGSGGGEILPAGLRVVTEVGAAGSGAP

DLTAAVAAMGDEAFDFIGLPFNDAASINMMMTEMNDSSGRWSYARQLYGHVYTAKLGTLSELVGAGDMHN

QQHITLAGYEKETQSPVDELVASRLAREAVFIRNDPARPTQTGELVGMLPAPKGKRFIMTEQQTLLSHGV

ATAYVEGGTLQIQRSVTTYKKNAYGVADNSYLDSETLHTSAYVLRKLKSVITSKYGRHKLANDGTRFGPG

QAIVTPAVIKGELLATYRQMERAGIVENYDLFKQYLIVERDADNPNRLNTLFPPDYVNQLRVFAVVNQFR

LQYSEESA

>gi|30063418|ref|NP_837589.1| hypothetical protein S2127 [Shigella flexneri 2a str. 2457T]

MAKIAGTCFFKVDGQQLSLTGGIEVPMNTNVRDDIVGMAGDVDYKETWRSPYVKGTFKVPKNFPVDKITT

SDQMTITAELANGMVYVLSAAWLHGEANHNAEEGTADLEFHGEEGGYQ

>gi|30063417|ref|NP_837588.1| bacteriophage protein [Shigella flexneri 2a str. 2457T]

MAMNVTEIVLKKPVTAHNEMLHVLELREPTYDEIEALGFPFIISGEGSIKLDSQVALKYIPLLAGIPCSS

AAQMAKLDIFKTSMQILRFFTQSETGSTSGNDSTMLPGSGN

>gi|30063416|ref|NP_837587.1| tail protein [Shigella flexneri 2a str. 2457T]

MADSFQLKAIITAVDKVSAPLKGMQRQLKGFKKEFASLSLGAAGAGTAVLGALALPVKSAIALESKMADV

RKVVDGLDTPEAFKAMTEQVRDLSTELPMSAEGIAEIVAAGGQAGIARDELMQFTDDAVKMGVAFDTTAE

ESGQMMAQWRTAFKLTQGEVAGLADKINYLGNTGPASAKKISDVVTRIGPLGSVAGVASGEIAAMGATIA

GMGVESEIAATGIKNFMLSLTARDSATKSQKKVLRSLRISPKKLAADMQKDARGAMLHVLDSLAKVPKEK

QAAVLKALFGKESLGAIAPLLTNLDLLRTNFNRVADAQQYGGSMQKEYAARAATTENQLLLLQNQINAIS

STLGETFLPSLNEGIKEMKPFLEEVRTFVRENPEVVKTIAKTGAALLTMGVAIGTLTRITKIMGSVMNMT

PAKGLIALLVGGAYLIIDNWETVGPVVKKVWQEVDQVVRAMGGWEQAVKTIATVSALYIGVKAVASIRAA

TVAQNQWTTAAGKTGLKLKGLGKISLIGGLLELGMMAQEFEKEHPWLVKNFVADALNSGFGLNDKFDEWG

KQFHDFVYDMTGWQMPRGDGYLSPDKRYTPNVSLERNQLLSLASSPATRSELKVTFDNAPPGMRVIDLPK

TGDPFMKITHDVGYSPFKR

>gi|30063415|ref|NP_837586.1| IS1 orfB [Shigella flexneri 2a str. 2457T]

MSRQCTHYGRWPQHGFTSLKKLRPQSVTSRIQPGSDVIVCAEMDEHWGYVGAKSRQRWLFYAYDRIRRTV

VAHVFGERTLATLERLLSLLSAFEVVVWMTDGWPLYESRLKGKLHVISKRYTQRIERHNLNLRQHLARLG

RKSLSFSKSVELHDKVIGHYLNIKHYQ

>gi|30063414|ref|NP_837585.1| IS1 orfA [Shigella flexneri 2a str. 2457T]

MASISIRCPSCSATEGVVRNGKSTAGHQRYLCSHCRKTWQLQFTYTASQPGTHQKIIDMAMNGVGCRASA

RIMGVGLNTVLRHLKNSGRSR

>gi|30063413|ref|NP_837584.1| hypothetical protein S2121 [Shigella flexneri 2a str. 2457T]

MTPEMLEAAREARRQEIEAWRTEQEAKPFTFEWNGRIWNAGPDSLGRLSPVVMLAKSVTAQTHMAWSDAD

NQQVKLSMPELEELAAAMVQAQVDRNDEIYRRQREMKEELSGLDDLASIRAFDVE

>gi|30063412|ref|NP_837583.1| invasion plasmid antigen [Shigella flexneri 2a str. 2457T]

MLPVNNPPLSTGNVSFYRTTSIDNVHNNYLSEWVEWTKNSISGENRETAFTRLQLCLENSETSLDLSCLG

LRSLPRLPDNLDEINVSNNQLSMLPELPRALKELNASSNQLSALPELPVSLEYINVSDNHLFALPELPAS
LEYINVSDNHLSVLPRLPMSLELLDAARNALEVIPDFPERDDHIIRIFWLNQNRITAIPESILGLSSDSV
VNLRENQLSPRIMQTLLQQTAQPDYHGPRIYFSMSDGQQNTLHRPLADAVTAWFPENKQSDVSQIWHAFE
HEEHANTFSAFLDRLSDTVSARNTSGFREQVAAWLEKLSASAELRQQSFAVAADATESCEDRVALTWNNL
RKTLLVHQASEGLFDNDTGALLSLGREMFRLEILEDIARDKVRTLHFVDEIEVYLAFQTMLAEKLQLSTA
VKEMRFYGVSGVTANDLRTAEAMVRSREENEFTDWFSLWGPWHAVLKRTEADRWAQAEEQKYEMLENEYP
QRVADRLKASGLSGDADAEREAGAQVMRETEQQIYRQLTDEVLALRLSENGSQLHHS

>gi|30063411|ref|NP_837582.1| bacteriophage protein [Shigella flexneri 2a str. 2457T]
MLPQHSDIEIAWYASIQQEPNGWKTVTTQFYIQGFSEHIAPLQDAVDLEIATEEENSLLEAWKKYRVLLN
RVDTSTAPDIEWPEEPDTM

>gi|30063410|ref|NP_837581.1| hypothetical protein S2117 [Shigella flexneri 2a str. 2457T]
MAIRLHKLAVALGVFIVSAPAFSHGHHSHGKPLTEVEQKAANGVFDDTNVQNRTLSDWDGVWQSVYPLLQ
SGKLDPVFQKKADADKTKTFAEIKDYYRKGYPTDIEMIGIEDGIVEFHRNNETTSCKYDYDGYKILTYKS
GKKGVRYLFECKDPESKAPKYIQFSDHIIAPRKSSHFHIFMGNDSQQSLLNEMENWPTYYPYQLSSEEVV
EEMMSH

>gi|30063409|ref|NP_837580.1| sulfite oxidase subunit YedZ [Shigella flexneri 2a str. 2457T]
MRLTAKQVIWLKVCLHLAGLLPFLWLVWAINHGGLGADPVKDIQHFTGRTALKFLLAALLITPLARYAKQ
PLLIRTRRLLGLWCFAWATLHLTSYALLELGVNNLALLGKELITRPYLTLGIISWVILLALAFTSTQSMQ
RKLGKHWQQLHNFVYLVAILAPIHYLWSVKIISPQPLIYAGLAVLLLALRYKKLLSLFNRLRKQAHNKLS
L

>gi|30063408|ref|NP_837579.1| hypothetical protein S2114 [Shigella flexneri 2a str. 2457T]

MLKRYLVLSVATAAFSLPSLVYAAQQNILSVHILNQQTGKPAADVTVTLEKKADNGWLQLNTAKTDKDGR

IKALWPEQTATTGDYRVVFKTGDYFKKQNLESFFPEIPVEFHINKVNEHYHVPLLLSQYGYSTYRGS

>gi|30063407|ref|NP_837578.1| transcriptional regulatory protein YedW [Shigella flexneri 2a str. 2457T]

MKILLIEDNQRTQEWVTQGLSEAGYVIDAVSDGRDGLYLALKDDYALIILDIMLPGMDGWQILQTLRTAK

QTPVICLTARDSVDDRVRGLDSGANDYLVKPFSFSELLARVRAQLRQHHALNSTLEISGLKMDSVSQSVS

RDNISITLTRKEFQLLWLLASRAGEIIPRTVIASEIWGINFDSDTNTVDVAIRRLRAKVDDPFPEKLIAT

IRGMGYSFVAVKK

>gi|30063406|ref|NP_837577.1| 2-component sensor protein [Shigella flexneri 2a str. 2457T]

MKRLSITVRLTLLFIFLQSVAGAGIVWTLYNGLASELKWRDDTTLINRTAQIKQLLIDGVNPDTLPVYFN

RMMDVSQDILIIHSDGINKIVNRTNVSDDMLNNIPASETISAAGIYRSIINDTEIDALRINIDEVSPSLT

VTVAKLASARHNMLEQYKINSIIICIVAIILCSVLSPLLIRTGVREIKKLSGVTEALNYNDSRVPVEVNA

LPRELKPLGQALNKMHHALVKDFERLSQFADDLAHELRTPINALLGQNQVTLSQIRSIAEYQKTIAGNIE

ELENISRLTENILFLARADKNNVLVKLDSLSLNKEVENLLDYLEYLSDEKEICFKVKCNQQIFADKILLQ

RMLSNLIVNAIRYSPEKSRIHITSFLDANGSLNIDIASPGTKINEPEKLFRRFWRGDNSRHSVGQGLGLS

LVKAIAELHGGSATYHYLSKHNVFRITLPQRN

>gi|30063405|ref|NP_837576.1| chaperone protein HchA [Shigella flexneri 2a str. 2457T]

MTVQTSKNPQVDIAEDNAFFPSEYSLSQYTSPVSDLDGVDYPKPYRGKHKILVIAADERYLPTDNGKLFS

TGNHPIETLLPLYHLHAAGFEFEVATISGLMTKFEYWAMPHKDEKVMPFFEQHKSLFRNPKKLADVVASL

NADSEYAAIFVPGGHGALIGLPESQDVAAALQWAIKNDRFVISLCHGPAAFLALRHSDNPLNGYSICAFP

DAADKQTPDIGYMPGHLTWYFGEELKKMGMNIINDDITGRVHKDRKRLTGDSPFAANALGKLAAQKCWQL

TRVNPCYIVM

>gi|30063404|ref|NP_837575.1| IS1 orfB [Shigella flexneri 2a str. 2457T]

MSRQCTHYGRWPQHGFTSLKKLRPQSVTSRIQPGSDVIVCAEMDEHWGYVGAKSRQRWLFYAYDRIRRTV

VAHVFGERTLATLERLLSLLSAFEVVVWMTDGWPLYESRLKGKLHVISKRYTQRIERHNLKLRQHLARLG

RKSLSFSKSVELHDKVIGHYLNIKHYQ

>gi|30063403|ref|NP_837574.1| IS1 orfA [Shigella flexneri 2a str. 2457T]

MASISIRCPSCSATEGVVRNGKSTAGHQRYLCSHCRKTWQLQFTYTASQPGTHQKIIDMAMNGVGCRASA

RIMGVGLNTVLRHLKNSGRSR

>gi|30063402|ref|NP_837573.1| outer membrane protein [Shigella flexneri 2a str. 2457T]

MSGDSGGQSTGYKDSDRLNYIEIGTWYYFNKNMNIYTAYQINLLDKSDYVLAHGLNTDDQLAVGIVYQF

>gi|30063399|ref|NP_837570.1| outer membrane pore protein [Shigella flexneri 2a str. 2457T]

MTLRCLLNPWRFSERILTDRENGEKGDNSQDTSYARVGVKGETQINPEMTGYGQFELDLEASNRHNPDQT

RLAYAGLSYKDFGSFDYSRNVGVAYDAEAFTDMFVEWGGDSWAGTDLFMTNRTNGVATYRNTDFFGMVEG

LNFALQYQGKNEGTGNYKANGDGHGLSATYTIDGFSFAGAYANSDRTDWQSGDGKGERAEVWALSTKYDA

NNVYAAVMYGESHNMNSDDGDVVNKTQNFEAVLQYQFDFGLRPSIGYSYSKALDVAGWICPYISRHLLSL

NPLQARCRRYSRGER

>gi|30063398|ref|NP_837569.1| ISEhe3 orfA [Shigella flexneri 2a str. 2457T]

MSGKRYPEEFKTEAVKQVVDRGYSVASVATRLDITTHSLYAWIKKYGPDSSTNKEQSDAQAEIRRLQKEL

KRVTDERDILKKAAAYFAKLSD

>gi|30063397|ref|NP_837568.1| ISEhe3 orfB [Shigella flexneri 2a str. 2457T]

MLDVHPSGFYAWLQQPHSQRHQADLRLTGQIKQFWLESGCVYGYRKIHLDLRDSGQQCGVNRVWRLMKRV

GIKAQVGYRSPRARKGEASIVSPNRLQRQFNPDAPDERWVTDITYIRTHEGWLYLAVVVDLFSRKIIGWS

MQSRMTKDIVLNALLMAVWWRNPEKQVLVHSDQGSQYTSHEWQSFLKSHGLEGSMSRRGNCHDNAVAESF

FQLLKRERIKKKIYGTREEARSDIFDYIEMFYNSKRRHGSSEQMSPTEYENQYYQRLGSV

>gi|30063396|ref|NP_837567.1| DNA cytosine methylase [Shigella flexneri 2a str. 2457T]

MQENISVTDSYSTGNAAQAMLEKLLQIYDVKMLVAQLNGVGENHWSAAILKRALANDSAWHRLSEKEFAH

LQTLLPKPPEHHPHYAFRFIDLFAGIGGIRRGFESIGGQCVFTSEWNKHAVRTYKANHYCDPATHHFNED

IRDITLSHQEGVSDEAAAEHIRQHIPEHDVLLAGFPCQPFSLAGVSKKNSLGRAHGFACDTQGTLFFDVV

RIIDARRPAMFVLENVKNLKSHDKGKTFRIIMQTLDELGYDVADAEDNGPDDPKIIDGKHFLPQHRERIV

LVGFRRDLNLKADFTLRDISECFPAQRVTLAQLLDPMVEAKYILTPVLWKYLYRYAKKHQARGNGFGYGM

VYPNNPQSVTRTLSARYYKDGAEILIDRGWDMATGEKDFDDPLNQQHRPRRLTPRECARLMGFEAPGEAK

FRIPVSDTQAYRQFGNSVVVPVFAAVAKLLEPKIKQAVALRQQEAQHGRRSR

>gi|30063395|ref|NP_837566.1| DNA mismatch endonuclease, patch repair protein [Shigella flexneri 2a str. 2457T]

MVDVHDKATRSKNMRAIATRDTAIEKRLASLLTGQGLAFRVQDASLPGRPDFVVDEYRCVIFTHGCFWHH

HHCYLFKVPATRTEFWLEKIGKNVERDRRDISRLQELGWRVLIVWECALRRREKLTDAALTERLEEWICG

EGASAQIDTQGIHLLA

>gi|30063394|ref|NP_837565.1| hypothetical protein S2098 [Shigella flexneri 2a str. 2457T]

MRFRQLLPLFGALFALYIIWGSTYFVIRIGVGSWPPLMMAGVRFQAAGILLLAFLLLRGHKLPPLRPLLN

AALIGLLLLAVGNGMVTVAEHQNVPSGIAAVVIATVPLFTLCFSRLFGIKTRKLEWVGIAIGLAGIIMLN

SGGNLSGNPWGAILILIGSISWAFGSVYGSRITLPVGMMAGAIEMLAAGVVLMIASMIAGEKLTALPSLS

GFLAVGYLALFGSIIAINAYMYLIRNVSPALATSYAYVNPVVAVLLGTGLGGETLSKIEWLALGVIVFAV

VLVTLGKYLFPAKPVVAPVIQDASSE

>gi|30063393|ref|NP_837564.1| hypothetical protein S2097 [Shigella flexneri 2a str. 2457T]

MLLAGSSLLTLLDDIATLLDDISVMGKLAAKKTAGVLGDDLSLNAQQVSGVRANRELPVVWGVAKGSLIN

KVILVPLALIISAFIPWAITPLLMIGGAFLCFEGVEKVLHMLEARKHKEDPAQSQQRLEKLAAQDPLKFE

KDKIKGAIRTDFILSAEIVAITLGIVAEAPLLNQVLVLSGIALVVTVGVYGLVGVIVKIDDLGYWLAEKS

SALMQALGKGLLIIAPWLMKALSIVGTLAMFLVGGGIVVHGIAPLHHAIEHIAGQQSAVVAMILPTVLNL

ILGFIIGGIVVLGVKAVAKMRGQAH

>gi|30063392|ref|NP_837563.1| hypothetical protein S2096 [Shigella flexneri 2a str. 2457T]

MSFMVSEEVTVKEGGPRMIVTGYSSGMVECRWYDGYGVKREAFHETDLVPGEGSRSAEEV

>gi|30063391|ref|NP_837562.1| hypothetical protein S2092 [Shigella flexneri 2a str. 2457T]

MKTAKVYSDTAKREVSVDVDALLAAINEISESEVHRSQNDSEHVSVDGREYHTWRELADAFELDIHDFSV

SEVNR

>gi|30063390|ref|NP_837561.1| hypothetical protein S2091 [Shigella flexneri 2a str. 2457T]

MKVNDRVTVKTDGGPRRPGVVLAVEEFSEGTMYLVALEDYPLGIWFFNEAGHQDGIFVEKAE

>gi|30063389|ref|NP_837560.1| positive regulator for ctr capsule biosynthesis, positive transcription factor [Shigella flexneri 2a str. 2457T]

MSTIIMDLCSYTRLGLTGYLLSRGVKKREINDIETVDDLAIACDSQRPSVVFINEDCFIHDASNSQRIKH

IINQHPNTLFIVFMAIANVHFDEYLLVRKNLLISSKSIKPESLDDILGDILKKETTITSFLNMPTLSLSR

TESSMLRMWMAGQGTIQISDQMNIKAKTVSSHKGNIKRKIKTHNKQVIYHVVRLTDNVTNGIFVNMR

>gi|30063388|ref|NP_837559.1| flagellar biosynthesis protein FliR [Shigella flexneri 2a str. 2457T]

MMQETSDQWLSWLSLYFWPLLRVLALISTAPILSERSVPKRVKLGLAMMITFAIAPSLPANDVPVFSFFA

LWLAVQQILIGIALGFTMQFAFAAVRTAGEIIGLQMGLSFATFVDPGSHLNMPVLARIMDMLALLLFLTF

NGHLWLISLLVDTFHTLPIGSEPLNSNAFLAPTKAGSLIFLNGLMLALPLITLLLTLNLALGLLNRMAPQ

LSIFVIGFPLTLTVGISLMAALMPLIAPFCEHLFSEIFNLLADIISELPLI

>gi|30063387|ref|NP_837558.1| flagellar biosynthesis protein FliQ [Shigella flexneri 2a str. 2457T]

MTPESVMMMGTEAMKVALALAAPLLLVALVTGLIISILQAATQINEMTLSFIPKIIAVFIAIIIAGPWML

NLLLDYVRTLFTNLPYIIG

>gi|30063385|ref|NP_837556.1| flagellar motor switch protein FliN [Shigella flexneri 2a str. 2457T]

MSDMNNPADDNNGAMDDLWAEALSEQKSTSEKSAADAVFQQFGGGDVSGTLQDIDLIMDIPVKLTVELGR

TRMTIKELLRLTQGSVVALDGLAGEPLDILINGYLIAQGEVVVVADKYGVRITDIITPSERMRRLSR

>gi|30063384|ref|NP_837555.1| flagellar motor switch protein FliM [Shigella flexneri 2a str. 2457T]

MGDSILSQAEIDALLNGDSEVKDEPTASISGESDIRPYDPNTQRRVVRERLQALEIINERFARHFRMGLF

NLLRRSPDITVGAIRIQPYHEFARNLPVPTNLNLIHLKPLRGTGLVVFSPSLVFIAVDNLFGGDGRFPTK

VEGREFTHTEQRVINRMLKLALEGYSDAWKAINPLEVEYVRSEMQVEFTNITTSPNDIVVNTPFHVEIGN

LTGEFNICLPFSMIEPLRELLVNPPLENSRNEDQNWRDNLVRQVQHSQLELVANFADISLRLSQILKLKP

GDVLPIEKPDRIIAHVDGVPVLTSQYGTLNGQYALRIEHLINPILNSLNEEQPK

>gi|30063383|ref|NP_837554.1| flagellar basal body-associated protein FliL [Shigella flexneri 2a str. 2457T]

MTDYAISKKSKRSLWIPILVFITLAACASAGYSYWHSHQVAADDKAQQRVVPSPVFYALDTFTVNLGDAD

RVLYIGITLRLKDEATRSRLSEYLPEVRSRLLLLFSRQDAAVLATEEGKKNLIAEIKTTLSTPLVAGQPK

QDVTDVLYTAFILR

>gi|30063382|ref|NP_837553.1| flagellar hook-length control protein [Shigella flexneri 2a str. 2457T]

MIRLAPLITADVDTTTLPGGKASDAAQDFLTLLSEALAGETTTDKAAPQLLVATDKPTTKGEPLVSDILA

DAQQADLLIPVDETLPVINDEQSTSTPLTTAQTMTLAAVADKNTTKDEKADDLNEDVTASLSALFAMLPG
FDNTPKVTDAPSTVLPAEKPTLFTKLTSAQLTTAQPDDAPGTPAQPLTPLVAEAQSKAEVISTPSPVTAD
ASPLITPHQTQPLPTVAAPVLSAPLGSHEWQQSLSQHISLFTRQGQQSAELRLHPQDLGEVQISLKVDDN
QAQIQMISPHQHVRAALEAALPVLRTQLAESGIQLGQSNISGESFSGQQQAASQQQQSQRTANHEPLAGE
DDDTLPVPVSLQGRVTGNSGVDIFA

>gi|30063381|ref|NP_837552.1| flagellum-specific ATP synthase [Shigella flexneri 2a str. 2457T]
MTTRLTRWLTTLDNFEAKMAQLPAVRRYGRLTRATGLVLEATGLQLPLGATCVIERQNGSETHEVESEVV
GFNGQRLFLMPLEEVEGVLPGARVYAKNISAEGLQSGKQLPLGPALLGRVLDGSGKPLDGLPFPDTTETG
ALITPPFNPLQRTPIEHVLDTGVRPINALLTVGRGQRMGLFAGSGVGKSVLLGMMARYTRADVIVVGLIG
ERGREVKDFIENILGAEGRARSVVIAAPADVSPLLRMQGAAYATRIAEDFRDRGQHVLLIMDSLTRYAMA
QREIALAIGEPPATKGYPPSVFAKLPALVERAGNGISGGGSITAFYTVLTEGDDQQDPIADSARAILDGH
IVLSRRLAEAGHYPAIDIEASISRAMTALISEQHYARVRTFKQLLSSFQRNRDLVSVGAYAKGSDPMLDK
AIALWPQLEGYLQQGIFERADWEASLQGLERIFPTVS

>gi|30063380|ref|NP_837551.1| flagellar assembly protein H [Shigella flexneri 2a str. 2457T]
MSDNLPWKTWTPDDLAPPPAEFVPMVESEETIIEEAEPSLEQQLAQLQMQAHEQGYQAGIAEGRQQGHEQ
GYQEGLAQGLEQGLAEAKAQQAPIHARMQQLVSEFQTTLDALDSVIASRLMQMALEAARQVIGQTPTVDN
SALIKQIQQLLQQEPLFSGKPQLRVHPDDLQRVDDMLGATLSLHGWRLRGDPTLHPGGCKVSADEGDLDA
SVATRWQELCRLAAPGVV

>gi|30063379|ref|NP_837550.1| flagellar motor switch protein G [Shigella flexneri 2a str. 2457T]
MSNLTGTDKSVILLMTIGEDRAAEVFKHLSQREVQTLSAAMANVTQISNKQLTDVLAEFEQEAEQFAALN
INANDYLRSVLVKALGEERAASLLEDILETRDTASGIETLNFMEPQSAADLIRDEHPQIIATILVHLRRA
QAADILALFDERLRHDVMLRIATFGGVQPAALAELTEVLNGLLDGQNLKRSKMGGVRTAAEIINLMKTQQ

EEAVITAVREFDGELAQKIIDEMFLFENLVDVDDRSIQRLLQEVDSESLLIALKGAEQPLREKFLRNMSQ

RAADILRDDLAKRGPVRLSQVENEQKAILLIVRRLAETGEMVIGSGEDTYV

>gi|30063378|ref|NP_837549.1| flagellar hook-basal body protein FliE [Shigella flexneri 2a str. 2457T]

MSAIQGIEGVISQLQATAMSARAQESLPQPTISFAGQLHAALDRISDTQTVARTQAEKFTLGEPGVALND

VMTDMQKASVSMQMGIQVRNKLVAAYQEVMSMQV

>gi|30063377|ref|NP_837548.1| multidrug efflux protein [Shigella flexneri 2a str. 2457T]

MNPYIYLGGAILAEVIGTTLMKFSEGFTRLWPSVGTIICYCASFWLLAQTLAYIPTGIAYAIWSGVGIVL

ISLLSWGFFGQRLDLPAIIGMMLICAGVLVINLLSRSAPH

>gi|30063376|ref|NP_837547.1| kinase inhibitor [Shigella flexneri 2a str. 2457T]

MKKLIVSSVLAFITFSAQAAAFQVTSNEIKTGEQLTTSHVFSGFGCEGGNTSPSLTWSGAPEGTKSFAVT

VYDPDAPTGSGWWHWTVANIPATVTYLPTDAGRRDGTKLPTGAVQGRNDFGYAGFGGACPPKGDKPHHYQ

FKVWALKTDKIPVDSNSSGALVGYMLNANKIATAEITPVYEIK

>gi|30063375|ref|NP_837546.1| regulator [Shigella flexneri 2a str. 2457T]

MLARDKSNLKIEEIRMHKHHEIHRVKPLMPALCRIRQGKKVINWETHSLTVDNNQIILFPCGYEFYIVNY

PEAGLYLAEMLYYPIDLIEKFQKFYAITDQIRNTTGFCLPQNPELIYCWEQLKTSISRGFSTQIQEHLAM

GVLLSLGAHHVNCLLLSDSKQSLISRCYNLMLSEPRTKWTANKVARYLYISVSTLHRRLASEGISFQSIL

DDVRLNNALSCYTNDSKTHQRDCQGKWLQVSFSFY

>gi|30063374|ref|NP_837545.1| outer membrane porin protein C precurser [Shigella flexneri 2a str. 2457T]

MKKLTVAISAVAASVLMAMSAQAAEIYNKDSNKLDLYGKVNAKHYFSSNDADDGDTTYVRLGFKGETQIN

DQLTGFGQWEYEFKGNRAESQGSSKDKTRLAFAGLKFGDYGSIDYGRNYGVAYDIGAWTDVLPEFGGDTW

TQTDVFMTGRTTGVATYRNNDFFGLVDGLNFAAQYQGKNDRTDVTEANGDGFGFSTTYEYEGFGVGATYA

KSDRTNDQVIYGNNSLNASGQNAEVWAAGLKYDANNIYLATTYSETQNMTVFGNNHIANKAQNFEVVAQY

QFDFGLRPSVAYLQSKGKDLGAWGDQDLIEYIDVGATYYFNKNMSTFVDYKINLIDKSDFTKASGVATDD

IVAVGLVYQF

>gi|30063373|ref|NP_837544.1| virulence protein [Shigella flexneri 2a str. 2457T]

MRYRNSRQVTGVVKNVTVSQSCGKWYISIQAESEVSTPVHPSASMVGLDAGVAKLASLSDGTVFEPVNSF

QKNQKTLARLQRQLSRKVKFSNNWQKQKRKIQRLHSCIANIRRDYLHKVTTTVSKNHVMIVIEDLKVSNM

SKSAAGTVSQPGRNVRAKSGLNRSILDQGWYEMRRQLEYKQLWRGGQVLAVPPAYTSQRCACCGHTAKEN

RLSQSKFRCQVCGYTANADVNGARNILAAGHAVLACGEMVQSGRSLKQEPTEMIQATA

>gi|30063372|ref|NP_837543.1| hypothetical protein S2069 [Shigella flexneri 2a str. 2457T]

MKNIVPDYRLDMVGEPCPYPAVATLEAMPQLKKGEILEVVSDCPQSINNIPLDARNHGYTVLDIQQDGPT

IRYLIQK

>gi|30063371|ref|NP_837542.1| inner membrane protein [Shigella flexneri 2a str. 2457T]

MSWQQFKHAWLIKFWAPIPAVIAAGILSTYYFGITGTFWAVTGEFTRWGGQLLQLFGVHAEEWGYFKIIH

LEGSPLTRIDGMMILGMFGGCFAAALWANNVKLRMPRSRIRIMQAIIGGIIAGFGARLAMGCNLAAFFTG

IPQFSLHAWFFAIATAIGSWFGARFTLLPIFRIPVKMQKVSAASPLTQKPDQARRRFRLGMLVFFGMLGW

ALLTAMNQPKLGLAMLFGVGFGLLIERAQICFTSAFRDMWITGRTHMAKAIIIGMAVSAIGIFSYVQLGV

EPKIMWAGPNAVIGGLLFGFGIVLAGGCETGWMYRAVEGQVHYWWVGLGNVIGSTILAYYWDDFAPALAT

DWDKINLLKTFGPMGGLLVTYLLLFAALMLIIGWEKRFFRREAPQTAKEIA

>gi|30063370|ref|NP_837541.1| hypothetical protein S2067 [Shigella flexneri 2a str. 2457T]

MKKLAIAGALMLLAGCAEVENYNNVVKTPAPDWLAGYWQTKGPQRALVSPEAIGSLIVTKEGDTLDCRQW

QRGIAVPGKLTLMSDDLTNVTVKRELYEVERDGNTIEYDGMTMERVDRPTAECAAALDKAPLPTPLP

>gi|30063369|ref|NP_837540.1| alpha-amylase [Shigella flexneri 2a str. 2457T]

MRNPTLLQCFHWYYPEGGKLWPELAERADGFNDIGINMVWLPPAYKGASGGYSVGYDSYDLFDLGEFDQK

GSIPTKYGDKAQLLAAIDALKRNDIAVLLDVVVNHKMGADEKEAIRVQRVNADDRTQIDEEIIECEGWTR

YTFPARAGQYSQFIWDFKCFSGIDHIEHPDEDGIFKIVNDYTGEGWNDQVDDELGNFDYLMGENIDFRNH

AVTEEIKYWARWVMEQTQCDGFRLDAVKHIPAWFYKEWIEHVQEVAPKPLFIVAECWSHEVDKLQTYIDQ

VEGKTMLFDAPLQMKFHEASRMGRDYDMTQIFTGTLVEADPFHAVTLVANHDTQPLQALEAPVEPWFKPL

AYALILLRENGVPSVFYPDLYGAHYEDVGGDGQTYPIDMPIIEQLDELILARQRFAHGVQTLFFDHPNCI

AFSRSGTDEYPGCVVVMSNGDDGEKTIHLGENYGNKTWRDFLGNRQESVVTDENGEATFFCNGGSVSVWV

IEEVI

>gi|30063368|ref|NP_837539.1| flagellar biosynthesis protein FliT [Shigella flexneri 2a str. 2457T]

MNNAPHLYFAWQQLVEKSQLMLRLATEEQWDELIASEMAYVNAVQEIAHLTEEVAPSTTMQEQLRPMLHL

ILDNESKVKQLLQIRMDELAKLVGQSSVQKSVLSAYGDQGGFVLAPQDNLF

>gi|30063367|ref|NP_837538.1| flagellar protein FliS [Shigella flexneri 2a str. 2457T]

MYAAKGTQAYAQIGVESTVMSASQQQLVTMLFDGVLSALVRARLFMQDNNQQGKGVSLSKAINIIENGLR

VSLDEESKDELTQNLIALYSYMVRRLLQANLRNDVSAVEEVEALMRNIADAWKESLLSPSSIQDPV

>gi|30063366|ref|NP_837537.1| flagellar capping protein [Shigella flexneri 2a str. 2457T]

MASISSLGVGSGLDLSSILDSLTAAQKATLTPISNQQSSFTAKLSAYGTLKSALTTFQTANTALSKADLF

SATSTTSSTTAFSATTAGNAIAGKYTISVTHLAQAQTLTTRTTRDDTKTAIATSDSKLTIQQGDDKDPIT

IDISAANSSLSGIRDAINNAKAGVSASIINVGNGEYRLSVTSNDTGLDNAMTLSVSGDDALQSFMGYDAS

ASSNGMEVSVAAQNAQLTVNNVAIENSSNTISDALENITLNLNDVTTGNQTLTITQDTSKVQTAIKDWVN

AYNSLIDTFSSLTKYTAVDAGADSQSSSNGALLGDSTLRTIQTQLKSMLSNTVSSSSYKTLAQIGITTDP

SDGKLELDADKLTAALKKDASGVGALIVGDGKKTGITTTIGSNLTSWLSTTGIIKAATDGVSKTLNKLTK

DYNAASDRIDAQVARYKEQFTQLDVLMTSLNSTSSYLTQQFENNSNSK

>gi|30063365|ref|NP_837536.1| flagellin [Shigella flexneri 2a str. 2457T]

MAQVINTNSLSLITQNNINKNQSALSSSIERLSSGLRINSAKDDAAGQAIANRFTSNIKGLTQAARNAND

GISVAQTTEGALSEINNNLQRIRELTVQASTGTNSDSDLDSIQDEIKSRLDEIDRVSGQTQFNGVNVLAK

DGSMKIQVGANDGQTITIDLKKIDSDTLGLNGFNVNGGGAVANTAASKADLVAANATVVGNKYTVSAGYD

AAKASDLLAGVSDGDTVQATINNGFGTAASATNYKYDSASKSYSFDTTTASAADVQKYLTPGVGDTAKGT

ITIDGSAQDVQISSDGKITASNGDKLYIDTTGRLTKNGSGASLTEASLSTLAANNTKATTIDIGGTSISF

TGNSTTPDTITYSVTGAKVDQAAFDKAVSTSGNNVDFTTAGYSVNGTTGAVTKGVDSVYVDNNEALTTSD

TVDFYLQDDGSVTNGSGKAVYKDADGKLTTDAETKAATTADPLKALDEAISSIDKFRSSLGAVQNRLDSA

VTNLNNTTTNLSEAQSRIQDADYATEVSNMSKAQIIQQAGNSVLAKANQVPQQVLSLLQG

>gi|30063364|ref|NP_837535.1| flagella biosynthesis protein FliZ [Shigella flexneri 2a str. 2457T]

MPHFNFDYQEFLMMVQHLKRRPLSRYLKDFKHSQTHCAHCRKLLDRITLVRDGKIVNKIEISRLDTLLDE

KGWQTEQQSWAALCRFCGDLHCKTQSDFFDIIGFKKFLFEQTEMSPGTVREYVVRLRRLGNHLHEQNISL

DQLQDGFLDEILAPWLPTTSTNNYRIALRKYQHYQRQTCTGLVQKSSSLPASDIY

>gi|30063362|ref|NP_837533.1| transport system permease (former yecC) [Shigella flexneri 2a str. 2457T]

MQESIQLVIDSLPFLLKGAGYTLQLSIGGMFFGLLLGFILALMRLSPIWPVRWLARFYISIFRGTPLIAQ

LFMIYYGLPQFGIELDPIPSAMIGLSLNTAAYAAETLRAAISSIDKGQWEAAASIGMTPWQTMRRAILPQ

AARVALPPLSNSFISLVKDTSLAATIQVPELFRQAQLITSRTLEVFTMYLAASLIYWIMATVLSTLQNHF

ENQLNRQEREPK

>gi|30063361|ref|NP_837532.1| amino-acid ABC transporter ATP-binding protein YecC [Shigella flexneri 2a str. 2457T]

MSAIEVKNLVKKFHGQTVLHGIDLEVKPGEVVAIIGPSGSGKTTLLRSINLLEQPEAGTITVGDITIDTA

RSLSQQKSLIRQLRQHVGFVFQNFNLFPHRTVLENIIEGPVIVKGEPKEEATARARELLAKVGLAGKETS

YPRRLSGGQQQRVAIARALAMRPEVILFDEPTSALDPELVGEVLNTIRQLAQEKRTMVIVTHEMSFARDV

ADRAIFMDQGRIVEQGAAKALFADPQQPRTRQFLEKLLLQ

>gi|30063360|ref|NP_837531.1| transcriptional regulator of ftsQAZ gene cluster [Shigella flexneri 2a str. 2457T]

MQDTDFFSWRRTMLLRFQRMEAAEEAYHEIELQAQQLEYDYYSLCVRHPVPFTRPKVAFYTNYPESWVSY

YQAKNFLAIDPVLNPENFSQGHLMWNDDLFSEAQPLWEAAHAHGLRRGVTQYLMLPNRALGFLSFSRCST

REIPILSDELQLKMQLLVRESLMALMRLNDEIVMTPEMNFSKREKEILKWTAEGKTSAEIAMILSITG

>gi|30063359|ref|NP_837530.1| hypothetical protein S2054 [Shigella flexneri 2a str. 2457T]

MSTPDFSTAENNQELANEVSCLKAMLTLMLQAMGQADAGRVMLKMEKQLALIEDETQAAVFSKTVKQIKQ

AYRQ

>gi|30063358|ref|NP_837529.1| response regulator [Shigella flexneri 2a str. 2457T]

MINVLLVDDHELVRAGIRRILEDIKGIKVVGEASCGEDAVKWCRTNAVDVVLMDMSMPGIGGLEATRKIA

RSTADVKIIMLTVHTENPLPAKVMQAGAAGYLSKGAAPQEVVSAIRSVYSGQRYIASDIAQQMALSQIEP

EKTESPFASLSERELQIMLMITKGQKVNEISEQLNLSPKTVNSYRYRMFSKLNIHGDVELTHLAIRHGLC

NAETLSSQ

>gi|30063356|ref|NP_837527.1| phosphatidylglycerophosphate synthetase [Shigella flexneri 2a str. 2457T]

MQFNIPTLLTLFRVILIPFFVLVFYLPVTWSPFAAALIFCVAAVTDWFDGFLARRWNQSTRFGAFLDPVA

DKVLVAIAMVLVTEHYHSWWVTLPAATMIAREIIISALREWMAELGKRSSVAVSWIGKVKTTAQMVALAW

LLWRPNIWVEYVGIALFFVAAVLTLWSMLQYLSAARADLLDQ

>gi|30063355|ref|NP_837526.1| hypothetical protein S2047 [Shigella flexneri 2a str. 2457T]

MKTGPLNESELEWLDDILTKYNTDHAILDVAELDGLLTAVLSSPQEIEPEQWLVAVWGGADYVPRWASEK

EMTRFMNLAFQHMADTAERLNEFPEQFEPLFGLREVDGSELTIVEEWCFGYMRGVALSDWSTLPDSLKPA

LEAIALHGTEENFERVEKMSPEAFEESVDAIRLAALDLHAYWMAHPQEKAVQQPIKAEEKPGRNDPCPCG

SGKKFKQCCLH

>gi|30063354|ref|NP_837525.1| tyrosine-specific transport protein [Shigella flexneri 2a str. 2457T]

MKNRTLGSVFIVAGTTIGAGMLAMPLAAAGVGFSVTLILLIGLWALMCYTALLLLEVYQHVPADTGLGTL

AKRYLGRYGQWLTGFSMMFLMYALTAAYISGAGELLASSISDWTGISMSATAGVLLFTFVAGGVVCVGTS

LVDLFNRFLFSAKIIFLVVMLVLLLPHIHKVNLLTLPLQQGLALSAIPVIFTSFGFHGSVPSIVSYMDGN

IRKLRWVFIIGSAIPLVAYIFWQVATLGSIDSTTFMGLLANHAGLNGLLQALREMVASPHVELAVHLFAD

LALATSFLGVALGLFDYLADLFQRSNTVGGRLQTGAITFLPPLAFALFYPRGFVMALGYAGVALAVLALI

IPSLLTWQSRKHNPQAGYRVKGGRPALVVVFLCGIAVIGVQFLIAAGLLPEVG

>gi|30063353|ref|NP_837524.1| hypothetical protein S2045 [Shigella flexneri 2a str. 2457T]

MDSIHGHEVLNMMIESGEQYTHASLEAAIKARFGEQARFHTCSAEGVTAGELVAFLAAKGKFIPSEEGFS

TDQSKICRH

>gi|30063352|ref|NP_837523.1| ferritin [Shigella flexneri 2a str. 2457T]

MLKPEMIEKLNEQMNLELYSSLLYQQMSAWCSYHTFEGAAAFLRRHAQEEMTHMQRLFDYLTDTGNLPRI

NTVESPFAEYSSLDELFQETYKHEQLITQKINELAHAAMTNQDYPTFNFLQWYVSEQHEEEKLFKSIIDK

LSLAGKSGEGLYFIDKELSTLDTQN

>gi|30063351|ref|NP_837522.1| hypothetical protein S2043 [Shigella flexneri 2a str. 2457T]

MRLLILTLSLITLAGCTVTRQAHVSEVDAATGIVRLVYDQAFLQHAHTDRYVSRGIADRACQQEGYTHAI

PFGQPVGNCSLFAGSLCLNTEFTLSYQCHHSAFPVFL

>gi|30063349|ref|NP_837520.1| ferritin-like protein [Shigella flexneri 2a str. 2457T]

MATAGMLLKLNSQMNREFYASNLYLHLSNWCSEQSLNGTATFLRAQAQSNVTQMMRMFNFMKSVGATPIV

KAIDVPGEKLNSLEELFQKTMEEYEQRSSTLAQLADEAKELNDDSTVNFLRDLEKEQQHDGLLLQTILDE

VRSAKLAGMCPVQTDQHVLNVVSHQLH

>gi|30063348|ref|NP_837519.1| L-arabinose transporter ATP-binding protein [Shigella flexneri 2a str. 2457T]

MQQSTPYLSFRGIGKTFPGVKALTDISFDCYAGQVHALMGENGAGKSTLLKILSGNYAPTTGSVVINGQE

MSFSDTTAALNAGVAIIYQELHLVPEMTVAENIYLGQLPHKGGIVNRSLLNYEAGLQLKHLGMDIDPDTP

LKYLAIGQWQMVEIAKALARNAKIIAFDEPTSSLSAREIGNLFRVIRELRKEGRVILYVSHRMEEIFALS

DAITVFKDGRYVKTFTDMQQVDHDALVQAMVGRDIGDIYGWQPRSYGEERLRLDAVKALGVRTPISLAVR

SGEIVGLFGLVGAGRSELMKGLFGGTQITAGQVYIDQQPIDIRKPSHAIAAGMMLCPEARKAEGIIPVHS

VRDNINISARRKHVLGGCVINNSWEENNADQHIRSLNIKTPGAEQLIMNLSGGNQQKAILGRWLSEEMKV

ILLDEPTRGIDVGAKHEIYNVIYALAAQGVAVLFASSDLPEVLGVADRIVVMREGEIAGELLHEQADERQ

ALSLAMPKVSQAVA

>gi|30063347|ref|NP_837518.1| L-arabinose transporter permease [Shigella flexneri 2a str. 2457T]

MMSSVSTSGSGAPKSSFSFGRIWDQYGMLVVFAVLFIACAIFVPNFATFINMKGLGLAISMSGMVACGML

FCLASGDFDLSVASVIACAGVTTAVVINLTESLWIGVAAGLLLGVLCGLVNGFVIAKLKINALITTLATM

QIVRGLAYIISDGKAVGIEDESFFALGYANWFGLPAPIWLTVACLIIFGLLLNKTTFGRNTLAIGGNEEA

ARLAGVPVVRTKIIIFVLSGLVSAIAGIILASRMTSGQPMTSIGYELIVISACVLGGVSLKGGIGKISYV

VAGILILGTVENAMNLLNISPFAQYVVRGLILLAAVIFDRYKQKAKRTV

>gi|30063346|ref|NP_837517.1| trehalose-6-phosphate phosphatase [Shigella flexneri 2a str. 2457T]

MTEPLTETPELSAKYAWFFDLDGTLAEIKPHPDQVVVPDNILQGLQLLATASDGALALISGRSMVELDAL

AKPYRFPLAGVHGAERRDINGKTHIVHLPDAIARDISVQLHTVIAQYPGAELEAKGMAFALHYRQAPQHE

DALMTLAQRITQIWPQMALQQGKCVVEIKPRGTSKGEAIAAFMQEAPFIGRTPVFLGDDLTDESGFAVVN

RLGGMSVKIGTGATQASWRLAGVPDVWSWLEMITAALQQKRENNRSDDYESFSRSI

>gi|30063345|ref|NP_837516.1| IS1 orfB [Shigella flexneri 2a str. 2457T]

MSRQCTHYGRWPQHGFTSLKKLRPQSVTSRIQPGSDVIVCAEMDEHWGYVGAKSRQRWLFYAYDRIRRTV

VAHVFGERTLATLERLLSLLSAFEVVVWMTDGWPLYESRLKGKLHVISKRYTQRIERHNLNLRQHLARLV

RKSLSFSKSVELHDKVIGHYLNIKHYQ

>gi|30063344|ref|NP_837515.1| IS1 orfA [Shigella flexneri 2a str. 2457T]

MASISIRCPSCSATEGVVRNGKSTAGHQRYLCSHCRKTWQLQFTYTASQPGTHQKIIDMAMNGVGCRASA

RIMGVGLNTVLRHLKNSGRSR

>gi|30063343|ref|NP_837514.1| transcriptional activator FlhC [Shigella flexneri 2a str. 2457T]

MSEKSIVQEARDIQLAMELITLGARLQMLESETQLSRGRLIKLYKELRGSPPPKGMLPFSTDWFMTWEQN

VHASMFCNAWQFLLKTGLCNGVDAVIKAYRLYLEQCPQAEEGPLLALTRAWTLVRFVESGLLQLSSCNCC

GGNFITHAHQPVGSFACSLCQPPSRAVKRRKLSQNPADIIPQLLDEQRVQAV

>gi|30063342|ref|NP_837513.1| flagellar motor protein MotA [Shigella flexneri 2a str. 2457T]

MLILLGYLVVLGTVFGGYLMTGGSLGALYQPAELVIIAGAGIGSFIVGNNGKAIKGTLKALPLLFRRSKY

TKAMYMDLLALLYRLMAKSRQMGMFSLERDIENPRESEIFASYPRILADSVMLDFIVDYLRLIISGHMNT

FEIEALMDEEIETHESEAEVPANSLALVGDSLPAFGIVAAVMGGVHALGSADRPAAELGALIAHAMVGTF

LGILLAYGFISPLASVLRQKSAETSKMMQCVKVTLLSNLNGYAPPIAVEFGRKTLYSSERPSFIELEEHV

RAVKNPQQQTTTEEA

>gi|30063341|ref|NP_837512.1| flagellar motor protein MotB [Shigella flexneri 2a str. 2457T]

MKNQAHPIIVVKRRKAKSHGAAHGSWKIAYADFMTAMMAFFLVMWLISISSPKELIQIAEYFRTPLATAV

TGGDRISNSESPIPGGGDDYTQSQGEVNKQPNIEELKKRMEQSRLRKLRGDLDQLIESDPKLRALRLHLK

IDLVQEGLRIQIIDSQNRPMFRTGSADVEPYMRDILRAIAPVLNGIPNRISLSGHTDDFPYASGEKGYSN

WELSADRANASRRELMVGGLDSGKVLRVVGMAATMRLSDRGPDDAVNRRISLLVLNKQAEQAILHENAES

QNEPVSALEKPEVAPQVSVPTMPSAEPR

>gi|30063340|ref|NP_837511.1| chemotaxis protein CheA [Shigella flexneri 2a str. 2457T]

MSIDISDFYQTFFDEADELLADMEQHLLVLQPEAPDAEQLNAIFRAAHSIKGGAGTFGFSVLQETTHLME

NLLDEARRGEMQLNTDIINLFLETKDIMQEQLDAYKQSQEPDAASYDYICQALRQLALEAKGETPSAVTR

LSVVAKSEPQDEQSRSQLPRRIILSRLKAGEVDLLEEELGHLTTLNDVVKGADSLSAILPGDIAEDDITA

VLCFVIEADQITFETVEVSPKIATPPVLKLAAEQAPTGRVEREKTTRSSESTSIRVAVEKVDQLINLVGE

LVITQSMLAQRSSELDPVNHGDLITSMGQLQRNARDLQESVMSIRMMPMEYVFSRYPRLVRDLAGKLGKQ

VELTLVGSSTELDKSLIERIIDPLTHLVRNSLDHGIELPEKRLAAGKNSVGNLILSAEHQGGNICIEVTD

DGAGLNRERILAKAASQGLTVSENMSDDEVAMLIFAPGFSTAEQVTDVSGRGVGMDVVKRNIQEMGGHVE

IQSKQGTGTTIRILLPLTLAILDGMSVRVADEVFILPLNAVMESLQPREADLHPLAGGERVLEVRGEYLP

IVELWKVFNVAGAKTEATQGIVVILQSGGRRYALLVDQLIGQHQVVVKNLESNYRKVPGISAATILGDGS

VALIVDVSALQAINREQRMANTAA

>gi|30063339|ref|NP_837510.1| purine-binding chemotaxis protein [Shigella flexneri 2a str. 2457T]

MTGMTNVTKLASEPSGQEFLVFTLGDEEYGIDILKVQEIRGYDQVTRIANTPAFIKGVTNLRGVIVPIVD

LRIKFSQVDVDYNDNTVVIVLNLGQRVVGIVVDGVSDVLSLTAEQIRPAPEFAVTLSTEYLTGLGALGDR

MLILVNIEKLLNSEEMALLDSAASEVA

>gi|30063338|ref|NP_837509.1| methyl-accepting chemotaxis protein II, aspartate sensor receptor [Shigella flexneri 2a str. 2457T]

MINRIRVVTLLVMVLGVFALLQLISGSLFFSSLHHSQKSFVVSNQLREQQGELTSTWDLMLQTRINLSRS

AVRMMMDSSNQQSNAKVELLDSARKTLAQAATHYKKFKSMAPLPEMVATSRNFDEKYKNYYTALTELIDY

LDYGNTGAYFAQPTQGMQNAMGEAFAQYALSSEKLYRDIVTDNADDYRFAQWQLAVIALVVVLILLVAWY

GIRRMLLTPLAKIIAHICEIAGGNLANTLTIDGRSEMGDLAQSVSHMQRSLTDTVTHVREGSDAIYAGTR

EIAAGNTDLSSRTEQQASALEETAASMEQLTATVKQNADNARQASQLAQSASDTAQHGGKVVDGVVKTMH

EIADSSKKIADIISVIDGIAFQTNILALNAAVEAARAGEQGRGFAVVAGEVRNLASRSALAAKEIKALIE

DSVSRIDTGSVLVESAGETMNNIVNAVTRVTDIMGEIASASDEQSRGIDQVALAVSEMDRVTQQNASLVQ

ESAAAAAALEEQASRLTQAVSAFRLAASPLTNKPQTPSRPASEQPPAQPRLRITEQDPNWETF

>gi|30063337|ref|NP_837508.1| methyl-accepting protein IV [Shigella flexneri 2a str. 2457T]

MFNRIRISTTLFLILILCGILQIGSNGMSFWAFRDDLQRLNHGEQSNQQRAALAQTRAVMLQASTALNKA

GTLTALSYPADDIKTLMTTARASLTQSTTLFKSFMAMTAGNEHVRALQKETEKSFARWHNDLEHQATWLE

SNQLSDFLTAPVQGSQNAFDVNFEAWQLEINHVLEAASAQSQRNYQISALVFISMIIVAAIYISSALWWT

RKMIVQPLAIIGSHFDSIAAGNLARPIAVYGRNEITAIFASLKTMQQALRGTVSDVRKGSHEMHIGIAEI

VAGNNDLSSRTEQQAASLAQTAASMEQLTATVGQNADNARQASELAKNAATTAQAGGVQVSTMTHTMQEI

ATSSQKIGDIISVIDGIAFQTNILALNAAVEAARAGEQGRGFAVVAGEVRNLASRSAQAAKEIKGLIEES

VNRVQQGSKLVNNAAATMTDIVSSVTRVNDIMGEIASASEEQRRGIEQVAQAVSQMDQVTQQNSSLVEEA

AVATEQLANQADHLSSRVAVFTLEEHEVARHESAQLQIAPVVS

>gi|30063336|ref|NP_837507.1| chemotaxis methyltransferase CheR [Shigella flexneri 2a str. 2457T]

MTSSLPCGQTSLLLQMTERLALSDAHFRRISQLIYQRAGIVLADHKRDMVYNRLVRRLRSLGLTDFGHYL

NLLESNQHSGEWQAFINSLTTNLTAFFREAHHFPLLADHARRRSGEDRVWSAAASTGEEPYSIAMTLADT

LGTVPGRWKVFASDIDTEVLEKARSGIYRHEELKNLTPQQLQRYFMRGTGPHEGLVRVRQELANYVDFAP
LNLLAKQYTVPGPFDAIFCRNVMIYFDQTTQQEILRRFVPLLKPDGLLFAGHSENFSHLERRFTLRGQTV
YALSKD

>gi|30063335|ref|NP_837506.1| chemotaxis-specific methylesterase [Shigella flexneri 2a str. 2457T]
MSKIRVLSVDDSALMRQIMTEIINSHSDMEMVATAPDPLVARDLIKKFNPDVLTLDVEMPRMDGLDFLEK
LMRLRPMPVVMVSSLTGKGSEVTLRALELGAIDFVTKPQLGIREGMLAYSEMIAEKVRTAAKASLAAHKP
LSAPTTLKAGPLLSSEKLIAIGASTGGTEAIRHVLQPLPLSSPALLITQHMPPGFTRSFADRLNKLCQIG
VKEAEDGERVLPGHAYIAPGDRHMELARSGANYQIKIHDGPAVNRHRPSVDVLFHSVAKQAGRNAVGVIL
TGMGNDGAAGMLAMRQAGAWTLAQNEASCVVFGMPREAINMGGVCEVVDLSQVSQQMLATISAGQAIRI

>gi|30063334|ref|NP_837505.1| chemotaxis regulatory protein CheY [Shigella flexneri 2a str. 2457T]
MADKELKFLVVDDFSTMRRIVRNLLKELGFNNVEEAEDGVDALNKLQAGGYGFVISDWNMPNMDGLELLK
TIRADGAMSALPVLMVTAEAKKENIIAAAQAGASGYVVKPFTAATLEEKLNKIFEKLGM

>gi|30063333|ref|NP_837504.1| chemotaxis regulator CheZ [Shigella flexneri 2a str. 2457T]
MMQPSIKPADEHSAGDIIARIGSLTHMLRDSLRELGLVQAIAEAAEAIPDARDRLYYVVQMTAQAAERAL
NSVEASQPHQDQMEKSAKALTQRWDDWFADPIDLADARELVTDTRQFLADVPAHTSFTNAQLLEIMMAQD
FQDLTGQVIKRMMDVIQEIERQLLMVLLENIPEQESRPKRENQSLLNGPQVDTSKAGVVASQDQVDDLLD
SLGF

>gi|30063332|ref|NP_837503.1| IS600 orfB [Shigella flexneri 2a str. 2457T]
MAHIRTRETYGTRRLQTELAENGIIVGRDRLARLRKELRLRCKQKRKFRATTNPNHNLPVAPNLLNQTFA
PTAPNQVWVADLTYVATQEGWLYLAGIKDVYTCEIVGYAMGERMTKELTGKALFMALRSQRPPAGLIHHS
DRGSQYCAYDYRVIQEQFGLKTSMSRKGNCYDNAPMESFWGTLKNESLSHYRFNNRDEAISVIREYIEIF

YNRQRRHSRLGNISPAAFREKYHQMAA

>gi|30063331|ref|NP_837502.1| hypothetical protein S2019 [Shigella flexneri 2a str. 2457T]

MNVVSNTQLLEQRIADFFTLSDEHKKARVLLDTLACSCPARIFGGMVRDLGLYGVDGFSSDLDIVIGRSR

EELFQTLAELPVKQLRFNKFGGIRFRYHDFEFDIWNLNETWAFQEKLIFCEDESSLLNEVA

>gi|30063330|ref|NP_837501.1| IS911 orfA [Shigella flexneri 2a str. 2457T]

MKKRNFSAEFKRESAQLVVDQKYTVADAAKAMDVGLSTMTRWVKQLRDERQGKTPKASPITPEQIEIREL

RKKLQRIEMENEILKKATALLMSDSLNSSR

>gi|30063329|ref|NP_837500.1| hypothetical protein S2015 [Shigella flexneri 2a str. 2457T]

MEWQQLLNCNRRKDNLKKTNIAFLHPLASGRQEIERDYDRLLFAAPTRRLADKTQVFPLDQNDSIRTRLT

HSHEVANFARGIGMRLAFEMKEHIFGEIPTGIVVERDVPALLAAIGLAHDLGNPPFGHLKWSTKTGHRVR

VFPVSIFRFVWG

>gi|30063328|ref|NP_837499.1| flagellar biosynthesis protein FlhB [Shigella flexneri 2a str. 2457T]

MSDESDDKTEAPTPHRLEKAREEGQIPRSRELTSLLILLVGVSVIWFGGVSLARRLSGMLSAGLHFDHSI

IKDPNLILGQIILLIREAMLALLPLISGVVLVALISPVMLGGLVFSGKSLQPKFSKLNPLPGIKRIFSAQ

TSAELLKAILKTILVGSVTGFFLWHHWPQMMRLMAESPITAMGNAMDLVGLCALLVVLGVIPMVGFDVFF

QIFSHLKKLRMSRQDIRDEFKQSEGDPHVKGRICQMQRAAARRRMMADVPKADVIVNNPTHYSVALQYDE

NKMSAPKVVAKGAGLVALRIREIGAENNVPTLEAPPLARALYRHAEIGQQIPGQLYAAVAEVLAWVWQLK

RWRLAGGQRPVHLLIFRCRQPWILLTRNRPMSNLAAMLRLPANLKST

>gi|30063327|ref|NP_837498.1| flagellar protein [Shigella flexneri 2a str. 2457T]

MRTLLAILLFPLLVQAAGEGMWQASSVGITLNHRGESMSSAPLSARQPASGLMTLVAWRYQLIGPTPAGL

RVRLCSQSRCVELEGQSGTTVAFSGIAAAEPLRFIWEVPGGG

>gi|30063326|ref|NP_837497.1| IS629 orfA [Shigella flexneri 2a str. 2457T]

MTKNTRFSPEVRQRAVRMVLESQGEYDSQWATICSIAPKIGCTPETLRVWGRQHERDTGGGDGGLITAER

QRLKEPERENRELRRSNNILRLASAYFAKAEFDRLWKK

>gi|30063325|ref|NP_837496.1| IS629 orfB [Shigella flexneri 2a str. 2457T]

MWRSSLMCLPDTSWGAMETTFVLDALEQALWARRPSGTVHHSDKGSQYVSLAYTQRLKEAGLLASTGSTG

DSYDNAMAESINGLYKAEVIHRKSWKNRAEVELATLTWVDWYNNRRLLERLGHTPPAEAEKAYYASIGND

DLAA

>gi|30063324|ref|NP_837495.1| arginyl-tRNA synthetase [Shigella flexneri 2a str. 2457T]

MNIQALLSEKVRQAMIAAGAPADCEPQIRQSAKVQFGDYQANGMMAVAKKLGMAPRQLAEQVLTHLDLNG

IASKVEIAGPGFINIFLDPAFLAEHVQQALASDRLGVATPEKQTIVVDYSAPNVAKEMHVGHLRSTIIGD

TAVRTLEFLGHKVIRANHVGDWGTQFGMLIAWLEKQQQENAGEMELADLEGFYRDAKKHYDEDEEFAERA

RNYVVKLQSGDEYFREMWRKLVDITMTQNQITYDRLNVTLTRDDVMGESLYNPMLPGIVADLKAKGLAVE

SEGATVVFLDVFKNKEGEPMGVIIQKKDGGYLYTTTDIACAKYRYETLHADRVLYYIDSRQHQHLMQAWA

IVRKAGYVPESVPLEHHMFGMMLGKDGKPFKTRAGGTVKLADLLDEALERARRLVAEKNPDMPADELEKL

ANAVGIGAVKYADLSKNRTTDYIFDWDNMLAFEGNTAPYMQYAYTRVLSVFRKAEINEEQLAAAPVIIRE

DREAQLAARLLQFEETLTVVAREGTPHVMCAYLYDLAGLFSGFYEHCPILSAENEEVRNSRLKLAQLTAK

TLKLGLDTLGIETVERM

>gi|30063323|ref|NP_837494.1| hypothetical protein S2006 [Shigella flexneri 2a str. 2457T]

MANWQSIDELQDIASDLPRFTHALDELSRRLGLDITPLTADHISLRCHQNVTAERWRRGFEQCGELLSEK

MINGRPICLFKLHEPVQVAHWQFSNVELPWPGEKRYPHEGWEHIEIVLPGDPETLNARALALLSDEGLSL

PGISVKTSSPKGEHERLPNPTLAVTDGKTTIKFHPWSIEEIVASEQSA

>gi|30063322|ref|NP_837493.1| copper homeostasis protein, partial [Shigella flexneri 2a str. 2457T]

MPRMEKIMAAAGPLAVTFHRAFDMCANPLYTLNNLAELGIARVLTSGQKSDALQGLSKIMELIAHRDAPI

IMAGAGVRAENLHHFLDAGVLEVHSSAGAWQASPMRYRNQGLSMSSDEHADEYSRYIVDGAAVAEMKGII

ERHQAK

>gi|30063321|ref|NP_837492.1| cytochrome C-type protein [Shigella flexneri 2a str. 2457T]

MRGKKRIGLLFLLIAVVVGGGGLLLAQKALHKTSDTAFCLSCHSMSKPFEEYQGTVHFSNQKGIRAECAD

CHIPKSGMDYLFAKLKASKDIYHEFVSGKIDSDDKFETHRQEMAETVWKELKATDSATCRSCHSFDAMDI

ASQSESAQKMHNKAQKGGETCIDCHKGIAHFPPEIKMDDNAAHELESQAATSVTNGAHIYPFKTSRIGEL

ATVNPGTDLTVVDASGKQPIVLLQGYQMQGSENTLYLAAGQRLALATLSEEGIKALTVNGEWQADEYGNQ

WRQASLQGALTDPALADRKPLWQYAEKLDDTYCAGCHASIAADHYTVNAWPSIAKGMGARTSMSENEPDI

LTRYFQYNAKDITEKQ

>gi|30063320|ref|NP_837491.1| biotin sulfoxide reductase [Shigella flexneri 2a str. 2457T]

MALTRREFIKHSGIAAGALVVTSAAPLPAWAEEKGGKILTAGRWGAMSVEVKDGKIVSSTGALAKTIPNS

LQSTAADQVHTTARIQHPMVRKSYLDNPLQPAKGRGEDTYVQVSWEQALKLIHEQHERIRKANGPSAIFA

GSYGWRSSGVLHKAQTLLQRYMNLAGGYSGHSGDYSTGAAQVIMPHVVGSVEVYEQQASWPLILENSQVV

VLWGMNPLNTLKIAWSSTDEQGLEYFHQLKKSGKPVIAIDPIRSETIEFFGDNATWIAPNMGTDVALMLG

IAHTLMTQGKHDKVFLEKYTTGYPQFEEYLTGKSDNTPKSAVWTAEITGVPEAQIVKLAELMAANRTMLM

AGWGIQRQQYGEQKHWMLVTLAAMLGQIGTPGGGFGFSYHYSNGGNPTRVGGVLPEMSAAIAGQASEAAD

DGGMTAIPVARIVDALENPGGKYQHNGKEQTYPNIKMIWWAGGGNFTHHQDTNRLIKAWQKPEMIVVSEC

YWTAAAKHADIVLPITTSFERNDLTMTGDYSNQHIVPMKQAVAPQFEARNDFDVFADLAELLKPGGKEIY

TEGKDEMAWLKFFYDAAQKGARAQRVTMPMFNAFWQQNKLIEMRRSEKNEQYVRYGDFRADPVKNALGTP

SGKIEIYSKTLEKFGYKDCPAHPTWLAPDEWKGTADEKQLQLLTAHPAHRLHSQLNYAELRKKYAVADRE

PITIHTEDAARFGIANGDLVRVWNKRGQILTGAVVTDGIKKGVVCVHEGAWPDLENGLCKNGSANVLTAD

IPSSQRANACAGNSALVYIEKYTGNAPKLTAFDKPAVQA

>gi|30063319|ref|NP_837490.1| enzyme [Shigella flexneri 2a str. 2457T]

MIDFGNFYSLIAKNHLSHWLETLPAQIANWQREQQHGLFKQWSNTVEFLPEIKPYRLDLLHSVTAESEEP

LSTGQIKRIETLMRNLMPWRKGPFSLYGVNIDTEWRSDWKWDRVLPHLSDLTGRTILDVGCGSGYHMWRM

IGAGAHLAVGIDPTQLFLCQFEAVRKLLGNDQRAHLLPLGIEQLPALKAFDTVFSMGVLYHRRSPLEHLW

QLKDQLVNEGELVLETLVIDGDENTVLVPGDRYAQMRNVYFIPSALALKNWLKKCGFVDIRIVDVCVTTT

EEQRRTEWMVTESLSDFLDPHDPSKTVEGYPAPKRAVLIARKP

>gi|30063318|ref|NP_837489.1| hypothetical protein S2001 [Shigella flexneri 2a str. 2457T]

MSHRDTLFSAPIARLGDWTFDERVAEVFPDMIQRSVPGYSNIISMIGMLAERFVQPGTQVYDLGCSLGAA

TLSVRRNIHHDNCKIIAIDNSPAMIERCRRHIDAYKAPTPVDVIEGDIRDIAIENASMVVLNFTLQFLEP

SERQALLDKIYQGLNPGGALVLSEKFSFEDAKVGELLFNMHHDFKRANGYSELEISQKRSMLENVMLTDS

VETHKARLHKAGFEHSELWFQCFNFGSLVALKAEDAA

>gi|30063317|ref|NP_837488.1| hypothetical protein S2000 [Shigella flexneri 2a str. 2457T]

MVSALYAVLSALLLMKFSFDVVRLRMQYRVAYGDGGFSELQSAICIHGNAVEYIPIAIVLMLFMEMNGAE

TWMVHICGIVLLAGRLMHYYGFHHRLFRWRRSGMSATWCALLLMVLANLWYMPWELVFSLR

>gi|30063316|ref|NP_837487.1| IS911 orfA [Shigella flexneri 2a str. 2457T]

MKKRNFSAEFKRESAQLVVDQKYTVADAAKAMDVGLSTMTRWVKQLRDERQGKTPKASPITPEQIEIREL

RKKLQRIEMENEILKKATALLMSDSLNSSR

>gi|30063315|ref|NP_837486.1| bacteriophage protein [Shigella flexneri 2a str. 2457T]

MNKAFELWVHQRYGNHYDLTRDVDGFYCREIVKRMFEVWCHCRGLSVV

>gi|30063314|ref|NP_837485.1| hypothetical protein S1994 [Shigella flexneri 2a str. 2457T]

MFALIQRGQIYTDRAGYPVEITRSTEHSVFFRRMDGRTGRVRIGEFSSLFEHIDHLEYHKILAETEQEKH

LKKLRAMQRK

>gi|30063313|ref|NP_837484.1| hypothetical protein S1992 [Shigella flexneri 2a str. 2457T]

MIIQSKLIRAALVCAAKNDVRYYLNGLHITPKHIEATNGSVALRMAHGIRTKKNIIVQFEGGVPAKAETT

ELIFSKEPIAVHRDQFQRRLSITGIKLVDGCFPDLDRIIPKKFDRCTHPVLQAGYLSYPEKMFGRERKFI

PVQLRPSGDGQAVRIQFDSIINSMYGNPEFVVMPCRDHGDFNVAQEHPE

>gi|30063312|ref|NP_837483.1| hypothetical protein S1991 [Shigella flexneri 2a str. 2457T]

MNTVTINNKQFPVIEYRGQRVVTLAMIDEVHQRPEGTARAAFNRNREHFISGVDYAELGADVIRTDLPEG

TFSKFAPSGIVLFESGYLMLTKPFNDALAWQVQRELVNSYFRTRTPLTEIEMIAAIAADAVRQQKRLNHV

EEQLETVTEAVETIKRGNMRAGYVGYRQVVAKSGMSDTKCRNLVNAYQIPTDTHEFMTPDGLLSRRAIVE

LEPFMAAFHQMMSEADPRGTRWYHPKMGLFQVIGWEDKA

>gi|30063311|ref|NP_837482.1| serine protease [Shigella flexneri 2a str. 2457T]

MKKVIPALALVLLTTGLVGCDRVEPGNVGIKVNKLGDDKGVGEVVGVGRYWTGWNTEVYIFPTFKQMKTY

DEPFSFQMSDGTTIGYHIGVAYKVDPSKVTTVFQTYRKGVDDITDTDLRQKIADALNRLASKMTTDKFID

GGKSELLDAALKDIQEEMTPIGIQVMSLSYVGKPEYPPTVIDSINAKVTANQKTLQCEQEVKQREAEANM

LRAEAAGQADAIRTKAQAEADAIRLRGEALRQNPNVMELEAINKWNGTLPQYMTSGANTPFIQVK

>gi|30063310|ref|NP_837481.1| IS600 orfB [Shigella flexneri 2a str. 2457T]

MAHIRTRETYGTRRLQTELAENGIIVGRDRLARLRKELRLRCKQKRKFRATTNPNHNLPVAPNLLNQTFA

PTAPNQVWVADLTYVATQEGWLYLAGIKDVYTCEIVGYAMGERMTKELTGKALFMALRSQRPPAGLIHHS

DRGSQYCAYDYRVIQEQFGLKTSMSRKGNCYDNAPMESFWGTLKNESLSHYRFNNRDEAISVIREYIEIF

YNRQRRHSRLGNISPAAFREKYHQMAA

>gi|30063309|ref|NP_837480.1| crossover junction endodeoxyribonuclease [Shigella flexneri 2a str. 2457T]

MRHEFILPYPPTVNTYWRRRGSTYFVSKAGERYRRDVALIVRQQRLKLSLSGRLAIKIIAEPPDKRRRDL

DNILKAPLDALTHAGLLIDDEQFDETNIVRGLPVPGGRLGIKITELECA

>gi|30063308|ref|NP_837479.1| Q antiterminator encoded by prophage CP-933P [Shigella flexneri 2a str. 2457T]

MNNQYLQFVREQLIIATADLSGATKGQLEAWQENAMFDTGRYRRKKIRYRDEVTGKMITRDNPPIPGKQS

LAKGASIPLVSQVAFSTSSWRRAVLSLEEHYKAWLLWCYSGSICWEYQITITHWAWEEFKAHSGTRKIAE

KTQERLKKLIWLAAQAVKAELFGGEGYEYQELALLAGVTTKNWSKTFTGHWVVMKHIFHRLDSEALLFVM

RTRSEQKAAFSKQSIAKVD

>gi|30063307|ref|NP_837478.1| integrase [Shigella flexneri 2a str. 2457T]

MIQLSSSHKLPAVYYLYQRNGVYYFRLRVRQSNNDRMTSISLRTKDRRTAMAYSRHIKAALKAIHADRPN

ATYEEMREHLKDIAECELSMGRSDLFEPDMRDIYRDQYGELGESLTDALASEPLSIDQHRYINEALKVLK

ACMRRIEAGDSQPLIDYVDLFNDIDRQDNQADSVSLSVNAPEVKPEVTPSITIASLFEQYEAENYQNWKP

ATLRENKASHAALIEIFDHLGLNADANRADMLRVRDVLQQLPRNRKQRFKDVPLADLLSREDKTDCLDVV

TINNKYLIKMAAVFRWAVRNDLIKKNMTEGLELKVPQRKASGARNAFSTEQVGQLLVAAKAYSQKTSGKP

YHYYVTALAAITGARLNEIAQLQVKDVRTTEAGTVYIHINEDDSSLPGKSIKNAHSDRCVPLVDGAYGFI

LADFMALVETRRGADGDDAMVFDGLRLMKNGYGEQVSKWFNRTLLPKVLVDRSGLAFHSFRHTVAAQLKQ

HGVELAYAQAIMGHSSGSITYDRYAKEVEVDRLVNVMADVYKET

>gi|30063306|ref|NP_837477.1| IS911 orfA [Shigella flexneri 2a str. 2457T]

MKKRNFSAEFKRESAQLVVDQKYTVADAAKAMDVGLSTMTRWVKQLRDERQGKTPKASPITPEQIEIREL

RKKLQRIEMENEILKKATALLMSDSLNSSR

>gi|30063305|ref|NP_837476.1| IS911 orfB, partial [Shigella flexneri 2a str. 2457T]

MKELGLVSCQQPTHRYKRGGHEHVAIPNYLERQFAVTEPNQVWCGDVTYIWTGKRWAYLAVVLDLFARKP

VGWAMSFSPDSRLTMKALEMAWETRGKPGGVMFHSDQGSHYTSRQFRQLLWRYQIRQSMSRRGNCWDNSP

MERFFRSLKNEWMPVVGYVSFSEAAHAITDYIVGYYSALRPHEYNGGLPPNESENRYWKNSNSVASFC

>gi|30063304|ref|NP_837475.1| IS1 orfA [Shigella flexneri 2a str. 2457T]

MASISIRCPSCSATEGVVRNGKSTAGHQRYLCSHCRKTWQLQFTYTASQPGTHQKIIDMAMNGVGCRASA

RIMGVGLNTVLRHLKNSGRSR

>gi|30063303|ref|NP_837474.1| IS1 orfB [Shigella flexneri 2a str. 2457T]

MTDGWPLYESRLKGKLHVISKRYTQRIERHNLNLRQHLARLVRKSLSFSKSVELHDKVIGHYLNIKHYQ

>gi|30063302|ref|NP_837473.1| IS600 orfB [Shigella flexneri 2a str. 2457T]

MAHIRTRETYGTRRLQTELAENGIIVGRDRLARLRKELRLRCKQKRKFRATTNPNHNLPVAPNLLNQTFA

PTAPNQVWVADLTYVATQEGWLYLAGIKDVYTCEIVGYAMGERMTKELTGKALFMALRSQRPPAGLIHHS

DRGSQYCAYDYRVIQEQSGLKTSMSRKGNCYDNAPMESFWGTLKNESLSHYRFNNRDEAISVIREYIEIF

YNRQRRHSRLGNISPAAFREKYHQMTA

>gi|30063301|ref|NP_837472.1| hypothetical protein S1971 [Shigella flexneri 2a str. 2457T]

MYWYGHEMYYSPGSNTVSWRFCAPSGHGLSGMAISDTGRNSADNVDGVYYRPLQKLINGTWYNVASI

>gi|30063300|ref|NP_837471.1| tail fiber assembly protein [Shigella flexneri 2a str. 2457T]

MTKRYSVTWLFSEDGKNWYEELKNFASDTIKIAYTGDGRVVWVGKDVTGIEPRNASVIEVPDITANRRIT

APGYWFYRNDEFVFDYRLKAEDERDALLAQVSARTGEWEEDLLLGLISDEDKEKLKACRIYAKSLQAMDF

STITDKSSYNAIEWPASPEGSS

>gi|30063299|ref|NP_837470.1| tail fiber protein [Shigella flexneri 2a str. 2457T]

MSVVISGALTDGAGIPMSGYHIILKSRVNTPEVVMNTVADVMTGNDGEYCFHARTGKYGVYLKQDWRNEY

NVGDIAVYEDSKPGTLNDFLIAPDEGDLKPDVVKRFEEMVAQAQQSAGAAAGNAQQTAQDVAAAAGYARA

AEQAKNDIDAALTGTLKTANHLSETAAAGEKAQQKSRDNLGLKSAATMEAQSDIYDRTKGRLAIPGAFGF

GCAFLPEDVIRFDTKSDFLAWVRNALPGEYSVAGPYGIIIPDTRFEGVLSIRWTDARPETTEPRYRAKSL

TFYGINGPIYHTRYRYWPISRLTDWVKINITTEDIIYRIVASSVRNRWGDPDIGGLIIAAYQGEADGDKV

IRLVRGQSYRGSRLGPVGISVPSTPTGTYIASPQFFITGCSEHSLPGSYCALSGVPDAHVSGAMPGLFIR

TS

>gi|30063298|ref|NP_837469.1| hypothetical protein S1968 [Shigella flexneri 2a str. 2457T]

MEIKKIINPRYTESGAVDCDVFFDDRDQAVPYTATADDVAPTGQQIWQELQSGKWGEIAPFTVTPEMLEA

AREARRQEIEAWRAEQEAKLFTFEWNGRIWNAGPDSLGRLSPVVMLAKSVTAQTHMAWSDADNQQVKLSM

PELEELAAAMVQAQVDRNDEIYRRQRELKEELNSLKDLNSVRNFIVE

>gi|30063297|ref|NP_837468.1| Iron transport protein, inner membrane component [Shigella flexneri 2a str. 2457T]

MMALLLEPLQFTFMSHALLISLVVSIPCALLSVFLVLKGWALMGDAMSHAVFPGIVLTWILGLPLATGAF

VAGVFCAVATGYLKDNSRIKQDTVMGIVFSGMFAAGLILYIAIKPDVHLDHILFGDMLGITIGDIIQTVI

IAGLVTLVISVKWRDFLLFSFDYQQAQVSGLHTRWLHYGLLCMVSLTIVATLKAVGIILSISLLIAPGAI

AVLLTQRFHIALLLATGISVIVSMTGVWLSFFIDSAPAPTIVVLFAVVFIMTFTVTSINARTKGNAHTQD

LLSPN

>gi|30063296|ref|NP_837467.1| Iron transport protein [Shigella flexneri 2a str. 2457T]

MNVLLEPFSYEYMLNAMWVSAMVGGLCAFLSCYLMLKGWSLIGDALSHSIVPGVAGAYMLGLPFSLGAFF

SGGLAAGSMLFLNQRTRLKEDAIIGLIFSSFFGLGLFMVSLNPTSVNIQTIVLGNILAIDPADILQLTII

GILSIIVLFFKWKDLMVTFFDENHARAIGLHPGRLKILFFTLLSVSTVAALQTVGAFLVICLVVTPGATA

WLLTDRFPRLLIIAVTIGSVTSFLGAWVSYFLDGATGGIIVVAQTLLFLLAFVFAPTHGLLANRRRAHKA

LEDRS

>gi|30063295|ref|NP_837466.1| Iron transport protein, ATP-binding component [Shigella flexneri 2a str. 2457T]

MMQSAGIVVNDVTVTWRNGHTALRDASFTVPSGSIAALVGVNGSGKSTLFKAIMGFVRLTSGKISVLGIP

TRQALQKNLVAYVPQSEEVDWSFPVLVEDVVMMGRYGHMGMLRIAKKRDRQIVTDALERVDMVDFRHRQI

GELSGGQKKRVFLARAIAQQGDVILLDEPFTGVDVKTEAKIISLLRELGSEGKTMLVSTHNLGSVTTFCD

YTVMVKGTVLASGPTDTTFTAENLELAFSGVLRHVTLNGSEESIITDDERPFVAHRPSAVQREAR

>gi|30063294|ref|NP_837465.1| Iron transport protein [Shigella flexneri 2a str. 2457T]

MLLGCLALTCSIAFQASATEKFKVITTFTIIADMAKNVAGDAAEVSSITKPGAEIHEYQPTPGDIKRAQG

AQLILANGMNLELWFQRFYQHLNGVPEVIVSSGVTPVGITEGPYEGKPNPHAWMSPDNALIYVDNIRDAL

IKYDPANAQTYQRNADTYKAKITQTLAPLRKQITELPENQRWMVTSEGAFSYLARDLGLKELYLWPINAD

QQGTPQQVRKVVDIVKKNHIPAVFSESTISDKPARQVARETGAHYGGVLYVDSLSTENGPVPTYIDLLKV

TTSTLVQGIKAGKREK

>gi|30063293|ref|NP_837464.1| integrase for prophage CP-933R [Shigella flexneri 2a str. 2457T]

MESEKGQSQNLWKFAVYSGLRHGELAALAWEDVDFEKGIVNVRRNLTILDMFGPPKTNAGIRTVALLQPA

LEALKEQYKLTGHHRKSEITFYHREYGRTEKQKLHFVFMPRVCNEKQKPYYSVSSLGTRWNAAVKRAGIR

RRNPYHTRHTFACWLLTAGANPAFIASQMGHETAQMVYEIYGMWIDDMNDEQIAMLNARLS

>gi|30063292|ref|NP_837463.1| IS1 orfA [Shigella flexneri 2a str. 2457T]

MASISIRCPSCSATEGVVRNGKSTAGHQRYLCSHCRKTWQLQFTYTASQPGTHQKIIDMAMNGVGCRASA

RIMGVGLNTVLRHLKNSGRSR

>gi|30063291|ref|NP_837462.1| IS1 orfB [Shigella flexneri 2a str. 2457T]

MSRQCTHYGRWPQHGFTSLKKLRPQSVTSRIQPGSDVIVCAEMDEHWGYVGAKSRQRWLFYAYDRIRRTV

VAHVFGERTLATLERLLSLLSAFEVVVWMTDGWPLYESRLKGKLHVISKRYTQRIERHNLNLRQHLARLV

RKSLSFSKSVELHDKVIGHYLNIKHYQ

>gi|30063290|ref|NP_837461.1| IS629 orfB [Shigella flexneri 2a str. 2457T]

MARCTVARLMAVMGLAGVLRGKKVRTTISRKAVAAGDRVNRQFVAERPDQLWVADFTYVSTWRGFVYVAF

IIDVFAGYIVGWRVSSSMETTFVLDALEQALWARRPSGTVHHSDKGSQYVSLAYTQRLKEAGLLASTGST

GDSYDNAMAESINGLYKAEVIHRKSWKNRAEVELATLTWVDWYNNRRLLERLGHIPPAEAEKAYYASIGN

DDLAA

>gi|30063289|ref|NP_837460.1| IS629 orfA [Shigella flexneri 2a str. 2457T]

MTKNTRFSPEVRQRAVRMVLESQGEYDSQWATICSIAPKIGCTPETLRVWVRQHERDTGGGDGGLTTAER

QRLKELERENRELRRSNDILRQASAYFAKAEFDRLWKK

>gi|30063288|ref|NP_837459.1| bacteriophage protein [Shigella flexneri 2a str. 2457T]

MGFAGDGKTYTASELAIGLVMLMRQRGIEAGNRPVMFLDTETGSDWVKPRFDAENIELFTAKTRSFVDLL

EAINEAESSGSVMIIDSISHFWTGLCDEYARRRNRKRGLEFSDWAWLKQEWRRFTDRFVNSQAHIIMCGR

AGYEYDFFEGDDGKRQLAKTGIKMKAETETGYEPSIRIQMEKQMNIETGQVWRTARILKDRSTRIDGQVF

SNPTFKNFLPHIESLNLGGEHPGIDTSRDNSELFANDGTPTWLKEKRAKEIALDEIIELLNKHHGGTSND

AKRAKADLLEKTFTSRSWERIKGMDWPTIKAGRNALWIELEGVEYAFPDPQTQNETQQAGYDENIPV

>gi|30063287|ref|NP_837458.1| host-nuclease inhibitor protein Gam [Shigella flexneri 2a str. 2457T]

MSVKLRLPQSAYPIFDRIEEMAWARHYQQIVREEKETELADDLEKGLPQHLFESLCIDHLQRHGANKQAI

SHAFDDDVEFQERMAEHIRYMAETIAHHQVDIDLED

>gi|30063286|ref|NP_837457.1| exodeoxyribonuclease VIII [Shigella flexneri 2a str. 2457T]

MNTDKQVYPLYYEAKNDKVRKRLGIKGGFYWAEAKKLSIAISRGAVAIDDAGYDEDDFKKPVRVNLPVVD

DLPPEGVFDTEFCNRYEKGGEDGITMVFIASSPSVQDKPASTDNTNVNGEDMTEIEENMLLPVSGQELPI

RWLAQHGSEKPVTHVSRDELQALHIARDEELPAVTALAVSHKTSLLDPLEIRDLHKLVRDTDRVFPNPGN

SSLGLMTAFFEAYMDADYTDRGLLTKEWMKGNRVSRITRTASGANAGGGNLTDRGEGFVHDLTSLARDVA

TGVLARSMDVDIYNLHPAHAKRVEEIIAENKPPFSVFRDKFITMPGGMDYSRAIVVASVKEAPIGIEVIP

AHVTEYLNKVLTETDHANPDPEIVDIACGRSSAPMPQRVTEEGKQDDEEKTQPSGAMADEQATAETMEPD

ATEHHQNTQPLDAQSQVNSVDEKYQELRAELHEARKNIPPKNPVDADKLLAALR

>gi|30063285|ref|NP_837456.1| hypothetical protein S1954 [Shigella flexneri 2a str. 2457T]

MITSYEATVVTTDDIVHEVNLEGKRIGYVIKTENKETPFTVVDIDGPSGNVKTLNDGVKKMCLVHIGKNL

PAEKKAEFLATLIAMKLKGEI

>gi|30063283|ref|NP_837454.1| insertion sequence 2 OrfA protein [Shigella flexneri 2a str. 2457T]

MIVLIPVFRLVIGEQIIDVLGPEKRRRRTTQEKIAIVQQSFEPGMTVSLVARQHGVAASQLFLWRKQYQE

GSLTAVAAGEQVVPASELAAAMKQIKELQRLLGKKTMENELLKEAVEYGRAKKWIAHAPLLPGDGE

>gi|30063282|ref|NP_837453.1| hypothetical protein S1950 [Shigella flexneri 2a str. 2457T]

MLNVAIENQNGWNYSASAPHKTGAGRGNPNVTGAHSRAEAVFLCVMHSSIQIMVGCAGQSQDWPGSGVTG

ISTPVRLTTLMVVENLGGELINLSLEDAIMATIPALSHPDVTIENGRAVTTSVAVAEFFHKRHDNVLRAI

ANTECSPEFNALNFEDVTYTDTKGEKRPMTKSPKTASFSW

>gi|30063281|ref|NP_837452.1| hypothetical protein S1949 [Shigella flexneri 2a str. 2457T]

MLMSLAQCPGFLFAHREECTVEIKKIINPRYTESGAVDCDVFFDDRDQAVPYTATADDVAPTGQQIWQEL

QSGKWGEIAPFTVTPEMLEAAREARRQEIEAWRTEQEAKPFTFEWNGRTWNADASSVARLSPVVMLAKSV

AAQTHMVWSDADNQQVKLSMPELEELAAAMVQAQVDRNDEIYRRQREMKEELSSLDDLASIRAFDVK

>gi|30063280|ref|NP_837451.1| hypothetical protein S1948 [Shigella flexneri 2a str. 2457T]

MNKPQLAESQKTALLTEAESVIRPPGCAVRLNRETDESGEARGRASVFPESVRSVVYAM

>gi|30063279|ref|NP_837450.1| invasion plasmid antigen [Shigella flexneri 2a str. 2457T]

MLPTNNNHRLISNSFSTYSIDTSRAYENYLTHWTEWKNNRIQEEQRDIAFQRLVSCLQNQETNLDLSELG

LTTLPEIPPGIKSINISKNNLSLIPPLPASLTQLNVSYNRLIELPALPQGLKLLNASHNQLITLPTLPIS

LKELHVSNNQLCSLPVLPELLETLDVSCNGLAVLPPLPFSLQEISAIGNLLSELPPLPHNIHSIWAIDNM

LTDIPYLPENLRNGYFDINQISHIPESILNLRNECSIDISDNPLSSHALQSLQRLTSSPDYHGPQIYFSM

SDGQQNTLHRPLADAVTAWFPENKQSDVSQIWHAFEHEEHANTFSAFLDRLSDTVSARNTSGFREQVAAW

LEKLSASAELRQQSFAVAADATESCEDRVALTWNNLRKTLLVHQASEGLFDNDTGALLSLGREMFRLEIL

EDIARDKVRTLHFVDEIEVYLAFQTMLAEKLQLSTAVKEMRFYGVSGVTANDLRTAEAMVRSREENEFTD

WFSLWGPWHAVLKRTEADRWALAEEQKYEMLENEYPQRVADRLKASGLSGDADAEREAGAQVMRETEQQI

YRQLTDEVLALRLPENGSQLHHS

>gi|30063278|ref|NP_837449.1| hypothetical protein S1946 [Shigella flexneri 2a str. 2457T]

MRFEICIAKEKMTKMPTGAVDALKEELTRRISKRYDDVEVIVKATSNDGLSVTRTADKDSAKTFVQETLK

DTWESADEWFVH

>gi|30063277|ref|NP_837448.1| ISSfl4 orf [Shigella flexneri 2a str. 2457T]

MTESSDYESVQVFIGVDVGKDTHHAVAINRSGKRLFDKALPNDENKLRSLISDLKQHGQILLVVDQPATI

GALPVAVARSEGVLVGYLPGLAMRRIADLHAGEAKTDARDAAIIAEAARTLPHALRTLKLADEQIAELSM

LCGFDDDLAAQTTQASNRILGLLTQIHPAPERVLGPRLEHPAVLDLLQRYPSPEKLASLGEKKLAAQLCK

LAPRLGKRLAADIAQALAEQTVVVPGTNAAAVVLPRLALQLITLRKQRDEVALEVEQRVLAHPLYPVLTS

MPGVGVRTAARLLTEVACRAFASAAHLAAYAGLAPVTRRSGSSIRGEHPSRRGNKALKRALFLSAFTALR

DPLSRAYYTRKMSQGKRHNQVLIALARRRCDVLFAMMRDGTFYTPQGS

>gi|30063276|ref|NP_837447.1| aspartyl-tRNA synthetase [Shigella flexneri 2a str. 2457T]

MRTEYCGQLRLSHVGQQVTLCGWVNRRRDLGSLIFIDMRDREGIVQVFFDPDRADALKLASELRNEFCIQ

VTGTVRARDEKNINRDMATGEIEVLASSLTIINRADVLPLDSNHVNTEEARLKYRYLDLRRPEMAQRLKT

RAKITSLVRRFMDDHGFLDIETPMLTKATPEGARDYLVPSRVHKGKFYALPQSPQLFKQLLMMSGFDRYY

QIVKCFRDEDLRADRQPEFTQIDVETSFMTAPQVREVMEALVRHLWLEVKGVDLGDFPVMTFAEAERRYG

SDKPDLRNPMELTDVADLLKSVEFAVFAGPANDPKGRVAALRVPGGASLTRKQIDEYGNFVKIYGAKGLA

YIKVNERAKGLEGINSPVAKFLNAEIIEAILDRTAAQDGDMIFFGADNKKIVADGMGALLLKVGKDLGLT

DESKWAPLWVIDFPMLGDDGEGGLTAMHHPFTSPKDMTAAELKAAPENAVANAYDMVINGYEVGGGSVRI

HNGDMQQTVFGILGINEEEQREKFGFLLDALKYGTPPHAGLAFGLDRLTMLLTGTDNIRDVIAFPKTTAA

ACLMTEAPSFANPTALAELSIQVVKKAENN

>gi|30063275|ref|NP_837446.1| dATP pyrophosphohydrolase [Shigella flexneri 2a str. 2457T]

MSEGSQRRGSVKDKVYKRPVSILVVIYAQDTKRVLMLQRRDDPDFWQSVTGSVEEGETAPQAAMREVKEE

VTIDVVAEQLTLIDCQRTVEFEIFSHLRHRYAPGVTRNTESWFCLALPHERQIVFTEHLAYKWLDAPAAA

ALTKSWSNRQAIEQFVINAA

>gi|30063274|ref|NP_837445.1| hypothetical protein S1940 [Shigella flexneri 2a str. 2457T]

MAGHSKWANTRHRKAAQDAKRGKIFTKIIRELVTAAKLGGGDPDANPRLRAAVDKALSNNMTRDTLNRAI

ARGVGGDDDANMETIIYEGYGPGGTAIMIECLSDNRNRTVAEVRHAFSKCGGNLGTDGSVAYLFSKKGVI

SFEKGDEDTIMEAALEAGAEDVVTYDDGAIDVYTAWEEMGKVRDALEAAGLKADSAEVSMIPSTKADMDA

ETAPKLMRLIDMLEDCDDVQEVYHNGEISDEVAATL

>gi|30063273|ref|NP_837444.1| Holliday junction resolvase [Shigella flexneri 2a str. 2457T]

MAIILGIDPGSRVTGYGVIRQVGRQLSYLGSGCIRTKVDDLPSRLKLIYAGVTEIITQFQPDYFAIEQVF

MAKNADSALKLGQARGVAIVAAVNQELPVFEYAARQVKQTVVGMGSAEKSQVQHMVRTLLKLPANPQADA

ADALAIAITHCHVSQNAMQMSESRLNLTRGRLR

>gi|30063272|ref|NP_837443.1| Holliday junction DNA helicase RuvA [Shigella flexneri 2a str. 2457T]

MIGRLRGIIIEKQPPLVLIEVGGVGYEVHMPMTCFYELPEAGQEAIVFTHFVVREDAQLLYGFNNKQERT

LFKELIKTNGVGPKLALAILSGMSAQQFVNAVEREEVGALVKLPGIGKKTAERLIVEMKDRFKGLHGDLF

TPAADLVLTSPASPATDDAEQEAVAALVALGYKPQEASRMVSKIARPDTSSETLIREALRAAL

>gi|30063271|ref|NP_837442.1| Holliday junction DNA helicase RuvB [Shigella flexneri 2a str. 2457T]

MIEADRLISAGTTLPEDVADRAIRPKLLEEYVGQPQVRSQMEIFIKAAKLRGDALDHLLIFGPPGLGKTT

LANIVANEMGVNLRTTSGPVLEKAGDLAAMLTNLEPHDVLFIDEIHRLSPVVEEVLYPAMEDYQLDIMIG

EGPAARSIKIDLPPFTLIGATTRAGSLTSPLRDRFGIVQRLEFYQVPDLQYIVSRSARFMGLEMSDDGAQ

EVARRARGTPRIANRLLRRVRDFAEVKHDGTISADIAAQALDMLNVDAEGFDYMDRKLLLAVIDKFFGGP

VGLDNLAAAIGEERETIEDVLEPYLIQQGFLQRTPRGRMATTRAWNHFGITPPEMP

>gi|30063270|ref|NP_837441.1| high-affinity zinc transporter membrane component [Shigella flexneri 2a str. 2457T]

MIELLFPGWLAGIMLACAAGPLGSFVVWRRMSYFGDTLAHASLLGVAFGLLLDVNPFYAVIAVTLLLAGG

LVWLEKRPQLAIDTLLGIMAHSALSLGLVVVSLMSNIRVDLMAYLFGDLLAVTPEDLISIAIGVVIVVAI

LFWQWRNLLSMTISPDLAFVDGVKLQQVKLLLMLVTALTIGVAMKFVGALIITSLLIIPAATARRFARTP

EQMAGVAVLVGMVAVTGGLTFSAFYDTPAGPSVVLCAALLFIISMMKKQAS

>gi|30063269|ref|NP_837440.1| high-affinity zinc transporter ATPase [Shigella flexneri 2a str. 2457T]

MTSLVSLENVSVSFGQRRVLSDVSLELKPGKILTLLGPNGAGKSTLVRVVLGLVTPDEGVIKRNGKLRIG

YVPQKLYLDTTLPLTVNRFLRLRPGTHKEDILPALKRVQAGHLINAPMQKLSGGETQRVLLARALLNCPQ

LLVLDEPTQGVDVNGQVALYDLIDQLRRELDCGVLMVSHDLHLVMAKTDEVLCLNHHICCSGTPEVVSLH

PEFISMFGPRGAEQLGIYRHHHNHRHDLQGRIVLRRGNDRS

>gi|30063267|ref|NP_837438.1| hypothetical protein S1932 [Shigella flexneri 2a str. 2457T]

MQQIARSVALAFNNLPRPHRVMLGSLTVLTLAVAVWRPYVYHRDATPIVKTIELEQNEIRSLLPEASEPI

DQAAQEDEAIPQDELDDKIAGEAGVHEYVVSTGDTLSSILNQYGIDMGDITQLAAADKELRNLKIGQQLS

WTLTADGELQRLTWEVSRRETRTYDRTAANGFKMTSEMQQGEWVNNLLKGTVGGSFVASARNAGLTSAEV

SAVIKAMQWQMDFRKLKKGDEFAVLMSREMLDGKREQSQLLGVRLRSEGKDYYAIRAEDGKFYDRNGTGL

AKGFLRFPTAKQFRISSNFNPRRTNPVTGRVAPHRGVDFAMPQGTPVLSVGDGEVVVAKRSGAAGYYVAI

RHGRSYTTRYMHLRKILVKPGQKVKRGDRIALSGNTGRSTGPHLHYEVWINQQAVNPLTAKLPRTEGLTG

SDRREFLAQAKEIVPQLRFD

>gi|30063266|ref|NP_837437.1| lipid A biosynthesis (KDO)2-(lauroyl)-lipid IVA acyltransferase [Shigella flexneri 2a str. 2457T]

METKKNNSEYIPEFDKSFRHPRYWGAWLGVAAMAGIALTPPKFRDPILARLGRFAGRLGKSSRRRALINL

SLCFPERSEAEREAIVDEMFATAPQAMAMMAELAIRGPEKIQPRVGWQGLEIIEEMRRNNEKVIFLVPHG

WAVDIPAMLMASQGQKMAAMFHNQGNPVFDYVWNTVRRRFGGRLHARNDGIKPFIQSVRQGYWGYYLPDQ

DHGPEHSEFVDFFATYKATLPAIGRLMKVCRARVVPLFPIYDGKTHRLTIQVRPPMDDLLEADDHTIERR

MNEEVEIFVGPRPEQYTWILKLLKTRKPGEIQPYKRKDLYPIK

>gi|30063265|ref|NP_837436.1| pyruvate kinase [Shigella flexneri 2a str. 2457T]

MSKRLRRTKIVTTLGPATDRDNNLEKVIAAGANVVRMNFSHGSPEDHKMRADKVREIAAKLGRHVAILGD

LQGPKIRVSTFKEGKVFLNIGDKFLLDANLGKGEGDKEKVGIDYKGLPTDVVPGDILLLDDGRVQLKVLE

VQGMKVFTEVTVGGPLSNNKGINKLGGGLSAEALTEKDKADIKTAALIGVDYLAVSFPRCGEDLNYARRL

ARDAGCDAKIVAKVERAEAVCSQDAMDDIILASDVVMVARGDLGVEIGDPELVGIQKALIRRARQLNRAV

ITATQMMESMITNPMPTRAEVMDVANAVLDGTDAVMLSAETAAGQYPSETVAAMARVCLGAEKIPSINVS

KHRLDVQFDNVEEAIAMSAMYAANHLKGVTAIITMTESGRTALMTSRISSGLPIFAMSRHERTLNLTALY

RGVTPVPFDSANDGVAAASEAVNLLRDKGYLMSGDLVIVTQGDVMSTVGSTNTTRILTVE

>gi|30063264|ref|NP_837435.1| DNA-binding transcriptional regulator HexR [Shigella flexneri 2a str. 2457T]

MNMLEKIQSQLEHLSKSERKVAEVILASPDNAIHSSIAALALEANVSEPTVNRFCRSMDTRGFPDFKLHL

AQSLANGTPYVNRNVNEDDSVESYTGKIFESAMATLDHVRHSLDKSAINRTVDLLTQAKKIAFFGLGSSA

AVAHDAMNKFFRFNVPVVYSDDIVLQRMSCMNCSDGDVVVLISHTGRTKNLVELAQLARENDAMVIALTS

AGTPLAREATLAITLDVPEDTDIYMPMVSRLAQLTVIDVLATGFTLRRGAKFRDNLKRSRKRR

>gi|30063263|ref|NP_837434.1| glucose-6-phosphate 1-dehydrogenase [Shigella flexneri 2a str. 2457T]

MAVTQTAQACDLVIFGAKGDLARRKLLPSLYQLEKVGQLNPDTRIIGVGRADWDKAAYTKVVREALETFM

KETIDEGLWDTLSARLDFCNLDVNDTAAFNRLGAMLDQKNRITINYFAMPPSTFGAICKGLGEAKLNAKP

ARVVMEKPLGTSLATSQEINDQVGEYFEECQVYRIDHYLGKETVLNLLALRFANSLFVNNWDNRTIDHVE

ITVAEEVGIEGRWGYFDKAGQMRDMIQNHLLQILCMIAMSPPSDLSADSIRDEKVKVLKSLRRIDRSNVR

EKTVRGQYTAGFAQGKKVPGYLEEEGANKSSNTETFVAIRVDIDNWRWAGVPFYLRTGKRLPTKCSEVVV

YFKTPELNLFKESWQDLPQNKLTIRLQPDEGVDIQVLNKVPGLDHKHNLQITKLDLSYSETFNQTHLADA

YERLLLETMRGIQALFVRRDEVEEAWKWVDSITEAWAMDNDAPKPYQAGTWGPVASVAMITRDGRSWNEF

E

>gi|30063262|ref|NP_837433.1| phosphogluconate dehydratase [Shigella flexneri 2a str. 2457T]

MNPQLLRVTNRIIERSRETRSAYLARIEQAKTSTVHRSQLACGNLAHGFAACQPENKASLKSMLRNNIAI

ITSYNDMLSAHQPYEHYPEIIRKALHEANAVGQVAGGVPAMCDGVTQGQDGMELSLLSREVIAMSAAVGL

SHNMFDGALFLGVCDKIVPGLTMAALSFGHLPAVFVPSGPMASGLPNKEKVRIRQLYAEGKVDRMALLES

EAASYHAPGTCTFYGTANTNQMVVEFMGMQLPGSSFVHPDSPLRDALTAAAARQVTRMTGNGNEWMPIGK

MIDEKVVVNGIVALLATGGSTNHTMHLVAMARAAGIQINWDDFSDLSDVVPLMARLYPNGPADINHFQAA

GGVPVLVRELLKAGLLHEDVNTVAGFGLSRYTLEPWLNNGELDWREGAEKSLDSNVIASFEQPFSHHGGT

KVLSGNLGRAVMKTSAVPVENQVIEAPAVVFESQHDVMPAFEAGLLDRDCVVVVRHQGPKANGMPELHKL

MPPLGVLLDRCFKIALVTDGRLSGASGKVPSAIHVTPEAYDGGLLAKVRDGDIIRVNGQTGELTLLVDEA

ELAAREPHIPDLSASRVGTGRELFSALREKLSGAEQGATCITF

>gi|30063261|ref|NP_837432.1| keto-hydroxyglutarate-aldolase/keto-deoxy-phosphogluconate aldolase [Shigella flexneri 2a str. 2457T]

MKNWKTSAESILTTGPVVPVIVVKKLEHAVPMAKALVAGGVRVLEVTLRTECAVDAIRAIAKEVPEAIVG

AGTVLNPQQLAEVTEAGAQFAISPGLTEPLLKAATEGTIPLIPGISTVSELMLGMDYGLKEFKFFPAEAN

GGVKALQAIAGPFSQVRFCPTGGISPANYRDYLALKSVLCIGGSWLVPADALEAGDYDRITKLAREAVEG

AKL

>gi|30063260|ref|NP_837431.1| phosphoribosylglycinamide formyltransferase 2 [Shigella flexneri 2a str. 2457T]

MTLLGTALRPAATRVMLLGSGELGKEVAIECQRLGVEVIAVDRYADAPAMHVAHRSHVINMLDGDALRRV

VELEKPHYIVPEIEAIATDMLIQLEEEGLNVVPCARATKLTMNREGIRRLAAEELQLPTSTYRFADSKSL

FREAVAAIGYPCIVKPVMSSSGKGQTFIRSAEQLAQAWEYAQQGGRAGAGRVIVEGVVKFDFEITLLTVS

AVDGVHFCAPVGHRQEDGDYRESWQPQQMSPLALERAQEIARKVVLALGGYGLFGVELFVCGDEVIFSEV

SPRPHDTGMVTLISQDLSEFALHVRAFLGLPVGGIRQYGPAASAVILPQLTSQNVTFDNVQNAVGADLQI
RLFGKPEIDGSRHLGVVLATSESVVDAIERAKHAAGQVKVQG

>gi|30063259|ref|NP_837430.1| hypothetical protein S1923 [Shigella flexneri 2a str. 2457T]
MKKRGAFLGRLLVSACASVFAANNETSKSVTFPKCEGLDAAGIAASVKRDYQQNRVARWADDQKIVGQAD
PVAWVSLQDIQGKDDKWSVPLTVRGKSADIHYQVCVDCKAGMAEYQRR

>gi|30063258|ref|NP_837429.1| hypothetical protein S1922 [Shigella flexneri 2a str. 2457T]
MANWLNQLQSLLGQSSSSTSSSADQGLGKLLVSGALGGLAGLLVANKSARKLLTKYGTNALLVGGGAVAG
TVLWNKYKDKIRAAHQDEPQFGAQSTPLDERTERLILALVFAAKSDGHIDANERAAIDQQLREAGVEEQG
RVLIEQAIEQPLDPQRLATGVRNEEEALEIYFLSCAAIDIDHFMERSYLNALGDALKIPQDVREGIERDL
EQQKRTLAE

>gi|30063257|ref|NP_837428.1| protease 2 [Shigella flexneri 2a str. 2457T]
MLPKAARIPHAMTLHGDTRIDNYYWLRDDTRSQPKVLDYLQQENSYGHRVMASQQALQDRILKEIIDRIP
QREVSAPYIKNGYRYRHIYEPGCEYAIYQRQSAFSEEWDEWETLLDANKRAAHSEFYSMGGMAITPDNTI
MALAEDFLSRRQYGIRFRNLETGNWYPELLDNVEPSFVWANDSWTFYYVRKHPVTLLPYQVWRHAIGTPA
SQDTLIYEEKDDTYYVSLHKTTSKHYVVIHLASATTSEVRLLDAEMADAEPFVFLPRRKDHEYSLDHYQH
RFYLRSNRHGKNFGLYRTRMRDEQQWEELIPPRENIMLEGFTLFTDWLVVEERQRGLTSLRQINRKTREV
IGIAFDDPAYVTWIAYNPEPETARLRYGYSSMTTPDTLFELDMDTGERRVLKQTEVPGFDAANYRSEHLW
IVARDGVEVPVSLVYHRKHFRKGHNPLLVYGYGSYGASIDADFSFSRLSLLDRGFVYAIVHVRGGGELGQ
QWYEDGKFLKKKNTFNDYLDACDALLKLGYGSPSLCYAMGGSAGGMLMGVAINQRPELFHGVIAQVPFVD
VVTTMLDESIPLTTGEFEEWGNPQDPQYYEYMKSYSPYDNVTAQAYPHLLVTTGLHDSQVQYWEPAKWVA
KLRELKTDDHLLLLCTDMDSGHGGKSGRFKSYEGVAMEYAFLVALAQGTLPATPAD

>gi|30063256|ref|NP_837427.1| exodeoxyribonuclease X [Shigella flexneri 2a str. 2457T]

MLRIIDTETCGLQGGIVEIASVDVIDGKIVNPMSLLVRPDRPISPQAMAIHRITEAMVADKPWIEDVIPH

YYGSEWYVAHNASFDRRVLPEMPGEWICTMKLARRLWPGLKYSNMALYKTRKLNVQTPLGLHHHRALYDC

YITAALLIDIMNTSGWTAEQMADITGRPSLMTTFTFGKYRGKAVSDVAERYPGYLRWLFNNLDSMSPELR

LTLKHYLENT

>gi|30063255|ref|NP_837426.1| hypothetical protein S1919 [Shigella flexneri 2a str. 2457T]

MTIIAGLPVEYNDRFIRGIAVFAPWRKTPGNYHQSHGACLGRRSRTITVVDEQPQGMDMDPTCSLFTTGQ

CLGEPDLLASARRLQFFSHQYSIAVLMANARGNSALWDEYGRLIVRADRGSLLLVGQRSSQGWQGDIIPL

R

>gi|30063254|ref|NP_837425.1| DNA polymerase III subunit theta [Shigella flexneri 2a str. 2457T]

MLKNLAKLDQTEMDKVNVDLAAAGVAFKERYNMPVIAEAVEREQPEHLRSWFRERLIAHRLASVNLSRLP

YEPKLK

>gi|30063253|ref|NP_837424.1| hypothetical protein S1917 [Shigella flexneri 2a str. 2457T]

MASTARSLRYALAILTTSLVTPSVWAHAHLTHQYPAANAQVTAAPQAITLNFSEGVETGFSGAKITGPKN

ENIKTLPAKRNEQDQKQLIVPLADSLKPGTYTVDWHVVSVDGHKTKGHYTFSVK

>gi|30063252|ref|NP_837423.1| hypothetical protein S1915 [Shigella flexneri 2a str. 2457T]

MMKKSILAFLLLTSSAAALAAPQVITVSRFEVGKDKWAFNREEVMLTCRPGNALYVINPSTLVQYPLNDI

AQKEVASGKTNAQPISVIQIDDPNNPGEKMSLAPFIERAEKLC

>gi|30063251|ref|NP_837422.1| IS629 orfA [Shigella flexneri 2a str. 2457T]

MTKNTRFSPEVRQRAVRMVLESQGEYDSQWATICSIAPKIGCTPETLRVWGRQHERDTGGGDGGLTTAER

QRLKEPERENRELRRSNNILRLASAYFAKAEFDRLWKK

>gi|30063250|ref|NP_837421.1| IS629 orfB [Shigella flexneri 2a str. 2457T]

MAVMGLAGVLRGKKVRTTISRKAVAAGDRVNRQFVAERPDQLWVADFTYVSTWRGFVYVAFIIDVFAGYI

VGWRVSSSMETTFVLDALEQALWARRPSGTVHHSDKGSQYVSLALHTAA

>gi|30063249|ref|NP_837420.1| IS629 orfB [Shigella flexneri 2a str. 2457T]

MAESINGLYKAEVIHRKSWKNRAEVELATLTWVDWYNNRRLQERLGHIPPAEAEKAYYASIGNDDLAA

>gi|30063248|ref|NP_837419.1| IS1294 transposase [Shigella flexneri 2a str. 2457T]

MPDALAAEGSSKREWNRFLDTHYRRGWNVNVSRVMDNATHVAVYFGSYLKKPPVPMSRLEHYAGQDEIGL

RYNSHRTKREEYLLMSGDEFMERFSWHVADKGFRMVIRGPESGEAAITGRCGVRHNGDSEKNGEANHKER

DVSAVTEG

>gi|30063247|ref|NP_837418.1| IS1294 transposase, partial [Shigella flexneri 2a str. 2457T]

MLPRFADIFQQGNRWLNWLEKQPEGAVRPVVIESVTKIMACGTTLMGYTQWCCSSPDCCHTKKVCFRCKS

RSCPHCGVKAGAQWIQYLLSLVPYCPWQHIVFTLPCQYWSLVFHNRWLLAEMSRIAADVILEICHQADVE

PGIFTVIHTWGRDQQWHPHIHLSTTAGGVTSGHTWKNLHFYARKVMSMWRYRITRLLSRNTLTW

>gi|30063246|ref|NP_837417.1| transport system permease [Shigella flexneri 2a str. 2457T]

MHSERAPFFLKLAAWGGVVFLHFPILIIAAYAFNTEDAAFSFPPQGLTLRWFSVAAQRSDILDAVTLSLK

VAALATLIALVLGTLAAAALWRRDFFGKNAISLLLLLPIALPGIVTGLALLTAFKTINLEPGFFTIVVGH

ATFCVVVVFNNVIARFRRTSWSLVEASMDLGANGWQTFRYVVLPNLSSALLAGGMLAFALSFDEIIVTTF

TAGHERTLPLWLLNQLGRPRDVPVTNVVALLVMLVTTLPNLGAWWLTREGDNGQ

>gi|30063245|ref|NP_837416.1| gamma-aminobutyraldehyde dehydrogenase [Shigella flexneri 2a str. 2457T]

MQHKLLINGERVSGEGEKQPVYNPATGDVLLEIAEVSAEQVDAAVRAADAAFAEWGQTTPKARAECLLKL

ADVIEENGQVFAELESRNCGKPLHSAFNDEIPAIVDVFRFFAGAARCLNGLAAGEYLEGHTSMIRRDPLG

VVASIAPWNYPLMMAAWKLAPALAAGNCVVLKPSEITPLTALKLAELAKDIFPAGVINILFGRGKTVGDP

LTGHPKVRMVSLTGSIATGEHIISHTASSIKRTHMELGGKAPVIVFDDADIEAVVEGVRTFGYYNAGQDC

TAACRIYAQKGIYDTLVEKLGAAVATLKSGAPDDESTELGPLSSLAHLERVSKAVEEAKATGHIKVITGG

EKRKGNGYYYAPTLLAGALQDDAIVQKEVFGPVVSVTPFDNEEQVVNWANDSQYGLASSVWTKDVGRAHR

VSARLQYGCTWVNTHFMLVSEMPHGGQKLSGYGKDMSLYGLEDYTVVRHVMVKH

>gi|30063244|ref|NP_837415.1| hypothetical protein S1904 [Shigella flexneri 2a str. 2457T]

MKKLDARQKMAHICARFIHMAGRPYMFLYQHMLVFYAVMAAIAFLITWFLSHDKKRIRFLSAFLVGATWP

MSFPVALLFSLF

>gi|30063243|ref|NP_837414.1| hypothetical protein S1903 [Shigella flexneri 2a str. 2457T]

MSHLDEVIARVDAAIEESVIAHMNELLIALSDDAELSREDRYTQQQRLRTAIAHHGRKHKEDMEARHEQL

TKGGTIL

>gi|30063242|ref|NP_837413.1| hypothetical protein S1902 [Shigella flexneri 2a str. 2457T]

MNQSLTLAFLIAAGIGLVVQNTLMARITQTSSTILIAMLLNSLVGIVLFVSILWFKQGMAGFGELVSSVR

WWTLIPGLLGSFFVFASISGYQNVGAATTIAVLVASQLIGGLVLDIFRSHGVPLRALFGPICGAILLVVG

AWLVARRSF

>gi|30063241|ref|NP_837412.1| resistance protein [Shigella flexneri 2a str. 2457T]

MSIRFARKADCAAIAEIYNHAVLYTAAIWNDQTVDADNRIAWFEARTIAGYPVLVSEEDGVVTGYASFGD

WRSFDGFRHTVEHSVYVHPDHQGKGLGRKLLSRLIDEARDCGKHVMVAGIESQNQASLHLHQSLGFVVTA

QMPQVGTKFGHWLDLTFMQLQLDERTEPDAIG

>gi|30063238|ref|NP_837409.1| oxidoreductase [Shigella flexneri 2a str. 2457T]

MLQRGCDGCKQNQSREPATIDGFMPKAQESIMGQQKQRNRRWVLASRPHGAPVPENFRLEEDDVATPGEG

QVLLRTVYLSLDPYMRGRMSDEPSYSPPVDIGGVMVGGTVSRVVESNHPDYQPGDWVLGYSGWQDYDISS

GDDLVKLGDHPQNPSWSLGVLGMPGFTAYMGLLDIGQPKEGETLVVAAATEPVGATVGQIGKLKGCRVVG

VAGGAEKCRHATEVLGFDVCLDHHADDFAEQLAKACPKGIDIYYENVGGKVFDAVLPLLNTSARIPVCGL

VSSYNATELPPGPDRLPLLMATVLKKRIRLQGFIIAQDYGHRIHEFQKEMGQWVKEDKIHYREDITDGLE

NAPQTFIGLLKGKNFGKVVIRVAGDD

>gi|30063237|ref|NP_837408.1| DNA-binding transcriptional regulator [Shigella flexneri 2a str. 2457T]

MNSFCHAGRVGQKTREGIKMPGTGKMKHVSLTLQVENDLKHQLSIGALKPGARLITKNLAEQLGMSITPV

REALLRLVSVNALSVAPAQAFTVPEVRQSQLDEINRIRYELELMAVALAVENLSSQDIAELQELLGKLQQ

AQEKGDMEQIINVNRLFRLAIYHRSNMPILCEMIEQLWIRMGPALHYLYEAINPAELRERIENYHLLLAA

LKAKDKEGCKHCLAEIMQQNIAILYQQYNR

>gi|30063236|ref|NP_837407.1| IS91 orf [Shigella flexneri 2a str. 2457T]

MLPRFADIFQQGNRWLNWLEKQPEGSVRPVVIESVTKIMACGTTLMGYTQWCCSSPDCCHTKKVCFRCKS

RSCPHCGVKAGAQWIQYLLSLVPYCPWQHIVFTLPCQYWSLVFHNRWLLAEMSRIAADVILEICHQADVE

PGIFTVIHTWGRDQQWHPHIHLSTTAGGVTSGHTWKNLHFYARKVMSMWRYRITRLLSRKYPDLVMPDAL

AAEGSSKREWNRFLDTHYRRGWNVNVSRVMDNTTHVAVYFGSYLKKPPVPMSRLEHYAGQDEIGLRYNSH

RTKREEYLLMSGDEFMERFSWHVADKGFRMVRYYGFLSPAKRRLLEEVVYIITETVRKTAMQIRWRGMYQ

RLLKVDPLKCILCGSQMRFTGLKRGYRLAEQVLMHEPLGPDAVVRLRAAEGKLRPF

>gi|30063235|ref|NP_837406.1| nitrite extrusion protein 2 [Shigella flexneri 2a str. 2457T]

MALQNEKNSRYLLRDWKPENPAFWENKGKHIARRNLWISVSCLLLAFCVWMLFSAVTVNLNKIGFNFTTD

QLFLLTALPSVSGALLRVPYSFMVPIFGGRRWPVFSTAILIIPCVWLGIPVQNPNTPFGIFIVIALLCGF

AGANFASSMGNISFFFPKAKQGSALGINGGLGNLGVSVMQLVAPLVIFVPVFAFLGVNGVPQADGSVMSL

ANAAWIWVPLLAIATIAAWSGMNDIASSRASIADQLPVLQRLHLWLLSLLYLATFGSFIGFSAGFAMLAK

TQFPDVNILRLAFFGPFIGAIARSVGGAISDKFGGVRVTLINFIFMAIFSALLFLTLPGTGSGNFIAFYA

VFMGLFLTAGLGSGSTFQMIAVIFRQITIYRVKMKGGSDEQAQREAVTETAATLGFISAIGAVGGFFIPQ

AFGMSLNMTGSPVGAMKVFLIFYIVCVLLTWLVYGRRKFSQK

>gi|30063234|ref|NP_837405.1| IS1 orfB [Shigella flexneri 2a str. 2457T]

MSRQCTHYGRWPQHGFTSLKKLRPQSVTSRIQPGSDVIVCAEMDEHWGYVGAKSRQRWLFYAYDRIRRTV

VAHVFGERTLATLERLLSLLSAFEVVVWMTDGWPLYESRLKGKLHVISKRYTQRIERHNLKLRQHLARLG

RKSLSFSKSVELHDKVIGHYLNIKHYQ

>gi|30063233|ref|NP_837404.1| IS1 orfA [Shigella flexneri 2a str. 2457T]

MASISIRCPSCSATEGVVRNGKSTAGHQRYLCSHCRKTWQLQFTYTASQPGTHQKIIDMAMNGVGCRASA

RIMGVGLNTVLRHLKNSGRSR

>gi|30063232|ref|NP_837403.1| glycoprotein [Shigella flexneri 2a str. 2457T]

MITDLILHNHPRMKTITLNDNHIAHLNAKNTTKLEYLNLSNNNLLPTNDIDQLISSKHLWHVLVNGINND

PLAQMQYWTAVRNIIDDTNEVTIDLSGLNLTTQPPGLQNFTSINLDNNQLTHFDATNYDRLVKLSLNSNA

LESINFPQGRNVSITHISMNNNALRNIDIDRLSSVTYFSAAHNQLEFVQLESCEWLQYLNLSHNQLTDIV

AGNKNELLLLDLSHNKLTSLHNVLFPNLNTLLINNNLLSEIKIFYSNFRNVQTLNAANNQLKYINLDFLT

YLPSIKSLRLDNNKITHIDTNNTSDIGTLFPIIKQSKNLNFLNVSGKNN

>gi|30063231|ref|NP_837402.1| outer membrane porin protein [Shigella flexneri 2a str. 2457T]

MKLKIVAVVVTGLLAANVAHAAEVYNKDGNKLDLYGKVTALRYFTDDKRDDGDKTYARLGFKGETQINDQ

MIGFGHWEYDFKGYNDEANGSRGNKTRLAYAGLKISEFGSLDYGRNYGVGYDIGSWTDMLPEFGGDTWSQ

KDVFMTYRTTGLATYRNYDFFGLIEGLNFAAQYQGKNKRTDNSHLYGADDTRANGDGFGISSTYVYDGFG

IGAVYTKSDRTNAQERAAANPLNASGKNAELWATGIKYDANNIYFAANYDETLNMTTYGDGYISNKAQSF

EVVAQYQFDFGLRPSLAYLKSKGRDLGRYADQDMIEYIDVGATYFFNKNMSTYVDYKINLIDESDFTRAV

DIRTDNIVATGITYQF

>gi|30063230|ref|NP_837401.1| hypothetical protein S1885 [Shigella flexneri 2a str. 2457T]

MTRQKATLIGLIAIVLWSTMVGLIRGVSEGLGPVGGAAAIYSLSGLLLIFTVGFPRIRQIPKGYLLAGSL

LFVSYEIYLALSLGYAATRHQAIEVGMVNYLWPSLTILFAILFNGQKTNWLIVPGLLLALVGVCWVLGGE

NGLHYDEIINNITTSPLSYFLAFIGAFIWAAYCTVTNKYARGFNGITVFVLLTGACLWIYYFLTPQPEMV

FSTPVMIKLISAAFTLGFAYTAWNVGILHGNVTIMAVGSYFTPVLSSALAAVLLSAPLSFSFWQGALMVC

GGSLLCWLATRRG

>gi|30063229|ref|NP_837400.1| formate dehydrogenase-N, nitrate-inducible, alpha subunit [Shigella flexneri 2a str. 2457T]

MDVSRRQFFKICAGGMAGTTVAALGFAPKQALAQARNYKLLRAKEIRNTCTYCSVGCGLLMYSLGDGAKN

AREAIYHIEGDPDHPVSRGALCPKGAGLLDYVNSENRLRYPKYRAPGSDKWQRISWEEAFSRIAKLMKAD

RDANFIEKNEQGVTVNRWLSTGMLCASGASNETGMLTQKFARSLGMLAVDNQARVUHGPTVASLAPTFGR

GAMTNHWVDIKNANVVMVMGGNAAEAHPVGFRWAMEAKNNNDATLIVVDPRFTRTASVADIYAPIRSGTD

ITFLSGILRYLIENNKINAEYVKHYTNASLLVRDDFAFEDGLFSGYDAEKRQYDKSSWNYQFDENGYAKR

DETLTHPRCVWNLLKAHVSRYTPDVVENICGTPKADFLKVCEVLASTSAPDRTTTFLYALGWTQHTVGAQ

NIRTMAMIQLLLGNMGMAGGGVNALRGHSNIQGLTDLGLLSTSLPGYLTLPSEKQVDLQSYLEANTPKAT

LADQVNYWSNYPKFFVSLMKSFYGDAAQKENNWGYDWLPKWDQTYDVIKYFNMMDEGKVTGYFCQGFNPV

ASFPDKNKVVSCLSKLKYMVVIDPLVTETSTFWQNHGESNDVDPASIQTEVFRLPSTCFAEEDGSIANSG

RWLQWHWKGQDAPGEARNDGEILAGIYHHLRELYQAEGGKGVEPLMKMSWNYKQPHEPQSDEVAKENNGY

ALEDLYDANGVLIAKKGQLLSSFAHLRDDGTTASSCWIYTGSWTEQGNQMANRDNSDPSGLGNTLGWAWA

WPLNRRVLYNRASADINGKPWDPKRMLIQWNGSKWTGNDIPDFGNAAPGTPTGPFIMQPEGMGRLFAINK

MAEGPFPEHYEPIETPLGTNPLHPNVVSNPVVRLYEQDALRMGKKEQFPYVGTTYRLTEHFHTWTKHALL

NAIAQPEQFVEISETQAAAKGINNGDRVTVSSKRGFIRAVAVVTRRLKPLNVNGQQVETVGIPIHWGFEG

VARKGYIANTLTPNVGDANSQTPEYKAFLVNIEKA

>gi|30063228|ref|NP_837399.1| formate dehydrogenase-N, nitrate-inducible, iron-sulfur beta subunit [Shigella flexneri 2a str. 2457T]

MAMETQDIIKRSATNSITPPSQVRDYKAEVAKLIDVSTCIGCKACQVACSEWNDIRDEVGHCVGVYDNPA

DLSAKSWTVMRFSETEQNGKLEWLIRKDGCMHCEDPGCLKACPSAGAIIQYANGIVDFQSENCIGCGYCI

AGCPFNIPRLNKEDNRVYKCTLCVDRVSVGQEPACVKTCPTGAIHFGTKKEMLELAEQRVAKLKARGYEH

AGVYNPEGVGGTHVMYVLHHADQPELYHGLPKDPKIDTSVSLWKGALKPLAAAGFIATFAGLIFHYIGIG

PNKEVDDDEEDHHE

>gi|30063227|ref|NP_837398.1| formate dehydrogenase-N subunit gamma [Shigella flexneri 2a str. 2457T]

MSKSKMIVRTKFIDRACHWTVVICFFLVALSGISFFFPTLQWLTQTFGTPQMGRILHPFFGIAIFVALMF

MFVRFVHHNIPDKKDIPWLLNIVEVLKGNEHKVADVGKYNAGQKMMFWSIMSMIFVLLVTGVIIWRPYFA

QYFPMQVVRYSLLIHAAAGIILIHAILIHMYMAFWVKGSIKGMIEGKVSRRWAKKHHPRWYREIEKAEAK

KESEEGI

>gi|30063226|ref|NP_837397.1| hypothetical protein S1881 [Shigella flexneri 2a str. 2457T]

MDYCCVGRNNNDSVPREVTLLRVKTLMKMANHPRPGDIIQESLDELNVSLREFARAMEIAPSTASRLLTG

KAALTPEMAIKLSVVIGSSPQMWLNLQNAWSLAEAEKTVVVSRLRRLVTQ

>gi|30063225|ref|NP_837396.1| alcohol dehydrogenase [Shigella flexneri 2a str. 2457T]

MKTAVVTKDHHVDVTDKTLRSLKHGEALLKMECCGVCHTDLHVKNGDFGDKTGVILGHEGIGVVAEVGPG

VTSLKPGDRASVAWFYEGCGHCEYCNSGNETLCRSVKNAGYSVDGGMEEECIVVADYAVKVPDGLDSAAP

SSITCAGVTTYKAVKLSKIRPGQWIAIYGLGGLGNLALQYAKNVFNAKVIAIDVNDEQLKLATEMDADLA

INSRTEDAAKIVQEKTGGAHAAVVTAVAKAAFNSAVDAVRAGGRVVAVGLPPESMSLDIPRLVLDGIEVV

GSLVGTRQDLTEAFQFAAEGKVVPKVALRPLADINTIFTEMEEGKIRGRMVIDFRH

>gi|30063224|ref|NP_837395.1| malate dehydrogenase [Shigella flexneri 2a str. 2457T]

MEPKTKKQRSLYIPYAGPVLLEFPLLNKGSAFSMEERRNFNLLGLLPEVVETIEEQAERAWIQYQGFKTE

IDKHIYLRNIQDTNETLFYRLVNNHLDEMMPVIYTPTVGAACERFSEIYRRSRGVFISYQNRHNMDDILQ

NVPNHNIKVIVVTDGERILGLGDQGIGGMGIPIGKLSLYTACGGISPAYTLPVVLDVGTNNQQLLNDPLY

MGWRNPRITDDEYYEFVDEFIQAVKQRWPDVLLQFEDFAQKNAMPLLNRYRNEICSFNDDIQGTAAVTVG

TLIAASRAAGGQLSEKKIVFLGAGSAGCGIAEMIIAQTQREGLSEEAARQKVFMVDRFGLLTDKMPNLLP

FQTKLVQKRENLSDWDTDSDVLSLLDVVRNVKPDILIGVSGQTGLFTEEIIREMHKHCPRPIVMPLSNPT

SRVEATPQDIIAWTEGNALVATGSPFNPVVWKDKIYPIAQCNNAFIFPGIGLGVIASGASRITDEMLMSA

SETLAQYSPLVLNGEGLVLPELKDIQKVSRAIAFAVGKMAQQQGVAVKTSAEALQQAIDDNFWQAEYRDY

RRTSI

>gi|30063223|ref|NP_837394.1| IS1 orfA [Shigella flexneri 2a str. 2457T]

MASISIRCPSCSATEGVVRNGKSTAGHQRYLCSHCRKTWQLQFTYTASQPGTHQKIIDMAMNGVGCRASA

RIMGVGLNTVLRHLKNSGRSR

>gi|30063222|ref|NP_837393.1| IS1 orfB [Shigella flexneri 2a str. 2457T]

MSRQCTHYGRWPQHGFTSLKKLRPQSVTSRIQPGSDVIVCAEMDEHWGYVGAKSRQRWLFYAYDRIRRTV

VAHVFGERTLATLERLLSLLSAFEVVVWMTDGWPLYESRLKGKLHVISKRYTQRIERHNLNLRQHLARLV

RKSLSFSKSVELHDKVIGHYLNIKHYQ

>gi|30063221|ref|NP_837392.1| osmotically inducible protein [Shigella flexneri 2a str. 2457T]

MTIHKKGQAHWEGDIKRGKGTVSTESGVLNQQPYGFNTRFEGEKGTNPEELIGAAHAACFSMALSLMLGE

AGFTPTSIDTTADVSLDKVDAGFAITKIALKSEVAVPGIDASTFDGIIQKAKAGCPVSQVLKAEITLDYQ

LKS

>gi|30063220|ref|NP_837391.1| ATP-binding component of a transport system [Shigella flexneri 2a str. 2457T]

MTQPVLDIQQLHLSFPGFNGDVHALNNVSLQINRGEIVGLVGESGSGKSVTAMLIMRLLPTGSYCVHRGQ

ISLLGEDVLNAREKQLRQWRGARVAMIFQEPMTALNPTRRIGLQMMDVIRHHQPISRREARAKAIALLEE

MQIPDAVEVMSRYPFELSGGMRQRVMIAPAFSCEPQLIIAGEPTTALDVTVQLQVLRLLKHKARASGTAV

LFISHDMAVVSQLCDSVYVMYAGSVIESGGTADVIHHPRHPYTIGLLQCAPEHGVPRQPLPAIPGTVPNL

THLPDGCAFRDRCYAAGAQCENVPALTACGDNNQHCTCWYPQQEVISV

>gi|30063219|ref|NP_837390.1| ATP-dependent peptide transporter membrane subunit [Shigella flexneri 2a str. 2457T]

MMLSEETSAVRPQKQTRFNGAKLVWMLKGSPLTVTGAVIIVLMRLMMIFSPWLATHDPNAIDLTARLLPP

SAAHWFGTDEVGRDLFSRVLVGSQQSILAGLVVVAIAGMIGSLLGCLSGVLGGRADAIIMRIMDIMLSIP

SLVLTMALAAALGPSLFNAMLAIAIVRIPFYVRLARGQALVVRQYTYVQAAKTFGASRWHLINWHILRNS

LPPLIVQASLDIGSAILMAATLGFIGLGAQQPSAEWGAMVANGRNYVLDQWWYCAFPGAAILLTSVGFNL

FGDGIRDLLDPKAGGKQS

>gi|30063218|ref|NP_837389.1| hypothetical protein S1869 [Shigella flexneri 2a str. 2457T]

MDICSRNKKLTIRRPAILVALALLLCSCKSTPPESMVTPPAGSKPPATTQQSSQPMRGIWLATVSRLDWP

PVSSVNISNPTSRARVQQQAMIDKLDHLQRLGINTVFFQVKPDGTALWPSKILPWSDLMTGKIGENPGYD

PLQFMLDEAHKRGMKVHAWFNPYRVSVNTKPGTIRELNSTLSQQPASVYVQHRDWIRTSGDRFVLDPGIP

EVQDWITSIVAEVVSRYPVDGVQFDDYFYTESPGSRLNDNETYRKYGGAFASKADWRRNNTQQLIAKVSH

TIKSIKPGVEFGVSPAGVWRNRSHDPLGSDTRGAAAYDESYADTRRWVEQGLLDYIAPQIYWPFSRSAAR

YDVLAKWWADVVKPTRTRLYIGIAFYKVGEPSKIEPDWMINGGVPELKKQLDLNDAVPEISGTILFREDY

LNKPQTQQAVSYLQSRWGS

>gi|30063217|ref|NP_837388.1| acid sensitivity protein, transporter [Shigella flexneri 2a str. 2457T]

MATSVQTGKAKQLTLLGFFAITASMVMAVYEYPTFATSGFSLVFFLLLGGILWFIPVGLCAAEMATVDGW

EEGGVFAWVSNTLGPRWGFAAISFGYLQIAIGFIPMLYFVLGALSYILKWPALNEDPITKTIAALIILWA

LALTQFGGTKYTARIAKVGFFAGILLPAFILIALAAIYLHSGAPVAIEMDSKTFFPDFSKVGTLVVFVAF

ILSYMGVEASATHVNEMSNPGRDYPLAMLLLMVAAICLSSVGGLSIAMVIPGNEINLSAGVMQTFTVLMS

HVAPEIEWTVRVISALLLLGVLAEIASWIVGPSRGMYVTAQKNLLPAAFAKMNKNGVPVTLVISQLVITS

IALIILTNTGGGNNMSFLIALALTVVIYLCAYFMLFIGYIVLVLKHPDLKRTFNIPGGKGVKLVVAIVGL

LTSIMAFIVSFLPPDNIQGDSTDMYVELLVVSFLVVLALPFILYAVHDRKGKANTGVTLEPINSQNAPKG

HFFLHPRARSPHYIVMNDKKH

>gi|30063216|ref|NP_837387.1| glutamate decarboxylase [Shigella flexneri 2a str. 2457T]

MDKKQVTDLRSELLDSRFGAKSISTIAESKRFPLHEMRDDVAFQIINDELYLDGNARQNLATFCQTWDDE

NVHKLMDLSINKNWIDKEEYPQSAAIDLRCVNMVADLWHAPAPKNGQAVGTNTIGSSEACMLGGMAMKWR

WRKRMEAAGKPTDKPNLVCGPVQICWHKFARYWDVELREIPMRPGQLFMDPKRMIEACDENTIGVVPTFG

VTYTGNYEFPQPLHDALDKFQADTGIDIDMHIDAASGGFLAPFVAPDIVWDFRLPRVKSISASGHKFGLA

PLGCGWVIWRDEEALPQELVFNVDYLGGQIGTFAINFSRPAGQVIAQYYEFLRLGREGYTKVQNASYQVA

AYLADEIAKLGPYEFICTGRPDEGIPAVCFKLKDGEDPGYTLYDLSERLRLRGWQVPAFTLGGEATDIVV

MRIMCRRGFEMDFAELLLEDYKASLKYLSDHPKLQGIAQQNSFKHT

>gi|30063215|ref|NP_837386.1| IS1 orfB [Shigella flexneri 2a str. 2457T]

MSRQCTHYGRWPQHGFTSLKKLRPQSVTSRIQPGSDVIVCAEMDEHWGYVGAKSRQRWLFYAYDRIRRTV

VAHVFGERTLATLERLLSLLSAFEVVVWMTDGWPLYESRLKGKLHVISKRYTQRIERHNLKLRQHLARLG

RKSLSFSKSVELHDKVIGHYLNIKHYQ

>gi|30063214|ref|NP_837385.1| IS1 orfA [Shigella flexneri 2a str. 2457T]

MASISIRCPSCSATEGVVRNGKSTAGHQRYLCSHCRKTWQLQFTYTASQPGTHQKIIDMAMNGVGCRASA

RIMGVGLNTVLRHLKNSGRSR

>gi|30063213|ref|NP_837384.1| IS1 orfA [Shigella flexneri 2a str. 2457T]

MASISIRCPSCSATEGVVRNGKSTAGHQRYLCSHCRKTWQLQFTYTASQPGTHQKIIDMAMNGVGCRASA

RIMGVGLNTVLRHLKNSGRSR

>gi|30063212|ref|NP_837383.1| IS1 orfB [Shigella flexneri 2a str. 2457T]

MSRQCTHYGRWPQHGFTSLKKLRPQSVTSRIQPGSDVIVCAEMDEHWGYVGAKSRQRWLFYAYDRIRRTV

VAHVFGERTLATLERLLSLLSAFEVVVWMTDGWPLYESRLKGKLHVISKRYTQRIERHNLNLRQHLARLV

RKSLSFSKSVELHDKVIGHYLNIKHYQ

>gi|30063211|ref|NP_837382.1| hypothetical protein S1861 [Shigella flexneri 2a str. 2457T]

MKRVLIPGVILCGADVAQAVDDKNMYMYFFEEMTVYAPVPVPVNGNTHYTSESIERLPTGNGNISDLLRT

NPAVRMDSTQSTSLNQGDIRPEKISIHGASPYQNAYLIDGISATNNLNPANESDASSATNISGMSQGYYL

DVSLLDNVTLYDSFVPVEFGRFNGGVIDAKIKRFNADDSKVKLGYRTTRLDWLTSHIDENNKSAFNQGSS

GSTYFSPDFKKNFYTLSFNQELADNFGVTAGLSRRQSDITRADYVSNDGIVAGRAQYKNVIDTALSKFTW

FASDRFTHDLTLKYTGSSRDYNTSTFPQSDREMGNKSYGLAWDMDTQLAWAKLRTTVGWDHISDYTRHDH

DIWYTELSCTYGDITGRCTRGGLGHISQAVDNYTFKTRLDWQKFAVGDVSHQPYFGAEYIYSDAWTERHN

QSESYVINAAGKKTNHTIYHKGKGSLGIDNYTLYMADHISWRNVSLMPGVRYDYDNYLSNHNISPRFMTE

WDIFADQTSMITAGYNRYYGGNILDMGLRDIRNSWTESVSGNKTLTRYQNLKTPYNDELAMGLQQKIDKN
VIARASEAHDQISKSSRTDSATKTTITEYNNDGKTKTHSFNLSFELAEPLHIRQVDINPQIVFSYIKSKG
NLSLNNGYEESNTGDNQVVYNGNLVSYDSVPVADFNNPLKISLNMDFTHQPSGLVWANTLAWQEARKARI
ILGKTNAQYISEYSDYKQYVDEKLDSSLTWDTRLSWTPQFLKQQNLTISADILNVLDSKTAVDTTNTGVA
TYASGRTFWLDVSMKF

>gi|30063210|ref|NP_837381.1| enzyme [Shigella flexneri 2a str. 2457T]
MHVTAKPSSFQCNLKCDYCFYLEKESQFTHEKWMDDSTLKEFIKQYIEASGNQVYFTWQGGEPTLAGLDF
FRKVIHYQQRYAGQKRIFNTLQTNGILLNNEWCAFLKEHEFLVGISIDGPQELHDRYRRSNSGNGTFAKV
IAAIERLKSYQVEFNTLTVINNVNVHYPLEVYHFLKSIGSKHMQFIELLETGTPNIDFSGHSENTFRIID
FSVPPTAYGKFMSTIFMQWVKNDVGEIFIRQFESFVSRFLGNGHTSCIFQESCKDNLVVESNGDIYECDH
FVYPQYKIGNINKSELKTMNSVQLTAQKKRISAKCQQCAYKPICNGGCPKHRITKVNNETVSYFCEGYKI
LFSTMVPYMNAMVELAKNRVPLYHIMDVAKQMENN

>gi|30063209|ref|NP_837380.1| IS1 orfA [Shigella flexneri 2a str. 2457T]
MASISIRCPSCSATEGVVRNSKSTAGHQRYLCSHCRKTWQLQFTYTASQPGTHQKIIDMAMNGVGCRASA
RIMGVGLNTVLRHLKNSGRSR

>gi|30063208|ref|NP_837379.1| IS1 orfB [Shigella flexneri 2a str. 2457T]
MSRQCTHYGRWPQHGFTSLKKLRPQSVTSRIQPGSDVIVCAEMDEHWGYVGAKSRQRWLFYAYDRIRRTV
VAHVFGERTLATLERLLSLLSAFEVVVWMTDGCPLYESRLKGKLHVISKRYTQRIERHNLNLRQHLARLV
RKSLSFSKSVELHDKVIGHYLNIKHYQ

>gi|30063207|ref|NP_837378.1| 3-dehydroquinate dehydratase [Shigella flexneri 2a str. 2457T]
MKTVTVKDLVIGAGAPKIIVSLMAKDIARVKSEALAYREADFDILEWRVDHFADLSNVESVMAAAKILRE

TMPEKPLLFTFRSAKEGGEQAISTEAYIALNRAAIDSGLVDMIDLELFTGDDQVKETVAYAHAHDVKVVM
SNHDFHKTPEAEEIIARLRKMQSFDADIPKIALMPQSTSDVLTLLAATLEMQEQYADRPIITMSMAKTGV
ISRLVGEVFGSAATFGAVKKASAPGQISVNDLRTVLTILHQA

>gi|30063206|ref|NP_837377.1| quinate/shikimate dehydrogenase [Shigella flexneri 2a str. 2457T]
MDVTAKYELIGLMAYPIRHSLSPEMQNKALEKAGLPFTYMAFEVDNDSFPGAIEGLKALKMRGTGVSMPN
KQLACEYVDELTPAAKLVGAINTIVNDDGYLRGYNTDGTGHIRAIKESGFDIKGKTMVLLGAGGASTAIG
AQGAIEGLKEIKLFNRRDEFFDKALAFAQRVNENTDCVVTDTDLADQQAFAEALASADILTNGTKVGMKP
LENESLVNDISLLHPGLLVTECVYNPHMTKLLQQAQQAGCKTIDGYGMLLWQGAEQFTLWTGKDFPLEYV
KQVMGFGA

>gi|30063205|ref|NP_837376.1| amino acid/amine transport protein [Shigella flexneri 2a str. 2457T]
MTSLAEKFSTDNAGIAYLISGIGLGRLISILFFGVISDKFGRRAVILMAVIMYLLFFFGIPACPNLTLAY
GLAVCVGIANSALDTGGYPALMECFPKASGSAVILVKAMVSFGQMFYPMLVSYMLLNNIWYGYGLIIPGI
LFVLITLMLLKSKFPSQLVDASVANELPQMNSKPLVWLEGVSSVLFGVAAFSTFYVIVVWMPKYAMAFAG
MSEAEALKTISYYSMGSLVCVFIFAALLKKMVRPIWANVFNSALATITAAIIYLYPSPLVCNAGAFVIGF
SAAGGILQLGVSVMSEFFPKSKAKVTSIYMMMGGLANFVIPLITGYLSNIGLQYIIVLDFTFALLALITA
IIVFSRYYRVFIIPENDVRFGERKFSTRLNTIKHRG

>gi|30063204|ref|NP_837375.1| transport system permease [Shigella flexneri 2a str. 2457T]
MGVILMSLNMASLETLWQTNAAGVSIVISSLGIGRLSVLLFAGLLSDRFGRRPFIMLGMCCYMAFFFDIL
QTNNIIIAYVFGFLAGMANSFLDAGTYPSLMEAFPRSPGTANILIKAFVSSGQFLLPLIISLLVWAELWF
GWSFMIAAGIMFINALFLYRCTFPPHPGRRLPVIKKTTSSTEHRCSIIDLASYTLYGYISMATFYLVSQW
LAQYGQFVAGMSYTMSIKLLSIYTVGSLLCVFITAPLIRNTVRPTTLLMLYTFISFIALFTVCLHPTFYV
VIIFAFVIGFTSAGGVVQIGLTLMAERFPYAKGKATGIYYSTGSIATFTIPLITAHLSQRSIADIMWFDT

AIGFLLALFIGLRSRKKTRHHSLKENVAPGG

>gi|30063203|ref|NP_837374.1| hypothetical protein S1851 [Shigella flexneri 2a str. 2457T]

MNAYELQALRHIFAMTIDECATWIAQTGDSESWRQWENGKCAIPDCVVEQLLAMRQQRKKHLHAIIEKIN

NRIGNNTMRFFPDLTAFQQVYPDGNFIDWKIYQSVAAELYAHDLERLC

>gi|30063202|ref|NP_837373.1| inner membrane protein [Shigella flexneri 2a str. 2457T]

MVNVRQPRDVAQILLSVLFLAIMIVACLWIVQPFILGFAWAGTVVIATWPVLLRLQKIMFGRRSLAVLVM

TLLLVMVFIIPIALLVNSIVDGSGPLIKAISSGDMTLPDLAWLNTIPVIGAKLYAGWHNLLDMGGTAIMA

KVRPYIGTTTTWFVGQAAHIGRFMVHCALMLLFSALLYWRGEQVAQGIRHFATRLAGVRGDAAVLLAAQA

IRAVALGVVVTALVQAVLGGIGLAVSGVPYATLLTVLMILSCLVQLGPLPVLIPAIIWLYWTGDTTWGTV

LLVWSGVVGTLDNVIRPMLIRMGADLPLILILSGVIGGLIAFGMIGLFIGPVLLAVSWRLFAAWVEEVPP

PTDQPEEILEELGEIEKPNK

>gi|30063201|ref|NP_837372.1| oxidase [Shigella flexneri 2a str. 2457T]

MIPQISLAPGVVQLVLNFLQELEQQGFTGDAATSYADRLTMSTDNSIYQLLPDAVVFPRSTADVALIARL

AAQERYSSLIFTPRGGGTGTNGQALNQGIIVDMSRHMNRIIEINPEEGWVRVEAGVIKDQLNQYLKPFGY

FFAPELSTSNRATLGGMINTDASGQGSLVYGKTSDHVLGVRAVLLGGDILDTQPLPVELAETLGKSNTTI

GRIYNTVYQRCRQQRQLIIDNFPKLNRFLTGYDLRHVFNDEMTEFDLTRILTGSEGTLAFITEARLDITR

LPKVRRLVNVKYDSFDSALRNAPFMVEARALSVETVDSKVLNLAREDIVWHSVSELITDMPDKEMLGLNI

VEFAGDDEALIDERVNALCVRLDELIASQQAGVIGWQVCRELAGVERIYAMRKKAVGLLGNAKGAAKPIP

FAEDTCVPPEHLADYIAEFRALLDSHGLSYGMFGHVDAGVLHVRPALDMCDPQQEILMKQISDDVVALTA

KYGGLLWGEHGKGFRAEYSPAFFGEELFAELRKVKAAFDPHNRLNPGKICPPEGLDAPMMKVDAVKRGTF

DRQIPIAVRQQWRGAMECNGNGLCFNFDARSPMCPSMKITQNRIHSPKGRATLVREWLRLLADRGVDPLK

LEQELPESGVSLRTLIARTRNSWHANKGEYDFSHEVKEAMSGCLACKACSTQCPIKIDVPEFRSRFLQLY

HTRYLRPLRDHLVATVESYAPLMARAPKTFNFFINQPLVRKLSEKHIGMVDLPLLSVPSLQQQMVGHRSA

NMTLEQLEALNAEQKARTVLVVQDPFTSYYDAQVVADFVRLVEKLGFQPVLLPFSPNGKAQHIKGFLNRF

AKTAKKTADFLNRMAKLGMPMVGVDPALVLCYRDEYKLALGEERGEFNVLLANEWLASALESHPVATVSG

ESWYFFGHCTEVTALPGAPAQWAAIFARFGAKLENVSVGCCGMAGTYGHEAKNHENSLGIYELSWHQAMQ

RLPRNRCLATGYSCRSQVKRVEGTGVRHPVQALLEIIK

>gi|30063200|ref|NP_837371.1| hypothetical protein S1848 [Shigella flexneri 2a str. 2457T]

MIWKRKITLEALNAMGEGNMVGFLDIRFEHIGDDTLEATMPVDSRTKQPFGLLHGGASVVLAESIGSVAG

YLCTEGEQKVVGLEINANHVRSAREGRVRGICKPLHLGSRHQVWQIEIFDEKGRLCCSSRLTTAIL

>gi|30063199|ref|NP_837370.1| hypothetical protein S1847 [Shigella flexneri 2a str. 2457T]

MTHIAFGHYLHSKRWYTVQVLSKSEDAMSTQLDPTQLAIEFLRRDQSNLSPAQYLKRLKQLELEFADLLT

LSSAELKEEIYFAWRLGVH

>gi|30063198|ref|NP_837369.1| iron-sulfur cluster assembly scaffold protein [Shigella flexneri 2a str. 2457T]

MDMHSGTFNPQDFAWQGLTLTPAAAIHIRELVAKQPGMVGVRLGVKQTGCAGFGYVLDSVSEPDKDDLLF

EHDGAKLFVPLQSMPFIDGTEVDFVREGLNQIFKFHNPKAQNECGCGESFRV

>gi|30063197|ref|NP_837368.1| cysteine desulfurase activator complex subunit SufB [Shigella flexneri 2a str. 2457T]

MSRNTEATDDVKTWTGGPLNYKEGFFTQLATDELAKGINEEVVRAISAKRNEPEWMLEFRLNAYRAWLEM

EEPHWLKAHYDKLNYQDYSYYSAPSCGNCDDTCASEPGAVQQTGANAFLSKEVEAAFEQLGVPVREGKEV

AVDAIFDSVSVATTYREKLAEQGIIFCSFGEAIHDHPELVRKYLGTVVPGNDNFFAALNAAVASDGTFIY

VPKGVRCPMELSTYFRINAEKTGQFERTILVADEDSYVSYIEGCSAPVRDSYQLHAAVVEVIIHKNAEVK

YSTVQNWFPGDNNTGGILNFVTKRALCEGENSKMSWTQSETGSAITWKYPSCILRGDNSIGEFYSVALTS

GHQQADTGTKMIHIGKNTKSTIISKGISAGHSQNSYRGLVKIMPTATNARNFTQCDSMLIGANCGAHTFP

YVECRNNSAQLEHEATTSRIGEDQLFYCLQRGISEEDAISMIVNGFCKDVFSELPLEFAVEAQKLLAISL

EHSVG

>gi|30063196|ref|NP_837367.1| cysteine desulfurase [Shigella flexneri 2a str. 2457T]

MLSIKDLHVSVEDKAILRGLSLDVRPGEVHAIMGPNGSGKSTLSATLAGREDYEVTGGTVEFKGKDLLAL

SPEDRAGEGIFMAFQYPVEIPGVSNQFFLQTALNAVRSYRGQETLDRFDFQDLMEEKIALLKMPEDLLTR

SVNVGFSGGEKKRNDILQMAVLEPELCILDESDSGLDIDALKVVADGVNSLRDGKRSFIIVTHYQRILDY

IKPDYVHVLYQGRIVKSGDFTLVKQLEEQGYGWLTEQQ

>gi|30063195|ref|NP_837366.1| cysteine desulfurase activator complex subunit SufD [Shigella flexneri 2a str. 2457T]

MAGLPNSSNALQQWHHLFEAEGTKRSPQAQQHLQQLLRTGLPTRKHENWKYTPLEGLTNSQFVSIAGDIS

PQQRDALALTLDAVRLVFVDGRYVPALSDAIEGSGYEVSINDDRQGLPDAIQAEVFLHLTESLAQSVTHI

AVKRGQRPAKPLLLMHITQGVAGEEVNTAHYRHHLDLAEGAEATVIEHFVSLNDARHFTGARFTINVAAN

AHLQHIKLAFENPVSHHFAHNDLLLADDATAFSHSFLLGGAVLRHNTSTQLNGENSTLRINSLAMPVKNE

VCDTRTWLEHNKGFCNSRQLHKTIVSDKGRAVFNGLINVAQHAIKTDGQMTNNNLLMGKLAEVDTKPQLE

IYADDVKCSHGATVGRIDDEQMFYLRSRGINQQDAQQMIIYAFAAELTEALRDEGLKQQVLARIGQRLPG

GAR

>gi|30063194|ref|NP_837365.1| bifunctional cysteine desulfurase/selenocysteine lyase [Shigella flexneri 2a str. 2457T]

MTFSVDKVRADFPVLSREVNGLPLAYLDSAASAQKPSQVIDAEAEFYRHGYAAVHRGIHTLSAQATEKME

NVRKRASLFINARSAEELVFVRGTTEGINLVANSWGNSNVRAGDNIIISQMEHHANIVPWQMLCARVGAE

LRVIPLNPDGTLQLETLPTLFDEKTRLLAITHVSNVLGTENPLAEMITLAHQHGAKVLVDGAQAVMHHPV

DVQALDCDFYVFSGHKLYGPTGIGILYVKEALLQEMPPWEGGGSMIATVSLSEGTTWTKAPWRFEAGTPN

TGGIIGLGAALEYVSALGLNSIAEYEQNLMHYALSQLESVPDLTLYGQQNRLGVIAFNLGKHHAYDVGSF

LDNYGIAVRTGHHCAMPLMAYYNVPAMCRASLAMYNTHEEVERLVTGLQRIHRLLG

>gi|30063193|ref|NP_837364.1| cysteine desufuration protein SufE [Shigella flexneri 2a str. 2457T]

MALLPDKEKLLRNFLRCANWEEKYLYIIELGQRLPELRAEDGSPQNSIQGCQSQVWIVMRQNAQGIIELQ

GDSDAAIVKGLIAVVFILYDQMTPQDIVNFDVRPWFEKMALTQHLTPSRSQGLEAMIRAIRAKAAALS

>gi|30063192|ref|NP_837363.1| hypothetical protein S1840 [Shigella flexneri 2a str. 2457T]

MKRASLLTLTLIGAFSAIQAAWAVDYPLPPTGSRLVGQNQTYTVQEGDKNLQAIARRFDTAAMLILEANN

TIAPVPKPGTTITIPSQLLLPDAPRQGIIVNLAELRLYYYPPGENIVQVYPIGIGLQGLETPVMETRVGQ

KIPNPTWTPTAGIRQRSLERGIKLPPVVPAGPNNPLGRYALRLAHGNGEYLIHGTSAPDSVGLRVSSGCI

RMNAPDIKALFSSVRTGTPVKVINEPVKYSVEPNGMRYVEVHRPLSAEEQQNVQTMPYTLPAGFTQFKDN

KAVDQKLVDKALYRRAGYPVSVSSGATPAASNAPSVESAQNGEPEQGNMLRATQ

>gi|30063191|ref|NP_837362.1| murein lipoprotein [Shigella flexneri 2a str. 2457T]

MKATKLVLGAVILGSTLLAGCSSNAKIDQLSSDVQTLNAKVDQLSNDVNAMRSDVQAAKDDAARANQRLD

NMATKYRK

>gi|30063190|ref|NP_837361.1| pyruvate kinase [Shigella flexneri 2a str. 2457T]

MKKTKIVCTIGPKTESEEMLAKMLDAGMNVMRLNFSHGDYAEHGQRIQNLRNVMSKTGKTAAILLDTKGP

EIRTMKLEGGNDVSLKAGQTFTFTTDKSVIGNSEMVAVTYEGFTTDLSVGNTVLVDDGLIGMEVTAIEGN

KVICKVLNNGDLGENKGVNLPGVSIALPALAEKDKQDLIFGCEKGVDFVAASFIRKRSDVIEIREHLKAH

GGENIHIISKIENQEGLNNFDEILEASDGIMVARGDLGVEIPVEEVIFAQKMMIEKCIRARKVVITATQM

LDSMIKNPRPTRAEAGDVANAILDGTDAVMLSGESAKGKYPLEAVSIMATICERTDRVMNSRLEFNNDNR

KLRITEAVCRGAVETAEKLDAPLIVVATQGGKSARAVRKYFPDATILALTTNEKTAHQLVLSKGVVPQLV

KEITSTDDFYRLGKELALQSGLAHKGDVVVMVSGALVPSGTTNTASVHVL

>gi|30063189|ref|NP_837360.1| IS1 orfB [Shigella flexneri 2a str. 2457T]

MSRQCTHYGRWPQHGFTSLKKLRPQSVTSRIQPGSDVIVCAEMDEHWGYVGAKSRQRWLFYAYDRIRRTV

VAHVFGERTLATLERLLSLLSAFEVVVWMTDGWPLYESRLKGKLHVISKRYTQRIERHNLNLRQHLARLG

RKSLSFSKSVELHDKVIGHYLNIKHYQ

>gi|30063188|ref|NP_837359.1| IS1 orfA [Shigella flexneri 2a str. 2457T]

MASISIRCPSCSATEGVVRNGKSTAGHQRYLCSHCRKTWQLQFTYTASQPGTHQKIIDMAMNGVGCRASA

RIMGVGLNTVLRHLKNSGRSR

>gi|30063187|ref|NP_837358.1| hypothetical protein S1835 [Shigella flexneri 2a str. 2457T]

MGNRTKEDELYREMCRVVGKVVLEMRDLGQEPKHIVIAGVLRTALANKRIQRSELEKQAMETVINALVK

>gi|30063186|ref|NP_837357.1| hypothetical protein S1834 [Shigella flexneri 2a str. 2457T]

MNPVDRPLLDIGLTRLEFLRISGKGLAGLTIAPALLSLLGCKQEDIDSGTVGLINTPKGVLVTQRARCTG

CHRCEISCTNFNDGSVGTFFSRIKIHRNYFFGDNGVGSGGGLYGDLNYTADTCRQCKEPQCMNVCPIGAI

TWQQKEGCITVDHKRCIGCSACTTACPWMMATVNTESKKSSKCVLCGECANACPTGALKIIEWKDITV

>gi|30063185|ref|NP_837356.1| hypothetical protein S1832 [Shigella flexneri 2a str. 2457T]

MNHQDELPLAKVSEVDEAKRQWLQGMRHPVDTVTEPEPAEILAEFIRQHSAAGQLVARTVFLSPPYSVAE

EELSVLLESIKQNGDYADIACMTGSQDDYYYSTQAMSENYAAMSLQVVEQDICRAIAHAVRFECQTYPRP

YKVAMLMQAPYYFQEAQIEAAIAAMDVAPEYADIRQVESSTAVLYLFSERFMTYGKAYGLCEWFEVEQFQ

NP

>gi|30063184|ref|NP_837355.1| oxidoreductase, Fe-S subunit [Shigella flexneri 2a str. 2457T]

MSWIGWTVAATALGDNQMSFTRRKFVLGMGTVIFFTGSASSLLANTRQEKEVRYAMIHDESRCNGCNICA

RACRKTNHVPAQGSRLSIAHIPVTDNDNETQYHFFRQSCQHCEDAPCIDVCPTGASWRDEQGIVRVEKSQ

CIGCSYCIGACPYLVRYLNPVTKVADKCDFCAESRLAKGFPPICVSACPEHALIFGREDSPEIQAWLQDN

KYYQYQLPGTGKPHLYRRFGQHLIKKENV

>gi|30063183|ref|NP_837354.1| hypothetical protein S1830 [Shigella flexneri 2a str. 2457T]

MNPSQHAEQFQSQLANYVPQFTPEFWPVWLIIAGVLLVGMWLVLGLHALLRARGVKKSATDHGEKVYLYS

KAVRLWHWSNALLFVLLLASGLINHFAMVGATAVKSLVAVHEVCGFLLLACWLGFVLINAVGDNGHHYRI

RRQGWLERAAKQTRFYLFGIMQGEEHPFPATTQSKFNPLQQVAYVGVMYGLLPLLLLTGLLCLYPQAVGD

VFPGVRYWLLQAHFALAFISLFFIFGHLYLCTTGRTPHETFKSMVDGYHRH

>gi|30063182|ref|NP_837353.1| hypothetical protein S1829 [Shigella flexneri 2a str. 2457T]

MIITRADLREWRIGAVMYRWFLRHFPRGGSYADIRHALIEEGYTDWAESLVEYAWKKWLADENFAHQEVS

SMQKLATDPGEIPFCSQFARSDDHARIGCCEDNARIATAGYAAQIASMGYSVRIGSVGFNSHIGSSGERA

RVAVTGNSSRISSAGDSSRIANTGMRVRVCTLGERCHVASNGDLVQIASFGANARIANSGDNVHIIASGE

NSTVVSTGVVDSIILGLGGSAALAYHDGERVRFAVAIEGENNIRTGVRYRLNEQHQFVEC

>gi|30063181|ref|NP_837352.1| hypothetical protein S1827 [Shigella flexneri 2a str. 2457T]

MATLLQLHFAFNGPFGDAMAEQLKPLAESINQEPGFLWKVWTESEKNHEAGGIYLFTDEKSALAYLEKHT

ARLKNLGVEEVVAKVFDVNEPLSQINQAKLA

>gi|30063180|ref|NP_837351.1| hypothetical protein S1824 [Shigella flexneri 2a str. 2457T]

MGSDAKNLMSDGNVQIVKTGEVLGATQLTEGELIVEAGGRAENTVVTGAGWLKVATGGIAKCTQYGNNGT

LSVSDGAIATDIVQSEGGAISLSTLATVNGRHPEGEFSVDQGYACGLLLENGGNLRVLEGHRAEKIILDQ

EGGLLVNGTTSAVVVDEGGELLVYPSGEASNCEINQGGVFMLAGKASDTLLAGGTMNNLGGEDSDTIVEN

GSIYRLGTDGLQLYSSGKTQNLSVNVGGRAEVHAGTLENAVIQGGTVILLSPTSADENFVVEEDRAPVEL

TGSVALLDGASMIIGYGAELQQSTITVQQGGVLILDGSTVKGDSVTFIVGNINLNGGKLWLITGAATHVQ

LKVKRLRGEGAICLQTSAKEISPDFINVKGEVNGDIRVEITDASRQTLCNALKLQPDEDGIGATLQPA

>gi|30063179|ref|NP_837350.1| multidrug efflux protein [Shigella flexneri 2a str. 2457T]

MQKYISEARLLLALAIPVILAQIAQTAMGFVDTVMAGGYSATDMAAVAIGTSIWLPAILFGHGLLLALTP

VIAQLNGSGRRERIAHQVRQGFWLAGFVSVLIMLVLWNAGYIIRYMENIDPALADKAVGYLRALLWGAPG

YLFFQVARNQCEGLAKTKPGMVMGFIGLLVNIPVNYIFIYGHFGMPELGGVGCGVATAAVYWVMFLAMVS

YIKRARSMRDIRNEKGTAKPEPAVMKRLIQLGLPIALALFFEVTLFAVVALLVSPLGIVDVAGHQIALNF

SSLMFVLPMSLAAAVTIRVGYRLGQGSTLDAQTAARTGLMVGVCMATLTAIFTVSLREQIALLYNDNPEV

VTLAAHLMLLAAVYQISDSIQVIGSGILRGYKDTRSIFYITFTAYWVLGLPSGYILALTDLVVEPMGPAG

FWIGFIIGLTSAAIMMMLRMRFLQRMPSAIILQRASR

>gi|30063178|ref|NP_837349.1| riboflavin synthase subunit alpha [Shigella flexneri 2a str. 2457T]

MFTGIVQGTAKLVSIDEKPNFRTHVVELPDHMLDGLETGASVAHNGCCLTVTEINGNHVSFDLMKETLRI

TNLGDLKVGDWVNVERAAKFSDEIGGHLMSGHIMTTAEVAKILTSENNRQIWFKVQDSQLMKYILYKGFI

GIDGISLTVGEVTPTRFCVHLIPETLERTTLGKKKLGARVNIEIDPQTQAVVDTVERVLAARENAMNQPG

TEA

>gi|30063177|ref|NP_837348.1| cyclopropane-fatty-acyl-phospholipid synthase [Shigella flexneri 2a str. 2457T]

MSSSCIEEVSVPDDNWYRIANELLSRAGIAINGSAPADIRVKNPDFFKRVLQEGSLGLGESYMDGWWECD

RLDMFFSKVLRAGLENQLPHHFKDTLRIAGARLFNLQSKKRAWIVGKEHYDLGNDLFSRMLDPFMQYSCA

YWKDADNLESAQQAKLKMICEKLQLKPGMRVLDIGCGWGGLAHYMASNYDVSVVGVTISAEQQKMAQERC

KGLDVTILLQDYRDLNDQFDRIVSVGMFEHVGPKNYDTYFAVVDRNLKPEGIFLLHTIGSKKTDLNVDPW

INKYIFPNGCLPSVRQIAQSSEPHFVMEDWHNFGADYDTTLMAWYERFLAAWPEIADNYSERFKRMFTYY

LNACAGAFRARDIQLWQVVFSRGVENGLRVAR

>gi|30063176|ref|NP_837347.1| inner membrane transport protein YdhC [Shigella flexneri 2a str. 2457T]

MQPGKRFLVWLAGLSVLGFLATDMYLPAFAAIQADLQTPASAVSASLSLFLAGFAAAQLLWGPLSDRYGR

KPVLLIGLTIFALGSLGMLWVENAATLLVLRFVQAVGVCAAAVIWQALVTDYYPSQKVNRIFATIMPLVG

LSPALAPLLGSWLLVHFSWQAIFATLFAITVVLILPIFWLKPTTKARNNSQDGLTFTDLLRSKTYRGNVL

IYAACSASFFAWLTGSPFILSEMGYSPAVIGLSYVPQTIAFLIGGYGCRAALQKWQGKQLLPWLLVLFAV

SVIATWAAGFISHVSLVEILIPFCVMAIANGAIYPIVVAQALRPFPHATGRAAALQNTLQLGLCFLASLV

VSWLISISTPLLTTTSVMLSTVVLVALGYMMQRCEEVDCPNHGNAEVAHSESH

>gi|30063175|ref|NP_837346.1| DNA-binding transcriptional regulator [Shigella flexneri 2a str. 2457T]

MWSEYSLEVVDAVARNGSFSAAAQELHRVPSAVSYTVRQLEEWLAVPLFERRHRDVELTAAGAWFLKEGR

SVVKKMQITRQQCQQIANGWRGQLAIAVDNIVRPERTRQMIVDFYRHFDDVELLVFQEVFNGVWDALSDG

RVELAIGATRAIPVGGRYAFRDMGMLSWSCVVASHHPLALMDGPFSDDTLRNWPSLVREDTSRTLPKRIT

WLLDNQKRLVVPDWESSATCISAGLCIGMVPTHFAKPWLNEGKWVALELENPFPDSACCLTWQQNDMSPA

LTWLLEYLSDSETLNKEWLREPEETPATGD

>gi|30063174|ref|NP_837345.1| DNA-binding transcriptional repressor PurR [Shigella flexneri 2a str. 2457T]

MATIKDVAKRANVSTTTVSHVINKTRFVAEETRNAVWAAIKELHYSPSAVARSLKVNHTKSIGLLATSSE

AAYFAEIIEAVEKNCFQKGYTLILGNAWNNLEKQRAYLSMMAQKRVDGLLVMCSEYPEPLLAMLEEYRHI

PMVVMDWGEAKADFTDAVIDNAFEGGYMAGRYLIERGHREIGVIPGPLERNTGAGRLAGFMKAMEEAMIK

VPESWIVQGDFEPESGYRAMQQILSQPHRPTAVFCGGDIMAMGALCAADEMGLRVPQDVSLIGYDNVRNA

RYFTPALTTIHQPKDSLGETAFNMLLDRIVNKREEPQSIEVHPRLIERRSVADGPFRDYRR

>gi|30063173|ref|NP_837344.1| transporter [Shigella flexneri 2a str. 2457T]

MKINYPLLALAIGAFGIGTTEFSPMGLLPVIARGVDVSIPAAGMLISAYAVGVMVGAPLMTLLLSHRARR

SALIFLMAIFTLGNVLSAIAPDYMTLMLSRILTSLNHGAFFGLGSVVAASVVPKHKQASAVATMFMGLTL

ANIGGVPAATWLGETIGWRMSFLATAGLGVISMVSLFFSLPKGGTGARPEVKKELAVLMRPQVLSALLTT

VLGAGAMFTLYTYISPVLQSITHATPVFVTAMLVLIGVGFSIGNYLGGKLADRSVNGTLKGFLLLLMVIM

LAIPFLARNEFGAAISMVVWGAATFAVVPPLQMRVMHVASEAPGLSSSVNIGAFNLGNALGAAAGGAVIS

AGLGYSFVPVTGAIVAGLALLLVFMSARKQPETVCVANS

>gi|30063172|ref|NP_837343.1| superoxide dismutase [Shigella flexneri 2a str. 2457T]

MSFELPALPYAKDALAPHISAETIEYHYGKHHQTYVTNLNNLIKGTAFEGKSLEEIIRSSEGGVFNNAAQ

VWNHTFYWNCLAPNAGGEPTGKVAEAIAASFGSFADFKAQFTDAAIKNFGSGWTWLVKNSDGKLAIVSTS

NAGTPLTTDATPLLTVDVWEHAYYIDYRNARPGYLEHFWALVNWEFVAKNLAA

>gi|30063171|ref|NP_837342.1| lipoprotein [Shigella flexneri 2a str. 2457T]

MARINRISITLCALLFTTLPLTPMAHASKQARESSATTHITKKADKKKSTATTKKTQKASQKTAKKAASK

SMTKSKTASSVKKSSITASKNAKTRSKHAVNKTASASFTEKCTKRKGYKSHCVKVKNAASGTLADAHKAK

VQKATKVAMNKLMQQIGKPYRWGGSSPRTGFDCSGLVYYAYKDLVKIRIPRTANEMYHLRDAGPIERSEL

KNGDLVFFRTQGRGTADHVGVYVGNGKFIQSPRTGQEIQITSLSEDYWQRHYVGARRVMTPKTLR

>gi|30063170|ref|NP_837341.1| hypothetical protein S1814 [Shigella flexneri 2a str. 2457T]

MSTTIEKIQRQIAENPILLYMKGSPKLPSCGFSAQAVQALAACGERFAYVDILQNPDIRAELPKYANWPT

FPQLWVDGELVGGCDIVIEMYQRGELQQLIKETAAKYKSEEPDAE

>gi|30063169|ref|NP_837340.1| virulence protein [Shigella flexneri 2a str. 2457T]

MRRFAGACRFVFNRALALQNENHEAGNKYIPYGKMASWLVEWKNATETQWLKDSPSQPLQQSLKDLERAY
KNFFQKRAAFPRFKKRGQNAAFRYPQDVKLDQENSRIFLPKLGWMRYRNSRQVTGVVKNVTVSQSCGTWY
ISIQTESEVSTPVHPSASMIGLDAGVAKLATLSDGTVFEPVNSFQKNQKTLARLQRQLSRKVKFSNNWQK
QKRKIQRLHSCIANIRRDYLHKVTTTVSKNHAMIVIEDLKVSNMSKSAAGTVSQPGRNVRAKSGLNRSIL
DQGWYEMRRQLEYKQLWSGGQVLAVPPAYTSQRCACCGHTAKENRLSQSKFRCQVCGYTANADVNGARNI
LAAGHAVLACGEMVQSGRSLKQEPTEMIQATA

>gi|30063168|ref|NP_837339.1| ribonuclease T [Shigella flexneri 2a str. 2457T]
MSDNAQLTGLCDRFRGFYPVVIDVETAGFNAKTDALLEIAAITLKMDEQGWLMPDTTLHFHVEPFVGANL
QPEALAFNGIDPNDPDRGAVSEYEALHEIFKVVRKGIKASGCNRAIMVAHNANFDHSFMMAAAERASLKR
NPFHPFATFDTAALAGLALGQTVLSKACQTAGMDFDSTQAHSALYDTERTAVLFCEIVNRWKRLGGWPLP
AAEEV

>gi|30063167|ref|NP_837338.1| glyoxalase I [Shigella flexneri 2a str. 2457T]
MRLLHTMLRVGDLQRSIDFYTKVLGMKLLRTSENPEYKYSLAFVGYGPETEEAVIELTYNWGVDKYELGT
AYGHIALSVDNAAEACEKIRQNGGNVTREAGPVKGGTTVIAFVEDPDGYKIELIEEKDAGRGLGN

>gi|30063166|ref|NP_837337.1| N-ethylmaleimide reductase [Shigella flexneri 2a str. 2457T]
MSSEKLYSPLKVGAITAANRIFMAPLTRLRSIEPGDIPTPLMAEYYRQRASAGLIISEATQISAQAKGYA
GAPGIHSPEQIAAWKKITAGVHAENGHMALQLWHTGRISHASLQPGGQAPVAPSALSAGTRTSLRDENGQ
AIRVETSMPRALELEEIPGIVNDFRQAIANAREAAFDLVELHSAHGYLLHQFLSPSSNHRTDQYGGSVEN
RARLVLEVVDAGIEEWGADRIGIRVSPIGTFQNTDNGPNEEADALYLIEQLGKRGIAYLHMSEPDWAGGE
PYTDAFREKVRARFHGPIIGAGAYTVEKAETLIGKGLIDAVAFGRDWIANPDLVARLQLKAELNPQRAES
FYGGGAEGYTDYPTL

>gi|30063165|ref|NP_837336.1| hypothetical protein S1807 [Shigella flexneri 2a str. 2457T]

MNKHTEHDTREHLLATGEQLCLQRGFTGMGLSELLKTAEVPKGSFYHYFRSKEAFGVAMLERHYAAYHQR

LAELLQSGEGNYRDRILAYYQQTLNQFCQHGTISGCLTVKLSAEVCDLSEDMRSAMDKGARGVIALLSQA

LENGRENHCLTFCGEPLQQAQVLYALWLGANLQAKISRSFEPLENALAHVKNIIATPAV

>gi|30063164|ref|NP_837335.1| hypothetical protein S1808 [Shigella flexneri 2a str. 2457T]

MFASIMFGVFVHALECSRPVGLVQAKLLLWTLTASIMVIILSEEGEVAEQLEFFPVQSPCRGICQSDERG

FCRGCFRSRDERFNWNKMSDGEKQEVLRLCRQRLMRKLRANKPASSDEPEQPSLF

>gi|30063163|ref|NP_837334.1| hypothetical protein S1806 [Shigella flexneri 2a str. 2457T]

MVQRITIAPEGPEFSRFVMGYWRLMDWNMSARQLVSFIEEHLDLGVTTVDHADIYGGYQCEAAFGEALKL

APHLRERMEIVSKCGIATTAREENVIGHYITDRNHIIKSAEQSLINLATDHLDLLLIHRPDPLMDADEVA

DAFKHLHQSGKVRHFGVSNFTPAQFALLQSRLPFTLATNQVEISPVHQPLLLDGTLDQLQQLRVRPMAWS

CLGGGRLFNDDYFQPLRDELAVVAEELNAGSIEQVVYAWVLRLPSQPLPIIGSGKIERVRAAVEAETLKM

TRQQWFRIRKAALGYDVP

>gi|30063162|ref|NP_837333.1| superoxide dismutase [Shigella flexneri 2a str. 2457T]

MNGGPMKRFSLAILALVVATGAQAASEKVEMNLVTSQGVGQSIGSVTITETDKGLEFSPDLKALPPGEHG

FHIHAKGSCQPATKDGKASAAESAGGHLDPQNTGKHEGPEGTGHLGDLPALVVNNDGKATDAVIAPRLKS

LDEIKDKALMVHVGGDNMSDQPKPLGGGGERYACGVIK

>gi|30063161|ref|NP_837332.1| hypothetical protein S1803 [Shigella flexneri 2a str. 2457T]

MVTMSIKTIKYFSTIIVAIVAVLAGWWLWNYYMQSPWTRDGKIRAEQVSITPQVSGRIVELNIKDNQLVN

AGELLLTIDKTPFQIAELNAQAQLAKAQSDLAKANNEANRRRHLSQNFIFAEELDTANLNVKAMQASVNA

AQATLKQTQWQLAQTEIRAPVSGWVTNLTTRIGDYADTGKPLFALVDSHSFYVIGYFEETKLRHIREGAP

AQITLYSDNKTLQGHVSSIGRAIYDQSVESDSSLIPDVKPNVPWVRLAQLVPVRFALDKVPGDVTLVSGT
TCSIAVGQ

>gi|30063160|ref|NP_837331.1| hypothetical protein S1802 [Shigella flexneri 2a str. 2457T]
MKFMLNATGLPLQDLVFGASVYFPPFFKAFAFGFVIWLVVHRLLRGWIYAGDIWHPLLMDLSLFAICVCL
ALAILIAW

>gi|30063158|ref|NP_837329.1| outer membrane protein [Shigella flexneri 2a str. 2457T]
MIKRVLVVSMVGLSLVGCVNNDTLSGDVYTASEAKQVQNVSYGTIVNVRPVQIQGGDDSNVIGAIGGAVL
GGFLGNTVGGGTGRSLATAAGAVAGGVAGQGVQSAMNKTQGVELEIRKDDGNTIMVVQKQGNTRFSPGQR
VVLASNGSQVTVSPR

>gi|30063157|ref|NP_837328.1| anhydro-N-acetylmuramic acid kinase [Shigella flexneri 2a str. 2457T]
MKSGRFIGVMSGTSLDGVDVVLATIDEHRVAQLASLSWPIPVSLKQAVLDICQGQQLTLSQFGQLDTQLG
RLFADAVNALLKEQNLQARDIVAIGCHGQTVWHEPTGVAPHTLQIGDNNQIVARTGITVVGDFRRRDIAL
GGQGAPLVPAFHHALLAHPTERRMVLNIGGIANLSLLIPGQLVGGYDTGPGNMLMDAWIWRQAGKPYDKD
AEWARAGKVILPLLQNMLSDPYFSQPAPKSTGREYFNYGWLERHLRHFPGVDPRDVQATLAELTAVTISE
QVLLSGGCERLMVCGGGSRNPLLMARLAVLLLGTEVTTTDAVGISGDDMEALAFAWLAWRTLAGLPGNLP
SVTGASQETVLGAIFPANS

>gi|30063154|ref|NP_837325.1| hypothetical protein S1796 [Shigella flexneri 2a str. 2457T]
MQTDTLEYQCDEKPLTVKLNNPRQEVSFVYDNQLLHLKQGLSASGARYSDGIYVFWSKGEEATVYKRDRI
VLNNCQLQNPQR

>gi|30063153|ref|NP_837324.1| pyridoxamine 5'-phosphate oxidase [Shigella flexneri 2a str. 2457T]

MSDNDELQQIAHLRREYTKGGLRRRDLPADPLTLFERWLSQACEAKLADPTAMVVATVDEHGQPYQRIVL

LKHYDEKGMVFYTNLGSRKAHQIENNPRVSLLFPWHTLERQVMVIGKAERLSTLEVMKYFHSRPRDSQIG

AWVSKQSSRISARGILESKFLELKQKFQQGEVPLPSFWGGFRVSLEQIEFWQGGEHRLHDRFLYQRENDA

WKIDRLAP

>gi|30063152|ref|NP_837323.1| tyrosyl-tRNA synthetase [Shigella flexneri 2a str. 2457T]

MASSNLIKQLQERGLVAQVTDEEALAERLAQGPIALYCGFDPTADSLHLGHLVPLLCLKRFQQAGHKPVA

LVGGATGLIGDPSFKAAERKLNTEETVQEWVDKIRKQVAPFLDFDCGENSAIAANNYDWFGNMNVLTFLR

DIGKHFSVNQMINKEAVKQRLNREDQGISFTEFSYNLLQGYDFACLNKQYDVVLQIGGSDQWGNITSGID

LTRRLHQNQVFGLTVPLITKADGTKFGKTEGGAVWLDPKKTSPYKFYQFWINTADADVYRFLKFFTFMSI

EEINALEEEDKNSGKAPRAQYVLAEQVTRLVHGEEGLQAAKRITECLFSGSLSALSEADFEQLAQDGVPM

VEMEKGADLMQALVDSELQPSRGQARKTIASNAITINGEKQSDPEYFFKEEDRLFGRFTLLRRGKKNYCL

ICWK

>gi|30063151|ref|NP_837322.1| pyridoxamine kinase [Shigella flexneri 2a str. 2457T]

MMKNILAIQSHVVYGHAGNSAAEFPMRRLGANVWPLNTVQFSNHTQYGKWTGCVMPPSHLTEIVQGIAAI

DKLHTCDAVLSGYLGSAEQGEHILGIVRQVKAANPQAKYFCDPVMGHPEKGCIVAPGVAEFHVRHGLPAS

DIIAPNLVELEILCEHAVNNVEEAVLAARELIEQGPQIVLVKHLARAGYSRDRFEMLLVTADEVWHISRP

LVDFGMRQPVGVGDVTSGLLLVKLLQGATLQEALEHVTAAVYEIMVTTKAMQEYELQVVAAQDRIAKPEH

YFSATKL

>gi|30063150|ref|NP_837321.1| glutathionine S-transferase [Shigella flexneri 2a str. 2457T]

MKLFYKPGACSLASHITLRESGKDFTLVSVDLMKKRLENGDDYFSVNPKGQVPALLLDDGTLLTEGVAIM

QYLADSVPDRQLLAPVNSISRYKTIEWLNYIATELHKGFTPLFRPDTPEEYKPTVRAQLDKKLQYVNEAL

KDEHWICGQRFTIADAYLFTVLRWAYAVKLNLEGLEHIAAFMQRMAERPEVQDALSAEGLK

>gi|30063149|ref|NP_837320.1| tripeptide transporter permease [Shigella flexneri 2a str. 2457T]

MSTANQKPTESVSLNAFKQPKAFYLIFSIELWERFGYYGLQGIMAVYLVKQLGMSEADSITLFSSFSALV

YGLVAIGGWLGDKVLGTKRVIMLGAIVLAIGYALVAWSGHDAGIVYMGMAAIAVGNGLFKANPSSLLSTC

YEKNDPRLDGAFTMYYMSVNIGSFFSMIATPWLAAKYGWSVAFALSVVGLLITIVNFAFCQRWVKQYGSK

PDFEPVNYRNLLLTIIGVVALIAVATWLLHNQEVARMALGVVAFGIVVIFGKEAFAMKGAARRKMIVAFI

LMLEAIIFFVLYSQMPTSLNFFAIRNVEHSILGLAVEPEQYQALNPFWIIIGSPILAAIYNKMGDTLPMP

TKFAIGMVMCSGAFLILPLGAKFASDAGIVSVSWLVASYGLQSIGELMISGLGLAMVAQLVPQRLMGFIM

GSWFLTTAGANLIGGYVAGMMAVPDNVTDPLMSLEVYGRVFLQIGVATAVIAVLMLLTAPKLHRMTQDDA

ADKAAKATVA

>gi|30063148|ref|NP_837319.1| endonuclease III [Shigella flexneri 2a str. 2457T]

MNKAKRLEILTRLRENNPHPTTELNFSSPFELLIAVLLSAQATDISVNKATAKLYPVANTPAAMLELGVE

GVKTYIKTIGLYNSKAENIIKTCRILLEQHNGEVPEDRAALEALPGVGRKTANVVLNTAFGWPTIAVDTH

IFRVCNRTQFAPGKNVEQVEEKLLKVVPAEFKVDCHHWLILHGRYTCIARKPRCGSCIIEDLCEYKEKVD

I

>gi|30063147|ref|NP_837318.1| electron transport complex RsxE subunit [Shigella flexneri 2a str. 2457T]

MSEIKDVIVQGLWKNNSALVQLLGLCPLLAVTSTATNALGLGLATTLVLTLTNLTISTLRHWTPAEIRIP

IYVMIIASVVSAVQMLINAYAFGLYQSLGIFIPLIVTNCIVVGRAEAFTAKKGPALSALDGFSIGTGATC

AMFVLGSLREIIGNGTLFDGADALLGSWAKVLRVEIFHTDSPFLLAMLPPGAFIGLGLMLAGKYLIDERM

KKRRAEAAAERALPNGETGNV

>gi|30063146|ref|NP_837317.1| electron transport complex protein RnfG [Shigella flexneri 2a str. 2457T]

MLKTIRKHGITLALFAAGSTGLTAAINQMTKTTIAEQASLQQKTLFDQVLPAERYNNALAQSCYLVTAPE

LGKGEHRVYIAKQDDKPVAAVLEATAPDGYSGAIQLLVGADFNGTVLGTRVTEHHETPGLGDKIELRLSD

WLTHFAGKKISGADDAHWAVKKDGGDFDQFTGATITPRAVVNAVKRAGLYAQTLPAQLSQLPACGE

>gi|30063145|ref|NP_837316.1| electron transport complex protein RnfD [Shigella flexneri 2a str. 2457T]

MVFRIASSPYTHNQRQTSRIMLLVLLAAVPGIAAQLRFFGWGTLVQILLASVSALLAEALVLKLRKQSVA

ATLKDNSALLTGLLLAVSIPPLAPWWMVVLGTVFAVIIAKQLYGGLGQNPFNPAMIGYVVLLISFPVQMT

SWLPPHEIAVNIPGFIDAIQVIFSGHTASGGDMNTLRLGIDGISQATPLDTFKTSVRAGHSVEQIMQYPI

YSGILAGAGWQWVNLAWLAGGVWLLWQKAIRWHIPLSFLVTLALCATLGWLFSPETLAAPQIHLLSGATM

LGAFFILTDPVTASTTNRGRLMFGALAGLLVWLIRSFGGYPDGVAFAVLLANITVPLIDYYTRPRVYGHR

KG

>gi|30063144|ref|NP_837315.1| electron transport complex protein RnfC [Shigella flexneri 2a str. 2457T]

MLKLFSAFRKNKIWDFNGGIHPPEMKTQTNGTPLRQVLLAQHFVIPLKQHIGAEGELCVSVGDKVLRGQP

LTRGRGKMLPVHAPTSGTVTAIAPHSTAHPSALAELSVIIDADGEDCWIPRDGWVDYRSRSREELIERIH

QFGVAGLGGAGFPTGVKLQGGGDKIETLIINAAECEPYITADDRLMQDCAAQVVEGIRILAHILQPREIL

IGIEDNKPQAISMLRAVLADSNDISLRVIPTKYPSGGAKQLTYILTGKQVPHGGRSSDIGVLMQNVGTAY

AVKRAVIDGEPITERVVTLTGEAIARPGNVWARLGTPVRHLLNDAGFCPSADQMVIMGGPLMGFTLPWLD

VPVVKITNCLLAPSANELGEPQEEQSCIRCSACADACPVDLLPQQLYWFSKGQQHDKATTHNIADCIECG

ACAWVCPSNIPLVQYFRQEKAEIAAIRQEEKRAAEAKARFEARQARLEREKATRLERHKSAAVQPAAKDK

DAIAAALARVKEKQAQATQPIVIKAGERPDNSAIIAEREARKAQARAKQAELEQTNDAATVAEPRKTAVE

AAIARSKARKLEQQQANAEPEEQVDPRKAAVEAAIARAKARKLEQQQANAEPEEQVDPRKAAVEAAIARA

KARKLEQQQANAEPEEQVDPRKAAVEAAIARAKARKLEQQQANAEPEEQVDPRKAAVEAAIARAKARKLE

QQQANAEPEEQVDPRKAAVAAAIARVQAKKAAQQKVVNED

>gi|30063143|ref|NP_837314.1| electron transport complex protein RnfB [Shigella flexneri 2a str. 2457T]

MNAIWIAVAAVSLLGLAFGAILGYASRRFAVEDDPVVEKIDEILPQSQCGQCGYPGCRPYAEAISCNGEK

INRCAPGGEAVMLKIAELLNVEPQPLDGEAQELTPARMVAVIDENNCIGCTKCIQACPVDAIVGATRVMH

TVMSDLCTGCNLCVDPCPTHCISLQPVAETPDSWKWDLNTIPVRIIPVEHHA

>gi|30063142|ref|NP_837313.1| Na(+)-translocating NADH-quinone reductase subunit E [Shigella flexneri 2a str. 2457T]

MTDYLLLFVGTVLVNNFVLVKFLGLCPFMGVSKKLETAMGMGLATTFVMTLTSICAWLIDTWILIPLNLI

YLRTLAFILVIAVVVQFTEMVVRKTSPVLYRLLGIFLPLITTNCAVLGVALLNINLGHNFLQSALYGFSA

AVGFSLVMVLFAAIRERLAVADVPAPFRGNAIALITAGLMSLAFMGFSGLVKL

>gi|30063141|ref|NP_837312.1| hypothetical protein S1783 [Shigella flexneri 2a str. 2457T]

MTTTTPQRIGGWLLGPLAWLLVALLSTTLALLLYTAALSSPQTFQTLGGQALTTQILWGVSFITAIAMWY

YTLWLTIAFFKRRRCVPKHYIIWLLISVLLAVKAFAFSPVEVGIAVRQLLFTLLATALIVPYFKRSSRVK

ATFVNP

>gi|30063140|ref|NP_837311.1| oriC-binding nucleoid-associated protein [Shigella flexneri 2a str. 2457T]

MTVQDYLLKFRKISSLESLEKLYDHLNYTLTDDQELINMYRAADHRRAELVSGGRLFDLGQVPKSVWHYV

Q

>gi|30063138|ref|NP_837309.1| adenosine deaminase [Shigella flexneri 2a str. 2457T]

MIDTTLPLTDIHRHLDGNIRPQTILELGRQYNISLPAQSLETLIPHVQVIANEPDLVSFLTKLDWGVKVL

ASLDACRRVAFENIEDAARNGLRYVELRFSPGYMAMAHKLPVAGVVEAVIDGVREGCRTFGVQAKLIGIM

SRTFGEAACQQELEAFLAHRDQITALDLAGDELGFPGSLFLSHFNRARDAGWHITVHAGEAAGPESIWQA

IRELGAERIGHGVKAIEDRALMDFLAEQQIGIESCLTSNIQTSTVAELAAHPLKTFLEHGIRASINTDDP

GVQGVDIIHEYTVAAPAAGLSREQIRQAQINGLEMAFLSAEEKRALREKVAAK

>gi|30063137|ref|NP_837308.1| cystathionine beta-lyase; maltose regulon modulator [Shigella flexneri 2a str. 2457T]

MFDFSKVVDRHGTWCTQWDYVADRFGTADLLPFTISDMDFATAPCIIEALNQRLMHGVFGYSRWKNDEFL

AAIAHWFSTQHYTAIDTQTVVYGPSVIYMVSELIRQWSETGEGVVIHTPAYDAFYKAIEGNQRTVMPVAL

EKQADGWFCDMGKLEAVLAKPECKIMLLCNPQNPTGKVWTCDELEIMADLCERHDVRVISDEIHMDMVWG

EQPHIPWSNVARGDWALLTSGSKSFNIPALTGAYGIIENSSSRDAYLSALKGRDGLSSPSVLALTAHIAA

YQQGAPWLDALRVYLKDNLTYISDKMNAAFPELNWQIPQSTYLAWLDLRPLNIDDNALQKALIEQEKVAI

MPGYTYGEEGRGFVRLNAGCPRSKLEKGVAGLINAIRAVR

>gi|30063136|ref|NP_837307.1| bifunctional maltose and glucose-specific PTS system components IICB [Shigella flexneri 2a str. 2457T]

MTAKTAPKVTLWEFFQQLGKTFMLPVALLSFCGIMLGIGSSLSSHDVITLIPVLGNPVLQAIFTWMSKIG

SFAFSFLPVMFCIAIPLGLARENKGVAAFAGFVGYAVMNLAVNFWLTNKGILPTTDAAVLKANNIQSILG

IQSIDTGILGAVIAGIIVWMLHERFHNIRLPDALAFFGGTRFVPIISSLVMGLVGLVIPLVWPIFAMGIS

GLGHMINSAGDFGPMLFGTGERLLLPFGLHHILVALIRFTDAGGTQEVCGQTVSGALTIFQAQLSCPTTH

GFSESVTRFLSQGKMPAFLGGLPGAALAMYHCARPENRHKIKGLLISGLIACVVGGTTEPLEFLFLFVAP

VLYVIHALLTGLGFTVMSVLGVTIGNTDGNIIDFVVFGILHGLSTKWYMVPVVAAIWFVVYYVIFRFAIT

RFNLKTPGRDSEVASSIEKAVAGAPGKSGYNVPAILEALGGADNIVSLDNCITRLRLSVEDMSLVNVQAV

KDNRAIGVVQLNQHNLQVVIGPQVQSVKDEMAGLMHTVQA

>gi|30063135|ref|NP_837306.1| DNA-binding transcriptional repressor MalI [Shigella flexneri 2a str. 2457T]

MATAKKITIHDVALAAGVSVSTVSLVLNGKGRISTATGERVNAAIEELGFVRNRQASALRGGQSGVIGLI

VRDLSAPFYAELTAGLTEALEAQGRMVFLLHGGKDGEQLAQRFSLLLNQGVDGVVIAGAAGSSDDLRRMA

EEKAIPVIFASRASYLDDVDTVRPDNMQAAQLLTEHLIRNGHQRIAWLGGQSSSLTRAERVGGYCATLLK

FGLPFHSDWVLECTSSQKQAAEAITALLRHNPTISAVVCYNETIAMGAWFGLLKAGRQSGESGVDRYFEQ

QVSLAAFTDATPTTLDDIPVTWASTPARELGITLADRMMQKITHEETHSRNLIIPARLIAAK

>gi|30063134|ref|NP_837305.1| IS1 orfA [Shigella flexneri 2a str. 2457T]

MASISIRCPSCSATEGVVRNGKSTAGHQRYLCSHCRKTWQLQFTYTASQPGTHQKIIDMAMNGVGCRASA

RIMGVGLNTVLRHLKNSGRSR

>gi|30063133|ref|NP_837304.1| IS1 orfB [Shigella flexneri 2a str. 2457T]

MSRQCTHYGRWPQHGFTSLKKLRPQSVTSRIQPGSDVIVCAEMDEHWGYVGAKSRQRWLFYAYDRIRRTV

VAHVFGERTLATLERLLSLLSAFEVVVWMTDGCPLYESRLKGKLHVISKRYTQRIERHNLNLRQHLARLV

RKSLSFSKSVELHDKVIGHYLNIKHYQ

>gi|30063132|ref|NP_837303.1| repressor for uid operon [Shigella flexneri 2a str. 2457T]

MMDNMQTEAQPTRTRILNAAREIFSENGFHSASMKAICKSCAISPGTLYHHFISKEALIQAIILQDQERA

LARFREPIEGIHFVDYMVESIVSLTHEAFGQRALVVEIMAEGMRNPQVAAMLKNKHMTITEFVAQRMRDA

QQKGEISPDINTAMTSRLLLDLTYGVLADIEAEDLAREASFAQGLRAMIGGILTAS

>gi|30063131|ref|NP_837302.1| beta-D-glucuronidase [Shigella flexneri 2a str. 2457T]

MLRPVETPTREIKKLDGLWAFSLDRENCGIDQRWCESALQESRAIAVPGSFNDQFADADIRNYVGNVWYQ

REVFIPKGWAGQRIVLRFDAVTHYGKVWVNNQEVMEHQGGYTPFEADVTPYVIAGKSVRITVCVNNELNW

QTIPPGMVITDENGKKKQSYFHDFFNYAGIHRSVMLYTTPNTWVDDITVVTHVAQDCNHASVDWQVVANG

DISVELRDADQQVVATGQGTSGTLQVVNPHLWQPGEGYLYELCVTAKSQTECDIYPLRVGIRSVAVKGEQ

FLINHKPFYFTGFGRHEDADLRGKGFDNVLMVHDHALMDWIGANSYRTSHYPYAEEMLDWADEHGIVVID

ETAAVGFNLSLGIGFEAGNKPKELYSEEAVNGETQQAHLQAIKELIARDKNHPSVVMWSIANEPDTRPQG

AREYFAPLAEATRKLDPTRPITCVNVMFCDAHTDTISDLFDVLCLNRYYGWYVQSGDLETAEKVLEKELL

AWQEKLHQPIIITEYGVDTLAGLHSMYTDMWSEEYQCAWLDMYHRVFDRVSAVVGEQVWNFADFATSQGI

LRVGGNN

>gi|30063129|ref|NP_837300.1| hypothetical protein S1768 [Shigella flexneri 2a str. 2457T]

MNKSLVAVGVIVALGVVWTGGAWYTGKKIETHLEDMVAQANAQLKLTAPESNLEVSYQNYHRGVFSSQLQ

LLVKPIAGKENPWIKSGQSVIFNESVDHGPFPLAQLKKLNLIPSMASIQTTLVNNEVSKPLFDMAKGETP

FEINSRIGYSGDSSSDISLNPLNYEQKDEKVAFSGGEFQLNADRDGKAISLSGEAQSGRIDAVNEYNQKV

QLTFNNLKTDGSSTLGSFGERVGNQKLSLEKMTISVEGKELALLEGMEISGKSDLVNDGKTINSQLDYSL

NSLKVQNQDLGSGKLTLKVGQIDGEAWHQFSQQYNAQTQALLAQPEIANNPELYQEKVTEAFFSALPLML

KGDPVITIAPLSWKNSQGESALNLSLFLKDPATTKEAPQTLAQEVDRSVKSLDAKLTIPVDMATELMTQV

AKLEGYQEDQAKKLAKQQVEGASAMGQMFRLTTLQDNTITTSLQYANGQITLNGQKMSLEDFVGMFADAS

S

>gi|30063128|ref|NP_837299.1| mannose-6-phosphate isomerase [Shigella flexneri 2a str. 2457T]

MQKLINSVQNYAWGSKTALTELYGMENPSSQPMAELWMGAHPKSSSRVQNAAGDIVSLRDVIESDKSTLL

GEAVAKRFGELPFLFKVLCAAQPLSIQVHPNKHNSEIGFAKENAAGIPMDAAERNYKDPNHKPELVFALT

PFLAMNAFREFSEIVSLLQPVAGAHPAIAHFLQQPHAERLSELFANLLNMQGEEKSRALAILKSALDSQQ

GEPWQTIRLISEFYPEDSGLFSPLLLNVVKLNPGEAMFLFAETPHAYLQGVALEVMANSDNVLRAGLTPK

YIDIPELVANVKFEAKPANQLLTQPVKQGAELDFPIPVDDFAFSLHDLSDKETTISQQSAAILFCVEGDA

TLWKGSQQLQLKPGESAFIAANESPVTVKGHGRLARVYNKL

>gi|30063127|ref|NP_837298.1| fumarase A, fumarate hydratase class I; aerobic isozyme [Shigella flexneri 2a str. 2457T]

MSNKPFHYQSPFPLKKNDTEYYLLTSEHVSVSEFEGQEILKVAPEALTLLARQAFHDASFMLRPAHQQQV

ADILRDPEASENDKYVALQFLRNSDIAAKGVLPTCQDTGTAIIVGKKGQRVWTGGGDEAALARGVYNTYI

EDNLRYSQNAPLDMYKEVNTGTNLPAQIDLYAVDGDEYKFLCIAKGGGSANKTYLYQETKALLTPGKLKN

YLVEKMRTLGTAACPPYHIAFVIGGTSAETNLKTVKLASAKYYDELPTEGNEHGQAFRDVELEKELLIEA

QNLGLGAQFGGKYFAHDIRVIRLPRHGASCPVGMGVSCSADRNIKAKINRQGIWIEKLEHNPGKYIPEEL

RKAGEGEAVRVDLNRPMKEILAQLSQYPVSTRLSLNGTIIVGRDIAHAKLKERMDNGEGLPQYIKDHPIY

YAGPAKTPEGYASGSLGPTTAGRMDSYVDQLQAQGGSMIMLAKGNRSQQVTDACKKHGGFYLGSIGGPAA

VLAQGSIKSLECVEYPELGMEAIWKIEVEDFPAFILVDDKGNDFFQQIQLTQCTRCVK

>gi|30063126|ref|NP_837297.1| fumarate hydratase [Shigella flexneri 2a str. 2457T]

MNTVRSEKDSMGAIDVPADKLWGAQTQRSLEHFRISTEKMPTSLIHALALTKRAAAKVNEDLDLLSEEKA

SAIRQAADEVLAGQHDDEFPLAIWQTGSGTQSNMNMNEVLANRASELLGGVRGMERKVHPNDDVNKSQSS

NDVFPTAMHVAALLALRKQLIPQLKTLTQTLSEKSRAFADIVKIGRTHLQDATPLTLGQEISGWVAMLEH

NLKHIEYSLPHVAELALGGTAVGTGLNTHPEYARRVADELAVITCAPFVTAPNKFEALATCDALVQAHGA

LKGLAASLMKIANDVRWLASGPRCGIGEISIPENEPGSSIMPGKVNPTQCEALTMLCCQVMGNDVAINMG

GASGNFELNVFRPMVIHNFLQSVRLLADGMESFNKHCAVGIEPNRERINQLLNESLMLVTALNTHIGYDK

AAEIAKKAHKEGLTLKAAALALGYLSEAEFDSWVRPEQMVGSMKAGR

>gi|30063125|ref|NP_837296.1| DNA replication terminus site-binding protein [Shigella flexneri 2a str. 2457T]

MARYDLVDRLNTTFRQMEQELATFAAHLEQHKLLVARVFSLPEVKKEDEHNPLNRIEVKQHLGNDAQSLA

LRHFRHLFIQQQSENRSSKAAVRLPGVLCYQVDNLSQAALVSHIQHINKLKTTFEHIVTVESELPTAARF

EWVHRHLPGLITLNAYRTLTVLHDPATLRFGWANKHIIKNLHRDEVLAQLEKSLKSPRSVAPWTREEWQR

KLEREYQDIAALPQNAKLKIKRPVKVQPIARVWYKGDQKQVQHACPTPLIALINRDNGAGVPDVGELLNY

DADNVQHRYKPQAQPLRLIIPRLHLYVAD

>gi|30063124|ref|NP_837295.1| sensor protein RstB [Shigella flexneri 2a str. 2457T]

MKKLFIQFYLLLFVCFLVMSLLVGLVYKFTAERAGKQSLDDLMNSSLYLMRSELREIPPHDWGKTLKEMD

LNLSFDLRVEPLSKYHLDDISMHRLRGGEIVALDDQYTFLQRIPRSHYVLAVGPVPYLYYLHQMRLLDIA

LIAFIAISLAFPVFIWMRPHWQDMLKLEAAAQRFGDGHLNERIHFDEGSSFERLGIAFNQMADNINALIA

SKKQLIDGIAHELRTPLVRLRYRLEMSDNLSAAESQALNRDISQLEALIEELLTYARLDRPQNELHLSEP

DLPLWLSTHLADIQAVTPDKTVRIKTLMQGHYAALDMRLMERVLDNLLNNALRYCHSTVETSLLLSGNRA

TLIVEDDGPGIAPENREHIFEPFVRLDPSRDRSTGGCGLGLAIVHSIALTMGGTVNCDISELGGARFSFS

WPLWHNIPQFTSA

>gi|30063123|ref|NP_837294.1| transporter [Shigella flexneri 2a str. 2457T]

MSLTIVACMLCLVMIAGLVGLAIASAIGLDASAFGHMQSSLTSDDVSKTAAVSLPRLITSLIPTNLFLDL

TGARSVSVIGIVIFTILAGIALLKVKKDAPAEGEKLSAGINAIQIWVMKMVRIVIALTPYGVMALMAKVF

STYQLSQFTNLLGFIVACYLAVFVMFIVHSGILLLIGKNPVNYYKTVWQVLTFAFVSRSSAASIPLAISA

LEKLGVPNTIANIAASFGSSMGQNGCAGIYPAVMVAMIAPTMGIDPFSLQFLATLLPAIALGSIGVAGVG

SGGTFAALIVLSTLNFPVALVVIFIAIEPIVDMARTALNVNGSIMSGVISARLLKDRETKEQQSVTLN

>gi|30063122|ref|NP_837293.1| DNA-binding transcriptional regulator RstA [Shigella flexneri 2a str. 2457T]

MNTIVFVEDDAEVGSLIAAYLAKHDMQVTVEPRGDQAEETILRENPDLVLLDIMLPGKDGMTICRDLRAK

WSGPIVLLTSLDSDMNHILALEMGACDYILKTTPPAVLLARLRLHLRQNEQATLTKGLQETSLTPYKALH

FGTLTIDPINRVVTLANTEISLSTADFELLWELATHAGQIMDRDALLKNLRGVSYDGLDRSVDVAISRLR

KKLLDNAAEPYRIKTVRNKGYLFAPHAWDSDNKQSDD

>gi|30063121|ref|NP_837292.1| hypothetical protein S1760 [Shigella flexneri 2a str. 2457T]

MGLVIKAALGALVVLLIGVLAKTKNYYIAGLIPLFPTFALIAHYIVASERGIEALRATIIFSMWSIIPYF

VYLVSLWYFTGMMRLPAAFVGSVACWGISAWVLIICWIKLH

>gi|30063120|ref|NP_837291.1| short chain dehydrogenase [Shigella flexneri 2a str. 2457T]

MGKTQPLPILITGGGRRIGLALAWHFINQKQPVIVSYRTHYPAIDGLINAGAQCIQADFSTNDGVMAFAD
EVLKTTHGLRAILHNASAWIAEKPGAPLADVLACMMQIHVNTPYLLNHALERLLRGHGHAASDIIHFTDY
VVERGSDKHVAYAASKAALDNMTRSFARKLAPEVKVNSIAPSLILFNEHDDAEYRQQALNKSLMKTAPGE
KEVIDLVDYLLTSCFVTGRSFPLDGGRHLR

>gi|30063119|ref|NP_837290.1| arginine/ornithine antiporter [Shigella flexneri 2a str. 2457T]

MEKKLGLSALTALVLSSMLGAGVFSLPQNMAAVASPAALLIGWGITGAGILLLAFAMLILTRIRPELDGG
IFTYAREGFGELIGFCSAWGYWLCAVIANVSYLVIVFSALSFFTDTPELRLFGDGNTWQSIVGASALLWI
VHFLILRGVQTAASINLVATLAKLLPLGLFVVLAMMMFKLDTFKLDFTGLALGVPVWEQVKNTMLITLWV
FIGVEGAVVVSARARNKRDVGKATLLAVLSALGVYLLVTLLSLGVVARPELAEIRNPSMAGLMVEMMGPW
GEIIIAAGLIVSVCGAYLSWTIMAAEVPFLAATHKAFPRIFARQNAQAAPSASLWLTNICVQICLVLIWL
TGSDYNTLLTIASEMILVPYFLVGAFLLKIATRPLHKAVGVGACIYGLWLLYASGPMHLLLSVVLYAPGL
LVFLYARKTHTHDNVLNRQEMVLIGMLLIASVPATWMLVG

>gi|30063118|ref|NP_837289.1| hypothetical protein S1757 [Shigella flexneri 2a str. 2457T]

MKLKNTLLASALLSATAFSVNAATELTPEQAAAVKPFDRVVVTGRFNAIGEAVKAVSRRADKEGAASFYV
VDTSDFGNSGNWRVVADLYKADAEKAEETSNRVINGVVELPKDQAVLIEPFDTVTVQGFYRSQPEVNDAI
TKAAKAKGAYSFYIVRQIDANQGGNQRITAFIYKKDAKKRIVQSPDVIPADSEAGRAALAAGGEAAKKVE
IPGVATTASPSSEVGRFFETQSSKGGRYTVTLPDGTKVEELNKATAAMMVPFDSIKFSGNYGNMTEVSYQ
VAKRAAKKGAKYYHITRQWQKRGNNLTVSADLYK

>gi|30063117|ref|NP_837288.1| NAD(P) transhydrogenase subunit alpha [Shigella flexneri 2a str. 2457T]

MEGNIMRIGIPRERLTNETRVAATPKTVEQLLKLGFTVAVESGAGQLASFDDKAFVQAGAEIVEGNSVWQ
SEIILKVNAPLDDEIALLNPGTTLVSFIWPAQNPELMQKLAERNVTVMAMDSVPRISRAQSLDALSSMAN
IAGYRAIVEAAHEFGRFFTGQITAAGKVPPAKVMVIGAGVAGLAAIGAANSLGAIVRAFDTRPEVKEQVQ

SMGAEFLELDFKEEAGSGDGYAKVMSDAFIKAEMELFAAQAKEVDIIVTTALIPGKPAPKLITREMVDSM

KAGSVIVDLAAQNGGNCEYIVPGEIFSTENGVKVIGYTDLPGRLPTQSSQLYGTNLVNLLKLLCKEKDGN

ITVDFDDVVIRGVTVIRAGEITWPAPPIQVSAQPQAAQKAAPEVKTEEKCACSPWRKYALMALAIILFGW

LSSVAPKEFLGHFTVFALACVVGYYVVWNVSHALHTPLMSVTNAISGIIVVGALLQIGQGGWVSFLSFIA

VLIASINIFGGFTVTQRMLKMFRKN

>gi|30063116|ref|NP_837287.1| pyridine nucleotide transhydrogenase [Shigella flexneri 2a str. 2457T]

MSGGLVTAAYIVAAILFIFSLAGLSKHETSRQGNNFGIAGMAIALIATIFGPDTGNVGWILLAMVIGGAI

GIRLAKKVEMTEMPELVAILHSFVGLAAVLVGFNSYLHHDAGMAPILVNIHLTEVFLGIFIGAVTFTGSV

VAFGKLCGKISSKPLMLPNRHKMNLAALVVSFLLLIVFVRTDSVGLQVLALLIMTAIALVFGWHLVASIG

GADMPVVVSMLNSYSGWAAAAAGFMLSNDLLIVTGALVGSSGAILSYIMCKAMNRSFISVIAGGFGTDGS

STGDDQEVGEHREITAEETAELLKNSHSVIITPGYGMAVAQAQYPVAEITEKLRARGINVRFGIHPVAGR

LPGHMNVLLAEAKVPYDIVLEMDEINDDFADTDTVLVIGANDTVNPAAQDDPKSPIAGMPVLEVWKAQNV

IVFKRSMNTGYAGVQNPLFFKENTHMLFGDAKASVDAILKAL

>gi|30063115|ref|NP_837286.1| transporter [Shigella flexneri 2a str. 2457T]

MAKPIITLNGLKIVIMLGMLVIILCGIRFAAEIIVPFILALFIAVILNPLVQHMVRWRVPRVLAVSILMT

IIVMAMVLLLAYLGSALNELTRTLPQYRNSIMTPLQALEPLLQRVGIDVSVDQLAHYIDPNAAMTLLTNL

LTQLSNAMSSIFLLLLTVLFMLLEVPQLPGKFQQMMARPVEGMAAIQRAIDSVSHYLVLKTAISIITGLV

AWAMLAALDVRFAFVWGLLAFALNYIPNIGSVLAAIPPIAQVLVFNGFYEALLVLAGYLLINLVFGNILE

PRIMGRGLGLSTLVVFLSLIFWGWLLGPVGMLLSVPLTIIVKIALEQTAGGQSIAVLLSDLNKE

>gi|30063114|ref|NP_837285.1| multidrug efflux system protein MdtJ [Shigella flexneri 2a str. 2457T]

MYIYWILLGLAIATEITGTLSMKWASVSEGNGGFILMLVMISLSYIFLSFAVKKIALGVAYALWEGIGIL

FITLFSVLLFDESLSLMKIAGLTTLVAGIVLIKSGTRKARKPELEVNHGAV

>gi|30063113|ref|NP_837284.1| multidrug efflux system protein MdtI [Shigella flexneri 2a str. 2457T]

MAQFEWVHAAWLALAIVLEIVANVFLKFSDGFRRKIFGLLSLAAVLAAFSALSQAVKGIDLSVVYALWGG

FGIAATLAAGWILFGQRLNRKGWIGLVLLLAGMIMVKLA

>gi|30063112|ref|NP_837283.1| hypothetical protein S1751 [Shigella flexneri 2a str. 2457T]

MRTTIAVVLGAISLTSAFVFADKPDVAKSANDEVSTLFFGHDDRVPVNDTTQSPWDAVGQLETASGNLCT

ATLIAPNLALTAGHCLLTPPKGKADKAVALRFVSNKGLWRYEIHDIEGRVDPTLGKRLKADGDGWIVPPA

AAPWDFGLIVLRNPPSGITPLPLFEGDKAALTAALKSAGRKVTQAGYPEDHLDTLYSHQNCEVTGWAQTS

VMSHQCDTLPGDSGSPLMLHTDDGWQLIGVQSSAPAAKDRWRADNRAISVTGFRDKLDQLSQK

>gi|30063110|ref|NP_837281.1| LYSR-type transcriptional regulator [Shigella flexneri 2a str. 2457T]

MNIELRHLRYFVAVAEELHFGRAAARLNISQPPLSQQIQALEQQIGARLLARTNRSVLLTAAGKQFLADS

RQILSMVDDAAARAERLHQGEAGELRIGFTSSAPFIRAVSDTLSLFRRDYPDVHLQTREMNTREQIAPLI

EGTLDMGLLRNTALPETLEHAVIVHEPLMAMIPYDHPLANNPSVTLAELAELAELAKEPFVFFDPHVGTG

LYDDILGLMRRYHLTPVITQEVGEAMTIIGLVSAGLGVSILPASFKRVQLNEMRWVPIAEEDAVSEMWLV

WPKHHEQSPAARNFRIHLLNALR

>gi|30063109|ref|NP_837280.1| NAGC-like transcriptional regulator [Shigella flexneri 2a str. 2457T]

MVAENQPGHIDQIKQTNAGAVYRLIDQLGPVSRIDLSRLAQLAPASITKIVREMLEAHLVRELEIKEAGN

RGRPAVGLVVETEAWHYLSLRISRGEIFLALRDLSSKLVVEESQELALKDDLPLLDRIISHIDQFFIRHQ

KKLERLTSIAITLPGIIDTENGIVHRMPFYEDVKEMPLGEALEQHTGVPVYIQHDISAWTMAEALFGASR

GARDVIQVVIDHNVGVGVITDGHLLHAGSSSLVEIGHTQVDPYGKRCYCGNHGCLETIASVDSILELAQL

RLNQSMSSMLHGQPLTVDSLCQAALRGDLLAKDIIIGVGAHVGRILAIMVNLFNPQKILIGSPLSKAADI

LFPVISDSIRQQALPAYSQHISVESTQFSNQGTMAGAALVKDAMYNGSLLIRLLQG

>gi|30063108|ref|NP_837279.1| dithiobiotin synthetase [Shigella flexneri 2a str. 2457T]

MLKRFFITGTDTSVGKTVVSRALLQALASQGKRVAGYKPVAKGSKETPEGLRNKDALVLQSVSTIELPYE

AVNPIALSEEESSVAHSCPINYTLISNGLANLTDKVDHVVVEGTGGWRSLMNDLRPLSEWVVQEQLPVLM

VVGIQEGCINHALLTAQAIANDGLPLIGWVANRINPGLAHYAEIIDVLGKKLPAPLIGELPYLPRAEQRE

LGQYIRLAMLRSVLAVDRVTV

>gi|30063106|ref|NP_837277.1| twin-argninine leader-binding protein DmsD [Shigella flexneri 2a str. 2457T]

MTHFSQQDNFSVAARVLGALFYYAPESAEAAPLVAVLTSDGWETQWPLPEASLAPLVTAFQTQCEETHAQ

AWQRLFVGPWALPSPPWGSVWLDRESVLFGDSTLALRQWMREKGIQFEMKQNEPEDHFGSLLLMAAWLAE

NGRQTECEELLAWHLFPWSTRFLDVFIEKAEHPFYRALGELARLTLAQWQSQLLIPVAVKPLFR

>gi|30063105|ref|NP_837276.1| DMSO reductase anchor subunit [Shigella flexneri 2a str. 2457T]

MGNGWHEWPLVIFTVLGQCVVGALIVSGIGWFAAKNDADRQHIVRGMFFLWLLMGIGFIASVMHLGSPLR

AFNSLNRIGASGLSNEIAAGSIFFAVGGLWWLVAVIGKMPQALGKLWLLVSMALGVIFVWMMTCVYQIDT

VPTWHNGYTTLAFFLTVLLSGPILAAAILRAARVTFNTTPFAIISVLALIACAEVIVLQGLSLASIHSSV

QQASALVPDYASLQVWRVVLLCAGLGCWLCPLIRRREPHVAGLVLGLILILVGEMIGRVLFYGLHMTVGM

AIAG

>gi|30063104|ref|NP_837275.1| oxidoreductase, Fe-S subunit [Shigella flexneri 2a str. 2457T]

MTTQYGFFIDSSRCTGCKTCELACKDFKDLGPEVSFRRIYEYAGGDWQEDNGIWHQNVFAYYLSISCNHC

DDPACTKVCPSGAMHKREDGFVVVDEDVCIGCRYCHMACPYGAPQYNAEKGHMTKCDGCYSRVAEGKQPI

CVESCPLRALEFGPIEELRQKHGTLAAVAPLPRAHFTKPNIVIKPNANSRPTGDTTGYLANPEEV

>gi|30063103|ref|NP_837274.1| integrase [Shigella flexneri 2a str. 2457T]

MRQRELAATAKAFMLDRPEVSLQELTEHLRSMAEQFLTDASDDYWNGLEVATLVDEKSNLKELAATQALS
LDQQKGIRLALEVLTAAQQRVDTGDTSGLIKLIDDNNLTDDSTIGDSTSILNNEQGDRPAVFTQERQSSV
VFSSLVSSLLAEKVQTLKTSSYKDLSSSLNTVSRFLPEDMDLMSRSGWLAVRDSMLASEVRPSTINKLLT
KAKMCLDYGLMNGQLEGRNPIERMKLTKDIDSKRRAFTDEELERLLVRVESE

>gi|30063102|ref|NP_837273.1| IS911 orfA [Shigella flexneri 2a str. 2457T]
MKKRNFSAEFKRESAQLVVDQKYTVADAAKAMDVGLSTMTRWVKQLRDERQGKTPKASPITPEQIEIREL
RKKLQRIEMENEILKKATALLMSDSLNSSR

>gi|30063101|ref|NP_837272.1| IS911 orfB, partial [Shigella flexneri 2a str. 2457T]
MKELGLVSCQQPTHRYKRGGHEHVAIPNYLERQFAVTEPNQVWCGDVTYIWTGKRWAYLAVVLDLFARKP
VGWAMSFSPDSRLTMKALEMAWETRGKPGGVMFHSDQGSHYTSRQFRQLLWRYQIRQSMSRRGNCWDNSP
MERFFRSLKNEWMPVVGYVSFSEAAHAITDYIVGYYSALRPHEYNGGLPPNESENRYWKNSNSVASFC

>gi|30063100|ref|NP_837271.1| hypothetical protein S1732 [Shigella flexneri 2a str. 2457T]
MARPIATHDNTFTKAYLQQHCGDLLSFDGQGDLSGWLDDVLTGAGRLNESMASNTKPVSPYLILTQLLTH
DTLTVSAVQESLSRKRVALGEPMVSTRYARYVYATVVSASKSVQYHASKAGS

>gi|30063099|ref|NP_837270.1| hypothetical protein S1731 [Shigella flexneri 2a str. 2457T]
MNTELPVTNRQFLIILLTNAESSVDCCRYNLDLLKLSRWYAQAEDWDYEKIREMYAEPIQSYLQTHTEEE
LKRGLQDIFYFNNPYTCEGDSEEMDWIASRSDADLKHISNAMMQVESKWSRRLQVLTEDYENGTFDKLVP
PDAERLQAKADQEQRFKSFAEALAGGASYDF

>gi|30063098|ref|NP_837269.1| hypothetical protein S1730 [Shigella flexneri 2a str. 2457T]
MTSDNKQEYTIHIFGQDITSQDGLFRLDEIRRVGIQIGKVEDNRSTEVARFMRTKAGRSLTNPENGFVKK

INMGHLGTSWLADPVAAIEFGRWLDLGYGLAVTRAFVSMTSSATVQSAVASAEDIKPETKEFIQRHGTGL

YIAGREVSQAEYSDYQAVTAGRCEWR

>gi|30063097|ref|NP_837268.1| hypothetical protein S1728 [Shigella flexneri 2a str. 2457T]

MMFNNNNWKLSVTDINLYENTVSLDGQPYPLSLAIKTLIPGYLSGLPSTSREAMELLEALAEAGVTIGNF

FSNDLMTAYGRRQMNKRAEAERIAKEQRLQAERMREENMTDAEWQKELQRREQVKAERRTYGESLRSATH

SAGRSRAAIVADLESGGNWMDSL

>gi|30063096|ref|NP_837267.1| hypothetical protein S1727 [Shigella flexneri 2a str. 2457T]

MTTIKDAFQFGIEPVRITDTDNIQVNEGLPTNADPQVYALQLAKTVKAMLNGVLKDAQENIPFPVEVLPT

RNSLPTPIIAHTLADRSVVVPVRGGKRPEVVTVPSGQEIVVEPIEQAILISEQTKLWDAKSSTGFTQGTL

QQDAMNICENVVRTINARMVDVLESSKLLKTVELPVLTGSLTAKADAIMDALYENTESSFGSEVSDYGII

AHESQLKALSRLAAKQGFSGEDAIVDMLGTDIAYYNGEDKGVFMLAKRFTALSFGCFRHDGENITVVLSR

DGDSQSHDLEILGKVFVVAEAATTIKMGTGSATAVLPVVKRLKFTKTEA

>gi|30063095|ref|NP_837266.1| hypothetical protein S1723 [Shigella flexneri 2a str. 2457T]

MKLSTCCAALLLALASPAVLAAPGSCERIQSDISQRIINNGVPESSFTLSIVPNDQVDQPDSQVVGHCAN

DTHKILYNRTTSGNVSAPAQSTQDGAPAEPQ

>gi|30063093|ref|NP_837264.1| hypothetical protein S1720 [Shigella flexneri 2a str. 2457T]

MKITLSKRIGLLAFLLPCALALSTTVHAETNKLVIESGDSAQSRQHAAMEKEQWNDTRNLRQKVNKRTEK

EWDKADAAFDNRDKCEQSANINAYWEPNTLRCLDRRTGRVITP

>gi|30063092|ref|NP_837263.1| hypothetical protein S1719 [Shigella flexneri 2a str. 2457T]

MIKTTLLFFATALCEIIGCFLPWLWLKRNASIWLLLPAGISLALFVWLLTLHPAASGRVYAAYGGVYVCT

ALMWLRVVDGVKLSLYDWTGALIALCGMLIIVAGWGRT

>gi|30063091|ref|NP_837262.1| starvation sensing protein [Shigella flexneri 2a str. 2457T]

MKIVKAEVFVTCPGRNFVTLKITTEDGITGLGDATLNGRELSVASYLQDHLCPRLIGRDAHRIEDIWQFF

YKGAYWRRGPVTMSAISAVDMALWDIKAKAANMPLYQLLGGASREGVMVYCHTTGHSIDEALDDYARHQE

LGFKAIRVQCGIPGMKTTYGMSKGKGLAYEPATKGQWPEEQLWSTEKYLDFMPKLFDAVRNKFGFNEHLL

HDMHHRLTPIEAARFGKSIEDYRMFWMEDPTPAENQECFRLIRQHTVTPIAVGEVFNSIWDCKQLIEEQL

IDYIRTTLTHAGGITGMRRIADFASLYQVRTGSHGPSDLSPVCMAAALHFDLWVPNFGVQEYMGYSEQML

EVFPHNWTFDNGYMHPGDKPGLGIEFDEKLAAKYPYEPAYLPVARLEDGTLWNR

>gi|30063090|ref|NP_837261.1| ISEhe3 orfA [Shigella flexneri 2a str. 2457T]

MSGKRYPEEFKTEAVKQVVDRGYSVASVATRLDITTHSLYAWIKKYGPDSSTNKEQSDAQAEIRRLQKEL

KRVTDERDILKKAAAYFAKLSD

>gi|30063089|ref|NP_837260.1| ISEhe3 orfB [Shigella flexneri 2a str. 2457T]

MLDVHPSGFYAWLQQPHSQRHQADLRLTGQIKQFWLESGCVYGYRKIHLDLRDSGQQCGVNRVWRLMKRV

GIKAQVGYRSPRARKGEASIVSPNRLQRQFNPDAPDERWVTDITYIRTHEGWLYLAVVVDLFSRKIIGWS

MQSRMTKDIVLNALLMAVWRRNPEKQVLVHSDQGSQYTSHEWQSFLKSHGLEGSMSRRGNCHDNAVAESF

FQLLKRERIKKKIYGTREEARSDIFDYIEMFYNSKRRHGSSEQMSPTEYENQYYQRLGSV

>gi|30063086|ref|NP_837257.1| transport system permease [Shigella flexneri 2a str. 2457T]

MRIRYGWELALAALLVIEIVAFGAINPRMLDLNMLLFSTSDFICIGIVALPLTMVIVSGGIDISFGSTIG

LCAIALGVLFQSGVPMPLAILLTLLLGALCGLINAGLIIYTKVNPLVITLGTLYLFAGSALLLSGMAGAT

GYEGIGGFPMAFTDFANLDVLGLPVPLIIFLICLLVFWLWLHKTHAGRNVFLIGQSPRVALYSAIPVNRT

LCALYAMTGLASAVAAVLLVSYFGSARSDLGASFLMPAITAVVLGGANIYGGSGSIIGTAIAVLLVGYLQ

QGLQMAGVPNQVSSALSGALLIVVVVGRSVSLHRQQIKEWLARRANNPLP

>gi|30063085|ref|NP_837256.1| transport system permease [Shigella flexneri 2a str. 2457T]

MLKFIQNNREITALLAVVLLFVLPGFLDRQYLSVQTLTMVYSSAQILILLAMGATLVMLTRNIDVSVGSI

TGMCAVLLGMLLNAGYSLPVACVATLLLGLLAGFFNGALVAWLKIPAIVATLGTLGLYRGIMLLWTGGKW

IEGLPAELKQLSAPLLLGISAIGWLTIILVAFMAWLLAKTAFGRSFYATGDNLQGARQLGVRTEAIRIVA

FSLNGCMAALAGIVFASQIGFILNQTGTGLEMKAIAACVLGGISLLGGSGAIIGAVLGAWFLTQIDSVLV

LLRIPAWWNDFIAGLVLLAVLVFDGRLRCALERNLRRQKYARFMTPPPSVKPASSGKKREAA

>gi|30063084|ref|NP_837255.1| ATP-binding component of a transport system [Shigella flexneri 2a str. 2457T]

MQTSDTRALPLLCARSVYKQYSGVNVLKGIDFTLHQGEVHALLGGNGAGKSTLMKIIAGITPADSGTLEI

GGNNYARLTPVHAHQLGIYLVPQEPLLFPSLSIKENILFGLAKKQLSMQKMKNLLAALGCQFDLHSLAGS

LDVADRQMVEILRGLMRDSRILILDEPTASLTPAETERLFSRLQELLATGVGIVFISHKLPEIRQIADRI

SVMRDGTIALSGKTSELSTDDIIQAITPAVREKSLSASQKLWLELPGNRPQHAVGTPVLTLENLTGEGFR

NVSLTLNAGEILGLAGLVGAGRTELAETLYGLRTLRGGRIMLNGKEINKLSTGERLLRGLVYLPEDRQSS

GLNLDASLAWNVCALTHNLRGFWAKTAKDNATLERYRRALNIKFNQPEQAARTLSGSNQQKILIAKCLEA

SPQALIVDEPTRGVDVSARNDIYQLLRSIAAQNVAVLFISSDMEEIELMADRVYVMHQGEITHSALTGRD

INVETIMRVAFGDSQRQEASC

>gi|30063083|ref|NP_837254.1| sorC family transcriptional regulator [Shigella flexneri 2a str. 2457T]

MTINDSAISEQGMCEEEQVARIAWFYYHDGLTQSEISDRLGLTRLKVSRLLEKGHQSGIIRVQINSRFEG

CLEYETQLRRQFSLQHVRVIPGLADADVGGRLGIGAAHMLMSLLQPQQMLAIGFGEATMNTLQRLSGFIS

SQQIRLVTLSGGVGSYMTGIGQLNAACSVNIIPAPLRASSADIARTLKNENCVKDVLLAAQAADVAIVGI

GAVSQQDDATIIRSGYISQGEQLMIGRKGAVGDILGYFFDAKGDVVTDIKIHNELIGLPLSALKTIPVRV

GVAGGENKAEAIAAAMKGGYINALVTDQDTAAAILRS

>gi|30063080|ref|NP_837251.1| aldolase [Shigella flexneri 2a str. 2457T]

MDWGMQSRLSWIFNPKTGKTVMLAFDHGYFQGPTIGLERIDINIAPLFEHADVLMCTRGILRSVVPPATN

KPVVLRASGANSILAELSNEAVALSMDDAVRLNSCAVAAQVYIGSEYEHQSIKNIIQLVDAGMKVGMPTM

AVTGVGKDMVRDQRYFSLATRIAAEMGAQIIKTYYVEKGFERIVAGCPVPIVIAGGKKLPERETLEMCWQ

AIDQGASGVDMGRNIFQSDHPVAMMKAVQAVVHHNETADRAYELYLSEKQ

>gi|30063079|ref|NP_837250.1| autoinducer-2 (AI-2) modifying protein LsrG [Shigella flexneri 2a str. 2457T]

MHVTLVEINVHEDKVDEFIEVFRQNHLGSVQEEGNLRFDVLQDPEVNSRFYIYEAYKDEDTVAFHKTTPH

YKTCVAKLESLMTGPRKKRLFNGLMP

>gi|30063078|ref|NP_837249.1| trans-aconitate 2-methyltransferase [Shigella flexneri 2a str. 2457T]

MSDWNPSLYLHFSAERSRPAVELLARVPLENVEYVADLGCGPGNSTALLHQRWPAARITGIDSSPAMIAE

ARSALPDCQFVEADIRNWQPEQALDLIFANASLQWLPDHYELFPHLVSLLNPQGVLAVQMPDNWLEPTHV

LMREVAWEQNYPDRGREPLAGVHAYYDILSEAGCEVDIWRTTYYHQMPSHQAIIDWVTATGLRPWLQDLT

ESEQQLFLTRYHQMLEEQYPLQENGQILLAFPRLFIVARRTE

>gi|30063077|ref|NP_837248.1| hypothetical protein S1701 [Shigella flexneri 2a str. 2457T]

MIVITFNRATFPRLKITMIVRPQQHWLRRIFVWHGSVLSKISSRLLLNFLFSIAVIFMLPWYTHLGIKFT

LAPFSILGVAIAIFLGFRNNAGYARYVEARKLWGQLMIASRSLLREVKTTLPDSASVREFARLQIAFAHC

LRMTLRKQPQAEVLAHYLKTGDLQRVLASNSPANRILLIMGEWLAVQRRNGQLSDILFISLNDRLNDISA

VLAGCERIAYTPIPFAYTLILHRTVYLFCIMLPFALVVDLHYMTPFISVLISYTFISLDCLAEELEDPFG

TENNDLPLDAICNAIEIDLLQMNDEAEIPAKILPDRHYQLT

>gi|30063076|ref|NP_837247.1| altronate oxidoreductase [Shigella flexneri 2a str. 2457T]

MKTLNRRDFPGAQYPERIIQFGEGNFLRAFVDWQIDLLNEHTDLNSGVVVVRPIETSFPPSLSTQDGLYT

TIIRGLNEKGEAVSDARLIRSVNREISVYSEYDEFLKLAHNPEMRFVFSNTTEAGISYHAGDKFDDAPAV

SYPAKLTRLLFERFSHFNGALDKGWIIIPCELIDYNGDALRELVLRYAQEWALPEAFIQWLDQANSFCST

LVDRIVTGYPRDEVAKLEEELGYHDGFLDTAEHFYLFVIQGPKSLATELRLDKYPLNVLIVDDIKPYKER

KVAILNGAHTALVPVAFQAGLDTVGEAMNDAEICAFVEKAIYEEIIPVLDLPRDELESFASAVTGRFRNP

YIKHQLLSIALNGMTKFRTRILPQLLAGQKANGTLPARLTFALAALIAFYRGERNGETYPVQDDAHWLER

YQQLWSQHRDRVIGTQELVAIVLAEKDHWEQDLTQVPGLVEQVANDLDAILEKGMREAVRPLC

>gi|30063075|ref|NP_837246.1| hypothetical protein S1698 [Shigella flexneri 2a str. 2457T]

MLADWFSEQFSTGVLIVPCMLTLAIPGVLPRFKAEQMMPAIAFIVSVIASVVIGGAGSLAFPLPALIWCA

VRYTPQVTCLLTFVTGAVEIVLVANSVIDISVGSPFSIPEMFSARLGIATMAICPIMVSFSVAAINLLMK

QVALRADFDFLTQVYSRSGLYEALKSPSLKQTQHLTVMLLDIDYFKSINDNYGHECGDKVLSVFARHIQK

IVGDKGLVARMGGEEFAVAVPSVNPVDGLLMAEKIRKGVELQPFTWQQKTLYLTVSIGVGSGRASYRTLT

DDFNKLMVEADTCLYRSKKDGRNRTSTMRYGEEVV

>gi|30063074|ref|NP_837245.1| hypothetical protein S1697 [Shigella flexneri 2a str. 2457T]

MQSLDPLFARLSRSKFRSRFRLGMKERQYCLEKGAPVIEQHAADFVAKRLAPALPANDGKQTPMRGHPVF

IAQHATATCCRGCLAKWHNIPQGVSLSEEQQRYIVAVIYHWLVV

>gi|30063073|ref|NP_837244.1| glutaminase [Shigella flexneri 2a str. 2457T]

MAVAMDNAILENILRQVRPLIGQGKVADYIPALATVDGSRLGIAICTVDGQLFQAGDAQERFSIQSISKV

LSLVVAMRHYSEEEIWQRVGKDPSGSPFNSLVQLEMEQGIPRNPFINTGALVVCDMLQGRLSAPRQRMLE

VVRGLSGVSDISYDTVVARSEFEHSARNAAIAWLMKSFGNFHHDVTTVLQNYFHYCALKMSCVELARTFV

FLANQGKAIHIDEPVVTPMQARQINALMATSGMYQNAGEFAWRVGLPAKSGVGGGIVAIVPHEMAIAVWS

PELDDAGNSLAGIAVLEQLTKQLGRSVY

>gi|30063072|ref|NP_837243.1| hypothetical protein S1694 [Shigella flexneri 2a str. 2457T]

MVTPVSICNYISLPDDFPARNIAPQVKEVLKDFIDALSTIICDEEWRTSLNTNSATKKIFNNLDNLSYIQ

RTSFRGNDTLYNEKVQFKLTYPVKNGRHKENIEFQVVINLSPIYLDNFRHDGEINIFCAPNPKPVTMGRV

FQTGVERVLFLFMNDFIEQFPMINLGAPIKRAHTPHIEPLPPDHHTAADYLRQFDLLVLNFISRGNFVIL

PRLWNNSEVHRWFVNKDPNLITAILDITDSELKEDLLQSLMDSLGSNKHVQPEVCICFLSLLAEQESPHF

QDLFLFFANMLLHYHQFMNPNESDLNDVLMPASLSDDKIIKHMARRTLKLFVKNETPPKVTHEDLVKNRP

RSPVRPPIPATAKTPDLPERH

>gi|30063071|ref|NP_837242.1| sugar efflux transporter [Shigella flexneri 2a str. 2457T]

MTTNTVSRKVAWLRVVTLAVAAFIFNTTEFVPVGLLSDIAQSFHMQTAQVGIMLTIYAWVVALMSLPFML

MTSQVERRKLLICLFVVFIASHVLSFLSWSFTVLVISRIGVAFAHAIFWSITASLAIRMAPAGKRAQALS

LIATGTALAMVLGLPLGRIVGQYFGWRMTFFAIGIGALVTLLCLIKLLPLLPSEHSGSLKSLPLLFRRPA

LMSIYLLTVVVVTAHYTAYSYIEPFVQNIAGFSANFATALLLLLGGAGIIGSVIFGKLGNQYASALVSTA

IALLLVCLALLLPAANSEIHLGVLSIFWGIAMMIIGLGMQVKVLALAPDATDVAMALFSGIFNIGIGAGA

LVGNQVSLHWSMSMIGYVGAVPAFAALIWSIIIFRRWPVTLEEQTQ

>gi|30063070|ref|NP_837241.1| multiple drug resistance protein MarC [Shigella flexneri 2a str. 2457T]

MLDLFKAIGLGLVVLLPLANPLTTVALFLGLAGNMNSAERNRQSLMASVYVFAIMMVAYYAGQLVMDTFG

ISIPGLRIAGGLIVAFIGFRMLFPQQKAIDSPEAKSKSEELEDEPSANIAFVPLAMPSTAGPGTIAMIIS

SASTVRQSSTFADWVLMVAPPLIFFLVAVILWGSLRSSGAIMRLVGKGGIEAISRLMGFLLVCMGVQFII

NGILEIIKTYH

>gi|30063068|ref|NP_837239.1| DNA-binding transcriptional activator MarA [Shigella flexneri 2a str. 2457T]

MSRRNTDAITIHSILDWIEDNLESPLSLEKVSERSGYSKWHLQRMFKKETGHSLGQYIRSRKMTEIAQKL

KESNEPILYLAERYGFESQQTLTRTFKNYFDVPPHKYRMTNMQGESRFLHPLNHYNS

>gi|30063067|ref|NP_837238.1| hypothetical protein S1689 [Shigella flexneri 2a str. 2457T]

MKPLSSAIAAALILFSAQSVAEQTTQPVVTSCANVVVVPPSQEQPPFDLNHMGTGSDKSDALGVPYYNQH

AM

>gi|30063065|ref|NP_837236.1| transporter [Shigella flexneri 2a str. 2457T]

MATLPFMTIYLSRQYSLSVDLIGYAMTIALTIGVVFSLGFGILADKFDKKRYMLLAITAFASGFIAIPLV

NNVTLVVLFFALINCAYSVFATVLKAWFADNLSSTSKTKIFSINYTMLNIGWTIGPPLGTLLVMQSINLP

FWLAAICSAFPMLFIQIWVKRSEKIIATETGSVWSPKVLLQDKALLWFTCSGFLASFVSGAFASCISQYV

MVIADGDFAEKVVAVVLPVNAAMVVTLQYSVGRRLNPANIRALMTAGTLCFVIGLVGFIFSGNNLLLWGM

SAAVFTVGEIIYAPGEYMLIDHIAPPGMKASYFSAQSLGWLGAAINPLVSGVVLTSLPPSSLFVILALVI

IAAWVLMLKGIRARPWGQPALC

>gi|30063064|ref|NP_837235.1| IS600 [Shigella flexneri 2a str. 2457T]

MAHIRTRETYGTRRLQTELAENGIIVGRDRLARLRKELRLRCKQKRKFRATTNPNHNLPVAPNLLNQTFA

PTAPNQVWVADLTYVATQEGWLYLAGIKDVYTCEIVGYAMGERMTKELTGKALFMALRSQRPPAGLIHHS

DRGSQYCAYDYRVIQEQSGLKTSMSRKGNCYDNAPMESFWGTLKNESLSHYRFNNRDEAISVIREYIEIF

YNRQRRHSRLGNISPAAFREKYHQMAA

>gi|30063063|ref|NP_837234.1| IS600 [Shigella flexneri 2a str. 2457T]

MSRKTQRYSKEFKAEAVRTVLENQLSISEGASRLSLPEGTLGQWVTAARKGLGTPGSRTVAELESEILQL

RKALNEARLERDILKKATAYFAQESLKNTR

>gi|30063062|ref|NP_837233.1| hypothetical protein S1684 [Shigella flexneri 2a str. 2457T]

MKFQSIVLASFLVMPYALADDQGGLKQDAAPPPPHAIEDGYRGTDDAKKMTVDFAKNMHDGASVSLRGNL

ISHKGVR

>gi|30063061|ref|NP_837232.1| competence damage-inducible protein A [Shigella flexneri 2a str. 2457T]

MNINKDKIVQLADTDTIENLTSALSQRLIADQLRLTTAESCTGGKLASALCAAEDTPKFYGAGFVTFTDQ

AKMKILSVSQQSLERYSAVSEKVAAEMATGAIERADADVSIAITGYGGPEGGEDGTPAGTVWFAWHIKGQ

NYTAVMHFAGDCETVLALAVRFALAQLLQLLL

>gi|30063060|ref|NP_837231.1| dipeptidyl carboxypeptidase II [Shigella flexneri 2a str. 2457T]

MTTMNPFLVQSTLPYLAPHFDQIANHHYRPAFDEGMQQKRAEIAAIALNPQTPDFNNTILALEQSGELIT

RVTSVFFAMTAAHTNDELQRLDEQFSAELAELANDIYLNGELFARVDAVWQRRESLGLDSESIRLVEVIH

QRFVLAGAKLAQADKAKLKVLNTEAATLTSQFNQRLLAANKSGGLVVNDIAQLAGMSEQEIALAAEAARE

KGLDNKWLIPLLNTTQQPALAEMRDRATREKLFIAGWTRAEKNDANDTRAIIQRLVEIRAQQAKLLGFPH

YAAWKIADQMAKTPEAALNFMREIVPAARQRASDELASIQAVIDKQQGGFSAQPWDWAFYAEQVRREKFD

LDEAQLRPYFELNTVLNEGVFWTANQLFGIKFVERFDIPVYHPDVRVWEIFDHNGVGLALFYGDFFARDS

KSGGAWMGNFVEQSTLNETHPVIYNVCNYQKPAAGEPALLLWDDVITLFHEFGHTLHGLFARQRYATLSG

TNTPRDFVEFPSQINEHWATHPQVFARYARHYQSGAAMPDELQQKMRNASLFNKGYEMSELLSAALLDMR

WHCLEENEAMQDVDDFELRALVAENMDLPAIPPRYRSSYFAHIFGGGYAAGYYAYLWTQMLADDGYQWFV

EQGGLTRENGQRFREAILSRGNSEDLERLHRQWRGKAPQIMPMLQHRGLNI

>gi|30063059|ref|NP_837230.1| 3-hydroxy acid dehydrogenase [Shigella flexneri 2a str. 2457T]

MIVLVTGATAGFGECITRRFIQQGHKVIATGRRQERLQELTDELGDNLYIAQLDVRNRAAIEEMLASLPA

EWSNIDILVNNAGLALGMEPAHKASVEDWETMIDTNNKGLVYMTRAVLPGMVERNHGHIINIGSTAGSWP

YAGGNVYGATKAFVRQFSLNLRTDLHGTAVRVTDIEPGLVGGTEFSNVRFKGDDGKAEKTYQNTVALTPE

DVSEAVWWVSTLPAHVNINTLEMMPVTQSYAGLNVHRQ

>gi|30063058|ref|NP_837229.1| hypothetical protein S1680 [Shigella flexneri 2a str. 2457T]

MTVETQLNPTQPVNQQIYRILRRDIVHCLIAPGTPLSEKEVSVRFNVSRQPVREAFIKLAENGLIQIRPQ

RGSYVNKISMAQVRNGSFIRQAIECAVARRAASMITESQCYQLEQNLHQQRIAIERKQLDDFFELDDNFH

QLLTQIADCQLAWDTIENLKATVDRVRYMSFDHVSPPEMLLRQHLDIFSALQKRDGDAVERAMTQHLQEI

SESVRQIRQENSDWFSEE

>gi|30063057|ref|NP_837228.1| hypothetical protein S1679 [Shigella flexneri 2a str. 2457T]

MTTYDRNRNAITTGSRVMVSGTGHTGKILSIDTEGLTAEQIRRGKTVVVEGCEEKLAPLDLIRLGMN

>gi|30063056|ref|NP_837227.1| oxidoreductase [Shigella flexneri 2a str. 2457T]

MGNNLLSAKATLPVYDRNNLAPRIVHLGFGAFHRAHQGVYADILATEHFSDWGYYEVNLIGGEQQIADLQ

QQDNLYTVAEMSADAWTARVVGVVKKALHVQMDGLETVLAAMCEPQIAIVSLTITEKGYFHSPATGQLML

EHPMVAADVQNPHQPKTATGVIVEALARRKAAGLPAFTVMSCDNMPENGHVMRDVVTSYAQAVDVKLAQW

IEDNVTFPSTMVDRIVPAVTEDTLAKIEQLTGVRDPAGVACEPFRQWVIEDNFVAGRPEWEKAGAELVSD

VLPYEEMKLRMLNGSHSFLAYLGYLAGYQHINDCMEDEHYRHAAYGMMLQEQAPTLKVQGVDLQDYANRL

IARYSNPALRHRTWQIAMDGSQKLPQRMLDSVRWHLAHDSKFDLLALGVAGWMRYVGGVDEQGNPIEISD

PLLPVIQKAVQSSAEGKARVQSLLAIKAIFGDDLPDNSLFTAKVTEAYLSLLAHGAKATVAKYSVK

>gi|30063055|ref|NP_837226.1| IS600 orfA [Shigella flexneri 2a str. 2457T]

MSRKTQRYSKEFKAEAVRTVLENQLSISEGASRLSLPEGTLGQWVTAARKGLGTPGSRTVAELESEILQL

RKALNEARLERDILKKATAYFAQESLKNTR

>gi|30063054|ref|NP_837225.1| IS600 orfB [Shigella flexneri 2a str. 2457T]

MAHIRTRETYGTRRLQTELAENGIIVGRDRLARLRKELRLRCKQKRKFRATTNPNHNLPVAPNLLNQTFA

PTAPNQVWVADLTYVATQEGWLYLAGIKDVYTCEIVGYAMGERMTKELTGKALFMALRSQRPPAGLIHHS

DRGSQYCAYDYRVIQEQSGLKTSMSRKGNCYDNAPMESFWGTLKNESLSHYRFNNRDEAISVIREYIEIF

YNRQRRHSRLGNISPAAFREKYHQMTA

>gi|30063053|ref|NP_837224.1| IS911 orfA [Shigella flexneri 2a str. 2457T]

MKKRNFSAEFKRESAQLVVDQKYTVADAAKAMDVGLSTMTRWVKQLRDERQGKTPKASPITPEQIEIREL

RKKLQRIEMENEILKKATALLMSDSLNSSR

>gi|30063052|ref|NP_837223.1| IS911 orfB, partial [Shigella flexneri 2a str. 2457T]

MKELGLVSCQQPTHRYKRGGHEHVAIPNYLERQFAVTEPNQVWCGDVTYIWTGKRWAYLAVVLDLFARKP

VGWAMSFSPDSRLTMKALEMAWETRGKPGGVMFHSDQGSHYTSRQFRQLLWRYQIRQSMSRRGNCWDNSP

MERFFRSLKNEWMPVVGYVSFSEAAHAITDYIVGYYSALRPHEYNGGLPPNESENRYWKNSNSVASFC

>gi|30063051|ref|NP_837222.1| bifunctional antitoxin/transcriptional repressor RelB [Shigella flexneri 2a str. 2457T]

MGSINLRIDDELKARSYAALEKMGVTPSEALRLMLEYIADNERLPFKQTLLSDEDAELVEIVKERLRNPK

PVRVTLDEL

>gi|30063050|ref|NP_837221.1| hypothetical protein S1669 [Shigella flexneri 2a str. 2457T]

MAYFLDFDERALKEWRKLGSTVREQLKKKLVEVLESPRIEANKLRGMPDCYKIKLRSSGYRLVYQVIDEK

VVVFVISVGKRERSEVYSEAVKRIL

>gi|30063049|ref|NP_837220.1| prophage maintenance protein [Shigella flexneri 2a str. 2457T]

MKQQKAMLIALIVICLTVIVTALVTRKDLCEVRIRTGQTEVAVFTAYEPEE

>gi|30063048|ref|NP_837219.1| hypothetical protein S1667 [Shigella flexneri 2a str. 2457T]

MNIEELRKIFCEDGLYAVCVENGNIVSHYRIVCLQKNGAALINFVDARVTDGFILRDGEFVTSLQALKEI

GIKAGFSAFSEE

>gi|30063047|ref|NP_837218.1| endodeoxyribonuclease RUS (Holliday junction resolvase) [Shigella flexneri 2a str. 2457T]

MLIDLVLPYPPTVNTYWRRRGSTYFISEEGKRYRRAVALIVRQQRLKLSLSGRLAIKVIAEPPDKRRRDL

DNILKAPLDALTHAGVLMDDEQFDEINIVRGQPVSGGRLGVKIYPIMH

>gi|30063046|ref|NP_837217.1| hypothetical protein S4818 [Shigella flexneri 2a str. 2457T]

MLERTMAFKHYDVVRAAPPSDLAEKLTHKLKEGWQPFGSPVAITPYTLMQAIAAEGDVVVSGATEPE

>gi|30063045|ref|NP_837216.1| lysis protein S of prophage CP-933V [Shigella flexneri 2a str. 2457T]

MDQMEKITTGVSYTTSAVGTGYWFLQLLDRVSPSQWAAIGVLGSLLFGLLTYLTEVYWQ

>gi|30063044|ref|NP_837215.1| IS600 orfB [Shigella flexneri 2a str. 2457T]

MAHIRTRETYGTRRLQTELAENGIIVGRDRLARLRKELRLRCKQKRKFRATTNPNHNLPVAPNLLNQTFA

PTAPNQVWVADLTYVATQEGWLYLAGIKDVYTCEIVGYAMGERMTKELTGKALFMALRSQRPPAGLIHHS

DRGSQYCAYDYRVIQEQSGLKTSMSRKGNCYDNAPMESFWGTLKNESLSHYRFNNRDEAISVIREYIEIF

YNRQRRHSRLGNISPAAFREKYHQMAA

>gi|30063043|ref|NP_837214.1| IS1 orfB [Shigella flexneri 2a str. 2457T]

MSRQCTHYGRWPQHGFTSLKKLRPQSVTSRIQPGSDVIVCAEMDEHWGYVGAKSRQRWLFYAYDRIRRTV

VAHVFGERTLATLERLLSLLSAFEVVVWMTDGCPLYESRLKGKLHVISKRYTQRIERHNLNLRQHLARLV

RKSLSFSKSVELHDKVIGHYLNIKHYQ

>gi|30063042|ref|NP_837213.1| IS1 orfA [Shigella flexneri 2a str. 2457T]

MASISIRCPSCSATEGVVRNGKSTAGHQRYLCSHCRKTWQLQFTYTASQPGTHQKIIDMAMNGVGCRASA
RIMGVGLNTVLRHLKNSGRSR

>gi|30063041|ref|NP_837212.1| oxidoreductase [Shigella flexneri 2a str. 2457T]
MKKKIESYQGAAGGWGAVKSVANAVRKQMDIRHDVIAMFDMNKPEGFDCPGCAWPDPKNSASFDICENGA
KAIAWEVTDKQVNASFFAENTVQSLLTWGDHELEAAGRLTQPLKYDAVSDCYKPLSWQQAFDEIGARLQS
YSDPNQVEFYTSGRTSNEAAFLYQLFAREYGSNNFPDCSNMCHEPTSVGLAASIGVGKGTVLLEDFEKCD
LVICIGHNPGTNHPRMLTSLRALVKRGAKMIAINPLQERGLERFTAPQNPFEMLTNSETQLASAYYNVRI
GGDMALLKGMMRLLIERDDAASAAGRPSLLDDEFIQTHTVGFDELRRDVLNSEWKDIERISGLSQTQIAE
LADAYAAAERTIICYGMGITQHEHGTQNVQQLVNLLLMKGNIGKPGAGICPLRGHSNVQGDRTVGITEKP
SAEFLARLGERYGFPPPHAPGHAAIASMQAICTGQARALICMGGNFALAMPDREASAVPLTQLDLAVHVA
TKLNRSHLLTARHSYILPVLGRSEIDMQKSGAQAVTVEDSMSMIHASRGVLKPAGVMLKSECAVVAGIAQ
AALPQSVVAWEYLVEDYDRIRNDIEAVLPEFADYNQRIRHPGGFHLINAAAERRWMTPSGKANFITSKGL
LEDPSSAFNSKLVMATVRSHDQYNTTIYGMDDRYRGVFGQRDVVFMSAKQAKICRVKNGERVNLIALTPD
GKRSSRRMDRLKVVIYPMADRSLVTYFPESNHMLTLDNHDPLSGIPGYKSIPVELEPSN

>gi|30063040|ref|NP_837211.1| hypothetical protein S1655 [Shigella flexneri 2a str. 2457T]
MHATTVKNKITQRDNYKEIMSVIVVVLLLTLTLIAIFSAIDQLSISEMGRIARDLTHFIINSLQDWK

>gi|30063039|ref|NP_837210.1| transcriptional regulator YdeO [Shigella flexneri 2a str. 2457T]
MSLVCSVIFIHHAFNANILDKDYAFSDGEILMVDNAVRTHFEPYERHFKEIGFNENTIKKYLQCTNIQTV
TVPVPAKFLRASNVPTGLLNEMIAYLNSEERNHHNFSELLLFSCLSIFAACKGFITLLTNGVLSVSGKVR
NIVNMKLAHPWKLKDICDCLYISESLLKKKLKQEQTTFSQILLDARMQHAKNLIRVEGSVNKIAEQCGYA
STSYFIYAFRKHFGNSPKRVSKEYRCQRHTGMNTGNTMSALAI

>gi|30063038|ref|NP_837209.1| IS1 [Shigella flexneri 2a str. 2457T]

MAHVFGERTLATLERLLSLLSAFEVVVWMTDGCPLYESRLKGKLHVISKRYTQRIERHNLNLRQHLARLV

RKSLSFSKSVELHDKVIGHYLNIKHYQ

>gi|30063037|ref|NP_837208.1| ARAC-type regulatory protein [Shigella flexneri 2a str. 2457T]

MYQRCFDNASETLFVAGKTPRLSRFAFSDDPKWESGHHVHDNETELIYVKKGVARFTIDSSLYVAHADDI

VVIERGRLHAVASDVNDPATTCTCALYGFQFQGAEENQLLQPHSCPVIAAGQGKEVIKTLFNELSVILPQ

SKNSQTSSLWDALAYTLAILVMLPTY

>gi|30063036|ref|NP_837207.1| electron transfer flavoprotein YdiQ [Shigella flexneri 2a str. 2457T]

MKIITCFKLVPEEQDIVVTPEYTLNFDNADAKISQFDLNAIEAASQLATDDDEIAALTVGGSLLQNSKVR

KDVLSRGPHSLYLVQDAQLEHALPLDTAKALAAAIEKIGFDLLIFGEGSGDLYAQQVGLLVGEILQLPVI

NAVSAIQRQGNTLVIERTLEDDVEVIELSVPAVLCVTSDINVPRIPSMKAILGAGKKPVNQWQASDIDWS

QSAPLAELVGIRVPPQTERKHIILDNDSPEAIAELAEHLKKALN

>gi|30063035|ref|NP_837206.1| electron transfer flavoprotein subunit YdiR [Shigella flexneri 2a str. 2457T]

MSQLNSVWVFSDNPERYAELFGGAQQWGQQVYAIVQNTDQAQAVMPYGPKCIYVLEQNDALQRTENYAES

IAALLKDKHPAMLLLAATKRGKALAARLSVQLNAALVNDATAVDIVDGHICAEHRMYGGLAFAQEKINSP

LAIITLAPGVQEPCTSDTSHQCPTETVPYVAPRHEILCRERRAKAASSVDLSKAKRVVGVGRGLAAQDDL

KMVHELAAVLNAEVGCSRPIAEGENWMERERYIGVSGVLLKSDLYLTLGISGQIQHMVGGNGVKVIVAIN

KDKNAPIFNYADYGLVGDIYKVVPALISQLSR

>gi|30063034|ref|NP_837205.1| hypothetical protein S1649 [Shigella flexneri 2a str. 2457T]

MSDDKFDAIVVGAGVAGSVAALVMARAGLDVLVIERGDSAGCKNMTGGRLYAHTLEAIIPGFAVSAPVER

KVTREKISFLTEESAVTLDFHRGQPDVPQHASYTVLRNRLDPWLMEQAEQAGAQFIPGVRVDALVREGNK

VTGVQAGDDILEANVVILADGVNSMLGRSLGMVPASDPHHYAVGVKEVIGLTPEQINDRFNITGEEGAAW
LFAGSPSDGLMAGGFLYTNKDSISLGLVCGLGDIAHAQKSVPQMLEDFKQHPAIRPLISGGKLLEYSAHM
VPEGGLAMVPQLVNEGVMIVGDAAGFCLNLGFTVRGMDLAIASAQAAATTVIAAKERADFSANSLAQYKR
ELEQSCVMRDMQHFRKIPALMENPRLFSQYPRMVADIMNEMFTIDGKPNQPVRKMIMGHAKKIGLINLLK
DGIKGATAL

>gi|30063033|ref|NP_837204.1| hypothetical protein S1648 [Shigella flexneri 2a str. 2457T]
MSQNATVNVDIKLGVNKFHVDEGHPHIILTENPDINEFRKLMKACPAGLYKQDDVGNIHFDPAGCLECGT
CRVLCGNTILEQWQYPAGTFGIDFRYG

>gi|30063032|ref|NP_837203.1| short chain acyl-CoA synthetase [Shigella flexneri 2a str. 2457T]
MHPTGPQLGPDVLFRESKMKVTLTFNEQRRAAYRQQGLWGDASLADYWQQTARAMPDKIAVVDNHGASYT
YSALDHAASCLANWMLAKGIESGDRIAFQLPGWCEFTVIYLACLKIGAVSVPLLPSWREAELVWVLNKCQ
AKMFFAPTLFKQTRPVDLILPLQNQLPQLQQIVGVDKLAPATSSLSLSQIIADNIPLTTAITTHGDELAA
VLFTSGTEGLPKGVMLTHNNILASERAYCARLNLTWLDVFMMPAPLGHATGFLHGVTAPFLIGARSVLLD
IFTPDACLALLEQQRCTCMLGATPFVYDLLNLLEKQPADLSALRFFLCGGTTIPKKVARECQQRSIKLLS
VYGSTESSPHAVVNLDNSLSRFMHTDGYAAAGVEIKVVDDARKTLPPGCEGEEASRGPNVFMGYFDEPEL
TARALDEEGWYYSGDLCRMDEAGYIKITGRKKDIIVRGGENISSREVEDILLQHPKIHDACVVAMPDERL
GERSCAYVVLKAPHHSLSLEEMAAFFSRKRVAKYKYPEHIVVIEKLPRTASDKIQKFLLRKDIIRRLTQD
VCEEIE

>gi|30063031|ref|NP_837202.1| phosphoenolpyruvate synthase [Shigella flexneri 2a str. 2457T]
MSNNGSSPLVLWYNQLGMNDVDRVGGKNASLGEMITNLSGMGVSVPNGFATTADAFNQFLDQSGVNQRIY
ELLDKTDIDDVTQLAKAGAQIRQWIIDTPFQPELENAIREAYAQLSADDENASFAVRSSATAEDMPDASF
AGQQETFLNVQGFDAVLVAVKHVFASLFNDRAISYRVHQGYDHRGVALSAGVQRMVRSDLASSGVMFSID

TESGFDQVVFITSAWGLGEMVVQGAVNPDEFYVHKPTLAANRPAIVRRTMGSKKIRMVYAPTQEHGKQVK

IEDVPQEQRDIFSLTNEEVQELAKQAVQIEKHYGRPMDIEWAKDGHTGKLFIVQARPETVRSRGQVMERY

TLHSQGKIIAEGRAIGHRIGAGPVKVIHDISEMNRIEPGDVLVTDMTDPDWEPIMKKASAIVTNRGGRTC

HAAIIARELGIPAVVGCGDATERMKDGENVTVSCAEGDTGYVYAELLEFSVKSSSVETMPDLPLKVMMNV

GNPDRAFDFACLPSEGVGLARLEFIINRMIGVHPRALLEFDDQEPQLQNEIREMMKGFDSPREFYVGRLT

EGIATLGAAFYPKRVIVRLSDFKSNEYANLVGGERYEPDEENPMLGFRGAGRYVSDSFRDCFALECEAVK

RVRNDMGLTNVEIMIPFVRTVDQAKAVVEELARQGLKRGENGLKIIMMCEIPSNALLAEQFLEYFDGFSI

GSNDMTQLALGLDRDSGVVSELFDERNDAVKALLSMAIRAAKKQGKYVGICGQGPSDHEDFAAWLMEEGI

DSLSLNPDTVVQTWLSLAELKK

>gi|30063030|ref|NP_837201.1| hypothetical protein S1645 [Shigella flexneri 2a str. 2457T]

MDNAVDRHVFYISDGTAITAEVLGHAVMSQFPVTISSITLPFVENESRARAVKDQIDAIYHQTGVRPLVF

YSIVLPEIRAIILQSEGFCQDIVQALVAPLQQEMKLDPTPIAHRTHGLNPNNLNKYDARIAAIDYTLAHD

DGISLRNLDQAQVILLGVSRCGKTPTSLYLAMQFGIRAANYPFIADDMDNLVLPASLKPLQHKLFGLTID

PERLAAIREERRENSRYASLRQCRMEVAEVEALYRKNQIPWINSTNYSVEEIATKILDIMGLSRRMY

>gi|30063029|ref|NP_837200.1| phospho-2-dehydro-3-deoxyheptonate aldolase [Shigella flexneri 2a str. 2457T]

MNRTDELRTARIESLVTPAELALRYPVTPGVATHVTDSRRRIEKILNGEDKRLLVIIGPCSIHDLTAAME

YATRLQSLRNQYQSRLEIVMRTYFEKPRTVVGWKGLISDPDLNGSYRVNHGLELARKLLLQVNELGVPTA

TEFLDMVTGQFIADLISWGAIGARTTESQIHREMASALSCPVGFKNGTDGNTRIAVDAIRAARASHMFLS

PDKNGQMTIYQTSGNPYGHIIMRGGKKPNYHADDIAAACDTLHEFDLPEHLVVDFSHGNCQKQHRRQLEV

CEDICQQIRNGSTEIAGIMAESFLREGTQKIVGGQPLTYGQSITDPCLGWEDTERLVEKLAFAVDTRF

>gi|30063028|ref|NP_837199.1| hypothetical protein S1643 [Shigella flexneri 2a str. 2457T]

MRYTDSRKLTPETDANHKTASPQPIRRITSQTLLGPDGKLIIDHDGQEYLLRKTQAGKLLLTK

>gi|30063027|ref|NP_837198.1| hypothetical protein S1642 [Shigella flexneri 2a str. 2457T]

MTLSFITRWRDELPETYTALSPTPLNNARLIWHNTELANTLSIPSSLFKNAAGVWGGETLLPGMSPLAQV

YSGHQFVVWAGQLGDGRGILLGEQLLADGTTMDWHLKGAGLTPYSRMGDGRAVLRSTIRESLASEAMHYL

GIPTTRALSIVTSDSPVYRETVEPGAMLMRVAPSHLRFGHFEHFYYRREPEKVRQLADFAIRHYWSHLED

DEDKYRLWFNDVVARTASLIAQWQTVGFAHGVMNTDNMSLLGLTLDYGPFGFLDDYEPGFICNHSDHQGR

YSFDNQPAVALWNLQRLAQTLSPFVAVDALNEALDSYQQVLLTHYGQRMRQKLGFMTEQKEDNALLNELF

SLMARERSDYTRTFRMLSLTEQHSAASPLREEFIDRAAFDDWFARYRGRLQQDEVSDSERQQLMQSVNPA

LVLRNWLAQRAIEAAEKGDMMELHRLHEALRNPFSDRDDDYVSRPPDWGKRLEVSCSS

>gi|30063026|ref|NP_837197.1| hypothetical protein S1641 [Shigella flexneri 2a str. 2457T]

MKIFLENLYHSDCYFLPIRDNQQLLVGVELITHFSSEDGTVRIPTSRVIAQLTEEQHWQLFSEQLELLKS

CQHFFIQHKLFAWLNLTPQVATLLLDRDNYAGELLKYPFIELLINENYPHLNEGKDNRDLLSLSQMYPLV

LGNLGAGNSTMKAVFDGLFTRVMLEKGFIQQQITHRSFEPFIRAIQAQISPCCNCIIAGGIDTPEILAQI

TPFDFHALQGCLWPAVPINQITTLVQR

>gi|30063025|ref|NP_837196.1| lipoprotein [Shigella flexneri 2a str. 2457T]

MRFCLILITALFLAGCSHHKAPPPNARLSDSITVIAGLNDQLQSWHGTPYRYGGMTRRGVDCSGFVVVTM

RDRFDLHLPRETKQQASIGTQIDKDELLPGDLVFFKTGSGQNGLHVGIYDTNNQFIHASTSKGVMRSSLD

NVYWQKNFWQARRI

>gi|30063024|ref|NP_837195.1| vitamin B12-transporter ATPase [Shigella flexneri 2a str. 2457T]

MSIVMQLQDVAESTRLGPLSGEVRAGEILHLVGPNGAGKSTLLARMAGMTSGKGSIQFAGQPLEAWSATK

LALHRAYLSQQQTPPFAMPVWHYLTLHQHDKTRTELLNDVAGALALDDKLGRSTNQLSGGEWQRVRLAAV

VLQITPQANPAGQLLLLDEPMNSLDVAQQSALDKILSALCQQGLAIVMSSHDLNHTLRHAHRAWLLKGGK

MLASGRREEVLTPPNLAQAYGMNFRRLDIEGHRMLISTI

>gi|30063023|ref|NP_837194.1| glutathione peroxidase [Shigella flexneri 2a str. 2457T]

MQDSILTTVVKDIDGEVTTLEKYAGNVLLIVNVASKCGLTPQYEQLENIQKAWADRGFVVLGFPCNQFLE

QEPGSDEEIKTYCTTTWGVTFPMFSKIEVNGEGRHPLYQKLIAAAPTTVAPEESGFYARMVSKGRAPLYP

DDILWNFEKFLVGRDGKVIQRFSPDMTPEDPIVMESIKLALAK

>gi|30063022|ref|NP_837193.1| vtamin B12-transporter permease [Shigella flexneri 2a str. 2457T]

MLTLARQQQRQNIRWLLCLSVLMLLALLLSLCAGEQWISPGDWFTPRGELFVWQIRLPRTLAVLLVGAAL

AISGAVMQALFENPLAEPGLLGVSNGAGVGLIAAVLLGQGQLPNWALGLCAIAGALIITLILLRFARRHL

STSRLLLAGVALGIICSALMTWAIYFSTSVDLRQLMYWMMGGFGGVDWRQSWLMLALIPVLLWICCQSRP

MNMLALGEISARQLGLPMWFWRNVLVAATGWMVGVSVALAGAIGFIGLVIPHILRLCGLTDHRVLLPGCA

LAGASALLLADIVARLALAAAELPIGVVTATLGAPVFIWLLLKAGR

>gi|30063021|ref|NP_837192.1| integration host factor subunit alpha [Shigella flexneri 2a str. 2457T]

MALTKAEMSEYLFDKLGLSKRDAKELVELFFEEIRRALENGEQVKLSGFGNFDLRDKNQRPGRNPKTGED

IPITARRVVTFRPGQKLKSRVENASPKDE

>gi|30063020|ref|NP_837191.1| phenylalanyl-tRNA synthetase subunit beta [Shigella flexneri 2a str. 2457T]

MKFSELWLREWVNPAIDSDTLANQITMAGLEVDGVEPVAGSFHGVVVGEVVECAQHPNADKLRVTKVNVG

GDRLLDIVCGAPNCRQGLRVAVATIGAVLPGDFKIKAAKLRGEPSEGMLCSFSELGISDDHSGIIELPAD

APIGTDIREYLKLDDNTIEISVTPNRADCLGIIGVARDVAVLNQLPLVEPEIVPVGATIDDTLPITVEAP

EACPRYLGRVVKGINVKAPTPLWMKEKLRRCGIRSIDAVVDVTNYVLLELGQPMHAFDKDRIEGGIVVRM

AKEGETLVLLDGTEAKLNADTLVIADHNKALAMGGIFGGEHSGVNDETQNVLLECAFFSPLSITGRARRH

GLHTDASHRYERGVDPVLQHKAMERATRLLIDICGGEAGPVIDITNEATLPKRATITLRRSKLDRLIGHH

IADEQVTDILRRLGCEVTEGKDEWQAVAPSWRFDMEIEEDLVEEVARVYGYNNIPDEPVQASLIMGTHRE

ADLSLKRVKTLLNDKGYQEVITYSFVDPKVQQMIHPGVEALLLASPISVEMSAMRLSLWTGLLATVVYNQ

NRQQNRVRIFESGLRFVPDTQAPLGIRQDLMLAGVICGNRYEEHWNLAKETVDFYDLKGDLESVLDLTGK

LNEVEFRAEANPALHPGQSAAIYLKGERIGFVGVVHPELERKLDLNGRTLVFELEWNKLADRVVPQAREI

SRFPANRRDIAVVVAENVPAADILSECKKVGVNQVVGVNLFDVYRGKGVAEGYKSLAISLILQDTSRTLE

EEEIAATVAKCVEALKERFQASLRD

>gi|30063019|ref|NP_837190.1| phenylalanyl-tRNA synthetase subunit alpha [Shigella flexneri 2a str. 2457T]

MSHLAELVASAKAAISQASDVAALDNVRVEYLGKKGHLTLQMTTLRELPPEERPAAGAVINEAKEQVQQA

LNARKAELESAALNARLAAETIDVSLPGRRIENGGLHPVTRTIDRIESFFGELGFTVATGPEIEDDYHNF

DALNIPGHHPARADHDTFWFDATRLLRTQTSGVQIRTMKAQQPPIRIIAPGRVYRNDYDQTHTPMFHQME

GLIVDTNISFTNLKGTLHDFLRNFFEEDLQIRFRPSYFPFTEPSAEVDVMGKNGKWLEVLGCGMVHPNVL

RNVSIDPEVYSGFAFGMGMERLTMLRYGVTDLRSFFENDLRFLKQFK

>gi|30063017|ref|NP_837188.1| 50S ribosomal protein L20 [Shigella flexneri 2a str. 2457T]

MARVKRGVIARARHKKILKQAKGYYGARSRVYRVAFQAVIKAGQYAYRDRRQRKRQFRQLWIARINAAAR

QNGISYSKFINGLKKASVEIDRKILADIAVFDKVAFTTLVEKAKAALA

>gi|30063016|ref|NP_837187.1| 50S ribosomal protein L35 [Shigella flexneri 2a str. 2457T]

MPKIKTVRGAAKRFKKTGKGGFKHKHANLRHILTKKATKRKRHLRPKAMVSKGDLGLVIACLPYA

>gi|30063015|ref|NP_837186.1| translation initiation factor IF-3 [Shigella flexneri 2a str. 2457T]

MSLREALEKAEEAGVDLVEISPNAEPPVCRIMDYGKFLYEKSKSSKEQKKKQKVIQVKEIKFRPSTDEGD

YQVKLRSLIRFLEEGDKAKITLRFRGREMAHQQIGMEVLNRVKDDLQELAVVESFPTKIEGRQMIMVLAP

KKKQ

>gi|30063014|ref|NP_837185.1| threonyl-tRNA synthetase [Shigella flexneri 2a str. 2457T]

MPVITLPDGSQRHYDHAVSPMDVALDIGPGLAKACIAGRVNGELVDACDLIENDAQLSIITAKDEEGLEI

IRHSCAHLLGHAIKQLWPHTKMAIGPVIDNGFYYDVDLDRTLTQEDVEALEKRMHELAEKNYDVIKKKVS

WHEARETFANRGESYKVSILDENIAHDDKPGLYFHEEYVDMCRGPHVPNMRFCHHFKLMKTAGAYWRGDS

NNKMLQRIYGTAWADKKALNAYLQRLEEAAKRDHRKIGKQLDLYHMQEEAPGMVFWHNDGWTIFRELEVF

VRSKLKEYQYQEVKGPFMMDRVLWEKTGHWDNYKDAMFTTSSENREYCIKPMNCPGHVQIFNQGLKSYRD

LPLRMAEFGSCHRNEPSGSLHGLMRVRGFTQDDAHIFCTEEQIRDEVNGCIRLVYDMYSTFGFEKIVVKL

STRPEKRIGSDEMWDRAEADLAVALEENNIPFEYQLGEGAFYGPKIEFTLYDCLDRAWQCGTVQLDFSLP

SRLSASYVGEDNERKVPVMIHRAILGSMERFIGILTEEFAGFFPTWLAPVQVVIMNITDSQSEYVNELTQ

KLSNAGIRVKADLRNEKIGFKIREHTLRRVPYMLVCGDKEVESGKVAVRTRRGKDLGSMDVNEVIEKLQQ

EIRSRSLKQLEE

>gi|30063013|ref|NP_837184.1| IS1 orfA [Shigella flexneri 2a str. 2457T]

MASISIRCPSCSATEGVVRNGKSTAGHQRYLCSHCRKTWQLQFTYTASQPGTHQKIIDMAMNGVGCRASA

RIMGVGLNTVLRHLKNSGRSR

>gi|30063012|ref|NP_837183.1| IS1 orfB [Shigella flexneri 2a str. 2457T]

MSRQCTHYGRWPQHGFTSLKKLRPQSVTSRIQPGSDVIVCAEMDEHWGYVGAKSRQRWLFYAYDRIRRTV

VAHVFGERTLATLERLLSLLSAFEVVVWMTDGWPLYESRLKGKLHVISKRYTQRIERHNLNLRQHLARLG

RKSLSFSKSVELHDKVIGHYLNIKHYQ

>gi|30063011|ref|NP_837182.1| hypothetical protein S1625 [Shigella flexneri 2a str. 2457T]

MLAGGMFASLNAAADDSVFTVMDDPASAKKPFEGNLNAGYLAQSGNTKSSSLTADTTMTWYGQTTAWSLW

GNASNTSSNDERSSEKYAAGGRSRFNLTDYDYLFGQASWLTDRYNGYRERDVLTAGYGRQFLNGPVHSFR

FEFGPGVRYDKYTDNASETQPLGYASGAYAWQLTDNAKFTQGVSVFGAEDTTLNSESALNVAINEHFGLK

VAYNVTWNSEPPESAPEHTDRRTTLSLGYSM

>gi|30063010|ref|NP_837181.1| 6-phosphofructokinase [Shigella flexneri 2a str. 2457T]

MVRIYTLTLAPSLDSATITPQIYPEGKLRCTAPVFEPGGGGINVARAIAHLGGSATAIFPAGGATGEHLV

SLLADENVPVATVEAKDWTRQNLHVHVEASGEQYRFVMPGAALNEDEFRQLEEQVLEIESGAILVISGSL

PPGVKLEKLTQLISAAQNQGIRCIIDSSGEALSAALAIGNIELVKPNQKELSALVNRELTQPDDVRKAAQ

EIVNSGKAKRVVVSLGPQGALGVDSENCIQVVPPPVKSQSTVGAGDSMVGAMTLKLAENASLEEMVRFGV

AAGSAATLNQGTRLCSHDDTQKIYAYLSR

>gi|30063009|ref|NP_837180.1| hypothetical protein S1623 [Shigella flexneri 2a str. 2457T]

MASGDLVHYVITVMLHEDTLTEINELNNYLTRDGFLLTMTDDEGNIHELGTNTFGLISTQSEEEIRELVS

GLTQSATGKDPEITITTWEEWNSNRK

>gi|30063008|ref|NP_837179.1| hypothetical protein S1622 [Shigella flexneri 2a str. 2457T]

MWQAISRLLSEQLGEGEIELRNELPGGEVHAAWHLRYAGHDFFVKCDERELLPGFTAEADQLELLSRSKT

VTVPKVWAVGADRDYSFLVMDYLPPRPLDAHSAFILGQQIARLHQWSDQPQFGLDFDNSLSTTPQPNTWQ

RRWSTFFAEQRIGWQLELAAEKGIAFGNIDAIVEHIQQRLASHQPQPSLLHGDLWSGNCALGPDGPYIFD

PACDWGDRECDLAMLPLHTEQPPQIYDGYQSVSPLPADFLERQPVYQLYTLLNRARLFGGQHLVIAQQSL

DRLLAA

>gi|30063007|ref|NP_837178.1| hypothetical protein S1621 [Shigella flexneri 2a str. 2457T]

MTYQQAGRIAVLKRILGWVIFIPALISTLISLLKFMNTRQENQEGINAVMLDFAHVMIDMMQANTPFLNL

FWYNSPTPNFNGGVNVMFWVIFILIFVGLALQDSGARMSRQARFLREGVEDQLILEKAKGEEGLTREQIE

SRIVVPHHTIFLQFFSLYILPVICIAAGYVFFSLLGFI

>gi|30063006|ref|NP_837177.1| 2-deoxyglucose-6-phosphatase [Shigella flexneri 2a str. 2457T]

MSTPRQILAAIFDMDGLLIDSEPLWDRAELDVMASLGVDISRRNELPDTLGLRIDMVVDLWYARQPWNGP

SRQEVVERVIARAISLVEETRPLLPGVREAVALCKEQGLLVGLASASPLHMLEKVLTMFDLRDSFDALAS

AEKLPYSKPHPQVYLDCAAKLGVDPLTCVALEDSVNGMIASKAARMRSIVVPAPEAQNDPRFVLANVKLS

SLTELTAKDLLG

>gi|30063005|ref|NP_837176.1| hypothetical protein S1619 [Shigella flexneri 2a str. 2457T]

MTAEGHLLFSIACAVFAKNAELTPVLAQGDWWHIVPSAILTCLLPDIDHPKSFLGQRLKWISKPIARAFG

HRGFTHSLLAVFALLATFYLKVPEGWFIPADALQGMVLGYLSHILADMLTPAGVPLLWPCRWRFRLPILV

PQKGNQLERFICMALFVWSVWMPHSLPENSAVRWSSQMINTLQIQFHRLIKHQVEY

>gi|30063004|ref|NP_837175.1| IS1 orfA [Shigella flexneri 2a str. 2457T]

MASISIRCPSCSATEGVVRNGKSTAGHQRYLCSHCRKTWQLQFTYTASQPGTHQKIIDMAMNGVGCRASA

RIMGVGLNTVLRHLKNSGRSR

>gi|30063003|ref|NP_837174.1| IS1 orfB [Shigella flexneri 2a str. 2457T]

MSRQCTHYGRWPQHGFTSLKKLRPQSVTSRIQPGSDVIVCAEMDEHWGYVGAKSRQRWLFYAYDRIRRTV

VAHVFGERTLATLERLLSLLSAFEVVVWMTDGWPLYESRLKGKLHVISKRYTQRIERHNLKLRQHLARLG

RKSLSFSKSVELHDKVIGHYLNIKHYQ

>gi|30063002|ref|NP_837173.1| cell division modulator [Shigella flexneri 2a str. 2457T]

MRLVKPVMKKPLRQQNRQIISYVPRTEPAPPEHAIKMDSFRDVWMLRGKYVAFVLMGESFLRSPAFTVPE

SAQRWANQIRQEGEVTE

>gi|30063001|ref|NP_837172.1| hydroperoxidase II [Shigella flexneri 2a str. 2457T]

MSQHNEKNPHQHQSPLHDSSEAKPGMDSLAPEDGSHRPAAEPTPPGAQPTAPGSLKAPDTRNEKLNSLED

VRKGSENYALTTNQGVRIADDQNSLRAGNRGPTLLEDFILREKITHFDHERIPERIVHARGSAAHGYFQP

YKSLSDITKADFLSDPNKITPVFVRFSTVQGGAGSADTVRDIRGFATKFYTEEGIFDLVGNNTPIFFIQD

AHKFPDFVHAVKPEPHWAIPQGQSAHDTFWDYVSLQPETLHNVMWAMSDRGIPRSYRTMEGFGIHTFRLI

NAEGKATFVRFHWKPLAGKASLVWDEAQKLTGRDPDFHRRELWEAIEAGDFPEYELGFQLIPEEDEFKFD

FDLLDPTKLIPEELVPVQRVGKMVLNRNPDNFFAENEQAAFHPGHIVPGLDFTNDPLLQGRLFSYTDTQI

SRLGGPNFHEIPINRPTCPYHNFQRDGMHRMGIDTNPANYEPNSINDNWPRETPPGPKRGGFESYQERVE

GNKVRERSPSFGEYYSHPRLFWLSQTPFEQRHIVDGFSFELSKVVRPYIRERVVDQLAHIDLTLAQAVAK

NLGIELTDDQLNITPPPDVNGLKKDPSLSLYAIPDGDVKGRVVAILLNDEVRSADLLAILKAMKAKGVHA

KLLYSRMGEVTADDGTVLPIAATFAGAPSLTVDAVIVPCGNIADIADNGDANYYLMEAYKHLKPIALAGD

ARKFKATIKVADQGEEGIVEADSTDGSFMDELLTLMAEHRVWSRIPKIDKIPA

>gi|30063000|ref|NP_837171.1| hypothetical protein S1612 [Shigella flexneri 2a str. 2457T]

MERLLIVNADDFGLSKGQNYGIIEACRNGIVTSTTALVNGQAIDHAVQLSRDEPSLAIGMHFVLTMGKPL

TAMPGLTRDGVLGKWIWQLAEEDALPLEEITQELASQYLRFIELFGRKPTHLDSHHHVHMFPQIFPIVAR

FAAEEGIALRIDRQPLSNAGDLPANLRSSQGFSSAFYGEEISEALFLQVLDDASHRGDPSLEVMCHPAFI

DNTIRQSAYCFPRLTELEVLTSASLKYAIAERGYRLGSYLDV

>gi|30062999|ref|NP_837170.1| cryptic phospho-beta-glucosidase; cryptic [Shigella flexneri 2a str. 2457T]

MSQKLKVVTIGGGSSYTPELLEGFIKRYHELPVSELWLVDVEGGKAKLDIIFDLCQRMIDNAGVPMKLYK

TLDCREALKDADFVTTQLRVGQLPARELDERIPLSHGYLGQETNGAGGLFKGLRTIPVIFDIVKDVEELC

PNAWVINFTNPAGMVTEAVYRHTGFKRFIGVCNIPIGMKMFIHDVLMLKDSDDLSIDLFGLNHMVFIKDV

LVNGKSRFAELLDGVASGQLKVSGVKNIFDLPFSEGLIRSLNLLPCSYLLYYFKQKEMLAIEMGEYYKGG

ARAQVVQKVEKQLFELYKNPELKVKPKELEQRGGAYYSDAACEVINAIYNDKQAEHYVNIPHHGHIDNIP

ADWAVEMTCKLGRDGATPHPRITHFDDKVMGLIHTIKGFEIAASNAALSGEFNDVLLALNLSPLVHSDRD

AELLAREMILAHEKWLPNFADCIAELKKAH

>gi|30062998|ref|NP_837169.1| IS1 orfA [Shigella flexneri 2a str. 2457T]

MASISIRCPSCSATEGVVRNGKSTAGHQRYLCSHCRKTWQLQFTYTASQPGTHQKIIDMAMNGVGCRASA

RIMGVGLNTVLRHLKNSGRSR

>gi|30062997|ref|NP_837168.1| IS1 orfB [Shigella flexneri 2a str. 2457T]

MSRQCTHYGRWPQHGFTSLKKLRPQSVTSRIQPGSDVIVCAEMDEHWGYVGAKSRQRWLFYAYDRIRRTV

VAHVFGERTLATLERLLSLLSAFEVVVWMTDGWPLYESRLKGKLHVISKRYTQRIERHNLNLRQHLARLG

RKSLSFSKSVELHDKVIGHYLNIKHYQ

>gi|30062996|ref|NP_837167.1| N,N'-diacetylchitobiose-specific PTS system transporter subunit IIA [Shigella flexneri 2a str. 2457T]

MMDLDNIPDTQTEAEELEEVVMGLIINSGQARSLAYAALKQAKQGDFAAAKAMMDQSRMALNEAHLVQTK

LIEGDAGEGKMKVSLVLVHAQDHLMTSMLARELITELIELHEKLKA

>gi|30062995|ref|NP_837166.1| N,N'-diacetylchitobiose-specific PTS system transporter subunit IIB [Shigella flexneri 2a str. 2457T]

MEKKHIYLFCSAGMSISLLVSKMRAQAEKYEVPVIIEAFPETLAGEKGQNADVVLLGPQIAYMLPEIQRL

LPNKPVEVIDSLLYGKVDGLGVLKAAVAAIKKAAAN

>gi|30062994|ref|NP_837165.1| DNA-binding transcriptional activator OsmE [Shigella flexneri 2a str. 2457T]

MNKNMAGILSAAAVLTMLAGCTAYDRTKDQFVQPVVKDVKKGMSRAQVAQIAGKPSSEVSMIHARGTCQT

YILGQRDGKAETYFVALDDTGHVINSGYQTCAEYDTDPQAAK

>gi|30062993|ref|NP_837164.1| NAD synthetase [Shigella flexneri 2a str. 2457T]

MTLQQQIIKALGAKPQINAEEEIRRSVDFLKSYLQTYPFIKSLVLGISGGQDSTLAGKLCQMAINELRQE

TRNESLQFIAVRLPYGVQADEQDCQDAIAFIQPDRVLTVNIKGAVLASEQALREAGIELSDFVRGNEKAR

ERMKAQYSIAGMTSGVVVGTDHAAEAITGFFTKYGDGGTDINPLYRLNKRQGKQLLTALGCPEHLYKKAP

TADLEDDRPSLPDEVALGVTYDNIDDYLEGKNLPEQVARTIENWYLKTEHKRRPPINVFDDFWKK

>gi|30062992|ref|NP_837163.1| nucleotide excision repair endonuclease [Shigella flexneri 2a str. 2457T]

MVRRLTSPRLEFEAAAIYEYPEHLHSFLNDLPTRPGVYLFHGESDTMPLYIGKSVNIRSRVLSHLRTPDE

AAMLRQSRRISWICTAGEIGALLLEARLIKEQQPLFNKRLRRNRQLCALQLNEKRVDVVYAKEVDFSRAP

NLFGLFANRRAALQALQSIADEQKLCYGLLGLEPLSRGRACFRSALKRCAGACCGKESHEEHALRLRQSL

ERLRVVCWPWQGAVALKEQHPEMTQYHIIQNWLWLGAVNSLEEATTLIRTPAGFDHDGYKILCKPLLSGN

YEITELDPANDQRAS

>gi|30062991|ref|NP_837162.1| hypothetical protein S1601 [Shigella flexneri 2a str. 2457T]

MEYFDMRKMSVNLWRNAAGETREICTFPPAKRDFYWRASIASIAANGEFSLFPGMERIVTLLEGGEMLLE

SADRFNHTLKPLQPFAFTADQVVKAKLTAGQMSMDFNIMTRLDVCKAKVRIAERTFTTFGSRGGVVFVIN

GAWQLGDKLLTTDQGVCWFDGRHTLRLLQPQGKLLFSEINWLAGHSPDQVQ

>gi|30062990|ref|NP_837161.1| periplasmic protein [Shigella flexneri 2a str. 2457T]

MRKLTALFVASTLALGAANLAHAADTTTAAPADAKPMMHHKGKFGPHQDMMFKDLNLTDAQKQQIHEIMK

GQRDQMKRPPLEERRAMHDIIASDTFDKAKAEAQIAKMEEQRKANMLAHMETQNKIYNILTPEQKKQFNA

NFEKRLTERPAAKGKMPATAE

>gi|30062989|ref|NP_837160.1| succinylglutamate desuccinylase [Shigella flexneri 2a str. 2457T]

MDNFLALTLTGKKPVITEREINGVRWRWLGDGVLELTPLTPPQGALVISAGIHGNETAPVEMLDALLGAI

SHGEIPLRWRLLVILGNPPALKQGKRYCHSDMNRMFGGRWQLFAESGETCRARELEQCLEDFYDQGKESV

RWHLDLHTAIRGSLHPQFGVLPQRDIPWDEKFLTWLGAAGLEALVFHQEPGGTFTHFSVRHFGALACTLE

LGKALPFGQNDLRQFAVTASAIAALLSGESVGIVRTPPLRYRVVSQITRHSPSFEMHMASDTLNFMPFEK

GTLLAQDGEERFTVTHDVEYVLFPNPLVALGLRAGLMLEKIS

>gi|30062988|ref|NP_837159.1| succinylglutamic semialdehyde dehydrogenase [Shigella flexneri 2a str. 2457T]

MTLWINGDWITGQGASRVKRNPVSGEVLWQGNDADAAQVEQACRAARAAFPRWARLSLAERQVVVERFAG

LLESNKAELTAIIARETGKPRWEAATEVTAMINKIAISIKAYHVRTGEQRSEMPDGAASLRHRPHGVLAV

FGPYNFPGHLPNGHIVPALLAGNTIIFKPSELTPWSGEAVMRLWQQAGLPPGVLNLVQGGRETGQALSAL

EDLDGLLFTGSANTGYQLHRQLSGQPEKILALEMGGNNPLIIDEVADIDAAVHLTIQSAFVTAGQRCTCA

RRLLLKSGAQGDAFLASLVAVSQRLTPGNWDDEPQPFIGGLISEQAAQQVVTAWQQLEAMGGRTLLAPRL

LQAGTSLLTPGIIEMTGVGGVPDEEVFGPFLRVWRYDTFDEAIRMANNTRFGLFCGLVSPEREKFDQLLL

EARAGIVNWNKPLTGAASTAPFGGIGASGNHRPSAWYAADYCAWPMASLESDSLTLPATLNPGLDFSDEV

VR

>gi|30062987|ref|NP_837158.1| arginine succinyltransferase [Shigella flexneri 2a str. 2457T]

MVIRPVERSDVSALMQLASKTGGGLTSLPANEATLSARIERAIKTWQGELPKSEQGYVFVLEDSETGTVA

GICAIEVAVGLNDPWYNYRVGTLVHASKELNVYNALPTLFLSNDHTGSSELCTLFLDPDWRKEGNGYLLS

KSRFMFMAAFRDKFNDKVVAEMRGVIDEHGYSPFWQSLGKRFFSMDFSRADFLCGTGQKAFIAELMPKHP

IYTHFLSQEAQDVIGQVHPQTAPARAVLEKEGFRYRNYIDIFDGGPTLECDIDRVRAIRKSRLVEVAEGQ

PAQGDFPACLVANENYHHFRVVLARTDPATERLILTAAQLDALKCHAGDRVRLVRLCAEEKTA

>gi|30062986|ref|NP_837157.1| exonuclease III [Shigella flexneri 2a str. 2457T]

MKFVSFNINGLRARPHQLEAIVEKHQPDVIGLQETKVHDDMFPLEEVAKLGYNVFYHGQKGHYGVALLTK

ETPIAVRRGFPGDDEEAQRRIIMAEIPSLLGNVTVINGYFPQGESRDHPIKFPAKAQFYQNLQNYLETEL

KRDNPVLIMGDMNISPTDLDIGIGEENRKRWLRTGKCSFLPEEREWMDRLMSWGVVDTFRHANPQTADRF

SWFDYRSKGFDDNRGLRIDLLLASQPLAECCVETGIDYEIRSMEKPSDHAPVWATFRR

>gi|30062985|ref|NP_837156.1| hypothetical protein S1593 [Shigella flexneri 2a str. 2457T]

MNAERNFLFACLIFALVIYAIHAFGLFDLLTDLPHLQTLIRQSGLFGYSLYILLFINAPLFLLPGSILVI

AGGIVFGPLLGTQLSLIAATLASSCSFLLARWLGRDLLLKYVGHSHTFQAIEKGIARNGIDFLILTRLIP

LFPYNIQNYAYGFTTIAFWPYTLISALTTLPGIVIYTVMASDLANEGITLRFILQLCLAGLALFILIQLA

KLYARHKHVDLSASRRSPLTHPKNEG

>gi|30062984|ref|NP_837155.1| hypothetical protein S1592 [Shigella flexneri 2a str. 2457T]

MKALRCALFYNSVWRAWRCLFSSSSQNSTPDTNMWICLLRAAAHLLTQKMKDRTMSQHYSVSWKKGLAAL

CLLAVAGLSGCDQKENAAAKVEYDGLSNSQPLRVDANNHTVTMLVQINGRFLTDDTRHGIVFKDGSNGHK

SLFMAYAPPKAFYEALKEAGGTPGENMTMDNKETTHVTGSKLDISVNWQGAAKAYSFDEVIVDSNGKKLD

MRFGGNLTAAEEKKTGCLVCLDSCPVGIVSNATYTYGAVEKHGEVKFKGNASVLPADNTLATVTFKITE

>gi|30062983|ref|NP_837154.1| hypothetical protein S1591 [Shigella flexneri 2a str. 2457T]

MKIQSRKIWYYRITLIILLFAMLLAWALLPGVHEFINRSVAAFAAVDQQGIERFIQSYGALAAVVSFLLM

ILQAIAAPLPAFLITFANASLFGAFWGGLLSWTSSMAGAALCFFIARVMGREVVEKLTGKTVLDSMDGFF

TRYGKHTILVCRLLPFVPFDPISYAAGLTSIRFRSFFIATGLGQLPATIVYSWAGSMLTGGTFWFVTELF

ILFALTVVIFMAKKIWLERQKRND

>gi|30062982|ref|NP_837153.1| hypothetical protein S1590 [Shigella flexneri 2a str. 2457T]

MGLPPLSKIPFILRPQAWLHRRHYGEVLSPIRWWGRIPFIFYLVSMFVGWLERKRSPLDPVVRSLVSARI

AQMCLCEFCVDITSMKVAERTGSTDKLLAVADWRQSPLFSDEERLALEYAEAASVTPPTVDDALRTRLAA

HFDAQALTELTALIGLQNLSARFNSAMDIPAQGLCRIPEKRS

>gi|30062981|ref|NP_837152.1| IS4 orf [Shigella flexneri 2a str. 2457T]

MHIGQALDLVSRYDSLRNPLTSLGDYLDPELISRCLAESGTVTLRKRRLPLEMMVWCIVGMALERKEPLH

QIVNRLDIMLPGNRPFVAPSAVIQARQRLGSEAVRRVFTKTAQLWHNATPHPHWCGLTLLAIDGVFWRTP

DTPENDAAFPRQTHAGNPALYPQVKMVCQMELTSHLLTAAAFGTMKNSENELAEQLIGQTGDNTLTLMDK

GYYSLGLLNGWSLAGEHRHWMIPLRKGAQYEELRKLGKGDHLVKLKTSPQARKKWPGLGNEVTARLLTVT

RKGKVCHLLTSMTDAMRFPGGEMADLYSNRWEIELGYREIKQTMQLSRLTLRSKKPELVEQELWGVLLAY

NLVRYQMIKMAEHLKGYWPNQLSFSESCGMVMRMLMTLQGASPGRIPELMRDLASMGQLVKLPTRRGRAF

PRVVKERPWKYPTAPKKSQSVA

>gi|30062980|ref|NP_837151.1| ATP-binding component of a transport system [Shigella flexneri 2a str. 2457T]

MLCVKNVSLRLPESRLLTNVNFTVDKGDIVTLMGPSGCGKSTLFSWMIGALAGQFSCTGELWLNEQRIDM

LPTAQRQIGILFQDALLFDQFSVGQNLLLALPATLKGNARRNAVNDALERSGLDGAFHQDPATLSGGQRA

RVALLRALLAQPKALLLDEPFSRLDVALRDNFRQWVFSEVRALAIPVVQVTHDLQDVPADSSVLDMAQWS

ENYNKLR

>gi|30062979|ref|NP_837150.1| cytochrome oxidase [Shigella flexneri 2a str. 2457T]

MLDRHLHPRIKPLLHQCVRVLDKPGITPDGLTLVGFAIGVLALPFLALGWYLAALVVILLNRLLDGLDGA

LARRRGLTDAGGFLDISLDFLFYALVPFGFILAAPEQNALAGGWLLFAFIGTGSSFLAFAALAAKHQIDN

PGYAHKSFYYLGGLTEGTETILLFVLGCLFPAWFAWFAWFAWIFGALCWMTTFTRVWSGYLTLKSLQRQ

>gi|30062978|ref|NP_837149.1| pyrimidine (deoxy)nucleoside triphosphate pyrophosphohydrolase [Shigella flexneri 2a str. 2457T]

MKMIEVVAAIIERDGKILLAQRPAQSDQAGLWEFAGGKVELDESQQQALVRELNEELDIEATVGEYVASH

QREVSGRIIHLHAWHVPDFHGTLQAHEHQALVWCSPEEALQYPLAPADIPLLEAFMALRAARAAD

>gi|30062977|ref|NP_837148.1| hypothetical protein S1580 [Shigella flexneri 2a str. 2457T]

MSRALFAVVLAFPLIALANPHYRPDVEVNVPPEVFSSGGQSAQPCTQCCVYQDQNYSEGAVIKAEGILLQ

CQRDDKTLSTNPLLWRRVKP

>gi|30062974|ref|NP_837145.1| DNA topoisomerase III [Shigella flexneri 2a str. 2457T]

MRLFIAEKPSLARAIADVLPKPHRKGDGFIECGNGQVVTWCIGHLLEQAQPDAYDSRYARWNLADLPIVP

EKWQLQPRPSVTKQLNVIKRFLHEASEIVHAGDPDREGQLLVDEVLDYLQLAPEKRQQVQRCLINDLNPQ

AVERAIDRLRSNSEFVPLCVSALARARADWLYGINMTRAYTILGRNAGYQGVLSVGRVQTPVLGLVVRRD

EEIENFVAKDFFEVKAHIVTPADERFTAIWQPSEACEPYQDEEGRLLHRPLAEHVVNRISGQPAIVTSYN

DKRESESAPLPFSLSALQIEAAKRFGLSAQNVLDICQKLYETHKLITYPRSDCRYLPEEHFAGRHAVMNA

ISVHAPDLLPQPVVDPDIRNRCWDDKKVDAHHAIIPTARSSAINLTENEAKVYNLIARQYLMQFCPDAVF

RKCVIELDIAKGKFVAKARFLAEAGWRTLLGSKERDEENDGTPLPVVAKGDELLCEKGEVVERQTQPPRH

FTDATLLSAMTGIARFVQDKDLKKVLRATDGLGTEATRAGIIELLFKRGFLTKKGRYIHSTDAGKALFHS

LPEMATRPDMTAHWESVLTQISEKQCRYQDFMQPLVGTLYQLIDQAKRTPVRQFRGIVAPGSGGSADKKK

AAPRKRSAKKSPPADEAGSGAIA

>gi|30062973|ref|NP_837144.1| selenophosphate synthetase [Shigella flexneri 2a str. 2457T]

MSENSIRLTQYSHGAGCGCKISPKVLETILHSEQAKFVDPNLLVGNETRDDAAVYDLGNGTSVISTTDFF

MPIVDNPFDFGRIAATNAISDIFAMGGKPIMAIAILGWPINKLSPEIAREVTEGGRYACRQAGIALAGGH

SIDAPEPIFGLAVTGIVPTERVKKNSTAQAGCKLFLTKPLGIGVLTTAEKKSLLKPEHQGLATEVMCRMN

IAGASFANIEGVKAMTDVTGFGLLGHLSEMCQGAGVQARVDYEAIPKLPGVEEYIKLGAVPGGTERNFAS

YGHLMGEMPREVRDLLCDPQTSGGLLLAVMPEAENEVKTTAAEFGIELTAIGELVPARGGRAMVEIR

>gi|30062972|ref|NP_837143.1| hypothetical protein S1573 [Shigella flexneri 2a str. 2457T]

MDALELLINRRSASRLAEPAPTGEQLQNILRAGMRAPDHKSMQPWHFFVIEGEGCERFSAVLEQGAIAAG

SDDKAIDKARNAPFRAPLIITVVAKCKENHKVPRWEQEMSAGCAVMAMQMAAVAQGFGGIWRSGALTESP

VVREAFGCREQDKIVGFLYLGTPQLKASTSINVPDPTPFVTYF

>gi|30062971|ref|NP_837142.1| protease 4 [Shigella flexneri 2a str. 2457T]

MRTLWRFIAGFFKWTWRLLNFVREMVLNLFFIFLVLVGVGIWMQVSGGDSKETASRGALLLDISGVIVDK

PDSSQRFSKLSRQLLGASSDRLQENSLFDIVNTIRQAKDDRNITGIVMDLKNFAGGDQPSMQYIGKALKE

FRDSGKPVYAVGENYSQGQYYLASFANKIWLSPQGVVDLHGFATNGLYYKSLLDKLKVSTHVFRVGTYKS

AVEPFIRDDMSPAAREADSRWIGELWQNYLNTVAANRQIPAQQVFPGAQGLLEGLTKTGGDTAKYALENK

LVDALASSAEIEKALTKEFGWSKTDKNYRAISYYDYALKTPADTGDSIGVVFANGAIMDGEETQGNVGGD

TTAAQIRDARLDPKVKAIVLRVNSPGGSVTASEVIRAELAAARAAGKSVVVSMGGMAASGGYWISTPANY

IVANPSTLTGSIGIFGVITTVENSLDSIGVHTDGVSTSPLADVSITRALPPEAQQMMQLSIENGYKRFIT

LVADARHSTPEQIDKIAQGHVWTGQDAKANGLVDSLGDFDDAVAKAAELAKVKQWHLEYYVDEPTFFDKV

MDNMSGSVRAMLPDAFQAMLPAPLASVASTVKSESDKLAAFNDPQNRYAFCLTCANVR

>gi|30062970|ref|NP_837141.1| asparaginase [Shigella flexneri 2a str. 2457T]

MQKKSIYVAYTGGTIGMQRSEQGYIPVSGHLQRQLALMPEFHRPEMPDFTIHEYTPLMDSSDMTPEDWQH

IAEDIKAHYDDYDGFVILHGTDTMAYTASALSFMLENLGKPVIVTGSQIPLAELRSDGQINLLNALYVAP

NYPINEVTLFFNNRLYRGNRTTKAHADGFDAFASPNLPPLLEAGIHIRRLNTPPAPHGEGELIVHPITPQ

PIGVVTIYPGISADVVRNFLRQPVKALILRSYGVGNAPQNKAFLQELQEASDRGIVVVNLTQCMSGKVNM

SGYATGNALAHAGVIDGADMTVEATLTKLHYLLSQELDTETIRKAMSQNLRGELTPDD

>gi|30062968|ref|NP_837139.1| transporter [Shigella flexneri 2a str. 2457T]

MEQYDQIGARLDRLPLARFHYRIFGIISFSLLLTGFLSYSGNVVLAKLVSNGWSNNFLYAAFTSALMFGY

FIGSLTGGFIGDYFGRRRAFRINLLIVGIAATGAAFVPDMYWLIFFRFLMGTGMGALIMVGYASFTEFIP

ATVRGKWSARLSFVGNWSPMLSAAIGVVVIAFFSWRIMFLLGGIGILLAWFLSGKYFIESPRWLEGKGQI

AGAESQLREVEQQIEREKSIRLPQLTLNQSNSKVKVIKGTFRLLFKGEMLRRTLVAITVLIAMNISLYTI

TVWIPTIFVNSGIDVDKSILMTAVIMIGAPVGIFIAALIIDHFPRRLFGSALLIIIAVLGYIYSSQTTEW

AILIYGLVMIFFLYMYVCFASAVYIPELWPTHLRLRGSGFVNAVGRIVAVFTPYGVAALLTHYGSITVFM

VLGVMLVLCALVLSIFGIETRKVSLEEISEVN

>gi|30062967|ref|NP_837138.1| DEOR-type transcriptional regulator [Shigella flexneri 2a str. 2457T]

MAAKDRIQAIKQMVANDKKVTVSNLSGIFQVTEETIRRDLEKLEDEGFLTRTYGGAVLNTAVLTENIHFY

KRASSFYEEKLLIARKALPFIDNKTTMAADSSSTVMELLKLLQDRSDLTLLTNSAEAIHVLAQSEIKVVS

TGGELNKNTLSLQGRITKEIISRYHVDIMVMSCKGLDINSGALDSNEAEAEIKKTMIRQATEVALLVDHS

KFDRKAFVQLADFSHINYIITDKSPGAEWIAFCKDNNIQLVW

>gi|30062966|ref|NP_837137.1| hypothetical protein S1567 [Shigella flexneri 2a str. 2457T]

MGLGTWAIGGGPAWNGDLDRQICIDTILEAHRCGINLIDTALGYNFGNSEVIVGQALKKLPREQVVVETK

CGIVWERKGSLFNKVGDRQLYKNLSPESIREEVEASLQRLGIDYIDIYMTHWQSVPPFFTPIAETVAVLN

ELKAEGKIRAIGAANVDADHIREYLQYGELDIIQAKYSILDRAMENELLPLCRDNGIVVQVYSPLEQGLL

TGTITRDYVPGGARANKVWFQRENMLKVIDMLEQWQPLCARYQCTIPTLALAWILKQSDLISILSGATAP

EQVRENVAALNINLSDADATLMREMAEALER

>gi|30062965|ref|NP_837136.1| kinase [Shigella flexneri 2a str. 2457T]

MDNLDVICIGAAIVDIPLQPVSKNIFDVDSYPLERIAMSTGGDAINEATIISRLGHRTALMSRIGKDASG

QFILDHCRKENIDIQSLKQDVSIDTSINVGLVTEDGERTFVTNRNGSLWKLNIDDVDFARFPQAKLLSLA

SIFNSPLLDGKALTEIFTQAKARQMIICADMIKPRLNETLDDICEALSYVDYLFPNFAEEKLLTGKETLD

EIADCFLACGVKTVVIKTGKDGCFIKRGDMTMKVPAVAGITAIDTIGAGDNFASGFIAALLEGKNLRECA

RFANATAAISVLSVGATTGVKNRKLVEQLLEEYEG

>gi|30062964|ref|NP_837135.1| aldolase [Shigella flexneri 2a str. 2457T]

MLADIRYWENDATNKYYAIAHFNVWNAEMLMGVKDAAEEAKSPVIISFSTGFVGNTSFEDFSHMMVSMAQ

KATVPVITHWDHGRSIEIIHNAWTHGMNSLMRDASAFDFEENIRLTKEAVDFFHPLGIPVEAELGHVGNE

TVYEEALAGYHYTDPDQAAEFVERTGCDSLAVAIGNQHGVYTSEPQLNFEVVKRVRDAVSVPLVLHGASG

ISDADIKTAISLGIAKINIHTELCQAAMVAVKENQDQPFLHLEREVRKAVKERALEKIELFGSDGKAE

>gi|30062963|ref|NP_837134.1| transporter [Shigella flexneri 2a str. 2457T]

MEQITKPHCGARLDRLPDCRWHSSMFAIVAFGLLVCWSNAVGGLILAQLKALGWTDNSTTATFSAITTAG

MFLGALVGGIIGDKTGRRNAFILYEAIHIASMVVGAFSPNMDFLIACRFVMDVGLGALLVTLFAGFTEYM

PGRNRGTWSSRVSFIGNWSYPLCSLIAMGLTPLISAEWNWRVQLLIPAILSLIATALAWRYFPESPRWLE

SRGRYQEAEKVMRSIEEGVIRQTGKPLPPVVIADDGKAPQAVPYSALLTGVLLKRVILGSCVLITMNVVQ

YTLINWLPTIFMTQGINLKDSIVLNTMSMFGAPFGIFIAMLVMDKIPRKTMGVGLLILIAVLGYIYSLQT

SMLLITLIGFFLITFVYMYVCYASAVYVPEIWPTEAKLRGSGLANAVGRISGIAAPYAVAVLLSSYGVTG

VFILLGAVSNIVAIAIATIGIETKGVSVESLSIDAVANK

>gi|30062962|ref|NP_837133.1| oxidoreductase [Shigella flexneri 2a str. 2457T]

MKALARFGKAFGGYKMIHVPQPICGPEDVVTEIKAAAICGADMKHYNVDSGSDEFNSIRGHEFAGCIAQV

GEKVKDWKVGQRVVSDNSGHVCGVCPACEQGDFLCCTEKVNLGLDNNTWGGGFSKYCLVPGEILKIHRHA

LWEIPDGVDYEDAAVLDPICNAYKSIAQQSKFLPGQDVVVIGTGPLGLFSVQMARIMGAVNIVVVGLQED

VAVRFPVAKELGATAVVNGSTEDVVARCQQICGKDNLGLVIECSGANIALKQAIEMLRPNGEVVRVGMGF

KPLDFSINDITAWNKSIIGHMAYDSTSWRNAIRLLASGAIKVKPMITHRIGLSQWREGFDAMVDKTAIKV

IMTYDFDE

>gi|30062961|ref|NP_837132.1| hypothetical protein S1561 [Shigella flexneri 2a str. 2457T]

MNLDDIINSMTPEVYQRLSTAVELGKWPDGVALTEEQKENCLQLVMLWQARHNTEAQHMTIDTNGQMVMK

SKQQLKEDFGISAKPIAMFK

>gi|30062960|ref|NP_837131.1| methionine sulfoxide reductase B [Shigella flexneri 2a str. 2457T]

MANKPSAEELKKNLSEMQFYVTQNHGTEPPFTGRLLHNKRDGVYHCLICDAPLFHSETKYDSGCGWPSFY

EPVSEESIRYIKDLSHGMQRIEIRCGNCDAHLGHVFPDGPQPTGERYCVNSASLRFTDGENGEEING

>gi|30062959|ref|NP_837130.1| glyceraldehyde-3-phosphate dehydrogenase [Shigella flexneri 2a str. 2457T]

MTIKVGINGFGRIGRIVFRAAQKRSDIEIVAINDLLDADYMAYMLKYDSTHGRFDGTVEVKDGHLIVNGK

KIRVTAERDPANLKWDEVGVDVVAEATGLFLTDETARKHITAGAKKVVMTGPSKDNTPMFVKGANFDKYA

GQDIVSNASCTTNCLAPLAKVINDNFGIIEGLMTTVHATTATQKTVDGPSHKDWRGGRGASQNIIPSSTG

AAKAVGKVLPELNGKLTGMAFRVPTPNVSVVDLTVRLEKAATYEQIKAAVKAAAEGEMKGVLGYTEDDVV

STDFNGEVCTSVFDAKAGIALNDNFVKLVSWYDNETGYSNKVLDLIAHISK

>gi|30062958|ref|NP_837129.1| hypothetical protein S1558 [Shigella flexneri 2a str. 2457T]

MIKKIFALPVIEQISPVLSRRKLDELDLIVVDHPQVKASFALQGAHLLSWKPAGEEEVLWLSNNTPFKNG

VAIRGGVPVCWPWFGPAAQQGLPAHGFARNLPWTLKSHHEDADGVALTFELTQSEETKKFWPHDFTLLAH

FRVGKTCEIDLESHGEFETTSALHTYFNVGDIAKVSVSGLGDRFIDKVNDAKEDVLTDGIQTFPDRTDRV

YLNPQDCSVINDEALNRIIAVGHQHHLNVVGWNPGPALSVSMGDMPDDGYKTFVCVETAYASETQKVTKE

KPAHLAQSIRVAKR

>gi|30062957|ref|NP_837128.1| aldehyde reductase [Shigella flexneri 2a str. 2457T]

MQQKMIQFSGDVSLPAIGQGTWYMGEDASQRKTEVAALRAGIELGLTLIDTAEMYADGGAEKVVGEALTG

LREKVFLVSKVYPWNAGGQKAINACEASLRRLNTDYLDLYLLHWSGSFAFEETVAAMEKLIAQGKIRRWG

VSNLGYADMQELWQLPGGNQCATNQVLYHLGSRGIEYDLLPWCQQQQMPVMAYSPLAQAGRLRNGLLKNA

VVNEIAHVQQHAAALEVELSSAELAMLDKAYPAPKGKTALDMV

>gi|30062956|ref|NP_837127.1| hypothetical protein S1556 [Shigella flexneri 2a str. 2457T]

MTKLKLLALGVLIATSAGVAHAEGKFSLGAGVGVVEHPYKDYDTDVYPVPVINYEGDNFWFRGLGGGYYL

WNDATDKLSITAYWSPLYFKAKDSGDHQMRHLDDRKSTMMAGLSYAHFTQYGYLRTTLAGDTLDNSNGIV

WDMAWLYRYTNGGLTVTPGIGVQWNSENQNEYYYGVSRKESARSGLRGYNPNDSWSPYLELSASYNFLGD

WSVYGTARYTRLSDEVTDSPMVDKSWTGLISTGITYKF

>gi|30062955|ref|NP_837126.1| hypothetical protein S1555 [Shigella flexneri 2a str. 2457T]

MNIFDHYRQRYEAAKDEEFTLQEFLTTCRQDRSAYANAAERLLMAIGEPVMVDTAQDPRLSRLFSNRVIA

RYPAFEEFYGMEDAIEQIVSYLKHAAQGLEEKKQILYLLGPVGGGKSSLAERLKSLMQLVPIYVLSANGE

RSPVNDHPFCLFNPQEDAQILEKEYGIPRRYLGTIMSPWAAKRLHEFGGDITKFRVVKVWPSILQQIAIA

KTEPGDENNQDISALVGKVDIRKLEHYAQNDPDAYGYSGALCRANQGIMEFVEMFKAPIKVLHPLLTATQ

EGNYNGTEGISALPFNGIILAHSNKSEWVTFRNNKNNEAFLDRVYIVKVPYCLRISEEIKIYEKLLNHSE

LTHAPCAPGTLETLSRFSILSRLKEPENSSIYSKMRVYDGESLKDTDPKAKSYQEYRDYAGVDEGMNGLS

TRFAFKILSRVFNFDHVEVAANPVHLFYVLEQQIEVYWQ

>gi|30062954|ref|NP_837125.1| IS600 orfB [Shigella flexneri 2a str. 2457T]

MAHIRTRETYGTRRLQTELAENGIIVGRDRLARLRKELRLRCKQKRKFRATTNPNHNLPVAPNLLNQTFA

PTAPNQVWVADLTYVATQEGWLYLAGIKDVYTCEIVGYAMGERMTKELTGKALFMALRSQRPPAGLIHHS

DRGSQYCAYDYRVIQEQSGLKTSMSRKGNCYDNAPMESFWGTLKNESLSHYRFNNRDEAISVIREYIEIF

YNRQRRHSRLGNISPAAFREKYHQMAA

>gi|30062953|ref|NP_837124.1| hypothetical protein S1552 [Shigella flexneri 2a str. 2457T]

MTEMAKGSVTHQQLIALLSQEGANFRVVTHEAVGKCEAVSEIRGTALGQGAKALVCKVKGNGVNQHVLAI

LAADQQADLSQLASHIGGLRASLASPAEVDELTGCVFGAIPPFSFHPKLKLVADPLLFERFDEIAFNAGM

LDKSVILKTADYLRIAQPELVNFRRTA

>gi|30062952|ref|NP_837123.1| hypothetical protein S1551 [Shigella flexneri 2a str. 2457T]

MHANNNVKTAISRGHAMNDQMFVETLIITSSFFAIAVVLVLSVLLIERIG

>gi|30062951|ref|NP_837122.1| hypothetical protein S1550 [Shigella flexneri 2a str. 2457T]

MFDVTLLILLGLAALGFISHNTTVAVSILVLIIVRVTPLSTFFPWIEKQGLSIGIIILTIGVMAPIASGT

LPPSTLIHSFLNWKSLVAIAVGVIVSWLGGRGVTLMGSQPQLVAGLLVGTVLGVALFRGVPVGPLIAAGL

VSLIVGKQ

>gi|30062950|ref|NP_837121.1| amino acid/amine transport protein [Shigella flexneri 2a str. 2457T]

MTCSTSLSGKNRIVLIAGILMIATTLRVTFTGAAPLLDTIRSAYSLTTAQTGLLTTLPLLAFALISPLAA

PVARRFGMERSLFAALLLICAGIAIRSLPSPYLLFGGTAVIGGGIALGNVLLPGLIKRDFPHSVARLTGA

YSLTMGAAAALGSAMVVPLALNGFGWQGALFMLMCFPLLALFLWLPQWRSQQHANLSTSRALHTRGIWRS

PLAWQVTLFLGINSLVYYVIIGWLPAILISHGYSEAQAGSLHGLLQLATAAPGLLIALFLHHVKDQRGIA

AFVALMCAVGAVGLCFMPAHAITWTLLFGFGSGATMILGLTFIGLRQVLRIRRRHSRGWHNPSGICWQPV

GRR

>gi|30062949|ref|NP_837120.1| hypothetical protein S1547 [Shigella flexneri 2a str. 2457T]

MKGRTNTMNIQCKRVYDPAEQSDGYRVLVDRLWPRGIKKTDLALDEWDKEITPSTELRKAFHGEVVDFAT

FREQYRAELAQHEQEGKRLADIAKKQPLTLLYAAKNTTQNHALVLADWLRSL

>gi|30062948|ref|NP_837119.1| hypothetical protein S1546 [Shigella flexneri 2a str. 2457T]

MKIISFVLPCLLVLAGCSTPSQPEAPKPPQIGMANPASVYCQQKGGTLIPVQTAQGVSNNCKLPGGETID

EWALWRRDHPAGEK

>gi|30062947|ref|NP_837118.1| hypothetical protein S1545 [Shigella flexneri 2a str. 2457T]

MSDQIIARVSQSLAKEQSLESLVRQLLEMLEMVTDMESTYLTKVDVEARLQHIMFARNSQKMHIPENFTV

LWDYSLCKRAIDENCFFSDEVPDRWGDCIAARNLGITTFLSTPIHLPDGSFYGTLCAASSEKRQWSERAE

QVLQLFAGLIAQYIQKEALVEQLHEANAALIAQSYTDSLTGLPNRRAIFENLTTLFSLARHLNHKIMIAF

IDLDNFKLINDRFGHNSGDLFLIQVGERLNTLQQNGEVIGRLGGDEFLVVSLNNENADISSLRERIQQQI

RGEYHLGDVDLYYPGASLGIVEVDPETTDADSALHAADIAMYQEKKHKQKTPFVAHPALHS

>gi|30062946|ref|NP_837117.1| IS600 orfB [Shigella flexneri 2a str. 2457T]

MAHIRTRETYGTRRLQTELAENGIIVGRDRLARLRKELRLRCKQKRKFRATTNPNHNLPVAPNLLNQTFA

PTAPNQVWVADLTYVATQEGWLYLAGIKDVYTCEIVGYAMGERMTKELTGKALFMALRSQRPPAGLIHHS

DRGSQYCAYDYRVIQEQSGLKTSMSRKGNCYDNAPMESFWGTLKNESLSHYRFNNRDEAISVIREYIEIF

YNRQRRHSRLGNISPAAFREKYHQMAA

>gi|30062945|ref|NP_837116.1| IS600 orfA [Shigella flexneri 2a str. 2457T]

MSRKTQRYSKEFKAEAVRTVLENQLSISEGASRLSLPEGTLGQWVTAARKGLGTPGSRTVAELESEILQL

RKALNEARLERDILKKATAYFAQESLKNTR

>gi|30062944|ref|NP_837115.1| repressor protein [Shigella flexneri 2a str. 2457T]

MPPEIMRFKERLEEAMNGESSRAFAAKCGLSDGVIRNYLSGKTYPSLDRLAQIAYATGRPIEWFIQSQSM

PMVEHKNNQENKNSQPDRSTLKTKLIAIVELLEDKELHSAIELFRKKGFEALMPEIFSSDDSIQADLQGI

SQQTLQTAKMLESLPTDTRKKILSKYGIYEQEGLVAPSQEPQDVKKAV

>gi|30062943|ref|NP_837114.1| diogenase beta subunit [Shigella flexneri 2a str. 2457T]

MSDYQMFEVQVSQVEPLTEQVKRFTLVATDGKPLPAFTGGSHILVQMSDGDNQYSNAYSLLSSPHNTSSY
QIAVRLEENSRGGSRFLHQQVKVGDRLTISTPNNLFALIPSARKHLFIAGGIGITPFLSHMAELQHSDID
WQLHYCSRNPESCAFRAELVQHPQAEKVHLHHSSTGTRLELARLLADIEPGTHVYTCGPEALIEAVRSEA
ARLDIAADTLHFEQFAIEDKTGDAFTLVLARSGKEFVVPEEMTILQVIENNKAAKVECLCREGVCGTCET
AILEGEADHRDQYFSDEERASQQSMLICCSRAKGKRLVLDL

>gi|30062942|ref|NP_837113.1| ribonuclease D [Shigella flexneri 2a str. 2457T]
MITTDDALASLCEAVRVFPAIALDTEFVRTRTYYPQLGLIQLFDGEHLALIDPLGITDWSPLKAILRDPS
ITKFLHAGSEDLEVFLNVFGELPQPLIDTQILAAFCGRPMSWGFASMVEEYSGVTLDKSESRTDWLARPL
TERQCEYAAADVWYLLPITAKLMVETEASGWLPAALDECRLMQMRRQEVVAPEDAWRDITNAWQLRTRQL
ACLQLLADWRLRKARERDLAVNFVVREEHLWSVARYMPGSLGELDSLGLSGSEIRFHGKTLLALVEKAQT
LPEDALPQPMLNLMDMPGYRKAFKAIKSLITDVSETHKISAELLASRRQINQLLNWHWKLKPQNNLPELI
SGWRGELMAEALHNLLQEYPQ

>gi|30062941|ref|NP_837112.1| long-chain-fatty-acid--CoA ligase [Shigella flexneri 2a str. 2457T]
MLTACISFGVAMTTNTHFRGEELKKVWLNRYPADVPTEINPDRYQSLVDMFEQSVARYADQPAFVNMGEV
MTFRKLEERSRAFAAYLQQGLGLKKGDRVALMMPNLLQYPVALFGILRAGIIVVNVNPLYTPRELEHQLN
DSGASAIVIVSNFAHTLEKVVDKTAVQHVILTRMGDQLSTAKGTVVNFVVKYIKRLVPKYHLPDAISFRS
ALHNGYRMQYVKPELVPEDLAFLQYTGGTTGVAKGAMLTHRNMLANLEQVNATYGPLLHPGKELVVTALP
LYHIFALTINCLLFIELGGQNLLITNPRDIPGLVKELAKYPFTAITGVNTLFNALLNNKEFQQLDFSSLH
LSAGGGMPVQQVVAERWVKLTGQYLLEGYGLTECAPLVSVNPYDIDYHSGSIGLPVPSTEAKLVDDDDNE
VPPGQPGELCVKGPQVMLGYWQRPDATDEIIKNGWLHTGDIAVMDEEGFLRIVDRKKDMILVSGFNVYPN
EIEDVVMQHPGVQEVAAVGVPSGSSGEAVKIFVVKKDPSLTEESLVTFCRRQLTGYKVPKLVEFRDELPK
SNVGKILRRELRDEARGKVDNKA

>gi|30062940|ref|NP_837111.1| outer membrane protein [Shigella flexneri 2a str. 2457T]

MAVQKNVIKGILAGTFALMLSGCVTVPDAIKGSSTTPQQDLVRVMSAPQLYVGQEARFGGKVVAVQNQQG

KTRLEIATVPLDSGARPTLGEPSRGRIYADVNGFLDPVDFRGQLVTVVGPITGAVDGKIGNTPYKFMVMQ

VTGYKRWHLTQQVIMPPQPIDPWFYGGRGWPYGYGGWGWYNPGPARVQTVVTE

>gi|30062939|ref|NP_837110.1| hypothetical protein S1536 [Shigella flexneri 2a str. 2457T]

MRILAIDTATEACSVALWNDGTVNAHFELCPREHTQRILPMVQDILTTSGTSLTDINALAYGRGPGSFTG

VRIGIGIAQGLALGAELPMIGVSTLMTMAQGAWRKNGATRVLSAIDARMGEVYWAEYQRDENGIWHGEET

EAVLKPELVHERMQQLSGEWVTVGTGWQAWPDLGKESGLVLRDGEVLLPAAEDMLPIACQMFAEGKTVAV

EHAEPVYLRNNVAWKKLPGKE

>gi|30062938|ref|NP_837109.1| enzyme [Shigella flexneri 2a str. 2457T]

MTDDFAPDGQLAKAIPGFKPREPQRQMAVAVTQAIEKGQPLVVEAGTGTGKTYAYLAPALRAKKKVIIST

GSKALQDQLYSRDLPTVSKALKYTGNVALLKGRSNYLCLERLEQQALAGGDLPVQILSDVILLRSWSNQT

VDGDISTCVSVAEDSQAWPLVTSTNDNCLGSDCPMYKDCFVVKARKKAMDADVVVVNHHLFLADMVVKES

GFGELIPEADVMIFDEAHQLPDIASQYFGQSLSSRQLLDLAKDITIAYRTELKDTQQLQKCADRLAQSAQ

DFRLQLGEPGYRGNLRELLANPQIQRAFLLLDDTLELCYDVAKLSLGRSALLDAAFERATLYRTRLKRLK

EINQPGYSYWYECTSRHFTLALTPLSVADKFKELMAQKPGSWIFTSATLSVNDDLHHFTSRLGIEQAESL

LLPSPFDYSRQALLCVPRNLPQTNQPGSARQLAAMLRPIIEANNGRCFMLCTSHAMMRDLAEQFRATMTL

PVLLQGETSKGQLLQQFVSAGNALLVATSSFWEGVDVRGDTLSLVIIDKLPFTSPDDPLLKARMEDCRLR

GGDPFDEVQLPDAVITLKQGVGRLIRDADDRGVLVICDYRLVMRPYGATFLASLPPAPRTRDIARAVRFL

AIPSSR

>gi|30062937|ref|NP_837108.1| hypothetical protein S1534 [Shigella flexneri 2a str. 2457T]

MAVFAARKWKYNEDKMMTIVRIDAEARWSDVVIHNNTLYYTGVPENLDADAFEQTANTLAQIDAVLEKQG

SNKSSILDATIFLADKNDFAAMNKAWDAWVVAGHAPVRCTVQAGLMNPKYKVEIKIVAAV

>gi|30062936|ref|NP_837107.1| hypothetical protein S1533 [Shigella flexneri 2a str. 2457T]

MQIKVIYSLIDNMVNFKDKNMPAVIDKALDFIGAMDVSAPTPSSMNESTAKGIFKYLKELGVPASAADIT

ARADQEGWNPGFTEKMVGWAKKMETGERSVIKNPEYFSTYMQEELKALV

>gi|30062935|ref|NP_837106.1| hypothetical protein S1532 [Shigella flexneri 2a str. 2457T]

MFAGLPSLIHEQQQKAVERIQELMAQGMSSGQAIALVAEELRANHSGELIVARFEDEDE

>gi|30062934|ref|NP_837105.1| para-aminobenzoate synthase component I [Shigella flexneri 2a str. 2457T]

MKTLSPTVITLPWRQDAAEFYFSRLSHLPWAMLLHSGYADHPYSRFDIVVADPICTLTTLGKETVVSESE

KRTTTTDDPLQVLQQVLDRADIRPTHNEDLPFQGGALGLFGYDLGRRFESLPEIAEQDIVLPDMAVGIYD

WALIVDHQRHTVSLLSHNDVNARRAWLESQQFSPQEDFTLTSDWQSNMTHEQYGEKFRQVQEYLHSGDCY

QVNLAQRFHATYSGDEWQAFLQLNQANRAPFSAFLRLEQGAILSLSPERFILCDNSEIQTRPIKGTLPRL

PDPQEDSKQAEKLANSAKDRAENLMIVDLMRNDIGRVAVAGSVKVPELFVVEPFPAVHHLVSTITARLPE

QLHASDLLRAAFPGGSITGAPKVRAMEIIDELEPQRRNAWCGSIGYLSFCGNMDTSITIRTLTAINGQIY

CSAGGGIVADSQEEAEYQETFDKVNKILRQLEK

>gi|30062933|ref|NP_837104.1| hypothetical protein S1530 [Shigella flexneri 2a str. 2457T]

MEYRSLTLDDFLSRFQLLRPQINREPLNHRQAAVLIPIVRRPQPGLLLTQRSIHLRKHAGQVAFPGGAVD

DTDASVIAAALREAEEEVAIPPSAVEVIGVLPPVDSVTGYQVTPVVGIIPPDLPYRASEDEVSAVFEMPL

AQALHLGRYHPLDIYRRGDSHRVWLSWYEQYFVWGMTAGIIRELALQIGVTP

>gi|30062932|ref|NP_837103.1| L-serine deaminase [Shigella flexneri 2a str. 2457T]

MISLFDMFKVGIGPSSSHTVGPMKAGKQFVDDLVEKGLLDSVTRVAVDVYGSLSLTGKGHHTDIAIIMGL

AGNEPATVDIDSIPGFIRDVKERERLLLAQGRHEVDFPRDNGMRFHNGNLPLHENGMQIHAYNGDEVVYS

KTYYSIGGGFIVDEEHFGQDAANEVSVPYPFKSATELLAYCNETGYSLSGLAMQNELALHSKKEIDEYFA

HVWQTMQACIDRGMNTEGVLPGPLRVPRRASALRRMLVSSDKLSNDPMNVIDWVNMFALAVNEENAAGGR

VVTAPTNGACGIVPAVLAYYDHFIESVSPDIYTRYFMAAGAIGALYKMNASISGAEVGCQGEVGVACSMA

AAGLAELLGGSPEQVCVAAEIGMEHNLGLTCDPVAGQVQVPCIERNAIASVKAINAARMALRRTSAPRVS

LDKVIETMYETGKDMNAKYRETSRGGLAIKVQCD

>gi|30062931|ref|NP_837102.1| hypothetical protein S1528 [Shigella flexneri 2a str. 2457T]

MQKAQRIIKTYRRNRMIVCTICALVTLASTLSVRFISQRNLNQQRVVQFANHAVEELDKVLLPLQAGSKV

LLPLIGLPCSVAHLPLRKQAAKLQTVRSIGLVQDGTLYCSSIFGYRNVPVVDILAELPAPQPLLRLTIDR

ALIKGSPVLIQWTPAAGSSNAGVIEMINIDLLTAMLLEPQLPQISSASLTVDKRHLLYGNGLVDSLPQPE

DNENYQVSSQSFPFTINVNGPGATALAWHYLPTQLPLAVLLSLLVGYIAWLATAYRMSFSREINLGLAQH

EFELFCQPLLNARSQQCIGVEILLRWNNPRQGWISPDVFIPIAEEHHLIVPLTHYVMAETIRQHHVFPMS

SQFHVGINVAPSHFRRGVLIKDLNQYWFSAHPIQQLILEITERDALLDVDYRIARELHRKNVKLAIDDFG

TGNSSFSWLETLRPDVLKIDKSFTAAIGSDAVNSTVTDIIIALGQRLNIELVAEGVETQEQAKYLRRHGV

HILQGYLYAQPMPLRDFPKWLAGSQPPPARHNGHITPIMSLR

>gi|30062930|ref|NP_837101.1| transporter [Shigella flexneri 2a str. 2457T]

MEFLMDPSIWAGLLTLVVLEIVLGIDNLVFIAILADKLPPKQRDKARLLGLSLALIMRLGLLSLISWMVT

LTKPLFTVMDFSFSGRDLIMLFGGIFLLFKATTELHERLENRDHDSGHGKGYASFWVVVTQIVILDAVFS

LDAVITAVGMVNHLPVMMAAVVIAMAVMLLASKPLTRFVNQHPTVVVLCLSFLLMIGLSLVAEGFGFHIP

KGYLYAAIGFSIIIEVFNQIARRNFIRHQSTLPLRARTADAILRLMGGKRQANVQHDADNPMPIPEGAFA

EEERYMINGVLTLASRSLRGIMTPRGEISWVDANLGVDEIREQLLSSPHSLFPVCRGELDEIIGIVRAKE

LLVALEEGVDVAAIASASPAIIVPETLDPINLLGVLRRARGSFVIVTNEFGVVQGLVTPLDVLEAIAGEF

PDADETPEIITDGNGWLVKGGTDLHALQQALDVEHLADDDDIATVAGLVISANGHIPRVGDVIDVGPLHI

TIFEANDYRVDLVRIVKEQPAHDEDE

>gi|30062929|ref|NP_837100.1| PTS enzyme IIAB [Shigella flexneri 2a str. 2457T]

MTIAIVIGTHGWAAEQLLKTAEMLLGEQENVGWIDFVPGENAETLIEKYNAQLAKLDTTKGVLFLVDTWG

GSPFNAASRIVVDKEHYEVIAGVNIPMLVETLMARDDDPSFDELVALAVETGREGVKALKAKPVEKAAPA

PAAAAPKAAPTPAKPMGPNDYMVIGLARIDDRLIHGQVATRWTKETNVSRIIVVSDEVAADTVRKTLLTQ

VAPPGVTAHVVDVAKMIRVYNNPKYAGERVMLLFTNPTDVERLVEGGVKITSVNVGGMAFRQGKTQVNNA

VSVDEKDIEAFKKLNARGIELEVRKVSTDPKLKMMDLISKIDK

>gi|30062928|ref|NP_837099.1| PTS enzyme IIC [Shigella flexneri 2a str. 2457T]

MEITTLQIVLVFIVACIAGMGSILDEFQFHRPLIACTLVGIVLGDMKTGIIIGGTLEMIALGWMNIGAAV

APDAALASIISTILVIAGHQSIGAGIALAIPLAAAGQVLTIIVRTITVAFQHAADKAADNGNLTAISWIH

VSSLFLQAMRVAIPAVIVALSVGTSEVQNMLNAIPEVVTNGLNIAGGMIVVVGYAMVINMMRAGYLMPFF

YLGFVTAAFTNFNLVALGVIGTVMAVLYIQLSPKYNRVAGAPAQAAGNNDLDNELD

>gi|30062927|ref|NP_837098.1| mannose-specific PTS system protein IID [Shigella flexneri 2a str. 2457T]

MSEMVDTTQTTTEKKLTQSDIRGVFLRSNLFQGSWNFERMQALGFCFSMVPAIRRLYPENNEARKQAIRR

HLEFFNTQPFVAAPILGVTLALEEQRANGAEIDDGAINGIKVGLMGPLAGVGDPIFWGTVRPVFAALGAG

IAMSGSLLGPLLFFILFNLVRLATRYYGVAYGYSKGIDIVKDMGGGFLQKLTEGASILGLFVMGALVNKW

THVNIPLVVSTITGQDGQTRVTTVQTILDQLMPGLVPLLLTFACMWLLRKKVNPLWIIVGFFVIGIAGYA

CGLLGL

>gi|30062926|ref|NP_837097.1| hypothetical protein S1523 [Shigella flexneri 2a str. 2457T]

MTITDLVLILFIAALLAFAIYDQFIMPRRNGPTLLAIPLLRRGRIDSVIFVGLIVILIYNNVTNHGALIT

TWLLSALALMGFYIFWIRIPKIIFKQKGFFFANVWIEYSRIKAMNLSEDGVLVMQLEQRRLLIRVRNIDD

LEKIYKLLVSGNAANLLI

>gi|30062925|ref|NP_837096.1| IS1 orfA [Shigella flexneri 2a str. 2457T]

MASISIRCPSCSATEGVVRNGKSTAGHQRYLCSHCRKTWQLQFTYTASQPGTHQKIIDMAMNGVGCRASA

RIMGVGLNTVLRHLKNSGRSR

>gi|30062924|ref|NP_837095.1| IS1 orfB [Shigella flexneri 2a str. 2457T]

MSRQCTHYGRWPQHGFTSLKKLRPQSVTSRIQPGSDVIVCAEMDEHWGYVGAKSRQRWLFYAYDRIRRTV

VAHVFGERTLATLERLLSLLSAFEVVVWMTDGWPLYESRLKEKLHVISKRYTQRIERHNLNLRQHLARLG

RMSLSFSKSVELHDKVIGHYLNIKHYQ

>gi|30062923|ref|NP_837094.1| hypothetical protein S1520 [Shigella flexneri 2a str. 2457T]

MFAGGDDVFYGYPGQDVVMNITATVLLAFGMSMDAFAASIGKGAPLHKPKFSEALRTGLIFGAVETLTPL

IGWGMGMLASRFVLEWNHWIAFVLLIFLGGRMIIEGFRGADDEDEEPRRRHGFWLLVTTAIATSLDAMAV

GVGLAFLQVNIIATALAIGCATLIMSTLGMMVGRFIGSIIGKKAEILGGLVLIGIGVQILWTHFHG

>gi|30062922|ref|NP_837093.1| 23S rRNA methyltransferase A [Shigella flexneri 2a str. 2457T]

MSFSCPLCHQPLSREKNSYICPQRHQFDMAKEGYVNLLPVQHKRSRDPGDSAEMMQARRAFLDAGHYQPL

RDAIVAQLRERLDEKATAVLDIGCGEGYYTHAFADALPEITTFGLDVSKVAIKAAAKRYPQVTLCVASSH

RLPFSDTSMDAIIRIYAPCKAEELARVVKPGGWVITATPGPRHLMELKGLIYNEVHLHAPHAEQLEGFTL

QQSAELCYPMRLRGDEAVALLQMTPFAWRAKPEVWQTLAAKEVFDCQTDFNIHLWQRSY

>gi|30062921|ref|NP_837092.1| cold shock-like protein CspC [Shigella flexneri 2a str. 2457T]

MAKIKGQVKWFNESKGFGFITPADGSKDVFVHFSAIQGNGFKTLAEGQNVEFEIQDGQKGPAAVNVTAI

>gi|30062920|ref|NP_837091.1| hypothetical protein S1517 [Shigella flexneri 2a str. 2457T]

MCGIFSKEVLSKHVDVEYRFSAEPYIGASCSNVSVLSMLCLRAKKTI

>gi|30062919|ref|NP_837090.1| hypothetical protein S1516 [Shigella flexneri 2a str. 2457T]

MNEVVNSGVMNIASLVVSVVVLLIGLILWFFINRASSRTNEQIELLEALLDQQKRQNALLRRLCEANEPE

KADKKTIESQKSVDDEDIIRLVAER

>gi|30062918|ref|NP_837089.1| hypothetical protein S1515 [Shigella flexneri 2a str. 2457T]

MKKFRWVVLVVVVLACLLLWAQVFNMMCDQDVQFFSGICAINQFIPW

>gi|30062917|ref|NP_837088.1| regulator [Shigella flexneri 2a str. 2457T]

MANADLDKQPDSVSSVLKVFGILQALGEEREIGITELSQRVMMSKSTVYRFLQTMKTLGYVAQEGESEKY

SLTLKLFELGARALQNVDLIRSADIQMREISRLTKETIHLGALDEDSIVYIHKIDSMYNLRMYSRIGRRN

PLYSTAIGKVLLAWRDRDEVKQILEGVEYKRSTERTITSTEALLPVLDQVREQGYGEDNEEQEEGLQCIA

VPVFDRFGVVIAGLSISFPTLRFSEERLQEYVAMLHTAARKISAQMGYHDYPF

>gi|30062916|ref|NP_837087.1| transporter [Shigella flexneri 2a str. 2457T]

MVAIHLLPVSYNSATSTVNISARIIPLLIIHQRYKIPMPKVQADGLPLPQRYGAILTIVIGISMAVLDGA

IANVALPTIATDLHATPASSIWVVNAYQIAIVISLLSFSFLGDMFGYRRIYKCGLVVFLLSSLFCALSDS

LQMLTLARVIQGFGGAALMSVNTALIRLIYPQRFLGRGMGINSFIVAVSSAAGPTIAAAILSIASWKWLF

LINVPLGIIALLLAMRFLPPNGSRASKPRFDLPSAVMNALTFGLLITALSGFAQGQSLTLIAAELVVMVV

VGIFFIRRQLSLPVPLLPVDLLRIPLFSLSICTSVCSFCAQMLAMVSLPFYLQTVLGRSEVETGLLLTPW

PLATMVMAPLAGYLIERVHAGLLGALGLFIMATGLFSLVLLPASPADINIIWPMILCGAGFGLFQSPNNH

TIITSAPRERSGGASGMLGTARLLGQSSGAALVALMLNQFGDNGTHVSLMAAAILAVIAACVSGLRITQP

RARA

>gi|30062915|ref|NP_837086.1| heat shock protein HtpX [Shigella flexneri 2a str. 2457T]

MRIALFLLTNLAVMVVFGLVLSLTGIQSSSVQGLMIMALLFGFGGSFVSLLMSKWMALRSVGGEVIEQPR

NERERWLVNTVATQARQAGIAMPQVAIYHAPDINAFATGARRDASLVAVSTGLLQNMSPDEAEAVIAHEI

SHIANGDMVTMTLIQGVVNTFVIFISRILAQLAAGFMGGNRDEGEESNGNPLIYFAVATVLELVFGILAS

IITMWFSRHREFHADAGSAKLVGREKMIAALQRLKTSYEPQEATSMMAFCINGKSKSLSELFMTHPPLDK

RIEALRTGEYLK

>gi|30062913|ref|NP_837084.1| hypothetical protein S1510, partial [Shigella flexneri 2a str. 2457T]

MKAGQNAMDATVLEITKDGVRVQLNSGMSLIVRAEHLVF

>gi|30062912|ref|NP_837083.1| hypothetical protein S1509 [Shigella flexneri 2a str. 2457T]

MNKTEFYADLNRDFNALMAGETSFLATLANTSALLYERLTDVNWAGFYLLEDDTLVLGPFQGKIACVRIP

VGRGVCGTAVARNQVQRIEDVHAFDGHIACDAASNSEIVLPLVVKNQIIGVLDIDSTVFARFTDEDEQGL

RQLVAQLEKVLATTDYKKFFASVAG

>gi|30062911|ref|NP_837082.1| hypothetical protein S1508 [Shigella flexneri 2a str. 2457T]

MALNTPQITPTKKITVRAIGEELPRGDYQRCPQCDMLFSLPEINSHQSAYCPRCQAKIRDGRDWSLTRLA

AMAFTMLLLMPFAWGEPLLHIWLLGIRIDANVMQGIWQMTKQGDAITGSMVFFCVIGAPLILVSSIAYLW

FGNRLGMNLRPVLLMLERLKEWVMLDIYLVGIGVASIKVQDYAHIQAGVGLFSFVALVILTTVTLSHLNV

EELWERYYPQRPATRRDEKLRVCLGCHFTGYPDQRGLCPRCHIPLRLRRRHSLQKCWAALLASIVLLLPA

NLLPISIIYLNGGRQEDTILSGIMSLASSNIAVAGIVFIASILVPFTKVIVMFTLLLSIHFKCQQGLRTR

ILLLRMVTWIGRWSILDLFVISLTMSLINRDQILAFTMGPAAFYFGAAVILTILAVEWLDSRLLWDAHES

GNARFDD

>gi|30062910|ref|NP_837081.1| hypothetical protein S1505 [Shigella flexneri 2a str. 2457T]

MKTSVRIGAFEIDDGELHGESPGDRTLTIPCKSDPDLCMQLDAWDAETSIPALLNGEHSVLYRTRYDQQS

DAWIMRLA

>gi|30062909|ref|NP_837080.1| hypothetical protein S1504 [Shigella flexneri 2a str. 2457T]

MAGYLSWLFPRCKISPKLNGTAPHFGDEMFALVLFVCYLDGGCEDIVVDVYNTEQQCLYSMSDQRIRHGS

CFPIEDFIDGFWQPAQEYGDF

>gi|30062908|ref|NP_837079.1| IS600 orfB [Shigella flexneri 2a str. 2457T]

MAHIRTRETYGTRRLQTELAENGIIVGRDRLARLRKELRLRCKQKRKFRATTNPNHNLPVAPNLLNQTFA

PTAPNQVWVADLTYVATQEGWLYLAGIKDVYTCEIVGYAMGERMTKELTGKALFMALRSQRPPAGLIHYS

DRGSQYCAYDYRVIQEQSGLKTSMSRKGNCYDNAPMESFWGTLKNESLSHYRFNNRDEAISVIREYIEIF

YNRQRRHSRLGNISPAAFREKYHQMAA

>gi|30062907|ref|NP_837078.1| IS600 orfA [Shigella flexneri 2a str. 2457T]

MSRKTQRYSKEFKAEAVRTVLENQLSISEGASRLSLPEGTLGQWVTAARKGLGTPGSRTVAELESEILQL

RKALNEARLERDILKKATAYFAQESLKNTR

>gi|30062906|ref|NP_837077.1| IS1 orfB [Shigella flexneri 2a str. 2457T]

MSCQCTHYGRWPQHGFTSLKKLRPQSVTSRIQPGSDVIVCAEMDEHWGYVGAKSRQRWLFYAYDRIRRTV

VAHVFGERTLATLERLLSLLSAFEVVVWMTDGWPLYESRLKGKLHVISKRYTQRIERHNLNLRQHLARLG

RKSLSFSKSVELHDKVIGHYLNIKHYQ

>gi|30062905|ref|NP_837076.1| IS1 orfA [Shigella flexneri 2a str. 2457T]

MASISIRCPSCSATEGVVRNGKSTAGHQRYLCSHCRKTWQLQFTYTASQPGTHQKIIDMAMNGVGCRASA

RIMGVGLNTVLRHLKNSGRSR

>gi|30062904|ref|NP_837075.1| IS91 orf [Shigella flexneri 2a str. 2457T]

MNRATLRAVHAAGADTGKNGRKLPLRAAGYFRIPFVINTYKPEDQLFVSGIHKEGNIQVCAALNGIVKII

KAQNFSVFFVKKIIRMKFHNIAGKFASLWCGFAFTGFS

>gi|30062903|ref|NP_837074.1| IS91 orf [Shigella flexneri 2a str. 2457T]

MLPRFADIFQQGNRWLNWLEKQPEGSVRPVVTESVTKIMACGTTLMGYTQWCCSSPDCCHTKKVCFRCKS

RSCPHCGVKAGAQWIQYLLSLVPDCPWQHIVFTLPCQYWSLVFHNRWLLAEMSRIAADVILEICHQTDVE

PGIFTVIHTWGRDQQWHPHIHLSTTAGGVTSGHTWKNLHFYARKVMSMWRYRITRLLSRKYPELVIPDEL

AVEGNSKRDWNRFLDTHYRRGWNVNVSRVMDNATHVAVYFGSYLKKPPVPMSRLEHYAGQDEIGLRYNSH

RTKREEYLLMSGDEFMERFSWHVADKGFRMVRYYGFLSPAKRRLLEEVVYIITETVRKTAMQITWRGMYQ

RLLKVDPLKCVLCGSQMRFTGLKRGYRLAEQVLMHEPLARMRWCG

>gi|30062902|ref|NP_837073.1| hypothetical protein S1495 [Shigella flexneri 2a str. 2457T]

MRTTSFAKVAALCGLLALSGCASKITQPDKYNNYSDLKETTSATGKPVLRWVDPSFDQSKYDSIVWNPIT

YYPVPKPSTQVGQKVLDKILNYTNTEMKEAIAQRKPVVTTAGPRSLIFRGAITGVDTSKEGLQFYEVVPV

ALVVAGTQMATGHRTMDTRLYFEGELIDAATNKPVIKVVRQGEGKDLNNESTPMAFENIKQVIDDMATDA

TMFDVNKK

>gi|30062901|ref|NP_837072.1| tellurite resistance protein TehB [Shigella flexneri 2a str. 2457T]

MIIRDENYFTDKYELTRTHSEVLEAVKVVKPGKTLDLGCGNGRNSLYLAANGYDVDAWDKNAMSIANVER

IKSIENLDNLHTRVVDLNNLTFDGQYDFILSTVVLMFLEAKTIPGLIANMQRCTKPGGYNLIVAAMDTAD

YPCTVGFTFAFKEGELRRYYEGWERVKYNEDVGELHRTDANGNRIKLRFATMLARKK

>gi|30062900|ref|NP_837071.1| potassium-tellurite ethidium and proflavin transporter [Shigella flexneri 2a str. 2457T]

MQSDKVLNLPAGYFGIVLGTIGMGFAWRYASQVWQVSHWLGDGLVILAMIIWGLLTSAFITRLIRFPHSV

LAEVRHPVLSSFVSLFPATTMLVAIGFVPWFRPLAVCMFSFGVVVQLAYAAWQTAGLWRGSHPEEATTPG

LYLPTVANNFISAMACGALGYTDAGLVFLGAGVFSWLSLEPVILQRLRSSGELPTALRTSLGIQLAPALV

ACSAWLSVNGGEGDTLAKMLFGYGLLQLLFMLRLMPWYLSQPFNASFWSFSFGVSALATTGLHLGSGSDN

GFFHTLAVPLFIFTNFIIAILLIRTFALLMQGKLLVRTERAVLMKAEDKE

>gi|30062899|ref|NP_837070.1| hypothetical protein S1492 [Shigella flexneri 2a str. 2457T]

MRKYRLSEEQRAFSYQEDGTKKSVLLRQIIAISDFNDVIAGTAGGWIDRETVLAQEGNCWIYDQNAIAFG

GTVISGNTRITGTSVLWGEVYATDNVWIDNSEISQGAYISDSVTIHDSLVYVQCRIFGHALIDQHSMIVA

AQGLTPDHQLLLQIYDRARVSASRIVHQAQIYGDAVVRYAFIEHRAEVFDFASVEGNEENNVWLCDCAKV

YGHAQVKAGIEEDAIPTIHYSSQVAEYAIVEGNCVLKHHVLIGGNAVVRGGPILLDEHVVIQGESRITGA

VIIENHVELTDHAVVEAFDGDTVHVRGPKVINGEERITRTPLAGLL

>gi|30062898|ref|NP_837069.1| ribosomal-protein-L7/L12-serine acetyltransferase [Shigella flexneri 2a str. 2457T]

MTETIKVSESLELHAVAESHVTPLYQLICKNKTWLQQSLNWPQFVQSEEDTRKTVQGNVMLHQRGYAKMF

MIFKEDELIGVISFNRIEPLNKTAEIGYWLDESHQGQGIISQALQTLIHHYAQSGELRRFVIKCRVDNPQ

SNQVALRNGFILEGCLKQAEFLNDAYDDVNLYARIIDS

>gi|30062897|ref|NP_837068.1| hypothetical protein S1490, partial [Shigella flexneri 2a str. 2457T]

MSLFDKHNKLDHEIARKEGSDGRGYNAEVVRMKKQKLQLKDEMLKILQQESVKEV

>gi|30062896|ref|NP_837067.1| hypothetical protein S1489 [Shigella flexneri 2a str. 2457T]

MADTVYLKYTPSDYSFNLGKNASGIVFNQTAPPEEGAEEKTINSSRGRQHTDVYPALAGNTDTAMFH

>gi|30062895|ref|NP_837066.1| glucan biosynthesis protein D [Shigella flexneri 2a str. 2457T]

MDRRRFIKGSMAMAAVCGTSGIASLFSQAAFAADSDIADGQTQRFDFSILQSMAHDLAQTAWRGAPRPLP

DTLATMTPQAYNSIQYDAEKSLWHNVENRQLDAQFFHMGMGFRRRVRMFSVDPATHLAREIHFRPELFKY

NDAGVDTKQLEGQSDLGFAGFRVFKAPELARRDVVSFLGASYFRAVDDTYQYGLSARGLAIDTYTDSKEE

FPDFTAFWFDTVKPGATTFTVYALLDSASITGAYKFTIHCEKSQVIMDVENHLYARKDIKQLGISPMTSM

FSCGTNERRMCDTIHPQIHDSDRLSMWRGNGEWICRPLNNPQKLQFNAYTDNNPKGFGLLQLDRDFSHYQ

DIMGWYNKRPSLWVEPRNKWGKGTIGLMEIPTTGETLDNIVCFWQPEKAVKAGDEFAFQYRLYWSAQPPV

HCPLARVMATRTGMGGFPEGWAPGEHYPEKWARRFAVDFVGGDLKAAAPKGIEPVITLSSGEAKQIEILY

IEPIDGYRIQFDWYPTSDSTDPVDMRMYLRCQGDAISETWLYQYFPPAPDKRQYVDDRVMS

>gi|30062894|ref|NP_837065.1| hypothetical protein S1487 [Shigella flexneri 2a str. 2457T]

MANSITADEIREQFSQAMSAMYQQEVPQYGTLLELVADVNLAVLENNPQLHEKMVNADELARLNVERHGA

IRVGTAQELATLRRMFAIMGMYPVSYYDLSQAGVPVHSTAFRPIDDASLARNPFRVFTSLLRLELIENEI

LRQKAAEILRQRDIFTPRCRQLLEEYEQQGGFNETQAQEFVQEALETFRWHQLATVDEETYRALHNEHRL

IADVVCFPGCHINHLTPRTLDIDRVQSMMPECGIEPKILIEGPPRREVPILLRQTSFKALEETVLFAGQK

QGTHTARFGEIEQRGVALTPKGRQLYDDLLRNAGTGQDNLTHQMHLQETFRTFPDSEFLMRQQGLAWFRY

RLTPSGEAHRQAIHPGDDPQPLIERGWVVAQPITYEDFLPVSAAGIFQSNLGNETQTRSHGNASREAFEQ

ALGCPVLDEFQLYQEAEERSKRRCGLL

>gi|30062893|ref|NP_837064.1| LYSR-type transcriptional regulator [Shigella flexneri 2a str. 2457T]

MISPRKHQYYPFCCNIIALIVNNILQSSYHNQTTSLVSDTASFLTSLMEKNSLFSQRIRLRHLHTFVAVA

QQGTLGRAAETLNLSQPALSNTLNELEQLTGARLFERGRQGAQLTLPGEQFLTHAVRVLDAINTAGQSLH

RKEGLNNDVVRVGALPTAALGILPSVIGQFHQQQKETTLQVATMSNPMILAGLKTGEIDIGIGRMSDPEL

MTGLNYELLFLESLKLVVRPNHPLLQENVTLSRVLEWPVVVSPEGTAPRQHSDALVQSQGCKIPSGCIET

LSTSLSRQLTVEYDYVWFVPSGAVKDDLRHATLVALPVPGHGAGEPIGILTRVDATFSSGCQLMINAIRK

SMPF

>gi|30062892|ref|NP_837063.1| methyl-accepting chemotaxis protein III, ribose sensor receptor [Shigella flexneri 2a str. 2457T]

MNTTPSQRLGFLHHIRLVPLFACILGGILVLFALSSALAGYFLWQADRDQRDVTAEIEIRTGLANSSDFL

RSARINMIQAGAASRIAEMEAMKRNIAQAESEIKQSQQGYRAYQNRPVKTPADEAFDTELNQRFQAYITG

MQPMLKYAKNGMFEAIINHESEQIRPLDNAYTDILNKAVKIRSTRANQLAELAHQRTRLGGMFMIGAFVL

ALVMTLITFMVLRRIVIRPLQHAAQRIEKIASGDLTMNDEPAGRNEIGRLSRHLQQMQHSLGMTVGTVRQ

GAEEIYRGTSEISAGNADLSSRTEEQAAAIEQTAASMEQLTATVKQNADNAHHASKLAQEASIKASDGGQ

TVSGVVKTMGAISTSSKKISEITAVINSIAFQTNILALNAAVEAARAGEQGRGFAVVASEVRTLASRSAQ

AAKEIEGLISESVRLIDLGSDEVATAGKTMSTIVDAVASVTHIMQEIAAASDEQSRGITQVSQAISEMDK

VTQQNASLVEEASAAAVSLEEQAARLTEAVNVFRLNKHSVSAEPRGAGEPVSFATV

>gi|30062891|ref|NP_837062.1| IS103 orf [Shigella flexneri 2a str. 2457T]

MSKPKYPFEKRLEVVNHYFTTDDGYRIISARFGVPRTQVRTWVALYEKHGEKGLIPKPKGVSADPELRIK

VVKAVIEQHMSLNQAAAHFMLAGSGSVARWLKVYEERGEAGLRALKIGTKRNIAISVDPEKAASALELSK

DRRIEDLERQVRFLETRLMYLKKLKALAHPTKK

>gi|30062890|ref|NP_837061.1| IS103 orf [Shigella flexneri 2a str. 2457T]

MKVLNVLRQFYPLDELLRAAEIPRSTFYYHLKALSKPDKYADVKKRIGEIYHENRGRYGYRRVTLSLHRE

GKQINHKAVQRLMGTLSLKAAIKVKRYRSYRGEVGQTAPNVLQRDFKATRPNEKWVTDVTEFAVNGRKLY

LSPVIDLFNNEVISYSLSERPVMNMVENMLDQAFKKLNPHEHPVLHSDQGWQYRMRRYQNILKEHGIKQS

MSRKGNCLDNAVVECFFGTLKSECFYLDEFSNISELKDAVTEYIEYYNSRRISLKLKGLTPNEYRNQTYM

PRV

>gi|30062889|ref|NP_837060.1| hypothetical protein S1479 [Shigella flexneri 2a str. 2457T]

MKKLALILFMGTLVSFYADAGRKPCSGSKGGISHCTAGGKFVCNDGSISASKKTCTN

>gi|30062887|ref|NP_837058.1| aldehyde dehydrogenase A [Shigella flexneri 2a str. 2457T]

MSVPVQHPMYIDGQFVTWRGDAWIDVVNPATEAVISRIPDGQAEDARKAIDAAERAQPEWEALPAIERAS

WLCKISAGIRERASEISALIVEEGGKIQQLAEVEVAFTADYIDYMAEWARRYEGEIIQSDRPGENILLFK

RALGVTTGILPWNFPFFLIARKMAPALLTGNTIVIKPSEFTPNNAIAFAKIVDEIGLPRGVFNLVLGRGE

TVGQELAGNPKVAMVSMTGSVSAGEKIMATAAKNITKVCLELGGKAPAIVMDDADLELAVKAIVDSRVIN

SGQVCNCAERVYVQKGIYDQFVNLLGEALQAVQFGNPAERNDIAMGPLINAAALERVEQKVARAVEEGAR

VALGGKAVEGKGYYYPPTLLLDVRQEMSIMHEETFGPVLPVVAFDTLEDAISMANDSDYGLTSSIYTQNL

NVAMKAIKGLKFGETYINRENFEAMQGFHAGWRKSGIGGADGKHGLHEYLQTQVVYLQS

>gi|30062886|ref|NP_837057.1| IS600 orfB [Shigella flexneri 2a str. 2457T]

MAHIRTRETYGTRRLQTELAENGIIVGRDRLARLRKELRLRCKQKRKFRATTNPNHNLPVAPNLLNQTFA

PTAPNQVWVADLTYVATQEGWLYLAGIKDVYTCEIVGYAMGERMTKELTGKALFMALRSQRPPAGLIHHS

DRGSQYCAYDYRVIQEQSGLKTSMSRKGNCYDNAPMESFWGTLKNESLSHYRFNNRDEAISVIREYIEIF

YNRQRRHSRLGNISPAAFREKYHQMAA

>gi|30062885|ref|NP_837056.1| ATP-dependent RNA helicase HrpA [Shigella flexneri 2a str. 2457T]

MLRDRLRFSRRLHCVKKVKNPDAQQAIFQEMAKEIDQAAGKVLLREAARPEITYPDNLPVSQKKQDILEA

IRDHQVVIVAGETGSGKTTQLPKICMELGRGIKGLIGHTQPRRLAARTVANRIAEELKTEPGGCIGYKVR

FSDHVSDNTMVKLMTDGILLAEIQQDRLLMQYDTIIIDEAHERSLNIDFLLGYLKELLPRRPDLKIIITS

ATIDPERFSRHFNNAPIIEVSGRTYPVEVRYRPIVEEADDTERDQLQAIFDAVDELSQESPGDILIFMSG

EREIRDTADALNKLNLRHTEILPLYARLSNSEQNRVFQSHSGRRIVLATNVAETSLTVPGIKYVIDPGTA

RISRYSYRTKVQRLPIEPISQASANQRKGRCGRVSEGICIRLYSEDDFLSRPEFTDPEILRTNLASVILQ

MTALGLGDIAAFPFVEAPDKRNIQDGVRLLEELGAITTDEQASAYKLTPLGRQLSQLPVDPRLARMVLEA
QKHGCVREAMIITSALSIQDPRERPMDKQQASDEKHRRFHDKESDFLAFVNLWNYLGEQQKALSSNAFRR
LCRTDYLNYLRVREWQDIYTQLRQVVKELGIPVNSEPAEYREIHIALLTGLLSHIGMKDADKQEYTGARN
ARFSIFPGSGLFKKPPKWVMVAELVETSRLWGRIAARIDPEWVEPVAQHLIKRTYSEPHWERAQGAVMAT
EKVTVYGLPIVAARKVNYSQIDSALCRELFIRHALVEGDWQTRHAFFRENLKLRAEVEELEHKSRRRDIL
VDDETLFEFYDQRISHDVISARHFDSWWKKVSRETPDLLNFEKSMLIKEGAEKISKLDYPNFWHQGNLKL
RLSYQFEPGADADGVTVHIPLPLLNQVEESGFEWQIPGLRRELVIALIKSLPKPVRRNFVPAPNYAEAFL
GRVTPLELPLLDSLERELRRMTGVTVDREDWHWDQVPDHLKITFRVVDDKNKKLKEGRSLQDLKDALKGK
VQETLSAVADDGIEQSGLHIWSFGQLPESYEQKRGNYKVKAWPALVDERDSVTIKLFDNPLEQKQAMWNG
LRRLLLLNISSPIKYLHEKLPNKAKLGLYFNPYGKVLELIDDCISCGVDQLIDANGGPVWTEEGFAALHE
KVRAELNDTVVDIAKQVEQILTAVFNINKRLKGRVDMTMALGLSDIKAQMGGLVYRGFVTGNGFKRLGDT
LRYLQAIEKRLEKLAVDPHRDRAQMLKVENVQQAWQQWINKLPPARREDEDVKEIRWMIEELRVSYFAQQ
LGTPYPISDKRILQAMEQISG

>gi|30062884|ref|NP_837055.1| azoreductase [Shigella flexneri 2a str. 2457T]
MSKVLVLKSSILAGYSQSNQLSDYFVEQWSEKHSADEITVRDLAANPIPVLDGELVGALRPSDAPLTPRQ
QEALALSDELIAELKAHDVIVIAAPMYNFNISTQLKNYFDLVARAGVTFRYTENGPEGLVTGKKAIVITS
RGGIHKDGPTDLVTPYLSTFLGFIGITDVKFVFAEGIAYGPEMAAKAQSDAKAAIDSIVAA

>gi|30062883|ref|NP_837054.1| IS600 orfA [Shigella flexneri 2a str. 2457T]
MSRKTQRYSKEFKAEAVRTVLENQLSISEGASRLSLPEGTLGQWVTAARKGLGTPGSRTLAELESEILQL
RKALNEARLERDILKKATAYFAQESLKNTR

>gi|30062882|ref|NP_837053.1| IS600 orfB [Shigella flexneri 2a str. 2457T]
MAHIRTRETYGTRRLQTELAENGIIVGRDRLARLRKELRLRCKQKRKFRATTNPNHNLPVAPNLLNQTFA

PTAPNQVWVADLTYVATQEGWLYLAGIKDVYTCEIVGYAMGERMTKELTGKALFMALRSQRPPAGLIHHS
DRGSQYCAYDYRVIQEQFGLKTSMSRKGNCYDNAPMESFWGTLKNESLSHYRFNNRDEAISVIREYIEIF
YNRQRRHSRLGNISPAAFREKYHQMTA

>gi|30062881|ref|NP_837052.1| hypothetical protein S1466 [Shigella flexneri 2a str. 2457T]
MENSRIPGEHFFTTSDNTALYYRHWPTLQPGAKKVIVLFHRGHEHSGRLQHLVDELAMPDTAFYAWDARG
HGKNSGPRGYSPSLMRSVQDVDEFVRFAASDSQVGLEEVVVIAQSVGAVLVATWVHDYAPAIRGLVLASP
AFKVKLYVPLARPALALWHRLRGLFFINSYVKGRYLTHDRQRVASFNNDPLITRAIAVNILLDLYKTSER
IVSDAAAITLPTQLLISGDDYVVHRQPQIDFYQRLRSPLKELHLLPGFYHDTLGEENRAQAFEKMQSFIC
RLYANKSQKFDYQHEDRTGPSADRWRLLSGGPVPLSPVDLAYRFMRKAMKLFGAHSAGLHLGMSTGFDSG
SSLDYVYQNQPQGSNAFGRLIDKIYLNSVGWRGIRQRKTHLQILIKQAVADLHAKGLAVRVVDIAAGHGR
YVLDALANEPAVSDILLRDYSELNVAQGQEMIAQRGMSGRVRFEQGDAFNPEELSALTPRPTLAIVSGLY
ELFPENEQVKNSLAGLANAIEPGGILIYTGQPWHPQLELIAGVLTSHKDGKPWVMRVRSQGEMDSLVRNA
GFDKCTQRIDVWGIFTVSMAVRRDN

>gi|30062880|ref|NP_837051.1| phosphatidate cytidylyltransferase [Shigella flexneri 2a str. 2457T]
MISGPRWMTLTFFTLISFLALKEYCTLISVHFSRWLYWVIPLNYLLIGFNCFELFLLFIPLTGFLILATW
RVFVGDPSGFQHTVSAIFWGWIMTVFALSHAAWLLMLPTINIQGGALLVLFLLALTESNDIAQYLWGKSC
GRRKVVPKVSPGKTLEGLLGGVITTMIASLIIGPLLTPLNTLQALLAGLLIGISGFCGDVVMSAIKRDIG
VKDSGKLLLGHGGLLDRIDSLIFTAPVFFYFIRYCCY

>gi|30062879|ref|NP_837050.1| hypothetical protein S1464 [Shigella flexneri 2a str. 2457T]
MTLYQIKPLFQSLLRPTMFWLYKHHVTANHITLTALALSLFTGLLLVLVAQPILFLLLPIVLFIRMALNA
LDGMLARECNQQTRLGAILNETGDVISDIALYLPFLFLPESNASLVILMLFCTILTEFCGLLAQTINGVR
SYAGPFGKSDRALIFGLWGLAVAIYPQWMQWNNLLWSIASILLICTAINRCRSVLLMSAER

>gi|30062878|ref|NP_837049.1| hypothetical protein S1463, partial [Shigella flexneri 2a str. 2457T]

MAGNVCNQMKKEALFRPKPSPELVQELQMLDEGNVAAFEGRDIATFDLAIMRTLPRLKGISANLRKQLIN

SNDEQTIESMARYMPDNEILELTDQQLGYQPVVLGLLDREPLSVEIMTRMSRLPDGVGPLNLALRENLPL

DIVMTLAKRDWDMIIQELYKDAWLLPESIIDGYIRSDDSSIRQVGAGGQLTYNQAMQLANDSSNNVVTSL

AFKLAEMKHHGQLLRMTPQESDKVAAYLYQKFENDDDLIRVLFLALPDNLQFNFVKRMEKKSPAYFCCRD

MQVIHSDAALQRLLTRFNDPEGWSNLAKNQYLSTSMKQKIWQRALSHRKNNPKADSDAYETSADMILSEL

ISHGEVDDQMLLNAAALIRLEDWDFLESALVSWDNLPAVVLKELQQNTPRNDIWAKFFLRQENSSRAQVD

EALRVYYALDPDALAQLDVLAKQPDRIWWSTLAKSNLTFFKFGALSNRHTPSAALAAEIDPEWWIVAMNN

PRFPVDVLKARLKRDPLLALELVNPELDLVRQLALNGKTRAIREQAMRKLDELY

>gi|30062877|ref|NP_837048.1| IS1 orfA [Shigella flexneri 2a str. 2457T]

MASISIRCPSCSATEGVVRNGKSTAGHQRYLCSHCRKTWQLQFTYTASQPGTHQKIIDMAMNGVGCRASA

RIMGVGLNTVLRHLKNSGRSR

>gi|30062876|ref|NP_837047.1| IS1 orfB [Shigella flexneri 2a str. 2457T]

MSRQCTHYGRWPQHGFTSLKKLRPQSVTSRIQPGSDVIVCAEMDEHWGYVGAKSRQRWLFYAYDRIRRTV

VAHVFGERTLATLERLLSLLSAFEVVVWMTDGWPLYESRLKGKLHVISKRYTQRIERHNLKLRQHLARLG

RKSLSFSKSVELHDKVIGHYLNIKHYQ

>gi|30062875|ref|NP_837046.1| D-lactate dehydrogenase [Shigella flexneri 2a str. 2457T]

MKLAVYSTKQYDKKYLQQVNESFGFELEFFDFLLTEKTAKTANGCEAVCIFVNDDGSHPVLEELKKHGVK

YIALRCAGFNNVDLDAAKELGLKVVRVPAYDPEAVAEHAIGMMMTLNRRIHRAYQRTRDANFSLEGLTGF

TMYGKTAGVIGTGKIGVAMLRILKGFGMRLLAFDPYPSAAALELGVEYVDLPTLFSESDVISLHCPLTPE

NYHLLNEAAFDQMKNGVMIVNTSRGALIDSQAAIEALKNQKIGSLGMDVYENERDLFFEDKSNDVIQDDV

FRRLSACHNVLFTGHQAFLTAEALTSISQTTLQNLSNLEKGETCPNELV

>gi|30062874|ref|NP_837045.1| hypothetical protein S1458 [Shigella flexneri 2a str. 2457T]

MLGKYKAVLALLLLIILVPLTLLMTLGLWVPTLADIWLPLGTRIALDESPRITRKGLIIPDLRYLVGDCQ

LAHITNASLSHPSRWLLNVGTVELDSACLAKLPQTEQSPAAPKTLAQWQSMLPNTWINIDKLIFSPWQEW

QGKLSLALTSDIQQLRYQGEKVKFQGQLKGQQLTVSELDVVAFENQPPVKLVGEFTMPLVPDGLPVSGHA

TATLNLPQEPSLVDAELDWQENSGQLIVLARDNGDPLLDLPWQITRQQLTVSDGRWSWLYAGFPLSGRLG

VKVDNWQAGLENALVSGRLNVLTQGQAGKGNAVLNFGPGKLSMDNSQLPLQLTGEAKQADLILYARLPAQ

LSGSLSDPTLTFEPGALLRSKGRVIDSLDIDEIRWPLAGVKVTQRGVDGRLQAILQAHENELGDFVLHMD

GLANDFLPDAGRWQWRYWGKGSFTPMNATWDVAGKGEWHDSTITLTDLSTGFDQLQYGTMTVEKPRLILD

KPVVWVRDAQHPSFSGALSLDAGQTLFTGGSVLPPSTLKFSVDGRNPTYFLFKGDLHAGEIGPVRVNGRW

DGIRLRGNAWWPKQSLTVFQPLVPPDWKMNLRDGELYAQVAFSAAPEQGFRAGGHGVLKGGSAWMPDNQV

NGVDFVLPFRFADGAWHLGTRGPVTLRIAEVINLVTAKNITADLQGRYPWTEEEPLLLTDVSVDVLGGNV

LMKQLRMPQHDPALLRLNNLSSSELVSAVNPKQFAMSGAFSGALPLWLNNEKWIVKDGWLANSGLMTLRL

DKDTADAVVKDNMTAGSAINWLRYMEISRSSTKINLDNLGLLTMQANITGTSRVDGKSGTVNLNYHHEEN

IFTLWRSLRFGDNLQAWLEQNARLPGNDCPQGKECEEKQ

>gi|30062873|ref|NP_837044.1| hypothetical protein S1457 [Shigella flexneri 2a str. 2457T]

MKILLAALTSSFMLVGCTPRIEVAAPKEPITINMNVKIEHEIIIKADKDVEELLETRSDLF

>gi|30062872|ref|NP_837043.1| hypothetical protein S1456 [Shigella flexneri 2a str. 2457T]

MMKKTLLLCAFLVGLVSSNVMALTLDEARTQGRVGETFYGYLVALKTDAETEKLVTEINAERKASYQQRA

KQNNVSVDDIAKLAGQKLVARAKPGEYVQGINGKWVRKF

>gi|30062871|ref|NP_837042.1| IS1 orfA [Shigella flexneri 2a str. 2457T]

MASISIRCPSCSATEGVVRNGKSTAGHQRYLCSHCRKTWQLQFTYTASQPGTHQKIIDMAMNGVGCRASA

RIMGVGLNTVLRHLKNSGRSR

>gi|30062870|ref|NP_837041.1| IS1 orfB [Shigella flexneri 2a str. 2457T]

MSRQCTHYGRWPQHGFTSLKKLRPQSVTSRIQPGSDVIVCAEMDEHWGYVGAKSRQRWLFYAYDRIRRTV

VAHVFGERTLATLERLLSLLSAFEVVVWMTDGWPLYESRLKGKLHVISKRYTQRIERHNLKLRQHLARLG

RKSLSFSKSVELHDKVIGHYLNIKHYQ

>gi|30062869|ref|NP_837040.1| oxidoreductase, Fe-S subunit [Shigella flexneri 2a str. 2457T]

MITIDGNGAVASVTFRISEVIAIYPITPSSTMAEQADAWAGNGLKNVWGDTPRVVEMQSEAGAIATVHGA

LQTGALSTSFTSSQGLLLMIPTLYKLAGELTPFVLHVAARTVATHALSIFGDHSDVMAVRQTGCAMLCAA

NVQEAQDFALISHIATLKSRVPFIHFFDGFRTSHEINKIVPLADDTILDLMPQAEIDAHRARALNPEHPV

IRGTSANPDTYFQSREATNPWYNAVYDHVEQAMNDFAAATGRQYQPFEYYGHPQAERVIILMGSAIGTCE

EVVDELLTRGEKVGVLKVRLYRPFSAKHLLQALPGSVRSVAVLDRTKEPGAQAEPLYLDVMTALAEAFNN

GERETLPRVIGGRYGLSSKEFGPDCVLAVFAELNAAKPKARFTVGIYDDVTNLSLPLPENTLPNSAKLEA

LFYGLGSDGSVSATKNNIKIIGNSTPWYAQGYFVYDSKKAGGLTVSHLRVSEQPIRSAYLISQADFVGCH

QLQFIDKYQMAERLKPSGIFLLNTPYSADEVWSRLPQEVQAVLNQKKARFYVINAAKIARECGLAARINT

VMQMAFFHLTQILPGDSALAELQGAIAKSYSSKGQDLVERNWQALALARESVEEVPLQPVNPHSANRPPV

VSDAAPDFVKTVTAAMLAGLGDALPVSALPPDGTWPMGTTRWEKRNIAEEIPIWKEELCTQCNHCVAACP

HSAIRAKVVPPEAMENAPASLHSLDVKSRDMRGQKYVLQVAPEDCTGCNLCVEVCPAKDRQNPEIKAINM

MSRQEHVEEEKINYDFFLNLPEIDRSKLERIDIRTSQLITPLFEYSGACPGCGETPYIKLLTQLYGDRML

IANATGCSSIYGGNLPSTPYTTDANGRGPAWANSLFEDNAEFGLGFRLTVDQHRVRVLRLLDQFADKIPA

ELLTALKSDATPEVRREQVAALRQQLNDVAEAHELLRDADALVEKSIWLIGGDGWAYDIGFGGLDHVLSL

TENVNILVLDTQCYSNTGGQASKATPLGAVTKFGEHGKRKARKDLGVSMMMYGHVYVAQISLGAQLNQTV

KAIQEAEAYPGPSLIIAYSPCEEHGYDLALSHDQMRQLTATGFWPLYRFDPRRADEGKLPLALDSRPPSE

ALEETLLHEQRFRRLNSQQPEVAEQLWKDAAANLQKRYDFLAQMAGKAEKSNTD

>gi|30062868|ref|NP_837039.1| filament protein [Shigella flexneri 2a str. 2457T]

MNSVITQKVSSGVTLYADTKTGGFMNRTILVPIDISDSELTQRVISHVEAEAKIDDAEVHFLTVIPSLPY

YASLGLAYSAELPAMDDLKAEAKSQLEEIIKKFKLPTDRVHVHVEEGSPKDRILELAKKIPAHMIIIASH

RPDITTYLLGSNAAAVVRHAECSVLVVR

>gi|30062867|ref|NP_837038.1| C32 tRNA thiolase [Shigella flexneri 2a str. 2457T]

MSQNQEISKKEQYNLNKLQKRLRRNVGEAIADFNMIEEGDRIMVCLSGGKDSYTMLEILRNLQQSAPINF

SLVAVNLDQKQPGFPEHVLPEYLEKLGVEYKIVEENTYGIVKEKIPEGKTTCSLCSRLRRGILYRTATEL

GTTKIALGHHRDDILQTLFLNMFYGGKMKGMPPKLMSDDGKHIVIRPLAYCREKDIQRFADAKAFPIIPC

NLCGSQPNLQRQVIADMLRDWDKRYPGRIETMFSAMQNVVPSHLCDTNLFDFKGITHGSEVVNGGDLAFD

REEIPLQPAGWQPEEDENQLDELRLNVVEVK

>gi|30062866|ref|NP_837037.1| ATP-dependent RNA helicase DbpA [Shigella flexneri 2a str. 2457T]

MTAFSTLNVLPPAQLTNLNELGYLTMTPVQAAALPAILAGKDVRVQAKTGSGKTAAFGLGLLQQIDASLF

QTQALVLCPTRELADQVAGELRRLARFLPNTKILTLCGGQPFGMQRDSLQHAPHIIVATPGRLLDHLQKG

TVSLDALNTLVMDEADRMLDMGFSDAIDDVIRFAPASRQTLLFSATWPEAIAAISGRVQRDPLAIEIDST

DALPPIEQQFYETSSKGKIPLLQRLLSLHQPSSCVVFCNTKKDCQAVCDALNEVEQSALSLHGDLEQRDQ

TLVRFANGSARVLVATDVAARGLDIKSLELVVNFELAWDPEVHVHRIGRTARAGNSGLAISFCAPEEAQR

ANIISDMLQIKLNWQTPPANSSIVTLEAEMATLCIDGGKKAKMRPGDVLGALTGDIGLDGADIGKIAVHP

AHVYVAVRQAVAHKAWKQLQGGKIKGKTCRVRLLK

>gi|30062865|ref|NP_837036.1| zinc transporter [Shigella flexneri 2a str. 2457T]

MEAIKGSDVNVPDAVFAWMLDGRGGVKPLENTDVIDEAHPCWLHLNYVHHDSAQWLATTPLLPNNVRDAL

AGESTRPRVSRFGEGTLITLRCINGSTDERPDQLVAMRVYMDGRLIVSTRQRKVLALDDVVSDLEEGTGP
TDCGGWLVDVCDALTDHSSEFIEQLHDKIIDLEDNLLDQQIPPRGFLALLRKQLIVMRRYMAPQRDVYAR
LASERLPWMSDDQRRRMQDIADRLGRGLDEIDACIARTGVMADEIAQVMQENLARRTYTMSLMAMVFLPS
TFLTGLFGVNLGGIPGGGWQFGFSIFCILLVVLIGGVALWLHRSKWL

>gi|30062864|ref|NP_837035.1| secretion protein [Shigella flexneri 2a str. 2457T]
MVRAPIDGIVANRSAHTGSWVEGGTSLVSLVPVSELWVDANYKENQIAGMKPGMKAEIRADILKGEVFHG
HIESLSPATGASFSLIPIENATGNFTKIVQRVPVRIAFDDAKELKQLLRPGLSVTVSVDER

>gi|30062863|ref|NP_837034.1| IS1294 transposase [Shigella flexneri 2a str. 2457T]
MLSAFTPRPLKRLFTANQCWTSFLDAGGLRDIEVEAVTKMLACGTRILGVKEYICDKPECPHVRYVTNSC
GSRACPSCGKKATDLWIATQLNRLPDCDWVHLVFTLPDTQWPVFESNRWLLNDVCRLAVENLLYAARKRG
QEPGIFCAIHTYGRRLNWHPHVHVSVTCGGLNKHGQWKKLSFLKDAMRSRWMWNMRQRLLKAWSEGLAMP
ESLSHITTESQRRSLVLKAGGKYWHVYMSKKTAGGRNTARYLGRYLKKPPIAASRLAHYNGGGA

>gi|30062862|ref|NP_837033.1| IS1294 transposase [Shigella flexneri 2a str. 2457T]
MVRYFGFLANRVCGEKLPQVYRALGMDKPEPVAKVCYAQMVKQFLSRDPFECVLCGGRMVYRRAIAGLNV
EGLKKNARDISLLRYMPA

>gi|30062861|ref|NP_837032.1| secretion protein [Shigella flexneri 2a str. 2457T]
MIISKKQLIGVVAIGILLTGVVFFIWRVSKGRFIQTTDDAYIGGNITTVASKVSGYISAIEVRDNQSVKK
GDIILRLDDRDYRANVARLEAKIKSSKANLEGIQATITMQQSIIQSASETWQAVKHEEQKRLRDTERYEK
LAQSAAISQQIIDNARFDYQQVAAKERKAANDFLVEKQRLAVLSAQEENVRASIEEVQAALTQALLDLEY
TLVHV

>gi|30062860|ref|NP_837031.1| hypothetical protein S1430, partial [Shigella flexneri 2a str. 2457T]

MGVKMSLELPWPNDERLQQLCENLLNNQGYLPTLDNLADKINVSSRTLMRLFVKETGLTFRHWVQQMHVI

SAVTLLDDGYSLTKIAHRLGYASAESFGNMFKRRTGYSPGKFTRRLTMHNYAITRQMI

>gi|30062859|ref|NP_837030.1| O-6-alkylguanine-DNA:cysteine-protein methyltransferase [Shigella flexneri 2a str. 2457T]

MLRLLEEKIATPLGPLWVICDEQFRLRAVEWEEYSERMVQLLDIHYRKEGYERISATNPGGLSDKLREYF

AGNLSIIDTLPTATGGTPFQREVWKTLRTIPCGQVMHYGQLAEQLGRPGAARAVGAANGSNPISIVVPCH

RVIGRNGTMTGYAGGVQRKEWLLRHEGYLLL

>gi|30062858|ref|NP_837029.1| fumarate/nitrate reduction transcriptional regulator [Shigella flexneri 2a str. 2457T]

MIPEKRIIRRIQSGGCAIHCQDCSISQLCIPFTLNEHELDQLDNIIERKKPIQKGQTLFKAGDELKSLYA

IRSGTIKSYTITEQGDEQITGFHLAGDLVGFDAIGSGHHPSFAQALETSMVCEIPFETLDDLSGKMPNLR

QQMMRLMSGEIKGDQDMILLLSKKNAEERLAAFIYNLSRRFAQRGFSPREFRLTMTRGDIGNYLGLTVET

ISRLLGRFQKSGMLAVKGKYITIENNDALAQLAGHTRNVA

>gi|30062857|ref|NP_837028.1| universal stress protein UspE [Shigella flexneri 2a str. 2457T]

MAMYQNMLVVIDPNQDDQPALRRAVYLHQRIGGKIKAFLPIYDFSYEMTTLLSPDERTAMRQGVISQRTA

WIHEQAKYYLNAGVPIEIKVVWHNRPFEAIIQEVISGGHDLVLKMAHQHDRLEAVIFTPTDWHLLRKCPS

PVWMVKDQPWPEGGKALVAVNLASEEPYHNALNEKLVKETIELAEQVNHTEVHLVGAYPVTPINIAIELP

EFDPSVYNDAIRGQHLLAMKALRQKFGINENMTHVEKGLPEEVIPDLAEHLQAGIVVLGTVGRTGISAAF

LGNTAEQVIDHLRCDLLVIKPDQYQTPVELDDEEDD

>gi|30062856|ref|NP_837027.1| IS911 orfB [Shigella flexneri 2a str. 2457T]

MKELGLVSCQQPTHRYKRGGHEHVAIPNYLERQFAVTEPNQVWCGDVTYIWTGKRWAYLAVVLDLFARKP

VGWAMSFSPDSRLTMKALEMAWETRGKPGGVMFHSDQGSHYTSRQFRQLLWRYQNEPARKLLG

>gi|30062855|ref|NP_837026.1| IS91 orf [Shigella flexneri 2a str. 2457T]

MARSAKPRKRKPASQRSKLPRYVVKLHEDDFFDEEDAEVLRFDSFDDAVECCADLNIPFFVDAGNKKLVF

WFVRVDDEGYPEIARCTEREFATILAGISAGGMYCPECGTVHWPDGVAPPF

>gi|30062854|ref|NP_837025.1| IS91 orf, partial [Shigella flexneri 2a str. 2457T]

MLPRFADIFQQGNRWLNWLEKQPEGAVRPVVIESVTKIMACGTTLMGYTQWCCSSPDCCHTKKVCFRCKS

RSCPHCGVKAGAQWIQYLLSLVPYCPWQHIVFTLPCQYWSLVFHNRWLLAEMSRIAADVILEICHQTDVE

PGIFTVIHTWGRDQQWHPHIHLSTTAGGVTSGHTWKNLHFYARKVMSMWRYRITRLLSRNTLTW

>gi|30062853|ref|NP_837024.1| IS91 orf [Shigella flexneri 2a str. 2457T]

MPDALAAEGSSKREWNRFLDTHYRRGWNVNVSRVMDNATHVAVYFGSYLKKPPVPMSRLEHYAGQDEIGL

RYNSHRTKREEYLLMSGDEFMERFSWHVADKGFRMVRYYGFLSPAKRRLLEEVVYIITETVRKTAMQITW

RGMYQRLLKVDPLKCVLCGSQMRFTGLKRGYRLAEQVLMHEPLARMRWCG

>gi|30062852|ref|NP_837023.1| IS911 orfA [Shigella flexneri 2a str. 2457T]

MKKRNFSAEFKRESAQLVVDQKYTVADAAKAMDVGLSTMTRWVKQLRDERQGKTPKASPITPEQIEIREL

RKKLQRIEMENEILKKATALLMSDSLNSSR

>gi|30062851|ref|NP_837022.1| hypothetical protein S1425 [Shigella flexneri 2a str. 2457T]

MIAELFTNNALNLVIIFGSCAALILMSFWFRRGNRKRKGFLFHAVQFLIYTIIISAVGSISNYVIENYKL

KFITPGVIDFICTSLIAVILTIKLFLLINQFEKQQIKKGRDITSARIMSRIIKITIIVVLVLLYGEHFGM

SLSGLLTFGGIGGLAVGMAGKDILSNFFSGIMLYFDRPFSIGDWIRSPDRNIEGTVAEIGWRITKITTFD

NRPLYVPNSLFSSISVENPGRMTNRRITTTIGLRYEDAAKVGVIVEAVREMLKNHPAIDQRQTLLVYFNQ

FADSSLNIMVYCFTKTTVWAEWLAAQQDVYLKIIDIVQSHGADFAFPSQTLYMDNITPPEQGR

>gi|30062850|ref|NP_837021.1| transport periplasmic protein [Shigella flexneri 2a str. 2457T]

MSVRGKLMKHSVSVTCCALLVSSISLSYAAEVPSGTVLAEKQELVRHIKDEPASLDPAKAVGLPEIQVIR

DLFEGLVNQNEKGEIVPGVATQWKSNDNRIWTFTLRDNAKWADGTPVTAQDFVYSWQRLVDPKTLSPFAW

FAALAGINNAQAIIDGKATPDQLGVTAVDAHTLKIQLDKPLPWFVNLTANFAFFPVQKANVESGKEWTKP

GNLIGNGAYVLKERVVNEKLVVVPNTHYWDNAKTVLQKVTFLPINQESAATKRYLAGDIDITESFPKNMY

QKLLKDIPGQVYTPPQLGTYYYAFNTQKGPTADQRVRLALSMTIDRRLMTEKVLGTGEKPAWHFIPDVTA

GFTPEPSPFEQMSQEELNAQAKTLLSAAGYGPQKPLKLTLLYNTSENHQKIAIAVASMWKKNLGVDVKLQ

NQEWKTYIDSRNTGNFDVIRASWVGDYNEPSTFLTLLTSMHSGNISRFNNPAYDKVLAQASTENTVKARN

ADYNAAEKILMEQAPIAPIYQYTNGRLIKPWLKGYPINNPEDVAYSRTMYIVKH

>gi|30062849|ref|NP_837020.1| IS600 orfA [Shigella flexneri 2a str. 2457T]

MSRKTQRYSKEFKAEAVRTVLENQLSISEGASRLSLPEGTLGQWVTAARKGLGTPGSRTLAELESEILQL

RKALNEARLERDILKKATAYFAQESLKNTR

>gi|30062848|ref|NP_837019.1| IS600 orfB [Shigella flexneri 2a str. 2457T]

MAHIRTRETYGTRRLQTELAENGIIVGRDRLARLRKELRLRCKQKRKFRATTNPNHNLPVAPNLLNQTFA

PTAPNQVWVADLTYVATQEGWLYLAGIKDVYTCEIVGYAMGERMTKELTGKALFMALRSQRPPAGLIHHS

DRGSQYCAYDYRVIQEQSGLKTSMSRKGNCYDNAPMESFWGTLKNESLSHYRFNNRDEAISVIREYIEIF

YNRQRRHSRLGNISPAAFREKYHQMAA

>gi|30062847|ref|NP_837018.1| LYSR-type transcriptional regulator [Shigella flexneri 2a str. 2457T]

MKREEIADLMAFVVVAEERSFTRAAARLSMVQSALSQIVRRIEERLGLRLLTRTTRSVVPTEAGEHLLSV

LGPILHDIDSALTSLSDLQNRPSGTIRITTVEHAAKTILLPAMRTFLKSHPEIDIQLTIDYGLTDVVSER

FDAGVLRCTGNSGYYHLFFF

>gi|30062846|ref|NP_837017.1| ISSfl4 orf [Shigella flexneri 2a str. 2457T]

MTESSDYESVQVFIGVDVGKDTHHAVAINRSGKRLFDKALPNDENKLRSLISDLKQHGQILLVVDQPATI

GALPVAVARSEGVLVGYLPGLAMRRIAALHAGEAKTDARDAAIIAEAARTLPHALRTLKLADEQIAELSM

LCGFDDDLAAQTTQASNRIRGLLTQIHPALERVLGPRLEHPAVLDLLQRYPSPEKLASLGEKKLAAQLCK

LAPRLGKRLAADIAQALAEQTVVVPGTNAAAVVLPRLALQLITLRKQRDEVALEVEQRVLAHPLYPVLTS

MPGVGVRTAARLLTEVACRAFASAAHLAAYAGLAPVTRRSGSSIRGEHPSRRGNKALKRALFLSAFTASP

VRSPGLTTPAK

>gi|30062845|ref|NP_837016.1| hypothetical protein S1416 [Shigella flexneri 2a str. 2457T]

MKNVLILGAGGQIARHVINQLADKQTIKQTLFARQPAKIHKPYPTNSQIIMGDVLNHAALKQAMQGQDIV

YANLTGEDLDIQANSVIAAMKACDVKRLIFVLSLGIYDEVPGKFVEWNNAVIGEPLKPFRRAADAIEASG

LEYTILRPAWLTDEDIIDYELTSRNEPFKGTIVSRKSVAALITDIIDKPEKHIGENIGINQPGTDGDKPF

FM

>gi|30062844|ref|NP_837015.1| murein peptide amidase A [Shigella flexneri 2a str. 2457T]

MTVTRSRAERGAFPPGTEHYGRSLLGAPLIWFPAPAASRESGLILAGTHGDENSSVVTLSCALRTLTPSL

RRHHVVLCVNPDGCQLGLRANANGVDLNRNFPAANWKEGETVYRWNSAAEERDVVLLTGDKPGSEPETQA

LCQLIHRIQPAWVVSFHDPLACIEDPRHNELGEWLAQSFELPLVTSVGYETPGSFGSWCADLNLHCITAE

FPPISSDEASEKYLFAMANLLRWHPKDAIRPS

>gi|30062843|ref|NP_837014.1| muconate cycloisomerase I [Shigella flexneri 2a str. 2457T]

MRIVKVFEEAWPLHTPFVIARGSRSEARVVVVELEEEGIKGTGECTPYPRYGESDASVMAQIMSVVPQLE

KGLTREELQKILPAGAARNALDCALWDLAARKQQQSLADLIGITLPETVITAQTVVIGTPDQMANSASTL

WQAGAKLLKVKLDNHLISERMVAIRTAVPDATLIVDANESWRAEGLAARCQLLADLGVAMLEQPLPAQDD

AALENFIHPLPICADESCHTRSNLKALKGRYEMVNIKLDKTGGLTEALALATEARAQGFSLMLGCMLCTS

RAISAALPLVPQVSFADLDGPTWLAVDVEPALQFTTGELHL

>gi|30062842|ref|NP_837013.1| thiol peroxidase [Shigella flexneri 2a str. 2457T]

MSQTVHFQGNPVTVANSIPQAGSKAQTFTLVAKDLSDVTLGQFAGKRKVLNIFPSIDTGVCAASVRKFNQ

LATEIDNTVVLCISADLPFAQSRFCGAEGLNNVITLSTFRNAEFLQAYGVAIADGPLKGLAARAVVVIDE

NDNVIFSQLVDEITTEPDYEAALAVLKA

>gi|30062841|ref|NP_837012.1| DNA-binding transcriptional regulator TyrR [Shigella flexneri 2a str. 2457T]

MRLEVFCEDRLGLTRELLDLLVLRGIDLRGIEIDPIGRIYLNFAELEFESFSSLMAEIRRIAGVTDVRTV

PWMPSEREHLALSALLEALPEPVLSVDMKSKVDMANPASCQLFGQKLDRLRNHTAAQLINGFNFLRWLES

EPQDSHNEHVVINGQNFLMEITPVYLQDENDQHVLTGAVVMLRSTIRMGRQLQNVAAQDVSAFSQIVAVS

PKMKHVVEQAQKLAMLSAPLLITGDTGTGKDLFAYACHQASPRAGKPYLALNCASIPEDAVESELFGHAP

EGKKGFFEQANGGSVLLDEIGEMSPRMQAKLLRFLNDGTFRRVGEDHEVHVDVRVICATQKNLVELVQKG

VFREDLYYRLNVLTLNLPPLRDCPQDIMPLTELFVARFADEQGVPRPKLAADLNTVLTRYAWPGNVRQLK

NAIYRALTQLDGYELRPQDILLPDYDAATVAVGEDAMEGSLDEITSRFERSVLTQLYRNYPSTRKLAKRL

GVSHTAIANKLREYGLSQKKNEE

>gi|30062840|ref|NP_837011.1| hypothetical protein S1411 [Shigella flexneri 2a str. 2457T]

MTEPLKPRIDFDGPLEVEQNPKFRAQQTFDENQAQNFAPATLDEAQEEEGQVEAVMDAALRPKRSLWRKM

VMGGLALFGASVVGQGIQWTMNAWQTQDWVALGGCAAGALIIGAGVGSVVTEWRRLWRLRQRAHERDEAR

DLLHSHGTGKGRAFCEKLAQQAGIDQSHPALQRWYASIHETQNDREVVSLYAHLVQPVLDAQARREISRS

AAESTLMIAVSPLALVDMAFIAWRNLRLINRIATLYGIELGYYSRLRLFKLVLLNIAFAGASELVREVGM

DWMSQDLAARLSTRAAQGIGAGLLTVRLGIKAMELCRPLPWIDDDKPRLGDFRRQLIGQVKETLQKGKTP

SEK

>gi|30062839|ref|NP_837010.1| enzyme [Shigella flexneri 2a str. 2457T]

MKRLKNELNALVNRGVDRHLRLAVTGLSRSGKTAFITAMVNQLLNIHAGARLPLLSAVREERLLGVKRIP

QRDFGIPRFTYDEGLAQLYGDPPAWPTPTRGVSEIRLALRFKSNDSLLRHFKDTSTLYLEIVDYPGEWLL

DLPMLAQDYLSWSRQMTGLLNGQRGEWSAKWRMMCEGLDPLAPADENRLADIAAVWTDYLHHCKQQGLHF

IQPGRFVLPGDMAGAPALQFFPWPDVDAWDESKLAQADKHTNAGMLRERFNYYCEKVVKGFYKNHFLRFD

RQIVLVDCLQPLNSGPQAFNDMRLALTQLMQSFHYGQRTLFRRLFSPVIDKLLFAAMKADHVTVDQHANM

VSLLQQLIQDAWQNAAFERISMDCLGLASVQATTSGIIDVNGEKIPALRGNRLSDGALLTVYPGEVPARL

PGQAFWDKQGFQFEAFRPQVMDVDKPLPHIRLDAALEFLIGDKLR

>gi|30062838|ref|NP_837009.1| LACI-type transcriptional regulator [Shigella flexneri 2a str. 2457T]

MSPTIYDIARVAGVSKSTVSRVLNKQTNISPEAREKVLRAIEELQYQPNKLARALTSSGFDAIMVISTRS

TKTTAGNPFFSEVLHAITAKAEEEGFDVILQTSHNPAEDLQKCESKIKQKMIKGIIMLSSPADESFFAQL

DKYDIPVVVIGKVEGQYAHVYSVDTDNFGDSIALTDALIESGHQNIACLHAPLDVHVSVDRVNGYKQSLS

AHNIAVRDKWIVDGGYTHETALQAARQLLSQSPLPEAVFATDSLKLMSIYRAAAEKNIAIPQQLAVVGYS

NETLSFILTPAPGGIDVPTQELGQQSCELLFRLISGKPSPQNITVATHMTLK

>gi|30062837|ref|NP_837008.1| ATP-binding component of a transport system [Shigella flexneri 2a str. 2457T]

MAQLSLQHIQKIYDNQVHVVKDFNLEIADKEFIVFVGPSGCGKSTTLRMIAGLEEISGGDLLIDGKRMND

VPAKARNIAMVFQNYALYPHMTVYDNMAFGLKMQKIAKEVIDERVNWAAQILGLREYLKRKPGALSGGQR

QRVALGRAIVREAGVFLMDEPLSNLDAKLRVQMRAEISKLHQKLNTTMIYVTHDQTEAMTMAARIVIMKD

GIVQQVGAPKTVYNQPANMFVAGFIGSPAMNFIRGTIDGDKFVTETLKLTILEEKLAVLKTQESLHKPIV

MGIRPEDIHPDAQEENNISAKISVAELTGAEFMLYTTVGGTS

>gi|30062836|ref|NP_837007.1| beta-phosphoglucomutase [Shigella flexneri 2a str. 2457T]

MKLQGVIFDLDGVITDTAHLHFQAWQQIAAEIGISIDAQFNESLKGISRDESLRRILQHGGKEGDFNPQE

RAQLAYRKNLLYVHSLRELTVNAVLPGIRNLLAELRAQQIPVGLASVSLNAPTILAALELREFFTFCADA

SQLKNSKPDPEIFLAACAGLGVPPQACIGIEDAQAGIDAINASGMRSVGIGAGLTGAQLLLPSTDSLTWS

RLSAFWQNV

>gi|30062835|ref|NP_837006.1| hypothetical protein S1405 [Shigella flexneri 2a str. 2457T]

MTRPVTLSEPHFSQHTLNKYASLMAQGNGYLGLRASHEEDYTRQTRGMYLAGLYHRAGKGEINELVNLPD

ILGMEIAINGEVFSLSREAWQRELDFASGELRRSVVWRTSNGAGYTIASRRFVSADQLPLIALEITIMPL

DADASVLISTGIDATQTNHGRQHLDETQVRVFGQHLMQGIYTTQDGRSDVAISCCCQVSGDVQQCYTAKE

RRLLQHTSTQLHAGETVTLQKLVWIDWRDDRQAALDEWGSVSLRQLEMCAQQSYDQHLAASTENWRQWWQ

KRRITVNGGDAHDQQALDYALYHLRIMTPAHGERSSIAAKGLTGEGYKGHVFWDTEVFLLPFHLFSDPTV

ARSLLRYRWHNLPGAQEKARRNGWQGALFPWESARSGEEETPEFAAINIRTGLRQKVASAQAEHHLVADI

AWAVIQYWQTTGDESFIAHEGMALLLETAKFWISRAVRVNDRLEIHDVIGPDEYTEHVNNNAFTSYMVYY

NVQQALSIARQFGCSDDAFIHRAEMFLKELRLPEIQPDGVLPQDDSFMTKPAINLAKYKAAAGKQTILLD

YSRAEVNEMQILKQADVVMLNYMLPEQFSSASCLANLQFYEPRTIHDSSLSKAIHGIVAARCGLLTQSYQ

FWREGTEIDLGADPHSCDDGIHAAATGAIWLGAIQGFAGVSVRDGELHLNPALLEQWQQLSFPLFWQGCE

LQVTLDAQRIAIRTSAPVSLRLDGQLISVAEESVFCLGDFILPFNGTATTHQEDE

>gi|30062834|ref|NP_837005.1| dehydrogenase [Shigella flexneri 2a str. 2457T]

MKSAMTSSPLRVAIIGAGQVADKVHASYYCTRNDLELVAVCDSRLSQAQALAEKYGNASVWDDPQAMLLA

VKPDVVSVGSPNRFHYEHTLMALEAGCHVMCEKPPAMTTEQAWEMCDTARKLGKVLAYDFHHRFALDTQQ

LREQVTNGVLGEIYVTTARALRRCGVPGWGVFTNKELQGGGLLIDIGIHMLDAAMYVLGFPAVKSVNAHS

FQKIGTQKSCGQFGEWDPATYSVEDSLFGTIEFHNGGILWLETSFALNIREQLIMNVSFCGDKAGATLFP

AHIYTDNNGELMTLMQREMADDNRHLRSMEAFINHVQGKPVMIADAEQGYIIQQLVAALYQSAETGTRVE

L

>gi|30062833|ref|NP_837004.1| oxidoreductase [Shigella flexneri 2a str. 2457T]

MKKLVATAPRVAALVEYEDRAILANEVKIRVRFGAPKHGTEVVDFRAASPFIDEDFNGEWQMFTPRPADA

PRGIEFGKFQLGNMVVGDIIECGSDVTDYAVGDSVCGYGPLSETVIINAVNNYKLRKMPQGSSWKNAVCY

DPAQFAMSGVRDANVRVGDFVVVVGLGAIGQIAIQLAKRAGASVVIGVDPIAHRCDIARRHGADFCLNPI

GTDVGKEIKTLTGKQGADVIIETSGYADALQSALRGLAYGGTISYVAFAKPFAEGFNLGREAHFNNAKIV

FSRACSEPNPDYPRWSRKRIEETCWELLMNGYLNCEDLIDPVVTFANSPESYMQYVDQHPEQSIKMGVTF

>gi|30062832|ref|NP_837003.1| transport system permease [Shigella flexneri 2a str. 2457T]

MATNKRTLSRIGFYCGLALFLIITLFPFFVMLMTSFKSAKEAISLHPTLLPQQWTLEHYVDIFNPMIFPF

VDYFRNSMVVSVVSSVVAVFLGILGAYALSRLRFKGRMTINASFYTVYMFSGILLVVPLFKIITALGIYD

TEMALIITMVTQTLPTAVFMLKSYFDTIPDEIEEAAMMDGLNRLQIIFRITVPLAMSGLISVFVYCFMVA

WNDYLFASIFLSSASNFTLPVGLNALFSTPDYIWGRMMAASLVTALPVVIMYALLERFIKSGLTAGGVKG

>gi|30062831|ref|NP_837002.1| binding-protein dependent transport protein [Shigella flexneri 2a str. 2457T]

MNRLFSGRSDMPFALLLLAPSLLLLGGLVAWPMVSNIEISFLRLPLNPNIESTFVGVSNYVRILSDPGFW

HSLWMTVWYTALVVAGSTVLGLAVAMFFNREFRLRKTARSLVILSYVTPSISLVFAWKYMFNNGYGIVNY

LGVDLLHLYEQAPLWFDNPGSSFVLVVLFAIWRYFPYAFISFLAILQTIDKSLYEAAEMDGANAWQRFRI

VTLPAIMPVLATVVTLRTIWMFYMFADVYLLTTKVDILGVYLYKTAFAFNDLGKAAAISVVLFIIIFAVI

LLTRKRVNLNGNK

>gi|30062830|ref|NP_837001.1| IS1 orfB [Shigella flexneri 2a str. 2457T]

MSRQCTHYGRWPQHGFTSLKKLRPQSVTSRIQPGSDVIVCAEMDEHWGYVGAKSRQRWLFYAYDRIRRTV

VAHVFGERTLATLERLLSLLSAFEVVVWMTDGWPLYESRLKGKLHVISKRYTQRIERHNLNLRQHLARLV

RKSLSFSKSVELHDKVIGHYLNIKHYQ

>gi|30062829|ref|NP_837000.1| IS1 orfA [Shigella flexneri 2a str. 2457T]

MASISIRCPSCSATEGVVRNGKSTAGHQRYLCSHCRKTWQLQFTYTASQPGTHQKIIDMAMNGVGCRASA

RIMGVGLNTVLRHLKNSGRSR

>gi|30062828|ref|NP_836999.1| thiosulfate:cyanide sulfurtransferase [Shigella flexneri 2a str. 2457T]

MAIFIHRRTLTCLKKGLLALALVFSLPVFAAEHWIDVRVPEQYQQEHVQGAINIPLKEVKERIATAVPDK

NDTVKVYCNAGRQSGQAKELLSEMGYTHVENAGGLKDIAMPKVKG

>gi|30062827|ref|NP_836998.1| peripheral inner membrane phage-shock protein [Shigella flexneri 2a str. 2457T]

MNTRWQQAGQKVKLGFKLAGKLVLLTALRYGPAGVAGWAIKSVARRPLKMLLAVALEPLLSRAANKLAQR

YKR

>gi|30062826|ref|NP_836997.1| DNA-binding transcriptional activator PspC [Shigella flexneri 2a str. 2457T]

MAGINLNKKLWRIPQQGMVRGVCAGIANYFDVPVKLVRILVVLSIFFGLALFTLVAYIILSFALDPMPDN

MAFGEQLPSSSELLDEVDRELAASETRLREMERYVTSDTFTLRSRFRQL

>gi|30062825|ref|NP_836996.1| phage shock protein B [Shigella flexneri 2a str. 2457T]

MSALFLAIPLTIFVLFVLPIWLWLHYSNRSGRSELSQSEQQRLAQLVDEAKRMRERIQALESILDAEHPN

WRDR

>gi|30062824|ref|NP_836995.1| phage shock protein PspA [Shigella flexneri 2a str. 2457T]

MGIFSRFADIVNANINALLEKAEDPQKLVRLMIQEMEDTLVEVRSTSARALAEKKQLTRRIEQASAREVE

WQEKAELALLKEREDLARAALIEKQKLTDLIKSLEHEVTLVDDTLARMKKEIGELENKLSETRARQQALM

LRHQAANSSRDVRRQLDSGKLDEAMARFESFERRIDQMEAEAESHSFGKQKSLDDQFAELKADDAISEQL

AQLKAKMKQDNQ

>gi|30062822|ref|NP_836993.1| 4-aminobutyrate transaminase [Shigella flexneri 2a str. 2457T]

MSNNEFHQRRLSATPRGVGVMCNFFAQSAENATLKDVEGNEYIDFAAGIAVLNTGHRHPDLVAAVEQQLQ

QFTHTAYQIVPYESYVTLAEKINTLAPVSGQAKTAFFTTGAEAVENAVKIARAHTGRPGVIAFSGGFHGR

TYMTMALTGKVAPYKIGFGPFPGSVYHVPYPSDLHGISTQDSLDAIERLFKSDIEAKQVAAIIFEPVQGE

CGFNVAPKELVAAIRRLCDEHGIVMIADEVQSGFARTGKLFAMDHYADKPDLMTMAKSLAGGMPLSGVVG

NANIMDAPAPGGLGGTYAGNPLAVAAAHAVLNIIDKESLCERANQLGQRLKNTLIDAKESVPAIAAVRGL

GSMIAAEFNDPQTGEPSAAIAQKIQQRALAQGLLLLTCGAYGNVIRFLYPLTIPDAQFDAAMKILQDALS

D

>gi|30062821|ref|NP_836992.1| oxidoreductase [Shigella flexneri 2a str. 2457T]

MTEHTSSYYAASANKYAPFDTLNESITCDVCVVGGGYTGLSSALHLAEAGFDVVVLEASRIGFGASGRNG

GQLVNSYSRDIDVIEKSYGMDTARMLGSMMFEGGEIIRERIKRYQIDCDYRPGGLFVAMNDKQLATLEEQ

KENWVRYGNKQLELLDVNAIRREVASDRYTGALLDHSGGHIHPLNLAIGEADAIRLNGGRVYELSAVTQI

QHTTPAVVRTANGQVTAKYVIVAGNAYLGDKVEPELAKRSMPCGTQVITTERLSEDLARSLIPKNYCVED

CNYLLDYYRLTADNRLLYGGGVVYGARNPDDVERLVVPKLLKTFPQLKGVKIDYRWTGNFLLTLSHMPQF

GRLDTNIYYMQGYSGHGVTCTHLAGRLIAELLRGDAERFDAFANLPHYPFPGGRTLRVPFTAMGAAYYSL

RDRLGV

>gi|30062820|ref|NP_836991.1| gamma-glutamyl-gamma-aminobutyraldehyde dehydrogenase [Shigella flexneri 2a str. 2457T]

MNFHHLAYWQDKALSLAIENRLFINGEYTAAAENETFETVEPVTQAPLAKIARGKSVDIDRAVSAARGVF

ERGDWSLSSPAKRKAVLNKLADLMEAHAEELALLETLDTGKPIRHSLRDDIPGAARAIRWYAEAIDKVYG

EVATTSSHELAMIVREPVGVIAAIVPWNFPLLLTCWKLGPALAAGNSVILKPSEKSPLSAIRLAGLAKEA

GLPDGVLNVVTGFGHEAGQALSRHNDIDAIAFTGSTRTGKQLLKDAGDSNMKRVWLEAGGKSANIVFADC

PDLQKAASATAAGIFYNQGQVRIAGTRLLLEESIADEFLALLKQQAQNWQPGHPLDPATTMGTLIDSAHA

DSVHTFIREGEGKGQLLLDGRNAGLAAAIGPTIFVDVDPNASLSREEIFGPVLVVTRFTSEDQALQLAND

SQYGLGAAVWTRDLSRAHRMSRRLKAGSVFVNNYNDGDMTVPFGGYKQSGNGRDKSLHALEKFTELKTIW

ISLEA

>gi|30062819|ref|NP_836990.1| DNA-binding transcriptional repressor PuuR [Shigella flexneri 2a str. 2457T]

MSDEGLAPGKRLSEIRQQQGLSQRRAAELSGLTHSAISTIEQDKVSPAISTLQKLLKVYGLSLSEFFSEP

EKPDEPQVVINQDDLIEMGSQGVSMKLVHNGNPNRTLAMIFETYQPGTTTGERIKHQGEEIGTVLEGEIV

LTINGQDYHLVAGQSYAINTGIPHSFSNTSAGICRIISAHTPTTF

>gi|30062818|ref|NP_836989.1| gamma-glutamyl-gamma-aminobutyrate hydrolase [Shigella flexneri 2a str. 2457T]

MRLSMENIMNNPVIGVVMCRNRLKGHATQTLQEKYLNAIIHAGGLPIALPHALAEPSLLEQLLPKLDGIY

LPSSPSNVQPHLYGENGDEPDADPGRDLLSMAIINAALERRIPIFAICRGLQELVVATGGSLHRKLCEQP

ELLEHREDPELPVEQQYAPSHEVQVEEGGLLSALLPECSNFWVNSLHGQGAKVVSPRLRVEARSPDGLVE

AVSVINHPFALGVQWHPEWNSSEYALSRILFEGFITAWQHHIAEKQRL

>gi|30062817|ref|NP_836988.1| glutamine synthetase [Shigella flexneri 2a str. 2457T]

METNIVEVENFVQQSEERRGSAFTQEVKRYLERYPNTQYVDVLLTDLNGCFRGKRIPVSSLKKLEKGCYF

PASVFAMDILGNVVEEAGLGQEMGEPDRTCVPVLGSLTPSAADPEFIGQMLLTMVDEDGAPFDVEPRNVL

NRLWQQLRQRGLFPVVAVELEFYLLDRQRDAEGDLQPPCAPGTDDRNTQSQVYSVDNLNHFADVLNDIDE

LAQLQLIPADGAVAEASPGQFEINLYHTDNVLEACDDALALKRLVRLMAEKHKMHATFMAKPYEEHAGSG

MHIHISMQNNRGENVLSDTEGEDSPLLKKMLAGMIDLMPSSMALLAPNVNSYRRFQPGMYVPTQASWGHN

NRTVALRIPCGDRHNHRVEYHVAGADANPYLVMAAIFAGILHGLDNELPLQEEVEGNGLEQEGLPFPIRQ

SDALGEFIENDHLRRYLGERFCHVYHACKNDELLQFERLITETEIEWMLKNA

>gi|30062816|ref|NP_836987.1| amino acid/amine transport protein [Shigella flexneri 2a str. 2457T]

MAINSPLNIAAQPGKTRLRKSLKLWQVVMMGLAYLTPMTVFDTFGIVSGISDGHVPASYLLALAGVLFTA

ISYGKLVRQFPEAGSAYTYAQKSINPHVGFMVGWSSLLDYLFLPMINVLLAKIYLSALFPEVPPWVWVVT

FVAILTAANLKSVNLVANFNTLFVLVQISIMVVFIFLVVQGLHKGEGVGTVWSLQPFISENAHLIPIITG

ATIVCFSFLGFDAVTTLSEETPDAARVIPKAIFLTAVYGGVIFIAASFFMQLFFPDISRFKDPDAALPEI

ALYVGGKLFQSIFLCTTFVNTLASGLASHASVSRLLYVMGRDNVFPERVFGYVHPKWRTPALNVIMVGIV

ALSALFFDLVTATALINFGALVAFTFVNLSVFNHFWRRKGMNKSWKDHFHYLLMPLVGALTVGVLWVNLE

STSLTLGLVWASLGGAYLWYLIRRYRKVPLYEGDRTPVSET

>gi|30062815|ref|NP_836986.1| peptide transport periplasmic protein [Shigella flexneri 2a str. 2457T]

MRQVLSSLLVIAGLVSGQAIAAPESPPHADIRDSGFVYCVSGQVNTFNPSKASSGLIVDTLAAQFYDRLL

DVDPYTYRLMPELAESWEVLDNGATYRFHLRRDVPFQKTDWFTPTRKMNADDVVFTFQRIFDRNNPWHNV

NGSNFPYFDSLQFADNVKSVRKLDNHTVEFRLAQPDASFLWHLATHYASVMSAEYARKLEKEDRQEQLDR

QPVGTGPYQLSEYRAGQFIRLQRHDDFWRGKPLMPQVVVDLGSGGTGRLSKLLTGECDVLAWPAASQLSI

LRDDPRLRLTLRPGMNVAYLAFNTAKPPLNNPAVRHALALAINNQRLMQSIYYGTAETAAFILPRASWAY

DNEAKITEYNPAKSREQLKALGLENLTLKLWVPTRSQAWNPSPLKTAELIQADMAQVGVKVVIVPVEGRF

QEARLMDMSHDLTLSGWATDSNDPDSFFRPLLSCAAIHSQTNLAHWCNPKFDSVLRKALSSQQLAARIEA

YDEAQSILAQELPILPLASSLRLQAYRYDIKGLVLSPFGNASFAGVYREKQDEVKKP

>gi|30062814|ref|NP_836985.1| peptide transport permease [Shigella flexneri 2a str. 2457T]

MIIFTLRRILLLIVTLFLLTFVGFSLSYFTPHAPLQGASLWNAWVFWFNGLIHWDFGVSSINGQPIAEQL

KELFPATIELCILAFGFALIVGIPVGMIAGITRHKWQDNLINAIALLGFSIPVFWLALLLTLFCSLTLGW

LPVSGRFDLLYEVKPITGFALIDAWLSDSPWRDEMIMSAIRHMILPVITLSVAPTTEVIRLMRISTIEVY

DQNYVKAAATRGLSRFTILRRHVLHNALPPVIPRLGLQFSTMLTLAMITEMVFSWPGLGRWLINAIRQQD

YAAISAGVMVCGSLVIIVNVISDILGAMANPLKHKEWYALR

>gi|30062813|ref|NP_836984.1| peptide transport permease [Shigella flexneri 2a str. 2457T]

MPYDSVYSEKRPPGTLRTAWRKFYSDASAMVGLYGCAGLAVLCIFGGWFAPYGIDQQFLGYQLLPPSWSR

YGEVSFFLGTDDLGRDVLSRLLSGAAPTVGGAFVVTLAATICGLVLGTFAGATHGLRSAVLNHILDTLLA

IPSLLLAIIVVAFAGPSLSHAMFAVWLALLPRMVRSIYSMVHDELEKEYVIAARLDGASTLNILWFAVMP

NITAGLVTEITRALSMAILDIAALGFLDLGAQLPSPEWGAMLGDALELIYVAPWTVMLPGAAIMISVLLV

NLLGDGVRRAIIAGVE

>gi|30062812|ref|NP_836983.1| ATP-binding protein of peptide transport system [Shigella flexneri 2a str. 2457T]

MPLLDIRNLTIEFKTGDEWVKAVDRVSMTLTEGEIRGLVGESGSGKSLIAKAICGVNKDNWRVTADRMRF

DDIDLLRLSARERRKLVGHNVSMIFQEPQSCLDPSERVGRQLMQNIPAWTYKGRWWQRFGWRKRRAIELL

HRVGIKDHKDAMRSFPYELTEGECQKVMIAIALANQPRLLIADEPTNSMEPTTQAQIFRLLTRLNQNSNT

TILLISHDLQMLSQWADKINVLYCGQTVETAPSKELVTMPHHPYTQALIRAIPDFGSAMPHKSRLNTLPG

AIPLLEQLPIGCRLGPRCPYAQRECIVTPRLTGAKNHLYACHFPLNMEKE

>gi|30062811|ref|NP_836982.1| ATP-binding protein of peptide transport system [Shigella flexneri 2a str. 2457T]

MIETLLEVRNLSKTFRYRTGWFRRQTVEAVKPLSFTLREGQTLAIIGENGSGKSTLAKMLAGMIEPTSGE

LLIDDHPLHFGDYSFRSQRIRMIFQDPSTSLNPRQRISQILDFPLRLNTDLEPEQRRKQIIETMRMVGLL

PDHVSYYPHMLAPGQKQRLGLARALILRPKVIIADEALASLDMSMRSQLINLMLELQEKQGISYIYVTQH

IGMMKHISDQVLVMHQGEVVERGSTADVLASPLHELTKRLIAGHFGEALTADAWRKDR

>gi|30062810|ref|NP_836981.1| hypothetical protein S1376 [Shigella flexneri 2a str. 2457T]

MMDKIKSNARDLRRNLTLQERKLWRYLRSRRFGDFKFRRQHPVGSYILDFACCSARVVVELDGGQHDLAV

AYDTRRTSWLESQGWTVLRFWNNEIDCNEEAVLEIILQELNRRSPSP

>gi|30062809|ref|NP_836980.1| enoyl-(acyl carrier protein) reductase [Shigella flexneri 2a str. 2457T]

MGFLSGKRILVTGVASKLSIAYGIAQAMHREGAELAFTYQNDKLKGRVEEFAAQLGSDIVLQCDVAEDAS

IDTMFAELGKVWPKFDGFVHSIGFAPGDQLDGDYVNAVTREGFKIAHDISSYSFVAMAKACRSMLNPGSA

LLTLSYLGAERAIPNYNVMGLAKASLEANVRYMANAMGPEGVRVNAISAGPIRTLAASGIKDFRKMLAHC

EAVTPIRRTVTIEDVGNSAAFLCSDLSAGISGEVVHVDGGFSIAAMNELELK

>gi|30062808|ref|NP_836979.1| oxidoreductase [Shigella flexneri 2a str. 2457T]

MEQRHITGKSHWYHETQSSTTEYDVLPLVPEAAKVSDPFLLDVILDEETLAPFLSWLVPARVLAVELFPD

QLTVTRSQTFTAYERLSTALTVAQVCGVQRLCNYYSARLTPLPGPDSSRESNHRLAQITQYARQLASSPS

IIDNRSRQHLNDVGLTVWDCVIINQIIGFIGFQARTIATFQAYLGHPVRWLPGLAIQNYADASLFADESI

RWRSSYEVEKLPEEHTKSSTAELCQLAETLSLHPISLSLLEKLLNSTRVNAQPDNQLAALLCARINGSPA

CFSTCMDSSNEYKKISTLLRKGENEINRWADRHSVERATVQAIQWLTRAPDRFSAAQFSPLLEHEKSSTQ

IINLLVWSGLCGWINRLKIALGETY

>gi|30062807|ref|NP_836978.1| exoribonuclease II [Shigella flexneri 2a str. 2457T]

MFQDNPLLAQLKQQLHSQTPRAEGVVKATEKGFGFLEVDAQKSYFIPPPQMKKVMHGDRIIAVIHSEKER

ESAEPEELVEPFLTRFVGKVQGKNDRLTIVPDHPLLKDAIPCRAARGLNHEFKEGDWAVAEMRRHPLKGD

RSFYAELTQYITFGDDHFVPWWVTLARHNLEKEAPDGVATEMLDEGLVREDLTSLDFVTIDSASTEDMDD

ALFAKALPDDKLQLIVAIADPTAWIAEGSKLDKAAKIRAFTNYLPGFNIPMLPRELSDDLCSLRANEVRP

VLACRMTLSADGTIEDNIEFFAATIESKAKLVYDQVSDWLENTGDWKPESEAIAEQVRLLAQICQRRGEW

RHNHALVFKDRPDYRFILGEKGEVLDIVAEPRRIANRIVEEAMIAANICAARVLRDKLGFGIYNVHMGFD

PANADALAALLKTHGLHVDAEEVLTLDGFCKLRRELDAQPTGFLDSRIRRFQSFAEISSEPGPHFGLGLE

AYATWTSPIRKYGDMINHRLLKAVIKGETATRPQDEITVQMAERRRLNRMAERDVGDWLYARFLKDKAGT

GTRFAAEIVDISRGGMRVRLVDNGAIAFIPAPFLHAVRDELVCSQENGTVQIKGETVYKVTDVIDVTIAE

VRMETRSIIARPVA

>gi|30062806|ref|NP_836977.1| DEOR-type transcriptional regulator [Shigella flexneri 2a str. 2457T]

MNSRQQTILQMVIDQGQVSVTDLAKATGVSEVTIRQDLNTLEKLSYLRRAHGFAVSLDSDDVETRMMSNY

TLKRELAEFAASLVQPGETIFIENGSSNALLARTLGEQKKNVTIITVSSYIAHLLKDAPCEVILLGGVYQ

KKSESMVGPLTRQCIQQVHFSKAFIGIDGWQPETGFTGRDMMRTDVVNAVLEKECEAIVLTDSSKFGAVH

SYSIGPVERFNRVITDSKIRASDLMHLEHSKLTVHVVDI

>gi|30062805|ref|NP_836976.1| lipoprotein [Shigella flexneri 2a str. 2457T]

MFVTSKKMTAAVLAITLAMSLSACSNWSKRDRNTAIGAGAGALGGAVLTDGSTLGTLGGAAVGGVIGHQV

GK

>gi|30062804|ref|NP_836975.1| translation initiation factor Sui1 [Shigella flexneri 2a str. 2457T]

MMSDSNSRLVYSTETGRIDEPKAAPVRPKGDGAVRIQRQTSGRKGKGVCLITGIDLDDAELTKLAAELKK

KCGCGGAVKDGVIEIQGDKRDLLKSLLEAKGMKVKLAGG

>gi|30062803|ref|NP_836974.1| orotidine 5'-phosphate decarboxylase [Shigella flexneri 2a str. 2457T]

MTLTASSSSRAVTNSPVVVALDYHNCDDALSFVDKIDPRDCRLKVGKEMFTLFGPQFVRELQQRGFDIFL

DLKFHDIPNTAAHAVAAAADLGVWMVNVHASGGARMMTAAREALVPFGKDAPLLIAVTVLTSMEASDLAD

LGVTLSPADYAERLAALTQKCDLDGVVCSAQEAVRFKQVFGQEFKLVTPGIRPQGSEAGDQRRIMTPEQA

LAAGVDYMVIGRPVTQSVDPAQTLKAINASLQRSA

>gi|30062802|ref|NP_836973.1| tetratricopeptide repeat protein [Shigella flexneri 2a str. 2457T]

MLELLFLLLPVAAAYGWYMGRRSAQQNKQDEANRLSRDYVAGVNFLLSNQQDKAVDLFLDMLKEDTGTVE

AHLTLGNLFRSRGEVDRAIRIHQTLMESASLTYEQRLLAIQQLGRDYMAAGLYDRAEDMFNQLTDETDFR

IGALQQLLQIYQATSEWQKAIDVAERLVKLGKDKQRVEIAHFYCELALQHMASDDLDRAMTLLKKGAAAD

KNSARVSIMMGRVFMAKGEYAKAVESLQRVISQDRELVSETLEMLQTCYQQLGKTAEWAEFLQRAVEENT

GADAELMLADIIEARDGSEAAQVYITRQLQRHPTMRVFHKLMDYHLNEAEEGRAKESLMVLRDMVGEKVR

SKPRYSCQKCGFTAYTLYWHCPSCRAWSTIKPIRGLDGL

>gi|30062801|ref|NP_836972.1| hypothetical protein S1366 [Shigella flexneri 2a str. 2457T]

MKYLLIFLLVLAIFVISVTLGAQNDQQVTFNYLLAQGEYRISTLLAVLFAAGFAIGWLICGLFWLRVRVS

LARAERKIKRLENQLSPATDVAVVPHSSAAKE

>gi|30062800|ref|NP_836971.1| phosphatidylglycerophosphatase B [Shigella flexneri 2a str. 2457T]

MRSIARRTAVGAALLLVMPVAVWISGWRWQPGEQSWLLKAAFWVTETVTQPWGVITHLILFGWFLWCLRF

RIKAAFVLFAILAAAILVGQGVKSWIKDKVQEPRPFVIWLEKTHHIPVDEFYTLKRAERGNLVKEQLAEE

KNIPQYLRSHWQKETGFAFPSGHTMFAASWALLAVGLLWPRRRTLTIAILLVWATGVMGSRLLLGMHWPR

DLVVATLISWALVAVATWLAQRICGPLTPPAEENREIAQREQES

>gi|30062799|ref|NP_836970.1| GTP cyclohydrolase II [Shigella flexneri 2a str. 2457T]

MQLKRVAEAKLPTPWGDFLMVGFEELATGHDHVALVYGDISGHTPVLARVHSECLTGDALFSLRCDCGFQ

LEAALTQIAEEGRGILLYHRQEGRNIGLLNKIRAYALQDQGYDTVEANHQLGFAADERDFTLCADMFKLL

GVNEVRLLTNNPKKVEILTEAGINIVERVPLIVGRNPNNEHYLDTKAEKMGHLLNK

>gi|30062798|ref|NP_836969.1| aconitate hydratase [Shigella flexneri 2a str. 2457T]

MSSTLREASKDTLQAKDKTYHYYSLPLAAKSLGDITRLPKSLKVLLENLLRWQDGNSVTEEDIHALAGWL

KNAHADREIAYRPARVLMQDFTGVPAVVDLAAMREAVKRLGGDTAKVNPLSPVDLVIDHSVTVDRFGDDE

AFEENVRLEMERNHERYVFLKWGKQAFSRFSVVPPGTGICHQVNLEYLGKAVWSELQDGEWIAYPDTLVG

TDSHTTMINGLGVLGWGVGGIEAEAAMLGQPVSMLIPDVVGFKLTGKLREGITATDLVLTVTQMLRKHGV

VGKFVEFYGDGLDSLPLADRATIANMSPEYGATCGFFPIDAVTLDYMRLSGRSEDQVELVEKYAKAQGMW

RNPGDEPIFTSMLELDMNDVEASLAGPKRPQDRVALPDVPKAFAASNELKVNATHKDRLPVDYVMNGHQY

QLPDGAVVIAAITSCTNTSNPSVLMAAGLLAKKAVTLGLKRQPWVKASLAPGSKVVSDYLAKAKLTPYLD

ELGFNLVGYGCTTCIGNSGPLPDPIETAIKKGDLTVGAVLSGNRNFEGRIHPLVKTNWLASPPLVVAYAL

AGNMNINLASEPIGHDRKGEPVYLKDIWPSAQEIARAVEQVSTEMFRKEYAEVFEGTAEWKEINVTRSDT

YGWQEDSTYIRLSPFFDEMQATPAPVEDIHGARILAMLGDSVTTDHISPAGSIKPDSPAGRYLQGRGVER

KDFNSYGSRRGNHEVMMRGTFANIRIRNEMVPGVEGGMTRHLPDSDVVSIYDAAMRYKQEQTPLAVIAGK

EYGSGSSRDWAAKGPRLLGIRVVIAESFERIHRSNLIGMGILPLEFPQGVTRKTLGLTGEEKIDIGDLQN

LQPGATVPVTLTRADGSQEVVPCRCRIDTATELTYYQNDGILHYVIRNMLK

>gi|30062797|ref|NP_836968.1| transcriptional regulator CysB [Shigella flexneri 2a str. 2457T]

MKLQQLRYIVEVVNHNLNVSSTAEGLYTSQPGISKQVRMLEDELGIQIFSRSGKHLTQVTPAGQEIIRIA

REVLSKVDAIKSVAGEHTWPDKGSLYIATTHTQARYALPNVIKGFIERYPRVSLHMHQGPPTQIADAVSK

GNADFAIATEALHLYEDLVMLPCYHWNRAIVVTPDHPLAGKKAITIEELAQYPLVTYTFGFTGRSELDTA

FNRAGLTPRIVFTATDADVIKTYVRLGLGVGVIASMAVDPVADPDLVRVDAHDIFSHSTTKIGFRRSTFL

RSYMYDFIQRFAPHLTRDVVDAAVALRSNEEIEVMFKDIKLPEK

>gi|30062796|ref|NP_836967.1| DNA topoisomerase I [Shigella flexneri 2a str. 2457T]

MGKALVIVESPAKAKTINKYLGCDYVVKSSVGHIRDLPTSGSAAKKSADSTSTKTAKKPKKDERGALVNR

MGVDPWHNWEAHYEVLPGKEKVVSELKQLAEKADHIYLATDLDREGEAIAWHLREVIGGDDARYSRVVFN

EITKNAIRQAFNKPGELNIDRVNAQQARRFMDRVVGYMVSPLLWKKIARGLSAGRVQSVAVRLVVERERE

IKAFVPEEFWEVDASTTTPSGEALALQVTHQNDKPFRPVNKEQTQAAVSLLEKARYSVLEREDKPTTSKP

GAPFITSTLQQAASTRLGFGVKKTMMMAQRLYEAGYITYMRTDSTNLSQDAVNMVRGYISDNFGKKYLPE
SPNQYASKENSQEAHEAIRPSDVNVMAESLKDMEADAQKLYQLIWRQFVACQMTPAKYDSTTLTVGAGDF
RLKARGRILRFDGWTKVMPALRKGDEDRILPAVDKGDALTLVELTPAQHFTKPPARFSEASLVKELEKRG
IGRPSTYASIISTIQDRGYVRVENRRFYAEKMGEIVTDRLEENFRELMNYDFTAQMENSLDQVANHEAEW
KAVLDHFFSDFTQQLDKAEKDPEEGGMRPNQMVLTSIDCPTCGRKMGIRTASTGVFLGCSGYALPPKERC
KTTINLVPENEVLNVLEGEDAETNALRAKRRCPKCGTAMDSYLIDPKRKLHVCGNNPTCDGYEIEEGEFR
IKGYDGPIVECEKCGSEMHLKMGRFGKYMACTNEECKNTRKILRNGEVAPPKEDPVPLPELPCEKSDAYF
VLRDGAAGVFLAANTFPKSRETRAPLVEELYRFRDRLPEKLRYLADAPQQDPEGNKTMVRFSRKTKQQYV
SSEKDGKATGWSAFYVDGKWVEGKK

>gi|30062795|ref|NP_836966.1| hypothetical protein S1360 [Shigella flexneri 2a str. 2457T]
MNKETQPIDRETLLKEANKIIREHEDTLAGIEATGVTQRNGVLVFTGDYFLDEQGLPTAKSTAVFNMFKH
LAHVLSEKYHLVD

>gi|30062794|ref|NP_836965.1| periplasmic protease [Shigella flexneri 2a str. 2457T]
MELLSEYGLFLAKIVTVVLAIAAIAAIIVNVAQRNKRQRGELRVNNLSEQYKEMKEELAAALMDSHQQKQ
WHKAQKKKHKQEAKAAKAKAKLGEVATDSKPRVWVLDFKGSMDAHEVNSLREEITAVLAAFKPQDQVVLR
LESPGGMVHGYGLAASQLQRLRDKNIPLTVTVDKVAASGGYMMACVADKIVSAPFAIVGSIGVVAQMPNF
NRFLKSKDIDIELHTAGQYKRTLTLLGENTEEGREKFREELNETHQLFKDFVKRMRPSLDIEQVATGEHW
YGQQAVEKGLVDEINTSDEVILSLMEGREVVNVRYMQRKRLIDRFTGSAAESADRLLLRWWQRGQKPLM

>gi|30062793|ref|NP_836964.1| short chain dehydrogenase [Shigella flexneri 2a str. 2457T]
MHYQPKQDLLNDRIILVTGASDGIGREAAMTYARYGATVILLGRNEEKLRQVASHINEETGRQPQWFILD
LLTCTSENCQQLAQRIVVNYPRLDGVLHNAGLLGDVCPMSEQNPQVWQDVMQINVNATFMLTQALLPLLL
KSDAGSLVFTSSSVGRQGRANWGAYAASKFATEGMMQVLADEYQQRLRVNCINPGGTRTAMRASAFPTED

PQKLKTPADIMPLYLWLMGDDSRRKTGMTFDAQPGRKPGISQ

>gi|30062792|ref|NP_836963.1| cob(I)yrinic acid a,c-diamide adenosyltransferase [Shigella flexneri 2a str. 2457T]

MSDERYQQRQQRVKEKVDARVAQAQDERGIIIVFTGNGKGKTTAAFGTATRAVGHGKKVGVVQFIKGTWP

NGERNLLEPHGVEFQVMATGFTWDTQNRESDTAACREVWQHAKRMLADSSLDMVLLDELTYMVAYDYLPL

EEVVQALNERPHQQTVIITGRGCHRDILELADTVSELRPIRHAFDAGVKAQIGIDY

>gi|30062791|ref|NP_836962.1| 23S rRNA pseudouridylate synthase B [Shigella flexneri 2a str. 2457T]

MSEKLQKVLARAGHGSRREIESIIEAGRVSVDGKIAKLGDRVEVTPGLKIRIDGHLISVRESAEQICRVL

AYYKPEGELCTRNDPEGRPTVFDRLPKLRGARWIAVGRLDVNTCGLLLFTTDGELANRLMHPSREVEREY

AVRVFGQVDDAKLRDLSRGVQLEDGPAAFKTIKFSGGEGINQWYNVTLTEGRNREVRRLWEAVGVQVSRL

IRVRYGDIPLPKGLPRGGWTELDLAQTNYLRELVELPPETSSKVAVEKDRRRMKANQIRRAVKRHSQVSG

SRRSGGRNNG

>gi|30062790|ref|NP_836961.1| hypothetical protein S1355 [Shigella flexneri 2a str. 2457T]

MGTAAEHHREGIMSQFFYIHPDNPQQRLINQAVEIVRKGGVIVYPTDSGYALGCKIEDKNAMERICRIRQ

LPDGHNFTLMCRDLSELSTYSFVDNVAFRLMKNNTPGNYTFILKGTKEVPRRLLQEKRKTIGMRVPSNPI

AQALLEALGEPMLSTSLMLPGSEFTESDPEEIKDRLEKQVDLIIHGGYLGQKPTTVIDLTDDTPVVVREG

VGDVKPFL

>gi|30062789|ref|NP_836960.1| enzyme [Shigella flexneri 2a str. 2457T]

MSDTNYAVIYDLHSHTTASDGCLTPEALVHRAVEMRVGTLAITDHDTTAAIAPAREEISRSGLALNLIPG

VEISTVWENHEIHIVGLNIDITHPLMCEFLAQQTERRNQRAQQIAERLEKAQIPGALEGAQRLAQGGAVT

RGHFARFLVECGKASSMADVFKKYLARGKTGYVPPQWCTIEQAIDVIHHSGGKAVLAHPGRYNLSAKWLK

RLVAHFAEHHGDAMEVAQCQQSPNERTQLATLARQHHLWASQGSDFHQPCPWIELGRKLWLPAGVEGVWQ

LWEQPQNTTEREL

>gi|30062787|ref|NP_836958.1| anthranilate synthase component I [Shigella flexneri 2a str. 2457T]

MQTQKPTLELLTCEGAYRDNPTALFHQLCGNRPATLLLESADIDSKDDLKSLLLIDSALRITALGDTVTI

QALSGNGEALLALLDNALPAGVESEQSPNCRVLRFPPVSPLLDEDARLCSLSVFDAFRLLQNLLNVPKEE

REAMFFGGLFSYDLVAGFEDLPQLSAENNCPDFCFYLAETLMVIDHQKKSTRIQASLFAPNEEEKQRLTA

RLNELRQQLTEAAPPLPVVSVPHMRCECNQSDEEFGGVVRLLQKAIRAGEIFQVVPSRRFSLPCPSPLAA

YYVLKKSNPSPYMFFMQDNDFTLFGASPESSLKYDATSRQIEIYPIAGTRPRGRRADGSLDRDLDSRIEL

EMRTDHKELSEHLMLVDLARNDLARICTPGSRYIADLTKVDRYSYVMHLVSRVVGELRHDLDALHAYRAC

MNMGTLSGAPKVRAMQLIAEAEGRRRGSYGGAVGYFTAHGDLDTCIVIRSALVENGIATVQAGAGVVLDS

VPQSEADETRNKARAVLRAIATAHHAQETF

>gi|30062786|ref|NP_836957.1| bifunctional glutamine amidotransferase/anthranilate phosphoribosyltransferase [Shigella flexneri 2a str. 2457T]

MADILLLDNIDSFTYNLADQLRSNGHNVVIYRNHIPAQTLIERLATMSNPVLMLSPGPGVPSEAGCMPEL

LTRLRGKLPIIGICLGHQAIVEAYGGYVGQAGEILHGKASSIEHDGQAMFAGLTNPLPVARYHSLVGSNI

PAGLTINAHFNGMVMAVRHDADRVCGFQFHPESILTTQGARLLEQTLAWAQQKLEPANTLQPILEKLYQA

QTLSQQESHQLFSAVVRGELKPEQLAAALVSMKIRGEHPNEIAGAATALLENAAPFPRPDYLFADIVGTG

GDGSNSINISTASAFVAAACGLKVAKHGNRSVSSKSGSSDLLAAFGINLDMNANKSRQALDELGVCFLFA

PKYHTGFRHAMPVRQQLKTRTLFNVLGPLINPAHPPLALIGVYSPELVLPIAETLRVLGYQRAAVVHSGG

MDEVSLHAPTIVAELHDGKIKSYQLTAEDFGLTPYHQEQLAGGTPEENRDILTRLLQGKGDAAHEAAVAA

NVAMLMRLHGHEDLQANAQTVLEVLRSGSAYDRVTALAARG

>gi|30062785|ref|NP_836956.1| bifunctional indole-3-glycerol phosphate synthase/phosphoribosylanthranilate isomerase [Shigella flexneri 2a str. 2457T]

MMQTVLAKIVADKAIWVETRKQQQPLASFQNEVQPSTRHFYDALQGARTAFILECKKASPSKGVIRDDFD

PARIAAIYKHYASAISVLTDEKYFQGSFDFLLIVSQIAPQPILCKDFIIDPYQIYLARYYQADACLLMLS

VLDDDQYRQLAAVAHSLEMGVLTEVSNEEELERAIALGAKVVGINNRDLRDLSIDLNRTRELAPKLGHNV

TVISESGINTYAQVRELSHFANGFLIGSALMAHDDLHAAVRRVLLGENKVCGLTRGQDAKAAYDAGAIYG

GLIFVATSPRCVNVEQAQEVMAAAPLQYVGVFRNHDIADVVDKAKVLSLAAVQLHGNEDQLYIDTLREAL

PAHVAIWKALSVGETLPAREFQHVDKYVLDNGQGGSGQRFDWSLLNGQSLGNVLLAGGLGADNCVEAAQT

GCAGLDFNSAVESQPGIKDARLLASVFQTLRAY

>gi|30062784|ref|NP_836955.1| tryptophan synthase subunit beta [Shigella flexneri 2a str. 2457T]

MTTLLNPYFGEFGGMYVPQILMPALRQLEEAFVSAQKDPEFQAQFNDLLKNYAGRPTALTKCQNITAGTN

TTLYLKREDLLHGGAHKTNQVLGQALLAKRMGKTEIIAETGAGQHGVASALASALLGLKCRIYMGAKDVE

RQSPNVFRMRLMGAEVIPVHSGSATLKDACNEALRDWSGSYETAHYMLGTAAGPHPYPTIVREFQRMIGE

ETKAQILEREGRLPDAVIACVGGGSNAIGMFADFINETNVGLIGVEPGGHGIETGEHGAPLKHGRVGIYF

GMKAPMMQTEDGQIEESYSISAGLDFPSVGPQHAYLNSTGRADYVSITDDEALEAFKTLCLHEGIIPALE

SSHALAHALKMMRENPDKEQLLVVNLSGRGDKDIFTVHDILKARGEI

>gi|30062783|ref|NP_836954.1| tryptophan synthase subunit alpha [Shigella flexneri 2a str. 2457T]

MERYESLFAQLKERKEGAFVPFVTLGDPGIEQSLKIIDTLIEAGADALELGIPFSDPLADGPTIQNATLR

AFAAGVTPAQCFEMLALIRQKHPTIPIGLLMYANLVFNKGIDEFYAQCEKVGVDSVLVADVPVEESAPFR

QAALRHNVAPIFICPPNADDDLLRQIASYGRGYTYLLSRAGVTGAENRAALPLNHLVAKLKEYNAAPPLQ

GFGISAPDQVKAAIDAGAAGAISGSAIVKIIEQHINEPEKMLAALKVFVQPMKAATRS

>gi|30062782|ref|NP_836953.1| hypothetical protein S1348 [Shigella flexneri 2a str. 2457T]

MSTHNTHINETMQRNGRNNMAEHRGSSGNFAEDREKASDAGRKGGQHSGGNFKNDPQRASEAGKKGGQQS

GGNKSGKS

>gi|30062781|ref|NP_836952.1| structural protein [Shigella flexneri 2a str. 2457T]

MNMKTIEDVFIQLLSDTYSAEKQLTRALAKLARATSNEKLSQAFHAHLEETHGQIERIDQVVESESNLKI

KRMKCVAMEGLIEEANEVIESTEKNEVRDAALIAAAQKVEHYEIASYGTLATLAEQLGYRKAAKLLKETL

EEEKATDIKLTDLALNNVNKKAENKA

>gi|30062780|ref|NP_836951.1| hypothetical protein S1346 [Shigella flexneri 2a str. 2457T]

MNRIEHYHDWLRDAHAMEKQAESMLESMASRIDNYPELRARIEQHLSETKNQIVQLETILDRNDISRSVI

KDSMSKMAALGQSIGGIFPSDEIVKGSIGGYVFEQFEITCYTSLLAAAKNAGDTVSIPIIEAILNEEKQM

ADWLIQHIPQTTEKFLIRSETDGVEAKK

>gi|30062779|ref|NP_836950.1| outer membrane protein W [Shigella flexneri 2a str. 2457T]

MKKLTVAALAVTTLLSGSAFAHEAGEFFMRAGSATVRPTEGAGGTLGSLGGFSVTNNTQLGLTFTYMATD

NIGVELLAATPFRHKIGTRATGDIATVHHLPPTLMAQWYFGDASSKFRPYVGAGINYTTFFDNGFNDHGK

EAGLSDLSLKDSWGAAGQVGVDYLINRDWLVNMSVWYMDIDTTANYKLGGAQQHDSVRLDPWVFMFSAGY

RF

>gi|30062778|ref|NP_836949.1| hypothetical protein S1344 [Shigella flexneri 2a str. 2457T]

MSITAQSVYRDTGNFFRNQFMTILLVSLLCAFITVVLGHVFSPSDAQLAQLNDGVPVSGSSGLFDLVQNM

SPEQQQILLQASAASTFSELIGNAILAGGVILIIQLVSAGQRVSALRAIGASAPILPKLFILIFLTTLLV

QIGIMLVVVPGIIMAILLALAPVMLVQDKMGIFASMRSSMRLTWANMRLVAPAVLSWLLAKTLLLLFASS

FAALTPEIGAVLANTLSNLISAILLIYLFRLYMLIRQ

>gi|30062777|ref|NP_836948.1| intracellular septation protein A [Shigella flexneri 2a str. 2457T]

MKQFLDFLPLVVFFAFYKIYDIYAATAALIVATAIVLIYSWVRFRKVEKMALITFVLVVVFGGLTLFFHN

DEFIKWKVTVIYALFAGALLVSQWVMKKPLIQRMLSKELTLPQPVWSKLNLAWAVFFILCGLANIYIAFW

LPQNIWVNFKVFGLTALTLIFTLLSGIYIYRHMPQEDKS

>gi|30062776|ref|NP_836947.1| acyl-CoA thioester hydrolase [Shigella flexneri 2a str. 2457T]

MSTTHNVPQGDLVLRTLAMPADTNANGDIFGGWLMSQMDIGGAILAKEIAHGRVVTVRVEGMTFLRPVAV

GDVVCCYARCVQKGTTSVSINIEVWVKKVASEPIGQRYKATEALFKYVAVDPEGKPRALPVE

>gi|30062775|ref|NP_836946.1| transporter [Shigella flexneri 2a str. 2457T]

MTSITLDLPRRFPWPTLLSVCIHGAVVAGLLYTSVHQVIELPAPAQPISVTMVAPADLEPPQAVQPPPEP

VVEPEPEPEPIPEPPKEAPVVIEKPKPKPKPKPKPVKKVQEQPKRDVKPVESRPASPFENTAPARPTSST

ATAATSKPVTSVASGPRALSRNQPQYPARAQALRIEGQVKVKFDVTPDGRVDNVQILSAKPANMFEREVK

NAMRRWRYEPGKPGSGIVVNILFKINGTTEIQ

>gi|30062773|ref|NP_836944.1| IS600 orfB [Shigella flexneri 2a str. 2457T]

MAHIRTCETYGTRRLQTELAENGIIVGRDRLARLRKELRLRCKQKRKFRATTNPNHNLPVAPNLLNQTFA

PTAPNQVWVADLTYVAGIKDVYTCEIVGYAMGERMTKELTGKALFMALRSQRPPAGLIHHSDRGSQYCAY

DYRVIQEQFGLKTSMSRKGNCYDNAPMESFWGTLKNESLSHYRFNNRDEAISVIREYIEIFYNRQRRHSR

LGNISPATFREKYHQMAA

>gi|30062772|ref|NP_836943.1| IS600 orfA [Shigella flexneri 2a str. 2457T]

MSRKTQRYSKEFKAEAVRTVLENQLSISEGASRLSLPEGTLGQWVTAARKGLGTPGSRTVAELESEILQL

RKALNEARLERDILKKATAYFAQESLKNTR

>gi|30062771|ref|NP_836942.1| voltage-gated potassium channel [Shigella flexneri 2a str. 2457T]

MSHWATFKQTATNLWVTLRHDILALAVFLNGLLIFKTIYGMSVNLLDIFHIKAFSELDLSLLANAPLFML

GVFLVLNSIGLLFRAKLAWAISIILLLIALIYTLHFYPWLKFSIGFCIFTLVFLLILRKDFSHSSAAAGT

IFAFISFTTLLFYSTYGALYLSEGFNPRIESLMTAFYFSIETMSTVGYGDIVPVSESARLFTISVIISGI

TVFATSMTSIFGPLIRGGFNKLLKGNNHTMHRKDHFIVCGHSILAINTILQLNQRGQNVTVISNLPEDDI

KQLEQRLGDNADVIPGDSNDSSVLKKAGIDRCRAILALSDNDADNAFVVLSAKDMSSDVKTVLAVSDSKN

LNKIKMVHPDIILSPQLFGSEILARVLNGEEINNDMLVSMLLNSGHGIFSDNDEQETKADSKESAQK

>gi|30062770|ref|NP_836941.1| cardiolipin synthetase [Shigella flexneri 2a str. 2457T]

MTTVYTLVSWLAILGYWLLIAGVTLRILMKRRAVPSAMAWLLIIYILPLVGIIAYLAVGELHLGKRRAER

ARAMWPSTAKWLNDLKACKHIFAEENSSVAAPLFKLCERRQGIAGVKGNQLQLMTESDDVMQALIRDIQL

ARHNIEMVFYIWQPGGMADQVAESLMAAARRGIHCRLMLDSAGSVAFFRSPWPELMRNAGIEVVEALKVN

LMRVFLRRMDLRQHRKMIMIDNYIAYTGSMNMVDPRYFKQDAGVGQWIDLMARMEGPIATAMGIIYSCDW

EIETGKRILPPPPDVNIMPFEQASGHTIHTIASGPGFPEDLIHQALLTAAYSAHEYLIMTTPYFVPSDDL

LHAICTAAQRGVDVSIILPRKNDSMLVGWASRAFFTELLAAGVKIYQFEGGLLHTKSVLVDGELSLVGTV

NLDMRSLWLNFEITLAIDDKGFGADLAAVQDDYISRSRLLDARLWLKRPLWQRVAERLFYFFSPLL

>gi|30062768|ref|NP_836939.1| ATP-binding protein of oligopeptide ABC transport system [Shigella flexneri 2a str. 2457T]

MNAVTEGRKVLLEIADLKVHFEIKDGKQWFWQPPKTLKAVDGVTLRLYEGETLGVVGESGCGKSTFARAI

IGLVKATDGHVAWLGKELLGMKPDEWRAVRSDIQMIFQDPLASLNPRMTIGEIIAEPLRTYHPKMSRQEV

RERVKAMMLKVGLLPNLINRYPHEFSGGQCQRIGIARALILEPKLIICDEPVSALDVSIQAQVVNLLQQL

QREMGLSLIFIAHDLAVVKHISDRVLVMYLGHAVELGTYDEVYHNPLHPYTRALMSAVPIPDPDLEKNKT

IQLLEGELPSPINPPSGCVFRTRCPIAGPECAKTRPVLEGSFRHAVSCLKVDPL

>gi|30062767|ref|NP_836938.1| oligopeptide transporter ATP-binding component [Shigella flexneri 2a str. 2457T]

MSVIETATVPLAQQQADALLNVKDLRVTFSTPDGDVTAVNDLNFSLRAGETLGIVGESGSGKSQTAFALM

GLLAANGRIGGSATFNGREILNLPERELNKLRAEQISMIFQDPMTSLNPYMRVGEQLMEVLMLHKNMSKA

EAFEESVRMLDAVKMPEARKRMKMYPHEFSGGMRQRVMIAMALLCRPKLLIADEPTTALDVTVQAQIMTL

LNELKREFNTAIIMITHDLGVVAGICDKVLVMYAGRTMEYGNARDVFYQPVHPYSIGLLNAVPRLDAEGE

TMLTIPGNPPNLLRLPKGCPFQPRCPHAMEICSSAPPLEEFTPGRLRACFKPVEELL

>gi|30062766|ref|NP_836937.1| oligopeptide transport permease [Shigella flexneri 2a str. 2457T]

MMLSKKNSETLENFSEKLEVEGRSLWQDARRRFMHNRAAVASLIVLVLIALFVILAPMLSQFAYDDTDWA

MMSSAPDMESGHYFGTDSSGRDLLVRVAIGGRISLMVGVAAALVAVVVGTLYGSLSGYLGGKVDSVMMRL

LEILNSFPFMFFVILLVTFFGQNILLIFVAIGMVSWLDMARIVRGQTLSLKRKEFIEAAQVGGVSTPGIV

IRHIVPNVLGVVVVYASLLVPSMILFESFLSFLGLGTQEPLSSWGALLSDGANSMEVSPWLLLFPAGFLV

VTLFCFNFIGDGLRDALDPKDR

>gi|30062765|ref|NP_836936.1| oligopeptide transporter permease [Shigella flexneri 2a str. 2457T]

MLKFILRRCLEAIPTLFILITISFFMMRLAPGSPFTGERTLPPEVMANIEAKYHLNDPIMTQYFSYLKQL

AHGDFGPSFKYKDYSVNDLVASSFPVSAKLGAAAFFLAVILGVSAGVIAALKQNTKWDYTVMGLAMTGVV

IPSFVVAPLLVMIFAIILHWLPGGGWNGGALKFMILPMVALSLAYIASIARITRGSMIEVLHSNFIRTAR

AKGLPMRRIILRHALKPALLPVLSYMGPAFVGIITGSMVIETIYGLPGIGQLFVNGALNRDYSLVLSLTI

LVGALTILFNAIVDVLYAVIDPKIRY

>gi|30062764|ref|NP_836935.1| periplasmic oligopeptide binding protein [Shigella flexneri 2a str. 2457T]

MTNITKRSLVAAGVLAALMAGNVALAADVPAGVTLAEKQTLVRNNGSEVQSLDPHKIEGVPESNISRDLF

EGLLVSDLDGHPAPGVAESWDNKDAKVWTFHLRKDAKWSDGTPVTAQDFVYSWQRSVDPNTASPYASYLQ

YGHIAGIDEILEGKKPITDLGVKAIDDHTLEVTLSEPVPYFYKLLVHPSTSPVPKAAIEKFGEKWTQPGN

IVTNGAYTLKDWVVNERIVLERSPTYWNNAKTVINQVTYLPIASEVTDVNRYRSGEIDMTYNNMPIELFQ

KLKKEIPDEVHVDPYLCTYYYEINNQKPPFNDVRVRTALKLGMDRDIIVNKVKAQGDMPAYGYTPPYTDG

AKLTQPEWFGWSQEKRNEEAKKLLAEAGYTADKPLTINLRYNTSDLHKKLAIAASSLWKKNIGVNVKLVN

QEWKTFLDTRHQGTFDVARAGWCADYNEPTSFLNTMLSNSSMNTAHYKSPAFDSIMAETLKVTDEAQRTA

LYTKAEQQLDKDSAIVPVYYYYVNARLVKPWVGGYTGKDPLDNTYTRNMYIVKH

>gi|30062763|ref|NP_836934.1| IS1 orfA [Shigella flexneri 2a str. 2457T]

MASISIRCPSCSATEGVVRNGKSTAGHQRYLCSHCRKTWQLQFTYTASQPGTHQKIIDMAMNGVGCRASA

RIMGVGLNTVLRHLKNSGRSR

>gi|30062762|ref|NP_836933.1| IS1 orfB [Shigella flexneri 2a str. 2457T]

MSRQCTHYGRWPQHGFTSLKKLRPQSVTSRIQPGSDVIVCAEMDEHWGYVGAKSRQRWLFYAYDRIRRTV

VAHVFGERTLATLERLLSLLSAFEVVVWMTDGWPLYESRLKGKLHVISKRYTQRIERHNLNLRQHLARPV

RKSLSFSKSVELHDKVIGHYLNIKHYQ

>gi|30062761|ref|NP_836932.1| hypothetical protein S1327 [Shigella flexneri 2a str. 2457T]

MIQTLFDFPVYFKFFIGLFALVNPVGIIPVFISMTSYQTAAARNKTNLTANLSVAIILWISLFLGDTILQ

LFGISIDSFRIAGGILVVTIAMSMISGKLGEDKQNKQEKSETAVRESIGVVPLALPLMAGPGAISSTIVW

GTRYHSISYLFGFFVAIALFALCCWGLFRMAPWLVRVLRQTGINVITRIMGLLLMALGIEFIVTGIKGIF

PGLLN

>gi|30062760|ref|NP_836931.1| bifunctional acetaldehyde-CoA/alcohol dehydrogenase [Shigella flexneri 2a str. 2457T]

MAVTNVAELNALVERVKKAQREYASFTQEQVDKIFRAAALAAADARIPLAKMAVAESGMGIVEDKVIKNH

FASEYIYNAYKDEKTCGVLSEDDTFGTITIAEPIGIICGIVPTTNPTSTAIFKSLISLKTRNAIIFSPHP

RAKDATNKAADIVLQAAIAAGAPKDLIGWIDQPSVELSNALMHHPDINLILATGGPGMVKAAYSSGKPAI

GVGAGNTPVVIDETADIKRAVASVLMSKTFDNGVICASEQSVVVVDSVYDAVRERFATHGGYLLQGKELK

AVQDVILKNGALNAAIVGQPAYKIAELAGFSVPENTKILIGEVTVVDESEPFAHEKLSPTLAMYRAKDFE

DAVEKAEKLVAMGGIGHTSCLYTDQDNQPARVSYFGQKMKTARILINTPASQGGIGDLYNFKLAPSLTLG

CGSWGGNSISENVGPKHLINKKTVAKRAENMLWHKLPKSIYFRRGSLPIALDEVITDGHKRALIVTDRFL

FNNGYADQITSVLKAAGVETEVFFEVEADPTLSIVRKGAELANSFKPDVIIALGGGSPMDAAKIMWVMYE

HPETHFEELALRFMDIRKRIYKFPKMGVKAKMIAVTTTSGTGSEVTPFAVVTDDATGQKYPLADYALTPD

MAIVDANLVMDMPKSLCAFGGLDAVTHAMEAYVSVLASEFSDGQALQALKLLKEYLPASYHEGSKNPVAR

ERVHSAATIAGIAFANAFLGVCHSMAHKLGSQFHIPHGLANALLICNVIRYNANDNPTKQTAFSQYDRPQ

ARRRYAEIADHLGLSAPGDRTAAKIEKLLAWLETLKAELGIPKSIREAGVQEADFLANVDKLSEDAFDDQ

CTGANPRYPLISELKQILLDTYYGRDYVEDETAAKKEAAPAKAEKKAKKSA

>gi|30062759|ref|NP_836930.1| IS4 orf [Shigella flexneri 2a str. 2457T]

MHIGQALDLVSRYDSLRNPLTSLGDYLDPELISRCLAESGTVTLRKRRLPLEMMVWCIVGMALERKEPLH

QIVNRLDIMLPGNRPFVAPSAVIQARQRLGSEAVRRVFTKTAQLWHNATPHPHWCGLTLLAIDGVFWRTP

DTPENDAAFPRQTHAGNPALYPQVKMVCQMELTSHLLTAAAFGTMKNSENELAEQLIGQTGDNTLTLMDK

GYYSLGLLNGWSLAGEHRHWMIPLRKGAQYEELRKLSKGDHLVKLKTSPQARKKWPGLGNEVTARLLTVT

RKGKVCHLLTSMTDAMRFPGGEMADLYSNRWEIELGYREIKQTMQLSRLTLRSKKPELVEQELWGVLLAY

NLVRYQMIKMAEHLKGYWPNQLSFSESCGMVMRMLMTLQGASPGRIPELMRDLASMGQLVKLPTRRERAF

PRVVKERPWKYPTAPKKSQSVA

>gi|30062758|ref|NP_836929.1| thymidine kinase [Shigella flexneri 2a str. 2457T]

MAQLYFYYSAMNAGKSTALLQSSYNYQERGMRTVVYTAEIDDRFGAGKVSSRIGLSSPAKLFNQNSSLFD

EIRAEHEQQAIHCVLVDECQFLTRQQVYELSEVVDQLDIPVLCYGLRTDFRGELFIGSQYLLAWSDKLVE

LKTICFCGRKASMVLRLDQAGRPYNEGEQVVIGGNERYVSVCRKHYKEALQVGSLTAIQERHRHD

>gi|30062757|ref|NP_836928.1| global DNA-binding transcriptional dual regulator H-NS [Shigella flexneri 2a str. 2457T]

MSEALKILNNIRTLRAQARECTLETLEEMLEKLEVVVNERREEESAAAAEVEERTRKLQQYREMLIADGI

DPNELLNSLAAVKSGTKAKRAQRPAKYSYVDENGETKTWTGQGRTPAVIKKAMDEQGKSLDDFLIKQ

>gi|30062756|ref|NP_836927.1| UTP--glucose-1-phosphate uridylyltransferase subunit GalU [Shigella flexneri 2a str. 2457T]

MAAINTKVKKAVIPVAGLGTRMLPATKAIPKEMLPLVDKPLIQYVVNECIAAGITEIVLVTHSSKNSIEN

HFDTSFELEAMLEKRVKRQLLDEVQSICPPHVTIMQVRQGLAKGLGHAVLCAHPVVGDEPVAVILPDVIL

DEYESDLSQDNLAEMIRRFDETGHSQIMVEPVADVTAYGVVDCKGVELAPGESVPMVGVVEKPKADVAPS

NLAIVGRYVLSADIWPLLAKTPPGAGDEIQLTDAIDMLIEKETVEAYHMKGKSHDCGNKLGYMQAFVEYG

IRHNTLGTEFKAWLEEEMGIKK

>gi|30062755|ref|NP_836926.1| response regulator of RpoS [Shigella flexneri 2a str. 2457T]

MTQPLVGKQILIVEDEQVFRSLLDSWFSSLGATTVLAADGVDALELLGGFTPDLMICDIAMPRMNGLKLL

EHIRNRGDQTPVLVISATENMADIAKALRLGVEDVLLKPVKDLNRLREMVFACLYPSMFNSRVEEEERLF

RDWDAMVDNPAAAAKLLQELQPPVQQVISHCRVNYRQLVAADKPGLVLDIAALSENDLAFYCLDVTRAGH

NGVLAALLLRALFNGLLQEQLAHQNQRLPELGALLKQVNHLLRQANLPGQFPLLVGYYHRELKNLILVSA

GLNATLNTGEHQVQISNGVPLGTLGNAYLNQLSQRCDAWQCQIWGTGGRLRLMLSAE

>gi|30062754|ref|NP_836925.1| hypothetical protein S1320 [Shigella flexneri 2a str. 2457T]

MRKIKIGLALGSGAARGWSHIGVINALKKVGIEIDIVAGCSIGSLVGAAYACDRLSALEDWVTSFSYWDV

LRLMDLSWQRGGLLRGERVFNQYREIMPETEIENCSRRFAAVATNLSTGRELWFTEGDLHLAIRASCSIP

GLMAPVAHNGYWLVDGAVVNPIPISLTRALGADIVIAVDLQHDAHLMQQDLLSFNVSEENSENGDSLPWH

ARLKERLGSITTRRAVTAPTATEIMTTSIQVLENRLKRNRMAGDPPDILIQPVCPQISTLDFHRAHAAIA

AGQLAVERKMDELLPLVRTNI

>gi|30062752|ref|NP_836923.1| formyltetrahydrofolate deformylase [Shigella flexneri 2a str. 2457T]

MHSLQRKVLRTICPDQKGLIARITNICYKHELNIVQNNEFVDHRTGRFFMRTELEGIFNDSTLLADLDSA

LPEGSVRELNPAGRRRIVILVTKEAHCLGDLLMKANYGGLDVEIAAVIGNHDTLRSLVERFDIPFELVSH

EGLSRNEHDQKMADAIDAYQPDYVVLAKYMRVLTPEFVARFPNKIINIHHSFLPAFIGARPYHQAYERGV

KIIGATAHYVNDNLDEGPIIMQDVIHVDHTYTAEDMMRAGRDVEKNVLSRALYKVLAQRVFVYGNRTIIL

>gi|30062751|ref|NP_836922.1| nitrate reductase 1, cytochrome b(NR), gamma subunit [Shigella flexneri 2a str. 2457T]

MQFLNMFFFDIYPYIAGAVFLIGSWLRYDYGQYTWRAASSQMLDRKGMNLASNLFHIGILGIFVGHFFGM

LTPHWMYEAWLPIEVKQKMAMFAGGASGVLCLIGGVLLLKRRMFSPRVRATTTGADILILSLLVIQCALG

LLTIPFSAQHMDGSEMMKLVGWAQSVVTFHGGASQHLDGVAFIFRLHLVLGMTLFLLFPFSRLVHIWSVP

VEYLTRKYQLVRARH

>gi|30062750|ref|NP_836921.1| nitrate reductase 1, delta subunit [Shigella flexneri 2a str. 2457T]

MIELVIVSRLLEYPDAALWQHQQEMFEAIAASKNLSKEDAHALGIFLRDLTAMDPLDAQVQYSELFDRGR

ATSLLLFEHVHGESRDRGQAMVDLLAQYEQHGLQLNSRELPDHLPLYLEYLSQLPQSEAVEGLKDIAPIL

ALLSARLQQRESRYAVMFDLLLKLANTAIDSDKVAEKIADEARDDTPQALDAVWEEEQVKFFADKGCGDS

AITAHQRRFAGAVAPQYLNITTGGQH

>gi|30062749|ref|NP_836920.1| nitrate reductase 1, beta subunit [Shigella flexneri 2a str. 2457T]

MKIRSQVGMVLNLDKCIGCHTCSVTCKNVWTSREGVEYAWFNNVETKPGQGFPTDWENQEKYKGGWIRKI

NGKLQPRMGNRAMLLGKIFANPHLPGIDDYYEPFDFDYQNLHTAPEGSKSQPIARPRSLITGERMAKIEK

GPNWEDDLGGEFDKLAKDKNFDNIQKAMYSQFENTFMMYLPRLCEHCLNPACVATCPSGAIYKREEDGIV

LIDQDKCRGWRMCITGCPYKKIYFNWKSGKSEKCIFCYPRIEAGQPTVCSETCVGRIRYLGVLLYDADAI

ERAASTENEKDLYQRQLDVFLDPNDPKVIEQAIKDGIPLSVIEAAQQSPVYKMAMEWKLALPLHPEYRTL

PMVWYVPPLSPIQSAADAGELGSNGILPDVESLRIPVQYLANLLTAGDTKPVLRALKRMLAMRHYKRAET

VDGKVDTRALEEVGLTEAQAQEMYRYLAIANYEDRFVVPSSHRELAREAFPEKNGCGFTFGDGCHGSDTK

FNLFNSRRIDAIDVTSKTEPHP

>gi|30062748|ref|NP_836919.1| nitrate reductase 1 alpha subunit [Shigella flexneri 2a str. 2457T]

MSKFLDRFRYFKQKGETFADGHGQLLNTNRDWEDGYRQRWQHDKIVRSTHGVNCTGSCSWKIYVKNGLVT

WETQQTDYPRTRPDLPNHEPRGCPRGASYSWYLYSANRLKYPMMRKRLMKMWREAKALHSDPVEAWASII

EDADKAKSFKQARGRGGFVRSSWQEVNELIAASNVYTIKNYGPDRVAGFSPIPAMSMVSYASGARYLSLI

GGTCLSFYDWYCDLPPASPQTWGEQTDVPESADWYNSSYIIAWGSNVPQTRTPDAHFFTEVRYKGTKTVA

VTPDYAEIAKLCDLWLAPKQGTDAAMALAMGHVMLREFHLDNPSQYFTDYVRRYTDMPMLVMLEERDGYY

AAGRMLRAADLVDALGQENNPEWKTVAFNTNGEMVAPNGSIGFRWGEKGKWNLEQRDGKTGEETELQLSL

LGSQDEIAEVGFPYFGGDGTEHFNKVELENVLLHKLPVKRLQLADGSTALVTTVYDLTLANYGLERGLND

VNCATSYDDVKAYTPAWAEQITGVSRSQIIRIAREFADNADKTHGRSMIIVGAGLNHWYHLDMNYRGLIN

MLIFCGCVGQSGGGWAHYVGQEKLRPQTGWQPLAFALDWQRPARHMNSTSYFYNHSSQWRYETVTAEELL

SPMADKSRYTGHLIDFNVRAERMGWLPSAPQLGTNPLTIAGEAEKAGMNPVDYTVKSLKEGSIRFAAEQP

ENGKNHPRNLFIWRSNLLGSSGKGHEFMLKYLLGTEHGIQGKDLGQQGGVKPEEVDWQDNGLEGKLDLVV

TLDFRLSSTCLYSDIILPTATWYEKDDMNTSDMHPFIHPLSAAVDPAWEAKSDWEIYKAIAKKFSEVCVG

YLGKETDIVTLPIQHDSAAELAQPLDVKDWKKGECDLIPGKTAPHIMVVERDYPATYERFTSIGPLMEKI

GNGGKGIAWNTQSEMDLLRKLNYTKAEGPAKGQPMLNTAIDAAEMILTLAPETNGQVAVKAWAALSEFTG

RDHTHLALNKEDEKIRFRDIQAQPRKIISSPTWSGLEDEHVSYNAGYTNVHELIPWRTLSGRQQLYQDHQ

WMRDFGESLLVYRPPIDTRSVKEVIGQKSNGNPEKALNFLTPHQKWGIHSTYSDNLLMLTLGRGGPVVWL

SEADAKDLGIADNDWIEVFNSNGALTARAVVSQRVPAGMTMMYHAQERIVNLPGSEITQQRGGIHNSVTR

ITPKPTHMIGGYAHLAYGFNYYGTVGSNRDEFVVVRKMKNIDWLDGEGNDQVQESVK

>gi|30062747|ref|NP_836918.1| nitrate transport protein nark [Shigella flexneri 2a str. 2457T]

MSHSSASERATGAVITDWRPEDPAFWQQRGQRIASRNLWISVPCLLLAFCVWMLFSAVAVNLPKVGFNFT

TDQLFMLTALPSVSGALLRVPYSFMVPIFGGRRWTAFSTGILIIPCVWLGFAVQDTSTPYSVFIIISLLC

GFAGANFASSMANISFFFPKQKQGGALGLNGGLGNMGVSVMQLVAPLVVSLSIFAVFGSQGVKQPDGTEL

YLANASWIWVPFLAIFTIAAWFGMNDLATSKASIKEQLPVLKRGHLWIMSLLYLATFGSFIGFSAGFAML

SKTQFPDVQILQYAFFGPFIGALARSAGGALSDRLGGTRVTLVNFILMAIFSGLLFLTLPTDGQGGSFMA

FFAVFLALFLTAGLGSGSTFQMISVIFRKLTMDRVKAEGGSDERAMREAATGTAAALGFISAIGAIGGFF

IPKAFGSSLALTGSPVGAMKVFLIFYIACVVITWAVYGRHSKK

>gi|30062746|ref|NP_836917.1| nitrate/nitrite sensor protein NarX [Shigella flexneri 2a str. 2457T]

MLKRCLSPLTLVNQVALIVLLSTAIGLAGMAVSGWLVQGVQGSAHAINKAGSLRMQSYRLLAAVPLSEKD

KPLIKEMEQTAFSAELTRAAERDGQLAQLQGLQDYWRNELIPALMRAQNRETVSADVSQFVAGLDQLVSG

FDRTTEMRIETVVLVHRVMAVFMALLLVFTIIWLRARLLQPWRQLLAMASAVSHRDFTQRANISGRNEMA

MLGTALNNMSAELAESYAVLEQRVQEKTAGLEHKNQILSFLWQANRRLHSRAPLCERLSPVLNGLQNLTL

LRDIELRVYDTDDEENHQEFTCQPDMTCDDKGCQLCPRGVLPVGDRGTTLKWRLADSHTQYGILLATLPQ

GRHLSHDQQQLVDTLVEQLTATLALDRHQERQQQLIVMEERATIARELHDSIAQSLSCMKMQVSCLQMQG

DALPESSRELLSQIRNELNASWAQLRELLTTFRLQLTEPGLRPALEASCEEYSAKFGFPVKLDYQLPPRL

VPSHQAIHLLQIAREALSNALKHSQASEVVVTVAQNDNQVKLTVQDNGCGVPENAIRSNHYGMIIMRDRA

QSLRGDCRVRRRESGGTEVVVTFIPEKTFTDVQGDTHE

>gi|30062745|ref|NP_836916.1| transcriptional regulator NarL [Shigella flexneri 2a str. 2457T]

MSNQEPATILLIDDHPMLRTGVKQLISMAPDITVVGEASNGEQGIELAESLDPDLILLDLNMPGMNGLET

LDKLREKSLSGRIVVFSVSNHEEDVVTALKRGADGYLLKDMEPEDLLKALHQAAAGEMVLSEALTPVLAA

SLRANRATTERDVNQLTPRERDILKLIAQGLPNKMIARRLDITESTVKVHVKHMLKKMKLKSRVEAAVWV

HQERIF

>gi|30062744|ref|NP_836915.1| hypothetical protein S1307 [Shigella flexneri 2a str. 2457T]

MAPENHDGEKHFAEIVKDFGETSMNDNGLDTGEQAKAFAWGKVRDALSQQVNQHVESWLSPWGNASVDVK

VDNEGHFTGSRGSWFVPLQDNDRYLTWSQLGLTQQDNGLVSNVGVGQRWARGNWLVGYNTFYDNLQDENL

QRAGFGAEAWGEYLRLSANFYQPFAAWHEQTATQEQRMARGYDLTARMRMPFYQHLNTSVSLEQYFGDRV

DLFNSGTGYHNPVALSLGLNYTPVPLVTVTAQHKQGESGENQNNLGLNLNYRFGVPLKKQLSAGEVAESQ

SLRGSRYDNPQRNNLPTLEYRQRKTLTVFLATPPWDLKPGETVPLKLQIRSRYGIRQLIWQGDTQILSLT

PGAQANSAEGWTLIMPDWQNGEGASNHWRLSVVVEDNQGQRVSSNEITLTLVEPFDALSNDELRWEP

>gi|30062743|ref|NP_836914.1| hypothetical protein S1306 [Shigella flexneri 2a str. 2457T]

MQKIVIVANGAPYGSESLFNSLRLAIALREQESNLDLRLFLMSDAVTAGLRGQKPGEGYNIQQMLEILTA

QNVPVKLCKTCTDGRGISTLPLIDGVEIGTLVELAQWTLSADKVLTF

>gi|30062742|ref|NP_836913.1| cation transport regulator [Shigella flexneri 2a str. 2457T]

MQWRRCKVITRDFLMNADCKTAFGAIEESLLWSAEQRAASLAATLACRPDEGPVWIFGYGSLMWNPALEF

TESCTGTLVGWHRAFCLRLTAGRGTAHQPGRMLALKEGGRTTGVAYRLPEETLEQELTLLWKREMITGCY

LPTWCQLDLDDGRTVNAIVFIMDPRHPEYESDTRAQVIAPLIAAASGPLGTNAQYLFSLEQELIKLGMQD

DGLNDLLVSVKKLLAENYPDGVLRPGFA

>gi|30062741|ref|NP_836912.1| cation transport regulator [Shigella flexneri 2a str. 2457T]

MPYKTKSDLPESVKHVLPSHAQDIYKEAFNSAWDQYKDKEDRRDDASREETAHKVAWAAVKHEYAKGDDD

KWHKKS

>gi|30062740|ref|NP_836911.1| calcium/sodium:proton antiporter [Shigella flexneri 2a str. 2457T]

MSNAQEAVKTRHKETSLIFPVLVLVVLFLWGSSQTLPVVIAINLLALIGILSSAFSVVRHADVLAHRLGE

PYGSLILSLSVVILEVSLISALMATGDAAPTLMRDTLYSIIMIVTGGLVGFSLLLGGRKFATQYMNLFGI

KQYLIALFPLAIIVLVFPMALPAANFSTGQALLVALISAAMYGVFLLIQAKTHQSLFVYEHEDDSDDDDP

HHGKTSAHSSLWHAIWLIIHLIAVIAVTKMNASSLETLLDSMNAPVAFTGFLVALLILSPEGLGALKAVL

NNQVQRAMNLFFGSVLATISLTVPVVTLIAFMTGNELQFALGAPEMVVMVASLVLCHISFSTGRTNVLNG

AAHLALFAAYLMTIFA

>gi|30062739|ref|NP_836910.1| 2-dehydro-3-deoxyphosphooctonate aldolase [Shigella flexneri 2a str. 2457T]

MKQKVVSIGDINVANDLPFVLFGGMNVLESRDLAMRICEHYVTVTQKLGIPYVFKASFDKANRSSIHSYR

GPGLEEGMKIFQELKQTFGVKIITDVHEPSQAQPVADVVDVIQLPAFLARQTDLVEAMAKTGAVINVKKP

QFVSPGQMGNIVDKFKEGGNEKVILCDRGANFGYDNLVVDMLGFSIMKKVSGNSPVIFDVTHALQCRDPF

GAASGGRRAQVAELARAGMAVGLAGLFIEAHPDPEHAKCDGPSALPLAKLEPFLKQMKAIDDLVKGFEEL

DTSK

>gi|30062738|ref|NP_836909.1| transcriptional regulator [Shigella flexneri 2a str. 2457T]

MRSLADFEFNKAPLCEGMILACEAIRRDFPSQDVYDELERLVSLAKEEISQLLPLEEQLEKLIALFYGDW

GFKASRGVYRLSDALWLDQVLKNRQGSAVSLGAVLLWVANRLDLPLLPVIFPTQLILRIECPDGEIWLIN

PFNGESLSEHMLDGWLKGNISPSAELFYEDLDEADNIEVIRKLLDTLKASLMEENQMELALRTSEALLQF

NPEDPYEIRDRGLIYAQLDCEHVALNDLSYFVERCPEDPISEMIRAQINNIAHKHIVLH

>gi|30062737|ref|NP_836908.1| transcriptional regulator [Shigella flexneri 2a str. 2457T]

MTSFSTLLSVHLISIALSVGLLTLRFWLRYQKHPQAFARWTRIVPPVVDTVLLLSGIALMAKAHILPFSG

QAQWLTEKLFGVIIYIVLGFIALDYRRMHSQQARIIAFPLALVVLYIIIKLATTKVPLLG

>gi|30062736|ref|NP_836907.1| N5-glutamine S-adenosyl-L-methionine-dependent methyltransferase [Shigella flexneri 2a str. 2457T]

MEYQHWLREAISQLQASESPRRDAEILLEHVTGRGRTFILAFGETQLTDEQCQQLDALLTRRRDGEPIAH

LTGVREFWSLPLFVSPATLIPRPDTECLVEQALARLPEQPCRILDLGTGTGAIALALASERPDCEIIAVD

RMPDAVSLAQRNAQHLAIKNIHILQSDWFSALAGQQFAMIVSNPPYIDEQDPHLQQGDVRFEPLTALVAA

DSGMADIVHIIEQSRNALVSGGFLLLEHGWQQGEAVRQAFILAGYHDVETCRDYGDNERVTLGRYYQ

>gi|30062735|ref|NP_836906.1| peptide chain release factor 1 [Shigella flexneri 2a str. 2457T]

MKPSIVAKLEALHERHEEVQALLGDAQTIADQERFRALSREYAQLSDVSRCFTDWQQVQEDIETAQMMLD

DPEMREMAQDELREAKEKSEQLEQQLQVLLLPKDPDDERNAFLEVRAGTGGDEAALFAGDLFRMYSRYAE

ARRWRVEIMSASEGEHGGYKEIIAKISGDGVYGRLKFESGGHRVQRVPATESQGRIHTSACTVAVMPELP

DAELPDINPADLRIDTFRSSGAGGQHVNTTDSAIRITHLPTGIVVECQDERSQHKNKAKALSVLGARIHA

AEMAKRQQAEASTRRNLLGSGDRSDRNRTYNFPQGRVTDHRINLTLYRLDEVMEGKLDMLIEPIIQEHQA

DQLAALSEQE

>gi|30062734|ref|NP_836905.1| glutamyl-tRNA reductase [Shigella flexneri 2a str. 2457T]

MTLLALGINHKTAPVSLRERVSFSPDKLDQALDSLLAQPMVQGGVVLSTCNRTELYLSVEEQDNLQEALI

RWLCDYHNLNEEDLRKSLYWHQDNDAVSHLMRVASGLDSLVLGEPQILGQVKKAFVDSQKGHMKASELER

MFQKSFSVAKRVRTETDIGASAVSVAFAACTLARQIFESLSTVTVLLVGAGETIELVARHLREHKVQKMI

IANRTRERAQILADEVGAEVIALSEIDERLREADIIISSTASPLPIIGKGMVERALKSRRNQPMLLVDIA

VPRDVEPEVGKLANAYLYSVDDLQSIISHNLAQRKAAAVEAETIVAQETSEFMAWLRAQSASETIREYRS

QAEHVRDELTAKALAALEQGGDAQAIMQDLAWKLTNRLIHAPTKSLQQAARDGDNERLNILRDSLGLE

>gi|30062733|ref|NP_836904.1| outer membrane lipoprotein LolB [Shigella flexneri 2a str. 2457T]

MPLPDFRLIRLLPLAALVLTACSVTTPKGPGKSPDSPQWRQHQQDVRNLNQYQTRGAFAYISDQQKVYAR

FFWQQTGQDRYRLLLTNPLGSTELELNAQPGNVQLVDNKGQRYTADDAEEMIGKLTGMPIPLNSLRQWIL

GLPGDATDYKLDDQYRLSEITYSQNGKNWKVVYGGYDTKTQPAMPANMELTDGGQRIKLKMDNWIVK

>gi|30062732|ref|NP_836903.1| 4-diphosphocytidyl-2-C-methyl-D-erythritol kinase [Shigella flexneri 2a str. 2457T]

MRTQWPSPAKLNLFLYITGQRADGYHTLQTLFQFLDYGDTISIELRDDGDIRLLTPVEGVEHEDNLIVRA

ARLLMKTAADSGRLPTGSGANISIDKRLPMGGGLGGGSSNAATVLVALNHLWQCGLSMDELAEMGLTLGA

DVPVFVRGHAAFAEGVGEILTPVDPPEKWYLVAHPGVSIPTPVIFKDPELPRNTPKRSIETLLKCEFSND

CEVIARKRFREVDAVLSWLLEYAPSRLTGTGACVFAEFDTESEARQVLEQAPEWLNGFVAKGVNLSPLHR

AML

>gi|30062731|ref|NP_836902.1| ribose-phosphate pyrophosphokinase [Shigella flexneri 2a str. 2457T]

MPDMKLFAGNATPVLAQRIANRLYTSLGDAAVGRFSDGEVSVQINENVRGGDIFIIQSTCAPTNDNLMEL

VVMVDALRRASAGRITAVIPYFGYARQDRRVRSARVPITAKVVADFLSSVGVDRVLTVDLHAEQIQGFFD

VPVDNVFGSPILLEDMLQLNLDNPIVVSPDIGGVVRARAIAKLLNDTDMAIIDKRRPRANVSQVMHIIGD

VAGRDCVLVDDMIDTGGTLCKAAEALKERGAKRVFAYATHPIFSGNAANNLRNSVIDEVVVCDTIPLSDE

IKSLPNVRTLTLSGMLAEAIRRISNEESISAMFEH

>gi|30062730|ref|NP_836901.1| sulfate transporter YchM [Shigella flexneri 2a str. 2457T]

MPFRALIDVCWKEKYTAARFTRDLIAGITVGIIAIPLAMALAIGSGVAPQYGLYTAAVAGIVIALTGGSR

FSVSGPTAAFVVILYPVSQQFGLAGLLVATLLSGIFLILMGLARFGRLIEYIPVSVTLGFTSGIGITIGT

MQIKDFLGLQMAHVPEHYLQKVGALFMALPTINVGDAAIGIVTLGILVFWPRLGIRLPGHLPALLAGCAV

MGIVNLLGGHVATIGSQFHYVLADGSQGNGIPQLLPQLVLPWDLPNSEFTLTWDSIRTLLPAAFSMAMLG

AIESLLCAVVLDGMTGTKHKANSELVGQGLGNIIAPFFGGITATAAIARSAANVRAGATSPISAVIHSIL

VILALLVLAPLLSWLPLSAMAALLLMVAWNMSEAHKVVDLLRHAPKDDIIVMLLCMSLTVLFDMVIAISV

GIVLASLLFMRRIARMTRLAPVVVDVPDDVLVLRVIGPLFFAAAEGLFTDLESRLEGKRIVILKWDAVPV

LDAGGLDAFQRFVKRLPEGCELRVCNVEFQPLRTMARAGIQPIPGRLAFFPNRRAAMADL

>gi|30062729|ref|NP_836900.1| hypothetical protein S1292 [Shigella flexneri 2a str. 2457T]

MKRKNASILGNVLMGLGLVVMVVGVGYSILNQLPQFNMPQYFAHGAVLSIFVGAILWLAGARVGGHEQVC

DRYWWVRHYDKRCRRSDNRRHS

>gi|30062728|ref|NP_836899.1| peptidyl-tRNA hydrolase [Shigella flexneri 2a str. 2457T]

MTIKLIVGLANPGAEYAATRHNAGAWFVDLLAERLRAPLREEAKFFGYTSRVTLGSEDVRLLVPTTFMNL

SGKAVAAMASFFRINPDEILVAHDELDLPPGVAKFKLGGGHGGHNGLKDIISKLGNNPNFHRLRIGIGHP

GDKNKVVGFVLGKPPVSEQKLIDEAIDEAARCTEMWFTDGLTKATNRLHAFKAQ

>gi|30062727|ref|NP_836898.1| GTP-dependent nucleic acid-binding protein EngD [Shigella flexneri 2a str. 2457T]

MGFKCGIVGLPNVGKSTLFNALTKAGIEAANFPFCTIEPNTGVVPMPDPRLDQLAEIVKPQRTLPTTMEF

VDIAGLVKGASKGEGLGNQFLTNIRETEAIGHVVRCFENDNIIHVSGKVNPADDIEVINTELALADLDTC

ERAIHRVQKKAKGGDKDAKAELAVLEKCLPQLENAGMLRALDLSAEEKAAIRYLSFLTLKPTMYIANVNE

DGFENNPYLDQVREIAAKEGSVVVPVCAAVEADIAELDDEERDEFMQELGLEEPGLNRVIRAGYKLLNLQ

TYFTAGVKEVRAWTIPVGATAPQAAGKIHTDFEKGFIRAQTISFEDFITYKGEQGAKEAGKMRAEGKDYI

VKDGDVMNFLFNV

>gi|30062726|ref|NP_836897.1| adhesion and penetration protein [Shigella flexneri 2a str. 2457T]

MGIKQHNGNTKADRLAELKIRSPSIQLIKFGAIGLNAIIFSPLLIAADTGSQYGTNITINDGDRITGDTA

DPSGNLYGVMTPAGNTPGNINLGNDVTVNVNDASGYAKGIIIQGKNSSLTANRLTVDVVGQTSAIGINLI

GDYTHADLGTGSTIKSNDDGIIIGHSSTLTATQFTIENSNGIGLTINDYGTSVDLGSGSKIKTDGSTGVY

IGGLNGNNANGAARFTATDLTIDVQGYSAMGINVQKNSVVDLGTNSTIKTNGDNAHGLWSFGQVSANALT

VDVTGAAANGVEVRGGTTTIGADSHISSAQGGGLVTSSSDATINFSGTAAQRNSIFSGGSYGASAQTATA

VINMQNTDITVDRNGSLALGLWALSGGRITGDSLAITGAAGARGIYAMTNSQIDLTSDLVIDMSTPDQMA

IATQHDDGYAASRINASGRMLINGSVLSKGGLINLDMHPGSVWTGSSLSDNVNGGKLDVAMNNSVWNVTS

NSNLDTLALSHSTVDFASHGSTAGTFTTLNVENLSGNSTFIMRADVVGEGNGVNNRGDLLNISGSSAGNH

VLAIRNQGSEATTGNEVLTVVKTTDGAASFSASSQVELGGYLYDVRKNGTNWELYASGTVPEPTPNPEPT

PAPAQPPIVNPDPTPEPAPTPKPTTTADAGGNYLNVGYLLNYVENRTLMQRMGDLRNQSKDGNIWLRSYG

GSLDSFASGKLSGFDMGYSGIQFGGDKRLSDVMPLYVGLYIDSTHASPDYSGGDGTARSDYMGMYASYMA

QNGFYSDLVIKASRQKNSFHVLDSQNNGVNANGTANGMSISLEAGQRFNLSPTGYGFYIEPQTQLTYSHQ

NEMAMKASNGLNIHLNHYESLLGRASMILGYDITAGNSQLNVYVKTGAIREFSGDTEYLLNDSREKYSFK

GNGWNNGVGVSAQYNKQHTFYLEADYTQGNLFDQKQVNGGYRFSF

>gi|30062725|ref|NP_836896.1| DNA-binding transcriptional regulator DhaR [Shigella flexneri 2a str. 2457T]

MRDMSGAFNNDGRGISPLIATSWERCNKLMKRETWNVPHQAQGVTFASIYRRKKAMLTLGQAALEDAWEY

MAPRECALFILDETACILSRNGDPQTLQQLSALGFNDGTYCAEGIIGTCALSLAAISGQAVKTMADQHFK

QALWNWAFCATPLFDSKGRLTGTIALACPVEQTTAADLPLTLAIAREVGNLLLTDSLLAETNRHLNQLNA

LLESMDDGVISWDEQGNLQFINAQAARVLRLDATASQGRAITELLTLPAVLQQAIKLAHPLKHVEATFES

QHQFIDAVITLKPIIETQGTSFILLLHPVEQMRQLMTSQLGKVSHTFAHMPQDDPQTRRLIHFGRQAARS

SFPVLLCGEEGVGKALLSQAIHNESERAAGPYIAVNCELYGDAALAEEFIGGDRTDNENGRLSRLELAHG

GTLFLEKIEYLAVELQSALLQVIKQGVITRLDARRLIPIDVKVIATTTADLAMLVEQNRFSRQLYYALHA

FEITIPPLRMRRGSIPALVNNKLRSLEKRFSTRLKIDDDALARLVSCAWPGNDFELYSVIENLALSSDNG

RIRVSDLPEHLFTEQATDDVSATRLSTSLSFAEVEKEAIINAAQVTGGRIQEMSALLGIGRTTLWRKMKQ

HGIDAGQFKRRV

>gi|30062724|ref|NP_836895.1| dihydroxyacetone kinase subunit DhaK [Shigella flexneri 2a str. 2457T]

MPYRNCWSKIMKKLINDVQDVLDEQLAGLAKAHPSLTLHQDPVYVTRADAPVAGKVALLSGGGSGHEPMH

CGYIGQGMLSGACPGEIFTSPTPDKIFECSMQIDGGEGVLLIIKNYTGDILNFETATELLHDSGVKVTTV

VIDDDVAVKDSLYTAGRRGVANTVLIEKLVGAAAERGDSLDACAELGRKLNNQGHSIGIALGACTVPAAG

KPSFTLADNEMEFGVGIHGEPGIDRRPFSSLDQTVDEMFDTLLEHGSYHRTLRFWDYQQGSWQEEPQTKQ

PLQSGDRVIALVNNLGATPLSELYGVYNRLTTRCQQAGLTIERNLIGAYCTSLDMTGFSITLLKVDDETL

ALWDAPVHTPALNWGK

>gi|30062723|ref|NP_836894.1| dihydroxyacetone kinase ADP-binding subunit [Shigella flexneri 2a str. 2457T]

MSLSRTQIVNWLTRCGDIFNTESEYLTGLDREIGDADHGLNMNRGFSKVVEKLPAIADKDIGFILKNTGM

TLLSSVGGASGPLFGTFFIRAAQATQARQSLTLEELYQMFRDGADGVISRGKAEPGDKTMCDVWVPVVES

LRQSSEQNLSVPVALEAASSIAESAAQSTITMQARKGRASYLGERSIGHQDPGATSVMFMMQMLALAAKE

>gi|30062722|ref|NP_836893.1| dihydroxyacetone kinase subunit M [Shigella flexneri 2a str. 2457T]

MVNLVIVSHSSRLGEGVGELARQMLMSDSCKIAIAAGIDDPQNPIGTDAVKVMEAIESVADADHVLVMMD

MGSALLSAETALELLAPEIAAKVRLCAAPLVEGTLAATVSAASGADIDKVIFDAMHALEAKREQLGLPSS

DTEISDTCPPYDEEARSLAVVIKNRNGLHVRPASRLVYTLSTFNADMLLEKNGKCVTPESINQIALLQVR

YNDTLRLIAKGPEAEEALIAFRHLAEDNFGETEEVAPPTLRPVLPVSGKAFYYQPVLCTVQAKSTLTAEE

EQDRLRQAIDFTLLDLMTLTAKAEASGLDDIAAIFSGHHTLLGDPELLAAASELLQHEHCTAEYAWQQVL

KELSQQYQQLDDEYLQARYIDVDDLLHRTLVHLTQTKEELPQFNSPTILLAENIYPSTVLQLDPAVVKGI

CLSAGSPVSHSALIARELGIGWICQQGEKLYAIQPEETLTLDVKTQRFNRQG

>gi|30062721|ref|NP_836892.1| trehalase [Shigella flexneri 2a str. 2457T]

MKSPAPSRPQKMALIPACIFLCFAALSVQAEETPVTPQPPDILLGPLFNDVQNVKLFPDQKTFADAVPNS

DPLMILADYRMQQNQSGFDLRHFVNVNFTLPKEGEKYVPPEGQSLREHIDGLWPVLTRSTENTEKWDSLL

PLPEPYVVPGGRFREVYYWDNYFTMLGLAESGHWDKIADMVANFAHEINTYGHIPNGNRSYYLSRSQPPF

FALMVELLAQHEGDAALKQYLPQMQKEYAYWMDGVENLQAGQQEKRVVKLQDSTLLNRYWDDRDTPRPES

WVEDIATAKSNPNRPATEIYRDLRSAAASGWDFSSRWMDNPQQLNTLRTTSIVPVDLNSLMFKMEKILAR

ASKAAGDNAMANQYETLANARQKGIEKYLWNDQQGWYADYDLKSHKVRNQLTAAALFPLYVNAAAKDRAN

KMATATKTHLLQPGGLNTTSVKSGQQWDAPNGWAPLQWVATEGLQNYGQKEVAMDISWHFLTNVQHTYDR

EKKLVEKYDVSTTGTGGGGGEYPLQDGFGWTNGVTLKMLDLICPKEQPCDNVPATHPTVKSATTQPSTKE

AQPTP

>gi|30062720|ref|NP_836891.1| IS1 orfA [Shigella flexneri 2a str. 2457T]

MASISIRCPSCSATEGVVRNGKSTAGHQRYLCSHCRKTWQLQFTYIASQPGTHQKIIDMAMNGVGCRASA

RIMGVGLNTVLRHLKNSGRSR

>gi|30062719|ref|NP_836890.1| IS1 orfB [Shigella flexneri 2a str. 2457T]

MSRQCTHYGRWPQHGFTSLKKLRPQSVTSRIQPGSDVIVCAEMDEHWGYVGAKSRQRWLFYAYDRIRRTV

VAHVFGERTLATLERLLSLLSAFEVVVWMTDGWPLYESRLKGKLHVISKRYTQRIERHNLNLRQHLARLG

RKSLSFSKSVELHDKVIGHYLNIKHYQ

>gi|30062718|ref|NP_836889.1| ABC transporter ATP-binding protein [Shigella flexneri 2a str. 2457T]

MPITRRTFAQALASTLLLQSLPSFSQTVNRFASQSLPEAQNITRIVSAGAPADLLLLAVAPEKMVGFSSF

DFARQALIPLPEHIRQLPRLGRLAGRASTLSLEGLMALHPDLVVDCGNTDETLISQARQVSEQTQIPWLL

LNGKLAQSAEQLTTLGKTLGEEHRAAEQANLASHFVGEAQAFATSPAANLRFYAARGPRGLETGLQGSLH

TEAAELLGLHNVAQIADRHGLTQVSMENLLRWQPDIILVQEAVTADFIRRDPLWQGVKAVAEQRILFLSG

LPFGWLDAPPGINRLLGLRRLHAWLDPAINRQFKSDMQHYAQLFWHCSLSDADYQKLVAS

>gi|30062717|ref|NP_836888.1| iron compound ABC transporter permease [Shigella flexneri 2a str. 2457T]

MRIVNGCILLLAAISITFAAVSGAYHLDMQQLLALILRQENESVQEQIVFWQIRVPRILAALLLGAALAG

AGTTYQGMLRNPLVSPDILGVSAGAGLGACAAILWGLPIVYIQLYAFCGGLMVVAGVWLITRRVTRHDPI

LTLVLVGIALGTLCGAGISLIKTLADPYTQLPSITFWLLGGLSTVTLRDLCYAAPIILIGSLPLFFLRWR

MNLLTLSDDEARSLGLNVTRLRFGLIVCATLITASTVAIAGIIGWIGLVVPHIARLLTGHNHQQLLPMAM

CTGAILLLLTDTLARSIGTTEIPLGILTAFVGAPFFLLLLLRGGRQ

>gi|30062716|ref|NP_836887.1| ferric enterobactin transport ATP-binding protein [Shigella flexneri 2a str. 2457T]

MTLLAVRHASLGYSRHPVLRDVSFTLSQGTICCLLGANGCGKTTLMRSILGVIPLLKGEILLDSLSVQTL

SHRQRAQAIAWVPQAHDGIFAFSVLDMVLMGLAPTIGAFSVPGKQERLKATEQLEKLGILHLATRRWNTL
SGGERQLALIARALVQQPRLLLLDEPASSLDFGHQIQLLDTLAQLKNNGMTMLMSTHHTLHANAIADSII
QVEPDGRVTQGLPTEQLTTNKLAALYRVSADQIHHHLSAISR

>gi|30062715|ref|NP_836886.1| hypothetical protein S1277 [Shigella flexneri 2a str. 2457T]
MLIDDIDFADLYLQQLKLAHRTEKTPDHWDQRAEKMAENCASPTDSYLQQLIAKIDLQGAQTLFDMGCGP
GTVSLALADKLTTVYGVDYSQGMLNVAARRAAALKADNVHWIQRAWEEDWSDLPRCDIAVASRSTLVADM
RHAMSKLNNQARLRVYTTHLVSTSFVSPAIQRAAGREVIELPNYIFALNVLYQMGIYAHVDFIRGQNCQQ
DNSTWERFEQNVSWSLGALNDDERERLYHWYQQQDARALAPASRDWALIWWDSVPQEAL

>gi|30062714|ref|NP_836885.1| molybdenum transport protein ModD [Shigella flexneri 2a str. 2457T]
MIFLSQAQIDALLLEDIQGGDLTTRALNIGHQPGYIAFFHRQGGCVSGVSVACKMLTTLGLTIDDAVSDG
SQANAGQRLIRAQGNAAALHQGWKAVQNVLEWSCGVSDYLAQMLALLRERYPDGNIACTRKAIPGTRLLA
SQAILAAGGLIHRAGCAETILLFANHRHFLHDNQGWSGAIAQLRRHAPEKKIVVEADTPKEAIAALRAQP
DVLQLDKFSPQQATEIAQIAPSLAPHCTLALTGGINLTTLKNYLDCGIRLFITSAPYYAAPADIKVSLQP
TASI

>gi|30062713|ref|NP_836884.1| hypothetical protein S1273 [Shigella flexneri 2a str. 2457T]
MGIIAWIIFGLIAGIIGKLIMPGRDGGGFFLTCILGIVGAVVGGWLATMFGIDGSISGFNLHSFLVAVVG
AILVLGVFRLLRRE

>gi|30062712|ref|NP_836883.1| hypothetical protein S1272 [Shigella flexneri 2a str. 2457T]
MSHYHEQFLKQNPLAVLGVLRDLHKAAIPLRLSWNGGQLISKILAITPDKLVLDFGSQAEDNIAVLKAQH
ITITAETQGAKVEFTVEQLQQSEYLQLPAFITVPPPTLWFVQRRRYFRISAPLHPPYFCQTKLADNSTLR
FRLYDLSLGGMGALLETAKPAELQEGMRFAQIEVNMGQWGVFHFDAQLISTSERKVIDGKNETITTPRLS

FRFLNVSPTVERQLQRIIFSLEREAREKADKVRD

>gi|30062711|ref|NP_836882.1| murein transglycosylase E [Shigella flexneri 2a str. 2457T]

MKQRHGNSLLTTETKCISCRLGVPLSPQRRFQAIRIEEVKLRWFAFLIVLLAGCSSKHDYTNPPWNAKVP

VQRAMQWMPISQKAGAAWGVDPQLITAIIAIESGGNPNAVSKSNAIGLMQLKASTSGRDVYRRMGWSGEP

TTSELKNPERNISMGAAYLNILETGPLAGIEDPKVLQYALVVSYANGAGALLRTFSSDRKKAISKINDLD

ADEFLDHVARNHPAPQAPRYIYKLEQALDAM

>gi|30062710|ref|NP_836881.1| L,D-carboxypeptidase A [Shigella flexneri 2a str. 2457T]

MSLFHLIAPSGYSIKQHAALRGIQRLTDAGHQVNNVEVIARRCERFAGTETERLEDLNSLARLTTPNTIV

LAVRGGYGASRLLADIDWQALVARQQHDPLLICGHSDFTAIQCGLLAQGNVITFSGPMLVANFGADELNA

FTEHHFWLALRNETFTIEWQGEGPTCQTEGTLWGGNLAMLISLIGTPWMPKIENGILVLEDINVHPFRVE

RMLLQLYHAGILPRQKAIILGSFSGSTPNDYDAGYNLESVYAFLRSRLSIPLITGLDFGHEQRTVTLPLG

AHAILNNTREGAQLTISGHPVLKM

>gi|30062708|ref|NP_836879.1| invasion plasmid antigen fragment, partial [Shigella flexneri 2a str. 2457T]

MKYLSPQKFSWGDAPWQIIDLSIAGKVNIQVDNNTIITLGTRLNQQHNEFMMVAKWCEWAIQQDGLQENL

QKNLYEILEENQQNKQSEIPQEDLKESLEEIKENILEENLPASRIENRAEALRRMKECLITRRSMLNLSN

LGLTSLPENLPPHLIEFYCSKNVLTALPKVMPKWLLVLDCTDNVLILLPEGAALKTDGTEVL

>gi|30062707|ref|NP_836878.1| alanine racemase [Shigella flexneri 2a str. 2457T]

MTRPIQASLDLQALKQNLSIVRQAAPHARVWSVVKANAYGHGIERIWSALGATDGFALLNLEEAITLRER

GWKGPILMLEGFFHAQDLEIYDQHRLTTCVHSNWQLKALQNARLKAPLDIYLKVNSGMNRLGFQSDRVLT

VWQQLRAMANVGEMTLMSHFAEAEHPDGISGAMARIEQAAEGLECRRSLSNSAATLWHPEAHFDWVRPGI

ILYGASPSGQWRDIANTGLRPVMTLSSEIIGVQTLKAGERVGYGGRYTARDEQRIGIVAAGYADGYPRHA

PTGAPVLVDGVRTMTVGTVSMDMLAVDLTPCPQAGIGTPVELWGKEIKIDDVAAAAGTVGYELMCALALR

VPVVTV

>gi|30062706|ref|NP_836877.1| D-amino acid dehydrogenase small subunit [Shigella flexneri 2a str. 2457T]

MRVVILGSGVVGVASAWYLNQAGHEVTVIDREPGAALETSAANAGQISPGYAAPWAAPGVPLKAIKWMFQ

RHAPLAVRLDGTQFQLKWMWQMLRNCDTSHYMENKGRMVRLAEYSRDCLKALRAETNIQYEGRQGGTLQL

FRTEQQYENATRDIAVLEDAGVPYQLLESSRLAEVDPALAEVAHKLTGGLQLPNDETGDCQLFTQNLARM

AEQAGVKFRFNTPVDQLLCDGEQIYGVKFGDEVIKADAYVMAFGSYSTAMLKGIVDIPVYPLKGYSLTIP

IAQEDGAPVSTILDETYKIAITRFDNRIRVGGMAEIVGFNTELLQPRRETLEMVVRDLYPRGGHVEQATF

WTGLRPMTPDGTPVVGSTRFKNLWLNTGHGTLGWTMACGSGQLLSDLLSGRTPAIPYEDLSVARYSRGFT

PSRPGHLHGAHS

>gi|30062705|ref|NP_836876.1| SpoVR family protein [Shigella flexneri 2a str. 2457T]

MATIDSMNKDTTRLSDGPDWTFDLLDVYLAEIDRVAKLYRLDTYPHQIEVITSEQMMDAYSSVGMPINYP

HWSFGKKFIETERLYKHGQQGLAYEIVINSNPCIAYLMEENTITMQALVMAHACYGHNSFFKNNCLFRSW

TDASSIVDYLIFARKYITECEERYGVDEVERLLDSCHALMNYGVDRYKRPQKISLQEEKARQKSREEYLQ

SQVNMLWRTLPKREEEKTVAEARRYPSEPQENLLYFMEKNAPLLESWQREILRIVRKVSQYFYPQKQTQV

MNEGCATFWHYTILNHLYDEGKVTERFMLEFLHSHTNVVFQPPYNSPWYSGINPYALGFAMFQDIKRICQ

SPTEEDKYWFPDIAGSDWLETLHFAMRDFKDESFISQFLSPKVMRDFRFFTVLDDDRHNYLEISAIHNEE

GYREIRNRLSSQYNLSNLEPNIQIWNVDLRGDRSLTLRYIPHNRAPLDRGRKEVLKHVHRLWGFDVMLEQ

QNEDGSIELLERCPPRMGNL

>gi|30062704|ref|NP_836875.1| fatty acid metabolism regulator [Shigella flexneri 2a str. 2457T]

MVIKAQSPAGFAEEYIIESIWNNRFPPGTILPAERELSELIGVTRTTLREVLQRLARDGWLTIQHGKPTK

VNNFWETSGLNILETLARLDHESVPQLIDNLLSVRTNISTIFIRTAFRQHPDKAQEVLATANEVADHADA

FAELDYNIFRGLAFASGNPIYGLILNGMKGLYTRIGRHYFANPEARSLALGFYHKLSALCSEGAHDQVYE

TVRRYGHESGEIWHRMQKNLPGDLAIQGR

>gi|30062703|ref|NP_836874.1| sodium/proton antiporter [Shigella flexneri 2a str. 2457T]

MEISWGRALWRNFLGQSPDWYKLALIIFLIVNPLIFLISPFVAGWLLVAEFIFTLAMALKCYPLLPGGLL

AIEAVFIGMTSAEHVREEVAANLEVLLLLMFMVAGIYFMKQLLLFIFTRLLLSIRSKMLLSLSFCVAAAF

LSAFLDALTVVAVVISVAVGFYGIYHRVASSRTEDTDLQDDSHIDKHYNVVLEQFRGFLRSLMMHAGVGT

ALGGVMTMVGEPQNLIIAKAAGWHFGDFFLRMSPVTVPVLICGLLTCLLVEKLRWFGYGETLPEKVREVL

QQFDDQSRHQRTRQDKIRLIVQAIIGVWLVTALALHLAEVGLIGLSVIILATSLTGVTDEHAIGKAFTES

LPFTALLTVFFSVVAVIIDQQLFSPIIQFVLQASEHAQLSLFYIFNGLLSSISDNVFVGTIYINEAKAAM

ESGAITLKQYELLAVAINTGTNLPSVATPNGQAAFLFLLTSALAPLIRLSYGRMVWMALPYTLVLTLVGL

LCVEFTLAPVTEWFMQMGWIATL

>gi|30062702|ref|NP_836873.1| disulfide bond formation protein B [Shigella flexneri 2a str. 2457T]

MLRFLNQCSQGRGAWLLMAFTALALELTALWFQHVMLLKPCVLCIYERCALFGVLGAALIGAIAPKTPLR

YVAMVIWLYSAFRGVQLTYEHTMLQLYPSPFATCDFMVRFPEWLPLDKWVPQVFVASGDCAERQWDFLGM

EMPQWLLGIFIAYLIVAVLVVISQPFKAKKRDLFGR

>gi|30062701|ref|NP_836872.1| DNA polymerase V subunit UmuC [Shigella flexneri 2a str. 2457T]

MFALCDVNAFYASCETVFRPDLWGKPVVVLSNNDGCVIARNAEAKALGVKMGDPWFKQKDLFRRCGVVCF

SSNYELYADMSNRVMSTLEELSPRVEIYSIDEAFCDLTGVRNCRDLTDFGREIRATVLQRTHLTVGVGIA

QTKTLAKLANHAAKKWQRQTGGVVDLSNLERQRKLMSALPVDDVWGIGRRISKKLDAMGIKTVLDLADTD

IRFIRKHFNVMLERTVRELRGEPCLQLEEFAPTKQEIICSRSFGERITDYTSMRQAICSYAARAAEKLRS

EHQYCRFISTFIKTSPFALNEPYYGNSASVKLLTPTQDSRDIINAATRSLDAIWQAGHRYQKAGVMLGDF

FSQGVAQLNLFDDNAPRPGSEQLMAVMDTLNAKEGRGTLYFAGQGIQQQWQMKRAMLSPRYTTRSSDLLR

VK

>gi|30062700|ref|NP_836871.1| DNA polymerase V subunit UmuD [Shigella flexneri 2a str. 2457T]

MLFIKPADLREIVTFPLFSDLVQCGFPSPAADYVEQRIDLNQLLIQHPSATYFVKASGDSMIDGGISDGD

LLIVDSAITASHGDIVIAAVDGEFTVKKLQLRPTVQLIPMNSAYSPITISSEDTLDVFGVVIHVVKAMR

>gi|30062698|ref|NP_836869.1| hypothetical protein S1257 [Shigella flexneri 2a str. 2457T]

MYQHHNWQGALLDYPVSKVVCVGSNYAKHIKEMGSAVPEEPVLFIKPETALCDLRQPLAIPSDFGSVHHE

VELAVLIGATLRQATEEHVRKAIAGYGVALDLTLRDVQGKMKKAGQPWEKAKAFDNSCPLSGFIPAAEFT

GDPQNTTLGLSVNGEKRQQGTTADMIHKIVPLIAYMSKFFTLKAGDVVLTGTPDGVGPLQSGDELTVTFD

GHSLTTRVL

>gi|30062697|ref|NP_836868.1| hypothetical protein S1256 [Shigella flexneri 2a str. 2457T]

MPKPGILKSKSMFCAIYRSSKRDQTYLYVEKKDDFSRVPEELMKGFGQPQLAMILPLDGRKKLVNADIEK

VKLALTEQGYYLQLPPPPEDLLKQHLSVMGQKTDDTNK

>gi|30062696|ref|NP_836867.1| IS3 orfB [Shigella flexneri 2a str. 2457T]

MKYVFIEKHQAEFSIKAMCRVLRVARSGWYTWCQRRTRISTRQQFRQHCDSVVLAAFTRSKQRYGAPRLT

DELRAQGYPFNVKTVAASLRRQGLRAKASRKFSPVSYRAHGLPVSENLLEQDFYASGPNQKWAGDITYLR

TDEGWLYLAVVIDLWSRAVIGWSMSPRMTAQLACDALQMALWRRKRPRNVIVHTDRGGQYCSADYQAQLK

RHNLRGSMSAKGCCYDNACVESFFHSLKVECIHGEHFISREIMRATVFNYIECDYNRWRRHSWCGGLSPE

QFEN

>gi|30062695|ref|NP_836866.1| IS3 orfA [Shigella flexneri 2a str. 2457T]

MTKTVSTSKKPRKQHSPEFRSEALKLAERIGVTAAARELSLYESQLYNWRSKQQNQQTSSERELEMSTEI

ARLKRQLAERDEELAILQKAATYFAKRLK

>gi|30062693|ref|NP_836864.1| septum formation inhibitor [Shigella flexneri 2a str. 2457T]

MSNTPIELKGSSFTLSVVHLHEAEPKVIHQALKDKIAQAPTFLKHAPVVLNVSALEDPVNWSAMHKAVSA

TGLRVIGVSGCKDAQLKAEIEKMGLPILTEGKEKAPRPAPAPQAPAQNTMPVTKTRLIDTPVRSGQRIYA

PQCDLIVTSHVSAGAELIADGNIHVYGMMRGRALAGASGDRETQIFCTNLMAELVSIAGEYWLSDQIPAE

FYGKAARLQLVENALTVQPLN

>gi|30062692|ref|NP_836863.1| cell division inhibitor MinD [Shigella flexneri 2a str. 2457T]

MARIIVVTSGKGGVGKTTSSAAIATGLAQKGKKTVVIDFDIGLRNLDLIMGCERRVVYDFVNVIQGDATL

NQALIKDKRTENLYILPASQTRDKDALTREGVAKVLDDLKAMDFEFIVCDSPAGIETGALMALYFADEAI

ITTNPEVSSVRDSDRILGILASKSRRAENGEEPIKEHLLLTRYNPGRVSRGDMLSMEDVLEILRIKLVGV

IPEDQSVLRASNQGEPVILDINADAGKAYADTVERLLGEERPFRFIEEEKKGFLKRLFGG

>gi|30062691|ref|NP_836862.1| cell division topological specificity factor MinE [Shigella flexneri 2a str. 2457T]

MALLDFFLSRKKNTANIAKERLQIIVAERRRSDAEPHYLPQLRKDILEVICKYVQIDPEMVTVQLEQKDG

DISILELNVTLPEAEELK

>gi|30062690|ref|NP_836861.1| hypothetical protein S1246 [Shigella flexneri 2a str. 2457T]

MRKEFVDDNRVKVNSDGNFVNDLSGRRGIYQAGIKASFSSTLSGHFGVGYSHGAGVESPWNAVAGVNWSF

>gi|30062689|ref|NP_836860.1| hypothetical protein S1245 [Shigella flexneri 2a str. 2457T]

MADMNRTTKGALLGAGVGLLTGNGVNGVLKGAAVGAGVSAVTEKGRDGKNARKGAKVGAAVGAVTGVLTG

NGLEGAIKGAVIGGTGGAILGKMK

>gi|30062688|ref|NP_836859.1| hypothetical protein S1244 [Shigella flexneri 2a str. 2457T]

MMMKKFALLAGLFVFAPMTWAQDYNIKNGLPSETYITCAEANEMAKTDSAQVAEIVAVMGNASVASRDLK

IEQSPELSAKVVEKLNQVCAKDPQMLLITAIDDTMRAIGKK

>gi|30062687|ref|NP_836858.1| hypothetical protein S1242 [Shigella flexneri 2a str. 2457T]

MQGKNTIVTTGDYSIGLLSQTSGNLNTDTIIRVNSDGSVTPSFSDGDDTFIVTAGNHAVGVLACASPGSA

CACVSSLDEESTADTGSNENNAIAKLDMAKGEITTHGTESYAAYANGTVVKAGDTLDYTNASVTLTDVDI

TTHGDNAHAIAARQGTVSFNQGEIYTTGPDAAIAKIYNGGTVTLKNTSAVAHQGSGIVLESSINGQEATV

DILSGSSLRSANEILYHKDETSNVTITDSEVSSAADVFINNIKGHLTVDATNSKITGSANISTDDNTHTY

LSLSDNSTWDIKADSTVSNLTVDNSTVYISRADGRDVEPTRLTITENYVGNNGVLHLRTELDDDNSATDK

VVINGNTSGTTRVKVTNAGGSGAYTLNGIEIISVEGESNGEFIKDSRIFAGAYEYSLTRGNTEATNKNWY

LTNFQATSGGETNSGGSSAPTVAPTPVLRPEAGSYVANLAAANTLFVMRLNDRAGETRYIDPVTEQERSS

RLWLRQIGGHNAWRDSNGQLRTTSHRYVSQLGGDLLTGGFTDSDSWRLGVMAGYARDYNLTHSSVSDYRS

KGSVRGYSAGLYATWFADDISKKGAYIDSWAQYSWFKNSVKGDELAYESYSAKGATVSLEAGYGFALNKS

FGLEAAKYTWIFQPQAQAIWMGVDHNAHTEANGSRIENDANNNIQTRLGFRTFIRTQEKNSGPHGDDFEP

FVEMNWIHNSKDFAVSMNGVKVEQDGVSNLGEIKLGVNGNLNPAASVWGNVGVQLGDNGYNDTAVMVGLK

YKF

>gi|30062686|ref|NP_836857.1| hypothetical protein S1241 [Shigella flexneri 2a str. 2457T]

MRNTLIPILVAICLFITGVAILNIQLWYSAKAEYLAGARYAANNINHILEEASQATQTAVNIAGKECDLE

EQYQLGTEAALKPHLRTIIILKQGIVWCTSLPGNRVLLSRIPVVPDSNLLLAPAIDTVNRLPILLYQSQF

ADTRILVTISDQHIRGALNVPLKGVRYVLRVADDIIGPTADVMTLNGHYPYTEKVHSTKYHFTIIFNPPP

LFSFYRLIDKGFGILIFILLIASAAAFLLDRYFNKSATPEEILRRAINNGEIVPFYQPVVNGREGTLRGV

EVLARWKQPHGGYISPAAFIPLAEKSGLIVPLTQSLMNQVARQMNAIASKLPEGFHIGINFSASHIISPT

FVDECLNFRDSFTRRDLNLVLEVTEREPLNVDESLVQRLNILHENGFVIALDDFGTGYSGLSYLHDLHID

YIKIDHSFVGRVNADPESTRILDCVLDLARKLSISIVAEGVETKEQLGYLNQNNITFQQGYYFYKPVTYI

DLVKIILSKPKVKVVVE

>gi|30062685|ref|NP_836856.1| IS1 orfA [Shigella flexneri 2a str. 2457T]

MASISIRCPSCSATEGVVRNGKSTAGHQRYLCSHCRKTWQLQFTYTASQPGTHQKIIDMAMNGVGCRASA

RIMGVGLNTVLRHLKNSGRSR

>gi|30062684|ref|NP_836855.1| IS1 orfB [Shigella flexneri 2a str. 2457T]

MSRQRTHYGRWPQHGFTSLKKLRPQSVTSRIQPGSDVIVCAEMDEHWGYVGAKSRQRWLFYAYDRIRRTV

VAHVFGERTLATLERLLSLLSAFEVVVWMTDGWPLYESRLKGKLHVISKRYTQRIERHNLNLRQHLARPV

RKSLSFSKSVELHDKVIGHYLNIKHYQ

>gi|30062683|ref|NP_836854.1| isocitrate dehydrogenase [Shigella flexneri 2a str. 2457T]

MESKVVVPAQGKKITLQNGKLNVPENPIIPYIEGDGIGVDVTPAMLKVVDAAVEKAYKGERKISWMEIYT

GEKSTQVYGQDVWLPAETLDLIREYRVAIKGPLTTPVGGGIRSLNVALRQELDLYICLRPVRYYQGTPSP

VKHPELTDMVIFRENSEDIYAGIEWKADSADAEKVIKFLREEMGVKKIRFPEHCGIGIKPCSEEGTKRLV

RAAIEYAIANDRDSVTLVHKGNIMKFTEGAFKDWGYQLAREEFGGELIDGGPWLKVKNPNTGKEIVIKDV

IADAFLQQILLRPAEYDVIACMNLNGDYISDALAAQVGGIGIAPGANIGDECALFEATHGTAPKYAGQDK

VNPGSIILSAEMMLRHMGWTEAADLIVKGMEGAINAKTVTYDFERLMEGAKLLKCSEFGDAIIKNM

>gi|30062682|ref|NP_836853.1| 23S rRNA pseudouridine synthase E [Shigella flexneri 2a str. 2457T]

MQKTSFRNHQVKRFSSQRSTRRKHENQPTRVILFNKPYDVLPQFTDETGRKTLKEFIPVQGVYAAGRLDR

DSEGLLVLTNNGALQARLTQPGKRTGKIYYVQVEGIPTQDALEALRNGVTLNDGPTLPAGAELVDEPAWL

WPRNPPIRERKSIPTSWLKITLYEGRNRQVRRMTAHVGFPTLRLIRYAMGDYSLDNLANGEWREVTD

>gi|30062681|ref|NP_836852.1| phosphohydrolase [Shigella flexneri 2a str. 2457T]

MFKPHVTVACVVHAEGKFLVVEETINGKALWNQPAGHLEADETLVEAAARELWEETGISAQPQHFIRMHQ

WIAPDKTPFLRFLFAIELEQICPTQPHDSDIDCCRWVSAEEILQASNLRSPLVAESIRCYQSGQRYPLEM

IGDFNWPFTKGVI

>gi|30062679|ref|NP_836850.1| hypothetical protein S1234 [Shigella flexneri 2a str. 2457T]

MAKNYYDITLALAGICQSARLVQQLAHQGHCDADALHVSLNSIIDMNPSSTLAVFGGSEANLRVGLETLL

GVLNASSCQGLNAELTRYTLSLMVLERKLSSAKGALDTLGNRINGLQRQLEHFDLQSETLMSAMAAIYVD

VISPLGPRIQVTGSPAVLQSPQVQAKVRATLLAGIRAAVLWHQVGGGRLQLMFSRNRLTTQAKQILAHLT

PEL

>gi|30062678|ref|NP_836849.1| adenylosuccinate lyase [Shigella flexneri 2a str. 2457T]

MELSSLTAVSPVDGRYGDKVSALRGIFSEYGLLKFRVQVEVRWLQKLAAHAAIKEVPAFAADAIGYLDAI

VANFSEEDAARIKTIERTTNHDVKAVEYFLKEKVAEIPELHAVSEFIHFACTSEDINNLSHALMLKTARD

EVILPYWRQLIDGIKDLAVQYRDIPLLSRTHGQPATPSTIGKEMANVAYRMERQHRQLNQVEILGKINGA

VGNYNAHIAAYPEVDWHQFSEEFVTSLGIQWNPYTTQIEPHDYIAELFDCVARFNTILIDFDRDVWGYIA

LNHFKQKTIAGEIGSSTMPHKVNPIDFENSEGNLGLSNAVLQHLASKLPVSRWQRDLTDSTVLRNLGVGI

GYALIAYQSTLKGVSKLEVNRDHLLDELDHNWEVLAEPIQTVMRRYGIEKPYEKLKELTRGKRVDAEGMK

QFIDGLALPEEEKARLKAMTPANYIGRAITMVDELK

>gi|30062677|ref|NP_836848.1| DNA-binding transcriptional regulator PhoP [Shigella flexneri 2a str. 2457T]

MRVLVVEDNALLRHHLKVQIQDAGHQVDDAEDAKEADYYLNEHLPDIAIVDLGLPDEDGLSLIRRWRSND

VSLPILVLTARESWQDKVEVLSAGADDYVTKPFHIEEVMARMQALMRRNSGLASQVISLPPFQVDLSRRE

LSINDEVIKLTAFEYTIMETLIRNNGKVVSKDSLMLQLYPDAELRESHTIDVLMGRLRKKIQAQYPQEVI

TTVRGQGYLFELR

>gi|30062676|ref|NP_836847.1| sensor protein PhoQ [Shigella flexneri 2a str. 2457T]

MKKLLRLFFPLSLRVRFLLATAAVVLVLSLAYGMVALIGYSVSFDKTTFRLLRGESNLFYTLAQWENNKL

HVELPENIDKQSPTMTLIYDENGQLLWAQRDVPWLMKMIQPDWLKSNGFHEIEADVNDTSLLLSGDHSIQ

QQLQEVREDDDDAEMTHSVAVNVYPATSRMPKLTIVVVDTIPVELKSSYMVWSWFIYVLSANLLLVIPLL

WVAAWWSLRPIEALAKEVRELEEHNRELLNPATTRELTSLVRNLNRLLKSERERYDKYRTTLTDLTHSLK

TPLAVLQSTLRSLRSEKMSVSDAEPVMLEQISRISQQIGYYLHRASMRGGTLLSRELHPVAPLLDNLTSA

LNKVYQRKGVNISLDISPEISFVGEQNDFVEVMGNVLDNACKYCLEFVEISARQTDEHLYIVVEDDGPGI

PLSKREVIFDRGQRVDTLRPGQGVGLAVAREITEQYEGKIVAGESMLGGARMEVIFGRQHSAPKDE

>gi|30062675|ref|NP_836846.1| hypothetical protein S1229 [Shigella flexneri 2a str. 2457T]

MTEAEILRLIRRVSGISQQADEQTTQPDSVTAENYARVVAEVMRRDGIQLNDADMRDIRIRVLEMLAYNR

RVELYREKEKITYHWKKPERLRR

>gi|30062674|ref|NP_836845.1| terminase of prophage CP-933C [Shigella flexneri 2a str. 2457T]

MRGRPIELEPWQQFAFACILGFKVKATGRRKYTSAFIEVPRKNAKSTTAAILANWFLIMENGQQDIYTAA

VSRDQARIVFDNARQMCLLSRPLRRRVNIQAHKVIHPKSNSLLKPLAAKAATIEGTNPSLSIVDEYHLHP

DNGVYSALELGMGARPEGLLFAITTSGSNVVSACKQHYDYCCQILDGEEVNDSIFVLIYELDDENEVDDP

AMWIKANPNIDVSVDREKLASTIQKARGIPSQWVEMMTKRFNIWCQGTTPWMGNGAWAECAGTFTEEDLH

GQECYAGLDLSSTSDISSVCYAFPVGKTIMLISRHYLPEFQLQNPANKNRAVYRQWAKAGWIRTTPGDCI

DYDRIRDDIMQDAEKFNIRLVGFDTWNATHLRTQLQGAGFEVEPFPQTYLRFSPAAKSFEVFVNRRVIVH

RGDPVLSWSMSNVVMQSDANANIKPNKKKSPNKIDPSVAALMAFGTFQAEHEDFAFDISDSHRQKLEEFS

GV

>gi|30062673|ref|NP_836844.1| hypothetical protein S1227 [Shigella flexneri 2a str. 2457T]

MKQWREKSRQLAERGDLTPADWSNLELYCVNYSIYRKAVADLAARGFSIVNSQGGESRNPALSAKSDAER

VMIKMASLLGFDPISRRKNPPETEEEDELDRLE

>gi|30062672|ref|NP_836843.1| holin protein of prophage CP-933C [Shigella flexneri 2a str. 2457T]

MVVDHIIPINGGDDVLFWPEWNHQPLCQACHNQKTKWLDPATKSKRAAGGFREEEERAANRNNWMYDADE

>gi|30062671|ref|NP_836842.1| hypothetical protein S1225 [Shigella flexneri 2a str. 2457T]

MTEELITLEEVKLHCRIDGDDEDQLISGYIAASLEACQIHIGRRFDDGLAFTPAIKIGCMMFIAHLYENR

QIVADNAKTRVPMTIGALWTAYRDVGGY

>gi|30062670|ref|NP_836841.1| head-tail adaptor [Shigella flexneri 2a str. 2457T]

MNIGRLRDRITIQTLKQTRDITGEILETWEDGHTLWASVNMVSSKEAISSGAELAIGTVRIWIRYRKDIN

ATSRIKVSTGPLAGRVLNIIGQPLPDAARTRLEILCREGAEK

>gi|30062669|ref|NP_836840.1| head portal protein [Shigella flexneri 2a str. 2457T]

MTLDEFMALAGTSNTGAGEYVSSGTAESLPAVMNAVTVISDAVATMPCYLYLVRNEKGKEAREWLDSHPV

DHILNERPNAWQTPYQFKRMMIRYCLLSGNAYAVIQWGRDGFPAALHPYPPQSVNVEQTGDHNWRYCITD

AYTGNIRNYLPWEVLHLRYSTDDGFMGRSPVTICRESLGLGLAQQRHGASVMRDGMMAAGVITSGEWLDG

VKGKQALAALERYKGARNAGKTPILEGGMSYQQLGMSNQDAEWLASRRFTIEDIARMFNVSPIFLQEYSN

STYSNFSEASRAFLTMTMRPWLANFEQQIKNALLVASPVPGIRYQVEFDSADLLRATPGERFATYERGIK

SDVMCPNEAREREGLSPRDGGDEFS

>gi|30062668|ref|NP_836839.1| head maturation protease of prophage CP-933C [Shigella flexneri 2a str. 2457T]

MGEKIMKSMEIRSSEITTSGAGTLTGYVVRWDKLSELLWGEFYEKFQRGAFTEWLAAGNDVRGLYEHDHS

MLLGRTRSGTLKLEEDETGLRFELTPPDTSTGRDVIELVKRGDISGMSFGFRSRKDVWDTTTDPCVRTVL

VAELYEITVTSVPAYPDSGVELARRSLYEQHPEKMPRADNRRWWADLAGV

>gi|30062667|ref|NP_836838.1| hypothetical protein S1219 [Shigella flexneri 2a str. 2457T]

MPMKFDEILKQRDKYHADNMETMNITDYRAFLETGALIEKDHHGFVRCALSGEMLAVNPEQTDALIEFLK

GIRD

>gi|30062666|ref|NP_836837.1| hypothetical protein S1218 [Shigella flexneri 2a str. 2457T]

MTAQIAAYGQLVGDPLVKQSSKGTPEPPTENYADLRNAVNRTYADMGLSKLAE

>gi|30062665|ref|NP_836836.1| hypothetical protein S1217 [Shigella flexneri 2a str. 2457T]

MSEPKFGEKFYKHNGRITILQISAATPGWWVETDEGSSPVASWELCVLSYPDRDVYQDILPVISTDKGMK

PVDIKNMGFQCVMLTEKMMEEMKKNNAGSLH

>gi|30062664|ref|NP_836835.1| hypothetical protein S1216 [Shigella flexneri 2a str. 2457T]

MTEHALKVAIRTIDRHAGEGYAKAHPELISAFMTTTAANFATLTEREIAEAEQVTTINVKTGEVES

>gi|30062663|ref|NP_836834.1| bacteriophage P4 DNA primase [Shigella flexneri 2a str. 2457T]

MKLAPNVKKQPRGIKHKDTEVIIFAGSDAWSHAKQWQEQDGPASGDNVPPVWLGPNQLAELDALKIVPDG

KKRVRLYQAGELDLVETKKIGQKLAAADIQDANFYPEGMHVQKCENWRRYLNAERENIAAGLTMPEQKNT

QLAQMADSERAQLLAERFDGVCVHQESEIVHVWRGGVWCPVSTMELSREMVVIYSEHRATFSKRVINNAV

EALKVIAEPMGEPSGDLLPFANGALDLKTGEFSPHTPENWITTHNGIEYTPPAPGENIRDNALNFHKWLE

HAAGKDQRKMMRICAALYMIMANRYDWQMFIEATGDGGSGKSTFTHIASLLAGKQNTVSAEMTSLDDAGG

RAQVVGSRLIVLADQPKYTGEGTGIKKITGGDPVEINPKYEKRFTAVIRAVVLATNNNPMIFTERAGGVA

RRRVIFRFDNIVSEAEKDRELPEKIAAEIPVIIRRLLANFTDSEKARALLLEQRDGDEALAIKQQTDPVI

EFCQFLNFLEEARGLMMGGGGDSVKYTTRNSLYRVYLAFMAYAGRSKPLNVNDFGKAMKPAAKVYGHEYI

TRKVKGVTQTNAITTDECDAFL

>gi|30062662|ref|NP_836833.1| hypothetical protein S1214 [Shigella flexneri 2a str. 2457T]

MKLKYSGLTASGNTHPKFTRGDIYRDQYGGMVTIKGVAERRITYRREGYEYDCVMPVYQFRRDFTLVQAA

PRSKPTSREKARANIQEIKKMLNVFRGKK

>gi|30062661|ref|NP_836832.1| hypothetical protein S1212 [Shigella flexneri 2a str. 2457T]

MCSDTLHTINTKSKAPHCRSSNGASNQQRKLGAVMVAVNHIPHLVHTQTAFVWRFLALSAGESQIIHVTA

WTEREARSRCPSGCVAVFAARIRQGETYAQ

>gi|30062660|ref|NP_836831.1| integrase of prophage CP-933C [Shigella flexneri 2a str. 2457T]

MSRALNKLSDTQLRKINGTPAQKTAFLNDGGNLSVRHSTSGLLTWYFTYRAGTGRGAPPERIKLGNYPDL

SLKSAREKAAQCRAWLAEGKNPRHELNYTVQEALKPVTVGDALTYWLESYAKENRVDYAALKKRLNNHVI

QHIGAMPLDKCELRHWLACFDQVAKRTPVTAGFLLQTCKQALKFCRRRRYAISNVLDDMSVADVGKKPDI

SERVLSTKELGELLQALDKKIFSPYYIALIRLLIVFGCRTVELRLSEISEWDFTEMLWTVPKEHSKTKVA

IFRPIPEAILPFVTQLVEQNRHTGLLLGEVKQETSVSQYGRLAHRRLNHPHWSLHDIRRTFTTMLNDLGV

DPHVVEQLTGHQMPGMQRVYNHSRYLDAKRNALDMWTERLGILAGTHENVTTLPVARRK

>gi|30062659|ref|NP_836830.1| hypothetical protein S1210 [Shigella flexneri 2a str. 2457T]

MLNMEYQLTLNWPDFLERHWQKRPVVLKRGFNNFIDPISPDELAGLAMESEVDSRLVSHQDGKWQVSHGP

FESYDHLGETNWSLLVQAVNHWHEPTAALMRPFRELPDWRIDDLMISFSVPGGGVGPHLDQYDVFIIQGT

GRRRWRVGEKLQMKQHCPHPDLLQVDPFEAIIDEELEPGDILYIPPGFPHEGYALENAMNYSVGFRAPNT

RELISGFADYVLQRELGGNYYSDPDVPPRAHPADVLPQEMDKLREMMLELINQPEHFKQWFGEFISQSRH

ELDIAPPEPPYQPDEIYDALKQGDVLVRLGGLRVLRIGDDVYANGEKIDSPHRPALDALASNIALTAENF

GDALEDPSFLAMLAALVNSGYWFFEG

>gi|30062658|ref|NP_836829.1| peptidase T [Shigella flexneri 2a str. 2457T]

MDKLLERFLNYVSLDTQSKAGVRQVPSTEGQWKLLHLLKEQLEEMGLINVTLSEKGTLMATLPANVPGDI

PAIGFISHVDTSPDCSGKNVNPQIVENYRGGDIALGIGDEVLSPVMFPVLHQLLGQTLITTDGKTLLGAD

DKAGIAEIMTALAVLQQKNIPHGDIRVAFTPDEEVGKGAKHFDVDAFDARWAYTVDGGGVGELEFENFNA

ASVNIKIVGNNVHPGTAKGVMVNALSLAARIHAEVPADESPEMTEGYEGFYHLASMKGTVERADMHYIIR

DFDRKQFEARKRKMMEIAKKVGKGLHPDCYIELVIEDSYYNMREKVVEHPHILDIAQQAMRDCDIEPELK

PIRGGTDGAQLSFMGLPCPNLFTGGYNYHGKHEFVILEGMEKAVQVIVRIAELTAQRK

>gi|30062657|ref|NP_836828.1| putrescine/spermidine ABC transporter ATPase [Shigella flexneri 2a str. 2457T]

MGQSKKLNKQPSSLSPLVQLAGIRKCFDGKEVIPQLDLTINNGEFLTLLGPSGCGKTTVLRLIAGLETVD

SGRIMLDNEDITHVPAENRYVNTVFQSYALFPHMTVFENVAFGLRMQKTPAAEITPRVMEALRMVQLETF

AQRKPHQLSGGQQQRVAIARAVVNKPRLLLLDESLSALDYKLRKQMQNELKALQRKLGITFVFVTHDQEE

ALTMSDRIVVMRDGRIEQDGTPREIYEEPKNLFVAGFIGEINMFNATVIERLDEQRVRANVEGRECNIYV

NFAVEPGQKLHVLLRPEDLRVEEINDDNHAEGLIGYVRERNYKGMTLESVVELENGKMVMVSEFFNEDDP

DFDHSLDQKMAINWVESWEVVLADEEHK

>gi|30062655|ref|NP_836826.1| spermidine/putrescine ABC transporter [Shigella flexneri 2a str. 2457T]

MIGRLLRGGFMTAIYAYLYIPIIILIVNSFNSSRFGINWQGFTTKWYSLLMNNDSLLQAAQHSLTMAVFS

ATFATLIGSLTAVALYRYRFRGKPFVSGMLFVVMMSPDIVMAISLLVLFMLLGIQLGFWSLLFSHITFCL

PFVVVTVYSRLKGFDVRMLEAAKDLGASEFTILRKIILPLAMPAVAAGWVLSFTLSMDDVVVSSFVTGPS

YEILPLKIYSMVKVGVSPEVNALATILLVLSLVMVIASQLIARDKTKGNTGDVK

>gi|30062654|ref|NP_836825.1| spermidine/putrescine ABC transporter substrate-binding protein [Shigella flexneri 2a str. 2457T]

MKKWSRHLLAAGALALGMSAAHADDNNTLYFYNWTEYVPPGLLEQFTKETGIKVIYSTYESNETMYAKLK

TYKDGAYDLVVPSTYYVDKMRKEGMIQKIDKSKLTNFSNLDPDMLNKPFDPNNDYSIPYIWGATAIGVNG

DAVDPKSVTSWADLWKPEYKGSLLLTDDAREVFQMALRKLGYSGNTTDPKEIEAAYNELKKLMPNVAAFN

SDNPANPYMEGEVNLGMIWNGSAFVARQAGTPIDVVWPKEGGIFWMDSLAIPANAKNKEGALKLINFLLR

PDVAKQVAETIGYPTPNLAARKLLSPEVANDKTLYPDAETIKNGEWQNDVGAASSIYEEYYQKLKAGR

>gi|30062653|ref|NP_836824.1| NAD-dependent deacetylase [Shigella flexneri 2a str. 2457T]

MLSRRGHRLSRFRKNKRRLRERLRQRIFFRDKVVPEAMEKPRVLVLTGAGISAESGIRTFRAADGLWEEH

RVEDVATPEGFDRDPELVQAFYNARRRQLQQPEIQPNAAHLALAKLQDALGDRFLLVTQNIDNLHERAGN

TNVIHMHGELLKVRCSQSGQVLDWTGDVTPEDKCHCCQFPAPLRPHVVWFGEMPLGMDEIYMALSMADVF

IAIGTSGHVVYPAAGFVHEAKLHGAHTVELNLEPSQVGNEFAEKYYGPASQVVPEFVEKLLEGL

>gi|30062652|ref|NP_836823.1| N-acetyl-D-glucosamine kinase [Shigella flexneri 2a str. 2457T]

MYYGFDIGGTKIALGVFDSGRQLQWEKRVPTPRDSYDAFLDAVCELVAEADQRFGCKGSVGIGIPGMPET

EDGKLYAANVPAASGKPLRADLSARLDRDVRLDNDANCFALSEAWDDEFTQYPLVMGLILGTGVGGGLVF

NGKPITGKSYITGEFGHMRLPVDALTMMGLDFPLRRCGCGQHGCIENYLSGRGFAWLYQHYYHQPLQAPE

IIALYDQGDEQARAHVERYLDLLAVSLGNILTIVDPDLVVIGGGLSNFPAITTQLADRLPCHLLPVARVP

RIERARHGDAGGMRGAAFLHLTD

>gi|30062651|ref|NP_836822.1| outer membrane-specific lipoprotein transporter subunit LolE [Shigella flexneri 2a str. 2457T]

MAMPLSLLIGLRFSRGRRRGGMVSLISVISTIGIALGVAVLIVGLSAMNGFERELNNRILAVVPHGEIEA

VNQPWTNWQEALDNVQKVPGIAAAAPYINFTGLVESGANLRAIQVKGVNPQQEQRLSALPSFVQGDAWRN

FKAGEQQIIIGKGVADALKVKQGDWVSIMIPNSNPEHKLMQPKRVRLHIAGILQLSGQLDHSFAMIPLAD

AQQYLDMGSSVSGIALKMTDVFNANKLVRDAGEVTNSYVYIKSWIGTYGYMYRDIQMIRAIMYLAMVLVI

GVACFNIVSTLVMAVKDKSGDIAVLRTLGAKDGLIRAIFVWYGLLAGLFGSLCGVVIGVVVSLQLTPIIE

WIEKLIGHQFLSSDIYFIDFLPSELHWLDVFYVLVTALLLSLLASWYPARRASNIDPARVLSGQ

>gi|30062650|ref|NP_836821.1| lipoprotein transporter ATP-binding subunit [Shigella flexneri 2a str. 2457T]

MNKILLQCDNLCKRYQEGSVQTDVLLNVSFSVGEGEMMAIVGSSGSGKSTLLHLLGGLDTPTSGDVIFNG

QPMSKLSSAAKAELRNQKLGFIYQFHHLLPDFTALENVAMPLLIGKKKPAEINSCALEMLKAVGLEHRAN

HRPSELSGGERQRVAIARALVNNPRLVLADEPTGNLDARNADSIFQLLGELNRLQGTAFLVVTHDLQLAK

RMSRQLEMRDGRLTAELSLMGAE

>gi|30062649|ref|NP_836820.1| outer membrane-specific lipoprotein transporter subunit LolC [Shigella flexneri 2a str. 2457T]

MYQPVALFIGLRYMRGRAVDRFGRFVSWLSTIGITLGVMALVTVLSVMNGFERELQNNILGLMPQAILSS

EHGSLNPQQLPETAVKLDGVNRVAPITTGDVVLQSARSVAVGVMLGIDPAQKDPLTPYLVNVKQTDLEPG

KYNVILGEQLASQLGVNRGDQIRVMVPSASQFTPMGRIPSQRLFNVIGTFAANSEVDGYEMLVNIEDASR

LMRYPAANITGWRLWLDEPLRVDSLSQQKLPEGSKWQDWRDRKGELFQAVRMEKNMMGLLLSLIVAVAAF

NIITSLGLMVMEKQGEVAILQTQGLTPRQIMMVFMVQGASAGIIGAILGAALGALLASQLNNLMPIIGVL

LDGAALPVAIEPLQVIVIALVAMAIALLSTLYPSWRAAATQPAEALRYE

>gi|30062648|ref|NP_836819.1| hypothetical protein S1199 [Shigella flexneri 2a str. 2457T]

MKQKELWINQIKGLCICLVVIYHSVITFYPHMTTFQHPLSEVLSKCWIYFNLYLAPFRMPVFFFISGYLI

RRYIDSVPWGNCLDKRIWNIFWVLALWGVVQWLALSALNQWLAPERDLSNASNAAYADSTGEFLHGMITA

STSLWYLYALIVYFVVCKIFSRLALPLFALFVLLSVAVNFVPTPWWGMNSVIRNLPYYSLGAWFGATIMT

CVKEVPLRRHLLMASLLTVLAVGAWLFTISLLLSLVSIVVIMKLFYQYEQRFGMRSTSLLNVIGSNTIAI

YTTHRILVEIFSLTLLAQMNAARWSPQVELTLLLVYPFVSLFICTVAGLLVRKLSQRAFSDLLFSPPSLP

AAVSYSR

>gi|30062647|ref|NP_836818.1| transcription-repair coupling factor [Shigella flexneri 2a str. 2457T]

MPEQYRYTLPVKAGEQRLLGELTGAACATLVAEIAERHAGPVVLIAPDMQNALRLHDEISQFTDQMVMNL

ADWETLPYDSFSPHQDIISSRLSTLYQLPTMQRGILIVPVNTLMQRVCPHSFLHGHALVMKKGQRLSRDA

LRTQLDSAGYRHVDQVMEHGEYATRGALLDLFPMGSELPYRLDFFDDEIDSLRVFDVDSQRTLEEVEAIN

LLPAHEFPTDKAAIELFRSQWRDTFEVKRDPEHIYQQVSKGTLPAGIEYWQPLFFSEPLPPLFSYFPANT

LLVNTGDLETSAERFQADTLARFENRGVDPMRPLLPPQSLWLRVDELFSELKNWPRVQLKTEHLPTKAAN

ANLGFQKLPDLAVQAQQKAPLDALRKFLETFDGPVVFSVESEGRREALGELLARIKIAPQRIMRLDEASD

RGRYLMIGAAEHGFVDKARNLALICESDLLGERVARRRQDSRRTINPDTLIRNLAELHIGQPVVHLEHGV

GRYAGMTTLEAGGITGEYLMLTYANDAKLYVPVSSLHLISRYAGGAEENAPLHKLGGDAWSRARQKAAEK

VRDVAAELLDIYAQRAAKEGFAFKHDREQYQLFCDSFPFETTPDQAQAINAVLSDMCQPLAMDRLVCGDV

GFGKTEVAMRAAFLAVDNHKQVAVLVPTTLLAQQHYDNFRDRFANWPVRIEMISRFRSAKEQTQILAEVA

EGKIDILIGTHKLLQSDVKFKDLGLLIVDEEHRFGVRHKERIKAMRANVDILTLTATPIPRTLNMAMSGM

RDLSIIATPPARRLAVKTFVREYDSLVVREAILREILRGGQVYYLYNDVENIQKAAERLAELVPEARIAI

GHGQMRERELERVMNDFHHQRFNVLVCTTIIETGIDIPTANTIIIERADHFGLAQLHQLRGRVGRSHHQA

YAWLLTPHPKAMTTDAQKRLEAIASLEDLGAGFALATHDLEIRGAGELLGEEQSGSMETIGFSLYMELLE

NAVDALKAGREPSLEDLTSQQTEVELRMPSLLPDDFIPDVNTRLSFYKRIASAKTENELEEIKVELIDRF

GLLPDPARTLLDIARLRQQAQKLGIRKLEGNEKGGVIEFAEKNHVNPAWLIGLLQKQPQHYRLDGPTRLK

FIQDLSERKTRIEWVRQFMRELEENAIA

>gi|30062646|ref|NP_836817.1| hypothetical protein S1197 [Shigella flexneri 2a str. 2457T]

MMIKTRFSRWLTFFTFAAAVALALPAKANTWPLPPASSRLVGENKFHVVENDGGSLEAIAKKYNVGFLAL

LQANPGVDPYVPRAGSVLTIPLQTLLPDAPREGIVINIAELRLYYYPPGKNSVTVYPIGIGQLGGDTLTP

TMVTTVSDKRANPTWTPTANIRARYKAQGIELPAVVPAGPDNPMGHHAIRLAAYGGVYLLHGTNADFGIG

MRVSSGCIRLRDDDIKTLFSQVTPGTKVNIINTPIKVSAEPNGARLVEVHQPLSEKIDDDPQLLPITLNS

AMQSFKDAAQTDAEVMQHVMDVRSGMPVDVRRHQVSPQTL

>gi|30062645|ref|NP_836816.1| hypothetical protein S1196, partial [Shigella flexneri 2a str. 2457T]

MSFANFAAVEVQSTPEGQQKVGTISANAGTNLGSLEEQLAQKADEMGAKSFRITSVTGPNTLHGTAVIYK

>gi|30062644|ref|NP_836815.1| hypothetical protein S1195 [Shigella flexneri 2a str. 2457T]

MGSGLVNGGDYFYNNLSFTVTRYNGIMATDSTQCVKKSRGRPKVFDRDAALDKAMKLFWQHGYEATSLAD

LVEATGAKAPTLYAEFTNKEGLFRAVLDRYIDRFAAKHEAQLFCEEKSVESALADYFAAIANCFTSKDTP

AGCFMINNCTTLSPDSGDIANTLKSRHAMQERTLQQFLCQRQARGEIPTHCDVTHLAEFLNCIIQGMSIS

AREGASLEKLMQIAGTTLRLWPELVK

>gi|30062643|ref|NP_836814.1| hypothetical protein S1194 [Shigella flexneri 2a str. 2457T]

MNKSMLAGIGIGVAAALGVAAVASLNVFERGPQYAQVVSATPIKETVKTPRQECRNVTVTHRRPVQDENR

ITGSVLGAVAGGVIGHQFGGGRGKDVATVVGALGGGYAGNQIQGSLQESDTYTTTQQRCKTVYDKSEKML

GYDVTYKIGDQQGKIRMDRDPGTQIPLDSNGQLILNNKV

>gi|30062642|ref|NP_836813.1| respiratory NADH dehydrogenase [Shigella flexneri 2a str. 2457T]

MTTPLKKIVIVGGGAGGLEMATQLGHKLGRKKKAKITLVDRNHSHLWKPLLHEVATGSLDEGVDALSYLA

HARNHGFQFQLGSVIDIDREAKTITIAELRDEKGELLVPERKIAYDTLVMALGSTSNDFNTPGVKENCIF

LDNPHQARRFHQEMLNLFLKYSANLGANGKVNIAIVGGGATGVELSAELHNAIKQLHSYGYKGLTNEALN

VTLVEAGERILPALPPRISAAAHNELTKLGVRVLTQTMVTSADEGGLHTKDGEYIEADLMVWAAGIKAPD

FLKDIGGLETNRINQLVVEPTLQTTRDPDIYAIGDCASCPRPEGGFVPPRAQAAHQMATCAMNNILAQMN

GKPLKNYQYKDHGSLVSLSNFSTVGSLMGNLTRGSMMIEGRIARFVYISLYRMHQIALHGYFKTGLMMLV

GSINRVIRPRLKLH

>gi|30062641|ref|NP_836812.1| hypothetical protein S1192 [Shigella flexneri 2a str. 2457T]

MIIYLHGFDSNSPGNHEKVLQLQFIDPDVRLISYSTRHPKHDMQHLLKEVDKMLQLNVDERPLICGVGLG
GYWAERIGFLCDIRQVIFNPNLFPYENMEGKIDRPEEYADIATKCVTNFREKNRDRCLVILSRNDEALNS
QRTSEELHHYYEIVWDEEQTHKFKNISPHLQRIKAFKTLG

>gi|30062640|ref|NP_836811.1| beta-hexosaminidase [Shigella flexneri 2a str. 2457T]
MGPVMLDVEGYELDAEEREILAHPLVGGLILFTRNYHDPAQLRELVRQIRAASRNHLVVAVDQEGGRVQR
FREGFTRLPAAQSFAALLGMEEGGKLAQEAGWLMASEMIAMDIDISFAPVLDVGHISAAIGERSYHADPQ
KALAIASRFIDGMHEAGMKTTGKHFPGHGAVTADSHKETPCDPRPQAEIRAKDMSVFSSLIRENKLDAIM
PAHVIYSDVDPRPASGSPYWLKTVLRQELGFDGVIFSDDLSMEGAAIMGSYAERGQASLDAGCDMILVCN
NRKGAVSVLDNLSPIKAERVTRLYHKGSFSRQELMDSARWKAISTRLNQLHERWQEEKVGH

>gi|30062639|ref|NP_836810.1| thiamine kinase [Shigella flexneri 2a str. 2457T]
MPFRSNNPITRDELLSRFFPQFHPVTTFNSGLSGGSFLIEHQGQRFVVRQPHDPDAPQSAFLRQYRALSQ
LPASIAPKPHLYLRDWMVVDYLPGAVKTYLPDTNELAGLLYYLHQQPRFGWRITLLPLLELYWQQSDPAR
RTVGWLRMLKRLRKAREPRPLRLSPLHMDVHAGNLVHSASGLKLIDWEYAGDGDIALELAAVWVENTEQH
RQLVNDYATRAKIYPAQLWRQVRRWFPWLLMLKGGWFEYRWRQTGDQQFIRLADDTWRQLLIKQ

>gi|30062638|ref|NP_836809.1| hypothetical protein S1189 [Shigella flexneri 2a str. 2457T]
MTKMSRYALITALAMFLAGCVGQREPAPVEEVKPAPEQPAEPQQPVPTVPSVPTIPQQPGPIEHEDRTAP
PAPHIRHYDWNGAMQPMVSKMLGADGVTAGSVLLVDSVNNRTNGSLNAAEATETLRNALANNGKFTLVSA
QQLSMAKQQLGLSPQDSLGTRSKAIGIARNVGAHYVLYSCASGNVNAPTLQMQLMLVQTGEIIWSGKGAV
SQQ

>gi|30062637|ref|NP_836808.1| hypothetical protein S1188 [Shigella flexneri 2a str. 2457T]
MRKGCFGLVSLVLLLLVGCRSHPEIPVNDEQSLVMESSLLAAGISAEKPVLSTSDIQPSASSTIYNERQE

PVTVHYRFYWYDARGLEMHPLERPRSVTIPAHSAVTLYGSANFLGAHKVRLYLYL

>gi|30062636|ref|NP_836807.1| purine nucleoside phosphoramidase [Shigella flexneri 2a str. 2457T]

MAEETIFSKIIRREIPSDIVYQDDLVTAFRDISPQAPTHILIIPNILIPTVNDVSAEHEQALGRMITVAA

KIAEQEGIAEDGYRLIMNTNRHGGQEVYHIHMHLLGGRPLGPMLAHKGL

>gi|30062635|ref|NP_836806.1| glucose-specific PTS system IIBC components [Shigella flexneri 2a str. 2457T]

MFKNAFANLQKVGKSLMLPVSVLPIAGILLGVGSANFSWLPAVVSHVMAEAGGSVFANMPLIFAIGVALG

FTNNDGVSALAAVVAYGIMVKTMAVVAPLVLHLPAEEIASKHLADTGVLGGIISGAIAAYMFNRFYRIKL

PEYLGFFAGKRFVPIISGLAAIFTGVVLSFIWPPIGSAIQTFSQWAAYQNPVVAFGIYGFIERCLVPFGL

HHIWNVPFQMQIGEYTNAAGQVFHGDIPRYMAGDPTAGKLSGGFLFKMYGLPAAAIAIWHSAKPENRAKV

GGIMISAALTSFLTGITEPIEFFFMFVAPILYIIHAILAGLAFPICILLGMRDGTSFSHGLIDFIVLSGN

SSKLWLLPIVGIGYAIVYYTIFRVLIKALDLKTPGREDATEDAKATGTSEMAPALVAAFGGKENITNLDA

CITRLRVSVADVSKVDQAGLKKLGAAGVVVAGSGVQAIFGTKSDNLKTEMDEYIRNH

>gi|30062634|ref|NP_836805.1| metallodependent hydrolase [Shigella flexneri 2a str. 2457T]

MFLVDSHCHLDGLDYESLHKDVDDVLAKAAARDVKFCLAVATTLPGYLHMRDLVGERDNVVFSCGVHPLN

QNDPYDVEDLRRLAAEEGVVALGETGLDYYYTPETKVRQQESFIHHIQIGRELNKPVIVHTRDARADTLA

ILREEKVTDCGGVLHCFTEDRETAGKLLDLGFYISFSGIVTFRNAEQLRDTARYVPLDRLLVETDSPYLA

PVPHRGKENQPAMVRDVAEYMAVLKGVAVEELAQVTTDNFARLFHIDASRLQSIR

>gi|30062633|ref|NP_836804.1| DNA polymerase III subunit delta' [Shigella flexneri 2a str. 2457T]

MRWYPWLRPDFEKLVASYQAGRGHHALLIQALPGMGDDALIYALSRYLLCQQPQGHKSCGHCRGCQLMQA

GTHPDYYTLAPEKGKNTLGIDAVREVTEKLNEHARLGGAKVVWVTDAALLTDAAANALLKTLEEPPAETW

FFLATREPERLLATLRSRCRLHYLAPPPEQYAVTWLSREVTMSQDALLAALRLSAGSPGAALALFQGDNW

QARETLCQALAYSVPSGDWYSLLAALNHEQAPARLHWLATLLMDALKRHHGAAQVTNVDVPGLVAELANH

LSPSRLQAILGDVCHIREQLMSVTGINRELLITDLLLRIEHYLQPGVVLPVPHL

>gi|30062632|ref|NP_836803.1| thymidylate kinase [Shigella flexneri 2a str. 2457T]

MRSKYIVIEGLEGAGKTTARNVVVETLEQLGIRDMVFTREPGGTQLAEKLRSLVLDIKSVGDEVITDKAE

VLMFYAARVQLVETVIKPALANGTWVIGDRHDLSTQAYQGGGRGIDQHMLATLRDAVLGDFRPDLTLYLD

VTPEVGLKRARARGELDRIEQESFDFFNRTRARYLELAAQDKSIHTIDATQPLEAVMDAIRTTVTHWVKE

LDA

>gi|30062631|ref|NP_836802.1| hypothetical protein S1181 [Shigella flexneri 2a str. 2457T]

MKKVLLIILLLLVVLGIAAGVGVWKVRHLADSKLLIKEETIFTLKPGTGRLALGEQLYADKIINRPRVFQ

WLLRIEPDLSHFKAGTYRFTPQMTVREMLKLLESGKEAQFPLRLVEGMRLSDYLKQLREAPYIKHTLSDD

KYATVAQALELENPEWIEGWFWPDTLMYTANTTDVALLKRVHKKMVKAVDSAWEGRADGLPYKDKNQLVT

MASIIEKETAVASERDQVASVFINRLRIGMRLQTDPTVIYGMGERYNGKLSRADLETPTAYNTYTITGLP

PGAIATPGADSLKAAAHPAKTPYLYFVADGKGGHTFNTNLASHNKSVQDYLKVLKEKNAQ

>gi|30062630|ref|NP_836801.1| 4-amino-4-deoxychorismate lyase [Shigella flexneri 2a str. 2457T]

MFLINGYKQESLAVSDRATQFGDGCFTTARVIDGKVSLLSAHILRLQDACQRLMISCDFWPQLEQEMKTL

AAEQQNGVLKVVISRGSGGRGYSTLNSGPATRILSVTAYPAHYDRLRNEGMTLALSPVRLGRNPHLAGIK

HLNRLEQVLIRSHLEQTNADEALVLDSEGWVTECCAANLFWRKGNVVYTPRLDQAGVNGIMRQFCIRLLA

QSSYQLVEVQASLEEALQADEMVICNALMPVMPVRACGDVTFSSATLYEYLAPLCERPN

>gi|30062629|ref|NP_836800.1| 3-oxoacyl-(acyl carrier protein) synthase II [Shigella flexneri 2a str. 2457T]

MSKRRVVVTGLGMLSPVGNTVESTWKALLAGQSGISLIDHFDTSAYATKFAGLVKDFNCEDIISRKEQRK

MDAFIQYGIVAGVQAMQDSGLEITEENATRIGAAIGSGIGGLGLIEENHTSLMNGGPRKISPFFVPSTIV

NMVAGHLTIMYGLRGPSISIATACTSGVHNIGHAARIIAYGDADVMVAGGAEKASTPLGVGGFGAARALS

TRNDNPQAASRPWDKERDGFVLGDGAGMLVLEEYEHAKKRGAKIYAELVGFGMSSDAYHMTSPPENGAGA

ALAMANALRDAGIEASQIGYVNAHGTSTPAGDKAEAQAVKTIFGEAASRVLVSSTKSMTGHLLGAAGAVE

SIYSILALRDQAVPPTINLDNPDEGCDLDFVPHEARQVSGMEYTLCNSFGFGGTNGSLIFKKI

>gi|30062628|ref|NP_836799.1| acyl carrier protein [Shigella flexneri 2a str. 2457T]

MSTIEERVKKIIGEQLGVKQEEVTNNASFVEDLGADSLDTVELVMALEEEFDTEIPDEEAEKITTVQAAI

DYINGHQA

>gi|30062627|ref|NP_836798.1| 3-ketoacyl-ACP reductase [Shigella flexneri 2a str. 2457T]

MNFEGKIALVTGASRGIGRAIAETLAARGAKVIGTATSENGAQAISDYLGANGKGLMLNVTDPASIESVL

EKIRAEFGEVDILVNNAGITRDNLLMRMKDEEWNDIIETNLSSVFRLSKAVMRAMMKKRHGRIITIGSVV

GTMGNGGQANYAAAKAGLIGFSKSLAREVASRGITVNVVAPGFIETDMTRALSDDQRAGILAQVPAGRLG

GAQEIANAVAFLASDEAAYITGETLHVNGGMYMV

>gi|30062626|ref|NP_836797.1| acyl carrier protein S-malonyltransferase [Shigella flexneri 2a str. 2457T]

MTQFAFVFPGQGSQTVGMLADMAASYPIVEETFAEASAALGYDLWALTQQGSAEELNKTWQTQPALLTAS

VALYRVWQQQGGKAPAMMAGHSLGEYSALVCAGVIDFADAVRLVEMRGKFMQEAVPEGTGAMAAIIGLDD

APIAKACEEAAEGQVVSPVNFNSPGQVVIAGHKEAVERAGAACKAAGAKRALPLPVSVPSHCALMKPAAD

KLAVELAKITFNAPTVSVVNNVDVKCETNGDAIRDALVRQLYNPVQWTKSVEYMAAQGVEHLYEVGPGKV

LTGLTKRIVDTLTASALNEPSAMAAALEL

>gi|30062625|ref|NP_836796.1| 3-oxoacyl-ACP synthase [Shigella flexneri 2a str. 2457T]

MYTKIIGTGSYLPEQVRTNADLEKMVDTSDEWIVTRTGIRERHIAAPNETVSTMGFEAATRAIEMAGIEK

DQIGLIVVATTSATHAFPSAACQIQSMLDIKGCPAFDVAAACAGFTYALSVADQYVKSGAVKYALVVGSD

VLARTCDPTDRGTIIIFGDGAGAAVLAASEEPGIISTHLHADGSYGELLTLPNADRVNPENSIHLTMAGN

EVFKVAVTELAHIVDETLAANNLDRSQLDWLVPHQANLRIISATAKKLGMSMDNVVVTLDRHGNTSAASV

PCALDEAVRDGRIKPGQLVLLEAFGGGFTWGSALVRF

>gi|30062623|ref|NP_836794.1| 50S ribosomal protein L32 [Shigella flexneri 2a str. 2457T]

MAVQQNKPTRSKRGMRRSHDALTAVTSLSVDKTSGEKHLRHHITADGYYRGRKVIAK

>gi|30062622|ref|NP_836793.1| hypothetical protein S1172 [Shigella flexneri 2a str. 2457T]

MQKVKLPLTLDPVRTAQKRLDYQGIYTPDQVERVAESVVSVDSDVECSMSFAIDNQRLAVLNGDAKVTVT

LECQRCGKPFTHQVYTTYCFSPVRSDEQAEALPEAYEPIEVNEFGEIDLLAMVEDEIILALPVVPVHDSE

HCEVSEADMVFGELPEEVQKPNPFAVLASLKRK

>gi|30062620|ref|NP_836791.1| 23S rRNA pseudouridylate synthase C [Shigella flexneri 2a str. 2457T]

MKTETPSVKIVAITADEAGQRIDNFLRTQLKGVPKSMIYRILRKGEVRVNKKRIKPEYKLEAGDEVRIPP

VRVAEREEEAVSPHLQKVAALADVILYEDDHILVLNKPSGTAVHGGSGLSFGVIEGLRALRPEARFLELV

HRLDRDTSGVLLVAKKRSALRSLHEQLREKGMQKDYLALVRGQWQSHVKSVQAPLLKNILQSGERIVRVS

QEGKPSETRFKVEERYAFATLVRCSPVTGRTHQIRVHTQYAGHPIAFDDRYGDREFDRQLTEAGTGLNRL

FLHAAALKFTHPGTGEVMRIEAPMDEGLKRCLQKLRNAR

>gi|30062619|ref|NP_836790.1| hypothetical protein S1169 [Shigella flexneri 2a str. 2457T]

MSSRVANLTVIVSSKRRRVSVARFSCGKTAQLSKKQTGYYSPEIFPSTGKDCNPQPANCLKDQYVLRHCC

VDDRSGKMGYSVKFLVLTRMDTETASLFHCKPCYSKMTFTIYHPLTHSFFTSCW

>gi|30062617|ref|NP_836788.1| flagellar rod assembly protein/muramidase FlgJ [Shigella flexneri 2a str. 2457T]

MISDSKLLASAAWDAQSLNELKAKASEDPAANIRPVARQVEGMFVQMMLKSMRDALPKDGLFSSEHTRLY

TSMYDQQIAQQMTAGKGLGLAEMMVKQMTPEQPLPEESTPAAPMKFPLETVVRYQNQTLSQLVQKAVPRN

YDDSLPGDSRAFLAQLSLPAQLASQQSGVPHHLILAQAALESGWGQRQIRRENGEPSYNLFGVKASGNWK

GQVTEITTTEYENGEAKKVKAKFRVYSSYLEALSDYVGLLTRNPRYAAVTTAASAEQGAQVLQDAGYATD

PHYARKLTNMIQQMKSISDKVSKTYSMNIDNLF

>gi|30062616|ref|NP_836787.1| flagellar basal body P-ring protein [Shigella flexneri 2a str. 2457T]

MIKFLSALILLLVTTAAQAERIRDLTSVQGVRQNSLIGYGLVVGLDGTGDQTTQTPFTTQTLNNMLSQLG

ITVPTGTNMQLKNVAAVMVTASLPPFGRQGQTIDVVVSSMGNAKSLRGGTLLMTPLKGVDSQVYALAQGN

ILVGGAGASAGGSSVQVNQLNGGRITNGAVIERELPSQFGVGNTLNLQLNDEDFSMAQQIADTINRVRGY

GSATALDARTIQVRVPSGNSSQVRFLADIQNMQVNVTPQDAKVVINSRTGSVVMNREVTLDSCAVAQGNL

SVTVNRQANVSQPDTPFGGGQTVVTPQTQIDLRQSGGSLQSVRSSASLNNVVRALNALGATPMDLMSILQ

SMQSAGCLRAKLEII

>gi|30062615|ref|NP_836786.1| flagellar basal body L-ring protein [Shigella flexneri 2a str. 2457T]

MQKNAAHTYAISSLLVLSLTGCAWIPSTPLVQGATSAQPVPGPTPVANGSIFQSAQPINYGYQPLFEDRR

PRNIGDTLTIVLQENVSASKSSSANASRDGKTNFGFDTVPRYLQGLFGNARADVEASGGNTFNGKGGANA

SNTFSGTLTVTVDQVLVNGNLHVVGEKQIAINQGTEFIRFSGVVNPRTISGSNTVPSTQVADARIEYVGN

GYINEAQNMGWLQRFFLNLSPM

>gi|30062614|ref|NP_836785.1| flagellar basal body rod protein FlgG [Shigella flexneri 2a str. 2457T]

MISSLWIAKTGLDAQQTNMDVIANNLANVSTNGFKRQRAVFEDLLYQTIRQPGAQSSEQTTLPSGLQIGT

GVRPVATERLHSQGNLSQTNNSKDVAIKGQGFFQVMLPDGSSAYIRDGSFQVDQNGQLVTAGGFQVQPAI

TIPANALSITIGRDGVVSVTQQGQAAPVQVGQLNLTTFMNDTGLESIGENLYTETQSSGAPNESTPGLNG

AGLLYQGYVETSNVNVAEELVNMIQVQRAYEINSKAVSTTDQMLQKLTQL

>gi|30062613|ref|NP_836784.1| flagellar hook protein FlgE [Shigella flexneri 2a str. 2457T]

MAFSQAVSGLNAAATNLDVIGNNIANSATYGFKSGTASFADMFAGSKVGLGVKVAGITQDFTDGTTTNTG

RGLDVAISQNGFFRLVDSNGSVFYSRNGQFKLDENRNLLNTQGLQLTGYPVTGTPPTIQQGANPTNISIP

NTLMAAKTTTTASMQINLNSSDPLPTVTPFSASNADSYNKKGSVTVFDSQGNAHDMSVYFVKTGDNNWQV

YTQDSSDPNSIAKTATTLEFNANGTLVDGAMANNIATGAINGAEPATFSLSFLNSMQQNTGANNIVATTQ

NGYKPGDLVSYQINDDGTVVGNNSNEQTQLLGQIVLANFANNEGLASEGDNVWSATQSSGVALLGTAGTG

NFGTLTNGALEASNVDLSKELVNMIVAQRNYKSNAQTIKTQDQILNTRVNLR

>gi|30062612|ref|NP_836783.1| flagellar basal body rod modification protein [Shigella flexneri 2a str. 2457T]

MSIAVTTTDPTNTGVSTTSSSSLTGSNAADLQSSFLTLLVAQLKNQDPTNPMENNELTSQLAQISTVSGI

EKLNTTLGSISGQIDNSQSLQASNLIGHGVMIPGTTVLAGTGSEEGAVTTTTPFGVELQQAADKVTATIT

DKNGAVVRTIDIGELTAGVHSFTWDGSLTDGSTAPNGSYNVAISASNGGTQLVAQPLQFALVQGVIRGNS

GNTLDLGTYGTTTLDEVRQII

>gi|30062611|ref|NP_836782.1| flagellar basal body rod protein FlgB [Shigella flexneri 2a str. 2457T]

MLDKLDAALRFQQEALNLRAQRQEVLAANIANADTPGYQARDIDFASELKKVMQRGRDATSVVALTMTST

QHIPAQALTPPTAELQYRIPDQPSLDGNTVDMDRERTQFADNSLQYQMSLSALSGQIKGMMNVLQSGN

>gi|30062610|ref|NP_836781.1| flagellar basal body P-ring biosynthesis protein FlgA [Shigella flexneri 2a str. 2457T]

MLTIKRSVAIIAILFSPLSTASNLTSQLHNFFSAQLAGVSDEVRVSIRTAPNLLPPCEQPLLSMSNNSRL

WGNVNVLARCGNDKRYLQVNVQATGNYVVAAMPIVRGGKLEAGNVKLKRGRLDTLPPRTVLDINQLVDAV

SLRDLSPDQPIQLTQFRQAWRVKAGQRVNVIASGDGFSANAEGQALNNAAVAQNARVRMVSEQVVSGVVD

ADGNILINL

>gi|30062609|ref|NP_836780.1| anti-sigma28 factor FlgM [Shigella flexneri 2a str. 2457T]

MSIDRTSPLKPVSTVQPRETTDAPVTNSRAAKTTASTSTSVTLSDAQAKLMQPGSSDINLERVEALKLAI

RNGELKMDTGKIADALINEAQQDLQSN

>gi|30062608|ref|NP_836779.1| flagella synthesis protein FlgN [Shigella flexneri 2a str. 2457T]

MTRLAEILDQMSAVLNDLKTVMDQEQQHLSMGQINGSQLQWITEQKSSLLATLDYLEQLRKKEPNTANSV

DISQRWQEITVKTQQLRQMNQHNGWLLEGQIERNQQALEMLKPHQEPTLYGANGQTSTPHRGSKKISI

>gi|30062607|ref|NP_836778.1| virulence factor [Shigella flexneri 2a str. 2457T]

MNLLKSLAAVSSMTMFSRVLGFARDAIVARIFGAGMATDAFFVAFKLPNLLRRIFAEGAFSQAFVPILAE

YKSKQGEDATRVFVSYVSGLLTLALAVVTVAGMLAAPWVIMVTAPGFADTADKFALTSQLLKITFPYILL

ISLASLVGAILNTWNRFSIPAFAPTLLNISMIGFALFAAPYFNPPVLALAWAVTVGGILQLVYQLPHLKK

IGMLVLPRINFHDAGAMRVVKQMGPAILGVSVSQISLIINTIFASFLASGSVSWMYYADRLMEFPSGVLG

VALGTILLPSLSKSFASGNHDEYNRLMDWGLRLCFLLALPSAVALGILSGPLTVSLFQYGKFTAFDALMT

QRALIAYSVGLIGLIVVKVLAPGFYSRQDIKTPVKIAIVTLILTQLMNLAFIGPLKHAGLSLSIGLAACL

NASLLYWQLRKQKIFTPQPGWMAFLLRLVVAVLVMSGVLLGMLHIMPEWSLGTMPWRLLRLMAVVLAGIA

AYFAALAVLGFKVKEFARRTV

>gi|30062606|ref|NP_836777.1| virulence factor [Shigella flexneri 2a str. 2457T]

MKKLRIGVVGLGGIAQKAWLPVLAAASDWTLQGAWSPTRAKALPICDSWRIPYADSLSSLAASCDAVFVH

SSTASHFDVVSTLLNAGVHVCVDKPLAENLRDAERLVELAARKKLTLMVGFNRRFAPLYGELKTQLATAA

SLRMDKHRSNSVGPHDLYFTLLDDYLHVVDTALWLSGGKASLDGGTLLTNDAGEMLFAEHHFSAGPLQIT

TCMHRRAGSQRETVQAVTDGALIDITDMREWREERGQGVVNKPIPGWQSTLEQRGFVGCARHFIECV

>gi|30062605|ref|NP_836776.1| hypothetical protein S1151 [Shigella flexneri 2a str. 2457T]

MKYQLTALEARVIGCLLEKQVTTPEQYPLSVNGVVTACNQKTNREPVMNLSESEVQEQLDNLVKRHYLRT

VSGFGNRVTKYEQRFCNSEFGDLKLSAAEVALITTLLLRGAQTPGELRSRAARMYEFSDMAEVESTLEQL

ANREDGPFVVRLAREPGKRESRYMHLFSGEVEDQPAVTAMSNMVDGDLQTRVEALEIEVAELKQRLDSLL

AHLGD

>gi|30062604|ref|NP_836775.1| ribosomal-protein-S5-alanine N-acetyltransferase [Shigella flexneri 2a str. 2457T]

MFGYRSNVPKVRLTTDRLVVRLVHDRDAWRLADYYAENRHFLKPWEPVRDESHCYPSGWQARLGMINEFH

KQGSAFYFGLFDPDEKEIIGVANFSNVVRGSFHACYLGYSIGQKWQGKGLMFEALTAAIRYMQRTQHIHR

IMANYMPHNKRSGDLLARLGFEKEGYAKDYLLIDGQWRDHVLTALTTPDWTPGR

>gi|30062603|ref|NP_836774.1| multidrug resistance protein MdtH [Shigella flexneri 2a str. 2457T]

MSRVSQARNLGKYFLLIDNMLVVLGFFVVFPLISIRFVDQMGWAAVMVGIALGLRQFIQQGLGIFGGAIA

DRFGAKPMIVTGMLMRAAGFATMGIAHEPWLLWFSCLLSGLGGTLFDPPRSALVVKLIRPQQRGRFFSLL

MMQDSASAVIGALLGSWLLQYDFRLVCATGAVLFVLCAAFNAWLLPAWKLSTVRTPVREGMTRVMRDKRF

VTYVLTLAGYYMLAVQVMLMLPIMVNDVAGAPSAVKWMYAIEACLSLTLLYPIARWSEKHFRLEHRLMAG

LLIMSLSMMPVGMVSGLQQLFTLICLFYIGSIIAEPARETLSASLADARARGSYMGFSRLGLAIGGAIGY

IGGGWLFDLGKSAHQPELPWMMLGIIGIFTFLALGWQFSQKRAARRLLERDA

>gi|30062602|ref|NP_836773.1| glutaredoxin [Shigella flexneri 2a str. 2457T]

MKLYIYDHCPYCLKARMIFGLKNIPVELHVLLNDDAETPTRMVGQKQVPILQKDDSRYMPESMDIVHYVD

KLDGKPLLTGKRSPAIEEWLRKVNGYVNKLLLPRFAKSAFDEFSTPAARKYFVDKKEASAGNFADLLAHS

DGLIKNISDDLRALDKLIVKPNAVNGELSEDDIQLFPLLRNLTLVAGINWPSRVANYRDNMAKQTQINLL

SSMAI

>gi|30062601|ref|NP_836772.1| hypothetical protein S1147 [Shigella flexneri 2a str. 2457T]

MNKFLFAAALIVSGLLVGCNQLTQYTITEQEINQSLAKHNNFSKDIGLPGVADAHIVLTNLTSQIGREEP
NKVTLTGDANLDMNSLFGSQKATMKLKLKALPVFDKEKGAIFLKEMEVVDATVQPEKMQTVMQTLLPYLN
QALRNYFNQQPAYVLREDGSQGEAMAKKLAKGIEVKPGEIVIPFTD

>gi|30062600|ref|NP_836771.1| dihydroorotase [Shigella flexneri 2a str. 2457T]
MTAPSQVLKIRRPDDWHLHLRDGDMLKTVVPYTSEIYGRAIVMPNLAPPVTTVEAAVAYRQRILDAVPAG
HDFTPLMTCYLTDSLDPNELERGFNEAVFTAAKLYPANATTNSSHGVTSIDAIMPVLERMEKIGMPLLVH
GEVTHADIDIFDREARFIESVMEPLRQRLTALKVVFEHITTKDAADYVRDGNERLAATITPQHLMFNRNH
MLVGGVRPHLYCLPILKRNIHQQALRELVASGFNRVFLGTDSAPHARHRKESSCGCAGCFNAPTALGSYA
TVFEEMNALQHFEAFCSVNGPQFYGLPVNDTFIELVREEQQVAESIALTDDTLVPFLAGETVRWSVKQ

>gi|30062599|ref|NP_836770.1| DNA damage-inducible protein I [Shigella flexneri 2a str. 2457T]
MRIEVTIAKTSPLPAGAIDALAGELSRRIQYAFPDNEGHVSVRYAAANNLSVIGATKEDKQRISEILQET
WESADDWFVSE

>gi|30062597|ref|NP_836768.1| N-methyltryptophan oxidase [Shigella flexneri 2a str. 2457T]
MKYDLIIIGSGSVGAAAGYYATRAGLNVLMTDAHMPPHQHGSHHGDTRLIRHAYGEGEKYVPLVLRAQTL
WDELSRHNEDDPIFVRSGVINLGPADSAFLANVAHSAEQWQLNVEKLDAQGIMARWPEIRVPDNYIGLFE
TDSGFLRSELAIKTWIQLAKEAGCAQLFNCPVTAIRHDDDGVTIETVDGEYQAKKAIVCAGTWVKDLLPE
LPVQPVRKVFAWYQADGRYSVKNKFPAFTGELPNGDQYYGFPAENDALKIGKHNGGQVIHSADERVPFAE
VASDGSEAFPFLRNVLPGIGCCLYGAACTYDNSPDEDFIIDTLPGHDNTLLITGLSGHGFKFASVLGEIA
ADFAQDQKSDFDLTPFRLSRFQ

>gi|30062596|ref|NP_836767.1| hypothetical protein S1142 [Shigella flexneri 2a str. 2457T]
MRRLLHYLINNIREHLMLYLFLWGLLAIMDLIYVFYF

>gi|30062595|ref|NP_836766.1| cytochrome [Shigella flexneri 2a str. 2457T]

MSFTNTPERYGVISAAFHWLSAIIVYGMFALGLWMVTLSYYDGWYHKAPELHKSIGILLMMGLVIRVLWR

VISPPPGPLPSYSPMTRLAAKAGHLALCLLLFAIGISGYLISTADGKQISVFGWFDVPATLADAGAQADF

AGALHFWLAWSVVVLSVMHGFMALKHHFIDKDDTLKRMLGKSSSDYGV

>gi|30062594|ref|NP_836765.1| hypothetical protein S1140 [Shigella flexneri 2a str. 2457T]

MKKSLLGLTFASLMFSAGSAVAADYKIDKEGQHAFVNFRIQHLGYSWLYGTFKDFDGTFTFDEKNPAADK

VNVTINTTSVDTNHAERDKHLRSADFLNTTKYPQATFTSTSVKKDGDELDITGDLTLNGVTKPVTLEAKL

IGQGDDPWGGKRAGFEAEGKIKLKDFNIKTDLGPASQEVDLIISVEGVQQK

>gi|30062593|ref|NP_836764.1| hypothetical protein S1139 [Shigella flexneri 2a str. 2457T]

MPVLHNRISNDALKAKMLAESEPRTTISFYKYFHIADPKATRDALYQLFTALNVFGRVYLAHEGINAQIS

VPASNVETFRAQLYAFDPALEGLRLNIALDDDGKSFWVLRMKVRDRIVADGIDDPHFDASNVGEYLQAAE

VNAMLDDPDALFIDMRNHYEYEVGHFENALEIPADTFREQLPKAVEMMQAHKDKKIVMYCTGGIRCEKAS

AWMKHNGFNKVWHIEGGIIEYARKAREQGLPVRFIGKNFVFDERMGERISDEIIAHCHQCGAPCDSHTNC

KNDGCHLLFIQCPVCAEKYKGCCSEICCEESALPPEEQRRRRAGRENGNKIFNKSRGRLNTTLGIPDPTE

>gi|30062592|ref|NP_836763.1| lipid A biosynthesis lauroyl acyltransferase [Shigella flexneri 2a str. 2457T]

MTNLPKFSTALLHPRYWLTWLGIGVLWLVVQLPYPIIYRLGCGLGKLALRFMKRRAKIVHRNLELCFPEM

SEQERRKMVVKNFESVGMGLMETGMAWFWPDRRIARWTEVIGMEHIRDVQAQKRGILLVGIHFLTLELGA

RQFGMQEPGIGVYRPNDNPLIDWLQTWGRLRSNKSMLDRKDLKGMIKALKKGEVVWYAPDHDYGPRSSVF

VPLFAVEQAATTTGTWMLARMSGACLVPFVPRRKPDGKGYQLIMLPPECSPPLDDAETTAAWMNKVVEKC

IMMAPEQYMWLHRRFKTRPEGVPSRY

>gi|30062591|ref|NP_836762.1| hypothetical protein S1136 [Shigella flexneri 2a str. 2457T]

MRRAISYIRFSSERQLKGDSVRRQSKLVTDWLDKNPEFYLDSSLSFKDLGKSAFSGKHLKGGLGDFLTAI

EKGLVKAGDTLLIESLDRLSRQDIDIASELLRRILRAGVDVVTLSDGEHYTRESLKDPLALIKSILIMQR

AHEESLRKSERVQAAWNRKKELISEGIKVSRRCPAWLRLNDDRRTFTIIPDKVEVVKRAFDLRLQGLSFW

AITRTLNDEGHLSLNQYTPKQKGWSDTAVKKLLRNRAVIGCFTPAGREEVQGYYPAIISESLFYRVQQLN

TGQYGRASVSSNPLSVNLFRGIIKCEVYWQ

>gi|30062590|ref|NP_836761.1| IS600 orfB [Shigella flexneri 2a str. 2457T]

MSRKGNCYDNAPMESFWGTLKNESLSHYRFNNRDEAISVIREYIEIFYNRQRRHSRLGNISPAAFREKYH

QIAA

>gi|30062589|ref|NP_836760.1| IS600 orfB [Shigella flexneri 2a str. 2457T]

MAHIRTRETYGTRRLQTELAENGIIVGRDRLARLRKELRLRCKQKRKFRATTNPNHNLPVAPNLLNQTFA

PTAPNQVWVADLTYVATQEGWLYLAGIKDVYTCEIVGYAMGERMTKELTGKALFMALRSQRPPAGLIHHS

DRGSSTAHTITGSYRSSLV

>gi|30062588|ref|NP_836759.1| IS600 orfA [Shigella flexneri 2a str. 2457T]

MSRKTQRYSKEFKAEAVRTVLENQLSISEGASRLSLPEGTLGQWVTAARKGLGTPGSRTVAELESEILQL

RKALNEARLERDILKKATAYFAQESLKNTR

>gi|30062587|ref|NP_836758.1| IS629 orfB [Shigella flexneri 2a str. 2457T]

MARCTVARLMAVMGLAGVLRGKKVRTTISRKAVAAGDRVNRQFVAERPEQLWVADFTYVSTWQGFVYVAF

IIDVFAGYIVGWRVSSSMETTFVLDALEQALWARRPSGTVHHSDKGSQYVSLAYTQRLKEAGLLASTGST

GDSYDNAMAESINGLYKAEVIHRKSWKNRAEVELATLTWVDWYNNRRLLERLGHIPPAEAEKAYYASIGN

DDLAA

>gi|30062586|ref|NP_836757.1| IS629 orfA [Shigella flexneri 2a str. 2457T]

MTKNTRFSPEVRQRAVRMVLESQGEYDSQWATICSIAPKIGCTPETLRVWVRQHERDTGGGDGGLTTAER

QRLKEPERENRELRRSNDILRQASAYFAKAEFDRLWKK

>gi|30062583|ref|NP_836754.1| hypothetical protein S1128 [Shigella flexneri 2a str. 2457T]

MENFIDTLENWRDQKQIISVICKDGIKIDDSIITSFKASKDVGISNGLRIQLTFQEINFKAIVGQTDVSA

ATGRTSTTNDGGATSKKNTGNTTTSLGSPMLTCKELFSYSASELSDEALKARVTCSKSVSVKNGESTFTA

>gi|30062582|ref|NP_836753.1| IS911 orfB, partial [Shigella flexneri 2a str. 2457T]

MATRRGYQMGRWLAGRLMKELGLVSCQQPTHRYKRGGHEHVAIPNYLERQFAVTEPNQVWCGDVTYIWTG

KRWAYLAVVLDLFARKPVGWAMSFSPDSRLTMKALEMAWETRGKPGGVMFHSDQGSHYTSRQFRQLLWRY

QIRQSMSRRGNCWDNSPMERFFRSLKNEWMPVVGYVSFSEAAHAITDYIVGYYSALRPHEYNGGLPPNES

ENRYWKNSNSVASFC

>gi|30062581|ref|NP_836752.1| IS911 orfA [Shigella flexneri 2a str. 2457T]

MKKRNFSAEFKRESAQLVVDQKYTVADAAKAMDVGLSTMTRWVKQLRDERQGKTPKASPITPEQIEIREL

RKKLQRIEMENEILKKATALLMSDSLNSSR

>gi|30062580|ref|NP_836751.1| IS629 orfA [Shigella flexneri 2a str. 2457T]

MTKNTRFSPEVRQRAVRMVLESQGEYDSQWATICSIAPKIGCTPETLRVWVRQHERDTGGGDGGLTTAER

QRLKEPERENRELRRSNDILRQASAYFAKAEFDRLWKK

>gi|30062579|ref|NP_836750.1| IS629 orfB [Shigella flexneri 2a str. 2457T]

MAVMGLAGVLRGKKVRTTISRKAVAAGDRVNRQFVAERPEQLWVADFTYVSTWRGFVYYVAFIIDVFAGYI

VGWRVSSSMETTFVLDALEQALWARRPSGTVHHSDKGSQYVSLAYTQRLKEAGLLASTGSTGDSYDNAMA
ESINGLYKAEVIHRKSWKNRAEVELATLTWVDWYNNRRLLERLGHIPPAEAEKAYYASIGNDDLAA

>gi|30062578|ref|NP_836749.1| hypothetical protein S4817, partial [Shigella flexneri 2a str. 2457T]
MNNFVNNTQSTPSAITGDMVNSNAQLAMAVSELGRKVDALTAETKKPLSVNITGEATVRPDAPSFFSFNQ
SANDQFTQDMLLSSSYPEEE

>gi|30062577|ref|NP_836748.1| hypothetical protein S4815 [Shigella flexneri 2a str. 2457T]
MTLPVFITVIADHDKPQPSGCLLDNLSSAAVKTAGVLISFQAVLSAYQRVMEVGLQRASAERSIDFVFSK
DANQVKDFTKSLAQTTGLDIAELQSQFAGFGASARESLGIQGSEELFRNMIGYSRLMGRSEEEIKRALTA
LSQMAGKGQIMAEELKGQLAEAVPGMVQVFAKATGKTEQELFDAMKKGALKSADTLQKVTQELNKQITAK
GGWKAISESTQAQLGNLKNSWNTTLDSIFRGSENGLQDFTRSLTNLLNALGGSGKSLGESLGSLMTSMSH
GVDSLTDISYRVRAFFDEVTLAYRPLNDTQRKIVDGLSDGLIDGLKMLAVAVTAQKAANVAGGLLNLANA
VTSFGDAVNKAKAGKAGSSGGALNKVGRVVLYAELVNFALDNLNPEAAKNPNLHQSGIGTETNFNPEYKG
DSSKPIVLSLTGLMVQLIKLLTSKNR

>gi|30062576|ref|NP_836747.1| hypothetical protein S4816 [Shigella flexneri 2a str. 2457T]
MVCNYRYKNRQCHCLSGGYMARSAKPRKRKPASQRSKLPRYVVKLHEDDFFDEEDAEVLRFDSFDDAVEC
CADLNIPFFVDAGNKKLVFWFVRVDDEGYPEIARCTEREFATILAGISAGGMYCPECGTVHWPDGVAPPF

>gi|30062575|ref|NP_836746.1| IS91 orf [Shigella flexneri 2a str. 2457T]
MLPRFADIFQQGNRWLNWLEKQPEGAVRPVVIESVTKIMACGTTLMGYTQWCCSSPDCCHTKKVCFRCKS
RSCPHCGVKAGAQWIQYLLSLVPYCPWQHIVFTLPCQYWSLVFHNRWLLAEMSRIAADVILEICHQADVE
PGIFTVIHTWGRDQQWHPHIHLSTTAGGVTSGHTWKNLHFYARKVMSMWRYRITRLLSRKYPYLVMPDAL
AAEGSSKREWNRFLDTHYRRGWNVNVSRVMDNATHVAVYFGSYLKKPPVPMSRLEHYAGQDEIGLRYNSH

RTKREEYLLMSGDESMERFSWHVADKGFRMVRYYVFLSPAKRRLLEEVVYIITETVRKTAMQITWRGMYQ
Q

>gi|30062574|ref|NP_836745.1| glucosyltransferase MdoH [Shigella flexneri 2a str. 2457T]
MNKTTEYIDAMPIAASEKAALPKTDIRAVHQALDAEHRTWAREDDSPQGSVKARLEQAWPDSLADGQLIK
DDEGRDQLKAMPEAKRSSMFPDPWRTNPVGRFWDRLRGRDVTPRYLARLTKEEQESEQKWRTVGTIRRYI
LLILTLAQTVVATWYMKTILPYQGWALINPMDMVGQDLWVSFMQLLPYMLQTGILILFAVLFCWVSAGFW
TALMGFLQLLIGRDKYSISASTVGDEPLNPEHRTALIMPICNEDVNRVFAGLRATWESVKATGNAKHFDV
YILSDSYNPDICVAEQKAWMELIAEVGGEGQIFYRRRRRRVKRKSGNIDDFCRRWGSQYSYMVVLDADSV
MTGDCLCGLVRLMEANPNAGIIQSSPKASGMDTLYARCQQFATRVYGPLFTAGLHFWQLGESHYWGHNAI
IRVKPFIEHCALAPLPGEGSFAGSILSHDFVEAALMRRAGWGVWIAYDLPGSYEELPPNLLDELKRDRRW
CHGNLMNFRLFLVKGMHPVHRAVFLTGVMSYLSAPLWFMFLALSTALQVVHALTEPQYFLQPRQLFPVWP
QWRPELAIALFASTMVLLFLPKLLSILLIWCKGTKEYGGFWRVTLSLLLEVLFSVLLAPVRMLFHTVFVV
SAFLGWEVVWNSPQRDDDSTSWGEAFKRHGSQLLLGLVWAVGMAWLDLRFLFWLAPIVFSLILSPFVSVI
SSRATVGLRTKRWKLFLIPEEYSPPQVLVDTDRFLEMNRQRSLDDGFMHAVFNPSFNALATAMATARHRA
SKVLEIARDRHVEQALNETPEKLNRDRRLVLLSDPVTMARLHFRVWNSPERYSSWVSYYEGIKLNPLALR
KPDAASQ

>gi|30062573|ref|NP_836744.1| glucan biosynthesis protein G [Shigella flexneri 2a str. 2457T]
MMKMRWLSAAVMLTLYTSSSWAFSIDDVAKQAQSLAGKGYEAPKSNLPSVFRDMKYADYQQIQFNHDKAY
WNNLKTPFKLEFYHQGMYFDTPVKINEVTATAVKRIKYSPDYFTFGDVQHDKDTVKDLGFAGFKVLYPIN
SKDKNDEIVSMLGASYFRVIGAGQVYGLSARGLAIDTALPSGEEFPRFKEFWIERPKPTDKRLTIYALLD
SPRATGAYKFVVMPGRDTVVDVQSKIYLRDKVGKLGVAPLTSMFLFGPNQPSPANNYRPELHDSNGLSIH
AGNGEWIWRPLNNPKHLAVSSFSMENPRGFGLLQRGRDFSRFEDLDDRYDLRPSAWVTPKGEWGKGSVEL
VEIPTNDETNDNIVAYWTPDQLPEPGKEMNFKYTITFSRDEDKLHAPDNAWVQQTRRSTGDVKQSNLIRQ

PDGTIAFVVDFTGAEMKKLPEDTPVTAQTSIGDNGEIVESTVRYNPVTKGWRLVMRVKVKDAKKITEMRA

ALVNADQTLSETWSYQLPANE

>gi|30062572|ref|NP_836743.1| glucans biosynthesis protein [Shigella flexneri 2a str. 2457T]

MNPVPAQREYFLDSIRAWLMLLGIPFHISLIYSSHTWHVNSAEPSLWLTLFNDFIHSFRMQVFFVISGYF

SYMLFLRYPLKKWWKVRVERVGIPMLTAIPLLTLPQFIMLQYVKGKAESWPGLSLYDKYNTLAWELISHL

WFLLVLVVMTTLCVWIFKRIRNNLENSDKTNKKFSMVKLSVIFLCLGIGYAVIRRTIFIVYPPILSNGMF

NFIVMQTLFYLPFFILGALAFIFPHLKALFTTPSRGCTLAAALAFVAYLLNQRYGSGDAWMYETESVITM

VLGLWMVNVVFSFGHRLLNFQSARVTYFVNASLFIYLVHHPLTLFFGAYITPHITSNWLGFLCGLIFVVG

IAIILYEIHLRIPLLKFLFSGKPVVKRENDKAPAR

>gi|30062571|ref|NP_836742.1| synthase [Shigella flexneri 2a str. 2457T]

MMKKTPTSTKDSLPNKEMNDLPRLASAVLPLCSQHPGQCGLFPLEKSLDAFAARYRLAEMAEHTLDVQYY

IWQDDMSGRLLFSALLAAAKRGVRVRLLLDDNNTPGLDDILRLLDSHPRIEVRLFNPFSFRLLRPLGYIT

DFSRLNRRMHNKSFTVDGVVTLVGGRNIGDAYFGAGEEPLFSDLDVMAIGPVVEDVADDFARYWYCKSVS

PLQQVLDVPEGEMADRIELPASWHNDAMTHRYLRKMESSPFINHLVDGTLPLIWAKTRLLSDDPAKGEGK

AKRHSLLPQRLFDIMGSPSERIDIISSYFVPTRAGVAQLLRMVRKGVKIAILTNSLAANDVAVVHAGYAR

WRKKLLRYGVELYELKPTREQSSTLHDRGITGNSGASLHAKTFSIDGKTVFIGSFNFDPRSTLLNTEMGF

VIESETLAQLIDKRFIQSQYDAAWQLRLDRWGRINWVDRHAKKEVILKKEPATSFWKRVMVRLASILPVE

WLL

>gi|30062570|ref|NP_836741.1| hypothetical protein S1115 [Shigella flexneri 2a str. 2457T]

MKTRIHVVQGDITKLAVDVIVNAANPSLMGGGGVDGAIHRAAGPALLDACLKVRQQQGDCPTGHAVITLA

GDLPAKAVVHTVGPVWRGGEQNEDQLLQDAYLNSLRLVAANSYTSVAFPAISTGVYSYPRAAAAEIAVKT

VSEFITRHALPEQVYFVCYDEENAHLYERLLTQQGDE

>gi|30062569|ref|NP_836740.1| hypothetical protein S1114 [Shigella flexneri 2a str. 2457T]

MFRPFLDSLMLGSLFFPFIAIAGSTAQGGVIHFYGQIVEPACDVSTQSSPVEMNCPQNGSIPGKTYSSKA

LMSGNVKNAQIASVKVQYLDKQKKLAVMNIEYN

>gi|30062568|ref|NP_836739.1| autoagglutination protein [Shigella flexneri 2a str. 2457T]

MNALLLLAALSSQITFNTTQQGDMYTIIPEVTLTQSCLCRVQILSLREGSSGQSQTKQEKTLSLPANQPI

ALTKLSLNISPEDRVKIVVTVSDGQALHLSQQWTPSSEKS

>gi|30062567|ref|NP_836738.1| IS600 ORF2 [Shigella flexneri 2a str. 2457T]

MAHIRTRETYGTRRLQTELAENGIIVGRDRLARLRKELRLRCKQKRKFRATTNPNHNLPVAPNLLNQTFA

PTAPNQVWVADLTYVATQEGWLYLAGIKDVYTCEIVGYAMGERMTKELTGKALFMALRSQRPPAGLIHHS

DRGSQYCAYDYRVIQEQSGLKTSMSRKGNCYDNAPMESFWGISPRQPSGKNIIRWLLKKEQMVVSAIAST

PQ

>gi|30062566|ref|NP_836737.1| IS600 [Shigella flexneri 2a str. 2457T]

MSRKTQRYSKEFKAEAVRTVLENQLSISEGASRLSLPEGTLGQWVTAARKGLGTPGSRTLAELESEILQL

RKALNEARLERDILKKATAYFAQESLKNTR

>gi|30062565|ref|NP_836736.1| curlin minor subunit [Shigella flexneri 2a str. 2457T]

MKNKLLFMMLTILGAPGIAAAAGYDLANSEYNFAVNELSKSSFNQAAIIGQAGTNNSAQLRQGGSKLLAV

VAQEGSSNRAKIDQTGDYNLAYIDQAGSANDASISQGAYGNTAMIIQKGSGNKANITQYGTQKTAVVVQR

QSQMAIRVTQR

>gi|30062564|ref|NP_836735.1| DNA-binding transcriptional regulator CsgD [Shigella flexneri 2a str. 2457T]

MFNEVHSIHGHTLLLITKPSLQATALLQHLKQSLAITGKLHNIQRSLDDISSGSIILLDMMEADKKLIHY

WQDTLSRKNNNIKILLLNTPEDYPYRDIENWPHINGVFYAMEDQERVVNGLQGVLRGECYFTQKLASYLI

THSGNYRYNSTESALLTHREKEILNKLRIGASNNEIARSLFISENTVKTHLYNLFKKIAVKNRTQAVSWA

NDNLRR

>gi|30062563|ref|NP_836734.1| curli assembly protein CsgE [Shigella flexneri 2a str. 2457T]

MKRYLRWIVAAEFLFAAGNLHAVEVEVPGLLTDHTVSSIGHDFYRAFSDKWESDYTGNLTINERPSARWG

SWITITVNQDVIFQTFLFPLKRDSEKTVVFALIQTEEALNRRQINQALLSTDDLAHDEF

>gi|30062562|ref|NP_836733.1| curli assembly protein CsgF [Shigella flexneri 2a str. 2457T]

MRVKHAVVLLMLISPLSWAGTMTFQFRNPNFGGNPNNGAFLLNSAQAQNSYKDPSYNDDFGIETPSALDN

FTQAIQSQILGGLLSNINTGKPGRMVTNDYIVDIANRDGQLQLNVTDRKTGQTSTIQVSGLQNNSTDF

>gi|30062561|ref|NP_836732.1| hypothetical protein S1103 [Shigella flexneri 2a str. 2457T]

MAAFSAIMRGMNILLSIAITTGILSGIWGWVAVSLGLLSWAGFLGCTAYFACPQGGLKGLAISAATLLSG

VVWAMVIIYGSALAPHLEILGYVITGIVAFLMCIQAKQLLLSFVPGTFIGACATFAGQGDWKLVLPSLAL

GLVFGYAMKNSGLWLAARSAKTAHREQEIKNKA

>gi|30062560|ref|NP_836731.1| oxidoreductase component [Shigella flexneri 2a str. 2457T]

MNEFSILCRVLGSLYYRQPQDPLLVPLFTLIREGKLAANWPLEQDELLTRLQKSCDMTQVSADYNALFIG

DECAVPPYRSAWVEDATEAEVRAFLSERGMPLADTPADHIGTLLLAASWLEDQSTEDESEALETLFSEYL

LPWCGAFLGKVEAHATTPFWRTMAPLTRDAISAMWDELEEDSEE

>gi|30062559|ref|NP_836730.1| hydrolase [Shigella flexneri 2a str. 2457T]

MYPVDLHMHTVASTHAYSTLSDYIAQAKQKGIKLFAITDHGPDMEDAPHHWHFINMRIWPRVVDGVGILR

GIEANIKNVDGEIDCSGKMFDSLDLIIAGFHEPVFAPHDKATNTQAMISTIASGNVHIISHPGNPKYEID
VKAVAEAAAKHQVALEINNSSFLHSRKGSEDNCRAVAAAVRDAGGWVALGSDSHTAFTMGEFEECLKILD
AVDFPLERILNVSPRRLLNFLESRGMAPIAEFADL

>gi|30062558|ref|NP_836729.1| hypothetical protein S1096 [Shigella flexneri 2a str. 2457T]
MIPDYLTFIRFQDKRNLIYIYAIGLILIGFYWKNAGFTFPSEDIGVVSGILALVLYNFIFDLKAYWAYKC
VTKNIDFSWFKKKQNHKIELFLTQPLVAGFLSLIMLSAMSWGLYQLLPSLYALFLISLLGPLVIFLLFRM
IRTSYVKQVAISVAKKVKYKSLTRYVLLSVCISTVVNLLTISPLRNSDSFVTEGQWLTFKSIIALLILCG
VVLAINLFFLRFSKRPAFLGRFFLQEIDLFFSSENTLSTFFAKPLWLRLFILRVIEMMWITLVSVLATLV
EWRIWFEAYFLLCYVPCLIYYFFHCRFLWHNDFMMACDMYFRWGHFNK

>gi|30062557|ref|NP_836728.1| IS3 orfA [Shigella flexneri 2a str. 2457T]
MTKTVSTSKKPRKQHSPEFRSEALKLAERIGVTAAARELSLYESQLYNWRSKQQNQQTSSERELEMSTEI
ARLKRQLAERDEELAILQKAATYFAKRLK

>gi|30062556|ref|NP_836727.1| IS3 orfB [Shigella flexneri 2a str. 2457T]
MKYVFIEKHQAEFSIKAMCRVLRVARSGWYTWCQRRTRISTRQQFRQHCDSVVLAAFTRSKQRYGAPRLT
DELRAQGYPFNVKTVAASLRRQGLRAKASRKFSPVSYRAHGLPVSENLLEQDFYASGPNQKWAGDITYLR
TDEGWLYLAVVIDLWSRAVIGWSMSPRMTAQLACDALQMALWRRKRPRNVIVHTDRGGQYCSADYQAQLK
RHNLRGSMSAKGCCYDNACVESFFHSLKVECIHGEHFISREIMRATVFNYIECDYNRWRRHSWCGGLSPE
QFENQNLA

>gi|30062555|ref|NP_836726.1| hypothetical protein S1091 [Shigella flexneri 2a str. 2457T]
MQYKDENGVNEPSRRRLLKGIGALAGSCPVAHAQKTQSAPGTLSPDARNEKQPFYGEHQAGILTPQQAAM
MLVAFDVLASDKADLERLFRLLTQRFAFLSQGGAAPETPNPRLPPLDSGILGGYIAPDNLTITLSVGHSL

FDERFGLAPQMPKKLQKMTRFPNDSLDAALCHGDVLLQICANTQDTVNHALRDIIKHTPDLLSVRWKREG
FISDHAARSKGKETPINLLGFKDGTANPDSQNDKLMQKVVWVTADQQEPAWTIGGSYQAVRLIQFRVEFW
DRTPLKEQQTIFGRDKQTGAPLGMQHEHDVPDYASDPEGKVIALDSHIRLANPRTAESESSLMLRRGYSY
SLGVTNSGQLDMGLLFVCYQHDLEKGFLTVQKRLNGEALEEYVKPIGGGYFFSLPGVKDANDYLGRALLQ
V

>gi|30062554|ref|NP_836725.1| hypothetical protein S1090 [Shigella flexneri 2a str. 2457T]
MTINFRRNALQLSVAALFSSAFMANAADVPQVKVTVTDKQCEPMTITVNAGKTQFIIQNHSQKALEWEIL
KGVMVVEERENIAPGFSQKMTANLQPGEYDMTCGLLTNPKGKLIVKGEATADAAQSDALLSLGGAITAYK
AYVMAETTQLVTDTKAFTDAIKAGDIEKAKALYAPTRQHYERIEPIAELFSDLDGSIDAREDDYEQKAAD
PKFTGFHRLEKALFGDNTTKGMDQYAEQLYTDVVDLQKRISELAFPPSKVVGGAAGLIEEVAASKISGEE
DRYSHTDLWDFQANVEGSQKIVDLLRPQLQKANPELLAKVDANFKKVDTILAKYRTKDGFETYDKLTDAD
RNALKGPITALAEDLAQLRGVLGLD

>gi|30062553|ref|NP_836724.1| cytochrome [Shigella flexneri 2a str. 2457T]
MTSRSRQAAYSGIFINETTGEFPQKEQELFEGIVAVIAVVILTWMVFWMRKVSRNVKVQLEQAVDSALQR
GNHHGWALVMMVFFAVAREGLESVFFLLAAFQQDVGIWPPLGAMLGLATAVVLGFLLYWGGIRLNLGAFF
KWTSLFILFVAAGLAAGAIRAFHEAGLWNHFQEIAFDMSAVLSTHSLFGTLMEGIFGYQEAPSVSEVAVW
FIYLIPALVAFALPPRAGATASRSA

>gi|30062552|ref|NP_836723.1| IS91 [Shigella flexneri 2a str. 2457T]
MARSAKPRKRKPASQRSKLPRYVVKLHEDDFFDEEDAEVLRFDNFDDAVECCADLNIPFFVDAGNKKLVF
WFVRVDDEGYPEIARCTEREFATILAGISAGGMYCPECGTVHWPD

>gi|30062551|ref|NP_836722.1| IS91 [Shigella flexneri 2a str. 2457T]

MLPRFADIFQQGNRWLNWLEKQPEGAVRPVVIESVTKIMACGTTLMGYTNRCCSSPDCCHTKKVCFRCKS
RSCPHCVVKAGAQWIQYLLSLVPYCPWQHIVFTLPCQYWSLVFHNRWLLAKMSRIAADVILEICHQADVE
PGIFTVIHTWGRDQQWHPHIHLSTTAGGVTSGHTWKNLHFYARKVMSMWRYRITRLLSRKYPYLVMPDAL
AAEGSSKREWNRFLDTHYRRGWNVNVSRVMDNATHVAVYFGSYLKKPPVPMSRLEHYAGQDEIGLRYNSH
RTKREEYLLMSGDESMERFSWHVADKGFRMVRYYVFLSPAKRRLLEEVVYIITETVRKTAMQITWRGMYQ
RLLKVDPLKCVLCGSQMRFTGLKRGYRLAEQVLMHEPLARMRWCG

>gi|30062550|ref|NP_836721.1| tet operon regulator [Shigella flexneri 2a str. 2457T]
MTQGAVKTTGKRSRAVSAKKKAILSAALDTFSQFGFHGTRLEQIAELAGVSKTNLLYYFPSKEALYIAVL
RQILDIWLAPLKAFREDFAPLAAIKEYIRLKLEVSRDYPQASRLFCMEMLAGAPLLMDELTGDLKALIDE
KSALIAGWVKSGKLAPIDPQHLIFMIWASTQHYADFAPQVEAVTGATLRDEVFFNQTVENVQRIIIEGIR
PR

>gi|30062549|ref|NP_836720.1| hypothetical protein S1085 [Shigella flexneri 2a str. 2457T]
MQDAAPRLTFTLRDEERLMMKIGVFVPIGNNGWLISTHAPQYMPTFELNKAIAQKAEHYHFDFALSMIKL
RGFGGKTEFWDHNLESFTLMAGLAAVTSRIQIYATAATLTLPPAIVARMAATIDSISGGRFGVNLVTGWQ
KPEYEQMGIWPGDDYFSRRYDYLTEYVQVLRDLWGTGKSDFKGDFFTMNDCRVSPQPSIPMKVICAGQSD
AGMAFSAQYADFNFCFGKGVNTPTAFAPTAARMKQATEQTGRDVGSYVLFMVIADETDDATRAKWEHYKA
GADEEALSWLTEQSQKDTRSGTDTNVRQMADPTSAVNINMGTLVGSYASVARMLDEVASVPGAEGVLLTF
DDFLSGIETFGERIQPLMQCRAHLSALTQEVA

>gi|30062548|ref|NP_836719.1| synthetase [Shigella flexneri 2a str. 2457T]
MPRPSLCADSGGGMMTTLTARPEAITFDPQQSAQIVVDMQNAYATPGGYLDLAGFDVSTTRPVIANIQTA
VTAARAAGMLIIWFQNGWDEQYVEAGGPGSPNFHKSNALKTMRKQPQLQGKLLAKGSWDYQLVDELVPQP
GDIVLPKPRYSGFFNTPLDSILRSRGIRHLVFTGIATNVCVESTLRDGFFLEHFGVVLEDATHQAGPEFA

QKAALFNIETFFGWVSDVETFCDALSPTSFARIA

>gi|30062547|ref|NP_836718.1| hypothetical protein S1083 [Shigella flexneri 2a str. 2457T]

MPKSVIIPAGSSAPLAPFVPGTLADGVVYVSGTLAFDQHNNVLFADDPKAQTRHVLETIRKVIETAGGTM

ADVTFNSIFITDWKNYAAINEIYAEFFPGDKPARFYIQCGLVKPDALVEIATIAHIAK

>gi|30062546|ref|NP_836717.1| acetyltransferase [Shigella flexneri 2a str. 2457T]

MKLSLSSPPYADAPVVVLISGLGGSGSYWLPQLAVLEQEYQVVCYDQRGTGNNPDTLAEDYSIAQMAAEL

HQALVAAGIERYAVVGHALGALVGMQLALDYPASVTMLVSVNGWLRINAHTRRCFQVRERLLYSGGAQAW

VEAQPLFLYPADWMAARAPRLEAEDALALAHFQGKNNLLRRLNALKRADFSHHADRIRCPVQIICASDDL

LVPTACSSELHAALPDSQKMVMPYGGHACNVTDPETFNALLLNGLASLLHHREAAL

>gi|30062545|ref|NP_836716.1| hypothetical protein S1081 [Shigella flexneri 2a str. 2457T]

MNEAVSPGALSTLFTDARTHNGWRETPVSDETLQELYALMKWGPTSANCSPARIVFIRTAEGKERLRPAL

SSGNLQKTLTAPVTAIVAWDSEFYERLPLLFPHGDARSWFTSSPQLAEETAFRNSSMQAAYLIVACRALG

LDTGPMSGFDRQHVDDAFFAGSTLKSNLLINIGYGDSSKLFARLPRLSFEEACGLL

>gi|30062544|ref|NP_836715.1| hypothetical protein S1080 [Shigella flexneri 2a str. 2457T]

MSCMGAAVNIITTDGPAGRAGFTASAVCSVTDTPPTLLVCLNRGASVWPVFNENRTLCVNTLSAGQEPLS

NLFGGKTPMEHRFAAARWQTGVTGCPQLEEALVSFDCRISQVVSVGTHDILFCAIEAIHRHTTPYGLVWF

DRSYHALMRPAC

>gi|30062543|ref|NP_836714.1| transporter [Shigella flexneri 2a str. 2457T]

MVRSQLSRADAPCLLTSLRRQLMAMFGFPHWQLKSTSTESGVVAPDERLPFAQTAVMGVQHAVAMFGATV

LMPILMGLDPNLSILMSGIGTLLFFFITGGRVPSYLGSSAAFVGVVIAATGFNGQGINPNISIALGGIIA

CGLVYTVIGLVVMKIGTRWIERLMPPVVTGAVVMAIGLNLAPIAVKSVSASAFDSWMAVMTVLCIGLVAV

FTRGMIQRLLILVGLIVACLLYGVMTNVLGLGKAVDFTLVSYAAWFGLPHFSTPGFNGQAMMLIAPVAVI

LVAENLGHLKAVAGMTGRNMDPYMGRAFVGDGLATMLSGSVGGSGVTTYAENIGVMAVTKVYSTLVFVAA

AVIAMLLGFSPKFGALIHTIPAAVIGGASIVVFGLIAVAGARIWVQNRVDLSQNANLIMVAVTFVLGAGD

FALTLGGFTLGGIGTATFGAILLNALLSRRLVDVPPPEVVHQEP

>gi|30062542|ref|NP_836713.1| hypothetical protein S1078 [Shigella flexneri 2a str. 2457T]

MISILAKKLLQDITKRGSVRVSSLSLVPRSENMAAAIRRVNSMQYDVYTQTEVSNGKPSRRFRQFCRRPR

KSIRSR

>gi|30062541|ref|NP_836712.1| TrpR binding protein WrbA [Shigella flexneri 2a str. 2457T]

MAKVLVLYYSMYGHIETMARAVAEGASKVDGAEVVVKRVPETMPPQLFEKAGGKTQTAPVATPQELADYD

AIIFGTPTRFGNMSGQMRTFLDQTGGLWASGALYGKLASVFSSTGTGGGQEQTITSTWTTLAHHGMVIVP

IGYAAQELFDVSQVRGGTPYGATTIAGGDGSRQPSQEELSIARYQGEYVAGLAVKLNG

>gi|30062540|ref|NP_836711.1| hypothetical protein S1076 [Shigella flexneri 2a str. 2457T]

MPTQEAKAHHVGEWASLRNTSPEIAEAIFEVAGYDEKMAEKIWEEGSDEVLVKAFAKTDKDSLFWGEQTI

ERKNV

>gi|30062539|ref|NP_836710.1| glucose-1-phosphatase/inositol phosphatase [Shigella flexneri 2a str. 2457T]

MNKTLIAATVAGIVLLASNAQAQTVPEGYQLQQVLMMSRHNLRAPLANNGSVLEQSTPNKWPEWDVPGGQ

LTTKGGVLEVYMGHYMREWLAEQGIVKSGECPPPDTVYAYANSLQRTVATAQFFITGAFPGCDIPVHHQE

KMGTMDPTFNPMITDDSAAFSEQAVAAMEKELSKLQLTDSYQLLEKIVNYKDSPACKEKQQCSLVDGKNT

FSAKYQQEPGVSGPLKVGNSLVDAFTLQYYEGFPMDQVAWGEIKSDQQWKVLSKLKNGYQDSLFTSPEVA

RNVAKPLVSYIDKALVTDRASAPKITVLVGHDSNIASLLTALDFKPYQLHDQNERTPIGGKIVFQRWHDS

KANRDLMKIEYVYQSAEQLRNADALTLQAPAQRVTLELSGCPIDANGFCPMDKFDSVLNEAVK

>gi|30062538|ref|NP_836709.1| hypothetical protein S1074 [Shigella flexneri 2a str. 2457T]

MGSNIHGISCTANNYLKQAWNDIKNEYEKNQTYSITLFENTLVCFMRLYNELRRKVNEEDTPCLECESLE

KEFEEMQNDNDLSLFMRTLRTNDTQIYSGVSGGITYTIQYVQDVDIVRVSLPGRGSESITDFKGYYWYGF

MEYIENINACDDVFSEYCLDNENMSIQPEQINMPGISDLDTGIDLSGISFIQSEINKTYGLKYAPVDGDG

YCLLRAILVLKEHEYSWALGSHKTQKQVYEEFIKIVDKQTIEALVDTAFYNLREDVKTLFGVDLQSDNKI

QGVGSFMSWSFLFFKKQFIDSCLNDKKCILHLPEFIFNDNKNLLALDTDTSDRIKAVKNFLAVLSDSICS

LFIVNSNVASISLGNESFSTDEDLEYGYLTNTGNHYDVYLPPELFSQAYKLKNKEMNAQLDYLNRYAT

>gi|30062537|ref|NP_836708.1| curved DNA-binding protein CbpA [Shigella flexneri 2a str. 2457T]

MELKDYYAIMGVKPTDDLKTIKTAYRRLARKYHPDVSKEPDAEARFKEVAEAWEVLSDEQRRAEYDQMWQ

HRNDPQFNRQFHHGDGQSFNAEDFDDIFSSIFGQHARQSRQRPAARGHDIEIEVAVFLEETLTEHKRTIS

YNLPVYNAFGMIEQEIPKTLNVKIPAGVGNGQRIRLKGQGTPGENGGPNGDLWLVIHIAPHPLFDIVGQD

LEIVVPVSPWEAALGTKVTVPTLKESILLTIPPGSQAGQRLRVKGKGLVSKKQTGDLYAVLKIVMPPKPD

ENTAALWQQLADAQSSFDPRKDWGKA

>gi|30062536|ref|NP_836707.1| chaperone-modulator protein CbpM [Shigella flexneri 2a str. 2457T]

MANVTVTFTITEFCLHTGISEEELNEIVGLGVVEPREIQETTWVFDDHAAIVVQRAVRLRHELALDWPGI

AVALTLMDDIAHLKQENRLLRQRLSRFVAHP

>gi|30062535|ref|NP_836706.1| IS1 orfA [Shigella flexneri 2a str. 2457T]

MASISIRCPSCSATEGVVRNGKSTAGHQRYLCSHCRKTWQLQFTYTASQPGTHQKIIDMAMNGVGCRASA

RIMGVGLYTVLRHLKNSGRSR

>gi|30062534|ref|NP_836705.1| IS1 orfB [Shigella flexneri 2a str. 2457T]

MSRQCTHYGRWPLHGFTSLKKLRPQSVTSRIQPGSDVIVCAEMDEHWGYVGAKSRQRWLFYAYDRIRRTV

VAHVFGERTLATLERLLSLLSAFEVVVWMTDGCPLYESRLKGKLHVISKRYTQRIERHNLNLRQHLARLV

RKSLSFSKSVELHDKVIGHYLNIKHYQ

>gi|30062533|ref|NP_836704.1| trimethylamine N-oxide reductase, cytochrome c-type subunit [Shigella flexneri 2a str. 2457T]

MRKLWNALRRPSARWSVLALVAIGIVIGIALIVLPHVGIKVTSTTEFCVSCHSMQPVYEEYKQSVHFQNA

SGVRAECHDCHIPPDIPGMVKRKLEASNDIYQTFIAHSIDTPEKFEAKRAELAEREWARMKENNSATCRS

CHNYDAMDHARQHPEAARQMKVAAKDNQSCIDCHKGIAHQLPDMSSGFRKQFDELRASANDSGDTLYSID

IKPIYAAKGDKEASGSLLPASEVKVLKRDGDWLQIEITGWTESAGRQRVLTQFPGKRIFVASIRGDVQQQ

VKTLEKTTVADTNTEWSKLQATAWMKKGDMVNDIKPIWAYADSLYNGTCNECHGAPEIAHFDANGWIGTL

NGMIGFTSLDKREERTLLKYLQMNASDTAGKAHGDKKEEK

>gi|30062532|ref|NP_836703.1| DNA-binding transcriptional regulator TorR [Shigella flexneri 2a str. 2457T]

MPHHIVIVEDKPVTQARLQSYFTQEGYTVSVTASGAGLREIMQNQPVDLILLDINLPDENGLMLTRALRE

RSTVGIILVTGRSDRIDRIVGLEMGADDYVTKPLELRELVVRVKNLLWRIDLARQAQPHTQDYCYRFAGY

CLNVSRHTLERDGEPIKLTRAEYEMLVAFVTNPGEILSRERLLRMLSARRVENPDLRTVDVLIRRLRHKL

SADLLVTQHGEGYFLAADVC

>gi|30062531|ref|NP_836702.1| TMAO reductase system periplasmic protein TorT [Shigella flexneri 2a str. 2457T]

MRVLLFLLLSLFMLSAFSADNLLRWHDAQHFTVQASMPLKAKRVWKLCALYPSLKDSYWLSLNYGMQEAA

RRYGVDLKVLEAGGYSQLATQQAQIDQCKQWGAEAILLGSSTTSFPDLQKQVANLPVIELVNALDDPQVK

SRVGVPWFQMGYQPGRYLVQWAHGKPLNVLLMPGPDNAGGSKEMVEGFRAAIAGSPVRIVDIALGDNDIE

IQRNLLQEMLERHPEIDVVAGTAIAAEAAMGEGRNLKTPLTVVSFYLSHQVYRGLKRGRVIMAASDQMVW

QGELAVEQAIRQLQGQSVSDNVSPPILVLTPKNADREHIRRSLSPGGFRPVYFYQHTSAAKK

>gi|30062530|ref|NP_836701.1| hypothetical protein S1062 [Shigella flexneri 2a str. 2457T]

MAEKKRTRWQRRPGTTGGKLPWNDWRNATTWRKATQFLLLAMNIYIAITFWYWVRYYETAGSTTFVARPG

GIEGWLPIAGLMNLKYSLATGQLPSVHAAAMLLLVAFIVISLLLKKAFCSWLCPVGTLSELIGDLGNKLF

GRQCVLPRWLDIPLRGVKYLLLSLFLYIALLMPAQAIHYFMLSPYSVVMDVKMLDFFRHMGTATLISVTV

LLIASLFIRHAWCRYLCPYGALMGVVSLLSPFKIRRNAESCIDCGKCAKKCPSRIPVDKLIQVRTVECTG

CMTCVESCPVASTLTFSLQKPAANKKAFALSGWLMTLLVLGIMFAVIGYAMYAGVWQSPVPEELYRRLIP

QAPMIGH

>gi|30062529|ref|NP_836700.1| cold shock gene [Shigella flexneri 2a str. 2457T]

MRRWISQNNIRLPRGAFFISALFFFNAVCIVSDNLLIIESFGKMAYNISYLTRVPGTNTLLACCCLSRPD

EVNSEY

>gi|30062528|ref|NP_836699.1| cold shock protein CspG [Shigella flexneri 2a str. 2457T]

MTGLVKWFNADKGFGFITPDDGSKDVFVHFTAIQSNEFRTLNENQKVEFSIEQGQRGPAAANVVTL

>gi|30062527|ref|NP_836698.1| IS1 orfB [Shigella flexneri 2a str. 2457T]

MSRQCTHYGRWPQHGFTSLKKLRPQSVTSRIQPGSDVIVCAEMDEHWGYVGAKSRQRWLFYAYDRIRRTV

VAHVFGERTLATLERLLSLLSAFEVVVWMTDGCPLYESRLKGKLHVISKRYTQRIERHNLNLRQHLARLG

RKSLSFSKSVELHDKVIGHYLNIKHYQ

>gi|30062526|ref|NP_836697.1| IS1 orfA [Shigella flexneri 2a str. 2457T]

MASISIRCPSCSATEGVVRNGKSTAGHQRYLCSHCRKTWQLQFTYTASQPGTHQKIIDMAMNGVGCRASA

RIMGVGLNTVLRHLKNSGRSR

>gi|30062525|ref|NP_836696.1| cold shock-like protein [Shigella flexneri 2a str. 2457T]

MSRKMTGIVKTFDRKSGKGFIIPSDGRKEVQVHISAFTPRDAEVLIPGLRVEFCRVNGLRGPTAANVYLS

>gi|30062524|ref|NP_836695.1| hypothetical protein S1056 [Shigella flexneri 2a str. 2457T]

MNKGKVMKHKLSAILMAFMLTTPAAFAAPEAANGTEATTGTTGTTTTTTGATTTAATTSGVAAGAVGTAT
VVGVATAVGVATLAVVAANDSGDGGSHNTSTTTSTTR

>gi|30062523|ref|NP_836694.1| regulator [Shigella flexneri 2a str. 2457T]

MRPLILSIFALFLAGCTHSQQSMVDTFRASLFDNQDITVAAQQIQALPYSTMYLRLNEGQRIFVVLGYIE
QEQSKWLSQDNAMLVTHNGRLLKTVKLNNNLLEVTNPGRAPLRNALAIKDGSRWTRDILWSEDNHFRSAT
LSSTFSFAGLETLNIAGHNVLCNVWQEEVTSTLPEKQWQNTFWVDSATGQVRQSRQMLGAGVIPVEMTFL
KPAP

>gi|30062522|ref|NP_836693.1| hypothetical protein S1054 [Shigella flexneri 2a str. 2457T]

MNKLQSYFIASVLYVMTPHAFAQGTVTIYLPSEQQTLSVGPVENVVQLVTQPQLRDRLWWPGALLTDSAA
KAKALKDYQHVMAQLASWEAEADDDVAATIKSVRQQLLNLNITGRLPVKLDPDFVRVDENSNPPLVGDYT
LYTVQRPVTITLLGAVSGAGQLPWQAGRSVTDYLQDHPRLAGADKNNVMVITPEGETVVAPVALWNKRHV
EPPPGSQLWLGFSAHVLPEKYADLNDQIVSVLTQRVPD

>gi|30062521|ref|NP_836692.1| hypothetical protein S1053 [Shigella flexneri 2a str. 2457T]

MKKNSYLLSCLAIAVSSACHAEVLTYPDPLGSSQSDFGGTGLLQMPNARIAPEGEFSVNYRDNDQYRFYS
TSVALFPWLEGTIRYTDVRTRKYSQWEDFSGDQSYKDKSFDFKLRLWEEGYWLPQVAFGKRDIAGTGLFD
GEYLVASKQAGPFDFTLGMAWGYAGNAGNITNPFCRVSDKYCHRAESHDAGDISFSDIFRGPASIFGGIE
YQTPWNPLRLKLEYDGNNYQNDFAGKLPQASHFNVGAVYRAASWADLNLSYERGNTLMFGFTLRTNFNDL

RPALRDTPKPAWQPAPESEGLQYTTVANQLTALKYNAGFDAPEIQLRDKTLYMSGQQYKYRDSREAVDRA
NRILVNNLPQGVEKISVTQKREHMAMVTTETDVASLRKQLAGTAPGKSEQLQQQRVEAEDLSAFGRCYRI
REDRFSYSFNPTLSQSLGGPEDFYMFQLGLMSSARYWFTDHLLLDDGIFTNIYNNYDKFKSSLLPADSTL
PRVRTHIRDYVRNDVYLNNLQANYFADLGNGFYGQVYGGYLETMYAGVGSELLYRPLDASWALGVDVNYV
KQRDWDNMMRFTDYSTPTGFVTAYWNPPTLNGVLMKLSVGQYLAKDKGATIDVAKLFDSGVAVGVWAAIS
NVSKDDYGEGGFSKGFYISIPFDLMTIGPNRNRAVVSWTPLTRDGGQMLSRKYQLDPMTAEREVPVGQ

>gi|30062520|ref|NP_836691.1| exopolysaccharide export protein [Shigella flexneri 2a str. 2457T]
MKKNIFKFSVLTLAVLSLTACTLVPGQNLSTSNKDVIELPDNQYDLDKMVNIYPVTPGLIDQLRAKPIMS
QANPELEQQIANYEYRIGIGDVLMVTVWDHPELTTPAGQYRSASDTGNWVNADGAIFYPYIGRLKVAGKT
LTQVRNEITARLDSVIESPQVDVSVAAFRSQKAYVTGEVSKSGQQPITNIPLTIMDAINAAGGLTADADW
RNVVLTQNGVKTKVNLYALMQRGDLRQNKLLHPGDILFIPRNDDLKVFVMGEVGKQSTLKMDRSGMTLAE
ALGNAEGMNQDVADATGIFVIRATQNKQNGKIANIYQLNAKDASAMILGTEFQLEPYDIVYVTTAPLARW
NRVISLLVPTISGVHDLTETSRWIQTWPN

>gi|30062518|ref|NP_836689.1| phosphoanhydride phosphorylase [Shigella flexneri 2a str. 2457T]
MKAILIPFLSLLIPLTPQSAFAQSEPELKLESMVIVSRHGVRAPTKATQLMQDVTPDAWPTWPVKLGWLT
PRGGELIAYLGHYQRQRLVADGLLAKKGCPQSAQVAIIADVDERTRKTGEAFTAGLAPDCAITVHTQADT
SSPDPLFNPLKTGVCQLDNANVTDAILCRAGGSIADFTGHRQTVFRELERVLNFPQSNLCLNREKQDESC
SLTQALPSELKVSADNVSLTGAVSLASMLTEIFLLQQAQGMPEPGWGRITDSHQWNTLLSLHNAQFYLLQ
RTPEVARSRATPLLDLIMAALTPHPPQKQAYGVTLPTSVLFIAGHDTNLANLGGALELNWTLPGQPDNTP
PGGELVFERWRRLSDNSQWIQVSLVFQTLQQMRDKTPLSLNTPPGEVKLTLAGCEERNAQGMCSLAGFTQ
IVNEARIPACSL

>gi|30062517|ref|NP_836688.1| third cytochrome oxidase, subunit II [Shigella flexneri 2a str. 2457T]

MFDYETLRFIWWLLIGVILVVFMISDGFDMGIGCLLPLVARNDDERRIVINSVGAHWEGNQVWLILAGGA

LFAAWPRVYAAAFSGFYVAMILVLCSLFFRPLAFDYRGKIADARWRKMWDAGLVIGSLVPPVVFGIAFGN

LLLGVPFAFTPQLRVEYLGSFWQLLTPFPLLCGLLSLGMVILQGGVWLQLKTVGVIHLRSQLATKRAALL

VMLCFLLAGYWLWVGIDGFVLLAQDANGPSHPLMKLVAVLPGAWMNNFVESPVLWIFPLLGFFCPLLTVM

AIYRGRPGWGFLMASLMQFGVIFTAGITLFPFVMPSSVSPISSLTLWDSTSSQLTLSIMLVIVLIFLPIV

LLYTLWSYYKMWGRMTTETLRRNKNELY

>gi|30062516|ref|NP_836687.1| third cytochrome oxidase, subunit I [Shigella flexneri 2a str. 2457T]

MWDVIDLSRWQFALTALYHFLFVPLTLGLIFLLAIMETIYVVTGKTIYRDMTRFWGKLFGINFALGVATG

LTMEFQFGTNWSFYSNYVGDIFGAPLAMEALMAFFLESTFVGLFFFGWQRLNKYQHLLVTWLVAFGSNLS

ALWILNANGWMQYPTGAHFDIDTLRMEMTSFSELVFNPVSQVKFVHTVMAGYVTGAMFIMAISAWYLLRG

RERDVALRSFAIGSVFGTLAIIGTLQLGDSSAYEVAQVQPVKLAAMEGEWQTEPAPAPFHVVAWPEQDQE

RNAFAIKIPALLGILATHSLDKPVPGLKNLMAETYPRLQRGRMAWLLMQEISQGNREPHVLQAFRELEGD

LGYGMLLSRYAPDMNHVTAAQYQAAMRGAIPQVAPVFWSFRIMVGCGSLLLLVMLIALVQTLRGKIDQHR

WVLKMALWSLPLPWIAIEAGWFMTEFGRQPWAIQDILPTYSAHSALTTGQLAFSLIMIVGLYTLFLIAEV

YLMQKYARLGPSAMQSEQPTQQQG

>gi|30062515|ref|NP_836686.1| hydrogenase-1 operon protein hyaF [Shigella flexneri 2a str. 2457T]

MSETFFHLLGPGTQPNDDSFSMNPLPITCQVNDEPSMAALEQCAHSPQVIALLNELQHQLSERQPPLGEV

LAVDLLNLNADDRHFINTLLGEGEVSVRIQQADDSKSEIQEAIFCGLWRVRRRRGEKLLEDKLEAGCAPL

ALWQAATQNLLPTDSLLPPPIDGLMNGLPLAHELLAHVRNPDAQPHSINLTQLPISEADRLFLSRLCGPG

NIQIRTIGYGESYINATGLRHVWHLRCTDTLKGPLLESYEICPIPEVVLAAPEDLVDSAQRLSEVCQWLA

EAAPT

>gi|30062514|ref|NP_836685.1| hydrogenase-1 operon protein HyaE [Shigella flexneri 2a str. 2457T]

MSNDTPFDALWQRMLARGWTPVSESRLDDWLTQAPDGVVLLSSDPKRTPEVSDNPVMIGELLHEFPDYTW

QVAIADLEQSEAIGDRFGAFRFPATLVFTGGNYRGVLNGIHPWAELINLMRGLVEPQQERAS

>gi|30062513|ref|NP_836684.1| hydrogenase 1 maturation protease [Shigella flexneri 2a str. 2457T]

MSEQHVVVMGLGNLLWADEGFGVRVAERLYAHYHWPEYVEIVDGGTQGLNLLGYVESASHLLILDAIDYG

LEPGTLRTYAGERIPAYLSAKKMSLHQNSFSEVLALADIRGHLPAHIALVGLQPAMLDDYGGSLSELARE

QLPAAEQSALAQLAAWGSVPQPANESRCLNYDCLSMENYEGVRLRQYRMTQEEQG

>gi|30062512|ref|NP_836683.1| hydrogenase 1 b-type cytochrome subunit [Shigella flexneri 2a str. 2457T]

MQQKSDNVVSHYVFEAPVRIWHWLTVLCMAVLMVTGYFIGKPLPSVSGEATYLFYMGYIRLIHFSAGMIF

TVVLLMRIYWAFVGNRYSRELFIVPVWRKSWWQGVWYEIRWYLFLAKRPSADIGHNPIAQAAMFGYFLMS

VFMIITGFALYSEHSQYAIFAPFRYVVEFFYWTGGNSMDIHSWHRLGMWLIGAFVIGHVYMALREDIMSD

DTVISTMVNGYRSHKFGKISNKERS

>gi|30062511|ref|NP_836682.1| hydrogenase 1 large subunit [Shigella flexneri 2a str. 2457T]

MSTQYETQGYTINNAGRRLVVDPITRIEGHMRCEVNINDQNVITNAVSCGTMFRGLEIILQGRDPRDAWA

FVERICGVCTGVHALASVYAIEDAIGIKVPDNANIIRNIMLATLWCHDHLVHFYQLAGMDWIDVLDALKA

DPRKTSELAQSLSSWPKSSPGYFFDVQNRLKKFVEGGQLGIFRNGYWGHPQYKLPPEANLMGFAHYLEAL

DFQREIVKIHAVFGGKNPHPNWIVGGMPCAINIDESGAVGAVNMERLNLVQSIITRTADFINNVMIPDAL

AIGQFNKPWSEIGTGLSDKCVLSYGAFPDIANDFGEKSLLMPGGAVINGDFNNVLPVDLVDPQQVQEFVD

HAWYRYPNDQVGRHPFDGITDPWYNPGDVKGSDTNIQQLNEQERYSWIKAPRWRGNAMEVGPLARTLIAY

HKGDAATVESVDRMMSALNLPLSGIQSTLGRILCRAHEAQWAAGKLQYFFDKLMTNLKNGNLATASTEKW

EPATWPTECRGVGFTEAPRGALGHWAAIRDGKIDLYQCVVPTTWNASPRDPKGQIGAYEAALMNTKMAIP

EQPLEILRTLHSFDPCLACSTHVLGDDGSELISVQVR

>gi|30062510|ref|NP_836681.1| hydrogenase-1 small subunit [Shigella flexneri 2a str. 2457T]

MNNEETFYQAMRRQGVTRRSFLKYCSLAATSLGLGAGMAPKIAWALENKPRIPVVWIHGLECTCCTESFI
RSAHPLAKDVILSLISLDYDDTLMAAAGTQAEEVFEDIITQYNGKYILAVEGNPPLGEQGMFCISSGRPF
IEKLKRAAAGASAIIAWGTCASWGCVQAARPNPTQATPIDKVITDKPIIKVPGCPPIPDVMSAIITYMVT
FDRLPDVDRMGRPLMFYGQRIHDKCYRRAHFDAGEFVQSWDDDAARKGYCLYKMGCKGPTTYNACSSTRW
NDGVSFPIQSGHGCLGCAENGFWDRGSFYSRVVDIPQMGTHSTADTVGLTALGVVAAAVGVHAVASAVDQ
RRRHNQQPTETEHQPGNEDKQA

>gi|30062509|ref|NP_836680.1| hypothetical protein S1038 [Shigella flexneri 2a str. 2457T]

MDRIVSSSHDRTSLLSTHKVLRNTYFLLSLTLAFSAITATASTVLMLPSPGLILTLVGMYGLMFLTYKTA
NKPTGIISAFAFTGFLGYILGPILNTYLSAGMGDVIAMALGGTALVFFCCSAYVLTTRKDMSFLGGMLMA
GIVVVLIGMIANIFLQLPALHLAISAVFILISSGAILFETSNIIHGGETNYIRATVSLYVSLYNIFVSLL
SILGFASRD

>gi|30062507|ref|NP_836678.1| hypothetical protein S1036 [Shigella flexneri 2a str. 2457T]

MSKICIIAWIYGRVQGVGFRYTTQYEAKRLGLTGYAKNLDDGSVEVVACGEEGQVEKLMQWLKSGGPRSA
RVERVLSEPHHPSGELTDFRIR

>gi|30062506|ref|NP_836677.1| oxidoreductase [Shigella flexneri 2a str. 2457T]

MEGKASLGETIDIVDHQGKWLARGAYSPASQIRARVWTFDPSESIDIAFFSRRLQQAQKWRDWLAQKDGL
DSYRLIAGESDGLPGITIDRFGNFLVLQLLSAGAEYQRAALISALQTLYPECAIYDRSDVAVRKKEGMEL
TQGLVTGELPPALLPIEEHGMKLLVDIQHGHKTGYYLDQRDSRLATRRYVENKRVLNCFSYTGGFAVSAL
MGGCSQVVSVDTSQEALDIARQNVELNKLDLSKAEFVRDDVFKLLRTYRDRGEKFDVIVMDPPKFVENKS
QLMGACRGYKDINMLAIQLLNEGGILLTFSCSGLMTSDLFQKIIADAAIDAGRDVQFIEQFRQAADHPVI
ATYPEGLYLKGFACRVM

>gi|30062505|ref|NP_836676.1| hypothetical protein S1034 [Shigella flexneri 2a str. 2457T]

MWNFTLISKVKISREVTMIASKFGIGQQVRHSLLGYLGVVVDIDPVYSLSEPSPDELAVNDELRAAPWYH
VVMEDDNGLPVHTYLAEAQLSSELQDEHPEQPSMDELAQTIRKQLQAPRLRN

>gi|30062504|ref|NP_836675.1| hypothetical protein S1033 [Shigella flexneri 2a str. 2457T]

MVGMSALSYTLLNSLEEIMKETDIAGILTSTHTIALVGASDKPDRPSYRVMKYLLDQGYHVIPVSPKVAG
KTLLGQQGYGTLADVPEKVDMVDVFRNSEAAWGVAQEAIAIGAKTLWMQLGVINEQAAVLARDAGLNVVM
DRCPAIEIPRLGLAK

>gi|30062503|ref|NP_836674.1| hypothetical protein S1032 [Shigella flexneri 2a str. 2457T]

MKTGIVTTLIALCLPVSVFATTLRLSTDVDLLVLDGKKVSSSLLRGADSIELDNGPHQLVFRVEKTIHLS
NSEERLYISPPLVVSFNTQLINQVNFRLPRLENEREANHFDAAPRLELLDGDATPIPVKLDILAITSTAK
TIDYEVEVERYNKSAKRASLPQFATMMADDSTLLSGVSELDAIPPQSQVLTEQRLKYWFKLADPQTRNTF
LQWAEKQPSS

>gi|30062501|ref|NP_836672.1| DNA helicase IV [Shigella flexneri 2a str. 2457T]

MELKATTLGKRLAQHPYDRAVILNAGIKVSGDRHEYLIPFNQLLAIHCKRGLVWGELEFVLPDEKVVRLH
GTEWGETQRFYHHLDAHWRRWSGEMSEIASGVLRQQLDLIATRTGENKWLTREQTSGVQQQIRQALSALP
LPVNRLEEFDNCREAWRKCQAWLKDIESARLQHNQAYTEAMLTEYADFFRQVESSPLNPAQARAVVNGEH
SLLVLAGAGSGKTSVLVARAGWLLARGEASPEQILLLAFGRKAAEEMDERIRERLHTEDITARTFHALAL
HIIQQGSKKVPIVSKLENDTAARHELFIAEWRKQCSEKKAQAKGWRQWLTEEMQWSVPEGNFWDDEKLQR
RLASRLDRWVSLMRMHGGAQAEMIASAPEEIRDLFSKRIKLMAPLLKAWKGALKAENAVDFSGLIHQAIV
ILEKGRFISPWKHILVDEFQDISPQRAALLAALRKQNSQTTLFAVGDDWQAIYRFSGAQMSLTTAFHENF
GEGDRCDLDTTYRFNSRIGEVANRFIQQNPGQLKKPLNSLTNGDKKAVTLLDESQLDALLDKLSGYAKPE

ERILILARYHHMRPASLEKAATRWPKLQIDFMTIHASKGQQADYVIIVGLQEGSGGFPAAARESIMEEAL

LPPVEDFPDAEERRLMYVALTRARHRVWALFNKENPSPFVEILKNLDVPVARKP

>gi|30062500|ref|NP_836671.1| hypothetical protein S1029 [Shigella flexneri 2a str. 2457T]

MRTVLNILNFVLGGFATTLGWLLATLVSIVLIFTLPLTRSCWEITKLSLVPYGNEAIHVDELNPAGKNVL

LNTGGTVLNIFWLIFFGWWLCLMHIATGIAQCISIIGIPVGIANFKIAAIALWPVGRRVVSVETAQAARE

ANARRRFE

>gi|30062499|ref|NP_836670.1| hypothetical protein S1028 [Shigella flexneri 2a str. 2457T]

MAFMLSPLLKRYTWNSAWLYYARIFIALCGTTAFPWWLGDVKLTIPLTLGMVAAALTDLDDRLAGRLRNL

IITLFCFFIASASVELLFPWPWLFAIGLTLSTSGFILLGGLGQRYATIAFGALLIAIYTMLGTSLYEHWY

QQPMYLLAGAVWYNVLTLIGHLLFPVRPLQDNLARCYEQLARYLELKSRMFDPDIEDESQAPLYDLALAN

GQLMATLNQTKLSLLTRLRGDRGQRGTRRTLHYYFVAQDIHERASSSHIQYQTLREHFRHSDVLFRFQRL

MSMQGQACQQLSRCILLRQSYQHDPHFERAFTHIDAALERIRDNGAPADLLKTLGFLLNNLRAIDAQLAT

IESEQAQALPRNNDENELADDSPHGLSDIWLRLSRHFTPESALFRHAVRMSLVLCFGYAIIQITGMHHGY

WILLTSLFVCQPNYNATRHRLKLRIIGTLVGIAIGIPVLWFVPSLEGQLVLLVITGVLFFAFRNVQYAHA

TMFITLLVLLCFNLLGEGFEVALPRVIDTLIGCAIAWAAVSYIWPDWKFRNLPRMLERATEANCRYLDAI

LEQYHQGRDNRLAYRIARRDAHNRDAELASVVSNMSSEPNVTPQIREAAFRLLCLNHTFTSYISALGAHR

EQLTNPEILAFLDDAVCYVDDALHHQPADEERVNQALAGLKQRMQQLEPRADSKEPLVVQQVGLLIALLP

EIGRLQRQITVPTSGRTGVFH

>gi|30062498|ref|NP_836669.1| IS2 orfB [Shigella flexneri 2a str. 2457T]

MARGWGVSLVSRCLLVSRAQLHVILRRTDDWMDGRRSRHTDDTDVLLRIHHVIGELPTYGYRRVWALFRR

QAELDGMPAINAKRVYRLMRQNALLLERKPAVPPSKRAHTGRVAVKESNQRWCSDGFEFCCDNGERLRVT

FALDCCDREALHWAGTTGGFNSETVQDVMLGAVERRFGNDLPSSPVEWLTDNGSCYRANETRQFARMLGL

>gi|30062496|ref|NP_836667.1| hypothetical protein S1025 [Shigella flexneri 2a str. 2457T]

MKSLSYKRIYKSQEYLATLGTIEYRSLFGSYSLTVDDTVFAMVSDGELYLRACEQSAQYCVKHPPVWLTY

KKCGRSVTLNYYRVDESLWRNQLKLVRLSKYSLDAALKEKSTRNTRERLKDLPNMSFHLEAILGEVGRLA

P

>gi|30062495|ref|NP_836666.1| SOS cell division inhibitor [Shigella flexneri 2a str. 2457T]

MYTSGYAHRSSSFSSAASKIARVSTENTTAGLISEVVYREDQPMMTQLLLLPLLQQLGQQSRWQLWLTPQ

QKLSREWVQASGLPLTKVMQISQLSPCHTVESMVRALRTGNYSVVIGWLTDDLTEEEHAELVDAANEGNA

MGFIMRPVSASSHTTRQLSGLKIHSNLYH

>gi|30062494|ref|NP_836665.1| outer membrane protein A [Shigella flexneri 2a str. 2457T]

MKKTAIAIAVALAGFATVAQAAPKDNTWYTGAKLGWSQYHDTGFIPNNGPTHENQLGAGAFGGYQVNPYV

GFEMGYDWLGRMPYKGDNINGAYKAQGVQLTAKLGYPITDDLDIYTRLGGMVWRADTKANVPGGASFKDH

DTGVSPVFAGGVEYAITPEIATRLEYQWTNNIGDANTIGTRPDNGLLSLGVSYRFGQGEAAPVVAPAPAP

EVQTKHFTLKSDVLFNFNKATLKPEGQAALDQLYSQLSNLDPKDGSVVVLGYTDRIGSDAYNQGLSERRA

QSVVDYLISKGIPADKISARGMGESNPVTGNTCDNVKQRAALIDCLAPDRRVEIEVKGIKDVVTQPQA

>gi|30062493|ref|NP_836664.1| hypothetical protein S1022 [Shigella flexneri 2a str. 2457T]

MKYQQLENLESGWKWKYLVKKHREGELITRYIEASAAQEAVDVLLSLENEPVLVNGWIDKHMNPELVNRM

KQTIRARRKRHFNAEHQHTRKKSIDLEFIVWQRLAGLAQRRGKTLSETIVQLIEDAENKEKYANKMSSLK

QDLQALLGKE

>gi|30062492|ref|NP_836663.1| ATP-dependent protease [Shigella flexneri 2a str. 2457T]

MTITKLAWRDLVPDTDSYQEIFAQPHLIDENDPLFSDTQPRLQFALEQLLHTRASSSFMLAKAPEESEYL

NLIADAARTLQSDAGQLVGDHYEVSGHSIRLRHAVSADDNFATLTQVVAADWVEAEQLFGCLRQFNGDIT

LQPGLVHQANGGILIISLRTLLAQPLLWMRLKNIVNRERFDWVAFDESRPLPVSVPSMPLKLKVILVGER

ESLADFQEMEPELSEQAIYSEFEDTLQIVDAESVSQWCRWVTFTARHNHLPAPGADAWPVLIREAARYTG

EQETLPLSPQWILRQCKEVASLCDGDTFSGEQLNLMLQQREWREGFLAERMQDEILQEQILIETEGERIG

QINALSVIEFPGHPRAFGEPSRISCVVHIGDGEFTDIERKAELGGNIHAKGMMIMQAFLMSELQLEQQIP

FSASLTFEQSYSEVDGDSASMAELCALISALADVPVNQSIAITGSVDQFGRVQPVGGLNEKIEGFFAICQ

QRELTGKQGVIIPTANVRHLSLHSELVKAVEEGKFTIWAVDDVTDALPLLLNLVWDGEGQTTLMQTIQER

IAQASQQEGRHRFPWPLRWLNWFIPN

>gi|30062491|ref|NP_836662.1| 3-hydroxydecanoyl-ACP dehydratase [Shigella flexneri 2a str. 2457T]

MVDKRESYTKEDLLASGHGELFGAKGPQLPAPNMLMIDRVVKMTETGGNFDKGYVEAELDINPDLWFFGC

HFIGDPVMPGCLGLDAMWQLVGFYLGWLGGEGKGRALGVGEVKFTGQVLPTAKKVTYRIHFKRIVNRRLI

MGLADGEVLVDCRLIYTASDLKVGLFQDTSAF

>gi|30062490|ref|NP_836661.1| ribosome modulation factor [Shigella flexneri 2a str. 2457T]

MKRQKRDRLERAHQRGYQAGIAGRSKEMCPYQTLSQRSQWLGGWREAMADRVVMA

>gi|30062489|ref|NP_836660.1| hypothetical protein S1018 [Shigella flexneri 2a str. 2457T]

MKKWLVTIAALWLAGCSSGEINKNYYQLPVVQSGTQSTASQGNRLLWVEQVTVPDYLAGNGVVYQTSDVK

YVIANNNLWASPLDQQLRNTLVANLSTQLPGWVVASQPLGSAQDTLNVTVTEFNGRYDGKVIVSGEWLLN

HQGQLIKRPFRLEGVQTQDGYDEMVKVLAGVWSQEAASIAQEIKRLP

>gi|30062488|ref|NP_836659.1| paraquat-inducible protein B [Shigella flexneri 2a str. 2457T]

MESNNGEAKIQKVKNWSPVWIFPIVTALIGAWVLFYHYSHQGPEVTLITANAEGIEGGKTTIKSRSVDVG

VVESATLADDLTHVEIKARLNSGMEKLLHKDTVFWVVKPQIGREGISGLGTLLSGVYIELQPGAKGSKMD

KYDLLDSPPLAPPDAKGIRVILDSKKAGQLSPGDPVLFRGYRVGSVETSTFDTQKRNISYQLFINAPYDR

LVTSNVRFWKDSGIAVDLTSAGMRVEMGSLTTLLSGGVSFDVPEGLDLGQPVAPKTAFVLYDDQKSIQDS

LYTDHIDYLMFFKDSVRGLQPGAPVEFRGIRLGTVSKVPFFAPNMRQTFNDDYRIPVLIRIEPERLKMQL

GENADVVEHLGELLKRGLRGSLKTGNLVTGALYVDLDFYPNTPAITGIREFNGYQIIPTVSGGLAQIQQR

LMEALDKINKLPLNPMIEQATSTLSESQRTMKNLQTTLDSMNKILASQSMQQLPTDMQSTLRELNRSMQG

FQPGSAAYNKMVADMQRLDQVLRELQPVLKTLNEKSNALVFEAKDKKDPEPKRAKQ

>gi|30062487|ref|NP_836658.1| paraquat-inducible protein A [Shigella flexneri 2a str. 2457T]

MCEHHHAAKHILCSQCDMLVALPRLEHGQKAACPRCGTTLTVAWDAPRQRPTAYALAALFMLLLSNLFPF

VNMNVAGVTSEITLLEIPGVLFSEDYASLGTFFLLFVQLVPAFCLITILLLVNRAELPVRLKEQLARVLF

QLKTWGMAEIFLAGVLVSFVKLMAYGSIGVGSSFLPWCLFCVLQLRAFQCVDRRWLWDDIAPMPELRQPL

KPGVTGIRQGLRSCSCCTAILPADEPVCPRCSTKGYVRRRNSLQWTLALLVTSIMLYLPANILPIMVTDL

LGSKMPSTILAGVILLWSEGSYPVAAVIFLASIMVPTLKMIAIAWLCWDAKGHGKRDSERMHLIYEVVEF

VGRWSMIDVFVIAVLSALVRMGGLMSIYPAMGALMFALVVIMTMFSAMTFDPRLSWDRQPESEHEES

>gi|30062486|ref|NP_836657.1| ABC transporter ATPase [Shigella flexneri 2a str. 2457T]

MSLISMHGAWLSFSDAPLLDNAELHIEDNERVCLVGRNGAGKSTLMKILNREQGLDDGRIIYEQDLIVAR

LQQDPPRNVEGSVYDFVAEGIEEQAEYLKRYHDISRLVMNDPSEKNLNELAKVQEQLDHHNLWQLENRIN

EVLAQLGLDPNVALSSLSGGWLRKAALGRALVSNPRVLLLDEPTNHLDIETIDWLEGFLKTFNGTIIFIS

HDRSFIRNMATRIVDLDRGKLVTYPGNYDQYLQEKEEALRVEELQNAEFDLKLAQEEVWIRQGIKARRTR

NEGRVRALKAMRRERGERREVMGTAKMQVEEASRSGKIVFEMEDVCYQVNGKQLVKDFSAQVLRGDKIAL

IGPNGCGKTTLLKLMLGQLQADSGRIHVGTKLEVAYFDQHRAELDPDKTVMDNLAEGKQEVMVNGKPRHV

LGYLQDFLFHPKRAMTPVRAFSGGERNRLLLARLFLKPSNLLILDEPTNDLDVETLELLEELIDSYQGTV

LLVSHDRQFVDNTVTECWIFEGGGKIGRYVGGYHDARGQQEQYVALKQPAVKKSEEAAAPKAETVKRSSS

KLSYKLQRELEQLPQLLEDLEAKLEALQTQVADASFFSQPHEQTQKVLADMAAAEQELEQAFERWEYLEA

LKNGG

>gi|30062485|ref|NP_836656.1| 23S rRNA m(2)G2445 methyltransferase [Shigella flexneri 2a str. 2457T]

MNSLFASTARGLEELLKTELENLGAVECQVVQGGVHFKGDTRLVYQSLMWSRLASRIMLPLGECKVYSDL

DLYLGVQAINWTEMFNPGATFAVHFSGLNDTIRNSQYGAMKVKDAIVDAFTRKNLPRPNVDRDAPDIRVN

VWLHKETASIALDLSGDGLHLRGYRDRAGIAPIKETLAAAIVMRSGWQSGTPLLDPMCGSGTLLIEAAML

ATDRAPGLHRGRWGFSGWAQHDEAIWQEVKAEAQTRARKGLAEYSSHFYGSDSDARVIQRARTNARLAGI

GELITFEVKDVAQLTNPLPKGPYGTVLSNPPYGERLDSEPALIALHSLLGRIMKNQFGGWNLSLFSASPD

LLSCLQLRADKQYKAKNGPLDCVQKNYHVAESTPDSKPAMVAEDYANRLRKNLKKFEKWARQEGIECYRL

YDADLPEYNVAVDRYADWVVVQEYAPPKTIDAHKARQRLFDIIAATISVLGIAPNKLVLKTRERHKGKNQ

YQKLGEKGEFLEVTEYNAHLWVNLTDYLDTGLFLDHRIARRMLGQMSKGKDFLNLFSYTGSATVHAGLGG

ARSTTTVDMSRTYLEWAERNLRLNGLTRRAHRLIQADCLAWLREANEQFDLIFIDPPTFSNSKRMEDAFD

VQRDHLALMKDLKRLLRAGGTIMFSNNKRGFRMDLDGLAKLGLKAQEITQKTLSQDFARNRQIHNCWLIT

AA

>gi|30062484|ref|NP_836655.1| hypothetical protein S1013 [Shigella flexneri 2a str. 2457T]

MATLTRLFIHPVKSMRGIGLTHALADVSGLAFDRIFMITEPDGTFITARQFPQMVRFTPSPVHDGLHLTA

PDGSSAYVRFADFATQDAPTEVWGTHFTARIAPDAINKWLSGFFSREVQLRWVGPQMTRRVKRHNTVPLS

FADGYPYLLANEASLRDLQQRCPASVKMEQFRPNLVVSGASAWEEDSWKVIRIGDVVFDVVKPCSRCIFT

TVSPEKGQKHPAGEPLKTLQSFRTAQDNGDVDFGQNLIARNSGVIRVGDEVEILATAPAKIYGAAAADDT

ANITQQPDANVDIDWQGQAFRGNNQQVLLEQLENQGIRIPYSCRAGICGSCRVQLLEGEVTPLKKSAMGD

DGTILCCSCVPKTALKLTR

>gi|30062483|ref|NP_836654.1| hypothetical protein S1012 [Shigella flexneri 2a str. 2457T]

MLLRFYRVGERQMRIKPDDNWRWYYDEEHDRMMLDLANGMLFRSRFARKMLTPDAFSPAGFCVDDAALYF

SFEEKCRDFNLSKDQKAELVLNALVAIRYLKPQMPKSWHFVSHGEMWVPMPGDAACVWLSDTHEQVNLLV

VESGENAALCLLAQPCVVIAGRAMQLGDAIKIMNDRLKPQVNVDSFSLEQAV

>gi|30062482|ref|NP_836653.1| dihydroorotate dehydrogenase 2 [Shigella flexneri 2a str. 2457T]

MYYPFVRKALFQLDPERAHEFTFQQLRRITGTPFEALVRQKVPAKPVNCMGLTFKNPLGLAAGLDKDGEC

IDALGAMGFGSIEIGTVTPRPQPGNDKPRLFRLVDAEGLINRMGFNNLGVDNLVENVKKAHYDGVLGINI

GKNKDTPVEQGKDDYLICMEKIYAYAGYIAINISSPNTPGLRTLQYGEALDDLLTAIKNKQNDLQAMHHK

YVPIAVKIAPDLSEDELIQVADSLVRHNIDGVIATNTTLDRSLVQGMKNCDQTGGLSGRPLQLKSTEIIR

RLSLELNGRLPIIGVGGIDSVIAAREKIAAGASLVQIYSGFIFKGPPLIKEIVTHI

>gi|30062481|ref|NP_836652.1| putativi pili assembly chaperone [Shigella flexneri 2a str. 2457T]

MPARHLYFIMTNTWNRLALLIFAVLSLLVAGELQAGVVVGGTRFIFPADRESISILLTNTSQESWLINSK

INRPTRWAGGEASTVPALLLAAPPLILLKPGTTGTLRLLRTESDILPVDRETLFELSIASVPSGKVENQS

VKVAMRSVFKLFWRPEGLPGDPLEAYQQLRWTRNSQGVQLTNPTPYYINLIQVSVNGKALSNAGVVPPKS

QRQTNWCQVIAPCHVAWRTINDYGGLSAKKEQNLP

>gi|30062480|ref|NP_836651.1| fimbrial-like protein [Shigella flexneri 2a str. 2457T]

MRRRFSILIWFTSEGVMLKRIIWILFLLGLTGGCELFAHDGTVNISGSFRRNTCVLAQDSKQINVQLGDV

SLTRFFHGNYGPEKSFIINLQDCGTDVSTVDVTFSGTPDGVQSEMLSIESGTDAASGLAIAILDDAKILI

PLNQASKDYSLHSGKVPLTFYAQLRPVNSDVQSGKVNASATFVLHYD

>gi|30062479|ref|NP_836650.1| fimbrial-like protein [Shigella flexneri 2a str. 2457T]

MKKKTIYQCVILFFSLLNIHVGMAGPEQVSMHIYGNVVDQGCDVATKSALQNIHIGDFNISDFQAANTVS

TAADLNIDITGCAAGITGADVLFSGEADTLAPTLLKLTDTGGSGGMATGIAVQILDAQSQQEIPLNQVQP

LTPLKAGDNTLKYQLRYKSTKAGATGGNATAVLYFDLVYQ

>gi|30062478|ref|NP_836649.1| fimbrial protein [Shigella flexneri 2a str. 2457T]

MQIIFGEKCVLLLRLFFAAVLMLWCAQTAAYIGQCHTTQGNPYIGVNFGVKTLDEEENTAGVVKDKFYQW

NESNDYYVSCDCDKDNVRSGRWAFAADSPLVYLGDNWYKINDYLAAKVLLQVKGSSPTAVPFENVGTGAD

TRWHICDPGGQRLGGQGASGNSGSFSLKILQPFVGSVVIPPMALARLFECYNIPAGDSCTTTGTPVLVYY

LSGTINSLGSCSVNAGETIEVDLGDVFAANFRVVGHKPLGARTAELAIPVRCNTGNAGLVNVNLSLTATS

DPSYPQAIKTSRPGVGVVVTDSQHNIISPAGGTLPLSIPDDADSIRE

>gi|30062477|ref|NP_836648.1| outer membrane protein [Shigella flexneri 2a str. 2457T]

MYRTHRQHSLLSSGGVPSFIGGLVVFVSAAFNAQAETWFDPAFFKDDPSMVADLSRFEKGQKITPGVYRV

DIVLNQTIVDTRNVNFVEITPEKGIAACLTTESLDAMGVNTDAFPAFKQLDKQVCVPLAEIIPDASVTFN

VNKLRLEISVPQIAIKSNARGYVPPERWDEGINALLLGYSFSGANSIHSSADSDSGDSYFLNLNSGVNLG

PWRLRNNSTWSRSSGQTAEWKNLSSYLQRAVIPLKGELTVGDDYTAGDFFDSVSFRGVQLASDDNMLPDS

LKGFAPVVRGIAKSNAQITIKQNGYTIYQTYVSPGAFEISDLYSTSSSGDLLVEIKEADGSVNSYSVPFS

SVPLLQRQGRIKYAVTLAKYRTNSNEQQESKFAQTTLQWGGPWGTTWYGGGQYAEYYRAAMFGLGFNLGD

FGAISFDATQAKSTLADQSEHKGQSYRFLYAKTLNQLGTNFQLMGYRYSTSGFYTLSDTMYKHMDGYEFN

DGDDEDTPMWSRYYNLFYTKRGKLQVNISQQLGEYGSFYLSGSQQTYWHTDQQDRLLQFGYNTQIKDLSL

GVSWNYSKSRGQPDADQVFALNFSLPLNLLLPRSNDSYTRKKNYAWMTSNTSIDNEGHITQNLGLTETLL

DDGNLSYSVQQGYNSEGKTANGSASMDYKGAFADARVGYNYSDNGSQQQLNYALSGSLVAHSQGITLGQS

LGETNVLIAAPGAENTRVANSTGLKTDWRGYTVVPYATSYRENRIALDAASLKRNVDLENAVVNVVPTKG

ALVLAEFNAHAGARVLMKTSKQGIPLRFGAIATLDGIQTNSGIIDDDGSLYMSGLPAQGAITVRWGEAPD

QICHISYQLTEQQINSAITRMDAICR

>gi|30062476|ref|NP_836647.1| IS1 orfA [Shigella flexneri 2a str. 2457T]

MASISIRCPSCSATEGVVRNGKSTAGHQRYLCSHCRKTWQLQFTYTASQPGTHQKIIDMAMNGVGCRASA

RIMGVGLNTVLRHLKNSGRSR

>gi|30062475|ref|NP_836646.1| IS1 orfB [Shigella flexneri 2a str. 2457T]

MSRQCTHYGRWPQHGFTSLKKLRPQSVTSRIQPGSDVIVCAEMDEHWGYVGAKSRQRWLFYAYDRIRRTV

VAHVFGERTLATLERLLSLLSAFEVVVWMTDGWPLYESRLKGKLHVISKRYTQRIERHNLNLRQHLARLV

RKSLSFSKSVELHDKAIGHYLNIKHYQ

>gi|30062474|ref|NP_836645.1| IS1 orfB, A [Shigella flexneri 2a str. 2457T]

MASISIRCPSCSATEGVVRNGKSTAGHQRYLCSHCRKTWQLQFTYTASQPGTHQKIIDMAMNGVGCRASA

RIMGVGLYTVLRHLKNSAESVTSRIQPGSDVIVCAEMDEHWGYVGAKSRQRWLFYAYDRIRRTVVAHVFG

ERTLATLERLLSLLSAFEVVVWMTDGCPLYESRLKGKLHVISKRYTQRIERHNLNLRQHLARLVRKSLSF

SKSVELHDKAIGHYLNIKHYQ

>gi|30062473|ref|NP_836644.1| NAD(P)H-dependent FMN reductase [Shigella flexneri 2a str. 2457T]

MRVITLAGSPRFPSRSSSLLEYAREKLNGLDVEVYHWNLQNFAPEDLLYARFDSPALKTFTEQLQQADGL

IVATPVYKAAYSGALKTLLDLLPERALQGKVVLPLATGGTVAHLLAVDYALKPVLSALKAQEILHGVFAD

DSQLIDYHHRPQFTPNLQTHLDTALETFWQALHRRDVQVPDLLSLRGNAHA

>gi|30062471|ref|NP_836642.1| alkanesulfonate monooxygenase [Shigella flexneri 2a str. 2457T]

MSLNMFWFLPTHGDGHYLGTEEGSRPVDHGYLQQIAQAADRLGYTGVLIPTGRSCEDAWLVAASMIPVTQ

RLKFLVALRPSVTSPTVAARQAATLDRLSNGRALFNLVTGSDPQELAGDGVFLDHSERYEASAEFTQVWR

RLLLGETVNFNGKHIHVRGAKLLFPPIQQPYPPLYFGGSSDVAQELAAEQVDLYLTWGEPPELVKEKIEQ

VRAKAAAYGRKIRFGIRLHVIVRETNDEAWQAAERLISHLDDETIAKAQAAFARTDSVGQQRMAALHNGK

RDNLEISPNLWAGVGLVRGGAGTALVGDGPTVAARINEYAALGIDSFVLSGYPHLEEAYRVGELLFPHLD

VAIPEIPQPQPLNPQGEAVANDFIPRKVAQS

>gi|30062468|ref|NP_836639.1| aliphatic sulfonates transport ATP-binding subunit [Shigella flexneri 2a str. 2457T]

MNTARLNQGTPLLLNAVSKHYAENIVLNQLDLHIPAGQFVAVVGRSGGGKSTLLHLLAGLETPTAGDVLA

GTTPLAEIQDDTRMMFQDARLLPWKSVIDNVGLGLKGQWRDAARRALAAAGLENRAGEWPAALSGGQKQR

VALARALIHRPGLLLLDEPLGALDALTRLEMQDLIVSLWQQHGFTVLLVTHDVSEAVAMADRVLLIEEGK

ISLDLTVDIPRPRRLGSVRLAELEAEVLQRVMRRGHSEQLIRRHG

>gi|30062467|ref|NP_836638.1| aminopeptidase N [Shigella flexneri 2a str. 2457T]

MTQQPQAKYRHDYRAPDYQITDIDLTFDLDAQKTVVTAVSQAVRHGASDAPLRLNGEDLKLVSVHINDEP

WTAWKEEEEGALVISNLPERFTLKIINEISLAANTALEGLYQSGDALCTQCEAEGFRHITYYLDRPDVLA

RFTTKIIADKIKYPFLLSNGNRVAQGELENGRHWVQWQDPFPKPCYLFALVAGDFDVLRDTFTTRSGREV

ALELYVDRGNLDRAPWAMTSLKNSMKWDEERFGLEYDLDIYMIVAVDFFNMGAMENKGLNIFNSKYVLAR

TDTATDKDYLDIERVIGHEYFHNWTGNRVTCRDWFQLSLKEGLTVFRDQEFSSDLGSRAVNRINNVRTMR

GLQFAEDASPMAHPIRPDMVIEMNNFYTLTVYEKGAEVIRMIHTLLGEENFQKGMQLYFERHDGSAATCD

DFVQAMEDASNVDLSHFRRWYSQSGTPVVTVKDDYNPETEQYTLTISQRTPATPDQAEKQPLHIPFAIEL

YDNEGKVIPLQKGGHPVNSVLNVTQAEQTFVFDNVYFQPVPALLCEFSAPVKLEYKWSDQQLTFLMRHAR

NGFSRWDAAQSLLATYIKLNVARHQQGQPLSLPVHVADAFRAVLLDEKIDPALAAEILTLPSVNEMAELF

DIIDPIAIAEVREALTRTLATELADELLAIYNANYQSEYRVEHEDIAKRTLRNACLRFLAFGETHLADVL

VSKQYHEANNMTDALAALSAAVAAQLPCRDALMQEYDDKWHQDGLVMDKWFILQATSPAANVLETVRGLL

QHRSFTMSNPNRIRSLIGAFAGSNPAAFHAEDGSGYQFLVEMLTDLNSRNPQVASRLIEPLIRLKRYDAK

RQEKMRAALEQLKGLENLSGDLYEKITKALA

>gi|30062466|ref|NP_836637.1| nicotinate phosphoribosyltransferase [Shigella flexneri 2a str. 2457T]

MTQFASPVLHSLLDTDAYKLHMQQAVFHHYYDVHVAAEFRCRGDDLLGIYADAIREQIQAMQHLRLQDDE

YQWLSALPFFKADYLNWLREFRFNPEQVTVSNDNGKLDIRLSGPWREVILWEVPLLAVISEMVHRYRSPQ

ADVAQALDTLESKLVDFSALTAGLDMSRFHLMDFGTRRRFSREVQETIVKRLQQESWFVGTSNYDLARRL
SLTPMGTQAHEWFQAHQQISPDLANSQRAALAAWLEEYPDQLGIALTDCITMDAFLRDFGVEFASRYQGL
RHDSGDPVEWGEKAIAHYEKLGIDPQSKTLVFSDNLDLRKAVELYRHFSSRVQLSFGIGTRLTCDIPQVK
PLNIVIKLVECNGKPVAKLSDSPGKTICHDKAFVRALRKAFDLPHIKKAS

>gi|30062465|ref|NP_836636.1| asparaginyl-tRNA synthetase [Shigella flexneri 2a str. 2457T]
MSVVPVADVLQGRVAVDSEVTVRGWVRTRRDSKAGISFLAVYDGSCFDPVQAVINNSLPNYNEDVLRLTT
GCSVIVTGKVVASPGQGQQFEIQASKVEVAGWVEDPDTYPMAAKRHSIEYLREVAHLRPRTNLIGAVARV
RHTLAQALHRFFNEQGFFWVSTPLITASDTEGAGEMFRVSTLDLENLPRNDQGKVDFDKDFFGKESFLTV
SGQLNGETYACALSKIYTFGPTFRAENSNTSRHLAEFWMLEPEVAFANLNDIAGLAEAMLKYVFKAVLEE
RADDMKFFAERVDKDAVSRLERFIEADFAQVDYTDAVTILENCGRKFENPVYWGVDLSSEHERYLAEEHF
KAPVVVKNYPKDIKAFYMRLNEDGKTVAAMDVLAPGIGEIIGGSQREERLDVLDERMLEMGLNKEDYWWY
RDLRRYGTVPHSGFGLGFERLIAYVTGVQNVRDVIPFPRTPRNASF

>gi|30062464|ref|NP_836635.1| outer membrane protein F [Shigella flexneri 2a str. 2457T]
MMKRNILAVIVPALLVAGTANAAEIYNKDGNKVDLYGKTVGLHYFSKGNGENSYGGNGDMTYARLGFKGE
TQINSDLTGYGQWEYNFQGNNSEGADAQTGNKTRLAFAGLKYADVGSFDYGRNYGVVYDALGYTDMLPEF
GGDTAYSDDFFVGRVGGVATYRNSNFFGLVDGLNFAVQYLGKNERDTARRSNGDGVGGSISYEYEGFGIV
GAYGAADRTNLQEAQPLGNGKKAEQWATGLKYDANNIYLAANYGETRNATPITNKFTNTSGFANKTQDVL
LVAQYQFDFGLRPSIAYTKSKAKDVEGIGDVDLVNYFEVGATYYFNKNMSTYVDYIINQIDSDNKLGVGS
DDTVAVGIVYQF

>gi|30062463|ref|NP_836634.1| aromatic amino acid aminotransferase [Shigella flexneri 2a str. 2457T]
MFENITAAPADPILGLADLFRADERPGKINLGIGVYKDETGKTPVLTSVKKAEQYLLENETTKNYLGIDG
ILEFGRCTQELLFGKGSALINDKRARTAQTPGGTGALRVAADFLAKNTSVKRVWVSNPSWPNHKSVFNSA

GLEVREYAYYDAENHTLDFDALINSLNEAQAGDVVLFHGCCHNPTGIDPTLEQWQTLAQLSVEKGWLPLF
DFAYQGFARGLEEDAEGLRAFAAMHKELIVASSYSKNFGLYNERVGACTLVAADSEIVDRAFSQMKAAIR
ANYSNPPAHGASVVATILSNDALRAIWEQELTDMRQRIQRMRQLFVNTLQEKGANRDFSFIIKQNGMFSF
SGLTKEQVLRLREEFGVYAVASGRVNVAGMTPDNMAPLCESIVAVL

>gi|30062462|ref|NP_836633.1| hypothetical protein S0988 [Shigella flexneri 2a str. 2457T]
MNYRIIPVTAFSQNCSLIWCEQTRLAALVDPGGDAEKIKQEVDDSGLTLMQILLTHGHLDHVGAAAELAQ
HYGVPVFGPEKEDEFWLQGLPAQSRMFGLEECQPLTPDRWLNEGDTISIGNVTLQVLHCPGHTPGHVVFF
DDRAKLLISGDVIFKGGVGRSDFPRGDHNQLISSIKDKLLPLGDDVTFIPGHGPLSTLGYERLHNPFLQD
EMPVW

>gi|30062461|ref|NP_836632.1| hypothetical protein S0987 [Shigella flexneri 2a str. 2457T]
MDKFDANRRKLLALGGVALGAAILPTPAFATLSTPRPRILTLNNLHTGESIKAEFFDGRGYIQEELAKLN
HFFRDYRANKIKSIDPGLFDQLYRLQGLLGTRKPVQLISGYRSIDTNNELRARSRGVAKKSYHTKGQAMD
FHIEGIALSNIRKAALSMRAGGVGYYPRSNFVHIDTGPARHW

>gi|30062460|ref|NP_836631.1| hypothetical protein S0986 [Shigella flexneri 2a str. 2457T]
MLLNMMCGRRLSAISLCLAVTFAPLFNAQADEPEVIPGDSPVAVSEQGEALPQAQATAIMAGIQPLPEGA
AEKARTQIESQLPAGYKPVYLNQLQLLYAARDMQPMWENRDAVKAFQQQLAEVAIAGFQPQFNKWVELLT
DPGVNGMARDVVLSDAMMGYLHFIANIPIKGTRWLYSSKPYALSTPPLSVINQWQLALDKGQLPTFVAGL
APQHPQYAAMHESLLVLLSDTKPWPQLTGKATLRPGQWSNDVPALREILQRTGMLDGGPKITLPGDDTPT
DAVVSPSAVTVETAETKPMDKQTTSRSKPAPAVRAAYDNELVEAVKRFQAWQGLGADGAIGPATRDWLNV
TPAQRAGVLALNIQRLRLLPTELSTGIMVNIPAYSLLYYQNGNQVLDSRVIVGRPDRKTPMMSSALNNVV
VNPPWNVPPTLARKDILPKVRNDPGYLESHGYTVMRGWNSREAIDPWEVDWSTITASNLPFRFQQAPGPR
NSLGRYKFNMPSSEAIYLHDTPNHNLFKRDTRALSSGCVRVNKASDLANMLLQDAGWNDKRISDALKQGD

TRYVNIRQSIPVNLYYLTAFVGADDRTQYRTDIYNYDLPARSSSQIVSKAEQLIR

>gi|30062459|ref|NP_836630.1| IS4 orf [Shigella flexneri 2a str. 2457T]

MHIGQALDLVSRYDSLRNPLTSLGDYLDPELISRCLAESGTVTLRKRRLPLEMMVWCIVGMALERKEPLH

QIVNRLDIMLPGNRPFVAPSAVIQARQRLGSEAVRRVFTKTAQLWHNATPHPHWCGLTLLAIDGVFWRTP

DTPENDAAFPRQTHAGNPALYPQVKMVCQMELTSHLLTAAAFGTMKNSENELAEQLIEQTGDNTLTLMDK

GYYSLGLLNGWSLAGEHRHWMIPLRKGAQYEELRKLGKGDHLVKLKTSPQARKKWPGLGNEVTARLLTVT

RKGKVCHLLTSMTDAMRFPGGEMADLYSNRWEIELGYREIKQTMQLSRLTLRSKKPELVEQELWGVLLAY

NLVRYQMIKMAEHLKGYWPNQLSFSESCGMVMRMLMTLQGVSPGRIPELMRDLASMGQLVKLPTRRGRAF

PRVVKERPWKYPTAPKKSQSVA

>gi|30062458|ref|NP_836629.1| cell division protein MukB [Shigella flexneri 2a str. 2457T]

MIERGKFRSLTLINWNGFFARTFDLDELVTTLSGGNGAGKSTTMAAFVTALIPDLTLLHFRNTTEAGATS

GSRDKGLHGKLKAGVCYSMLDTINSRHQRVVVGVRLQQVAGRDRKVDIKPFAIQGLPMSVQPTQLVTETL

NERQARVLPLNELKDKLEAMEGVQFKQFNSITDYHSLMFDLGIIARRLRSASDRSKFYRLIEASLYGGIS

SAITRSLRDYLLPENSGVRKAFQDMEAALRENRMTLEAIRVTQSDRDLFKHLISEATNYVAADYMRHANE

RRVHLDKALEFRRELHTSRQQLAAEQYKHVDMARELAEHNGAEGDLEADYQAASDHLNLVQTALRQQEKI

ERYEVDLDELQIRLEEQNEVVAEAIERQEENEARAEAAELEVDELKSQLADYQQALDVQQTRAIQYNQAI

AALNRAKELCHLPDLTADSAAEWLETFQAKELEATEKMLSLEQKMSMAQTAHSQFEQAYQLVVAINGPLA

RNEAWDVARELLREGVDQRHLAEQVQPLRMRLSELEQRLREQQEAERLLADFCKRQGKNFDIDELEALHQ

ELEARIASLSDSVSNAREERMALRQEQEQLQSRIQSLMQRAPVWLAAQNSLNQLSEQCGEEFSSSQDVTE

YLQQLLEREREAIVERDEVGARKNAVDEEIERLSQPGGSEDQRLNALAERFGGVLLSEIYDDVSLEDAPY

FSALYGPSRHAIVVPDLSQVTEHLEGLTDCPEDLYLIEGDPQSFDDSVFSVDELEKAVVVKIADRQWRYS

RFPEVPLFGRAARESRIESLHAEREVLSERFATLSFDVQKTQRLHQAFSRFIGSHLAVAFESDPEAEIRQ

LNSRRVELERALSNHENDNQQQRIQFEQAKEGVTALNRILPRLNLLADDSLADRVDEIRERLDEAQEAAR

FVQQFGNQLAKLEPIVSVLQSDPEQFEQLKEDYAYSQQMQRDARQQAFALTEVVQRRAHFSYSDSAEMLS

GNSDLNEKLRERLEQAEAERTRAREALRGHAAQLSQYNQVLASLKSSYDTKKELLNDLQRELQDIGVRAD

SGAEERARIRRDELHAQLSNNRSRRNQLEKALTFCEAEMDNLTRKLRKLERDYFEMREQVVTAKAGWCAV

MRMVKDNGVERRLHRRELAYLSADDLRSMSDKALGALRLAVADNEHLRDVLRMSEDPKRPERKIQFFVAV

YQHLRERIRQDIIRTDDPVEAIEQMEIELSRLTEELTSREQKLAISSRSVANIIRKTIQREQNRIRMLNQ

GLQNVSFGQVNSVRLNVNVRETHAMLLDVLSEQHEQHQDLFNSNRLTFSEALAKLYQRLNPQIDMGQRTP

QTIGEELLDYRNYLEMEVEVNRGSDGWLRAESGALSTGEAIGTGMSILVMVVQSWEDESRRLRGKDISPC

RLLFLDEAARLDARSIATLFELCERLQMQLIIAAPENISPEKGTTYKLVRKVFQNTEHVHVVGLRGFAPQ

LPETLLGRDEAPSQAS

>gi|30062457|ref|NP_836628.1| condesin subunit E [Shigella flexneri 2a str. 2457T]

MPVKLAQALANPLFPALDSALRSGRHIGLDELDNHAFLMDFQEYLEEFYARYNVELIRAPEGFFYLRPRS

TTLIPRSVLSELDMMVGKILCYLYLSPERLANEGIFTQQELYDELLTLADEAKLLKLVNNRSTGSDVDRQ

KLQEKVRSSLNRLRRLGMVWFMGHDSSKFRITESVFRFGADVRAGDDPREAQRRLIRDGEAMPIENHLQL

NDETEESQPDSGEEE

>gi|30062456|ref|NP_836627.1| condesin subunit F [Shigella flexneri 2a str. 2457T]

MSEFSQTVPELVAWARKNDFSISLPVDRLSFLLAVATLNGERLDGEMSEGELVDAFRHVSDAFEQTSETI

GVRANNAINDMVRQRLLNRFTSEQAEGNAIYRLTPLGIGITDYYIRQREFSTLRLSMQLSIVAGELKRAA

DAAEEGGDEFHWHRNVYAPLKYSVAEIFDSIDLTQRLMDEQQQQVKDDIAQLLNKDWRAAISSCELLLSE

TSGTLRELQDTLEAAGDKLQANLLRIQDATMTHDDLHFVDRLVFDLQSKLDRIISWGQQSIDLWIGYDRH

VHKFIRTAIDMDKNRVFAQRLRQSVQTYFDEPWALTYANADRLLDMRDEEMALRDEEVTGELPEDLEYEE

FNEIREQLAAIIEEQLAVYKTRQVPLDLGLVVREYLSQYPRARHFDVARIVIDQAVRLGVAQADFTGLPA

KWQPINDYGAKVQAHVIDKY

>gi|30062455|ref|NP_836626.1| metallothionein SmtA [Shigella flexneri 2a str. 2457T]

MQDRNFDDIAEKFSRNIYGTTKGQLRQAILWQDLDRVLAEMGPQKLRVLDAGGGEGQTAIKMAERGHQVI

LCDLSAQMIDRAKQAAEAKGVSDNMQFIHCAAQDVASHLETPVDLILFHAVLEWVADPRSVLQTLWSVLR

PGGVLSLMFYNAHGLLMHNMVAGNFDYVQAGMPKKKKRTLSPDYPRDPAQVYLWLEEAGWQIMGKTGVRV

FHDYLREKYQQRDCYEALLELETRYCRQEPYITLGRYIHVTARKPQSKDKV

>gi|30062454|ref|NP_836625.1| hypothetical protein S0980 [Shigella flexneri 2a str. 2457T]

MLFTLKKVIGNMLLPLPLMLLIIGAGLALLWFSRFQKTGKIFISIGWLALLLLSLQPVADRLLRPIESTY

PTWNNSQKVDYIVVLGGGYTWNPQWAPSSNLINNSLPRLNEGIRLWRENPGSKLIFTGGVAKTNTVSTAE

VGARVAQSLGVPREQIITLDLPKDTEEEAAAVKQAIGDAPFLLVTSASHLPRAMIFFQQEGLNPLPAPAN

QLAIDSPLNPWERAIPSPVWLMHSDRVGYETLGRIWQWLKGPSGEPRQE

>gi|30062453|ref|NP_836624.1| hypothetical protein S0979 [Shigella flexneri 2a str. 2457T]

MEQLRAELSHLLGEKLSRIECVNEKADTALWALYDSQGNPMPLMARSFSTPGKARQLAWKTTMLARSGTV

RMPTIYGVMTHEEHPGPDVLLLERMRGVSVEAPARTPERWEQLKDQIVEALLAWHRQDSRGCVGAVDNTQ

ENFWPSWYRQHVEVLWTTLNQFNNTGLTMQDKRILFRTRECLPALFEGFNDNCVLIHGNFCLRSMLKDSR

SDQLLAMVGPGLMLWAPREYELFRLMDNSLAEDLLWSYLQRAPVAESFIWRRWLYVLWDEVAQLVNTGRF

SRRNFDLASKSLLPWLA

>gi|30062452|ref|NP_836623.1| 3-deoxy-manno-octulosonate cytidylyltransferase [Shigella flexneri 2a str. 2457T]

MSFVVIIPARYASTRLPGKPLVDINGKPMIVHVLERARESGAERIIVATDHDDVARAVEAAGGEVCMTRA

DHQSGTERLAEVVEKCAFSDDTVIVNVQGDEPMIPATIIRQVADNLAQRQVGMATLAVPIHNAEEAFNPN

AVKVVLDAEGYALYFSRATIPWDRDRFAEGLETVGDNFLRHLGIYGYRAGFIRRYVNWQASPLEHIEMLE

QLRVLWYGEKIHVAVAQEVPGTGVDTPEDLERVRAEMR

>gi|30062451|ref|NP_836622.1| hypothetical protein S0977 [Shigella flexneri 2a str. 2457T]

MDHRLLEIIACPVCNGKLWYNQEKQELICKLDNLAFPLRDGIPVLLETEARVLTADESKS

>gi|30062450|ref|NP_836621.1| hypothetical protein S0976 [Shigella flexneri 2a str. 2457T]

MSLPHLSLADARNLHLAAQGLLNKPRRRASLEDIPATISRMSLLQIDTINIVARSPYLVLFSRLGNYPAQ

WLDESLARGELMEYWAHEACFMPRSDFRLIRHRMLAPEKMGWKYKDAWMQEHEAEIAQLIQHIHDKGPVR

SADFEHPRKGASGWWEWKPHKRHLEGLFTAGKVMVIERRNFQRVYDLTHRVMPDWDDERDLVSQTEAEII

MLDNSARSLGIFREQWLADYYRLKRPALAAWREARAEQQQIIAVHVEKLGNLWLHADLLPLLERALAGKL

TATHSAVLSPFDPVVWDRKRAEQLFDFSYRLECYTPAPKRQYGYFVLPLLHRGQLVGRMDAKMHRQTGIL

EVISLWLQEGIKPTTMLQKGLRQAITDFASWQQATRVTLGRCPQGLFTDCRTGWEIDPVA

>gi|30062449|ref|NP_836620.1| tetraacyldisaccharide 4'-kinase [Shigella flexneri 2a str. 2457T]

MIEKIWSGESPLWRLLLPLSWLYGLVSGAIRLCYKLKLKRAWRAPVPVVVVGNLTAGGNGKTPVVVWLVE

QLQQRGIRVGVVSRGYGGKAESYPLLLSADTTTAQAGDEPVLIYQRTDAPVAVSPVRSDAVKAILAQHPD

VQIIVTDDGLQHYRLARDVEIVVIDGVRRFGNGWWLPAGPMRERAGRLKSVDAVIVNGGVPRSGEIPMHL

LPGQAVNLRTGTRCDVAQLEHVVAMAGIGHPPRFFATLKMCGVQPEKCVPLADHQSLNHADVSALVSAGQ

TLVMTEKDAVKCRAFAEENWWYLPVDAQLSGDEPAKLLTQLTSLASGN

>gi|30062448|ref|NP_836619.1| lipid transporter ATP-binding/permease [Shigella flexneri 2a str. 2457T]

MHNDKDLSTWQTFRRLWPTIAPFKAGLIVAGVALILNAASDTFMLSLLKPLLDDGFGKTDRSVLVWMPLV

VIGLMILRGITSYVSSYCISWVSGKVVMTMRRRLFGHMMGMPVSFFDKQSTGTLLSRITYDSEQVASSSS

GALITVVREGASIIGLFIMMFYYSWQLSIILIVLAPIVSIAIRVVSKRFRNISKNMQNTMGQVTTSAEQM

LKGHKEVLIFGGQEVETKRFDKVSNRMRLQGMKMVSASSISDPIIQLIASLALAFVLYAASFPSVMDNLT

AGTITVVFSSMIALMRPLKSLTNVNAQFQRGMAACQTLFTILDSEQEKDEGKRVIERATGDVEFRNVTFT

YPGRDVPALRNINLKIPAGKTVALVGRSGSGKSTIASLITRFYDIDEGEILMDGHDLREYTLASLRNQVA

LVSQNVHLFNDTVANNIAYARTEQYSREQIEEAARMAYAMDFINKMDNGLDTVIGENGVLLSGGQRQRIA
IARALLRDSPILILDEATSALDTESERAIQAALDELQKNRTSLVIAHRLSTIEKADEIVVVEDGVIVERG
THNDLLEHRGVYAQLHKMQFGQ

>gi|30062447|ref|NP_836618.1| integration host factor subunit beta [Shigella flexneri 2a str. 2457T]
MTKSELIERLATQQSHIPAKTVEDAVKEMLEHMASTLAQGERIEIRGFGSFSLHYRAPRTGRNPKTGDKV
ELEGKYVPHFKPGKELRDRANIYG

>gi|30062446|ref|NP_836617.1| 30S ribosomal protein S1 [Shigella flexneri 2a str. 2457T]
MTESFAQLFEESLKEIETRPGSIVRGVVVAIDKDVVLVDAGLKSESAIPAEQFKNAQGELEIQVGDEVDV
ALDAVEDGFGETLLSREKAKRHEAWITLEKAYEDAETVTGVINGKVKGGFTVELNGIRAFLPGSLVDVRP
VRDTLHLEGKELEFKVIKLDQKRNNVVVSRRAVIESENSAERDQLLENLQEGMEVKGIVKNLTDYGAFVD
LGGVDGLLHITDMAWKRVKHPSEIVNVGDEITVKVLKFDRERTRVSLGLKQLGEDPWVAIAKRYPEGTKL
TGRVTNLTDYGCFVEIEEGVEGLVHVSEMDWTNKNIHPSKVVNVGDVVEVMVLDIDEERRRISLGLKQCK
ANPWQQFAETHNKGDRVEGKIKSITDFGIFIGLDGGIDGLVHLSDISWNVAGEEAVREYKKGDEIAAVVL
QVDAERERISLGVKQLAEDPFNNWVALNKKGAIVTGKVTAVDAKGATVELADGVEGYLRASEASRDRVED
ATLVLSVGDEVEAKFTGVDRKNRAISLSVRAKDEADEKDAIATVNKQEDANFSNNAMAEAFKAAKGE

>gi|30062445|ref|NP_836616.1| cytidylate kinase [Shigella flexneri 2a str. 2457T]
MTAIAPVITIDGPSGAGKGTLCKAMAEALQWHLLDSGAIYRVLALAALHHHVDVASEDALVPLASHLDVR
FVSTNGNLEVILEGEDVSGEIRTQEVANAASQVAAFPRVREALLRRQRAFRELPGLIADGRDMGTVVFPD
APVKIFLDASSEERAHRRMLQLQEKGFSVNFERLLAEIKERDDRDRNRAVAPLVPAADALVLDSTTLSIE
QLIEKALQYARQKLALA

>gi|30062444|ref|NP_836615.1| heat shock protein [Shigella flexneri 2a str. 2457T]

MKNTKLLLAIATSAALLTGCQNTHGIDTNMAISSGLNAYKAATLSDADAKAIANQGCAEMDSGNQVASKS

SKYGKRLAKIAKALGNNINGTPVNYKVYMTSDVNAWAMANGCVRVYSGLMDMMNDNEIEGVLGHELGHVA

LGHSLAEMKASYAIVAARDAISATSGVASQLSRSQLGDIAEGAINAKYSRDKESEADDFSFDLLKKRGIS

TQGLVGSFEKLASLDGGRTQSMFDSHPPSTERAQHIRDRIASGK

>gi|30062443|ref|NP_836614.1| IS4 orf [Shigella flexneri 2a str. 2457T]

MHIGQALDLVSRYDSLRNPLTSLGDYLAPELISRCLAESGTVTLRKRRLPLEMMVWCIVGMVLERKEPLH

QIVNRLDIMLPGNRPFVAPSAVIQARQRLGSEAVRRVFTKTAQLWHNATPHPHWCGLTLLAIDGVFWRTP

DTPENDAAFPRQTHAGNPALYPQVKMVCQMELTSHLLTAAAFGTMKNSENELAEQLIEQTGDNTLTLMDK

GYYSLGLLNGWSLAGEHRHWMIPLRKGAQYEELRKLGKGDHLVKLKTSPQARKKWPGLGNEVTARLLTVT

RKGKVCHLLTSMTDAMRFPGGEMADLYSNRWEIELGYREIKQTMQLSRLTLRSKKPELVEQELWGVLLAY

NLVRYQMIKMAEHLKGYWPNQLSFSESCGMVMRMLMTLQGASPGRIPELMRDLASMGQLVKLPTRRGRAF

PRVVKERPWKYPTAPKKSQSVA

>gi|30062442|ref|NP_836613.1| 3-phosphoshikimate 1-carboxyvinyltransferase [Shigella flexneri 2a str. 2457T]

MESLTLQPIARVDGTINLPGSKSVSNRALLLAALAHGKTVLTNLLDSDDVRHMLNALTALGLSYTLSADR

TRCEIIGNGGPLHAEGALELFLGNAGTAMRPLAAALCLDSNDIVLTGEPRMKERPIGHLVDALRLGGAKI

TYLEQENYPPLRLQGGFTGGNVDVDGSVSSQFLTALLMTAPLAPEDTVIRIKGDLVSKPYIDITLNLMKT

FGVEIENQHYQQFVVKGGQSYQSPGTYLVEGDASSASYFLAAAAIKGGTVKVTGIGRNSMQGDIRFADVL

EKMGATICWGDDYISCTRGELNAIDMDMNHIPDAAMTIATAALFAKGTTTLRNIYNWRVKETDRLFAMAT

ELRKVGAEVEEGHDYIRITPPEKLNFAEIATYNDHRMAMCFSLVALSDTPVTILDPKCTAKTFPDYFEQL

ARISQAA

>gi|30062441|ref|NP_836612.1| phosphoserine aminotransferase [Shigella flexneri 2a str. 2457T]

MAQIFNFSSGPAMLPAEVLKQAQQELRDWNGLGTSVMEVSHRGKEFIQVAEEAEKDFRDLLNVPSNYKVL

FCHGGGRGQFAAVPLNILGDKTTADYVDAGYWAASAIKEAKKYCTPNVFDAKVTVDGLRAVKPMREWQLS

DNAAYMHYCPNETIDGIAIDETPDFGKDVVVAADFSSTILSRPIDVSRYGVIYAGAQKNIGPAGLTIVIV

REDLLGKANIACPSILDYSILNDNGSMFNTPPTFAWYLSGLVFKWLKANGGVAEMDKINQQKAELLYGVI

DNSDFYRNDVAKANRSRMNVPFQLADSALDKLFLEESFAAGLHALKGHRVVGGMRASIYNAMPLEGVKAL

TDFMVEFERRHG

>gi|30062440|ref|NP_836611.1| hypothetical protein S0964 [Shigella flexneri 2a str. 2457T]

MTQTFIPGKDAALEDSIARFQQKLSDLGFQIEEASWLNPVPNVWSVHIRDKECALCFTNGKGATKKAALA

SALGEYFERLSTNYFFADFWLGETIANGPFVHYPNEKWFPLSENDDVPEGLLDDRLRAFYDPENELTGSM

LIDLQSGNEDRGICGLPFTRQSDNQTVYIPMNIIGNLYVSNGMSAGNTRNEARVQGLSEVFERYVKNRII

AESISLPEIPADVLARYPAVVEAIETLETEGFPIFAYDGSLGGQYPVICVVLFNPANGTCFASFGAHPDF

GVALERTVTELLQGRGLKDLDVFTPPTFDDEEVAEHTNLETHFIDSSGLISWDLFKQDADYPFVDWNFSG

TTEEEFATLMAIFNKEDKEVYIADYEHLGVYACRIIVPGMSDIYPAEDLWLANNSMGSHLRETILSLPGS

EWEKEDYLNLIEQLDEEGFDDFTRVRELLGLATGSDNGWYTLRIGELKAMLALAGGDLEQALVWTEWTME

FNSSVFSPERANYYRCLQTLLLLAQEEDRQPLQYLNAFVRMYGADAVEAASAAMSGEAAFYGLQPVDSDL

HAFAAHQSLLKAYEKLQRAKAAFWAK

>gi|30062439|ref|NP_836610.1| formate transporter [Shigella flexneri 2a str. 2457T]

MKADNPFDLLLPAAMAKVAEEAGVYKATKHPLKTFYLAITAGVFISIAFVFYITATTGTGTMPFGMAKLV

GGICFSLGLILCVVCGADLFTSTVLIVVAKASGRITWCQLAKNWLNVYFGNLVGALLFVLLMWLSGEYMT

ANGQWGLNVLQTADHKVHHTFIEAVCLGILANLMVCLAVWMSYSGRSLMDKAFIMVLPVAMFVASGFEHS

IANMFMIPMGIVIRDFASPEFWTAVGSAPENFSHLTVMNFITDNLIPVTIGNIIGGGLLVGLTYWVIYLR

ENDHH

>gi|30062438|ref|NP_836609.1| formate acetyltransferase 1 [Shigella flexneri 2a str. 2457T]

MSELNEKLATAWEGFTKGDWQNEVNVRDFIQKNYTPYEGDESFLAGATEATTTLWDKVMEGVKLENRTHA

PVDFDTAVASTITSHDAGYINKALEKVVGLQTEAPLKRALIPFGGIKMIEGSCKAYNRELDPMIKKIFTE

YRKTHNQGVFDVYTPDILRCRKSGVLTGLPDAYGRGRIIGDYRRVALYGIDYLMKDKYAQFTSLQADLEN

GVNLEQTIRLREEIAEQHRALGQMKEMAAKYGYDISGPATNAQEAIQWTYFGYLAAVKSQNGAAMSFGRT

STFLDVYIERDLKAGKITEQEAQEMVDHLVMKLRMVRFLRTPEYDELFSGDPIWATESIGGMGLDGRTLV

TKNSFRFLNTLYTMGPSPEPNMTILWSEKLPLNFKKFAAKVSIDTSSLQYENDDLMRPDFNNDDYAIACC

VSPMIVGKQMQFFGARANLAKTMLYAINGGVDEKLKMQVGPKSEPIKGDLLSYDEVMERMDHFMDWLAKQ

YITALNIIHYMHDKYSYEASLMALHDRDVIRTMACGIAGLSVAADSLSAIKYAKVKPIRDEDGLAIDFEI

EGEYPQFGNNDPRVDDLAVDLVERFMKKIQKLHTYRDAIPTQSVLTITSNVVYGKKTGNTPDGRRAGAPF

GPGANPMHGRDQKGAVASLTSVAKLPFAYAKDGISYTFSIVPNALGKDDEVRKTNLAGLMDGYFHHEASI

EGGQHLNVNVMNREMLLDAMENPEKYPQLTIRVSGYAVRFNSLTKEQQQDVITRTFTQSM

>gi|30062437|ref|NP_836608.1| pyruvate formate lyase-activating enzyme 1 [Shigella flexneri 2a str. 2457T]

MSVIGRIHSFESCGTVDGPGIRFITFFQGCLMRCLYCHNRDTWDTHGGKEVTVEDLMKEVVTYRHFMNAS

GGGVTASGGEAILQAEFVRDWFRACKKEGIHTCLDTNGFVRRYDPVIDELLEVTDLVMLDLKQMNDEIHQ

NLVGVSNHRTLEFAKYLANKNVKVWIRYVVVPGWSDDDDSAHRLGEFTRDMGNVEKIELLPYHELGKHKW

VAMGEEYKLDGVKPPKKETMERVKGILEQYGHKVMF

>gi|30062436|ref|NP_836607.1| IS600 orfA [Shigella flexneri 2a str. 2457T]

MSRKTQRYSKEFKAEAVRTVLENQLSISEGASRLSLPEGTLGQWVTAARKGLGTPGSRTVAELESEILQL

RKALNEARLERDILKKATAYFAQESLKNTR

>gi|30062435|ref|NP_836606.1| IS600 orfB [Shigella flexneri 2a str. 2457T]

MAHIRTRETYGTRRLQTELAENGIIVGRDRLARLRKELRLRCKQKRKFRATTNPNHNLPVAPNLLNQTFA

PTAPNQVWVADLTYVATQEGWLYLAGIKDVYTCEIVGYAMGERMTKELTGKALFMALRSQRPPAGLIHHS

DRGSQYCAYDYRVIQEQSGLKTSMSRKGNCYDNAPMESFWGTLKNESLSHYRFNNRDEAISVIREYIEIF

YNRQRRHSRLGNISPAAFREKYHQMAA

>gi|30062434|ref|NP_836605.1| IS1 orfB [Shigella flexneri 2a str. 2457T]

MSRQCTHYGRWPQHGFTSLKKLRPQSVTSRIQPGSDVIVCAEMDEHWGYVGAKSRQRWLFYAYDRIRRTV

VAHVFGERTLATLERLLSLLSAFEVVVWMTDGWPLYESRLKGKLHVISKRYTQRIERHNLNLRQHLARLV

RKSLSFSKSVELHDKVIGHYLNIKHYQ

>gi|30062433|ref|NP_836604.1| IS600 orfB [Shigella flexneri 2a str. 2457T]

MAHIRTRETYGTRRLQTELAENGIIVGRDRLARLRKELRLRCKQKRKFRATTNPNHNLPVAPNLLNQTFA

PTAPNQVWVADLTYVATQEGWLYLAGIKDVYTCEIVGYAMGERMTKELTGKALFMALRSQRPPAGLIHHS

DRGSQYCAYDYRVIQEQSGLKTSMSRKGNCYDNAPMESFWGTLKNESLSHYRFNNRDEAISVIREYIEIF

YNRQRRHSRLGNISPAAFREKYHQMAA

>gi|30062432|ref|NP_836603.1| IS600 orfA [Shigella flexneri 2a str. 2457T]

MSRKTQRYSKEFKAEAVRTVLENQLSISEGASRLSLPEGTLGQWVTAARKGLGTPGSRTVAELESEILQL

RKALNEARLERDILKKATAYFAQESLKNTR

>gi|30062431|ref|NP_836602.1| IS1 orfB [Shigella flexneri 2a str. 2457T]

MSRQCTHYGRWPQHGFTSLKKLRPQSVTSRIQPGSDVIVCAEMDEHWGYVGAKSRQRWLFYAYDRIRRTV

VAHVFGERTLATLERLLSLLSAFEVVVWMTDGWPLYESRLKGKLHVISKRYTQHIERHNLNLRQHLARLG

RKSLSFSKSVELHDKVIGHYLNIKHYQ

>gi|30062430|ref|NP_836601.1| IS1 orfA [Shigella flexneri 2a str. 2457T]

MASISIRCPSCSATEGVVRNGKSTAGHQRYLCSHCRKTWQLQFTYIASQPGTHQKIIDMAMNGVGCRASA

RIMGVGLNTVLRHLKNSGRSR

>gi|30062429|ref|NP_836600.1| hypothetical protein S0950 [Shigella flexneri 2a str. 2457T]

MIWQPEFTDKTLSRKPGAVQLVTCKQNEVEPEDWLREVIEKLNDWPSNQVHELLPWNFSSVK

>gi|30062428|ref|NP_836599.1| IS629 orfB [Shigella flexneri 2a str. 2457T]

MPLLDKLREQYRVGPLCSELHIAPSTYYHCQQQRHHPDKRSARAQRDDWLKKEIQRVYDENHKVYGVRKV

WRQLLREGIRVARCTVARLMEVMGLAGVLRGKKVRTTISRKAVAAGDRVNRQFVAERPDQLWVADFTYVS

TWQGFVYVAFIIDVFAGYIVGWRVSSSMETTFVLDALEQALWARRPSGTVHHSDKGSQYVSLAYTQRLKE

AGLLASTGSTGDSYDNAMAESINGLYKAEVIHRKSWKNRAEVELATLTWVDWYNNRRLLERLGHIPPAEA

EKAYYASIGNDDLAA

>gi|30062427|ref|NP_836598.1| IS629 orfA [Shigella flexneri 2a str. 2457T]

MTKNTRFSPEVRQRAVRMVLESQGEYDSQWATICSIAPKIGCTPETLRVWVRQHERDTGGGDGGLATAER

QRLKELERENRELRRSNDILRQASAYFAKAEFDRLWKK

>gi|30062426|ref|NP_836597.1| ISSfl3 orfC [Shigella flexneri 2a str. 2457T]

MNNELPDDIELLKAMLRKQQSRLRQYACQVAGYEQEIERLKAQLDRLRRMLFGQSSEKKRHKLENQIRQA

EKRLSELENRLNTARNLLEDASSVTDSPDTSPPSENPIASKPEFPGRKSSRKPLPAELPRETHRLLPAET

SCPACGGVLKEMGETISEQLDIINTEPPRESWRLNFLRKR

>gi|30062425|ref|NP_836596.1| ISSfl3 orfB [Shigella flexneri 2a str. 2457T]

MITLPTGTKIWIIAGITDMRCGFNGLASKVQNTLKDDPFSGHIFVFRGRSGKMVKILWADRDGLCLFTKR

LAGDPGRESAPDASSVIHATGGDRVATSQTDRTAWHPDITRDKTRE

>gi|30062424|ref|NP_836595.1| ISSfl3 orfA, partial [Shigella flexneri 2a str. 2457T]

MPDSVSFAQLDAILYANRKKELTEPQIREGSWRKERRTSYSREFKVRLAKQALQPGAVVARIAREHDINN

NLLFKWKSQYEDGLLSDDDIQECMPVPVALTDTPEPTRPVTNPFWRNKHDERPEGAPGNVPRCELHLKSG

VVKLSDPLTPELLRALIREMKGGIR

>gi|30062423|ref|NP_836594.1| lysozyme protein R of prophage CP-933K [Shigella flexneri 2a str. 2457T]

MSPSLRKAVAVAIGGGAVAIASVLITGPGGNDGLEGVSYIPYKDIIGVWTVCHGHTGKDIMPGKTYTEAE

CKALLNKDLVTVARQINPYIKVDIPETTVSAPSATSCENCPVWQQSRPSILMDTNDEFPDNKRYSLLPFL

FA

>gi|30062422|ref|NP_836593.1| IS600 orfA [Shigella flexneri 2a str. 2457T]

MSRKTQRYSKEFKAEAVRTVLENQLSISEGASRLSLPEGTLGQWVTAARKGLGTPGFRTVAELESEILQL

RKALNEARLERDILKKATAYFAQESLKNTR

>gi|30062421|ref|NP_836592.1| IS600 orfB [Shigella flexneri 2a str. 2457T]

MAHIRTRETYGTRRLQTELAENGIIVGRDRLARLRKELRLRCKQKRKFRATTNPNHNLPVAPNLLNQTFA

PTAPNQVWVADLTYVATQEGWLYLAGIKDVYTCEIVGYAMGERMTKELTGKALFMALRSQRPPAGLIHHS

DRGSQYCAYDYRVIQEQSGLKTSMSRKGNCYDNAPMESFWGTLKNESLSHYRFNNRDEAISVIREYIEIF

YNRQRRHSRLGNISPAAFREKYHQMTA

>gi|30062420|ref|NP_836591.1| integrase encoded by prophage CP-933K; partial, partial [Shigella flexneri 2a str. 2457T]

MSKIKAIRRGLPDAPLEDITTKEIAAMLNGYIDEGKAASAKLIRSTLSDAFREAIAEGHITTNPVAATRA

AKSEVRRSRLTADEYLKIYQAAESSPCWLRLAMELAVVTGQRVGDLCEMKWSDIVDGYLYVEQSKTGVKI

AIPTTLHVDALGISMKETLDKCKEILGGETIIASTRREPLSSGTVSRYFMRARKASGLSFEGDPPTFHEL

RSLSARLYEKQISDKFAQHLLGHKSDTMASQYRDDRGREWDKIEIK

>gi|30062419|ref|NP_836590.1| pectinesterase [Shigella flexneri 2a str. 2457T]

MNTFSVSRLALALAFGVTLTACSSTPPDQRPSDQTAPGTSSRPILSAKEALNFDAQHYFASLTPGAAAWN

PSPITLPAQPDFVVGPAGTQGVTHTTIQAAVDAAIIKRTNKRQYIAVMPGEYQGTVYVPAAPGGITLYGT

GEKPIDVKIGLSLDGGMSPADWRHDVNPRGKYMPGKPAWYMYDSCQSKRSDSIGVLCSAVFWSQNNGLQL

QNLTIENTLGDSVDAGNHPAVALRTDGDQVQINNVNILGRQNTFFVTNSGVQNRLETNRQPRTLVTNSYI

EGDVDIVSGRGAVVFDNTEFRVVNSRTQQEAYVFAPATLSNIYYGFLAVNSRFNASGDGVAQLGRSLDVD

ANTNGQVVIRDSAINEGFNTAKPWADAVISNRPFAGNTGSVDDNDEIQRNLNDTNYNRMWEYNNRGVGSK

VVAEEKK

>gi|30062418|ref|NP_836589.1| enzyme [Shigella flexneri 2a str. 2457T]

MIKLSEKGVFLASNNEIIAEEHFTGEIKKEEAKKGTIAWSILSSHNTSGNMDKLKIKFDSLASHDITFVG

IVQTAKASGMERFPLPYVLTNCHNSLCAVGGTINGDDHVFGLSAAQRYGGIFVPPHIAVIHQYMREMMAG

GGKMILGSDSHTRYGALGTMAVGEGGGELVKQLLNDTWDIDYPGVVAVHLTGKPAPYVGPQDVALAIIGA

VFKNGYVKNKVMEFVGPGVSALSTDFRNSVDVMTTETTCLSSVWQTDEEVHNWLALHGRGQDYCQLNPQP

MAYYDGCISVDLSAIKPMIALPFHPSNVYEIDTLNQNLTDILREIEIESERVAHGKAKLSLLDKVENGRL

KVQQGIIAGCSGGNYENVIAAANALRGQSCGNDTFSLAVYPSSQPVFMDLAKKGVVADLIGAGAIIRTAF

CGPCFGAGDTPINNGLSIRHTTRNFPNREGSKPANGQMSAVALMDARSIAATAANGGYLTSASELDCWDN

VPEYAFDVTPYKNRVYQGFVKGATQQPLIYGPNIKDWPELGALTDNIVLKVCSKILDEVTTTDELIPSGE

TSSYRSNPIGLAGNVSELTEVFARIKQIAGQEHIDPLQTEIGSMVYAVKPGDGSAREQAASCQRVIGGLA

NIAEEYATKRYRSNVINWGMLPLQMAEVPTFEVGDYIYIPGIKAALDNPGTTFKGYVIHEDAPVTEITLY

MESLTAEEREIIKAGSLINFNKNRQM

>gi|30062417|ref|NP_836588.1| membrane pump protein [Shigella flexneri 2a str. 2457T]

MPAPAGLSELAWVLFGIYLAAIVGLVIKPFPEPVVLLIAVAASMVVVGNLSDGAFKTTAVLSGYSSGTTW
LVFSAFTLSAAFVTTGLGKLIAYLLIGKIGNTMLGLGYVTVFLDLVLAPATPSNTARAGGIVLPIINSVA
VALGSEPEKSPRRVGHYLMMSIYMVTKTTSYMFFTAMAGNILALKMINDILHLQISWGGWALAAGLPGII
MLLVTPLVIYTMYPPEIKKVDNKTIAKAGLAELGPMKIREKMLLGVFVLALLGWIFSKSLGVDESTVAIV
VMATMLLLGIVTWEDVVKNKGGWNTLIWYGGIIGLSSLLSKVKFFEWLAEVFKNNLAFDGHGNVAFFVII
FLSIIVRYFFASGSAYIVAMLPVFAMLANVSGAPLMLTALALLFSNSYGGMVTHYGGAAGPVIFGVGYND
IKSWWLVGAVLTILTFLVHITLGVWWWNMLIGWNML

>gi|30062416|ref|NP_836587.1| hypothetical protein S0937 [Shigella flexneri 2a str. 2457T]
MAIMGSGNALEIDGIGGGNPLTSKVAIISRSSDPRADVDYLFAQVIVHEQRVDTTPNCGNMLSGVGAFAI
ENGLIAATSPVTRVRIRNVNTGTFIEADVQTPNGVVEYEGSARIDGVPGTAAPVALTFLNAAGTKTGKVF
PTDNQIDYFDDVPVTCIDMAMPVVIIPAEYLGKTGYELPAELDADKALLARIESIRLQAGKAMGLGDVSN
MVIPKPVLISPAQKGGAINVRYFMPHSCHRALAITGAIAISSSCALEGTVTRQIVPSVGYGNINIEHPSG
ALDVHLSNEGQDATTLRASVIRTTRKIFSGEVYLP

>gi|30062415|ref|NP_836586.1| 6-phosphogluconolactonase [Shigella flexneri 2a str. 2457T]
MKQTVYIASPESQQIHVWNLNHEGALTLTQVVDVPGQVQPMVVSPDKRYLYVGVRPEFRVLAYRIAPDDG
ALTFAAESVLPGSPTHISTDHQGQFVFVGSYNAGNVSVTRLEDGLPVGVVDVVEGLDGCHSANISPDNRT
LWVPALKQDRICLFTVSDDGHLVAQDPAEVTTVEGAGPRHMVFHPNEQYAYCVNELNSSVDVWELKDPHG
NIECVQTLDMMPENFSDTRWAADIHITPDGRHLYACDRTASLITVFSVSEDGSVLSKEGFQPTETQPRGF
NVDYSGKYLIAAGQKSHHISVYEIVGEQGLLHEKGRYAVGQGPMWVVVNAH

>gi|30062414|ref|NP_836585.1| invasion plasmid antigen [Shigella flexneri 2a str. 2457T]
MREINMLRNISSCLFPHISTITSPNHYLSEWDDWEKQGLPEEQRTEAVRRLRACLTSKGHKLDLRALALS
SLPVLPACIKKLDVSCNKLTILTDLPENIKELIARDNFLTHISALPHYLITLDVSENQLENLPLLPDTIK

SLSAEYNRLSTLPSLPLNLKKLEVRNNELQTLPSLPSNLKILKVAHNHLTELPPLPRRLQLLFAYSNRLS

NLPNIQENIIMRRFFYFENNQITTIPTNLFRLDPHITIEIANNPLSDQTLLFLIQQTSVPNFNGPQFRIS

LSDQNRLFLRQMLPQNLHSRHIRVITEGGQNFQIPPLPETVAAWFPEADRREVSTQWTSFSTEENSRAFS

AFLDRLSDTVSARNTSGFREQVAAWLEKLSASAELRQQSFAVAADATESCEDRVALTWNNLRKTLLVHQA

SEGLFDNDTGALLSLGREMFRLEILEDIARDKVRTLHFVDEIEVYLAFQTMLAEKLQLSTAVKEMRFYGV

SGVTANDLRTAEAMVRSREENEFTDWFSLWGPWHAVLKRTEADRWAQAEEQKYEMLENEYPQRVADRLKA

SGLSDDADAEREAGAQVMRETEQQIYRQLTDEVLALRLSENGSQLHHS

>gi|30062413|ref|NP_836584.1| hypothetical protein S0932 [Shigella flexneri 2a str. 2457T]

MSLAQCPGFLFAHREECTVEIKKIINPRYTESGAVDCDVFFDDRDQAVPYTATADDVAPTGQRIWQELQS

GKWGEIAPFTVTPEMLEAAREARRQEIEAWRTEQEAKPFTFEWNGRIWNAGPNSLGRLYPVVMAAKSDIV

RDVMTWGDADNQQVKLSMPELEELAAAMVQAQVDRNDEIYRRQREMKEELSGLDDLASIRAFDVE

>gi|30062412|ref|NP_836583.1| IS600 orfA [Shigella flexneri 2a str. 2457T]

MSRKTQRYSKEFKAEAVRTVLENQLSISEGASRLSLPEGTLGQWVTAARKGLGTPGSRTVAELESEILQL

RKALNEARLERDILKKATAYFAQESLKNTR

>gi|30062411|ref|NP_836582.1| IS600 orfB [Shigella flexneri 2a str. 2457T]

MYLAGIKDVYTCEIVGYAMGERMTKELTGKALFMALRSQRPPAGLIHHSDRGSQYCAYDYRVIQEQSGLK

TSMSRKGNCYDNAPMESFWGTLKNESLSHYRFNNRDEAISVIREYIEIFYNRQRRHSRLGNISPAAFREK

YHQMAA

>gi|30062410|ref|NP_836581.1| exodeoxyribonuclease VIII of prophage CP-933R [Shigella flexneri 2a str. 2457T]

MSTKPLFLLRKAKKSSGEPDVVLWASDDFESTSTTLDYLLVKSGKKLSNYFKAVATNFPVVNDLPPEGEI

DFTWSERYQLSKDSMTWELKPGAAPDDVHHQDNAQETKELAGGQEENAQADAHEDCQDCEVSVATLRFTQ

RLLHIFTYAAGDLKYLHHATREQRKHITALEMDQENSYVQNLLLAIRGMAEPTTLDNAALLRLTDAIKAE

VYWQ

>gi|30062409|ref|NP_836580.1| IS600 orfB [Shigella flexneri 2a str. 2457T]

MAHIRTRETYGTRRLQTELAENGIIVGRDRLARLRKELRLRCKQKRKFRATTNPNHNLPVAPNLLNQTFA

PTAPNQVWVADLTYVATQEGWLYLAGIKDVYTCEIVGYAMGERMTKELTGKALFMALRSQRPPAGLIHHS

DRGSQYCAYDYRVIQEQSGLKTSMSRKGNCYDNAPMESFWGTLKNESLSHYRFNNRDEAISVIREYIEIF

YNRQRRHSRLGNISPAAFREKYHQMAA

>gi|30062408|ref|NP_836579.1| IS600 orfA [Shigella flexneri 2a str. 2457T]

MSRKTQRYSKEFKAEAVRTVLENQLSISEGASRLSLPEGTLGQWVTAARKGLGTPGSRTVAELESEILQL

RKALNEARLERDILKKSTAYFAQESLKNTR

>gi|30062407|ref|NP_836578.1| hypothetical protein S0925, partial [Shigella flexneri 2a str. 2457T]

MYQITKNGFVFLVMGFTGKKAAAFKEAYIAEFDRMEAELRQNNTPPADKMIPGDGRTLVVHFDKFGNVEF

TETVPDGALVCTLETFRFYLEKQGWTLVNRGAIKNMTVEQLLSLK

>gi|30062406|ref|NP_836577.1| bacteriophage protein [Shigella flexneri 2a str. 2457T]

MLNVAIENQNGWNYSAPAPHKTGAGIATPTMTTAHNRAQAVFLCVKHSHIQIMVGRAGQPQGWPVSVVTG

CSNPVRLTTHEIATSGGESFKLTIEAAIMATILTLSHPDATIENGRAVTTSVAVAEFFRKMHKTLFRRSK

LSNARRSLTGLILSPSPIPTPKAKNAQCTKSPKTASFSW

>gi|30062403|ref|NP_836574.1| hypothetical protein S0921 [Shigella flexneri 2a str. 2457T]

MNETELKHVIALLLEDAKRLQQLEPNAGTGARIWLAKEALESGDYDSEEAFYKAEGRAGYSPGLGGV

>gi|30062402|ref|NP_836573.1| hypothetical protein S0920 [Shigella flexneri 2a str. 2457T]

MNTIDLGNNESPVYGVFPNQDGTFTAITYTKSKTFKTEAGARRWLGRHSGE

>gi|30062401|ref|NP_836572.1| DNA-binding transcriptional regulator DicC [Shigella flexneri 2a str. 2457T]

MLKIDAIAFFGSKTKLANAAGVRLASIAAWGELVPEGRAMRLQEASGGELQYDPKVYDEYRKAKRPGKVI

HENQA

>gi|30062400|ref|NP_836571.1| bacteriophage protein [Shigella flexneri 2a str. 2457T]

MKIRHEHIESVLLALAAEKGQAWVANAITEEYLRQGGGELSLVPGKDWNNQQNIYHRWLKGETKAQREKI

QKLIPAVLAILPRELRHRLCIFDTLERRALLAAQEALSTAIDAHDDAVQAVYRKAHFSGGGSPGDSVVVH

>gi|30062399|ref|NP_836570.1| bacteriophage protein [Shigella flexneri 2a str. 2457T]

MASNWIKLEVITPDKPEIFRLAEILNIDPDAALGKVIRFWAWADQQMIDGNADCNARGVTKSAIDRITFM

SGFADALIQVGWLVENDGGLSLPNFERHNGKSSKKRAVTNERVTKIRELKRKGNAASVTQTDQKALPVEE

EEEDLNTDLPLNPPRQKRASKKFEPEAIELPDWLPETLWHEWVRFRQALRKPIRTEQGDTGTGKIPSAGF

YT

>gi|30062398|ref|NP_836569.1| bacteriophage protein [Shigella flexneri 2a str. 2457T]

MAKPFTPEQREELKTRIVELVHQDGRVTIRQLSDETGISRASVGRLCIELVASGDVYNSGYGLFPSEQAR

KDWQSARKKLSRVKVRKPVVVDPDLIWSLPDGEIRRYDRRLNIICRECRKSEAMQRVLAFYQGNFQEAIL

>gi|30062397|ref|NP_836568.1| hypothetical protein S0913 [Shigella flexneri 2a str. 2457T]

MEALQASEIDYTIFFYNPNIHPQKEYLIRKDENIRFAEQHGVPFIDADYDTDNWFERAKGMEWEPERGIR

CTMCFDMRFERTALYAAENGFSVISSSLGISRWKNMQQVNDCGRRAVAHYPGMVYWDYNWRKQGGSSRMI

EISKREKFYQQEYCGCVYSLRDTNLHRKSQGRPLIKIGQLHYGKEEKE

>gi|30062396|ref|NP_836567.1| host cell-killing modulation protein, partial [Shigella flexneri 2a str. 2457T]

MTALVTRKDLCEVRIRTGQTEVAVFVDYESRK

>gi|30062395|ref|NP_836566.1| hypothetical protein S0911, partial [Shigella flexneri 2a str. 2457T]

MAHIQLVKQTSSGLLLPATPESCDFLHQIKIGEWIHADFKRVRNYAFHKRFFKLLQLGFDYWTPVGGAIT

PRERKLVSGFVDYLCESVGREHTPALSEAAEQYLNTVATRRTRDTALLKSFEA

>gi|30062394|ref|NP_836565.1| IS600 orfB [Shigella flexneri 2a str. 2457T]

MAHIRTRETYGTRRLQTELAENGIIVGRDRLARLRKELRLRCKQKRKFRATTNPNHNLPVAPNLLNQTFA

PTAPNQVWVADLTYVATQEGWLYLAGIKDVYTCEIVGYAMGERMTKELTGKALFMALRSQRPPAGLIHHS

DRGSQYCAYDYRVIQEQSGLKTSMSRKGNCYDNAPMESFWGTLKNESLSHYRFNNRDEAISVIREYIEIF

YNRQRRHSRLGNISPAAFREKYHQMTA

>gi|30062393|ref|NP_836564.1| IS600 orfA [Shigella flexneri 2a str. 2457T]

MSRKTQRYSKEFKAEAVRTVLENQLSISEGASRLSLPEGTLGQWVTAARKGLGTPGSRTVAELESEILQL

RKALNEARLERDILKKATAYFAQESLKNTR

>gi|30062392|ref|NP_836563.1| hypothetical protein S0908, partial [Shigella flexneri 2a str. 2457T]

MDETEFQQVYKSVLNVLWNWILFRKFSSPEQVENVAAQLLEFA

>gi|30062391|ref|NP_836562.1| bacteriophage protein [Shigella flexneri 2a str. 2457T]

MVDLLKAARGQMCTVRIPGYCNHDPETSVLAHYRLAGTCGTAIKPHDMQAAIACSSCHDLIDGRVKTSDY

TKEELRLMHAEGVFRTQEIWRKEGYL

>gi|30062390|ref|NP_836561.1| bacteriophage protein [Shigella flexneri 2a str. 2457T]

MIYPTNTGKSGEHLRLTTLESVWIQGKLRMWGRWSYIGVKWHTEFGHLNRGDMLTSEQHRCSNEKKKF

>gi|30062389|ref|NP_836560.1| IS911 orfA [Shigella flexneri 2a str. 2457T]

MKKRNFSAEFKRESAQLVVDQKYTVADAAKAMDVGLSTMTRWVKQLRDERQGKTPKASPITPEQIEIREL

RKKLQRIEMENEILKKATALLMSDSLNSSR

>gi|30062388|ref|NP_836559.1| ISSfl3 orfC,D [Shigella flexneri 2a str. 2457T]

MNNELPDDIELLKAMLRKQQSRLRQYACQVAGYEQEIERLKAQLDRLRRMLFGQSSEKKRHKLENQIRQA

EKRLSELENRLNTARNLLEDASSVTDSPDTSPPSENPIASKPEFPGRKSSRKPLPAELPRETHRLLPAET

SCPACGGVLKEMGETISEQLDIINTAFKVIETIRPKLACSRCDVIVQAPLPPKPIERGYASAGLLARILV

SKYMEHIPLYRQSEIYARQGVELSRNTMVRWVSEMADKLRPLYIALNDYVLEAGKVHADDTPVKVLAPGN

GKTKTGRLWVYVRDDRNAGSSLPAAVWFAYSADRKGEHPQLHLAKYQGVLQADAYAGYNVLYETGRVKEA

GCLAHARRKIHDEDVRRPTEMTQEALRRIAELYDIEAEIRGSPAEERLAVRKARSVQLMQSLYDWIQLQR

KTLSKHAEMAKAFDYILNHWNALNEFCRDGRVEIDNNIGENALRSVAVGRKNYLFFGSDKGGESAAIIYS

LLVTCKQNEVEPEDWLREVIEKLNDWPSNQVHELLPWNFSSVK

>gi|30062387|ref|NP_836558.1| ISSfl3 orfB [Shigella flexneri 2a str. 2457T]

MITLPTGTKIWIIAGITDMRCGFNGLASKVQNTLKDDPFSGHIFVFRGRSGKMVKILWADRDGLCLFTKR

LAGDPGRESAPDASSVIHATGGDRVATSQTDRTAWHPDITRDKTRE

>gi|30062386|ref|NP_836557.1| ISSfl3 orfA [Shigella flexneri 2a str. 2457T]

MNSQTTKDIPCFRSYLPDALRLRFEDKLTIRAIAQRLGLSHSTIHTLFQRFLASGIAWPLPDSVSFAQLD

AILYANRKKELTEPQIREGSWRKERRTSYSREFKVRLAKQALQPGAVVARIAREHDINNNLLFKWKSQYE

DGLLSDDDIQECMPVPVALTDTPEPTRPVTNPFWRNKHDERPEGAPGNVPRCELHLKSGVVKLSDPLTPE

LLRALIREMKGGIR

>gi|30062385|ref|NP_836556.1| MFS family transporter protein [Shigella flexneri 2a str. 2457T]

MSTYTRPVMLLLSGLLLLTLAIAVLNTLVPLWLAQEHMSTWQVGVVSSSYFTGNLVGTLLTGYVIKRIGF

NRSYYLSSFIFAAGCAGLGLMIGFWSWLAWRFVAGVGCAMIWVVVESALMCSGTSRNRGRLLAAYMMVYY

VGTFLGQLLVSKVSTELMSVLPWVTGLTLAGILPLLFTRVLNQQAENHDSTSITAMLKLRQARLGVNGCI

ISGIVLGSLYGLMPLYLNHKGVSNASIGFWMAVLVSAGILGQWPIGRLADKFGRLLVLRVQVFVVILGSI

AMLSQAAMAPALFILGAAGFTLYPVAMAWACEKVEHHQLVAMNQALLLSYTVGSLLGPSFSAMLMQNFSD

NLLFIMIASVSFIYLLMLLRNAGHTPKPVAHV

>gi|30062384|ref|NP_836555.1| hypothetical protein S0897 [Shigella flexneri 2a str. 2457T]

MTKPYVRLDKNDAAVLLVDHQAGLLSLVRDIEPDKFKNNVLALGDLAKYFNLPTILTTSFETGPNGPLVL

ELKAQFPDAPYIARPGNINAWDNEDFVKAVKATGKKQLIIAGVVTEVCVAFPALSAIEEGFDVFVVTDAS

GTFNEITRHSAWDRMSQAGAQLMTWFGVACELHRDWRNDIEGLATLFSNHIPDYRNLMTSYDTLTKQK

>gi|30062383|ref|NP_836554.1| anaerobic dimethyl sulfoxide reductase subunit C [Shigella flexneri 2a str. 2457T]

MGSGWHEWPLMIFTVFGQCVAGGFIVLALALLKGDLRAEAQQRVIACMFGLWVLMGIGFIASMLHLGSPM

RAFNSLNRVGASALSNEIASGSIFFAVGGIGWLLAMLKKLSPALRTLWLVVTMVLGVVFVWMMVRVYNSI

DTVPTWYSIWTPMGWAMRLLPAISVLALVVSGVMSVMQGTELATIHSSVQQAAALVPDYGALMSWRIVLL

AVALCLWIAPQLKGYQPAVPLLSVSFILLLAGELIGRGVFYGLHMTVGMAVAS

>gi|30062382|ref|NP_836553.1| anaerobic dimethyl sulfoxide reductase subunit B [Shigella flexneri 2a str. 2457T]

MTTQYGFFIDSSRCTGCKTCELACKDYKDLTPEVSFRRIYEYAGGDWQEDNGVWHQNVFAYYLSISCNHC

EDPACTKVCPSGAMHKREDGFVVVDEDVCIGCRYCHMACPYGAPQYNETKGHMTKCDGCYDRVAEGKKPI

CVESCPLRALDFGPIDELRKKHGDLAAVAPLPRAHFTKPNIVIKPNANSRPTGDTTGYLANPKEV

>gi|30062381|ref|NP_836552.1| anaerobic dimethyl sulfoxide reductase subunit A [Shigella flexneri 2a str. 2457T]

MASSALTLPFSRIAHAVDSAIPTKSDEKVIWSACTVNCGSRCPLRMHVVDGEIKYVETDNTGDDNYDGLH

QVRACLRGRSMRRRVYNPDRLKYPMKRVGARGEGKFERISWEEAYDIIATNMQRLIKEYGNESIYLNYGT

GTLGGTMTRSWPPGNTLVARLMNCCGGYLNHYGDYSSAQIAEGLNHTYGGWADGNSPSDIENSKLVVLFG

NNPGETRMSGGGVTYYLEQARQKSNARMIIIDPRYTDTGAGREDEWIPIRPGTDAALVNGLAYVMITKNL

VDQAFLDKYCVGYDEKTLPASAPKNGHYKAYILGEGPDGVAKTPEWASQITGVPADKIIKLAREIGSTKP

AFISQGWGPQRHANGEIATRAISMLAILTGNVGINGGNSGAREGSYSLPFVRMPTLENPIQTSISMFMWT

DAIERGPEMTALRDGVRGKDKLDVPIKMIWNYAGNCLINQHSEINRTHEILQDDKKCELIVVIDCHMTSS

AKYADILLPDCTASEQMDFALDASCGNMSYVIFNDQVIKPRFECKTIYEMTSELAKRLGVEQQFTEGRTQ

EEWMRHLYAQSREAIPELPTFEEFRKQGIFKKRDPQGHHVAYKAFREDPQANPLTTPSGKIEIYSQALAD

IAATWELPEGDVIDPLPIYTPGFESYQDPLNKQYPLQLTGFHYKSRVHSTYGNVDVLKAACRQEMWINPL

DAQKRGIHNGDKVRIFNDRGEVHIEAKVTPRMMPGVVALGEGAWYDPDAKRVDKGGCINVLTTQRPSPLA

KGNPSHTNLVQVEKV

>gi|30062380|ref|NP_836551.1| seryl-tRNA synthetase [Shigella flexneri 2a str. 2457T]

MLDPNLLRNEPDAVAEKLARRGFKLDVDKLGALEERRKVLQVKTENLQAERNSRSKSIGQAKARGEDIEP

LRLEVNKLGEELDAAKAELDALQAEIRDIALTIPNLPADEVPVGKDENDNVEVSRWGTPREFDFEVRDHV

TLGEMHSGLDFAAAVKLTGSRFVVMKGQIARMHRALSQFMLDLHTEQHGYSENYVPYLVNQDTLYGTGQL

PKFAGDLFHTRPLEEEADTSNYALIPTAEVPLTNLVRGEIIDEDDLPIKMTAHTPCFRSEAGSYGRDTRG

LIRMHQFDKVEMVQIVRPEDSMAALEEMTGHAEKVLQLLGLPYRKIILCTGDMGFGACKTYDLEVWIPAQ

NTYREISSCSNVWDFQARRMQARCRSKSDKKTRLVHTLNGSGLAVGRTLVAVMENYQQADGRIEVPEVLR

PYMNGLEYIG

>gi|30062379|ref|NP_836550.1| recombination factor protein RarA [Shigella flexneri 2a str. 2457T]

MSNLSLDFSDNTFQPLAARMRPENLAQYIGQQHLLAAGKPLPRAIEAGHLHSMILWGPPGTGKTTLAEVI

ARYANADVERISAVTSGVKEIREAIERARQNRNAGRRTILFVDEVHRFNKSQQDAFLPHIEDGTITFIGA

TTENPSFELNSALLSRARVYLLKSLSTEDIEQVLTQAMEDKTRGYGGQDIVLPDDTRRAIAELVNGDARR

ALNTLEMMADMAEVYDSGKRVLKPELLTEIAGERSARFDNKGDRFYDLISALHKSVRGSAPDAALYWYAR

IITAGGDPLYVARRCLAIASEDVGNADPRAMQVAIAAWDCFTRVGPAEGERAIAQAIVYLACAPKSNAVY

TAFKAALADARERPDYDVPVHLRNAPTKLMKEMGYGQEYRYAHDEANAYAAGEVYFPPEIAQTRYYFPTN

RGLEGKIGEKLAWLAEQDQNSPIKRYR

>gi|30062378|ref|NP_836549.1| outer-membrane lipoprotein carrier protein [Shigella flexneri 2a str. 2457T]

MMKKIAITCALLSSLVASSVWADAASDLKSRLDKVSSFHASFTQKVTDGSGAAVQEGQGDLWVKRPNLFN

WHMTQPDESILVSDGKTLWFYNPFVEQATATWLKDATGNTPFMLIARNQSSDWQQYNIKQNGDDFVLTPK

ASNGNLKQFTINVGRDGTIHQFSAVEQDDQRSSYQLKSQQNGAVDAAKFTFTPPQGVTVDDQRK

>gi|30062377|ref|NP_836548.1| DNA translocase FtsK [Shigella flexneri 2a str. 2457T]

MSQEYTEDKEVTLTKLSSGRRLLEALLILIVLFAVWLMAALLSFNPSDPSWSQTAWHEPIHNLGGMPGAW

LADTLFFIFGVMAYTIPVIIVGGCWFAWRHQSSDEYIDYFAVSLRIIGVLALILTSCGLAAINADDIWYF

ASGGVIGSLLSTTLQPLLHSSGGTIALLCVWAAGLTLFTGWSWVTIAEKLGGWILNILTFASNRTRRDDT

WVDEDEYEDDEEYEDENHGKQHESRRARILRGALARRKRLAEKFINPMGRQTDAALFSGKRMDDDEEITY

TARGVAADPDDVLFSGNRATQPEYDEYDPLLNSAPITEPVAVAAAATTATQSWAAPVEPVTQTPPVASVD

VPPSQPTVAWQPVPGPQTGEPVIAPAPEGYPQQSQYAQPAVQYNEPLQQPVQPQQPYYAPAAEQPAQQPY

YAPAPEQPVAGNAWQAEEQQSTFAPLSTYQTEQTYQQPAAQEPLYQQPQPVEQQPVVEPEPVVEETKPAR

PPLYYFEEVEEKRAREREQLAAWYQPIPEPVKEPEPIKSSLKAPSVAAVPPVEAAAAVSPLASGVKKATL

ATGAAATVAAPVFSLANSGGPRPQVKEGIGPQLPRPKRIRVPTRRELASYGIKLPSQRAAEEKAREAQRN

QYDSGDQYNDDEIDAMQQDELARQFAQTQQQRYGEQYQHDVPVNAEDADAAAEAELARQFAQTQQQRYSG

EQPAGANPFSLDDFEFSPMKALLDDGPHEPLFTPIVEPVQQPQQPVAPQQQYQQPQQPVAPQQQYQQPQQ

QVAPQPQYQQPQQPVAPQQQYQQPQQPVAPQPQYQQPQQPVAPQPQYQQPQQPVAPQPQDTLLHPLLMRN

GDSRPLHKPTTPLPSLDLLTPPPSEVEPVDTFALEQMARLVEARLADFRIKADVVNYSPGPVITRFELNL

APGVKAARISNLSRDLARSLSTVAVRVVEVIPGKPYVGLELPNKKRQTVYLREVLDNAKFRDNPSPLTVV

LGKDIAGEPVVADLAKMPHLLVAGTTGSGKSVGVNAMILSMLYKAQPEDVRFIMIDPKMLELSVYEGIPH

LLTEVVTDMKDAANALRWCVNEMERRYKLMSALGVRNLAGYNEKIAEADRMMRPIPDPYWKPGDSMDAQH

PVLKKEPYIVVLVDEFADLMMTVGKKVEELIARLAQKARAAGIHLVLATQRPSVDVITGLIKANIPTRIA

FTVSSKIDSRTILDQAGAESLLGMGDMLYSGPNSTLPVRVHGAFVRDQEVHAVVQDWKARGRPQYVDGIT

SDSESEGGAGGFDGAEELDPLFDQAVQFVTEKRKASISGVQRQFRIGYNRAARIIEQMEAQGIVSEQGHN

GNREVLAPPPFD

>gi|30062376|ref|NP_836547.1| leucine-responsive transcriptional regulator [Shigella flexneri 2a str. 2457T]

MVDSKKRPGKDLDRIDRNILNELQKDGRISNVELSKRVGLSPTPCLERVRRLERQGFIQGYTALLNPHYL

DASLLVFVEITLNRGAPDVFEQFNTAVQKLEEIQECHLVSGDFDYLLKTRVPDMSAYRKLLGETLLRLPG

VNDTRTYVVMEEVKQSNRLVIKTR

>gi|30062375|ref|NP_836546.1| thioredoxin reductase [Shigella flexneri 2a str. 2457T]

MGTTKHSKLLILGSGPAGYTAAVYAARANLQPVLITGMEKGGQLTTTTEVENWPGDPNDLTGPLLMARMH

EHATKFETEIIFDHINKVDLQNRPFRLNGDNGEYTCDALIIATGASARYLGLPSEEAFKGRGVSACATCD

GFFYRNQKVAVIGGGNTAVEEALYLSNIASEVHLIHRRDGFRAEKILIKRLMDKVENGNIILHTNRTLEE

VTGDQMGVTGVRLRDTQNSDNIESLDVAGLFVAIGHSPNTAIFEGQLELENGYIKVQSGIHGNATQTSIP

GVFAAGDVMDHIYRQAITSAGTGCMAALDAERYLDGLADAK

>gi|30062374|ref|NP_836545.1| cysteine/glutathione ABC transporter membrane/ATP-binding component [Shigella flexneri 2a str. 2457T]

MNKSRQKELTRWLKQQSVISQRWLNISRLLGFVSGILIIAQAWFMARILQHMIMENIPREALLLPFTLLV

LTFVLRAWVVWLRERVGYHAGQHIRFAIRRQVLDRLQQAGPAWIQGKPAGSWATLVLEQIDDMHDYYARY

LPQMALAVSVPLLIVVAIFPSNWAAALILLGTAPLIPLFMALVGMGAADANRRNFLALARLSGHFLDRLR

GMETLRIFGRGEAEIESIRSASEDFRQRTMEVLRLAFLSSGILEFFTSLSIALVAVYFGFSYLGELDFGH

YDTGVTLAAGFLALILAPEFFQPLRDLGTFYHAKAQAVGAADSLKTFMETPLAHPQRGEAELALTDPLTI

EAEDLFITSPEGKTLAGPLNFTLPAGQRAVLVGRSGSGKSSLLNALSGFLSYQGSLRINGIELRDLSPES

WRKHLSWVGQNPQLPAAKLRDNVLLARPDASEQELQAALDNAWVSEFLPLLPQGVDTPVGDQAARLSVGQ

AQRVAVARALLNPCSLLLLDEPAASLDAHSEQRVMEALNAASLRQTTLMVTHQLEDLADWDVIWVMQDGR

IIEQGRYAELSVAGGPFATLLAHRQEEI

>gi|30062373|ref|NP_836544.1| cysteine/glutathione ABC transporter membrane/ATP-binding component [Shigella flexneri 2a str. 2457T]

MRALLPYLALYKRHKWMLSLGIVLAIVTLLASIGLLTLSGWFLSASAVAGVAGLYSFNYMLPAAGVRGAA

ITRTAGRYFERLVSHDATFRVLQHLRIYTFSKLLPLSPAGLARYRQGELLNRVVADVDTLDHLYLRVISP

LVGAFVVIMVVTIGLSFLDFTLAFTLGSVMLLTLFLMPPLFYRAGKSTGQNLTHLRGQYRQQLTAWLQGQ

AELTIFGASDRYRTQLENTEIQWLEAQRRQSELTALSQAIMLLIGALAVILMLWMASGGVGGNAQPGALI

ALFVFCALAAFEALAPVTGAFQHLGQVIASAVRISDLTDQKPEVTFPDTQTRVADRVSLTLRDVQFTYPE

QSQQALKGISLQVNAGEHIAILGRTGCGKSTLLQLLTRAWDPQQGEILLNDSPIASLNEAALRQTISVVP

QRVHLFSATLRDNLLLASPGSSDEALSEILRRVGLEKLLEDAGLNSWLGEGGRQLSGGELRRLAIARTLL

HDAPLVLLDEPTEGLDATTESQILELLAKMMREKTVLMVTHRLRGLSRFQQIIVMDNGQIIEQGTHAELL

ARQGRYYQFKQGL

>gi|30062372|ref|NP_836543.1| leucyl/phenylalanyl-tRNA--protein transferase [Shigella flexneri 2a str. 2457T]

MRLVQLSRHSIAFPSPEGALREPNGLLALGGDLSPARLLMAYQRGIFPWFSPGDPILWWSPDPRAVLWPE

SLHISRSMKRFHKRSPYRVTMNYAFGQVIEGCASDREEGTWITRGVVEAYHRLHELGHAHSIEVWREDEL

VGGMYGVAQGTLFCGESMFSRMENASKTALLVFCEEFIGHGGKLIDCQVLNDHTASLGACEIPRRDYLNY

LNQMRLGRLPNNFWVPRCLFSPQE

>gi|30062371|ref|NP_836542.1| translation initiation factor IF-1 [Shigella flexneri 2a str. 2457T]

MAKEDNIEMQGTVLETLPNTMFRVELENGHVVTAHISGKMRKNYIRILTGDKVTVELTPYDLSKGRIVFR

SR

>gi|30062370|ref|NP_836541.1| ATP-dependent Clp protease ATP-binding subunit [Shigella flexneri 2a str. 2457T]

MLNQELELSLNMAFARAREHRHEFMTVEHLLLALLSNPSAREALEACSVDLVALRQELEAFIEQTTPVLP

ASEEERDTQPTLSFQRVLQRAVFHVQSSGRNEVTGANVLVAIFSEQESQAAYLLRKHEVSRLDVVNFISH

GTRKDEPTQSSDPGSQPNSEEQAGGEERMENFTTNLNQLARVGGIDPLIGREKELERAIQVLCRRRKNNP

LLVGESGVGKTAIAEGLAWRIVQGDVPEVMADCTIYSLDIGSLLAGTKYRGDFEKRFKALLKQLEQDTNS

ILFIDEIHTIIGAGAASGGQVDAANLIKPLLSSGKIRVIGSTTYQEFSNIFEKDRALARRFQKIDITEPS

IEETVQIINGLKPKYEAHHDVRYTAKAVRAAVELAVKYINDRHLPDKAIDVIDEAGARARLMPVSKRKKT

VNVADIESVVARIARIPEKSVSQSDRDTLKKLSDRLKMLVFGQDKAIEALTEAIKMARAGLGHEHKPVGS

FLFAGPTGVGKTEVTVQLSKALGIELLRFDMSEYMERHTVSRLIGAPPGYVGFDQGGLLTDAVIKHPHAV

LLLDEIEKAHPDVFNILLQVMDNGTLTDNNGRKADFRNVVLVMTTNAGVRETERKSIGLIHQDNSTDAME

EIKKIFTPEFRNRLDNIIWFDHLSTDVIHQVVDKFIVELQVQLDQKGVSLEVSQEARNWLAEKGYDRAMG

ARPMARVIQDNLKKPLANELLFGSLVDGGQVTVALDKEKNELTYGFQSAQKHKAEAAH

>gi|30062369|ref|NP_836540.1| ATP-dependent Clp protease adaptor protein ClpS [Shigella flexneri 2a str. 2457T]

MGKTNDWLDFDQLAEEKVRDALKPPSMYKVILVNDDYTPMEFVIDVLQKFFSYDVERATQLMLAVHYQGK

AICGVFTAEVAETKVAMVNKYARENEHPLLCTLEKA

>gi|30062368|ref|NP_836539.1| stationary phase/starvation inducible regulatory protein CspD [Shigella flexneri 2a str. 2457T]

MEKGTVKWFNNAKGFGFICPEGGGEDIFAHYSTIQMDGYRTLKAGQSVQFDVHQGPKGNHASVIVPVEVE

AAVA

>gi|30062367|ref|NP_836538.1| macrolide transporter ATP-binding /permease [Shigella flexneri 2a str. 2457T]

MTPLLELKDIRRSYPAGDEQVEVLKGISLNIYAGEMVAIVGASGSGKSTLMNILGCLDKATSGTYRVAGQ

DIATLDADALAQLRREHFGFIFQRYHLLPHLTVEQNVEVPAVYAGLERKQRLLRAQELLQRLGLEDRTEY

YPAQLSGGQQQRVSIARALMNGGQVILADEPTGALDSHSGEEVMAILHQLRDRGHTVIIVTHDPQVAAQA

ERVIEIRDGEIVRNPPAIEKVNVAGGTEPVVNTVSGWRQFVSGFNEALTMAWRALAANKMRTLLTMLGII

IGIASVVSIVVVGDAAKQMVLADIRSIGTNTIDVYPGKDFGDDDPQYQQALKYDDLIAIQKQPWVASATP

AVSQNLRLRYNNVDVAASANGVSGDYFNVYGMTFSEGNTFNQEQLNGRAQVVVLDSNTRRQLFPHKADVV

GEVILVGNMPARVIGVAEEKQSMFGSSKVLRVWLPYSTMSGRVMGQSWLNSITVRVKEGFDSAEAEQQLT

RLLSLRHGKKDFFTWNMDGVLKTVEKTTRTLQLFLTLVAVISLVVGGIGVMNIMLVSVTERTREIGIRMA

VGARASDVLQQFLIEAVLVCLVGGALGITLSLLIAFTLQLFLPGWEIGFSPLALLLAFLCSTATGILFGW

LPARNAARLDPVDALARE

>gi|30062366|ref|NP_836537.1| macrolide transporter subunit MacA [Shigella flexneri 2a str. 2457T]

MKKRKTVKKRYVIALVIVIAGLITLWRILNAPVPTYQTLIVRPGDLQQSVLATGKLDALRKVDVGAQVSG

QLKTLSVAIGDKVKKDQLLGVIDPEQAENQIKEVEATLMELRAQRQQAEAELKLARVTYSRQQRLAQTQA

VSLQDLDTAATEMAVKQAQIGTIDAQIKRNQASLDTAKTNLDYTRIVAPMAGEVTQITTLQGQTVIAAQQ

APNILTLADMSTMLVKAQVSEADVIHLKPGQKAWFTVLGDPLTRYEGQIKDVLPTPEKVNDAIFYYARFE

VPNPNGLLRLDMTAQVHIQLTDVKNVLTIPLSALGDPVGDNRYKVKLLRNGETREREVTIGARNDTDVEI

VKGLEAGDEVVIGEAKPGAAQ

>gi|30062365|ref|NP_836536.1| enzyme [Shigella flexneri 2a str. 2457T]

MVKSTSCITIDFMNMSQLTERTFTPSESLSSLSLFLSLARGQCRPGKFWHRCSFRQKFLLRSLIMPRLSV

EWMNELSHWPNLNVLLTRQPRLPVRLHRPYLAANLSRKQLLEALRYHYALLRGCMSAEEFSLYLNTPGLQ

LAKLEGKNGEQFTLELTMMISMDKEGDSTILFRNSEGIPLAEITFTLCEYQGKRTMFIGGLQGAKWEIPH

QEIQNATKACHGLFPKRLVMEAACLFAQRLQVEQIIAVSNETHIYRSLRYRDKEGKIHADYNAFWESVGG

VCDAERHYRLPAQIARKEIAEIASKKRAEYRRRYEMLDAIQPQMATMFRG

>gi|30062364|ref|NP_836535.1| hypothetical protein S0876 [Shigella flexneri 2a str. 2457T]

MILERVEIVGFRGINRLSLMLEQNNVLIGENAWGKSSLLDALTLLLSPESDLYHFERDDFWFPPGDINGR

EHHLHIILTFRESLPGRHRVRRYRPLEACWTPCTDGYHRIFYRLEGESAEDGSVMTLRSFLDKDGHPIDV

EDINDQARHLVRLMPVLRLRDARFMRRIRNGTVPNVPNVEVTARQLDFLARELSSHPQNLSDGQIRQGLS

AMVQLLEHYFSEQGAGQARYRLMRRRASNEQRSWRYLDIINRMIDRPGGRSYRVILLGLFATLLQAKGTL

RLDKDARPLLLIEDPETRLHPIMLSVAWHLLNLLPLQRIATTNSGELLSLTPVEHVCRLVRESSRVAAWR

LGPSGLSTEDSRRISFHIRFNRPSSLFARCWLLVEGETETWVINELARQCGHHFDAEGIKVIEFAQSGLK

PLVKFARRMGIEWHVLVDGDEAGKKYAATVRSLLNNDREAEREHLTALPALDMEHFMYRQGFSDVFHRVA

QIPENVPMNLRKIISKAIHRSSKPDLAIEVAMEAGRRGVDSVPTLLKKMFSRVLWLARGRAD

>gi|30062363|ref|NP_836534.1| IS1 orfA [Shigella flexneri 2a str. 2457T]

MASISIRCPSCSATEGVVRNGKSTAGHQRYLCSHCRKTWQLQFTYTASQPGTHQKIIDMAMNGVGCRASA

RIMGVGLNTVLRHLKNSGRSR

>gi|30062362|ref|NP_836533.1| IS1 orfB [Shigella flexneri 2a str. 2457T]

MSRQCTHYGRWPQHGFTSLKKLRPQSVTSRIQPGSDVIVCAEMDEHWGYVGAKSRQRWLFYAYDRIRRTV

VAHVFGERTLATLERLLSLLSAFEVVVWMTDGWPLYESRLKGKLHVISKRYTQRIERHNLNLRQHLARLG

RKSLSFSKSVELHDKVIGHYLNIKHYQ

>gi|30062361|ref|NP_836532.1| aquaporin Z [Shigella flexneri 2a str. 2457T]

MFRKLAAECFGTFWLVFGGCGSAVLAAGFPELGIGFAGVALAFGLTVLTMAFAVGHISGGHFNPAVTIGL

WAGGRFPAKEVVGYVIAQVVGGIVAAALLYLIASGKTGFDAAASGFASNGYGEHSPGGYSMLSALVVELV

LSAGFLLVIHGATDKFAPAGFAPIAIGLALTLIHLISIPVTNTSVNPARSTAVAIFQGGWALEQLWFFWV

VPIVGGIIGGLIYRTLLEKRD

>gi|30062358|ref|NP_836529.1| surface protein [Shigella flexneri 2a str. 2457T]

MCHRAFRLLLCKDWIFMFSGLLIILVPLIVGYLIPLRQQAALKAINQLLSWMVYLILFFMGISLAFLDNL

ASNLLAILHYSAVSITVILLCNIAALMWLERGLPWRNHHQQEKLPSRIAMALESLKLCGVVVIGFAIGLS

GLAFLQHATEASEYTLILLLFLVGIQLRNNGMTLKQIVLNRRGMIVAVVVVVSSLIGGLINAFILDLPIN

TALAMASGFGWYSLSGILLTESFGPVIGSAAFFNDLARELIAIMLIPGLIRRSRSTALGLCGATSMDFTL

PVLQRTGGLDMVPAAIVHGFILSLLVPILIAFFSA

>gi|30062356|ref|NP_836527.1| HCP oxidoreductase, NADH-dependent [Shigella flexneri 2a str. 2457T]

MTMPTNQCPWRIQVHHITQETPDVWTISLICHDHYPYRAGQYALVSVRNSAETLRAYTISSTPGVSEYIT

LTVRRIDDGVGSQWLTRDVKRGDYLWLSDAMGEFTCDDKAEDKFLLLAAGCGVTPIMSMRRWLAKNRPQA

DVRVIYNVRTPQDVIFADEWRNYPVTLVAENNVTEGFIAGRLTRELLADVPDLASRTVMTCGPAPYMDWV

EQEVKALGVTRFFKEKFFTPVAEAATSGLKFTKLQPAREFYAPVGTTLLEALESNNVPVVAACRAGVCGC

CKTKVVSGEYTVSSTMTLTDAEIAEGYVLACSCHPQGDLVLA

>gi|30062355|ref|NP_836526.1| pyruvate dehydrogenase [Shigella flexneri 2a str. 2457T]

MKQTVAAYIAKTLESAGVKRIWGVTGDSLNGLSDSLNRMGTIEWMSTRHEEVAAFAAGAEAQLSGELAVC

AGSCGPGNLHLINGLFDCHRNNVPVLAIAAHIPSSEIGSGYFQETHPQELFRECSHYCELVSSPEQIPQV

LAIAMRKAVLNRGVSVVVLPGDVALKPAPEGATTHWYHAPQPVVMPEEEELRKLAQLLRYSSNIALMCGS

GCAGAHKELVEFAGKIKAPIVHALRGKEHVEYDNPYDVGMTGLIGFSSGFHTMMNADTLVLLGTQFPYRA

FYPTDAKIIQIDINPASIGAHSKVDMALVGDIKSTLRALLPLVEEKTDRKFLDKALEDYRDARKGLDDLA

KPSEKAIHPQYLAQQISHFAADDAIFTCDVGTPTVWAARYLKMNGKRRLLGSFNHGSMANAMPQALGAQA

TEPERQVVAMCGDGGFSMLMGDFLSVVQMKLPVKIIVFNNSVLGFVAMEMKAGGYLTDGTELHDTNFARI

AEACGITGIRVEKASEVDEALQRAFSIDGPVLVDVVVAKEELAIPPQIKLEQAKGFSLYMLRAIISGRGD

EVIELAKTNWLR

>gi|30062354|ref|NP_836525.1| L-threonine aldolase [Shigella flexneri 2a str. 2457T]

MIDLRSDTVTRPSRAMLEAMMAAPVGDDVYGDDPTVNALQDYAAELSGKEAAIFLPTGTQANLVALLSHC

ERGEEYIVGQAAHNYLFEAGGAAVLGSIQPQPIDAAADGTLPLDKVAMKIKPDDIHFARTKLLSLENTHN

GKVLPREYLKEAWEFTRERNLALHVDGARIFNAVVAYGCELKEITQYCDSFTICLSKGLGTPVGSLLVGN

RDYIKRAIRWRKMAGGGMRQSGILAAAGMYALENNVARLQEDHDNAAWMAEQLREAGADVMRQDTNMLFV

RVGEENAAALGEYMKARNVLINASPIVRLVTHLDVSREQLAEVAAHWRAFLAR

>gi|30062353|ref|NP_836524.1| dTDP-glucose enzyme [Shigella flexneri 2a str. 2457T]

MPQRILVLGASGYIGQHLVRTLSQQGHQILAAARHVDRLAKLQLANVSCHKVDLSWPDNLPALLQDIDTV

YFLVHSMGEGGDFIAQERQLALNVRDALREVPVKQLIFLSSLQAPPHEQSDHLRARQATADILREANVPV

TELRAGIIVGAGSAAFEVMRDMVYNLPVLTPPRWVRSRTTPIALENLLHYLVALLDHPANEHRIFEAAGP

EVLSYQQQFEHFMAVSGKRRWLIPIPLPTRWISVWFLNVITSVPPTTARALIQGLKHDLLADDTALRALI

PQRLIAFDDAVRSTLKEEEKLVNSSDWGYDAQAFARWRPEYGYFAKQAGFTVKTSASLAALWLVVNQIGG

KERYFFGNILWQTRALMDRAIGHNLAKGRPEREYLQTGDAVDSWKVIVVEPKKQLTLLFGMKAPGLGRLC

FTLEDKGDYRTIDVRAFWHPHGMPGLFYWLLMIPAHLFIFRGMAKQIARLAEQSTD

>gi|30062352|ref|NP_836523.1| nucleotide di-P-sugar epimerase or dehydratase [Shigella flexneri 2a str. 2457T]

MRTSFSQQQWIDMKVLVTGATSGLGRNAVEFLCQKGISVRASGRNEAMGKLLEKMGAEFVPTDLTELVSS

QAKVMLAGIDTLWHCSSFTSPWGTQQAFDLANVRATRRLGEWAVAWGVRNFIHISSPSLYFDYHHHRNIK
EDFRPHRFANEFARSKAASEEVINMLSQANPQTRFTILRPQSLFGPHDKVFIPRLAHMMHHYGSILLPHG
GSALVDMTYYENAVHAMWLASQEACDKLPSGRVYNITNGEHRTLRSIVQKLIDELNIDCRIRSVPYPMLD
MIARSMERLGRKSAKEPPLTHYGASKLNFDFTLDITRAQEELGYQPVITLDEGIEKTAAWLRDHGKLPR

>gi|30062351|ref|NP_836522.1| regulator [Shigella flexneri 2a str. 2457T]
MRRVFWLVAAALLLAGCAGEKGIVEKEGYQLDTRHQAQAAYPRIKVLVIHYTADDFDSSLATLTDKQVSS
HYLIPAVPPRYNGKPRIWQLVPEQELAWHAGISAWRGATRLNDTSIGIELENRGWQKSSGVKYFAPFEQA
QIQALIPLAKDIIARYHIKPENVVAHADIAPQRKDDPGPLFPWQQLAQQGIGAWPDAQRVNFYLAGRAPH
TPVDTASLLELLARYGYDVKPDMTPREQRRVIMAFQMHFRPTLYNGEADAETQAIAEALLEKYGQD

>gi|30062350|ref|NP_836521.1| hypothetical protein S0862 [Shigella flexneri 2a str. 2457T]
MQFSTTPTLEGLTIVEYCGVVTGEAILGANIFRDFFAGIRDIVGGRSGAYEKELRKAREIAFEELGSQAR
ALGADAVVGIDIDYETVGQNGSMLMVSVSGTAVKTRR

>gi|30062349|ref|NP_836520.1| hypothetical protein S4814 [Shigella flexneri 2a str. 2457T]
MINNISDQASSFPGTQLNQSDNFLDSLREFFAILNPSRKGELSTWDTIYLHLILAINADSDLIKNDVLLA
ENIPSANYQFNTFFSNTFEIDVKKYLGKSEDNEVEIKAGNERISIGIRNVSNGKLERQQFLFPLDYENKL
QEQLDKYFTIESHPLLYRYTIGSKIANVIFEKLYSRIDFNKEQYISFIKDAFIHFYDYSRRYAISENIDK
DAVTNNIALMSTFYDSDNTSGEVLNNDFTEEESFETALDVEHAIVLGFADDNFETKPVHYQDLLTRFSAF
QDTVFNLFPEMHSSHYHDICSVSVDMTKGTQCMIHLMVNEEVFMSLPVPVATMVREDASNLVNLKTLLND
GCFIKYSHFNDVALIKQNISNLYLSHTVVNESILKKCCFENGSLGDVKITNSNVINSAFKNISFRSVKIN
NVNTHSLKFINCNFFNVDMIRVNLSKCLFHECSMHGVKIKPWLPVKWTKELISDYLYGCLLSLYSICARD
IYNMNAGNNVKVAADAFLEIIFSLKNKYCIKLLSAQDRAFIYEFARMIFAYINDKSIEILLLSCFAAADQ
KAIQRYRPQSQDGEDFRSHLQYKLPLSAH

>gi|30062348|ref|NP_836519.1| hypothetical protein S4813 [Shigella flexneri 2a str. 2457T]

MVYTVYCLGNLSKSATSNLEWVASNGLKPNQVFRLFQNCIKNNVLNNLIAFGRSVTQTAS

>gi|30062347|ref|NP_836518.1| lipoprotein [Shigella flexneri 2a str. 2457T]

MRYRSLSLLIPCALLLSACTTVTPAYKDNGPRTGSCVQGGPDSVAQQFYDYRIQHRSNDITALRPYLSDK

LATQLSDASRDNSHRELLSSDPFSSRTTLPDSAHVASASTIPNRDARNIPLRVDLKQGDQGWQDEVLMIQ

EGQCWVIDDVRYLGGSVHATAGTLRQSIENR

>gi|30062346|ref|NP_836517.1| arginine transporter ATP-binding subunit [Shigella flexneri 2a str. 2457T]

MSIQLNGINCFYGAHQALFDITLDCPQGETLVLLGPSGAGKSSLLRVLNLLEMPRSGTLNIAGNHFDFTK

TPSDKAIRDLRRNVGMVFQQYNLWPHLTVQQNLIEAPCRVLGLSKDQALARAEKLLERLRLKPYSDRYPL

HLSGGQQQRVAIARALMMEPQVLLFDEPTAALDPEITAQIVSIIRELAETNITQVIVTHEVEVARKTASR

VVYMENGHIVEQGDASCFTEPQTEAFKNYLSH

>gi|30062345|ref|NP_836516.1| arginine 3rd transport system periplasmic binding protein [Shigella flexneri 2a str. 2457T]

MKKVLIAALIAGFSLSATAAETIRFATEAPYPPFESIDANNQIVGFDVDLAQALCKEIDATCTFSNQAFD

SLIPSLKFRRVEAVMAGMDITPEREKQVLFTTPYYDNSALFVGQQGKYTSVDQLKGKKVGVQNGTTHQKF

IMDKHPEITTVPYDSYQNAKLDLQNGRIDSVFGDTAVVTEWLKDNPKLAAVGDKVTDKDYFGTGLGIAVR

QGNTELQQKLNTALEKVKKDGTYETIYNKWFQK

>gi|30062344|ref|NP_836515.1| arginine transporter permease subunit ArtQ [Shigella flexneri 2a str. 2457T]

MNEFFPLASAAGMTVGLAVCALIVGLALAMFFAVWESAKWRPVAWAGSALVTILRGLPEILVVLFIYFGS

SQLLLTLSDGFTINLGFVQIPVQMDIENFDVSPFLCGVIALSLLYAAYASQTLRGALKAVPVGQWESGQA

LGLSKSAIFFRLVMPQMWRHALPGLGNQWLVLLKDTALVSLISVNDLMLQTKSIATRTQEPFTWYIVAAA

IYLVITLLSQYILKRIDLRATRFERRPS

>gi|30062343|ref|NP_836514.1| arginine transporter permease subunit ArtM [Shigella flexneri 2a str. 2457T]

MFEYLPELMKGLHTSLTLTVASLIVALILALIFTIILTLKTPVLVWLVRGYITLFTGTPLLVQIFLIYYG

PGQFPTLQEYPALWHLLSEPWLCALIALSLNSAAYTTQLFYGAIRAIPEGQWQSCSALGMSKKDTLAILL

PYAFKRSLSSYSNEVVLVFKSTSLAYTITLMEVMGYSQLLYGRTYDVMVFGAAGIIYLVVNGLLTLMMRL

IERKALAFERRN

>gi|30062342|ref|NP_836513.1| arginine 3rd transport system periplasmic binding protein [Shigella flexneri 2a str. 2457T]

MKKLVLAALLASFTFGASAAEKINFGVSATYPPFESIGANNEIVGFDIDLAKALCKQMQAECTFTNHAFD

SLIPSLKFRKYDAVISGMDITPERSKQVSFTTPYYENSAVVISKKDTYKTFADLKGKRIGMENGTTHQKY

IQDQHPEVKTVSYDSYQNAFIDLKNGRIDGVFGDTAVVNEWLKTNPQLGVATEKVTDPQYFGTGLGIAVR

PDNKALLEKLNNALAAIKADGTYQKINDQWFPQ

>gi|30062341|ref|NP_836512.1| 23S rRNA methyluridine methyltransferase [Shigella flexneri 2a str. 2457T]

MQCALYDAGRCRSCQWITQPIPEQLSAKTADLKNLLADFPVEEWCAPVSGPEQGFRNKAKMVVSGSVEKT

LLGMLHRDGTPEDLCDCPLYPASFAPVFAALKPFIARAGLTPYNVARKRGELKYILLTESQSDGGMMLRF

VLRSDTKLAQLRKALPWLHEQLPQLKVITVNIQPVHMAIMEGETEIYLTEQQALAERFNDVPLWIRPQSF

FQTNPAVASQLYATARDWVRQLPVKHMWDLFCGVGGFGLHCATPDMQLTGIEIAPEAIACAKQSAAELGL

TRLQFQALDSTQFATAQGEVPELVLVNPPRRGIGKPLCDYLSTMAPRFIIYSSCNAQTMAKDIRELPGYR

IERVQLFDMFPHTAHYEVLTLLVKQ

>gi|30062340|ref|NP_836511.1| hypothetical protein S0854 [Shigella flexneri 2a str. 2457T]

MEDETLGFFKKTSSSHARLNVPALVQVAALAIIMIRGLDVLMIFNTLGVRGIGEFIHRSVQTWSLTLVFL

SSLVLVFIEIWCAFSLVKGRRWARWLYLLTQITAASYLWAASLGYGYPELFSIPGESKREIFHSLMLQKL

PDMLILMLLFVPSTSRRFFQLQ

>gi|30062339|ref|NP_836510.1| putrescine transporter subunit: membrane component of ABC superfamily [Shigella flexneri 2a str. 2457T]

MNNLPVVRSPWRIVILLLGFTFLYAPMLMLVIYSFNSSKLVTVWGGWSTRWYGELLRDDAMMSAVGSSLT

IAACAATAAAILGTIAAVVLVRFGRFRGSNGFAFMITAPLVMPDVITGLSLLLLFVALAHAIGWPADRGM

LTIWLAHVTFCTAYVAVVISSRLRELDRSIEEAAMDLGATPLKVFFVITLPMIMPAIISGWLLAFTLSLD

DLVIASFVSGPGATTLPMLVFSSVRMGVNPEINALATLILGAVGIVGFIAWYLMARAEKQRIRDIQRARR

G

>gi|30062338|ref|NP_836509.1| putrescine transporter subunit: membrane component of ABC superfamily [Shigella flexneri 2a str. 2457T]

MNTLEPAAQSKPPGGFKLWLSQLQMKHGRKLVIALPYIWLILLFLLPFLIVFKISLAEMARAIPPYTELM

EWADGQLSITLNLGNFLQLTDDPLYFDAYLQSLQVAAISTFCCLLIGYPLAWAVAHSKPSTRNILLLLVI

LPSWTSFLIRVYAWMGILKNNGVLNNFLLWLGVIDQPLTILHTNLAVYIGIVYAYVPFMVLPIYTALIRI

DYSLVEAALDLGARPLKTFFTVIVPLTKGGIIAGSMLVFIPAVGEFVIPELLGGPDSIMIGRVLWQEFFN

NRDWPVASAVAIIMLLLLIVPIMWFHKHQQKSVGEHG

>gi|30062336|ref|NP_836507.1| putrescine transporter subunit: periplasmic-binding component of ABC superfamily [Shigella flexneri 2a str. 2457T]

MTALNKKWLSGLVAGALMAVSVGTLAAEQKTLHIYNWSDYIAPDTVANFEKETGIKVVYDVFDSNEVLEG

KLMAGSTGFDLVVPSASFLERQLTAGVFQPLDKSKLPEWKNLDPELLKLVAKHDPDNKFAMPYMWATTGI

GYNVDKVKAVLGENAPVDSWDLILKPENLEKLKSCGVSFLDAPEEVFATVLNYLGKDPNSTKADDYTGPA

TDLLLKLRPNIRYFHSSQYINDLANGDICVAIGWAGDVWQASNRAKEAKNGVNVSFSIPKEGAMAFFDVF

AMPADAKNKDEAYQFLNYLLRPDVVAHISDHVFYVNANKAATPLVSAEVRENPGIYPPADVRAKLFTLKV

QDPKIDRVRTRAWTKVKSGK

>gi|30062335|ref|NP_836506.1| sensory transduction regulator [Shigella flexneri 2a str. 2457T]

MTSLVVPGLDTLRQWLDDLGMSFFECDNCQALHLPHMQNFDGVFDAKIDLIDNTILFSAMAEVRPSAVLP

LAADLSAINASSLTVKAFLDMQDDNLPKLVVCQSLSVMQGVTYEQFAWFVRQSEEQISMVILEANAHQLL

LPTDDEGQNNVTENYFLH

>gi|30062334|ref|NP_836505.1| ribosomal protein S6 modification protein [Shigella flexneri 2a str. 2457T]

MKIAILSRDGTLYSCKRLREAAIQRGHLVEILDPLSCYMNINPAASSIYYKGRKLPHFDAVIPRIGTAIT

FYGTAALRQFEMLGSYPLNESVAIARARDKLRSMQLLARQGIDLPVTGIAHSPDDTSDLIDMVGGAPLVV

KLVEGTQGIGVVLAETRQAAESVIDAFRGLNAHILVQEYIKEAQGCDIRCLVVGDEVVAAIERRAKEGYF

RSNLHRGGAASVASITPQEREIAIKAARTMALDVAGVDILRANRGPLVMEVNASPGLEGIEKTTGIDIAG

KMIRWIERHATTEYCLKTGG

>gi|30062333|ref|NP_836504.1| nitroreductase A [Shigella flexneri 2a str. 2457T]

MTPTIELICGHRSIRHFTDEPISEAQCEAIINSARATSSSSFLQCSSIIRITDKALREELVTLTGGQKHV

AQAAEFWVFCADFNRHLQICPDAQLGLAEQLLLGVVDTAMMAQNALTAAESLGLGGVYIGGLRNNIEAVT

KLLKLPQHVLPLFGLCLGWPADNPDLKPRLPASILVHENSYQPLDKDALAQYDEQLAEYYLTRGSNNRWD

TWSDHIRRTIIKESRPFILDYLHKQGWATR

>gi|30062332|ref|NP_836503.1| hypothetical protein S0846 [Shigella flexneri 2a str. 2457T]

MRAIGKLPKGVLILEFIGMMLLAVALLSVSDSLSLPEPFSRPEVQILMIFLGVLLMLPAAVVVILQVAKR

LAPQLMNCPPQYSRSEREKDNDANH

>gi|30062331|ref|NP_836502.1| glutaredoxin [Shigella flexneri 2a str. 2457T]

MQTVIFGRSGCPYCVRAKDLAEKLSNERDDFQYQYVDIRAEGITKEDLQQKAGKPVETVPQIFVDQQHIG
GYTDFAAWVKENLDA

>gi|30062330|ref|NP_836501.1| hypothetical protein S0844 [Shigella flexneri 2a str. 2457T]
MKHKQRWAGAICCFVLFIVVCLFLATHMKGAFRAAGHPEIGLLFFILPGAVASFFSQRREVLKPLFGAML
AAPCSMLIMRLFFSPTRSFWQELAWLLSAVFWCALGALCFLFISSLFKPQHRKNQ

>gi|30062329|ref|NP_836500.1| hypothetical protein S0843 [Shigella flexneri 2a str. 2457T]
MNINVAELLNGNYILLLFVVLALGLCLGKLRLGSIQLGNSIGVLVVSLLLGQQHFSINTDALNLGFMLFI
FCVGVEAGPNFFSIFFRDGKNYLMLALVMVGSALVIALGLGKLFGWDIGLTAGMLAGSMTSTPVLVGAGD
TLHHSGMESRQLSLALDNLSLGYALTYLIGLVSLIVGARYLPKLQHQDLQTSAQQIARERGLDTDANRKV
YLPVIRAYRVGPELVAWTDGKNLRELGIYRQTGCYIERIRRNGILANPDGDAVLQMGDEIALVGYPDAHA
RLDPSFRNGKEVFDRDLLDMRIVTEEVVVKNHNAVGKRLAQLKLTDHGCFLNRVIRSQIEMPIDDNVVLN
KGDVLQVSGDARRVKTIADRIGFISIHSQVTDLLAFCAFFVIGLMIGMITFQFSTFSFGMGNAAGLLFAG
IMLGFMRANHPTFGYIPQGALSMVKEFGLMVFMAGVGLSAGSGINNGLGAIGGQMLIAGLIVSLVPVVIC
FLFGAYVLRMNRALLFGAMMGARTCAPAMEIISDTARSNIPALGYAGTYAIANVLLTLAGTIIVMVWPGL
G

>gi|30062328|ref|NP_836499.1| DEOR-type transcriptional regulator [Shigella flexneri 2a str. 2457T]
MRRANDPQRREKIIQATLEAVKLYGIHAVTHRKIATLAGVPLGSMTYYFSGIDELLLEAFSSFTEIMSRQ
YQAFFSDVSDAQGACQAITDMIYSSQVATPDNMELMYQLYALASRKPLLKTVMQNWMQRSQQTLEQWFEP
GTARALDAFIEGMTLHFVTDRKPLSREEILRMVERVAG

>gi|30062327|ref|NP_836498.1| DEOR-type transcriptional regulator [Shigella flexneri 2a str. 2457T]
MTVNSSRNALKRRTWALFMFFFLPGLLMASWATRTPAIRDILSVSIAEMGGVLFGLSIGSMSGILCSAWL

VKRFGTRNVILVTMSCALIGMMILSLALWLTSPLLFAVGLGVFGASFGSAEVAINVEGAAVEREMNKTVL

PMMHGFYSLGTLAGAGVGMALTAFGVPATVHILLAALVGIAPIYIAIQAIPDGTGKNAADGTQHGEKGIP

FYRDIQLLLIGVVVLAMAFAEGSANDWLPLLMVDGHGFSPTSGSLIYAGFTLGMTVGRFTGGWFIDSYSR

VAVVRASALMGALGIGLIIFVDSAWVAGVSVVLWGLGASLGFPLTISAASDTGPDAPTRVSVVATTGYLA

FLVGPPLLGYLGEHYGLRSAMLVVLALVILAAIVAKAVAKPDTKTQTAMENS

>gi|30062326|ref|NP_836497.1| hypothetical protein S0840 [Shigella flexneri 2a str. 2457T]

MDGTFLSDQKTYNRERFMAQYQQMKAQGIRFVVASGNQYYQLISFFPEIANEIAFVAENGGWVMSEGKDV

FNGELSKDAFATVVEHLLTRPEVEIIACGKNSAYTLKKYDDAMKTVAEMYYHRLEYVDNFDNLEDIFFKF

GLNLSDELIPQVQKALHEAIGDIMVPVHTGNGSIDLIIPGVHKANGLRQLQKLWGIDDSEVVVFGDGGND

IEMLRQAGFSFAMENAGSAVVAAAKYRAGYNNREGVLDVIDKVLKHEAPFDQ

>gi|30062325|ref|NP_836496.1| hypothetical protein S0839 [Shigella flexneri 2a str. 2457T]

MIMKNCLLLGALLMGFTGVAMAQSVTVDVPSGYKVVVVPDSVSVPQAVSVATVPQTVYVAPAPAPAYRPH

PYVRHLASVGEGMVIEHQIDDHHH

>gi|30062324|ref|NP_836495.1| chloramphenicol resistance pump Cmr [Shigella flexneri 2a str. 2457T]

MQNKLASGARLGRQALLFPLCLVLYEFSTYIGNDMIQPGMLAVVEQYQAGIDWVPTSMTAYLAGGMFLQW

LLGPLSDRIGRRPVMLAGVVWFIVTCLAILLAQNIEQFTLLRFLQGISFCFIGAVGYAAIQESFEEAVCI

KITALMANVALIAPLLGPLVGAAWIHVLPWEGMFVLFAALAAISFFGLQRAMPETAMRIGEKLSLKELGR

DYKLVLKNGRFVAGALGFVSLPLLAWIAQSPIIIITGEQLSSYEYGLLQVPIFGALIAGNLLLARLTSRR

TVRSLIIMGGWPIMIGLLVAAAATVISSHAYLWMTAGLSIYAFGIGLANAGLVRLTLFASDMSKGTVSAA

MGMLQMLIFTVGIEISKHAWLNGGNGLFNLFNLVNGILWLSLMVIFLKDKQMGNSHEG

>gi|30062323|ref|NP_836494.1| undecaprenyl pyrophosphate phosphatase [Shigella flexneri 2a str. 2457T]

MLENLNLSLFSLINATPDSAPWMISLAIFIAKDLITVVPLLAAVLWLWGLTAQRQLVIKIAIALAVSLFV

SWTMGHLFPHDRPFVENIGYNFLHHAADDSFPSDHGTVIFTFALAFLCWHRLWSGSLLMVLAVVIAWSRV

YLGVHWPLDMLGGLLAGMIGCLSAQIIWQAMGHKLYQRLQSWYRVCFALPIRKGWVRD

>gi|30062322|ref|NP_836493.1| DNA-binding transcriptional repressor DeoR [Shigella flexneri 2a str. 2457T]

METRREERIGQLLQELKRSDKLHLKDAAALLGVSEMTIRRDLNNHSAPVVLLGGYIVLEPRSASHYLLSD

QKSRLVEEKRRAAKLAATLVEPDQTLFFDCGTTTPWIIEAIDNEIPFTAVCYSLNTFLALKEKPHCRAFL

CGGEFHASNAIFKPIDFQQTLNNFCPDIAFYSAAGVHVSKGATCFNLEELPVKHWAMSMAQKHVLVVDHS

KFGKVRPARMGDLKRFDIVVSDCCPEDEYVKYAQTHRIKLMY

>gi|30062321|ref|NP_836492.1| IS600 orfB [Shigella flexneri 2a str. 2457T]

MAHIRTRETYGTRRLQTELAENGIIVGRDRLARLRKELRLRCKQKRKFRATTNPNHNLPVAPNLLNQTFA

PTAPNQVWVADLTYVATREGWLYLAGIKDVYTCEIVGYAMGERMTKELTGKALFMALRSQRPPAGLIHHS

DRGSQYCAYDYRVIQEQSGLKTSMSRKGNCYDNAPMESFWGTLKNESLSHYRFNNRDEAISVIREYIEIF

YNRQRRHSRLGNISPAAFREKYHQMAA

>gi|30062320|ref|NP_836491.1| IS600 orfA [Shigella flexneri 2a str. 2457T]

MSRKTQRYSKEFKAEAVRTVLENQLSISEGASRLSLPEGTLGQWVTAARKGLGTPGSRTVAELESEILQL

RKALNEARLERDILKKATAYFAQESLKNTR

>gi|30062319|ref|NP_836490.1| transferase [Shigella flexneri 2a str. 2457T]

MSMITLWGRNNSTNVKKVLLTLEELELPYEQILAGREFGINHDADFLAMNPNGLVPLLRDDESDLILWES

NAIVRYLAAQYGQKRLWIDSPARRAEAEKWMDWANQTLSNAHRGILMGLVRTPPEERDQAAIDASCKECD

ALFALLDAELAKVKWFSGDEFGVGDIAIAPFIYNLFNVGLTWTPRPNLQRWYQQLTERPAVRKVVMIPVS

>gi|30062318|ref|NP_836489.1| biofilm formation regulatory protein BssR [Shigella flexneri 2a str. 2457T]

MFVDRQRIDLLNRLIDARVDLAAYVQLRKAKGYMSVSESNHLRDNFFKLNRELHDKSLRLNLHLDQEEWS

ALHHAEEALATAAVCLMSGHHDCPTVITVNADKLENCLMSLTLSIQSLQKHAMLEKA

>gi|30062317|ref|NP_836488.1| hypothetical protein S0828 [Shigella flexneri 2a str. 2457T]

MSKVTPQPKIGFVSLGCPKNLVDSERILTELRTEGYDVVPSYDDANMVIVNTCGFIDSAVQESLEAIGEA

LNENGKVIVTGCLGAKEDQIREVHPKVLEITGPHSYEQVLEHVHHYVPKPKHNPFLSLVPEQGVKLTPRH

YAYLKISEGCNHRCTFCIIPSMRGDLMSRPIGEVLSEAKRLVDAGVKEILVISQDTSAYGVDVKHRTGFH

NGEPVKTSMVSLCEQLSKLGIWTRLHYVYPYPHVDDVIPLMAEGKILPYLDIPLQHASPRILKLMKRPGS

VDRQLARIKQWREICPELTLRSTFIVGFPGETEEDFQMLLDFLKEARLDRVGCFKYSPVEGADANALPDQ

VPEEVKEERWNRFMQLQQQISAERLQEKVGREILVIIDEVDEEGAIGRSMADAPEIDGAVYLNGETNVKP

GDILRVKVEHADEYDLWGSRV

>gi|30062316|ref|NP_836487.1| hypothetical protein S0827 [Shigella flexneri 2a str. 2457T]

MSRINKFVLTVSLLIFIMISAVACGIYTQMVKERVYSLKQSVIDTAFAVANIAEYRRSVAIDLINTLNPT

EEQLLVGLRTAYADSVSPSYLYDVGPYLISSDECIQVKEFEKNYCADIMQVVKYRHVKNTGFISFDGKTF

VYYLYPVTHNRSLIFLLGLERFSLLSKSLVMDSENLMFSLFKNGKPVTGDEYNAKNAIFTVSEAMEHFAY

LPTGLYVFAYKKDVYLRVCTLIIFFAALVAVILGASCLYLVRRVINRGIVEKEAIINNHFERVLDGGLFF

SAADVKKLYSMYNSAFLDDLTKAMGRKSFDEDLKALPEKGGYLCLFDVDKFKNINDTFGHLLGDEVLMKV

VKILKSQIPVDKGKVYRFGGDEFAVIYTGGTLEELLSILKEIVHFQVGSINLSTSIGVAHSNECTTVERL

KMLADERLYKSKKNGRAQISWQ

>gi|30062315|ref|NP_836486.1| transport system permease [Shigella flexneri 2a str. 2457T]

MRLFNWRRQAVLNAMPLVKPDQVRTPWHEFWRRFRRQHMAMTAALFVILLIVVAIFARWIAPYDAENYFD

YDNLNNGPSLQHWFGVDSLGRDIFSRVLVGAQISLAAGVFAVFIGVAIGTLLGLLAGYYEGWWDRLIMRI

CDVLFAFPGILLAIAVVAVLGSGIANVIIAVAIFSIPAFARLVRGNTLVLKQQTFIESARSIGASDMTIL

LRHILPGTVSSIVVFFTMRIGTSIISAASLSFLGLGAQPPTPEWGAMLNEARADMVIAPHVAVFPALAIF

LTVLAFNLLGDGLRDALDPKIKG

>gi|30062314|ref|NP_836485.1| transport system permease [Shigella flexneri 2a str. 2457T]

MLYYVIKRLLGLIPTLFIVSVLVFLFVHMLPGDPARLIAGPEADAQVIELVRQQLGLDQPLYHQFWHYIS

NAVQGDFGLSMVSRRPVADEIASRFMPTLWLTITSMVWAVIFGMAAGIIAAVWRNRWPDRLSMTIAVSGI

SFPAFALGMFLIQVFSVELGWLPTVGADSWQHYILPSLTLGAAVAAVMARFTRASFVDVLSEDYMRTARA

KGVSETWVVLKHGLRNAMIPVVTMMGLQFGFLLGGSIVVEKVFNWPGLGRLLVDSVEMRDYPVIQAEILL

FSLEFILINLVVDVLYAAINPAIRYK

>gi|30062313|ref|NP_836484.1| transporter [Shigella flexneri 2a str. 2457T]

MARAVHRSGLVALGIATALMASCAFAAKDVVVAVGSNFTTLDPYDANDTLSQAVAKSFYQGLFGLDKEMK

LKNVLAESYTVSDDGITYTVKLREGIKFQDGTDFNAVAVKANLDRASDPANHLKRYNLYKNIAKTEAIDP

TTVKITLKQPFSAFINILAHPATAMISPTALEKYGKEIGFHPVGTGPYELDTWNQTDFVKVKKFAGYWQP

GLPKLDSITWRPVADNNTRAAMLQTGEAQFAFPIPYEQATLLEKNKNIELMASPSIMQRYISMNVTQKPF

DNPKVREALNYAINRPALVKVAFAGYATPATGVVPPSIAYAQSYKPWPYDPVKARELLKEAGYPNGFSTT

LWSSHNHSTAQKVLQFTQQQLAQVGIKAQVTAMDAGQRAAEVEGKGQKESGVRMFYTGWSASTGEADWAL

SPLFASQNWPPTLFNTAFYSNKQVDDFLAQALKTNDPAEKTRLYKAAQDIIWQESPWIPLVVEKLVSAHS

KNLTGFWIMPDTGFSFEDADLQ

>gi|30062312|ref|NP_836483.1| glutathione transporter ATP-binding protein [Shigella flexneri 2a str. 2457T]

MLAVENLNIAFMQDQQKIAAVRNLSFSLQRGETLAIVGESGSGKSVTALALMRLLEQAGGLVQCDKMLLR

RRSRDVIELSEQSAAQMRHVRGADMAMIFQEPMTSLNPVFTVGEQIAESIRLHQNASREEAMVEAKRMLD

QVRIPEAQTILSRYSHQLSGGMRQRVMIAMALSCRPAVLIADEPTTALDVTIQAQILQLIKVLQKEMSMG

VIFITHDMGVVAEIADRVLVMYQGEAVETGTVEQIFHAPQHPYTRALLAAVPQLGAMKGLDYPRRFPLIS

LEHPAKQEPPIEQKTVVDGEPVLRVRNLVTRFPLRSGLLNRVTREVHAVEKVSFDLWPGETLSLVGESGS

GKSTTGRALLRLVESQGGEIIFNGQRIDTLSPGKLQALRRDIQFIFQDPYASLDPRQTIGDSIIEPLRVH

GLLPGKDAAARVAWLLERVGLLPEHAWRYPHEFSGGQRQRICIARALALNPKVIIADEAVSALDVSIRGQ

IINLLLDLQRDFGIAYLFISHDMAVVERISHRVAVMYLGQIVEIGSRCAVFENPQHPYTRKLLAAVPVAE

PSRQRPQRVLLSDDLPSNIHLRGEEVAAVSLQCVGPGHYVAQPQSEYAFMRR

>gi|30062311|ref|NP_836482.1| L-asparaginase [Shigella flexneri 2a str. 2457T]

MGKAVIAIHGGAGAISRAQMSLQQELRYIEALSAIVETGQKMLEAGESALDVVTEAVRLLEECPLFNAGI

GAVFTRDETHELDACVMDGNTLKAGAVAGVSHLRNPVLAARLVMEQSPHVMMIGEGAENFAFAHGMERVS

PEIFSTPLRYEQLLAAREEGATVLDHSGAPLDEKQKMGTVGAVALDLDGNLAAATSTGGMTNKLPGRVGD

SPLVGAGCYANNASVAVSCTGTGEVFIRALAAYDIAALMDYGGLSLAEACERVVMEKLPALGGSGGLIAI

DHEGNVALPFNTEGMYRAWGYAGDTPTTGIYREKGDTVATQ

>gi|30062310|ref|NP_836481.1| molybdopterin biosynthesis protein MoeA [Shigella flexneri 2a str. 2457T]

MEFTTGLMSLDTALNEMLSRVTPLTTQETLPLVQCFGRILASDVVSPLDVPGFDNSAMDGYAVRLADIAS

GQPLPVAGKSFAGQPYHGEWPAGTCIRIMTGAPVPEGCEAVVMQEQTEQTDNGVRFTAEVRSGQNIRRRG

EDISAGAVVFPAGTRLTTAELPVIASLGIAEVPVIRKVRVALFSTGDELQLPGQPLGDGQIYDTNRLAVH

LMLEQLGCEVINLGIIRDDPHALRAAFIEADSQADVVISSGGVSVGEADYTKMILEELGEIAFWKLAIKP

GKPFAFGKLSNSWFCGLPGNPVSATLTFYQLVQPLLAKLSGNTASGLPARQRVRTASRLKKTPGRLDFQR

GVLQRNADGELEVTTTGHQGSHIFSSFSLGNCFIVLERDRGNVDVREWVEVEPFNALFGGL

>gi|30062309|ref|NP_836480.1| molybdopterin biosynthesis protein MoeB [Shigella flexneri 2a str. 2457T]

MAELSDQEMLRYNRQIILRGFDFDGQEALKDSRVLVVGLGGLGCAASQYLASAGVGNLTLLDFDTVSLSN

LQRQTLHSDATVGQPKVESARDALTRINPHIAITPVNALLDDAELAALIAEHDLVLDCTDNVAVRNQLNA

GCFAAKVPLVSGAAIRMEGQITVFTYQDGEPCYRCLSRLFGENALTCVEAGVMAPLIGVIGSLQAMEAIK

LLAGYGKPASGKIVMYDAMTCQFREMKLMRNPGCEVCGQ

>gi|30062307|ref|NP_836478.1| pyruvate formate-lyase 2 activating enzyme [Shigella flexneri 2a str. 2457T]

MFERNREATMIFNIQRYSTHDGPGIRTVVFLKGCSLGCRWCQNPESRARTQDLLYDARLCLEGCELCAKA

APEVIERALNGLLIHREKLTPEHLTALTDCCPTQALTVCGEVKSVEEIMATVLRDKPFYDRSGGGLTLSG

GEPFMQPEMATALLQACHEAGIHTAVETCLHVPWKYIAPSLPYIDLFLADLKHVADAPFKQWTDGNAARV

LDNLKKLAAAGKKIIIRVPLIQGFNADETSVKAITDFAADELHVGEIHFLPYHTLGINKYHLLNLPYDAP

EKPLDAPELLDFAQQYACQKGLTATLRG

>gi|30062306|ref|NP_836477.1| formate acetyltransferase [Shigella flexneri 2a str. 2457T]

MTTLKLDTLSDRIKAHKNALVHIVKPPVCTERAQHYTEMYQQHLDKPIPVRRALALAHHLANRTIWIKHD

ELIIGNQASEVRAAPIFPEYTVSWIEKEIDDLADRPGAGFAVSEENKRVLHEVCPWWRGQTVQDRCYGMF

TDEQKGLLATGIIKAEGNMTSGDAHLAVNFPLLLEKGLDGLREKVAERRSRINLTVLEDLHGEQFLKAID

IMLVAVSEHIERFAALARAMAATETRESRRDELLAMAENCDLIAHQPPQTFWQALQLCYFIQLILQIESN

GHSVSFGRMDQYLYPYYRRDVELNQTLDREHAIELLHSCWLKLLEVNKIRSGSHSKASAGSPLYQNVTIG

GQNLVDGQPMDAVNPLSYAILESCGRLRSTQPNLSVRYHAGMSNDFLDACVHVIRCGFGMPAFNNDEIVI

PEFIKLGIEPQDAYDYAAIGCIETAVGGKWGYRCTGMSFINFARVMLAALEGGRDATSGKVFLPQEKALS

AGNFNNFDEVMDAWDTQIRYYTRKSIEIEYVVDTMLEENVHDILCSALVDDCIERAKSIKQGGAKYDWVS

GLQVGIANLGNSLAAVKKLVFEQGAIGQQQLAAALADDFDGLTHEQLRQRLINGAPKYGNDDDTVDTLLA

RAYQTYIDELKQYHNPRYGRGPVGGNYYAGTSSISANVPFGAQTMATPDGRKAHTPLAEGASPASGTDHL

GPTAVIGSVGKLPTAAILGGVLLNQKLNPATLENESDKQKLMILLRTFFEVHKGWHIQYNIVSRETLLEA

KKHPDQYRDLVVRVAGYSAFFTALSPDAQDDIIARTEHML

>gi|30062305|ref|NP_836476.1| hypothetical protein S0815 [Shigella flexneri 2a str. 2457T]

MSVKVIVTDMDGTFLNDAKTYNQPRFMAQYQELKKRGIKFVVASGNQYYQLISFFPELKDEISFVAENGA

LVYEHGKQLFHGELTRHESRIVIGELLKDKQLNFVACGLQSAYVSENAPEAFVALMAKHYHRLKPVKDYQ

EIDDVLFKFSLNLPDEQIPLVIDKLHIALDGIMKPVTSGFGFIDLIIPGLHKANGISRLLKRWDLSPQNV

VAIGDSGNDAEMLKMARYSFAMGNAAENIKQIARYATDDNNHEGALNVIQAVLDNTSPFNS

>gi|30062304|ref|NP_836475.1| hypothetical protein S0814 [Shigella flexneri 2a str. 2457T]

MASTFTSDTLPADHKAAIRQMKHALRAQLGDVQQIFNQLSDDIATRVAEINALKAQGDAVWPVLSYADIK

AGHVTAEQREQIKRRGCAVIKGHFPREQALGWDQSMLDYLNRNRFDEVYKGPGDNFFGTLSASRPEIYPI

YWSQAQMQARQSEEMANAQSFLNRLWTFESDGKQWFNPDVSVIYPDRIRRLPPGTTSKGLGAHTDSGALE

RWLLPAYQHVFANVFNGNLAKYDPWHAAHRTEVEEYTVDNTTKCSVFRTFQGWTALSDMLPGQGLLHVVP

IPEAMAYVLLRPLLDDVPEDELCGVAPGRVLPVSEQWHPLLIEALTSIPKLEAGDSVWWHCDVIHSVAPV

ENQQGWGNVMYIPAAPMCEKNLAYAHKVKAALEKGASPGDFPREDYETNWEGRFTLADLNIHGKRALGMD

V

>gi|30062303|ref|NP_836474.1| ATP-binding component of a transport system [Shigella flexneri 2a str. 2457T]

MLVSSNVTMQFGSKPLFENISVKFGGGNRYGLIGANGSGKSTFMKILGGDLEPTLGNVSLDPNERIGKLR

QDQFAFEEFTVLDTVIMGHKELWEVKQERDRIYALPEMSEEDGYKVADLEVKYGEMDGYSAEARAGELLL

GVGIPVEQHYGPMSEVAPGWKLRVLLAQALFAEPDILLLDEPTNNLDIDTIRWLEQVLNERDSTMIIISH

DRHFLNMVCTHMADLDYGELRVYPGNYDEYMTAATQARERLLADNAKKKAQIAELQSFVSRFSANASKSR

QATSRARQIDKIKLEEVKASSRQNPFIRFEQDKKLFRNALEVEGLTKGFDNGPLFKNLNLLLEVGEKLAV

LGTNGVGKSTLLKTLVGDLQPDSGTVKWSENARIGYYAQDHEYEFENDLTVFEWMSQWKQEGDDEQAVRS

ILGRLLFSQDDIKKPAKVLSGGEKGRMLFGKLMMQKPNILIMDEPTNHLDMESIESLNMALELYQGTLIF

VSHDREFVSSLATRILEITPERVIDFSGNYEDYLRSKGIE

>gi|30062302|ref|NP_836473.1| hypothetical protein S0812 [Shigella flexneri 2a str. 2457T]

MNMKLKTLFAAAFAVVGFCSTASAVTYPLPTDGSRLVGQNQVITIPEGNTQPLEYFAAEYQMGLSNMMEA

NPGVDTFLPKGGTVLNIPQQLILPDTVHEGIVINSAEMRLYYYPKGTNTVIVLPIGIGQLGKDTPINWTT

KVEHKKAGPTWTPTAKMHAEYRAAGEPLPAVVPAGPDNPMGLYALYIGRLYAIHGTNANFGIGLRVSHGC

VRLRNEDIKFLFEKVPVGTRVQFIDEPVKATTEPDGSRYIEVHNPLSTTEAQFEGQEIVPITLTKSVQTV

TGQPDVDQVVLDEAIKNRSGMPVRLN

>gi|30062301|ref|NP_836472.1| hypothetical protein S0811 [Shigella flexneri 2a str. 2457T]

MSLPFLRTLQGDRFFQLLILVGIGLSFFVPFAPKSWPAAIDWHTIITLSGLMLLTKGVELSGYFDVLGRK

MVRRFATERRLAMFMVLAAALLSTFLTNDVALFIVVPLTITLKRLCEIPVNRLIIFEALAVNAGSLLTPI

GNPQNILIWGRSGLSFAGFIAQMAPMAGAMMLTLLLLCWCCFPGKALQYHTGVQTPEWKPRLVWNCLGLY

IVFLTALEFKQELWGLVIVAAGFVLLARRVVLSVDWTLLLVFMAMFIDVHLLTQLPALQGVLGNVSHLSE

PGLWLTAVGLSQVISNVPSTILLLNYVPPSLLLAWAVNVGGFGLLPGSLANLIALRMANDRRIWWRFHLY

SIPMLLWAALVGYVLLVMIPAW

>gi|30062300|ref|NP_836471.1| manganese transport regulator MntR [Shigella flexneri 2a str. 2457T]

MSRRAGTPIAKKVTQLVNVEEHVEGFRQVREAHRRELIDDYVELISDLIREVGEARQVDMAARLGVSQPT

VAKMLKRLATMGLIEMIPWRGVFLTAEGEKLAQESRERHQIVENFLLVLGVSPEIARRDAEGMEHHVSEE

TLDAFRLFTQKHGAK

>gi|30062299|ref|NP_836470.1| enzyme [Shigella flexneri 2a str. 2457T]

MNLTLKESLVTRSRVFSPWTAFYFLQSLLINLGLGYPFSLLYTAAFTAILLLLWRTLPRVQKVLVGVSSL

VAACYFPFAQAYGAPNFNTLLALHSTNMEESTEILTIFPWYSYLVGLFIFALGVIAIRRKKENEKARWNT

FDSLCLVFSVATFFVAPVQNLAWGGVFKLKDTGYPVFRFAKDVIVNNNEVIEEQERMAKLSGMKDTWTVT

AVKPKYQTYVVVIGESARRDALGAFGGHWDNTPFASSVNGLIFADYIAASGSTQKSLGLTLNRVVDGKPQ

FQDNFVTLANRAGFQTWWFSNQGQIGEYDTAIASIAKRADEVYFLKEGNFEADKNTKDEALLDMTAQVLA

QEHSQPQLIVLHLMGSHPQACDRTQGKYETFVQSKETSCYLYTMTQTDDLLRKLYDQLRNSGSSFSLVYF

SDHGLAFKERGKDVQYLAHDDKYQQNFQVPFMVISSGDKAHRVIKARRSANDFLGFFSQWTGIKAKEINI

KYPFISEKKAGPIYITNFQLQKVDYNHLGTDIFDPKP

>gi|30062298|ref|NP_836469.1| outer membrane protein X [Shigella flexneri 2a str. 2457T]

MKKIACLSALAAVLAFTAGTSVAATSTVTGGYAQSDAQGQMNKMGGFNLKYRYEEDNSPLGVIGSFTYTE

KSRTASSGDYNKNQYYGITAGPAYRINDWASIYGVVGVGYGKFQTTEYPTYKHDTSDYGFSYGAGLQFNP

MENVALDFSYEQSRIRSVDVGTWIAGVGYRF

>gi|30062297|ref|NP_836468.1| threonine and homoserine efflux system [Shigella flexneri 2a str. 2457T]

MPGSLRKMPVWLPIVILLVAMASIQGGASLAKSLFPLVSAPGVTALRLALGTLILIAFFKPWRLRFAKEQ

RLPLLFYGVSLGGMNYLFYLSIQTVPLGIAVALEFTGPLAVALFSSRRPVDFVWVVLAVLGLWFLLPLGQ

DVSHVDLTGCALALGAGACWAIYILSGQRAGAEHGPATVAIGSLIAALIFVPIGALQAGEALWHWSVIPL

GLAVVILSTALPYSLEMIALTRLPTRTFGTLMSMEPALAAVSGMIFLGETLTPIQLLALGAIIAASMGST

LTVRKESKIKELDIN

>gi|30062296|ref|NP_836467.1| DNA starvation/stationary phase protection protein Dps [Shigella flexneri 2a str. 2457T]

MSTAKLVKSKATNLLYTRNDVSDSEKKATVELLNRQVIQFIDLSLITKQAHWNMRGANFIAVHEMLDGFR

TALIDHLDTMAERAVQLGGVALGTTQVINSKTPLKSYPLDIHNVQDHLKELADRYAIVANDVRKAIGEAK

DDDTADILTAASRDLDKFLWFIESNIE

>gi|30062295|ref|NP_836466.1| glutamine ABC transporter periplasmic protein [Shigella flexneri 2a str. 2457T]

MKSVLKVSLAALTLAFAVSSHAADKKLVVATDTAFVPFEFKQGDKYVGFDVDLWAAIAKELKLDYELKPM

DFSGIIPALQTKNVDLALAGITITDERKKAIDFSDGYYKSGLLVMVKANNNDVKSVKDLDGKVVAVKSGT

GSVDYAKANIKTKDLRQFPNIDNAYMELGTNRADAVLHDTPNILYFIKTAGNGQFKAVGDSLEAQQYGIA

FPKGSDELRDKVNGALKTLRENGTYNEIYKKWFSTEPK

>gi|30062294|ref|NP_836465.1| glutamine ABC transporter permease [Shigella flexneri 2a str. 2457T]

MQFDWSAIWPAIPLLIEGAKMTLWISVLGLAGGLVIGLLAGFARTFGGWIANHVALVFIEVIRGTPIVVQ

VMFIYFALPMAFNDLRIDPFTAAVVTIMINSGAYIAEITRGAVLSIHKGFREAGLALGLSRWETIRYVIL

PLALRRMLPPLGNQWIISIKDTSLFIVIGVAELTRQGQEIIAGNFRALEIWSAVAVFYLIITLVLSFILR

RLERRMKIL

>gi|30062293|ref|NP_836464.1| glutamine ABC transporter ATP-binding protein [Shigella flexneri 2a str. 2457T]

MIEFKNVSKHFGPTQVLHNIDLNIAQGEVVVIIGPSGSGKSTLLRCINKLEEITSGDLIVDGLKVNDPKV

DERLIRQEAGMVFQQFYLFPHLTALGNVMFGPLRVRGANKEEAEKLARELLAKVGLAERAHHYPSELSGG

QQQRVAIARALAVKPKMMLFDEPTSALDPELRHEVLKVMQDLAEEGMTMVIVTHEIGFAEKVASRLIFID

KGRIAEDGNPQVLIKNPPSQRLQEFLQHVS

>gi|30062292|ref|NP_836463.1| SAM-dependent methyltransferase [Shigella flexneri 2a str. 2457T]

MGNDKRSQIFTLSRYAGILRRCLKENAMSAQKPGLHPRNRHHSRYDLATLCQVNPELRQFLTLTPAGEQS

VDFANPLAVKALNKALLAHFYAVANWDIPDGFLCPPVPGRADYIHHLADLLAEASGTIPANASILDIGVG

ANCIYPLIGVHEYGWRFTGSETSSQALSSAQAIISANPGLNRAIRLRRQKESGAIFNGIIHKNEQYDATL

CNPPFHDSAAAARAGSERKRRNLGLNKDDALNFGGQQQELWCEGGEVNFIKKMIEESKGFAKQVMWFTSL

VSRGENLPPLYRALTDVGAVKVVKKEMAQGQKQSRFIAWTFMNDEQRRRFVNRQR

>gi|30062291|ref|NP_836462.1| hypothetical protein S0799 [Shigella flexneri 2a str. 2457T]

MHVIDLSYFVKIYRSKNKHFSVNFLRHLHRAGDLHLSGTTRCIYEDVIMKKCLTLLIATVLSGISLTAYA

AQPMSNLDSGQLRPAGTVSATGASNLSDLEDKLAEKAREQGAKGYVINSAGGNDQMLGTATIYK

>gi|30062290|ref|NP_836461.1| IS4 orf [Shigella flexneri 2a str. 2457T]

MHIGQALDLVSRYDSLRNPLTSLGDYLDPELISRCLAESGTVTLRKRRLPLEMMVWCIVGMALERKEPLH

QIVNRLDIMLPGNRPFVAPSAVIQARQRLGSEAVRRVFTKTAQLWHNATPHPHWCGLTLLAIDGVFWRTP

DTPENDAAFPRQTHAGNPALYPQVKMVCQMELTSHLLTAAAFGTMKNSENELAEQLIGQTGDNTLTLMDK

GYYSLGLLNGWSLAGEHRHWMIPLRKGAQYEELRKLSKGDHLVKLKTSPQARKKWPGLGNEVTARLLTVT

RKGKVCHLLTSMTDAMRFPGGEMADLYSNRWEIELGYREIKQTMQLSRLTLRSKKPELVEQELWGVLLAY

NLVRYQMIKMAEHLKGYWPNQLSFSESCGMVMRMLMTLQGASPGRIPELMRDLASMGQLVKLPTRRGRAF

PRVVKERPWKYPTAPKKSQSVA

>gi|30062288|ref|NP_836459.1| hypothetical protein S0794 [Shigella flexneri 2a str. 2457T]

MASAWANDDAVNEQINSTIEDAIARARGEIPRGESLYECEECGAPIPQARREAIPGVRLCIHCQQEKDLQ

KPAYTGYNRRGSKDRQLR

>gi|30062287|ref|NP_836458.1| hypothetical protein S0793 [Shigella flexneri 2a str. 2457T]

MKTINTVVAAMALSTLSFGVFAAEPVTASQAQNMNKIGVVSADGASTLDALEAKLAEKAAAAGASGYSIT

SATNNNKLSGTAVIYK

>gi|30062286|ref|NP_836457.1| hypothetical protein S0792 [Shigella flexneri 2a str. 2457T]

MESGHRFDAQTLHSFIQAVFRQMGSEEQEAKLVADHLIAANLAGHDSHGIGMIPSYVRSWSQGHLQINHH

AKIVKEAGAAVTLDGDRAFGQVAAHEAMALGIEKAHQHGIAAVALHNSHHIGRIGYWAEQCSAAGFVSIH

FVSVVGIPMVAPFHGRDSRFGTNPFCVVFPRKDNFPLLLDYATSAIAFGKTRVAWHKGVPVPPGCLIDVN

GVPTTNPAVMQESPLGSLLTFAEHKGYALAAMCEILGGALSGGKTTHQETLQTSPDAILNCMTTIIINPE

LFGAPDCNAQTEAFAEWVKASPHDDDKPILLPGEWEVNTRRERQEQGIPLDAGSWQAICDAARQIGMSEE

TLQAFCQQLAS

>gi|30062285|ref|NP_836456.1| glycosyl transferase family protein [Shigella flexneri 2a str. 2457T]

MDYRKIIKEIGRGKNHARDLDRDTARGLYAHMLNGEVPDLELGGVLIALRIKGEGEAEMLGFYEAMQNHT

IKLTPPAGKPMPIVIPSYNGARKQANLTPLLAILLHKLGFPVVVHGVSEDPTRVLTETIFELMGITPTLH

GGQAQAKLDEHQPVFMPVGAFCPPLEKQLAMRWRMGVRNSAHTLAKLATPFAEGEALCLSSVSHPEYIGR

VAKFFSDIGGWALLMHGTEGEVYANPQRCPQINLIDREGMRVLYEKQDPAGSELLPQAKDPETTAQWTER

CLAGSEAIPESLKIQMACCLVATGEAATISDGLARVNQAF

>gi|30062284|ref|NP_836455.1| ATP-dependent DNA helicase DinG [Shigella flexneri 2a str. 2457T]

MALTAALKAQIAAWYKALQEQIPDFIPRAPQRQMIADVAKTLAGEEGRHLAIEAPTGVGKTLSYLIPGIA

IAREEQKTLVVSTANVALQDQIYSKDLPLLKKIIPDLKFTAAFGRGRYVCPRNLTALASTEPTQQDLLAF

LDDELTPNNQEEQKRCAKLKGDLDTYKWDGLRDHTDIAIDDDLWRRLSTDKASCLNRNCYYYRECPFFVT

RREIQEAEVVVANHALVMAAMESEAVLPDPKNLLLVLDEGHHLPDVARDALEMSAEITVPWYRLQLDLFT

KLVATCMEQFRPKTIPPLAIPERLNAHCEELYELIASLNNILNLYMPAGQEAEHRFAMGELPDELLEICQ

RLAKLTEMLRGLAELFLNDLSEKTGSHDIVRLHRLILQMNRALGMFEVQSKLWRLASLAQSSGAPVTKWA

TREEREGQLHLWFHCVGIRVSDQLERLLWRSIPHIIVTSATLRSLNSFSRLQEMSGLKEKAGDRFVALDS

PFNHCEQGKIVIPRMRFEPSIDNEEQHIAEMAAFFREQVESKKYLGMLVLFASGRAMQRFLDYVTDLRLM

LLVQGDQPRYRLVELHRKRVANGERSVLVGLQSFAEGLDLKGDLLSQVHIHKIAFPPIDSPVVITEGEWL

KSLNRYPFEVQSLPSASFNLIQQVGRLIRSHGCWGEVVIYDKRLLTKNYGKRLLDALPVFPIEQPEVPEG

IVKKKEKTKSPRRRRR

>gi|30062283|ref|NP_836454.1| hypothetical protein S0789 [Shigella flexneri 2a str. 2457T]

MPVRAQRIQHVMQDTIINFYSTSDDYGDFSNFAARPIKVDGNTWPTSEHYFQAQKFLDEKYREEIRRVSS

PMVAARMGRNRSKPLRKNWESVKEQVMRKALRAKFEQHAELRVLLLATAPAKLVEHTENDAYWGDGGNGK

GKNRLGYLLMELREQLAIEK

>gi|30062282|ref|NP_836453.1| ATP-dependent RNA helicase RhlE [Shigella flexneri 2a str. 2457T]

MSFDSLGLSPDILRAVAEQGYREPTPIQQQAIPAVLEGRDLMASAQTGTGKTAGFTLPLLQHLITRQPHA

KGRRPVRALILTPTRELAAQIGENVRDYSKYLNIRSLVVFGGVSINPQMMKLRGGVDVLVATPGRLLDLE

HQNAVKLDQVEILVLDEADRMLDMGFIHDIRRVLTKLPAKRQNLLFSATFSDDIKALAEKLLHNPLEIEV

ARRNTASDQVTQHVHFVDKKRKRELLSHMIGKGNWQQVLVFTRTKHGANHLAEQLNKDGIRSVAIHGNKS

QGARTRALADFKSGDIRVLVATDIAARGLDIEELPHVVNYELPNVPEDYVHRIGRTGRAAATGEALSLVC

VDEHKLLRDIEKLLKKEIPRIAIPGYEPDPSIKAEPIQNGRQQRGGIRLMNKRKTA

>gi|30062281|ref|NP_836452.1| DNA-binding transcriptional regulator [Shigella flexneri 2a str. 2457T]

MDCAMNNPAMTIKGEQAKKQLIAAALAQFGEYGMNATTREIAAQAGQNIAAITYYFGSKEDLYLACAQWI

ADFIGEQFRPHAEEAERLFAQPQPDRAAIRELILRACRNMIKLLTQDDTVNLSKFISREQLSPTAAYHLV

HEQVISPLHSHLTRLIAAWTGCDANDTRMILHTHALIGEILAFRLGKETILLRTGWTAFDEEKTELINQT

VTCHIDLILQGLSQRSL

>gi|30062280|ref|NP_836451.1| hypothetical protein S0786 [Shigella flexneri 2a str. 2457T]

MMKKPVVIGLAVVVLAAVVAGGYWWYQSRQDNGLTLYGNVDIRTVNLSFRVGGRVESLAVDEGDAIKAGQ

VLGELDHKPYEIALMQAKAGVSVAQAQYDLMLAGYRDEEIAQAAAAVKQAQAAYDYAQNFYNRQQGLWKS

RTISANDLENARSSRDQAQATLKSAQDKLRQYRSGNREQDIAQAKASLEQAQAQLAQAELNLQDSTLIAP

SDGTLLTRAVEPGTVLNEGGTVFTVSLTRPVWVRAYVDERNLDQAQPGRKVLLYTDGRPDKPYHGQIGFV

SPTAEFTPKTVETPDLRTDLVYRLRIVVTDADDALRQGMPVTVQFGDEAGHE

>gi|30062279|ref|NP_836450.1| ATP-binding component of a transport system [Shigella flexneri 2a str. 2457T]

MTRQDMNDAVITLNGLEKRFPGMDKPAVAPLDCTIHAGYVTGLVGPDGEGKTTLMRMLAGLLKPDSGSAT

VIGFDPIKNDGALHAVLGYMPQKFGLYEDLTVMENLNLYADLRSVTGEARKQTFARLLEFTSLGPFTGRL

AGKLSGGMKQKLGLACTLVGEPKVLLLDEPGVGVDPISRRELWQMVHELAGEGMLILWSTSYLDEAEQCR

DVLLMNEGELLYQGEPKALTQTMAGRSFLMTSPHEGNRKLLQRALKLPQVSDGMIQGKSVRLILKKEATP

DDIRHADGMPEININETTPRFEDAFIDLLGGAGTSESPLGAILHTVEGTPGETVIEAKELTKKFGDFAAT

DHVNFAVKRGEIFGLLGPNGAGKSTTFKMMCGLLVPTSGQALVLGMDLKESSGKARQHLGYMAQKFSLYG

NLTVEQNLRFFSGVYGLRGRAQNEKISRMSEAFGLKSIASHATDELPLGFKQRLALACSLMHEPDILFLD

EPTSGVDPLTRREFWLHINSMVEKGVTVMVTTHFMDEAEYCDRIGLVYRGKLIASGTPDDLKAQSANDEQ

PDPTMEQAFIQLIHDWDKEHSNE

>gi|30062278|ref|NP_836449.1| hypothetical protein S0784 [Shigella flexneri 2a str. 2457T]

MSNPILSWHRVRALCVKETRQIVRDPSSWLIAVVIPLLLLFIFGYGINLDSSKLRVGILLEQRSEAALDF

THTMTGSPYIDATISDNRQELIAKMQSGKIRGLVVIPVDFAEQMERANATAPIQVITDGSEPNTANFVQG

YVEGIWQIWQMQRAEDNGQTFEPLIDVQTRYWFNPAAISQHFIIPGAVTIIMTVIGAILTSLVVAREWER

GTMEALLSTEITRTELLLCKLIPYYFLGMLAMLLCMLVSVFILGVPYRGSLLILFFISSLFLLSTLGMGL

LISTITRNQFNAAQVALNAAFLPSIMLSGFIFQIDSMPAVIRAVTYIIPARYFVSTLQSLFLAGNIPVVL

VVNVLFLIASAVMFIGLTWLKTKRRLD

>gi|30062277|ref|NP_836448.1| hypothetical protein S0783 [Shigella flexneri 2a str. 2457T]

MFHRLWTLIRKELQSLLREPQTRAILILPVLIQVILFPFAATLEVTNATIAIYDEDNGEHSVELTQRFAR

ASAFTHVLLLKSPQEIRPTIDTQKALLLVRFPADFSRKLDTFQTAPLQLILDGRNSNSAQIAANYLQQIV

KNYQQELLEGKPKPNNSELVVRNWYNPNLDYKWFVVPSLIAMITTIGVMIVTSLSVAREREQGTLDQLLV

SPLTTWQIFIGKAVPALIVATFQATIVLAIGIWAYQIPFAGSLALFYFTMVIYGLSLVGFGLLISSLCST

QQQAFIGVFVFMMPAILLSGYVSPVENMPVWLQNLTWINPIRHFTDITKQIYLKDASLDIVWNSLWPLLV

ITATTGSAAYAMFRRKVM

>gi|30062276|ref|NP_836447.1| hypothetical protein S0782 [Shigella flexneri 2a str. 2457T]

MKWQQRVRVATGLSCWQIMLHLLVVALLVVGWMSKTLVHVGVGLCALYCVTVVMMLVFQRHPEQRWREVA

DVLEELTTTWYFGAALIVLWLLSRVLENNFLLAIAGLAILAGPAVVSLLAKDKKLHHLTSKHRVRR

>gi|30062275|ref|NP_836446.1| hypothetical protein S0781 [Shigella flexneri 2a str. 2457T]

MPDQTQQFSFKVLTINIHKGFTAFNRRFILPELRDAVRTVSADIVCLQEVMGAHEVHPLHVENWPDTSHY

EFLADTMWSDFAYGRNAVYPEGHHGNAVLSRYPIEHYENRDVSVDGAEKRGVLYCRIVPPMTGKAIHVMC

VHLGLREAHRQAQLAMLAEWVNELPDGEPVLVAGDFNDWRQKANHPLKVQAGLDEIFTRAHGRPARTFPV

QFPLLRLDRIYVKNASASAPTALPLRTWRHLSDHAPLSAEIHL

>gi|30062274|ref|NP_836445.1| cardiolipin synthase 2 [Shigella flexneri 2a str. 2457T]

MKCSWREGNKIQLLENGEQYYPAVFKAIGEAQERIILETFIWFEDNVGKQLHAALLAAAQRGVKAEVLLD

GYGSPDLSDEFVNELTAAGVVFRYYDPRPRLFGMRTNVFRRMHRKIVVIDARIAFIGGLNYSAEHMSSYG

PEAKQDYAVRLEGPIVEDILQFELENLPGQSAARRWWRRHHKAEENRQPGEAQVLLVWRDNEEHRDDIER

HYLKMLTQAQREVIIANAYFFPGYRFLHALRKAARRGVRIKLIIQGEPDMPIVRVGARLLYNYLVKGGVQ

VFEYRRRPLHGKVALMDDHWATVGSSNLDPLSLSLNLEANVIIHDRHFNQTLRDNLNGIIAADCQQVDET

MLPKRTWWNLTKSVLAFHFLRHFPALVGWLPAHTPRLTQVDPPAQPTMETQDRVETENTGVNP

>gi|30062273|ref|NP_836444.1| hypothetical protein S0779 [Shigella flexneri 2a str. 2457T]

MSKSHPRWRLAKKILTWLFFIAVIVLLVVYAKKVDWEEVWKVIRDYNRVALLSAVGLVVVSYLIYGCYDL

LARFYCGHKLAKRQVMLVSFICYAFNLTLSTWVGGIGMRYRLYSRLGLPGSTITRISSLSITTNWLGYIL

LAGIIFTAGVVELPDYWYVDQTTLRILGIGLLMIIAVYLWFCAFAKHRHMTIKGQKLVLPSWKFALAQML

ISSVNWMVMGAIIWLLLGQSVNYFFVLGVLLVSSIAGVIVHIPAGIGVLEAVFIALLAGEHTSKGSIIAA

LLAYRVLYYFIPLLLALICYLLLESQAKKLRAKNEAAM

>gi|30062272|ref|NP_836443.1| hypothetical protein S0778 [Shigella flexneri 2a str. 2457T]

MESYSQNSNKLDFQHEARILNGIWLITALGLVATAGLAWGAKYLEITATKYDSPPMYVAIGLLLLCMYGL

SKDINKINAAIAGVIYLFLLSLVAIVVASLVPVYAIIIVFSTAGAMFLISMLAGLLFNVDPGSHRFIIMM

TLTGLALVIIVNAALMSERPIWVISCLMIVLWPGIISHGRNKLLELAGKCHSEELWSPVRCAFTGALTLY

YYFIGFFGILAAIAITLVWQRHTRFFH

>gi|30062271|ref|NP_836442.1| hypothetical protein S0777 [Shigella flexneri 2a str. 2457T]

MDRFPRSDSIVQPWAGLQTYMAQVYGWMTVGLLLTAFVAWYAANSAAVMELLFTNRVFLIGLIIAQLALV

IVLSAMIQKLSAGVTTMLFMLYSALTGLTLSSIFIVYTAASIASTFVVTAGMFGAMSLYGYTTKRDLSGF

GNMLFMALIGIVLASLVNFWLKSEALMWAVTYIGVIVFVGLTAYDTQKLKNMGEQIDTRDTSNLRKYSIL

GALTLYLDFINLFLMLLRIFGNRR

>gi|30062270|ref|NP_836441.1| molybdopterin synthase large subunit [Shigella flexneri 2a str. 2457T]

MAETKIVVGPQPFSVGEEYPWLAERDEDGAVVTFTGKVRNHNLGDSVKALTLEHYPGMTEKALAEIVDEA

RNRWPLGRVTVIHRIGELWPGDEIVFVGVTSAHRSSAFEAGQFIMDYLKTRAPFWKREATPEGDRWVEAR

ESDLQAAKRW

>gi|30062269|ref|NP_836440.1| molybdopterin synthase small subunit [Shigella flexneri 2a str. 2457T]

MIKVLFFAQVRELVGTDATEVAADYPTVEALRQHLAAQSDRWALALEDGKLLAAVNQTLVSFDHPLTDGD

EVAFFPPVTGG

>gi|30062268|ref|NP_836439.1| molybdenum cofactor biosynthesis protein C [Shigella flexneri 2a str. 2457T]

MSQLTHINAAGEAHMVDVSAKAETVREARAEAFVTMRSETLAMIIDGRHHKGDVFATARIAGIQAAKRTW

DLIPLCHPLMLSKVEVNLQAEPEHNRVRIETLCRLTGKTGVEMEALTAASVAALTIYDMCKAVQKDMVIG

PVRLLAKSGGKSGDFKVEADD

>gi|30062267|ref|NP_836438.1| molybdopterin biosynthesis protein B [Shigella flexneri 2a str. 2457T]

MSQVSTEFIPTRIAILTVSNRRGEEDDTSGHYLRDSAQEAGHHVVDEAIVKENRYAIRAQVSAWIASDDV

QVVLITGGTGLTEGDQAPEALLPLFDREVEGFGEVFRMLSFEEIGTSTLQSRAVAGVANKTLIFAMPGST

KACRTAWENIIAPQLDARTRPCNFHPHLKK

>gi|30062266|ref|NP_836437.1| molybdenum cofactor biosynthesis protein A [Shigella flexneri 2a str. 2457T]

MASQLTDVFARKFYYLRLSITDVCNFRCTYCLPDGYKPSGVTNKGFLTVDEIRRVTRAFASLGTEKVRLT

GGEPSLRRDFTDIIAAVRENDAIRQIAVTTNGYRLERDVANWRDAGLTGINVSVDSLDARQFHAITGQDK

FNQVMAGIDAAFEAGFEKVKVNTVLMRDVNHHQLDTFLNWIQHRPIQLRFIELMETGEGSELFRKHHISG

QVLRDELLRRGWIHQLRQRSDGPAQVFCHPDYAGEIGLIMPYEKDFCATCNRLRVSSIGKLHLCLFGEGG

VNLRDLLEGDTQQQALEARISAALREKKQTHFLHQNNTGITQNLSYIGG

>gi|30062265|ref|NP_836436.1| structural protein [Shigella flexneri 2a str. 2457T]

MRNRTLADLDRVVALGGGHGLGRVLSSLSSLGSRLTGIVTTTDNGGSTGRIRRSEGGIAWGDMRNCLNQL

ITEPNVASAMFEYRFGGNGELSGHNLGNLMLKALDHLSVRPLEAINLIRNLLKVDTHLIPMSEHPVDLMA

IDDQGHEVYGEVNIDQLTTPIQELLLTPNVPATREAVHAINEADLIIIGPGSFYTSLMPILLLKEIAQAL

RRTPAPMVYIGNLGRELSLPAANLKLESKLAIMEQYVGKKVIDAVIVGPKVDVSAVKERIVIQEVLEASD

IPYRHDRQLLHNALEKALQALG

>gi|30062264|ref|NP_836435.1| excinuclease ABC subunit B [Shigella flexneri 2a str. 2457T]

MSKPFKLNSAFKPSGDQPEAIRRLEEGLEDGLAHQTLLGVTGSGKTFTIANVIADLQRPTMVLAPNKTLA

AQLYGEMKEFFPENAVEYFVSYYDYYQPEAYVPSSDTFIEKDASVNEHIEQMRLSATKAMLERRDVIVVA

SVSAIYGLGDPDLYLKMMLHLTVGMIIDQRAILRRLAELQYARNDQAFQRGTFRVRGEVIDIFPAESDDI

ALRVELFDEEVERLSLFDPLTGQIVSTIPRFTIYPKTHYVTPRERIVQAMEEIKEELAARRKVLLENNKL

LEEQRLTQRTQFDLEMMDELGYCSGIENYSRFLSGRGPGEPPPTLFDYLPADGLLVVDESHVTIPQIGGM

YRGDRARKETLVEYGFRLPSALDNRPLKFEEFEALAPQTIYVSATPGNYELEKSGGDVVDQVVRPTGLLD

PIIEVRPVATQVDDLLSEIRQRAAINERVLVTTLTKRMAEDLTEYLEEHGERVRYLHSDIDTVERMEIIR

DLRLGEFDVLVGINLLREGLDMPEVSLVAILDADKEGFLRSERSLIQTIGRAARNVNGKAILYGDRSTPS

MAKAIGETERRREKQQKYNEEHGITPQGLNKKVVDILALGQNIAKTKAKGRGKSRPIVEPDNVPMDMLPK

ALQQKIHELEGLMMQHAQNLEFEEAAQIRDQLHQLRELFIAAS

>gi|30062263|ref|NP_836434.1| dithiobiotin synthetase [Shigella flexneri 2a str. 2457T]

MSKRYFVTGTDTEVGKTVASCALLQAAKAVGYRTAGYKPVASGSEKTPEGLRNSDALALQHNSSLQLDYA

TVNPYTFAESTSPHIISAQEGRPIESLVMSAGLRALEQQADWVLVEGAGGWFTPLSDTFTFADWVTQEQL

PVILVVGVKLGCINHAMLTAQAIQHAGLTLAGWVANDVTPPGKRHAEYMTTLTRMIPAPLLGEIPWLAEN

PENAATGKYINLALL

>gi|30062262|ref|NP_836433.1| biotin biosynthesis protein BioC [Shigella flexneri 2a str. 2457T]

MATVNKQAIAAAFGRAAAHYEQHADLQRQSADALLAMLPQRKYTHVLDAGCGPGWMSRHWRERHAQVTAL

DLSPPMLAQARHKDAADHYLAGDIESLPLATATFDLAWSNLAVQWCGNLSTALRELYRVVRPGGVVAFTT

LVQGSLPELHQARQAVDERPHANRFLPPDEIEESLNGVRYQHHIQLITLWFDDALSAMRSLKGIGATHLH

EGRDPRILTRSQLQRLQLAWPQQQGRYPLTYHLFLGVIARE

>gi|30062261|ref|NP_836432.1| 8-amino-7-oxononanoate synthase [Shigella flexneri 2a str. 2457T]

MSWQEKINAALDARRAADALRRRYPVAQGAGRWLVADDRQYLNFSSNDYLGLSHHPQIIRAWKQGAEQFG

IGSGGSGHVSGYSVVHQALEEELAEWLGYSRALLFISGFAANQAVIAAMMAKEDRIVADRLSHASLLEAA

SLSPSQLRRFTHNDVAHLARLLASPCPGQQLVVTEGVFSMDGDSAPLAEIQQVTQQHNGWLMVDDAHGTG

VIGEQGRGSCWLQKVKPELLVVTFGKGFGVSGAAVLCSSTVADYLLQFARHLIYSTSMPPAQAQALRASL

AVIRRDEGDARREKLVSLIARFRAGVQDLPFTLADSCSAIQPLIVGDNSRALQLAEKLRQQGCWVTAIRP

PTVPAGTARLRLTLTAAHEMQDIDRLLEVLHGNG

>gi|30062260|ref|NP_836431.1| biotin synthase [Shigella flexneri 2a str. 2457T]

MAHRPRWTLSQVTELFEKPLLDLLFEAQQVHRQHFEPRQVQVSTLLSIKTGACPEDCKYCPQSSRYKTGL

EAERLMEVEQVLESARKAKAAGSTRFCMGAAWKNPHERDMPYLEQMVQGVKAMGLEACMTLGTLSESQAQ

RLANAGLDYYNHNLDTSPEFYGNIITTRTYQERLDTLEKVRDAGIKVCSGGIVGLGETVKDRAGLLLQLA

NLPTPPESVPINMLVKVKGTPLADNDDVDAFDFIRTIAVARIMMPTSYVRLSAGREQMNEQTQAMCFMAG

ANSIFYGCKLLTTPNPEEDKDLQLFRKLGLNPQQTAVLAGDNEQQQRLGQALMTPDTDEYYNAAAL

>gi|30062259|ref|NP_836430.1| adenosylmethionine-8-amino-7-oxononanoate aminotransferase [Shigella flexneri 2a str. 2457T]

MTTDDLAFDQRHIWHPYTSMTSPLPVYPVASAEGCELILSDGRRLVDGMSSWWAAIHGYNHPQLNAAMKT

QIDAMSHVMFGGITHAPAIELCRKLVAMTPQPLECVFLADSGSVAVEVAMKMALQYWQAKGEARQRFLTF

RNGYHGDTFGAMSVCDPDNSMHSLWKGYLPENLFAPAPQSRMDGEWDERDMVGFARLMAAHRHEIAAVII

EPIVQGAGGMRMYHPEWLKRIRKMCDREGILLIADEIATGFGRTGKLFACEYAEIAPDILCLGKALTGGT

MTLSATLTTREVAETISNGEAGCFMHGPTFMGNPLACAAANASLAIIESGEWQQQVAAIEVQLREQLAPA

RDAEMVADVRVLGAIGVVETTRPVNMAALQKFFVEQGVWIRPFGKLIYLMPPYIILPQQLQRLTAAVNRA

VQDETFFCQ

>gi|30062258|ref|NP_836429.1| kinase inhibitor protein [Shigella flexneri 2a str. 2457T]

MKLISNDLRDGDKLPHRHVFNGMGYDGDNISPHLAWDDVPAGTKSFVVTCYDPDAPTGSGWWHWVVVNLP

ADTRVLPQGFGSGLVAMPDGVLQTRTDFGKTGYDGAAPPKGETHRYIFTVHALDVERIDVDEGASGAMVG

FNVHFHSLASASITAMFS

>gi|30062257|ref|NP_836428.1| hypothetical protein S0763 [Shigella flexneri 2a str. 2457T]

MWLAENEGVKFWLNVLTELKKRGWSPYLFIKPVVCPPQHHFSALIEILSMVVNSANPVR

>gi|30062256|ref|NP_836427.1| tail component of prophage CP-933K [Shigella flexneri 2a str. 2457T]

MHMSLAQCPGFLFAHREECTVEIKKIINPRYTESGAVDCDVFFDDRDQAVPYTATADDVAPTGQQIWQEL

QSGKWGEIAPFTVTPEMLEAAREARRQEIEAWRTEQEAKPFTFEWNGRTWNADASSVARLSPVVMLAKSV

AAQTHMVWSDADNQQVKLSMPELEELAAAMVQAQVDRNDEIYRRQREMKEELSSLDDLASIRAFDVK

>gi|30062255|ref|NP_836426.1| tail component encoded by cryptic prophage CP-933M [Shigella flexneri 2a str. 2457T]

MAVKISGVLKDGAGKPVVNCAIELRARRTSPTVVAHVVATCVTDNNGAYVIEAEPGYYEVALHCNGWQPT

RVGDIDVAPTDAPGTLNAFLNAPKDGDLRPEVMKRFEEMVAQAQQSAGAAAGNAQQTAQDVAAAATARDD

AQRFAEKARQDATVTAEDRKATAEDVTSTGANAAAAGQSAQDAAGYARAAEQAKNDIDAALTGTLKMANH

LSEIAAAGEKAQQKSRDNLGLKSAATMEAQSDIYDRTKGRLAIPGAFGFGCAFLPEDVIRFDTKSDFLAW

VRNALPGEYSVAGPYGIIIPDTRFEGVLSIRWTDARPETTEPRYRAKSLTFYGINGPIYHTRYRYWPISR

LTG

>gi|30062254|ref|NP_836425.1| membrane protein precursor [Shigella flexneri 2a str. 2457T]

MRKLYAAILSAAICLAVSGAPAWASEHRSTLSAGYLHASTNAPGSDDLNGINVKYRYEFTDTLGLVTSFS

YANAEDEQKTHYSDTRWHEDSVRNRWFSVMAGLSVRVNEWFSAYAMAGVAYSRVSTFSGDYLRVTDNKGK

THDVLTGSDDNRHSNTSLAWGAGVQFNPTESVAIDLAYEGSGSGDWRTDGFIVGVGYKF

>gi|30062253|ref|NP_836424.1| host specificity protein [Shigella flexneri 2a str. 2457T]

MGKGSSKGHTPREAKDNLKSTQLLSVIDAISEGPVDGPVDGLKSVLLNGTPVLDSEGKTNFSGVTVVFRA

GEQEQTPPEGFESSGSETVLGTEVKYDTPITRTITSANIDRLRLTFGVQALVETTSKGDRNPSEVRLLVQ

IQRNGGWVTEKDITIKGKTTSQYLASVVVGNLPPRPFNIRMRRMTPDSTTDQLQNKTLWSSYTEIIDVKQ

CYPNTALVGVQVDSEQFGNQQVSRNYHLRGRILQVPSNYNPQTRQYSGIWDGTLKPAYSNNMAWCLWDML

THPRYGMGKRLGAADVDKWALYVIGQYCDQSVPDGFGGTEPRITCNAYLTTQRKAWDVLSDFCSAMRCMP

VWNGQTLTFVQDRPSDKVWTYNRSNVVMPDDGAPFRYSFSALKDRHNVVEVNWIDPDNGHETATELVEDT

QAIVRYGRNVTKMDAFGCTSRGQAHRAGLWLIKTELLETQTVDFSVGAEGLRHVPGDVIEICDDDYAGIS

TGGRVLAVNSQTRTLTLDREITLPSSGTTLISLVDGNGNPVSVEVQSVTDGVKVKVSRVPDGVAEYSVWG

LKLPTLRQRLFRCVSIRENDDGTYAITAVQHVPEKEAIVDNGAHFDGDRRGTVNGVTPPAVQHLTAEVTA

DSGEYQVLARWDTPKVVKGVSFLLRLTVAADDGSERLVSTARTTETTYRFRQLALGNYSLTVRAVNARGQ

QGDPASVSFRIAAPAAPVTIELIPGYFQITVVPKLAVYDPTVQFEFWFSEKRIADIRQVETSARYLGTAL

YWIAASINIRPGHDYYFYVRSVNTVGKSAFVEAVGRASDDAEGYLSFYKGLINKTHLGKELWTQIDNGQL

APDLTEIRTSITNVSNEITQTVNKKLENQSAAIQQIQKVQVDTNNNLNSMWAVKLQQMKDGRLYIAGIGA

GIENTPAGMQSQVLLAADRIAMINPANGNTKPMFVGQGDQIFMNDVFLKRLTAPTITSGGNPPAFSLTPG

GRLTAKNADISGNVNANSGTLNNVTINKNCRVLGKLSANQIEGDLVKTVGKPFPRDSRAPERWPSGTITV

RVYDDQPFDRQIVIPAVAFRGAKHERKNNNIYSSCRLIVKKNGAEIYNRTTLDNTLIYTGVIDMPAGHGH

MTLEFSVSAWLVNGWYPTASISDLLVVVMKKSTAGISIS

>gi|30062252|ref|NP_836423.1| tail component [Shigella flexneri 2a str. 2457T]

MKTGAEAIRALATQLPAFRQKLSDGWYQVRIAGRDAGETELSARLNEPLANGAVIHIVPRLVGAKSGGVF

QAVLGAAVMAVAIWMPGVGIMASNLLFSLGASMTLGGVAQMLAPKARTPRTQTTDNGKQNTCFSSLDNMV

AQGNVLPVLYGEMRVGSRVVSQEISTADEGDGGQVVVIGR

>gi|30062251|ref|NP_836422.1| tail assembly protein [Shigella flexneri 2a str. 2457T]

MAPEDWLRAQMQGEIVALVHSHPGGLPWLSEADRRLQVQSDLPWWLVCRGTIHKFRCVPHLTGRRFEHGV

TDCYTLFRDAYHLAGIEMPDFHRGDDWWRHGQNLYLDNLEATGLYQVPLSSAQPGDVLLCCFGSSVPNHA

AIYCGDGELLHHIPEQLSKRERYTDKWQRRTHSLWRHREWHASAFTGICNDLAAASTFV

>gi|30062250|ref|NP_836421.1| minor tail protein [Shigella flexneri 2a str. 2457T]

MQDIRQETLNECTRAEQSARVELWEIDLTEVGGERYFFCNEQNEKGEPVTWQGRQYQAYPIQGSGFELNG

RGCAARPTLTVSNLHGMVTGMAEDLQSLVGGTVVRRKVYARFLDAVNFVNGNSDADPEQEVISRWRIEQC

SELSAVSASFVLATPTETDGAVFPGRIMLANTCMWTYRSDECGYTGRAVADEFDKPTTDIRKDKCSKCMR

GCELRNNTGNFGGFLSINKLSQ

>gi|30062249|ref|NP_836420.1| minor tail protein [Shigella flexneri 2a str. 2457T]

METFHWKVRPDMNVVSEPKVVTVKLGDGYEQRRAAGLNNQLSTYSVTIRVRKGEHPSLKAFLERHGGVRA

FQWTPPYDWKLIRVVCRKWSASVGALWVTVTADFEQVVN

>gi|30062248|ref|NP_836419.1| tail length tape measure protein precursor [Shigella flexneri 2a str. 2457T]

MDQIANLVIDLGIDAAEFKNEIPRIKNLLNGAASDAERSSARMQRFMERQTQAARQTMQAASSAATAASA

HAQTVEKNARAHERMAREVEQTRLRVDALNQKMREEQAQARALAEAQDKAAAAFYRQIDSVKQAGAGLQE

LQRIQQQIRQARNSGGVGQQDYLALISEITAKTRALTQAEEQATRQKAAFIRQLKEQATRQNLSSSELLR

ARAAQLGVSSAAEVYIRKMERAGKATHSLGLKSAAARRELGVLISQMARGNFGALRGSGITLANRAGWTG

ALMSPKGMMTGGVIGGLVAAVLGLGKAWHDGRKEGEKFNRQLALTGHYAGVTVGQLWKLSRAISGNGITQ

HAAAGALAQVVGSGAFHGNDIGMVAKAAAQMERSVGQSVSDTINQFKRLKDDPVNAAKALDNALHFLNAT

QLEQIRVLGEQGRSSDAARIAMSALAEETGKRTSDIDNNLNALGSTLQTLSDWWKQFWDAAMNIGREDSL

DAQIDALQEKIQRAKKYPWTNASTQVEYDQQRLNDLQEKKRRKDLQDAKAQAERNYQEQQKRRNAENAAL

NRMNETEAARHQREIARINAMQYADQAVRDAAIQRENERYEKAIKKNTRATRNDEATRLLLQYSQQQAQV

EGQIAAARQSAGIATERMTEAHKQLLALQQRISDLDGKKLTADEKSVLARKNELIQALTLLDVKQQELQK

QTALNDLRKKTVQLTSQLADKERALREQHNLDIATAGMGDKQRQRYQAQLRIRQEYRQQLQQLENDSRQK

GTYGTEDYRRAEEVLKGSLKRQLNENKRYWQELEVAQGDWKNGAMRAFQNFTADADNAAGTAEQMFTVAF

SSAGNALATFCTTGKLNFKSFTSSLLSDMARIMAQMAMMQAVKGVGSLFGFTTNADGGVYQSADLSRYSG

TVVNRPTFFAFAKGAGVMGEAGPEAILPLRRGADGKLGVVADTGGSGMVMFAPQYNIEINNDGTNGQIGP

AALKVVYDLGKKAAADFMQQQARDGGRLSGAYR

>gi|30062247|ref|NP_836418.1| tail component of prophage CP-933K [Shigella flexneri 2a str. 2457T]

MLSDMSATELGEWGDYFRMQSFSDVWMDAQFASLKALIVRMVSGSSDAAVADFSLLPEENGIPERTDEEL

MHLGEGISGGVRYGPDSQPGH

>gi|30062246|ref|NP_836417.1| tail component of prophage CP-933K [Shigella flexneri 2a str. 2457T]

MFLKQDTFNYEKQSVVLSELSGLQRIEYLTFVQQRTAKFDAQEGELPEAERQIAFLRMGMDINAWLVSRS

LWNAEQSQDVETLCASIMTTWSYDALGAGAERVLSLSGMGTIENAGDDDHEALTPEKS

>gi|30062245|ref|NP_836416.1| tail component of prophage CP-933K [Shigella flexneri 2a str. 2457T]

MTTPNPLAKTKGAGTTFWMYTGNGDAFANPLSDTDWLRLAMVKDLQPGEMTADAEDDTYLDDEDADWKTT

TQGQKSVGDTSATLACRPGDSGQKKLVQLFDSGEVCAFRIKYPNGTVDVFRGWLSSLGKTIASKDVMTRT

VKISGVGRPYLAEEGTETVGVTGLTVAPASVSVKAGATTTLTFTVKPDGASDKAISVHSSDPQTASVTLS

GLVATVKGVKQGSVSIVGMTSDGEFVAVAAVTVSAP

>gi|30062244|ref|NP_836415.1| tail component of prophage CP-933K [Shigella flexneri 2a str. 2457T]

MNRHTQIRQSVLARLREQCGDSATFFDGLPAFIDAQELPAVAVWLSDAQYTGKMTDEDDWQAVLHIAVFI

RAQAPDSELDMWMESTIFPALNDVPALSGLIDTLNPLGFNYQRDNEMATWAMAEITYQITYTN

>gi|30062243|ref|NP_836414.1| tail component of prophage CP-933K [Shigella flexneri 2a str. 2457T]

MKGLENAIRNLNSLDTRMVPQTSAWAVNRVAAKIVSVATRQVAQNTVAGDNQVKGIPLKTVRERVRLLKA

SPSGKMYARMRVNRGNLPAIKLGTAQVRLARSRHGSNSRHRGSVLKVGKYLFRDAFIQQLANGRWHVMRR

IDGKNRYPIDVVKIPMSGPLTQAFEDARDRIIAAEMPKQLGYALKQQLRLHLSK

>gi|30062242|ref|NP_836413.1| tail attachment protein [Shigella flexneri 2a str. 2457T]

MDGFDNLFDAALAGVNEVILRDMGISAVITSGELEGTHLTGVFDDPESISFVAGGIRLEDSSPCLFVKTA

DISQLRRQDTLTIGDDSFFVDRITPDDGGCCYIRLRRGSPAPQNNARMRRYDEGT

>gi|30062241|ref|NP_836412.1| DNA-packaging protein [Shigella flexneri 2a str. 2457T]

MATKEENLKRLCELAERLGREPDVSGSAADIAQRVAELEEELGDAGEVAEPDTYSPSKDDPTAHKGESVP

EQPGIVTGDETGQVTVVALATLHTKCNGSVIFVSPGTSFRVSAEVAASMAAGGLAKRQ

>gi|30062240|ref|NP_836411.1| major capsid protein [Shigella flexneri 2a str. 2457T]

MGLFTTRQLLGYTEQKVKFRALFLELFFRRTVNFHTEEVMLDKITGKTPVAAYVSPVVEGKVLRHRGGET

RVLRPGYVKPKHEFNYQQAVERLPGEDPAQLNGPAYRRLRIITDNLKQEEHAIVQVEEMQAVNAVLYGKY

TMEGEQFETVEVDFGRSVGNNITQAGGTEWSAQDRDTFDPTHDLDAYCDFASGTINIAIMDGKVWRLLNG

FKLFREKLDTRRGSNSQLETAVKDLGAVVSFKGYYGDLAIVVAKTSYVAEDGTEKRYLPDGTLVLGNTAA

EGIRCYGAIQDAQALSEGVVASSRYPKHWLTVGDPAREFTMTQSAPLMVLPDPDEFVVVQVK

>gi|30062239|ref|NP_836410.1| capsid protein small subunit [Shigella flexneri 2a str. 2457T]

MVTKTITEQRAEVRIFAGNDPAHTATGCSGISSATPALTPLMLDEDTGKLVVWDGQKAGHVVGILVLPLE

GTETVLTYYKSGTFATEAIHWPERVDTHKKANAFAGSALSHAALP

>gi|30062238|ref|NP_836409.1| head-tail preconnector gp5 [Shigella flexneri 2a str. 2457T]

MRRNLSHIIAAAFNEPLLLEPAYARVFFCALGREMGAASLSVPQQQVQLDAPGMLAETDEYMAGGKRPAR

VYRVVNGIAVLPVTGTLVHRLGGMRPFSGMTGYDGIVACLQQAMADTQVRGVLLDIDSSGGQATGAFDCA

DMIYRLRQLKPVWALCNDTACSAAMLLASACSRRLVTQTSRIGSIGVMMGHVSYAGHLAQAGVDITLIYA

GSHKVDGNQFESLPAKVRQDMQQRIDAARRMFAEKVAMYTGLSVDAVMGTEAAVFEGQSGIEAELADELI

NASDAISVMAATLNTHDTGGTMPQLTATEAAAQENQRVMGILTCQEAKGREQLATMLAGQQGMSVEQARA

ILAVAAPQQSGVSAQSEADRIMACEEAKGREQLAATLAAMPEMTVEKARPILAASPQANAGPSLRDQIMA

LDEAKGAEAQAEKLAACPGMTVENARAVLAAASGRAEPVSASTTAMFEHFMANHSPAAVQGGVPQTSADG

DADVKMLMAMP

>gi|30062237|ref|NP_836408.1| head-tail preconnector gp5, partial [Shigella flexneri 2a str. 2457T]

MLKFRTFFPGDALNLQTAQDSDNGFSALEQALLRYIAAGLGVSYEQLSRDYSKVSYSSARASANESWRYF

MGRRKFIAARLATQMFSCWLEEALLRGIIRPPRARFDFYQARSAWSRAEWISSGRMAIDGLKEVQESVMR

IEAGLSTYEKELALMGEDYQDIFRQQVRESAEREKAGLSRPVWIAQAYQQQIAESRRPEEETTSRET

>gi|30062236|ref|NP_836407.1| head-tail preconnector gp5 [Shigella flexneri 2a str. 2457T]

MKRTPVLIDVNGVPLRESLSYNGGGAGFGGQMAEWLPPAQSADAALLPALRLGNARADDLVRNNGIAANA

VALHKDHIVGHMFLISYRLNWRWLGMRETTAKSFVGEVEAAWSEYAEGMFGEIDVEGKRTFTEFIREGVG

VHAFNGEIFVQPVWDTETTQLFRTRFKAVSPKRVDTPGHGMGNHFLRAGVEVDRYGRAIAYHICEDDFPF

SGAGRWERIPRELPTGRPAMLHIFEPVEDGQTRGANQFYSVMERMKMLDSLQATQLQSAIVKAMYAATIE

SELDTEKAFEYIAGAPQGQQDNPLINILEKFTRWYDTNNVTLGGVKIPHLFSWRCAEPADSAGFRQWIFG

A

>gi|30062235|ref|NP_836406.1| head completion protein gp3 [Shigella flexneri 2a str. 2457T]

MVTVAELQALRQARLDLLTGKRVVSVQKDGRRIEYTAASLNELNRAINDAESVLGTTRRRRPLGVRL

>gi|30062234|ref|NP_836405.1| DNA packaging protein of prophage CP-933R; terminase large subunit [Shigella flexneri 2a str. 2457T]

MNISEQQLSNMMVAVTIALQPLVRVLPVTAVEWADQNYYLPKESSYGEGEWKTLQFQVAIMNCMGNDEIR

TVNLIKSARVGYTKMVLGVIGYFIEHKSRNTLLFQPTDSAAEDFMKSHVEATIRDVPCLKKLSPWLGRKH

RDNTLTLKRFSSGVGFWCLGGAAAKNYREKSVDVVCYDELSSFEPDVEKEGSPTLLGDKRIEGSVWPKSI

RGSTPKIKGSCQIEKAANESAHFMRFYVPCPHCGEEQYLKFGDDATSFGLKWEKGKPETVYYLCEHNGCV

IRQSELDQTGGRWICDNTGMWTCDGLTFFSASGNEIPPPRSITFHIWTAYSPFTTRVQIVCDWLDALKDP

NGVKTFVNTTLGETWEEAVGEKLDHQVLMDKVVPYTATVPVRVVYLTAGIDSQRNRFEMYVWGWAPGEEA

FLVDKIIIMGRPDEEETLLRVDAAINKKYRHADGTEMTISRVCWDIGGIDGEIVYQRSKKHGIFRVLPVK

GASVYGKPVITMPKTRNQRGVYLCEVGTDTAKEILYARMKADPTPVNEATSYAIRFPDNPEIFSQTEAQQ

LVAEELVEKWEKGKMRLLWDNKKRRNEALDCLVYAYAALRVSVQRWQLDLAVQLHPARMQLHNRPLKNWQ

RSCPEE

>gi|30062233|ref|NP_836404.1| IS600 orfB [Shigella flexneri 2a str. 2457T]

MAHIRTRETYGTRRLQTELAENGIIVGRDRLARLRKELRLRCKQKRKFRATTNPNHNLPVAPNLLNQTFA

PTAPNQVWVADLTYVATQEGWLYLAGIKDVYTCEIVGYAMGERMTKELTGKALFMALRSQRPPAGLIHHS

DRGSQYCAYDYRVIQEQSGLKTSMSRKGNCYDNAPMESFWGTLKNESLSHYRFNNRDEAISVIREYIEIF

YNRQRRHSRLGNISPAAFREKYHQMAA

>gi|30062232|ref|NP_836403.1| IS600 orfA [Shigella flexneri 2a str. 2457T]

MSRKTQRYSKEFKAEAVRTVLENQLSISEGASRLSLPEGTLGQWVTAARKGLGTPGSRTLAELESEILQL

RKALNEARLERDILKKATAYFAQESLKNTR

>gi|30062231|ref|NP_836402.1| endopeptidase [Shigella flexneri 2a str. 2457T]

MSRVTAIISALVICIIVCLSWAVNHYRDNAITYKEQRDKNAGELKLANATITDMQQRQRDADALDAKYTK

ELADAKAENDALRRKLDNGGRVLVKGKCPVPSSAETSSASGMGNDATVELSPVAGRNVLGIRDG

>gi|30062230|ref|NP_836401.1| endolysin R of prophage CP-933V [Shigella flexneri 2a str. 2457T]

MSAKIKYGLSAAVLALIAAGASAPQILDQFLNEKEGNHTTAYRDGSGIWTICRGATMVDGKPVIPGMKLS

KEKCDQVNAIERDKALAWVERNIKVPLTEPQKAGIASFCPYNIGPGKCFPSTFYKRLNAGDRKGACEAIR

WWIKDVGRDCRIRSNNCYGQVIRRDQESALACWGIDQ

>gi|30062229|ref|NP_836400.1| hypothetical protein S0732 [Shigella flexneri 2a str. 2457T]

MKHEEMNQRFNHLENEITELNKKLSALVSSEDENKRRDEHYAAIYDYCHKVAHETFMKFLQEKFLPAALS
EKEAAYLRPEYVITVNSAGEEEHKSDFIASAPDKDQEPHRPFRVSCEEGEFVVYENGKPVRASHHHCLKI
INLAIRCLKDENTRVMKRIGRCMGYLQVAAEIEALASGADMDAAVREALLRDFNTPPLRKSLMTGSSRG

>gi|30062228|ref|NP_836399.1| S protein [Shigella flexneri 2a str. 2457T]
MKSMDKISTGIAYGTSAGSAGYWFLQWLDQVRPSQWAAIGVLGSLLLGLLTYLTNLYFKIREDKRKAARG
E

>gi|30062227|ref|NP_836398.1| Q protein [Shigella flexneri 2a str. 2457T]
MRDIQQVLERWGAWVANNHEDVTWSSIAAGFKGLIPSKVKSRPQCCDDDAMVICGCMARLNRNNNDLHDL
LVDYYVFGMTFMTLARKHGCSDGYIGKKLQKAEGVVEGMLMMLGVRLEMDKYIEHP

>gi|30062226|ref|NP_836397.1| bacteriophage protein [Shigella flexneri 2a str. 2457T]
MSVKIQTIPELLIQTRGNMTEVSRMLNCNRATVRKYAEDKEGKGHAIVDGVLIVHRGWDRGKDSDA

>gi|30062225|ref|NP_836396.1| bacteriophage protein [Shigella flexneri 2a str. 2457T]
MNNQTMTFTPEQLRKHAQEMLRHAEQLEKTGITKDAIRKDMVPALRELMQAKHRAQKAVDELVDCVAELE
TRVGKFEKLVQEALR

>gi|30062224|ref|NP_836395.1| helicase [Shigella flexneri 2a str. 2457T]
MTTPVWRNDDLEGAVIGAFFLRGADPEVMDILATLPADVFSVRAYQDIYTGICRQARVSGVIDPVLLCNE
MPELAPVITDTGRKTWVKSSLEHYVAALRRNAALRDAEKTLNEALQKLRDAHTCEAAEDALKDAQNMMVT
LSTGKGVIQPVHIDDVLPEVVERVECRNQGLEKSRTLMTGIDELDAKTGGMEPGDLVFIAARPSMGKTEL
ALDIIDKVTEQGHGVLLFTMEMANIQIGERMVSAAGGMPVSRLKSVAHFEDEDWTRFSQGVGRMTGRNIW
MVDQANLAIDEICATTKHHLIKYPETALVVVDYLGLIKTRTTGRHDLAVGEISKGLKGLAKSGGFPLIAL

SQLSRGVESRPNKRPMNSDLKNSGEIEADADIILMLYRDEVYNPDTQARGIAEINITKQRNGSLGTIYRR

FYNGHFLPVDQESARVLSTPMKPGNPRRYSNKRTDSSKMERFF

>gi|30062223|ref|NP_836394.1| replication protein DnaC [Shigella flexneri 2a str. 2457T]

MMTFNLREQQKRLQARMDELRAEIAFAQKGEKPWPYRSCLMREGRGYCEKHGEYHTHILVWSDRNGEDRE

EISCCPDCLIAEANDLTMELSSIKAEELTDNAGIALRFRDCEFDNYLEVNPGAARNLAACRRYAENWPDM

LENGTSLVMTGSCGTGKNHLAVAMAKHIIRNYLASVEITDVMRLTRAVKNCWRNDSEKTADEVIERYASM

DLLIIDEVGVQFGSAAEMAILQEIINARYESILPTILISNLSPEELWAFISPRIADRITDGGRNWLSFNW

PSYRSRIRGVAA

>gi|30062222|ref|NP_836393.1| IS911 orfA [Shigella flexneri 2a str. 2457T]

MKKRNFSAEFKRESAQLVVDQKYTVADAAKAMDVGLSTMTRWVKQLRDERQGKTPKASPITPEQIEIREL

RKKLQRIEMENEILKKATALLMSDSLNSSR

>gi|30062221|ref|NP_836392.1| IS911 orfB, partial [Shigella flexneri 2a str. 2457T]

MCSGFIAAATDTGKTVLKNQTADGLYYAVRYLSYMASATVRPEQEASPQWQPGEATRWDAGLLAGS

>gi|30062220|ref|NP_836391.1| IS911 orfB, partial [Shigella flexneri 2a str. 2457T]

MKELGLVSCQQPTHRYKRGGHEHVAIPNYLERQFAVTEPNQVWCGDVTYIWTGKRWAYLAVVLDLFARKP

VGWAMSFSPDSRLTMKALEMAWETRGKPGGVMFHSDQGSHYTSRQFRQLLWRYQIRQSMSRRGNCWDNSP

MERFFRSLKNEWMPVVGYVSFSEAAHAITDYIVGYYSALRPHEYNGGLPPNESENRYWKNSNSVASFC

>gi|30062219|ref|NP_836390.1| bacteriophage protein [Shigella flexneri 2a str. 2457T]

MRPSELSDLLWAQVDRVAPHLLPNGKIEGHEWVAGNVNGDKGNSLKVNLIGKKKWADFAEGDGGDMLDLW

MACRGINLHQAMQEAKAFLGIKDDDHHFDARREKKFSRPDRKKIARYVTRTESHLEYLQSRGISPEVVKR

YEVVSGKVWNGERELDALVLPYKRDGELLQVKRISTERPDGKKVIMAEGDCEPCLFGWQALDAGVRVVVL

CEGEIDCMSYAQYGISALSVPFGGGKGAKQQWIEFEYHNLDRFEEIFISMDVDDVGREAAREIVSRLGEH

RCRLVTLPYKDINECLMNGVTEDEIWQYIGTASYFDPEELYSAREFYQDTINAFYGKQQYLFNPPWESLA

DKFQFREAELTLVNGVHGHGKACPLNEPILLADGTWTTHGNVKIGDQVASVDGNPSTVTGIFPQGVRDVY

RVTFEDGRYVDCAGDHLWEVTSRGFTKGEKRRVIDTFGLKRLSETKRHKNGVRIPEITGDFGDHSEPLAW

VIGSLLGDGSLSNGSVKFSNVEPYMIERMKAELPDYNFSGDGKDWLISTARGQVNPLMETLRGYGLMGCT

AKNKFIPRVFFSANKSTRIGMLCGLLETDGYVEKDGTLVFSSASEELRNEVVNKNWPPS

>gi|30062218|ref|NP_836389.1| IS600 orfA [Shigella flexneri 2a str. 2457T]

MSRKTQRYSKEFKAEAVRTVLENQLSISEGASRLSLPEGTLGQWVTAARKGLGTPGSRTVAELESEILQL

RKALNEARLERDILKKATAYFAQESLKNTR

>gi|30062217|ref|NP_836388.1| IS600 orfB [Shigella flexneri 2a str. 2457T]

MAHIRTRETYGTRRLQTELAENGIIVGRDRLARLRKELRLRCKQKRKFRATTNPNHNLPVAPNLLNQTFA

PTAPNQVWVADLTYVAGIKDVYTCEIVGYAMGERMTKELTGKALFMALRSQRPPAGLIHHSDRGSQYCAY

DYRVIQEQSGLKTSMSRKGNCYDNAPMESFWGTLKNESLSHYRFNNRDEAISVIREYIEIFYNRQRRHSR

LGNISPAAFREKYHQMAA

>gi|30062216|ref|NP_836387.1| hypothetical protein S0714, partial [Shigella flexneri 2a str. 2457T]

MYQMEKISTGIAYGTSAGSAGYWFLQWLDQ

>gi|30062215|ref|NP_836386.1| bacteriophage protein [Shigella flexneri 2a str. 2457T]

MFNLMLTSKKLTKTAINEALRRMKKAGLNKSELEAFLRDMINGKQKSWLAHCTDAEALCIDRVISEVLAE

HPGLISVLRQRYEGRGMTKCKMAELLNDAHPKWSLRTCERRIEHWLKVAEFILYKPMVMAFGIEKKVIAF

>gi|30062214|ref|NP_836385.1| IS911 orfB [Shigella flexneri 2a str. 2457T]

MKALEMAWETRGKPGGVMFHSDQGSHYTSRQFRQLLWRYQIRQSMSRRGNCWDNSPMERFFRSLKNEWMP

VVGYVSFSEAAHAITDYIVGYYSALRPHEYNGGLPPNESENRYWKNSNSVASFC

>gi|30062213|ref|NP_836384.1| IS911 orfB [Shigella flexneri 2a str. 2457T]

MKELGLVSCQQPTHRYKRGGHEHVAIPNYLERQFSVTEPNQVWSVCDLYLDG

>gi|30062212|ref|NP_836383.1| IS911 orfB, partial [Shigella flexneri 2a str. 2457T]

MCSGFIAAATDTGKTVLKNQTADGLYYAVRYLSYMASATVRPEQEASPQWQPGEATRWDAGLLAGS

>gi|30062211|ref|NP_836382.1| IS911 orfA [Shigella flexneri 2a str. 2457T]

MKKRNFSAEFKRESAQLVVDQKYTVADAAKAMDVGLSTMTRWVKQLRDERQGKTPKASPITPEQIEIREL

RKKLQRIEMENEILKKATALLMSDSLNSSR

>gi|30062210|ref|NP_836381.1| hypothetical protein S0704, partial [Shigella flexneri 2a str. 2457T]

MPSSAETSSASGMGNDATVELSPVAGRNVLGIRDGIISDQTALRMLQEYIRIQCLGG

>gi|30062209|ref|NP_836380.1| IS911 orfA [Shigella flexneri 2a str. 2457T]

MKKRNFSAEFKRESAQLVVDQKYTVADAAKAMDVGLSTMTRWVKQLRDERQGKTPKASPITPEQIEIREL

RKKLQRIEMENEILKKATALLMSDSLNSSR

>gi|30062208|ref|NP_836379.1| IS911 orfB, partial [Shigella flexneri 2a str. 2457T]

MCSGFIAAATDTGKTVLKNQTADGLYYAVRYLSYMASATVRPEQEASPQWQPGEATRWDAGLLAGS

>gi|30062207|ref|NP_836378.1| IS911 orfB, partial [Shigella flexneri 2a str. 2457T]

MKELGLVSCQQPTHRYKRGGHEHVAIPNYLERQFAVTEPNQVWCGDVTYIWTGKRWAYLAVVLDLFARKP

VGWAMSFSPDSRLTMKALEMAWETRGKPGGVMFHSDQGSHYTSRQFRQLLWRYQIRQSMSRRGNCWDNSP

MERFFRSLKNEWMPVVGYVSFSEAAHAITDYIVGYYSALRPHEYNGGLPPNESENRYWKNSNSVASFC

>gi|30062206|ref|NP_836377.1| bacteriophage protein [Shigella flexneri 2a str. 2457T]

MAKPDWEAIESAYRAGVLSLRDIGEKYGVTEGAIRKRAKKLDWARSGGTQVCKNGTQKRKVRTSRKPAIT

GLTQKSTQLKTESTPDTKPIRGMRTDPPTNPFQPGNQQALKHGGYARRLLLKDEVIEDAKALTLEDELFR

LRANNLVAAENIGRWLVSLEDANGDQERKMLIENISAAEKAMMRNTVRIESIVGTLATVGKIFADTAYRK

AATDKVSLEADRLRRDAGIDDGNGERDLNDFYSDIQTDAESGFT

>gi|30062205|ref|NP_836376.1| bacteriophage protein [Shigella flexneri 2a str. 2457T]

MRARNKVLYGGRSSSKSWDAAGIAIFLSNKYTLRFCCARQIQNKIEESVYTLLKIQIDRFGLRHRFRILN

NKIINRVTGSEFVFYGLWRNIEEIKSLEGIDMLWLEEAHALTEYQWKILEPTIRKEGSECWFIFNPGLVT

DFVWRNFVVDPPEGTLIRKINYDENPFLSDTMLKVIDAARRRDPDGFKHVYEGVPESDDDAAIRLAP

>gi|30062202|ref|NP_836373.1| bacteriophage protein [Shigella flexneri 2a str. 2457T]

MSGNIGANPAIIKLSWIEAAVDAHKTLNFEPSGRKRIGFDVADSGTDKCANVYRHGSVVFWADEWKAKED

ELLKSCQRTYQAALEREADIVYDSIGVGASAGAKFSEINADRKSENAYARRVNYQRFNAGAGVHEPDDEY

NGIPNKDFFANLKAQAWWLTVSEIRLTPLTTENSILWMS

>gi|30062201|ref|NP_836372.1| hypothetical protein S0695 [Shigella flexneri 2a str. 2457T]

MDELISIDSRCPLLEKLKLELTTPHRDFDRNGRVMVESKKDLAKREIPSPNVADAFIMAFAPIDTSLDIW

EQLGRQA

>gi|30062200|ref|NP_836371.1| bacteriophage protein [Shigella flexneri 2a str. 2457T]

MARNKQALRRTVQATADGYENFIARVGMQTPNQHSASTYRANFTSRNRMLVEWSYRSSWIIGEAVDAIPD
DMTRKGIRITSEIDAKDRGILESQLDELQIWDALNDVLKWSRLYGGAVGFIMIEGQAPMTPLRPETIGKG
KFKGILPLDRWMVDPALTRRIKDMGPDLGKPEFYDVVTTATGIPAWRIHHSRLIRFDGVTLPFQQKMTEN
EWGMSVVERIWDRLTAFDSATVGAAQLVYKAHLRTYSVEKLRELIALGGPAYEALLKNIDLIRQFQSNEG
MTLMDSRDKFETHQYSFSGLDDILSQFAEQISGAVGIPLVRLFGQSPKGFSTGDADLANYYDRISSLQER
RLRLPVRRILDIMHRSELGKPLPDDFTFEFNPLWQMSDVDRSTVALNTTNAISTALGDGLMTLKAAMTDL
RENSDVTGIGASITNEDIENAEDEAPPGIGEPDDEPQEPSGGNPVSNQPTQDSEGGRRHRKWSLRWFK

>gi|30062199|ref|NP_836370.1| bacteriophage protein [Shigella flexneri 2a str. 2457T]
MGQVMQSIIAEQVKYIKSLPLEAADRVYDIQNKAIEAVVTGGRAEQFAKEIASTGDVAKSRADLIARTEL
GRATGALDMARAIAIGSDGYIWRTADDGDVRDSHDHMKGKFVRWDSPPTLDGMTGHAGELPNCRCYKEIV
FVRVPFAMKRAA

>gi|30062198|ref|NP_836369.1| bacteriophage protein [Shigella flexneri 2a str. 2457T]
MKYFFETRLGETRYRLADGSLLCKDVPIGRTGKQLYGADDLPKLKPDKFVEIVVTRSPEQVFHPATLASF
EGMSITILHPEDENGNVRLVNPENWKVLAVGHLQNVRRGTGEQSDLMLADLIVKDESAIQLIEDGLREVS
CGYDAEYEQTEPGKAEQVDITGNHVALVPKGRAGNRCAIGDRDTMANQKKNWWNRMRAAIKTGDADTMNE
LVESAPASVTGDEGDLPQGVNLNINLSPQQPLPDKAPEMGGDPTGDSDDDLKTLLKALLAKLERNATGDN
DNKPDDNPTGDGEDDEEETTITGDSAWRAEVIVPGIDLSRKMKPTAFKREVLASADKTLVRQIVGDADIR
KLPKQSVDMAFNAVSEVAKGRNTRATTGDAQRLNMGMTSIASLNKQNAEFWANRKG

>gi|30062197|ref|NP_836368.1| bacteriophage protein [Shigella flexneri 2a str. 2457T]
MNNVFLYRMPVGIAGAVSRPQDLTVEPVVLKSDNAFAAYGLAGKYDDDGFFVPLADGDTADKVKGIYVRP
YPTTSQPDMVRQVGTGKNFPGDAMKRGYVTVNLGSDFDASTIKKGDLH

>gi|30062196|ref|NP_836367.1| hypothetical protein S0690 [Shigella flexneri 2a str. 2457T]

MSTSVSGKNVVLTNAEFTGAGDADGNAEISWKI

>gi|30062195|ref|NP_836366.1| bacteriophage protein [Shigella flexneri 2a str. 2457T]

MITFDQATVDSSGAFLIGELERLDQTLNLPLVGYTWSRDIQLREDASIADDISSWTNTSFAAAGTGANPN

GKNWVGKDSTAIAGVNVDIGKSGNPLNLWGMELGWTVIELQAAQQVGRPIDTQKYDGMQLKWQMDNDEQV

YVGDSALNLKGLVTLDGVPVNNAAKTWATSTPDEIRASINQVLSDAWAASGYSVVPRDLLIPPEQFALLS

SIIVSSAGNQSLLTNLQTNTISYHQNGVPLNIRAVKWLKGRGVGNKDRIVAYTNDKKYVRYPLVPLQSVP

VQYRGLYQIVTYYGKLGAVEPVYKETISYVDGI

>gi|30062194|ref|NP_836365.1| hypothetical protein S0688 [Shigella flexneri 2a str. 2457T]

MAKNDAVIHVHTPFVLTLPDGSRREFVKGRHVVEEDVATHWFTRAHAEVSVGKATDARNEVKNAKELRCT

GNSGHYHLFFF

>gi|30062193|ref|NP_836364.1| IS600 orfB [Shigella flexneri 2a str. 2457T]

MAHIRTRETYGTRRLQTELAENGIIVGRDRLACLRKELRLRCKQKRKFRATTNPNHNLPVAPNLLNQTFA

PTAPNQVWVADLTYVATQEGWLYLAGIKDVYTCEIVGYAMGERMTKELTGKALFMALRSQRPPAGLIHHS

DRGSQYCAYDYRVIQEQSGLKTSMSRKGNCYDNAPMESFWGTLKNESLSHYRFNNRDEAISVIREYIEIF

YNRQRRHSRLGNISPAAFREKYHQMAA

>gi|30062192|ref|NP_836363.1| IS600 orfA [Shigella flexneri 2a str. 2457T]

MSRKTQRYSKEFKAEAVRTVLENQLSISEGASRLSLPEGTLGQWVTAARKGLGTPGSRTVAELESEILQL

RKALNEARLERDILKKATAYFAQESLKNTR

>gi|30062191|ref|NP_836362.1| phage transposase [Shigella flexneri 2a str. 2457T]

MLQQGKFKTSQGCFEIARPTLEAHDYDREALWSKWDKASDSQRSLAEKWLPSIQATDEMLNQGISTKTAF

ATVAGHYQVSASTLRDKYYQVQKFAKPDWAAALVDGRGASRRNVHKSEFDEDAWQFLIADYLRPEKPAFR

KCYERLELAAREHGWSIPSRATAFRRIQQLDEAMVVACREGEHALMHLIPAQQRTVEHLDAMQWINGDGY

LHNVFVRWFNGDVIRPKTWFWQDVKTRKILGWRCDVSENIDSIRLSFMDVVTRYSIPEDFHITIDNTRGA

ANKWLTGGAPNRYRFKVKEDDPKGLFLLMGAKMHWTSVVAGKGWGQAKPVERAFGVGGLEEYVDKHPALA

GAYTGPNPQAKPDNYGDHAVDAELFLKTFAEGVAMFNARTGRETEMCGGKLSFDDVFEREYARTIVRKPT

EEQKRMLLLPAEAVNVSRKGEFTLKVGGSLKGAKNVYYNMALMNAGVKKVVVRFDPQQLHSTVY

>gi|30062190|ref|NP_836361.1| repressor protein [Shigella flexneri 2a str. 2457T]

MKSANEINQWMTPKQITELDGMPGTIQGVHKRAKKEGWPKRSQEGRRGPGVEYIPAIPAMQKIAIEEMNA

DTLKMFSLLINKLGENEVREIELNMAKYGLSGLMQERPSISVDTLLATLGIDRQTLQTALALHKLPPETR

QEILSMYGVHKQEEPVAPLLEPQDAKKAV

>gi|30062189|ref|NP_836360.1| C4-dicarboxylate transporter DcuC [Shigella flexneri 2a str. 2457T]

MLTFIELLIGVVVIVGVARYIIKGYSATGVLFVGGLLLLIISAIMGHKVLPSSQASTGYSATDIVEYVKI

LLMSRGGDLGMMIMMLCGFAAYMTHIGANDMVVKLASKPLQYINSPYLLMIAAYFVACLMSLAVSSATGL

GVLLMATLFPVMVNVGISRGAAAAICASPAAIILAPTSGDVVLAAQASEMSLIDFAFKTTLPISIAAIIG

MAIAHFFWQRYLDKKEHISHEMLDVSEITTTAPAFYAILPFTPIIGVLIFDGKWGPQLHIITILVICMLI

ASILEFLRSFNTQKVFSGLEVAYRGMADAFANVVMLLVAAGVFAQGLSTIGFIQSLISIATSFGSASIIL

MLVLVILTMLAAVTTGSGNAPFYAFVEMIPKLAHSSGINPAYLTIPMLQASNLGRTLSPVSGVVVAVAGM

AKISPFEVVKRTSVPVLVGLVIVIVATELMVPGTAAAVTGK

>gi|30062188|ref|NP_836359.1| palmitoyl transferase [Shigella flexneri 2a str. 2457T]

MNVSKYVAIFSFVFIQLISVGKVFANADERMTTFRENIAQTWQQPEHYDLYIPAITWHARFAYDKEKTDR

YNERPWGGGFGLSRWDEKGNWHGLYAMAFKDSWNKWEPIAGYGWESTWRPLADENFHLGLGFTAGVTARD

NWNYIPLPVLLPLASVGYGPVTFQMTYIPGTYNNGNVYFAWMRFQF

>gi|30062187|ref|NP_836358.1| cold shock protein CspE [Shigella flexneri 2a str. 2457T]

MSKIKGNVKWFNESKGFGFITPEDGSKDVFVHFSAIQTNGFKTLAEGQRVEFEITNGAKGPSAANVIAL

>gi|30062186|ref|NP_836357.1| camphor resistance protein CrcB [Shigella flexneri 2a str. 2457T]

MLQLLLAVFIGGGTGSVARWLLSMRFNPLHQAIPLGTLAANLIGAFIIGMGFAWFSRMTNIDPVWKVLIT

TGFCGGLTTFSTFSAEVVFLLQEGRFGWALLNVFVNLLGSFAMTALAFWLFSASTVH

>gi|30062185|ref|NP_836356.1| amidase [Shigella flexneri 2a str. 2457T]

MMTTILTIHLPSTPGRAWNMLVALQAGNIVARYAKLHLYDAFAIQESRHVDAGNEIAPLLEVEGMKVGLM

TCYDLRFPELALAQALQGAEILVLPAAWVRGPLKEHHWSTLLAARALDTTCYMVAAGECGNKNIGQSRII

DPFGVTIAAASEMPALIMAEVTPERVRQVRAQLPILNNRRFAPPQLL

>gi|30062184|ref|NP_836355.1| twin arginine translocase E [Shigella flexneri 2a str. 2457T]

MGEISITKLLVVAALVVLLFGTKKLRTLGGDLGAAIKGFKKAMNDDDAAAKKGADVDLQAEKLSHKE

>gi|30062183|ref|NP_836354.1| lipoyl synthase [Shigella flexneri 2a str. 2457T]

MSKPIVMERGVKYRDADKMALIPVKNVATEREALLRKPEWMKIKLPADSTRIQGIKAAMRKNGLHSVCEE

ASCPNLAECFNHGTATFMILGAICTRRCPFCDVAHGRPVAPDANEPVKLAQTIADMALRYVVITSVDRDD

LRDGGAQHFADCITAIREKSPQIKIETLVPDFRGRMDRALDILTATPPDVFNHNLENVPRIYRQVRPGAD

YNWSLKLLERFKEAHPEIPTKSGLMVGLGETNEEIIEVMRDLRRHGVTMLTLGQYLQPSRHHLPVQRYVS

PDEFDEMKAEALAMGFTHAACGPFVRSSYHADLQAKGMEVK

>gi|30062182|ref|NP_836353.1| DNA-binding transcriptional regulator [Shigella flexneri 2a str. 2457T]

MLNLTPSAISQSIQKLRVIFPDPLFIRKGQGVTPTAFAMHLHEYISQGLESILGALDIEGSYDKQRTITI
ATTPSVGALVLPVIYRAIKTHYPQLLLRNPPISDAENQLSQFQTDLIIDNMFCTNRTVQHHVLFTDNMVL
ICRKGNPLLSLEDDRETIDNAAHVLLLPEGQNFSGLRQRVQEMFPDRQINFTSYNILTIAALVANSDMLA
IIPSRFYNLFSRCWPLEKLPFPSLNEEQIDFSIHYNKFILRDPILHGVIDVIRNAF

>gi|30062180|ref|NP_836351.1| hypothetical protein S0672 [Shigella flexneri 2a str. 2457T]
MKTKLNELLEFPTPFTYKVMGQALPELVDQVVEVVQRHAPGDYTPTVKPSSKGNYHSVSITINATHIEQV
ETLYEELGKIDIVRMVL

>gi|30062179|ref|NP_836350.1| D-alanyl-D-alanine carboxypeptidase [Shigella flexneri 2a str. 2457T]
MNTIFSARIMKRLALTTALCTAFISAAHADDLNIKTMIPGVPQIDAESYILIDYNSGKVLAEQNADVRRD
PASLTKMMTSYVIGQAMKAGKFKETDLVTIGNDAWATGNPVFKGSSLMFLKPGMQVPVSQLIRGINLQSG
NDACVAMADFAAGSQDAFVGLMNSYVNALGLKNTHFQTVHGLDADGQYSSARDMALIGQALIRDVPNEYS
IYKEKEFTFNGIRQLNRNGLLWDNSLNVDGIKTGHTDKAGYNLVASATEGQMRLISAVMGGRTFKGREAE
SKKLLTWGFRFFETVNPLKVGKEFASEPVWFSDSDRASLGVDKDVYLTIPRGRMKDLKASYVLNSSELHA
PLQKNQVVGTINFQLDGKTIEQRPLVVLQEIPEGNFFGKIIDYIKLMFHHWFG

>gi|30062178|ref|NP_836349.1| rare lipoprotein A [Shigella flexneri 2a str. 2457T]
MRKQWLGICIAAGMLAACTSDDGQQQTVSVPQPAVCNGPIVEISGADPRFEPLNATANQDYQRDGKSYKI
VQDPSRFSQAGLAAIYDAEPGSNLTASGEAFDPTKLTAAHPTLPIPSYARITNLANGRMIVVRINDRGPY
GNDRVISLSRAAADRLNTSNNTKVRIDPIIVAQDGSLSGPGMACTTVAKQTYALPAPPDLSGGAGTSSVS
GPQGDILPVSNSTLKSEDPTGAPVTSSGFLGAPTTLAPGVLEGSEPTPAPQPVVTASSTTPATSPAMVTP
QAASQSASGNFMVQVGAVSDQARAQQYQQQLGQKFGVPGRVTQNGAVWRIQLGPFASKAEASTLQQRLQT
EAQLQSFITTAQ

>gi|30062177|ref|NP_836348.1| cell wall shape-determining protein [Shigella flexneri 2a str. 2457T]

MTDNPNKKTFWDKVHLDPTMLLILLALLVYSALVIWSASGQDIGMMERKIGQIAMGLVIMVVMAQIPPRV

YEGWAPYLYIICIILLVAVDAFGAISKGAQRWLDLGIVRFQPSEIAKIAVPLMVARFINRDVCPPSLKNT

GIALVLIFMPTLLVAAQPDLGTSILVALSGLFVLFLSGLSWRLIGVAVVLVAAFIPILWFFLMHDYQRQR

VMMLLDPESDPLGAGYHIIQSKIAIGSGGLRGKGWLHGTQSQLEFLPERHTDFIFAVLAEELGLVGILIL

LALYILLIMRGLWIAARAQTTFGRVMAGGLMLILFVYVFVNIGMVSGILPVVGVPLPLVSYGGSALIVLM

AGFGIVMSIHTHRKMLSKSV

>gi|30062176|ref|NP_836347.1| penicillin-binding protein 2 [Shigella flexneri 2a str. 2457T]

MKLQNSFRDYTAESALFVRRALVAFLGILLLTGVLIANLYNLQIVRFTDYQTRSNENRIKLVPIAPSRGI

IYDRNGIPLALNRTIYQIEMMPEKVDNVQQTLDALRSVVDLTDDDIAAFRKERARSHRFTSIPVKTNLTE

VQVARFAVNQYRFPGVEVKGYKRRYYPYGSALTHVIGYVSKINDKDVERLNNDGKLANYAATHDIGKLGI

ERYYEDVLHGQTGYEEVEVNNRGRVIRQLKEVPPQAGHDIYLTLDLKLQQYIETLLAGSRAAVVVTEPRT

GGVLALVSTPSYDPNLFVDGISSKDYSALLNDPNTPLVNRATQGVYPPASTVKPYVAVSALSAGVITRNT

TLFDPGWWQLPGSEKRYRDWKKWGHGRLNVTRSLEESADTFFYQVAYDMGIDRLSEWMGKFGYGHYTGID

LAEERSGNMPTREWKQKRFKKPWYQGDTIPVGIGQGYWTATPIQMSKALMILINDGIVKVPHLLMSTAED

GKQVPWVQPHEPPVGDIHSGYWELAKDGMYGVANRPNGTANKYFASAPYKIAAKSGTAQVFGLKANETYN

AHKIAERLRDHKLMTAFAPYNNPQVAVAMILENGGAGPAVGTLMRQILDHIMLGDNNTDLPAENPAVAAA

EDH

>gi|30062175|ref|NP_836346.1| rRNA large subunit methyltransferase [Shigella flexneri 2a str. 2457T]

MKLQLVAVGTKMPDWVQTGFTEYLRRFPKDMPFELIEIPAGKRGKNADIKRILDKEGELMLAAAGKNRIV

TLDIPGKPWDTPQLAAELERWKLDGRDVSLLIGGPEGLSPACKAAAEQSWSLSALTLPHPLVRVLVAESL

YRAWSITTNHPYHRE

>gi|30062174|ref|NP_836345.1| hypothetical protein S0666, partial [Shigella flexneri 2a str. 2457T]

MIICTGTSSRHVMSIADHVVQESRAAGLLPLGVEGENSADWIVVDLGDVIVHVMQEESRRLYELEKLWS

>gi|30062173|ref|NP_836344.1| alpha-ribazole phosphatase [Shigella flexneri 2a str. 2457T]

MRLWLIRHGETQANVDGLYSGHAPTPLTARGIEQAQNLHTLLDDVSFDLVLCSELERAQHTARLVLSDRQ

LPVHIIPELNEMFFGDWEMRHHRDLMQEDAENYSAWCNDWQHAIPTNGEGFQAFSQRVERFIARLSEYQH

YQNILIVSHQGVLSLLIARLIGMPAESMWHFRVDQGCWSAIDINQKFATLRVLNSRAIGVENA

>gi|30062172|ref|NP_836343.1| nicotinic acid mononucleotide adenylyltransferase [Shigella flexneri 2a str. 2457T]

MKSLQALFGGTFDPVHYGHLKPVETLANLIGLTRVTIIPNNVPPHRPQPEANSVQRKHMLELAIADKPLF

TLDERELKRNAPSYTAQTLKEWRQEQGPDVPLAFIIGQDSLLTFPTWYEYETILDNAHLIVCRRPGYPLE

MAQPQYQQWLEDHLTHNPEDLHLQPAGKIYLAETPWFNISATIIRERLQNGESCEDLLPEPVLTYINQQG

LYR

>gi|30062171|ref|NP_836342.1| DNA polymerase III subunit delta [Shigella flexneri 2a str. 2457T]

MIRLYPEQLRAQLNEGLRAAYLLLGNDPLLLQESQDAVRQVAAAQGFEEHHTFSIDPNTDWNAIFSLCQA

MSLFASRQTLLLLLPENGPNAAINEQLLTLTGLLHDDLLLIVRGNKLSKAQENAAWFTALANRSVQVTCQ

TPEQAQLPRWVAVRAKQLNLELDDAANQVLCYCYEGNLLALAQALERLSLLWPDGKLTLPRVEQAVNDAA

HFTPFHWVDALLMGKSKRALHILQQLRLEGSEPVILLRTLQRELLLLVNLKRQSAHTPLRALFDKHRVWQ

NRRGMMGEALNRLSQPQLRQAVQLLTRTELTLKQDYGQSVWAELEGLSLLLCHKPLADVFIDG

>gi|30062170|ref|NP_836341.1| LPS-assembly lipoprotein RlpB [Shigella flexneri 2a str. 2457T]

MRYLATLLLSLAVLITAGCGWHLRDTTQVPSTMKVMILDSGDPNGPLSRAVRNQLRLNGVELLDKETTRK

DVPSLRLGKVSIAKDTASVFRNGQTAEYQMIMTVNATVLIPGRDIYPISAKVFRSFFDNPQMALAKDNEQ

DMIVKEMYDRAAEQLIRKLPSIRAADIRSDEEQTSTTTDTPATPARVSTMLGN

>gi|30062169|ref|NP_836340.1| leucyl-tRNA synthetase [Shigella flexneri 2a str. 2457T]

MQEQYRPEEIESKVQLHWDEKRTFEVTEDESKEKYYCLSMLPYPSGRLHMGHVRNYTIGDVIARYQRMLG

KNVLQPIGWDAFGLPAEGAAVKNNTAPAPWTYDNIAYMKNQLKMLGFGYDWSRELATCTPEYYRWEQKFF

TELYKKGLVYKKTSAVNWCPNDQTVLANEQVIDGCCWRCDTKVERKEIPQWFIKITAYADELLNDLDKLD

HWPDTVKTMQRNWIGRSEGVEITFNVNDYDNTLTVYTTRPDTFMGCTYLAVAAGHPLAQKAAENNPELAA

FIDECRNTKVAEAEMATMEKKGVDTGFKAVHPLTGEEIPVWAANFVLMEYGTGAVMAVPGHDQRDYEFAS

KYGLNIKPVILAADGSEPDLSQQALTEKGVLFNSGEFNGLDHEAAFNAIADKLTAMGVGERKVNYRLRDW

GVSRQRYWGAPIPMVTLEDGTVMPTPDDQLPVILPEDVVMDGITSPIKADPEWAKTTVNGMPALRETDTF

DTFMESSWYYARYTCPEYKEGMLDSEAANYWLPVDIYIGGIEHAIMHLLYFRFFHKLMRDAGMVNSDEPA

KQLLCQGMVLADAFYYVGENGERNWVSPVDAIVERDEKGRIVKAKDAAGHELVYTGMSKMSKSKNNGIDP

QVMVERYGADTVRLFMMFASPADMTLEWQESGVEGANRFLKRVWKLVYEHTAKGDVAALNVDALTEDQKA

LRRDVHKTIAKVTDDIGRRQTFNTAIAAIMELMNKLAKAPTDGEQDRALMQEALQAVVRMLNPFTPHICF

TLWQELKGEGDIDNAPWPVADEKAMVEDSTLVVVQVNGKVRAKITVPVDATEEQVRERAGQEHLVAKYLD

GVTVRKVIYVPGKLLNLVVG

>gi|30062168|ref|NP_836339.1| hypothetical protein S0660 [Shigella flexneri 2a str. 2457T]

MNKVAQYYRELVASLNERLRNGERDIDALVEQARERVIKTGELTRTEIDELTRAVRRDLEEFAMSYEESL

KEESDSVFMRVIKESLWQELADITDKTQLEWREIFQDLNHHGVYHSGEVVGLGNLVCEKCHFHLPIYTPE

VLTLCPKCGYDQFQRRPFEP

>gi|30062167|ref|NP_836338.1| hypothetical protein S0659 [Shigella flexneri 2a str. 2457T]

MIMIFTSSCCDNLSIDEIIERAEKGDCEAQYIVGFYYNRDSAIDSPDDEKAFYWLKLAAEQGHCEAQYSL

GQKYTEDKSRHKDNEQAIFWLKKAALQGHTFASNALGWILDRGEDPNYKEAVTWYQIAAESGMSYAQNNL

GWMYRNGNGVAQDYTLAFFWYKQAALQGHSYAQDNLADLYKDGEGVAQNKTLAAFWYLKSAQQGNRHAQF

QIAWDYNAGEGVDQDYKQAMYWYLKAAAQESVGAYVNIGYMYKHGQGVEKDYQAAFEWFTKAAECNDATA
WYNLAIMYHYGEGRPVDLRQALDLYRKVQSSGTRDVSQEIRETEALL

>gi|30062166|ref|NP_836337.1| hypothetical protein S0658 [Shigella flexneri 2a str. 2457T]
MDMGSQKILFALSTPMEIRNECCLPSHSSPKMYLGTCFFDLSSSWGIDDRDDLLRIIHRMIENGHAARLA
GFYHRWFRYSPCEWRDYLAELNEQGQAYAQFVASTAECCGEGGIKAWDYVRMGFLSRMGVLNNWLSEEES
LWIQSRIHLRALRYYSNWRQYFAGYTFGRQYWQSPEDDHLPLLREFLARKEYDDSGNDMFYQLFASDDAY
YPTLSWQPLADYPTCPETLKDMSDL

>gi|30062165|ref|NP_836336.1| enzyme [Shigella flexneri 2a str. 2457T]
MKNCWKILDIEETTDVDIIRRAYLALLPSFHPETDPQGFKQLRQAYEEALRIAQSPAKSVWQPEEYEVAE
HEILLAFRALLASDSERFLPSAWQRFIQQLNYCSMEEIDELRWSLCTIAMNTAHLSFECVVLLAERLRWL
QEENVGEIDEEELESFLYAIAKGNVFNFQTILHLPVAVQNDTIDFYQMFARIWSSHPEWLTLYLAQHRAV
IIPDDAKLHRNLLRWYSASRLGIPELLDYARSWREAEPDNEDARYYEYAQRVYCGEGESLLAELCYYWRE
YPSTQADALMLQWCRQHRVYYYPLVVMMIEARDLVNDQGKPLLYVPGDSARTRFHLYEILSDEKLSALGR
SLVEMVLHKGRKPRISLTRDTEHPLWPLYLVAKQLVQASQPTEESLMPIVSRLDAEDRCPLEALIIRRLL
IQAANFTGQETVEPEPQPQPMPVDDGGLGCLGVIKIIFYIFIFAGLIGKILHLFG

>gi|30062164|ref|NP_836335.1| hypothetical protein S0656 [Shigella flexneri 2a str. 2457T]
MNKKLMYIFAIFIVAAITCISQPKKTMLRDKAMVNYAFDYLRSPGSLPLTTAATELSAIHGHSTSQYRLG
EFYLHGSDGKPLDYTQARYWYEQSAEQENPRAQSKLGWIYLKGLGVKPDTRKAILWYKEAAEQGYAHAQY
TLGLIYRNGTGINVNHYESQKWLKLAAKQHYKNAERLLAGLPAH

>gi|30062163|ref|NP_836334.1| tRNA ligase [Shigella flexneri 2a str. 2457T]
MNKEEQYLLFALSAPMEILNQSCKPAHDSPKMYTGIKEFDLSSSWGINNRDDLIQTIYQMTDDGHANDLA

GLYLTWQRSSPEEWKALIAGGSERGLIYTQFVAQTAMCCGEGGIKAWDYVRMGFLSRAGVLNNWLTEEES

LWLQSRVYARAHHYYHSWMHYFSAYSLGRLYWQSSQCEDNASLREALTLYKYDSAGSRMFEELAAGSDRF

YATLPWQPLTVQPECPVTLKDVSDL

>gi|30062162|ref|NP_836333.1| hypothetical protein S0654 [Shigella flexneri 2a str. 2457T]

MKTCWQILEIESTTQIDIIRQAYLARLPLCHPETDPQGFKALRQAYEEALRLAVNPVEEADDEEKDATAE

HEILRAFRTLLDSESDRFQPSAWQKFIQQLNTWNMEDVDQLRWPLCAIAIEARYLSLNCASLLAERLNWH

SFNDSEGMDEEEREAFLEAIQAGDCFDFLSLLEYPVALQNQTVEYYFALERCCRYHPDYVTAFLAMEGPW

FIPDDAKLHRKLLRWYSSVQTGMVELIPVAKQWQAEEPESEDARYYQCAQRLYCGEGESLLADLGAYWES

YPSTQADNLLLQWSKRHCPDYFALLVMVIEARSMVDAQGQPLKYVPGESARTRLLWAEILHSGKLSPLGQ

SFIESLFFKRKAWAWWKSRVGSETEEDSPLLDLYRVAEQVVLEAFPKQEMLARLNTRLEGGDAHPLEAII

TRMLLTKVKLEPEDEDVDEPTPENHEEKNDEGEKPQSITSIIKISLTVLVIGYALGKIAMLFS

>gi|30062161|ref|NP_836332.1| ribonucleoside hydrolase 1 [Shigella flexneri 2a str. 2457T]

MALPILLDCDPGHDDAIAIVLALASPELDVKAITSSAGNQTPEKTLRNVLRMLTLLNRTDIPVAGGAVKP

LMRELIIADNVHGESGLDGPALPEPTFAPQNCTAVELMAKTLRESAEPVTIVSTGPQTNVALLLNSHPEL

HSKIARIVIMGGAMGLGNWTPAAEFNIYVDPEAAEIVFQSGIPVVMAGLDVTHKAQIHVEDTERFRAIGN

PVSTIVAELLDFFLEYHKDEKWGFVGAPLHDPCTIAWLLKPELFTTVERWVGVETQGKYTQGMTVVDYYY

LTGNKPNATVIVDVDRQGFVDLLADRLKFYA

>gi|30062160|ref|NP_836331.1| ATP-binding protein of glutamate/aspartate transport system [Shigella flexneri 2a str. 2457T]

MITLKNVSKWYGHFQVLTDCSTEVKKGEVVVVCGPSGSGKSTLIKTVNGLEPVQQGEITVDGIVVNDKKT

DLAKLRSRVGMVFQHFELFPHLSIIENLTLAQVKVLKRDKAPAREKALKLLERVGLSAHANKFPAQLSGG

QQQRVAIARALCMAPIAMLFDEPTSALDPEMINEVLDVMVELANEGMTMMVVTHEMGFARKVANRVIFMD

EGKIVEDSPKDAFFDDPKSDRAKDFLAKILH

>gi|30062159|ref|NP_836330.1| glutamate/aspartate transport system permease [Shigella flexneri 2a str. 2457T]

MYEFDWSSIVPSLPYLLDGLVITLKITVTAVVIGILWGTMLAVMRLSSFAPVSWFAKAYVNVFRSIPLVM

VLLWFYLIVPGFLQNVLGLSPKNDIRLISAMVAFSMFEAAYYSEIIRAGIQSISRGQSSAALALGMTHWQ

SMKLIILPQAFRAMVPLLLTQGIVLFQDTSLVYVLSLADFFRTASTIGERDGTQVEMILFAGFVYFVISL

SASLLVSYLKRRTA

>gi|30062158|ref|NP_836329.1| glutamate/aspartate transport system permease [Shigella flexneri 2a str. 2457T]

MSIDWNWGIFLQQAPFGNTTYLGWVWSGFQVTIALSICAWIIAFLVGSFFGILRTVPNRFLSGLGTLYVE

LFRNVPLIVQFFTWYLVIPELLPEKIGMWFKAELDPNIQFFLSSMLCLGLFTAARVCEQVRAAIQSLPRG

QKNAALAMGLTLPQTYRYVLLPNAYRVIVPPMTSEMMNLVKNSAIASTIGLVDMAAQAGKLLDYSAHAWE

SFTAITLAYVLINAFIMLVMTLVERKVRLPGNMGGK

>gi|30062157|ref|NP_836328.1| glutamate and aspartate transporter subunit [Shigella flexneri 2a str. 2457T]

MQLRKPATAILALALSAGLAQADDAAPAAGSTLDKIAKNGVIVVGHRESSVPFSYYDNQQKVVGYSQDYS

NAIVEAVKKKLNKPDLQVKLIPITSQNRIPLLQNGTFDFECGSTTNNVERQKQAAFSDTIFVVGTRLLTK

KGGDIKDFADLKGKAVVVTSGTTSEVLLNKLNEEQKMNMRIISAKDHGDSFRTLESGRAVAFMMDDALLA

GERAKAKKPDNWDIVGKPQSQEAYGCMLRKDDPQFKKLMDDTIAQVQTSGEAEKWFDKWFKNPIPPKNLN

MNFELSDEMKALFKEPNDKALN

>gi|30062156|ref|NP_836327.1| apolipoprotein N-acyltransferase [Shigella flexneri 2a str. 2457T]

MDFASLIERQRIRLLLALLFGACGTLAFSPYDVWPAAIISLMGLQALTFNRRPLQSAAIGFCWGFGLFGS

GINWVYYVSIATFGGMPGPVNIFLVVLLAAYLSLYTGLFAGVLSRLWPKTTWLRVAIATPALWQVTEFLRG

WVLTGFPWLQFGYSQIDGPLKGLAPLMGVEAINFLLMMVSGLLALALVKRNWRPLVVAVVLFALPFPLRY

IQWFTPQPEKTIQVSMVQGDIPQSLKWDEGQLLNTLKIYYNATAPLMGKSSLIIWPESAITDLEINQQPF

LKALDGELRDKGSSLVTGIVDARLNKQNRYDTYNTIITLGKGAPYSYESADRYNKNHLVPFGEFVPLESI

LRPLAPFFDLPMSSFSRGPYIQPPLSANGIELTAAICYEIILAEQVRDNFRPDTDYLLTISNDAWFGKSI

GPWQHFQMARMRALELARPLLRSTNNGITAVIGPQGEIQAMIPQFAREVLTTNVTPTTGLTPYARTGNWP

LWVLTALFGFAAVLMSLRQRRK

>gi|30062155|ref|NP_836326.1| transporter [Shigella flexneri 2a str. 2457T]

MSDDNSHSSDTISNKKGFFSLLLSQLFHGEPKNRDELLALIRDSGQNDLIDEDTRDMLEGVMDIADQRVR

DIMIPRSQMITLKRNQTLDECLDVIIESAHSRFPVISEDKDHIEGILMAKDLLPFMRSDAEAFSMDKVLR

QAVVVPESKRVDRMLKEFRSQRYHMAIVIDEFGGVSGLVTIEDILELIVGEIEDEYDEEDDIDFRQLSRH

TWTVRALASIEDFNEAFGTHFSDEEVDTIGGLVMQAFGHLPARGETIDIDGYQFKVAMADSRRIIQVHVK

IPDDSPQPKLDE

>gi|30062154|ref|NP_836325.1| metalloprotease [Shigella flexneri 2a str. 2457T]

MSQVILDLQLACEDNSGLPEESQFQTWLNAVIPQFQEESEVTIRVVDTAESHSLNLTYRGKDKPTNVLSF

PFEVPPGMEMSLLGDLVICRQVVEKEAQEQGKPLEAHWAHMVVHGSLHLLGYDHIEDDEAEEMEALETEI

MLALGYEDPYIAEKE

>gi|30062153|ref|NP_836324.1| ATP-binding protein in pho regulon [Shigella flexneri 2a str. 2457T]

MFSFRRHTDKRNGLNIDTREITLEPADNARLLSLCGPFDDNIKQLERRLGIEINRRDNHFKLTGRPICVT

AAADILRSLYVDTAPMRGQIQDIEPEQIHLAIKEARVLEQSAESVPEYGKAVNIKTKRGVIKPRTPNQAQ

YIANILDHDITFGVGPAGTGKTYLAVAAAVDALERQEIRRILLTRPAVEAGEKLGFLPGDLSQKVDPYLR

PLYDALFEMLGFEKVEKLIERNVIEVAPLAYMRGRTLNDAFIILDESQNTTIEQMKMFLTRIGFNSKAVI

TGDVTQIDLPRNTKSGLRHAIEVLADVEEISFNFFHSEDVVRHPVVARIVNAYEAWEEAEQKRKAALAAE

RKREEQEQK

>gi|30062152|ref|NP_836323.1| hypothetical protein S0643 [Shigella flexneri 2a str. 2457T]

MTKKLHIKTWGCQMNEYDSSKMADLLDATHGYQLTDVAEEADVLLLNTCSIREKAQEKVFHQLGRWKLLK

EKNPDLIIGVGGCVASQEGEHIRQRAHYVDIIFGPQTLHRLPEMINSVRGDRSPVVDISFPEIEKFDRLP

EPRAEGPTAFVSIMEGCNKYCTYCVVPYTRGEEVSRPSDDILFEIAQLAAQGVREVNLLGQNVNAWRGEN

YDGTTGSFADLLRLVAAIDGIDRIRFTTSHPIEFTDDIIEVYRDTPELVSFLHLPVQSGSDRILNLMGRT

HTALEYKAIIRKLRAARPDIQISSDFIVGFPGETTEDFEKTMKLIADVNFDMSYSFIFSARPGTPAADMV

DDVPEEEKKQRLYILQERINQQAMAWSRRMLGTTQRILVEGTSRKSIMELSGRTENNRVVNFEGTPDMIG

KFVDVEITDVYPNSLRGKVVRTEDEMGLRMAETPESVIARTRKENDLGVGYYQP

>gi|30062151|ref|NP_836322.1| 2-octaprenyl-3-methyl-6-methoxy-1,4-benzoquinol hydroxylase [Shigella flexneri 2a str. 2457T]

MTNQPTEIAIVGGGMVGGALALGLAQHGFSVTVIEHAEPAPFVADSQPDVRISAISAASVSLLKGLGVWD

AVQAMRCHPYRRLETWEWETAHVVFDAAELKLPLLGYMVENTVLQQALWQALEAHPKVTLRVPTSLIALH

RDNDLQELELKGGETIRAKLVIGADGANSQVRQMAGIGVHAWQYAQSCMLISVQCENDPGDSTWQQFTPD

GPRAFLPLFDNWASLVWYDSPTRIRQLQNMNMAQLQAEIAKHFPSRLGYVTPLAAGAFPLTRRHALQYVQ

PGLALVGDAAHTIHPLAGQGVNLGYRDVDALIDVLVNARSYGEAWASYPVLKRYQMRRMADNFIMQSGMD

LFYAGFSNNLPPLRFVRNLGLMAAERAGVLKRQALKYALGL

>gi|30062150|ref|NP_836321.1| asparagine synthetase B [Shigella flexneri 2a str. 2457T]

MCSIFGVFDIKTDAVELRKKALELSRLMRHRGPDWSGIYASDNAILAHERLSIVDVNAGAQPLYNQQKNH

VLAVNGEIYNHQALRAEYGDRYQFQTGSDCEVILALYQEKGPEFLDDLQGMFAFALYDSEKDAYLIGRDH

LGIIPLYMGYDEHGQLYVASEMKSLVPVCRTIKEFPAGSYLWSQDGEIRSYYHRDWFDFDAVKDNVTDKN

ELRQALEDSVKSHLMSDVPYGVLLSGGLDSSIISAITKKYAARRVEDQERSEAWWPQLHSFAVGLPGSPD

LKAAQEVANHLGTVHHEIHFTVQEGLDAIRDVIYHIETYDVTTIRASTPMYLMSRKIKAMGIKMVLSGEG

SDEVFGGYLYFHKAPNVKELHEETVRKLLALHMYDCARANKAMSAWGVEARVPFLDKKFLDVAMRINPQD

KMCGNGKMEKHILRECFESYLPASVAWRQKEQFSDGVGYSWIDTLKEVAAQQVSDQQLETARFRFPYNTP

TSKEAYLYREIFEELFPLPSAAECVPGGPSVACSSAKAIEWDEAFKKMDDPSGRAVGVHQSAYK

>gi|30062149|ref|NP_836320.1| transcriptional repressor of nag (N-acetylglucosamine) operon [Shigella flexneri 2a str. 2457T]

MTPGGQAQIGNVDLVKQLNSAAVYRLIDQYGPISRIQIAEQSQLAPASVTKITRQLIERGLIKEVDQQAS

TGGRRAISIVTETRNFHAIGVRLGRHDATITLFDLSSKVLAEEHYPLPERTQQTLEHALLNAIAQFIDSY

QRKLRELIAISVILPGLVDPDSGKIHYMPHIQVENWGLVEALEERFKVTCFVGHDIRSLALAEHYFGASQ

DCEDSILVRVHRGTGAGIISNGRIFIGRNGNVGEIGHIQVEPLGERCHCGNFGCLETIAANAAIEQRVLN

LLKQGYQSRVPLDDCTIKTICKAANKGDSLASEVIEYVGRHLGKTIAIAINLFNPQKIVIAGEITEADKV

LLPAIESCINTQALKAFRTNLPVVRSELDHRSAIGAFALVKRAMLNGILLQHLLEN

>gi|30062148|ref|NP_836319.1| N-acetylglucosamine-6-phosphate deacetylase [Shigella flexneri 2a str. 2457T]

MYALTQGRIFTGHEFLDDHAVVIADGLIKSVCPVAELPPEIEQRSLNGAILSPGFIDVQLNGCGGVQFND

TAEAVSVETLEIMQKANEKSGCTNYLPTLITTSDELMKQGVRVMREYLAKHPNQALGLHLEGPWLNLVKK

GTHNPNFVRKPDAALVDFLCENADVITKVTLAPEMVPAEVISKLANAGIVVSAGHSNATLKEAKAGFRAG

ITFATHLYNAMPYITGREPGLAGAILDEADIYCGIIADGLHVDYANIRNAKRLKGDKLCLVTDATAPAGA

NIEQFIFAGKTIYYRNGLCVDENGTLSGSSLTMIEGVRNLVEHCGIALDEVLRMATLYPARAIDVEKRLG

TLAAGKVANLTAFTPDFKITKTIVNGNEVVTQ

>gi|30062147|ref|NP_836318.1| glucosamine-6-phosphate deaminase [Shigella flexneri 2a str. 2457T]

MRLIPLTTAEQVGKWAARHIVNRINAFKPTADRPFVLGLPTGGTPMTTYKALVEMHKAGQVSFKHVVTFN

MDEYVGLPKEHPESYYSFMHRNFFDHVDIPAENINLLNGNAPDIDAECRQYEEKIRSYGKIHLFMGGVGN

DGHIAFNEPASSLASRTRIKTLTHDTRVANSRFFDNDVNQVPKYALTVGVGTLLDAEEVMIMVLGSQKAL

ALQAGGEGCVNHMWTISCLQLHPKAIMVCDEPSTMELKVKTLRYFNELEAENIKGL

>gi|30062146|ref|NP_836317.1| N-acetyl glucosamine specific PTS system components IIABC [Shigella flexneri 2a str. 2457T]

MNILGFFQRLGRALQLPIAVLPVAALLLRFGQPDLLNVAFIAQAGGAIFDNLALIFAIGVASSWSKDSAG

AAALAGAVGYFVLTKAMVTINPEINMGVLAGIITGLVGGAAYNRWSDIKLPDFLSFFGGKRFVPIATGFF

CLVLAAIFGYVWPPVQHAIHAGGEWIVSAGALGSGIFGFINRLLIPTGLHQVLNTIAWFQIGEFTNAAGT

VFHGDINRFYAGDGTAGMFMSGFFPIMMFGLPGAALAMYFAAPKERRPMVGGMLLSVAVTAFLTGVTEPL

EFLFMFLAPLLYLLHALLTGISLFVATLLGIHAGFSFSAGAIDYALMYNLPAASQNVWMLLVMGVIFFAI

YFVVFSLVIRMFNLKTPGREDKEDEIVTEEANSNTEEGLTQLATNYIAAVGGTDNLKAIDACITRLRLTV

ADSARVNDTMCKRLGASGVVKLNKQTIQVIVGAKAESIGDAMKKVVARGPVAAASAEATPATAAPVAKPQ

AVPNAVSIAELVSPITGDVVALDQVPDEAFASKAVGDGVAVKPTDKIVVSPAAGTIVKIFNTNHAFCLET

EKGAEIVVHMGIDTVALEGKGFKRLVEEGAQVSAGQPILEMDLDYLNANARSMISPVVCSNIDDFSGLLI

KAQGHVVAGQTPLYEIKK

>gi|30062145|ref|NP_836316.1| glutaminyl-tRNA synthetase [Shigella flexneri 2a str. 2457T]

MSEAEARPTNFIRQIIDEDLASGKHITVHTRFPPEPNGYLHIGHAKSICLNFGIAQDYKGQCNLRFDDTN

PVKEDIEYVDSIKNDVEWLGFHWSGNVRYSSDYFDQLHAYAIELINKGLAYVDELTPEQIREYRGTLTQP

GKNSPYRDRSVEENLALFEKMRAGGFEEGKACLRAKIDMASPFIVMRDPVLYRIKFAEHHQTGNKWCIYP

MYDFTHCISDALEGITHSLCTLEFQDNRRLYDWVLDNITIPVHPRQYEFSRLNLEYTVMSKRKLNLLVTD

KHVEGWDDPRMPTISGLRRRGYTAASIREFCKRIGVTKQDNTIEMASLESCIREDLNENAPRAMAVIDPV

KLVIENYQGEGEMVTMPNHPNKPEMGSRQVPFSGEIWIDRADFREEANKQYKRLVLGKEVRLRNAYVIKA

ERVEKDAEGNITTIFCTYDADTLSKDPADGRKVKGVIHWVSAAHALPVEIRLYDRLFSVPNPGAADDFLS

VINPESLVIKQGFAEPSLKDAVAGKAFQFEREGYFCLDSRHSTAEKPVFNRTVGLRDTWAKVGE

>gi|30062144|ref|NP_836315.1| hypothetical protein S0623 [Shigella flexneri 2a str. 2457T]

MRTFSGKRSTLALAIAGVTAMSGFMAMPEARAEGFIDDSTLTGGIYYWQRERDRKDVTDGDKYKTNLSHS

TWNANLDFQSGYAADMFGLDIAAFTAIEMAENGDSSHPNEIAFSKSNKAYDEDWSGDKSGISLYKAAAKF

KYGPVWARAGYIQPTGQTLLAPHWSFMPGTYQGAEAGANFDYGDAGALSFSYMWTNEYKAPWHLEMDEFY

QNDKTTKVDYLHSIGAKYDFKNNFVLEAAFGQAEGYIDQYFAKASYKFDIAGSPLTTSYQFYGTRDKVDD

RSVNDLYDGTAWLQALTFGYRAADVVDLRLEGTWIKADSQQGYFLQRMTPTYASSNGRLDIWWDNRSDFN

ANGEKAVFFSAMYDLKNWNLPGFAIGASYVYAWDAKPATWQSNPDAYYDKNRTIEESAYSLDAVYTIQDG

RAKGTMFKLHFTEYDNHSDIPSWGGGYGNIFQDERDVKFMVIAPFTIF

>gi|30062143|ref|NP_836314.1| hypothetical protein S0622 [Shigella flexneri 2a str. 2457T]

MKKLILIAMMASGLVACAQSTAPQEDSRLKEAYSACINTAQGSPEKIEACQSVLNVLKKEKQHQQFADQE

SVRVLDYQQCLRATQTGHDQAVKADCDKVWQEIRSNNK

>gi|30062142|ref|NP_836313.1| ferric uptake regulator [Shigella flexneri 2a str. 2457T]

MTDNNTALKKAGLKVTLPRLKILEVLQEPDNHHVSAEDLYKRLIDMGEEIGLATVYRVLNQFDDAGIVTR

HNFEGGKSVFELTQQHHHDHLICLDCGKVIEFSDDSIEARQREIAAKHGIRLTNHSLYLYGHCAEGDCRE

DEHAHEGK

>gi|30062141|ref|NP_836312.1| flavodoxin FldA [Shigella flexneri 2a str. 2457T]

MAITGIFFGSDTGNTENIAKMIQKQLGKDVADVHDIAKSSKEDLEAYDILLLGIPTWYYGEAQCDWDDFF

PTLEEIDFNGKLVALFGCGDQEDYAEYFCDALGTIRDIIEPRGATIVGHWPTAGYHFEASKGLADDDHFV

GLAIDEDRQPELTAERVEKWVKQISEELHLDEILNA

>gi|30062140|ref|NP_836311.1| LexA regulated protein [Shigella flexneri 2a str. 2457T]

MYYGALSIRAEAWLIVSPEVTKIMAKEQTDRTTLDLFAHERRPGRPKTNPLSRDEQLRINKRNQLKRDKV

RGLKRVELKLNAEAVEALNELAESRNMSRSELIEEMLMQQLAALRSQGIV

>gi|30062139|ref|NP_836310.1| hypothetical protein S0618 [Shigella flexneri 2a str. 2457T]

MKLNIRAQTAQNQHNNSPIVLVHGLFGSLDNLGVLARDLVNDHNIIQVDMRNHGLSPREPVMNYPAMAQD

LVDTLDALQIDKATFIGHSMGGKAVMALTALAPDRIDKLVAIDIAPVDYHVRRHDEIFAAINAVSESDAQ

TRQQAAAIMRQHLNEEGVIQFLLKSFVDGEWRFNVPVLWDQYPHIVGWEKIPAWDHPALFIPGGNSPYVS

EQYRDDLLAQFPQARAHVIAGAGHWVHAEKPDAVLRAIRRYLND

>gi|30062138|ref|NP_836309.1| replication initiation regulator SeqA [Shigella flexneri 2a str. 2457T]

MKTIEVDDELYSYIASHTKHIGESASDILRRMLKFSAASQPAAPVTKEVRVASPAIVEAKPVKTIKDKVR

AMRELLLSDEYAEQKRAVNRFMLLLSTLYSLDAQAFAEATESLHGRTRVYFAADEQTLLKNGNQTKPKHV

PGTPYWVITNTNTGRKCSMIEHIMQSMQFPAELIEKVCGTI

>gi|30062137|ref|NP_836308.1| phosphoglucomutase [Shigella flexneri 2a str. 2457T]

MAIHNRAGQPAQQSDLINVAQLTAQYYVLKPEAGNAEHAVKFGTSGHRGSAARHSFNEPHILAIAQAIAE

ERAKNGITGPCYVGKDTHALSEPAFISVLEVLAANGVDVIVQENNGFTPTPAVSNAILVHNKKGGPLADG

IVITPSHNPPEDGGIKYNPPNGGPADTNVTKVVEDKANALMADGLKGVKRISLDEAMASGYVKEQDLVQP

FVEGLADIVDMAAIQKAGLTLGVDPLGGSGIEYWKRIGEYYNLNLTIVNDQVDQTFRFMHLDKDGAIRMD

CSSECAMAGLLALRDKFDLAFANDPDYDRHGIVTPAGLMNPNHYLAVAINYLFQHRPQWGKDVAVGKTLV

SSAMIDRVVNDLGRKLVEVPVGFKWFVDGLFDGSFGFGGEESAGASFLRFDGTPWSTDKDGIIMCLLAAE

ITAVTGKNPQEHYNELAKRFGAPSYNRLQAAATSAQKAALSKLSPEMVSASTLAGDPITARLTAAPGNGA

SIGGLKVMTDNGWFAARPSGTEDAYKIYCESFLGEEHRKQIEKEAVEIVSEVLKNA

>gi|30062136|ref|NP_836307.1| putrescine transporter [Shigella flexneri 2a str. 2457T]

MSQAKSNKMGVVQLTILTMVNMMGSGIIMLPTKLAEVGTISIISWLVTAVGSMALAWAFAKCGMFSRKSG

GMGGYAEYAFGKSGNFIANYTYGVSLLIANVAIAISAVGYGTELLGASLSPVQIGLATIGVLWICTVANF

GGARITGQISSITVWGGIIPVVGLCIIGWFWFSPTLYVDSWNPHHAPFFSAVGSSIAMTLWAFLGLESAC

ANTDVVENPERNVPIAVLGGTLGAAVIYIVSTNVIAGIVPNMELANSTAPFGLAFAQMFTPEVGKVIMAL

MVMSCCGSLLGWQFTIAQVFKSSSDEGYFPKIFSRVTKVDAPVQGMLTIVIIQSGLALMTISPSLNSQFN

VLVNLAVVTNIIPYILSMAALVIIQKVANVPPSKAKVANFVAFVGAMYSFYALYSSGEEAMLYGSIVTFL

GWTLYGLVSPRFELKNKHG

>gi|30062135|ref|NP_836306.1| potassium-transporting ATPase subunit C [Shigella flexneri 2a str. 2457T]

MSGLRPALSTFLFLLLITGGVYPLLTTALGQWWYPWQANGSLIREGDTVRGSALIGQNFTGNGYFHGRPS

ATAEMPYNPQASGGSNLAVSNPELDKQIAARVAALRAANPDASTNVPVELVTASASGLDNNITPQAAAWQ

IPRIAKARNLSVEQLTQLIAKYSQQPLVKYIGQPVVNIVELNLALDKLDE

>gi|30062134|ref|NP_836305.1| potassium-transporting ATPase subunit A [Shigella flexneri 2a str. 2457T]

MAAQGFLLIATFLLVLMVLARPLGSGLARLINDIPLPGTAGVERILFRLPGVSDHEMNWKQYLCAILGLN

MLGLAVLFFMLLGQHYLPLNPQQLPGLSWDLALNTAVSFVTNTNWQSYSGETTLSYFSQMAGLTVQNFLS

AASGIAVIFAFIRAFTRQSMSTLGNAWVDLLRITLWVLVPVALLIALFFIQQGAQQNFLPYQAVNTVEGA

QQLLPMGPVASQEAIKMLGTNGGGFFNANSSHPFENPTALTNFVQMLAIFLIPTALCFAFGEVTGDRRQG

RMLLWAMSVIFVICVGVVMWAEVQGNPHLLALGADSSINMEGKESRFGVLVSSLFAVVTTAASCGAVIAM

HDSFTALGGMVPMWLMQIGEVVFGGVGSGLYGMMLFVLLAVFIAGLMIGRTPEYLGKKIDVREMKLTALA

ILVTPTLVLMGAALAMMTDAGRSAMLNPSPHGFSEVLYAVSSAANNNGSAFAGLSANSPFWNCLLAFCMF

VGRFGVIIPVMAIAGSLVSKKSQPASSGTLPTHGPLFVGLLIGTVLLVGALTFIPALALGPVAEYLS

>gi|30062133|ref|NP_836304.1| hypothetical protein S0608 [Shigella flexneri 2a str. 2457T]

MELYREYPAWLIFLRRTYAVAAGVLALPFMLFWKDRARFYSYLHRVWSKTSDKPVWMDQAEKATGDFY

>gi|30062132|ref|NP_836303.1| IS1 orfA [Shigella flexneri 2a str. 2457T]

MLAWLPFPSDVLPAPLLKAWCVTGKSTAGHQRYLCSHCRKTWQLQFTYTASQPGTHQKIIDMAMNDVGCR
ASARIMGVGLNTVLRHLKNSGRSR

>gi|30062131|ref|NP_836302.1| IS1 orfB [Shigella flexneri 2a str. 2457T]
MSRQCTHYGRWPQHGFTSLKKLRPQSVTSRIQPGSDVIVCAEMDEHWGYVGAKSRQRWLFYAYDRIRRTV
VAHVFGERTLATLERLLSLLSAFEVVVWMTDGWPLYESRLKGKLHVISKRYTQRIERHNLNLRQHLARLV
RKSLSFSKSVELHDKVIGHYLNIKHYQ

>gi|30062130|ref|NP_836301.1| Rhs-family protein [Shigella flexneri 2a str. 2457T]
MEEAFWAARQGDALLHTSFMADVLGAVLEVAANVVIDALIVGTTSLLATSVGITMGCTAVVLTGFVAGVA
MVYTGVTEKVSEACSALANMLFPPQIEGYILTGSGDTWINSKPAARAAATAASRQDIEAQEAQAKAEQEE
AQRQEDARTFRDVAGEYLEMAGAIALAVNPVTGPVLMSRALHDQLSTEEGRSEMVDGVTHFVSELWQPTV
ASAAPGSTPTPDDKIDCHKHPSSLTQFLAQKKAAFLDAPLGTALNLLNPGGMLETAFQGVNAVIGSISNL
FKGDDEPPAAEYIAEGARDVRINSQPATRSGARCTCEARVVNEPGNGAFVSPDVRIGGPLLVVRDIRSGR
SQITLVATVALMFLRPGKMLSKIACFAASVGMSMLTQKATSALPHPVNAATGAKYLADDDDFDFSLPGHF
PLDWQRVYSSRDERTEGMFGQGWSVMYEVSLVCTPGSADENCMTFVSGMGRRLDMEAVLPGGGFYSPGEG
LAVRRGEQGHWLISSDDGQCFLFEADPHHPQRQRLKMLGDRNSNCLNLYYDDRGRIVEISGEQQRPCIRL
YYELAAHPRRVTQIYQHFPETAPLLLRRYSYDEAGHLNGVYDSTGHLLREFAYDENHCMTLHRQPGGEGY
YYQWGWYEGPDDAAWRVTGHHTDSGEQYRLAWDLAQRRLCVTDGLGRTRYHQWDAQNQVTAYQDEAGQVT
TFRWSDEERLLLGMTDPQGGKWRYVYDLQGHITETHDPLGRVEQAQWHPVWHQPETEVDAAGNLLGRRAG
ERATAENSVVPFNRLMSYRGVHYRYDEFGRAVEKEGRSGTQSYRYDAEHRMVEVTTARGTYRYVYDALGR
RTEKQHISPDGKPYNRTKFLWDGMRLAQESRPEGTGSLYIYRDQGSYEPLARVDKAGKEGPNRILYFHTD
VNGAPEEMTDSDGKIVWETGYQVWGNTIQEKDHGGVEQNLRYQGQYLDRETGLHYNLHRYYDPDVGRFMV
TDPIGLRGGLNLYSYAPNPLKYADPLGLTPCAVSNQKANRLLDSSETKVTVRSRSDAEQLFMDRYLGHNY
KNMTGESGPSTKNLMEYLTENKTKAGSYHWDDIKDPSVTKPSYRVSGHGPGNPDGDLPHLQVHQHGGSVR

HIFFPWET

>gi|30062129|ref|NP_836300.1| hypothetical protein S0604 [Shigella flexneri 2a str. 2457T]

MAEHDITPEVTISKTQRRYKVGYVRARHQDRKTGIARYYSQHPSLHLKGDWLKEAGFDTGRGVTVKISEG

GLTIIADSDEVQELRDGMFSVLNES

>gi|30062128|ref|NP_836299.1| hypothetical protein S0603 [Shigella flexneri 2a str. 2457T]

MNLQRFDDSTLIRIFALHELHRLKEHGLTRGALLDYHSRYKLVFLAHSQPEYRKLGPFVADIHQWQNLDD

FYNQYRQRVIVLLSHPANPRDHTNVLMHVQGYFRPHIDSTERQQLAALIDSYRRGEQPLLAPLMRIKHYM

ALYPDAWLSGQRYFELWPRVINLRHSGVL

>gi|30062127|ref|NP_836298.1| deoxyribodipyrimidine photolyase [Shigella flexneri 2a str. 2457T]

MTTHLAWFRQDLRLHDNLALAAACRNSSARVLALYIATPRKWETHNMSPRQAELINAQLNGLQIALAEKG

IPLLFREVDDFVASVEIVKQVCAENSVTHLFYNYQYEVNERARDVDVERALRNVVCEGFDDSVILPPGAV

MTGNHEMYKVFTPFKNAWLKRLREGMPECVAAPKVRSSGSIKPAPSITLNYPRQSFDTAHFPVEEKAAIA

QLRQFCENGAGEYEQQRDFPAVEGTSRLSASLATGGLSPRQCLHRLLAEQPQALDGGAGSVWLSELIWRE

FYRHLMTYYPSLCKHRPFIAWTDRVQWQSNPAHLQAWQKGKTGYPIIDAAMRQLNSTGWMHNRLRMITAS

FLVKDLLIDWREGERYFMSQLIDGDLAANNGGWQWAASTGTDAAPYFRIFNPITQGEKFDREGEFIRRWL

PELRDVPGKAVHEPWKWAQKAGVKLDYPQPIVEHKEARVQTLAAYEAARKGK

>gi|30062126|ref|NP_836297.1| transporter [Shigella flexneri 2a str. 2457T]

MNKHASQPRAIYYVVALQIWEYFSFYGMRALLILYLTNQLKYNDTHAYELFSAYCSLVYVTPILGGFLAD

KVLGNRMAVMLGALLMAIGHVVLGASEIHPSFLYLSLAIIVCGYGLFKSNVSCLLGELYEPTDPRRDGGF

SLMYAAGNVGSIIAPIACGYAQEEYSWAMGFGLAAVGMIAGLVIFLCGNRHFTHTRGVNKKVLRATNFLL

PNWGWLLVLLVATPALITVLFWKEWSVYALIVATIIGLGVLAKIYRKAENQKQRKELGLIVTLTFFSMLF

WAFAQQGGSSISLYIDRFVNRDMFGYTVPTAMFQSINAFAVMLCGVFLAWVVKESVAGNRTVRIWGKFAL

GLGLMSAGFCILTLSARWSAMYGHSSLPLMVLGLAVMGFAELFIDPVAMSQITRIEIPGVTGVLTGIYML

LSGAIANYLAGVIADQTSQASFDASGAINYSINAYIEVFDQITWGALACVGLVLMIWLYQALKFRNRALA

LES

>gi|30062125|ref|NP_836296.1| hydrolase-oxidase [Shigella flexneri 2a str. 2457T]

MKNTELEQLINEKLNSAAISDYAPNGLQVEGKETVQKIVTGVTASQALLDEAVRLGADAVIVHHGYFWKG

ESPVIRGMKRNRLKTLLANDINLYGWHLPLDAHPELGNNAQLAALLGITVMGEIEPLVPWGELTMPVPGM

ELASWIEARLGRKPLWCGDTGPEVVQRVAWCTGGGQSFIDSAARFGVDAFITGEVSEQTIHSAREQGLHF

YAAGHHATERGGIRALSEWLNENTDLDVTFIDIPNPA

>gi|30062124|ref|NP_836295.1| carboxylase [Shigella flexneri 2a str. 2457T]

MQRARCYLIGETAVVLELEPPVTLASQKRIWRLAQRLVDMPNVVEAIPGMNNITVILRNPESLALDAIER

LQRWWEESEALEPESRFIEIPVVYGGAGGPDLAVVAAYCGLSEKQVVELHSSVEYVVWFLGFQPGFPYLG

SLPEQLHTPRRAEPRLLVPAGSVGIGGPQTGVYPLATPGGWQLIGHTSLSLFDPARDEPILLRPGDSVRF

VPQKEGIC

>gi|30062123|ref|NP_836294.1| LamB/YcsF family protein [Shigella flexneri 2a str. 2457T]

MKIDLNADLGEGCASDAELLTLVSSANIACGFHAGDAQTMQAFVREAIKNGVAIGAHPSFPDRENFGRSA

MQLPPETVYAQTLYQIGALATIARAQGGVMRHVKPHGMLYNQAAKEAQLADAIARAVYACDPALVLVGLA

GSELIRAGKQYGLTTREEVFADRGYQADGSLVPRSQPGALIENEEQALAQTLEMVQHGRVKSITGEWATV

AAQTVCLHGDGEHALAFARRLRATFAKKGIVVAA

>gi|30062122|ref|NP_836293.1| endonuclease VIII [Shigella flexneri 2a str. 2457T]

MPEGPEIRRAADNLEAAIKGKPLTDVWFAFPQLKTYQSQLIGQHVTHVETRGKALLTHFPNGLTLYSHNQ

LYGVWRVVDTGEEPQTTRVLRVKLQTADKTILLYSASDIEMLRPEQLTTHPFLQRVGPDVLDPNLTPEVV
KERLLSPRFRNRQFAGLLLDQAFLAGLGNYLRVEILWQVGLTGNHKAKDLNAAQLDALAHALLEIPRFSY
ATRGQVDENKHHGALFRFKVFHRDGELCERCGGIIEKTTLSSRPFYWCPGCQH

>gi|30062121|ref|NP_836292.1| transporter [Shigella flexneri 2a str. 2457T]
MLYRYTKEKIMRERRMPVLQWGMLCVLSLLLSIGFLAVHLPAALLLGPMIAGIIFSMRGITLQLPRSAFL
AAQAILGCMIAQNLTGSILTTLAANWPIVLAILLVTLLSSAIVGWLLVRYSSLPGNTGAWGSSPGGAAAM
VAMAQDYGADIRLVAFMQYLRVLFVAGAAVLVTRMMLGDNAEAVNQQIVWFPPVSINLLLTILLAVVAGT
AGCMLRLPSGTMLIPMLAGAVLQSGQLITIELPEWLLAMAYMAIGWRIGLGFDKQILLRALRPLPQILLS
IFALLAICAGMAWGLTRFMHIDFMTAYLATSPGGLDTVAVIAAGSNADMALIMAMQTLRLFSILLTGPAI
ARFISTYAPKRSA

>gi|30062120|ref|NP_836291.1| chaperone [Shigella flexneri 2a str. 2457T]
MTFMKGLPLLLLVASLCSHAALQPDRTRIVFNANDKATSLRVDNRSDKLPYLAYSWLENEKGEKSDDLLV
ALPPIQRLEPKATTQVRIVKQASTTKLPGDRETLFFYNMREIPPAPEKNSDHAVLQVAIQSRIKVFWRPA
ALRKKAGEKVELQLQVSQQGNQLTLKNPTAYYLTIAYLGRNEKGVLPGFKTVMVAPFSTVNTNTGNYSGS
QFYLGYMDDYGALRMTTLNCSGQCHLQAVEAKK

>gi|30062119|ref|NP_836290.1| outer membrane protein [Shigella flexneri 2a str. 2457T]
MDTVNIYRLSFVSCLVVAMPCALAVEFNLNVLDKSMRDRIDISLLKEKGVIAPGEYFVSVAVNNNQISNE
QKINWHKNDDKTIPCINDLLVDKFGLKPEVRQSLPLINQCVDFSSRPEMLFNFDQANQQLNISIPQAWLA
WHSENWTPPSTWKEGVAGVLMDYNLFASSYRPQDGSSSTNLNAYGTAGINTGAWRLRSDYQLNHTDSDDN
HEQSGEISRTYLFRPLPQLGSKLTLGETDFSPNIFDGFSYTGAALASDDRMLPWELRGYAPQISGIAQTN
ATVTISQSGRVIYQKKVPPGPFIIDDLNQSVQGTLDVKVTEEDGRVNNFQVSAASTPFLTRQGQVRYKLA
AGQPRPSMSHQTENETFFSNEVSWGMLSNTSLYSGLLLSGDDYHSAAMGIGQNMLWLGALSFDVTWASSH

FDTQQDERGLSYRFNYSKQVDATNSTISLAAYRFSDRHFHSYANYLDHKYNDSDAQDEKQTISLSVGQPI

TPLNLNLYANLLHQTWWNADASTTANITAGFNVDIGDWRDISISTSFNTTHYEDKDRDNQIYLSISLPFG

NGGRVGYDMQNSSHSTTHRMSWNDTLDERNSWGMSAGLQSDRPDNGAQVSGNYQHLSSAGEWDISGTYAA

NDYSSVSSSWSGSFTATQYGAAFHRRSSTNEPRLMVSTDGVADIPVQGNLDYTNHFGIAVVPLISSYQPS

TVAVNMNDLPDGVTVAENVIKETWIEGAIGYKSLASRSGKDVNVIIRNASGQFPPLGADIRQDDSGISVG

MVGEEGHAWLSGVAENQKFTVVWGDSQHCSLHLPEHMEDTANRLILPCH

>gi|30062118|ref|NP_836289.1| fimbrial-like protein [Shigella flexneri 2a str. 2457T]

MFKGQKTLAALAVSLLFTAPVYAADEGSGEIHFKGEVIEAPCEIHQDDIDKEVELGQVTTSHINQSHHSD

AVAVDLLLVNCDLENSSNGSGGKISKVAVTFDSSAKTTGADPILNNTSTGEATGVGVRLMNKDQSNIVLG

TATPDIDLAPTSSEQTLNFFAWMEQIDQATPVTPGAVTANATYVLDYK

>gi|30062117|ref|NP_836288.1| type II citrate synthase [Shigella flexneri 2a str. 2457T]

MADTKAKLNLNGDTAVELDVLKGTLGQDVIDIRTLGSKGVFTFDPGFTSTASCESKITFIDGDEGILLHR

GFPIDQLATDSNYLEVCYILLNGEKPTQEQYDEFKTTVTRHTMIHEQITRLFHAFRRDSHPMAVMCGITG

ALAAFYHDSLDVNNPRHREIAAFRLLSKMPTMAAMCYKYSIGQPFVYPRNDLSYAGNFLNMMFSTPCEPY

EVNPILERAMDRILILHADHEQNASTSTVRTAGSSGANPFACIAAGIASLWGPAHGGANEAALKMLEEIS

SVKHIPEFVRRAKDKNDSFRLMGFGHRVYKNYDPRATVMRETCHEVLKELGTKDDLLEVAMELENIALND

PYFIEKKLYPNVDFYSGIILKAMGIPSSMFTVIFAMARTVGWIAHWSEMHSYGMKIARPRQLYTGYEKRD

FKSDIKR

>gi|30062115|ref|NP_836286.1| succinate dehydrogenase cytochrome b556 small membrane subunit [Shigella flexneri 2a str. 2457T]

MVSNASALGRNGVHDFILVRATAIVLTLYIIYMVGFFATSGELTYEVWIGFFASAFTKVFTLLALFSILI

HAWIGMWQVLTDYVKPLALRLMLQLVIVVALVVYVIYGFVVVWGV

>gi|30062114|ref|NP_836285.1| succinate dehydrogenase flavoprotein subunit [Shigella flexneri 2a str. 2457T]

MKLPVREFDAVVIGAGGAGMRAALQISQSGQTCALLSKVFPTRSHTVSAQGGITVALGNTHEDNWEWHMY

DTVKGSDYIGDQDAIEYMCKTGPEAILELEHMGLPFSRLDDGRIYQRPFGGQSKNFGGEQAARTAAAADR

TGHALLHTLYQQNLKNHTTIFSEWYALDLVKNQDGAVVGCTALCIETGEVVYFKARATVLATGGAGRIYQ

STTNAHINTGDGVGMAIRAGVPVQDMEMWQFHPTGIAGAGVLVTEGCRGEGGYLLNKHGERFMERYAPNA

KDLAGRDVVARSIMIEIREGRGCDGPWGPHAKLKLDHLGKEVLESRLPGILELSRTFAHVDPVKEPIPVI

PTCHYMMGGIPTKVTGQALTVNEKGEDVVVPGLFAVGEIACVSVHGANRLGGNSLLDLVVFGRAAGLHLQ

ESIAEQGALHDASESDVEASLDRLNRWNNNRNGEDPVAIRKALQECMQHNFSVFREGDAMAKGLEQLKVI

RERLKNARLDDTSSEFNTQRVECLELDNLMETAYATAVSANFRTESRGAHSRFDFPDRDDENWLCHSLYL

PESESMTRRSVNMEPKLRPAFPPKIRTY

>gi|30062113|ref|NP_836284.1| succinate dehydrogenase iron-sulfur subunit [Shigella flexneri 2a str. 2457T]

MRLEFSIYRYNPDVDDAPRMQDYTLEAEEGRDMMLLDALIQLKEKDPSLSFRRSCREGVCGSDGLNMNGK

NGLACITPISALNQPGKKIVIRPLPGLPVIRDLVVDMGQFYAQYEKIKPYLLNNGQNPPAREHLQMPEQR

EKLDGLYECILCACCSTSCPSFWWNPDKFIGPAGLLAAYRFLIDSRDTETDSRLDGLSDAFSVFRCHSIM

NCVSVCPKGLNPTRAIGHIKSMLLQRNA

>gi|30062112|ref|NP_836283.1| hypothetical protein S0585 [Shigella flexneri 2a str. 2457T]

MNALSGLQVITRRPDKRSASGVTKKCRKSLKTAPDTKTVFKGSFASHYVDKSSQVNPGTHITVRGSIHGE

VSIKKMLKGSRCRTAL

>gi|30062111|ref|NP_836282.1| 2-oxoglutarate dehydrogenase E1 component [Shigella flexneri 2a str. 2457T]

MQNSALKAWLDSSYLSGANQSWIEQLYEDFLTDPDSVDANWRSTFQQLPGTGVKPDQFHSQTREYFRRLA

KDASRYSSTISDPDTNVKQVKVLQLINAYRFRGHQHANLDPLGLWQQDKVADLDPSFHDLTEADFQETFN

VGSFASGKETMKLGELISALKQTYCGPIGAEYMHITSTEEKRWIQQRIESGRATFNSEEKKRFLSELTAA

EGLERYLGAKFPGAKRFSLEGGDALIPMLKEMIRHAGNSGTREVVLGMAHRGRLNVLVNVLGKKPQDLFD

EFAGKHKEHLGTGDVKYHMGFSSDFQTDGGLVHLALAFNPSHLEIVSPVVIGSVRARLDRLDEPNSNKVL

PITIHGDAAVTGQGVVQETLNMSKARGYEVGGTVRIVINNQVGFTTSNPLDARSTPYCTDIGKMVQAPIF

HVNADDPEAVAFVTRLALDFRNTFKRDVFIDLVCYRRHGHNEADEPSATQPLMYQKIKKHPTPRKIYADK

LEQEKVATLEDATEMVNLYRDALDAGDCVVAEWRPMNMHSFTWSPYLNHEWDEEYPNKVEMKRLQELAKR

ISTVPEAVEMQSRVAKIYGDRQAMAAGEKLFDWGGAENLAYATLVDEGIPVRLSGEDSGRGTFFHRHAVI

HNQSNGSTYTPLQHIHNGQGAFRVWDSVLSEEAVLAFEYGYATAEPRTLTIWEAQFGDFANGAQVVIDQF

ISSGEQKWGRMCGLVMLLPHGYEGQGPEHSSARLERYLQLCAEQNMQVCVPSTPAQVYHMLRRQALRGMR

RPLVVMSPKSLLRHPLAVSSLEELANGTFLPAIGEIDELDPKGVKRVVMCSGKVYYDLLEQRRKNNQHDV

AIVRIEQLYPFPHKAMQEVLQQFAHVKDFVWCQEEPLNQGAWYCSQHHFREVIPFGASLRYAGRPASASP

AVGYMSVHQKQQQDLVNDALNVE

>gi|30062110|ref|NP_836281.1| dihydrolipoamide succinyltransferase [Shigella flexneri 2a str. 2457T]

MSSVDILVPDLPESVADATVATWHKKPGDAVVRDEVLVEIETDKVVLEVPASADGILDAVLEDEGTTVTS

RQILGRLREGNSAGKETSAKSEEKASTPAQRQQASLEEQNNDALSPAIRRLLAEHNLDASAIKGTGVGGR

LTREDVEKHLAKAPAKESAPAAAAPAAQPALAARSEKRVPMTRLRKRVAERLLEAKNSTAMLTTFNEVNM

KPIMDLRKQYGEAFEKRHGIRLGFMSFYVKAVVEALKRYPEVNASIDGDDVVYHNYFDVSMAVSTPRGLV

TPVLRDVDTLGMADIEKKIKELAVKGRDGKLTVEDLTGGNFTITNGGVFGSLMSTPIINPPQSAILGMHA

IKDRPMAVNGQVEILPMMYLALSYDHRLIDGRESVGFLVTIKELLEEPTRLLLDV

>gi|30062109|ref|NP_836280.1| succinyl-CoA synthetase subunit beta [Shigella flexneri 2a str. 2457T]

MNLHEYQAKQLFARYGLPAPVGYACTTPREAEEAASKIGAGPWVVKCQVHAGGRGKAGGVKVVNSKEDIR

AFAENWLGKRLVTYQTDANGQPVNQILVEAATDIAKELYLGAVVDRSSRRVVFMASTEGGVEIEKVAEET

PHLIHKVALDPLTGPMPYQGRELAFKLGLEGKLVQQFTKIFMGLATIFLERDLALIEINPLVITKQGDLI

CLDGKLGADGNALFRQPDLREMRDQSQEDPREAQAAQWELNYVALDGNIGCMVNGAGLAMGTMDIVKLHG

GEPANFLDVGGGATKERVTEAFKIILSDDKVKAVLVNIFGGIVRCDLIADGIIGAVAEVGVNVPVVVRLE

GNNAELGAKKLADSGLNIIAAKGLTDAAQQVVAAVEGK

>gi|30062108|ref|NP_836279.1| succinyl-CoA synthetase subunit alpha [Shigella flexneri 2a str. 2457T]

MSILIDKNTKVICQGFTGSQGTFHSEQAIAYGTKMVGGVTPGKGGTTHLGLPVFNTVREAVAATGASASV

IYVPAPFCKDSILEAIDAGIKLIITITEGIPTLDMLTVKVKLDEAGVRMIGPNCPGVITPGECKIGIQPG

HIHKPGKVGIVSRSGTLTYEAVKQTTDYGFGQSTCVGIGGDPIPGSNFIDILEMFEKDPQTEAIVMIGEI

GGSAEEEAAAYIKEHVTKPVVGYIAGVTAPKGKRMGHAGAIIAGGKGTADEKFAALEAAGVKTVRSLADI

GEALKTVLK

>gi|30062107|ref|NP_836278.1| DNA-binding transcriptional repressor MngR [Shigella flexneri 2a str. 2457T]

MGHKPLYRQIADRIREQIARGELKPGDALPTESALQTEFGVSRVTVRQALRQLVEQQILESIQGSGTYVK

EERVNYDIFQLTSFDEKLSDRHVDTHSEVLIFEVIPADDFLQQQLQITVQDRVWHVKRVRYRKQKPMALE

ETWMPLALFPDLTWQVMENSKYHFIEEVKKMVIDRSEQEIIPLMPTEEMSRLLNISQTKPILEKVSRGYL

VDGRVFEYSRNAFNTDDYKFTLIAQPKIIAISTKRPSYDGQPCGNRQPFAIPVEGSGEGYFLLQIFISEQ

FSVLHRYPPGCERFSRLRLPEQRISHPQSLSHRQ

>gi|30062106|ref|NP_836277.1| 2-O-a-mannosyl-D-glycerate specific PTS transporten components IIABC [Shigella flexneri 2a str. 2457T]

MVLFCRAHWRDYKNDQVRIMMNLTTLTHRDALCLNARFTSREEAIHALTQRLAALGKISSTEQFLKEVYR

RESLGPTALSEGLAVPHGKTAAVKEAAFAVATLSEPLQWEGVDGPEAVDLVVLLAIPLNEAGTTHMQLLT

ALTTRLADDEIRARIQSATTPDELLSALDDKGGTQPSASFSNAPTIVCVTACPAGIAHTYMAAEYLEKAG

RKLGVNVYVEKQGANGIEGRLTADQLNSATACIFAAEVAIKESERFNGIPALSVPVAEPIRHAEALIQQS

LTLERGDETRTLQQDTQPVKSVKTELKQALLSGISFAVPLIVAGGTVLAVAVLLSQIFGLQDLFNEENSW

LWMYRKLGGGLLGILMVPVLAAYTAYSLADKPALAPGFAAGLAANMIGSGFLGAVVGGLIAGYLIRWMKN

HLRLSSKFNGFLTFYLYPVLGTLGAGSLMLFVVGEPVAWINNSLTAWLNGLSGSNALLLGAILGFMCSFD

LGGPVNKAAYAFCLGAMANGVYGPYAIFASVKMVSAFTVTASTMLAPRLFKEFEIETGKSTWLLGLAGIT

EGAIPMAIEDPLRVIGSFVLGSMVTGAIVGAMNIGLSTPGAGIFSLFLLHDNGAGGVMAAIGWFGAALVG

AAISTAILLIWRRHAVKHGNYLTDGVMP

>gi|30062105|ref|NP_836276.1| alpha-mannosidase [Shigella flexneri 2a str. 2457T]

MKAVSRVHITPHMHWDREWYFTTEESRILLVNNMEEILCRLEQDNEYKYYVLDGQTAILEDYFAVKPENK

DRLKKLVEAGKLIIGPWYTQTDTTIVSAESIVRNLMYGMRDCLAFGEPMKIGYLPDSFGMSGQLPHIYNG

FGITCTMFWRGCSERHGTDKTEFLWQSSDGSEVTAQVMPLGYAIGKYLPADENGLRKRLDSYFDVLEKAS

VTKEILLPNGHDQMPLQQNIFEVMEKLREIYPQRKFVMSRFEEVFEKIEAQRDNLATLKGEFIDGKYMRV

HRTIGSTRMDIKVAHARIENKIVNLLEPLATLARTLGFGYHHGLLEKMWKEILKNHAHDSIGCCCRDKVH

REIVARFELAEDMADNLLRFYMRKIADNMPQSDADKLVLFNLMPWPREEVINTTVRLRASQFNLRDDRGQ

PVPYFIRHAREIDPGLIDRQIVHYGNYDPFMEFDIQINQIVPSMGYRTLYIEANQPGNVIAAKSDAEGIL

ENAFWQIALNEDGTLQLVDKDSGVRYDRVLQIEESSDDGDEYDYSPAKEEWVITSANAKPQCEIIHEAWQ

SRAVIRYEMAVPRNLQQRSARQSTGRVDVELVVTLSHNNRRIDVDINLDNQADDHRLRVLIPTSFNTDSV

LADTQFGSLTRPVNDSAMNNWQQESWKEAPVPVWNMLNYAALQEGRNGMAVFSEGLREFEVIGEEKKTFA

ITLLRGVGLLGKEDLLLRPRRPSGIKMPVPDSQLRGLLSCRLSLLSYTGTPTAAGVAQQARAWLTPVQCY

NKIPWDAMKLNKAGFNVPESYSLLKMPPVGCLISALKKAEDRQEVILRLFNPAESATCDATVAFSREVIS

CSETMMDEHITTEENQGSNLSGPFLPGQSRTFSYRLA

>gi|30062104|ref|NP_836275.1| cytochrome d terminal oxidase, polypeptide subunit I [Shigella flexneri 2a str. 2457T]

MMLDIVELSRLQFALTAMYHFLFVPLTLGMAFLLAIMETVYVLSGKQIYKDMTKFWGKLFGINFALGVAT

GLTMEFQFGTNWSYYSHYVGDIFGAPLAIEGLMAFFLESTFVGLFFFGWDRLGKVQHMCVTWLVALGSNL

SALWILVANGWMQNPIASDFNFETMRMEMVSFSELVLNPVAQVKFVHTVASGYVTGAMFILGISAWYMLK

GRDFAFAKRSFAIAASFGMAAVLSVIVLGDESGYEMGDVQKTKLAAIEAEWETQPAPAAFTLFGIPDQEE

ETNKFAIQIPYALGIIATRSVDTPVIGLKELMVQHEERIRNGMKAYSLLEQLRSGSTDQTVRDQFNSMKK

DLGYGLLLKRYTPNVADATEAQIQQATKDSIPRVAPLYFAFRIMVACGFLLLAIIALSFWSVIRNRIGEK

KWLLRAALYGIPLPWIAVEAGWFVAEYGRQPWAIGEVLPTAVANSSLTAGDLIFSMVLICGLYTLFLVAE

LFLMFKFARLGPSSLKTGRYHFEQSSTTTQPAR

>gi|30062103|ref|NP_836274.1| cytochrome d terminal oxidase polypeptide subunit II [Shigella flexneri 2a str. 2457T]

MIDYEVLRFIWWLLVGVLLIGFAVTDGFDMGVGMLTRFLGRNDTERRIMINSIAPHWDGNQVWLITAGGA

LFAAWPMVYAAAFSGFYVAMILVLASLFFRPVGFDYRSKIEETRWRNMWDWGIFIGSFVPPLVIGVAFGN

LLQGVPFNVDEYLRLYYTGNFFQLLNPFGLLAGVVSVGMIITQGATYLQMRTVGELHLRTRATAQVAALV

TLVCFALAGVWVMYGIDGYVVKSTMDHYAASNPLNKEVVREAGAWLVNFNNTPILWAIPALGVVLLLLTI

LTARMDKAAWAFVFSSLTLACIILTAGIAMFPFVMPSSTMMNASLTMWDATSSQLTLNVMTWVAVVLVPI

ILLYTAWCYWKMFGRITKEDIERNTHSLY

>gi|30062102|ref|NP_836273.1| hypothetical protein S0575 [Shigella flexneri 2a str. 2457T]

MSKIIATLYAVMDKRPLRALSFVMALLLAGCMFWDPSRFAAKTSELEIWHGLLLMWAVCAGVIHGVGFRP

QKVLWQGIFCPLLADIVLIVGLIFFFF

>gi|30062101|ref|NP_836272.1| acyl-CoA thioester hydrolase YbgC [Shigella flexneri 2a str. 2457T]

MNTTLFRWPVRVYYEDTDAGGVVYHASYVAFYERARTEMLRHHHFSQQALMAERVAFVVRKMTVEYYAPA

RLDDMLEIQTEITSMRGTSLVFTQRIVNAENTLLNEAEVLVVCVDPLKMKPRALPKSIVAEFKQ

>gi|30062100|ref|NP_836271.1| colicin uptake protein TolQ [Shigella flexneri 2a str. 2457T]

MTDMNILDLFLKASLLVKLIMLILIGFSIASWAIIIQRTRILNAAAREAEAFEDKFWSGIELSRLYQESQ

GKRDNLTGSEQIFYSGFKEFVRLHRANSHAPEAVVEGASRAMRISMNRELENLETHIPFLGTVGSISPYI

GLFGTVWGIMHAFIALGAVKQATLQMVAPGIAEALIATAIGLFAAIPAVMAYNRLNQRVNKLELNYDNFM

EEFTAILHRQAFTVSESNKG

>gi|30062099|ref|NP_836270.1| colicin uptake protein TolR [Shigella flexneri 2a str. 2457T]

MARARGRGRRDLKSEINIVPLLDVLLVLLLIFMATAPIITQSVEVDLPDATESQAVSSNDNPPVIVEVSG

IGQYTVVVEKDRLERLPPEQVVAEVSSRFKANPKTVFLIGGAKDVPYDEIIKALNLLHSAGVKSVGLMTQ

PI

>gi|30062098|ref|NP_836269.1| cell envelope integrity inner membrane protein TolA [Shigella flexneri 2a str. 2457T]

MSKATEQNDKLKRAIIISAVLHVILFAALIWSSFDENIEASAGGGGGSSIDAVMVDSGAVVEQYKRMQSQ

ESSAKRSDEQRKMKEQQAAEELREKQAAEQERLKQLEKERLAAQEQKKQAEEAAKQAELKQKQAEVAAAK

AAADAKAAEEAAKKAAADAKKKAEAEAAKAAAEAQKKAEVAAAALKKKAEAAEAAAAEARKKAATEAAEK

AKAEAEKKAAAEKAAADKKAAAEKAAADKKAAEKAAADKAAADKKAAAEKAAADKKAAAAKAAAEKAAAA

KAAAEADDIFGELSSGKNAPKTGGGAKGNNASPAGSGNTKNNGASGADINNYAGQIKSAIESKFYDASSY

AGKTCTLRIKLAPDGMLLDIKPEGGDPALCQAALAAAKLAKIPKPPSQAVYEVFKNAPLDFKP

>gi|30062097|ref|NP_836268.1| translocation protein TolB [Shigella flexneri 2a str. 2457T]

MKQALRVAFGFLILWASVLHAEVRIVIDSGVDSGRPIGVVPFQWAGPGAAPEDIGGIVAADLRNSGKFNP

LDRARLPQQPGSAQEVQPAAWSALGIDAVVVGQVTPNPDGSYNVAYQLVDTGGAPGTVLAQNSYKVNKQW

LRYAGHTASDEVFEKLTGIKGAFRTRIAYVVQTNGGQFPYELRVSDYDGYNQFVVHRSPQPLMSPAWSPD

GSKLAYVTFESGRSALVIQTLANGAVRQVASFPRHNGAPAFSPDGSKLAFALSKTGSLNLYVMDLASGQI

RQVTDGRSNNTEPTWFPDSQNLAFTSDQAGRPQVYKVNINGGAPQRITWEGSQNQDADVSSDGKFMVMVS

SNGGQQHIAKQDLATGGVQVLSSTFLDETPSLAPNGTMVIYSSSQGMGSVLNLVSTDGRFKARLPATDGQ

VKFPAWSPYL

>gi|30062096|ref|NP_836267.1| peptidoglycan-associated outer membrane lipoprotein [Shigella flexneri 2a str. 2457T]

MQLNKVLKGLMIALPVMAIAACSSNKNASNDGSEGMLGAGTGMDANGGNGNMSSEEQARLQMQQLQQNNI

VYFDLDKYDIRSDFAQMLDAHANFLRSNPSYKVTVEGHADERGTPEYNISLGERRANAVKMYLQGKGVSA

DQISIVSYGKEKPAVLGHDEAAYSKNRRAVLVY

>gi|30062095|ref|NP_836266.1| tol-pal system protein YbgF [Shigella flexneri 2a str. 2457T]

MSSNFRHQLLSLSLLVGIAAPWAAFAQAPISSVSSGSVEDRVTQLERISNAHSQLLTQLQQQLSDNQSDI

DSLRGQIQENQYQLNQVVERQKQILLQIDSLSSGGAAAQSTSSDQSGATASTTPTADAGTANAGAPVKSG

DANTDYNAAIALVQDKSRQDDAMVAFQNFIKNYPDSTYLPNANYWLGQLNYNKGKKDDAAYYFASVVKNY

PKSPKAADAMFKVGVIMQDKGDTAKAKAVYQQVISKYPGTDGAKQAQKRLNAM

>gi|30062094|ref|NP_836265.1| quinolinate synthetase [Shigella flexneri 2a str. 2457T]

MSVMFDPDTAIYPFPPKPTPLSIDEKAYYREKIKRLLKERNAVMVAHYYTDPEIQQLAEETGGCISDSLE

MARFGAKHPASTLLVAGVRFMGETAKILSPEKTILMPTLQVECSLDLGCPVEEFNAFYDAHPDRTVVVYA

NTSAAVKARADWVVTSSIAVELIDHLDSLGEKIIWAPDKHLGRYVQKQTGGDILCWQGACIVHDEFKTQA

LTRLQEEYPDAAILVHPESPQAIVDMADAVGSTSQLIAAAKALPHQRLIVATDRGIFYKMQQAVPDKELL

EAPTAGEGATCRSCAHCPWMAMNDLQAIAEALEQEGSNHEVHVDERLRERALVPLNRMLDFAATLRG

>gi|30062093|ref|NP_836264.1| PnuC protein [Shigella flexneri 2a str. 2457T]

MDFFSVQNILVHIPIGAGGYDLSWIEAVGTIAGLLCIGLASLEKISNYFFGLINVTLFGIIFFQIQLYAS

LLLQVFFFAANIYGWYAWSRQTSQNEAELKIRWLPLPKALSWLAVCVVSIGLMTVFINPVFAFLTRVAVM

IMQALGLQVVMPELQPDAFPFWDSCMMVLSIVAMILMTRKYVENWLLWVIINVISVVIFALQGVYAMSLE

YIILTFIALNGSRMWINSARERGSRALSH

>gi|30062092|ref|NP_836263.1| zinc transporter ZitB [Shigella flexneri 2a str. 2457T]

MAHSHSHTSSHLPEDNNARRLLYAFGVTAGFMLVEVVGGFLSGSLALLADAGHMLTDTAALLFALLAVQF

SRRPPTIRHTFGWLRLTTLAAFVNAIALVVITILIVWEAIERFRTPRPVEGGMMMAIAVAGLLANILSFW

LLHHGSEEKNLNVRAAALHVLGDLLGSVGAIIAALIIIWTGWTPADPILSILVSLLVLRSAWRLLKDSVN

ELLEGAPVSLDIAELKRRMCREIPEVRNVHHVHVWMVGEKPVMTLHVQVIPPHDHDALLDQIQHYLMDHY

QIEHTTIQMEYQPCHRPDCHLNEGVSGHSHHHH

>gi|30062091|ref|NP_836262.1| homeobox protein [Shigella flexneri 2a str. 2457T]

MKMTKLATLFLTATLSLASGAALAADSGAQTNNGQANAAADAGQVAPDARENVAPNNVDNNGVNTGSGGT

MLHSDGSSMNNDGMTKDEEHKNTMCKDGRCPDINKKVQTGDGINNDVDTKTDGTTQ

>gi|30062090|ref|NP_836261.1| phospho-2-dehydro-3-deoxyheptonate aldolase [Shigella flexneri 2a str. 2457T]

MNYQNDDLRIKEIKELLPPVALLEKFPATENAANTVAHARKAIHKILKGNDDRLLVVIGPCSIHDPVAAK

EYATRLLALREELKDELEIVMRVYFEKPRTTVGWKGLINDPHMDNSFQINDGLRIARKLLLDINDSGLPA

AGEFLDMITPQYLADLMSWGAIGARTTESQVHRELASGLSCPVGFKNGTDGTIKVAIDAINAAGAPHCFL

SVTKWGHSAIVNTSGNGDCHIILRGGKEPNYSAKHVAEVKEGLNKAGLPAQVMIDFSHANSSKQFKKQMD

VCADVCQQIAGGEKAIIGVMVESHLVEGNQSLDSGEPLAYGKSITDACIGWEDTDALLRQLVNAVKARRG

>gi|30062089|ref|NP_836260.1| phosphoglyceromutase [Shigella flexneri 2a str. 2457T]

MAVTKLVLVRHGESQWNKENRFTGWYDVDLSEKGVSEAKAAGKLLKEEGYSFDFAYTSVLKRAIHTLWNV

LDELDQAWLPVEKSWKLNERHYGALQGLNKAETAEKYGDEQVKQWRRGFAVTPPELTKDDERYPGHDPRY

AKLSEKELPLTESLALTIDRVIPYWNETILPRMKSGERVIIAAHGNSLRALVKYLDNMSEEEILELNIPT

GVPLVYEFDENFKPLKRYYLGNADEIAAKAAAVANQGKAK

>gi|30062088|ref|NP_836259.1| aldose 1-epimerase [Shigella flexneri 2a str. 2457T]

MLNETPALAPDGQPYRLLTLRNNAGMVVTLMDWGATLLSARIPLSDGSVREALLGCASPECYQDQAAFLG

ASIGRYANRIANSRYTFDGETVTLSPSQGVNQLHGGPEGFDKRRWQIVNQNDRQVLFALSSDDGDQGFPG

NLGATVQYRLTDDNRISITYRATVDKPCPVNMTNHVYFNLDGEQSDVRNHKLQILADEYLPVDEGGIPHD

GLKSVAGTSFDFRSAKIIASEFLADDDQRKVKGYDHAFLLQAKGDGKKVAAHVWSADEKLQLKVYTTAPA

LQFYSGNFLGGTPSRGTEPYADWQGLALESEFLPDSPNHPEWPQPDCFLRPGEEYSSLTEYQFIAE

>gi|30062087|ref|NP_836258.1| galactokinase [Shigella flexneri 2a str. 2457T]

MSLKEKTQSLFANAFGYPATHTIQAPGRVNLIGEHTDYNDGFVLPCAIDYQTVISCAPRDDRKVRVMAAD

YENQLDEFSLNAPIVAHENYQWANYVRGVVKHLQLRNNSFGGVDMVISGNVPQGAGLSSSASLEVAVGTV

LQQLYHLPLDGAQIALNGQEAENQFVGCNCGIMDQLISALGKKDHALLIDCRSLGTKAVSMPKGVAVVII

NSNFKRTLVGSEYNTRREQCETGARFFQQPALRDVTIEEFNAVAHELDPIVAKRVRHILTENARTVEAAS

ALEQGDLKRMGELMAESHASMRDDFEITVPQIDTLVEIVKAVIGDKGGVRMTGGGFGGCIVALIPEELVP

AVQQAVAEQYEAKTGIKETFYVCKPSQGAGQC

>gi|30062086|ref|NP_836257.1| galactose-1-phosphate uridylyltransferase [Shigella flexneri 2a str. 2457T]

MTQFNPVDHPHRRYNPLTGQWILVSPHRAKRPWQGAQETPAKQVLPAHDPDCFLCAGNVRVTGDKNPDYT

GTYVFTNDFAALMSDTPDAPESNDPLMRCQSARGTSRVICFSPDHSKTLPELSVAALTEIVKTWQEQTAE

LGKTYPWVQVFENKGAAMGCSNPHPHGQIWANSFLPNEAECEDRLQKEYFAEQKSPMLVDYVQRELADGS

RTVVETEHWLAVVPYWAAWPFETLLLPKAHVLRITDLTDAQRSDLALALKKLTSRYDNLFQCSFPYSMGW

HGAPFNGEENQHWQLHAHFYPPLLRSATVRKFMVGYEMLAETQRDLTAEQAAERLRAVSDIHFRESGV

>gi|30062085|ref|NP_836256.1| UDP-galactose-4-epimerase [Shigella flexneri 2a str. 2457T]

MRVLVTGGSGYIGSHTCVQLLQNGHDVIILDNLCNSKRSVLPVIERLGGKHPTFVEGDIRNEALMTEILH

DHAIDTVIHFAGLKAVAESVQKPLEYYDNNVNGTLRLISAMRAANVKNFIFSSSATVYGDQPQIPYVESF

PTGTPQSPYGKSKLMVEQILTDLQKAQPDWSIALLRYFNPVGAHPSGDMGEDPQGIPNNLMPYIAQVAVG

RRDSLAIFGNDYPTEDGTGVRDYIHVMDLADGHVVAMEKLANKPGVHIYNLGAGVGSSVLDVVNAFSKAC

GKPVNYHFAPRREGDLPAYWADASKADRELNWRVTRTLDEMAQDTWHWQSRHPQGYPD

>gi|30062084|ref|NP_836255.1| molybdenum transport ATP-binding protein ModF [Shigella flexneri 2a str. 2457T]

MSSLQILQGTFRLSDTKTLQLPQLTLNAGDSWAFVGSNGSGKSALARALAGELPLLKGERQSQFSHITRL

SFEQLQKLVSDEWQRNNTDMLGPSEDYTGRTTAEIIQDEVKDAPRCMQLAQQFGITALLDRRFKYLSTGE

TRKTLLCQALMSEPDLLILDEPFDGLDVASRQQLAERLASLHQSGITLVLVLNRFDKIPEFVQFAGVLAD

CTLAETGAKEELLQQALVAQLAHSEQLEGVQLPEPDEPSARHALPANESRIVLNNGVVSYNDRPILNNLS

WQVNPGEHWQIVGPNGAGKSTLLSLITGDHPQGYSNDLTLFGRRRGSSETIWDIKKHIGYVSSSLHLDYR

VSTTVRNVILSGYFDSIGIYQVVSDRQQKLVQQWLDILGIDKHTADAPFHSLSWGQQRLALIVRALVKHP

TLLILDEPLQGLDPLNRQLIRRFVDVLISEGETQLLFVSHHAEDAPACITHRLEFVPDGGLYRYVLTKIY

>gi|30062083|ref|NP_836254.1| DNA-binding transcriptional regulator ModE [Shigella flexneri 2a str. 2457T]

MQAEILLTLKLQQKLFADPRRISLLKHIALSGSISQGAKDAGISYKSAWDAINEMNQLSEHILVERATGG

KGGGGAVLTRYGQRLIQLYDLLAQIQQKAFDVLSDDDALPLNSLLAAISRFSLQTSARNQWFGTITARDH

DDVQQHVDVLLADGKTRLKVAITAQSGARLGLDEGKEVLILLKAPWVGITQDEAVAQNADNQLPGIISHI

ERGAEQCEVLMALPDGQTLCATVPVNEATSLQQGQNVTAYFNADSVIIATLC

>gi|30062082|ref|NP_836253.1| hypothetical protein S0550 [Shigella flexneri 2a str. 2457T]

MLELLKSLVFAVIMVPVVMAIILGLIYGLGEVFNIFSGVGKKDQPGQNH

>gi|30062081|ref|NP_836252.1| molybdate transporter periplasmic protein [Shigella flexneri 2a str. 2457T]

MARKWLNLFAGAALSFAVAGNALADEGKITVFAAASLTNAMQDIATQYKKEKGVDVVSSFASSSTLARQI

EAGAPADLFISADQKWMDYAVDKKAIDTATRQTLLGNSLVVVAPKASEQKDFTIDSKTNWTSLLNGGRLA

VGDPEHVPAGIYAKEALQKLGAWDTLSPKLAPAEDVRGALALVERNEAPLGIVYGSDAVASKGVKVVATF

PEDSHKKVEYPVAVVEGHNNATVKAFYDYLKGPQAAEIFKRYGFTTK

>gi|30062080|ref|NP_836251.1| molybdate ABC transporter permease [Shigella flexneri 2a str. 2457T]

MILTDPEWQAVLLSLKVSSLAVLFSLPFGIFFAWLLVRCTFPGKALLDSVLHLPLVLPPVVVGYLLLVSM

GRRGFIGERLYDWFGITFAFSWRGAVLAAAVMSFPLMVRAIRLALEGVDVKLEQAARTLGAGRWRVFFTI

TLPLTLPGIIVGTVLAFARSLGEFGATITFVSNIPSETRTIPSAMYTLIQTPGGESGAARLCIISIALAM

ISLLISEWLARISRERAGR

>gi|30062079|ref|NP_836250.1| molybdate transporter ATP-binding protein [Shigella flexneri 2a str. 2457T]

MLELNFSQTLGNHCLTINETLPANGITAIFGVSGAGKTSLINAISGLTRPQKGRIVLNGRVLNDAEKGIC

LTPEKRRVGYVFQDARLFPHYKVRGNLRYGMSKSMVDQFDKLVALLGIEPLLDRLPGSLSGGEKQRVAIG

RALLTAPELLLLDEPLASLDIPRKRELLPYLQRLTREINIPMLYVSHSLDEILHLADRVMVLENGQVKAF

GALEEVWGSSVMNPWLPKEQQSSILKVTVLEHHPHYAMTALALGDQHLWVNKLGEPLQAALRIRIQASDV

SLVLQPPQQTSIRNVLRAKVVNSYDDNGQVEVELEVGGKTLWARISPWARDELAIKPGLWLYAQIKSVSI

TA

>gi|30062078|ref|NP_836249.1| phosphotransferase [Shigella flexneri 2a str. 2457T]

MTTRVIALDLDGTLLTPKKTLLPSSIEALARAREAGYQLIIVTGRHHVAIHPFYQALALDTPAICCNGTY

LYDYHAKTVLEADPMPVNKALQLIEMLNEHHIHGLMYVDDAMVYEHPTGHVIRTSNWAQTLPPEQRPTFT

QVASLAETTQQVNAVWKFALTHDDLPQLQHFGKHVEHELGLECEWSWHDQVDIAHGGNSKGKRLTKWVEA

QGWSMENVVAFGDNFNDISMLEAAGTGVAMGNADDAVKARANIVIGDNTTDSIAQFIYSHLI

>gi|30062077|ref|NP_836248.1| IS1 orfA [Shigella flexneri 2a str. 2457T]

MASISIRCPSCSATEGVVRNGKSTAGHQRYLCSHCRKTWQLQFTYTASQPGTHQKIIDMAMNGVGCRASA

RIMGVGLNTVLRHLKNSGRSR

>gi|30062076|ref|NP_836247.1| IS1 orfB [Shigella flexneri 2a str. 2457T]

MSRQCTHYGRWPQHGFTSLKKLRPQSVTSRIQPGSDVIVCAEMDEHWGYVGAKSRQRWLFYAYDRIRRTV

VAHVFGERTLATLERLLSLLSAFEVVVWMTDGWPLYESRLKGKLHVISKRYTQRIERHNLNLRQHLARLV

RKSLSFSKSVELHDKVIGHYLNIKHYQ

>gi|30062075|ref|NP_836246.1| citrate lyase synthetase (citrate (pro-3S)-lyase ligase [Shigella flexneri 2a str. 2457T]

MKIKGYQFATLNNIRCKSITTCTFRILFIMFGNDIFTRVKRSENKKMAEIAQFLHENDLSVDTTVEVFIT

VTRDEKLIACGGIAGNIIKCVAISESVRGEGLALTLATELINLAYERHSTHLFIYTKTEYEALFRQCGFS

TLTCVPGVMVLMENSATRLKRYAESLKKFRHPGNKIGCIVMNANPFTNGHRYLIQQAAAQCDWLHLFLVK

EDSSRFPYEDRLDLVLKGTADIPRLTVHRGSEYIISRATFPCYFIKEQSVINHCYTEIDLKIFRQYLAPA

LGVTHRFVGTEPFCRVTAQYNQDMRYWLETPTISAAPIELVEIERLRYQEMPISASRVRQLLAKNDLTAI

APLVPAVTLHYLQNLLEHSRQDAAARQKTPA

>gi|30062074|ref|NP_836245.1| citrate lyase subunit gamma [Shigella flexneri 2a str. 2457T]

MKINQPAVAGTLESGDVMIRIAPLDTQDIDLQINSSVEKQFGDAIRTTILDVLARYNVRGVQLNVDDKGA

LDCILHARLEALLARASGIPALPWEDCQ

>gi|30062073|ref|NP_836244.1| citrate lyase beta chain (acyl lyase subunit) [Shigella flexneri 2a str. 2457T]

MISASLQQRKTRTRRSMLFVPGANAAMVSNSFIYPADALMFDLEDSVALREKDTARRMVYHALQHPLYRD

IETIVRVNALDSEWGVNDLEAVVRGGADVVRLPKTDTAQDVLDIEKEILRIEKACGREPGSTGLLAAIES

PLGITRAVEIAHASERLIGIALGAEDYVRNLRTERSPEGTELLFARCSILQAARSAGIQAFDTVYSDANN

EAGFLQEAAHIKQLGFDGKSLINPRQIDLLHNLYAPTQKEVDHAHRVVEAAEAAAREGLGVVSLNGKMVD

GPVIDRARLVLSRAELSGIREE

>gi|30062072|ref|NP_836243.1| citrate lyase subunit alpha [Shigella flexneri 2a str. 2457T]

MTQKIEQSQRQERVAAWNRRTECDLAAFQNSPKQTYQAEKARDRKLCANLEEAIRRSGLQDGMTVSFHHA

FRGGDLTVNMVMDVIAKMGFKNLTLASSSLSDCHAPLVEHIRQGVVTRIYTSGLRGPLAEEISRGLLAEP

VQIHSHGGRVHLVQSGELNIDVAFLGVPSCDEFGNANGYTGKACCGSLGYAMVDADNAKQVVMLTEELLP

YPHNPASIEQDQVDLIVKVNRVGDAAKIGAGTTRMTTNPRELLIARSAADVIVNSGYFKEGFSMQTGTGG

ASLAVTRFLEDKMRSRDIRADFALGGITATMVDLHEKGLIRKLLDVQSFDSHAAQSLARNPNHIEISANQ

YANWGSKGASVDRLDVVVLSALEIDRQFNVNVLTGSDGVLRGASGGHCDTAIASALSIIVAPLVRGRIPT

LVDNVLTCITPGSSVDILVTDHGIAVNPARPELAERLQEAGIKVVSIEWLRERARLLTGEPQPIEFTDRV

VAVVRYRDGSVIDVVHQVKE

>gi|30062071|ref|NP_836242.1| 2-(5''-triphosphoribosyl)-3'-dephosphocoenzyme-A synthase [Shigella flexneri 2a str. 2457T]

MHLLPELASHHAVSIPELLVSRDERQARQHAWLKHHPVPLVSFTVVAPGPIKDSEVTRRIFNHRVTALRA

LATKQGWQIQEQAALVSASGPEGMLSIAAPARDLKLATIELEHSHPLGRLWDIDVLTPEGEILSRRDYSL

RLAAACCASKAQPSARVEKPIN

>gi|30062070|ref|NP_836241.1| triphosphoribosyl-dephospho-CoA synthase [Shigella flexneri 2a str. 2457T]

MSMPATSTKTTKLATSLIDEYALLGWRAMLTEVNLSPKPGLVDRINCGAHKDMALEDFHRSALAIQGWLP

RFIEFGACSAEMAPEAVLHGLRPIGMACEGDMFRATAGVNTHKGSIFSLGLLCAAIGRLLQLNQPVTPTT

VCSTAASFCRGLTDRELRTNNSRLTAGQRLYQQLGLTGARGEAEAGYPLVINHALPHYLTLLDQGLDPEL

ALLDTLLLLMATNGDTNVASRGGEGGLRWLQREAQTLLQKGGIRTPTDLDYLRQFDRECIERNLSPGGSA

DLLILTCFLAQI

>gi|30062069|ref|NP_836240.1| ribonuclease I [Shigella flexneri 2a str. 2457T]

MSSTPIMKAFCRNAALLAVSLLPLSSANAVALQAKQYGDFDRYVLALSWQTGFCQSQHDRNRNERDECRL

QTETTNKTDFLTVHGLWPGLPKSVAARGVDERRWMRFGCATRPIPNLPEARASRMCSSPETGLSLETAAK

LSEVMPGAGGRSCLERYEYAKHGACFGFDPDAYFGTMVRLNQEIKESEAGKFLADNYGKTVSRRDFDAAF

AKSWGKENVKAVKLTCQGNPAYLTEIQISIKADAINAPLSANSFLPQPHPGNCGKTFVIDKAGY

>gi|30062068|ref|NP_836239.1| nucleoside diphosphate kinase regulator [Shigella flexneri 2a str. 2457T]

MSRPTIIINDLDAERIDILLEQPAYAGLPIADALNAELDRAQMCSPEEMPHDVVTMNSRVKFRNLSDGEV

RVRTLVYPAKMTDSNTQLSVMAPVGAALLGLRVGDSIHWELPGGVATHLEVLELEYQPEAAGDYLL

>gi|30062067|ref|NP_836238.1| hypothetical protein S0533 [Shigella flexneri 2a str. 2457T]

MLLPWLFPQTKRNNFAAKVNSENQEKLLILRKEDASASSGQSELSLVQMGLKQVVASSFWLVFEFQHFKV

GCNAARKFPVNGIANAQPQQCGTYRSHNGKLSIAIGHFCRIHQRAHTHFAIAKIAEFNPAVHCHHVVWHL

FWRTHLGAIQLCV

>gi|30062066|ref|NP_836237.1| oxidoreductase [Shigella flexneri 2a str. 2457T]

MKALTYHGPHHVQVENVPDPGIEQADDIILRITVTAICGSDLHLYRGKIPQVKHGDIFGHEFMGEVVETG

KDVKNLQKGDRVVIPFVIACGDCFFCRLQQYAACENTNAGKGAALNKKQIPAPAALFGYSHLYGGVPGGQ

AEYVRVPKGNVGPFKVPPLLSDDKALFLSDILPTAWQAAKNVQIQQGSSVAVYGAGPVGLLTIACARLLG

AEQIFVVDHHPYRLRFAADRYGAIPINFDEDSDPAQSIIEQTAGHRGVDAVIDAVGFEAKGSTTETVLTN

LKLEGSSGKALRQCIAAVRRGGIVSVPGVYAGFIHGFLFGDAFDKGLTFKMGQTHVHAWLGELLPLIEKG

LLKPEEIVTHYMPFEEAARGYEIFEKREEECRKVILVPGAQSAEAAQKAVSGLVNAMPGGTI

>gi|30062065|ref|NP_836236.1| hypothetical protein S0531 [Shigella flexneri 2a str. 2457T]

MYKAIIMPVDVFEMELSDKAVRHAEFLAQDDGVIHLLHVLPGSASLSLHRFAADVRRFEEHLQHEAEERL

QTMVSHFTIDPSRIKQHVRFGSVRDEVNELAEELGADVVVIGSRNPSISTHLLGSNASSVIRHANLPVLV

VR

>gi|30062064|ref|NP_836235.1| alkyl hydroperoxide reductase, F52a subunit [Shigella flexneri 2a str. 2457T]

MMMFKAQEINMLDTNMKTQLKAYLEKLTKPVELIATLDDSAKSAEIKELLAEIAELSDKVTFKEDNSLPV

RKPSFLITNPGSNQGPRFAGSPLGHEFTSLVLALLWTGGHPSKEAQSLLEQIRHIDGDFEFETYYSLSCH

NCPDVVQALNLMSVLNPRIKHTAIDGGTFQNEITDRNVMGVPAVFVNGKEFGQGRMTLTEIVAKIDTGAE

NRAAEELNKRDAYDVLIVGSGPAGAAAAIYSARKGIRTGLMGERFGGQILDTVDIENYISVPKTEGQKLA

GALKVHVDEYDVDVIDSQSASKLIPAAVEGGLHQIETASGAVLKARSIIVATGAKWRNMNVPGEDQYRTK

GVTYCPHCDGPLFKGKRVAVIGGGNSGVEAAIDLAGIVEHVTLLEFAPEMKADQVLQDKLRSLKNVDIIL

NAQTTEVKGDGSKVVGLEYRDRVSGNIHNIELAGIFVQIGLLPNTNWLEGAVERNRMGEIIIDAKCETNV

KGVFAAGDCTTVPYKQIIIATGEGAKASLSAFDYLIRTKTA

>gi|30062063|ref|NP_836234.1| alkyl hydroperoxide reductase subunit C [Shigella flexneri 2a str. 2457T]

MSLINTKIKPFKNQAFKNGEFIEITEKDTEGRWSVFFFYPADFTFVCPTELGDVADHYEELQKLGVDVYA

VSTDTHFTHKAWHSSSETIAKIKYAMIGDPTGALTRNFDNMREDEGLADRATFVVDPQGIIQAIEVTAEG

IGRDASDLLRKIKAAQYVASHPGEVCPAKWKEGEATLAPSLDLVGKI

>gi|30062061|ref|NP_836232.1| ISEhe3 orfB [Shigella flexneri 2a str. 2457T]

MLDVHPSGFYAWLQQPHSQRHQADLRLTGQIKQFWLESGCVYGYRKIHLDLRDSGQQCGVNRVWRLMKRV

GIKAQVGYRSPRARKGEASIVSPNRLQRQFNPDAPDERWVTDITYIRTHEGWLYLAVVVDLFSRKIIGWS

MQSRMTKDIVLNALLMAVWRRNPEKQVLVHSDQGSQYTSHEWQSFLKSHGLEGSMSRRGNCHDNAVAESF

FQLLKRERIKKKIYGTREEARSDIFDYIEMFYNSKRRHGSSEQMSPTEYENQYYQRLGSV

>gi|30062060|ref|NP_836231.1| ISEhe3 orfA [Shigella flexneri 2a str. 2457T]

MSGKRYPEEFKTEAVKQVVDRGYSVASVATRLDITTHSLYAWIKKYGPDSSTNKEQSDAQAEIRRLQKEL

KRVTDERDILKKAAAYFAKLSD

>gi|30062059|ref|NP_836230.1| IS911 orfA [Shigella flexneri 2a str. 2457T]

MKKRNFSAEFKRESAQLVVDQKYTVADAAKAMDVGLSTMTRWVKQLRDERQGKTPKASPITPEQIEIREL

RKKLQRIEMENEILKKATALLMSDSLNSSR

>gi|30062058|ref|NP_836229.1| IS911 orfB, partial [Shigella flexneri 2a str. 2457T]

MKELGLVSCQQPTHRYKRGGHEHVAIPNYLERQFAVTEPNQVWCGDVTYIWTGKRWAYLAVVLDLFARKP

VGWAMSFSPDSRLTMKALEMAWETRGKPGGVMFHSDQGSHYTSRQFRQLLWRYQIRQSMSRRGNCWDNSP

MERFFRSLKNEWMPVVGYVSFSEAAHAITDYIVGYYSALRPHEYNGGLPPNESENRYWKNSNSVASFC

>gi|30062057|ref|NP_836228.1| aminotransferase [Shigella flexneri 2a str. 2457T]

MTNNPLIPQSKLPQLGTTIFTQMSALAQQHQAINLSQGFPDFDGPRYLQERLAYHVAQGANQYAPMTGVQ

ALREAIAQKTERLYGYQPDADSDITVTAGATEALYAAITALVRNGDEVICFDPSYDSYAPAIALSGGIVK

RMALQPPHFRVDWQEFAALLSERTRLVILNTPHNPSATVWQQADFAALWQAIAGHEIFVISDEVYEHINF

SQQGHASVLAHPQLRERAVAVSSFGKTYHMTGWKVGYCVAPAPISAEIRKVHQYLTFSVNTPAQLALADM

LRAEPEHYLALPDFYRQKRDILVNALNESRLEILPCEGTYFLLVDYSAVSTLDDVEFCQWLTQEHGVAAI

PLSVFCADPFPHKLIRLCFAKKESTLLAAAERLRQL

>gi|30062056|ref|NP_836227.1| hypothetical protein S0519 [Shigella flexneri 2a str. 2457T]

MPHNPIRVVVGPANYFSHPGSFNHLHNFFTDEQLSRAVWIYGKRAIAAAQTKLPPAFGLPGAKHILFRGH

CSESDVQQLAAESGDDRSVVIGVGGGALLDTAKALARRLGLPFVAAPTIAATCAAWTPLSVWYNDAGQAL

HYEIFDDANFMVLVEPEIILNAPQQYLLAGIGDTLAKWYEAVVLAPQPETLPLTVRLGINNAQAIRDVLL

NSSEQALADQQNQQLTQSFCDVVDAIIAGGGMVGGLGDRFTRVAAAHAVHNGLTVLPQTEKFLHGTKVAY

GILVQSALLGQDDVLAQLTGAYQRFHLPTTLAELEVDINNQAEIDKVIAHTLRPVESIHYLPVTLTPDTL

RAAFEKVESFKA

>gi|30062055|ref|NP_836226.1| carbon starvation protein [Shigella flexneri 2a str. 2457T]

MNKSGKYLVWTVLSVMGAFALGYIALNRGEQINALWIVVASVCIYLIAYRFYGLYIAKNVLAVDPTRMTP

AVRHNDGLDYVPTDKKVLFGHHFAAIAGAGPLVGPVLAAQMGYLPGMIWLLAGVVLAGAVQDFMVLFVST

RRDGRSLGELVKEEMGPTAGVIALVACFMIMVIILAVLAMIVVKALTHSPWGTYTVAFTIPLAIFMGIYL

RYLRPGRIGEVSVIGLVFLIFAIISGGWVAESPTWAPYFDFTGVQLTWMLVGYGFVAAVLPVWLLLAPRD

YLSTFLKIGTIVGLAVGILIMRPTLTMPALTKFVDGTGPVWTGNLFPFLFITIACGAVSGFHALISSGTT

PKMLANEGQACFIGYGGMLMESFVAIMALVSACIIDPGVYFAMNSPMAVLAPAGTADVVASAAQVVSSWG

FSITPDTLNQIASEVGEQSIISRAGGAPTLAVGMAYILHGALGGMMDVAFWYHFAILFEALFILTAVDAG

TRAARFMLQDLLGVVSPGLKRTDSLPANLLATALCVLAWGYFLHQSVVDPLGGINTLWPLFGIANQMLAG

MALMLCAVVLFKMKRQRYAWVALVPTAWLLICTLTADWLKAFSPDAKVGFLVIANKFQAMIDSGNIPSQY

TESQLAQLVFNNRLDAGLTIFFMVVVVVLALFSIKTALAALKDPKPTAKETPYEPMPENVEEIVAQAKGA

H

>gi|30062054|ref|NP_836225.1| hypothetical protein S0517 [Shigella flexneri 2a str. 2457T]

MIWKRHLTLDELNATSDNTMVAHLGIVYTLLGDDVLEAEMPVDTRTHQPFGLLHGGASAALAETLGSMAG

FMMTRDGQCVVGTELNATHHRPVSEGKVRGVCQPLHLGRQNQSWEIVVFDEQGRRCCTCRLGTAVLG

>gi|30062053|ref|NP_836224.1| 2,3-dihydroxybenzoate-2,3-dehydrogenase [Shigella flexneri 2a str. 2457T]

MDFSGKNVWVTGAGKGIGYATALTFVEAGAKVTGFDQAFTQEQYPFATEVMDVADAGQVAQVCQRLLAET

ERLDVLINAAGILRMGATDQLSKEDWQQTFAVNVGGAFNLFQQTMNQFRRQRGGAIVTVASDAAHTARIG

MSAYGASKAALKSLALSVGLELAGSGVRCNVVSPGSTDTDMQRTLWVSDDAEEQRIRGFGEQFKLGIPLG

KIARPQEIANTILFLASDLASHITLQDIVVDGGSTLGA

>gi|30062052|ref|NP_836223.1| 2,3-dihydro-2,3-dihydroxybenzoate synthetase [Shigella flexneri 2a str. 2457T]

MAIPKLQAYALPESHDIPQNKVDWAFELQRAALLIHDMQDYFVSFWGENCPMMEQVIANIAALRDYCKQH

NIPVYYTAQPKEQSDEDRALLNDMWGPGLTRSPEQQKVVDRLTPDADDTVLVKWRYSAFHRSPLEQMLKE

SGRNQLIITGVYAHIGCMTTATDAFMRDIKPFMVADALADFSRDEHLMSLKYVAGRSGRVVMTEELLPAP

IPASKAALREVILPLLDESDEPFDDDNLIDYGLDSVRMMALAARWRKVHGDIDFVMLAKNPTIDAWWKLL

SREVK

>gi|30062051|ref|NP_836222.1| enterobactin synthase subunit E [Shigella flexneri 2a str. 2457T]

MSIPFTRWPEEFARRYREKGYWQDLPLTDILTRHAASDSIAVIDGERQLSYRELNQAADNLACSLRRQGI

KPGETALVQLGNVAELYITFFALLKLGVAPVLALFSHQRSELNAYASQIEPALLIADRQHALFSGDDFLN

TFVAEHSSIRVVQLHNDSGEHNLQDAINHPAEGFTATPSPADEVAYFQLSGGTTGTPKLIPRTHNDYYYS

VRRSAEICQFTQQTRYLCSIPAAHNYAMSSPGSLGVFLAGGTVVLAADPSATLCFPLIAKHQVNVTALVP

PAVSLWLQALAEGESRAQLASLKLLQVGGARLSATLAARIPAEIGCQLQQVFGMAEGLVNYTRLDDSAEK

IIHTQGYPMCPDDEVWVADAEGNPLPQGEVGRLMTRGPYTFRGYYKSPQHNASAFDANGFYCSGDLISID

PEGYITVQGREKDQINRGGEKIAAEDIENLLLSHPAVIYAALVSMENELMGEKSCAYLVVKEPLRAVQVR

RFLREQGIAEFKLPDRVECVDSLPLTAVGKVDKKQLRQWLASRASA

>gi|30062050|ref|NP_836221.1| iron-enterobactin transporter periplasmic binding protein [Shigella flexneri 2a str. 2457T]

MRLAPLYRNALLLTGLLLSGIAVVQAADWPRQITDSRGTHTLESQPQRIVSTSVTLTGSLLAIDAPVIAS

GATTPNNRVADDQGFLRQWSKVAKERKLQRLYIGEPSAEAVAAQMPDLILISATGGDSALALYDQLSTIA

PTLIINYDDKSWQSLLTQLGEITGHEKQAAERIAQFDKQLAAAKEQIKLPPQPVTAIVYTAAAHSANLWT

PESAQGQMLEQLGFTLAKLPAGLNASQSQGKRHDIIQLGGENLAAGLNGESLFLFAGDQKDADAIYANPL

LAHLPAVQNKQVYALGTETFRLDYYSAMQVLERLKALF

>gi|30062049|ref|NP_836220.1| enterobactin exporter EntS [Shigella flexneri 2a str. 2457T]

MNKQSWLLNLSLLKTHPAFRAVFLARFISIVSLGLLGVAVPVQIQMMTHSTWQVGLSVTLTGGAMFVGLM

VGGVLADRYERKKVILLARGTCGIGFIGLCLNALLPEPSLLAIYLLGLWDGFFASLGVTALLAATSALVG

RENLMQAGAITMLTVRLGSVISPMIGGLLLATGGVAWNYGLAAAGTFITLLPLLSLPELPPPPQPLEHPL

KSLLAGFRFLLASPLLGGLLTMASAVLVLYPALADNWQMSAAQIGFLYAAIPLGAAIGALTSGKLAHSAR

PGLLMLLSTLGSFLAIGLFGLMPMWILGVVCLALFGWLSAVSSLLQYTMLQTQTPEAMLGRINGLWTAQN

VTGDAIGAALLGGLGAMMTPVASASASGFGLLIIGVLLLLVLVELRRFRQTPPQVTASDS

>gi|30062048|ref|NP_836219.1| iron-enterobactin transporter membrane protein [Shigella flexneri 2a str. 2457T]

MEVRISGSVAVTRAIAVPGLLLLLIIATALSLLIGAKSLPASVVLEAFSGTCQSADCTIVLDARLPRTLA

GLLAGGALGLAGALMQTLTRNPLADPGLLGVNAGASFAIVLGAALFGYSSAQEQLAMAFAGALVASLIVA

FTGSQGGGQLSPVRLTLAGVALAAVLEGLTSGIALLNPDVYDQLRFWQAGSLDIRNLHTLKVVLIPVLIA

GATALLLSRALNSLSLGSDTATALGSRVARTQLIGLLAITVLCGSATAVVGPIAFIGLMMPHMARWLVGA

DHRWSLPVTLLATPALLLFADIIGRVIVPGELRVSVVSAFIGAPVLIFLVRRKTRGGA

>gi|30062047|ref|NP_836218.1| iron-enterobactin transporter permease [Shigella flexneri 2a str. 2457T]

MIYVSRRLLITCLLLISACVVAGIWGLRSGAVTLEISQVFAALMGDAPRSMTMVVTEWRLPRVLMALLIG

AALGVSGAIFQSLMRNPLGSPDVMGFNTGAWSGVLVAMVLFGQDLTAIALAAMVGGIITSLLVWLLAWRN

GIDTFRLIIIGIGVRAMLVAFNTWLLLKASLETALTAGLWNAGSLNGLTWAKTSPSAPIIILMLIAATLL

VRRMRLLEMGDDTACALGVSVERSRLLMMLVAVVLTAAATALAGPISFIALVAPHIARRISGTARWGLTQ

AALCGALLLLAAVLCAQQLFMPYQLPVGVVTVSLGGIYLIVLLIQESRKK

>gi|30062046|ref|NP_836217.1| iron-enterobactin transporter ATP-binding protein [Shigella flexneri 2a str. 2457T]

MTESVARLRGEQLTLGYGKYTVAENLTVEIPDGHFTAIIGPNGCGKSTLLRTLSRLMTPAHGHVWLDGEH
IQHYASKEVARRIGLLAQNATTPGDITVQELVARGRYPHQPLFTRWRKEDEEAVTKAMQATGITHLADQS
VDTLSGGQRQRAWIAMVLAQETAIMLLDEPTTWLDISHQIDLLELLSELNREKGYTLAAVLHDLNQACRY
ASHLIALREGKIVAQGAPKEIVTAELIERIYGLRCMIIDDPVAGMPLVVPLGRTAPSTANS

>gi|30062045|ref|NP_836216.1| IS1 orfB [Shigella flexneri 2a str. 2457T]
MSRQCTHYGRWPQHGFTSLKKLRPQSVTSRIQPGSDVIVCAEMDEHWGYVGAKSRQRWLFYAYDRIRRTV
VAHVFGERTLATLERLLSLLSAFEVVVWMTDGWPLYESRLKGKLHVISKRYTQRIERHNLNLRQHLARLV
RKSLSFSKSVELHDKVIGHYLNIKHYQ

>gi|30062044|ref|NP_836215.1| IS1 orfA [Shigella flexneri 2a str. 2457T]
MASISIRCPSCSATEGVVRNGKSTAGHQRYLCSHCRKTWQLQFTYTASQPGTHQKIIDMAMNGVGCRASA
RIMGVGLNTVLRHLKNSGRSR

>gi|30062043|ref|NP_836214.1| enterobactin synthase subunit F [Shigella flexneri 2a str. 2457T]
MSQHLPLVAAQPGIWMAEKLSDLPSAWSVAHYVELTGEVDAPLLARAVVAGLAQADTLRMRFTEDNGEVW
QWVDDALIFELPEIIDLRTNIDPHGTAQALMLADLQQDLRVDSGKPLVFHQLIQVADNRWYWYQRYHHLL
VDGFSFPAITRQIANIYCALLRGEPTPASPFTPFADVVEEYQQYRESEAWQRDAAFWVEQRRQLPPPASL
SPAPLAGRSASADILRLKMEFTDGEFRQLATQLSGVQRTDLALALTALWLGRLCNRMDYAAGFIFMRRLG
SAALTATGPVLNVLPLGIHIAAQETLPELATRLATRLAAQLKKMRRHQRYDAEQIVRDSGRAAGDEPLFG
PVLNIKVFDYQLDIPGVQAQTHTLATGPVNDLELALFPDEHGDLSIEILANKQRYDEPTLIQHAERLKML
IAQFAADPALLCGDVDIMLPGEYAQLAQINATQVEIPETTLSALVAEQAAKTPDAPALADARYLFSYREM
HEQVVALANLLRERGVKPGDSVAVALPRSVFLTLALHAIVEAGAAWLPLDTGYPDDRLKMMLEDARPSLL
ITTDDQLPRFSDVPNLTNLCYNAPLTPQGSAPLQLSQPHHTAYIIFTSGSTGRPKGVMVGQTAIVNRLLW
MQNHYPLTGEDVVAQKTPCSFDVSVWEFFWPFIAGAKLVMAEPEAHRDPLAMQQFFAEYGVTTTHFVPSM

LAAFVASLTPQTARQSCVTLKQVFCSGEALPADLCREWQQLTGAPLHNLYGPTEAAVDVSWYPAFGEELA

QVRGSSVPIGYPVWNTGLRILDAMMHPVPPGVAGDLYLTGIQLAQGYLGRPDLTASRFIADPFAPGERMY

RTGDVARWLDNGAVEYLGRSDDQLKIRGQRIELGEIDRVMQALPDVKQAVTHACVINQAAATGGDARQLV

GYLVSQSGLPLDTSALQAQLRETLPPHMVPVVLLQLPQLPLSANGKLDRKALPLPELKAQAPGRAPKAGS

ETIIAAAFASLLGCDVQDADADFFALGGHSLLAMKLAAQLSRQFARQVTPGQVMVASTVAKLATIIDGEE

DSSRRMGFETILPLREGNGPTLFCFHPASGFAWQFSVLSRYLDPQWSIIGIQSPRPHGPMQTATNLDEVC

EAHLATLLEQQPHGIAARLRARGEQVAFLGLLDTWPPETQNWQEKEANGLDPEVLAEINREREAFLAAQQ

GSTSTELFTTIEGNYADAVRLLTTAHSVPFDGKATLFVAERTLQEGMSPERAWSPWIAELDIYRQDCAHV

DIISPGAFVKIGPIIRATLNR

>gi|30062042|ref|NP_836213.1| enterobactin/ferric enterobactin esterase [Shigella flexneri 2a str. 2457T]

MTALKVGSESWWQSKHGPEWQRLNDEMFEVTFWWRDPQGSEEYSTIKRVWVYITGVTDHHQNSQPQSMQR

IAGTDVWQWTTQLNANWRGSYCFIPTERDDIFSAPSPDRLELREGWRKLLPQAIADPLNPQSWKGGLGHA

VSALEMPQAPLQPGWDCPQAPEIPAKEIIWKSERLKNSRRVWIFTTGDVTAEERPLAVLLDGEFWAQSMP

VWPVLTSLTHRQQLPPAVYVLIDAIDTTHRAHELPCNADFWLAVQQELLPLVKVIAPFSDRADRTVVAGQ

SFGGLSALYAGLHWPERFGCVLSQSGSYWWPHRGGQQEGVLLEKLKAGEVSAEGLRIVLEAGIREPMIMR

ANQALYAQLHPIKESIFWRQVDGGHDALCWRGGLMQGLIDLWQPLFHDRS

>gi|30062041|ref|NP_836212.1| outer membrane receptor FepA [Shigella flexneri 2a str. 2457T]

MNKKIHSLALLVNLGIYGVAQAQEPTDTPVSHDDTIVVTAAEQNLQAPGVSTITADEIRKNPVARDVSEI

IRTMPGVNLTGNSTSGQRGNNRQIDIRGMGPENTLILIDGKPVSSRNSVRQGWRGERDTRGDTSWVPPEM

IERIEVLRGPAAARYGNGAAGGVVNIITKKGSGEWHGSWDAYFNAPEHKEEGATKRTNFSLTGPLGDEFS

FRLYGNLDKTQADAWDINQGHQSARAGTYATTLPAGREGVINKDINGVVRWDFAPLQSLELEAGYSRQGN

LYAGDTQNTNSDAYTRSKYGDETNRLYRQNYALTWNGGWDNGVTTSNWVQYEHTRNSRIPEGLAGGTEGK

FNEKATQDFVDIDLDDVMLHSEVNLPIDFLVNQTLTLGTEWNQQRMKDLSSNTQALTGTNTGGAIDGVSA

TDRSPYSKAEIFSLFAENNMELTDSTIVTPGLRFDHHSIVGNNWSPALNISQGLGDDFTLKIGIARAYKA

PSLYQTNPNYILYSKGQGCYASAGGCYLQGNDDLKVETSINKEIGLEFKRDGWLAGVTWFRNDYRNKIEA

GYVAVGQNAVGTDLYQWDNVPKAVVEGLEGSLNVPVSETVMWTNNITYMLKSENKTTGDRLSIIPEYTLN

STLSWQAREDLSMQTTFTWYGKQQPKKYNYKGQPAVGPETKEISPYSIVGLSATWDVTKNVSLTGGVDNL

FDKRLWRAGNAQTTGDLAGANYIAGAGAYTYNEPGRTWYMSINTHF

>gi|30062040|ref|NP_836211.1| phosphopantetheinyltransferase component of enterobactin synthase multienzyme complex [Shigella flexneri 2a str. 2457T]

MVDMKTTHTSLPFAGHTLHFVEFDPANFCEQDLLWLPHYAQLQHAGRKRKTEHLAGRIAAVYALREYGYK

CVPAIGELRQPVWPAEVYGSISHCGATALAVVSRQPIGVDIEEIFSAQTATELTDNIITPAEHERLADCG

LAFSLALTLAFSAKESAFKASEIQTDAGFLDYQIISWNKQQVIIHRENEMFAVHWQIKEKIVITLCQHD

>gi|30062039|ref|NP_836210.1| hypothetical protein S0500 [Shigella flexneri 2a str. 2457T]

MKPQEIFLSILGLPLRTSTTRMVVPSSHGGNINMLTKYALVAVIVLCLTVLGFTLLVGDSLCEFTVKERN

IEFKAVLAYEPKK

>gi|30062038|ref|NP_836209.1| hypothetical protein S0499 [Shigella flexneri 2a str. 2457T]

MLTKYALVAVIVLCLTVLGFTLLAGDSLCEFTVKERNIEFRAVLAYEPKK

>gi|30062037|ref|NP_836208.1| IS150 orfB [Shigella flexneri 2a str. 2457T]

MKVLNELRQFYPLDELLRAAEIPRSTFYYHLKALSKPDKYADVKKRIGEIYHENRGRYGYRRVTLSLHRE

GKQINHKAVQRLMGTLSLKAAIKVKRYRSYRGEVGQTAPNVLQRDFKLRGQTRSGLPMLLNLQSMGASCI

CLQ

>gi|30062036|ref|NP_836207.1| IS103 orf [Shigella flexneri 2a str. 2457T]

MSKPKYPFEKRLEVVNHYFTTDDGYRIISARFGVPRTQVRTWVALYEKHGEKGLIPKPKGVSADPELRIK

VVKAVIEQHMSLNQAAAHFMLAGSGSVARWLKVYEERGEAGLRALKIGTKRNIAISVDPEKAASALELSK
DRRIEDLERQVRFLETRLMYLKKLKALAHPTKK

>gi|30062035|ref|NP_836206.1| carboxylate-amine ligase [Shigella flexneri 2a str. 2457T]
MPLPDFHVSEPFTLGIELEMQVVNPPGYDLSQDSSMLIDAVKNKITAGEVKHDITESMLELATDVCRDIN
QAAGQFSAMQKVVLQAAADHHLEICGGGTPPFQKWQRQEVCDNERYQRTLENFGYLIQQATVFGQHVHVG
CASGDDAIYLLHGLSRFVPHFIALSAASPYMQGTDTRFAPSRPNIFSAFPDNGPMPWVSNWQQFEALFRC
LSYTTMIDSIKDLHWDIRPSPHFGTVEVRVMDTPLTLSHAVNMAGLIQATAHWLLTERPFKHQEKDYLLY
KFNRFQACRYGLEGVITDPHTGDRRPLTEDTLRLLEKIAPSAHKIGASSAIEALHRQVVSGLNEAQLMRD
FVADGGSLIGLVKKHCEIWAGD

>gi|30062034|ref|NP_836205.1| hypothetical protein S0495 [Shigella flexneri 2a str. 2457T]
MKHPLETLTTAAGILLMAFLSCLLLPAPALGLTLAQKLVTMFHLMDLSQLYTLLFCLWFLVLGAIEYFVL
RFIWRRWFSLAD

>gi|30062033|ref|NP_836204.1| hypothetical protein S0494 [Shigella flexneri 2a str. 2457T]
MDKQSLHETAKRLALELPFVELCWPFGPEFDVFKIGGKIFMLSSELRGVPFINLKSDPQKSLLNQQIYPS
IKPGYHMNKKHWISVYSGEEISEALLRDLINDSWNLVVDGLAKRDQKRVRPG

>gi|30062032|ref|NP_836203.1| dihydropteridine reductase [Shigella flexneri 2a str. 2457T]
MDIISVALKRHSTKAFDASKKLTPEQAEQIKTLLQYSPSSTNSQPWHFIVASTEEGKARVAKSAAGNYVF
NKRKILDASHVVVFCAKTAMDDAWLKLVVDQEDADGRFATPEAKAANDKGRKFFAYMHRKDLHDDAEWMA
KQVYLNVGNFLLGVAALGLDAVPIEGFDAAILDAEFGLKEKGYTSLVVVPVGHHSVEDFNATLPKSRLPQ
NTTLTEV

>gi|30062031|ref|NP_836202.1| transporter [Shigella flexneri 2a str. 2457T]

MQDLISQVEDLAGIEIDHTTSMVMIFGIIFLTAVVVHIILHWVVLRTFEKRAIASSRLWLQIITQNKLFH

RLAFTLQGIIVNIQAVFWLQKGTEAADILTTCAQLWIMMYALLSVFSLLDVILNLAQKFPAASQLPLKGI

FQGIKLIGAILVGILMISLLIGQSPAILISGLGAMAAVLMLVFKDPILGLVAGIQLSANDMLKLGDWLEM

PKYGADGAVIDIGLTTVKVRNWDNTITTIPTWSLVSDSFKNWSGMSASGGRRIKRSISIDVTSIRFLDED

EMQRLNKAHLLKPYLTSRHQEINEWNRQQGSTESVLNLRRMTNIGTFRAYLNEYLRNHPRIRKDMTLMVR

QLAPGDNGLPLEIYAFTNTVVWLEYESIQADIFDHIFAIVEEFGLRLHQSPTGNDIRSLAGAFKQ

>gi|30062030|ref|NP_836201.1| phenylalanine transporter [Shigella flexneri 2a str. 2457T]

MKNASTVSEDTASNQEPTLHRGLHNRHIQLIALGGAIGTGLFLGIGPAIQMAGPAVLLGYGVAGIIAFLI

MRQLGEMVVEEPVSGSFAHFAYKYWGPFAGFLSGWNYWVMFVLVGMAELTAAGIYMQYWFPDVPTWIWAA

AFFIIINAVNLVNVRLYGETEFWFALIKVLAIIGMIGFGLWLLFSGYGGEKASIDNLWRYGGFFATGWNG

LILSLAVIMFSFGGLELIGITAAEARDPEKSIPKAVNQVVYRILLFYIGSLVVLLALYPWVEVKSNSSPF

VMIFHNLDSNVVASALNFVILVASLSVYNSGVYSNSRMLFGLSVQGNAPKFLTRVSRRGVPINSLMLSGA

ITSLVVLINYLLPQKAFGLLMALVVATLLLNWIMICLAHLRFRAAMRRQGRETQFKALLYPFGNYLCIAF

LGMILLLMCTMDDMRLSAILLPVWIVFLFVAFKTLRRK

>gi|30062029|ref|NP_836200.1| inner membrane component for iron transport [Shigella flexneri 2a str. 2457T]

MIEWIIRRSVANRFLVLMGALFLSIWGTWTIINTPVDALPDLSDVQVIIKTSYPGQAPQIVENQVTYPLT

TTMLSVPGAKTVRGFSQFGDSYVYVIFEDGTDPYWARSRVLEYLNQVQGKLPAGVSAELGPDATGVGWIY

EYALVDRSGKHDLADLRSLQDWFLKYELKTIPDVAEVASVGGVVKEYQVVIDPQRLAQYGISLAEVKSAL

DASNQEAGGSSIELAEAEYMVRASGYLQTLDDFNHIVLKASENGVPVYLRDVAKVQIGPKMRRGIAELNG

EGEVAGGVVILRSGKNAREVIAAVKDKLETLKSSLPEGVEIVTTYDRSQLIDRAIDNLSGKLLEEFIVVA

VVCAPFLWHVRSALVAIISLPLGLCIAFIVMPFQGLNANIMSLGGIAIAVGAMVDAAIVMIENAHKRLEE

WQHQHPDATLDNKTRWQVITDASVEVGPALFISLLIITLSFIPIFTLEGQEGRLFGPLAFTKTYAMAGAA

LLAIVVIPILMGYWIRGKIPPESSNPLNRFLIRVYHPLLLKVLHWPKTTLLVAALSVLTVLWPLNKVGGE

FLPQINEGDLLYMPSTLPGISAAEAASMLQKTDKLIMSVPEVARVFGKTGKAETATDSAPLEMVETTIQL

KPQDQWRPGMTMDKIIEELDNTVRLPGLANLWVPPIRNRIDMLSTGIKSPIGIKVSGTVLADIDAMAEQI

EEVARTVPGVASALAERLEGGRYINVEINREKAARYGMTVADVQLFVTSAVGGAMVGETVEGIARYPINL

RYPQSWRDSPQALRQLPILTPMKQQITLADVADVKVSTGPSMLKTENARPTSWIYIDARDRDMVSVVHDL

QKAIAEKVQLKPGTSVAFSGQFELLERANHKLKLMVPMTLMIIFVLLYLAFRRVGEALLIISSVPFALVG

GIWLLWWMGFHLSVATGTGFIALAGVAAEFGVVMLMYLRHAIEAEPSLNNPQTFSEQKLDEALYHGAVLR

VRPKAMTVAVIIAGLLPILWGTGAGSEVMSRIAAPMIGGMITAPLLSLFIIPAAYKLMWLHRHRVRN

>gi|30062028|ref|NP_836199.1| insertion sequence 2 OrfA protein [Shigella flexneri 2a str. 2457T]

MIVLILVFRLVIGEQIIDVLGPEKRRRRTTQEKIAIVQQSFEPGMTVSLVARQHGVAASQLFLWRKQYQE

GSLTAVAAGEQVVPASELAAAMKQIKELQRLLGKKTMKNELLKEAVEYGRAKKWIAHAPLLPGDGE

>gi|30062027|ref|NP_836198.1| insertion sequence 2 OrfB protein [Shigella flexneri 2a str. 2457T]

MDGRRSRHTDDTDVLLRIHHVIGELPTYGYRRVWALLRRQAELDGMPAINAKRVYRLMRQNALLLERKPA

VSPSKRAHTGRVAVKESNQRWCSDGFEFCCDNGERLRVTFALDCCDREALHWARTTGGFNSETVQDVMLG

AVERRFGNDLPSSPVEWLTDNGSCYRANETRQFARMLGLEPKNTAVRSPESNGIAESFVKTIKRDYISIM

PKPDGLTAAKNLAEAFEHYNEWHPHSALGYRSPREYLRQRACNGLSDNRCLEI

>gi|30062026|ref|NP_836197.1| IS629 orfA [Shigella flexneri 2a str. 2457T]

MTKNTRFSPEVRQRAVRMVLESQGEYDSQWATICSIAPKIGCTPETLRVWVRQHERDTGGDDGGLTTAER

QRLKEPERENRELRRSNDILRQASAYFAKAEFDRLWKK

>gi|30062025|ref|NP_836196.1| IS629 orfB [Shigella flexneri 2a str. 2457T]

MAVMGLAGVLRGKKVRTTISRKAVAAGDRVNRQFVAERPDQLWVADFTYVSTWRGFVYVAFIIDVFAGYI

VGWRVSSSMETTFVLDALEQALWARRPSGTVHHSDKGSQYVSLAYTQRLKEAGLLASTGSTGDSYDNAMA

ESINGLYKAEVIHRKSWKNRAEVELATLTWVDWYNNRRLLERLGHIPPAEAEKAYYASIGNDDLAA

>gi|30062024|ref|NP_836195.1| IntA, partial [Shigella flexneri 2a str. 2457T]

MAISDTKLRTIYGKPYSGPQEVADADGLSVRISPKGVIQFQYRYRWHGKPNRLGLGRYPSLSLKDARQIT

ADLRKLYFSGTDPRTYFEEKVENSMTVAQCLDYWFDN

>gi|30062023|ref|NP_836194.1| periplasmic copper-binding protein [Shigella flexneri 2a str. 2457T]

MKKALQVAMFSLFTVIGFNAQANEHHHETMSEAQPQVISATGVVKGVDLESKKITIHHDPIAAVNWPEMT

MRFTITPQTKMSEIKTGDKVAFNFVQQGNLSLLQDIKVSQ

>gi|30062022|ref|NP_836193.1| copper/silver efflux system outer membrane protein CusC [Shigella flexneri 2a str. 2457T]

MSPCKLLPFCVALALTGCSLAPDYQRPVMPVPQQFSLSQNGLVNAADNYQNVGWRTFFVDNQVKTLISEA

LVNNRDLRMATLKVQEARAQYRLTDADRYPQLNGEGSGSWSGNLKGDSATTREFSTGLNASFDLDFFGRL

KNMSEAERQNYLATEEAQRAVHILLVSNVAQSYFNQQLAYAQLQIAEETLRNYQQSYAFVEKQLLTGSSN

VLALEQARGVIESTRSDIAKRQGELAQANNALQLLLGSYGKLPQAQTVNSGSLQSVKLPAGLSSQILLQR

PDIMEAEHALMAANANIGAARAAFFPSISLTSGISTASSDLSSLFNASSGMWNFIPKIEIPIFNAGRNQA

NLDIAEIRQQQSVVNYEQKIQNAFKEVADALALRQSLDDQISAQQRYLASLQITLQRARALYQHGAVSYL

EVLDAERSLFATRQTLLDLNYARQVNEIYLYTALGGGWQQ

>gi|30062021|ref|NP_836192.1| DNA-binding transcriptional activator CusR [Shigella flexneri 2a str. 2457T]

MKLLIVEDEKKTGEYLTKGLTEAGFVVDLADNGLNGYHLAMTGDYDLIILDIMLPDVNGWDIVRMLRSAN

KGMPILLLTALGTIEHRVKGLELGADDYLVKPFAFAELLARVRTLLRRGAAVIIESQFQVADLMVDLVSR

KVTRSGTRITLTSKEFTLLEFFLRHQGEVLPRSLIASQVWDMNFDSDTNAIDVAVKLLRGKIDNDFEPKL

IQTVRGVGYMLEVPDGQ

>gi|30062020|ref|NP_836191.1| sensor kinase CusS [Shigella flexneri 2a str. 2457T]

MVSKPFQRPFSLATRLTFFISLATIAAFFAFAWIIIHSVKVHFAEQDINDLKEISATLERVLNHPDETQA

RRLMTLEDIVSGYSNVLISLADSHGKTVYHSPGAPDIREFARDAIPDKDARGGEVYLLSGPTIMMPGHGH

GHMEHSNWRMINLPVGPLVDGKPIYTLYIALSIDFHLHYINDLMNKLIMTASVISILIVFIVLLAVHKGH

APIRSVSRQIQNITSKDLDVRLDPQTVPIELEQLVLSFNHMIERIEDVFTRQSNFSADIAHEIRTPITNL

ITQTEIALSQSRSQKELEDVLYSNLEELTRMAKMVSDMLFLAQADNNQLIPEKKMLNLADEVGKVFDFFE

ALAEDRGVELRFVGDKRQVAGDPLMLRRALSNLLSNALRYTPTRETIVVRCQTVDHQVQVSVENPGTPIA

PEHLPRLFDRFYRVDPSRQRKGEGSGIGLAIVKSIVVAHKGTVAVTSDARGTRFVITLPA

>gi|30062019|ref|NP_836190.1| hypothetical protein S0476 [Shigella flexneri 2a str. 2457T]

MRKFIFVLLTLLLVSPFSFAMKGIIWQPQNRDSQVTDTQWQGLMSQLRFQGFDTLVLQWTRYGDAFTQPE

QRTLLFKRAAAAQQTGLKLIVGLNADPEFFMHQKQSSAALESYLNRLLAADLQQARLWSAAPGITPDGWY

ISAEIDDLNWRSEAARQPLLTWLNNAQRLISDVSAKPVYISSFFAGNMSPDGYRQLLEQVKATGVNVWVQ

DGSGVDKLTAEQRERYLQASADCQSSAPASGIVYELFVAGKGKTFTAKPKPDAEIASLLAKRSSCGKDML

YFSLRYLPVAHGILEY

>gi|30062018|ref|NP_836189.1| envelope protein [Shigella flexneri 2a str. 2457T]

MQLSSSEPCVVILTEKEVEVSVNNHATFTLPKNYLAAFACNNNVIELSTLNHVLITHINRNIINDYLLFL

NKKLTCVKPWSRLATPVIACHSRTPEVFRLATNHSKQQSSKPCEAELTRALLFTVLSNFLEQSRFIALLM

YILRSSVRDSVCRIIQSDIQHYWNLRIVASSLCLSPSLLKKKLKNENTSYSQIVTECRMRYAVQMLLMDN

KNITQVAQLCGYSSTSYFISVFKAFYSLTPLNYLAKQRQKVMW

>gi|30062017|ref|NP_836188.1| IS1 orfA [Shigella flexneri 2a str. 2457T]

MASISIRCPSCSATEGVVRNGKSTAGHQRYLCSHCRKTWQLQFTYTASQPGTHQKIIDMAMNGVGCRASA

RIMGVGLNTVLRHLKNSGRSR

>gi|30062016|ref|NP_836187.1| IS1 orfB [Shigella flexneri 2a str. 2457T]

MSRQCTHYGRWPQHGFTSLKKLRPQSVTSRIQPGSDVIVCAEMDEHWGYVGAKSRQRWLFYAYDRIRRTV

VAHVFGERTLATLERLLSLLSAFEVVVWMTDGWPLYESRLKGKLHVISKRYTQRIERHNLNLRQHLARLG

RKSLSFSKSVELHDKVIGHYLNIKHYQ

>gi|30062015|ref|NP_836186.1| chaperone [Shigella flexneri 2a str. 2457T]

MMTKIKLLMLMIFYLIISASAHAAGGIALGATRIIYPADAKQTAVWIKNSHTNELFLVNSWIENSSGVKE

KSFIITPPLFVSEPKSENTLRIIYTGPPLAADRESLFWMNVKTIPSVDKNALNGRNVLQLAILSRMKLFL

RPIQLQELPAEAPDTLKFSRSGNYINVHNPSPFYVTLVNLQVGSQKLGNAMAAPRVSSQIPLPSGVQGKL

KFQTVNDYGSVTPVREVNLN

>gi|30062014|ref|NP_836185.1| fimbrial-like protein [Shigella flexneri 2a str. 2457T]

MESINEIEGIYMKLRFISSALAAALFAATGSYAAVVDGGTIHFEGELVNAACSVNTDSADQVVTLGQYRT

DIFNAVGNTSALIPFTIQLNDCDPVVAANAAVAFSGQADAINDNLLAIVSSTNTTTATGVGIEILDNTSA

ILKPDGNSFSTNQNLIPGTNVLHFSARYKGTGTSASAGQANADATFIMRYE

>gi|30062013|ref|NP_836184.1| bifunctional 5,10-methylene-tetrahydrofolate dehydrogenase/ 5,10-methylene-tetrahydrofolate cyclohydrolase [Shigella flexneri 2a str. 2457T]

MAAKIIDGKTIAQQVRSEVAQKVQARIAAGLRAPGLAVVLVGSNPASQIYVASKRKACEEVGFVSRSYDL

PETTSEAELLELIDALNADNTIDGILVQLPLPAGIDNVKVLERIHPDKDVDGFHPYNVGRLCQRAPRLRP

CTPRGIVTLLERYNIDTFGLNAVVIGASNIVGRPMSMELLLAGCTTTVTHRFTKNLRHHVENADLLIVAV

GKPGFIPGDWIKEGAIVIDVGINRLENGKVVGDVVFEDAAKRASYITPVPGGVGPMTVATLIENTLQACV

EYHDPQGE

>gi|30062011|ref|NP_836182.1| hypothetical protein S0466 [Shigella flexneri 2a str. 2457T]

MPTVITHAAVPLCIGLGLGSKGLLFAGIILAMLPDADVLSFKFGVAYGNVFGHRGFTHSLVFAFVVPLLC

VLIGRRWFRAGLIRCWLFLTVSLLSHSLLDSVTTGGKGVGWLWPWSDERFFAPWQVIKVAPFALSRYTTA

YGHQVIISELMWVWLPGMLLMGMLWWRRR

>gi|30062010|ref|NP_836181.1| cysteinyl-tRNA synthetase [Shigella flexneri 2a str. 2457T]

MLKIFNTLTRQKEEFKPIHAGEVGMYVCGITVYDLCHIGHGRTFVAFDVVARYLRFLGYKLKYVRNITDI

DDKIIKRANENGESFVALVDRMIAEMHKDFDALNILRPDMEPRATHHIAEIIELTEQLIAKGHAYVADNG

DVMFDVPTDPTYGVLSRQDLDQLQAGARVDVVDDKRNRMDFVLWKMSKEGEPSWPSPWGAGRPGWHIECS

AMNCKQLGNHFDIHGGGSDLMFPHHENEIAQSTCAHDGQYVNYWMHSGMVMVDREKMSKSLGNFFTVRDV

LKYYDAETVRYFLMSGHYRSQLNYSEENLKQARAALERLYTALRGTDKTVAPAGGEAFEARFIEAMDDDF

NTPEAYSVLFDMAREVNRLKAEDMAAANAMASHLRKLSAVLGLLEQEPEAFLQSGAQADDSEVAEIEALI

QQRLDARKAKDWAAADAARDRLNEMGIVLEDGPQGTTWRRK

>gi|30062009|ref|NP_836180.1| peptidyl-prolyl cis-trans isomerase B (rotamase B) [Shigella flexneri 2a str. 2457T]

MVTFHTNHGDIVIKTFDDKAPETVKNFLDYCREGFYNNTIFHRVINGFMIQGGGFEPGMKQKATKEPIKN

EANNGLKNTRGTLAMARTQAPHSATAQFFINVVDNDFLNFSGESLQGWGYCVFAEVVDGMDVVDKIKGVA

TGRNGMHQDVPKEDVIIESVTVSE

>gi|30062008|ref|NP_836179.1| UDP-2,3-diacylglucosamine hydrolase [Shigella flexneri 2a str. 2457T]

MATLFIADLHLCVEEPAITAGFLRFLAGEARKADALYILGDLFEAWIGDDDPNPLHRQMAAAIKAVSDSG

VPCYFIHGNRDFLLGKRFARESGMTLLPEEKVLELYGRRVLIMHGDTLCTDDAGYQAFRAKVHKPWLQML

FLALPLFVRKRIAARMRANSKEANSSKSLAIMDVNQNAVVSAMEKHQVQWLIHGHTHRPAVHELIANQQP

AFRVVLGAWHTEGSMVKVTADDVELIHFPF

>gi|30062007|ref|NP_836178.1| phosphoribosylaminoimidazole carboxylase catalytic subunit [Shigella flexneri 2a str. 2457T]

MSSRNNPARVAIVMGSKSDWATMQFAVEIFEILNVPHHVEVVSAHRTPDKLFSFAESAEENGYQVIIAGA

GGAAHLPGMIAAKTLVPVLGVPVQSAALSGVDSLYSIVQMPRGIPVGTLAIGKAGAANAALLAAQILATH

DKELHQRLNGWRKAQTDEVLENPDPRGAA

>gi|30062006|ref|NP_836177.1| phosphoribosylaminoimidazole carboxylase ATPase subunit [Shigella flexneri 2a str. 2457T]

MKQVCVLGNGQLGRMLRQAGEPLGIAVWPVGLDAEPAAVPFQQSVITAEIERWPETALTRELARHPAFVN

RDVFPIIADRLTQKQLFDKLHLPTAPWQLLADRSEWPAVFDRLGELAIVKRRTGGYDGRGQWSLRANETE

QLPAECYGECIVEQGINFSGEVSLVGARGFDGSTVFYPLTHNLHQDGILRTSVAFPQANAQQQAQAEEML

SAIMQELGYVGVMAMECFVTPQGLLINELAPRVHNSGHWTQNGASISQFELHLRAITDLPLPQPVVNNPS

VMINLIGSDVNYDWLKLPLVHLHWYDKEVRPGRKVGHLNLTDSDTSRLTATLEALIPLLPPEYASGVIWA

QSKFS

>gi|30062005|ref|NP_836176.1| carbamate kinase [Shigella flexneri 2a str. 2457T]

MKTLVVALGGNALLQRGEALTAENQYRNIASAVPALARLARSYRLAIVHGNGPQVGLLALQNLAWKEVEP

YPLDVLVAESQGMIGYMLAQSLSAQPQMPPVTTVLTRIEVSPDDPAFLQPEKFIGPVYQPEEQEALEAAY

GWQMKRDGKYLRRVVASPQPRKILDSEAIELLLKEGHVVICSGGGGVPVTEDGAGSEAVIDKDLAAALLA

EQINADGLVILTDADAVYENWGTPQQRAIRHATPDELAPFAKADGSMGPKVTAVSGYVRSRSKPAWIGAL

SRIEETLAGEAGTCISL

>gi|30062003|ref|NP_836174.1| hypothetical protein S0457 [Shigella flexneri 2a str. 2457T]

MGYLNNVTGYREDLLANRAIVKHGNFALLTPDGLIKNIIPDFENCDATILSTPKLGASFVDYLVTLHQNG

GNQQGFGGEGIETFLYVISGNITAKAEGKTFALSEGGYLYCPPGSLMTFVNAQAEDSQIFLYKRRYVPVE

GHAPWLFSGNASELERIHYEGMDDVILLDFLPKELGFDMNMHILSFAPGASHGYIETHVQEHGAYILSGQ
GVYNLGNAANLLI

>gi|30062002|ref|NP_836173.1| IS1 orfA [Shigella flexneri 2a str. 2457T]
MASISIRCPSCSATEGVVRNGKSTAGHQRYLCSHCRKTWQLQFTYIASQPGTHQKIIDMAMNGVGCRASA
RIMDVGLNTVLRHLKNSGRSR

>gi|30062001|ref|NP_836172.1| IS1 orfB [Shigella flexneri 2a str. 2457T]
MSRQCTHYGRWPQHGFTSLKKLRPQSVTSRIQPGSDVIVCAEMDEHWGYVGAKSRQRWLFYAYDRIRRTV
VAHVFGERTLATLERLLSLLSAFEVVVWMTDGWPLYESRLKGKLHVISKRYTQHIERHNLNLRQHLARLG
RKSLSFSKSVELHDKVIGHYLNIKHYQ

>gi|30062000|ref|NP_836171.1| glyoxylate-induced protein [Shigella flexneri 2a str. 2457T]
MLRFSANLSMLFGEYDFLARFEKAAQCGFRGVEFMFPYDYDIEELKQVLASNKLEHTLHNLPAGDWAAGE
RGIACIPGREEEFRDGVAAAIRW

>gi|30061999|ref|NP_836170.1| glyoxylate carboligase [Shigella flexneri 2a str. 2457T]
MAKMRAVDAAMYVLEKEGITTAFGVPGAAINPFYSAMRKHGGIRHILARHVEGVSHMAEGYTRATAGNIG
VCLGTSGPAGTDMITALYSASADSIPILCITGQAPRARLHKEDFQAVDIEAIAKPVSKMAVTVREAALVP
RVLQQAFHLMRSGRPGPVLVDLPFDVQVAEIEFDPDMYEPLPVYKPAASRMQIEKAVEMLIQAERPVIVA
GGGVINADAAALLQQFAELTSVPVIPTLMGWGCIPDDHELMAGMVGLQTAHRYGNATLLASDMVFGIGNR
FANRHTGSVEKYTEGRKIVHIDIEPTQIGRVLCPDLGIVSDAKAALTLLVEVAQEMQKAGRLPCRKEWVA
DCQQRKRTLLRKTHFDNVPVKPQRVYEEMNKAFGRDVCYVTTIGLSQIAAAQMLHVFKDRHWINCGQAGP
LGWTIPAALGVCAADPKRNVVAISGDFDFQFLIEELAVGAQFNIPYIHVLVNNAYLGLIRQSQRAFDMDY
CVQLAFENINSSEVNGYGVDHVKVAEGLGCKAIRVFKPEDIAPAFEQAKALMAQYRVPVVVEVILERVTN

ISMGSELDNVMEFEDIADNAADAPTETCFMHYE

>gi|30061998|ref|NP_836169.1| DNA-binding transcriptional repressor AllR [Shigella flexneri 2a str. 2457T]

MTEVRRRGRPGQAEPVAQKGAQALERGIAILQYLEKSGGSSSVSDISLNLDLPLSTTFRLLKVLQAADFV

YQDSQLGWWHIGLGVFNVGAAYIHNRDVLSVAGPFMRRLMLLSGETVNVAIRNGNEAVLIGQLECKSMVR

MCAPLGSRLPLHASGAGKALLYPLAEEELMSIILQTGLQQFTPTTLVDMPTLLKDLEQARELGYTVDKEE

HVVGLNCIASAIYDDVGRVVAAISISGPSSRLTEDRFVSQGELVRDTARDISTALGLKAHP

>gi|30061997|ref|NP_836168.1| ureidoglycolate hydrolase [Shigella flexneri 2a str. 2457T]

MKLQVLPLSQEAFSAYGDVIETQQRDFFHINNGLVERYHDLALVEILEQDRTLISINRAQPANLPLTIHE

LERHPLGTQAFIPMKGEVFVVVVALGDDKPDLSTLRAFITNGEQGVNYHRNVWHHPLFAWQRVTDFLTID

RGGSDNCDVESIPEQELCFA

>gi|30061996|ref|NP_836167.1| DNA-binding transcriptional activator AllS [Shigella flexneri 2a str. 2457T]

MFDPETLRTFIAVAETGSFSKAAERLCKTTATISYRIKLLEENTGVALFFRTTRSVTLTAAGEHLLSQAR

DWLSWLESMPSELQQVNDGVERQVNIVINNLLYNPQAVAQLLAWLNERYPFTQFHISRQIYMGVWDSLLY

EGFSLAIGVTGTEALANTFSLDPLGSVQWRFVMAADHPLANVEEPLTEAQLRRFPAVNIEDSAGTLTKRV

AWRLPGQKEIIVPDMETKIAAHLAGVGIGFLPKSLCQSMIDNQQLVSRVIPTMRPPSPLSLAWRKFGSCK

AVEDIVTLFTQHRPEISGFLEIFGTPRS

>gi|30061995|ref|NP_836166.1| tRNA 2-selenouridine synthase [Shigella flexneri 2a str. 2457T]

MQERHTEQDYRALLIADTPIIDVRAPIEFEQGAMPAAINLPLMDNDERASVGTCYKQQGSDAALALGHKL

VAGEIRQQRMYAWRAACLQNPQGILCCARGGQRSHIVQRWLHEAGIDYPLVEGGYKALRQTAIQATIELA

QKPIVLIGGCTGSGKTLLVQQQPNGVDLEGLARHRGSAFGRTLQPQLSQASFENLLAAEMLKTDARQDLR

LWVLEDESRMIGSNHLPECLRERMTQAAIAVVEDPFEIRLERLNEEYFLRMHHDFTHAYGDEQGWQEYCE

YLHHGLSAIKRRLGLQRYNELAAQLDTALTTQLTTGSTDGHLAWLVPLLKEYYDPMYRYQLEKKAEKVVF

RGEWAEVAVWVKAQ

>gi|30061994|ref|NP_836165.1| ABC transporter ATP-binding protein [Shigella flexneri 2a str. 2457T]

MPAENIVEVHHLKKSVGQGEHELSILTGVELVVKRGETIALVGESGSGKSTLLAILAGLDDGSSGEVSLV

GQPLHNMDEEARAKLRAKHVGFVFQSFMLIPTLNALENVELPALLRGESSAESRNGAKALLEQLGLGKRL

DHLPAQLSGGEQQRVALARAFNGRPDVLFADEPTGNLDRQTGDKIADLLFSLNREHGTTLIMVTHDLQLA

ARCDRCLRLVNGQLQEEA

>gi|30061993|ref|NP_836164.1| short chain dehydrogenase [Shigella flexneri 2a str. 2457T]

MTHKATEILTGKVMQKSVLITGCSSGIGLESALELKRQGFHVLAGCRKPNDVERMNNMGFTGVLIDLDSP

ESVNRAADEVIALTDNCLYGIFNNAGFGMYGPLSTISRAQMEQQFSANFFGAHQLTMRLLPAMLPHGEGR

IVMTSSVMGLISTPGRGAYAASKYALEAWSDALRMELRHSGIKVSLIEPGPIRTRFTDNVNQTQSDKPVE

NPGIAARFTLGPEAVVDKVRHAFISEKPKMRYPVTLVTWAVMVLERLLPGRVMDKILQG

>gi|30061992|ref|NP_836163.1| thioredoxin-like protein [Shigella flexneri 2a str. 2457T]

MSKKISTTESDSMSVENIVNINESNLQQVLEQSMTTPVLFYFWSERSQHCLQLTPILESLAAQYNGQFIL

AKLDCDAEQMIAAQFGLRAIPTVYLFQNGQPVDGFQGPQPEEAIRALLDKVLPREEELKAQQAMQLMQEG

NYTDALPLLKDAWQLSNQNGEIGLLLAETLIALNRSEDAEAVLKTIPLQDQDTRYQGLVAQIELLKQAAD

KPEIQQLQQQVAENPEDAALATQLALQLHQVGRNEEALELLFGHLRKDLTAADGQTRKTFQEILAALGTG

DALASKYRRQLYALLY

>gi|30061991|ref|NP_836162.1| metal resistance protein [Shigella flexneri 2a str. 2457T]

MPEKCRKHAMNSHNITNESLALALMLVVVAILISHKEKLALEKDILWSVGRAIIQLIIVGYVLKYIFSVD

DASLTLLMVLFICFNAAWNAQKRSKYIAKAFISSFIAITVGAGITLAVLILSGAIEFIPMQVIPVAGMIA

GNAMVAVGLCYNNLGQRVISEQQQIQEKLSLGATPKQASAILIRDSIRAALIPTVDSAKTVGLVSLPGMM

SGLIFAGIDPVKAIKYQIMVTFMLLSTASLSTIIACYLTYRKFYNSRHQLVVTQLKKK

>gi|30061990|ref|NP_836161.1| ABC transporter ATP-binding protein [Shigella flexneri 2a str. 2457T]

MQENSPLLQLQNVGYLAGDTKILNNINFSLRAGEFKLITGPSGCGKSTLLKIVASLISPTSGTLLFEGED

VSTLKPEIYRQQVSYCAQTPTLFGDTVYDNLIFPWQIRDQQPDPAIFLDFLERFALPDSILTKNIAELSG

GEKQRISLIRNLQFMPKVLLLDEITSALDESNKHNVNEMIHRYVREQNIAVLWVTHDKDEINHADKVITL

QPHAGEMQEARYELA

>gi|30061989|ref|NP_836160.1| protease [Shigella flexneri 2a str. 2457T]

MLIFIPILIFVALVIVGAGVKIVPQGYQWTVERFGRYTKTLQPGLSLVVPFMDRIGRKINMMEQVLDIPS

QEVISKDNANVTIDAVCFIQVIDAPRAAYEVSNLELAIINLTMTNIRTVLGSMELDEMLSQRDSINSRLL

RIVDEATNPWGIKVTRIEIRDVRPPAELISSMNAQMKAERTKRAYILEAEGIRQAEILKAEGEKQSQILK

AEGERQSAFLQAEARERSAEAEARATKMVSEAIASGDIQAVNYFVAQKYTEALQQIGSSSNSKVVMMPLE

ASSLMGSIAGIAELVKDSANKRTQP

>gi|30061988|ref|NP_836159.1| hypothetical protein S0440 [Shigella flexneri 2a str. 2457T]

MELMVVHPHIFWLSLGGLLLAAEMLGGNGYLLWSGVAAVITGLVVWLVPLGWEWQGVMFAILTLLAAWLW

WKWLSRRVREQKHSDSHLNQRGQQLIGRRFVLESPLVNGRGHMRVGDSSWPVSASEDLGAGTHVEVIAIE

GITLHIRAVSS

>gi|30061987|ref|NP_836158.1| DNA-binding transcriptional regulator CueR [Shigella flexneri 2a str. 2457T]

MNISDVAKITGLTSKAIRFYEEKGLVTPPMRSENGYRTYTQQHLNELTLLRQARQVGFNLEESGELVNLF

NDPQRHSADVKRRTLEKVAEIERHIEELQSMRDQLLALANACPGDDSADCPIIENLSGCCHHRAG

>gi|30061986|ref|NP_836157.1| amino acid/amine transport protein [Shigella flexneri 2a str. 2457T]

MMNTEGNNGNKPLGLWNVVSIGIGAMVGAGIFALLGQAALLMEASTWGAFAFGGIVAMFSGYAYARLGAS

YPSNGGIIDFFRRGLGNGVFSLALSLLYLLTLAVSIAMVARAFGAYAVQFLHEGSQEEHLILLYALGIIA

VMTLFNSLSNHAVGRLEVILVGIKMMILLLLIIAGVWSLQPAHISVSAPPSSGAFFSCIGITFLAYAGFG

MMANAADKVKDPQVIMPRAFLVAIGVTTLLYISLALVLLSDVSALELEKYADTAVAQAASPLLGHVGYVI

VVIGALLATASGINANLFAVFNIMDNMGSERELPKLMNKSLWRQSTWGNIIVVVLIMLMTAALNLDSLAS

VASATFLICYLAVFVVAIRLRHDIHASLPILIVGTLVMLLVIVGFIYSLWSQGSRALIWIIGAILLSLIV

AMVMKRNKTV

>gi|30061985|ref|NP_836156.1| glutaminase [Shigella flexneri 2a str. 2457T]

MLDANKLQQAVDQAYTQFHSLNGGQNADYIPFLANVPGQLAAVAIVTCDGNVYSAGDSDYRFALESISKV

CTLALALEDVGPQAVQDKIGADPTGLPFNSVIALELHGGKPLSPLVNAGAIATTSLINAENAEQRWQRIL

HIQQQLAGEQVALSDEVNQSEQTTNFHNRAIAWLLYSAGYLYCDAMEACDVYTRQCSTLINTVELATLGA

TLAAGGVNPLTHKRVLQADNVPYILAEMMMEGLYGRSGDWAYRVGLPGKSGVGGGILAVVPGVMGIAAFS

PPLDEEGNSVRGQKMVASVAKQLGYNVFKG

>gi|30061984|ref|NP_836155.1| copper exporting ATPase [Shigella flexneri 2a str. 2457T]

MSQTIDLNLDGLSCGHCVKRVKESLEQRPDVEQADVSITEAHVTGTASAEQLIETIKQAGYDASVSHPKA

KPLAESSIPSEALTAVSEALPAATADDDDSQQLLLSGMSCASCVTRVQNALQSVPGVTQARVNLAERTAL

VMGSASPQDLVQAVEKAGYGAEAIEDDAKRRERQQETAVATMKRFRWQAIVALAVGIPVMVWGMIGDNMI

VTADNRSLWLVIGLITLAVMVFASGHFYRSAWKSLLNGAATMDTLVALGTGVAWLYSMSVNLWPQWFPME

ARHLYYEASAMIIGLINLGHMLEARARQRSSKALEKLLDLTPPTARLVTDEGEKSVPLAEVQPGMLLRLT

TGDRVPVDGEITQGEAWLDEAMLTGEPIPQQKGEGDSVHAGTVVQDGSVLFRASAVGSHTTLSRIIRMVR

QAQSSKPEIGQLADKISAIFVPVVVVIALVSAAIWYFFGPAPQIVYTLVIATTVLIIACPCALGLATPMS

IISGVGRAAEFGVLVRDADALQRASTLDTVVFDKTGTLTEGKPQVVAVKTFADVDEAQALRLAAALEQGS

SHPLARAILDKAGDMQLPQVNGFRTLRGLGVSGEAEGHALLLGNQALLNDQQVDTKAIEADISAQASQGA

TPVLLAIDGKAVALLAVRDPLRSDSVAALQRLHKAGYRLVMLTGDNPTTANAIAKEAGIDEVIAGVLPDG

KAEAIKRLQSEGRQVAMVGDGINDAPALAQADVGIAMGGGSDVAIETAAITLMRHSLMGVADALAISRAT

LRNMKQNLLGAFIYNSIGIPVAAGILWPFTGTLLNPVVAGAAMALSSITVVSNANRLLRFKPKE

>gi|30061983|ref|NP_836154.1| hypothetical protein S0435 [Shigella flexneri 2a str. 2457T]

MIQYVLASLFTGKQQLKTMKQATRKPTTPGDILLYEYLEPLDLKINELAELLHVHRNRVSALINNNRKLT

TEMAFRLAKVFDTTVDFRLNLQAAVDLWEVENNMRTQEELGRIETVAEYLARREERAKKVA

>gi|30061982|ref|NP_836153.1| ligase [Shigella flexneri 2a str. 2457T]

MDLLYRVKTLWAALRGNHYTWPAIDITLPGNRHFHLIGSIHMGSHDMAPLPTRLLKKLKNADALIVEADV

STSDTPFANLPACEALEERISEEQLQNLQHISQEMGISPSLFSTQPLWQIAMVLQATQAQKLGLRAEYGI

DYQLLQAAKQQHKPVIELEGAENQIAMLLQLPDKGLALLDDTLTHWHTNARLLQQMMSWWLNAPPQNNDI

TLPNTFSQSLYDVLMHQRNLAWRDKLRAMPPGRYVVAVGALHLYGEGNLPQMLR

>gi|30061981|ref|NP_836152.1| hypothetical protein S0433 [Shigella flexneri 2a str. 2457T]

MTPAVKLLEKNKISFQIHTYEHDPAETNFGDEVVKKLGLNPDQVYKTLLVAVNGDMKHLAVAVTPVAGQL

DLKKVAKALGAKKVEMADPMVAQRSTGYLVGGISPLGQKKRLPTIIDAPAQEFATIYVSGGKRGLDIELA

AGDLAKILDAKFADIARRD

>gi|30061980|ref|NP_836151.1| bifunctional UDP-sugar hydrolase/5'-nucleotidase periplasmic precursor [Shigella flexneri 2a str. 2457T]

MKLLQRGVALALLTTFTLASETALAYEQDKTYKITVLHTNDHHGHFWRNEYGEYGLAAQKTLVDGIRKEV

AAEGGSVLLLSGGDINTGVPESDLQDAEPDFRGMNLVGYDAMAIGNHEFDNPLTVLRQQEKWAKFPLLSA

NIYQKSTGERLFKPWALFKRQDLKIAVIGLTTDDTAKIGNLEYFTDIEFRKPADEAKLVIQELQQTEKPD

IIIAATHMGHYDNGEHGSNAPGDVEMARALPAGSLAMIVGGHSQDPVCMAAENKKQVDYVPGTPCKPDQQ

NGIWIVQAHEWGKYVGRADFEFRNGEMKMVNYQLIPVNLKKKVTWEDGKSERVLYTPEIAENQQMISLLS

PFQNKGKAQLEVKIGKTNGRLEGDRDKVRFVQTNMGRLILAAQMDRTGADFAVMSGGGIRDSIEAGDISY

KNVLKVQPFGNVVVYADMTGKEVIDYLTAVAQMKPDSGAYPQFANVSFVAKDGKLNDLKIKGEPVDPAKT

YRMATLNFNATGGDGYPRLDNKPGYVNTGFIDAEVLKAYIQKSSPLDVSVYEPKGEVSWQ

>gi|30061979|ref|NP_836150.1| fosmidomycin resistance protein [Shigella flexneri 2a str. 2457T]

MAMSEQTQPVAGAAASTTKARTSFGILGAISLSHLLNDMIQSLILAIYPLLQSEFSLTFMQIGMITLTFQ

LASSLLQPVVGYWTDKYPMPWSLPIGMCFTLSGLVLLALAGSFGAVLLAAALVGTGSSVFHPESSRVARM

ASGGRHGLAQSIFQVGGNFGSSLGPLLAAVIIAPYGKGNVAWFVLAALLAIVVLAQISRWYSAQHRMNKG

KPKATIINPLPRNKVVLAVSILLILIFSKYFYMASISSYYTFYLMQKFGLSIQNAQLHLFAFLFAVAAGT

VIGGPVGDKIGRKYVIWGSILGVAPFTLILPYARLHWTGVLTVIIGFILASAFSAILVYAQELLPGRIGM

VSGLFFGFAFGMGGLGAAVLGLIADHTSIELVYKICAFLPLLGMLTIFLPDNRHKD

>gi|30061978|ref|NP_836149.1| cation:proton antiport protein [Shigella flexneri 2a str. 2457T]

MHHATPLITTIVGGLVLAFILGMLANKLRISPLVGYLLAGVLAGPFTPGFVADTKLAPELAELGVILLMF

GVGLHFSLKDLMAVKSIAIPGAIAQIAVATLLGMALSAVLGWSLMTGIVFGLCLSTASTVVLLRALEERQ

LIDSQRGQIAIGWLIVEDLVMVLTLVLLPAVAGMMEQGDVGFATLAVDMGITIGKVIAFIAIMMLVGRRL

VPWIMARSAATGSRELFTLSVLALALGIAFGAVELFDVSFALGAFFAGMVLNESELSHRAAHDTLPLRDA

FAVLFFVSVGMLFDPLILIQQPLAVLATLAIILFGKSVAALFLVRLFGHSQRTALTIAASLAQIGEFAFI

LAGLGMALNLLPQAGQNLVLAGAILSIMLNPVLFALLEKYLAKTETLEEQTLEEAIEEEKQIPVDICNHA

LLVGYGRVGSLLGEKLLASDIPLVVIETSRTRVDELRERGVRAVLGNAANEEIMQLAHLECAKWLILTIP

NGYEAGEIVASARAKNPDIEIIARAHYDDEVTYITERGANQVVMGEREIARTMLELLETPPAGEVVTG

>gi|30061977|ref|NP_836148.1| inosine-guanosine kinase [Shigella flexneri 2a str. 2457T]

MKFPGKRKSKHYFPVNARDPLLQQFQPENETSAAWVVGIDQTLVDIEAKVDDEFIERYGLSAGHSLVIED

DVAEVLYQELKQKNLITHQFAGGTIGNTMHNYSVLADDRSVLLGVMCSNIEIGSYAYRYLCNTSSRTDLN

YLQGVDGPIGRCFTLIGESGERTFAISPGHMNQLRAESIPEDVIAGASALVLTSYLVRCKPGEPMPEATM

KAIEYAKKYNVPVVLTLGTKFVIAENPQWWQQFLKDHVSILAMNEDEAEALTGESDPLLASDKALDWVEL

VLCTAGPIGLYMAGFTEDEAKRKTQHPLLPGAIAEFNQYEFSRAMRHKDCQNPLRVYSHIAPYMGGPEKI

MNTNGAGDGALAALLHDITANSYHRSNVPNSSKHKFTWLTYSSLAQVCKYANRVSYQVLNQHSPRLTRGL

PEREDSQEESYWDR

>gi|30061976|ref|NP_836147.1| lipase [Shigella flexneri 2a str. 2457T]

MKPENKLPVLDLISAEMKTVVNTLQPDLPSWPATGTIAEQRQYYTLERRFWNAGAPEMATRAYMVPTKYG

QVETRLFCPQPDSPATLFYLHGGGFILGNLDTHDRIMRLLASYSQCTVIGINYTLSPEARFPQAIEEIVA

ACCYFHQQAEDYQINMSRIGFAGDSAGAMLALASALWLRDKQIDCGKIAGVLLWYGLYGLRDSVTRRLLG

GVWDGLTQQDLQMYEEAYLSNDADRESPYYCLFNNDLTREVPPCFIAGAEFDPLLDDSRLLY

>gi|30061975|ref|NP_836146.1| ferrochelatase [Shigella flexneri 2a str. 2457T]

MRQTKTGILLANLGTPDAPTPEAVKRYLKQFLSDRRVVDTSRLLWWPLLRGVILPLRSPRVAKLYASVWM

EDGSPLMVYSRQQQQALAQRLPDTPVALGMSYGSPSLESSVDELLAEHVDHIVVLPLYPQFSCSTVGAVW

DELARILARKRSIPGISFIRDYADNHDYINALANSVRASFAKHGEPDLLLLSYHGIPQRYADEGDDYPQR

CRTTTRELASALGMVPEKVMMTFQSRFGREPWLMPYTDETLKMLGEKGVGHIQVMCPGFAADCLETLEEI

AEQNREVFLGAGGKKYEYIPALNATPEHIEMMANLVAAYR

>gi|30061974|ref|NP_836145.1| adenylate kinase [Shigella flexneri 2a str. 2457T]

MRIILLGAPGAGKGTQAQFIMEKYGIPQISTGDMLRAAVKSGSELGKQAKDIMDAGKLVTDELVIALVKE

RIAQEDCRNGFLLDGFPRTIPQADAMKEAGINVDYVLEFDVPDELIVDRIVGRRVHAPSGRVYHVKFNPP

KVEGKDDVTGEELTTRKDDQEETVRKRLVEYHQMTAPLIGYYSKEAEAGNTKYAKVDGTKQVAEVRADLE

KILG

>gi|30061973|ref|NP_836144.1| heat shock protein 90 [Shigella flexneri 2a str. 2457T]

MKGQETRGFQSEVKQLLHLMIHSLYSNKEIFLRELISNASDAADKLRFRALSNPDLYEGDGELRVRVSFD

KDKRTLTISDNGVGMTRDEVIDHLGTIAKSGTKSFLESLGSDQAKDSQLIGQFGVGFYSAFIVADKVTVR

TRAAGEKPENGVFWESAGEGEYTVADITKEDRGTEITLHLREGEDEFLDDWRVRSIISKYSDHIALPVEI

EKREEKDGETVISWAKINKAQALWTRNKSEITDEEYKEFYKHIAHDFNDPLTWSHNRVEGKQEYTSLLYI

PSQAPWDMWNRDHKHGLKLYVQRVFIMDDAEQFMPNYLRFVRGLIDSSDLPLNVSREILQDSTVTRNLRN

ALTKRVLQMLEKLAKDDAEKYQTFWQQFGLVLKEGPAEDFANQEAIAKLLRFASTYTDSSAQTVSLEDYV

SRMKEGQEKIYYITADSYAAAKSSPHLELLRKKGIEVLLLSDRIDEWMMNYLTEFDGKPFQSVSKVDESL

EKLADEVDESAKEAEKALTPFIDRVKALLGERVKDVRLTHRLTDTPAIVSTDADEMSTQMAKLFAAAGQK

VPEVKYIFELNPDHVLVKRAADTEDEAKFSEWVELLLDQALLAERGTLEDPNLFIRRMNQLLVS

>gi|30061972|ref|NP_836143.1| recombination protein RecR [Shigella flexneri 2a str. 2457T]

MQTSPLLTQLMEALRCLPGVGPKSAQRMAFTLLQRDRSGGMRLAQALTRAMSEIGHCADCRTFTEQEVCN

ICSNPRRQENGQICVVESPADIYAIEQTGQFSGRYFVLMGHLSPLDGIGPDDIGLDRLEQRLAEEKITEV

ILATNPTVEGEATANYIAELCAQYDVEASRIAHGVPVGGELEMVDGTTLSHSLAGRHKIRF

>gi|30061971|ref|NP_836142.1| hypothetical protein S0423 [Shigella flexneri 2a str. 2457T]

MFGKGGLGNLMKQAQQMQEKMQKMQEEIAQLEVTGESGAGLVKVTINGAHNCRRVEIDPSLLEDDKEMLE

DLVAAAFNDAARRIEETQKEKMASVSSGMQLPPGFKMPF

>gi|30061970|ref|NP_836141.1| DNA polymerase III subunits gamma and tau [Shigella flexneri 2a str. 2457T]

MSYQVLARKWRPQTFADVVGQEHVLTALANGLSLGRIHHAYLFSGTRGVGKTSIARLLAKGLNCETGITA

TPCGVCDNCREIEQGRFVDLIEIDAASRTKVEDTRDLLDNVQYAPARGRFKVYLIDEVHMLSRHSFNALL

KTLEEPPEHVKFLLATTDPQKLPVTILSRCLQFHLKALDVEQIRHQLEHILNEEHIAHEPRALQLLARAA
EGSLRDALSLTDQAIASGDGQVSTQAVSAMLGTLDDDQALSLVEAMVEGNGERVMALINEAAARGIEWEA
LLVEMLGLLHRIAMVQLSPAALGNDMAAIELRMRELARTIPPTDIQLYYQTLLIGRKELPYAPDRRMGVE
MTLLRALAFHPRMPLPEPEVPRQSFAPVAPTAVMTPTLVPPQSAPQQAPTVPLPETTSQVLAARQQLQRV
QGATKAKKSEPAAATRARPVNNAALERLASVTDRVQARPVPSALEKAPAKKEAYRWKATTPVMQQKEVVA
TPKALKKALEHEKTPELAVKLAAEAIERDPWAAQVSQLSLPKLVEQVALNAWKEESDNAVCLHLRSSQRH
LNNRGAQQKLAEALSMLKGSTVELTIVEDDNPAVRTPLEWRQAIYEEKLAQARESIIADNNIQTLRRFFD
AELDEESIRPI

>gi|30061969|ref|NP_836140.1| adenine phosphoribosyltransferase [Shigella flexneri 2a str. 2457T]
MTATAQQLEYLKNSIKSIQDYPKPGILFRDVTSLLEDPKAYALSIDLLVERYKNAGITKVVGTEARGFLF
GAPVALGLGVGFVPVRKPGKLPRETISENYDLEYGTDQLEIHVDAIKPGDKVLVVDDLLATGGTIEATVK
LIRRLGGEVADAAFIINLFDLGGEQRLEKQGITSYSLVPFPGH

>gi|30061968|ref|NP_836139.1| hypothetical protein S0420 [Shigella flexneri 2a str. 2457T]
MQRIILIIIGWLAVVLGTLGVVLPVLPTTPFILLAAWCFARSSPRFHAWLLYRSWFGSYLRFWQKHHAMP
RGVKPRAILLILLTFAISLWFVQMPWVRIMLLVILACLLFYMWRIPVIDEKQEKH

>gi|30061967|ref|NP_836138.1| primosomal replication protein N'' [Shigella flexneri 2a str. 2457T]
MKTALLLEKLEGQLATLRQRCAPVSQFATLSARFDRHLFQTRATTLQSCLDEAGDNLAALRHAVEQQQLP
QVAWLAEHLAAQLEAIAREASAWSLREWDSAPPKISRWQRKRIQHQDFERRLREMVAERRARLARVTDLV
EQQTLHREVKAYEARLARCRHALEKIENRLARLTR

>gi|30061966|ref|NP_836137.1| hypothetical protein S0418 [Shigella flexneri 2a str. 2457T]
MSLENAPDDVKLAVDLIVLLEENQIPASTVLRALDIVKRDYEKKLTHDDEAEK

>gi|30061965|ref|NP_836136.1| potassium efflux protein KefA [Shigella flexneri 2a str. 2457T]

MTMFQYYKRSRHFVFSAFIAFVFVLLCQNTAFARASSNGDLPTKADLQAQLDSLNKQKDLSAQDKLVQQD

LTDTLATLDKIDRIKEETVQLRQKVAEAPEKMRQATAALTALSDVDNDEETRKILSTLSLRQLETRVAQA

LDDLQNAQNDLASYNSQLVSLQTQPERVQNAMYNASQQLQQIRSRLDGTDVGETALRPSQKVLMQAQQAL

LNAEIDQQRKSLEGNTVLQDTLQKQRDYVTANSARLEHQLQLLQEAVNSKRLTLTEKTAQEAVSPDEAAR

IQANPLVKQELEINQQLSQRLITATENGNQLMQQNIKVKNWLERALQSERNIKEQIAVLKGSLLLSRILY

QQQQTLPSADELENMTNRIADLRLEQFEVNQQRDALFQSDAFVNKLEEGHTNEVNSEVHDALLQVVDMRR

ELLDQLNKQLGNQLMMAINLQINQQQLMSVSKNLKSILTQQIFWVNSNRPMDWDWIKAFPQSLKDEFKSM

KITVNWEKAWPAVFIAFLAGLPLLLIAGLIHWRLGWLKAYQQKLASAVGSLRNDSQLNTPKAILIDLIRA

LPVCLIILAVGLILLTMQLNISELLWSFSKKLAIFWLVFGLCWKVLEKNGVAVRHFGMPEQQTSHWRRQI

VRISLALLPIHFWSVVAELSPLHLMDDVLGQAMIFFNLLLIAFLVWPMCRESWRDKESHTMRLVTITVLS

IIPIALMVLTATGYFYTTLRLSGRWIETVYLVIIWNLLYQTVLRGLSVAARRIAWRRALARRQNLVKEGA

EGAEPPEEPTIALEQVNQQTLRITMLLMFALFGVMFWAIWSDLITVFSYLDSITLWHYNGTEAGAAVVKN

VTMGSLLFAIIASMVAWALIRNLPGLLEVLVLSRLNMRQGASYAITTILNYIIIAVGAMTVFGSLGVSWD

KLQWLAAALSVGLGFGLQEIFGNFVSGLIILFERPVRIGDTVTIGSFSGTVSKIRIRATTITDFDRKEVI

IPNKAFVTERLINWSLTDTTTRLVIRLGVAYGSDLEKVRKVLLKAATEHPRVMHEPMPEVFFTAFGASTL

DHELRLYVRELRDRSRTVDELNRTIDQLCRENDINIAFNQLEVHLHNEKGDEVTEVKRDYKGDDPTPAVG

>gi|30061964|ref|NP_836135.1| DNA-binding transcriptional repressor AcrR [Shigella flexneri 2a str. 2457T]

MARKTKQEAQETRQHILDVALRLFSQQGVSSTSLGEIAKAAGVTRGAIYWHFKDKSDLFSEIWELSESNI

GELELEYQAKFPGDPLSVLREILIHVLESTVTEERRRLLMEIIFHKCEFVGEMAVVQQAQRNLCLESYDR

IEQTLKHCIEAKMLPADLMTRRAAIIMRGYISGLMENWLFAPQSFDLKKEARDYVAILLEMYLLCPTLRN

PATNE

>gi|30061963|ref|NP_836134.1| acriflavin resistance protein AcrA precursor [Shigella flexneri 2a str. 2457T]

MNKNRGFTPLAVVLMLSGSLALTGCDDKQAQQGGQQMPAVGVVTVKTEPLQITTELPGRTSAYRIAEVRP

QVSGIILKRNFKEGSDIEAGVSLYQIDPATYQATYDSAKGDLAKAQAAANIAQLTVNRYQKLLGTQYISK

QGYDQALADAQQANAAVTAAKAAVETARINLAYTKVTSPISGRIGKSNVTEGALVQNGQATVLATVQQLD

PIYVDVTQSSNDFLRLKQELANGTLKQENGKAKVSLITSDGIKFPQDGTLEFSDVTVDQTTGSITLRAIF

PNPDHTLLPGMFVRARLEEGLNPNAILVPQQGVTRTPRGDATVLVVGADDKVETRPIVASQAIGDKWLVT

EGLKAGDRVVISGLQKVRPGVQVKAQEVTADNNQQAASGAQPEQSKS

>gi|30061962|ref|NP_836133.1| acriflavine resistance protein [Shigella flexneri 2a str. 2457T]

MPNFFIDRPIFAWVIAIIIMLAGGLAILKLPVAQYPTIAPPAVTISASYPGADAKTVQDTVTQVIEQNMN

GIDNLMYMSSNSDSTGTVQITLTFESGTDADIAQVQVQNKLQLAMPLLPQEVQQQGVSVEKSSSSFLMVV

GVINTDGTMTQEDISDYVAANMKDAISRTSGVGDVQLFGSQYAMRIWMNPNELNKFQLTPVDVITAIKAQ

NAQVAAGQLGGTPPVKGQQLNASIIAQTRLTSTEEFGKILLKVNQDGSRVLLRDVAKIELGGENYDIIAE

FNGQPASGLGIKLATGANALDTAAAIRAELAKMEPFFPSGLKIVYPYDTTPFVKISIHEVVKTLVEAIIL

VFLVMYLFLQNFRATLIPTIAVPVVLLGTFAVLAAFGFSINTLTMFGMVLAIGLLVDDAIVVVENVERVM

AEEGLPPKEATRKSMGQIQGALVGIAMVLSAVFVPMAFFGGSTGAIYRQFSITIVSAMALSVLVALILTP

ALCATMLKPIAKGDHGEGKKGFFGWLNRMFEKSTHHYTDSVGGILRSTGRYLVLYLIIVVGMAYLFVRLP

SSFLPDEDQGVFMTMVQLPAGATQERTQKVLNEVTHYYLTKEKNNVESVFAVNGFGFAGRGQNTGIAFVS

LKDWADRPGEENKVEAITMRATRAFSQIKDAMVFAFNLPAIVELGTATGFDFELIDQAGLGHEKLTQARN

QLLAEAAKHPDMLTSVRPNGLEDTPQFKIDIDQEKAQALGVSINDINTTLGAAWGGSYVNDFIDRGRVKK

VYVMSEAKYRMLPDDIGDWYVRAADGQMVPFSAFSSSRWEYGSPRLERYNGLPSMEILGQAAPGKSTGEA

MELMEQLASKLPTGVGYDWTGMSYQERLSGNQAPSLYAISLIVVFLCLAALYESWSTPFSVMLVVPLGVI

GALLAATFRGLTNDVYFQVGLLTTIGLSAKNAILIVEFAKDLMDKEGKGLIEATLDAVRMRLRPILMTSL

AFILGVMPLVISTGAGSGAQNAVGTGVMGGMVTATVLAIFFVPVFFVVVRRRFSRKNEDIEHSHTVDHH

>gi|30061961|ref|NP_836132.1| hypothetical protein S0413 [Shigella flexneri 2a str. 2457T]

MDEYSPKRHDIAQLKFLCETLYHDCLANLEESNHGWVNDPTSAINLQLNELIEHIATFALNYKIKYNEDN

KLIEQIDEYLDDTFMLFSSYGINMQALQKWRKSGNRLFRCFVNATKENPASLSC

>gi|30061960|ref|NP_836131.1| hemolysin expression-modulating protein [Shigella flexneri 2a str. 2457T]

MSEKPLTKTDYLMRLRRCQTIDTLERVIEKNKYELSDNELAVFYSAADHRLAELTMNKLYDKIPSSVWKF

IR

>gi|30061959|ref|NP_836130.1| hypothetical protein S0410 [Shigella flexneri 2a str. 2457T]

MTEIQRLLTETIESLNTREKRDNKPRFSISFIRKHPGLFIGMYVAFFATLAVMLQSETLSGSVWLLVVLF

ILLNGFFFFDVYPRYRYEDIDVLDFRVCYNGEWYNTRFVPAALVEAILNSPRVADVHKEQLQKMIVRKGE

LSFYDIFTLARAESTS

>gi|30061958|ref|NP_836129.1| hypothetical protein S0409 [Shigella flexneri 2a str. 2457T]

MRTRHLVGLISGVLILSVLLPVGLSIWLAHQQVETSFIEELDTYSSRVAIRANKVATQGKDALQELERWQ

GAACSEAHLMEMRRVSYSYRYIQEVVYIDNNVPQCSSLEHESPPDTFPELGKISKDGYRVWLTSHNDLGI

IRYMVAMGTAHYVVMIDPASFIDVIPYSSWQIDAAIIGNAHNVVITSSDEIAQGIITRLQKTPGEHIENN

GIIYDILPFLEMNISIITWASTKMLQKGWHRQVFIWLPLGLVIGLLAAMFVLRILRRIQSPHHRLQDAIE

NRDICVHYQPIVSLANGKIVGAEALARWPQTDGSWLSPDSFIPLAQQTGLSEPLTLLIIRSVFEDMGDCL

RQHPQQHISINLESTVLTSEKIPQLLREMINHYQVNPRQIALELTEREFADPKTSAPIISRYREAGHEIY

LDDFGTGYSSLSYLQDLDVDILKIDKSFVDALEYKNVTPHIIEMAKTLKLKMVAEGIETSKQEEWLRQHG

VHYGQGWLYSKALPKEDFLRWAEQHL

>gi|30061957|ref|NP_836128.1| hypothetical protein S0408 [Shigella flexneri 2a str. 2457T]

MKYVDGFVVAVPADKKDAYREMAAKAAPLFKEFGALRIVECWASDVPDGKVTDFRMAVKAEENEEVVFSW

IEYPSKEVRDAANQKMMSDPRMKEFGESMPFDGKRMIYGGFESIIDE

>gi|30061956|ref|NP_836127.1| hypothetical protein S0406 [Shigella flexneri 2a str. 2457T]

MEKEDSFPQRVWQIVAAIPEGYVTTYGDVAKLAGSPRAARQVGGVLKRLPEGSTLPWHRVVNRHGTISLT

GPDLQRQRQALLAEGVMVSGSGQIDLQRYRWNY

>gi|30061955|ref|NP_836126.1| IS4 orf [Shigella flexneri 2a str. 2457T]

MHIGQALDLVSRYDSLRNPLTSLGDYLAPELISRCLAESGTVTLRKRRLPLEMMVWCIVGMVLERKEPLH

QIVNRLDIMLPGNRPFVAPSAVIQARQRLGSEAVRRVFTKTAQLWHNATPHPHWCGLTLLAIDGVFWRTP

DTPENDAAFPRQTHAGNPALYPQVKMVCQMELTSHLLTAAAFGTMKNSENELAEQLIEQTGDNTLTLMDK

GYYSLGLLNGWSLAGEHRHWMIPLRKGAQYEELRKLGKGDHLVKLKTSPQARKKWPGLGNEVTARLLTVT

RKGKVCHLLTSMTDAMRFPGGEMADLYSHRWEIELGYREIKQTMQLSRLTLRSKKPELVEQELWGVLLAY

NLVRYQMIKMAEHLKGYWPNQLSFSESCGMVMRMLMTLQGASPGRIPELMRDLASMGQLVKLPTRRGRAF

PRVVKERPWKYPTAPKKSQSVA

>gi|30061954|ref|NP_836125.1| hypothetical protein S0404 [Shigella flexneri 2a str. 2457T]

MKLVHMASGLAVAIALAACADKSADIQTPAPAANTSISATQQPAIQQPNVSGTVWIRQKVALPPDAVLTV

TLSDASLADAPSKVLAQKAVRTEGKQSPFSFVLPFNPADVQPNARILLSAAITVNDKLVFITDTVQPVIN

QGGTKADLTLVPVQQTAVPVQASGGATTTVPSTSPTQVNPSSAVPAPTQY

>gi|30061953|ref|NP_836124.1| acyl-CoA thioesterase II [Shigella flexneri 2a str. 2457T]

MSQALKNLLTLLNLEKIEEGLFRGQSEDLGLRQVFGGQVVGQALYAAKETVPEERLVHSFHSYFLRPGDS

KKPIIYDVETLRDGNSFSARRVAAIQNGKPIFYMTASFQAPEAGFEHQKTMPSAPAPDGLPSETQIAQSL

AHLLPPVLKDKFICDRPLEVRPVEFHNPLKGHVAEPHRQVWIRANGSVPDDLRVHQYLLGYASDLNFLPV

ALQPHCIGFLEPGIQIATIDHSMWFHRPFNLNEWLLYSVESTSASSARGFVRGEFYTQDGVLVASTVQEG

VMRNHN

>gi|30061952|ref|NP_836123.1| ammonium transporter [Shigella flexneri 2a str. 2457T]

MKIATIKTGLASLAMLPGLVMAAPAVADKADNAFMMICTALVLFMTIPGIALFYGGLIRGKNVLSMLTQV

TVTFALVCILWVVYGYSLAFGEGNNFFGNINWLMLKNIELTAVMGSIYQYIHVAFQGSFACITVGLIVGA

LAERIRFSAVLIFVVVWLTLSYIPIAHMVWGGGLLASHGALDFAGGTVVHINAAIAGLVGAYLIGKRVGF

GKEAFKPHNLPMVFTGTAILYIGWFGFNAGSVGTANEIAALAFVNTVVATAAAILGWIFGEWALRGKPSL

LGACSGAIAGLVGVTPACGYIGVGGALIIGVVAGLAGLWGVTMLKRLLRVDDPCDVFGVHGVCGIVGCIM

TGIFAASSLGGVGFAEGVTMGHQLLVQLESIAITIVWSGVVAFIGYKLADLTVGLRVPEEQEREGLDVNS

HGENAYNA

>gi|30061951|ref|NP_836122.1| nitrogen regulatory protein P-II 2 [Shigella flexneri 2a str. 2457T]

MKLVTVIIKPFKLEDVREALSSIGIQGLTVTEVKGFGRQKGHAELYRGAEYSVNFLPKVKIDVAIADDQL

DEVIDIVSKAAYTGKIGDGKIFVAELQRVIRIRTGEADEAAL

>gi|30061950|ref|NP_836121.1| multidrug transporter membrane\ATP-binding components [Shigella flexneri 2a str. 2457T]

MRSFSQLWPTLKRLLAYGSPWRKPLGIAVLMMWVAAAAEVSGPLLISYFIDNMVAKNNLPLKVVAGLAAA

YVGLQLFAAGLHYAQSLLFNRAAVGAVQQLRTDVMDAALRQPLSEFDTQPVGQVISRVTNDTEVIRDLYV

TVVATVLRSAALVGAMLVAMFSLDWRMALVAIMIFPVVLVVMVIYQRYSTPIVRRVRAYLADINDGFNEI

INGMSVIQQFRQQARFGERMGEASRSHYMARMQTLRLDGFLLRPLLSLFSSLILCGLLMLFGFSASGTIE

VGVLYAFISYLGRLNEPLIELTTQQAMLQQAVVAGERVFELMDGPRQQYGNDDYPLQSGTIEVDNVSFAY

RDDNLVLKNINLSVPSRNFVALVGHTGSGKSTLASLLMGYYPLTEGEIHLDGRPLSSLSHSALRQGVAMV

QQDPVVLADTFLANVTLGRDISEERVWQALETVQLAELARSMSDCIYTPLGEQGNNLSVGQKQLLALARV

LVETPQILILDEATASIDSGTEQAIQHALAAVREHTTLVVIAHRLSTIIDADTILVLHRGQAVEQGTHQQ

LLAAQGRYWQMYQLQLAGEELAASVREEESLSA

>gi|30061949|ref|NP_836120.1| multidrug transporter membrane\ATP-binding components [Shigella flexneri 2a str. 2457T]

MRLFAQLSWYFRREWRRYLGAVALLVIIAMLQLVPPKVVGIVVDGVTEQHFTTGLSLMWIATMVLIAVVV

YLLRYVWRVLLFGASYQLAVELREDYYRQLSRQHPEFYLRHRTGDLMARATNDVDRVVFAAGEGVLTLVD

SLVMGCAVLIMMSTQISWQLTLFALLPMPVMAIMIKRNGDALHERFKLAQAAFSSLNDRTQESLTSIRMI

KAFGLEDRQSALFAADAEDTGKKNMRVARIDARFDPTIYIAIGMANLLAIGGGSWMVVQGSLTLGQLTSF

MMYLGLMIWPMLALAWMFNIVERGSAAYSRIRAMLAEAPVVNDGSEPVPEGRGELDVNIHQFTYPQTDHP

ALENVNFALKPGQMLGICGPTGSGKSTLLSLIQRHFDVSEGDIRFHDIPLTKLQLDSWRSRLAVVSQTPF

LFSDTVANNIALGCPNATQQEIEHVARLASVHDDILRLPQGYDTEVGERGVMLSGGQKQRISIARALLVN

AEILILDDALSAVDGRTEHQILHNLRQWGQGRTVIISAHRLSALTEASEIIVMQHGHIAQRGNHDELAQQ

SGWYRDMYRYQQLEAALDDVPEIREEAVDA

>gi|30061948|ref|NP_836119.1| LRP-like transcriptional regulator [Shigella flexneri 2a str. 2457T]

MVFKPHRINCRDFTFKIELKEKKILRGRAMLDKIDRKLLALLQQDCTLSLQALAEAVNLTTTPCWKRLKR

LEDDGILIGKVALLDPEKIGLGLTAFVLIKTQHHSSEWYCRFVTVVTEMPEVLGVWRMAGEYDYLMRVQV

ADMKRYDEFYKRLVNSVPGLSDVTSSFAMEQIKYTTSLPIE

>gi|30061947|ref|NP_836118.1| hypothetical protein S0397 [Shigella flexneri 2a str. 2457T]

MEKEMARLVAFDMDGTLLMPDHHLGEKTLSTLARLRERDITLTFATGRHALEMQHILGALSLDAYLITGN

GTRVHSLEGELLHRDDLPADVAELVLYQQWDTRASMHIFNDDGWFTGKESPALLQVFVYSGFRYQIIDVK

KMPLGSVTKICFCGDHDDLTRLQIQLYEALGERAHLCFSATDCLEVLPVGCNKGAALTVRTQHLGLSLRD

CMAFGDAMNDREMLGSVGSGFIMGNAMPQLRAELPHLPVIGHCRNQAVSHYLTHWLDYPHLPYSPE

>gi|30061946|ref|NP_836117.1| queuosine biosynthesis protein QueC [Shigella flexneri 2a str. 2457T]

MKRAVVVFSGGQDSTTCLVQALQQYDEVHCVTFDYGQRHRAEIDVARELALKLGARAHKVLDVTLLNELA

VSSLTRDSIPVPDYEPEADGIPNTFVPGRNILFLTLAAIYAYQVKAEAVITGVCETDFSGYPDCRDEFVK

ALNHAVSLGMAKDIRFETPLMWIDKAETWALADYYGKLDLVRNETLTCYNGIKGDGCGHCAACNLRANGL

NHYLADKPTVMAAMKQKTGLR

>gi|30061945|ref|NP_836116.1| hypothetical protein S0394 [Shigella flexneri 2a str. 2457T]

MQTQIKVRGYHLDVYQHVNNARYLEFLEEARWDGVENSDSFQWMTAHNIAFVVVNININYRRPAVLSDLL

TITSQLQQLNGKSGILNQVITLEPEGQVVADALITFVCIDLKTQKALALEGELREKLEQMVK

>gi|30061944|ref|NP_836115.1| hypothetical protein S0393 [Shigella flexneri 2a str. 2457T]

MKHGIKALLITLSLACAGMSHSALAAASVAKPTAVETKAEAPAAQSKAAVPAKASDEEGTRVSINNASAE

ELARAMNGVGLKKAQAIVSYREEYGPFKTVEDLKQVPGMGNSLVERNLAVLTL

>gi|30061943|ref|NP_836114.1| peptidyl-prolyl cis-trans isomerase (rotamase D) [Shigella flexneri 2a str. 2457T]

MMDSLRTAANSLVLKIIFGIIIVSFILTGVSGYLIGGGNNYAAKVNDQEISRGQFENAFNSERNRMQQQL

GDQYSELAANEGYMKTLRQQVLNRLIDEALLDQYARELKLGISDEQVKQAIFATPAFQVDGKFDNSRYNG

ILNQMGMTADQYAQALRNQLTTQQLINGVAGTDFMLKGETDELAALVAQQRVVREATIDVNALAAKQPVT

EQEIASYYEQNKNNFMTPEQFRVSYIKLVAATMQQPVSDADIQSYYDQHQDQFTQPQRTRYSIIQTKTED

EAKAVLDELNKGGDFAALAKEKSADIISARNGGDMGWLEDATIPDELKNAGLKEKGQLSGVIKSSVGFLI

VRLDDIQPAKVKSLDEVRDDIAAKVKHEKALDAYYALQQKVSDAASNDTESLAGAEQAAGVKATQTGWFS

KDNLPEELNFKPVADAIFNGGLVGENGASGINSDIITVDGDRAFVLRVSEHKPEAVKPLADVQEQVKALV

QHNKAEQQAKVDAEKLLVDLKAGKGAEAMQAAGLKFGEPKTLSRSGRDPISQAAFALPLPAKDKPSYGMA

TDMQGNVVLLALDEVKQGSMPEDQKKAMVQGITQNNAQIVFEALMSNLRKEAKIKIGDALEQQ

>gi|30061942|ref|NP_836113.1| transcriptional regulator HU subunit beta [Shigella flexneri 2a str. 2457T]

MNKSQLIDKIAAGADISKAAAGRALDAIIASVTESLKEGDDVALVGFGTFAVKERAARTGRNPQTGKEIT

IAAAKVPSFRAGKALKDAVN

>gi|30061941|ref|NP_836112.1| DNA-binding ATP-dependent protease La [Shigella flexneri 2a str. 2457T]

MNPERSERIEIPVLPLRDVVVYPHMVIPLFVGREKSIRCLEAAMDHDKKIMLVAQKEASTDEPGVNDLFT

VGTVASILQMLKLPDGTVKVLVEGLQRARISALSDNGEHFSAKAEYLESPTIDEREQEVLVRTAISQFEG

YIKLNKKIPPEVLTSLNSIDDPARLADTIAAHMPLKLADKQSVLEMSDVNERLEYLMAMMESEIDLLQVE

KRIRNRVKKQMEKSQREYYLNEQMKAIQKELGEMDDVPDENEALKRKIDAAKMPKEAKEKAEAELQKLKM

MSPMSAEATVVRGYIDWMVQVPWNARSKVKKDLRQAQEILDTDHYGLERVKDRILEYLAVQSRVNKIKGP

ILCLVGPPGVGKTSLGQSIAKATGRKYVRMALGGVRDEAEIRGHRRTYIGSMPGKLIQKMAKVGVKNPLF

LLDEIDKMSSDMRGDPASALLEVLDPEQNVAFSDHYLEVDYDLSDVMFVATSNSMNIPAPLLDRMEVIRL

SGYTEDEKLNIAKRHLLPKQIERNALKKGELTVDDSAIIGIIRYYTREAGVRGLEREISKLCRKAVKQLL

LDKSLKHIEINGDNLHDYLGVQRFDYGRADNENRVGQVIGLAWTEVGGDLLTIETACVPGKGKLTYTGSL

GEVMQESIQAALTVVRARAEKLGINPDFYEKRDIHVHVPEGATPKDGPSAGIAMCTALVSCLTGNPVRAD

VAMTGEITLRGQVLPIGGLKEKLLAAHRGGIKTVLIPFENKRDLEEIPDNVIADLDIHPVKRIEEVLTLA

LQNEPSGMQVVTAK

>gi|30061940|ref|NP_836111.1| ATP-dependent protease ATP-binding subunit ClpX [Shigella flexneri 2a str. 2457T]

MTDKRKDGSGKLLYCSFCGKSQHEVRKLIAGPSVYICDECVDLCNDIIREEIKEVAPHRERSALPTPHEI

RNHLDDYVIGQEQAKKVLAVAVYNHYKRLRNGDTSNGVELGKSNILLIGPTGSGKTLLAETLARLLDVPF

TMADATTLTEAGYVGEDVENIIQKLLQKCDYDVQKAQRGIVYIDEIDKISRKSDNPSITRDVSGEGVQQA

LLKLIEGTVAAVPPQGGRKHPQQEFLQVDTSKILFICGGAFAGLDKVISHRVETGSGIGFGATVKAKSDK

ASEGELLAQVEPEDLIKFGLIPEFIGRLPVVATLNELSEEALIQILKEPKNALTKQYQALFNLEGVDLEF

RDEALDAIAKKAMARKTGARGLRSIVEAALLDTMYDLPSMEDVEKVVIDESVIDGQSKPLLIYGKPEAQQ

ASGE

>gi|30061939|ref|NP_836110.1| ATP-dependent Clp protease proteolytic subunit [Shigella flexneri 2a str. 2457T]

MSYSGERDNFAPHMALVPMVIEQTSRGERSFDIYSRLLKERVIFLTGQVEDHMANLIVAQMLFLEAENPE

KDIYLYINSPGGVITAGMSIYDTMQFIKPDVSTICMGQAASMGAFLLTAGAKGKRFCLPNSRVMIHQPLG

GYQGQATDIEIHAREILKVKGRMNELMALHTGQSLEQIERDTERDRFLSAPEAVEYGLVDSILTHRN

>gi|30061938|ref|NP_836109.1| trigger factor [Shigella flexneri 2a str. 2457T]

MQVSVETTQGLGRRVTITIAADSIETAVKSELVNVAKKVRIDGFRKGKVPMNIVAQRYGASVRQDVLGDL

MSRNFIDAIIKEKINPAGAPTYVPGEYKLGEDFTYSVEFEVYPEVELQGLEAIEVEKPIVEVTDADVDGM

LDTLRKQQATWKEKDGAVEAEDRVTIDFTGSVDGEEFEGGKASDFVLAMGQGRMIPGFEDGIKGHKAGEE

FTIDVTFPEEYHAENLKGKAAKFAINLKKVEERELPELTAEFIKRFGVEDGSVEGLRAEVRKNMERELKS

AIRNRVKSQAIEGLVKANDIDVPAALIDSEIDVLRRQAAQRFGGNEKQALELPRELFEEQAKRRVVVGLL

LGEVIRTNELKADEERVKGLIEEMASAYEDPKEVIEFYSKNKELMDNMRNVALEEQAVEAVLAKAKVTEK

ETTFNELMNQQA

>gi|30061935|ref|NP_836106.1| muropeptide transporter [Shigella flexneri 2a str. 2457T]

MSSQYLRIFQQPRSAILLILGFASGLPLALTSGTLQAWMTVENIDLKTIGFFSLVGQAYVFKFLWSPLMD

RYTPPFFGRRRGWLLATQILLLVAIAAMGFLEPGTQLRWMAALAVVIAFCSASQDIVFDAWKTDVLPAEE

RGAGAAISVLGYRLGMLVSGGLALWLADKWLGWQGMYWLMAALLIPCIIATLLAPEPTDTIPVPKTLEQA

VVAPLRDFFGRNNAWLILLLIVLYKLGDAFAMSLTTTFLIRGVGFDAGEVGVVNKTLGLLATIVGALYGG

ILMQRLSLFRALLIFGILQGASNAGYWLLSITDKHLYSMGAAVFFENLCGGMGTSAFVALLMTLCNKSFS

ATQFALLSALSAVGRVYVGPVAGWFVEAHGWSTFYLFSVAAAVPGLILLLVCRQTLEYTRVNDNFISRTE

YPAGYAFAMWTLAAGVSLLAVWLLLLTMDALDLTHFSFLPALLEVGVLVALSGVVLGGLLDYLALRKTHL

T

>gi|30061934|ref|NP_836105.1| ISSfl3 orfA, partial [Shigella flexneri 2a str. 2457T]

MPDSVSFAQLDAILYANRKKELTEPQIREGSWRKERRTSYSREFKVRLAKQALQPGAVVARIAREHDINN

NLLFKWKSQYEDGLLSDDDIQECMPVPVALTDTPEPTRPVTNPFWRNKHDERPEGAPGNVPRCELHLKSG

VVKLFDPLTPELLRALIREMKGGIR

>gi|30061933|ref|NP_836104.1| ISSfl3 orfB [Shigella flexneri 2a str. 2457T]

MITLPTGTKIWIIAGITDMRCGFNGLASKVQNTLKDDPFSGHIFVFRGRSGKMVKILWADRDGLCLFAKR

LERGRFVWPVTREGKVHLTPAQLSMLLEGIAWPHPKRTERPGIRI

>gi|30061932|ref|NP_836103.1| ISSfl3 orfC [Shigella flexneri 2a str. 2457T]

MNNELPDDIELLKAMLRKQQSRLRQYACQVAGYEQERCTQMTLR

>gi|30061930|ref|NP_836101.1| ISSfl3 orfD [Shigella flexneri 2a str. 2457T]

MSSAPLPPKPIERGYASAGLLARILVSKYMEHIPLYRQSEIYARQGVELSRNTMVRWVSEMADKLRPLYI

ALNDYVLEAGKVHADDTPVKVLAPGNGKTKTGRLWVYVRDDRNAGSSLPAAVWFAYSADRKGEHPQLHLA

KYQGVLQADAYAGYNVLYETGRVKEAGCLAHARRKIHDEDVRRPTEMTQEALRRIAELYDIEAEIRGSPA

EERLAVRKARSVQLMQSLYDWIQLQRKTLSKHAEMAKAFDYILNHWNALNEFCRDGRVEIDNNIGENALR

SVAVGRKNYLFFGSDKGGESAAIIYSLLVTCKQNEVEPEDWLREVIEKLNDWPSNQVHELLPWNFSSVK

>gi|30061929|ref|NP_836100.1| cytochrome o ubiquinol oxidase subunit III [Shigella flexneri 2a str. 2457T]

MATDTLTHATAHAHEHGHHDAGGTKIFGFWIYLMSDCILFSILFATYAVLVNGTAGGPTGKDIFELPFVL

VETFLLLFSSITYGMAAIAMYKNNKSQVISWLALTWLFGAGFIGMEIYEFHHLIVNGMGPDRSGFLSAFF

ALVGTHGLHVTSGLIWMAVLMVQIARRGLTSTNRTRIMCLSLFWHFLDVVWICVFTVVYLMGAM

>gi|30061928|ref|NP_836099.1| cytochrome o ubiquinol oxidase subunit IV [Shigella flexneri 2a str. 2457T]

MSHSTDHSGASHGSVKTYMTGFILSIILTVIPFWMVMTGAASPAVILGTILAMAVVQVLVHLVCFLHMNT

KSDEGWNMTAFVFTVLIIAILVVGSIWIMWNLNYNMMMH

>gi|30061927|ref|NP_836098.1| protoheme IX farnesyltransferase [Shigella flexneri 2a str. 2457T]

MFKQYLQVTKPGIIFGNLISVIGGFLLASKGSIDYPLFIYTLVGVSLVVASGCVFNNYIDRDIDRKMERT

KNRVLVKGLISPAVSLVYATLLGIAGFMLLWFGANPLACWLGVMGFVVYVGVYSLYMKRHSVYGTLIGSL

SGAAPSVIGYCAVTGEFDSGAAILLAIFSLWQMPHSYAIAIFRFKDYQAANIPVLPVVKGISVAKNHITL

YIIAFAVATLMLSLGGYAGYKYLVVAAAVSVWWLGMALRGYKVADDRIWARKLFGFSIIAITALSVMMSV

DFMVPDSHTLLAAVW

>gi|30061926|ref|NP_836097.1| hypothetical protein S0373 [Shigella flexneri 2a str. 2457T]

MPGKTAALYDVDKTLKNARVELKTSPDAKNKLREAAQAVGVDLSAFILSAAMERAESVLDNQRRRELSNQ

SWELMNQLIAEPAQPTLALKALMKRKNSDGRQA

>gi|30061925|ref|NP_836096.1| hypothetical protein S4812 [Shigella flexneri 2a str. 2457T]

MLGGARKYQKRGFGQDLLCDFFEHVKKIHPALPIKGVYLDADPAAINFYARLGFVQLSATPNAFGAVPMF

LAIQHILAA

>gi|30061924|ref|NP_836095.1| IS1 orfB [Shigella flexneri 2a str. 2457T]

MAHVFGERTLATLERLLSLLSAFEVVVWMTDGWPLYESRLKGKLHVISKRYTQRIERHNLNLRQHLARLG

RKSLSFSKSVELHDKVIGHYLNIKHYQ

>gi|30061923|ref|NP_836094.1| IS1 orfA [Shigella flexneri 2a str. 2457T]

MASISIRCPSCSATEGVVRNGKSTAGHQRYLCSHCRKTWQLQFTYTASQPGTHQKIIDMAMNGVGCRASA

RIMGVGLNTVLRHLKNSGRSR

>gi|30061922|ref|NP_836093.1| hypothetical protein S4810 [Shigella flexneri 2a str. 2457T]

MYKSLFTIIILLYSASSYAKSESPPASTITFIAQIDHVELNKTALSHDMKLVLADRFYFKTSNPCETNSI

SARPESMDITDKAYADKLKKLETPFLDDRYFFMTINQCDGNGTPMLTDIKVCTTELCGAEYTKKMSTVWL

DETLKPTAKRLAIYFIQSPLPYDEEKKQWKVTGWYISSTDNQSSTKQIAFEGYTDTDNLSDSKFISSFKS

WFESGKIKNIANYNPKGERDGKYESYYNSNGNVYESLSFSNGLINGEFITYYENGAIESKRHFNNDKIKD

GECPHFYEDGKIKQQHSYLNSKLDGPAYEYYPDGKLMQENFYQQSVLIGNDTSYYQSGKISSIHNRNSRG

QYDGANERYSEEGKLLSKSVYKDGKQISVQTWYENGQKEEEKHFDEQGQLNGLVKQWYKNGNLAKSQNYK

HDILDGNSEEWYENGIPESLYPYKNGKTDGIAKSWNKYGKLTYSIEYKNGVENGAYRNWSKNTGKLTKET

LYVNGIRQGVEKEFNDRTGKLLTSTQYVNNKRHGTEETYDQNGIKYITCYQNDQKLSSLDNPTQIKDNAT

TGDSSAQFALGKYEFICANIDEGIKWLTKSAEQKNTDAIYFLATAYKGNGIPANNEKYLAYLQQAATLGS

SNAQAEIGYLYLIGKELPQNLPDAGVWFKKAAAQGNFAAHFYLGRMYQNGDGVERNMEKARFHLSNAAEG

GIEPALKALNELEHQTK

>gi|30061921|ref|NP_836092.1| hypothetical protein S4809 [Shigella flexneri 2a str. 2457T]

MKAKSTLLLMILSVFYSHQVLSSEPTQPDVYSVVEKKLNSALPLEENTTFRSQAEWFNLQYELLVSGYPE

RAYNLLSKLETESKNAKYYLDLTASRVAEEEAPETALTFLSKIGLNKPATLTSFESYINSWIKSKQPEKA

MQLLSLDENALNYYLPTVLVAYQIVPDKAISIYNSVYGDNVVIPYQQVTMLLTVAEGYQTKGDIKNAIHY

ADKALNMFDKAIAVDSSRESHYSDEYLKLMNIYAANGNKEKAVVLSQRLHKAITESQSNYLSTLEGILLF

YRNNDMQQDYQNSLSNYIVFIDKIFAFSPSTRSELSLINLLNSLNEVELMRKRLAFLMTAPEYACYNSQY

CYEDKVSALKILYRHHDNELMDKYFTTLLKEISNQPLEDWDIIISNTGEKFSEVGLTKYAQHLASFAEEF

YIKEQKSHSESEMRGLFSSLAELYSFGNDTASANRVFHQHVPAFNTDHMIQYFINAKEWNNARELLIKEE

MLGMHNLILLENICMQKNAECMAHITFTLNKLTTQPAITKIDSVGNEQLYQIGKIYHKLEIKPEPEQQVL

IQSLYDRASGSASATQ

>gi|30061920|ref|NP_836091.1| nucleotide-binding protein [Shigella flexneri 2a str. 2457T]

MPSFDIVSEVDLQEARNAVDNASREVESRFDFRNVEASFELNDASKTIKVLSESDFQVNQLLDILRAKLL

KRGIEGSSLDVPENIVHSGKTWFVEAKLKQGIESATQKKIVKMIKDSKLKVQAQIQGDEIRVTGKSRDDL

QAVMAMVRGGDLGQPFQFKNFRD

>gi|30061919|ref|NP_836090.1| 2-dehydropantoate 2-reductase [Shigella flexneri 2a str. 2457T]

MKITVLGCGALGQLWLTALCKQGHEVQGWLRVPQPYCSVNLVETDGSIFNESLTANDPDFLATSDLLLVT

LKAWQVSDAVKSLASTLPVTTPILLIHNGMGTIEELQNIQQPLLMGTTTHAARRDGNVIIHVANGITHIG

PARQQDGDYSYLADILQTVLPDVAWHNNIRAELWRKLAVNCVINPLTAIWNCPNGELRHHPQEIMQICEE

VAAVIEREGHHTSAEDLRDYVMQVIDATAENISSMLQDIRALRHTEIDYINGFLLRRARAHGIAVPENTR

LFEMVKRKESEYERIGTGLPRPW

>gi|30061918|ref|NP_836089.1| hypothetical protein S0369 [Shigella flexneri 2a str. 2457T]

MNMSASALVCLAPGSEETEAVTTINLLVRGGIKVTTASVDSDGNLAITCSRGVKLLADAPLVEVADGEYD

VIVLPGGIKGTECFRDSTLLVETVKQFHRSGRIVAAICAAPATVLVPHDIFPIGNMTGFPTLKDKIPAEQ

WQDKRVVWDARVKLLTSQGPGTAIDFGLKIIDLLVGREKAHEVASQLVMAAGIYNYYE

>gi|30061917|ref|NP_836088.1| thiamine biosynthesis protein ThiI [Shigella flexneri 2a str. 2457T]

MKFIIKLFPEITIKSQSVRLRFIKILTGNIRNVLKHYDETLAVVRHWDNIEVRAKDENQRLTIRDALTRI

PGIHHILEVEDVPFTDMHDIFEKALVQYRDQLEGKTFCVRVKRRGKHDFSSIDVERYVGGGLNQHIESAR

VKLTNPDVTVHLEVEDDRLLLIKGRYEGIGGFPIGTQEDVLSLISGGFDSGVSSYMLMRRGCRVHYCFFN

LGGAAHEIGVRQVAHYLWNRFGSSHRVRFVAINFEPVVGEILEKIDDGQMGVILKRMMVRAASKVAERYG

VQALVTGEALGQVSSQTLTNLRLIDNVSDTLILRPLISYDKEHIINLARQIGTEDFARTMPEYCGVISKS

PTVKAVKSKIEAEEEKFDFSILDKVVEEANNVDIREIAQQTEQEVVEVETVNGFGPNDVILDIRSVDEQE

DKPLKVEGIDVVSLPFYKLSTKFGDLDQNKTWLLWCERGVMSRLQALYLREQGFNNVKVYRP

>gi|30061916|ref|NP_836087.1| exodeoxyribonuclease VII small subunit [Shigella flexneri 2a str. 2457T]

MPKKNEAPASFEKALSELEQIVTRLESGDLPLEEALNEFERGVQLARQGQAKLQQAEQRVQILLSDNEDA

SLTPFTPDNE

>gi|30061915|ref|NP_836086.1| geranyltranstransferase [Shigella flexneri 2a str. 2457T]

MDFPQQLEACVKQANQALSRFIAPLPFQNTPVVETMQYGALLGGKRLRPFLVYATGHMFGVSTNTLDAPA

AAVECIHAYSLIHDDLPAMDDDDLRRGLPTCHVKFGEANAILAGDALQTLAFSILSDANMPEVSDRDRIS

MISELASASGIAGMCGGQALDLDAEGKHVPLDALERIHRHKTGALIRAAVRLGALSAGDKGRRALPVLDK

YAESIGLAFQVQDDILDVVGDTATLGKRQGADQQLGKSTYPALLGLEQARKKARDLIDDARQALKQLAEQ

SLDTSALEALADYIIQRNK

>gi|30061914|ref|NP_836085.1| 1-deoxy-D-xylulose-5-phosphate synthase [Shigella flexneri 2a str. 2457T]

MSFDIAKYPTLALVDSTQELRLLPKESLPKLCDELRRYLLDSVSRSSGHFASGLGTVELTVALHYVYNTP

FDQLIWDVGHQAYPHKILTGRRDKIGTIRQKGGLHPFPWRGESEYDVLSVGHSSTSISAGIGIAVAAEKE

GKNRRTVCVIGDGAITAGMAFEAMNHAGDIRPDMLVVLNDNEMSISENVGALNNHLAQLLSGKLYSSLRE

GGKKVFSGVPPIKELLKRTEEHIKGMVVPGTLFEELGFNYIGPVDGHDVLGLITTLKNMRDLKGPQFLHI

MTKKGRGYEPAEKDPITFHAVPKFDPSSGCLPKSSGGLPSYSKIFGDWLCETAAKDNKLMAITPAMREGS

GMVEFSRKFPDRYFDVAIAEQHAVTFAAGLAIGGYKPIVAIYSTFLQRAYDQVLHDVAIQKLPVLFAIDR

AGIVGADGQTHQGAFDLSYLRCIPEMVIMTPSDENECRQMLYTGYHYNDGPSAVRYPRGNAVGVELTPLE

KLPIGKGIVKRRGEKLVILNFGTLMPETAKVAESLNATLVDMRFVKPLDEALILEMAASHEALVTVEENA

IMGGAGSGVNEVLMAHRKPVPVLNIGLPDFFIPQGTQEEMRAELGLDAAGMEAKIKAWLA

>gi|30061913|ref|NP_836084.1| phosphatidylglycerophosphatase A [Shigella flexneri 2a str. 2457T]

MTILSRHKDVAKSRLKMSNPWHLLAVGFGSGLSPIVPGSMGSLAAIPFWYLMTFLPWQLYSLVVMLGICI

GVYLCHQTAKDMGVHDHGSIVWDEFIGMWITLMALPTNDWQWVAAGFVIFRILDMWKPWPIRWFDRNVHG

GMGIMIDDIVAGVISAGILYFIGHHWPLGILS

>gi|30061912|ref|NP_836083.1| thiamine monophosphate kinase [Shigella flexneri 2a str. 2457T]

MACGEFSLIARYFDRVRSSRLDVELGIGDDCALLNIPEKQTLAISTDTLVAGNHFLPDIDPADLAYKALA

VNLSDLAAMGADPAWLTLALTLPDVDEAWLESFSDSLFDLLNYYDMQLIGGDTTRGPLSMTLGIHGFVPM

GRALTRSGAKPGDWIYVTGTPGDSAAGLAILQNRLQVADAKDADYLIKRHLRPSPRILQGQALRDLANSA

IDLSDGLISDLGHIVKASDCGARIDLALLPFSDALSRHVEPEQALRWALSGGEDYELCFTVPELNRGALD

VALGHLGVPFTCIGQMTADIEGLCFIRDGEPVTFDWKGYDHFVTP

>gi|30061911|ref|NP_836082.1| transcription antitermination protein NusB [Shigella flexneri 2a str. 2457T]

MKPAARRRARECAVQALYSWQLSQNDIADVEYQFLAEQDVKDVDVLYFRELLAGVATNTAYLDGLMKPYL

SRLLEELGQVEKAVLRIALYELSKRSDVPYKVAINEAIELAKSFGAEDSHKFVNGVLDKAAPVIRPNKK

>gi|30061910|ref|NP_836081.1| 6,7-dimethyl-8-ribityllumazine synthase [Shigella flexneri 2a str. 2457T]

MNIIEANVATPDARVAITIARFNNFINDSLLEGAIDALKRIGQVKDENITVVWVPGAYELPLAAGALAKT

GKYDAVIALGTVIRGGTAHFEYVAGGASNGLAHVAQDSEIPVAFGVLTTESIEQAIERAGTKAGNKGAEA

ALTALEMINVLKAIKA

>gi|30061909|ref|NP_836080.1| bifunctional diaminohydroxyphosphoribosylaminopyrimidine deaminase/5-amino-6-(5-phosphoribosylamino)uracil reductase [Shigella flexneri 2a str. 2457T]

MQDEYYMARALKLAQRGRFTTHPNPNVGCVIVKDGEIVGEGYHQRAGEPHAEVHALRMAGEKAKGATAYV

TLEPCSHHGRTPPCCDALIAAGVARVVAAMQDPNPQVAGRGLYRLQQAGIDVSHGLMMSEAEQLNKGFLK

RMRTGFPYIQLKLGASLDGRTAMASGESQWITSPQARRDVQRQRAQSHAILTSSATVLADDPALTVRWSE

LDEQTQALYPQQNLRQPVRIVIDSQNRVTPEHRIVQQPGETWFARTQEDSREWPETVRTLLIPEHKGHLD

LVVLMMQLGKQQINSIWVEAGPTLAGVLLQAGLVDELIVYIAPKLLGSDARGLCSLPGLEKLADAPQFKF

KEIRHVGPDVCLHLVGA

>gi|30061908|ref|NP_836079.1| transcriptional regulator NrdR [Shigella flexneri 2a str. 2457T]

MHCPFCFAVDTKVIDSRLVGEGSSVRRRRQCLVCNERFTTFEVAELVMPRVVKSNDVREPFNEEKLRSGM

LRALEKRPVSSDYVEMAINHIKSQLRATGEREVPSKMIGNLVMEQLKKLDKVAYIRFASVYRSFEDIKEF

GEEIARLED

>gi|30061906|ref|NP_836077.1| outer membrane protein Tsx [Shigella flexneri 2a str. 2457T]

MKKTLLAAGAVLALSSSFTVNAAENDKPQYLSDWWHQSVNVVGSYHTRFGPQIRNDTYLEYEAFAKKDWF

DFYGYADAPVFFGGNSDAKGIWNHGSPLFMEIEPRFSIDKLTNTDLSFGPFKEWYFANNYIYDMGRNKDG

RQSTWYMGLGTDIDTGLPMSLSMNVYAKYQWQNYGAANENEWDGYRFKIKYFVPITDLWGGQLSYIGFTN

FDWGSDLGDDSGNAINGIKTRTNNSIASSHILALNYDHWHYSVVARYWHDGGQWNDDAELNFGNGNFNVR

STGWGGYLVVGYNF

>gi|30061905|ref|NP_836076.1| hypothetical protein S0355 [Shigella flexneri 2a str. 2457T]

MAIIPKNYARLESGYREKALKIYPWVCGRCSREFVYSNLRELTVHHIDHDHTNNPEDGSNWELLCLYCHD

HEHSKYTEADQYGTTVIAGEDAQKDVGEAKYNPFADLKAMMNKKK

>gi|30061904|ref|NP_836075.1| preprotein translocase subunit SecF [Shigella flexneri 2a str. 2457T]

MAQEYTVEQLNHGRKVYDFMRWDYWAFGISGLLLIAAIVIMGVRGFNWGLDFTGGTVIEITLEKPAEIDV

MRDALQKAGFEEPMLQNFGSSHDIMVRMPPAEGETGGQVLGSQVLKVINESTNQNAAVKRIEFVGPSVGA

DLAQTGAMALMAALLSILVYVGFRFEWRLAAGVVIALTHDVIITLGILSLFHIEIDLTIVASLMSVIGYS

LNDSIVVSDRIRENFRKIRRGTPYEIFNVSLTQTLHRTLITSGTTLMVILMLYLFGGPVLEGFSLTMLIG

VSIGTASSIYVASALALKLGMKREHMLQQKVEKEGADQPSILP

>gi|30061903|ref|NP_836074.1| preprotein translocase subunit SecD [Shigella flexneri 2a str. 2457T]

MLNRYPLWKYVMLIVVIVIGLLYALPNLFGEDPAVQITGARGVAASEQTLIQVQKTLQEEKITAKSVALE

EGAILARFDSTDTQLRAREALMGVMGDKYVVALNLAPATPRWLAAIHAEPMKLGLDLRGGVHFLMEVDMD

TALGKLQEQNIDSLRSDLREKGIPYTTVRKENNYGLSITFRDAKARDEAIAYLSKRHPDLVISSQGSNQL

RAVMSDARLSEAREYAVQQNINILRNRVNQLGVAEPVVQRQGADRIVVELPGIQDTARAKEILGATATLE

FRLVNTNVDQAAAASGRVPGDSEVKQTREGQPVVLYKRVILTGDHITDSTSSQDEYNQPQVNISLDSAGG

NIMSNFTKDNIGKPMATLFVEYKDSGKKDANGRAVLVKQEEVINIANIQSRLGNSFRITGINNPNEARQL

SLLLRAGALIAPIQIVEERTIGPTLGMQNIEQGLEACLAGLLVSILFMIIFYKKFGLIATSALIANLILI

VGIMSLLPGATLSMPGIAGIVLTLAVAVDANVLINERIKEELSNGRTVQQAIDEGYRGAFSSIFDANITT

LIKVIILYAVGTGAIKGFAITTGIGVATSMFTAIVGTRAIVNLLYGGKRVKKLSI

>gi|30061902|ref|NP_836073.1| preprotein translocase subunit YajC [Shigella flexneri 2a str. 2457T]

MSFFISDAVAATGAPAQGSPMSLILMLVVFGLIFYFMILRPQQKRTKEHKKLMDSIAKGDEVLTNGGLVG

RVTKVAENGYIAIALNDTTEVVIKRDFVAAVLPKGTMKAL

>gi|30061901|ref|NP_836072.1| queuine tRNA-ribosyltransferase [Shigella flexneri 2a str. 2457T]

MKFELDTTDGRARRGRLVFDRGVVETPCFMPVGTYGTVKGMTPEEVEATGAQIILGNTFHLWLRPGQEIM

KLHGDLHDFMQWKGPILTDSGGFQVFSLGDIRKITEQGVHFRNPINGDPIFLDPEKSMEIQYDLGSDIVM

IFDECTPYPADWDYAKRSMEMSLRWAKRSRERFDSLGNKNALFGIIQGSIYEDLRDISVKGLVDIGFDGY

AVGGLAVGEPKADMHRILEHVCPQIPADKPRYLMGVGKPEDLVEGVRRGIDMFDCVMPTRNARNGHLFVT

DGVVKIRNAKYKSDTGPLDPECDCYTCRNYSRAYLHHLDRCNEILGARLNTIHNLRYYQRLMAGLRKAIE

EGKLESFVTDFYQRQGREVPPLNVD

>gi|30061900|ref|NP_836071.1| S-adenosylmethionine:tRNA ribosyltransferase-isomerase [Shigella flexneri 2a str. 2457T]

MRVTDFSFELPESLIAHYPMPERSSCRLLSLDGPTGALTHGTFTDLLDKLNPGDLLVFNNTRVIPARLFG

RKASGGKIEVLVERMLDDKRILAHIRASKAPKPGAELLLGDDESINATMTARHGALFEVEFNDERSVLDI

LNSIGHMPLPPYIDRPDEDADRELYQTVYSEKPGAVAAPTAGLHFDEPLLEKLRAKGVEMAFVTLHVGAG

TFQPVRVDTIEDHIMHSEYAEVPQDVVDAVLAAKARGNRVIAVGTTSVRSLESAAQAAKNDLIEPFFDDT

QIFIYPGFQYKVVDALVTNFHLPESTLIMLVSAFAGYQHTMNAYKAAVEEKYRFFSYGDAMFITYNPQAI

NERVGE

>gi|30061899|ref|NP_836070.1| maltodextrin glucosidase [Shigella flexneri 2a str. 2457T]

MMLNAWHLPVPPFVKQSKDQLLITLWLTGEDPPQRIMLRTEHDNEEMSVPMHKQRSQPQPGVTAWRAAID

LSSGQPRRRYSFKLLWHDRQRWFTPQGFSRMPPARLEQFAVDVPDIGPQWAADQIFYQIFPDRFARSLPR

EAEQDHVYYHHAAGQEIILRDWDEPVTAQAGGSTFYGGDLDGISEKLPYLKKLGVTALYLNPVFKAPSVH

KYDTEDYRHVDPQFGGDGALLRLRHNTQQLGMRLVLDGVFNHSGDSHAWFDRHNLGTGGACHNSESPWRD

WYSFSDDGTALDWLGYASLTKLDYQSESLVNEIYRGEDSIVRHWLKAPWNMDGWRLDVVHMLGEAGGARN

NMQHVAGITEAAKETQPEAYIVGEHFGDARQWLQADVEDAAMNYRGFTFPLWGFLANTDISYDPQQIDAQ

TCMAWMDNYRAGLSHQQQLRMFNQLDSHDTARFKTLLGRDIARLPLAVVWLFTWPGVPCIYYGDEVGLDG

KNDPFCRKPFPWQVEKQDTALFALYRRMIVLRKKSQALRRGGCQVLYAEDNVVVFVRVLNQQRVLVAINR

GEASEVVLPASPFLNAVQWQCKEGHGQLTDGILALPAISATVWMN

>gi|30061898|ref|NP_836069.1| proline-specific permease [Shigella flexneri 2a str. 2457T]

MESKNKLKRGLSTRHIRFMALGSAIGTGLFYGSADAIKMAGPSVLLAYIIGGIAAYIIMRALGEMSVHNP

AASSFSRYAQENLGPLAGYITGWTYCFEILIVAIADVTAFGIYMGVLFPTVPHWIWVLSVVLIICAVNLM

SVKVFGELEFWFSFFKVATIIIMIVAGFGIIIWGIGNGGQPTGIHNLWSNGGFFSNGWLGMVMSLQMVMF

AYGGIEIIGITAGEAKDPEKSIPRAINSVPMRILVFYVGTLFVIMSIYPWNQVGTAGSPFVLTFQHMGIT

FAASILNFVVLTASLSAINSDVFGVGRMLHGMAEQGSAPKIFSKTSRRGIPWVTVLVMTTALLFAVYLNY

IMPENVFLVIASLATFATVWVWIMILLSQIAFRRRLPPEEVKALKFKVPGGVATTIGGLIFLLFIIGLIG

YHPDTRISLYVGFAWIVVLLIGWMFKRRHDRQLAENQ

>gi|30061897|ref|NP_836068.1| branched-chain amino acid aminotransferase [Shigella flexneri 2a str. 2457T]

MTHQLRSRDIIALGFMTFALFVGAGNIIFPPMVGLQAGEHVWTAAFGFLITAVGLPVLTVVALAKVGGGV

DSLSTPIGKVAGVLLATVCYLAVGPLFATPRTATVSFEVGIAPLTGDSALPLFIYSLVYFAIVILVSLYP

GKLLDTVGNFLAPLKIIALVILSVVAIVWPAGSVSTATEAYQNAAFSNGFVNGYLTMDTLGAMVFGIVIV

NAARSRGVTEARLLTRYTVWAGLMAGVGLTLLYLALFCLGSDSASLVDQSANGAAILHAYVQHTFGGGGS

FLLAALIFIACLVTAVGLTCACAEFFAQYVPLSYRTLVFILGGFSMVVSNLGLSQLIQISVPVLTAIYPP

CIALVVLSFTRSWWHNSSRVIAPPMFISLLFGILDGIKASAFSDILPSWAQRLPLAEQGLAWLMPTVVMV

VLAIIWDRAAGRQVTSSAH

>gi|30061896|ref|NP_836067.1| phosphate regulon sensor protein [Shigella flexneri 2a str. 2457T]

MLERLSWKRLVLELLLCCLPAFILGAFFGYLPWFLLASVTGLLIWHFWNLLRLSWWLWVDRSMTPPPGRG

SWEPLLYGLHQMQLRNKKRRRELGNLIKRFRSGAESLPDAVVLTTEEGGIFWCNGLAQQILGLRWPEDNG

QNILNLLRYPEFTQYLKTRDFSRPLNLVLNTGRHLEIRVMPYTHKQLLMVARDVTQMHQLEGARRNFFAN

VSHELRTPLTVLQGYLEMMDEQPLEGAVREKALHTMREQTQRMEGLVKQLLTLSKIEAAPTQLLNEKVDV

PMMLRVVEREAQTLSQKKQTFTFEIDNGLKVSGNEDQLRSAISNLVYNAVNHTPEGTHITVRWQRVPHGA

EFSVEDNGPGIAPEHIPRLTERFYRVDKARSRQTGGSGLGLAIVKHAVNHHESRLNIESTVGKGTRFSFV

IPERLIAKNSD

>gi|30061895|ref|NP_836066.1| transcriptional regulator PhoB [Shigella flexneri 2a str. 2457T]

MARRILVVEDEAPIREMVCFVLEQNGFQPVEAEDYDSAVNQLNEPWPDLILLDWMLPGGSGIQFIKHLKR

ESMTRDIPVVMLTARGEEEDRVRGLETGADDYITKPFSPKELVARIKAVMRRISPMAVEEVIKMQGLSLN

PTSHRVMAGEEPLEMGPTEFKLLHFFMTHPERVYSREQLLNHVWGTNVYVEDRTVDVHIRRLRKALEPGG
HDRMVQTVRGTGYRFSTRF

>gi|30061894|ref|NP_836065.1| exonuclease subunit SbcD [Shigella flexneri 2a str. 2457T]
MRILHTSDWHLGQNFYSKSREAEHQAFLDWLLETAQTHQVDAIIVAGDVFDTGSPPSYARTLYNRFVVNL
QQTGCHLVVLAGNHDSVATLNESRDIMAFLNTTVVASAGHAPQILPRRDGTPGAVLCPIPFLRPRDIISS
QAGLNGIEKQQHLLAAITDYYQQHYADACKLRGDQPLPIIATGHLTTVGSSKSDAVRDIYIGTLDAFPAQ
NFPPADYIALGHIHRAQIIGGMEHVRYCGSPIPLSFDECGKSKYVHLVTFSNGKLESVENLNVPVTQPMA
VLKGDLASITAQLEQWRDVSQEPPVWLDIEITTDEYLHDIQRKIQALTESLPVEVLLVRRSREQRERVLA
SQQRETLSELSVEEVFNRRLALEDLDESQQQRLQHLFTTTLRTLAGEHEA

>gi|30061893|ref|NP_836064.1| exonuclease subunit SbcC [Shigella flexneri 2a str. 2457T]
MKILSLRLKNLNSLKGEWKIDFTREPFASNGLFAITGPTGAGKTTLLDAICLALYHETPRLSNVSQSQND
LMTRDTAECLAEVEFEVKGEAYRAFWSQNRARNQPDGNLQVPRVELARCADGKILADKVKDKLELTATLT
GLDYGRFTRSMLLSQGQFAAFLNAKPKERAELLEELTGTEIYGQISAMVFEQHKSARTELEKLQAQASGV
TLLTPEQVQSLTASLQVLTDEEKQLITAQQQEQQSLNWLTRQDELQQEASRRQQALQQALAEEEKAQPQL
AALSLAQPARNLRPHWERIAEHSAALAHIRQQIEEVNTRLQSTMALRASIRHHAAKQSAELQQQQQSLNT
WLQEHDRFRQWNNELAGWRAQFSQQTSDREHLRQWQQQLTHAEQKLNALAAITLTLTADEVATALAQHAE
QRPLRQRLVALHGQIVPQQKRLAQLMVTIQNVTLEQTQRNVALNEMRQRYKEKTQQLADVKTICEQEARI
KTLEAQRAQLQAGQPCPLCGSTSHPAVEAYQALEPGVNQSRLLALENEVKKLGEEGAALRGQLDALTKQL
QRDENEAQSLRQDEQALTQQWQAVTASLNITLQPQDDIQPWLDAQDEHERQLRLLSQRHELQGQIAAHNQ
QIIQYQQQIEQRQQQLLTALAGYALTLPQEDEEESWLATRQQEAQSWQQRQNELTALQNRIQQLTPILET
LPQSDDLPHSEETVALDNWRQVHEQCLALHSQQQTLQQQDVLAAQSLQKAQAQFDTALQASVFDDQQAFL
AALMDEQTLTQLEQLKQNLENQRRQAQTLVTQTAETLAQHQQHRPDGLALTVTVEQIQQELAQTHQKLRE
NTTSQGEIRQQLKQDADNRQQQQTLMQQIAQMTQQVEDWGYLNSLIGSKEGDKFRKFAQGLTLDNLVHLA

NQQLTRLHVRYLLQRKASEALEVEVVDTWQADAVRDTRTLSGGESFLVSLALALALSDLVSHKTRIDSLF
LDEGFGTLDSETLDTALDALDALNASGKTIGVISHVEAMKERIPVQIKVKKINGLGYSKLESTFAVK

>gi|30061892|ref|NP_836063.1| MFS transport protein AraJ [Shigella flexneri 2a str. 2457T]
MKKVILSLALGTFGLGMAEFGIMSVLTELAHNVGISIPAAGHMISYYALVVVVGAPIIALFSSRYSLKHI
LLFLVALCVIGNAMFTLSSSYLMLAIGRLVSGFPHGAFFGVGAIVLSKIIKPGKVTAAVAGMVSGMTVAN
LLGIPLGTYLSQECWRYTFLLIAVFNIAVMASVYFWVPDIRDEAKGNLREQFHFLRSPAPWLIFAATMFG
NAGVFAWFSYVKPYMMFISGFSETAMTFIMMLVGLGMVLGNMLSGRISGRYSPLRIAAVTDFIIVLALLM
LFFCGGMKTTSLIFAFICCAGLFALSAPLQILLLQNAKGGELLGAAGGQIAFNLGSAVGAYCGGMMLTLG
LAYNYVALPAALLSFAAMSSLLLYGRYKRQQAADSPVLAKPLG

>gi|30061890|ref|NP_836061.1| recombination associated protein [Shigella flexneri 2a str. 2457T]
MLWFKNLMVYRLSREISLRAEEMEKQLASMAFTPCGSQDMAKMGWVPPMGSHSDALTHVANGQIVICARK
EEKILPSPVIKQALEAKIAKLEAEQARKLKKTEKDSLKDEVLHSLLPRAFSRFSQTMMWLDTVNGLIMVD
CASAKKAEDTLALLRKSLGSLPVVPLSMENPIELTLTEWVRSGSAAQGFQLLDEAELKSLLEDGGVIRAK
KQDLTSEEITNHIEAGKVVTKLALDWQQRIQFVMCDDGSLKRLKFCDELRDQNEDIDREDFAQRFDADFI
LMTGELAALIQNLIEGLGGEAQR

>gi|30061889|ref|NP_836060.1| hypothetical protein S0337 [Shigella flexneri 2a str. 2457T]
MTQRPWSKLQREIYDLLTPTINLQIHCTRYPMRSQNGGSTDLPRYWITLDKNVIWDYPKDFIAGNAGVRN
FHGETCWYPYLTDICSISDLLREYIDTPKAELLTKQFTSDKWGLVNILRAADRRIGMRRLDQLRRKTHNI
AALKIIARRSE

>gi|30061888|ref|NP_836059.1| hypothetical protein S0336 [Shigella flexneri 2a str. 2457T]
MLQSRNDHLRQTALRNAHTPASLLTTLTEPQDRSLAINNPQLAADVKTAWLKEDPSLLLFVEQPDLSQLR

DLVKTGATRKIRSEARHRLEEKQ

>gi|30061887|ref|NP_836058.1| hypothetical protein S0335 [Shigella flexneri 2a str. 2457T]

MLQSNEYFSGKVKSIGFSSSSTGRASVGVMVEGEYTFSTAEPEEMTVISGALNVLLPDATDWQVYEAGSV

FNVPGHSEFHLQVAEPTSYLCRYL

>gi|30061886|ref|NP_836057.1| hypothetical protein S0334 [Shigella flexneri 2a str. 2457T]

MSASLAILTIGIVPMQEVLPLLTEYIDEDNISHHSLLGKLSREEVMAEYAPEAGEDTILTLLNDNQLAHV

SRRKVERDLQGVVEVLDNQGYDVILLMSTANISSMTARNTIFLEPSRILPPLVSSIVEDHQVGVIVPVEE

MLPVQAQKWQILQKSPVFSLGNPIHDSEQKIIDAGKELLAKGADVIMLDCLGFHQRHRDLLQKQLDVPVL

LSNVLIARLAAELLV

>gi|30061885|ref|NP_836056.1| hypothetical protein S0333 [Shigella flexneri 2a str. 2457T]

MPTKPPYPREAYIVTIEKGKPGQTVTWYQLRADHPKPDSLISEHPTAQEAMDAKKRYEDPDKE

>gi|30061884|ref|NP_836055.1| shikimate kinase [Shigella flexneri 2a str. 2457T]

MTQPLFLIGPRGCGKTTVGMALADSLNRRFVDTDQWLQSQLNMTVAEIVEREEWAGFRARETAALEAVTA

ASTVIATGGGIILTEFNRHFMQNNGIVVYLCAPVSVLVNRLQAAPEEDLRPTLTGKPLSEEVQEVLEERD

ALYREVAHIIIDATNEPSQVISEIRSALAQTINC

>gi|30061882|ref|NP_836053.1| pyrroline-5-carboxylate reductase [Shigella flexneri 2a str. 2457T]

MEKKIGFIGCGNMGKAILGGLIASGQVLPGQIWVYTPSPDKVAALHDQFGINAAESAQEVAQIADIIFAA

VKPGIMIKVLSEITSSLNKDSLVVSIAAGVTLDQLARALGHDRKIIRAMPNTPALVNAGMTSVTPNALVT

PEDTADVLNIFRCFGEAEVIAEPMIHPVVGVSGSSPAYVFMFIEAMADAAVLGGMPRAQAYKFAAQAVMG

SAKMVLETGEHPGALKDMVCSPGGTTIEAVRVLEEKGFRAAVIEAMTKCMEKSEKLSKS

>gi|30061881|ref|NP_836052.1| diguanylate cyclase AdrA [Shigella flexneri 2a str. 2457T]

MFPKIMNDENFFKKAAAHGEEPPLTPQNEHQRSGLRFARRVRLPRAVGLAGMFLPIASTLVSHPPPGWWW

LVLVGWAFVWPHLAWQIASRAVDPLSREIYNLKTDAVLAGMWVGVMGVNVLPSTAMLMILCLNLMGAGGP

RLFVAGLVLMVVSCLVTLELTGITVSFNSAPLEWWLSLPIIVVYPLMFGWVSYQTATKLAEHKRRLQVMS

TRDGMTGVYNRRHWETMLRNEFDNCRRHNRDATLLIIDIDHFKSINDTWGHDVGDEAIVALTRQLQITLR

GSDVIGRFGGDEFAVIMSGTPAESAITAMLRVREGLNTLRLPNTPQVTLRISVGVAPLNPQMSHYREWLK

SADLALYKAKKAGRNRTEVAA

>gi|30061880|ref|NP_836051.1| IS629, orfA [Shigella flexneri 2a str. 2457T]

MTKNTRFSPEVRQRAVRMVLESQGEYDSQWATICSIAPKIGCTPETLRVWVRQHERDTGGGDGGLTTAER

QV

>gi|30061879|ref|NP_836050.1| IS629 orfB [Shigella flexneri 2a str. 2457T]

MAVMGLAGVLRGKKVRTTISRKAVAAGDRVNRQFVAERPDQLWVADFTYVSTWRGFVYVAFIIDVFAGYI

VGWRVSSSMETTFVLDALEQALWARRPSGTVHHSDKGSQYVSLAYTQRLKEAGLLASTGSTGDSYDNAMA

ESINGLYKAEVIHRKSWKNRAEVELATLTWVDWYNNRRLLERLGHIPPAEAEKAYYASIGNDDLAA

>gi|30061878|ref|NP_836049.1| IS600 orfB [Shigella flexneri 2a str. 2457T]

MYLAGIKDVYTCEIVGYAMGERMTKELTGKALFMALRSQRPPAGLIHHSDRGSQYCAYDYRVIQEQFGLK

TSMSRKGNCYDNAPMESFWGTLKNESLSHYRFNNRDEAISVIREYIEIFYNRQRRHSRLGNISPAAFREK

YHQMAA

>gi|30061877|ref|NP_836048.1| hypothetical protein S4804 [Shigella flexneri 2a str. 2457T]

MHMIEINSLLLITSVILMSLLAVGLFDKISPINLVEHGRNNQIDGMRGFLAIFVLIHHAAIWNGYLSSGV

WEAPSSNLLANLGQVGVSFFFMITGYLFFSKIISGDQDWTRLYVSRLLRLTPMFIVSLCLIFIIVGFKSG

WRMQVSTEELFVSIMKWLPFTALGMPNINDVKDSFTINAAVTWTLVYEWFFYFSLPVISALIKRKVSIYM

VMISAISLFVFILFFSKIHIASFLFGLLAFLLNKSKIVNGIAKAKVTPIIITAIMVFEMTYFKTTYAPLP

LILCGITFIIIASGCDLYGILRLNITRKLGETTYSVYLLHGIFLYCLMTWIIPNNYTENTFIILVSTTAF

LITFTSCLTFKLIETPFIKLTKQTTTLVKELIPTLTNNNQ

>gi|30061876|ref|NP_836047.1| phage integrase [Shigella flexneri 2a str. 2457T]

MSLFRRGEIWYASFTLPNGKRFKQSLGTKDKRQATELHDKLKAEAWRVSKLGEIPDITFEEACVRWLEEK

AHKKSLDDDKSRIGFWLQHFAGMQLRDITESKIYSAMQKMTNRRHEENWRLRAEACRKKGKPVPEYTPKP

ASVATKATHLSFIKALLRAAEREWKMLDKAPIIKVPQPKNKRIRWLEPHEAQRLIDECPEPLKSVVEFAL

ATGLRRSNIINLEWQQIDMQRRVAWINPEESKSNRAIGVALNDTACRVLKKQIGNHHRWVFVYKESCTKP

DGTKAPTVRKMRYDANTAWKAALRRAGIDDFRFHDLRHTWASWLVQAGVPLSVLQEMGGWESIEMVRRYA

HLAPNHLTEHARQIDSILNPSVPNSSQSKNKEGTNDV

>gi|30061875|ref|NP_836046.1| IS600 orfA [Shigella flexneri 2a str. 2457T]

MSRKTQRYSKEFKAEAVRTVLENQLSISEGASRLSLPEGTLGQWVTAARKGLGTPGSRTVAELESEILQL

RKALNEARLERDILKKATAYFAQESLKNTR

>gi|30061874|ref|NP_836045.1| IS600 orfB [Shigella flexneri 2a str. 2457T]

MAHIRTRETYGTRRLQTELAENGIIVGRDRLARLRKELRLRCKQKRKFRATTNPNHNLPVAPNLLNQTFA

PTAPNQVWVADLTYVATQEGWLYLAGIKDVYTCEIVGYAMGERMTKELTGKALFMALRSQRPPAGLIHHS

DRGSQYCAYDYRVIQEQFGLKTSMSRKGNCYDNAPMESFWGTLKNESLSHYRFNNRDEAISVIREYIEIF

YNRQRRHSRLGNISPAAFREKYHQMAA

>gi|30061873|ref|NP_836044.1| integrase fragment, partial [Shigella flexneri 2a str. 2457T]

MRTDSNKAWKGALKRAGISNFRFHDLRHTWASWLVQSGVSLLALKEMGGWETLEMVQRYAHLSAGHLTEH

ASKIDAIISRNGTNTAQEENVVYLNAR

>gi|30061872|ref|NP_836043.1| flippase [Shigella flexneri 2a str. 2457T]

MLKLFVKYTSIGVLNTLIHWVVFGVCIYAAHTSQALANFTGFVVAVSFSFFANARFTFKALTTAMRYMLY

VGFMGILSVIVGWAADKCSLPPIVTLITFSAISLVCGFVYSKFIVFRDAK

>gi|30061871|ref|NP_836042.1| bactoprenol glucosyl transferase [Shigella flexneri 2a str. 2457T]

MKISLVVPVFNEEEAIPVFYKTVREFQELKPYEVEIVFINDGSKDATESIINALAVSDPLVVPLSFTRNF

GKEPALFAGLDHASGDAVIPIDVDLQDPIEVIPHLIEKWQAGADMVLAKRSDRSTDGRLKRKTAEWFYKL

HNKISTPKIEENVGDFRLMSREVVENIKLLPERNLFMKGILSWVGGQTDVVEYVRAERVAGISKFNGWKL

WNLALEGITSFSTFPLRVWTYIGLFVASISFLYGAWMIIDTLVFGNPVRGYPSLLVSILFLGGVQLIGIG

VLGEYIGRIYIEVKNRPKYIIKKSHRGNP

>gi|30061870|ref|NP_836041.1| glucosyl tranferase II [Shigella flexneri 2a str. 2457T]

MIKINLFKNANLLAFISCFAISIYCYWGWLYDGTLNIDGEFTNNFYQTITLGRWFHTFLRHYFLPEPFSL

YITPLIALSFIIISAFIICRSLKLESYELLIGMLVFITFPQISYQLEFLNQADTVGIAFLLAAISAIIFH

SQKNRIVIFSGIVLSILSMAIYQTFVTYIIAFVIGLQINSIIRNEKNIRESFYSSCLSLSLIALSTLIYL

LLTKAIKHYFSLESNEYISNYIQNASDIKWLVKSAIDNIYNFYNNPPTGLNLYKWLLIPLLILMFTLTYK

LKTRSIYLISSIIFIYILPVIFIVVVGSGAPPRLFVLMPIVAVILFSCLSNFRSIKYLNCMFFLFIIFNG

VSTSKNLFLNDTLARQKDISLAKEISYTSQTKGISLNGKYIYIYGSNDSGNMLSMSADTFGKSFFWWDGG

NYFRMVAFMNYYGICNCKPANKEQIEKIYPIVKSLPSWPNPDSIAEINGLVIIKLSEKKGWLPFNI

>gi|30061869|ref|NP_836040.1| hypothetical protein S4808 [Shigella flexneri 2a str. 2457T]

MPARKVCQTFFRNALASTHQYRQNAIIDSAAALVGGASLSLTSIGRHLPGPARVKDKIKRVDCLLGNKRL

HNDIPLIFKNITSMMTNKLSWCVIAVDWSGYPFQEYHVLRASLLCDGRSIPLMSQVFPSKKNNEAVEIAF
LDALSGAISPRTRVVIVTDAGFQSAWFRHIKSQGWDFIGRIRGVVKFRLDSDKDKWLDIKMCRGSSEAKY
LGTGTLARKKRSQCEGHFYLYKHSPKGRKSRRARGRPGLPTTEKEQKAAGREPWLIFSNTAEFNAKKIMK
LYSRRMQIEQNFRDEKSERFGFGLRASRSRRGERFLVLSLLVTLASIVLWLLGYHFENKGFHLKYQANSL
KKRRVLSFLTLAENVLRFNPELLRRAQPEKILCRLASTYRSMVLAY

>gi|30061868|ref|NP_836039.1| hypothetical protein S4802 [Shigella flexneri 2a str. 2457T]
MYNNKIDSNRKIRHDWVDALKFLGIFAIYLGHLGLGAGKLYPFVFSYHVPLFFFAAGFFTIKKNDLSVFD
YIKSKFYRLMIPYFTFAFSILIINTINSGETIDYIYNHIYDIIYGVRNNQFVGTIWFINCLFVIIAIDAI
FREIVKNNIVILIISLLSFMLSQTVLNHNPLLDPQWFWNIDSAMAYWWLLPLGRCMFLELTRDRFFGKSK
IGFIVFSITAIMSAYQLLNQKPLLFKIISIFNADIISSSYIQAINTIITTVGLIIFNIFIAKIICGNDFI
VRAGRNTLNICGMEYITKLFIPMALAIIGFSVTIPNPICAIIYTCICVYVSDKIGHWLSRTVGGPFLIK

>gi|30061867|ref|NP_836038.1| phage tail fibre protein [Shigella flexneri 2a str. 2457T]
MSRTLDLILLCRPVQDTVHLLMRITLQWDINKMSYFYSASTNGFYSTEFHGTNIPDDAVEISESEWKTLI
NAQSVTKMITCGENGHPVIVDRPSPTPEQLALINDEKKSALIAEATNVIAPLQDAVDLGMATDDETKLYW
HGKNIGCCLCVLM

>gi|30061866|ref|NP_836037.1| hypothetical protein S0319 [Shigella flexneri 2a str. 2457T]
MHRIDTKTAKKDKFGAGKNGFTRGNPQTGTPATDLDDDYFDMLQEELCSVVEASGASLEKGRHDQLLTAL
RALLLSRKNPFGDIKSDGTVKTALENLGLGEAAKRNVGTGANQIPDMSLFASINTVTAAAQKFPSGLILQ
CGQLNGAPNVSSTYGMRFPMTFSRVIAVVVTLNVTGAAGQPTVSATSVQNTGFNITVSPGSGYGSSADAY
YIAMGY

>gi|30061865|ref|NP_836036.1| IS600 orfB [Shigella flexneri 2a str. 2457T]

MAHIRTRETYGTRRLQTELAENGIIVGRDRLARLRKELRLRCKQKRKFRATTNPNHNLPVAPNLLNQTFA

PTAPNQVWVADLTYVATQEGWLYLAGIKDVYTCEIVGYAMGERMTKELTGKALFMALRSQRPPAGLIHHS

DRGSQYCAYDYRVIQEQFGLKTSMSRKGNCYDNAPMESFWGTLKNESLSHYRFNNRDEAISVIREYIEIF

YNRQRRHSRLGNISPAAFREKYHQMAA

>gi|30061864|ref|NP_836035.1| IS600 orfA [Shigella flexneri 2a str. 2457T]

MSRKTQRYSKEFKAEAVRTVLENQLSISEGASRLSLPEGTLGQWVTAARKGLGTPGSRTLAELESEILQL

RKALNEARLERDILKKATAYFAQESLKNTR

>gi|30061863|ref|NP_836034.1| phage integrase [Shigella flexneri 2a str. 2457T]

MSLFRRGEIWYASFTLPNGKRFKQSLGTKDKRQATELHDKLKAEAWRVSKLGEIPDITFEEACVRWLEEK

AHKKSLDDDKSRIGFWLQHFAGMQLRDITESKIYSAMQKMTNRRHEENWRLRAEACRKKGKPVPEYTPKP

ASVATKATHLSFIKALLRAAEREWKMLDKAPIIKVPQPKNKRIRWLEPHEAQRLIDECPEPLKSVVEFAL

ATGLRRSNIINLEWQQIDMQRRVAWINPEESKSNRAIGVALNDTACRVLKKQIGNHHRWVFVYKESCTKP

DGTKAPTVRKMRYDANTAWKAALRRAGIDDFRFHDLRHTWASWLVQAGVPLSVLQEMGGWESIEMVRRYA

HLAPNHLTEHARQIDLILNPSVPNLSQSRNKEGTNDV

>gi|30061862|ref|NP_836033.1| gamma-glutamyl phosphate reductase [Shigella flexneri 2a str. 2457T]

MLEQMGIAAKQASYKLAQLSSREKNRVLEKIADELEAQSEIILNANAQDVADARANGLSEAMLDRLALTP

ARLKGIADDVRQVCNLADPVGQVIDGGVLDSGLRLERRRVPLGVIGVIYEARPNVTVDVASLCLKTGNAV

ILRGGKETCRTNAATVAVIQDALKSCGLPAGAVQAIDNPDRALVSEMLRMDKYIDMLIPRGGAGLHKLCR

EQSTIPVITGGIGVCHIYVDESAEIAEALKVIVNAKTQRPSTCNTVETLLVNKNIADSFLPALSKQMAES

GVTLHADAAALTQLQAGPAKVVAVKAEEYDDEFLSLDLNVKIVSDLDDAIAHIREHGTQHSDAILTRDMR

NAQRFVNEVDSSAVYVNASTRFTDGGQFGLGAEVAVSTQKLHARGPMGLEALTTYKWIGIGDYTIRA

>gi|30061861|ref|NP_836032.1| gamma-glutamyl kinase [Shigella flexneri 2a str. 2457T]

MSDSQTLVVKLGTSVLTGGSRRLNRAHIVELVRQCAQLHAAGHRIVIVTSGAIAAGREHLGYPELPATIA

SKQLLAAVGQSRLIQLWEQLFSIYGIHVGQMLLTRADMEDRERFLNARDTLRALLDNNIVPVINENDAVA

TAEIKVGDNDNLSALAAILAGADKLLLLTDQKGLYTADPRRNPQAELIKDVYGIDDALRAIAGDSVSGLG

TGGMSTKLQAADVACRAGIDTIIAAGSKPGVIGDVMEGISVGTLFHAQATPLENRKRWIFGAPPAGEITV

DEGATAAILERGSSLLPKGIKSVTGNFSRGEVIRICNLEGRDIAHGVSRYNSDALRRIAGHHSQEIDAIL

GYEYGPVAVHRDDMITR

>gi|30061860|ref|NP_836031.1| IS1 orfB [Shigella flexneri 2a str. 2457T]

MSRQCTHYGRWPQHGFTSLKKLRPQSVTSRIQPGSDVIVCAEMDEHWGYVGAKSRQRWLFYAYDRIRRTV

VAHVFGERTLATLERLLSLLSAFEVVVWMTDGWPLYESRLKGKLHVISKRYTQRIERHNLNLRQHLARLV

RKSLSFSKSVELHDKVIGHYLNIKHYQ

>gi|30061859|ref|NP_836030.1| IS1 orfA [Shigella flexneri 2a str. 2457T]

MASISIRCPSCSATEGVVRNGKSTAGHLRYLCSHCRKTWQLQFTYTASQPGKHQKIIDMAMNGVGCRASA

RIMGVGLNTVLRHLKNSGRSR

>gi|30061858|ref|NP_836029.1| outer membrane phosphoporin protein E [Shigella flexneri 2a str. 2457T]

MKKSTLALVVMGIVASASVQAAEIYNKDGNKLDVYGKVKAMHYMSDNASKDGDQSYIRFGFKGETQINDQ

LTGYGRWEAEFAGNKAESDTAQQKTRLAFAGLKYKDLGSFDYGRNLGALYDVEAWTDMFPEFGGDSSAQT

DNFMTKRASGLATYRNTDFFGVIDGLNLTLQYQGKNENRDVKKQNGDGFGTSLTYDFGGSDFAISGAYTN

SDRTNEQNLQSRGTGKRAEAWATGLKYDANNIYLATFYSETRKMTPITGGFANKTQNFEAVAQYQFDFGL

RPSLGYVLSKGKDIEGIGDEDLVNYIDVGATYYFNKNMSAFVDYKINQLDSDNKLNINNDDTVAVGMTYQ

F

>gi|30061857|ref|NP_836028.1| DNA-binding transcriptional regulator Crl [Shigella flexneri 2a str. 2457T]

MTLPSGHPKSRLIKKFTALGPYIREGKCEDNRFFFDCLAVCVNVKPAPEVREFWGWWMELEAQESRFTYS

YQFGLFDKAGDWKSVPVKDTEVVERLEHTLREFHEKLRELLTTLNLKLEPANDFRDEPVKLTA

>gi|30061856|ref|NP_836027.1| fermentation/respiration switch protein [Shigella flexneri 2a str. 2457T]

MTQANLSETLFKPRFKHPETSTLVRRFNHGAQPPVQSALDGKTIPHWYRMINRLMWIWRGIDPREILDVQ

ARIVMSDAERTDDDLYDTVIGYRGGNWIYEWATQAMVWQQKACTEEDPQLSGRHWLHAATLYNIAAYPHL

KGDDLAEQAQALSNRAYEEAAQRLPGTMRQMEFTVPGGAPITGFLHMPKGDGPFPTVLMCGGLDAMQTDY

YSLYERYFAPRGIAMLTIDMPSVGFSSKWKLTQDSSLLHQHVLKALPNVPWVDHTRVAAFGFRFGANVAV

RLAYLESPRLKAVACLGPVVHTLLSDFKCQQQVPEMYLDVLASRLGMHDASDEALRVELNRYSLKVQGLL

GRRCPTPMLSGYWKNDPFSPEEDSRLITSSSADGKLLEIPFNPVYRNFDKGLQEITGWIEKRLC

>gi|30061855|ref|NP_836026.1| xanthine-guanine phosphoribosyltransferase [Shigella flexneri 2a str. 2457T]

MSEKYIVTWDMLQIHARKLASRLMPSEQWKGIIAVSRGGLVPGALLARELGIRHVDTVCISSYDHDNQRE

LKVLKRAEGDGEGFIVIDDLVDTGGTAVAIREMYPKAHFVTIFAKPAGRPLVDDYVVDIPQDTWIEQPWD

MGVVFVPPISGR

>gi|30061854|ref|NP_836025.1| aminoacyl-histidine dipeptidase [Shigella flexneri 2a str. 2457T]

MSELSQLSPQPLWDIFAKICSIPHPSYHEEQLAEYIVGWAKEKGFHVERDQVGNILIRQPATAGMENRKP

VVLQAHLDMVPQKNNDTVHDFTKDPIQPYIDGEWVKARGTTLGADNGIGMASALAVLADENVVHGPLEVL

LTMTEEAGMDGAFGLQSNWLQADILINTDSEEEGEIYMGCAGGIDFTSNLHLDREAVPAGFETFKLTLKG

LKGGHSGGEIHVGLGNANKLLVRFLAGHAEELDLRLIDFNGGTLRNAIPREAFATIAVAADKVDALKSLV

NTYQEILKNELAEKEKNLALLLDSVANDKAALIAKSRDTFIRLLNATPNGVIRNSDVAKGVVETSLNVGV

VTMTDNNVEIHCLIRSLIDSGKDYVVSMLDSLGKLAGAKTEAKGAYPGWQPDANSPVMHLVRETYQRLFN

KTPNIQIIHAGLECGLFKKPYPEMDMVSIGPTITGPHSPDEQVHIESVGHYWTLLTELLKEIPAK

>gi|30061853|ref|NP_836024.1| peptide chain release factor 2, partial [Shigella flexneri 2a str. 2457T]

MKKALDRLIKEAARQDVAVTVLETETGRYSDTLRSALISLDGDNAWALSESWCGTIQWICPSPYRPHHGR

KNWFLGIGRFTADEQEQSDAIRYETLRSSGPGGQHVNKTDSAVRATHLASGISVKVQSERSQHANKRLAR

LLIAWKLDQQQ

>gi|30061852|ref|NP_836023.1| hypothetical protein S0304 [Shigella flexneri 2a str. 2457T]

MGNYIRPLSDAVFTIASDDLWIESLAIQQLHTTANLPNMQRVVGMPDLHPGRGYPIGAAFFSAGRFYPAL

VGNDIGCGMALWQTDILARKYNADKFEKRLSALDDVAEESWLEENLPSAFAQHPWRSSLGSIGGGNHFAE

LQQVDQIINAELFALAGLDAQHLQLLVHSGSRGLGQSILQRHIASFSHHGLPEGSDDALAFARFNRHLIA

LRIMQQVKATGSPVLDVAHNFVSACRIGDQQGVLHRKGATPDDCGLVVIPGSRGDYSRLVQPVRSEETLH

SLAHGAGRKWGRTECKGRLAAKYTATQLSRTELGSRVICRDKQLIFEEAPQAYKSAESVVQCQGRPD

>gi|30061851|ref|NP_836022.1| hypothetical protein S0303 [Shigella flexneri 2a str. 2457T]

MNNIQIRDYQPGDFQQLCAIFIRAVTMTVSQHYSLQQIAAWAQIDESRWKEKLAKSQVRVAVINAKLVGF

ITCVEHYIDMLFVDPEYTRRGVASALLKPFIKSESELTVDASITAKPFFERYGFQTVKQQRVECRGAWFI

NFYMRYKPQH

>gi|30061850|ref|NP_836021.1| toxin YafO [Shigella flexneri 2a str. 2457T]

MRVFKTKLIRLQLTAEELDALTADFISYKRDGVLPDIFGRDALYDDSFTWPLIKFERVAHIHLANVNNPF

PPQLRQFSRTNDEAHLVYCQGAFDEQAWLLIAILKPEPHKLARDNNQMHKIGKMAEAFRMRF

>gi|30061849|ref|NP_836020.1| antitoxin of the YafO-YafN toxin-antitoxin system [Shigella flexneri 2a str. 2457T]

MHRILAEKSVNITELRKNPAKYFIDQPVAVLSNNRPAGYLLSASAFEALMDMLAEQEEKKPIKARFRPSA

ARLEEITRRAKQYLNDMTDDDFNDFKE

>gi|30061848|ref|NP_836019.1| DNA polymerase IV [Shigella flexneri 2a str. 2457T]

MRKIIHVDMDCFFAAVEMRDNPALRDIPIAIGGSRERRGVISTANYPARKFGVRSAMPTGMALKLCPHLT

LLPGRFDAYKEASNHIREIFSRYTSRIEPLSLDEAYLDVTDSVHCHGSATLIAQEIRQTIFNELQLTASA

GVAPVKFLAKIASDMNKPNGQFVITPAEVPAFLQTLPLEKIPGVGKVSAAKLEAMGLRTCGDVQKCDLVI

LLKRFGKFGRILWERSQGIDERDVNSERLRKSVGVERTMAEDIHHWSECEAIIERLYPELERRLAKVKPD

LLIARQGVKLKFDDFQQTTQEHVWPRLNKADLIATARKTWDERRGGRGVRLVGLHVTLLDPQMERQLVLG

L

>gi|30061847|ref|NP_836018.1| hypothetical protein S0298 [Shigella flexneri 2a str. 2457T]

MTTIKLIVNSVSKSERESIIAALHGQSIFNGGGLSPLNKISPSHPPKPATVAVPEETEKKARDVNEKTAL

LKKKSATELGELATSINTIARDAHMEANLEMEIVPQGLRVLIKDDQNRNMFERGSAQIMPFFKTLLVELA

PVFDSLYNKIIITGHTDAMAYKNNIYNNWNLSGDRALSARRVLEEAGMPEDKVMQVSAMADQMLLDAKNP

QSAGNRRIEIMVLTKSASDTLYQYFGQHGDKVVQPLVQKLDKQQVLSQRMR

>gi|30061846|ref|NP_836017.1| flagellar biosynthetic protein FlhA [Shigella flexneri 2a str. 2457T]

MLSRSDLLTLLTINFIVVTKEAERISEVSARFTLDAMPGKQMAIDADLNAGLINQAQAQTRRKDVASEAD

FYGAMDGASKFVRGDAIAGMMILAINLSGGVCIGIFKYNLSADAAFQQYVLMTIGDGLVAQIPSLLLSTA

AAIIVTRVSDNGDIAHDVRHQLLASPSVLYTATGIMFVLAVVPGMPHLPFLLFSALLGFTGWRMSKRPQA

AKAEEKSLETLTRTITETSEQQVSWETIPLIEPISLSLGYKLVALVDKAQGNPLTQRIRGVRQVISDGNG

VLLPEIRIRENFRLKPSQYAIFINGIEADEADIPADKLMALPSSETYGEIDGVLGNDPAYGIPVTWIQPA

QKAKALNMGYQVIDSASVIATHVNKIVRSYIPDLFNYDDITQLHNRLSSMAPRLAEDLSAALNYSQLLKV

YRALLTEGVSLRDIVTIATVLVASSTVTKDHILLAADVRLALRRSITHPFVRKQELTVYTLNNELENLLT

NVVNQAQQAGKVMLDSVPVDPNMLNQFQSTMPQVKEQMKAAGKDPVLLVPPQLRPLLPRYARLFAPGLHV

LSYNEVPDELELKIMGALM

>gi|30061845|ref|NP_836016.1| hypothetical protein S0297 [Shigella flexneri 2a str. 2457T]

MSEYRRYYIKGGTWFFTVNLRNRRSHLLTTQFQTLRNAIINVKRDRPFEINAWVVLPEHMHCIWTLPESD

DDFSSRWREIKKQFTHACGLKNIWQPRFWEHAIRNTKDYRHHVDYIYINPEKHGWVKQVSDWPFSTFHRD

VARGLYPIDWAGDITDLSAGERIIL

>gi|30061844|ref|NP_836015.1| lipoprotein [Shigella flexneri 2a str. 2457T]

MRFAPFTRQRHHNREQIKQHPEWFPAPLKASDRRWQALAENNHFLSSDHLHNITEVAIHRLEQQLGKPYV

WGGTRPDQGFDCSGLVFYAYNKILEAKLPRTANEMYHYRRATIVANNDLRRGDLLFFHIHSREIADHMGG

YLGDGQFIESLRTGETIRVSRLAEPFWQDHFLGARRILTEEMIL

>gi|30061843|ref|NP_836014.1| hypothetical protein S0295 [Shigella flexneri 2a str. 2457T]

MRKIALILAMLLIPCVSFAGLLGSSSSTTPVSKEYKQQLMGSPVYIQIFKEERTLDLYVKMGEQYQLLDS

YKICKYSGGLGPKQRQGDFKSPEGFYSVQRNQLKPDSRYYKAINIGFPNAYDRAHGYEGKYLMIHGDCVS

IGCYAMTNQGIDEIFQFVTGALVFGQPSVQVSIYPFRMTDANMKRHKYSNFKDFWEQLKPGYDYFEQTRK

PPTVSVANGRYVVSKPLSHEVVQPQLASNYTLPEAK

>gi|30061842|ref|NP_836013.1| amidotransferase [Shigella flexneri 2a str. 2457T]

MCELLGMSANVPTDICFSFTGLVQRGGGTGPHKDGWGITFYEGKGCRTFKDPQPSFNSPIAKLVQDYPIK

SCSVVAHIRQANRGEVALENTHPFTRELWGRNWTYAHNGQLTGYKSLETGNFRPVGETDSEKAFCWLLHK

LTQRYPRTPGNMAAVFKYIASLADELRAKGVFNMLLSDGRYVMAYCSTNLHWITRRAPFGVATLLDQDVE

IDFSSQTTPNDVVTVIATQPLTGNETWQKIMPGEWRLFCLGERVV

>gi|30061841|ref|NP_836012.1| phosphoheptose isomerase [Shigella flexneri 2a str. 2457T]

MYQDLIRNELNEAAETLANFLKDDANIHAIQRAAVLLADSFKAGGKVLSCGNGGSHCDAMHFAEELTGRY
RENRPGYPAIAISDVSHISCVGNDFGFNDIFSRYVEAVGREGDVLLGISTSGNSANVIKAIAAAREKGMK
VITLTGKDGGKMAGTADIEIRVPHFGYADRIQEIHIKVIHILIQLIEKEMVK

>gi|30061839|ref|NP_836010.1| C-lysozyme inhibitor [Shigella flexneri 2a str. 2457T]
MGRISSGGMMFKAITTVAALVIATSAMAQDDLTISSLAKGETTKAAFNQMVQGHKLPAWVMKGGTYTPAQ
TVTLGDETYQVMSACKPHDCGSQRIAVMWSEKSNQMTGLFSAIDEKTSQEKLTWLNVNDALSIDGKTVLF
AALTGSLENHPDGFNFK

>gi|30061838|ref|NP_836009.1| hypothetical protein S0290 [Shigella flexneri 2a str. 2457T]
MPSLKITLLQQPLVWVDGPANLRHFDRQLEGITGRDVIVLPEMFTSGFAMEAAASSLAQDDVVNWMTAKA
QQCNALIAGSVALQTESGSVNRFLLVEPGGTVHFYDKRHLFRMADEHLHYKAGNARVIVEWRGWRILPLV
CYDLRFPVWSRNLNDYDLALYVANWPAPRSLHWQALLTARAIENQAYVAGCNRVGSDGNGCHYRGDSRVI
NPQGEIIATADAHKATRIDAELSMVALREYREKFPAWRDADEFRLW

>gi|30061837|ref|NP_836008.1| Rhs-family protein [Shigella flexneri 2a str. 2457T]
MTARDYIWNADGEVGGINDKLRGCLVFSYDRSGWLTSRTGQMYDHDHYYYDKAGNLLTDEYQGAVMDNRL
PGYGRDRYRYNEWGELTERRDQQLEWNAQGQLTRVISSNSETRYQYDALGRRISKATSNLHTDRGERSRT
TFVWEGFRLLQETTWQGKRTYLYDAEQPYTPVAAITGRGESQKIWYYHTDLTGTVHEVTAPDGTLVWAGY
QAGFGENRGDISNSGAYFEQPQRLPGQYFDEETGLHYNLFRYYAPECGRFVSQDPIGLNGGLNLYAYAPN
PLGWIDPLGLNSLGIDSNNIFRGDGNYKGGGIGQPKSGTISAQDIIDHL

>gi|30061836|ref|NP_836007.1| Rhs-family protein [Shigella flexneri 2a str. 2457T]
MIEDGSPNVFLGGGTQTVLEISPEIPDWLRQVVDVLYVVAGMLGGLAGAWRQAAKMGSKFGTKCAAKFIG
GEMVGMAISDTVMGLFSNPVDVTTGQKILLPETDFTLPGRLPVTCSRFYASHLETEGLLGRGWRLNWEIN

LREDETYITFIGVQGRELSYPKEMLIPGHQIFDPEEQFYLSRLHDGCYVLHYTDCSYYVFDEFDDHGVAP

LLFMETPYRQRIAFGRENGRLVRVASSSGHHLLLHRTMTQAGERLSHIELLKGGRPGNLVEYRYDDNGQL

TGVVNRAGVTVRQFAYENGLMTEHRNATGFTCTYHWEEIEGFPRVVEHTTSDGEDYRFHYDFAGGQTVVT

GRPEQKWQWWFDEETYVTAHRTPGGGMYRFTYNENHFPVAVELPGERRVTLEYDTLSRVVKETDPAGRVT

QTQWNGSFAEITRRALDDDHVWKADYNEHGQVIRETDPEGRVTRYGYDDQGLPETVCHPGKQQDRYTWNA

LGLLSSHRRITGSVQSWQYTQRGMLARHTDEEKRETRWQYTPEGLVASLSNGNGAQYRFSYDGDGRLTGE

QRPDGLIRMFALNADGFPVIIPTQGTEGGVRNEQQERDALGRLLRSDTQHSTRTFSYNRLDQITEVTLTP

TEEGERLHHMQADTVRFAYDRSGWLTAEHSVHGSIKYRRDALGNPTDITLPDGQHLSHLYYGSGHLLQTA

LDGITVSEYERDSLHRQVIRTQGKLATFSGYNADNRLSWQRSLPGGSQNPQQAVLPRRRTTA

>gi|30061835|ref|NP_836006.1| Rhs-family protein [Shigella flexneri 2a str. 2457T]

MYEAARVDDPIYHTSALAGFLIGAIIGIAIIAVASFAFFTCGFLAGLILSFLADQIASGVLQLGEAIGRS

IHSTVGKILTGSDNVSTNSRPAARAVLSTVICEDHSPEIRIAQGSGNIYINSQLPVRMTTLSVMQ

>gi|30061834|ref|NP_836005.1| hypothetical protein S0286 [Shigella flexneri 2a str. 2457T]

MTGEQILATHRSGKTEVYQRQAGFITGPAKVLMLTLTTQRPFDDHTDQLWTAWLTSFQPAKS

>gi|30061833|ref|NP_836004.1| hypothetical protein S0285 [Shigella flexneri 2a str. 2457T]

MTSLYRIQEGCFALPETFLDRTVNIFVPSGNERATPSLNIFRDTLRPDENLTTYIDRQIALMKKKT

>gi|30061832|ref|NP_836003.1| Rhs-family protein [Shigella flexneri 2a str. 2457T]

MSFVSTGNKPVGNGGPVITTPPIAGESGGMSTGSAVTDVSGAAEEMAEQAAADLFGALPEPSGLVKAAVA

AAQAAAAAAGISDMAGAVQDAAASLAAGAPGAHNVTVSGSAVPPQMLLFAGMNGSEGLGNLFSYTVQLKT

LDALNLGYVSPAANLPLQLMVGKDLCVSIELDGGGKRYISGLVTAARVVGHESRSVTYELRIEPWLKLLT

HTSDYKAFQNKTVVDILDEVLDEYSFPVEKRLVENYPPRAWQVQYGETDFDFIQRLMQEWGIYWWFEHSE

NSHTLVLVDAINGHKACPDSPLVEWHQEGLKLDKEFIHTITASERLRTGKWVMDDFDFMKPRSLLKSTVA
SPRDTGHAEYEHYEWPGDYFTTGEGEMLTRIRMEAQRSPGSRAHGAGHIRTLMTGYTFTLMNHPTAEINQ
EYLLVQTTLFLRDNAQHSGQDQHFTYVTTFELHPTSEVYRPQRTLSKPHTKGPQSAIVTGPAGQEIWTDK
YGRVKVQFGWDRYGKNDENSSCWIRVSYPWAGKGFGMIQIPRIGQEVLVDFKNGDPDLPIIVGRTYNQDT
MPPWGLPGMASQSGIFSHSLQGGPTNGNMLRFDDKTGAEEVKFHAEKDLNTTVKNNETHTVMVDRIKTIV
KNETISVGEDRNATITKNDGLSVKLAQTINVGTTYRLDVGDQFTLRCGNAALVLHKDGSIEFCGKQLLLH
TSDVMQLIGSGIDMNPDGGTAMTADDIAPLPTPDVSE

>gi|30061831|ref|NP_836002.1| IS1 orfB [Shigella flexneri 2a str. 2457T]
MSRQCTHYGRWPQHGFTSLKKLRPQSVTSRIQPGSDVIVCAEMDEHWGYVGAKSRQRWLFYAYDRIRRTV
VAHVFGERTLATLERLLSLLSAFEVVVWMTDGWPLYESRLKGKLHVISKRYTQRIERHNLNLRQHLARLG
RKSLSFSKSVELHDKVIGHYLNIKHYQ

>gi|30061830|ref|NP_836001.1| IS1 orfA [Shigella flexneri 2a str. 2457T]
MASISIRCPSCSATEGVVRNGKSTAGHQRYLCSHCRKTWQLQFTYTASQPGTHQKIIDMAMNGVGCRASA
RIMGVGLNTVLRHLKNSGRSR

>gi|30061829|ref|NP_836000.1| dehydrogenase subunit [Shigella flexneri 2a str. 2457T]
MNVNFFVTCIGDALKSRMARDSVLLLEKLGCRVNFPEKQGCCGQPAINSGYIKEAIPGMKNLIAALEDND
DPIISPAGSCTYAIKSYPTYLADEPEWASRAEKVAARMQDLTSFIVNKLGVVDVGASLQGRAVYHPSCSL
ARKLGVKDEPLTLLKNVRGLELLTFAEQDTCCGFGGTFSVKMAEISGEMVKEKVVHLMEVRPEYLIGADV
SCLLNISGRLQREGQKVKVMHIAEVLMSR

>gi|30061828|ref|NP_835999.1| hypothetical protein S0280 [Shigella flexneri 2a str. 2457T]
MSIKTSNTDFKTRIRQQIEDPIMRKAVASAQQRIGANRQKMVDELGHWEEWRDRAAQIRDHVLSNLDAYL

YQLSEKVTQNGGHVYFAKTKEDATRYILQVAQRKNARKVVKSKSMVTEEIGVNHVLQDAGIQVIETDLGE

YILQLDQDPPSHVVVPAIHKDRHQIRRVLHERLGYEGPETPEAMTLFIRQKIREDFLSAEIGITGCNFAV

AETGSVCLVTNEGNARMCTTLPKTHIAVMGMERIAPTFAEVDVLITMLARSAVGARLTGYNTWLTGPREA

GHVDGPEEFHLVIVDNGRSEVLASEFRDVLRCIRCGACMNTCPAYRHIGGHGYGSIYPGPIGAVISPLLG

GYKDFKDLPYACSLCTACDSVCPVRIPLSKLILRHRRVMAEKGITAKAEQRAIKMFAYANSHPGLWKVGM

MAGAHAASWFINGGKTPLKFGAISDWMEARDLPEADGESFRSWFKKHQAQEKKNG

>gi|30061826|ref|NP_835997.1| hypothetical protein S0278 [Shigella flexneri 2a str. 2457T]

MDACLFHCKYPGITNAWIFTEEQHNHDGTGLSQVVLNAIFNLVCLLQVYVQISYLSQQSSIIRYTAFTGP

>gi|30061825|ref|NP_835996.1| hypothetical protein S0277 [Shigella flexneri 2a str. 2457T]

MSEQIKQDIDLIEILFHLKKKIRVILFIMAICMAMVLLFLYINKDNTKVIYSLKINQTTPGILVSCDSNN

NFACQTTMTEDVIQRITTFFQTSPDIKNREIRLEWSGDKRDLPTAEEEISRVQASIIKWYASEYHNGRQV

LDEIQTPSAINSELYTKMIYLTRNWSLYPNGDGCVTISSPEIKNKYPAAICLALGFFLSIVISVMFCLVK

KMVDEYQQNSGQ

>gi|30061824|ref|NP_835995.1| IS1 orfA [Shigella flexneri 2a str. 2457T]

MASISIRCPSCSATEGVVRNGKSTAGHLRYLCSHCRKTWQLQFTYTASQPGTHQKIIDMAMNGVGCRASA

RIMGVGLNTVLRHLKNSGRSR

>gi|30061823|ref|NP_835994.1| IS1 orfB [Shigella flexneri 2a str. 2457T]

MSRQCTHYGRWPQHGFTSLKKLRPQSVTSRIQPGSDVIVCAEMDEHWGYVGAKSRQRWLFYAYDRIRRTV

VAHVFGERTLATLERLLSLLSAFEVVVWMTDGWPLYESRLKGKLHVISKRYTQRIERHNLNLRQHLARLG

RKSLSFSKSVELHDKVIGHYLNIKHYQ

>gi|30061822|ref|NP_835993.1| taurine transporter substrate binding subunit [Shigella flexneri 2a str. 2457T]

MNVTVAYQTSAEPAKVAQADNTFAKESGASVDWRKFDSGASIVRALASGDVQIGNLGSSPLAVAASQQVP

IEVFLLASKLGNSEALVVKKTISKPEDLIGKRIAVPFISTTHYSLLAALKHWGIKPGLVEIVNLQPPAII

AAWQRGDIDGAYVWAPAVNALEKDGKVLTDSEQVGQWGAPTLDVWVVRKDFAEKHPEVVKAFAKSAIDAQ

QPYIANPDAWLKQPENISKLARLSGVPEGDVPGLVKGNTYLTPQQQTAELTGPVNKAIIDTAQFLKEQGK

VPAVANDYSQYVTSRFVQ

>gi|30061821|ref|NP_835992.1| taurine transporter ATP-binding subunit [Shigella flexneri 2a str. 2457T]

MLQISHLYADYGGKPALEDINLTLESGELLVVLGPSGCGKTTLLNLIAGFVPYQHGSIQLAGKRIEGPGA

ERGVVFQNEGLLPWRNVQDNVAFGLQLAGIEKMQRLEIAHQMLKKVGLEGAEKRYIWQLSDGQRQRVGIA

RALAANPQLLLLDEPFGALDAFTRDQMQTLLLKLWQETGKQVLLITHDIEEAVFMATELVLLSSGPGRVL

ERLRLNFARRFVAGESSRSIKSDPQFIAMREYVLSRVFEQREAFS

>gi|30061820|ref|NP_835991.1| taurine transporter subunit [Shigella flexneri 2a str. 2457T]

MSVLINEKLHSRRLKWRWPLSRQVTLSIGTLAVLLTVWWTVAALQLISPLFLPPPQQVLAKLLTIAGPQG

FMDATLWQHLAASLTRIVLALLAAVVIGIPVGIAMGLSPTVRGILDPVIELYRPVPPLAYLPLMVIWFGI

GETSKILLIYLAIFAPVAMSALAGVKSAQQVRIRAAQSLGASRAQVLWFVILPGALPEILTGLRIGLGVG

WSTLVAAELIAATRGLGFMVQSAGEFLATDVVLAGIAVIAIIAFLLELGLRALQRRLTPWHGEVQ

>gi|30061819|ref|NP_835990.1| taurine dioxygenase [Shigella flexneri 2a str. 2457T]

MSERLSITPLGPYIGAQISGADLTRPLSDNQFEQLYHAVLRHQVVFLRDQAITPQQQRALAQRFGELHIH

PVYPHAEGVDEIIVLDTHNDNPPDNDNWHTDVTFIETPPAGAILAAKELPSTGGDTLWTSGIAAYEALSV

PFRQLLSWLRAEHDFRKSFPEYKYRKTEEEHQRWREAVAKNPPLLHPVVRTHPVSGKQALFVNEGFTTRI

VDVSEKESEALLGFLFAHITKPEFQVRWRWQPNDIAIWDNRVTQHYANADYLPQRRIMHRATILGDKPFY

RAG

>gi|30061818|ref|NP_835989.1| delta-aminolevulinic acid dehydratase [Shigella flexneri 2a str. 2457T]

MHLNSTNIRQTMTDLIQRPRRLRKSPALRAMFEETTLSLNDLVLPIFVEEEIDDYKAVEAMPGVMRIPEK

HLAREIERIANAGIRSVMTFGISHHTDETGSDAWREDGLVARMSRICKQTVPEMIVMSDTCFCEYTSHGH

CGVLCEHGVDNDATLENLGKQAVVAAAAGADFIAPSAAMDGQVQAIRQALDAAGFKDTAIMSYSTKFASS

FYGPFREAAGSALKGDRKSYQMNPMNRREAIRESLLDEAQGADCLMVKPAGAYLDIVRELRERTELPIGA

YQVSGEYAMIKFAALAGAIDEEKVVLESLGSIKRAGADLIFSYFALDLAEKKILR

>gi|30061817|ref|NP_835988.1| hypothetical protein S0269 [Shigella flexneri 2a str. 2457T]

MLRYRRMVQLFFPLSPNVDIYNLWRKTRIKLIKGRYFSMLQIMLFLMHTRYKYQKYKPDKYLINIQQSLN

YSIYSSTTKRQKIITKSYK

>gi|30061814|ref|NP_835985.1| IS600 orfA [Shigella flexneri 2a str. 2457T]

MSRKTQRYSKEFKAEAVRTVLENQLSISEGASRLSLPEGTLGQWVTAARKGLGTPGSRTVAELESEILQL

RKALNEARLERDILKKATAYFAQESLKNTR

>gi|30061813|ref|NP_835984.1| IS600 orfB [Shigella flexneri 2a str. 2457T]

MAHIRTRETYGTRRLQTELAENGIIVGRDRLARLRKELRLRCKQKRKFRATTNPNHNLPVAPNLLNQTFA

PTAPNQVWVADLTYVATQEGWLYLAGIKDVYTCEIVGYAMGERMTKELTGKALFMALRSQRPPAGLIHHS

DRGSQYCAYDYRVIQEQSGLKTSMSRKGNCYDNAPMESFWGTLKNESLSHYRFNNRDEAISVIREYIEIF

YNRQRRHSRLGNISPAAFREKYHQMAA

>gi|30061812|ref|NP_835983.1| ISEhe3 orfB [Shigella flexneri 2a str. 2457T]

MLDVHPSGFYAWLQQPHSQRHQADLRLTGQIKQFWLESGCVYGYRKIHLDLRDSGQQCGVNRVWRLMKRV

GIKAQVGYRSPRARKGEASIVSPNRLQRQFNPDAPDERWVTDITYIRTHEGWLYLAVVVDLFSRKIIGWS

MQSRMTKDIVLNALLMAVWRRNPEKQVLVHSDQGSQYTSHEWQSFLKSHGLEGSMSRRGNCHDNAVAESF

FQLLKRERIKKKIYGTREEARSDIFDYIEMFYNSKRRHGSSEQMSPTEYENQYYQRLGSV

>gi|30061811|ref|NP_835982.1| beta-lactam binding protein AmpH [Shigella flexneri 2a str. 2457T]

MKRSLLFSAVLCAASLTSVHAAQPITEPEFASDIVDRYADHIFYGSGATGMALVVIDGNQRVFRSYGETR

PGNNVRPQLDSVVRIASLTKLMTSEMLVKLLDQGTVKLNDPLSKYAPPGARVPTYNGTPITLVNLATHTS

ALPREQPGGAAHRPVFVWPTREQRWKYLSTAKLKAAPGSQAAYSNLAFDLLADALANASGKPYTQLFEEQ

ITHPLGMKDTTYTPSPDQCRRLMVAERGASPCNNTLAAIGSGGVYSTPGDMMRWMQQYLSSDFYQRSNQA

DRMQTLIYQRAQFTKVIGMDVPGKADALGLGWVYMAPKEGRPRIIQKTGGGGGFITYMAMIPQKNIGAFV

VVTRSPLTRFKNMSDGINDLVTELSGNKPLVIPAS

>gi|30061810|ref|NP_835981.1| transporter [Shigella flexneri 2a str. 2457T]

MFKSFFPKPGTFFLSAFVWALIAVIFWQAGGGDWVARITGASGQIPISAARFWSLDFLIFYAYYIVCVGL

FAFFWFIYSPHRWQYWSILGTALIIFVTWFLVEVGVAVNAWYAPFYDLIQTALSSPHKVTIEQFYREVGV

FLGIALIAVVISVLNNFFVSHYVFRWRTAMNEYYMANWQQLRHIEGAAQRVQEDTMRFASTLENMGVSFI

NAIMTLIAFLPVLVTLSAHVPELPIIGHIPYGLVIAAIVWSLMGTGLLAVVGIKLPGLEFKNQRVEAAYR

KELVYGEDDATRATPPTVRELFSAVRKNYFRLYFHYMYFNIARILYLQVDNVFGLFLLFPSIVAGTITLG

LMTQITNVFGQVRGAFQYLINSWTTLVELMSIYKRLRSFEHELDGDKIQEVTHTLS

>gi|30061809|ref|NP_835980.1| hypothetical protein S0257 [Shigella flexneri 2a str. 2457T]

MSRVNHLSSLSLLAVLVLAGCSSQAPQPLKKGEKAIDVASVVRQKMPASVKDRDAWAKDLATTFESQGLA

PTLENVYSVLAVAQQESNYQADPAVPGLSKIAWQEIDRRAERMHIPAFLVHTALKIKSPNGKSYSERLDS

VRTEKQLSAIFDDLISMVPMGQTLFGSLNPVRTGGPMQVSIAFAEQHTKGYPWKMDGTVRQEVFSRRGGL

WFGTYHLLNYPASYSAPIYRFADFNAGWYASRNAAFQNAVSKASGVKLALDGDLIRYDSKEPGKTELATR

KLAGKLGMSDSEIRRQLEKGDSFSFEETALYKKVYQLAEAKTGKSLPREMLPGIQLESPKITRNLTTAWF
AKRVDERRARCMKQ

>gi|30061808|ref|NP_835979.1| hypothetical protein S0256 [Shigella flexneri 2a str. 2457T]
MADFTLSKSLFSGKYRNASSTPGNIAYALFVLFCFWAGAQLLNLLVHAPGVYERLMQVQETGRPRVEIGL
GVGTIFGLIPFLAGCLIFAVVALWLHWRHRRQ

>gi|30061807|ref|NP_835978.1| hypothetical protein S0255 [Shigella flexneri 2a str. 2457T]
MNLRCESQNGNLKLNLLIITKMLSTPQTVVTEYARMLFFVNGAAMNLPVKIRRDWHYYAFAIGLIFILNG
VVGVLGFEAKGWQTYAVGLVTWVISFWLAGLIIRRRDEETENAQ

>gi|30061806|ref|NP_835977.1| D-alanyl-alanine synthetase A [Shigella flexneri 2a str. 2457T]
MEKLRVGIVFGGKSAEHEVSLQSAKNIVDAIDKSRFDVVLLGIDKQGQWHVSDASNYLLNADDPAHIALR
PSATSLAQVPGKHEHQLIDAQNGQPLPTVDVIFPIVHGTLGEDGSLQGMLRVANLPFVGSDVLASAACMD
KDVTKRLLRDAGLNIAPFITLTRANRHNISFAEVESKLGLPLFVKPANQGSSVGVSKVTSEEQYAIAVDL
AFEFDHKVIVEQGIKGREIECAVLGNDNPQASTCGEIVLTSDFYAYDTKYIDEDGAKVVVPAAIAPEIND
KIRAIAVQAYQTLGCAGMARVDVFLTPENEVVINEINTLPGFTNISMYPKLWQASGLGYTDLITRLIELA
LERHAADNALKTTM

>gi|30061805|ref|NP_835976.1| hypothetical protein S0253 [Shigella flexneri 2a str. 2457T]
MKNLIAELLFKLAQKEEESKELCAQVEALEIIVTAMLRNMAQNDQQRLIDQVEGALYEVKPDASIPDDDT
ELLRDYVKKLLKHPCQ

>gi|30061804|ref|NP_835975.1| alkaline phosphatase [Shigella flexneri 2a str. 2457T]
MSRPRLIVALFLFFNVFVHGENKVKQSTIALALLPLLFTPVTKARTPEMPVLENRAAQGDITAPGGARRL

TGDQTAALRDSLSDKPAKNIILLIGDGMGDSEITAARNYAEGAGGFFKGIDALPLTGQYTHYALNKKTGK
PDYVTDSAASATAWSTDVKTYNGALGVDIHEKDHPTILEMAKAAGLATGNVSTAELQDATPAALVAHVTS
RKCYGPSATSEKCPGNALEKGGKGSITEQLLNARADVTLGGGAKTFAETATAGEWQGKTLREQAQARGYQ
LVNDAASLNSVTEANQQKPLLGLFADGNMPVRWLGPKATYHGNIDKPAVTYTPNPQRNDSVPTLAQMTDK
AIELLSKNEKGFFLQVEGASIDKQDHAANPCGQIGETVDLDEAVQRALEFAKKEGNTLVIVTADHAHASQ
VVAPDTKAPGLTQALNTKDGAVMVMSYGNSEEDSQEHTGSQLRIAAYGPHAANVVGLTDQTDLFYTMKAA
LGLK

>gi|30061803|ref|NP_835974.1| hypothetical protein S0251 [Shigella flexneri 2a str. 2457T]
MKRDGAMKITLLVTLLFGLVFLTTVGAAERTLTPQQQRMTSCNQQATAQALKGDARKTYMSDCLKNSKSA
PGEKSLTPQQQKMRECNNQATQQALKGDDRNKFMSACLKKAA

>gi|30061802|ref|NP_835973.1| IS629 orfB [Shigella flexneri 2a str. 2457T]
MARCTVARLMAVMGLAGVLRGKKVRTTISRKAVAAGDRVNRQFVAERPDQLWVADFTYVSTWRGFVYVAF
IIDVFAGYIVGWRVSSSMETTFVLDALEQALWARRPSGTVHHSDKGSQYVSLAYTQRLKEAGLLASTGST
GDSYDNAMAESINGLYKAKVIHRKSWKNRAEVELATLTWVDWYNNRRLPERLGHIPPAEAEKAYYASIGN
DDLAA

>gi|30061801|ref|NP_835972.1| IS629 orfA [Shigella flexneri 2a str. 2457T]
MTKNTRFSPEVRQRAVRMVLESQGEYDSQWATICSIAPKIGCTPETLRVWGRQHERDTGGGDGGLTTAER
QRLKELERENRELRRSNDILRQASAYFAKAEFDRLWKK

>gi|30061800|ref|NP_835971.1| DNA transfer protein [Shigella flexneri 2a str. 2457T]
MANYRDLLEQAGARYGVPEGLMTALGAKESSYNPAAVSSAGAVGLTQVMPGTWRDMGYTDEQMQNPEYQA
DAGARYLAKMYQQFGNWRDSLQAYHDGPGNVMKAKRGEYTPGPEGRGYVDDRFAQWAGDPVTDSTVEQRA

TSAKVHPQQDPNNPFAQLEVQSSEQVSASGVQSDPNNPFAQIEQQAASQQPPQPVSSVAPKPVQQQTVNQ
ANNAPTREEPSLMQQAGDWLTGGQSAGQIAEQAGRGLVNIPFDVLQGGASLINAISQGLGGPKVLDDVYR
PVDRPTDPYAQAGESIGGYLVPGAGVAGNMVIGSLADAANQRGDFAENAAINAGLSIATHGLINGVTRGV
RGASNIISGNKTSAQRATTAPTETSPFSGDAAAATNPAVHAAEARVAQGVPMTPATKNPEEVVRTVAAQK
RPNLASSLDELDINPQAEVLESAERLNVDSLLPSHFSGNEQYKAVEQAIKSRAGSALQVQENEAIRQLAQ
GAGEIIDRVSGAKDALGMSDKFIDTVNGRMSALMKRSDQLYRNVEKAMPAGAKIDAPSTRSMLKQVAEDL
GGMKNLDPIEKRVEVA

>gi|30061799|ref|NP_835970.1| prophage DNA injection protein [Shigella flexneri 2a str. 2457T]
MATWQGTNGGLLAGIGGVNSNAPSVNDIGNTLQLIRQNNDIERSGANNVGLTALQGLSGIAGVFQQEKQA
QRQKEFQQAYANAYASGDRGALRQLATQYPDQIESVRKGMGFIDEEQRNSIGTLAAGARLASSSPEAMQS
WLQNNAGELARVGVNPHDVAQMYQQNPRQFGEFVDHLGMNSLGPEKYFDLQDKMQGRQVTMRGQDLDSQT
AARNQAITMRGQDIQANLGQQRINLDAETNRINNENKRLDRMLSAETNDLKRQEIQSRIAANNQQLQQKQ
QALNDGYKDGINTLTTSMFTLNDIVSSPSLKSITGLRGVIPNVPGSQAADTQARLDTFKSQAYLTAVQAM
RGMGALSDAEGKKLDQAVGSLQNSQSEESFRRNAGVILNTLNQKRNEAVGKYVQQNGIKRVEAPQASIDY
LKQHPELSIDFINRYGYLPSLGQ

>gi|30061798|ref|NP_835969.1| DNA transfer protein [Shigella flexneri 2a str. 2457T]
MLYAFKLGRKLRGEEPYCPEKGGKGGSSDKSAKYAAEAQKYAADLQNQQWQTIMKNLAPFTPLAEQYVNQ
LQNLSSLEGQGQALNQYYNSKQYKDLAGQARYQSLAAAEATGGLGSTATSNQLATIAPTLGQSWLSNQMS
NYNNLANVGLGALQGQANAGQTYANNMSSIAQQSAALAAANANKPSSLQTAISGGTSGAIAGAGLASLLG
TSTPWGAGIGAGIGLLGSLF

>gi|30061797|ref|NP_835968.1| head assembly protein [Shigella flexneri 2a str. 2457T]
MITFKPTRNIDLIEAVGNHPDIIAGSNNGDGYDYKPECRYFEVNVHGQFGGIVYYQEIQPLTLDCHAMYL

PEIRGFSKEIGLAFWRYILTNTTVQCVTSFAARKFRHGQMYCAMIGLKRVGTIKKYFKGVDDVTFYSATR
EELIDFLNHGR

>gi|30061796|ref|NP_835967.1| packaged DNA stabilization protein [Shigella flexneri 2a str. 2457T]
MADSNLNVPVIIQATRLDTSVLPRNIFSQSYLLYVIAQGTDVGNVANKANEAGQGAYDAQVRNDEQDVIL
ADHEQRISAAEATLVNHEERIRQAESTLQDHETRIAQNESDISSLDTRVQSLESQVSDHETRIDALEYAT
TRKKSEVVYSGVSVTIPTAPTNLVSLLKTLTPSSGSLAPFFDTVNNKMVVFNENKTLLFKLSIVGTWPSG
TANRSMQLTFSGSVPDTLVSSRNAATTTDNILLATFFSVDKDGFLATNGSTLTIQSNGAAFTATTIKIIA
EQ

>gi|30061795|ref|NP_835966.1| packaged DNA stabilization protein [Shigella flexneri 2a str. 2457T]
MPIQQLPLMKGVGKDFRNADYIDYLPVNMLATPKEILNSSGYLRSFPGIAKRSDVNGVSRGVEYNMAQNA
VYRVCGGKLYKGESEVGDVAGSGRVSMAHGRTSQAVGVNGQLVEYRYDGTVKTVSNWPTDSGFTQYELGS
VRDITRLRGRYAWSKDGTDSWFITDLEDESHPDRYSAQYRAESQPDGIIGIGTWRDFIVCFGSSTIEYFS
LTGATTVGAALYVAQPSLMVQKGIAGTYCKTPFADSYAFISNPATGAPSVYIIDSGQVSPIASASIEKIL
RSYTADELADGVMESLRFDAHELLIIHLPRHVLVYDASSIANGPQWCVLKTGLYDDVYRAIDFVYEGNQI
TCGDKLESVTGKLQFDISSQYDKQQEHLLFTPLFKADNARVFDLEVESSTGVAQYADRLFLSATTDCINY
GREQMIEQNEPFVYDKRVLWKRVGRIRKNVGFKLRVITKSPVTLSGAQIRIE

>gi|30061794|ref|NP_835965.1| DNA stabilization protein [Shigella flexneri 2a str. 2457T]
MNLTTKGDLVLAALRKLGVASNATLTDVEPQSMEDGVNDLEMMMAEWLGGDASPGINVGYIFADADVAPD
PGDEHGLSNNAINAVIFNLACRIAPDYALEASAKLITTARYGKERLVKLSAMDRAKAAKCKSGYPNRMPV
GSGNQLAKWNGWNYFHRKEPCDNGSE

>gi|30061793|ref|NP_835964.1| hypothetical protein S0241 [Shigella flexneri 2a str. 2457T]

MTHMIFRHGDMKKWKGVGYDFEIVKAEELQEYLDAGWFSHPDDLLKDVAEPEPEEKQRKKPGRKPKAAAD
EPDNEG

>gi|30061792|ref|NP_835963.1| coat protein [Shigella flexneri 2a str. 2457T]
MALNEGQLVTYALDEIIGTVQNLTPMASKVTKYPPPAESMQRSSNTVWMPVEQEAPTQTGWDLTGNATGI
LELSVKCNMGDPDNDFFELRADDLRDERSYRRRIQASAKKLANNIESAIAKQATEMGSLVVHDTRAIGPS
TGLPGWDFVSDAERLMFSRELNRDMGISYFLNPDDYRKAGRNLVDGDIFGRVPEEAYRNGTIQRQIAGFD
EILRSPKLPAVTKSTATGVTVSGAQKFKPQAYTLDTDGNKENVDNRVATVTVSSTTGFKRGDKISFIGVK
FLSQMAKNVLTDDATFSITRVIDGTHIEITPKPIALDDASLTKEEKAYANVNTSLADTTPVNVLNVATTT
ANVFWADDSIRLLSQPIPVTHELFAGMKTSSFSIPGIGVNGIFATQGDINTLSGKCRIAVWYSACAVRPE
AIGVGLPNQTA

>gi|30061791|ref|NP_835962.1| scaffolding protein [Shigella flexneri 2a str. 2457T]
MDQTTDIQASEELTLPGNHAAASADGLVVDNANDSAGQEEGFEIVRKDDEKPKQDPATNAEFARRRIERK
RQRELEQQMEAVKRGELPEHLRVNPELPKQPDPNDYLSEDALAKYDYDQSRALAAFQQANSEWQIKAMDA
RSQAVAEQGRKTQEFTQQSAQYVEAARKHYEAAEKLNIPDYQEKEDAFMQLVPPAVGADIMRLFPEKSAA
LMYHLGANPEKTRQLLAMDGQSALIELTRLSERLTLKPRAKPVSEAPLPDEPIQGHAVAANISAIEKQME
AAANKGDVETYRKLKAQLNKGIR

>gi|30061790|ref|NP_835961.1| packaging glycoprotein [Shigella flexneri 2a str. 2457T]
MADENRLNSILCKFDADWMASDEARTEATNDLYFSRVSQWDDWLSNYTTLQYRGQFDVVRPEVRKLVAEM
RRNPVDVLFRPKDGANPDAADVLMGMYRTDMRHNTAKIAVNVGVREQIESGVGAWRLVTQYEDNDPTSNN
QVIRRLPIHEACSHVIWDANSKQMDKSDAKHCTVINALSRNGWKEFAEDYGIDPDTLPSFQNPNDTWLFP
WVSNDVVYVAEYYEVEEEKEKVFIYRDPLTGEPVSYYQQDIKDVIDDLANRGFIKVAERKVKRRRVYKSI
ITCTQILKDREKIAGEHIPIVPVYGEWSFAGDKECYEGVVRLTKDGQRLRNMIMSFNADIVARSPKKKPT

FFPEQIEGYEYMYGGNDDYPYYLQNRTDENGNDLPIGPISYMENPEVPQANAYMLEAATNAVKEVASLGV

DAQAANGQVAFDTVNQLNMRADLETYVFQDNLATAMRRDGEIYASMVNDIYDVPRHVTLTLEDGSEKDVQ

LYAQVVDYQSGNVVTLNDIRGRYECYTDVGPSFQSMKEQNRAEIQELLTKVPQGTPEFQMLMLQYFTLLD

GKGVEMMREYANKQLVMMGLKKPETPEEMEMVQQAQQQPQQPSAEQIQAQGILLQGQAELLKAENQQAQI

QVEAAKVEAQNQLNAAKIAEIFNNMDLDKQAELREYLRLVGQFQQQRSKDARANAELLLKDADQTHSQRM

DFANLMRQVQIPSGGVAETPQ

>gi|30061789|ref|NP_835960.1| terminase large subunit [Shigella flexneri 2a str. 2457T]

METSTRRAGGEQLKGQYADAGFMMLQEHATWPDGGNAVEPGITELRDMMLDGRFKVFNTCEPFFEEFRLY

HRDENGKIVKLNDDVLSAVRYAYMMRRFAKMMRDIKKPKEKKIPAPIRPIARRT

>gi|30061788|ref|NP_835959.1| terminase large subunit, partial [Shigella flexneri 2a str. 2457T]

MWFDEEPPYSIYGEGLTRTNKYGQFSILTFTPLMGMSDVVTKFLKNPSKSQKVVNMTIYDAEHYTDEQKE

QIIASYPEHEREARARGIPTMGSGRIFQIPEETIKCQPFECPDHFYVIGGMDFGWDHPQAQVQLWWDKEA

DTIYVSRVWKAKEKTAVQAWGAVKSWAHKVPTAWPHDGNQHEKGRR

>gi|30061787|ref|NP_835958.1| IS911 orfB [Shigella flexneri 2a str. 2457T]

MVTLCHVFGVHRSSYRYWKNRPEKPDGRRAVLRSQVLELHGISHGSAGARSIATMATRRGYQMGRWLAGR

LMKELGLVSCQQPTHRYKRGGHEHVAIPNYLERQFAVTEPNQVWCGDVTYIWTGKRWAYLAVVLDLFARK

PVGWAMSFSPDSRLTMKALEMAWETRGKPGGVMFHSDQGSHYTSRQFRQLLWRYQIRQSMSRRGNCWDNS

PMERFFRSLKNEWMPVVGYVSFSEAAHAITDYIVGYYSALRPHEYNGGLPPNESENRYWKNSNSVASFC

>gi|30061786|ref|NP_835957.1| IS911 orfA [Shigella flexneri 2a str. 2457T]

MTRWVKQLRDERQGKTPKASPITPEQIEIRELRKKLQRIEMENEILKRLRALDVRLPEQFSIIGKLRAHY

PVVTLCHVFGVHRSSYRYWKNRPEKPDGRRAVLRSQVLELHGISHGSAGARSIATMATRRGYQMGRWLAG

RLMKELGLVSCQQPTHRYKRGGHEHVAIPNYLERQFAVTEPNQVWCGDVTYIWTGKRWAYLAVVLDLFAR

KPVGWAMSFSPDSRLTMKALEMAWETRGKPGGVMFHSDQGSHYTSRQFRQLLWRYQIRQSMSRRGNCWDN

SPMERFFRSLKNEWMPVVGYVSFSEAAHAITDYIVGYYSALRPHEYNGGLPPNESENRYWKNSNSVASFC

>gi|30061785|ref|NP_835956.1| hypothetical protein S0233, partial [Shigella flexneri 2a str. 2457T]

MTTSNTLSDYAVLVPERLEAILDRAAQRAGHEDISELLGCTVCSYAGDDTGIYLLPKRFASISFRSTKDA

KTVDVKVTRNSNTAGYDLELISVVDVLATGSVKVKAGEFDVEKDASFPLIHVIRFTTPRVTINPE

>gi|30061784|ref|NP_835955.1| hypothetical protein S0232 [Shigella flexneri 2a str. 2457T]

MMNPCGYSYGYTLIYLWSSPLVTLERVTEVLTLRQWPQSSRLERGQGGRGTDLCLLDTYRDFCEIFLIQG

HNLPPLAPTDRKVKTCKHQSNSYCGHTADCYYLSLPVEHLADQVDTEDELKQLL

>gi|30061783|ref|NP_835954.1| lysis protein S [Shigella flexneri 2a str. 2457T]

MKSMDKISTGIAYGTSSGSAGYWFLQWLDQVSPSQWAAIGVLGSLVLGFLTYLTNLYFKIREDKRKAARG

E

>gi|30061782|ref|NP_835953.1| hypothetical protein S0230 [Shigella flexneri 2a str. 2457T]

MTQKYELIVKGIRNFENKVTVTLALRDKKRFDGEIFDLDISLDRVEGAALEFYEAAARRSIRQVFLDVAA

GLCEGDELLPETRPCSEARYTIKINSSDNSITGC

>gi|30061781|ref|NP_835952.1| endolysin R of prophage CP-933V, partial [Shigella flexneri 2a str. 2457T]

MERNIKVPLTEPQKAGIASFCPYNIGPGKCFPSTFYKRLNAGDRKGACEAIRWWIKDGGRDCRIRSNNCY

GQVIRRDQESALACWGIDQ

>gi|30061779|ref|NP_835950.1| IS911 orfB [Shigella flexneri 2a str. 2457T]

MVTLCHVFGVHRSSYRYWKNRPEKPDGRRAVLRSQVLELHGISHGSAGARSIATMATRRGYQMGRWLAGR

LMKELGLVSCQQPTHRYKRGGHEHVAIPNYLERQFAVTEPNQVWCGDVTYIWTGKRWAYLAVVLDLFARK

PVGWAMSFSPDSRLTMKALEMAWETRGKPGGVMFHSDQGSHYTSRQFRQLLWRYQIRQSMSRRGNCWDNS

PMERFFRSLKNEWMPVVGYVSFSKAAHAITDYIVGYYSALRPHEYNGGLPPNESENRYWKNSNSVASFC

>gi|30061778|ref|NP_835949.1| IS911 orfA [Shigella flexneri 2a str. 2457T]

MKKRNFSAEFKRESAQLVVDQKYTVADAAKAMDVGLSTMTRWGKQLRDERQGKTPKASPITPEQIEIREL

RKKLQRIEMENEILKKATALLMSDSLNSSR

>gi|30061777|ref|NP_835948.1| insertion element IS2 transposase InsD [Shigella flexneri 2a str. 2457T]

MDSARALIARGWGVSLVSRCLRVSRAQLHVILRRTDDWMDGRRSRHTDDTDVLLRIHHVIGELPTYGYRR

VWALLRRQAELDGMPAINAKRVYRLMRQNALLLERKPAVPPSKRAHTGRVAVKESNQRWCSDGFEFCCDN

GERLRVTFALDCCDREALHWAGTTGGFNSETVQDVMLGAVERRFGNDLPSSPVEWLTDNGSCYRANETRQ

FARMLGLEPKNTAVRSPESNGIAESFVKTIKRDYISIMPKPDGLTAAKNLAEAFEHYNEWHPHSALGYRS

PREYLRQRACNGLSDNRCLEI

>gi|30061776|ref|NP_835947.1| insertion sequence 2 OrfA protein [Shigella flexneri 2a str. 2457T]

MIVLILVFRLVIGEQIIDVLGPEKRRRRTTQEKIAIVQQSFEPGMTVSLVARQHGVAASQLFLWRKQYQE

GSLTAVAAGEQVVPASELAAAMKQIKELQRLLGKKTMKNELLKEAVEYGRAKKWIAHAPLLPGDGE

>gi|30061775|ref|NP_835946.1| hypothetical protein S0219, partial [Shigella flexneri 2a str. 2457T]

MKPNITWGLQKIQAHGIADNTIYATEGTQIIFTVTSPWIPVFKVNDDVKIALTLTRHEEAWWIINQSTEY

CCTVNDQIVEPHHRMRLNEGDLIEWGLSS

>gi|30061774|ref|NP_835945.1| hypothetical protein S0218 [Shigella flexneri 2a str. 2457T]

MLSPEQDLNRHADPFPFNEEPSSQDLDSLRDLVTEAETLQDMVTGGLSIDAILNVLDATGEDETIWPVEK

TPPDILHLLSPEYAPETAHNTVLPDLTRKEHRIIGIDSHYRINPAQHGEIYHDKQ

>gi|30061773|ref|NP_835944.1| hypothetical protein S0217 [Shigella flexneri 2a str. 2457T]

MTNNNELPITLSALLRDYSVVEGIQMAEQQVRMHPAQASRRHSLFQLLCVAGDWSRALQQIQLCARMDAN

YTREAQVFGELIRCEIYRHACFQGEQRPGVILPPPAWMEDLLTALACNARGEAQEADAHRSRALEAITDT

SGQWNGGAFDWISDSDSRTGPVLELIAGGAYIWLPFSQICSLKSPRPAHLIDLIWKPVNVTLNNGDTHSA

>gi|30061772|ref|NP_835943.1| hypothetical protein S0215 [Shigella flexneri 2a str. 2457T]

MSASRQRPGNHLLPTLFDRLCDDAPNQKRDHGISVSPVQIKEIIRRDLSFLLNTVSHEDDIDAARYPYAA

ASVLNYGLPPLAGSFLYEHKWDDIRRAILRTITRFEPRLKASTLQIIPLQDERRQSGHNTLQFEIRGEIL

TQPYPTAFRVRSALDMEQSRITFF

>gi|30061771|ref|NP_835942.1| outer membrane usher protein [Shigella flexneri 2a str. 2457T]

MDPRLLEYYNRELSYLRETGAEFAARHPKVAARLGMQGTDIADPYVERMVEAFSFLTARTQLKIDAEFPR

FTQRLLEVVSPNYVTPTPSMSVAQLHPDTEEGDLAKGFTVPRDTAFFSAIPEGESTACQFRSSQDVTLWP

LAIEEARLTAAPPDMPALHRYLPANIHVAGALRITLRTFGELTFSQLAGLDRLPFYLCGEERTASHLLEL

LHTSAIAPLAGIPGHFDGALDVNLQQPVMYEGLEPDQGLLPLAWNVFHGHNLLHEYFACPERFYFFTPTG

LSAGLQKIDGGVAEIVILLNRLPPDWLIHQTNAAQFSLFCTPVINLFPRVTARIDVTHSTTEQHLVVDRT

HPLDYEVFSVQEVEGLETDTTRKMAFRPLYHTRNNDEGNHGRYFSLRREPRRLSENARRYGTRTPYTGSE

VFLSLVDQYEAPYPENLRHITITAMVTNRDLPCLIARNGRDDLTVDAAIPVAGVGLIRPPRSPQPPMAER

EMAWRLIRQLSFNYLPLADLDHRTGGQALRDLLNLFIPAHDSPQSRQVRSLIGCKTTPVTRRLPGSGLLV

YGRGVSCELTVDEEGFSGISPYLFGLVLEHYIARHVSINTFSQMTLHSMQRGKIMTWPVRAGQRGSV

>gi|30061770|ref|NP_835941.1| periplasmic chaperone of fimbral assembly machinery [Shigella flexneri 2a str. 2457T]

MSNDFTQAQAPPWRYGFLNLMRRVDVQLCTVPAGNTWQPRMEKFRLGQTPALTFAPREIASVGWQEGRLH
ISLYSLVLWGPNGPLPLHYTELARNRTESRR

>gi|30061769|ref|NP_835940.1| hypothetical protein S0212, partial [Shigella flexneri 2a str. 2457T]
MRESADSPLPDHARLAASAHLVRESRNPDGLATTLAH

>gi|30061768|ref|NP_835939.1| hypothetical protein S0211 [Shigella flexneri 2a str. 2457T]
MEIFLSDPISAECPCGPDLEYDPEYLLLFTRAAPREEAQYGDFVSTPENINWAELERDAHRLLMRSKDIR
ILVVLLRCRIQQAGARGLSEALTLLETLCSTYPDAIHPQLLATEDITAEDAAVARSNALAALLDHEGVMA
DIRGITLSNNAAMRLQVRDVERSLSALRPADALAPESVRQQLADLEARGTLPLDAFRQAAETTERLQRHA
RETLNDQAPDFSRLTQLLALLPGAVQSTTPEILPQPQAEQPENATIVHTEQMQAEQIALPVHIAEEISPM
TGAEPRQIRDRNDALERLRVIRRWFEHSEPSSPTIPLLRQAERLVGKRVSEVINEIPVELLEKWDALE

>gi|30061767|ref|NP_835938.1| DNA polymerase III subunit epsilon [Shigella flexneri 2a str. 2457T]
MSTAITRQIVLDTETTGMNQIGAHYEGHKIIEIGAVEVVNRRLTGNNFHVYLKPDRLVDPEAFGVHGIAD
EFLLDKPTFAEVADEFMDYIRGAELVIHNAAFDIGFMDYEFSLLKRDIPKTNTFCKVTDSLAVARKMFPG
KRNSLDALCARYEIDNSKRTLHGALLDAQILAEVYLAMTGGQTSMAFAMEGETQQQQGEATIQRIVRQAS
KLRVVFATDEELAAHEARLDLVQKKGGSCLWRA

>gi|30061766|ref|NP_835937.1| ribonuclease H [Shigella flexneri 2a str. 2457T]
MLKQVEIFTDGSCLGNPGPGGYGAILRYRGREKTFSAGYTRTTNNRMELMAAIVALEALKEHCEVILSTD
SQYVRQGITQWIHNWKKRGWKTADKKPVKNVDLWQRLDAALGQHQIKWEWVKGHAGHPENERCDELARAA
AMNPTLEDTGYQVEV

>gi|30061765|ref|NP_835936.1| hypothetical protein S0207 [Shigella flexneri 2a str. 2457T]

MMKPARVPQTVVAPDCWGDLPWGELYRKALERQLNPWFTKMYGFHLLKIGNLSAEINCEACAVSHQVNVS

AQGMPVQVQADPLHLPFADKSVDICLLAHTLPWCTDPHRLLREADRVLIDDGWLVISGFNPISLMGLRKL

VPVLRKTSPYNSRMFTLMRQLDWLSLLNFEVLHASRFHVLPWNKHGGKLLNAHIPALGCLQLIVARKRTI

PLTLNPMKQSKNKPRIRQAVGATRQCRKPQA

>gi|30061764|ref|NP_835935.1| hydroxyacylglutathione hydrolase [Shigella flexneri 2a str. 2457T]

MNLNSIPAFDDNYIWVLNDEAGRCLIVDPGDAEPVLNAITANNWQPEAIFLTHHHHDHVGGVKELVEKFP

QIVVYGPQETQDKGTTQVVKDGETAFVLGHEFSVITTPGHTLGHICYFSKPYLFCGDTLFSGGCGRLFEG

TALQMYQSLKKLSALPDDTLVCCAHEYTLSNMKFALSIFPHDLSINDYYRKVKELRAKNQITLPVILKNE

RQINVFLRTEDIDLINGINEETLLQQPEERFAWLRSKKDRF

>gi|30061763|ref|NP_835934.1| membrane-bound lytic murein transglycosylase D [Shigella flexneri 2a str. 2457T]

MKAKAILLASVLLVGCQSTGNVQQHAQSLSAAGQGEAAKFTSQARWMDDGTSIAPDGDLWAFIGDELKMG

IPENDRIREQKQKYLRNKSYLHDVTLRAEPYMYWIAGQVKKRNMPMELVLLPIVESAFDPHATSGANAAG

IWQIIPSTGRNYGLKQTRNYDARRDVVASTTAALNMMQRLNKMFDDDWLLTVAAYNSGEGRVMKAIKTNK

ARGKSTDFWSLPLPQETKQYVPKMLALSDILKNSKRYGVRLPTTDESRALARVHLSSPVEMAKVADMAGI

SVSKLKTFNAGVKGSTLGASGPQYVMVPKKHADQLRESLASGEIAAVQSTLVANNTPLNSRVYTVRSGDT

LSSIASRLGVSTKDLQQWNKLRGSKLKPGQSLTIGAQRLANNSDSITYRVRKGDSLSSIAKRHGVNIKDV

MRWNSDTANLQPGDKLTLFVKNNNMPDS

>gi|30061762|ref|NP_835933.1| biotin synthesis protein [Shigella flexneri 2a str. 2457T]

MGCGAGHASFVAAQNVSAVVAYDLSAQMLDVVAQAAEARQLKNITTRQGYAESLPFADNAFDIVISRYSA

HHWHDVGAALREVNRILKPGGRMIVMDVMSPGHPVRDIWLQTVEALRDTSHVRNYASGEWLRLINEANLI

VDNLITDKLPLEFCSWVARMRTPEALVDAIRIYQQSASTEVRTYFALQNDGSFTSDIIMVEAHKAA

>gi|30061761|ref|NP_835932.1| hypothetical protein S0203 [Shigella flexneri 2a str. 2457T]

MRKNTYAMRYVAGQPAERILPPGSFASIGQALPPGEPLSTEERIRILVWNIYKQQRAEWLSVLKNYGKDA

HLVLLQEAQTTPELVQFATANYLAADQVPAFVLPQHPSGVMTLSAAHPVYCCPLREREPILRLAKSALVT

VYPLPDTRLLMVVNIHAVNFSLGVDVYSKQLLPIGDQIAHHSGPVIMAGDFNAWSRRRMNALYRFAREMS

LRQVRFTDDQRRRAFGRPLDFVFYRGLNVSEASVLVTRASDHNPLLVEFSPGKPDK

>gi|30061760|ref|NP_835931.1| transcriptional regulator LYSR-type [Shigella flexneri 2a str. 2457T]

MKATSEELAIFVSVVESGSFSRAAEQLGQANSAVSRAVKKLEMKLGVSLLNRTTRQLSLTEEGERYFRRV

QSILQEMAAAESEIMETRNTPRGLLRIDAATPVVLHFLMPLIKPFRERYPEVTLSLVSSETIINLIERKV

DVAIRAGTLTDSSLRARPLFNSYRKIIASPDYISRYGKPETIDDLKQHVCLGFTEPASLNTWPIACSDGQ

LHEVKYGLSSNSGETLKQLCLSGNGIACLSDYMIDKEIARGELVELMADKVLPVEMPFSAVYYSDRAVST

RIRAFIDFLSEHVKTAPGGVMPIS

>gi|30061759|ref|NP_835930.1| IS4 orf [Shigella flexneri 2a str. 2457T]

MHIGQALDLVSRYDSLRNPLTSLGDYLDPELISRCLAESGTVTLRKRRLPLEMMVWCIVGMALERKEPLH

QIVNRLDIMLPGNRPFVAPSAVIQARQRLGSEAVRRVFTKTAQLWHNATPHPHWCGLTLLAIDGVFWRTP

DTPENDAAFPRQTHAGNPALYPQVKMVCQMELTSHLLTAAAFGTMKNSENELAEQLIEQTGDNTLTLMDK

GYYSLGLLNGWSLAGEHRHWMIPLRKGAQYEELRKLGKGDHLVKLKTSPQARKKWPGLGNEVTARLLTVT

RKGKVCHLLTSMTDAMRFPGGEMADLYSNRWEIELGYREIKQTMQLSRLTLRSKKPELVEQELWGVLLAY

NLVRYQMIKMAEHLKGYWPNQLSFSESCGMVMRMLMTLQGASPGRIPELMSDLASMGQLVKLPTRRGRAF

PRVVKERPWKYPTAPKKSQSVA

>gi|30061758|ref|NP_835929.1| 2,5-diketo-D-gluconate reductase B [Shigella flexneri 2a str. 2457T]

MAIPAFGLGTFRLKDDVVISSVKTALELGYRAIDTAQIYDNEAAVGQAIAESGVPRHELYITTKIWIENL

SKDKLIPSLKESLQKLRTDYVDLTLIHWPSPNDEVSVEEFMQALLEAKKQGLTREIGISNFTIPLMEKVI

AAVGAENIATNQIELSPYLQNRKVVAWAKQHGIHITSYMTLAYGKALKDEVIARIAAKHNAIPAQVILAW

AMGEGYSVIPSSTKRKNLESNLKAQNLQLDAEDKKAIAALDCNDRLVSPEGLAPEWD

>gi|30061757|ref|NP_835928.1| D,D-heptose 1,7-bisphosphate phosphatase [Shigella flexneri 2a str. 2457T]

MAKSVPAIFLDRDGTINVDHGYVHEIDNFEFIDGVIDAMRELKKMGFALVVVTNQSGIARGKFTEAQFET

LTEWMDWSLADRDVDLDGIYYCPHHPQGSVEEFRQVCDCRKPHPGMLLSARDYLHIDMAASYMVGDKLED

MQAAVAANVGTKVLVRTGKPITPEAENAADWVLNSLADLPQAIKKQQKPAQ

>gi|30061756|ref|NP_835927.1| DL-methionine transporter ATP-binding subunit [Shigella flexneri 2a str. 2457T]

MIKLSNITKVFHQGTRTIQALNNVSLHVPAGQIYGVIGASGAGKSTLIRCVNLLERPTEGSVLVDGQELT

TLSESELTKARRQIGMIFQHFNLLSSRTVFGNVALPLELDNTPKDEIKRRVTELLSLVGLGDKHDSYPSN

LSGGQKQRVAIARALASNPKVLLCDEATSALDPATTRSILELLKDINRRLGLTILLITHEMDVVKRICDC

VAVISNGELIEQDTVSEVFSHPKTPLAQKFIQSTLHLDIPEDYQERLQTEPFTDCVPMLRLEFTGQSVDA

PLLSETARRFNVNNNIISAQMDYAGGVKFGIMLTEMHGTQQDTQAAIAWLQEHHVKVEVLGYV

>gi|30061755|ref|NP_835926.1| DL-methionine transporter permease subunit [Shigella flexneri 2a str. 2457T]

MSEPMMWLLVRGVWETLAMTFVSGFFGFVIGLPVGVLLYVTRPGQIIANAKLYRTVSAIVNIFRSIPFII

LLVWMIPFTRVIVGTSIGLQAAIVPLTVGAAPFIARMVENALLEIPTGLIEASRAMGATPMQIVCKVLLP

EALPGLVNAATITLITLVGYSAMGGAVGAGGLGQIGYQYGYIGYNATVMNTVLVLLVILVYLIQFAGDRI

VRAVTRK

>gi|30061754|ref|NP_835925.1| DL-methionine transporter substrate-binding subunit [Shigella flexneri 2a str. 2457T]

MAFKFKTFAAVGALIGSLALVGCGQDEKDPNHIKVGVIVGAEQQVAEVAQKVAKDKYGLDVELVTFNDYV

LPNEALSKGDIDANAFQHKPYLDQQLKDRGYKLVAVGNTFVYPIAGYSKKIKSLDELQDGSQVAVPNDPT
NLGRSLLLLQKVGLIKLKDGVGLLPTVLDVVENPKNLKIVELEAPQLPRSLDDAQIALAVINTTYASQIG
LTPAKDGIFVEDKDSPYVNLIVTREDNKDAENVKKFVQAYQSDEVYEAANKVFNGGAVKGW

>gi|30061753|ref|NP_835924.1| outer membrane lipoprotein [Shigella flexneri 2a str. 2457T]
MRALPICLVALMLSGCSMLSRSPVEPVQSTAPQPKAEPAKPKAPRATPVRIYTNAEELVGKPFRDLGEVS
GDSCQASNQDSPPSIPTARKQMQINASKMKANAVLLHSCEVTSGTPGCYRQAVCIGSALNITAK

>gi|30061752|ref|NP_835923.1| hypothetical protein S0188 [Shigella flexneri 2a str. 2457T]
MSSFQFEQIGVIRSPYKEKFAVPRQPGLVKSANGELHLIASYNQADAVRGLEAFSHLWILFVFHQTMEGG
WRPTVRPPRLGGNARMGVFATRSTFRPNPIGMSLVELKEVVCHKGSVILKLGSLDLVDGTPVVDIKPYLP
FAESLPDASASYAQSAPAAEMAVSFTAEVEKQLLTLEKRYPQLTLFIREVLAQDPRPAYRKGEETGKTYA
VWLHDFNVRWRVTDAGFEVFALEPR

>gi|30061751|ref|NP_835922.1| prolyl-tRNA synthetase [Shigella flexneri 2a str. 2457T]
MRTSQYLLSTLKETPADAEVISHQLMLRAGMIRKLASGLYTWLPTGVRVLKKVENIVREEMNNAGAIEVS
MPVVQPADLWQESGRWEQYGPELLRFVDRGERPFVLGPTHEEVITDLIRNELSSYKQLPLNFYQIQTKFR
DEVRPRFGVMRSREFLMKDAYSFHTSQESLQETYDAMYAAYSKIFSRMGLDFRAVQADTGSIGGSASHEF
QVLAQSGEDDVVFSDTSDYAANIELAEAIAPKEPRAAATQEMTLVDTPNAKTIAELVEQFNLPIEKTVKT
LLVKAVEGSSFPLVALLVRGDHELNEVKAEKLPQVASPLTFATEEEIRAVVKAGPGSLGPVNMPIPVVID
RTVAAMSDFAAGANIDGKHYFGINWDRDVATPEVADIRNVVAGDPSPDGQGTLLIKRGIEVGHIFQLGTK
YSEALKASVQGEDGRNQILTMGCYGIGVTRVVAAAIEQNYDERGIVWPDAIAPFQVAILPMNMHKSFRVQ
ELAEKLYSELRAQGIEVLLDDRKERPGVMFADMELIGIPHTIVLGDRNLDNDDIEYKYRRNGEKQLIKTG
DIVEYLVKQIKG

>gi|30061750|ref|NP_835921.1| hypothetical protein S0186 [Shigella flexneri 2a str. 2457T]

MDKPKAYCRLLLPSFLLLSACTVDISQPDPSATAVDAEAKTWAVKFQHQSSFTEQSIKEITAPDLKPGDL

LFSSSLGVTSFGIRVFSTSSVSHVAIFLGDNNVAEATGAGVQIVSLKKAMKHSDKLFVLRVPDLTPQQAT

DITAFANKIKDSGYNYRGIVEFIPFMVTRQMCSLNPFSEDFRQQCVSDLAKAQLSSVCEGDKKSWFCSEF

VTDAFAKSGHPLTLAQSGWISPADLMHMRIGDVSAFKPETQLQYVGHLKPGIYIKAGRFVGLTQ

>gi|30061749|ref|NP_835920.1| lipoprotein involved with copper homeostasis and adhesion [Shigella flexneri 2a str. 2457T]

MVKKAIVTAMAVISLFTLMGCNNRAEVDTLSPAQAAELKPMPQSWRGVLPCADCEGIEISLFLEKDGTWV

MNERYLGAREEPSSFASYGTWARTADKLVLTDSKGEKSYYRAKGDALEMLDREGNPIESQFNYTLEPAQS

SLPMTPMTLRGMYFYMADAATFTDCATGKRFMVANNAELERSYLAARGHSEKPVLLSVEGHFTLEANPDT

GAPTKVLAPDTAGKFYPNKDCSSLGQ

>gi|30061748|ref|NP_835919.1| peptidyl-tRNA hydrolase domain protein [Shigella flexneri 2a str. 2457T]

MIVISRHVAIPDGELEITAIRAQGAGGQHVNKTSTAIHLRFDIRASSLPEYYKERLLAASHHLISSDGVI

VIKAQEYRSQELNREAALARLVAVIKDLTKEQKARRPTRPTRASKERRLASKAQKSSVKAMRGKVRSGRE

>gi|30061747|ref|NP_835918.1| hypothetical protein S0183 [Shigella flexneri 2a str. 2457T]

MALKATIYKATVNVADLDRNQFLDASLTLARHPSETQERMMLRLLAWLKYADERLQFTRGLCADDEPEAW

LRNDHLGIDLWIELGLPDERRIKKACTQAAEVALFTYNSRAAQIWWQQNQSKCVQFANLSVWYLDDEQLA

KVSAFADRTMTLQATIQDGVIWLSDDKNNLEVNLTAWQQPS

>gi|30061746|ref|NP_835917.1| Rho-binding antiterminator [Shigella flexneri 2a str. 2457T]

MSMNDTYQPINCDDYDNLELACQHHLMLTLELKDGEKLQAKASDLVSRKNVEYLVVEAAGETRELRLDKI

TSFSHPEIGTVVVSES

>gi|30061745|ref|NP_835916.1| tRNA(Ile)-lysidine synthetase [Shigella flexneri 2a str. 2457T]

MTLTLNRQLLTSRQILVAFSGGLDSTVLLHQLVQWRTENPGVTLRAIHVHHGLSANADAWVTHCENVCQQ

WQVPLVVERVQLAQEGLGIEAQARQARYQAFARTLLPGEVLVTAQHLDDQCETFLLALKRGSGPAGLSAM

AEVSEFAGTRLIRPLLARTRGELEQWALAHGLRWIEDESNQDDSYDRNFLRLRVVPLLQQRWPHFAEATA

RSATLCAEQESLLDELLADDLAHCQTSQGTLQIAPMLAMSDARRAAIIRRWLAGQNAPMPSRDALVRIWQ

EVALAREDASPCLRLGAFEIRRYQSQLWWIKSVTGQSETIVLWQTWLQPLELPAGLGTVQLTAGGDIRPP

RADEAVSVRFKAPGLLHIVGRNGGRKLKKIWQELGVPPWLRDTTPLLFYGETLIAAAGVFVTQEGVAEGE

NGVSFVWQKTLS

>gi|30061743|ref|NP_835914.1| lysine decarboxylase 2 [Shigella flexneri 2a str. 2457T]

MNIIAIMGPHGVFYKDEPIKELESALVAQGFQIIWPQNSVDLLKFIEHNPRICGVIFDWDEYSLDLCSDI

NQLNEYLPLYAFINTHSTMDVSVQDMRMALWFFEYALGQAEDIAIRMRQYTNEYLDNITPPFTKALFTYV

KERKYTFCTPGHMGGTAYQKSPVGCLFYDFFGGNTLKADVSISVTELGSLLDHTGPHLEAEEYIARTFGA

EQSYIVTNGISTSNKIVGMYAAPSGSTLLIDRNCHKSLAHLLMMNDVVPVWLKPTRNALGILGGIPRREF

TRDSIEEKVAATTQAQWPVHAVITNSTYDGLLYNTDWIKQTLDVPSIHFDSAWVPYTHFHPIYQGKSGMS

GERVAGKVFFETQSTHKMLAALSQASLIHIKGEYDEEAFNEAFMMHTTTSPSYPIVASVKTAAAMLRGNP

GKRLINRSVERALHFRKEVQRLREESDGWFFDIWQPPQVDEAECRPVAPGEQWHGFNDADADHMFLDPVK

VTILTPGMDEQGNMSEEGIPAALVAKFLDERGIVVEKTGPYNLLFLFSIGIDKTKAMGLLRGLTEFKRSY

DLNLRIKNMLPDLYAEDPDFYRNMRIQDLAQGIHKLIRKHDLSGLMLRAFDTLPEMIMTPHQAWQRQIKG

EVETIALEQLVGRVSANMILPYPPGVPLLMPGEMLTKESRTVLDFLLMLCSVGQHYPGFETDIHGAKQDE

DGVYRVRVLKMAG

>gi|30061742|ref|NP_835913.1| acetyl-CoA carboxylase carboxyltransferase subunit alpha [Shigella flexneri 2a str. 2457T]

MSLNFLDFEQPIAELEAKIDSLTAVSRQDEKLDINIDEEVHRLREKSVELTRKIFADLGAWQIAQLARHP

QRPYTLDYVRLAFDEFDELAGDRAYADDKAIVGGIARLDGRPVMIIGHQKGRETKEKIRRNFGMPAPEGY
RKALRLMQMAERFKMPIITFIDTPGAYPGVGAEERGQSEAIARNLREMSRLGVPVVCTVIGEGGSGGALA
IGVGDKVNMLQYSTYSVISPEGCASILWKSADKAPLAAEAMGIIAPRLKELKLIDSIIPEPLGGAHRNPE
AMAASLKAQLLADLADLDVLSTEDLKNRRYQRLMSYGYA

>gi|30061741|ref|NP_835912.1| DNA polymerase III subunit alpha [Shigella flexneri 2a str. 2457T]
MSEPRFVHLRVHSDYSMIDGLAKTAPLVKKAAALGMPALAITDFTNLCGLVKFYGAGHGAGIKPIVGADF
NVQCDLLGDELTHLTVLAANNTGYQNLALLISKAYQRGYGAAGPIIDRDWLIELNEGLILLSGGRMGDVG
RSLLRGNSALVDECVAFYEEHFPDRYFLELIRTGRPDEESYLHAAVELAEARGLPVVATNDVRFIDSSDF
DAHEIRVAIHDGFTLDDPKRPRNYSPQQYMRSEEEMCELFADIPEALANTVEIAKRCNVTVRLGEYFLPQ
FPTGDMSTEDYLVKRAKEGLEERLAFLFPDEEERLKRRPEYDERLETELQVINQMGFPGYFLIVMEFIQW
SKDNGVPVGPGRGSGAGSLVAYALKITDLDPLEFDLLFERFLNPERVSMPDFDVDFCMEKRDQVIEHVAD
MYGRDAVSQIITFGTMAAKAVIRDVGRVLGHPYGFVDRISKLIPPDPGMTLAKAFEAEPQLPEIYEADEE
VKALIDMARKLEGVTRNAGKHAGGVVIAPTKITDFAPLYCDEEGKHPVTQFDKSDVEYAGLVKFDFLGLR
TLTIINWALEMINKRRAKNGEPPLDIAAIPLDDKKSFDMLQRSETTAVFQLESRGMKDLIKRLQPDCFED
MIALVALFRPGPLQSGMVDNFIDRKHGREEISYPDVQWQHESLKSVLEPTYGIILYQEQVMQIAQVLSGY
TLGGADMLRRAMGKKKPEEMAKQRSVFAEGAEKNGINAELAMKIFDLVEKFAGYGFNKSHSAAYALVSYQ
TLWLKAHYPAEFMAAVMTADMDNTEKVVGLVDECWRMGLKILPPDINSGLYHFHVNDDGEIVYGIGAIKG
VGEGPIEAIIEARNKGGYFRELFDLCARTDTKKLNRRVLEKLIMSGAFDRLGPHRAALMNSLGDALKAAD
QHAKAEAIGQADMFGVLAEEPEQIEQSYASCQPWPEQVVLDGERETLGLYLTGHPINQYLKEIERYVGGV
RLKDMHPTERGKVITAAGLVVAARVMVTKRGNRIGICTLDDRSGRLEVMLFTDALDKYQQLLEKDRILIV
SGQVSFDDFSGGLKMTAREVMDIDEAREKYARGLAISLTDRQIDDQLLNRLRQSLEPHRSGTIPVHLYYQ
RADARARLRFGATWRVSPSDRLLNDLRGLIGSEQVELEFD

>gi|30061740|ref|NP_835911.1| ribonuclease HII [Shigella flexneri 2a str. 2457T]

MIEFVYPHTQLVAGVDEVGRGPLVGAVVTAAVILDPARPIAGLNDSKKLSEKRRLALYEEIKEKALSWSL

GRAEPHEIDELNILHATMLAMQRAVAGLHIAPEYVLIDGNRCPKLPMPAMAVVKGDSRVPEISAASILAK

VTRDAEMAALDIVFPQYGFAQHKGYPTAFHLEKLAEYGATEHHRRSFGPVKRALGLAS

>gi|30061739|ref|NP_835910.1| lipid-A-disaccharide synthase [Shigella flexneri 2a str. 2457T]

MTDQRPLTIALVAGETSGDILGAGLIRALKERVPNARFVGVAGPRMQAEGCEAWYEMEELAVMGIVEVLG

RLRRLLHIRADLTKRFGELKPDVFVGIDAPDFNITLEGNLKKQGIKTIHYVSPSVWAWRQKRVFKIGRAT

DLVLAFLPFEKAFYDKYNVPCRFIGHTMADAMPLDPDKNGARDVLGIPYDAHCLALLPGSRGAEVEMLSA

DFLKTAQLLRQTYPDLEIVVPLVNAKRREQFERIKAEVAPDLSVHLLDGMGREAMVASDAALLASGTAAL

ECMLAKCPMVVGYRMKPFTFWLAKRLVKTDYVSLPNLLAGRELVKELLQEECEPQKLAAALLPLLANGKT

SHAMHDTFRELHQQIRCNADEQAAQAVLELAQ

>gi|30061738|ref|NP_835909.1| UDP-N-acetylglucosamine acyltransferase [Shigella flexneri 2a str. 2457T]

MIDKSAFVHPTAIVEEGASIGANAHIGPFCIVGPHVEIGEGTVLKSHVVVNGHTKIGRDNEIYQFASIGE

VNQDLKYAGEPTRVEIGDRNRIRESVTIHRGTVQGGGLTKVGSDNLLMINAHIAHDCTVGNRCILANNAT

LAGHVSVDDFAIIGGMTAVHQFCIIGAHVMVGGCSGVAQDVPPYVIAQGNHATPFGVNIEGLKRRGFSRE

AITAIRNAYKLIYRSGKTLDEVKPEIAELAETYPEVKAFTDFFARSTRGLIR

>gi|30061737|ref|NP_835908.1| (3R)-hydroxymyristoyl-ACP dehydratase [Shigella flexneri 2a str. 2457T]

MTTNTHTLQIEEILELLPHRFPFLLVDRVLDFEEGRFLRAVKNVSVNEPFFQGHFPGKPIFPGVLILEAM

AQATGILAFKSVGKLEPGELYYFAGIDEARFKRPVVPGDQMIMEVTFEKTRRGLTRFKGVALVDGKVVCE

ATMMCARSREA

>gi|30061736|ref|NP_835907.1| UDP-3-O-[3-hydroxymyristoyl] glucosamine N-acyltransferase [Shigella flexneri 2a str. 2457T]

MPSIRLADLAQQLDAELHGDGDIVITGVASMQSAQTGHITFMVNPKYREHLGLCQASAVVMTQDDLPFAK

SAALVVKNPYLTYARMAQILDTTPQPAQNIAPSAVIDATAKLGNNVSIGANAVIESGVELGDNVIIGAGC

FVGKNSKIGAGSRLWANVTIYHEIQIGQNCLIQSGTVVGADGFGYANDRGNWVKIPQIGRVIIGDRVEIG

ACTTIDRGALDDTVIGNGVIIDNQCQIAHNVVIGDNTAVAGGVIMAGSLKIGRYCMIGGASVINGHMEIC

DKVTVTGMGMVMRPITEPGVYSSGIPLQPNKVWRKTAALVMNIDDMSKRLKSLERKVNQQD

>gi|30061735|ref|NP_835906.1| periplasmic chaperone [Shigella flexneri 2a str. 2457T]

MKKWLLAAGLGLALATSAQAADKIAIVNMGSLFQQVAQKTGVSNTLENEFKGRASELQRMETDLQAKMKK

LQSMKAGSDRTKLEKDVMAQRQTFAQKAQAFEQDRARRSNEERGKLVTRIQTAVKSVANSQDIDLVVDAN

AVAYNSSDVKDITADVLKQVK

>gi|30061734|ref|NP_835905.1| outer membrane protein assembly factor YaeT [Shigella flexneri 2a str. 2457T]

MAMKKLLIASLLFSSATVYGAEGFVVKDIHFEGLQRVAVGAALLSMPVRTGDTVNDEDISNTIRALFATG

NFEDVRVLRDGDTLLVQVKERPTIASITFSGNKSVKDDMLKQNLEASGVRVGESLDRTTIADIEKGLEDF

YYSVGKYSASVKAVVTPLPRNRVDLKLVFQEGVSAEIQQINIVGNHAFTTDELISHFQLRDEVPWWNVVG

DRKYQKQKLAGDLETLRSYYLDRGYARFNIDSTQVSLTPDKKGIYVTVNITEGDQYKLSGVEVSGNLAGH

SAEIEQLTKIEPGELYNGTKVTKMEDDIKKLLGRYGYAYPRVQSMPEINDADKTVKLRVNVDAGNRFYVR

KIRFEGNDTSKDAVLRREMRQMEGAWLGSDLVDQGKERLNRLGFFETVDTDTQRVPGSPDQVDVVYKVKE

RNTGSFNFGIGYGTESGVSFQAGVQQDNWLGTGYAVGINGTKNDYQTYAELSVTNPYFTVDGVSLGGRLF

YNDFQADDADLSDYTNKSYGTDVTLGFPINEYNSLRAGLGYVHNSLSNMQPQVAMWRYLYSMGEHPSTSD

QDNSFKTDDFTFNYGWTYNKLDRGYFPTDGSRVNLTGKVTIPGSDNEYYKVTLDTATYVPIDDDHKWVVL

GRTRWGYGDGLGGKEMPFYENFYAGGSSTVRGFQSNTIGPKAVYFPHQASNYDPDYDYECATQDGAKDLC

KSDDAVGGNAMAVASLEFITPTPFISDKYANSVRTSFFWDMGTVWDTNWDSSQYSGYPDYSDPSNIRMSA

GIALQWMSPLGPLVFSYAQPFKKYDGDKAEQFQFNIGKTW

>gi|30061733|ref|NP_835904.1| zinc metallopeptidase RseP [Shigella flexneri 2a str. 2457T]

MLSFLWDLASFIVALGVLITVHEFGHFWVARRCGVRVERFSIGFGKALWRRTDKLGTEYVIALIPLGGYV

KMLDERAEPVVPELRHHAFNNKSVGQRAAIIAAGPVANFIFAIFAYWLVFIIGVPGVRPVVGEIAANSIA

AEAQIAPGTELKAVDGIETPDWDAVRLQLVDKIGDESTTITVAPFDSDQRRDVKLDLRHWAFEPDKEDPV

SSLGIRPRGPQIEPVLENVQPNSAASKAGLQAGDRIVKVDGQPLTQWVTFVMLVRDNPGKSLALEIERQG

SPLSLTLIPESKPGNGKAIGFVGIEPKVIPLPDEYKVVRQYGPFNAIVEATDKTWQLMKLTVSMLGKLIT

GDVKLNNLSGPISIAKGAGMTAELGVVYYLPFLALISVNLGIINLFPLPVLDGGHLLFLAIEKIKGGPVS

ERVQDFCYRIGSILLVLLMGLALFNDFSRL

>gi|30061731|ref|NP_835902.1| undecaprenyl pyrophosphate synthase [Shigella flexneri 2a str. 2457T]

MMLSATQPLSEKLPAHGCRHVAIIMDGNGRWAKKQGKIRAFGHKAGAKSVRRAVSFAANNGIEALTLYAF

SSENWNRPAQEVSALMELFVWALDSEVKSLHRHNVRLRIIGDTSRFNSRLQERIRKSEALTAGNTGLTLN

IAANYGGRWDIVQGVRQLAEKVQQGNLQPDQIDEEMLNQHVCMHELAPVDLVIRTGGEHRISNFLLWQIA

YAELYFTDVLWPDFDEQDFEGALNAFANRERRFGGTEPGDETA

>gi|30061730|ref|NP_835901.1| 1-deoxy-D-xylulose 5-phosphate reductoisomerase [Shigella flexneri 2a str. 2457T]

MKQLTILGSTGSIGCSTLDVVRHNPEHFRVVALVAGKNVTRMVEQCLEFSPRYAVMDDEASAKLLKTMLQ

QQGSRTEVLSGQQAACDMAALEEVDQVMAAIVGAAGLLPTLAAIRAGKTILLANKESLVTCGRLFMDAVK

QSKAQLLPVDSEHNAIFQSLPQPIQHNLGYADLEQNGVVSILLTGSGGPFRETPLRDLATMTPDQACRHP

NWSMGRKISVDSATMMNKGLEYIEARWLFNASASQMEVLIHPQSVIHSMVRYQDGSVLAQLGEPDMRTPI

AHTMAWPNRVNSGVKPLDFCKLSALTFAAPDYERYPCLKLAMEAFEQGGQAATTALNAANEITVAAFLAQQ

IRFTDIAALNLSVLEKMDMREPQCVDDVLSVDANASEVARKEVMRLAS

>gi|30061729|ref|NP_835900.1| ribosome recycling factor [Shigella flexneri 2a str. 2457T]

MISDIRKDAEVRMDKCVEAFKTQISKIRTGRASPSLLDGIVVEYYGTPTPLRQLASVTVEDSRTLKINVF

DRSMSPAVEKAIMASDLGLNPNSAGSDIRVPLPPLTEERRKDLTKIVRGEAEQARVAVRNVRRDANDKVK

ALLKDKEISEDDDRRSQDDVQKLTDAAIKKIEAALADKEAELMQF

>gi|30061728|ref|NP_835899.1| uridylate kinase [Shigella flexneri 2a str. 2457T]

MATNAKPVYKRILLKLSGEALQGTEGFGIDASILDRMAQEIKELVELGIQVGVVIGGGNLFRGAGLAKAG

MNRVVGDHMGMLATVMNGLAMRDALHRAYVNARLMSAIPLNGVCDSYSWAEAISLLRNNRVVILSAGTGN

PFFTTDSAACLRGIEIEADVVLKATKVDGVFTADPAKDPTATMYEQLTYSEVLEKELKVMDLAAFTLARD

HKLPIRVFNMNKPGALRRVVMGEKEGTLITE

>gi|30061727|ref|NP_835898.1| elongation factor Ts [Shigella flexneri 2a str. 2457T]

MAEITASLVKELRERTGAGMMDCKKALTEANGDIELAIENMRKSGAIKAAKKAGNVAADGVIKTKIDGNY

GIILEVNCQTDFVAKDAGFQAFADKVLDAAVAGKITDVEVLKAQFEEERVALVAKIGENINIRRVAALEG

DVLGSYQHGARIGVLVAAKGADEELVKHIAMHVAASKPEFIKPEDVSAEVVEKEYQVQLDIAMQSGKPKE

IAEKMVEGRMKKFTGEVSLTGQPFVMEPSKTVGQLLKEHNAEVTGFIRFEVGEGIEKVETDFAAEVAAMS

KQS

>gi|30061726|ref|NP_835897.1| 30S ribosomal protein S2 [Shigella flexneri 2a str. 2457T]

MATVSMRDMLKAGVHFGHQTRYWNPKMKPFIFGARNKVHIINLEKTVPMFNEALAELNKIVSRKGKILFV

GTKRAASEAVKDAALSCDQFFVNHRWLGGMLTNWKTVRQSIKRLKDLETQSQDGTFDKLTKKEALMRTRE

LEKLENSLGGIKDMGGLPDALFVIDADHEHIAIKEANNLGIPVFAIVDTNSDPDGVDFVIPGNDDAIRAV

TLYLGAVAATVREGRSQDLASQAEESFVEAE

>gi|30061725|ref|NP_835896.1| methionine aminopeptidase [Shigella flexneri 2a str. 2457T]

MAISIKTPEDIEKMRVAGRLAAEVLEMIEPYVKPGVSTGELDRICNDYIVNEQHAVSACLGYHGYPKSVC

ISINEVVCHGIPDDAKLLKDGDIVNIDVTVIKDGFHGDTSKMFIVGKPTIMGERLCRITQESLYLALRMV

KPGINLREIGAAIQKFVEAEGFSVVREYCGHGIGRGFHEEPQVLHYDSRETNVVLKPGMTFTIEPMVNAG

KKEIRTMKDGWTVKTKDRSLSAQYEHTIVVTDNGCEILTLRKDDTIPAIISHDE

>gi|30061724|ref|NP_835895.1| PII uridylyl-transferase [Shigella flexneri 2a str. 2457T]

MNTLPEQYANTALSTLPGQPQNPCAWPRDELTVCGIKAHIDTFQRWLGDAFDNGISAEQLIEARTEFIDQ

LLQRLWIEAGFSQIADLALVAVGGYGRGELHPLSDIDLLILSRKKLPDDQAQKVGELLTLLWDVKLEVGH

SVRTLEECMLEGLSDLTVATNLIESRLLIGDVALFLELQKHIFSEGFWPSDKFYAAKVEEQNQRHQRYHG

TSYNLEPDIKSSPGGLRDIHTLQWVARRHFGATSLDEMVGFGFLTSAERAELNECLHILWRIRFALHLVV

SRYDNRLLFDRQLSVAQRLNYSGEGNEPVERMMKDYFRVTRRVSELNQMLLQLFDEAILALPADEKPRPL

DDEFQLRGTLIDLRDETLFMRQPEAILRMFYTMVRNSAITGIYSTTLRQLRHARRHLQQPLCNIPQARKL

FLSILRHPGAVRRGLLPMHRHSVLGAYMPQWSHIVGQMQFDLFHAYTVDEHTIRVMLKLESFASEETRQR

HPLCVDVWPRLPSTELIFIAALFHDIAKGRGGDHSILGAQDVVHFAELHGLNSRETQLVAWLVRQHLLMS

VTAQRRDIQDPEVIKQFAEEVQTENRLRYLVCLTVADICATNETLWNSWKQSLLRELYFATEKQLRRGMQ

NTPDMRERVRHHQLQALALLRMDNIDEEALHQIWSRCRANYFVRHSPNQLAWHARHLLQHDLSKPLVLLS

PQATRGGTEIFIWSPDRPYLFAAVCAELDRRNLSVHDAQIFTTRDGMAMDTFIVLEPDGSPLSADRHEVI

RFGLEQVLTQSSWQPPQPRRQPAKLRHFTVETEVTFLPTHTDRKSFLELIALDQPGLLARVGKIFADLGI

SLHGARITTIGERVEDLFIIATADRRALNNELQQEVHQRLTEALNPNDKG

>gi|30061723|ref|NP_835894.1| 2,3,4,5-tetrahydropyridine-2,6-carboxylate N-succinyltransferase [Shigella flexneri 2a str. 2457T]

MQQLQNIIETAFERRAEITPANADTVTREAVNQVIALLDSGALRVAEKIDGQWVTHQWLKKAVLLSFRIN

DNQVIEGAESRYFDKVPMKFADYDEARFQKEGFRVVPPAAVRQGAFIARNTVLMPSYVNIGAYVDEGTMV

DTWATVGSCAQIGKNVHLSGGVGIGGVLEPLQANPTIIEDNCFIGARSEVVEGVIVEEGSVISMGVYIGQ

STRIYDRETGEIHYGRVPAGSVVVSGNLPSKDGKYSLYCAVIVKKVDAKTRGKVGINELLRTID

>gi|30061722|ref|NP_835893.1| hypothetical protein S0158 [Shigella flexneri 2a str. 2457T]

MYDNLKSLGITNPEEIDRYSLRQEANNDILKIYFQKDKGEFFAKSVKFKYPRQRKTVVADGVGQGYKEVQ

EISPNLRYIIDELDQICQRDRSEVDLKRKILDDLRHLESVVTNKISEIEADLEKLTRK

>gi|30061721|ref|NP_835892.1| serine endoprotease [Shigella flexneri 2a str. 2457T]

MKKNTLALSALALSLGLALSPLSATAAETSSATTAQQMPSLAPMLEKVMPSVVSINVEGSTTVNTPRMPR

NFQQFFGDDSPFCQEGSPFQSSPFCQGGQGGNGGGQQQKFMALGSGVIIDADKGYVVTNNHVVDNATVIK

VQLSDGRKFDAKMVGKDPRSDIALIQIQNPKKLTAIKMADSDALRVGDYTVAIGNPFGLGETVTSGIVSA

LGRSGLNAENYENFIQTDAAINRGNSGGALVNLNGELIGINTAILAPDGGNIGIGFAIPSNMVKNLTSQM

VEYGQVKRGELGIMGTELNSELAKAMKVDAQRGAFVSQVLPNSSAAKAGIKAGDVITSLNGKPISSFAAL

RAQVGTMPVGSKLTLGLLRDGKQVNVNLELQQSSQNQVDSSSIFNGIEGAEMSNKGKDQGVVVNNVKTGT

PAAQIGLKKGDVIIGANQQAVKNIAELRKVLDSKPSVLALNIQRGDSTIYLLMQ

>gi|30061720|ref|NP_835891.1| deoxyguanosinetriphosphate triphosphohydrolase [Shigella flexneri 2a str. 2457T]

MAQIDFRKKINWHRRYRSPQGVKTEHEILRIFESDRGRIINSPAIRRLQQKTQVFPLERNAAVRTRLTHS

MEVQQVGRYIAKEILSRLKELKLLEAYGLDELTGPFESIVEMSCLMHDIGNPPFGHFGEAAINDWFRQRL

HPEDAESQPLTDDRCSVAALRLRDGEEPLNELRRKIRQDLCHFEGNAQGIRLVHTLMRMNLTWAQVGGIL

KYTRPAWWRGETPETHHYLMKKPGYYLSEEAYIARLRKELNLALYSRFPLTWIMEAADDISYCVADLEDA

VEKRIFTVEQLYHHLHEAWGQHEKGSLFSLVVENAWEKSRSNSLSRSTEDQFFMYLRVNTLNKLVPYAAQ

RFIDNLPAIFAGTFNHALLEDASECSDLLKLYKNVAVKHVFSHPDVEQLELQGYRVISGLLEIYRPLLSL

SLSDFTELVEKERVKRFPIESRLFHKLSTRHRLAYVEAVSKLPSDSPEFPLWEYYYRCRLLQDYISGMTD

LYAWDEYRRLMAVEQ

>gi|30061719|ref|NP_835890.1| 5'-methylthioadenosine/S-adenosylhomocysteine nucleosidase [Shigella flexneri 2a str. 2457T]

MKIGIIGAMEEEVTLLRDKIENRQTISLGGCEIYTGQLNGTEVALLKSGIGKVAAALGATLLLEHCKPDV

IINTGSAGGLAPTLKVGDIVVSDEARYHDADVTAFGYEYGQLPGCPAGFKADDKLIAAAEACIAELNLNA

VRGLIVSGDAFINGSVGLAKIRHNFPQAIAVEMEATAIAHVCHNFNVPFVVVRAISDVADQQSHLSFDEF

LAVAAKQSSLMVESLVQKLAHG

>gi|30061718|ref|NP_835889.1| vitamin B12-transporter protein BtuF [Shigella flexneri 2a str. 2457T]

MAKSLFRALVALSFLAPLWLNAAPRVITLSPANTELAFAAGITPVGVSSYSDYPPQAQKIEQVSTWQGMN

LERIVALKPDLVIAWRGGNAERQVDQLASLGIKVMWVDATSIEQIANALRQLAPWSPQPDKAEQAAQSLL

DQYVQLKAQYADKPKKRVFLQFGINPPFTSGKESIQNQVLEVCGGENIFKDSRVPWPQVSREQVLARSPQ

AIVITGGPDQIPKIKQYWGEQLKIPVIPLTSDWFERASPRIILAAQQLCNALSQVD

>gi|30061717|ref|NP_835888.1| hypothetical protein S0152 [Shigella flexneri 2a str. 2457T]

MLVYWLDIVGTAVFAIYGVLLAGKLRMDPFGVLVLGVVTAVGDGTIRDMALDHGPVFWVKDPTDLVVAMV

TSMLTIVLVRQPRRLPKWMLPVLDAVGLAVFVGISVNKAFNAEAGPLIAVCMGVITGVGGGIIRDVLVRE

IPMILRTEIYATACIIGGIVHATAYYTFSVPLETASMMGMVVTLLIRLAAIRWHLKLPTFALDENGR

>gi|30061716|ref|NP_835887.1| iron-sulfur cluster insertion protein ErpA [Shigella flexneri 2a str. 2457T]

MSDDVALPLEFTDAAANKVKSLIADEDNPNLKLRVYITGGGCSGFQYGFTFDDQVNEGDMTIEKQGVGLV

VDPMSLQYLVGGSVDYTEGLEGSRFIVTNPNAKSTCGCGSSFSI

>gi|30061715|ref|NP_835886.1| chloride channel protein [Shigella flexneri 2a str. 2457T]

MKTDTPSLETQQAARLRRRQLIRQLLERDKTPLAILFMAAVVGTLVGLAAVAFDKGVAWLQNQRMGALVH

TADNYPLLLTVAFLCSAVLAMFGYFLVRKYAPEAGGSGIPEIEGALEDQRPVRWWRVLPVKFFGGLGTLG

GGMGLGREGPTVQIGGNISRMVLDIFRLKGDEARHTLLATGAAAGLAAAFNAPLAGILFIIEEMRPQFRY

TLISIKAVFIGVIMSTIMYRIFNHEVALIDVGKLSDAPLNTLWLYLILGIIFGIFGPIFNKWVLGMQDLL

HRVHGGNITKWVLMGGAIGGLCGLLGFVAPATSGGGFNLIPIATAGNFSMGMLVFIFVARVITTLLCFSS

GAPGGIFAPMLALGTVLGTAFGMVAVELFPQYHLEAGTFAIAGMGALLAASIRAPLTGIILVLEMTDNYQ

LILPMIITGLGATLLAQFTGGKPLYSAILARTLAKQEAEQLARSKAASASENT

>gi|30061714|ref|NP_835885.1| glutamate-1-semialdehyde aminotransferase [Shigella flexneri 2a str. 2457T]

MSKSENLYSAARELIPGGVNSPVRAFTGVGGTPLFIEKADGAYLYDVDGKAYIDYVGSWGPMVLGHNHPA

IRNAVIEAAERGLSFGAPTEMEVKMAQLVTELVPTMDMVRMVNSGTEATMSAIRLARGFTGRDKIIKFEG

CYHGHADCLLVKAGSGALTLGQPNSPGVPADFAKHTLTCTYNDLASVRAAFEQYPQEIACIIVEPVAGNM

NCVPPLPEFLPGLRALCDEFGALLIIDEVMTGFRVALAGAQDYFAVVPDLTCLGKIIGGGMPVGAFGGRR

DVMDALAPTGPVYQAGTLSGNPIAMAAGFACLNEVAQPGIHETLDELTTRLAEGLLEAAEEAGIPLVVNH

VGGMFGIFFTDAESVTCYQDVMACDVERFKRFFHMMLDEGVYLAPSAFEAGFMSVAHSMEDINNTIDAAR

RVFAKL

>gi|30061713|ref|NP_835884.1| iron-hydroxamate transporter permease subunit [Shigella flexneri 2a str. 2457T]

MSKRIALFPALLLALLVIVATALTWMNFSQALPRSQWAQAAWSPDIDVIEQMIFHYSLLPRLAISLLVGA

GLGLVGVLFQQVLRNPLAEPTTLGVATGAQLGITVTTLWAIPSAMASQFAALAGACVVGLIVFGVAWGKR

LSPVTLIFAGLVVSLYCGAINQLLVIFHHDQLQSMFLWSTGTLTQTDWGGVERLWPQLLGGVMLTLLLLR

PLTLMGLDDGVARNLGLALSLARLAALSLAIVISALLVNAVGIIGFIGLFAPLLAKMLGARRLLPRLMLA

SLIGALILWLSDQIILWLTRVWMEVSTGSVTALIGAPLLLWLLPRLRSISAPDMKVNDRVATERQHVLAF

ALAGGVLLLMAVVVALSFGRDAHGWTWASGALLEDLMPWRWPRIMAALFAGVMLAVAGCIIQRLTGNPMA

SPEVLGISSGAAFGVVLMLFLVPGNAFGWLLPAGSLGAAVTLLIIMIAAGRGGFSPHRMLLAGMALSTAF

TMLLMMLQASGDPRMAQVLTWISGSTYNATDAQVWRTGIVMVILLAITPLCRRWLTILPLGGDTARAVGM

ALTPTRIALLLLAACLTATATMTIGPLSFVGLMAPHIARMMGFRRTMPHIVISALVGGLLLVFADWCGRM

VLFPFQIPAGLLSTFIGAPYFIYLLRKQSR

>gi|30061712|ref|NP_835883.1| iron-hydroxamate transporter substrate-binding subunit [Shigella flexneri 2a str. 2457T]

MSGLPLISRRRLLTAMALSPLLWQMNTAHAAAIDPNRIVALEWLPVELLLALGIVPYGVADTINYRLWVS

EPPLPDSVIDVGLRTEPNLELLTEMKPSFMVWSAGYGPSSEMLARIAPGRGFNFSDGKHPLAMARKSLTE

MADLLNLQSAAETHLAQYEDFIRSMKPRFVKRGARPLLLTTLIDPRHMLVFGPNSLFQEILDEYGIPNAW

QGETNFWGSTAVSIDRLAAYKDVDVLCFDHDNSKDMDALMATPLWQAMPFVRTGRFQRVPAVWFYGATLS

AMHFVRVLDNAIGGKA

>gi|30061711|ref|NP_835882.1| iron-hydroxamate transporter ATP-binding subunit [Shigella flexneri 2a str. 2457T]

MQEYTNHSDTTFALRNISFRVPGRTLLHPLSLTFPAGKVTGLIGHNGSGKSTLLKMLGRHQPPSEGEILL

DAQPLESWSSKAFARKVAYLPQQLPPAEGMTVRELVAIGRYPWHGALGRFGAADREKVEEAISLVGLKPL

AHRLVDSLSGGERQRAWIAMLVAQDSRCLLLDEPTSALDIAHQVDVLALVHRLSQERGLTVIAVLHDINM

AARYCDYLVALRSGEMIAQGTPAEIMRGETLEMIYGIPMGILPHPAGAAPVSFVY

>gi|30061710|ref|NP_835881.1| ferrichrome outer membrane transporter [Shigella flexneri 2a str. 2457T]

MARSKTAQPKHSLRKIAVVVATAVSGMSVYAQAAVEPKEDTITVTAAPAPQESAWGPAATIAARQSATGT

KTDTPIQKVPQSISVVTAEEMALHQPKSVKEALSYTPGVSVGTRGASNTYDHLIIRGFAAEGQSQNNYLN

GLKLQGNFYNDAVIDPYMLERAEIMRGPVSVLYGKSSPGGLLNMVSKRPTTEPLKEVQFKAGTDSLFQTG

FDFSDALDDDGVYSYRLTGLARSANAQQKGSEEQRYAIAPAFTWRPDDKTNFTFLSYFQNEPETGYYGWL

PKEGTVEPLPNGKRLPTDFNEGAKNNTYSRNEKMVGYSFDHEFNDTFTVRQNLRFAENKTSQNSVYGYGV

CSDPANAYSKQCAALAPADKGHYLARKYVVDDEKLQNFSVDTQLQSKFATGDIDHTLLTGVDFMRMRNDI

NAWFGYDDSVPLFNLYNPVNPDFDFNAKDPANSGPYRILNKQKQTGVYVQDQAQWDKVLVTLGGRYDWAD

QESLNRVAGTTDKRDDKQFTWRGGVNYLFDNGVTPYFSYSESFEPSSQVGKDGNIFAPSKGKQYEVGVKY

VPEDRPIVVTGAVYNLTKTNNLMADPEGSFFSVEGGEIRARGVEIEAKAALSASVNVVGSYTYTDAEYTT

DTTYKGNTPAQVPKHMASLWADYTFFDGPLSGLTLGTGGRYTGSSYGDPANSFKVGSYTVVDALVRYDLA

RVGMAGSNVALHVNNLFDREYVASCFNTYGCFWGAERQVVATATFRF

>gi|30061709|ref|NP_835880.1| penicillin-binding protein 1b [Shigella flexneri 2a str. 2457T]

MAGNDREPIGRKGKPTRPVKQKVSRRRYEDDDDYDDYDDYEDEEPMPRKGKGKGKGRKPRGKRGWLWLLL

KLAIVFAVLIAIYGVYLDQKIRSRIDGKVWQLPAAVYGRMVNLEPDMTISKNEMVKLLEATQYRQVSKMT

RPGEFTVQANSIEMIRRPFDFPDSKEGQVRARLTFDGDHLATIVNMENNRQFGFFRLDPRLITMISSPNG

EQRLFVPRSGFPDLLVDTLLATEDRHFYEHDGISLYSIGRAVLANLTAGRTVQGASTLTQQLVKNLFLSS

ERSYWRKANEAYMALIMDARYSKDRILELYMNEVYLGQSGDNEIRGFPLASLYYFGRPVEELSLDQQALL

VGMVKGASIYNPWRNPKLALERRNLVLRLLQQQQIIDQELYDMLSARPLGVQPRGGVISPQPAFMQLVRQ

ELQAKLGDKVKDLSGVKIFTTFDSVAQDAAEKAAVEGIPALKKQRKLSDLETAIVVVDRFSGEVRAMVGG

SEPQFAGYNRAMQARRSIGSLAKPATYLTALSQPKIYRLNTWIADAPIALRQPNGQVWSPQNDDRRYSES

GRVMLVDALTRSMNVPTVNLGMALGLPAVTETWIKLGVPKDQLHPVPAMLLGALNLTPIEVAQAFQTIAS

GGNRAPLSALRSVIAEDGKVLYQSFPQAERAVPAQAAYLTLWTMQQVVQRGTGRQLGAKYPNLHLAGKTG

TTNNNVDTWFAGIDGSTVTITWVGRDNNQPTKLYGASGAMSIYQRYLANQTPTPLNLVPPEDIADMGVDY

DGNFVCSGGMRVLPVWTSDPQSLCQQSEMQQQPSGNPFDQSSQPQQQPQQQPAQQEQKDSDGVAGWIKDM

FGSN

>gi|30061708|ref|NP_835879.1| ATP-dependent RNA helicase HrpB [Shigella flexneri 2a str. 2457T]

MLQCGAKNVNPLERFVSSLPVAAVLPELLTALDCAPQVLLSAPTGAGKSTWLPLQLLAHPGINGKIILLE

PRRLAARNVAQRLAELLNEKPGDTVGYRMRAQNCVGLNTRLEVVTEGVLTRMIQRDPELSGVGLVILDEF

HERSLQADLALALLLDVQQGLRDDLKLLIMSATLDNDRLQQMLPEAPVVISEGRSFPVERRYLPLPAHQR

FDEAVAVATAEMVRQESGSLLLFLPGVGEIQRVQEQLASRIGSDVVLCPLYGVLSLNDQRKAILPAPQGM

RKVVLATNIAETSLTIEGIRLVVDCAQERVARFDPRTGLTRLITQRVSQASMTQRAGRAGRLEPGISLHL

IAKEQAERAAAQSEPEILQSDLSGLLMELLQWGCSDPAQMSWLDQPPTVNLLAAKRLLQMLGALEGERLS

AQGQKMAALGNDPRLAAMLVNAKSDDEAATAAKIAAILEEPPRMGNSDLGVAFSRNQPAWQQRSQQLLKR

LNVRGGEADSSLIAPLLAGAFADRIAHRRGQDGRYQLANSMGAMLDADDALSRHEWLIAPLLLQGSASPD

ARILLALPVDIDELVQRCPQLVQQSDTVEWDDAQGTLKAWRRLQIGQLTVKVQPLAKPSEDELHQAMLNG

IRDKGLSVLNWTAEAEQLRLRLLCAAKWLPEYDWPAVDDESLLATLETWLLPHMTGVHSLRGLKSLDIYQ

ALRGLLDWGMQQRLDSELPAHYTVPTGSRIAIRYHEDNPPALAVRMQEMFGEATNPTIAQGRVPLVLKLL

SPAQRPLQITRDLGAFWKGAYREVQKEMKGRYPKHVWPDDPANTAPTRRTKKYS

>gi|30061707|ref|NP_835878.1| hypothetical protein S0142 [Shigella flexneri 2a str. 2457T]

MPHMSEPQRLFFAIDLPAEIREQIIHWRATHFPPEAGRPVAADNLHLTLAFLGEVSAEKEKALSLLAGRI

RQPGFTLTLDDAGQWLRSRVVWLGMRQPPRGLIQLANMLRSQAARSGCFQSNRPFHPHITLLRDASEAVT

FPPPGFNWSYAVTEFTLYASSFARGRTRYTPLKRWALTQ

>gi|30061706|ref|NP_835877.1| sugar fermentation stimulation protein A [Shigella flexneri 2a str. 2457T]

MEFSPPLQRATLIQRYKRFLADVITPDGRELTLHCPNTGAMTGCATPGDTVWYSTSDNTKRKYPHTWELT

QSQSGAFICVNTLWANRLTKEAILNESISELSGYSSLKSEVKYGSERSRIDFMLQADSRPDCYIEVKSVT

LAENEQGYFPDAVTERGQKHLRELMSVAAEGQRAVIFFAVLHSAITRFSPARHIDEKYAQLLSEAQQRGV

EILAYKAEISAEGMALKKSLPVTL

>gi|30061705|ref|NP_835876.1| DnaK transcriptional regulator DksA [Shigella flexneri 2a str. 2457T]

MQEGQNRKTSSLSILAIAGVEPYQEKPGEEYMNEAQLAHFRRILEAWRNQLRDEVDRTVTHMQDEAANFP

DPVDRAAQEEEFSLELRNRDRERKLIKKIEKTLKKVEDEDFGYCESCGVEIGIRRLEARPTADLCIDCKT

LAEIREKQMAG

>gi|30061702|ref|NP_835873.1| 2-amino-4-hydroxy-6-hydroxymethyldihydropteridine pyrophosphokinase [Shigella flexneri 2a str. 2457T]

MTVAYIAIGSNLASPLEQVNAALKALGDIPESRILAVSSFYRTPPLGPQDQPDYLNAAVALETSPAPEEL

LNHTQRIELQQGRVRKAERWGPRTLDLDIMLFGNEVINTERLTVPHYDMKNRGFMLWPLFEIAPELAFPD

GETLREVLHTRAFDKLSKW

>gi|30061701|ref|NP_835872.1| IS1 orfB [Shigella flexneri 2a str. 2457T]

MSRQCTHYGRWPQHGFTSLKKLRPQSVTSRIQPGSDVIVCAEMDEHWGYVGAKSRQRWLFYAYDRIRRTV

VAHVFGERTLATLERLLSLLSAFEVVVWMTDGWPLYESRLKGKLHVISKRYTQRIERHNLNLRQHLARLV

RKSLSFSKSVELHDKVIGHYLNIKHYQ

>gi|30061700|ref|NP_835871.1| IS1 orfA [Shigella flexneri 2a str. 2457T]

MASISIRCPSCSATEGVVRNGKSTAGHQRYLCSHCRKTWQLQFTYTASQPGKHQKIIDMAMNGVGCRASA

RIMGVGLNTVLRHLKNSGRSR

>gi|30061699|ref|NP_835870.1| 3-methyl-2-oxobutanoate hydroxymethyltransferase [Shigella flexneri 2a str. 2457T]

MKPTTIASLQKYKQDKKRFATITAYDYSFAKLFADEGLNVMLVGDSLGMTVQGHDSTLPVTVADIAYHTA

AVRRGAPNCLLLADLPFMAYATPEQAFENAATVMRAGANMVKIEGGEWLVETVKMLTERAVPVCGHLGLT

PQSVNIFGGYKVQGRGNEASDRLLSDALALEAAGAQLLVLECVPVELAKRITEALAIPVIGIGAGNVTDG

QILVMHDAFGITGGHIPKFAKNFLAETGDIRAAVRQYMAEVESGVYPGEEHSFH

>gi|30061698|ref|NP_835869.1| pantoate--beta-alanine ligase [Shigella flexneri 2a str. 2457T]

MLIIETLPLLRQQIRRLRMEGKRVALVPTMGNLHNGHMKLVDEAKARADVVVVSIFVNPMQFDRPEDLAR

YPRTLQEDCEKLNKRKVDLVFAPSVKEIYPNGTETHTYVDVPGLSTMLEGASRPGHFRGVSTIVSKLFNL

VQPDIACFGEKDFQQLALIRKMVADMGFDIEIVGVPIMRAKDGLALSSRNGYLTAEQRKIAPGLYKVLSS

IADKLQAGERDLDEIITIAGQELNEKGFRADDIQIRDADTLLEVSETSKRAVILVAAWLGDARLIDNKMV

ELA

>gi|30061697|ref|NP_835868.1| hypothetical protein S0131 [Shigella flexneri 2a str. 2457T]

MDAPSTTPHDAVFKQFLMHAETARDFLEIHLPVELRELCDLNTLHLESGSFIEECLKGHSTDVLYSMQMQ

GNPGYLHVVIEHQSKPDKKMAFRMMRYSIAAMHRHLEAGHDKLPLVVPILFYQGEATPYPLSMCWFDMFY

SPELARRVYNSPFPLVDITITPDDEIMQHRRIAILELLQKHIRQRDLMLLLEQLVTLIDEGYTSGSQLVA

MQNYMLQRGHTEQADLFYGVLRDRETGGKSMMTLAQWFEEKGIEKGIQQGRQEERQEFALRLLSKGMSRE

DVAEMANLPLAEIDKVINLI

>gi|30061696|ref|NP_835867.1| aspartate alpha-decarboxylase [Shigella flexneri 2a str. 2457T]

MIRTMLQGKLHRVKVTHADLHYEGSCAIDQDFLDAAGILENEAIDIWNVTNGKRFSTYAIAAERGSRIIS

VNGAAAHCASVGDIVIIASFVTMPDEEARTWRPNVAYFEGDNEMKRTAKAIPVQVA

>gi|30061695|ref|NP_835866.1| hypothetical protein S0129 [Shigella flexneri 2a str. 2457T]

MYKQAVILLLMLFTASVSAALPARYMQTIENAAVWAQISDKMVTVGNIRAGQIIAVEPTAASYYAFNFGF

GKGFIDKGHLEPVQGRQKVEDGLGDLNKPLSNQNLITWKDTPVYNAPSVGSAPFGVLADNLRYPILHKLK

DRLNQTWYQIRIGDRLAYISALDAQPDNGLPVLTYHHILRDEENTRFRHTSTTTSVRAFNNQMAWLRDRG

YATLSMAQLEGYVKNTINLPARAVVITFDDGLKSVSRYAYPVLKQYGMKATVFIVTSRIKRHPQKWNPKS

LQFMSVSELNEIRDVFDFQSHTHFLHRVDGYRRPILLSRSEHNILFDFARSRRALAQFNPHVWYLSYPFG

GFNDKAVKAANDAGFHLAVTTMKGKVKPGDNPLLLKRLYILRTDSLEKMSRLVSNQPQG

>gi|30061694|ref|NP_835865.1| PTS enzyme II B component [Shigella flexneri 2a str. 2457T]

MLGWVITCHDDRAQEILDALEKKHGALLQCRAVNFWRGLSSNMLSRMMCDALHEADSGEGVIFLTDIAGA

PPYRVASLLSHKHSRCEVISGVTLPLIEQMMACRETMTSSEFRECIVELGGPEVSSLWHQQQKNPPFVLK

HNLYEY

>gi|30061693|ref|NP_835864.1| hypothetical protein S0127 [Shigella flexneri 2a str. 2457T]

MMHLYWVALKSIWAKEIHRFMRIWVQTLVPPVITMTLYFIIFGNLIGSRIGDMHGFSYMQFIVPGLIMMS

VITNAYANVASSFFGAKFQRNIEELLVAPVPTHVIIAGYVGGGVARGLFVGILVTAISLFFVPFQVHSWV

FVALTLVLTAVLFSLAGLLNGVFAKTFDDISLVPTFVLTPLTYLGGVFYSLTLLPPFWQGLSHLNPIVYM

ISGFRYGFLGINDVPLVTTFGVLVVFIVAFYLICWSLIQRGRGLRS

>gi|30061692|ref|NP_835863.1| ATP-binding component of a transport system [Shigella flexneri 2a str. 2457T]

MTIALELQQLKKNYPGGVQALRGIDLQVEAGDFYALLGPNGAGKSTTIGIISSLVNKTSGRVSVFGYDLE

KDVVNAKRQLGLVPQEFNFNPFETVQQIVVNQAGYYGVERKEAYIRSEKYLKQLDLWGKRNERARMLSGG

MKRRLMIARALMHEPKLLILDEPTAGVDIELRRSMWGFLKDLNDKGTTIILTTHYLEEAEMLCRNIGIIQ

HGELVENTSMKALLAKLKSETFILDLAPKSPLPKLDGYQYRLVDTATLEVEVLREQGINSVFTQLSEQGI

QVLSMRNKANRLEELFVSLVNEKQGDRA

>gi|30061691|ref|NP_835862.1| carbonic anhydrase [Shigella flexneri 2a str. 2457T]

MKDIDTLISNNALWSKMLVEEDPGFFEKLAQAQKPRFLWIGCSDSRVPAERLTGLEPGELFVHRNVANLV

IHTDLNCLSVVQYAVDVLEVEHIIICGHYGCGGVQAAVENPELGLINNWLLHIRDIWFKHSSLLGEMPQE

RRLDTLCELNVMEQVYNLGHSTIMQSAWKRGQKVTIHGWAYGIHDGLLRDLDVTATNRETLEQRYRHGIS

NLKLKHANHK

>gi|30061689|ref|NP_835860.1| glucose dehydrogenase [Shigella flexneri 2a str. 2457T]

MAINNTASRRLLVTLTALFAALCGLYLLIGGGWLVAIGGSWYYPIAGLVMLGVAWMLWRSKRAALWLYAA

LLLGTMIWGVWEVGFDFWALTPRSDILVFFGIWLILPFVWRRLVIPASGAVAALVVALLISGGILTWAGF

NDPQEINGTLSADATPAEAISPVADQDWPAYGRNQEGQRFSPLKQIHADNVHKLKEAWVFRTGDVKQPND

PGEITNEVTPIKVGDTLYLCTAHQRLFALDAASGKEKWHYDPELKTNESFQHVTCRGVSYHEAKAETASP

EVMADCPRRIILPVNDGRLIAINAENGKLCETFANKGVLNLQSNMPDTKPGLYEPTSPPIITDKTIVMAG

SVTDNFSTRETSGVIRGFDVNTGELLWAFDPGAKDPNAIPSDEHTFTFNSPNSWAPAAYDAKLDLVYLPM

GVTTPDIWGGNRTPEQERYASSILALNATTGKLAWSYQTVHHDLWDMDLPAQPTLADITVNGQKVPIIYA

PAKTGNIFVLDRRNGELVVPAPEKPVPQGAAKGDYVTPTQPFSELSFRPTKDLSGADMWGATMFDQLVCR

VMFHQMRYEGIFTPPSEQGTLVFPGNLGMFEWGGISVDPNREVAIANPMALPFVSKLLPRGPGNPMEQPK

DAKGTGTESGIQPQYGVPYGVTLNPFLSPFGLPCKQPAWGYISALDLKTNEVVWKKRIGTPQDSMPFPMP

VPVPFNMGMPMLGGPISTAGNVLFIAATADNYLRAYNMSNGEKLWQGRLPAGGQATPMTYEVNGKQYVVI

SAGGHGSFGTKMGDYIVAYALPDDVK

>gi|30061688|ref|NP_835859.1| multicopper oxidase [Shigella flexneri 2a str. 2457T]

MQRRDFLKYSVALGVASALPLWSRAVFAAERPTLPIPDLLTTDARNRIQLTIGAGQSTFGEKTATTWGYN

GNLLGPAVKLQRGKAVTVDIYNQLAEETTLHWHGLEVPGDVDGGPQGIIPPGGKRSVTLNVDQPAATCWF

HPHQHGKTGRQVAMGLAGLVVIEDDEILKLMLPKQWGIDDVPVIVQDKKFSADGQIDYQLDVMTAAVGWF

GDTLLTNGAIYPQHAAPRGWLRLRLLNGCNARSLNFATSDNRPLYVIASDGGLLPEPVKVSELPVLMGER

FEVLVEVNDNKPFDLVTLPVSQMGMAIAPFDKPHPVMRIQPIAISASGALPDTLSSLPALPSLEGLTVRK

LQLSMDPMLDMMGMQMLMEKYGDQAMAGMDHSQMMGHMGHGNMNHMNHGGKFDFHHANKINGQAFD
MNKP

MFAAAKGQYERWVISGVGDMMLHPFHIHGTQFRILSENGKPPAAHRAGWKDTVKVEGNVSEVLVKFNHNA

PKEHAYMAHCHLLEHEDTGMMLGFTV

>gi|30061687|ref|NP_835858.1| hypothetical protein S0121 [Shigella flexneri 2a str. 2457T]

MSIVLPLTGRSSRRHNLIDNNGRRLARSVLTFIFFKPLVEAMKTFFRTVLFGSLIAVCANSYALSESEAE

DMADLTAVFVFLKNDCGYQNLSNGQIRRALVFFAQQNQWDLSNYDTFDMKALGEDSYRDLSGIGIPVAKK

CKALARDSLSLLAYVK

>gi|30061686|ref|NP_835857.1| spermidine synthase [Shigella flexneri 2a str. 2457T]

MAEKKQWHETLHDQFGQYFAVDNVLYHEKTDHQDLIIFENAAFGRVMALDGVVQTTERDEFIYHEMMTHV

PLLAHGHAKHVLIIGGGDGAMLREVTRHKNVESITMVEIDAGVVSFCRQYLPNHNAGSYDDPRFQLVIDD

GVNFVNQTSQTFDVIISDCTDPIGPGESLFTSAFYEGCKRCLNPGGIFVAQNGVCFLQQEEAIDSHRKLS

HYFSDVGFYQAAIPTYYGGIMTFAWATDNDALRHLSTEIIQARFLASGLKCRYYNPAIHTAAFALPQYLQ

DALASQPS

>gi|30061685|ref|NP_835856.1| S-adenosylmethionine decarboxylase [Shigella flexneri 2a str. 2457T]

MKKLKLHGFNNLTKSLSFCIYDICYAKTAEERDGYIAYIDELYNANRLTEILSETCSIIGANILNIARQD

YEPQGASVTILVSEEPVDPKLIDKTEHPGPLPETVVAHLDKSHICVHTYPESHPEGGLCTFRADIEVSTC

GVISPLKALNYLIHQLESDIVTIDYRVRGFTRDINGMKHFIDHEINSIQNFMSDDMKALYDMVDVNVYQE

NIFHTKMLLKEFDLKHYMFHTKPEDLTDSERQEITAALWKEMREIYYGRNMPAV

>gi|30061683|ref|NP_835854.1| bifunctional aconitate hydratase 2/2-methylisocitrate dehydratase [Shigella flexneri 2a str. 2457T]

MLEEYRKHVAERAAEGIAPKPLDANQMAALVELLKNPPAGEEEFLLDLLTNRVPPGVDEAAYVKAGFLAA

IAKGEAKSPLLTPEKAIELLGTMQGGYNIHPLIDALDDAKLAPIAAKALSHTLLMFDNFYDVEEKAKAGN

EYAKQVMQSWADAEWFLNRPALAEKLTVTVFKVTGETNTDDLSPAPDAWSRPDIPLHALAMLKNAREGIE

PDQPGVVGPIKQIEALQQKGFPLAYVGDVVGTGSSRKSATNSVLWFMGDDIPHVPNKRGGGLCLGGKIAP

IFFNTMEDAGALPIEVNVSNLNMGDVIDVYPYKGEVRNHETGELLATFELKTDVLIDEVRAGGRIPLIIG

RGLTTKAREALGLPHSDVFRQAKDVAESDRGFSLAQKMVGRACGVKGIRPGAYCEPKMTSVGSQDTTGPM

TRDELKDLACLGFSADLVMQSFCHTAAYPKPVDVNTHHTLPDFIMNRGGVSLRPGDGVIHSWLNRMLLPD

TVGTGGDSHTRFPIGISFPAGSGLVAFAAATGVMPLDMPESVLVRFKGKMQPGITLRDLVHAIPLYAIKQ

GLLTVEKKGKKNIFSGRILEIEGLPDLKVEQAFELTDASAERSAAGCTIKLNKEPIIEYLSSNIVLLKWM

IAEGYGDRRTLERRIQGMEKWLANPELLEADADAEYAAVIDIDLAEIKEPILCAPNDPDDARPLSAVQGE

KIDEVFIGSCMTNIGHFRAAGKLLDAHKGQLPTRLWVAPPTRMDAAQLTEEGYYSVFGKSGARIEIPGCS

LCMGNQARVADGATVVSTSTRNFPNRLGTGANVFLASAELAAVAALIGKLPTPEEYQTYVAQVDKTAVDT

YRYLNFNQLSQYTEKADGVIFQTAV

>gi|30061682|ref|NP_835853.1| dihydrolipoamide dehydrogenase [Shigella flexneri 2a str. 2457T]

MSTEIKTQVVVLGAGPAGYSAAFRCADLGLETVIVERYNTLGGVCLNVGCIPSKALLHVAKVIEEAKALA

EHGIVFGEPKTDIDKIRTWKEKVINQLTGGLAGMAKGRKVKVVNGLGKFTGANTLEVEGENGKTVINFDN

AIIAAGSRPIQLPFIPHEDPRIWDSTDALELKEVPERLLVMGGGIIGLEMGTVYHALGSQIDVVEMFDQV

IPAADKDIVKVFTKRISKKFNLMLETKVTAVEAKEDGIYVTMEGKKAPAEPQRYDAVLVAIGRVPNGKNL

DAGKAGVEVDDRGFIRVDKQLRTNVPHIFAIGDIVGQPMLAHKGVHEGHVAAEVIAGKKHYFDPKVIPSI

AYTEPEVAWVGLTEKEAKEKGISYETATFPWAASGRAIASDCADGMTKLIFDKESHRVIGGAIVGTNGGE

LLGEIGLAIEMGCDAEDIALTIHAHPTLHESVGLAAEVFEGSITDLPNPKAKKK

>gi|30061681|ref|NP_835852.1| dihydrolipoamide acetyltransferase [Shigella flexneri 2a str. 2457T]

MAIEIKVPDIGADEVEITEILVKVGDKVEAEQSLITVEGDKASMEVPSPQAGIVKEIKVSVGDKTQTGAL

IMIFDSADGAADAAPAQAEEKKEAAPAAAPAAAAAKDVNVPDIGSDEVEVTEILVKVGDKVEAEQSLITV

EGDKASMEVPAPFAGTVKEIKVNVGDKVSTGSLIMVFEVAGEAGAAAPAAKQEATPAATPAPAAGVKEVN

VPDIGGDEVEVTEVMVKVGDKVAAEQSLITVEGDKASMEVPAPFAGVVKELKVNVGDKVKTGSLIMIFEV

EGAAPAAAPAKQEAAAPAPAAKAEAPAAKAEGKSEFAENDAYVHATPLIRRLAREFGVNLAKVKGTGRKG

RILREDVQAYVKEAIKRAEAAPAATGGGIPGMLPWPKVDFSKFGEIEEVELGRIQKISGANLSRNWVMIP

HVTHFDKTDITELEAFRKQQNEEAAKRKLDVKITPVVFIMKAVAAALEQMPRFNSSLSEDGQRLTLKKYI

NIGVAVDTPNGLVVPVFKDVNKKGIIELSRELMTISKKARDGKLTAGEMQGGCFTISSIGGLGTTHFAPI

VNAPEVAILGVSKSAMEPVWNGKEFVPRLMLPISLSFDHRVIDGADGARFITIINNTLSDIRRLVM

>gi|30061680|ref|NP_835851.1| pyruvate dehydrogenase subunit E1 [Shigella flexneri 2a str. 2457T]

MSERFPNDVDPIETRDWLQAIESVIREEGVERAQYLIDQLLAEARKGGVNVAAGTGISNYINTIPVEEQP

EYPGNLELERRIRSAIRWNAIMTVLRASKKDLELGGHMASFQSSATIYDVCFNHFFRARNEQDGGDLVYF

QGHISPGVYARAFLEGRLTQEQLDNFRQEVHGNGLSSYPHPKLMPEFWQFPTVSMGLGPIGAIYQAKFLK

YLEHRGLKDTSKQTVYAFLGDGEMDEPESKGAITIATREKLDNLVFVINCNLQRLDGPVTGNGKIINELE

GIFEGAGWNVIKVMWGSRWDELLRKDTSGKLIQLMNETVDGDYQTFKSKDGAYVREHFFGKYPETAALVA

DWTDEQIWALNRGGHDPKKIYAAFKKAQETKGKATVILAHTIKGYGMGDAAEGKNIAHQVKKMNMDGVRH

IRDRFNVPVSDADIEKLPYITFPEGSEEHTYLHAQRQKLHGYLPSRQPNFTEKLELPSLQDFGALLEEQS

KELSTTIAFVRALNVMLKNKSIKDRLVPIIADEARTFGMEGLFRQIGIYSPNGQQYTPQDREQVAYYKED

EKGQILQEGINELGAGCSWLAAATSYSTNNLPMIPFYIYYSMFGFQRIGDLCWAAGDQQARGFLIGGTSG

RTTLNGEGLQHEDGHSHIQSLTIPNCISYDPAYAYEVAVIMHDGLERMYGEKQENVYYYITTLNENYHMP

AMPEGAEEGIRKGIYKLETIEGSKGKVQLLGSGSILRHVREAAEILAKDYGVGSDVYSVTSFTELARDGQ

DCERWNMLHPLETPRVPYIAQVMNDAPAVASTDYMKLFAEQVRTYVPADDYRVLGTDGFGRSDSRENLRH

HFEVDASYVVVAALGELAKRGEIDKKVVADAIAKFNIDADKVNPRLA

>gi|30061679|ref|NP_835850.1| transcriptional regulator PdhR [Shigella flexneri 2a str. 2457T]

MAYSKIRQPKLSDVIEQQLEFLILEGTLRPGEKLPPERELAKQFDVSRPSLREAIQRLEAKGLLLRRQGG

GTFVQSSLWQSFSDPLVELLSDHPESQYDLLETRHALEGIAAYYAALRSTDEDKERIRELHHAIELAQQS

GDLDAESNAVLQYQIAVTEAAHNVVLLHLLRCMEPMLAQNVRQNFELLYSRREMLPLVSSHRTRIFEAIM

VGKPEEAREASHRHLAFIEEILLDRSREESRRERSLRRLEQRKN

>gi|30061678|ref|NP_835849.1| aromatic amino acid transporter [Shigella flexneri 2a str. 2457T]

MMEGQQHGEQLKRGLKNRHIQLIALGGAIGTGLFLGSASVIQSAGPGIILGYAIAGFIAFLIMRQLGEMV

VEEPVAGSFSHFAYKYWGSFAGFASGWNYWVLYVLVAMAELTAVGKYIQFWYPEIPTWVSAAVFFVVINA

INLTNVKVFGEMEFWFAIIKVIAVVAMIIFGGWLLFSGNGGPQASVSNLWDQGGFLPHGFTGLVMMMAII

MFSFGGLELVGITAAEADNPEQSIPKATNQVIYRILIFYIGSLAVLLSLMPWTRVTADTSPFVLIFHELG

DTFVANALNIVVLTAALSVYNSCVYCNSRMLFGLAQQGNAPKALASVDKRGVPVNTILVSALVTALCVLI

NYLAPESAFGLLMALVVSALVINWAMISLAHMKFRRAKQEQGVVTRFPALLYPLGNWICLLFMAAVLVIM

LMTPGMAISVYLIPVWLIVLGIGYLFKEKTAKAVKAH

>gi|30061677|ref|NP_835848.1| regulatory protein AmpE [Shigella flexneri 2a str. 2457T]

MTLFTTLLVLIFERLFKLGEHWQLDHRLEAFFRRVKHFSLGRTLGMTIIAMGVTFLLLRALQGVLFNVPT

LLVWLLIGLLCIGAGKVRLHYHAYLTAASRNDSHARATMAGELTMIHGVPAGCDEREYLRELQNALLWIN

FRFYLAPLFWLIVGGTWGPVTLMGYAFLRAWQYWLARYQTPHHRLQSGIDAVLHVLDWVPVRLAGVVYAL

IGHGEKALPAWFASLGDFHTSQYQVLTRLAQFSLAREPHVDKVETPKAAVSMAKKTSFVVVVVIALLTIY

GALV

>gi|30061676|ref|NP_835847.1| N-acetyl-anhydromuranmyl-L-alanine amidase [Shigella flexneri 2a str. 2457T]

MLLEQGWLVGARRVPSPHYDCRLDDETPTLLVVHNISLPPGEFGGPWIDALFTGTIDPQAHPFFAEIAHL

RVSAHCLIRRDGEIVQYVPFDKRAWHAGVSQYQGRERCNDFSIGIELEGTDTLAYTDAQYQQLAAVTRAL

IDRYPDIANNMTGHCDIAPDRKTDPGPAFDWARFRALVSKETT

>gi|30061675|ref|NP_835846.1| quinolinate phosphoribosyltransferase [Shigella flexneri 2a str. 2457T]

MPPRRYNPDTRRDELLERINLDIPGAVAQALREDLGGTVDANNDITAKLLPENSRSHATVITRENGVFCG

KRWVEEVFIQLAGDDVTIIWHVDDGDVINANQPLFELEGPSRVLLTGERTALNFVQTLSGVASKVRHYVE

LLEGTNTQLLDTRKTLPGLRSALKYAVLCGGGANHRLGLSDAFLIKENHIIASGSVRQAVEKASWLHPDA

PVEVEVENLEELDEALKAGADIIMLDNFETEQMREAVKRTNGKALLEVSGNVTDKTLREFAETGVDFISV

GALTKHVQALDLSMRFR

>gi|30061674|ref|NP_835845.1| major pilin subunit [Shigella flexneri 2a str. 2457T]

MDKQRGFTLIELMVVIGIIAILSSIGIPAYQNYLRKAALTDMLQTFVPYRTAVELCALEHGGLDTCDGGS

NGIPSPTTTRYVSAMSVAKGVVSLTGQESLNGLSVVMTPGWDNANGVTGWARNCNIQSDSALQQACEDVF

RFDDAN

>gi|30061673|ref|NP_835844.1| type IV pilin biogenesis protein [Shigella flexneri 2a str. 2457T]

MASKQLWRWHGITGDGNAQDGMLWAESRTLLLMALQQQMVTPLSLKRIAINSAQWRGDKSAEVIHQLATL

LKAGLTLSEGLALLAEQHPSKQWQALLQSLAHDLEQGIAFSNALLPWSEAFPPLYQAMIRTGELTGKLDE

CCFELARQQKSQRQLTDKVKSALRYPIIILAMAIMVVVAMLHFVLPEFAAIYKTFNTPLPALTQGIMTLA

DFSGEWGWLLVLFGFLLAIANKLLMRRPTWLIARQKLLLRIPIMGSLMRGQKLTQIFTILALTQSAGITF

LQGVESVRETMRCPYWVQLLTQIQHDISNGHPIWLALKNAGEFSPLCLQLVRTGEASGSLDLMLDNLAHH

HRDNTMALADNLAALLEPALLIITGGIIGTLVVAMYLPIFHLGDAMSGMG

>gi|30061672|ref|NP_835843.1| hypothetical protein S0104 [Shigella flexneri 2a str. 2457T]

MLLHASVGRLQVVEDAVFLFQHTNKRGALGQLFQA

>gi|30061671|ref|NP_835842.1| guanosine 5'-monophosphate oxidoreductase [Shigella flexneri 2a str. 2457T]

MRIEEDLKLGFKDVLIRPKRSTLKSRSDVELERQFTFKHSGQSWSGVPIIAANMDTVGTFSMASALASFD

ILTAVHKHFSVEEWQAFINNSSADVLKHVMVSTGTSDADFEKTKQILDLNPALNFVCIDVANGYSEHFVQ

FVAKAREAWPTKTICAGNVVTGEMCEELILSGADIVKVGIGPGSVCTTRVKTGVGYPQLSAVIECADAAH

GLGGMIVSDGGCTTPGDVAKAFGGGADFVMLGGMLAGHEESGGRIVEENGEKFMLFYGMSSESAMKRHVG

GVAEYRAAEGKTVKLPLRGQVENTARDILGGLRSACTYVGASRLKELTKRTTFIRVLEQENRIFNNL

>gi|30061670|ref|NP_835841.1| dephospho-CoA kinase [Shigella flexneri 2a str. 2457T]

MGYIVALTGGIGSGKSTVANAFADLGINVIDADIIARQVVEPGAPALHAIADHFGENMIAADGTLQRRAL

RERIFANPEEKNWLNDLLHPLIQQETQHQIQQATSPYVLWVVPLLVENSLYKKANRVLVVDVSPETQLKR

TMQRDDVTREHVEQILAAQATREARLAVADDVIDNNGAPDAIASDVARLHAHYLQLASQFVSQEKP

>gi|30061669|ref|NP_835840.1| hypothetical protein S0101 [Shigella flexneri 2a str. 2457T]

MQTQVLFEHPLNEKMRTWLRIEFLIQQLTVNLPIVDHAGALHFFRNVSELLDVFERAEVRTELLKELNRQ

QRKLQTWIGVPGVDQSRIEALIQQLKAAVSVLISAPRIGQFLREDRLIALVRQRLSIPGGCCSFDLPTLH

IWLHLPQAQRDSQVETWIASLNPLTQALTMVLDLIRQSAPFRKQTSLNGFYQDNGGDADLLRLNLSLDSQ

LYPQISGHKSRFAIRFMPLDTENGQVPERLDFELACC

>gi|30061668|ref|NP_835839.1| zinc-binding protein [Shigella flexneri 2a str. 2457T]

MSETITVNCPTCGKTVVWGEISPFRPFCSKRCQLIDLGEWAAEEKRIPSSGDLSESDDWSEEPKQ

>gi|30061667|ref|NP_835838.1| hypothetical protein S0099 [Shigella flexneri 2a str. 2457T]

MAIFPKAMTGAKSQSSDICLMPHVGLIRRGQRRIRHLVQMSDAA

>gi|30061666|ref|NP_835837.1| nucleoside triphosphate pyrophosphohydrolase [Shigella flexneri 2a str. 2457T]

MKKLQIAVGIIRNENNEIFITRRAADAHMANKLEFPGGKIEMGETPEQAVVRELQEEVGITPQHFSLFEK

LEYEFPDRHITLWFWLVESWEGVPWGKEGQPGEWMSLVGLNADDFPPANEPVIAKLKRL

>gi|30061665|ref|NP_835836.1| preprotein translocase subunit SecA [Shigella flexneri 2a str. 2457T]

MLIKLLTKVFGSRNDRTLRRMRKVVNIINAMEPEMEKLSDEELKGKTAEFRARLEKGEVLENLIPEAFAV

VREASKRVFGMRHFDVQLLGGMVLNERCIAEMRTGEGKTLTATLPAYLNALTGKGVHVVTVNDYLAQRDA

ENNRPLFEFLGLTVGINLPGMPAPAKREAYAADITYGTNNEYGFDYLRDNMAFSPEERVQRKLHYALVDE

VDSILIDEARTPLIISGPAEDSSEMYKRVNKIIPHLIRQEKEDSETFQGEGHFSVDEKSRQVNLTERGLV

LIEELLVKEGIMDEGESLYSPANIMLMHHVTAALRAHALFTRDVDYIVKDGEVIIVDEHTGRTMQGRRWS

DGLHQAVEAKEGVQIQNENQTLASITFQNYFRLYEKLAGMTGTADTEAFEFSSIYKLDTVVVPTNRPMIR

KDLPDLVYMTEAEKIQAIIEDIKERTAKGQPVLVGTISIEKSELVSNELTKAGIKHNVLNAKFHANEAAI

VAQAGYPAAVTIATNMAGRGTDIVLGGSWQAEVAALENPTAEQIEKIKADWQVRHDAVLEAGGLHIIGTE

RHESRRIDNQLRGRSGRQGDAGSSRFYLSMEDALMRIFASDRVSGMMRKLGMKPGEAIEHPWVTKAIANA

QRKVESRNFDIRKQLLEYDDVANDQRRAIYSQRNELLDVSDVSETINSIREDVFKATIDAYIPPQSLEEM

WDIPGLQERLKNDFDLDLPITEWLDKEPELHEETLRERILAQSIEVYQRKEEVVGAEMMRHFEKGVMLQT

LDSLWKEHLAAMDYLRQGIHLRGYAQKDPKQEYKRESFSMFAAMLESLKYEVISTLSKVQVRMPEEVEEL

EQQRRMEAERLAQMQQLSHQDDDSAAAAALAAQTGERKVGRNDPCPCGSGKKYKQCHGRLQ

>gi|30061663|ref|NP_835834.1| UDP-3-O-[3-hydroxymyristoyl] N-acetylglucosamine deacetylase [Shigella flexneri 2a str. 2457T]

MIKQRTLKRIVQATGVGLHTGKKVTLTLRPAPANTGVIYRRTDLNPPVDFPADAKSVRDTMLCTCLVNEH

DVRISTVEHLNAALAGLGIDNIVIEVNAPEIPIMDGSAAPFVYLLLDAGIDELNCAKKFVRIKETVRVED

GDKWAEFKPYNGFSLDFTIDFNHPAIDSSNQRYAMNFSADAFMRQISRARTFGFMRDIEYLQSRGLCLGG

SFDCAIVVDDYRVLNEDGLRFEDEFVRHKMLDAIGDLFMCGHNIIGAFTAYKSGHALNNKLLQAVLAKQE

AWEYVTFQDDAELPLAFKAPSAVLA

>gi|30061662|ref|NP_835833.1| cell division protein FtsZ [Shigella flexneri 2a str. 2457T]

MFEPMELTNDAVIKVIGVGGGGGNAVEHMVRERIEGVEFFAVNTDAQALRKTAVGQTIQIGSGITKGLGA

GANPEVGRNAADEDRDALRAALEGADMVFIAAGMGGGTGTGAAPVVAEVAKDLGILTVAVVTKPFNFEGK

KRMAFAEQGITELSKHVDSLITIPNDKLLKVLGRGISLLDAFGAANDVLKGAVQGIAELITRPGLMNVDF

ADVRTVMSEMGYAMMGSGVASGEDRAEEAAEMAISSPLLEDIDLSGARGVLVNITAGFDLRLDEFETVGN

TIRAFASDNATVVIGTSLDPDMNDELRVTVVATGIGMDKRPEITLVTNKQVQQPVMDRYQQHGMAPLTQE

QKPVAKVVNDNAPQTAKEPDYLDIPAFLRKQAD

>gi|30061661|ref|NP_835832.1| cell division protein FtsA [Shigella flexneri 2a str. 2457T]

MIKATDRKLVVGLEIGTAKVAALVGEVLPDGMVNIIGVGSCPSRGMDKGGVNDLESVVKCVQRAIDQAEL

MADCQISSVYLALSGKHISCQNEIGMVPISEEEVTQEDVENVVHTAKSVRVRDEHRVLHVIPQEYAIDYQ

EGIKNPVGLSGVRMQAKVHLITCHNDMAKNIVKAVERCGLKVDQLIFAGLASSYSVLTEDERELGVCVVD

IGGGTMDIAVYTGGALRHTKVIPYAGNVVTSDIAYAFGTPPSDAEAIKVRHGCALGSIVGKDESVEVPSV

GGRPPRSLQRQTLAEVIEPRYTELLNLVNEEILQLQEKLRQQGVKHHLAAGIVLTGGAAQIEGLAACAQR

VFHTQVRIGAPLNITGLTDYAQEPYYSTAVGLLHYGKESHLNGEAEVEKRVTASVGSWIKRLNSWLRKEF

>gi|30061660|ref|NP_835831.1| cell division protein FtsQ [Shigella flexneri 2a str. 2457T]

MSQAALNTRNSEEEVSSRRNNGTRLAGILFLLTVLTTVLVSGWVVLSWMEDAQRLPLSKLVLTGERHYTR

NDDIRQSILALGEPGTFMTQDVNIIQTQIEQRLPWIKQVSVRKQWPDELKIHLVEYVPIARWNDQHMVDA

EGNTFSVPPDRTSKQVLPMLYGPEGSANEVLQGYREMGQMLAKDRFTLKEAAMTARRSWQLTLNNDIKLN

LGRGDTMKRLVRFVELYPVLQQQAQTDGKRISYVDLRYDSGAAVGWAPLPPEESTQQQNQAQAEQQ

>gi|30061659|ref|NP_835830.1| D-alanine--D-alanine ligase [Shigella flexneri 2a str. 2457T]

MTDKIAVLLGGTSAEREVSLNSGAAVLAGLREGGIDAYPVDPKEVDVTQLKSMGFQKVFIALHGPGGEDG

TLQGMLELMGLPYTGSGVMASALSIDKLRSKLLWQGAGLPVAPWVALTRAEFEKGLSDKQLAEISALGLP

VIVKPSREGSSVGMSKVVAENALQDALRLAFQHDEEVLIEKWLSGPEFTVAILGEEILPSIRIQPSGTFY

DYEAKYLSDETQYFCPAGLEASQEANLQALVLKAWTTLGCKGWGRIDVMLDSDGQFYLLEANTSPGMTSH

SLVPMAARQAGMSFSQLVVRILELAD

>gi|30061658|ref|NP_835829.1| UDP-N-acetylmuramate--L-alanine ligase [Shigella flexneri 2a str. 2457T]

MNTQQLAKLRSIVPEMRRVRHIHFVGIGGAGMGGIAEVLANEGYQISGSDLAPNPVTQQLLNLGATIYFN

HRPENVRDASVVVVSSAISADNPEIVAAHEARIPVIRRAEMLAELMRFRHGIAIAGTHGKTTTTAMVSSI

YAEAGLDPTFVNGGLVKAAGVHARLGHGRYLIAEADESDASFLHLQPMVAIVTNIEADHMDTYQGDFENL

KQTFINFLHNLPFYGRAVMCVDDPVIRELLPRVGRQTTTYGFSEDADVRVEDYQQIGPQGHFTLLRQDKE

PMCVTLNAPGRHNALNAAAAVAVATEEGIDDEAILRALESFQGTGRRFDFLGEFPLEPVNGKSGTAMLVD

DYGHHPTEVDATIKAARAGWPDKNLVMLFQPHRFTRTRDLYDDFANVLTQVDTLLMLEVYPAGEAPIPGA

DSRSLCRTIRGRGKIDPILVPDPAQVAEMLAPVLTGNDLILVQGAGNIGKIARSLAEIKLKPQTPEEEQH

D

>gi|30061657|ref|NP_835828.1| undecaprenyldiphospho-muramoylpentapeptide beta-N-acetylglucosaminyltransferase [Shigella flexneri 2a str. 2457T]

MSGQGKRLMVMAGGTGGHVFPGLAVAHYLMAQGWQVRWLGTADRMEADLVPKHGIEIDFIRISGLRGKGI

KALIAAPLRIFNAWRQARAIMKAYKPDVVLGMGGYVSGPGGLAAWSLGIPVVLHEQNGIAGLTNKWLAKI

ATKVMQAFPGAFPNAEVVGNPVRTDVLALPLPQQRLAGREGPVRVLVVGGSQGARILNQTMPQVAAKLGD

SVTIWHQSGKGSQQSVEQAYAEAGQPQHKVTEFIDDMAAAYAWADVVVCRSGALTVSEIAAAGLPALFVP

FQHKDRQQYWNALPLEKAGAAKIIEQPQLSVDAVANTLAGWSRETLLTMAERARAASIPDATERVANEVS

RAARA

>gi|30061656|ref|NP_835827.1| cell division protein FtsW [Shigella flexneri 2a str. 2457T]

MRLSLPRLKMPRLPGFSILVWISTALKGWVMGSREKDTDSLIMYDRTLLWLTFGLAAIGFIMVTSASMPI

GQRLTNDPFFFAKRDGVYLILAFILAIITLRLPMEFWQRYSATMLLGSIILLMIVLVVGSAVKGASRWID

LGLLRIQPAELTKLSLFCYIANYLVRKGDEVRNNLRGFLKPMGVILVLAVLLLAQPDLGTVVVLFVTTLA

MLFLAGAKLWQFIAIIGMGISAVVLLILAEPYRIRRVTAFWNPWEDPFGSGYQLTQSLMAFGRGELWGQG

LGNSVQKLEYLPEAHTDFIFAIIGEELGYVGVVLALLMVFFVAFRAMSIGRKALEIDHRFSGFLACSIGI

WFSFQALVNVGAAAGMLPTKGLTLPLISYGGSSLLIMSTAIMMLLRIDYETRLEKAQAFVRGSR

>gi|30061655|ref|NP_835826.1| UDP-N-acetylmuramoyl-L-alanyl-D-glutamate synthetase [Shigella flexneri 2a str. 2457T]

MADYQGKNVVIIGLGLTGLSCVDFFLARGVTPRVMDTRMTPPGLDKLPEAVERHTGSLNDEWLMAADLIV

ASPGIALAHPSLSAAADAGIEIVGDIELFCREAQAPIVAITGSNGKSTVTTLVGEMAKAAGVNVGVGGNI

GLPALMLLDDECELYVLELSSFQLETTSSLQAVAATILNVTEDHMDRYPFGLQQYRAAKLRIYENAKVCV

VNADDALTMPIRGADERCVSFGVNMGDCHLNHQQGETWLRVKGEKVLNVKEMKLSGQHNYTNALVALALA

DAAGLPRASSLKALTTFTGLPHRFEVVLEHNGVRWINDSKATNVGSTEAALNGLHVDGTLHLLLGGDGKS

ADFSPLVRYLNGDNVRLYCFGRDGAQLAALRPEVAEQTETMEQAMRLLAPRVQPGDMVLLSPACASLDQF

KNFEQRGNEFARLAKELG

>gi|30061654|ref|NP_835825.1| phospho-N-acetylmuramoyl-pentapeptide-transferase [Shigella flexneri 2a str. 2457T]

MLVWLAEHLVKYYSGFNVFSYLTFRAIVSLLTALFISLWMGPRMIAHLQKLSFGQVVRNDGPESHFSKRG

TPTMGGIMILTAIVISVLLWAYPSNPYVWCVLVVLVGYGVIGFVDDYRKVVRKDTKGLIARWKYFWMSVI

ALGVAFALYLAGKDTPATQLVVPFFKDVMPQLGLFYILLAYFVIVGTGNAVNLTDGLDGLAIMPTVFVAG

GFALVAWATGNMNFASYLHIPYLRHAGELVIVCTAIVGAGLGFLWFNTYPAQVFMGDVGSLALGGALGII

AVLLRQEFLLVIMGGVFVVETLSVILQVGSFKLRGQRIFRMAPIHHHYELKGWPEPRVIVRFWIISLMLV

LIGLATLKVR

>gi|30061653|ref|NP_835824.1| UDP-N-acetylmuramoyl-tripeptide--D-alanyl-D-alanine ligase [Shigella flexneri 2a str. 2457T]

MISVTLSQLTDILNGELQGADITLDAVTTDTRKLTPGCLFVALKGERFDAHDFADQAKAGGAGALLVSRP

LDIDLPQLIVKDTRLAFGELAAWVRQQVPALVVALTGSSGKTSVKEMTAAILSQCGNTLYTAGNLNNDIG

VPMTLLRLTPEYDYAVIELGANHQGEIAWTVSLTRPEAALVNNLAAAHLEGFGSLAGVAKAKGEIFSGLP

ENGIAIMNADNNDWLNWQSVIGSRKVWRFSPNAANSDFTATNIHVTSHGMEFTLQTPTGSVDVLLPLPGR

HNIANALAAAALSMSVGATLDAIKAGLANLKAVPGRLFPIQLAENQLLLDDSYNANVGSMTAAVQVLAEM

PGYRVLVVGDMAELGAESEACHVQVGEAAKAAGIDRVLSMGKQSHAISTASGVGEHFADKTALITRLKSL

IAEQQVITILVKGSRSAAMEEVVRALQENGTC

>gi|30061652|ref|NP_835823.1| UDP-N-acetylmuramoylalanyl-D-glutamate--2,6-diaminopimelate ligase [Shigella flexneri 2a str. 2457T]

MADRNLRDLLAPWVPDAPSRALREMTLDSRVAAAGDLFVAVVGHQADGRRYIPQAIAQGVAAIIAEAKDE

ATDGEIREMHGVPVIYLSQLNERLSALAGRFYHEPSDNLRLVGVTGTNGKTTTTQLLAQWSQLLGETSAV

MGTVGNGLLGKVIPTENTTGSAVDVQHELAGLVDQGATFCAMEVSSHGLVQHRVAALKFPASVFTNLSRD

HLDYHGDMEHYEAAKWLLYSEHHCGQAIINADDEVGRRWLAKLPDAVAVSMEDHINPNCHGRWLKATEVN

YHDSGATIRFSSSWGDGEIESHLMGAFNVSNLLLALATLLALGYPLADLLKTAARLQPVCGRMEVFTAPG

KPTVVVDYAHTPDALEKALQAARLHCAGKLWCVFGCGGDRDKGKRPLMGAIAEEFADVAVVTDDNPRTEE

PRAIINDILAGMLDAGHAKVMEGRAEAVTCAVMQAKENDVVLVAGKGHEDYQIVGNQRLDYSDRVTVARL

LGGIA

>gi|30061651|ref|NP_835822.1| penicillin-binding protein 3; peptidoglycan synthetase [Shigella flexneri 2a str. 2457T]

MKAAAKTQKPKRQEEHANFISWRFALLCGCILLALAFLLGRVAWLQVISPDMLVKEGDMRSLRVQQVSTS

RGMITDRSGRPLAVSVPVKAIWADPKEVHDAGGISVGDRWKALANALNIPLDQLSARINANPKGRFIYLA

RQVNSDMADYIKKLKLPGIHLREESRRYYPSGEVTAHLIGFTNVDSQGIEGVEKSFDKWLTGQPGERIVR

KDRYGRVIEDISSTDSQAAHNLALSIDERLQALVYRELNNAVAFNKAESGSAVLVDVNTGEVLAMANSPS

YNPNNLSGTPKEAMRNRTITDVFEPGSTVKPMVVMTALQRGVVRENSVLNTIPYRINGHEIKDVARYSEL

TLTGVLQKSSNVGVSKLALTMPSSALVDTYSRFGLGKATNLGLVGERSGLYPQKQRWSDIERATFSFGYG

LMVTPLQLARVYATIGSYGIYRPLSITKVDPPVPGERVFPESIVRTVVHMMESVALPGGGGVKAAIKGYR

IAIKTGTAKKVGPDGRYINKYIAYTAGVAPASQPRFALVVVINDPQAGKYYGGAVSAPVFGAIMGGVLRT

MNIEPDALTTGDKNEFVINQGEGTGGRS

>gi|30061650|ref|NP_835821.1| cell division protein FtsL [Shigella flexneri 2a str. 2457T]

MISRVTEALSKVKGSMGSHERHALPGVIGDDLLRFGKLPLCLFICIILTAVTVVTTAHHTRLLTAQREQL

VLERDALDIEWRNLILEENALGDHSRVERIATEKLQMQHVDPSQENIVVQK

>gi|30061649|ref|NP_835820.1| S-adenosyl-methyltransferase MraW [Shigella flexneri 2a str. 2457T]

MMENYKHTTVLLDEAVNGLNIRPDGIYIDGTFGRGGHSRLILSQLGEEGRLLAIDRDPQAIAVAKTIDDP

RFSIIHGPFSALGEYVAERDLIGKIDGILLDLGVSSPQLDDAERGFSFMRDGPLDMRMDPTRGQSAAEWL

QTAEEADIAWVLKTYGEEHFAKRIARAIVERNREQPMTRTKELAEVVAAATPVKDKFKHPATRTFQAVRI

WVNSELEEIEQALKSSLNVLAPGGRLSIISFHSLEDRIVKRFMRENSRGPQVPAGLPMTEEQLKKLGGRQ

LRALGKLMPGEEEVAENPRARSSVLRIAERTNA

>gi|30061648|ref|NP_835819.1| cell division protein MraZ [Shigella flexneri 2a str. 2457T]

MFRGATLVNLDSKGRLSVPTRYREQLLENAAGQMVCTIDIHHPCLLLYPLPEWEIIEQKLSRLLSMNPVE

RRVQRLLLGHASECQMDGAGRLLIAPVLRQHAGLTKEVMLVGQFNKFELWDETTWHQQVKEDIDAEQLAT

GDLSERLQDLSL

>gi|30061647|ref|NP_835818.1| IS1 orfB [Shigella flexneri 2a str. 2457T]

MSCQCTHYGRWPQHGFTSLKKLRPQSVTSRIQPGSDVIVCAEMDEHWGYVGAKSRQRWLFYAYDRIRRTV

VAHVFGERTLATLERLLSLLSAFEVVVWMTDGWPLYESRLKGKLHVISKRYTQRIERHNLNLRQHLARLV

RKSLSFSKSVELHDKVIGHYLNIKHYQ

>gi|30061646|ref|NP_835817.1| IS1 orfA [Shigella flexneri 2a str. 2457T]

MASISIRCPSCSATEGVVRNGKSTAGHQRYLCSHCRKTWQLQFTYTASQPGTHQKIIDMAMNGVGCRASA

RIMGVGLNTVLRHLKNSGRSR

>gi|30061645|ref|NP_835816.1| DNA-binding transcriptional regulator FruR [Shigella flexneri 2a str. 2457T]

MKLDEIARLAGVSRTTASYVINGKAKQYRVSDKTVEKVMAVVREHNYHPNAVAAGLRAGRTRSIGLVIPD

LENTSYTRIANYLERQARQRGYQLLIACSEDQPDNEMRCIEHLLQRQVDAIIVSTSLPPEHPFYQRWAND

PFPIVALDRALDREHFTSVVGADQDDAEMLAEELRKFPAETVLYLGALPELSVSFLREQGFRTAWKDDPR

EVHFLYANSYEREAAAQLFEKWLETHPMPQALFTTSFALLQGVMDVTLRRDGKLPSDLAIATFGDNELLD

FLQCPVLAVAQRHRDVAERVLEIVLASLDEPRKPKPGLTRIKRNLYRRGVLSRS

>gi|30061643|ref|NP_835814.1| acetolactate synthase 3 regulatory subunit [Shigella flexneri 2a str. 2457T]

MRRILSVLLENESGALSRVIGLFSQRGYNIESLTVAPTDDPTLSRMTIQTVGDEKVLEQIEKQLHKLVDV

LRVSELGQGAHVEREIMLVKIQASGYGRDEVKRNTEIFRGQIIDVTPSLYTVQLAGTSDKLDAFLASIRD

VAKIVEVARSGVVGLSRGDKIMR

>gi|30061640|ref|NP_835811.1| leu operon leader peptide [Shigella flexneri 2a str. 2457T]

MTHIVRFIGLLLLNASSLRGRRVSGIQH

>gi|30061639|ref|NP_835810.1| 2-isopropylmalate synthase [Shigella flexneri 2a str. 2457T]

MSQQVIIFDTTLRDGEQALQASLSVKEKLQIALALERMGVDVMEVGFPVSSPGDFESVQTIARQVKNSRV

CALARCVEKDIDVAAESLKVAEAFRIHTFIATSPMHIATKLRSTLDEVIERAIYMVKRARNYTDDVEFSC

EDAGRTPIADLARVVEAAINAGATTINIPDTVGYTMPFEFAGIISGLYERVPNIDKAIISVHTHDDLGLA

VGNSLAAVHAGARQVEGAMNGIGERAGNCSLEEVIMAIKVRKDILNVHTAINHQEIWRTSQLVSQICNMP

IPANKAIVGSGAFAHSSGIHQDGVLKNRENYEIMTPESIGLNQIQLNLTSRSGRAAVKHRMDEMGYKESE

YNLDNLYDAFLKLADKKGQVFDYDLEALAFIGKQQEEPEHFRLDYFSVQSGSNDIATAAVKLACGEEVKA

EAANGNGPVDAVYQAINRITDYNVELVKYSLTAKGHGKDALGQVDIVTNYNGRRFHGVGLATDIVESSAK

AMVHVLNNIWRAAEVEKELQRKTQHNENNKETV

>gi|30061637|ref|NP_835808.1| isopropylmalate isomerase large subunit [Shigella flexneri 2a str. 2457T]

MAKTLYEKLFDAHVVYEAENETPLLYIDRHLVHEVTSPQAFDGLRAHGRPVRQPGKTFATMDHNVSTQTK

DINACGEMARIQMQELIKNCKEFGVELYDLNHPYQGIVHVMGPEQGVTLPGMTIVCGDSHTATHGAFGAL

AFGIGTSEVEHVLATQTLKQGRAKTMKIEVQGKAAPGITAKDIVLAIIGKTGSAGGTGHVVEFCGEAIRD

LSMEGRMTLCNMAIEMGAKAALVAPDETTFNYVKGRLHAPKGKDFDDAVAYWKTLQTDEGATFDTVVTLQ

AEEISPQVTWGTNPGQVISVNDNIPDPASFADPVERASAEKALAYMGLKPGIPLTEVAIDKVFIGSCTNS

RIEDLRAAAEIAKGRKVAPGVQALVVPGSGPVKAQAEAEGLDKIFIEAGFEWRLPGCSMCLAMNNDRLNP

GERCASTSNRNFEGRQGRGGRTHLVSPAMAAAAAVTGHFADIRNIK

>gi|30061636|ref|NP_835807.1| isopropylmalate isomerase small subunit [Shigella flexneri 2a str. 2457T]

MAEKFIKHTGLVVPLDAANVDTDAIIPKQFLQKVTRTGFGAHLFNDWRFLDEKGQQPNPDFVLNFPQYQG

ASILLARENFGCGSSREHAPWALTDYGFKVVIAPSFADIFYGNSFNNQLLPVKLSDAEVDELFALVKANP

GIHFDVDLEAQEVKAGEKTYRFTIDAFRRHCMMNGLDSIGLTLQHDDAIASYEAKQPAFMN

>gi|30061635|ref|NP_835806.1| transporter [Shigella flexneri 2a str. 2457T]

MIWIMTMARRMNGVYAAFMLVAFMMGVAGALQAPTLSLFLSREVGAQPFWIGLFYTVNAIAGIGVSLWLA

KRSDSQGDRRKLIIFCCLMAIGNALLFAFNRHYLTLITCGVLLASLANTAMPQLFALAREYADNSAREVV

MFSSVMRAQLSLAWVIGPPLAFMLALNYGFTVMFSIAAGIFTLSLVLIAFMLLSVARVELPSENALSMQG

DWQDSNVRMLFVASTLMWTCNTMYIIDMPLWISSDLGLPDKLAGFLMGTAAGLEIPAMILAGYYVKRYGK

RRMMVIAVVAGVLFYTGLIFFHSRMALMTLQLFNAVFIGIVAGIGMLWFQDLMPGRAGAATTLFTNTIST

GVILAGVIQGAIAQSWGHFAVYWVIAIISVVALFLTAKVKDV

>gi|30061634|ref|NP_835805.1| transcriptional regulator SgrR [Shigella flexneri 2a str. 2457T]

MPSARLQQQFIRLWQCCEGKSQDTTLNELAALLSCSRRHMRTLLNTMQDRGWLTWEAEVGRGKRSRLTFL

YTGLALQQQRAEDLLEQDRIDQLVQLVGDKATVRQMLVSHLGRSFRQGRHILRVLYYRPLRNLLPGSALR

RSETHIARQIFSSLTRINEENGELEADIAHHWQQISPLHWRFFLRPGVHFHHGRELEMDDVIASLKRINT

LPLYSHITDIVSPTPWTLDIHLTQPDRWLPLLLGQVPAMILPCEWETLSNFASHPIGTGPYAVIRNSTNQ

LKIQAFDDFFGYRALIDEVNVWVLPEIADEPAGGLMLKGPQGEKKEIESRLEEGCYYLLFDSRTHRGANQ

QVRDWVSYVLSPTNLVYFAEEQYQQLWFPAYGLLPRWHHARTIKSEKPAGLESLTLTFYQDHSEHRVIAG

IMQQILASHQVTLEIKEISYDQWHEGEIESDIWLNSANFTLPLDFSLFAHLCEVPLLQHCIPIDWQADAA

RWRNGEMNLANWCQQLVASKAMVPLIHHWLIIQGQRSMRGLRMNTLGWFDFKSAWFAPPDP

>gi|30061633|ref|NP_835804.1| thiamine transporter substrate binding subunit [Shigella flexneri 2a str. 2457T]

MLKKCLPLLLLCTAPVFAKPVLTVVYTYDSFAADWGPGPKIKKAFEANCNCELKLVALEDGVSLLNRLRME

GKNSKADVVLGLDNNLLDAASKTGLFAKSGVAADAVNVPGGWNNDTFVPFDYGYFAFVYDKNKLKNPPQS

LKELVESDQNWRVIYQDPRTSTPGLGLLLWMQKVYGDDAPQAWQKLAKKTVTVTKGWSEAYGLFLKGESD

LVLSYTTSPAYHILEEKKDNYAAANFSEGHYLQVEVAARTAASKQPELAQKFLQFMVSPAFQNAIPTGNW

MYPVANVTLPAGFEQLTKPATTLEFTPAEVAAQRQAWISEWQRAVSR

>gi|30061632|ref|NP_835803.1| thiamine transporter membrane protein [Shigella flexneri 2a str. 2457T]

MATRRQPLIPGWLIPGVSAATLVVAVALAAFLALWWNAPQGNWVAVWQDSYLWHVVRFSFWQAFLSALLS

VVPAIFLARALYRRRFPGRLALLRLCAMTLILPVLVAVFGILSVYGRQGWLASLCQSLGLEWTFSPYGLQ

GILLAHVFFNLPMASRLLLQALENIPGEQRQLAAQLGMRGWHFFRFVEWPWLRRQIPPVAALIFMLCFAS

FATVLSLGGGPQATTIELAIYQALSYDYDPARAAMLALLQMVCCLGLVLLSQRLSKAIAPGTTLLQGWRD

PDDRLHSRICDTVLIVLALLLLLPPLLAVIVDGVNRQLPEVLAQPVLWQALWTSLRIALAAGVLCVVLTM

MLLWSSRELRARQKMLAGQALEMSGMLILAMPGIVLATGFFLLLNNTIGLPQSADGIVIFTNALMAIPYA

LKVLENPMRDITARYSMLCQSLGIEGWSRLKVVELRALKRPLAQALAFACVLSIGDFGVVALFGNDDFRT

LPFYLYQQIGSYRSQDGAVTALILLLLCFLLFTVIEKLPGRNVKTD

>gi|30061631|ref|NP_835802.1| thiamine transporter ATP-binding subunit [Shigella flexneri 2a str. 2457T]

MLKLTDITWLYHHLPMRFSLTVERGEQVAILGPSGAGKSTLLNLIAGFLTPASGSLTIDGVDHTTTPPSR

RPVSMLFQENNLFSHLTVAQNIGLGLNPGLKLNAAQQEKMHAIARQMGIDNLMARLPGELSGGQRQRVAL

ARCLVREQPILLLDEPFSALDPALRQEMLTLVSTSCQQQKMTLLMVSHSVEDAARIATRSVVVADGRIAW

QGKTEELLSGKASASAILGITG

>gi|30061630|ref|NP_835801.1| hypothetical protein S0062 [Shigella flexneri 2a str. 2457T]

MQALLEHFITQSTVYSLMAVVLVAFLESLALVGLILPGTVLMAGLGALIGSGELSFWHAWLAGIVGCLLG

DWISFWLGWRFKKPLHRWSFLKKNKALLDKTEHALHQHSMFTILVGRFVGPTRPLVPMVAGMLDLPVAKF

ITPNIIGCLLWPPFYFLPGILAGAAIDIPAGMQSGEFKWLLLATAVFLWVGGWLCWRLWRSGKATDRLSH

YLSRGRLLWLTPLISAIGVVALVVLIRHPLMPVYIDILRKVVGV

>gi|30061629|ref|NP_835800.1| DNA-binding transcriptional regulator AraC [Shigella flexneri 2a str. 2457T]

MAEAQNDPLLPGYSFNAHLVAGLTPIEANGYLDFFIDRPLGMKGYILNLTIRGQGVVKNQGREFVCRPGD

ILLFPPGEIHHYGCHPEAREWYHQWVYFRPRAYWHEWLNWPSIFANTGFFRPDEAHQPYFSDLFGQIINA

GQGEGRYSELLAMNLLEQLLLRRMEAINESLHPPMDNRVREACQYISDHLADSNFDIASVAQHVCLSPSR

LSHLFRQQLGISVLSWREDQRISQAKLLLSTTRMPIATVGRNVGFDDQLYFSRVFKKCTGASPSEFRAGC

EEKVNDVAVKLS

>gi|30061628|ref|NP_835799.1| ribulokinase [Shigella flexneri 2a str. 2457T]

MAIAIGLDFGSDSVRALAVDCASGEEIATSVEWYPRWQKGQFCDAPNNQFRHHPRDYIESMEAALKTVLA

ELSVEQRAAVVGIGVDSTGSTPAPIDADGNVLALRPEFAENPNAMFVLWKDHTAVEEAEEITRLCHAPGN

VDYSRYIGGIYSSEWFWAKILHVTRQDSAVAQSAASWIELCDWVPALLSGTTRPQDIRRGRCSAGHKSLW

HESWGGLPPASFFDELDPILNRHLPSPLFTDTWTADIPVGTLCPEWAQRLGLPESVVISGGAFDCHMGAV

GAGAQPNALVKVIGTSTCDILIADKQSVGERAVKGICGQVDGSVVPGFIGLEAGQSAFGDIYAWFGRVLG

WPLEQLAAQHPELKAQINASQKQLLPALTEAWAKNPSLDHLPVVLDWFNGRRTPNANQRLKGVITDLNLA

TDTPLLFGGLIAATAFGARAIMECFTDQGIAVNNVMALGGIARKNQVIMQACCDVLNRPLQIVASDQCCA

LGAAIFAAVAAKVHADIPSAQQKMASAVEKTLQPRSEQAQRFEQLYRRYQQWAMSAEQHYLPTSAPAQAA

QAVATL

>gi|30061627|ref|NP_835798.1| L-arabinose isomerase [Shigella flexneri 2a str. 2457T]

MAIFDNYEVWFVIGSQHLYGPETLRQVTQHAEHVVNALNTEAKLPYKLVLKPLGTTPDEITAICRDANYD

DRCAGLVVWLHTFSPAKMWINGLTMLNKPLLQFHTQFNAALPWDSIDMDFMNLNQTAHGGREFGFIGARM

RQQHAVVTGHWQDKQAHERIGSWMRQAVSKQDTRHLKVCRFGDNMREVAVTDGDKVAAQIKFGFSVNTWA

VGYLVQVVNSISDGDVNALVDEYESCYTMTPATQIHGEKRQNVLEAARIELGMKRFLEQGGFHAFTTTFE

DLHGLKQLPGLPVQRLMQQGYGFAGEGDWKTAALLRIMKVMSTGLQGGTSFMEDYTYHFEKGNDLVLGSH

MLEVCPSIAVEEKPILDVQHLGIGGKDDPARLIFNTQTGPAIVASLIDLGDRYRLLVNCIDTVKTPHSLP

KLPVANALWKAQPDLPTASEAWILAGGAHHTVFSHALNLNDMRQFAEMHDIEITVIDNDTRLPAFKDALR

WNEVYYGFRR

>gi|30061626|ref|NP_835797.1| L-ribulose-5-phosphate 4-epimerase [Shigella flexneri 2a str. 2457T]

MLKDLKRQVLEANLALPKHNLVTLTWGNVSAVDRERGVFVIKPSGVDYSVMTADDMVVVSIETGEVVEGT

KKPSSDTPTHRLLYQAFPSIGGIVHTHSRHATIWAQAGQSIPATGTTHADYFYGTIPCTRKMTDAEINGE

YEWETGNVIVETFEKQGIDAAQMPGVLVHSHGPFAWGKNAEDAVHNAIVLEEVAYMGIFCRQLAPQLPDM

QQTLLDKHYLRKHGAKAYYGQ

>gi|30061625|ref|NP_835796.1| DNA polymerase II [Shigella flexneri 2a str. 2457T]

MAQAGFILTRHWRDTPQGTEVSFWLATDNGPLQVTLAPQESVAFIHADQVPRAQHILQGEQGFRLTPLAL

KDFHRQPVYGLYCRAHRQLMNYEKRLREGGVTVYEADVRPPERYLMERFITSPVWVEGDMHNGTIVNARL

KPHPDYRPPLKWVSIDIETTRHGELYCIGLEGCGQRIVYMLGPENGDASSLDFELEYVASRPLLLEKLNA

WFANHDPDVIIGWNVVQFDLRMLQKHAERYRLPLRLGRDNSELEWREHGFKNGVFFAQAKGRLIIDGIEA

LKSAFWNFSSFSLETVAQELLGEGKSIDNPWDRMDEIDRRFAEDKPALATYNLKDCELVTQIFHKTEIMP

FLLERATVNGLPVDRHGGSVAAFGHLYFPRMHRAGYVAPNLGEVPPHASPGGYVMDSRPGLYDSVLVLDY

KSLYPSIIRTFLIDPVGLVEGMAQPDPEHSTEGFLDAWFSREKHCLPEIVTNIWHGRDKAKRQGNKPLSQ

ALKIIMNAFYGVLGTTACRFFDPRLASSITMRGHQIMRQTKALIEAQGYDIIYGDTDSTFVWLKGAHSEE

EAAKIGRALVQHVNAWWAETLQKQRLTSALELEYETHFCRFLMPTIRGADTGSKKRYAGLIQEGDKQRMV

FKGLETVRTDWTPLAQQFQQELYLRIFRNEPYQEYVRQTIDKLMAGELDARLVYRKRLRRPLSEYQRNVP

PHVRAARLADEENQKRGRPLQYQNRGTIKYVWTTNGPEPLDYQRSPLDYEHYLTRQLQPVAEGILPFIED

NFATLMTGQLGLF

>gi|30061624|ref|NP_835795.1| ATP-dependent helicase HepA [Shigella flexneri 2a str. 2457T]

MPFTLGQRWISDTESELGLGTVVAVDARTVTLLFPSTGENRLYARSDSPVTRVMFNPGDTITSHDGWQMQ

VEEVKEENGLLTYIGTRLDTEESGVALREVFLDSKLVFSKPQDRLFAGQIDRMDRFALRYRARKYSSEQF

RMPYSGLRGQRTSLIPHQLNIAHDVGRRHAPRVLLADEVGLGKTIEAGMILHQQLLSGAAERVLIIVPET

LQHQWLVEMLRRFNLRFALFDDERYAEAQHDAYNPFDTEQLVICSLDFARRSKQRLEHLCEAEWDLLVVD

EAHHLVWSEDAPSREYQAIEQLAEHVPGVLLLTATPEQLGMESHFARLRLLDPNRFHDFAQFVEEQKNYR

PVADAVAMLLAGNKLSNDELNMLGEMIGEQDIEPLLQAANSDSEDAQSARQELVSMLMDRHGTSRVLFRN

TRNGVKGFPKRELHTIKLPLPTQYQTAIKVSGIMGARKSAEDRARDMLYPERIYQEFEGDNATWWNFDPR

VEWLMGYLTSHRSQKVLVICAKAATALQLEQVLREREGILAAVFHEGMSIIERDRAAAWFAEEDTGAQVL

LCSEIGSEGRNFQFASHMVMFDLPFNPDLLEQRIGRLDRIGQAHDIQIHVPYLEKTAQSVLVRWYHEGLD

AFEHTCPTGRTIYDSVYNDLINYLASPDQTEGFDDLIKSCREQHEALKAQLEQGRDRLLEIHSNGGEKAQ

ALAESIEEQDDDTNLIAFAMNLFDIIGINQDDRGDNMIVLTPSDHMLVPDFPGLSEDGITITFDREVALA

REDAQFITWEHPLIRNGLDLILSGDTGSSTISLLKNKALPVGTLLVELIYVVEAQAPKQLQLNRFLPPTP

VRMLLDKNGNNLAAQVEFETFNRQLNAVNRHTGSKLVNAVQQDVHAILQLGEAQIEKSARALIDAARNEA

DEKLSAELSRLEALRAVNPNIRDDELTAIESNRQQVMESLDQAGWRLDALRLIVVTHQ

>gi|30061623|ref|NP_835794.1| 23S rRNA/tRNA pseudouridine synthase A [Shigella flexneri 2a str. 2457T]

MGMENYNPPQEPWLVILYQDDHIMVVNKPSGLLSVPGRLEEHKDSVMTRIQRDYPQAESVHRLDMATSGV

IVVALTKAAERELKRQFREREPKKQYVARVWGHPSPAEGLVDLPLICDWPNRPKQKVCYETGKPAQTEYE

VVEYAADNTARVVLKPITGRSHQLRVHMLALGHPILGDRFYASPEARAMAPRLLLHAEMLTITHPAYGNS

MTFKAPADF

>gi|30061622|ref|NP_835793.1| Dna-J like membrane chaperone protein [Shigella flexneri 2a str. 2457T]

MQYWGKIIGVAVALLMGGGFWGVVLGLLIGHMFDKARSRKMAWFANQRERQALFFATTFEVMGHLTKSKG

RVTEADIHIASQLMDRMNLHGASRTAAQNAFRVGKSDNYPLREKMRQFRSVCFGRFDLIRMFLEIQIQAA

FADGSLHPNERAVLYVIAEELGISRAQFDQFLRMMQGGAQFGGGYQQQSGGGNWQQAQRGPTLEDACNVL

GVKPTDDATTIKRAYRKLMSEHHPDKLVAKGLPPEMMEMAKQKAQEIQQAYELIKQQKGFK

>gi|30061621|ref|NP_835792.1| organic solvent tolerance protein [Shigella flexneri 2a str. 2457T]

MKKRIPTLLATMIATALYSQQGLAADLASQCMLGVPSYDRPLVQGDTNDLPVTINADHAKGDYPDDAVFT

GSVDIMQGNSRLQADEVQLHQKEAPGQPEPVRTVDALGNVHYDDNQVILKGPKGWANLNTKDTNVWEGDY

QMVGRQGRGKADLMKQRGENRYTILDNGSFTSCLPGSDTWSVVGSEIIHDREEQVAEIWNARFKVGPVPI

FYSPYLQLPVGDKRRSGFLIPNAKYTTTNYFEFYLPYYWNIAPNMDATITPHYMHRRGNIMWENEFRYLS

QAGAGLMELDYLPSDKVYEDEHPNDDSSRRWLFYWNHSGVMDQVWRFNVDYTKVSDPSYFNDFDNKYGSS

TDGYATQKFSVGYAVQNFNATVSTKQFQVFSEQNTSSYSAEPQLDVNYYQNDVGPFDTRIYGQAVHFVNT

RDDMPEATRVHLEPTINLPLSNNWGSINTEAKFLATHYQQTNLDWYNSRNTTKLDESVNRVMPQFKVDGK

MVFERDMEMLAPGYTQTLEPRAQYLYVPYRDQSDIYNYDSSLLQSDYSGLFRDRTYGGLDRIASANQVTT

GVTSRIYDDAAVERFNISVGQIYYFTESRTGDDNITWENDDKTGSLVWAGDTYWRISERWGLRGGIQYDT

RLDNVATSNSSIEYRRDEDRLVQLNYHYASPEYIQATLPKYYSTAEQYKNGISQVGAVASRPIADRWSIV

GAYYYDTNANKQADSMLGVQYSSCCYAIRVGYERKLNGWDNDKQHAVYDNAIGFNIELRGLSSNYGLGTQ

EMLRSNILPYQNTL

>gi|30061620|ref|NP_835791.1| peptidyl-prolyl cis-trans isomerase SurA [Shigella flexneri 2a str. 2457T]

MKNWKTLLLGIAMIANTSFAAPQVVDKVAAVVNNGVVLESDVDGLMQSVKLNAAQARQQLPDDATLRHQI

MERLIMDQIILQMGQKMGVKISDEQLDQAIANIAKQNNMTLDQMRSRLAYDGLNYNTYRNQIRKEMIISE

VRNNEVRRRITILPQEVESLAQQVGNQNDASTELNLSHILIPLPENPTSDQVNEAESQARAIVDQARNGA

DFGKLAIAHSADQQALNGGQMGWGRIQELPGIFAQALSTAKKGDIVGPIRSGVGFHILKVNDLRGESKNI

SVTEVHARHILLKPSPIMTDEQARVKLEQIAADIKSGKTTFAAAAKEFSQDPGSANQGGDLGWATPDIFD

PAFRDALTRLNKGQMSAPVHSSFGWHLIELLDTRNVDKTDAAQKDRAYRMLMNRKFSEEAASWMQEQRAS

AYVKILSN

>gi|30061619|ref|NP_835790.1| 4-hydroxythreonine-4-phosphate dehydrogenase [Shigella flexneri 2a str. 2457T]

MVKTQRVVITPGEPAGIGPDLVVQLAQREWPVELVVCADATLLTDRAAMLGLPLTLRPYSPNSPAQPQTT

GTLTLLPVALRESVTAGQLAIENGHYVVETLARACDGCLNGEFAALITGPVHKGVINDASIPFTGHTEFF

EERSQAKKVVMMLATEELRVALATTHLPLRDIADAITPALLHEVIAILHHDLRTKFGIAEPRILVCGLNP

HAGEGGHMGTEEIDTIIPVLDELRAQGMKLNGPLPADTLFQPKYLDNADAVLAMYHDQGLPVLKYQGFGR

GVNITLGLPFIRTSVDHGTALELAGRGEADVGSFITALNLAIKMIVNTQ

>gi|30061618|ref|NP_835789.1| dimethyladenosine transferase [Shigella flexneri 2a str. 2457T]

MNNRVHQGHLARKRFGQNFLNDQFVIDSIVSAINPQKGQAMVEIGPGLAALTEPVGERLDQLTVIELDRD

LAARLQTHPFLGPKLTIYQQDAMTFNFGELAEKMGQPLRVFGNLPYNISTPLMFHLFSYTDAIADMHFML

QKEVVNRLVAGPNSKAYGRLSVMAQYYCNVIPVLEVPPSAFTPPPKVDSAVVRLVPHATMPHPVKDIRVL

SRITTEAFNQRRKTIRNSLGNLFSVEVLTGMGIDPAMRAENISVAQYCQMANYLAENAPLQES

>gi|30061617|ref|NP_835788.1| ApaG protein [Shigella flexneri 2a str. 2457T]

MINSPRVCIQVQSVYIEAQSSPDNERYVFAYTVTIRNLGRAPVQLLGRYWLITNGNGRETEVQGEGVVGV

QPLIAPGEEYQYTSGAIIETPLGTMQGHYEMIDENGVPFSIDIPVFRLAVPTLIH

>gi|30061616|ref|NP_835787.1| diadenosine tetraphosphatase [Shigella flexneri 2a str. 2457T]

MATYLIGDVHGCYDELIALLHKVEFTPGKDTLWLTGDLVARGPGSLDVLRYVKSLGDSVRLVLGNHDLHL

LAVFAGISRNKPKDRLTPLLEAPDADELLNWLRRQPLLQIDEEKKLVMAHAGITPQWDLQTAKECARDVE

AVLSSDSYPFFLDAMYGDMPNNWSPELRGLGRLRFITNAFTRMRFCFPNGQLDMYSKESPEEAPAPLKPW

FAIPGPVAEEYSIAFGHWASLEGKGTPEGIYALDTGCCWGGSLTCLRWEDKQYFVQPSNRHKDLGEAAAS

>gi|30061615|ref|NP_835786.1| dihydrofolate reductase [Shigella flexneri 2a str. 2457T]

MISLIAALAVDRVIGMENAMPWNLPADLAWFKRNTLNKPVIMGRHTWESIGRPLPGRKNIILSSQPGTDD

RVTWVKSVDEAIAACGDVPEIMVIGGGRVYEQFLPKAQKLYLTHIDAEVEGDTHFPDYEPDDWESVFSEF

HDADAQNSHSYCFEILERR

>gi|30061614|ref|NP_835785.1| glutathione-regulated potassium-efflux system protein KefC [Shigella flexneri 2a str. 2457T]

MDSHTLVQALIYLGSAALIVPIAVRLGLGSVLGYLIAGCIIGPWGLRLVTDAESILHFAEIGVVLMLFII

GLELDPQRLWKLRAAVFGGGALQMVICGGLLGLFCMLLGLRWQVAELIGMTLALSSTAIAMQAMNERNLM

VTQMGRSAFAVLLFQDIAAIPLVAMIPLLAASSASTTMGAFALSALKVAGALVLVVLLGRYVTRPALRFV

ARSGLREVFSAVALFLVFGFGLLLEEVGLSMAMGAFLAGVLLASSEYRHALESDIEPFKGLLLGLFFIGV

GMSIDFGTLIENPLRIVILLLGFLIIKIAMLWLIARPLQVPNKQRRWFAVLLGQGSEFAFVVFGAAQMAN

VLEPEWAKSLTLAVALSMAATPILLVILNRLEQSSTEEAREADEIDEEQPRVIIAGFGRFGQITGRLLLS

SGVKMVVLDHDPDHIETLRKFGMKVFYGDATRMDLLESAGAAKAEVLINAIDDPQTNLQLTEMVKEHFPH

LQIIARARDVDHYIRLRQAGVEKPERETFEGALKTGRLALESLGLGPYEARERADVFRRFNIQMVEEMAM

VENDTKARAAVYKRTSAMLSEIITEDREHLSLIQRHGWQGTEEGKHTGNMADEPETKPSS

>gi|30061613|ref|NP_835784.1| glutathione-regulated potassium-efflux system ancillary protein KefF [Shigella flexneri 2a str. 2457T]

MILIIYAHPYPHHSHANKRMLEQARTLEGVEIRSLYQLYPDFNIDIAAEQEALSRADLIVWQHPMQWYSI

PPLLKLWIDKVLSHGWAYGHGGTALHGKHLLWAVTTGGGESHFEIGAHPGFDVLSQPLQATAIYCGLNWL

PPFAMHCTFICDDETLEGQARHYKQRLLEWQEAHHG

>gi|30061612|ref|NP_835783.1| transporter [Shigella flexneri 2a str. 2457T]

MQPSRNFDDLKFSSIHRRILLWGSGGPFLDGYVLVMIGVALEQLTPALKLDADWIGLLGAGTLAGLFVGT

SLFGYISDKVGRRKMFLIDIIAIGVISVATMCVSSPVELLVMRVLIGIVIGADYPIATSMITEFSSTRQR

AFSISFIAAMWYVGATCADLVGYWLYDVEGGWRWMLGSAAIPCLLILIGRFELPESPRWLLRKGRVKECE

EMMIKLFGEPVAFDEEQPQQTRFRDLFNRRHFPFVLFVAAIWTCQVIPMFAIYTFGPQIVGSLGLGVGKN

AALGNVVISLFFMLGCIPPMLWLNTAGRRPLLIGSFAMMTLALAVLGLIPDMGIWLVVMAFAVYAFFSGG

PGNLQWLYPNELFPTDIRASAVGVIMSLSRIGTIVSTWALPIFINNYGISNTMLMGAGISLFGLLISVAF

APETRGMSLAQTSNMTIRGQRMGLFVQISLFSESILLTISRVNI

>gi|30061611|ref|NP_835782.1| ferredoxin [Shigella flexneri 2a str. 2457T]

MTSPVNVDVKLGVNKFNVDEEHPHIVVKADADKQVLELLVKACPAGLYKKQDDGSVRFDYAGCLECGTCR

ILGLGSALEQWEYPRGTFGVEFRYG

>gi|30061610|ref|NP_835781.1| oxidoreductase FixC [Shigella flexneri 2a str. 2457T]

MSEDIFDAIIVGAGLAGSVAALVLAREGAQVLVIERGNSAGAKNVTGGRLYAHSLEHIIPGFADSAPVER

LITHEKLAFMTEKSAMTMDYCNGDETSPSQRSYSVLRSKFDAWLMEQAEEAGAQLITGIRVDNLVQRDGK

VVGVEADGDVIEAKTVLLADGVNSILAEKLGMAKRVKPTDVAVGVKELIELPKSVIEDRFQLQGNQGAAC

LFAGSPTDGLMGGGFLYTNENTLSLGLVCGLHHLHDAKKSVPQMLEDFKQHPAVAPLIAGGKLVEYSAHV

VPEAGINMLPELVGDGVLIAGDAAGMCMNLGFTIRGMDLAIAAGEAAAKTVLSAMKSDDFSKQKLAEYRQ

HLESGPLRDMRMYQKLPAFLDNPRMFSGNPELAVGVARDLFTIDGSAPELMRKKILRHGKKVGFINLIKD

GMKGVTVL

>gi|30061608|ref|NP_835779.1| L-carnitine/gamma-butyrobetaine antiporter [Shigella flexneri 2a str. 2457T]

MKNEKRKTGIEPKVFFPPLIIVGILCWLTVRDLDAANVVINAVFSYVTNVWGWAFEWYMVVMLFGWFWLV

FGPYAKKRLGNEPPEFSTASWIFMMFASCTSAAVLFWGSIEIYYYISTPPFGLEPNSTGAKELGLAYSLF

HWGPLPWATYSFLSVAFAYFFFVRKMEVIRPSSTLVPLAGEKHAKGLFGTIVDNFYLVALIFAMGTSLGL

ATPLVTECMQWLFGIPHTLQLDAIIITCWIILNAICVACGLQKGVRIASDVRSYLSFLMLGWVFIVSGAS

FIMNYFTDSVGMLLMYLPRMLFYTDPIAKGGFPQGWTVFYWAWWVIYAIQMSIFLARISRGRTVRELCFG

MVMGLTASTWILWTVLGSNTLLLMDKNIINIPNLIEQYGVARAIIETWAALPLSTATMWGFFILCFIATV

TLVNACSYTLAMSTCREVRDGEEPPLLVRIGWSILVGIIGIVLLALGGLKPIQTAIIAGGCPLFFVNIMV

TLSFIKDAKQNWKD

>gi|30061607|ref|NP_835778.1| crotonobetainyl-CoA dehydrogenase [Shigella flexneri 2a str. 2457T]

MDFNLNDEQELFVAGIRELMASENWEAYFAECDRDSVYPERFVKALADMGIDSLLIPEEHGGLDAGFVTL

AAVWMELGRLGAPTYVLYQLPGGFNTFLREGTQEQIDKIMAFRGTGKQMWNSAITEPGAGSDVGSLKTTY

TRRNGKIYLNGSKCFITSSAYTPYIVVMARDGASPDKPVYTEWFVDMSKPGIKVTKLEKLGLRMDSCCEI

TFDDVELDEKDMFGREGNGFNRVKEEFDHERFLVALTNYGTAMCAFEDAARYANQRVQFGEAIGRFQLIQ

EKFAHMAIKLNSMKNMLYEAAWKADNGTITSGDAAMCKYFCANAAFEVVDSAMQVLGGVGIAGNHRISRF

WRDLRVDRVSGGSDEMQILTLGRAVLKQYR

>gi|30061606|ref|NP_835777.1| crotonobetainyl-CoA:carnitine CoA-transferase [Shigella flexneri 2a str. 2457T]

MDHLPMPKFGPLAGLRVVFSGIEIAGPFAGQMFAEWGAEVIWIENVAWADTIRVQPNYPQLSRRNLHALS

LNIFKDEGREAFLKLMETTDIFIEASKGPAFARRGITDEVLWQHNPKLVIAHLSGFGQYGTEEYTNLPAY

NTIAQAFSGYLIQNGDVDQPMPAFPYTADYFSGLTATTAALAALHKVRETGKGESIDIAMYEVMLRMGQY

FMMDYFNGGEMCPRMSKGKDPYYAGCGLYKCADGYIVMELVGITQIEECFKDIGLAHLLGTPEIPEGTQL

IHRIECPYGPLVEEKLDAWLAAHTIAEVKERFAELNIACAKVLTVPELESNPQYVARESITQWQTMDGRT

CKGPNIMPKFKNNPGQIWRGMPSHGMDTAAILKNIGYSENDIQELVSKGLAKVED

>gi|30061605|ref|NP_835776.1| crotonobetaine/carnitine-CoA ligase [Shigella flexneri 2a str. 2457T]

MDRGAMDIIGGQHLRQMWDDLADVYGHKTALICESSGGVVNRYSYLELNQEINRTANLFYTLGIRKGNKV

ALHLDNCPEFIFCWFGLAKIGAIMVPINARLLREESEWILQNSQACLLVTSAQFYPMYQQIQQEDATQLR

HICLTDVALPADDGVSSFTQLKNQQPATLCYAPPLSTDDTAEILFTSGTTSRPKGVVITHYNLRFAGYYS

AWQCALRDDDVYMTVMPAFHIDCQCTAAMAAFSAGATFVLVEKYSARAFWGQAQKYRATITECIPMMIRT

LMVQPPSANDRQHRLREVMFYLNLSEQEKDAFCERFGVRLLTSYGMTETIVGIIGDRPSDKRRWPSIGRA

GFCYEAEIRDDHNRPLPAGEIGEICIKGVPGKTIFKEYFLNPKATAKVLEADGWLHTGDTGYRDEEGFFY

FVDRRCNMIKRGGENVSCVELENIIATHPKIQDIVVVGIKDSIRDEAIKAFVVLNEGETLSEEEFFRFCE

QNMAKFKVPSYLEIRKDLPRNCSGKIIRKNLK

>gi|30061604|ref|NP_835775.1| carnitinyl-CoA dehydratase [Shigella flexneri 2a str. 2457T]

MKRQGTTLPANNHALKQYAFFAGMLSSLKKQKWRKGMSESLHLTRNGSILEITLDRPKANAIDAKTSFEM

GEVFLNFRDDPQLRVAIITGAGEKFFSAGWDLKAAAEGEAPDADFGPGGFAGLTEIFNLDKPVIAAVNGY

AFGGGFELALAADFIVCADNASFALPEAKLGIVPDSGGVLRLPKILPPTIVNEMVMTGRRMGAEEALRWG

IVNRVVSQAELMDNARELAQQLVNSAPLAIAALKEIYRTTSEMPVEEAYRYIRSGVLKHYPSVLHSEDAI

EGPLAFAEKRDPVWKGR

>gi|30061603|ref|NP_835774.1| carnitine operon protein CaiE [Shigella flexneri 2a str. 2457T]

MERTLTTVSYYAFEGLIPVVHPTAFVHPSAVLIGDVIVGAGVYIGPLASLRGDYGRLIVQAGANIQDGCI

MHGYCDTDTIVGENGHIGHGAILHGCVIGRDALVGMNSVIMDGAVIGEESIVAAMSFVKAGFSGEKRQLL

MGTPARAVRSVSDDELHWKRLNTKEYQDLVGRCHASLHETQPLRQMEENRPRLQGTTDVTPKR

>gi|30061601|ref|NP_835772.1| carbamoyl phosphate synthase large subunit [Shigella flexneri 2a str. 2457T]

MPKRTDIKSILILGAGPIVIGQACEFDYSGAQACKALREEGYRVILVNSNPATIMTDPEMADATYIEPIH

WEVVRKIIEKERPDAVLPTMGGQTALNCALELERQGVLEEFGVTMIGATADAIDKAEDRRRFDVAMKKIG

LETARSGIAHTMEEALAVAADVGFPCIIRPSFTMGGSGGGIAYNREEFEEICARGLDLSPTKELLIDESL

IGWKEYEMEVVRDKNDNCIIVCSIENFDAMGIHTGDSITVAPAQTLTDKEYQIMRNASMAVLREIGVETG

GSNVQFAVNPKNGRLIVIEMNPRVSRSSALASKATGFPIAKVAAKLAVGYTLDELMNDITGGRTPASFEP

SIDYVVTKIPRFNFEKFAGANDRLTTQMKSVGEVMAIGRTQQESLQKALRGLEVGATGFDPKVSLDDPEA

LTKIRRELKDAGAERIWYIADAFRAGLSVDGVFNLTNIDRWFLVQIEELVRLEEKVAEVGITGLNAEFLR

QLKRKGFADARLAKLAGVREAEIRKLRDQYDLHPVYKRVDTCAAEFATDTAYMYSTYEEECEANPSTDRE

KIMVLGGGPNRIGQGIEFDYCCVHASLALREDGYETIMVNCNPETVSTDYDTSDRLYFEPVTLEDVLEIV

RIEKPKGVIVQYGGQTPLKLARALEAAGVPVIGTSPDAIDRAEDRERFQHAVDRLKLKQPANATVTAIEM

AVEKAKEIGYPLVVRPSYVLGGRAMEIVYDEADLRRYFQTAVSVSNDAPVLLDHFLDDAVEVDVDAICDG

EMVLIGGIMEHIEQAGVHSGDSACSLPAYTLSQEIQDVMRQQVQKLAFELQVRGLMNVQFAVKNNEVYLI

EVNPRAARTVPFVSKATGVPLAKVAARVMAGKSLAEQGVTKEVIPPYYSVKEVVLPFNKFPGVDPLLGPE

MRSTGEVMGVGRTFAEAFAKAQLGSNSTMKKHGRALLSVREGDKERVVDLAAKLLKQGFELDATHGTAIV

LGEAGINPRLVNKVHEGRPHIQDRIKNGEYTYIINTTSGRRAIEDSRVIRRSALQYKVHYDTTLNGGFAT

AMALNADATEKVISVQEMHAQIK

>gi|30061600|ref|NP_835771.1| carbamoyl phosphate synthase small subunit [Shigella flexneri 2a str. 2457T]

MIKSALLVLEDGTQFHGRAIGATGSAVGEVVFNTSMTGYQEILTDPSYSRQIVTLTYPHIGNVGTNDADE

ESSQVHAQGLVIRDLPLIASNFRNTEDLSSYLKRHNIVAIADIDTRKLTRLLREKGAQNGCIIAGDNPDA

ALALEKARAFPGLNGMDLAKEVTTAEAYSWTQGSWTLTGGLPEAKKEDELPFHVVAYDFGAKRNILRMLV

DRGCRLTIVPAQTSAEDVLKMNPDGIFLSNGPGDPAPCDYAITAIQKFLETDIPVFGICLGHQLLALASG

AKTVKMKFGHHGGNHPVKDVEKNVVMITAQNHGFAVDEATLPANLRVTHKSLFDGTLQGIHRIDKPAFSF

QGHPEASPGPHDAAPLFDHFIKLIEQYRKTAK

>gi|30061599|ref|NP_835770.1| dihydrodipicolinate reductase [Shigella flexneri 2a str. 2457T]

MHDANIRVAIAGAGGRMGRQLIQAALALEGVQLGAALEREGSSLLGSDAGELAGAGKTGVTVQSSLDAIK

DDFDVFIDFTRPEGTLNHLAFCRQHGKGMVIGTTGFDEAGKQAIRDAAADIAIVFAANFSVGVNVMLKLL

EKAAKVMGDYTDIEIIEAHHRHKVDAPSGTALAMGEAIAHALDKDLKDCAVYSREGHTGERVPGTIGFAT

VRAGDIVGEHTAMFADIGERLEITHKASSRMTFANGAVRSALWLSGKESGLFDMRDVLDLNNL

>gi|30061598|ref|NP_835769.1| ribonucleoside hydrolase RihC [Shigella flexneri 2a str. 2457T]

MRLPIFLDTDPGIDDAVAIAAAIFAPELDLQLMTTVAGNVSVEKTTRNALQLLHFWNAEIPLAQGAAVPL

VRAPRDAASVHGESGMAGYDFVEHNRKPLGIPAFLAIRDALMRAPEPVTLVAIGPLTNIALLLLQCPECK

PYIRRLVIMGGSAGRGNCTPNAEFNIAADPEAAACVFRSGIEIVMCGLDVTNQAILTPDYLATLPELNRT

GKMLHALFSHYRSGSMQSGLRMHDLCAIAWLVRPDLFTLKPCFVAVETQGEFTSGTTVVDIDGCLGKPAN

VQVALDLNVKGFQQWVAEVLALVP

>gi|30061597|ref|NP_835768.1| 4-hydroxy-3-methylbut-2-enyl diphosphate reductase [Shigella flexneri 2a str. 2457T]

MQILLANPRGFCAGVDRAISIVENALAIYGAPIYVRHEVVHNRYVVDSLRERGAIFIEQISEVPDGAILI

FSAHGVSQAVRNEAKSRDLTVFDATCPLVTKVHMEVARASRRGEESILIGHAGHPEVEGTMGQYSNPEGG

MYLVESPDDVWKLTVKNEEKLSFMTQTTLSVDDTSDVIDALRKRFPKIVGPRKDDICYATTNRQEAVRAL

AEQAEVVLVVGSKNSSNSNRLAELAQRMGKHAFLIDDAKDIQEEWVKEVKCVGVTAGASAPDILVQNVVA

RLQQLGGGEAIPLEGREENIVFEVPKELRVDIREVD

>gi|30061596|ref|NP_835767.1| FKBX-type 16kD peptidyl-prolyl cis-trans isomerase (a rotamase) [Shigella flexneri 2a str. 2457T]

MSESVQSNSAVLVHFTLKLDDGTTAESTRNNGKPALFRLGDASLSEGLEQHLLGLKVGDKTTFSLEPDAA

FGVPSPDLIQYFSRREFMDAGEPEIGAIMLFTAMDGSEMPGVIREINGDSITVDFNHPLAGQTVHFDIEV

LEIDPALEA

>gi|30061595|ref|NP_835766.1| lipoprotein signal peptidase [Shigella flexneri 2a str. 2457T]

MSQSICSTGLRWLWLVVVVLIIDLGSKYLILQNFALGDTVPLFPSLNLHYARNYGAAFSFLADSGGWQRW

FFAGIAIGISVLLAVMMYRSKATQKLNNIAYALIIGGALGNLFDRLWHGFVVDMIDFYVGDWHFATFNLA

DTAICVGAALIVLEGFLPSKAKKQ

>gi|30061594|ref|NP_835765.1| isoleucyl-tRNA synthetase [Shigella flexneri 2a str. 2457T]

MSDYKSTLNLPETGFPMRGDLAKREPGMLARWTDDDLYGIIRAAKKGKKTFILHDGPPYANGSIHIGHSV

NKILKDIIVKSKGLSGYDSPYVPGWDCHGLPIELKVEQEYGKPGEKFTAAEFRAKCREYAATQVDGQRKD

FIRLGVLGDWSHPYLTMDFKTEANIIRALGKIIGNGHLHKGAKPVHWCVDCRSALAEAEVEYYDKTSPSI

DVAFQAVDQDALKAKFGVSNVNGPISLVIWTTTPWTLPANRAISIAPDFDYALVQIDGQAVILAKDLVES

VMQRIGVTDYTILGTVKGAELELLRFTHPFMGFDVPAILGDHVTLDAGTGAVHTAPGHGPDDYVIGQKYG

LETANPVGPDGTYLPGTYPTLDGVNVFKANDIVVALLQEKGALLHVEKMQHSYPCCWRHKTPIIFRATPQ

WFVSMDQKGLRAQSLKEIKGVQWLPDWGQARIESMVANRPDWCISRQRTWGVPMSLFVHKDTEELHPRTL

ELMEEVAKRVEVDGIQAWWDLDAKEILGDEADQYVKVPDTLDVWFDSGSTHSSVVDVRPEFAGHAADMYL

EGSDQHRGWFMSSLMISTAMKGKAPYRQVLTHGFTVDGQGRKMSKSIGNTVSPQDVMNKLGADILRLWVA

STDYTGEMAVSDEILKRAADSYRRIRNTARFLLANLNGFDPAKDMVKPEEMVVLDRWAVGCAKAAQEDIL

KAYEAYDFHEVVQRLMRFCSVEMGSFYLDIIKDRQYTAKADSVARRSCQTALYHIAEALVRWMAPILSFT

ADEVWGYLPGECEKYVFTGEWYEGLFGLADSEAMNDAFWDELLKVRGEVNKVIEQARADKKVGGSLEAAV

TLYAEPELAAKLTALGDELRFVLLTSGATVADYNDAPADAQQSEVLKGLKVALSKAEGEKCPRCWHYTQD

VGKVAEHAEICGRCVSNVAGDGEKRKFA

>gi|30061593|ref|NP_835764.1| bifunctional riboflavin kinase/FMN adenylyltransferase [Shigella flexneri 2a str. 2457T]

MKLIRGIHNLSQAPQEGCVLTIGNFDGVHRGHRALLQGLQEEGRKRNLPVMVMLFEPQPLELFATDKAPA

RLTRLREKLRYLAECGVDYVLCVRFDRRFAALTAQNFISDLLVKHLRVKFLAVGDDFRFGAGREGDFLLL

QKAGMEYGFDITSTQTFCEGGVRISSTAVRQALADDNLALAESLLGHPFAISGRVVHGDELGRTIGFPTA

NVPLRRQVSPVKGVYAVEVLGLGEKPLPGVANIGTRPTVAGIRQQLEVHLLDVAMDLYGRHIQVVLRKKI

RNEQRFASLDELKAQIARDELTAREFFGLTKPA

>gi|30061592|ref|NP_835763.1| hypothetical protein S0023 [Shigella flexneri 2a str. 2457T]

MCRHSLRSDGAGFYQLAGCEYSFSAIKIAAGGQFLPVICSMAMKSHFFLISVLNRRLTLTAVQGILGRFS

LF

>gi|30061590|ref|NP_835761.1| IS1 orfA [Shigella flexneri 2a str. 2457T]

MASISIRCPSCSATEGVVRNGKSTAGHQRYLCSHCRKTWQLQFTYTASQPDTHQKIIDMAMNGVGCRASA

RIMGVGLNTVLRHLKNSGRSR

>gi|30061589|ref|NP_835760.1| IS1 orfB [Shigella flexneri 2a str. 2457T]

MSRQCTHYGRWPQHGFTSLKKLRPQSVTSRIQPGSDVIVCAEMDEHWGYVGAKSRQRWLFYAYDRIRRTV

VAHVFGERTLATLERLLSLLSAFEVVVWMTDGWPLYESRLKGKLHVISKRYTQRIERHNLNLRQHLARLG

RKSLSFSKSVELHDKVIGHYLNIKHYQ

>gi|30061588|ref|NP_835759.1| transcriptional activator NhaR [Shigella flexneri 2a str. 2457T]

MSHINYNHLYYFWHVYKEGSVVGAAEALYLTPQTITGQIRALEERLQGKLFKRKGRGLEPSELGELVYRY

ADKMFTLSQEMLDIVNYRKESNLLFDVGVADALSKRLVSSVLNAAVVEGEPIHLRCFESTHEMLLEQLSQ

HKLDMIISDCPIDSTQQEGLFSVRIGECGVSFWCTNPPPEKPFPACLEERRLLIPGRRSMLGRKLLNWFN

SQGLNVEILGEFDDAALMKAFGAMHNAIFVAPTLYAYDFYADKTVVEIGRVENVMEEYHAIFAERMIQHP

AVQRICNTDYSALFSPAVR

>gi|30061587|ref|NP_835758.1| pH-dependent sodium/proton antiporter [Shigella flexneri 2a str. 2457T]

MKHLHRFFSSDASGGIILIIAAILAMIMANSGATSGWYHDFLETPVQLRVGSLEINKNMLLWINDALMAV

FFLLVGLEVKRELMQGSLASLRQAAFPVIAAIGGMIVPALLYLAFNYADPITREGWAIPAATDIAFALGV

LALLGSRVPLVLKIFLMALAIIDDLGAIIIIALFYTNDLSMASLGVAAVAIAVLAVLNLCGVRRTGVYIL

VGVVLWTAVLKSGVHATLAGVIVGFFIPLKEKHGRSPAKRLEHVLHPWVAYLILPLFAFANAGVSLQGVT

LDGLTSILPLGIIAGLLIGKPLGISLFCWLALRLKLAHLPEGTTYQQIMVVGILCGIGFTMSIFIASLAF

GSVDPELINWAKLGILVGSISSAVIGYSWLRVRLRPSV

>gi|30061586|ref|NP_835757.1| membrane toxin [Shigella flexneri 2a str. 2457T]

MLNTCRVPLTDRKVKEKRAMKQHKAMIVALIVICITAVVAALVTRKDLCEVHIRTGQTEVAVFTAYESE

>gi|30061585|ref|NP_835756.1| chaperone protein DnaJ [Shigella flexneri 2a str. 2457T]

MAKQDYYEILGVSKTAEEREIRKAYKRLAMKYHPDRNQGDKEAEAKFKEIKEAYEVLTDSQKRAAYDQYG

HAAFEQGGMGGGGFGGGADFSDIFGDVFGDIFGGGRGRQRAARGADLRYNMELTLEEAVRGVTKEIRIPT

LEECDVCHGSGAKPGTQPQTCPTCHGSGQVQMRQGFFAVQQTCPHCQGRGTLIKDPCNKCHGHGRVERSK

TLSVKIPAGVDTGDRIRLAGEGEAGEHGAPAGDLYVQVQVKQHPIFEREGNNLYCEVPINFAMAALGGEI

EVPTLDGRVKLKVPGETQTGKLFRMRGKGVKSVRGGAQGDLLCRVVVETPVGLNEKQKQLLQELQESFGG

PTGEHNSPRSKSFFDGVKKFFDDLTR

>gi|30061584|ref|NP_835755.1| molecular chaperone DnaK [Shigella flexneri 2a str. 2457T]

MGKIIGIDLGTTNSCVAIMDGTIPRVLENAEGDRTTPSIIAYTQDGETLVGQPAKRQAVTNPQNTLFAIK

RLIGRRFQDEEVQRDVSIMPFKIIAADNGDAWVEVKGQKMAPPQISAEVLKKMKKTAEDYLGEPVTEAVI

TVPAYFNDAQRQATKDAGRIAGLEVKRIINEPTAAALAYGLDKGTGNRTIAVYDLGGGTFDISIIEIDEV

DGEKTFEVLATNGDTHLGGEDFDSRLINYLVEEFKKDQGIDLRNDPLAMQRLKEAAEKAKIELSSAQQTD

VNLPYITADATGPKHMNIKVTRAKLESLVEDLVNRSIEPLKVALQDAGLSVSDIDDVILVGGQTRMPMVQ

KKVAEFFGKEPRKDVNPDEAVAIGAAVQGGVLTGDVKDVLLLDVTPLSLGIETMGGVMTTLIAKNTTIPT

KHSQVFSTAEDNQSAVTIHVLQGERKRAADNKSLGQFNLDGINPAPRGMPQIEVTFDIDADGILHVSAKD

KNSGKEQKITIKASSGLNEDEIQKMVRDAEANAEADRKFEELVQTRNQGDHLLHSTRKQVEEAGDKLPAD

DKTAIESALTALETALKGEDKAAIEAKMQELAQVSQKLMEIAQQQHAQQQTAGADASANNAKDDDVVDAE

FEEVKDKK

>gi|30061583|ref|NP_835754.1| hypothetical protein S0013 [Shigella flexneri 2a str. 2457T]

MKSVFTISASLAISLMLCCTAQANDHKLLGVIAMPRNETNDLALKLPVCRIVKRIQLSADHGDLQLSGAS

VYFKAARSASQSLNIPSEIKEGQTTDWININSDNDNKRCVSKITFSGHTVNSSDMATLKIIGDD

>gi|30061581|ref|NP_835752.1| hypothetical protein S0012 [Shigella flexneri 2a str. 2457T]

MNVNYLNDSDLDFLQHCSEEQLANFARLLTHNEKGKTRLSSILMRNELFKSMEGHPEQHRRNWQLIAGEL

QHFGGDSIANKLRGHGKLYRAILLDVSKRLKLKADKEMSTFEIEQQLLEQFLRNTWKKMDEEHKQEFLHA

VDARVNELEELLPLLMKDKLLAKGVSHLLSSQLTRILRTHAAMSVLGHGLLRGAGLGGPVGAALNGVKAV

SGSTYRVTIPAVLQIACLRRMVSATQV

>gi|30061580|ref|NP_835751.1| hypothetical protein S0010 [Shigella flexneri 2a str. 2457T]

MGNTKLANPAPLGLMGFGMTTILLNLHNVGYFALDGIILAMGIFYGGIAQIFAGLLEYKKGNTFGLTAFT

SYGSFWLTLVAILLMPKLGLTDAPNAQFLGVYLGLWGVFTLFMFFGTLKGARVLQFVFFSLTVLFALLAI

GNIAGNAAIIHFAGWIGLICGASAIYLAMGEVLNEQFGRTVLPIGESH

>gi|30061579|ref|NP_835750.1| molybdenum cofactor biosynthesis protein [Shigella flexneri 2a str. 2457T]

MNTLRIGLVSISDRASSGVYQDKGIPALEEWLTSALTTPFELETRLIPDEQAIIEQTLCELVDEMSCHLV

LTTGGTGPARRDVTPDATLAVADREMPGFGEQMRQISLHFVPTAILSRQVGVIRKQALILNLPGQPKSIK

ETLEGVKDAEGNVVVHGIFASVPYCIQLLEGPYVETAPEVVAAFRPKSARRDVSE

>gi|30061578|ref|NP_835749.1| transaldolase B [Shigella flexneri 2a str. 2457T]

MTDKLTSLRQYTTVVADTGDIAAMKLYQPQDATTNPSLILNAAQIPEYRKLIDDAVAWAKQQSNDRAQQI

VDATDKLAVNIGLEILKLVPGRISTEVDARLSYDTEASIAKAKRLIKLYNDAGISNDRILIKLASTWQGI

RAAEQLEKEGINCNLTLLFSFAQARACAEAGVFLISPFVGRILDWYKANTDKKEYAPAEDPGVVSVSEIY

QYYKEHGYETVVMGASFRNIGEILELAGCDRLTIAPALLKELAESEGAIERKLSYTGEVKARPARITESE
FLWQHNQDPMAVDKLAEGIRKFAIDQEKLEKMIGDLL

>gi|30061577|ref|NP_835748.1| hypothetical protein S0006 [Shigella flexneri 2a str. 2457T]
MLILISPAKTLDYQSPLTTTRYTLPELLDNSQQLIHEARKLTPPQISTLMRISDKLAGINAARFHDWQPD
FTPANARQAILAFKGDVYTGLQAETFSEDDFDFAQQHLRMLSGLYGVLRPLDLMQPYRLEMGIRLENARG
KDLYQFWGDIITNKLNEALAAQGDNVVINLASDEYFKSVKPKKLNAEIIKPVFLDEKNGKFKIISFYAKK
ARGLMSRFIIENRLTKPEQLTGFNSEGYFFDEDSSSNGELVFKRYEQR

>gi|30061576|ref|NP_835747.1| hypothetical protein S0005 [Shigella flexneri 2a str. 2457T]
MQSIVLALSLVLVAPMAAQAAEITLVPSVKLQIGDRDNRGYYWDGGHWRDHGWWKQHYEWRGNRWHPHGP
PPPPRHHKKAPHDHHGGHGPGKHHR

>gi|30061575|ref|NP_835746.1| threonine synthase [Shigella flexneri 2a str. 2457T]
MKLYNLKDHNEQVSFAQAVTQGLGKNQGLFFPHDLPEFSLTEIDEMLKLDFVTRSAKILSAFIGDEIPQE
ILEERVRAAFAFPAPVANVESDVGCLELFHGPTLAFKDFGGRFMAQMLTHIADDKPVTILTATSGDTGAA
VAHAFYGLPNVKVVILYPRGKISPLQEKLFCTLGGNIETVAIDGDFDACQALVKQAFDDEELKVALGLNS
ANSINISRLLAQICYYFEAVAQLPQEARNQLVVSVPSGNFGDLTAGLLAKSFGLPVKRFIAATNVNDTVP
RFLHDGQWSPKATQATLSNAMDVSQPNNWPRVEELFRRKIWQLKELGYAAVDDETTQQTMRELKELGYTS
EPHAAVAYRALRDQLNPGEYGLFLGTAHPAKFKESVEAILGETLDLPKELAERADLPLLSHNLPADFSAL
RKLMMNHQ

>gi|30061574|ref|NP_835745.1| homoserine kinase [Shigella flexneri 2a str. 2457T]
MVKVYAPASSANMSVGFDVLGAAVAPVDGALLGDVVTVEAAETFSLNNLGRFADKLPSEPRENIVYQCWE
RFCQELGKQIPVAMTLEKNMPIGSGLGSSACSVVAALMAMNEHCGKPLNDTRLLALMGELEGRISGSIHY

DNVAPCFLGGMQLMIEENDIISQQVPGFDEWLWVLAYPGIKVSTAEARAILPAQYRRQDCIAHGRHLAGF

IHACYSRQPELAAKLMKDVIAEPYRERLLPGFRQARQAVAEIGAVASGISGSGPTLFALCDKPDTAQRVA

DWLGKNYLQNQEGFVHICRLDTAGARVLEN

>gi|30061573|ref|NP_835744.1| bifunctional aspartokinase I/homeserine dehydrogenase I [Shigella flexneri 2a str. 2457T]

MRVLKFGGTSVANAERFLRVADILESNARQGQVATVLSAPAKITNHLVAMIEKTISGQDALPNISDAERI

FAELLTGLAAAQPGFPLAQLKTFVDQEFAQIKHVLHGISLLGQCPDSINAALICRGEKMSIAIMAGVLEA

RGHNVTVIDPVEKLLAVGHYLESTVDIAESTRRIAASRIPADHMVLMAGFTAGNEKGELVVLGRNGSDYS

AAVLAACLRADCCEIWTDVDGVYTCDPRQVPDARLLKSMSYQEAMELSYFGAKVLHPRTITPIAQFQIPC

LIKNTGNPQAPGTLIGASRDEDELPVKGISNLNNMAMFSVSGPGMKGMVGMAARVFAAMSRARISVVLIT

QSSSEYSISFCVPQSDCVRAERAMQEEFYLELKEGLLEPLAVTERLAIISVVGDGMRTLRGISAKFFAAL

ARANINIVAIAQGSSERSISVVVNNDDATTGVRVTHQMLFNTDQVIEVFVIGVGGVGGALLEQLKRQQSW

LKNKHIDLRVCGVANSKALLTSVHGLNLENWQEELAQAKEPFNLGRLIRLVKEYHLLNPVIVDCTSSQAV

ADQYADFLREGFHVVTPNKKANTSSMDYYHQLRYAAEKSRRKFLYDTNVGAGLPVIENLQNLLNAGDELV

KFSGILSGSLSYIFGKLDEGMSFSEATTLAREMGYTEPDPRDDLSGMDVARKLLILARETGRELELADIE

IEPVLPAEFNAEGDVAAFMANLSQLDDLFAARVAKARDEGKVLRYVGNIDEDGVCRVKIAEVDGNDPLFK

VKNGENALAFYSHYYQPLPLVLRGYGAGNDVTAAGVFADLLRTLSWKLGV

>gi|30061572|ref|NP_835743.1| thr operon leader peptide [Shigella flexneri 2a str. 2457T]

MKRISTTITTTITITTGNGAG

>gi|145588047|gb|AAP15908.2| ISSfl3 orfC [Shigella flexneri 2a str. 2457T]

MKVLAPGNGKTKTGRLWVYYVRDDRNAGSSLPAAVWXAYSXDRXXXXPQJHLAXXXGRKPDRQ

>gi|30043903|gb|AAP19622.1| hypothetical protein S4706 [Shigella flexneri 2a str. 2457T]

MRITIILVAPARAENIGAAARAMKTMGFSELRIVDSQAHLEPATRWVAHGSGDIIDNIKVFPTLAESLHD

VDFTVATTARSRAKYHYYATPVELVPLLEEKSSWMSHAALVFGREDSGLTNEELALADVLTGVPMVADYP

SLNLGQAVKVYCYQLATLIQQPAKSDTTADQHQLQALRERVMALLTTLAVADDIKLVDWLQQRLGLLEQR

DTAMLHRLLHDIEKNITK

>gi|30043902|gb|AAP19621.1| hypothetical protein S4705 [Shigella flexneri 2a str. 2457T]

MTKVRNCVLDALSINVNNIISLVVGTFPQDPTVSKTAVILTILTAT

>gi|30043901|gb|AAP19620.1| negative response regulator of genes in aerobic pathways (sensors, ArcB and CpxA) [Shigella flexneri 2a str. 2457T]

MQTPHILIVEDELVTRNTLKSIFEAEGYDVFEATDGAEMHQILSEYDINLVIMDINLPGKNGLLLARELR

EQANVALMFLTGRDNEVDKILGLEIGADDYITKPFNPRELTIRARNLLSRTMNLGTVSEERRSVESYKFN

GWELDINSRSLIGPDGEQYKLPRSEFRAMLHFCENPGKIQSRAELLKKMTGRELKPHDRTVDVTIRRIRK

HFESTPDTPEIIATIHGEGYRFCGDLED

>gi|30043900|gb|AAP19619.1| inner membrane protein CreD [Shigella flexneri 2a str. 2457T]

MLKSPLFWKMTTLFGAVLLLLIPIMLIRQVIVERADYRSDVEDAIRQSTSGPQKLVGPLIAIPVTELYTV

QEEDKTVERKRSFIHFWLPESLMVDGNQNVEERKIGIYTGQVWHSDLTLKADFDVSRLSELNAPNIILGK

PFIVISVGDARGIGVVKAPEVNGTALTIEPGTGLEQGGQGVHIPLPEGDWRKQNLQLNMALNLSGTGDLS

VVPAGRNSEMTLTSNWPHPSFLGDFLPAKREASESGFQAQWQSSWFANNLGERFASGNDTGWENFPAFSV

AVTTPADQYQLTDRATKYAILLIALTFMAFFVFETLTAQRLHPMQYLLVGLSLVMFYLLLLALSEHIGFT

VAWIIASLIGALMNGIYLQAVLKGWRNSMLFTLALLLLDGVMWGLLNSADSALLLGTSVLVVALAGMMFV

TRNIDWYAFSLPKMKASKEVTTDDELRIWK

>gi|30043899|gb|AAP19618.1| catabolite repression sensor kinase for PhoB, alternative sensor for pho regulon [Shigella flexneri 2a str. 2457T]

MRIGMRLLLGYFLLVAVAAWFVLAIFVKEVKPGVRRATEGTLIDTATLLAELARPDLLSGDPTHGQLAQA
FNQLQHRPFRANIGGINKVRNEYHVYMTDAHGKVLFDSANKAVGQDYSRWNDVWLTLRGQYGARSTLQNP
ADPESSVMYVAAPIMGGSRLIGVLSVGKPNAAMAPVIKRSERRILWASAILLGIALVIGAGMVWWINRSI
ARLTRYADSVTDNKPVPLPDLGSSELRKLAQALESMRVKLEGKNYIEQYVYALTHELKSPLAAIRGAAEI
LREGPPPEVVARFTDNILTQNARMQALVETLLRQARLENRQEVVLTAVDVAALFRRVSEARTVQLAEKNI
TLHVMPTEVNVAAEPALLDQALGNLLDNAIDFTPESGCITLSAEVDQEHVTLKVLDTGSGIPDYALSRIF
ERFYSLPRANGQKSSGLGLAFVSEVARLFNGEVTLRNVQEGGVLASLRLHRHFT

>gi|30043898|gb|AAP19617.1| catabolic regulation response regulator [Shigella flexneri 2a str. 2457T]
MQRETVWLVEDEQGIADTLVYMLQQEGFAVEVFERGLPVLDKARQQVPDVMILDVGLPDISGFELCRQLL
ALHPALPVLFLTARSEEVDRLLGLEIGADDYVAKPFSPREVCARVRTLLRRVKKFSTPSPVIRIGHFELN
EPAAQISWFDTPLTLTRYEFLLLKTLLKSPGRVWSRQQLMDSVWEDAQDTYDRTVDIHIKTLRAKLRAIN
PDLSPINTHRGMGYSLRGL

>gi|30043897|gb|AAP19616.1| hypothetical protein S4700 [Shigella flexneri 2a str. 2457T]
MKYKHLILSLSLIMLGPLAHAEEIGSVDTVFKMIGPDHKIVVEAFDDPDVKNVTCYVSRAKTGGIKGGLG
LAEDTSDAAISCQQVGPIELSDRIKNGKAQGEVVFKKRTSLVFKSLQVVRFYDAKRNALAYLAYSDKVVE
GSPKNAISAVPVMPWRQ

>gi|30043896|gb|AAP19615.1| right origin-binding protein [Shigella flexneri 2a str. 2457T]
MDQAGIIRDLLIWLEGHLDQPLSLDNVAAKAGYSKWHLQRMFKDVTGHAIGAYIRARRLSKSAVALRLTA
RPILDIALQYRFDSQQTFTRAFKKQFAQTPALYRRSPEWSAFGIRPPLRLGEFTMPEHKFVTLEDTPLIG
VTQSYSCSLEQISDFRHEMRYQFWHDFLGNAPTIPPVLYGLNETRPSQDKDDEQEVFYTTALAQDQADGY
VLTGHPVMLQGGEYVMFTYEGLGTGVQEFILTVYGTCMPMLNLTRRKGQDIERYYPAEDAKAGDRPINLR
CELLIPIRR

>gi|30043895|gb|AAP19614.1| phosphoglyceromutase 2 [Shigella flexneri 2a str. 2457T]

MLQVYLVRHGETQWNAERRIQGQSDSPLTAKGEQQAMQVATRAKELGITHIISSDLGRTRRTAEIIAQAC

GCDIIFDSRLRELNMGVLEKRHIDSLTEEEENWRRQLVNGTVDGRIPEGESMQELSDRVNAALESCRDLP

QGSRPLLVSHGIALGCLVSTILGLPAWAERRLRLRNCSISRVDYQESLWLASGWVVETAGDISHLDAPAL

DELQR

>gi|30043894|gb|AAP19613.1| regulator for trp operon and aroH; trp aporepressor [Shigella flexneri 2a str. 2457T]

MAQQSPYSAAMAEQRHQEWLRFVDLLKNAYQNDLHLPLLNLMLTPDEREALGTRVRIVEELLSGEMSQRE

LKNELGAGIATITRGSNSLKAAPVELRQWLEEVLLKSD

>gi|30043893|gb|AAP19612.1| soluble lytic murein transglycosylase [Shigella flexneri 2a str. 2457T]

MYLHLEDALVEKAKQVTWRLLAAGVCLLTVSSVARADSLDEQRSRYAQIKQAWDNRQMDVVEQMMPGLKD

YPLYPYLEYRQITDDLMNQPAVTVTNFVRANPTLPPARTLQSRFVNELARREDWRGLLAFSPEKPGTTEA

QCNYYYAKWNTGQSEEAWQGAKELWLTGKSQPNACDKLFSVWRASGKQDPLAYLERIRLAMKAGNTGLVT

VLAGQMPADYQTIASAIISLANNPNTVLTFARTTGATDFTRQMAAVAFASVARQDAENARLMIPSLAQAQ

QLNEDQIQELRDIVAWRLMGNDVTDEQAKWRDDAIMRSQSTSLIERRVRMALGTGDRRGLNTWLARLPME

AKEKDEWRYWQADLLLERGREAEAKEILHQLIQQRGFYPMVAAQRIGEEYELKIDKAPQNVDSTLTQGPE

MARVRELMYWNLDNTARSEWANLVKSKSKTEQAQLARYAFNNQWWDLSVQATIAGKLWDHLEERFPLAYN

DLFKRYTSGKEIPQSYAMAIARQESAWNPKVKSPVGASGLMQIMPGTATHTVKMFSIPGYSSPGQLLDPE

TNINIGTSYLQYVYQQFGNNRIFSSAAYNAGPGRVRTWLGNSAGRIDAVAFVESIPFSETRGYVKNVLAY

DAYYRYFMGDKPTLMSATEWGRRY

>gi|30043892|gb|AAP19611.1| putative ATP-binding component of a transport system [Shigella flexneri 2a str. 2457T]

MAQFVVYTMHRVGKVVPPKRHILKNISLSFFPGAKIGVLGLNGAGKSTLLRIMAGIDKDIEGEARPQPDIK

IGYLPQEPQLNPEHTVRESIEEAVSEVVNALKRLDEVYALYADPDADFDKLAAEQGRLEEIIQAHDGHNL

NVQLERAADALRLPDWDAKIANLSGGERRRVALCRLLLEKPDMLLLDEPTNHLDAESVAWLERFLHDFEG

TVVAITHDRYFLDNVAGWILELDRGEGIPWEGNYSSWLEQKDQRLAQEASQEAARRKSIEKELEWVRQGT

KGRQSKGKARLARFEELNSTEYQKRNETNELFIPPGPRLGDKVLEVSNLRKSYGDRLLIDDLSFSIPKGA

IVGIIGPNGAGKSTLFRMISGQEQPDSGTITLGETVKLASVDQFRDSMDNSKTVWEEVSGGLDIMKIGNT

EMPSRAYVGRFNFKGVDQGKRVGELSGGERGRLHLAKLLQVGGNMLLLDEPTNDLDIETLRALENALLEF

PGCAMVISHDRWFLDRIATHILDYQDEGKVEFFEGNFTEYEEYKKRTLGADALEPKRIKYKRIAK

>gi|30043891|gb|AAP19610.1| probable nadAB transcriptional regulator [Shigella flexneri 2a str. 2457T]

MRDTEGDMSSFDYLKTAIKQQGCTLQQVADASGMTKGYLSQLLNAKIKSPSAQKLEALHRFLGLEFPRQK

KTIGVVFGKFYPLHTGHIYLIQRACSQVDELHIIMGFDDTRDRALFEDSAMSQQPTVPDRLRWLLQTFKY

QKNIRIHAFNEEGMEPYPHGWDVWSNGIKKFMAEKGIQPDLIYTSEEADAPQYMEHLGIETVLVDPKRTF

MSISGAQIRENPFRYWEYIPTEVKPFFVRTVAILGGESSGKSTLVNKLANIFNTTSAWEYGRDYVFSHLG

GDEIALQYSDYDKIALGHAQYIDFAVKYANKVAFIDTDFVTTQAFCKKYEGREHPFVQALIDEYRFDLVI

LLENNTPWVADGLRSLGSSVDRKEFQNLLVEMLEENNIEFVRVEEDDYDSRFLRCVELVREMMGEQG

>gi|30043890|gb|AAP19609.1| probable ATP-dependent protease [Shigella flexneri 2a str. 2457T]

MAKAPKRAFVCNECGADYPRWQGQCSACHAWNTITEVRLAASPTVARNERLSGYAGSAGVAKVQKLSDIS

LEELPRFSTGFKEFDRVLGGGVVPGSAILIGGNPGAGKSTLLLQTLCKLAQQMKTLYVTGEESLQQVAMR

AHRLGLPTDNLNMLSETSIEQICLIAEEEQPKLMVIDSIQVMHMADVQSSPGSVAQVRETAAYLTRFAKT

RGVAIVMVGHVTKDGSLAGPKVLEHCIDCSVLLDGDADSRFRTLRSHKNRFGAVNELGVFAMTEQGLREV

SNPSAIFLSRGDEVTSGSSVMVVWEGTRPLLVEIQALVDHSIMANPRRVAVGLEQNRLAILLAVLHRHGG

LQMADQDVFVNVVGGVKVTETSADLALLLAMVSSLRDRPLPQDLVVFGEVGLAGEIRPVPSGQERISEAA

KHGFRRAIVPAANVPKKAPEGMQIFGVKKLSDALSVFDDL

>gi|30043889|gb|AAP19608.1| 3-phosphoserine phosphatase [Shigella flexneri 2a str. 2457T]

MPNITWCDLPEDVSLWPGLPLSLSGDEVMPLDYHAGRSGWLLYGRGLDKQRLTQYQSKLGAAMVIVAAWC

VEDYQVIRLAGSLTARATRLAHEAQLDVAPLGKIPHLRTPGLLVMDMDSTAIQIECIDEIAKLAGTGEMV

AEVTERAMRGELDFTASLRSRVATLKGADANILQQVRENLPLMPGLTQLVLKLETLGWKVAIASGGFTFF

AEYLRDKLRLTAVVANELEIMDGKFTGNVIGDIVDAQYKAKTLTRLAQEYEIPLAQTVAIGDGANDLPMI

KAAGLGIAYHAKPKVNEKAEVTIRHADLMGVFCILSGSLNQK

>gi|30043888|gb|AAP19607.1| hypothetical protein S4690 [Shigella flexneri 2a str. 2457T]

MARTKLKFRLHRAVIVLFCLALLVALMQGASWFSQNHQRQRNPQLEELARTLARQVTLNVAPLMRTDSPD

EKRIQAILDQLTDESRILDAGVYDEQGDLIARSGESVEVRDRLALDGKKAGGYFNQQIVEPIAGKNGPLG

YLRLTLDTHTLATEAQQVDNTTNILRLMLLLSLAIGVVLTRTLLQGKRTRWQQSPFLLTASKPVPEEEES

EKKE

>gi|30043887|gb|AAP19606.1| hypothetical protein S4688 [Shigella flexneri 2a str. 2457T]

MIDLENQEREIINLMFSQRISWLAAVRIRHKLSLAEVSKMLGISINSLKQIEKTERLSSNIKSKMAEIYG

CPPELLICPSWMTAEHK

>gi|30043886|gb|AAP19605.1| purine-nucleoside phosphorylase [Shigella flexneri 2a str. 2457T]

MATPHINAEMGDFADVVLMPGDPLRAKYIAETFLEDAREVNNVRGMLGFTGTYKGRKISVMGHGMGIPSC

SIYTKELITDFGVKKIIRVGSCGAVLPHVKLRDVVIGMGACTDSKVNRIRFKDHDFAAIADFDMVRNAVD

AAKALGVDARVGNLFSADLFYSPDGEMFDVMEKYGILGVEMEAAGIYGVAAEFGAKALTICTVSDHIRTH

EQTTAAERQTTFNDMIKIALESVLLGDKE

>gi|30043885|gb|AAP19604.1| phosphopentomutase [Shigella flexneri 2a str. 2457T]

MKRAFIMVLDSFGIGATEDAERFGDVGADTLGHIAEACAKGEADNGRKGPLNLPNLTRLGLAKAHEGSTG
FIPAGMDGNAEVIGAYAWAHEMSSGKDTPSGHWEIAGVPVLFEWGYFSDHENSFPQELLDKLVERANLPG
YLGNCHSSGTVILDQLGEEHMKTGKPIFYTSADSVFQIACHEETFGLDKLYELCEIAREELTNGGYNIGR
VIARPFIGDKAGNFQRTGNRHDLAVEPPAPTVLQKLVDEKHGQVVSVGKIADIYANCGITKKVKATGLDA
LFDATIKEMKEAGDNTIVFTNFVDFDSSWGHRRDVAGYAAGLELFDRRLPELMSLLRDDDILILTADHGC
DPTWTGTDHTREHIPVLVYGPKVKPGSLGHRETFADIGQTLAKYFGTSDMEYGKAMF

>gi|30043884|gb|AAP19603.1| thymidine phosphorylase [Shigella flexneri 2a str. 2457T]
MFLAQEIIRKKRDGHALSDEEIRFFINGIRDNTISEGQIAALAMTIFFHDMTMPERVSLTMAMRDSGTVL
DWKSLHLNGPIVDKHSTGGVGDVTSLMLGPMVAACGGYIPMISGRGLGHTGGTLDKLESIPGFDIFPDDN
RFREIIKDVGVAIIGQTSSLAPADKRFYATRDITATVDSIPLITASILAKKLAEGLDALVMDVKVGSGAF
MPTYELSEALAEAIVGVANGAGVRTTALLTDMNQVLASSAGNAVEVREAVQFLTGEYRNPRLFDVTMALC
VEMLISGKLAKDDAEARAKLQAVLDNGKAAEVFGRMVAAQKGPTDFVENYAKYLPTAMLTKAVYADTEGF
VSEMDTRALGMAVVAMGGGRRQASDTIDYSVGFTDMARLGDQVDGQRPLAVILAKDENSWQEAAKAVKAA
IKLADNAPESTPTVYRRISE

>gi|30043883|gb|AAP19602.1| 2-deoxyribose-5-phosphate aldolase [Shigella flexneri 2a str. 2457T]
MTDLKASSLRALKLMDLTTLNDDDTDEKVIALCHQAKTPVGNTAAICIYPRFIPIARKTLKEQGTPEIRI
ATVTNFPHGNDDIEIALAETRAAIAYGADEVDVVFPYRALMAGDEQVGFDLVKACKEACAAANVLLKVII
ETGELKDEALIRKASEISIKAGADFIKTSTGKVAVNATPESARIMMEVIRDMGVEKTVGFKPAGGVRTAE
DAQKYLAIADELFGADWADARHYRFGASSLLASLLKALGHGDGKSASSY

>gi|30043882|gb|AAP19601.1| hypothetical protein S4683 [Shigella flexneri 2a str. 2457T]
MPTSHENALQQRCQQIVTSPVLSPEQKRHFLALEAENNLPYPQLPAEARRALDEGVICDMFEGHAPYKPR
YVLPDYARFLANGSEWLELEGAKDLDDALSLLTILYHHVPSVTSMPVYLGQLDALLQPYVRILTQNEIDV

RIKRFWRYLDRTLPDAFMHANIGPSDSPITRAILRADAQVSPNLTFIYDPEITPDDLLLEVAKNICECSK
PHIANGPVHDKIFTKGGYGIVSCYNSLPLAGGGSTLVRLNLKAIAERSESLDDFFTRTLPHYCQQQIAII
DARCEFLYQQSHFFENSFLVKEGLINPERFVPMFGMYGLAEAVNLLCEKEGIAARYGKEAAANEVGYRIS
AQLAEFVANTPVKYGWQKRAMLHAQSGISSDIGTTPGARLPYGDEPDPITHLQTVAPHHAYYYSGISDIL
TLDETIKRNPQALVQLCLGAFKAGMREFTANVSGNDLVRVTGYMVRLSDLEKYRAEGSRTNTTWLGEEAA
RNTRILERQPRVISHEQQMRFSQ

>gi|30043881|gb|AAP19600.1| putative activating enzyme [Shigella flexneri 2a str. 2457T]
MNSRCALVSKIIPFSCVDGPGSRLALFLQGCNLRCKNCHNPWTMGRCNDCGECVPQCPHQALQIVDGKVV
WNAVVCEQCDTCLKMCPQHATPMAQSMSVDEVLSHVRKAVLFIEGITVSGGEATTQLPFVVALFTAIKND
PQLRHLTCLVDSNGMLSETGWEKLLPVCDGAMLDLKAWGSECHQQLTGRDNQQIKRSIYLLAERGKLAEL
RLLMIPGQVDYLQHIEELAAFIKGLGDVPVRLNAFHAHGVYGEAQSWASATPEDVEPLADALKVRGVSRL
IFPALYL

>gi|30043880|gb|AAP19599.1| hypothetical protein S4681 [Shigella flexneri 2a str. 2457T]
MQALAENYQPLYAVLGLHPGMLEKHSDVSLEQLQQALERRPAKVVAVGEIGLDLFGDDPQFERQQWLLDE
QLKLAKRYDLPVILHSRRTHDKLAMHLKRHDLPRAGVVHGFSGSQQQAERFVQLGYKIGVGGTITYPRAS
KTRDVIAKLPLASLLLETDAPDMPLNGFQGQPNRPEQAARVFAVLCELRPEPADEIAEVLLNNTYAVFNV
RG

>gi|30043879|gb|AAP19598.1| hypothetical protein S4680 [Shigella flexneri 2a str. 2457T]
MGQRIPVTLGNIAPLSLRPFQPGRIALVCEGGGQRGIFTAGVLDEFMRAQFNPFDLYLGTSAGAQNLSAF
ICNQPGYARKVIMRYTTKREFFDPLRFVRGGNLIDLDWLVEATASQMPLQMDTAARLFDSGKSFYMCACR
QDDYAPNYFLPTKQNWLDVIRASSAIPGFYRSGVSLEGINYLDGGISDAIPVKEAARQGAKTLVVIRTVP
SQMYYTPQWFKRMERWLGDSSLQPLVNLVQHHETSYRDIQQFIEKPPGKLRISEIYPPKPLHSIALGSRI

PALREDYKLGRLCGRYFLATVGKLLTEKAPLTRHLVPVVTPESIVIPPAPVANDTLVAEVSDAPQANDPT

FNNEDLA

>gi|30043878|gb|AAP19597.1| hyperosmotically inducible periplasmic protein [Shigella flexneri 2a str. 2457T]

MTMTRLKISKTLLAVMLTSAVATGSAYAENNAQTTNESAGQKVDSSMNKVGNFMDDSAITAKVKAALVDH

DNIKSTDISVKTDQKVVTLSGFVESQAQAEEAVKVAKGVEGVTSVSDKLHVRDAKEGSVKGYAGDTATTS

EIKAKLLADDIVPSRKVKVETTDGVVQLSGTVDSQAQSDRAESIAKAVDGVKSVKNDLKTK

>gi|30043877|gb|AAP19596.1| peptide chain release factor RF-3 [Shigella flexneri 2a str. 2457T]

MTLSPYLKEVAKRRTFAIISHPDAGKTTITEKVLLFGQAIQTAGTVKGRGSNQHAKSDWMEMEKQRGISI

TTSVMQFPYHDCLVNLLDTPGHEDFSEDTYRTLTAVDCCLMVIDAAKGVEDRTRKLMEVTRLRDTPILTF

MNKLDRDIRDPMELLDEVENELKIGCAPITWPIGCGKLFKGVYHLYKDETYLYQSGKGHTIQEVRIVKGL

NNPDLDAAVGEDLAQQLRDELELVKGASNEFDKELFLAGEITPVFFGTALGNFGVDHMLNGLVEWAPAPM

PRQTDTRTVEASEDKFTGFVFKIQANMDPKHRDRVAFMRVVSGKYEKGMKLRQVRTAKDVVISDALTFMA

GDRSHVEEAYPGDILGLHNHGTIQIGDTFTQGEMMKFTGIPNFAPELFRRIRLKDPLKQKQLLKGLVQLS

EEGAVQVFRPISNNDLIVGAVGVLQFDVVVARLKSEYNVEAVYESVNVATARWVECADAKKFEEFKRKNE

SQLALDGGDNLAYIATSMVNLRLAQERYPDVQFHQTREH

>gi|30043876|gb|AAP19595.1| putative phosphatase [Shigella flexneri 2a str. 2457T]

MKWDWIFFDADETLFTFDSFTGLQRMFLDYSVTFTAEDFQDYQAVNKPLWVDYQNGAITSLQLQHGRFES

WAERLKVEAGLLNDAFINAMAEICTPLPGAVSLLNAIRSNAKIGIITNGFSALQQVRLERTGLRDYFDLL

VISEEVGVAKPNKKIFDYALEQAGNPDRSRVLMVGDTAESDILGGINAGLATCWLNAHNREQPEGIAPTW

TVSSLHELEQLLCKH

>gi|30043875|gb|AAP19594.1| acyltransferase for 30S ribosomal subunit protein S18 [Shigella flexneri 2a str. 2457T]

MNTISSLETTDLPAAYHIEQRAHAFPWSEKTFASNQGERYLNFQLTQNGKMAAFAITQVVLDEATLFNIA

VDPDYQRQGLGRALLEHLIDELEKRGVATLWLEVRASNAAAIALYESLGFNEATIRRNYYPTTDGREDAI

IMALPISM

>gi|30043874|gb|AAP19593.1| DNA polymerase III, psi subunit [Shigella flexneri 2a str. 2457T]

MTSRRDWQLQQLGITQWSLRRPGALQGEIAIASPAHVRLVMVANDLPALTDPLVSDVLRALTVSPDQVLQ

LTPEKIAMLPQGSRCNSWRLGTDEPLSLEGAQVASPALTELRANPTARAALWQQICTYEHDFFPRND

>gi|30043873|gb|AAP19592.1| putative enzyme [Shigella flexneri 2a str. 2457T]

MSAFTPASEVLLRHSDDFEQSRILFAGDLQDDLPARLDTAASRAHTQQFHHWQVLSCQMGDNARFSLVAT

ANDVADCDTLIYYWPKNKPEAQFQLMNLLSLLPVGTDIFVVGENRSGVRSAEQMLADYAPLNKVDSARRC

GLYFGRLEKQPVFDADKFWGEYSVDGLTVKTLPGVFSRDGLDVGSQLLLSTLTPHTKGKVLDVGCGAGVL

SVAFARHSPKIRLTLCDVSAPAVEASRATLAANGVEGEVFASNVFSEVKGCFDMIISNPPFHDGMQTSLD

AAQTLIRGAVRHLNSGGELRIVANAFLPYPDVLDETFGFHEVIAQTGRFKVYRAIMTRQAKKG

>gi|30043872|gb|AAP19591.1| hypothetical protein S4670 [Shigella flexneri 2a str. 2457T]

MAYRSAPLYEDVIWRTHLQPQDAGLAQAVRAMIAKHREHLLEFIRLDEPAPLNAMTLAQWSSPNALSSLL

AVYSDHIYRNQPTMIRENKPLISLWAQWYIGLMVPPLMLALLTQEKALDVSPEHFHAEFHETGRVACFWV

DVCEDKNATPHSPQQRMETLISQALVPVVQALEATGEINGKLIWSNTGYLINWYLTEMKQLLGEATVESL

RHALFFEKTLTNGEDNPLWRTVVLRDGLLVRRTCCQRYRLPDVQQCGYCTLK

>gi|30043871|gb|AAP19590.1| 2-component transcriptional regulator [Shigella flexneri 2a str. 2457T]

MSRISFAAVIFSFSAMRSERREGLSCLTELAIKFPRTRRLVIVDDDIEARLIGSLSPSPLDGVLSKASTL

EIFHQELFLSLNGVRQATDRLNNQWYINQSRTLSPTEREILRFMSRGYSMTQIAEQLKRNIKTIRAHKFN

VMSKLGVSSDAGLLEAADILLCMRHCEASNVLHPY

>gi|30043870|gb|AAP19589.1| IS1 orfB [Shigella flexneri 2a str. 2457T]

MSRQCTHYGRWPQHGFTSLKKLRPQSVTSRIQPGSDVIVCAEMDEHWGYVGAKSRQRWLFYAYDRIRRTV

VAHVFGERTLATLERLLSLLSAFEVVVWMTDGWPLYESRLKGKLHVISKRYTQRIERHNLNLRQHLARLG

RKSLSFSKSVELHDKVIGHYLNIKHYQ

>gi|30043869|gb|AAP19588.1| IS1 orfA [Shigella flexneri 2a str. 2457T]

MASISIRCPSCSATEGVVRNGKSTAGHQRYLCSHCRKTWQLQFTYTASQPGTHQKIIDMAMNGVGCRASA

RIMGVGLNTVLRHLKNSGRSR

>gi|30043868|gb|AAP19587.1| hypothetical protein S4664 [Shigella flexneri 2a str. 2457T]

MILMTSGLNIEWSTFMASMLVGTIGIQWSRWYLAHPKVFTVAAVIPMFPGISAYTAMISSVKISQLGYSE

PLMITLLTNFLTASSIVGALSIGLSIPGLWLYRKRPRV

>gi|30043867|gb|AAP19586.1| primosomal protein i [Shigella flexneri 2a str. 2457T]

MSSRVLTPDVVGIDALVHDHQTVLAKAEGGVVAVFANNAPAFYAVTPARLAELLALEEKLARPGSDVALD

DQLYQEPQAAPVAVPMGKFAMYPDWQPDADFIRLAALWGVALREPVTTEELASFIAYWQAEGKVFHHVQW

QQKLARSLQIGRASNGGLPKRDVNTVSEPDSQIPPGFRG

>gi|30043866|gb|AAP19585.1| chromosome replication protein DnaC [Shigella flexneri 2a str. 2457T]

MKNVGDLMQRLQKMMPAHIKPAFKTGEELLAWQKEQGAIRSAALERENRAMKMQRTFNRSGIRPLHQNCS

FENYRVECEGQMNALSKARQYVEEFDGNIASFIFSGKPGTGKNHLAAAICNELLLRGKSVLIITVADIMS

AMKDTFRNSGTSEEQLLNDLSNVDLLVIDEIGVQTESKYEKVIINQIVDRRSSSKRPTGMLTNSNMEEMT

KLLGERVMDRMRLGNSLWVIFNWDSYRSRVTGKEY

>gi|30043865|gb|AAP19584.1| putative glycoprotein/receptor [Shigella flexneri 2a str. 2457T]

MMMKTIKHLLCCAIAASALISTGVHAASWKDALSSAASELGNQNSTTQEGGWSLASLTNLLSSGNQALSA

DNMNNAAGILQYCAKQKLASVTDAENIKNQVLEKLGLNSEEQKEDTNYLDGIQGLLKTKDGQQLNLDNIG

TTPLAEKVKTKACDLVLKQGLNFIS

>gi|30043864|gb|AAP19583.1| phosphoglycerol transferase I [Shigella flexneri 2a str. 2457T]

MLIYAWKAGRNTWWFAATLTVLGLFVVLNITLFASDYFTGDGINDAVLYTLTNSLTGAGVSKYILPGIGI

VLGLTAVFGALGWILRRRRHHPHHFGYSLLALLLALGSVDASPAFRQITELVKSQSRDGDPDFAAYYKEP

SKTIPDPKLNLVYIYGESLERTYFDNEAFPDLTPELGALKNEGLDFSHTQQLPGTDYTIAGMVASQCGIP

LFAPFEGNASASVSSFFPQNICLGDILKNSGYQNYFVQGANLRFAGKDVFLKSHGFDHLYGSEELKSVVA

DPHYRNDWGFYDDTVLDEAWKKFEELSRSGQRFSLFTLTVDTHHPDGFISRTCNRKKYDFDGKPNQSFSA

VSCSQENIATFINKIKASPWFKDTVIVVSSDHLAMNNTAWKYLNKQDRNNLFFVIRGDKPQQKTLAVKRN

TMDNGATVLDILGGDNYLGLGRSSLSGQSMSEIFLNIKEKTLAWKPDIIRLWKFPKEMKEFTIDQQKNMI

AFSGSHFRLPLLLRVSDKRVEPLPESEYSAPLRFQLADFAPRDNFVWVDSCYKMAQLWAPELALSTDWCV

SQGQLGGQQIVQHVDKTTWKSKTAFKDTVIDMARYKGNVDTLKIVDNDIRYKADSFIFNVAGAPEEVKQF

SGISRPESWGRWSNAQLGDEVKIEYKHPLPKKFDLVITAKAYGNNASRPIPVRVGNEEQTLVLGNEVTTT

TLHFDNPTDADTLVIVPPEPVSTNEGNILGHSPRKLGIGMVEIKVVEREG

>gi|30043863|gb|AAP19582.1| hypothetical protein S4658 [Shigella flexneri 2a str. 2457T]

MYNISRTTVRHILSQLRECGVLTQVGNDYVIARKPDHDDGFACTTASMSEQNKVFEQAFFTMINQRQLRP

RETFSELQLARAAGVSPVVVREYLLKFGRYNLIQSEKRGQWSMKQFDQSYAEQLFELREMLETHSLQHFL

NLPDHDPRWLQAKTMLERHRLLRDNIGNSFRMFSQLDRDFHSLLLSAADNIFFDQSLEIISVIFHFHYQW

DESDLKQRNIIAVDEHMTILSALICRSDLDATLALRNHLNSAKQSMIRSINENTRYAH

>gi|30043862|gb|AAP19581.1| putative transport protein [Shigella flexneri 2a str. 2457T]

MEKENITLDPRSSFTPSSSADIPVPPDGLVQRSTRIKRIQTTAMLLLFFAAVINYLDRSSLSVANLTIRE

ELGLSATEIGALLSVFSLAYGIAQLPCGPLLDRKGPRLMLGLGMFFWSLFQAMSGMVHSFTQFVLVRIGM

GIGEAPMNPCGVKVINDWFNIKERGRPMGFFNAASTIGVAVSPPILAAMMLVMGWRGMFITIGVLGIFLA

IGWYMLYRNREHVELTAVEQAYLNAGSVNARRDPLNFAEWRSLFRNRTMWGMMLGFSGINYTAWLYLAWL

PGYLQTAYNLDLKSTGLMAAIPFLFGAAGMLVNGYVTDWLVKGGMAPIKSRKICIITGMFCSAAFTLIVP

QATTSMMAVLLIGMALFCIHFAGTSCWGLIHVAVASRMTASVGSIQNFASFICASFAPIITGFIVDTTHS

FRLALIICGCVTAAGALAYIFLVRQPINDPRKD

>gi|30043861|gb|AAP19580.1| methyl-accepting chemotaxis protein I, serine sensor receptor [Shigella flexneri 2a str. 2457T]

MLKRIKIVTSLLLVLAVFGLLQLTSGGLFFNALKNDKENFTVLQTIRQQQPTLNGSWVALLQTRNTLNRA

GIRYMMDQNNIGSGSTVAELMQSASISLKQAEKNWADYEALPRDPRQSTAAAAEIKRNYDIYHNALAELI

QLLGAGKINEFFDQPTQGYQDGFEKQYVAYMEQNDRLYDIAVSDNNASYSQAMWILVGVMIVVLAVIFAV

WFGIKASLVAPMNRLIDSIRHIAGGDLVKPIEVDGSNEMGQLAESLRHMQGELMRTVGDVRNGANAIYSG

ASEIATGNNDLSSRTEQQAASLEETAASMEQLTATVKQNAENARQASHLALSASETAQRGGKVVDNVVQT

MRDISTSSQKIADIISVIDGITFQTNILALNAAVEAARAGEQGRGFAVVAGEVRNLAQRSAQAVREIKSL

IEDSVGKVDVGSTLVESAGETMAEIVSAVTRVTDIMGEIASASDEQSRGIDQVGLAVAEMDRVTQQNAAL

VEESAAAAAALEEQASRLTEAVAVFRIQQQQQQQRETSAVVKTVTPATPRKMAVADSGENWETF

>gi|30043860|gb|AAP19579.1| regulator of the 4HPA-hydroxylase operon [Shigella flexneri 2a str. 2457T]

MHDSLTIALLQAREAAMSYFRPIVKRHNLTEQQWRIVRILAESPSMDFHDLAYRACILRPSLTGILTRME

RDGLVLRLKPINDQRKLYISLTKEGQALYNRAQTQIEEAYRQIEAQFTAEKMQQLTHLLEEFIALGNSRQ

EDIPGDNE

>gi|30043859|gb|AAP19578.1| 5-oxo-1,2,5-tricarboxilic-3-penten acid decarboxilase/isomerase [Shigella flexneri 2a str. 2457T]

MKGTIFAVALNHRSQLDAWQEAFQQSPYKAPPKTAVWFIKPRNTVIGCGEPIPFPQGEKVLSGATVALIV

GKTATKVREEDAAEYIAGYALDNDVSLPEESFYRPAIKAKCRDGFCPIGETVALSNVDNLTIYTEINGRP

ADHWNTADLQRNAAQLLSALSEFATLNPGDAILLGTPQARVEIQPSDRVRVLAEGFPPLENPVVDEREVT

TRKSFPTLPHPHGTLFALGLNYADHASELEFKPPEEPLVFLKAPNTLTGDNQTSVRPNNIEYMHYEAELV

VVIGKQARNVSEADAMDYVAGYTVCNDYAIRDYLENYYRPNLRVKSRDGLTPMLSTIVPKEAIPDPHNLT

LRTFVNGELRQQGTTADLIFSVPFLIAYLSEFMTLNPGDMIATGTPKGLSDVVPGDEVVVEVEGVGRLVN

RIVSEETAK

>gi|30043858|gb|AAP19577.1| 5-carboxy-2-hydroxymuconate semialdehyde dehydrogenase [Shigella flexneri 2a str. 2457T]

MKKVNHWINGKNVAGNDYFQTTNPATGEVLADVASGGEAEINQAVAAAKEAFPKWADLPMKERARLMRRL

GDLIDQNVPEIAAMETADTGLPIHQTKNVLIPRASHNFEFFAEVCQQMNGKTYPVDDKMLNYTLVQPVGV

CALASPWNVPFMTATWKVAPCLALGNTAVLKMSELSPLTADRLGELALEAGIPAGVLNVVQGYGATAGDA

LVRHHDVRAVSFTGGTATGRNIMKNAGLKKYSMELGGKSPVLIFEDADIERALDAALFTIFSINGERCTA

GSRIFIQQSIYPEFVKRFAERANRLRVGDPNDPNTQVRALISQQHWEKVSGYIRLGIEEGATLLAGGPDK

PSDLPAHLKGGNFLRPTVLADVDNRMRVAQEEIFGPVACLLPFKDEAEGLRLANDVEYGLASYIWTQDVS

KVLRLARGIEAGMVFVNTQNVRDLRQPFGGVKASGTGREGGEYSFEVFAEMKNVCISMGDHPIPKWGV

>gi|30043857|gb|AAP19576.1| homoprotocatechuate dyoxygenase [Shigella flexneri 2a str. 2457T]

MGKLALAAKITHVPSMYLSELPGKNHGCRQGAIDGHKEISKRCREMGVDTIIVFDTHWLVNSAYHINCAD

HFEGVYTSNELPHFIRDMTYNYEGNPELGQLIAEEALKLGVRAKAHNIPSLKLEYGTLVPMRYMNEDKHF

KVVSISAFCTVHDFADSRKLGEAILKAIEQYDGTVAVLASGSLSHRFIDDQRAEEGMNSYTREFDRQMDE

RVVKLWREGQFKEFCNMLPEYADYCYGEGNMHDTVMLLGMLGWDKYDGKVEFITELFPSSGTGQVNAVFP

LPA

>gi|30043856|gb|AAP19575.1| 5-carboxymethyl-2-hydroxymuconate delta-isomerase [Shigella flexneri 2a str. 2457T]

MPHFIVECTENIREEARLPELFASVNTALAATGIFPLGGIRSRAHWIDTWQMADGKHDYAFVHMTLKIGS

GRSLESRQEVGEMLFDLIKTHFASLMESRYLALSFEIAELHPTLNFKQNNVHALFK

>gi|30043855|gb|AAP19574.1| 2-oxo-hept-3-ene-1, 7-dioate hydratase [Shigella flexneri 2a str. 2457T]

MFDKHTHTLIAQRLDQAEKQREQIRAISLDYPEITIEDAYAVQREWVRLKIAESRTLKGHKIGLTSKAMQ

ASSQISEPDYGALLDDMFFHDGSDIPTDRFIVPRIEVELAFVLAKPLRGPNCTLFDVYNATDYVIPALEL

IDARCHNIDPETQRPRKVFDTISDNAANAGVILGGRPIKPDELDLRWISALMYRNGVIEETGVAAGVLNH

PANGVAWLANKLAPYDVQLETGQIILGGSFTLPVPARKGDTFHVDYGNMGSISCRFV

>gi|30043854|gb|AAP19573.1| 2,4-dihydroxyhept-2-ene-1,7-dioic acid aldolase [Shigella flexneri 2a str. 2457T]

MENSFKAALKAGRPQIGLWLGLSSSYSAELLAGPGFDWLLIDGEHAPNNVQTVLTQLQAIAPYPSQPVVR

PSWNDPVQIKQLLDVGTQTLLVPMVQNADEARKAVRATRYPPAGIRGVGSALARASRWNRIPDYLQKAND

QMCVLVQIETREAMKNLPQILDVEGVDGVFIGPADLSADMGYAGNPQHPEVQAAIEQAIVQIREAGKAPG

ILIANEQLAKRYLELGALFVAVGVDTTLLARAAEALAARFGNSSSISMAKQNNNSVY

>gi|30043853|gb|AAP19572.1| putative 4-hydroxyphenylacetate permease [Shigella flexneri 2a str. 2457T]

MTTSTLQYKDNKTVEIEHRVINKLFRRLIIFLFILFVFSFLDRINIGFAGLTMGKDLGLTSTMFGLAATL

FYVTYVMCGIPSNMMLAKIGARRWIAGIMVVWGIASTCTMFATSPETLYLLRMLVGIAEAGFLPGILVYL

TWWFPAYHRARANALFMIAMPVTMMLGLILSGYILALDGLWNLKGWQWLFLLEGLPSVILGVVTWFYLND

TPDQATWLDENEKQALKTMILREQESAFAHASVPRSTLREVLTPAIMLYTLAYFCLTNTLSAINIWTPQI

LQSFNTGSSNIMIGLLAAIPQFCTIFGMIWWSRRSDRLKERKKHTILPYLFAAAGWLLASATDHSLIQLS

SIIMASMGSFTAMTIFWTTPDQVISLQSRAVAMAVINAVGNVGSAVSPLLIGILKDATGSFSSGLWFVAG

LLVLGALVMTRIPMGSREHSLSSELIAPKPQ

>gi|30043852|gb|AAP19571.1| putative 4-hydroxyphenylacetate 3-monooxygenase operon regulatory protein [Shigella flexneri 2a str. 2457T]

MSSEAIFEHGYENIDLVREYDARYAGEDVHYETFARLANFFGRDMRPHWHDRYFQLHYLVTGRITLQLDE

HFYSVRAPLFVLTPPSVPHTFFTNPDSDGHVLTVRQELIWPLVEKLWPGSGDAAMLQGICLSLEGMEHTL

AALNHYWPIISLEFQQNKQGREILLQSLAQSIFTILLREAPPNDISTCSVRGEMCLFQKFNRLIDENYRQ

HLMVPEYATMLGMSESRLTELCRRFANQSPKRLIFERVSREARRLLLYSEQSINQIALDLGYKDPAYFAR

FFNRMMGCSPTQFRGR

>gi|30043851|gb|AAP19570.1| hypothetical protein S4646 [Shigella flexneri 2a str. 2457T]

MATALIIGNKTWLIDAGYGASLQLVKNNIPLRQIDNILLTHLHSDHILDYPSVLMNAWASGLQDKTIHVF

GPPGTRAMTAASWQVFDRDITLRMEEEGKPDPRKLVTAQDVGEGVVYKDDTVTVTALKVPHSPFPDGEAF

AYRFDTQGKRIVFSGDTSYFPPLATFSEGADILVHEAVHEPSVAKLADSIGNGKTLAKAIASHHTTIEDV

GKIANEAKVKKLILSHLVPITVPDETWKQEAQKTIQVLSSLVMKI

>gi|30043850|gb|AAP19569.1| component C of the 4HPA-hydroxylase [Shigella flexneri 2a str. 2457T]

MQLDEQRLRFHDAMASLSAAVNIITTEGDAGQCGITATAVCSVTDTPPSLMVCINANSAMNPVFQGNGKL

CVNVLNHEQELMARHFAGMTGMAMEERFSLSCWQKGPLAQPVLKGSLASLEGEIRDVQAIGTHLVYLVEI

KNIILSAEGHGLIYFKRRFHPVMLEMEAAI

>gi|30043849|gb|AAP19568.1| putative carbon starvation protein [Shigella flexneri 2a str. 2457T]

MPGFTMDTKKLFKHIPWVILGIIGAFCLAVVALRRGEHVSALWIVVASVSVYLVAYRYYSLYIAQKVMKL

DPTRATPAVINNDGLNYVPTNRYVLFGHHFAAIAGAGPLVGPVLAAQMGYLPGTLWLLAGVVLAGAVQDF

MVLFISSRRNGASLGEMIKEEMGPVPGTIALFGCFLIMIIILAVLALIVVKALAESPWGVFTVCSTVPIA

LFMGIYMRFIRPGRVGEVSVIGIVLLVASIYFGGVIAHDPYWGPALTFKDTTITFALIGYAFVSALLPVW

LILAPRDYLATFLKIGVIVGLALGIVVLNPELKMPAMTQYIDGTGPLWKGALFPFLFITIACGAVSGFHA

LISSGTTPKLLANETDARFIGYGAMLMESFVAIMALVAASIIEPGLYFAMNTPPAGLGITMPNLHEMGGE

NAPIIMAQLKDVTAHAAATVSSWGFVISPEQILQTAKDIGEPSVLNRAGGAPTLAVGIAHVFHKVLPMAD

MGFWYHFGILFEALFILTALDAGTRSGRFMLQDLLGNFIPFLKKTDSLVAGIIGTAGCVGLWGYLLYQGV

VDPLGGVKSLWPLFGISNQMLAAVALVLGTVVLIKMKRTQYIWVTVVPAVWLLICTTWALGLKLFSTNPQ

MEGFFYMASQYKEKIANGTDLTAQQIANMNHIVVNNYTNAGLSILFLIVVYSIIFYGFKTWLAVRNSDKR

TDKETPYVPIPEGGVKISSHH

>gi|30043848|gb|AAP19567.1| hypothetical protein S4641 [Shigella flexneri 2a str. 2457T]

MFGNLGQAKKYLGQAAKMLIGIPDYDNYVEHMKTNHPDKPYMSYEEFFRERQNARYGGDGKGGMRCC

>gi|30043847|gb|AAP19566.1| hypothetical protein S4640 [Shigella flexneri 2a str. 2457T]

MIENEFGEVSVDDQLIGDRATQIKTLTNGCICCSRSNELEDALLDLLDNLDKGNIQFDRLVIECTGMADP

GPIIQTFFSHEILCQLYLLDGVIALVDAVHADEQMNQFTIAQSQVGYADRILLTKTDVAGEAEKLRERLA

RINARAPVYTVTHGDIDLGLLFNTNGFMLEENVVSTKPRFHFIADKQNDISSIVVELDYPVDISEVSRVM

ENLLLESADKLLRYKGMLWIDGEPNRLLFQGVQRLYSADWDRPWGDEKPHSTMVFIGIQLPEEEIRAAFA

GLRK

>gi|30043846|gb|AAP19565.1| IS1 orfB [Shigella flexneri 2a str. 2457T]

MSRQCTHYGRWPQHGFTSLKKLRPQSVTSRIQPGSDVIVCAEMDEHWGYVGAKSRQRWLFYAYDRIRRTV

VAHVFGERTLATLERLLSLLSAFEVVVWMTDGWPLYESRLKGKLHVISKRYTQRIERHNLNLRQHLARLV

RKSLSFSKSVELHDKVIGHYLNIKHYQ

>gi|30043845|gb|AAP19564.1| IS1 orfA [Shigella flexneri 2a str. 2457T]

MASISIRCPSCSATEGVVRNGKSTAGHQRYLCSHCRKTWQLQFTYTASQPGTHQKIIDMAMNGVGCRASA

RIMGVGLNTVLRHLKNSGRSR

>gi|30043844|gb|AAP19563.1| hypothetical protein S4635 [Shigella flexneri 2a str. 2457T]

MSVEKLIVDHMETWTSALQTRSTAGRGSSGKIDLYGIKKLRELILELAVRGKLVPQDPNDEPASDLLKRI

AAEKAELVKQGKIKKQKPLPEISEEEKPFELPEGWEWVHLPDIYCSISESSRKIKSSEILPEGKYPVIEQ

SQEFISGYCNNECLLIKLNNPVIVFGDHTRNIKFIDFDFVVGADGVKILSPILICERFFFWQLRSFKLDV

RGYARHFKVLNSCLFALPPIAEQERIVEKVSSLMSLCDQLEQQSLTSLDAHQQLVETLLGTLTDSQNTAE

LAENWARISEHFDTLFTTEASVDALKQTILQLAVMGKLVPQDPNDEPASELLKRIAQEKAQLVKEGKIQK

PLPPISDEEKPFELPEGWEWCRIGNIVNIKSELVSPKDYLNLYQVAPDIIEKGTGRVISKRTVKESGVKG

PNSRFYKGQIVYSKIRPSLSKVFLAEYNGLCSADMYPLDCYINPNYLLKYILSIPFLMQVKKAENRIKMP

KLNSDSFYNIIVAIPPYNEQQAIFDKINSIEAVCNGLISYIGIYHKTQLHLADALTDAAIN

>gi|30043843|gb|AAP19562.1| hypothetical protein S4633 [Shigella flexneri 2a str. 2457T]

MLFDHLRDEVMRLDAGITQEVLKLYIAFKAETNFVDVVPQKSRLRLSLNMQFHELVDPKGIAKDVTNVGR

WGNGDVEIGFSDLAQLPYIMGLIRQAFEKQMESALV

>gi|30043842|gb|AAP19561.1| IS600 orfA [Shigella flexneri 2a str. 2457T]

MSRKTQRYSKEFKAEAVRTVLENQLSISEGASRLSLPEGTLGQWVTAARKGLGTPGSRTVAELESEILQL

RKALNEARLERDILKKATAYFAQESLKNTR

>gi|30043841|gb|AAP19560.1| IS600 orfB [Shigella flexneri 2a str. 2457T]

MAHIRTRETYGTRRLQTELAENGIIVGRDRLARLRKELRLRCKQKRKFRATTNPNHNLPVAPNLLNQTFA

PTAPNQVWVADLTYVATQEGWLYLAGIKDVYTCEIVGYAMGERMTKELTGKALFMALRSQRPPAGLIHHS

DRGSQYCAYDYRVIQEQFGLKTSMSRKGNCYDNAPMESFWGTLKNESLSHYRFNNRDEAISVIREYIEIF

YNRQRRHSRLGNISPAAFREKYHQMAA

>gi|30043840|gb|AAP19559.1| ISEhe3 orfB [Shigella flexneri 2a str. 2457T]

MLDVHPSGFYAWLQQPHSQRHQADLRLTGQIKQFWLESGCVYGYRKIHLDLRDSGQQCGVNRVWRLMKRV

GIKAQVGYRSPRARKGEASIVSPNRLQRQFNPDAPDERWVTDITYIRTHEGWLYLAVVVDLFSRKIIGWS

MQSRMTKDIVLNALLMAVWRRNPEKQVLVHSDQGSQYTSHEWQSFLKSHGLEG

>gi|30043839|gb|AAP19558.1| ISEhe3 orfA [Shigella flexneri 2a str. 2457T]

MSGKRYPEEFKTEAVKQVVDRGYSVASVATRLDITTHSLYAWIKKYGPDSSTNKEQSDAQAEIRRLQKEL

KRVTDERDILKKAAAYFAKLSD

>gi|30043838|gb|AAP19557.1| 50S ribosomal subunit protein L9 [Shigella flexneri 2a str. 2457T]

MQVILLDKVANLGSLGDQVNVKAGYARNFLVPQGKAVPATKKNIEFFEARRAELEAKLAEVLAAANARAE

KINALETVTIASKAGDEGKLFGSIGTRDIADAVTAAGVEVAKSEVRLPNGVLRTTGEHEVSFQVHSEVFA

KVIVNVVAE

>gi|30043837|gb|AAP19556.1| 30S ribosomal subunit protein S18 [Shigella flexneri 2a str. 2457T]

MARYFRRRKFCRFTAEGVQEIDYKDIATLKNYITESGKIVPSRITGTRAKYQRQLARAIKRARYLSLLPY
TDRHQ

>gi|30043836|gb|AAP19555.1| primosomal replication protein N [Shigella flexneri 2a str. 2457T]

MTNRLVLSGTVCRTPLRKVSPSGIPHCQFVLEHRSVQEEAGFHRQAWCQMPVIVSGHENQAITHSITVGS

RITVQGFISCHKAKNGLSKMVLHAEQIELIDSGD

>gi|30043835|gb|AAP19554.1| 30S ribosomal subunit protein S6 [Shigella flexneri 2a str. 2457T]

MRHYEIVFMVHPDQSEQVPGMIERYTAAITGAEGKIHRLEDWGRRQLAYPINKLHKAHYVLMNVEAPQEV

IDELETTFRFNDAVIRSMVMRTKHAVTEASPMVKAKDERRERRDDFANETADDAEAGDSEE

>gi|30043834|gb|AAP19553.1| hypothetical protein S4624 [Shigella flexneri 2a str. 2457T]

MFSRVLALLAVLLLSANTWAAIEINNHQARNMDDVQSLGVIYINHNFATESEARQALNEETDAQGATYYH

VILMREPGSNGNMHASADIYR

>gi|30043833|gb|AAP19552.1| putative epimerase/aldolase [Shigella flexneri 2a str. 2457T]

MQKLKQQVFEANMDLPRYGLVTFTWGNVSAIDRERGLVVIKPSGVAYETMKADDMVVVDMSGKVVEGEYR

PSSDTATHLELYRRYPSLGGIVHTHSTHATAWAQAGLAIPALGTTHADYFFGDIPCTRGLSEEEVQGEYE

LNTGKVIIETLGNAEPLHTPGIVVYQHGPFAWGKDAHDAVHNAVVMEEVAKMAWIARSINPQLNHIDSYL

MNKHFMRKHGPNAYYGQK

>gi|30043832|gb|AAP19551.1| putative hexulose-6-phosphate isomerase [Shigella flexneri 2a str. 2457T]

MLSKQIPLGIYEKALPAGECWLERLRLAKTLGFDFVEMSVDETNERLSRLDWSREQRLALVNAIVETGVR

VPSMCLSAHRRFPLGSEDDAVRAQGLEIMRKAIQFAQDVGIRVIQLAGYDVYYQEANNETRRRFRDGLKE

SVEMASRAQVTLAMEIMDYPLMNSISKALGYAHYLNNPWFQLYPDIGNLSAWDNDVQMELQAGIGHIVAV

HVKDTKPGVFKNVPFGEGVVDFERCFETLKQSGYCGPYLIEMWSETAEDPAAEVVKACDWVKARMAKAGM

VEAA

>gi|30043831|gb|AAP19550.1| probable hexulose-6-phosphate synthase [Shigella flexneri 2a str. 2457T]

MSLPMLQVALDNQTMDSAYETTRLIAEEVDIIEVGTILCVGEGVRAVRDLKALYPHKIVLADAKIADAGK

ILSRMCFEANADWVTVICCADINTAKGALDVAKEFNGDVQIELTGYWTWEQAQQWRDAGIQQVVYHRSRD

AQAAGVAWGEADITAIKRLSDMGFKVTVTGGLALEDLPLFKGIPIHVFIAGRSIRDAASPVEAARQFKRS

IAELWG

>gi|30043830|gb|AAP19549.1| putative PTS system enzyme II A component [Shigella flexneri 2a str. 2457T]

MKLHDSLAENKSIRLQAEAETWQDAVKIGVDLLVAADVVEPRYYQAILDAVEQHGPYFVLAPGLAMPHGR

PEEGVKKTGFALVTLKKPLEFNHEDNDPVDILITMAAVDANTHQEVGIMQIVNLFEDEENFDRLRACRTE

QEVLDLIDRTNAAA

>gi|30043829|gb|AAP19548.1| hypothetical protein S4619 [Shigella flexneri 2a str. 2457T]

MTVRILAVCGNGQGSSMIMKMKVDQFLTQSNIDHTVNSCAVGEYKSELSGADIIIASTHIAGEITVTGNK

YVVGVRNMLSPADFGPKLLEVIKAHFPQDVK

>gi|30043828|gb|AAP19547.1| hypothetical protein S4618 [Shigella flexneri 2a str. 2457T]

MHNIPGVRNTRLPLLQEIVMEILYNIFTVFFNQVMTNAPLLLGIVTCLGYILLRKSVSVIIKGTIKTIIG

FMLLQAGSGILTSTFKPVVAKMSEVYGINGAISDTYASMMATIDRMGDAYSWVGYAVLLALALNICYVLL

RRITGIRTIMLTGHIMFQQAGLIAVTLFIFGYSMWTTIICTAILVSLYWGITSNMMYKPTQEVTDGCGFS

IGHQQQFASLIAYKVAPFLGKKEESVEDLKLPGWLNIFHDNIVSTAIVMTIFFGAILLSFGIDTVQAMAG

KVHWTVYILQTGFSFAVAIFIITQGVRMFVAELSEAFNGISQRLIPGAVLAIDCAAIYSFAPNAVVWGFM

WGTIGQLIAVGILVACGSSILIIPGFIPMFFSNATIGVFANHFGGWRAALKICLVMGMIEIFGCVWVVKL

TGMSAWMGMADWSILAPPMMQGFFSIGIAFMAVIIVIALAYMFFAGRALRAEEDAEKQLAEQSA

>gi|30043827|gb|AAP19546.1| hypothetical protein S4617 [Shigella flexneri 2a str. 2457T]

MAMSKVKSITRESWILSTFPEWGSWLNEEIEQEQVAPGTFAMWWLGCTGIWLKSEGGTNVCVDFWCGTGK

QSHGNPLMKQGHQMQRMAGVKKLQPNLRTTPFVLDPFAIRQIDAVLATHDHNDHIDVNVAAAVMQNCADD

VPFIGPKTCVDLWIGWGVPKERCIVVKPGDVVKVKDIEIHALDAFDRTALITLPADQKAAGVLPDGMDDR

AVNYLFKTPGGSLYHSGDSHYSNYYAKHGNEHQIDVALGSYGENPRGITDKMTSADMLRMGEALNAKVVI

PFHHDIWSNFQADPQEIRVLWEIKKDRLKYGFKPFIWQVGGKFTWPLDKDNFEYHYPRGFDDCFTIEPDL

PFKSFL

>gi|30043826|gb|AAP19545.1| putative DEOR-type transcriptional regulator [Shigella flexneri 2a str. 2457T]

MTEAQRHQILLEMLAQLGFVTVEKVVERLGISPATARRDINKLGESGKLKKVRNGAEAITQQRPRWTPMN

LHQAQNHDEKVRIAKAASQLVNPGESVVINCGSTAFLLGREMCGKPVQIITNYLPLANYLIDQEHDSVII

MGGQYNKSQSITLSPQGSENSLYAGHWMFTSGKGLTAEGLYKTDMLTAMAEQKMLSVVGKLVVLVDSSKI

GERAGMLFSRADQIDMLITGKNANPEILQQLEAQGVSILRV

>gi|30043825|gb|AAP19544.1| hypothetical protein S4615 [Shigella flexneri 2a str. 2457T]

MIEIESRELADIPVLHAYPVGQKDTPLPCVIFYHGFTSSSLVYSYFAVALAQAGLRVIMPDAPDHGSRFS

GDEARRLNQFWQILLQSMQEFTTLRAAIAEENWLLDDRLAVGGASMGAMTALGITARHPTVKCTASMMGS

GYFTSLARSLFPPLIPETAAQQNEFNKIVAPLAEWEATNHLEQLGDRPLLLWHGLDDDVVPADESLRLQQ

ALSETGRDKLLTCSWQPGVRHRITPEALDAAVTFFRQHL

>gi|30043824|gb|AAP19543.1| hypothetical protein S4614 [Shigella flexneri 2a str. 2457T]

MWVTLSSRFHSKIAYQVTIREHSPKVKLIRHYTMVSRKRNSVIYRFASLLLVLMLSACSALQGTPQPAPP

VTDHPQEIRRDQTQGLQRIGSVSTMVRGSPDDALAEIRAKAVAAKADYYVVVMVDETIVTGQWYSQAILY

RK

>gi|30043823|gb|AAP19542.1| hypothetical protein S4613 [Shigella flexneri 2a str. 2457T]

MGKRKMELTMKQLLASPSLQLVTYPASATAQSAEFASADCVTGLNEIGQISVSNISGDPQDVERIVALKA

DEQGASWYRIITMYEDQQPDNWRVQAILYA

>gi|30043822|gb|AAP19541.1| IS1 orfA [Shigella flexneri 2a str. 2457T]

MASISIRCPSCSATEGVVRNGKSTAGHQRYLCSHCRKTWQLQFTYTASQPGKHQKIIDMAMNGVGCRASA

RIMGVGLNTVLRHLKNSGRSR

>gi|30043821|gb|AAP19540.1| IS1 orfB [Shigella flexneri 2a str. 2457T]

MSRQCTHYGRWPQHGFTSLKKLRPQSVTSRIQPGSDVIVCAEMDEHWGYVGAKSRQRWLFYAYDRIRRTV
VAHVFGERTLATLERLLSLLSAFEVVVWMTDGWPLYESRLKGKLHVISKRYTQRIERHNLNLRQHLARLV
RKSLSFSKSVELHDKVIGHYLNIKHYQ

>gi|30043820|gb|AAP19539.1| putative synthetase/amidase [Shigella flexneri 2a str. 2457T]

MLRHNVPVRRDLDQIAANNGFDFHIIDNEIYWDESRAYRFTLRQIEEQIEKPTAELHQMCLEVVDRAVKD
EEILTQLAIPPLYWDVIAESWRARDPSLYGRMDFAWCGNAPVKLLEYNADTPTSLYESAYFQWLWLEDAR
RSGIIPRDADQYNAIQERLISRFSELYSRELFYFCCCQDTDEDRSTVLYLQDCAQQAGQESRFIYIEDLG
LGVGGVLTDLDDNVIQRAFKLYPLEWMMRDDNGPLLRKRREQWVEPLWKSILSNKGLMPLLWRFFPGHPN
LLASWFEGEKSQIAAGESYVRKPIYSREGGNVTIFDGQNNVVDHADGDYADEPMIYQAFQPLPRFGDSYT
LIGSWIVDDEACGMGIREDNTLITKDTSRFVPHYIAG

>gi|30043819|gb|AAP19538.1| hypothetical protein S4607 [Shigella flexneri 2a str. 2457T]

MARKRKSRNNSKIGHGAISRIGRPNNPFEPRRNRYAQKYLTLALMGGAAFFVLKGCSDSSDVDNDGDGTF
YATVQDCIDDGNNADICARGWNNAKAAFYADVPKNMTQQNCQSKYENCYYDNVEQSWIPVVSGFLLSRVI
RKDRDEPFVYNSGGSSFASRPVWRNTSGDYSWRFGSGKKESYSSGGFTTRKASTVSRGGYGRSSSARGHW
GG

>gi|30043818|gb|AAP19537.1| IS1 orfB [Shigella flexneri 2a str. 2457T]

MSRQCTHYGRWPQHGFTSLKKLRPQSVTSRIQPGSDVIVCAEMDEHWGYVGAKSRQRWLFYAYDRIRRTV
VAHVFGERTLATLERLLSLLSAFEVVVWMTDGCPLYESRLKGKLHVISKRYTQRIERHNLNLRQHLARLV
RKSLSFSKSVELHDKVIGHYLNIKHYQ

>gi|30043817|gb|AAP19536.1| IS1 orfA [Shigella flexneri 2a str. 2457T]

MASISIRCPSCSATEGVVRNGKSTAGHQRYLCSHCRKTWQLQFTYTASQPGTHQKIIDMAMNGVGCRASA

RIMGVGLNTVLRHLKNSGRSR

>gi|30043816|gb|AAP19535.1| hypothetical protein S4603 [Shigella flexneri 2a str. 2457T]

MSEMIYGIHAVQALLERAPERFQEVFILKGREDKRLLPLIHALESQGVVIQLANRQYLDEKSDGAVHQGI

IARVKPGRQYQENDLPDLIASLDQPFLLILDGVTDPHNLGACLRSADAAGVHAVIVPKDRSAQLNATAKK

VACGAAESVPLIRVTNLARTMRMLQEENIWIVGTAGEADHTLYQSKMTGRLALVMGAEGEGMRRLTREHC

DELISIPMAGSVSSLNVSVATGICLFEAVRQRS

>gi|30043815|gb|AAP19534.1| putative enzyme [Shigella flexneri 2a str. 2457T]

MTKIFIRDDNGGTEMSQDPFQEREAEKYANPIPSREFILEHLTKREKPASRDELAVELHIEGEEQLEGLR

RRLRAMERDGQLVFTRRQCYALPERLDLVKGTVIGHRDGYGFLRVEGRKDDLYLSSEQMKTCIHGDQVLA

QPLGADRKGRREARIVRVLVPKTSQIVGRYFTEAGVGFVVPDDSRLSFDILIPPDQIMGARMGFVVVVEL

TQRPTRRTKAVGKIVEVLGDNMGTGMAVDIALRTHEIPYIWPQAVEQQVAGLKEEVPEEAKAGRVDLRDL

PLVTIDGEDARDFDDAVYCEKKRGGGWRLWVAIADVSYYVRPPTPLDREARNRGTSVYFPSQVIPMLPEV

LSNGLCSLNPQVDRLCMVCEMTVSSKGRLTGYKFYEAVMSSHARLTYTKVWHILQGDQDLREQYAPLVKH

LEELHNLYKVLDKAREERGGISFESEEAKFIFNAERRIERIEQTQRNDAHKLIEECMILANISAARFVEK

AKEPALFRIHDKPSTEAITSFRSVLAELGLELPGGNKPEPRDYAELLESVADRPDAEMLQTMLLRSMKQA

IYDPENRGHFGLALQSYAHFTSPIRRYPDLTLHRAIKYLLAKEQGHQGNTTETGGYHYSMEEMLQLGQHC

SMAERRADEATRDVADWLKCDFMLDQVGNVFKGVISSVTGFGFFVRLDDLFIDGLVHVSSLDNDYYRFDQ

VGQRLMGESSGQTYRLGDRVEVRVEAVNMDERKIDFSLISSERAPRNVGKTAREKAKKGDAGKKGGKRCQ

VGKKVNFEPDSAFRGEKKTKPKAAKKDARKAKKPSAKTQKIAAATKAKRAAKKKVAE

>gi|30043814|gb|AAP19533.1| hypothetical protein S4601 [Shigella flexneri 2a str. 2457T]

MQLTSFTDYGLRALIYMASLPEGRMTSISEVTDVYGVSRNHMVKIINQLSRAGYVTAVRGKNGGIRLGKP

ASAIRIGDVVRELEPLSLVNCSSEFCHITPACRLKQALSKAVQSFLTELDNYTLADLVEENQPLYKLLLV

E

>gi|30043813|gb|AAP19532.1| adenylosuccinate synthetase [Shigella flexneri 2a str. 2457T]

MGNNVVVLGTQWGDEGKGKIVDLLTERAKYVVRYQGGHNAGHTLVINGEKTVLHLIPSGILRENVTSIIG

NGVVLSPAALMKEMKELEDRGIPVRERLLLSEACPLILDYHVALDNAREKARGAKAIGTTGRGIGPAYED

KVARRGLRVGDLFDKETFAEKLKEVMEYHNFQLVNYYKAEAVDYQKVLDDTMAVADILTSMVVDVSDLLD

QARQRGDFVMFEGAQGTLLDIDHGTYPYVTSSNTTAGGVATGSGLGPRYVDYVLGILKAYSTRVGAGPFP

AELFDETGEFLCKQGNEFGATTGRRRRTGWLDTVAVRRAVQLNSLSGFCLTKLDVLDGLKEVKLCVAYRM

PDGREVTTTPLAADDWKGVEPIYETMPGWSESTFGVKDRSGLPQAALNYIKRIEELTGVPIDIISTGPDR

TETMILRDPFDA

>gi|30043812|gb|AAP19531.1| hypothetical protein S4599 [Shigella flexneri 2a str. 2457T]

MNSTIWLALALVLVLEGLGPMLYPKAWKKMISAMTNLPDNILRRFGGGLVVAGVVVYYMLRKTIG

>gi|30043811|gb|AAP19530.1| protease specific for phage lambda cII repressor [Shigella flexneri 2a str. 2457T]

MRKSVIAIIIIVLVVLYMSVFVVKEGERGITLRFGKVLRDDDNKPLVYEPGLHFKIPFIETVKMLDARIQ

TMDNQADRFVTKEKKDLIVDSYIKWRISDFSRYYLATGGGDISQAEVLLKRKFSDRLRSEIGRLDVKDIV

TDSRGRLTLEVRDALNSGSAGTEDEVTTPAADNAIAEAAERVTAETKGKVPVINPNSMAALGIEVVDVRI

KQINLPTEVSEAIYNRMRAEREAVARRHRSQGQEEAEKLRATADYEVTRTLAEAERQGRIMRGEGDAEAA

KLFADAFSKDPDFYAFIRSLRAYENSFSGNQDVMVMSPDSDFFRYMKTPTSATR

>gi|30043810|gb|AAP19529.1| protease specific for phage lambda cII repressor [Shigella flexneri 2a str. 2457T]

MAWNQPGNNGQDRDPWGSSKPGGNSEGNGNKGGRDQGPPDLDDIFRKLSKKLGGLGGGKGTGSGGGSSSQ

GPRPQLGGRVVTIAAAAIVIIWAASGFYTIKEAERGVVTRFGKFSHLVEPGLNWKPTFIDEVKPVNVEAV

RELAASGVMLTSDENVVRVEMNVQYRVTNPEKYLYSVTSPDDSLRQATDSALRGVIGKYTMDRILTEGRT

VIRSDTQRELEETIRPYDMGITLLDVNFQAARPPEEVKAAFDDAIAARENEQQYIREAEAYSNEVQPRAN

GQAQRILEEARAYKAQTILEAQGEVARFAKLLPEYKAAPEITRERLYIETMEKVLGNTRKVLVNDKGGNL

MVLPLDQMLKGGNAPAAKSDNGASNLLRLPPASSSTTSGASNTSSTSQGDIMDQRRANAQRNDYQRQGE

>gi|30043809|gb|AAP19528.1| GTP-binding subunit of protease specific for phage lambda cII repressor [Shigella flexneri 2a str. 2457T]

MFDRYDAGEQAVLVHIYFTQDKDMEDLQEFESLVSSAGVEALQVITGSRKAPHPKYFVGEGKAVEIAEAV

KAMGASVVLFDHALSPAQERNLERLCECRVIDRTGLILDIFAQRARTHEGKLQVELAQLRHLATRLVRGW

THLERQKGGIGLRGPGETQLETDRRLLRNRIVQIQSRLERVEKQREQGRQSRIKADVPTVSLVGYTNAGK

STLFNRITEARVYAADQLFATLDPTLRRIDVADVGETVLADTVGFIRHLPHDLVAAFKATLQETRQATLL

LHVIDAADVRVQENIEAVNTVLEEIDAHEIPTLLVMNKIDMLEDFEPRIDRDEENKPIRVWLSAQTGAGI

PQLFQALTERLSGEVAQHTLRLPPQEGRLRSRFYQLQAIEKEWMEEDGSVSLQVRMPIVDWRRLCKQEPA

LIDYLI

>gi|30043808|gb|AAP19527.1| host factor I for bacteriophage Q beta replication [Shigella flexneri 2a str. 2457T]

MAKGQSLQDPFLNALRRERVPVSIYLVNGIKLQGQIESFDQFVILLKNTVSQMVYKHAISTVVPSRPVSH

HSNNAGGGTSSNYHHGSSAQNTSAQQDSEETE

>gi|30043807|gb|AAP19526.1| delta(2)-isopentenylpyrophosphate tRNA-adenosine transferase [Shigella flexneri 2a str. 2457T]

MSDISKASLPKAIFLMGPTASGKTALAIELRKILPVELISVDSVLIYKGMDIGTAKPNAEELLAAPHRLL

DIRDPSQAYSAADFRRDALAEMADITAAGRIPLLVGGTMLYFKALLEGLSPLPSADPEVRARIEQQAAEQ

GWESLHRQLQEVDPVAAARIHPNDPQRLSRALEVFFISGKTLTELTQTSGDALPYQVHQFAIAPASRELL

HQRIEQRFHQMLASGFEAEVRALFARGDLHTDLPSIRCVGYRQMWSYLEGEISYDEMVYRGVCATRQLAK

RQITWLRGWEGVHWLDSEKPEQARDEVLQVVGAIAG

>gi|30043806|gb|AAP19525.1| enzyme in methyl-directed mismatch repair [Shigella flexneri 2a str. 2457T]

MPIQVLPPQLANQIAAGEVVERPASVVKELVENSLDAGATRIDIDIERGGAKLIRIRDNGCGIKKDELAL

ALARHATSKIASLDDLEAIISLGFRGEALASISSVSRLTLTSRTAEQQEAWQAYAEGRDMDVTVKPAAHP

VGTTLEVLDLFYNTPARRKFLRTEKTEFSHIDEIIRRIALARFDVTINLSHNGKIVRQYRAVPEGGQKER

RLGAICGTAFLEQALAIEWQHGDLTLRGWVADPNHTTPALAEIQYCYVNGRMMRDRLINHAIRQACEDKL

GADQQPAFVLYLEIDPHQVDVNVHPAKHEVRFHQSRLVHDFIYQGVLSVLQQQLETPLPLDDEPQPAPRA

IPENRVAAGRNHFAEPAAREPVAPRYSPAPASGSRPAAPWPNAQPGYQKQQGEVYRQLLQTPAPMQKPKA

PEPQEPALAANSQSFGRVLTIVHSDCALLERDGNISLLSLPVAERWLRQAQLTPGEAPVCAQPLLIPLRL

KVSGEEKSALEKAQSALAELGIDFQSDAQHVTIRAVPLPLRQQNLQILIPELIGYLAKQSVFEPGNIAQW

IARNLMSEHAQWSMAQAITLLADVERLCPQLVKTPPGGLLQSVDLHPAIKALKDE

>gi|30043805|gb|AAP19524.1| N-acetylmuramoyl-l-alanine amidase II, murein hydrolase [Shigella flexneri 2a str. 2457T]

MMYRIRNWLVATLLLLCTPVGAATLSDIQVSNGNQQARITLSFIGDPDYAFSHQSKRTVALDIKQTGMIQ

GLPLLFSGNNLVKAIRSGTPKDAQTLRLVVDLTENGKTEAVKRQNGSNYTVVFTINADVPPPPPPPPVVA

KRVETPAVVAPRVSEPARNPFKTESNRTTGVISSNTVTRPAARATANTGDKIIIAIDAGHGGQDPGAIGP

GGTREKNVTIAIARKLRTLLNDDPMFKGVLTRDGDYFISVMGRSDVAHKQNANFLVSIHADAAPNRSATG

ASVWVLSNRRANSEMASWLEQHEKLSELLGGAGDVLANSQSDPYLSQAVLDLQFGHSQRVGYDVATSMIS

QLQRIGEIHKRRPEHASLGVLRSPDIPSVLVETGFISNNSEERLLASDDYQQQLAEAIYKGLRNYFLAHP

MQSAPQGATAQTASTVTTPDRTLPN

>gi|30043804|gb|AAP19523.1| hypothetical protein S4591 [Shigella flexneri 2a str. 2457T]

MMNRVIPLPDEQATLDLGERVAKACDGATVIYLYGDLGAGKTTFSRGFLQALGHQGNVKSPTYTLVEPYT

LDNLMVYHFDLYRLADPEELEFMGIRDYFANDAICLVEWPQQGTGVLPDPDVEIHIDYQAQGREARVSAV

SSAGELLLARLAG

>gi|30043803|gb|AAP19522.1| hypothetical protein S4589 [Shigella flexneri 2a str. 2457T]

MTDHTMKKNPVSIPHTVWYADDIRRGEREAADVLGLTLYELMLRAGEAAFQVCRSAYPDARHWLVLCGHG

NNGGDGYVVARLAKAVGIEVTLLAQESDKPLPEEAALAREAWLNAGGEIHASNIVWPESVDLIVDALLGT

GLQQAPRESISQLIDHANSHPAPIVAVDIPSGLLAETGATPGAVINADHTITFIALKLGLLTGKARDVTG

QLHFDSLGLDSWLAGQETKIQRFSAEQLSHWLKPRRPTSHKGDHGRLVIIGGDHGTAGAIRMTGEAALRA

GAGLVRVLTRSENIAPLLTARPELMVHELTMDSLTESLEWADVVVIGPGLGQQEWGKKALQKVENFRKPM

LWDADALNLLAINPDKRHNRVITPHPSEAARLLGCSVAEIESDRLHCAKRLVQRYGGVAVLKGAGTVVAA

HPDALGIIDAGNAGMAIGGKGDVLSGIIGALLGQKLSPYDAACAGCVAHGAAADVLAARFGTRGMLATDL

FSTLQRIVNPEVTDKNHDESSNSAP

>gi|30043802|gb|AAP19521.1| hypothetical protein S4590 [Shigella flexneri 2a str. 2457T]

MSEPLDLNQLAQKIKQWGLELGFQQVGITDTDLSESEPKLQAWLDKQYHGEMDWMARHGMLRARPHELLP

GTLRVISVRMNYLPANAAFASTLKNPKLGYVSRYALGRDYHKLLRNRLKKLGEMIQQHCVSLNFRPFVDS

APILERPLAEKAGLGWTGKHSLILNREAGSFFFLGELLVDIPLPVDQPVEEGCGKCVACMTICPTGAIVE

PYTVDARRCISYLTIELEGALPEELRPLMGNRIYGCDDCQLICPWNRYSQLTTEDDFSPRKPLHAPELIE

LFAWSEEKFLKVTEGSAIRRIGHLRWLRNIAVALGNAPWDETILTALESRKGEHPLLDEHIAWAIAQQIE

RRNACIVEVQLPKKQRLVRVIEKGLPRDA

>gi|30043801|gb|AAP19520.1| hypothetical protein S4584 [Shigella flexneri 2a str. 2457T]

MGKTSMIHAIVDQYSHCEWVENSMSANENNLIWIDLEMTGLDPERDRIIEIATLVTDANLNILAEGPTIA

VHQSDEQLALMDDWNVRTHTASGLVERVKASTMGDREAELATLEFLKQWVPAGKSPICGNSIGQDRRFLF

KYMPELEVYFHYRYLDVSTLKELARRWKPEILDGFTKQGTHQAMDDIRESVAELAYYREHFIKL

>gi|30043800|gb|AAP19519.1| hypothetical protein S4583 [Shigella flexneri 2a str. 2457T]

MNANHQRRLKTSKEKPDYDDNLFGEPDEGIVISRFGMHADVESADGDVHRCNIRRTIRSLVTGDRVVWRP

GKPAAEGVNVKGIVEAVHERTSVLTRPDFYDGVKPIAANIDQIVIVSAILPELSLNIIDRYLVACETLQI

EPIIVLNKIDLLDDEGMAFVNEQMDIYRNIGYRVLMVSSHTQDGLKPLEEALTGRISIFAGQSGVGKSSL

LNALLGLQKEILTNDVSDNSGLGQHTTTAARLYHFPHGGDVIDSPGVREFGLWHLEPEQITQGFVEFHDY

LGLCKYRDCKHDTDPGCAIREAVEEGKIAETRFENYHRILESMAQVKTRKNFSDTDD

>gi|30043799|gb|AAP19518.1| phosphatidylserine decarboxylase [Shigella flexneri 2a str. 2457T]

MLNSFKLSLQYILPKLWLTRLAGWGASKRAGWLTKLVIDLFVKYYKVDMKEAQKPDTASYRTFNEFFVRP

LRDEVRPIDTDPNVLVMPADGVISQLGKIEEDKILQAKGHNYSLEALLAGNYLMADLFRNGTFVTTYLSP

RDYHRVHMPCNGILREMIYVPGDLFSVNHLTAQNVPNLFARNERVICLFDTEFGPMAQILVGATIVGSIE

TVWAGTITPPREGIIKRWTWPAGENDGSVALLKGQEMGRFKLGSTVINLFAPGKVNLVEQLESLSVTKIG

QPLAVSTETFVTPDAEPAPLPAEEIEAEHDASPLVDDKKDQV

>gi|30043798|gb|AAP19517.1| putative periplasmic binding protein [Shigella flexneri 2a str. 2457T]

MRLIITFLMAWCLSWGAYAATAPDSKQITQELEQAKAAKPAQPEVVEALQSALNALEERKGSLERIKQYQ

QVIDNYPKLSATLRAQLNNIRDEPRSVSPGMSTDALNQEILQVSSQLLDKSRQAQQEQERAREIADSLNQ

LPQQQTDARRQLNEIERRLGTLTGNTPLNQAQNFALQSDSARLKALVDELELAQLSANNRQELARLRSEL

AEKESQQLDAYLQALRNQLNSQRQLEAERALESTELLAENSADLPKDIVAQFKINRELSAALNQQAQRME

LVASQQRQAASQTLQVRQALNTLREQSQWLGSSNLLGEALRAQVARLPEMPKPQQLDTEMAQLRVQRLRY

EDLLNKQPLLRQIHQADGQPLTAEQNRILEAQLRTQRELLNSLLQGGDTLLLELTKLKVSNRQLEDALKE

VNEATHRYLFWASDVRPMTIAWPLEIAQDLRRLISLDTFSQLGKASVMMLTSKETILPLFGALILVGCSI

YSRRYFTRFLERSAAKVGKVTQDHFWLTLRTLFWSILVASPLPVLWMTLGYGLREAWPYPLAVAIGDGVT

ATVPLLWVVMICATFARPNGLFIAHFGWPRERVSRGMRYYLMSIGLIMALMMFDNLDDREFSGSLGRLCF

ILICGALAVVTLSLKKAGIPLYLNKEGSGDNITNHMLWNMMIGAPLVAILASAVGYLATAQALLARLETS

VAIWFLLLVVYHVIRRWMLIQRRRLAFDRAKHRRAEMLAQRARGEEEAHHHSSPEGAIEVDESEVDLDAI

SAQSLRLVRSILMLIALLSVIVLWSEIHSAFGFLENISLWDVTSTVQGVESLEPITLGAVLIAILVFIIT

TQLVRNLPALLELAILQHLDLTPGTGYAITTITKYLLMLIGGLVGFSMIGIEWSKLQWLVAALGVGLGFG

LQEIFANFISGLIILFEKPIRIGDTVTIRDLTGSVTKINTRATTISDWDRKEIIVPNKAFITEQFINWSL

SDSVTRVVLTIPAPADANSEEVTEILLTAARRCSLVIDNPAPEVFLVDLQQGIQIFELRIYAAEMGHRMP

LRHEIHQLILAGFHAHGIDMPFPPFQMRLESLNGKQTGRTLTSAGKGRQAGSL

>gi|30043797|gb|AAP19516.1| IS1 orfA [Shigella flexneri 2a str. 2457T]

MASISIRCPSCSATEGVVRNGKSTAGHQRYLCSHCRKTWQLQFTYTASQPDTHQKIIDMAMNGVGCRASA

RIMGVGLNTVLRHLKNSGRSR

>gi|30043796|gb|AAP19515.1| putative lysyl-tRNA synthetase [Shigella flexneri 2a str. 2457T]

MSETASWQPSASIPNLLKRAAIMAEIRRFFADRGVLEVETPCMSQATVTDIHLVPFETRFVGPGHSQGMN

LWLMTSPEYHMKRLLVAGCGPVFQLCRSFRNEEMGRYHNPEFTMLEWYRPHYDMYRLMNEVDDLLQQVLD

CPAAESLSYQQAFLRYLEIDPLSADKTQLREVAAKLDLSNVADTEEDRDTLLQLLFTFGVEPNIGKEKPT

FVYHFPASQASLAQISTEDHRVAERFEVYYKGIELANGFHELTDAREQQQRFEQDNRKRAARGLPQHPID

QNLIEALKVGMPDCSGVALGVDRLVMLALGAETLAEVIAFSVDRA

>gi|30043795|gb|AAP19514.1| fumarate reductase, anaerobic, flavoprotein subunit [Shigella flexneri 2a str. 2457T]

MQTFQADLAIVGAGGAGLRAAIAAAQANPNAKIALISKVYPMRSHTVAAEGGSAAVAQDHDSFEYHFHDT

VAGGDWLCEQDVVDYFVHHCPTEMTQLELWGCPWSRRPDGSVNVRRFGGMKIERTWFAADKTGFHMLHTL

FQTSLQFPQIQRFDEHFVLDILVDDGHVRGLVAINMMEGTLVQIRANAVVMATGGAGRVYRYNTNGGIVT

GDGMGMALSHGVPLRDMEFVQYHPTGLPGSGILMTEGCRGEGGILVNKNGYRYLQDYGMGPETPLGEPKN

KYMELGPRDKVSQAFWHEWRKGNTISTPRGDVVYLDLRHLGEKKLHERLPFICELAKAYVGVDPVKEPIP

VRPTAHYTMGGIETDQNCETRIKGLFAVGECSSVGLHGANRLGSNSLAELVVFGRLAGEQATERAATAGN

GNEAAIEAQAAGVEQRLKDLVNQDGGENWAKIRDEMGLAMEEGCGIYRTPELMQKTIDKLAELQERFKRV

RITDTSSVFNTDLLYTIELSHGLNVAECMAHSAMARKESRGAHQRLDEGCTERDDVNFLKHTLAFRDADG

TTRLEYSDVKITTLPPAKRVYGGEADAADKAEAANNKEKANG

>gi|30043794|gb|AAP19513.1| fumarate reductase, anaerobic, iron-sulfur protein subunit [Shigella flexneri 2a str. 2457T]

MAEMKNLKIEVVRYNPEVDTAPHSAFYEVPYDATTSLLDALGYIKDNLAPDLSYRWSCRMAICGSCGMMV

NNVPKLACKTFLRDYTDGMKVEALANFPIERDLVVDMTHFIESLEAIKPYIIGNSRTADQGTNIQTPAQM

AKYHQFSGCINCGLCYAACPQFGLNPEFIGPAAITLAHRYNEDSRDHGKKERMAQLNSQNGVWSCTFVGY

CSEVCPKHVDPAAAIQQGKVESSKDFLIATLKPR

>gi|30043793|gb|AAP19512.1| fumarate reductase, anaerobic, membrane anchor polypeptide [Shigella flexneri 2a str. 2457T]

MTTKRKPYVRPMTSTWWKKLPFYRFYMLREGTAVPAVWFSIELIFALFALKNGPEAWAGFVDFLQNPVIV

IINLITLTAALLHTKTWFELAPKAANIIVKDEKMGPEPIIKSLWAVTVVATIVILFVALYW

>gi|30043792|gb|AAP19511.1| fumarate reductase, anaerobic, membrane anchor polypeptide [Shigella flexneri 2a str. 2457T]

MINPNPKRSDEPVFWGLFGAGGMWSAIIAPVMILLVGILLPLGLFPGDALSYERVLAFAQSFIGRVFLFL

MIVLPLWCGLHRMHHAMHDLKIHVPAGKWVFYGLAAILTVVTLIGVVTI

>gi|30043791|gb|AAP19510.1| beta-lactamase [Shigella flexneri 2a str. 2457T]

MFKTTLCALLITASYSTFAAPQQINDIVHRTITPLIEQQKIPGMAVAVIYQGKPYYFTWGYADIAKKQPV

TQQTLFELGSVSKTFTGVLGGDAIARGEIKLSDPATKYWPELTAKQWNGITLLHLATYTAGGLPLQVPDE
VKSSSDLLRFYQNWQPAWAPGTQRLYANSSIGLFGALAVKPSGLSFEQAMQTRVFQPLKLNHTWINVPPP
EEKNYAWGYREGKAVHVSPGALDAEAYGVKSTIEDMARWVQSNLPPLDINEKTLQQGIQLAQSRYWQTGD
MYQGLGWEMLDWPVNPDSIINGSDNKIALAARPVKAIKPPTPAVRASWVHKTGATGGFGSYVAFIPEKEL
GIVMLANKNYPNPARVAAACQILNALQ

>gi|30043790|gb|AAP19509.1| outer membrane lipoprotein (lipocalin) [Shigella flexneri 2a str. 2457T]
MRLLPLVAAATAAFLVVACSFPTPPRGVTVVNNFDAKRYLGTWYEIARFDHRFERGLEKVTATYSLRDDG
GLNVINKGYNPDRGMWQQSEGKAYFTGAPTRAALKVSFFGPFYGGYNVIALDREYRHALVCGPDRDYLWI
LSRTPTISDEVKQEMLAVATREGFDVSKFIWVQQPGS

>gi|30043789|gb|AAP19508.1| suppresser of groEL [Shigella flexneri 2a str. 2457T]
MPFVFSAIVIKVIVEIPLPPGKISVQPSALQDDLQTPLFTGDGPKFPEPDMSWIILVIAGLLEVVWAVGL
KYTHGFSRLTPSVITVTAMIVSMALLAWAMKSLPVGTAYAVWTGIGAVGAAITGIVLLGESANPMRLASL
ALIVLGIIGLKLSTH

>gi|30043788|gb|AAP19507.1| elongation factor P (EF-P) [Shigella flexneri 2a str. 2457T]
MATYYSNDFRAGLKIMLDGEPYAVEASEFVKPGKGQAFARVKLRRLLTGTRVEKTFKSTDSAEGADVVDM
NLTYLYNDGEFWHFMNNETFEQLSADAKAIGDNAKWLLDQAECIVTLWNGQPISVTPPNFVELEIVDTDP
GLKGDTAGTGGKPATLSTGAVVKVPLFVQIGEVIKVDTRSGEYVSRVK

>gi|30043787|gb|AAP19506.1| hypothetical protein S4569 [Shigella flexneri 2a str. 2457T]
MAHIVTLNTPSREDWLTQLADVVTDPDELLRLLNIDADEKLLAGRSAKKLFALRVPRSFIDRMEKGNPDD
PLLRQVLTSQDEFVVASGFSTDPLEEQHSVVPGLLHKYHNRALLLVKGGCAVNCRYCFRRHFPYAENQGN
KRNWQTALEYVAAHPELDEMIFSGGDPLMAKEHELDWLLTQLEAIPHIKRLRIHSRLPIVIPARITEALV

ERFARSTLQILLVNHINHANEVDETFRQAMAKLRRVGVTLLNQSVLLRGVNDNAQTLANLSNALFDAGVM

PYYLHVLDKVQGAAHFMVSDDEARQIMRELLTLVSGYLVPKLAREIGGEPSKTPLDLQLRQQ

>gi|30043786|gb|AAP19505.1| IS1 orfB [Shigella flexneri 2a str. 2457T]

MSRQCTHYGRWPQHGFTSLKKLRPQSVTSRIQPGSDVIVCAEMDEHWGYVGAKSRQRWLFYAYDRIRRTV

VAHVFGERTLATLERLLSLLSAFEVVVWMTDGWPLYESRLKGKLHVISKRYTQRIERHNLNLRQHLARLV

RKSLSFSKSVELHDKVIGHYLNIKHYQ

>gi|30043785|gb|AAP19504.1| IS1 orfA [Shigella flexneri 2a str. 2457T]

MASISIRCPSCSATEGVVRNGKSTAGHQRYLCSHCRKTWQLQFTYTASQPGTHQKIIDMAMNGVGCRASA

RIMGVGLNTVLRHLKNSGRSR

>gi|30043784|gb|AAP19503.1| hypothetical protein S4566 [Shigella flexneri 2a str. 2457T]

MAISIKGVNTGVIRKSNNFIALALKIKEPRNKESLFFMSVMELRDLLIALESRLHQKHKLDAAARLQYEQ

ARDKVIKKMAENIPEILVDELKNADINRRVNTLELTDNQGENLTFVLTLHDGSKCELVVNELQIEMLARA

IIHAINNAEMRELALRITSLLDFLPLYDVDCQDNGNLEYDTYSQPEWKHNLFDHYLAVLYRFKDESGKEQ

FSGAVVKTREATPGKEIEAITRRMLDFSPRLKKLAGVPCQVYVRTVAANNAQPLTQDQCLRALHHLRVQS

TSKTAPQAK

>gi|30043783|gb|AAP19502.1| hypothetical protein S4565 [Shigella flexneri 2a str. 2457T]

MASSSLIMGNNMHVKYLAGIVGAALLMAGCSSSNELSAAGQSVRIVDEQPGAECQLIGTATGKQSNWLSG

QHGEEGGSMRGAANDLRNQAAAMGGNVIYGISSPSQGMLSSFVPTDSQIIGQVYKCPN

>gi|30043782|gb|AAP19501.1| GroEL, chaperone Hsp60, peptide-dependent ATPase, heat shock protein [Shigella flexneri 2a str. 2457T]

MAAKDVKFGNDARVKMLRGVNVLADAVKVTLGPKGRNVVLDKSFGAPTITKDGVSVAREIELEDKFENMG

AQMVKEVASKANDAAGDGTTTATVLAQAIITEGLKAVAAGMNPMDLKRGIDKAVTAAVEELKALSVPCSD
SKAIAQVGTISANSDETVGKLIAEAMDKVGKEGVITVEDGTGLQDELDVVEGMQFDRGYLSPYFINKPET
GAVELESPFILLADKKISNIREMLPVLEAVAKAGKPLLIIAEDVEGEALATLVVNTMRGIVKVAAVKAPG
FGDRRKAMLQDIATLTGGTVISEEIGMELEKATLEDLGQAKRVVINKDTTTIIDGVGEEAAIQGRVAQIR
QQIEEATSDYDREKLQERVAKLAGGVAVIKVGAATEVEMKEKKARVEDALHATRAAVEEGVVAGGGVALI
RVASKLADLRGQNEDQNVGIKVALRAMEAPLRQIVLNCGEEPSVVANTVKGGDGNYGYNAATEEYGNMID
MGILDPTKVTRSALQYAASVAGLMITTECMVTDLPKNDAADLGAAGGMGGMGGMGGMM

>gi|30043781|gb|AAP19500.1| GroES, 10 Kd chaperone [Shigella flexneri 2a str. 2457T]
MNIRPLHDRVIVKRKEVETKSAGGIVLTGSAAAKSTRGEVLAVGNGRILENGEVKPLDVKVGDIVIFNDG
YGVKSEKIDNEEVLIMSESDILAIVEA

>gi|30043780|gb|AAP19499.1| putative transport protein [Shigella flexneri 2a str. 2457T]
MSGLKQELGLAQGIGLLSTSLLGTGVFAVPALAALVAGNNSLWAWPVLIILVFPIAIVFAILGRHYPSAG
GVAHFVGMAFGSRLERVTGWLFLSVIPVGLPAALQIAAGFGQAMFGWHSWQLLLAELGTLALVWYIGTLG
ASSSANLQTVIAGLIVALIVAIWWAGDIKPASIPFPAPGNIELTGLFAALSVMFWCFVGLEAFAHLASEF
KNPERDFPRALMIGLLLAGLVYWGCTVVVLHFDAYGEQMAAAASLPKIVVQLFGVGALWIACVIGYLACF
ASLNIYIQSFARLVWSQAQHNPDHYLARLSSRHIPNNALNAVLGCCVVSTLVIHALEINLDALIIYANGI
FIMIYLLCMLAGCKLLQGRYRLLAVVGGLLCVLLLAMVGWKSLYALIMLVGLWLFLPKRKTPENGITT

>gi|30043779|gb|AAP19498.1| hypothetical protein S4561 [Shigella flexneri 2a str. 2457T]
MLVIFTSVIGMSLVRNQGFKNFVLMQQKMAAGENPAAEMIKSVSLIIAGLLLLLPGFFTDFLGLLLLLPP
VQKHLTVKLMPHLRFSRMPGGGFSAGTGGGNTFDGEYQRKDDDRDRLDHKDDRQD

>gi|30043778|gb|AAP19497.1| aspartate ammonia-lyase (aspartase) [Shigella flexneri 2a str. 2457T]

MCLKQIIGSLKKKVHMSNNIRIEEDLLGTREVPADAYYGVHTLRAIENFYISNNKISDIPEFVRGMVMVK

KAAAMANKELQTIPKSVANAIIAACDEVLNNGKCMDQFPVDVYQGGAGTSVNMNTNEVLANIGLELMGHQ

KGEYQYLNPNDHVNKCQSTNDAYPTGFRIAVYSSLIKLVDAINQLREGFERKAVEFQDILKMGRTQLQDA

VPMTLGQEFRAFSILLKEEVKNIQRTAELLLEVNLGATAIGTGLNTPKEYSPLAVKKLAEVTGFPCVPAE

DLIEATSDCGAYVMVHGALKRLAVKMSKICNDLRLLSSGPRAGLNEINLPELQAGSSIMPAKVNPVVPEV

VNQVCFKVIGNDTTVTMAAEAGQLQLNVMEPVIGQAMFESVHILTNACYNLLEKCINGITANKEVCEGYV

YNSIGIVTYLNPFIGHHNGDIVGKICAETGKSVREVVLERGLLTEAELDDIFSVQNLMHPAYKAKRYTDE

SEQ

>gi|30043777|gb|AAP19496.1| anaerobic dicarboxylate transport protein [Shigella flexneri 2a str. 2457T]

MLVVELIIVLLAIFLGARLGGIGIGFAGGLGVLVLAAIGVKPGNIPFDVISIIMAVIAAISAMQVAGGLD

YLVHQTEKLLRRNPKYITILAPIVTYFLTIFAGTGNISLATLPVIAEVAKEQGVKPCRPLSTAVVSAQIA

ITASPISAAVVYMSSVMEGHGISYLHLLSVVIPSTLLAVLVMSFLVTMLFNSKLSDDPIYRKRLEEGLVE

LRGEKQIEIKSGAKTSVWLFLLGVVGVVIYAIINSPSMGLVEKPLMNTTNAILIIMLSVATLTTVICKVD

TDNILNSSTFKAGMSACICILGVAWLGDTFVSNNIDWIKDTAGEVIQGHPWLLAVIFFFASALLYSQAAT

AKALMPMALALNVSPLTAVASFAAVSGLFILPTYPTLVAAVQMDDTGTTRIGKFVFNHPFFIPGTLGVAL

AVCFGFVLGSFML

>gi|30043776|gb|AAP19495.1| divalent cation tolerance protein [Shigella flexneri 2a str. 2457T]

MLDEKSSNTTSVVVLCTAPDEATAQDLAAKVLAEKLAACATLIPGATSLYYWEGKLEQEYEVQMILKTTV

SHQQALLECLKSHHPYQTPELLVLPVTHGDTDYLSWLNASLR

>gi|30043775|gb|AAP19494.1| thiol:disulfide interchange protein [Shigella flexneri 2a str. 2457T]

MAQRIFTLILLLLCSTSVFAGLFDAPGRSQFVPADQAFAFDFQQNQHDLNLTWQIKDGYYLYRKQIRITPE

HAKIADEQLPQGVWHEDEFYGKSEIYRDRLTLPVTINQASAGATLTVTYQGCADAGFCYPPETKTVPLSE

VVANNAASQPVSVSQQEQHTAQLPFSALWALLIGIGIAFTPCVLPMYPLISGIVLGGKQRLSTARALLLT

FIYVQGMALTYTALGLVVAAAGLQFQAALQHPYVLIGLAIVFTLLAMSMFGLFTLQLPSSLQTRLTLMSN

RQQGGSPSGVFVMGAIAGLICSPCTTAPLSAILLYIAQSGNMWPGGGTLYLYALGMGLPLMLITVFGNRL

LPKSGPWMEQVKTAFGFVILALPVFLLERVIGDVWGLRLWSALGVAFFGWAFITSLQAKRGWMRVVQIIL

LAAALVSVRPLQDWAFGATHTAQTQTHLNFTQIKTVDELNQALVEAKGKPVMLDLYADWCVACKEFEKYT

FSDPQVQKALADTVLLQANVTANDAQDVALLKHLNVLGLPTILFFDGQGQEHPQARVTGFMDAETFSAHL

RDRQP

>gi|30043774|gb|AAP19493.1| hypothetical protein S4556 [Shigella flexneri 2a str. 2457T]

MQREDVLGEALKLLELQGIANTTLEMVAERVDYPLDELRRFWPDKEAILYDALRYLSQQIDVWRRQLMLD

ETQTAEQKLLARYQALSECVKNNRYPGCLFIAACTFYPDPGHPIHQLADQQKSAAYDFTHELLTTLEVDD

PAMVAKQMELVLEGCLSRMLVNRSQADVDTAHRLAEDILRFARCRQGGALT

>gi|30043773|gb|AAP19492.1| IS1 orfB [Shigella flexneri 2a str. 2457T]

MSRQCTHYGRWPQHGFTSLKKLRPQSVTSRIQPGSDVIVCAEMDEHWGYVGAKSRQRWLFYAYDRIRRTV

VAHVFGERTLATLERLLSLLSAFEVVVWMTDGWPLYESRLKGKLHVISKRYTQRIERHNLNLRQHLARLV

RKSLSFSKSVELHDKVIGHYLNIKHYQ

>gi|30043772|gb|AAP19491.1| IS1 orfA [Shigella flexneri 2a str. 2457T]

MASISIRCPSCSATEGVVRNGKSTAGHQRYLCSHCRKTWQLQFTYTASQPGKHQKIIDMAMNGVGCRASA

RIMGVGLNTVLRHLKNSGRSR

>gi|30043771|gb|AAP19490.1| transport of lysine/cadaverine [Shigella flexneri 2a str. 2457T]

MSSAKKIGLFACTGVVAGNMMGSGIALLPANLASIGGIAIWGWIISIIGAMSLAYVYARLATKNPQQGGP

IAYAGEISPAFGFQTGVLYYHANWIGNLAIGITAVSYLSTFFPVLNDPVPAGIACIAIVWVFTFVNMLGG

TWVSRLTTIGLVLVLIPVVMTAIVGWHWFDAATYAANWNTADTTDGHAIIKSILLCLWAFVGVESAAVST

GMVKNPKRTVPLATMLGTGLAGIVYIAATQVLSGMYPSSVMAASGAPFAISASTILGNWAAPLVSAFTAF

ACLTSLGSWMMLVGQAGVRAANDGNFPKVYGEVDSNGIPKKGLLLAAVKMTALMILITLMNSAGGKASDL

>gi|30043770|gb|AAP19489.1| IS600 orfB [Shigella flexneri 2a str. 2457T]

MCQVFGVSRSGYYDRVQHAPSDRKQSDERLKLEIKVAHIRTRETYGTRRLQTELAENGIIVGRDRLARLR

KELRLRCKQKRKFRATTNPNHNLPVAPNLLNQTFAPTAPNQVWVADLTYVATQEGWLYLAGIKDVYTCEI

VGYAMGERMTKELTGKALFMALRSQRPPAGLIHHSDRGSQYCAYDYRVIQEQSGLKTSMSRKGNCYDNAP

MESFWGTLKNESLSHYRFNNRDEAISVIREYIEIFYNRQRRHSRLGNISPAAFREKYHQMAA

>gi|30043769|gb|AAP19488.1| IS1 orfA [Shigella flexneri 2a str. 2457T]

MASISIRCPSCSATEGVVRNGKSTAGHQRYLCSHCRKTWQLQFTYTASQPGTHQKIIDMAMNGVGCRASA

RIMGVGLNTVLRHLKNSGRSR

>gi|30043768|gb|AAP19487.1| IS1 orfB [Shigella flexneri 2a str. 2457T]

MSRQCTHYGRWPQHGFTSLKKLRPQSVTSRIQPGSDVIVCAEMDEHWGYVGAKSRQRWLFYAYDRIRRTV

VAHVFGERTLATLERLLSLLSAFEVVVWMTDGWPLYESRLKGKLHVISKRYTQRIERHNLNLRQHLARLG

RKSLSFSKSVELHDKVIGHYLNIKHYQ

>gi|30043767|gb|AAP19486.1| hypothetical protein S4548 [Shigella flexneri 2a str. 2457T]

MRDLLQRPDLFSINTATLGYKTPLPAIIDACAARGIGAIAPWRRELQGEDLQQITRQLAASNMSVSGLCR

STYYTAPTLAERKLAIDDNRRALDDAAVLNAACYMQVVGGLPTGTKDLYEAREQVKQGIRQLLPHSKDVG

VPIALEPLHPMTAADRSCLCTLRQALDWCDELDPDGEFGLGVAVDVYHVWWDPDLASQILRAGKRILAFH

VSDWLMPTTDLVNDRGMPGDGVINIPSIRRLVENAGFNGAIELEIFSPYWWQKDINSTGNDSNLLIVFYV

QIMPDDLVMQLHRF

>gi|30043766|gb|AAP19485.1| hypothetical protein S4547 [Shigella flexneri 2a str. 2457T]

MKSTRPVAVITGATRGIGKGCAQELARGGFNLLINDRPDADSVEKLHITQQECLAEGVEVICFPADVGDL

SLHEEMLDAAQNQWGRLDCLLNNAGISVKKRGDLLDLEPDSFDQNIAINTRAPFFLAQAFSKRLLAQPKP

EAELPHRSIIFVSSINAIMLAMNRGEYTIAKTAVSAAARLFAARLCNEQIGVYEVRPGLIKTDMTIPATA

YYDELIAKGLVPWGRWGYPADIASTVRAMAEGKLIYTCGQAVAIDGGLSMPRF

>gi|30043765|gb|AAP19484.1| hypothetical protein S4546 [Shigella flexneri 2a str. 2457T]

MHSENQSKGVHYAKSQRLLEINHAHLQLMESLLDEGKKYNIFKPGIDPLQVNINIAALGGYYLINQHTLG

LVYHISMVSPQALEARRKVIKETILSWLLVDPSSTAHE

>gi|30043764|gb|AAP19483.1| hypothetical protein S4545 [Shigella flexneri 2a str. 2457T]

MIEEQDVEQEETMPGRFELKPTLEKVWHAPDNFRFMDPLPPMHRRGIIIAAIVLVVGFLLPSDDTPNASV

VTREAQLDIQSQSQPPTEEQPQTTQTQPFQPDSGIDNQWRSYRVEPGKTMAQLFRDHGLPATDVYAMAQV

EGAGKPLSNLQNGQMVKIRQNASGVVTGLTIDTGNNQQVLFTRQPDGSFIRAR

>gi|30043763|gb|AAP19482.1| transport protein for D-alanine, D-serine, and glycine [Shigella flexneri 2a str. 2457T]

MVDQVKVVADDQAPAEQSLRRNLTNRHVQLIAIGGAIGTGLFMGSGKTISLAGPSIIFVYMIIGFMLFFV

MRAMGELLLSNLEYKSFSDFASDLLGPWAGYFTGWTYWFCWVVTGMADVVAITAYAQFWFPDLSDWVASL

AVIVLLLTLNLATVKMFGEMEFWFAMIKIVAIVSLIVVGLVMVAMHFQSPTGVEASFAHLWNDGGWFPKG

LSGFFAGFQIAVFAFVGIELVGTTAAETKDPEKSLPRAINSIPIRIIMFYVFALIVIMSVTPWSSVVPEK

SPFVELFVLVGLPAAASVINFVVLTSAASSANSGVFSTSRMLFGLAQEGVAPKAFAKLSKRAVPAKGLTF

SCICLLGGVVMLYVNPSVIGAFTMITTVSAILFMFVWTIILCSYLVYRKQRPHLHEKSIYKMPLGKLMCW

VCMAFFVFVVVLLTLEDDTRQALLVTPLWFIALGLGWLFIGKKRAAELRK

>gi|30043762|gb|AAP19481.1| hypothetical protein S4542 [Shigella flexneri 2a str. 2457T]

MAYRDQPLGELALSIPRASALFRKYDMDYCCGGKQTLARAAARKELDVEVIEAELAKLAEQPIEKDWRSA

PLAEIIDHIIVRYHDRHREQLPELILQATKVERVHADKPSVPKGLTKYLTMLHEELSSHMMKEEQILFPM

IKQGMGSQAMGPISVMESEHDEAGELLEVIKHTTNNVTPPPEACTTWKAMYNGINELIDDLMDHISLENN

VLFPRALAGE

>gi|30043761|gb|AAP19480.1| putative transmembrane subunit [Shigella flexneri 2a str. 2457T]

MISGVLYALLAGLMWGLIFVGPLIVPEYPAMLQSMGRYLALGLIALPIAWLGRVRLRQLARRDWLTALML

TMMGNLIYYFCLASAIQRTGAPVSTMIIGTLPVVIPVFANLLYSQRDGKLAWGKLAPALICIGIGLASVN

IAELNHGLPDFDWARYTSGIVLALVSVVCWAWYALRNARWLRENPDKHPMMWATAQALVTLPVSLIGYLV

ACYWLNIQTPDFSLPFGPRPLVFISLMVAIAVLCSWVGALCWNVASQRLPTVILGPLIVFETLAGLLYTF

LLRQQMPPLMTLSGIALLVIGVVIAVRAKPEKPLTESVSES

>gi|30043760|gb|AAP19479.1| putative oxidoreductase [Shigella flexneri 2a str. 2457T]

MIAITGATGQLGHYIIESLMKTVPASQIVAIVRNPAKAQALAAQGITVRQADYSDEAALTSALQGVEKLL

LISSSEVGQRAPQHRNVINAAKAAGVKFIAYTSLLHADTSPLGLADEHIETEKMLADSGIVYTLLRNGWY

SENYLASAPAALEHGVFIGAAGDGKIASATRADYATAAARVISEAGHEGKVYELAGDSAWTLTQLAAELT

KQSGKQVTYQNLSEADFAAALKSVGLPDGLADMLADSDVGASKGGLFDNSKTLSKLIGCPTTTLAESVSH

LFNVNN

>gi|30043759|gb|AAP19478.1| hypothetical protein S4540 [Shigella flexneri 2a str. 2457T]

MGSLHRFVLCLNTLTPKLTFSKYVQKGKYEMSQVSLSQQLKEGNLFAEQCPSREVLKHVTSRWGVLILVA

LREGTHRFSDLRRKIGGVSEKMLAQSLQALEQDGFLNRIAYPVVPPHVEYSLTPLGEQVSEKVAALADWI

ELNLPEVLAVRDERAA

>gi|30043758|gb|AAP19477.1| 2:3-cyclic-nucleotide 2-phosphodiesterase [Shigella flexneri 2a str. 2457T]

MIKFSATLLATLIAASVNAATVDLRIMETTDLHSNMMDFDYYKDTATEKFGLVRTASLINDARNEVKNSV

LVDNGDLIQGSPLADYMSAKGLKAGDVHPVYKALNTLDYTVGTLGNHEFNYGLDYLKNALAGAKFPYVNA

NVIDARTKQPMFTPYLIKDTEVVDKDGKKQTLKIGYIGVVPPQIMGWDKANLSGKVTVNDITETVRKYVP

EMREKGADVIVVLAHSGLSADPYKVMAENSVYYLSEIPGVNAIMFGHAHAVFPGKDFADIEGADITKGTL

NGVPAVMPGMWGDHLGVVDLQLSNDSGKWQVTQAKAEARPIYDIANKKSLAAEDSKLVETLKADHDATRQ

FVSKPIGKSADNMYSYLALVQDDPTVQVVNNAQKAYVEHYIQGDPDLAKLPVLSAAAPFKVGGRKNDPAS

YVEVEKGQLTFRNAADLYLYPNTLIVVKASGKEVKEWLECSAGQFNQIAPNSTKPQSLINWDGFRTYNFD

VIDGVNYQIDVTQPARYDGECQMINANAERIKNLTFNGKPIDPNAMFLVATNNYRAYGGKFAGTGDSHIA

FASPDENRSVLAAWIADESKRAGEIHPAADNNWRLAPIAADKKLDIRFETSPSDKAAAFIKEKGQYPMNK

VATDDIGFAIYQVDLSK

>gi|30043757|gb|AAP19476.1| ammonium transport system structural protein [Shigella flexneri 2a str. 2457T]

MLDQVCQLARNAGDAIMQVYDGTKPMDVVSKADNSPVTAADIAAHTVIMDGLRTLAPDIPVLSEEDPPGW

EVRQHWQRYWLVDPLDGTKEFIKRNGEFTVNIALIDHGKPILGVVYAPVMNVMYSAAEGKAWKEECGVRK

LIQVRDARPPLVVISRSHADAELKEYLQQLGEHQTTSIGSSLKFCLVAEGQAQLYPRFGPTNIWDTAAGH

AVAAAAGAHVHDWQGKPLDYTPRESFLNPGFRVSIY

>gi|30043756|gb|AAP19475.1| hypothetical protein S4535 [Shigella flexneri 2a str. 2457T]

MTLRKILALTCLLLPMMASTHQFEIGQRVPPIGITDRGELVLDKDQFSYKTWNSAQLVGKVRVLQHIAGR

TSAKEKNATLIEAIKSAKLPHDRYQTTTIVNTDDAIPGSGMFVRSSLESNKKLYPWSQFIVDSNGVARGA

WQLDEESSAVVVLDKDGRVQWAKDGALTPEEVQQVMDLLQKLLK

>gi|30043755|gb|AAP19474.1| hypothetical protein S4534 [Shigella flexneri 2a str. 2457T]

MAPHRRNTQQEITMKIFQRYNPLQVAKYVKILFRGRLYIKDIGAFEFDKGKILIPKVKDKLHLSVMSEVN
RQVMRLQTEMA

>gi|30043754|gb|AAP19473.1| putative transport protein [Shigella flexneri 2a str. 2457T]
MLNSILVILCLIAVSAFFSMSEISLAASRKIKLKLLADEGNINAQRVLNMQENPGMFFTVVQIGLNAVAI
LGGIVGDAAFSPAFHSLFSRYMSAELSEQLSFILSFSLVTGMFILFADLTPKRIGMIAPEAVALRIINPM
RFCLYVCTPLVWFFNGLANMIFRIFKLPMVRKDDITSDDIYAVVEAGALAGVLRKQEHELIENVFELESR
TVPSSMTPRENVIWFDLHEDEQSLKNKVAEHPHSKFLVCNEDIDHIIGYVDSKDLLNRVLANQSLALNSG
VQIRNTLIVPDTLTLSEALESFKTAGEDFAVIMNEYALVVGIITLNDVMTTLMGDLVGQGLEEQIVARDE
NSWLIDGGTPIDDVMRVLDIDEFPQSGNYETIGGFMMFMLRKIPKRTDSVKFAGYKFEVVDIDNYRIDQL
LVTRIDSKATALSPKLPDAKDKEESVA

>gi|30043753|gb|AAP19472.1| peptide methionine sulfoxide reductase [Shigella flexneri 2a str. 2457T]
MSLFDKKHLVSPADALPGRNTPMPVATLHAVNGHSMTNVPDGMEIAIFAMGCFWGVERLFWQLPGVYSTA
AGYTGGYTPNPTYREVCSGDTGHAEAVRIVYDPSVISYEQLLQVFWENHDPAQGMRQGNDHGTQYRSAIY
PLTPEQDAAARASLERFQAAMLAADDDRHITTEIANATPFYYAEDDHQQYLHKNPYGYCGIGGIGVCLPP
EA

>gi|30043752|gb|AAP19471.1| hypothetical protein S4531 [Shigella flexneri 2a str. 2457T]
MRYIRQLCCVSLLCLSGSAVAANVRLQVEGLSGQLEKNVRAQLSTIESDEVTPDRRFRARVDDAIREGLK
ALGYYQPTIEFDLRPPPKKGRQVLIAKVTPGVPVLIGGTDVVLRGGARTDKDYLKLLDTRPAIGTVLNQG
DYENFKKSLTSIALRKGYFDSEFTKAQLGIALGLHKAFWDIDYNSGERYRFGHVTFEGSQIRDEYLQNLV
PFKEGDEYESKDLAELNRRLSATGWFNSVVVAPQFDKARETKVLPLTGVVSPRTENTIETGVGYSTDVGP
RVKATWKKPWMNSYGHSLTTSTSISAPEQTLDFSYKMPLLKNPLEQYYLVQGGFKRTDLNDTESDSTTLV
ASRYWDLSSGWQRAINLRWSLDHFTQGEITNTTMLFYPGVMISRTRSRGGLMPTWGDSQRYSIDYSNTAW

GSDVDFSVFQAQHVWIRTLYDRHRFVTRGTLGWIETGDFDKVPPDLRFFVGGDRSIRGYKYKSIAPKYAN
GDLKGASKLITGSLEYQYNVTGKWWGAVFVDSGEAVSDIRRSDFKTGTGVGVRWESPVGPIKLDFAVPVA
DKDEHGLQFYIGLGPEL

>gi|30043751|gb|AAP19470.1| hypothetical protein S4530 [Shigella flexneri 2a str. 2457T]
MSLWKKISLGVVIVILLLLGSVAFLVGTTSGLHLVFKAADRWVPGLDIGKVTGGWRDLTLSDVRYEQLGV
AVKAGNLHLGVGLECLWNSSVCINDLALKDIQVNIDSKKMPPSEQVEEEEDSGPLDLSTPYPITLTRVAL
DNVNIKIDDTTVSVMDFTSGLNWQEKTLTLKPTSLKGLLIALPKVVEVAQEEVVEPKIENPQPEEKPLGE
TLKDLFSRPVLPEMTDVHLPLNLNIEEFKGEQLRVTGDTDITVRTMLLKVSSIDGNTKLDALDIDSNQGI
VNASGTAQLSDNWPVDITLNSTLNVEPLKGEKVKLKVGGALREQLEIGVNLSGPVDMDLRAQTRLAEAGL
PLNVEVNSKQLYWPFTGEKQYQADDLKLKLTGKMTDYTLSMRTAVKGQEIPPATITLDAKGNEQQVNLDK
LTVAALEGKTELKALLDWQQAISWRGELTLNGINTAKEFPEWPSKLNGLIKTRGSLYGGTWQMEVPELKL
TGNVKQNKVNVDGTLKGNSYMQWMIPGLHLELGPNSAEVKGELGVKDLNLDATINAPGLDNALPGLGGTA
KGLVKVRGTVEAPQLLADITARGLRWQELSVAQVRVEGDIKSTDQIAGKLDVRVEQISQPDVNINLVTLN
AKGSEKQHELQLRIQGEPVSGQLNLAGSFDRKEERWKGTLSNTRFQTPVGPWSLTRDIALDYRNKEQKIS
IGPHCWLNPNAELCVPQTIDAGAEGRAVVNLNRFDLAMLKPFMPETTQASGIFTGKADVAWDTTKEGLPQ
GSITLSGRNVQVTQTVNDAALPVAFQTLNLTAELRNNRAELGWTIRLTNNGQFDGQVQVTDPQGRRNLGG
NVNIRNFNLAMINPIFTRGEKAAGMVSANLRLGGDVQSPQLFGQLQVTGVDIDGNFMPFDMQPSQLAVNF
NGMRSTLAGTVRTQQGEIYLNGDADWSQIENWRARVTAKGSKVRITVPPMVRMDVSPDVVFEATPNLFTL
DGRVDVPWARIVVHDLPESAVGVSSDVVMLNDNLQPEEPKTASIPINSNLIVHVGNNVRIDAFGLKARLT
GDLNVVQDKQGLGLNGQINIPEGRFHAYGQDLIVRKGELLFSGPPDQPYLNIEAIRNPDATEDDVIAGVR
VTGLADEPKAEIFSDPAMSQQAALSYLLRGQGLESDQSDSAAMTSMLIGLGVAQSGQIVGKIGETFGVSN
LALDTLGVGDSSQVVVSGYVLPGLQVKYGVGIFDSIATLTLRYRLMPKLYLEAVSGVDQALDLLYQFEF

>gi|30043750|gb|AAP19469.1| hypothetical protein S4529 [Shigella flexneri 2a str. 2457T]

MRIFVYGSLRHKQGNSHWMTNAQLLGDFSIDNYQLYSLGHYPGAVPGNGTVHGEVYRIDNATLAELDALR

TRGGEYARQLIQTPYGSAWMYVYQRPVDGLKLIESGDWLDRDK

>gi|30043749|gb|AAP19468.1| hypothetical protein S4528 [Shigella flexneri 2a str. 2457T]

MITYPCLTSRRFQLALIHRRVVDKRTSMHSHTASESTGARIHRPWCARHQVRPAWRCQYDKLHRVPFRSP

ELRLDSGPGYTTGSYRY

>gi|30043748|gb|AAP19467.1| inorganic pyrophosphatase [Shigella flexneri 2a str. 2457T]

MSLLNVPAGKDLPEDIYVVIEIPANADPIKYEIDKESGALFVDRFMSTAMFYPCNYGYINHTLSLDGDPV

DVLVPTPYPLQPGSVIRCRPVAVLKMTDEAGEDAKLVAVPHSKLSKEYDHIKDVNDLPELLKAQIAHFFE

HYKDLEKGKWVKVEGWENAEAAKAEIVASFERAKNK

>gi|30043747|gb|AAP19466.1| putative ATP-binding component of a transport system [Shigella flexneri 2a str. 2457T]

MTTDQHQEILRTEGLSKFFPGVKALDNVDFSLRRGEIMALLGENGAGKSTLIKALTGVYHADRGTIWLEG

QTISPKNTAHAQQLGIGTVYQEVNLLPNMSVADNLFIGREPKRFGLLRRKEMEKRATELMASYGFSLDVR

EPLNRFSVAMQQIVAICRAIDLSAKVLILDEPTASLDTQEMELLFGLMRQLRDRGVSLIFVTHFLDQVYQ

VSDRITVLRNGSFVGCRETRELPQIELVKMMLGRELDTHALQRAGRTLLSDKPVAAFKNYGKKGTIAPFD

LEVRPGEIVGLAGLLGSGRTETAEVIFGIKPADSGTALIKGKPQTLRSPHQASVLGIGFCPEDRKTDGII

AAASVRENIILALQAQRGWLRPISRKEQQEIAERFIRQLGIRTPSTEQPIEFLSGGNQQKVLLSRWLLTR

PQFLILDELTRGIDVGAHAEIIRLIETLCADGLALLVISSELEELVGYADRVIIMRDRKQVAEIPLAQLS

VPAIMNAIAA

>gi|30043746|gb|AAP19465.1| putative transport system permease protein [Shigella flexneri 2a str. 2457T]

MMPQSLPDTTPPKRRFHWPTGMPQLAALLLVLLVDSLVAPHFWQVVLQDGRLFGSPIDILNRAAPVALLA

IGITLVIATGGIDLSVGAVMAIAGATTAAMTVAGFSLPIVLLSALGTGILAGLWNGILVAILKIQPFVAT
LILMVAGRGVAQLITAGQIVTFNSPDLSWFGSGSLLFLPTPVIIAVLTLLLFWLLTRKTALGMFIEVVGI
NIRAAKNAGVNTRIIVMLTYVLSGLCAAIAGIIVAADIRGADANNAGLWLELDAILAVVIGGGSLMGGRF
NLLLSVVGALIIQGMNTGILLSGFPPEMNQVVKAVVVLCVLIVQSQRFISLIKGVRSHDKT

>gi|30043745|gb|AAP19464.1| IS4 orf [Shigella flexneri 2a str. 2457T]
MHIGQALDLVSRYDSLRNPLTSLGDYLDPELISRCLAESGTVTLRKRRLPLEMMVWCIVGMVLERKEPLH
QIVNRLDIMLPGNRPFVAPSAVIQARQRLGSEAVRRVFTKTAQLWHNATPHPHWCGLTLLAIDGVFWRTP
DTPENDAAFPRQTHAGNPALYPQVKMVCQMELTSHLLTAAAFGTMKNSENELAEQLIEQTGDNTLTLMDK
GYYSLGLLNGWSLAGEHRHWMIPLRKGAQYEELRKLGKGDHLVKLKTSPQARKKWPGLGNEVTARLLTVT
RKGKVCHLLTSMTDAMRFPGGEMADLYSNRWEIELGYREIKQTMQLSRLTLRSKKPELVEQELWGVLLAY
NLVRYQMIKMAEHLKGYWPNQLSFSESCGMVMRMLMTLQGASPGRIPELMRDLASMGQLVKLPTRRGRAF
PRVVKERPWKYPTAPKKSQSVA

>gi|30043744|gb|AAP19463.1| fructose-bisphosphatase [Shigella flexneri 2a str. 2457T]
MKTLGEFIVEKQHEFSHATGELTALLSAIKLGAKIIHRDINKAGLVDILGASGAENVQGEVQQKLDLFAN
EKLKAALKARDIVAGIASEEEDEIVVFEGCEHAKYVVLMDPLDGSSNIDVNVSVGTIFSIYRRVTPVGTP
VTEEDFLQPGNKQVAAGYVVYGSSTMLVYTTGCGVHAFTYDPSLGVFCLCQERMRFPEKGKTYSINEGNY
IKFPNGVKKYIKFCQEEDKSTNRPYTSRYIGSLVADFHRNLLKGGIYLYPSTASHPDGKLRLLYECNPMA
FLAEQAGGKASDGKERILDIIPETLHQRRSFFVGNDHMVEDVERFIREFPDA

>gi|30043743|gb|AAP19462.1| putative ligase [Shigella flexneri 2a str. 2457T]
MRIHILGICGTFMGGLAMLARQLGHEVTGSDANVYPPMSTLLEKQGIELIQGYDASQLEPQPDLVIIGNA
MTRGNPCVEAVLEKNIPYMSGPQWLHDFVLRDRWVLAVAGTHGKTTTAGMATWILEQCGYKPGFVIGGVP
GNFEVSARLGESDFFVIEADEYDCAFFDKRSKFVHYCPRTLILNNLEFDHADIFDDLKAIQKQFHHLVRI

VPGQGRIIWPENDINLKQTMAMGCWSEQELVGEQGHWQAKKLTTDASEWEVLLDGEKVGEVKWSLVGEHN

MHNGLMAIAAARHVGVAPADAANALGSFINARRRLELRGEANGVTVYDDFAHHPTAILATLAALRGKVGG

TARIIAVLEPRSNTMKMGICKDDLAPSLGRADEVFLLQPAHIPWQVAEVAEACVQPAHWSGDVDTLADMV

VKTAQPGDHILVMSNGGFGGIHQKLLDGLAKKAEAAQ

>gi|30043742|gb|AAP19461.1| putative alpha helix protein [Shigella flexneri 2a str. 2457T]

MTKQPEDWLDDVPGDDIEDEDDEIIWVSKSEIKRDAEELKRLGAEIVDLGKNALDKIPLDADLRPAIELA

QRIKMEGRRRQLQLIGKMLRQRDVEPIRQALDKLKNRHNQQVVLFHKLENLRDRLIDQGDDAIAEVLNLW

PDADRQQLRTLIRNAKKEKEGNKPPKSARQIFQYLRELAENEG

>gi|30043741|gb|AAP19460.1| putative peptide maturation protein [Shigella flexneri 2a str. 2457T]

MALAMKVISQVEAQRKILEEAVSTALELASGKSDGAEVAVSKTTGISVSTRYGEVENVEFNSDGALGITV

YHQNRKGSASSTDLSPQAIARTVQAALDIARYTSPDPCAGVADKELLAFDAPNLDLFHPAEVSPDEAIEL

AARAEQAALQADKRITNTEGGSFNSHYGVKVFGNSHGMLQGYCSTRHSLFSCVIAEENGDMERDYAYTIG

RAMSDLQTPEWVGADCARRTLSRLSPRKLSTMKAPVIFANEVATGLFGHLVGAIAGGSVYRKSTFLLDSL

GKQILPDWLTIEEHPHLLKGLASTPFDSEGVRTERRDIIKDGILTQWLLTSYSARKLGLKSTGHAGGIHN

WRIAGQGLSFEQMLKEMGTGLVVTELMGQGVSAITGDYSRGAAGFWVENGEIQYPVSEITIAGNLKDMWR

NIVTVGNDIETRSNIQCGSVLLPEMKIAGQ

>gi|30043740|gb|AAP19459.1| anaerobic ribonucleotide reductase activating protein [Shigella flexneri 2a str. 2457T]

MNYHQYYPVDIVNGPGTRCTLFVSGCVHECPGCYNKSTWRVNSGQPFTKAMEDKIIKDLNDTRIKRQGIS

LSGGDPLHPQNVPDILKLVKRIRAECPGKDIWVWTGYKLDELNAAQMQVVDLINVLVDGKFVQDLKDPSL

IWRGSSNQVVHHLR

>gi|30043739|gb|AAP19458.1| anaerobic ribonucleoside-triphosphate reductase [Shigella flexneri 2a str. 2457T]

MTPHVMKRDGCKVPFKSERIKEAILRAAKAAEVDDADYCATVAAVVNEQMQGRNQVDINEIQTAVENQLM

SGPYKQLARAYIEYRHDRDIEREKRGRLNQEIRGLVEQTNASLLNENANKDSKVIPTQRDLLAGIVAKHY

ARQHLLPRDVVQAHERGDIHYHDLDYSPFFPMFNCMLIDLKGMLTQGFKMGNAEIEPPKSISTATAVTAQ

IIAQVASHIYGGTTINRIDEVLAPFVTASYNKHRKTAEEWNIPDAEGYANSRTIKECYDAFQSLEYEVNT

LHTANGQTPFVTFGFGLGTSWESRLIQESILRNRIAGLGKNRKTAVFPKLVFAIRDGLNHKKGDPNYDIK

QLALECASKRMYPDILNYDQVVNVTGSFKTPMGCRSFLGVWENENGEQIHDGRNNLGVISLNLPRIALEA

KGDEATFWKLLDERLVLARKALMTRIARLEGVKARVAPILYMEGACGVRLNADDDVSEIFKNGRASISLG

YIGIHETINALFSGEHVYDNEKLRAKGIAIVERLRQAVDQWKEETGYGFSLYSTPSENLCDRFCRLDTAE

FGVVPGVTDKGYYTNSFHLDVEKKVNPYDKIDFEAPYPPLANGGFICYGEYPNIQHNLKALEDVWDYSYQ

HVPYYGTNTPIDECYECGFTGEFECTSKGFTCPKCGNHDASRVSVTRRVCGYLGSPDARPFNAGKQEEVK

RRVKHLGNGQIG

>gi|30043738|gb|AAP19457.1| trehalase 6-P hydrolase [Shigella flexneri 2a str. 2457T]

MTNLPHWWQNGVIYQIYPKSFQDTTGSGTGDLRGVIQRLDYLHKLGVDAIWLTPFYVSPQVDNGYDVANY

TAIDPTYGTLDDFDELVTQAKSRGIRIILDMVFNHTSTQHAWFREALNKESPYRQFYIWRDGEPETPPNN

WRSKFGGSAWRWHAESEQYYLHLFAPEQADLNWENPAVRAELKKVCEFWADRGVDGLRLDVVNLISKDPR

FPEDLDGDGRRFYTDGPRAHEFLHEMNRDVFTPRGLMTVGEMSSTSLEHCQRYAALTGSELSMTFNFHHL

KVDYPGGEKWTLAKPDFVALKTLFRHWQQGMHNVAWNALFWCNHDQPRIVSRFGDEGEYRVPAAKMLAMV

LHGMQGTPYIYQGEEIGMTNPHFTRITDYRDVESLNMFAELRNDGRDADELLAILASKSRDNSRTPMQWS

NGDNAGFTAGEPWIGLGDNYQQINVEAALADDSSVFYTYQKLIALRKQEAILTWGNYQDLLPNSPVLWCY

RREWKGQTLLVIANLSREIQPWQPGQMRGNWQLVMHNYEEASPQPCAMNLRPFEAVWWLQK

>gi|30043737|gb|AAP19456.1| PTS system enzyme II [Shigella flexneri 2a str. 2457T]

MSKINQTDIDRLIELVGGRGNIATVSHCITRLRFVLNQPANARPKEIEQLPMVKGCFTNAGQFQVVIGTN

VGDYYQALIASTGQAQVDKEQVKKAARQNMKWHEQLISHFAEIFFPLLPALISGGLILGFRNVIGDLPMS
NGQTLAQMYPSLQTIYDFLWLIGEAIFFYLPVGICWSAVKKMGGTPILGIVLGVTLVSPQLMNAYLLGQQ
LPEVWNFGMFSIAKVGYQAQVIPALLAGLALGVIETRLKRIVPDYLYLVVVPVCSLILAVFLAHALIGPF
GRMIGDGVAFAVRHLMTGSFAPIGAALFGFLYAPLVITGVHQTTLAIDLQMIQSMGGTPVWPLIALSNIA
QGSAVIGIIISSRKHNEREISVPAAISAWLGVTEPAMYGINLKYRFPMLCAMIGSGLAGLLCGLNGVMAN
GIGVGGLPGILSIQPSYWQVFALAMAIAIIIPIVLTSFIYQRKYRLGTLDIV

>gi|30043736|gb|AAP19455.1| repressor of treA,B,C [Shigella flexneri 2a str. 2457T]
MQNRLTIKDIARLSGVGKSTVSRVLNNESGVSQRTRERVEAVMNQHGFSPSRSARAMRGQSDKVVAIIVT
RLDSLSENLAVQTMLPAFYEQGYDPIMMENQFSPQLVAEHLGVLKRRNIDGVVLFGFTGITEEMLAHWQS
SLVLLARDAKGFASVCYDDEGAIKILMQRLYDQGHRNISYLGVPHSDVTTGKRRHEAYLAFCKAHKLHPV
AALPGLAMKQGYENVAKVITPETTALLCATDTLALGASKYLQEQRIDTLQLASVGNTPLMKFLHPEIVTV
DPGYAEAGRQAACQLIAQVTGRSEPQQIIIPATLS

>gi|30043735|gb|AAP19454.1| Mg2+ transport ATPase, P-type 1 [Shigella flexneri 2a str. 2457T]
MFKEIFTRFIRHLPSRLVHRDPLPGAQQTVNTAVPPSLSAHCLKMAVMPEEELWKTFDTHPEGLNQAEVE
SAREQHGDNKLPAQQPSPWWVHLWVCYRNPFNILLTILGAISYATEDLFAAGVIALMVAISTLLNFIQEA
RSTKAADALKAMVSNTATVLRVINDKGENGWLEIPIDQLVPGDIIKLAAGDMIPADLRILQARDLFVAQA
SLTGESLPVEKAATTRQPEHSNPLECDTLCFMGTTVVSGTAQAMVIATGANTWFGQLAGRVSEQESEPNA
FQQGISRVSMLLIRFMLVMAPVVLLINGYTKGDWWEAALFALSVAVGLTPEMLPMIVTSTLARGAVKLSK
QKVIVKHLDAIQNFGAMDILCTDKTGTLTQDKIVLENHTDISGKTSERVLHSAWLNSHYQTGLKNLLDTA
VLEGTDEESARSLASRWQKIDEIPFDFERRRMSVVVAENTEHHQLVCKGALQEILNVCSQVRHNGEIVPL
DDTMLRKIKRVTDTLNRQGLRVVAVATKYLPAREGDYQRADESDLILEGYIAFLDPPKETTAPALKALKA
SGITVKILTGDSELVAAKVCHEVGLDAGEVVIGSDIETLSDDELANLAQRTTLFARLTPMHKERIVTLLK
REGHVVGFMGDGINDAPALRAADIGISVDGAVDIAREAADIILLEKSLMVLEEGVIEGRRTFANMLKYIK

LTASSNFGNVFSVLVASAFLPFLPMLPLHLLIQNLLYDVSQVAIPFDNVDDEQIQKPQRWNPADLGRFMV

FFGPISSIFDILTFCLMWWVFHANTPETQTLFQSGWFVVGLLSQTLIVHMIRTRRVPFIQSCASWPLMIM

TVIVMIVGIALPFSPLASYLQLQALPLSYFPWLVAILAGYMTLTQLVKGFYSRRYGWQ

>gi|30043734|gb|AAP19453.1| hypothetical protein S4509 [Shigella flexneri 2a str. 2457T]

MSQTFYRCNKGEIMSKTIATENAPAAIGPYVQGVDLGNMIITSGQIPVNPKTGEVPADVAAQARQSLDNV

KAIVEAAGLKVGDIVKTTVFVKDLNDFATVNATYEAFFTEHNATFPARSCVEVARLPKDVKIEIEAIAVR

R

>gi|30043733|gb|AAP19452.1| aspartate carbamoyltransferase, regulatory subunit [Shigella flexneri 2a str. 2457T]

MTHDNKLQVEAIKRGTVIDHIPAQIGFKLLSLFKLTETDQRITIGLNLPSGEMGRKDLIKIENTFLSEEQ

VDQLALYAPQATVNRIDNYEVVGKSRPSLPERIDNVLVCPNSNCISHAEPVSSSFAVRKRANDIALKCKY

CEKEFSHNVVLAN

>gi|30043732|gb|AAP19451.1| aspartate carbamoyltransferase, catalytic subunit [Shigella flexneri 2a str. 2457T]

MANPLYQKHIISINDLSRDDLNLVLATAAKLKANPQPELLKHKVIASCFFEASTRTRLSFETSMHRLGAS

VVGFSDSANTSLGKKGETLADTISVISTYVDAIVMRHPQEGAARLATEFSGNVPVLNAGDGSNQHPTQTL

LDLFTIQETQGRLDNLHVAMVGDLKYGRTVHSLTQALAKFDGNRFYFIAPDALAMPQYILDMLDEKGIAW

SLHSSIEEVMAEVDILYMTRVQKERLDPSEYANVKAQFVLRASDLHNAKANMKVLHPLPRVDEIATDVDK

TPHAWYFQQAGNGIFARQALLALVLNRDLVL

>gi|30043731|gb|AAP19450.1| pyrBI operon leader peptide [Shigella flexneri 2a str. 2457T]

MVQCVRHFVLPRLKKDAGLPFFFPLITHSQPLNRGAFFCLGVRR

>gi|30043730|gb|AAP19449.1| hypothetical protein S4506 [Shigella flexneri 2a str. 2457T]

MSNTLNHTSSRQIVRHYTHSQKRCKHLMQYFVSANGLFELKVKIYAFLFSMILEGKCRSVSIIADISCFF

LFHFHAIRNAFYSIHPTYRAEGENERLTLLLIAQGYALSL

>gi|30043729|gb|AAP19448.1| hypothetical protein S4504 [Shigella flexneri 2a str. 2457T]

MVERTAVFPAGRHSLYAEHRYSAAIRSGDLLFVSGQVGSREDGTPEPDFQQQVRLAFDNLHATLAAAGCT

FDDIIDVTSFHTDPEKQSEDIMTVKNEIFSAPPYPNWTAVGVTWLAGFDFEIKVIARIPEL

>gi|30043728|gb|AAP19447.1| putative oxidoreductase [Shigella flexneri 2a str. 2457T]

MGAFTGKTVLILGGSRGIGAAIVRRFVTDGANVRFTYAGSKDAAERLAQETGATAVFTDSADRDAVIDVV

RKSGALDILVVNAGIGVFGDALELNADDIDRLFKINIHAPYHASVEAARQMPEGGRILIIGSVNGDRMPV

AGMAAYAASKSALQGMARGLARDFGPRGITINVVQPGPIDTDVNPANGPMRDMLHSFMAIKRHGQPEEVA

GMVAWLAGPEASFVTGAMHTIDGAFGA

>gi|30043727|gb|AAP19446.1| hypothetical protein S4502 [Shigella flexneri 2a str. 2457T]

MSLKLLIILVLTPRVSTRLLAVRLGYLAVYSMNMSVRKLFRLPIFFVMIVR

>gi|30043726|gb|AAP19445.1| hypothetical protein S4501 [Shigella flexneri 2a str. 2457T]

MVLEGIHSHDPQARDIAIQYYHAAETTIYDYIARRHPQSAQCVTDFMSTVMSGLSAKAREGHSIEQLCAT

AALAGEAIKTLLKE

>gi|30043725|gb|AAP19444.1| hypothetical protein S4500 [Shigella flexneri 2a str. 2457T]

MIIGNIHNLQPWLPQELRQAIEHIKAHVTAETPKGKHDIEGNRLFYLISEDMTEPYEARRAEYHARYLDI

QIVLKGQEGMTFSTQPAGAPDTDWLADKDIAFLPEGVDEKTVILNEGDFVVFYPGEVHKPLCAVGAPAQV

RKAVVKMLMA

>gi|30043724|gb|AAP19443.1| IS1 orfB [Shigella flexneri 2a str. 2457T]

MSRQCTHYGRWPQHGFTSLKKLRPQSVTSRIQPGSDVIVCAEMDEHWGYVGAKSRQRWLFYAYDRIRRTV

VAHVFGERTLATLERLLSLLSAFEVVVWMTDGWPLYESRLKGKLHVISKRYTQRIERHNLNLRQHLARLV

RKSLSFSKSVELHDKVIGHYLNIKHYQ

>gi|30043723|gb|AAP19442.1| IS1 orfA [Shigella flexneri 2a str. 2457T]

MASISIRCPSCSATEGVVRNGKSTAGHQRYLCSHCRKTWQLQFTYTDSQPGTHQKIIDMAMNGVGCRASA

RIMGVGLNTVLRHLKNSGRSR

>gi|30043722|gb|AAP19441.1| ornithine carbamoyltransferase 1 [Shigella flexneri 2a str. 2457T]

MSGFYHKHFLKLLDFTPAELNSLLQLAAKLKADKKSGKEEAKLTGKNIALIFEKDSTRTRCSFEVAAYDQ

GARVTYLGPSGSQIGHKESIKDTARVLGRMYDGIQYRGYGQEIVETLAEYAGVPVWNGLTNEFHPTQLLA

DLLTMQEHLPGKAFNEMTLVYAGDARNNMGNSMLEAAALTGLDLRLVAPQACWPEAALVTECRALAQQNG

GNITLTEDVAKGVEGADFIYTDVWVSMGEAKEKWAERIALLRDYQVNSKMMQLTGNPEVKFLHCLPAFHD

DQTTLGKKMAEEFGLHGGMEVTDEVFESAASIVFDQAENRMHTIKAVMVATLSKLNN

>gi|30043721|gb|AAP19440.1| hypothetical protein S4495 [Shigella flexneri 2a str. 2457T]

MANPEQLEEQREETRLIIEELLEDGSDPDALYTIEHHLSADDLETLEKAAVEAFKLGYEVTDPEELEVED

GDIVICCDILSECALNADLIDAQVEQLMTLAEKFDVEYDGWGTYFEDPNGEDGDDEDFVDEDDDGVRH

>gi|30043720|gb|AAP19439.1| hypothetical protein S4493 [Shigella flexneri 2a str. 2457T]

MAQVINEMDVPSHSFVFHGTGERYFLICVVNVLLTIITLGIYLPWALMKCKRYLYANMEVNGQRFSYGIT

GGNVFVSCLVFVFFYFAILMTVSADMPLVGCVLTLLLLVLLIFMAAKGLRYQALMTSLNGVRFSFNCSLK

GFWWVTFFLPILMAIGMGTVFFISTKMLHANSSSSVIISVVLMAIVGIVSIGIFNGTLYSLVMSFLWSNT

SFGIHRFKVKLDTAYCIKYAILAFLALLPFLAVAGYIIFDQILNEYDSSGYANDDIENLQQFMEMQRKMI

IAQLIYYFGIAVSTSYLTVSLRNHFMSNLSLNDGRIRFRSTLTYHGMLYRMCALVVISGITGGLAYPLLK

IWMIDWQAKNTYLLGDLDDLPLINKEEQPDKGFLASISRGVMPSLPFL

>gi|30043719|gb|AAP19438.1| valine tRNA synthetase [Shigella flexneri 2a str. 2457T]

MEKTYNPQDIEQPLYEHWEKQGYFKPNGDESQESFCIMIPPPNVTGSLHMGHAFQQTIMDTMIRYQRMQG

KNTLWQVGTDHAGIATQMVVERKIAAEEGKTRHDYGREAFIDKIWEWKAESGGTITRQMRRLGNSVDWER

ERFTMDEGLSNAVKEVFVRLYKEDLIYRGKRLVNWDPKLRTAISDLEVENRESKGSMWHIRYPLADGAKT

ADGKDYLVVATTRPETLLGDTGVAVNPEDPRYKDLIGKYVILPLVNRRIPIVGDEHADMEKGTGCVKITP

AHDFNDYEVGKRHALPMINILTFDGDIRESAQVFDTKGNESDVYSSEIPAEFQKLERFAARKAVVAAVDA

LGLLEEIKPHDLTVPYGDRGGVVIEPMLTDQWYVRADVLAKPAVEAVENGDIQFVPKQYENMYFSWMRDI

QDWCISRQLWWGHRIPAWYDEAGNVYVGRNEEEVRKENNLGANVALRQDEDVLDTWFSSALWTFSTLGWP

ENTDALRQFHPTSVMVSGFDIIFFWIARMIMMTMHFIKDENGKPQVPFHTVYMTGLIRDDEGQKMSKSKG

NVIDPLDMVDGISLPELLEKRTGNMMQPQLADKIRKRTEKQFPNGIEPHGTDALRFTLAALASTGRDINW

DMKRLEGYRNFCNKLWNASRFVLMNTEGQDCGFNGGEMTLSLADRWILAEFNQTIKAYREALDSFRFDIA

AGILYEFTWNQFCDWYLELTKPVMNGGTEAELRGTRHTLVTVLEGLLRLAHPIIPFITETIWQRVKVLCG

ITADTIMLQPFPQYDASQVDEAALADTEWLKQAIVAVRNIRAEMNIAPGKPLELLLRGCSADAERRVNEN

RGFLQTLARLESITVLPADDKGPVSVTKIIDGAELLIPMAGLINKEDELARLAKEVAKIEGEISRIENKL

ANEGFVARAPEAVIAKEREKLEGYAEAKAKLIEQQAVIAAL

>gi|30043718|gb|AAP19437.1| DNA polymerase III, chi subunit [Shigella flexneri 2a str. 2457T]

MKNATFYLLDNDTTVDGLSAVEQLVCEIAAERWRSGKRVLIACEDEKQAYRLDEALWARPAESFVPHNLA

GEGPRGSAPVEIAWPQKRSSSPRDILISLRTSFADFATAFTEVVDFVPYEDSLKQLARERYKAYRVAGFN

LNTATWK

>gi|30043717|gb|AAP19436.1| aminopeptidase A/I [Shigella flexneri 2a str. 2457T]

MEFSVKSGSPEKQRSACIVVGVFEPRRLSPIAEQLDKISDGYISALLRRGELEGKPGQTLLLHHVPNVLS

ERILLIGCGKERELDERQYKQVIQKTINTLNDTGSMEAVCFLTELHVKGRNNYWKVRQAVETAKETLYSF

DQLKTNKSEPRRPLRKMVFNVPTRRELTSGERAIQHGQAIAAGIKAAKDLGNMPPNICNAAYLASQARQL

ADSYSKNVITRVIGEQQMKELGMHSYLAVGQGSQNESLMSVIEYKGNASEDARPIVLVGKGLTFDSGGIS

IKPSEGMDEMKYDMCGAAAVYGVMRMVAELQLPINVIGVLAGCENMPGGRAYRPGDVLTTMSGQTVEVLN

TDAEGRLVLCDVLTYVERFEPEAVIDVATLTGACVIALGHHITGLMTNHNPLAHELIAASEQSGDRAWRL

PLGDEYQEQLESNFADMANIGGRPGGAITAGCFLSRFTRKYNWAHLDIAGTAWRSGKAKGATGRPVALLA

QFLLNRAGFNGEE

>gi|30043716|gb|AAP19435.1| hypothetical protein S4489 [Shigella flexneri 2a str. 2457T]

MIIIRYLVRETLKSQLAILFILLLIFFCQKLVRILGAAVDGDIPANLVLSLLGLGVPEMAQLILPLSLFL

GLLMTLGKLYTESEITVMHACGLSKAVLVKAAMILAVFTAIVAAVNVMWAGPWSSRHQDEVLAEAKANPG

MAALAQGQFQQATNGSSVLFIESVDGSDFKDVFLAQIRPKGNARPSVVVADSGHLTQLRDGSQVVTLNQG

TRFEGTALLRDFRITDFQDYQAIIGHQAVALDPNDTDQMDMRTLWNTDTDRARAELNWRITLVFTVFMMA

LMVVPLSVVNPRQGRVLSMLPAMLLYLLFFLIQTSLKSNGGKGKLDPTLWMWTVNLIYLALAIVLNLWDT

VPVRRLRASFSRKGAV

>gi|30043715|gb|AAP19434.1| hypothetical protein S4488 [Shigella flexneri 2a str. 2457T]

MQPFGVLDRYIGKTIFTTIMMTLFMLVSLSGIIKFVDQLKKAGQGSYDALGAGMYTLLSVPKDVQIFFPM

AALLGALLGLGMLAQRSELVVMQASGFTRMQVALSVMKTAIPLVLLTMAIGEWVAPQGEQMARNYRAQAM

YGGSLLSTQQGLWAKDGNNFVYIERVKGDEVLGGISIYAFNENRRLQSVRYAATAKFDPEHKVWRLSQVD

ESDLTNPKQITGSQTVSGTWKTDLTPDKLGVVALDPDALSISGLHNYVKYLKSSGQDAGRYQLNMWSKIF

QPLSVAVMMLMALSFIFGPLRSVPMGVRVVTGISFGFVFYVLDQIFGPLTLVYGIPPIIGALLPSASFFL

ISLWLLMRKS

>gi|30043714|gb|AAP19433.1| hypothetical protein S4487 [Shigella flexneri 2a str. 2457T]

MSEPLLIARTPDTELFLLPGMANRHGLITGATGTGKTVTLQKLAESLSEIGVPVFMADVKGDLTGIAQAG

TASEKLLTRLKNIGVNDWQPHANPVVVWDIFGEKGHTVRATVSDLGPLLLARLLNLNDVQSGVLNIIFRI

ADDQGLLLLDFKDLRAITQYIGDNAKSFQNQYGNISSASVGAIQRGLLSLEQQGAAHFFGEPMLDIKDWM

RTDANGKGVINILSAEKLYQMPKLYAASLLWMLSELYEQLPEAGDLEKPKLVFFFDEAHLLFNDAPQVLL

DKIEQVIRLIRSKGVGVWFVSQNPSDIPDNVFGQLGNRVQHALRAFTPKDQKAVKTAAQTMRANPAFDTE

KAIQELGTGEALISFLDAKGSPSVVERAMVIAPCSRMGPVTEDERNGLINHSPVYGKYEDEVDRESAYEM

LQKGFQASTEQQNNPPVKGKEVAVDDGILGGLKDILFGTTGPRGGKKDGVVQTMAKSAARQVTNQIVRGD

VGEFAGGEKKISQT

>gi|30043713|gb|AAP19432.1| putative oxidoreductase [Shigella flexneri 2a str. 2457T]

MLYTSQTTPEKAPKMSMIKSYAAKEAGGELEVYEYDPGELKPQDVEVQVDYCGICHSDLSMIDNEWGFSQ

YPLVAGHEVIGRVVALGSAAQDKGLQVGQRVGIGWTARSCGHCDACISGNQINCEQGAVPTIMNRGGFAE

KLRADWQWVIPLPENIDIESAGPLLCGGITVFKPLLMHHITATSRVGVIGIGGLGHIAIKLLHAMGCEVT

AFSSNPAKEQEVLAMGADKVVNSRDPQALKALSGQFDLIINTVNVSLDWQPYFEALTYGGNFHTVGAVLT

PLSVPAFTLIAGDRSISGSATGTPYELRKLMRFAARSKVAPTTELFPMSKINDAIQHVRDGKARYRVVLK

ADF

>gi|30043712|gb|AAP19431.1| putative P4-type integrase [Shigella flexneri 2a str. 2457T]

MALSDVKVRSAKPEAKAYKLTDGEGMVLLVHPNGSKYWRLRYRFGGKEKMLALGKYPEVSLADARARRDE

ARKLLANGVDPSENKKAVKVEQEQEAITFEVVARDWHASNQKWSASHSARVLKSLEDNLFTAIGKRNIAE

LKTRDLLVPIKAVESSGRLEVAARLQQRTTAIMRFAVQSGLIDYNPAQEIAGAVATAKRQHRAALELNRI

PELLHRIDHYSGRPLTRLAVELTLLVFIRSSELRFARWSEIDFETAMWTIPGEREQLEGVKHSQRGSKMR

TPHLVPLSRQALSILEKIKSMSGNRELIFVGDHDPRKPMSENTVNKALRVMGYDTKVEVCGHGFRTMACS

SLIESGLWSRDAVERQMSHQERSSVRAAYIHKAEHLGERRLMFEVVNKNWPPS

>gi|30043711|gb|AAP19430.1| IS911 orfB [Shigella flexneri 2a str. 2457T]

MKELGLVSCQQPTHRYKRGGHEHVAIPNYLERQFAVTEPNQVWCGDVTYIWTGKRWAYLAVVLDLFARKP

VGWAMSFSPDSRLTMKALEMAWETRGKPGGVMFHSDQGSHYTSRQFRQLLWRYQIRQSMSRRGNCWDNSP

MERFFRSLKNEWMPVVGYVSFSEAAHAITDYIVGYYSALRPHEYNGGLPPNESENRYWKNSNSVASFC

>gi|30043710|gb|AAP19429.1| IS911 orfA [Shigella flexneri 2a str. 2457T]

MKKRNFSAEFKRESAQLVVDQKYTVADAAKAMDVGLSTMTRWVKQLRDERQGKTPKASPITPEQIEIREL

RKKLQRIEMENEILKKATALLMSDSLNSSR

>gi|30043709|gb|AAP19428.1| IS1 orfB [Shigella flexneri 2a str. 2457T]

MSRQCTHYGRWPQHGFTSLKKLRPQSVTSRIQPGSDVIVCAEMDEHWGYVGAKSRQRWLFYAYDRIRRTV

VAHVFGERTLATLERLLSLLSAFEVVVWMTDGWPLYESRLKGKLHVISKRYTQRIERHNLNLRQHLARLV

RKSLSFSKSVELHDKVIGHYLNIKHYQ

>gi|30043708|gb|AAP19427.1| IS1 orfA [Shigella flexneri 2a str. 2457T]

MASISIRCPSCSATEGVVRNGKSTAGHQRYLCSHCRKTWQLQFTYTASQPGTHQKIIDMAMNGVGCRASA

RIMGVGLNTVLRHLKNSGRSR

>gi|30043707|gb|AAP19426.1| IS600 orfB [Shigella flexneri 2a str. 2457T]

MAHIRTRETYGTRRLQTELAENGIIVGRDRLARLRKELRLRCKQKRKFRATTNPNHNLPVAPNLLNQTFA

PTAPNQVWVADLTYVATQEGWLYLAGIKDVYTCEIVGYAMGERMTKELTGKALFMALRSQRPPAGLIHHS

DRGSQYCAYDYRVIQEQSGLKTSMSRKGNCYDNAPMESFWGTLKNESLSHYRFNNRDEAISVIREYIEIF

YNRQRRHSRLGNISPAAFREKYHQMAA

>gi|30043706|gb|AAP19425.1| IS600 orfA [Shigella flexneri 2a str. 2457T]

MSRKTQRYSKEFKAEAVRTVLENQLSISEGASRLSLPEGTLGQWVTAARKGLGTPGSRTVAELESEILQL

RKALNEARLERDILKKATAYFAQESLKNTR

>gi|30043705|gb|AAP19424.1| ISEhe3a orf [Shigella flexneri 2a str. 2457T]

MSGKRYPEEFKTEAVKQVVDRGYSVASVATRLDITTHSLYAWIKKYGPDSSTNKEQSDAQAEIRRLQKEL

KRVTDERDILKKAAAYFAKLSD

>gi|30043704|gb|AAP19423.1| hypothetical protein S4474 [Shigella flexneri 2a str. 2457T]

MWAFARRALNVEDILCLPIQDCDKSHIWLLVKDDQRLE

>gi|30043703|gb|AAP19422.1| putative deoR family transcriptional regulator [Shigella flexneri 2a str. 2457T]

MLEVSVMTIRRDLHQEDEPLPLTLLGGYIVMVNKPAPSMPVIHDVPKNHRDDLPIAILAAGMVNENDLIF

FDNGQEIPLVISMIPDAITFTGICYSHRVFVALNEKPNVTAILCGGTYRARSDAFYDASNSSPLDSLNPR

KIFISASGVHNHFGVSWFNPEDLATKRKVMNRGLRKILLARHALFDEVASASLAPISAFDVLISDRPLPA

DYVTHCQNGSVKIITPDSEDE

>gi|30043702|gb|AAP19421.1| IS1 orfA [Shigella flexneri 2a str. 2457T]

MASISIRCPSCSATEGVVRNGKSTAGHLRYLCSHCRKTWQLQFTYTASQPGTHQKIIDMAMNGVGCRASA

RIMGVGLNTVLRHLKNSGRSR

>gi|30043701|gb|AAP19420.1| IS1 orfB [Shigella flexneri 2a str. 2457T]

MSRQCTHYGRWPQHGFTSLKKLRPQSVTSRIQPGSDVIVCAEMDEHWGYVGAKSRQRWLFYAYDRIRRTV

VAHVFGERTLATLERLLSLLSAFEVVVWMTDGWPLYESCLKGKLHVISKRYTQRIERHNLNLRQHLARLV

RKSLSFSKSVELHDKVIGHYLNIKHYQ

>gi|30043700|gb|AAP19419.1| hypothetical protein S4470 [Shigella flexneri 2a str. 2457T]

MQGYHHPLATNHQTQYGTVGQALHIARKLLPFIPDNAGILIVPCCRGGSAFTAGSEGTYSERHGASHDAC

RWGTDTPLYQDLVSRTRAALAKNPQNKFLGVCWMQGEFDLMTSDYASHPQHFNHMVEAFRRDLKQYHSQL

NNITDAPWFCGDTTWYWKENFPHSYEAIYGNYQNNVLANIIFVDFQQQGERGLTNAPDEDPDDLSTGYYG

SAYRSPENWTTALRSSHFSTAARRGIISDRFVEAILQFWRER

>gi|30043699|gb|AAP19418.1| hypothetical protein S4469 [Shigella flexneri 2a str. 2457T]

MTTLTARVFTTAEIIYRKTVIALVCHLNCSRQETVTMNKTIMALAIMMASFAANASVLPETPVPFKSGTG

AIDNDTVYIGLGSAGTAWYKLDTQAKDKKWTALAAFPGGPREQATSAFIDGNLYVFGGIGKNSEGLTQVF

NDVHKYNPKTNSWVKLMSHAPMGMAGHVTFVHNGKAYVTGGVNQNIFNGYFEDLNEAGKDSTAIDKINAH

YFDKKAEDYFFNKFLLSFDPSTQQWSYAGESPWYGTAGAAVVNKGDKTWLINGEAKPGLRTDAVFELDFT

GNNLKWNKLDPVSSPDGVAGGFAGISNDSLIFAGGAGFKGSRENYQNGKNYAHEGLKKSYSTDIHLWHNG

KWDKSGELSQGRAYGVSLPWNNSLLIIGGETAGGKAVTDSVLISVKDNKVTVQN

>gi|30043698|gb|AAP19417.1| hypothetical protein S4468 [Shigella flexneri 2a str. 2457T]

MESNTWNTIHDNKKENAALNDVQVEVNYAIKLDDQWTVRPGMLTHFSSNGTRYGPYVKLSWDATKDLNFG

IRYRYDWKAYRQQDLSGDMSRDNVHRWDGYVTYHINSDFTFAWQTTLYSKQNDYRYANHKKWATENAFVL

QYHMTPDITPYIEYDYLDRQGVYNGRDNLSENSYRIGVSFKL

>gi|30043697|gb|AAP19416.1| recombinase; regulator for fimA [Shigella flexneri 2a str. 2457T]

MRNKADNKKRNFLTHSEIESLLKAANTGPHATRNYCLILLCFIHGFRASEICRLRISDIDLKAKCIYIHR

LKKGFSTTHPLLNKEVQALKNWLSIRTSYPHAESEWVFLSRKGNPLSRQQFYHIISTSGGNAGLSLEIHP

HMLRYSCGFALANMGIDTRLI

>gi|30043696|gb|AAP19415.1| recombinase; regulator for fimA [Shigella flexneri 2a str. 2457T]

MSKRRYLTGKEVQAMMQAVCYGATGARDYCLILLAYRHGMRISELLDLHYQDLDLNEGRINIRRLKNGFS

TVHPLRFDEREAVERWTQERANWKGADRTDAIFISRRGSRLSRQQAYRIIRDAGIEAGTVTQTHPHMLRH

ACGYELAERGADTRLIQDYLGHRNIRHTVRYTASNAARFAGLWERNNLINEKLKREEV

>gi|30043693|gb|AAP19413.1| major type 1 subunit fimbrin (pilin) [Shigella flexneri 2a str. 2457T]

MKIKTLAIVVLSALSLSSAAALADTTTVNGGTIHFKGEVVNAACAVDAGSVDQTVQLGQVRTASLKQAGA

TSSAVGFNIQLNDCDTTVATKAAVAFLGTAIDATRTDVLALQSSAAGSATNVGVQILDRTGNALTLDGAT

FSAQTTLNNGTNTIPFQARYYAIGEATPGAANADATFKVQYQ

>gi|30043692|gb|AAP19412.1| fimbrial protein [Shigella flexneri 2a str. 2457T]

MRMQPSRFSINNLPRFKDVITGRDAHPCAIKITMKRKRLFLLASLLPMFALAGNKWNTTLPGGNMQFQGV

IIAETCRIEAGDKQMTVNMGQISSNRFHAAGEDSAPVPFVIHLRECSTVVSERVGVAFHGVADGKNPDVL

SVGEGPGIASNIGVALFDDEGNLVPINRPPANWKRLYSGSTSLHFIAKYRATGRRVTGGIANAQAWFSLT

YQ

>gi|30043691|gb|AAP19411.1| periplasmic chaperone [Shigella flexneri 2a str. 2457T]

MSNKNVNVRKSQEITFCLLAGILMFMAMMVAGRAEAGVALGATRVIYPAGQKQVQLAVTNNDENSTYLIQ

SWVENADGVKDGRFIVTPPLFAMKGKKENTLRILDATNNQLPQDRESLFWMNVKAIPSMDKSKLTENMLQ

LAIISRIKLYYRPAKLALPPDQAAEKLRFRRSANSLTLINPTPYYLTVTELNAGTRVLENALVPPMGEST

VKLPSDAGSNITYRTINDYGALTPKMTGVME

>gi|30043690|gb|AAP19410.1| IS1 orfA [Shigella flexneri 2a str. 2457T]

MASISIRCPSCSATEGVVRNGKSTAGHQRYLCSHCRKTWQLQFTYTASQPGKHQKIIDMAMNGVGCRASA

RIMGVGLNTVLRHLKNSGRSR

>gi|30043689|gb|AAP19409.1| IS1 orfB [Shigella flexneri 2a str. 2457T]

MSRQCTHYGRWPQHGFTSLKKLRPQSVTSRIQPGSDVIVCAEMDEHWGYVGAKSRQRWLFYAYDRIRRTV

VAHVFGERTLATLERLLSLLSAFEVVVWMTDGCPLYESRLKGKLHVISKRYTQRIERHNLNLRQHLARLV

RKSLSFSKSVELHDKVIGHYLNIKHYQ

>gi|30043688|gb|AAP19408.1| minor fimbrial subunit, precursor polypeptide [Shigella flexneri 2a str. 2457T]

MRNKPFYLLCAFLWLAVSRVLAADSTITIRGYVRDNGCSVAAESTNFTVDLMENAAKQFNNIGATTPVVP

FRILLSPCGNAVSAVKVGFTGVADSHNANLLALENTVSAAAGLGIQLLNEQQNQIPLNAPSSAISWTTLT

PGKPNTLNFYARLMATQVPVTAGHINATATFTLEYQ

>gi|30043687|gb|AAP19407.1| minor fimbrial subunit, precursor polypeptide [Shigella flexneri 2a str. 2457T]

MKWCKRGYVLAAMLALASATIQAADVTITVNGKVVAKPCTVSTTNATVDLGDLYSFSLMSAGAASAWHDV

ALELTNCPVGTSRVTASFSGAADSTGYYKNQGTAQNIQLELQDDSGNTLNTGATKTVQVDDSSQSAHFPL

QVRALTVNGGATQGTIQAVISITYTYS

>gi|30043686|gb|AAP19406.1| minor fimbrial subunit, D-mannose specific adhesin [Shigella flexneri 2a str. 2457T]

MKRAITLFAVLLMGWSVNAWSFACKTANGTAIPIGGGSANVYVNLAPVVNVGQNLVVDLSTQIFCHNDYP

ETITDYVTLQRGSAYGGVLSNFSGTVKYSGSSYPFPTTSETPRVVYNSRTDKPWPVALYLTPVSSAGGVA

IKAGSLIAVLILRQTNNYNSDDFQFVWNIYANNDVVVPTGGCDVSARDVTVTLPDYPGSVPIPLTVYCAK

SQNLGYYLSGTTADAGNSIFTNTASFSPAQGVGVQLTRNGTIIPANNTVSLGAVGTSAVSLGLTANYART

GGQVTAGNVQSIIGVTFVYQ

>gi|30043685|gb|AAP19405.1| gluconate transport system permease 3 [Shigella flexneri 2a str. 2457T]

MHVLNILWVVFGIGLMLILNLKFKINSMVALLVAALSVGMLAGMDLMSLLHTMKAGFGNTLGELAIIVVF

GAVIGKLMVDSGAAHQIAHTLLARLGLRYVQLSVIIIGLIFGLAMFYEVAFIMLAPLVIVIAAEAKIPFL

KLAIPAVAAATTAHSLFPPQPGPVALVNAYGADMGMVYIYGVLVTIPSVICAGLILPKFLGNLERPTPSF

LKADQPVDMNNLPSFGVSILVPLIPAIIMISTTIANIWLVKDTPAWEVVNFIGSSPIAMFIAMVVAFVLF

GTARGHDMQWVMNAFESAVKSIAMVILIIGAGGVLKQTIIDTGIGDTIGMLMSHGNISPYIMAWLITVLI

RLATGQGVVSAMTAAGIISAAILDPATGQLVGVNPALLVLATAAGSNTLTHINDASFWLFKGYFDLSVKD

TLKTWGLLELVNSVVGLIIVLIISMVA

>gi|30043684|gb|AAP19404.1| mannonate hydrolase [Shigella flexneri 2a str. 2457T]

MEQTWRWYGPNDPVSLADVRQAGATGVVTALHHIPNGEVWSVEEILKRKAIVEDAGLVWSVVESVPIHED

IKTHTGNYEQWIANYQQTLRNLAQCGIRTVCYNFMPVLDWTRTDLEYVLPDGSKALRFDQIEFAAFEMHI

LKRPGAEADYTEEEIAQAAERFATMSDEDKARLTRNIIAGLPGAEEGYTLDQFRKHLELYKDIDKAKLRE

NFAVFLKAIIPVAEEVGVRMAVHPDDPPRPILGLPRIVSTIEDMQWMVDTVNSMANGFTMCTGSYGVRAD

NDLVDMIKQFGPRIYFTHLRSTMREDNPKTFHEAAHLNGDVDMYKVVKAIVEEEHRRKAEGKEDLIPMRP

DHGHQMLDDLKKKTNPGYSAIGRLKGLAEVRGVELAIQRAFFSR

>gi|30043683|gb|AAP19403.1| D-mannonate oxidoreductase [Shigella flexneri 2a str. 2457T]

MTTIVDSNLPVARPSWDHSRLESRIVHLGCGAFHRAHQALYTHHLLESTDSDWGICEVNLMPGNDRMLIE

NLKKQQLLYTVAEKGAESTELKIIGSMKEALHPEIDGCEGILNAMARPQTAIVSLTVTEKGYCADAASGQ

LDLNNPLIKHDLENPTAPKSAIGYIVEALRLRREKGLKAFTVMSCDNVRENGHVAKVAVLGLAQARDPQL

AAWIEENVTFPCTMVDRIVPAATPETLQEIADQLGVYDPCAIACEPFRQWVIEDNFVNGRPDWDKVGTQF

VADVVPFEMMKLRMLNGSHSFLAYLGYLGGYETIADTMTNPDYRKAAFALMMQEQAPTLSMPEGTDLNAY

ATLLIERFSNPSLRHRTWQIAMDGSQKLPQRLLDPVRLHLQNGGSWRHLALGVAGWMGYTQGVDEQGNAI

DVVDPMLAEFQKINAQYQGADRVKALLGLSGIFADDLPQNADFVGAVTSAYQQLCERGARECVAAL

>gi|30043682|gb|AAP19402.1| regulator for uxu operon [Shigella flexneri 2a str. 2457T]

MKSVTSAQRPYQEVGAMIHDLIIKTPYNPGERLPPEREIAEMLDVTRTVVREALIMLEIKGLVEVRRGAG

IYVLDSSGSHNTDSPDANVCNDAGPFELLQARQLLESNIAEFAALQATREDIVKMRQALQLEERELASSA

PGSSESGDMQFHLAIAEATHNSMLVELFRQSWQWRENNPMWIQLHSHLDDSLYRKEWLGDHKQILAALIK

KDARAAKLAMWQHLENVKQRLLEFSNVDDIYFDGYLFDSWPLDKVDA

>gi|30043681|gb|AAP19401.1| IS1 orfA [Shigella flexneri 2a str. 2457T]

MASISIRCPSCSATEGVVRNGKSTAGHQRYLCSHCRKTWQLQFTYTASQPGTHQKIIDMAMNGVGCRASA

RIMGVGLNTVLRHLKNSGRSR

>gi|30043680|gb|AAP19400.1| IS1 orfB [Shigella flexneri 2a str. 2457T]

MSRQRTHYGRWPQHGFTSLKKLRPQSVTSRIQPGSDVIVCAEMDEHWGYVGAKSRQRWLFYAYDRIRRTV

VAHVFGERTLATLERLLSLLSAFEVVVWMTDGWPLYESRLKGKLHVISKRYTQRIERHNLNLRQHLARLV

RKSLSFSKSVELHDKVIGHYLNIKHYQ

>gi|30043679|gb|AAP19399.1| hypothetical protein S4448 [Shigella flexneri 2a str. 2457T]

MRKMMRQSLQAVLPEISGNKTSLLRKSVCSDLLTLFNSPHSALPSLLVSGMPEWQVHNPSDKHLQSWYCR

QLRSALLFHEPRIAALQVNLKEAYCHTLAISLEIMLYHDDEPLTFDLVWDNGGWRSATLENVS

>gi|30043678|gb|AAP19398.1| putative LYSR-type transcriptional regulator [Shigella flexneri 2a str. 2457T]

MDDCGAVLHNIETKWLYDFLTLEKCRNFSQAAVSRNVSQPAFSRRIRALEQAIGVELFNRQVTPLQLSEQ

GKIFHSQIRHLLQQLESNLAELRGGSDYAQRKIKIAAAHSLSLGLLPSIISQMPPLFTWAIEAIDVDEAV

DKLREGQSDCIFSFHDEDLLEAPFDHIRLFESQLFPVCASDEHGEALFNLAQPHFPLLNYSRNSYMVRLI

NRTLTRHSELSFSTFFVSSMSELLKQVALDGCGIAWLPEYAIQQEIRSGQLVVLNRDEQVIPIQAYAYRM

NTRMNPVAERFWRELRELEIVLS

>gi|30043677|gb|AAP19397.1| isoaspartyl dipeptidase [Shigella flexneri 2a str. 2457T]

MIDYTAAGFTLLQGAHLYAPEDRGICDVLVANGKIIAVASNIPSDIVPNCTVVDLSGQILCPGFIDQHVH

LIGGGGEAGPTTRTPEVALSRLTEAGVTSVVGLLGTDSISRHPESLLAKTRALNEEGISAWMLTGAYHVP

SRTITGSVEKDVAIIDRVIGVKCAISDHRSAAPDVYHLANMAAESRVGGLLGGKPGVTVFHMGDSKKALK

PIYDLLENCDVPISKLLPTHVNRNVPLFEQALEFARKGGTIDITSSIDEPVAPAEGIARAVQAGIPLARV

TLSSDGNGSQPFFDDEGNLTHIGVAGFETLLETVQVLVKDYDFSISDALRPLTSSVAGFLNMSGKGEILP

GNDADLLVMTPELRIEQVYARGKLMVKDGKACVKGTFETA

>gi|30043676|gb|AAP19396.1| hypothetical protein S4445 [Shigella flexneri 2a str. 2457T]

MTTQVRKNVMDMFIDGARRGFTIATTNLLPNVVMAFVIIQALKITGLLDWVGHICEPVMALWGLPGEAAT

VLLAALMSMGGAVGVAASLATADALTGHDVTVLLPAMYLMGNPVQNVGRCLGTAEVNAKYYPHIITVCVI

NALLSIWVMQLIV

>gi|30043675|gb|AAP19395.1| hypothetical protein S4444 [Shigella flexneri 2a str. 2457T]

MGIVMTQQGDAVAGELATEKVGIKGYLAFFLTIIFFPGVFSGTDSWWRVFDFSVLNGSFGQLPGANGATT

SFRGVGGAGAKDGFLFALELAPSVILSLGIISITDGLGGLRAAQQLMTPVLKPLLGIPGICSLALIANLQ

NTDAAAGMTKELAQEGEITERDKVIFAAYQTSGSAIITNYFSSGVAVFAFLGTSVIVPLAVILVFKFVGA

NILRVWLNFEERRNPTQGAQA

>gi|30043674|gb|AAP19394.1| hypothetical protein S4443 [Shigella flexneri 2a str. 2457T]

MWKRFCAGLSCRKFINPVTMTLVNKNRKSRDENAMAKYNEKELADTSKFLSFVLRHKPEAIGIVLDREGW

ADINKLILCAQKAGKRLTRALLDTVVATSDKKRFSYSSDGRCIRAVQGHSTSQVAISFAEKTPPQFLYHG

TASRFLDEIKKQGLIAGDRHYVHLSADEATARKVGARHGSPVVLTVKAQEMAKRGIPFWQAENGVWLTST

VAVEFLEWSFMPTGVQKQ

>gi|30043673|gb|AAP19393.1| IS2 orfB [Shigella flexneri 2a str. 2457T]

MARGWGVSLVSRCLRVSRAQLHVILRRTDDWMDGRRSRHTDDTDVLLRIHHVIGELPTYGYRRVWALLRR

QAELDGMPAINAKRVYRLMRQNALLLERKPAVPPSKRAHTGRVAVKESNQRWCSDGFEFCCDNGERLRVT

FALDCCDREALHWAVTTGGFNSETVQDVMLGAVERRFGNDLPSSPVEWLTDNGSCYRANETRQFARMLGL

EPKNTAVRSPESNGIAESFVKTIKRDYISIMPKPDGLTAAKNLAEAFEHYNEWHPHSALGYRSPREYLRQ

RACNGLSDNRCLEI

>gi|30043672|gb|AAP19392.1| IS2 orfA [Shigella flexneri 2a str. 2457T]

MIVLILVFRLVIGEQMIDVLGPEKRRRRTTQEKIAIVQQSFEPGMTVSLVARQHGVAASQLFLWRKQYQE

GSLTAVAAGEQVVPASELAAAMKQIKELQRLLGKKTMENELLKEAVEYGRAKKWIAHAPLLPGDGE

>gi|30043671|gb|AAP19391.1| ISSfl3 orfD [Shigella flexneri 2a str. 2457T]

MEHIPLYRQSEIYARQGVELSRNTMVRWVSEMADKLRPLYIALNDYVLEAGKVHADDTPVKVLAPGNGKT

KTGRLWVYVRDDRNAGSSLPAAVWFAYSADRKGEHPQLHLAKYQGVLQADAYAGYNVLYETGRVKEAGCL

AHARRKIHDEDVRRPTEMTQEALRRIAELYDIEAEIRGSPAEERLAVRKARSVQLMQSLYDWIQLQRKTL

SKHAEMAKAFDYILNHWNALNEFCRDGWVEIDNNIGENALRSVAVGRKNYLFFGSDKGGESAAIIYSLLV

TCKQNEVEPEDWLREVIEKLNDWPSNQVHKLLPWNFSSVK

>gi|30043670|gb|AAP19390.1| ISSfl3 orfC [Shigella flexneri 2a str. 2457T]

MNNELPDDIELLKAMLRKQQSRLRQYACQVAGYEQEIERLKAQLDRLRRMLFGQSSEKKRHKLENQIRQA

EKRLSELENRLNTARNLLEDASSVTDSPDTSPPSENPIASKPESPGRKSSRKPLPAELPRETHRLLPAET

SCPACGGVLKEMGETISEQLDIINTAFKVIETIRPKLACSRCDVIGTTSS

>gi|30043669|gb|AAP19389.1| ISSfl3 orfB [Shigella flexneri 2a str. 2457T]

MITLPTGTKIWIIAGITDMRCGFNGLASKVQNTLKDDPFSGHIFVFRGRSGKMVKILWADRDGLCLFTKR

LAGDPGRESAPDASSVIHATGGDRVATSQTDRTAWHPDITRDKTRE

>gi|30043668|gb|AAP19388.1| ISSfl3 orfA [Shigella flexneri 2a str. 2457T]

MNSQTTKDIPCFRSYLPDALRLRFEDKLTIRAIAQRLGLSHSTIHTLFQRFLASGIAWPLPDSVSFAQLD

AILYANRKKELTEPQIREGSWRKERRTSYSREFKVRLAKQALQPGAVVARIAREHDINDNLLFKWKSQYE

DGLLSDDDIQECMPVPVALTDTPEPTRPVTNPFWRNKHDERPEGAPGNVPRCELHLKSGVVKLFDPLTPE

LLRALIREMKGGIR

>gi|30043667|gb|AAP19387.1| 50S ribosomal subunit protein L17 [Shigella flexneri 2a str. 2457T]

MRHRKSGRQLNRNSSHRQAMFRNMAGSLVRHEIIKTTLPKAKELRRVVEPLITLAKTDSVANRRLAFART

RDNEIVAKLFNELGPRFASRAGGYTRILKCGFRAGDNAPMAYIELVDRSEKAEAAAE

>gi|30043666|gb|AAP19386.1| RNA polymerase, alpha subunit [Shigella flexneri 2a str. 2457T]

MQGSVTEFLKPRLVDIEQVSSTHAKVTLEPLERGFGHTLGNALRRILLSSMPGCAVTEVEIDGVLHEYST

KEGVQEDILEILLNLKGLAVRVQGKDEVILTLNKSGIGPVTAADITHDGDVEIVKPQHVICHLTDENASI

SMRIKVQRGRGYVPASTRIHSEEDERPIGRLLVDACYSPVERIAYNVEAARVEQRTDLDKLVIEMETNGT

IDPEEAIRRAATILAEQLEAFVDLRDVRQPEVKEEKPEFDPILLRPVDDLELTVRSANCLKAEAIHYIGD

LVQRTEVELLKTPNLGKKSLTEIKDVLASRGLSLGMRLENWPPASIADE

>gi|30043665|gb|AAP19385.1| 30S ribosomal subunit protein S4 [Shigella flexneri 2a str. 2457T]

MGPKLKLSRREGTDLFLKSGVRAIDTKCKIEQAPGQHGARKPRLSDYGVQLREKQKVRRIYGVLERQFRN

YYKEAARLKGNTGENLLALLEGRLDNVVYRMGFGATRAEARQLVSHKAIMVNGRVVNIASYQVSPNDVVS

IREKAKKQSRVKAALELAEQREKPTWLEVDAGKMEGTFKRKPERSDLSADINEHLIVELYSK

>gi|30043664|gb|AAP19384.1| 30S ribosomal subunit protein S11 [Shigella flexneri 2a str. 2457T]

MAKAPIRARKRVRKQVSDGVAHIHASFNNTIVTITDRQGNALGWATAGGSGFRGSRKSTPFAAQVAAERC

ADAVKEYGIKNLEVMVKGPGPGRESTIRALNAAGFRITNITDVTPIPHNGCRPPKKRRV

>gi|30043663|gb|AAP19383.1| 30S ribosomal subunit protein S13 [Shigella flexneri 2a str. 2457T]

MARIAGINIPDHKHAVIALTSIYGVGKTRSKAILAAAGIAEDVKISELSEGQIDTLRDEVAKFVVEGDLR

REISMSIKRLMDLGCYRGLRHRRGLPVRGQRTKTNARTRKGPRKPIKK

>gi|30043662|gb|AAP19382.1| 50S ribosomal subunit protein L36 [Shigella flexneri 2a str. 2457T]

MKVRASVKKLCRNCKIVKRDGVIRVICSAEPKHKQRQG

>gi|30043661|gb|AAP19381.1| putative ATPase subunit of translocase [Shigella flexneri 2a str. 2457T]

MAKQPGLDFQSAKGGLGELKRRLLFVIGALIVFRIGSFIPIPGIDAAVLAKLLEQQRGTIIEMFNMFSGG

ALSRASIFALGIMPYISASIIIQLLTVVHPTLAEIKKEGESGRRKISQYTRYGTLVLAIFQSIGIATGLP

NMPGMQGLVINPGFAFYFTAVVSLVTGTMFLMWLGEQITERGIGNGISIIIFAGIVAGLPPAIAHTIEQA

RQGDLHFLVLLLVAVLVFAVTFFVVFVERGQRRIVVNYAKRQQGRRVYAAQSTHLPLKVNMAGVIPAIFA

SSIILFPATIASWFGGGTGWNWLTTISLYLQPGQPLYVLLYASAIIFFCFFYTALVFNPRETADNLKKSG

AFVPGIRPGEQTAKYIDKVMTRLTLVGALYITFICLIPEFMRDAMKVPFYFGGTSLLIVVVVIMDFMAQV

QTLMMSSQYESALKKANLKGYGR

>gi|30043660|gb|AAP19380.1| 50S ribosomal subunit protein L15 [Shigella flexneri 2a str. 2457T]

MRLNTLSPAEGSKKAGKRLGRGIGSGLGKTGGRGHKGQKSRSGGGVRRGFEGGQMPLYRRLPKFGFTSRK

AAITAEVRLSDLAKVEGGVVDLNTLKAANIIGIQIEFAKVILAGEVTTPVTVRGLRVTKGARAAIEAAGG

KIEE

>gi|30043659|gb|AAP19379.1| 50S ribosomal subunit protein L30 [Shigella flexneri 2a str. 2457T]

MAKTIKITQTRSAIGRLPKHKATLLGLGLRRIGHTVEREDTPAIRGMINAVSFMVKVEE

>gi|30043658|gb|AAP19378.1| 30S ribosomal subunit protein S5 [Shigella flexneri 2a str. 2457T]

MAHIEKQAGELQEKLIAVNRVSKTVKGGRIFSFTALTVVGDGNGRVGFGYGKAREVPAAIQKAMEKARRN

MINVALNNGTLQHPVKGVHTGSRVFMQPASEGTGIIAGGAMRAVLEVAGVHNVLAKAYGSTNPINVVRAT

IDGLENMNSPEMVAAKRGKSVEEILGK

>gi|30043657|gb|AAP19377.1| 50S ribosomal subunit protein L18 [Shigella flexneri 2a str. 2457T]

MDKKSARIRRATRARRKLQELGATRLVVHRTPRHIYAQVIAPNGSEVLVAASTVEKAIAEQLKYTGNKDA

AAAVGKAVAERALEKGIKDVSFDRSGFQYHGRVQALADAAREAGLQF

>gi|30043656|gb|AAP19376.1| 50S ribosomal subunit protein L6 [Shigella flexneri 2a str. 2457T]

MSRVAKAPVVVPAGVDVKINGQVITIKGKNGELTRTLNDAVEVKHADNTLTFGPRDGYADGWAQAGTARA

LLNSMVIGVTEGFTKKLQLVGVGYRAAVKGNVINLSLGFSHPVDHQLPAGITAECPTQTEIVLKGADKQV

IGQVAADLRAYRRPEPYKGKGVRYADEVVRTKEAKKK

>gi|30043655|gb|AAP19375.1| 30S ribosomal subunit protein S8, and regulator [Shigella flexneri 2a str. 2457T]

MSMQDPIADMLTRIRNGQAANKAAVTMPSSKLKVAIANVLKEEGFIEDFKVEGDTKPELELTLKYFQGKA

VVESIQRVSRPGLRIYKRKDELPKVMAGLGIAVVSTSKGVMTDRAARQAGLGGEIICYVA

>gi|30043654|gb|AAP19374.1| 30S ribosomal subunit protein S14 [Shigella flexneri 2a str. 2457T]

MAKQSMKAREVKRVALADKYFAKRAELKAIISDVNASDEDRWNAVLKLQTLPRDSSPSRQRNRCRQTGRP

HGFLRKFGLSRIKVREAAMRGEIPGLKKASW

>gi|30043653|gb|AAP19373.1| 50S ribosomal subunit protein L5 [Shigella flexneri 2a str. 2457T]

MAKLHDYYKDEVVKKLMTEFNYNSVMQVPRVEKITLNMGVGEAIADKKLLDNAAADLAAISGQKPLITKA

RKSVAGFKIRQGYPIGCKVTLRGERMWEFFERLITIAVPRIRDFRGLSAKSFDGRGNYSMGVREQIIFPE

IDYDKVDRVRGLDITITTTAKSDEEGRALLAAFDFPFRK

>gi|30043652|gb|AAP19372.1| 50S ribosomal subunit protein L24 [Shigella flexneri 2a str. 2457T]

MAAKIRRDDEVIVLTGKDKGKRGKVKNVLSSGKVIIEGINLVKKHQKPVPALNQPGGIVEKEAAIQVSNV

AIFNAATGKADRVGFRFEDGKKVRFFKSNSETIK

>gi|30043651|gb|AAP19371.1| 50S ribosomal subunit protein L14 [Shigella flexneri 2a str. 2457T]

MIQEQTMLNVADNSGARRVMCIKVLGGSHRRYAGVGDIIKITIKEAIPRGKVKKGDVLKAVVVRTKKGVR

RPDGSVIRFDGNACVLLNNNSEQPIGTRIFGPVTRELRSEKFMKIISLAPEVL

>gi|30043650|gb|AAP19370.1| 30S ribosomal subunit protein S17 [Shigella flexneri 2a str. 2457T]

MTDKIRTLQGRVVSDKMEKSIVVAIERFVKHPIYGKFIKRTTKLHVHDENNECGIGDVVEIRECRPLSKT

KSWTLVRVVEKAVL

>gi|30043649|gb|AAP19369.1| 50S ribosomal subunit protein L29 [Shigella flexneri 2a str. 2457T]

MKAKELREKSIEELNTELLNLLREQFNLRMQAASGQLQQSHLLKQVRRDVARVKTLLNEKAGA

>gi|30043648|gb|AAP19368.1| 50S ribosomal subunit protein L16 [Shigella flexneri 2a str. 2457T]

MLQPKRTKFRKMHKGRNRGLAQGTDVSFGSFGLKAVGRGRLTARQIEAARRAMTRAVKRQGKIWIRVFPD

KPITEKPLAVRMGKGKGNVEYWVALIQPGKVLYEMDGVPEELAREAFKLAAAKLPIKTTFVTKTVM

>gi|30043647|gb|AAP19367.1| 30S ribosomal subunit protein S3 [Shigella flexneri 2a str. 2457T]

MGQKVHPNGIRLGIVKPWNSTWFANTKEFADNLDSDFKVRQYLTKELAKASVSRIVIERPAKSIRVTIHT

ARPGIVIGKKGEDVEKLRKVVADIAGVPAQINIAEVRKPELDAKLVADSITSQLERRVMFRRAMKRAVQN

AMRLGAKGIKVEVSGRLGGAEIARTEWYREGRVPLHTLRADIDYNTSEAHTTYGVIGVKVWIFKGEILGG

MAAVEQPEKPAAQPKKQQRKGRK

>gi|30043646|gb|AAP19366.1| 50S ribosomal subunit protein L22 [Shigella flexneri 2a str. 2457T]

METIAKHRHARSSAQKVRLVADLIRGKKVSQALDILTYTNKKAAVQVKKVLESAIANAEHNDGADIDDLK

VTKIFVDEGPSMKRIMPRAKGRADRILKRTSHITVVVSDR

>gi|30043645|gb|AAP19365.1| 30S ribosomal subunit protein S19 [Shigella flexneri 2a str. 2457T]

MPRSLKKGPFIDLHLLKKVEKAVESGDKKPLRTWSRRSTIFPNMIGLTIAVHNGRQHVPVFVTDEMVGHK

LGEFAPTRTYRGHAADKKAKKK

>gi|30043644|gb|AAP19364.1| 50S ribosomal subunit protein L2 [Shigella flexneri 2a str. 2457T]

MAVVKCKPTSPGRRHVVKVVNPELHKGKPFAPLLEKNSKSGGRNNNGRITTRHIGGGHKQAYRIVDFKRN

KDGIPAVVERLEYDPNRSANIALVLYKDGERRYILAPKGLKAGDQIQSGVDAAIKPGNTLPMRNIPVGST

VHNVEMKPGKGGQLARSAGTYVQIVARDGAYVTLRLRSGEMRKVEADCRATLGEVGNAEHMLRVLGKAGA

ARWRGVRPTVRGTAMNPVDHPHGGGEGRNFGKHPVTPWGVQTKGKKTRSNKRTDKFIVRRRSK

>gi|30043643|gb|AAP19363.1| 50S ribosomal subunit protein L23 [Shigella flexneri 2a str. 2457T]

MIREERLLKVLRAPHVSEKASTAMEKSNTIVLKVAKDATKAEIKAAVQKLFEVEVEVVNTLVVKGKVKRH

GQRIGRRSDWKKAYVTLKEGQNLDFVGGAE

>gi|30043642|gb|AAP19362.1| 50S ribosomal subunit protein L4 [Shigella flexneri 2a str. 2457T]

MELVLKDAQSALTVSETTFGRDFNEALVHQVVVAYAAGARQGTRAQKTRAEVTGSGKKPWRQKGTGRARS

GSIKSPIWRSGGVTFAARPQDHSQKVNKKMYRGALKSILSELVRQDRLIVVEKFSVEAPKTKLLAQKLKD

MALEDVLIITGELDENLFLAARNLQKVDVRDATGIDPVSLIAFDKVVMTADAVKQVEEMLA

>gi|30043641|gb|AAP19361.1| 50S ribosomal subunit protein L3 [Shigella flexneri 2a str. 2457T]

MIGLVGKKVGMTRIFTEDGVSIPVTVIEVEANRVTQVKDLANDGYRAIQVTTGAKKANRVTKPEAGHFAK

AGVEAGRGLWEFRLAEGEEFTVGQSISVELFADVKKVDVTGTSKGKGFAGTVKRWNFRTQDATHGNSLSH

RVPGSIGQNQTPGKVFKGKKMAGQMGNERVTVQSLDVVRVDAERNLLLVKGAVPGATGSDLIVKPAVKA

>gi|30043640|gb|AAP19360.1| 30S ribosomal subunit protein S10 [Shigella flexneri 2a str. 2457T]

MQNQRIRIRLKAFDHRLIDQATAEIVETAKRTGAQVRGPIPLPTRKERFTVLISPHVNKDARDQYEIRTH

LRLVDIVEPTEKTVDALMRLDLAAGVDVQISLG

>gi|30043639|gb|AAP19359.1| bacterioferrin [Shigella flexneri 2a str. 2457T]

MKGDVKIINYLNKLLGNELVAINQYFLHARMFKNWGLMRLNDVEYHESIDEMKHADKYIERILFLEGIPN

LQDLGKLGIGEDVEEMLQSDLRLELEGAKDLREAIAYADSVHDYVSRDMMIEILADEEGHIDWLETELDL

IGKIGLQNYLQSQIKVKD

>gi|30043638|gb|AAP19358.1| hypothetical protein S4406 [Shigella flexneri 2a str. 2457T]

MYVCLCNGVSDKKIRQAVRQFHPQSFQQLRKFIPVGNQCGKCIRAAREVMQDELMQMPEFKEIA

>gi|30043637|gb|AAP19357.1| protein chain elongation factor EF-Tu (duplicate of tufB) [Shigella flexneri 2a str. 2457T]

MSKEKFERTKPHVNVGTIGHVDHGKTTLTAAITTVLAKTYGGAARAFDQIDNAPEEKARGITINTSHVEY

DTPTRHYAHVDCPGHADYVKNMITGAAQMDGAILVVAATDGPMPQTREHILLGRQVGVPYIIVFLNKCDM

VDDEELLELVEMEVRELLSQYDFPGDDTPIVRGSALKALEGDAEWEAKILELAGFLDSYIPEPERAIDKP

FLLPIEDVFSISGRGTVVTGRVERGIIKVGEEVEIVGIKETQKSTCTGVEMFRKLLDEGRAGENVGVLLR

GIKREEIERGQVLAKPGTIKPHTKFESEVYILSKDEGGRHTPFFKGYRPQFYFRTTDVTGTIELPEGVEM

VMPGDNIKMVVTLIHPIAMDDGLRFAIREGGRTVGAGVVAKVLG

>gi|30043636|gb|AAP19356.1| GTP-binding protein chain elongation factor EF-G [Shigella flexneri 2a str. 2457T]

MDWMEQEQERGITITSAATTAFWSGMAKQYEPHRINIIDTPGHVDFTIEVERSMRVLDGAVMVYCAVGGV

QPQSETVWRQANKYKVPRIAFVNKMDRVGANFLKVVNQIKTRLGANPVPLQLAIGAEEHFTGVVDLVKMK

AINWNDADQGVTFEYEDIPADMVELANEWHQNLIESAAEASEELMEKYLGGEELTEAEIKGALRQRVLNN

EIILVTCGSAFKNKGVQAMLDAVIDYLPSPVDVPAINGILDDGKDTPAERHASDDEPFSALAFKIATDPF

VGNLTFFRVYSGVVNSGDTVLNSVKAARERFGRIVQMHANKREEIKEVRAGDIAAAIGLKDVTTGDTLCD

PDAPIILERMEFPEPVISIAVEPKTKADQEKMGLALGRLAKEDPSFRVWTDEESNQTIIAGMGELHLDII

VDRMKREFNVEANVGKPQVAYRETIRQKVTDVEGKHAKQSGGRGQYGHVVIDMYPLEPGSNPKGYEFIND

IKGGVIPGEYIPAVDKGIQEQLKAGPLAGYPVVDMGIRLHFGSYHDVDSSELAFKLAASIAFKEGFKKAK

PVLLEPIMKVEVETPEENTGDVIGDLSRRRGMLKGQESEVTGVKIHAEVPLSEMFGYATQLRSLTKGRAS

YTMEFLKYDEAPSNVAQAVIEARGK

>gi|30043635|gb|AAP19355.1| 30S ribosomal subunit protein S7 [Shigella flexneri 2a str. 2457T]

MPRRRVIGQRKILPDPKFGSELLAKFVNILMVDGKKSTAESIVYSALETLAQRSGKSELEAFEVALENVR

PTVEVKSRRVGGSTYQVPVEVRPVRRNALAMRWIVEAARKRGDKSMALRLANELSDAAENKGTAVKKRED

VHRMAEANKAFAHYRW

>gi|30043634|gb|AAP19354.1| 30S ribosomal subunit protein S12 [Shigella flexneri 2a str. 2457T]

MATVNQLVRKPRARKVAKSNVPALEACPQKRGVCTRVYTTTPKKPNSALRKVCRVRLTNGFEVTSYIGGE

GHNLQEHSVILIRGGRVKDLPGVRYHTVRGALDCSGVKDRKQARSKYGVKRPKA

>gi|30043633|gb|AAP19353.1| hypothetical protein S4401 [Shigella flexneri 2a str. 2457T]

MLHTLHRSPWLTDFAALLRLLSEGDELLLLQDGVTAAVDGNRYLESLRNAPIKVYALNEDLIARGLTGQI

LNDIIPIDYTDFVRLTVKHSSQMAW

>gi|30043632|gb|AAP19352.1| hypothetical protein S4400 [Shigella flexneri 2a str. 2457T]

MKRIAFVFSTAPHGTAAGREGLDALLATSALTDDLAVFFIADGVFQLLSGQKPDAVLARDYIATFKLLSL

YDIEQCWVCAASLRERGLDPQTPFVVEATPLEADALRRELANYDVILRF

>gi|30043631|gb|AAP19351.1| hypothetical protein S4399 [Shigella flexneri 2a str. 2457T]

MRFAIVVTGPAYGTQQASSAFQFAQALIAEGHKLSSVFFYREGVYNANQLTSPASDEFDLVRGWQQLNAQ

HGVALNICVAAALRRGIVDETEAGRLGLASSNLQPGFTLSGLGALAEASLTCDRVVQF

>gi|30043630|gb|AAP19350.1| hypothetical protein S4398 [Shigella flexneri 2a str. 2457T]

MSRSLLTNETSELDLLDQRPFDQTDFDILKSYEAVVDGLAMLIGSHCEIVLHSLQDLKCSAIRIANGEHT

GRKIGSPITDLALRMLHDMTGADSSVSKCYFTRAKSGVLMKSLTIAIRNREQRVIGLLCINMNLDVPFSQ

IMSTFVPPETPDVGSSVNFASSVEDLVTQTLEFTIEEVNADRNVSNNAKNRQIVLNLYEKGIFDIKDAIN

QVADRLNISKHTVYLYIRQFKSGDFQGQDK

>gi|30043629|gb|AAP19349.1| FKBP-type peptidyl-prolyl cis-trans isomerase (rotamase) [Shigella flexneri 2a str. 2457T]

MKSLFKVTLLATTMAVALHAPITFAAEAAKPATTADSKASFKNDDQKSAYALGASLGRYMENSLKEQEKL

GIKLDKDQLIAGVQDAFADKSKLSDQEIEQTLQAFEARVKSSAQAKMEKDAADNEAKGKEYREKFAKEKG

VKTSSTGLVYQVVEAGKGEAPKDSDTVVVNYKGTLIDGKEFDNSYTRGEPLSFRLDGVIPGWTEGLKNIK

KGGKIKLVIPPELAYGKAGVPGIPPNSTLVFDVELLDVKPAPKADAKPEADAKAADSAKK

>gi|30043628|gb|AAP19348.1| host factor for lysis of phiX174 infection [Shigella flexneri 2a str. 2457T]

MQDLSLEARLAELESRLAFQEITIEELNVTVAAHEMEMAKLRDHLRLLTEKLKASQPSNIASQAEETPPP

HY

>gi|30043627|gb|AAP19347.1| FKBP-type peptidyl-prolyl cis-trans isomerase (rotamase) [Shigella flexneri 2a str. 2457T]

MKVAKDLVVSLAYQVRTEDGVLVDESPVSAPLDYLHGHGSLISGLETALEGHEVGDKFDVAVGANDAYGQ

YDENLVQRVPKDVFMGVDELQVGMRFLAETDQGPVPVEITAVEDDHVVVDGNHMLAGQNLKFNVEVVAIR

EATEEELAHGHVHGAHDHHHDHDHDGCCGGHGHDHGHEHGGEGCCGGKGNGGCGCH

>gi|30043626|gb|AAP19346.1| K+ efflux; NEM-activable K+/H+ antiporter [Shigella flexneri 2a str. 2457T]

MLAGIAIGPWGLGFISDVDEILHFSELGVVFLMFIIGLELNPSKLWQLRRSIFGVGAAQVLLSAALLAGL

LMLTDFAWQAAVVGGIGFAMSSTAMALQLMREKGMNRSESGQLGFSVLLFQDLAVIPALALVPLLAGSAD

EHFDWMKIGMKVLEFVGMLIGGRYLLRPVFRFIAASGVREVFTAATLLLVLGSALFMDALGLSMALGTFI

AGVLLAESEYRHELETAIDPFKGLLLGLFFISVGMSLNLGVLYTHLLWVVISVVVLVAVKILVLYLLARL

YGVRSSERMQFAGVLSQGGEFAFVLFSTASSQRLFQGDQMAPLLMKLVDKWLSRQFNGPEEEDEKPWVND

DKPQVIVVGFGRFGQVIGRLLMANKMRITVLERDISAVNLMRKYGYKVYYGDATQVDLLRSAGAEAAESI

VITCNEPEDTMKLVEICQQHFPHLHILARARGRVEAHELLQAGVTQFSRETFSSALELGRKTLVTLGMHP

HQAQRAQLHFRRLDMRMLRELIPMHADTVQISRAREARRELEEIFQREMQQERRQLDGWDEFE

>gi|30043625|gb|AAP19345.1| putative NAD(P)H oxidoreductase [Shigella flexneri 2a str. 2457T]

MSQPAKVLLLYAHPESQDSVANRVLLKPATQLSNVTVHDLYAHYPDFFIDIPREQALLREHEVIVFQHPL

YTYSCPALLKEWLDRVLSRGFASGPGGNQLAGKYWRNVITTGEPESAYRYDALNRYPMSDVLRPFELAAG

MCRMHWLSPIIIYWARRQSAKELASHARAYGDWLANPLSPGGR

>gi|30043624|gb|AAP19344.1| putative ATP-binding component of a transport system [Shigella flexneri 2a str. 2457T]

MIVFSSLQIRRGVRVLLDNATATINPGQKVGLVGKNGCGKSTLLALLKNEISADGGSYTFPGSWQLAWVN
QETPALPQAALEYVIDGDREYRQLEAQLHDANERNDGHAIATIHSKLDAIDAWSIRSRAASLLHGLGFSN
EQLERPVSDFSGGWRMRLNLAQALICRSDLLLLDEPTNHLDLDAVIWLEKWLKSYQGTLILISHDRDFLD
PIVDKIIHIEQQSMFEYTGNYSSFEVQRATRLAQQQAMYESQQERVAHLQSYIDRFRAKATKAKQAQSRI
KMLERMELIAPAHVDNPFRFSFRAPESLPNPLLKMEKVSAGYGDRIILDSIKLNLVPGSRIGLLGRNGAG
KSTLIKLLAGELAPVSGEIGLAKGIKLGYFAQHQLEYLRADESPIQHLARLAPQELEQKLRDYLGGFGFL
GDKVTEETRRFSGGEKARLVLALIVWQRPNLLLLDEPTNHLDLDMRQALTEALIEFEGALVVVSHDRHLL
RSTTDDLYLVHDRKVEPFDGDLEDYQQWLSDVQKQENQTDEAPKENANSAQARKDQKRREAELRAQTQPL
RKEIARLEKEMEKLNAQLAQAEEKLGDSELYDQSRKAELTACLQQQASAKSGLEECEMAWLEAQEQLEQM
LLEGQSN

>gi|30043623|gb|AAP19343.1| hypothetical protein S4391 [Shigella flexneri 2a str. 2457T]
MAQITTTDANEFSSSAEFTPMRGFSNCHLQTMLPRLFRRQVKFTPYWQRLELPDGDFVDLAWSEDPAQAK
HKPRLVVFHGLEGSLNSPYPHGLVEAAQKRGWLGVVMHFRGCSGEPNRMHRIYHSGETEDASWFLRWLQR
EFGHAPTAAVGYSLGGNMLACLLAKEGNDLPVDAAVIVSAPFMLEACSYHMEKGFSRVYQRYLLNLLKAN
AARKLAAYPGTLPINLAQLKSVRRIREFDDLITARIHGYADAIDYYRQCSAMPMLNRIAKPTLIIHAKDD
PFMDHQVIPKPESLPSQVEYQLTEHGGHVGFIGGTLLHPQMWLESRIPDWLTTYLEAKSC

>gi|30043622|gb|AAP19342.1| hypothetical protein S4390 [Shigella flexneri 2a str. 2457T]
MLIPWQDLSPETLENLIESFVLREGTDYGEHERTLEQKVADVKRQLQCGEAVLVWSELHETVNIMPRSQF
RE

>gi|30043621|gb|AAP19341.1| probable phosphoribulokinase [Shigella flexneri 2a str. 2457T]
MSAKHPVIAVTGSSGAGTTTTSLAFRKIFAQLNLHAAEVEGDSFHRYTRPEMDMAIRKARDAGRHISYFG
PEANDFGLLEQTFIEYGQSGKGKSRKYLHTYDEAVPWNQVPGTFTPWQPLPEPTDVLFYEGLHGGVVTPQ

HNVAQHVDLLVGVVPIVNLEWIQKLIRDTSERGHSREAVMDSVVRSMEDYINYITPQFSRTHLNFQRVPT
VDTSNPFAAKGIPSLDESFVVIHFRNLEGIDFPWLLAMLQGSFISHINTLVVPGGKMGLAMELIMLPLVQ
RLMEGKKIE

>gi|30043620|gb|AAP19340.1| hypothetical protein S4388 [Shigella flexneri 2a str. 2457T]
MQARVKWVEGLTFLGESASGHQILMDGNSGDKAPSPMEMVLMAAGGCSAIDVVSILQKGRQDVVDCEVKL
TSERREEAPRLFTHINLHFIVTGRDLKDAAVARAVDLSAEKYCSVALMLEKAVNITHSYEVVAA

>gi|30043619|gb|AAP19339.1| cyclic AMP receptor protein [Shigella flexneri 2a str. 2457T]
MVLGKPQTDPTLEWFLSHCHIHKYPSKSTLIHQGEKAETLYYIVKGSVAVLIKDEEGKEMILSYLNQGDF
IGELGLFEEGQERSAWVRAKTACEVAEISYKKFRQLIQVNPDILMRLSAQMARRLQVTSEKVGNLAFLDV
TGRIAQTLLNLAKQPDAMTHPDGMQIKITRQEIGQIVGCSRETVGRILKMLEDQNLISAHGKTIVVYGTR

>gi|30043618|gb|AAP19338.1| hypothetical protein S4386 [Shigella flexneri 2a str. 2457T]
MWRRLIYHPDINYALRQTLVLCLPVAVGLMLGELRFGLLFSLVPACCNIAGLDTPHKRFFKHLIIGASLF
ATCSLLTQLLLVKDVPLPFLLTGLTLVLGVTAELGPLHAKLLPASLLAAIFTLSLAGYMPVWEPLLIYAL
GTLWYGLFNWFWFWIWREQPLRESLSLLYRELADYCEAKYSLLTQHTDPEKALPPLLVRQQKAVDLITQC
YQQMHMLSAQNNTDYKRMLRIFQEALDLQEHISVSLHQPEEVQKLVERSHAEEVIRWNAQTVAARLRVLA
DDILYHRLPTRFTMEKQIGALEKIARQHPDNPVGQFCYWHFSRIARVLRTQKPLYARDLLADKQRRMPLL
PALKSYLSLKSPALRNAGRLSVMLSVASLMGTVLHLPKSYWILMTVLLVTQNGYGATRLRIVNRSVGTVV
GLIIAGVALHFKIPESYTLTLMLITTLASYLILRKNYGWATVGFTITAVYTLQLLWLNGEQYILPRLIDT
IIGCLIAFGGTVWLWPQWQSGLLRKNAHDALEAYQEAIRLILSEDPQPTPLAWQRMRVNQAHNTLYNSLN
QAMQEPAFNSHYLADMKLWVTHSQFIVEHINAMTTLAREHRALPPELAQEYLQSCEIAIQRCQQRLEYDE
PGSSGDANIMDAPEMQPHEGAAGTLEQHLQRVIGHLNTMHTISSMAWRQRPHHGIWLSRKLRDSKA

>gi|30043617|gb|AAP19337.1| acetylornithine delta-aminotransferase [Shigella flexneri 2a str. 2457T]

MAIEQTAITRATFDEVILPIYAPAEFIPVKGQGSRIWDQQGKEYVDFAGGIAVTALGHCHPALVNALKTQ

GETLWHISNVFTNEPALRLGRKLIEATFAERVVFMNSGTEANETAFKLARHYACVRHSPFKTKIIAFHNA

FHGRSLFTVSVGGQPKYSDGFGPKPADIIHVPFNDLHAVKAVMDDHTCAVVVEPIQGEGGVTAATPEFLQ

GLRELCDQHQALLVFDEVQCGMGRTGDLFAYRYYGVTPDILTSAKALGGGFPISAMLTTAEIASAFHPGS

HGSTYGGNPLACAVAGAAFDIINTPEVLEGIQAKRQRFVDHLQKIDQQYDVFSDIRGMGLLIGAELKPHY

KGRARDFLYAGAEVGVMVLNAGPDVMRLAPSLVVEDADIDEGMQRFAHAVAKVVGA

>gi|30043616|gb|AAP19336.1| p-aminobenzoate synthetase, component II [Shigella flexneri 2a str. 2457T]

MILLIDNYDSFTWNLYQYFCELGADVLVKRNDALTLADIDALKPQKIVISPGPCTPDEAGISLDVIRHYA

GRLPILGVCLGHQAMAQAFGGKVVRAAKVMHGKTSPITHSGEGVFRGLANPLTVTRYHSLVVEPDSLPAC

FDVTAWSETREIMGIRHRQWDLEGVQFHPESILSEQGHQLLANFLHL

>gi|30043615|gb|AAP19335.1| protein induced in stationary phase [Shigella flexneri 2a str. 2457T]

MSDKFGEGRDLYLYPGLDIMRNRLNIRQQQRLEQAAYEMTALRAATIELGPLVRGLPHLRTIHRQLYQDI

FDWAGQLREVDIYQGDTPFCHFAYIEKEGNALMQDLEEEGYLVGLEKAKFVERLAHYYCEINVLHPFRVG

SGLAQRIFFEQLAIHAGYQLSWQGIEKEAWNQANQSGAMGDLTALQMIFSKVVSEAGESE

>gi|30043614|gb|AAP19334.1| hypothetical protein S4382 [Shigella flexneri 2a str. 2457T]

MKKLTDKQKSRLWELQRNRNFQASRRLEGVEMPLVTLTAAEALARLEELRRHYER

>gi|30043613|gb|AAP19333.1| peptidyl-prolyl cis-trans isomerase A (rotamase A) [Shigella flexneri 2a str. 2457T]

MFKSTLAAMAAVFALSALSALSPAAMAAKGDPHVLLTTSAGNIELELDKQKAPVSVQNFVDYVNSGFYNN

TTFHRVIPGFMIQGGGFTEQMQQKKPNPPIKNEADNGLRNTRGTIAMARTADKDSATSQFFINVADNAFL

DHGQRDFGYAVFGKVVKGMDVADKISQVPTHDVGPYQNVPSKPVVILSAKVLP

>gi|30043612|gb|AAP19332.1| putative transport protein [Shigella flexneri 2a str. 2457T]

MTNSNRIKLTWISFLSYALTGALVIVTGMVMGNIADYFNLPVSSMSNTFTFLNAGILISIFLNAWLMEIV

PLKTQLRFGFLLMVLAVAGLMFSHSLALFSTAMFILGVVSGITMSIGTFLITQMYEGRQRGSRLLFTDSF

FSMAGMIFPMIAAFLLARSIEWYWVYACIGLVYVAIFILTFGCEFPALGKRAPKTDAPVEKEKWGIGVLF

LSVAALCYILGQLGFISWVPEYAKGLGMSLNDAGTLVSNFWMSYMVGMWAFSFILRFFDLQRILTVLAGL

AAILMYVFNTGTPVHMAWSILALGFFSSAIYTTIITLGSQQTKVPSPKLVNFVLTCGTIGTMLTFVVTGT

IVEHSGPQAALLTANGLYAVVFVMCFLLGFVSRHRQHNTLTSH

>gi|30043611|gb|AAP19331.1| nitrite reductase (NAD(P)H) subunit [Shigella flexneri 2a str. 2457T]

MSKVRLAIIGNGMVGHRFIEDLLDKSDAANFDITVFCEEPRIAYDRVHLSSYFSHHTAEELSLVREGFYE

KHGIKVLVGERAITINRQEKVIHSSAGRTVFYDKLIMATGSYPWIPPIKGSDTQDCFVYRTIEDLNAIES

CARRSKRGAVVGGGLLGLEAAGALKNLGIETHVIEFAPMLMAEQLDQMGGEQLRRKIESMGVRVHTSKNT

LEIVQEGVEARKTMRFADGSELEVDFIVFSTGIRPRDKLATQCGLDVAPRGGIVINDSCQTSDPDIYAIG

ECASWNNRVFGLVAPGYKMAQVAVDHILGSENAFEGADLSAKLKLLGVDVGGIGDAHGRTPGARSYVYLD

ESKEIYKRLIVSEDNKTLLGAVLVGDTSDYGNLLQLVLNAIELPENPDSLILPAHSGSGKPSIGVDKLPD

SAQICSCFDVTKGDLIAAINKGCHTVAALKAETKAGTGCGGCIPLVTQVLNAELAKQGIEVNNNLCEHFA

YSRQELFHLIRVEGIKTFEELLAKHGKGYGCEVCKPTVGSLLASCWNEYILKPEHTPLQDSNDNFLANIQ

KDGTYSVIPRSPGGEITPEGLMAVGRIAREFNLYTKITGSQRLAMFGAQKDDLPEIWRQLIEAGFETGHA

YAKALRMAKTCVGSTWCRYGVGDSVGLGVELENRYKGIRTPHKMKFGVSGCTRECSEAQGKDVGIIATEK

GWNLYVCGNGGMKPRHADLLAADIDRETLIKYLDRFMMFYIRTADKLTRTAPWLENLEGGIDYLKAVIID

DKLGLNAHLEEEMARLREAVVCEWTETVNTPSAQTRFKHFINSDKRDPNVQMVPEREQHRPATPYERIPV

TLVEDNA

>gi|30043610|gb|AAP19330.1| nitrite reductase (NAD(P)H) subunit [Shigella flexneri 2a str. 2457T]

MSQWKDICKIDDILPETGVCALLGDKQVAIFRPYHSDQVFAISNIDPFFESSVLSRGLIAEHQGELWVAS

PLKKQRFRLSDGLCMEDEQFSVKHYEARVKDGVVQLRG

>gi|30043609|gb|AAP19329.1| nitrite reductase, NirC protein [Shigella flexneri 2a str. 2457T]

MFLTFGVKAGSISHGQMWAILPQTWLGNLVGSVFVAMLYSWGGGSLLPVDTSIVHSVALAKTTAPAMVLF

FKGALCNWLVCLAIWMALRTEGAAKFIAIWWCLLAFIASGYEHSIANMTLFALSWFGNHSEAYTLAGIGH

NLLWVTLGNTLSGAVFMGLGYWYATPKANRPVADKFNQTETAAG

>gi|30043608|gb|AAP19328.1| uroporphyrinogen III methylase [Shigella flexneri 2a str. 2457T]

MDHLPIFCQLRDRDCLIVGGGDVAERKARLLLDAGARLTVNALAFIPQFTAWADAGMLTLVEGPFDESLL

DTCWLAIAATDDDTLNQRVSEAAEARRIFCNVVDAPKAASFIMPSIIDRSPLMVAVSSGGTSPVLARLLR

EKLESLLPLHLGQVAKYAGQLRGRVKQQFATMSERRRFWEKLFVNDRLAQSLANNDQKAITETTEQLINE

PLDHRGEVVLVGAGPGDAGLLTLKGLQQIQQADVVVYDRLVSDDIMNLVRRDADRVFVGKRAGYHCVPQE

EINQILLREAQKGKRVVRLKGGDPFIFGRGGEELETLCNAGIPFSVVPGITAASGCSAYSGIPLTHRDYA

QSVRLITGHLKTGGELDWENLAAEKQTLVFYMGLNQAATIQQKLIEHGMPGEMPVAIVENGTAVTQRVID

GTLTQLGELAQQMNSPSLIIIGRVVGLRDKLNWFSNH

>gi|30043607|gb|AAP19327.1| hypothetical protein S4375 [Shigella flexneri 2a str. 2457T]

MNKFIKVALVGAVLATLTACTGHIENRDKNCSYDYLLHPAISISKIIGGCGPTAQ

>gi|30043606|gb|AAP19326.1| putative transport protein [Shigella flexneri 2a str. 2457T]

MLDIDKSTVDFLVTENMVQEVEKVLSHDVPLVHAIVEEMVKRDIDRIYFVACGSPLNAAQTAKHLADRFS

DLQVYAISGWEFCDNTPYRLDDRCAVIGVSDYGKTEEVIKALELGRACGALTAAFTKRADSPITSAAEFS

IDYQADCIWEIHLLLCYSVVLEMITRLAPNAEIGKIKNDLKQLPNALGHLVRTWEEKGRQLGELASQWPM

IYTVAAGPLRPLGYKEGIVTLMEFTWTHGCVIESGEFRHGPLEIVEPGVPFLFLLGNDESRHTTERAINF

VKQRTDNVIVIDYAEISQGLHPWLAPFLMFVPMEWLCYYLSIYKDHNPDERRYYGGLVEY

>gi|30043605|gb|AAP19325.1| hypothetical protein S4371 [Shigella flexneri 2a str. 2457T]

MKTGMFTCGHQRLPIEHAFRDASELGYDGIEIWGGRPHAFAPDLKAGGIKQIKALAQTYQMPIIGYTPET

NGYPYNMMLGDEHMRRESLDMIKLAIDMAKEMNAGYTLISAAHAGYLTPPNVIWGRLAENLSELCEYAEN

IGMDLILEPLTPYESNVVCNANDVLHALALVPSPRLFSMVDICAPYVQAEPVMSYFDKLGDKLRHLHIVD

SDGASDTHYIPGEGKMPLRELMRDIIDRGYEGYSTVELVTMYMNEPRLYARQALERFRALLPEDER

>gi|30043604|gb|AAP19324.1| putative kinase [Shigella flexneri 2a str. 2457T]

MKTLATIGDNCVDIYPQLNKAFSGGNAVNVAVYCTRYGIQPGCITWVGDDDYGTKLKQDLARMGVDISHV

HTKHGVTAQTQVELHDNDRVFGDYTEGVMADFALSEEDYAWLAQYDIVHAAIWGHAEDAFPQLHAAGKLT

AFDFSDKWDSPLWQTLVPHLDFAFASAPQEDEALRLKMKAIVARGAGTVIVTLGENGSIAWDGAQFWRQA

PEPVTVIDTMGAGDSFIAGFLCGWSAGMTLPQAMAQGTACAAKTIQYHGAW

>gi|30043603|gb|AAP19323.1| putative transcriptional regulator [Shigella flexneri 2a str. 2457T]

MLLLAGKRPYNPSNSFFISGSIMSATDRYSHQLLYATVRQRLLDDIAQGVYQAGQQIPTENELCTQYNVS

RITIRKAISDLVADGVLIRWQGKGTFVQSQKVENALLTVSGFTDFGVSQGKSTKEKVIEQERVSAAPFCE

KLNIPGNSEVFHLCRVMYLDKEPLFIDSSWIPLSRYPDFDEIYVEGSSTYQLFQERFDTRVVSDKKTIDI

FAATRPQAKWLKCELGEPLFRISKIAFDQNDKPVHVSELFCRANRITLTIDNKRH

>gi|30043602|gb|AAP19322.1| hypothetical protein S4368 [Shigella flexneri 2a str. 2457T]

MKTFPLQSLTLAEAQQKQFALVDTICRHFPGSEFLAGGDLGLTPSLNQPRITQRVEQVLADAFHAQAAAL

VQGAGTGAIRAGLAALLKPGQRLLVHDAPVYPTTRVIIEQMGLTLITADFNDLSALKQVVDEQQPDAALV

QHTRQQPQDSYVLADVLATLRAAGVPALTDDNYAVMKVARIGCECGANVSTFSCFKLFGPEGVGAVVGDA

DVISRIRATLYSGGSQIQGSQALEVLRGLVFAPLIHAVKAGVSERLLALLNGGAVAEVKSAVIANAQSKV

LIVEFHQLIAARVLEEAQKRGALPYPVGAESKYEIPPLFYRLSGTFRQANPQLEHCAIRINPNRSGEETV

LRILRESIASI

>gi|30043601|gb|AAP19321.1| putative transport system permease protein [Shigella flexneri 2a str. 2457T]

MDLYIQIIVVACLTGMTSLLAHRSAAVFHDGIHPILPQLIEGYMNRREAGSIAFGLSIGFVASVGISFTL

KTGLLNAWLLFLPTDILGVLAINSLMAFGLGAIWGVLILTCLLPVNQLLTALPVDVLGSLGELSSPVVSA

FALFPLVAIFYQFGWKQSLVAAVVVLMTRVVVVRYFPHLNPESIEIFIGMVMLLGIAITHDLRHRDENDI

DASGLSVFEERTSRIIKNLPYIAIVGALIAAVASMKIFAGSEVSIFTLEKAYSAGVTPEQSQTLINQAAL

AEFMRGLGFVPLIATTALATGVYAVAGFTFVYAVGYLSPNPMVAAVLGAVVISAEVLLLRSIGKWLGRYP

SVRNASDNIRNAMNMLMEVALLVGSIFAAIKMAGYTGFSIAVAIYFLNESLGRPVQKMAAPVVAVMITGI

LLNVLYWLGLFVPA

>gi|30043600|gb|AAP19320.1| hypothetical protein S4366 [Shigella flexneri 2a str. 2457T]

MKIPLNFSNKDRLMKKIGVAGLQREQIKKTIEATAPGCFEVFIHNDMEAAMKVKSGQLDYYIGACNTGAG

AALSIAIAVIGYNKSCTIAKPGIKAKDEHIAKMIAEGKVAFGLSVEHVEHAIPMLINHLK

>gi|30043599|gb|AAP19319.1| putative hydrolase [Shigella flexneri 2a str. 2457T]

MSFDPTGYTLAHEHLHIDLSGFKNNVDCRLDQYAFICQEMNDLMTRGVRNVIEMTNRYMGRNAQFMLDVM

RETGINVVACTGYYQDAFFPEHVVTRSVQELAQEMVDEIEQGIDGTELKAGIIAEIGSSEGKITPLEEKV

FIAAALAHNQTGRPISTHTSFSTMGLEQLALLQAHGVDLSRVTVGHCDLKDNLDNILKMIDLGAYVQFDT

IGKNSYYPDEKRIAMLHALRDRGLLNRVMLSMDITRRSHLKANGGYGYDYLLTTFIPQLRQSGFSQADVD

VMLRENPSQFFQ

>gi|30043598|gb|AAP19318.1| putative mutase [Shigella flexneri 2a str. 2457T]

MARFVVLVIDSFGVGAMKDVTLVRPQDAGANTCGHILSQLPHLQLPTLEKLGLINTLGYAPGDMQPSDSA

TWGVAELQHEGGDTFMGHQEILGTRPLPPLRMPFRDVIDRVEQALVSAGWQVERRGDDLQFLSVNQAVAI

GDNLEADLGQVYNITANLSVISFDDAIKIGRIVRDQVQVGRVITFGGLLTNSQRILDAAESKEGRFIGIN

APRSGAYDNGFQVVHMGYGVDEKVQVPQKLYEAGVPTVLVGKVADIVSNPYGVSWQNLVDSQRIMDITLD

EFNTHPTAFICINIQETDLAGHAEDVARYAERLQVVDRNLARLVEAMQPDDCLVVMADHGNDPTIGHSHH

TREVVPVLVYQQGLVATQLGVRTTLSDVGATVCEFFRAPPPQNGRSFLSSLRFAGDTL

>gi|30043597|gb|AAP19317.1| hypothetical protein S4363 [Shigella flexneri 2a str. 2457T]

MFVEALKRQNPALISAALSLWQQGKIAPDSWVIDVDQILENGKRLIETARLYGIELYLMTKQFGRNPWLA

EKLLALGYSGIVAVDYKEARVMRRAGLPVAHQGHLVQIPCHQVADAVEQGTDIITVFTLDKAREVSAAAV

KAGRVQPVLLKVYSDDDFLYPGQESGFVQHSLHEVIAEIKKLPGLHLAGLTHFPCLLWDEAAGKVLPTPN

LHTLVQARDQLAKSGIAIEQLNVPSATSCTSLPLLAEYGATHAEPGHALTGTIPANQQGDQPERIAMLWL

SEISHHFRGDSYCYGGGYYRRGHAQHALVFTPENQKITETNLKTVDDSSIDYTLPLAGEYPVSSAVVLCF

RTQIFVTRSDVVLVSGIQRGEPEIVGRYDSLGNPLEA

>gi|30043596|gb|AAP19316.1| hypothetical protein S4362 [Shigella flexneri 2a str. 2457T]

MGNRNPVIKRKASDMETRLNLLCEAGVIDKDVCKGMMQVVNVLEKECHLPVRSEQGTMAMTHMASALMRS

RRGEEIEPLDNELLAELAQSSHWQAVVQLHQELLKEFALEVNPCEEGYFLANLYGLWMAANEEV

>gi|30043595|gb|AAP19315.1| hypothetical protein S4361 [Shigella flexneri 2a str. 2457T]

MDNKALLSHVDINNVVCAMPLPYTRLYEGLASGLKAQFDGIPFYYAHMRGADIRVECLLNGVYDMAVVSR

LAAESYLSQNNLCIALELGPHTYVGEHQLICRKGESGNVKRVGLDSRSADQKIMTDVFFGDSDVERVDLS

YHESLQRIVKGDVDAVIWNVVAENELTMLGLEATPLTDDPRFLQATEAVVLTRVDDYPMQQLLRAVVDKH

ALLAHQQRVVSGEQEPSY

>gi|30043594|gb|AAP19314.1| tryptophan tRNA synthetase [Shigella flexneri 2a str. 2457T]

MTKPIVFSGAQPSGELTIGNYMGALRQWVNMQDDYHCIYCIVDQHAITVRQDAQKLRKATLDTLALYLAC

GIDPEKSTIFVQSHVPEHAQLGWALNCYTYFGELSRMTQFKDKSARYAENINAGLFGYPVLMAADILLYQ

TNLVPVGEDQKQHLELSRDIAQRFNALYGEIFKVPEPFIPKSGARVMSLLEPTKKMSKSDDNRNNVIGLL

EDPKSVVKKIKRAVTDSDEPPVVRYDVQNKAGVSNLLDILSAVTGQSIPELEKQFEGKMYGHLKGEVADA

VSGMLTELQERYHRFRNDEAFLQQVMKDGAEKASVHASRTLKAVYEAIGFVAKP

>gi|30043593|gb|AAP19313.1| phosphoglycolate phosphatase [Shigella flexneri 2a str. 2457T]

MNKFEDIRGVAFDLDGTLVDSAPGLAAAVDMALYALELPVAGEERVITWIGNGADVLMERALTWARQERA

TLRKTMGKPPVDDDIPAEEQVRILRKLFDRYYGEVAEEGTFLFPHVADTLGALQAKGLPLGLVTNKPTPF

VAPLLEALDIAKYFSVVIGGDDVQNKKPHPDPLLLVAERMGIAPQQMLFVGDSRNDIQAAKAAGCPSVGL

TYGYNYGEAIDLSQPDVIYQSINDLLPALGLPHSENQESKND

>gi|30043592|gb|AAP19312.1| D-ribulose-5-phosphate 3-epimerase [Shigella flexneri 2a str. 2457T]

MKQYLIAPSILSADFARLGEDTAKALAAGADVVHFDVMDNHYVPNLTIGPMVLKSLRNYGITAPIDVHLM

VKPVDRIVPDFAAAGASIITFHPEASEHVDRTLQLIKENGCKAGLVFNPATPLSYLDYVMDKLDVILLMS

VNPGFGGQSFIPQTLDKLREVRRRIDESGFDIRLEVDGGVKVNNIGEIAAAGADMFVAGSAIFDQPDYKK

VIDEMRSELAKVSHE

>gi|30043591|gb|AAP19311.1| DNA adenine methylase [Shigella flexneri 2a str. 2457T]

MKKNRAFLKWAGGKYPLLDDIKRHLPKGECLVEPFVGAGSVFLNTDFSRYILADINSDLISLYNIVKMRT

DEYVQAARELFVPETNCAEVYYQFREEFNKSQDPFRRAVLFLYLNRYGYNGLCRYNLRGEFNVPFGRYKK

PYFPEAELYHFAEKAQNAFFYCESYADSMERADDASVVYCDPPYAPLSATANFTAYHTNSFTLEQQAHLA

EIAEGLVERHIPVLISNHDTMLTREWYQRAKLHVVKVRRSISSNGGTRKKVDELLALYKPGVVSPAKK

>gi|30043590|gb|AAP19310.1| putative membrane protein [Shigella flexneri 2a str. 2457T]

MDEFKPEDELKPDPSDRRTGRSRQSSERSERTERGEPQINFDDIELDDTDDRRPTRAQKERNEEPEIEEE

IDESEDETVDEERVERRPRKRKKAASKPASRQYMMMGVGILVLLLLIIGIGSALKAPSTTSSDQTASGEK

SIDLAGNATDQANGVQPAPGTTSAENTQQDVSLPPISSTPTQGQTPVATDGQQRVEVQGDLNNALTQPQN

QQQLNNVAVNSTLPTEPATVAPVRNGNASRDTAKTQTAERPSTTRPVRQQAVIEPKKPQATVKTEPKPVA

QTPKRTEPEPAAPVASTKAPAATSTPAPKETATTAPVQTASPAQTTATPAAGGKTAGNVGSLKSAPSSHY

TLQLSSSSNYDNLNGWAKKENLKNYVVYETTRNGQPWYVLVSGVYASKEEAKKAVSTLPADVQAKNPWAK

PLRQVQADLK

>gi|30043589|gb|AAP19309.1| 3-dehydroquinate synthase [Shigella flexneri 2a str. 2457T]

MERIVVTLGERSYPITIASGLFNEPASFLPLKSGEQVMLVTSETLAPLYLDKVRGVLEQAGVNVDSVILP

DGEQYKSLAVLDTVFTALLQKPHGRDTTLVALGGGVVGDLTGFAAASYQRGVRFIQVPTTLLSQVDSSVG

GKTAVNHPLGKNMIGAFYQPASVVVDLDCLKTLPPRELASGLAEVIKYGIILDGAFFNWLEENLDALLRL

DGPAMAYCIRRCCELKAEVVAADERETGLRALLNLGHTFGHAIEAEMGYGNWLHGEAVAAGMVMAARTSE

RLGQFSSAETQRIITLLTRAGLPVNGPREMSAQAYLPHMLRDKKVLAGEMRLILPLAIGKSEVRSGVSHE

LVLNAIADCQSA

>gi|30043588|gb|AAP19308.1| shikimate kinase I [Shigella flexneri 2a str. 2457T]

MVARQRACSDQICRGMGDLFSCQTRWSIEIIFSLTLAISYEVSVHVLRRSLSEAGLSLTNSLSSTEKMAE

KRNIFLVGPMGAGKSTIGRQLAQQLNMEFYDSDQEIEKRTGADVGWVFDLEGEEGFRDREEKVINELTEK

QGIVLATGGGSVKSRETRNRLSARGVVVYLETTIEKQLARTQRDKKRPLLHVETPPREVLEALANERNPL

YEEIADVTIRTDDQSAKVVANQIIHMLESN

>gi|30043587|gb|AAP19307.1| putative transport portein [Shigella flexneri 2a str. 2457T]

MKQWIAALLLMLIPGVQAAKPQKVTLMVDDVPVAQVLQALAEQEKLNLVVSPDVSGTVSLHLTDVPWKQA

LQTVVKSAGLITRQEGNILSVHSIAWQNDNIARQEAEQARAQANLPLENRSITLQYADAGELAKAGEKLL

SAKGSMTVDKRTNRLLLRDNKTALSALEQWVAQMDLPVGQVELSAHIVTINEKSLRELGVKWTLADAQHA

GGVGQVTTLGSDLSVATATTHVGFNIGRINGRLLDLELSALEQKQQLDIIASPRLLASHLQPASIKQESE

IPYQVSSGESGATSVEFKEAVLGMEVTPTVLQKGRIRLKLHISQNVPGQVLQQADGEVLAIDKQEIETQV

EVKSGETLALGGIFTRKNKSGQDSVPLLGDIPWFGQLFRHDGKEDERRELVVFITPRLVSSE

>gi|30043586|gb|AAP19306.1| hypothetical protein S4351 [Shigella flexneri 2a str. 2457T]

MNMFFDWWFATSPRLRQFCWAFWLLMLVTLIFLSSTHHEERDALIRLRASHHQQWAALYRLVDTTPFSEE

KTLPFSPLDFQLSGAQLVSWHPSAQGGELALKTLWEAVPSAFTRLAERNVSVSRFSLSVEGDDLLFTLQL

EMPHEG

>gi|30043585|gb|AAP19305.1| hypothetical protein S4350 [Shigella flexneri 2a str. 2457T]

MNPPINFLPWRQQRRTAFLRFWLLMFVAPLLLAVGITLILRLTGSAEARIDAVLLQAEQQLARSLQITKP

RLLEQQQLREQRSQRQRQRQFTRDWQSALEALAALLPEHAWLTTISWQQGTLEIKGLTTSITALNALETS

LRQDASFHLNQRGATQQDAQGRWQFEYQLTRKVSDEHVL

>gi|30043584|gb|AAP19304.1| hypothetical protein S4349 [Shigella flexneri 2a str. 2457T]

MTGDKEIITMTFKIWQIGLHLQQQEAAAVAIVRGAKECFLQRWWRLPLAHDIIKDGRIVDAQQLAKTLLP

WSRELPQRHHIMLAFPASRTLQRSFPRPSMSLGEREQTAWLSGTMARELDMDPDSLRFDYSEDSLSPAYN

VTAAQSKELATLLTLAERLRVHVSAITPDASALQRFLPFLPSHQQCLAWRDNEQWLWATRYSWGRKLAVG

MTSAKELAAALSVDPDSVAICGEGGFDPWEAVSVRQPPLPPPGGDFAIALGLALGKAY

>gi|30043583|gb|AAP19303.1| peptidoglycan synthetase, penicillin-binding protein 1A [Shigella flexneri 2a str. 2457T]

MKFVKYFLILAVCCILLGAGSIYGLYRYIEPQLPDVVTLKDVRLQIPMQIYSADGELIAQYGEKRRIPVT

LDQIPPEMVKAFIATEDSRFYEHHGVDPVGIFRAASVALFSGHASQGASTITQQLARNFFLSPERTMMRK

IKEVFLAIRIEQLLTKDEILELYLNKIYLGYRAYGVGAAAQVYFGKTVDQLTLNEMAVIAGLPKAPSTFN

PLYSMDRAVARRNVVLSRMLDEGYITQQQFDQTRTEAINANYHAPEIAFSAPYLSEMVRQEMYNRYGESA

YEDGYRIYTTITRKVQQAAQQAVRNNVLDYDMRHGYRGPANVLWKVGESAWDNNKITDTLKALPTYGPLL

PAAVTSANPQGATAMLADGSTVALSMEGVRWARPYRSDTQQGPTPRKVTDVLQTGQQIWVRQVGDAWWLA

QVPEVNSALVSINPQNGAVMALVGGFDFNQSKFNRATQALRQVGSNIKPFLYTAAMDKGLTLASMLNDVP

ISRWDAGAGSDWQPKNSPPQYAGPIRLRQGLGQSKNVVMVRAMRAMGVDYAAEYLQRFGFPAQNIVHTES

LALGSASFTPMQVARGYAVMANGGFLVDPWFISKIENDQGGVIFEAKPKVACPECDIPVIYGDTQKSNVL

ENNDVEDVAISREQQNVSVPMPQLEQANQALVAKTGAQEYAPHVINTPLAFLIKSALNTNIFGEPGWQGT

GWRAGRDLQRRDIGGKTGTTNSSKDAWFSGYGPGVVTSVWIGFDDHRRNLGHTTASGAIKDQISGYEGGA

KSAQPAWDAYMKAVLEGVPEQPLTPPPGIVTVNIDRSTGQLANGGNSREEYFIEGTQPTQQAVHEVGTTI

IDNGEAQELF

>gi|30043582|gb|AAP19302.1| hypothetical protein S4347 [Shigella flexneri 2a str. 2457T]

MNKSLQKPTILNVETVARSRLFTVESVDLEFSNGVRRVYERMRPTNREAVMIVPIVDDHLILIREYAVGT

ESYELGFSKGLIDPGESVYEAANRELKEEVGFGANDLTFLKKLSMAPSYFSSKMNIVVAQDLYPESLEGD

EPEPLPQVRWPLAHMMDLLEDPDFNEARNVSALFLVREWLKGQGRV

>gi|30043581|gb|AAP19301.1| putative dehydrogenase [Shigella flexneri 2a str. 2457T]

MSTIVIFLAALLACSLLAGWLIKVRSRRRQLPWTNAFADAQTRKLTPEERSAVENYLESLTQVLQVPGPT

EASAAPISLALNAESNNVMMLTHAITRYGISTDDPNKWRYYLDSVEVHLPPFWEQYINDENTVELIHTDS

LPLVISLNGHTLQEYMQETRGYALQPVPSTQASIRGEESEQIELLNIRKETHEEYALSRPRGLREALLIV

ASFLMFFFCLITPDVFVPWLAGGALLLLGAGLWGLFAPPAKSSLREIHCLRGTPRRWGLFGENDQEQINN

ISLGIIDLVYPAHWQPYIAQDLGQQTDIDIYLDRHVVRQGRYLSLHDEVKNFPLQHWLRSTIIAAGSLLV

LFMLLFWIPLDMPLKFTLSWMKGAQTIEATSVKQLADAGVRVGDTLRISGTGMCNIRTSGTWSAKTNSPF

LPFDCSQIIWNDARSLPLPESELVNKATALTEAVNRQLHPKPEDESRVSASLRSAIQKSGMVLLDDFGDI

VLKTADLCSAKDDCVRLKNALVNLGNSKDWDALVKRANAGKLDGVNVLLRPVSAESLDNLVATSTAPFIT

HETARAAQSLNSPAPGGFLIVSDEGSDFVDQPWPSASLYDYPPQEQWNAFQKLAQMLMHTPFNAEGIVTK

IFTDANGTQHIGLHPIPDRSGLWRYLSTTLLLLTMLGSAIYNGVQAWRRYQRHRTRMMEIQAYYESCLNP

QLITPSESLIE

>gi|30043580|gb|AAP19300.1| putative phosphatase [Shigella flexneri 2a str. 2457T]

MLPCRANCFTLEISLMHINIAWQDVDTVLLDMDGTLLDLAFDNYFWQKLVPETWGAKNGVTPQEAMEYMR

QQYHDVQHTLNWYCLDYWSEQLGLDICAMTTEMGPRAVLREDTIPFLEALKASGKQRILLTNAHPHNLAV

KLEHTGLDAHLDLLLSTHTFGYPKEDQRLWHAVAEATGLKAERTLFIDDSEAILDAAAQFGIRYCLGVTN

PDSGIAEKQYQRHPSLNDYRRLIPSLM

>gi|30043579|gb|AAP19299.1| hypothetical protein S4344 [Shigella flexneri 2a str. 2457T]

MKEKPAVEVRLDKWLWAARFYKTRALAREMIEGGKVHYNGQRSKPSKIVELNATLTLRQGNDERTVIVKA

ITEQRRPASEAALLYEETAESVEKREKMAMARKLNALTMPHPDRRPDKKERRDLLRFKHGDSE

>gi|30043578|gb|AAP19298.1| hypothetical protein S4343 [Shigella flexneri 2a str. 2457T]

MPQHDQLHRYLFENFAVRGELVTVSETLQQILENHDYPQPVKNVLAELLVATSLLTATLKFDGDITVQLQ

GDGPMNLAVINGNNNQQMRGVARVQGEIPENADLKTLVGNGYVVITITPSEGERYQGVVGLEGDTLAACL

EDYFMRSEQLPTRLFIRTGDVDGKPAAGGMLLQVMPAQNTQQDDFDHLATLTETIKTEELLTLPANEVLW

RLYHEEEVTVYDPQDVEFKCTCSRERCADALKTLPDEEVDSILAEDGEIDMHCDYCGNHYLFNAMDIAEI

RNNASPADP

>gi|30043577|gb|AAP19297.1| putative transport protein [Shigella flexneri 2a str. 2457T]

MDNVELSPATRWGMIATGLLQGMIATGLLQGLVCYLLIAWLAGKNHSWIVYGVPATVAFSSVLLFSVISF

KQKRLWGWLALVFIATTGMSGWLKWQTDGMNPWRAEKAIWDFGCYLLLMAMLLLPWIQQSLRIRNGSSRY

SYFYQSVWHNVLILLVIFLSNGLTWLVLLLWGELFKLVGITFFNTLFFATDWFMYLTFGLVTALAVILAR

TQSRLIDSIQKLFTLIATGLLPLVSLLTLMFIITLPFTGLSAISRHISAAGLLLTLAFLQLILMAIVRDP

QKASLPWTGPLRCLIKTALLVAPLYVFVATWGLWLRVAQYGWTVDRLQGALAELVLLVWSLGYFVSIVWR

NGQNPLVLQGKVNLAVSLLVLVILVLLNSPVLDSMRISVNSHMARYQSGKNTPDQVTIYMLEQSGRYGRA

ALESLKSDAGFMKDPKRARDLLMALDGEQHLQEQVSEKVFAENVLIAPGSVKPDATFWSALIQDRYNVMT

CIEKDACVLVEQDLNSDGQAERILFAFNDDRVIVYGFDSDRKEWDALDMSLLPNEITKEKLLTAAKDGKL

GTRPKAWRDLTVDGETLEINLSK

>gi|30043576|gb|AAP19296.1| phosphoenolpyruvate carboxykinase [Shigella flexneri 2a str. 2457T]

MRVNNGLPPQELEAYGISDVHDIVYNPSYDLLYQEELDPSLTGYERGVLTNLGAVAVDTGIFTGRSPKDK

YIVRDDTTRDTFWWADKGKGKNDNKPLSPETWQHLKGLVTRQLSGKRLFVVDAFCGANPDTRLSVRFITE

VAWQAHFVKNMFIRPSDEELAGFKPDFIVMNGAKCTNPQWKEQGLNSENFVAFNLTERMQLIGGTWYGGE

MKKGMFSMMNYLLPLKGIASMHCSANVGEKGDVAVFFGLSGTGKTTLSTDPKRRLIGDDEHGWDDDGVFN

FEGGCYAKTIKLSKEAEPEIYNAIRRDALLENVTVREDGTIDFDDGSKTENTRVSYPIYHIDNIVKPVSK

AGHATKVIFLTADAFGVLPPVSRLTADQTQYHFLSGFTAKLAGTERGITEPTPTFSACFGAAFLSLHPTQ

YAEVLVKRMQAAGAQAYLVNTGWNGTGKRISIKDTRAIIDAILNGSLDNAETFTLPMFNLAIPTELPGVD

TKILDPRNTYASPEQWQEKAETLAKLFIDNFDKYTDTPAGAALVAAGPKL

>gi|30043575|gb|AAP19295.1| protein histidine kinase/phosphatase sensor for OmpR, modulates expression of ompF and ompC [Shigella flexneri 2a str. 2457T]

MRRLRFSPRSSFARTLLLIVTLLFASLVTTYLVVLNFAILPSLQQFNKVLAYEVRMLMTDKLQLEDGTQL

VVPPAFRREIYRELGISLYSNEAAEEAGLRWAQHYEFLSHQMAQQLGGPTEVRVEVNKSSPVVWLKTWLS

PNIWVRVPLTEIHQGDFSPLFRYTLAIMLLAIGGAWLFIRIQNRPLVDLEHAALQVGKGIIPPPLREYGA

SEVRSVTRAFNHMAAGVKQLADDRTLLMAGVSHDLRTPLTRIRLATEMMSEQDGYLAESINKDIEECNAI

IEQFIDYLRTGQEMPMEMADLNAVLGEVIAAESGYEREIETALYPGSIEVKMHPLSIKRAVANMVVNAAR

YGNGWIKVSSGTEPNRAWFQVEDDGPGIAPEQRKHLFQPFVRGDSARTISGTGLGLAIVQRIVDNHNGML

ELGTSERGGLSIRAWLPVPVTRAQGTTKEG

>gi|30043574|gb|AAP19294.1| response regulator (sensor, EnvZ) [Shigella flexneri 2a str. 2457T]

MQENYKILVVDDDMRLRALLERYLTEQGFQVRSVANAEQMDRLLTRESFHLMVLDLMLPGEDGLSICRRL

RSQSNPMPIIMVTAKGEEVDRIVGLEIGADDYIPKPFNPRELLARIRAVLRRQANELPGAPSQEEAVIAF

GKFKLNLGTREMFREDEPMPLTSGEFAVLKALVSHPREPLSRDKLMNLARGREYSAMERSIDVQISRLRR

MVEEDPAHPRYIQTVWGLGYVFVPDGSKA

>gi|30043573|gb|AAP19293.1| transcription elongation factor and transcript cleavage factor [Shigella flexneri 2a str. 2457T]

MRIIKQTKGINEMKTPLVTREGYEKLKQELNYLWREERPEVTKKVTWAASLGDRSENADYQYNKKRLREI

DRRVRYLTKCLENLKIVDYSPQQEGKVFFGAWVEIENDDGVTHRFRIVGYDEIFGRKDYISIDSPMARAL

LKKEVGDLAVVNTPAGEANWYVNAIEYVKP

>gi|30043572|gb|AAP19292.1| hypothetical protein S4337 [Shigella flexneri 2a str. 2457T]

MPFIARYRKEITGGLDDTQLRNLETRLSYLRELEERRQAILKSISEQGKLTDDLAKAINATLSKTELEDL

YLPYKPKRRTRGQIAIEAGLEPLADLLWSDPSHTPEVAAAQYVDADKGVADTKAALDGARYILMERFAED

AALLAKVRDYLWKNAHLVSTVVSGKEEEGAKFRDYFDHHEPLSTVPSHRALAMFRGRNEGVLQLSLNADP

QFDEPPKESYCEQIIMDHLGLRLNNAPADSWRKSVVSWTWRIKVLMHLETELMGTVRERAEDEAINVFAR

NLHDLLMAAPAGLRATMGLDPGLRTGVKVAVVDATGKLVATDTIYPHTGQAAKAAMTVAALCEKHNVELV

AIGNGTASRETERFYLDVQKQFPKVTAQKVIVSEAGASVYSASELAAQEFPDLDVSLRGAVSIARRLQDP

LAELVKIDPKSIGVGQYQHDVSQTQLARKLDAVVEDCVNAVGVDLNTASVPLLTRVAGLTRMMAQNIVAW

RDENGQFQNRQQLLKVSRLGPKAFEQCAGFLRINHGDNPLDASTVHPEAYPVVERILAATQQALKDLMGN

SSELRNLKASDFTDEKFGVPTVTDIIKELEKPGRDPRPEFKTAQFADGVETMNDLQPGMILEGAVTNVTN

FGAFVDIGVHQDGLVHISSLSNKFVEDPHTVVKAGDIVKVKVLEVDLQRKRIALTMRLDEQPGETNSRRG

GGNERPQNNRPAAKPRGREAQPAGNSAMMDALAAAMGKKR

>gi|30043571|gb|AAP19291.1| ferrous iron transport protein A [Shigella flexneri 2a str. 2457T]

MQYTPDTAWKITGFSREISPAYRQKLLSLGMLPGSSFDVVRVAPLGDPIHIETRRVSLVLRKKDLALLEV

EAVSC

>gi|30043570|gb|AAP19290.1| ferrous iron transport protein B [Shigella flexneri 2a str. 2457T]

MKKLTIGLIGNPNSGKTTLFNQLTGARQRVGNWAGVTVERKEGQFSTTDHQVTLVDLPGTYSLTTISSQT

SLDEQIACHYILSGDADLLINVVDASNLERNLYLTLQLLELGIPCIVALNMLDIAEKQNIRIEIDALSAR

LGCPVIPLVSTRGRGIEALKLAIDRYKANENVELVHYAQPLLNEADSLAKVMPSDIPLKQRRWLGLQMLE

GDIYSRAYAGEASQHLDATLARLRNEMDDPALHIADARYQCIAAICDVVSNTLTAEPSRFTTAVDKIVLN

RFLGLPIFLFVMYLMFLLAINIGGALQPLFNVGSVALFVHGIQWIGYTLHFPDWLTIFLAQGLGGGINTV

LPLVPQIGMMYLFLSFLEDSGYMARAAFVMDRLMQALGLPGKSFVPLIVGFGCNVPSVMGARTLDAPRER

LMTIMMAPFMSCGARLAIFAVFAAAFFGQNGALAVFSLYMLGIVMAVLTGLMLKYTIMRGEATPFVMELP

VYHVPHVKSLIIQTWQRLKGFVLRAGKVIIIVSIFLSAFNSFSLSGKIVDNINDSALASVSRVITPVFKP

IGVHEDNWQATVGLFTGAMAKEVVVGTLNTLYTAENIQDEEFNPAEFNLGEELFSAVDETWQSLKDTFSL

SVLMNPIEASKGNGEMGTGAMGVMDQKFGSAAAAYSYLIFVLLYVPCISVMGAIARESSRGWMGFSILWG

LNIAYSLATLFYQVASYSQHPTYSLVCILAVILFNIVAIGLLRRARSRVDIELLATRKSVSSCCAASTTG

DCH

>gi|30043569|gb|AAP19289.1| hypothetical protein S4334 [Shigella flexneri 2a str. 2457T]

MASLIQVRDLLALRGRMEATQISQTLNTPQPMINAMLQQLESMGKAVRIQEEPDGCLSGSCKSCPEGKAC

LREWWALR

>gi|30043568|gb|AAP19288.1| hypothetical protein S4333 [Shigella flexneri 2a str. 2457T]

MSKKQSSTPHDALFKLFLRQPETARDFLAFHLPAPIHALCDMKTLKLESRSFIDDDLRESYSDVLWSVKT

EQGPGYIYCLIEHQSTSNKLIAFRMMRYAIAAMQNHLDAGYKTLPMVVPLLFYHGIESPYPYSLCWLDCF

ADPKLARQLYASAFPLIDVTVMPDDEIMQHRRMALLELIQKHIRQRDLMGLVEQMACLLSSGYANDRQIK

GLFNYILQTGDAVRFNDFIDGVAERSPKHKESLMTIAERLRQEGEQSKALHIAKIMLESGVPLADIMRFT

GLSEEELAAASRLAP

>gi|30043567|gb|AAP19287.1| IS2 orfA [Shigella flexneri 2a str. 2457T]

MIVLILVFRLVIGVQMIDVLGPEKRRRRTTQEKIAIVQQSFEPGMTVSLVARQHGVAASQLFLWRKQYQE

GSLTAVAAGEQVVPASELAAAMKQIKELQRLLGKKTMENELLKEAVEYGRAKKWIAHAPLLPGDGE

>gi|30043566|gb|AAP19286.1| IS2 orfB [Shigella flexneri 2a str. 2457T]

MARGWGVSLVSRCLRVSRAQLHVILRRTDDWMDGRRSRHTDDTDVLLRIHHVIGELPTYGYRRVWALLRR

QAELDGMPAINAKRVYRLMRQNALLLERKPAVPPSKRAHTGRVAVKESNQRWCSDGFEFCCDNGERLRVT

FALDCCDREALHWAVTTGGFNSETVQDVMLGAVERRFGNDLPSSPVEWLTDNGSCYRANETRQFARMLGL

EPKNTAVRSPESNGIAESFVKTIKRDYISIMPKPDGLTAAKNLAEAFEHYNEWHPHSALGYRSPREYLRQ

RACNGLSDNRCLEI

>gi|30043565|gb|AAP19285.1| putative enzyme in biotin precursor biosynthesis [Shigella flexneri 2a str. 2457T]

MNNIWWQTKGQGNVHLVLLHGWGLNAEVWRCIDEELSSHFTLHLVDLPGFGRSRGFGALSLADMAEAVLQ

QAPDKAIWLGWSLGGLVASQIALTHPERVQALVTVASSPCFSARDEWPGIKPDVLAGFQQQLSDDFQRTV

ERFLALQTMGTETARQDARALKKTVLALPMPEVDVLNGGLEILKTVDLRQPLQNVSMPFLRLYGYLDGLV

PRKVVPMLDKLWPHSESYIFAKAAHAPFISHPVEFCHLLVALKQRVLVVSES

>gi|30043564|gb|AAP19284.1| hypothetical protein S4330 [Shigella flexneri 2a str. 2457T]

MLFIRYCSLLTYKDEHMLTVPGLCWLCRMPLALGHWGICSVCSRATRTDKTLCPQCGLPATHSHLPCGRC

LQKPPPWQRLVTVADYAPPLSPLIHQLKFSRRSEIASALSRLLLLEVLHARRTTGLQLPDRIISVPLWQR

RHWRRGFNQSDLLCQPLSRWLHCQWDSEAVTRTRATATQHFLSARLRKRNLKNAFRLELPVQGRHMVIVD

DVVTTGSTVAEIAQLLLRNGAATVQVWCLCRTL

>gi|30043563|gb|AAP19283.1| hypothetical protein S4328 [Shigella flexneri 2a str. 2457T]

MIRISDAAQAHFAKLLANQEEGTQIRVFVINPGTPNAECGVSYCPPDAVEATDTALKFDLLTAYVDELSA

PYLEDAEIDFVTDQLGSQLTLKAPNAKMRKVADDAPLMERVEYMLQSQINPQLAGHGGRVSLMEITEDGY

AILQFGGGCNGCSMVDVTLKEGIEKQLLNEFPELKGVRDLTEHQRGEHSYY

>gi|30043562|gb|AAP19282.1| high-affinity transport permease for gluconate [Shigella flexneri 2a str. 2457T]

MPLVIVAIGVILLLLLLMIRFKMNGFIALVLVALAVGLMQGMPLDKVIGSIKAGVGGTLGSLALIMGFGAM

LGKMLADCGGAQRIATTLIAKFGKKHIQWAVVLTGFTVGFALFYEVGFVLMLPLVFTIAASANIPLLYVG

VPMAAALSVTHGFLPPHPGPTAIATIFNADMGKTLLYGTILAIPTVILAGPVYARVLKGIDKPIPEGLYS

AKTFSEEEMPSFGVSVWTSLVPVVLMAMRAIAEMILPKGHAFLPVAEFLGDPVMATLIAVLIAMFTFGLN

RGRSMDQINDTLVSSIKIIAMMLLIIGGGGAFKQVLVDSGVDKYIASMMHETNISPLLMAWSIAAVLRIA

LGSATVAAITAGGIAAPLIATTGVSPELMVIAVGSGSVIFSHVNDPGFWLFKEYFNLTIGETIKSWSMLE

TIISVCGLVGCLLLNMVI

>gi|30043561|gb|AAP19281.1| 4-alpha-glucanotransferase (amylomaltase) [Shigella flexneri 2a str. 2457T]

MESKRLDNAALAAGISPNYINAHGKPQSISAETKRRLLDAMHQRTATKVAVTPVPNVMVYTSGKKMPMVV

EGSGEYSWQLTTEEGTQYKGHVTGGKAFNLPTKLPEGYHTLTLTQDDQRAHCRVIVAPKRCYEPQALLNK

QKLWGACVQLYTLRSEKNWGIGDFGDLKAMLVDVAKRGGSFIGLNPIHALYPANPESASPYSPSSRRWLN

VIYIDVNAVEDFHLSEEAQAWWQLPTTQQTLQQARDADWVDYSTVTALKMTALRMAWKGFAQRDDEQMAA

FRQFVAEQGDSLFWQAAFDALHAQQVKEDEMRWGWPAWPEMYKNVDSPEVRQFCEEHRDDVDFYLWLQWL

AYSQFAACWEISQGYEMPIGLYRDLAVGVAEGGAETWCDRELYCLKASVGAPPDILGPLGQNWGLPPMDP

HIITARAYEPFIELLRANMQNCGALRIDHVMSMLRLWWIPYGETADQGAYVHYPVDDLLSILALESKRHR

CMVIGEDLGTVPVEIVGKLRSSGVYSYKVLYFENDHEKTFRAPKAYPEQSMAVAATHDLPTLRGYWESGD

LTLGKTLGLYPDEVVLRGLYQDRELAKQGLLDALHKYGCLPKRAGHKASLMSMTPTLNRGLQRYIADSNS

ALLGLQPEDWLDMAEPVNIPGTSYQYKNWRRKLSATLESMFADDGVNKLLKDLDRRRRAAAKKK

>gi|30043560|gb|AAP19280.1| maltodextrin phosphorylase [Shigella flexneri 2a str. 2457T]

MSQPIFNDKQFQEVLSRQWQRYGLNSAAEMTPRQWWLAVSEALAEMLRAQPFAKPVANQRHVNYISMEFL

IGRLTGNNLLNLGWYQDVQDSLKAYDINLTDLLEEEIDPALGNGGLGRLAACFLDSMATVGQSATGYGLN

YQYGLFRQSFVDGKQVEAPDDWHRSNYPWFRHNEALDVQVGIGGKVMKDGRWEPEFTITGQAWDLPVVGY

RNGVAQPLRLWQATHAHPFDLTKFNDGDFLRAEQQGINAEKLTKVLYPNDNHTAGKKLRLMQQYFQCACS

VADILRRHHLAGRKLHELADYEVIQLNDTHPTIAIPELLRVLTDEHQMSWDDAWAITSKTFAYTNHTLMP

EALERWDVKLVKGLLPRHMQIINEINTRFKTLVEKTWPGDEKVWAKLAVVHDKQVHMANLCVVGGFAVNG

VAALHSDLVVKDLFPEYHQLWPNKFHNVTNGITPRRWIKQCNPALAALLDKSLQKEWANDLDQLINLEKF

ADDAKFRQQYREIKQANKVCLAEFVKVRTGIEINPQAIFDIQIKRLHEYKRQHLNLLHILALYKEIRENL

QADRVPRVFLFGAKAAPGYYLAKNIIFAINKVADVINNDPLVGDKLKVVFLPDYCVSAAEKLIPAADISE

QISTAGKEASGTGNMKLALNGALTVGTLDGANVEIAEKVGEENIFIFGHTVEQVKAILAKGYDPVKWRKK

DKVLDAVLKELESGKYSDGDKHAFDQMLHSIGKQGGDPYLVMADFAAYVEAQKQVDVLYRDQEAWTRAAI

LNTARCGMFSSDRSIRDYQARIWQAKR

>gi|30043559|gb|AAP19279.1| hypothetical protein S4323 [Shigella flexneri 2a str. 2457T]

MLEPLLAKIGIHQQTTLLRHGFYPAGGGVVATEVSPVASFSTLQLGERGNIVQMRGEVLLAGVPRHVAER

EIATLAGIFSLHEQNIHNLPRDQGPGNTVSLEVESENITERFFVVGEKRVSAEVVAAQLVKEVKRYLASP

AAVGEYLADQLVLPMALAGAGEFTVAHPSCHLLTNIAVVERFLPVRFGLIETDGVTRVSIE

>gi|30043558|gb|AAP19278.1| hypothetical protein S4321 [Shigella flexneri 2a str. 2457T]

MNYELLTTENAPVKMWTKGVPVEADARQQLINTAKMPFIFKHIAVMPDVHLGKGSTIGSVIPTKGAIIPA

AVGVDIGCGMNALRTALTAADLPENLAELRQAIETAVPHGRTTGRCKRDKGAWENPPFNVDAKWAELEAG

YQWLTQKYPRFLNTNNYKHLGTLGTGNHFIEICLDESEQVWIMLHSGSRGIGNAIGTYFIDLAQKEMQDQ

LETLPSRDLAYFMEGTEYFDDYLRAVAWAQLFASLNRDAMMENVVTALQSITQKTVRQPQTLAMEEINCH

HNYVQKEQLFGEEIYVTRKGAVSARAGQYGIIPGSMGAKSFIVRGLGNEESFCSCSHGAGRVMSRTKAKK

LFSVEDQIRATAHVECRKDAEVIDEIPMAYKDIDAVMAAQSDLVEVIYTLRQVVCVKG

>gi|30043557|gb|AAP19277.1| putative 2-component regulator [Shigella flexneri 2a str. 2457T]

MRKTVAFGFVGTVLDYAGRGSQRWSKWRPSLCIYQQESLVIDRLELLHDARSRSLFETLKRDIASVSPET

EVVGVEIELHNPWDFEEVYACLHDFARGYEFQPEKEDYLIHITTGTHVAQICWFLLAEARYLPARLIQSS

TPRKKEQPRGPGEVTIIDLDLSRYNAIASRFAEERQQTLDFLKSGIATRNPHFNRMIEQIEKVAIKSRAP

ILLNGPTGAGKSFLARRIFELKQARHQFSGAFVEVNCATLRGDTAMSTLFGHVKGAFTGARESREGLLRS

ANGGMLFLDEIGELGADEQAMLLKAIEEKTFYPFGSDRQVSSDFQLIAGTVRDLRQLVAEGKFREDLYAR

INLWTFTLPGLRQRQEDIEPNLDYEVERHATLTGDSVRFNTEARRAWLAFATSPQATWRGNFRELSASVT

RMATFATSGRITLDVVEDEINRLRYNWQESRPSALTALLGAEAENIDLFDRMQLEHVIAICRQAKSLSAA

GRQLFDVSRQGKASVNDADRLRKYLARFGLTWEAVQDQHSSS

>gi|30043556|gb|AAP19276.1| repressor of the glp operon [Shigella flexneri 2a str. 2457T]

MKQTQRHNGIIELVKQQGYVSTEELVEHFSVSPQTIRRDLNELAEQNLILRHHGGAALPSSSVNTPWHDR

KATQTEEKERIARKVAEQIPNGSTLFIDIGTTPEAVAHALLNHSNLRIVTNNLNVANTLMVKEDFRIILA

GGELRSRDGGIIGEATLDFISQFRLDFGILGISGIDSDGSLLEFDYHEVRTKRAIIENSRHVMLVVDHSK

FGRNAMVNMGSISMVDAVYTDAPPPVSVMQVLTDHHIQLELC

>gi|30043555|gb|AAP19275.1| protein of glp regulon [Shigella flexneri 2a str. 2457T]

MITSFANPRVAQAFVDYMATQGVILTIQQHNQSDVWLADESQAERVRAELARFLENPADPRYLAASWLAG

HTGSGLHYRRYPFFAALRERAGPVTWVMMIACVVVFIAMQILGDQEVMLWLAWPFDPTLKFEFWRYFTHA

LMHFSLMHILFNLLWWWYLGGAVEKRLGSGKLIVITLISALLSGYMQQKFSGPWFGGLSGVVYALMGYVW

LRGERDPQSGIYLQRGLIIFALIWIVAGWFDLFGMSMANGAHIAGLAVGLAMAFVDSLNARKRK

>gi|30043554|gb|AAP19274.1| protein of glp regulon [Shigella flexneri 2a str. 2457T]

MDQFECINVADAHQKLQEKEAVLVDIRDPQSFAMGHAVQAFHLTNDTLGAFMRDNDFDTPVMVMCYHGNS

SKGAAQYLLQQGYDVVYSIDGGFEAWQRQFPAEVAYGA

>gi|30043553|gb|AAP19273.1| sn-glycerol-3-phosphate dehydrogenase (aerobic) [Shigella flexneri 2a str. 2457T]

MAASMVLVSRADAAGRGLSVLMLEAQDLACATSSASSKLIHGGLRYLEHYEFRLVSEALAEREVLLKMAP

HIAFPMRFRLPHRPHLRPAWMIRIGLFMYDHLGKRTSLPGSTGLRFGANSVLKPEIKRGFEYSDCWVDDA

RLVLANAQMVVRKGGEVLTRTRATSARRENGLWIVEAEDIDTGKKYSWQARGLVNATGPWVKQFFDEGMH

LPSPYGIRLIKGSHIVVPRVHTQKQAYILQNEDKRIVFVIPWMDEFSIIGTTDVEYKGDPKAVKIEESEI

NYLLKVYNAHFKKQLSRDDIVWTYSGVRPLCDDESDSPQAITRDYTLDIHDENGKAPLLSVFGGKLTTYR

KLAEHALEKLTPYYQGIGPAWTKESVLPGGAIEGDRDDYAARLRRRYPFLTESLARHYTRTYGSNSELLL

GNAGTVSDLGEDFGHEFYEAELKYLVDHEWVRRTDDALWRRTKQGMWLNADQQSRVSQWLVEYTQQKLSL

AS

>gi|30043552|gb|AAP19272.1| IS4 orf [Shigella flexneri 2a str. 2457T]

MTDAMRFPGGEMADLYSHRWEIELGYREIKQTMQLSRLTLRSKKPELVEQELWGVLLAYNLVRYQMIKMA

EHLKGYWPNQLSFSESCGMVMRMLMTLQGASPGRIPELMRDLASMGQLVKLPTRRGRAFPRVVKERPWKY

PTAPKKSQSVA

>gi|30043551|gb|AAP19271.1| IS4 orf [Shigella flexneri 2a str. 2457T]

MHIGQALDLVSRYDSLRNPLTSLGDYLDPELISRCLAESGTVTLRKRRLPLEMMVWCIVGMALERKEPLH

QIVNRLDIMLPGNRPFVAPSAVIQARQRLGSEAVRRVFTKTAQLWHNATPHPHWCGLTLLAIDGVFWRTP

DTPENDAAFPRQTHAGNPALYPQVKMVCQMELTSHLLTAAAFGTMKNSENELAEQLIEQTGDNTLTLMDK

GYYSLGLLNGWSLAGEHRHWMIPLRKGAQYEELRKLGKGDHLVKLKTSPQARKKWPGLGNEVTARLLTVR

AKEKSAIC

>gi|30043550|gb|AAP19270.1| glycogen phosphorylase [Shigella flexneri 2a str. 2457T]

MNAPFTYSSPTLSVEALKHSIAYKLMFTIGKDPVVANKHEWLNATLFAVRDRLVERWLRSNRAQLSQETR

QVYYLSMEFLIGRTLSNAMLSLGIYEDVQGALEAMGLNLEELIDEENDPGLGNGGLGRLAACFLDSLATL

GLPGRGYGIRYDYGMFKQNIVNGSQKESPDYWLEYGNPWEFKRHNTRYKVRFGGRIQQEGKKTRWIETEE

ILGVAYDQIIPGYDTDATNTLRLWSAQASSEINLGKFNQGDYFAAVEDKNHSENVSRVLYPDDSTYSGRE

LRLRQEYFLVSSTIQDILSRHYQLHKTYDNLADKIAIHLNDTHPVLSIPEMMRLLIDEHQFSWDDAFEVC

CQVFSYTNHTLMSEALETWPVDMLGKILPRHLQIIFEINDYFLKTLQEQYPNDTDLLGRASIIDESNGRR

VRMAWLAVVVSHKVNGVSELHSNLMVQSLFADFAKIFPGRFTNVTNGVTPRRWLAVANPSLSAVLDEHLG

RNWRTDLSLLNELQQHCDFPMVNHAVHQAKLENKKRLAEYIAQQLNVVVNPKALFDVQIKRIHEYKRQLM

NVLHVITRYNRIKADPDAKWVPRVNIFGGKAASAYYMAKHIIHLINDVAKVINNDPQIGDKLKVVFIPNY

SVSLAQLIIPAADLSEQISLAGTEASGTSNMKFALNGALTIGTLDGANVEMLDHVGADNIFIFGNTAEEV

EELRRQGYKPREYYEKDEELHQVLTQIGSGVFSPEDPGRYRDLVDSLINFGDHYQVLADYRSYVDCQDKV

DELYELQEEWTAKAMLNIANMGYFSSDRTIKEYADHIWHIDPVRL

>gi|30043549|gb|AAP19269.1| glycogen synthase [Shigella flexneri 2a str. 2457T]

MQVLHVCSEMFPLLKTGGLADVIGALPAAQIADGVDARVLLPAFPDIRRGVTDAQVVSRRDTFAGHITLL

FGHYNGVGIYLIDAPHLYDRPGSPYHDTNLFAYTDNVLRFALLGWVGAEMASGLDPFWRPDVVHVHDWHA

GLAPAYLAARGRPAKSVFTVHNLAYQGMFYAHHMNDIQLPWSFFNIHGLEFNGQISFLKAGLYYADHITA

VSPTYAREITEPQFAYGMEGLLQQRHREGRLSGVLNGVDEKIWSPETDLLLASRYTRDTLEDKAENKSQL

QIAMGLKVDDKVPLFAVVSRLTSQKGLDLVLEALPGLLEQGGQLALLGAGDPVLQEGFLAAAAEYPGQVG

VQIGYHEAFSHRIMGGADVILVPSRFEPCGLTQLYGLKYGTLPLVRRTGGLADTVSDCSLENLADGVASG

FVFEDSNAWSLLRAIRRAFVLWSRPSLWRFVQRQAMAMDFSWQVAAKSYRELYYRLK

>gi|30043548|gb|AAP19268.1| glucose-1-phosphate adenylyltransferase [Shigella flexneri 2a str. 2457T]

MVSLEKNDHLMLARQLPLKSVALILAGGRGTRLKDLTNKRAKPAVHFGGKFRIIDFALSNCINSGIRRMG

VITQYQSHTLVQHIQRGWSFFNEEMNEFVDLLPAQQRMKGENWYRGTADAVTQNLDIIRRYKAEYVVILA

GDHIYKQDYSRMLIDHVEKGARCTVACMPVPIEEASAFGVMAVDENDKIIEFVEKPANPPSMPNDPSKSL

ASMGIYVFDADYLYELLEEDDRDENSSHDFGKDLIPKITEAGLAYAHPFPLSCVQSDPDAEPYWRDVGTL

EAYWKANLDLASVVPELDMYDRNWPIRTYNESLPPAKFVQDRSGSHGMTLNSLVSGGCVISGSVVVQSVL

FSRVRVNSFCNIDSAVLLPEVWVGRSCRLRRCVIDRACVIPEGMVIGENAEEDARRFYRSEEGIVLVTRE

MLRKLGHKQER

>gi|30043547|gb|AAP19267.1| glycosyl hydrolase, debranching enzyme [Shigella flexneri 2a str. 2457T]

MTQLAIGKPAPLGAHYDGQGVNFTLFSAHAERVELCVFDANGQEHRYDLPGNSGDIWHGYLPDARPGLRY

GYRVHGPWQPAEGHRFNPAKLLIDPCARQIDGEFKDNPLLHAGHNEPDYRDNAAIAPKCVVVVDHYDWED

DAPPRTPWGSTIIYEAHVKGLTYLHPEIPVEIRGTYKALGHPVMINYLKQLGITALELLPVAQFASEPRL

QRMGLSNYWGYNPVAMFALHPAYACSPETALDEFRDAIKALHKAGIEVILDIVLNHSAELDLDGPLFSLR

GIDNRSYYWIREDGDYHNWTGCGNTLNLSHPAVVDYASACLRYWVETCHVDGFRFDLAAVMGRTPEFRQD

APLFTAIQNCPVLSQVKLIAEPWDIAPSGYQVGNFPPLFAEWNDHFRDAARRFWLHYDLPLGAFAGRFAA

SSDVFKRNGRLPSAAINLVTAHDGFTLRDCVCFNHKHNEANGEENRDGTNNNYSNNHGKEGLGGTLDLVE

RRRDSIHALLTTLLLSQGTPMLLAGDEHGHSQHGNNNAYCQDNQLNWLDWSQASSGLTAFTAALIHLRKR

IPALVENRWWEEGDGNVRWLNRYAQPLSTDEWQNGPKQLQILLSDRFLIAINATLEVTEIVLPAGEWHAI

PPFAGEDNPVITVVWQGPAHGLCVFQR

>gi|30043546|gb|AAP19266.1| 1,4-alpha-glucan branching enzyme [Shigella flexneri 2a str. 2457T]

MSDRIDRDVINALIAGHFADPFSVLGMHKTTAGLEVRALLPDATDVWVIEPKTGRKLAKLECLDSRGFFS

GVIPRRKNFFRYQLAVVWHGQQNLIDDPYRFGPLIQEMDAWLLSEGTHLRPYETLGAHADTMDGVTGTRF

SVWAPNARRVSVVGQFNYWDGRRHPMRLRKESGIWELFIPGAHNGQLYKYEMIDANGNLRLKSDPYAFEA

QMRPETASLICGLPEKVVQTEERKKANQFDAPISIYEVHLGSWRRHTDNNFWLSYRELADQLVPYAKWMG

FTHLELLPINEHPFDGSWGYQPTGLYAPTRRFGTRDDFRYFIDAAHAAGLNVILDWVPGHFPTDDFALAE

FDGTNLYEHSDPREGYHQDWNTLIYNYGRREVSNFLVGNALYWIERFGIDALRVDAVASMIYRDYSRKEG

EWIPNEFGGRENLEAIEFLRNTNRILGEQVSGAVTMAEESTDFPGVSRPQDMGGLGFWYKWNLGWMHDTL

DYMKLDPIYRQYHHDKLTFGMLYNYTENFVLPLSHDEVVHGKKSILDRMPGDAWQKFANLRAYYGWMWAF

PGKKLLFMGNEFAQGREWNHDASLDWHLLEGGDNWHHGVQRLVRDLNLTYRHHKAMHELDFDPYGFEWLV

VDDKERSVLIFVRRDKEGNEIIVASNFTPVPRHDYRFGINQPGKWREILNTDSIHYHGSNAGNGGTVHSD

EIASHGRQHSLSLTLPPLATIWLVREAE

>gi|30043545|gb|AAP19265.1| aspartate-semialdehyde dehydrogenase [Shigella flexneri 2a str. 2457T]

MKNVGFIGWRGMVGSVLMQRMVEERDFDAIRPVFFSTSQLGQAAPSFGGTTGTLQDAFDLEALKALDIIV

TCQGGDYTNEIYPKLRESGWQGYWIDAASSLRMKDDAIIILDPVNQDVITDGLNNGIRTFVGGNCTVSLM

LMSLGGLFANDLVDWVSVATYQAASGGGARHMRELLTQMGHLYGHVADELATPSSAILDIERKVTTLTRS

GELPVDNFGVPLAGSLIPWIDKQLDNGQSREEWKGQAETNKILNTSSVIPVDGLCVRVGALRCHSQAFTI

KLKKDVSIPTVEELLAAHNPWAKVVPNDREITMRELTPAAVTGTLTTPVGRLRKLNMGPEFLSAFTVGDQ

LLWGAAEPLRRMLRQLA

>gi|30043544|gb|AAP19264.1| hypothetical protein S4306 [Shigella flexneri 2a str. 2457T]

MNEIISAAVLLILIMDPLGNLPIFMSVLKHTEPKRRRAIMVRELLIALLVMLVFLFAGEKILAFLSLRAE

TVSISGGIILFLIAIKMIFPSASGNSSGLPAGEEPFIVPLAIPLVAGPTILATLMLLSHQYPNQMGHLVI

ALLLAWGGTFVILLQSSLFLRLLGEKGVNALERLMGLILVMMATQMFLDGIRMWMKG

>gi|30043543|gb|AAP19263.1| low-affinity gluconate transport permease protein [Shigella flexneri 2a str. 2457T]

MKARMHAFLALMVVSMGAGLFSGMPLDKIAATMEKGMGGTLGFLAVVVALGAMFGKILYETGAVDQIAVK

MLKSFGHSRAHYAIGLAGLVCALPLFFEVAIVLLISVAFSMARHTGTNLVKLVIPLFAGVAAAAAFLVPG

PAPMLLASQMNADFGWMILIGLCAAIPGMIIAGPLWGNFISRYVELHIPDDISEPHLGEGKMPSFGFSLS

LILLPLVLVGLKTIAARFVPEGSTAYEWFEFIGHPFTAILVACLVAIYGLAMRQGMPKDKVMEICGHALQ

PAGIILLVIGAGGVFKQVLVDSGVGPALGEALTGMGLPIAITCFVLAAAVRIIQGAATVACLTAVGLVMP

VIEQLNYSGAQMAALSICIAGGSIVVSHVNDAGFWLFGKFTGATEAETLKTWTMMETILGTVGAIVGMIA

FQLLS

>gi|30043542|gb|AAP19262.1| gluconokinase 2 [Shigella flexneri 2a str. 2457T]

MGVSGSGKSAVASEVAHQLHAAFLDGDFLHPRRNIEKMASGEPLNDDDRKPWLQALNDSAFAMQRTNKVS

LIACSALKKHYRDLLREGNPNLSFIYLKGDFDVIESRLKARKGHFFKTQMLVTQFETLQEPGADETDVLV

VDIDQPLEGVVASTIEVIKKGK

>gi|30043541|gb|AAP19261.1| regulator of gluconate (gnt) operon [Shigella flexneri 2a str. 2457T]

MTVSRFLRNPEQVSVALRGKIAAALDELGYIPNRAPDILSNATSRAIGVLLPSLTNQVFAEVLRGIESVT

DAHGYQTMLAHYGYKPEMEQERLESMLSWNIDGLILTERTHTPRTLKMIEVAGIPVVELMDSQSPCLDIA

VGFDNFEAARQMTTAIIARGHRHIAYLGARLDERTIIKQKGYEQAMLDAGLVPYSVMVEQSSSYSSGIEL

IRQARREYPQLDGVFCTNDDLAVGAAFECQRLGLKVPDDMAIAGFHGHDIGQVMEPRLASVLTPRERMGS

IGAERLLARIRGESVTPKMLDLGFTLSPGGSI

>gi|30043540|gb|AAP19260.1| hypothetical protein S4301 [Shigella flexneri 2a str. 2457T]

MIYLRKANERGHANHGWLDSWHTFSCANYYDPNFMGFSALRVINDDVIEAGQGFGTHPHKDMEILTYVLE

GTVEHQDSMGNKEQVPAGEFQIMSAGTGIRHSEYNPSSTERLHLYQIWIMPEENGITPRYEQRRFDAVQG

KQLVLSPDARDGSLKVHQDMELYRWALLKDEQSVHQIAAERRVWIQVVKGNVTINGVKASTSDGLAIWDE

QAISIHADSDSEVLLFDLPPV

>gi|30043539|gb|AAP19259.1| putative regulator [Shigella flexneri 2a str. 2457T]

MVINCAFIGFGKSTTRYHLPYVLNRKDSWHVAHIFRRHAKPEEQAPIYSHIHFTSDLDEVLNDPDVKLVV

VCTHADSHFEYAKRALEAGKNVLVEKTFTPTLAQAKELFALAKSKGLTVTPYQNRRFDSCFLTAKKAIES

GKLGEIVEVESHFDYYRPVAETKPGLPQDGAFYGLGVHTMDQIISLFGRPDHVAYDIRSLRNKANPDDTF

EAQLFYGDLKAIVKTSHLVKIDYPKFIVHGKKGSFIKYGIDQQETSLKANIMPGEPGFAADDSVGVLEYV

NDEGVTVREEMKPEMGDYGRVYDALYQTITHGAPNYVKESEVLTNLEILERGFEQASPSTVTLAN

>gi|30043538|gb|AAP19258.1| hypothetical protein S4299 [Shigella flexneri 2a str. 2457T]

MSEIVIRHAETRDYEAIRQIHAQPEVYCNTLQVPHPSDHMWQERLADRPGIKQLVACIDGDVVGHLTIDV

QQRPRRSHVADFGICVDSRWKNRGVASALMREMIEMCDNWLRVDRIELTVFVDNAPAIKVYKKFGFEIEG

TGKKYALRNGEYVDAYYMARVK

>gi|30043537|gb|AAP19257.1| gamma-glutamyltranspeptidase [Shigella flexneri 2a str. 2457T]

MIKPTFLRRVAIAALLSGSCFSAAAAPPAPPVSYGVEEDVFHPVRAKQGMVASVDATATQVGVDILKEGG

NAVDAAVAVGYALAVTHPQAGNLGGGGFMLIRSKNGNTTAIDFREMAPAKATRDMFLDDQGNPDSKKSLT

SHLASGTPGTVAGFSLALDKYGTMPLNKVVQPAFKLARDGFIVNDALADDLKTYGSEVLPNHENSKAIFW

KEGEPLKKGDTLVQANLAKSLEMIAENGPDEFYKGTIAEQIAQEMQKNGGLITKEDLAAYKAVERTPISG

DYRGYQVYSMPPPSSGGIHIVQILNILENFDMKKYGFGSADAMQIMAEAEKYAYADRSEYLGDPDFVKVP

WQALTNKAYAKSIADQIDIKKAKPSSEIRPGKLAPYESNQTTHYSVVDKDGNAVAVTYTLNTTFGTGIVA

GESGILLNNQMDDFSAKPGVPNVYGLVGGDANAVGPNKRPLSSMSPTIVVKDGKTWLVTGSPGGSRIITT

VLQMVVNSIDYGMNVAEATNAPRFHHQWLPDELRVEKGFSPDTLKLLEAKGQKVALKEAMGSTQSIMVGP

DGELYGASDPRSVDDLTAGY

>gi|30043536|gb|AAP19256.1| hypothetical protein S4297 [Shigella flexneri 2a str. 2457T]

MKRLLILTALLPFVGFAQPINTLNNPNQPGYQIPSQQRMQTQMQTQQIQQKGMLNQQLKTQTQLQQQHLE

NQINNNSQRVLQSQPGERNPARQQMLPNTNGGMLNSNRNPDSSLNQQHMLPERRNGDMLNQPSTPQPDIP

LKTIGP

>gi|30043535|gb|AAP19255.1| glycerophosphodiester phosphodiesterase [Shigella flexneri 2a str. 2457T]

MSNWPYPRIVAHRGGGKLAPENTLAAIDVGAKYGHKMIEFDAKLSKDGEIFLLHDDNLERTSNGWGVAGE

LNWQDLLRVDAGSWYSKAFKGEPLPLLSQVAERCREHGMMANIEIKPTTGTGPLTGKMVALAARQLWAGM

TPPLLSSFEIDALEAAQQAAPELPRGLLLDEWRDDWRELTARLGCVSIHLNHKLLDKARVMQLKDAGLRI

LVYTVNKPQHAAELLRWGVDCICTDAIDVIGPNFTAQ

>gi|30043534|gb|AAP19254.1| sn-glycerol 3-phosphate transport protein, integral membrane protein [Shigella flexneri 2a str. 2457T]

MIENRPWLTIFSHTMLILGIAVILFPLYVAFVAATLDKQAVYAAPMTLIPGTHLLENIHNIWVNGVGTNS

APFWRMLLNSFVMAFSITLGKITVSMLSAFAIVWFRFPLRNLFFWMIFITLMLPVEVRIFPTVEVIANLK

MLDSYAGLTLPLMASATATFLFRQFFMTLPDELVEAARIDGASPMRFFCDIVFPLSKTNLAALFVITFIY

GWNQYLWPLLIITDVDLGTTVAGIKGMIATGEGTTEWNSVMAAMLLTLIPPVVIVLVMQRAFVRGLVDSE

K

>gi|30043533|gb|AAP19253.1| sn-glycerol 3-phosphate transport protein, integral membrane protein [Shigella flexneri 2a str. 2457T]

MSSSRPVFRSRWLPYLLVAPQLIITVIFFIWPAGEALWYSLQSVDPFGFSSQFVGLDNFVTLFHDSYYLD

SFWTTIKFSTFVTVSGLLVSLFFAALVEYIVRGSRFYQTLMLLPYAVAPAVAAVLWIFLFNPGRGLITHF

LAEFGYDWNHAQNSGQAMFLVVFASVWKQISYNFLFFYAALQSIPRSLIEAAAIDGAGPIRRFFKIALPL

IAPVSFFLLVVNLVYAFFDTFPVIDAATSGGPVQATTTLIYKIYREGFTGLDLASSAAQSMVLMFLVIVL

TVVQFRYVESKVRYQ

>gi|30043532|gb|AAP19252.1| sn-glycerol 3-phosphate transport protein, periplasmic binding protein [Shigella flexneri 2a str. 2457T]

MKPLHYTASALALGLALMGNAQAVTTIPFWHSMEGELGKEVDSLAQRFNAENPDYKIVPTYKGNYEQNLS

AGIAVFRTGNAPAILQVYEVGTATMMASKAIKPVYDVFKEAGIQFDESQFVPTVSGYYSDSKTGHLLSQP

FNSSTSVLYYNKDAFKKAGLDPEQPPKTWQDLADYSAKLKASGIKCGYASGWQGWIQLENFSAWNGLPFA

SKNNGFDGTDAVLEFNKPEQVKHIAMLEEMNKKGDFSYVGRKDESTEKFYNGDCAMTTASSGSLANIREY

AKFNYGVGMMPYDADAKDAPQNAIIGGASLWVMQGKDKETYTGVAKFLDFLAKPENAAEWHQKTGYLPIT

KAAYDLTREQGFYEKNPGADTATRQMLNKPPLPFTKGLRLGNMPQIRVIVDEELESVWTGKKTPQQALDT

AVERGNQLLRRFEKSTKS

>gi|30043531|gb|AAP19251.1| ATP-binding component of leucine transport [Shigella flexneri 2a str. 2457T]

MEKVMLSFDKVSAHYGKIQALHEVSLHINQGEIVTLIGANGAGKTTLLGTLCGDPRATSGRIVFDDKDIT

DWQTAKIMREAVAIVPEGRRVFSRMTVEENLAMGGFFAERDQFQERIKWVYELFPRLHERRVQRAGTMSG

GEQQMLAIGRALMSNPRLLLLDEPSLGLAPIIIQQIFDTIEQLREQGMTIFLVEQNANQALKLADRGYVL

ENGHVVLSDTGDALLANEAVRSAYLGG

>gi|30043530|gb|AAP19250.1| ATP-binding component of high-affinity branched-chain amino acid transport system [Shigella flexneri 2a str. 2457T]

MSQPLLSVNGLMMRFGGLLAVNNVNLELYPQEIVSLIGPNGAGKTTVFNCLTGFYKPTGGTILLRDQHLE

GLPGQQIARMGVVRTFQHVRLFREMTVIENLLVAQHQQLKTGLFSGLLKTPSFRRAQSEALDRAATWLER

IGLLEHANRQASNLAYGDQRRLEIARCMVTQPEILMLDEPAAGLNPKETKELDELIAELRNHHNTTILLI

EHDMKLVMGISDRIYVVNQGTPLANGTPEQIRNNPDVIRAYLGEA

>gi|30043529|gb|AAP19249.1| high-affinity branched-chain amino acid transport protein, membrane component [Shigella flexneri 2a str. 2457T]

MKPMHIAMALLSAAMFFVLAGVFMGVQLELDGTKLVVDTASDIRWQWMFIGTAVVFFFQLLRPAFQKGLK

SVSGPKFILPAIDGSTVKQKLFLVALLVLVVAWPFMVSRGTVDIATLTMIYIILGLGLNVVVGLSGLLVL

GYGGFYAIGAYTFALLNHYYGLGFWTCLPIAGLMAAAAGFLLGFPVLRLRGDYLAIVTLGFGEIVRILLL

NNTEITGGPNGISQIPKPTLFGLEFSRTAREGGWDTFSNFFGLKYDPSDRVIFLYLVALLLVVLSLFVIN

RLLRMPLGRAWEALREDEIACRSLGLSPRRIKLTAFTISAAFAGFAGTLFAARQGFVSPESFTFAESAFV

LAIVVLGGMGSQFAVILAAVLLVVSRELMRDFNEYSMLMLGGLMVLMMIWRPQGLLPMTRPQLKLKNGAA

KGEQA

>gi|30043528|gb|AAP19248.1| high-affinity branched-chain amino acid transport protein, membrane component [Shigella flexneri 2a str. 2457T]

MSEQFLYFLQQMFNGVTLGSTYALIAIGYTMVYGIIGMINFAHGEVYMIGSYVSFMIIAALMMMGIDTGW

LLVAAGFVGAIVIASAYGWSIERVAYRPVRNSKRLIALISAIGMSIFLQNYVSLTEGSRDVALPSLFNGQ

WVVGHSENFSASITTMQAVIWIVTFLAMLALTIFIRYSRMGRACRACAEDLKMASLLGINTDRVIALTFV

IGAAMAAVAGVLLGQFYCVINPYIGFMAGMKAFTAAVLGGIGSIPGAMIGGLILGIAEALSSAYLSTEYK

DVVSFALLILVLLVMPTGILGRPEVEKV

>gi|30043527|gb|AAP19247.1| high-affinity leucine-specific transport protein, periplasmic binding protein [Shigella flexneri 2a str. 2457T]

MKRNAKTIIAGMIALAISHTAMADDIKVAVVGAMSGPIAQWGDMEFNGARQAIKDINVKGGIKGDKLVGV

EYDDACDPKQAVAVANKIVNDGIKYVIGHLCSSSTQPASDIYEDEGILMISPGATNPELTQRGYQHIMRT

AGLDSSQGPTAAKYILETVKPQRIAIIHDKQQYGEGLARSVQDGLKAANANVVFFDGITAGEKDFSALIA

RLKKENIDFVYYGGYYPEMGQMLRQARSVGLKTQFMGPEGVGNASLSNIAGDAAEGMLVTMPKRYDQDPA

NQGIVDALKADKKDPSGPYVWITYAAVQSLATALERTGSDEPLALVKDLKANGANTVIGPLNWDEKGDLK

GFDFGVFQWHADGSSTAAK

>gi|30043526|gb|AAP19246.1| hypothetical protein S4286 [Shigella flexneri 2a str. 2457T]

MKLTIIRLENFSDQDRIDLQKIWPEYSPSSLQVDDNHRIYAARFNERLLAAVRVTLSGTEGALDSLRVRE

VTRRRGVGQYLLEEVLRNNPGVSCWWMADAGVEDRGVMTAFMQALGFTAQQGGWEKR

>gi|30043525|gb|AAP19245.1| high-affinity amino acid transport protein, periplasmic binding protein [Shigella flexneri 2a str. 2457T]

MLNNNITTHVITRRMGILRMNIKGKALLAGCIALAFSNMALAEDIKVAVVGAMSGPVAQYGDQEFTGAEQ

AVADINAKGGIKGNKLQIVKYDDACDPKQAVAVANKVVNDGIKYVIGHLCSSSTQPASDIYEDEGILMIT

PAATAPELTARGYQLILRTTGLDSDQGPTAAKYILEKVKPQRIAIVHDKQQYGEGLARAVQDGLKKGNAN

VVFFDGITAGEKDFSTLVARLKKENIDFVYYGGYHPEMGQILRQARAAGLKTQFMGPEGVANVSLSNIAG

ESAEGLLVTKPKNYDQVPANKPIVDAIKAKKQDPSGAFVWTTYAALQSLQAGLNQSDDPAEIAKYLKANS

VDTVMGPLTWDEKGDLKGFEFGVFDWHANGTATDAK

>gi|30043524|gb|AAP19244.1| sigma(32) factor of RNA polymerase [Shigella flexneri 2a str. 2457T]

MTDKMQSLALAPVGNLDSYIRAANAWPMLSADEERALAEKLHYHGDLEAAKTLILSHLRFVVHIARNYAG

YGLPQADLIQEGNIGLMKAVRRFNPEVGVRLVSFAVHWIKAEIHEYVLRNWRIVKVATTKAQRKLFFNLR

KTKQRLGWFNQDEVEMVARELGVTSKDVREMESRMAAQDMTFDLSSDDDSDSQPMAPVLYLQDKSSNFAD

GIEDDNWEEQAANRLTDAMHGLDERSQDIIRARWLDEDNKSTLQELADRYGVSAERVRQLEKNAMKKLRA

AIEA

>gi|30043523|gb|AAP19243.1| cell division membrane protein [Shigella flexneri 2a str. 2457T]

MNKRDAINHIRQFGGRLDRFRKSVGGSGDGGRNAPKRAKSSPKPVNRKTNVFNEQVRYAFHGALQDLKSK

PFATFLTVMVIAISLTLPSVCYMVYKNVNQAATQYYPSPQITVYLQKTLDDDAAAGVVAQLQAEQGVEKV

NYLSREDALGEFRNWSGFGGALDMLEENPLPAVAVVIPKLDFQGTESLNTLRDRITQINGIDEVRMDDSW

FARLAALTGLVGRVSAMIGVLMVAAVFLVIGNSVRLSIFARRDSINVQKLIGATDGFILRPFLYGGALLG

FSGALLSLILSEILVLRLSSAVAEVAQVFGTKFDINGLSFDECLLLLLVCSMIGWVAAWLATVQHLRHFT

PE

>gi|30043522|gb|AAP19242.1| ATP-binding component of a membrane-associated complex involved in cell division [Shigella flexneri 2a str. 2457T]

MIRFEHVSKAYLGGRQALQGVTFHMQPGEMAFLTGHSGAGKSTLLKLICGIERPSAGKIWFSGHDITRLK

NREVPFLRRQIGMIFQDHHLLMDRTVYDNVAIPLIIAGASGDDIRRRVSAALDKVGLLDKAKNFPIQLSG

GEQQRVGIARAVVNKPAVLLADEPTGNLDDALSEGILRLFEEFNRVGVTVLMATHDINLISRRSYRMLTL

SDGHLHGGVGHE

>gi|30043521|gb|AAP19241.1| cell division membrane protein [Shigella flexneri 2a str. 2457T]

MAKEKRRGFFSWLGFGQKEQTPEKETEVQNEQPVVEEIVQAQEPAKASEQAVEEQPQAHTEAEAETFAAD

VVEVTEQVVESEKAQPEAEVVAQPEPVVEETPEPVAIEREELPLPEDVNAEEVSPEEWQAEAETVEIVEA

AEEEAAKEEITDEELEAQALAAEAAEEAVMVVPPAEEEQPVAEIAQEQEKPTKEGFFARLKRSLLKTKEN

LGSGFISLFRGKKIDDDLFEELEEQLLIADVGVETTRKIITNLTEGASRKQLRDAEALYGLLKEEMGEIL

AKVDEPLNVEDKTPFVILMVGVNGVGKTTTIGKLARQFEQQGKSVMLAAGDTFRAAAVEQLQVWGQRNNI

PVIAQHTGADSASVIFDAIQAAKARNIDVLIADTAGRLQNKSHLMEELKKIVRVMKKLDVEAPHEVMLTI

DASTGQNAVSQAKLFHEAVGLTGITLTKLDGTAKGGVIFSVADQFGIPIRYIGVGERIEDLRPFKADDFI

EALFARED

>gi|30043520|gb|AAP19240.1| hypothetical protein S4280 [Shigella flexneri 2a str. 2457T]

MKKPNHSGSGQIRIIGGQWRGRKLPVPDSPGLRPTTDRVRETLFNWLAPVIVDAQCLDCFAGSGALGLEA

LSRYAAGATLIEMDRVVSQQLIKNLATLKAGNARVVNSNAMSFLAQKGTPHNIVFVDPPFRRGLLEETIN

LLEDNGWLADEALIYVESEVENGLPTVPANWSLHREKVAGQVAYRLYQHEAQGESDAD

>gi|30043519|gb|AAP19239.1| hypothetical protein S4279 [Shigella flexneri 2a str. 2457T]

MLINIGRLLMLCVWGFLILNLVHPFPRPLNIFVNVALIFTVLMHGMQLALLKSTLPKDGPQMTTAEKVRI

FLFGVFELLVWQKKFKVKK

>gi|30043518|gb|AAP19238.1| putative receptor [Shigella flexneri 2a str. 2457T]

MSKPPLFFIVIIGLIVVAASFRFMQQRREKADNDMAPLQQKLVVVSNKREKPINDRRSRQQEVTPAGTSM

RYEASFKPQSGGMEQTFRLDAQQYHALTVGDKGTLSYKGTRFVSFVGEQ

>gi|30043517|gb|AAP19237.1| putative enzyme [Shigella flexneri 2a str. 2457T]

MLWSFIAVCLSAWLSVDASYRGPTWQRWVFKPLTLLLLLLLAWQAPMFDAISYLVLAGLCASLLGDALTL

LPRQRLMYAIGAFFLSHLLYTIYFASQMTLSFFWPLPLVLLVLGALLLAIIWTRLEEYRWPICTFIGMTL

VMVWLAGELWFFRPTAPALSAFVGASLLFISNFVWLGSHYRRRFRADNAIAAACYFAGHFLIVRSLYL

>gi|30043516|gb|AAP19236.1| zinc-transporting ATPase [Shigella flexneri 2a str. 2457T]

MSTPDNHGKKAPQFAAFKPLTTVQNANDCCCDGACSSSPTLSENVSGTRYSWKVSGMDCAACARKVENAV

RQLAGVNQVQVLFATEKLVVDADNDIRAQVESAVQKAGYSLRDEQAADEPQASRLKENLPLITLIVMMAI

SWGLEQFNHPFGQLAFIATTLVGLYPIARQALRLIKSGSYFAIETLMSVAAIGALFIGATAEAAMVLLLF

LIGERLEGWAASRARQGVSALMALKPETATRLRNGEREEVAINSLRPGDVIEVAAGGRLPADGKLLSPFA

SFDESALTGESIPVERATGDKVPAGATSVDRLVTLEVLSEPGASAIDRILKLIEEAEERRAPIERFIDRF

SRIYTPAIMAVALLVTLVPPLLFAASWQEWIYKGLTLLLIGCPCALVISTPAAITSGLAAAARRGALIKG

GAALEQLGRVTQVAFDKTGTLTVGKPRVTAIHPATGISESELLTLAAAVEQGATHPLAQAIVREAQVAEL

AIPTAESQRALVGSGIEAQVNGERVLICAAGKHPADAFAGLINELESAGQTVVLVVRNDDVLGIIALQDT

LRADATTAISELNALGVKGVILTGDNPRAAAAIAGELGLEFKAGLLPEDKVKAVTKLNQHAPLAMVGDGI

NDAPAMKAAAIGIAMGSGTDVALETADAALTHNHLRGLVQMIELARATHANIRQNITIALGLKGIFLVTT

LLGMTGLWLAVLADTGATVLVTANALRLLRRR

>gi|30043515|gb|AAP19235.1| hypothetical protein S4275 [Shigella flexneri 2a str. 2457T]

MTDLFSSPDHTLDALGLRCPEPVMMVRKTVRNMQPGETLLIIADDPATTRDIPGFCTFMEHELVAKETDG

LPYRYLIRKGG

>gi|30043514|gb|AAP19234.1| hypothetical protein S4274 [Shigella flexneri 2a str. 2457T]

MNVFSQTQRYKALFWLSLFHLLVITSSNYLVQLPVSIFGFHTTWGAFSFPFIFLATDLTVRIFGAPLARR

IIFAVMIPALLISYVISSLFYMGSWQGFGALAHFNLFVARIATASFMAYALGQILDVHVFNRLRQSRRWW

LAPTASTLFGNVSDTLAFFFIAFWRSPDAFMAEHWMEIALVDYCFKVLISIVFFLPMYGVLLNMLLKRLA

DKSEINALQAS

>gi|30043513|gb|AAP19233.1| hypothetical protein S4273 [Shigella flexneri 2a str. 2457T]

MIRWMNEPLWPFIERKKSMRNLVKYVGIGLLVMGLAACDDKDTNATAQGSVAESNATGNPVNLLDGKLSF

SLPADMTDQSGKLGTQANNMHVWSDATGQKAVIVIMGDDPKEDLAVLAKRLEDQQRSRDPQLQVVTNKAI

ELKGHKMQQLDSIISAKGQTAYSSVILGNVGNQLLTMQITLPADDQQKAQTTAENIINTLVIQ

>gi|30043512|gb|AAP19232.1| putative transport [Shigella flexneri 2a str. 2457T]

MVKMKHCCKNVVILMPEPVAEPALNGLRLNLRIVSIVMFNFASYLTIGLPLAVLPGYVHDVMGFSAFWAG

LVISLQYFATLLSRPHAGRYADLLGPKKIVVFGLCGCFLSGLGYLTAGLTASLPVISLLLLCLGRVILGI

GQSFAGTGSTLWGVGVVGSLHIGRVISWNGIVTYGAMAMGAPLGVVFYHWGGLQALALIIMGVALVAILL

AIPRPTVKASKGKPLPFRAVLGRVWLYGMALALASAGFGVIATFITLFYDAKGWDGAAFALTLFSCAFVG

TRLLFPNGINRIGGLNVAMICFSVEIIGLLLVGVATMPWMAKIGVLLAGAGFSLVFPALGVVAVKAVPQQ

NQGAALATYTVFMDLSLGVTGPLAGLVMSWAGVPVIYLAAAGLVAIALLLTWRLKKRPPEHVPEAASSS

>gi|30043511|gb|AAP19231.1| hypothetical protein S4271 [Shigella flexneri 2a str. 2457T]

METPQPDKTGMHILLKLASLVVILAGIHAAADIIVQLLLALFFAIVLNPLVTWFIRRGVQRPVAITIVVV

VMLIALTALVGVLAASFNEFISMLPKFNKELTRKLFKLQEMLPFLNLHMSPERMLQRMDSEKVVTFTTAL

MTGLSGAMASVLLLVMTVVFMLFEVRHVPYKMRFALNNPQIHIAGLHRALKGVSHYLALKTLLSLWTGVI

VWLGLALMGVQFALMWAVLAFLLNYVPNIGAVISAVPPMIQVLLFNGVYECILVGALFLVVHMVIGNILE

PRMMGHRLGMSTMVVFLSLLIWGWLLGPVGMLLSVPLTSVCKIWMETTKGGSKLAILLGPGRPKSRLPG

>gi|30043510|gb|AAP19230.1| hypothetical protein S4270 [Shigella flexneri 2a str. 2457T]

MYRIVLGKVSTLSAAPLPPGLREQAPQGPRRERWLAGRTLLSHTLSPLPEIIYGEQGKPAFAPETPLWFN

LSHSGDDIALLLSDEGEVGCDIEVIRPRVNWRWLANAVFSLGEHAEMDAVHPDQQLEMFWRIWTRKEAIV

KQRGGSAWQIVSVDSTYHSSLSVSHCQLENLSLAICTPTPFTLTADSVQWIDSVN

>gi|30043509|gb|AAP19229.1| periplasmic binding protein for nickel [Shigella flexneri 2a str. 2457T]

MLSTLRRTLFALLACASFIVHAAAPDEITTAWPVNVGPLNPHLYTPNQMFAQSMVYEPLVKYQADGSVIP

WLAKSWTHSEDGKTWTFTLRDDVKFSNGEPFDAEAAAENFRAVLDNRQRHAWLELANQIVDVKALNKTEL

QITLKSAYYPFLQELALPRPFRFIAPSQFKNHETMNGIKAPIGTGPWILQESKLNQYDVFVRNENYWGEK

PAIKKITFNVIPDPTTRAVAFETGDIDLLYGNEGLLPLDTFARFSQNPAYHTQLSQPIETVMLALNTAKA

PTNELAVREALNYAVNKKSLIDNALYGTQQVADTLFAPSVPYANLGLKPRQYDPQKAKELLEKAGWTLPA

GKDIREKNGQPLRIELSFIGTDALSKSMAEIIQADMRQIGADVSLIGEEESSIYARQRDGRFGMIFHRTW

GAPYDPHAFLSSMRVPSHADFQAQQGLADKPLIDKEIGEVLATHDETQRQALYRDILTRLHDEAVYLPIS

YISMMVVSKPELGNIPYAPIATEIPFEQIKPVKP

>gi|30043508|gb|AAP19228.1| transport of nickel, membrane protein [Shigella flexneri 2a str. 2457T]

MLRYVLRRFLLLIPMVLAASVIIFLMLRLGTGDPALDYLRLSNLPPTPEMLASTRTMLGLDQPLYVQYGT

WLWKALHLDFGISFASQRPVLDDMLNFLPATLELAGAALVLILLTSVPLGIWAARHRDRLPDFAVRFIAF

LGVSMPNFWLAFLLVMAFSVYLQWLPAMGYGSWQHIILPAVSIAFMSLAINARLLRASMLDVAGQRHVTW

ARLRGLNDKQTERRHILRNASLPMITAVGMHIGELIGGTMIIENIFAWPGVGRYAVSAIFNRDYPVIQCF

TLMMVVVFVVCNLIVDLLNAALDPRIRRHEGAHA

>gi|30043507|gb|AAP19227.1| transport of nickel, membrane protein [Shigella flexneri 2a str. 2457T]

MNFFLSSRWSVRLALIIIALLALIALTSQWWQPYDPQAIDLPSCLLSPDAQHWLGTDHLGRDIFSRLMAA

TRVSLGSVMACLLLVLTLGLVIGGSAGLIGGHVDQATMRVADMFMTFPTSILSFFMVGVLGTGLTNVIIA

IALSHWAWYARMVRSLVISLRQREFVLASRLSGAGHVRVFVDHLAGAVIPSLLVLATLDIGHMMLHVAGM

SFLGLGVTAPTAEWGVMINDARQYIWTQPLQMFWPGLALFISVMAFNLVGDALRDHLDPHLVTEHAH

>gi|30043506|gb|AAP19226.1| ATP-binding protein of nickel transport system [Shigella flexneri 2a str. 2457T]

MPQQIELRNITLQAAQPLVHGVSLTLQRGRVLALVGGSGSGKSLTCAATLGILPAGVRQTAGEILADGKP

VSPYALRGIKIATIMQNPRSAFNPLHTMHTHARETCLALGKPADDATLTAAIEAVGLENAARVLKLYPFE

MSGGMLQRMMIAMAVLCESPFIIADEPTTDLDVVAQARILDLLESIMQKQAPGMLLVTHDMGVVARLADD

VAVMSQGKIVEQGDVETLFNAPKHTVTRSLVSAHLALYGMELAS

>gi|30043505|gb|AAP19225.1| ATP-binding protein of nickel transport system [Shigella flexneri 2a str. 2457T]

MTLLNVCGLSHHYAHGGFSGKHQHQAVLNNVSLTLKSGETVALLGRSGCGKSTLARLLVGLESPSQGNIS

WHGESLAKLNRAQRKAFRRDIQMVFQDSISAVNPRKTVREILREPMRHLLSLKKSEQLARASEMLKAVDL

DDSVLDKRPPQLSGGQLQLVCLARALAVEPKLLILDEAVSNLDLVLQAGVIRLLKKLQQQFGTACLFITH

DLRLVERFCQRVMVMDNGQIAETQAVGDKLTFSSDAGRVLQNAVLPAFPVRRRTSEKV

>gi|30043504|gb|AAP19224.1| hypothetical protein S4264 [Shigella flexneri 2a str. 2457T]

MQRVTITLDDDLLETLDSLSQRRGYNNRSEAIRDILRSALAQEATQQHGTQGFAVLSYVYEHEKRDLASR

IVSTQHHHHDLSVATLHVHINHDDCLEIAVLKGDMGDVQHFADDVIAQRGVRHGHLQCLPKED

>gi|30043503|gb|AAP19223.1| hypothetical protein S4263 [Shigella flexneri 2a str. 2457T]

MIKLKTPNSMEIAEQPAVITYVPELNAFRGKFLGLSGYCDFVSDSIQGLQKEGELSLREYLEDCKAAGIE

PYARTEKIKTFTLRYPESLSERLNNAAAQQQVSVNTYIIETLNERLNHL

>gi|30043502|gb|AAP19222.1| putative transporter [Shigella flexneri 2a str. 2457T]

MRHLRNIFNLGIKELRSLLGDKAMLTLIVFSFTVSVYSSATVTPGSLNLAPIAIADMDQSQLSNRIVNSF

YRPWFLPPEMITADEMDAGLDAGRYTFAINIPPNFQRDVLAGRQPDIQVNVDATRMSQAFTGNGYIQNII

NGEVNSFVARYRDNSEPLVSLETRMRFNPNLDPAWFGGVMAIINNITMLAIVLTGSALIREREHGTVEHL

LVMPITPFEIMMAKIWSMGLVVLVVSGLSLVLMVKGVLGVPIEGSIPLFMLGVALSLFATTSIGIFMGTI

ARSMPQLGLLVILVLLPLQMLSGGSTPRESMPQMVQDIMLTMPTTHFVSLAQAILYRGAGFEIVWPQFLT

LMAIGGAFFTIALLRFRKTIGTMA

>gi|30043501|gb|AAP19221.1| putative ATP-binding component of a transport system [Shigella flexneri 2a str. 2457T]

MTHLELVPVPPVAQLAGVSQHYGKTVALNNITLDIPARCMVGLIGPDGVGKSSLLSLISGARVIEQGNVV

VLGGDMHDPKHRRDVCPRIAWMPQGLGKNLYHTLSVYENVDFFARLFGHDKAEREVRINELLTSTGLAPF

RDRPAGKLSGGMKQKLGLCCALIHDPELLILDEPTTGVDPLSRAQFWDLIDSIRQRQSNMSVLVATAYME

EAERFDWLVAMNAGEVLATGSAEELRQQTQSATLEEAFINLLPQAQRQAHQAVVIPPYQPENAEIAIEAR

DLTMRFGSFVAVDHVNFRIPRGEIFGFLGSNGCGKSTTMKMLTGLLPASEGEAWLFGQPVDPKDIDTRRR

VGYMSQAFSLYNELTVRQNLELHARLFHIPEAEIPARVAEMSERFKLNDVEDVLPESLPLGIRQRLSLAV

AVIHRPEMLILDEPTSGVDPVARDMFWQLMVDLSRQDKVTIFISTHFMNEAERCDRISLMHAGKVLASGT

PQELVEKRGAASLEEAFIAYLQEAAGQSNEAEAPPVVHDTTHAPRQGFSLRRLFSYSRREALELRRDPVR

STLALMGTVILMLIMGYGISMDVENLRFAVLDRDQTVSSQAWTLNLSGSRYFIEQPPLTSYDELDRRMRA

GDITVAIEIPPNFGRDIARGTPVELGVWIDGAMPSRAETVKGYVQAMHQSWLQDVASRQSTPASQSGLMN

IETRYRYNPDVKSLPAIVPAVIPLLLMMIPSMLSALSVVREKELGSIINLYVTPTTRSEFLLGKQLPYIA

LGMLNFFLLCGLSVFVFGVPHKGSFLTLTLAALLYIIIATGMGLLISTFMKSQIAAIFGTAIITLIPATQ

FSGMIDPVASLEGPGRWIGEVYPTSHFLTIARGTFSKALDLTDLWQLFIPLLIAIPLVMGLSILLLKKQE

G

>gi|30043500|gb|AAP19220.1| putative membrane protein [Shigella flexneri 2a str. 2457T]

MDKSKRHLAWWVVGALAVAAVVAWWLLRPAGVPEGFAVSNGRIEATEVDIASKIAGRIDTILVKEGQFVR

EGEVLAKMDTRVLQEQRLEAIAQIKEAQSAVAAAQALLEQRQSETRAAQSLVNQRQAELDSVAKRHTRSR

SLAQRGAISAQQLDDDRAAAESARAALESAKAQVSASKAAIEAARTNIIQAQTRVEAAQATERRIAADID

DSELKAPRDGRVQYRVAEPGEVLAAGGRVLNMVDLSDVYMTFFLPTEQAGTLKLGGEARLILDAAPDLRI

PATISFVASVAQFTPKTVETSDERLKLMFRVKARIPPELLQQHLEYVKTGLPGVAWVRVNEELPWPDDLV

VRLPQ

>gi|30043499|gb|AAP19219.1| hypothetical protein S4259 [Shigella flexneri 2a str. 2457T]

MKIGTVAGTNGSTTTIATNDMVQEHVTNFTKELFGYIANGIGDDISSIARTMLGEVVEKIDDWQIERFQQ

SIQDDKISFTIQTNHSEKYSMLSGMRAHILRRNNCYQFIVTINSKNYGCPLDNTDINWCSIVYLLNNMTV

NDNANDVAVTESYKPIWNWEISQYNVFDIKFETIIKPQFADRTYFSNCSPVDPTSTRPTYFGDTDGSVGA

VLYALFATGHLGIMAEGENFLSQLLNIEDEVLNVLLRENFNEQLDTNVNTIISILNRRDNVLESLQPYLV

INKDAVTPCTFLGDQTGDRFSNICGDQFIIDLLKRIMSINENVHVLAGNHETNCNGNYMQNFTRMKPLDE

DTYDGIKDYPVCFYDPKYKIMANHHGITFDDQRKRYIIGPITVSIDEMTNALDPVELAEIINKKHHAIIN

GKKFKTSRAISCRSFNRYFSVSTDYRPKLEALLACSQMLGINQVVAHNGNGGRERIGETGTVLGLNARDS

KHAGRMFSMHNCQINPGAGPEITTPWKSYQHEKNRNGLMPLIRRRTMLQL

>gi|30043498|gb|AAP19218.1| hypothetical protein S4838 [Shigella flexneri 2a str. 2457T]

MTKLMQFVQRCYYMTNKKMYFILILVFTLLQVCFFALWKARDGSTTSLECTSTLTRNAKTDHSLYYSANL

SVILKKDGSGSFTIVGLTDEDTPRKFSHSYFFTYKIDSNGRISGNAKAKVSGLENQIKDENFRLNFLDAS

LTGKGNARLSKFNNVYIFSIPGLIINTCAPI

>gi|30043497|gb|AAP19217.1| hypothetical protein S4837 [Shigella flexneri 2a str. 2457T]

MERRCFFIINDAIIFDPNKCSLSNVNTQDEINFPATAGCILAVLLESNGGAVDRNSIIDSVLERFSFDLS

NNTLNQYISLLRKSFKNIGVEENVILTIPKVGFYISENITISKQSYDDSHLQEDVVEKVERKRSIPLFRI

LISLVVVIVIIEMFAVAFKDTYSITDYPLVKSGKIDTCDLYLSENLNEIGLAKSQIEAAEISKKHLPCVP

GANFIYDVNSMHYLLGEGQRYLSRCIKSSNDETYAICSEVLLYD

>gi|30043496|gb|AAP19216.1| hypothetical protein S4836 [Shigella flexneri 2a str. 2457T]

MLSKITQELLKMNNVLLMRPDDSEFMIPDDWSGLNAKGITYEQDNRVTESFLPFKSFYCSPVCTTVELIS

QNNCFAMGIRYLLEKKRDLRLNHFKAFSHKCETKPDVLIMTGCTVADTYSIFSRIVEENIMKTMTPIVII

TGHHSEDLCSLASKLGGVKIISPTEKIAKIENAINDAFNSKPKKIRFLTDILSPQQIETMKMVAQGRSVN

EISAAMNISIKTVYAHKVRAINKLGIKKKVYEALFYKALIQFN

>gi|30043495|gb|AAP19215.1| hypothetical protein S4255 [Shigella flexneri 2a str. 2457T]

MKKTLLAIMLAGTAFASQAGTLVSQGTEASANLTLTKPIVVNNTIQPVKGVYSGTLTAWTPLATGIVGAS

DGQSHDYAVTFPDDIYAESSTSADAVISGDNNPDHKLKVSLTTLEQDPPSAASEEIGGKRYMMLKNTGTG

GAYRVVSHMKEQVVEPDSYTIRTQAYIYAE

>gi|30043494|gb|AAP19214.1| IS2 orfA [Shigella flexneri 2a str. 2457T]

MIVLILVFRLVIGEQMIDVLGPEKRRRRTTQEKIAIVQQSFEPGMTVSLVARQHGVAASQLFLWRKQYQE

GSLTAVAAGEQVVPASELAAAMKQIKELQRLLGKKTMENELLKEAVEYGRAKKWIAHAPLLPGDGE

>gi|30043493|gb|AAP19213.1| IS2 orfB [Shigella flexneri 2a str. 2457T]

MARGWGVSLVSRCLRVSRAQLHVILRRTDDWMDGRRSRHTDDTDVLLRIHHVIGELPTYGYRRVWALLRR

QAELDGMPAINAKRVYRLMRQNALLLERKPAVPPSKRAHTGRVAVKESNQRWCSDGFEFCCDNGERLRVT

FALDCCDREALHWAVTTGGFNSETVQDVMLGAVERRFGNDLPSSPVEWLTDNGSCYRANETRQFARMLGL

EPKNTAVRSPESNGIAESFVKTIKRDYISIMPKPDGLTAAKNLAEAFEHYNEWHPHSALGYRSPREYLRQ

RACNGLSDNRCLEI

>gi|30043492|gb|AAP19212.1| probable fimbrial protein remnant [Shigella flexneri 2a str. 2457T]

MFYRLSLILIMALLAGQLSAQEWSFDSSQLEGNVSADTVAMFNQGEQLPGNYRVEIYLNGEKVDVGEFPF

HRPESPEEKELVPCLTVDDLIHYGIKIDKSSSDTDNKKNQCFKWNSIEGLKVNYDFDSQRVQITVPQLYL

QDKKSSLAPVSLWNEGVAAFRMVYQTNIDISKQNDNQSTTRNSRYGRFTPGFNLGAWRFRSSVTWSKELG

QSERWQRGYMWFERGINAIKSRLTLGESYTSSEVFDSIPFRGGMLATDDAMTPPEDSYYTPVVHGIAQSE

AQVIIKQNGQIIFTRSVPPGPFALDNLPTLAVGGELDVTVRESNGEEQYFSVPFQTPAIALHEGYFKYSV

MGGNIKKKV

>gi|30043491|gb|AAP19211.1| possible chaperone [Shigella flexneri 2a str. 2457T]

MLVQSKVFEEDKVTAAPFIVTPPLFRLNPGQQNKMWVVQTAPALQDREKLHWLCATGITPKKSDVWAEKD

KEKIAENVNLNIQVNVHTCIKLITRPSGIEGKMVNASEKLSWKKQGDKIVAVNQSPFYINLSSLQVGGSK

IEKLDYIAPFSQHEFVMPKTSGQDIKWSVITDEGGESRLFISHLQS

>gi|30043490|gb|AAP19210.1| hypothetical protein S4249 [Shigella flexneri 2a str. 2457T]

MLAALADRPRWRGRPLSGLLRLIFVASLLCWCVGISNYAMPGETTADFTHSSSNDQRIRILKGAISLIQQ

RPLTGWGLGSFEATFPQALEDVGVTNIESDTATHPHNELLFVTAEGGIVALFGFLLLSGVWLWPCICRLK

MTNNNAHHQCEKWFIALMGLPLVIHMMTEYPLYTSVPHLMLLLLLFRIGMPENVMNQVRIPKTVRLGILP

VIVLIMTAALVILVAGFDAQRELTRAEADMNEGFIPRLPEHSWKYLTQAERLERDQHLLIANKPGFVRDT

ESMSLFTVWGNKWLAIHNDADVSAAMIFIAHRRGDYLTAEKIRKRAARVFVNDERFTRGGN

>gi|30043489|gb|AAP19209.1| IS600 orfA [Shigella flexneri 2a str. 2457T]

MSRKTQRYSKEFKAEAVRTVLENQLSISEGASRLSLPEGTLGQWVTAARKGLGTPGSRTVAELESEILQL

RKALNEARLERDILKKATAYFAQESLKNR

>gi|30043488|gb|AAP19208.1| IS1 orfB [Shigella flexneri 2a str. 2457T]

MSRQCTHYGRWPQHGFTSLKKLRPQSVTSRIQPGSDVIVCAEMDEHWGYVGAKSRQRWLFYAYDRIRRTV

VAHVFGERTLATLERLLSLLSAFEVVVWMTDGWPLYESRLKGKLHVISKRYTQRIERHNLNLRQHLARLG

RKSLSFSKSVELHDKVIGHYLNIKHYQ

>gi|30043487|gb|AAP19207.1| IS1 orfA [Shigella flexneri 2a str. 2457T]

MAFISIRCPSCSATEGVVRNGKSTAGHQRYLCSHCRKTWQLQFTYTASQPGTHQKIIDMAMNGVGCRASA

RIMGVGLNTVLRHLKNSGRSR

>gi|30043486|gb|AAP19206.1| hypothetical protein S4245 [Shigella flexneri 2a str. 2457T]

MERFDAIIIGAGAAGMFCSALAGQVGRRVLLIDNGKILGRKILMSGGGRCNFTNLYVEPGAYLSQNPHFC

KSALARFTQWDFIDLVNKHGIAWHEKTLGQLFCDDSAQQIVDMLVDECEKGNVTFRLRSEVLSVAKDETG

FTLDLNGMTVGCEKLVIATGGLSMPGLGASPFGYKIAEQFGLNVLPTRAGLVPFTLHKPLLEELQVLAGV

AVPSVITAENGTVFRENLLFTHRGLSGPAVLQISSYWQPGEFVSINLLPDVDLETFLNEQRNAHPNQSLK

NTLAVHLPKRLVERLQQLGQIPDVSLKQLNVCDQQALISTLTDWRVQPNGTEGYRTAEVTLGGVDTNELS

SRTMEARKVPGLYFIGEVMDVTGWLGGYNFQWAWSSAWACAQDLASIVK

>gi|30043485|gb|AAP19205.1| low-affinity phosphate transport protein [Shigella flexneri 2a str. 2457T]

MLHLFAGLDLHTGLLLLLALAFVLFYEAINGFHDTANAVATVIYTRAMRSQLAVVMAAVFNFLGVLLGGL

SVAYAIVHMLPTDLLLNMGSSHGLAMVFSMLLAAIIWNLGTWYFGLPASSSHTLIGAIIGIGLTNALMTG

TSVVDALNIPKVLSIFGSLIVSPIVGLVFAGGLIFLLRRYWSGTKKRARIHLTPAEREKKDGKKKPPFWT

RIALILSAIGVAFSHGANDGQKGIGLVMLVLIGVAPAGFVVNMNATGYEITRTRDAINNVEAYFEQHPAL

LKQATGADQLVPAPEAGATQPAEFHCHPSNTINALNRLKGMLTTDVESYDKLSLDQRSQMRRIMLCVSDT

IDKVVKMPGVSADDQRLLKKLKSDMLSTIEYAPVWIIMAVALALGIGTMIGWRRVATTIGEKIGKKGMTY

AQGMSAQMTAAVSIGLASYTGMPVSTTHVLSSSVAGTMVVDGGGLQRKTVTSILMAWVFTLPAAVLLSGG
LYWFSLQFL

>gi|30043484|gb|AAP19204.1| hypothetical protein S4243 [Shigella flexneri 2a str. 2457T]
MISTVALFWALCVVCIVNMARYFSSLRALLVVLRNCDPLLYQYVDGGGFFTSHGQPNKQVRLVWYIYAQR
YRDHHDDEFIRRCERVRRQFILTSALCGLVVVSLIALMIWH

>gi|30043483|gb|AAP19203.1| universal stress protein [Shigella flexneri 2a str. 2457T]
MAYKHILIAVDLSPESKVLVEKAVSMARPYNAKVSLIHVDVNYSDLYTGLIDVNLGDMQKRISEETHHAL
TELSTNAGYPITETLSGSGDLGQVLVDAIKKYDMDLVVCGHHQDFWSKLMSSARQLINTVHVDMLIVPLR
DEEE

>gi|30043482|gb|AAP19202.1| hypothetical protein S4240 [Shigella flexneri 2a str. 2457T]
MKICLIDETGAGDGALSVLAARWGLEHDEDNLMALVLTPEHLELRKRDEPKLGGIFVDFVGGAMAHRRKF
GGGRGEAVAKAVGIKGDYLPDVVDATAGLGRDAFVLASVGCRVRMLERNPVVAALLDDGLARGYADAEIG
GWLQERLQLIHASSLTALTDITPRPQVVYLDPMFPHKQKSALVKKEMRVFQSLVGPDLDADGLLEPARLL
ATKRVVVKRPDYAPPLANVATPNAVVTKGHRFDIYAGTPV

>gi|30043481|gb|AAP19201.1| oligopeptidase A [Shigella flexneri 2a str. 2457T]
MTNPLLTPFELPPFSKILPEHVVPAVTKALNDCRENVERVVAQGAPYTWENLCQPLAEVDDVLGRIFSPV
SHLNSVKNSPELREAYEQTLPLLSEYSTWVGQHEGLYKAYRDLRDGDHYATLNTAQKKAVDNALRDFELS
GIGLPKEKQQRYGEIATRLSELGNQYSNNVLDATMGWTKLVTDEAGLAGMPESALAAAKAQAEAKELEGY
LLTLDIPSYLPVMTYCDNQALREEMYRAYSTRASDQGPNAGKWDNSKVMEEILALRHELAQLLGFENYAF
KSLATKMAENPQQVLDFLTDLAKRARPQGEKELEQLRAFAKAEFGVDELQPWDIAYYSEKQKQHLYSISD
EQLRPYFPENKAVNGLFEVVKRIYGITAKERKDVDVWHPDVRFFELYDENNELRGSFYLDLYARENKRGG

AWMDGCVGQMRKADGSLQKPVAYLTCNFNRPVNGKPALFTHDEVITLFHEFGHGLHHMLTRIETAGVSGI
SGVPWDAVELPSQFMENWCWEPEALAFISGHYETGEPLPKELLDKMLAAKNYQAALFILRQLEFGLFDFR
LHAEFRPDQGAKILETLAEIKKLVAVVPSPSWGRFPHAFSHIFAGGYAAGYYSYLWADVLAADAFSRFEE
EGIFNRETGQSFLDNILSRGGSEEPMDLFKRFRGREPQLDAMLEHYGIKG

>gi|30043480|gb|AAP19200.1| hypothetical protein S4238 [Shigella flexneri 2a str. 2457T]
MLSYRHSFHAGNHADVLKHTVQSLIIESLKEKDKPFLYLDTHAGAGRYQLGSEHAERTGEYLEGIARIWQ
QDDLPAELEAYINVVKHFNRSGQLRYYPGSPLIARQLLREQDSLQLTELHPSDYPLLRSGFQKDSRARVE
KADGFQQLKAKLPPVSRRGLILIDPPYEMKTDYQAVVSGIAEGYKRFATGTYALWYPVVLRQQIKRMIHD
LEATGIRKILQIELAVLPDSDRRGMTASGMIVINPPWKLEQQMNNVLSWLHSKLVPAGTGHATVSWIVPE

>gi|30043479|gb|AAP19199.1| glutathione oxidoreductase [Shigella flexneri 2a str. 2457T]
MTKHYDYIAIGGGSGGIASINRAAMYGQKCALIEAKELGGTCVNVGCVPKKVMWHAAQIREAIHMYGPDY
GFDTTINKFNWETLIASRTAYIDRIHTSYENVLGKNNVDVIKGFARFVDAKTLEVNGETITADHILIATG
GRPSHPDIPGVEYGIDSDGFFALPALPERVAVVGAGYIAVELAGVINGLGAKTHLFVRKHAPLRSFDPMI
SETLVEVMNAEGPQLHTNAIPKAVVKNADGSLTLELEDGRSETVDCLIWAIGREPANDNINLEAAGVKTN
EKGYIVVDKYQNTNIEGIYAVGDNTGAVELTPVAVAAGRRLSERLFNNKPDEHLDYSNIPTVVFSHPPIG
TVGLTEPQAREQYGDDQVKVYKSSFTAMYTAVTTHRQPCRMKLVCVGPEEKIVGIHGIGFGMDEMLQGFA
VALKMGATKKDFDNTVAIHPTAAEEFVTMR

>gi|30043478|gb|AAP19198.1| IS1 orfB [Shigella flexneri 2a str. 2457T]
MSRQCTHYGRWPQHGFTSLKKLRPQSVTSRIQPGSDVIVCAEMDEHWGYVGAKSRQRWLFYAYDRIRRTV
VAHVFGERTLATLERLLSLLSAFEVVVWMTDGWPLYESRLKGKLHVISKRYTQRIERHNLNLRQHLARLG
RKSLSFSKSVELHDKVIGHYLNIKHYQ

>gi|30043477|gb|AAP19197.1| IS1 orfB [Shigella flexneri 2a str. 2457T]

MASISIRCPSCSATEGVVRNGKSTAGHQRYLCSHCRKTWQLQFTYTASQPGTHQKIIDMAMNGVGCRASA

RIMGVGLNTVLRHLKNSGRSR

>gi|30043476|gb|AAP19196.1| arsenate reductase [Shigella flexneri 2a str. 2457T]

MSNITIYHNPACGTSRNTLEMIRNSGTEPTIIHYLETPPTRDELVKLIADMGISVRALLRKNVEPYEELG

LAEDKFTDDRLIDFMLQHPILINRPIVVTPLGTRLCRPSEVVLEILPDAQKGAFSKEDGEKVVDEAGKRL

K

>gi|30043475|gb|AAP19195.1| arsenical pump membrane protein [Shigella flexneri 2a str. 2457T]

MLLAGAIFVLTIVLVIWQPKGLGIGWSATLGAVLALVTGVVHPGDIPVVWNIVWNATAAFIAVIIISLLL

DESGFFEWAALHVSRWGNGRGRLLFTWIVLLGAAVAALFANDGAALILTPIVIAMLLALGFSKGTTLAFV

MAAGFIADTASLPLIVSNLVNIVSADFFGLGFREYASVMVPVDIAAIVATLVMLHLYFRKDIPQNYDMAL

LKSPAEAIKDPATFKTGWVVLLLLLVGFFVLEPLGIPVSAIAAVGALILFVVAKRGHAINTGKVLRGAPW

QIVIFSLGMYLVVYGLRNAGLTEYLSGVLNVLADNGLWAATLGTGFLTAFLSSIMNNMPTVLVGALSIDG

STASGVIKEAMVYANVIGCDLGPKITPIGSLATLLWLHVLSQKNMTISWGYYFRTGIIMTLPVLFVTLAA

LALRLSFTL

>gi|30043474|gb|AAP19194.1| transcriptional repressor of chromosomal ars operon [Shigella flexneri 2a str. 2457T]

MLPIQLFKILADETRLGIVLLLSELGELCVCDLCTALDQSQPKISRHLALLRESGLLLDRKQGKWVHYRL

SPHIPAWAAKIIDEAWRCEQEKIQAIVRNLARQNCSGDSKNICS

>gi|30043473|gb|AAP19193.1| IS1 orfA [Shigella flexneri 2a str. 2457T]

MASISIRCPSCSATEGVVRNGKSTAGHQRYLCSHCRKTWQLQFTYTASQPGTHQKIIDMAMNGVGCRASA

RIMGVGLNTVLRHLKNSGRSR

>gi|30043472|gb|AAP19192.1| IS1 orfB [Shigella flexneri 2a str. 2457T]

MSRQCTHYGRWPQHGFTSLKKLRPQSVTSRIQPGSDVIVCAEMDEHWGYVGAKSRQRWLFYAYDRIRRTV

VAHVFGERTLATLERLLSLLSAFEVVVWMTDGWPLYESRLKGKLHVISKRYTQRIERHNLNLRQHLARLV

RKSLSFSKSVELHDKVIGHYLNIKHYQ

>gi|30043471|gb|AAP19191.1| hypothetical protein S4229 [Shigella flexneri 2a str. 2457T]

MFLAVELSLLFIVISAGVSLIRQKVPDHKIQQMMGARKGRGYLLAALLGAVTPFCSCSTIPMLRGLLSAK

AGFGPTLTFLFVSPLLNPIIVGLMWMTFGWKVTLLYAIIAAGVSVLASIILDSLGFERHIIASKSSSANC

CAPAKTSPGTTYTPIKVSCCSPAAKAIENPVVTCCSTKAVVSINPIKLATKDALQQFKDVLPYLLLGVLI

GSFIYGFIPSAWIAAHAGADNPFAIPLSAVVGIPLYIRAEAVIPLASVLMTKGMGLGALMALIIGSAGAS

LTEVILLKSMFRMPMIAAFLTVILGMAILMGYLTQLLF

>gi|30043470|gb|AAP19190.1| outer membrane protein induced after carbon starvation [Shigella flexneri 2a str. 2457T]

MVLIFVDKDSNMNMTKGALILSLSFLLAACSSIPQNIKGNNQPDIQKSFVAVHNQPGLYVGQQARFGGKV

INVINGKTDTLLEIAVLPLDSYAKPDIEANYQGRLLARQSGFLDPVNYRNHFVTILGTIQGEQPGFINKV

PYNFLEVNMQGIQVWHLREVVNTTYNLWDYGYGAFWPEPGWGAPYYTNAVSQVTPELVK

>gi|30043469|gb|AAP19189.1| hypothetical protein S4226 [Shigella flexneri 2a str. 2457T]

MFLIITRDTMFFTAMKNILSKGNVVHIQNEEEIDVMLHQNAFVIIDTLMNNVFHSNFLTQIERLKPVHVI

VFSPFNIKRCLGKVPVTFVPRTITIIDFVALINGSYCSVPEANVSLSRKQHQVLSCIANQMTTEDILEKL

KISLKTFYCHKHNIMMILNLKRINELVRHQHIDYLV

>gi|30043468|gb|AAP19188.1| hypothetical protein S4224 [Shigella flexneri 2a str. 2457T]

MGYKMNISSLRKAFIFMGAVAALSLVNAQSALAANESAKDMTCQEFIDLNPKAMTPVAWWMLHEETVYKG

GDTVTLNETDLTQIPKVIEYCKKNPQKNLYTFKNQASNDLPN

>gi|30043467|gb|AAP19187.1| hypothetical protein S4223 [Shigella flexneri 2a str. 2457T]

MKKVLGVILGGLLLLPVVSNAADAQKAADNKKPVNSWTCEDFLAVDESFQPTAVGFAEALNNKDKPEDAV

LDVQGIATVTPAIVQACTQDKQANFKDKVKGEWDKIKKDM

>gi|30043466|gb|AAP19186.1| hypothetical protein S4222 [Shigella flexneri 2a str. 2457T]

MLYIDKATILKFDLEMLKKHRRAIQFIAVLLFIVGLLCISFPFVSGDILSTVVGALLICSGIALIVGLFS

NRSHNFWPVLSGFLVAVAYLLIGYFFIRAPELGIFAIAAFIAGLFCVAGVIRLMSWYRQRSMKGSWLQLV

IGVLDIVIAWIFLGATPMVSVTLVSTLVGIELIFSAASLFSFASLFVKQQ

>gi|30043465|gb|AAP19185.1| hypothetical protein S4221 [Shigella flexneri 2a str. 2457T]

MIFLMTKDSFLLQGFWQLKDNHEMIKINSLSEIKKVGNKPFKVIIDTYHNHILDEEAIKFLEKLDAERII

VLAPYHISKLKAKAPIYFVSRKESIKNLLEITYGKHLPHKNSQLCFSHNQFKIMQLILKNKNESNITSTL

NISQQTLKIQKFNIMYKLKLRRMSDIVTLGITSYF

>gi|30043464|gb|AAP19184.1| IS1 orfB [Shigella flexneri 2a str. 2457T]

MSRQCTHYGRWPQHGFTSLKKLRPQSVTSRIQPGSDVIVCAEMDEHWGYVGAKSRQRWLFYAYDRIRRTV

VAHVFGERTLATLERLLSLLSAFEVVVWMTDGWPLYESRLKGKLHVISKRYTQRIERHNLNLRQHLARLV

RKSLSFSKSVELHDKVIGHYLNIKHYQ

>gi|30043463|gb|AAP19183.1| IS1 orfA [Shigella flexneri 2a str. 2457T]

MASISIRCPSCSATEGVVRNGKSTAGHQRYLCSHCRKTWQLQFTYTASQPGTHQKIIDMAMNGVGCRASA

RIMGVGLNTVLRHLKNSGRSR

>gi|30043462|gb|AAP19182.1| cytoplasmic trehalase [Shigella flexneri 2a str. 2457T]

MLNQKIQNPNPDELMIEVDLCYELDPYELKLDEMIEAEPEPEMIEGLPASDALTPADRYLELFEHVQSAK

IFPDSKTFPDCAPKMDPLDILIRYRKVRRHRDFDLRKFVENHFWLPEVYSSEYVSDPQNSLKEHIDQLWP

VLTREPQDHIPWSSLLALPQSYIVPGGRFSETYYWDSYFTMLGLAESGREDLLKCMADNFAWMIENYGHI

PNGNRTYYLSRSQPPVFALMVELFEEDGVRGARRYLDHLKMEYAFWMDGAESLIPNQAYRHVVRMPDGSL

LNRYWDDRDTPRDESWLEDVETAKHSGRPPNEVYRDLRAGAASGWDYSSRWLRDTGRLASIRTTQFIPID

LNAFLFKLESAIANISALKGEKETEALFRQKASARRDAVNRYLWDDENGIYRDYDWRREQLALFSAAAIV

PLYVGMANHEQADRLANAVRSRLLTPGGILASEYETGEQWDKPNGWAPLQWMAIQGFKMYGDDLLGDEIA

RSWLKTVNQFYLEQHKMIEKYHIADGVPREGGGGEYPLQDGFGWTNGVVRRLIGLYGEP

>gi|30043461|gb|AAP19181.1| IS4 orf [Shigella flexneri 2a str. 2457T]

MHIGQALDLVSRYDSLRNPLTSLGDYLDPELISRCLAESGTVTLRKRRLPLEMMVWCIVGMALERKEPLH

QIVNRLDIMLPGNRPFVAPSAVIQARQRLGSEAVRRVFTKTAQLWHNATPHPHWCGLTLLAIDGVFWRTP

DTPENDAAFPRQTHAGNPALYPQVKMVCQMELTSHLLTAAAFGTMKNSENELAEQLIEQTGDNTLTLMDK

GYYSLGLLNGWSLAGEHRHWMIPLRKGAQYEELRKLGKGDHLVKLKTSPQARKKWPGLGNEVTARLLTVT

RKGKVCHLLTSMTDAMRFPGGEMADLYSHRWEIELGYREIKQTMQLSRLTLRSKKPELVEQELWGVLLAY

NLVRYQMIKMAEHLKGYWPNQLSFSESCGMVMRMLMTLQGASPGRIPELMRDLASMGQLVKLPTRRGRAF

PRVVKERPWKYPTAPKKSQSVA

>gi|30043460|gb|AAP19180.1| putative regulator [Shigella flexneri 2a str. 2457T]

MQIVMFDRQSIFIHGIKISLQQRIPGVSIQGVSQADELWQKLESYPEALVMLDGDQDGEFCYWLLQKTVV

QFPEVKVLITATDCNKRWLQEVIHFNVLAIVPRDSTVETFALAVNSAAMGMMFLPGDWRTTPEKDIKDLK

SLSARQREILTMLAAGESNKEIGRALNISTGTVKAHLESLYRRLEVKNRTQAAMMLNISS

>gi|30043459|gb|AAP19179.1| putative transcriptional regulator LYSR-type [Shigella flexneri 2a str. 2457T]

MLEKTINNAICALLFRCEQQSVKEMDKIHAMQLFIKVAELESFSRAADFFALPKGSVSRQIQALEHQLGT
QLLQRTTRRVKLTPEGMTYYQRAKDVLSNLSELDGLFQQDATSISGKLRIDIPSGIAKNLLLPRLSEFLY
QHPGIELELSSHNRPVDILHDGFDCVIRTGALPEDGVIARPLGKLTMVNCASPHYLTRFGYPQSPDDLTS
HAIVRYTPHLGVHPLGFEVASVNGVQWFKSGGMLTVNSSENYLAAGLAGLGIIQIPRIAVREALRAGRLI
EVLPGYRAEPLSLSLVYPQRRELSRRVNLFMQWLAGVMKEHLD

>gi|30043458|gb|AAP19178.1| hypothetical protein S4213 [Shigella flexneri 2a str. 2457T]
MTQENEIKRPTQDLEHEPIKQLDNSEKGGKVSQALETVTTTAEKVQRQPVIAHLIRATERFNDRLGNQFG
AAITYFSFLSMIPILMVSFAAGGFVLASHPMLLQDIFDKILQNISDPTLAATLKNTINTAVQQRTTVGLV
GLAVALYSGINWMGNLREAIRAQSRDVWERSPQDQEKFWVKYLRDFISLIGLLIALIVTLSITSVAGSAQ
QMIISALHLNSIEWLKPTWRLIGLAISIFANYLLFFWIFWRLPRHRPRKKALIRGTFLAAIGFEVIKIVM
TYTLPSLMKSPSGAAFGSVLGLMAFFYFFARLTLFCAAWIATAEYKDDPRMPGKTQP

>gi|30043457|gb|AAP19177.1| putative transport protein [Shigella flexneri 2a str. 2457T]
MQATATTLDHEQEYTPINSRNKVLVASLIGTAIEFFDFYIYATAAVIVFPHIFFPQGDPTAATLQSLATF
AIAFVARPIGSAVFGHFGDRVGRKATLVASLLTMGISTVVIGLLPGYATIGIFAPLLLALARFGQGLGLG
GEWGGAALLATENAPPRKRALYGSFPQLGAPIGFFFANGTFLLLSWLLTDEQFMSWGWRVPFIFSAVLVI
IGLYVRVSLHESPVFEKVAKAKKQVKIPLGTLLTKHVRVTVLGTFIMLATYTLFYIMTVYSMTFSTAAAP
VGLGLPRNEVLWMLMMAVIGFGVMVPVAGLLADAFGRRKSMVIITTLIILFALFAFNPLLGSGNPILVFA
FLLLGLSLMGLTFGPMGALLPELFPTEVRYTGASFSYNVSSILGASVAPYIAAWLQANYGLGAVGLYLAA
MAGLTLIALLLTHETRHQSL

>gi|30043456|gb|AAP19176.1| hypothetical protein S4211 [Shigella flexneri 2a str. 2457T]
MSKAGKITAAISGAFLLLIVVAIILIATFDWNRLKPTINQKVSAELNRPFAIRGNLGVVWERQKQETGWR
SWVPWPHVHAEDIILGNPPDIPEVTMVHLPRVEATLAPLALLTKTVWLPWIKLEKPDARLIRLSEKNNNW

TFNLANDDNKDANAKPSAWSFRLDNILFDQGRIAIDDKVSKADLEIFVDPLGKPLPFSEVTGSKGKADKE
KVGDYIFGLKAQGRYNGEPLTGTGKIGGMLALRGEGTPFPVQADFRSGNTRVAFDGVVNDPMKMGGVDLR
LKFSGDSLGDLYELTGVLLPDTPPFETDGRLVAKIDTEKSSVFDYRGFNGRIGDSDIHGSLVYTTGKPRP
KLEGDVESRQLRLADLGPLIGVDSGKGAEKSKRSEQKKGEKSVQPAGKVLPYDRFETDKWDVMDADVRFK
GRRIEHGSSLPISDLSTHIILKNADLRLQPLKFGMAGGSIAANIHLEGDKKPMQGRADIQARRLKLKELM
PDVELMQKTLGEMNGDAELRGSGNSVAALLGNSNGNLKLLMNDGLVSRNLMEIVGLNVGNYIVGAIFGDD
EVRVNCAAANLNIANGVARPQIFAFDTENALINVTGTASFASEQLDLTIDPESKGIRIITLRSPLYVRGT
FKNPQAGVKAGPLIARGAVAAALATLVTPAAALLALISPSEGEANQCRTILSQMKK

>gi|30043455|gb|AAP19175.1| hypothetical protein S4210 [Shigella flexneri 2a str. 2457T]
MIRQVIQRISNPEASIESLQERRFWLQCERAYTWQPIYQTCGRLMAVELLTVVTHPLNPSQRLPPDRYFT
EITVSHRMEVVKEQIDLLAQKADFFVEHGLLASVNIDGPTLIALRQQPKILRQIERLPWLRFELVEHIRL
PKDSTFASMCEFGPLWLDDFGTGMANFSALSEVRYDYIKIARELFVMLRQSPEGRTLFSQLLHLMNRYCR
GVIVEGVETPEEWRDVQNSPAFAAQGWFLSRPAPMETLNTAVLAL

>gi|30043454|gb|AAP19174.1| ketodeoxygluconokinase [Shigella flexneri 2a str. 2457T]
MFKSDAARTRQRLSARKGRTLPPGRGKFPHSTTESFRNTFWLKKIMEHCFNMVDQQTTTAQTNANFLQIR
FTTMSKKIAVIGECMIELSEKGADVKRGFGGDTLNTSVYIARQVDPAALTVHYVTALGTDSFSQQMLDAW
HGENVDTSLTQRMENRLPGLYYIETDSTGERTFYYWRNEAAAKFWLASEQSAAICEELANFDYLYLSGIS
LAILSPTSREKLLSLLRECRAKGGKVIFDNNYRPRLWASKEETQQVYQQMLECTDIAFLTLDDEDALWGQ
QPVEDVIARTHNAGVKEVVVKRGADSCLVSIAGEALVDVPAVKLPKEKVIDTTAAGDSFSAGYLAVRLTG
GSAENAAKRGHLTASTVIQYRGAIIPREAMPA

>gi|30043453|gb|AAP19173.1| hypothetical protein S4208 [Shigella flexneri 2a str. 2457T]
MQGIKIRLLAGGLLMMATAGYVQADALQPDPAWQQGTLSNGLQWQVLTTPQRPSDRVEIRLLVNTGSLAE

STQQSGYSHAIPRIALTQSGGLDAAQARSLWQQGIDPKRPMPPVIVSYDTTLFNLSLPNNRNDLLKEALS

YLANATGKLTITPETINHALQSQDMVATWPADTKEGWWRYRLKGSTLLGHDPADPLKQPVEAEKIKDFYQ

KWYTPDAMTLLVVGNVDARSVVDQINKTFGELKGKRETPAPVPTLSPLRAEAVSIMTDAVRQDRLSIMWD

TPWQPIRESAALLRYWRADLAREALFWHVQQALSASDSKDIGLGFDCRVLYLRAQCAINIESPNDKLNSN

LNLVARELAKVRDKGLPEEEFNALVAQKKLELQKLFAAYARADTDILMGQRMRSLQNQVVDIAPEQYQKL

RQDFLNSLTVEMLNQDLRQQLSNDMALILLQPKGEPEFNMKALQAAWDQIMAPSTAAATTSVATDDVHPE

VTDIPPAQ

>gi|30043452|gb|AAP19172.1| C4-dicarboxylate transport protein [Shigella flexneri 2a str. 2457T]

MKTSLFKSLYFQVLTAIAIGILLGHFYPEIGEQMKPLGDGFVKLIKMIIAPVIFCTVVTGIAGMESMKAV

GRTGAVALLYFEIVSTIALIIGLIIVNVVQPGAGMNVDPATLDAKAVAVYADQAKDQGIVAFIMDVIPAS

VIGAFASGNILQVLLFAVLFGFALHRLGSKGQLIFNVIESFSQVIFGIINMIMRLAPIGALGAMAFTIGK

YGVGTLVQLGQLIICFYITCILFVVLVLGSIAKATGFSIFKFIRYIREELLIVLGTSSSESALPRMLDKM

EKLGCRKSVVGLVIPTGYSFNLDGTSIYLTMAAVFIAQATNSQMDIVHQITLLIVLLLSSKGAAGVTGSG

FIVLAATLSAVGHLPVAGLALILGIDRFMSEARALTNLVGNGVATIVVAKWVKELDHKKLDDVLNNRAPD

GKTHELSS

>gi|30043451|gb|AAP19171.1| hypothetical protein S4206 [Shigella flexneri 2a str. 2457T]

MVAAVVLVFVFIFCTVLLFHLVQQNRYNTATQLESIARSVREPLSSAILKGDIPEAEAILASIKPAGVVS

RADVVLPNQFQALRKSFIPERPVPVMVTRLFELPVQISLGVYSLERPANPQPIAYLVLQADSFRMYKFVM

STLSTLVTIYLLLSLILTVAISWCINRLILHPLRNIARELNAIPAQELVGHQLALPRLHQDDEIGMLVRS

YNLNQQLLQRHYEEQNENAMRFPVSDLPNKALLMEMLEQVVARKQTTALMIITCETLRDTAGVLKEAQRE

ILLLTLVEKLKSVLSPRMILAQISGYDFAVIANGVQEPWHAITLGQQVLTIMSERLPIERIQLRPHCSIG

VAMFYGDLTAEQLYSRAISAAFTARHKGKNQIQFFDPQQMEAAQKRLTEESDILNALENHQFAIWLQPQV

EMTSGKLVSAEVLLRIQQPDGSWDLPDGLIDRIECCGLMVTVGHWVLEESCRLLAAWQERGIMLPLSVNL

SALQLMHPNMVADMLELLTRYRIQPGTLILEVTESRRIDDPHAAVAILRPLRNAGVRVALDDFGMGYAGL
RQLQHMKSLPIDVLKIDKMFVEGLPEDSSMIAAIIMLAQSLNLQMIAEGVETEAQRDWLAKAGVGIAQGF
LFARPLPIEIFEESYLEEK

>gi|30043450|gb|AAP19170.1| putative endoglucanase [Shigella flexneri 2a str. 2457T]
MNVLRSGIVTMLLLAAFSVQAACTWPAWEQFKKDYISQEGRVIDPSDARKITTSEGQSYGMFFALAANDR
AAFDNILDWTQNNLAQGSLKERLPAWLWGKKENSKWEVLDSNSASDGDVWMAWSLLEAGRLWKEQRYTDI
GSALLKRIAREEVVTVPGLGSMLLPGKVGFAEDNSWRFNPSYMPPTLAQYFTRFGAPWTTLRETNQHLLL
ETAPKGFSPDWVRYEKDKGWQLKAEKTLISSYDAIRVYMWVGMMPDSDPQKARMLNRFKPMATFTEKNGY
PPEKVDVATGKAQGKGPVGFSAAMLPFLQNRDAQAVQRQRVADNFPGSDTYYNYVLTLFGQGWDQHRFRF
STKGELLPDWGQECANSH

>gi|30043449|gb|AAP19169.1| hypothetical protein S4203 [Shigella flexneri 2a str. 2457T]
MAMGMSAFPSFMTQATPATQPLINAEPAVAAQTEQNPQVGQVMSGEQGADAPIVAQNGPSRDVKLTFAQI
APPPGSMVLRGINPNGSIEFGMRSDEVVTKAMLNFEYTPSPSLLPVQSQLKVYLNDELMGVLPVTKEQLG
KKTLAQMPINPLFITDFNRVRLEFVGHYQDVCENPASTTLWLDVGRSSGLDLTYQTLNVKNDLSHFPVPF
FDPRDNRTNTLPMVFAGAPDVGLQQASAIVASWFGSRSGWRGQNFPVLYNQLPDRNAIVFATNDKRPDFL
RDHPAVKAPVIEMINHPQNPYVKLLVVFGRDDKDLLQAAKGIAQGNILFRGESVVVNEVKPLLPRKPYDA
PNWVRTDRPVTFGELKTYEEQLQSSGLEPAAINVSLNLPPDLYLMRSTGIDMDINYRYTMPPVKDSSRMD
ISLNNQFLQSFNLSSKQEANRLLLRIPVLQGLLDGKTDVSIPALKLGATNQLRFDFEYMNPMPGGSVDNC
ITFQPVQNHVVIGDDSTIDFSKYYHFIPMPDLRAFANAGFPFSRMADLSQTITVMPKAPNEAQMETLLNT
VGFIGAQTGFPAINLTVTDDGSTIQGKDADIMIIGGIPDKLKDDKQIDLLVQATESWVKTPMRQTPFPGI
VPDESDRAAETQSTLTSFGAMAAVIGFQSPYNDQRSVIALLADSPRGYEMLNDAVNDSGKRATMFGSVAV
IRESGINSLRVGDVYYVGHLPWFERLWYALANHPILLAVLATISVILLAWVLWRLLRIISRRRLNPDNE

>gi|30043448|gb|AAP19168.1| hypothetical protein S4201 [Shigella flexneri 2a str. 2457T]

MAVLGLQGVRGGVGTTTITAALAWSLQMLGENVLVVDACPDNLLRLSFNVDFTHRQGWARAMLDGQDWRD

AGLRYTSQLDLLPFGQLSIEEQENPQHWQTRLSDICSGLQQLKASGRYQWILIDLPRDASQITHQLLSLC

EHSLAIVNVDANCHIRLHQQALPDGAHILINDFRIGSQVQDDIYQLWLQSQRRLLPMLIHRDEAMAECLA

AKQPVGEYRSDALAAEEILTLANWCLLNYSGLKTPVGSAS

>gi|30043447|gb|AAP19167.1| hypothetical protein S4200 [Shigella flexneri 2a str. 2457T]

MNNNEPDTLPDPAIGYIFQNDIVALKQAFSLPDIDYADISQREQLAAALKRWPLLAEFAQQK

>gi|30043446|gb|AAP19166.1| putative protease [Shigella flexneri 2a str. 2457T]

MRDIVDPVFSIGISSLWDELRHMPAGGVWWFNVDRHEDAISLANQTIASQAETAHVAVISMDSDPAKIFQ

LDDSQGPEKIKLFSMLNHEKGLYYLARDLQCSIDPHNYLFILVCANNAWQNIPAERLRSWLDKMNKWSRL

NHCSLLVINPGNNNDKQFSLLLEEYRSLFGLASLRFQGDQHLLDIAFWCNEKGVSARQQLSVQQQNGIWT

LVQSEEAEIQPRSDEKRILSNVAVLEGAPPLSEHWQLFNNNEVLFNEARTAQAATVVFSLQQNAQIEPLA

RSIHTLRRQRGSAMKILVRENTASLRATDERLLLACGANMVIPWNAPLSRCLTMIESVQGQKFSRYVPED

ITTLLSMTQPLKLRGFQKWDVFCNAVNNMMNNPLLPAHGKGVLVALRPVPGIRVEQALTLCRPNRTGDIM

TIGGNRLVLFLSFCRINDLDTALNHIFPLPTGDIFSNRMVWFEDDQISAELVQMRLLAPEQWGMPLPLTQ

SSKPVINAEHDGRHWRRIPEPMRLLDDAVERSS

>gi|30043445|gb|AAP19165.1| hypothetical protein S4198 [Shigella flexneri 2a str. 2457T]

MMTISDIIEIIIVCALIFFPLGYLARHSLRRIRDTLRLFFAKPRYVKPAGTLRRTEKARATKK

>gi|30043444|gb|AAP19164.1| hypothetical protein S4197 [Shigella flexneri 2a str. 2457T]

MTQFTQNTAMPSSLWQYWRGLSGWNFYFLVKFGLLWAGYLNFHPLLNLVFAAFLLMPIPRYSLHRLRHWI

ALPIGFALFWHDIWLPGPESIMSQGSQVAGFSTDYLIDLVTRFINWQMIGAIFVLLVAWLFLSQWIRITV

FVVAILLWLNVLTLAGPSFSLWPAGQPTTTVTTTGGNAAATVAATGGAPVVGDMPAQTAPPTTANLNAWL

NNFYNAEAKRKSTFPSSLPADAQPFELLVINICSLSWSDIEAAGLMSHPLWSHFDIDFKNFNSATSYSGP

AAIRLLRASCGQTSHTNLYQPANNDCYLFDNLSKLGFTQHLMMGHNGQFGGFLKEVRENGGMQTELMDQT

NLPVILLGFDGSPVYDDTAVLNRWLDVTEKDKNSRSATFYNTLPLHDGNHYPGVSKTADYKARAQKFFDE

LDAFFTELEKSGRKVMVVVVPEHGGALKGDRMQVSGLRDIPSPSITDVPVGVKFFGMKAPHQGAPIVIDQ

PSSFLAISDLVVRVLDGKIFTEDNVDWKKLTSGLPQTAPVSENSNAVVIQYQDKPYVRLNGGDWVPYPQ

>gi|30043443|gb|AAP19163.1| putative transporter protein [Shigella flexneri 2a str. 2457T]

MQHNTLSKHNQKLPFTRYDFGWVLLCIGMAIGAGTVLMPVQIGLKGIWVFITAAIIAYPVTWVVQDIYLK

TLSESDSCNDYTDIISHYLGKNWGIFLGVIYFLMIIHGIFIYSLSVVFDSASYLKTFGLTDADLSQSLLY

KVAIFAVLIAIASGGERLLFKISGPMVVVKVGIIVVFGFAMIPHWNFANITAFPQASVFFRDVLLTIPFC

FFSAVFIQVLNPMNIAYRKREADKVLATRLALRTHRISYITLIAVILFFAFSFTFSISHEEAVSAFEQNI

SALALAAQVIPGHIIHITSTVLNIFAVLTAFFGIYLGFHEAIKGIILNLLSRIIDTKKINSRMLTLAICA

FIVITLTIWVSFRVSVLVFFQLGSPLYGIVSCLIPFFLIYKVAQLEKLRGFKAWLILLYGILLCLSPLLK

LIE

>gi|30043442|gb|AAP19162.1| putative ATP-binding component of dipeptide transport system [Shigella flexneri 2a str. 2457T]

MSTQEATSQQPLLQAIDLKKHYPVKKGMFVPERLVKALDGVSFNLERGKTLAVVGESGCGKSTLGRLLTM

IETPTGGELYYQGQDLLKHDPQAQKLRRQKIQIVFQNPYGSLNPRKKVGQILEEPLLINTSLSKEQRREK

ALSMMAKVGLKTEHYDRYPHMFSGGQRQRIAIARGLMLDPDVVIADEPVSALDVSVRAQVLNLMMDLQQE

LGLSYVFISHDLSVVEHIADEVMVMYLGRCVEKGTKDQIFNNPRHPYTQALLSATPRLNPDDRRERIKLT

GELPSPLNPPPGCAFNARCRRRFGPCTQLQPQLKDYGGQLVACFAVDQDENPQR

>gi|30043441|gb|AAP19161.1| putative ATP-binding component of dipeptide transport system [Shigella flexneri 2a str. 2457T]

MALLNVDKLSVHFGDESAPFRAVDRISYSVKQGEVVGIVGESGSGKSVSSLAIMGLIDYPGRVMAEKLEF

NGQDLQRISEKERRNLVGAEVAMIFQDPMTSLNPCYTVGFQIMEAIKVHQGGNKSTRRQRAIDLLNQVGI

PDPASRLDVYPHQLSGGMSQRVMIAMAIACRPKLLIADEPTTALDVTIQAQIIELLLELQQKENMALVLI

THDLALVAEAAHKIIVMYAGQVVETGDAHAIFHAPRHPYTQALLRALPEFAQDKERLASLPGVVPGKYDR

PNGCLLNPRCPYATDRCRAEEPALNMLADGRQSKCHYPLDDAGRPTL

>gi|30043440|gb|AAP19160.1| dipeptide transport system permease protein 2 [Shigella flexneri 2a str. 2457T]

MSQVTENKVISAPVPMTPLQEFWHYFKRNKGAVVGLVYVAIVLFIAIFANWIAPYNPADQFRDALLAPPA

WQEGGSMAHLLGTDDVGRDVLSRLMYGARLSLLVGCLVVVLSLIMGVILGLIAGYFGGLVDNIIMRVVDI

MLALPSLLLALVLVAIFGPSIGNAALALTFVALPHYVRLTRAAVLVEVNRDYVTASRVAGAGAMRQMFIN

IFPNCLAPLIVQASLGFSNAILDMAALGFLGMGAQPPTPEWGTMLSDVLQFAQSAWWVVTFPGLAILLTV

LAFNLMGDGLRDALDPKLKQ

>gi|30043439|gb|AAP19159.1| dipeptide transport system permease protein 1 [Shigella flexneri 2a str. 2457T]

MLQFILRRLGLVIPTFIGITLLTFAFVHMIPGDPVMIMAGERGISPERHVQLLAELGLDKPMWQQYLHYI

WGVMHGDLGISMKSRIPVWEEFVPRFQATLELGVCAMIFATAVGIPVGVLAAVKRGSIFDHTAVGLALTG

YSMPIFWWGMMLIMLVSVHWNLTPVSGRVSDMVFLDDSNPLTGFMLIDTAIWGEDGNFIDAVAHMILPAI

VLGTIPLAVIVRMTRSSMLEVLGEDYIRTARAKGLTRMRVIIVHALRNAMLPVVTVIGLQVGTLLAGAIL

TETIFSWPGLGRWLIDALQRRDYPVVQGGVLLVATMIILVNLLVDLLYGVVNPRIRHKK

>gi|30043438|gb|AAP19158.1| dipeptide transport protein [Shigella flexneri 2a str. 2457T]

MRISLKKSGMLKLGLSLVAMTVAASVQAKTLVYCSEGSPEGFNPQLFTSGTTYDASSVPLYNRLVEFKIG

TTEVIPGLAEKWEVSEDGKTYTFHLRKGVKWHDNKEFKPTREMNADDVVFSFDRQKNAQNPYHKVSGGSY

EYFEGMGLPELISEVKKVDDNTVQFVLTRPEAPFLADLAMDFASILSKEYADAMMKAGTPEKLDLNPIGT

GPFQLQQYQKDSRIRYKAFDGYWGTKPQIDTLVFSITPDASVRYAKLQKNECQVMPYPNPADIARMKQDK

SINLMEMPGLNVGYLSYNVQKKPLDDVKVRQALTYAVNKDAIIKAVYQGAGVSAKNLIPPTMWGYNDDVQ

DYTYDPEKAKALLKEAGLEKGFSIDLWAMPVQRPYNPNARRMAEMIQADWAKVGVQAKIVTYEWGEYLKR

AKDGEHQTVMMGWTGDNGDPDNFFATLFSCAASEQGSNYSKWCYKPFEDLIQPARATDDHNKRVELYKQA

QVVMHDQAPALIIAHSTVFEPVRKEVKGYVVDPLGKHHFENVSIE

>gi|30043437|gb|AAP19157.1| hypothetical protein S4189 [Shigella flexneri 2a str. 2457T]

MLSPVCPGFVCMRYIKSITQQKLSFLLAIYIGLFMNGAVFYRRFGSYAHDFTVWKGVSAVVELAATVLVT

FFLLRLLSLFGRRSWRILASLVVLFSAGASYYMTFLNVVIGYGIIASVMTTDIDLSKEVVGLNFILWLIA

VSALPLILIWNNRCRYTLLRQLRTPGQRIRSLVVVVLAGIMVWAPIRLLDIQQKKVERATGVDLPSYGGV

VANSYLPSNWLSALGLYAWARVDESSDNNSLLNPAKKFTYQAPQNVDDTYVVFIIGETTRWDHMGIFGYE

RNTTPKLAQEKNLAAFRGYSCDTATKLSLRCMFVRQGGAEDNPQRTLKEQNIFAVLKQLGFSSDLYAMQS

EMWFYSNTMADNIAYREQIGAEPRNRGKPVDDMLLVDEMQQSLGRNPDGKHLIILHTKGSHFNYTQRYPR

SFAQWKPECIGVDSGCTKAQMINSYDNSVTYVDHFISSVIDQVRDKKAIVFYAADHGESINEREHLHGTP

RELAPPEQFRVPMMVWMSDKYLENPANAQAFAQLKKEADMKVPRRHVELYDTIMGCLGYTSPDGGINENN

NWCHIPQTKVAAAN

>gi|30043436|gb|AAP19156.1| putative resistance protein [Shigella flexneri 2a str. 2457T]

MTPSNYQRTRWLTLIGTIITQFALGSVYTRSLFNGALSAKLDAPVSQVAFSFGLLSLGLAISSSVAGKLQ

ERFGVKRVTVASGILLGLGFFLTAHSNNLMMLWLSAGVLVGLADGAGYLLTLSNCVKWFPERKGLISAFA

IGSYGLGSLGFKFIDTQLLETVGLEKTFVIWGAIALVMIVFGATLMKDAPKQEVKTSNGVVEKDYTLAES

MRKPQYWMLAVMFLTACMSGLYVIGVAKDIAQSLAHLDVVSAANAVTVISIANLSGRLVLGILSDKIARI

RVITIGQVISLVGMAALLFAPLNAVTFFAAIACVAFNFGGTITVFPSLVSEFFGLNNLAKNYGVIYLGFG

IGSIFGSIIASLFGGFYVTFYVIFALLILSLALSTTIRQPEQKMLREAHGSL

>gi|30043435|gb|AAP19155.1| putative lipase [Shigella flexneri 2a str. 2457T]

MIIKKSGGRWQLSLLASVVISAFFLNTAYAWQQEYIVDTQPGHSTERYTWDSDHQPDYNDILSQRIQSSQ

RALGLEVNLAEETPVDVTSSMSMGWNFPLYEQVTTGPVAALHYDGTTTSMYNEFGDSTTTLTDPLWHASV

SSLGWRVDSRLGDLRPWAQISYNQQFGENIWKAQSGLSRMTATNQNGNWLDVTVGADMLLNQNIAAYAAL

TQAENTTNNSDYLYTMGVSARF

>gi|30043434|gb|AAP19154.1| 3-methyl-adenine DNA glycosylase I [Shigella flexneri 2a str. 2457T]

MERCGWVSQGPLYIAYHDNEWGVPETDSKKLFEMICFEGQQAGLSWITVLKKRENYRAYFHQFDPVKVAA

MQEEDVERLVQDAGIIRHRGKIQAIIGNARAYLQMEQNGEPFPDFVWSFVNHQPQVTQATTLSEIPTSTS

ASDALSKALKKRGFKFVGTTICYSFMQACGLVNDHVVGCCCYLGNKP

>gi|30043433|gb|AAP19153.1| hypothetical protein S4185 [Shigella flexneri 2a str. 2457T]

MIREAQRSELPAILELWLESTTWGHPFIKANYWRDCIPLVRDAYLANAQNWVWEEDGKLLGFVSIMEGRF

LAAMFVAPKAVRRGIGKALMQYVQQRYPHLMLEVYQKNQPAIDFYRAQGFHIVDCAWQDETQLPTWIMSW

PVVQTL

>gi|30043432|gb|AAP19152.1| biotin sulfoxide reductase [Shigella flexneri 2a str. 2457T]

MLVETDGETVFSSRGALATGMENSLQSAVRDQVHSNTRVRFPMVRKGFLASPENPQGIRGQDEFVRVSWD

EALDLIHQQHKRIREAYGPASIFAGSYGWRSNGVLHKASTLLQRYMALAGGYTGHLGDYSTGAAQAIMPY

VVGGSEVYQQQTSWPLVLEHSDVVVLWSANPLNTLKIAWNASDEQGLSYFSALRDSGKKLICIDPMRSET

VDFFGDKMEWVAPHMGTDVALMLGIAHTLVENGWHDEAFLARCTTGYAVFASYLLGESDGIAKNAEWAAE

ICGVGAAKIRELAAIFHQNTTMLMAGWGMQRQQFGEQKHWMIVTLAAMLGQIGTPGGGFGLSYHFANGGN

PTRRSAVLSSMQGSLPGGCDAVDKIPVARIVEALENPGGAYQHNGMNRHFPDIRFIWWAGGANFTHHQDT

NRLIRAWQKPELVVISECFWTAAAKHADIVLPATTSFERNDLTMTGDYSNQHLVPMKQVVPPRYEARNDF

DVFAELSERWEKGGYARFTEGKSQLQWLETFYNVARQRGASQQVELPPFAEFWQANQLIEMPENPDSERF

IRFADFCRDPLAHPLKTASGKIEIFSQRIADYGYPDCPGHPMWLEPDEWQGNAEPEQLQVLSAHPAHRLH

SQLNYSSLRELYAVANREPVTIHPDDAQARGITEGDMVRVWNSRGQILAGAVISEGIKPGVICIHEGAWP

DLDLTADGICKNGAVNVLTKDLPSSRLGNGCAGNTTLAWLEKYNGPELTLTAFEPPASS

>gi|30043431|gb|AAP19151.1| putative outer membrane protein [Shigella flexneri 2a str. 2457T]

MSGALAVSGCTTNPYTGEREAGKSAIGAGLGSLVGAGIGALSSSKKDRGKGALIGAAAGAALGGGVGYYM

DVQEAKLRDKMRGTGVSVTRSGDNIILNMPNNVTFDSSSAPLKPAGANTLTGVAMVLKEYPKTAVNVIGY

TDSTGGHDLNMRLSQQRADSVASALITQGVDASRIRTQGLGPANPIASNSTAEGKAQNRRVEITLSPL

>gi|30043430|gb|AAP19150.1| putative dehydrogenase [Shigella flexneri 2a str. 2457T]

MKPSVILYKALPDDLLQRLQAHFTVHQVANLSPQTVVQNAAIFAEAEGLLGSNENVDAALLEKMPKLRAT

STISVGYDNFDVDALTARKILLMHTPTVLTETVADTLMALVLSTARRVVEVAERVKAGEWTASIGPDWYG

TDVHHKTLGIVGMGRIGMALAQRVHFGFNMPILYNARRHHKEAEERFNARYCDLDTLLQESDFVCLILPL

TDETHHLFGAEQFAKMKSSAIFINAGRGPVVDENALIAALQKGEIHAAGLDVFEQEPLSVDSPLLSMANV

VAVPHIGSATHETRYGMAACAVDNLIDALQGKVEKNCVNPHVAD

>gi|30043429|gb|AAP19149.1| hypothetical protein S4181 [Shigella flexneri 2a str. 2457T]

MILPGRLRRKGILQACPGLSLSRQTRVCRCALFLGERSKKMATGKSCSRWFAPLAALLMVVSLSGCFDKE

GDQRKAFIDFLQNTVMRSGERLPTLTADQKKQFGPFVSDYAILYGYSQQVNQAMDSGLRPVVDSVNAIRV

PQDYVTQSGPLREMNGSLGVLAQQLQNAKLQADAAHSALKQSDDLKPVFDQAFTKVVTTPADALQPLIPA

AQTFTQQLVMVGDYIAQQGTQVSFVANGIQFPTSQQASEYNKLIAPLPAQHQAFNQAWTTAVTATQ

>gi|30043428|gb|AAP19148.1| hypothetical protein S4180 [Shigella flexneri 2a str. 2457T]

MEYKDPMHELLSSLEQIVFKDETQKITLTHRTTSCTEIEQL

>gi|30043427|gb|AAP19147.1| cold shock protein 7.4, transcriptional activator of hns [Shigella flexneri 2a str. 2457T]

MSGKMTGIVKWFNADKGFGFITPDDGSKDVFVHFSAIQNDGYKSLDEGQKVSFTIESGAKGPAAGNVTSL

>gi|30043425|gb|AAP19146.1| IS150 putative transposase [Shigella flexneri 2a str. 2457T]

MKVLNELRQFYPLDELLRAAEIPRSTFYYHLKALSKPDKYADVKKRIGEIYHENRGRYGYRRVTLSLHRE

GKQINHKAVQRLMGTLSLKAAIKVKRYRSYRGEVGQTAPNVLQRDFKATRPNEKWVTDVTEFAVNGRKLY

LSPVIDLFNNEVISYSLSERPVMNMVENMLDQAFKKLNPHEHPVLHSDQGWQYRMRRYQNILKEHGIKQS

MSRKGNCLDNAVVECFFGTLKSECFYLDEFSNISELKDAVTEYIEYYNSRRISLKLKGLTPIEYRNQTYM

PRV

>gi|30043424|gb|AAP19145.1| IS1 orfA [Shigella flexneri 2a str. 2457T]

MASISIRCPSCSATEGVVRNGKSTAGHQRYLCSHCRKTWQLQFTYTASQPGTHQKIIDMAMNGVGCRASA

RIMGVGLNTVLRHLKNSGRSR

>gi|30043423|gb|AAP19144.1| IS1 orfB [Shigella flexneri 2a str. 2457T]

MSRQCTHYGRWPQHGFTSLKKLRPQSVTSRIQPGSDVIVCAEMDEHWGYVGAKSRQRWLFYAYDRIRRTV

VAHVFGERTLATLERLLSLLSAFEVVVWMTDGWPLYESRLKGKLHVISKRYTQRIERHNLNLRQHLARLV

RKSLSFSKSVELHDKVIGHYLNIKHYQ

>gi|30043422|gb|AAP19143.1| putative cytochrome C peroxidase [Shigella flexneri 2a str. 2457T]

MKMVSRITAIGLAGVAICYLGLSGYVWYHDNKRSKQADVQASAVSENNKVLGFLREKGCDYCHTPSAELP

AYYYIPGAKQLMDYDIKLGYKSFNLEAVRAALLADKPVSQSDLNKIEWVMQYETMPPTRYTALHWAGKVS

DEERAEILAWIAKQRAEYYASNDTAPEHRNEPVQPIPQKLPTDAQKVALGFALYHDPRLSADSTISCAHC

HTLNAGGVDGRKTSIGVGGAVGPINAPTVFNSVFNVEQFWDGRAATLQDQAGGPPLNPIEMASKSWDEII

AKLEKDPQLKAQFLEVYPQGFSGENITDAIAEFEKTLITPDSPFDKWLRGDENALTAQQKKGYQLFKDNK

CATCHGGIILGGRSFEPLGLKKDFNFGEITAADIGRMNVTKEERDKLRQKVPGLRNVALTAPYFHRGDVP

TLDGAVKLMLRYQVGKELPQEDVDDIVAFLHSLNGVYTPYMQDKQ

>gi|30043421|gb|AAP19142.1| glutamate decarboxylase isozyme [Shigella flexneri 2a str. 2457T]

MDQKLLTDFRSELLDSRFGAKAISTIAESKRFPLHEMRDDVAFQIINDELYLDGNARQNLATFCQTWDDE

NVHKLMDLSINKNWIDKEEYPQSAAIDLRCVNMVADLWHAPAPKNGQAVGTNTIGSSEACMLGGMAMKWR

WRKRMEAAGKPTDKPNLVCGPVQICWHKFARYWDVELREIPMRPGQLFMDPKRMIEACDENTIGVVPTFG

VTYTGNYEFPQPLHDALDKFQADTGIDIDMHIDAASGGFLAPFVAPDIVWDFRLPRVKSISASGHKFGLA

PLGCGWVIWRDEEALPQELVFNVDYLGGQIGTFAINFSRPAGQVIAQYYEFLRLGREGYTKVQNASYQVA

AYLADEIAKQGPYEFICTGRPDEGIPAVCFKLKDGEDPGYTLYDLSERLRLRGWQVPAFTLGGEATDIVV

MRIMCRRGFEMDFAELLLEDYKASLKYLSDHPKLQGIAQQNSFKHT

>gi|30043420|gb|AAP19141.1| putative ARAC-type regulatory protein [Shigella flexneri 2a str. 2457T]

MQSLHGNCLIAYARHKYILTMVNGEYRYFNGGDLVFADASQIRVDKCVENFVLVSRDTLSLFLPMLKEEA

LNLHAHKKISSLLVHHCSRDIPVFQEVAQLSQNKNLRYAEMLRKRALIFALLSVFLEDEHFIPLLLNVLQ

PNMRTRVCTVINNNIAHEWTLARIASELLMSPSLLKKKLREEETSYSQLLTECRMQRALQLIVIHGFSIK

RVAVSCGYHSVSYFIYVFRNYYGMTPTEYQERSAQGLPNRDSAASIVAQGNFYGTNRSAEGIRL

>gi|30043419|gb|AAP19140.1| putative ARAC-type regulatory protein [Shigella flexneri 2a str. 2457T]

MTHVCSVILIRRSFDIYHEQQKISLHNESILLLEKNLADDFAFCSPDTRRLDIDELTVCHYLQNIRQLPR

NLGLHSKDRLLINQSPPMPLVTAIFDSFNESGVNSPILSNMLYLSCLSMFSHKKELIPLLFNSISTVSGK

VERLISFDIAKRWYLRDIAERMYTSESLIKKKLQDENTCFSKILLASRMSMARRLLELRQIPLHTIAEKC

GYSSTSYFINTFRQYYGVTPHQFAQHSPGTFS

>gi|30043418|gb|AAP19139.1| hypothetical protein S4169 [Shigella flexneri 2a str. 2457T]

MIFKNILNNFARFILLVPQEGVTHNAQGKATALILDKDDVVQLREIEASKAIGDQWVVTSGLQAGDRVIV

SGLQRIRPGIKARAISSSQENASTESKQ

>gi|30043417|gb|AAP19138.1| IS1 orfA [Shigella flexneri 2a str. 2457T]

MASISIRCPSCSATEGVVRNGKSTAGHQRYLCSHCRKTWQLQFTYTASQPGTHQKIIDMAMNGVGCRASA

RIMGVGLNTVLRHLKNSGRSR

>gi|30043416|gb|AAP19137.1| IS1 orfB [Shigella flexneri 2a str. 2457T]

MSCQCTHYGRWPQHGFTSLKKLRPQSVTSRIQPGSDVIVCAEMDEHWGYVGAKSRQRWLFYAYDRIRRTV

VAHVFGERTLATLERLLSLLSAFEVVVWMTDGWPLYESRLKGKLHVISKRYTQRIERHNLNLRQHLARLV

RKSLSFSKSVELHDKVIGHYLNIKHYQ

>gi|30043415|gb|AAP19136.1| glycine tRNA synthetase, beta subunit [Shigella flexneri 2a str. 2457T]

MSEKTFLVEIGTEELPPKALRSLAESFAANFTAELDNAGLAHGTVQWFAAPRRLALKVANLAEAQPDREI

EKRGPAIAQAFDAEGKPSKAAEGWARGCGITVDQAERLTTDKGEWLLYRAHVKGESTEALLPNMVATSLA

KLPIPKLMRWGASDVHFVRPVHTVTLLLGDKVIPATILGIQSDRVIRGHRFMGEPEFTIDNADQYPEILR

ERGKVIADYEERKAKIKADAEEAARKIGGNADLSESLLEEVASLVEWPVVLTAKFEEKFLAVPAEALVYT

MKGDQKYFPVYANDGKLLPNFIFVANIESKDPQQIISGNEKVVRPRLADAEFFFNTDRKKRLEDNLPRLQ

TVLFQQQLGTLRDKTDRIQALAGWIAEQIGADVNHATRAGLLSKCDLMTNMVFEFTDTQGVMGMHYARHD

GEAEDVAVALNEQYQPRFAGDDLPSNPVACALAIADKMDTLAGIFGIGQHPKGDKDPFALRRAALGVLRI

IVEKNLNLDLQTLTEEAVRLYGDKLTNANVVDDVIDFMLGRFRAWYQDEGYTVDTIQAVLARRPTRPADF

DARMKAVSHFRTLEAAAALAAANKRVSNILAKSDEVLSDRVNASTLKEPEEIKLAMQVVVLRDKLEPYFA

EGRYQDALVELAELREPVDAFFDKVMVMVDDKELRLNRLTMLEKLRELFLRVADISLLQ

>gi|30043414|gb|AAP19135.1| glycine tRNA synthetase, alpha subunit [Shigella flexneri 2a str. 2457T]

MQKFDTRTFQGLILTLQDYWARQGCTIVQPLDMEVGAGTSHPMTCLRALGPEPMAAAYVQPSRRPTDGRY
GENPNRLQHYYQFQVVIKPSPDNIQELYLGSLKELGMDPTIHDIRFVEDNWENPTLGAWGLGWEVWLNGM
EVTQFTYFQQVGGLECKPVTGEITYGLERLAMYIQGVDSVYDLVWSDGPLGKTTYGDVFHQNEVEQSTYN
FEYADVDFLFTCFEQYEKEAQQLLALENPLPLPAYERILKAAHSFNLLDARKAISVTERQRYILRIRTLT
KAVAEAYYASREALGFPMCNKDK

>gi|30043413|gb|AAP19134.1| hypothetical protein S4164 [Shigella flexneri 2a str. 2457T]
MQPKIYWIDNLRGIACLMVVMIHTTTWYVTNAHSVSPVTWDIANVLNSASRVSVPLFFMISGYLFFGERS
AQPRHFLRIGLCLIFYSAIALLYIALFTSINMELALKNLLQKPVFYHLWFFFAIAVIYLVSPLIQVKNVG
GKMLLVLMVVIGIIANPNTVPQKIDGFEWLPINLYINGDTFYYILYGMLGRAIGMMDTQHKALSWVSAAL
FATGVFIISRGTLYELQWRGNFADTWYLYCGPMVFICAIALLTLVKNTLDTRTIRGLGLISRHSLGIYGF
HALIIHALRTRGIELKNWPILDIIWIFCATLAASLLLSMLVQRIDRNRLVS

>gi|30043412|gb|AAP19133.1| hypothetical protein S4163 [Shigella flexneri 2a str. 2457T]
MDNKISTYSPAFSIVSWIALVGGIVTYLLGLWNAEMQLNEKGYYFAVLVLGLFSAASYQKTVRDKYEGIP
TTSIYYMTCLTVFIISVALLMVGLWNATLLLSEKGFYGLAFFLSLFGAVAVQKNIRDAGINPPKETQVTQ
EEYSE

>gi|30043411|gb|AAP19132.1| hypothetical protein S4162 [Shigella flexneri 2a str. 2457T]
MKTSKTVAKLLFVVGALVYLVGLWISCPLLSGKGYFLGVLMTATFGNYAYLRAEKLGQLDNFFTHICQLV
ALITIGLLFIGVLNAPINAYEMVIYPIAFFVCLFGQMRLFRSV

>gi|30043410|gb|AAP19131.1| xylulokinase [Shigella flexneri 2a str. 2457T]
MYIGIDLGTSGVKVILLNEQGEVVASQTEKLTVSRPHPLWSEQDPEQWWQATDRAMKALGDQHSLQDVKA
LGIAGQMHGATLLDAQQRVLRPAILWNDGRCAQECTLLEARVPQSRVITGNLMMPGFTAPKLLWVQRHEP

EIFRQIDKVLLPKDYLRLRMTGEFASDMSDAAGTMWLDVAKRDWSDVMLQACDLSRDQMPALYEGSEITG

ALLPEVAKAWGMATVPVVAGGGDNAAGAVGVGMVDANQAMLSLGTSGVYFAVSEGFLSKPESAVHSFCHA

LPQRWHLMSVMLSAASCLDWAAKLTGLCNVPALIAAAQQADESAEPVWFLPYLSGERTPHNNPQAKGVFF

GLTHQHGPNELARAVLEGVGYALADGMDVVHACGIKPQSVTLIGGGARSEYWRQMLADISGQQLDYRTGG

DVGPALGAARLAQIAANPEKSLIELLPQLPLEQSHLPDAQRYAAYQPRRETFRRLYQQLLPLMA

>gi|30043409|gb|AAP19130.1| xylose binding protein [Shigella flexneri 2a str. 2457T]

MKIKNILLTLCTSLLLTNVAAHAKEVKIGMAIDDLRLERWQKDRDIFVKKAESLGAKVFVQSANGNEETQ

MSQIENMINRGVDVLVIIPYNGQVLSNVVKKAKQEGIKVLAYDRMINDADIDFYISFDNEKVGELQAKAL

VDIVPQGNYFLMGGSPVDNNAKLFRAGQMKVLKPYVDSGKIKVVGDQWVDGWLPENALKIMENALTANNN

KIDAVVASNDATAGGAIQALSAQGLSGKVAISGQDADLAGIKRIAAGTQTMTVYKPITLLANTAAEIAVE

LGNGQEPKADTSLNNGLKDVPSRLLTPIDVNKNNIKDTVIKDGFHKESEL

>gi|30043408|gb|AAP19129.1| putative ATP-binding protein of xylose transport system [Shigella flexneri 2a str. 2457T]

MPYLLEMKNITKTFGSVKAIDNVSLRLNAGEIVSLCGENGSGKSTLMKVLCGIYPHGSYEGEIIFAGEEI

QASHIRDTERKGIAIIHQELALVKELTVLENIFLGNEITHNGIMDYDLMTLRYQKLLAQVSLSISPDTRV

GDLGLGQQQLVEIAKALNKQVRLLILDEPTASLTEQETSVLLDIIRDLQQHGIACIYISHKLNEVKAISD

TICVIRDGQHIGTRDAAGMSEDDIITMMVERELTAIYPNEPHTTGDEILRIEHLTAWHPVNRHIKRVNDV

SFSLKRGEILGIAGLVGAGRTETIQCLFGVWPGQWEGKIYIDGKQVDIRNCQQAITQGIAMVPEDRKRDG

IVPVMAVGKNITLAALNKFTGGISQLDDAAEQKCILESIQQLKVKTSSPDLAIGRLSGGNQQKAILARCL

LLNPRILILDEPTRGIDIGAKYEIYKLINQLVQQGIAVIVISSELPEVLGLSDRVLVMHEGKLKANLINH

NLTQEQVMEAALRSEHHVEKQSV

>gi|30043407|gb|AAP19128.1| putative xylose transporter, membrane component [Shigella flexneri 2a str. 2457T]

MSKSNPSEVKLAVPTSGGFSGLKSLNLQVFVMIAAIIAIMLFFTWTTDGAYLSARNVSNLLRQTAITGIL
AVGMVFVIISAEIDLSVGSMMGLLGGVAAICDVWLGWPLPLTIIVTLVLGLLLGAWNGWWVAYRKVPSFI
VTLAGMLAFRGILIGITNGTTVSPTSAAMSQIGQSYLPASTGFIIGALGLMAFVGWQWRGRMRRQALGLQ
SPASTAVVGRQALTAIIVLGAIWLLNDYRGVPTPVLLLTLLLLGGMFMATRTAFGRRIYAIGGNLEAARL
SGINVERTKLAVFAINGLMVAIAGLILSSRLGAGSPSAGNIAELDAIAACVIGGTSLAGGVGSVAGAVMG
AFIMASLDNGMSMMDVPTFWQYIVKGAILLLAVWMDSATKRRS

>gi|30043406|gb|AAP19127.1| putative ATP-binding protein [Shigella flexneri 2a str. 2457T]
MILTPIRRYGAMILMLLTLVFSSEVLAKTHTTTASQKSHLTKASNKQVSSKQEYSRNSAKSSSLPDLRKY
PSGTPRKKAFLRTVMPYITSQNAAITAERNWLISKQYQGQWAPAERARLKDIAKRYKVKWSGNTRKIPWN
TLLERVDIIPTSMVATMAAAESGWGTSKLARNNNNLFGMKCMKGRCTNAPGKVKGYSQFSSVKESVSAYV
TNLNTHPAYSSFRKSRAQLRKADQEVTATAMIHKLKGYSTKGKSYNNYLFAMYQDNQRLIAAHM

>gi|30043405|gb|AAP19126.1| alpha-amylase [Shigella flexneri 2a str. 2457T]
MKLAACFLTLLPGFAVAASWTSPGFPAFSEQGTGTFVSHAQLPKGTRPLTLNFDQQCWQPADAIKLNQML
SLQPCSNTPPQWRLFRDGKYTLQIDTRSGTPTLMISIQNAAEPVANLVRECPKWDGLPLTLDVSATFPEG
AAVRDYYSQQIAIVKNGQITLQPAATSNGLLLLERAETDTSAPFDWHNATVYFVLTDRFENGDPSNDQSY
GRHKDGMAEIGTFHGGDLRGLTNKLDYLQQLGVNALWISAPFEQIHGWVGGGTKGDFPHYAYHGYYTQDW
TNLDANMGNEADLRTLVDSAHQRGIRILFDVVMNHTSYATLADMQEYQFGALYLSGDEVKKTLGERWSDW
KPAAGQTWHSFNDYINFSDKTGWDKWWGKNWIRTDIGDYDNPGFDDLTMSLAFLPDIKTESTTASGLPVF
YKNKTDTHAKVIEGFTPRDYLTHWLSQWVRDYGIDGFRVDTAKHVELPAWQQLKTEASSALREWKKTNPD
KALDDKPFWMTGEAWGHGVMQSDYYRHGFDAMINFDYQEQAAKAVDCLAQMDTTWQQMAEKLQGFNVLSY
LSSHDTRLFREGGDKAAELLLLAPGAVQIFYGDESSRPFGPTGSDPLQGTRSDMNWQDVSGKSAANVAHW
QKISQFRARHPAIGAGKQTTLSLKQGYGFVREHGDDKVLVIWAGQQ

>gi|30043404|gb|AAP19125.1| alanine-alpha-ketoisovalerate/valine-pyruvate transaminase C [Shigella flexneri 2a str. 2457T]

MTFSLFGDKFTRHSGITLLMEDLNDGLRTPGAIMLGGGNPAQIPEMQDYFQTLLTDMLESGKATDALCNY

DGPQGKTELLTLLAGMLREKLGWDIEPQNIALTNGSQSAFFYLFNLFAGRRADGRVKKVLFPLAPEYIGY

ADAGLEEDLFVSARPNIELLPEGQFKYHVDFEHLHIGEETGMICVSRPTNPTGNVITDEELLKLDALANQ

HGIPLVIDNAYGVPFPGIIFSEGRPLWNPNIVLCMSLSKLGLPGSRCGIIIANEKIITAITNMNGIISLA

PGGIGPAMMCEMIKRNDLLRLSETVIKPFYYQRVQETIAIIRRYLPENRCLIHKPEGAIFLWLWFKDLPI

TTEQLYQRLKARGVLMVPGHNFFPGLDKPWPHTHQCMRMNYVPEPEKIEAGVKILAEEIERAWAESH

>gi|30043403|gb|AAP19124.1| hypothetical protein S4152 [Shigella flexneri 2a str. 2457T]

MNRFIIADATKCIGCRTCEVACAVSHQENQDCAALSPDEFISRIRVIKDHSWTTPVACHQCEDAPCANVC

PVDAISREHGHIFVEQTRCIGCKSCMLACPFGAMEVVSSRKKARAIKCDLCWHRETGPACVEACPTKALQ

CMDVEKVQRHRLQQQPV

>gi|30043402|gb|AAP19123.1| putative regulator [Shigella flexneri 2a str. 2457T]

MGKEVMGKKENEMAQEKERPAGSQSLFRGLMLIEILSNYPNGCPLAHLSELAGLNKSTVHRLLQGLQSCG

YVTTAPAAGSYRLTTKFIAVGQKALSSLNIIHIAAPHLEALNIATGETINFSSREDDHAILIYKLEPTTG

MLRTRAYIGQHMPLYCSAMGKIYMAFGHPDYVKSYWENHQHEIQPLTRNTITELPAMFDELAHIRESGAA

MDREENELGVSCIAVPVFDIHGRVPYAVSISLSTSRLKQVGEKNLLKPLRETAQAISNELGFTVRDDLGA

IT

>gi|30043401|gb|AAP19122.1| putative dehydrogenase [Shigella flexneri 2a str. 2457T]

MKVTFEQLKAAFNRVLISRGVDSETADACAEMFARTTESGVYSHGVNRFPRFIQQLENGDIIPDAQPKRI

TSLGAIEQWDAQRSIGNLTAKKMMDRAIELAADHGIGLVALRNANHWMRGGSYGWQAAEKGYIGICWTNS

IAVMPPWGAKECRIGTNPLIVAIPSTPITMVDMSMSMFSYGMLEVNRLAGRQLPVDGGFDDEGNLTKEPG

VIEKNRRILPMGYWKGSGMSIVLDMIATLLSDGASVAEVTQDNSDEYGISQIFIAIEVDKLIDGPTRDAK

LQRIMDYVTTAERADENQAIRLPGHEFTTLLAENRRNGITVDDSVWAKIQAL

>gi|30043400|gb|AAP19121.1| IS1 orfA [Shigella flexneri 2a str. 2457T]

MASISIRCPSCSATEGVVRNGKSTAEHQRYLCSHCRKTWQLQFTYTASQPGTHQKIIDMAMNGVGCRASA

RIMGVGLNTVLRHLKNSGRSR

>gi|30043399|gb|AAP19120.1| IS1 orfB [Shigella flexneri 2a str. 2457T]

MSRQCTHYGRWPQHGFTSLKKLRPQSVTSRIQPGSDVIVCAEMDEHWGYVGAKSRQRWLFYAYDRIRRTV

VAHVFGERTLATLERLLSLLSAFEVVVWMTDGWPLYESRLKGKLHVISKRYTQRIERHNLNLRQHLARLG

RKSLSFSKSVELHDKVIGHYLNIKHYQ

>gi|30043398|gb|AAP19119.1| putative membrane protein [Shigella flexneri 2a str. 2457T]

MAVLIFLGCLLGGIAIGLPIAWALLLCGAALMFWLDMFDVQIMAQTLVNGADSFSLLAIPFFVLAGEIMN

AGGLLKRIVDLPMKLVGHKPGGLGYVGVLAAMIMASLSGSAVADTAAVAALLVPMMRSANYPVNRAAGLI

ASGGIIAPIIPPSIPFIIFGVSSGLSISKLFMAGIAPGIMMGATLMLTWWRQASRLNLPRQQKATMQEIW

HSFVSGIWALFLPVIIIGGFRSGLFTPTEAGAVAAFYALFVATVIYREMTFATLWHVLIGAAKTTSVVMF

LVASAQVSAWLITIAELPMMVSDLLQPLVDSPRLLFIVIMVAILIVGMVMDLTPTVLILTPVLMPLVKEA

GIDPIYFGVMFIINCSIGLITPPIGNVLNVISGVAKLKFDDAVRGVFPYVLVLYSLLVVFVFIPDLIILP

LKWMVMLPTY

>gi|30043397|gb|AAP19118.1| IS1 orfA [Shigella flexneri 2a str. 2457T]

MASISIRCPSCSATEGVVRNGKSTAGHQRYLCSHCRKTWQLQFTYTASQPGTHQKIIDMAMNGVGCRASA

RIMGVGLNTVLRHLKNSGRSR

>gi|30043396|gb|AAP19117.1| IS1 orfB [Shigella flexneri 2a str. 2457T]

MSCQCTHYGRWPQHGFTSLKKLRPQSVTSRIQPGSDVIVCAEMDEHWGYVGAKSRQRWLFYAYDRIRRTV

VAHVFGERTLATLERLLSLLSAFEVVVWMTDGWPLYESRLKGKLHVISKRYTQRIERHNLNLRQHLARLG

RKSLSFSKSVELHDKVIGHYLNIKHYQ

>gi|30043395|gb|AAP19116.1| aldehyde dehydrogenase B (lactaldehyde dehydrogenase) [Shigella flexneri 2a str. 2457T]

MIAVVKLLPTGEDFASHVYPCYTSHRKETIMTNNPPSAQIKPGEYGFPIKLKARYDNFIGGEWVAPADGE

YYQNLTPVTGQLLCEVASSGKRDIDLALDAAHKVKDKWAHTSVQDRAAILFKIADRMEQNLELLATAETW

DNGKPIRETSAADVPLAIDHFRYFASCIRAQEGGISEVDSETVAYHFHEPLGVVGQIIPWNFPLLMASWK

MAPALAAGNCVVLKPARLTPLSVLLLMEIVGDLLPPGVVNVVNGAGGEIGEYLATSKRIAKVAFTGSTEV

GQQIMQYATQNIIPVTLELGGKSPNIFFADVMDEEDAFFDKALEGFALFAFNQGEVCTCPSRALVQESIY

ERFMERAIRRVESIRSGNPLDSVTQMGAQVSHGQLETILNYIDIGKKEGADVLTGGRRKLLEGELKDGYY

LEPTILFGQNNMRVFQEEIFGPVLAVTTFKTMEEALELANDTQYGLGAGVWSRNGNLAYKMGRGIQAGRV

WTNCYHAYPAHAAFGGYKQSGIGRETHKMMLEHYQQTKCLLVSYSDKPLGLF

>gi|30043394|gb|AAP19115.1| putative oxidoreductase [Shigella flexneri 2a str. 2457T]

MASSTFFIPSVNVIGADSLTDAMNMMADYGFTRTLIVTDNMLTKLGMAGDVQKALEERNIFSVIYDGTQP

NPTTENVAAGLKLLKENNCDSVISLGGGSPHDCAKGIALVAANGGDIRDYEGVDRSAKPQLPMIAINTTA

GTASEMTRFCIITDEARHNKMAIVDKHVTPLLSVNDSSLMIGMPKSLTAATGMDALTHAIEAYVSIAATP

ITDACALKAVTMIAENLPLAVEDGSNAKAREAMAYAQFLAGMAFNNASLGYVHAMAHQLGGFYNLPHGVC

NAVLLPHVQVFNSKVAAARLRDCAAAMGVNVTGKNDAEGAEACINAIRELAKKVDIPAGLRDLNVKEEDF

AVLATNALKDACGFTNPIQATHEEIVVIYRAAM

>gi|30043393|gb|AAP19114.1| selenocysteinyl-tRNA-specific translation factor [Shigella flexneri 2a str. 2457T]

MIIATAGHVDHGKTTLLQAITGVNADRLPEEKKRGMTIDLGYAYWPQPDGRVPGFIDVPGHEKFLSNMLA

GVGGIDHALLVVACDDGVMAQTREHLAILQLTGNPMLTVALTKADRVDEARVDEVERQVKEVLREYGFAE

AKLFITAATEGRGIDALREHLLQLPEREHASQHSFRLAIDRAFTVKGAGLVVTGTALSGEVKVGDSLWLT

GVNKPMRVRALHAQNQPTETANAGQRIALDIAGDAEKEQINRGDWLLADVPPEPFTRVIVELQTHAPLTQ

WQPLHIHHAASHVTGRVSLLEDNLAELVFDTPLWLADNDRLVLRDISARNTLAGARVVMLNPPRRGKRKP

EYLQWLASLARAQNDSEALAVHLQRGAVNLPDFAWARQLNGEGMRELLQQPGYIQAGYSLLNAPVAARWQ

RKILDTLATYHEQHRDEPGPGRERLRRMALPMEDEALVLLLIEKMRESGDILSHHGWLHLPDHKAGFSEE

QQAIWQKAEPLFGDEPWWVRDLAKETGTDEQAMRLTLRQAAQQGIITAIVKDRYYRNDRIVEFANMIRDL

DQECGSTCAADFRDRLGVGRKLAIQILEYFDRIGFTRRRGNDHLLRDALLFPEK

>gi|30043392|gb|AAP19113.1| selenocysteine synthase: L-seryl-tRNA (Ser) selenium transferase [Shigella flexneri 2a str. 2457T]

MTTETRSLYSQLPAIDRLLRDSSFLSLRDTYGHTRVVELLRQMLDEAREVIRGSQTLPAWCENWAQEVDA

RLTKEAQSALRPVINLTGTVLHTNLGRALQAEAAVEAVAQAMRSPVTLEYDLDDAGRGHRDRALAQLLCR

ITGAEDACIVNNNAAAVLLMLAATASGKEVVVSRGELVEIGGAFRIPDVMRQAGCTLHEVGTTNRTHAND

YRQAVNENTALLMKVHTSNYSIQGFTKAIDEAELVALGKELDVPVVTDLGSGSLVDLSQYGLPKEPMPQE

LIAAGVSLVSFSGDKLLGGPQAGIIVGKKEMIARLQSHPLKRALRADKMTLAALEATLRLYLHPEALSEK

LPTLRLLTRSAEVIQIQAQRLQAPLVAHYGAEFAVQVMPCLSQIGSGSLPVDRLPSAALTFTPHDGRGSH

LESLAARWRELPVPVIGRIYDGRLWLDLRCLEDEQRFLEMLLK

>gi|30043391|gb|AAP19112.1| putative S-transferase [Shigella flexneri 2a str. 2457T]

MKLVGSYTSPFVRKLFILLLEKGITFEFINELPYNADNGVAQFNPLGKVPVLVTEEGECWFDSPIIAEYI

ELMNVAPAMLPRDPLESLRVRKIEALADGIMDAGLVSVREQARPAAQQSEDELLRQREKINRSLDVLEGY

LVDGTLKTDTVNLATIAIACAVGYLNFRRVAPGWCVDRPHLVKLVENLFSRESFARTEPPKA

>gi|30043390|gb|AAP19111.1| putative membrane protein [Shigella flexneri 2a str. 2457T]

MDLLIVLTYVALATAALGGVFLVSGLILLMNYNHPYTFTAQKAVIAIPITPQVTGIVTEVTDKNNQLIQK

GEVLFKLDPVRYQARVDRLQADLMTATHNIKTLRAQLTEAQANTTQVSAERDRLFKNYQRYLKGSQAAVN

PFSERDIDDARQNFLAQDALVKGSVAEQAQIQSQLDSMVNGEQSQIVSLRAQLTEAKYNLEQTVIRAPSN

GYVTQVLIRPGTYAAALPLRPVMVFIPEQKRQIVAQFRQNSLLRLKPGDDAEVVFNALPGQVFHGKLTSI

LPVVPGGSYQAQGVLQSLTVVPGTDGVLGTIELDPNDDIDALPDGIYAQVAVYSDHFSHVSVMRKVLLRM

TSWMHYLYLDH

>gi|30043389|gb|AAP19110.1| hypothetical protein S4136 [Shigella flexneri 2a str. 2457T]

MFLNYFALGVLIFVFLVIFYGIIAIHDIPYLIAKKRNHPHADAIHTAGWVSLFTLHVIWPFLWIWATLYQ

PERGWGMQSHVASQEKATEPEIAALSDRISRLEHQLAAEKKTDYSTFPEI

>gi|30043388|gb|AAP19109.1| PTS system, mannitol-specific enzyme IIABC components [Shigella flexneri 2a str. 2457T]

MSSDIKIKVQSFGRFLSNMVMPNIGAFIAWGIITALFIPTGWLPNETLAKLVGPMITYLLPLLIGYTGGK

LVGGKRGGVVGAITTMGVIVGADMPMFLGSMIAGPLGGWCIKHFDRWVDGKIKSGFEMLVNNFSAGIIGM

ILAILAFLGIGPIVEALSKMLAAGVNFMVVHDMLPLASIFVEPAKILFLNNAINHGIFSPLGIQQSHELG

KSIFFLIEANPGPGMGVLLAYMFFGRGSAKQSAGGAAIIHFLGGIHEIYFPYVLMNPRLILAVILGGMTG

VFTLTILGGGLVSPASPGSILAVLAMTPKGAYFANIAGVCAAMAVSFVVSAILLKTSKVKEEDDIEAATR

RMQDMKAESKGTSPLSAGDVTNDLSHVRKIIVACDAGMGSSAMGAGVLRKKIQDAGLSQISVTNSAINNL

PPDVDLVITHRDLTERAMRQVPQAQHISLTNFLDSGLYTSLTERLVAAQRHTENEVKVKDSLKDSFDDSS

ANLFKLGAENIFLGRKAATKEEAIRFAGEQLVKGGYVEPEYVQAMLDREKLTPTYLGESIAVPHGTVEAK

DRVLKTGVVFCQYPEGVRFGEEEDDIARLVIGIAARNNEHIQVITSLTNALDDESVIERLAHTTSVDEVL

ELLAGRK

>gi|30043387|gb|AAP19108.1| mannitol-1-phosphate dehydrogenase [Shigella flexneri 2a str. 2457T]

MKALHFGAGNIGRGFIGKLLADAGIQLTFADVNQVVLDALNARHSYQVHVVGETEQVDTVSGVNAVSSIG

DDVVDLIAQVDLVTTAVGPVVLERIAPAIAKGLVKRKEQGNESPLNIIACENMVRGTTQLKGHVMNALPE

DAKAWVEEHVGFVDSAVDRIVPPSASATNDPLEVTVETFSEWIVDKTQFKGALPNIPGMELTDNLMAFVE

RKLFTLNTGHAITAYLGKLAGHQTIRDAILDEKIRAVVKGAMEESGAVLIKRYGFDADKHAAYIQKILGR

FENPYLKDDVERVGRQPLRKLSAGDRLIKPLLGTLEYSLPHKNLIQGIAGAMHFRSEDDPQAQELAALIA

DKGPQAALAQISGLDANSEVVSEAVTAYKAMQ

>gi|30043386|gb|AAP19107.1| repressor for mtl [Shigella flexneri 2a str. 2457T]

MVDQAQDTLRPNNRLSDMQATMEQTQAVENRVLERLNAGKTVRSFLITAVELLTEAVNLLVLQVFRKDDY

AVKYAVEPLLDGDGPLGDLSVRLKLIYGLGVINRQEYEDAELLMALREELNHDGNEYAFTDDEILGPFGE

LHCVAALPPPPQFEPADSSLYAMQIQRYQQAVRSTMVLSLTELISKISLKKAFQK

>gi|30043385|gb|AAP19106.1| hypothetical protein S4132 [Shigella flexneri 2a str. 2457T]

MKEVEKNEIKRLSDRLDAIRHQQADLSLVEAADKYAELEKEKATLEAEIARLREVHSQKLSKEAQKLMKM

PFQRAITKKEQADMGKLKKSVRGLVVVHPMTALGREMGLEEMTGFSKTTF

>gi|30043384|gb|AAP19105.1| IS1 orfB [Shigella flexneri 2a str. 2457T]

MSCQCTHYGRWPQHGFTSLKKLRPQSVTSRIQPGSDVIVCAEMDEHWGYVGAKSRQRWLFYAYDRIRRTV

VAHVFGERTLATLERLLSLLSAFEVVVWMTDGWPLYESRLKGKLHVISKRYTQRIERHNLNLRQHLARLV

RKSLSFSKSVELHDKVIGHYLNIKHYQ

>gi|30043383|gb|AAP19104.1| IS1 orfA [Shigella flexneri 2a str. 2457T]

MASISIRCPSCSATEGVVRNGKSTAGHQRYLCSHCRKTWQLQFTYTASQPGTHQKIIDMAMNGVGCRASA

RIMGVGLNTVLRHLKNSGRSR

>gi|30043382|gb|AAP19103.1| L-lactate permease [Shigella flexneri 2a str. 2457T]

MNLWQQNYDPAGNIWLSSLIASLPILFFFFALIKLKLKGYVAASWTVAIALAVALLFYKMPVANALASVV

YGFFYGLWPIAWIIIAAVFVYKISVKTGQFDIIRSSILSITPDQRLQMLIVGFCFGAFLEGAAGFGAPVA

ITAALLVGLGFKPLYAAGLCLIVNTAPVAFGAMGIPILVAGQVTGIDSFEIGQMVGRQLPFMTIIVLFWI

MAIMDGWRGIKETWPAVVVAGGSFAIAQYLSSNFIGPELPDIISSLVSLLCLTLFLKRWQPVRVFRFGDL

GASQVDMTLAHTGYTAGQVLRAWTPFLFLTATVTLWSIPPFKALFASGGALYEWVINIPVPYLNKLVARM

PPVVSEATAYAAVFKFDWFSATGTAILFAALLSIVWLKMKPSDAISTFGSTLKELALPIYSIGMVLAFAF

ISNYSGLSSTLALALAHTGHAFTFFSPFLGWLGVFLTGSDTSSNALFAALQATAAQQIGVSDLLLVAANT

TGGVTGKMISPQSIAIACAAVGLVGKESDLFRFTVKHSLIFTCMVGVITTLQAYVLTWMIP

>gi|30043381|gb|AAP19102.1| transcriptional regulator [Shigella flexneri 2a str. 2457T]

MIVLPRRLSDEVADRVRALIDEKNLEAGMKLPAERQLAMQLGVSRNSLREALAKLVSEGVLLSRRGGGTF

IRWRHDTWSEQNIVQPLKTLMADDPDYSFDILEARYAIEASTAWHAAMRATPGDKEKIQLCFEATLSEDP

DIASQADVRFHLAIAEASHNIVLLQTMRGFFDVLQSSVKHSRQRMYLVPPVFSQLTEQHQAVIDAIFAGD

ADGARKAMMAHLSFVHTTMKRFDEDQARHARITRLPGEHNEHSREKNA

>gi|30043380|gb|AAP19101.1| L-lactate dehydrogenase [Shigella flexneri 2a str. 2457T]

MIISAASDYRAAAQRILPPFLFHYMDGGAYSEYTLRRNVEDLSEVALRQRILKNMSDLSLETTLFNEKLS

MPVALAPVGLCGMYARRGEVQAAKAADAHGIPFTLSTVSVCPIEEVAPAIKRPMWFQLYVLRDRGFMRNA

LERAKAAGCSTLVFTVDMPTPGARYRDAHSGMSGPNAAMRRYLQAVTHPQWAWDVGLNGRPHDLGNISAY

LGKPTGLEDYIGWLGNNFDPSISWKDLEWIRDFWDGPMVIKGILDPEDARDAVRFGADGIVVSNHGGRQL

DGVLSSARALPAIADAVKGDIAILADSGIRNGLDVVRMIALGADTVLLGRAFLYALATAGQAGVANLLNL

IEKEMKVAMTLTGAKSISEITQDSLVQGLGKELPTALAPMAKGNAA

>gi|30043379|gb|AAP19100.1| hypothetical protein S4123 [Shigella flexneri 2a str. 2457T]

MLNIVLYEPEIPPNTGNIIRLCANTGFRLHIIEPMGFAWDDKRLRRAGLDYHEFTAVTRHHDYRAFLEAE

NPQRLFALTTKGTPAHSAVSYQDGDYLMFGPETRGLPASILDALPAEQKIRIPMVPDSRSMNLSNAVSVV

VYEAWRQLGYPGALLRD

>gi|30043378|gb|AAP19099.1| serine acetyltransferase [Shigella flexneri 2a str. 2457T]

MSCEELEIVWNNIKAEARTLADCEPMLASFYHATLLKHENLGSALSYMLANKLSSPIMPAIAIREVVEEA

YAADPEMIASAACDIQAVRTRDPAVDKYSTPLLYLKGFHALQAYRIGHWLWNQGRRALAIFLQNQVSVTF

QVDIHPAAKIGRGIMLDHATGIVVGETAVIENDVSILQSVTLGGTGKSGGDRHPKIREGVMIGAGAKILG

NIEVGRGAKIGAGSVVLQPVPPHTTAAGVPARIVGKPDSDKPSMDMDQHFNGINHTFEYGDGI

>gi|30043377|gb|AAP19098.1| glycerol-3-phosphate dehydrogenase (NAD+) [Shigella flexneri 2a str. 2457T]

MNQRNASMTVIGAGSYGTALAITLARNGHEVVLWGHDPEHIATLERDRCNAAFLPDVPFPDTLHLESDLA

TALAASRNILVVVPSHVFGEVLRQIKPLMRPDARLVWATKGLEAETGRLLQDVAREALGDQIPLAVISGP

TFAKELAAGLPTAISLASTDQTFADDLQQLLHCGKSFRVYSNPDFIGVQLGGAVKNVIAIGAGMSDGIGF

GANARTALITRGLAEMSRLGAALGADPATFMGMAGLGDLVLTCTDNQSRNRRFGMMLGQGMDVQSAQEKI

GQVVEGYRNTKEVRELAHRFGVEMPITEEIYQVLYCGKNAREAALTLLGRARKDERSSH

>gi|30043376|gb|AAP19097.1| molecular chaperone in protein export [Shigella flexneri 2a str. 2457T]

MSEQNNTEMTFQIQRIYTKDISFEAPNAPHVFQKDWQPEVKLDLDTASSQLADDVYEVVLRVTVTASLGE

ETAFLCEVQQGGIFSIAGIEGTQMAHCLGAYCPNILFPYARECITSMVSRGTFPQLNLAPVNFDALFMNY

LQQQAGEGTEEHQDA

>gi|30043375|gb|AAP19096.1| glutaredoxin 3 [Shigella flexneri 2a str. 2457T]

MANVEIYTKETCPYCHRAKALLSSKGVSFQELPIDGNAAKREEMIKRSGCTTVPQIFIDAQHIGGCDDLY

ALDARGGLDPLLK

>gi|30043374|gb|AAP19095.1| hypothetical protein S4118 [Shigella flexneri 2a str. 2457T]

MQEIMQFVGRHPILSIAWIALLVAVLVTTFKSLTSKVKVITRGEATRLINKEDAVVVDLRQRDDFRKGHI

AGSINLLPSEIKANNVGELEKHKDKSVIVVDGSGMQCQEPANALTKAGFAQVFVLKEGVAGWAGENLPLV

RGK

>gi|30043373|gb|AAP19094.1| putative 2,3-bisphosphoglycerate-independent phosphoglycerate mutase [Shigella flexneri 2a str. 2457T]

MSVSKKPMVLVILDGYGYREEQQDNAIFSAKTPVMDALWANRPHTLIDASGLEVGLPDRQMGNSEVGHVN

LGAGRIVYQDLTRLDVEIKDRAFFANPVLAGAVHKAKNAGKAVHIMGLLSAGGVHSHEDHIMAMVELAAE

RGAEKIYLHAFLDGRDTPPRSAESSLKKFEEKFAALGKGRVASIIGRYYAMDRDNRWDRVEKAYDLLTLA

QGEFQADTAVAGLQAAYARDENDEFVKATVIRAEGQPDAAMEDGDALIFMNFRADRAREITRAFVNADFD

GFARKKVVNVDFVMLTEYAADIKTAVAYPPASLVNTFGEWMAKNDKTQLRISETEKYAHVTFFFNGGVEE

SFKGEDRILINSPKVATYDLQPEMSSAELTEKLVAAIKSGKYDTIICNYPNGDMVGHTGVMEAAVKAVEA

LDHCVEEVAKAVESVGGQLLITADHGNAEQMRDPATGQAHTAHTNLPVPLIYVGDKNVKAVAGGKLSDIA

PTMLSLMGMEIPQEMTGKPLFIVE

>gi|30043372|gb|AAP19093.1| putative membrane protein [Shigella flexneri 2a str. 2457T]

MTRAVKPRRFAIRPIIYASVLSAGVLLCAFSAHADERDQLKSIQADIAAKERAVRQKQQQRASLLAQLKK

QEEAISEATRKLRETQNTLNQLNKQIDEMNASIAKLEQQKAAQERSLAAQLDAAFRQGEHTGIQLILSGE

ESQRGQRLQAYFGYLNQARQETIAQLKQTREEVTMQHAELEEKQSEQQTLLYEQRAQQAKLTQALNERKK

TLAGLESSIQQGQQQLSELRANESRLRNSIARAEAAAKARAEREAREAQAVRERQKEATRKGTTYKPTES

EKSLMSRTGGLGAPRGQAFWPVRGPTLHRYGEQLQGELRWKGMVIGASEGTEVKAIADGRVILADWLQGY

GLVVVVEHGKGDMSLYGYNQSALVSVGSQVRAGQPIALVGSSGGQGRPSLYFEIRRQGQAVNPQPWLGR

>gi|30043371|gb|AAP19092.1| hypothetical protein S4115 [Shigella flexneri 2a str. 2457T]

MLAMPSAISVAVLPDSPHAREMATKAHNSGHEVLIHLPMAPLSKQPLEKNTLRPEMSSDEIERIIRSAVN

NVPYAVGINNHMGSKMTSNLFGMQKVMQALERYNLYFLDSVTIGNTQAMRAAQGTGVKVIKRKVFLDDSQ

NEADIRVQFNRAIDLARRNGSTIAIGHPHPSTVRVLQQMVYNLPPDITLVKASSLLNEPQVDTSTPPKNA

VPDAPRNPFRGVKLCKPKKPIEPVYANRFFEVLSESISQSTLIVYFQHQWQGWGKQPEAAKFNASAN

>gi|30043370|gb|AAP19091.1| putative regulator [Shigella flexneri 2a str. 2457T]

MYKRLITNVRSVKVGYQALLWSFRLWQWRDKTRSHHRITRSAFNLR

>gi|30043369|gb|AAP19090.1| IS1 orfB [Shigella flexneri 2a str. 2457T]

MSRQCTHYGRWPQHGFTSLKKLRPQSVTSRIQPGSDVIVCAEMDEHWGYVGAKSRQRWLFYAYDRIRRTV

VAHVFGERTLATLERLLSLLSAFEVVVWMTDGWPLYESRLKGKLHVISKRYTQRIERHNLNLRQHLARLV

RKSLSFSKSVELHDKVIGHYLNIKHYQ

>gi|30043368|gb|AAP19089.1| IS1 orfA [Shigella flexneri 2a str. 2457T]

MASISIRCPSCSATEGVVRNGKSTAGHQRYLCSHCRKTWQLQFTYTASQPGTHQKIIDMAMNGVGCRASA

RIMGVGLNTVLRHLKNSGRSR

>gi|30043367|gb|AAP19088.1| threonine dehydrogenase [Shigella flexneri 2a str. 2457T]

MKALSKLKAEEGIWMTDVPVPELGHNDLLIKIRKTAICGTDVHIYNWDEWSQKTIPVPMVVGHEYVGEVV

GIGQEVKGFKIGDRVSGEGHITCGHCRNCRGGRTHLCRNTIGVGVNRPGCFAEYLVIPAFNAFKIPDNIS

DDLASIFDPFGNAVHTALSFDLVGEDVLVSGAGPIGIMAAAVAKHVGARNVVITDVNEYRLELARKMGIT

RAVNVAKENLNDVMAELGMTEGFDVGLEMSGAPPAFRTMLDTMNHGGRIAMLGIPPSDMSIDWTKVIFKG

LFIKGIYGREMFETWYKMAALIQSGLDLSPIITHRFSIDDFQKGFDAMRSGQSGKVILSWD

>gi|30043366|gb|AAP19087.1| 2-amino-3-ketobutyrate CoA ligase (glycine acetyltransferase) [Shigella flexneri 2a str. 2457T]

MRGEFYQQLTNDLETARAEGLFKEERIITSAQQADITVADGSHVINFCANNYLGLANHPDLIAAAKAGMD

SHGFGMASVRFICGTQDSHKELEQKLAAFLGMEDAILYSSCFDANGGLFETLLGAEDAIISDALNHASII

DGVRLCKAKRYRYANNDMQELEARLKEAREAGARHVLIATDGVFSMDGVIANLKGVCDLADKYDALVMVD

DSHAVGFVGENGRGSHEYCDVMGRVDIITGTLGKALGGASGGYTAARKEVVEWLRQRSRPYLFSNSLAPA

IVAASIKVLEMVEAGSELRDRLWANARQFREQMSAAGFTLAGADHAIIPVMLGDAVVAQKFARELQKEGI

YVTGFFYPVVPKGQARIRTQMSAAHTPEQITRAVEAFTRIGKQLGVIA

>gi|30043365|gb|AAP19086.1| ADP-L-glycero-D-mannoheptose-6-epimerase [Shigella flexneri 2a str. 2457T]

MIIVTGGAGFIGSNIVKALNDKGITDILVVDNLKDGTKFVNLVDLDIADYMDKEDFLIQIMAGEEFGDVE

AIFHEGACSSTTEWDGKYMMDNNYQYSKELLHYCLEREIPFLYASSAATYGGRTSDFIESREYEKPLNVY

GYSKFLFDEYVRQILPEANSQIVGFRYFNVYGPREGHKGSMASVAFHLNTQLNNGESPKLFEGSENFKRD

FVYVGDVADVNLWFLENGVSGIFNLGTGRAESFQAVADATLAYHKKGQIEYIPFPDKLKGRYQAFTQADL

TNLRAAGYDKPFKTVAEGVTEYMAWLNRDA

>gi|30043364|gb|AAP19085.1| ADP-heptose--lps heptosyltransferase II [Shigella flexneri 2a str. 2457T]

MKILVIGPSWVGDMMMSQSLYRTLQARYPQAIIDVMAPAWCRPLLSRMPEVNEAIPMPLGHGALEIGERR

KLGHSLREKRYDRAYVLPNSFKSALVPFFAGIPHRTGWRGEMRYGLLNDVRVLDKEAWPLMVERYVALAY

DKGIMRTAQDLPQPLLWPQLQVSEGEKSYTCNQFSLSSERPMIGFCPGAEFGPAKRWPHYHYAELAKQLI

DEGYQVVLFGSAKDHEAGNEILAALNTEQQAWCRNLAGETQLDQAVILIAACKAIVTNDSGLMHVAAALN

RPLVALYGPSSPDFTPPLSHKARVIRLITGYHKVRKGDAAEGYHQSLIDITPQRVLEELNALLLQEEA

>gi|30043363|gb|AAP19084.1| heptosyl transferase I [Shigella flexneri 2a str. 2457T]

MGDVLHTLPALTDAQQAIPGIKFDWVVEEGFAQIPSWHAAVERVIPVAIRRWRKAWFSAPIKAERKAFRE

ALQAENYDAVIDAQGLVKSAALVTRLAHGVKHGLDWQTAREPLASLFYNCKHHIAKQQHAVERTRELFAK

SLGYSKPQTQGDYAIAQHFLTNLPTDAGEYAVFLHATTRDDKHWPEEHWRELIGLLADSGIRIKLPWGAP

HEEERAKRLAEGFAYVEVLPKMSLEGVARVLAGAKFVVSVDTGLSHLTAALDRPNITVYGPTDPGLIGGY

GKNQVECRSTSMSLADLPAQTVFQNLNLEIITNKLTSEIR

>gi|30043362|gb|AAP19083.1| lipid A-core, surface polymer ligase [Shigella flexneri 2a str. 2457T]

MTSTLFFSLEKKNWIAYWNRALVFLFITTYFLGGITRYKHLIVILMTITTIVYLCKRPKHYLSLFKTFLF

GSVAILTIAALLSLLQSPDAGASMKEVFKAIIENTLLCTIAIPVILRDEKREDVEKIVFFSFISALGLRC

FSELITYYKDYQQGIMPFADYRHRSISDSMVFLFPALLNLWLIKSAKYRISFVVLSVIFIFLILGTLSRG

AWLSVLVIGLIWILMFKQWKLLLVGVMVSIIALSVIFTHKEMTAKLTYKLQQTNSSYRYANGTQGSALDL

ILENPVIGYGYGNVAYKDVYNKRVIDYPEWTFRQSIGPHNFALFIWFGTGLLGLVSLMMLYCAILKECIK

NGVKNKYRSPYNAYYIILLSFIGYFVIRGNVEQIEPNLLGVYAGLLLAMKNK

>gi|30043361|gb|AAP19082.1| lipopolysaccharide 1,2-N- acetylglucosaminetransferase [Shigella flexneri 2a str. 2457T]

MVDKIIFTVTPIFSIPPRGAAAVETWMYQVAQRTNFPNRIVCIKNPGYSNYTFVNDNCSIHRVGFSRIYK

RLFQKWTRLDPLPYSQRILNIAHDFPITKESVIVVHNSMKLYTQIRKLAPQARVVIHMHNAFEPKLLEQN

VKMIVPSLYLKKYYQSYLANADIEIVPNGIDLETYQSNFQPITRSELNISPEEKIIFYAGRIVPDKGILL

LMQSFEKLAAAHKNLKLVVIGDYTEMSKSDKGAYQRNVREIAKRLKDRCIMLGSIPPEKMYCYYPLADLV

VIPSQFQEPFCMVAIEAMGAGKPVLVSTRGGMTEFVKENTTGFHLKEPMTADSISSDILKTLANPELTAV

AKQGQDFVFDHYSWDGVTQRFEEVIHNWFE

>gi|30043360|gb|AAP19081.1| putative UDP-glucose:(galactosyl) LPS alpha1,2-glucosyltransferase [Shigella flexneri 2a str. 2457T]

MDFKHLTQFKDIIELDKRPVKLDERETFNVSWGIDENYQVGAAISIASILEKNKQNKFTFHIIADYLDKE

YIELLSQLATKYQTVIKLYHIDSEPLKALPQSNIWPVSIYYRLLSFDYFSARLDSLLYLDADIVCKGSLN

ELIALEFKDEYGAVVIDVDAMQSKSAERLCNEDFNGSYFNSGVMYINLREWLKQRLTEKFFDLLSDESII

KKLKYPDQDILNLMFLHHAKILPRKYNCIYTIKSEFEEKNSEYYTRFINDDTVFIHYTGITKPWHDWANY

ASADYFRNIYNISPWRNIPYKKAVKKHEHKEKYKHLLYQKKFLDGVFTAIKYNVMKG

>gi|30043359|gb|AAP19080.1| putative LPS biosynthesis protein [Shigella flexneri 2a str. 2457T]

MIYNKTINGLKVFIKDNDPFYEQVLNDFLTCRVKTLKVFRSIDDTKVILIDTARGPLVLKVYAPKHKMTE

RFLKSCIKKDYYENLIYQTDRVRGEGIQSINDYFLLAERKTLNFAHYYIMLIEYIEGVGLNEYLEISEDL

KDQLSESIKELHQHGMVSGDPHKGNFIVSEKGLRLIDLSGKKTTAVLKAKDRIDLERHYNIKNELKDFGY

TYLIFKKKIKKVIRDVKVKLGLKSK

>gi|30043358|gb|AAP19079.1| UDP-D-galactose:(glucosyl)lipopolysaccharide- alpha-1,3-D-galactosyltransferase [Shigella flexneri 2a str. 2457T]

MSQLNDSDIILFEYNFHYQNIRSKNTLDIAFGIDRNFLFGCGVAIASILLNNREISCEFHVFTDYISDKD

KLYFSDLAKQYNSRINIYVINCDKLKSLPSTKNWTYATYFRFIIADYFYHKHEKILYLDADIACKGSIKE

LLDYQFSTNEIAAVVAERDIEWWQNRASVLTTPQLASGYFNAGFLLINIDEWNLNNISSKAIEMLRDPDW

VSKITHLDQDVLNVLLNGKVKFISEKYNTRYSINYELKDKVDNPVNDDTVFIHYVGPTKPWHEWADYPVS

RSFLIAKAASPWSKEDLLKPVNSNQYRYCAKHKFKQKHYMAGIFNYLKYYKEKCF

>gi|30043357|gb|AAP19078.1| lipopolysaccharide core biosynthesis protein [Shigella flexneri 2a str. 2457T]

MVWMVELKEPFATLWRGKDPFEEVKTLQGEVFRELETRRTLRFEMAGKSYFLKWHRGTTLKEIIKNLLSL

RMPVLGADREWNAIHRLRDVGVDTMYGVAFGEKGMNPLTRTSFIITEDLTPTISLEDYCADWATNPPDVR

VKRMLIKRVATMVRDMHAAGINHRDCYICHFLLHLPFSGKEEELKISVIDLHRAQLRTRVPGRWRDKDLI

GLYFSSMNIGLTQRDIWRFMKVYFAAPLKDILKQEQGLLSQAEAKATKIRERTIRKSL

>gi|30043356|gb|AAP19077.1| LPS alpha1,3-glucosyltransferase [Shigella flexneri 2a str. 2457T]

MIVAFCLYKYFPFGGLQRDFMRIAQTVAARGHHVRVYTQSWEGECPDVFEVIKVPVKSHTNHGRNAEYFA

WVQKHLREHPVDKVVGFNKMPGLDVYYAADVCYAEKVAQEKGFFYRLTSRYRHYAAFERATFEQGKPTQL

LMLTDKQIADFQKHYQTEAERFHILPPGIYPDRKYSQQPANSREIFRKKNGITEQQYLLLQVGSDFTRKG

ADRSIEALASLPDSLRHNTLLYVVGQDKPRKFEALAEKRGVRSNVHFFSGRNDVSELMAAADLLLHPAYQ

EAAGIVLLEAITAGLPVLTTAVCGYAHYIVDANCGEAIAEPFRQETLNEILRKALTQSSLRQAWAENARH

YADTQDLYSLPEKAADIITGGLDG

>gi|30043355|gb|AAP19076.1| lipopolysaccharide core biosynthesis protein [Shigella flexneri 2a str. 2457T]

MDKPFRRILLIKMRFHGDMLLTTPVISSLKKNYPDAKIDVLLYQDTIPILSENPEINALYGIKNKKAKAS

EKIANFFHLIKVLRANKYDLIVNLTDQWMVAILVRLLNARVKISQDYHHRQSAFWRKSFTHLVPLQGGNV

VESNLSVLTPLGLDSLVKQTTMSYPPASWKRMRRELDHAGVGQNYVVIQPTARQIFKCWDNAKFSAVIDA

LHARGYEVVLTSGPDKDDLACVNEIAQGCQTPPVTALAGKVTFPELGALIDHAQLFIGVDSAPAHIAAAV

NTPLISLFGATDHIFWRPWSNNMIQFWAGDYREMPTRDQRDRNEMYLSVIPAADVIAAVDKLLPSSTTGT

SL

>gi|30043354|gb|AAP19075.1| IS1 orfB [Shigella flexneri 2a str. 2457T]

MSRQCTHYGRWPQHGFTSLKKLRPQSVTSRIQPGSDVIVCAEMDEHWGYVGAKSRQRWLFYAYDRIRRTV

VAHVFGERTLATLERLLSLLSAFEVVVWMTDGWPLYESRLKGKLHVISKRYTQRIERHNLNLRQHLARLV

RKPLSFSKSVELHDKVIGHYLNIKHYQ

>gi|30043353|gb|AAP19074.1| IS1 orfA [Shigella flexneri 2a str. 2457T]

MASISIRCPSCSATEGVVRNGKSTAGHQRYLCSHCRKTWQLQFTYTASQPGTHQKIIDMAMNGVGCRASA

RIMGVGLNTVLRHLKNSGRSR

>gi|30043352|gb|AAP19073.1| 3-deoxy-D-manno-octulosonic-acid transferase (KDO transferase) [Shigella flexneri 2a str. 2457T]

MLELLYTALLYLIQPLIWIRLWVRGRKAPAYRKRWGERYGFYRHPLKPGGIMLHSVSVGETLAAIPLVRA

LRHRYPDLPITVTTMTPTGSERVQSAFGKDVQHVYLPYDLPDALNRFLNKVDPKLVLIMETELWPNLIAA

LHKRKIPLVIANARLSARSAAGYAKLGKFVRRLLRRITLIAAQNEEDGARFVALGAKNNQVTVTGSLKFD

ISVTPQLAAKAVTLRRQWAPHRPVWIATSTHEGEESVVIAAHQALLQQFPNLLLILVPRHPERFPDAINL

VRQAGLSYITRSSGEVPSTSTQVVVGDTMGELMLLYGIADLAFVGGSLVERGGHNPLEAAAHAIPVLMGP

HTFNFKDICARLEQASGLITVTDATTLAKEVSSLLTDAVYRSFYGRHAVEVLYQNQGALQRLLQLLEPYL

PPKTH

>gi|30043351|gb|AAP19072.1| putative enzyme of lipopolysaccharide synthesis [Shigella flexneri 2a str. 2457T]

MQKRAIYPGTFDPITNGHIDIVTRATQMFDHVILAIAASPSKKPMFTLEERVALAQQATAHLGNVEVVGF

SDLMANFARNQHATVLIRGLRAVADFEYEMQLAHMNRHLMPELESVFLMPSKEWSFISSSLVKEVARHQG

DVTHFLPENVHQALMAKLA

>gi|30043350|gb|AAP19071.1| formamidopyrimidine DNA glycosylase [Shigella flexneri 2a str. 2457T]

MPELPEVETSRRGIEPHLVGATILHAVVRNGRLRWPVSEEIYRLSDQPVLSVQRRAKYLLLELPEGWIII

HLGMSGSLRILPEELPPEKHDHVDLVMSNGKVLRYTDPRRFGAWLWTKELEGHNVLAHLGPEPLSDDFNG

EYLHQKCAKKKTAIKPWLMDNKLVVGVGNIYASESLFAAGIHPDRLASSLSLAECELLARVIKAVLLRSI

EQGGTTLKDFLQSDGKPGYFAQELQVYGRKGEPCRVCGTPIVATKHAQRATFYCRQCQK

>gi|30043349|gb|AAP19070.1| 50S ribosomal subunit protein L33 [Shigella flexneri 2a str. 2457T]

MAKGIREKIKLVSSAGTGHFYTTTKNKRTKPEKLELKKFDPVVRQHVIYKEAKIK

>gi|30043348|gb|AAP19069.1| 50S ribosomal subunit protein L28 [Shigella flexneri 2a str. 2457T]

MSRVCQVTGKRPVTGNNRSHALNATKRRFLPNLHSHRFWVESEKRFVTLRVSAKGMRVIDKKGIDTVLAE

LRARGEKY

>gi|30043347|gb|AAP19068.1| DNA repair protein [Shigella flexneri 2a str. 2457T]

MKNNAQLLMPREKMLKFGISALTDVELLALFLRTGTRGKDVLTLAKEMLENFGSLYGLLTSEYEQFSGVH

GIGVAKFAQLKGIAELARRYYNVRMREESPLLSPEMTREFLQSQLTGEEREIFMVIFLDSQHRVITHSRL
FSGTLNHVEVHPREIIREAIKINASALILAHNHPSGCAEPSKADKLITERIIKSCQFMDLRVLDHIVIGR
GEYVSFAERGWI

>gi|30043346|gb|AAP19067.1| flavoprotein [Shigella flexneri 2a str. 2457T]
MKARQQKYCDKIANFWCHPTGKIIMSLAGKKIVLGVSGGIAAYKTPELVRRLRDRGADVRVAMTEAAKAF
ITPLSLQAVSGYPVSDSLLDPAAEAAMGHIELGKWADLVILAPATADLIARVAAGMANDLVSTICLATPA
PVAVLPAMNQQMYRAAATQHNLEVLASRGLLIWGPDSGSQACGDIGPGRMLDPLTIVDMAVAHFSPVNDL
KHLNIMITAGPTREPLDPVRYISNHSSGKMGFAIAAAAARRGANVTLVSGPVSLPTPPFVKRVDVMTALE
MEAAVNASVQQQNIFIGCAAVADYRAATVAPEKIKKQATQGDELTIKMVKNPDIVAGVAALKDHRPYVVG
FAAETNNVEEYARQKRIRKNLDLICANDVSQPTQGFNSDNNALHLFWQDGDKVLPLERKELLGQLLLDEI
VTRYDEKNRR

>gi|30043345|gb|AAP19066.1| deoxyuridinetriphosphatase [Shigella flexneri 2a str. 2457T]
MMKKIDVKILDPRVRKEFPLPTYATSGSAGLDLRACLDDAVELAPGDTTLVPTGLAIHIADPSLAAMMLP
RSGLGHKHGIVLGNLVGLIDSDYQGQLMISVWNRGQDSFTIQPGERIAQMIFVPVVQAEFNLVEDFDATD
RGEGGFGHSGRQ

>gi|30043344|gb|AAP19065.1| putative transcriptional regulator [Shigella flexneri 2a str. 2457T]
MPPGKCLFSGVFCNMAEKQTAKRNRREEILQSLALMLESSDGSQRITTAKLAASVGVSEAALYRHFPSKT
RMFDSLIEFIEDSLITRINLILKDEKDTTARLRLIVLLLLGFGERNPGLTRILTGHALMFEQDRLQGRIN
QLFERIEAQLRQVLREKRMREGEGYTTDETLLASQILAFCEGMLSRFVRSEFKYRPTDDFDARWPLIAAQ
LQ

>gi|30043343|gb|AAP19064.1| orotate phosphoribosyltransferase [Shigella flexneri 2a str. 2457T]

MKSYQRQFIEFALSKQVLKFGEFTLKSGRKSPYFFNAGLFNTGRDLALLGRFYAEALVDSGIEFDLLFGP

AYKGIPIATTTAVALAEHHDLDLPYCFNRKEAKDHGEGGNLVGSALQGRVMLVDDVITAGTAIRESMEII

QANGATLAGVLISLDRQERGRGEISAIQEVERDYNCKVISIITLKDLIAYLEEKPEMAEHLAAVKAYREE

FGV

>gi|30043342|gb|AAP19063.1| RNase PH [Shigella flexneri 2a str. 2457T]

MRPAGRSNNQVRPVTLTRNYTKHAEGSVLVEFGDTKVLCTASIEEGVPRFLKGQGQGWITAEYGMLPRST

HTRNAREAAKGKQGGRTMEIQRLIARALRAAVDLKALGEFTITLDCDVLQADGGTRTASITGACVALADA

LQKLVENGKLKTNPMKGMVAAVSVGIVNGEAICDLEYVEDSAAETDMNVVMTEDGRIIEVQGTAEGEPFT

HEELLTLLALARGGIESIVATQKAALAN

>gi|30043341|gb|AAP19062.1| putative alpha helix protein [Shigella flexneri 2a str. 2457T]

MIRSMTAYARREIKGEWGSATWEMRSVNQRYLETYFRLPEQFRSLEPVVRERIRSRLTRGKVECTLRYEP

DVSAQGELILNEKLAKQLVTAANWVKMKSDEGEINPVDILRWPGVMAAQEQDLDAIAAEILAALDGTLDD

FIVARETEGQALKALIEQRLEGVTAEVVKVRAHMPEILQWQRERLVAKLEDAQVQLENNRLEQELVLLAQ

RIDVAEELDRLEAHVKETYNILKKKEAVGRRLDFMMQEFNRESNTLASKSINAEVTNSAIELKVLIEQMR

EQIQNIE

>gi|30043340|gb|AAP19061.1| DNA-damage-inducible protein [Shigella flexneri 2a str. 2457T]

MNEHHQPFEEIKLINANGAEQWSARQLGKLLGYSEYRHFIPVLTRAKEACENSGHTIDDHFEEILDMVKI

GSNAKRALKDIVLSRYACYLVVQNGDPAKPVIAAGQTYFTIQTRRQELADDEAFKQLREDEKRLFLRNEL

KEHNKQLVEAAQQAGVATATDFAIFQNHGYQGLYGGLDQKAIHQLKGLKKSQKILDHMGSTELAANLFRA

TQTEEKLKRDSVNSKQQANTTHFDVGSKVRQTIQELGGTIPEELPTPQVSIKQLENSVKITEKK

>gi|30043339|gb|AAP19060.1| hypothetical protein S4083 [Shigella flexneri 2a str. 2457T]

MTMRTNRYPARQIFRERTMLLHILYLVGITAEAMTGALAAGRRRMDTFGVIIIATATAIGGGSVRDILLG

HYPLGWVKHPEYVIIVATAAVLTTIVAPVMPYLRKVFLVLDALGLVVFSIIGAQVALDMGHGPIIAVVAA

VTTGVFGGVLRDMFCKRIPLVFQKELYAGVSFASAVLYIALQHYVSNHDVVIISTLVFGFFARLLALRLK

LGLPVFYYSHEGH

>gi|30043338|gb|AAP19059.1| putative enzyme [Shigella flexneri 2a str. 2457T]

MKVWMAILISILCWQSSVWAVCPAWSPARAQEEISRLQQQIKQWDDDYWKEGKSEVEDGVYDQLSARLTQ

WQRCFVSEPRDVMMPPLNGAVMHPVAHTGVRKMADKNALSLWMRERSDLWVQPKVDGVAVTLVYRDGKLN

KAISRGNGLKGEDWTQKVSLISAVLQTVSGPLANSTLQGEIFLQREGHIQQQMGGINARAKVAGLMMRQG

NSDTLNSLAVFVWAWPDGPQLMTDRLKELATAGFTLTQRYTRAVKNADEVARVRNEWWKAKLPFVTDGVV

VRGAKEPESRHWLPGQAEWLVAWKYQPVAQVAEVKAIQFAVGKSGKISVVASLAPVMLDDKKVQRVNIGS

VRRWQEWDIAPGDQILVSLAGQGIPRIDDVVWRGAERTKPTPPENRFNPLTCYFASDVCQEQFISRLVWL

GSKQVLGLDGIGEAGWRALHQTHRFEHIFSWLLLTPEQLQNTPGIAKSKSAQLWHQFNLARNQPFTRWVM

AMGIPLTRAALNASDERSWSQLLFSTEQFWQQLPGTGSGRARQVIEWKENAQIKKLGSWLAAQQITGFEP

>gi|30043337|gb|AAP19058.1| guanylate kinase [Shigella flexneri 2a str. 2457T]

MAQGTLYIVSAPSGAGKSSLIQALLKTQPLYDTQVSVSHTTRQPRPGEVHGEHYFFVNHDEFKEMISRDA

FLEHAEVFGNYYGTSREAIEQVLATGVDVFLDIDWQGAQQIRQKMPHARSIFILPPSKIELDRRLRGRGQ

DSEEVIAKRMAQAVAEMSHYAEYDYLIVNDDFDTALTDLKTIIRAERLRMSRQKQRHDALISKLLAD

>gi|30043336|gb|AAP19057.1| RNA polymerase, omega subunit [Shigella flexneri 2a str. 2457T]

MARVTVQDAVEKIGNRFDLVLVAARRARQMQVGGKDPLVPEENDKTTVIALREIEEGLINNQILDVRERQ

EQQELEAAELQAVTAIAEGRR

>gi|30043335|gb|AAP19056.1| (p)ppGpp synthetase II and guanosine-3,5-bis pyrophosphate 3-pyrophosphohydrolase [Shigella flexneri 2a str. 2457T]

MYLFESLNQLIQTYLPEDQIKRLRQAYLVARDAHEGQTRSSGEPYITHPVAVACILAEMKLDYETLMAAL
LHDVIEDTPATYQDMEQLFGKSVAELVEGVSKLDKLKFRDKKEAQAENFRKMIMAMVQDIRVILIKLADR
THNMRTLGSLRPDKRRRIARETLEIYSPLAHRLGIHHIKTELEELGFEALYPNRYRVIKEVVKAARGNRK
EMIQKILSEIEGRLQEAGIPCRVSGREKHLYSIYCKMVLKEQRFHSIMDIYAFRVIVNDSDTCYRVLGQM
HSLYKPRPGRVKDYIAIPKANGYQSLHTSMIGPHGVPVEVQIRTEDMDQMAEMGVAAHWAYKEHGETSTT
AQIRAQRWMQSLLELQQSAGSSFEFIESVKSDLFPDEIYVFTPEGRIVELPAGATPVDFAYAVHTDIGHA
CVGARVDRQPYPLSQPLTSGQTVEIITAPGARPNAAWLNFVVSSKARAKIRQLLKNLKRDDSVSLGRRLL
NHALGGSRKLNEIPQENIQRELDRMKLATLDDLLAEIGLGNAMSVVVAKNLQHGDASIPPATQSHGHLPI
KGADGVLITFAKCCRPIPGDPIIAHVSPGKGLVIHHESCRNIRGYQKEPEKFMAVEWDKETAQEFITEIK
VEMFNHQGALANLTAAINTTTSNIQSLNTEEKDGRVYSAFIRLTARDRVHLANIMRKIRVMPDVIKVTRN
RN

>gi|30043334|gb|AAP19055.1| putative RNA methylase [Shigella flexneri 2a str. 2457T]
MNPTRYARICEMLARRQPDLTVCMEQVHKPHNVSAIIRTADAVGVHEVHAVWPGSRMRTMASAAAGSNSW
VQVKTHRTIGDAVAHLKGQGMQILATHLSDNAVDFREIDYTRPTCILMGQEKTGITQEALALADQDIIIP
MIGMVQSLNVSVASALILYEAQRQRQNAGMYLRENSMLPEAEQQRLLFEGGYPVLAKVAKRKGLPYPHVN
QQGEIEADADWWATMQAAG

>gi|30043333|gb|AAP19054.1| DNA helicase [Shigella flexneri 2a str. 2457T]
MKGRLLDAVPLSSLTGVGAALSNKLAKINLHTVQDLLLHLPLRYEDRTHLYPIGELLPGVYATVEGEVLN
CNISFGGRRMMTCQISDGSGILTMRFFNFSAAMKNSLATGRRVLAYGEAKRGKYGAEMIHPEYRVQGDLS
TPELQETLTPVYPTTEGVKQATLRKLTDQALDLLDTCAIEELLPPELSQGMMTLPEALRTLHRPPPTLQL
SDLETGQHPAQRRLILEELLAHNLSMLALRAGAQRFHAQPLSANDTLKNKLLAALPFKPTGAQARVVAEI
EHDMALDVPMMRLVQGDVGSGKTLVAALAALRAIAHGKQVALMAPTELLAEQHANNFRNWFAPLGIEVGW
LAGKQKGKARLSQQEAIASGQVQMIVGTHAIFQEQVQFNGLALVIIDEQHRFGVHQRLALWEKGQQQGFH

PHQLIMTATPIPRTLAMTAYADLDTSVIDELPPGRTPVTTVAIPDTRRTDIIDRVRHACITEGRQAYWVC
TLIEESELLEAQAAEATWEELKLALPELNVGLVHGRMKPAEKQAVMASFKQGELHLLVATTVIEVGVDVP
NASLMIIENPERLGLAQLHQLRGRVGRGAVASHCVLLYKTPLSKTAQIRLQVLRDSNDGFVIAQKDLEIR
GPGELLGTRQTGNAEFKVADLLRDQAMIPEVQRLARHIHERYPQQAKALIERWMPETERYSNA

>gi|30043332|gb|AAP19053.1| glutamate transport protein [Shigella flexneri 2a str. 2457T]
MFHLDTLATLVAATLTLLLGRKLVHSVSFLKKYTIPEPVAGGLLVALALLVLKKSMGWEVNFDMSLRDPL
MLAFFATIGLNANIASLRAGGRVVGIFLIVVVGLLVMQNAIGIGMASLLGLDPLMGLLAGSITLSGGHGT
GAAWSKLFIERYGFTNATEVAMACATFGLVLGGLIGGPVARYLVKHSTTPNGIPDDQEVPTAFEKPDVGR
MITSLVLIETIALIAICLTVGKIVAQLLAGTAFELPTFVCVLFVGVILSNGLSIMGFYRVFERAVSVLGN
VSLSLFLAMALMGLKLWELASLALPMLAILVVQTIFMALYAIFVTWRMMGKNYDAAVLAAGHCGFGLGAT
PTAIANMQAITERFGPSHMAFLVVPMVGAFFIDIVNALVIKLYLMLPIFAG

>gi|30043331|gb|AAP19052.1| putative transport protein [Shigella flexneri 2a str. 2457T]
MSVSTLESENAQPVAQTQNSELIYRLEDRPPLPQTLFAACQHLLAMFVAVITPALLICQALGLPAQDTQH
IISMSLFASGVASIIQIKAWGPVGSGLLSIQGTSFNFVAPLIMGGTALKTGGADVPTMMAALFGTLMLAS
CTEMVISRVLHLARRIITPLVSGVVVMIIGLSLIQVGLTSIGGGYAAMSDNTFGAPKNLLLAGVVLALII
LLNRQRNPYLRVASLVIAMAAGYALAWFMGMLPESNEPMTQELIMVPTPLYYGLGIEWSLLLPLMLVFMI
TSLETIGDITATSDVSEQPVSGPLYMKRLKGGVLANGLNSFVSAVFNTFPNSCFGQNNGVIQLTGVASRY
VGFVVALMLIVLGLFPAVSGFVQHIPEPVLGGATLVMFGTIAASGVRIVSREPLNRRAILIIALSLAVGL
GVSQQPLILQFAPEWLKNLLSSGIAAGGITAIVLNLIFPPEKQ

>gi|30043330|gb|AAP19051.1| hypothetical protein S4074 [Shigella flexneri 2a str. 2457T]
MKFIGKLLLYILIALLVVIAGLYFLLQTRWGAEHISAWVSENSDYHLAFGAMDHRFSAPSHIVLENVTFG
RDGQPAPLVAKSVDIALSSRQLTEPRHVDTILLENGTLNLTDQTAPLPFKADRLQLRDMAFNSPNSEWKL

SAQRVNGGVVPWSPEAGKVLGTKAQIQFSAGSLSLNDVPATNVLIEGSIDNDRVTLTNLGADIARGTLTG

NAQRNADGSWQVENLRMADIRLQSEKSLTDFFAPLRSVPSLQIGRLEVIDARLQGPDWAVTDLDLSLRNM

TFSKDDWQTQEGKLSMNASEFIYGSLHLFDPIINAEFSPQGVALRQFTSRWEGGMVRTSGNWLRDGKTLI

LDDAAIAGLEYTLPKNWQQLWMETTPGWLNSLQLKRFSASRNLIIDIDPDFPWQLTALDGYGANLTLVTD

HKWGVWSGSANLNAAAATFNRVDVRRPSLALTANSSTVNISELSAFTEKGILEATASVSQTPQRQTHISL

NGRGVPVNILQQWGWPELPLTGDGNIQLTASGDIQANAPLKPTVSGQLHAVNAAKQQVTQTMNAGVVSSG

EVTSTEPVR

>gi|30043329|gb|AAP19050.1| hypothetical protein S4073 [Shigella flexneri 2a str. 2457T]

MKISDGNWLIQPGLNLIHPLQVFEVEQQDNEMVVYAAPRDVRERTWQLDTPLFTLRFFSPQEGIVGVRIE

HFQGALNNGPHYPLNILQDVKVTIENTERYAEFKSGNLSARVSKGEFWSLDFLRNGERITGSQVKNNGYV

QDTNNQRNYMFERLDLGVGETVYGLGERFTALVRNGQTVETWNRDGGTSTEQAYKNIPFYMTNRGYGVLV

NHPQCVSFEVGSEKVSKVQFSVESEYLEYFVIDGPTPKAVLDRYTRFTGRPALPPPWSFGLWLTTSFTTN

YDEATVNSFIDGMAERNLPLHVFHFDCFWMKAFQWCDFEWDPLTFPDPEGMIRRLKAKGLKICVWINPYI

GQKSPVFKELQEKGYLLKRPDGSLWQWDKWQPGLAIYDFTNPDACKWYADKLKGLVAMGVDCFKTDFGER

IPIDVQWFDGSDSQKMHNHYAYIYNELVWNVLKDTVGEEEAVLFARSASVGAQKFPVHWGGDCYANYESM

AESLRGGLSIGLSGFGFWSHDIGGFENTAPAHVYKRWCAFGLLSSHSRLHGSKSYRVPWAYDDESCDVVR

FFTQLKCRMMPYLYREAARANARGTPMMRAMMMEFPDDPACDYLDRQYMLGDNVMVAPVFTEAGDVQFYL

PEGRWTHLWHNDELDGSRWHKQQHGFLSLPVYVRDNTLLALGNNDQRPDYVWHEGTAFHLFNLQDGHEAV

CEVPAADGSVIFTLKAARTGNTITVTGAGEAKNWTLCLRNVVKVNGLQGGSQAESEQGLVVTPQGNALTI

TL

>gi|30043328|gb|AAP19049.1| putative integrase [Shigella flexneri 2a str. 2457T]

MNSSKAGGCHGINDIKVKTAKPKDKPYKLADGGGMYLLINTNGSKYWRMKYRFAGKEKMLSIGVYPDVTL

ADAREKRSEARKLLAAGGDPGEAKKEEKIAQQMSLKNTFEAIAREWHQLKADRWSLRYRDEIIDTFEKDI

FPYIGKRPIAEIKPMKLLEALRKMEKRGALEKMRKVRQRCGEVFRYAIVTGRADYNPAPDLASALATPKK

VHFPFLTANELPHFLNDLAGYTGSIITKTATQIIMLTGVRTQELRFARWEDIDFETKLWEIPAEVMKMKR

PHIVPLSEQVIMLFKQLEPISKHHPLVFIGRNDPRKPISKESINQVIELLGYKGRLTGHGFRHTMSTILH

EQGFNSAWIEMQLAHVDKNSIRGTYNHALYLDGRREMMQWYADYIDSLSSRES

>gi|30043327|gb|AAP19048.1| hypothetical protein S4834 [Shigella flexneri 2a str. 2457T]

MNDRLCFEVHDNKGYFVFPDTWFGPLLGEFEEVLDAYDADEISETSYINKLRRLAQREPDFIDIHAHLAY

AFLEQNAPRKALNAALKGLAAGNRIIPESFCGEIIWMHPENRPYLRALYAAILANVHLQRHQDAVMLTDK

ILAYNPEDNQGARWLLGSELLRTGDHERAFSVLKEHADEFSPYWYELGLLHFLNGEHVKAATAFRHGFAT

NTYIAEMLCGNLHPFPLAVWHDFSGSLDTAEDYYATYSPLWGQYPEALLFVNWLYNHSSVLHERSEIIKC

AEMLIQEDDFEICESILRQQEHLWKRIDKTLSEEIVQKCRNMNGEYIWPWILPFSAAGMKHSSIQYQ

>gi|30043326|gb|AAP19047.1| hypothetical protein S4833 [Shigella flexneri 2a str. 2457T]

MDENALRFASYWRNSLADAESGKGCFERKDAQNFTHWHGIAAGRLDEAIVNKFFKGEKDDVETVNVILRP

KVYFRLLQHGKDRSAGTPDIVTPIVTPALLSREGFLYPTPATSIPRDLLEPLPKGAFSIGEIGQYDKYKT

THTDPTHVMWTRP

>gi|30043325|gb|AAP19046.1| IS3 orfB [Shigella flexneri 2a str. 2457T]

MKYVFIENHRAEFSIKAMCRVLRVARSGWYVWLRRRHQMSLRQQFRLTCDAAVHKAFFEAKQRYGAPRLA

DELPEFNIKTIAASLRRQGLRAKASRKFSPVSYRAHGLPVLENLLEQDFSASGPKPEVGG

>gi|30043324|gb|AAP19045.1| IS3 orfA [Shigella flexneri 2a str. 2457T]

MTKPVSISKKPRKQHTPEFRNEALKLAERIGVAAAARELSLYESQLYAWRSKQQQQMSSSERESELAAEN

VRLKRQLAEQAEELSILQKAATYFAKRLK

>gi|30043323|gb|AAP19044.1| hypothetical protein S4831 [Shigella flexneri 2a str. 2457T]

MMLFSIIIMLLSPKITHANEKVICQNSASFVTKEFICGTILVSLILSSLFCYISISPSIFMHTYGLSTFS

YSIIFSFSVLFFIIGNQLSKIEKLSNLWFLLPNSQFYRITNKWFKNRICRIYYRFYSNNKRWYNQFPVSN

RKRQRCWSRNYFRSWFTNNFITVNSRNRHCRK

>gi|30043322|gb|AAP19043.1| IS629 orfB [Shigella flexneri 2a str. 2457T]

MARCTVARLMAVMGLAGVLRGKKVRTTISRKAVAAGDRVNRQFVAERPDQLWVADFTYVSTWRGFVYVAF

IIDVFAGYIVGWRVSSSMETTFVLDALEQALWARRPSGTVHHSDKGSQYVSLVYTQRLKEAGLLASTGST

GDSYDNAMAESINGLYKAEVIHRKSWKNRAEVELATLTWVDWYNNRRLLERLGHIPPAEAEKAYYASIGN

DDLAA

>gi|30043321|gb|AAP19042.1| IS629 orfA [Shigella flexneri 2a str. 2457T]

MTKNTRFSPEVRQRAVRMVLESQGEYDSQWATICSIAPKIGCTPETLRVRVRQHERDTGGGDGGLTTAER

QRLKELERENRELRRSNDILRQASAYFAKAEFDRLWKK

>gi|30043320|gb|AAP19041.1| IS2 orfB [Shigella flexneri 2a str. 2457T]

MARGWGVSLVSRCLRVSRAQLHVILRRTDDWMDGRRSRHTDDTDVLLRIHHVIGELPTYGYRRVWALLRR

QAELDGMPAINAKRVYRIMRQNALLLERKPAVPPSKRAHTGRVAVKESNQRWCSDGFEFCCDNGERLRVT

FALDCCDREALHWAVTTGGFNSETVQDVMLGAVERRFGNDLPSSPVEWLTDNGSCYRANETRQFARMLGL

EPKNTAVRSPESNGIAESFVKTIKRDYISIMPKPDGLTAAKNLAEAFEHYNEWHPHSALGYRSPREYLRQ

RACNGLSDNRCLEI

>gi|30043319|gb|AAP19040.1| IS2 orfA [Shigella flexneri 2a str. 2457T]

MIVLILVFRLVIGVQMIDVLGPEKRRRRTTQEKIAIVQQSFEPGMTVSLVARQHGVAASQLFLWRKQYQE

GSLTAVAAGEQVVPASELAAAMKQIKELQRLLGKKTMENELLKEAVEYGRAKKWIAHAPLLPGDGE

>gi|30043318|gb|AAP19039.1| colV-immunity protein [Shigella flexneri 2a str. 2457T]

MKRWFKYPVTFDLHYTVSHRLIFAPARFLFLLVVTILNAYDHSILAPVWLCFNSAFRADKQLLAGILLVL

MSVPLFTSADFHIPSNRAYSLSLDYSIVRLKFFLNNNRSTAGRSSPFFITHCTDNHNLISRN

>gi|30043317|gb|AAP19038.1| IS1 orfB [Shigella flexneri 2a str. 2457T]

MSRQCTHYGRWPQHGFTSLKKLRPQSVTSRIQPGSDVIVCAEMDEHWGYVGAKSRQRWLFYAYDRIRRTV

VAHVFGERTLATLERLLSLLSAFEVVVWMTDGWPLYESRLKGKLHVISKRYTQRIERHNLNLRQHLARLV

RKSLSFSKSVELHDKVIGHYLNIKHYQ

>gi|30043316|gb|AAP19037.1| IS1 orfA [Shigella flexneri 2a str. 2457T]

MASISIRCPSCSATEGVVRNGKSTAGHQRYLCSHCRKTWQLQFTYTASQPGTHQKIIDMAMNGVGCRASA

RIMGVGLNTVLRHLKNSGRSR

>gi|30043315|gb|AAP19036.1| hypothetical protein S4829 [Shigella flexneri 2a str. 2457T]

MPCFTAMRAEIALMSGSAFAVTHHAFSSGAGRTSDGNGSHASTLKSPSYTKSVSWQHYPWSETKAAYRCF

SNDKVSANKIMTPHKENITERAQQFKRVLVLQDTTELNYSGQKEKQGVGPKKHKDERNLFLHPQLVISES

GVCLGVYDDYQWFRDELKTSKNTRQEICNDLLHKKHVSEKETWRWVEGYNKATELARQCPDTHVLSISDR

EGDFYDLFERAAQTPGIKADWLVRMKFNNRATLNINGKRDHRLLHERIMEITPQQLVEFTIPDGRGQQAR

TLPDMLSSDAGPLSYPLIYSSKVMIISLFDSTVMPDKYRHNGK

>gi|30043314|gb|AAP19035.1| putative membrane transport protein [Shigella flexneri 2a str. 2457T]

MSPGIEDTPQKPLSCWPLAFSAGLLGIGQNGLLVVLPVLVIQTNLSLSVWAALLMLGSMLFLPSSPWWGK

QISRTGSKPVVLWALGGYGISFTLLGLGSVLMATSAITTAVGLGILIIARIAYGLTVSAMVPACQVWALQ

RAGEGNRMAALATISSGLSCGRLFGPLCAAAMLAIHPLAPLGLLMAAPVLALLMLLRLPGTPPQPTPECK

SVSLKRDCLPYLLCAILLAAAVSMMQLGLSPALTRQFATDTTAISQQVAWLLGLSAVAALIAQFGVLRPQ

RLTPVALLLSAGVLMSGGLAIMLSEQLWLFYPGCAVLSFGAALATPAYQLLLNDKLADGAGAGWLATSHT

LGYGLCALLVPLVSKTGVAIALIMAALFATILFTIVSVFIWHYRTIK

>gi|30043313|gb|AAP19034.1| siderophore biosynthesis protein [Shigella flexneri 2a str. 2457T]

MRIIIDIIVIILLCGSCLTMTLPSEKPATDVAAQCFLNALIRETTDWKLTEYPPDELLIPLDEQKSLHFR

VAYFSPTQHHRFAFPARLVTASGSYPVDFTTLSRLIIDKLRHQLFLPVPLCETFHQRVLESHVHTQQAID

ARHDWAALREKALNFGEAEQALLTGHAFHPAPKSHEPFNRREAERYLPDMAPHFPLRWFSVDKTQIAGES

LHLNLQQRLTRFAAENAPQLLNELSDNQWLFPLHPWQGEYLLQQGWCQALVAKGLIKDLGEAGTSWLPTT

SSRSLYCATSRDMIKFSLSVRLTNSIRTLSVKEVKRGMRLARLAQTDGWQMLQVRFPTFRVMQEDGWAGL

LDLNGNIMQESLFALRENLLVDQPKSQTNVLVSLTQAAPDGGDSLLVSAVKRLSDRLGITVQQAAHAWVD

AYCQQVLKPLFTAEADYGLVLLAHQQNILVQMLGDLPVGFIYRDCQGSAFMPHATDWLDSIGEAQAENIF

THEQLLRYFPYYLLVNSTFAVTAALGAAGLDSESNLMARVRASLAEVRDQVTHKTCLNYVLESPYWNVKG

NFFCYLNDHNENTIVDPSVIYFDFANPLQAQEV

>gi|30043312|gb|AAP19033.1| siderophore biosynthesis protein [Shigella flexneri 2a str. 2457T]

MSEANIIHSRYGLRCEKLDKPLNLGWGLDNSAVLHCPGELPTGWLCDALDQIFIAAPQLSAVALPWAEWR

EEPQALTLFGQVKSDIIHRTAFWQLPLWLSSPANRASGEMVFDAEREIYFPQRPPRPQGEVYRRYDPRIR

RMLSFRIADPVSDAERFTRWMNDPRVEYFWEQSGSLEVQTAYLERQLTGKHAFPLIGCFDDRPFSYFEIY

WAAEDRIGRHYSWQPFDRGLHLLVGEQQWRGAHYVQSWLRGLTHYLLLDEPRTQRTVLEPRTDNQRLFRH

LEPAGYRTIKEFDFPHKRSRMVMADRHHFFTEVGL

>gi|30043311|gb|AAP19032.1| siderophore biosynthesis protein [Shigella flexneri 2a str. 2457T]

MNHKDWDFVNRRLVAKMLSEMEYEQVFHAESQGDDHYCINLPGAQWRFIAERGIWGWLWIDAQTLRCTDE

PVLAQTLLMQLKPVLSMSDATVAEHMQDLYATLLGDLQLLKARRGLSASDLINLDADRLQCLLSGHPKFV

FNKGRRGWGKEALERYAPEYTNTFRLHWLAVKREHMIWRCDNDLDIQQLLTAAMDPQEFTRFSQVWQENG
LDHNWLPLPVHPWQWQQKIATDFIADFAEGRMVSLGEFGDQWLAQQSLRTLTNASRRGGLDIKLPLTIYN
TSCYRGIPGRYIAAGPLASRWLQQVFATDATLVQSGAVILGEPAAGYVSHEGYAALARAPYRYQEMLGVI
WRENPCRWLKPDESPVLMATLMECDENNQPLAGAYIDRSGLDAETWLTQLFRVVVVPLYHLLCRYGVALI
AHGQNITLAMKEGVPQRVLLKDFQGDMRLVKEAFPEMDSLPQEVRDVTSRLSADYLIHDLQTGHFVTVLR
FISPLMVRLGVPERRFYQLLAAVLSDYMNKHPQMAERFALFSLFRPQIIRVVLNPVKLTWPDLDGGSRML
PNYLENLQNPLWLVTQEYES

>gi|30043310|gb|AAP19031.1| lysine:N6-hydroxylase [Shigella flexneri 2a str. 2457T]
MKKSVDFIGVGTGPFNLSIAALSHQIEELNCLFFDEHPHFSWHPGMLVPDCHMQTVFLKDLVSAVAPTNP
YSFVNYLVKHKKFYRFLTSRLRTVSREEFSDYLRWAAEDMNNLYFSHTVENIDFDKKSRLFLVQTSRGEY
FAHNICLGTGKQPYLPPCVKHVTQSCFHASEMNLRRPDLSGKRITVVGGGQSGADLFLNALRGEWGEAAE
INWVSRRNNFNALDEAAFADEYFTPEYISGFSGLKEDIRHQLLDEQKMTSDGITADSLLTIYRELYHRFE
VLRKPRNIRLLPSRSVTTLESSGPGWRLLMEHHLDQGRESLESDVVIFATGYRSALPQILPSLMPLITMH
DKNTFKVRDDFTLEWSGPKENNIFAVNASMQTHGIAEPQLSLMAWRSARILNRVLGRDLFDLSMPPALIQ
WRSGSRKKPQPEAASLTHYTANIQE

>gi|30043309|gb|AAP19030.1| putative ferric siderophore receptor [Shigella flexneri 2a str. 2457T]
MRKKYMPRALGPLLLVVLSPAVAQQNDDNEIIVSASRSNRTVAEMAQTTWVIENAELEQQIQGGKELKDA
LAQLIPGLDVSSQSRTNYGMNMRGRPLVVLIDGVRLNSSRSDSRQLDSVDPFNIDHIEVISGATALYGGG
STGGLINIVTKKGQPETMMEFEAGTKSGFNSSKDHDERIAGAVSGGNDHISGRLSVAYQKFGGWFDGNGD
ATLLDNTQTGLQHSNRLDIMGTGTLNIDESRQLQLITQYYKSQGDDNYGLNLGKGFSAISGSSTPYVSKG
LNSDRIPGTERHLISLQYSDSDFLGQELVGQVYYRDESLRYYPFPTVNANKQATAFSSSQQDTDQYGMKL
TLNSQLMDGWQITWGLDAEHERFTSNQMFFDLAQASASGGLNNHKIYTTGRYPSYDITNLAAFLQSSYDI
NDIFTVSGGVRYQYTENRVDDFIDYTQQQKIATGKAISADAIPGGSVDYDNFLFNAGLLMHITERQQAWF

NFSQGVALPDPGKYYGRGIYGAAVNGHLPLTKSVNVSDSKLEGVKVDSYELGWRFIGDNLRTQIAAYYSL

SNKSVERNKDLTISVKDDRRRIYGVEGAVDYLIPDTDWSTGVNFNVLKTESKVNGQWQKYDVKESSPSKA

TAYINWAPEPWSLRVQSTTSFDVSDAEGNDINGYTTVDFISSWQLPVGTLSFSVENLFDRDYTTVWGQRA

PLYYSPGYGPASLYDYKGRGRTFGLNYSVLF

>gi|30043308|gb|AAP19029.1| IS2 orfB [Shigella flexneri 2a str. 2457T]

MARGWGVSLVSRCLRVSRAQLHVILRRTDDWMDGRRSRHTDDTDVLLRIHHVIGELPTYGYRRVWALLRR

QAELDGMPAINAKRVYRLMRQNALLLERKPAVPPSKRAHTGRVAVKESNQRWCSDGFEFCCDNGERLRVT

FALDCCDREALHWAVTTGGFNSETVQDVMLGAVERRFGNDLPSSPVEWLTDNGSCYRANETRQFARMLGL

EPKNTAVRSPESNGIAESFVKTIKRDYISIMPKPDGLTAAKNLAEAFEHYNEWHPHSALGYRSPREYLRQ

RACNGLSDNRCLEI

>gi|30043307|gb|AAP19028.1| IS2 orfA [Shigella flexneri 2a str. 2457T]

MIVLILVFRLVIGEQMIDVLGPEKRRRRRTTQEKIAIVQQSFEPGMTVSLVARQHGVAASQLFLWRKQYQE

GSLTAVAAGEQVVPASELAAAMKQIKELQRLLGKKTMKNELLKEAVEYGRAKKWIAHAPLLPGDGE

>gi|30043306|gb|AAP19027.1| putative long polar fimbriae [Shigella flexneri 2a str. 2457T]

MMTTRIVVGLTAGTCLIFSQNLMAEVSVFNPALLEINHQSGVDIRQFNRANLMPPGVYSVDIFINGKMFE

RQDVTFVQDNPDADLHACFIAIKKTLSSFGIKVDALKSFNDVDETVCLDPAPRIEGSSWQFDSDKLQLNI

SIHQIYMDAMAYDYISPTRWDEGINALTINYDFSGSHTLRSDYGSQETDTSYLNLRNGLNIGPWRLRNYS

TLNTSDGRAEYNSISTWIQRDIAALRSQIMIGDTWTASDIFDSTQIRGARLYTDNDMLPASQNGFAPVVR

GIAKSNATVIIRQNGYVIYQSAVPQGAFEITDLNTASTGGDLDVTIKEEDGSEQRFTQPYASLAILKREG

LTDVDVSVGELRDEDGFTPDVLQAQILHGFSHGITLYGGMQAAENYGSAALGVGKDLGALGAISFDVTHA

RANFSHDDTETGQSYRFLYSKLFDDTDTSLRLVGYRYSTEGYYTLNEWASRRNSPEDFWETGNRRSRVEG

TLTQSLGRDYGNLYLTLSRQQYWHTDDVERLMQFGYSSSWKRLSWNVSWSYSNTARQGTGNNHASDNTSE

QIYMLSLSVPLSGWWGNSYATYSVSQNDNSGSSHQLGLSGTALERNNLSWNLMQSYNSHDDEVGGNMSLT

YDGSYGTVNGSYNYSQNSQRLNYGIRGGILAHSEGVTLSQELGETIALVKAPGAAGLEIDNMRGAATDWR

GYTVKTQLNPYDENRVAISDNYFSKSNIELDNTVVTMVPTRGAVVKAEFVTHVGYRVLFRVLNANGKPVP

FGAIAAIQDASLADSGIVGDRGELYLSGLPEKGQVTLSWGENASTKCIFNYSFSTPESESGLIEQGVTCH

>gi|30043305|gb|AAP19026.1| IS1 orfA [Shigella flexneri 2a str. 2457T]

MASISIRCPSCSATEGVVRNGKSTAGHQRYLCSHCRKTWQLQFTYTASQPGTHQKIIDMAMNGVGCRASA

RIMGVGLNTVLRHLKNSGRSR

>gi|30043304|gb|AAP19025.1| high-affinity phosphate-specific transport protein; periplasmic phosphate-binding protein [Shigella flexneri 2a str. 2457T]

MKVMRTTVATVVAATLSMSAFSVFAEASLTGAGATFPAPVYAKWADTYQKETGNKVNYQGIGSSGGVKQI

IANTVDFGASDAPLSDEKLAQEGLFQFPTVIGGVVLAVNIPGLKSGELVLDGKTLGDIYLGKIKKWDDEA

IAKLNPGLKLPSQNIAVVRRADGSGTSFVFTSYLAKVNEEWKNNVGTGSTVKWPIGLGGKGNDGIAAFVQ

RLPGAIGYVEYAYAKQNNLAYTKLISADGKPVSPTEENFANAAKGADWSKTFAQDLTNQKGEDAWPITST

TFILIHKDQKKPEQGTEVLKFFDWAYKTGAKQANDLDYASLPDSVVEQVRAAWKTNIKDSSGKPLY

>gi|30043303|gb|AAP19024.1| high-affinity phosphate-specific transport protein, cytoplasmic membrane component [Shigella flexneri 2a str. 2457T]

MAATKPAFNPPGKKGDIIFSVLVKLAALIVLLMLGGIIVSLIISSWPSIQKFGLAFLWTKEWDAPNDIYG

ALVPIYGTLVTSFIALLIAVPVSFGIALFLTELAPGWLKRPLGIAIELLAAIPSIVYGMWGLFIFAPLFA

VYFQEPVGNIMSNIPIVGALFSGPAFGIGILAAGVILAIMIIPYIAAVMRDVFEQTPVMMKESAYGIGCT

TWEVIWRIVLPFTKNGVIGGIMLGLGRALGETMAVTFIIGNTYQLDSASLYMPGNSITSALANEFAEAES

GLHVAALMELGLILFVITFIVLA

>gi|30043302|gb|AAP19023.1| high-affinity phosphate-specific transport protein [Shigella flexneri 2a str. 2457T]

MAMVEMQTTAALAESRRKMQARRRLKNRIALTLSMATMAFGLFWLIWILMSTITRGIDGMSLALFTEMTP

PPNTEGGGLANALAGSGLLILWATVFGTPLGIMAGIYLAEYGRKSWLAEVIRFINDILLSAPSIVVGLFV

YTIVVAQMEHFSGWAGVIALALLQVPIVIRTTENMLKLVPDSLREAAYALGTPKWKMISAITLKASVSGI

MTGILLAIARIAGETAPLLFTALSNQFWSTDMMQPIANLPVTIFKFAMSPFAEWQQLAWAGVLIITLCVL

LLNILARVVFAKNKHG

>gi|30043301|gb|AAP19022.1| ATP-binding component of high-affinity phosphate-specific transport system [Shigella flexneri 2a str. 2457T]

MSMVETAPSKIQVRNLNFYYGKFHALKNINLDIAKNQVTAFIGPSGCGKSTLLRTFNKMFELYPEQRAEG

EILLDGDNILTNSQDIALLRAKVGMVFQKPTPFPMSIYDNIAFGVRLFEKLSRADMDERVQWALTKAALW

NETKDKLHQSGYSLSGGQQQRLCIARGIAIRPEVLLLDEPCSALDPISTGRIEELITELKQDYTVVIVTH

NMQQAARCSDHTAFMYLGELIEFSNTDDLFTKPAKKQTEDYITGRYG

>gi|30043300|gb|AAP19021.1| negative regulator for pho regulon and putative enzyme in phosphate metabolism [Shigella flexneri 2a str. 2457T]

MDSLNLNKHISGQFNAELESIRTQVMTMGGMVEQQLSDAITAMHNQDSDLAKRVIEGDKNVNMMEVAIDE

ACVRIIAKRQPTASDLRLVMVISKTIAELERIGDVADKICRTALEKFSQQHQPLLVSLESLGRHTIQMLH

DVLDAFARMDIDEAVRIYREDKKVDQEYEGIVRQLMTYMMEDSRTIPSVLTALFCARSIERIGDRCQNIC

EFIFYYVKGQDFRHVGGDELDKLLAGKDSDK

>gi|30043299|gb|AAP19020.1| positive regulation of bgl operon [Shigella flexneri 2a str. 2457T]

MNMQITKILNNNVVVVIDDQQREKVVMGRGIGFQKRAGERINSSGIEKEYALSSHELNGRLSELLSHIPL

EVMATCDRLISLAQERLGKLQDSIYISLTDHCQFAIKRFQQNVLLWDIQRLYPKEFQLGEEALTIIDKRL

GVQLPKDEVGFIAMHLVSAQMSGNMEDVAGVTQLMREMLQLIKFQFSLNYQEESLSYQRLVTHLKFLSWR

ILEHASINDSDESLQQAVKQNYPQAWQCAERIAIFIGLQYQRKISPAEIMFLAINIERVRKEH

>gi|30043298|gb|AAP19019.1| PTS system beta-glucosides, enzyme II, cryptic [Shigella flexneri 2a str. 2457T]

MTELARKIVAGVGGADNIVSLMHCATRLRFKLKDESKAQAEVLKKTPGIIMVVESGGQFQVVIGNHVADV

FLAVNSVAGLGEKAQQAPENDEKGNLLNRFVYVISGIFTPLIGLMAATGILKGMLALALTFQWTTEQSGT

YLILFSASDALFGFFPIILGYTAGKRFGGNPFTAMVIGGALVHPLILTAFENGQKADAPGLDFLGIPVTL

LNYSSSVIPIIFSAWLCSILERRLNAWLPSAIKNFFTPLLCLMVITPVTFLLVGQLSTWISEMIAAGYLW

LYQAVPAFAGAVMGGFWQIFVMFGLHRGLVPLCINNFTVLGYDTMIPLLMPAIMAQVGAALGVFLCERDA

QKKVVAGSAALTGLFGITEPAVYGVNLPRKYPFVIACISGALGATIIGYAQTKVYSFGLPSIFTFMQTIP

STGIDFTVWASVIGGVIAIGCAFVGTLMLHFITSKRQPAQGAPQEKTPEVITPPEQGGICSPMTGEIVPL

IHVADTTFASGLLGKGIAILPSVGEVRSPVAGRIASLFATLHAIGIESDDGVEILIHVGIDTVKLDGKFF

SAHVNVGDKVNTGDRLISFDIPAIREAGFDLTTPVLISNSDDFTDVLPHGTAQISAGEPLLSIIR

>gi|30043297|gb|AAP19018.1| phospho-beta-glucosidase B cryptic [Shigella flexneri 2a str. 2457T]

MKAFPETFLWGGATAANQVEGAWLEDGKGITTSDLQPHGVMGKMEPRILGKENIKDVAIDFYYRYPEDIA

LFAEMGFTCLRISIAWARIFPQGDEAEPNEAGLAFYDRLFDEMAQAGIKPLVTLSHYEMLYGLVKNYGGW

ANRAVIDHFEHYARTVFTRYQHKVALWLTFNEINMSLHAPFTGVGLAEESGEAEVYQAIHHQLVASARAV

KAYHSLIPEAKIGNMLLGGLVYPLTCQPQDMLQAMEENRRWMFFGDVQARGQYPGYMQRFFRDHNITIEM

TESDAEDLKHTVDFISFSYYMTGCVSHDESINKNAQGNILNMIPNPHLKSSEWGWQIDPVGLRVMLPTY

>gi|30043296|gb|AAP19017.1| IS1 orfA [Shigella flexneri 2a str. 2457T]

MASISIRCPSCSATEGVVRNGKSTAGHQRYLCSHCRKTWQLQFTYTASQPGTHQKIIDMAMNGVGCRASA

RIMGVGLNTVLRHLKNSGRSR

>gi|30043295|gb|AAP19016.1| IS1 orfB [Shigella flexneri 2a str. 2457T]

MSRQCTHYGRWPQHGFTSLKKLRPQSVTSRIQPGSDVIVCAEMDEHWGYVGAKSRQRWLFYAYDRIRRTV

VAHVFGERTLATLERLLSLLSAFEVVVWMTDGWPLYESRLKGKLHVISKRYTQRIERHNLNLRQHLARLG

RKSLSFSKSVELHDKVIGHYLNIKHYQ

>gi|30043294|gb|AAP19015.1| putative receptor protein [Shigella flexneri 2a str. 2457T]

MAPLAFSAQSLAESLTVEQRLELLEKALRETQSELKKYKDEEKKKYTPATVNRSVSTNDQGYAANPFPTS

SAAKPDAVLVKNEEKNASETGSIYSSMTLKDFSKFVKDEIGFSYNGYYRSGWGTASHGSPKSWAIGSLGR

FGNEYSGWFDLQLKQRVYNENGKRVDAVVMIDGNVGQQYSTGWFGDNAGGENFMQFSDMYVTTKGFLPFA

PEADFWVGKHGAPKIEIQMLDWKTQRTDAAAGVGLENWKVGPGKIDIALVREDVDDYDRSLQNKQQINTH

TIDLRYKDIPLWDKATLMVSGRYVTANESASEKDNQDNNGYYDWKDTWMFGTSLTQKFDKGGFNEFSFLV

ANNSIARNFGRYAGASPFTTFNGRYYGDHTGGTAVRLTSQGEAYIGDHFIVANAIVYSFGNNIYSYETGA

HSDFESIRAVVRPAYIWDQYNQTGVELGYFTQQNKDANSNKFNESGYKTTLFHTFKVNTSMLTSRLEIRF

YATYIKALENELDGFTFEDNKDAQFAVGAQAEIWW

>gi|30043293|gb|AAP19014.1| putative xylanase [Shigella flexneri 2a str. 2457T]

MNIKIAALTLAIASGISAQWAIAADMPASPAPTIPVKQYVTQVNADNSVTFRYFAPGAKNVSVVVGVPVP

DNIHPMTKDEAGVWSWRTPILKGNLYEYFFNVDGVRSIDTGTAMTKPQRQVNSSMILVPGSYLDTRSVAH

GDLIAITYHSNALQSERQMYVWTPPGYTGMGEPLPVLYFYHGFGDTGRSAIDQGRIPQIMDNLLAEGKIK

PMLVVIPDTETDAKGIIPEDFVPQERRKVFYPLNAKAADRELMNDIIPLISKRFSVRKDADGRALAGLSQ

GGYQALVSGMNHLESFGWLATFSGVTTTTVPDEGVAARLNDPAAINQQLRNFTVVVGDKDVVTGKDIAGL

KTELEQKKIKFDYQEYPGLNHEMDVWRPAYAAFVQKFKIRH

>gi|30043292|gb|AAP19013.1| putative isomerase [Shigella flexneri 2a str. 2457T]

MKLIITEDYQEMSRVAAHHLLGYMSKTRRVNLAITAGSTPKGMYEYLTTLVKGKPWYDNCYFYNFDEIPF

RGKEGEGVTITNLRNLFFTPAGIKEENIQKLTIDNYREHDQKLAREGGLDLVVLGLGADGHFCGNLPNTT

HFHEQTVEFPIQGEMVDIVAHGELGGDFSLVPDSYVTMGPKSIMAAKNLLIIVSGAGKAQALKNVLQGPV

TEDVPASVLQLHPSLMVIADKAAAAELAFG

>gi|30043291|gb|AAP19012.1| ISSfl3 orfC [Shigella flexneri 2a str. 2457T]

MNNELPDDIELLKAMLRKQQSRLRQYACQVAGYEQEIERLKAQLDRLRRMLFGQSSEKKRHKLENQIRQA

EKRLSELENRLNTARNLLEDASSVTDSPDTSPPSENPIASKPESPGRKSSRKPLPAELPRETHRLLPAET

SCPACGGVLKEMGETISEQLDIINTAFKVIETIRPKLACSRCDVIVQAPLPPKPIERGYASAGLLARILV

SKYMEHIPLYRQSEIYARQGVELSRNTMVRWVSEMADKLRPLYIALNDYVLEAGKVHADDTPVKVLAPGN

GKTKTGRLWVYVRDDRNAGSSLPAAVWFAYSADRKGEHPQLHLAKYQGVLQADAYAGYNVLYETGRVKEA

GCLAHARRKIHDEDVRRPTEMTQEALRRIAELYDIEAEIRGSPAEERLAVRKARSVQLMQSLYDWIQLQR

KTLSKHAKMAKAFDYILNHWNALNEFCRDGRVEIDNNIGENALRSVAVGRKNYLFFGSDKGGESAAIIYS

LLVTCKQNEVEPEDWLREVIEKLNDWPSNQVHELLPWNFSSVK

>gi|30043290|gb|AAP19011.1| ISSfl3 orfB [Shigella flexneri 2a str. 2457T]

MITLPTGTKIWIIAGITDMRCGFNGLASKVQNTLKDDPFSGHIFVFRGRSGKMVKILWADRDGLCLFTKR

LAGDPGRESAPDASSVIHATGGDRVATSQTDRTAWHPDITRDKTRE

>gi|30043289|gb|AAP19010.1| ISSfl3 orfA [Shigella flexneri 2a str. 2457T]

MNSQTTKDIPCFRSYLPDALRLRFEDKLTIRAIAQRLGLSHSTIHTLFQRFLASGIAWPLPDSVSFAQLD

AILYANRKKELTEPQIREGSWRKERRTSYSREFKVRLAKQALQPGAVVARIAREHDINNNLLFKWKSQYE

DGLLSDDDIQECMPVPVALTDTPEPTRPVTNPFWRNKHDERPEGAPGNVPRCELHLKSGVVKLFDPLTPE

LLRALIREMKGGIR

>gi|30043288|gb|AAP19009.1| hypothetical protein S4028 [Shigella flexneri 2a str. 2457T]

MSVSRRVIHHGLYFAVLGPLIGVLFLVLYIFFAKEPLVLLVIIQVLPLFLLLSITTGAIPALLTGVMVAC

LPEKIGSQKNYRCLAGGIGGVVITEIYCAVIVHIKGMASSELFENILSGDSLVVRIIPALLAGVVMSRII

TRLPGLDISCPETDSLS

>gi|30043287|gb|AAP19008.1| putative phosphatase [Shigella flexneri 2a str. 2457T]

MSRIEAVFFDCDGTLVDSEVICSRAYVTMFQEFGITLDPEEVFKRFKGVKLYEIIDIVSLEHGVTLAKTE

AEHVYRAEVARLFDSAIEGAGALLSAITAPMCVVSNGPNNKMQHSMGKLNMLHYFPDKLFSGYDIQRWKP

DPALMFHAAKAMNVNVENCILVDDSVAGAQSGIDAGMEVFYFCADPHNKPIVHPKVTTFTHLSQLPELWK

ARGWDITA

>gi|30043286|gb|AAP19007.1| putative membrane / transport protein [Shigella flexneri 2a str. 2457T]

MSHQHTTQTSGQRMLERVFKLREHGTTARTEVIAGFTTFLTMVYIVFVNPQILGVAGMDTSAVFVTTCLI

AAFGSIMMGLFANLPVALAPAMGLNAFFAFVVVQAMGLPWQIGMGAIFWGAVGLLLLTIFRVRYWMIANI

PVSLRVGITSGIGLFIGMMGLKNAGVIVANSETLVSIGNLTSHSVLLGILGFFIIAILASRNIHAAVLVS

IVVTTLLGWMLGDVHYNGIVSAPPSVMTVVGHVDLAGSFNLGLAGVIFSFMLVNLFDSSGTLIGVTDKAG

LADEKGKFPRMKQALYVDSISSVTGSFIGTSSVTAYIESSSGVSVGGRTGLTAVVVGLLFLLVIFLSPLA

GMVPDYAAAGALIYVGVLMTSSLARVNWQDLTESVPAFITAVMMPFSFSITEGIALGFISYCVMKIGTGR

LRDLSPCVIIVALLFILKIVFIDAH

>gi|30043285|gb|AAP19006.1| hypothetical protein S4025 [Shigella flexneri 2a str. 2457T]

MSEKLQVVTLLGSLRKGSFNGMVARTLPKIAPASMEVNALPSIADIPLYDADVQQEEGFPATVEALAEQI

RQADGVVIVTPEYNYSVPGGLKNAIDWLSRLPDQPLAGKPVLIQTSSMGVIGGARCQYHLRQILVFLDAM

VMNKPEFMGGVIQNKVDPQTGEVIDQGTLDHLTGQLTAFGEFIQRVKI

>gi|30043284|gb|AAP19005.1| hypothetical protein S4024 [Shigella flexneri 2a str. 2457T]

MATHFARGILTEGHLISVRLPSQCHQEARNIPPHRQSRFLASRGLLAELMFMLYGIGELPEIVTLPKGKP

VFSDKNLPSFSISYAGNMVGVALTTEGECGLDMELQRATRGFHSPHAPDNHTFSSNESLWISKQNDPNEA

RAQLITLRRSVLKLTGDVLNDDPRDLQLLPIAGRLKCAHVNHVEALCDAEDVLVWSVAVTPAIEKLSVWE

LDGKHSWKSLPDIHSRANNPTSRMMRFAQLSTVKSFSPN

>gi|30043283|gb|AAP19004.1| IS600 orfA [Shigella flexneri 2a str. 2457T]

MSRKTQRYSKEFKAEAVRTVLENQLSISEGASRLSLPEGTLGQWVTAARKGLGTPGSRTLAELESEILQL

RKALNEARLERDILKKATAYFAQESLKNTR

>gi|30043282|gb|AAP19003.1| IS600 orfB [Shigella flexneri 2a str. 2457T]

MAHIRTRETYGTRRLQTELAENGIIVGRDRLARLRKELRLRCKQKRKFRATTNPNHNLPVAPNLLNQTFA

PTAPNQVWVADLTYVATQEGWLYLAGIKDVYTCEIVGYAMGERMTKELTGKALFMALRSQRPPAGLIHHS

DRGSQYCAYDYRVIQEQFGLKTSMSRKGNCYDNAPMESFWGTLKNESLSHYRFNNRDEAISVIREYIEIF

YNRQRRHSRLGNISPAAFREKYHQMAA

>gi|30043281|gb|AAP19002.1| putative transport protein [Shigella flexneri 2a str. 2457T]

MSRFLICSFALVLLYPAGIDMYLVGLPRIAADLNASEAQLHIAFSVYLAGMAAAMLFAGKVADRSGRKPV

AIPGAALFIIASVFCSLAETSTLFLAGRFLQGLGAGCCYVVAFAILRDTLDDRRRAKVLSLLNGITCIIP

VLAPVLGHLIMLKFPWQSLFWAMAMMGIAVLMLSLFILKETRPASPAASDKPRENSESLLNRFFLSRVVI

TTLSVSVILTFVNTSPVLLMEIMGFERGEYATIMALTAGVSMTFSFSTPFALGIFKPRTLMITSQVLFLA

AGITLAVSPSHAVSLFGITLICAGFSVGFGVAMSQALGPFSLRAGVASSTLGIAQVCGSSLWIWLAAVVG

IGAWNMLIGILIACSIVSLLLIMFVAPGRPVAAHEEIHHHA

>gi|30043280|gb|AAP19001.1| low-affinity tryptophan permease [Shigella flexneri 2a str. 2457T]

MTDQAEKKHSAFWGVMVIAGTVIGGGMFALPVDLAGAWFFWGAFILIIAWFSMLHSGLLLLEANLNYPVG

SSFNTITKDLIGNTWNIISGITVAFVLYILTYAYISANGAIISETISMNLGYHANPRIVGICTAIFVASV

LWISSLAASRITSLFLGLKIISFVIVFGSFFFQVDYSILRDATSTTAGTSYFPYIFMALPVCLASFGFHG

NIPSLIICYGKRKDKLIKSVVFGSLLALVIYLFWLYCTMGNIPRESFKAIISSGGNIDSLVKSFLGTKQH

GIIEFCLLVFSNLAVASSFFGVTLGLFDYLADLFKIDNSHGGRFKTVLLTFLPPALLYLIFPNGFIYGIG

GAGLCATIWAVIIPAVLAIKARKKFPNQMFTVWGGNLIPAIVILFGITVILCWFGNVFNVLPKFG

>gi|30043279|gb|AAP19000.1| tryptophanase [Shigella flexneri 2a str. 2457T]

MENFKHLPEPFRIRVIEPVKRTTRAYREEAIIKSGMNPFLLDSEDVFIDLLTDSGTGAVTQSMQAAMMRG

DEAYSGSRSYYALAESVKNIFGYQYTIPTHQGRGAEQIYIPVLIKKREQEKGLDRSKMVAFSNYFFDTTQ

GHSQINGCTVRNVYIKEAFDTGVRYDFKGNFDLEGLERGIEEVGPNNVPYIVATITSNSAGGQPVSLANL

KAMYSIAKKYDIPVVMDSARFAENAYFIKQREAEYKDWTIEQITRETYKYADMLAMSAKKDAMVPMGGLL

CMKDDSFFDVYTECRTLCVVQEGFPTYGGLEGGAMERLAVGLYDGMNLDWLAYRIAQVQYLVDGLEEIGV

VCQQAGGHAAFVDAGKLLPHIPADQFPAQALACELYKVAGIRAVEIGSFLLGRDPKTGKQLPCPAELLRL

TIPRATYTQTHMDFIIEAFKHVKENAANIKGLTFTYEPKVLRHFTAKLKEV

>gi|30043278|gb|AAP18999.1| tryptophanase leader peptide [Shigella flexneri 2a str. 2457T]

MNILHVCVTSKWFNIDNKIVDHRP

>gi|30043277|gb|AAP18998.1| IS1 orfA [Shigella flexneri 2a str. 2457T]

MASISIRCPSCSATEGVVRNGKSTAGHQRYLCSHCRKTWQLQFTYTASQPGTHQKIIDMAMNGVGCRASA

RIMGVGLNTVLRHLKNSGRSR

>gi|30043276|gb|AAP18997.1| IS1 orfB [Shigella flexneri 2a str. 2457T]

MSRQCTHYGRWPQHGFTSLKKLRPQSVTSRIQPGSDVIVCAEMDEHWGYVGAKSRQRWLFYAYDRIRRTV

VAHVFGERTLATLERLLSLLSAFEVVVWMTDGWPLYESRLKGKLHVISKRYTQRIERHNLNLRQHLARLG

RKSLSFSKSVELHDKVIGHYLNIKHYQ

>gi|30043275|gb|AAP18996.1| GTP-binding protein in thiophene and furan oxidation [Shigella flexneri 2a str. 2457T]

MSDNDTIVAQATPPGRGGVGILRISGFKAREVAETVLGKLPKPRYADYLPFKDADGSVLDQGIALWFPGP

NSFTGEDVLELQGHGGPVILDLLLKRILTIPGLRIARPGEFSERAFLNDKLDLAQAEAIADLIDASSEQA

ARSALNSLQGAFSARVNHLVEALTHLRIYVEAAIDFPDEEIDFLSDGKIEAQLNDVMADLDAVRAEARQG

SLLREGMKVVIAGRPNAGKSSLLNALAGREAAIVTDIAGTTRDVLREHIHIDGMPLHIIDTAGLREASDE

VERIGIERAWQEIEQADRVLFMVDGTTTDAVDPAEIWPEFIARLPAKLPITVVRNKADITGETLGMSEVN

GHALIRLSARTGEGVDVLRNHLKQSMGFDTNMEGGFLARRRHLQALEQAAEHLQQGKAQLLGAWAGELLA

EELRLAQQNLSEITGEFTSDDLLGRIFSSFCIGK

>gi|30043274|gb|AAP18995.1| 60 KD inner-membrane protein [Shigella flexneri 2a str. 2457T]

MDSQRNLLVIALLFVSFMIWQAWEQDKNPQPQAQQTTQTTTTAAGSAADQGVPASGQGKLISVKTDVLDL

TINTRGGDVEQALLPAYPKELNSTQPFQLLETSPQFIYQAQSGLTGRDGPDNPANGPRPLYNVEKDAYVL

AEGQNELQVPMTYTDAAGNTFTKTFVLKRGDYAVNVNYNVQNAGEKPLEISSFGQLKQSITLPPHLDTGS

SNFALHTFRGAAYSTPDEKYEKYKFDTIADNENLNISSKGGWVAMLQQYFATAWIPHNDGTNNFYTANLG

NGIVAIGYKSQPVLVQPGQTGAMNSTLWVGPEIQDKMAAVAPHLDLTVDYGWLWFISQPLFKLLKWIHSF

VGNWGFSIIIITFIVRGIMYPLTKAQYTSMAKMRMLQPKIQAMRERLGDDKQRISQEMMALYKAEKVNPL

GGCFPLLIQMPIFLALYYMLMGSVELRQAPFALWIHDLSAQDPYYILPILMGVTMLFIQKMSPTTVTDPM

QQKIMTFMPVIFTVFFLWFPSGLVLYYIVSNLVTIIQQQLIYRGLEKRGLHSREKKKS

>gi|30043273|gb|AAP18994.1| RNase P, protein component; protein C5 [Shigella flexneri 2a str. 2457T]

MVKLAFPRELRLLTPSQFTFVFQQPQRAGTPQITILGRLNSLGHPRIGLTVAKKNVRRAHERNRIKRLTR

ESFRLRQHELPAMDFVVVAKKGVADLDNRALSEALEKLWRRHCRLARGS

>gi|30043272|gb|AAP18993.1| 50S ribosomal subunit protein L34 [Shigella flexneri 2a str. 2457T]

MKRTFQPSVLKRNRSHGFRARMATKNGRQVLARRRAKGRARLTVSK

>gi|30043271|gb|AAP18992.1| replication initiation protein DnaA [Shigella flexneri 2a str. 2457T]

MSLSLWQQCLARLQDELPATEFSMWIRPLQAELSDNTLALYAPNRFVLDWVRDKYLNNINGLLTSFCGAD

APQLRFEVGTKPVTQTPQAAVTSNVAAPAQVAQTQPQRAAPSTRSGWDNVPAPAEPTYRSNVNVKHTFDN

FVEGKSNQLARAAACQVADNPGGAYNPLFLYGGTGLGKTHLLHAVGNGIMARKPNAKVVYMHSERFVQDM

VKALQNNAIEEFKRYYRSVDALLIDDIQFFANKERSQEEFFHTFNALLEGNQQIILTSDRYPKEINGVED

RLKSRFGWGLTVAIEPPELETRVAILMKKADENDIRLPGEVAFFIAKRLRSNVRELEGALNRVIANANFT

GRAITIDFVREALRDLLALQEKLVTIDNIQKTVAEYYKIKVADLLSKRRSRSVARPRQMAMALAKELTNH

SLPEIGDAFGGRDHTTVLHACRKIEQLREESHDIKEDFSNLIRTLSS

>gi|30043270|gb|AAP18991.1| DNA polymerase III, beta-subunit [Shigella flexneri 2a str. 2457T]

MKFTVEREHLLKPLQQVSGPLGGRPTLPILGNLLLQVADGTLSLTGTDLEMEMVARVALVQPHEPGATTV

PARKFFDICRGLPEGAEIAVQLEGERMLVRSGRSRFSLSTLPAADFPNLDDWQSEVEFTLPQATMKRLIE

ATQFSMAHQDVRYYLNGMLFETEGEELRTVATDGHRLAVCSMPIGQSLPSHSVIVPRKGVIELMRMLDGG

DNLLRVQIGSNNIRAHVGDFIFTSKLVDGRFPDYRRVLPKNPDKHLEAGCDLLKQAFARAAILSNEKFRG

VRLYVSENQLKITANNPEQEEAEEILDVTYSGAEMEIGFNVSYVLDVLNALKCENVRMMLTDSVSSVQIE

DAASQSAAYVVMPMRL

>gi|30043269|gb|AAP18990.1| Rec protein [Shigella flexneri 2a str. 2457T]

MSLTRLLIRDFRNIETADLALSPGFNFLVGANGSGKTSVLEAIYTLGHGRAFRSLQIGRVIRHEQEAFVL

HGRLQGEERETAIGLTKDKQGDSKVRIDGTDGHKVAELAHLMPMQLITPEGFTLLNGGPKYRRAFLDWGC

FHNEPGFFTAWSNLKRLLKQRNAALRQVTRYEQLRPWDKELIPLAEQISTWRAEYSAGIAADMDDTCKQF

LPEFSLTFSFQRGWEKETEYAEVLERNFERDRQLTYTAHGPHKADLRIRADGAPVEDTLSRGQLKLLMCA

LRLAQGEFLTRESGRRCLYLIDDFASELDDERRGLLASRLKATQSQVFVSAISAEHVIDMSDENSKMFTV

EKGKITD

>gi|30043268|gb|AAP18989.1| DNA gyrase subunit B, type II topoisomerase [Shigella flexneri 2a str. 2457T]

MSNSYDSSSIKVLKGLDAVRKRPGMYIGDTDDGTGLHHMVFEVVDNAIDEALAGHCKEIIVTIHADNSVS

VQDDGRGIPTGIHPEEGVSAAEVIMTVLHAGGKFDDNSYKVSGGLHGVGVSVVNALSQKLELVIQREGKI

HRQIYEHGVPQAPLAVTGETEKTGTMVRFWPSLETFTNVTEFEYEILAKRLRELSFLNSGVSIRLRDKRD

GKEDHFHYEGGIKAFVEYLNKNKTPIHPNIFYFSTEKDGIGVEVALQWNDGFQENIYCFTNNIPQRDGGT

HLAGFRAAMTRTLNAYMDKEGYSKKAKVSATGDDAREGLIAVVSVKVPDPKFSSQTKDKLVSSEVKSAVE

QQMNELLAEYLLENPTDAKIVVGKIIDAARAREAARRAREMTRRKGALDLAGLPGKLADCQERDPALSEL

YLVEGDSAGGSAKQGRNRKNQAILPLKGKILNVEKARFDKMLSSQEVATLITALGCGIGRDEYNPDKLRY

HSIIIMTDADVDGSHIRTLLLTFFYRQMPEIVERGHVYIAQPPLYKVKKGKQEQYIKDDEAMDQYQISIA

LDGATLHTNASAPALAGEALEKLVSEYNATQKMINRMERRYPKAMLKELIYQPTLTEADLSDEQTVTRWV

NALVSELNDKEQHGSQWKFDVHTNAEQNLFEPIVRVRTHGVDTDYPLDHEFITGGEYRRICTLGEKLRGL

LEEDAFIERGERRQPVASFEQALDWLVKESRRGLSIQRYKGLGEMNPEQLWETTMDPESRRMLRVTVKDA

IAADQLFTTLMGDAVEPRRAFIEENALKAANIDI

>gi|30043267|gb|AAP18988.1| hypothetical protein S4005 [Shigella flexneri 2a str. 2457T]

MGLFDEVVGAFLKGDAGKYQAILSWVEEQGGIQVLLEKLQSGGLGAILSTWLSNQQRNQSVSGEQLESAL

GTNAVSDLGQKLGVDTSTASSLLAEQLPKIIDALSPQGEVSAQANNDLLSAGMELLKGKLFR

>gi|30043266|gb|AAP18987.1| hypothetical protein S4004 [Shigella flexneri 2a str. 2457T]

MAIKLIAIDMDGTLLLPDHTISPAVKNAIAAARARGVNVVLTTGRPYAGVHNYLKELHMEQPGDYCITYN

GALVQKAADGSTVAQTALSYDDYRFLEKLSREVGSHFHALDRTTLYTANRDISYYTVHESFVATIPLVFC

EAEKMDPNTQFLKVMMIDEPAILDQAIARIPQEVKEKYTVLKSAPYFLEILDKRVNKGTGVKSLADVLGI

KPEEIMAIGDQENDIAMIEYAGVGVAMDNAIPSVKEVANFVTKSNLEDGVAFAIEKYVLN

>gi|30043265|gb|AAP18986.1| putative FADA-type transcriptional regulator [Shigella flexneri 2a str. 2457T]

MIANNQDREAFNEADIRYHEAVLQSVHNPVLQQLSIAISSLQRAVFERTWMGDEANMPQTLQEHKALFDA

IRHQDGDAAEQAALTMIASSTRRLKEIT

>gi|30043264|gb|AAP18985.1| regulator protein for dgo operon [Shigella flexneri 2a str. 2457T]

MTLNKTDRIVITLGKQIVHGKYVPGSPLPAEAELCEEFATSRNIIREVFRSLMAKRLIEMKRYRGAFVAP

RNQWNYLDTDVLQWVLENDYDPRLISAMSEVRNLVEPAIARWAAERATSSDLAQIESALNEMIANNQDRE

AFNEADIRYHEAVLQSVHNPVLQQLSIAISSLQRAVFERTWMGDEANMPQTLQEHKALFDAIRHQDGDAA

EQAALTMIASSTRRLKEIT

>gi|30043263|gb|AAP18984.1| 2-oxo-3-deoxygalactonate kinase [Shigella flexneri 2a str. 2457T]

MTARYIAIDWGSTNLRAWLYQGDHCLESRQSEAGVTRLNGKSPAAVLAEVTTDWREENTPVVMAGMVGSN

VGWKVAPYLSVPARFSSIGEQLTSVGDNIWIIPGLCVSHDDNHNVMRGEETQLIGARALAPSSLYVMPGT

HCKWVQADSQQINDFRTVMTGELHHLLLNHSLIGAGLPPQENSADAFAAGLERGLNAPAILPQLFEVRAS

HVLGTLPREQVSEFLSGLLIGAEVASMRDYVTHQHAITLVAGTSLTARYQQAFQAMGCDVAAVAGDTAFQ

AGIRSIAHAVAN

>gi|30043262|gb|AAP18983.1| D-galactonate transport protein [Shigella flexneri 2a str. 2457T]

MVSGFAMPKIWRKLAMDIPVNAAKPGRRRYLTLVMIFITVVICYVDRANLAVASAHIQEEFGITKAEMGY

VFSAFAWLYTLCQIPGGWFLDRVGSRVTYFIAIFGWSVATLFQGFATGLMSLIGLRAITGIFEAPAFPTN

NRMVTSWFPEHERASAVGFYTSGQFVGLAFLTPLLIWIQEMLSWHWVFIVTGGIGIIWSLIWFKVYQPPR

LTKGISKAELDYIRDGGGLVDGDAPVKKEARQPLTAKDWKLVFHRKLIGVYLGQFAVASALWFFLTWFPN

YLTQEKGITALKAGFMTTVPFLAAFVGVLLSGWVADLLVRKGFSLGFARKTPIICGLLISTCIMGANYTN

DPMMIMCLMALAFFGNGFASITWSLVSSLAPMRLIGLTGGVFNFAGGLGGITVPLVVGYLAQGYGFAPAL

VYISAVALIGALSYILLVGDVKRVG

>gi|30043261|gb|AAP18982.1| hypothetical protein S3997 [Shigella flexneri 2a str. 2457T]

MEHFDVAIIGLGPAGSALARKLAGKMQVIALDKKHQCGTEGFSKPCGGLLAPDAQRSFIRDGLTLPVDVI

ANPQIFSVKTVDVAASLTRNYQRSYININRHAFDLWMKSLIPASVEVYHDSLCRKIWREDDKWHVIFRAD

GWEQHITARYLVGADGANSMVRRHLYPDHQIRKYVAIQQWFAEKHPVPFYSCIFDNAITDCYSWSISKDG

YFIFGGAYPMKDGQTRFTTLKEKMSAFQFQFGKAVKSEKCTVLFPSRWQDFVCGKDNAFLIGEAAGFISA

SSLEGISYALDSAEILRAVFLKQPEKSNAAYWRATHKLRLKLFGKIVKSRCLTAPALRKWIMRSGVAHIP

QLKDYPTRFTSPTSRM

>gi|30043260|gb|AAP18981.1| hypothetical protein S3996 [Shigella flexneri 2a str. 2457T]

MMAGTVLYQDRAMKQITFAPRNHLLTNTNTNTNTNTNTWTPDSQWLVFDVRPSGASFTGETIERVNIHTG

EVEVIYRASQGAHVGVVTVHPKSEKYVFIHGPENPDETWHYDFHHRRGVIAEGGKVSNLDAMDITAPYTP

GALRGGSHVHVFSPNGERGSFTYNDHVMHQLDPALDLRNVGVAAPFGPVNVQKQHPREYSGSHWCVLVSK

TTPTPQPGSDEINRAYEEGWVGNHALAFIGDTLSPKGEKVPELFIVELPQDEASWKSAGDAPLSGTETTL

PAPPRGVVQRRLTFTHHRAYPGLVNVPRHWVRCNPQGTQIAFLMRDDNGIVQLWLISPQGGEPRKLTHNK

TDIQSAFNWHPSGEWLGFVLDNRIVCAHAQSGEVEYLTENHANPPSADAVVFSPDGQWLAWMEGGQLWIT

ETDR

>gi|30043259|gb|AAP18980.1| hypothetical protein S3995 [Shigella flexneri 2a str. 2457T]

MMMLFSARDRYNSSGQHPRSTDEFIMIRNVLLAFMICSGMTLLGGCSSVMSHTGGKEGTYPGTRASATMI

GDDETNWGTKSLAILDMPFTAVMDTLLLPWDVFRKDSSVRSRVEKSEANAQATNAVIPPARMPDN

>gi|30043258|gb|AAP18979.1| heat shock protein [Shigella flexneri 2a str. 2457T]

MRNFDLSPLYRSAIGFDRLFNHLENNQSQSNGGYPPYNVELVDENHYRIAIAVAGFAESELEITAQDNLL

VVKGAHADEQKERTYLYQGIAERNFERKFQLAENIHVRGANLVNGLLYIDLERVIPEAKKPRRIEIN

>gi|30043257|gb|AAP18978.1| heat shock protein [Shigella flexneri 2a str. 2457T]

MRNFDLSPLMRQWIGFDKLANALQNAGESQSFPPYNIEKSDDNHYRITLALAGFRQEDLEIQLEGTRLSV

KGTPEQPKEEKKWLHQGLMNQPFSLSFTLAENMEVSGATFVNGLLHIDLIRNEPEPIAAQRIAISERPAL

NS

>gi|30043256|gb|AAP18977.1| putative transport protein [Shigella flexneri 2a str. 2457T]

MSDIALTVSILALVAVVGLFIGNVKFRGIGLGIGGVLFGGIIVGHFVSQAGMTLSSDMLHVIQEFGLILF

VYTIGIQVGPGFFASLRVSGLRLNLFAVLIVIIGGLVTAILHKLFDIPLPVVLGIFSGAVTNTPALGAGQ

QILRDLGTPMEMVDQMGMSYAMAYPFGICGILFTMWMLRVIFRVNVETEAQQHESSRTNGGALIKTINIR

VENPNLHDLAIKDVPILNGDKIICSRLKREETLKVPSPDTIIQLGDLLHLVGQPADLHNAQLVIGQEVDT

SLSTKGTDLRVERVVVTNENVLGKRIRDLHFKERYDVVISRLNRAGVELVASGDISLQFGDILNLVGRPS

AIDAVANVLGNAQQKLQQVQMLPVFIGIGLGVLLGSIPVFVPGFPAALKLGLAGGPLIMALILGRIGSIG

KLYWFMPPSANLALRELGIVLFLSVVGLKSGGDFVNTLVNGEGLSWIGYGALITAVPLITVGILARMLAK

MNYLTMCGMLAGSMTDPPALAFANNLHPTSGAAALSYATVYPLVMFLRIITPQLLAVLFWSIG

>gi|30043255|gb|AAP18976.1| putative transcriptional regulator [Shigella flexneri 2a str. 2457T]

MIYKSIAERLRIRLNSADFTLNSLLPGEKKLAEEFAVSRMTIRKAIDLLVAWGLVVRRHGSGTYLVRKDV

LHQTASLTGLVEVLKRQGKTVTSQVLIFEIMPAPPAIASQLRIQINEQIYFSRRVRFVEGKPLMLEDSYM

PVKLFRNLSLQHLEGSKFEYIEQECGILIGGNYESLMPVLADRLLARQMKVAEHTPLLRITSLSYSESGE

FLNYSVMFRNASEY

>gi|30043254|gb|AAP18975.1| PTS system, arbutin-like IIB component [Shigella flexneri 2a str. 2457T]

MFSNHADMMLTQIAIGLCFTLLYFVVFRTLILQFNMCTPGREDAEVKLYSKAEYKASRGQTTAAEPKKEL

DQAAGILQALGGVGNISSINNCATRLRIALHDMSQTLDDEVFKKLGAHGVFRSGDAIQVIIGLHVSQLRE

QLDSLINSHQSAENVAITEAV

>gi|30043253|gb|AAP18974.1| putative ARAC-type regulatory protein [Shigella flexneri 2a str. 2457T]

MNGKLQSSDVKNETPYNIPLLINENVISSGISLISLWHTYADEHYRVIWPRDKKKPLIANSWVAVYTVQG

CGKILLKNGEQITLHSNCIIFLKPMDIHSYHCEGLVWEQYWMEFTPTSMMDIPVGQQSVIYNGEIYNQEL

TEVAELITSPEAIKNNLAVAFLTKIIYQWICLMSADGKKDPQRRQIEKLIATLHASLQQRWSVADMAATI

PCSEAWLRRLFLRYTGKTPKEYYLDARLDLALSLLKQQGNSVGEVADTLNFFDSFHFSKAFKHKFGYAPS

AVLKNTDQHPTDASPHN

>gi|30043252|gb|AAP18973.1| putative cotransporter [Shigella flexneri 2a str. 2457T]

MNSLQILSFVGFTLLVAVITWWKVRKTDTGSQQGYFLAGRSLKAPVIAASLMLTNLSTEQLVGLSGQAYK

SGMSVMGWEVTSAVTLIFLALIFLPRYLKRGIATIPDFLEERYDKTTRIIIDFCFLIATGVCFLPIVLYS

GALALNSLFHVGESLQISHGAAIWLLVILLGLAGILYAVIGGLRAMAVADSINGIGLVIGGLMVPVFGLI

AIGKGSFMQGIEQITTVHAEKLNSVGGPTDPLPIGAAFTGLILVNTFYWCTNQGIVQRTLASKSLAEGQK

GALLTAVLKMLDPLVLVLPGLIAFHLYQDLPKADMAYPTLVNNVLPVPLEGFFGAVLFGAVISTFNGFLN

SASTLFSMGIYRRIINQNAEPQQLVTVGRKFGFFIAIVSVLVAPWIANAPQGLYSWMKQLNGIYNVPLVT

IIIMGFFFPRIPALAAKVAMGIGIISYITINYLVKFDFHFLYVLACTFCINVVVMLVIGFIKPRATPFTF

KDAFAVDMKPWKNVKIASIGILFAMIGVYVGLAEFGGYGTRWLAMISYFIAAVVIVYLIFDSWWHRHDPA

VTFTPDAKDSL

>gi|30043251|gb|AAP18972.1| hypothetical protein S3984 [Shigella flexneri 2a str. 2457T]

MGIIAQNKISSLGMLFGAIALMMGIIHFSFGPFSAPPPTLESIVADKTAEIKRGLLAGIKGEKITTVEKK

EDMDIDKILDQSGIALAIAALLCAFIGGMRKENRWGIRGALVFGIGIVCSILLIFLIFSFLTGGSLV

>gi|30043250|gb|AAP18971.1| hypothetical protein S3983 [Shigella flexneri 2a str. 2457T]

MKISRLGEAPDYRFSLANERTFLAWIRTALGFLAAGVGLDQLAPDFATPVIRELLALLLCLFSGGLAMYG

YLRWLRNEKAMRLKEDLPYTNSLLIISLILMVVAVIVMGLVLYAG

>gi|30043249|gb|AAP18970.1| hypothetical protein S3982 [Shigella flexneri 2a str. 2457T]

MPDSRKARRIADPGLQPERTSLAWFRTMLGYGALMALAIKHNWHQAGMLFWISIGILAIVALILWHYTRN

RNLMDVTNSDFSQFHVVRDKFLISLAVLSLAILFAVTHIHQLIVFIERVA

>gi|30043248|gb|AAP18969.1| putative transcriptional regulator [Shigella flexneri 2a str. 2457T]

MTGSQVIDAEEDRHKLVVEYKDALQPADFYHNFKQRGIRSVQLIPYLEFDDRGDLTAASVTAELWGKFLI

ALFECWVRADISRISIELFDATLQKWCGSENPQPRRDCQACDWHRLCPHARQETPDSVLCAGYQAFYSYS

APHMRVMRDLIKQHRSPMELMTMLR

>gi|30043247|gb|AAP18968.1| multidrug resistance protein D [Shigella flexneri 2a str. 2457T]

MLVLLVAVGQMAQTIYIPAIADMARDLNVREGAVQSVMGAYLLTYGVSQLFYGPISDRVGRRPVILVGMS

IFMLATLVAVTTSSLTVLIAASAMQGMGTGVGGVMARTLPRDLYERTQLRHANSLLNMGILVSPLLAPLI

GGLLDTMWNWRACYLFLLVLCAGVTFSMARWMPETRPVDAPRTRLLTSYKTLFGNSGFNCYLLMLIGGLA

GIAAFEACSGVLMGAVLGLSSMTVSILFILPIPAAFFGAWFAGRPNKRFSTLMWQSVICCLLAGLLMWIP

DWFGVMNVWTLLVPAALFFFGAGMLFPLATSGAMEPFPFLAGTAGALVGGLQNIGSGVLASLSAMLPQTG

QGSLGLLMTLMGLLIVLCWLPLATRMSHQGQPV

>gi|30043246|gb|AAP18967.1| ilvB operon leader peptide [Shigella flexneri 2a str. 2457T]

MTTSMFNAKLLPTAPSAAVVVVRVVVVVGNAP

>gi|30043245|gb|AAP18966.1| acetolactate synthase I, valine-sensitive, large subunit [Shigella flexneri 2a str. 2457T]

MASSGTTSTRKRFTGAEFIVHFLEQQGIKIVTGIPGGSILPVYDALSQSTQIRHILARHEQGAGFIAQGM

ARTDGKPAVCMACSGPGATNLVTAIADARLDSIPLICITGQVPASMIGTDAFQEVDTYGISIPITKHNYL

VRHIEELPQVMSDAFRIAQSGRPSPVWIDIPKDVQTAVFEIEAQPAVAEKAVAPAFSEESIRDAATMINA

AKRPVLYLGGGVINAPARVRELAEKAQLPTTMTLMALGMLPKAHPLSLGMLGMHGVRSTNYILQEADLLI

VLGARFDDRAIGKTEQFCPNAKIIHVDIDRAELGKIKQPHVAIQADVDDVLAQLIPRVEAQPRAEWHQLV

ADLQREFPCPIPKACDPLSHYGLINAVAACVDDNAIITTDVGQHQMWTAQAYPLNRPRQWLTSGGLGTMG

FGLPAAIGAALANPDRKVLCFSGDGSLMMNIQEMATASENQLDVKIILMNNEALGLVHQQQSLFYEQGVF

AATYPGKINFMQIAAGFGLETCDLNNEADPQAALQEIINRPGPALIHVRIDAEEKVYPMVPPGAANTEMV

GE

>gi|30043244|gb|AAP18965.1| acetolactate synthase I, valine sensitive, small subunit [Shigella flexneri 2a str. 2457T]

MQNTTHNNVILELTVRNHPGVMTHVCGLFARRAFNVEGILCLPIQDSDKSHIWLLVNDDQRLEQMISQIA

KLEDVVKVQRNQSDPTMFNKIAVFFQ

>gi|30043243|gb|AAP18964.1| response regulator [Shigella flexneri 2a str. 2457T]

MITVALIDDHLIVRSGFAQLLGLEPDLQVVAEFGSGREALAGLPGRGVQVCICDISMPDISGLELLSQLP

KGMATIMLSVHDSPALVEQALNAGARGFLSKRCSPDELIAAVHTVATGGCYLTPDIAIKLASGRQDPLTK

RERQVAEKLAQGMAVKEIAAELGLSPKTVHVHRANLMEKLGVSNDVELARRMFDGW

>gi|30043242|gb|AAP18963.1| sensor histidine protein kinase, phosphorylates UhpA [Shigella flexneri 2a str. 2457T]

MKTLFSRLITVIACFFIFSAAWFCLWSISLHLVERPDMAVLLFPFGLRLGLMLQCPRGYWPVLLGAEWLL

IYWLTQAVGLTHFPLLMIGSLLTLLPVALISRYRHQRDWRTLLLQGAALTAAALLQSLPWLWHGKESWNA

LLLTLTGGLTLAPICLVFWHYLANNTWLPLGPSLVSQPINWRGRHLVWYLLLFVISLWLQLGLPDELSRF

TPFCLALPIIALAWHYGWQGALIATLMNAIALIASQTWRDHLVDLLLSLLVQSLTGLLLGAGIQRLRELN

QSLQKELARNQHLAERLLETEESVRRDVARELHDDIGQTITAIRTQAGIVQRLAADNASVKQSGQLIEQL

SLGVYDAVRRLLGRLRPRQLDDLTLEQAIRSLMREMELEGRGIVSHLEWRIDESALSENQRVTLFRVCQE

GLNNIVKHADASAVTLQGWQQDERLMLVIEDDGSGLPPGSGQQGFGLTGMRERVTALGGTLHISCLHGTR

VSVSLPQRYV

>gi|30043241|gb|AAP18962.1| regulator of uhpT [Shigella flexneri 2a str. 2457T]

MLPFLKAPADAPLMTDKHEIDARYRYWRRHILLTIWLGYALFYFTRKSFNAAVPEILANGVLSRSDIGLL

ATLFYITYGVSKFVSGIVSDRSNARYFMGIGLIATGIMNILFGFSTSLWAFAVLWVLNAFFQGWGSPVCA

RLLTAWYSRTERGGWWALWNTAHNVGGALIPIVMAASALHYGWRAGMMIAGCMAIVVGIFLCWRLRDRPQ

ALGLPAVGEWRHDALEIAQQQEGAGLTRKEILTKYVLLNPYIWLLSFCYVLVYVVRAAINDWGNLYMSET

LGVDLVTANTAVTMFELGGFIGALVAGWGSDKLFNGNRGPMNLIFAAGILLSVGSLWLMPFASYVMQATC

FFTIGFFVFGPQMLIGMAAAECSHKEAAGAATGFVGLFAYLGASLAGWPLAKVLDTWHWSGFFVVISIAA

GISALLLLPFLNAQTPREA

>gi|30043240|gb|AAP18961.1| hexose phosphate transport protein [Shigella flexneri 2a str. 2457T]

MLAFLNQVRKPTLDLPLEVRRKMWFKPFMQSYLVVFIGYLTMYLIRKNFNIAQNDMISTYGLSMTQLGMI

GLGFSITYGVGKTLVSYYADGKNTKQFLPFMLILSAICMLGFSASMGSGSVSLFLMIAFYALSGFFQSTG

GSCSYSTITKWTPRRKRGTFLGFWNISHNLGGAGAAGVALFGANYLFDGHVIGMFIFPSIIALIVGFIGL

RYGSDSPESYGLGKAEELFGEEISEEDKETESTDMTKWQIFVEYVLKNKVIWLLCFANIFLYVVRIGIDQ

WSTVYAFQELKLSKAVAIQGFTLFEAGALVGTLLWGWLSDLANGRRGLVACIALALIIATLGVYQHASNE

YIYLASLFALGFLVFGPQLLIGVAAVGFVPKKAIGAADGIKGTFAYLIGDSFAKLGLGMIADGTPVFGLT

GWAGTFAALDIAAIGCICLMAIVAVMEERKIRREKKIQQLTVA

>gi|30043239|gb|AAP18960.1| probable adenine deaminase (synthesis xanthine) [Shigella flexneri 2a str. 2457T]

MNNSINHKFHHISRAEYQELLAVSRGDAVADYIIDNVSILDLINAGEISGPIVIKGRYIAGVGAEYADTP

ALQRIDARGATAVPGFIDAHLHIESSMMTPVTFETATLPRGLTTVICDPHEIVNVMGEAGFAWFARCAEQ

ARQNQYLQVSSCVPALEGCDVNGASFTLEQMLAWRDHPQVTGLAEMMDYPGVINGQNALLDKLDAFRYLT

LDGHCPGLGGKELNAYIAAGIENCHESYQLEEGRRKLQLGMSLMIREGSAASNLNALAPLINEFNSPQCM

LCTDDRNPWEIAHEGHIDALIRRLIEQHNVPLHVAYRVASWSTARHFGLNHLGLLAPGKQADIVLLSDAR

KVTVQQVLVKGEPIDAQTLQAEESARLALSAPPYGNTIARQPVSASDFALQFTPGKRYRVIDVIHNELIT

HSRSSVYSENGFDRDDVCFIAVLERYGQRLAPACGLLGGFGLNEGALAATVSHDSHNIVVIGRSAEEMAL

AVNQVIQDGGGLCVVRNGQVQSHLPLPIAGLMSTDTAQSLAEQIDALKAAARECGPLPDEPFIQMAFLSL

PVIPALKLTSQGLFDGEKFAFTTLEVTE

>gi|30043238|gb|AAP18959.1| hypothetical protein S3971 [Shigella flexneri 2a str. 2457T]

MIIITEPLLSFVLQKQGIKSPPMDKKMNNDNTDYVSNESGTLSRLFKLPQHGTTVRTELIAGMTTFLTMV

YIVFVNPQILGAAQMDPKVVFVTTCLIAGIGSIAMGIFANLPVALAPAMGLNAFFAFVVVGAMGISWQTG

MGAIFWGAIGLFLLTLFRIRYWMISNIPLSLRIGITSGIGLFIALMGLKNTGVIVANKDTLVMIGDLSSH

GVLLGILGFFIITVLSSRHFHAAVLVSIVVTSCCGLFFGDVHFSGVYSIPPDISGVIGEVDLSGALTLEL

AGIIFSFMLINLFDSSGTLIGVTDKAGLIDSNGKFPNMNKALYVDSVSSVAGAFIGTSSVTAYIESTSGV

AVGGRTGLTAVVVGVMFLLVMFFSPLVAMVPPYATAGALIFVGVLMTSSLARVNWDDFTESVPAFITTVM

MPFTFSITEGIALGFMSYCIMKVCTGRWRDLNLCVVVVAALFALKIILVD

>gi|30043237|gb|AAP18958.1| hypothetical protein S3970 [Shigella flexneri 2a str. 2457T]

MSSDYAGELMIWIMLATLAVVFVVGFRVLTSGARKAIRRLSDRLNIDVVPVESMVDQMGKSAGDEFLRYL

HRPDESHLQNAAQVLLIWQIVIVDDSEQNLLQWHRILQKARLSAPITDAQVRLALGFLRETEPEMQDINA

FQMRYNAFFQPAEGVHWLH

>gi|30043236|gb|AAP18957.1| putative transport protein [Shigella flexneri 2a str. 2457T]

MDKTFTPHPVIFALFRFDLNSSSSNTVKVAKTLNSSHISMLKWSAFPLKHATGNTMSEFIAENRGADAIT

RPNWSAVFSVAFCVACLIIVEFLPVSLLTPMAQDLGISEGVAGQSVTVTAFVAMFASLFITQTIQATDRR

YVVILFAVLLTLSCLLVSFANSFSLLLIGRACLGLALGGFWAMSASLTMRLVPPRTVPKALSVIFGAVSI
ALVIAAPLGSFLGELIGWRNVFNAAAVMGVLCIFWIIKSLPSLPGEPSHQKQNTFRLLQRPGVMAGMIAI
FMSFAGQFAFFTYIRPVYMNLAGFGVDGLTLVLLSFGIASFIGTSLSSFILKRSVKLALAGAPLILAVSA
LVLTLWGSDKIVATGVAIIWGLTFALVPVGWSTWITRSLADQAEKAGSIQVAVIQLANTCGAAIGGYALD
NIGLTSPLMLSGTLMLLTALLVTAKVKMKKS

>gi|30043235|gb|AAP18956.1| putative transport protein [Shigella flexneri 2a str. 2457T]
MKPTTLLLIFTFFAMPGIVYAESPFSSLQSAKEKTTVLQDLRKICTPHASLSDEAWEKLMLSDENNKQHI
REAIVAMERNNQSNYWEALGKVECPDM

>gi|30043234|gb|AAP18955.1| IS600 orfA [Shigella flexneri 2a str. 2457T]
MSRKTQRYSKEFKAEAVRTVLENQLSISEGASRLSLPEGTLGQWVTAARKGLGTPGSRTLAELESEILQL
RKALNEARLERDILKKATAYFAQESLKNTR

>gi|30043233|gb|AAP18954.1| IS600 orfB [Shigella flexneri 2a str. 2457T]
MAHIRTRETYGTRRLQTELAENGIIVGRDRLARLRKELRLRCKQKRKFRATTNPNHNLPVAPNLLNQTFA
PTAPNQVWVADLTYVATQEGWLYLAGIKDVYTCEIVGYAMGERMTKELTGKALFMALRSQRPPAGLIHHS
DRGSQYCAYDYRVIQEQFGLKTSMSRKGNCYDNAPMESFWGTLKNESLSHYRFNNRDEAISVIREYIEIF
YNRQRRHSRLGNISPAAFREKYHQMAA

>gi|30043232|gb|AAP18953.1| putative secreted autotransporter toxin [Shigella flexneri 2a str. 2457T]
MFTTKGIDISGELTMMGIPEQNSKTVTPGLHYAADGFRLSGGNANFIARNMASVTGNIYADDAATITLGQ
PETETPTISSAYQAWAETLLYGFDTAYRGAITAPKATVSMNNAIWHLNSQSSINRLETKDSMVRFTGDNG
KFTTLTVDNLTIDDSAFVLRANLAQADQ

>gi|30043231|gb|AAP18952.1| IS2 orfA [Shigella flexneri 2a str. 2457T]

MIVLILVFRLVIGEQMIDVLGPEKRRRRTTQEKIAIVQQSFEPGMTVSLVARQHGVAASQLFLWRKQYQE

GSLTAVAAGEQVVPASELAAAMKQIKELQRLLGKKTMENELLKEAVEYGRAKKWIAHAPLLPGDGE

>gi|30043230|gb|AAP18951.1| IS2 orfB [Shigella flexneri 2a str. 2457T]

MARGWGVSLVSRCLRVSRAQLHVILRRTDDWMDGRRSRHTDDTDVLLRIHHVIGELPTYGYRRVWALLRR

QAELDGMPAINAKRVYRLMRQNALLLERKPAVPPSKRAHTGRVAVKESNQRWCSDGFEFCCDNGERLRVT

FALDCCDREALHWAVTTGGFNSETVQDVMLGAVERRFGNDLPSSPVEWLTDNGSCYRANETRQFARMLGL

EPKNTAVRSPESNGIAESFVKTIKRDYISIMPKPDGLTAAKNLAEAFEHYNEWHPHSALGYRSPREYLRQ

RACNGLSDNRCLEI

>gi|30043229|gb|AAP18950.1| putative major fimbrial subunit [Shigella flexneri 2a str. 2457T]

MKRNIIGGAFTLASLMLAGHALAEDGVVNFVGEIVDTTCEVTSDTADQIVPLGKVSKNAFSGVGSLASPQ

KFSIKLENCPATYTQAAVRFDGTEAPGGDGDLKVGTPLTAGNPGDFTGTGQAIAATGVGIRIFNQSDNSQ

VKLYNDSAYTAIDAEGKAEMKFIARYVATNATVTAGTANADSQFTVEYKK

>gi|30043228|gb|AAP18949.1| IS4 orf [Shigella flexneri 2a str. 2457T]

MHIGQALDLVSRYDSLRNPLTSLGDYLDPELISRCLAESGTVTLRKRRLPLEMMVWCIVGMALERKEPLH

QIVNRLDIMLPGNRPFVAPSAVIQARQRLGSEAVRRVFTKTAQLWHNATPHPHWCGLTLLAIDGVFWRTP

DTPENDAAFPRQTHAGNPALYPQVKMVCQMELTSHLLTAAAFGTMKNSENELAEQLIEQTGDNTLTLMDK

GYYSLGLLNAWSLAGEHRHWMIPLRKGAQYEELRKLGKGDHLVKLKTSPQARKKWPGLGNEVTARLLTVT

RKGKVCHLLTSMTDAMRFPGGEMADLYSHRWEIELGYREIKQTMQLSRLTLRSKKPELVEQELWGVLLAY

NLVRYQMIKMAEHLKGYWPNQLSFSESCGMVMRMLMTLQGASPGRIPELMSDLASMGQLVKLPTRRGRAF

PRVVKERPWKYPTAPKKSQSVA

>gi|30043227|gb|AAP18948.1| L-glutamine:D-fructose-6-phosphate aminotransferase [Shigella flexneri 2a str. 2457T]

MCGIVGAIAQRDVAEILLEGLRRLEYRGYDSAGLAVVDAEGHMTRLRRLGKVQMLAQAAEEHPLHGGTGI

AHTRWATHGEPSEANAHPHVSEHIVVVHNGIIENHEPLREELKARGYTFVSETDTEVIAHLVNWELKQGG

TLREAVLRAIPQLRGAYGTVIMDSRHPDTLLAARSGSPLVIGLGMGENFIASDQLALLPVTRRFIFLEEG

DIAEITRRSVNIFDKTGAEVKRQDIESNLQYDAGDKGIYRHYMQKEIYEQPNAIKNTLTGRISHGQVDLS

ELGPNADELLSKVEHIQILACGTSYNSGMVSRYWFESLAGIPCDVEIASEFRYRKSAVRRNSLMITLSQS

GETADTLAGLRLSKELGYLGSLAICNVPGSSLVRESDLALMTNAGTEIGVASTKAFTTQLTVLLMLVAKL

SRLKGLDASIEHDIVHGLQALPSRIEQMLSQDKRIEALAEDFSDKHHALFLGRGDQYPIALEGALKLKEI

SYIHAEAYAAGELKHGPLALIDADMPVIVVAPNNELLEKLKSNIEEVRARGGQLYVFADQDAGFVSSDNM

HIIEMPHVEEVIAPIFYTVPLQLLAYHVALIKGTDVDQPRNLAKSVTVE

>gi|30043226|gb|AAP18947.1| N-acetyl glucosamine-1-phosphate uridyltransferase [Shigella flexneri 2a str. 2457T]

MLNNAMSVVILAAGKGTRMYSDLPKVLHTLAGKAMVQHVIDAANELGAAHVHLVYGHGGDLLKQALKDDN

LNWVLQAEQLGTGHAMQQAAPFFADDEDILMLYGDVPLISVETLQRLRDAKPQGGIGLLTVKLDDPTGYG

RITRENGKVTGIVEHKDATDEQRQIQEINTGILIANGADMKRWLAKLTNNNAQGEYYITDIIALAYQEGR

EIVAVHPQRLSEVEGVNNRLQLSRLERVYQSEQAEKLLLAGVMLRDPARFDLRGTLTHGRDVEIDTNVII

EGNVTLGHRVKIGTGCVIKNSVIGDDCEISPYTVVEDANLAAACTIGPFARLRPGAELLEGAHVGNFVEM

KKARLGKGTKAGHLTYLGDAEIGDNVNIGAGTITCNYDGANKFKTIIGDDVFVGSDTQLVAPVTVGKGAT

IAAGTTVTRNVGENALAISRVPQTQKEGWRRPVKKK

>gi|30043225|gb|AAP18946.1| membrane-bound ATP synthase, F1 sector, epsilon-subunit [Shigella flexneri 2a str. 2457T]

MTYHLDVVSAEQQMFSGLVEKIQVTGSEGELGIYPGHAPLLTAIKPGMIRIVKQHGHEEFIYLSGGILEV

QPGNVTVLADTAIRGQDLDEARAMEAKRKAEEHISSSHGDVDYAQASAELAKAIAQLRVIELTKKAM

>gi|30043224|gb|AAP18945.1| membrane-bound ATP synthase, F1 sector, beta-subunit [Shigella flexneri 2a str. 2457T]

MATGKIVQVIGAVVDVEFPQDAVPRVYDALEVQNGNERLVLEVQQQLGGGIVRTIAMGSSDGLRRGLDVK

DLEHPIEVPVGKATLGRIMNVLGEPVDMKGEIGEEERWAIHRAAPSYEELSNSQELLETGIKVIDLMCPF

AKGGKVGLFGGAGVGKTVNMMELIRNIAIEHSGYSVFAGVGERTREGNDFYHEMTDSNVIDKVSLVYGQM

NEPPGNRLRVALTGLTMAEKFRDEGRDVLLFVDNIYRYTLAGTEVSALLGRMPSAVGYQPTLAEEMGVLQ

ERITSTKTGSITSVQAVYVPADDLTDPSPATTFAHLDATVVLSRQIASLGIYPAVDPLDSTSRQLDPLVV

GQEHYDTARGVQSILQRYQELKDIIAILGMDELSEEDKLVVARARKIQRFLSQPFFVAEVFTGSPGKYVS

LKDTIRGFKGIMEGEYDHLPEQAFYMVGSIEEAVEKAKKL

>gi|30043223|gb|AAP18944.1| membrane-bound ATP synthase, F1 sector, gamma-subunit [Shigella flexneri 2a str. 2457T]

MAGAKEIRSKIASVQNTQKITKAMEMVAASKMRKSQDRMAASRPYAETMRKVIGHLAHGNLEYKHPYLED

RDVKRVGYLVVSTDRGLCGGLNINLFKKLLAEMKTWTDKGVQCDLAMIGSKGVSFFNSVGGNVVAQVTGM

GDNPSLSELIGPVKVMLQAYDEGRLDKLYIVSNKFINTMSQVPTISQLLPLPASDDDDLKHKSWDYLYEP

DPKALLDTLLRRYVESQVYQGVVENLASEQAARMVAMKAATDNGGSLIKELQLVYNKARQASITQELTEI

VSGAAAV

>gi|30043222|gb|AAP18943.1| membrane-bound ATP synthase, F1 sector, alpha-subunit [Shigella flexneri 2a str. 2457T]

MQLNSTEISELIKQRIAQFNVVSEAHNEGTIVSVSDGVIRIHGLADCMQGEMISLPGNRYAIALNLERDS

VGAVVMGPYADLAEGMKVKCTGRILEVPVGRGLLGRVVNTLGAPIDGKGPLDHDGFSAVEAIAPGVIERQ

SVDQPVQTGYKAVDSMIPIGRGQRELIIGDRQTGKTALAIDAIINQRDSGIKCIYVAIGQKASTISNVVR

KLEEHGALANTIVVVATASESAALQYLAPYAGCAMGEYFRDRGEDALIIYDDLSKQAVAYRQISLLLRRP

PGREAFPGDVFYLHSRLLERAARVNAEYVEAFTKGEVKGKTGSLTALPIIETQAGDVSAFVPTNVISITD

GQIFLETNLFNAGIRPAVNPGISVSRVGGAAQTKIMKKLSGGIRTALAQYRELAAFSQFASDLDDATRKQ

LDHGQKVTELLKQKQYAPMSVAQQSLVLFAAERGYLADVELSKIGSFEAALLAYVDRDHAPLMQEINQTG

GYNDEIEGKLKGILDSFKATQSW

>gi|30043221|gb|AAP18942.1| membrane-bound ATP synthase, F1 sector, delta-subunit [Shigella flexneri 2a str. 2457T]

MSEFITVARPYAKAAFDFAVEHQSVERWQDMLTFAAEVTKNEQMAELLSGALAPETLAESFIAVCGEQLD

ENGQNLIRVMAENGRLNALPDVLEQFIHLRAVSEATAEVDVISAAALSEQQLAKISAAMEKRLSRKVKLN

CKIDKSVMAGVIIRSGDMVIDGSVRGRLERLADVLQS

>gi|30043220|gb|AAP18941.1| membrane-bound ATP synthase, F0 sector, subunit b [Shigella flexneri 2a str. 2457T]

MNLNATILGQAIAFVLFVLFCMKYVWPPLMAAIEKRQKEIADGLASAERAHKDLDLAKASATDQLKKAKA

EAQVIIEQANKRRSQILDEAKAEAEQERTKIVAQAQAEIEAERKRAREELRKQVAILAVAGAEKIIERSV

DEAANSDIVDKLVAEL

>gi|30043219|gb|AAP18940.1| membrane-bound ATP synthase, F0 sector, subunit c [Shigella flexneri 2a str. 2457T]

MENLNMDLLYMAAAVMMGLAAIGAAIGIGILGGKFLEGAARQPDLIPLLRTQFFIVMGLVDAIPMIAVGL

GLYVMFAVA

>gi|30043218|gb|AAP18939.1| membrane-bound ATP synthase, F0 sector, subunit a [Shigella flexneri 2a str. 2457T]

MASENMTPQDYIGHHLNNLQLDLRTFSLVDPQNPPATFWTINIDSMFFSVVLGLLFLVLFRSVAKKATSG

VPGKFQTAIELVIGFVNGSVKDMYHGKSKLIAPLALTIFVWVFLMNLMDLLPIDLLPYIAEHVLGLPALR

VVPSADVNVTLSMALGVFILILFYNIKMKGIGGFTKELTLQPFNHWAFIPVNLILEGVSLLSKPVSLGLR

LFGNMYAGELIFILIAGLLPWWSQWILNVPWAIFHILIITLQAFIFMVLTIVYLSMASEEH

>gi|30043217|gb|AAP18938.1| membrane-bound ATP synthase [Shigella flexneri 2a str. 2457T]

MKNVMSVSLVSRNVARKLLLVQLLVVIASGLLFSLKDPFWGVSAISGGLAVFLPNVLFMIFAWRHQAHTP

AKGRVAWTFAFGEAFKVLAMLVLLVVALAVLKAVFLPLIVTWVLVLVVQILAPAVINNKG

>gi|30043216|gb|AAP18937.1| glucose-inhibited division protein GidB [Shigella flexneri 2a str. 2457T]

MLNKLSLLLKDAGISLTDHQKNQLIAYVNMLHKWNKAYNLTSVRDPNEMLVRHILDSIVVAPYLQGERFI

DVGTGPGLPGIPLSIVRPEAHFTLLDSLGKRVRFLRQVQHELKLENIEPVQSRVEEFPSEPPFDGVISRA

FASLNDMVSWCHHLPGEQGRFYALKGQMPEDEIALLPEEYQVESVVKLQVPALDGERHLVVIKANKI

>gi|30043215|gb|AAP18936.1| glucose-inhibited division protein GidA [Shigella flexneri 2a str. 2457T]

MFYPDPFDVIIIGGGHAGTEAAMAAARMGQQTLLLTHNIDTLGQMSCNPAIGGIGKGHLVKEVDALGGLM

AKAIDQAGIQFRILNASKGPAVRATRAQADRVRYRQAVRTALENQPNLMIFQQAVEDLIVENDRVVGAVT

QMGLKFRAKAVVLTVGTFLDGKIHIGLDNYSGGRAGDPPSIPLSRRLRELPLRVGRLKTGTPPRIDARTI

DFSVLAQQHGDNPMPVFSFMGNASLHPQQVPCYITHTNEKTHDVIRSNLDRSPMYAGVIEGVGPRYCPSI

EDKVMRFADRNQHQIFLEPEGLTSNEIYPNGISTSLPFDVQMQIVRSMQGMENAKIVRPGYAIEYDFFDP

RDLKPTLESKFIQGLFFAGQINGTTGYEEAAAQGLLAGLNAARLSADKEGWAPARSQAYLGVLVDDLCTL

GTKEPYRMFTSRAEYRLMLREDNADLRLTEIGRELGLVDDERWARFNEKLENIERERQRLKSTWVTPSAE

AAAEVNAHLTAPLSREASGEDLLRRPEMTYEKLTTLTPFAPALTDEQAAEQVEIQVKYEGYIARQQDEIE

KQLRNENTLLPATLDYRQISGLSNEVIAKLNDHKPASIGQASRISGVTPAAISILLVWLKKQGMLRRSA

>gi|30043214|gb|AAP18935.1| MioC protein [Shigella flexneri 2a str. 2457T]

MADITLISGSTLGGAEYVAEHLAEKLEEVGFTTETLHGPLLEDLSASGIWLVISSTHGAGDIPDNLSPFY

EALQEQKPDLSAVRFGTIGIGSREYDTFCGAIDKLEAELKNSGAKQTGETLKINILDHDIPEDPAEEWLG

SWINLLK

>gi|30043213|gb|AAP18934.1| regulator for asnA, asnC and gidA [Shigella flexneri 2a str. 2457T]

MENYLIDNLDRGILEALMGNARTAYAELAKQFGVSPGTIHVRVEKMKQAGIITGARIDVSPKQLGYDVGC
FIGIILKSAKDYPSALAKLESLDEVTEAYYTTGHYSIFIKVMCRSIDALQHVLINKIQTIDEIQSTETLI
VLQNPIMRTIKP

>gi|30043212|gb|AAP18933.1| asparagine synthetase A [Shigella flexneri 2a str. 2457T]
MKTAYIAKQRQISFVKSHFSRQLEERLGLIEVQAPILSRVGDGTQDNLSGCEKAVQVKVKALPDAQFEVV
HSLAKWKRQTLGQHDFSAGEGLYTHMKALRPDEDRLSPLHSVYVDQWDWERVMGDGERQLSTLKSTVEAI
WAGIKATEAAVNEEFGLAPFLPDQIHFVHSQELLSRYPDLDAKGRERAIAKDLGAVFLVGIGGKLSDGHR
HDVRAPDYDDWSTPSELGHAGLNGDILVWNPVLEDAFELSSMGIRVDADTLKHQLALTGDEDRLQLEWHQ
ALLRGEMPQTIGGGIGQSRLTMLLLQLPHIGQVQCGVWPAAVRESVPSLL

>gi|30043211|gb|AAP18932.1| hypothetical protein S3943 [Shigella flexneri 2a str. 2457T]
MRSRLKDARVPPELTEEVMCYQQSQLLSTPQFIVQLPQILDLLHRLNSPWAEQARQLVDANSTITSALHT
LFLQRWRLSLIVQATTLNQQLLEEEREQLLSEVQERMTLSGQLEPILADNNTAAGRLWDMSAGQLKRGDY
QFIVKYGEFLNEQPELKRLAEQLGRSREAKSIPRNDAQMETFRTMVREPATVPEQVDGLQQSDDILRLLP
PELATLGITELEYEFYRRLVEKQLLTYRLHGESWREKVFERPVVHKDYDEQPRGPFIVCVDTSGSMGGFN
EQCAKAFCLALMRIALAENRRCYIMLFSTEIVRYELSGPQGIEQAIRFLSQQFRGGTDLASCFRAIMERL
QSREWFDADAVVISDFIAQRLPDDVTSKVKELQRVHQHRFHAVAMSAHGKPGIMRIFDHIWRFDTGMRSR
LLRRWRR

>gi|30043210|gb|AAP18931.1| putative 2-component regulator [Shigella flexneri 2a str. 2457T]
MTKGRHFIMAHPHLLAERISRLSSSLEKGLYERSHAIRLCLLAALSGESVFLLGPPGIAKSLIARRLKFA
FQNARAFEYLMTRFSTPEEVFGPLSIQALKDEGRYERLTSGYLPEAEIVFLDEIWKAGPAILNTLLTAIN
ERQFRNGAHVEKIPMRLLVAASNELPEADSSLEALYDRMLIRLWLDKVQDKANFRSMLTSQQDENDNPVP
DALQVTDEEYERWQKEIGEITLPDHVFELIFILRQQLDKLPDAPYVSDRRWKKAIRLLQASAFFSGRSAV

APVDLILLKDCLWYDAQSLNLIQQQIDVLMTGHAWQQQGMLTRLGAIVQRHLQLQQQQSDKTALTLIRLG

GIFSRRQQYQLPVNVTASTLTLLLQKPLKLHDMEVVHISFERSALEQWLSKGGEIRGKLNGIGFAQKLNL

EVDSTQHLVVRDVSLQGSTLALPGSSAEGLPGEIKQQLEELESDWRKQHALFSEQQKCLFIPGDWLGRIE

DSLQDVGAQIRQAQQC

>gi|30043209|gb|AAP18930.1| IS4 orf [Shigella flexneri 2a str. 2457T]

MHIGQALDLVSRYDSLRNPLTSLGDYLDPELISRCLAESGTVTLRKRRLPLEMMVWCIVGMALERKEPLH

QIVNRLDIMLPGNRPFVAPSAVIQARQRLGSEAVRRVFTKTAQLWHNATPHPHWCGLTLLAIDGVFWRTP

DTPENDAAFPRQTHAGNPALYPQVKMVCQMELTSHLLTAAAFGTMKNSENELAEQLIEQTGDNTLTLMDK

GYYSLGLLNGWSLAGEHRHWMIPLRKGAQYEELRKLGKGDHLVKLKTSPQARKKWPGLGNEVTARLLTVT

RKGKVCHLLTSMTDAMRFPGGEMADLYSNRWEIELGYREIKQTMQLSRLTLRSKKPELVEQELWGVLLAY

NLVRYQMIKMAEHLKGYWPNQLSFSESCGMVMRMLMTLQGASPGRIPELMRDLASMGQLVKLPTRRGRAF

PRVVKERPWKYPTAPKKSQSVA

>gi|30043208|gb|AAP18929.1| low-affinity potassium transport protein [Shigella flexneri 2a str. 2457T]

MSTDNKQSLPAITLAAIGVVYGDIGTSPLYTLRECLSGQFGFGVERDAVFGFLSLIFWLLIFVVSIKYLT

FVMRADNAGEGGILTLMSLAGRNTSARTTSMLVIMGLIGGSFFYGEVVITPAISVMSAIEGLEIVAPQLD

TWIVPLSIIVLTLLFMIQKHGTAMVGKLFAPIMLTWFLILAGLGLRSIIANPEVLHALNPMWAVHFFLEY

KTVSFIALGAVVLSITGGEVLYADMGHFGKFSIRLAWFTVVLPSLTLNYFGQGALLLKNPEAIKNPFFLL

APDWALIPLLIIAALATVIASQAVISGVFSLTRQAVRLGYLSPMRIIHTSEMESGQIYIPFVNWMLYVAV

VIVIVSFEHSSNLAAAYGIAVTGTMVLTSILSTTVARQNWHWNKYFVALILIAFLCVDIPLFTANLDKLL

SGGWLPLSLGTVMFIVMTTWKSERFRLLRRMHEHGNSLEAMIASLEKSPPVRVPGTAVYMSRAINVIPFA

LMHNLKHNKVLHERVILLTLRTEDAPYVHNVRRVQIEQLSPTFWRVVASYGWRETPNVEEVFHRCGLEGL

SCRMMETSFFMSHESLILGKRPWYLRLRGKLYLLLQRNALRAPDQFEIPPNRVIELGTQVEI

>gi|30043207|gb|AAP18928.1| D-ribose high-affinity transport system; membrane-associated protein [Shigella flexneri 2a str. 2457T]

MKKGTVLNSDISSVISRLGHTDTLVVCDAGLPIPKSTTRIDMALTQGVPSFMQVLGVVTNEMQVEAAIIA

EEIKQHNPQLHETLLTHLEQLQKHQGNTIEIRYTTHEQFKQQTAESQAVIRSGECSPYANIILCAGVTF

>gi|30043206|gb|AAP18927.1| IS1 orfA [Shigella flexneri 2a str. 2457T]

MASISIRCPSCSATEGVVRNGKSTAGHQRYLCSHCRKTWQLQFTYTASQPGTHQKIIDMAMNGVGCRASA

RIMGVGLNTVLRHLKNSGRSR

>gi|30043205|gb|AAP18926.1| IS1 orfB [Shigella flexneri 2a str. 2457T]

MSRQCTHYGRWPQHGFTSLKKLRPQSVTSRIQPGSDVIVCAEMDEHWGYVGAKSRQRWLFYAYDRIRRTV

VAHVFGERTLATLERLLSLLSAFEVVVWMTDGWPLYESRLKGKLHVISKRYTQRIERHNLNLRQHLARLG

RKSLSFSKSVELHDKVIGHYLNIKHYQ

>gi|30043204|gb|AAP18925.1| D-ribose high-affinity transport protein [Shigella flexneri 2a str. 2457T]

MITQTVSGRRYFTKAWLMEQKSLIALLVLIAIVSTLSPNFFTINNLFNILQQTSVNAIMAVGMTLVILTS

GIDLSVGSLLALTGAVAASIVGIEVNALVAVAAALTLGAAIGAVTGVIVAKGRVQAFIATLVMMLLLRGV

TMVYTNGSPVNTGFTENADLFGWFGIGRPLGVPTPVWIMGIVFLAAWYMLHHTRLGRYIYALGGNEAATR

LSGINVNKIKIIVYSLCGLLASLAGIIEVARLSSAQPTAGTGYELDAIAAVVLGGTSLAGGKGRIVGTLI

GALILGFLNNGLNLLGVSSYYQMIVKAVVILLAVLVDNKKQ

>gi|30043203|gb|AAP18924.1| ribokinase [Shigella flexneri 2a str. 2457T]

MDIPNMQNAGSLVVLGSINADHILNLQSFPTPGETVTGNHYQVAFGGKGANQAVAAGRSGANIAFIACTG

DDSIGESVRQQLATDNIDITPVSVIKGESTGVALIFVNGEGENVIGIHAGANAALSPSLVEAQRERIANA

SALLMQLESPLESVMAAAKIAHQNKTIVALNPAPARELPDELLALVDIITPNETEAEKLTGIRVENDEDA

AKAAQVLHEKGIRTVLITLGSRGVWASVNGEGQRVPGFRVQAVDTIAAGDTFNGALITALLEEKPLPEAI

RFAHAAAAIAVTRKGAQPSVPWREEIDAFLDRQR

>gi|30043202|gb|AAP18923.1| regulator for rbs operon [Shigella flexneri 2a str. 2457T]

MKDVARLAGVSTSTVSHVINKDRFVSEAITAKVEAAIKELNYAPSALARSLKLNQTHTIGMLITASTNPF

YSELVRGVERSCFERGYSLVLCNTEGDEQRMNRNLETLMQKRVDGLLLLCTETHQPSREIMQRYPTVPTV

MMDWAPFDGDSDLIQDNSLLGGDLATQYLIDKGHTRIACITGPLDKTPARLRLEGYRAAMKRAGLNIPDG

YEVTGDFEFNGGFDAMRQLLSHPLRPQAVFTGNDAMAVGVYQALYQAELQVPQDIAVIGYDDIELASFMT

PPLTTIHQPKDELGELAIDVLIHRITQPTLQQQRLQLTPILMERGSA

>gi|30043201|gb|AAP18922.1| putative transport protein [Shigella flexneri 2a str. 2457T]

MSGLPWIAAMAFFMQALDATILNTALPAIAHSLNRSPLAMQSAIISYTLTVAMLIPVSGWLADRFGTRRI

FTLAVSLFTLGSLACALSNSLPQLVVFRVIQGIGDAMMMPVARPALLRAYPRNELLPVLNFVAMPGLVGP

ILGPVLGGVLVTWATWHWIFLINIPIGIAGLLYARKHMPNFTTARRRFDITGFLLFGLSLVLFSSGIELF

GEKIVASWIALTVIVTSIGLLLLYILHARRTPNPLISLDLFKTRTFSIGIVGNIATRLGTGCVPFLMPLM

LQVGFGYQAFIAGCMMAPTALGSIIAKSMVTQVLRRLGYRHTLVGITVIIGLMIAQFSLQSPAMAIWMLI

LPLFILGMAMSTQFTAMNTITLADLTDDNASSGNSVLAVTQQLSISLGVAVSAAVLRIYEGMEGTTTVEQ

FHYTFITMGIITVASAAMFMLLKTTDGNNLIKRQRKSMPNRVPSESE

>gi|30043200|gb|AAP18921.1| hypothetical protein S3929 [Shigella flexneri 2a str. 2457T]

MSRTAVREAVKTLTAKGMVLPRPRIGTRVMPQSNWNFLDQELLTWWITEENFHQVIDHFLVMRICLEPQA

CLLAATVGTAEQKAHLNTLMAEMAALKENFRRERWIEVDMAWHEHIYEMSANPFLTSFASLFHSVYHTYF

TSITSDTVIKLDLHQAIVDAIIQSDGDAAFKACQALLRSPDK

>gi|30043199|gb|AAP18920.1| hypothetical protein S3921 [Shigella flexneri 2a str. 2457T]

MDTELLKTFLEVSRTRHFGRAAESLYLTQSAVSFRIRQLENQLGVNLFTRHRNNIRLTAAGEKLLPYAET

LMSTWQAARKEVAHTSRHNEFSIGASASLWECMLNQWLGRLYQNQDAHTGLQFEARIAQRQSLVKQLHER

QLDLLITTEAPKMDEFSSQLLGYFTLALYTSAPSKLKGDLNYLRLEWGPDFQQHEAGLIGADEVPILTTS

SAELAQQQIAMLNGCTWLPVSWARKKGGLHTVVDSTTLSRPLYAIWLQNSDKNALIRDLLKINVLDEVY

>gi|30043198|gb|AAP18919.1| hypothetical protein S3920 [Shigella flexneri 2a str. 2457T]

MAESFTTTNRYFDNKHYPRGFSRHGDFTIKEAQLLERHGYAFNELDLGKREPVTEEEKLFVAVCRGEREP

VTEAERVWSKYMTRIKRPKRFHTLSGGKPQVEGAEDYTDSDD

>gi|30043197|gb|AAP18918.1| ilvGEDA operon leader peptide [Shigella flexneri 2a str. 2457T]

MTALLRVISLVVISVVVIIIPPCGAALGRGKA

>gi|30043196|gb|AAP18917.1| acetolactate synthase II, large subunit [Shigella flexneri 2a str. 2457T]

MNGAQWVVHALRAQGVNTVFGYPGGAIMPVYDALYDGGVEHLLCRHEQGAAMAAIGYARATGKTGVCIAT

SGPGATNLITGLADALLDSIPVVAITGQVSAPFIGTDAFQEVDVLGLSLACTKHSFLVQSLEELPRIMAE

AFDVASSGRPGPVLVDIPKDIQLASGDLEPWFTTVENEVTFPHAEVEQARQMLAKAQKPMLYVGGGVGMA

QAVPALREFLAATKMPATCTLKGLGAVEADYPYYLGMLGMHGTKAANFAVQECDLLIAVGARFDDRVTGK

LNTFAPHASVIHMDIDPAEMNKLRQAHVALQGDLNALLPALQQPLNINDWQQHCAQLRDEHSWRYDHPGD

AIYAPLLLKQLSDRKPADCVVTTDVGQHQMWAAQHIAHTRPENFITSSGLGTMGFGLPAAVGAQVARPND

TVVCISGDGSFMMNVQELGTVKRKQLPLKIVLLDNQRLGMVRQWQQLFFQERYSETTLTDNPDFLMLASA

FGIPGQHITRKDQVEAALDTMLNSDGPYLLHVSIDELENVWPLVPPGASNSEMLEKLS

>gi|30043195|gb|AAP18916.1| acetolactate synthase II, small subunit [Shigella flexneri 2a str. 2457T]

MQHQVNVSARFNPETLERVLRVVRHRGFHVCSMNMAAASDAQNINIELTVASPRSVDLLFSQLNKLVDVA

HVAICQSTTTSQQIRA

>gi|30043194|gb|AAP18915.1| branched-chain amino-acid aminotransferase [Shigella flexneri 2a str. 2457T]

MTTKKADYIWFNGEMVRWEDAKVHVMSHALHYGTSVFEGIRCYDSHKGPVVFRHREHMQRLHDSAKIYRF

PVSQSIDELMEACRDVIRKNNLTSAYIRPLIFVGDVGMGVNPPAGYSTDVIIAAFPWGAYLGAEALEQGI

DAMVSSWNRAAPNTIPTAAKAGGNYLSSLLVGSEARRHGYQEGIALDVNGYISEGAGENLFEVKDGVLFT

PPFTSSALPGITRDAIIKLAKELGIEVREQVLSRESLYLADEVFMSGTAAEITPVRSVDGIQIGEGRCGP

VTKRIQQAFFGLFTGETEDKWGWLDQVNQ

>gi|30043193|gb|AAP18914.1| dihydroxyacid dehydratase [Shigella flexneri 2a str. 2457T]

MPKYRSATTTHGRNMAGARALWRATGMTDADFGKPIIAVVNSFTQFVPGHVHLRDLGKLVAEQIEAAGGV

AKEFNTIAVDDGIAMGHGGMLYSLPSRELIADSVEYMVNAHCADAMVCISNCDKITPGMLMASLRLNIPV

IFVSGGPMEAGKTKLSDQIIKLDLVDAMIQGADPKVSDSQSDQVERSACPTCGSCSGMFTANSMNCLTEA

LGLSQPGNGSLLATHADRKQLFLNAGKRIVELTKRYYEQNDESALPRNIASKAAFENAMTLDIAMGGSTN

TVLHLLAAAQEAEIDFTMSDIDKLSRKVPQLCKVAPSTQKYHMEDVHRAGGVIGILGELDRAGLLNRDVK

NVLGLTLPQTLEQYDVMLTQDDAVKNMFRAGPAGIRTTQAFSQDCRWDSLDDDRANGCIRSLEHAYSKDG

GLAVLYGNFAENGCIVKTAGVDDSILKFTGPAKVYESQDDAVEAILGGKVVAGDVVVIRYEGPKGGPGMQ

EMLYPTSFLKSMGLGKACALITDGRFSGGTSGLSIGHVSPEAASGGSIGLIEDGDLIAIDIPNRGIQLQV

SDAELAARREAQEARGDKAWTPKNRERQVSFALCAYASLATSADKGAVRDKSKLGG

>gi|30043192|gb|AAP18913.1| threonine deaminase (dehydratase) [Shigella flexneri 2a str. 2457T]

MADSQPLSGAPEGAEYLRAVLRAPVYEAAQVTPLQKMEKLSSRLDNVILVKREDRQPVHSFKLRGAYAMM

AGLTEEQKAHGVITASAGNHAQGVAFSSARLGVKALIVMPTATADIKVDAVRGFGGEVLLHGANFDEAKA

KAIELSQQQGFTWVPPFDHPMVIAGQGTLALELLQQDAHLDRVFVPVGGGGLAAGVAVLIKQLMPQIKVI

AVEAEDSACLKAALDAGHPVDLPRVGLFAEGVAVKRIGDETFRLCQEYLDDIITVDSDAICAAMKDLFED

VRAVAEPSGALALAGMKKYIAQHNIRGERLAHILSGANVNFHGLRYVSERCELGEQREALLAVTIPEEKG

SFLKFCQLLGGRSVTEFNYRFADAKNACIFVGVRLSRGLEERKEILQMLNDGGYSVVDLSDDEMAKLHVR

YMVGGRPSHPLQERLYSFEFPESPGALLRFLNTLGTHWNISLFHYRSHGTDYGRVLAAFELGDHEPDFET

RLNELGYDCHDETNNPAFRFFLAG

>gi|30043191|gb|AAP18912.1| ilvC positive regulator [Shigella flexneri 2a str. 2457T]

MDLRDLKTFLHLAESRHFGRSARAMHVSPSTLSRQIQRLEEDLGQPLFVRDNRTVTLTEAGEELRVFAQQ

TLLQYQQLRHTIDQQGPLLYGELHIFCSVTAAYSHLPPILDRFRAEHPSVEIKLTTGDAADAMEKVVTGE

ADLAIAGKPETLPGAVAFSMLENLAVVLIAPALPCPVRNQVSVEKPDWSTVPFIMADQGPVRRRIELWFR

RNKISNPMIYATVGGHEAMVSMVALGCGVALLPEVVLENSPEPVRNRVMILERSDEKTPFELGVCAQKKR

LHEPLIEAFWKILPNH

>gi|30043190|gb|AAP18911.1| ketol-acid reductoisomerase [Shigella flexneri 2a str. 2457T]

MANYFNTLNLRQQLAQLGKCRFMGRDEFADGASYLQGKKVVIVGCGAQGLNQGLNMRDSGLDISYALRKE

AIAEKRASWRKATENGFKVGTYEELIPQADLVVNLTPDKQHSDVVRTVQPLMKDGAALGYSHGFNIVEVG

EQIRKDITVVMVAPKCPGTEVREEYKRGFGVPTLIAVHPENDPKGEGMAIAKAWAAATGGHRAGVLESSF

VAEVKSDLMGEQTILCGMLQAGSLLCFDKLVEEGTDPAYAEKLIQFGWETITEALKQGGITLMMDRLSNP

AKLRAYALSEQLKEIMAPLFQKHMDDIISGEFSSGMMADWANDDKKLLTWREETGKTAFETAPQYEGKIG

EQEYFDKGVLMIAMVKAGVELAFETMVDSGIIEESAYYESLHELPLIANTIVRKRLYEMNVVISDTAEYG

NYLFSYACVPLLKPFMAELQPGDLGKAIPEGAVDNAQLRDVNEAIRSHAIEQVGKKLRGYMTDMKRIAVA

G

>gi|30043189|gb|AAP18910.1| peptidyl-prolyl cis-trans isomerase C (rotamase C) [Shigella flexneri 2a str. 2457T]

MAKTAAALHILVKEEKLALDLLVQIKNGADFGKLAKKHSICPSGKRGGDLGEFRQGQMVPAFDKVVFSCP

VLEPTGPLHTQFGYHIIKVLYRN

>gi|30043188|gb|AAP18909.1| rep helicase [Shigella flexneri 2a str. 2457T]

MRLNPGQQQAVEFVTGPCLVLAGAGSGKTRVITNKIAHLIRGCGYQARHIAAVTFTNKAAREMKERVGQT

LGRKEAHGLMISTFHTLGLDIIKREYAALGMKANFSLFDDTDQLALLKELTEGLIEDDKVLLQQLISTIS

NWKNDLKTPSQAAASAIGERDRIFAHCYGLYDAHLKACNVLDFDDLILLPTLLLQRNEEVRERWQNKIRY

LLVDEYQDTNTSQYELVKLLVGSRARFTVVGDDDQSIYSWRGARPQNLVLLSQDFPALKVIKLEQNYRSS

GRILKAANILIANNPHVFEKRLFSELGYGTELKVLSANNEEHEAERVTGELIAHHFVNKTQYKDYAILYR

GNYQSRVFEKFLMQNRIPYKISGGTSFFSRPEIKDLLAYLRVLTNPDDDSAFLRIVNTPKREIGPATLKK

LGEWAMTRNKSMFTASFDMGLSQTLSGRGYEALTRFTHWLAEIQRLAEREPIAAVRDLIHGMDYESWLYE

TSPSTKAAEMRMKNVNQLFSWMTEMLEGSELDEPMTLTQVVTRFTLRDMMERGESEEELDQVQLMTLHAS

KGLEFPYVYMVGMEEGFLPHQSSIDEDNIDEERRLAYVGITRAQKELTFTLCKERRQYGELVRPEPSRFL

LELPQDDLIWEQERKVVSAEERMQKGQSHLANLKAMMAAKRGK

>gi|30043187|gb|AAP18908.1| guanosine pentaphosphatase, exopolyphosphatase [Shigella flexneri 2a str. 2457T]

MGSTSSLYAAIDLGSNSFHMLVVREVAGSIQTLTRIKRKVRLAAGLNSENDLSNEAMERGWQCLRLFAER

LQDIPPSQIRVVATATLRLAVNAGDFIAKAQEILGCPVQVISGEEEARLIYQGVAHTTGGADQRLVVDIG

GASTELVTGTGAQTTSLFSLSMGCVTWLERYFADRNLGQENFDAAEKAAREVLRPVADELRYHGWKVCVG

ASGTVQALQEIMMAQGMDERITLEKLQQLKQRAIHCGRLEELEIDGLTLERALVFPSGLAILIAIFTELN

IQCMTLAGGALREGLVYGMLHLAVEQDIRSRTLRNIQRRFMIDIDQAQRVAKVAANFFDQVENEWHLEAI

SRDLLISACQLHEIGLSVDFKQAPQHAAYLVRNLDLPGFTPAQKKLLATLLLNQTNPVDLSSLHQQNAVP

PRVAEQLCRLLRLAIIFASRRRDDLVPEMTLQANHELLTLTLPQGWLTQHPLGKEIIAQENQWQSYVHWP

LEVH

>gi|30043186|gb|AAP18907.1| putative ATP-dependent RNA helicase [Shigella flexneri 2a str. 2457T]

MSKTHLTEQKFSDFALHPKVVEALEKKGFHNCTPIQALALPLTLAGRDVAGQAQTGTGKTMAFLTSTFHY

LLSHPAIADRKVNQPRALIMAPTRELAVQIHADAEPLAEATGLKLGLAYGGDGYDKQLKVLESGVDILIG

TTGRLIDYAKQNHINLGAIQVVVLDEADRMYDLGFIKDIRWLFRRMPPANQRLNMLFSATLSYRVRELAF

EQMNNAEYIEVEPEQKTGHRIKEELFYPSNEEKMRLLQTLIEEEWPDRAIIFANTKHRCEEIWGHLAADG

HRVGLLTGDVAQKKRLRILDEFTRGDLDILVATDVAARGLHIPAVTHVFNYDLPDDCEDYVHRIGRTGRA

GASGHSISLACEEYALNLPAIETYIGHSIPVSKYNPDALMTDLPKPLRLTRPRTGNGPRRTGAPRNRRRS

G

>gi|30043185|gb|AAP18906.1| thioredoxin 1 [Shigella flexneri 2a str. 2457T]

MLHQQRNQHARLIPVELYMSDKIIHLTDDSFDTDVLKADGAILVDFWAEWCGPCKMIAPILDEIADEYQG

KLTVAKLNIDQNPGTAPKYGIRGIPTLLLFKNGEVAATKVGALSKGQLKEFLDANLA

>gi|30043184|gb|AAP18905.1| rho operon leader peptide [Shigella flexneri 2a str. 2457T]

MRSEQISGSSLNPSCRFSSAYSPVTRQRKDMSR

>gi|30043183|gb|AAP18904.1| transcription termination factor Rho, polarity suppressor [Shigella flexneri 2a str. 2457T]

MNLTELKNTPVSELITLGENMGLENLARMRKQDIIFAILKQHAKSGEDIFGDGVLEILQDGFGFLRSADS

SYLAGPDDIYVSPSQIRRFNLRTGDTISGKIRPPKEGERYFALLKVNEVNFDKPENARNKILFENLTPLH

ANSRLRMERGNGSTEDLTARVLDLASPIGRGQRGLIVAPPKAGKTMLLQNIAQSIAYNHPDCVLMVLLID

ERPEEVTEMQRLVKGEVVASTFDEPASRHVQVAEMVIEKAKRLVEHKKDVIILLDSITRLARAYNTVVPA

SGKVLTGGVDANALHRPKRFFGAARNVEEGGSLTIIATALIDTGSKMDEVIYEEFKGTGNMELHLSRKIA

EKRVFPAIDYNRSGTRKEELLTTQEELQKMWILRKIIHPMGEIDAMEFLINKLAMTKTNDDFFEMMKRS

>gi|30043182|gb|AAP18903.1| UDP-GlcNAc:undecaprenylphosphate GlcNAc-1-phosphate transferase [Shigella flexneri 2a str. 2457T]

MNLLTVSTDLISIFLFTTLFLFFARKVAKKVGLVDKPNFRKRHQGLIPLVGGISVYAGICFTFGIVDYYI

PHASLYLACAGVLVFIGALDDRFDISVKIRATIQAAVGIVMMVFGKLYLSSLGYIFGSWEMVLGPFGYFL

TLFAVWAAINAFNMVDGIDGLLGGLSCVSFAAIGMILWFDGQTSLAIWCFAMIAAILPYIMLNLGILGRR

YKVFMGDAGSTLIGFTVIWILLETTQGKTHPISPVTALWIIAIPLMDMVAIMYRRLRKGMSPFSPDRQHI

HHLIMRAGFTSRQAFVLITLAAALLASIGVLAEYSHFVPEWVMLVLFLLAFFLYGYCIKRAWKVARFIKR

VKRRLRRNRGGSPNLTK

>gi|30043181|gb|AAP18902.1| putative transport protein [Shigella flexneri 2a str. 2457T]

MTQPMPGKPAEDAENELDIRGLFRTLWAGKLWIIGMGLAFALIALAYTFFARQEWSSTAITDRPTVNMLG

GYYSQQQFLRNLDVRSNMASADQPSVMDEAYKEFVMQLASWDTRREFWLQTDYYKQRMVGNSKADAALLD

EMINNIQFIPGDFTRAVNDSVKLIAETAPDANNLLRQYVAFASQRAASHLNDELKGAWAARTIQMKAQVK

RQEEVAKAIYDRRMNSIEQALKIAEQHNISRSATDVPAEELPDSEMFLLGRPMLQARLENLQAVGPAFDL

DYDQNRAMLNTLNVGPTLDPRFQTYRYLRTPEEPVKRDSPRRAFLMIMWGIVGGLIGAGVALTRRCSK

>gi|30043180|gb|AAP18901.1| UDP-N-acetyl glucosamine-2-epimerase [Shigella flexneri 2a str. 2457T]

MSAKALAAYSKRIDVKVLTVFGTRPEAIKMAPLVHALAKDPFFEAKVCVTAQHREMLDQVLKLFSIVPDY

DLNIMQPGQGLTEITCRILEGLKPILAEFKPDVVLVHGDTTTTLATSLAAFYQRIPVGHVEAGLRTGALY

SPWPEEANRTLTGHLAMYHFSPTETSRQNLLRENVADSRIFITGNTVIDALLWVRDQVMSSDTLRSELAA

NYPFIAPDKKMILVTGHRRESFGRGFEEICHALADIATTHQDIQIVYPVHLNPNVREPVNRILGHVKNVI

LIDPQEYLPFVWLMNHAWLILTDSGGIQEEAPSLGKPVLVMRDTTERPEAVTAGTVRLVGTDKQRIVEEV

TRLLKDENEYQAMSRAHNPYGDGQACSRILEALKNNRISL

>gi|30043179|gb|AAP18900.1| UDP-N-acetyl-D-mannosaminuronic acid dehydrogenase [Shigella flexneri 2a str. 2457T]

MSFATISVIGLGYIGLPTAAAFASRQKQVIGVDINQHAVDTINRGEIHIVEPDLASVVKTAVEGGFLRAS

TTPVEADAWLIAVPTPFKGDHEPDMTYVESAARSIAPVLKKGALVILESTSPVGSTEKMAEWLAEMRPDL

TFPQQVGEQADVNIAYCPERVLPGQVMVELIKNDRVIGGMTPVCSARASELYKIFLEGECVVTNSRTAEM

CKLTENSFRDVNIAFANELSLICADQGINVWELIRLANRHPRVNILQPGPGVGGHCIAVDPWFIVAQNPQ

QARLIRTAREVNDHKPFWVIDQVKAAVADCLAATDKRASELKIACFGLAFKPNIDDLRESPAMEIAELIA

QWHSGETLVVEPNIHQLPKKLTGLCTLAQLDEALATADVLVMLVDHSQFKVINGDNVHQQYVVDAKGVWR

>gi|30043178|gb|AAP18899.1| dTDP-glucose 4,6-dehydratase [Shigella flexneri 2a str. 2457T]

MRKILITGGAGFIGSALVRYIINETSDAVVVVDKLTYAGNLMSLAPVAQSERFAFEKVDICDRAELARVF

TEHQPDCVMHLAAESHVDRSIDGPAAFIETNIVGTYTLLEAARAYWNTLTEDKKSAFRFHHISTDEVYGD

LHSTDDFFTETTPYAPSSPYSASKASSDHLVRAWLRTYGLPTLITNCSNNYGPYHFPEKLIPLMILNALA

GKPLPVYGNGQQIRDWLYVEDHARALYCVATTGKVGETYNIGGHNERKNLDVVETICELLEELASNKPHG

VAYYRDLITFVADRPGHDLRYAIDASKIARELGWLPQETFESGMRKTVQWYLANESWWKQVQDGSYQGER

LGLKG

>gi|30043177|gb|AAP18898.1| glucose-1-phosphate thymidylyltransferase [Shigella flexneri 2a str. 2457T]

MKGIILAGGSGTRLHPITRGVSKQLLPIYDKPMIYYPLSVLMLAGIREILIITTPEDKGYFQRLLGDGSE

FGIQLEYAEQPSPDGLAQAFIIGETFLNGEPSCLVLGDNIFFGQGFSPKLRHVAARTEGATVFGYQVMDP

ERFGVVEFDDNFRAISLEEKPKQPKSNWAVTGLYFYDSKVVEYAKQVKPSERGELEITSINQMYLEAGNL

TVELLGRGFAWLDTGTHDSLIEASTFVQTVEKRQGFKIACLEEIAWRNGWLDDEGVKRAASSLAKTGYGQ

YLLELLRARPRQY

>gi|30043175|gb|AAP18897.1| hypothetical protein S3896 [Shigella flexneri 2a str. 2457T]

MQAKIAASNTGELDALQQLGFSLVEGEVDLALPVNNVSDSGAVVAQETDIPALRQLASAAFAQSRFRAPW

YAPDASGRFYAQWIENAVRGTFDHQCLILRAASGDIRGYVSLRELNATDARIGLLAGRGAGAELMQTALN

WAYARGKTTLRVATQMGNTAALKRYIQSGANVESTAYWLYR

>gi|30043174|gb|AAP18896.1| putative regulator [Shigella flexneri 2a str. 2457T]

MIPFNAPPVVGTELDYMQSAMGSGKLCGDGGFTRRCQQWLEQRFGSAKVLLTPSCTASLEMAALLLDIQP

GDEVIMPSYTFVSTANAFVLRGAKIVFVDVRPDTMNIDETLIEAAITDKTRVIVPVHYAGVACEMDTIMA

LAKKHNLFVVEDAAQGVMSTYKGRALGTIGHIGCFSFHETKNYTAGGEGGATLINDKALIERAEIIREKG
TNRSQFFRGQVDKYTWRDIGSSYLMSDLQAAYLWAQLEAADRINQQRLALWQNYYDTLAPLAKAGRIELP
SIPDGCVQNAHMFYIKLRDIDDRSALINFLKEAEIMAVFHYIPLHGCPAGEHFGEFHGEDRYTTKESERL
LRLPLFYNLSPVNQRTVIATLLNYFS

>gi|30043173|gb|AAP18895.1| putative cytochrome [Shigella flexneri 2a str. 2457T]
MSLAKASLWTAASTLVKIGAGLLVGKLLAVSFGPAGLGLAANFRQLITVLGVLAGAGIFNGVTKYVAQYH
DNPQQLRRVVGTSSAMVLGFSTLMALVFVLAAAPISQGLFGNTDYQGLVRLVALVQMGIAWGNLLLALMK
GFRDAAGNALSLIVGSLIGVLAYYVSYRLGGYEGALLGLALIPALVVIPAAIMLIKRGVIPLSYLKPSWD
NGLAGQLSKFTLMALITSVTLPVAYIMMRKLLAAQYSWDEVGIWQGVSSISDAYLQFITASFSVYLLPTL
SRLTEKRDITREVVKSLKFVLPAVAAASFTVWLLRDFAIWLLLSNKFTAMRDLFAWQLVGDVLKVGAYVF
GYLVIAKASLRFYILAEVSQFTLLMVFAHWLIPAHGALGAAQAYMATYIVYFSLCCGVFLLWRRRA

>gi|30043172|gb|AAP18894.1| 4-alpha-l-fucosyltransferase [Shigella flexneri 2a str. 2457T]
MTVLIHVLGSDIPHHNRTVLRFFNDALAATSEHAREFMVVGKDDGLSDSCPALSVQFFPGKKSLAEAVIA
KAKANRQQRFFFHGQFNPTLWLALLSGGIKPSQFFWHIWGADLYELSSGLRYKLFYPLRRLAQKRVGCVF
ATRGDLSFFAKTHPKVRGELLYFPTRMDPSLNTMANDRQREGKMTILVGNSGDRSNEHVAALRAVHQQFG
DTVKVVVPMGYPPNNEAYIEEVRQAGLELFSEENLQILSEKLEFDAYLALLRQCDLGYFIFARQQGIGTL
CLLIQAGIPCVLNRENPFWQDMTEQHLPVLFTTDDLNEDIVREAQRQLASADKNTIAFFSPNYLQGWQRA
LAIAAGEVA

>gi|30043171|gb|AAP18893.1| TDP-Fuc4NAc:lipidII transferase [Shigella flexneri 2a str. 2457T]
MSLLQFSGLFVVWLLCTLFIATLTWFEFRRVRFNFNVFFSLLFLLTFFFGFPLTSVLVFRFDVGVAPPEI
LLQVLLSAGCFYAVYYVTYKTRLRKRVADVPRRPLFTMNRVETNLTWVILMGIALVSVGIFFMHNGFLLF
RLNSYSQIFSSEVSGVALKRFFYFFIPAMLVVYFLRQDSKAWLFFLVSTVAFGLLTYMIVGGTRANIIIA

FAIFLFIGIIRGWISLWMLAAAGVLGIVGMFWLALKRYGMNVSGDEAFYTFLYLTRDTFSPWENLALLLQ

NYDNIDFQGLAPIVRDFYVFIPSWLWPGRPSMVLNSANYFTWEVLNNHSGLAISPTLIGSLVVMGGALFI

PLGAIVVGLIIKWFDWLYELGNRETNRYKAAILHSFCFGAIFNMIVLAREGLDSFVSRVVFFIVVFGACL

MIAKLLYWLFESAGLIHKRTKSSLRTQVEG

>gi|30043170|gb|AAP18892.1| probable UDP-N-acetyl-D-mannosaminuronic acid transferase [Shigella flexneri 2a str. 2457T]

MNNNTTAPTYTLRGLQLIGWRDMQHALDYLFADGQLKQGTLVAINAEKMLTIEDNAEVRELINAAEFKYA

DGISIVRSVRKKYPQAQVSRVAGADLWEELMARAGKEGTPVFLVGGKPEVLAQTEAKLRNQWNVNIVGSQ

DGYFKPEQRQALFERIHASGAQIVTVAMGSPKQEIFMRDCRLVHPDALYMGVGGTYDVFTGHVKRAPKIW

QTLGLEWLYRLLSQPSRIKRQLRLLRYLRWHYTGNL

>gi|30043169|gb|AAP18891.1| putative amino acid/amine transport protein [Shigella flexneri 2a str. 2457T]

MADNKPELQRGLEARHIELIALGGTIGVGLFMGAASTLKWAGPSVLLAYIIAGLFVFFIMRSMGEMLFLE

PVTGSFAVYAHRYMSPFFGYLTAWSYWFMWMAVGISEITAIGVYVQFWSPEMAQWIPALIAVALVALANL

AAVRLYGEIEFWFAMIKVTTIIVMIVIGLGVIFFGFGNGGQSIGFSNLTEHGGFFAGGWKGFLTALCIVV

ASYQGVELIGITAGEAKNPQVTLRSAVGKVLWRILIFYVGAIFVIVTIFPWNEIGSNGSPFVLTFAKIGI

TAAAGIINFVVLTAALSGCNSGMYSCGRMLYALAKNRQLPAAMAKVSRHGVPVAGVAVSIAILLIGSCLN

YIIPNPQRVFVYVYSASVLPGMVPWFVILISQLRFRRAHKAAIASHPFRSILFPWANYVTMAFLICVLIG

MYFNEDTRMSLFVGIIFMLAVTAIYKVFGLNRHGKAHKLEE

>gi|30043168|gb|AAP18890.1| putative arylsulfatase regulator [Shigella flexneri 2a str. 2457T]

MLQQVPTRAFHVIAKPSGSDCNLNCDYCFYLEKQSLYREKPVTHMDDDTLEAYIRHYIAASELQNEVAFT

WQGGEPTLLGLEFYRRAVALQAKYGAGRKISNSFQTNGVLLDDEWCAFLAENNFLVGLSLDGPAEIHNQY

RVTKGGRPTHKLVMRALTLLQKHHVDYNVLVCVNRTSAQQPLQVYDFLCDAGVEFIQFIPVVERLADETA

VRDGLKLHAPGDIQGELTEWSVRPDEFGEFLVAIFDHWIKRDVGKIFVMNIEWAFANFVGAPGAVCHHQP

TCGRSVIVEHNGDVYACDHYVYPQYRLGNMHQQTIAEMIDSPQQQVFGEDKFKQLPAQCRSCNVLKACWG

GCPKHRFMLDASGKPGLNYLCAGYQRYFRHLPPYLKAMADLLAHGRPASDIMQAHLMVVNK

>gi|30043167|gb|AAP18889.1| IS2 orfB [Shigella flexneri 2a str. 2457T]

MARGWGVSLVSRCLRVSRAQLHVILRRTDDWMDGRRSRHTDDTDVLLRIHHVIGELPTYGYRRVWALLRR

QAELDGMPAINAKRVYRLMRQNALLLERKPAVPPSKRAHTGRVAVKESNQRWCSDGFEFCCDNGERLRVT

FALDCCDREALHWAVTTGGFNSETVQDVMLGAVERRFGNDLPSSSVEWLTDNGSCYRANETRQFARMLGL

EPKNTAVRSPESNGIAESFVKTIKRDYISIMPKPDGLTAAKNLAEAFEHYNEWHPHSALGYRSPREYLRQ

RACNGLSDNRCLEI

>gi|30043166|gb|AAP18888.1| IS2 orfA [Shigella flexneri 2a str. 2457T]

MIVLILVFRLVIGEQMIDVLGPEKRRRRTTQEKIAIVQQSFEPGMTVSLVARQHGVAASQLFLWRKQYQE

GSLTAVAAGEQVVPASELAAAMKQIKELQRLLGKKTMENELLKEAVEYGRAKKWIAHAPLLPGDGE

>gi|30043165|gb|AAP18887.1| HemY protein [Shigella flexneri 2a str. 2457T]

MLKVLLLFVLLIAGIVVGPMIAGHQGYVLIQTDNYNIETSVTGLAIILILAMVVLFAIEWLLRRIFRTGA

HTRGWFVGRKRRRARKQTEQALLKLAEGDYQQVEKLMAKNADHAEQPVVNYLLAAEAAQQRGDEARANQH

LERAAELAGNDTIPVEITRVRLQLARNENHAARHGVDKLLEVTPRHPEVLRLAEQAYIRTGAWSSLLDII

PSMAKAHVGDEEHRAMLEQQAWIGLMDQARADNGSEGLRNWWKNQSRKTRHQVALQVAMAEHLIECDDHD

TAQQIIIDGLKRQYDDRLLLPIPRLKTNNPEQLEKVLRQQIKNVGDRPLLWSTLGQSLMKHGEWQEASLA

FRAALKQRPDAYDYAWLADALDRLHKPEEAAAMRRDGLMLTLQNNPPQ

>gi|30043164|gb|AAP18886.1| uroporphyrinogen III methylase [Shigella flexneri 2a str. 2457T]

MTEQEKTSAVVEETREAVDTTSQPVATEKKSKNNTALILSAVAIAIALAAGVGLYGWGKQQAVNQTATSD

ALANQLTALQKAQESQKAELEGIIKQQAAQLKQANRQQETLAKQLDEVQQKIATISGSDAKTWLLAQADF

LVKLAGRKLWSDQDVTTSAALLKSADASLADMNDPSLITVRRAITDDIASLSAVSQVDYDGIILKLNQLS

NQVDNLRLADNDSDGSPMDSDGEELSSSISEWRINLQKSWQNFMDNFITIRRRDDTAVPLLAPNQDIYLR

ENIRSRLLVAAQAVPRHQEETYRQALENVSTWVRAYYDTDDATTKAFLDEVDQLSQQNISMDLPETLQSQ

AMLEKLMQTRVRNLLAQPAAGTTEAKPAPAPAPAPAPQGDTPAAAPQGE

>gi|30043163|gb|AAP18885.1| uroporphyrinogen III synthase [Shigella flexneri 2a str. 2457T]

MSILVTRPSPAGEELVSRLRTLGQVAWHFPLIEFSPGRQLPQLADQLAALGESDLLFALSQHAVAFAQSQ

LHQQDRKWPQLPTYFAIGRTTALALHTVSGQKILYPQDREISEVLLQLPELQNIAGKRALILRGNGGREL

IGDTLTVRGAEVTFCECYQRCAIHYDGAEEAMRWQSREVTMVVVTSGEMLQQLWSLIPQWYREHWLLHCR

LLVVSERLAKLARELGWQDIKVADNADNDALLRALQ

>gi|30043162|gb|AAP18884.1| porphobilinogen deaminase, hydroxymethylbilane synthase [Shigella flexneri 2a str. 2457T]

MLDNVLRIATRQSPLALWQAHYVKDKLMASHPGLVVELVPMVTRGDVILDTPLAKVGGKGLFVKELEVAL

LENRADIAVHSMKDVPVEFPQGLGLVTICEREDPRDAFVSNNYDSLDALPAGSIVGTSSLRRQCQLAERR

PDLIIRSLRGNVGTRLSKLDNGEYDAIILAVAGLKRLGLESRIRAALPPEISLPAVGQGAVGIECRLDDT

RTRELLAALNHHETALRVTAERAMNTRLEGGCQVPIGSYAELIDGEIWLCALVGAPDGSQIIRGERRGAP

QDAEQMGISLAEELLNNGAREILAEVYNGEAPA

>gi|30043161|gb|AAP18883.1| adenylate cyclase [Shigella flexneri 2a str. 2457T]

MYLYIETLKQRLDAINQLRVDRALAAMGPAFQQVYSLLPTLLHYHHPRMPGYLDGNVPKGICLYTPDETQ

RHYLNELELYRGMSVQDPPKGELPITGVYTMGSTSSVGQSCSSDLDIWVCHQSWLDSEERQLLQRKCSLL

ESWAASLGVEVSFFLIDENRFRHNESGSLGGEDCGSTQHILLLDEFYRTAVRLAGKRILWNMVPCDEEEH

YDDYVMTLYAQGVLTPNEWLDLGGLSSLSAEEYFGASLWQLYKSIDSPYKAVLKTLLLEAYSWEYPNPRL

LAKDIKQRLHDGEIVSFGLDPYCMMLERVTEYLTAIEDFTRLDLVRRCFYLKVCEKLSRERACVGWRRAV

LSQLVSEWGWDEARLAMLDNRANWKIDQVREAHNELLDAMMQSYRNLIRFARRNNLSVSASPQDIGVLTR
KLYAAFEALPGKVTLVNPQISPDLSEPNLTFIYVPPGRANRSGWYLYNRAPNIESIISHQPLEYNRYLNK
LVAWAWFNGLLTSRTRLYIKGNGIVDLPKLQEMVADVSHHFPLRLPAPTPKALYSPCEIRHLAIIVNLEY
DPTAAFRNQVVHFDFRKLDVFSFGENQNCLVGSVDLLYRNSWNEVRTLHFNGEQSMIEALKTILGKMHQD
AAPPDSVEVFCYSQHLRGLIRTRVQQLVSECIELRLSSTRQETGRFKALRVSGQTWGLFFERLNVSVQKL
ENAIEFYGAISHNKLHGLSVQVETNHVKLPAVVDGFASEGIIQFFFEETQDENGFNIYILDESNRVEVYH
HCEGSKEELVRDVSRFYSSSHDRFTYGSSFINFNLPQFYQIVKVDGREQVIPFRTKSIGNLPPANQDHDT
PLLQQYFS

>gi|30043160|gb|AAP18882.1| hypothetical protein S3876 [Shigella flexneri 2a str. 2457T]
MNDSEFHRLADQLWLTIEERLDDRDGDSDIDCEINGGVLTITFENGSKIIINRQEPLHQVWLATKQGGYH
FDLKGDEWICDRSGETFWDLLEQAATQQAGETVSFR

>gi|30043159|gb|AAP18881.1| IS1 orfA [Shigella flexneri 2a str. 2457T]
MASISIRCPSCSATEGVVRNGKSTAGHQRYLCSHCRKTWQLQFTYTASQPGTHQKIIDMAMNGVGCRASA
RIMGVGLNTVLRHLKNSGRSR

>gi|30043158|gb|AAP18880.1| IS1 orfB [Shigella flexneri 2a str. 2457T]
MSRQRTHYGRWPQHGFTSLKKLRPQSVTSRIQPGSDVIVCAEMDEHWGYVGAKSRQRWLFYAYDRIRRTV
VAHVFGERTLATLERLLSLLSAFEVVVWMTDGWPLYESRLKGKLHVISKRYTQRIERHNLNLRQHLARPV
RKSLSFSKSVELHDKVIGHYLNIKHYQ

>gi|30043157|gb|AAP18879.1| hypothetical protein S3871 [Shigella flexneri 2a str. 2457T]
MVYLRPYKETNLSLPDVNYKSLRRLPNLLIDPTTLDEWDKEPPLTDLTTDYLYEGAQAWYPHYSWHSDGR
NILYAGEVVQNPPGTPPVDVASFKAWGDFAADKHSLYFEGKRTDDNGGGNSLDIKTLHQVEFRPPWDPDL

LGLILRDANFLYINGHRLADPESFRVLAQKSWDQRGKFSTTFNPCIAVPFGPWDTLARTRTKILLNGEQL

DADPDTFSVVRWMPGSLLTWRDKNGLQRKVLDKENLAWDEDLTKHCLDFSLLEKKVFWRKGPACKQEELP

GLDPEQFQPISDAVAQHQDSLYTIIETESGNRKLEIVKLDDPNLIINKRFNAGKRHGYLLTRAEG

>gi|30043156|gb|AAP18878.1| putative outer membrane lipoprotein [Shigella flexneri 2a str. 2457T]

MKNVFKALTVLLTLFSLTGCGLKGPLYFPPADKNAPPPTKPVETQTQSTVPDKNDRATGDGPSQVNY

>gi|30043155|gb|AAP18877.1| diaminopimelate epimerase [Shigella flexneri 2a str. 2457T]

MMQFSKMHGLGNDFMVVDAVTQNVFFSPELIRRLADRHLGVGFDQLLVVEPPYDPELDFHYRIFNADGSE

VAQCGNGARCFARFVRLKGLTNKRDIRVSTANGRMVLTVTDDDLVRVNMGEPNFEPSAVPFRANKAEKTY

IMRAAEQTILCGVVSMGNPHCVIQVDDVDTAAVETLGPVLESHERFPERANIGFMQVVKREHIRLRVYER

GAGETQACGSGACAAVAVGIQQGLLDEEVRVELPGGRLDIAWKGPGHPLYMTGPAVHVYDGFIHL

>gi|30043154|gb|AAP18876.1| hypothetical protein S3868 [Shigella flexneri 2a str. 2457T]

MKQPGEELQETLTELDDRAVVDYLIKNPEFFIRNARAVEAIRVPHPVRGTVSLVEWHMARARNHIHVLEE

NMALLMEQAIANEGLFYRLLYLQRSLTAASSLDDMLMRFHRWARDLGLAGASLRLFPDRWRLGAPSNHTH

LALSRQSFEPLRIQRLGQEQHYLGPLNGPELLVVLPEAKAVGSVAMSMLGSDADLGVVLFTSRDASHYQQ

GQGTQLLHEIALMLPELLERWIERV

>gi|30043153|gb|AAP18875.1| site-specific recombinase [Shigella flexneri 2a str. 2457T]

MTDLHTDVERYLRYLSVERQLSPITLLNYQRQLEAIINFASENGLQSWQQCDAAMVRNFAVRSRRKGLGA

ASLALRLSALRSFFDWLVSQNELKANPAKGVSAPKAPRHLPKNIDVDDMNRLLDIDINDPLAVRDRAMLE

VMYGAGLRLSELVGLDIKHLDLESGEVWVMGKGSKERRLPIGRNAVAWIEHWLDLRDLFGSEDDALFLSK

LGKRISARNVQKRFAEWGIKQGLNNHVHPHKLRHSFATHMLESSGDLRGVQELLGHANLSTTQIYTHLDF

QHLASVYDAAHPRAKRGK

>gi|30043152|gb|AAP18874.1| putative phosphatase [Shigella flexneri 2a str. 2457T]

MRFYRPLGRISALTFDLDDTLYDNRPVILRTEREALTFVQNYHPALRSFQNEDLQRLRQAVREAEPEIYH
DVTRWRFRSIEQAMLDAGLSAEEASAGAHAAMINFAKWRSRIDVPQQTHDTLKQLAKKWPLVAITNGNAQ
PELFGLGDYFEFVLRAGPHGRSKPFSDMYFLAAEKLNVPIGEILHVGDDLTTDVGGAIRSGMQACWIRPE
NGDLMQTWDSRLLPHLEISRLASLTSLI

>gi|30043151|gb|AAP18873.1| DNA-dependent ATPase I and helicase II [Shigella flexneri 2a str. 2457T]

MDVSYLLDSLNDKQREAVAAPRSNLLVLAGAGSGKTRVLVHRIAWLMSVENCSPYSIMAVTFTNKAAAEM
RHRIGQLMGTSQGGMWVGTFHGLAHRLLRAHHMDANLPQDFQILDSEDQLRLLKRLIKAMNLDEKQWPPR
QAMWYINSQKDEGLRPHHIQSYGNPVEQTWQKVYQAYQEACDRAGLVDFAELLLRAHELWLNKPHILQHY
RERFTNILVDEFQDTNNIQYAWIRLLAGDTGKVMIVGDDDQSIYGWRGAQVENIQRFLNDFPGAEIIRLE
QNYRSTSNILSAANALIENNNGRLGKKLWTDGADGEPISLYCAFNELDEARFVVNRIKTWQDNGGALAEC
AILYRSNAQSRVLEEALLQASMPYRIYGGMRFFERQEIKDALSYLRLIANRNDDAAFERVVNTPTRGIGD
RTLDVVRQTSRDRQLTLWQACRELLQEKALAGRAASALQRFMELIDALAQETADMPLHVQTDRVIKDSGL
RTMYEQEKGEKGQTRIENLEELVTATRQFSYNEEDEDLMPLQAFLSHAALEAGEGQADTWQDAVQLMTMH
SAKGLEFPQVFIVGMEEGMFPSQMSLDEGGRLEEERRLAYVGVTRAMQKLTLTYAETRRLYGKEVYHRPS
RFIGELPEECVEEVRLRATVSRPVSHQRMGTPMVENDSGYKLGQRVRHAKFGEGTIVNMEGSGEHSRLQV
AFQGQGIKWLVAAYARLESV

>gi|30043150|gb|AAP18872.1| hypothetical protein S3864 [Shigella flexneri 2a str. 2457T]

MQSGPMLMENGVINPRIHPNVASRKIRNGVGINKHGNAVFLLSQQATNFYDFACYAKAKLNVEQLLYLGG
TISHMYMKGGAIPWQRYPFVTMISVERKG

>gi|30043149|gb|AAP18871.1| IS4 orf [Shigella flexneri 2a str. 2457T]

MHIGQALDLVSRYDSLRNPLTSLGDYLDPELISRCLAESGTVTLRKRRLPLEMMVWCIVGMALERKEPLH
QIVNRLDIMLPGNRPFVAPSAVIQARQRLGSEAVRRVFTKTAQLWHNATPHPHWCGLTLLAIDGVFWRTP
DTPENDAAFPRQTHAGNPALYPQVKMVCQMELTSHLLTAAAFGTMKNSENELAEQLIEQTGDNTLTLMDK
GYYSLGLLNGWSLAGEHRHWMIPLRKGAQYEELRKLGKGDHLVKLKTSPQARKKWPGLGNEVTARLLTVT
RKGKVCHLLTSMTDAMRFPGGEMADLYSNRWEIELGYREIKQTMQLSRLTLRSKKPELVEQELWGVLLAY
NLVRYQMIKMAEHLKGYWPNQLSFSESCGMVMRMLMTLQGASPGRIPELMSDLASMGQLVKLPTRRGRAF
PRVVKERPWKYPTAPKKSQSVA

>gi|30043148|gb|AAP18870.1| hypothetical protein S3862 [Shigella flexneri 2a str. 2457T]
MAHRLLIGKGMITLNLKRIFLTLTLLPLFAVAADDCALSDPTLTVQAYTVNPQTERVKMYWQKANGEAWG
TLHALLADINSQGQVQMAMNGGIYDESYAPLGLYIENGQQKV

>gi|30043147|gb|AAP18869.1| Mg2+ transport, system I [Shigella flexneri 2a str. 2457T]
MLSAFQLENNRLTRLEVEESQPLVNAVWIDLVEPDDDERLRVQSELGQSLATRPELEDIEASARFFEDDD
GLHIHSFFFFEDAEDHAGNSTVAFTIRDGRLFTLRERELPAFRLYRMRARSQSMVDGNAYELLLDLFETK
IEQLADEIENIYSDLEQLSRVIMEGHQGDEYDEALSTLAELEDIGWKVRLCLMDTQRALNFLVRKARLPG
GQLEQAREILRDIESLLPHNESLFQKVNFLMQAAMGFINIEQNRIIKIFSVVSVVFLPPTLVASSYGMNF
EFMPELKWSFGYPGAIIFMILAGLAPYLYFKRKNWL

>gi|30043146|gb|AAP18868.1| hypothetical protein S3860 [Shigella flexneri 2a str. 2457T]
MSKEYMNDDSLSEKWKYRFNFYDQHGFPGFWGATPEYKAAFKALKVRQRLTIQMNFIAFFCSWIYLFVLG
LWKKAIIVLLLGILSLFVGALIGVNILGIAVAAYVAVNTNKWFYEKEVKGLNTWSL

>gi|30043145|gb|AAP18867.1| hypothetical protein S3859 [Shigella flexneri 2a str. 2457T]
MLTGDSHKDVKFMLRMFIPTSNGKISRHRYIFLFILINFIFAFLIIFFNDGEAGFLVIVSTIALHYLVIN

MNCQRLRDSGFIYIKIYVFGTLAVYIISIITMIAEDFACSGNGSMIFLICYFSTFSMLMLAPTDSSKQ

>gi|30043144|gb|AAP18866.1| hypothetical protein S3858 [Shigella flexneri 2a str. 2457T]

MDAKQTRQGVLLALAAYFIWGIAPAYFKLIYYVPADEILTHRVIWSFFFMVVLMSICRQWSYLKTLIQTP

QKIFMLAVSAVLIGGNWLLFIWAVNNHHMLEASLGYFINPLVNIVLGMIFLGERFRRMQWLAVILAICGV

LVQLWTFGSLPIIALGLAFSFAFYGLVRKKIAVEAQTGMLIETMWLLPVAAIYLFAIADSSTSHMGQNPM

SLNLLLIAAGIVTTVPLLCFTAAATRLRLSTLGFFQYIGPTLMFLLAVTFYGEKPGADKMVTFTFIWVAL

AIFVMDAIYTQRRTSK

>gi|30043143|gb|AAP18865.1| hypothetical protein S3857 [Shigella flexneri 2a str. 2457T]

MKCKDDMSAVLTAEQALKLVGEMFVYHMPFNRALGMELERYEKEFAQLAFKNQPMMVGNWAQSILHGGVI

ASALDVAAGLVCVGSTLTRHETISEDELRQRLSRMGTIDLRVDYLRPGRGERFTATSSLLRAGNKVAVAR

VELHNEEQLYIASATATYMVG

>gi|30043142|gb|AAP18864.1| outer membrane phospholipase A [Shigella flexneri 2a str. 2457T]

MRTLQGWLLPVFMLPMAVYAQEATVKEVHDAPAVRGSIIANMLQEHDNPFTLYPYDTNYLIYTQTSDLNK

EAIASYDWAENARKDEVKFQLSLAFPLWRGILGPNSVLGASYTQKSWWQLSNSEESSPFRETNYEPQLFL

GFATDYRFAGWTLRDVEMGYNHDSNGRSDPTSRSWNRLYTRLMAENGNWLVEVKPWYVVGNTDDNPDITK

YMGYYQLKIGYHLGDAVLSAKGQYNWNTGYGGAELGLSYPITKHVRLYTQVYSGYGESLIDYNFNQTRVG

VGVMLNDLF

>gi|30043141|gb|AAP18863.1| ATP-dependent DNA helicase [Shigella flexneri 2a str. 2457T]

MNVAQAEVLNLESGAKQVLQETFGYQQFRPGQEEIIDTVLSGRDCLVVMPTGGGKSLCYQIPALLLNGLT

VVVSPLISLMKDQVDQLQANGVAAACLNSTQTREQQLEVMTGCRTGQIRLLYIAPERLMLDNFLEHLAHW

NPVLLAVDEAHCISQWGHDFRPEYAALGQLRQRFPTLPFMALTATADDTTRQDIVRLLGLNDPLIQISSF

DRPNIRYMLMEKFKPLDQLMRYVQEQRGKSGIIYCNSRAKVEDTAARLQSRGISAAAYHAGLENNVRADV
QEKFQRDDLQIVVATVAFGMGINKPNVRFVVHFDIPRNIESYYQETGRAGRDGLPAEAMLFYDPADMAWL
RRCLEEKPQGQLQDIERHKLNAMGAFAEAQTCRRLVLLNYFGEGRQEPCGNCDICLDPPKQYDGSTDAQI
ALSTIGRVNQRFGMGYVVEVIRGANNQRIRDYGHDKLKVYGMGRDKSHEHWVSVIRQLIHLGLVTQNIAQ
HSALQLTEAARPVLRGESSLQLAVPRIVALKPKAMQKSFGGNYDRKLFAKLRKLRKSIADESNVPPYVVF
NDATLIEMAEQMPITASEMLSVNGVGMRKLERFGKPFMALIRAHVDGDDEE

>gi|30043140|gb|AAP18862.1| hypothetical protein S3854 [Shigella flexneri 2a str. 2457T]
MGYQMLRGALKKEAVSAPAPQVELAKSGRSFLKGLLTNLANPKAIIYFGSVFSLFVGDNVGTTERWGIFA
LIIVETLAWFTVVASLFALPQMRRGYQRLAKWIDGFAGALFAGFGIHLIISR

>gi|30043139|gb|AAP18861.1| hypothetical protein S3853 [Shigella flexneri 2a str. 2457T]
MIAFEVLKWAGAAYLIWLGIQQWRAAGAIDLKSLASTQSRRHLFQRAVFVNLTNPKSIVFLAALFPQFIM
PQQPQLMQYIVLGVTTIVVDIIVMIGYATLAQRIALWIKGPKQMKALNKIFGSLFMLVGALLASARHA

>gi|30043138|gb|AAP18860.1| lysophospholipase L(2) [Shigella flexneri 2a str. 2457T]
MFQQQKDWETRENAFAAFTMGPLTDFWRQRDEAEFTGVDDIPVRFVRFRAQHHDRVVVICPGRIESYVKY
AELAYDLFHLGFDVLIIDHRGQGRSGRLLADPHLGHVNRFNDYVDDLAAFWQQEVQPGPWRKRYILAHSM
GGAISTLFLQRHPGVCDAIALTAPMFGIVIRMPSFMARQILNWAEAHPRFRDGYAIGTGRWRALPFAINV
LTHSRQRYRRNLRFYADDPTIRVGGPTYHWVRESILAGEQVLAGAGDDATQTLLLQAEEERVVDNRMHDR
FCELRTAAGHPVEGGRPLVIKGAYHEILFEKDAMRSVALHAIVDFFNRHNSPSGNRSTEV

>gi|30043137|gb|AAP18859.1| hypothetical protein S3851 [Shigella flexneri 2a str. 2457T]
MYQVVASDLDGTLLSPDHTLSPYAKETLKLLTARGINFVFATGRHHVDVGQIRDNLEIKSYMITSNGARV
HDLDGNLIFAHNLDRDIASDLFGVVNDNPDIITNVYRDDEWFMNRHRPEEMRFFKEAVFKYALYEPGLLE

PEGISKVFFTCDSHEQLLPLEQAINARWGDRVNVSFSTLTCLEVMAGGVSKGHALEAVAKKLGYSLKDCI

AFGDGMNDAEMLSMAGKGCIMGSAHQRLKDLHPELEVIGTNADDAVPHYLRKLYLS

>gi|30043136|gb|AAP18858.1| hypothetical protein S3850 [Shigella flexneri 2a str. 2457T]

MALLIITTILWAFSFSFYGEYLAGHVDSYFAVLVRVGLAALVFLPFLRTRSNSLKTVGLYMLVGAMQLGV

MYMLSFRAYLYLTVSELLLFTVLTPLYITLIYDIMSKRRLRWGYAFSALLAVIGAGIIRYDQVTDHFWTG

LLLVQLSNITFAIGMVGYKRLMETRPMPQYNAFAWFYLGAFLVAVIAWFLLGNAQKMPQTTLQWGILVFL

GVVASGIGYFMWNYGATQVDAGTLGIMNNMHVPAGLLVNLAIWHQQPHWPTFITGALVILASLWVHRKWV

APRSSQTADDRRRDCALSE

>gi|30043135|gb|AAP18857.1| regulator for metE and metH [Shigella flexneri 2a str. 2457T]

MIEVKHLKTLQALRNCGSLAAAAATLHQTQSALSHQFSDLEQRLGFRLFVRKSQPLRFTPQGEILLQLAN

QVLPQISQALQACNEPQQTRLRIAIECHSCIQWLTPALGNFHKNWPQVEMDFKSGVTFDPQPALQQGELD

LVMTSDILPRSGLHYSPMFDYEVRLVLAPDHPLAAKTRITPEDLASETLLIYPVQRSRLDVWRHFLQPAG

VSPSLKSVDNTLLLIQMVAARMGIAALPHWVVESFERQGLVVTKTLGEGLWSRLYAAVRDGEQRQPVTEA

FIRSARNHACDHLPFVKSAERPTYDAPTVRPGSPARL

>gi|30043134|gb|AAP18856.1| tetrahydropteroyltriglutamate methyltransferase [Shigella flexneri 2a str. 2457T]

MTILNHTLGFPRVGLRRELKKAQESYWAGNSTREELLTVGRELRARHWDQQKQAGIDLLPVGDFAWYDHV

LTTSLLLGNVPPRHQNKDGSVDIDTLFRIGRGRAPTGEPAAAAEMTKWFNTNYHYMVPEFVKGQQFKLTW

TQLLEEVDEALALSHNVKPVLLGPVTYLWLGKVKGEQFDRLSLLNDILPVYQQVLAELAKRGIEWVQIDE

PALVLELPQAWLDAYKPAYDALQGQVKLLLTTYFEGVTPNLDTITALPVQGLHVDLVHGKDNVVELHKRL

PSDWLLSAGLINGRNVWRADLTEKYAQIKDIVGKRDLWVASSCSLLHSPIDLSVETRLDAEVKSWFAFAL

QKCHELALLRDALNSGDTAALAEWSAPIQARRHSTRVHNPAVEKRLAAITAQDSQRANVYEVRAEAQRAR

FKLPAWPTTTIGSFPQTTEIRTLRLDFKKGNLDANNYRTGIAEHIKQAIVEQERLGLDVLVHGEAERNDM

VEYFGEHLDGFVFTQNGWVQSYGSRCVKPPIVIGDISRPAPITVEWAKYAQSLTDKPVKGMLTGPVTILC
WSFPREDVSRETIAKQIALALRDEVADLEAAGIGIIQIDEPALREGLPLRRSDWDAYLQWGVEAFRINAA
VAKDDTQIHTHMCYCEFNDIMDSIAALDADVITIETSRSDMELLESFEEFDYPNEIGPGVYDIHSPNVPS
VEWIEALLKKAAKRIPAERLWVNPDCGLKTRGWPETRAALANMVQAAQNLRRG

>gi|30043133|gb|AAP18855.1| hypothetical protein S3847 [Shigella flexneri 2a str. 2457T]
MNIAAVAGSPTGDMIEIGQHLRVRHFCYQTAKQRGDIGKIIRIAFAEVKFRCDSQIPLQRQTATNVADMF
MHAKNFLHYNDHWQWAIALLWSGMVSRHVIPL

>gi|30043132|gb|AAP18854.1| uridine phosphorylase [Shigella flexneri 2a str. 2457T]
MSKSDVFHLGLTKNDLQGATLAIVPGDPDRVEKIAALMDKPVKLASHREFTTWRAELDGKPVIVCSTGIG
GPSTSIAVEELAQLGIRTFLRIGTTGAIQPHINVGDVLVTTASVRLDGASLHFAPLEFPAVADFECTTAL
VEAAKSIGATTHVGVTVSSDTFYPGQERYDTYSGRVVRHFKGSMEEWQAMGVMNYEMESATLLTMCASQG
LRAGMVAGVIVNRTQQEIPNAETMKQTESHAVKIVVEAARRLL

>gi|30043131|gb|AAP18853.1| putative alpha helix chain [Shigella flexneri 2a str. 2457T]
MVYAVIALVGVAIGWLFASYQHAQQKAEQLAEREEMVAELSAAKQQITQSEHWRAECELLNNEVRSLQSI
NTSLEADLREVTTRMEAAQQHADDKIRQMINSEQRLSEQFENLANRIFEHSNRRVDEQNRQSLNSLLSPL
REQLDGFRRQVQDSFGKEAQERHTLTHEIRNLQQLNAQMAQEAINLTRALKGDNKTQGNWGEVVLTRVLE
ASGLREGYEYETQVSIENDARSRMQPDVIVRLPQGKDVVIDAKMTLVAYERYFNAEDDYTRESALQEHIA
SVRNHIRLLGRKDYQQLPGLRTLDYVLMFIPVEPAFLLALDRQPELITEALKNNIMLVSPTTLLVALRTI
ANLWRYEHQSRNAQQIADRASKLYDKMRLFIDDMSAIGQSLDKAQDNYRQAMKKLSSGRGNVLAQAEAFR
GLGVEIKREINPDLAEQAVSQDEEYRLRSVPEQPNDEAYQRDDEYNQQSR

>gi|30043130|gb|AAP18852.1| ubiquinone/menaquinone biosynthesis methyltransferase [Shigella flexneri 2a str. 2457T]

MVDKSQETTHFGFQTVAKEQKADMVAHVFHSVASKYDVMNDLMSFGIHRLWKRFTIDCSGVRRGQTVLDL
AGGTGDLTAKFSRLVGETGKVVLADINESMLKMGREKLRNIGVIGNVEYVQANAEALPFPDNTFDCITIS
FGLRNVTDKDKALRSMYRVLKPGGRLLVLEFSKPIIEPLSKAYDAYSFHVLPRIGSLVANDADSYRYLAE
SIRMHPDQDTLKAMMQDAGFESVDYYNLTAGVVALHRGYKF

>gi|30043129|gb|AAP18851.1| hypothetical protein S3842 [Shigella flexneri 2a str. 2457T]
MPFKPLVTAGIESLLNTFLYRSPALKTARSRLLGKVLRVEVKGFSTSLILVFSERQVDVLGEWAGDADCT
VIAYASVLPKLRDRQQLTALIRSGELEVQGDIQVVQNFVALADLAEFDPAELLAPYTGDIAAEGISKAMR
GGAKFLHHGIKRQQRYVAEAITEEWRMAPGPLEVAWFAEETAAVERAVDALTKRLEKLEAK

>gi|30043128|gb|AAP18850.1| hypothetical protein S3841 [Shigella flexneri 2a str. 2457T]
MTPGEVRRLYFIIRTFLSYGLDELIPKMRITLPLRLWRYSLFWMPNRHKDKLLGERLRLALQELGPVWIK
FGQMLSTRRDLFPPHIADQLALLQDKVAPFDGKLAKQQIEAAMGGLPVEAWFDDFEIKPLASASIAQVHT
ARLKSNGKEVVIKVIRPDILPVIKADLKLIYRLARWVPRLLPDGRRLRPTEVVREYEKTLIDELNLLRES
ANAIQLRRNFEDSPMLYIPEVYPDYCSEGMMVMERIYGIPVSDVAALEKNGTNMKLLAERGVQVFFTQVF
RDSFFHADMHPGNIFVSYEHPENPKYIGIDCGIVGSLNKEDKRYLAENFIAFFNRDYRKVAELHVDSGWV
PPDTNVEEFEFAIRTVCEPIFEKPLAEISFGHVLLNLFNTARRFNMEVQPQLVLLQKTLLYVEGVGRQLY
PQLDLWKTAKPFLESWIKDQVGIPALVRAFKEKAPFWVEKMPELPELVYDSLRQGKYLQHSVDKIARELQ
SNHVRQGQSRYFLGIGATLVLSGTFLLVSRPEWGLMPGWLMAGGLIAWFVGWRKTR

>gi|30043127|gb|AAP18849.1| hypothetical protein S3840 [Shigella flexneri 2a str. 2457T]
MRLCLIIIYHRGTCMGGISIWQLLIIAVIVVLLFGTKKLGSIGSDLGASIKGFKKAMSDDEPKQDKTSQD
ADFTAKTIADKQADTNQEQAKTEDAKRHDKEQV

>gi|30043126|gb|AAP18848.1| hypothetical protein S3838 [Shigella flexneri 2a str. 2457T]

MFDIGFSELLLVFIIGLVVLGPQRLPVAVKTVAGWIRALRSLATTVQNELTQELKLQEFQDSLKKVEKAS

LTNLTPELKASMDELRQAAESMKRSYVANDPEKASDEAHTIHNPVVKDNEAAHEGVTPAAAQTQASSPEQ

KPETTPEPVVKPAADAEPKTAAPSPSSSDKP

>gi|30043125|gb|AAP18847.1| hypothetical protein S3837 [Shigella flexneri 2a str. 2457T]

MSVEDTQPLITHLIELRKRLLNCIIAVIVIFLCLVYFANDIYHLVSAPLIKQLPQGSTMIATDVASPFFT

PIKLTFMVSLILSAPVILYQVWAFIAPALYKHERRLVVPLLVSSSLLFYIGMAFAYFVVFPLAFGFLANT

APEGVQVSTDIASYLSFVMALFMAFGVSFEVPVAIVLLCWMGITSSEDLRKKRPYVLVGAFVVGMLLTPP

DVFSQTLLAIPMYCLFEIGVFFSRFYVGKGRNREEENDAEAESEKTEE

>gi|30043124|gb|AAP18846.1| hypothetical protein S3836 [Shigella flexneri 2a str. 2457T]

MFDIGVNLTSSQFAKDRDDVVARAFDAGVNGLLITGTNLRESQQAQKLARQYSSCWSTAGVHPHDSSQWQ

AATEEAIIELAAQPEVVAIGECGLDFNRNFSTPEEQERAFVAQLRIAAELNMPVFMHCRDAHERFMTLLE

PWLDKLPGAVLHCFTGTREEMQACVARGIYIGITGWVCDERRGLELRELLPLIPAEKLLIETDAPYLLPR

DLTPKPSSRRNEPAHLPHILQRIAHWRGEDAAWLAATTDANVKTLFGIAF

>gi|30043123|gb|AAP18845.1| transcriptional activator [Shigella flexneri 2a str. 2457T]

MQSWYLLYCKRGQLQRAQEHLERQAVNCLAPMITLEKIVRGKRTAVSEPLFPNYLFVEFDPEVIHTTTIS

ATRGVSHFVRFGASPAIVPSAVIHQLSVYKPKDIVDPAPPYPGDKVIITEGAFEGFQAIFTEPDGEARSM

LLLNLINKEIKHSVKNTEFRKL

>gi|30043122|gb|AAP18844.1| putative oxidoreductase [Shigella flexneri 2a str. 2457T]

MDAMKYNDLRDFLTLLEQQGELKRITLPVDPHLEITEIADRTLRAGGPALLFENPKGYSMPVLCNLFGTP

KRVAMGMGQEDVSALREVGKLLAFLKEPEPPKGFRDLFDKLPQFKQVLNMPTKRLRGAPCQQKIVSGDDV

DLNRIPIMTCWPEDAAPLITWGLTVTRGPHKERQNLGIYRQQLIGKNKLIMRWLSHRGGALDYQEWCAAH

PGERFPVSVALGADPATILGAVTPVPDTLSEYAFAGLLRGTKTEVVKCISNDLEVPASAEIVLEGYIDPG

EMAPEGPYGDHTGYYNEVDNFPVFTVTHITQREDAIYHSTYTGRPPDEPAVLGVALNEVFVPILQKQFPE

IVDFYLPPEGCSYRLAVVTIKKQYAGHAKRVMMGVWSFLRQFMYTKFVIVCDDDVNARDWNDVIWAITTR

MDPARDTVLVENTPIDYLDFASPVSGLGSKMGLDATNKWPGETQREWGRPIKKDPDVVAHIDAIWDELAI

FNNGKSA

>gi|30043121|gb|AAP18843.1| ferrisiderophore reductase, flavin reductase (NADPH:flavin oxidoreductase) [Shigella flexneri 2a str. 2457T]

MTTLSCKVTSVAAITDTVYRVRIVPDAAFSFRAGQYLMVVMDERDKRPFSMASTPDEKGFIELHIGASEI

NLYAKAVMDRILKDHQIVVDLPHGEAWLRDDEERPMILIAGGTGFSYARSILLTALARNPNRDITIYWGG

REEQHLYDLCELEALSLKHLGLQVVPVVEQPEAGWRGRTGTVLTAVLQDHGTLAEHDIYIAGRFEMAKIA

RDLFCSERNAREDRLFGDAFAFI

>gi|30043120|gb|AAP18842.1| thiolase I, 3-ketoacyl-CoA thiolase, acetyl-CoA transferase [Shigella flexneri 2a str. 2457T]

MEQVVIVDAIRTPMGRSKGGAFRNVRAEDLSAHLMRSLLARNPALEAAALDDIYWGCVQQTLEQGFNIAR

NAALLAEVPHSVPAVTVNRLCGSSMQALHDAARMIMTGDAQACLVGGVEHIGHVPMSHGVDFHPGLSRNV

AKAAGMMGLTAEMLARMHGISREMQDAFAARSHARAWAATQSAAFKNEIIPTGGHDADGVLKQFNYDEVI

RPETTVEALATLRPAFDPVNGTVTAGTSSALSDGAAAMLVMSESRAHELGLKPRARVRSMAVVGCDPSIM

GYGPVPASKLALKKAGLSASDIGVFEMNEAFAAQILPCIKDLGLMEQIDEKINLNGGAIALGHPLGCSGA

RISTTLLNLMERKDVQFGLATMCIGLGQGIATVFERV

>gi|30043119|gb|AAP18841.1| 4-enzyme protein, 3-hydroxyacyl-CoA dehydrogenase; 3-hydroxybutyryl-CoA epimerase; delta(3)-cis-delta(2)-trans-enoyl-CoA isomerase; enoyl-CoA hydratase [Shigella flexneri 2a str. 2457T]

MLYKGDTLYLDWLEDGIAELVFDAPGSVNKLDTATVASLGEAIGVLEQQSDLKGLLLRSNKAAFIVGADI

TEFLSLFLVPEEQLSQWLHFANSVFNRLEDLPVPTIAAVNGYALGGGCECVLATDYRLATPDLRIGLPET

KLGIMPGFGGSVRMPRMLGADSALEIIAAGKDVGADQALKIGLVDGVVKAEKLIEGAMAILRQAINGDLD

WKAKRQPKQEPLKLSKIEATMSFTIAKGMVAQTAGKHYPAPITAVKTIEAAARFGREEALNLENKSFVPL

AHTNEARALVGIFLNDQYVKGKAKKLTKDVETPKQAAVLGAGIMGGGIAYQSAWKGVPVVMKDINDKSLT

LGMTEAAKLLNKQLERGKIDGLKLAGVISTIHPTLDYAGFDRVDIVVEAVVENPKVKKAVLAETEQKVRQ

DTVLASNTSTIPISELANALERPENFCGMHFFNPVHRMPLVEIIRGEKSSDETIAKVVAWASKMGKTPIV

VNDCPGFFVNRVLFPYFAGFSQLLRDGADFRKIDKVMEKQFGWPMGPAYLLDVVGIDTAHHAQAVMAAGF

PQRMQKDYRDAIDALFDANRFGQKNGLGFWRYKEDSKGKPKKEEDAAVEDLLAEVSQPKRDFSEEEIIAR

MMIPMVNEVVRCLEEGIIATPAEADMALVYGLGFPPFHGGAFRWLDTLGSAKYLDMAQQYQHLGPLYEVP

EGLRNKARHNEPYYPPVEPARPVGDLKTA

>gi|30043118|gb|AAP18840.1| proline dipeptidase [Shigella flexneri 2a str. 2457T]

MESLASLYKNHIATLQERTRDALARFKLDALLIHSGELFNVFLDDHPYPFKVNPQFKAWVPVTQVPNCWL

LVDGVNKPKLWFYLPVDYWHNVEPLPTSFWTEDVEVIALPKADGIGSLLPAARGNIGYIGPVPERALQLG

IEASNINPKGVIDYQHYYRSFKTEYELACMREAQKMAVNGHRAAEEAFRSGMSEFDINIAYLTATGHRDT

DVPYSNIVALNEHAAVLHYTKLDHQAPEEMRSFLLDAGAEYNGYAADLTRTWSAKSDNDYAQLVKDVNDE

QLALIATMKAGVSYVDYHIQFHQRIAKLLRKHQIITDMSEEAMVENDLTGPFMPHGIGHPLGLQVHDVAG

FMQDDSGTHLAAPAKYPYLRYTRILQPGMVLTIEPGIYFIESLLAPWREGQFSKHFNWQKIEALKPFGGI

RIEDNVVIHENNVENMTRDLKLA

>gi|30043117|gb|AAP18839.1| hypothetical protein S3828 [Shigella flexneri 2a str. 2457T]

MESWLIPAAPVTVVEEIKKSRFITMLAHTDGVEAAKAFVESVRAEHPDARHHCVAWVAGAPDDSQQLGFS

DDGEPAGTAGKPMLAQLMGSGVGEITAVVVRYYGGILLGTGGLVKAYGGGVNQALRQLTTQRKTPLTEYT

LQCEYSQLTGIEALLGQCDGKIINSDYQAFVLLRVALPAAKVAEFSAKLADFSRGSLQLLAIEE

>gi|30043116|gb|AAP18838.1| potassium uptake protein [Shigella flexneri 2a str. 2457T]

MHFRAITRIVGLLVILFSGTMIIPGLVALIYRDGAGRAFTQTFFVALAIGSMLWWPNRKEKGELKSREGF

LIVVLFWTVLGSVGALPFIFSESPNLTITDAFFESFSGLTTTGATTLVGLDSLPHAILFYRQMLQWFGGM

GIIVLAVAILPILGVGGMQLYRAEMPGPLKDNKMRPRIAETAKTLWLIYVLLTVACALALWFAGMDAFDA

IGHSFATIAIGGFSTHDASIGYFDSPTINTIIAIFLLISGCNYGLHFSLLSGRSLKVYWRDPEFRMFIGV

QFTLVVICTLVLWFHNVYSSALMTINQAFFQVVSMATTAGFTTDSIARWPLFLPVLLLCSAFIGGCAGST

GGGLKVIRILLLFKQGNRELKRLVHPNAVYSIKLGNRALPERILEAVWGFFSAYALVFIVSMLAIIATGV

DDFSAFASVVATLNNLGPGLGVVADNFTSMNPVAKWILIANMLFGRLEVFTLLVLFTPTFWRE

>gi|30043115|gb|AAP18837.1| protoporphyrin oxidase [Shigella flexneri 2a str. 2457T]

MKTLILFSTRDGQTREIASYLASELKELGIQTDVANVHRIEEPQWENYDRVVIGASIRYGHYHSAFQEFV

KKHATRLNSMPSAFYSVNLVARKPEKRTPQTNSYARKFLMNSQWRPDRCAVIAGALRYPRYRWYDRFMLK

LIMKMSGGETDTRKEVVYTDWEQVANFAREIAHLTDKPTLK

>gi|30043114|gb|AAP18836.1| molybdopterin-guanine dinucleotide biosynthesis protein B [Shigella flexneri 2a str. 2457T]

MAGKTMIPLLAFAAWSGTGKTTLLKKLIPALCARGIRPGLIKHTHHDMDVDKPGKDSYELRKAGAVQTIV

ASQQRWALMTETPDEEELDLHFLASRMDTSKLDLILVEGFKHEEIAKIVLFRDGAGHRPEELVIDRHVIA

VASDVPLNLDVALLDINDVEGLADFVVEWMQKQNG

>gi|30043113|gb|AAP18835.1| molybdopterin-guanine dinucleotide biosynthesis protein A [Shigella flexneri 2a str. 2457T]

MNLMTTITGVVLAGGKARRMGGVDKGLLDLNGKPLWQHVADALKTQLSHVVVNANRHQEIYQAGGLKVIE

DSLADYPGPLAGMLSVMQQEAGEWFLFCPCDTPYIPHDLAARLNHQRKDAPVVWVHDGERDHPTIALVNR

AIEPLLLEYLQAGERRVMAFMRLAGGHAVDFSDHKDAFVNVNTPEELARWQEKR

>gi|30043112|gb|AAP18834.1| hypothetical protein S3818 [Shigella flexneri 2a str. 2457T]

MKCKRLNEVIELLQPAWQKEPDFNLLQFLQKLAKESGFDGELADLTDDILIYHLKMRDSAKDAVIPGLQK
DYEEDFKTALLRARGVIKE

>gi|30043111|gb|AAP18833.1| hypothetical protein S3817 [Shigella flexneri 2a str. 2457T]
MNNSAFTFQTLHPDTIMDALFKQGIRVDSGLTPLNSYENRVYQFQDEERRRFVVKFYRPERWTADQILEE
HQFALQLVNDEVPVAAPVAFNGQTLLNHQGFYFAVFPSVGGRQFEADNIDQMEAVGRYLGRMHQTGRKQL
FIHRPTIGLNEYLIEPRKLFEDATLIPSGLKAAFLKATDELIAAVTAHWREDFTVLRLHGDCHAGNILWR
DGPMFVDLDDARNGPAIQDLWMLLNGDKAQQRMQLETIIEAYEEFSEFDTAEIGLIEPLRAMRLVYYLAW
LMRHWADPAFPKNFPWLTGEDYWLRQTATFIEQAKVLQEPPLQLTPMY

>gi|30043110|gb|AAP18832.1| protein disulfide isomerase I [Shigella flexneri 2a str. 2457T]
MKKIWLALAGLVLAFSASAAQYEDGKQYTTLEKPVAGAPQVLEFFSFFCPHCYQFEEVLHISDNVKKKLP
EGVKMTKYHVNFMGGDLGKDLTQAWAVAMALGVEDKVTVPLFEGVQKTQTIRSASDIRDVFINAGIKGEE
YDAAWNSFVVKSLVAQQEKAAADVQLRGVPAMFVNGKYQLNPQGMDTSNMDVFVQQYADTVKYLSEEK

>gi|30043109|gb|AAP18831.1| putative GTP-binding protein [Shigella flexneri 2a str. 2457T]
MDIQSFAVLSGNIYMIRKSATGVIVALAVIWGGGTWYTGTQIQPGIEKFIKDFNDAKKKGEHAYDMTLSY
KNFDKGFFNSRFQMQMTFDNGAPDLNIKPGQKVVFDVDVEHGPLPITMLMHGNVIPALAAAKVNLVNNEL
TQPLFIAAKNKSPVEATLRFAFGGSFSTTLDVAPAEYGKFSFGEGQFTFNGDGSSLSNLDIEGKVEDIVL
QLSPMNKVTAKSFTIDSLARLEEKKFPVGESESKFNQINIINHGEDVAQIDAFVAKTRLDRVKDKDYINV
NLTYELDKLTKGNQQLGSGEWSLIAESIDPSAVRQFIIQYNIAMQKQLAAHPELANDEVALQEVNAALFK
EYLPLLQKSEPTIKQPVRWKNALGELNANLDISIADPAKSSSSTNKDIKSLNFDVKLPLNVVTETAKQLN
LSEGMDAEKAQKQADKQISGMMTLGQMFQLITIDNNTASLQLRYTPGKVVFNGQEMSEEEFMSRAGRFVH

>gi|30043108|gb|AAP18830.1| putative endonuclease [Shigella flexneri 2a str. 2457T]

MANLLNKFIMTRILAAITLLLSIVLTILVTIFCSVPIIIAGIVKLLLPVPVIWRKVSRFCDFMMYCWCEG
LAVLLHLNPHLQWEVHGLEGLSKKNWYLLICNHRSWADIVVLCVLFRKHIPMNKYFLKQQLAWVPFLGLA
CWALDMPFMKRYSRAYLLRHPERRGQDVETTRRSCEKFRLHPTTIVNFVEGSRFTQEKHQQTHSTFQNLL
PPKAAGIAMALNVLGKQFDKLLNVTLCYPDNNRQPFFDMLSGKLTRIVVHVDLQPIADELHGDYINDKSF
KRHFQQWLNSLWQEKDRLLTSLMSSQRQNK

>gi|30043107|gb|AAP18829.1| DNA polymerase I [Shigella flexneri 2a str. 2457T]
MVQIPQNPLILVDGSSYLYRAYHAFPPLTNSAGEPTGAMYGVLNMLRSLIMQYKPTHAAVVFDAKGKTFR
DELFEHYKSHRPPMPDDLRAQIEPLHAMVKAMGLPLLAVSGVEADDVIGTLAREAEKAGRPVLISTGDKD
MAQLVTPNITLINTMTNTILGPEEVVNKYGVPPELIIDFLALMGDSSDNIPGVPGVGEKTAQALLQGLGG
LDTLYAEPEKIAGLSFRGAKTMAAKLEQNKEVAYLSYQLATIKTDVELELTCEQLEVQQPAAEELLGLFK
KYEFKRWTADVEVGKWLQAKGAKTAAKPQETSVADEAPEVTATVISYDNYVTILDEETLKAWIAKLEKAP
VFAFDTETDSLDNISANLVGLSFAIEPGVAAYIPVAHDYLDAPDQISHERALELLKPLLEDEKALKVGQN
LKYDRGILANYGIELRGIAFDTMLESYILNSVAGRHDMDSLAERWLKHKTITFEEIAGKGKNQLTFNQIA
LEEAGRYAAEDADVTLQLHLKMWPDLQKHKGPLNVFENIEMPLVPVLSRIERNGVKIDPKVLHNHSEELT
LRLAELEKKAHEIAGEEFNLSSTKQLQTILFEKQGIKPLKKTPGGAPSTSEEVLEELALDYPLPKVILEY
RGLAKLKSTYTDKLPLMINPKTGRVHTSYHQAVTATGRLSSTDPNLQNIPVRNEEGRRIRQAFIAPEDYV
IVSADYSQIELRIMAHLSRDKGLLTAFAEGKDIHRATAAEVFGLPLETVTSEQRRSAKAINFGLIYGMSA
FGLARQLNIPRKEAQKYMDLYFERYPGVLEYMERTRAQAKELGYVETLDGRRLYLPDIKSSNGARRAAAE
RAAINAPMQGTAADIIKRAMIAVDAWLQAEQPRVRMIMQVHDELVFEVHKDDVDAVAKQIHQLMENCTRL
DVPLLVEVGSGENWDQAH

>gi|30043106|gb|AAP18828.1| hypothetical protein S3811 [Shigella flexneri 2a str. 2457T]
MSAPDIRHLPSDTGIEVAFAGRSNAGKSSALNTLTNQKSLARTSKTPGRTQLINLFEVADGKRLVDLPGY
GYAEVPEEMKRKWQRALGEYLEKRQSLQGLVVLMDIRHPLKDLDQQMIEWAVDSNIAVLVLLTKADKLAS

GARKALLNMVREAVLAFNGDVQVETFSSLKKQGVDKLRQKLDTWFSEMQPVEETQDGE

>gi|30043105|gb|AAP18827.1| hypothetical protein S3810 [Shigella flexneri 2a str. 2457T]

MKPSSSNSRSKGHAKARRKTREELDQEARDRKRQKKRRGHAPGSRAAGGNTTSGSKGQNAPKDPRIGSKT

PIPLGVTEKVTKQHKPKSEKPMLSPQAELELLETDERLDALLERLEAGETLSAEEQSWVDAKLDRIDELM

QKLGLSYDDDEEEEEDEKQEDMMRLLRGN

>gi|30043104|gb|AAP18826.1| O2-independent coproporphyrinogen III oxidase [Shigella flexneri 2a str. 2457T]

MSVQQIDWDLALIQKYNYSGPRYTSYPTALEFSEDFGEQAFLQAVARYPERPLSLYVHIPFCHKLCYFCG

CNKIVTRQQHKADQYLDALEQEIVHRAPLFAGRHVSQLHWGGGTPTYLNKAQISRLMKLLRENFHFNTDA

EISIEVDPREIELDVLDHLRAEGFNRLSMGVQDFNKEVQRLVNREQDEEFIFALLNHAREIGFTSTNIDL

IYGLPKQTPESFAFTLKRVAELNPDRLSVFNYAHLPTIFAAQRKIKDADLPSPQQKLDILQETIAFLTQS

GYQFIGMDHFARPDDELAVAQREGVLHRNFQGYTTQGDTDLLGMGVSAISMIGDCYAQNQKELKQYYQQV

YEQGNALWRGIALTRDDCIRRDVIKSLICNFRLDYAPLEKQWDLHFADYFAEDLKLLAPLAKDGLVDVDE

KGIQVTAKGRLLIRNICMCFDTYLRQKARMQQFSRVI

>gi|30043103|gb|AAP18825.1| sensor glnL response regulator (nitrogen regulator I, NRI) [Shigella flexneri 2a str. 2457T]

MQRGIVWVVDDDSSIRWVLERALAGAGLTCTTFENGAEVLEALASKTPDVLLSDIRMPGMDGLALLKQIK

QRHPMLPVIIMTAHSDLDAAVSAYQQGAFDYLPKPFDIDEAVALVERAISHYQEQQQPRNIQLNGPTTDI

IGEAQAMQDVFRIIGRLSRSSISVLINGESGTGKELVAHALHRHSPRTKAPFIALNMAAIPKDLIESELF

GHEKGAFTGANTIRQGRFEQADGGTLFLDEIGDMPLDVQTRLLRVLADGQFYRVGGYAPVKVDVRIIAAT

HQNLEQRVQEGKFREDLFHRLNVIRVHLPPLRERREDIPRLARHFLQVAARELGVEAKLLHPETEAALTR

LAWPGNVRQLENTCRWLTVMAAGQEVLIQDLPGELFESTVAESTSQMQPDSWATLLAQWADRALRSGHQN

LLSEAQPELERTLLTTALRHTQGHKQEAARLLGWGRNTLTRKLKELGME

>gi|30043102|gb|AAP18824.1| histidine protein kinase sensor for GlnG regulator (nitrogen regulator II, NRII) [Shigella flexneri 2a str. 2457T]

MATGTQPDAGQILNSLINSILLIDDNLAIHYANPAAQQLLAQSSRKLFGTPLPELLSYFSLNIELMQESL

EAGQGFTDNEVTLVIDGRSHILSVTAQRMPDGMILLEMAPMDNQRRLSQEQLQHAQQVAARDLVRGLAHE

IKNPLGGLRGAAQLLSKALPDPSLLEYTKVIIEQADRLRNLVDRLLGPQLPGTRVTESIHKVAERVVTLV

SMELPDNVRLIRDYDPSLPELAHDPDQIEQVLLNIVRNALQALGPEGGEIILRTRTAFQLTLHGERYRLA

ARIDVEDNGPGIPPHLQDTLFYPMVSGREGGTGLGLSIARNLIDQHSGKIEFTSWPGHTEFSVYLPIRK

>gi|30043101|gb|AAP18823.1| glutamine synthetase [Shigella flexneri 2a str. 2457T]

MSAEHVLTMLNEHEVKFVDLRFTDTKGKEQHVTIPAHQVNAEFFEEGKMFDGSSIGGWKGINESDMVLMP

DASTAVIDPFFADSTLIIRCDILEPGTLQGYDRDPRSIAKRAEDYLRSTGIADTVLFGPEPEFFLFDDIR

FGSSISGSHVAIDDIEGAWNSSTQYEGGNKGHRPAVKGGYFPVPPVDSAQDIRSEMCLVMEQMGLVVEAH

HHEVATAGQNEVATRFNTMTKKADEIQIYKYVVHNVAHRFGKTATFMPKPMFGDNGSGMHCHMSLSKNGV

NLFAGDKYAGLSEQALYYIGGVIKHAKAINALANPTTNSYKRLVPGYEAPVMLAYSARNRSASIRIPVVS

SPKARRIEVRFPDPAANPYLCFAALLMAGLDGIKNKIHPGEAMDKNLYDLPPEEAKEIPQVAGSLEEALN

ELDLDREFLKAGGVFTDEAIDAYIALRREEDDRVRMTPHPVEFELYYSV

>gi|30043100|gb|AAP18822.1| putative GTP-binding factor [Shigella flexneri 2a str. 2457T]

MIEKLRNIAIIAHVDHGKTTLVDKLLQQSGTFDSRAETQERVMDSNDLEKERGITILAKNTAIKWNDYRI

NIVDTPGHADFGGEVERVMSMVDSVLLVVDAFDGPMPQTRFVTKKAFAYGLKPIVVINKVDRPGARPDWV

VDQVFDLFVNLDATDEQLDFPIVYASALNGIAGLDHEDMAEDMTPLYQAIVDHVPAPDVDLDGPFQMQIS

QLDYNSYVGVIGIGRIKRGKVKPNQQVTIIDSEGKTRNAKVGKVLGHLGLERIETDLAEAGDIVAITGLG

ELNISDTVCDTQNVEALPALSVDEPTVSMFFCVNTSPFSGKEGKFVTSRQILDRLNKKLVHNVALRVEET

EDADAFRVSGRGELHLSVLIENMRREGFELAVSRPKVIFREIDGRKQEPYENVTLDVEEQHQGSVMQALG

ERKGDLKNMNPDGKGRVRLDYVIPSRGLIGFRSEFMTMTSGTGLLYSTFSHYDDVRPGEVGQRQNGVLIS

NGQGKAVAFALFGLQDRGKLFLGHGAEVYEGQIIGIHSRSNDLTVNCLTGKKLTNMRASGTDEAVVLVPP

IRMTLEQALEFIDDDELVEVTPTSIRIRKRHLTENDRRRANRAPKDD

>gi|30043099|gb|AAP18821.1| putative transcriptional regulator [Shigella flexneri 2a str. 2457T]

MAENQSTVENAKEKLDRWLKDGITTPGGKLPSERELGELLGIKRMTLRQALLNLEAESKIFRKDRKGWFV

TQPRFNYSPELSASFQRAAIEQGREPSWGFTEKNRTSDIPETLAPLIAVTPSTELYRITGWGALEGHKVF

YHETYINPEVAPGFIEQLENHSFSAVWEKCYQKETVVKKLIFKPVRMPGDISKYLGGSAGMPAILIEKHR

ADQQGNIVQIDIEYWRFEAVDLIINL

>gi|30043098|gb|AAP18820.1| hypothetical protein S3803 [Shigella flexneri 2a str. 2457T]

MVTINNARKILQRVDTLPLYLHAYAFHLNMRLERVLPADLLDIASENNLRGVKIHVLDGERFSLGNMDDK

ELSAFGDKARRLNLDIHIETSASDKASIDEAVAIALKTGATSVRFYPRYEGNLRDVLSIIANDIAYVRET

YQDSGLTFTIEQHEDLKSHELVSLVKESEMESLSLLFDFANMINANEHPIDALKTMAPHITQVHIKDALI

VKEQGGLGHKACISGQGDMPFKALLTHLICLGDDEPQVTAYGLEEEVDYYASAFRFEDEDDNPWIPYRQM

SETPLPENHLLDARLRKEKEDAINQINHVRNVLQQIKQEANHLLNH

>gi|30043097|gb|AAP18819.1| putative resistance protein [Shigella flexneri 2a str. 2457T]

MLTKKKWALFSLLTLCGGTIYKLPSLKDAFYIPMQEYFHLTNGQIGNAMSVNSFVTTVGFFLSIYFADKL

PRRYTMSFSLIATGLLGVYLTTMPGYWGILFVWALFGVTCDMMNWPVLLKSVSRLGNSEQQGRLFGFFET

GRGIVDTVVAFSALAVFTWFGSGLLGFKAGIWFYSLIVIAVGIIIFFVLNDKEEAPSVEVKKEDGASQNT

SMTSVLRDKTIWLIAFNVFFVYAVYCGLTFFIPFLKNIYLLPVALVGAYGIINQYCLKMIGGPIGGMISD

KILKSPSKYLCYTFIISTAALVLLIMLPHESMPVYLGMACTLGFGAIVFTQRAVFFAPIGEAKIGNAANL

LI

>gi|30043096|gb|AAP18818.1| IS1 orfA [Shigella flexneri 2a str. 2457T]

MASISIRCPSCSATEGVVRNGKSTAGHQRYLCSHCRKTWQLQFTYTASQPGTHQKIIDMAMNGVGCRASA
RIMGVGLNTVLRHLKNSGRSR

>gi|30043095|gb|AAP18817.1| IS1 orfB [Shigella flexneri 2a str. 2457T]
MSRQCTHYGRWPQHGFTSLKKLRPQSVTSRIQPGSDVIVCAEMDEHWGYVGAKSRQRWLFYAYDRIRRTV
VAHVFGERTLATLERLLSLLSAFEVVVWMTDGWPLYESRLKGKLHVISKRYTQRIERHNLNLRQHLARLG
RKSLSFSKSVELHDKVIGHYLNIKHYQ

>gi|30043094|gb|AAP18816.1| putative permease [Shigella flexneri 2a str. 2457T]
MSDHNPLTLKLNLREKIAYGMGDVGSNLMLCIGTLYLLKLYTDELGMPAYYGGIIFLVAKFFTAFTDMLT
GFLLDSRKNIGPKGKFRPFILYAAVPAALIATLQFIATTFCLPVKTTIATTLFMMFGLSYSLMNCSYGAM
IPAITKNPNERAQLAAYRQGGATIGLLICTVAFIPLQSLFSDSTVGYACAALMFSIGGFIFMMLCYRGVK
EHYVDTAPTGHKASILKSFCAIFRNPPLLVLCIANLCTLAAFNIKLAIQVYYTQYVLNDINLLSWMGFFS
MGCILVGVLLVPVTVKCFGKKQVYLAGMVLWAVGDILNYFWGSNSFTFVMFSCVAFFGTAFVNSLNWALV
PDTVDYGEWKTGIRAEGSVYTGYTFFRKISAALAGFLPGIMLTQIGYVPNIAQSDATLQGLRQLIFIWPC
ALAIIAALTMGFFYTLNEKRFALIIEEINQRKNKEMATEKKTASVTL

>gi|30043093|gb|AAP18815.1| putative glycosidase [Shigella flexneri 2a str. 2457T]
MDTPRPQLLDFQFHQNNDSFTLHFQQRLILTHNKDNPCLWIGSGIADIDMFRGNFSIKDKLQEKIALTDA
IVSQSPDGWLIHFSRGSDISATLNISADDQGRLLLELQNDNLNHNRIWLRLAAQPEDHIYGCGEQFSYFD
LRGKPFPLWTSEQGVGRNKQTYVTWQADCKENAGGDYYWTFFPQPTFVSTQKYYCHVDNSCYMNFDFSAP
EYHELALWEDKATLRFECADTYISLLEKLTALLGRQPELPDWIYDGVTLGIQGGTEVCQKKLDTMRNAGV
KVNGIWAQDWSGIRITSFGKRVMWNWKWNSENYPQLDSRIKQWYQEGVQFLAYINPYVASDKDLCEEAAQ
HGYLAKDASGGDYLVEFGEFYGGVVDLTNPEAYAWFKEVIKKNMIELGCGGWMADFGEYLPTDTYLHNGV
SAEIMHNAWPALWAKCNYEALEETGKLGEILFFMRAGSTGSQKYSTMMWAGDQNVDWSLDDGLASVVPAA

LSLAMTGHGLHHSDIGGYTTLFEMKRSKELLLRWCDFSAFTPMMRTHEGNRPGDNWQFDGDAETIAHFAR
MTTVFTTLKPYLKEAVALNAKSGLPVMRPLFLHYEDDAHTYSLKYQYLLGRDILVAPVHEEGRSDWTLYL
PEDNWVHAWTGEAFRGGEVTVNAPIGKPPVFYRADSEWAALFASLNSI

>gi|30043092|gb|AAP18814.1| putative aldose-1-epimerase [Shigella flexneri 2a str. 2457T]
MQITNMHCSGQTVSLAAGDYHATIVTVGAGLAELTFQGCHLVIPHKPEEMPLAHLGKVLIPWPNRIANGC
YRYQGQEYQLPINEHSSKAAIHGLLAWRDWQISELTTTSVTLTAFLPPSYGYPFMLASQVVYSLNAHTGL
SVEIASQNIGTVAAPYGVGIHPYLTCNLTSVDEYLFQLPANQVYAVDEHVNPTTLHHVDELDLNFTQAKK
IAATKIDHTFKTANDLWEMTITHPQQALSVSLCSDQPWVQVYSGEKLQRQGLAVEPMSCPPNAFNSGIDL
LLLEPGKPHRLFFNIYGQRK

>gi|30043091|gb|AAP18813.1| hypothetical protein S3794 [Shigella flexneri 2a str. 2457T]
MKWFNTLSHNRWLEQETDRIFDFGKNSVVPTGFGWLGNKGQIKEEMGTHLWITARMLHVYSVAAAMGRPG
AYALVDHGIKAMNGALRDKKYGGWYACVNDEGVVDASKQGYQHFFALLGAASAVTTGHPEARKLLDYTIE
IIEKYFWSEEEQMCLESWDEAFSKTEEYRGGNANMHAVEAFLIVYDVTHDKKWLDRAIRVASVIIHDVAR
NNHYRVNEHFDTQWNPLPDYNKDNPAHRFRAFGGTPGHWIEWGRLMLHIHAALEARCEQPPAWLLEDAKG
LFNATVRDAWAPDGADGIVYTVDWEGKPVVRERVRWPIVEAMGTAYALYTVTGDRQYETWYQTWWEYCIK
YLMDYENGSWWQELDADNKVITKVWDGKQDIYHLLHCLVIPRIPLAPGMAPAVAAGLLDINAK

>gi|30043090|gb|AAP18812.1| putative aldolase [Shigella flexneri 2a str. 2457T]
MNKYTINDITRASGGFAMLAVDQREAMRMMFAAAGAPAPVADSVLTDFKVNAAKALSPYASAILVDQQFC
YRQVVEQNAIAKSCAMIVAADEFIPGNGIPVDSVVIDRKINPLQIKQDGGKALKLLVLWRSDEDAQQRLD
MVKEFNELCHSHGLVSIIEPVVRPPRRGDKFDREQAIIDAAKELGDSGADLYKVEMPLYGKGPQQELLCA
SQRLNDHINMPWVILSSSVDEKLFPRAVRVAMTAGASGFLAGRAVWASVVGLPDNELMLRDVCAPKLQQL
GDIVDEMMAKRR

>gi|30043089|gb|AAP18811.1| putative dehydrogenase [Shigella flexneri 2a str. 2457T]

MAAIAFIGLGQMGSPMASNLLQQGHQLRVFDVNAEAVRHLVDKGATPAANPAQAAKDAEFIITMLPNGDL

VRNVLFGENGVCEGLSTDALVIDMSTIHPLQTDKLIADMQAKGFSMMDVPVGRTSANAITGTLLLLAGGT

AEQVERATPILMAMGSELINAGGPGMGIRVKLINNYMSIALNALSAEAAVLCEALNLPFDVAVKVMSGTA

AGKGHFTTSWPNKVLSGDLSPAFMIDLAHKDLGIALDVANQLHVPMPLGAASREVYSQARAAGRGRQDWS

AILEQVRVSAGMTAKVKM

>gi|30043088|gb|AAP18810.1| putative kinase [Shigella flexneri 2a str. 2457T]

MIRVACVGITVMDRIYYVEGLPTESGKYVARNYTEVGGGPAATAAVAAARLGAQVDFIGRVGDDDAGNSL

LAELESWGVNTRYTKRYNQAKSSQSAIMVDTKGERIIINYPSPDLLPDAEWLEEIDFSQWDVVLADVRWH

DGAKKAFTLARQAGVMTVLDGDITPQDISELVALSDHAAFSEPGLARLTGVKEMASALKQAQTLTNGHVY

VTQGSAGCDWLENGGRQHQPAFKVDVVDTTGAGDVFHGALAVALATSGDLAESVRFASGVAALKCTRPGG

RAGIPDCDQTRSFLSLFV

>gi|30043087|gb|AAP18809.1| putative DEOR-type transcriptional regulator [Shigella flexneri 2a str. 2457T]

MSLTELTGNPRHDQLLMLIAERGYMNIDELANLLDVSTQTVRRDIRKLSEQGLITRHHGGAGRASSVVNT

AFEQREVSQTEKKKAIAEAVADYIPDGSTIFITIGTTVEHVARALLNHNHLRIITNSLRVAHILYHNPRF

EVMVPGGTLRSHNSGIIGPSAASFVADFRADYLVTSVGAIESDGALMEFDVNEANVVKTMMAHARNILLV

ADHTKYHASAAVEIGNVAQVTALFTDELPPAALKSRLQDSQIEIILPQEDA

>gi|30043086|gb|AAP18808.1| putative phosphatase [Shigella flexneri 2a str. 2457T]

MLYIFDLGNVIVDIDFNRVLGAWSDLTRIPLASLKKSFHMGEAFHQHERGEISDEAFAEALCHEMALPLS

YEQFSHGWQAVFVALRPEVIAIMHKLREQGHRVVVLSNTNRLHTTFWPEEYPEIRDAADHIYLSQDLGMR

KPEARIYQHVLQAEGFSPSDTVFFDDNADNIEGANQLGITSILVKDKTTIPDYFAKVLC

>gi|30043085|gb|AAP18807.1| tRNA processing exoribonuclease BN [Shigella flexneri 2a str. 2457T]

MLKTIQDKARHRTRPLWAWLKLLWQRIDEDNMTTLAGNLAYVSLLSLVPLVAVVFALFAAFPMFSDVSIQ

LRHFIFANFLPATGDVIQRYIEQFVANSNKMTAVGACGLIVTALLLMYSIDSALNTIWRSKRARPKIYSF

AVYWMILTLGPLLAGASLAISSYLLSLRWASDLNTVIDNVLRIFPLLLSWISFWLLYSIVPTIRVPNRDA

IVGAFVAALLFEAGKKGFALYITMFPSYQLIYGVLAVIPILFVWVYWTWCIVLLGAEITVTLGEYRKLKQ

AAEQEEDDEP

>gi|30043084|gb|AAP18806.1| hypothetical protein S3787 [Shigella flexneri 2a str. 2457T]

MIALIQRVTRASVTVEGEVTGEIGAGLLVLLGVEKDDDEQKANRLCERVLGYRIFSDAEGKMNLNVQQAG

GSVLVVSQFTLAADTERGMRPSFSKGASPDRAEALYDYFVERCRQQEMNTQTGRFAADMQVSLVNDGPVT

FWLQV

>gi|30043083|gb|AAP18805.1| putative acetyltransferase [Shigella flexneri 2a str. 2457T]

MSQLPGLSRETRESIAMYHLQVPQTEEELERYYHFRWEMLRKPLHQPKGSERDAWDAMAHHQMVVDEQGN

LVAVGRLYINADNEASIRFMVVHPDVQDKGLGTLMAMTLESVARQEGVKRVTCSAREDAVEFFAKLGFVN

QGEITTPTTTPIRHFLMIKPVATLDDILHRGDWCAQLQQAWYEHIPLSEKMGVRIQQYTGQKFITTMPET

GNQNPHHTLFAGSLFSLATLTGWGLIWLMLRERHLGGTIILADAHIRYSKPISGKPHAVADLGALSGDLD

RLARGRKARVQMQVEIFGDETPGAVFEGTYIVLPAKPFGPYEEGGNEEE

>gi|30043082|gb|AAP18804.1| IS1 orfB [Shigella flexneri 2a str. 2457T]

MSRQCTHYGRWPQHGFTSLKKLRPQSVTSRIQPGSDVIVCAEMDEHWGYVGAKSRQRWLFYAYDRIRRTV

VAHVFGERTLATLERLLSLLSAFEVVVWMTDGWPLYESRLKGKLHVISKRYTQRIERHNLKLRQHLARLG

RKSLSFSKSVELHDKVIGHYLNIKHYQ

>gi|30043081|gb|AAP18803.1| IS1 orfA [Shigella flexneri 2a str. 2457T]

MASISIRCPSCSATEGVVRNGKSTAGHLRYLCSHCRKTWQLQFTYTASQPGTHQKIIDMAMNGVGCRASA

RIMGVGLNTVLRHLKNSGRSR

>gi|30043080|gb|AAP18802.1| hypothetical protein S4828 [Shigella flexneri 2a str. 2457T]

MTGTVYKLFGNRLLNARNQLDKNLLEMAMQMVYGKDMVTKSGDILIDIQLNKDGSLQSKHYTLDVGEVIN

SYNIDPLCECFKY

>gi|30043079|gb|AAP18801.1| hypothetical protein S4827 [Shigella flexneri 2a str. 2457T]

MCERMKGGISMFNHTTKTADNIEHNLQLSFLVGSRPSITNKYSAKDVGPDILALPVNVYHADFKIKINNA

VEKASSIVLCAVQKNLHAARKVLSGIIGNVPKNNKPKTIIFDLRFLSAIKQKVFLGSEKPLFVKHTAMMR

EACKELPQSVEYVPLACEAHSHRSVALAKTVAAPIMATGVGLALMYATSSGPYYAQSLSGSPDIEMIKNR

MAELFNQSDISVRNKYSPAFDKWNELVDKLNHERNTVPFACLTTILATAINETLEGDTNVAVVMGCKSAK

DRTISIVLGNSMLQTLLEKRLADSGEIENLFDQQGYFSCDSLTPEELMMLKDLFDIRVLHVSNKFNVGLQ

GNINTDVLQDSFFKNVDFIHEYNSYTVGMGV

>gi|30043078|gb|AAP18800.1| hypothetical protein S3783 [Shigella flexneri 2a str. 2457T]

MSNICKVVVMAMNTVFLHLSEEAIKRLNKLRGWRKVSRSAILREAVEQYLERQQFPVRKAKGGRQKGEVV

GVDDQCKEHKE

>gi|30043077|gb|AAP18799.1| hypothetical protein S3782 [Shigella flexneri 2a str. 2457T]

MNSLAGIDMGRILLDLSNEVIKQLDDLEVQRNLPRADLLREAVDQYLINQSQTARTSVPGIWQGCEEDGV

EYQRKLREEW

>gi|30043076|gb|AAP18798.1| FdhE protein [Shigella flexneri 2a str. 2457T]

MSIRIIPQDELGSSEKRTADMIPPLLFPRLKNLYNRRAERLRELAENNPLGDYLRFAALIAHAQKVVLYD

HPLEMDLTARIKEASAQGKPPLDIHVLPRDKHWQKLLMALIAELKPEMSGPALAVIENLEKASTQELEDM

ASALFASDFSSVSSDKAPFIWAALSLYWAQMANLIPGKARAEYGEQRQYCPVCGSMPVSSMVQIGTTQGL

RYLHCNLCETEWHVVRVKCSNCEQSGKLHYWSLDDEQAAIKAESCDDCGTYLKILYQEKEPKVEAVADDL

ASLVLDARMEQEGYARSSINPFLFPGEGE

>gi|30043075|gb|AAP18797.1| formate dehydrogenase, cytochrome B556 (FDO) subunit [Shigella flexneri 2a str. 2457T]

MKRRDTIVRYTAPERINHWITAFCFILAAVSGLGFLFPSFNWLMQIMGTPQLARILHPFVGVVMFASFII

MFFRYWHQNLINRDDIFWAKNIRKIVVNEEVGDTGRYNFGQKCVFWAAIIFLVLLLVSGVIIWRPYFAPA

FSIPVIRFALMLHSFAAVALIVVIMVHIYAALWVKGTITAMVEGWVTSAWAKKHHPRWYREVRKTTEKKA

E

>gi|30043074|gb|AAP18796.1| formate dehydrogenase-O, iron-sulfur subunit [Shigella flexneri 2a str. 2457T]

MAYQSQDIIRRSATNGLTPAPQARDFQEEVAKLIDVTTCIGCKACQVACSEWNDIRDTVGNNIGVYDNPN

DLSAKSWTVMRFSEVEQNDKLEWLIRKDGCMHCSDPGCLKACPAEGAIIQYANGIVDFQSEQCIGCGYCI

AGCPFDIPRLNPEDNRVYKCTLCVDRVVVGQEPACVKTCPTGAIHFGTKESMKTLASERVAELKTRGYDN

AGLYDPAGVGGTHVMYVLHHADKPNLYHGLPENPEISETVKFWKGIWKPLAAVGFAATFAASIFHYVGVG

PNRADEEENNLHEEKDEERK

>gi|30043073|gb|AAP18795.1| formate dehydrogenase-O, major subunit [Shigella flexneri 2a str. 2457T]

MQVSRRQFFKICAGGMAGTTAAALGFAPSVALAETRQYKLLRTRETRNTCTYCSVGCGLLMYSLGDGAKN

AKASIFHIEGDPDHPVNRGALCPKGAGLVDFIHSESRLKFPEYRAPGSDKWQQISWEEAFDRIAKLMKED

RDANYIAQNAEGVTVNRWLSTGMLCASASSNETGYLTQKFSRALGMLAVDNQARVUHGPTVASLAPTFGR

GAMTNHWVDIKNANLVVVMGGNAAEAHPVGFRWAMEAKIHNGAKLIVIDPRFTRTAAVADYYAPIRSGTD

IAFLSGVLLYLLNNEKFNHEYTEAYTNASLIVREDYGFEDGLFTGYDAEKRKYDKSSWTYELDENGFAKR
DTTLQHPRCVWNLLKQHVSRYTPDVVENICGTPKDAFLKVCEYIAETSAHDKTASFLYALGWTQHSVGAQ
NIRTMAMIQLLLGNMGMAGGGVNALRGHSNIQGLTDLGLLSQSLPGYMTLPSEKQTDLQTYLTANTPKPL
LEGQVNYWGNYPKFFVSMMKAFFGDKATAENSWGFDWLPKWDKGYDVLQYFEMMKEGKVNGYICQGFNPV
ASFPNKNKVIGCLSKLKFLVTIDPLNTETSNFWQNHGELNEVDSSKIQTEVFRLPSTCFAEENGSIVNSG
RWLQWHWKGADAPGIALTDGEILSGIFLRLRKMYAEQGGANPDQVLNMTWNYAIPHEPKSEEVAMESNGK
ALADITDPATGAVIVKKGQQLSSFAQLRDDGTTSCGCWIFAGSWTPEGNQMARRDNADPSGLGNTLGWAW
AWPLNRRILYNRASADPQGNPWDPKRQLLKWDGTKWTGWDIPDYSAAPPGSGVGPFIMQQEGMGRLFALD
KMAEGPFPEHYEPFETPLGTNPLHPNVISNPAARIFKDDAEALGKADKFPYVGTTYRLTEHFHYWTKHAL
LNAILQPEQFVEIGESLANKLGIAQGDTVKVSSNRGYIKAKAVVTKRIRTLKANGKDIDTIGIPIHWGYE
GVAKKGFIANTLTPFVGDANTQTPEFKSFLVNVEKV

>gi|30043072|gb|AAP18794.1| FdhD protein [Shigella flexneri 2a str. 2457T]
MKKTQRKEIENVTNITGVRQIELWRRDDLQHPRLDEVAEEVPVALVYNGISHVVMMASPKDLEYFALGFS
LSEGIIESPRDIFGMDVVPSCNGLEVQIELSSRRFMGLKERRRALAGRTGCGVCGVEQLNDIGKPVQPLP
FTQTFDLNKLDDALRHLNDFQPVGRLTGCTHAAAWMLPSGELVGGHEDVGRHVALDKLLGRRSQEGESWQ
QGAVLVSSRASYEMVQKSAMCGVEILFAVSAATTLAVEVAERCNLTLVGFCKPGRATVYTHPQRLSN

>gi|30043071|gb|AAP18793.1| IS1 orfB [Shigella flexneri 2a str. 2457T]
MSRQCTHYGRWPQHGFTSLKKLRPQSVTSHIQPGSDVIVCAEMDEHWGYVGAKSRQRWLFYAYDRIRRTV
VAHVFGERTLATLERLLSLLSAFEVVVWMTDGWPLYESRLKGKLHVISKRYTQRIERHNLNLRQHLARLG
RKSLSFSKSVELHDKVIGHYLNIKHYQ

>gi|30043070|gb|AAP18792.1| IS1 orfA [Shigella flexneri 2a str. 2457T]
MASISIRCPSCSATEGVVRNGKSTAGHQRYLCSHCRKTWQLQFTYTASQPDTHQKIIDMAMNGVGCRASA

RIMGVGLNTVLRHLKNSGRSR

>gi|30043069|gb|AAP18791.1| putative frv operon regulatory protein [Shigella flexneri 2a str. 2457T]

MLNERQLKIVDLLEQQPRTPGELAQQTGVSGRTILRDIDYLNFTLNGKARIFASGSAGYQLEIFERRSFF

QLLQKHDNDDRLLALLLLNTFTPRAQLASALNLPETWVAERLPQLKQRYERTCCLASRPGLGHFIDETEE

KLVILLANLLRKDPFLIPLAGITRDNLQHLSTACDNQHRWPLIQGDYLSSLILAIYALRNQLTDEWPQYP

VMLPTY

>gi|30043068|gb|AAP18790.1| hypothetical protein in frv operon [Shigella flexneri 2a str. 2457T]

MNIELLQQLCEASAVSGDEQEVRDILINTLEPCVNEITFDGLGSFVARKGNKGPKVAVVGHMDEVGFMVT

HIDESGFLRFTTIGGWWNQSMLNHRVTIRTHKGVKIPGVIGFVAPHALTEKQRQQPLSFDEMFIDIGANS

RDEVEKRGVAMGDFISPEANFACWGEDKVVGKALDNRIGCAMMAELLQTVNNPEITLYGVGSVEEEVGLR

GAQTSAEHIKPDVVIVLDTAVAGDVPGIDNIKYPLKLGQGPGLMLFDKRYFPNQKLVAALKNCAAHNDLP

LQFSTMKTGATDGGRYNVMGGGRPVVALCLPTRYLHANSGMISKADYDALLTLIRGFLTTLTAEKVNAFS

QFRQVD

>gi|30043067|gb|AAP18789.1| PTS system, fructose-like enzyme IIBC component [Shigella flexneri 2a str. 2457T]

MESSLRIVAITNCPAGIAHTYMVAEALEQKARSLGHTIKVETQGSSGVENRLSSEEIAAADYVILATGRG

LSGDDRARFAGKKVYEIAISLALKNIDQIFSELPTNSQLFAADSGVKLGKQEVQSGSVMSHLMAGVSAAL

PFVIGGGILVALANMLVQFGLPYTDMSKGAPSFTWVVESIGYLGFTFMIPIMGAYIASSIADKPAFAPAF

LVCYLANDKALLGTQSGAGFLGAVVLGLAIGYFVFWFRKVRLGKALQPLLGSMLIPFVTLLVFGVLTYYV

IGPVMSDLMGGLLHFLNTIPPSMKFAAAFLVGAMLAFDMGGPINKTAWFFCFSLLEKHIYDWYAIVGVVA

LMPPVAAGLATFIAPKLFTRQEKEAASSAIVVGATVATEPAIPYALAAPLPMITANTLAGGITGVLVIAF

GIKRLAPGLGIFDPLIGLMSPVGSFYLVLAIGLALNISFIIVLKGLWLRRKAKAAQQELVHEH

>gi|30043066|gb|AAP18788.1| PTS system, fructose-specific IIA component [Shigella flexneri 2a str. 2457T]

MAALTASCIDLNIQGNGAYSVLKQLATIALQNGFITDSHQFLQTLLLREKMHSTGFGSGVAVPHGKSACV

KQPFVLFARKAQAIDWKASDGEDVNCWICLGVPQSGEEDQVKIIGTLCRKIIHQEFIHQLQQGDTDQVLA

LLNQTLSS

>gi|30043065|gb|AAP18787.1| hypothetical protein S3770 [Shigella flexneri 2a str. 2457T]

MIRKAFVMQVNPDAHEEYQRRHNPIWPELEAVLKSHGVHNYAIYLDKARNLLFAMVEIESEERWNAVAST

DVCQRWWKYMTDVMPANPDNSPVSSELQEVFYLP

>gi|30043064|gb|AAP18786.1| rhamnulose-phosphate aldolase [Shigella flexneri 2a str. 2457T]

MPLLANTPFIVTGSGKFFRNVQLDPAANLGVVKVDSDGAGYHILWGLTNEAVPTSELPAHFLSHCERIKA

TNGKDRVIMHCHATNLIALTYVLENDTAVFTRQLWEGSTECLVVFPDGVGILPWMVPGTDEIGQATAQEM

QKHSLVLWPFHGVFGSGPTLDEAFGLIDTAEKSAQVLVKVYSMGGMKQTISREELIALGKRFGVTPLASA

LAL

>gi|30043063|gb|AAP18785.1| L-rhamnose isomerase [Shigella flexneri 2a str. 2457T]

MTTQLEQAWELAKQRFAAVGIDVEEALRQLDRLPVSMHCWQGDDVSGFENPEGSLTGGIQATGNYPGKAR

NASELRADLEQAMRLIPGPKRLNLHAIYLESDTPVSRDQIKPEHFKNWVEWAKANQLGLDFNPSCFSHPL

SADGFTLSHPDDSIRQFWIDHCKASRRVSAYFGEQLGTPSVMNIWIPDGMKDITVDRLAPRQRLLAALDE

VISEKLNPAHHIDAVESKLFGIGAESYTVGSNEFYMGYATSRQTALCLDAGHFHPTEVISDKISAAMLYV

PQLLLHVSRPVRWDSDHVVLLDDETQAIASEIVRHDLFDRVHIGLDFFDASINRIAAWVIGTRNMKKALL

RALLEPTAELRKLEAAGDYTARLALLEEQKSLPRQAVWEMYCQHHDTPAGSEWLESVRAYEKAILSQRG

>gi|30043062|gb|AAP18784.1| rhamnulokinase [Shigella flexneri 2a str. 2457T]

MTFRNCVAVDLGASSGRVMLARYERECRSLTLREIHRFNNGLHSQNGYVTWDVDSLESAIRLGLNKVCEE

GIRIDSIGIDTWGVDFVLLDQQGQRVGLPVAYRDSRTNGLMPQAQQQLGKRDIYQRSGIQFLPFNTLYQL

RALTEQQPELIPHIAHALLMPDYFSYRLTGKMNWEYTNATTTQLVNINSDDWDESLLAWSGANKAWFGRP

THPGNVIGHWICPQGNEIPVVAVASHDTASAVIASPLNGSRAAYLSSGTWSLMGFESQTPFTNDTALAAN

ITNEGGAEGRYRVLKNIMGLWLLQRVLQERQINDLPALIAATQALPACRFIINPNDDRFINPDEMCSEIQ

AACRETAQPIPGSDAELARCIFDSLALLYADVLHELAQLRGEDFSQLHIVGGGCQNTLLNQLCADACGIR

VIAGPVEASTLGNIGIELMTLDELNNVDDFRQVVSTTANLTTFTPNPDSEIAHYVAQIHSTRQTKELCA

>gi|30043061|gb|AAP18783.1| positive regulator for rhaBAD operon [Shigella flexneri 2a str. 2457T]

MTVLHSVDFFPSCNASVAIEPRLPQADFPEHHHDFHEIVIVEHGTGIHVFNGQPYTITGGTVCFVRDHDR

HLYEHTDNLCLTNVLYRSPDRFQFLAGLNQLLPQELDGQYPSHWRVNHSVLQQVRQLVAQMEQQEGENDL

PSTASREILFMQLLLLLRKSSLQENLENSASRLNLLLAWLEDHFADEVNWDAVADQFSLSLRTLHRQLKQ

QTGLTPQRYLNRLRLMKARHLLRHSEASVTDIAYRCGFSDSNHFSTLFRREFNWSPRDIRQGRDGFLQ

>gi|30043060|gb|AAP18782.1| positive regulator for rhaRS operon [Shigella flexneri 2a str. 2457T]

MAFCNNANLLNVFVRHIANNQLRSLAEVATVAHQLKLLKDDFFASDQQAVAVADRYPQDVFAEHTHDFCE

LVIVWRGNGLHVLNDRPYRITRGDLFYIHADDKHSYASVNDLVLQNIIYCPERLKLNLDWQGAIPGFSAS

AGQPHWRLGSVGMAQARQVIGQLEHESSQHVPFANEMAELLFGQLVMLLNRHRYTSDSLPPTSSETLLDK

LITRLAASLKSPFALDKFCDEASCSERVLRQQFRQQTGMTINQYLRQVRVCHAQYLLQHSRLLISDISTE

CGFEDSNYFSVVFTRETGMTPSQWRHLNSQKD

>gi|30043059|gb|AAP18781.1| superoxide dismutase, manganese [Shigella flexneri 2a str. 2457T]

MSYTLPSLPYAYDALEPHFDKQTMEIHHTKHHQTYVNNANAALESLPEFANLPVEELITKLDQLPADKKT

VLRNNAGGHANHSLFWKGLKKGTTLQGDLKAAIERDFGSVDNFKAEFEKAAASRFGSGWAWLVLKGDKLA

VVSTANQDSPLMGEAISGASGFPILGLDVWEHAYYLKFQNRRPDYIKEFWNVVNWDEAAARFAAKK

>gi|30043058|gb|AAP18780.1| 2-keto-3-deoxy-D-gluconate transport protein [Shigella flexneri 2a str. 2457T]

MQIKRSIEKIPGGMMLVPLFLGALCHTFSPGAGKYFGSFTNGMITGTVPILAVWFFCMGASIKLSATGTV

LRKSGTLVVTKIAVAWVVAAIASRIIPEHGVEVGFFAGLSTLALVAAMDMTNGGLYASIMQQYGTKEEAG

AFVLMSLESGPLMTMIILGTAGIASFEPHVFVGAVLPFLVGFALGNLDPELREFFSKAVQTLIPFFAFAL

GNTIDLTVIAQTGLLGILLGVAVIIVTGIPLIIADKLIGGGDGTAGIAASSSAGAAVATPVLIAEMVPAF

KPMAPAATSLVATAVIVTSILVPILTSIWSRKVKARAAKIEILGTVK

>gi|30043057|gb|AAP18779.1| IS2 orfB [Shigella flexneri 2a str. 2457T]

MARGWGVSLVSRCLRVSRAQLHVILRRTDDWMDGRRSRHTDDTDVLLRIHHVIGELPTYGYRRVWALLRR

QAELDGMPAINAKRVYRLMRQNALLLERKPAVPPSKRAHTGRVAVKESNQRWCSDGFEFCCDNGERLRVT

FALDCCDREVLHWAVTTGGFNSETVQDVMLGAVERRFGNDLPSSPVEWLTDNGSCYRANETRQFARMLGL

EPKNTAVRSPESNGIAESFVKTIKRDYISIMPKPDGLTAAKNLAEAFEHYNEWHPHSALGYRSPREYLRQ

RACNGLSDNRCLEI

>gi|30043056|gb|AAP18778.1| IS2 orfA [Shigella flexneri 2a str. 2457T]

MIVLILVFRLVIGEQMIDVLGPEKRRRRTTQEKIAIVQQSFEPGMTVSLVARQHGVAASQLFLWRKQYQE

GSLTAVAAGEQVVPASELAAAMKQIKELQRLLGKKTMENELLKEAVEYGRAKKWIAHAPLLPGDGE

>gi|30043055|gb|AAP18777.1| hypothetical protein S3759 [Shigella flexneri 2a str. 2457T]

MRYPVDVYTGKIQAYPEGKPSAIAKIQVDGELMLTELGLEGDEQAEKKVHGGPDRALCHYPREHYLYWVR

EFPEQAELFVAPAFGENLSTDGLTESNVHMGDIFRWGEALIQVSQPRSPCYKLNYHFDISDIAQLMQNTG

KVGWLYSVIAPGKVSADAPLELVSRVSDVTVQEAAAIAWHMPFDDDQYHRLLSAAGLSKSWTRTMQKRRL

SGKIEDFSRRLWGK

>gi|30043054|gb|AAP18776.1| probable sensor protein (histidine protein kinase) [Shigella flexneri 2a str. 2457T]

MIGSLTARIFAIFWLTLVLVLMLVLMLPKLDSRQMTELLDSEQRQGLMIEQHVEAELANDPPNDLMWWRR

LFRAIDKWAPPGQRLLLVTTEGRVIGAERSEMQIIRNFIGQADNADHPQKKKYGRVELVGPFSVRDGEDN

YQLYLIRPASSSQSDFINLLFDRPLLLLIVTMLVSTPLLLWLAWSLAKPARKLKNAADEVAQGNLRQHPE

LEAGPQEFLAAGASFNQMVTALERMMTSQQRLLSDISHELRTPLTRLQLGTALLRRRSGESKELERIETE

AQRLDSMINDLLVMSRNQQKNALVSETIKANQLWSEVLDNAAFEAEQMGKSLTVNFPPGPWPLYGNPNAL

ESALENIVRNALRYSHTKIEVGFAVDKDGITITVDDDGPGVSPEDREQIFRPFYRTDEARDRESGGTGLG

LAIVETAIQQHRGWVKAEDSPLGGLRLVIWLPLYKRS

>gi|30043053|gb|AAP18775.1| transcriptional regulator in 2-component system [Shigella flexneri 2a str. 2457T]

MNKILLVDDDRELTSLLKELLEMEGFNVIVAHDGEQALDLLDDSIDLLLLDVMMPKKNGIDTLKALRQTH

QTPVIMLTARGSELDRVLGLELGADDYLPKPFNDRELVARIRAILRRSHWSEQQQNNDNGSPTLEVDALV

LNPGRQEASFDGQTLELTGTEFTLLYLLAQHLGQVVSREHLSQEVLGKRLTPFDRAIDMHISNLRRKLPD

RKDGHPWFKTLRGRGYLMVSAS

>gi|30043052|gb|AAP18774.1| hypothetical protein S3755 [Shigella flexneri 2a str. 2457T]

MFDGISLTEHQRQQMRDLMQQARHEQPPVNVSELETMHRLVTAENFDENAVRAQAEKMANEQIARQVEMA

KVRNQMYRLLTPEQQAVLNEKHQQRMEQLRDVTQWQKSSSLKLLSSSNSRSQ

>gi|30043051|gb|AAP18773.1| putative transport system permease protein [Shigella flexneri 2a str. 2457T]

MNQSYGRLVSRAAIAATAMASLLLLIKIFAWWYTGSVSILAALVDSLVDIGASLTNLLVVRYSLQPADDN

HSFGHGKAESLAALAQSMFISGSALFLFLTGIQHLVSPTPMTDPGVGVIVTIVALICTIILVSFQRWVVR

RTQSQAVRADMLHYQSDVMMNGAILLALGLSWYGWHRADALFALGIGIYILYSALRMGYEAVQSLLDRAL

PDEERQEIIDIVTSWPGVSGAHDLRTRQSGPTRFIQIHLEMEDSLPLVQAHMVADQVEQAILRRFPGSDV

IIHQDPCSVVPREGKRSMLS

>gi|30043050|gb|AAP18772.1| 6-phosphofructokinase I [Shigella flexneri 2a str. 2457T]

MLTSGGDAPGMNAAIRGVVRSALTEGLEVMGIYDGYLGLYEDRMVQLDRYSVSDMINRGGTFLGSARFPE

FRDENIRAVAIENLKKRGIDALVVIGGDGSYMGAMRLTEMGFPCIGLPGTIDNDIKGTDYTIGFFTALST

VVEAIDRLRDTSSSHQRISVVEVMGRYCGDLTLAAAIAGGCEFVVVPEVEFSREDLVNEIKAGIAKGKKH

AIVAITEHMCDVDELAHFIEKETGRETRATVLGHIQRGGSPVPYDRILASRMGAYAIDLLLAGYGGRCVG

IQNEQLVHHDIIDAIENMKRPFKGDWLDCAKKLY

>gi|30043049|gb|AAP18771.1| periplasmic sulfate-binding protein [Shigella flexneri 2a str. 2457T]

MNKWGVGLTFLLAATSVMAKDIQLLNVSYDPTRELYEQYNKAFSAHWKQQTGDNVVIRQSHGGSGKQATS

VINGIEADVVTLALAYDVDAIAERGRIDKEWIKRLPDNSAPYTSTIVFLVRKGNPKQIHDWNDLIKPGVS

VITPNPKSSGGARWNYLAAWGYALHHNNNDQAKAQDFVRALYKNVEVLDSGARGSTNTFVERGIGDVLIA

WENEALLAANELGKDKFEIVTPSESILAEPTVSVVDKVVEKKGTKEVAEAYLKYLYSPEGQEIAAKNYYR

PRDAEVAKKYENAFPKLKLFTIDEEFGGWTKAQKEHFANGGTFDQISKR

>gi|30043048|gb|AAP18770.1| CDP-diacylglycerol phosphotidylhydrolase [Shigella flexneri 2a str. 2457T]

MKKAGLLFLVMIVIAVVAAGIGYWKLTGEESDTLRKIVLEECLPNQQQNQNPSPCAEVKPNAGYVVLKDL

NGPLQYLLMPTYRINGTESPLLTDPSTPNFFWLAWQARDFMSKKYGQPVPDRAVSLAINSRTGHTQNHFH

IHISCIRPDVREQLDKNLANISSRWLPLPGGLRGHEYLARRVTESELVQRSPFMMLAEEVPEAREHMGSY

GLAMVRQSDNSFVLLATQRNLLTLNRASAEEIQDHECEILR

>gi|30043047|gb|AAP18769.1| triosephosphate isomerase [Shigella flexneri 2a str. 2457T]

MRHPLVMGNWKLNGSRHMVHELVSNLRKELAGVAGCAVAIAPPEMYIDMAKREAEGSHIMLGAQNVDLNL

SGAFTGETSAAMLKDIGAQYIIIGHSERRTYHKESDELIAKKFAVLKEQGLTPVLCIGETEAENEAGKTE

EVCARQIDAVLKTQGAAAFEGAVIAYEPVWAIGTGKSATPAQAQAVHKFIRDHIAKVDANIAEQVIIQYG

GSVNASNAAELFAQPDIDGALVGGASLKADAFAVIVKAAEAAKQA

>gi|30043046|gb|AAP18768.1| hypothetical protein S3749 [Shigella flexneri 2a str. 2457T]

MKPGCTLFFLLCSALTVTTTAHAQTPDTATTAPYLLAGAPTFDLSISQFREDFNSQNPSLPLNEFRAIDS

SPDKANLTRAASKINENLYASTALERGTLKIKSIQMTWLPIQGPEQKAAKAKAQEYMAAVIRTLTPLMTK

TQSQKKLQSLLTAGKNKRYYTETEGALRYVVADNGEKGLTFAVEPIKLALSESLEGLNK

>gi|30043045|gb|AAP18767.1| hypothetical protein S3747 [Shigella flexneri 2a str. 2457T]

MKDVVDKCSTKGCAIDIGTVIDNDNCTSKFSRFFATREEAESFMTKLKELAAAASSADEGASVAYKIKDL

EGQVELDAAFTFSCQAEMIIFELSLRSLA

>gi|30043044|gb|AAP18766.1| putative regulator [Shigella flexneri 2a str. 2457T]

MAYKHIGVAISGNEEDALLVNKALELARHNDAHLTLIHIDDGLSELYPGIYFPATEDILQLLKNKSDNKL

YKLTKNIQWPKTKLRIERGEMPETLLEIMQKEQCDLLVCGHHHSFINRLMPAYRGMINKMSADLLIVPFI

DK

>gi|30043043|gb|AAP18765.1| ferredoxin-NADP reductase [Shigella flexneri 2a str. 2457T]

MADWVTGKVTKVQNWTDALFSLTVHAPVLPFTAGQFTKLGLEIDGERVQRAYSYVNSPDNPDLEFYLVTV

PDGKLSPRLAALKPGDEVQVVSEAAGFFVLDEVPDCETLWMLATGTAIGPYLSILQLGKDLDRFKNLVLV

HAARYAADLSYLPLMQELEKRYEGKLRIQTVVSRETAAGSLTGRIPALIESGELESAIGLPMNKETSHVM

LCGNPQMVRDTQQLLKETRQMTKHLRRRPGHMTAEHYW

>gi|30043042|gb|AAP18764.1| hypothetical protein S3744 [Shigella flexneri 2a str. 2457T]

MRRELAIEFSRVTESAALAGYKWLGRGDKNTADGAAVNAMRIMLNQVNIDGTIVIGEGEIDEAPMLYIGE

KVGTGRGDAVDIAVDPIEGTRMTAMGQANALAVLAVGDKGCFLNAPDMYMEKLIVGPGAKGTIDLNLPLA
DNLRNVAAALGKPLSELTVTILAKPRHDAVIAEMQQLGVRVFAIPDGDVAASILTCMPDSEVDVLYGIGG
APEGVVSAAVIRALDGDMNGRLLARHDVKGDNEENRRIGEQELARCKAMGIEAGKVLRLGDMARSDNVIF
SATGITKGDLLEGISRKGNIATTETLLIRGKSRTIRRIQSIHYLDRKDPEMQVHIL

>gi|30043041|gb|AAP18763.1| glycerol kinase [Shigella flexneri 2a str. 2457T]
MTEKKYIVALDQGTTSSRAVVMDHDANIISVSQREFEQIYPKPGWVEHDPMEIWATQSSTLVEVLAKADI
SSDQIAAIGITNQRETTIVWEKETGKPIYNAIVWQCRRTAEICEHLKRDGLEDYIRSNTGLVIDPYFSGT
KVKWILDHVEGSHERARRGELLFGTVDTWLIWKMTQGRVHVTDYTNASRTMLFNIHTLDWDDKMLEVLDI
PREMLPEVRRSSEVYGQTNIGGKGGTRIPISGIAGDQQAALFGQLCVKEGMAKNTYGTGCFMLMNTGEKA
VKSENGLLTTIACGPTGEVNYALEGAVFMAGASIQWLRDEMKLINDAYDSEYFATKVQNTNGVYVVPAFT
GLGAPYWDPYARGAIFGLTRGVNANHIIRATLESIAYQTRDVLEAMQADSGIRLHALRVDGGAVANNFLM
QFQSDILGTRVERPEVREVTALGAAYLAGLAVGFWQNLDELQEKAVIEREFRPGIETTERNYRYAGWKKA
VKRAMAWEEHDE

>gi|30043040|gb|AAP18762.1| glycerol diffusion facilitator protein [Shigella flexneri 2a str. 2457T]
MSQTSTLKGQCIAEFLGTGLLIFFGVGCVAALKVAGASFGQWEISVIWGLGVAMAIYLTAGVSGAHLNPA
VTIALWLFACFDKRKVIPFIVSQVAGAFCAAALVYGLYYNLFFDFEQTHHIVRGSVESVDLAGTFSTYPN
PHINFVQAFAVEMVITAILMGLILALTDDGNGVPRGPLAPLLIGLLIAVIGASMGPLTGFAMNPARDFGP
KVFA

>gi|30043039|gb|AAP18761.1| hypothetical protein S3741 [Shigella flexneri 2a str. 2457T]
MTMSLEVFEKLEAKVQQAIDTITLLQMEIEELKEKNNSLSQEVQNAQHQREELERENNHLKEQQNGWQER
LQALLGRMEEV

>gi|30043038|gb|AAP18760.1| 2-demethylmenaquinone 2-C-methyltransferase [Shigella flexneri 2a str. 2457T]

MKYDTSELCDIYQEDVNVVEPLFSNFGGRASFGGQIITVKCFEDNGLLYDLLEQNGRGRVLVVDGGGSVR

RALVDAELARLAVQNEWEGLVIYGAVRQVDDLEELDIGIQAMAAIPVGAAGEGIGESDVRVNFGGVTFFS

GDHLYADNTGIILSEDPLDIE

>gi|30043037|gb|AAP18759.1| 1,4-dihydroxy-2-naphthoate octaprenyltransferase [Shigella flexneri 2a str. 2457T]

MTEQQISRTQAWLESLRPKTLPLAFAAIIVGTALAWWQGHFDPLVALLALITAGLLQILSNLANDYGDAV

KGSDKPDRIGPLRGMQKGVITQQKMKRALIITVVLICLSGLALVAVACHTLADFVGFLILGGLSIIAAIT

YTVGNRPYGYIGLGDISVLVFFGWLSVMGSWYLQAHTLIPALILPATACGLLATAVLNINNLRDINSDRE

NGKNTLVVRLGEVNARRYHACLLMGSLVCLALFNLFSLHSLWGWLFLLAAPLLVKQARYVMREMDPVAMR

PMLERTVKGALLTNLLFVLGIFLSQWAA

>gi|30043036|gb|AAP18758.1| heat shock protein HslVU, ATPase subunit [Shigella flexneri 2a str. 2457T]

MSEMTPREIVSELDKHIIGQDNAKRSVAIALRNRWRRMQLNEELRHEVTPKNILMIGPTGVGKTEIARRL

AKLANAPFIKVEATKFTEVGYVGKEVDSIIRDLTDAAVKMVRVQAIEKNRYRAEELAEERILDVLIPPAK

NNWGQTEQQQEPSAARQAFRKKLREGQLDDKEIEIDLAAAPMGVEIMAPPGMEEMTSQLQSMFQNLGGQK

QKARKLKIKDAMKLLIEEEAAKLVNPEELKQDAIDAVEQHGIVFIDEIDKICKRGESSGPDVSREGVQRD

LLPLVEGCTVSTKHGMVKTDHILFIASGAFQIAKPSDLIPELQGRLPIRVELQALTTSDFERILTEPNAS

ITVQYKALMATEGVNIEFTDSGIKRIAEAAWQVNESTENIGARRLHTVLERLMEEISYDASDLSGQNITI

DADYVSKHLDALVADEDLSRFIL

>gi|30043035|gb|AAP18757.1| heat shock protein HslVU, proteasome-related peptidase subunit [Shigella flexneri 2a str. 2457T]

MTTIVSVRRNGHVVIAGDGQATLGNTVMKGNVKKVRRLYNDKVIAGFAGGTADAFTLFELFERKLEMHQG

HLVKAAVELAKDWRTDRMLRKLEALLAVADETASLIITGNGDVVQPENDLIAIGSGGPYAQAAARALLEN

TELSAREIAEKALDIAGDICIYTNHFHTIEELSYKA

>gi|30043034|gb|AAP18756.1| essential cell division protein [Shigella flexneri 2a str. 2457T]

MAQRDYVRRSQPAPSRRKKSTSRKKQRNLPAVSPAMVAIAAAVLVTFIGGLYFITHHKKEESETLQSQKV

TGNGLPPKPEERWRYIKELESRQPGVRAPTEPSAGGEVKTPEQLTPEQRQLLEQMQADMRQQPTQLVEVP

WNEQTPEQRQQTLQRQRQAQQLAEQQRLAQQSRTTEQSWQQQTRTSQAAPVQAQPRQSKPASTQQPYQDL

LQTPAHTTAQSKPQQAAPVARAADAPKPTAEKKDERRWMVQCGSFRGAEQAETVRAQLAFEGFDSKITTN

NGWNRVVIGPVKGKENADSTLNRLKMAGHTNCIRLAAGG

>gi|30043033|gb|AAP18755.1| regulator for deo operon, udp, cdd, tsx, nupC, and nupG [Shigella flexneri 2a str. 2457T]

MKAKKQETAATMKDVALKAKVSTATVSRALMNPDKVSQATRNRVEKAAREVGYLPQPMGRNVKRNESRTI

LVIVPDICDPFFSEIIRGIEVTAANHGYLVLIGDCAHQNQQEKTFIDLIITKQIDGMLLLGSRLPFDASI

EEQRNLPPMVMANEFAPELELPTVHIDNLTAAFDAVNYLYEQGHKRIGCIAGPEEMPLCHYRLQGYVQAL

RRCGIMVDPQYIARGDFTFEAGSKAMQQLLDLPQPPTAVFCHSDVMALGALSQAKRQGLKVPEDLSIIGF

DNIDLTQFCDPPLTTIAQPRYEIGREAMLLLLDQMQGQHVGSGSRLMDCELIIRGSTRALP

>gi|30043032|gb|AAP18754.1| primosomal protein N (factor Y), putative helicase [Shigella flexneri 2a str. 2457T]

MPVAHVALPVPLPRTFDYLLPEGMTVKAGCRVRVPFGKQQERIGIVVSVSDASELPLTELKAVVEVLDGE

PVFTHSVWRLLLWAADYYHHPIGDVLFHALPILLRQGRPAANAPMWYWFATEQGQAVDLNSLKRSPKQQQ

ALAALRQGKIWRDQVATLEFNDAALQALRKKGLCDLASETPEFSDWRTNYAVSGERLRLNTEQATAVGAI

HSAADTFSAWLLAGVTGSGKTEVYLSVLENVLAQGKQALVMVPEIGLTPQTIARFRERFNAPVEVLHSGL

NDSERLSAWLKAKNGEAAIVIGTRSALFTPFKNLGVIVIDEEHDSSYKQQEGWRYHARDLAVYRAHSEQI

PIILGSATPALETLCNVQQKKYRLLRLTRRAGNARPAIQHVLDLKGQKVQAGLAPALITRMRQHLQANNQ

VILFLNRRGFAPALLCHDCGWIAECPRCDHYYTLHQAQQHLRCHHCDSQRPVPRQCPSCGSTHLVPVGLG
TEQLEQTLAPLFPDVPISRIDRDTTSRKGALEQQLAEVHRGGARILIGTQMLAKGHHFPDVTLVALLDVD
GALFSADFRSAERFAQLYTQVAGRAGRAGKQGEVVLQTHHPEHPLLQTLLYKGYDAFAEQALAERRMMQL
PPWTSHVIVRAEDHNNQHAPLFLQQLRNLILSSPLADDKLWVLGPVPALAPKRGGRWRWQILLQHPSRVR
LQHIISGTLALINTIPDSRKVKWVLDVDPIEG

>gi|30043031|gb|AAP18753.1| 50S ribosomal subunit protein L31 [Shigella flexneri 2a str. 2457T]
MKKDIHPKYEEITASCSCGNVIKIRSTVGHDLNLDVCSKCHPFFTGKQRDVATGGRVDRFNKRFNIPGSK

>gi|30043030|gb|AAP18752.1| hypothetical protein S3732 [Shigella flexneri 2a str. 2457T]
MKNRLLILSLLVSVPAFAWQPQTGDIIFQISRSSQSKAIQLATHSDYSHTGMLVMRNKKPYVFEAVGPVK
YTPLKQWIAHGEKGNYVVRRVEGGLSVEQQQKLTQTAKRYLGKPYDFSFSWSDDRQYCSEVVWKVYQNAL
GMRVGEQQKLKEFDLSNPLVQAKLKERYGKNIPLEETVVSPQAVFDAPQLTTVAKEWPLFSW

>gi|30043029|gb|AAP18751.1| repressor of all met genes but metF [Shigella flexneri 2a str. 2457T]
MAEWSGEYISPYAEHGKKSEQVKKITVSIPLKVLKILTDERTRRQVNNLRHATNSELLCEAFLHAFTGQP
LPDDADLRKERSDEIPEAAKEIMREMGINPETWEY

>gi|30043028|gb|AAP18750.1| cystathionine gamma-synthase [Shigella flexneri 2a str. 2457T]
MTRKQATIAVRSGLNDDEQYGCVVPPIHLSSTYNFTGFNEPRAHDYSRRGNPTRDVVQRALAELEGGAGA
VLTNTGMSAIHLVTTVFLKPGDLLVAPHDCYGGSYRLFDSLAKRGCYRVLFVDQGDEQALRAALAEKPKL
VLVESPSNPLLRVVDIAKICHLAREVGAVSVVDNTFLSPALQNPLALGADLVLHSCTKYLNGHSDVVAGV
VIAKDPDVVTELAWWANNIGVTGGAFDSYLLLRGLRTLVPRMELAQRNAQAIVKYLQTQPLVKKLYHPSL
PENQGHEIAARQQKGFGAMLSFELDGDEQTLCRFLGGLSLFTLAESLGGVESLISHAATMTHAGMAPEAR
AAAGISETLLRISTGIEDGEDLIADLENGFRAANKG

>gi|30043027|gb|AAP18749.1| aspartokinase II and homoserine dehydrogenase II [Shigella flexneri 2a str. 2457T]

MSVIAQAGAKGRQLHKFGGSSLADVKCYLRVAGIMAEFSQPDDMMVVSAAGSTTNQLINWLKLSQTDRLS

AHQVQQTLRRYQCDLISGLLPAEEADSLISAFVSDLERLAALLDSGINDAVYAEVVGHGEVWSARLMSAV

LNQQGLPAAWLDAREFLRAERAAQPQVDEGLSYPLLQQLLVQHPSKRLVVTGFISRNNAGETVLLGRNGS

DYSATQIGALAGVSRVTIWSDVAGVYSADPRKVKDACLLPLLRLDEASELARLAAPVLHARTLQPVSGSE

IDLQLRCSYTPDQGSTRIERVLASGTGARIVTSHDDVCLIEFQVPASQDFKLAHKEIDQILKRAQVRPLA

VGVHNDRQLLQFCYTSEVADSALKILDEAGLPGELRLRQGLALVAMVGAGVTRNPLHCHRFWQQLKGQPV

EFTWQSDDGISLVAVLRTGPTESLIQGLHQSVFRAEKRIGLVLFGKGNIGSRWLELFAREQSTLSARTGF

EFVLAGVVDSRRSLLSYDGLDASRALAFFNDEAVEQDEESLFLWMRAHPYDDLVVLDVTASQQLADQYLD

FASHGFHVISANKLAGASDSNKYRQIHDAFEKTGRHWLYNATVGAGLPINHTVRDLIDSGDTILSISGIF

SGTLSWLFLQFDGSVPFTELVDQAWQQGLTEPDPRDDLSGKDVMRKLVILAREAGYNIEPDQVRVESLVP

AHCEGGSIDHFFENGDELNEQMVQRLEAAREMGLVLRYVARFDANGKARVGVEAVREDYPLASLLPCDNV

FAIESRWYRDNPLVIRGPGAGRDVTAGAIQSDINRLAQLL

>gi|30043026|gb|AAP18748.1| 5,10-methylene-tetrahydrofolate reductase [Shigella flexneri 2a str. 2457T]

MSFFHASQRDALNQSLAEVQGQINVSFEFFPPRTSEMEQTLWNSIDRLSSLKPKFVSVTYGANSGERDRT

HSIIKGIKDRTGLEAAPHLTCIDATPDELRTIARDYWNNGIRHIVALRGDLPPGSGKPEMYASDLVTLLK

EVADFDISVAAYPEVHPEAKSAQADLLNLKRKVDAGANRAITQFFFDVESYLRFRDRCVSAGIDVEIIPG

ILPVSNFKQAKKFADMTNVRIPAWMAQMFDGLDDDAETRKLVGANIAMDMVKILSREGVKDFHFYTLNRA

EMSYAICHTLGVRPGL

>gi|30043025|gb|AAP18747.1| catalase; hydroperoxidase HPI(I) [Shigella flexneri 2a str. 2457T]

MSTSDDIHNTTATGKCPFHQGGHDQSAGAGTTTRDWWPNQLRVDLLNQHSNRSNPLGEDFDYRKEFSKLD

YYGLKKDLKALLTESQPWWPADWGSYAGLFIRMAWHGAGTYRSIDGRGGAGRGQQRFAPLNSWPDNVSLD

KARRLLWPIKQKYGQKISWADLFILAGNVALENSGFRTFGFGAGREDVWEPDLDVNWGDEKAWLTHRHPE

ALAKAPLGATEMGLIYVNPEGPDHSGEPLSAAAAIRATFGNMGMNDEETVALIAGGHTLGKTHGAGPTSN

VGPDPEAAPIEEQGLGWASTYGSGVGADAITSGLEVVWTQTPTQWSNYFFENLFKYEWVQTRSPAGAIQF

EAVDAPEIIPDPFDPSKKRKPTMLVTDLTLRFDPEFEKISRRFLNDPQAFNEAFARAWFKLTHRDMGPKS

RYIGPEVPKEDLIWQDPLPQPIYNPTEQDIIDLKFAIADSGLSVSELVSVAWASASTFRGGDKRGGANGA

RLALMPQRDWDVNAAAVRALPVLEKIQKESGKASLADIIVLAGVVGVEKAASAAGLSIHVPFAPGRVDAR

QDQTDIEMFELLEPIADGFRNYRARLDVSTTESLLIDKAQQLTLTAPEMTALVGGMRVLGANFDGSKNGV

FTDRVGVLSNDFFVNLLDMRYEWKATDESKELFEGRDRETGEVKYTASRADLVFGSNSVLRAVAEVYASS

DAHEKFVKDFVAAWVKVMNLDRFDLL

>gi|30043024|gb|AAP18746.1| PTS system, fructose-like enzyme II component [Shigella flexneri 2a str. 2457T]

MNELVQILKNTRQHLMTGVSHMIPFVVSGGILLAVSVMLYGKGAVPDAVADPNLKKLFDIGVAGLTLMVP

FLAAYIGYSIAERSALAPCAIGAWVGNSFGAGFFGALIAGIIGGIVVHYLKKIPVHKVLRSVMPIFIIPI

IGTLITAGIMMWGLGEPVGALTNSLTQWLQGMQQGSIVMLAVIMGLMLAFDMGGPVNKVAYAFMLICVAQ

GVYTVVAIAAVGICIPPLGMGLATLIGRKNFSAEERETGKAALVMGCVGVTEGAIPFAAADPLRVIPSIM

VGSVCGAVTAALVGAQCYAGWGGLIVLPVVEGKLGYIAAVAVGAVVTAVCVNVLKSLARKNGSSTDEKED

DLDLDFEIN

>gi|30043023|gb|AAP18745.1| PTS system fructose-like IIB component 1 [Shigella flexneri 2a str. 2457T]

MTKIIAVTACPSGVAHTYMAAEALESAAKAKGWEVKVETQGSIGLENELTAEDVASADMVILTKDIGIKF

EERFAGKTIVRVNISDAVKRADAIMSKIEAHLAQTA

>gi|30043022|gb|AAP18744.1| formate acetyltransferase 2 [Shigella flexneri 2a str. 2457T]

MTNRISRLKTALFANTREISLERALLYTASHRQTEGEPVILRRAKATAYILEHVEISIRDEELIAGNRTV

KPRAGIMSPEMDPYWLLKELDQFPTRPQDRFAISEEDKRIYREELFPYWEKRSMKDFINGQMTDEVKAAT

NTQIFSINQTDKGQGHIIIDYPRLLNHGLGELVAQMQQHCQQQPENHFYQAALLLLEASQKHILRYAELA

ETMAANCTDAQRREELLTIAEISRHNAQHKPQTFWQACQLFWYMNIILQYESNASSLSLGRFDQYMLPFY

QASLTQGEDPAFLKELLESLWVKCNDIVLLRSTSSARYFAGFPTGYTALLGGLTENGRSAVNVLSFLCLD

AYQSVQLPQPNLGVRTNALIDTPFLMKTAETIRLGTGIPQIFNDEVVVPAFLNRGVSLEDARDYSVVGCV

ELSIPGRTYGLHDIAMFNLLKVMEICLHENEGNAALTYEGLLEQIRAKISHYITLMVEGSNICDIGHRDW

APVPLLSSFISDCLEKGRDITDGGARYNFSGVQGIGIANLSDSLHALKGMVFEQQRLSFDELLSVLKANF

ATPEGEKVRARLINRFEKYGNDIDEVDNISAELLRHYCKEVEKYQNPRGGYFTPGSYTVSAHVPLGSVVG

ATPDGRFAGEQLADGGLSPMLGQDAQGPTAVLKSVSKLDNTLLSNGTLLNVKFTPATLEGEAGLRKLADF

LRVFTQLKLQHIQFNVVNADTLREAQQRPQDYAGLVVRVAGYSAFFVELSKEIQDDIIRRTAHQL

>gi|30043021|gb|AAP18743.1| probable pyruvate formate lyase activating enzyme 2 [Shigella flexneri 2a str. 2457T]

MTSSAGQRISCNVVETRRDDVARIFNIQRYSLNDGEGIRTVVFFKGCPHLCPWCANPESISGKIQTVRRE

AKCLHCAKCLRDADECPSGAFERIGRDISLDALEREVMKDDIFFRTSGGGVTLSGGEVLMQAEFATRFLQ

RLRLWGVSCAIETAGDAPASKLLPLAKLCDEVLFDLKIMDATQARDVVKMNLPRVLENLRLLVSEGVNVI

PRLPLIPGFTLSRENMQQALDVLIPLKIRQIHLLPFHQYGEPKYRLLGKTWSMKEVPAPSSADVTTMREM

AERAGFQVTVGG

>gi|30043020|gb|AAP18742.1| PTS system fructose-like IIB component 2 [Shigella flexneri 2a str. 2457T]

MAYLVAVTACVSGVAHTYMAAERLEKLCQLEKWGVSIETQGALGTENRLADEDIRRADVALLITDIELAG

AERFEHCRYVQCSIYAFLREPQRVMSAVRKVLSAPQQTHLILE

>gi|30043019|gb|AAP18741.1| putative ARAC-type regulatory protein [Shigella flexneri 2a str. 2457T]

MYHDVSYLLSRLINGPLSLRQIYFASSNGPVPDLAYQVDFPRLEIVLEGEFVDTGAGATLVPGDVLYVAA
GGWNFPQWKTPATTFSVLFGKQQLGFSVVQWDGKQYQNLAKQHVARRGPRIGSFLLQTLNEMQMQPQEQQ
TARLIVASLLSHCRDLLGSQIQTASRSQALFEAIRDYIDERYASALTRESVAQAFYISPNYLSHLFQKTG
AIGFNEYLNHTRLEHAKTLLKGYDLKVKEVAHACGFVDSNYFCRLFRKNTEHSPSEYRRQYHSQLTEKPT
TPE

>gi|30043018|gb|AAP18740.1| hypothetical protein S3714 [Shigella flexneri 2a str. 2457T]
MHSTEVQAKPLSSWKALGWALLYFWFFSTLLQAIIYISGYSGTNGIRDSLLFSSLWLIPVFLFPKRIKII
AAVIGVVLWTASLAALCYYVIYGQEFSQSVLFVMFETNTNEASEYLSQYFSLKIVLIALAYTAVAVLLWT
RLRPVYIPKPWRYVVSFALLYGLILHPIAMNTFIKNKPFEKTLDNLASRMEPAAPWQFLTGYYQYRQQLN
SLTKLLNENNALPPLANFKDESGNEPRTLVLVIGESTQRGRMSLYGYPRETTPELDALHKTDPNLTVFNN
VVTSRPYTIEILQQALTFANEKNPDLYLTQPSLMNMMKQAGYKTFWITNQQTMTARNTMLTVFSRQTDKQ
YYMNQQRTQSAREYDTNVLKPFQDVLNDPAPKKLIIVHLLGTHIKYKYRYPENQGKFDGNTDHVPPGLSA
EELESYNDYDNANLYNDHVVASLIKDFKAADPNGFLVYFSDHGEEVYDTPPHKTQGRNEDNPTRHMYTIP
FLLWTSEKWQATHPRDFSQDVDRKYSLAELIHTWSDLAGLSYDGYDPTRSVVNPQFKETTRWIGNPYKKN
ALIDYDTLPYGDQVGNQ

>gi|30043017|gb|AAP18739.1| phosphoenolpyruvate carboxylase [Shigella flexneri 2a str. 2457T]
MNEQYSALRSNVSMLGKVLGETIKDALGEHILERVETIRKLSKSSRAGNDANRQELLTTLQNLSNDELLP
VARAFSQFLNLANTAEQYHSISPKGEAASNPEVIARTLRKLKNQPELSEDTIKKAVESLSLELVLTAHPT
EITRRTLIHKMVEVNACLKQLDNKDIADYERNQLMRRLRQLIAQSWHTDEIRKLRPSPVDEAKWGFAVVE
NSLWQGVPNYLRELNEQLEENLGYKLPVEFVPVRFTSWMGGDRDGNPNVTADITRHVLLLSRWKATDLFL
KDIQVLVSELSMVEATPELLALVGEEGAAEPYRYLMKNLRSRLMATQAWLEARLKGEELPKPEGLLTQNE
ELWEPLYACYQSLQACGMGIIANGDLLDTLRRVKCFGVPLVRIDIRQESTRHTEALGELTRYLGIGDYES
WSEADKQAFLIRELNSKRPLLPRNWQPSAETREVLDTCQVIAEAPQGSIAAYVISMAKTPSDVLAVHLLL

KEAGIGFAMPVAPLFETLDDLNNANDVMTQLLNIDWYRGLIQGKQMVMIGYSDSAKDAGVMAASWAQYQA

QDALIKTCEKAGIELTLFHGRGGSIGRGGAPAHAALLSQPPGSLKGGLRVTEQGEMIRFKYGLPEITVSS

LSLYTGAILEANLLPPPEPKESWRRIMDELSVISCDLYRGYVRENKDFVPYFRSATPEQELGKLPLGSRP

AKRRPTGGVESLRAIPWIFAWTQNRLMLPAWLGAGTALQKVVEDGKQSELEAMCRDWPFFSTRLGMLEMV

FAKADLWLAEYYDQRLVDKALWPLGKELRNLQEEDIKVVLAIANDSHLMADLPWIAESIQLRNIYTDPLN

VLQAELLHRSRQAEKEGQEPDPRVEQALMVTIAGIAAGMRNTG

>gi|30043016|gb|AAP18738.1| acetylornithine deacetylase [Shigella flexneri 2a str. 2457T]

MKNKLPPFIEIYRALIATPSISATEEALDQSNADLITLLADWFKDLGFNVEVQPVPGTRNKFNMLASYGQ

GAGGLLLAGHTDTVPFDDGRWTRDPFTLTEHDGKLYGLGTADMKGFFAFILDALRDVDVTKLKKPLYILA

TADEETSMAGARYFAETTALRPDCAIIGEPTSLQPVRAHKGHISNAIRIQGQSGHSSDPARGINAIELMH

DAIGHILQLRDNLKERYHYEAFTVPYPTLNLGHIHGGDASNRICACCELHMDIRPQPGMTLNELNGLLND

ALAPVSERWPGRLTVDELHPPIPGYECPPNHQLVEVVEKLLGAKTEVVNYCTEAPFIQTLCPTLVLGPGS

INQAHQPDEYLETRFIKPTRELIIQVIHHFCWH

>gi|30043015|gb|AAP18737.1| N-acetyl-gamma-glutamylphosphate reductase [Shigella flexneri 2a str. 2457T]

MLNTLIVGASGYAGAELVTYVNRHPHMNITALTVSAQSNDAGKLISDLHPQLKGIVELPLQPMSDISEFS

PGVDVVFLATAHEVSHDLAPQFLEAGCVVFDLSGAFRVNDATFYEKYYGFTHQYPELLEQAAYGLAEWCG

NKLKEANLIAVPGCYPTAAQLALKPLIDADLLDLNQWPVINATSGVSGAGRKAAISNSFCEVSLQPYGVF

THRHQPEIATHLGADVIFTPHLGNFPRGILETITCRLKSGVTQAQVAQALQQAYAHKPLVRLYDKGVPAL

KNVVGLPFCDIGFAVQGEHLIIVATEDNLLKGAAAQAVQCANIRFGYAETQSLI

>gi|30043014|gb|AAP18736.1| acetylglutamate kinase [Shigella flexneri 2a str. 2457T]

MNPLIIKLGGVLLDSEEALERLFSALVNYRESHQRPLVIVHGGGCVVDELMKGLNLPVKKKNGLRVTPAD

QIDIITGALAGTANKTLLAWAKKHQIAAVGLFLGDGDSVKVTQLDEELGHVGLAQPGSPKLINSLLENGY

LPVVSSIGVTDEGQLMNVNADQAATALAATLGADLILLSDVSGILDGKGQRIAEMTAAKAEQLIEQGIIT

DGMIVKVNAALDAARTLGRPVDIASWRYAEQLPALFNGMPMGTRILA

>gi|30043013|gb|AAP18735.1| argininosuccinate lyase [Shigella flexneri 2a str. 2457T]

MALWGGRFTQAADQRFKQFNDSLRFDYRLAEQDIVGSVAWSKALVTVGVLTAEEQAQLEEALNVLLEDVR

ARPQQILESDAEDIHSWVEGKLIDKVGQLGKKLHTGRSRNDQVATDLKLWCKDTVSELLTANRQLQSALV

ETAQNNQDAVMPGYTHLQRAQPVTFAHWCLAYVEMLARDESRLQDALKRLDVSPLGCGALAGTAYEIDRE

QLAGWLGFASATRNSLDSVSDRDHVLELLSAAAIGMVHLSRFAEDLIFFNTGEAGFVELSDRVTSGSSLM

PQKKNPDALELIRGKCGRVQGALTGMMMTLKGLPLAYNKDMQEDKEGLFDALDTWLDCLHMAALVLDGIQ

VKRPRCQEAAQQGYANATELADYLVAKGVPFREAHHIVGEAVVEAIRQGKPLEDLPLSELQKFSQVIGED

VYPILSLQSCLEKRAAKGGVSPQQVAQAIAFAQARLE

>gi|30043012|gb|AAP18734.1| IS4 orf [Shigella flexneri 2a str. 2457T]

MHIGQALDLVSRYDSLRNPLTSLGDYLDPELISRCLAESGTVTLRKRRLPLEMMVWCIVGMVLERKEPLH

QIVNRLDIMLPGNRPFVAPSAVIQARQRLGSEAVRRVFTKTAQLWHNATPHPHWCGLTLLAIDGVFWRTP

DTPENDAAFPRQTHAGNPALYPQVKMVCQMELTSHLLTAAAFGTMKNSENELAEQLIEQTGDNTLTLMDK

GYYSLGLLNGWSLAGEHRHWMIPLRKGAQYEELRKLGKGDHLVKLKTSPQARKKWPGLGNEVTARLLTVT

RKGKVCHLLTSMTDAMRFPGGEMADLYSNRWEIELGYREIKQTMQLSRLTLRSKKPELVEQELWGVLLAY

NLVRYQMIKMAEHLKGYWPNQLSFSESCGMVMRMLMTLQGASPGRIPELMSDLASMGQLVKLPTRRGRAF

PRVVKERPWKYPTAPKKSQSVA

>gi|30043011|gb|AAP18733.1| putative sensor protein [Shigella flexneri 2a str. 2457T]

MKIIAADVFVTCPGRNFVTLKITTESGLCGLGDATLNGRELSVASYLKDHLCPQLIGRDASRIEDIWQFF

YKGAYWRRGPVTMSAISAIDMALWDIKAKAANMPLYQLLGGASREGVMVYCHTTGRTIDEVLEDYAKHQQ

MGFKAIRVQCGVPGMQTTYGLAKGKGLAYEPATKGLWPEEQLWSSEKYLDFTPKLFEAVRNKFGFNEHLL

HDMHHRLTPIEAARFGKSIEDYRLFWMEDPTPAENQECFRLIRQHTVTPIAVGEVFNSIWDCKQLIEEQL

IGYIRTTITHAGGITGMRRIADFASLYQVRTGSHGPSDLSPVCHAAALHFDLWVPNFGVQEYMGYSEQML

EVFPYSWRFEEGYMHPGDEPGLGISFDEKLAAKYPYDPEYLPVARLEDGTLLNW

>gi|30043010|gb|AAP18732.1| IS1 orfA [Shigella flexneri 2a str. 2457T]

MASISIRCPSCSATEGVVRNGKSTAGHQRYLCSHCRKTWQLQFTYTASQPGTHQKIIDMAMNGVGCRASA

RIMGVGLNTVLRHLKNSGRSR

>gi|30043009|gb|AAP18731.1| IS1 orfB [Shigella flexneri 2a str. 2457T]

MSRQRTHYGRWPQHGFTSLKKLRPQSVTSRIQPGSDVIVCAEMDEHWGYVGAKSRQRWLFYAYDRIRRTV

VAHVFGERTLATLERLLSLLSAFEVVVWMTDGWPLYESRLKGKLHVISKRYTQRIERHNLNLRQHLARLV

RKSLSFSKSVELHDKVIGHYLNIKHYQ

>gi|30043008|gb|AAP18730.1| activator of hydrogen peroxide-inducible genes OxyR [Shigella flexneri 2a str. 2457T]

MNIRDLEYLVALAEHRHFRRAADSCHVSQPTLSGQIRKLEDELGVMLLERTSRKVLFTQAGMLLVDQART

VLREVKVLKEMASQQGETMSGPLHIGLIPTVGPYLLPHIIPMLHQTFPKLEMYLHEAQTHQLLAQLDSGK

LDCVILALVKESEAFIEVPLFDEPMLLAIYEDHPWANRECVLMADLAGEKLLMLEDGHCLRDQAMGFCFE

AGADEDTHFRATSLETLRNMVAAGSGITLLPALAVPPERKRDGVVYLPCIKPEPCRTIGLVYRPGSPLRS

RYEQLAEAIRARMDGHFDKVLKQAV

>gi|30043007|gb|AAP18729.1| putative oxidoreductase [Shigella flexneri 2a str. 2457T]

MGLVKQGARVAVIERYQNVGGGCTHWGTIPSKALRHAVSRIIEFNQNPLYSDHSRLLRSSFADILNHADN

VINQQTRMRQGFYERNHCEILQGNARFVDEHTLALDCPDGSVETLTAEKFVIACGSRPYHPTDVDFTHPR

IYDSDSILSMHHEPRHVLIYGAGVIGCEYASIFRGMDVKVDLINTRDRLLAFLDQEMSDSLSYHFWNSGV

VIRHNEEYEKIESCDDGVIMHLKSGKKLKADCLLYANGRTGNTDSLALQNIGLETDSRGQLKVNSMYQTA

QPHVYAVGDVIGYPSLASAAYDQGRIAAQALVKGEATAHLIEDIPTGIYTIPEISSVGKTEQQLTAMKVP

YEVGRAQFKHLARAQIVGMNVGTLKILFHRETKEILGIHCFGERAAEIIHIGQAIMEQKGGGNTIEYFVN

TTFNYPTMAEAYRVAALNGLNRLF

>gi|30043006|gb|AAP18728.1| hypothetical protein S3699 [Shigella flexneri 2a str. 2457T]

MFILWYSASSTFGKDSDIVMGVRAQQKEKTRRSLVEAAFSQLSAERSFASLSLREVAREAGIAPTSFYRH

FRDVDELGLTMVDESGLMLRQLMRQARQRIAKGGSVIRTSVSTFMEFIGNNPNAFRLLLRERSGTSAAFR

AAVAREIQHFIAELADYLELENHMPRAFTEAQAEAMVTIVFSAGAEALDVGVEQRRQLEERLVLQLRMIS

KGAYYWYRREQEKTAIIPGNVKDE

>gi|30043005|gb|AAP18727.1| hypothetical protein S3698 [Shigella flexneri 2a str. 2457T]

MKQANQDRGTLLLALVAGLSINGTFAALFSSIVPFSVFPIISLVLTVYCLHQRYLNRTMSVGLPGLAAAC

FILGVLLYSTVVRAEYPDIGSNFFPAVLSVIMVFWIGAKMRNRKQEVAE

>gi|30043004|gb|AAP18726.1| tRNA (uracil-5-)-methyltransferase [Shigella flexneri 2a str. 2457T]

MTPEHLPTEQYEAQLAEKVVRLQSMMAPFSDLVPEVFRSPVSHYRMRAEFRIWHDGDDLYHIIFDQQTKS

RIRVDSFPAASELINQLMTAMIAGVRNNPVLRHKLFQIDYLTTLSNQAVVSLLYHKKLDDEWRQEAEALR

DALRAQNLNVHLIGRATKTKIALDQDYIDERLPVAGKEMIYRQVENSFTQPNAAMNIQMLEWALDVTKGS

KGDLLELYCGNGNFSLALARNFDRVLATEIAKPSVAAAQYNIAANHIDNVQIIRMAAEEFTQAMNGVREF

NRLQGIDLKSYQFETIFVDPPRSGLDSETEKMVQAYPRILYISCNPETLCKNLETLSQTHKVERLALFDQ

FPYTHHMECGVLLTAK

>gi|30043003|gb|AAP18725.1| outer membrane receptor for transport of vitamin B12, E colicins, and bacteriophage BF23 [Shigella flexneri 2a str. 2457T]

MIKKASLLTACSVTAFSAWAQDTSPDTLVVTAIRFEQPRSTVLAPTTVVTRQDIDRWQSTSVNDVLRRLP

GVDITQNGGSGQLSSIFIRGTNASHVLVLIDGVRLNLAGVSGSADLSQFPIALVQRIEYIRGPRSAVYGS

DAIGGVVNIITTRDEPGTEISAGWGSNSYQNYDVSTQQQLGDKTRVTLLGDYAHTHGYDVVAYGNTGTQA

QTDNDGFLSKTLYGALEHNFTDAWSGFVRGYGYDNRTNYDAYYSPGSPLLDTRKLYSQSWDAGLRYNGEL

IKSQLITSYSHSKDYNYDPHYGRYDSSATLDEMKQYTVQWANNVIVGHGSIGAGVDWQKQTTTPGTGYVE

DGYDQRNTGIYLTGLQQVGDFTFEGAARSDDNSQFGRHGTWQTSAGWEFIEGYRFIASYGTSYKALNLGQ

LYGFYGNPNLDPEKSKQWEGAFEGLTAGVNWRISGYRNDVSDLIDYDDHTLKYYNEGKARIKGVEATANF

DTGPLTHTVSYDYVDARNAITDTPLLRRAKQQVKYQLDWQLYDFDWGITYQYLGTRYDKDYSSYPYQTVK

MGGVSLWDLAVAYPVTSHLTVRGKIANLFDKDYETVYGYQTAGREYTLSGSYTF

>gi|30043002|gb|AAP18724.1| glutamate racemase [Shigella flexneri 2a str. 2457T]

MLVFDSGVGGLSVYDEIRHLLPDLHYIYAFDNVAFPYGEKSEAFIVERVVAIVTAVQERYPLALAVVACN

TASTVSLPALREKFDFPVVGVVPAIKPAARLTANGIVGLLATRGTVKRSYTHELIARFANECQIEMLGSA

EMVELAEAKLHGEDVSLDALKRILRPWLRMKEPPDTVVLGCTHFPLLQEELLQVLPEGTRLVDSGAAIAR

RTAWLLEHEAPDAKSADANIAFCMAMTPEAEQLLPVLQRYGFETLEKLAVLG

>gi|30043001|gb|AAP18723.1| UDP-N-acetylenolpyruvoylglucosamine reductase [Shigella flexneri 2a str. 2457T]

MNHSLKPWNTFGIDHNAQHIVCAEDEQQLLNAWQHATAEGQPVLILGEGSNVLFLEDYRGTVIINRIKGI

EIHDEPDAWYLHVGAGENWHRLVKYTLQEGMPGLENLALIPGCVGSSPIQNIGAYGVELQRVCAYVDCVE

LATGKQVRLTAKECRFGYRDSIFKHEYQDRFAIVAVGLRLPKEWQPVLTYGDLTRLDPTTVTPQQVFNAV

CHMRTTKLPDPKVNGNAGSFFKNPVVSAETAKALLAQFPTAPNYPQAGGSVKLAAGWLIDQCQLKGMQMG

GAAVHRQQALVLINEDNAKSEDVVQLAHHVRQKVGEKFNVWLEPEVRFIGASGEVSAVETIS

>gi|30043000|gb|AAP18722.1| biotin-[acetyl CoA carboxylase] holoenzyme synthetase and biotin operon repressor [Shigella flexneri 2a str. 2457T]

MKDNTVPLKLIALLANGEFHSGEQLGETLGMSRAAINKHIQTLRDWGVDVFTVPGKGYSLPEPIQLLNAE

QILGQLDGGSVAVLPVIDSTNQFLLDRIGELKSGDACVAEYQQAGRGRRGRKWFSPFGANLYLSMFWRLE

QGPAAAIGLSLVIGIVMAEVLRKLGADKVRVKWPNDLYLQDRKLAGILVELTGKTGDAAQIVIGAGINMA

MRRVEESVVNQGWITLQEAGINLDRNTLAAMLIRELRAALELFEQEGLAPYLSRWEKLDNFINRPVKLII

GDKEIFGISRGIDKQGALLLEQDGIIKPWMGGEISLRSAEK

>gi|30042999|gb|AAP18721.1| pantothenate kinase [Shigella flexneri 2a str. 2457T]

MTPYLQFDRNQWAALRDSVPMTLSEDEIARLKGINEDLSLEEVAEIYLPLSRLLNFYISSNLRRQAVLEQ

FLGTNGQRIPYIISIAGSVAVGKSTTARVLQALLSRWPEHRRVELITTDGFLHPNQVLKERGLMKKKGFP

ESYDMHRLVKFVSDLKSGVPNVTAPVYSHLIYDVIPDGDKTVVQPDILILEGLNVLQSGMDYPHDPHHVF

VSDFVDFSIYVDAPEDLLQTWYINRFLKFREGAFTDPDSYFHNYAKLTKEEAIKTAMTLWKEINWLNLKQ

NILPTRERASLILTKSANHVVEEVRLRK

>gi|30042998|gb|AAP18720.1| hypothetical protein S3687 [Shigella flexneri 2a str. 2457T]

METCEFMQLKNCIICFIGHILCYTMKKQPNGGQISTLFQHHCAQQCAPAKP

>gi|30042997|gb|AAP18719.1| protein chain elongation factor EF-Tu [Shigella flexneri 2a str. 2457T]

MSKEKFERTKPHVNVGTIGHVDHGKTTLTAAITTVLAKTYGGAARAFDQIDNAPEEKARGITINTSHVEY

DTPTRHYAHVDCPGHADYVKNMITGAAQMDGAILVVAATDGPMPQTREHILLGRQVGVPYIIVFLNKCDM

VDDEELLELVEMEVRELLSQYDFPGDDTPIVRGSALKALEGDAEWEAKILELAGFLDSYIPEPERAIDKP

FLLPIEDVFSISGRGTVVTGRVERGIIKVGEEVEIVGIKETQKSTCTGVEMFRKLLDEGRAGENVGVLLR

GIKREEIERGQVLAKPGTIKPHTKFESEVYILSKDEGGRHTPFFKGYRPQFYFRTTDVTGTIELPEGVEM

VMPGDNIKMVVTLIHPIAMDDGLRFAIREGGRTVGAGVVAKVLS

>gi|30042996|gb|AAP18718.1| preprotein translocase [Shigella flexneri 2a str. 2457T]

MSANTEAQGSGRGLEAMKWVVVVALLLAAIVGNYLYRDIMLPLRALAVVILIAAAGGVALLTTKGKATIA

FAREARTEVRKVIWPTRQETLHTTLIVAAVTAVMSLILWGLDGILVRLVSFITGLRF

>gi|30042995|gb|AAP18717.1| component in transcription antitermination [Shigella flexneri 2a str. 2457T]

MSEAPKKRWYVVQAFSGFEGRVATSLREHIKLHNMEDLFGEVMVPTEEVVEIRGGQRRKSERKFFPGYVL

VQMVMNDASWHLVRSVPRVMGFIGGTSDRPAPISDKEVDAIMNRLQQVGDKPRPKTLFEPGEMVRVNDGP

FADFNGVVEEVDYEKSRLKVSVSIFGRATPVELDFSQVEKA

>gi|30042994|gb|AAP18716.1| 50S ribosomal subunit protein L11 [Shigella flexneri 2a str. 2457T]

MAKKVQAYVKLQVAAGMANPSPPVGPALGQQGVNIMEFCKAFNAKTDSIEKGLPIPVVITVYADRSFTFV

TKTPPAAVLLKKAAGIKSGSGKPNKDKVGKISRAQLQEIAQTKAADMTGADIEAMTRSIEGTARSMGLVV

ED

>gi|30042993|gb|AAP18715.1| 50S ribosomal subunit protein L1 [Shigella flexneri 2a str. 2457T]

MAKLTKRMRVIREKVDATKQYDINEAIALLKELATAKFVESVDVAVNLGIDARKSDQNVRGATVLPHGTG

RSVRVAVFTQGANAEAAKAAGAELVGMEDLADQIKKGEMNFDVVIASPDAMRVVGQLGQVLGPRGLMPNP

KVGTVTPNVAEAVKNAKAGQVRYRNDKNGIIHTTIGKVDFDADKLKENLEALLVALKKAKPTQAKGVYIK

KVSISTTMGAGVAVDQAGLSASVN

>gi|30042992|gb|AAP18714.1| 50S ribosomal subunit protein L10 [Shigella flexneri 2a str. 2457T]

MALNLQDKQAIVAEVSEVAKGALSAVVADSRGVTVDKMTELRKAGREAGVYMRVVRNTLLRRAVEGTPFE

CLKDAFVGPTLIAYSMEHPGAAARLFKEFAKANAKFEVKAAAFEGELIPASQIDRLATLPTYEEAIARLM

ATMKEASAGKLVRTLAAVRDAKEAA

>gi|30042991|gb|AAP18713.1| 50S ribosomal subunit protein L7/L12 [Shigella flexneri 2a str. 2457T]

MSITKDQIIEAVAAMSVMDVVELISAMEEKFGVSAAAAVAVAAGPVEAAEEKTEFDVILKAAGANKVAVI

KAVRGATGLGLKEAKDLVESAPAALKEGVSKDDAEALKKALEEAGAEVEVK

>gi|30042990|gb|AAP18712.1| RNA polymerase, beta subunit [Shigella flexneri 2a str. 2457T]

MVYSYTEKKRIRKDFGKRPQVLDVPYLLSIQLDSFQKFIEQDPEGQYGLEAAFRSVFPIQSYSGNSELQY

VSYRLGEPVFDVQECQIRGVTYSAPLRVKLRLVIYEREAPEGTVKDIKEQEVYMGEIPLMTDNGTFVING

TERVIVSQLHRSPGVFFDSDKGKTHSSGKVLYNARIIPYRGSWLDFEFDPKDNLFVRIDRRRKLPATIIL

RALNYTTEQILDLFFEKVIFEIRDNKLQMELVPERLRGETASFDIEANGKVYVEKGRRITARHIRQLEKD

DVKLIEVPVEYIAGKVVAKDYIDESTGELICAANMELSLDLLAKLSQSGHKRIETLFTNDLDHGPYISET

LRVDPTNDRLSALVEIYRMMRPGEPPTREAAESLFENLFFSEDRYDLSAVGRMKFNRSLLREEIEGSGIL

SKDDIIDVMKKLIDIRNGKGEVDDIDHLGNRRIRSVGEMAENQFRVGLVRVERAVKERLSLGDLDTLMPQ

DMINAKPISAAVKEFFGSSQLSQFMDQNNPLSEITHKRRISALGPGGLTRERAGFEVRDVHPTHYGRVCP

IETPEGPNIGLINSLSVYAQTNEYGFLETPYRKVTDGVVTDEIHYLSAIEEGNYVIAQANSNLDEEGHFV

EDLVTCRSKGESSLFSRDQVDYMDVSTQQVVSVGASLIPFLEHDDANRALMGANMQRQAVPTLRADKPLV

GTGMERAVAVDSGVTAVAKRGGVVQYVDASRIVIKVNEDEMYPGEAGIDIYNLTKYTRSNQNTCINQMPC

VSLGEPVERGDVLADGPSTDLGELALGQNMRVAFMPWNGYNFEDSILVSERVVQEDRFTTIHIQELACVS

RDTKLGPEEITADIPNVGEAALSKLDESGIVYIGAEVTGGDILVGKVTPKGETQLTPEEKLLRAIFGEKA

SDVKDSSLRVPNGVSGTVIDVQVFTRDGVEKDKRALEIEEMQLKQAKKDLSEELQILEAGLFSRIRAVLV

AGGVEAEKLDKLPRDRWLELGLTDEEKQNQLEQLAEQYDELKHEFEKKLEAKRRKITQGDDLAPGVLKIV

KVYLAVKRRIQPGDKMAGRHGNKGVISKINPIEDMPYDENGTPVDIVLNPLGVPSRMNIGQILETHLGMA

AKGIGDKINAMLKQQQEVAKLREFIQRAYDLGADVRQKVDLSTFSDEEVMRLAENLRKGMPIATPVFDGA

KEAEIKELLKLGDLPTSGQIRLYDGRTGEQFERPVTVGYMYMLKLNHLVDDKMHARSTGSYSLVTQQPLG

GKAQFGGQRFGEMEVWALEAYGAAYTLQEMLTVKSDDVNGRTKMYKNIVDGNHQMEPGMPESFNVLLKEI

RSLGINIELEDE

>gi|30042989|gb|AAP18711.1| RNA polymerase, beta prime subunit [Shigella flexneri 2a str. 2457T]

MKDLLKFLKAQTKTEEFDAIKIALASPDMIRSWSFGEVKKPETINYRTFKPERDGLFCARIFGPVKDYEC

LCGKYKRLKHRGVICEKCGVEVTQTKVRRERMGHIELASPTAHIWFLKSLPSRIGLLLDMPLRDIERVLY

FESYVVIEGGMTNLERQQILTEEQYLDALEEFGDEFDAKMGAEAIQALLKSMDLEQECEQLREELNETNS

ETKRKKLTKRIKLLEAFVQSGNKPEWMILTVLPVLPPDLRPLVPLDGGRFATSDLNDLYRRVINRNNRLK

RLLDLAAPDIIVRNEKRMLQEAVDALLDNGRRGRAITGSNKRPLKSLADMIKGKQGRFRQNLLGKRVDYS

GRSVITVGPYLRLHQCGLPKKMALELFKPFIYGKLELRGLATTIKAAKKMVEREEAVVWDILDEVIREHP

VLLNRAPTLHRLGIQAFEPVLIEGKAIQLHPLVCAAYNADFDGDQMAVHVPLTLEAQLEARALMMSTNNI

LSPANGEPIIVPSQDVVLGLYYMTRDCVNAKGEGMVLTGPKEAERLYRSGLASLHARVKVRITEYEKDAN

GELVAKTSLKDTTVGRAILWMIVPKGLPYSIVNQALGKKAISKMLNTCYRILGLKPTVIFADQIMYTGFA

YAARSGASVGIDDMVIPEKKHEIISEAEAEVAEIQEQFQSGLVTAGERYNKVIDIWAAANDRVSKAMMDN

LQTETVINRDGQEEKQVSFNSIYMMADSGARGSAAQIRQLAGMRGLMAKPDGSIIETPITANFREGLNVL

QYFISTHGARKGLADTALKTANSGYLTRRLVDVAQDLVVTEDDCGTHEGIMMTPVIEGGDVKEPLRDRVL

GRVTAEDVLKPGTADILVPRNTLLHEQWCDLLEENSVDAVKVRSVVSCDTDFGVCAHCYGRDLARGHIIN

KGEAIGVIAAQSIGEPGTQLTMRTFHIGGAASRAAAESSIQVKNKGSIKLSNVKSVVNSSGKLVITSRNT

ELKLIDEFGRTKESYKVPYGAVLAKGDGEQVAGGETVANWDPHTMPVITEVSGFVRFTDMIDGQTITRQT

DELTGLSSLVVLDSAERTAGGKDLRPALKIVDAQGNDVLIPGTDMPAQYFLPGKAIVQLEDGVQISSGDT

LARIPQESGGTKDITGGLPRVADLFEARRPKEPAILAEISGIVSFGKETKGKRRLVITPVDGSDPYEEMI

PKWRQLNVFEGERVERGDVISDGPEAPHDILRLRGVHAVTRYIVNEVQDVYRLQGVKINDKHIEVIVRQM

LRKATIVNAGSSDFLEGEQVEYSRVKIANRELEANGKVGATYSRDLLGITKASLATESFISAASFQETTR

VLTEAAVAGKRDELRGLKENVIVGRLIPAGTGYAYHQDRMRRRAAGEAPAAPQVTAEDASASLAELLNAG

LGGSDNE

>gi|30042988|gb|AAP18710.1| thiamin biosynthesis protein, thiazole moiety [Shigella flexneri 2a str. 2457T]

MKTFSNRWRQLDWDDIRLRINGKTAADVERALNASQLTRDDMMALLSPAASGYLEQLAQQAQRLTRQRFG

NTVSFYVPLYLSNLCANDCTYCGFSMSNRIKRKTLDEADIARESAAIREMGFEHLLLVTGEHQAKVGMDY

FRRHLPALREQFSSLQMEVQPLAETEYAELKQLGLDGVMVYQETYHEATYTRHHLKGKKQDFFWRLETPD

RLGRAGIDKIGLGALIGLSDNWRVDCYMVAEHLLWLQQHYWQSRYSVSFPRLRPCTGGIEPASIMDERQL

VQTICAFRLLAPEIELSLSTRESPWFRDRVIPLAINNVSAFSKTQPGGYADNHPELEQFSPHDDRRPEAV

AAVLTAQGLQPVWKDWDSYLGRASQRL

>gi|30042987|gb|AAP18709.1| thiamin biosynthesis protein, thiazole moiety [Shigella flexneri 2a str. 2457T]

MSSGRNISCRMATRSCFFRLLQGVEMLRIADKTFDSHLFTGTGKFASSQLMVEAIRASGSQLVTLAMKRV

DLRQHNDAILEPLIAAGVALLPNTSGAKTAEEAIFAAHLAREALGTNWLKLEIHPDARWLLPDPIETLKA

AETLVQQGFVVLPYCGADPVLCKRLEEVGCAAVMPLGAPIGSNQGLETRAMLEIIIQQATVPVVVDAGIG

VPSHATQALEMGADAVLVNTAIAVADDPVNMAKAFRLAVEVGLLARQSGPGSRSYFAHATSPLTGFLEAS

A

>gi|30042986|gb|AAP18708.1| thiamin biosynthesis protein, thiazole moiety [Shigella flexneri 2a str. 2457T]

MNDRDFMRYSRQILLDDIALDGQQKLLDSQVLIIGLGGLGTPAALYLAGAGVGTLVLADDDDVHLSNLQR

QILFTTEDINSPKSQVSQQRLTQLNPDIQLTALQQRLTGEALKDAVAQADVVLDCTDNMATRQEINAACV

ALNTPLITASAVGFGGQLMVLTPPWEQGCYRCLWPDNQQPERNCRTAGVVGPVVGVMGTLQALEAIKLLS

GIETPAGELRLFDGKSSQWRSLALRRASGCPVCGGSNADPV

>gi|30042985|gb|AAP18707.1| thiamin biosynthesis protein, thiazole moiety [Shigella flexneri 2a str. 2457T]

MYQPDFPPVPFRLGLYPVVDSVQWIERLLDAGVRTLQLRIKDRRDEEVEADVVAAIALGRRYNARLFIND

YWRLAIKHQAYGVHLGQEDLQATDLNAIRAAGLRLGVSTHDDMEIDVALAARPSYIALGHVFPTQTKQMP

SAPQGLEQLARHVERLADYPTVAIGGISLARAPAVIATGVGSIAVVSAITQAADWRLATAQLLEIAGVGD

E

>gi|30042984|gb|AAP18706.1| thiamine biosynthesis protein, pyrimidine moiety [Shigella flexneri 2a str. 2457T]

MSATKLTRREQRARAQHFIDTLEGTAFPNSKRIYITGTHPGVRVPMREIQLSPTLIGGSKEQPQYEENEA

IPVYDTSGPYGDPQIAINVQQGLAKLRQPWIDARADTEELTVRSSDYTRTRLADDGLDELRFSGLLTPKR

AKTGRRVTQLHYARQGIITPEMEFIAIRENMGRERIRSEVLRHQHPGMSFGAHLPENITAEFVRDEVAAG

RAIIPANINHPESEPMIIGRNFLVKVNANIGNSAVTSSIEEEVEKLVWSTRWGADTVMDLSTGRYIHETR

EWILRNSPVPIGTVPIYQALEKVNGIAEDLTWEAFRDTLLEQAEQGVDYFTIHAGVLLRYVPMTAKRLTG

IVSRGGSIMAKWCLSHHQENFLYQHFREICEICAAYDVSLSLGDGLRPGSIQDANDEAQFAELHTLGELT

KIAWEYDVQVMIEGPGHVPMQMIRRNMTEELEHCHEAPFYTLGPLTTDIAPGYDHFTSGIGAAMIGWFGC

AMLCYVTPKEHLGLPNKEDVKQGLITYKIAAHAADLAKGHPGAQIRDNAMSKARFEFRWEDQFNLALDPF

TARAYHDETLPQESGKVAHFCSMCGPKFCSMKISQEVRDYAAAQTIEVGMADMSENFRARGGEIYLHKEE

A

>gi|30042983|gb|AAP18705.1| putative transcriptional regulator [Shigella flexneri 2a str. 2457T]

MLNQLDNLTERVRGSNKLVDRWLHVRKHLLVAYYNLVGIKPGKESYMRLNEKALDDFCQSLVDYLSAGHF

SIYERILHKLEGNGQLARAAKIWPQLEANTQQIMDYYDSSLETAIDHDNYLEFQQVLSDIGEALEARFVL

EDKLILLVLDAARVKHPA

>gi|30042982|gb|AAP18704.1| hypothetical protein S3667 [Shigella flexneri 2a str. 2457T]

MDRIIEKLDHGWWVVSHEQKLWLPKGELPYGEAANFDLVGQRALQIGEWQGEPVWLIQQQRRHDMGSVRQ

VIDLDVGLFQLAGRGVQLAEFYRSHKYCGYCGHEMYPSKTEWAMLCSHCRERYYPQIAPCIIVAIRRDDS

LLLAQHTRHRNGVHTVLAGFVEVGETLEQAVAREVMEESGIKVKNLRYVTSQPWPFPQSLMTAFMAEYDS

GDIVIDPKELLEANWYRYDDLPLLPPPGTVARRLIEDTVAMCRAEYE

>gi|30042981|gb|AAP18703.1| uroporphyrinogen decarboxylase [Shigella flexneri 2a str. 2457T]

MTELKNDRYLRALLRQPVDVTPVWMMRQAGRYLPEYKATRAQAGDFMSLCKNAELACEVTLQPLRRYPLD

AAILFSDILTVPDAMGLGLYFEAGEGPRFTSPVTCKADVDKLPIPDPEDELGYVMNAVRTIRHELKGEVP

LIGFSGSPWTLATYMVEGGSSKAFTVIKKMMYADPQALHALLDKLAKSVTLYLNAQIKAGAQAVMIFDTW

GGVLTGRDYQQFSLYYMHKIVDGLLRENDGRRVPVTLFTKGGGQWLEAMAETGCDALGLDWTTDIADARR

RVGNKVALQGNMDPSMLYAPPARIEEEVATILAGFGHGEGHVFNLGHGIHQDVPPEHAGVFVEAVHRLSE

QYHR

>gi|30042980|gb|AAP18702.1| endonuclease V (deoxyinosine 3 endonuclease) [Shigella flexneri 2a str. 2457T]

MDLASLRAQQIELASSVICEDRLDKDPPDLIAGADVGFEQGGEVTRAAMVLLKYPSLELVEYKVARIATT

MPYIPGFLSFREYPALLAAWEMLSQKPDLVFVDGHGISHPRRLGVASHFGLLVDVPTIGVAKKRLCGKFE

PLSSKPGALAPLMDKGEQLAWVWRSKARCNPLFIATGHRVSVDSALAWVQRCMKGYRLPEPTRWADAVAS

ERPAFVRYTANQP

>gi|30042979|gb|AAP18701.1| hypothetical protein S3664 [Shigella flexneri 2a str. 2457T]

MLQNPIHLRLERLESWQHVTFMACLCERMYPNYAMFCQQTGFGDGQIYRRILDLIWETLTVKDAKVNFDS

QLEKFEEAIPSADDFDLYGVYPAIDACVALSELVHSRLSGETLEHAVEVSKTSITTVAMLEMTQAGREMG

DEELKENPAVEQEWDIQWEIFRLLAECEERDIELIKGLRADLRESGESNIGIIFQQ

>gi|30042978|gb|AAP18700.1| DNA-binding protein HU-alpha (HU-2) [Shigella flexneri 2a str. 2457T]

MNKTQLIDVIAEKAELSKTQAKAALESTLAAITESLKEGDAVQLVGFGTFKVNHRAERTGRNPQTGKEIK

IAAANVPAFVSGKALKDAVK

>gi|30042977|gb|AAP18699.1| hypothetical protein S3662 [Shigella flexneri 2a str. 2457T]

MNSFNEGVVSPLLSFWRRSLMLAGALFLTACSHNSSLPPFTASGFAEDQGAVRIWRKDSGDNVHLLAVFS

PWRSGDTTTREYRWQGDNLTLININVYSKPPVNIRARFDDRGDLSFMQRESDGEKQQLSNDQIDLYRYRA

DQIRQISDALRQGRVVLRQGRWHAMEQTVTTCEGQTIKPDLDSQAIAHIERRQSRSSVDVSVAWLEAPEG

SQLLLVANSDFCRWQPNEKTF

>gi|30042976|gb|AAP18698.1| hypothetical protein S3661 [Shigella flexneri 2a str. 2457T]

MSKNDSLPAAGESFLLVYHARLPVISAFHRWHGRCNTRSKTTTGGLTMKRNTKIALVMMALSAMAMGSTS

AFAHGGHGMWQQNAAPLTSEQQTAWQKIHNDFYAQSSALQQQLVTKRYEYNALLAANPPDSSKINAVAKE

MENLRQSLDELRVKRDIAMAEAGIPRGAGMGMGYGGCGGGGHMGMGHW

>gi|30042975|gb|AAP18697.1| sensor kinase for HydG, hydrogenase 3 activity [Shigella flexneri 2a str. 2457T]

MRFMQRSKDSLAKWLSAILPVVIVGLVGLFAVTVIRDYGRETAAARQTLLEKGSVLIRALESGSRVGMGM

RMHHAQQQALLEEMAGQPGVRWFAVTDEQGTIVMHSNSGMVGKQLYSPQEMQQLHPGDEEAWRRIDSADG

EPVLEIYRQFQPMFAAGMYRMRHMQQYAATPQAIFIAFDASNIVSAEDREQRNTLIILFALATVLLASVL

SFFWYRRYLRSRQLLQDEMKRKEKLVALGHLAAGVAHEIRNPLSSIKGLAKYFAERAPAGGEAHQLAQVM

AKEADRLNRVVSELLELVKPTHLALQAVDLNTLINHSLQLVSQDANSREIQLRFTANDTLPEIQADPDRL

TQVLLNLYLNAIQAIGQHGVISVTASESGAGVKISVTDSGKGIAADQLEAIFTPYFTTKAEGTGLGLAVV

HNIVEQHGGTIQVASQEGKGSTFTLWLPVNITRKDPQG

>gi|30042974|gb|AAP18696.1| response regulator of hydrogenase 3 activity (sensor HydH) [Shigella flexneri 2a str. 2457T]

MTHDNIDILVVDDDISHCTILQALLRGWGYNVALANSGRQALEQVREQVFDLVLCDVRMAEMDGIATLKE

IKALNPAIPVLIMTAYSSVETAVEALKTGAQDYLIKPLDFDNLQATLEKALAHTHSIDAETPAVTASQFG

MVGKSPAMQHLLSEIALVAPSEATVLIHGDSGTGKELVARAIHASSARSEKPLVTLNCAALNESLLESEL

FGHEKGAFTGADKRREGRFVEADGGTLFLDEIGDISPMMQVRLLRAIQEREVQRVGSNQTISVDVRLIAA

THRDLAAEVNAGRFRQDLYYRLNVVAIEVPSLRQRREDIPLLAGHFLQRFAERNRKAVKGFTPQAMDLLI

HYDWPGNIRELENAVERAVVLLTGEYISERELPLAIASTPIPLGQSQDIQPLVEVEKEVILAALEKTGGN

KTEAARQLGITRKTLLAKLSR

>gi|30042973|gb|AAP18695.1| phosphoribosylglycinamide synthetase, GAR synthetase [Shigella flexneri 2a str. 2457T]

MKVLVIGNGGREHALAWKAAQSPLVETVFAAPGNAGTALEPALQNVAIGVTDIPALLDFAQNEKIDLTIV

GPEAPLVKGVVDTFRAAGLKIFGPTAGAAQLEGSKAFTKDFLARHKIPTAEYQNFTEVEPALVYLREKGA

PIVIKADGLAAGKGVIVAMTLEEAEAAVHDMLAGNAFGDAGHRIVIEEFLDGEEASFIVMVDGEHVLPMA

TSQDHKRVGDKDNGPNTGGMGAYSPAPVVTDEVHQRTMERIIWPTVKGMAAEGNTYTGFLYAGLMIDKQG

NPKVIEFNCRFGDPETQPIMLRMKSDLVELCLAACEGKLDEKTSEWDERASLSVVMAAGGYPGDYRTGDV

IHGLPLEEVADGKVFHAGTKLADDEQVVTSGGRVLCVTALGHTVAEAQKRAYALMTDIHWDDCFCRKDIG

WRAIEREQN

>gi|30042972|gb|AAP18694.1| phosphoribosylaminoimidazolecarboxamide formyltransferase, AICAR formyltransferase; IMP cyclohydrolase [Shigella flexneri 2a str. 2457T]

MQQRRPVRRALLSVSDKAGIVEFAQALSARGVELLSTGGTARLLAEKGLPVTEVSDYTGFPEMMDGRVKT

LHPKVHGGILGRRGQDDAIMEEHQIQPIDMVVVNLYPFAQTVAREGCSLEDAVENIDIGGPTMVRSAAKN

HKDVAIVVKSSDYDAIIKEMDDNEGSLTLATRFDLAIKAFEHTAAYDSMIANYFGSMVPAYHGESKEAAG

RFPRTLNLNFIKKQDMRYGENSHQQAAFYIEENVKEASVATATQVQGKALSYNNIADTDAALECVKEFAE

PACVIVKHANPCGVAIGNSILDAYDRAYKTDPTSAFGGIIAFNRELDAETAQAIISRQFVEVIIAPSASE

EALKITAAKQNVRVLTCGQWGERVPGLDFKRVNGGLLVQDRDLGMVGAEELRVVTQRQPTEQELRDALFC

WKVAKFVKSNAIVYAKNNMTIGIGAGQMSRVYSAKIAGIKAADEGLEVKGSSMASDAFFPFRDGIDAAAA

AGVTCVIQPGGSIRDDEVIAAADEHGIAMLFTDMRHFRH

>gi|30042971|gb|AAP18693.1| hypothetical protein S3652 [Shigella flexneri 2a str. 2457T]

MVISIRRSRHEEGEELVAIWCRSVDATHDFLSAEYRAELEELVRSFLPEAPLWVAVNEREQPVGFMLLSG

QHMDALFIDPDVCGCGVGRMLVEHALSMAPELTTNVNEQNEQAVGFYKKVGFKVTGRSEVDDLGKPYPLL

NLAYVGE

>gi|30042970|gb|AAP18692.1| homoserine transsuccinylase [Shigella flexneri 2a str. 2457T]

MPIRVPDELPAVNFLREENVFVMTTSRASGQEIRPLKVLILNLMPKKIETENQFLRLLSNSPLQVDIQLL

RIDSRESRNTPAEHLNNFYCNFEDIQEQNFDGLIVTGAPLGLVEFNDVAYWPQIKQVLEWSKDHVTSTLF

VCWAVQAALNILYGIPKQTRTDKLSGVYEHHILHPHALLTRGFDDSFLAPHSRYADFPAALIRDYTDLEI

LAETEEGDAYLFASKDKRIAFVTGHPEYDAQTLAQEYLRDVEAGLDPDVPYNYFPHNDPQNKPRASWRSH

GNLLFTNWLNYYVYQITPYDLRHMNPTLD

>gi|30042969|gb|AAP18691.1| isocitrate lyase [Shigella flexneri 2a str. 2457T]

MKTRTQQIEELQKEWTQPRWEGITRPYSAEDVVKLRGSVNPECTLAQLGAAKMWRLLHGESKKGYINNLG

ALTGGQALQQAKAGIEAVYLSGWQVAADANLAASMYPDQSLYPANSVPAVVERINNTFRRADQIQWSVGI

EPGDPRYVDYFLPIVADAEAGFGGVLNAFELMKAMIEAGAAAVHFEDQLASVKKCGHMGGKVLVPTQEAI

QKLVAARLAADVTGVPTLLVARTDADAADLITSDCDPYDSEFITGERTSEGFFRTHAGIEQAISRGLAYA

PYADLVWCETSTPDLELARRFAQAIHAKYPGKLLAYNCSPSFNWQKNLDDKTIASFQQQLSDMGYKFQFI

TLAGIHSMWFNMFDLANAYAQGEGMKHYVEKVQQPEFAAAKDGYTFVSHQQEVGTGYFDKVTTIIQGGTS

SVTALTGSTEESQF

>gi|30042968|gb|AAP18690.1| repressor of aceBA operon [Shigella flexneri 2a str. 2457T]

MKIISTIQKKEAVMVAPIPAKRGRKPAVATAPATGQVQSLTRGLKLLEWIAESNGSVALTELAQQAGLPN

STTHRLLTTMQQQGFVRQVGELGHWAIGAHAFMVGSSFLQSRNLLAIVHPILRNLMEESGETVNMAVLDQ

SDHEAIIIDQVQCTHLMRMSAPIGGKLPMHASGAGKAFLAQLSEEQVTKLLHRKGLHAYTHATLVSPVHL

KEDLAQTRKRGYSFDDEEHALGLRCLAACIFDEHREPFAAISISGPISRITDDRVTEFGAMVIKAAKEVT

LAYGGMR

>gi|30042967|gb|AAP18689.1| B12-dependent homocysteine-N5-methyltetrahydrofolate transmethylase [Shigella flexneri 2a str. 2457T]

MSSKVEQLRAQLNERILVLDGGMGTMIQSYRLNEADFRGERFADWPCDLKGNNDLLVLSKPEVIAAIHNA

YFEAGADIIETNTFNSTTIAMADYQMESLSAEINFAAAKLARACADEWTARTPEKPRYVAGVLGPTNRTA

SISPDVNDPAFRNITFDQLAAAYRESTKALVEGGADLILIETVFDTLNAKAAVFAVKTEFEALGVELPIM

ISGTITDASGRTLSGQTTEAFYNSLRHAEALTFGLNCALGPDELRQYVQELSRIAECYVTAHPNAGLPNA

FGEYDLDADTMAKQIREWAQAGFLNIVGGCCGTTPQHIAAMSRAVEGLAPRKLPEIPVACRLSGLEPLNI

GDDSLFVNVGERTNVTGSAKFKRLIKEEKYSEALDVARQQVENGAQIIDINMDEGMLDAEAAMVRFLNLI

AGEPDIARVPIMIDSSKWDVIEKGLKCIQGKGIVNSISMKEGVDAFIHHAKLLRRYGAAVVVMAFDEQGQ

ADTRARKIEICRRAYKILTEEVGFPPEDIIFDPNIFAVATGIEEHNNYAQDFIGACEDIKRELPHALISG

GVSNVSFSFRGNDPVREAIHAVFLYYAIRNGMDMGIVNAGQLAIYDDLPAELRDAVEDVILNRRDDGTER

LLELAEKYRGSKADDTANAQQAEWRSWDVNKRLEYSLVKGITEFIEQDTEEARQQATRPIEVIEGPLMDG

MNVVGDLFGEGKMFLPQVVKSARVMKQAVAYLEPFIEASKEQGKTNGKMVIATVKGDVHDIGKNIVGVVL

QCNNYEIVDLGVMVPAEKILRIAKEVNADLIGLSGLITPSLDEMVNVAKEMERQGFAIPLLIGGATTSKA

HTAVKIEQNYSGPTVYVQNASRTVGVVAALLSDTQRDDFVARTCKEYETVRIQHGRKKPRTPPVTLEAAR

DNDFAFDWQAYTPPVAHRLGVQEVEASIETLRNYIDWTPFFMTWSLAGKYPRILEDEVVGVEAQRLFKDA

NDMLDKLSAEKTLNPRGVVGLFPANRVGDDIEIYRDETRTHVINVSHHLRQQTEKTGFANYCLADFVAPK

LSGKADYIGAFAVTGGLEEDALADAFEAQHDDYNKIMVKALADRLAEAFAEYLHERVRKVYWGYAPNENL

SNEELIRENYQGIRPAPGYPACPEHTEKATIWELLEVEKHTGMKLTESFAMWPGASVSGWYFSHPDSKYY

AVAQIQRDQVEDHARRKGMSVSDVERWLAPNLGYDAD

>gi|30042966|gb|AAP18688.1| putative alpha helix protein [Shigella flexneri 2a str. 2457T]

MLTLLHLLSAVALLVWGTHIVRTGVMRVFGARLRTVLSRSVEKKPLAFCAGIGVTALVQSSNATTMLVTS

FVAQDLVALAPALVIVLGADVGTALMARILTFDLSWLSPLLIFIGVIFFLGRKQSRAGQLGRVGIGLGLI

LLALELIVQAVTPITQANGVQVIFASLTGDILLDALIGAMFAIISYSSLAAVLLTATLTAAGIISFPVAL

CLVIGANLGSGLLAMLNNSAANAAARRVALGSLLFKLVGSLIILPFVHLLAETMGKLSLPKAELVIYFHV

FYNLVRCLVMLPFVDPMARFCKTIIRDEPELDTQLRPKHLDVSALDTPTLALANAARETLRIGDAMEQMM

EGLNKVMHGEPRQEKELRKLADDINVLYTAIKLYLARMPKEELAEEESRRWAEIIEMSLNLEQASDIVER

MGSEIADKSLAARRAFSLDGLKELDALYEQLLSNLKLAMSVFFSGDVTSARRLRRSKHRFRILNRRYSHA

HVDRLHQQNVQSIETSSLHLGLLGDMQRLNSLFCSVAYSVLEQPDEDEGRDEY

>gi|30042965|gb|AAP18687.1| peptidase E [Shigella flexneri 2a str. 2457T]

MELLLLSNSTLPGKAWLEHALPLIAEQLQGRRSAVFIPFAGVTQTWDDYTEKTAAVLAPLGVSVTGIHSV

VDPVAAIENAEIVIVGGGNTFQLLKQCRERGLLAPITDVVKRGALYIGWSAGANLACPTIRTTNDMPIVD

PQGFDALNLFPLQINPHFTNALPEGHKGETREQRIRELLVVAPELTIIGLPEGNWITVSKGHATLGGPNT

TYVFKAGEEAVPLEAGHRF

>gi|30042964|gb|AAP18686.1| putative L-sorbose-1-P-reductase [Shigella flexneri 2a str. 2457T]

MKTTALRLYGKRDLRLETFDLPEMQEDEILATVVTDSLCLSSWKEANLGENHKKVPDDVTTNPIIIGHEF

CGDILAVGKKWQHKFQPGQRYVIQANLQLPDRPDCPGYSFPWVDGEATHVVIPNEVMEQYCLLAYDGETY

FEGSLVEPLSCVIGAFNANYHLQEGSYNHTMGIRPQGHTLILGGTGPMGLLAIDYALHGPVNPSLLVITD

TDNDKLSYARKHYPSEPQTLIHYLNATDAAFDTLMALSGGHGFDDIFVFVPNEGLVTLASSLLATDGCLN

FFAGPQDKHFSAPINFYDVHYAFTHYVGTSGGNTDDMRAAVKLIEEKKVQAAKVVTHILGLNAAGETTLE

LPAVGGGKKLVYTGKYLPLTSLTQIQDQALAAILARHQGIWSGEVEQYLLAHAEAISHD

>gi|30042963|gb|AAP18685.1| putative sorbose-permease PTS system IID component [Shigella flexneri 2a str. 2457T]

MEQRKITRSDLVSMFLRSNLQQASFNFERIHGLGFCYDMIPAIKRLYPLKEDQVAALRLHLVFFNTTPAV

CGPVIGAEIDDGTINGIKVGLMGPLAGVGDPLVWGTLRPITAALGASLALSGNILGPLLFFFIFNAVRLA

MKWYGLQLGFRKGVNIVSDMGGNVLQKLTEGASILGLFVMGVLVTKWTSINVPLVVSQTHAADGSTVTMT

VQNILDQLCPGLLALGLTLLMVRLLNKKINPVWLIFALFGLGIIGNALGFLS

>gi|30042962|gb|AAP18684.1| putative sorbose-permease PTS system IIC component [Shigella flexneri 2a str. 2457T]

MGSVLDEFQTHRPLIACTVIGLILGDLKTGIILGGTLELIALCWMNVGAAQSPDSALASIISAILVIVGQ

QSIATGIAIALPVAAAGQVLTVFARTITVVFQHAADKAAEEARFRTLDILHVSALGVQALRVAIPALIVS

LFVSADMVSNMLNAIPEFVTRGLQIAGGFIVVVGYAMVLRMMGVKYLMPFFFLGFLAGGYLDLSLLAFGG

VGVIMALVYIQLNPQWCKAEPQLQATASTALDQLDD

>gi|30042961|gb|AAP18683.1| putative sorbose-permease PTS system IIB component [Shigella flexneri 2a str. 2457T]

MNITLARIDDRLIHGQVTTVWSKVANAQRIIICNDEVYNDEVRRTLLRQAAPPGMKVNVVNIEKAVAVYH

NPQYQDETVFYLFTRPQDALAMVRQGVKIGTLNIGGMAWRPGKKQLTKAVSLDDDDINAFHELNNLGVIL

DLRVVASDPSINIIDKINEQLIAN

>gi|30042960|gb|AAP18682.1| putative sorbose-permease PTS system IIA component [Shigella flexneri 2a str. 2457T]

MVNAIFCAHGKLACAMLESVQMVYGNANVEAVAFVPGENAGDIAVKLEKLVSIHNQDEWLIAVDLQCGSP

WNAAAMLAMRNPRLRVISGLSLPLALELVDNQDSMNVDELCEHLTQIAKQTCVVWKQLATTEEDF

>gi|30042959|gb|AAP18681.1| putative sorbitol-6-phosphate 2-dehydrogenase [Shigella flexneri 2a str. 2457T]

MQTWLNLQDKIIIVTGGASGIGLAIVEELLAQGANVQMVDIHGGDGQYEGHKGYQFWPTDISSTKEVNHT

VAEIIQRFGRIDGLVNNAGVNFPRLLVDEKAPAGQYELNEAAFEKMVNINQKGVFLMSQAVARQMVKQHD

GVIVNVSSESGLEGSEGQSCYAATKAALNSFTRSWSKELGKHGIRVVGIAPGILEKTGLRTPEYEEALAW

TRNITVEQLREGYTKNAIPIGRAGRLAEVADFVCYLLSERASYITGVTTNIAGGKTRG

>gi|30042958|gb|AAP18680.1| probable sor-operon regulator [Shigella flexneri 2a str. 2457T]

MENSDDIRLIVKIAQLYYEQDMTQAQIARELGIYRTTISRLLKRGRDQGMVTIAINYDYNENLWLEQQLK

QKFGLKDVVVVSGNDEDEETQLAMMGLHGAQLLDRLLEPGDIVGFSWGRAVSALVENLPQAGQSRQLICV

PIIGGPSGKLESRYHVNTLTYSAAAKLKGESHLADFPALLDNPLIRNGIMQSQHFKTISAYWDNLDIALV

GIGSPAIRDGANWHAFYGGEESDDLNARQVAGDICSRFFDIHGAMVETNMSEKTLSIEMNKLKQARYSIG

IAMSEEKYSGIIGALRGKYINCLVTNSSTAELLLK

>gi|30042957|gb|AAP18679.1| hypothetical protein S3635 [Shigella flexneri 2a str. 2457T]

MLPDSSVRLNKYISESGICSRREADRYIEQGNVFLNGKRATIGDQVKPGDIVKVNGQLIEPREAEDLVLI

ALNKPVGIVSTTEDGERDNIVDFVNHSKRVFPIGRLDKDSQGLIFLTNHGDLVNKILRAGNDHEKEYLVT

VDKPITDEFIRGMGAGVPILGTVTKKCKVKKEAPFVFRITLVQGLNRQIRRMCEHFGYEVKKLERTRIMN

VSLSGIPLGEWRDLTDDELIDLFKLIENSSSEAKPKAKAKPKTAGIKRPVVKMEKTAEKGGRPASNGKRF

TSPGRKKKGR

>gi|30042956|gb|AAP18678.1| IS2 orfB [Shigella flexneri 2a str. 2457T]

MARGWGVSLVSRCLRVSRAQLHVILRRTDDWMDGRRSRHTDDTDVLLRIHHVIGELPTYGYRRVWALLRR

QAELDGMPAINAKRVYRLMRQNALLLERKPAVPPSKRAHTGRVAVKESNQRWCSDGFEFCCDNGERLRVT

FALDCCDREALHWAVTTGGFNSETVQDVMLGAVERRFGNDLPSSPVEWLTDNGSCYRANETRQFARMLGL

EPKNTAVRSPESNGIAESFVKTIKRDYISIMPKPDGLTAAKNLAEAFEHYNEWHPHSALGYRSPREYLRQ

RACNGLSDNRCLEI

>gi|30042955|gb|AAP18677.1| IS2 orfA [Shigella flexneri 2a str. 2457T]

MIVLILVFRLVIGVQMIDVLGPEKRRRRTTQEKIAIVQQSFEPGMTVSLVARQHGVAASQLFLWRKQYQE

GSLTAVAAGEQVVPASELAAAMKQIKELQRLLGKKTMENELLKEAVEYGRAKKWIAHAPLLPGDGE

>gi|30042954|gb|AAP18676.1| putative 2-component sensor protein [Shigella flexneri 2a str. 2457T]

MRHSLPYHILRKRPMKLSTTVILMVSAVLFSVLLVVHLIYFSQISDMTRDGLANKALAVARTLADSPEIR

QGLQKKPQESGIQAIAEAVRKRNDLLFIVVTDMQSLRYSHPEAQRIGQPFKGDDILNALNGEENVAINRG

FLAQALRVFTPIYDENHKQIGVVAIGLELSRVTQQINDSRWSIIWSVLFGMLVGLIGTCILVKVLKKILF

GLEPYEISTLFEQRQAMLQSIKEGVVAVDDRGEVTLINDAAQELLNYRKSQDDEKLSTLSHSWSQVVDVS

EVLRDGTPRRDEEITIKDRLLLINTVPVRSNGVIIGAISTFRDKTEVRKLMQRLDGLVNYADALRERSHE

FMNKLHVILGLLHLKSYKQLEDYILKTANNYQEEIGSLLGKIKSPVIAGFLISKINRATDLGHTLILNSE

SQLPDSGSEDQVATLITTLGNLIENALEALGPEPGGEISVTLHYRHGWLHCEVNDDGPGIAPDKIDHIFD

KGVSTKGSERGVGLALVKQQVENLGGSIAVESEPGIFTQFFVQIPWDGERSNR

>gi|30042953|gb|AAP18675.1| putative 2-component transcriptional regulator [Shigella flexneri 2a str. 2457T]

MINVLIIDDDAMVAELNRRYVAQIPGFQCCGTASTLEKAKEIIFNSDAPIDLILLDIYMQKENGLDLLPV

LHNARCKSDVIVISSAADAATIKDSLHYGVVDYLIKPFQASRFEEALTGWRQKKMALEKHQYYDQAELDQ

LIHGSSSNEQDPRRLPKGLTPQTLRTLCQWIDAHQDYEFSTDELANEVNISRVSCRKYLIWLVNCHILFT

SIHYGVTGRPVYRYRIQAEHYSLLKQYCQ

>gi|30042952|gb|AAP18674.1| anaerobic dicarboxylate transport [Shigella flexneri 2a str. 2457T]

MLFTIQLIIILICLFYGARKGGIALGLLGGIGLVILVFVFHLQPGKPPVDVMLVIIAVVAASATLQASGG

LDVMLQIAEKLLRRNPKYVSIVAPFVTCTLTILCGTGHVVYTILPIIYDVAIKNNIRPERPMAASSIGAQ

MGIIASPVSVAVVSLVAMLGNVTFDGRHLEFLDLLAITIPSTLIGILAIGIFSWFRGKDLDKDEEFQKFI

SVPENREYVYGDTATLLDKKLPKSNWLAMWIFLGAIAVVALLGADSDLRPSFGGKPLSMVLVIQMFMLLT

GALIIIILTKTNPASISKNEVFRSGMIAIVAVYGIAWMAETMFGAHMSEIQGVLGEMVKEYPWAYAIVLLL

VSKFVNSQAAALAAIVPVALAIGVDPAYIVASAPACYGYYILPTYPSDLAAIQFDRSGTTHIGRFVINHS

FILPGLIGVSVSCVFGWIFAAMYGFL

>gi|30042951|gb|AAP18673.1| fumarase B, fumarate hydratase Class I [Shigella flexneri 2a str. 2457T]

MSNKPFIYQAPFPMGKDNTEYYLLTSDYVSVADFDGETILKVEPEALTLLAQQAFHDASFMLRPAHQKQV

AAILHDPEASENDKYVALQFLRNSEIAAKGVLPTCQDTGTAIIVGKKGQRVWTGGGDEEALSKGVYNTYI
EDNLRYSQNAALDMYKEVNTGTNLPAQIDLYAVDGDEYKFLCVAKGGGSANKTYLYQETKALLTPGKLKN
FLVEKMRTLGTAACPPYHIAFVIGGTSAENNLKTVKLASAHYYDELPTEGNEHGQAFRDVQLEQELLEEA
QKLGLGAQFGGKYFAHDIRVIRLPRHGASCPVGMGVSCSADRNIKAKINREGIWIEKLEHNLGQYIPQEL
RQAGEGEAVKVDLNRPMKEILAQLSQYPVSTRLSLTGTIIVGRDIAHAKLKELIDAGKELPQYIKDHPIY
YAGPAKTPAGYPSGSLGPTTAGRMDSYVDLLQSHGGSMIMLAKGNRSQQVTDACHKHGGFYLGSIGGPAA
VLAQQSIKHLECVAYPELGMEAIWKIEVEDFPAFILVDDKGNDFFQQIVNKQCANCTK

>gi|30042950|gb|AAP18672.1| hypothetical protein S3628 [Shigella flexneri 2a str. 2457T]
MTRTLKPLILNTGALALTLILIYTGISAHDKLTWLLEVTPVIIVVPLLLATARRYPLTLLLYTLIFFHAI
ILMVGGQYTYAKVPVGFEVQEWLGLSRNPYDKLGHFFQGLVPALVAREILVRGMYVRGRKMVAFLVCCVA
LAISAMYELIEWWAALAMGQGADDFLGTQGDQWDTQSDMFCALLGALTTVILLARFHCRQLRRYGLITG

>gi|30042949|gb|AAP18671.1| melibiose permease II [Shigella flexneri 2a str. 2457T]
MTTKLSYGFGAFGKDFAIGIVYMYLMYYYTDVVGLSVGLVGTLFLVARIWDAINDPIMGWIVNATRSRWG
KFKPWILIGTLANSVILFLLFSAHLFEGTTQIVFVCVTYILWGMTYTIMDIPFWSLVPTITLDKREREQL
VPYPRFFASLAGFVTAGVTLPFVNYVGGGDRGFGFQMFTLVLIAFFIVSTIITLRNVHEVFSSDNQSSAE
GSHLTLKAIVALIYKNDQLSCLLGMALAYNVASNIITGFAIYYFSYVIGDADLFPYYLSYAGAANLVTLV
FFPRLVKSLSRRILWAGASILPVLSCGVLLLMALMSYHNVVLIVIAGILLNVGTALFWVLQVIMVADTVD
YGEYKLHVRCESIAYSVQTMVVKGGSAFAAFFIAVVLGMIGYVPNVEQSTQALLGMQFIMIALPTLFFMV
TLILYFRFYRLNGDTLRRIQIHLLDKYRKVPPEPVHADIPVGAVSDVKA

>gi|30042948|gb|AAP18670.1| alpha-galactosidase [Shigella flexneri 2a str. 2457T]
MMSAPKITFIGAGSTIFVKNILGDVFHREALKTAHIALMDIDPTRLEESHIVVRKLMDSAGASGKITCHT
QQKEALEDADFVVVAFQIGGYEPCTVTDFEVCKRHGLEQTIADTLGPGGIMRALRTIPHLWQICENMTEV

CPDATMLNYVNPMAMNTWAMYARYPYIKQVGLCHSVQGTAEELARDLNIDPATLRYRCAGINHMAFYLEL
ERKTADGSYVNLYPELLAAYEAGQAPKPNIHGNTRCQNIVRYEMFKKLGYFVTESSEHFAEYTPWFIKLG
REDLIERYKVPLDEYPKRCVEQLANWHKELEEYKNASRIDIKPSREYASTIMNAIWTGEPSVIYGNVRND
GLIDNLPQGCCVEVACLVDANGIQPTKVGTLPSHLAALMQTNINVQTLLTEAILTENRDRVYHAAMMDPH
TAAVLGIDEIYALVDDLIAAHGDWLPSWLHR

>gi|30042947|gb|AAP18669.1| regulator of melibiose operon [Shigella flexneri 2a str. 2457T]
MNTDTFMCSSDEKQTRSPLSLYSEYQRMEIEFRAPHIMPTSHWHGQVEVNVPFDGDVEYLINNEKVNINQ
GHITLFWACTPHQLTDTGTCQSMAIFNLPMHLFLSWPLDKDLINHVTHGMVIKSLATQQLSPFEVRRWQQ
ELNSPNEQIRQLAIDEIGLMLKRFSLSSWEPILVNKTSRTHKNSVSRHAQFYVSQMLGFIAENYDQALTI
NDVAEHVKLNANYAMGIFQRVMQLTMKQYITAMRINHVRALLSDTDKSILDIALTAGFRSSSRFYSTFGK
YVGMSPQQYRKLSQQRRQTFPG

>gi|30042946|gb|AAP18668.1| biodegradative arginine decarboxylase [Shigella flexneri 2a str. 2457T]
MKVLIVESEFLHQDTWVGNAVERLADALSQQNVTVIKSTSFDDGFAILSSNEAIDCLMFSYQMEHPDEHQ
NVRQLIGKLHERQQNVPVFLLGDREKALAAMDRDLLELVDEFAWILEDTADFIAGRAVAAMTRYRQQLLP
PLFSALMKYSDIHEYSWAAPGHQGGVGFTKTPAGRFYHDYYGENLFRTDMGIERTSLGSLLDHTGAFGES
EKYAARVFGADRSWSVVVGTSGSNRTIMQACMTDNDVVVVDRNCHKSIEQGLMLTGAKPVYMVPSRNRYG
IIGPIYPQEMQPETLQKKISESPLTKDKAGQKPSYCVVTNCTYDGVCYNAKEAQDLLEKTSDRLHFDEAW
YGYARFNPIYADHYAMRGEPGDHNGPTVFATHSIHKLLNALSQASYIHVREGRGAINFSRFNQAYMMHAT
TSPLYAICASNDVAVSMMDGNSGLSLTQEVIDEAVDFRQAMARLYKEFTADGSWFFKPWNKEVVTDPQTG
KTYDFADAPTKLLTTVQDCWVMHPGESWHGFKDIPDNWSMLDPIKVSILAPGMGEDGELEETGVPAALVT
AWLGRHGIVPTRTTDFQIMFLFSMGVTRGKWGTLVNTLCSFKHHYDANTPLAQVMPELVEQYPDTYANMG
IHDLGDTMFAWLKENNPGARLNEAYSGLPVAEITPREAYNAIVDNNVELVSIENLPGRIAANSVIPYPPG
IPMLLSGENFGDKNSPQVSYLRSLQSWDHHFPRFEHETEGTEIIDGIYHVMCVKA

>gi|30042945|gb|AAP18667.1| putative ARAC-type regulatory protein [Shigella flexneri 2a str. 2457T]

MRICSDQPCIVLLTEKDVWIRVNGKEPISLKANHMALLNCENNIIDVSSLNNTLVAHISHDIIKDYLRFL

NKDLSQIPVWQRSATPILTLPCLTPDVFRVAAQHSMMPAETESEKERTRALLFTVLSRFLDSKKFLSLMM

YMLRNCVSDSVYQIIESDIHKDWNLSMVASCLCLSPSLLKKKLKSENTSYSQIITTCRMRYAVNELMMDG

KNISQVSQSCGYNSTSYFISVFKDFYGMTPLHYVSQHRERTVA

>gi|30042944|gb|AAP18666.1| putative amino acid/amine transport protein [Shigella flexneri 2a str. 2457T]

MSSDADAHKVGLIPVTLMVSGNIMGSGVFLLPANLASTGGIAIYGWLVTIIGALGLSMVYAKMSFLDPSP

GGSYAYARRCFGPFLGYQTNVLYWLACWIGNIAMVVIGVGYLSYFFPILKDPLVLTITCVVVLWIFVLLN

IVGPKMITRVQAVATVLALIPIVGIAVFGWFWFRGETYMAAWNVSGLGTFGAIQSTLNVTLWSFIGVESA

SVAAGVVKNPKRNVPIATIGGVLIAAVCYVLSTTAIMGMIPNAALRVSASPFGDAARMALGDTAGAIVSF

CAAAGCLGSLGGWTLLAGQTAKAAADDGLFPPIFARVNKAGTPVAGLIIVGILMTIFQLSSISPNATKEF

GLVSSVSVIFTLVPYLYTCAALLLLGHGHFGKARPAYLAVTTIAFLYCIWAVVGSGAKEVMWSFVTLMVI

TAMYALNYNRLHKNPYPLDAPISKD

>gi|30042942|gb|AAP18665.1| hypothetical protein S3621 [Shigella flexneri 2a str. 2457T]

MRTLFDGNTVMLKRLLKRPSLNLLAWLLLAAFYISICLNIAFFKQVLQALPLDSLHNVLVFLSMPVVAFS

VINIVLTLSSFLWLNRPLACLFILIGAAAQYFIMTYGIVIDRSMIANIIDTTPAESYALMTPQMLLTLGF

SGVLAELIACWLKIKPATSRLRSVLFRGANILVSVLLILLVAALFYKDYASLFRNNKELVKSLSPSNSIV

ASWSWYSHQRLANLPLVRIGEDAHRNPLMQNEKRKNLTILIVGETSRAENFSLNGYPRETNPRLAKDNVV

YFPNTASCGTATAVSVPCMFSDMPREHYKEELAQHQEGVLDIIQRAGINVLWNDNDGGCKGACDRVPHQN

VTALNLPDQCINGECYDEVLFHGLEEYINNLQGDGVIVLHTIGSHGPTYYNRYPPQFRKFTPTCDTNEIQ

TCTKEQLVNTYDNTLVYVDYIVDKAINLLKEHQDKFTTSLVYLSDHGESLGENGIYLHGLPYAIAPDSQK

QVPMLLWLSEDYQKRYQVDQNCLQKQAQTQHYSQDNLFSTLLGLTGVETKYYQAADDILQTCRRVSK

>gi|30042941|gb|AAP18664.1| transcriptional regulatory protein [Shigella flexneri 2a str. 2457T]

MKILIVEDDTLLLQGLILAAQTEGYACDGVTTARMAEQSLEDGHYSLVVLDLGLPDEDGLHFLARIRQKK

YTLPVLILTARDTLTDKIAGLDVGADDYLVKPFALEELHARIRALLRRHNNQGESELIVGNLTLNMGRRQ

VWMGGEELILTPKEYALLSRLMLKAGSPVHREILYNDIYNWDNEPSTNTLEVHIHNLRDKVGKARIRTVR

GFGYMLVANEEN

>gi|30042940|gb|AAP18663.1| sensor protein for BasR [Shigella flexneri 2a str. 2457T]

MNLMRFLRRPISLRQRLILTIGAILLVFELISVFWLWHESTEQIQLFEQALRDNRNNDRHIMREIREAVA

SLIVPGVFMVSLTLFICYQAVRRITRPLAELQKELEARTADNLTPIAIHSATLEIEAVVSALNDLVSRLT

STLDNERLFTADVAHELRTPLAGVRLHLELLAKTHHIDVAPLVARLDQMMESVSQLLQLARAGQSFSSGN

YQHVKLLEDVILPSYDELSTMLDQRQQTLLLPESAADITVQGDATLLRMLLRNLVENAHRYSPQGSNIMI

KLQEDGGAVMAVEDEGPGIDESKCGELSKAFVRMDSRYGGIGLGLSIVSRITQLHHGQFFLQNRQETSGT

RAWVRLKKDQNVANQI

>gi|30042939|gb|AAP18662.1| low-affinity transport protein; proline permease II [Shigella flexneri 2a str. 2457T]

MLKRKKVKPITLRDVTIIDDGKLRKAITAASLGNAMEWFDFGVYGFVAYALGKVFFPGADPSVQMVAALA

TFSVPFLIRPLGGLFFGMLGDKYGRQKILAITIVIMSISTFCIGLIPSYDTIGIWAPILLLICKMAQGFS

VGGEYTGASIFVAEYSPDRKRGFMGSWLDFGSIAGFVLGAGVVVLISTIVGEANFLDWGWRIPFFIALPL

GIIGLYLRHALEETPAFQQHVDKLEQGDREGLQDGPKVSFKEIATKYWRSLLTCIGLVIATNVTYYMLLT

YMPSYLSHNLHYSEDHGVLIIIAIMIGMLFVQPVMGLLSDRFGRRPFVLLGSVALFVLAIPAFILINSNV

IGLIFAGLLMLAVILNCFMGVMASTLPAMFPTHIRYSALAAAFNISVLVAGLTPTLAAWLVESSQNLMMP

AYYLMVVAVIGLITGVTMKETANRPLKGATPAASDIQEAKEILVEHYDNIEQKIDNIDHEIADLQAKRTR

LVQQHPRIDE

>gi|30042938|gb|AAP18661.1| hypothetical protein S3617 [Shigella flexneri 2a str. 2457T]

MLESVHPRFLVDLAQGDDARHPQAHQQQFRERLMQELLSRVQLQTWTNGGMLNAPLSLRLTMVEKLASML

DPGHLALTQIAQHLALLQKMDHRQHSAFPELPQQIAALYEWFSARCRWKEKALTQRGLLVQAGDQSEQIF

TRWRAGAYNAWSLPGRCFIVLEELRWGAFGDACRLGSPQAVVLLLGDLREKATQHLAESINAAPTTRHYY

HQWFASSTVPTGGDHADFLSWLGKWTTADKQPVCWSVTQRWQTVALGMPRLCSAQRLVGAMVEEIFSVNL

A

>gi|30042937|gb|AAP18660.1| putative vimentin [Shigella flexneri 2a str. 2457T]

MYTQTLYELSQEAERLLQLSRQQLQLLEKMPLSVPGDDAPQLALPWSQPNIAERHAMLNNELCKISRLEM

VLAIVGTMKAGKSTTINAIVGTEVLPNRNRPMTALPTLIRHTPGQKEPVLHFSHVAPIDCLIQQLQQRLR

DCDIKHLTDVLEIDKDMRALMQRIENGVAFEKYYLGAQPIFHCLKSLNDLVRLAKALDVDFPFSAYAAIE

HIPVIEVEFVHLAGLESYPGQLTLLDTPGPNEAGQPHLQKMLNQQLARASAVLAVLDYTQLKSISDEEVR

EAILAVGQSVPLYVLVNKFDQQDRNSDDADQVRALISGTLMKGCITPQQIFPVSSMWGYLANRARYELAN

NGKLPAPEQQRWVEDFAHAALGRRWRHADLADLEHIRHAADQLWEDSLFAQPIQALLHAAYANASLYALR

SAAHKLLNYAQQAREYLDFRAHGLNVACEQLRQNIHQVEESLQLLQLNQAQVSGEIKHEIELALTSANHF

LRQQQDAVNAQLAALFQDDSEPLSEIRTRCETLLQTAQNTISRDFTLRFAEMESTLCRVLTDVIRPIEQQ

VKMELSESGFRPGFHFPVFHGVVPHFNTRQLFSEVISRQEATDEQSTRLGVVRETFSRWLNQPDWGRGNE

KSPTETVDYSVLQRALSAEVDLYCQQMAKVLAEQVDESVTAGMNTFFAEFASCLTELQTRLRESLALRQQ

NESVVRLMQQQLQQTVMTHSWIYTDAQLLRDDIQTLFTAERY

>gi|30042936|gb|AAP18659.1| hypothetical protein S3615 [Shigella flexneri 2a str. 2457T]

MSLPHCPKCNSEYTYEDNGMYICPECAYEWNDAEPAQESDELIVKDANGNLLADGDSVTIIKDLKVKGSS

SMLKIGTKVKNIRLVEGDHNIDCKIDGFGPMKLKSEFVKKN

>gi|30042935|gb|AAP18658.1| hypothetical protein S3614 [Shigella flexneri 2a str. 2457T]

MPLSPYLSFAGNCSDAIAYYQRTLGAELLYKISFGEMPKSAQDSAENCPSGMQFPDTAIAHANVRIAGSD

IMMSDAIPSGKASYSGFTLVLDSQQVEEGKRWFDNLAANGKIEMAWQETFWAHGFGKVTDKFGVPWMINV

VKQQPTQ

>gi|30042934|gb|AAP18657.1| ATP-binding component of phosphonate transporter [Shigella flexneri 2a str. 2457T]

MQTIIRVEKLAKTFNQHQALHAVDLNIHHSEMVALLGPSGSGKSTLLRHLSGLITGDKSAGSHIELLGRT

VQREGRLARDIRKSRANTGYIFQQFNLVNRLSVLENVLIGALGSTPFWRTCFSWFTGEQKQRALQALTRV

GMVHFAHQRVSTLSGGQQQRVAIARALMQQAKVILADEPIASLDPESARIVMDTLRDINQNDGITVVVTL

HQVDYALRYCERIVALRQGHVFYDGSSQLFDNERFDHLYRSINRIEENAKAA

>gi|30042933|gb|AAP18656.1| periplasmic binding protein component of phosphonate transporter [Shigella flexneri 2a str. 2457T]

MNAKIIASLAFTSMFSLSTLLTPAHAEEQEKALNFGIISTESQQNLKPQWTPFLQDMEKKLGVKVNAFFA

PDYAGIIQGMRFNKVDIAWYGNLSAMEAVDRANGQVFAQTVAADGSPGYWSVLIVNKDSPINNLNDLLAK

RKDLTFGNGDPNSTSGFLVPGYYVFAKNNISASDFKRTVNAGHETNALAVANKQVDVATNNTENLDKLKT

SAPEKLKELKVIWKSPLILGDPIVWRKNLSETTKDKIYDFFMNYGKTPEEKAVLERLGWAPFRASSDLQL

VPIRQLALFKEIQSVKGNKGLNEQDKLAKTTEIQAQLDDLDRLNNALSAMSSVSKAVQ

>gi|30042932|gb|AAP18655.1| membrane channel protein component of phosphonate transporter [Shigella flexneri 2a str. 2457T]

MPDAVQAPYPAYRPEPNMQTITIAPPKRSWFSLLSWAVVLDVLVVSWQGAEMAPLTLIKDGGNMATFAVM

TPTY

>gi|30042931|gb|AAP18654.1| IS1 orfB [Shigella flexneri 2a str. 2457T]

MSRQCTHYGRWPQHGFTSLKKLRPQSVTSRIQPGSDVIVCAEMDEHWGYVGAKSRQRWLFYAYDRIRRTV

VAHVFGERTLATLERLLSLLSAFEVVVWMTDGWPLYESRLKGKLHVISKRYTQHIERHNLNLRQHLARLV

RKSLSFSKSVELHDKVIGHYLNIKHYQ

>gi|30042930|gb|AAP18653.1| IS1 orfA [Shigella flexneri 2a str. 2457T]

MASISIRCPSCSATEGVVRNGKSTAGHQRYLCSHCRKTWQLQFTYTASQPGTHQKIIDMAMNGVGCRASA

RIMGVGLNTVLRHLKNSGRSR

>gi|30042929|gb|AAP18652.1| putative membrane protein [Shigella flexneri 2a str. 2457T]

MESTPKKAPRSKFPALLVVALALVALVFVIWRVDSAPSTNDAYASADTIDVVPEVSGRIVELAVTDNQAV

KQGDLLFRIDPRPYEANLAKAEASLAALDKQIMLTQRSVDAQQFGADSVNATVEKARAAAKQATDTLRRT

EPLLKEGFVSAEDVDRARTAQRAAEADLNAVLLQAQSAASAVSGVDALVAQRAAVEADIALTKLHLEMTT

VRAPFDGRIISLKTSVGQFASAMRPIFTLIDTRHWYVIANFRETDLKNIRSGTPATIRLMSDSGKTFEGK

VDSIGYGVLPDDGGLVLGGLPKVSRSINWVRVAQRFPVKIMVDKPDPEMFRIGASAVANLEPQ

>gi|30042928|gb|AAP18651.1| putative enzyme [Shigella flexneri 2a str. 2457T]

MTFEIPFVALSLAVLFYGIQSNAFYTKFVAILFVVATVLEIGSLFLIYKWSYGEPLIRLIIAGPILMGCM

FLMRTHRLGLVFFAVAIYGQTFPAMLDYPEVVVRLTLWCIVVGLYPTLLMTLIGVLWFPNRAITQMHQAL

NDRLDDAISHLTDSLAPLPETRIEREALALQKLNVFCLADDANWRTQSAWWQSCVATVTYIYSTLNRYDP

TSFADSQAIIEFRQKLASEINKLQHAVAEGQCWQSDWRLSESEAVAARECNLENICQTLLQLGQMNPNTP

PTPAAKPPSMVADAFTNPDYIRYAVKTLLACLICYTFYSGVDWEGIHTCMLTCVIVANPNVGSSYQKMVL

RFGGAFCGAILALLFTLLVMPWLDNIVELLFVLAPIFLLGAWIATSSERSSYIGTQMVVTFALATLENVF

GPVYDLVEIRDRALGIIIGTVVSAMIYTFVWPESEARTLPQKLAGALGMLSKVMRIPRQQEVTALRTYLQ

IRIGLHAAFNACEEMCQRVALERQLDSEERALLIERSQTVIRQGRDILHAWDATWNSAQALDNALQPDRA

GQFADALEKYAAGLATALSRSPQITLEETPTSQAILPTLLKQEQHVCQLFARLPDWTAPALTPATEQAQG

ATQ

>gi|30042927|gb|AAP18650.1| putative enzyme [Shigella flexneri 2a str. 2457T]

MINRQLSRLLLCSILGSTTLISGCALVRKDSAPHQQLKPEQIKLADDIHLASSGWPQAQWWKQLNDPQLD

ALIQRTLSGSHTLAEAKLREEKAQSQADLLDAGSQLQVAALGMLNRQRVSANGFLSPYAMDAPALGMDGP

YYTEATVGLFAGLDLDLWGVHRSAVAAAIGAHNAALAETAAVELSLTTGVAQLYYSMQASYQMLDLLEQT

RDVIDYAVKAHQSKVAHGLEAQVPFHGARAQILAVDKQIAAVKGQITETRESLRALIGAGASDMPEIKPV

ALPRVQTGIPATLSYELLARRPDLQAMRWYVQASLDQVDSARALFYPSFDIKAFFGLDAIHLDTLFKKTS

RQFNFIPGLKLPLFDGGRLNANLEGTRAASNMMIERYNQSVLNAVRDVAVNGTRLQTLNDEREMQAEHVE

ATRFTQRAAEAAYQRGLTSRLQATEARLPVLAEEMSLLMLDSRRVIQSIQLMKSLGGGYQAAPVVEKK

>gi|30042926|gb|AAP18649.1| hypothetical protein S3605 [Shigella flexneri 2a str. 2457T]

MLFLTFFAHANDSEPGSQYLKAAEAGDRRAQYFLADSWFSSGDLSKAEYWAQKAADSGDADACALLAQIK

ITNPVSLDYPQAKVLAEKAAQAGSKEGEVTLAHILVNTQAGKPDYPKAISLLENASEDLENDSAVDAQML

LGLIYANGVGIKADDDKATWYFKRSSAISRTGYSEYWAGMMFLNGEEGFIEKNKQKALHWLNLSCMEGFD

TGCEEFEKLTNG

>gi|30042925|gb|AAP18648.1| glutamate-aspartate symport protein [Shigella flexneri 2a str. 2457T]

MKNIKFSLAWQILFAMVLGILLGSYLHYHSDSRDWLVVNLLSPAGDIFIHLIKMIVVPIVISTLVVGIAG

VGDAKQLGRIGAKTIIYFEVITTVAIILGITLANVFQPGAGVDMSQLATVDISKYQSTTEAVQSSSHGIM

GTILSLVPTNIVASMAKGEMLPIIFFSVLFGLGLSSLPATHREPLVTVFRSISETMFKVTHMVMRYAPVG

VFALIAVTVANFGFSSLWPLAKLVLLVHFAILFFALVVLGIVARLCGLSVWILIRILKDELILAYSTASS

ESVLPRIIEKMEAYGAPASITSFVVPTGYSFNLDGSTLYQSIAAIFIAQLYGIDLSIWQEIILVLTLMVT

SKGIAGVAGVSFVVLLATLGSVGIPLEGLAFIAGVDRILDMARTALNVVGNALAVLVIAKWEHKFDRKKA

LAYEREVLGKFDKTADQ

>gi|30042924|gb|AAP18647.1| component of formate-dependent nitrite reductase complex [Shigella flexneri 2a str. 2457T]

MKQPQIPVKMLTTLTILMVFLCVGSYLLSPKWQAVRAEYQRQRDPLHQFASQQTPEAQLQALQDKIRANP

QNSEQWALLGEYYLWQNDYSNSLLAYRQALQLRGENAELYAALATVLYYQASQHMTAQTRAMIDKALALD

SNEITALMLLASDAFMQANYAQAIELWQKVMDLNSPRINRTQLVESINMAKLLQRRSD

>gi|30042923|gb|AAP18646.1| component of formate-dependent nitrite reductase complex [Shigella flexneri 2a str. 2457T]

MNKGLLTLLLLFTCFARAQVVDTWQFANPQQQQQALNIASQLRCPQCQNQNLLESNAPVAVSMRHQVYSM

VSEGKNEVEIIGWMTERYGDFVRYNPPLTGQTLVLWALPVVLLLLMALILWRVRAKR

>gi|30042922|gb|AAP18645.1| formate-dependent nitrite reductase [Shigella flexneri 2a str. 2457T]

MLTPLTAFAGVRLRWPAMMRLTCIGILAQFALLLLAFGVLTYCFLISDFSVIYVAQHSYSLLSWELKLAA

VWGGHEGSLLLWVLLLSAWSALFAWHYRQQSDPLFPLTLAVLSLMLAALLLFVVLWSDPFVRIFPPAIEG

RDLNPMLQHPGLIFHPPLLYLGYGGLMVAASVALASLLRGEFDAASARICWRWALPGWSALTAGIILGSW

WAYCELGWGGWWFWDPVENASLLPWLSATALLHSLSLTRQRGIFRHWSLLLAIVTLMLSLLGTLIVRSGI

LVSVHAFALDNVRAVPLFSLFALISLASLALYGWRARDGGPAVRFSGLSREMLILATLLLFCAVLLIVLV

GTLYPMIYGLLGWGRLSVGAPYFNRATLPFGLLMLVVIVLATFVSGKRVQLPALVAHAGVLLFAAGIVVS

SVSRQEISLNLQPGQQVTLAGYTFRFERLDLQARGNYTSEKAIVALFDHQQRIGELTPERRFYEARRQQM

MEPSIRWNGIHDWYAVMGEKTRADRYAFRLYVQSGVRWIWGGGLLMIAGALLSGWRGKKRDE

>gi|30042921|gb|AAP18644.1| formate-dependent nitrate reductase, transmembrane protein [Shigella flexneri 2a str. 2457T]

MTQTSAFHFESLVWDWPIAIYLFLIGISAGLVTLAVLLRRFYPQAGGADSTLLRTTLIVGPGAVILGLLI

LVFHLTRPWTFWKLMFHYSFTSVMSMGVMLFQLYMVVLVLWLAKIFEHDLLALQQRWLPKLGIVQKVLSL

LTPVHRGLETLMLVLAVLLGAYTGFLLSALKSYPFLNNPSLPVLLLFSGISSGAAVALIAMAIRQRSNPH

STEAHFVHRMEIPVVWGEIFLLVAFFVGLALGDDGKVRALVAALGGGFWTWWFWLGVAGLGLIVPMLLKP

WVNRSSGIPAVLAACGASLVGVLMLRFFILYAGQLTVA

>gi|30042920|gb|AAP18643.1| formate-dependent nitrite reductase [Shigella flexneri 2a str. 2457T]

MTWSRRQFLTGVGVLAAVSGTAGRVVAKTLNINGVRYGMVHDESLCIGCTACMDACREVNKVPEGVSRLT

IIRSEPQGEFPDVKYRFFRKSCQHCDHAPCVDVCPTGASFRDAASGIVDVNPDLCVGCQYCIAACPYRVR

FIHPVTKTADKCDFCRKTNLQAGKLPACVEACPTKALTFGNLDDPNSEISQLLRQKPTYRYKLALGTKPK

LYRVPFKYGEVSQ

>gi|30042919|gb|AAP18642.1| formate-dependent nitrite reductase [Shigella flexneri 2a str. 2457T]

MSVLRSLLTAGVLASGLLWSLNGITATPAVQASGDRYEVTQQRNPDAACLDCHKPDTEGMHGKHASVINP

NNKLPVTCTNCHGQASPQHREGVKDVMRFNEPMYKVGEQNSVCMSCHLPEQLQKAFWPHDVHVTKVACAS

CHSLHPQQDTMQTLSDKGRIKICVDCHSDQRTNPNFNPASVPLLKEQP

>gi|30042918|gb|AAP18641.1| periplasmic cytochrome c(552) [Shigella flexneri 2a str. 2457T]

MTRIKINARRIFSLLIPFFFFTSVHAEQTAAPAKPVTVEAKNETFAPQHHDQYLSWKATSEQSERVDALA

EDPRLVILWAGYPFSRDYNKPRGHAFAVTDVRETLRTGAPKNAEDGPLPMACWSCKSPDVARLIQKDGED

GYFHGKWARGGPEIVNNLGCADCHNTASPEFAKGKPELTLSRPYAARAMEAIGKPFEKAGRFDQQSMVCG

QCHVEYYFDGKNKAVKFPWDDGMKVENMEQYYDKIAFSDWTNSLSKTPMLKAQHPEYETWTAGIHGKNNV

TCIDCHMPKVQNAEGKLYTDHKIGNPFDNFAQTCANCHTQDKAALQKVVAERKQSINDLKIKVEDQLVHA

HFEAKAALDAGATEAEMKPIQDDIRHAQWRWDLAIASHGIHMHAPEEGLRMLGTAMDKAADARTKLARLL

ATKGITHEIQIPDISTKEKAQQAIGLNMEQIKAEKQDFIKTVIPQWEEQARKNGLLSQ

>gi|30042917|gb|AAP18640.1| hypothetical protein S3595 [Shigella flexneri 2a str. 2457T]

MNGTIYQRIEDNAHFRELVEKRQRFATILSIIMLAVYIGFILLIAFAPGWLGTPLNPNTSVTRGIPIGVG

VIVISFVLTGIYIWRANGEFDRLNNEVLHEVQAS

>gi|30042916|gb|AAP18639.1| putative transport protein [Shigella flexneri 2a str. 2457T]

MKRVLTALAATLPFAANAADAISGAVERQPTNWQAIIMFLIFVVFTLGITYWASKRVRSRSDYYTAGGNI

TGFQNGLAIAGDYMSAASFLGISALVFTSGYDGLIYSLGFLVGWPIILFLIAERLRNLGRYTSADVASYR

LKQGPIRILSACGSLVVVALYLIAQMVGAGKLIELLFGLNYHIAVVLVGVLMMMYVLFGGMLATTWVQII

KAVLLLFGASFMAFMVMKHVGFSFNNLFSEAMAVHPKGVDIMKPGGLVKDPISALSLGLGLMFGTAGLPH

ILMRFFTVSDAREARKSVFYATGFMGYFYILTFIIGFGAIMLVGANPEYKDAAGHLIGGNNMAAVHLANA

VGGNLFLGFISAVAFATILAVVADLTLAGASAVSHDLYANVFKKGATEREELRVSKITVLILGVIAIILG

VLFENQNIAFMVGLAFAIAASCNFPIILLSMYWSKLTTRGAMLGGWLGLITAVVLMILGPTIWVQILGHE

KAIFPYEYPALFSISVAFLGIWFFSATDNSAEGARERELFRAQFIRSQTGFGVEQGRAH

>gi|30042915|gb|AAP18638.1| hypothetical protein S3593 [Shigella flexneri 2a str. 2457T]

MRYNGLNNMFFPLCQINDNHSITSSSHTKKTKSDNYSKHHKNTLIDNKALSLFKIDDHEKVIGLIQKMKR

IYDSLPSGKITKETDRKIHKYFIDIALYANNKCDDRITRRVYLSKEKDVSIKVVYFINNVAVHNNTIEIP

QIVNGGYDFSHLSLKGIVIKDEDLSNSNFAGCRLQNAIFQDCNMYKTNFYYAIMEKILFDNCILDDSNFA

QIKMADGTLNACSAMHVQFYNAAMNRANIKNTFLDYSNFYMAYMAEVNLYKVIAPYVNLFKADLSFSKLD

LINFEHADLSRVNLNKAILQNINLIDSKLFCTWLTNTFLEMVICTGSNMANVNFNNANLSNCHFNCSILT

KACMFNTRLYRVNFDEASVQGMGISILRGEENIPIDSDTLVTLQKFFEEDCTSHTGMSQTEDNINAVAMK

ITADIMQHAD

>gi|30042914|gb|AAP18637.1| hypothetical protein S3592 [Shigella flexneri 2a str. 2457T]

MEIFFTILIMTLVVSLSGVVTRVMPFQIPLPLMQIAIGALLAWPTFGLHVEFDPELFLVLFIPPLLFADG

WKTPTREFLEHGREIFGLALALVVVTVVGIGFLIYWVVPGIPLIPAFALAAVLSPTDAVALSGIVGEGRI

PKKIMGILQGEALMNDASGLVSLKFAVAVAMGTMIFTVGGATVEFMKVAIGGILAGFVVSWLYGRSLRFL

SRWGGDEPATQIVLLFLLPFASYLIAEHIGVSGILAAVAAGMTITRSGVMRRAPLAMRLRANSTWAMLEF

VFNGMVFLLLGLQLPGILETSLMAAEIDPNVEIWMLFTDIILIYAALMLVRFGWLWTMKKFSNRFLKKKP

MEFGSWTTREILIASFAGVRGAITLAGVLSIPLLLPDGNVFPARYELVFLAAGVILFSLFVGVVMLPILL

QHIEVADHSQQLKEERIARAATAEVAIVAIQKMEERLAADTEENIDNQLLTEVSSRVIGNLRRRADGRND

VESSVQEENLERRFRLAALRSERAELYHLRATREISNETLQKLLHDLDLLEALLIEENQ

>gi|30042913|gb|AAP18636.1| hypothetical protein S3591 [Shigella flexneri 2a str. 2457T]

MSTPSARTGGSLDAWFKISQRGSTVRQEVVAGLTTFLAMVYSVIVVPGMLGKAGFPPAAVFVATCLVAGL

GSIVMGLWANLPLAIGCAISLTAFTAFSLVLGQHISVPVALGAVFLMGVLFTVISATGIRSWILRNLPHG

VAHGTGIGIGLFLLLIAANGVGLVIKNPLDGLPVALGDFATFPVIMSLAGLAVIIGLEKLKVPGGILLTI

IGISIVGLIFDPNVHFSGVFAMPSLSDENGNSLIGSLDIMGALNPVVLPSVLALVMTAVFDATGTIRAVA

GQANLLDKDGQIIDGGKALTTDSMSSVFSGLVGAAPAAVYIESAAGTAAGGKTGLTAITVGVLFLLILFL

SPLSYLVPGYATAPALMYVGLLMLSNVAKIDFADFVDAMAGLVTAVFIVLTCNIVTGIMIGFATLVIGRL

VSGEWRKLNIGTVVIAVALVTFYAGGWAI

>gi|30042912|gb|AAP18635.1| redox-sensing activator of soxS [Shigella flexneri 2a str. 2457T]

MEKKLPRIKALLTPGEVAKRSGVAVSALHFYESKGLITSIRNSGNQRRYKRDVLRYVAIIKIAQRIGIPL

ATIGEAFGVLPEGHTLSAKEWKQLSSQWREELDRRIHTLVALRDELDGCIGCGCLSRSDCPLRNPGDRLG

EEGTGARLLEDEQN

>gi|30042911|gb|AAP18634.1| regulation of superoxide response regulon [Shigella flexneri 2a str. 2457T]

MSHQKIIQDLIAWIDVHIDQPLNIDVVAKKSGYSKWYLQRMFRTVTHQTLGDYIRQRRLLLAAVELRTTE

RPIFDIAMDLGYVSQQTFSRVFRRQFDRTPSDYRHRL

>gi|30042910|gb|AAP18633.1| hypothetical protein S3587 [Shigella flexneri 2a str. 2457T]

MYHASREQRRAVQGIGSFQRNCLMATLTTGVVLLRWQLLSAVMMFLASTLNIRFRRSDYVGLAVISSGLG

VVSACWFAMGLLGITMADITAIWHNIESVMIEEMNQTPPQWPMILT

>gi|30042909|gb|AAP18632.1| IS1 orfA [Shigella flexneri 2a str. 2457T]

MASISIRCPSCSATEGVVRNGKSTAGHQRYLCSHCRKTWQLQFTYTASQPGTHQKIIDMAMNGVGCRASA

RIMGVGLNTVLRHLKNSGRSR

>gi|30042908|gb|AAP18631.1| IS1 orfB [Shigella flexneri 2a str. 2457T]

MSRQCTHYGRWPQHGFTSLKKLRPQSVTSRIQPGSDVIVCAEMDEHWGYVGAKSRQRWLFYAYDRIRRTV

VAHVFGERTLATLERLLSLLSAFEVVVWMTDGWPLYESRLKGKLHVISKRYTQHIERHNLNLRQHLARLV

RKSLSFSKSVELHDKVIGHYLNIKHYQ
[truncated: 321,564 more chars]
